
JOURNAL INFORMATION
==============================
NLM Title Abbreviation: Innov Aging
Journal ID: Innov Aging
NLM Journal ID: 101703706
Journal ID: innovateage

Innovation in Aging

EISSN: 2399-5300
Publisher: Oxford University Press

ARTICLE INFORMATION
==============================
PMCID: PMC11688754
DOI: 10.1093/geroni/igae098
Article ID: igae098
Article version: 1
Subjects: Full GSA 2024 Abstract Book Pdf, AcademicSubjects/SOC02600

Full GSA 2024 Abstract Book PDF

Publication date: 2024 Dec
Electronic publication date: 2024 Dec 31
Volume: 8
Issue: Suppl 1
Issue title: Program Abstracts from the GSA 2024 Annual Scientific Meeting, “The Fortitude Factor”
First page: NA
Last page: NA
Copyright: © The Author(s) 2024. Published by Oxford University Press on behalf of The Gerontological Society of America.
Copyright year: 2024
License: This is an Open Access article distributed under the terms of the Creative Commons Attribution License (https://creativecommons.org/licenses/by/4.0/), which permits unrestricted reuse, distribution, and reproduction in any medium, provided the original work is properly cited.
License URL: https://creativecommons.org/licenses/by/4.0/

Erratum in: Correction to: CHALLENGES AND KEY ASPECTS OF COLLABORATION BETWEEN VISITING NURSES AND CARE MANAGERS IN JAPAN 9 6 12 6 2025 igaf060 Innovation in Aging 10.1093/geroni/igaf060 PMC12160806 40510270
Page count: 1

==============================

### INNOVATION IN AGING®

GERONTOLOGICAL SOCIETY OF AMERICA®

Volume 8 • Issue S1

### Editorial Board - Innovation in Aging

**Steven M. Albert, PhD, MS, FGSA, Editor-in-Chief**
University of Pittsburgh, USA
E-mail: smalbert@pitt.edu
**Jennifer Tehan Stanley, PhD, FGSA, Deputy Editor-in-Chief**
University of Akron, USA
**Karen J. Jung, MSW, Managing Editor**
The Gerontological Society of America, USA
Email: ia@geron.org
**Christine E. Bishop, PhD, FGSA, Associate Editor, Economics of Aging**
Brandeis University, USA
**Julie Blaskewicz Boron, PhD, FGSA, Associate Editor, Technology**
University of Nebraska – Omaha, USA
**Howard B. Degenholtz, PhD, FGSA, Associate Editor, Long-Term Services and Supports**
University of Pittsburgh, USA
**Nancy W. Glynn, PhD, FGSA, Associate Editor, Epidemiology of Aging**
University of Pittsburgh, USA
**Min-Ah Lee, PhD, Associate Editor, Relationships & Well-being**
Chung-Ang University, South Korea
**Amanda N. Leggett, PhD, FGSA, Associate Editor, Dementia and Caregiving**
Wayne State University, USA
**Joshua D. Niznik, PharmD, PhD, Associate Editor, Pharmaceutical Outcomes and Policy**
UNC Chapel Hill School of Medicine, USA
**Lena K. Makaroun, MD, MS, Associate Editor, Geriatrics**
VA Pittsburgh Healthcare System, USA
University of Pittsburgh, USA
**Alison Phinney, PhD, RN, Associate Editor, Qualitative Research**
University of British Columbia, Canada
**J. Tina Savla, PhD, FGSA, Associate Editor, Methodology**
Virginia Tech, USA
**Roland J. Thorpe, Jr., PhD, FGSA, Associate Editor, Minority Health/Health Disparities**
Johns Hopkins Bloomberg School of Public Health, USA
**Noah J. Webster, PhD, FGSA, Associate Editor, Health Demography**
University of Michigan, USA
**W. Quin Yow, PhD, Associate Editor, Technology**
Singapore University of Technology & Design, Singapore

### Editorial Board

**Kathleen Abrahamson, RN, PhD, FGSA**
Purdue University, USA
**Tamara A. Baker, PhD, FGSA**
University of North Carolina – Chapel Hill, USA
**Scott R. Beach, PhD, FGSA**
University of Pittsburgh, USA
**Sara J. Czaja, PhD, FGSA**
Weill Cornell Medicine, USA
**Adam Davey, PhD, FGSA**
University of Delaware – Newark, USA
**Nancy R. Gee, PhD**
Virginia Commonwealth University, USA
**Megan Gilligan, PhD, FGSA**
Iowa State University, USA
**Allen Glicksman, PhD, FGSA**
NewCourtland, USA
**Ellen L. Idler, PhD, FGSA**
Emory University, USA
**Mary Ann Johnson, PhD**
University of Nebraska - Lincoln, USA
**Giyeon Kim, PhD, FGSA**
Chung-Ang University, South Korea
**TsuAnn Kuo, PhD**
Chung Shan Medical University, Taiwan
**Yeonjung Lee, PhD, MSW**
Chung-Ang University, South Korea
**Darren Liu, DrPH, FGSA**
West Virginia University, USA
**Hui Liu, PhD, FGSA**
Michigan State University, USA
**Mariana López-Ortega, PhD, MPP**
National Institute of Geriatrics, National Institutes of Health, Mexico
**Meghan K. Mattos, PhD, RN**
University of Virginia, USA
**Anne Collins McLaughlin, PhD**
North Carolina State University, USA
**David A. Nace, MD, MPH**
University of Pittsburgh, USA
**Lycia Tramujas Vasconcellos Neumann, PhD, MPA**
Alzheimer's Association, USA
**Marcia Ory, PhD, FGSA**
Texas A&M University, USA
**Richard Pak, PhD**
Clemson University, USA
**Karl Pillemer, PhD, FGSA**
Cornell University, USA
**Karen A. Roberto, PhD, FGSA**
Virginia Tech, USA
**Laura P. Sands, PhD, FGSA**
Virginia Tech, USA
**J. Jill Suitor, PhD, FGSA**
Purdue University, USA
**Robert Joseph Taylor, MSW, PhD**
University of Michigan, USA
**Jasmine L. Travers, PhD, RN**
New York University, USA
**Jennifer R. Turner, PhD**
University of Hawai'i at Hilo, USA
**Kaipeng Wang, PhD**
University of Denver, USA
**Richard Winett, PhD**
Virginia Tech, USA
**Yijin Wu, PhD**
Shandong University, China
**Yao Yao, MD**
Peking University, China
**Jeremy B. Yorgason, PhD, FGSA**
Brigham Young University, USA
**Anna Zajacova, PhD, FGSA**
Western University, Canada

### Honorary Editor-In-Chief

**Laura P. Sands, PhD, FGSA** (2017-2020)

### Program, Publications, and Products Committee

**Jennifer Ailshire, PhD, FGSA**
University of Southern California, USA
**Debra Bakerjian, FAAN, FGSA, MSN, NP, PhD**
University of California – Davis, USA
**Kara Dassel, PhD, FAGHE, FGSA**
University of Utah, USA
**Christine Fruhauf, PhD, FAGHE, FGSA**
Colorado State University, USA
**Rita Hu, MSW**
University of Michigan, USA
**Jung Kwak, PhD, MSW, FGSA**
University of Texas at Austin, USA
**Nathan K. LeBrasseur, PT, PhD**
Mayo Clinic, USA
**Judie Lieu, BA**
The Gerontological Society of America, USA
**Karen Lincoln, PhD, MA, MSW, FGSA**
University of California – Irvine, USA
**Elham Mahmoudi, PhD**
University of Michigan, USA
**Brianna Morgan, MSN, CRNP, ACHPN**
University of Pennsylvania, USA
**Tina Newsham, PhD, FAGHE**
University of North Carolina – Wilmington, USA
**Brandt Pence, PhD**
University of Memphis, USA
**Karon Phillips, MPH, PhD, FGSA**
Trust for America's Health, USA
**Blanka Rogina, PhD, FGSA**
University of Connecticut Health, USA
**Tetyana Shippee, PhD, FGSA**
University of Minnesota, USA
**Mo-kyung Sin, PhD, FGSA**
Seattle University, USA
**Kaipeng Wang, MSW, PhD**
University of Denver, USA

Fellow of the Gerontological Society of America (FGSA)

### Program Abstracts from the GSA 2024 Annual Scientific Meeting, "The Fortitude Factor"

Abstracts are arranged numerically by session and in the order of presentation within each session.
Abstracts are published as received.

### SESSION 1000 (SYMPOSIUM)

Abstract citation ID: igae098.0001

### AD BLOOD BIOMARKERS IN DIVERSE COMMUNITY SETTINGS: A LONGITUDINAL PERSPECTIVE FROM THE ARIC STUDY

Chair: Priya Palta
Co-Chair: Kevin Sullivan
Discussant: Natascha Merten

Alzheimer's disease and related dementias (ADRD) feature a prolonged preclinical stage spanning decades, with the transition from mid- to late-life marking the critical period for onset and accumulation of pathological brain changes that may lead to physical and cognitive disability. Identifying individuals at risk for cognitive and physical decline during preclinical stages when interventions or disease modifying treatments are more likely to be effective is needed. Blood biomarkers of ADRD pathology and neurodegeneration are promising cost-effective and non-invasive options to fill this gap. To date, however, there are limited data on temporal changes in blood biomarkers and their associations with cognitive, mobility, and neuroimaging outcomes in diverse community-based cohorts. This symposium will feature 23 years of data from the well-established community-based Atherosclerosis Risk in Communities (ARIC) Study cohort which assayed blood biomarkers of amyloid-β (Aβ)42/40, phosphorylated tau at threonine 181 (p-Tau181), neurofilament light (NfL), and glial fibrillary acidic protein (GFAP) using Quanterix Simoa assays on stored specimens at up to 3 timepoints in midlife and late-life in ~1,800 participants. This session will present findings on temporal blood biomarker changes from mid- to late-life and associated risk and protective factors of biomarker changes; mid- to late-life changes in blood biomarkers and associations with late-life neuroimaging measures of neurodegeneration, cerebrovascular disease, and amyloid deposition; and the associations of late-life blood biomarkers with prevalent and incident mobility and cognitive impairment.

Abstract citation ID: igae098.0002

### DESIGN, MEASURES, AND CORRELATES OF CHANGES IN AD BLOOD BIOMARKERS AND ASSOCIATIONS WITH NEUROIMAGING OUTCOMES

Priya Palta[1], James Pike[2], Yifei Lu[3], Jinyu Chen[1], Keenan Walker[4], Kevin Sullivan[5], and Thomas Mosley[6], *1. University of North Carolina, Chapel Hill, North Carolina, United States, 2. New York University, New York, New York, United States, 3. Merck, Landsdale, Pennsylvania, United States, 4. National Institute on Aging, Baltimore, Maryland, United States, 5. University of Mississippi Medical Center, Jackson, Mississippi, United States, 6. University of Mississippi Medical Center - The MIND Center, Jackson, Mississippi, United States*

Few studies have examined changes in blood-based biomarkers of AD pathology and neurodegeneration particularly during the mid- to late-life transition period and associations with brain pathology. Among 1525 (59.9% women, 25.8% Black) ARIC participants, we measured blood-based biomarkers of Aβ42, Aβ40, p-Tau181, NfL, and GFAP using stored specimens from midlife (1993-1995, mean age 58.3 years) and late-life (2011-2013, mean age 76.0 years followed to 2016-2019, mean age 80.7 years). Using linear mixed effects models, we examined whether known dementia-related risk factors in midlife (i.e., hypertension, diabetes, lipids, coronary heart disease, cigarette use, and physical activity) are associated with 20-year biomarker changes. We also examined the associations of midlife, late-life, and mid- to late-life changes in blood biomarkers with measures of whole brain cortical thickness, brain volume, and white matter hyperintensity volume (WMH) from a 3T brain MRI scan in multivariable linear regression models. All models were adjusted for age, sex, race-center, education, APOE, eGFR, body mass index, cardiovascular risk factors, and intracranial volume for volumetric analyses. Decreasing Aβ42/Aβ40 and increasing p-Tau181, NfL, and GFAP were observed from midlife to late-life. Midlife hypertension and diabetes were associated with a steeper rise in NfL and GFAP. Lower late-life levels of Aβ42/Aβ40 and higher late-life levels of p-Tau181, p-Tau181/Aβ42, NfL, and GFAP were associated with smaller cortical thickness and total brain volume, and larger WMH volume. Blood biomarkers of AD neuropathology, neuronal injury, and astrogliosis rise with age and are associated with known dementia risk factors and markers of neurodegeneration and cerebrovascular disease.

Abstract citation ID: igae098.0003

### BLOOD-BASED BIOMARKERS FOR ALZHEIMER'S DISEASE AND NEURODEGENERATION IN RELATION TO INCIDENT COGNITIVE STATUS

Kevin Sullivan[1], Chad Blackshear[1], James Pike[2], Keenan Walker[3], B. Gwen Windham[4], Thomas Mosley[4], Michael Griswold[4], and Priya Palta[5], *1. University of Mississippi Medical Center, Jackson, Mississippi, United States, 2. New York University, New York, New York, United States, 3. National Institute on Aging, Baltimore, Maryland, United States, 4. University of Mississippi Medical Center - The MIND Center, Jackson, Mississippi, United States, 5. University of North Carolina, Chapel Hill, North Carolina, United States*

Blood-based biomarkers for Alzheimer's Disease (AD) and neurodegeneration, including amyloid-beta (AB), ptau-181, glial fibrillary acidic protein (GFAP), and neurofilament

© The Author(s) 2024. Published by Oxford University Press on behalf of the Gerontological Society of America.
This is an Open Access article distributed under the terms of the Creative Commons Attribution License (https://creativecommons.org/licenses/by/4.0/), which permits unrestricted reuse, distribution, and reproduction in any medium, provided the original work is properly cited.

light (NFL), could advance the monitoring and detection of individuals at high risk for adverse cognitive outcomes. At visit 5 (2011-13) of the community-based ARIC study, AB42, AB40, ptau-181, GFAP, and NFL were measured in plasma using ultrasensitive single-molecule array (Simoa; Quanterix) assays in a sample of 919 cognitively unimpaired participants (mean age 76±5 years, 61% female, 31% Black). Cognitive outcomes included adjudicated dementia and mild cognitive impairment (MCI) ascertained using a neuropsychological battery, functional status questionnaires, and informant interviews across three in-person clinic exams [median follow-up of 6.5 years (IQR:5.8-6.9)]. Multinomial logistic regressions estimated Relative Risk Ratios (RRR) of the four-category outcome of MCI, dementia, and death vs. cognitively unimpaired status for each base(2) log-transformed biomarker separately, adjusted for age, sex, education, site-race, hypertension, diabetes, obesity, APOEε4, and estimated glomerular filtration rate. All blood biomarkers were associated with incident dementia risk [AB42:40 (RRR=0.57, 95% CI: 0.40,0.83); ptau-181:AB42 (RRR=2.40, 95% CI: 1.74,3.30); GFAP (RRR=2.35, 95% CI: 1.22,4.53); NFL (RRR= 1.95, 95% CI: 1.29, 2.94)] and incident MCI risk [AB42:40 (RRR=0.77, 95% CI: 0.62,0.95); ptau-181:AB42 (RRR=1.61, 95% CI: 1.31,1.99); GFAP (RRR=1.24, 95% CI: 0.99,1.56); NFL (RRR= 1.44, 95% CI: 1.11, 1.87)]. Blood biomarkers indicative of varying neuropathological processes show potential utility in cognitive risk assessment and disease characterization in Black and White older adults with up to a decade of follow-up.

Abstract citation ID: igae098.0004

### CHANGE IN ALZHEIMER'S DISEASE BLOOD-BASED BIOMARKERS AND ASSOCIATIONS WITH BRAIN AMYLOID DEPOSITION

James Pike[1], Yifei Lu[2], Jinyu Chen[3], Keenan Walker[4], Kevin Sullivan[5], Michael Griswold[6], Thomas Mosley[6], and Priya Palta[3], *1. New York University, New York, New York, United States, 2. Merck, Landsdale, Pennsylvania, United States, 3. University of North Carolina, Chapel Hill, North Carolina, United States, 4. National Institute on Aging, Baltimore, Maryland, United States, 5. University of Mississippi Medical Center, Jackson, Mississippi, United States, 6. University of Mississippi Medical Center - The MIND Center, Jackson, Mississippi, United States*

Blood-based biomarkers show promise as a noninvasive, inexpensive method for measuring Alzheimer's disease pathology throughout the lifecourse. However, the prospective associations and discriminatory accuracy of these biomarkers at different stages of the lifecourse and among diverse, community-dwelling populations requires further investigation. Between 2014 and 2015, 329 dementia-free participants from the Atherosclerosis Risk in Communities Study underwent brain MRI and PET scans. Amyloid positivity was defined as a standardized uptake value ratio greater than 1.2. Stored plasma samples collected in midlife (1993-95, mean age 58.5 years) and late-life (2011-13, mean age 76.2 years) from a subsample of 259 participants were assayed in 2022. The assay quantified amyloid-β (Aβ)42/40, phosphorylated tau (p-Tau181), neurofilament light (NfL), and glial fibrillary acidic protein (GFAP). Logistic regression models estimated the association between plasma biomarkers from midlife and late-life and amyloid positivity in late-life. Receiver operating characteristic curves documented the discriminatory accuracy of the biomarkers. Assays from midlife were not associated with amyloid positivity in late-life. However, late-life measurements of Aβ42/40, p-Tau181, and GFAP and change per decade from midlife to late-life in p-Tau181 and GFAP were associated with greater odds of amyloid positivity. The greatest discriminatory accuracy was achieved by using all assays measured in midlife and late-life (AUC = 0.754), although the accuracy when using only late-life measures was comparable (AUC = 0.737). Additional longitudinal research is needed to determine whether changes in plasma biomarkers measured before an individual is amyloid positive can identify older adults at risk of developing Alzheimer's disease.

Abstract citation ID: igae098.0005

### AD/NEURODEGENERATION BLOOD BIOMARKER ASSOCIATIONS WITH PREVALENT AND INCIDENT MOBILITY AND COGNITIVE IMPAIRMENT

B. Gwen Windham[1], Chad Blackshear[2], Kevin Sullivan[2], James Pike[3], Keenan Walker[4], Thomas Mosley[1], Priya Palta[5], and Michael Griswold[1], *1. University of Mississippi Medical Center - The MIND Center, Jackson, Mississippi, United States, 2. University of Mississippi Medical Center, Jackson, Mississippi, United States, 3. New York University, New York, New York, United States, 4. National Institute on Aging, Baltimore, Maryland, United States, 5. University of North Carolina, Chapel Hill, North Carolina, United States*

Blood biomarkers of Alzheimer Disease pathology (Aβ42, Aβ40, p-tau181) and neurodegeneration (neurofilament light (NFL), glial fibrillary acidic protein (GFAP)) may identify individuals at risk for mobility and cognitive impairment. Among 1751 ARIC study participants at Visit 5 (V5, 2011-13, mean age 76.2 years (5.3), 41% men, 28% Black), usual gait speed and cognitive status were assessed over 8 years at V5, V6 (2016-17), and V7 (2017-18). Impairment outcomes were mobility-only (gait speed <0.8m/s, n=430), cognitive-only (dementia, n=38), dual (n=54), or neither (referent, n=1229). Multinomial regression models examined relative risk ratios (RRR) per higher IQR of V5 log-transformed biomarkers with cross-sectional impairments and, among those with neither impairment at V5, incident impairments or death without impairment (n=168) through V7. All models adjusted for V5 age, sex, self-reported race (Black or White), renal function, and body mass index. Aβ42 was reversed and adjusted for Aβ40 (-Aβ42|40) for consistent directions of associations (higher=worse). Higher biomarkers were associated with greater prevalence of mobility, cognitive, and dual impairments. Incident impairments occurred in mobility-only (n=471), cognitive-only (n=39), and dual domains (n=55). All biomarkers were associated with incident dual impairment: p-tau181 (RRR=2.54; 95% CI: 1.45, 4.44), -Aβ42|40 (RRR=1.66; 1.31, 2.09), NfL (RRR=2.69; 1.22, 5.91) and GFAP (RRR=2.13; 1.07, 4.25). P-tau181, -Aβ42|40, and GFAP were also associated with incident cognitive-only impairment; P-tau181, NFL and GFAP were associated with incident mobility-only impairment. AD-pathology versus neurodegeneration blood biomarkers may help identify older adults at differential risk of functional decline, potentially facilitating earlier targeted interventions to preserve mobility and cognitive outcomes.

### SESSION 1005 (SYMPOSIUM)

Abstract citation ID: igae098.0006

### ADVANCING HOME MODIFICATION SERVICE DELIVERY THROUGH SYNTHESIZING DATA AND EVIDENCE

Chair: Bernard A. Steinman
Co-Chair: Scott Trudeau
Discussant: Maria Henke

Supportive housing options are a cornerstone to aging in place. Whereas most older people prefer to remain in their homes as they age, challenges associated with aging, including declines in physical health and functioning, often act as barriers to meeting this goal. Additionally, the home's condition can affect a person's ability to care for themselves and the ability of caregivers to provide quality care. Home modifications (HM) that increase safety and support daily activities play a key role in helping people stay in their homes and communities. Indeed, evidence-based interventions with HM components have been shown to reduce falls-related accidents, address a range of caregiving needs, and delay or prevent institutionalization–outcomes that otherwise come with high costs to individuals and society. In this symposium we have three aims: 1) to summarize previous research supporting the efficacy of programs with HM components in promoting aging in place via improved safety and access in the home; 2) to present three original studies that incorporated data from the National Health and Aging Trends Study (NHATS) to reveal HM use patterns (e.g., whether HMs are implemented proactively in advance of acquiring disability, or reactively in response to acquiring disability); and 3) to introduce a current HM service delivery model, describe its development, and discuss implications and next steps, including how current research findings can inform service delivery strategies in the future.

Abstract citation ID: igae098.0007

### HEALTH DIMENSIONS AS PREDICTORS OF HOME MODIFICATION IMPLEMENTATION: A LONGITUDINAL ANALYSIS OF NHATS DATA

Bernard A. Steinman[1], Mengzhao Yan[2], and Jon Pynoos[2], *1. University of Wyoming, Laramie, Wyoming, United States, 2. University of Southern California, Los Angeles, California, United States*

A significant proportion of older Americans experience chronic conditions, functional impairments, participation limitations, activities of daily living (ADL) and instrumental activities of daily living (IADL) disabilities that could impact their capacity to live independently in their homes. Home modifications (HM) implemented to promote safety and access can support aging in place by improving environmental fit with health characteristics of older individuals. We conceptualized health dimensions using the International Classification of Functioning, Disability, and Health (ICF) to estimate risk profiles for implementing shower/tub and toilet HMs. We analyzed data from the National Health and Aging Trends Study (NHATS) using Cox regression, to examine associations between each ICF health dimension (i.e., personal characteristics, chronic conditions, functional impairments, social participation, ADL and IADL disability) and the probability of community dwelling older adults implementing HMs over a five-year period (2011 to 2015). Results indicated that being older age, married, having more functional impairments, greater social participation, and greater ADL and IADL disabilities are each independently associated with a higher probability of acquiring both types of HM. For example, each additional functional limitation was associated with a 12% increased risk of implementing toilet modifications and a 4% increased risk of implementing shower/tub modifications. Additionally, compared to non-Hispanic Whites, Hispanics are less likely to implement either type of HM. Implications of this study are that older individuals choose to implement HMs in response to different need profiles and that some age-related changes may be more impactful in decision making than others.

Abstract citation ID: igae098.0008

### SUPPORTIVE HOME FEATURES AS PROACTIVE/REACTIVE ASSOCIATES OF DAILY FUNCTIONING IN OLDER ADULTS

Daniel Barbakoff[1], Kristen Glennie[2], and Bernard A. Steinman[3], *1. Weill Cornell Medicine, New York, New York, United States, 2. Colorado State University, Fort Collins, Colorado, United States, 3. University of Wyoming, Laramie, Wyoming, United States*

Supportive home features (SHF) include attributes of living spaces meant to help people overcome physical limitations that may be barriers to living independently. Evidence suggests that SHFs, including home modifications (HM), may help older adults to age in place at home. However, real-life implementation of HMs is not fully understood. The purpose of this study was to clarify associations between functioning in daily-living activities and the presence of SHFs. We analyzed two waves (2011 and 2015) of the National Health and Aging Trends Study (NHATS) to examine the directionality of this relationship. SHFs were operationalized using indicators of home accessibility (e.g., presence of a bedroom, kitchen, and full bathroom with a shower or tub on the same floor). HMs were operationalized based on whether bathroom fixtures were modified to accommodate disability with features such as grab bars. Daily activities were assessed using measures of activities of daily living (ADL; e.g., dressing) and instrumental ADLs (IADL; e.g., preparing a meal). Changes in ADL/IADL functioning and SHF/HM variables were assessed over the 5-year period. Regression models also included covariates for sociodemographics, chronic conditions, mobility functioning, and participation. Results show that, whereas ADL limitations statistically predict SHF/HM outcomes, the implementation of SHF/HMs does not predict any ADL/IADL outcomes. These findings suggest that in real life, older adults implement SHF/HMs reactively in response to functional needs, as opposed to planning proactively to meet needs in the future.

Abstract citation ID: igae098.0009

### DO RECOMMENDATIONS FROM REHABILITATION THERAPISTS MOTIVATE OLDER ADULTS TO INSTALL HOME MODIFICATIONS?

Mengzhao Yan[1], Bernard A. Steinman[2], and Jon Pynoos[1], *1. University of Southern California, Los Angeles,*

*California, United States, 2. University of Wyoming, Laramie, Wyoming, United States*

With the rapid growth of the aging population, private homes have increasingly become an alternative setting for the provision of long-term care (LTC). For many, LTC needs increase during recovery from acute medical events and subsequent rehabilitation services. An estimated 20% of older Americans undergo physical rehabilitation services, including physical therapy, occupational therapy, and speech therapy, that can help to promote function and improve their ability to carry out daily activities. Often, rehabilitation therapists may recommend that older adults receiving their services make home modifications (HMs) to facilitate their recovery and improve home accessibility and safety. Nevertheless, it is unclear to what extent receiving recommendations is associated with decisions by older adults to install HMs. Guided by Andersen's healthcare utilization model, which assesses factors that lead to the use of health services, we hypothesized that those who received recommendations from rehabilitation therapists would be more likely to modify their homes to accommodate disability. We analyzed data from the most recent round (2022) of the National Health and Aging Trends Study (NHATS). We used multivariate logistic regression to estimate the relationship between the recommendation and implementation of shower/tub and toilet HMs. Results indicated that older adults who received recommendations to modify their showers/tubs had 158% higher odds of installing shower/tub HMs, whereas those who received recommendations to modify areas around their toilets had 160% higher odds of installing toilet HMs. Findings suggest that older adults may be especially motivated to implement HM regimens when directed by rehabilitation professionals.

Abstract citation ID: igae098.0010

### INTRODUCING A SERVICE DELIVERY MODEL TO OPTIMIZE HOME MODIFICATION OUTCOMES

Emily Nabors[1], Kathleen Cameron[1], Mengzhao Yan[2], Jon Pynoos[2], and Scott Trudeau[3], *1. National Council on Aging, Arlington, Virginia, United States, 2. University of Southern California, Los Angeles, California, United States, 3. American Occupational Therapy Association, Stow, Massachusetts, United States*

Home modification services have become more widely available in response to the aging population's growing need for accessible home environments. Despite this growth, the home modification service delivery process is not well-defined, leading to services that do not adequately meet the needs of older adults. To address this gap, the Service Delivery Subcommittee of the National Home Safety and Home Modification Work Group, led by the National Council on Aging and funded by the Administration for Community Living, developed a Home Modification Service Delivery Model. Using an iterative, consensus building approach, this group of interdisciplinary practitioners and researchers developed a model to describe the ideal home modification service delivery process based on evidence and expert perspectives. The model outlines the value of working in a transdisciplinary team, describes key providers and professionals to involve on the team, and their respective roles in the home modification service delivery process (i.e., intake, assessment, completion of modifications, and follow-up). An understanding of the complexity of home modification and awareness of the types of providers that can and should be involved across all phases of home modification can increase the likelihood that consumers will receive the right services at the right time to adequately meet their current and future needs, regardless of their background and point of entry to the process. The Home Modification Service Delivery Model sets a critical foundation for future research and implementation efforts in the community. This session will review the model, describe the development process, and highlight next steps.

### SESSION 1010 (SYMPOSIUM)

Abstract citation ID: igae098.0011

### AGEISM EVERYWHERE ALL AT ONCE: DIFFERENT MANIFESTATIONS AND RESEARCH APPROACHES

Chair: Alison Chasteen

In the over 50 years since ageism was defined, the impact of age prejudice has never been clearer, with the increased expression of age bias both during and after the COVID-19 pandemic. This symposium aims to highlight the pervasive nature of ageism through a range of methodological approaches, including in-depth interviews, social media scraping, and experimental surveys, to underscore the ubiquity of ageism in contemporary society. We delve into various facets of ageism, exploring its manifestations in ageist group labels, media representations, beauty standards, and even its correlation with suicidal ideation. We also investigate ways to challenge ageist comments through confrontation. Vale assesses the impact of using ageist terms such as 'elderly' and 'senior citizen' on perceptions of older people. Fraser and colleagues explore online reactions to "The Golden Bachelor," the first older adult-focused version of the reality tv show. Soulie et al. investigate older women's responses to ageist beauty norms, including when they accept or resist them. Gendron and colleagues examine the role of internalized ageism in suicidal ideation among older people. Gans et al. test whether reactions to ageism confrontation vary by the intersecting identities (gender and race) of the older adult confronter as well as by the confrontation technique that is used. Altogether, these talks shed light on the multifaceted nature of ageism in North American society and offer valuable insights into strategies for combatting age bias. International Association of Gerontology & Geriatrics North American Regional Committee Sponsored Symposium

Abstract citation ID: igae098.0012

### REFRAMING AGING: THE IMPACT OF LABELS ON ATTITUDES TOWARD OLDER ADULTS

Michael Vale, and Ava Bjelka, *Sacred Heart University, Fairfield, Connecticut, United States*

Efforts to combat ageism advocate for avoiding labels, such as "old", "elderly", and "senior citizen"; however, these labels persist in everyday speech, and new terms like "Ok Boomer" have recently gained popularity. To examine the perception and impact of such labels on older adults, we had young, middle-aged, and older participants complete an online experiment in which they read a vignette. More specifically, we manipulated the label (i.e., older, old, elderly, senior citizen, Boomer, in their 70s) and gender (i.e., woman, man)

of the main character. Preliminary findings from a sample of 512 participants (ages 18-91) were analyzed using 6x2x3 ANOVAs. A three-way interaction was significant for ratings of offensiveness (p<.001, ηP2=.07), with middle-aged participants finding the labels most offensive. Older participants, particularly when the target was described as a woman, were most offended by labels like "elderly" or "old." Similarly, the appropriateness of labels varied across gender and label types (p=.07, ηP2=.02), with "old" rated more appropriate for women and "Boomer" for men. Differences in labels also affected perceptions of competence (p=.06, ηP2=.02). Meanwhile warmth perceptions varied across age groups (p<.001, ηP2=.11), with young adults viewing targets most warmly. These results suggest that labels matter in shaping perceptions of older adults, especially among the demographic they represent. Our findings can guide initiatives aimed at reframing aging perceptions and highlight how language can shape attitudes towards older adults. The results also underscore how ageism intersects with sexism. Further analysis will be conducted on the full sample (N<900).

Abstract citation ID: igae098.0013

### THE INFLUENCE OF MEDIA PORTRAYALS ON AGEISM: A CASE STUDY OF THE GOLDEN BACHELOR

Sarah Fraser[1], Alixe Menard[1], Alison Chasteen[2], Mateja Van Gameren[3], and Chris Belanger[1], *1. University of Ottawa, Ottawa, Ontario, Canada, 2. University of Toronto, Toronto, Ontario, Canada, 3. Lisgar Collegiate Institute, Ottawa, Ontario, Canada*

Background: Age-based discrimination or ageism is often propagated through media platforms (TV, news, social media). Focusing on the reality television show "The Golden Bachelor," which features older adult contestants seeking romance, this study explored ageism spread via social media posts related to the program. Methods: Using social astronomy software we scraped Reddit's English-language corpus for posts containing "golden bachelor" and extracted a sample of 1000 posts shared between August and September 2023. Qualitative content analysis of these posts revealed four forms of ageism (1) personal, (2) explicit, (3) implicit, and (4) benevolent. Results: Personal ageism emerged through comments devaluing older contestants' appearance: "Hey at 75 you can go on The Golden Bachelor; wouldn't that be fun. Wrinkles upon wrinkles…" Explicit ageism was evident in derogatory remarks targeting contestants' age and limited physical capabilities: "Can the Golden Bachelor still get it up?". Implicit ageism was discernible in subtle reinforcement of age-related stereotypes: "The guy who's going to be the Golden Bachelor is 71 and he looks much younger than I remember 71-year-olds looking. He seems like a pretty normal guy." and benevolent ageism manifested through the infantilization of older contestants: "cute old people love". Discussion and Implications: While "The Golden Bachelor" may help some understand the capabilities of older adults and counter the misperception of them as asexual, existing ageist stereotypes emerge and are propagated on social media platforms. These findings underscore the pervasive nature of ageism in social media and highlight the importance of addressing age-related biases in media representation.

Abstract citation ID: igae098.0014

### "JUST DON'T LOOK OLD; THAT'S WHAT'LL KILL YOU": EXAMINING OLDER WOMEN'S RESPONSE TO AGEIST BEAUTY NORMS

Brianna Soulie, Anne Barrett, and Hope Mimbs, *Florida State University, Tallahassee, Florida, United States*

Aging beyond youth presents challenges for women, who often struggle to remain visible and valued in an ageist society. Much of the literature has focused on women's engagement with age-related beauty work, with less attention given to the encounters with ageist beauty norms that motivate it. Studies also have given limited attention to the range of women's responses to these norms, which can include elements of acceptance or acquiescence, as well as resistance. Our study examines these issues using in-depth interviews with 20 women, ranging in age from 57 to 83. Analyses revealed that ageist beauty norms are woven into women's everyday interactions, often with co-workers, friends, or acquaintances. Our analyses unpack these norms to reveal the dominant age ideologies they reflect. They center on the assumption that faces and bodies that have aged beyond youth are unattractive and that the morally and practically appropriate response is to engage in beauty work to mask signs of aging. Women differed, however, in how they responded to these pressures, with some engaging in age-related beauty work and others opting out of it. Our analyses revealed that this choice often hinged on women's perceived security in their social roles – in particular, their employment and marital or partnership statuses. While many studies have examined how women engage in beauty work to remain competitive in the youth-oriented dating and job markets, our analyses revealed how security in these realms can lead women to disengage from beauty work aimed at masking signs of aging.

Abstract citation ID: igae098.0015

### INTERNALIZED AGEISM AS A RISK FACTOR FOR SUICIDAL IDEATION IN LATER LIFE

Tracey Gendron, Alyssa Camp, and Amateau Gigi, *Virginia Commonwealth University, Richmond, Virginia, United States*

The Interpersonal Theory of Suicide proposes that suicidal ideation is based on the immutability of two constructs – perceived burdensomeness and thwarted belongingness. This study examined the influence of ageism on suicidal ideation using the framework of the Interpersonal Theory of Suicide, investigating whether internalized and relational ageism contribute to suicidal ideation beyond the variance already explained by demographic factors, thwarted belonging, and perceived burdensomeness. Using Research Match volunteers 65 and older in the United States were recruited to participate in an online survey. A total of 454 individuals from over 30 states participated in the study. Twelve percent of respondents reported that they had experienced suicidal ideation in the past month. Hierarchical Logistic Regression analysis demonstrated that age (OR = 1.07, 95% CI [1.00, 1.15]), and internalized ageism (OR = 1.19, 95% CI [1.07, 1.32]) had significant associations with suicidal ideation. Among all independent variables, internalized ageism had the strongest association with suicidal ideation. Ageism can result in people feeling their needs being ignored or dismissed due to their age or feeling isolated from a world that does not recognize or value their contributions. Findings from this study further amplify the critical importance of understanding the real-world impact of ageism.

Abstract citation ID: igae098.0016

### CALLING OUT AGEISM: HOW INTERSECTING IDENTITIES AND CONFRONTATION TONE AFFECT PERCEPTIONS OF OLDER CONFRONTERS

Hannah Gans[1], Alison Chasteen[1], and Michelle Horhota[2], *1. University of Toronto, Toronto, Ontario, Canada, 2. Furman University, Greenville, South Carolina, United States*

The few studies that have examined confrontations of ageism have shown that older adults are viewed differently after challenging the behavior. What remains unclear is how older people's intersecting identities, as well as their confrontation tone, might influence reactions to their countering ageism. To address this question, we surveyed 500 young and older adults regarding their perceptions of warmth and competence towards older adult confronters who varied by race (Black, White) and gender (man, woman). Participants evaluated the older individuals both before and after they either moderately or strongly confronted an ageist backhanded compliment ("You don't look that old"). The older confronter's race and their response to the ageist comment moderated how they were perceived by participants. Specifically, both young and older adult participants rated older Black confronters as warmer when they used a moderate compared to a strong confrontation tone but did not make that distinction for White confronters. However, when the tone of the confrontation was not considered, young, and older participants generally differed in their perceptions of confronters. Older adult participants rated White confronters lower on warmth and competence after confronting compared to Black confronters. This pattern was not observed among young adult participants, underscoring the significance of intersectional identities in shaping perceptions among older adult perceivers. This study shows that older adult confronters' intersecting identities influence how they are perceived when they challenge ageist comments. This suggests that customized confrontation techniques may be needed for older adults whose gender and racial identities vary.

### SESSION 1015 (PAPER)

### AGING IN PLACE (PAPERS)

Abstract citation ID: igae098.0017

### UNMET COMMUNITY CARE NEEDS AND THE WELL-BEING OF OLDER ADULTS IN CHINA

Shibin Yan, *Rutgers, The State University of New Jersey, Camden, New Jersey, United States*

As China has experienced rapid population aging, the demand for elder care services has increased dramatically. The gap in service demand has become concerning because the traditional provision of eldercare heavily relies on informal care provided by the family. Social and demographic transformations have made the family-reliant care model unsustainable. Community care has become a reasonable and practical option to balance the care demand. However, there is a huge gap between community care provision and need. This prevailing unmet needs in community care services necessitated a deeper understanding of its impact on the well-being of older adults. Using data from the Chinese Longitudinal Healthy Longevity Study, we employ fixed-effect regressions to examine the effects of unmet community care needs on older adults' health status and subjective well-being (SWB), including specific insights on disabled and childless subpopulations. We find a negative association between unmet needs and both health and SWB, with no significant different effect among subpopulations. Interestingly, Its effect on life satisfaction is moderated by place of residence, with a stronger negative impact in urban areas. These findings highlight the urgent need for more equitable and accessible community care services, particularly for urban older adults, to improve their overall well-being.

Abstract citation ID: igae098.0018

### RACIAL/ETHNIC DISPARITIES IN THE NEIGHBORHOOD AMENITY ENVIRONMENTS OF US OLDER ADULTS

Calley Fisk[1], Hyungmin Cha[1], Eunyoung Choi[1], Yeon Jin Choi[2], and Jennifer Ailshire[1], *1. University of Southern California, Los Angeles, California, United States, 2. University of Kentucky, Lexington, Kentucky, United States*

Living in neighborhoods with diverse amenity environments, including a variety of facilities and services, may impact the health and well-being of older residents by providing increased access to essential services, fostering social networks, and building social capital within communities. Such environments, however, may not be uniformly experienced across racial/ethnic groups, potentially creating and reinforcing racial/ethnic disparities in aging-related outcomes. Our study provides rich descriptive information on the distribution of neighborhood amenities of U.S. adults ages 50 and older across and by different racial and ethnic groups. We link census tract-level data on amenities from the National Neighborhood Data Archive to population-level data from the Health and Retirement Study for the 2004-2016 data waves (N=26,194 respondents, 107,254 observations). Neighborhood amenity diversity was measured as a total amenity diversity score (range: 0-119), calculated by top-coding (at two) and summing counts of amenities across four categories: food (e.g., restaurants, grocery stores), retail (e.g., clothing, convenience stores), general services (e.g., banks, gyms), and civic services (e.g., senior facilities, community centers). On average, Black (mean=40.4, SE=0.80) and Hispanic (mean=43.2, SE=0.97) older adults lived in neighborhoods with significantly lower total amenity diversity scores compared to their White counterparts (mean=46.8, SE=0.49). In linear regression models, adjusting for clustering at the individual and tract levels and including demographic and socioeconomic variables, these differences remained significant for Black (b=-7.1, $p < 0.001$), but not Hispanic (b=-2.0, p=0.062) older adults. Finally, supplementary analyses stratifying by urbanicity and examining racial/ethnic differences in category-specific amenities revealed similar patterns as the total amenity diversity score.

Abstract citation ID: igae098.0019

### UNDERSTANDING THE MEANING OF HOME THROUGH A CO-DESIGNED VIRTUAL PHOTOVOICE STUDY

Joyce Weil, and Gitanjali Iyer, *Towson University, Towson, Maryland, United States*

Co-designed photovoice is a way for older adults to express what a place means to them from their own point of view using images and narrative. The goal of this study is to understand the concept of "home" using photovoice through the lens of older adults living in Baltimore, Maryland. As part of this co-designed, IRB-approved study, 14 older adults were interviewed twice via Zoom, a video calling platform, about the meaning of home and their community or neighborhood. Participants shared and explained the meaning of 10 photographs regarding what home meant to them. In the second Zoom interview, participants explained the meaning behind each photo and why they selected them. After analyzing the transcripts and photos of each participant, some common themes were found highlighting the meaning of home for the participants. Themes were about symbolism and meaning of objects around their home, neighborhood accessibility and walkability, daily routines and practices, and how "home" had changed during the pandemic. The ways in which virtual co-designed photovoice were based on older adults' reframing and designing the study will be discussed.

Abstract citation ID: igae098.0020

### A SPACE OF OUR OWN: RECLAIMING PUBLIC OPEN SPACES FOR SELF-EXPRESSION AND COMMUNITY

Ad Maulod, Malcolm Ravindran, and Yunjie Wong, *Duke-NUS Medical School, Singapore, Singapore*

Public open spaces give older persons opportunities to build social capital and community, and are important sites in promoting self-expression, meaning- and place-making–attributes crucial to successful ageing-in-place. In Singapore, residents have little influence over the design and production of public open spaces, while regulations on the use of these spaces may dissuade utilization by older persons. Despite 'age-friendly' principles undergirding Singapore's built environment, contestations arise in terms of top-down planned activity-usage, and older persons' actualization of what aspirations they have in using such spaces. Recently, the government has increased the provision of 'active ageing centres' nationwide, intended to be inclusive, low barrier-to-entry spaces within residential neighbourhoods, to encourage social engagement and community support among older persons. However, older persons' perceptions of designated senior-spaces as catering to the sick and vulnerable, leads to hesitance in utilizing those spaces. Drawing from ethnographic interviews across multiple qualitative research studies with over 200 older persons in Singapore, we found that participants expressed a lack of control over their community spaces, programme offerings, and are not empowered to organize activities with fellow residents. Yet, their narratives also highlight creative strategies to resist regulatory practices and inhabit public open spaces with activities that align with their desires to age well. Public open spaces are crucial to older persons' freedom and autonomy. Accessibility, in terms of physical and social infrastructure, needs to be reframed in ways that value older persons' diverse interests, capacity to self-organize, and potential for building community.

Abstract citation ID: igae098.0021

### SUPPORTING AGING IN PLACE THROUGH IWISH: IMPACTS FROM THE SUPPORTIVE SERVICES DEMONSTRATION

Ian Breunig[1], Melissa Vandawalker[2], Kimberly Groover[2], and Derek Hoodin[2], *1. Abt Global, Nashville, Tennessee, United States, 2. Abt Global, Cambridge, Massachusetts, United States*

The U.S. Congress sponsored the Supportive Services Demonstration (SSD) to learn whether structured health and wellness support can help older adults remain in HUD-assisted housing developments longer. The demonstration tested the Integrated Wellness in Supportive Housing (IWISH) model, which funds both an onsite Resident Wellness Director and part-time Wellness Nurse at multifamily properties to address health, housing, and social service needs of adults ages 62 and older. Through a cluster-randomized controlled trial including properties in seven states, SSD tests whether IWISH affects: (1) the length of stay in housing by reducing transitions to long-term care, (2) unplanned hospitalizations and other acute healthcare utilization, and (3) primary care utilization. We linked individual resident data with Medicare and Medicaid data and compared utilization and tenure between IWISH and control residents. During the first three years of the demonstration (October 2017–September 2020), we found no evidence that IWISH affected residents' healthcare utilization or caused residents to remain in their homes longer; but there are reasons to be optimistic about the longer-term impact of the IWISH model. Subgroup analyses show IWISH reduced rates that residents aged 62-64 and residents 85 or older used acute care services. We also found two aspects of the IWISH model seemed to improve outcomes: working individually with residents on meeting their health and wellness goals and providing transitional care to residents returning home from a hospital or long-term care stay. Outcomes were worse at properties that are isolated or lacking access to nutritional food.

Abstract citation ID: igae098.0022

### UNDERSTANDING INCLUSIVE PHYSICAL PLACES AND VIRTUAL SPACES: THE FORTITUDE OF OLDER MARGINALIZED COMMUNITIES

Mei Lan Fang[1], Judith Sixsmith[2], Susan Buell[2], Darren Chadwick[3], Kathryn Almack[4], Richard Vytniorgu[5], and Joe Tai[6], *1. Simon Fraser University, Vancouver, British Columbia, Canada, 2. University of Dundee, Dundee, Scotland, United Kingdom, 3. Liverpool John Moores University, Dundee, Scotland, United Kingdom, 4. University of Hertsfordshire, Hertfordshire, Scotland, United Kingdom, 5. University of Hertsfordshire, Hertfordshire, England, United Kingdom, 6. University of Dundee, University of Dundee, Scotland, United Kingdom*

The World Health Organization (WHO) Age-Friendly Cities and Communities (AFCC) agenda emphasizes collaboration among older individuals, local groups, councils, and businesses to enhance communities. This involves improvements in several social and environmental domains including transportation, outdoor spaces, volunteering, employment opportunities, and leisure and community services. IncludeAge, a UK Economic and Social Research Council funded project, addresses multifaceted aspects of age-friendly communities beyond the emphasis on social and physical environments, highlighting the importance of belonging and identity alongside cultural and life course experiences. Focusing on physical and online places and spaces, IncludeAge takes intersectional and life course perspectives to understanding the inclusion of mid-older (40+) people who are often marginalized: those with intellectual disabilities and LGBT+ individuals. Employing a transdisciplinary, innovative mixed-methods approach and guided by Community-Based Participatory Research principles, our longitudinal study uses life course interviews, GIS story mapping and social network analysis to document experiences of inclusion and exclusion in past lives and current real-time. Preliminary insights underscore the importance of the cultural and historical dynamics of experienced stigma and discrimination, the value of 'hidden spaces and places' and 'experiences on the periphery'. We use the Intersectional Place Perspective (IPP) theoretical model to aid interpretation. Through co-developed story maps and individual narratives, we aim to amplify the voices of marginalized groups in designing potential solutions to exclusionary experiences and practices, fostering empowerment and social inclusion. Our research will advance understanding of AFCC and community inclusion as a human right, thereby supporting meaningful everyday lives for our focal populations.

### SESSION 1020 (SYMPOSIUM)

Abstract citation ID: igae098.0023

### ALL IN HOW YOU LOOK AT IT: ATTITUDES, AFFECT, AND ABILITY IN THE ASSESSMENT OF SUBJECTIVE COGNITION

Chair: Emily Bower
Co-Chair: Melissa Zammitti
Discussant: Claudia Jacova Chenoweth

Subjective cognitive decline (SCD)—self-perceived persistent cognitive decline in the absence of objective cognitive impairment—is a risk factor for dementia. Despite the potential for SCD to aid in the detection of future dementia, differentiating trajectories of normative from non-normative decline among people with SCD is complicated for several reasons, including that self-perceived cognition is associated with socioemotional and general health factors in addition to objective abilities. Moreover, operational definitions of self-perceived cognition vary across the research literature and overlap with subjective aging. A robust understanding of the relationships among self-perceived cognition and aging, objective cognition, mood, attitudes, and health will strengthen the characterization and differentiation of SCD. This symposium will explore measures of perceived cognition and describe their relationships with mood, health, and aging attitudes among healthy older adults. The first presenter will describe the differential impacts of negative and positive affect on perceived memory difficulties, highlighting the complex relationships among depression, affective states, and subjective memory decline. The second presenter will expand on these findings by contrasting a measure of subjective memory decline with related measures of subjective memory and subjective age, and explore differential impacts of perceived health and objective memory. The third presenter will focus on perceived cognitive ability more broadly to describe its relationship with attitudes toward aging, depression, and objective cognition. The Discussant will compare and contrast findings with regard to operational definitions of self-perceived cognition and consider implications of the findings as they relate to emerging issues of clinical classification and management of SCD.

Abstract citation ID: igae098.0024

### UNVEILING THE DIFFERENTIAL IMPACT OF NEGATIVE AND POSITIVE AFFECT ON SUBJECTIVE COGNITIVE PERCEPTIONS

Jacklyn Gehling, Hannah Marx, Ravneet Dhaliwal,
Steven Harris, Frank Robertson, and Claudia Jacova Chenoweth, *Pacific University, Hillsboro, Oregon, United States*

Subjective Cognitive Decline (SCD) is a state of perceived decline in cognitive abilities that may mark Alzheimer's disease onset and may be influenced by affective and personality factors. This study aimed to examine the distinct impact of negative and positive affect on SCD burden in older adults. The sample included 279 cognitively healthy, community-dwelling participants, aged 60-96 (M=73.8), 52% female, education 10-20 years (M=14.9). Participants completed an online survey including demographics, depression (PHQ-2, M=.51), perceived memory difficulties (ECog-Memory, M=12.4), and affect (Positive and Negative Affect Schedule, PANAS, M=13.7 and M=7.1 respectively). Separate regression analyses of ECog-Memory showed that PANAS-Negative (B=.35, $\Delta R^2$=.05, p<.001) but not PANAS-Positive (B=-.08, $\Delta R^2$=.008, p=.12), added explained variance beyond demographics and depression. Further exploration of PANAS-Positive, incorporating its interaction with education due to trending significance (p=.08), showed that PANAS-Negative (B=.35, p<.001), but not PANAS-Positive (B=.52) or the interaction (B=-.04), contributed explained variance ($\Delta R^2$=.07). Our findings highlight the significant influence of negative affect on the subjective cognitive perceptions of older adults. Elevated negative affect is correlated with diminished subjective cognitive perceptions, and notably, this association remains independent of endorsed depression. Interestingly, positive affect, a known protective factor in AD-related cognitive decline, does not seem to have an impact on subjective cognition.

Abstract citation ID: igae098.0025

### BRIDGING PERSPECTIVES: A COMPARATIVE EXAMINATION OF SUBJECTIVE MEMORY AND AGING MEASURES

Steven Harris, Jacklyn Gehling, Hannah Marx,
Ravneet Dhaliwal, Frank Robertson, and
Claudia Jacova Chenoweth, *Pacific University, Hillsboro, Oregon, United States*

Understanding subjective cognition is imperative as it offers valuable insights into individuals' perceptions, beliefs, and interpretations regarding their cognitive abilities, exerting a profound influence on their overall well-being and behavior. This study sought to explore perceptions of subjective cognition and aging among a national sample of community-dwelling older adults (n=279, M=73.8, education M=14.9, 52% female). In particular, we evaluated relationships between three measures of subjective cognition and aging: subjective memory complaints, subjective memory age, and subjective age. Everyday Cognition-Memory (ECog-mem) items were summed to measure memory complaints; memory age identity (MAI) was obtained by subtracting subjective memory age from chronological age; Subjective age identity (SAI) was calculated by subtracting subjective age from chronological age. We also assessed subjective health and objective memory using AVLT-Delayed recall. We conducted three hierarchical regression analyses to predict ECog-mem, MAI, and SAI. In each model, we entered age and education in the first step, subjective health and objective memory (AVLT) in the second, and depression (PHQ-2) in the third. Our sample reported a low burden of memory complaints (M=12.35) and a younger subjective than chronological age: MAI (M=-10.82) and SAI (M=-9.86). These measures were only moderately correlated (range r.29-.64). Better health ratings predicted lower ECog-mem (B=1.387, p<.001), younger MAI (B=4.16, p<.001), and younger SAI (B=4.72, p<.001), and these contributions remained after PHQ2 entry. Objective memory did not contribute to the prediction. Subjective cognition and aging measures appear to capture somewhat different experiences, but these are all influenced by perceptions of health.

Abstract citation ID: igae098.0026

### ATTITUDES TOWARD AGING AND SUBJECTIVE COGNITIVE COMPLAINTS: DOES DEPRESSION MODERATE THEIR RELATIONSHIP?

Fatema Colombowala, and Emily Bower, *Pacific University, Hillsboro, Oregon, United States*

Subjective cognitive decline (SCD) is a risk factor for Alzheimer's disease and related dementias and affects a significant portion of the population, with prevalence ranging from 12.3% to 57% of older adults. Although some people with SCD will later develop dementia, others will not, suggesting that factors other than objective cognitive ability impact subjective cognition. Attitudes towards aging (ATA) may influence how individuals interpret age-related cognitive changes, potentially leading to heightened subjective cognitive complaints (SCC) among those who hold more negative attitudes toward aging. We investigated the relationship between ATA and SCC in a sample of older adults with SCD. We hypothesized negative attitudes would be associated with more SCC. Additionally, we examined if depression moderated the relationship between ATA and SCC, predicting that more severe depression would strengthen the relationship. There is a scarcity of studies that directly compare the strength of association between SCC and various outcomes such as depression and ATA. Preliminary analyses included 51 participants, predominantly female (72.5%), with an average age of 72 (SD = 8.5) who had SCD. They completed validated measures of SCC, aging attitudes (ATA), and depression as part of a larger study. Hypotheses were tested using linear regression models. We found more negative attitudes on the ATA psychosocial loss subscale were associated with higher SCC (B = 26.50, p =.007). Additionally, a diagnosis of depression moderated the relationship by strengthening it (B = 35.80, p =.0096). Findings suggest intervening to reduce depression and negative ATA could reduce SCC burden.

### SESSION 1025 (SYMPOSIUM)

Abstract citation ID: igae098.0027

### ALZHEIMER'S DISEASE GENOMIC, BIOLOGIC, AND BEHAVIORAL PATHWAYS AMONG EXCEPTIONAL LONGEVITY FAMILIES

Chair: Mary Wojczynski
Co-Chair: Stephanie Cosentino
Discussant: Nalini Raghavachari

Alzheimer's disease (AD) is the most common cause of dementia, and its prevalence rates increase sharply in older adults aged 65 and above. Examining whether long-lived families are protected against dementia, and the predictors of dementia within these families, can inform genetic, environmental, and behavioral factors associated with dementia-free survival and the delay of pathological aging. The Long Life Family Study (LLFS), funded by the National Institute on Aging, is an international collaborative study of the genetics and familial components of exceptional longevity and healthy aging. We phenotyped 4,953 individuals from 539 two-generational families (1,727 proband; 3,226 offspring) at baseline (2006-2009), with up to two follow-up in-person visits. These longitudinal, comprehensive in-person visits measured domains of healthy aging, including physical performance, cognition, and blood markers. Extensive genetic analyses were performed using the baseline blood draw, including GWAS chip, linkage analyses, WGS, metabolomics, and transcriptomics. Collectively, this symposium will present novel findings that examined genomic, biological, and behavioral pathways to AD. Specifically, Dr. Xicota will discuss results of the impact of sampling on the limitations of population polygenic risk scores for dementia. Then, Dr. Arbeev will share findings on the longitudinal changes of lysophosphatidylcholine and risk of incident AD. Next, Dr. Cheng will explain results of a multi-omic analysis of the effect of lipids on AD. Lastly, Dr. Gu will discuss analyses of sleep duration in relation to cognition in LLFS. As Discussant, Dr. Nalini Raghavachari from the NIA will share insights and propose future directions for LLFS.

Abstract citation ID: igae098.0028

### LIMITATIONS OF POLYGENIC RISK SCORES FOR DEMENTIA IN FAMILIAL EXCEPTIONAL AGING: IMPACT OF SAMPLING

Laura Xicota[1], Rong Cheng[2], Sandra Barral Rodriguez[2], Lawrence Honig[1], and Joseph Lee[2], *1. Columbia University Irving Medical Center, New York, New York, United States, 2. Columbia University, New York, New York, United States*

Genetic risk factors for late-onset Alzheimer's disease (LOAD) have been extensively studied in the general population of European background. Indeed, significant genetic variants with low to moderate contributions to LOAD have been used to calculate Polygenic Risk Scores (PRS) to identify individuals with a higher genetic risk of LOAD. The Long Life Family Study (LLFS) is characterized by having exceptionally long-lived individuals at lower risk of LOAD. With this study, we will uncover whether the described genetic risk factors for LOAD impact AD risk in LLFS, limited to US sites. In LLFS, PRS based on two large GWASs in the general population did not predict clinical AD status. The LLFS population did not have lower allele frequencies of risk alleles, but rather lacked putative effects in these risk alleles, with only 6.7% of SNPs from GWAS 1 and 7.7% from GWAS 2 were associated with AD. We then examine the relations between PRS and endophenotype, namely AD biomarkers (Aβ40, Aβ42, Aβ42/Aβ40, NfL, and GFAP). Levels of AD biomarkers were not positively associated with PRS, which can be explained by the low frequencies of SNPs associated with biomarker concentrations (3.3%-6.7% for GWAS 1; 2.6%-6.5% for GWAS 2). Here, we show that genetic risk factors for AD in the general population do not necessarily explain AD risk in a cohort of long-lived individuals; therefore, genetic risk factors based on large-scale GWASs ascertained from the general population need to be examined with caution when applied to a cohort ascertained for exceptional longevity.

Abstract citation ID: igae098.0029

### AGE TRAJECTORIES OF LYSOPHOSPHATIDYLCHOLINES IN RELATION TO ALZHEIMER'S DISEASE: FINDINGS FROM LLFS

Konstantin Arbeev[1], Olivia Bagley[1], Svetlana Ukraintseva[1], Stephanie Cosentino[2], Alexander Kulminski[1], Eric Stallard[1], and Anatoliy Yashin[1], *1. Duke University, Durham, North Carolina, United States, 2. Columbia University Irving Medical Center, New York, New York, United States*

Lysophosphatidylcholines (LPC) are phospholipids involved in apoptosis and demyelination, among other functions. They have been linked to cancer, atherosclerosis, and neurodegeneration (with potential trade-offs) and explored as early markers of Alzheimer's disease (AD) and accelerated aging. However, relationships between longitudinal changes in LPC levels and AD risk remain poorly understood. We applied statistical approaches for joint analyses of longitudinal and time-to-event outcomes, joint models (JM) and stochastic process models (SPM), to study longitudinal dynamics of LPC in relation to AD incidence in the Long Life Family Study (LLFS) participants. In total, 4,017 measurements of 23 LPC species in 2,858 LLFS participants from the U.S., who had 93 incident AD cases and up to two LPC measurements per individual, were analyzed. In JM applications, we found sex-specific relationship (for males only) between the dynamics of LPC species and AD incidence in the model adjusted for baseline age, education, smoking, medication use, and two genetic principal components. The strongest association was found for LPC(14:0/0:0) in males (regression coefficient: 0.739, p=2.3e-23). In SPM applications, we demonstrated that various hidden biomarkers of aging, embedded within the model's structure and indirectly evaluated from individual LPC trajectories and data on AD incidence, significantly contribute to the associations between LPC and AD. We also observed sex differences in 'optimal' and 'equilibrium' LPC trajectories, as well as in vulnerability to LPC deviations from these trajectories, and in how these relations change with age. Our findings call for further investigation into the biological mechanisms underlying the observed associations.

Abstract citation ID: igae098.0030

### GENES, LIPIDS, AND ALZHEIMER DISEASE RISK IN FAMILIES WITH EXCEPTIONAL LONGEVITY

Rong Cheng[1], Laura Xicota[2], Sandra Barral Rodriguez[1], Lawrence Honig[2], Gary Patti[3], and Joseph Lee[1], *1. Columbia University, New York, New York, United States, 2. Columbia University Irving Medical Center, New York, New York, United States, 3. Washington University in St. Louis, St. Louis, Missouri, United States*

Clinical studies have consistently shown that various classes of lipids are involved in the early stages of Alzheimer's Disease (AD). To better understand the biological processes involved, we examined three layers of omic data from the Long Life Family Study (LLFS), which aimed to investigate the biological basis of familial exceptional longevity. Using 3,059 US participants, we performed a multi-stage analysis examining whole genome sequencing, transcriptomic, and lipidomic data. In addition, we examined two independent datasets for confirmation: the Alzheimer's Disease Neuroimaging Initiative (ADNI) cohort, representing the general population; and the Multiomic Studies of Alzheimer's Disease in Adults with Down Syndrome (omicsADDS), representing the high-risk Down Syndrome cohort. First, to identify biologically relevant lipids, we identified 18 lipids of 188 were shown to be highly heritable and were associated with AD risk. Our genome search of 18 lipids yielded two loci: 11q12.2 included MYRF, TMEM258, FEN1, FADS1, FADS2, and FADS3 as candidate genes; while 20p12.1 included SPTLC3 and LINC01723. After excluding genes not differentially expressed between affected and unaffected individuals, genes on 11q12.2 were associated with five lipids, including classes of triacylglycerol, phosphatidylcholine, and lysophophatidylcholine. In contrast, genes on 20p12.1 were associated with the class of ceramide non-hydroxy fatty acid-sphingosine. Lastly, we confirmed the 11q12.2 associations observed in LLFS in the ADNI and omicsADDS datasets. In sum, we showed that 11 AD-associated lipids were associated with genes on 11q12.2 in LLFS and were confirmed in the general population and the high-risk cohort.

Abstract citation ID: igae098.0031

### SLEEP DURATION AND COGNITION IN THE LONG LIFE FAMILY STUDY

Emma Gordon[1], Yian Gu[2], Stephanie Cosentino[3], Stacy Anderson[4], and Svetlana Ukraintseva[5], *1. Albert Einstein College of Medicine, Bronx, New York, United States, 2. Columbia University, New York, New York, United States, 3. Columbia University Irving Medical Center, New York, New York, United States, 4. Boston University, Boston, Massachusetts, United States, 5. Duke University, Durham, North Carolina, United States*

Numerous studies demonstrate associations between sleep and cognition. This study includes a subset of 2344 participants from the Long Life Family Study, a cohort with slower overall rates of cognitive decline, and seeks to examine how sleep and cognition are related in this population. Our neuropsychological battery measured episodic, semantic, and working memory at baseline (2006-2009), and in a subset of 1633 participants at the follow-up visits 6.9 (SD=2.7) years later. Sleep habits were assessed once, using the Health Habits (HH) Questionnaire, between the two cognitive measures. Associations between sleep duration and memory were examined using generalized estimating equations models, accounting for relationship with long-lived families. Compared to those with 6.5-8.5 hours of sleep, those with > 8.5 hours of sleep had lower episodic memory scores at baseline (b=-0.705, p=0.021), but not at follow-up (b=-0.627, p=0.149), after controlling for age, sex, field center, APOE, and generation. Sleep duration was not associated with semantic or working memory. Those who had > 8.5 hours of sleep had faster decline in all memory scores, although not significantly. There was no difference in memory scores comparing < 6.5 hours of sleep to 6.5-8.5 hours of sleep. Lower memory scores at baseline predicted longer sleep time subsequently, while sleep time did not predict memory performance on follow-up. Overall, the current study did not support sleep duration as a significant contributor to memory decline, contradicting some earlier findings in the literature, and prompting further investigation into the connection between sleep and cognition, especially within this population.

### SESSION 1030 (SYMPOSIUM)

Abstract citation ID: igae098.0032

### BEST PROGRAMS FOR CAREGIVING: NEW PUBLIC VERSION FOR FAMILY AND FRIEND CAREGIVERS

Chair: Rachel Cannon

A new public version of Best Programs for Caregiving (BPC) launched in 2024 for family and friend caregivers to find and compare evidence-based, dementia caregiving programs available to them in their communities. Over 200 organizations were verified to be delivering one or more of the 47 programs in BPC. Data collected from surveys of developers of BPC programs and the organizations delivering BPC programs were used to understand the reach and characteristics of programs being delivered to caregivers, including information on how caregivers can enroll, types of help and support provided, cost and eligibility requirements, how they are delivered, and adaptations made for diverse communities. Survey results suggest there are more BPC programs available, and these programs have wider reach, than expected, with 25 programs offered to caregivers living anywhere in the US via remote delivery. Additionally, nearly one-fourth of programs (23.2%) were adapted for diverse communities. Along with demonstrating the new version of BPC for caregivers, discussion focuses on how caregivers can use BPC to find programs available to them, challenges encountered with verifying delivery organizations, the importance of developing culturally adapted programs, and how BPC can be used by healthcare systems, medical providers, and community organizations to strengthen implementations of comprehensive dementia care required by the Center for Medicare and Medicaid Innovation's new payment model "Guiding an Improved Dementia Experience" (GUIDE Model).

Abstract citation ID: igae098.0033

### HOW CAREGIVERS USE BEST PROGRAMS FOR CAREGIVING TO FIND INTERVENTIONS IN THEIR COMMUNITIES

Rachel Cannon[1], David Bass[1], Morgan Minyo[2], Sara Powers[1], Zoe Fete[1], and Megan Huth[1], *1. Benjamin Rose, Cleveland, Ohio, United States, 2. Benjamin Rose Institute on Aging, Cleveland, Ohio, United States*

A new public version of Best Programs for Caregiving (BPC) launched in 2024 to help family and friend caregivers find evidence-based, dementia caregiving support programs that are available to them in their communities. The new version of BPC profiles healthcare and community organizations that are verified delivery sites of the 47 programs that are included in the professional version that launched in 2020. More than 200 organizations were verified to be delivering these programs and completed a survey requesting information about their offering of the program. The new version of BPC displays organization's information on how caregivers can enroll in the program, types of help the program provides, program costs and eligibility requirements, how the program is delivered, and whether the program has been adapted for diverse groups. Caregivers can access Best Programs for Caregiving at bpc.caregiver.org and use their zip code to search for programs available to them both in-person and online. This presentation provides an overview of the interventions included in Best Programs for Caregiving and the data collection process, including a demonstration of how to use the site to find and compare evidence-based dementia caregiving programs.

Abstract citation ID: igae098.0034

### PROGRAM ADAPTATIONS FOR DIVERSE COMMUNITIES IN BEST PROGRAMS FOR CAREGIVING

Megan Huth[1], Sara Powers[1], Zoe Fete[1], Rachel Cannon[1], Morgan Minyo[2], and David Bass[1], *1. Benjamin Rose, Cleveland, Ohio, United States, 2. Benjamin Rose Institute on Aging, Cleveland, Ohio, United States*

To better understand the role of cultural adaptations among evidence-based dementia caregiving programs included on Best Programs for Caregiving (BPC), online surveys were distributed and collected from organizations delivering BPC programs in the community in 2023. Surveys collected data on: 1) Implementation of a culturally adapted program, 2) Diverse groups the program was adapted for, (e.g., racial and ethnic groups, LGBTQ+ groups), and 3) Types of cultural adaptations made (e.g., marketing materials revised, program delivered in language, consultation with diverse group members). Results indicated that 23.2% of organizations delivering BPC programs made adaptations for diverse groups, most commonly: 1) Hispanic/Latino (61.5%), 2) LGBTQ+ (53.8%), 3) Black/African American (46.2%). Among the programs that made adaptations, 100% consulted with group members or experts on the diverse group, 92.8% adapted content for members of the group, 53.8% provided staff training and began delivering the program in the group's language, and 46.2% created translations of the materials and adapted marketing and outreach tools for the diverse group. The least common adaptation was conducting research to show that the program benefited the diverse groups (25%). These findings show that while a larger than expected number of BPC have made modifications for diverse groups, the majority of programs (76.8%) were not adapted for any diverse community. The discussion will center on the importance of developing culturally adapted programs that better cater to the needs of diverse family and friend caregivers.

Abstract citation ID: igae098.0035

### USING BEST PROGRAMS FOR CAREGIVING TO MEET THE DIVERSE NEEDS OF GUIDE MODEL SITES AND BENEFICIARIES

Morgan Minyo[1], Katie Maslow[2], David Bass[3], Rachel Cannon[3], Zoe Fete[3], Megan Huth[3], and Sara Powers[3], *1. Benjamin Rose Institute on Aging, Cleveland, Ohio, United States, 2. Retired, Bethesda, Maryland, United States, 3. Benjamin Rose, Cleveland, Ohio, United States*

In 2023, the Center for Medicare and Medicaid Innovation announced a new alternative payment model for comprehensive dementia care called the Guiding an Improved Dementia Experience (GUIDE) Model. The GUIDE Model is intended to improve quality of life for people living with dementia (GUIDE Beneficiaries) and reduce strain on unpaid caregivers through a package of care coordination and management, caregiver education and services, and respite care. Health systems and other medical or community-based organizations that applied and were selected as GUIDE Model Sites must offer specific program components and care deliver requirements to identify services and supports needed by both Guide Beneficiaries and their caregivers. This requires GUIDE Model Sites to potentially add new supports to their dementia care or be able to link Beneficiaries and their caregivers to services that meet the diverse and changing needs of both individuals. The Best Programs for Caregiving (BPC) online database is a valuable resource that can benefit GUIDE Participating Sites and others, ensuring that family/friend caregivers are appropriately recognized and included within Beneficiary's dementia care. The dementia support programs in BPC meet GUIDE Model care delivery requirements including one-on-one and group caregiver education, care coaching, and ongoing monitoring and support. Additionally, BPC programs have proven positive outcomes for dementia family/friend caregivers, are actively being delivered by organizations across the US, and describe if/how a program has been adapted or delivered to caregivers from diverse communities. BPC's extensive database can help provide the necessary information for high-quality dementia care under the Guide Model.

Abstract citation ID: igae098.0036

### NUMBER AND REACH OF INTERVENTIONS IN BEST PROGRAMS FOR CAREGIVING

David Bass[1], Zoe Fete[1], Megan Huth[1], Rachel Cannon[1], Morgan Minyo[2], and Sara Powers[1], *1. Benjamin Rose, Cleveland, Ohio, United States, 2. Benjamin Rose Institute on Aging, Cleveland, Ohio, United States*

A major advance in Gerontology is the development of numerous evidence-based support programs for dementia family and friend caregivers. However, a common untested assumption is that these proven programs are not widely available to caregivers. Data collected for the new version of Best Programs for Caregiving (BPC) for family and friend caregivers afforded an opportunity to carefully document the scaling and reach of the 47 evidence-based programs in BPC. The first phase of this research surveyed program-developers to identify all healthcare and community organizations delivering their programs in 2022/2023. 600 service organizations were identified as offering a BPC program (nearly twice the number identified in 2019). The second phase of research surveyed the 600 delivery organizations to verify that programs were being delivered and to collect related data, such as geographic areas served. 252 (42%) of the 600 organizations were verified as delivering a BPC program. The geographic areas served ranged from a single city or county to the entire U.S. Notably, 25 organizations served caregivers nationally via remote program delivery. Reasons the 348 (58%) delivery organizations that were identified by developers, but not verified as delivering programs, included: stopped the program because of funding or staffing challenges, never delivered the program, or could not be reached. Results suggest more programs are available and have a wider reach to caregivers than expected, but the distribution of programs could be improved, if developers had more capacity or assistance with marketing, tracking, and monitoring program implementations.

Abstract citation ID: igae098.0037

### NEW APPROACH METHODOLOGIES (NAMS) TO STUDY AGING

Tiziana Cogliati, and Jennifer Fox, *NIA-NIH, Bethesda, Maryland, United States*

New Approach Methodologies (NAMs) include human-relevant cell-based (in vitro), cell-free (in chemico), and computational-based (in silico) models that are complementary, not a replacement, to existing animal models. This workshop focuses on 3-dimentional (3-D) in vitro human models including tissue chips, organoids, explants, known as microphysiological systems (MPS). The adoption of human in vitro NAMs for basic and translational research is expanding, recently promoted by the FDA Modernization Act 2.0 that authorizes their use to investigate drug safety and efficacy. Extending the use of human in vitro NAMs to the study of aging and the development of geroprotective interventions presents challenges recognized by the research community. These challenges can be addressed by leveraging our knowledge of the biology of aging and fostering interdisciplinary collaborations. This interdisciplinary session is designed to educate basic scientists, physician-scientists, clinicians, and a general audience of industry representatives interested in adopting NAMs for aging research. One of the goals is to promote a dialog about the challenges and opportunities posed by human in vitro NAMS and inspire new collaborative research initiatives bringing together basic scientists, bioengineers, physician-scientists and clinicians, and members of other GSA sections. At the conclusion of the session, attendees will be able to: (1) describe the different types of human in vitro NAMs and their applications in basic and translational research; (2) explain the challenges and current solutions to adopting human 3-D in vitro NAMs in research on aging; and (3) discuss how data from 3-D in vitro NAMs and from animal models are complementary.

### SESSION 1040 (SYMPOSIUM)

Abstract citation ID: igae098.0038

### DESIGN, EVALUATION, AND IMPLEMENTATION OF THE PRISM SOFTWARE SYSTEM: A SOFTWARE SYSTEM DESIGNED FOR OLDER ADULTS

Chair: Sara Czaja
Co-Chair: Hans-Werner Wahl
Discussant: Neil Charness

The PRISM system is an integrated software system designed, using a user-centered design approach, to foster social connectivity, cognitive engagement, and resource access among aging adults. Versions of PRISM have been evaluated in diverse contexts and geographic regions, and with diverse populations. This session will discuss how PRISM has been adapted, and present data on the impact of the use of PRISM across diverse contexts, on outcomes related to technology acceptance and proficiency, social isolation and loneliness, and quality of life. Walter R. Boot will discuss the evolution of PRISM and highlight how the findings from the PRISM 1.0 trial led to the development of PRISM 2.0 and PRISM CI for aging adults with a cognitive impairment. He will also discuss how PRISM was adapted for implementation in Germany. Sara J. Czaja will present the primary outcomes from the PRISM 2.0 trial, which evaluated a refined version of PRISM with older adults in diverse settings (rural locations, senior housing, assisted living facilities). She will focus on outcomes related to loneliness, social isolation, and quality of life. Wendy A. Rogers will focus on the acceptability and usability of PRISM. She will present data from the PRISM 1.0 and PRISM 2.0 trials and highlight findings regarding acceptability according to context of implementation. Nicole Memmer will address long-term usage of PRISM Germany. She will discuss differential patterns and preferences of use in relation to gender, technology generation, and technology skills and attitudes. N. Charness will serve as a discussant for the session.

Abstract citation ID: igae098.0039

### THE ROLE OF TECHNOLOGY IN SUPPORTING ENGAGEMENT IN OLDER ADULTS: FINDINGS FROM THE PRISM 2.0 TRIAL

Sara Czaja[1], Walter Boot[2], Neil Charness[3], and Wendy Rogers[4], *1. Weill Cornell Medicine/Center on Aging and Behavioral Research, New York, New York, United States, 2. Weill Cornell Medicine, New York, New York, United States, 3. Florida State University, Tallahassee, Florida, United States, 4. University of Illinois Urbana-Champaign, Champaign, Illinois, United States*

Technology applications can play a key role in enhancing social and cognitive engagement, decreasing loneliness, and facilitating access to resources among older adults. This presentation will focus on data from the cross-site randomized field trial that evaluated the PRISM 2.0 system, an integrated software system (developed by the Center for Research and Education on Aging and Technology Enhancement), designed using a user-centered design approach, that fostered social and cognitive engagement and access to information and resources. PRISM 2.0 was compared to a Standard Tablet Control condition, where participants received a computer tablet without the PRISM 2.0 software and the same amount of training and contact as those in the PRISM 2.0 condition. The trial was conducted in diverse living contexts including rural locations, senior housing, and assisted living communities (ALC). The sample included 245 adults aged 64-99 yrs. that were randomized within living context to either the PRISM 2.0 or the Standard Tablet Control condition. Data were collected at baseline, and 6-and 9-months post randomization. Findings will be presented on the impact of access to the PRISM 2.0 software system on social isolation, loneliness, social support, quality of life, and technology proficiency. Information will also be presented regarding the unique challenges of implementing PRISM 2.0 in the various contexts. Overall, the findings provide strong evidence that access to technology is beneficial to aging adults and underscore the importance of adapting technology to the needs and preferences of the user and providing adequate instructional support.

Abstract citation ID: igae098.0040

### THE EVOLUTION OF THE PERSONAL REMINDER INFORMATION AND SOCIAL MANAGEMENT (PRISM) SYSTEM

Walter Boot[1], Neil Charness[2], Sara Czaja[3], and Wendy Rogers[4], *1. Weill Cornell Medicine, New York, New York, United States, 2. Florida State University, Tallahassee, Florida, United States, 3. Weill Cornell Medicine/Center on Aging and Behavioral Research, New York, New York, United States, 4. University of Illinois Urbana-Champaign, Champaign, Illinois, United States*

The development of the Personal Reminder Information and Social Management (PRISM) System began fifteen years ago with a user-centered design process that resulted in PRISM 1.0, a desktop application that ran on a personal computer and included a keyboard, mouse, and printer. However, as the technological landscape changed, so did PRISM. Following the successful implementation of PRISM 1.0, PRISM 2.0 adapted the platform to be mobile and run on a tablet. Features such as videoconferencing and multiplayer games were added based on feedback from PRISM 1.0 trial participants and the availability of new applications. To broaden the reach of PRISM 2.0, it was also translated into Spanish. Building off the successful implementation of PRISM 2.0, versions of PRISM have been adapted to serve older adults experiencing cognitive impairment. Adapted versions of PRISM, with new features, are also currently being deployed in Germany to examine their impact in two different cities. This talk will focus on how PRISM has evolved over time and has been adapted to serve different, diverse populations of older people. Throughout this talk, we will emphasize how a user-centered design process, involving older adults in diverse contexts, has served the design and deployment of PRISM, and how what has been learned from each iteration of PRISM has informed the next to maximize the usefulness and usability of the system. How advances in virtual and augmented reality, artificial intelligence, and wearables might shape future iterations of PRISM will be discussed.

Abstract citation ID: igae098.0041

### UNDERSTANDING PRISM ACCEPTANCE THROUGH QUANTITATIVE AND QUALITATIVE MEASURES

Wendy Rogers[1], Tracy Mitzner[2], Sara Czaja[3], Walter Boot[4], and Neil Charness[5], *1. University of Illinois Urbana-Champaign, Champaign, Illinois, United States, 2. Person in Design, Atlanta, Georgia, United States, 3. Weill Cornell Medicine/Center on Aging and Behavioral Research, New York, New York, United States, 4. Weill Cornell Medicine, New York, New York, United States, 5. Florida State University, Tallahassee, Florida, United States*

Long-term adoption of technology is influenced both by the design of the technology itself as well by the users' attitudes towards the technology. Technology acceptance models have long indicated that individual differences in perceptions of ease of use and perceptions of usefulness are critical predictors of acceptance and use. Additional predictive factors include perceived enjoyment, facilitating conditions (e.g., instructional materials, social support), demographics (e.g., age, sex) and psychographics (e.g., personality, technology readiness). For PRISM 1.0 and PRISM 2.0 we implemented mixed methods to assess technology acceptance factors. We adapted standard survey measures of technology acceptance that were administered at baseline and after extended system use. We developed an interview that was administered at the end of the study to explore usage patterns, preferences, facilitators and barriers. Data from the technology acceptance assessments from PRISM 1.0 informed redesign decisions for the PRISM 2 system. We will present an overview of the quantitative and qualitative data to illustrate the value of the mixed methods approach and to provide insights about acceptance and adoption of novel technology system to older adults.

Abstract citation ID: igae098.0042

### EARLY ADOPTION AND LONG-TERM USE: ANALYZING OLDER ADULTS' USAGE PATTERNS AND PREDICTORS OF PRISM GERMANY

Nicole Memmer[1], Anna Schlomann[2], Hans-Werner Wahl[1], and Leon Radeck[3], *1. Heidelberg University, Heidelberg, Baden-Wurttemberg, Germany, 2. Network Aging Research, Heidelberg, Baden-Wurttemberg, Germany, 3. Institute of Computer Science, Heidelberg, Baden-Wurttemberg, Germany*

This study introduces PRISM-Germany, the German version of the PRISM-App originally developed by North American research consortium CREATE (Czaja et al. 2018), aiming to support social participation among older adults. Addressing the challenge of long-term adherence, we explored changes in app usage over 3 months and identified predictors of use, focusing on socio-demographic and technology-related variables. The analytical sample consisted of 171 community-dwelling adults aged 67–93 (mean age=73.7 years; female=55%) with medium digital competencies as part of the ongoing SMART-AGE complex intervention trial. Usage behavior in PRISM-Germany was assessed through a cloud-based data log procedure, providing real-time recording of individual usage behaviors. A baseline data protocol collected data on relevant predictors via the Redcap online software. Usage analyses indicated that on average 11 out of 32 available categories (e.g. email, video-chat, health information) were accessed in PRISM-Germany per week. A significant decline in average usage over time was observed, ranging from 1st month: m = 15.3 to 2nd month: m = 9.7, and finally to 3rd month: m = 7.5. ANCOVA revealed the decline as robust, controlling for socio-demographic and technology-related variables. At the multivariate level, chronological age, health, general technology acceptance, and previous technology competencies did not predict frequency of use, while female gender, not living alone, and being lower educated did (Adjusted R2 = 0.108). Concluding, 50% reduction in app use across three months as well as the sole predictive role of sociodemographic factors seem remarkable.

### SESSION 1045 (SYMPOSIUM)

Abstract citation ID: igae098.0043

### ADRD IN CONTEXT: SOCIAL, NEIGHBORHOOD, AND NATIONAL INFLUENCES

Chair: Toni Antonucci
Co-Chair: Laura Zahodne
Discussant: Erin Harrell

This symposium showcases innovative work from scholars in the National Institute on Aging (NIA)-funded Alzheimer's Disease Resource Center for Minority Aging Research, the Michigan Center for Contextual Factors in Alzheimer's Disease. Each paper examines ecological contexts (e.g., interpersonal relationships, neighborhoods, countries) that influence the experiences of dementia in diverse populations. Dr. Sol and colleagues examine how social resources differentially relate to cognitive health among Black and White older adults living in different types of neighborhoods. Dr. Roche-Dean et al. consider social factors that influence dementia diagnosis awareness across race/ethnicity in a national sample. Although a number of factors did not influence dementia diagnosis awareness in the aggregate sample, analyses stratified by race indicate that married Black participants had greater dementia diagnosis awareness. Dr. Alhasan and colleagues examine community livability and dementia in different South Carolinian counties. Dementia prevalence was higher in less livable counties among women but lower among men. Finally, Dr. Lopez-Anuarbe and colleagues explore differences in health care costs and dementia status among Mexicans and Mexican Americans. They find opposing patterns of association between dementia status and health care costs, as well as contrasting urban-rural differences, among Mexicans in Mexico versus Mexican Americans in the U.S. In sum, each of these papers addresses the critical role of social, neighborhood and national contexts across the dementia continuum and additionally considers variation across sociodemographic groups. Finally, Dr. Erin Harrell from the NIA will offer perspectives on how examining contextual factors can advance NIA's goal of understanding and eliminating ADRD inequalities.

Abstract citation ID: igae098.0044

### SOCIAL RESOURCES ARE ASSOCIATED WITH COGNITIVE FUNCTION ACROSS BLACK AND WHITE OLDER ADULTS AND NEIGHBORHOOD TYPES

Ketlyne Sol, Toni Antonucci, Philippa Clarke, and Laura Zahodne, *University of Michigan, Ann Arbor, Michigan, United States*

Few studies examine neighborhood resources in healthy cognitive aging despite evidence that living in neighborhoods with lower socioeconomic status (NSES) is associated with increased risk for Alzheimer's Disease and Related Diseases (ADRD) with persistent race disparities in ADRD and NSES. Fewer studies examine which social resources buffer against the detrimental effects of living in lower-resourced neighborhoods. In this cross-sectional study, multiple linear regressions examined associations between structural and functional social resources and cognition stratified by intersectional groups of participant self-reported race and neighborhood type. Participants were drawn from the Michigan Cognitive Aging Project, a longitudinal epidemiological study of cognitive aging in southeastern Michigan (Mage=64). Global cognition was a composite of five comprehensively-assessed cognitive domains. Participant addresses were linked to year 2017 measures of neighborhood resources from the National Neighborhood Data Archive. Latent class analysis of 2,813 Michigan census tracts identified that most participants lived in tracts characterized as low-resourced (Bn=162=Wn=128) or moderately-resourced (Wn=80>Bn=64). Only among White residents of low-resourced neighborhoods, a larger social network (β=.257, p=.001) was associated with better cognition. Among Black residents of low-resourced neighborhoods, more support in their closest relationship (β=.150, p=.044) was associated with better cognition. Among Black residents of moderately-resourced neighborhoods, more strain from friends (β=-.255, p=.087) was associated with worse cognition. Support from relatives of Black, but not White, residents in both neighborhood types was associated with better cognition. Nuanced, culturally-sensitive interventions may support racially diverse older adults to foster and broaden desired relationships to buffer against adverse cognitive outcomes of neighborhood stressors.

Abstract citation ID: igae098.0045

### FACTORS INFLUENCING DEMENTIA DIAGNOSIS AWARENESS AMONG RACIALLY/ETHNICALLY DIVERSE OLDER ADULTS

Maria Roche-Dean[1], Sol Baik[2], Noah Webster[3], Heehyul Moon[4], and Laura Zahodne[3], *1. University of Kansas, Kansas City, Kansas, United States, 2. University of Virginia, Charlottesville, Virginia, United States, 3. University of Michigan, Ann Arbor, Michigan, United States, 4. University of Louisville, Louisville, Kentucky, United States*

Black and Hispanic adults arrive in later life with a greater risk for Alzheimer's Disease and Related Dementias (ADRD). Untimely dementia diagnoses continue to be a challenge in non-Hispanic Black and Hispanic-identified older individuals, delaying symptom management and access to health services. In this study, we examine whether social factors are associated with awareness of dementia diagnosis among older adults with probable dementia. Data were from the National Health and Aging Trends Study round 1, a nationally representative sample of Medicare beneficiaries aged 65 and older assessed in 2011. Those selected for analysis met algorithmic criteria for probable dementia (N=561). Logistic regression was performed to assess the influence of marital status, social network size, and number of helpers on the odds of reporting a dementia diagnosis, controlling for demographics. In the whole sample, younger individuals and women had greater odds of dementia diagnosis awareness. Race/ethnicity, marital status, the number of helpers, and social network size were not associated with dementia diagnosis awareness. Analyses stratified by race/ethnicity indicated that being married was only associated with greater odds of dementia diagnosis awareness among Black participants. The lack of association between social network, number of helpers, and dementia awareness could reflect limitations on how probable dementia and dementia awareness were operationalized in this dataset. Furthermore, marital support may be important for timely diagnosis for Black individuals with dementia.

Abstract citation ID: igae098.0046

### LOWER COMMUNITY LIVABILITY IS ASSOCIATED WITH HIGHER DEMENTIA PREVALENCE AMONG WOMEN IN SOUTH CAROLINA COUNTIES

Dana Alhasan[1], Marilyn Wende[2], Toni Antonucci[3], Florence Dallo[4], and Maggi Miller[5], *1. University of North Carolina, Charlotte, Charlotte, North Carolina, United States, 2. University of Florida, Gainesville, Florida, United States, 3. University of Michigan, Ann Arbor, Michigan, United States, 4. Oakland University, Rochester, Michigan, United States, 5. University of South Carolina, Columbia, South Carolina, United States*

Community livability may be associated with lower dementia prevalence through access to healthcare professionals, for example. Given the high rates of dementia among older adults with a disproportionate impact on women, there is a need to understand how community livability may buffer against dementia and mitigate gender disparities. Yet, few studies have considered community livability as an exposure. Therefore, our exploratory study determined county-level associations between community livability and dementia prevalence by gender in South Carolina (SC). We retrieved county-level dementia prevalent cases in 2016 among ≥50 years old (N=46) from the SC Alzheimer's Disease Registry. Community livability data (categorized into tertiles) were obtained from the American Association of Retired Persons' Livability Index in 2018, which included seven domains: housing, neighborhood, transportation, physical environment, healthcare access, social engagement, and economic as well as educational opportunity. We conducted OLS regression analysis adjusting for county-level rurality to estimate beta coefficients and p-values. The study included 48.6% women (n=622) and 51.4% men (n=656). In adjusted analyses, low vs. high livability was associated with higher dementia prevalence (B=0.38; p-value=0.31). Although not statistically significant, the findings are in the expected direction. Low vs. high livability was associated with higher dementia among women (B=0.11; p-value=0.69), but lower dementia among men (B= -0.78; p-value=0.07). Our novel findings regarding communities with higher livability being associated with lower dementia highlight the potential health benefits of enhancing the livability of communities and is a first step towards future research to better understand this understudied relationship.

Abstract citation ID: igae098.0047

### HEALTH CARE COST DIFFERENCES BY GEOGRAPHIC CONTEXT AND DEMENTIA STATUS FOR MEXICANS AND MEXICAN AMERICANS

Mónika López Anuarbe[1], and Angela Gutierrez[2], *1. Connecticut College, New London, Connecticut, United States, 2. Western University of Health Sciences, Pomona, California, United States*

Research documents geographic differences in healthcare costs among Latinés living in the U.S. Yet healthcare costs by geographic context and dementia status for Latiné subpopulations residing in different countries, such as Mexicans in the U.S. and in México, have been overlooked. Utilizing pooled cross-sectional and weighted data of Mexicans ages 50+ from the 2012-2015 Mexican Health and Aging Study (n=15,723) and the 2012-2016 Health and Retirement Study (n=5,136), we investigated: 1) Associations between geographic context and healthcare costs; 2) Whether dementia status moderates the geographic context-healthcare costs relationship. We hypothesized that persons with dementia incurred higher healthcare costs in both countries, and that residents in less populated areas had higher healthcare costs. Preliminary findings from our two-part model (logit; GLM), which accounted for personal and healthcare characteristics, propose that persons with dementia in México had 60.3% higher average healthcare costs than individuals without dementia. Respondents with dementia also had higher odds (b=0.12; p=0.04) of incurring any healthcare costs in less densely populated areas (under 15,000 persons). However, their U.S. counterparts with dementia had lower, but insignificant, odds of incurring healthcare costs, with the dementia-rural interaction suggesting that individuals with dementia in areas with at 250,000-1 million persons were less likely to incur healthcare costs compared to areas with at least 1 million inhabitants (p<0.001), and that their healthcare costs were lower (p=0.05).These results underscore how geographical context varies across countries and how dementia status leaves some individuals more financially vulnerable than others, affecting healthcare access and overall health.

### SESSION 1050 (PAPER)

### EDUCATION: GERONTOLOGY/GERIATRIC EDUCATION (PAPERS)

### GLOBAL AGING AND EDUCATION; WORKFORCE

Abstract citation ID: igae098.0048

### IMPACT OF A KINESIOLOGY COURSE ON EXPECTATIONS REGARDING AGING AND AGEIST BEHAVIORS

Kate Hamel, *San Francisco State University, San Francisco, California, United States*

Students majoring in Kinesiology often aspire to work in healthcare or fitness professions where they will frequently work with older adults. Many students don't recognize this opportunity however, and are often initially drawn to Kinesiology through their own experience with exercise or as athletes and generally assume that they will work with a young, active population in their future careers. Younger adults have also been shown to have negative perceptions and attitudes toward aging and this may shape the way they interact with older adults and could impact their career choice. The purpose of this study was to examine the expectations regarding aging (ERA) and the prevalence of ageist behaviors in students taking a Kinesiology elective course on aging, prior to and after the completion of the course. Students taking the course in two different semesters completed the ERA-12, the Relating to Older People Evaluation and open-ended questions to assess their perceptions, attitudes and behaviors toward aging and older adults. No differences were found between the two cohorts of students in any of the baseline or end of semester measures. Following the completion of the course, students had significantly higher ERA-12 scores and expectations regarding physical health, cognitive functioning and mental health in older adults all improved. There was no difference in positive ageist behaviors, but there was a reduction in negative ageist behaviors. These findings suggest that course-based interventions such as this one, may significantly improve attitudes toward aging and increase student's interest in working with older adults.

Abstract citation ID: igae098.0049

### COLLABORATIVE INTERNATIONAL EXPERIENTIAL LEARNING IN GERONTOLOGY: AGING GLOBALLY INITIATIVE

Aleksandra Zecevic[1], and Anne-Marie Boström[2], *1. University of Western Ontario, London, Ontario, Canada, 2. Karolinska Institutet, Stockholm, Stockholms Lan, Sweden*

Population aging is greatly impacting future generations of healthcare providers tasked with improving the healthcare systems they are inheriting. In this presentation, we introduce an innovative example of how students, researchers, educators and community partners from Canada and Scandinavia came together to comparatively explore models of health, social care, and welfare to advance global health. Collaborative international experiential learning in gerontology is an approach that broadens the application of academic theory outside classroom in an international context. It includes authentic reflection, develops transferable skills and strengthens employability. Our network supports future generations of healthcare professionals in becoming global-ready graduates. We highlight a collaborative course called Aging Globally: Lessons from Scandinavia, initiated at Western University, Canada in 2018, and delivered in partnership with OsloMet University (Norway), Karolinska Institutet (Sweden) and seven non-academic partners, such as Socialstyrelsen and Silviahemmet (Sweden) and Cycling without Age (Denmark). Over the past seven years, the course involved 425 students and 24 professors from Health Studies, Occupational Therapy, Physiotherapy, Nursing, and Technology, Science and Design programs. Student outcomes include expended knowledge, cultural competencies, and transferable skills. The course was a catalyst for three curriculum development grants totaling CAD $2 million, enrichment of the curriculum with 57 international internships and 13 exchanges, 12 summer courses, and new research partnerships, lifting international education in gerontology to a new level. Presenters will share experiences, evidence of impact on students, and describe joint efforts to inspire social change and sustainability of improved quality of life and well-being for older adults everywhere.

Abstract citation ID: igae098.0050

### EXPLORING OPPORTUNITIES TO FOSTER GERONTOLOGY EDUCATION AND GLOBAL ENGAGEMENT

Ramraj Gautam[1], Linda Hollis[2], and Jennifer Mendez[3], *1. University of Massachusetts Lowell, Lowell, Massachusetts, United States, 2. University of Arizona, Tucson, Arizona, United States, 3. Wayne State University School of Medicine, Detroit, Michigan, United States*

The Academy for Gerontology in Higher Education (AGHE) helps advance gerontology, geriatrics, and age-friendly instruction through the development of curriculum, educational, and training materials. Much of this work is done in the context of the United States. GSA/AGHE being an international organization there remains a further need to bring global gerontology educators and experts together. Using the UN Decade of Healthy Aging framework, the purpose of this study was a) to learn more about international gerontology education programs in order to collaborate and share materials, and b) to explore opportunities in increasing international aging programs. The study protocol was IRB-approved. International gerontology educators were recruited through announcements in aging newsletters, GSA Connect, interest groups, AGHExchange, and through GSA 2023 international presenters' listserv. Twenty-six participants representing academic institutions from ten different countries responded to the Qualtrics survey. Several strategies were suggested for sharing gerontology education resources: curricula and syllabi sharing through cloud servers, inviting international guest lectures, involving students in international and intergenerational projects, and collaborations with international educators. Among the four World Health Organization decades of healthy aging 2022-23 focus areas, participants ranked Age-Friendly Environments as number one, followed by combating ageism, long-term care, and integrated care. 61.5% of respondents were not a member of AGHE – GSA and 85.7% of participants showed interest in joining. Plans to incorporate the suggested strategies and future collaboration international gerontology educator opportunities will be discussed.

Abstract citation ID: igae098.0051

### IPE CASE STUDY ON COMMUNICATION AND ASSESSMENT OF OLDER ADULTS: A COMPARISON OF THREE ONLINE MODALITIES

Tara Sharpp[1], and Laura Gaeta[2], *1. California State University Sacramento, Sacramento, California, United States, 2. California State University Sacramento, California State University, Sacramento, California, United States*

Interprofessional education (IPE) is critical for health profession students to learn teamwork, shared experiences, and communication. In this mixed-method study, we compared an IPE activity for Nursing, Audiology, Communication Sciences, Gerontology, and Health Science students using three online modalities: (1) Synchronous online, (2) Asynchronous online, and (3) Bichronous online (combination of asynchronous and synchronous). In IPE groups, the students examined the same unfolding case study about an older adult with hearing loss, depression, and dementia. Over three terms, 168 students completed pretest and posttest measures, including a standardized IPE scale (IPAS subscale), a knowledge quiz, and an evaluation. Knowledge quiz scores significantly improved in all three groups (synchronous: $t = -2.586$, 2-sided $p = 0.013$; asynchronous: $t = -4.129$, 2-sided $p < 0.001$; bichronous: $t = -3.748$, 2-sided $p < 0.001$). Students also demonstrated significantly increased attitudes toward working in teams in the IPAS subscale in the posttest in all three groups. ANOVA and Kruskal Wallis tests demonstrated no statistically significant differences in the IPAS scale and evaluation scores between groups, indicating that students in all three modalities had similar levels of learning regarding their IPE. In the open-ended responses of the evaluation, students reported they learned practical examples of how to work with team members from their group and that they valued the online experience. The results indicate that health professions students can effectively learn from a case study activity how to communicate with older adults and work in interprofessional teams in various forms of online modalities.

### SESSION 1055 (SYMPOSIUM)

Abstract citation ID: igae098.0052

### EFFECTS OF HEARING INTERVENTION ON BRAIN HEALTH: FINDINGS OF THE ACHIEVE TRIAL AND AND ACHIEVE-MRI SUBSTUDY

Chair: Alison Huang
Co-Chair: Frank Lin
Discussant: Caterina Rosano

This session will present the primary findings of the ACHIEVE-MRI (Aging and Cognitive Health Evaluation in Elders Magnetic Resonance Imaging) study, a substudy of the ACHIEVE randomized controlled trial (Clinicaltrials.gov Identifier: NCT03243422). Prior observational studies have linked hearing loss with changes in brain structure and function. Hearing loss may directly modify brain morphology and exacerbate mechanistic pathways such as greater cognitive load (diversion of cognitive resources to effortful listening to the detriment of other cognitive functions) and reduced social connection. Whether treatment of hearing loss can effectively reduce age-related brain atrophy is unknown and has yet to be tested in randomized trials. The ACHIEVE trial (2018-2022) tested the effect of a best-practices hearing intervention versus health education control on three-year change in cognition (primary outcome) among 977 adults aged 70-84 years with untreated hearing loss and without substantial cognitive impairment. MRI imaging data was gathered on a subset of participants (n=445) in the ACHIEVE-MRI substudy. This session will first describe the overall study design of the ACHIEVE study and ACHIEVE-MRI substudy and then present the primary findings of the ACHIEVE- MRI substudy – the effect of the hearing intervention vs. health education control on 3-year change in brain structure and function. This session will also examine the effect of hearing intervention on speech understanding in the presence of noise. The session will close with an investigation of for whom hearing intervention may be most effective for, examining moderation by baseline levels of dementia risk in the ACHIEVE study.

Abstract citation ID: igae098.0053

### STUDY DESIGN OF THE AGING AND COGNITIVE HEALTH EVALUATION IN ELDERS (ACHIEVE) AND ACHIEVE-MRI STUDY

Alison Huang, *Johns Hopkins Bloomberg School of Public Health, Baltimore, Maryland, United States*

This presentation describes the study design of the Aging and Cognitive Health Evaluation in Elders (ACHIEVE) study and the ACHIEVE-MRI sub-study. The ACHIEVE study (n=977) is a multicenter, randomized controlled trial testing the effect of a best-practice hearing intervention vs. health education control on the primary outcome of cognitive decline over 3 years. Participants in the ACHIEVE study were 977 community-dwelling adults aged 70-84 years with untreated hearing loss and without substantial cognitive impairment. The ACHIEVE study was partially nested within the Atherosclerosis Risk in Communities [ARIC] study; participants were recruited from the ARIC study (n=238) and de novo (n=739) from the surrounding communities of four U.S. sites. Participants were randomized 1:1 to hearing intervention or health education control and followed for 3 years. ACHIEVE study participants (n=977) were median age 76.4 years, 53.5% female, 87.8% White, and 53.3% held a Bachelor's degree or higher. The ACHIEVE-MRI study is a sub-study testing the effects of hearing intervention on 3-year change in brain MRI characteristics and cognitive function with speech-in-noise performance. The ACHIEVE-MRI study included a subset of ACHIEVE study participants (n=445) who received three-dimensional magnetic resonance imaging at baseline and at 3-year follow-up. Participants were slightly younger and had higher income and greater memory function compared to ACHIEVE study participants. The ACHIEVE-MRI study also had a larger proportion of participants recruited de novo (83%). This presentation provides important background for contextualization of ACHIEVE study and ACHIEVE-MRI study findings on brain structure and function and speech-in-noise performance presented in this symposium.

Abstract citation ID: igae098.0054

### THE ACHIEVE TRIAL: EFFECT OF HEARING LOSS INTERVENTION ON WHITE MATTER INTEGRITY

Jennifer Deal, *Johns Hopkins University, Baltimore, Maryland, United States*

Hearing treatment delays cognitive decline in at-risk older adults. Pathways linking hearing loss to cognitive decline are unknown but may include central effects on brain structure/function. The ACHIEVE randomized controlled trial tested hearing intervention (amplification and rehabilitation) versus health education control on cognitive decline in 977 adults aged 70-84 years. Here we describe the effect of treatment on the pre-specified outcomes of white matter hyperintensity (WMH) volume and microstructural integrity in 445 participants who underwent 3T brain magnetic resonance imaging scans at baseline and Year 3. Fractional anisotropy (FA) and mean diffusivity (MD) were derived from diffusion tensor imaging. Participants were 76.4±4.0 years, 50% female and 12% self-reported Black race. Control (vs. hearing intervention) participants were older and had lower baseline memory scores, but otherwise treatment groups were balanced on measured characteristics. Among 303 participants who completed both MRI scans (n=141 treatment, n=162 control), using linear mixed effects models in an intention-to-treat analysis adjusted for baseline hearing loss severity, recruitment source, study site, age, sex, education, interaction terms between each covariate and time, and time-varying total intracranial volume, hearing treatment slowed rates of WMH volumetric change in whole brain (difference in 3-year change comparing intervention to control: -0.077 cm3, 95% confidence interval [CI]: -0.152, -0.002), with the strongest lobar effect in the occipital lobe (-0.151, 95% CI: -0.284, -0.017). No consistent associations were observed for FA and MD. Follow-up of ACHIEVE participants is ongoing to determine if intervention effects on white matter integrity require longer follow-up time.

Abstract citation ID: igae098.0055

### EFFECT OF A HEARING INTERVENTION ON THREE-YEAR CHANGE IN BRAIN MORPHOLOGY: ACHIEVE RANDOMIZED TRIAL

James Pike, *New York University, New York, New York, United States*

Prior studies among older adults have documented associations between hearing loss and changes in brain morphology. Whether interventions involving hearing aids can reduce age-related atrophy is unknown. A substudy within the Aging and Cognitive Health Evaluation in Elders (ACHIEVE, Clinicaltrials.gov Identifier: NCT03243422) randomized controlled trial tested the effect of a best-practices hearing intervention versus health education control on three-year change in cortical thickness among older adults with hearing loss. The study enrolled 977 community-dwelling adults aged 70-84 years at baseline (2018-2019) with untreated hearing loss (better ear pure tone average [0.5-4 kHz] ≥30 and <70 dB HL) and without substantial cognitive impairment. Participants were randomized to a hearing intervention (provision of hearing aids and related technologies, counseling, and education) or a health education control (individual sessions with an educator covering health-related topics). Three-dimensional magnetic resonance imaging was performed on 3 Tesla Siemens scanners in a subsample of 445 participants (50.3% women, 11.7% Black) at the baseline and three-year follow-up. Mixed effects models were used in intention-to-treat analyses to estimate three-year change in cortical thickness. Compared to the health education control, the hearing intervention exhibited a nominally protective effect on three-year change in average cortical thickness. The greatest effect size was observed in the occipital lobe, while the smallest effect size was detected in the temporal lobe. Statistically significant effects were detected in the pars orbitalis, rostral anterior cingulate, posterior cingulate, and isthmus cingulate. The results suggest that a hearing intervention may reduce cortical thinning among older adults.

Abstract citation ID: igae098.0056

### HEARING AID USE, COGNITIVE MEASURES, AND SPEECH-IN-NOISE PERFORMANCE: FINDINGS FROM THE ACHIEVE STUDY

Nicholas Reed[1], Kening Jiang[1], Alison Huang[2], and Emmanuel Garcia Morales[3], *1. Johns Hopkins University, Baltimore, Maryland, United States, 2. Johns Hopkins*

*Bloomberg School of Public Health, Baltimore, Maryland, United States, 3. Cochlear Center for Hearing & Public Health, Baltimore, Maryland, United States*

There is a paucity of information on how measures of cognition, brain structure, audition, and sociodemographic (e.g., education) affect changes in speech-in-noise performance, a priority for communication, with hearing aids over time. The ACHIEVE study is a randomized control trial of older adults with hearing loss enrolled to a hearing intervention or health education control over 3-years. Self-report demographic and health measures, social participation, hearing (pure-tone audiometry, Hearing Handicap Inventory for The Elderly[HHIE]), and a cognitive test battery (global and domain-specific [language; memory; executive function]) were collected at baseline. In addition, a subsample completed Magnetic Resonance Imaging (MRI) as part of an ancillary. Speech-in-noise performance (Quick Speech-in-Noise test; scores range 0-30 with higher being better) was measured at baseline without hearing aids and at 2-months and 3-years with hearing aids. Among 220 participants (median age=75.5; 50.9% Female) in the intervention arm, median unaided speech-in-noise scores were 19.0 (IQR=16.0-22.5) at baseline. At ~2-months and 3-years, aided scores were 21.3 (18.0-23.5) and 20.0 (16.0-22.5), respectively. Using linear mixed-effects models, age, education, martial status, social network, depressive symptoms, hypertension, hearing loss, HHIE, global, language domain and executive cognitive domains, temporal lobe volume, white matter hyperintensities, white matter integrity (fractional anisotropy, and mean diffusivity) were associated with baseline speech-in-noise performance. However, 2-month change was only associated with baseline hearing loss (B=0.28;95%CI=0.19-0.36), HHIE (B=0.12;95%CI=0.05-0.18), temporal lobe volume (B=0.93;95%CI=0.40-1.45), white matter hyperintensities (B=1.09;95%CI=0.43-1.76), fractional anisotrophy (B=-0.72;95%CI=-1.40--0.05), and mean diffusivity (B=0.70;95%CI=0.03-1.37) while 3-year change was only associated with baseline hearing (B=0.15;95%CI=0.04-0.26), HHIE (B=0.09;95%CI=0.01-0.19), and fractional anisotrophy (B=-1.14;95%CI=-2.08--0.20).

Abstract citation ID: igae098.0057

**EFFECT OF HEARING INTERVENTION ON OLDER ADULTS AT RISK OF COGNITIVE DECLINE: ACHIEVE RANDOMIZED TRIAL**

James Pike, *New York University, New York, New York, United States*

The Aging and Cognitive Health Evaluation in Elders (ACHIEVE, Clinicaltrials.gov Identifier: NCT03243422) randomized trial investigated the effect of a hearing intervention versus a health education control on 3-year cognitive change among dementia-free older adults with untreated hearing loss. Participants were recruited from the Atherosclerosis Risk in Communities (ARIC) study (n=238) or de novo from the community (n=739). The hearing intervention slowed cognitive decline by 48% in ARIC participants, but not in healthy de novo volunteers. A possible explanation for this difference is that the cognitive benefits of the hearing intervention could be observed only for individuals at increased risk for cognitive decline. To evaluate whether risk of cognitive decline moderated the effect of the hearing intervention, we used a sample of dementia-free ARIC participants (N=2,692) who did not participate in ACHIEVE to develop a model that explained 80.7% of the variance in cognitive decline over a 6-year period. The model was applied to baseline measures of ACHIEVE participants (N=977) to calculate their predicted risk of cognitive decline. The covariate-adjusted, intention-to-treat effect of the hearing intervention on 3-year cognitive change was examined in a mixed effects model that included a three-way interaction between time, randomization, and predicted risk of cognitive decline. Among participants in the top quartile of risk, cognitive decline in the hearing intervention group was 58.1% (95% CI 31.4%-90.9%) slower than the control group. The results suggest the effect of a hearing intervention on 3-year cognitive change was greatest among individuals at higher risk of cognitive decline.

### SESSION 1060 (SYMPOSIUM)

Abstract citation ID: igae098.0058

**ENHANCING SUPPORT SYSTEMS FOR OLDER ADULTS' HEALTH AND WELL-BEING: A HEALTH EQUITY APPROACH**

Chair: Ann Nguyen
Co-Chair: Katrina Ellis

This symposium examines psychosocial factors that shape the health and well-being of older adults using a health equity lens. The first paper examines the interplay between racial discrimination, social relationships and cellular aging among older African Americans. The findings from latent class analysis demonstrate significant heterogeneity in experiences of discrimination, social relationships, and susceptibility to molecular stress among African Americans. The second paper investigates protective factors and adverse outcomes associated with elder abuse among persons living with dementia (PLWD) using a systematic review methodology. The results identify several caregiver-related factors that protected against elder abuse and adverse outcomes related to medical conditions and medication adherence. The third paper reports the findings for the effect of urbanicity of residence on the health of caregivers of PLWD using data from the 2020-2022 Behavioral Risk Factor Surveillance Systems surveys. This analysis indicates that caregivers who lived in rural areas were more likely to self-report poor physical health than caregivers who lived in urban areas. The fourth paper examines barriers for caregivers to engage in fall risk management from the perspective of health professionals who work with PLWD and their caregivers using semi-structured, qualitative interview data. Thematic analysis identifies the several themes related to barriers. Overall, these papers will provide important insights into modifiable psychosocial factors that influence health and well-being. Modifiable psychosocial factors have broad translational importance in promoting health equity. The findings will be discussed with attention to important socio-cultural and contextual factors and highlight implications for gerontological practice and research.

Abstract citation ID: igae098.0059

### DISCRIMINATION, SOCIAL RELATIONSHIPS, AND ACCELERATED AGING AMONG MIDDLE-AGED AND OLDER AFRICAN AMERICANS

Karen Lincoln[1], Bryan Gaines[1], Hawa Mariko[1], Kaushik Golithadka[2], and Steve Cole[3], *1. University of California Irvine, Irvine, California, United States, 2. University of Southern California, Los Angeles, California, United States, 3. University of California Los Angeles, Los Angeles, California, United States*

This study addresses the limited exploration of the interplay between racial discrimination, social relationships and cellular aging among African Americans. Utilizing latent class analysis, distinct risk and protective profiles were defined by perceptions of discrimination, social support, and negative interactions among middle-aged and older African Americans. Findings revealed four distinct profiles, demonstrating significant heterogeneity in experiences of discrimination, social relationships, and susceptibility to molecular stress among African Americans. The largest profile (32.09%) was characterized by high everyday and major discrimination, low family and friend support, and minimal strain. The second profile (25.37%) had wide variation, including moderate everyday discrimination, high major discrimination, very low family support, moderate friend support, moderate family and high friend strain. The smallest profile (19.4%) was characterized by high everyday and major discrimination, high family support, moderate friend support, and high strain from family and friends. Respondents in this profile had significantly elevated molecular stress compared to the reference profile (23.13%), which was characterized by low discrimination, high support, and high strain from family and friends. Regression analysis revealed that African Americans with more health conditions were more likely to belong to the high-risk profile than the reference profile. This study identified a subgroup of African Americans with an elevated risk of accelerated aging, marked by substantial racial discrimination and ambivalent social relationships. Findings provide insights into health disparities by identifying genes activated in response to discrimination and the potential influence of social factors in mitigating or exacerbating the effects of racial discrimination on cellular aging.

Abstract citation ID: igae098.0060

### COMPARING PHYSICAL HEALTH CHALLENGES OF RURAL AND URBAN CAREGIVERS OF PEOPLE WITH DEMENTIA IN THE UNITED STATES

Fei Wang[1], E-Shien Chang[2], and Phoebe Tran[1], *1. University of Tennessee Knoxville, Knoxville, Tennessee, United States, 2. Weill Cornell Medical College, New York, New York, United States*

Increased dependence on informal caregiving and limited access to healthcare services in rural U.S. communities may contribute to worse physical health in rural informal caregivers of persons with dementia (PWD) relative to urban counterparts. We used nationally representative 2020-2022 Behavioral Risk Factor Surveillance Systems surveys to examine the association between rural/urban residence and poor physical health days in caregivers of PWD (N=17405). Self-reported physical health within previous month was categorized as follows: 0 poor physical health days (PPHD), 1-13 PPHD, 14+ PPHD. Information on rural/urban county of residence, sociodemographic characteristics, and caregiving factors were obtained from the surveys. Our results showed that 1 in 4 rural caregivers (25%) reported 14+ PPHD in the previous month as compared to 1 in 10 urban caregivers (10%). Findings from multinomial logistic models indicated that rural caregivers had 2.16 times the odds (OR: 2.16, 95% CI:1.03-4.53) of having 14+ vs 0 PPHD compared to urban caregivers after adjusting for sociodemographic characteristics and caregiving factors. These findings suggested prolonged physical health challenges for rural caregivers. In contrast, the odds of having 1-13 vs 0 PPHD were not significantly different between rural and urban caregivers (OR: 0.92, 95% CI: 0.53-1.62). Efforts to enhance long-term physical health for rural caregivers are warranted. Given that geographical isolation and limited resources in rural settings may impede physical health, community-based activities promoting physical activity and social connections should be prioritized. Policy advocacy is crucial to raise awareness of urban-rural health disparities and support targeted interventions for rural caregivers of PWD.

Abstract citation ID: igae098.0061

### ENGAGING DEMENTIA CARE PARTNERS IN FALL RISK MANAGEMENT: HEALTH PROFESSIONALS' PERSPECTIVES

Yuanjin Zhou[1], Amanda Kilcrease[1], and Elizabeth Phelan[2], *1. The University of Texas Austin, Austin, Texas, United States, 2. University of Washington, Seattle, Washington, United States*

Older people with dementia are at higher risk of falls than those without dementia. Informal care partners are well-positioned to assist them in reducing their fall risk. However, little is known about what support care partners may need to accomplish this. Health professionals with experience working with older people with dementia and their care partners may have important insights. This study thus aimed to characterize barriers for care partners to engage in fall risk management (FRM) from health professionals' perspectives and identify strategies to overcome these barriers. Using snowball sampling, we conducted semi-structured, qualitative interviews via Zoom with 22 health professionals from six disciplines (geriatric medicine, nursing, occupational therapy, physical therapy, social work, and pharmacy). These health professionals had an average of 22 (range: 5-44) years of clinical experience in dementia care. Thematic analysis identified the following themes related to barriers: care partners' care burden, a low perceived need for FRM, a tendency to prioritize the preferences of older people, resistance to making changes, disagreement about care arrangement, and challenges accessing needed resources (e.g., food, home safety evaluation, mobility devices). Suggested strategies included addressing care partners' primary concerns first (e.g., behavioral issues of older people with dementia), assessing care partners' motivation, existing strategies, and potential support for FRM, linking recommendations to priorities and goals of the dyads, and connecting them with appropriate resources. This study offers new perspectives on challenges dementia care partners may face in managing fall risk. These perspectives warrant further exploration with dementia care partners.

Abstract citation ID: igae098.0062

**ELDER ABUSE IN OLDER ADULTS WITH DEMENTIA: PROTECTIVE FACTORS AND ADVERSE EFFECTS**

Wenxing Wei[1], sarah Balser[1], Ann Nguyen[1], and Weidi Qin[2], *1. Case Western Reserve University, Cleveland, Ohio, United States, 2. University of Wisconsin Madison, Madison, Wisconsin, United States*

With the rapid increase in the aging population, more attention has been paid to studying older adults with dementia. Despite the fact that older adults with dementia are more likely to be abused compared to their cognitively intact counterparts, little attention has been paid to abuse within this population. It is important to recognize the unique characteristics and vulnerabilities of this specific group. This systematic review, conducted using the PRISMA model, aims to critically examine, evaluate, and synthesize literature on protective factors and adverse effects of elder abuse among individuals with dementia. A search was undertaken using the Ageline, Medline, CINAHL, and PsycINFO databases for peer-reviewed articles published in English up to June 2023. A total of 291 articles were identified by the systematic search, and eight articles were included in the review. The results showed that protective factors related to elder abuse are mainly examined at a perpetrator level, including caregiver-related factors (e.g., caregiver coping strategies), relational factors (e.g., premorbid relationship rewards), and contextual factors (e.g., informal support from family and friends during the COVID-19 pandemic). Adverse effects, specifically an increased risk of various medical conditions and poor medication adherence, were identified but less frequently discussed. This review highlights the need for better support and training for caregivers of older adults with dementia, as well as comprehensive policies to prevent elder abuse. It also calls for future research to find ways to protect these older adults and mitigate the adverse effects of elder abuse.

### SESSION 1065 (SYMPOSIUM)

Abstract citation ID: igae098.0063

**GERIATRIC WORKFORCE TRAINING TO ADVANCE AGE-FRIENDLY CARE IN RURAL COMMUNITIES**

Chair: Jennifer Severance
Co-Chair: Sara Murphy

Texas has one of the highest rates of rural hospital closures and a scarcity of healthcare professionals, including primary care physicians and registered nurses, with 82% of Texas counties designated as primary care shortage areas. Critical workforce shortages require innovative training models. To address challenges in rural communities across Texas and the surrounding region, a HRSA-funded Geriatrics Workforce Enhancement Program (GWEP) leveraged partnerships with academic, primary care, long-term care, and community-based organizations and expertise in geriatric clinical care. This symposium explores innovative approaches to geriatrics training by sharing critical resources, responding to community needs, and applying technology to transform clinical training environments for medical students, primary care residents, practicing health care teams, and older adults and caregivers in rural communities; expand organizational capacity to provide quality geriatrics care; and replicate evidence-based practices to impact older adults at the individual, community, and population levels. First, presenters will provide an overview of the overall strategy involving collaborators as key connections and subject matter experts for the unique needs and opportunities in rural communities. This is followed by individual projects engaging medical students, residents, practitioners, and older adults and caregivers as learners and change agents for Age-Friendly care. This symposium presents cross-sector recruitment strategies using technology and partnerships as well as methods of adapting these models to a local context while expanding training opportunities for rural areas and underserved populations.

Abstract citation ID: igae098.0064

**EVIDENCE-BASED FALL PREVENTION TRAINING FOR OLDER ADULTS IN RURAL COMMUNITIES**

Sara Murphy[1], Andrew Crocker[2], Susanna Luk-Jones[3], and Bergen Lemack[1], *1. University of North Texas Health Science Center, Fort Worth, Texas, United States, 2. Texas A&M AgriLife Extension Service, Amarillo, Texas, United States, 3. University of North Texas Health Science Center at Fort Worth, Fort Worth, Texas, United States*

Falls among older adults is a public health issue affecting one-fourth of adults aged 65 and older annually and is a leading cause of unintentional injury death. Nearly 22% of older Texans live in one of the 199 rural counties, many without easy healthcare access. Texas has 71 counties with no hospital access, and only 83 rural counties have hospitals that provide 24/7 emergency care services. In response to these challenges, the University of North Texas Health Science Center Geriatric Workforce Enhancement Program (GWEP) partnered with Texas A&M Agrilife Extension Services (AgriLife) to expand A Matter of Balance (AMOB) fall prevention program for older adults in rural communities throughout Texas. Delivered as eight sessions, AMOB is designed to improve participant confidence, physical activity, and awareness through group activities in exercise, safety, assertiveness training, and problem-solving. Pre and post-surveys were administered to rate participant knowledge and confidence in managing fall-related situations on a scale from 1 (not sure at all) to 4 (absolutely sure). In 2023, AMOB sessions were held in 11 rural communities. Participants (n=182) were predominantly female (90.1%) aged 35 to 96 (average age: 75.88). A paired t-test comparing pre- and post-intervention scores revealed significant enhancements in knowledge about fall prevention strategies, improved ability to protect themselves during falls, enhanced physical strength, steadier balance, and better fall recovery skills. Evidence-based programs, like AMOB can be broadly disseminated to address challenges of falls among older adults at the individual and community level.

Abstract citation ID: igae098.0065

**WORKFORCE ENHANCEMENTS FOR HEALTHY AGING AND INDEPENDENT LIVING: COLLABORATION TO IMPROVE HEALTH CARE**

Janice Knebl, Jennifer Severance, and Sara Murphy, *University of North Texas Health Science Center, Fort Worth, Texas, United States*

There are 48 Geriatric Workforce Enhancement Programs (GWEP) funded by the Health Resources and Services Administration in 2019 to integrate geriatrics education into primary care training, increase patient and family engagement, and improve health outcomes. The University of North Texas Health Science Center (UNTHSC) GWEP collaborates with a county hospital, a private university, an Area Agency on Aging, and other community-based organizations to align education and training enhancements and activities with community needs. Using a multipronged strategy focused on engaging local primary care and long-term care teams to identify community needs and learner preferences, a GWEP team of interprofessional faculty and community and health care partners combined critical resources to develop and enhance curricula in geriatric best practices using the Age-Friendly Health Systems 4Ms framework. Partners also developed an infrastructure to reach rural and underserved communities using learning technologies, social media platforms, and community health educators. This presentation will address the impact on medical professions students and professionals, the community partnerships that were built, and the advancement of interprofessional geriatric education.

Abstract citation ID: igae098.0066

### INTEGRATING AGE-FRIENDLY QUALITY IMPROVEMENT INTO RURAL CLINICAL ROTATION FOR MEDICAL STUDENTS

Keri Christensen, Jennifer Severance, and Lesca Hadley, *University of North Texas Health Science Center, Fort Worth, Texas, United States*

Early learning experiences in quality improvement (QI) prepare medical students for practice in complex health systems and for improving processes of care for older adult patients. We developed a curriculum using the Institute for Healthcare Improvement (IHI) Age-Friendly Health Systems 4Ms framework to train third-year medical students (n=75) in geriatrics-focused QI during a twelve-week family medicine clinical rotation in rural communities. Prior to the rotation, students completed asynchronous online training introducing QI models, Plan-Do-Study-Act (PDSA) cycles, and the Age-Friendly 4Ms framework. During the rotation, students met with their preceptor to identify a goal and conduct project activities to enhance Age-Friendly care for older adults within their practice setting. Students also collected information about the local community and patient outcome data related to CMS quality measures. Geriatrics faculty conducted weekly virtual meetings with students to discuss projects and challenges. A final written assignment summarizing their project results, clinical implications and next steps for the primary care setting in a standard abstract format that can be used for a poster presentation at a professional conference. Students (n=71) completed evaluation surveys to rate the usefulness of training and to self-assess knowledge and skills using five-point Likert-type items. Students (n=50) reported increased knowledge and skills in conducting QI activities and improved understanding of the importance of improving processes and patient care. Integrating the Age-Friendly Health Systems framework into QI training can support competency development in system-based practice while disseminating Age-Friendly practices and resources within a health care setting.

Abstract citation ID: igae098.0067

### USING PROJECT ECHO FOR GERIATRIC TRAINING IN RURAL COMMUNITIES

Sarah Ross[1], Susanna Luk-Jones[2], Sara Murphy[1], Matthew Biggerstaff[1], and Jennifer Severance[1], *1. University of North Texas Health Science Center, Fort Worth, Texas, United States, 2. .University of North Texas Health Science Center at Fort Worth, Fort Worth, Texas, United States*

Expanding geriatric education and training to health care teams using the Project ECHO (Extension for Community Healthcare Outcomes) model can improve access to quality geriatric care in rural communities. The UNTHSC Center for Older Adults applied the Project ECHO model to deliver interactive videoconferencing sessions and connect interprofessional teams of geriatric specialists with primary care providers and the nursing home workforce. Recruitment included social media marketing and mailing campaigns to target rural and other underserved communities in Texas and the surrounding regions. Training was offered as weekly, bi-weekly, or monthly ECHO sessions featuring specialist-led didactic presentations followed by interactive breakout discussions across a variety of geriatric topics in dementia care, workforce considerations, and age-friendly healthcare. Participants completed post-session surveys to track changes in geriatric care knowledge, skills, and abilities using a five-point Likert scale. From September 2019 to September 2022, a total of 157 ECHO sessions were conducted with 437 unique participants from 77 counties in 14 states. Of these 77 counties, 23 (29.9%) were rural. The most common job titles were nursing (36.2%), administration and leadership (24.9%), physician (12.1%) and student (7.9%). Out of the 437 unique attendees, 158 (36.2%) participated in at least 25% of the sessions. Participants completed post-session surveys to track changes in geriatric care knowledge, skills, and abilities using a five-point Likert scale, with positive scores on all measures.

### SESSION 1070 (PAPER)

### HEALTH CARE AND LONG-TERM CARE

Abstract citation ID: igae098.0068

### PERSON-CENTERED CARE PRACTICES PREDICT STAFF BURNOUT AND ENGAGEMENT IN LONG-TERM CARE STAFF

Sophia Geisser[1], Kate Smith[1], A. Lynn Snow[1], Amber Collins[2], Kimberly Curyto[3], Christine Hartmann[4], Cameron Camp[5], and Michelle Hilgeman[2], *1. University of Alabama, Tuscaloosa, Alabama, United States, 2. Tuscaloosa VA Medical Center, Tuscaloosa, Alabama, United States, 3. Western New York VA Healthcare System, Batavia, New York, United States, 4. VA Bedford Healthcare System & University of Massachusetts Lowell, Bedford, Massachusetts, United States, 5. Center for Applied Research in Dementia, Solon, Ohio, United States*

Long-term care (LTC) staff experience above-average rates of burnout, which have personal and professional consequences. Little is known about the effects on staff of implementing person-centered care practices to improve LTC residents' quality of life. We examined the relationship between staff perceptions of person-centered care and staff burnout and engagement in Veterans Affairs Community Living Centers (CLCs). We used baseline staff survey data (N=205) from a randomized Montessori implementation-effectiveness clinical trial in eight CLCs across five states. Data were collected anonymously online across all shifts (day/evening/night) from RNs (37.6%), nursing assistants (31.2%), and a variety of staff job types (31.3%). GLM models were used to examine the association between Person-Centered Practices in Assisted Living subscales (PC-PAL; Workplace Practices, Social Connectedness, Individualized Care and Services) and burnout and engagement measures from existing VA administrative data. Omnibus tests significantly predicted indicators of employee burnout and engagement ($p$=.05-$p$<.001). Individualized Care and Services predicted all three burnout indicators: 1) feeling burned out ($F$=4.289, $p$=.04); 2), considering leaving the job this year ($F$=5.477, $p$=.02), and 3) the job is emotionally hardening ($F$=4.026, $p$=.046); and one of the three engagement indicators ($F$=4.793, $p$=.03). Workplace Practices predicted all three measures of positive engagement: 1) seeing job as more than a paycheck ($F$=5.167, $p$=.02); job-related accomplishments ($F$=5.772, $p$=.017); and "doing more than is actually required" ($F$=8.888, $p$=.003). Social Connectedness was not a predictor in any model. Results demonstrated positive outcomes of person-centered care practices include impacts on LTC staff burnout and engagement.

Abstract citation ID: igae098.0069

### THE ROLE OF PERCEIVED CARING AND PARTNERSHIP IN PATIENT–PHYSICIAN RELATIONSHIPS

Yulri Kim[1], JeongEun Lee[1], Joseph Svec[2], and Eva Kahana[3], *1. Iowa State University, Ames, Iowa, United States, 2. St. Joseph's University, Patchogue, New York, United States, 3. Case Western Reserve University, Cleveland, Ohio, United States*

The rise of health care consumerism, facilitated by the internet use, empowers patients to seek information beyond their physicians (Martijn et al., 2020). Extant studies have shown that this shift toward healthcare consumerism, characterized by self-care movements such as proactive attitude and seeking health information (Haug, 1979), could challenge the patient-physician relationship (Haluza et al., 2017). On the other hand, the patient-physician relationship could be strengthened when patients feel a connection with their physician who show caring attitudes (Takeshita et al., 2020). Drawing data from 232 older adults ($M$= 76.16 years, $SD$= 8.27) in Miami, we examined the association between healthcare consumerism and patient-physician relationships using multiple linear regression. Respondents were randomly sampled from a probability-based research panel of community-dwelling older residents of Miami, Florida. The data showed that higher healthcare consumerism was linked with lower levels of patient-physician relationships. A moderation analysis revealed that greater perceived care from physicians and partnership attenuated the association between health consumerism (e.g., proactive attitude, health information seeking) and the patient-physician relationship. The findings highlight the significance of perceived caring and partnership in fostering strong patient-physician relationships. Findings suggest that healthcare consumerism, a growing trend, does not have to come at the expense of patient-physician relationships. By cultivating a sense of caring and patient-physician partnerships, healthcare providers can help build trust and collaboration, culminating into a more positive healthcare experience for patients.

Abstract citation ID: igae098.0070

### PERSPECTIVES ON SENSORIZATION IN THE NURSING HOME SETTING AMONG LEADERS, STAFF, RESIDENTS, AND FAMILY MEMBERS

Sarah Holmes[1], Kelly Doran[2], Zainab Balogun[3], Yoon Chung Kim[2], Lewis Davis[3], Luke Zimmerman[3], Roberto Yus[3], and Tera Reynolds[3], *1. University of Maryland School of Nursing, Baltimore, Maryland, United States, 2. University of Maryland Baltimore, Baltimore, Maryland, United States, 3. University of Maryland Baltimore County, Baltimore, Maryland, United States*

Sensorization, the process of adding smart devices (e.g., wearable or motion sensors) to spaces that can sense the environment and connect to the Internet, has numerous benefits such as improving patient outcomes, reducing clinical errors, and measuring provider stress to offer real-time interventions. However, decisions about which sensors to use can involve trade-offs including cost, accuracy, usability, and privacy. We sought to describe stakeholder perspectives toward sensorization in the nursing home. Guided by community-based participatory principles, this descriptive study included place-based observations of the nursing home environment, structured surveys to identify a priority goal that can be addressed with sensors, and qualitative interviews. The purposive sample of 25 participants included nursing home leaders (n=2), care staff (n=5), residents (n=10) and family members (n=8). Surveys were analyzed using descriptive statistics. The top priority goal identified was staff stress reduction and additional goals were resident fall prevention and staff workflow and efficiency. Sensorization plans were developed and tailored to address this goal, and interviews were conducted with participants (n=25) to understand their perceptions of the plans. Interviews were audio recorded, transcribed, and thematically analyzed in NVivo12. Themes were identified in four topic areas: 1) information types collected by sensors; 2) data sharing privacy concerns; 3) sensor location and usability; and 4) implementation feasibility. Findings shed light on the unique perspectives of the stakeholders on how they view and value the use of sensors which will inform additional research on sensorization in nursing homes to overcome implementation barriers using a systematic, participatory process.

Abstract citation ID: igae098.0071

### PERCEPTIONS OF THE "GOOD LIFE" AMONG NURSING HOME RESIDENTS WITH DEMENTIA, FAMILY, AND STAFF

Jessica Benfer[1], Gretchen Tucker[2], and John Cagle[2], *1. Meros Center, Milwaukee, Wisconsin, United States, 2. University of Maryland Baltimore, Baltimore, Maryland, United States*

Providing high quality care to nursing home residents with dementia and enhancing their quality of life is complicated by a variety of noteworthy challenges (e.g., staff turnover, difficult transitions, quarantines). Our objective was to characterize perceptions of the "Good Life" among nursing home residents with dementia, families, and staff from a nationally derived sample of nursing homes. Using Gitlin's "Good Life" model as a conceptual framework, 46 mixed-method interviews were conducted over the phone among 11 residents with dementia, 19 family members, and 16 staff from seven nursing homes across the United States. For resident interviews, family members or staff were present to help facilitate the interviews. Data were analyzed thematically. Overall, efforts were viewed as positive in nursing homes to support engaging environments for residents with dementia. Residents in our sample had better days when there was an easy-to-follow schedule of daily events and they felt connected with others, including having positive interactions with staff. Family members reported better days when they were reassured that they were doing what is right for their loved one and could balance caretaking with the rest of their lives. Staff were primarily concerned that residents were well cared for and their needs were met. Findings provide insight into several potential avenues to better promote the Good Life among nursing home residents with dementia, their families, and staff.

Abstract citation ID: igae098.0072

### IMPLEMENTING EPIC-VR IN HEALTH CARE: ALIGNING VIRTUAL REALITY TRAINING WITH ORGANIZATIONAL CONTEXT AND READINESS

Marie Savundranayagam[1], Grace Norris[1], Grace Malheiro[1], Annette Schumann[1], Jennifer Campos[2], and Joseph Orange[1], *1. Western University, London, Ontario, Canada, 2. The KITE Research Institute, University Health Network, Toronto, Ontario, Canada*

Be EPIC-VR is a virtual reality (VR)-based person-centered communication training for frontline healthcare workers in dementia care. The current study investigated factors influencing the successful implementation of Be EPIC-VR in home care and long-term care, using the Consolidated Framework for Implementation Research. Participants included eight managers from four care settings. Managers were chosen for their decision-making roles in enabling their teams to take Be EPIC-VR. Semi-structured interviews were conducted before and after Be EPIC-VR's implementation and analyzed using framework analysis. Two themes emerged: organizational context and organizational readiness. Organizational context had two subthemes: increased training needs and staffing resources. Managers identified dementia-specific training needs evolving from limited training during the COVID-19 pandemic. Staffing-related resources, such as scheduling and backfilling, were essential for implementation. This organizational context shaped the organizational readiness for Be EPIC-VR, which was evidenced by three subthemes: relative priority for Be EPIC-VR, relative advantage of Be EPIC-VR, and tailoring strategies for successful implementation. Managers prioritized Be EPIC-VR's implementation because it aligned with organizational goals to support communication and address responsive behaviours. Managers reported on the relative advantage of Be EPIC-VR compared with existing training programs, emphasizing its interactive VR simulations, the ability to practice new skills in a safe environment, and personalized feedback. Tailored strategies affecting implementation included having sufficient resources to support remote delivery, piloting with small groups, and promoting an openness to VR technology. The findings underscore the value of aligning innovations with organizational goals and tailoring implementation plans to specific organizational contexts.

### SESSION 1075 (SYMPOSIUM)

Abstract citation ID: igae098.0073

### POLICY SERIES: INEQUITABLE IMPACTS OF POLICY AND PAYMENT CHANGES ON CARE USE AND OUTCOMES FOR OLDER ADULTS WITH HIGH CARE NEEDS

Chair: Jason Falvey
Discussant: Amit Kumar

Over the past decade, the CMS has introduced numerous value-based policy initiatives. While these changes aim to reduce cost of care, they often have disparate effects on patient care and clinical outcomes particularly among minority communities and older adults with high-needs. The first presentation explores the disproportionate impacts of Medicare Advantage (MA) penetration among skilled nursing facility (SNF) users across various racial and ethnic identities, shedding light on the disparities perpetuated within the healthcare system. Another will then examine the ramifications of the Patient-Driven Groupings Model (PDGM) on older adults with dementia receiving home care, scrutinizing the changes in care and potential impacts on outcomes associated with this shift. Following this, the discussion shifts to the inequitable access to adult day services in urban areas, particularly highlighting the barriers faced by many older adults seeking non-institutional long-term care options that are growing in number around the country. Last, the symposium will discuss findings from qualitative work exploring how neighborhood disadvantage affects community mobility and successful aging in place among older adults recovering from lower extremity surgery, and the structural inequities experienced by this population with changes to home health care benefits. We will specifically highlight factors such as community cohesion and exposures to violent crime as key deterrents for recovery among older adults living with new mobility disability. Overall, this symposium will stimulate current debate on merits and demerits of various federal policies and growth of MA plans for older adults with high-needs.

Abstract citation ID: igae098.0074

### COMMUNITY MOBILITY IN OLDER ADULTS RECOVERING FROM DISABILITY IN DISINVESTED NEIGHBORHOODS: A QUALITATIVE STUDY

Jason Falvey[1], Lindsey Mathis[2], and Jasmine Cooper-Williams[2], *1. University of Maryland School of Medicine, Baltimore, Maryland, United States, 2. University of Maryland Baltimore, Baltimore, Maryland, United States*

In socioeconomically disadvantaged areas, crumbling infrastructure and perceptions of limited safety can reduce opportunities for older adults to safely get around outside and engage meaningfully in social activities. This study seeks to understand how older adults in disinvested communities meaningfully recover community mobility after disabling injuries, and evaluates how recent payment reforms have challenged individualized rehabilitation delivery in home and outpatient settings. We recruited 13 adults over the age of 55, residing in neighborhoods with an Area Deprivation Index (ADI) of ≥9/10. Participants engaged in one-hour semi-structured interviews, coded by two independent researchers. The reliability between coders was verified using a kappa statistic. Five critical themes describe these mobility experiences: 1) exacerbating impact of chronic diseases and physical limitations on community mobility 2) environmental and infrastructural barriers restricting access to community resources; 3) influence of perceived safety and violent crime on participation in outdoor activities; 4) essential role of social support networks in enhancing mobility and 5) power of individual and community resilience in mitigating mobility challenges. One participant noted: "I never know when they going to start shooting and stuff. So when it start getting dark, I get up pretty early and get out early, try to get my stuff done [before dark]." –a vivid illustration of modifications in mobility forced by the fear of gun violence, complicating the recovery process Our study suggests new care pathways are needed to enhance the support for older adults navigating the challenges of recovery within socioeconomically disadvantaged neighborhoods.

Abstract citation ID: igae098.0075

### PAYMENT POLICY IMPACT ON HOME HEALTH ACCESS FOR PEOPLE LIVING WITH DEMENTIA

Sara Knox[1], Daniel Brinton[2], Jada Johnson[2], and Kit Simpson[2], *1. Medical University of South Carolina, Charleston, South Carolina, United States, 2. Medical University of South Carolina, Charleston, South Carolina, United States*

Home health has been recommended as a strategy to prevent institutionalizations and promote aging in place for people living with dementia (PLWD). Longer home health episodes have been shown to optimize home health outcomes for PLWD. Recent payment policy reforms threaten the viability of this approach. The Patient-Driven Grouping Model (PDGM) uses patient characteristics to determine payment. Concerns have been raised that this model disincentivizes home health agencies from admitting patients, such as PLWD, who are admitted from the community and require longer episodes of care. We applied the PDGM to 100% home health files from 2019 to examine differences in PDGM components for PLWD and their peers before PDGM implementation. Two propensity score-matched groups of 153,957 individuals with and without dementia were created based on patient demographics, caregiver support, cognitive status, prior functional status, prior health care utilization, and Charlson comorbidity scores. Multivariate regression was used to examine the relationship between dementia and admission source/episode timing, functional limitations, and comorbidity level controlling for age, sex, and race. PLWD had greater odds of early community admissions (1.2 p<.0001), high functional limitation (1.09 p<.001), and of having a high comorbidity adjustment (1.49, p<.001). The primary clinical grouping for both groups was cardiac and circulatory. However, people with ADRD had twice as many Behavioral Health clinical group records. These findings indicate that people living with dementia did access and use home health differently before the implementation of the PDGM and may be experiencing negative repercussions of the new payment model.

Abstract citation ID: igae098.0076

### GEOGRAPHIC PATTERNS IN THE LOCATIONS OF ADULT DAY CENTERS AND NURSING HOMES IN TEXAS

Brian Downer, *The University of Texas Medical Branch, Galveston, Texas, United States*

Most older adults want to live at home in a community setting and avoid moving to a nursing home. Adult day centers (ADCs) are a valuable resource that can help older adults live at home. However, a concern is older adults may not have access to ADCs to benefit from their services. This analysis describes geographic patterns in the locations of ADCs and nursing homes in Texas. We used data from the Texas Department of Health and Human Services and the Centers for Medicare and Medicaid Services (CMS) to identify all licensed ADCs and CMS certified nursing homes, respectively, in Texas. Data for county characteristics (population, % aged >65) came from the US Census. Texas has 425 ADCs and 1,195 nursing homes. Among the 254 Texas counties, 48 (18.9%) have at least one ADC and 217 (85.4%) have at least one nursing home. Of the 37 counties with no nursing home, 1 county had an ADC while 170 of the 206 counties without an ADC had at least one nursing home. Approximately 6% (n=73) of nursing homes and 54.8% of ADCs (n=233) were in the thirteen counties boarding Mexico, with Hidalgo County (total population n=855,175; 11.5% aged >65) having nearly one-third of all ADCs (n=132). Conversely, the five most populous counties in Texas (total population n=12,482,916; 12.0% aged >65) had 26.1% of ADCs (n=111) and 30.0% of nursing homes (n=354). These findings suggest that older adults living in large metropolitan areas of Texas may have inadequate access to ADCs.

Abstract citation ID: igae098.0077

### COMMUNITY DISCHARGE AFTER SKILLED NURSING FACILITIES BETWEEN MEDICARE FEE-FOR-SERVICE AND MEDICARE ADVANTAGE

Maricruz Rivera-Hernandez[1], Lin-Na Chou[2], and Amit Kumar[2], *1. Brown University School of Public Health, Providence, Rhode Island, United States, 2. University of Utah, Salt Lake City, Utah, United States*

The purpose of this study was to assess differences in rates of successful community discharge among Medicare Advantage (MA) and fee-for-service (FFS) enrollees by race/ethnicity. We linked the Master Beneficiary Summary File, Medicare Provider and Analysis Review data, and the Minimum Data Set. We identified 2,412,731 Medicare enrollees who were admitted to a skilled nursing facility in 2020. We assessed community discharge within 100 days remaining out of the skilled nursing facility for 30 days (LTC focus; Rivera-Hernandez et al., 2019). Approximately, 34.48% beneficiaries were enrolled in a MA plan at admission. A higher proportion of MA beneficiaries were discharged to the community compared to FFS beneficiaries (53.64% vs. 42.70%). Among MA beneficiaries, 54.50% of Hispanic beneficiaries and 47.51% of Black beneficiaries were discharged to the community compared to 53.31% among white enrollees. However, 45.85% of white FFS beneficiaries were discharged to the community compared to 38.71% Hispanic and 34.60% of Black FFS beneficiaries. After adjusting for clinical, demographic characteristics and facility fixed-effects, rates of discharge to the community remained approximately 4.87 percentage points (p.p.) higher (95% CI, 4.72 to 5.02) among MA compared to FFS enrollees. In addition, this difference among FFS and MA beneficiaries remained wider among Black (3.43 p.p., 95% CI, 3.05 to 3.81) enrollees when compared to the difference among white FFS and MA enrollees. The observed differences in successful discharge to the community among Medicare enrollees suggest the need to promote equity in the transition of care among the underserved adult population.

### SESSION 1080 (SYMPOSIUM)

Abstract citation ID: igae098.0078

### NURSING HOME STAFF PERSPECTIVES ON SUPPORTING PERSON-CENTERED CARE IN THE FACE OF RISK TO MAINTAIN QUALITY OF LIFE

Chair: Kimberly Curyto
Discussant: Philip Taylor

Nursing home (NH) quality of care efforts emphasize person-centered care as a best practice. Evaluations of person-centered interventions report positive outcomes including improved resident psychological wellbeing, quality of life, and staff job satisfaction. However, prioritizing NH resident personal preferences and values can lead to choices that increase risk, and thus staff must act in ways that balance resident autonomy and safety. Best practices in person-centered care in the NH should honor choice and maintain safety in support of overall resident wellbeing through shared decision making among residents, families and the whole clinical team. The COVID-19 pandemic placed NH residents and staff at risk of decline and death and created barriers to implementing person-centered care. A primary focus of this symposium is to share a range of staff perspectives on supporting NH resident autonomy and safety in the face of risk, including those of NH leaders, mental health providers, and direct care team members such as nursing staff, recreation/activities staff, and social workers. Presentations will highlight the impact of a focus on safety during the increased risk from the pandemic on resident and staff wellbeing, and staff adaptation efforts to ensure person-centered care. Understanding NH staff and leadership perspectives on response to risk while supporting person-centered care will inform future care management during crises and other times of increased concern for safety in NH settings. Important perspectives of paid care workers during the pandemic will be discussed in the context of workforce development efforts.

Abstract citation ID: igae098.0079

### MENTAL HEALTH PROVIDERS' PERSPECTIVES OF BALANCING RISK AND RESPONSE AND THE PANDEMIC'S IMPACT IN VHA NURSING HOMES

Kimberly Curyto[1], Kyle Page[2], Kristy Shoji[3], HyeRim Ryu[2], Tracey Washington[4], and Michele Karel[5], *1. Western New York VA Healthcare System, Batavia, New York, United States, 2. Edward Hines, Jr. VA Hospital, Oak Park, Illinois, United States, 3. Eastern Colorado Health Care System Rocky Mountain Regional VA, Aurora, Colorado, United States, 4. Captain James A. Lovell Federal Health Care Center, North Chicago, Illinois, United States, 5. VA Central Office, Washington, District of Columbia, United States*

The COVID-19 pandemic forced a reconceptualization of how mental health services were offered to older adults in nursing homes, affecting residents and staff, as each experienced stringent and evolving infection control precautions. An anonymous national survey of Veterans Health Administration Community Living Center (CLC) mental health professionals in October 2022 included questions about the impact of the pandemic on CLC residents and healthcare teams. Respondents (N=107) included psychologists (82%) and psychiatric providers (18%). Sixty-six percent of respondents worked in the CLC before the pandemic and reported providing mostly in-person clinical care (97%). In comparison, across the entire sample two-years later, 72% reported providing in-person clinical care, 23% a mix of in-person and virtual care, and 5% entirely virtual care. An expert panel coded responses to the question, please describe your work with Veterans and teams in the CLC during the pandemic, in rotating groups of two to identify qualitative themes, and reviewed as a group to achieve consensus. Several themes emerged: 1) varied and changing methods/modes of work, 2) continued psychotherapy and assessment services, 3) increased education and support for the team, 4) declines in resident engagement, mental health, and cognition, and 5) negative impact on providers/the team (e.g., barriers to teamwork and person-centered care). Results underscore the impact the pandemic/precautions had on residents' and teams' well-being, as well as the adaptability of mental health providers, and may inform ongoing and future efforts to balance risk and support person-centered care.

Abstract citation ID: igae098.0080

### RISK PERCEPTIONS OF DIRECT CARE NURSING HOME STAFF ASSOCIATED WITH RESTRICTED SOCIAL ACTIVITIES

Nahida Akter[1], Melissa McClean[2], Kalei Kowalchik[1], Jacqueline Mogle[3], Joan Carpenter[2], and LIza Behrens[1], *1. The Pennsylvania State University, University Park, Pennsylvania, United States, 2. University of Maryland Baltimore, Baltimore, Maryland, United States, 3. Clemson University, Clemson, South Carolina, United States*

Recognized globally, social activities support better quality of life in older adulthood. The COVID-19 pandemic presented unprecedented challenges for nursing homes (NHs) in balancing infection prevention control (IPC) measures with honoring resident preferences for social activities. The purpose of this study was to explore NH staff's risk perceptions around facilitating resident preferences amidst pandemic restrictions. Guided by the Preference-Based Person-Centered Risk Engagement Model, this study used mixed methods to achieve an in-depth understanding NH staff risk perception. Quantitative data included demographics and risk perceptions using the Risk Propensity Scale. Participants (N=24) were 39 years on average (range: 18-62), mostly female (88%) and white (79%). Purposive sampling reflected a range of roles within the NH context: certified nursing assistants

(29%), activities directors (25%), social workers (25%), and nurses (21%). Average risk perceptions scores were in the risk avoider range at 3.53 (SD = 1.43; range: 1.00-7.14) and most participants (58%) self-identified as risk avoiders. Those who identified as risk seekers were significantly more likely to be male (chi-sq(2)=17.14, p<.01) and African American (chi-sq(4)=20.42, p<.01). Qualitatively, semi-structured individual interviews collected staff perceptions of risk related to residents' social activities. Guided content analysis revealed two main themes supported by six sub-themes: Factors of decision-making (family influence, organizational, staff and resident characteristics) and Direct care staff influencing preference-based care (cognitive skills and technical skills). Findings will be discussed considering implications for how best to support direct-care staff with varied perceptions of risk (risk seekers/risk avoiders) to encourage social activities when IPC measures are indicated.

Abstract citation ID: igae098.0081

### LONGITUDINAL QUALITATIVE RESULTS FROM STAFF IMPLEMENTING MONTESSORI DURING THE PANDEMIC

Michelle Hilgeman[1], Katherine Kennedy[2], Whitney Mills[3], A. Snow[1], Teddy Bishop[1], Nathalie McIntosh[4], and Kimberly Curyto[5], *1. Tuscaloosa VA Medical Center, Tuscaloosa, Alabama, United States, 2. VA Providence Healthcare System, Center of Innovation in Long-Term Services and Supports, Providence, Rhode Island, United States, 3. VA Providence Health Care System, VA Providence Health Care System, Rhode Island, United States, 4. Providence VA Medical Center, Providence, Rhode Island, United States, 5. Western New York VA Healthcare System, Batavia, New York, United States*

Promoting autonomy, dignity, and independence are core values of Montessori approaches to person-centered care in nursing homes. Yet infection control precautions restricted staff and resident freedoms during the COVID-19 pandemic. We describe longitudinal data from a stepped-wedge, randomized clinical trial examining implementation and effectiveness of Montessori approaches in eight Department of Veterans Affairs nursing homes (2021-2023). COVID-rates were calculated from electronic health records (EHRs) and graphed over 18 months around the intervention. Staff (N=168) participated in qualitative individual or group interviews at baseline, 3-, 6-, 9-, and 12-months. Normalization Process Theory informed questions and qualitative content analysis about the effects of COVID-19 on Montessori implementation and staff experiences. Transcripts were coded by members of the study team using NVivo. There was variability in COVID rates: most NHs concentrated below 1% of resident-days with COVID and occurred after 3-months of Montessori training/implementation, while two sites experienced peaks of 5% and 15%. Qualitative themes across implementation included: 1) Staff described fluctuating waves of COVID, changes in precaution protocols, and variable policy interpretations across sites to be associated with negative impact on residents and staff (isolation, loneliness, burden, burnout, low morale). 2) Staff identified creative adaptations (e.g. walkie-talkies) to implement Montessori within precaution protocols. 3) Staff reported that Montessori principals improved morale, mitigated negative effects of the pandemic and related precautions, and created a pathway for increased advocacy for person-centered care and resident wellbeing despite risks. Findings highlight the importance of reinforcing person-centered care in the midst of safety concerns during crisis.

Abstract citation ID: igae098.0082

### NURSING HOME LEADER PERSPECTIVES OF DELIVERING QUALITY CARE AT TRANSITION TO ENDEMIC PHASE OF COVID-19

LIza Behrens[1], Kimberly VanHaitsma[1], Marie Boltz[1], William Calo[2], Lauren Van Scov[3], and Jennifer Kraschnewski[3], *1. The Pennsylvania State University, University Park, Pennsylvania, United States, 2. Penn State College of Medicine, Hershey, Pennsylvania, United States, 3. The Pennsylvania State University College of Medicine, Hershey, Pennsylvania, United States*

Nursing Home (NH) leaders are challenged to deliver quality person-centered care (PCC), despite the COVID-19 transition to an endemic phase. This study aimed to describe NH leadership perspectives on preparing and maintaining quality care during the transition point between the COVID-19 pandemic and endemic to identify how best to support NHs in balancing infection risks with PCC. Fifteen (n=15) NH leaders enrolled in the Project Extension for Community Health Outcomes (ECHO) study participated in semi-structured interviews between March and April of 2022. Interviews lasted approximately 60 minutes, were recorded, transcribed verbatim, and verified for accuracy. Multiple coders used constant comparative method to code remaining transcripts (final kappa was.76). Final descriptive themes and subthemes were created via group consensus. NH Leaders reported an average of 18.2 years of experience and held various roles as leaders including Administrator (n=7), Director of Clinical/Health Services (n=3), Director of Nursing (n=2), Director of Infection Prevention and Control (n=1), and Staff Development Director/Coordinator (n=2). Leaders described their perspectives in six themes: Facilitators to operations during COVID-19, Barriers to operations during COVID-19, Resources, Infection control and vaccine uptake, Handling residents' preferences during COVID-19, and ECHO participation. Results confirmed continued challenges in delivering quality PCC impacted by enforcement issues with vaccination processes and uptake for staff, supply gaps, and lack of knowledge in how to handle residents' preferences for social activities. Results demonstrate the need for more resources to support staff delivery of quality PCC while balancing risks related to managing the COVID-19 endemic.

### SESSION 1085 (SYMPOSIUM)

Abstract citation ID: igae098.0083

### OLDER ADULTS' EXPERIENCES WITH INNOVATIONS IN HEALTH CARE DELIVERY: FINDINGS FROM THE NPHA AND AARP SURVEYS

Chair: Erica Solway
Co-Chair: Teresa Keenan
Discussant: Teresa Keenan

Innovations in health care delivery have resulted in dramatic shifts in the experience of seeking and receiving health care services. Many of these innovations, like telehealth and patient portals, predated the COVID-19 pandemic, but the pandemic quickly spurred rapid changes in availability and use. It is important to understand how older adults view and experience these innovations and the opportunities and challenges these innovations present. Since its launch in 2017, the National Poll on Healthy Aging (NPHA) has surveyed a nationally representative sample of adults ages 50-80 to gauge their perspectives on health care and health policy-related topics, including health care innovations, and assess trends and changes in views and experiences over time. In addition, AARP regularly conducts surveys to offer additional timely insights on important related topics. This symposium will focus on lessons learned from the NPHA related to telehealth, patient portals, direct-to-consumer health care services, and use of alternative sites for health care, like urgent care and retail clinics. In addition, it will highlight key findings from recent AARP surveys related to views on innovations in health care delivery among adults and older adults and will share how these insights can inform policy and practice.

Abstract citation ID: igae098.0084

### USE OF AND EXPERIENCES WITH TELEHEALTH VISITS AND PATIENT PORTALS IN A NATIONALLY REPRESENTATIVE SAMPLE OF OLDER ADULTS

Erica Solway, Dianne Singer, Matthias Kirch, Scott Roberts, and , Jeff Kullgren, *University of Michigan, Ann Arbor, Michigan, United States*

The University of Michigan National Poll on Healthy Aging (NPHA) has explored older adults' changing views and experiences related to telehealth and other health-related technologies over the past several years. NPHA surveys are fielded twice per year to a nationally representative sample of over 2,500 adults ages 50-80 (completion rates: 50-60%). When the poll first included questions about telehealth use and perceptions in 2019, just 4% of U.S. adults ages 50-80 reported having a telehealth visit in the past year. Telehealth expanded rapidly during the pandemic and by mid-2020, 26% of older adults reported having had a telehealth visit within the prior three months. In addition, the NPHA has explored patient portal use, documenting an increase in portal uptake over time as well as older adults' experiences with, and barriers to, portal use. Recently, the NPHA explored older adults' experiences related to sending portal messages to their health care providers and the out-of-pocket costs associated with this. Other recent NPHA research has demonstrated use and interest in direct-to-consumer telehealth services provided by online-only companies. As of 2023, the poll found that 7.5% of people ages 50-80 had used at least one direct-to-consumer health care service from an online-only provider, and 32% said they would be interested in these types of health care services in the future. The NPHA provides timely insights on older adults' changing perspectives and experiences related to emerging areas of health care that can help to inform efforts to improve access, quality, and integration of health care.

Abstract citation ID: igae098.0085

### USE OF AND EXPERIENCES WITH ALTERNATIVE SITES FOR HEALTH CARE BY US ADULTS AGES 50–80

Teresa Keenan[1], Harriet Komisar[1], Olivia Dean[2], Erica Solway[3], and Cheryl Lampkin[1], *1. AARP, Washington, District of Columbia, United States, 2. AARP, Oakland, California, United States, 3. University of Michigan, Ann Arbor, Michigan, United States*

In recent years, "alternative" sites for health care – such as urgent care and retail health clinics – have expanded to meet the growing demand for more convenient and accessible care. In August 2023, the University of Michigan National Poll on Healthy Aging asked a national sample of 2,687 adults ages 50-80 (completion rate: 50%) about their use of and opinions about alternative sites of care. In all, 60% of adults ages 50-80 reported using an alternative site in the past two years: urgent care clinic (47%), retail clinic (28%), worksite clinic (9%), and mobile health clinic (5%). Use of these alternative sites was more common among those ages 50-64, women, and those with higher incomes. Among older adults who visited an alternative care site in the past two years, 91% reported having a primary care provider (PCP). Of those with a PCP, 71% contacted their PCP about their visit to an alternative site. The most common reasons reported for visiting an alternative site were to avoid the hospital emergency department (44%) or to get a vaccine, specific test or to get a health exam (35%). The majority (62%) said they were likely (11% very likely, 51% somewhat likely) to use an alternative site for health care in the future, including 43% who had not used in alternative site in the past two years. Poll findings suggest that these sites will continue to play a significant role in the delivery of health care in the future.

Abstract citation ID: igae098.0086

### A NEW NORMAL: AARP SURVEY FINDINGS ON OLDER ADULTS' EXPERIENCES AND PERSPECTIVES ON TELEHEALTH AND AI IN HEALTH CARE

Cheryl Lampkin, and Teresa Keenan, *AARP, Washington, District of Columbia, United States*

The COVID-19 pandemic ushered in a "new normal" as it relates to receiving health care--especially for older adults. This presentation will include insights on how new technology and attitudes about technology have impacted how older adults receive health care services. For example, findings from a recent survey of 1,700 older adults conclude that while over half say they are likely to attend a phone or video session with a mental health professional (53%) or their primary care provider (52%) for a mental health concern, most are skeptical of telehealth believing they get better quality care for both physical (82%) and mental health (69%) concerns in person. Similarly, another AARP study found older adults believe in-person visits are better than telehealth visits when it comes to providing a personal touch (73%), diagnosis accuracy (72%), and thoroughness (68%). However, many said in-person and telehealth are about the same when it comes to keeping personal information safe (60%) and private (52%). These and other insights are based on data obtained from multiple recent AARP surveys fielded among nationally representative samples of midlife and older adults. Studies also included oversamples of Black/African American and Hispanic/Latino respondents, enabling enhanced analyses. This multi-analysis presentation will provide unprecedented insights on familiarity and use of technology in receiving health care as well as opinions and attitudes on the use of artificial intelligence (AI) in health care. Additionally, a preview of new data collected in late fall 2024 will provide timely insights on current views on health care innovations.

### SESSION 1090 (PAPER)

### ORAL AND SENSORY CARE

Abstract citation ID: igae098.0087

### RELATIONSHIP OF MULTISENSORY IMPAIRMENT WITH PHYSICAL PERFORMANCE IN OLDER ADULTS IN SOMMA

Atalie Thompson[1], Tyler Mansfield[2], Eileen Johnson[3], Peggy Cawthon[4], Elsa Strotmeyer[5], Jeff Williamson[6], Steve Cummings[4], and Stephen Kritchevsky[7], *1. Wake Forest University School of Medicine, Winston-Salem, North Carolina, United States, 2. University of California San Francisco, San Francisco, California, United States, 3. Sutter Health, San Francisco, California, United States, 4. California Pacific Medical Center, San Francisco, California, United States, 5. University of Pittsburgh, Pittsburgh, Pennsylvania, United States, 6. Atrium Health Wake Forest Baptist, Winston-Salem, North Carolina, United States, 7. Wake Forest University School of Medicine, Wake Forest University School of Medicine, North Carolina, United States*

There is growing evidence that various sensory and cognitive functions can impact physical performance but the relationship of multisensory impairment (MSI) and cognition with mobility in older adults is not well understood. We hypothesized that MSI would be associated with worse performance on timed physical mobility assessments, but that the association would be attenuated by controlling for tests of cognitive processing speed. Participants (N=799) were older adults (mean age 76.25+/-4.95 years; 59.82% female; 84.46% Non-Hispanic White) who completed tests of physical performance, cognitive function, and multiple sensory domains (vision, olfaction, peripheral sensation, self-reported hearing). An 8-point MSI scale was created: 1 point for moderate and 2 points for severe impairment in each sense. Separate linear regression models examined the association of MSI with 400-m gait speed, extended short physical performance battery (eSPPB), 4-square step test (FSST) and stair climb test, adjusting for demographic and clinical covariates. Each additional point on the MSI scale was associated with 0.009 m/s slower walking speed (p=0.049), 0.05 lower eSPPB score (p=0.0001), and 0.2 s longer FSST time (p=0.008), but there was no association with stair climb time(p=0.59). After adjusting for DSST and Trails B, the association of MSI with 400-m gait speed and FSST were no longer significant (p>0.05) and the association with eSPPB was partially attenuated (-0.03 units/MSI point, p=0.0037). Older adults with MSI had worse mobility function which may be partially related to cognitive processing speed. Future studies should investigate if older adults with MSI would benefit from interventions to improve mobility.

Abstract citation ID: igae098.0088

### MEDIATION OF THE NUTRITIONAL STATUS BETWEEN THE NUMBER OF TEETH AND DEPRESSION AMONG OLDER ADULTS IN WESTERN CHINA

Xin Tian[1], Yuexia Hu[2], Yuqing Xie[2], Xin Xia[3], and Yanyan Wang[4], *1. Sichuan University, Chengdu, China (People's Republic), 2. Sichuan university, Chengdu, Sichuan, China (People's Republic), 3. Center of Gerontology and Geriatrics, West China Hospital, Sichuan University/ National Clinical Research Center for Geriatrics, West China Hospital, Sichuan University, Chengdu, Sichuan, China (People's Republic), 4. West China School of Nursing, Sichuan University, Chengdu, Sichuan, China (People's Republic)*

Depression is a common mental disorder. Studies have shown that depression is associated with the number of teeth, but underlying mechanisms deserve further research. Our study aimed to explore the mediating effect of nutritional status between the number of teeth and depression. We analyzed cross-sectional baseline data from the 2018 West China Health and Aging Trend study (WCHAT), including 6632 multi-ethnic adults aged 50-95. The survey tool for depressive symptoms is 15-item Geriatric Depression Scale (DGS-15), and nutritional status was evaluated through Mini Nutritional Assessment-Short Form (MNA-SF). We conducted a multiple linear regression to estimate relationships between the number of teeth, nutritional status, and depression. Mediation models and pathway analysis were constructed to figure out the mediating role of nutritional status. Of 6632 participants, the depressive symptoms proportion was 17.3%. Multiple linear regression proved a total association between the number of teeth and depression ($\beta$ = -0.148; 95% confidence interval: -0.218 to -0.078), the associations remained significant after adjusting MNA-SF scores. Mediation analysis verified nutritional status partially mediates the associations between the number of teeth and depression (indirect effect estimate = -0.059, 95% CI: -0.076 to -0.044; direct effect estimate = -0.088, 95% CI: -0.155 to -0.017). Structural equation model framework pathway analysis confirmed the association between the number of teeth, nutritional status, and depression. Our data suggested that nutritional status partially mediated the relationship between the number of teeth and depression, early identification of nutritional status and emphasis on oral hygiene are necessary to mitigate the risk of depression.

Abstract citation ID: igae098.0089

### THE ASSOCIATION BETWEEN ORAL HEALTH/ HYGIENE AND COGNITIVE IMPAIRMENT AMONG MULTIETHNIC OLDER ADULTS IN WEST CHINA

Yuqing Xie[1], Xin Xia[2], Yun Li[3], Yanyan Wang[4], Xin Tian[5], and Yuexia Hu[1], *1. Sichuan university, Chengdu, Sichuan, China (People's Republic), 2. Center of Gerontology and Geriatrics, West China Hospital, Sichuan University/ National Clinical Research Center for Geriatrics, West China Hospital, Sichuan University, Chengdu, Sichuan, China (People's Republic), 3. Sichuan university, Chendu, Sichuan, China (People's Republic), 4. West China School of Nursing, Sichuan University, Chengdu, Sichuan, China (People's Republic), 5. Sichuan University, Chengdu, China (People's Republic)*

The prevalence of cognitive impairment increases with age. Meanwhile, older adults with cognitive impairment typically have deteriorating oral health and poor oral hygiene. However, the distribution of oral health and cognitive impairment varies among different ethnic groups. The aim of this cross-sectional, multi-center study was to investigate the association between oral health and hygiene and cognitive impairment in a sample of multi-ethnic older adults aged 50 years and older from four provinces in western China (N=6529, mean age 62.4 ±8.3 years). Oral health and hygiene were assessed by specialized oral tests and a self-made questionnaire. Cognitive status was assessed using the Chinese version of the Short Portable Mental Status Questionnaire (SPMSQ). Three multiple regression models were used to examine the association between oral health and hygiene and cognitive impairment, adjusting for relevant variables. Statistical analysis showed that the prevalence of cognitive impairment was 15.4%, with the Yi group having the highest prevalence (28.9%), followed by Tibetans and others. Poorer self-rated oral health, fewer residual teeth, less frequency of using toothbrush and toothpaste, and rare dental visits increased the risk of cognitive impairment. The SPMSQ scores and correlations between oral health and hygiene and cognitive impairment were heterogeneous across the multi-ethnic groups. These findings highlight that declining oral health and inadequate oral hygiene may increase the risk of cognitive impairment. The potential value of this study is to illustrate that implementing and promoting oral health and hygiene is essential to prevent cognitive impairment in multi-ethnic older populations.

Abstract citation ID: igae098.0090

### RELATIONSHIP OF VISUAL IMPAIRMENT AND POOR MOBILITY TO MORTALITY IN OLDER ADULTS

Atalie Thompson[1], Joseph Rigdon[1], Michael Miller[1], Rebecca Neiberg[1], Jeff Williamson[2], and Stephen Kritchevsky[3], *1. Wake Forest University School of Medicine, Winston-Salem, North Carolina, United States, 2. Atrium Health Wake Forest Baptist, Winston-Salem, North Carolina, United States, 3. Wake Forest University School of Medicine, Wake Forest University School of Medicine, North Carolina, United States*

Older adults with visual impairment (VI) have greater risk of mortality but the reasons are poorly understood. We have shown that older adults with VI are more likely to have poor mobility performance on the short physical performance battery (SPPB). In this analysis we examined whether VI predicted mortality over 10 years and if this was related to poor baseline mobility (SPPB< 9). We included 2457 older adults who completed three vision tests at the year 3 visit in the Health, Aging and Body Composition study. VI was defined by visual acuity < 20/40, log contrast sensitivity < 1.55 or stereoacuity >85. Cox proportional hazard models for mortality were adjusted for age, sex, black race, education, body mass index, smoking, depression, hypertension, heart disease, stroke, and diabetes. In separate models, VI (Hazard ratio (HR) 1.475, 95% CI (1.313-1.658), p< 0.0001) and SPPB< 9 (HR 1.469, 95% CI (1.233-1.75), p< 0.0001) each predicted mortality. When adding poor mobility (as a main effect and interaction with VI) and using unimpaired vision and mobility as the reference, those with only VI (HR 1.451, 95% CI(1.28-1.645), p< 0.0001) or only poor mobility (HR 1.495, 95% CI(1.117-2.0), p=0.0068) had similar HRs while those with both VI and poor mobility had an increased mortality risk (HR 1.943, 95% CI(1.562-2.418), p< 0.001), but the interaction was not significant (p=0.55). After accounting for major comorbidities, older adults with VI and poor mobility are at a greater risk of mortality. Future studies should consider if interventions to improve visual function and mobility improve survival in older adults.

### SESSION 1095 (PAPER)

### PREDICTORS AND CONSEQUENCES OF ADVERSITY AND STRESS

Abstract citation ID: igae098.0091

### RACIAL AND ETHNIC DIFFERENCES IN AGEISM AND HEALTH: EVIDENCE FROM THE EXPERIENCES OF AGING IN SOCIETY PROJECT

Julie Ober Allen, *University of Wisconsin, Madison, Wisconsin, United States*

Age-based stereotypes, prejudices, and discrimination (ageism) are implicated in a variety of poor health outcomes, yet it is unclear if all segments of the older adult population are affected similarly. This study examined racial and ethnic differences in ageism and health using data from the Experiences of Aging in Society project (2021-2023) with a sample equally divided between NonHispanic Black, White, and Asian American and Hispanic US adults age 50+ (N=236; Mage 65; 72% female). We identified variations in amount and types of routine ageism (Everyday Ageism Scale and subscales) and associations with health using ANCOVA and z-tests of differences in race/ethnicity-stratified regression parameters. While all racial/ethnic groups reported comparable amounts of routine ageism overall, differences were detected in types: exposure to ageist messages, White=most, Black=least; age discrimination, Black=most, White=least; and internalized ageism, Hispanic=most, Black=least. In the full sample, ageism was associated with more chronic physical health conditions and greater odds of a mental health disorder (p-values<.02); of the three types, age discrimination was most closely associated with physical health outcomes and internalized ageism with mental. Relationships differed by race/ethnicity: ageism was only associated with physical health among Black (association driven by age discrimination) and White adults (driven by internalized ageism) and with mental health among White adults (driven by internalized ageism) (p-values<.05). Exposure to routine ageism may be universal; yet, healthy aging initiatives should attend to nuances at the intersection of age, race, and ethnicity that contribute to divergent experiences of ageism and consequences for mental and physical health.

Abstract citation ID: igae098.0092

### EFFECTS OF A CANCER DIAGNOSIS ON ECONOMIC PROSPERITY IN THE LONG RUN: EVIDENCE FROM LINKED SURVEY AND CREDIT DATA

John Warren[1], Amanda Thomas[1], Eric Grodsky[2], and Chandra Muller[3], *1. University of Minnesota, Minneapolis, Minnesota, United States, 2. University of Wisconsin Madison, Madison, Wisconsin, United States, 3. The University of Texas Austin, Austin, Texas, United States*

Although 40% of Americans will experience cancer in their lifetime, little is known about the long-term impacts of cancer diagnosis on debt, wealth, and financial precarity later in life. Using data from High School and Beyond (HSB)—a large (n~25,500) nationally representative study of high school students followed from 1980 through 2022—linked to consumer credit records (2004-2023), we estimate changes in debt, credit, and other economic measures resulting from after a cancer diagnosis. We also consider how these processes vary across demographic, educational, and family background groups. Approximately 13,900 individuals from the original cohort responded to the 2021/2022 survey, with more than 1,000 indicating a lifetime diagnosis other than skin cancer; another 490 cohort members died from various forms of cancer by 2021/2022. The most common cancers reported were breast (n=350) and prostate/vaginal (n=150 each) with more than one-third diagnosed in early adulthood (≤ 45 years old). Preliminary results using cross-sectional measures of financial well-being indicate reduced annual income earnings and heightened financial precarity as compared to otherwise similar people without a cancer diagnosis. With linked consumer credit data, we are well-equipped to expand knowledge about the effects of a cancer diagnosis on financial health by analyzing fluctuations in indicators such as credit score, medical and other forms of debt, bankruptcy, and estimated wealth in the months and years after a cancer diagnosis. Our analyses will model these outcomes, inclusive of key confounders (age, education, type of cancer, etc.) both for the full sample of demographic and socioeconomic subgroups.

Abstract citation ID: igae098.0093

### INEQUALITIES IN OLDER ADULTS' LIFELONG LEARNING: HOW SOCIO-ECONOMIC STATUS SHAPES PARTICIPATION AND BENEFITS

Shu Yee Chin[1], Jolin Chua[1], Nathan Widjaja[1], Shannon Ang[1], and Rahul Malhotra[2], *1. Nanyang Technological University, Singapore, Singapore, 2. Duke-NUS Medical School, Singapore, Singapore*

Although lifelong learning has been increasingly utilized to promote active ageing, research increasingly suggests that older adults with lower socio-economic statuses (SES) may face unique barriers to participation in these programs. Simultaneously, we know little about whether SES moderates the well-being benefits of lifelong learning programs. Using two waves of data (2016-17; 2019) from a nationally representative sample of Singaporeans aged 60 and above (n = 2,508), this study investigates whether older adults' SES shapes their participation and benefits reaped from lifelong learning programs. Firstly, we used a multinomial logistic regression model to examine the relationships between SES and participation in job-related and nonjob-related lifelong learning programs. Secondly, we used lagged dependent variable regression analyses with inverse probability weight adjustments to examine whether SES moderated the relationship between lifelong learning participation and quality of life, while addressing potential selection biases into lifelong learning participation. Findings indicate that older adults with higher educational attainment and occupational prestige had greater participation in both job- and nonjob-related lifelong learning. Moderation analyses further showed that the effects of nonjob-related learning on quality of life were only significant for those with lower SES. In summary, these findings suggest that although lower-SES older adults benefit more from participation in lifelong learning programs, they are also less likely to participate in such programs. Results highlight the need to make programs more accessible to lower-SES older adults. Future research should continue to investigate the mechanisms through which SES moderates the benefits of lifelong learning for well-being outcomes.

Abstract citation ID: igae098.0094

### STAYING GOLDEN: PERSPECTIVES FROM THE AGE 85+ ON THE REALITIES OF AGEISM

Taylor Patskanick[1], Sophia Ashebir[1], Lisa D'Ambrosio[2], and Joseph Coughlin[2], *1. Massachusetts Institute of Technology, Cambridge, Massachusetts, United States, 2. MIT AgeLab, Cambridge, Massachusetts, United States*

The conceptualization, prevalence, and consequences of ageism are increasingly well-documented in research, but our understanding of how older people may personally experience this phenomenon during specific age periods in their older adulthood is less understood (e.g., Chasteen et al., 2021). The 85+ age cohort may experience unique forms of ageism and age-based discrimination relative to other older age groups and can comment on how they experience these biases relative to other ages in their own lifespan. This paper shares findings from a qualitative, cross-sectional study with the MIT AgeLab 85+ Lifestyle Leaders panel, a longitudinal panel of U.S. octogenarians and nonagenarians. Utilizing four virtual focus groups (N=17) conducted in January 2024, findings were analyzed in MAXQDA2024 using a team-based, inductive, thematic analysis approach. Results characterize Lifestyle Leaders' experiences with felt forms of ageism (e.g., covert vs overt, positive vs negative, etc). The Lifestyle Leaders articulated experiences with ageism in specific domains such as product design, healthcare, and in the community-based built environment. Benevolent or compassionate ageism may be particularly amplified at age 85+. Lifestyle Leaders noted an inextricable intersectionality between their chronological age and social identities like physical ability, digital nativity, and gender. A plurality described having a longitudinal lens on the shifting institutional context for age-based discrimination over their life courses (e.g., ADEA). These findings and their implications for gerontological professionals and future research on ageism, including planned follow up to this study examining this topic via participatory action research methods, will be discussed.

Abstract citation ID: igae098.0095

### VICTIMIZATION EXPERIENCES AND COGNITIVE HEALTH: VARIATION BY SEXUAL MINORITY STATUS

Harry Barbee, *Johns Hopkins Bloomberg School of Public Health, Baltimore, Maryland, United States*

Studies have revealed how experiences of victimization generate stress and harm cognitive health outcomes. For minority populations, such as sexual minorities, victimization experiences may occur more frequently, thus posing a greater risk to their cognitive health outcomes in later life stages. This project explores this possibility by analyzing how different types of victimization experiences, including being treated with less respect, receiving poorer services, and being threatened or harassed influence cognitive health outcomes among older adults in the United States. To achieve this goal, I examined data from the Health and Retirement Study, a nationally representative survey of middle-age and older adults in the United States. Specifically, I employed adjusted logistic regression models and adjusted Poisson regression models to test the extent to which experiencing different forms of victimization influenced subjective cognitive health, via self-rated memory, as well as a variety of objective markers of cognitive health. All models controlled for key sociodemographic variables. Preliminary results indicate that respondents who experienced different types of victimization also reported significantly worse cognitive health outcomes. Additionally, sexual minorities who reported victimization experiences had worse cognitive health outcomes than the heterosexual respondents who also reported victimization experiences. Together, these results demonstrate how perceived experiences of victimization can harm cognitive health, and also reveals potential explanations for why older sexual minorities face greater risk of cognitive impairment than their heterosexual counterparts.

### SESSION 1100 (SYMPOSIUM)

Abstract citation ID: igae098.0096

### RESILIENCE AND SOCIAL CONNECTION AMONG MARGINALIZED OLDER ADULTS: CHALLENGES AND OPPORTUNITIES

Chair: Melanie Levasseur
Co-Chair: Andrew Wister

Resilience, i.e., the ability to cope and thrive in the face of adversity, and social connection are recognized factors of older adults' health, well-being and community integration. Improving resilience and social connection is especially challenging in marginalized older adults, who face multiple barriers to social participation. This symposium will present first theoretical contribution followed by empirical evidence where resilience and social connection were enhanced in marginalized older adults. The presentations will provide a forum to discuss the challenges and opportunities for better supporting resilience and social connection among marginalized older adults from Canada. Levasseur and colleagues examined the associations between vulnerability and life satisfaction, and the moderating effect of social support. Wister and colleagues discuss a resilience model designed to support recovery from social isolation and loneliness, which relies on internal and external resources across a socio-ecological spectrum. Majón-Valpuesta and colleagues considered the determinants regarding social participation opportunities for older transgender baby boomers in Canada. Meynet and colleagues identified and described studies assessing the efficacy of home-based and collective interventions on older adults' social participation, isolation, loneliness, and social connectivity. Li and colleagues verified the association of loneliness in long-term spousal caregivers with social participation and social support. Together, these presentations provide insights to understand how resilience and social connection can alleviate the risk of marginalization and promote health.

Abstract citation ID: igae098.0097

### VULNERABILITY, LIFE SATISFACTION, AND SOCIAL SUPPORT AMONG OLDER ADULTS: RESULTS FROM THE CLSA

Melanie Levasseur[1], Daniel Naud[1], Volker Cihlar[2], Frank Micheel[2], Andreas Mergenthaler[2], and Lise Trottier[3], *1. Université de Sherbrooke, Sherbrooke, Quebec, Canada, 2. Federal Institute for Population Research, Wiesbaden, Hessen, Germany, 3. Centre de recherche sur le vieillissement, CIUSSS Estrie - CHUS, Sherbrooke, Quebec, Canada*

When experiencing, at a specific moment, multidimensional difficulties (physiological, psychological, socioeconomic or social) increasing the probability of being harmed or having coping challenges that negatively impact their lives, individuals in situations of vulnerability are at risk of reduced life satisfaction. Although not demonstrated yet, social support may mitigate this impact in older adults. This study aimed to examine the associations between situations of vulnerability, life satisfaction and social support, as well as the moderating effect of social support on the association between situations of vulnerability and life satisfaction among older women and men. Secondary analyses of the cross-sectional data on 21,491 respondents aged ≥65 from the Canadian Longitudinal Study on Aging were conducted, stratified by sex. Confirmatory factor analysis was used for identifying a latent vulnerability variable using physiological, psychological, socioeconomic and social indicators. Linear regression estimated the association between vulnerability and life satisfaction, and the moderating effect of social support. Respondents were aged between 65 and 89 (mean=73.4; standard deviation=5.8). For both sexes, reporting more depressive symptoms, chronic conditions and insufficient income best explained vulnerability, followed by dependence in basic activities of daily living, less social participation and living with fewer people. Vulnerability was associated with lower life satisfaction ($p<0.001$) for both sexes, and social support acted as a buffer against vulnerability ($p<0.001$) for women. Results confirm the multidimensionality of vulnerability. The buffering effect of social support in women reinforces recommendations concerning policies and interventions designed to increase opportunities of social connections, and further research is warranted for men.

Abstract citation ID: igae098.0098

### SOCIAL ISOLATION AMONG MARGINALIZED OLDER ADULTS: APPLYING A UNIFIED MODEL OF RESILIENCE

Andrew Wister, *Simon Fraser University, Vancouver, British Columbia, Canada*

Social isolation is associated with reduced psychological well-being; increased anxiety and depression; compromised physical health; and higher mortality among older adults. Moreover, those who experience various forms of vulnerability and marginalization in society are particularly at risk. We apply the Unified Model of Resilience and Aging (UMRA), an interdisciplinary model that integrates psychological, physiological, and social dimensions of resilience, the latter of which embodies social participation, social isolation, and community belonging. Resilience models, like the UMRA, seek to understand why some individuals, families, and communities recover from adversity better than others, in this case, social isolation and loneliness. The UMRA integrates individual and system-level processes that occur over the life course. According to the UMRA, resilience depends on the activation of internal resources (positive attitude), and activation of external resources (e.g., support from family, friends or organization) which also vary with age and other factors. The UMRA thus includes four domains of environmental resilience situated across the socio-ecological spectrum from individual to global. It also includes four system-level (organizational) functions used by the National Academy of Sciences Resilience Model: 1) Planning for adverse events requires reductions in risk in response to an identified threat. 2) Absorption of stressors and outcomes associated with an adversity is necessary to initiate resilience through recovery and adaptation. 3) Recovery occurs through various forms of short and long-term strength-based resilience. 4) Adaptation relates to changes in the system to promote future resilience. We apply the UMRA to frame causes and consequences of social isolation.

Abstract citation ID: igae098.0099

### SOCIAL PARTICIPATION OF TRANSGENDER BABY BOOMERS IN QUÉBEC: RESULTS FROM A CASE STUDY OF ACTIVISTS

Dolores Majón Valpuesta, *Université de Sherbrooke, Sherbrooke, Quebec, Canada*

The systematic discrimination linked to the intersection of gender identity and advanced age in transgender older adults results in further social participation restrictions and higher risk of disability (65% of transgender adults report poor mental health). Despite the importance of participation for health, little is known about the social participation of transgender older adults, especially among the new generation, the baby boomers. This study thus aimed to explore the social participation for transgender older adults. Based on the Iridescent Life Course theory, a case study was carried out with 2 transgender baby boomer community-dwelling from Quebec (Canada). Two French-speaking transgender women, 60 and 67 years old, with more than 5 years of activism. According to the experience of these activists, participation was positive and, with the gender transition in old age, a form of liberation, overcoming fears and denunciation, having a favourable impact on the community (e.g., sharing life experience for empowering other generations). Despite their positive experience, gender transition also involved a networks restriction, and, in some situations, they considered their rights were not respect. Presenting themselves with pride allows them to overcome the stigmatization of others as a barrier to participate. Boomers are perceived as a prejudiced generation and, consequently, activists were more comfortable interacting with younger generations (more naturalization, less judgmental). When trying to be included in heteronormative and cisgender contexts that did not consider the specificity of their life trajectories as they age, transgender older adults perceived the invisibility of their realities conditioned their social participation.

Abstract citation ID: igae098.0100

### SOCIAL PARTICIPATION OF OLDER ADULTS AT RISK OF MARGINALIZATION: RESULTS FROM A SCOPING REVIEW OF INTERVENTIONS

Stéphanie Meynet[1], Trang Nguyen[2], Alexia Alaux[1], and Melanie Levasseur[1], *1. Université de Sherbrooke, Sherbrooke, Quebec, Canada, 2. Centre de recherche sur le vieillissement, Sherbrooke, Quebec, Canada*

Considering demographic aging, it is essential to better consider the heterogeneity of older adults in promoting the health of all, especially by fostering social participation of the population at risk of marginalization and social exclusion. Despite vulnerability to isolation and loneliness this population, knowledge about social participation interventions lack integration, and this group is under-represented in studies. This study thus aimed to identify, describe, and integrate knowledge about interventions that foster the social participation of older adults at risk of marginalization and social exclusion. Using 61 keywords in 6 databases, a scoping review identified 24 studies that assessed the influence of interventions on older adults' social participation, isolation, loneliness, social interactions, and social connectivity. While almost half of the interventions were individualized home-based, the others involved collective activities in the community. Most studies focused on older adults having low income, and only two studies documented interventions with older adults from minority groups of different gender identity or sexual orientation. Interpersonal relationships with peers, and free group activities based on older adults' interests and life experience were identified as favoring social participation. Perceived isolation, lack of access to transportation and the diversity of older participants represented challenges to successful interventions. While some initiatives were promising, further studies are needed to better develop and evaluate these interventions including assess their efficacy for this population. Providing integrated knowledge on the current initiatives, this exploratory study is the first step towards better crafted interventions that support healthy aging and more inclusive societies.

Abstract citation ID: igae098.0101

### LONELINESS AMONG LONG-TERM SPOUSAL CAREGIVERS: A GENDER-BASED ANALYSIS USING THE CLSA

Lun Li[1], Andrew Wister[2], and Yeonjung Lee[3], *1. MacEwan University, Edmonton, Alberta, Canada, 2. Simon Fraser University, Vancouver, British Columbia, Canada, 3. Chung-Ang University*

Spousal caregivers tend to undertake the most care for their loved ones. As a result, spousal caregivers also experience worse caregiving outcomes, including loneliness, than other types of caregivers. This study used three waves of data from the Canadian Longitudinal Study on Aging (2011 to 2021), and longitudinal analyses with the Linear mixed model were performed to examine the loneliness (measured by UCLA 3-item loneliness scale) of spousal caregivers over time. A total of 1569 participants were identified as long-term spousal caregivers (849 male and 720 female). The results showed that female spousal caregivers reported both a higher level of loneliness at the beginning and a greater increase of loneliness over time than male spousal caregivers. Besides participants' demographic, social-economic and health-related factors, caregiving hours, social participation, and social support are the key predictors of loneliness. At the same time, female spousal caregivers experience a steeper increase in caregiver hours, and a greater decrease in social participation and social support over time. These disparities in changes over time amplify the negative impacts of caregiving on female spousal caregivers. The findings reveal the greater caregiver burden taken by female spousal caregivers than male ones over time. The study supports future programs and services for female spousal caregivers to manage caregiving tasks better and maintain active social interaction to balance caregiving and social life.

### SESSION 1105 (SYMPOSIUM)

Abstract citation ID: igae098.0102

### SOCIAL ISOLATION AND SOCIAL ENGAGEMENT IN OLDER ADULTS: DETERMINANTS AND INTERVENTIONS

Chair: Jinyu Liu

This symposium brings together five studies to examine determinants of social isolation and social engagement and to explore ways to enhance social engagement and well-being among older Americans. Focusing on marital status, the first study finds that the risk of social isolation, from low to high, is coupled, widowed, divorced, and never married. Further, divorced and never-married men are at particularly high risk of social isolation. The second study is concerned with the co-evolution of loneliness and companionship among residents in assisted living communities. It finds that increased loneliness is associated with decreased companionship ties subsequently, but not vice versa. Using a community-based participatory research approach, the third study identifies issues related to transportation, health, and family support, that influence social re-engagement among older black Americans after the COVID-19 pandemic. The fourth and fifth studies are randomized controlled trials. The fourth study reports that older adults with cognitive impairment perceive decreased feelings of loneliness after participating in an intergenerational engagement program with trained young adults. The fifth study finds that an intervention that trains former dementia caregivers to provide volunteering support to current caregivers is feasible in the Chinese American community. Further, the intervention is effective in reducing the stress of current caregivers and increasing the sense of purpose of former caregivers. Together, the five studies show that the individual, familial, and community contexts shape social isolation and social engagement in later life and suggest that group- and context-specific interventions have promise to improve social well-being of older adults.

Abstract citation ID: igae098.0103

### VOLUNTEERING OF FORMER DEMENTIA CAREGIVERS: A PILOT INTERVENTION STUDY IN THE CHINESE AMERICAN COMMUNITY

Jinyu Liu[1], Yifan Lou[2], and Ethan Siu Leung Cheung[3], *1. Baylor University, Waco, Texas, United States, 2. Virginia Commonwealth University, New Haven, Virginia, United States, 3. University of Utah, Salt Lake City, Utah, United States*

To promote social engagement of elders of color and mental health of caregivers, we developed a three-month peer mentoring program (PMP) to train and supervise older Chinese Americans who used to be family caregivers of people with dementia (former caregivers) to provide weekly one-on-one mentoring to current caregivers in the same ethnic community. This pilot study aims to test the feasibility and preliminary effect of the PMP. A pilot randomized control trial was conducted among 38 current caregivers and 8 former caregivers in New York City. The current caregivers were randomly assigned to the PMP and control groups. Quantitative and qualitative data were collected at baseline, 3-month, and 9-month from former and current caregivers. T-test and difference-in-difference analyses were used to analyze quantitative data. A thematic analysis was conducted to understand former caregivers' volunteering experience. The feasibility of the intervention was high, as indicated by an acceptable retention rate, fidelity, and positive feedback from former caregivers. Compared with the control group, the current caregivers in the PMP group had greater reductions in scores for loneliness, caregiver burden and depressive symptoms. Though former caregivers did not report significant changed depressive symptoms and loneliness after participating in the PMP due to their minimal symptoms at baseline, they highlighted that the intervention enhanced their sense of purpose, fulfillment, growth, and self-efficacy in narrative responses. The results indicated the feasibility and efficacy of empowering existing human resources of former caregivers in the same ethnic community to improve the mental health of Chinese caregivers.

Abstract citation ID: igae098.0104

### MARITAL STATUS DIFFERENCES IN SOCIAL ISOLATION IN LATER LIFE

Lydia Li[1], and Sara McLaughlin[2], *1. University of Michigan, Ann Arbor, Michigan, United States, 2. Miami University, Oxford, Ohio, United States*

Social isolation, characterized by infrequent social contact, is associated with morbidity, mortality, and healthcare costs. Using 9 waves of data from the National Health and Aging Trends Study (NHATS) that has conducted annual interviews with a probability sample of Medicare beneficiaries since 2011, this study examines how social isolation differs by marital status and whether the association between marital status and social isolation varies by gender. The sample includes 7,077 respondents aged 65+. We examine 4 categories of marital status (coupled, widowed, divorced, and never married.) Social isolation is assessed by an established index that uses four domains (living arrangements, close networks, religious service attendance, and participation in other activities) to determine social isolation (yes and no). Covariates include age, gender, race, education, community/facility residence, and time. Multilevel logistic regression is conducted. Results show that the risk of social isolation, from low to high, is coupled, widowed, divorced, and never married. The difference between all pairs, except divorced and unmarried, is statistically significant. The interaction effects of marital status and gender on social isolation are statistically significant [$F (3, 54) = 3.43$, $p=.02$]. Specifically, the probability of social isolation for coupled, widowed, divorced, and never married is.19,.35,.49, and.52 in men and.09,.25,.29, and.32 in women. The findings imply that divorced and never-married men should be prioritized for social isolation reduction interventions.

Abstract citation ID: igae098.0105

### THE CO-EVOLUTION OF LONELINESS AND COMPANIONSHIPS IN AN ASSISTED LIVING COMMUNITY: A SOCIAL NETWORK ANALYSIS

Rebecca Mauldin[1], Jaclyn Kirsh[2], and Kayo Fujimoto[3], *1. University of Texas at Arlington, Arlington, Texas, United States, 2. The University of Texas Arlington, Arlington,*

*Texas, United States, 3. The University of Texas Health Science Center Houston, Houston, Texas, United States*

Loneliness and social isolation are distinct constructs that tend to be moderately correlated. The Evolutionary Theory of Loneliness posits that loneliness can motivate people to increase or improve their social ties. However, interrelated factors including loneliness, desired relationship quality/quantity, actual relationships, and appraisals of existing social networks may affect whether loneliness leads to increased social ties. In assisted living communities (ALCs) most opportunities for socializing with non-family members occur within the community, restricting residents' pool of potential friends and companions. This study investigates the co-evolution of loneliness and companionship ties among older residents in an ALC using three waves of secondary data from the Social Experiences in Assisted Living (SEAL) study. We estimated a stochastic actor-oriented network-behavioral co-evolution model for companionship and loneliness dynamics in a network of 38 ALC residents to understand the reciprocal effects of loneliness and companionships. We found that increased levels of loneliness were significantly associated with a decreased likelihood of subsequently maintaining or adding companionship ties among other residents ($\Theta = -.131$, $p < .01$). Changes in companionship ties were not significantly associated with changes in levels of loneliness. Greater frequency of contact with family members was significantly negatively associated with the likelihood of companionship ties with other residents ($\Theta = -.113$, $p < .05$), but was not significantly associated with levels of loneliness. These findings do not support the hypothesis that increased levels of loneliness lead to maintaining or adding companions in the ALC and have implication for both theory and practice.

Abstract citation ID: igae098.0106

### RE-ENGAGING AFRICAN AMERICAN AND BLACK OLDER ADULTS DURING THE COVID-19 ERA: A FOCUS GROUP STUDY

Rupal Parekh[1], Kelsi Carolan[2], Georgia Powell[3], and Christine Tocchi[2], *1. University of Connecticut, New York, Connecticut, United States, 2. University of Connecticut, Storrs, Connecticut, United States, 3. Powell Consulting, Windsor, Connecticut, United States*

Churches and senior centers are social engagement hubs for older Black and African Americans (BAA) that can positively impact well-being. Although these facilities re-opened after the COVID-19 pandemic, engagement with these facilities has not returned to pre-pandemic levels. Our long-term goal is to develop a behavioral intervention to address the re-engagement needs/concerns of BAA older adults as a means of preventing/reducing depressive symptoms and disability. In the first phase of this work, we used a community-based participatory research approach to determine barriers to, and facilitators of, activity reengagement through the perspectives of leaders of BAA churches and senior centers around Hartford, Connecticut. We conducted four focus groups with key stakeholders (n=4 senior center staff; n=4 senior, center staff; n=5 church staff; n=4 church staff) between May 2023 and July 2023. Using a Framework Method approach participants were asked to define engagement as it relates to senior center and church involvement since re-opening and to quantify, in their own words, the problem of disengagement. Participants were also asked about the effectiveness of online engagement vs. in-person engagement and members experience(s) with social isolation and loneliness. Line-by-line thematic analysis of the transcriptions was used to identify and group meaningful phrases and responses into major themes. Three organizing themes emerged: "transportation" concerns impacting members during COVID-19 pandemic, increased health issues among members; and issues and concerns related to family support. Leaders from churches and senior centers provided valuable insights into the overarching issues affecting re-engagement among older BAA senior center and church members.

Abstract citation ID: igae098.0107

### INTERGENERATIONAL REMINISCENCE ENGAGEMENT TO IMPROVE THE WELL-BEING OF OLDER ADULTS WITH COGNITIVE IMPAIRMENT

Ling Xu[1], Noelle Fields[1], Kathryn Daniel[2], Daisha Cipher[2], and Brooke Troutman[3], *1. The University of Texas at Arlington, Arlington, Texas, United States, 2. The University of Texas Arlington, Arlington, Texas, United States, 3. Air Force Academy, Air Force Academy, Colorado, United States*

Reminiscence and/or digital storytelling (DST) have been recognized as potentially effective intervention strategies for enhancing the well-being of older adults. When combined with an intergenerational engagement, they may also offer significant social and mental health benefits for this population. This study reported the efficacy of an intergenerational reminiscence engagement between trained young adult volunteers and community dwelling older adults living with cognitive impairment. A randomized control trial was used to assess the effects of the intervention. Participants of young and older adults were randomly paired and assigned to Reminiscence ($n = 54$) or sham (Social Wellness, $n = 49$) group. Data were collected at baseline, mid- intervention, at the end of the intervention. Linear mixed models (LMM) were performed to compare the Social Wellness group with the Reminiscence group on levels of loneliness, quality of life, affect, and resilience among the older adult participants. Results from LMM revealed no significant effects for group or time on loneliness, negative affect, and resilience. However, the groups significantly differed on their changes over time in quality of life ($t(101) = -3.06$, $p = .003$). Qualitative interviews with selected dyads showed that weekly intergenerational engagements with young adults decreased the feeling of loneliness among older adults. Results suggest that weekly intergenerational engagements with young adults benefit older adults with quality of life and loneliness. Implications for research and programs on reminiscence and DST in improving the well-being of older adults are discussed.

### SESSION 1110 (SYMPOSIUM)

Abstract citation ID: igae098.0108

### STUDYING LGBT OLDER ADULTS

Chair: Linda Waite
Discussant: Hui Liu

The size of the lesbian, gay, bisexual and transgender (LGBT) population in the U.S. is estimated to be at 9 to 11 million people (about 3.5–4.5%) and is expected to grow in the future.

LGBT populations persistently experience health disparities across multiple physical and mental, which widen over the life course. The study of the older LGBT population would benefit from a nationally-representative, longitudinal study with information on health outcomes but also on the social mechanisms at work that may exacerbate or ameliorate these disparities. The National Academy of Medicine (NAM) has recently called for more targeted research aimed at understanding the sources and consequences of these health disparities, particularly among older LGBT adults. The papers in this session focus on strategies to carry out such as study. O'Muircheartaigh and Schumm describe a novel strategy for recruiting such a sample; Lauderdale and Meyer present recent data mental and physical health in a small pilot study using this strategy; Johns et al. study differences in social characteristics and health for LGBT versus other older adults in two nationally-representative samples; and Azar et al. present international comparisons of these differences in the UK and the US. Together these papers add to our understanding of health and health disparities in older sexual minorities and how we might learn more about them.

Abstract citation ID: igae098.0109

### TESTING A RECRUITMENT STRATEGY FOR A NATIONAL SAMPLE OF OLDER LGBT ADULTS

Phil Schumm, and Colm O'Muircheartaigh, *University of Chicago, Chicago, Illinois, United States*

This presentation describes a recently completed feasibility study (SAMLAP) of procedures to screen and recruit a nationally-representative, probability sample of older LGBT adults, intended to serve as the basis for a large-scale, longitudinal study of their health trajectories as they age. SAMLAP used push-to-web with an ABS sample of 70,000 addresses in order (i) to identify LGBT household members aged 40 or more, and (ii) to follow up in-person with a sample of about 300 identified individuals. The sample was stratified by region, urbanicity, and legislative environment. We conducted several randomized experiments to test the effectiveness of different recruitment strategies, including: 1) varying incentive amounts and timings, 2) including an SAQ with the first web-push mailing, and 3) implementing a last-ditch contact via FedEx. We also varied invitation wording to test the effectiveness of different approaches to increase participation by sexual and gender minorities (SGM). We discuss the effectiveness of these strategies and suggest best practices to recruit a high-quality sample of older LGBT adults. The overall response rate to the screener was 15%. 13% of screened individuals who were over 40 years of age identified as LGBT; TK3% of households containing an age-eligible LBGT individual contained 2 or more eligible people. Of these eligible individuals, 47% were successfully interviewed either in person or by phone on a range of health and related social factors. We conclude that it is possible to identify a high-quality, U.S. national probability sample of older LGBT adults and to recruit them for follow-up interviews.

Abstract citation ID: igae098.0110

### DIFFERENCES IN SOCIAL CHARACTERISTICS AND HEALTH STATUS BY SEXUAL IDENTITY AMONG OLDER ADULTS: COMPARISON OF TWO COHORTS

James Iveniuk[1], Michelle Johns[2], Selena Zhong[2], and Kristen Schilt[3], *1. The National Opinion Research Center, Chicago, Illinois, United States, 2. NORC at the University of Chicago, Chicago, Illinois, United States, 3. University of Chicago, Chicago, Illinois, United States*

Sexual minority women experience poorer social, mental, and physical health than their heterosexual counterparts; yet limited work has been done to describe the scope of these health differences. Challenges to such analyses include a dearth of large-scale national surveys that collect sexual orientation data and small numbers of cases among those studies that do ask. To advance our understanding of the health and wellbeing of sexual minority women, we compare key indicators of health and wellbeing (e.g., diabetes, high blood pressure, asthma, depression, loneliness) by sexual identity in the National Social Life, Health, and Aging Project, a nationally representative study of older adults, and in SAMLAP, a nationally representative sample of older LGBT adults. We employ OLS and logistic regressions to assess how health outcomes differ between sexual minority women and heterosexual women in NSHAP as well as health differences among sexual minority women in SAMLAP. We controlled for age, marital status, race, education, and network size in our models. In NSHAP, we found that sexual minority women had higher depressive symptoms than heterosexual women but there were no other health differences between these two groups. In SAMLAP, bisexual women had higher odds of developing diabetes and more depressive symptoms compared to lesbian women. We will discuss our results within the context of the minority stress model which details the impact of social stigma on the health of sexual minority populations.

Abstract citation ID: igae098.0111

### MENTAL AND PHYSICAL HEALTH CONDITIONS LIMITING ACTIVITIES IN A NATIONAL SAMPLE OF OLDER LGBT ADULTS

Ilan Meyer[1], Lauren Bouton[2], and Diane Lauderdale[3], *1. University of California Los Angeles, Los Angeles, California, United States, 2. University of California Los Angeles School of Law, Los Angeles, California, United States, 3. Public Health Sciences University of Chicago, Chicago, Illinois, United States*

AIMS: Research suggests that the proportion of LGBT older adults experiencing disabilities and limitations in activities may be high compared with cisgender straight older adults, but we know little about these limitations. We aimed to understand the type and reasons for limitations in LGBT older adults. METHODS: Using a national probability sample of 210 LGBT adults 45 years and older we conducted in-person or zoom interviews and asked respondents if they were "limited in activities because of physical, mental, or emotional problems?" We followed with an open-ended question about the reason for endorsing having limitations. RESULTS: 36% of respondents reported 1 or more limitations. We categorized these limitations as related to mental health (17%), mobility (72%), pulmonary conditions (7%), sensory/cognition (8%), or cardiovascular including diabetes and obesity (8%). Overall limitations were more prevalent among respondents 65 years or older vs. 45-64 years old (47% vs. 30%), women vs. men (48% vs. 28%), White vs. POC (38% vs. 31%), bisexual and other sexual identities vs. LG (50% vs. 31%), and people with incomes below $49K vs. $50k or higher (48% vs. 32%). Reporting a chronic disease diagnosis (e.g., asthma, hypertension) vs. none was not associated with prevalence of limitations (37% vs. 36%) but self-rated poor/fair health was (58% vs. 31%). In age-adjusted analyses, being a woman, identifying as other than LG, lower income, and reporting poor/fair health were all associated with having limitations (RRR = 2.7, 3.3, 2.1, and 3.5, respectively). We will discuss predictors of observed high prevalence of limitations.

Abstract citation ID: igae098.0112

### SEXUAL ORIENTATION AND AGING: A US–ENGLAND COMPARATIVE STUDY OF SAME-SEX AND DIFFERENT-SEX HOUSEHOLDS

Ariel Azar[1], Laia Becares[2], and Dylan Kneale[3], *1. Purdue University, West Lafayette, Indiana, United States, 2. King's College London, London, England, United Kingdom, 3. University College London, london, England, United Kingdom*

This study compares the health, income, and housing situation of same-sex and opposite-sex households in the United States and England. The research uses data from the English Longitudinal Study of Ageing (ELSA) and the U.S. Health and Retirement Study (HRS) to examine how these inequalities change over time. The study shows that individuals in same-sex households in both countries face distinct challenges in chronic health conditions, mental health status, and economic well-being. We argue that these challenges are due to the interaction between life transitions and varying policy, legislative, and societal attitudes toward same-sex relationships. For example, disparities in housing tenure and retirement income, which are more pronounced in the U.S. than in England, reflect these dynamic, divergent social and policy landscapes. The study takes into account relationship quality and satisfaction, gender, age, urban and rural living environments, and employment status. The findings highlight the cumulative impact of national contexts on the experiences of aging individuals in same-sex relationships. The study acknowledges data limitations due to the underrepresentation of sexual minorities in large nationally representative studies. However, the insights gained are essential for understanding how different policy contexts across the life course can shape the specific challenges and inequalities faced by this demographic group. The study emphasizes the need for informed policies and interventions.

### SESSION 1115 (PAPER)

### TECHNOLOGY AND AGING (BSS)

Abstract citation ID: igae098.0113

### MESSAGE FRAMING AND INTENTION TO USE WEARABLE ROBOTS AMONG OLDER ADULTS: A PARALLEL AND MODERATED MEDIATION ANALYSIS

Clio Yuen Man Cheng, and Vivian Weiqun Lou, *The University of Hong Kong, Hong Kong, China (People's Republic)*

Little is known about the intention to use wearable robots among older adults, an emerging assistive technology to compensate for age-related functional decline. Informed by the prospect theory and senior technology acceptance model, we examined the effect of persuasive health messages in enhancing technology acceptance of wearable robots among older adults. A 2 (message framing: gain versus loss) x 2 (temporal framing: distal versus proximal) randomized experiment was conducted with 176 older adults aged 50 to 74 in Hong Kong. Non-parametric bootstrapping analyses were used to test the mediation model of attitudinal beliefs, performance, and perceived health benefits as parallel mediators of the relationship between message framing and behavioral intention to use wearable robots. Additionally, a moderated mediation model where the indirect effects of temporal framing are explored on perceived benefits in the mediation model. The results showed that gain-framed messages enhanced behavioral intention, through increased perceived health benefits (IE = -.242, 95% CI: LL = -.435 to UL = -.085, p <.01). Findings also provided evidence on the moderating role of temporal framing. Specifically, we observed that, for participants who were exposed to distally/gain-framed conditions, the IE is statistically significant (IE = -.301, 95% CI: LL = -.601 to UL = -.050, p =.032). For participants who were exposed to proximally/gain-framed conditions, the IE is higher and statistically significant (IE = -.432, 95% CI: LL = -.786 to UL = -.157, p =.007). This study suggests using proximally/gained-framed messages to increase wearable robots' acceptance is promising.

Abstract citation ID: igae098.0114

### INTERNET UTILIZATION AMONG OLDER ADULTS WITH VISUAL IMPAIRMENT

Rachel Mumby[1], Philippa Clarke[2], Yunshu Zhou[2], David Rein[3], and Joshua Ehrlich[2], *1. University of Michigan, Lansing, Michigan, United States, 2. University of Michigan, Ann Arbor, Michigan, United States, 3. NORC at the University of Chicago, Chicago, Illinois, United States*

Internet is increasingly used by older adults for communication, socialization, household tasks, and health information/care. Although the internet is most often accessed by visual means, little is known about whether vision impairment is associated with internet use in older adults. To address this question, we used nationally representative data from round 12 (collected in 2022) of the National Health and Aging Trends Study (NHATS), which included measures of visual function (distance visual acuity, near visual acuity, contrast sensitivity) and a series of questions about computer and internet use among US adults aged 65 and older. Data were analyzed using survey weighted multivariable logistic models adjusted for socioeconomic, demographic, and health-related covariates. There were 5,220 participants included (weighted: 45.6% male, 79.9% non-Hispanic White). Among those with any type of vision impairment, there were significantly lower odds of internet use (OR=0.59, 95% CI 0.47-0.73), email and texting (OR=0.52, 95% CI 0.40-0.68), online household activities (e.g., banking, grocery shopping; OR=0.69, 95% CI 0.57-0.84), online social networking (OR=0.64), 95% CI 0.52-0.80), and online health activities (e.g., prescription refills, telehealth; OR=0.64, 95% CI 0.50-0.82) compared to those without any vision impairment. Most of these associations were driven by near vision impairment. Although internet use has increased among older adults overall, there is a gap in utilization between older adults based on vision impairment status. Findings suggest a need for interventions to make the internet more accessible to older adults with impaired vision.

Abstract citation ID: igae098.0115

**FEASIBILITY AND ACCEPTABILITY OF IMPLEMENTING AN AI-ENABLED SERVICE ROBOT IN LONG-TERM CARE**

Lillian Hung, Haopu (Lily) Ren, Karen Lok Yi Wong, Katharine Davies, and Arisa Kinugawa, *University of British Columbia, Vancouver, British Columbia, Canada*

In long-term care (LTC), staff members are responsible for the complex needs of people with disabilities. This task can be enhanced by integrating service robots equipped with Artificial Intelligence (AI). These robots offer personalized service for residents and empower the staff with efficient support. Despite growing interest, the implementation of service robots in LTC remains under-investigated. This study examines the feasibility and acceptability of implementing a service robot, named Aether, in a Canadian LTC home, guided by the underpinnings of Collaborative Action Research. We included interdisciplinary staff in pre- and post-intervention focus groups and conducted conversational interviews with residents, staff members, and managers. Consolidated Framework for Implementation Research (CFIR) informed our implementation, data collection and analysis. We identified key facilitators (staff engagement and training) and barriers (environmental dynamics and resource limitations). Our results underscore the imperative of structural support at micro-, meso- and macro-levels for staff in LTC to implement technology effectively. This study contributes valuable insights into the future development and deployment of AI-enabled service robots in LTC.

Abstract citation ID: igae098.0116

**UNDERSTANDING OLDER ADULTS' DIGITAL EXPERIENCES DURING AND AFTER THE PANDEMIC: A MIXED-METHODS STUDY**

Zhan Yu[1], Xiayu Chen[2], Qingwen Xu[3], and Chenxi Huang[1], *1. East China Normal University, Shanghai, Shanghai, China (People's Republic), 2. University of Illinois Urbana-Champaign, Champaign, Illinois, United States, 3. New York University, New York, New York, United States*

During the COVID-19 pandemic, China adopted the zero-case policy and made full use of digital technology, such as mandatory QR code using; consequently, older adults were forced into the experiences of digital technology. This study aims to explore the psychological and behavioral effects of policy actions on older Chinese, particularly to address the long-term effects on their technology use. We employed a sequential mixed-methods approach. Using data from the CHRLS, generalized estimating equations was performed and the study found a significant increase in internet use among older adults in 2020 compared to 2015 (OR = 12.04, p <.001). Then three focus group interviews were conducted with older adults in Shanghai. Content analysis reveals three themes: the feeling of being "compelled" to use digital technologies during the pandemic to meet daily basic needs and maintain social connections; a perception of "digital invasion" in the post-pandemic era; and an increased consciousness of the importance of self-efficacy for continued technology use. Further codes network analysis and cross-group comparisons show that both positive and negative experiences of digital use during the pandemic related to the increased use after the pandemic; prior community technology education programs and support received resulted in varied experiences and perceptions of digital use. This study offers insights into older adults' adaptations to technology, which would help inform policies and practices for addressing the challenges of aging and technology use. It also highlights the need for tailored strategies that recognize the diverse needs of older adults in the digital age.

Abstract citation ID: igae098.0117

**DIFFERENCES IN BEHAVIOR CAPTURE USING ELECTRONIC MEDICAL RECORD OF NURSING HOMES ACROSS RESIDENT RACE AND ETHNICITY**

Hyunkyung Yun[1], and Ellen McCreedy[2], *1. Brown University, Providence, Rhode Island, United States, 2. Veterans' Administration, Providence, Rhode Island, United States*

Agitated behaviors are under-reported in the Minimum Data Set (MDS), a standardized assessment of nursing home (NH) residents, which poses challenges for pragmatic trialists hoping to use routinely collected data to assess study eligibility and outcomes. Our recent study found that using Interventions to Reduce Acute Care Transfers (eINTERACT) and physician orders from EMR increases behavior identification over the MDS alone by 25.7% relative increase (14.8% (SD 35.6) by MDS only vs. 18.6% (SD 38.9) by supplemented by EMR) among NH long-stay residents with dementia. We extended that work to consider variations in behavioral capture by race and ethnicity. We used MDS and EMR from one large, non-profit corporation serving 19,705 residents from 312 NHs between January 2020 and August 2022. We found Black residents were more likely (11.2% vs. 10.5% in total sample) to be in NHs regularly using EMR, defined as the top quartile NHs based on the percent of residents in the NH with eINTERACT form; Latinx residents were less likely to be in NHs regularly using EMR (3.5% vs. 6.0% in total sample). Among NHs regularly using EMR (n=5,192 residents, 81 NHs), Black residents were less likely to have behaviors documented in both MDS (Blacks 9.7% vs. Whites 16.5%) and EMR (eINTERACT: Blacks 1.9% vs. Whites 3.7%; Orders: Blacks 2.6% vs. Whites 3.9%) despite higher share of moderate/severe cognitive impairment (63.1% vs. Whites 60.0%) and less antipsychotic use (16.4% vs. Whites 26.2%). This has implications for inclusion in pragmatic trials and receipt of possibly advantageous treatments.

### SESSION 1120 (SYMPOSIUM)

Abstract citation ID: igae098.0118

**THE ASSOCIATIONS BETWEEN THE SOCIAL NETWORK, THE CARE NETWORK, AND THE WELL-BEING OF OLDER ADULTS IN THE NETHERLANDS**

Chair: Marjolein Broese Van Groenou
Discussant: Norah Keating

The increased demand for long-term care (LTC) and the drastic reforms of the LTC systems in western societies create uncertainty among families. It raises the question of whether and how families can provide appropriate LTC for their older relatives and maintain the wellbeing of the care recipient (CR). One solution is for core family members (partners and children) to organize care together with other informal and formal caregivers as much as possible in large and diverse care networks. Well functioning care networks could then possibly contribute to the wellbeing of the CR. The first contribution of this symposium focuses on the informal care potential of the older care recipient and investigates which characteristics of the social network are associated with a care network with many informal helpers. The second paper examines how different types of (informal) care networks contribute to CR's psychological well-being, and to what extent loneliness, perceived control and care sufficiency play a role in this. The third paper links the theoretical models of both papers and investigates whether and how the associations between the social network, the care network and wellbeing vary in different societal and LTC contexts. All contributions use the Longitudinal Aging Study Amsterdam (LASA), a long-term study into the functioning and care use of older people in the Netherlands. Data are used from ten three-yearly waves between 1992 and 2022. The discussion focuses on which directions the studies provide for improving long-term care for the older care recipients and their informal caregivers.

Abstract citation ID: igae098.0119

### THE ASSOCIATIONS BETWEEN SOCIAL NETWORKS, CARE NETWORKS, AND WELL-BEING COMPARED IN DIFFERENT TIME PERIODS

Marjolein Broese Van Groenou[1], Silvia Klokgieters[1], Jens Abbing[1], and Joukje Swinkels[2], *1. Vrije Universiteit Amsterdam, Amsterdam, Noord-Holland, Netherlands, 2. .VU Amsterdam, Amsterdam, Noord-Holland, Netherlands*

Objectives A well-functioning LTC network is important for the psychological well-being of older CRs. Yet, the use of formal and informal care depends on the allocation of LTC which may vary over historic time. This raises the question whether the links between social networks, care networks and psychological wellbeing differ between various LTC policy contexts in the Netherlands. Methods Data are from 65-plus in four waves of the LASA study that are ten years apart: 1992 (N=1794), 2002 (N=1284), 2012 (N=1183), 2022 (1011). Five care network types were considered (no care, partner care, informal, formal and privately paid care). Per wave regression analyses are used to analyze the associations between social networks, care networks and depressive symptoms, and effects are compared across waves using multigroup analysis. Results Preliminary results show that the prevalence of care network types differ over time, with a decrease in partner and informal care types and stability in formal and privately paid care types. Many of the effects appeared stable over time: in all waves social network composition contributed to informal care use; and partner care reduced depressive symptoms where formal care increased this. Care network types that fostered relatedness and sufficient care were important for wellbeing in all waves. Discussion Although the four waves represent varying LTC contexts, the findings support the robustness of the associations between social networks, care networks and wellbeing. Moreover, those who lack a partner and a social network risk dependency on formal care resulting in lower wellbeing in all times.

Abstract citation ID: igae098.0120

### THE ROLE OF SOCIAL NETWORK CHARACTERISTICS IN LONG-TERM CARE NETWORKS AMONG OLDER ADULTS IN THE NETHERLANDS

Jens Abbing[1], Joukje Swinkels[2], and Marjolein Broese Van Groenou[1], *1. Vrije Universiteit Amsterdam, Amsterdam, Noord-Holland, Netherlands, 2. VU Amsterdam, Amsterdam, Noord-Holland, Netherlands*

Objectives The increasing demand for long-term care (LTC) among western societies calls for a better understanding of how older adults build LTC networks consisting of informal and formal caregivers. Having supportive relationships is generally associated with receiving informal care, yet how specific characteristics of individuals' social networks relate to LTC networks is not fully understood. Therefore, the present study investigated how the size, composition, and relationship quality of individuals' social network influences what type of LTC network they have. Methods Data was used from the Longitudinal Aging Study Amsterdam that measured LTC use and social network characteristics of older adults (average age=70.88) in three-year intervals from 1992 to 2022. The sample contained 18,252 complete observations from 4,878 participants. Results Using multilevel latent class analysis, six LTC network types characterized by the main caregiver (co-residents, children, relatives, non-kin, publicly paid care and privately paid care) were identified. Cluster-robust multinomial logistic regression analyses revealed that social network size, composition and relationship quality all were associated with membership in the various informal care network types. In particular having a partner, and children and relatives living in proximity and/or with strong bonds contributed to an informal LTC network. Discussion Network composition was most important while the impact of relationship quality was relatively small. This suggests that older adults may refrain from using the full potential of their social network to build informal care networks, possibly because they prefer not to involve relatives or non-kin into caregiving or prefer formal or privately paid care.

Abstract citation ID: igae098.0121

### WHY IS PSYCHOLOGICAL WELL-BEING ASSOCIATED WITH THE COMPOSITION OF OLDER ADULT'S CARE NETWORK?

Joukje Swinkels[1], Jens Abbing[2], and Marjolein Broese Van Groenou[2], *1. VU Amsterdam, Amsterdam, Noord-Holland, Netherlands, 2. Vrije Universiteit Amsterdam, Amsterdam, Noord-Holland, Netherlands*

Objectives Older care recipients differ in types of care used: some receive mostly partner or other types of informal care, whereas others use mostly formal or privately paid care. This is mainly due to variation in health impairment, but it may also add to differences in psychological wellbeing. Applying Self-Determination Theory (SDT) to the care context, we hypothesize that care network types differ to the extent that they foster feelings of relatedness, autonomy and competence, resulting in different levels of psychological well-being. Methods Data are from ten three-yearly waves of the Longitudinal Aging Study Amsterdam (N= 18,434 observations from 4,837 older Dutch adults, from 1992 until 2022). Five care network types are created a priori composed of no care, (mainly) partner, informal, formal or privately paid care. Mixed-model regression analysis of depressive symptoms as measure of well-being is applied on care network type and loneliness (indicator of relatedness), mastery (autonomy) and care sufficiency (competence). Hybrid models disentangle between and within subjects effects. Results Receiving no care or care from a partner care network contributes the most to psychological wellbeing. Those with a formal care network type are worse off. Loneliness and care sufficiency partly explain these differences. Results of between and within effects are comparable. Discussion The findings support that applying SDT concepts of basic needs helps to understand how care impact psychological wellbeing. Results imply that decreasing loneliness by supporting informal care and increasing care sufficiency by improving the quality of formal care facilitates the functioning of care networks.

### SESSION 1125 (SYMPOSIUM)

Abstract citation ID: igae098.0122

### THE MEN IN DEMENTIA CAREGIVING: UNDERSTANDING MEN'S EXPERIENCES

Chair: Allison Lindauer
Discussant: Kenneth Hepburn

About 30% of caregivers for family members with dementia are men, and their numbers are rising with the increasing prevalence of dementia. However, there is limited information in the extant literature about the number of men providing care to family members with dementia, their experiences, and their engagement in caregiving research. What is known is concerning. Men, like women, experience burden in the caregiving role and are at higher risk for health problems than non-caregivers. In addition, men may experience role confusion and feel unprepared to assume the caregiving role. Finally, little is known about what motivates men to participate in dementia caregiving research. Here we describe men's experiences from three groups: Black American men, men caring for family members with primary progressive aphasia, and men caring for family members with advanced dementia. Along with quantitative and qualitative data, we will present findings from focus groups which center on men's motivations for joining research studies, perceived benefits of research participation, and men's concerns about research, including biospecimen collection. Unique insights and needs from each group will be addressed, along with strategies to promote recruitment and retention of men in caregiving research.

Abstract citation ID: igae098.0123

### CLINICAL APPROACHES TO SUPPORT MALE DEMENTIA FAMILY CAREGIVERS

Carolyn Clevenger, *Emory University, Atlanta, Georgia, United States*

Integrated Memory Care (IMC) is a primary care practice for people living with dementia and their family caregivers. The practice integrates geriatric medicine, dementia specialty care/cognitive neurology, and palliative care along with caregiver services including support groups, classes, and psychotherapy for individuals and families. Finally, the practice hosts various opportunities to participate in caregiving research. Since 2015, the practice has treated over 2500 people living with dementia (PLWD) and supported their family caregivers. The mean age of patients is 78.6 years, the majority (66%) are female, and 42% identify as a racial or ethnic minoritized group. Caregiver relationship to patients (PLWD) is divided evenly between spouse (48%) and adult child (48%) and their mean age is 65.3 years. Men serving as primary family caregiver differ from their female counterparts in choice of clinical services, engagement in caregiver-specific programs, and participation in research. Successful marketing approaches include emphasis on caregiver as hero archetype, a clear message of the return-on-investment of services and activities. This presentation will describe the experience of supporting and engaging men as dementia family caregivers using aggregated data from multiple pilot studies as well as direct input from current active caregivers and IMC program staff.

Abstract citation ID: igae098.0124

### CARING FOR PERSONS LIVING WITH PRIMARY PROGRESSIVE APHASIA: AN INVESTIGATION OF THE MALE EXPERIENCE

Lauren Dowden[1], Darby Morhardt[2], and Kaitlyn Lucca[2], *1. Northwestern University Feinberg School of Medicine, Chicago, Illinois, United States, 2. Northwestern University, Evanston, Illinois, United States*

Primary progressive aphasia (PPA) is a clinical syndrome that begins with language difficulties at symptom onset and for the initial phases of the disease. Multiple domains are affected over time, including behavior and personality changes. There are heterogeneous neuropathologic causes; namely, frontotemporal lobar degeneration or Alzheimer's disease. While there is evidence that women comprise the majority of caregivers for persons living with dementia, little is known regarding sex and gender differences in the experience of caregiving. Studies on caregivers in persons with PPA are rare and none have explored gender differences in the PPA caregiving role. This study explores the male experience of caring for persons living with PPA analyzing in depth interviews from a sample of 17 male caregiving spouses who attend a monthly PPA care partner support group. Open-ended questions explore coping strategies, approaches to the caregiving role, help-seeking behaviors, use of services, and overall caregiver experience. This analysis describes the male experience of caregiving for persons with PPA, highlights their unique needs over the illness course and provides a nuanced understanding of the gendered caregiver role within the context of a specific clinical profile versus broader dementia categories.

Abstract citation ID: igae098.0125

### EXPLORING THE UNIQUE NEEDS AND EXPERIENCES OF BLACK MALE CAREGIVERS

Robert Turner, *The George Washington University School of Medicine and Health Sciences, Washington, District of Columbia, United States*

An emerging area of public health concern for policymakers, caregivers and families, and health service workers is the growing number of older adults with Alzheimer's disease and related dementia (ADRD). The number of older Black Americans with ADRD who will require some care is projected to triple in the upcoming decade. According to survey data, 30% to 40% of informal caregivers are elderly husbands often caring for spouses experiencing dementia; however, this estimation may be low simply because many men – especially Black American men – do not formally identify as caregivers. Previous research also suggests that Black American men often feel unprepared as caregivers, are disinclined to discuss this role, and rely on informal support resources due to hesitation or unawareness. Although Black Americans are at greater risk of caregiver burden (CB), the body of research that focuses on adult Black American men as primary caregivers of a person with dementia (PWD) is sparse. Knowledge about dementia CB in this understudied sub-population is critical for several reasons, including the rapid projected growth that puts their health and well-being at risk. The Black Male Dementia Caregiver Burden Study examines the impact of dementia CB on the cognitive function and physiological health of this underrepresented population. Additionally, this project incorporates focus group and in-depth interview data that explores Black males' unique needs and experiences with caregiving while addressing their concerns about participating in caregiver research, including collecting biospecimen samples (e.g., urine, blood, saliva).

Abstract citation ID: igae098.0126

### CARING FOR FAMILY MEMBERS WITH ADVANCED DEMENTIA: MEN'S EXPERIENCES

Aimee Mooney[1], Charles Boardman[1], Sarah Gothard[2], Emily Tupper[2], Christina Zonker[2], and Allison Lindauer[2], *1. Oregon Health & Science University, Portland, Oregon, United States, 2. Oregon Alzheimer's Disease Research Center, Oregon Health & Science University, Portland, Oregon, United States*

About one-third of dementia family caregivers are men, and this number is increasing due to societal changes, shifting family patterns, and financial drivers. Caregiving men are an understudied group, representing about 30% of participants in caregiving research. A better understanding of men in caregiving can facilitate personalization of interventions, increase research participation, and sustain retention. Here we describe a subgroup of men who participated in the intervention study, Tele-STELLA (Support via Technology: Living and Learning with Advancing dementia, R01AG067546). Tele-STELLA is a multi-component, psychoeducational intervention designed to facilitate effective management of behavioral symptoms in those with advanced dementia. Of the 150 Tele-STELLA caregivers in the sample, 37 (25%) were men. Burden was measured using the Revised Memory and Behavior Problems Checklist reactivity subscale. Using a two-tailed t test at 0.1 $\alpha$, we found that there was significant improvement in reactivity scores (burden) for the men that completed the intervention (n=27). Depression did not change over the 8-week intervention period despite disease progression in their family members with dementia. Ten Tele-STELLA men participated in focus groups post-intervention. Employing thematic analysis, three important themes emerged. First, the men signed up for the study because it was recommended by their health care professionals. Second, men found the solution-focused curriculum increased their sense of "capability" as caregivers, and third, Tele-STELLA provided a sense of camaraderie with their caregiving men peers. Future studies may focus recruitment strategies on informing health care providers, tailor interventions to be solution-focused, and foster communication between men to build solidarity.

### SESSION 1130 (SYMPOSIUM)

Abstract citation ID: igae098.0127

### THE PROMISE OF FITNESS, HEALTH, AND DIGNITY FOR OLDER ADULTS: A SHOWCASE OF EARLY CAREER RESEARCH IN THE OAIC NETWORK

Chair: Karen Bandeen-Roche

The NIH-funded Older Americans Independence Centers (OAICs) aim to produce scientific knowledge allowing older adults to maintain or restore their independence. This national network has a key mission to develop the next generation of investigators and leaders in aging research. Individual OAICs achieve this mission through pilot funding and career development grants, activities and mentorship. The OAIC Coordinating Center additionally provides funding, programming, and an Early Career Faculty Working Group dedicated to fostering emerging scholars. This proposed first-annual GSA showcase features three OAIC-affiliated early career faculty nominated by the Early Career Faculty Working Group based on their receipt of national multi-center pilot awards or invited presentation of their work at monthly OAIC Directors virtual meetings or at the OAIC National Meeting. The talks selected for this symposium illustrate the highly interdisciplinary nature of the OAICs. Juan Pablo Palavicini (UTHSA) will present cross-species pilot work with Andrew Murton (UTMB) to evaluate whether myriocin may inhibit ceramide synthesis, and ultimately preserve muscle and insulin sensitivity in obese older adults. Emma Barber (Northwestern) will present work demonstrating association of heart and respiratory parameters with frailty—and of preoperative physical activity with post-operative complications—in older gynecologic oncology patients, laying groundwork for interventions. Cameron Gettel (Yale) will present findings documenting substantial emergency department use and expenditures by older adults in the last 180 days of life, using Medicare Current Beneficiary Survey data. The symposium concludes with a panel and open discussion addressing aging research career and science topics.

Abstract citation ID: igae098.0128

### REVERSING INSULIN RESISTANCE AND SARCOPENIA BY LOWERING TOXIC CERAMIDES IN OBESE NONHUMAN PRIMATES

Juan Palavicini[1], and Andrew Murton[2], *1. UT Health SA, San Antonio, Texas, United States, 2. UTMB Health, Galveston, Texas, United States*

Obese and old individuals are at heightened risk of developing insulin resistance, sarcopenia, and frailty. Specifically, we found that older obese men experience a diminished capacity to synthesize new muscle proteins in response to exogenous amino acids, a process pivotal for the maintenance of muscle mass and quality. Our ability to recreate this "anabolic resistance" to nutrition in lean young individuals through the acute infusion of lipid to induce obese-like ectopic lipid accumulation, suggests that the accumulation of lipid represents a pivotal mechanism by which the responsiveness of muscle to dietary amino acids diminishes with obesity. Ceramides are a class of lipids that have been associated with multiple negative metabolic effects and hallmarks of aging including insulin resistance, apoptosis, mitochondria disfunction, senescence, and inflammation. Using a variety of lipidomics approaches in combination with mouse models of obesity and diabetes, we have consistently found that ceramides accumulate in insulin sensitive organs, including muscle. Given prior evidence that protein synthesis is inhibited in human muscle cells subjected to ceramide treatment, which occurs in concert with the inhibition of mTOR signaling, a key driver of protein translation, we hypothesized that ceramides are major drivers of both insulin resistance and frailty. Here we will report how myriocin, an inhibitor of ceramide synthesis, restores insulin resistance in obese and diabetic mice. Findings from our ongoing pilot study in non-human primates to examine if myriocin improves insulin resistance and the anabolic response of muscle to diet in obese marmosets also will be reported.

Abstract citation ID: igae098.0129

### PHYSICAL ACTIVITY, HEART RATE VARIABILITY, FRAILTY, AND COMPLICATIONS IN GYNECOLOGIC ONCOLOGY

Emma Barber[1], Olivia Foley[2], Connor Wang[2], Allison Grubbs[3], and Karl Bilimoria[4], *1. Northwestern University, Chicago, Illinois, United States, 2. Northwestern University, Evanston, Illinois, United States, 3. Rush University, Chicago, Illinois, United States, 4. University of Indiana, Indianapolis, Indiana, United States*

Gynecologic oncology patients undergo complex operations with high rates of postoperative complication. Frailty is consistently associated with complication but is not routinely measured in clinical practice. This study aimed to examine the association of physiologic and activity data with frailty and postoperative complications in presurgical gynecologic oncology patients. We performed a prospective cohort study of gynecologic oncology surgery patients who wore wearable actigraphy rings before and after surgery from 03/2021-11/2023. Exposures were physiologic data (heart and respiratory rates, heart rate variability), preoperative activity intensity and level. Primary outcomes included frailty (Pajewski frailty score) and 30-day complications. Multivariable models were adjusted for age, cancer and surgery type. We enrolled 96 patients and 87 met inclusion criteria. 41.3% were not frail, 44% were pre-frail and 14.7% were frail. Average heart rate and respiratory rate differed between frail and non-frail patients (HR 78.6 vs 68.3bpm, aOR=1.10,95%CI1.01-1.20; RR 18.6 vs 16.7b/m,aOR=2.1,95%CI1.20–3.55). Heart rate variability (RMSSD) also differed (24 vs 30.9ms,aOR=0.81,95%CI0.68-0.97). Preoperative physical activity was also associated with postoperative complications. Overall, 29.9% experienced a complication and higher activity was associated with fewer complications: 500-1000 MET-mins (OR=0.26,95%CI=0.07-0.92) and >1000 MET-mins (OR=0.25,95%CI=0.07-0.94) vs <500 MET-mins; ≥8000 steps (OR=0.25,95%CI=0.08-0.73) vs <8000 steps. In conclusion, physiologic data and activity levels differ in frail compared to not frail patients independent of age. Wearable devices may be useful in the preoperative assessment of frailty. Furthermore, higher preoperative activity levels are associated with fewer postoperative complications. Physical activity may be a modifiable risk factor for adverse postoperative outcomes.

Abstract citation ID: igae098.0130

### EMERGENCY DEPARTMENT USE AND HEALTH CARE EXPENDITURES AMONG OLDER ADULTS AT END-OF-LIFE

Cameron Gettel[1], Courtney Kitchen[1], Craig Rothenberg[1], Yuxiao Song[1], Arjun Venkatesh[1], and Susan Hastings[2], *1. Yale University School of Medicine, New Haven, Connecticut, United States, 2. Durham VA, Durham, North Carolina, United States*

Emergency department (ED) visits at end-of-life (EOL) may cause financial strain to older adults and serve as a marker of inadequate access to community services and health care. We sought to examine EOL ED use, total health care spending, and out-of-pocket (OOP) spending among Medicare beneficiaries. Using data from the Medicare Current Beneficiary Survey, we conducted a pooled cross-sectional analysis including Medicare beneficiaries aged 65 years and older with a date of death between July 1, 2015 and December 31, 2021. Our primary outcomes were ED visits, total healthcare spending, and OOP healthcare spending in the 7, 30, 90, and 180 days preceding death. We compared total and OOP healthcare costs for those with at least 1 ED visit prior to death vs. without any ED visits within each timeframe and estimated a series of zero-inflated negative binomial regression models to identify associations between key beneficiary characteristics and the primary outcomes. Among 3,812 older adult decedents, 610 (16%), 1,207 (31.7%), 1,582 (41.5%), and 1,787 (46.9%) Medicare beneficiaries, respectively, had ED visits in the final 7, 30, 90, and 180 days of life. For Medicare beneficiaries with at least one ED visit in the final 30 days of life, the median total and OOP costs were respectively $12,366 and $160, compared respectively to $160 and $0 for those without any ED visits (P <0.001 for both comparisons). These estimates of ED use and costs during the terminal phase of life highlight the importance of appropriate and timely EOL care.

### SESSION 1135 (SYMPOSIUM)

Abstract citation ID: igae098.0131

### THROUGH THE LENS OF THEORY: APPROACHES TO BEHAVIORAL INTERVENTION OPTIMIZATION AND EVALUATION IN AGING ADULTS

Chair: Ashley Curtis
Discussant: Christina McCrae

A shortage of geriatric mental health providers, limited efficacy of pharmacological agents for cognition, sleep and associated functioning, as well as polypharmacy-related side effects, has prompted research on behavioral interventions.

Several behavioral interventions show promise for improving cognition and associated functioning, but optimization of treatments according to target end-user needs and characteristics is needed. This symposium describes psychosocial theory-based approaches for optimization and evaluation of behavioral interventions for aging adults. Dr. Ashley Curtis (Chair) will introduce the session. Paper 1 applies a biopsychosocial model to evaluation of cognitive training benefits and describes how intervention expectations and game engagement impact training benefits in middle-aged/older adults. Paper 2 discusses an iterative optimization process for a multi-modal positive psychiatry intervention for older adults that targets psychological wellness, health, and resilience. Paper 3 describes a user-centered mixed-methods evaluation of needs/preferences of grandparents raising grandchildren for behavioral sleep treatment and proposes recommendations for optimization of cognitive behavioral therapy for insomnia (CBT-I). Paper 4 describes mixed-methods optimization of web-based CBT-I for rural dementia caregivers and presents preliminary efficacy for sleep, arousal, cognition, and associated outcomes. Dr. Christina McCrae (Discussant) will integrate findings and discuss areas for future investigation. In sum, this symposium applies a theoretical lens to the optimization and evaluation of behavioral interventions for improving cognition, sleep and associated functioning in mid-to-late life. Thus, this session will provide recommendations for precision medicine-based tailoring of digital/multi-modal interventions that can be easily disseminated (online/in the community), to maximize improvement of psychological and behavioral wellbeing of aging adults.

Abstract citation ID: igae098.0132

### EXPECTATION AND ENGAGEMENT IMPACT COGNITIVE TRAINING BENEFITS IN MID-TO-LATE LIFE

Ashley Curtis[1], Amy Costa[1], Madison Musich[2], Anthony Schmiedeler[2], Kenda Eberhardt[3], and Christina McCrae[1], *1. University of South Florida, Tampa, Florida, United States, 2. University of Missouri, Columbia, Missouri, United States, 3. University of Kansas Medical Center, Overland Park, Kansas, United States*

Lack of pharmacological agents for cognition has prompted research on behavioral treatments such as cognitive training (CT). Evidence regarding CT benefit is mixed. In accordance with the biopsychosocial model, we examined whether individual characteristics such as expectation for improvement and engagement levels impact CT benefits in middle-aged/older adults. In Study 1, older adults with insomnia (N=24) completed 6-week CT (Nintendo DS-Big Brain Academy; 60 mins/day, 3x/week) and waitlist control (WLC) in a cross-over design. At baseline and post-CT/post-WLC, participants completed NIH toolbox-cognitive tasks. In Study 2, middle-aged adults with generalized anxiety disorder (GAD; N=17) completed 8-week CT (Cognifit; 45 mins 3x/week). At baseline/post-CT, participants completed State-Trait Anxiety Inventory, Cognitive Failures Questionnaire, and computerized cognitive tasks. At mid-point, participants evaluated expectations of cognitive/anxiety improvement (0:none-100:extreme improvement). At post-treatment for both studies, participants completed the Game Engagement Questionnaire. Bivariate correlations evaluated associations between game engagement and improvement expectations on post-training proportional changes in cognitive (Study 1&2) and anxiety (Study 2) outcomes. In Study 1, greater perceived competence was associated with greater CT-related inhibition improvement (r=-.57, p=.008). In Study 2, greater expected level of cognitive improvement (r=-.58, p=.047) and sensory game engagement (r=-.730, p=.002) were associated with less CT-related working memory improvement. Greater CT perceived skill/competence was associated with more anxiety reduction (r=.59, p=.02). A biopsychosocial approach to intervention evaluation may be useful in CT trials in aging adults with insomnia and GAD. Psychological user characteristics such as expectation for improvement and perceived competence, as well as biological sensory engagement impact CT benefits.

Abstract citation ID: igae098.0133

### IMPROVING WELL-BEING IN OLDER ADULTS: USING AN ITERATIVE PROCESS TO OPTIMIZE THE DESIGN OF A GROUP INTERVENTION

Erin Cassidy-Eagle[1], Christine Gould[2], Feng Vankee Lin[1], Maryam Makowski[1], and Dolores Gallagher-Thompson[1], *1. Stanford University School of Medicine, Stanford, California, United States, 2. VA Palo Alto Healthcare System, Palo Alto, California, United States*

Psychological health and well-being definitions are moving beyond the goal of simply decreasing symptoms and disorder rates and moving towards a life enhancing and positive focus. Our multi-modal group program targets a broad range of well-being dimensions including cognitive, behavioral, emotional, social, and physical health parameters. The changes to design and implementation made to the intervention as part of an iterative process will be discussed. Participants were older adults (65+), absent a dementia diagnosis, referred from providers across the healthcare system. The intervention focuses on a collection of topics that support optimal psychological wellness and promote health and resilience (i.e. digital tools and mobile apps, value-based behavioral activation to improve your mood, eating for a healthy brain, introduction to mindfulness, improving your sleep and strategies for managing anxiety) that are held weekly, with modules ranging in duration from 2-4 weeks, over a 7-month period. The results across 2 groups (N=40), both process and outcome, will be presented. Positive psychiatry interventions have great potential to support and strengthen the psychological well-being of older adults who are faced with the myriad of challenges that come with increasing age. Considering the geriatric mental health provider shortages and the overwhelmed healthcare system, this program has improved access to psychiatric care. Program goals also include aims of boosting positive emotions, life satisfaction, purpose and meaning, along with decreasing loneliness, depression, and anxiety.

Abstract citation ID: igae098.0134

### MIXED METHODS OPTIMIZATION OF BEHAVIORAL SLEEP TREATMENT FOR GRANDPARENTS RAISING GRANDCHILDREN

Melanie Stearns, Ashley Curtis, and Christina McCrae, *University of South Florida, Tampa, Florida, United States*

Grandparents raising grandchildren (GRG) report greater sleep problems compared to non-caregiving grandparents.

Cognitive Behavioral Therapy for Insomnia (CBT-I) is an evidence-based treatment for insomnia; however, the needs of older individuals caring for young children differ from the typical adult who receives CBT-I. Thus, we examined behavioral sleep treatment preferences and collected qualitative and quantitative sleep data from GRG to inform the adaptation of CBT-I for them. The sample consisted of 99 grandparents (Mage=53.62, 70% female) who reported being the primary caregiver of their grandchild (>17 years old). Participants reported on treatment preferences and provided open-ended responses to what negatively impacts their sleep and what they think might improve their sleep. Participants also completed the Pittsburg Sleep Quality Index (PSQI). Based on SOL (Mean=43min) reported on the PSQI, 55% (N=56) of GRG met insomnia criteria. Most GRG (54%) preferred online treatment (27%-in person, 18%-telehealth) and 60% reported they would rather have a behavioral treatment instead of sleep medication to improve their sleep. GRG reported that their stress, anxiety, family issues, and poor grandchild sleep negatively impact their sleep and that stress reduction, greater social support, bedtime routines, and better sleep habits for their grandchildren would likely improve their sleep. These results suggest that GRG prefer online behavioral sleep treatment compared to other modalities (in-person, telehealth) and sleep medication. They may also benefit from a CBT-I which also targets grandchild sleep, reduces stress/anxiety, and helps promote more positive family relationships. Thus, there is a need to adapt CBT-I for their needs.

Abstract citation ID: igae098.0135

### OPTIMIZATION OF WEB-BASED COGNITIVE BEHAVIORAL THERAPY FOR INSOMNIA NITECAPP IN RURAL DEMENTIA CAREGIVERS

Christina McCrae, *University of South Florida, Tampa, Florida, United States*

Informal dementia caregivers (CGs) often experience chronic insomnia that contributes to stress and poor health. Rural CGs are particularly vulnerable but have limited access to CBT for insomnia (CBT-I), a recommended treatment for chronic insomnia. Web delivery holds promise for improving outcomes and access to this frontline treatment. Here we describe the iterative development of web CBT-I for rural dementia CGs. Qualitative and quantitative evaluations were conducted to explore user needs and adapt/validate content using a stepwise approach: 1- advisory panel of rural CGs (n=5) and primary care providers (PCPs; n=5) gave feedback used to adapt NiteCAPP, 2-focus groups of CGs (n=5) and PCPs (n=7) reviewed the newly adapted intervention and gave additional feedback, 3-NiteCAPP CARES was piloted in CGs (n=5) to test feasibility and establish preliminary effects. Fourteen daily electronic sleep diaries and questionnaires assessing arousal, health, mood, burden, subjective cognition, and interpersonal processes were collected pre/post treatment. Feedback recommended streamlining content, eliminating jargon, and including CG focused content. Multiple features were evaluated positively (comprehensive and engaging information, CG focus, layout, easy-to-access material, easy-to-understand sleep graphs). Suggestions for improvement included giving day/night viewing options, collapsible text, font size options, tabbed video access, and a glossary. Preliminary effects on CG sleep, arousal, health, mood, burden, cognition, and interpersonal processes (ps<.05) were promising. Targeted web CBT-I may increase access and improve CG outcomes. NiteCAPP CARES is now being evaluated in NIA-funded RCT.

### SESSION 1140 (PAPER)

### UNDERSTANDING MENTAL HEALTH DYNAMICS IN LATER LIFE

Abstract citation ID: igae098.0136

### MENTAL HEALTH IMPACT OF BULLYING BY ETHNIC PEERS IN SENIOR HOUSING: A STUDY WITH OLDER KOREAN AMERICAN RESIDENTS

Yuri Jang[1], Juyoung Park[2], Min-Kyoung Rhee[1], Hi-Woo Lee[3], Nan Sook Park[4], and Soondool Chung[5], *1. University of Southern California, Los Angeles, California, United States, 2. USC Suzanne Dworak-Peck School of Social Work, Los Angeles, California, United States, 3. Little Tokyo Service Center, Los Angeles, California, United States, 4. University of South Florida, Tampa, Florida, United States, 5. Ewha Womans University, Seoul, Republic of Korea*

Bullying, a persistent negative interpersonal behavior directed at a specific individual or a group of individuals, is often associated with youth in school settings and young adults in the workplace. However, peer bullying is also common among older populations. Particularly in senior housing, where individuals with diverse backgrounds and characteristics reside in close proximity and where relocation is often not feasible, peer-to-peer aggressive behaviors are of concern. The purpose of the present study is to examine the experience of peer bullying and its mental health effects among ethnic minority older residents in subsidized senior housing. Surveys were administered to Korean Americans aged 65 and older (N = 343) in six senior housing facilities in the greater Los Angeles area. Multivariate regression models were used to examine the effect of being either a target or a witness of bullying on mental health outcomes, after controlling for covariates. About 18% of the sample had been a target of bullying, and over 31% had witnessed someone being bullied. Being a target of bullying was a significant predictor for both depressive symptoms and anxiety, whereas witnessing other residents being bullied was a significant predictor for anxiety only. These findings shed light on the common experience of bullying among ethnic minority older residents in senior housing as well as the adverse mental health impacts of peer bullying. Findings highlight the need for effective programs (e.g., awareness program, bystander education, and assertive communication training) to promote healthy interpersonal relationships in senor housing.

Abstract citation ID: igae098.0137

### DETERMINANTS OF AFFECT-BALANCE IN MONGOLIAN OLDER ADULTS

Saranchuluun Otgon[1], Denise Burnette[2], Zuchi Lkhamsuren[3], Fabio Casati[1], Yerkyebulan Mukhtar[4], and Sugarmaa Myagmarjav[4], *1. University of Trento, Trento, Trentino-Alto Adige, Italy, 2. Virginia*

*Commonwealth University, Richmond, Virginia, United States, 3. Asia University, Taichung, Taichung, Taiwan (Republic of China), 4. Mongolian National University of Medical Sciences, Ulaanbaatar, Ulaanbaatar, Mongolia*

Objectives: In Mongolia, the number of persons aged ≥ 60 years is projected to increase from 125,000 (7%) in 2000 to more than 800,000 (25%) by 2050. Current policies and programs are inadequate to support integrated health services that ensure the physical, functional, and psycho-social components of healthy active aging. The purpose of this study was to assess levels and correlates of affect-balance, an indicator of life satisfaction and general well-being, in older adults in Mongolia. Methods: Data are from an in-person survey with urban and rural adults aged 55 and 88 (N=304). Affect-balance scores were derived from the Organization for Economic Cooperation Development (OECD) scale for measuring psychological well-being. Results: Both male and female study participants reported high (positive) affect-balance. Self-rated health, educational attainment, loneliness, and engagement in volunteer activities were significantly associated with affect-balance. Volunteering, a key component of 'productive aging,' notably contributed to high levels of positive emotions and low levels of negative emotions. Conclusion: Findings highlight the importance of fostering positive emotions in later life and can inform the development of targeted policies and interventions to enhance older Mongolians' quality of life and well-being.

Abstract citation ID: igae098.0138

### COMPASSIONATE LOVE AND INTERSECTIONAL INEQUALITIES IN LATER-LIFE MENTAL HEALTH IN THE US

Tirth Bhatta[1], Jeffrey Albert[2], Stacy Torres[3], Eva Kahana[2], Nirmala Lekhak[1], and Timothy Goler[4], *1. University of Nevada Las Vegas, Las Vegas, Nevada, United States, 2. Case Western Reserve University, Cleveland, Ohio, United States, 3. University of California San Francisco, San Francisco, California, United States, 4. Norfolk State University, Norfolk, Virginia, United States*

Objectives: Despite the emerging evidence that compassionate love has a positive effect on mental health, its distribution across race, gender, and socioeconomic status and its subsequent role in mediating the effect of socioeconomic status on later life mental health across race and gender have yet to be examined. Methods: Based on our nationwide web-based survey of adults aged 50 years and older (n=1861), we conducted a mediation analysis to estimate the direct and indirect effects (via feeling love) of financial strain on depressive symptoms and anxiety across race and gender. Results: Our regression estimates indicate an average.09-point (p=.002) lower feeling of compassionate love among individuals experiencing increased financial strain. Women reported greater feeling of love (β=.22, p<.001) than men. Despite experiencing higher levels of financial strain, Black adults reported greater feelings of love (β=.42, p<.001) than white adults. Findings show an average.14-point (p<.001) and.21-point (p<.001) higher depressive symptoms and anxiety, respectively, among individuals experiencing increased financial strain. We found a statistically significant path-specific effect on both depressive symptoms (indirect effect=.018, p-value=.003) and anxiety (indirect effect=.196, p<.001) (from financial strain) through love. Statistically significant indirect effects through love on both depressive symptoms and anxiety were found for women and white adults, but not for men and Black adults. Discussion: Our study opens up a new avenue of research by investigating the role of compassionate love in transmitting the effects of financial strain on later-life mental health across race and gender.

Abstract citation ID: igae098.0139

### MINDFULNESS-BASED COGNITIVE THERAPY STUDY FOR DEPRESSION AMONG KOREAN SPEAKING OLDER ADULTS

Jenny Kim[1], Eun Jeong Lee[2], Amanda Shallcross[3], Seon Ah Ahn[4], Pallavi Visvanathan[5], Bohye Park[1], Hwa Young Chae[1], and Linda Ko[1], *1. University of Washington, Seattle, Washington, United States, 2. Asian American Resource and Information Network Inc, Wood-Ridge, New Jersey, United States, 3. Cleveland Clinic, Cleveland, Ohio, United States, 4. Ahn Counseling Center, Westwood, New Jersey, United States, 5. Manhattan Center for Mindfulness-Based Therapy, New York, New York, United States*

Korean American (KA) older adults experience higher risk for depression compared to Non-Hispanic whites. Healthy Mind Healthy Living (HMHL) is a single arm, pre/post pilot intervention study to test and evaluate the effect of an abbreviated telephone-based mindfulness-based cognitive therapy (MBCT-brief) protocol to reduce depression and improve sleep quality and stress. MBCT-brief was adapted culturally and linguistically for older KA adults; participants attended one of three MBCT-brief groups (8-10 participants, N=24), delivered virtually over the span of 8 weeks by a licensed bilingual/bicultural (English/Korean) clinical therapist. Survey data were collected at baseline and 9 weeks post intervention and measured depressive symptoms (QIDS-SR16), sleep quality (PROMIS Sleep Disturbance 4a), and stress (Perceived Stress Scale). The differences in the three outcome measures from pre and post were calculated using paired T-tests. We observed significant differences for changes in mean±SD for depressive symptoms and sleep quality. Post MBCT-brief, participants had a significant reduction in depressive symptoms (8.04±4.70) compared to baseline (10.29±4.22, t(23)=2.87, p=0.004). Similarly, participants had a significant improvement in sleep quality post MBCT-brief (51.03±2.82) compared to baseline (53.23±4.14, t(23)=2.53, p=0.009). Participants also had reduction in stress (17.54±4.56) compared to the baseline (17.21±3.99, t(23)=0.28, p=0.39), but it was not statistically significant. These findings indicate that culturally and linguistically adapted MBCT-brief has potential to improve symptoms of depression, sleep quality, and stress among older KA adults with limited English proficiency. Future larger trials may want to explore the efficacy and effectiveness of MBCT-brief on depression, sleep quality, and stress with a control group.

Abstract citation ID: igae098.0140

### HAPPINESS LOVES COMPANY? OLDER ADULTS' POSITIVE AFFECT ACROSS LAYERS OF THE MOMENTARY SOCIAL CONTEXT

Alyssa Goldman, and Sara Moorman, *Boston College, Chestnut Hill, Massachusetts, United States*

Positive affect supports physical and mental health as well as longevity. Ecological momentary assessments (EMAs) have been used to demonstrate that positive affect is significantly shaped by the quality and quantity of momentary social interactions that take place during everyday activities. We consider that positive affect may also be a function of the broader momentary interpersonal social environment, including the presence of acquaintances and unfamiliar others in everyday social spaces – social actors whose presence is typically not explored in studies of the social context. Using 15,379 EMAs from 378 older adults in the Chicago Health and Activity Space in Real-Time study in mixed effects regression models, we find that older adults experience more positive affect when they are with a social companion versus being alone, consistent with prior research. More novel, however, we find that when older adults spend time in social spaces where other people are present, they report significantly higher levels of momentary positive affect, regardless of whether they are with a personal social companion or alone, and regardless of the activity in which they are engaged. The magnitude of the effect of being in more occupied social spaces is approximately half the size of the effect of being in the presence of a social companion. We discuss the implications of the results for ongoing efforts to address social isolation among the older adult population and consider how the relevance of social actors beyond interpersonal ties could inform theory on the social climate of well-being and aging.

### SESSION 1145 (PAPER)

### ADULT PROTECTION I

Abstract citation ID: igae098.0141

### ELDER ABUSE PROSECUTION WITHOUT VICTIM COOPERATION: A DECISION-MAKING MODEL FOR PROSECUTORS

Catheryn Koss, *California State University Sacramento, Sacramento, California, United States*

Victims of elder abuse frequently refuse to cooperate with the prosecution of their abusers, especially if the accused is a spouse or other family member. What little guidance there is on how prosecutors should proceed in these situations tends to focus on evidentiary challenges and strategies to overcome them. While victims and prosecutors share the common overarching goal of addressing the problem of abuse, they may differ on how best to achieve that goal or at what cost. Victims may prioritize maintaining their relationships with their abusers and protecting their loved ones from the consequences of their actions. They may also fear loss of independence or placement in a long-term care facility. Prosecutors tend to prioritize preventing future abuse by keeping the abuser away from the victim and/or by reforming the abuser's behavior. Prosecution sends a message that abuse is a serious crime that will not be tolerated, potentially having a deterrent effect by putting other potential abusers on notice that they too will likely be punished. This presentation will examine the common barriers to participation elder abuse victims face and the concerns elder abuse victims may have about prosecuting their abusers. Then, drawing on existing elder abuse scholarship as well as debates about this issue from the intimate partner violence literature, the presenter will share a novel decision-making framework - grounded in trauma-informed practices - that prosecutors and others may use to weigh the victim's interests against the state's interests in prosecuting the accused.

Abstract citation ID: igae098.0142

### MEETING NEEDS: LINKING CLIENTS WITH A SELF-NEGLECT ALLEGATION TO HOME- AND COMMUNITY-BASED SERVICES

Samantha Tuft[1], Farida Ejaz[2], and Courtney Reynolds[1], *1. Benjamin Rose, Cleveland, Ohio, United States, 2. Benjamin Rose Institute on Aging, Cleveland, Ohio, United States*

This paper describes results from a federal grant in which Oklahoma Adult Protective Services (APS) collaborated with the Oklahoma City Indian Clinic and gerontological researchers to enhance APS self-neglect practice. A total of 188 APS clients from 11 counties were interviewed by phone at baseline during the COVID pandemic. This paper focuses on a subset of 45 randomly selected clients who were offered extensive case management services for a period of four months beginning shortly after they were reported to APS. The clients were primarily white and female with a mean age of 62 years and average annual income between $12,000-$15,000. During the study, 24% of the clients were screened as experiencing self-neglect based on the Recognizing Abuse Tool (Ejaz, et al., 2017). More importantly, 76% of the 45 clients reported that they needed help in at least one area, resulting in a total of 175 identified needs. The top five most identified needs were home repairs/maintenance, in-home care, public benefits, transportation, and case management. We will describe whether client needs were met during the study along with barriers to service utilization. Findings will be explored in light of their implications for APS practice and policy, particularly the role time-limited, intensive case management can play in preventing further reports to APS.

Abstract citation ID: igae098.0143

### APPROACHES FOR INCORPORATING OLDER ADULT PERSPECTIVES IN ELDER MISTREATMENT PREVENTION

Ruthann Froberg[1], and Randi Campetti[2], *1. Virginia Tech, Waltham, Massachusetts, United States, 2. Education Development Center, Waltham, Massachusetts, United States*

This presentation discusses an approach for engaging older adult feedback on elder mistreatment (EM). EM can be a challenging topic to engage communities with due to the stigma and the sense of futility around solving this social issue. Despite these obstacles, a local community coalition to address EM successfully elicited feedback from older adults and service providers to create recommendations for their ongoing work. Collaboratively, member organizations developed three focus groups composed of participants aged 60 and above who were familiar with existing community resources for older adults. Member organizations also identified service providers to participate in an additional focus group in order to improve understanding of the services available to older adults. Interviewers conducted hour-long sessions with each group to gather feedback and insights on EM and related issues. Participants highlighted the importance of community engagement, addressing isolation, increasing digital literacy, and improving access to social services. Healthcare providers were recognized for their role in detecting EM, but challenges in accessing services persist. Service providers underscored the importance of collaboration and addressing barriers such as staffing and funding. The resulting recommendations, including accessible healthcare services, digital literacy initiatives, and integrated community care, reflect practical feedback to guide the coalition's endeavors. This feedback has spurred renewed commitment within the coalition to address these recommendations in their ongoing work, highlighting the actionable insights garnered through community engagement.

Abstract citation ID: igae098.0144

### LESSONS LEARNED FROM A FIVE-YEAR STUDY ON PREVENTING SELF-NEGLECT AMONG AT-RISK HEALTH CARE PATIENTS IN TEXAS

Farida Ejaz[1], and Courtney Reynolds[2], *1. Benjamin Rose Institute on Aging, Cleveland, Ohio, United States, 2. Benjamin Rose, Cleveland, Ohio, United States*

This presentation focuses on lessons learned from a study on preventing self-neglect among healthcare patients that is currently featured as a Success on the Administration for Community Living's Elder Justice webpage. Collaborators included researchers, a large healthcare system, and Adult Protective Services. We focused on preventing self-neglect by identifying older and disabled patients from 19 primary care clinics with risk factors for self-neglect such as depression and dementia. Clinics were randomized to intervention (patients received 4-months of case management) and control (usual care) conditions, and 477 randomly selected patients participated. The sample was predominantly Hispanic (68%) and low income (< $1,361). The lessons learned have important implications for practice, policy, and future research. For example, language mattered when recruiting study participants and the term 'self-neglect' needed to be softened. Despite paying $25-$35 for an interview, the response rate was approximately 30%, leading to timeline delays. Intervention group patients (285) who were interviewed at home had higher response rates than control patients (192) interviewed at the clinic/other settings. Thus, conducting home assessments for self-neglect and offering case management to at-risk patients was feasible. In general, at-risk patients were not self-neglecting but had a variety of needs (823 total needs) such as food insecurity and home modifications and needed service referrals (total 1,226 referrals). Needs were met 65% of the time. Barriers to service utilization included refusals. Discussion will focus on how the lessons learned can help research-practice collaborations prevent self-neglect among low-income, minority populations and prepare for challenges in the field.

Abstract citation ID: igae098.0145

### A COMMUNITY APPROACH TO REDUCE AND PREVENT ELDER ABUSE

Sarah Marrs, Shannon Arnette, Madeline McIntyre, and Aisling Clardy, *Virginia Commonwealth University, Richmond, Virginia, United States*

Research on elder mistreatment mostly focuses on improving individuals' skills in recognizing, screening, and reporting abuse that has already occurred, typically considered secondary prevention. This is critical for effective and timely intervention. However, increasing awareness of and accessibility to resources and supports that safeguard older adults is an effective and necessary strategy for primary prevention. Unlike child abuse victims and their families, who have access to various wrap-around resources and services, these resources are highly limited for older adult victims. The purpose of this study was to conduct focus groups with older adults, caregivers of older adults, and various aging service providers to inform the development of the Safety Connector, an online tool designed to link older adults, caregivers, and professionals to services, resources, and educational materials to prevent elder mistreatment. A mock-up of the Safety Connector was created by the research team and presented during focus groups to garner feedback and perceptions of usability and effectiveness. Findings revealed a need for simplicity, accessibility and clarity on the purpose and intended users of the Safety Connector; and to distinguish it from similar websites. The resulting Safety Connector has been integrated into our state's existing No Wrong Door suite of websites. In this session, we will discuss research methods and results, and introduce the Safety Connector. We will also showcase other resources available on the Safety Connector's website and discuss this as a model for linking systems of care beyond our home state.

### SESSION 1150 (SYMPOSIUM)

Abstract citation ID: igae098.0146

### ADVANCES IN INTERDISCIPLINARY SCIENCE: BRAIN CONTROL OF MOBILITY IN AGING AND DISEASE POPULATIONS

Chair: Roee Holtzer
Discussant: Teresa Liu-Ambrose

Interdisciplinary research has demonstrated that cognitive and mobility performance and decline are inter-related and potentially coupled implicating the brain as a key common denominator shared by both domains of function. Novel studies in the current symposium utilize advanced neuroimaging methods to elucidate how structural and functional measures of brain integrity are utilized to predict mobility outcomes and mitigate against risk of developing mobility decline and impairments. Mark Wagshul demonstrates that whole brain latent patterns have differential associations with gait speed in older adults with and without multiple sclerosis. Paul Laurienti uses brain networks connectivity data to define cognitive reserve demonstrating that associations between amyloid beta and mobility decline are attenuated among older adults with high cognitive reserve. Chun Liang Hsu uses functional neural networks to define a novel construct - physical reserve. He demonstrates that among older adults with subcortical ischemic vascular cognitive impairment (SIVCI), high physical reserve attenuates the negative effect of white matter hyperintensities on postural stability. Roee Holtzer uses functional-near-infrared-spectroscopy (fNIRS) to assess oxygenated hemoglobin (HbO) in the prefrontal cortex during walking under single and dual-task conditions. He shows that higher fNIRS-derived HbO during dual-task walking is protective against future falls in older adults with relapsing-remitting but not progressive MS subtype. Combined, these studies shed light on novel discoveries concerning brain control of mobility in aging and disease populations. Furthermore, emphasis is placed on demonstrating how brain data are utilized to define constructs (cognitive and physical reserve) and elucidate theoretical models (compensatory reallocation) vis-à-vis mobility outcomes. Brain Interest Group Sponsored Symposium

Abstract citation ID: igae098.0147

### USING FUNCTIONAL BRAIN IMAGING DATA TO PREDICT FALLS IN OLDER ADULTS WITH MULTIPLE SCLEROSIS

Roee Holtzer, *Yeshiva University and Albert Einstein College of Medicine, Bronx, New York, United States*

Background: Falls are common and debilitating among older adults with multiple sclerosis. However, literature concerning modifiable risk factors of falls in this population is scarce. The current study was designed to address this gap in knowledge. We examined whether brain hemodynamic responses during dual-task-walking predict falls during longitudinal follow-up in older adults with multiple sclerosis with relapsing-remitting and progressive subtypes. Methods: Participants with relapsing-remitting (n=53, mean age=65.02±4.17ys, %female=75.5) and progressive (n=28, mean age=64.64±4.31ys, %female=50) multiple sclerosis subtypes completed a dual-task-walking paradigm. In addition, participants reported falls during longitudinal follow-up using a well-validated structured monthly telephone interview. Functional-near-infrared-spectroscopy (fNIRS) was utilized to assess oxygenated hemoglobin (HbO) in the prefrontal cortex during active walking under single and dual-task conditions. Results: General Estimating Equations models, adjusted for confounders, indicated that higher HbO under dual-task-walking was significantly associated with a reduction in the odds of reporting falls among participants with relapsing-remitting (OR=.472, p=.004, 95%CI=.284 to.785), but not progressive (OR=1.056, p=.792, 95%CI=.703 to 1.588) MS. In contrast, faster stride velocity under dual-task-walking was significantly associated with a reduction in the odds of reporting falls among progressive (OR=.658, p=.004, 95%CI=.495 to.874), but not relapsing-remitting (OR=.998, p =.995, 95%CI=.523 to 1.905) MS. Conclusion: Compensatory reallocation of brain resources under dual-task walking may provide protection against falls for older adults with multiple sclerosis, relapsing-remitting subtype. These findings have important implications for fall risk assessment and intervention procedures in older adults with multiple sclerosis.

Abstract citation ID: igae098.0148

### SELECTIVE GREY MATTER PATTERNS OF MOBILITY IN OLDER ADULTS WITH AND WITHOUT MULTIPLE SCLEROSIS

Mark Wagshul, *Albert Einstein College of Medicine, Bronx, New York, United States*

Background: Both healthy aging and multiple sclerosis (MS) are associated with changes in cortical grey matter (GM), notably frontal grey matter atrophy consistent with the last in-first out hypothesis, but its relationship to mobility impairments is unknown. We sought to identify brain patterns of cortical GM associated with walking performance, as measured by single- and dual-task walking speed, in older adults with and without MS. Methods: In a cohort of 97 MS and 105 healthy older adults, we measured cortical volume from T1-weighted MRI images and Freesurfer software, along with quantitative gait assessment during natural walking (single-task) and walking while reciting alternate letters of the alphabet (dual-task) conducted within a few weeks of the MRI. Principal components (PC) analysis identified significant latent patterns of GM volume, from the bilateral 68 Freesurfer cortical regions, and pattern scores were entered into regression analyses of gait velocity vs pattern scores, unadjusted and adjusted for demographics variables. Results: The first two PCs showed a significant association with single-task (p = 0.027/0.0025), but only PC2 was associated with dual-task (p=0.082/0.027). In group-stratified analyses, only PC1 was associated with single-task in healthy controls but just below the significance level (p=0.057), and only PC2 was associated with single-task in MS participants (p=0.0052). PC1 consisted of mostly posterior regions (medial occipital, inferior parietal), while PC2 consisted of mostly anterior regions (frontal, cingulate). Conclusion: Grey matter pattern analysis may distinguish between older adults with and without MS, corresponding to anterior, cognitive-control vs posterior, visual/automated control of mobility.

Abstract citation ID: igae098.0149

### ASSOCIATIONS BETWEEN FUNCTIONAL NETWORKS OF PHYSICAL RESERVE, POSTURAL INSTABILITY, AND WHITE MATTER LESIONS

Chun-Liang Hsu[1], Roee Holtzer[2], Roger Tam[3], Walid Alkeridy[4], and Teresa Liu-Ambrose[3], *1. The Hong Kong Polytechnic University, Hong Kong, Hong Kong, China (People's Republic), 2. Yeshiva University and Albert Einstein College of Medicine, Bronx, New York, United States, 3. University of British Columbia, Vancouver, British Columbia, Canada, 4. King Saud University, Riyadh, Ar Riyad, Saudi Arabia*

White matter hyperintensities (WMH) are clinical markers of subcortical ischemic vascular cognitive impairment (SIVCI) associated with impaired postural balance and falls. Physical reserve (PR) is a recently established construct that reflects one's capacity to maintain physical function despite brain pathology. This cross-sectional study aims to map functional networks associated with PR, and examining the relationship between PR, WMH, and postural balance. Physical reserve was defined in 22 community-dwelling older adults with SIVCI as the unexplained residual variance in Timed-Up-and-Go test (TUG) after accounting for age, global cognitive function measured by Alzheimer's Disease Assessment Scale-Cognitive-13 (ADAS-Cog-13), and hippocampal volume. Functional neural networks associated with PR were extrapolated as TUG-correlated network connectivity maps computed using general linear models that removed the effects of age, ADAS-Cog-13, and hippocampus volume. Subsequent analyses examined whether PR and its associated brain networks moderated the relationship between WMH and postural balance under two conditions – eyes open while standing on foam (EOF) and eyes open while standing on floor (EONF). Physical reserve and its associated functional neural networks - frontoparietal network (FPN) and default mode network (DMN) - significantly moderated the association between WMH and postural balance. Specifically, in those with high PR, postural balance was maintained regardless of WMH load while in those with low PR, postural balance worsened as WMH load increased. These results suggest the attenuated effects of WMH on postural stability due to PR may be underpinned by functional neural network reorganization in the FPN and DMN as a part of compensatory processes.

Abstract citation ID: igae098.0150

### BRAIN NETWORKS AND FUNCTIONAL RESERVE IN THE PRESENCE OF AMYLOID PATHOLOGY

Paul Laurienti, *Wake Forest University School of Medicine, Winston-Salem, North Carolina, United States*

Background: Amyloid beta (Aβ) is a biomarker of Alzheimer's disease and has been linked to cognitive and physical function decline. However, significant inter-individual variability exists with respect to the influence of Aβ on cognitive and motoric outcomes. The current study examined the potential contribution of brain networks on this inter-individual variability. Methods: Community-dwelling older adults (n=81, mean age=76ys, %female=51) from a larger longitudinal brain network study were included. Participants were adjudicated as cognitively normal at baseline. Each had resting-state functional magnetic resonance and Aβ positron emission tomography scans. A statistical interaction model was used to determine if network integrity moderated the effects of global Aβ on physical function as measured with the expanded short physical performance battery (eSPPB). Results: At baseline the central executive network (CEN) and the basal ganglia network (BGN) significantly ($p<0.05$) moderated Aβ associations with eSPPB. Physical function was highest in those individuals with high network integrity and low Aβ. Function was lowest in those with both low network integrity and high Aβ. Neither Aβ-network interaction was significant for longitudinal eSPPB change scores. Conclusion: Integrity in the CEN and BGN may underlie reserve in the presence of Aβ pathology as those individuals with high network integrity maintained their physical function. Given that these brain networks exhibit plasticity, they are a potential intervention target in older adults exhibiting functional decline in the presence of Aβ. Future work will need to replicate the findings in a larger and more diverse sample of older adults.

### SESSION 1155 (SYMPOSIUM)

Abstract citation ID: igae098.0151

### AGING, HEALTH, AND CAREGIVING IN ARAB AND ARAB IMMIGRANT POPULATIONS

Chair: Carlos Mendes de Leon
Discussant: Carlos Mendes de Leon

There has been a rapid increase in the number of older adults in Arab countries and among Arab immigrants in the US, leading to growing attention to the health and associated care needs due to impairments in physical and cognitive function. Although traditionally, the expectation may have been that the care for these needs would be provided by next of kin, family care resources are becoming less available due to significant social change, such as migration of offspring generations to larger urban centers or other countries. Because formal sources of care are often insufficient or lacking, older Arab adults face an increasingly challenging care environments for their basic care needs. The presentations in this symposium will reflect on aging and caregiving drawing from four geographic sources of information: Egypt, Lebanon, Qatar, and Detroit, Michigan. The Egyptian data come from a pilot study testing the feasibility of conducting a population-based longitudinal study of older adults based on the HRS Around the World Framework, documenting initial findings regarding cognitive impairment. The Lebanon study provides data from a new population-based survey study on caregiving burden and its correlates among older Lebanese adults that provide care to others. The Qatar data come from a qualitative study describing the Muslim religion-inspired belief systems that shape caregiving expectations and practices. The Michigan study presents findings from a culturally adapted intervention for caregivers of older Arab American patients with dementia. Together, these data provide an overview of caregiving needs and practices in Arab and Arab-American populations.

Abstract citation ID: igae098.0152

### EXPLORING AGING DYNAMICS IN EGYPT: INSIGHTS FROM THE AL-SEHA PILOT STUDY

Sara Moustafa, Nada Gaballah, Reem Deif, and Mohamed Salama, *The American University in Cairo, Cairo, Al Qahirah, Egypt*

The AL-SEHA (A Longitudinal Study of Egyptian Healthy Aging) pilot study is an HRS-Around the World (ATW) longitudinal sister study in Egypt. This foundational phase aims to uncover critical insights into cognitive impairment, the prevalence of non-communicable diseases (NCDs), and the pivotal role of family support for older adults in Egypt. The cross-sectional pilot was conducted in five Egyptian governorates: Cairo, Dakahlia, Beni Suef, Suez, and Fayoum. Participant selection was based on convenient sample of Egypt's population aged 50+. Data collection combined computer-aided personal interviewing (CAPI) with a Self-Completion Questionnaire (SCQ), and included comprehensive coverage of economic, health, and social domains including caregiving needs and arrangements. Initial findings highlight a significant association between cognitive impairment and NCDs, such as diabetes (62.3%), hypertension (75.8%), and musculoskeletal issues (61.3%). As in other populations, lower educational attainment had a significant negative association with cognitive impairment ($p=0.0001$), emphasizing socioeconomic factors' impact on cognitive health. Strong family support networks were identified as key in mitigating cognitive decline, underscoring the cultural importance of family in the Egyptian context. The AL-SEHA pilot study provides a vital foundation for understanding the aging process in Egypt, highlighting the interplay between cognitive health, physical well-being, and socio-economic and cultural factors. Findings advocate for integrated healthcare strategies that address the multifaceted needs of the older population. Future research, building on this pilot phase, is essential to develop evidence-based policies and interventions to enhance the quality of life for Egypt's aging population.

Abstract citation ID: igae098.0153

### IMPACT OF RELIGION ON FRAMING AND CARING FOR DEMENTIA AMONG MUSLIM ARABS IN QATAR: A QUALITATIVE STUDY

Suhad Daher-Nashif[1], Suzanne Hammad[2], and Tanya Kane[3], *1. Keele University, Newcastle, England, United Kingdom, 2. North Western Qatar, Doha, Ad Dawhah, Qatar, 3. Qatar University, Doha, Ad Dawhah, Qatar*

Studies have examined how religion plays a major role in framing mental disorders and in shaping caregiving practices. This presentation sheds light on dementia care among Muslim Arabs in Qatar. The presentation will examine how old age and dementia are framed in the Islamic religious resources, i.e., the Quran and Sunnah Nabawiyyah (statements and behaviours of Prophet Muhammad). Then it explores how these texts feed into the belief systems distributed within the community by sharia scholars, and later uncovers how these both sources are transformed to social behaviours in caring for older adults with dementia. The study uses descriptive qualitative methodology and is based on three data sources: Islamic religious texts i.e., the Quran and Sunnah, eight semi-structured interviews with Sharia scholars, and semi-structured interviews with 37 Arab-Muslim families from different Arab countries living in the state of Qatar. Thematic analysis has been applied to analyse the texts and the interviews. Compassion and honouring parents are commandments in the Islamic holy texts. The Quran and Hadith include definition and characteristics of ageing, of cognitive deterioration and give clear instructions on how and why older adults must be cared for. These values are expressed in the beliefs system constructed by sharia scholars that play a major role as motivators to caring for dementia patients by the families. Families used religious practices as diagnostic and treatment tools. Gaps between the holy text and the social beliefs were found in explaining dementia and Alzheimer. Moreover, religious practices played role in coping with caregiving-burden.

Abstract citation ID: igae098.0154

### CAREGIVER BURDEN AMONG LEBANESE OLDER ADULTS: FINDINGS FROM LSAHA (LEBANON STUDY ON AGEING AND HEALTH)

Sawsan Abdulrahim[1], Monique Chaaya[2], Aya El Sammak[2], Alexandra Abi Nassif[2], Mayssan Kabalan[2], and Carlos Mendes de Leon[3], *1. American University of Beirut, Cambridge, Massachusetts, United States, 2. American University of Beirut, Beirut, Beyrouth, Lebanon, 3. Georgetown University, Washington, District of Columbia, United States*

This study examines correlates of caregiver burden in a sample of older adults (≥60years) in a rural district in Lebanon who provide care to other older adults. Utilizing baseline data from the new Lebanon Study on Ageing and Health (LSAHA), we assessed associations of the Zarit Caregiver Burden Scale with sociodemographic characteristics of the care provider, relationship to care recipient, and type of care provided. Of a total of 1,345 participants, 216 (16%) reported providing care. A higher percentage of women (17%) than men (14%) were caregivers, while a higher percentage of men (47%) than women (37%) provided care to their spouses. After dichotomizing the Caregiving Burden Scale, high burden was more common among women (37%) then men (20%), and among the non-married/widowed (41%) than the married (28%). High burden was lowest among participants with more than 12th grade education. In multivariable logistic regression analysis, high education was significantly associated with lower odds of high burden (OR=0.18; %95 CI=0.04; 0.73) while caregiver gender and marital status were not. Those who provided care for healthcare-related issues were also significantly more likely to report high burden (OR=2.78; 95% CI=1.01; 7.65). In the setting of a mostly rural Lebanese population, the findings point to the burden associated with providing care to other older adults dealing with healthcare issues, possibly due to lack of access or proximity to healthcare facilities. Further analysis will focus on burden associated with characteristics of the care recipient and care provided to others inside versus outside the household.

Abstract citation ID: igae098.0155

### SUPPORTING ARAB AMERICANS FAMILIES LIVING WITH DEMENTIA: TESTING A CULTURALLY ADAPTED INTERVENTION

Kristine Ajrouch, Fatmeh Barada, Mary Janevic, and Toni Antonucci, *University of Michigan, Ann Arbor, Michigan, United States*

Culturally adapted interventions are needed to address unique challenges faced by minoritized groups who care for people living with Alzheimer's disease and related dementias (ADRD). Among Arab Americans, limited access to support services due to language and cultural barriers often results in a belief that family alone is the only option during times of need. We test 1) whether participation in an adapted ADRD care intervention is associated with positive outcomes for Arab American care partners (N=58) and 2) evaluate program satisfaction. We present a single group design using paired t-tests that demonstrate reduced care burden and increased care satisfaction from pre- to post-intervention. A measure of family conflict trended downward after program participation. Following the program, each participant completed a short evaluation survey sent vias email where they (N=43) rated their experience with the program on multiple dimensions: usefulness of the program, materials, language of delivery, and Alzheimer's Association resources. Respondents were also asked to explain their ratings, which were coded into four themes: Resources, Information regarding diseases, caregiving, and self-care. Data were analyzed descriptively and showed strong positive reactions to the program as well as areas for improvement. Findings provide preliminary data to illustrate that culturally adapted ADRD care intervention programs hold promise to help family members caring for a person living with dementia even when such programs not generally accepted by cultural norms.

### SESSION 1160 (SYMPOSIUM)

Abstract citation ID: igae098.0156

### APPLYING IMPLEMENTATION SCIENCE FRAMEWORKS TO DELIVER INTERVENTIONS IN THE NURSING HOME SETTING

Chair: Katherine Abbott
Discussant: Kathleen Unroe

Approximately 1.2 million people reside in more than 15,000 nursing homes (NH) in the United States. The 2022 NASEM

report on NH quality identified persistent issues to the provision of high-quality NH care in the United States. To improve NH quality, implementation science (IS) theories, methods, and frameworks are necessary to close the gap in delays of evidence based therapies and models of care reaching NH residents. This symposium highlights IS work in the NH setting that seeks to improve the quality of care delivered. Our first presentation reports findings on the pre-implementation work of a five-year VA funded study to ensure documentation of life sustaining treatment with over n=50 clinical and administrative staff. The second presentation utilizes the Practical, Robust Implementation and Sustainability Model (PRISM) to identify factors to consider before intervention implementation with results from n=5 focus groups with 27 NH Staff. The third presentation describes the modifications n=23 NH providers made to the Individualized Positive Psychosocial Interaction as well as their motivations behind the modifications via the Framework for Reporting Adaptations and Modifications to Evidence-Based Implementation Strategies. The fourth presentation reports findings from n=6 focus group sessions representing n=23 people living with dementia, their care partners and NH staff using the Readiness Assessment Pragmatic Trial Model to inform what mattered to them when implementing and evaluating a remote format of Me & My Wishes. The Discussant, Dr. Kathleen Unroe, will discuss the implications to advancing IS as well as the applicability of IS in the NH setting. Research in Quality of Care Interest Group Sponsored Symposium

Abstract citation ID: igae098.0157

### PREIMPLEMENTATION OF A QUALITY IMPROVEMENT PROGRAM FOR VETERANS LIVING IN COMMUNITY NURSING HOMES

Joan Carpenter[1], Ann Kutney-Lee[2], Andrew Murray[2], and Mary Ersek[3], *1. University of Maryland Baltimore, Baltimore, Maryland, United States, 2. Corporal Michael J. Crescenz VA Medical Center, Philadelphia, Pennsylvania, United States, 3. University of Pennsylvania, Philadelphia, Pennsylvania, United States*

We designed Preferences Elicited and Respected for Seriously Ill Veterans through Enhanced Decision-Making (PERSIVED), a five-year VA-funded quality improvement program, to support VA clinicians with data and tools to ensure consistent documentation of life sustaining treatment (LST) preferences for Veterans who receive care in community nursing homes (CNHs). Guided by the VA Quality Enhancement Research Initiative (QUERI) Roadmap for Implementation and Quality Improvement, we designed the pre-implementation phase. Over 5 months, we conducted virtual meetings with partners at six VA CNH programs to: 1) engage site leadership and recruit champions; 2) assess barriers and facilitators to documenting LST preferences; 3) develop process maps of existing workflows; and 4) evaluate baseline knowledge, skills and resources related to these clinical practices. Through individual interviews (n=41) and 14 meetings with clinical and administrative partners, we recruited 19 champions. Common barriers included lack of knowledge/training and confusion about roles/responsibilities among VA CNH program clinicians. Facilitators included access to VA and CNH electronic medical records and positive relationships among VA and CNH staff. Across the programs in this sample, process maps revealed variability in formal systems for ensuring LST documentation in CNHs and VA. Using these findings, we tailored educational resources, developed site-specific audit tools for tracking VA and CNH LST documentation, and created processes that integrated LST documentation into existing VA CNH program workflows. Pre-implementation proved to be a critical step to ensure success and guided us in developing individualized implementation plans and addressing potential challenges to evidence-based practice adoption.

Abstract citation ID: igae098.0158

### SUPPORTING THE UPTAKE OF PERSON-CENTERED COMMUNICATION STRATEGIES IN NURSING HOMES USING THE PRISM

Natalie Douglas, *University of Louisiana at Lafayette, Lafayette, Louisiana, United States*

The implementation of quality, person-centered care into nursing home (NH) settings is challenging due to the complexity of NH organizational structures, nationwide staffing shortages, and significant federal regulations. We used the Practical, Robust Implementation and Sustainability (PRISM) Model to assess key contextual factors that may influence the implementation of Dementia Collaborative Coaching, an evidence-informed communication coaching program designed to decrease negative, responsive behaviors and improve quality of life for people living with dementia in NHs. Employees (n=27) from three urban NHs including corporate leaders, managers, and direct care providers across a variety of disciplines engaged in n=5 focus group sessions to deepen understanding of barriers and facilitators to the implementation of Dementia Collaborative Coaching. Participants identified barriers to Dementia Collaborative Coaching implementation including the need to improve dementia care overall, inadequate leadership support, staffing concerns, and misinterpretation of NH federal regulations. Facilitators included enthusiasm for the program as well an agreed upon need for an NH-wide implementation and sustainability plan. Outcomes most relevant to the participants included decreased behavioral incidents and falls as well as increased engagement in both leisure and activities of daily living. Pre-implementation guided by the PRISM proved to be a critical step in maximizing the success of implementation of this intervention and developing relationships with key players in the NH. During this process, site-specific system and clinician level factors will inform future Dementia Collaborative Coaching implementation efforts which may also contribute to the implementation of other quality interventions for NH residents.

Abstract citation ID: igae098.0159

### ENGAGING PEOPLE LIVING WITH DEMENTIA, CARE PARTNERS, AND STAFF TO INFORM REMOTE ME AND MY WISHES

Gail Towsley, *University of Utah, Salt Lake City, Utah, United States*

Me & My Wishes, is a video recording approach for documenting, viewing, listening, and discussing nursing home (NH) residents' preferences for daily and end-of-life care. This project addressed the alignment and measurement domains of the Readiness Assessment Pragmatic Trial Model. Using a qualitative descriptive design, we conducted focus groups with experts representing persons living with dementia (PLWD) (n=6), care partners (n=8), and NH staff (n=9) to inform us of what mattered to them when implementing and evaluating a remote format of Me & My Wishes. We coded and analyzed audio-recorded transcripts, resolved coding discrepancies in team meetings, and derived domains from finalized codes. Three domains for implementing Me & My Wishes remotely was shared across groups. Prioritizing Care Conferences included leadership providing staff resources and support to prioritize care conferences utilizing Me & My Wishes. Addressing Competing Demands that Prevent Key People Attending involved identifying times for care conferences that allow residents, care partners, and staff to participate. Utilizing Technology and Learning to Communicate in a Virtual Environment encompassed recommendations to have a trained facilitator whom engages attendees and additional support (e.g. technical, emotional) for the PLWD. Experts provided input on content, structure, and design of a template for capturing video highlights, changes in preferences, and results of conversations that could be included in the medical record. Outcomes most important to them included person-centered care, dignity, feeling heard and understood, and improved knowledge. Experts identified key alignment and measurement aspects that informed modifications for delivering Me & My Wishes remotely.

Abstract citation ID: igae098.0160
### "YOU HAVE TO USE YOUR IMAGINATION": NURSING HOME PROVIDER MODIFICATIONS TO IMPLEMENTING THE IPPI

Miranda Kunkel[1], Nahida Akter[2], Kamryn Kasler[3], Cassie Keiser[3], Molly Noble[3], Alexis Talmage[3], Kimberly Van Haitsma[2], and Katherine Abbott[4], *1. Collaborative Consulting, Oxford, Ohio, United States, 2. The Pennsylvania State University, University Park, Pennsylvania, United States, 3. Miami University, Oxford, Ohio, United States, 4. Miami University's, Oxford, Ohio, United States*

Understanding modification motive can help improve intervention protocols and future implementation efforts. The core components of the IPPI that should not be modified include that staff approach one-to-one resident interactions with a positive intent, use good communication skills, and base interactions on a preferred activity of the resident living with dementia. We sought to understand what was modified and motivations behind the modifications from implementation champions. We utilized the Framework for Reporting Adaptations and Modifications to Evidence-Based Implementation Strategies (FRAME-IS) to track and characterize modifications using data from a total of n=78 interviews with n=23 NH provider champions who were implementing the IPPI in their community as part of a quality improvement project. Interviews were audio recorded, transcribed, and coded via the FRAME-IS in Dedoose. Over half of champions (61%; n=14) reported unplanned, fidelity-consistent modifications to IPPI content (e.g., loosening structure) or implementation context (e.g., setting). Motivation ranged from organizational (e.g., time constraints) to recipient (e.g., cognitive capacity), with many citing resident preferences. Modifications are a necessary aspect of evidence-based implementation efforts and should be embraced by implementation researchers. Many champions in the present study reported creative modifications previously unexplored by the IPPI research team. Available resources, COVID-19 regulations, and resident preferences can highly impact implementation efforts in the NH setting. Future implementation efforts should build flexibility into intervention protocols and welcome the influence of recipient preferences in order to reach a wider range of users and achieve greater satisfaction.

### SESSION 1165 (PAPER)

### CAREGIVER SUPPORTS AND RESOURCES

Abstract citation ID: igae098.0161
### LATENT CLASSES OF FAMILY CAREGIVERS OF PATIENTS RECEIVING PALLIATIVE ONCOLOGY

Todd Becker[1], Kyle Pitzer[2], Elizabeth Bryant[2], and Karla Washington[2], *1. Washington University School of Medicine in St. Louis, St. Louis, Missouri, United States, 2. Washington University in St. Louis, St. Louis, Missouri, United States*

Family caregivers (FCGs) are recognized as the "invisible backbone" of cancer care, where the national median age of new diagnosis is 66 years. Although research on caregiver retention has prioritized examination of the demands placed on FCGs, research on the resources mitigating or exacerbating these demands is limited. Understanding this interplay is key to supporting FCGs' continued partnership in care provision. Drawing on the job demands–resources model, we aimed to identify latent classes of FCGs of patients receiving palliative oncology by exploring how caregiving burden and resources overlap. As part of an ongoing multisite clinical trial on problem-solving therapy, we recruited 352 FCGs of patients receiving outpatient palliative care. We collected baseline data through established measures on FCGs' resources (area deprivation index, disposable income, self-rated health, formal education) and caregiving demands (caregiving distance, number of work-related changes, frequency of caregiving). We, then, used these grouping variables to identify latent classes of FCGs. Results indicated that a four-class model provided the best fit (AIC = 5,337.97, BIC = 5,658.66, entropy =.70). These four classes corresponded to shifting combinations of resources and demands: low resources–low demands (12%), low resources–high demands (31%), high resources–low demands (17%), and high resources–high demands (40%). To our knowledge, these results provide the first assessment of distinguishable subgroups of FCGs of patients receiving outpatient palliative oncology by demands and resources. Identification of these subgroups represents a crucial first step toward developing and refining supportive caregiver interventions to facilitate well-being and retention in service of patient care.

Abstract citation ID: igae098.0162
### "DIAGNOSE AND ADIOS": MULTIPERSPECTIVE INSIGHTS ON FORMAL SERVICE USE IN DEMENTIA FAMILY CAREGIVERS

Annabelle Greenfield[1], Hannah Mason[2], Francesco Osso[3], Katerina Bumbalova[4], Rachel Bloom[3], and Francesca Falzarano[5], *1. University of Southern California, San Marcos, California, United States, 2. Weill Cornell Medicine, New York, New York, United States, 3. Fordham University, New York, New York, United States, 4. University of Washington, Seattle, Washington, United States, 5. University of Southern California, Los Angeles, California, United States*

Over 11 million unpaid family caregivers currently provide care to 6.7 million individuals with Alzheimer's disease and related dementias (ADRD). The diverse roles associated with ADRD caregiving range from physical care responsibilities, providing emotional support, in addition to more complex roles such as medical/legal/financial decision-making. Formal and informal supports (e.g., respite, training/education, social support) may be protective against adverse stress-related outcomes such as depression, anxiety, and burden. Despite the existence of such supports, caregivers' utilization rates remain remarkably low. Using focus group methodology, the current study explored ADRD caregivers' experiences with the formal services system and identified barriers that hinder service access and utilization. Five semi-structured focus groups were conducted with ADRD family caregivers (n=13) and Dementia Care Community Partners (n=17). Qualitative findings identified three overarching concepts: 1) "Pathways to Preparedness," categorized primary domains of unmet needs of support, specifically regarding education, emergency preparedness, financial/legal guidance, and mental health. 2) "Multi-Level Barriers" examines obstacles identified across the individual, provider, and systemic levels that hinder service identification and access. 3) "Bridging the Gap" illustrates technology's potential in mending the broken needs-to-services continuum by providing tailored and personalized support to caregivers. Findings underscore the complex disconnect between caregivers' needs and existing services, leading to a lack of preparedness for caregiving roles and stress-related adverse outcomes. Yet, a viable solution lies in web-based behavioral interventions, creating a clear pathway for caregivers to identify, access, and utilize formal services, thus improving their overall experiences and outcomes.

Abstract citation ID: igae098.0163

### THE TIMING OF SUPPORT UTILIZATION AND ITS IMPLICATIONS FOR THE EFFECTIVENESS OF FORMAL CAREGIVING SUPPORT

Joseph Svec[1], Naomi Meinertz[2], and JeongEun Lee[3], *1. St. Joseph's University, Patchogue, New York, United States, 2. University of Missouri, Columbia, Missouri, United States, 3. Iowa State University, Ames, Iowa, United States*

Current literature on caregiving support indicates a mixed association between formal support (e.g., paid help, respite, training) and caregiver well-being. Compared to informal support from family and friends, formal support often comes with additional costs to caregivers in the form of financial, knowledge, and time barriers to access. This study seeks to examine those barriers, particularly the issue of when caregivers seek and utilize formal support as a predictor of its effectiveness. We utilize four waves of data from the National Study of Caregivers (NSOC) toward a series of fixed-effect linear regression models for caregiver emotional distress and physical health on formal support usage ($N = 2,083$). The dependent variable, experiencing any financial, emotional, or physical difficulties ($M = 0.83$, $SD = 0.87$), is regressed on whether the caregiver received support from paid help, financial help, or respite ($M = 0.51$, $SD = 0.77$). The findings show that formal support is positively associated with higher levels of caregiving difficulty (coeff = 0.22, $p < 0.05$); however, when controlling for past levels of difficulty (previous wave difficulty) and past usage of formal support (previous wave formal support), the positive association of formal support is mitigated. This suggests that the timing of adopting formal support can play a role in the challenges faced by caregivers, with earlier adoption potentially leading to reduced burdens of informal caregivers. While formal support remains positively associated with perceived difficulty, the mitigated coefficient when including lagged variables suggests that formal support effectiveness is also time sensitive.

Abstract citation ID: igae098.0164

### EXPLORING SECONDARY STRESSORS ON ADRD CAREGIVERS' EXPERIENCES WITH HOME AND COMMUNITY-BASED SERVICES (HCBS)

Francesca Falzarano[1], Megan McCarthy[2], Hannah Mason[3], Annabelle Greenfield[4], and Francesco Osso[5], *1. University of Southern California, Los Angeles, California, United States, 2. University of Colorado Firearm Injury Prevention Initiative, Denver, Colorado, United States, 3. Weill Cornell Medicine, New York, New York, United States, 4. University of Southern California, San Marcos, California, United States, 5. Fordham University, New York, New York, United States*

Family caregivers are the primary care providers for the growing population with Alzheimer's Disease and Related Dementias (ADRD), yet they often feel unprepared and ill-equipped to carry out their role. While caregivers report high unmet needs, the low uptake of home- and community-based services (HCBS) underscores major gaps between the family care unit and the formal healthcare system. Further, little research has examined how stressors outside of direct care provision influence how caregivers perceive HCBS. Thus, using the stress process model as a theoretical framework, the current study examines the differential influences of secondary stressors on caregivers' experiences with and attitudes toward HCBS, and its subsequent impact on psychosocial outcomes. ADRD caregivers (n=63) completed an online survey of measures assessing multiple dimensions of care-related stress. Path analyses were used to test caregivers' perceived experiences with HCBS as mediators influencing the relationship between care-related stress and psychosocial outcomes. Results showed that those with greater family conflict reported more negative experiences with HCBS, which subsequently predicted greater levels of burden. The magnitude of the direct effect was significantly reduced and became non-significant (from $\beta=.26$, $p<.05$ to $\beta=.16$, $p=.24$), providing evidence of full mediation. Additionally, difficulty finding services partially mediated the relationship between cost-of-care and role captivity on burden, self-efficacy, and depression. Findings underscore the need for additional research focused on gaining a more holistic understanding of factors that influence how caregivers interface with the HCBS to promote advancement and continuity across the needs-to-service-use continuum.

Abstract citation ID: igae098.0165

### DIGITAL HEALTH LITERACY AND SELF-EFFICACY IN USING E-HEALTH RESOURCES AMONG CAREGIVERS OF INDIVIDUALS WITH ADRD

Kyeung Mi Oh[1], and Jung-Ah Lee[2], *1. George Mason University, Fairfax, Virginia, United States, 2. University of California Irvine, Irvine, California, United States*

This study explored digital health literacy skills, self-efficacy in utilizing e-health resources, and self-efficacy in managing personal health, with a focus on caregivers of individuals with Alzheimer's disease and related dementias (ADRD). Data from the Health Information National Trends Survey 2022 were analyzed, involving a sample of 96 family and unpaid caregivers of individuals with ADRD. Among these caregivers, 49.9% lacked confidence in utilizing e-health resources. While over 70% utilized certain digital health tools, such as accessing medical information and viewing medical test results, usage of health or wellness apps (57%) and wearable devices (48%) was relatively lower. Engagement in sharing health information (21%) or interacting with individuals with similar health problems (33%) on social media was low, while watching health-related videos on social media (72%) was relatively high. Telehealth (59%) and patient portal usage (87% for own health and 34% for care recipient) were moderate. Controlling for demographic characteristics, each digital health literacy skill was assessed in relation to self-efficacy in utilizing e-health resources. The skill for using health or wellness apps (AOR=111.13, 95%CI: 109.45-112.83) showed the strongest association with self-efficacy, followed by the use of patient portals (AOR=15.77, 95% CI:15.58-15.97) and accessing test results online (AOR=5.64, 95% CI: 5.59-5.69). Significant correlations were found between self-efficacy in using e-health resources and managing personal health among ADRD caregivers (r=.166, P< 0.001). The insights from this study can guide the development of tailored e-health interventions to provide enhanced support for caregivers in their crucial role.

### SESSION 1170 (PAPER)

### COGNITION AND AGING

Abstract citation ID: igae098.0166

### A NEW ERA IN ASSESSING THE BURDEN OF HEARING LOSS IN LATER LIFE USING PATIENT RECORDS AND IMPLICATIONS FOR HEALTHY AGING

Dialechti Tsimpida, *University of Southampton, Southampton, England, United Kingdom*

Hearing loss is a significant global health concern and a leading cause of disability in later life. While conventionally considered an inevitable consequence of aging, recent evidence highlights modifiable risk factors challenging this notion. Nevertheless, epidemiological estimates, including Global Burden of Disease studies, overlook the actual hearing health needs of populations, relying instead on projected age demographics. This study aimed to provide a fresh perspective, quantifying hearing loss burden and distribution in older adults by analysing anonymised longitudinal medical records of 2.7 million registered patients in the Cheshire and Merseyside Integrated Care System (ICS) in England from 2013 to 2022. Using advanced spatial analysis techniques, including Cluster and Outlier Analysis and Geographically Weighted Regression, socio-spatial inequalities in hearing loss prevalence and incidence were investigated. Surprisingly, findings revealed hearing health disparities unrelated to population age, with residents of similar age experiencing worse hearing outcomes in different areas of the ICS. Deprivation emerged as a significant determinant, explaining up to 35% of the variance in hearing loss. These findings underscore the need for a paradigm shift in health policy-making, moving beyond age-based projections to more accurately estimate and address hearing health needs. Hearing health indicators are already available in existing patient records and should be systematically reported, analysed, and interpreted to create equitable long-term care models according to populations' actual needs. This novel data-driven approach is particularly important in the context of healthy aging initiatives, given that hearing capacity lies at the core of the Integrated Care for Older People (ICOPE) approach.

Abstract citation ID: igae098.0167

### SUBJECTIVE COGNITIVE DECLINE IN OLDER ADULTS BY SEXUAL ORIENTATION AND GENDER IDENTITY

Ellesse-Roselee Akre[1], Katherine Miller[2], Harry Barbee[1], and Tara McKay[3], *1. Johns Hopkins Bloomberg School of Public Health, Baltimore, Maryland, United States, 2. Johns Hopkins University, Baltimore, Maryland, United States, 3. Vanderbilt University, Nashville, Tennessee, United States*

LGBTQ+ adults experience a high risk of subjective cognitive decline (SCD) due to increased exposure to stressors that threaten cognitive health. Yet, researchers know little about how the risk of SCD varies by sexual orientation and gender identity (SOGI). We examine how SCD differs by SOGI using Wave 1 of the Vanderbilt University Social Networks, Aging, and Policy Study survey of LGBTQ+ adults aged 50-76 residing in the South (n=1256). We assess subjective cognitive health using an index of self-reported cognitive difficulties, many of which have been used in validated instruments. Using linear probability models, we examine the association of SCD with SOGI. To identify significant differences, we use standardized differences >10% in the average predicted probability of reporting ≥1 cognitive difficulty across SOGI, after adjusting for sociodemographic characteristics. Forty percent of respondents reported ≥1 cognitive difficulty. Bisexual individuals have the highest likelihood of SCD (54%), while lesbian/gay respondents had the lowest (39%). Across gender identities, gender non-conforming persons have the highest predicted probability of SCD (57%), followed by transgender men (54%), transgender women (48%), females/assigned female at birth (40%), and males/assigned male at birth (39%). Standardized differences are >10% across all comparisons. We find evidence that the prevalence of ≥1 cognitive difficulty in the sample of LGBTQ+ adults varies significantly by SOGI. Comprehensive SOGI health data from all US states is essential to accurately gauge SCD prevalence among LGBTQ+ older adults and to ensure inclusive clinical and public health interventions for populations at higher risk of cognitive impairment.

Abstract citation ID: igae098.0168

### ELDER MISTREATMENT AND COGNITIVE FUNCTIONING: FINDINGS AMONG CHINESE OLDER ADULTS IN THE US

Guoping Jin[1], and Fengyan Tang[2], *1. Adelphi University, Pittsburgh, Pennsylvania, United States, 2. University of Pittsburgh, Wexford, Pennsylvania, United States*

Elder mistreatment disproportionately affects racial and ethnic minority older adults, particularly Chinese older adults in the U.S., who face increased risks due to cultural disparities, intergenerational conflicts, and socioeconomic disparities. While elder mistreatment is known to compromise cognitive health as a stressful life event, research in racial/ethnic minority populations is limited. This study aimed to address this gap by investigating the longitudinal effects of elder mistreatment on cognitive decline over time among older Chinese Americans. Latent growth curve modeling (LGCM) was applied in a sample of Chinese older adults residing in the Greater Chicago area, drawn from the population Study of Chinese Elderly in Chicago (2011-2019), comprising 2,811 participants across four waves of assessment. Elder mistreatment was assessed using a comprehensive instrument at baseline and follow-ups to evaluate its prevalence and incidence, respectively. Cognitive performance tests were administered across four waves to track change trajectory. Compared with respondents without mistreatment reports at baseline, those reporting mistreatment exhibited better cognitive functioning (B = 0.07, p<.05). Those who reported being mistreated at follow-ups showed a faster cognitive decline (B = -0.04, p<.05). Neither mistreatment prevalence nor incidence was associated with an accelerated rate of cognitive decline. The results indicate that recent and potentially cumulative experiences of elder mistreatment have a negative effect on cognitive decline, underscoring the critical need for prevention, detection, and mitigation of elder mistreatment. Furthermore, culturally tailored practices and policies are needed to address elder mistreatment issues within the Chinese minority community.

Abstract citation ID: igae098.0169

**CROSS-NATIONAL DIFFERENCES IN AGE AND EDUCATION GRADIENTS FOR DECLINE IN VERBAL FUNCTIONING**

Chengming Han, Octavio Bramajo, and Brian Downer, *The University of Texas Medical Branch, Galveston, Texas, United States*

Background. Despite the well-established associations of education and higher cognition in old age, the patterns of education gradient in different social contexts have not been adequately explored. Objective. This paper explores cross national differences in cognitive decline between adults older than 50 years in the U.S., Mexico, and China. Methods. Data were drawn from Health and Retirement Study (HRS, 2012-2020), Mexican Health and Aging Study (MHAS, 2012-2021), and China Health and Retirement Longitudinal Study (CHARLS, 2011-2020). Multilevel models were used to estimate the change in verbal function across six age groups (50-55, 56-60, 61-65, 66-70, 71-75, and 76+) and six education groups. We compared the education gradients adjusting for physical health, residence, and gender. Results. HRS respondents had the highest levels of education, whereas respondents from CHARLS had the lowest. Higher educational attainment was associated with higher verbal functioning, but the education gradient was the most pronounced in CHARLS. The slopes of verbal functioning decline were steeper in respondents aged over 60 years in MHAS and CHARLS compared to HRS. However, MHAS and CHARLS respondents in the age groups of 50-60 and 76+ showed slower cognitive decline after adjusting for education. Conclusion. Middle-aged and older adults in Mexico and China experienced faster decline in verbal functioning after age 50 than in the US. These differences can be partly explained by the lower levels of education in Mexico and China. The education gradient displayed different patterns across countries. Future studies should pinpoint heterogeneity and make policies according to different social contexts.

Abstract citation ID: igae098.0170

**THE LONGITUDINAL INFLUENCE OF MENTAL DEMANDS OF OCCUPATION ON COGNITIVE FUNCTION AMONG MEXICAN OLDER ADULTS**

Mariela Gutierrez, *The University of Texas Medical Branch, Texas City, Texas, United States*

This study addresses the role of occupation as a determinant of cognitive health in low-middle income countries (LMICs), focusing on Mexico. While high-income countries have established links between cognitively stimulating jobs and preserved cognitive functioning, research in LMICs is limited. The aim is to investigate the association between lifetime mental work demands and memory changes from 2001 to 2018 in Mexican older adults. We used data from the Mexican Health and Aging Study (MHAS), a nationally representative study of individuals aged 50 and above. Participants main lifetime occupation is classified according to the Mexican Classification of Occupations. MHAS data was linked to the Occupational Information Network (O*NET), which provides descriptors for various job domains, including cognitive abilities. A mental demand index was created from ten O*NET items, and a memory score was derived from immediate and delayed recall of an 8-word list. Linear mixed effect models with random intercepts were conducted to analyze the association between mental demands of work and memory scores, adjusting for demographic, health, and job-related variables. While the association between mental work demands and memory changes was not statistically significant, estimates indicate a decline in memory scores over time. The findings reveal more memory decline among agricultural and domestic/service workers compared to professionals and administrative workers. Other work factors associated declines in memory are receiving benefits from work, age at which individuals began their work. This research contributes to understanding the complex interplay between occupation, cognitive health, and aging in LMICs, particularly in the Mexican context.

### SESSION 1175 (PAPER)

Abstract citation ID: igae098.0171

**EDUCATION AND TRAINING I: COMPETENCY-BASED AND INTERGENERATIONAL APPROACHES**

Competency-based training; Gerontology and Geriatrics training; Intergenerational programs and training

Abstract citation ID: igae098.0172

**AACN COMPETENCY-BASED ESSENTIAL REVISIT: EVIDENCE-BASED VALIDATION VIA RANDOMIZED CONTROL TRIAL**

Hsueh-Fen Kao[1], and Shirin Kiani[2], *1. The University of Texas El Paso, El Paso, Texas, United States, 2. Green Mountain Hospice, El Paso, Texas, United States*

In April 2021, American Association of Colleges of Nursing (AACN) approved The Essential for Professional Nursing Education, which calls for preparing future nurses using a competency-based approach in designing curricula for schools of nursing. Taiwan's nursing education system follows the American Model. This study, conducted in fall 2022 to spring 2023, intended to validate the competency-based approach through entrustable professional activities (EPAs) in the yet widely used nursing education arena. EPAs are practical approaches in assessing competence in real-world settings. A randomized control trial was used in Registered Nurse to Bachelor of Science in Nursing (RN-BSN) students at a college in southern Taiwan. 'Wound care for older adults with diabetes' was chosen to be the EPA intervention. After random assignment, students in the experimental group (n=31) received extra EPA simulation laboratory practice with feedback, and those in the control group (n=34) received usual practice and evaluation without simulation laboratory practice and feedback. In the end of semester, data in Competency Inventory of Nursing Students, Learning Satisfaction, Objective Structured Clinical Examinations were collected using analysis of variance (ANOVA) and Student's t test for analysis. The experimental group demonstrated significantly higher scores in competency and clinical examinations than the control group. However, both groups experienced an increase in learning satisfaction without reaching a significant difference, probably due to receiving more attention. Results of the study indicate that evidence-based pedagogy, e.g., EPAs, can serve as effective strategies in designing competency-based curricula in nursing education to meet the new AACN standards with compelling evidence.

Abstract citation ID: igae098.0173

### HANDS AND HEALTH AT HOME: THE IMPACT OF INTERGENERATIONAL SERVICE-LEARNING ON STUDENTS' PREHEALTH COMPETENCIES

Anna Schwartz[1], Rachel Logue Cook[1], Courtney Vanderlaan[2], and Susan Brown[1], *1. University of Michigan, Ann Arbor, Michigan, United States, 2. Michigan Medicine, Ann Arbor, Michigan, United States*

There is a critical need for developing the geriatric healthcare workforce, yet pre-health students are rarely given the opportunity to engage with older adults during their coursework. In addition, many health-related professional programs require students to demonstrate proficiency with a number of professional and analytical competencies. The purpose of this study was to determine the impact of Hands and Health at Home, an intergenerational hand-training program, as a gerontology-focused educational experience. In this program, upper level and graduate pre-health students were trained to deliver hand-based exercises to homebound older adults twice a week for 8 weeks. Students completed the Geriatric Attitudes Scale (GAS), Coping Self-Efficacy Scale (CSES), and a program evaluation survey. Forty students completed Hands and Hand at Home across three semesters. After the program, scores on the GAS ($p< 0.05$) and CSES ($p< 0.05$) significantly improved. Feedback about the program was overwhelmingly positive, where students indicated high satisfaction, said they would recommend the program to a friend, and felt prepared for their home training sessions. When asked to express any skills learned during the program, students often reported communication, teamwork, compassion, confidence, and creativity. The results from Hands and Health at Home demonstrate that this intergenerational service-learning program was a mutually beneficial way to deliver hand training to homebound older adults while also providing valuable hands-on experience for pre-health students. The program also resulted in reductions in ageist attitudes, improved self-efficacy, and the development of several important pre-health competencies, underscoring the need to offer more intergenerational and gerontology-based educational opportunities.

Abstract citation ID: igae098.0174

### TRAUMA-INFORMED PERSPECTIVES AND PRACTICES FOR TEACHING AND LEARNING IN GERONTOLOGY

Jennifer Sasser, *Portland Community College, PORTLAND, Oregon, United States*

With the increasing incidence of individual and shared traumatic events, as well as the complexity and poignancy of many of the issues considered in social gerontology and aging-related courses, there is a concomitant need for expanded awareness of the ways in which trauma shows up, both for our students as well as for ourselves as educators. What's more, for students preparing for careers in the field of aging, especially those who intend to work directly with older people and their families, developing a trauma-informed disposition and skill-set is particularly important. In this interactive session, an ongoing initiative in the context of a community college applied social gerontology program to infuse the curriculum with trauma-informed perspectives will be shared. In addition, a framework for trauma-informed andragogical practices for teaching and learning about aging issues will be introduced. Enacting a trauma-informed approach, the presenter will facilitate an opportunity for participants to think together about ways to apply this powerful approach in the context of their own professional practices.

Abstract citation ID: igae098.0175

### DESIGNING AND DISSEMINATING AN EDUCATIONAL PROGRAM IN OVER-THE-COUNTER HEARING TECHNOLOGY

Yi Shen[1], Dana Urbanski[2], Susan Anderson[1], and Adrian Lee[1], *1. University of Washington, Seattle, Washington, United States, 2. University of Tennessee Health Science Center, Knoxville, Tennessee, United States*

Over-the-Counter (OTC) hearing aids have been available to the US market since October 2022. These "do-it-yourself" hearing devices were introduced to promote accessibility and affordability of hearing healthcare to primarily older adults with perceived mild-to-moderate hearing loss. To date, many new delivery models of hearing healthcare have emerged in response to the availability of OTC hearing aids. However, without the assistance of a licensed hearing care professional, it is unclear whether older adults who purchase OTC hearing aids will receive sufficient support and assistance to achieve successful OTC hearing aid use. In this presentation, we review recent research to identify areas of unmet need in currently available delivery models for OTC hearing aids. The available evidence suggests the need for and potential benefits of an educational program on OTC hearing technology independent from any device manufacturer that could be delivered to professionals who are not hearing healthcare professionals but who may interact with older adult OTC hearing aid candidates (e.g., community health workers, long-term care staff, pharmacists, and other allied health professionals). To address this need, we conducted an instructional design process with a team of healthcare educators, researchers, audiologists, and instructional designers to create the blueprint of an educational program on OTC hearing technology. Here, we describe our design process for transforming the identified unmet needs into (1) the target audience; (2) the appropriate learning modes for the intended audience; and (3) a list of learning objectives. Additionally, we highlight our plans for implementation and dissemination of the program.

### SESSION 1180 (SYMPOSIUM)

Abstract citation ID: igae098.0176

### EMANCIPATORY GERONTOLOGY IN THE 21ST CENTURY—STRUCTURE, ACTION, AND IDEOLOGIES: A SYMPOSIUM HONORING CARROLL ESTES

Chair: Dale Dannefer
Co-Chair: Chris Phillipson
Discussant: Jessica Kelley

Emancipatory gerontology (Estes, 2020) envisions possibilities of moving toward a society where citizens of all ages experience dignity and access to essential resources. Such possibilities require transforming institutional configurations and dominant ideological narratives around class, race, gender, and age. Yet the longstanding resistance to such ideals has been further aggravated recently by the effects of neoliberal policies and by renewed forms of racist and other exclusionary ideologies, as well as ageism itself. The symposium presents critical work across a range of key gerontological domains. Papers explore the stratification of longevity and related issues, especially in urban settings, ideological and practical consequences of the intersection of age and race, and ongoing conceptual and ideological issues in life-course and gerontological theorizing. Maya Rockeymoore Cummings elaborates the fresh concept of rageism - the intersection of ageism and racism - and the ways in which various levers of power are used to undermine life chances of non-European racial and ethnic groups across the life course and generations. Jan Baars explores inequalities affecting ageing over the life course, arguing that long lives are increasingly a privilege for wealthier members of society. Chris Phillipson and Tine Buffel consider new social divisions emerging between and across age groups, with large cities driving distinctive forms of inequality associated with gentrification and class and race segregation. Dale Dannefer interrogates the continuing uncritical use of the concept of agency, exploring how discourses of life-course and gerontological scholarship may have ideological components that contributes to the legitimation of destructive social arrangements.

Abstract citation ID: igae098.0177

### LANGE/PAROLE, STRUCTURE/ACTION: TOWARD A REFRAMING OF AGENCY IN GERONTOLOGY AND LIFE COURSE STUDIES

Dale Dannefer, and Nan Zhou, *Case Western Reserve University, Cleveland, Ohio, United States*

Almost by definition, "Emancipatory Gerontology" carries implications like "choice" and "freedom" which in gerontology have often presupposed what is often termed "agency". Agency remains a popular concept in gerontology and related fields such as life-course studies when, e.g., exploring such matters as life transitions and decision-making in areas like family formation or retirement. Yet its often-glib and superficial invocation reflects daunting problems identified in recent scholarship, concerning agency's conceptualization and measurement. For any construct, adequate measurement presupposes a sound conceptual definition. The very definition of agency remains unresolved and problematic, despite numerous recent efforts at theoretical reformulation and refinement. Some issues revolve around nuances in the history of the term and its dimensions, others around issues of measurement. We contend that an even more fundamental set of problems arise because of a continued lack of recognition of the force of culture-specific symbol systems and social structure to frame the categories and nuances of thought within which agentic intentions are externalized in action. For example, no one chooses their native language, yet even freely chosen and passionately pursued "agentic" acts represent an expression of conscious intent formulated within perceptions, aesthetics, etc., structured by that language and its accompanying culture. We suggest that the structure-agency tension corresponds closely to the distinction between language and speech (langue/parole) articulated by de Saussure, and to related insights offered by Bourdieu. We contend that emancipation ultimately requires rigorous attention to constraint, and hence recognition of the structuring of agency by unrecognized and naturalized features of context.

Abstract citation ID: igae098.0178

### EMANCIPATORY GERONTOLOGY AND THE POLITICAL ECONOMY OF THE CITY: NEW INEQUALITIES AND POLICY RESPONSES

Chris Phillipson[1], and Tine Buffel[2], *1. University of Manchester, Manchester, England, United Kingdom, 2. The University of Manchester, Manchester, England, United Kingdom*

Carroll Estes identifies a central aim of an 'emancipatory gerontology' as 'advancing knowledge and the realization of dignity, access, equity and social justice through individual and collective agency and social institutions.' Wright argues that the word 'emancipation' signals a central moral purpose in the production of knowledge, with 'the elimination of oppression and the creation of conditions for human flourishing'. But the achievement of these aims has been undermined with the dismantling of the welfare state, extremes of economic inequality, and the expansion of precarious employment. The impact of such trends on groups such as older people continue to be the subject of extensive research. However, of additional concern are divisions created by economic and social forces operating within urban environments. Savage argues that large cities are not just products but drivers of inequality, with many European and North American cities no longer serving people from a wide range of communities, reflected in the decline of affordable housing, and the impact of gentrification. The presentation will consider how a political economy of the city, drawing on research in urban sociology, can re-vitalise a critical gerontology, bringing new perspectives on ageing and the life course. The paper will explore how this approach can be used as a framework for understanding new forms of inequality affecting later life. It will conclude by arguing that different ideas are needed for 'thinking about' ageing within cities, highlighting how innovations produced by urban environments can serve as a catalyst for emancipation and liberation from oppression.

Abstract citation ID: igae098.0179

### AGE, AGING, AND SOCIAL JUSTICE

Jan Baars, *University for Humanistic Studies, Haarlem, Noord-Holland, Netherlands*

The concept of Emancipatory Gerontology goes back to the early work of Habermas. An emancipatory human interest was presented as constitutive for a critical social science that reflects on its own social and methodological presuppositions and on the practical consequences of scientifically based actions. This program has lost nothing of its urgency. Studies of aging need to consider that they are part of a society where Long Lives are for the Rich (Baars, 2024). Even in a relatively egalitarian society as The Netherlands persons with a low SES live eight years shorter than the more advantaged and live 23 years longer with chronic disease. This widely spread phenomenon in Western countries qualifies not only institutionalized aging processes as deeply unequal but also changes the significance of 'middle age', education or work. Studies of aging cohorts need to include those who have already died of causes that can hardly be seen as autonomous choice or genetic misfortune. This profound inequality over the life course is constituted by a neoliberal society in which the rich become richer and the life course is increasingly organized as an entrepreneurial challenge. Besides the injustice that is inflicted upon disadvantaged citizens these structural inequalities are beginning to rip societies apart. However, social justice theories of aging, with their naturalization of 'age' and 'age groups' have hardly noticed the effects of social inequality on aging processes. These shortcomings will be analyzed and confronted with more adequate theories of social justice and aging.

Abstract citation ID: igae098.0180

**RAGEISM: RACISM, AGEISM, AND THE QUEST FOR LIBERATION DEMOCRACY**

Maya Rockeymoore Cummings, *Johns Hopkins University, Baltimore, Maryland, United States*

Structural discrimination subjects people from low-income and historically marginalized racial and ethnic groups in the United States to an anomalous aging process that exacerbates their exposure to social and environmental harms, deprives them of essential resources, and increases their levels of health-harming chronic stress. Although not typically referred to as such, this can also be considered a form of ageism. But the combination of racism and ageism, or "rageism," should be considered a unique phenomenon because of the compounded effects of both forms of discrimination on the lives of those who experience it. In this formulation racism still means a system of oppression enacted against people with darker skin—and can be extended to the intersecting isms associated with class, gender, sexuality, etc.—but the term ageism, which typically refers to discrimination against older adults aged fifty and over, is redefined to describe the systematic effort to deprive black and brown people by deliberately sabotaging their quality of life at every stage of the life course. This reconceptualization helps us to understand age discrimination in its most complete and pernicious form. The official definition of rageism, therefore, is a systematic and persistent form of race, ethnic, and age-based discrimination that uses political institutions, public policy, and other levers of power to undermine the quality and length of life of low-income and non-European racial and ethnic groups from pre-birth to death and across generations. This talk will describe and provide examples of rageism while offering a framework to overcome it.

### SESSION 1185 (SYMPOSIUM)

Abstract citation ID: igae098.0181

**ESPO AND ACADEMY FOR GERONTOLOGY IN HIGHER EDUCATION SECTION SYMPOSIUM: INTERGENERATIONAL ENGAGEMENT IN HIGHER EDUCATION: SUCCESSES, SETBACKS, AND BEST PRACTICES**

Chair: Abigail Stephan
Co-Chair: Janelle Fassi
Discussant: Carson De Fries

Connecting generations through experiential learning opportunities in higher education provides promise for mutually beneficial outcomes. While the theoretical underpinnings (i.e., the "why") are clear, the practical application (i.e., the "how") of bringing undergraduate students and older adults together can be more messy. This session focuses on lessons learned, strategies for success, and setbacks to be aware of, with the goal of enhancing our fortitude as educators who value and strive to incorporate impactful intergenerational experiences into the student experience. The first presentation explores the perspectives of students and older adult participants engaged in an intergenerational team-based learning course for first-year undergraduate students, framed by Age-Friendly University principles. The second presentation shares lessons learned from a new service-learning model in which students assessed the transportation needs of older adults engaged with the university-affiliated Osher Lifelong Learning Institute. The third presentation examines the experience of undergraduate students engaged in hands-on gerontology research and highlights takeaways based on students' intergenerational connections with older adult participants. The final presentation sheds light on sustainable program development through describing their creation of a checklist that aids in intergenerational technology program implementation within higher education. In addition to drawing on the perspectives of scholars across the academic career-span, this symposium session highlights the many contexts within higher education in which intergenerational connections can be facilitated. Aligning with the Annual Scientific Meeting 2024 theme, these presentations demonstrate fortitude in refining processes to ensure we are building optimal experiences that connect students and older adults.

Abstract citation ID: igae098.0182

**UNDERGRADUATE STUDENTS CONDUCTING GERONTOLOGY RESEARCH: AN AVENUE FOR INTERGENERATIONAL CONNECTION**

Abigail Stephan[1], Vanessa Martinez[1], Daniel Moss[1], Mary Walton[2], Ava McVey[1], Kalvry Cooper[1], Christine Phillips[3], and Lesley Ross[1], *1. Clemson University, Clemson, South Carolina, United States, 2. Furman University, Greenville, South Carolina, United States, 3. Clemson University Institute for Engaged Aging, Seneca, South Carolina, United States*

With the proportion of older adults and subsequent rates of age-related assets (e.g., wisdom) and challenges (e.g., Alzheimer's disease/related dementias), projected to rise over the coming decades, it is imperative that professionals across disciplines understand how physical, cognitive, and everyday function in older adulthood impact individuals, families, and communities. As educators, we have a responsibility to train professionals to be competent and aware of older adults' needs, whether or not students plan to pursue careers directly related to gerontology or geriatrics.

Many forms of experiential learning have been employed to accomplish this goal, though students' direct involvement in gerontology research has not been examined in detail. Our investigation debriefs the experiences of students and faculty involved in a pilot gerontology study where undergraduate students conducted in-person visits with older adult participants. Feedback was collected through anonymous online surveys, guided focus groups, and unstructured team conversations. A salient theme, and the focus of this presentation, was meaningful engagement between students and study participants. Students described gaining personal insights and professional skills from working with participants. Notably, we found that several structures supported positive connections (e.g., in-person interactions, pairing two student testers with a participant, shared goals, balance of structured activities and wait time for conversation). Areas for improvement included additional training for managing participants' study task pacing. Although these findings reflect lessons learned from one case, they can guide future endeavors of faculty who want to include undergraduate students in their research while promoting intergenerational learning.

Abstract citation ID: igae098.0183

### DESIGNING INTERGENERATIONAL LEARNING PLACES: STRATEGIES AND OUTCOMES AT AN AGE-FRIENDLY UNIVERSITY

Tamar Shovali, *Eckerd College, St Petersburg, Florida, United States*

The idea that universities play a vital role in promoting intergenerational learning and reciprocal sharing of knowledge among learners of all ages is a key principle of the Age Friendly University model. Although there is abundant research on the benefits of intergenerational relationships and learning in the classroom there is less information about details of how to transform a classroom space into a place where these relationships can occur. This suggests a need for reports of practical approaches in this area. I will describe an intergenerational team-based strategy employed with first year undergraduates in an intensive 3-week course. Students collaborated in teams across the semester to design an intergenerational activity tied to Age Friendly University principles to execute at the end of the semester. To assess the quality of intergenerational learning I surveyed students and older participants about the impact of the project on themselves, the College, and the local community. Prior to engaging in the projects the majority of students (66.7%) and older adults (66.7%) reported occasionally interacting with the other. Both reported an increase in the quality of their intergenerational interactions, students by 50% and older adults by 22.3%. Older and younger learners agreed that intergenerational projects benefit the College and the wider community beyond the College. Qualitative results about outcomes of this programming highlighted friendships and intergenerational communication and learning from younger learners and enjoyment and hope from older learners. Necessary to success in developing effective course-based programming are intentional design and ongoing evaluation.

Abstract citation ID: igae098.0184

### STUDENTS HELPING OLLI BUILD A MORE DIVERSE MEMBERSHIP WITH AN ASSESSMENT OF AVAILABLE TRANSPORTATION OPTIONS

Laura Donorfio, *University of Connecticut, Waterbury, Connecticut, United States*

An undergraduate adulthood and aging class partnered with Osher Lifelong Learning Institute's (OLLI) Diversity Committee to identify transportation resources available for seniors in their community to potentially increase the diversity of the OLLI membership. A needs assessment research framework was used to put a semester long, four-phase research project into place, each with specific goals and milestones. General milestones included researching available transportation resources and organizations for seniors; documenting target audiences and associated difficulties using said resources; exploring potential solutions and providing recommendations for future pilot projects. The Director of the OLLI program attended monthly report outs to assist in the creation of a doable pilot project, helping identify key constraints and logistics. The semester ended with a formal research report out, executive summary, and the university career counselor attending to help students brainstorm and create solid resume entries. The presentation will finish by describing what was learned via best practices and practices to avoid, particularly intergenerational "gaps" that surfaced on how to collect research, the types of research, and the breadth and depth of the research. Lastly lessons learned as a teacher on how to be in the "middle" of both generations and the OLLI director to help make the project a success and how to get student "buy in" by continually reviewing the importance of participating in a service-learning class project for future career aspirations.

Abstract citation ID: igae098.0185

### DEVELOPING THE INTERGENERATIONAL TECHNOLOGY ENGAGEMENT CHECKLIST FOR HIGHER EDUCATION (IG TECH)

Jill Juris[1], Skye Leedahl[2], Athena Sexton[1], Josie Santilli[2], and Meaghan Colvin[2], *1. Appalachian State University, Boone, North Carolina, United States, 2. University of Rhode Island, Kingston, Rhode Island, United States*

Intergenerational programs, as part of service learning initiatives in higher education, have shown to greatly benefit students in gaining professional experience that connects with classroom learning. In recent years, intergenerational technology programs in the United States and Canada have worked to enhance digital inclusion for older adults and provide service learning opportunities for students. Funded by the RRF Foundation for Aging, our multi-university research team set out to examine the implementation of programs that utilized the Cyber-Seniors reverse mentoring model within higher education. This presentation will describe the creation of a checklist to aid in the development of intergenerational technology programs within higher education, with the goal of building sustainable programs. The checklist was developed following a systematic review of the literature and interviews with 12 stakeholders. The systematic review included the analysis of 17 sources that addressed key elements of intergenerational programs such as planning, technology, accessibility, inclusivity, recruitment, marketing, safety, implementation, staffing, and training. Interviews were conducted with a purposive sample of ten higher education representatives and two Cyber-Seniors staff members, all with extensive knowledge of barriers and facilitators to program implementation. This resulted in the development of the InterGenerational Technology Engagement Checklist for Higher education (IG TECH). The IG TECH includes a series of questions to help faculty and/or staff make decisions about key elements of the program, including program planning, student recruitment, older adult recruitment and onboarding, and implementation/monitoring. Future research will evaluate the checklist through a Delphi Panel.

### SESSION 1190 (PAPER)

### EVALUATION AND INTERVENTION RESEARCH ON DIVERSE ISSUES IN AGING

Abstract citation ID: igae098.0186

### SHORT EDUCATION INTERVENTION MITIGATES AGEISM IN EARLY ADOLESCENCE

Assaf Suberry[1], Sarit Okun[2], and Liat Ayalon[3], *1. School of Social Work, Bar-Ilan University, Saad, HaDarom, Israel, 2. The Impact Center for Study Ageism and Old Age, Ramat Gan, HaMerkaz, Israel, 3. Bar Ilan University, Bar Ilan University, HaMerkaz, Israel*

This study explored the effectiveness of a 90-minute workshop in reducing ageism and fostering social activism among 318 Israeli teenagers (11-15 years old, 73.4% female). The workshop aimed to provide accurate information, counter-stereotypes, and peer discussion through activities like talks, games, videos, and meme creation to promote a more inclusive society. The intervention significantly improved familiarity with the concept of ageism, with teenagers drawing parallels to discrimination and racism. The Children's Attitudes Towards Elderly (CATE; Seefeldt et al., 1977) was administered before and after the intervention. A dependent samples t-test indicated a significant mean difference in age stereotypes within the same participants, with a medium effect size, ($t(317)=-11.91$, $p<.001$, Cohen's $d=.51$). Building on prior findings, this study adds to the evidence that educational interventions can improve attitudes towards older adults. Interestingly, while a wider range of age stereotypes emerged post-workshop, a shift towards positive perceptions was observed, particularly among females. Importantly, two-thirds of the teenagers' created memes targeted ageism against older adults, highlighting its prevalence. However, nearly 20% promoted age-inclusivity, and 17% addressed ageism towards younger generations. These findings suggest the intervention's potential in fostering social change and highlight the need for further exploration of ageism in youth populations.

Abstract citation ID: igae098.0187

### IMPLEMENTING ISUPPORT IN UGANDA: A PILOT RCT FOR REDUCING CAREGIVER STRESS.

Joy Louise Gumikiriza, and Kamada Lwere, *Makerere University, Kampala, Kampala, Uganda*

Background: Dementia is a significant public health issue in low- and middle-income countries, and Uganda has seen a notable increase in cases. The iSUPPORT program, adapted into AiSUPPORT, provides tailored interventions that address the sociocultural and economic context of Uganda, aiming to alleviate psychological distress among caregivers of patients with AD and dementia. Methods: This pilot randomized controlled trial (RCT) was conducted in the Wakiso District, enrolling 65 caregivers of Alzheimer's disease and related dementia (ADRD) patients. Participants were randomly assigned to either an intervention group that received AiSUPPORT sessions, or a control group that received standard care. This study aimed to assess the feasibility, fidelity, acceptability, and impact of the intervention on caregivers' psychological distress, quality of life, and depression severity, as measured using the Kessler Psychological Distress Scale and other tools. Results: In the intervention group, psychological distress scores reduced significantly, averaging 29.2 to 23.7 ($p< 0.001$), and depression severity scores dropped from 33.4 to 25.6 ($p< 0.001$). Quality of life scores improved substantially, from an average of 81.1 to 89.4 ($p< 0.001$), compared with the control group. The AiSUPPORT program demonstrated effectiveness and cultural appropriateness, with a 95% retention rate and high engagement. Conclusion: The AiSUPPORT program for dementia caregivers in Uganda's successful implementation in low-resource settings demonstrates its potential. The program's effectiveness, feasibility, and cultural sensitivity underscore the importance of cultural considerations in intervention design, supporting the integration of culturally adapted evidence-based interventions in national health systems to address the complex needs of dementia caregivers.

Abstract citation ID: igae098.0188

### A COMPREHENSIVE EVALUATION OF END-OF-LIFE CARE EDUCATION NEEDS FOR HEALTH CARE PROFESSIONAL STUDENTS

Quynh Phung[1], Angelique King[2], Autumn Decker[3], Anna Roman[2], Logan Patterson[4], Raven Weaver[5], and Cory Bolkan[5], *1. Washington State University, Vancouver, Washington, United States, 2. Washington State University, Spokane, Washington, United States, 3. Pacific University, Hillsboro, Oregon, United States, 4. Washington State University Elson S. Floyd College of Medicine, Spokane, Washington, United States, 5. Washington State University, Pullman, Washington, United States*

People are living longer than ever before, ushering in a need for more healthcare professionals to engage older patients about aging and end-of-life (EOL) values. Despite 99% of healthcare providers acknowledging the importance of advance care planning discussions, over two-thirds of physicians lack adequate training (Fulmer et al., 2018). To address this gap, rigorous evaluations of existing EOL education in professional health programs are needed. This study was conducted at a large public university's Health Science campus; 84 healthcare students (i.e., medical, nursing, and "other" programs) completed a survey, exploring their educational and socio-emotional needs regarding current death education curricula. Findings revealed deficiencies in EOL education in professional training, with only 32% reporting any curricular exposure. Individuals with prior exposure to death exhibited less negative feelings about death and greater familiarity with EOL care topics ($p< 0.05$). Compared to other programs' students, nursing students demonstrated a significantly higher overall familiarity of EOL topics ($p< 0.05$). However, there was still consistently lower familiarity in dementia directives and community resources across all programs. Over 83% expressed a strong desire for more death education. Though students favor comprehensive elective courses, time constraints make workshops and seminars more feasible. Important components in training include real exposure, reflection, and debriefs around EOL conversations or deaths. These findings underscore the urgent need for earlier and more comprehensive training in navigating complex EOL discussions and caring for older adults. Addressing these barriers has the potential to enhance professionals' skills and wellbeing, improving quality of care provided for older adults.

Abstract citation ID: igae098.0189

### MODERATORS OF TREATMENT EFFICACY IN SOCIAL INTELLIGENCE TRAINING WITH CUSTODIAL GRANDMOTHERS

Gregory Smith[1], Megan Dolbin-MacNab[2], Frank Infurna[3], and Luxin Hu[1], *1. Kent State University, Kent, Ohio, United States, 2. Virginia Tech, Blacksburg, Virginia, United States, 3. Arizona State University, Tempe, Arizona, United States*

In a previously reported randomized clinical trial (RCT), we found that online social intelligence training (SIT) was superior to an attention control (AC) condition at improving psychological and relational well-being within a national sample of 349 custodial grandmothers (CGM). However, because the corresponding effect sizes (ES) were modest (range =.22 -.41) and effects waned over time, the present investigation used the same RCT data to investigate moderators of differential treatment efficacy for psychological and relational outcomes at both immediate (T1) and nine-months (T5) post intervention. Multiple potential moderators (including CGM adverse childhood experiences, key contextual/demographic factors, and baseline values of all study outcomes) were first entered simultaneously into classification and regression tree analyses to identify predictors of treatment efficacy within the SIT condition at T1 and T5. Less favorable baseline values of the study outcomes emerged as the only significant predictors of treatment-related change in the SIT condition, with generally large effect sizes (.50 - 1.44) observed. To establish whether these predictors were true moderators of differential treatment efficacy, a series of MANOVAs were then conducted to see if these predictors interacted significantly with RCT condition. At T1 and T5, moderation was scarce across psychological outcomes (i.e., depressive and anxious symptoms, self-esteem, and loneliness) but plentiful across relational outcomes (i.e., prosocial behavior, attachment with grandchild, and peer and grandchild relational quality). We conclude that, SIT is a promising intervention for enhancing the relational well-being of CGM, especially for those with poorer baseline levels. [Funded by R01AG054571]

Abstract citation ID: igae098.0190

### EFFECTIVENESS OF FRIENDLY CALLING TO IMPROVE SOCIAL ENGAGEMENT AMONG MEALS ON WHEELS CLIENTS

Matthew Smith[1], Laura Belazis[2], and L. Carter Florence[2], *1. Texas A&M University, College Station, Texas, United States, 2. Meals on Wheels America, Arlington, Virginia, United States*

Socially vulnerable older adults are at increased risk for social disconnectedness. Friendly calling, defined as regularly scheduled check-in calls by a trained staff member or volunteer, is believed to provide companionship to older adult clients and ensure they are safe. However, less is known about the elements of success for coordinated friendly calling initiatives across multiple sites. The purpose of this pilot was to assess the effectiveness of friendly calling among Meals on Wheels (MOW) clients at seven sites across the country. To collect and manage data, a technology platform designed to coordinate meal delivery to MOW clients was integrated with a friendly calling scheduling solution and a risk assessment for social disconnectedness. All friendly callers underwent extensive training about meaningful engagement and the data platform before contacting clients. The 13-item Upstream Social Interaction Risk Scale (U-SIRS-13) was used to assess the effectiveness of the 7-site pilot between January to November 2023. A total of 10,400 calls were placed to MOW clients, totaling over 114,300 minutes of talk time. Of the 316 clients receiving friendly calls, 80.4% were at risk for social disconnectedness at baseline. On average, from baseline to 3-month follow-up, MOW clients significantly reduced their social disconnectedness risk ($t=-3.15$, $P=0.002$), with 52% of participants reporting reduced social disconnectedness risk ($z=-2.92$, $P=0.003$). Findings suggest that coordinated friendly calling initiatives can reduce social disconnectedness risk among MOW clients within short durations (i.e., 3 months), which highlights the feasibility of telephone-based intervention strategies for socially vulnerable older adults.

### SESSION 1195 (PAPER)

### GLOBAL AGING

Abstract citation ID: igae098.0191

### ANALYZING LONGEVITY TRENDS: AGE-SPECIFIC CONTRIBUTIONS TO LIFE EXPECTANCY GAINS ACROSS EUROPE

Yeonjung Lee[1], Iona Tsui[2], and Yongjie Yon[2], *1. Chung-Ang University, Seoul, Republic of Korea, 2. World Health Organization Regional Office for Europe, Copenhagen, Hovedstaden, Denmark*

The increase in life expectancy at birth has been apparent as mortality has decreased over the past decades. However, as people live longer, it is important to know which age groups have seen the greatest gains in life expectancy. The objective of this study is to examine how different age groups contribute to longer life expectancy. We applied Arriaga's (1984) life expectancy decomposition method to life tables for the total population of 50 out of the 53 Member States of the WHO European Region comparing two different time periods. Average life expectancy gains between 1999 and 2019 are 5.08 years, compared to 3.50 years between 1980 and 2000. Decomposing the differences in life expectancy showed that the gains in total life expectancy was increasingly driven by reduced mortality at older ages in many countries, especially those with the highest current life-expectancy, though there are variation among the countries. There are also differences between men and women. The differences in life expectancy gains between men and women range from 3.05 to 10.43 years and there are the largest differences in Eastern European countries. There is still room for improvement in health beyond age 70, as older age groups are contributing more to increasing longevity. The contribution of increased longevity after age 70 to total improvement in life expectancy at birth is above 30% for more than half of the countries, and this measure is expected to rise as population ageing continues to accelerate.

Abstract citation ID: igae098.0192

### DEMENTIA KNOWLEDGE IN CHINESE NEWSPAPERS (2005–2020): A TOPIC MODELING ANALYSIS

Lin Chen[1], Felicia F. Tian[2], and Yu Fu[2], *1. Fudan University, Somerville, Massachusetts, United States, 2. Fudan University, Shanghai, Shanghai, China (People's Republic)*

How public policies convey dementia is an important source of the public's understanding of dementia, and newspapers are critical to depicting and disseminating this information to the public. The present study used topic modeling strategies to analyze Chinese newspaper portrayals of dementia from 2005 to 2020 to trace changes in key areas of dementia knowledge in relevant policies. Using WiseNews, the largest Chinese media database, we chose 45 newspapers from mainland China and identified 12,719 articles related to dementia. Using latent Dirichlet allocation (LDA), we performed a topic modeling analysis and identified the six most prevalent topics on dementia across articles: lifestyle recommendations, neighborhood life, foundational scientific research, celebrity and media portrayals, dementia caregiving, and pharmaceutical innovations—all related to the dementia knowledge scale's four dimensions. Findings suggest a steady increase in the number of articles on dementia caregiving and a decline in lifestyle recommendations from 2005 to 2020. However, newspapers continued to stigmatize aging by regularly co-depicting dementia and old age and by using biased terminology. Among the first to investigate dementia's portrayals in mainland Chinese newspapers, this study illuminates the need for expanding mass media campaigns to raise the country's dementia knowledge to foster a dementia-inclusive society.

Abstract citation ID: igae098.0193

### DEVELOPING COMMUNITY-BASED AGING SERVICES IN NORWAY: LESSONS FROM THE US

Andrew Scharlach[1], and Laila Tingvold[2], *1. University of California Berkeley, Alamo, California, United States, 2. Norwegian University of Science and Technology, Trondheim, Sor-Trondelag, Norway*

Ongoing transformations in the Norwegian welfare state reflect decreasing reliance on government and an increasing role for civil society. Norway's current "Live Safely at Home" policy, for example, aims to expand community-based supports to enable older adults to live at home for as long as possible, while simultaneously promoting community engagement and social participation. In an effort to learn from the US' well-established mix of governmental and non-governmental aging support services, we began a study of the experiences of community-based aging service providers in the US. In the first phase, we conducted six weeks of ethnographic fieldwork and 31 in-depth interviews with staff and participants at aging service provider organizations in Northern California. Data were analyzed following thematic analysis procedures proposed by Braun and Clarke (2006, 2019). An overarching theme emerging from the data was tension between the goals of personal support and social inclusion. Organizations focused primarily on community engagement (e.g., senior centers, Villages) appeared to have limited ability to serve individuals as their physical or cognitive functioning declined, while organizations focused primarily on personal care typically provided little attention to fostering community engagement or social participation. These findings reflect a tension between the goals of "aging-in-place" and "aging-in-community." As Norway develops a more comprehensive community-based aging services system, it will be important to ground services in existing social institutions and informal social networks, enabling older Norwegians with functional disabilities to live safely at home while maintaining meaningful social and community engagement.

Abstract citation ID: igae098.0194

### DOES EDUCATIONAL ATTAINMENT IMPROVE COGNITIVE FUNCTIONING OF OLDER TRIBAL POPULATION IN INDIA?

Neha Kumari, and Nandita Saikia, *International Institute for Population Sciences, Mumbai, Maharashtra, India*

Enhanced cognitive functioning is closely linked with the overall health and well-being of the elderly population. The pivotal role of education in determining cognitive capabilities is widely recognized. Particularly, tribal communities, who often endure vulnerability and marginalization due to limited educational access, predominantly reside in rural Indian regions, magnifying their susceptibility to cognitive decline. Our study delves into the influence of education on cognitive functioning among tribal older adults in India. Our investigation draws on secondary data from the nationally representative Longitudinal Ageing Study in India (2017-18), encompassing 62,322 adults aged 45+ years. We employed continuous assessments of five cognitive domains to gauge cognitive functioning, adapted from the Mini-Mental State Examination (MMSE). Linear regression analysis and decomposition analysis unveiled the major associates and contributors of cognitive disparities between tribal and non-tribal cohorts. Regression results revealed significantly lower odds of better cognitive functioning among individuals in the tribal category compared to non-tribal counterparts (Coefficient: -1.33; $p < 0.001$). Decomposition analysis illuminated that a considerable 77.5% (Coefficient: -2.73; p-value < 0.001) of the divergence attributed to caste-based differences was elucidated by varying characteristics. Addressing the educational chasm between tribal and non-tribal adults could potentially ameliorate cognitive inequity by 39%, while narrowing the rural-urban residence divide could contribute an additional 8% reduction. Notably, among others, the absence of education emerges as a pivotal factor in the cognitive underdevelopment within India's tribal populace. Tailored educational policies targeted at tribal communities promise to foster cognitive growth and defer cognitive health deterioration.

Abstract citation ID: igae098.0195

### MEDICARE ENROLLEES WITH COMPLEX HEALTH NEEDS FROM PUERTO RICO

Maricruz Rivera-Hernandez, and Matthew Campos, *Brown University School of Public Health, Providence, Rhode Island, United States*

Older adults in Puerto Rico have higher rates of chronic conditions and poverty rates compared to adults in the US mainland (Matos-Moreno et al., 2022; U.S. Census Bureau, 2019). In addition, the island of Puerto Rico has faced unique challenges related to infrastructure damages as a result of Hurricane Maria in 2017 (Florido, 2020), and further continues to face disparities in quality of care for older adult populations (Rivera-Hernandez et al., 2021). While the proportion of older adults with health insurance in Puerto Rico is comparable to older adults in the U.S. mainland, disparities in quality and access to care exist for older adults in Puerto Rico (Matos-Moreno et al., 2022). Thus, the purpose of this study was to characterize older adults with complex care needs enrolled in Medicare Advantage (MA) from Puerto Rico. We used data from the Medicare Health Outcomes Survey and the Master Beneficiary Summary File from 2010 to 2019. We classified enrollees into high-need and non-high-need adults (DuGoff et al., 2019). We identified about 46,699 beneficiaries from Puerto Rico. Approximately, 57.09% were identified as high-need adults. A higher proportion of high-need older adults were female (56.59% vs. 50.99%), married (50.95% vs. 58.68%), had high school education (28.09% vs. 22.88) and household income of less than $10,000 (35.48% vs. 28.83%) compared to non-high-need beneficiaries. With physicians and health care providers leaving Puerto Rico, older adults with high-needs may be experiencing health care challenges. These issues may be contributing to the exodus of older adults from Puerto Rico.

### SESSION 1200 (SYMPOSIUM)

Abstract citation ID: igae098.0196

### POLICY SERIES: HEALTH AND AGING POLICY FELLOWS: CONNECTING EXPERIENCE TO POLICY

Chair: Maureen Henry
Discussant: Pi Ju Liu

For health and aging policy to be effective, it must link an understanding of how systems impact individuals to an understanding of how policy influences the systems. Systems such as health care and social service delivery, family caregiving, lifelong learning, zoning laws, and the housing market influence older people and their families. Policy can make these systems better - or worse - for supporting the health and wellbeing for older people. The Health and Aging Policy Fellows program provides experts with real-life experience the opportunity to learn how policy works so they can better support older people. This symposium will describe how current fellows are addressing federal policy within congress and administrative agencies, state policy through a non-profit organization comprising state aging officials, and across government through a non-profit organization focused on caregiving policy. The 2024 reauthorization of the Older Americans Act is an area of focus across all settings. Fellows are also working on care and payment models in primary care and geriatrics; assisted living facilities; direct care workforce; family caregiving policy and research agendas; elder justice, including Adult Protective Services, the Long-Term Care Ombudsman, guardianship). During the symposium, fellows will discuss these and related topics the context of their fellowship experiences.

Abstract citation ID: igae098.0197

### EMBRACING OPPORTUNITIES TO ENGAGE IN WORK ON POLICIES AND PROGRAMS ON ELDER RIGHTS

Pamela Teaster, *Virginia Tech., BLACKSBURG, Virginia, United States*

The Health and Aging Policy Fellowship (HAPF) provides fellows with an unprecedented opportunity to understand, to experience, and to delve into various dimensions of policy making with the goal of improving the health and well-being of present and future older adults and their families. The purpose of this presentation is to discuss opportunities that my fellowship placement afforded to work on elder rights policies with staff of the Administration for Community Living. First, I will provide an overview of ACL activities and responsibilities. Then, specifically, I will discuss policy making challenges and opportunities related to the projects on which I worked: Adult Protective Services, the Long-Term Care Ombudsman Program, and guardianship. I will conclude the presentation with how the experiences I had with the HAPF have shaped my present and future research, instruction, and outreach.

Abstract citation ID: igae098.0198

### ADVANCING FAMILY CAREGIVER HEALTH AS A KEY PUBLIC HEALTH PRIORITY

Courtney Polenick, *University of Michigan, Ann Arbor, Michigan, United States*

The Health and Aging Policy Fellowship (HAPF) offers exposure to the development and implementation of health policies at both the state and national levels. As a family caregiving researcher and the first Ralph C. Wilson, Jr. Foundation HAPF Caregiving Policy Track Fellow, my HAPF placements included the Office of U.S. Congresswoman Debbie Dingell and the National Alliance for Caregiving (NAC). These placements provided various opportunities to learn more about translating research findings into policies and actions that improve services and supports for older adults and their family caregivers. Based on extensive research showing that caregiving can lead to negative health effects, it is critical to consider family caregiver health as an important public health priority. My placement with the Office of Congresswoman Dingell focused on advancing legislation to enhance home and community-based services (HCBS), including supports for family caregivers. My placement with NAC focused on building upon their public health framework and further promoting family caregiver health as a key public health issue. We synthesized common priorities within existing public health frameworks that are applicable to family caregiving across the lifespan. We also identified upstream intervention and system change approaches for implementing parts of the 2022 National Strategy to Support Family Caregivers to advance family caregiver health within the public health landscape.

Abstract citation ID: igae098.0199
**DEEP DIVES INTO STATE POLICY: LOCATING LEVERS TO IMPROVE CARE FOR ADULTS AGING ALONE**
Jane Lowers, *Emory University, Atlanta, Georgia, United States*

The Health and Aging Policy Fellowship facilitates fellows' exposure to the intricacies of policy development and implementation in a variety of settings. My HAPF placement at ADvancing States provided an opportunity to investigate how individual states define "family" in regard to caregiving for older adults, both through regulations and Medcaid waivers. For state aging and disability directors, this analysis offered insight into how state policies aligned with federal policies, including the Older Americans Act, and opportunities to adapt other states' efforts to support unpaid caregiving networks. As a researcher, this fellowship work provided insight into the types of evidence needed to advocate for equitable services and supports for adults aging without biological family. This presentation will provide an overview of HAPF placement focused on state-level policies.

Abstract citation ID: igae098.0200
**LEARNING ABOUT THE PROCESS OF REVISION OF POLICIES AROUND INNOVATIVE CARE MODELS AT THE VHA**
Quratulain Syed, *Joseph Maxwell Cleland Atlanta VA Medical Center, Atlanta, Georgia, United States*

Having practiced as a geriatrician in underserved neighborhoods and post-acute care in the United States, I have witnessed older adults and individuals with multimorbidity facing the brunt of health policies that favor the healthy and the well-resourced. These observations have formulated my vision for age friendly health policies, and the need for front line clinicians to get a seat at the health policy table and influence the policies. The VA track of the Health and Aging Policy Fellows' Program is an opportunity for professionals involved in healthcare and research focused on older Veterans to nurture skill necessary for development and implementation of health policies that impact older Veterans. During the session, I will discuss my placement at the Veterans Health Administration Central Office with the Home & Community Based Programs. This opportunity has allowed me to learn about the process of updating and revision of policies and directives around innovative care models with demonstrated clinical outcomes.

Abstract citation ID: igae098.0201
**HEALTH AND AGING POLICY FELLOWSHIP: AN INCREDIBLE JOURNEY**
Natalie Baker, *University of Alabama at Birmingham School of Nursing, Birmingham, Alabama, United States*

Dr. Natalie Baker is a geriatric nurse practitioner and nursing professor. Her passion for improving the quality of care delivered in the long-term care setting led her to apply for the Health and Aging Policy Fellowship (HAPF). She will describe her previous national experience in health policy and desire to gain an in-depth immersion experience in policymaking at the federal level. As a nonresidential HAPF with the U.S. Senate Special Committee on Aging, Dr. Baker has had the opportunity to interact with national aging policymakers, research topics of concern, assist in drafting legislative bills, and plan committee hearings. Through the HAPF program, she experienced an unforgettable opportunity to have conversations with affected individuals, health care workers, and professional organizations. She observed skillful negotiations for bipartisan support in a deeply divided Congress while working alongside Senator Bob Casey's staff. From this incredible journey, Dr. Baker has gained a new perspective on aging challenges and pathways for meeting the needs of this vulnerable population.

### SESSION 1205 (PAPER)

### HEALTH BEHAVIOR CHANGES (PAPERS)

Abstract citation ID: igae098.0202
**DANCE AND EMOTION: THE IMPACT OF OLDER PERFORMERS ON AUDIENCE ENGAGEMENT**
Atsuko Miyazaki, *The University of Tokyo, Meguro-ku, Tokyo, Japan*

This study explores the emotional connections between dancers of advanced age and their audience, investigating the role of dance in promoting happiness and social engagement among older adults. The Federation International of Dancers Association Japan (FIDA JAPAN), a general incorporated association supporting dance activities for those aged 65 and above, has been actively launching hip-hop dance teams for older adults across Japan since 2021. In September 2023, the FIDA GOLD CUP 2023 dance competition was held, featuring 13 teams from various regions in Japan. Among the dancers who participated in the competition and responded to the post-performance survey ($n=148$, mean age=70.99 years, $SD=5.46$), 62.8% started dancing at the age of 65 or older. Audience members ($n=81$, mean age=51.69 years, $SD=15.85$) were also surveyed. Both surveys assessed various aspects of emotional experiences using a 5-point scale. Analysis revealed that dancers reported significantly higher levels of emotional resonance ($M=5.24$, $SD=0.82$) compared to the audience ($M=4.68$, $SD=0.62$), $t(227)=5.58$, $p< 0.001$, Cohen's $d=0.77$, indicating a large effect size. Additionally, a statistically significant positive correlation was found between dancers' feelings of exhilaration on stage and the audience's applause ($\rho=0.31$, $p< 0.01$), suggesting that the emotional experiences of dancers and their audience are interconnected. The results highlight the emotional interplay between dancers of advanced age and their audience, emphasizing the potential for dance to foster a shared emotional experience. The findings contribute to understanding how dance can enhance emotional well-being and social engagement, particularly for those who take up dancing in later life.

Abstract citation ID: igae098.0203
**CHARACTERIZATION OF DYNAMIC ADAPTATION TO STRESSORS USING MULTISYSTEM STIMULUS–RESPONSE DATA**
Karen Bandeen-Roche[1], Jiafeng Zhu[2], Qian-Li Xue[1], Brian Buta[1], Thomas Laskow[1], Jeremy Walston[1], and Ravi Varadhan[1], *1. Johns Hopkins University, Baltimore, Maryland, United States*, *2. Northwestern University, Evanston, Illinois, United States*

Resilience has emerged as a major gerontological concept aiming to promote more consistently positive outcomes following stressors. Achieving this aim relies on determining mechanisms underlying capacity to respond resiliently. This paper addresses the hypothesis that physical aspects of said capacity arise considerably from the fitness of one's physiology governing stress response, conceptualized as a dynamical system. We leverage the Study of Physical Resilience and Aging ("SPRING") pilot and Phase II studies, which implemented stimulus-response experiments to characterize physiological fitness in older adults scheduled for major stressors of either total knee replacement, incident hemodialysis, or bone marrow transplant for hematological cancers. We develop physiologic resilience capacity measures using time series from Holter monitor assessment, cortisol response to adrenocorticotropic hormone stimulation, and repeated diurnal salivary cortisol assessment. Principal components (PCs) of the multivariable summaries from these tests were derived using SPRING pilot data. The first two components represented "steady state" and "adaptation" mechanisms. Steady state and adaptation PC scores were separately regressed on major stressor type, hypothesized static physiologic indicators (physical frailty phenotype, self-report of health), and personal characteristics. Substantial association was manifest by stressor type and the static physiologic indicators: for both PCs, average scores were 0.6 SD higher for persons deemed robust versus prefrail/frail after stressor type adjustment ($p< 0.05$). Cross-validation analyses using Phase II data, as well as analyses to discriminate resilience phenotypes and outcomes post-stressor with these data, will be reported. Our study lays groundwork to better forecast and foster resilience of older adults to clinical stressors.

Abstract citation ID: igae098.0204

### THE OPIOID USE IN OLDER ADULTS COMPENDIUM OF RESOURCES: A USER-FRIENDLY TOOL FOR PRIMARY CARE

Latrice Vinson[1], Vonetta Dotson[2], and Arlene Bierman[2], *1. Agency for Healthcare Research and Quality, Rockville, Maryland, United States*, *2. Georgia State University, Atlanta, Georgia, United States*

Older adults are at greater risk of harm from opioid use, yet there is limited understanding of how to manage opioid use, misuse, and opioid use disorder among older adults, particularly in primary care practices (PCPs). AHRQ launched an initiative to synthesize the literature and create resources and tools to support PCPs in improving opioid management for older adults. This presentation describes the development of the initiative's final product, the "2024 Opioid Use in Older Adults Compendium of Resources" and recommendations for using the tool in practice and future research. The Compendium was created though an iterative, three-stage process: environmental scan; technical expert panel feedback; and testing and refining the tool by two learning collaboratives (LCs) across 14 PCPs implementing quality improvement (QI) projects. Resources from the scan and data derived from the LCs were packaged into a driver diagram, which was iteratively revised by the panel and LCs. The resulting Compendium identified seven high-leverage changes (e.g., risk assessment, patient engagement), each associated with a change strategy, key activities, and existing and newly developed tools/resources to bring about the desired change practices can use to improve opioid management and prevent misuse in older adults. PCPs can access the user-friendly Compendium on AHRQ's website to plan and implement QI activities related to opioid management. The Compendium identifies innovative screening measures, interventions, and patient-facing tools that present promising targets for future research.

Abstract citation ID: igae098.0205

### A META-COGNITIVE GROUP INTERVENTION FOR OLDER ADULTS WITH SCD OR MCI: RCT AND QUALITATIVE EVALUATION

Shlomit Rotenberg[1], Nicole Anderson[2], and Deirdre Dawson[1], *1. University of Toronto, Toronto, Ontario, Canada*, *2. Baycrest Academy for Research & Education, Toronto, Ontario, Canada*

We explored ASPIRE, a meta-cognitive group intervention aimed to improve engagement in daily activities of older adults with subjective cognitive decline (SCD) or mild cognitive impairment (MCI). We performed a double-blind randomized controlled trail, and complementary qualitative study. Participants were randomly assigned to ASPIRE (n=131) or a Brain Education control group (n=133). The primary quantitative outcome was improvement in performance of daily activities not trained in either intervention, using the Canadian Occupational Performance Measure (COPM). A linear mixed effects regression model found clinically significant improvement (change ≥ 2 points on the COPM) post intervention in 32.5% of untrained activities in ASPIRE; and 30.6% in Brain Education, with no significant group effects (exp(ß)=0.96, z=-0.15, p=.879). Improvements from pre- to post-intervention were found in both arms on secondary measures of subjective cognition and self-efficacy, with no significant group effects. Qualitative data were written responses about the experience of ASPIRE or Brain-Education collected post intervention, analysed using inductive thematic content analysis. ASPIRE participants were pleased with the Group Context, that included learning from, and helping others with shared experiences of cognitive concerns. They described ASPIRE as applicable to Everyday Life, providing practical strategies and motivating action through accountability to the group. Brain-Education participants were pleased with the Content, specifically the topic areas, described as interesting and stimulating. Both groups were pleased with the facilitation approach. We conclude that ASPIRE and Brain-Education have similar potential to improve subjective cognition, self-efficacy, and engagement in daily activities, but the mechanisms by which they work are different.

### SESSION 1210 (SYMPOSIUM)

Abstract citation ID: igae098.0206

### HEALTH RESILIENCE AND FORTITUDE FACTORS IN THE HRS

Chair: Rebecca Allen
Discussant: Jacqui Smith

This symposium presents data from recent waves of the Health and Retirement Study, a nationally representative sample of older adults in the United States, examining environmental transition and health variables as they relate to fortitude. Using stress process models and the Meikirch model of health as theoretical underpinning, these presentations examine fortitude by focusing on 1) transitions in place of residence and loneliness (inverse of fortitude), 2) relationship quality and pain intensity, and 3) sleep disorders, relationship quality, and life satisfaction. The first presenter found that subjective health, transitional threat, and social and community engagement all were significantly related to self-reported loneliness. However, the interaction between engagement and transitional threat did not explain additional variance in the model. The second presenter found that the association between perceived spousal support (personally acquired potential) and well-being significantly decreased when controlling for the intensity of pain (biologically given potential) experienced by the respondent. Similarly, using the Meikirch model of health, the third presenter conducted simple linear regression analyses to assess the relations between sleep disorders, perceived life satisfaction, and perceived spousal relationship quality. Having a sleep disorder significantly predicted life satisfaction and perceived problems in spousal relationships. Thus, perceived stress due to environmental transition and the interplay between biologically given potential and personally acquired potential explain significant variance in fortitude, as measured by loneliness (inversely), well-being, and life satisfaction. The discussion will address strengths and challenges in examining fortitude as a construct of interest in the nationally representative Health and Retirement Study dataset.

Abstract citation ID: igae098.0207

### THEY'VE GOT MY BACK: THE ROLE OF PAIN INTENSITY AS A MEDIATOR BETWEEN PERCEIVED RELATIONSHIP QUALITY AND RESILIENCE

Erick Carranza[1], Landon Peeples[1], Michael Buxton[1], M. Lindsey Jacobs[2], and Rebecca Allen[1], *1. University of Alabama, Tuscaloosa, Alabama, United States*, *2. The University of Alabama, Tuscaloosa, Alabama, United States*

Recent increases in the population of aging adults reflect a dire need to examine the social contexts that influence their experiences in relation to physical and mental health, especially during the ongoing coronavirus pandemic. Previous work identifies how perceived care partner relationships play a role in reducing psychological distress for aging adults facing a chronic illness (Schroepfer, 2008). Evidence also suggests that perceived relationship quality is associated with experiencing greater pain and disability (Campbell, 2012). However, little is known about the dynamic interplay between perceived relationship quality, pain intensity, and resilience. This presentation aims to draw upon the Meikirch model of health examining pain intensity as a biologically given potential and relationship quality as personally acquired potential while experiences during COVID-19 may be interpreted as environmental determinants of health. The objective is to understand how relationship quality influences levels of resilience (i.e., psychological well-being) and is then mediated by the intensity of pain. The mediation analysis was conducted using a secondary data approach with responses (N = 2686) from the 2020 wave of the Health and Retirement Study, a nationally representative sample of older adults in the United States. Results revealed a significant indirect effect, indicating that the association between perceived spousal support and resilience decreased when controlling for the intensity of pain experienced by the respondent. Future directions include examining responses from spouses to compare dyadically the perceptions of relationship quality and how these perceptions relate to pain intensity within one partner and dyadic perceptions of couple resilience.

Abstract citation ID: igae098.0208

### RESILIENCE IN HEALTH ADVERSITY: THE ROLE OF COMMUNITY ENGAGEMENT AND TRANSITIONAL THREAT

Ashley Fromenthal, and Rebecca Allen, *University of Alabama, Tuscaloosa, Alabama, United States*

Older adulthood is a time of many transitions and oftentimes comes with challenges such as financial difficulties, functional health decline, and a decrease in social support. Together, these challenges may accentuate feelings of loneliness in older adulthood. These challenges may be exacerbated when older adults face threats within their environment such as having housing issues or moving to a "worse" neighborhood. The current study aimed to explore community-based resilience factors for older adults facing transitional threats and functional health struggles centered around Lazarus and Folkman's Transactional Model of Stress and Coping. Using 2020 data from 4,688 participants in the Health and Retirement Study (HRS), multiple linear regressions were conducted to examine whether the relationship between subjective health and loneliness is mediated by transitional threats, and if social and community engagement moderate the mediating relationship. Results of the regressions show that the overall model is significant $F(2,4546) = 78.11$, $p < .001$, and that each of the predictor variables were significantly related to feelings of loneliness. However, results show that the interaction term (engagement x threat) did not explain a significant amount of variance in the model. This may be due to the measurement of these constructs within the HRS. Specifically, trait-dependent measurement of dynamic constructs such as feelings of loneliness and behavioral engagement may not be sensitive enough to detect moderated mediation effects within the context of transitional threats.

Abstract citation ID: igae098.0209

### SLEEP DISORDERS AND THEIR IMPACTS ON OLDER ADULT SPOUSAL RELATIONSHIP

Sophia Geisser[1], Ashley Fromenthal[1], Erick Carranza[1], Michelle Hilgeman[2], and A. Lynn Snow[1], *1. University of Alabama, Tuscaloosa, Alabama, United States*, *2. Tuscaloosa VA Medical Center, Tuscaloosa, Alabama, United States*

Aging is associated with biological and psychosocial impairments that affect individuals' sleep and quality of life. Approximately half of older adults suffer from a sleep disorder. Daytime consequences such as fatigue, excessive sleepiness, irritability, mood swings, and/or cognitive difficulties may be present with sleep disorder diagnoses, impacting interpersonal relationships. There is limited research on associations between sleep disorder and spousal relationship quality in older adults. The need to expand the field of research in sleep disorders is urgent, as sleep disorder diagnoses are expected to rise with the growing older adult population. The objective of this presentation is to describe a conceptual model of sleep disorders and relationship quality informed by the Meikirch model of health: sleep disorders represent biologically given potential, relationship quality represents personally acquired potential, and the COVID-19 pandemic represents the environmental determinants of health. Applying this theoretical underpinning, the Health and Retirement Study data from 2020 was analyzed via simple linear regression analyses to assess the relationships between sleep disorders and perceived life satisfaction in spousal relationships. Results show that having a sleep disorder significantly predicted life satisfaction: F(1,4647) = 40.49, p <.001. (b =.09, S.E. =.02, t = 6.36, p <.001, 95%CI[.06,.13]); and having a sleep disorder significantly predicted perceived problems in spousal relationships: F(1,4560) = 15.49, p <.001 (b = -.12, S.E. =.03, t = -3.94, p <.001, 95%CI[-.17,.06]). Understanding sleep-related associations may inform interventions to promote well-being and life satisfaction for older adult couples.

### SESSION 1215 (PAPER)

### LATE-LIFE EXPERIENCES: IDENTITY, MIGRATION, DECISION-MAKING, AND WELL-BEING

Abstract citation ID: igae098.0210

### DIFFERENCES BETWEEN LIFELONG SINGLES AND PARTNERED INDIVIDUALS IN PERSONALITY TRAITS AND LIFE SATISFACTION

David Richter[1], Julia Stern[2], Michael Krämer[3], Alexander Schumacher[4], and Geoff MacDonald[5], *1. SHARE Berlin Institute, Berlin, Berlin, Germany*, *2. University of Bremen, Bremen, Bremen, Germany*, *3. University of Zurich, Zurich, Zurich, Switzerland*, *4. Munich Research Institute for the Economics of Aging and SHARE Analyses (MEA-SHARE), Munich, Bayern, Germany*, *5. University of Toronto, Toronto, Ontario, Canada*

Being romantically partnered is widely seen as societal norm, and has been shown to be positively associated with important life outcomes, such as physical and mental health. However, the percentage of singles is steadily increasing, with more people staying single for life. We used the Survey of Health, Ageing and Retirement in Europe (N = 77,064 in retirement age from 27 countries) to investigate the Big Five personality traits and life satisfaction in lifelong singles as compared to (previously) partnered individuals. Specification curve analyses suggested that lifelong singles were less extraverted, conscientious, open to experiences, and less satisfied with their lives. Effects were stronger for never partnered than for never cohabitating or never married individuals, and partly moderated by gender, age, country-level singlehood, and gender ratio. Our study provides insights into characteristics of lifelong singles, and has implications for understanding mental health and structures of social support in older individuals.

Abstract citation ID: igae098.0211

### DETERMINANTS OF SETTLEMENT INTENTIONS AMONG OLDER CHINESE INTERNAL MIGRANTS: AN INTEGRATED PERSPECTIVE

Rui Kang[1], and Jia-jia Zhou[2], *1. The Hong Kong Polytechnic University, Hong Kong*, *2. Hong Kong Baptist University, Hong Kong, Hong Kong*

Understanding factors driving settlement intention of older migrants is important for policies to promote successful integration. This research examined to what extent the adaption, attachment to hometown, and environmental factors affect the settlement intentions among older internal migrants in China. A cross-sectional survey was conducted among 598 older migrants aged 50 and above in Shenzhen and Guangzhou, two large cities in China. Data on settlement intention, Hukou transfer intention, multidimensional adaption, environmental factors and attachment to hometown were collected using questionnaire survey. Multinomial logistic regression modelling was employed to identify the determinants of settlement intention. The findings revealed that better psychological adaptation and frequent participation in community activities increased the likelihood of settling in the host city and transferring Hukou registration. Regular involvement in voluntary activities and increased access to welfare services decreased the probability of expressing reluctance to settle and transfer their Hukou status. Conversely, maintaining connections with friends from their hometown reduced the likelihood of settlement in host city. Older migrants with living parents in their hometown are more likely to resist settle in the host city. However, a higher perceived quality of physical environments in the neighborhood elevated their likelihood of not deciding to settle and transfer their Hukou status. It is crucial for policy and service interventions to focus on enhancing the welfare and service environments in neighborhoods. Such improvements can facilitate the successful integration of older migrants into their new communities, thereby promoting their well-being and contributing to the overall social cohesion.

Abstract citation ID: igae098.0212

### IT'S BEYOND PERSONAL: MULTILEVEL FACTORS AFFECTING THE WILLINGNESS FOR PALLIATIVE CARE UTILIZATION

Juncheng Tong[1], and Jiawei Cao[2], *1. Nanjing University of Chinese Medicine, Nanjing, Jiangsu, China (People's Republic)*, *2. Nanjing University Of Chinese Medicine, Nanjing, Jiangsu, China (People's Republic)*

With so much effort devoted to the development of palliative care, significant progress has been achieved in the past few years in China. One of the foci is the patient education and public advocacy. However, evaluation research on this aspect is scarce, and little is known regarding people's willingness to engage in conversations about palliative care and potentially use such services if needed. Thus, this survey study aims to examine the public's willingness of palliative care utilization and associated factors from micro, meso, and macro levels. Ordinal regression analyses were performed to achieve this goal. Among 2,645 adult participants (aged 18-95), approximately 78% either 'agree' or 'strongly agree' with the statement that they are willing to use palliative care services if they are terminally ill. Regression results showed that at the micro-level (individual), willing to pay out-of-pocket and believing palliative care can improve end-of-life quality were associated with higher odds of willing to use palliative care. Meso-level measurement of family support and macro-level measurement of filial piety were also significant factors. Specifically, more family support was associated with an increase in the odds of willing to use palliative care (OR = 1.040, p = 0.034); and the odds of willing to use palliative care for people who considered palliative care not to be in line with filial piety was 0.014 (p < 0.001) times that of those who did. Married people were also more likely to be willing to use palliative care. Implications for research, policy, and practice are discussed.

Abstract citation ID: igae098.0213

### NO ONE REMEMBERS MY NAME: (RE)NEGOTIATING IDENTITY, AGENCY, AND RESILIENCE IN LATE-LIFE IMMIGRANT SOUTH ASIAN WOMEN

Mushira Khan, *Mather Institute, Evanston, Illinois, United States*

Despite a growing South Asian (SA) population in the US, little is known about the lived experiences of late-life SA immigrant women who may face unique challenges as they navigate their later years in a new cultural and social space. This qualitative study explored how late-life SA immigrant women negotiate issues around identity, faith, and the continuity/rupture of norms and familial bonds in the context of migration and multigenerational living. Using an intersectional lifecourse perspective, in-depth interviews were conducted with twelve SA women who immigrated to the US at age 65 or older and co-resided with their adult children and/or grandchildren in a large cosmopolitan US city. Thematic analysis of the interview data uncovered the resilience and strength of the study participants as they navigated settlement challenges, including culture shock, language barriers, health and social care access issues, and the intricate dynamics of multigenerational living. Drawing from their cultural heritage, they navigated these transitions while also (re)interpreting cultural traditions to find meaning and cope with a perceived loss of status in the family. Furthermore, despite migration challenges, study participants found solace in embracing their grandmother role and through support from their religious and cultural enclaves. These findings highlight the salience of faith, family, and community in the lives of late-life immigrant SA women–a group that often remains invisible in policy and practice–and can help policymakers and stakeholders, including community service providers, faith leaders, researchers, and family members, to provide culturally appropriate support and promote their health and well-being.

Abstract citation ID: igae098.0214

### PERSON-ENVIRONMENT FIT UNDERLYING PERSONALITY DEVELOPMENT IN MIDDLE AND LATE ADULTHOOD

Christopher Beam[1], and Emily Schoenhofen Sharp[2], *1. University of Southern California, Los Angeles, California, United States*, *2. Yale Medicine, New Haven, Connecticut, United States*

The corresponsive principle and the niche-picking principle have been used to explain personality development across adulthood. The corresponsive principle posits that reciprocal effects between people and their environments intensify personality traits because environments are selected based on these traits while the niche-picking principle posits that these reciprocal effects simply maintain existing trait levels. Using a longitudinal sample of twins from the Swedish Adoption/Twin Study of Aging (SATSA), we tested whether the corresponsive principle or the niche-picking principle better explained longitudinal within-family differences in neuroticism, extraversion, and openness to experience across two stages of adulthood development: middle adulthood (36--56 years) and older adulthood (56--91 years). Using exploratory data analysis and multilevel structural equation modeling, we show that niche-picking rather coresponsivity tends to provide a better overall description of the development of neuroticism, extraversion, and openness in middle and older adulthood. Within families, twins generally preserve their rank-order stability but do not diverge over time. Instead, there is a generally tendency for twins to maintain within-family differences over time or even become more alike, regardless of whether twins are identical or fraternal. Nevertheless, the role that niche-picking might play in the personality development across adulthood generally is low, as the majority of the total variance of neuroticism, extraversion, and openness is attributed to stable genetic and environmental variance, particularly in older adulthood. Taken together, these findings suggest that environmental variance - whether independent of or correlated with genotype - takes on an increasingly stronger role in personality development over the lifespan.

### SESSION 1220 (SYMPOSIUM)

Abstract citation ID: igae098.0215

### LIFE AT AGE 100 IN SWITZERLAND: FINDINGS FROM THE SWISS100 CENTENARIAN STUDY

Chair: Daniela Jopp

Switzerland is among the countries with the highest life expectancy and has seen a strong increase of very old individuals over the past century. Yet very little is known about its oldest-old population, including those aged 100 and older. This symposium presents findings from the first national centenarian study conducted in Switzerland, SWISS100. The presentations offer findings from the SWISS100 Main Study, allowing due to its interdisciplinary nature a comprehensive perspective on life at age 100 in Switzerland. Jopp and colleagues will present findings on key characteristics in centenarians (e.g., health, cognition, social resources, psychological strengths) and their associations with quality of life. Stahlmann and colleagues will share findings on the use of ambulatory assessment in centenarians, more specifically sound recordings which allow to investigate everyday life activities, showing that such innovative tools are applicable in this population and that these methods lead to important insights, complementing self-report. Masotti and colleagues will present results on the caregiving setting in centenarians, illustrating that Swiss centenarians show a notable variation in needs and support. Hoffman and colleagues will present results on centenarians' goals, coded by content and orientation, and associations to well-being outcomes, illustrating that goals remain important adaptation features in very old age. Overall, SWISS100 findings help to better understand the specific life phase centenarians are experiencing, including its challenges, but also show notable psychological resilience and well-being.

Abstract citation ID: igae098.0216

**LIFE SATISFACTION IN CENTENARIANS: FINDINGS FROM THE SWISS100 MAIN STUDY**

Daniela Jopp[1], Adar Hoffman[1], Armin von Gunten[2], Francois Herrmann[3], Christina Roecke[4], Mike Martin[4], and Stefano Cavalli[5], *1. University of Lausanne, Lausanne, Vaud, Switzerland*, *2. Lausanne University Hospital, Prilly, Vaud, Switzerland*, *3. Geneva University Hospitals, Thônex, Geneve, Switzerland*, *4. University of Zurich, Zurich, Zurich, Switzerland*, *5. University of Applied Sciences and Arts of Southern Switzerland, Manno, Ticino, Switzerland*

With increasing numbers of centenarians world-wide, quality of life in these very old individuals has become a topic of high interest, expanding prior biomedical research. Switzerland is not only among the countries with very high life expectancy but is also among the leading countries in terms life satisfaction. In the present study, we investigated the levels and predictors of life satisfaction in Swiss centenarians. The present analysis included a total of 148 centenarians of the SWISS100 Main Study (Mage = 101.8; range: 100-106; 74.3% female). After identification through the national address registry, centenarians were visited for in-person interviews. Centenarians' health was quite limited: they had an average of six health issues and over half of them lived in care facilities (57.4%). Their cognitive level was moderate to high. Most were widowed, 84% had children, and they reported a substantial number of social contacts. The majority of centenarians (82.4 %) were satisfied with their lives. Correlations indicated that sociodemographic and health variables had only few associations with life satisfaction, but that psychological aspects including optimistic outlook and meaning in life showed notable links. A regression analysis combining significant variables from domain-specific regressions revealed that optimistic outlook was the strongest predictor of interindividual differences in life satisfaction, followed by meaning in life. In sum, findings highlight that Swiss centenarians present poor levels of health yet manage to maintain high life satisfaction through psychological mechanisms, suggesting high resilience in these very old individuals, which replicates and extends results from prior centenarian studies.

Abstract citation ID: igae098.0217

**DECODING CENTENARIAN DAILY ACTIVITIES: A REAL-LIFE SENSOR APPROACH**

Melanie Stahlmann[1], Tabea Buehrer[1], Christina Roecke[1], Daniela Jopp[2], Stefano Cavalli[3], Armin von Gunten[4], Francois Herrmann[5], and Mike Martin[1], *1. University of Zurich, Zurich, Zurich, Switzerland*, *2. University of Lausanne, Lausanne, Vaud, Switzerland*, *3. University of Applied Sciences and Arts of Southern Switzerland, Manno, Ticino, Switzerland*, *4. Lausanne University Hospital, Prilly, Vaud, Switzerland*, *5. Hopitaux Universitaires Geneve, Thonex, Geneve, Switzerland*

In response to the decade of healthy aging, understanding daily activities in older adults has become increasingly important in aging research. While ambulatory assessment is now quite common, it often misses the oldest age groups. The SWISS100 study is the first to fill this gap by employing real-life sensors to explore Swiss centenarians' lives, blending questionnaire data with actigraphs for physical activity assessment, and smartphones equipped with the Electronically Activated Recorder (EAR) to capture everyday sounds and conversations. Out of N = 44 German-speaking centenarians who consented to the EAR study, technical issues and other challenges led to a final dataset from 35 participants. Over a time period of four days, more than 10,000 audio clips were collected, equating to roughly 2.7 hours of recordings per participant, which is about 4% of their waking hours. These recordings were then transcribed and analyzed to identify activities and social interactions. The analyses revealed that centenarians primarily spend their time alone (85%), with TV watching and resting as their main activities. When interacting, centenarians mostly engage with one familiar person, with a significant portion of conversations being "substantive talk" (44%), followed by "practical talk" (34%), though notable variations exist among individuals. These findings offer a deeper understanding of Centenarians' social and leisure patterns, enriching our knowledge of their daily lives. This study also demonstrates the feasibility and value of mobile sensing in researching very advanced ages, encouraging future studies to integrate such innovative methods for a more comprehensive view of participants' real-life experiences.

Abstract citation ID: igae098.0218

**PROVIDERS, FUNCTIONS, AND ADEQUACY OF CARE AMONG CENTENARIANS: INSIGHTS FROM SWISS100**

Barbara Masotti[1], Daniele Zaccaria[1], Francois Herrmann[2], Daniela Jopp[3], Armin von Gunten[4], and Stefano Cavalli[1], *1. University of Applied Sciences and Arts of Southern Switzerland, Manno, Ticino, Switzerland*, *2. Geneva University Hospitals, Thônex, Geneve, Switzerland*, *3. University of Lausanne, Lausanne, Vaud, Switzerland*, *4. Lausanne University Hospital, Prilly, Vaud, Switzerland*

At age 100 and beyond, individuals deal with increasing support needs, especially when their social network is at risk due to the death of spouses, siblings, and friends, while children may face own age-related health issues. However, little is known about the specific aids received by centenarians. Drawing from the care convoy framework, this study explored the diversity of care among Swiss centenarians. We investigated the composition and the functions of their care convoys and analysed the impact of individual and contextual characteristics. Additionally, we examined participants' perception of the adequacy of the aids received. The analyses are based on centenarian and/or proxy reports from the study SWISS100 (N = 275; Mage = 101.789) using descriptive statistics and logistic regression models. Subjective adequacy is examined through qualitative content analysis. Findings indicate that centenarians are part of intricate convoys of care. The diversity of types of care and providers was high among community-dwelling, where informal and formal caregivers both provide help with a wide range of tasks (e.g., personal care, monitoring). In institutional settings there was a more defined division of roles, with family and friends providing administrative and socioemotional support. The impact of individual and contextual factors varied depending on the type of care and/or the care provider. Participant satisfaction did not always align with the quantity of care received. Considering, in conclusion, the diversity of care observed, understanding centenarians' needs and resources is essential for developing tailored policies and services for them and their families.

Abstract citation ID: igae098.0219

### EXPLORING GOAL CONTENT AND ORIENTATION OF SWISS CENTENARIANS

Adar Hoffman[1], Daniela Jopp[1], Stefano Cavalli[2], Armin von Gunten[3], Christina Roecke[4], Mike Martin[4], and Francois Herrmann[5], *1. University of Lausanne, Lausanne, Vaud, Switzerland*, *2. University of Applied Sciences and Arts of Southern Switzerland, Manno, Ticino, Switzerland*, *3. Lausanne University Hospital, Prilly, Vaud, Switzerland*, *4. University of Zurich, Zurich, Zurich, Switzerland*, *5. Geneva University Hospitals, Thônex, Geneve, Switzerland*

In very old age, goal orientation is expected to shift towards maintenance-oriented goals, as adaptive mechanism (e.g., Ebner, Freund, & Baltes, 2006). For centenarians, opportunities can be especially limited due to physical and contextual constraints, calling for adjustment in goal content and orientation. This study investigated the number, content, and orientation of goals among a subsample of 135 centenarians (Mage = 101.6 years) in the SWISS100 study. Within this sample, 78 (58%) centenarians mentioned at least one goal, and 57 (42%) centenarians did not mention any. Specifically, 27 (35%) specified one goal, 24 (30%) two, and 27 (35%) three distinct goals. Goals were coded for content and orientation. Leisure and family contents were most prevalent domains for goal setting, covering half of the goals mentioned. Subsequently came contents of health and home-related, while domains of social, well-being, independence and religion were also mentioned but to a lesser extent. Moreover, approach and maintenance orientations were the most widespread, representing 75 percent of all goal orientations. Some improvement goals were mentioned, but avoidance goals were rare, particularly for second and third goals. A higher number of goals was associated with more meaning in life and higher subjective well-being. Overall, results show that centenarians still have goals, the most prevalent contents reflect their life stage, and the many approach goals suggest a positive orientation. As goals seem to remain important mechanism of adaptation among centenarians, more research is needed to better understand the role of goals as a potential means of successful aging.

### SESSION 1225 (SYMPOSIUM)

Abstract citation ID: igae098.0220

### LONELINESS AND SOCIAL ISOLATION AMONG VULNERABLE, DIVERSE, AND MINORITY GROUPS: NOVEL RESEARCH AND PRACTICAL INSIGHTS

Chair: Roger O'Sullivan
Co-Chair: Christina Victor
Discussant: Thomas Cudjoe

Loneliness and social isolation are global public health challenges with concerning implications for mental, cognitive, and physical health. Extensive evidence documents the prevalence, incidence, and negative health outcomes associated with loneliness and social isolation. In contrast, there is a dearth of research offering focused insights into three under-researched 'at-risk' populations: those with severe mental health illness, different racial and minority groups, and people who identify as LGBT. This symposium will address this gap by examining the experience of loneliness in these three populations and providing evidence to inform the development of future public health, policy, and service interventions. Pomeroy provides context with an overview of issues constraining progress in the field, including measurement inconsistency and the lack of diversity in studies of loneliness and social isolation. O'Sullivan, Taylor, and Victor highlight the importance of studying loneliness and social isolation within subpopulations and at-risk groups. O'Sullivan describes qualitative interviews with mental health patients to examine their conceptualization and lived experience of loneliness. Taylor's paper examines the issue of race, neighborhoods and their confluence on feelings of loneliness among older adults in the United States. Victor using longitudinal data reports that rates of chronic loneliness over three years are twice as high amongst LGB and minority ethnic groups compared with the general population aged 50+. Cudjoe, as discussant, highlights the implications for policy, services, and future research in this area and the need for focused public health solutions to tackle loneliness and social isolation.

Abstract citation ID: igae098.0221

### WHY MEASUREMENT MATTERS IN UNDERSTANDING SOCIAL CONNECTIONS: REFLECTIONS ON MEASURING SOCIAL ISOLATION

Mary Louise Pomeroy[1], Fereshteh Mehrabi[2], Emerald Jenkins[1], Roger O'Sullivan[3], Jim Lubben[4], and Thomas Cudjoe[5], *1. Johns Hopkins University, Baltimore, Maryland, United States*, *2. Concordia University, Montreal, Quebec, Canada*, *3. Ageing Research And Development Division, Institute Of Public Health, Dublin, Dublin, Ireland*, *4. Boston College, Chestnut Hill, Massachusetts, United States*, *5. Johns Hopkins University School of Medicine, Baltimore, Maryland, United States*

Social isolation has a negative impact on society and increases the risk of morbidity and mortality. Despite recent strides in this literature, methodological issues remain in how social isolation is measured and subsequently described. Fundamentally, most measures are not validated, do not measure objective social isolation, or interchange assessments of social isolation with loneliness. Many studies continue to rely on a single item to measure social isolation, most likely resulting in biased estimates. Researchers are faced with a variety of unvalidated metrics that differ in their theoretical framing and composition. We believe that this heterogeneity stems in part from the lack of psychometric testing, impeding the advancement of work in this area. The lack of consistency perpetuates misunderstandings in population-based estimates or other rigorous characterizations of social isolation, precludes the comparison of findings across studies, and undercuts our understanding of intervention effectiveness. We call for the research community to employ psychometric testing of social isolation measures and to solicit input from a wide array of stakeholders to substantiate a proposed measure. This information could then be used to generate broad support for use of evidence-based measures of social isolation, promoting consistency and uptake in clinical, research, and community settings. Establishing acceptability and validity of social isolation measures is critical to developing screening and prevention strategies, designing effective interventions, and adopting practice and policies that are relevant to stakeholders.

Abstract citation ID: igae098.0222

**THE LANGUAGE OF LONELINESS: DEFINITIONS, EXPERIENCES, AND CONCEPTUALIZATION: THE VOICE OF MENTAL HEALTH SERVICE USERS**

Roger O'Sullivan, *Institute of Public Health in Ireland, Belfast, Dublin, Ireland*

Loneliness is an important public health issue that describes a complex subjective personal experience of one's desired and actual or perceived social and/or emotional connections. In recent years we have seen a rise in the study of loneliness especially from a quantitative perspective. However, our qualitative understanding of what it means to be lonely and the everyday language best used to capture the personal experience of loneliness remains limited. This presentation based on life story interviews, with older adult mental health service users (18), objectively defined as lonely, gives voice to their definitions, personal experiences and overall conceptualization of loneliness. It sets out a thematic analysis of responses to the question "What does loneliness mean to you? Responses include subjective and objective aspects, frequency, duration and intensity as well as social; emotional and existential loneliness. Loneliness was not defined by participants in a singular manner and was discussed alongside social isolation, depression, grief, as well as feelings of emptiness, boredom and hopelessness. The insights are important to inform how we frame loneliness in research, policy and practice and highlights that our language needs to be sufficiently diverse to capture the complexity of loneliness not only for work with mental health service users but in society as a whole.

Abstract citation ID: igae098.0223

**LONELINESS TRANSITIONS AMONG MINORITY ETHNIC AND LGB POPULATIONS IN THE UK**

Christina Victor, and Isla Rippon, *Brunel University London, London, England, United Kingdom*

Loneliness has been identified as a major public health problem. Although there is a substantial body of research about loneliness in older adults in the UK, there is a significant evidence gap reporting experiences of loneliness among older people from ethnic minorities and those who identify as lesbian, gay, or bisexual (LGB). We focus upon the experiences of loneliness for adults aged 50+, from LGB and minority ethnic communities. Using waves 9-12 of the annual UK Household Longitudinal Study (UKHLS/Understanding Society) we measured loneliness using the three-item UCLA scale with a score of 6+ out of 9 defining loneliness. 7,646 respondents completed the loneliness measure at each wave. 1.7% of participants identified as LGB and 4.3% as Asian, 2.5% as black and 1.4% as mixed /other ethnicity. We grouped respondents into three categories: (a) consistently lonely; (b) consistently not lonely and (c) fluctuating loneliness. A higher proportion of LGB respondents were consistently lonely (16.4% vs 9.2%), compared to heterosexual respondents. Respondents from black (14.1%), Asian (11.2%) and other ethnic minorities (17.5%) were more likely to be consistently lonely in comparison to white respondents (8.9%). Shortcomings in data available on these groups of interest limit our analytical power to examine the importance of micro, meso and macro-level risk factors. Preliminary findings suggest that socio-demographic predictors for fluctuating or persistent loneliness differ in our groups of interest in comparison with white or heterosexual respondents. The higher level of persistent loneliness in the two groups has potential implications for their health and wellbeing.

### SESSION 1230 (PAPER)

### LONG-TERM CARE WORKFORCE

Abstract citation ID: igae098.0224

**TRENDS AND VARIATION OF MEDICARE ADVANTAGE AMONG LONG-STAY NURSING HOME RESIDENTS**

Hyunkyung Yun, and Cyrus Kosar, *Brown University, Providence, Rhode Island, United States*

Despite the large growth of the Medicare Advantage (MA) to now comprise over 50% of all Medicare beneficiaries, the role of MA in long-term care has been understudied. We examined trends in MA enrollment among long-stay nursing home (NH) residents and geographic variation in these trends (2010 to 2021). We compared the individual and NH characteristics between beneficiaries of traditional Medicare (TM), general MA, and MA plans specialized for long-term care, Institutional Special Needs Plans (I-SNPs). Among long-stay residents, MA enrollment grew from 13.0% in 2010 to 31.6% in 2021, while MA enrollment increased from 23.6% to 40.8% for all other Medicare beneficiaries. I-SNP enrollment grew substantially; in 2010, 34 I-SNPs enrolled 15.7% of MA long-stay residents. By 2021, 126 I-SNPs enrolled 30.8% of them. The individual- and facility- characteristics for TM and non-I-SNP MA enrollees were broadly comparable. I-SNP enrollees, however, were more likely to be Black (20.4% of residents vs 14.5% for TM, 13.5% for other MA) and dually enrolled in Medicaid (I-SNP 97.7%, TM 83.6%, other MA 83.1%). I-SNP enrollees were more likely to be in NH with 1-2 (low) star-ratings (I-SNP 38.7%, TM 38.4%, other MA 38.2%) and less likely to reside in facilities with 4-5 (high) star-ratings (I-SNP 38.7%, TM 42.1%, other MA 42.4%). MA enrollment has grown substantially among long-stay residents. As new models such as I-SNPs become more prevalent, future research and monitoring are needed to ensure that the care needs of this high-need population are being adequately met.

Abstract citation ID: igae098.0225

**USE OF A NOVEL STAFFING PROGRAM IN NURSING HOMES: A MIXED METHODS STUDY**

Lindsay Peterson, and Kelly Smith, *University of South Florida, Tampa, Florida, United States*

Staffing in nursing homes (NHs) has long been a critical policy issue. Faced with NH staffing challenges, Florida legislators approved a 2021 measure allowing lesser trained staff (personal care attendants [PCAs]) to work as certified aides for four months, with the expectation the PCA would become certified in that time. We used mixed methods, aiming to assess Florida NH administrators' views on this novel staffing program. We distributed a Qualtrics survey of closed- and open-ended questions to 695 administrators via email between July 2022 and September 2022, receiving 55 unduplicated, complete responses. Logistic regression was used to estimate relationships between independent variables (e.g. facility characteristics, federal five-star rating, survey responses) and the outcome, whether respondents found the PCA program beneficial (yes/no). To build on quantitative results, we used thematic analysis to assess four open-ended survey questions. Results found the average bed size of respondents' NHs was 135, 75% were nonprofit and 62% were part of a chain. Regression results found a significant relationship between rating the program as beneficial and hiring more PCAs (p=.003) and having a higher quality rating (p=.02). We identified three qualitative themes, 1) benefits of the program, 2) barriers to success, 3) how to make the program work, with an overarching theme of administrative proactivity. Analysis of quantitative and qualitative results together found administrators who rated the PCA program as beneficial were more likely to describe proactive efforts (e.g. establishing PCA mentorship programs). Results provide guidance on practices required to make a novel staffing program succeed.

Abstract citation ID: igae098.0226

### THE IMPACT OF THE COVID-19 PANDEMIC ON TEMPORARY AGENCY CARE STAFF USE IN OREGON ASSISTED LIVING

Sarah Dys, Ozcan Tunalilar, and Yuliana Levchenko, *Portland State University, Portland, Oregon, United States*

Temporary agency staff (TAS) use in long-term care has become a spotlight issue due to workforce shortages and increased dependence during the COVID-19 pandemic. Nursing homes (NH) and assisted living communities (AL) rely on TAS to maintain compliance with staffing standards and meet residents' needs. Settings with high TAS utilization have been associated with more deficiency citations and lower quality ratings. While recent NH studies show a substantial increase in chronic use of TAS, no similar studies document ALs' use and change of TAS. Using data from a representative sample of Oregon ALs in 2016 and 2023, we compare differences in and organizational (license type, size, Medicaid contracts, profit status, rurality) correlates of TAS use by estimating linear and logistic regression models. Share of ALs with any TAS use increased significantly from 13% in 2016 to 22% in 2023 (p=0.012). In 2016, there were no differences in the likelihood of ALs using any TAS, except for size. In 2023, ALs with Medicaid contracts and in rural areas were 46% and 44% less likely to report using any TAS, respectively. TAS mix used also differed between 2016 and 2023. TAS use decreased for registered nurses (53.8% vs. 25.9%; p=0.005) and increased for licensed practical nurses (0.8% vs. 10.2%; p=0.060) and certified nursing assistants (6.5% vs. 38.2%; p<.001), with no significant difference in personal care aides. Findings contribute to understanding how ALs utilize TAS and can potentially add to policy debates around more stringent regulations for NH and AL staffing.

Abstract citation ID: igae098.0227

### CHURN: TRANSITIONS OUT OF HEALTH SECTOR EMPLOYMENT FOR LOW-PREPARATION WORKERS, 2017–2022

Christine Bishop, and Karen Donelan, *Brandeis University, Waltham, Massachusetts, United States*

Shortages and turnover for front-line paid caregivers and support staff in long-term services and supports for older adults undermine quality of care. These workers are part of the health sector workforce, and also participate in a larger labor market of jobs requiring minimal formal education and brief training. The Brandeis Front-line Health Worker Survey queried a random sample of U.S. residents screened for holding patient-facing jobs in the health sector between the survey date (Summer 2022) and five years prior (2017), generating information on a retrospective longitudinal cross section of US health sector workers. Respondents reporting occupations in Bureau of Labor Statistics Job Zones 1 and 2 were identified as participating in low-preparation labor markets. About half of low-preparation health sector workers reported work in nursing homes, residential settings, or home care. We estimated annual rates of transition into and out of heath sector employment for all low-preparation health workers for years prior to and during the pandemic. Descriptive logistic regressions showed the relationship of job churn to gender, caregiver status, and previous employment pattern. Despite concerns about health workforce stability as the pandemic wound down, low-preparation workers exhibited about the same year-to-year transition rates out of (and back into) the health sector throughout the period. This suggests that the Great Resignation in healthcare is a continuing pattern rather than a pandemic phenomenon. Employers and public policy makers setting wages and working conditions should recognize that front-line workers have, and have always had, alternatives beyond the health sector.

Abstract citation ID: igae098.0228

### NURSING HOME STAFFING DISPARITIES IN DISTRESSED COMMUNITIES DURING PANDEMIC

Yu Jin Kang, Karen Nielsen, Jacqueline Jordan, and Blake McGee, *Georgia State University, Atlanta, Georgia, United States*

This repeated measures study aims to examine the extent of US nursing home staffing disparities in distressed communities during the COVID-19 pandemic. The study used 2020-2022 Nursing Home Compare, 2021 LTCFocus, and the Distressed Community Index from the Economic Innovation Group. Mixed-effects models were used for data analysis. Outcomes were quarterly case-mix adjusted hours per resident day (HPRD) of all nursing staff in the last three quarters of 2020 and all of 2021: registered nurses (RN), licensed practical and vocational nurses (LP/VN), and certified nursing aides (CNA). Predictors included 2020 first quarter nurse staffing levels and distressed community index quintile. Covariates were nursing home size (greater or less than 100 beds), 5-star overall rating, Medicaid payer percentage, and non-profit status. Of the 13,845 nursing homes, 70% were for-profit and 50% had over 100 beds. On average, 5-star overall rating of nursing homes was 3.19 (sd=1.41) and Medicaid payer percentage was 60% (sd=23.5%). Compared to pre-pandemic nurse HPRD, average HPRD of the subsequent 7 quarters declined (differences: Total=-0.67, 17%; RN=-0.30, 43%; LP/VN=-0.13, 15%; CNA=-0.26, 11%). Pandemic HPRD were negatively associated with pre-pandemic HPRD (slopes: Total= -0.078; RN= -0.039; LP/VN= -0.010; CNA= -0.062, all p< 0.001). Modeled interactions suggest that declines in HPRD were greatest in the most distressed communities. Analyses showed declines in nursing home staffing levels during the pandemic, with the lowest HPRD and greatest declines observed in the most distressed communities and RNs. Practical significance of the predictors and resident outcomes requires further examination.

Abstract citation ID: igae098.0229

### HOW TO TRAIN ENOUGH PROFESSIONAL DEMENTIA CAREGIVERS IN CHINA TO MEET THE FAST GROWING NEED?

Zengliang Gong, Gege Cai, and Chunjuan Xu, *Taikang Community, Beijing, Beijing, China*

**Purpose:** This paper try to establish an effective system for training dementia caregivers basing on the practices of a leading provider in China. Method: 1. Establish a holistic memory care program and curriculum system, and deliver them through a set of training programs to cover both first-line caregivers and managers, such as an 8-hour orientation session for newcomers, 23-hours on-job training per year, 40-hour training for new managers. 2. Estabilish a case competition among all communities of the provider across China, so that the team could learn from the specific care practices and from each other. 3. Test different training programs empirically to improve training effectiveness.

**Results:** 1. The memory care team in the provider has grown substantically, and the number of certified trainee of 8-hour orientation session has increased to 1400 since 2021. 2. The turnover rate of caregivers in the memory care area is 5% lower than the overall turnover rate of the care building. The turnover rate for memory care managers dropped from 57% in 2021 to 21% in 2023. 3. The experimental group that learned the concept and values of "person-centered care" first was significantly better than the control group in learning knowledge and skills in dementia care.

**Conclusion:** Systematic and practice-based training programs are conducive to quickly cultivating professional caregivers for dementia care. The Chinese government and the senior care industry should invest more resources in establishing similar training programs to meet the urgent needs of people with dementia and their families.

### SESSION 1235 (PAPER)

### MOBILITY/DISABILITY I (PAPERS)

Abstract citation ID: igae098.0230

### OLDER ADULT FALLS AND LIMITATIONS IN ACTIVITIES OF DAILY LIVING

Yara Haddad, Ketra Rice, Jufu Chen, and Ramakrishna Kakara, *Centers for Disease Control and Prevention, Atlanta, Georgia, United States*

Falls among older adults (65+) often lead to injury and loss of independence. Our study aimed to estimate the odds of developing limitations in activities of daily living (ADL) and instrumental activities of daily living (IADL) after a fall. We used 2015-2020 Medicare Current Beneficiary Survey data to estimate the odds of developing ADL/IADL limitations in the year following a fall and examined risk and protective factors for developing them. Using logistic regression, we calculated crude and adjusted odds ratios (OR) and 95% Confidence Intervals (CI), adjusting for demographic, economic, comorbidity, and physical activity variables. Older adults reporting a fall had twice the odds of developing limitations in ADLs (OR=2.02; CI:1.70-2.39) or IADLs (OR=2.01; CI:1.72-2.34) the following year compared with those who did not fall. Among those who fell, adjusted ORs for developing ADL or IADL limitations were significantly higher for adults aged 75+ compared with those 65-74 but were significantly lower for adults who engaged in moderate or vigorous activity for ≥ 2 hours per week during baseline compared with those who did not. Although the likelihood of developing limitations after a fall may increase with age, results suggest engaging in moderate or vigorous physical activity of ≥ 2 hours per week may be protective. Regular physical activity with structured strength and balance exercises has been shown to reduce falls risk. The Stopping Elderly Accidents Deaths and Injuries (STEADI) initiative encourages health care providers to screen and assess for falls risk and intervene using strategies such as physical therapy.

Abstract citation ID: igae098.0231

### MOBILITY ABILITIES MEDIATE THE ASSOCIATION BETWEEN A MORE ACTIVE LIFESTYLE WITH MOBILITY DISABILITY

Brittney Lange-Maia[1], Tianhao Wang[1], Shahram Oveisgharan[1], Jeffrey Hausdorff[2], David Bennett[1], and Aron Buchman[1], *1. Rush University Medical Center, Chicago, Illinois, United States*, *2. Tel Aviv Sourasky Medical Center, Tel Aviv, Tel Aviv, Israel*

Few studies have analyzed sensor-derived metrics of both mobility abilities and total daily physical activity (TDPA). We sought to determine if sensor-derived mobility metrics and quantitative indices of TDPA were independently associated with prevalent and incident mobility disability. Quantitative mobility metrics from 724 ambulatory adults (mean age 82 years, 77.4% female) were derived from a belt-worn sensor. A wrist-worn sensor worn quantified TDPA (up to 10 days). Mobility disability was characterized at baseline and longitudinally using the Rosow-Breslau scale. We examined cross-sectional association of the mobility metrics and TDPA with the odds baseline of disability using logistic regression, and incident disability using Cox models. Several mobility metrics (e.g. step time variability, turning angular velocity) and TDPA were significantly related to prevalent and incident mobility disability when examined in separate models. When examined together, however, the association of TDPA with mobility disability was attenuated. Mediation analysis indicated that the mobility metrics individually mediated between 7%-47% (p< 0.001) of the association between TDPA

and cross-sectional mobility disability, and between 20%-40% of the association between TDPA and incident mobility disability. As a group, mobility metrics mediated 58% of the association between TDPA and cross-sectional mobility disability, and 53% of the association with TDPA and incident mobility disability (p< 0.001 for all). Sensor-derived mobility metrics may distinguish different aspects of mobility disability and partially link a more active lifestyle with reduced disability. These findings can inform the design of intervention studies to develop targeted therapies to maintain ambulation and independent living in late life.

Abstract citation ID: igae098.0232

### SEX-RELATED DIFFERENCES IN PHYSICAL ACTIVITY PATTERNS OF OLDER INDIVIDUALS LIVING IN THE SARDINIAN BLUE ZONE

Massimiliano Pau, Benedetta Brandas, and Maria Chiara Fastame*, University of Cagliari, Cagliari, Sardegna, Italy*

Previous studies demonstrated that individuals born and resident in the Blue Zones (i.e, areas of exceptional longevity) exhibit reduced levels of depressive symptoms, better life satisfaction levels, and adopt more effective coping strategies to deal negative life events. However, few data is available regarding their physical activity (PA) profiles and, in particular, it is not known whether they are sex-related. Based on these considerations, in the present study we explored the effect of sex on a set of metrics which describe amount and intensity of physical activity (PA) in older people living in the Blue Zone of Sardinia (Italy). To this aim, 109 community-dwelling participants (Mage = 81.7 years old, SD = 8.1 years) were monitored for 7 days using a clinically validated wearable accelerometer which provided raw body accelerations as well as percentage of time spent in activities of different intensity namely sedentary behavior (SB), light PA (LPA) and moderate-to-vigorous PA (MVPA). The results showed that women exhibit a better PA profile with respect to men, characterized by significantly higher time spent in MVPA (+37%, p =.008) and reduced SB (-10%, p =.001). Also, those living in the Blue Zone exhibit higher PA levels with respect to peers resident in urban areas of Sardinia, thus suggesting that they adopt a more active lifestyle, which is thought to play a pivotal role in promoting functional and cognitive health in late adulthood. At the same time, futher studies are needed to clarify the nature of sex-related differences here observed.

Abstract citation ID: igae098.0233

### ACTIVITIES OF DAILY LIVING (ADL/IADL) ASSISTANCE AND DEPRESSION AMONG COMMUNITY-DWELLING OLDER ADULTS IN THE US

Debasree Das Gupta[1], and Uma Kelekar[2], *1. Utah State University, Logan, Utah, United States*, *2. Marymount University, Arlington, Virginia, United States*

Limitations in daily and instrumental activities of daily living (ADL/IADL) are among the strongest predictors of late-life depression, while ADL/IADL assistance has the potential to reduce depression among older adults. Prior research in this area has predominantly focused on proximate (e.g., going without food when hungry, wetting/soiling bed or clothes) or healthcare (e.g., hospitalizations, nursing home placements) outcomes of unmet ADL/IADL needs. Thus, the risk/protective association of ADL/IADL assistance with late-life depression remains unclear. To address this gap, we evaluated the longitudinal association between ADL/IADL assistance and subsequent depression using data from a nationally representative sample of community-dwelling Americans age 65 and older (n=5,176) who participated in the 2016-2018 Health and Retirement Study. We conducted bivariate and multivariable (logistic regression) analyses to estimate: 1) the proportion of older adults reporting ADL/IADL difficulties but not getting ADL/IADL help, and 2) the odds of subsequent depression among those reporting no versus one or more help with ADL/IADL (reference: those reporting no ADL/IADL difficulties). Results indicated a higher proportion of older adults did not get help with ADL (6.4%) compared to no help with IADL (1.9%). However, the adjusted odds of subsequent depression associated with no IADL assistance (adjusted odds ratio [aOR]: 3.22; 95% confidence interval [CI]: 2.19,4.78) was 1.66 times higher than that associated with no ADL assistance (aOR: 1.94; 95% CI: 1.35,2.78). Given these findings, enhancing long-term care services for older adults would be essential for meeting the current national goal of reducing mental distress among persons living with disabilities.

Abstract citation ID: igae098.0234

### MOBILITY DEVICE USE MODERATED BIDIRECTIONAL ASSOCIATION OF FEAR OF FALLING WITH HOMEBOUND STATUS

Qianyuan Li[1], Wenting Peng[2], Shuomin Wang[1], Huili Yang[3], Haihong Zhang[3], and Minhui Liu[4], *1. Central South University, Changsha, Hunan, China (People's Republic)*, *2. University of Washington, Seattle, Washington, United States*, *3. Ningxia Medical University, Yinchuan, Ningxia, China (People's Republic)*, *4. Ningxia Medical University, Yinchuan, Ningxia, China*

Previous studies showed fear of falling (FOF) is associated with homebound status, given that they have shared risk factors such as falls. However, the bidirectional relationship between fear of falling and homebound status and possible moderators remain unknown. Using the Year 2015-2019 waves of the National Health and Aging Trends Study, our sample included 3,983 community-dwelling older adults who had completed data on FOF and homebound status. We aimed to examine 1) whether FOF was associated with homebound status bidirectionally and 2) whether mobility device use moderated this bidirectional association. FOF was categorized as no FOF, FOF without fear-related activity restriction (FAR) and FAR. Based on the frequency, difficulty, and independence of outdoor mobility, homebound status was classified as non-homebound, semi-homebound, and homebound. The lagged generalized estimation equation models revealed that FOF without FAR (OR=1.34, 95% CI=1.19-1.52) and FAR (OR=1.62, 95% CI=1.38-1.91) increased the risks of semi-homebound/homebound status. Mobility device use moderated the longitudinal effects of FAR on homebound status with a statistical significance. Additionally, semi-homebound predicted the risks of FOF (OR=1.36, 95% CI=1.21-1.53), and its association was moderated by mobility device use. Overall, FOF is a risk factor for homebound status, and vice versa, and the risks may differ by the use of mobility devices. To prevent the progression of homebound status or FOF, future targeted strategies or community-based interventions should consider the fear of falling and homebound status simultaneously and identify the high risks of older adults who do not use mobility devices.

### SESSION 1240 (SYMPOSIUM)

Abstract citation ID: igae098.0235

### PERCEIVED AND PERFORMANCE FATIGABILITY IN UNDERSTANDING HEALTHY AGING: STUDY OF MUSCLE, MOBILITY AND AGING (SOMMA)

Chair: Nancy Glynn
Co-Chair: Peggy Cawthon
Discussant: Jennifer Schrack

Characterizing fatigability, the quantification of an individual's susceptibility to fatigue in the context of standardized physical task(s), is a more sensitive approach to assess the presence and severity of fatigue, a critical prodromal trait of chronic and acute health conditions. We have validated measures of perceived physical fatigability (Pittsburgh Fatigability Scale, PFS) and performance fatigability (Performance Deterioration [PD, measured during a fast-paced 400m walk by calculating gait speed of lap2-lap9/lap2*100] and the Pittsburgh Performance Fatigability Index [PPFI, measured during a usual-paced 400m by using wrist-worn raw accelerometry data to quantify percent cadence decline). Our symposium will capitalize on the extensive phenotype data collected at baseline in SOMMA (N=879, mean age 76.3±5.0 years; walking speed 1.04±0.20 m/s; 59.2% women; 85% white), to further elucidate the correlates and consequences associated with greater perceived and/or performance fatigability in order to inform potential interventions to promote healthy aging. First, Ms. Reagan Garcia will examine whether time spent in activPAL measured sedentary behaviors are related to higher perceived physical fatigability (PFS). Next, Dr. Kyle Moored will share findings on the role of maximal treadmill cardiorespiratory fitness (VO2peak) and PFS on life-space mobility and whether these associations are moderated by perceived neighborhood factors. Dr. Caterina Rosano will explore the association between nigrostriatal dopaminergic integrity and performance fatigability (PD). Lastly, Ms. Emma Gay will be comparing skeletal muscle energetics and walking energetics profiles of those with lower PFS/lower PPFI vs. higher PFS/higher PPFI. Discussant, Dr. Jennifer Schrack will offer insights for new directions for fatigability research.

Abstract citation ID: igae098.0236

### ASSOCIATION OF SEDENTARY BEHAVIORS WITH PERCEIVED PHYSICAL FATIGABILITY

Reagan Garcia[1], Eileen Johnson[2], Peggy Cawthon[3], Barbara Nicklas[4], Bret Goodpaster[5], Anne Newman[6], and Nancy Glynn[1], *1. University of Pittsburgh School of Public Health, Pittsburgh, Pennsylvania, United States*, *2. Sutter Health, San Francisco, California, United States*, *3. California Pacific Medical Center, San Francisco, California, United States*, *4. Wake Forest University School of Medicine, Winston-Salem, North Carolina, United States, 5. Advent Health, Orlando, Florida, United States*, *6. University of Pittsburgh, University of Pittsburgh, Pennsylvania, United States*

Greater perceived fatigability is a phenotype of poor energy regulation and is associated with physical activity, cardiovascular fitness, and mitochondrial energetics. However, it is unclear whether time spent in low energy expenditure activities, i.e. sedentary behaviors, contribute to greater perceptual feelings of fatigue. We examined whether sedentary behaviors were associated with perceived physical fatigability using cross-sectional data in the Study of Muscle, Mobility and Aging (SOMMA). Participants wore an activPAL on their right thigh for 7 consecutive 24-hour periods (valid wear=≥1 day with ≥10 hours). Total min/day sedentary time, time per sedentary bout (min/day), and number of sit-to-stand transitions were calculated using mean of out-of-bed intervals. Perceived fatigability was measured using the Pittsburgh Fatigability Scale Physical subscale (PFS, score 0-50, higher=greater fatigability, more severe=≥15). Logistic regressions were adjusted for age, sex, body mass index, Short Physical Performance Battery (range 0-12), and cost-capacity ratio (VO2 from 0.67 m/s 5-min walk/VO2peak *100). Participants (n=685; 76.4±5.1 years, 58% Women, 85.1% Non-Hispanic White, 54.9% PFS Physical score ≥15) had mean 619.1±113.1 min/day sedentary time, mean 15.2±5.8 min/day per sedentary bout, and 45.9±13.4 sit-to-stand transitions/day. One standard deviation higher mean sedentary time was associated with 26% greater odds of reporting more severe perceived physical fatigability after full adjustment (OR=1.26, 95% CI: 1.05-1.52). Mean time per sedentary bout and sit-to-stand transitions were not associated with perceived physical fatigability. Sedentary behavior has modest associations with perceived fatigability. Our work supports that interventions to reduce fatigability severity could be modestly enhanced if overall sedentary time is reduced.

Abstract citation ID: igae098.0237

### PHYSICAL FITNESS, FATIGABILITY, AND LIFE-SPACE MOBILITY: DIFFERENCES BY NEIGHBORHOOD CONTEXT

Kyle Moored[1], Kate Duchowny[2], Andrea Rosso[3], Peggy Cawthon[4], Michelle Carlson[5], and Nancy Glynn[6], *1. Johns Hopkins Bloomberg School of Public Health, Baltimore, Maryland, United States*, *2. University of Michigan, Ann Arbor, Michigan, United States*, *3. University of Pittsburgh, Pittsburgh, Pennsylvania, United States*, *4. California Pacific Medical Center, San Francisco, California, United States*, *5. Johns Hopkins University, Baltimore, Maryland, United States*, *6. University of Pittsburgh School of Public Health, Pittsburgh, Pennsylvania, United States*

Physical capacity (e.g., objective fitness, perceived fatigability) contributes independently to life-space mobility, the extent and independence of travel within the community. This relationship may differ by neighborhood context, where higher physical capacity may be especially necessary to overcome environmental barriers to mobility. We examined baseline associations between physical capacity and life-space mobility in community-dwelling older adults, and whether these associations were moderated by perceived neighborhood factors. Participants were from the

Study of Muscle, Mobility and Aging (SOMMA; N=775, Mean±Standard Deviation age=76.1±4.9). Life-space mobility was self-reported using the Life-Space Assessment (higher=greater life-space, 83.6±19.3). Physical capacity measures included maximal treadmill cardiorespiratory fitness (VO2peak, 20.3±4.8 mL/min/kg) and perceived physical fatigability (Pittsburgh Fatigability Scale (PFS) Physical score, higher=more fatigability, 15.4±8.5). Self-reported neighborhood physical disorder (e.g., trash/vandalism) and social cohesion (e.g., neighborhood trust) scores were stratified into tertiles. Linear regressions estimated associations adjusted for demographic, health, and socioeconomic confounders. Each 1-SD higher VO2peak was associated with 2.5-point higher (95% CI: 0.84,4.2) life-space score, with associations significantly greater for the highest physical disorder tertile (b=3.8, 95% CI: 1.5,6.2, p-interaction=.018) and lowest social cohesion tertile (b=5.7, 95% CI: 2.9,8.6, p-interaction=.046). Each 1-SD higher PFS Physical score was associated with 3.2-point lower (95% CI: -4.6,-1.7) life-space score, with no differences by neighborhood measures (p-interactions>.15). Unlike perceived physical fatigability, the impact of objective fitness on life-space mobility may be greatest for those living in more disordered and less socially integrated neighborhoods. Findings reinforce the importance of considering individual-level contributors to life-space mobility within the environmental context (i.e., person-environment fit).

Abstract citation ID: igae098.0238

### NIGROSTRIATAL DOPAMINE AND PHYSICAL PERFORMANCE FATIGABILITY IN COMMUNITY-DWELLING OLDER ADULTS

Caterina Rosano[1], Emma Gay[2], Paul Coen[3], Andrea Rosso[1], Anne Newman[4], Stephen Kritchevsky[5], and Nancy Glynn[2], *1. University of Pittsburgh, Pittsburgh, Pennsylvania, United States*, *2. University of Pittsburgh School of Public Health, Pittsburgh, Pennsylvania, United States*, *3. AdventHealth, Orlando, Florida, United States*, *4. University of Pittsburgh, University of Pittsburgh, Pennsylvania, United States*, *5. Wake Forest University School of Medicine, Wake Forest University School of Medicine, North Carolina, United States*

Increasing nigrostriatal dopaminergic signaling can ameliorate performance fatigability in healthy young adults, but its role in community-dwelling older adults is not known. We hypothesized higher nigrostriatal dopaminergic integrity would be associated with lower performance fatigability, independent of other factors that impact physical performance fatigability, including cardiorespiratory fitness and muscle energetics. In 125 participants of the Study of Muscle, Mobility and Aging (SOMMA), performance fatigability was measured as performance deterioration during a fast-paced 400 meter walk (% slowing down from the 2nd to the 9th lap, higher = more performance deterioration). Nigrostriatal DA integrity was measured using (+)-[11C]dihydrotetrabenazine (DTBZ) PET imaging. The binding signal was obtained separately for the subregions regulating sensorimotor (posterior putamen), reward (ventral striatum) and executive control processes (dorsal striatum). Multivariable linear regression models of performance fatigability (dependent variable) estimated the coefficients of dopamine integrity in striatal subregions (in separate models), adjusted for demographics, cognition, mitochondrial respiration (assayed in muscle biopsies) and cardiorespiratory fitness (via cardiopulmonary exercise testing). In this sample (mean age 75.4±4.0 years, 59% women), mean performance deterioration was 2.5%±4.7%. Higher [11C]-DTBZ binding in the posterior putamen was significantly associated with lower performance fatigability (demographic-adjusted standardized Beta= -1.18, 95% CI:-2.11, -0.25); results remained independent of further adjustment for other covariates, including cognition, cardiorespiratory fitness, and muscle energetics (standardized Beta= -1.03, 95% CI:-2.02, -0.10). Association with other striatal subregions were not significant. Dopaminergic integrity in the sensorimotor striatum may influence performance fatigability in older adults regardless of clinically overt diseases, and independent of other aging systems.

Abstract citation ID: igae098.0239

### DISCOVERING BIOENERGETIC AND FITNESS PROFILES OF COMBINED PERCEIVED AND PERFORMANCE FATIGABILITY

Emma Gay[1], Caterina Rosano[2], Paul Coen[3], Reagan Garcia[1], Bret Goodpaster[4], David Marcinek[5], Anne Newman[6], and Nancy Glynn[1], *1. University of Pittsburgh School of Public Health, Pittsburgh, Pennsylvania, United States*, *2. University of Pittsburgh, Pittsburgh, Pennsylvania, United States*, *3. AdventHealth, Orlando, Florida, United States*, *4. Advent Health, Orlando, Florida, United States*, *5. University of Washington, Seattle, Washington, United States*, *6. University of Pittsburgh, University of Pittsburgh, Pennsylvania, United States*

Demographic characteristics, skeletal muscle energetics, cardiorespiratory fitness (VO2peak), walking energetics, physical activity, and mobility are all independently associated with perceived and performance fatigability. Perceived and performance fatigability are distinct measurements that are complimentary to each other, and together could provide novel insight into potential targets for healthy aging interventions. We hypothesized that individuals with lower scores on both fatigability measurements (i.e., lower perceived/lower performance) will exhibit better cellular energetics and physical performance profiles compare to individuals with higher scores on both fatigability measurements (i.e., higher perceived/higher performance). Using a sample from SOMMA (n=799), we classified individuals using established severity cutpoints for the perceived-based Pittsburgh Fatigability Scale (PFS, range 0-50, lower<15, higher≥15) and the performance-based Pittsburgh Performance Fatigability Index (PPFI, 0-100%, men: lower<5.4%, higher≥5.4%, women: lower<3.5%, higher≥3.5%). Skeletal muscle energetics were measured in vivo via magnetic resonance spectroscopy and ex vivo (biopsies) via high-resolution respirometry. Daily activity count was measured with wrist-worn accelerometry. We assessed VO2peak during a maximal treadmill cardiopulmonary test and walking energetics (cost-capacity ratio [VO2 from 0.67 m/s 5-min walk/VO2peak*100]). Compared to those with higher PFS/higher PPFI (n=73, 9.1%), individuals classified as lower PFS/lower PPFI (n=331, 41.4%) were 2 years younger, 33% more likely a man, 16% more activity, had 18.5% (0.19 m/s) faster 4m gait speed, 15-25% higher muscle energetics, 26% (5.2 mL/kg/min) higher VO2peak, and 11% lower cost-capacity ratio, all p<0.05. Combining fatigability status revealed striking differences between lower PFS/lower PPFI vs higher PFS/higher PPFI, providing insight into targets (e.g., physical activity, bioenergetics, fitness) for intervention.

### SESSION 1245 (PAPER)

### PHYSICAL ACTIVITY AND EXERCISE (PAPERS)

Abstract citation ID: igae098.0240

### CAN DETERIORATION IN BALANCE BE EASILY DETECTED IN MIDDLE AGE?

Shmuel springer, Roee Hayek, Silvi Frenkel Toledo, and Guy Baranes, *Ariel University, Ariel, HaMerkaz, Israel*

Balance and physical performance tend to start declining in middle-age. However, most existing tests are generally not sensitive enough to detect subtle balance disorders in this population. We aimed to evaluate the utility of the Brief Balance Evaluation Systems Test (Brief-BESTest) and accelerometry data to identify middle-aged individuals with excessive balance deterioration. Young adults (YA, 25.3±2.3 years), early middle-aged (EMA, 47.7±2.6 years), and late middle-aged (LMA, 60.6±3.6 years) individuals, 25 subjects in each group, were tested using the Brief-BESTest with accelerometer (Delsys, USA) placed over their lower-back to measure the trajectory of the center-of-mass during standing tasks (95% confidence ellipse of trajectory, 95%CE). Significant differences in Brief-BESTest scores were found between the young and the two middle-aged cohorts ($P < 0.05$) and between EMA and LMA ($p < 0.05$). The 95%CE showed significant differences between YA and LMA in all standing conditions ($P < 0.05$) and between EMA and LMA ($p < 0.05$) when standing on a foam with eyes closed. Both, the Brief-BESTest scores and the 95%CE values demonstrated increased variation with age, allowing to identify subjects in the lower interquartile range (LIQR) defined as having accelerated balance deterioration. Significant moderate correlation ($R2=0.44$, $P=0.001$) was found between Brief-BESTest and 95%CE of single-leg-stance, and 46% of individuals in the LIQR were identified by both tests. These results enhance the utility of balance testing in midlife with the long-term goal of minimizing age-related functional decline. In addition, the results suggest that balance assessment using accelerometry motion data, available on any smartphone, can further facilitate early detection of balance decline.

Abstract citation ID: igae098.0241

### LEARNING YOGA ONLINE: PERSPECTIVES FROM OLDER WOMEN ENROLLED IN A CLINICAL TRIAL FOR URINARY INCONTINENCE

Francesca Nicosia, Margaret Chesney, Celia Kaplan, Berty Arreguin, Giselle Perez-Aguilar, and Alison Huang, *University of California San Francisco, San Francisco, California, United States*

Yoga is increasingly used as a therapeutic modality for aging-related health conditions but the feasibility of engaging older adults in yoga is unclear. We conducted a multisite randomized trial of a therapeutic yoga intervention for ambulatory older women with urinary incontinence. The trial included twice weekly group instruction in Hatha yoga techniques delivered via interactive, synchronous, videoconference-based instruction. To assess barriers and facilitators to online instruction for this population, we used mixed methods. Structured questionnaires administered at 12-weeks measured convenience, audio-visual elements, and social connection, each on a 4-pt scale (1: Poor, 4: Excellent). Interviews examined the experience of learning yoga through synchronous video instruction at home. A hybrid deductive/inductive thematic approach was used to analyze interview transcripts. Among the 51 participants completing questionnaires (age range 49-81; 16% Asian, 16% Latina, 71% White), ratings of convenience were high (90.3% "good-excellent"); ratings of perceptions of personal connection with other participants were lower (60.8% "good-excellent"). Among the 24 participant interviews (age range 45-90; 21% Asian, 17% multiracial; 17% Latina, 54% White), common challenges were audio-visual and physical space limitations. For some, privacy of the home setting mitigated incontinence stigma. Increased body awareness and perceived effectiveness in reducing incontinence and stress facilitated sustained participation. Participants identified effective online teaching strategies including reinforcement of verbal instructions with visual demonstrations about body position during yoga postures and individualized attention. Despite challenges with online delivery, the convenience and privacy of the at-home setting provides opportunities to engage diverse, older women in movement-based therapeutic yoga interventions.

Abstract citation ID: igae098.0242

### UNVEILING ASSOCIATIONS BETWEEN THE WORK-RELATED AND LEISURE-TIME PHYSICAL ACTIVITY WITH BIOLOGICAL AGE

Mingyue Hu, *Central South University, Changsha, Hunan, China (People's Republic)*

**Objectives:** This study aimed to examine the association and dose-relationship between different intensities of work-related and leisure-time physical activity (PA) and biological age (BA). Design: A cross-sectional study. Setting and participants: We used data of 5,216 participants aged 45 and over in the National Health and Nutrition Examination Surveys (NHANES). Measurements: PA was self-reported using the Global Physical Activity Questionnaire (GPAQ), and BAs were calculated by the PhenoAge and Klemera-Doubal method (KDM). In the analyses, survey-weighted generalized linear models were used to explore associations between PA and BAs. Restricted cubic spline characterized the dose-response relationship of PA and BAs. Subgroup analyses were conducted by age, sex, and ethnicity.

**Results:** Work-related light-intensity PA (LPA) was significantly associated with younger PhenoAge ($\beta=0.507$, 95%CI: -0.957, -0.056, $p = 0.031$) and KDM ($\beta=0.908$, 95%CI: -1.774, -0.042, $p = 0.042$). There was a U-shaped dose-response relationship between LPA and BAs (all $p < 0.05$). Leisure-time moderate-intensity PA (MPA) was associated with younger PhenoAge ($\beta=0.789$, 95%CI: -1.573, -0.006, $p=0.049$) and KDM ($\beta=1.910$, 95%CI: -3.417, -0.402, $p = 0.017$). There was a U-shaped dose-response relationship between MPA and BAs (all $p < 0.05$). In subgroup analyses for older adults, all intensities of PA were significantly associated with decreased BAs (all p < 0.05). Conclusions: Doing PA could delay the BAs, specifically the work-related LPA and leisure-time MPA. Older adults can benefit from all intensities of PA regardless of the PA of different environments and intensities.

Abstract citation ID: igae098.0243

### PATTERNS OF SEDENTARY BEHAVIORS IN OLDER ADULTS IN CHINESE CARE HOMES: A LATENT PROFILE ANALYSIS

Qi Li, Sihan Dong, Xueying Liu, Mengjiao Xu, and Yuting Song, *Qingdao University, Qingdao, Shandong, China (People's Republic)*

Sedentary behaviors remain common among older adults. Previous studies in this area are mostly focused on community settings and used variable-centered approach. In this study, we aimed to identify the pattern of sedentary behaviors among older adults in residential care homes in China. This cross-sectional analysis used data from 283 older adults who lived in 11 residential care homes in China. Eligible participant aged 65 years or older, had lived in the current care home for ≥3 months, and was able to communicate verbally or in writing. Sedentary behaviors were assessed with the Elderly Sedentary Behavior Questionnaire. Participants self-reported the average daily time spent in the past week on each of eight behaviors, such as watching TV, socializing, and doing nothing. Patterns of sedentary behaviors were identified using latent profile analysis and verified by associating with depressive symptoms using a regression model. The average daily sedentary time of older people was 11.6 hours (SD=2.6). Four patterns of sedentary behaviors emerged, which we labeled the high sedentism group (23%), the balanced group (50%), the high mentally-active group (8%), and the high mentally-passive group (19%). Compared to older adults in the balanced group, those in the high sedentism group and high mentally-passive group had higher risks of depressive symptoms and those in the high mentally-active group had lower risks of depressive symptoms. Targeted interventions are warranted for older adults of different patterns of sedentary behaviors, particularly older adults in the high sedentism group and high mentally-passive group.

Abstract citation ID: igae098.0244

### TWENTY-FOUR-HOUR MOVEMENT AND BLOOD PRESSURE: SIX PART COMPOSITIONAL DATA ANALYSIS STUDY FROM THE PROPASS CONSORTIUM

Joanna Blodgett[1], Matthew Ahmadi[2], Andrew Atkin[3], Annemarie Koster[4], Emmanuel Stamatakis[2], and Mark Hamer[1], *1. University College London, London, England, United Kingdom, 2. University of Sydney, Sydney, New South Wales, Australia, 3. University of East Anglia, Norwich, England, United Kingdom, 4. Maastricht University, Maastricht, Limburg, Netherlands*

Physical activity has widely known benefits for cardiovascular health. However, the influence of movement across the entire 24-hour behavioural spectrum (e.g. sleeping, sedentarism, activity type, etc.) on blood pressure remains underexplored. This study investigated associations between free-living activities that comprise a 24-hour day and systolic and diastolic BP (SBP; DBP). Data from six cohorts within the Prospective Physical Activity, Sitting and Sleep consortium (ProPASS) consortium (n=14,761; mean age 54.2±9.6 years) were analysed. Using thigh-worn accelerometers, all activity over a 7-day period and nurse-assessed BP were collected. Individual participant meta-analyses, employing compositional data analysis, were conducted. Reallocation plots were generated to estimate BP reductions by reallocating time from one behaviour to another, highlighting the potential benefits of minimal and optimal behavioural changes.

**Results:** On average, participants spent significant proportions of their day of sleeping (7.3hrs;31%), sedentary(10.9hrs;45%), standing(3.1hrs;13%), slow walking(1.5hrs;6%), fast walking(1.1hrs;5%), and exercise-like activity(10.9min;0.01%). Increased time spent in exercise or sleep, relative to other activities, correlated with lower BP. An additional 5 minutes/day of exercise-like activity was associated with estimated reductions of -0.68mmHg (-0.15,-1.21; SBP) and -0.54 mmHg (-0.19,0.89; DBP). Clinically meaningful improvements in SBP and DBP (2 & 1 mmHg) were estimated after reallocating 20-27 minutes and 10-15 minutes, respectively, from other activities to exercise.

**Conclusion:** This study highlight the significance of exercise in BP management, even in small doses within everyday life. It suggests that minor adjustments to daily routines, favouring exercise, could contribute to significant BP reductions in free-living contexts.

### SESSION 1250 (SYMPOSIUM)

Abstract citation ID: igae098.0245

### PSYCHOSOCIAL AND NEIGHBORHOOD-RELATED HEALTH FACTORS FOR BLACK AND WHITE ADULTS OF THE HANDLSLEEP PROJECT

Chair: Alyssa Gamaldo
Discussant: Orfeu Buxton

Disparities in cognition and sleep have been observed in middle-aged and older Black and White adults. However, limited research has examined the association between cognition and sleep as well as the underlying psychosocial, contextual, and biomarker factors that explain this association. This symposium will include presentations using data from Healthy Aging in Neighborhood of Diversity Across the Life Span Sleep Study (HANDLSleep), which was conducted to explore psychosocial and contextual factors related to sleep and cognition in a sample of Black and White middle-aged and older adults in Baltimore, MD. The objectives of the proposed symposium are the following: (1) discuss the associations observed between sleep and cognition using cross-sectional and ambulatory data (2) discuss how psychosocial, contextual, and inflammatory markers relate to sleep and cognition. Master and colleagues explored the daily coupling associations between actigraphic sleep and working memory. Allan and colleagues explored the association between different dimensions of health (e.g., sleep and cognition) and objective and subjective measures of neighborhood quality. Linying and colleagues examined the extent that mean and variability of actigraphic sleep relate to global mental status. Lastly, Shen and colleagues explored the association between actigraphic sleep and inflammatory markers and the extent this association varies by race and sex.

Abstract citation ID: igae098.0246

### DAILY ASSOCIATIONS OF ACTIGRAPHIC SLEEP AND NEXT-DAY WORKING MEMORY IN MIDLIFE AND OLDER ADULTS

Lindsay Master[1], Orfeu Buxton[1], Alexa Allan[1], Courtney Fine[2], Jacqueline Mogle[2], Alan Zonderman[3], Michele Evans[3], and Alyssa Gamaldo[2], *1. The Pennsylvania State University, University Park, Pennsylvania, United States*, *2. Clemson University, Clemson, South Carolina, United States*, *3. National Institute on Aging, Baltimore, Maryland, United States*

This study evaluates the day-to-day associations between actigraphic nighttime sleep and next day working memory in a sample of diverse midlife and older adults. The study used a subsample of the HANDLSleep Study which provided complete information of the variables of interest. Preliminary data from wave 5 (2022-current, n=104, 72% female, 55% Black adults, mean age: 62) were collected via a wrist-worn accelerometer and daily mobile phone cognitive tasks over one week, from which nighttime sleep measures and next-day working memory outcomes were derived. Multilevel linear models tested within-person associations of nightly sleep duration, timing, and quality with next-day mean reaction time calculated from the Multiple Object Tracking (MOT) Task via a mobile device, which evaluates working memory. Analyses were stratified by poverty status (above/below 125% federal poverty line) and adjusted for age, sex, race, WRAT3 literacy scores, average sleep measures, and weekend/weekday. Within individuals who live below the poverty line (n=47), on nights when participants fell asleep earlier (p=.013) and slept longer (p=.021) but had more minutes of wake (p=.0013) and less efficient sleep (p=.018) than their average, next-day mean reaction time was faster. Sleep measures were not associated with next-day working memory for individuals who live equal/above the poverty line. Our results demonstrate that within-person improvements to nightly sleep timing and duration may help next-day reaction time on working memory in adults who live below the poverty line.

Abstract citation ID: igae098.0247

### ASSOCIATIONS BETWEEN MEASURES OF HEALTH AND OBJECTIVE AND SUBJECTIVE NEIGHBORHOOD QUALITY

Alexa Allan[1], Roland Thorpe, Jr.[2], Orfeu Buxton[1], Lesley Ross[3], May Beydoun[4], Alan Zonderman[4], Michele Evans[4], and Alyssa Gamaldo[3], *1. The Pennsylvania State University, University Park, Pennsylvania, United States*, *2. Johns Hopkins University, Baltimore, Maryland, United States*, *3. Clemson University, Clemson, South Carolina, United States*, *4. National Institute on Aging, Baltimore, Maryland, United States*

The current study examined the cross-sectional associations between self-reported mental/emotional (e.g., patient health questionnaire), sleep (e.g., Pittsburgh Sleep Quality Index), physical (e.g., medical conditions), and cognitive health (e.g., Montreal Cognitive Assessment) and subjective and objective measures of neighborhood quality. This preliminary analysis included 77 community-dwelling socioeconomically diverse Black and White adults (Mage = 62.17, SDage = 9.71; 70% female) from the HANDLSleep study. Neighborhood quality was assessed using self-reported measures of physical built disorder (e.g., graffiti), social cohesion (e.g., close-knit neighborhood), and social control (e.g., neighbors act if children disrespecting an adult). Area Deprivation Index (ADI) National and State values were extracted from the Neighborhood Atlas using participant addresses. Multivariable linear regression analyses were conducted using health measures as the outcome of interest. Models were adjusted for age, sex, race, education, and poverty status. In the adjusted models, participants living in neighborhoods of greater deprivation (i.e., State ADI) reported higher levels of depressive symptomology ($p < .05$) and worse sleep quality ($p < .05$). Likewise, higher reports of physical built disorder were associated with poorer cognition (i.e., attention, $p < .05$). Contrastingly, reports of better social cohesion were associated with lower insomnia severity scores ($p < .05$) and better performance on a measure of executive function ($p < .01$). Reports of more social control was also associated with better executive function ($p < .05$). These findings highlight the importance of examining both objective and subjective neighborhood characteristics as they pertain to different dimensions of health.

Abstract citation ID: igae098.0248

### ASSOCIATIONS OF MEAN AND VARIABILITY OF ACTIGRAPHIC SLEEP AND GENERAL COGNITIVE PERFORMANCE

Linying Ji[1], Yuqi Shen[2], Orfeu Buxton[2], May Beydoun[3], Alan Zonderman[3], Michele Evans[3], and Alyssa Gamaldo[4], *1. Montana State University, Bozeman, Montana, United States*, *2. The Pennsylvania State University, University Park, Pennsylvania, United States*, *3. National Institute on Aging, Baltimore, Maryland, United States*, *4. Clemson University, Clemson, South Carolina, United States*

Sleep health is associated with cognitive function in midlife and older adults. Little research investigated the relationship between cognitive performance and variability, in addition to mean sleep measures. Using a subsample HANDLSleep study (n=81; 72% female, 52% Black adults; age range: 46-81), this study examined the cross-sectional relationship between cognitive function and both variability and mean sleep with a Bayesian variability model. Cognitive function was measured using Montreal Cognitive Assessment (MoCA). Sleep measures were estimated using one week of wrist actigraphy (mean ± SD = 6.8 ± 0.4 day), including nighttime sleep time (TST), wake after sleep onset (WASO), nighttime sleep midpoint (Timing), Sleep maintenance efficiency (SMEff), and nap minutes (NAP). All models controlled for age, race, sex, WRAT3 literacy scores, and poverty status. Results showed that more variability in sleep Timing ($b = -2.75$, CI [-5.18, -0.45], $p = 0.02$) and longer mean nap minutes ($b = -3.58$, CI [-6.52, -0.93], $p = 0.006$) were associated with worse cognitive performance (lower MoCA score). No significant associations were observed for TST, WASO, SMEff and MoCA. Findings suggest the importance of considering both variability and mean sleep measures in understanding how sleep relates to cognitive function in middle-age and older adults.

Abstract citation ID: igae098.0249

### GENDER AND RACE AS MODERATORS BETWEEN ACTIGRAPHY SLEEP AND INFLAMMATORY MARKERS

Yuqi Shen[1], Linying Ji[2], Claudette Kessler[1], Christopher Engeland[1], Orfeu Buxton[1], Alan Zonderman[3], Michele Evans[3], and Alyssa Gamaldo[4], *1. The Pennsylvania State University, University Park, Pennsylvania, United States*, *2. Montana State University, Bozeman, Montana, United States*, *3. National Institute on Aging, Baltimore, Maryland, United States*, *4. Clemson University, Clemson, South Carolina, United States*

This cross-sectional study investigated gender and race differences in the association between actigraphic sleep (mean levels and variability) and inflammatory markers (circulating interleukin [IL]-6, IL-10, tumor necrosis factor [TNF]-$\alpha$ and C-reactive protein [CRP]). The study included participants from the HANDLSleep study (n=99; 74% female, 54% Black adults; age range: 46-82). Within-person mean and standard deviation (iSD) of sleep were estimated using one week of wrist actigraphy (mean ± SD = 6.8 ± 0.5 day), including nighttime sleep time, 24-hr sleep time, wake after sleep onset, nighttime sleep midpoint (Timing), and sleep maintenance efficiency. Linear regression models were conducted including interaction terms (race*sleep; or sex*sleep) and covariates (age, WRAT3 literacy scores, poverty status). Results indicated that race significantly moderated the association between variability in sleep maintenance efficiency and circulating IL-6 (interaction term p =.002). Race-stratified analyses revealed that among White adults, greater variability in sleep maintenance efficiency was significantly associated with higher circulating IL-6 (b = 0.10, p =.003). No significant association was found among Black adults. Results also indicated that gender significantly moderated the relationship between mean level 24-hr total sleep time and circulating IL-6 (interaction term p =.001). Stratification analyses revealed that longer mean 24-hr total sleep time was associated with higher levels of IL-6 among men (b = 0.003, p =.008). No significant association was found among women. Consistent with prior studies, the current study observed associations between actigraphic sleep and inflammation primarily among White adults and among men.

### SESSION 1255 (SYMPOSIUM)

Abstract citation ID: igae098.0250

### SLEEP AND ITS TREATMENT: EFFECTS ON PHYSICAL AND PSYCHOSOCIAL WELL-BEING

Chair: Christopher Kaufmann
Co-Chair: Soomi Lee
Discussant: Katie Stone

Sleep disturbances, dysregulated circadian rhythms, and related disorders can impact multiple aspects of life, including physical health, mobility, mental well-being, and other outcomes of relevance to aging populations. Treatments for specific disorders, such as Continuous Positive Airway Pressure (CPAP) for sleep apnea, are also important for promoting positive health outcomes. Therefore, it is crucial to conduct research that clarifies the relationships among sleep, its treatments, and overall health. In this symposium, we examine the complex interplay of sleep, sleep disorders, and circadian rhythms, with physical and psychosocial well-being in older populations and their caregivers. Our first presentation uses data from the National Health and Aging Trends Study to evaluate sleep health characteristics as risk factors for physical frailty, including gender differences in this relationship. Our second presentation evaluates the influence of disrupted rest/activity circadian rhythms (measured by wrist actigraphy) on metabolic syndrome among older adult participants in the Baltimore Longitudinal Study of Aging. Our third presentation evaluates sleep quality in family caregivers and whether psychological resilience moderates the association of caregiving status with sleep quality. Our fourth and final presentation, using data from the Health and Retirement Study, identifies the comorbid chronic health conditions that may predict CPAP discontinuation in older adults with sleep apnea to identify populations requiring greater monitoring to improve treatment effectiveness. Overall, this symposium emphasizes the importance of sleep and its role in promoting positive health and well-being in aging and diverse populations.

Abstract citation ID: igae098.0251

### POORER SLEEP HEALTH MAY INCREASE THE RISK OF DEVELOPING PHYSICAL FRAILTY, PARTICULARLY AMONG WOMEN

Muhammad Thalil[1], Soomi Lee[1], and David Almeida[2], *1. The Pennsylvania State University, University Park, Pennsylvania, United States*, *2. Pennsylvania State University, University Park, Pennsylvania, United States*

Longitudinal evidence on the association between sleep and physical frailty is limited, especially using a multidimensional measure of sleep health. In this study, we examined the prospective association between sleep health and physical frailty among U.S. older adults. We also examined how this association differs by age, sex and race. We used data from the National Health and Aging Trends Study (NHATS), with a sample of 929 older adults (65+) who completed a sleep module during 2013 and 2014. Self-reported items on the SATED model (i.e., satisfaction, daytime alertness, timing, efficiency, and duration) were used to create a sleep health composite and Fried's frailty phenotype was used to assess frailty. Multiple multinomial logistic regression models adjusted for baseline frailty status, socio-demographics, and health correlates revealed that having poorer sleep health increased the risk of onset of frailty at follow-up (RR = 1.30, 95% CI: 1.02–1.64). Among specific sleep health dimensions, lack of daytime alertness and short (< 6 hours) or long (≥ 9 hours) sleep duration were significantly associated with frailty, however, poor sleep satisfaction, timing and efficiency were not significantly associated with frailty. Stratified analyses revealed that the association between sleep health and frailty was significant among women, those aged <80 years, and non-Hispanic Whites after adjusting for socio-demographics, but only significant among women (RR = 1.44, 95% CI: 1.04–1.98) after adding health correlates. Findings suggest that having poorer sleep health across multiple dimensions is associated with increased risk of frailty among older adults, especially among women.

Abstract citation ID: igae098.0252

### ASSOCIATIONS OF CIRCADIAN REST/ACTIVITY RHYTHMS WITH METABOLIC SYNDROME IN MIDDLE-AGED AND OLDER ADULTS

Yiwei Yue[1], Jill Rabinowitz[2], Yang An[3], Marc Kaizi-Lutu[1], Chunyu Liu[1], Eleanor Simonsick[4], Jennifer Schrack[5], and Adam Spira[1], *1. Johns Hopkins University, Baltimore, Maryland, United States*, *2. Rutgers, The State University of New Jersey, New Brunswick, New Jersey, United States*, *3. National Institute on Aging, Baltimore, Maryland, United States*, *4. National Institute on Aging, Baltimore City 0400 Baltimore City, Maryland, United States*, *5. Johns Hopkins Bloomberg School of Public Health, Baltimore, Maryland, United States*

Circadian rhythm disturbances often co-occur with adverse cardiometabolic outcomes, including high blood pressure, obesity, and diabetes. However, the links between disrupted circadian rhythms and metabolic syndrome (MetS) are understudied in later life. This study examined associations between actigraphic circadian rest/activity rhythm (CRAR) metrics and MetS in 388 Baltimore Longitudinal Study of Aging participants who completed 6.6±1.0 nights of wrist actigraphy (Mean age=72.6±10.1, 20.6% Black adults, 54.1% women) and measures of MetS. Predictors were relative amplitude (RA), interdaily stability, intradaily variability, midpoint of total activity during the least active continuous 5 hours, and midpoint of total activity during the most active continuous 10 hours (M10 time). Participants were classified as having MetS if they had ≥3 of the following: triglycerides >150 mg/dL; fasting blood glucose ≥100 mg/dL; high-density lipoproteins <40 mg/dL for men and <50 mg/dL for women; blood pressure ≥130/85 mmHg; waist circumference ≥102 cm for men and ≥88 cm for women. Overall, 39 participants had MetS at baseline and 24 developed MetS over 4.5±2.0 years of follow-up. Adjusting for demographics, physical activity, depressive symptoms, time, and interactions of time with covariates, higher RA was cross-sectionally associated with 29% lower odds of MetS (per SD, B=0.71, p=0.046). Longitudinally, a later M10 time was associated with a faster rate of increase in the odds of developing MetS (per SD, B=1.14, p=0.014). Findings link weaker and more phase-delayed CRARs to higher odds of MetS in middle-aged and older adults. Studies are needed to identify mechanisms linking disrupted CRARs to metabolic disorders.

Abstract citation ID: igae098.0253

### PSYCHOLOGICAL RESILIENCE MAY IMPROVE SLEEP QUALITY OF FAMILY CAREGIVERS OF OLDER ADULTS

Rahul Malhotra[1], Ha-Linh Quach[2], and Jeremy Lim-Soh[1], *1. Duke-NUS Medical School, Singapore, Singapore*, *2. Duke-NUS CARE, Singapore, Singapore*

Given the increasing focus on the importance of sleep for health and well-being, we assessed the (a) association of caregiving status (family caregiver/non-caregiver) with sleep quality and its moderation by psychological resilience and (b) factors influencing sleep quality among caregivers. Cross-sectional data on subjective sleep quality (Pittsburgh Sleep Quality Index (PSQI); higher global or component scores represent worse sleep quality) and psychological resilience (Connor-Davidson resilience scale) from Singapore on 167 family caregivers of older adults and 135 non-caregivers was analyzed. Multivariable linear regression models assessed the association of caregiving status with global PSQI score and it's moderation by psychological resilience and identified caregiver and care-recipient characteristics associated with global PSQI score among caregivers. Mean global PSQI score (6.0 versus 4.5), proportion with poor sleep quality (global PSQI score>5; 46.1% versus 28.1%) and mean component scores (for sleep latency, sleep quality, sleep duration, and daytime dysfunction) were higher for caregivers, versus non-caregivers, reflecting their worse sleep quality. In multivariable analysis, caregivers had higher global PSQI score than non-caregivers (beta-coefficient: 1.04, p<0.05). However, this association was moderated by psychological resilience (beta-coefficient: -0.70, p<0.05) – the deficit in sleep quality for caregivers versus non-caregivers reduced with higher psychological resilience. Finally, among caregivers, those with higher education, fewer chronic conditions, fewer functional limitations, and higher psychological resilience had lower global PSQI score, i.e., better sleep quality. Our findings reveal the detrimental effect of being a family caregiver (versus not) on sleep quality and identify psychological resilience as a potential modifiable factor for mitigating it.

Abstract citation ID: igae098.0254

### HEALTH CONDITIONS AND CONTINUOUS POSITIVE AIRWAY PRESSURE DISCONTINUATION IN US MIDDLE-AGED AND OLDER ADULTS

Vishaldeep Sekhon[1], David Roth[1], Jennifer Albrecht[2], Emerson Wickwire[2], Adam Spira[1], Halima Amjad[3], Chien-Yu Tseng[4], and Christopher Kaufmann[5], *1. Johns Hopkins University, Baltimore, Maryland, United States*, *2. University of Maryland School of Medicine, Baltimore, Maryland, United States*, *3. Johns Hopkins University School of Medicine, Baltimore, Maryland, United States*, *4. University of Florida College of Pharmacy, Gainesville, Florida, United States*, *5. University of Florida College of Medicine, Gainesville, Florida, United States*

Although continuous positive airway pressure (CPAP) is an efficacious treatment for obstructive sleep apnea (OSA), suboptimal adherence is common, reducing its effectiveness. OSA co-occurs with chronic health conditions that may impact adherence to CPAP. This study evaluated health conditions as predictors of CPAP discontinuation over 2 years. Data came from the 2016 and 2018 Health and Retirement Study, a longitudinal cohort study of a nationally representative sample of 20,000 older adults in the U.S. In both years, participants were asked if "a doctor had ever told them they have a sleep disorder," and if so, which disorder and any treatments received (including CPAP). In 2016, participants also reported whether a doctor ever told them they had several health conditions (high blood pressure, diabetes, cancer, lung disease, heart disease, stroke, psychiatric problems, and arthritis). We identified CPAP users in 2016 with available data in 2018. Participants not listing CPAP in 2018 were considered to have discontinued CPAP. We used logistic regression to determine which health conditions predicted CPAP discontinuation after controlling for age, sex, race/ethnicity, education, BMI, and marital status. Of N=902 CPAP users in 2016, 38% did not indicate CPAP use in 2018. Participants diagnosed with diabetes in 2016 had 36% increased odds of CPAP discontinuation (AOR=1.36, 95% CI=1.02-1.80) compared to those without diabetes after accounting for other covariates. CPAP discontinuation did not differ across other health conditions. Overall, CPAP users with diabetes were at increased likelihood of CPAP discontinuation, necessitating efforts to encourage CPAP adherence in this vulnerable group.

### SESSION 1260 (SYMPOSIUM)

Abstract citation ID: igae098.0255

### SUSTAINABLE DEMENTIA CARE: CLINICAL, PROGRAMMATIC, AND PUBLIC HEALTH PERSPECTIVES

Chair: Joseph Gaugler
Co-Chair: Elma Johnson
Discussant: Laura Gitlin

When considering the dissemination and implementation of evidence-based innovations in aging, sustainability is a driving concern but understudied outcome. Sustainability refers to "is the extent to which a newly implemented intervention is maintained or institutionalized within a service setting's ongoing, stable operations" (Lewis et al., 2017). Although attention in dementia care is beginning to pivot to issues related to successful dissemination and implementation of a growing number of evidence-based innovations, concerns related to the maintenance of long-term care adoption of dementia care interventions in clinical, community, and public health contexts remain pressing. Beyond dementia care and in the broader field of implementation science, available research on sustainability is limited with challenges ranging from heterogeneity in definitions, measurement, follow-up periods and similar issues. The objective of this symposium is to initiate a scientific and practice agenda as it relates to sustainability for dementia care interventions. We will feature presentations on dementia care sustainability from public health, community, clinical, and scientific perspectives. Following this symposium, we aim to establish a framework to better achieve sustainability of evidence-based and evidence-informed dementia care innovations across key settings and communities.

Abstract citation ID: igae098.0256

### CONSIDERATIONS FOR SUSTAINING DEMENTIA CARE INTERVENTIONS IN THE COMMUNITY

Sari Shuman, *RTI International, Research Triangle Park, North Carolina, United States*

As community organizations implement dementia care interventions, they are tasked with the day-to-day intricacies of disseminating their chosen program. However, to ensure dementia services and supports remain in the community, they must assess how they can sustain the intervention into the future. The ability to continue dementia programs hinges on careful planning and consideration of several factors. Community organizations need to consider and assess the immediate and longer-term demand for the program in the geographic region being served, determine if they have or need to build organizational support, assess staffing needs and training abilities, and decide how they will continue to fund the dementia services and supports offered through the program if it is successful. In terms of funding, estimating the present and future costs of the dementia care intervention can help community organizations determine if the full program or only portions can be sustained using current or future funding mechanisms. Community organizations also need to secure the commitment from organizational decision makers to continue dementia care programs that have proven effective. Collecting and analyzing program data to demonstrate the program's impact on participants can assist in garnering support from key organizational stakeholders. In this session, these considerations and others for sustaining dementia care interventions will be presented and discussed.

Abstract citation ID: igae098.0257

### SUSTAINABILITY OF COMPREHENSIVE DEMENTIA CARE INTERVENTIONS FROM A CLINICAL PERSPECTIVE

Gary Epstein-Lubow, Ellen Tambor, Maddie Johnson, Kristin Lees Haggerty, and Rebecca Stoeckle, *Education Development Center, Waltham, Massachusetts, United States*

Multiple evidence-based comprehensive dementia care programs are currently available for adoption by healthcare systems and providers. However, barriers to adoption and long-term sustainability remain. The need for effective payment policies to incentivize implementation of comprehensive dementia care has long been recognized and the recently announced Center for Medicare and Medicaid (CMS) Guiding an Improved Dementia Care Experience (GUIDE) Model is an important step forward in improving access to comprehensive dementia care for Medicare beneficiaries and family caregivers. The GUIDE Model seeks to confirm that comprehensive dementia care can be widely implemented without raising health care costs. Similar work is needed under Medicare Advantage and other health plans. The National Dementia Care Collaborative (NDCC) seeks to accelerate dissemination of six effective comprehensive dementia care programs. The NDCC provides a forum for addressing barriers to adopting and sustaining comprehensive dementia care and seeks to promote that GUIDE Model participants use at least one evidence-based program. Existing evidence-based dementia care models, developed and tested within large health systems and academic medical centers, are now demonstrating ability to adapt to the characteristics of diverse service-delivery organizations leading to widespread adoption and sustainability. To succeed, one high priority is expanding the dementia care workforce to deliver high quality, comprehensive dementia care. Also essential is consensus on a core set of metrics for monitoring the quality and fidelity of comprehensive dementia care.

Abstract citation ID: igae098.0258

### PUBLIC HEALTH APPROACHES FOR SUSTAINABILITY OF DEMENTIA EVIDENCE-BASED PROGRAMS

Kellie Waugh, and Talyah Sands Leavitt, *Association of State and Territorial Health Officials, Arlington, Virginia, United States*

ASTHO, with support from CDC, has gathered strategies, examples, and resources to inform the development of sustainability focused capacity-buildings materials for public health departments, including an actionable resource for integrating sustainability into Healthy Brain Initiative (HBI) implementation. Attendees will be provided with a guided look through this tool, developed to elevate the importance of sustainability and support health departments in sustaining their programmatic dementia work. The public health-driven strategies included provide a unique guide for embedding sustainability into dementia initiatives and evidence-based programs from initial planning to implementation. Subsequently, the guidance is extended to ensure health equity is operationalized throughout the entirety of this process. Attendees will enhance their ability to ensure positive outcomes and provide long-term program benefits by thoughtfully considering complex factors that determine the sustainability of evidence-based programs, such as duration of funding, partnership, and organizational support. To encourage practical application, the presentation will showcase two state public health department examples of evidence-based programs that have effectively prioritized sustainability. The presenter will offer reflective insights on what sustainability means for public health practitioners involved with dementia and older adult health programs and shed light on how these efforts sustain positive outcomes across the life span and advance health equity.

### SESSION 1265 (PAPER)

### TESTING INTERVENTIONS TO IMPROVE OUTCOMES FOR OLDER ADULTS

Abstract citation ID: igae098.0259

### STROKE PREVENTION EDUCATION IN AFRICAN AMERICANS: A COMMUNITY-BASED PARTICIPATORY FEASIBILITY STUDY

Niloufar Hadidi[1], Clarence Jones[2], Zachary Taylor[3], Emily Gorzycki[4], Allison Pasdo[1], Olga Gurvich[1], and Susan Everson-Rose[5], *1. University of Minnesota, Minneapolis, Minnesota, United States*, *2. Hue-MAN Partnership, Minneapolis, Minnesota, United States*, *3. Allina Health, Minneapolis, Minnesota, United States*, *4. Abbott Northwestern Hospital, Minneapolis, Minnesota, United States*, *5. University of Minnesota Medical School, Minneapolis, Minnesota, United States*

**Research aim:** To assess the feasibility and acceptability of a Stroke Champion "train-the-trainer" program to support implementation of stroke prevention education among African Americans (AA) residing in the Minneapolis-St. Paul metropolitan area. Significance: AAs are fifty percent more likely to experience stroke than Whites. This disparity is partially attributed to higher prevalence of modifiable stroke risk factors such as hypertension, diabetes, and obesity among AA communities. Training community members to disseminate health information is an evidence-based, community-specific health promotion strategy. Stroke Champions - individuals from the community with a desire to help improve the health of their community – could be trained and empowered to deliver stroke recognition and prevention education to peers.
**Methods:** Twelve AA Stroke Champions were recruited to assess the feasibility of educating community on 1) stroke warning signs and risk factors; and 2) strategies to mitigate stroke risk factors. A one arm pre-post-test design was used. Findings: We successfully developed and implemented a stroke education website for community education. Stroke Champions reported the website was helpful, easy to use, and a valuable health information resource They stated that they needed more support from the research team to deliver the education to peers in the community.
**Conclusion:** Engaging Stroke Champions is potentially beneficial in increasing stroke prevention knowledge. However, Stroke Champions need more knowledge and facilitation support from the research team to effectively engage and educate peers. Implication: Community engagement is an important way to empower individuals to take charge of their health and reduce stroke burden.

Abstract citation ID: igae098.0260

### CHANGES IN SEDENTARY PATTERNS AND SELF-REPORTED HEALTH IN A RANDOMIZED CONTROLLED TRIAL OF SITTING REDUCTION

Dori Rosenberg[1], Stefani Florez Acevedo[2], Mikael Anne Greenwood-Hickman[1], Weiwei Zhu[1], David Arterburn[1], Jennifer McClure[1], and Bev Green[1], *1. Kaiser Permanente Washington Health Research Institute, Seattle, Washington, United States*, *2. University of Washington, Seattle, Washington, United States*

**Background:** Interventions to reduce sedentary time in older adults have been limited to feasibility studies. Building off a series of pilot studies, we aimed to test the effects of the I-STAND sitting reduction intervention to reduce sedentary patterns and improve self-reported health outcomes.
**Methods:** 283 participants were randomized to the I-STAND sitting reduction arm or an attention control condition. I-STAND included 10 brief health coach sessions, a tabletop standing desk, and fitness tracker to prompt sitting breaks. The attention control received 10 brief general healthy living focused sessions. The activPAL accelerometer worn for 7-days at baseline, 3, and 6 months measured sitting metrics. We measured pain interference, depression, loneliness and sleep disturbance by self-report. Linear regression models included baseline outcomes, covariates (e.g. demographics, health status), and whether randomization occurred pre- or post-pandemic.
**Results:** I-STAND (N = 140) and control (N = 143) participants mean age was 68.8 (SD 6.2) years, mean BMI was 34.9 (SD 4.7) kg/m2, 51.9% had hypertension. Participants were 65.7% women, 68.9% White, 14.5% Black, 5.7% Hispanic/Latinx, 4.2% Multiracial, and 3.2% Asian. Sitting time (-31.85 mins/day, p = 0.003), prolonged sitting bouts (-.57 bouts, p = 0.004), and mean sitting bout duration (-1.8 mins, p = 0.006) reduced while standing time increased (+27.7 minutes, p = 0.003) favoring the intervention. Loneliness reduced favoring I-STAND (-0.27, 95%CI: -0.52, -0.02, p=0.032).

**Conclusion:** I-STAND changed sitting patterns and improved loneliness. Uptake of sitting reduction interventions by older adults could improve physical and mental health.

Abstract citation ID: igae098.0261

### MITOFIT: A FORMATIVE/SUMMATIVE EVALUATION OF A NOVEL INTERVENTION ON MITOCHONDRIAL FITNESS

Cathy Maxwell[1], Brandon Grubbs[2], Jeffrey Boon[3], John Dunavan[4], Kelly Knickerbocker[5], and Maulik Patel[5], *1. University of Utah College of Nursing, Salt Lake City, Utah, United States*, *2. Middle Tennessee State University, Murfreesboro, Tennessee, United States*, *3. Vanderbilt University Medical Center, Nashville, Tennessee, United States*, *4. Peer-Tree, Franklin, Tennessee, United States*, *5. Vanderbilt University, Nashville, Tennessee, United States*

A key driver that leads to age-associated decline and chronic disease is mitochondrial dysfunction. Our prior work revealed strong community interest in the concept of mitochondrial fitness that led to development of a video-based intervention to prompt behavior change in adults aged 50+.

**Aim:** To conduct formative and summative evaluations of MitoFit, an instructional, biologically-based communication intervention aimed at improving physical activity (PA) in older adults, aged 50+.

**Methods:** Phase 1 formative evaluation- Community-dwelling older adults (N=101), rated the acceptability, appropriateness and helpfulness of our MitoFit video series, titled, "How to Slow Down Aging Through Mitochondrial Fitness." (4 out of five on a Likert-scale survey). Phase II summative evaluation- A subgroup of phase I participants (N=19) participated in a 1-month MitoFit intervention prototype to evaluate intervention and data collection feasibility (70% completion).

**Results:** Phase I: Participants (mean age: 67.8 [SD 8.9]; 75% female) rated the MitoFit videos as acceptable (agree: 97%-100%), appropriate (agree: 100%) and helpful (agree: 95%-100%) to support adaptation and continued work on our novel approach. Phase II: Participants (mean age: 71.4; 72% female) demonstrated MitoFit competencies (obtaining pulse, calculating maximum and zone 2 heart rate, demonstration of exercises). At one-month post-instruction, 13 participants (68.4%) had completed a self-initiated daily walking/exercise plan and submitted a daily activity log. Feasibility scores ranged from 89.4% to 94.7%. Fifteen participants (78.9%) stated an intention to continue the MitoFit intervention.

**Conclusion:** MitoFit was enthusiastically embraced, and is a cost-effective, scalable, and efficacious intervention to advance with community-dwelling adults.

Abstract citation ID: igae098.0262

### DEVELOPING AND TESTING A PROTOCOL FOR TELEPHONE REASSURANCE CALLS: A RANDOMIZED PILOT STUDY

Morgan Minyo[1], David Bass[2], Katherine Judge[3], Kate McCarthy[2], Christine Terry[4], Crysta Willis[4], Douglas Beach[4], and Fatima Perkins[4], *1. Benjamin Rose Institute on Aging, Cleveland, Ohio, United States*, *2. Benjamin Rose, Cleveland, Ohio, United States*, *3. Cleveland State University, Cleveland, Ohio, United States*, *4. Western Reserve Area Agency on Aging, Cleveland, Ohio, United States*

Telephone reassurance call programs that provide conversation and check-ins to isolated older adults have increased since the COVID-19 pandemic. The goals of these programs are to give older adults opportunities to express unmet needs, receive service referrals, and increase their use of home-and community-based services. Few empirical studies have examined the efficacy of these programs, including whether different approaches to telephone reassurance calls lead to different outcomes. This 24-month randomized controlled pilot study recruited older adults from an Area Agency on Aging to compared two approaches to telephone reassurance calls: 1) usual care, unstructured friendly check-in calls from a social worker (n=20) and 2) a newly developed protocol of semi-structured calls from a social worker (n=23), which was adapted from an evidence-based program. Multivariate results found significant differences in multiple outcomes. Compared to usual care (M=0.7, SD=1.0), participants who received the new semi-structured calls (M=2.1, SD=2.8) identified 1.75 more unmet service needs (t=2.69, p=.01). Participants who received the new semi-structured calls (M=1.1, SD=1.3) also received 1.23 more service referrals (t=4.07, p<.01) than usual care participants (M=0.3, SD=0.6). Additionally, participants who received the new semi-structured calls (M=3.7, SD=2.6) started or continued using 1.39 more services (t=2.02, p=.05) than usual care participants (M=2.4, SD=1.7). Findings suggest that the semi-structured protocol led to more beneficial outcomes while requiring only a seven-minute increase in the length of each telephone reassurance call. Discussion will focus on next steps for expanding the semi-structured protocol and sample in a multisite randomized trial.

Abstract citation ID: igae098.0263

### EFFECTS AND DIRECTIONALITY OF A HANDICRAFT INTERVENTION ON HAND FUNCTION AND HIGH-LEVEL COGNITION IN OLDER ADULTS

Kimi Kobayashi-Cuya, Susumu Ogawa, Ai Iizuka, Tomoya Takahashi, and Hiroyuki Suzuki, *Tokyo Metropolitan Institute for Geriatrics and Gerontology, Tokyo, Japan*

A longitudinal bidirectional association of hand dexterity with executive function and processing speed in high-functioning older adults has been reported, suggesting that community-level intervention programs using activities that demand dexterous abilities may help prevent the decline in these cognitive functions. Therefore, the present single-blind crossover randomized controlled trial examined the intervention effects of a 12-week manual dexterity program in community-dwelling older adults on hand sensorimotor and high-level cognitive functions, and assessed the direction of their associations. Fifty-three participants (mean age±SD, 75.2±5.6, 83% female) were randomly assigned to the intervention (handicrafts and creative cooking) or active control period (lectures on health maintenance). The primary outcome was hand dexterity, and the secondary outcomes were handgrip strength and hand sensation for hand function, and for high-level cognition, executive function, which was assessed with the trail making test (TMT)B, ΔTMT (TMTA-B) and the letter and category fluency tests (LFT and CFT, respectively), and processing speed, evaluated with TMTA and digit symbol. Linear mixed-effects models, testing for carry-over effects, showed intervention effects in hand dexterity ($p< 0.05$) and executive function (TMTB, ΔTMT and LFT, $p< 0.05$), whereas no effects were found for handgrip, hand sensation, nor processing speed. Also, cross-lagged panel models, adjusting for potential confounders, further revealed moderate and significant associations between baseline hand dexterity and subsequent increase in TMTB, LFT ($p< 0.001$) and ΔTMT ($p< 0.05$). Our results indicate that the implementation of a short-term manual dexterity program may contribute to the prevention of decline in executive function in community-dwelling older adults.

### SESSION 1270 (SYMPOSIUM)

Abstract citation ID: igae098.0264

### THE NATIONAL COLLABORATORY TO ADDRESS ELDER MISTREATMENT MENTORSHIP PROGRAM: KEY LEARNINGS AND OPPORTUNITIES

Chair: Lauren Bangerter
Co-Chair: Kristin Lees Haggerty
Discussant: Laura Mosqueda

The National Collaboratory to Address Elder Mistreatment (NCAEM) recognizes that improving elder mistreatment identification, intervention, and prevention requires diverse mentorship. The NCAEM Mentorship program is a one-year program designed to mentor clinicians, service providers, researchers, policymakers, and advocates who are focusing on improving care for older adults experiencing or at risk of experiencing elder mistreatment. This symposium will begin by an overview of the NCAEM mentoring program history and goals and presentations from current and past mentees. Dr. Troutman-Jordan's presentation will discuss their mentee project that included focus groups with home health nurses, as well as the process of then serving as a mentor and highlight opportunities for collaborative mentoring to address elder abuse. Dr. Simley will describe a mentee project focused on developing a survey for the Parkinson's Foundation to help generate data to inform the development of a continuing education module on elder mistreatment among individuals living with Parkinson's disease. Dr. Roberts will will focus on the role of the NCAEM in catalyzing interdisciplinary elder abuse research in long-term care settings, including nursing homes and assisted living. Dr. Steinman's presentation will describe a mentee project focused on the conceptualization and development of a national study of racial/ethnic group differences in older adults' involvement in Adult Protective Services. Dr. Marrs's presentation will focus on the links between ageism and elder abuse and describe the development of an ageism public awareness project funded by the Administration for Community Living. Our discussant, Dr. Mosqueda, will provide summary remarks and moderate discussion.

Abstract citation ID: igae098.0265

### BRIDGING LONG-TERM CARE AND ELDER ABUSE RESEARCH IN A MENTOR–MENTEE COMMUNITY OF PRACTICE

Amy Roberts[1], E-Shien Chang[2], Tina Kilaberia[3], Anthony Rosen[4], Gary Epstein-Lubow[5], and Mark Lachs[6], *1. Miami University, Oxford, Ohio, United States*, *2. Weill Cornell Medical College, New York, New York, United States*, *3. New York University, New York, New York, United States*, *4. New York Presbyterian Hospital/Weill Cornell Medical Center, Weill Cornell Medicine, New York, New York, United States*, *5. Education Development Center, Waltham, Massachusetts, United States*, *6. Weill Cornell Medicine, New York, New York, United States*

The National Collaboratory to Address Elder Mistreatment (NCAEM) Mentorship Program serves as an emerging hub to learn from thought leaders, build partnerships, and forge collaborations in elder mistreatment research, policy, and advocacy efforts. This one-year program brings together clinicians, service providers, researchers, policymakers, and advocates from many disciplines who share a common goal of eradicating elder mistreatment. Our session will focus on the critical role of the NCAEM in catalyzing interdisciplinary elder abuse research in long-term care settings, including nursing homes and assisted living. We will first identify approaches, mechanisms, and lessons learned in building successful and sustainable mentoring experiences through the support of the NCAEM. Perspectives from both mentees and mentors will be highlighted to reflect the range of experiences, such as mentor-mentee meetings, peer-to-peer mentoring within and across mentee cohorts, and national convenings. To demonstrate the rich collaborative experience of the NCAEM community of practice, we will then present three completed and ongoing impact-driven research projects on resident-to-resident mistreatment in long-term care settings, including: (1) characterizing racial/ethnic conflicts among residents in long-term care, (2) recognizing resident-to-resident mistreatment as a key barrier to an age-friendly environment, and (3) identifying the training and program needs for directors of social services in nursing homes regarding resident-to-resident mistreatment. Lastly, we will discuss learning using the ongoing experience of the NCAEM as a model research network model to advance ongoing work, generate new areas of inquiry, and grow interest in a focused gerontology topic.

Abstract citation ID: igae098.0266

### GUARDING AGAINST HARM: UNDERSTANDING AND ADDRESSING ELDER MISTREATMENT RISKS IN THE PARKINSON'S POPULATION

Adrianne Smiley, *University of Pittsburgh, Pittsburgh, Pennsylvania, United States*

Parkinson's disease (PD), particularly in moderate-to-advanced stages, poses significant challenges, including cognitive and physiological decline, leading to reduced independence in daily activities. Functional dependence—to any degree—is a primary predictor of mistreatment. Despite the recognized predictors of elder mistreatment risks associated with functional impairment, the understanding of its impact within the PD community remains limited. PD research often focuses on physiological changes and pharmacological and rehabilitative interventions; however, the risks for mistreatment are often unaddressed. As such, this project centers on advancing and disseminating knowledge about abuse and mistreatment within the PD population. An evidence-based, community-engaged, participatory action research approach with collaborative analysis methodology was employed with a primary focus on PD-specific organizational partnership, intra-organizational structural and procedural analysis, interdisciplinary collaboration, and relationship-building for reflection on themes, patterns, gaps, and needs for improving patient safety resources as a primary prevention method for reducing risks for mistreatment in the PD community. Outcomes: This action-based approach resulted in 1) the development of a survey instrument to gauge knowledge gaps about mistreatment in the PD community and inform the development of educational offerings for healthcare recipients, caregivers, and licensed medical providers, 2) the development of a patient safety research staffer responsible for analyzing reports submitted to the organization's helpline to assess the prevalence of mistreatment risks according to self or caregiver reported data, and 3) establishment of a research agenda and population-centered plan for advancing knowledge about PD and elder mistreatment risks for harm prevention.

Abstract citation ID: igae098.0267

### A PUBLIC AWARENESS MESSAGE TO DISRUPT AGEISM AND PREVENT ELDER ABUSE

Sarah Marrs, *Virginia Commonwealth University, Richmond, Virginia, United States*

To date, much of the research on elder mistreatment has focused on achieving improved skills for recognizing, screening for, and reporting abuse that has already occurred, typically considered secondary prevention. Knowing how to recognize when abuse has occurred is indeed critical for effective and timely intervention. However, engaging in efforts that challenge how we as a society perceive aging and older adults is an important and necessary primary prevention strategy, which targets stopping abuse from occurring in the first place by focusing on the source of abuse, such as underlying beliefs or attitudes. Given the linkages between ageism and abuse, our team has engaged in public education and awareness campaigns aimed at changing the narrative we as individuals associate with aging and older adults. In this multimedia presentation, we will begin by briefly discussing the link between ageism (both individual and structural) and elder abuse, citing current scientific literature as well as our own research, and how this led to our receipt of funding from the Administration for Community Living (ACL) to conduct innovative elder justice training and research. Then, we will briefly discuss the methods and evaluation feedback for one small component of this work, an ageism and abuse public awareness video. Specifically, we will highlight the methods (i.e., qualitative data collected, literature reviewed) that informed the creation of the video, present the video, and then share the feedback we have collected as part of our overall project evaluation activities.

Abstract citation ID: igae098.0268

### COLLABORATIVE MENTORING OPPORTUNITIES VIA THE NATIONAL COLLABORATORY TO ADDRESS ELDER MISTREATMENT

Meredith Troutman-Jordan, *University of North Carolina Charlotte, Charlotte, North Carolina, United States*

Elder abuse is a silent public health issue; with nearly 5 million cases of abuse annually, its prevention is a high priority. Nurses and social workers are the largest groups of healthcare professionals to witness, report and advocate for elder abuse. They have both ethical and legal obligations as mandated reporters. However, a majority of elder abuse is not reported. Home health nurses and social workers are in key positions to identify and intervene when elder abuse is suspected. This presentation shares how collaborative mentoring has worked to address elder abuse. In collaborative mentoring, mentees have the opportunity to engage with a mentor and peers, present ideas, exchange knowledge and experience, and be actively engaged. As a NCAEM mentee the author was guided by a mentor in the development and implementation of a focus groups with home health nurses. Pilot data was used to secure funding for an ongoing mixed-methods study. Qualitative findings from phase one include uncertainty, advocacy, and fear, with some overlap in themes from social work and nursing. This data will inform intervention and resource development for home health nurses and social workers. Positive outcomes of this experience were inspiration to serve as a mentor for a subsequent NCAEM mentee, whose project deals with identification and intervention for elder abuse identified within VA homecare services. This presentation will share outcomes from the presenter's NCAEM project, the process of then serving as a mentor, and highlight opportunities through collaborative mentoring in addressing the issue of elder abuse.

Abstract citation ID: igae098.0269

### RACIAL/ETHNIC GROUP DIFFERENCES IN OLDER ADULTS' INVOLVEMENT WITH ADULT PROTECTIVE SERVICES

Kenneth Steinman, *The Ohio State University, Columbus, Ohio, United States*

Child protective services has long wrestled with concerns about Black and Hispanic families being disproportionately more likely to be investigated. In contrast, only one, small, recent study has quantified racial/ethnic group differences in adult protective services (APS). A paucity of research and inconsistent findings in this area have limited the generalizability of studies and complicated efforts to develop culturally sensitive practices for specific groups. The proposed project would represent the first national study of racial/ethnic group differences in older adults' involvement in APS. It would provide the several key findings, including: a conceptual framework that explains why racial/ethnic group differences in APS may exist; benchmarks for the "typical" magnitude of racial/ethnic group differences in key APS metrics across most US states; identification of mistreatment types, genders, and age groups where racial/ethnic group differences in APS metrics are especially large; identification of the types of racial/ethnic group differences in APS metrics that vary markedly from state to state or county to county; and descriptions of the community (e.g., poverty rate) and organizational (e.g., APS agency) characteristics that help explain this variation. The project will also engage researchers, practitioners, and community leaders in three advisory teams. Their regular involvement will help ensure that the project employs the best available theories and methods, provides useful guidance for APS professionals, and reflects the priorities and lived experiences of the communities being studied.

### SESSION 1275 (SYMPOSIUM)

Abstract citation ID: igae098.0270

### THE STARTING A TESTOSTERONE AND EXERCISE PROGRAM AFTER HIP INJURY (STEP-HI) TRIAL: STUDY RESULTS AND IMPLICATIONS

Chair: Ellen Binder
Discussant: Douglas Kiel

Hip fractures are common among older women and can have a devastating impact on their ability to remain independent. Many women who were high functioning before the hip fracture do not return to their pre-fracture level of function, have persistent weakness and mobility impairments, and may require ongoing supportive services. Age-associated androgen deficiency may contribute to deficits in muscle mass, strength and power that are common in older female hip fracture patients. The typical approach to hip fracture rehabilitation routinely includes a limited course of physical therapy but the precise role of exercise and the use of an anabolic therapy like testosterone has not been determined. The Starting a Testosterone and Exercise Program after Hip Injury (STEP-HI) Study is a three-group, randomized, double-blinded, placebo-controlled Phase III clinical trial that was conducted at 8 clinical sites in the USA. The trial was designed to evaluate a multi-modal intervention aimed at improving functional outcomes in older female hip fracture patients with persistent mobility impairment. 129 female hip fracture patients, age 65 yrs. and older were randomly assigned to one of three groups: supervised exercise plus 1% testosterone topical gel (EX+T); supervised exercise plus placebo gel (EX+P); or Enhanced Usual Care (EUC). This symposium will present the results of the trial, including recruitment and retention, primary and secondary outcome measures. The program will also address strategies to ensure fidelity with exercise in a multicenter study like STEP-HI. Implications of study findings for future research and clinical practice will be discussed.

Abstract citation ID: igae098.0271

### THE STEP-HI STUDY: RECRUITMENT, ADHERENCE, AND RESULTS FOR PRIMARY STUDY OUTCOME

Ellen Binder[1], Sarah Berry[2], Jenna Bartley[3], Robin Marcus[4], Christine McDonough[5], Dominic Reeds[1], Jay Magaziner[6], and Kenneth Schechtman[1], *1. Washington University School of Medicine, St. Louis, Missouri, United States*, *2. Hebrew Rehabilitation Center for the Aged, Boston, Massachusetts, United States*, *3. UConn Health, Farmington, Connecticut, United States*, *4. University of Utah Health, Salt Lake City, Utah, United States*, *5. University of Pittsburgh, Pittsburgh, Pennsylvania, United States*, *6. University of Maryland School of Medicine, Baltimore, Maryland, United States*

For STEP-HI women aged ≥65 years with a recent hip fracture were recruited from the community at large and Assisted Living facilities. Women who qualified for the study were assigned to one of three groups for 6 months: exercise plus testosterone (EX+T), exercise plus placebo (EX+P), or Enhanced Usual Care (EUC). EX+T were prescribed a dose of 1% testosterone topical gel intended to maintain serum total testosterone levels between 110-160 ng/dL. Both EX groups performed a supervised exercise program; EUC participants performed home exercise. To identify eligible patients 4695 chart reviews were completed, 207 women consented for screening, 153 were eligible, 129 were randomized to study groups, 122 included in the intention-to-treat (ITT) analyses. Mean age of the sample was 79.3 ± 8.4. Women assigned to the EX+T group achieved a mean serum total testosterone level of 170.1 ± 82.7 ng/dL, compared with 17.8 ± 13.3 ng/dL in the EX+P group ($p < 0.001$). Adherence to the supervised exercise program (number completed/number of expected sessions) was 82.5% in the EX+T group and 81.1% in the EX+P group ($p = 0.68$). The pre-specified primary outcome measure was Six Minute Walk Distance (SMWD). In the ITT analysis, SMWD increased over the 6-month intervention period in all three groups, but the changes in EX+T (n=53) vs. EX+P (n=51) vs. EUC (n=18) did not differ significantly by group ($p > 0.39$ for all comparisons). We conclude that in older women with a recent hip fracture, testosterone replacement therapy does not independently improve SMWD greater than exercise.

Abstract citation ID: igae098.0272

### THE STEP-HI STUDY: IMPACT OF TESTOSTERONE AND RESISTANCE EXERCISE ON SPPB, LEG STRENGTH, AND APPENDICULAR LEAN MASS

Elena Volpi[1], Douglas Kiel[2], Rocco Paluch[3], Peter Dore[4], Richard Fortinsky[5], George Kuchel[5], Denise Orwig[6], and Ellen Binder[4], *1. University of Texas Health San Antonio, San Antonio, Texas, United States*, *2. Harvard Medical School, Boston, Massachusetts, United States*, *3. University at Buffalo, SUNY, Buffalo, New York, United States*, *4. Washington University School of Medicine, St. Louis, Missouri, United States*, *5. University of Connecticut, Farmington, Connecticut, United States*, *6. University of Maryland School of Medicine, Baltimore, Maryland, United States*

The STEP-HI Study tested if testosterone replacement therapy in combination with resistance exercise training for 6 months could improve functional recovery from hip fracture in older women more than resistance exercise alone or standard of care. Here we present the results of the intention to treat analyses (N=122) of the effect of testosterone replacement plus exercise (EX+T, n=53) vs. exercise plus placebo (EX+P, n=51) vs. enhanced usual care (EUC, n=18) on the Short Physical Performance Battery (SPPB) scores, maximal leg press strength, and appendicular lean mass. Group differences were analyzed with contrasts using a mixed model repeated analysis of variance that included the following covariates for each outcome: clinical site, age, days since hip surgery, BMI, hip fracture type, general depression score, lung disease, comorbidity index and baseline level. EX+T had a greater improvement in SPPB score than EX+P ($p<0.009$). Maximal leg press strength increased in all groups by the end of the intervention period with no differences between groups ($p>0.05$). Appendicular lean mass increased to the same extent in the EX+T and EX+P groups, but slightly decreased in the EUC group as compared to EX+T (p=0.020) and EX+P (p=0.029). These findings suggest that testosterone may independently impact a composite measure of physical performance, while exercise significantly improves appendicular lean mass independent of testosterone replacement. The implications of these findings will be discussed.

Abstract citation ID: igae098.0273

### DELIVERING HIGH-INTENSITY STRENGTH TRAINING AND MAINTAINING INTERVENTION FIDELITY: THE STEP-HI STUDY

Michelle Rauzi[1], Melissa Tran[2], Kelly Monroe[3], and Jennifer Stevens-Lapsley[2], *1. Department of Veterans Affairs, Aurora, Colorado, United States*, *2. University of Colorado Anschutz Medical Campus, Aurora, Colorado, United States*, *3. Washington University School of Medicine, St. Louis, Missouri, United States*

Hip fractures in women can result in significant functional decline. The STEP-HI study evaluated the effect of testosterone therapy versus placebo combined with supervised exercise training on functional recovery following hip fracture. Maximizing internal validity was essential to maintain rigor of this study. Further, knowledge of both the intervention protocol and the fidelity process are essential to understand threats to internal validity, identify barriers to the protocol, and implement appropriate solutions. Our purpose is to provide an overview of the supervised exercise training intervention and discuss the advantages of and barriers to a remote intervention fidelity monitoring process. Twenty-eight exercise interventionists (EI) across 8 sites delivered the supervised exercise training protocol. EI training was included during the initial step of a 3-step fidelity process: Step 1 Training Certification, Step 2 Initial Fidelity, and Step 3 Ongoing Fidelity. The fidelity process was managed from one site that assessed intervention fidelity through videos submitted approximately every 6 months by each site. Supervising physical therapists oversaw fidelity at their respective site and completed in-person fidelity assessments once per month. The primary advantage of this process was cost savings that occurred because there were no site visits with associated travel costs. A significant barrier was technological challenges related to recording and uploading videos to a secure file-sharing website. Across all sites, 140 fidelity assessments were completed; the mean (SD) score was 96.3% (3.4%) indicating excellent intervention fidelity. Our procedures and lessons learned will guide planning for future clinical trials of complex interventions.

### SESSION 1280 (SYMPOSIUM)

Abstract citation ID: igae098.0274

### UNPACKING THE RELATIONSHIP BETWEEN EDUCATION AND COGNITIVE DECLINE AND DEMENTIA

Chair: Pamela Herd
Co-Chair: Katrina Walsemann

This session, which brings together a set of papers from participants in the Network on Education, Biosocial Pathways, and Dementia in Diverse Populations (EdDem), attends to the relationship between education and cognitive function and dementia in later life. The papers in this session explore two major themes. First, they examine the role of early and mid-life influences on these relationships. How do exposures to poor health in early life influence both educational attainment and subsequent risk for poor cognitive outcomes in later life? How does the content of early life schooling, including the rigor of the curriculum, influence both educational attainment and subsequent risk for poor cognitive outcomes in later life? The papers also consider the role of midlife factors, including occupation, health behaviors, and health conditions, in mediating the relationship between education and later life cognitive outcomes. Finally, these papers explore how the relationship between education and later life cognition varies within and across middle- and high-income countries. Overall, this set of papers helps unpack the persistent and robust relationship between education and cognitive function and dementia in later life. Session attendees will also have the opportunity to learn about the NIH-funded EdDem network, including funding and network related opportunities.

Abstract citation ID: igae098.0275

### HIGH SCHOOL CURRICULUM AND COGNITIVE FUNCTION IN THE EIGHTH DECADE OF LIFE

Sara Moorman, and Saber Khani, *Boston College, Chestnut Hill, Massachusetts, United States*

Formal educational attainment, or years of schooling, has a well-established positive effect on cognitive health across the life course. If educators and policymakers could imbue education with even more of the vital ingredients for sustained cognitive health, then rates of cognitive impairment might continue to decline in the population as cohorts age. We hypothesized that the content and difficulty of the curriculum in high school lead to higher levels of socioeconomic attainment in adulthood and, in turn, to better cognitive outcomes in older adulthood. We estimated multilevel structural equation models (MSEMs) in data from 2,405 individuals who attended one of 1,312 U.S. high schools in 1960 and participated in the Project Talent Aging Study in 2018. Curricular difficulty (i.e., college preparatory track) and content (i.e., a greater number of semesters of math and science) in high school were positively related to cognitive function (i.e., word recall and verbal fluency) at average age 75. Effects were robust to controlling for adolescent cognitive ability, academic performance, socioeconomic background, and school characteristics. Overall educational attainment significantly mediated the association: High school curricular content and difficulty were associated with higher degree attainment, which was associated with better cognitive function in later life. We discuss implications of these findings for educational policy, where the results suggest that interventions into the content and difficulty of the high school curriculum could potentially reap cognitive benefits decades later.

Abstract citation ID: igae098.0276

### UNDERSTANDING THE ROLE OF EDUCATION IN COGNITIVE FUNCTIONING AMONG OLDER BRAZILIANS

Mateo Farina, *University of Texas at Austin, Austin, Texas, United States*

Education has been linked to cognitive functioning in older adulthood across time and place. However, how education is linked to later life cognitive functioning may differ across country contexts. The role of education has been widely debated with evidence for both direct and indirect pathways (i.e. occupation, health behaviors, etc.). However, much of the research has been limited in low- and middle-income countries that, in general, have lower levels of education and greater levels of social inequity. As one of the fastest-aging countries that has seen substantial improvements in education levels over the last 60 years but with significant social inequality, Brazil provides a prime example to understand how education as an exposure or the sequelae of pathways from education may impact cognitive health in older adulthood. This study uses nationally representative data from the ELSI-Brazil Study (50+) to examine the association of education with older adult cognitive functioning (global and domain-specific). I used a series of linear regression models to assess the sensitivity of the association of education with cognitive functioning after adjustments for health behaviors, health conditions, and wealth. I found that education was positively associated with cognitive functioning; for the total population, it remained relatively unchanged with downstream adulthood factors. Nevertheless, among those with more than 8 years of education, differences were attenuated by downstream factors. This suggests that education as an exposure may drive future trends in cognitive health in countries with low levels of overall education.

Abstract citation ID: igae098.0277

### EDUCATION, OCCUPATIONAL ENVIRONMENT, AND COGNITIVE DECLINE IN LATE ADULTHOOD

Katy (Qiuchang) Cao, Dawn Carr, and Miles Taylor, *Florida State University, Tallahassee, Florida, United States*

Education and occupational environment shape cognitive decline and Alzheimer's Disease and Related dementias (ADRDs) in late adulthood. However, few studies have explored both factors simultaneously. This study aims to (1) Identify occupational environmental factors impacting cognitive decline and ADRD; (2) Analyze the separate or combined contributions of education and occupational environment to cognitive decline and ADRD; (3) Identify whether occupational environment mediates the association between education and cognitive decline/ADRD in late life. Structural Equation Modeling was conducted in Mplus using nationally representative data from the Health and Retirement Study (HRS) and Occupation Information Network (O*NET) linked dataset, incorporating new race/ethnicity adjusted ADRD measures. Confirmatory Factor Analyses suggested that hazardous exposure and cognitive complexity capture two separate but connected components of the occupational environment. Both the measurement (N=7301) and structural model (N=6345) demonstrated good model fit (TLI, CFI >=.95, SRMR<.08). Education had a positive direct effect on cognitive function (standardized direct effect = 0.08, p=0.002). Although the indirect effect of cognitive complexity was non-significant, hazardous occupational environment mediated education's link to cognitive function (standardized indirect effect= 0.018, p=0.03). A one-unit increase in the hazardous occupational environment was associated with a 0.06 decline in cognitive function accounting for education, gender, race/ethnicity, depressive symptoms, baseline cognition, and age of the latest cognitive assessment. No statistically significant relationships between occupational exposure and ADRD were identified due to the limited waves of the new ADRD measures. Findings may inform interventions addressing occupational hazards to modify the association between early-life education and late-life cognitive outcomes.

Abstract citation ID: igae098.0278

### CAUSAL EFFECTS OF SCHOOLING ON MEMORY AT OLDER AGES IN FIVE LOW- AND-MIDDLE-INCOME COUNTRIES

Shana Stites[1], Vikesh Amin[2], Jere Behrman[1], Jason Fletcher[3], Carlos Flores[4], Alfonso Flores-Lagunes[5], Iliana Kohler[1], and Hans-Peter Kohler[1], *1. University of Pennsylvania, Philadelphia, Pennsylvania, United States*, *2. Central Michigan University, Mount Pleasant, Michigan, United States*, *3. University of Wisconsin Madison, Madison, Wisconsin, United States*, *4. California Polytechnic State University, San Luis Obispo, California, United States*, *5. Syracuse University, Syracuse, New York, United States*

We investigate the relationships between early life schooling and performances on memory tests at older ages in China, Ghana, India, Mexico, and South Africa using harmonized data from the World Health Organization Study on Global Ageing and Adult Health. We apply a nonparametric partial-identification approach that bounds causal effects of increasing schooling at different parts of the schooling distributions under relatively weak assumptions. We find that an extra grade of schooling is associated with memory scores that are higher by at most 0.12 standard deviations (SDs) in China, 0.06 SDs in Ghana, 0.10 SDs in India, 0.09 SDs in Mexico, and 0.07 SDs in South Africa, between never-attending school and completing primary schooling. The bounds statistically rule out null effects for all countries except South Africa. At higher parts of the schooling distributions (e.g., high-school or university completion) the bounds cannot statistically rule out null effects.

Abstract citation ID: igae098.0279

### CHILDHOOD HEALTH AND COGNITIVE FUNCTION AT MIDLIFE: EVALUATING THE MEDIATING ROLE OF EDUCATION

Nicole Hair, *University of South Carolina, Columbia, South Carolina, United States*

Educational attainment is one of the strongest predictors of dementia among older adults. It is unclear, however, whether this association reflects the benefits of education per se or early life factors that influence educational attainment. We leverage the introduction of the first measles vaccine in 1963, which contributed to a pronounced and sustained reduction in infectious disease morbidity, to evaluate the effects of better overall health in childhood on cognitive function at midlife and, further, to assess the role of educational attainment in the causal pathway from early life health to later life cognition. Our project links early-life contextual data, e.g., state-level socioeconomic, epidemiologic and policy measures, to the Health and Retirement Study, a longitudinal population-based study of individuals over age 50 with validated cognitive measures. Our analytic sample includes older adults in the War Baby (1942-47), Early Baby Boomer (1948-53), Mid Baby Boomer (1954-59), and Late Baby Boomer (1960-65) cohorts who were born and attended school in the United States. Our analytic approach compares outcomes across birth cohorts with more or less exposure to measles vaccination campaigns in the 1960s and 1970s and across states with higher or lower baseline measles incidence. We find that mass vaccination against measles had positive and significant impacts on both educational attainment (e.g., years completed) and cognitive function (e.g., global cognition and episodic memory) at midlife and that education mediated the association between better overall health in childhood and cognitive function later in life.

### SESSION 1285 (SYMPOSIUM)

Abstract citation ID: igae098.0280

### WHEN CONTEXT GETS UNDER THE SKIN: HOW ATTITUDES SHAPE CONTEXTUAL IMPACT ON AGING WELL IN LATE LIFE

Chair: Oliver Schilling
Co-Chair: Björn Slaug
Discussant: Oliver Schilling

In late life, increasing risks of loss of resources may aggravate vulnerability to contextual conditions that challenge habitual life conduct. However, evidence of older adults' capabilities to deal with adversity suggests that subjective accounts of enabling or challenging contexts and corresponding attitudes, rather than objective context per se, shape conditions for aging well. This symposium highlights recent findings from studies that examined the role of subjective attitudes in associations between contextual conditions and aging well in very old adults: Sabatini et al. analyze associations between changes in depressive symptoms, attitudes to aging, and indicators of aging well (life satisfaction, informant-rated disability) in an Australian sample, their findings suggest a crucial role of attitudes to aging in mediating effects of poor mental health on aging well. Slaug et al. show that effects of functional limitations on active aging were moderated by how older Swedes perceived their housing (i.e., meaning of home). Zimmermann & Demirer show that impacts of neighborhood socioeconomic deprivation in Germany on functional health were mediated by social cohesion (i.e., trusting people in the neighborhood). Using multiple German very old samples, Kaspar et al. propose and test how a contextually enriched model of successful aging, comprising perceptions of contextually driven opportunity structures could better account for the realities of aging well with disability. Schilling will discuss these findings, focusing on needs to understand the interplay between subjective attitudes, contexts and aging well in late life.

Abstract citation ID: igae098.0281

### TESTING A CONTEXT-ENRICHED MODEL OF SUCCESSFUL AGING IN MULTIPLE REPRESENTATIVE SAMPLES OF VERY OLD ADULTS

Roman Kaspar[1], and Hans-Werner Wahl[2], *1. Charlotte Fresenius Hochschule, Cologne, Nordrhein-Westfalen, Germany*, *2. Heidelberg University, Heidelberg, Baden-Wurttemberg, Germany*

Objectively measurable outcomes of successful aging (SA) as used in Rowe and Kahn's model of SA always operate in multiple contexts that may or may not be perceived as providing opportunity structures for aging well. Context may become most critical in advanced old age. We posit that so far, no empirical test of a contextually enriched model of SA in advanced old age exists. We use multiple representative survey samples of very old individuals, i.e., the D80+ study (study 1; N = 3.233) and the NRW80+ study (study 2; N [baseline] = 1.863; N [wave 2, new independent sample] = 950; N [2-year panel sample] = 912). Study protocols were highly similar across studies and included a multidimensional self-report- based assessment of perceived aging context. Whereas rates of SA according to Rowe and Kahn decreased from 9.1% in those 80-84 years to 0.7% in those 90 years or older, rates for those with many contextually driven opportunity structures at their disposal even if not fulfilling Rowe and Kahn's criteria were much higher and decreased less steeply from 54.9% (80-84 years) to 44.4% (90 years and older). Also, greater stability of SA across 2 years was observed in supportive contexts. Decontextualizing SA may result in misleadingly low rates of SA in very old adults. Our findings underline the demand to go beyond SA as a process/outcome only at the individual level and aim future policies for aging well much more at strengthening opportunity structures.

Abstract citation ID: igae098.0282

### ATTACHING MORE MEANING TO THE HOME SUPPORTS ACTIVE AGING DESPITE FUNCTIONAL DECLINE

Björn Slaug[1], Marianne Granbom[2], Jonas Björk[1], Taina Rantanen[3], Steven Schmidt[1], and Susanne Iwarsson[1], *1. Lund University, Lund, Skane Lan, Sweden*, *2. Centre of Ageing and Supportive Environments (CASE), Lund University, Skane Lan, Sweden*, *3. University of Jyvaskyla, Jyvaskyla, Keski-Suomi, Finland*

When functional limitations increase with age, perceived qualities of the home may influence the level of activity among older adults. Active aging is the process through which people strive to maintain wellbeing when growing old. The aim of this study was to investigate whether perceived housing moderates the relationship between functional limitations and active aging. Cross-sectional data from a sub-sample (N=820; mean age=69.7; 54% women) of the Prospective RELOC-AGE was used. Functional limitations were captured by ten dichotomous questions. Active aging was assessed with the University of Jyvaskyla Active Aging Scale (UJACAS; 17 items, self-rated for four perspectives). Perceived housing was self-rated with four usability questions and the meaning of home (MOH; 28 items) questionnaire. Associations and interactions were analysed using linear regression models, adjusting for gender and educational level. Each functional limitation decreased the active aging score by almost five points (p<0.001). Usability did not moderate the relationship while MOH significantly attenuated the association between functional limitations and active aging (p=0.039). Those with high MOH had two points less decrease in active aging score compared to those with low MOH. Having a home with more personal meaning attached to it seems to provide more ability and opportunity for meaningful activities, thus supporting active aging despite functional limitations. This sheds new light on the known association between MOH and different aspects of wellbeing in old age and has relevance for further theory development.

Abstract citation ID: igae098.0283

### PERCEIVED NEIGHBORHOOD RESOURCES AND MEDIATING ROLE OF SOCIAL COHESION ON FUNCTIONAL HEALTH AMONG VERY OLD ADULTS

Jaroslava Zimmermann[1], and Ibrahim Demirer[2],
*1. University of Cologne, Cologne, Nordrhein-Westfalen, Germany*, *2. University of Cologne, Faculty of Medicine and University Hospital Cologne, Institute of Medical Sociology, Health Services Research, and Rehabilitation Science (IMVR), Cologne, Nordrhein-Westfalen, Germany*

Functional health is not only a key indicator of physical health in old age, but it also reflects the individual ability to self-care and participate independently in important areas of life. The evidence shows that older adults living in socioeconomically deprived neighborhoods have higher likelihood to experience functional limitations. Nevertheless, little is known about the underlying mechanisms explaining this effect. Following the social disorganization theory, the aim of this contribution was to test the hypothesis that the link between neighborhood socioeconomic resources and functional health is mediated by social cohesion. Data from the German representative study "Old age in Germany" were used. The study sample included 10,578 persons aged ≥80 years. Functional health was measured by instrumental activities of daily living (IADL). Neighborhood socioeconomic resources were operationalized by perceived walkability of neighborhood and building condition. Social cohesion referred to trusting people in neighborhood. Considering the complex sample design, path analysis based on structure equation modeling was conducted. Adjusting for individual and regional characteristics, the results showed that low walkability and poor building condition (independently of each other as well as in a combined form) were associated with decreased ability in IADL and lower social cohesion, while higher social cohesion increased IADL. The findings suggest that unfavorable socioeconomic conditions in neighborhoods negatively affect social cohesion and functional health. Moreover, social cohesion in neighborhoods have protective effect on functional health. Both, socioeconomic and social conditions should be targeted when planning interventions to promote or maintain functional health of the very old.

Abstract citation ID: igae098.0284

### SELF-PERCEPTIONS OF AGING MEDIATE THE ASSOCIATIONS OF CHANGE IN DEPRESSION WITH LIFE SATISFACTION AND DISABILITY

Serena Sabatini, *University of Surrey, Guildford, England, United Kingdom*

Self-perceptions of ageing (SPAs) significantly influence individuals' health trajectories and satisfaction with life. This study explores (1) the associations between 14-year change in depressive symptoms as predictor of follow-up scores on SPAs, satisfaction with life, and informant-rated disability and (2) whether SPAs mediate the associations of 14-year change in depressive symptoms with follow-up scores on satisfaction with life and informant-rated disability. Participants were 174 older adults (Mean age= 87.41; SD= 3.67; 60% women) from the Sydney Memory and Ageing Study (MAS) with complete wave 7 (14-year follow-up) data. Measures used were the Geriatric Depression Scale (GDS); the informant-rated World Health Organisation Disability Assessment Schedule (WHODAS); the Diener Satisfaction with Life Sclae (SwL); and the Laidlaw' Attitudes to Aging Questionnaire (AAQ) as indicator of SPAs. We calculated a change score for depressive symptoms. Linear regression models and structural equation models were used. After controlling for covariates of age, sex, marital status, occupation, and number of chronic health conditions, greater 14-year increase in depressive symptoms predicted more negative scores on SPAs (Standardised beta, $\beta = 0.41$; p<.001), lower SwL ($\beta = 0.30$; p<.001), and greater informant-rated disability ($\beta = 0.26$; p=.001). Participants' scores on SPAs partially mediated the association of 14-year change in depressive symptoms as predictors of follow-up scores on satisfaction with life and informant-rated disability. Negative SPAs may be one of the reasons why increased depressive symptoms in old and very old age can be related to lower satisfaction with life and informant-rated disability.

### SESSION 1290 (PAPER)

### ADDRESSING LONELINESS AND MENTAL HEALTH IN AGING POPULATIONS

Abstract citation ID: igae098.0285

### MEDIATING ROLE OF DEPRESSION IN THE ASSOCIATION BETWEEN CUMULATIVE LONELINESS AND MEMORY FUNCTION, 2006–2020

Xuexin Yu[1], Laura Zahodne[1], Alden Gross[2], Belinda Needham[1], Kenneth Langa[1], and Lindsay Kobayashi[1], *1. University of Michigan, Ann Arbor, Michigan, United States*, *2. Johns Hopkins Bloomberg School of Public Health, Baltimore, Maryland, United States*

**Objective:** To investigate the mediating effect of depressive symptoms in the association between cumulative loneliness and memory function.

**Methods:** Data were from 4,764 adults aged 50+ in the Health and Retirement Study in two random sub-cohorts from 2006-2018 (Cohort A) and 2008-2020 (Cohort B). Participants were categorized as experiencing loneliness at 0, 1, 2, or 3 time points according to the UCLA Loneliness Scale, administered every four years from 2006-2014 (Cohort A) or 2008-2016 (Cohort B), creating an 8-year exposure period in each cohort. Depressive symptoms were measured using the Center for Epidemiologic Studies Depression Scale in 2016 (Cohort A) or 2018 (Cohort B). Episodic memory function was assessed in 2018 (Cohort A) or 2020 (Cohort B). Causal mediation analysis was performed to investigate the mediating role of depressive symptoms in the cumulative loneliness-memory association, in the pooled cohorts.

**Results:** Mean age (SD) at baseline was 65 (7.6), and 62% were female (2,934/4,764). Greater cumulative loneliness over the 8-year exposure period was associated with lower memory function at the follow-up in a dose-response relationship. This association was mediated by depressive symptoms, with natural indirect effects ranging from -0.009 (95% CI: -0.016 to -0.002, loneliness at one time point), -0.016 (95% CI: -0.028 to -0.003, loneliness at two time points), to -0.020 (95% CI: -0.037 to -0.004, loneliness at three time points, 20% mediated).

**Discussion:** Depression could be a psychological mechanism through which cumulative loneliness affects memory function among middle-aged and older adults in the United States.

Abstract citation ID: igae098.0286

### OLDER ADULTS' PERCEPTIONS AND ATTITUDES TOWARD TECHNOLOGIES TO ADDRESS LONELINESS: AN INTEGRATIVE REVIEW

Emma Cho, Charlotte Weiss, and George Demiris, *University of Pennsylvania, Philadelphia, Pennsylvania, United States*

Older adults are at increased risk for loneliness and social isolation, which can affect healthy aging and well-being. This study aimed to understand how older adults perceive technology interventions that address loneliness and social isolation. Databases (PubMed, CINAHL, APApsychInfo, and Abstracts in Social Gerontology) were searched for articles reporting on perceptions of technologies that address social isolation and loneliness in older adults (65 years of age or older). Thirteen studies with English language, and peer-reviewed articles in various countries met the quality review and inclusion criteria. The findings of the review were synthesized into themes and sub-themes. The emergent themes suggest that older adults may positively perceive technologies (e.g., video phones, avatar pets, social assistive robots) to facilitate increased social connectedness, emotional attachment, companionship, and comfort, and connection to resources in the wider community. Older adults may also experience negative perceptions regarding technology: dissatisfaction with digital social communication style or content (e.g., repetitive conversations with digital pet) and technical barriers (e.g., audio delay on videophone, multiple steps to follow to use telephone). Technologies can offer solutions to address social connectedness in older adults, by mitigating loneliness and social isolation. Yet opportunities exist to partner with older adults to understand the barriers to using technology and co-create acceptable and feasible digital strategies to improve their overall well-being by reducing perceived loneliness and providing opportunities for improving social connectedness.

Abstract citation ID: igae098.0287

### TRAJECTORIES OF LONELINESS IN LATER LIFE: EVIDENCE FROM A 10-YEAR ENGLISH PANEL STUDY

Giorgio Di Gessa, *University College London, London, England, United Kingdom*

Loneliness is generally defined as the discrepancy between individuals' desired and actual social interactions and emotional support. Although the prevalence of loneliness is high among older people and is projected to rise, few studies have examined longitudinal patterns of loneliness. Moreover, most studies have focused on more "objective" risk factors for loneliness such as partnership status and frequency of contact, overlooking the quality of the relationship with and support from family and friends. Using data from six waves of the English Longitudinal Study of Ageing (2008/09 to 2018/19, N=4740), we used group-based trajectory modelling to identify distinctive trajectories of loneliness. Multinomial regression models were then used to examine characteristics associated with these trajectories, with a particular focus on size, support, closeness, and frequency of contact with social network members. We identified 5 groups of loneliness trajectories in later life, representing "stable low" (40% of the sample), "medium/low" (26%), "stable high" (11%), and "increasing" (14%) or "decreasing" (9%) levels of loneliness over time. Although there are socioeconomic and demographic differences across these trajectories of loneliness, health and relationship quality are their main drivers. Respondents with poor and deteriorating health were more likely to be classified as having "stable high" or "increasing" loneliness. Even if not having social networks is undoubtedly associated with higher risks of persistent loneliness, having friends and family is not enough: Respondents with low-quality relationships with both friends and family were also significantly more likely to be classified as having "stable high" or "increasing" levels of loneliness.

Abstract citation ID: igae098.0288

### SOCIAL ISOLATION, LONELINESS, AND DEPRESSIVE SYMPTOMS AMONG OLDER ADULTS: THE MODERATING EFFECT OF RESILIENCE

Ke Li[1], Fengyan Tang[2], Steven Albert[3], Mary Rauktis[3], and Mary Ohmer[3], *1. University of New Hampshire, Durham, New Hampshire, United States*, *2. University of Pittsburgh, Wexford, Pennsylvania, United States*, *3. University of Pittsburgh, Pittsburgh, Pennsylvania, United States*

Social isolation has been recognized as a social problem with negative health consequences. Using data from three waves of the Health and Retirement Study, this study aimed to examine the long-term impact of social isolation on loneliness and depressive symptoms and to explore the moderating effect of resilience. This study comprised 3,681 U.S. adults aged 60 and older at the baseline wave. Social isolation index was constructed using five indicators, including not married or cohabitating with a partner, no social participation, and less than monthly contacts with children, family members, or friends. Loneliness was measured by the UCLA loneliness scale and depressive symptoms were measured by the Center for Epidemiological Studies-Depression scale (CES-D). The moderator of resilience was assessed by the simplified resilience score (SRS). Latent growth curve models with robust standard errors were estimated. The results of latent growth curve models showed that social isolation was significantly associated with more initial loneliness and depressive symptoms. However, social isolation was associated with a slower increasing rate of loneliness, but no significant relationship with the change rate of depressive symptoms. Furthermore, resilience significantly buffered the negative effect of social isolation on the initial level of depressive symptoms. The findings underscore the importance of enacting strategies and interventions targeting resilience to address social isolation and its negative consequences among older adults.

Abstract citation ID: igae098.0289

### SOCIAL DISCONNECTION CORRELATES OF A "WISH TO DIE" AMONG A LARGE COMMUNITY-DWELLING COHORT OF OLDER ADULTS

Mark Ward, *Trinity College Dublin, Dublin, Dublin, Ireland*

Social disconnection and deaths by suicide among older adults are both important public health concerns. In response to the "Epidemic of loneliness and isolation", the Surgeon General's Advisory has identified social connection as a cross-disciplinary research priority and developed a national strategy to advance greater social connection. A 'Wish to Die' (WTD) is predictive of future suicide attempt(s) and social disconnection has been identified as an important predictor of suicide-related thoughts and behavior. Data were from The Irish Longitudinal Study on Ageing (TILDA), a sister cohort of the Health and Retirement Study (HRS). TILDA is a large cohort of 8500 community-dwelling older adults aged 50+. Cross-sectional analyses of the association between numerous markers of social disconnection (loneliness, social isolation, living alone, marital status, social participation, volunteering, and attending religious service) and WTD were conducted. Four percent reported a WTD. Both functional and structural markers of social disconnection were associated with WTD. Among these, loneliness was the strongest risk factor while attendance of religious services was an important protective behavior. There is a strong association between social disconnection and a WTD among older adults. There is also a strong association between depression and a WTD, while attending religious services or similarly prosocial settings may protect older adults from experiencing negative thoughts about dying.

### SESSION 1295 (SYMPOSIUM)

Abstract citation ID: igae098.0290

### ADVANCING AGING IN THE RIGHT PLACE THROUGH MULTIPLE ECOLOGICAL DOMAINS

Chair: Anthony Traver
Discussant: Amanda Lehning

Individuals experiencing housing insecurity encounter service gaps and environmental inadequacies that compromise their health, social inclusion, and resilience in later life. The Aging in the Right Place (AIRP) framework provides a model for recognizing and addressing these gaps and inadequacies across multiple ecological domains. This symposium brings together five papers to examine the interplay of housing, community context, and AIRP. The first paper uses photovoice and qualitative geospatial methods to investigate the social challenges of aging faced by older people experiencing homelessness (PEH). The second paper analyzes interviews to examine how the usage and perceptions of transportation resources differ between younger and older PEH. The third paper uses data from the National Health and Aging Trends Study to examine the influence of various housing typologies on participation in social and physical activities among older adults with functional limitations. The fourth paper profiles the implementation of emergency preparedness strategies by a senior housing advocacy group to enhance the resilience of affordable housing communities. Finally, the fifth paper presents results from a randomized controlled trial to demonstrate the effect of guaranteed basic income on housing access among older PEH. The symposium will conclude with a discussion of innovative community-level strategies to enhance health, inclusion, and resilience among adults who are attempting to age in their right place while economically and socially marginalized.

Abstract citation ID: igae098.0291

### THE EFFECT OF GUARANTEED BASIC INCOME ON HOUSING ACCESS AND HEALTH AMONG OLDER PEOPLE EXPERIENCING HOMELESSNESS

Anthony Traver, and Katie Calhoun, *The Ohio State University, Columbus, Ohio, United States*

The confluence of population aging and rising housing costs across the United States has resulted in an unprecedented number of older people experiencing homelessness (OPEH). Worldwide, guaranteed basic income programs are being explored as strategies to prevent or end an episode of homelessness. The Denver Basic Income Project (DBIP), which began in 2022, provided a guaranteed income to adults experiencing homelessness in Denver, CO. DBIP used a randomized controlled design to compare the impact of two payment methods, monthly ($1,000 per month), and lump sum ($6,500 the first month, $500 per month after that), to an active control group ($50 per month). This study used a subsample of DBIP participants aged 50 and older at the time of enrollment (n=221) to analyze the 12-month impact of guaranteed income on housing access, overall health (measured by the SF-36), and emergency department visits among OPEH in Denver, CO. Generalized linear mixed-effects models were used to estimate the effect of intention to treat on outcomes of interest. The increase in the likelihood of obtaining housing was significantly larger for the monthly and lump sum groups than the control group. Group assignment was not associated with a difference in the likelihood of visiting an emergency department or a change in overall health. Results indicate that guaranteed income programs can support housing access among OPEH but may not alter health status over twelve months.

Abstract citation ID: igae098.0292

### COMPARISON OF TRANSPORTATION USE AMONG OLDER AND YOUNGER PERSONS EXPERIENCING HOMELESSNESS

Sarah Canham[1], Jeff Rose[1], Shannon Jones[1], and Ivis Garcia[2], *1. University of Utah, Salt Lake City, Utah, United States*, *2. Texas A&M University, College Station, Texas, United States*

Regardless of age, transportation determines access to necessary services and supports, including food, healthcare, shelter, and social connection for persons experiencing homelessness (PEH). This study compared experiences and perceptions of transportation among PEH

aged <49 years old to PEH aged 50+ years. We conducted a secondary qualitative data analysis of semi-structured interviews with 18 PEH staying in emergency shelters in Salt Lake County (Utah). Using thematic analysis, we identified three points of comparison between older and younger PEH: 1) Modes of transportation used; 2) Reasons for using public transit; and 3) Perceptions of public transit. While there are some similarities across transportation modes used (e.g., public transit, dedicated homeless-services shuttle, walking), older PEH also reported using taxi services when it could be afforded or shared with others. In addition, older PEH more often discussed using public transit to get to healthcare appointments, while younger PEH reported needing transit to get to places of employment. Though both participant groups described the prohibitive costs of transit, OPEH recommended that to use transit most successfully, there need to be places to sit and rest when walking, as well as more publicly available bathrooms and crosswalks for safety. This age-based comparison offers insight into ways to increase transportation equity and support PEH of all ages who similarly have unmet mobility needs.

Abstract citation ID: igae098.0293

### ADVANCING HEALTHY AND SOCIAL LIVES BEYOND LATE-LIFE FUNCTIONAL LIMITATIONS: THE ROLE OF CONGREGATE SENIOR HOUSING

Byeongju Ryu[1], BoRin Kim[2], Sojung Park[1], and Jihye Baek[1], *1. Washington University in St. Louis, St. Louis, Missouri, United States*, *2. University of New Hampshire, Durham, New Hampshire, United States*

Older adults with functional limitations often move into congregate senior housing (e.g., retirement communities and independent living) to maintain social and healthy lifestyles. Research showed mixed findings: some indicated improved social and physical activity from proximity to neighbors and organized activities in housing, while others highlighted negative outcomes such as disruption of existing social networks by relocation and social exclusion of frailer individuals within senior housing. Drawing on the National Health and Aging Trends Study Disability Conceptual Framework, this study examined the association between living in congregate senior housing and social/physical activity participation and whether perceived social cohesion moderates the association. We employed weighted logistic regression models to analyze NHATS Round 9 participants (n=2918) who reported at least one limitation with self-care (e.g., eating), mobility (e.g., getting around inside), or household activity (e.g., preparing meals), excluding those with dementia or in long-term care settings. Our findings indicated that residing in congregate senior housing was positively associated with participation in clubs/organized events [OR=1.43, p<.05], volunteering [OR=1.59, p<.05], and walking for exercise [OR=1.72, p<.01]. Housing types showed no differential influence on the residents' activities, including visiting friends/family, attending religious services, and going out for enjoyment. We also found that senior housing residents were more likely to participate in organized events when they had stronger perceived social cohesion [OR=1.27, p<.05]. These findings suggest that congregate senior housing potentially enhances access to certain activities for those with functional limitations and that fostering perceived social cohesion is vital for organized activities in senior housing.

Abstract citation ID: igae098.0294

### A HOUSING COALITION IN DETROIT MICHIGAN PLANS AHEAD FOR INDIVIDUAL AND COMMUNITY-LEVEL EMERGENCIES

Tam Perry[1], Zach Kilgore[2], Michele Watkins[3], Claudia Sanford[4], Brenda Price[5], and Dennis Archambault[6], *1. Wayne State University, Detroit, Michigan, United States*, *2. CSI Support & Development, Warren, Michigan, United States*, *3. Volunteers of America Michigan, Southfield, Michigan, United States*, *4. United Community Housing Coalition, Detroit, Michigan, United States*, *5. AARP Michigan, Lansing, Minnesota, United States*, *6. Authority Health, Detroit, Michigan, United States*

Since its founding in 2013, Senior Housing Preservation-Detroit has worked tirelessly to address the displacement of older adults, the loss of senior housing as the city gentrifies; and ways to improve quality of life to those living in senior housing. As we turn the corner on the acute phase of the COVID-19 pandemic, the members of Senior Housing Preservation-Detroit are compelled to take stock of the lessons learned from the urgent, emergency context of serving older adults in the city of Detroit, and particularly those living in senior buildings. Whether the next emergency is isolated to a single building (in case of power outages or flooding) or affects many buildings (due to another infectious disease, our changing climate, etc.), we know that preparation must continue to happen on an individual and building level. Building staff and residents alike must be ready to respond to multi-faceted problems, and there must be better plans for serving a diverse senior population. SHP-D has explored ways to reduce risks to seniors in the next emergency, including improving communication, implementing preparedness plans, and enhancing the physical resiliency of buildings. This presentation will focus on the coalition's work on emergency planning to share knowledge which may benefit other communities planning for older adults in emergencies.

### SESSION 1300 (SYMPOSIUM)

Abstract citation ID: igae098.0295

### ADVANCING OLDER ADULTS' QUALITY OF LIFE AND INDEPENDENCE WITH FITNESS: INNOVATIVE APPROACHES AND STRATEGIES

Chair: Patricia Heyn
Co-Chair: James Dowd
Discussant: James Dowd

This symposium by the Fitness, Exercise, and Wellness (FEW) Interest Group showcases diverse approaches to enhancing well-being and quality of life for older adults through innovative exercise interventions and research methodologies. Dr. Sara Pappa's work outlines the success of the Northern Virginia Falls Prevention Alliance in deploying community-based evidence-based fall prevention programs, highlighting the effectiveness of academic-community partnerships in implementing and scaling such initiatives. Dr.

James Dowd delves into the motivations of older runners, revealing that while health benefits play a role, the habit and joy of running are fundamental factors in their persistence, shedding light on the fortitude of this demographic and the new insights that this research is contributing to the field. Dr. Julie Ries and colleagues address research redundancy in meta-analyses evaluating exercise interventions for older adults with cognitive impairments, advocating for more strategic and ethical research practices to optimize resources and knowledge accumulation. Finally, Dr. Patricia C. Heyn and colleagues present a case study of implementing the 4Ms framework in dementia care in Brazil, emphasizing personalized approaches and the role of technology in maintaining family engagement and improving caregiving quality and connectivity from afar. Together, these innovative presentations highlight the importance of interdisciplinary collaborations, personalized care approaches, and technological innovations in promoting older adults' functional well-being and autonomy, particularly in the realms of fall prevention, physical activity performance, cognitive health, and dementia care.

Abstract citation ID: igae098.0296

### EMPOWERING FALLS PREVENTION IN OLDER ADULTS: INTENTIONAL ACADEMIC–COMMUNITY PARTNERSHIPS FOR SUCCESS

Sara Pappa, *Marymount University, Arlington, Virginia, United States*

The Northern Virginia Falls Prevention Alliance exemplifies a robust academic-community partnership for deploying evidence-based falls prevention programs (EBFPP) within a metropolitan setting. The project provides accessible EBFPP such as "Stay Active and Independent for Life" (SAIL) for those at low to moderate risk, "A Matter of Balance" (MOB) aimed at alleviating the fear of falling, and the "Otago Exercise Program" (OEP) for high-risk individuals. Over eight years, the program has engaged more than 6,000 older adults, spread across many Northern Virginia community-based locations, showcasing a significant breadth of reach and adaptability. Adding to this, the recent incorporation of the "Saving Claire" Falls Prevention Project documentary heightens awareness and education, bolstering the program's outreach. This presentation will emphasize the pivotal roles of collaborative community organization, sustainable program delivery, leader preparation, and fostering trust with older adults, thereby reinforcing public health endeavors to mitigate fall-related injuries. With over a decade of program evolution, this session will outline key strategies for sustainability, accessibility, and adoption. Emphasizing the practicality and impact of academic-community alliances, it will underscore how such partnerships are instrumental in advocating the successful uptake and sustainability of EBFPP

Abstract citation ID: igae098.0297

### RUNNING PAST 50: THE FORTITUDE OF THE OLDER RUNNER

James Dowd, *University of Georgia, Athens, Georgia, United States*

Considering the low numbers of physically active older Americans (Elgaddal, Kramarow, Reuben, 2022), those over the age of 50 who belie this generalization are of interest for their commitment to fitness. Despite their regret over their loss of running speed, any comparison with their more sedentary age peers would favor the older runner: they are healthier, fitter, and stronger. The question I address in this paper is why the older runner continues to run. Based on a sample of over 60 interviews conducted from July to December of 2023, I found that it is not simply a matter of striving to be healthy, although that is part of their motivation. As important as health are (1) running has been a habit and consequently a foundational part of their identity; and (2) running brings joy and a sense of profound well-being that older runners experience following a race, almost regardless of their performance. When asked about their motivation, runners acknowledge the positive effects of exercise on their health. But as one 72-year-old runner explained "Well, physical health and conditioning is important. But I wouldn't say. I mean, that's a benefit. I wouldn't say that's why I run." The remainder of this paper probes more deeply into what might best be described as the fortitude of the older runner. The data confirm that older runners persist both because their running has become a habitual part of their everyday lives and also because their habit is a source of considerable joy for them.

Abstract citation ID: igae098.0298

### META-ANALYSES OF THE EFFECTS OF EXERCISE ON ALZHEIMER'S DISEASE: EVALUATING SYNTHESIS REDUNDANCY AND RESEARCH WASTE

Mahederemariam Dagne, *Marymount University, Arlington, Virginia, United States*

Research redundancy emerges when studies are unnecessarily duplicated, which not only depletes resources but also inflates research waste. In the last twenty years, we have been seeing a significant redundancy in the number of systematic reviews and meta-analyses evaluating exercise and physical activity interventions for cognitive function in older adults with cognitive impairments, particularly those with Alzheimer's disease. This presentation aims to dissect the prevalence of repeated studies within published meta-analyses through a living umbrella systematic review. It scrutinizes the overlap of randomized controlled trials (RCTs) across these analyses to elucidate the extent of redundancy and its implications on the accumulation of knowledge. Our analysis provides a critical assessment of the current landscape, charting a path toward more strategic, impactful research practices. By pinpointing specific instances of excessive duplication, we aim to foster a dialogue on refining research

Abstract citation ID: igae098.0299

### ENHANCING DIGNITY IN DEMENTIA CARE IN BRAZIL: A CAREGIVER REMOTE 4MS FRAMEWORK APPLICATION FOR MOBILITY SOLUTIONS

Patricia Heyn, *Marymount University, Fairfax Station, Virginia, United States*

This abstract describes a case study based on the personal experience of an Alzheimer's researcher who unexpectedly turned into a caregiver and crafted a novel care paradigm for her father with dementia living in a nursing home in Brazil. Confronting Brazil's traditional care models, which often exacerbate frailty by confining older adults to wheelchairs, she championed the Age-Friendly Health Systems movement and its core 4Ms framework—Medication, Mobility, Mentation, and What Matters the Most. This personalized approach, advocating for autonomy rather than restraint, sparked a systemic shift toward recognizing and nurturing the functional capabilities of older adults with dementia. Implementing this framework virtually, we are capable of training a caregiver remotely through WhatsApp to deliver tailored physical and cognitive exercises for a physically restrained person with dementia, demonstrating the feasibility of technology-facilitated caregiving. This intervention led to significant functional improvements: the transition from wheelchair to walker, then to cane, and finally, to unassisted ambulation, and improved strength and walking speed within a year. Highlighting this success, the presentation will discuss the transformative influence of the Aging Friendly 4Ms framework on person-centered geriatric care in an international context and the potent role of technology in maintaining family engagement and oversight in caregiving quality from afar. This case underscores the necessity of personalized care that regenerates and maintains active mobility, ultimately preserving the dignity, independence, and quality of life for older adults with dementia in underdeveloped institutional settings.

### SESSION 1305 (SYMPOSIUM)

Abstract citation ID: igae098.0300

### AGE-FRIENDLY PROGRESS WITH THE HEALTHYAGINGDATAREPORTS.ORG: NOTES FROM MA, RI, NH, CT, ME, MS, AND WY

Chair: Elizabeth Dugan

This symposium celebrates ten years of collaboration to translate data into high impact tools for stakeholders forming coalitions, identifying priorities, and working to make their state or community age-friendly. The session will review the tools included in the healthyagingdatareports.org website and highlight the exciting and replicable age-friendly work underway in Massachusetts, New Hampshire, Mississippi, Maine, Connecticut, Rhode Island, and Wyoming. We have found that identifying health disparities by gender, race, or geography may be a key step in addressing health equity. The process involved in each state and how the reports were launched to generate awareness and enthusiasm for each state's age-friendly priorities are described. Dugan (chair) will present an overview of the data reports (community-engagement, data sources, indicator calculation methods, hierarchical approach to reporting, data visualization tools, dissemination efforts, and on-going technical support). Then leaders from Massachusetts (Fuccione), New Hampshire (Rabalais), Mississippi (White), and Wyoming (Vincenti) will share an overview of their state's efforts to create longevity-ready communities and to promote healthy aging. They will also describe how their coalitions or collaboratives were formed and supported, how to identify the key stakeholders and various outreach approaches. State leaders will describe how the data report tools support their advocacy and/or outreach. Reflections on how age-friendly efforts are sustained and grow will be discussed. Attendees and presenters will then have a question and answer time to discuss how others could join the movement.

Abstract citation ID: igae098.0301

### LOOKING FORWARD TO A NEW DECADE OF TRANSLATING DATA INTO TOOLS TO SUPPORT STATE LEADERS ADVANCING HEALTH EQUITY

Elizabeth Dugan, *University of Massachusetts Boston, Boston, Massachusetts, United States*

This presentation will describe how the healthy aging data reports started and how the work has evolved. The importance of community-engagement, collaboration, and shared ownership has driven progress. The partnership of the Point32Health Foundation has been critical to our growth and success. Opening ways for other funders to support the work has allowed us to partner with other states to expand the reach and impact of the reports. How graduate gerontology students have contributed innovations and improvements to our process and products is highlighted.

Abstract citation ID: igae098.0302

### STATE LEADERS DESCRIBE EXCITING AND REPLICABLE AGE-FRIENDLY WORK

Nina Silverstein, *University of Massachusetts Boston, Needham, Massachusetts, United States*

State leaders describe age-friendly work in their state and how tools like the healthyagingdatareports.org can be leveraged to expand and accelerate progress. James Fuccione (Massachusetts), Jennifer Rabalais (New Hampshire), Kina White (Mississippi), and Virginia Vincenti (Wyoming) will provide insights about their state. Silverstein will identify common themes and lessons learned.

### SESSION 1310 (AWARD LECTURE (INVITATION ONLY))

Abstract citation ID: igae098.0303

### AGHE FACULTY AWARD PRESENTATIONS AND LECTURES

Chair: Joann Montepare

The AGHE Faculty Award Presentations and Lectures will feature the Distinguished Faculty Award recipient lecture and the Rising Star Early-Career Faculty award lecture. The 2024 Distinguished Faculty Award recipient is Lynn M. Holley, PhD, FAGHE of the University of Nebraska at Omaha. This award recognizes persons whose teaching stands out as exemplary, innovative, of impact, or any combination thereof. The 2024 Rising Star Early-Career Faculty Award Lecture recipient is Yeonjung Jane Lee, PhD, of the University of Hawai'i at Mānoa. The Rising Star Early-Career Faculty Award acknowledges new faculty whose teaching and leadership stand out as influential and innovative.

Abstract citation ID: igae098.0304

### TEACHING AND LEARNING IN TURBULENT TIMES

Lyn Holley, *University of Nebraska at Omaha, Omaha, Nebraska, United States*

Teachers now face circumstances in which everything, everywhere, seems to be changing all at once. The ongoing techno-communications revolution is continuous and relentless. Many agree that its impacts will equal those of the Industrial Revolution. With particular emphasis on Lifelong Learning and Intergenerational engagement, the recipient of the 2024 AGHE Distinguished Faculty Award will lead discussion of ongoing changes in traditional education, and then will describe some strategies for engaging existing resources and developing approaches to pedagogy and andragogy that are useful for coping with the continuous change of the current foundations of formal education. Finally, learning opportunities provided by GSA and AGHE annual conferences and by membership in GSA and AGHE will be discussed.

Abstract citation ID: igae098.0305

### UTILIZING A LENS OF DIVERSITY, EQUITY, DECOLONIZATION, AND INTERSECTIONALITY IN GERONTOLOGY EDUCATION

Yeonjung Jane Lee, *University of Hawai'i at Manoa, Honolulu, Hawaii, United States*

Educational transformation can be catalyzed by engaging in critical self-reflection. In light of the COVID-19 pandemic and deeply ingrained histories of racism and ageism, educators must reflect on their roles and encourage students to critically evaluate the impact of their identities on how they serve older adults. With the aging population becoming more diverse, it is imperative for students to be equipped to work with diverse older adults who come from various cultural backgrounds and life experiences. This session will delve into the presenter's teaching philosophy and experiences gleaned from instructing and conducting research at a minority-serving institute. With the aim of nurturing high-quality education to support a diverse student body, this session seeks to inspire meaningful dialogue and impart valuable insights about gerontology education. The presentation will cover the discussion integrating diversity, equity, decolonization, and intersectionality perspectives into gerontology education.

### SESSION 1315 (SYMPOSIUM)

Abstract citation ID: igae098.0306

### POLICY SERIES: AGING AND PUBLIC POLICY: NAVIGATING THE INTERSECTIONS

Chair: Michael Lepore
Co-Chair: Tara McMullen
Discussant: Karon Phillips

Contemporary politics and public policy are rife with matters of aging and inequality. While an impressive history of public health achievements over infectious diseases has enhanced the possibility for populations to enjoy longer and healthier lives, these benefits have not been enjoyed equally. Rather than overcome disparities in longevity and health, existing public policies designed for the younger and less diverse populations of earlier generations have potential to further entrench age-related inequalities in current populations into the future. The Gerontological Society of America's policy journal, Public Policy & Aging Report, has a rich history of directly tackling the thorny political issues at the heart of these inequalities, including national voting patterns of older White Americans supporting conservative political candidates and values that clash with their support of policies and programs from which they directly benefit. This symposium draws on such historic trends and contemporary research to shed light on the intersections of aging and public policy that we are navigating today, including federal and state legislative and regulatory initiatives to improve the economic security of low-income older adults, local programs in numerous states that use property tax levies for social care services to assist in maintaining community independence, and issues of voting access in the context of aging, disability, and cognitive impairment. Contributors to this session will build on recent publications in Public Policy & Aging Report and will engage in discussion addressing the 2024 US election and looking ahead to the future of aging and public policy.

Abstract citation ID: igae098.0307

### UNDERSTANDING VOTING TRENDS AMONG AGING AMERICANS

Goldie Byrd, Capri Foy, Allison Caban-Hol, TanYa Gwathmey, Patricia Smyre, and Kelvin Williams, *Wake Forest University School of Medicine, Winston-Salem, North Carolina, United States*

The right to vote, also known as "the franchise", is a fundamental right of United States (US) citizens, and is essential to preserve the democratic republic of the US. However, voting often requires significant planning, time and effort, and there are considerable and persistent challenges and threats to voting for several demographic groups in the US, including older adults. In this analysis, we will examine voting trends in this population in four recent general Presidential Elections (2008, 2012, 2016, and 2020) using data from the Current Population Survey (CPS). Specifically, we will examine self-reported voting percentages among noninstitutionalized older US adults (ages 60 years or older) who report having disabilities compared to older adults without disabilities, after adjustment for demographic variables. In addition, we will compare engagement in early voting, as well as voting in person or via mail, among older adults with and without disabilities. Lastly, we will examine whether these results are moderated by race, gender or other demographic factors (e.g. education). Results will be discussed in the context of the extant literature, as well as the history of legislative facilitators and barriers to voting for American adults with disabilities. Finally, we discuss voting trends among adults with disabilities for the 2024 Presidential Election, and we provide suggestions for future education and research and additional facilitators to enhance voting rates for older Americans with or without disabilities.

Abstract citation ID: igae098.0308

### LOCAL FUNDING FOR SOCIAL CARE FOR OLDER COMMUNITY MEMBERS: A BOTTOM-UP STRATEGY THAT IS ALSO GOOD POLITICS

Robert Applebaum[1], and Bailee Brekke[2], *1. Miami University, Oxford, Ohio, United States*, *2. Miami University, W. College Corner, Indiana, United States*

The national response to providing home and community-based services for older people with disability relies almost exclusively on the Medicaid program. Because 90% of older community members are not eligible for Medicaid this means that until a crisis the vast majority of older individuals are not eligible for public support. In 15 states across the nation, communities have raised local funds to support social care services to assist in maintaining community independence. Raising more than $400 million, primarily through property tax levies, the more than 400 communities using this approach fund an array of social care services, such as personal care, home modification, meals, and transportation targeted toward a population above the Medicaid eligibility threshold. This strategy is particularly salient in Ohio, where 74 or the state's 88 counties have levies totaling more than $200 million. This presentation will describe both the programs and politics associated with this funding approach. The presentation will highlight how funds are use and the target population served. Local funding has been successful at the polls, and a review of voting patterns found a more than 95% passage rate in the Ohio communities, with no differences in rates between red and blue counties of the state.

Abstract citation ID: igae098.0309

### CHANGES IN THE AGING POLICY LANDSCAPE SINCE 2020: HITS AND MISSES

Debra Lipson[1], and Christine Bishop[2], *1. Mathematica, Washington, District of Columbia, United States*, *2. Brandeis University, Waltham, Massachusetts, United States*

Despite deep political divisions after the 2020 election, Congress passed several major statutes to address the acute economic and health fallout of the COVID-19 pandemic, including provisions that helped older adults cover large and unexpected health care costs and expanded access to home and community-based care (HCBS). States used federal funds disbursed under these statutes to strengthen the long-term services and supports (LTSS) system and respond to the COVID-19 health emergency. But as the pandemic faded into the distance, so did the impetus to address the fundamental problems that pose the greatest long-term risk to the health and economic security of older adults. This paper reviews major legislative and regulatory initiatives adopted at the federal and state level since 2020 to improve the economic security of low-income older adults, expand Medicare and Medicaid coverage, strengthen nursing home quality and the direct care workforce, increase access to HCBS, and provide greater support to family caregivers. Despite these achievements, progress has been incremental. Little has been done to fix flaws in Medicare and Social Security financing, and policymakers have yet to address LTSS financing, all of which pose major threats to the long-term financial wellbeing of older adults and their families. These issues belong at the top of the aging policy agenda and urgently call for action by the next Administration, Congress, and state and local governments.

### SESSION 1320 (SYMPOSIUM)

Abstract citation ID: igae098.0310

### AGING IN LATIN AMERICA: HEALTH, DISPARITIES, AND OUTCOMES AMONG OLDER ADULTS

Chair: Margarita Maria Osuna
Discussant: Rafael Samper-Ternent

Older adults in Latin America are living in contexts of high levels of poverty, low education, poor health literacy and reduced healthcare access. This symposium is focused on heath-disparities among older adults living in Brazil, Costa Rica, Mexico, Puerto Rico, and Colombia. The symposium explores connections between aging and health such as longevity, cognition, disability, and wellbeing in the context of Latin America, aiming to shed light on health-related dimensions and disparities among older Latinos. Using data from the Brazilian Longitudinal Study of Aging, Gomes examines the relationship between hearing loss and cognitive decline. Using the longitudinal Puerto Rican Elderly Health Conditions Project and the Puerto Rico Contextual Data Resource, García, examines how the relationships between racial identity, neighborhood-level racial density, and neighborhood-level socioeconomic status are related to the probability of experiencing all-cause mortality among island-dwelling Puerto Ricans. Using four nationally representative surveys of older adults in Latin America, Osuna studies how disability prevalence varies among older adults in Mexico, Costa Rica, Colombia and Brazil. Lastly, Saenz studies evaluates how level of exposure to a major drought Mexico is related with health among older adults, for this study they use data from the Mexican Health and Aging Study. Results underline what populations in Latin American have increased risk of experiencing poorer health outcomes and disparities that may exist. The findings highlight the importance of understanding the conditions under which Latin American older adults are aging and the implications this can have in the future.

Abstract citation ID: igae098.0311

### CROSS-NATIONAL COMPARISON OF DISABILITY IN LATIN AMERICAN OLDER ADULTS

Margarita Maria Osuna[1], and Jennifer Ailshire[2], *1. University of Southern California Leonard Davis School of Gerontology, Los Angeles, California, United States*, *2. University of Southern California, Los Angeles, California, United States*

Latin America's population is aging rapidly. Little is known about disability prevalence among older adults in these countries. We estimate and compare disability prevalence from nationally representative surveys of older adults from Mexico (2015 Mexican Health and Aging Study, MHAS, N=6,699), Brazil (2015 Brazilian Longitudinal Study of Aging, ELSI, N=5,367), Colombia (2015 National Survey of Health and Wellbeing, SABE-Col, N=23,598), and Costa Rica (2005 Costa Rican Longevity and Healthy Aging, CRELES, N=2,607). Disabilities were defined using three harmonized measures: any limitations on (i) instrumental activities of daily living (IADL), (ii) activities of daily living (ADL), and (iii) mobility. Age-adjusted prevalence estimates were calculated by gender for each country, using WHO 2000-2025 Standard Population age categories. Findings reveal significant differences in disability prevalence across measures and countries. Prevalence of IADL disability in men ranged from 18% (Costa Rica) to 31% (Brazil). For women, IADL disability ranged from 24% (Colombia) to 30% (Brazil). ADL disability for men ranged from 10% (Brazil) to 20% (Mexico). Whereas women's prevalence range was 12% (Costa Rica) to 23% (Mexico), mobility disability exhibited the highest prevalence and had the largest disparities. Prevalence in men ranged from 27% (Colombia) to 78%

(Mexico). Women's disability prevalence ranged from 43% (Colombia to 83% in Mexico. Findings highlight the heterogeneity of health inequalities and raise policy implications. Aging and healthcare policies should focus on supporting older adults, with stronger social protections and home improvements to support disabled older adults. Our study provides insights on disability patterns in Latin America.

Abstract citation ID: igae098.0312

### POTENTIAL IMPLICATIONS OF THE 2011–2012 DROUGHT IN MEXICO ON OLDER ADULT HEALTH

Joseph Saenz[1], Jorge Peniche[2], Laura Tanner[1], Emma Aguila[2], Brian Downer[3], and Rebeca Wong[4], *1. Arizona State University, Phoenix, Arizona, United States*, *2. University of Southern California, Los Angeles, California, United States*, *3. The University of Texas Medical Branch, Galveston, Texas, United States*, *4. The University of Texas Health Sciences Center in San Antonio, San Antonio, Texas, United States*

**Background:** Climate change will increase the frequency and intensity of extreme weather events such as heatwaves, wildfires, hurricanes, and droughts. Few studies have evaluated the health impacts of drought exposure in low- and middle-income countries, which are most vulnerable to effects of climate change. This study evaluates how level of exposure to a major drought that affected Mexico from 2011-2012 related with post-drought health in older adults. **Methods:** We use the 2012 Mexican Health and Aging Study (n=8,954 respondents age 50+) linked with municipality-level data from the Drought Monitor of Mexico to identify droughts in MHAS respondents' community of residence from October 2010 to October 2012. Health domains evaluated included stress/mental health, physical health, and cognitive health. Linear, logistic, and multinomial regressions are used to estimate associations between the longest streak of consecutive months that one's community was in a drought and health, controlling for sociodemographic characteristics. **Results:** Six months of additional community-level drought exposure related with worse mental health and stress (higher depressive symptoms [β=-0.04, p<0.001], poorer self-rated health [OR: 1.11, p<0.01], poorer self-rated finances [OR: 1.09, p<0.05]) worse cognitive health (lower Verbal Learning [β=-0.03, p<0.01] and Verbal Recall [β=-0.04, p<0.01]), and increased likelihood of decreasing weight more than 5 kg during the drought (OR: 1.07, p<0.05). **Discussion:** Longer exposure to drought throughout the 2011-2012 drought was associated with worse health across multiple health domains. Future studies should seek to identify factors that may increase vulnerability or facilitate resilience in communities affected by drought.

Abstract citation ID: igae098.0313

### RACE, NEIGHBORHOOD DYNAMICS, AND MORTALITY PATTERNS IN OLDER PUERTO RICANS

Catherine García[1], Maria Aranda[2], and Michael Crowe[3], *1. Syracuse University, Syracuse, New York, United States*, *2. University of Southern California, Los Angeles, California, United States*, *3. University of Alabama at Birmingham, Birmingham, Alabama, United States*

This study explores the relationships between race, neighborhoods, and mortality among older Puerto Ricans, drawing upon theoretical frameworks in sociology, gerontology, and public health. We combined data from the longitudinal Puerto Rican Elderly Health Conditions Project (PREHCO; 2002-2021) and the Puerto Rico Contextual Data Resource (PR-CDR) to examine how the relationships between racial identity, neighborhood-level racial density, and neighborhood-level socioeconomic status are related to the probability of experiencing all-cause mortality among island-dwelling Puerto Ricans aged 60 and older (n=4,243). Findings show that young-old Black Puerto Ricans residing in high socioeconomic neighborhoods have a lower risk of mortality relative to their same aged Black counterparts residing in low socioeconomic neighborhoods, and White Puerto Ricans who reside in either high or low socioeconomic neighborhoods. However, this mortality advantage diminishes when they reach the age of 75 years and over. We hypothesize that in Puerto Rico, Black communities with access to economic capital are especially beneficial for its residents as they are in spaces where they feel welcomed and are surrounded by amenities that are essential for healthy aging or survival into old age. As Puerto Ricans in these communities age, this advantage may diminish, due to a ceiling effect, meaning that for older Puerto Ricans who survive to age 60 in these communities, there are fewer advantages to accumulate. Our research provides a foundation for informed policy decisions and targeted interventions to promote aging in place in Puerto Rico and offers a framework for understanding similar dynamics in diverse Latin American contexts.

Abstract citation ID: igae098.0314

### HEARING LOSS AND COGNITIVE DECLINE: EVIDENCE FROM THE LONGITUDINAL STUDY OF ADULT HEALTH (ELSA-BRASIL)

Natalia Gomes Goncalves[1], Alessandra Samelli[2], Paulo Lotufo[2], Isabela Bensenor[2], and Claudia Suemoto[2], *1. University of São Paulo Medical School, São Paulo, Sao Paulo, Brazil*, *2. University of São Paulo, São Paulo, Sao Paulo, Brazil*

Hearing loss is common in older adults and its prevalence increases with age, being present in 40% of adults aged 50 years or older and increasing to 70% in adults aged 70 years or older. Hearing loss in middle age is one of the main risk factors for dementia, and it is estimated that 7% of dementia cases in Brazil are due to hearing loss. Therefore, this study aimed to study the association between hearing loss and cognitive decline during 8 years of follow-up. Data included participants from the São Paulo center of the Longitudinal Study of Adult Health (ELSA-Brasil), who were evaluated in three study waves (2008-10, 2012-14, and 2017-19). Hearing loss was defined as pure-tone audiometry above 25 dB in either ear. Six tests evaluated cognitive performance in the memory, verbal fluency, and executive function domains. A global cognitive z-score was derived from these tests. The association between hearing loss and cognitive decline was evaluated with linear mixed-effects models adjusted for sociodemographic, lifestyle, and clinical variables. Of 805

participants (mean age 50.6±9.4 years, 52% women, 60% White), 62 had hearing loss. During follow-up, hearing loss was associated with faster global cognitive decline (β=-0.012, 95% CI=-0.023; 0.000, p=0.039). These results suggest that prevention of hearing loss in middle age could contribute to slowing cognitive decline in this population.

### SESSION 1325 (SYMPOSIUM)

Abstract citation ID: igae098.0315

### ALIGNING MENTAL HEALTH CARE FOR MARGINALIZED OLDER ADULTS: CLINICAL AND PROFESSIONAL DEVELOPMENT CONSIDERATIONS

Chair: Brenna Renn
Discussant: Kristopher Kern

As the older adult population grows and diversifies, the urgency mounts for clinicians and academics to provide inclusive, culturally responsive, and anti-racist services and training. Culturally responsive practice can improve care engagement and optimize patient outcomes. Attending to such issues across training ensures retention of students from marginalized backgrounds and prepares clinicians who reflect diversity of the aging population. This symposium convenes speakers from the Society of Clinical Geropsychology to discuss the challenges and opportunities in provider training and mental health service provision with a focus on inclusivity. Each presentation pays close attention to actionable steps. The first presenter outlines strategies to strengthen mentorship by fostering equity, diversity, and inclusivity. The second speaker presents qualitative findings regarding challenges, opportunities, and concrete strategies to improve culturally responsive and anti-racist care from a sample of geropsychologists and trainees across the U.S. The third presenter describes a national survey of geropsychology workforce diversification and presents inclusive strategies to support diverse learners at all stages of training. Finally, our Discussant synthesizes these findings in the broader context of training and clinical practice that is responsive to the needs of individuals from historically marginalized backgrounds and intersectional identities. The Discussant will comment on the pressing need to transform both how training is conducted and how services are delivered to ensure optimal outcomes. The insights gleaned from these three initiatives will lead to a rich discussion among the panel and audience on applications and future directions in geriatric mental health care with relevance across settings.

Abstract citation ID: igae098.0316

### INCLUSIVE MENTORSHIP IN GEROPSYCHOLOGY: CULTIVATING SAFETY, TRUST, HUMILITY, AUTHENTICITY, AND COMMUNICATION

Ira Yenko[1], Flora Ma[1], Anna Blanken[2], Claudia Son[3], and Laurie Chin[4], *1. Stanford University School of Medicine, Stanford, California, United States*, *2. San Francisco VA Healthcare System, San Francisco, California, United States*, *3. Biola University, La Mirada, California, United States*, *4. University of Indianapolis, Indianapolis, Indiana, United States*

Attention to equity, diversity, and inclusion (EDI) in mentorship is paramount; as such, the Society of Clinical Geropsychology's Mentorship and Diversity Committees have joined forces to outline concrete methods to improve such practices. Mentoring across differences can promote intercultural mentoring relationships that embrace diversity and support inclusion. This presentation explores the significance of EDI and offers practical strategies to shift towards a more inclusive perspective in mentorship, with special attention to the development of geropsychology trainees. This includes recognizing and valuing the unique perspectives, experiences, and identities that both mentors and mentees bring to the table. By fostering an inclusive mentorship environment, we can maximize opportunities for individuals with marginalized identities and ensure equal access to resources, support, and opportunities. This presentation will review the following strategies: (1) Creating a safe and welcoming space for mentees and maximizing trainee comfort in expressing their thoughts, concerns, and questions; (2) Being reliable, consistent, and supportive by establishing a mentor-mentee agreement outlining expectations, goals, and boundaries to set the stage for a trusting relationship; (3) Authenticity and acknowledging there is no one "right" way to be a geropsychologist; (4) Humility to create a culture of feedback and growth and remain open to diverse perspectives; (5) Actively listening to demonstrate empathy and validate mentee experiences; (6) Encouragement. By fostering a culture of mentorship to uplift, support, and empower the next generation, we pave the way for a future where geriatric mental health is inclusive and responsive to the diverse needs of older adults.

Abstract citation ID: igae098.0317

### QUALITATIVE INSIGHT ON PROFESSIONAL EXPERIENCES WORKING WITH DIVERSE OLDER ADULT POPULATIONS

Rakshitha Mohankumar[1], Kristopher Kern[2], and Brenna Renn[1], *1. University of Nevada Las Vegas, Las Vegas, Nevada, United States*, *2. VA North Las Vegas Health Care System, North Las Vegas, Nevada, United States*

The older adult population in the United States is expanding not only in number but also in diversity. Historically marginalized older adults face persistent and pervasive health disparities and barriers, spanning social, structural, and clinical factors, hindering access to services to help them age successfully and in a dignified manner. Regarding clinician barriers, training in the principles and practices of providing culturally respectful care is inconsistent. We sought to better understand ways that professional geropsychologists and trainees address diverse and intersectional identities during clinical practice and research with older adults. An online survey was distributed to the American Psychological Association Society of Clinical Geropsychology listserv. Questions included open-ended prompts asking about the ways in which respondents consider and engage in culturally responsive care to older adults with intersectional identities and those who have been historically marginalized by society, which were coded using qualitative thematic analysis. Respondents (n=28) were majority (81%) professional geropsychologists and primarily worked in the Department of Veterans Affairs, private practice, and/or academic settings. A common theme emerged of implementing a client-centered approach when working with this population, including adjusting existing measures and interventions to the needs of the individual and engaging in anti-discriminatory practices. Common limitations included desire for concrete strategies to increase access to care, working with LBTQIA+ older adults, and reducing stigma for seeking services. These results provide qualitative insight into how such issues are conceptualized in clinical practice across the U.S. and point to opportunities for training and professional development.

Abstract citation ID: igae098.0318

### BUILDING BRIDGES TOWARD WORKFORCE DIVERSITY: STRATEGIES TO SUPPORT RESILIENT LEARNERS

Rebecca Allen[1], Timothy Ly[1], Flora Ma[2], Brian Carpenter[3], and Jennifer Moye[4], *1. University of Alabama, Tuscaloosa, Alabama, United States*, *2. Stanford University School of Medicine, Stanford, California, United States*, *3. Washington University in St. Louis, St. Louis, Missouri, United States*, *4. VA Boston Healthcare System, Boston, Massachusetts, United States*

The Building Bridges national virtual conference, held in March 2021, aimed to provide opportunities for psychologists to discuss the dramatic geropsychological workforce shortage, identify means to address this shortage, and discover opportunities and challenges in ongoing efforts to diversify this workforce moving forward. Fifty-six individuals at all stages of training completed a pre-conference survey about workforce diversification and identified advertising and outreach (12.6%), student and faculty recruitment (11.5%), and culturally aware mentorship (10.3%) as strategies for recruiting diverse individuals into geropsychology. Four active workgroups identified enhancing diversity awareness and cultural humility as the most important target areas for change. Qualitative memos from these workgroups were analyzed by a two-member coding team working with an expert in qualitative analyses. Across workgroups, eight emergent themes included: 1) need for financial support, 2) recruitment efforts for diverse students and faculty, 3) intentional awareness emphasizing strength in diversity, 4) creation of inclusive environments, 5) creation of safe spaces for honest and proactive dialog, 6) creation of intersectional and intergenerational learning opportunities, 7) outreach and networking opportunities, and 8) didactic and experiential training. The prioritization of these themes varied by workgroup, with the academia workgroup prioritizing intentional awareness and creation of safe learning environments and the post-licensure clinical workgroup prioritizing recruitment of diverse learners and faculty as well as networking. Strategies for implementing changes to practice that support resilient and diverse learners at all stages of training will be discussed.

### SESSION 1330 (BIOLOGICAL SCIENCES INVITED SYMPOSIUM)

Abstract citation ID: igae098.0319

### TESTING THE GEROSCIENCE HYPOTHESIS

Chair: Monty Montano

This session explores the geroscience hypothesis through a series of cutting-edge talks examining the intersection of aging and chronic conditions, novel gerotherapeutics, and complex muscle, bone and adipose interactions.

Abstract citation ID: igae098.0320

### REFRAMING GEROSCIENCE TO IMPROVE OUTCOMES IN PEOPLE LIVING WITH CHRONIC INFECTION

Monty Montano, *Harvard Medical School, Boston, Massachusetts, United States*

This talk will highlight recent advancements in aging science as applied to individuals living with chronic conditions associated with infections, such as HIV-1 and SARS-CoV-2. The discussion will focus on aging biomarkers and health trajectories in individuals with chronic morbidities related to infection. Additionally, the talk will address the importance of considering both physiological and holistic outcomes, taking into account allostatic load and the life history of stressors, both biological and psychosocial in strategies designed to improve quality of life.

Abstract citation ID: igae098.0321

### XPRIZE HEALTHSPAN: A PATH TO ACCELERATE TRANSLATIONAL GEROSCIENCE

Jamie Justice, *XPRIZE Foundation, Winston-Salem, North Carolina, United States*

By targeting biological aging pathways with a single or combination of therapeutic treatments, it may be possible to restore function lost to age-related degradation of multiple organ systems. Interventions targeting aging pathways are being translated to humans, yet public perception, poor alignment and testing guidelines, and unclear regulatory and commercial pathways are barriers to clinical translation. XPRIZE Healthspan is a 7-yr $101M global competition to incentivize scientific teams to develop and test gerotherapeutics, accelerating progress from research & development to clinical trials. The winning team will demonstrate that their therapeutic treatment restores muscle, cognitive, and immune function in adults aged 50-80 years. The XPRIZE Healthspan concept was created through a deliberate process of scientific engagement and collaboration by 22 scientific advisors and technical experts across commercial and non-profit scientific sectors coupled with an open public comment period, and registration opened officially in July 2024. To date >420 investigative teams and companies from 53 countries have submitted an intent to compete. Teams span academic investigators, research networks or collaboratives, commercial start-ups, established biotech and pharmaceutical companies. A wide variety of potential therapeutics are proposed by teams for testing, including but not limited to AI-driven small molecule discovery, novel and repurposed pharmacologics, nutraceuticals, lifestyle interventions, devices, immunomodulatory agents, stem cell therapies, other biologics, and partial reprogramming – administered alone or in combination. In this talk we will review design of the global competition, describe frameworks for early-stage clinical trials, and provide an overview of the rapidly emerging field of translational geroscience.

Abstract citation ID: igae098.0322
### OSTEOSARCOPENIA: A GEROSCIENCE APPROACH
Gustavo Duque, *Bone, Muscle & Geroscience Group, Research Institute of the McGill University Health Centre, Montreal, Quebec, Canada*

In older persons, the combination of osteopenia/osteoporosis and sarcopenia - known as osteosarcopenia - has been proposed as a subset of individuals at higher risk of adverse outcomes. The pathophysiology of osteosarcopenia results from a complex set of interactions between bone, muscle, and fat influenced by genetic, mechanical, and biochemical factors. There is a complex crosstalk system between muscle and bone, in which fat has become a new player. This communication system comprises osteokines, myokines, adipokines and other secreted factors (i.e., extracellular vesicles, exosomes and microRNAs), some of which have positive or negative effects on muscle and/or bone metabolism and function. These effects include alterations in most of the hallmarks of aging, triggered by conditions such as sedentarism, obesity, malnutrition, inflammation, and menopause, which induce tissue loss and dysfunction. In contrast, anabolic factors could be stimulated to prevent muscle and bone loss of mass and function. Therefore, osteosarcopenia is an optimal condition in which geroscience-based approaches could target several hallmarks of aging to have a beneficial dual effect on bone and muscle. This session will discuss the biological characteristics of osteosarcopenia shared by muscle and bone, their relevance in the pathogenesis of the condition, and their potential use as therapeutic targets in osteosarcopenia.

Abstract citation ID: igae098.0323
### OBESITY AND ACCELERATED AGING
Dennis Villareal, *Baylor College of Medicine, Houston, Texas, United States*

Obesity in the older population has increased in prevalence and incidence over the last several decades and has been associated with a decline in physical function, quality of life, and increased risk of complications from cardiometabolic diseases. Several interrelated hallmarks of aging have been proposed. The pathophysiologic changes that occur with obesity are similar or contribute to those that occur with aging. In fact, the positive energy balance typical of obesity worsens the excess deposition of ectopic fat with aging. Obesity and aging are characterized by chronic low-grade inflammation, which contributes to reduced muscle quality and protein control mechanisms. Obesity causes oxidative stress and inflammation, which can increase telomere shortening. Obesity induces epigenetic alteration, which can accelerate age-related dysfunction. Calorie excess increases formation of reactive oxygen species (ROS), which damages nucleus, endoplasmic reticulum, and mitochondria. DNA damage from ROS upregulates p16 and p21, resulting in chromatic reorganization, cellular senescence, and production of proinflammatory factors (senescence-associated secretory phenotypes [SASP]). Persistent DNA damage associated with inefficient DNA repair with both obesity and aging may be the principal molecular event that underlies accelerated aging. Both obesity and aging deregulate cellular mechanisms related to nutrient signaling. Caloric restriction in combination with exercise slows biologic aging by protecting against the molecular and cellular damages that occur in obesity and aging.

### SESSION 1335 (PAPER)

### CAREGIVING FOR PEOPLE WITH DEMENTIA

Abstract citation ID: igae098.0324
### UNHEALTHY COPING: SMOKING AND DRINKING BEHAVIORS AND RELATED RISK FACTORS AMONG DEMENTIA CAREGIVERS
Jiaming Liang[1], and Maria Aranda[2], *1. University of Texas Health Science Center at Houston, SAN ANTONIO, Texas, United States*, *2. University of Southern California, Los Angeles, California, United States*

Dementia caregivers may adopt unhealthy lifestyle behaviors as a form of stress relief. While some interventions promoting healthier behaviors have been developed for dementia caregivers, their smoking and drinking behaviors have received limited attention. This study aimed to explore (1) the disparities in smoking and drinking behaviors between dementia caregivers and non-caregivers, and (2) potential risk factors associated with these behaviors among dementia caregivers. Utilizing data from the 2017 Behavioral Risk Factor Surveillance System, we conducted cross-sectional analyses on 280 dementia caregivers and 9,878 non-caregivers. Multinomial regression revealed that dementia caregivers are more likely to be daily e-cigarette users compared to never-users (RRR = 0.33, p = 0.04). Additionally, zero-inflated negative binomial regression indicated that dementia caregivers tend to consume 0.22 more alcoholic drinks per occasion than non-caregivers (p = 0.03). Interaction analyses demonstrated that younger age and lower income are associated with daily tobacco use. In contrast, factors such as older age, being unmarried, home ownership, higher income, employment, fewer chronic conditions, and absence of depression are related to increased alcohol consumption among dementia caregivers (i.e., drinking on more days in the past 30 days, and consuming more drinks per occasion). This study underscores the increased risk of unhealthy smoking and drinking behaviors among dementia caregivers and highlights the need for targeted interventions to address these risk factors and promote healthier lifestyle choices among this population.

Abstract citation ID: igae098.0325
### ENJOYMENT OF DAILY ACTIVITIES IN OLDER CAREGIVERS: AN ECOLOGICAL MOMENTARY ASSESSMENT
Abel Pichardo[1], and Brent Mausbach[2], *1. San Diego State University, San Diego, California, United States*, *2. University of California San Diego, La Jolla, California, United States*

Behavioral Activation (BA) theory posits that depression stems from poor exposure to positive environmental rewards, with increased positive activities linked to mood improvement. BA theory may explain the high prevalence of depressive symptoms observed in informal caregivers. The current study used an Ecological Momentary Assessment approach to investigate the association between daily variability in activities with variability in daily mood. Participants were 64 informal caregivers of Alzheimer's Disease or related disorders. Using a digital diary, participants recorded daily their mood, number of activities, and enjoyment ranging from 2 to 335 days. Enjoyment of activities and daily mood were rated on scales ranging from 1 to 5 (1 being least pleasant). A linear mixed-model analysis examined the associations between variability in mood with variability in both frequency and enjoyment of daily activities. Results indicated that a greater number of activities was associated with more positive mood for the day (B=0.045, t=13.58, p<.001). Greater average enjoyment of activities was also associated with more positive mood for the day (B=0.5, t=33.40, p<.001). There was a significant interaction of total activities by average enjoyment (B=-0.02, t=-8.49, p<.001). Post-hoc analyses indicated that increasing activation by 8 daily activities indicated an improvement in mood of approximately 0.3 points. However, replacing two moderately enjoyable activities with highly enjoyed activities indicated an improvement in mood of nearly 0.7 points. These findings suggest that modifying types of activities to focus on enjoyment rather than total activity may provide greater impact to overall mood.

Abstract citation ID: igae098.0326

### CAREGIVER NETWORK BURDENS FOR OLDER ADULTS WITH DEMENTIA RISK: FINDINGS FROM NHATS AND NSOC DATA

Joseph Svec[1], Natasha Nemmers[2], and JeongEun Lee[3], *1. St. Joseph's University, Patchogue, New York, United States*, *2. Wayne State University, Detroit, Michigan, United States*, *3. Iowa State University, Ames, Iowa, United States*

A growing body of research indicates that caregiver networks constitute an important resource for older adult well-being as well as the adult caregivers themselves. Among dementia patients, caregiver networks often face heightened strain and burdens which correspond with poor overall health of all caregivers. However, the condition of a caregiver network is also shown in the literature to have a direct impact on the quality of care that is provided. This study examines the bi-directional nature of caregiver and care-recipient outcomes by examining the extent to which lagged measures of caregiver network burden corresponds with current dementia risk for older adults. We utilize data from the National Health and Aging Studies (NHATS) and the National Study of Caregiving (NSOC) to test associations of lagged caregiver burden within networks (e.g., financial, emotional, or physical difficulties) with care recipient dementia classifications (e.g. no dementia, possible dementia, probable dementia). Results from multinomial logistic regressions of four NSOC-NHATS data waves (N = 1,343) indicate that the odds of older adult possible dementia increase by 1% (OR = 1.01, p < 0.01) with each additional percentage increase in emotional difficulty shares within networks from the previous wave. Moreover, the odds of probable dementia increase by 2% (OR = 1.02, p < 0.01) with regards to both emotional and physical difficulty shares within caregiver networks. These findings suggest the importance of developing interventions that address diverse burdens by strengthening care coordination, communication, and support across network members to improve the cognitive health outcomes of older adults.

Abstract citation ID: igae098.0327

### UNDERSTANDING SOCIAL ISOLATION AMONG MEXICAN AMERICAN ALZHEIMER'S CAREGIVERS: ROLE OF TECHNOLOGY

Lin Jiang, *University of Texas-Rio Grande Valley, Edinburg, Texas, United States*

The projected increase in Latinos affected by Alzheimer's disease and related dementia (ADRD) to 1.3 million by 2050 underscores the importance of understanding the experiences of Mexican American family caregivers. Despite recognized benefits of information communication technology (ICT) for caregivers, current research predominantly focuses on White American caregivers, leaving a gap in understanding the unique challenges faced by Mexican American caregivers. This study addresses this gap by conducting semi-structured, in-depth interviews with 24 Mexican American family caregivers of ADRD patients along the South Texas and Mexico Border. Participants were recruited through various channels, and Zoom interviews were conducted from January to Fall 2023, followed by thematic analysis. Participants ranged in age from 24 to 73, with an average of a college degree. Fourteen identified as Catholic, while seven identified as Christian. The average caregiving duration ranges from 1 to 13 years. Key findings reveal distinct experiences of social isolation and loneliness, aggravated by a lack of understanding from family members, with ICT playing a significant role in reducing social isolation, particularly during the COVID-19 pandemic. Additionally, religion emerged as a vital coping mechanism, alleviating social isolation and loneliness through online activities. The study highlights disparities in social isolation and emphasizes the potential of technology in addressing these challenges among Mexican American family caregivers of ADRD patients. Recognizing their unique obstacles and leveraging technology can inform interventions to enhance well-being and support. Future research should prioritize culturally sensitive ICT interventions to improve the quality of life for both caregivers and patients.

Abstract citation ID: igae098.0328

### PERSONALITY AND PREDEATH GRIEF IN FAMILY CAREGIVERS OF PERSONS WITH DEMENTIA

Elisabeth McLean[1], Lauren Elliott[1], Sydnie Schneider[1], Volker Neugebauer[2], and Jonathan Singer[1], *1. Texas Tech University, Lubbock, Texas, United States*, *2. Texas Tech University Health Sciences Center, Lubbock, Texas, United States*

Caring for a family member with Alzheimer's Disease or Alzheimer's Disease Related Dementias (AD/ADRD) is associated with psychosocial difficulties, including predeath grief (PDG; grief symptoms that can develop while an individual with a chronic, life-limiting illness is still living). PDG can be debilitating and is a robust predictor of prolonged grief disorder (PGD) once the person with AD/ADRD passes away. Past research has demonstrated several personality factors related to PGD in bereaved individuals; for example, low extraversion and agreeableness and high neuroticism have been found to confer greater risk of developing prolonged grief disorder after a death. However, it is presently unknown whether personality factors are related to the development of PDG prior to the death. Data were analyzed from a sample of family caregivers of individuals with AD/ADRD (Mage = 68.68, SD age = 11.89). Participants answered a battery of questions regarding pre-death grief (Prolonged Grief—12; PG-12), perceived social support (Oslo Social Support Scale; OSSS), and the NEO Five Factor Inventory (NEO-FFI-3). Bivariate correlations revealed non-significant relationships between all personality dimensions except for neuroticism (r =.441, p <.001). Additionally, a multiple linear regression examining the impact of neuroticism and perceived social support on PDG revealed a significant main effect of neuroticism (F(2, 52) = 6.53, p =.002, R2 =.17) on pre-death grief. Thus, family caregivers who have high levels of neuroticism may be at increased risk of experiencing pre-death grief and clinical intervention might be more warranted for this population.

### SESSION 1340 (SYMPOSIUM)

Abstract citation ID: igae098.0329

### CELEBRATING 50 YEARS OF AGING RESEARCH: A CHAT WITH NIA SENIOR LEADERSHIP

Chair: Amy Kelley
Discussant: Amy Kelley

The National Institute on Aging (NIA) at the National Institutes of Health, Department of Health and Human Services, is the federally designated lead agency on aging research and supports research to understand the nature of aging and to extend the healthy, active years of life. 2024 marks NIA's 50th anniversary, a milestone which prompts reflection on both past and present discoveries resulting from NIA-funded research. In the decades since its establishment, NIA has propelled advances across multiple domains of aging research, including basic, clinical, neuroscience, and behavioral and social research. Looking to the future, NIA seeks to drive further advances in these areas – with input on strategic priorities from investigators, advocates, and the public – to enable healthier aging for all. This symposium will provide a forum for exploration of NIA-funded research advances, as well as current and future research priorities of NIA's scientific divisions, and review of the NIA budget and its implications for the research community. A question-and-answer session with NIA senior staff will follow these remarks.

Abstract citation ID: igae098.0330

### OVERVIEW OF NIA BUDGET AND RESEARCH

Richard Hodes, *National Institute on Aging, Baltimore, Maryland, United States*

Dr. Hodes will discuss the NIA budget, overall research priorities of the Institute, NIA-funded research advances, and key programs and initiatives.

Abstract citation ID: igae098.0331

### NIA DIVISION OF GERIATRICS AND CLINICAL GERONTOLOGY

Basil Eldadah, *National Institute on Aging, Baltimore, Maryland, United States*

Dr. Eldadah will discuss research priorities of the NIA Division of Geriatrics and Clinical Gerontology. Dr. Eldadah will also be available for small group discussion.

Abstract citation ID: igae098.0332

### NIA DIVISION OF AGING BIOLOGY

Stacy Carrington-Lawrence, *National Institute on Aging, Baltimore, Maryland, United States*

Dr. Carrington-Lawrence will discuss research priorities of the NIA Division of Aging Biology. Dr. Carrington-Lawrence will also be available for small group discussion.

Abstract citation ID: igae098.0333

### NIA DIVISION OF NEUROSCIENCE

Molly Wagster, and Eliezer Masliah, *National Institute on Aging, Baltimore, Maryland, United States*

Dr. Masliah will discuss research priorities of the NIA Division of Neuroscience. Dr. Masliah will also be available for small group discussion.

Abstract citation ID: igae098.0334

### NIA DIVISION OF BEHAVIORAL AND SOCIAL RESEARCH

Lisbeth Nielsen, *National Institute on Aging, Baltimore, Maryland, United States*

Dr. Nielsen will discuss research priorities of the NIA Division of Behavioral and Social Research. Dr. Nielsen will also be available for small group discussion.

Abstract citation ID: igae098.0335

### NIA DIVISION OF EXTRAMURAL ACTIVITIES

Kenneth Santora, *National Institute on Aging, Baltimore, Maryland, United States*

Dr. Santora will discuss the work of the NIA Division of Extramural Activities. Dr. Santora will also be available for small group discussion.

Abstract citation ID: igae098.0336

### NIA OFFICE OF SPECIAL POPULATIONS

Patricia Jones, *National Institute on Aging, Baltimore, Maryland, United States*

Dr. Jones will discuss the work of the NIA Office of Special Populations. Dr. Jones will also be available for small group discussion.

### SESSION 1345 (SYMPOSIUM)

Abstract citation ID: igae098.0337

### DEVELOPMENT AND OPTIMIZATION OF MECHANISTICALLY SOUND DEMENTIA CARE INTERVENTIONS

Chair: Ana-Maria Vranceanu
Co-Chair: Talea Cornelius
Discussant: Elise Rice

Optimizing care for persons living with dementia (PLWD) and their caregivers (CGs) is an urgent priority. A recent AHRQ report highlights the poor quality of existent dementia interventions emphasizing the need to develop methodologically sound interventions that test theory-based mechanisms hypothesized to underlie intervention effects. This symposium will describe methodology and applications of the NIH Stage Model and the Science Of Behavior Change (SOBC) experimental medicine approach and how they inform interventions for PLWD and their CGs. Dr. Cornelius will introduce SOBC and discuss complexities of specifying conceptual models, measuring mechanisms, and study methodology in a dyadic context. Dr. Vranceanu will introduce the NIH stage model and discuss the development and refinement of the Mindfulness and Self-Compassionate Care Program for stressed CGs of PLWD who endorse behavioral symptoms (NIH Stage 1A), showcasing mechanism and measure selection, feasibility testing, and first test of mechanistic target engagement. Dr. Mroz will describe a Stage 0 Appreciative Inquiry approach, in partnership with PLWD (mild-moderate), to define promising pathways for measuring and intervening to bolster purpose in the wake of a dementia diagnosis. Dr. Plys will present feasibility, mechanism engagement, and clinical outcome results of an NIH Stage 1B trial comparing a mindfulness mobile app to a podcast control for stressed CGs of PLWD. Dr. Berridge will describe an intervention's non-linear pathway to Stage 1B mechanistic research to support empowered shared decision making about technology use for dementia care. The discussant, Dr. Rice will contextualize these findings within the larger literature and NIA funding opportunities.

Abstract citation ID: igae098.0338

### BOLSTERING SENSE OF PURPOSE FOR INDIVIDUALS LIVING WITH EARLY DEMENTIA: AN APPRECIATIVE INQUIRY APPROACH

Emily Mroz[1], Joan Monin[2], Kate Keefe[3], Erica DeFrancesco[3], and Heidi Gil[3], *1. Emory University, Decatur, Georgia, United States*, 2. *Yale School of Public Health, New Haven, Connecticut, United States*, 3. *LiveWell Institute, Southington, Connecticut, United States*

Maintenance of a sense of purpose is a hallmark of wellbeing. Individuals consistently report a diminished sense of purpose in the wake of a diagnosis of Alzheimer's disease or related dementias (ADRD). One recently-proposed hypothesis (Sutin et al., 2023) is that this trend is a consequence of early, ADRD-related neurodegeneration, implying that adults living with early ADRD may be less cognitively able to pursue purpose. In contrast, our team hypothesizes that these declines are the result of a lack of evidence-based practices in clinical and community settings focused on bolstering purpose following this life-changing diagnosis. Our team pursued Stage 0 research given the lack of existing interventions. In partnership with people (N = 17) living with mild-moderate ADRD recruited from the Empowering Partnership Network, we completed a Design Thinking workshop using an Appreciative Inquiry method (Stowell, 2013). Workshop insights were recorded, and the first author completed qualitative thick descriptions to synthesize insights. Findings establish that, with the availability of support, adults with mild-moderate ADRD can generate meaningful purposes. Findings also described how the ADRD diagnosis journey can undermine purpose and suggest opportunities for multicomponent, community-based interventions to bolster purpose following diagnosis. This presentation will end with recommendations for partnering with people living with ADRD and applying the Appreciative Inquiry method at Stage 0. We will also discuss how our findings help to re-define living well with purpose and ADRD, and to refine the Healthy Lifestyle Course, a novel community-based resource that shows promise for bolstering purpose in this population.

Abstract citation ID: igae098.0339

### FEASIBILITY AND MECHANISM ENGAGEMENT OF A MINDFULNESS MOBILE APP FOR CAREGIVERS: FINDINGS FROM AN NIH STAGE 1B TRIAL

Evan Plys[1], Emily Woodworth[1], Morgan Seward[1], Elizabeth Allen[1], Isabella Carroll[1], Jen Huberty[2], and Ana-Maria Vranceanu[1], *1. Massachusetts General Hospital, Boston, Massachusetts, United States*, 2. *Fit Minded Inc., Phoenix, Arizona, United States*

A large body of research has tested psychological interventions with dementia caregivers. Yet, this literature still lacks clear understanding of mechanisms of action for psychological outcomes. This NIH Stage 1B study tested the feasibility and mechanism engagement of a theory-based mindfulness mobile-app (Healthy Minds Program [HMP]) for stress, depression, and anxiety among caregivers; mindfulness skill was the proposed mechanism for each outcome. Stressed caregivers were randomized to HMP (n=40) or a podcast control (n=40). Groups did not vary by age, gender, race, or caregiver type. Among all participants (N=80), benchmarks were met for recruitment (90% eligible caregivers enrolled), data collection (98% with no questionnaires fully missing), and attrition (95% completed post-tests). For caregivers completing HMP, benchmarks were met for treatment satisfaction and credibility, but not for expectancy or adherence (63% completed 52.5 minutes weekly). Using Wilcoxon signed-rank/paired-samples t-tests, caregivers receiving HMP evidenced significant within-group improvements in mindfulness (Applied Mindfulness Process Scale; Cohen's d=1.16). Significant within-group differences were observed for stress (Perceived Stress Scale; Cohen's d=.91), depression (Hospital Anxiety and Depression Scale [HADS]; Cliff's delta=.34), and anxiety (HADS; Cohen's d=.78). Change in mindfulness significantly related with change in stress (r=.38), depression (r=.42), anxiety (r=.33). Findings suggest the HMP mobile-app was feasible and led to moderate to large within-group effects for mindfulness and clinical outcomes among caregivers. Moderate correlations between change scores confirmed the conceptual model with mindfulness as a proposed mechanism of action. We discuss protocol refinements (e.g., changes to dose for mobile-app) and preparation for a Stage 2 efficacy trial.

Abstract citation ID: igae098.0340

### LET'S TALK TECH: NAVIGATING TECHNOLOGY USE DECISIONS AND AN INDIRECT ROUTE TO MECHANISTIC RESEARCH

Clara Berridge, *University of Washington, Seattle, Washington, United States*

Grounded in the Theory of Dyadic Illness Management, Let's Talk Tech is the first tool to support informed, shared decision making about a range of technologies commonly used in dementia care. This presentation will describe the non-linear pathway taken to incorporate SOBC to examine the mechanisms and outcomes of Let's Talk Tech. In a Stage 1A pilot with people living with mild Alzheimer's disease (AD) and care partners, 88% of recruited dyads (n=29

dyads) completed 100% of the self-administered intervention delivered as a web-application. This pilot established promising preliminary feasibility and efficacy on post-test outcomes, with statistically significant improvements in care partner preparedness to make decisions about technology use, knowledge of the person living with AD's preferences, alignment, mutual understanding, and understanding of each featured technology category, as well as improvements for two categories for people living with AD. Strong interview themes clarified person-centered outcomes and surfaced additional potential mechanisms of change. By completing the intervention, all dyads developed a documented summary of the person living with AD's choices, available for future reference and updating. Participants wanted to share these choices beyond the dyads to prevent familial conflict and obtain technology use support from their broader care networks. A Stage 1A study through the Penn AITC enabled enhancements responsive to this feedback, including electronic person-directed sharing of the documented choices using FHIR and the SMART Health Links protocol. A current Stage1B study is powered to evaluate the impact of LTT on an expanded set of hypothesized mechanisms and outcomes.

Abstract citation ID: igae098.0341

### THE SCIENCE OF BEHAVIOR CHANGE: MECHANISTIC RESEARCH IN PEOPLE WITH DEMENTIA AND THEIR CARE PARTNERS

Talea Cornelius, *Columbia University Irving Medical Center, New York, New York, United States*

**Background:** Interventions to support health and well-being people with dementia (PWD) and their care partners (CPs) lack efficacy and potency. Understanding why these interventions do—or do not—produce desired change has potential to improve outcomes in PWD and CPs. The NIH-funded Science Of Behavior Change (SOBC) promotes a mechanism-focused approach to developing rigorous, transparent, scalable, and effective interventions.

**Methods:** In the SOBC experimental medicine approach, researchers identify a mechanism hypothesized to underlie behavior change, measure the mechanism, influence the mechanism, and test whether change in the mechanism explains subsequent change in the outcome of interest.

**Results:** The SOBC website hosts instructional materials, a measures repository, a checklist for designing, conducting, reporting, and evaluating mechanistic research across a range of study designs, specific aims templates, and more. International efforts are underway to rigorously link measures to potential mechanisms of action and encourage deep thinking about how to operationalize (measure) theoretical constructs. The challenges of moving from theory to study implementation are compounded in dyadic research (use of self v. partner report, analyzing individual reports v. dyad means, etc.).

**Conclusions:** Study methodology must correspond to conceptual models, and valid measurement of mechanisms is essential. Casting a spotlight on the intricacies of dyadic research, particularly in PWD and CPs where one dyad member experiences progressive cognitive decline and the dyad must cope with these changes together, promotes more careful thinking around these important issues, and the SOBC method and resources can facilitate a more rigorous, transparent, and cumulative science.

Abstract citation ID: igae098.0342

### ADDRESSING STRESS AMONG CAREGIVERS OF PEOPLE LIVING WITH DEMENTIA (PLWD) WITH BEHAVIORAL SYMPTOMS

Ana-Maria Vranceanu[1], Natalia Giraldo Santiago[2], Arden O'Donnel[3], and Christine Ritchie[4], *1. Massachusetts General Hospital, Boston, Massachusetts, United States*, *2. CHOIR-CASI, Boston, Massachusetts, United States*, *3. MGH Institute of Health Professions, Boston, Massachusetts, United States*, *4. Mass General Hospital and Harvard Medical School, Boston, Massachusetts, United States*

Current stress management interventions for caregivers of PLWD are primarily focused on providing support or education targeting general caregiver stress. Here we showcase the development and refinement of MASC, a theoretically informed program that integrates emotional regulation skills (mindfulness, compassion and self-compassion) with practical strategies to manage stress from challenging behavioral symptoms in PLWD. First, we report on a mixed methods study (N=24 caregivers who endorse stress from PLWD's behavioral symptoms; NIH stage 1A). Qualitative analyses provided a nuanced understanding of caregiver stress, highlighted the need for MASC, and showcased opportunities for tailoring of skills, program format and content. Quantitative analyses supported the need for the program and our theoretically informed conceptual model. Next, we report on an open pilot with exit interviews (N=10 caregivers; 2 groups; NIH stage 1A). MASC was feasible and acceptable (89% of eligible caregivers agreed to participate; 92% attended > 4/6 sessions; 70% reported at least moderate program satisfaction). Across the entire sample participation in MASC was associated with improvement in outcomes (stress, depression, and quality of life; ES=.3-.6) and mechanisms (self-compassion, compassion, and mindfulness; ES=.2-.7). Change in mechanisms was associated with change in outcomes, consistent with our conceptual model. Exit interviews supported the quantitative findings and provided recommendations for refinement and improvement. An NIH stage 1B pilot RCT of the refined MASC and protocol is ongoing. Integration of the Science of Behavior Change with the NIH stage model throughout this work will be detailed.

### SESSION 1350 (SYMPOSIUM)

Abstract citation ID: igae098.0343

### DIVERSITY OF DAILY EXPERIENCES: SOCIOECONOMIC AND PSYCHOLOGICAL CORRELATES AND IMPLICATIONS FOR HEALTHY AGING

Chair: Soomi Lee
Co-Chair: Rachel Koffer
Discussant: David Almeida

As intensive longitudinal data collection becomes more widespread, innovative methods to quantify daily and momentary experiences are needed to advance theoretical understanding of daily life and its implications for successful aging. A particularly promising area of advancement is in quantitative summaries of rich categorical data regarding daily experiences (e.g., types of activities including physical activity, chores, volunteering, etc.; types of stressors including work stressors, arguments, social network stressors, etc.; types of social engagements, including with church groups, neighbors, family, etc.). This symposium will discuss a framework for understanding antecedents and outcomes of diversity of daily experiences and provide exemplary studies within this framework. First, Koffer will present a new X-versity theoretical perspective, an innovative approach to understanding the richness and balance inherent in diverse daily experiences and its significance in the context of healthy aging. Three domains of daily experiences (activities, stressors, and social interactions) will then be represented in empirical presentations to discuss socioeconomic and psychological correlates of diversity of daily experiences. Santos finds higher income relates to higher activity diversity between 2011-2022, with differences by race/ethnicity. Surachman finds more financial hardship relates to higher stressor concentration (narrow and unbalanced stressor experiences in daily life), particularly for older adults. Jeon finds COVID-19 pandemic-driven decreases in social activity diversity related to increased loneliness. The Discussant, Almeida will provide an overview of the advancements in daily diary and intensive longitudinal study of everyday life and discuss implications of diversity of daily experiences for health and well-being across the adult life span. Health Behavior Change Interest Group Sponsored Symposium

Abstract citation ID: igae098.0344

### INTRODUCING "X-VERSITY" THEORY: EXPERIENTIAL DIVERSITY IN DAILY LIFE AND ITS IMPLICATIONS FOR HEALTHY AGING

Rachel Koffer[1], Soomi Lee[2], and Johanna Drewelies[3], *1. Arizona State University, Phoenix, Arizona, United States*, *2. The Pennsylvania State University, University Park, Pennsylvania, United States*, *3. Max Planck Institute for Human Development, Berlin, Berlin, Germany*

Lifespan developmental theories highlight that aging is characterized by gains and losses across different domains (e.g., social, emotional, physical, cognitive). Such gains and losses translate into aging-related change in experiences across various facets of daily life. However, much of present theory and empirical evidence has focused on overall quantity of experiences (e.g., total number of social activities) rather than how such experiences are spread across different types (e.g., whether a person spends relatively more of their time in one social activity compared to other activities). This presentation introduces the X-versity theory, in which we propose that experiential diversity (X-versity), conceptualized as rich and balanced experiences, is an important component of healthy and resilient aging. We present a conceptual framework that demonstrates the theoretical underpinnings associating person-environment interactions with differences in X-versity, and links X-versity with aging-related outcomes. Empirical evidence representing scientific advances in this field is then presented, particularly within the domains of activity, stressor, and emotion diversity. The empirical evidence will be used to demonstrate associations within the conceptual framework, and how diversity of experiences declines with age. We further provide methodological (e.g., quantification, measurement, timescales), theoretical (e.g., moderators, cross-domain considerations, contexts of ontogenetic and sociohistorical change processes) considerations for future studies of experiential diversity beyond the daily domains. X-versity theory provides a theoretical leap in lifespan socioemotional aging, taking advantage of rich categorical data measured in many studies of daily life, and allowing for more nuanced consideration of the role of diverse experiences for healthy aging.

Abstract citation ID: igae098.0345

### ACTIVITY DIVERSITY ASSOCIATED WITH INCOME LEVEL AMONG US ADULTS BUT LESS SO FOR RACIAL/ETHNIC MINORITIES

Alexis Santos-Lozada, and Soomi Lee, *The Pennsylvania State University, University Park, Pennsylvania, United States*

Active lifestyle is a key to healthy aging. Emerging research has shown that individuals with greater activity diversity are happier and healthier. Activity diversity, defined as the breadth and evenness of activity engagement across different types, may also allow opportunities to build human capital. This study examined whether activity diversity is associated with family income and its interaction with race/ethnicity. Data come from adults (n=115,771; Mage=45.62; 51% female; 37% racial/ethnic minorities) who participated in the American Time Use Survey between 2011 and 2022, excluding for 2020 due to the COVID-19 pandemic. An activity diversity index was calculated as conceptualized by Blau, using responses to a time-use questionnaire that covered all activities in which the respondent participated during the previous day. Generalized linear models revealed higher income to be associated with higher activity diversity. Further, there were differences in family income and activity diversity by race/ethnicity. Compared to non-Hispanic whites, racial/ethnic minorities reported earning less and engaging in less diverse activities. Moreover, the association between activity diversity and family income was moderated by race/ethnicity, such that higher family income was not as predictive of activity diversity among racial/ethnic minorities as it was among non-Hispanic Whites. Findings suggest that activity diversity may not only indicate one's lifestyle, but also relate to one's resources and capacity to explore new things in their life. Less resources available for racial/ethnic minorities may limit their opportunity to engage in diverse activities thought to improve human capital.

Abstract citation ID: igae098.0346

### FINANCIAL HARDSHIP IS ASSOCIATED WITH REPEATED STRESSORS AS MEASURED BY STRESSOR DIVERSITY

Agus Surachman, *Drexel University, Philadelphia, Pennsylvania, United States*

Financial hardship supplements traditional measures of socioeconomic status (i.e., education, income) to elucidate how the insufficiency of socioeconomic resources is experienced. Financial hardship may impact health by differentiating exposure to stressors in daily life. This study examined how three domains of financial hardship (i.e., material, behavioral, and psychological) relate to daily stressor concentration (e.g., experiencing many stressors that are all arguments versus fewer stressors spread across arguments, work stress, home overloads). Higher stressor concentration has been associated with poorer health and well-being across adulthood. The present analyses used data from the Midlife in the United States study wave 3 (2016-2020), in which 1,024 participants (ages = 43-90, 57% female) completed 8-day daily diary interviews. Measures of financial hardship include material indicators of poverty, healthcare, and financial aid; psychological perceptions of financial situation; and behaviors including payments, earnings, and spending. Daily stressor concentration was calculated using 1- the Gini diversity index. In a regression model accounting for age, sex, daily stressor exposure, and education, a higher total score of financial hardship was associated with higher stressor concentration ($b = 0.07$, $SE = 0.03$, $p < .01$). This result replicated across material ($b = 0.26$, $SE = 0.09$, $p < .01$) and psychological domains ($b = 0.11$, $SE = 0.04$, $p < .01$) of financial hardship, and the findings were exacerbated with older age ($ps < .05$). Experiencing repeated stressors in specific domains may be a mechanism by which insufficient economic resources can worsen health outcomes for adults as they age.

Abstract citation ID: igae098.0347

### RELATIONSHIP BETWEEN CHANGE IN FACE-TO-FACE SOCIAL ACTIVITY AND LONELINESS DURING COVID PANDEMIC IN US ADULTS

Sangha Jeon, and Susan Charles, *University of California Irvine, Irvine, California, United States*

**Objectives:** Participation in diverse face-to-face social activities is linked to low levels of loneliness, but social restrictions during the pandemic were restricted in-person interactions. As a result, many people turned to technology to stay connected to others. The current study examined whether shifts in face-to-face social activity variety correlate with pandemic-related loneliness, adjusting for baseline loneliness and sociodemographic factors. We also examined whether this relationship varies depending on online social activity variety. Method: Using data from the Health and Retirement Study (HRS), we included 1,412 adults aged 50 and older who completed questions about social activity participation in 2016 (Wave1; W1) and 2020 (Wave2; W2).

**Results:** Regression analyses revealed that those who experienced a greater decrease in face-to-face social activity variety during the pandemic compared to four years prior had higher levels of loneliness compared to those who experienced less change in in their pre-pandemic level of face-to-face social activity variety, after adjusting for demographic, health-related factors and loneliness at W1. In addition, online social activity variety does not moderate the relationship between change in face-to-face social activity variety and later loneliness. Conclusions: Findings suggest that engagement in diverse face-to-face social activities is associated with low level of loneliness, but online social activity variety cannot compensate the loss of opportunities for face-to-face social activities in middle-aged and older adults even in the situation with the restricted availability of face-to-face social interactions.

### SESSION 1355 (SYMPOSIUM)

Abstract citation ID: igae098.0348

### EAST MEETS WEST : APPLICATION OF AI TO SUPPORT HEALTHY AGING AND EFFECTIVE CARE IN DIVERSE CULTURES

Chair: Fei Sun
Co-Chair: Guifang Guo
Discussant: Yong Ding

This East Meets West symposium showcases five studies focused on the utilization of Artificial Intelligence (AI) in fostering healthy aging and effective care for older adults in the U.S.A. and mainland China. The first study delves into older Chinese adults' preferences for assistive robots, highlighting their need for safety, physical functioning, and emotional support, alongside concerns regarding cost and user-friendliness. The second study introduces an innovative approach by integrating assistive robots with reminiscence music therapy to aid stroke recovery, showing promising outcomes in enhancing self-efficacy, daily living activities, positive emotions, and upper limb functionality. The subsequent three studies explore the feasibility, usefulness, and efficacy of AI-based technologies in interpreting medical records, monitoring disease progression, and providing customized health information to family caregivers. The study from the U.S. reveals that lab results interpretation generated by ChatGPT-4 demonstrated higher accuracy, relevance, and helpfulness. Similarly, the research from China is pioneering modules for early detection and comprehensive monitoring of neurodegenerative disease progression. Despite encouraging evidence, the final study points out the limitations in using GPT-3.5 Turbo for health information classification (with a focus on daily care category) into three levels with the accuracy rate falling short of expectations. In summary, these studies offer promising evidence of the benefits of AI-based innovations in promoting healthy aging and supporting caregivers in the U.S.A and mainland China. They also underline the existing limitations and adopting a person-centered approach to cater to the specific needs of older adults. After individual presentations, a discussant will provide critical feedback.

Abstract citation ID: igae098.0349

### ENHANCING HOME-BASED CARE WITH ASSISTIVE ROBOTS: ACCEPTANCE AND PREFERENCES OF OLDER CHINESE

Yilin Wang[1], Hongxiu Chen[2], Xiuying Hu[1], and Yan Qi[3], *1. Innovation Center of Nursing Research and Nursing Key Laboratory of Sichuan Province, West China Hospital, Sichuan University, Chengdu, Sichuan, China (People's Republic), 2. Innovation Center of Nursing Research and Nursing Key Laboratory of Sichuan Province, chengdu, Sichuan, China (People's Republic), 3. China Rehabilitation Research Center, beijing, Beijing, China (People's Republic)*

This study aimed to assess the acceptance and preferences of the older Chinese towards assistive robots, focusing on their potential to aid in mobility, provide emergency response, and monitor daily activities. A structured survey questionnaire was distributed to evaluate their difficulties in daily activities, the use and expenditure on current eldercare assistive devices, and their interest in and concerns regarding assistive robots. Among 109 who responded, 85 were valid. More than half were over age 75, including those aged between 75 and 84 years old (37.7%) and those aged 85 and above (30.6%). Participants reported a strong interest in assistive robots with mobility aid, emergency response, and daily activity monitoring. As most of the care is provided at home (96.5%), significant difficulties were reported in activities such as stair navigation (67.1%), outdoor transportation (63.5%), bathing (55.3%), and toileting (47.1%). Canes (69.4%) and wheelchairs (60%) were the most used eldercare assistive devices, with expenditures mostly below 5,000 Chinese RMB (50.7%). Safety (81.2%) and ease of use (61.2%) were the primary considerations. Approximately 60% of respondents prioritized mobility assistance, while around 40% valued the ability of robots to provide companionship. Despite the interest in assistive robots, concerns over cost and the need for user-friendly interfaces were highlighted as potential barriers to adoption. The study underscores the potential of assistive robots in elder care, particularly in enhancing mobility and ensuring safety. To facilitate wider adoption, it is crucial to ensure that these technologies are affordable and accessible.

Abstract citation ID: igae098.0350

### EFFECTS OF REMINISCENT MUSIC THERAPY WITH ROBOT-ASSISTED REHABILITATION FOR POSTSTROKE RECOVERY OLDER CHINESE

Qian Liu[1], Li Liu[1], Huiping Wu[2], and Yujin Liu[3], *1. Innovation Center of Nursing Research and Nursing Key Laboratory of Sichuan Province, West China Hospital, Sichuan University, Chengdu, Sichuan, China (People's Republic)*, *2. Shenzhen Nurses Association, Shenzhen, Guangdong, China (People's Republic)*, *3. Changchun Humanities and Sciences College, Changchun, Jilin, China (People's Republic)*

This study aimed to investigate the impacts of reminiscent music therapy combined with robot-assisted rehabilitation on older stroke patients' rehabilitation self-efficacy, activities of daily living, self-esteem, positive emotion, and upper limb function. A single-blind, three-arm randomized controlled trial was conducted in a Chinese tertiary hospital from November 2022 to January 2024. Experimental group A received reminiscent music therapy combined with robot-assisted rehabilitation and usual care, experimental group B received robot-assisted rehabilitation and usual care, and the control group only received usual care. Songs released from 1935 to 1980 were obtained via three music apps (QQ Music, KuGou and NetEase) commonly used in China. Robot-assisted rehabilitation with five game modes was carried out by occupational therapists. Outcomes included rehabilitation self-efficacy, activities of daily living, self-esteem, positive emotion, and upper limb function, which were evaluated at baseline and post intervention. A total of 126 older stroke patients completed the study. Baseline balance was established across the three groups. After the intervention, participants in the experimental group A reported greater rehabilitation self-efficacy, activities of daily living, self-esteem, positive emotion, and upper limb function than did those in experimental group B and the control group ($p < 0.05$). All outcome measurements were significantly greater than those at baseline ($p < 0.05$). Reminiscent music therapy combined with robot-assisted rehabilitation is a promising method for improving mental health and rehabilitation outcomes in older stroke patients. It may be incorporated in routine rehabilitation training.

Abstract citation ID: igae098.0351

### USING INFORMATICS AND GENERATIVE AI TO SUPPORT OLDER ADULTS' UNDERSTANDING OF LAB TEST RESULTS

Zhe He[1], Balu Bhasuran[1], Qiao Jin[2], Shubo Tian[2], Karim Hanna[3], and Zhiyong Lu[2], *1. Florida State University, Tallahassee, Florida, United States*, *2. National Center for Biotechnology Information (NCBI), National Library of Medicine (NLM), National Institutes of Health, Bethesda, Maryland, United States*, *3. University of South Florida, Tampa, Florida, United States*

Viewing laboratory test results is the most frequently used function of patient portals, but lab results can be confusing for patients. In this project, we aim to gain insights into older adults' challenges in understanding lab test results. To achieve the goals, we first conducted an online survey with 276 patients with one or more chronic conditions. We found that younger adults and patients who had never used patient portals had significantly lower lab test comprehension scores compared to older adults, and those with higher numeracy or with no multiple chronic conditions. Even though older adults performed better on lab test comprehension tests, most reported they have difficulty understanding lab results online and prefer that their doctors explain these to them. In the second study, we selected 53 lab results-related questions from a community Q&A website, Yahoo! Answers, and evaluated the answers to these questions from four generative large language models. We utilized a ChatGPT-based evaluator Win Rate to judge whether a target model has higher quality in terms of relevance, correctness, helpfulness, and safety than the baseline model. Then, we performed a manual evaluation with medical experts for all the responses to seven selected questions on the same four aspects. The results of Win Rate and medical expert evaluation both showed that GPT-4's responses achieved better scores than all the other LLM responses from LLaMa 2, ORCA_mini, and MedAlpaca and human responses on all four aspects. However, LLM responses occasionally suffer from incorrect statements.

Abstract citation ID: igae098.0352

### MANAGEMENT OF NEURODEGENERATIVE DISEASES: APPLICATION OF MULTIMODAL, EXPERT-INTERACTIVE AI VERTICAL MODELS

Junyan Mao[1], Jialiang Tong[1], Weidong Huang[2], and Hongyu Li[3], *1. Shanghai Izhaohu Medical Technology Co. Ltd, Shanghai, Shanghai, China (People's Republic)*, *2. Changchun University of Chinese Medicine, Changchun,*

*Jilin, China (People's Republic)*, 3. *Jinzhou Medical University, Jin Zhou, Liaoning, China (People's Republic)*

This study proposes a comprehensive management model for neurodegenerative diseases, leveraging multimodal, expert-interactive AI vertical models to tackle the challenges of environmental dependency, temporal constraints, and data singularity. The model aims to enhance patient prognosis, quality of life, and survival. Our research methodology unfolds in three phases: The first step involves conducting a bibliometric analysis to integrate findings from existing studies. The second phase "Early Detection and Prediction," leverages computer vision (CV) and natural language processing to gather multimodal biomarker data, including voice, gait, vision, hearing, and PET scans. Data is analyzed using machine learning (such as SustaIn, Random Forests, and Support Vector Machines) and deep learning techniques for robust data modeling. The final step, "Effectiveness Verification," entails executing randomized controlled trials (RCTs) with a triple-blind setup—ensuring blindness among participants, researchers, and assessors—to rigorously evaluate the developed model's effectiveness. The research involved five-year data from 304 individuals aged 65 and older with neurodegenerative diseases. Objective biomarker data were collected through CV and sensors to facilitate early identification and diagnosis of neurodegenerative diseases. A sophisticated platform facilitated real-time monitoring of mental and behavioral symptoms and activities of daily living at disease onset, integrating both pharmacological and non-pharmacological interventions via advanced language models for holistic disease management. Utilizing dynamic video analysis and machine learning evaluations, the model significantly mitigated the progression of cognitive and physical impairments, enhanced quality of life, and decreased mortality rates, affirming the preliminary efficacy of the comprehensive management model.

Abstract citation ID: igae098.0353

### USING A LARGE LANGUAGE MODEL TO CLASSIFY DEMENTIA CAREGIVERS' HEALTH INFORMATION WANTS

Zhuochun Li[1], Zhimeng Luo[1], Ning Zou[1], Daqing He[1], Bo Xie[2], Robin Hilsabeck[3], and Alyssa Aguirre[2],
1. *University of Pittsburgh, Pittsburgh, Pennsylvania, United States*, 2. *The University of Texas Austin, Austin, Texas, United States*, 3. *The University of Texas Health Sciences Center San Antonio, San Antonio, Texas, United States*

Many caregivers of persons living with dementia use social media to express dementia care-related health information wants (HIWs), i.e., types of information caregivers seek that can help them manage challenges and concerns. Recent developments in large language models (LLMs) hold promise in automatic classification of HIWs expressed in large quantities of social media posts. As a first step, we evaluated GPT-3.5 Turbo's performance in classifying 60 social media posts based on our previously developed HIW framework, which includes 7 first-level categories, including daily care. Under daily care, there are 7 second-level categories and a total of 32 third-level subcategories. All 60 posts were previously verified by 3 experienced clinicians as being in the daily care category; 40 posts were in the second-level category of mood and behavior management and 20 in the second-level category of safety; and 20 posts in each of the 3 third-level categories: agitation; memory loss and confusion; and driving safety. We asked GPT-3.5 Turbo to classify the posts on the first level; 14 (23%) were accurately classified to daily care. Next, we asked GPT-3.5 Turbo to classify those 14 posts into one of the second-level categories in daily care; 10 (71%) were accurately classified into mood and behavior management or safety. Finally, we asked GPT-3.5 Turbo to classify the 10 remaining posts into the third-level subcategories. Seven (70%) were accurately classified: agitation 5/5 (100%), driving safety 2/2 (100%) and memory loss and confusion 0/3 (0%). Disappointed by GPT-3.5 Turbo's performance, we discuss plausible reasons and solutions.

### SESSION 1360 (SYMPOSIUM)

Abstract citation ID: igae098.0354

### EDITORS' PERSPECTIVES ON GLOBAL PUBLISHING OPPORTUNITIES IN GERONTOLOGY

Chair: Edward Miller
Co-Chair: Elizabeth Simpson

Global aging has proceeded at an unprecedented and accelerating rate. The aging of the population creates both opportunities and challenges for older adults, their families, and society. Importantly, there is substantial variation in the effects of and response to global aging both within and across nations depending, in part, on prevailing cultural expectations and values, political and economic imperatives, and social and demographic characteristics. Thus, while some regions and countries have responded with innovative policies and programs to better enable the growing cohort of older adults to remain active and engaged in the community, other regions and countries have struggled with their response or barely begun to plan for the rising population of older adults. This symposium assembles editors at five leading gerontological journals to demonstrate the role that peer-reviewed scholarship can play in disseminating knowledge that informs gerontological research, policy, and practice internationally. The editors include: Edward Alan Miller, PhD, Journal of Aging & Social Policy; Kyungmin Kim, PhD, Research on Aging; Debra Dobbs, PhD, Journal of Applied Gerontology; Danielle Nadorff, PhD, International Journal of Aging and Human Development; and Sandra Torres, PhD, Ageing & Society. Each presenter will review the scope, content, and focus of their journals and the role and opportunities for international scholarship.

Abstract citation ID: igae098.0355

### INTERNATIONAL PUBLISHING OPPORTUNITIES AT THE JOURNAL OF AGING & SOCIAL POLICY

Edward Miller[1], Elizabeth Simpson[1], Michael Gusmano[2], and Pamela Nadash[1], 1. *University of Massachusetts Boston, Boston, Massachusetts, United States*, 2. *Lehigh University, Bethlehem, Pennsylvania, United States*

Policymakers, practitioners, and researchers need a balanced, thoughtful, and analytical resource to meet the challenge of global aging at a rate that's historically unprecedented. The Journal of Aging & Social Policy (JASP), which was founded in 1989, serves this role by drawing contributions from an international panel of policy analysts and scholars who assume an interdisciplinary perspective in examining and analyzing critical phenomena that affect aging and the development and implementation of programs for older adults from a global perspective. Study settings extend beyond the United States to include Europe, the Middle East, Australia, Latin America, Asia, and the Asia-Pacific rim. This presentation will document the scope, content, and focus of JASP, including the rise of international submissions, which now account for approximately half of articles published. Opportunities for publishing in JASP will be discussed; so too will strategies for navigating the peer-review process successfully.

Abstract citation ID: igae098.0356

### RESEARCH ON AGING: THE INTERNATIONAL VIEW FROM THE EDITORS' DESKS

Kyungmin Kim[1], Jeffrey Burr[2], Jeffrey Stokes[2], and Changmin Peng[2], *1. Seoul National University, Seoul, Republic of Korea*, *2. University of Massachusetts Boston, Boston, Massachusetts, United States*

We review the scope, content, and focus of the peer-reviewed journal, Research on Aging (SAGE), publishing its 46th volume this year. We will discuss how scholarship produced from researchers around the globe has changed over the years. Data on submissions, acceptance rates, and the important role of an international editorial board will be presented. The review process will be described, along with suggestions on how to increase chances of success when submitting original research. Although Research on Aging is sometimes considered to focus primarily on social gerontology, the scope in recent years has widened considerably, with manuscripts in aging studies published from such fields as economics, psychology, demography, public health, and public policy, as well as from sociology, and social work, among others. One of several special issues forthcoming in the journal will be described to demonstrate the possibilities for international impact.

Abstract citation ID: igae098.0357

### THE JOURNAL OF APPLIED GERONTOLOGY: AN INTERNATIONAL PERSPECTIVE

Debra Dobbs, *University of South Florida, Tampa, Florida, United States*

The mission of applied gerontology is to bridge science and practice to benefit the health and well-being of older people, their families, their communities, and other contexts. Journal of Applied Gerontology features original investigations or meta-analyses/systematic reviews with significant clinical, policy, and/or practice implications. This presentation will provide insights from Journal of Applied Gerontology and its commitment to publish and disseminate scholarship with international application. Following an overview of the growing internationalization of peer-reviewed submissions to Journal of Applied Gerontology on a variety of topics and from a range of perspectives, the presentation will highlight opportunities to apply gerontological scholarship to aging contexts worldwide. Concluding comments will examine how outlets for dissemination and authors themselves can better position their work to enhance their influence on aging in an international context.

Abstract citation ID: igae098.0358

### PUBLISHING WITH THE INTERNATIONAL JOURNAL OF AGING AND HUMAN DEVELOPMENT

Danielle Nadorff[1], and Julie Patrick[2], *1. Mississippi State University, Mississippi State, Mississippi, United States*, *2. West Virginia University, Morgantown, West Virginia, United States*

For more than 50 years, under the leadership of four editors and two publishers, The International Journal of Aging and Human Development (IJAHD) has featured multidisciplinary scholarship related to aging processes and older adults. With the publication of eight issues a year and over 1000 pages of scientific content, the IJAHD places emphasis upon psychological and social studies of aging and the aged. However, the Journal also publishes research that integrates observations from other disciplines that illuminate the "human" side of gerontology. A more recent focus includes midlife development, as well. About half (47%) of the publications in the IJAHD are from international colleagues. IJAHD is delighted to support new investigators as they navigate dissemination. This presentation will discuss tips for both international and US-based scholars for ensuring timely reviews and positive decisions for manuscript submissions, including such areas as key words, suggesting unbiased reviewers, formatting, writing mechanics, clearly-articulated methods, and a sound theoretical basis.

Abstract citation ID: igae098.0359

### TOP TEN TIPS FROM THE EDITOR-IN-CHIEF OF AGEING & SOCIETY

Philip Taylor[1], Sandra Torres[2], and Dialechti Tsimpida[3], *1. University of Warwick, Coventry, England, United Kingdom*, *2. Uppsala University, Uppsala, Uppsala Lan, Sweden*, *3. University of Southampton, Southampton, England, United Kingdom*

Understanding the nuances of academic writing genres is crucial to nailing down the craft that is writing for peer-review journal publication. In this presentation, we will leverage an Editor-in-Chief's expertise, to explore the routines of esteemed international peer-review journals like Ageing & Society. By uncovering editors' expectations, this session offers invaluable insights into mastering the art of writing for prestigious publications. More specifically, this presentation will shed light on different publication outlets, the different readers these cater to, and how these differences impact how we craft manuscripts about the research we conduct. Building upon this foundational understanding, we will delve into strategies for presenting data collected in specific national contexts, contributing to global discussions in social gerontology.The ultimate goal is to provide attendees with an editor-in-chief's top 10 tips by focusing on the routines that established international peer-review journals, such as Ageing & Society utilize, and the expectations editors place on the different sections of a manuscript.

### SESSION 1365 (PAPER)

### FALLS (PAPERS)

Abstract citation ID: igae098.0360

### SLEEP QUALITY AND DURATION AND RISK OF FALLS IN OLDER ADULT WOMEN: THE STUDY OF WOMEN'S HEALTH ACROSS THE NATION (SWAN)

Jillian Baker[1], Michelle Hood[2], Leslie Swanson[2], Christopher Kline[3], Kelly Ylitalo[4], Jane Cauley[5], Dr. Robin Green[6], and Carrie Karvonen-Gutierrez[2], *1. University of Michigan School of Public Health, Ann Arbor, Michigan, United States*, *2. University of Michigan, Ann Arbor, Michigan, United States*, *3. University of Pittsburgh, Pittsburgh, Pennsylvania, United States*, *4. Baylor University, Waco, Texas, United States*, *5. University of Pittsburgh School of Public Health, Pittsburgh, Pennsylvania, United States*, *6. Einstein College of Medicine, Bronx, New York, United States*

**Background:** As the leading cause of injury in American older adults, falls can be consequential for function and mortality, but are preventable. Sleep may be a modifiable risk factor for falls. We hypothesized poorer sleep quality and shorter sleep duration would be associated with increased risk of falls and a greater number of falls.

**Methods:** The Study of Women's Health Across the Nation is a multi-ethnic, community-based longitudinal study of women followed through and beyond the menopausal transition. In 2010-2011, participants self-reported measures of sleep quality and duration. In 2015-2016, participants reported number of falls in the year prior. Adjusted models included demographic and socioeconomic factors, and health conditions.

**Results:** Women who reported frequent (5+ times/week) trouble falling asleep and frequent waking in the middle of the night in 2010-2011 had a 27% (RR=1.27, 95% CI=1.01,1.62) and 24% (RR=1.24, 95% CI=1.01,1.48) increased risk of a subsequent fall. Frequent trouble falling asleep and short sleep duration (< 6 hours) were both associated with higher likelihood of recurrent falls (>=3 falls vs. <=1 fall) (OR=2.54, 95% CI=1.30,4.96; OR=1.80, 95% CI=1.07,3.05). Conclusions: Multiple indicators of poor sleep including trouble falling asleep, frequent waking, and short duration were associated with increased fall burden in older adult women. As falls pose a major risk factor for decline in health and mortality, promoting adequate, high-quality sleep may be an essential component in fall prevention.

Abstract citation ID: igae098.0361

### GAIT SPEED MODERATES EFFICACY OF HOME-BASED EXERCISE ON FALLS IN OLDER ADULTS WITH PREVIOUS FALL

Jordyn Rice, Ryan Falck, Jennifer Davis, Larry Dian, Kenneth Madden, Wendy Cook, Karim Miran-Khan, and Teresa Liu-Ambrose, *University of British Columbia, Vancouver, British Columbia, Canada*

Exercise is an evidence-based strategy for preventing falls. However, its efficacy may vary based on individual characteristics, like gait speed. We performed a secondary analysis of a randomized controlled trial, to assess if baseline gait speed moderated the effects of an exercise program on subsequent falls among older adults. Community-dwelling adults >70 years who had fallen within the previous 12 months were randomized to either 12 months of home-based exercise (EX; n=172) or standard of care (SC; n=172). For this study, we examined intervention effects on fall rates at 6 and 12 months stratified by baseline gait speed (slow < 0.80m/s or normal >0.80 m/s) using negative binomial regressions. We also investigated whether baseline gait speed moderated the intervention effects on mobility and cognitive function using linear mixed modeling. At baseline, 134 participants had slow (EX=70; SC=64) and 210 had normal (EX=102; SC=108) gait speed. For participants with slow gait speed, EX reduced fall rates by 44% at 6 months (IRR=0.56; 95% CI:[0.33, 0.95]; p= 0.03) compared with SC; for participants with normal gait speed, there was no significant effect of exercise on fall rates at 6 or 12 months. Gait speed moderated intervention effects; EX participants with slow gait improved: 1) Timed Up-and-Go at 6 months (estimated mean difference= -4.05; 95% CI:[-6.82, -1.27]; p= 0.00); and 2) Digit Symbol Substitution Test at 12 months (estimated mean difference= 2.51; 95% CI:[0.81, 4.21]; p=0.00). Our study suggests older adults with slow gait speed may be a target population for exercise-based falls prevention.

Abstract citation ID: igae098.0362

### THE MEDIATING ROLE OF SOCIAL ISOLATION BETWEEN FALLS AND COGNITIVE FUNCTION AMONG OLDER ADULTS IN THE UNITED STATES

Wonkyung Jung[1], and Janiece Taylor[2], *1. Boston College, Baltimore, Maryland, United States*, *2. Johns Hopkins University, Johns Hopkins University, Maryland, United States*

Social isolation and falls are significant concerns affecting the cognitive health of older adults. However, little is known about the interrelationships between social isolation, falls, and cognitive function among older adults in the United States (U.S.). This study aims to investigate the interrelationships between these factors and the mediating role of social isolation on the relationship between fall experiences in the past 12 months and cognitive function among older adults in the U.S. Utilizing Round 5 data from the National Health and Aging Trends Study (NHATS), we employed linear regression models using Hayes's PROCESS macro with 5,000 bootstrapped mediation analyses in SPSS. Of the 947 participants, 60.8% were younger than 75 years, 38.6% were women, and most (77.1%) were White. Our findings revealed that social isolation did not mediate the relationship between falls and cognitive function. Additionally, we found no significant association between fall experiences and both social isolation and cognitive function. However, our analysis identified that socially isolated older adults were more likely to experience cognitive impairments ($\beta$ =.84, P <.001). These results highlight the complex nature of the relationships among social isolation, falls, and cognitive function in older adults. Further research is warranted to explore potential mechanisms underlying the observed associations and to develop targeted interventions to address cognitive impairments among socially isolated individuals. Strategies targeting social connectedness may have promising implications for promoting cognitive health in older adults in the U.S.

Abstract citation ID: igae098.0363

**EFFECTS OF DIFFERENT EXERCISE INTERVENTION MODALITIES ON BALANCE AND PHYSICAL FUNCTION AMONG OLDER ADULTS**

Xiaodong Chen, Lingxiao He, and Ya Fang, *Xiamen University, Xiamen, Fujian, China (People's Republic)*

**Abstract Objective:** To compare the effects of on-site team (OST) exercise and live-streamed home (LSH) exercise model on balance and physical function among community-dwelling older adults. Design, Setting, and Participants: A 24 week, randomized, controlled, assessor blinded trial was conducted from February 25, 2023 to November 30, 2023. Participants were 297 older adults aged 65 years and older from 11 communities across Xiamen, China.

**Interventions:** Participants were block randomized to OST and LSH groups with multimodal elastic band-based exercise offline and online (n=99), and a control group with no exercise intervention (n=99). Main outcome measures: The primary outcomes were Berg balance scale scores and the short physical performance battery (SPPB). Secondary outcomes were muscle mass, muscle strength (knee extension strength and grip strength), sleep quality, adherence, and adverse events.

**Results:** Among 297 randomized older adults (mean age, 71.8[SD, 5.5] years; 52.7% female), 280 (93.8%) completed the trial. Both intervention groups showed increase in Berg balance scale scores and SPPB scores relative to control (adjusted mean differences: OST group vs. control, 1.26 [95% CI, 0.74 to 1.79] and 3.76 [95% CI, 2.25 to 5.26]; P< 0.001; LSH group vs. control: 1.00 [95% CI, 0.44 to 1.56] and 3.42 [95% CI, 1.83 to 5.02]; P< 0.001; OST vs. LSH group, 0.261 [95% CI, -0.26 to 0.78] and 0.33 [95% CI, -1.11 to 1.78]; P=0.463 and 0.853). Similar results were seen in terms of muscle strength.

**Conclusion:** Live-streamed home exercise is an effective approach to improving balance and physical function among older adults.

Abstract citation ID: igae098.0364

**PILOT OUTCOMES OF A FALL PREVENTION PROGRAM "LIVE-LIFE" FOR COMMUNITY-DWELLING OLDER ADULTS IN CHINA**

Yuqian Luo[1], Cen Mo[2], Wenting Peng[3], Sarah Szanton[4], and Minhui Liu[5], *1. Hong Kong Polytechnic University, Hong Kong, Hong Kong, China (People's Republic)*, *2. Central South University, Changsha, Hunan, China (People's Republic)*, *3. University of Washington, Seattle, Washington, United States*, *4. Johns Hopkins University, Baltimore, Maryland, United States*, *5. Ningxia Medical University, Yinchuan, Ningxia, China*

More than 70 million older adults fall at least once each year in China, which may lead to disability, fear of falling, social isolation and even death. We conducted a pilot randomized controlled trial to evaluate the preliminary effects of the multi-component LIVE-LiFE program on health-related outcomes among community-dwelling older adults in China. Thirty-Eight participants, average 70.32 years old, 76.3% of female, were randomized to either the intervention group (n=20) or the control group (n=18) for 11 weeks. In the intervention group, participants received the LIVE-LiFE program (individualized strength and balance training, vision screening, medication review, and home hazards assessment and modification), whereas the control group received equal amounts of health education on fall prevention. To compare the differences of pre-post scores between two groups, we employed two independent sample t-test and independent sample Wilcoxon signed-rank test. Results indicated significant differences and moderate effect sizes on 3-meter walking speed (P< 0.001, d =-0.603) and home hazards (P=0.043, d=-0.504). The paired t-test and paired Wilcoxon signed-rank test were used to examine the paired LiFE assessment data of the intervention group. Significant improvements were observed on the total scores (P< 0.001, d=1.260), balance function dimension scores (P< 0.001, d=0.962), and strength function dimension scores (P=0.002, d=0.776). Our study showed positive preliminary effects of the LIVE-LiFE program on improving both personal function and environmental safety. Future research should consider both internal and external factors to prevent falls among community-dwelling older adults with larger sample sizes and longer follow-up.

### SESSION 1370 (SYMPOSIUM)

Abstract citation ID: igae098.0365

**FOOL ME ONCE: FRESH PERSPECTIVES ON FINANCIAL VULNERABILITY AND FINANCIAL EXPLOITATION**

Chair: Julie Miller
Discussant: Julie Miller

Threats associated with fraud and financial exploitation of older adults have grown exponentially. To surface and scale the most meaningful approaches to preventing financial exploitation among older adults, we need innovative approaches that challenge our collective understandings of which factors are at play and what works, why, and how. This symposium will bring together leading scholars to share emerging understandings of mechanisms that fuel financial exploitation, as well as novel approaches with important implications for prevention. The first presentation in the symposium will share new perspectives on cognitive underpinnings of scam susceptibility by exploring individual's abilities to discern credibility of information and their susceptibility to pseudo-profound statements. The second presentation will describe a randomized control trial with older adult victims of mail fraud designed to evaluate the efficacy of a mailed intervention intended to prevent revictimization. The third and final presentation will highlight an intersectional model of financial exploitation vulnerability that combines interpersonal factors and brain mechanisms, ultimately underscoring the importance of considering multiple factors and different levels of analysis when considering financial exploitation vulnerability in older age. Together, the three presentations will demonstrate the importance of reframing professionals'

and non-professionals' experiences with – and expectations about – financial vulnerability and financial exploitation.

Abstract citation ID: igae098.0366

**ABSTRACT THOUGHT LEADS TO UNPARALLELED BEAUTY: A STUDY ON BULLSHIT RECEPTIVITY AND SCAM SUSCEPTIBILITY**

Stacey Wood[1], Sagrika Jawadi[1], and David Hengerer[2], *1. Scripps College, Claremont, California, United States, 2. Claremont Graduate University, Claremont, California, United States*

In the age of disinformation, the ability to discern the credibility of information is more vital than ever. To assess an individual's susceptibility to pseudo-profound statements, or "bullshit", the Bullshit Receptivity (BSR) Scale, a list of meaningless, mundane, and profound statements, was devised in 2015. In Study 1, a lifespan sample of 213 individuals (age range 24-78, M = 46.05, SD = 17.21) participated in the study via MTurk and were asked to rate the profundity of various bullshit and mundane statements, complete a vocabulary test, and complete a subset of the CART (rational thought). Older adults performed significantly better than did younger and middle-aged adults on the overall BSR scale. The relationship between age and BSR score was partially mediated by performance on the vocabulary test, with a greater vocabulary leading to lower susceptibility to bullshit ($\beta = -.0248$, $p < .001$, SE =.002). In Study 2, we introduced scam susceptibility to investigate the relationship between scam susceptibility and bullshit receptivity. We replicated the study in person with 92 neurotypical participants (M = 19.96, SD = 5.42). Participants also completed subtests from the NIH Toolbox Cognition Battery and rated the appeal of fraudulent investment pitches and red-flag persuasion tactics. Scam susceptibility was positively related to bullshit receptivity, as measured by rated profundity of pseudo-profound statements ($r = .27$, $p < .05$). This information can be used to better understand the cognitive underpinnings of scam susceptibility, especially among older adults who are targeted for scams.

Abstract citation ID: igae098.0367

**A FRAUD AWARENESS MAIL CAMPAIGN SIGNIFICANTLY REDUCES RATES OF REVICTIMIZATION AMONG OLDER VICTIMS**

Marguerite DeLiema[1], M. Daniel Brannock[2], Edward Preble[2], and Lynn Langton[2], *1. University of Minnesota Twin Cities, St. Paul, Minnesota, United States, 2. RTI International, Research Triangle Park, North Carolina, United States*

Older adults are disproportionately targeted by scams, with direct cost estimates ranging from $5.9 to 48.4 billion per year (Federal Trade Commission, 2023). Much of these costs are driven by repeat and chronic victimization. We conducted a randomized control trial to evaluate the efficacy of a mailed intervention to prevent revictimization among known older adult mail fraud victims. Participants' addresses were randomly assigned to one of two treatment groups or to a control. Treatment groups received either (1) a single letter from the US Postal Inspection Service (USPIS) informing them that they paid money in response to a mail scam and may be targeted by additional scams, as well as education on how to spot mail scams, or (2) the same letter along with five additional "Fraud Fighter" awareness materials mailed two weeks apart. The control group received no materials. The effectiveness of the intervention was assessed through actual victim behavior. USPIS collected return addresses from detained victim mail for 4 months following the initial intervention mailing. We identified revictimization by matching the return addresses from the newly detained victim mail to the addresses of victims in the experiment. Compared to the control (no intervention), the single letter condition reduced the rate of revictimization by 8.6% and the multiple mailing condition reduced the rate of revictimization by 22.4% over 4 months. This represents the first test of a simple, scalable, mailed intervention to reduce the incidence and cost of mass marketing fraud among older Americans.

Abstract citation ID: igae098.0368

**FINANCIAL EXPLOITATION VULNERABILITY IN OLDER AGE: INTERPERSONAL AND BRAIN MECHANISMS**

Duke Han, *University of Southern California, Los Angeles, California, United States*

Financial exploitation vulnerability in older age can be viewed as an intersectional model of multiple potentially contributing features that can vary from individual predisposing characteristics to interpersonal dynamic factors. Here we present recent work from our lab focused on two mechanisms. In Study 1, thickness of the entorhinal cortex as measured by a 7-Tesla brain MRI scan was associated with a measure of perceived financial vulnerability in 97 older adults without any significant cognitive impairment in models adjusted for demographics and cognition (beta=-0.05, SE=0.02, $p<0.05$). The entorhinal cortex is well-known for its role as the first brain region to show signs of deterioration in preclinical Alzheimer's Disease; therefore, findings suggest early age-associated pathological brain changes may underlie financial vulnerability. In Study 2, financial vulnerability was associated with depth of relationships, but not breadth of relationships, in 115 older adults without any significant cognitive impairment after covarying for demographics, depressive/anxious symptoms, and cognition (beta=-.08, SE=.03), $p<.01$). Findings argue for the importance of considering interpersonal relationship dynamics when assessing financial vulnerability. Together, these studies argue for the importance of considering multiple factors and different levels of analysis when considering financial exploitation vulnerability in older age.

### SESSION 1375 (SYMPOSIUM)

Abstract citation ID: igae098.0369

**IN SEARCH OF TIME: TIME PERSPECTIVE AND LIFE'S FINITUDE**

Chair: Yaeji Kim-Knauss
Co-Chair: Clara de Paula Couto
Discussant: David Ekerdt

Human existence is inherently bounded by a finite timeline, and this reality shapes our perceptions, attitudes, and behaviors towards the time. This awareness of life's finitude prompts individuals to prioritize meaningful pursuits or to adapt to the constraints of time, as elucidated in theories in gerontology, and validated through numerous empirical studies. Against this background, this symposium delves into the impact of time perspectives on psychological outcomes, as well as on individuals' approaches to their own mortality. N. Fung and H. Fung will offer a comprehensive examination of how motivation changes as time left in life is perceived to decrease. Wirth et al. will present findings about the influences of different subsets of time perspective on life satisfaction using longitudinal data. Incorporating ideas from construal-level theory, de Paula Couto et al. will delve into age-specific factors associated with desired longevity under impairment conditions. Kim-Knauss et al. will discuss behaviors associated with perceived engagement in end-of-life planning and examine potential cultural differences by comparing the findings from Germany and South Korea. Selovin et al. will explore psychological and behavioral factors contributing to a sense of preparedness for death and dying among older adults. Finally, Ekerdt will discuss the findings from these presentations, offering comprehensive insights into the interplay between time perspective and human behavior in confronting mortality.

Abstract citation ID: igae098.0370

### END-OF-LIFE PLANNING IN GERMANY AND SOUTH KOREA: PERCEIVED ENGAGEMENT AND ACTUAL BEHAVIOR

Yaeji Kim-Knauss[1], Yumi Shin[2], Jung-Hwa Ha[3], and Frieder Lang[1], *1. Friedrich-Alexander-Universität Erlangen-Nürnberg, Nuremberg, Bayern, Germany, 2. Daegu University, Gyeongsan, Republic of Korea, 3. Seoul National University, Seoul, Republic of Korea*

End-of-life planning could manifest differently across cultures, shaped by prevailing social norms. This study examines behaviors associated with perceived engagement in end-of-life planning and explores potential cultural variations. We focus on two distinct cultures, Germany and South Korea, drawing on data from the 'Ageing as Future' project's online survey conducted in 2023 for the German sample (aged 60+, n=708) and data collected between 2023 and 2024 for the Korean sample (aged 60+, n=519). We identify two outcome behaviors of interest: advance care planning (ACP) and discussions with relatives about one's death, commonly known as formal and informal end-of-life planning, respectively. Logistic regression models revealed that perceiving oneself as actively engaged in end-of-life planning increased the likelihood of completing ACP, a finding consistent across cultures. Additionally, being German, older, female, and having higher education levels were associated with higher odds of ACP completion. Concerning discussions with relatives, on the other hand, perceiving engagement in end-of-life planning increased the likelihood of this informal planning, with a significantly stronger effect observed in the Korean sample compared to the German sample. Being German and having at least one child were also positively associated with engaging in these discussions. These findings suggest a greater reliance on informal discussions about death with relatives in the Korean context. Given the cultural differences and the lower prevalence of end-of-life discussions in Korea compared to Germany, the normalization of such discussions in Korea is needed, but with a culturally tailored approach.

Abstract citation ID: igae098.0371

### WILLINGNESS TO LIVE UNDER IMPAIRMENT: FACTORS ASSOCIATED WITH DESIRED LONGEVITY

Clara de Paula Couto[1], Fiona Rupprecht[2], Jana Nikitin[2], and Klaus Rothermund[1], *1. Friedrich Schiller University Jena, Jena, Thuringen, Germany, 2. University of Vienna, Vienna, Wien, Austria*

This study delves into the complexities of individuals' desires for longevity and their willingness to live with impairment. When deciding about their longevity desires, some individuals may focus on present-oriented, concrete aspects of their lives, like their current state of health, whereas others may weigh up more future-oriented, abstract aspects, such as how important it is to be healthy. We investigated what factors are associated with individuals' willingness to live with impairment. Factors associated with willingness to live with impairment were examined in a sample of N = 790 German participants aged 39–90 years (Mage = 63.38, SD = 14.30, 51% female). To examine age-specific factors related to desired longevity under impairment conditions, chronological age was added as a moderator in the analyses. Findings indicated that for middle-aged adults, fear of becoming a burden and higher importance of health were associated with lower willingness to live with impairment. For older adults, lower willingness to live with impairment was associated with higher levels of instrumental preparation for the end of life. The obtained age differences can be understood as reflecting a shift in time perspective and personal experiences. Among middle-aged adults, willingness to live with impairment is more strongly influenced by distant events that pertain to the future (e.g., fear of becoming a burden). Older adults, however, place more importance on their current life situation (e.g., instrumental preparation), and their willingness to live with impairment seems to be more conditional on practical aspects in the present.

Abstract citation ID: igae098.0372

### EMOTIONALLY MEANINGFUL GOALS IN LATE ADULTHOOD

Nicole Fung, and Helene H. Fung, *The Chinese University of Hong Kong, Hong Kong, China (People's Republic)*

Our perception of time greatly affects what kinds of goals we value and pursue. The socioemotional selectivity theory (SST; Carstensen, 1992; Carstensen et al., 1999) posits that people rearrange their goals in response to a limited future time perspective as they become older. Rather than investing in a limited and uncertain future, older adults will value and prioritize present-oriented emotionally meaningful goals over future-oriented goals. The SST literature thrived in the past three decades and emotionally meaningful goals were operationalized in various ways under different contexts. To better understand the changes in motivation when future time perspective becomes limited, we reviewed the empirical articles that formulated hypotheses according to the SST framework published since 1992. We identified different types of emotionally meaningful goals in the literature. The most prevalent type of emotionally meaningful goal in the literature is the pro-hedonic goal (preference towards positivity over negativity). Other types of emotionally meaningful goals include the pro-closeness goal (preference towards emotional closeness in social relationships), the prosocial and generativity goal (increased motivation to contribute to others), the social goal (increased importance in the social domain), the emotional goal (increased importance in the emotional domain), the self-maintenance goal (increased motivation for the intrinsic and self-concordant goal) and the meaningfulness goal (increased importance in meaningfulness). The empirical evidence of each goal will be reviewed and their relations to SST and a limited future time perspective will be discussed.

Abstract citation ID: igae098.0373

### LONGITUDINAL ASSOCIATIONS BETWEEN TIME PERSPECTIVE AND LIFE SATISFACTION ACROSS ADULTHOOD

Maria Wirth[1], Markus Wettstein[2], and Klaus Rothermund[1], *1. Friedrich Schiller University Jena, Jena, Thuringen, Germany*, *2. Humboldt University Berlin, Berlin, Berlin, Germany*

Subjective representations of time are an important predictor of well-being. How time is structured and represented in individual behavior and experience, is itself subject to developmental change. Young adults focus on preparing for their future, whereas during midlife, shifts towards a more present-oriented time perspective should arise. Realizing that important life goals may no longer be achievable should lead older adults to detach themselves from these goals and focus more on present and past experiences. These age-related changes in time perspective supposedly are associated with greater well-being. Previous studies, however, yielded heterogeneous results. However, these studies mostly investigated only one dimension of time perspective and did not include younger and/or middle-aged adults. To overcome these limitations, we investigated how changes in four facets of time perspective (orientation towards the past, concreteness of the future, obsolescence, and finitude of life) are related to changes in life satisfaction and whether these associations are moderated by chronological age. We used data from an age-diverse sample comprising 459 participants (30 – 80 years) of the “Aging as Future” (AAF) study comprising three measurement occasion over 10 years. Longitudinal multilevel regressions indicated that individuals with overall higher concreteness and lower obsolescence reported higher life satisfaction. Additionally, individuals reported higher life satisfaction on measurement occasions when they scored higher on concreteness. These associations were not moderated by age, which questions the idea of age-differential associations between time perspective and well-being. Our study provides a deeper understanding of the relation between time perspective and well-being across adulthood.

Abstract citation ID: igae098.0374

### THE ROLE OF PSYCHOLOGICAL AND BEHAVIORAL FACTORS IN FEELING WELL-PREPARED FOR DEATH AND DYING

Fabio Selovin[1], Fiona Rupprecht[2], and Jana Nikitin[2], *1. Institute of Psychology / Friedrich Schiller University Jena, Jena, Thuringen, Germany*, *2. University of Vienna, Vienna, Wien, Austria*

In previous research, death preparation has usually been defined as the amount and type of formal death arrangements an individual has undertaken (e.g., having written a last will). In contrast to such formal preparation, our study focuses on the subjective feeling of being prepared for death and dying. Based on construal-level theory, we assume that different behavioral and psychological factors matter for this subjective feeling of preparedness depending on the subjective temporal distance to death. The sample consisted of 121 participants aged 51 to 95 years (M = 63, SD = 9; 48% female). Participants were asked how prepared they felt for death and dying and how extensive they experienced their remaining future. Furthermore, we assessed aspects of formal death preparation, informal death preparation (i.e., death communication with partner), generativity and death attitudes (i.e., fear of death, belief in an afterlife). Results showed that neither formal nor informal death preparation were predictive of the subjective feeling of being prepared. Instead, the fear of death emerged as the strongest (negative) predictor across the sample. Interaction analyses further showed that individuals who felt close to death, felt prepared when their generativity and opportunities for being generative were high. Findings are discussed using a life-span perspective, illuminating what being prepared for death and dying might mean for the individual.

### SESSION 1380 (SYMPOSIUM)

Abstract citation ID: igae098.0375

### INVESTIGATING SOCIAL DETERMINANTS OF HEALTH IN ASIAN AMERICANS AND PACIFIC ISLANDERS

Chair: Yaguang Zheng
Co-Chair: Yanping Jiang
Discussant: Bei Wu

Asian Americans and Pacific Islanders (AAPI) are one of the fastest-growing racial minority groups in the United States, who disproportionately experience some unfavorable social determinants of health (SDoH), such as limited English proficiency, unemployment, lack of health insurance, low health literacy, lack of culturally and linguistically sensitive healthcare providers and programs, and reduced healthcare access. However, limited research has explored the impacts of SDoH on health outcomes among this population. The objective of this symposium is to address knowledge gaps by examining the association of SDoH with various health outcomes in AAPI. Jiang and colleagues explore the immigration stressors and sleep health among older Chinese immigrants using the longitudinal data from the Population Study of Chinese Elderly in Chicago. Qi and colleagues examine the prospective associations of multiple SDoH with premature all-cause mortality and investigate their respective contributions among a nationally representative cohort of Asian Americans. Further, Wang et al. discuss the challenges and successes in recruiting Chinese and Korean American dementia caregivers living in minority and underrepresented communities for a large health intervention study. Lastly, Kim & colleagues examine the end-of-life care disparities for

AAPI Medicare beneficiaries with dementia using Medicare data. Following the paper presentations, Dr. Wu will lead a discussion. The novel findings from this symposium will provide a comprehensive understanding of how SDoH impacts health outcomes in AAPI and how unique SDoH can be particularly targeted to improve health equity for this underserved ethnic minority group.

Abstract citation ID: igae098.0376

### PSYCHOSOCIAL AND IMMIGRATION DETERMINANTS OF SLEEP HEALTH AMONG OLDER CHINESE IMMIGRANTS

Yanping Jiang[1], and Fengyan Tang[2], *1. Rutgers, The State University of New Jersey, New Brunswick, New Jersey, United States*, *2. University of Pittsburgh, Wexford, Pennsylvania, United States*

Good sleep is critical for maintaining physical and cognitive health among older adults. Few studies, however, have examined factors related to sleep among older Asian immigrants, who face unique challenges that may put them at elevated risk of experiencing poor sleep. This study aimed to examine the effect of various psychosocial and immigrant factors on sleep outcomes over 2 years in a large sample of older Chinese American immigrants. Data were drawn from a subsample (N = 2,228) from the Population Study of Chinese Elderly in Chicago. Participants self-reported multiple psychosocial and immigrant factors, including neighborhood cohesion, neighborhood disorder, social disconnectedness, loneliness, perceived racial discrimination, perceived social support, negative social interactions, acculturation, perceived stress, depression, length of living in the U.S., income, and education. Participants also completed questionnaires on sleep quality and insomnia. Multivariate regression analyses showed that of the psychosocial and immigrant factors, depressive symptoms significantly predicted poorer sleep quality and worse insomnia over 2 years, controlling for baseline sleep quality and insomnia. Also, neighborhood disorder predicted worse insomnia over 2 years but not sleep quality. In addition, exploratory analyses for subdomains of sleep quality showed that perceived stress and negative social interactions predicted lower sleep efficiency over time. Neighborhood disorder and depressive symptoms appear to be salient risk factors for poor sleep health among older Chinese immigrants. Interventions targeting neighborhood environment and mental health services may be critical in improving sleep health and promoting healthy aging of older Chinese immigrants.

Abstract citation ID: igae098.0377

### PREMATURE DEATH ATTRIBUTABLE TO SOCIAL DETERMINANTS OF HEALTH AMONG ASIAN AMERICANS: A POPULATION-BASED COHORT

Xiang Qi[1], Katherine Wang[2], and Bei Wu[1], *1. New York University, New York, New York, United States*, *2. Duke University, Durham, North Carolina, United States*

Asian adults are one of the fastest growing ethnic minority groups in the US and have higher levels of unfavorable social determinants of health (SDoH), including limited English fluency, unemployment, and reduced healthcare access, yet the impact of these SDoH on mortality remains understudied in this population. This study aims to examine the prospective associations of multiple SDoH with premature all-cause mortality, and to investigate their respective contribution among a nationally-representative cohort of Asian Americans, using data from 3,888 Asian adults aged 20-74 who participated in the National Health and Nutrition Examination Survey 2011-2018. Cox proportional hazards regression models were employed to examine the relationships of SDoH factors with mortality; and population attributable fractions (PAFs) were calculated to assess the contributions of each individual SDoH on premature death. We found that unemployment, limited English proficiency, social isolation, no health insurance, in poverty, housing instability, less education, and food insecurity were significantly associated with mortality (HRs range from 1.20 to 2.35). SDoH explain up to 38.6% of mortality in the Asian Americans, among them, unemployment (18.5%), limited English fluency (9.0%), and social isolation (8.3%) showed the highest relative contributions to mortality. These results challenge the "Model Minority" stereotype, often perpetuated by English-only national survey data, and highlight the unique SDoH challenges faced by Asian Americans. Future research disentangling SDOHs on health outcomes by Asian subgroup is necessary to dispel the "model minority" stereotype that remains prevalent and provide better informed preventive practices and interventions for this population.

Abstract citation ID: igae098.0378

### ENGAGING CHINESE AND KOREAN AMERICAN COMMUNITIES IN DEMENTIA RESEARCH: A JOURNEY OF INCLUSIVITY AND PARTNERSHIP

Jing Wang[1], Mary Mittelman[2], I. Tek Leong[2], Sung Ji Park[2], Xiang Qi[3], Yaolin Pei[4], Cynthia Epstein[2], and Bei Wu[3], *1. University of New Hampshire, Durham, New Hampshire, United States*, *2. New York University, New York, New York, United States*, *3. New York University, New York, New York, United States*, *4. University of Texas at Austin, Texas, United States*

The NYUCI-ES Project, designed to deliver a family-based psychosocial intervention to enhance social support and reduce cardiometabolic disease risk for Chinese and Korean American dementia caregivers, has developed and undertaken a culturally sensitive recruitment strategy. Our navigation through the journey has shed light on the challenges and successes faced when recruiting in these minority and underrepresented communities. Common challenges include reluctance of involving other family members, stigma towards dementia and the concept of family counseling. Encountered challenges prompted us towards multiple efforts to engage the communities, including leveraging online communities, building strategic partnerships with local community organizations, participate in local events and make presentations at local service agencies, and working with community partners to adapt recruitment messages and methods to better align with cultural norms and preferences. For example, we replaced the term "counseling" with "family meetings" and used "memory issues" rather than "dementia" and emphasized the practical benefits and the opportunity for children to practice filial piety. For the Chinese community, our strategies focused on initiating the conversations with practical skills rather than directly discussing emotions. For the Korean community, framing the intervention in terms of discussing caregiving strategies while highlighting the personal and social benefits of participating in the study has helped lower the rejection rate. Our journey highlights the importance of culturally sensitive recruitment strategies in dementia research and demonstrates the potential for greater engagement and participation when communities are approached with respect, sensitivity, and an understanding of their unique cultural dynamics.

Abstract citation ID: igae098.0379

### END-OF-LIFE CARE DISPARITY FOR ASIAN AMERICAN PACIFIC ISLANDER AND WHITE AMERICANS WITH DEMENTIA

Hyosin Kim[1], Haiqun Lin[2], Anum Zafar[3], Bei Wu[4], Paul Duberstein[5], and Olga Jarrín[3], *1. Oregon State University, Corvallis, Oregon, United States*, *2. Rutgers, The State University of New Jersey, Newark, New Jersey, United States*, *3. Rutgers, The State University of New Jersey, New Brunswick, New Jersey, United States*, *4. New York University, New York, New York, United States*, *5. Rutgers, The State University of New Jersey, Piscataway, New Jersey, United States*

Alzheimer's disease and related dementias (ADRD) disproportionately affects Asian American and Pacific Islander (AAPI) older adults, similar to other minoritized groups. People living with ADRD have significant caregiving and end-of-life care needs. Little is known about racial disparities in end-of-life care for the AAPI Medicare beneficiaries with dementia. Using Medicare administrative, claims, and assessment from inpatient, hospice, and post-acute and long-term care settings for Medicare beneficiaries who died in 2019, differences in end-of-life care and place of death for AAPI (n= 20,301) and non-Hispanic White (n=762,443) Medicare beneficiaries with dementia and comparable levels of multimorbidity (median 6, IQR 5-8) were studied. Compared to White beneficiaries with dementia, AAPI beneficiaries with dementia were more likely to be dually enrolled in Medicare and Medicaid (60% vs. 32%), yet less likely to use home health care (52% vs. 55%) or nursing home care (54% vs. 64%) during the last 3 years of life; less likely to use hospice care during the last 6 months of life (52% vs. 65%); and more likely to die in an inpatient setting (34% vs. 26%) compared to their white counterparts. Differences were magnified among beneficiaries with high chronic disease burden (four or more comorbidities in addition to dementia). Underuse of hospice services and the high rates of Medicare-Medicaid dual enrollees among AAPI beneficiaries with dementia and multiple comorbidities warrant further attention, as the disease burden particularly increases with multiple chronic conditions.

### SESSION 1385 (SYMPOSIUM)

Abstract citation ID: igae098.0380

### LONGEVITY IN CULTURAL CONTEXT: FINDINGS FROM FOUR CENTENARIAN STUDIES

Chair: Peter Martin
Discussant: Leonard Poon

Life expectancy varies across cultural and international groups, but each culture contains long-lived individuals. To what extent survivors in different cultural groups have common or divergent characteristics is still being determined. The purpose of the symposium is to compare and contrast physical, functional, and mental health characteristics among four different cultural groups of centenarians: African American centenarians from the Health and Retirement Study, Hawaii centenarians of Japanese ancestry from the Kuakini Hawaii Centenarian Study, Japanese centenarians, and Native Americans from the Oklahoma Centenarian Study. The first study showcases longevity predictors for African Americans in the Health and Retirement Study, highlighting family longevity, individual and family resources, health behaviors, and physical, functional, and mental health. The second study utilizes centenarian data from the Kuakini Hawaii Centenarian Study, featuring their unique survivorship characteristics as participants age from 70 to 100 years. The third presentation highlights Japanese centenarians and reveals the diversity of physical and mental well-being of centenarians in Japan. Findings highlight the high bed-bound ratio among centenarians in Japan. Finally, the Oklahoma Centenarian Study identifies themes pertaining to underlying cultural influences using oral narratives of centenarian descendants of indigenous ancestry. The themes of "transmission of historical trauma," "shared collectivism," "leisure and sporting," and "educational attainment"signify themes of adaptation and survival. The four centenarian studies suggest similarities and essential differences when comparing these exceptional cultural groups of survivors. Practitioners and policymakers should be responsive to the diverse needs of centenarians who provide cultural individuality to their communities.

Abstract citation ID: igae098.0381

### UNVEILING LONGEVITY INSIGHTS: AFRICAN AMERICAN OLDER ADULTS IN THE HEALTH AND RETIREMENT STUDY

Meneka Johnson Nicholson[1], and Peter Martin[2], *1. Rural Health Medical Program, Inc., Selma, Alabama, United States*, *2. Iowa State University, Ames, Iowa, United States*

Although there is considerable longevity literature, few studies have focused on African American older adults. The current study assessed different domains of longevity predictors for African American participants in the Health and Retirement Study. A total of 723 African Americans (Mage = 68.2) who participated in wave 2 of the HRS were included in this study. Family longevity, individual and social resources, health behaviors and health were used to predict their age at death (Mage = 79.5). A blockwise multiple regression was used for the analyses. Results indicate that father's and mother's age at death did not predict longevity. Education and number of children were significant predictors when added as a second block ($\beta$ = -.24, p <.05 and $\beta$ =-.13, p <.05, respectively). Of the next block, body-mass index and smoking ($\beta$ = -.10, p <.05 and $\beta$ =-.26, p <.05, respectively), but not alcohol intake, added significantly to explaining longevity. Four specific health markers (i.e., self-rated health, high blood pressure, diabetes, and multimorbidity, $\beta$ = -.08, p <.05, $\beta$ =.30, p <.05, $\beta$ = -.26, p <.05, $\beta$ =-.29, p <.05, respectively) all added significantly to the explanation of longevity. The two final blocks (ADL functioning and level of depression), did not add significantly to the equation. On the whole, the model explained 35 percent of the variance. The results are important for African Americans, policymakers and practitioners as they can help identify risk and resilience factors of longevity.

Abstract citation ID: igae098.0382
### LONGEVITY IN THE PACIFIC: FINDINGS FROM THE KUAKINI HAWAII CENTENARIAN STUDY

Bradley Willcox[1], Kamal Masaki[1], Peter Martin[2], Gina Lee[3], Hardeep Obhi[4], and Leonard Poon[5], *1. Kuakini Medical Center, Honolulu/Kuakini Medical Center, Hawaii, United States*, *2. Iowa State University, Ames, Iowa, United States*, *3. University of Wisconsin Madison, Madison, Wisconsin, United States*, *4. University of California Davis, Davis, California, United States*, *5. University of Georgia, Athens, Georgia, United States*

The immigration of Japanese citizens to Hawaii more than one hundred years ago profoundly changed the culture of these islands. Their diet, exercise, and spirituality may have influenced the longevity of Hawaii residents. This presentation highlights longevity predictors among Hawaiian men of Japanese descent. A sample of 3,734 participants (Mage = 77.82 years at baseline) from the Kuakini Hawaii Centenarian Study was included in this research. With follow-up longitudinal data, we compared participants who survived into their 70s, their 80s, their 90s, or to 100 years of age. Results indicated that cognitive function, $F(3,3,733) = 52.59$, $p < .001$, and self-reported health, $F(3,3,733) = 18.72$, $p < .001$, at baseline distinguished those who survived to centenarian status compared to those who died in their 70s, 80s, or 90s. Centenarians were more likely to carry the protective FOXO3 allele, $\chi 2$ (N=3,352) = 8.94, $p < .05$, and less likely to be diagnosed with diabetes, $\chi 2$ (N=3,560) = 12.43, $p < .01$. Regression analyses suggested that father's age death ($\beta = .04$, $p < .05$), fluid intelligence ($\beta = .12$, $p < .001$), ADL impairment ($\beta = -.23$ $p < .001$), health behaviors, (i.e., BMI, smoking and drinking, $\beta = .55$, $p < .01$, $\beta = -.10$, $p < .001$, and $\beta = -.08$, $p < .01$, respectively), and diabetes ($\beta = -.06$, $p < .01$), were significantly associated with age at death. The results support policymakers and practitioners in promoting healthy longevity among a culturally distinct group.

Abstract citation ID: igae098.0383
### WHY CULTURAL CONTEXT IS VITAL TO STUDY LONGEVITY IN JAPAN

Yasuyuki Gondo, *Osaka University, Suita, Osaka, Japan*

Due to the worldwide expansion of the aging population, the number of centenarians has been increasing over the last decade. However, the centenarian population ratio is highly diverse among countries. The cause of diversity tends to be attributed to differences in the country's economic situation. However, cultural differences in attitudes toward aging and deference to older people are potentially influential factors. In the case of Japan, regardless of its higher centenarian ratio in the world, there is a larger bed-bound population of centenarians. This unique situation is caused by combining a universal insurance system supported by a strong economy, a traditional healthy diet, and motivation to maintain good health. However, rather than those, a conventional attitude of deference to older people would be an essential cause of longevity in Japan. In Japan, Respect for the Aged Day is a national holiday. It was enacted in 1963 based on the Act on Social Welfare for the Elderly. With this timing, many local governments start presenting cash to older people. Currently, these cultural-based policies seem to have reached a turning point. A multi-country survey reported that only 25% of Japanese people wish to live to 100, the lowest among countries. At the same time, the main reason not to wish for a long life was "dislike to be a burden to people around" by observing a more significant number of bed-bound older people daily. This negative motivation might accelerate healthy behavior and cause further longer life, ironically.

Abstract citation ID: igae098.0384
### RECONSTRUCTING CULTURAL IDENTITY: THE ORAL NARRATIVES OF CENTENARIAN DESCENDANTS OF INDIGENOUS ANCESTRY

Alex Bishop[1], Tonya Finchum[1], and Melinda Heinz[2], *1. Oklahoma State University, Stillwater, Oklahoma, United States*, *2. University of Northern Iowa, Cedar Falls, Iowa, United States*

This study examined storytelling narratives of indigenous centenarians from the Oklahoma 100 Year Life Project. Qualitative content analysis was conducted using oral history narratives of N = 16 centenarian descendants (n = 5 men; n = 11 women) of American Indian ancestry (n = 8 White/Caucasian; n = 4 African-American; n = 4 Indigenous). Of interest was identifying themes reflecting cultural influences. Analyses resulted in four thematic classifications. The first theme included "transmission of historical trauma," and involved resettlement, slavery, and race relations. One centenarian man reflected, "That's the reason they're called sooners... that use to belong to the Cherokees, and they took all the land..." A second theme involved "mutual interdependence." One centenarian woman remarked, "I never enjoyed anything if I had it and didn't share with somebody else. That's the way I've been all my life. I just love to share things with people" A third theme concerned "leisure and sporting." One centenarian man recounted training with the varsity track team, "... everybody was blowing just like a steam engine except me...I had been running all summer long out there in the buffalo grass pastures." The fourth theme entailed "lifelong learning." One centenarian woman recalled, "I organized a club... we studied Indian customs... our families and traditions... we called ourselves 'Tsa-la-gi,' which means Indian ladies." Results will further highlight differential influences of American Indian cultural heritage on socioemotional well-being in human longevity.

### SESSION 1390 (SYMPOSIUM)

Abstract citation ID: igae098.0385
### LONG-TERM IMPACTS OF A LIFESTYLE INTERVENTION IN OLDER ADULTS WITH DIABETES AND OVERWEIGHT OR OBESITY: LOOK AHEAD

Chair: Peter Huckfeldt
Co-Chair: Charles Semelka
Discussant: John Jakicic

Mid-life weight loss and maintenance is often recommended as a means to improve health later in life, however its long-term benefits have not been rigorously established.

The Action for Health in Diabetes (Look AHEAD) randomized controlled trial compared an intensive lifestyle intervention (ILI) designed to induce and maintain a 7% weight loss through caloric restriction and increased physical activity with a control condition of diabetes support and education (DSE) in 5,145 individuals aged 45-76 years with type 2 diabetes and overweight/obesity over up to 11 years of follow-up beginning in 2001. Although the ILI group had greater initial improvements in cardiovascular disease risk factors compared to DSE, there were no overall differences in the incidence of the trial's primary and secondary cardiovascular outcomes between the ILI and DSE groups. However, ILI did have a positive impact on diabetes control and complications, depression, physical health–related quality of life, and health care use and costs. Subsequently, the Look AHEAD trial transitioned to an in-person observational study in 2012 to assess the long-term benefits and potential harms of an ILI, which continued for 10 years before transitioning to phone-only observational period in 2022 – the Look AHEAD Aging study. This symposium will describe the long-term impact of the lifestyle intervention and the effect of diabetes-related risk factors on self-reported vision and hearing (Espeland and colleagues), nutritional risk (Houston and colleagues), self-reported disability in basic activities of daily living, instrumental activities of daily living, and mobility (Semelka and colleagues), and multi-morbidity (Davis and colleagues).

Abstract citation ID: igae098.0386

### LATE-LIFE DISABILITY FOLLOWING THE ACTION FOR HEALTH IN DIABETES (LOOK AHEAD) TRIAL

Charles Semelka[1], Rebecca Neiberg[1], Peter Huckfeldt[2], Kathleen Hayden[1], Haiying Chen[1], Lynne Wagenknecht[1], Mark Espeland[1], and Denise Houston[3], *1. Wake Forest University School of Medicine, Winston-Salem, North Carolina, United States*, *2. University of Minnesota School of Public Health, Minneapolis, Minnesota, United States*, *3. Wake Forest University School of Medicine, Winston Salem, North Carolina, United States*

The Look AHEAD trial intensive lifestyle intervention (ILI) was associated with better mobility in adults with type 2 diabetes and overweight/obesity. The objective of this study is to describe the post-intervention impact on disability in older adults. Disability was assessed using the Pepper Assessment Tool for Disability (PAT-D) at the first post-trial visit and three subsequent study visits during the observational phase. Disability was defined by self-reported responses 'of a lot of difficulty' or 'unable to do' on any item within the PAT-D subscales for Basic Activities of Daily Living (BADL), Instrumental Activities of Daily Living (IADL), and mobility. Regression models using generalized estimating equations were used to examine the association between intervention assignment and BMI across post-trial visits with disability prevalence, adjusting for age, sex, race/ethnicity, CVD history, and repeated measures. The mean±SD age and BMI of participants (n=1299) at the first post-trial visit was 68.7±5.6 years and 34.4±6.1 kg/m2, 61% were female, 69% were White, 8% had CVD history at baseline, and 51% were assigned to ILI. Over a median 9 years of post-trial follow-up, disability increased in BADLs from 3.1% to 12.6%, IADLs from 3.5% to 22.7%, and mobility from 21.9% to 46.7%. Participants with a higher BMI (35+ vs. <30 kg/m2) had increased odds of disability in IADLs (OR [95% CI]: 2.0 [1.5-2.8]) and mobility (2.2 [1.7-2.9]). There was no association between prior assignment to ILI and disability (p≥0.78). In conclusion, we found clinically relevant differences in late-life disability by post-trial BMI status.

Abstract citation ID: igae098.0387

### LONG-TERM ASSOCIATION OF DIABETES CONTROL WITH SELF-REPORTED VISION AND HEARING

Mark Espeland[1], Michael Walkup[1], Lynne Wagenknecht[1], Kathleen Hayden[1], Charles Semelka[1], Rena Wing[2], Tara Beckner[1], and Denise Houston[3], *1. Wake Forest University School of Medicine, Winston-Salem, North Carolina, United States*, *2. Miriam Hospital, Providence, Rhode Island, United States*, *3. Wake Forest University School of Medicine, Winston Salem, North Carolina, United States*

Older adults with type 2 diabetes are at increased risk for vision and hearing loss. We examined whether better control of glycosylated hemoglobin (HbA1c), blood pressure, and weight was associated with better self-reported vision and hearing acuity among older adults with type 2 diabetes and overweight or obesity. The Look AHEAD Aging study administered a standardized questionnaire to N=1,008 individuals, mean (SD) age 77.8 (5.7) years, who had been enrolled in the Look AHEAD trial of intensive lifestyle intervention (ILI). We examined how mean levels of HbA1c, systolic blood pressure (SBP), and body mass index (BMI) over the prior 10 years were associated with markers of vision and hearing acuity, controlling for age, gender, and diabetes duration. Hearing and vision deficits were assessed by self-report of fair, poor, or worse abilities. Multisensory deficits were defined as reported deficits for both vision and hearing. Relatively better 10-year control of HbA1c was associated with less prevalent vision deficits (OR=0.72 per IQR lower HbA1c, p=0.002) and with less prevalent multisensory deficits (OR=0.65 per IQR lower HbA1c, p=0.002). Better 10-year SBP control was also associated with less prevalent vision deficits (OR=0.72 per IQR lower SBP, p=0.005). Prior random assignment to ILI and 10-year BMI levels were not associated with self-reported vision. HbA1c, SBP, BMI, and intervention assignment were not significantly associated with hearing acuity. Long-term control of HbA1c and SBP are important to maintaining vision in older individuals with type 2 diabetes. HbA1c control may also reduce the prevalence of multisensory deficits.

Abstract citation ID: igae098.0388

### LONG-TERM EFFECTS OF AN INTENSIVE LIFESTYLE INTERVENTION ON NUTRITIONAL RISK IN OLDER ADULTS WITH DIABETES

Denise Houston[1], Andrea Anderson[2], Charles Semelka[2], Kathleen Hayden[2], Peter Huckfeldt[3], Haiying Chen[2], Lynne Wagenknecht[2], and Mark Espeland[2], *1. Wake Forest University School of Medicine, Winston Salem, North Carolina, United States*, *2. Wake Forest University School of Medicine, Winston-Salem, North Carolina, United States*, *3. University of Minnesota School of Public Health, Minneapolis, Minnesota, United States*

Malnutrition is common in older adults and is associated with morbidity, mortality and decreased quality of life. We examined the post-intervention impact of the Look AHEAD intensive lifestyle intervention (ILI) on nutritional risk – the presence of risk factors such as food insecurity, poor appetite, and low food intake which can lead to malnutrition – in older adults with type 2 diabetes and overweight/obesity in the Look AHEAD Aging study (~9 years after the intervention was terminated). Nutritional risk scores (range 0-21) were calculated from the DETERMINE checklist, a 10-item questionnaire of factors linked to poor nutrition in older age, and categorized as low (0-2), moderate (3-5) and high (6+). Multinomial logistic regression was used to examine the association between intervention assignment, post-intervention weight change, unintentional weight loss, and current BMI with nutritional risk adjusted for current age, sex, race/ethnicity, and education in 1032 participants. Forty percent of participants were at high nutritional risk and 48% at moderate nutritional risk. There was no association between prior assignment to ILI (p=0.65) or post-intervention weight loss or gain of ≥5% (p=0.09) and nutritional risk. However, unintentional weight loss of ≥10 lbs over the past year was associated with high nutritional risk (OR [95% CI]: 2.18 [1.16-4.09]) and current BMI of ≥30 kg/m2 was similarly associated with both moderate (1.62 [1.01-2.60]) and high (1.68 [1.03-2.74]) nutritional risk. In conclusion, current BMI and recent unintentional weight loss, but not past assignment to ILI or post-intervention weight loss/gain, were associated with nutritional risk.

Abstract citation ID: igae098.0389

### THE IMPACT OF AN INTENSIVE LIFESTYLE INTERVENTION IN TYPE 2 DIABETES ON LONG-TERM MULTIMORBIDITY

Ian Davis[1], Peter Huckfeldt[2], Mark Woodhouse[3], Mark Espeland[4], Lynne Wagenknecht[4], Dana Goldman[1], Marcel Salive[5], and Ann Harada[6], *1. University of Southern California, Los Angeles, California, United States*, *2. University of Minnesota School of Public Health, Minneapolis, Minnesota, United States*, *3. University of Minnesota, Minneapolis, Minnesota, United States*, *4. Wake Forest University School of Medicine, Winston-Salem, North Carolina, United States*, *5. National Institute on Aging, Baltimore, Maryland, United States*, *6. Leonard D. Schaeffer Center for Health Policy & Economics, Los Angeles, California, United States*

Multimorbidity, or the accumulation of multiple chronic diseases, is an important clinical issue in aging and is associated with geriatric syndromes, physical and cognitive function declines, increased health care costs, and poorer health-related quality of life. This study aims to better understand multimorbidity occurrence and accumulation over the postintervention period (2013-2019) of Look AHEAD, which randomized 5145 individuals aged 45-76 in 2001-2004 with type 2 diabetes and overweight/obesity to an intensive lifestyle intervention (ILI) or a control arm receiving diabetes support and education (DSE). The ILI was previously found to reduce weight, improve diabetes control, and reduce diabetes complications. The ILI was also associated with relative improvements in self-reported multimorbidity during the intervention period. We included 2940 participants consenting to linkage of their trial data with Medicare data, focusing on participant years with Medicare fee-for-service coverage during the postintervention period. We identified conditions from the list developed by the "Department of Health and Human Services Initiative on 21 Multiple Chronic Conditions (MCC)". We estimated ILI versus control differences in numbers of conditions using linear models that control for baseline demographic and clinical characteristics and study site. We found similar levels of multimorbidity for the ILI and DSE groups (4.21 versus 4.31, p=0.41). This result suggests that the positive impacts of the ILI on diabetes control did not translate to long-term reductions in multimorbidity.

### SESSION 1395 (SYMPOSIUM)

Abstract citation ID: igae098.0390

### NAVIGATING PAYMENT, POLICY, AND HEALTH SYSTEMS: HOW THE ORGANIZATION OF PAID CARE AFFECTS WORKERS AND FAMILIES

Chair: Emily Franzosa

The complex structure of paid personal and home care services can cause confusion and frustration for families as they navigate multiple payers and providers to get the care they need. At the same time, the fragmented industry structure, combined with low reimbursement for paid care services, leads to poor quality jobs and high workforce turnover for paid caregivers themselves. While the COVID-19 pandemic laid bare existing fault lines in paid care delivery, it also suggested promising avenues for intervention. This symposium explores organizational factors that may improve stability of the paid care workforce. First, we explore the impact of wages. Miller et al describe the impact of state policies such as hazard pay in stabilizing the workforce shortage during COVID-19, while Jiyeon Kim's innovative analysis examines whether holding multiple direct care jobs, which is common given the often part-time nature of paid care work, can lift workers out of poverty. Next, Patricia Kim and colleagues use integrated electronic medical record and interview data to explore the organization of paid care at end of life through the experience of patients receiving hospice care during the pandemic. Finally, Walkner et al share a promising pilot in the Veterans Health Administration to integrate paid caregivers into health systems by hiring them directly into health care teams. Together, this research highlights innovative avenues to improve pay, job stability and other key measures that may stabilize the workforce and reduce complexity in the paid care system.

Abstract citation ID: igae098.0391

### DIRECT CARE WORKER RETENTION DURING THE COVID-19 PANDEMIC AND THE IMPACT OF STATE POLICIES

Katherine Miller, Yang Yang, and Karen Shen, *Johns Hopkins University, Baltimore, Maryland, United States*

Due to persistently low wages and poor working conditions, direct care worker shortages and high turnover existed in long-term care (LTC) industries prior to COVID-19, but became more visible during the pandemic. Some states responded to the threat of a large exodus of workers through wage-support policies (e.g., hazard pay). However, little research has examined whether these state policies were effective in mitigating the effects of the pandemic on direct care worker exits. We use the Annual Social and Economic Supplement of the Current Population Survey 2016-2022 to decompose direct care worker exits based on where they exited to: (1) unemployment or exiting the labor force, (2) a different healthcare job or industry, and (3) non-healthcare job (e.g., retail). We compare the rates of these components for direct care workers pre-pandemic (2016-2019) and during the pandemic (examining 2020 and 2021-2022 separately), and the association of these rates with state wage-support policies. Direct care worker exits were highest in 2020, with workers primarily becoming unemployed or leaving the labor force. Yet, in 2021-2022, exits decreased relative to pre-pandemic periods, primarily because fewer people switched jobs. Among those who did switch jobs, we find that workers are more likely to stay in a healthcare industry in 2021 relative to 2016-2019. We find no evidence wage support policies were associated with direct care worker retention/exits during or post-pandemic. Together, our findings suggest that broader economic trends of a shrinking workforce may be the primary driver of direct care worker exits during the pandemic.

Abstract citation ID: igae098.0392

### THE EFFECT OF SECONDARY JOBHOLDING ON INCOME MOBILITY OF DIRECT CARE WORKERS

Jiyeon Kim, *PHI, New York, New York, United States*

Recent studies show that many direct care workers acquire secondary jobs because their primary employment does not offer sufficient wages or work hours. While prior research has emphasized how secondary jobs protect direct care workers from poverty, it is unclear how these jobs affect these workers' subsequent employment outcomes. This paper analyzes longitudinal data to investigate whether secondary jobs can serve as a pathway for direct care workers to obtain a primary job that provides income above the poverty-line and test if some secondary occupations are more beneficial than others. I find that whereas a secondary job in a non-direct care occupation helps direct care workers later attain a primary job that pays above poverty-level, working in two direct care jobs restrains them from doing so. In addition, these effects vary by race and gender such that the benefits to holding a non-direct care secondary job are greater among men and whites, compared to their women and racial minority counterparts. These findings raise concerns about unequal opportunities for wage mobility among direct care workers and the implications this has for the retention of this workforce.

Abstract citation ID: igae098.0393

### NAVIGATING PAID CAREGIVING IN HOSPICE DURING COVID-19: AN INTEGRATED MULTIPLE METHODS ANALYSIS

Patricia Kim[1], Emily Franzosa[2], Jonelle Boafo[1], Margaret McDonald[3], Laura Moreines[4], Daniel David[5], and Melissa Aldridge[1], *1. Icahn School of Medicine at Mount Sinai, New York, New York, United States*, *2. James J Peters VA Medical Center, Bronx, New York, United States*, *3. Visiting Nurse Service of New York, New York, New York, United States*, *4. New York University, New York, New York, United States*, *5. NYU College of Nursing, New York, New York, United States*

Paid caregivers are integral members of the end-of-life care team, but navigating paid care services is complex. Families must often work across multiple providers including hospice, home health, private pay, and government to coordinate the care they need to help patients die at home. We examined experiences with end-of-life paid caregiving among 61 patients in a large, New York City health system during the first year of the COVID-19 pandemic. We analyzed care trajectories of patients who enrolled in hospice and died between March 1, 2020-March 31, 2021, integrating data from real-time linked medical and hospice electronic health records with reflective interviews with 30 interdisciplinary team members at 3 organizations providing end-of-life care during the pandemic. We found: 1) Paid caregivers were the "eyes and ears" of the hospice team and paid caregivers' work helped families keep patients at home; 2) Patients' increasing end-of-life needs required additional paid caregivers, heightening patients' infection risk; 3) Conflicting organizational policies across providers and payers, such as screening, vaccination, and visitation restrictions, disrupted aide care; and 4) Scope of practice restrictions for some types of paid caregivers reduced their ability to help. Our findings highlight both the essential support role of paid caregiving in end-of-life care, and regulatory/policy burdens that may reduce the benefit of paid caregiving at this critical juncture. Supporting stability and continuity in caregiving teams and building more flexibility into paid caregivers' scope of practice and patients' care plans may assist aides and families in managing end-of-life care.

Abstract citation ID: igae098.0394

### CHALLENGES AND FACILITATORS TO IMPLEMENTING A PILOT HOME HEALTH PROGRAM IN THE VETERANS HEALTH ADMINISTRATION

Tammy Walkner[1], Aaron Seaman[2], Mary Good[1], Heather Benzel[3], Irene San Roman[1], and Heather Davila[1], *1. Iowa City VA Health Care System, Iowa City, Iowa, United States*, *2. Veterans Rural Health Resource Center, Iowa City, Iowa, United States*, *3. VA Midwest Healthcare Network (VISN 23), St. Paul, Nebraska, United States*

The Veterans Health Administration (VA) provides access to skilled home health (e.g. rehabilitation therapy, nursing) and home health aide services to >200,000 Veterans annually to support aging in place. Historically, these services have been provided by community-based agencies contracted with VA. In 2021, the Iowa City VA implemented the VA's first "VA Home Health" (VAHH) program. The goals were to increase Veterans' access to home health and improve care coordination. Given early evidence of the program's success, VAHH expanded in 2022 to three additional sites in Iowa and South Dakota. Our team conducted semi-structured interviews with VAHH leaders (n=13) and frontline staff (interviews in progress) at the 4 program sites about their implementation experiences. Implementation barriers include: a steep learning curve; the need to define roles and create operating procedures; recruiting and hiring sufficient staff to administer and deliver the program; and challenges obtaining space and vehicles for staff. Facilitators include: staff commitment to the program; existing relationships within and outside VA; and flexibility to tailor VAHH to meet site needs. Despite similarities across sites, there were also implementation differences, primarily related to recruiting staff, hiring processes, and needs of Veterans targeted for the program (e.g. social/medical complexity). Continued evaluation is part of the implementation process, and our team will be interviewing primary care staff next to gain their perspectives. Our findings provide insights into developing and implementing a promising home health program with lessons for VA sites that may also serve as a guide for other large healthcare organizations.

### SESSION 1400 (SYMPOSIUM)

Abstract citation ID: igae098.0395

### PHYSICAL AND MENTAL HEALTH NEEDS OF OLDER ADULTS EXPERIENCING HOMELESSNESS: CHALLENGES AND INNOVATIONS IN CARE

Chair: Anita Souza
Discussant: Jenny Tsai

Adults over the age of 50 are the largest growing demographic of individuals experiencing homelessness. They enter a continuum of housing services with a myriad of chronic and acute health conditions that have often been unaddressed due to sporadic and episodic care utilization primarily within emergency services. The compounding health impacts of chronic homelessness accelerate aging within this demographic. Housing services offer an opportunity to address unmitigated health issues. Housing services are seeking research and programmatic interventions to meet this need. This symposium will explore the physical and mental health needs of older adults experiencing homelessness across the continuum of housing services from shelter entry to permanent supportive housing though a variety of research methodologies. The first presenter (Souza) will present results of a study applying latent class analysis to administrative shelter services data to identify unobservable subgroups within the population of older adults living in shelter. The second presenter (Berg) will discuss the implementation of shelter-based care services to older adults with complex medical needs in a low barrier, harm reduction focused hotel shelter. The third presenter (Robinson) will describe a qualitative investigation examining barriers to mental health care for older adults in permanent supportive housing. The discussant (Tsai) will lead symposium attendees in a discussion of future considerations for research, policy, and practice to meet the needs of this growing demographic of older adults.

Abstract citation ID: igae098.0396

### A LATENT CLASS APPROACH TO UNDERSTANDING HOMELESSNESS AMONG OLDER ADULTS

Anita Souza, Jenny Tsai, and Kenneth Pike, *University of Washington, Seattle, Washington, United States*

The complex characteristics that differentiate adults over 50 experiencing homelessness are understudied. Latent class analysis (LCA) is a novel approach to identifying unobservable subgroups within populations. Housing providers have little information to adopt tailored service approaches. Capitalizing on the strength of LCA, this paper identifies the heterogeneity within older adults utilizing housing. Using a retrospective cohort study design, we extracted data from the Homeless Management Information System to describe the health conditions of adults utilizing services of a leading housing provider in Washington. These data captured developmental disability, physical health, disabling conditions, chronic conditions, mental health problems, and substance use. Among 1,971 homeless individuals, 76% were male, 49% white, and 70% homeless 6 months or more. Forty percent were 50 years or older; 17% were veterans. Probability calculated by LCA found that a 4-class solution best represented the data (fit indices: AIC=11,688, BIC=11839, entropy=0.85). Class 1 (n=519) reported higher than average proportions of mental health problems and substance abuse along with average proportions of members with a chronic health condition and slightly higher than average proportion of members with a disabling condition. Class 2 (n=254) was dominated by individuals with a disabling condition and physical disability. Class 3 (n=827) reported the lowest proportion of members with all conditions. Class 4 (n=371) was higher than average on all conditions. This knowledge builds a foundation for further research and policy endeavors to support older adults experiencing homelessness.

Abstract citation ID: igae098.0397

### EXAMINING AND ADDRESSING BARRIERS TO MENTAL HEALTH CARE FOR OLDER ADULTS IN PERMANENT SUPPORTIVE HOUSING

Randee Robinson, *University of Washington, Seattle, Washington, United States*

Permanent supportive housing is an evidence-based intervention to support individuals exiting from chronic homelessness. Increasingly, the demographic of adults prioritized for PSH are over the age of 50. Despite the higher prevalence of mental health concerns amongst older PSH residents their engagement with mental health services remains limited. The purpose of this investigation was to examine barriers to mental health care for older adults living in PSH and provide suggestions to address the identified barriers. A one-hour semi-structured focus group protocol was developed. Question sets focused on individual and system level barriers to mental health services. The focus group was digitally recorded, and responses were transcribed. Eight PSH residents from one housing provider participated in the focus group session. Thematic analysis utilizing inductive and deductive approaches was completed by two individuals. Themes of case manager role and limitations, stigma, service navigation, and trustworthiness emerged from the data. The mean age of participants was 63.8 years (SD 9.78). The mean length of tenancy was 6.6 years (SD 3.29). Addressing mental health access is critically important for older adults in PSH. Interventions to improve mental health care access at both the individual and systems level should target building trust, reinforcing available services, clarifying the care manager's role, and supporting staff consistency. Future research should target effective interventions to address these barriers.

Abstract citation ID: igae098.0398

### HEALTH CARE UTILIZATION OF AGING INDIVIDUALS EXPERIENCING CHRONIC HOMELESSNESS: A CASE STUDY APPROACH

Russell Berg, *Harborview Medical Center UW Medicine, Seattle, Washington, United States*

The population of individuals experiencing homelessness in the United States is both growing and aging. Late baby boomers are overrepresented amongst those experiencing chronic homelessness and this cohort is now of geriatric age. Many challenges interfere with providing medical care to this group including mental health, substance use disorders, carceral history, lack of surrogate decision makers and dementia. In some cities, the COVID 19 pandemic led to transition of congregate emergency homeless shelters to hotels, providing an opportunity to co-localize enhanced services such as embedded medical services. This session will present three medical cases from a low barrier, harm reduction focused hotel shelter for chronically homeless individuals. The cases focus on selected individuals with high hospital utilization and demonstrate how the hotel shelter model with embedded medical clinic can function as a "medical home" reducing unnecessary service utilization while pursuing placement for those requiring a higher level of care. While residing at the shelter, important medical conditions such as dementia and Parkinson's disease can be diagnosed that qualify residents for additional benefits. For some patients with severely limited life expectancy, the hotel shelter space can provide a setting for patient centered palliative care and even home hospice services. The challenges to providing care for an aging population experiencing homelessness require novel models for the provision of health care and hotel shelters with embedded medical care can fill the role of "medical home".

### SESSION 1405 (PAPER)

### PSYCHOSOCIAL AND BEHAVIORAL INFLUENCES ON COGNITIVE HEALTH

Abstract citation ID: igae098.0399

### PAIN AND MEMORY LANE: SEX-SPECIFIC INSIGHTS INTO STRESS REACTIVITY, PAIN, AND COGNITION IN MID- TO LATE LIFE

Ashley Curtis[1], Amy Costa[1], Melanie Stearns[1], Kevin McGovney[2], Mary Beth Miller[2], and Christina McCrae[1], *1. University of South Florida, Tampa, Florida, United States*, 2. *University of Missouri, Columbia, Missouri, United States*

Pain and cognition are bidirectionally associated. Perceived stress reactivity is linked to pain and cognitive perception, but interactive relationships are unclear. Sex differences are established in all factors (pain/stress reactivity/cognition), but studies in aging and chronic pain populations are limited. This study examined sex differences in interactive associations of pain and stress reactivity on memory in middle-aged/older adults with chronic pain. Cognitively healthy aging adults (N=678; 52%-women; Mage=62.4±11.3) experiencing frequent pain in past 3 months completed assessments of pain intensity (average past week), stress reactivity (Perceived Stress Reactivity Scale), and memory (Everyday Memory Questionnaire). Multiple regressions evaluated interactions between sex, pain, and stress reactivity on memory, controlling for age, education, #medical conditions, sleep/pain medication, and depression. Sex interacted with pain in associations between stress reactivity and memory (R-squared=.005, p=.04). Greater stress reactivity was associated with worse memory for women with lowest pain (~22/100; B=.59±.24, p=.01), both women (B=.63±.18, p<.001) and men (B=.76±.20, p<.001) with intermediate pain (~48/100), and both women (B=.67±.23, p=.003) and men (B=1.30±.28, p<.001) with highest pain (~73/100). Findings suggest relatively consistent associations between stress reactivity and memory across pain levels in aging women. In aging men, stress reactivity impacts memory at moderate pain levels, but this is exacerbated (two-fold) as pain increases. Sex-specific tailoring of behavioral pain treatments is encouraged. Techniques to reduce stress reactivity (cognitive control, mindfulness) may benefit memory. Prospective studies and objective assessments of stress reactivity, pain, cognition, and circulating sex hormones are needed to understand sex-specific modifiable risk factors of cognitive decline.

Abstract citation ID: igae098.0400

### THE COMBINED EFFECTS OF SMOKING, DRINKING, AND PHYSICAL ACTIVITIES ON THE COGNITIVE HEALTH OF OLDER ADULTS

Yan-Jhu Su, Elizabeth Dugan, and Jaqueline Avila, *University of Massachusetts Boston, Boston, Massachusetts, United States*

**Background:** Poor health behaviors such as smoking tobacco, alcohol consumption, and limited physical activity may be associated with poor cognitive health. Unhealthy behaviors often co-occur, and little research has examined the combined or cumulative effects of unhealthy behaviors on cognitive health. This examines how common health behaviors may co-occur and their associations with cognitive health among older U.S. adults.

**Methods:** This cross-sectional study used 2018's Health and Retirement Study (HRS) data, including 7,467 respondents aged 65 and older. Cognitive function scores were assessed with the Telephone Interview of Cognitive Status-Modified. Health behavior combinations were assessed, including smoking status, drinking amount, and frequency of physical activities. Linear regression was used to examine the association between different health behavior combinations and cognitive function.

**Results:** The most common combinations were healthy ones: never smoking, no drinking, and high-level physical activity (15.47%; reference group). All combinations with a prevalence of less than 2% were combined as "other" combinations. Compared to the reference group, respondents who never smoked, had no drinking, and had no physical activities had lower cognitive function (b = -1.13, 95% CI: -1.56, -0.71). Other unhealthy combinations that impacted cognition were never-smoked, had no drink, and low physical activities; and former smokers had no drink and low physical activities.

**Conclusions:** This cross-sectional study found that high levels of physical activity and some drinking are associated with better cognitive health, while smoking was associated with poorer cognitive health. Policies to promote physical activity across the lifespan may be promising avenues to pursue.

Abstract citation ID: igae098.0401

### HELPING BEHAVIORS AND COGNITIVE FUNCTIONING IN LATER LIFE: A FOCUS ON DOSE AND ASYMMETRIC EFFECTS

Sae Hwang Han[1], Jeffrey Burr[2], and Julia Tucker[1], *1. University of Texas at Austin, Austin, Texas, United States*, *2. University of Massachusetts Boston, Boston, Massachusetts, United States*,

Despite the burgeoning literature linking prosocial helping behaviors and cognitive functioning, empirical evidence indicating whether and how much ‘prescribing’ helping behaviors to older adults would confer cognitive benefits is limited. Further, earlier studies mostly focused on formal volunteering, while little is known about the cognitive benefits of informal helping given directly to non-household individuals. This study aims to bridge these gaps by identifying the cognitive benefits of transitioning into a formal volunteer and informal helper role, respectively, and to uncover the optimal dose-response parameters linking helping and cognition. Using longitudinal data from the Health and Retirement Study (1998–2020), we followed over 30,000 individuals aged 51 and older during the observation period spanning two decades. Cognitive functioning was assessed with the modified Telephone Interview for Cognitive Status (m-TICS; range: 0–27). Two forms of helping behaviors (i.e., formal volunteering and informal helping) were considered as status and time commitment. We used a multilevel modeling approach that allowed for estimating within–person effects specific to transitioning into (and out of) the respective helper role, as well as across different dosages of helping. Results indicated that transitioning into formal volunteering and informal helping were both associated with better cognitive functioning and slower cognitive decline. Further, moderate levels of helping (approximately 2–4 weekly hours) were consistently linked to robust cognitive benefits for both forms of helping. The study findings provide unique evidence supporting the view that social engagement through helping others in need represents modifiable lifestyle factors conducive to healthy cognitive aging.

Abstract citation ID: igae098.0402

### THE IMPACT OF CHILDHOOD READING ENVIRONMENTS ON COGNITIVE HEALTH IN LATER LIFE AMONG OLDER EUROPEANS

Haosen Sun[1], and Yueming Xi[2], *1. University of Nevada, Reno, Reno, Nevada, United States*, *2. McMaster Univeristy, Hamilton, Ontario, Canada*

While educational achievement helps to accumulate cognitive reserve and promote cognitive function in later life, the influence of the early living environment before completing formal education remains unclear. This study aims to investigate how childhood reading environments, alongside educational achievements, jointly contribute to cognitive health in older adulthood. Analyzing life history data from 123,199 older Europeans aged 50 and above, sourced from the Survey of Health, Ageing and Retirement in Europe, this research examines the interplay between childhood reading environments and educational achievement and their joint effect on cognitive health at older ages. Having more books in the household at age 10 is positively associated with higher cognitive functioning in later life, independent of educational achievements and other childhood socioeconomic status (SES) factors like household size and number of rooms. Additionally, access to books in childhood partially alleviates the cognitive disparities associated with educational achievement. These findings indicate that a rich reading environment at home in childhood offers enduring benefits for cognitive health, which is particularly valuable for individuals who ended up with lower educational achievements. This underscores the potential of interventions focused on enhancing early reading environments to promote long-term cognitive resilience.

Abstract citation ID: igae098.0403

### CONFLICT MONITORING AND INHIBITORY CONTROL IN OLDER ADULTS WITH AGE-RELATED HEARING LOSS

Shraddha Shende[1], and Raksha Mudar[2], *1. Illinois State University, Normal, Illinois, United States*, *2. University of Illinois, Champaign, Illinois, United States*

Various processes of cognitive control, including conflict monitoring and inhibitory control, are critical for listening in complex environments (e.g., speech-in-noise [SiN] recognition). However, few have examined both conflict monitoring and inhibitory control in individuals with age-related hearing loss (ARHL) despite their known challenges in SiN recognition. In 16 individuals with mild ARHL and 23 normal-hearing controls, we examined conflict monitoring using two picture-word interference tasks, “Animals” and “Objects” tasks; inhibitory control in two Go/NoGo tasks, “Single-Car” and “Object-Animal” task; and SiN using QuickSiN test. The picture-word interference tasks consisted of pictures of animals/objects that were superimposed with labels that either matched (matched condition) or did not match the pictures (unmatched condition). Participants responded by pushing the “matched” or “unmatched” button. Reaction time and accuracy were recorded. The Go/NoGo tasks consisted of pictures of cars/dogs in the Single-Car task and objects/animals in the Object-Animal task. Participants responded by pushing a button to some trials, the Go trials (cars/objects), and withholding button push to others, NoGo trials (dog/animals). Reaction time for Go trials and accuracy for Go/NoGo trials were recorded. Relative to controls, ARHL group had worse accuracy on unmatched condition of “Objects” task ($p = .006$) and Go trials of “Object-Animal” task ($p = .018$). Partial correlations revealed that worse SiN recognition was associated with worse accuracy on Go trials of the “Object-Animal” task, and no other correlations were significant. Our findings reveal alterations in both conflict monitoring and inhibitory control in mild ARHL, with inhibitory control linked to SiN recognition.

### SESSION 1410 (SYMPOSIUM)

Abstract citation ID: igae098.0404

### RECRUITMENT OF OLDER ADULTS FOR CLINICAL RESEARCH: STRATEGIES, COSTS, AND REPRESENTATION

Co-Chair: Barbara Nicklas
Co-Chair: Paul Coen
Discussant: Jay Magaziner

Clinical research is crucial for identifying new preventative strategies and treatments to address the diverse health care needs of older adults. A necessary component of conducting ethical and unbiased research is to successfully enroll enough study participants who represent the population being studied in a cost-effective and timely manner. Despite the NIH policy mandating that investigators include participants of all ages or specify an acceptable reason for excluding participants based on age, many studies still lack appropriate representation of older adults. Furthermore, an important objective of the NIA's Strategic Directions for Research for 2020-2025 is to "Develop and implement strategies to increase inclusion of underrepresented populations in aging research". To implement appropriate strategies for enhancing participation of historically excluded subpopulations, additional data are needed regarding the degree of underrepresentation of these individuals in the research being conducted and reasons for lack of inclusion. This symposium includes three presentations regarding recruitment of older adults for clinical research. These include a compiled analyses of studies conducted over the past 20 years at the Wake Forest OAIC, and data on recruiting older adults for the national Molecular Transducers of Physical Activity Consortium and the two-site observational Study of Muscle, Mobility and Aging. The data presented in the first two talks provide objective observations of the most effective strategies, and the costs, for recruiting older adults. The third presentation compares basic demographics of those enrolled to those who show interest and are screened for participation, and compared to the characteristics of the local population.

Abstract citation ID: igae098.0405

### THE STUDY OF MUSCLE, MOBILITY AND AGING (SOMMA): LESSONS LEARNED RECRUITING OLDER ADULTS

Kimberly Kennedy[1], Stephen Kritchevsky[2], Barbara Nicklas[1], Anne Newman[3], Nancy Glynn[4], Elsa Strotmeyer[5], and Michelle Danielson[5], *1. Wake Forest University School of Medicine, Winston-Salem, North Carolina, United States, 2. Wake Forest University School of Medicine, Wake Forest University School of Medicine, North Carolina, United States, 3. University of Pittsburgh, University of Pittsburgh, Pennsylvania, United States, 4. University of Pittsburgh School of Public Health, Pittsburgh, Pennsylvania, United States, 5. University of Pittsburgh, Pittsburgh, Pennsylvania, United States*

SOMMA is a two-site (Wake Forest University School of Medicine [Wake] and University of Pittsburgh [Pitt]) ongoing observational study of 879 participants ≥70 years investigating the role of muscle biology in age-related mobility decline. Those eligible were free from any life-threatening disease, able to walk ≥0.6 m/s (4m) and complete a 400m walk, and safely undergo muscle tissue biopsies and magnetic resonance imaging. Recruitment was from April 2018 to December 2021 and, despite partially spanning the COVID-19 pandemic, exceeded its goal of 875 (Total enrollment: Wake=440; Pitt=439). SOMMA phone screened 4225 persons (Wake=1887; Pitt=2338) with 33.1% (n=1398) eligible for further screening. Advertisement strategies for SOMMA were evaluated and direct mail postcards were most successful and cost-effective; 75% of those phone screened were from direct mailings. At Wake, advertising costs totaled $52k or ~$28/phone screen and $118/enrolled participant. At Pitt, advertising costs totaled $41k or ~$17/phone screen and $93/enrolled participant. Participants eligible after phone screening were invited to attend an in-person information session in which the comprehensive multi-day exam and detailed consent form were reviewed and 4m gait speed was assessed. Sessions were held virtually during pandemic, at which point the gait speed screening was discontinued. A total of 210 information sessions took place (Wake=90; Pitt=120), with an average attendance of ~15 individuals/session and ~80% of those who attended the session were eligible and 63% were ultimately enrolled. SOMMA demonstrated the successful recruitment of a large older adult cohort study for an exam-intensive protocol which included tissue sampling and MR measures.

Abstract citation ID: igae098.0406

### SUCCESSFUL APPROACHES TO ENHANCING OLDER ADULT REPRESENTATION IN MOTRPAC TRIAL

Sara Espinoza[1], Paul Coen[2], Jennifer Talton[3], Joseph Houmard[4], Kerrie Moreau[5], and Barbara Nicklas[6], *1. Cedars-Sinai Medical Center, Los Angeles, California, United States, 2. AdventHealth, Orlando, Florida, United States, 3. Wake Forest University, Winston-Salem, North Carolina, United States, 4. East Carolina University, Greenville, North Carolina, United States, 5. Colorado University, Aurora, Colorado, United States, 6. Wake Forest University School of Medicine, Winston-Salem, North Carolina, United States*

NIH mandates that clinical research include participants of all ages (or specify a reason for excluding based on age); yet, older adults (OAs) are often underrepresented even in studies without an age exclusion. Barriers to their inclusion are multi-factorial and include lack of recruitment strategies targeted to this demographic and eligibility criteria associated with age that reduce representation. Supported by multiple NIH Institutes, including NIA, the purpose of MoTrPAC is to characterize the molecular changes that underlie the benefits of exercise in humans. The goal is to randomize approximately 1980 sedentary, healthy participants (across 10 sites) to a 12-week control group or a supervised, moderate-intensity resistance or aerobic exercise intervention. The target is to balance enrollment across three groups (18-39, 40-59, and 60+ years). Recruitment is ongoing; as of 3/1/24, the number randomized is n=1043 with 20.6% OAs, compared to 40.5% younger and 38.9% middle-aged. Recruitment is monitored regularly, and early in recruitment a large age differential in eligibility yields was discovered, with only 4.5% of screened OAs being randomized, compared to 13.8% of middle-aged and 18.3% of younger individuals. Since the use of lipid-lowering medication was the single largest reason OAs did not qualify (36% of exclusions involved statin use in those >60 yrs), the protocol was altered to drop this exclusion to enhance OA representation. Also, successful recruitment marketing approaches are tracked closely and vary across age groups. This presentation will discuss the strategies which provide the most success for attracting OAs to inquire about the study.

Abstract citation ID: igae098.0407

**ARE UNDERSERVED GROUPS INCLUDED IN AGING RESEARCH? RESULTS ACROSS MANY CLINICAL RESEARCH STUDIES**

Rebecca Neiberg[1], Xiaoyan Leng[1], Denise Houston[2], Michael Miller[1], Kristen Beavers[1], W. Jack Rejeski[3], Stephen Kritchevsky[4], and Barbara Nicklas[1], *1. Wake Forest University School of Medicine, Winston-Salem, North Carolina, United States*, *2. Wake Forest University School of Medicine, Winston Salem, North Carolina, United States*, *3. Wake Forest University, Winston-Salem, North Carolina, United States*, *4. Wake Forest University School of Medicine, Wake Forest University School of Medicine, North Carolina, United States*

Research can be inherently biased when results cannot be generalized across different subsets of a population that differ from those enrolled in the study. Thus, it is critical to recruit a diverse range of individuals who represent the entire population and who represent the disease or health condition being studied. The purpose of this study was to examine the demographics of 3,454 individuals enrolled in 11 clinical studies conducted from 2002-2023 and supported by the Wake Forest Older Americans Independence Center. Studies included recruited >80 participants and had a minimum age eligibility of 55 years with no upper age exclusion. The total number of individuals phone screened for participation was 19,937. The characteristics of those enrolled were compared to those who were initially screened for participation to assess whether eligibility criteria enabled participants to approximately match the characteristics of those who inquired about participation. The demographics of the screened participants were: Age (mean±SD), 69.8±7.0 yrs; 69% female; 76.4% non-Hispanic white/23.6% black; and 79.3% with at least some college education. The enrolled participants were: Age, 69.8±6.6 yrs; 64% female; 76.1% non-Hispanic white/23.9% black; and 80.5% with some college education. The age distribution of those screened ranged from 23-98 years with 43.7% being >70 yrs, 19.9%> 75 yrs, and 6.8%> 80 yrs while the distribution of those enrolled ranged from 55-92 years with 45.2% being >70 yrs, 20.7%> 75 yrs, and 7.6%> 80 yrs. Further analyses will compare these demographics to those of the 5 counties surrounding the research center.

### SESSION 1415 (SYMPOSIUM)

Abstract citation ID: igae098.0408

**REIMAGINING WORK: TAPPING INTO THE STRENGTHS OF OLDER ADULTS**

Chair: Kathryn Nearing
Co-Chair: Katy Terveer
Discussant: Ronica Rooks

This session will focus on inspiring and informing the audience about how we can reimagine the way we work in order to benefit from the experience, skills, and wisdom of older workers. We will explore the modern trends that are enabling us to work longer and the history of ageism in the workplace. We will help aging advocates to understand and promote the benefits of working longer from an individual, employer, and societal level. Finally, we will look at some of the strategies that organizations can leverage in order to recruit and retain older workers as well as leverage the full potential of an age-diverse workforce.

Abstract citation ID: igae098.0409

**PROMOTING AND LEVERAGING THE FULL POTENTIAL OF AN AGE-INCLUSIVE WORKFORCE**

Katy Terveer, *Age Wave, Orinda, California, United States*

This session will focus on inspiring and informing the audience about how we can reimagine the way we work in order to benefit from the experience, skills, and wisdom of older workers. This session will elaborate on the Harvard Business Review article, “Redesigning Retirement: It’s time for a new deal between employers and older workers” written by the speaker (Katy Terveer) and her co-authors, Ken Dychtwald, PhD, and Bob Morison. We will explore the modern trends that are enabling longer workspans (i.e. the average number of years people spend in the workforce), such as the shift towards knowledge-based and service-based work and greater work flexibility. We will also explore the history of work and aging with an eye towards how ageism is holding us back in today’s workforce and what ageist myths we need to challenge. We will help aging advocates to understand and promote the benefits of increasing our workspans for individuals, employer organizations, and society. Finally, we will look at five key strategies that organizations can leverage to recruit and retain older workers as well as leverage the full potential of an age-inclusive workforce.

Abstract citation ID: igae098.0410

**ADVOCATING FOR AN AGE-INCLUSIVE WORKFORCE AND WORKSPACES: THE COLORADO EXPERIENCE**

Jodi Waterhouse, *CU Anschutz Multidisciplinary Center on Aging, Aurora, Colorado, United States*

This session will focus on the advocacy work that is required at the state level to ensure that older adult worker rights are protected, both as they are looking for meaningful work with a livable wage or once in the workplace. We will describe a step-by-step process to advocate for and successfully implement policies to promote an age-inclusive workforce in Colorado – a state with one of the fastest-growing older adult populations and one of the most robust age-friendly community movements in the US. To help drive change within our state, we started by building community consortiums focused on education, advocacy, and sponsoring legislation to address identified workforce needs and opportunities. We will highlight the importance of a comprehensive needs assessment process in which community consortiums engaged as the basis for their advocacy work. Examples of consortiums, created by the Colorado Governor’s Office, will be shared as a model for symposium participants to create similar working groups to engage key constituents in their own states. We will also share our experience of crafting bills and seeing them through the legislative process, highlighting bills that we have sponsored and that have been passed in Colorado. Attendees will leave this session with more insights about how to work with their state's elected officials to drive more support for an age-inclusive workforce and workplaces.

Abstract citation ID: igae098.0411

### OLDER ADULTS PURSUE ENCORE CAREERS IN RESEARCH TO PROMOTE INCLUSION OF OLDER ADULT PEERS IN CLINICAL TRIALS

Kathryn Nearing[1], Leslie Wright[2], Christin Williamson[3], and Cory Sedey-Seitz[4], *1. University of Colorado Anschutz Medical Campus, Denver, Colorado, United States*, *2. Kaiser Permanente Colorado, Aurora, Colorado, United States*, *3. Clinical Research Administration, Office of Regulatory Compliance, Aurora, Colorado, United States*, *4. Clinical Research Support Center, University of Colorado Anschutz Medical Campus, Aurora, Colorado, United States*

We created an innovative pathway to encore careers in research for older adults at the University of Colorado Anschutz Medical Campus. Our program trains older adults from the community (average age 68; range: 56-82) for specialized community-facing and participant-centered roles on clinical trial teams to increase the recruitment and retention of older adult peers, as well as other underrepresented populations, in clinical trials. Older Adult Research Specialists' roles include: 1) developing effective communication and outreach strategies to engage older adults from diverse backgrounds in research; 2) recruiting and consenting participants for trials; 3) supporting retention by identifying barriers to study participation and connecting study participants with needed resources to address social needs; 4) educating research teams regarding key considerations for including older adults in research, anchored by the Geriatric 5Ms, to inform trial design and implementation; and, 5) catalyzing research innovations to increase representation in, and the relevance and translational potential of, research. OARS prepare for their essential roles on clinical trial teams by completing a 14-week, 140-hour, virtual, competency-based training program. Health Navigation training is the foundation. To date, we have trained four cohorts (43 graduates). OARS are making the research workforce and associated teams more age-inclusive in the Division of Geriatric Medicine, Department of Neurology, Department of Physical Medicine and Rehabilitation Sciences, Center for Inclusive Design and Engineering, and at our healthcare affiliate, the Rocky Mountain Regional VA Medical Center. By promoting intergenerational learning, encore careers and research innovation, OARS are advancing our priorities as an Age-Friendly University.

Abstract citation ID: igae098.0412

### SHAPING, AND BEING SHAPED BY, POLICIES AND PROGRAMS THAT PROMOTE AN AGE-INCLUSIVE WORKFORCE: A PERSONAL JOURNEY

James Westcott, *University of Colorado Anschutz Medical Campus, Aurora, Colorado, United States*

In this session, I will share my personal journey as someone who has both shaped and been shaped by the innovative age-inclusive workforce policies and programming in Colorado. I will describe my state-wide leadership role as part of the Colorado Older Worker Policy Collaborative successfully advocating for the elimination of graduation dates and other age-identifying sections of job applications, which disadvantaged older adults in the job application process. I will also share my experience as an Older Adult Research Specialist who completed a novel pathway to encore careers in research program at the University of Colorado Anschutz Medical Campus. I will share how I apply my dual training as a health navigator and research navigator to recruit and retain older Veterans in clinical trials for the VA Eastern Colorado Geriatric Research Education and Clinical Center – one of 20 centers of excellence focused on aging in the Department of Veterans Affairs. I will describe the meaning of this work to me personally and professionally, as a culmination of many work and life experiences. I will also share the difference I make as an older adult peer for the Veterans with whom I engage in research and on a multi-disciplinary and intergenerational team comprised of an early career investigator and research assistant. My personal and professional journey highlights the importance of a multi-pronged approach, encompassing research, policy and action/application (e.g., education/training and hiring) to promoting the longevity dividend through meaningful work and gainful employment.

### SESSION 1420 (SYMPOSIUM)

Abstract citation ID: igae098.0413

### RESTORING GLUTATHIONE HAS BENEFITS FOR OLDER ADULTS AND MICE

Chair: George Taffet

The role and importance of mitochondrial function and glutathione is not well recognized by clinicians Glutathione is the most abundant intracellular anti-oxidant and is responsible for detoxifying many reactive, potentially harmful molecules and is most older adults are glutathione deficient because of low free Cysteine and especially Glycine. Supplementation with Glycine and N-Acetyl Cysteine is able to restore cellular glutathione. This symposium will fill these gaps by providing knowledge about glutathione, its role in defending anti-oxidant status, the deficient precursors, and the results when glutathione is restored by providing the precursors and the potential sex differences in these effects.

Abstract citation ID: igae098.0414

### GLYNAC AND THE AGING MOUSE HEART: SEX-RELATED DIFFERENCES AND OUTCOMES

Katarzyna Cieslik[1], Aude Angellini[2], Grecia Garcia Marquez[2], Marta Fiorotto[2], and George Taffet[1], *1. Houston Methodist Hospital, Houston, Texas, United States*, *2. BCM, Houston, Texas, United States*

Aging is associated with increased reactive oxygen species (ROS) resulting from an imbalance in antioxidant defenses and a rise in proinflammatory milieu. Glutathione (GSH) is a naturally occurring antioxidant which concentration is reduced in aging. Studying hearts of 21 month old male and female mice, we found that the enzymes of the GSH pathway synthesis were not downregulated however, supplementing a diet with GSH precursors (termed GlyNAC) for 3 months, improved cardiac diastolic function (measured longitudinally) and exercise performance in males. A much smaller GlyNAC effect on cardiac function and exercise was seen in females. Unbiased cardiac proteome mass spectrometry (MS) analysis validated by protein expression and functional assays demonstrated that a GlyNAC-supplemented diet led to an increase of Ndufb8 expression (a subunit of the mitochondrial respiratory chain complex), and higher enzymatic activities for CPT1b and CrAT, two carnitine acyltransferases, critical to cardiomyocyte fatty acid metabolism. Crosslinking and expression of fibrillar collagens is directly linked with extracellular matrix (ECM) stiffness, and although diastolic function was improved with GlyNAC, no significant change in fibrillar collagen was detected in the hearts of GlyNAC-fed old males. MS analysis determined that GlyNAC increased fibromodulin (Fmod) expression, which can inhibit collagen fibril formation. In summary, these data suggest that there are phenotypic sex-dimorphism differences in old mice and that these differences may affect the response to pharmacological and diet interventions, including GlyNAC. Furthermore, a simple diet intervention has been proven to ameliorate age-related cardiac diastolic dysfunction in old males.

Abstract citation ID: igae098.0415

### GLYNAC SUPPLEMENTATION LOWERS POSTPRANDIAL INFLAMMATION AND OXIDATIVE STRESS TO PROMOTE HEALTH IN OLDER HUMANS

Rajagopal Sekhar[1], Premranjan Kumar[1], George Taffet[2], and Charles Minard[1], *1. Baylor College of Medicine, Houston, Texas, United States*, *2. Houston Methodist Hospital, Houston, Texas, United States*

Elevated oxidative stress (OS) and inflammation are linked to several age-related abnormalities, but factors contributing to these defects are not well understood. Meal consumption is a known stimulus of OS and inflammation. In a randomized clinical trial (RCT) we investigated the impact of a single carbohydrate test-meal on postprandial OS and inflammation in older adults (OA) and young adults (YA), and tested the effect of supplementing GlyNAC (glycine and N-acetylcysteine) vs. placebo. 20 OA and 10 YA received a 75g oral glucose-test-meal with assessment of 2-hour postprandial change in plasma markers of OS (as TBARS) and inflammation (as IL-6). These measures were repeated and compared after OA and YA received their assigned supplements. Pre-supplementation: After consuming the glucose-meal, OA had significantly higher 2-hour postprandial increases in OS (change in plasma concentrations of TBARS: YA 0.02 ± 0.19 vs. 1.09 ± 0.96 in OA, p=0.03) and inflammation (change in plasma concentrations of IL-6: YA 0.03 ± 0.03 vs. 0.52 ± 0.22 OA, p<0.001). Post-supplementation: GlyNAC protected OA from the glucose-meal driven increases in OS (TBARS pre: 1.03 ± 0.44 vs. post -0.04 ± 0.19, p<0.001) and inflammation (IL-6 pre: 0.52 ± 0.20 vs. post 0.05 ± 0.03, p<0.001). No effects occurred in OA receiving placebo or YA receiving GlyNAC. These data indicate that carbohydrate intake contributes to significant increases in postprandial OS and inflammation in OA. By protecting from the postprandial (and overall) rise in OS and inflammation after carbohydrate consumption, GlyNAC supplementation promotes postprandial health in older humans.

Abstract citation ID: igae098.0416

### BRAIN GLUTATHIONE, MITOCHONDRIA, GLUCOSE METABOLISM, AND COGNITION IN AGED MICE: IMPACT OF GLYNAC SUPPLEMENTATION

Premranjan Kumar, and Rajagopal Sekhar, *Baylor College of Medicine, Houston, Texas, United States*

Alzheimer's disease (AD) remains unsolved, and is associated with brain defects including impaired glucose uptake, mitochondrial dysfunction, inflammation and glutathione deficiency. These defects also occur in age-related cognitive decline (ACD). Because mice do not naturally develop AD, mouse models of AD have been developed and studied. However, such mouse models are highly artificial because defects do not occur naturally and are artificially induced in mice. To understand the mechanistic basis of cognitive decline in aging, it is important to investigate these defects directly in the brain, which is only possible in animal models as performing brain biopsies in humans is not practical. To overcome these challenges, we studied glutathione deficiency, oxidative stress, glucose uptake, mitochondrial function, inflammation, genomic damage and neurotropic factors directly in the brains of old-mice (OM, 90-weeks)) and young-mice (YM, 20-weeks), and also measured cognition (maze-running). Compared to YM, OM had the following defects in the brain: (a) lower glutathione concentrations; (b) elevated oxidative stress (TBARS); (c) lower immunoblot expressions of: GLUT1 and GLUT3 (brain and neuronal glucose uptake); PGC1a; electron-chain complexes and PINK1 (mitochondria), TSPO (inflammation), pH2AX (genomic damage), and BDNF, GDNF and NGF (neurotrophic factors); (d) impaired mitochondrial respiration (high-resolution respirometry); (e) poor cognition (maze-running time). After 8-weeks of supplementation with GlyNAC (combination of glutathione precursors glycine and N-acetylcysteine), all of these defects improved/corrected in the brains of OM and cognitive performance improved significantly. The results of this study have important implications for improving brain health and cognition in human aging and in AD.

### SESSION 1425 (PAPER)

### RURAL AGING

Abstract citation ID: igae098.0417

### MORE THAN JUST A RIDE: TRANSPORTATION PROGRAMS TO MEET THE NEEDS OF OLDER ADULTS IN RURAL COMMUNITIES

Alycia Bayne, Alexa Siegfried, Skyla Chitwood, and Nathaly Valdivia, *NORC at the University of Chicago, Chicago, Illinois, United States*

Most older adults (age 65+) living in rural areas rely on personal vehicles for transportation. However, transportation is a significant challenge for older adults who need alternative forms of transportation, including those who do not drive or lack access to a vehicle. Fixed route transportation is limited in rural communities and may not meet the needs of older adults, particularly those who require assistance. Transportation barriers affect older adults' health, mobility, independence, and quality of life. Recognizing these challenges, the Health Resources and Services Administration

Federal Office of Rural Health Policy funded the NORC Walsh Center for Rural Health Analysis and the Rural Health Information Hub to develop the Rural Transportation Toolkit. The toolkit includes 15 promising program models to increase access to transportation, overcome barriers, and improve safety. To develop the toolkit, NORC conducted an environmental scan to identify programs and resources and interviewed representatives from nine rural transportation programs and four experts in rural transportation. The toolkit was first released in 2019, updated in 2024, and has had over 50,000 page views. This session describes toolkit findings, including transportation needs and challenges for rural older adults, and features program models practitioners can adapt. Program models include the Ride Sharing Model, Volunteer Driver Model, Voucher Model, and Coordinated Services Model. This session also describes how technology can improve rural transportation for older adults. This toolkit has the potential to help rural communities meet the transportation needs of older adults.

Abstract citation ID: igae098.0418

### FINANCIAL HORIZONS: EXAMINING RURAL AND URBAN NURSING HOME FINANCIAL PERFORMANCE

Gregory Orewa[1], Akbar Ghiasi[2], Shivani Gupta[3], Rohit Pradhan[4], and Robert Weech-Maldonado[5], *1. The University of Texas San Antonio, San Antonio, Texas, United States*, *2. University of the Incarnate Word, San Antonio, Texas, United States*, *3. University of Houston - Clear Lake, Houston, Texas, United States*, *4. Texas State University, San Marcos, Texas, United States*, *5. University of Alabama at Birmingham, Birmingham, Alabama, United States*

Rural nursing homes (NH) struggle with financial sustainability due to sparse populations, high costs, and scarce healthcare resources. Urban counterparts face increased operational costs and resource competition, despite larger populations and better infrastructure. Addressing these distinct challenges is essential for the sector's financial health and strategic relevance. This study examines financial performance differences between rural and urban nursing homes. The study utilized secondary data from 2017 to 2022 from the Medicare Cost Report, LTC Focus, Care Compare, and Area Health Resource File. The data were modeled using hierarchical linear regression controlling for year and state fixed effects. The dependent variable was operating margin, and the independent variable was nursing home location (Rural vs. Urban). Two models were run, the first controlling for organizational factors (size, quality, ownership, occupancy rate, payer mix, and percent minorities) and the second for organizational and environmental factors (population 65 and older, competition, and Medicare Advantage) that may be associated with financial performance. Results suggest that urban nursing homes had significantly higher operating margins compared to rural facilities ($\beta$=1.95, $p< 0.01$) after controlling for organizational factors. After controlling for environmental factors, urban nursing homes still had higher operating margins ($\beta$=0.90, $p< 0.01$) than rural facilities, but to a lesser extent. As such, rural nursing homes have lower financial performance than urban, even after accounting for organizational and environmental differences. To the extent that lower financial performance may affect quality and access to long-term care, policymakers should explore reimbursement strategies to address these financial disparities.

Abstract citation ID: igae098.0419

### NOMADIC GERONTOLOGY: AGING AS A NOMAD IN MONGOLIA

Reuben Ng, and Nicole Indran, *National University of Singapore, Singapore, Singapore*

Gerontologists have made remarkable strides in understanding the experiences of older adults in various contexts. Yet, amid this progress, some communities remain overlooked, such as older nomads in Mongolia. Notably, Mongolia is one of the last bastions of nomadism in the world. Today, many of the remaining nomads are individuals in their later years. This study aims to illuminate the lived experiences of older nomads in Mongolia. Specifically, we explore how they navigate the duality of being old and nomadic, particularly amid a changing social, political, economic and environmental setup. We interviewed a total of 32 Mongolian nomads whose average age was 65.3 years. Thematic analysis was employed to identify themes related to life as an older nomad. Three themes surfaced: 'Navigating Health Challenges Arising from Aging Bodies and Climate Change' (Theme 1), 'Desire to Preserve a Disappearing Heritage' (Theme 2) and 'Experiencing Neglect and Discrimination' (Theme 3). This study marks an important foray into research on older nomads, airing the voice of a community that is not only under-represented, but also fast vanishing. In today's context, their nomadic ways stand as an in bulwark against the tide of modernization. Our findings make a compelling case for continued research and policy design to enhance the quality of life of older nomads, even as their cultural heritage is safeguarded.

Abstract citation ID: igae098.0420

### RURAL–URBAN RESIDENCE, EDUCATION, AND SUBJECTIVE COGNITIVE DECLINE AMONG MIDDLE AGED AND OLDER ADULTS IN THE US

Roger Wong[1], and Amer Mansour[2], *1. State University of New York Upstate Medical University, New York, United States*, *2. State University of New York Upstate Medical University, Syracuse, New York, United States*

Subjective cognitive decline (SCD) is defined as an individual's self-reported experience of increased confusion or memory loss, and it is a known risk factor for dementia. There is no existing research on SCD disparities by rural-urban residence, and how education may play a role in this relationship. To address this gap, the 2022 Behavioral Risk Factor Surveillance System data was analyzed to elucidate the interplay between rurality, education, and SCD. The SCD measure was only collected from respondents aged 45 years and older, resulting in a sample size of 63,948 U.S. adults. SCD was significantly more common among rural (12.0%) compared to urban (10.7%) residents [X2(1)=15.5, p<.001]. In a multiple logistic regression model, rural residents had a 16% significantly higher odds of SCD compared to urban residents after adjusting for sociodemographic and health covariates (adjusted odds ratio [aOR]=1.16, 95% CI=1.01-1.33, p<.05). There was a negative relationship between education level and SCD, but insignificant. For example, a college degree was associated with 12.4% lower odds of SCD compared to those with less than high school education (aOR=0.88, 95% CI=0.70-1.09, p=.24). Education did not significantly moderate the association between rurality and SCD. Our results suggest rural residents have higher odds of experiencing SCD, but no level of education may buffer this relationship despite extensive research on the benefits of education on cognitive health. Future research is needed to examine the mechanisms contributing to higher levels of SCD among rural residents, and effective interventions that may be used to mitigate further cognitive decline.

### SESSION 1430 (SYMPOSIUM)

Abstract citation ID: igae098.0421

### STATEWIDE PROGRAMS TO IMPROVE DEMENTIA DETECTION AND CARE

Chair: Freddi Segal-Gidan
Discussant: Soo Borson

States are serving as sites for innovation in dementia care and practice. Representatives from three states, Washington, Georgia and California, will discuss their respective statewide dementia programs. Each state will provide an overview of their program, its goals and accomplishments. In California, the Dementia Care Aware program focused on reaching primary care teams with a training and support program for screening, especially for Medi-Cal (Medicaid) beneficiaries. In Georgia, Georgia Memory Net, has developed a statewide network of diagnostic clinics aimed at improving the standard of dementia care in people with a diagnosis.. Finally, Washington will discuss the Cognition in Primary Care program, which developed as a quality improvement initiative for primary care that provides a framework for evaluation of cognitive concerns and tools to assist in managing dementia as it progresses..

Abstract citation ID: igae098.0422

### A STATEWIDE PROGRAM FOR SCREENING FOR DEMENTIA IN MEDI-CAL PRIMARY CARE IN CALIFORNIA: DEMENTIA CARE AWARE

Anna Chodos, *University of California San Francisco School of Medicine, San Francisco, California, United States*

In California, the Department of Health Care Services (Medi-Cal) expanded benefits for older beneficiaries to include screening for dementia by trained primary providers starting in July 2022. In addition, they created a program to promote this screening and provide the training called Dementia Care Aware. The University of California, San Francisco was awarded the contract to realize this program through the end of September 2024 and brought together 4 other University of California campuses and statewide Alzheimer's organizations to stand up the program. During the symposium, attendees will hear about the key accomplishments from creating and disseminating this program. These include: the development of the screening approach, called the cognitive health assessment, with state and national experts; the creation of interactive on-line learning and live trainings; success in dissemination to California's 58 counties and overall uptake among providers and progress to the program's goals of 4000 trained in two years; and lessons learned. Implementation of dementia screening based on the cognitive health assessment in two public health systems (in San Francisco and Los Angeles) provide evidence of how screening is possible in safety net primary care. Finally, a summary of key challenges will show how common barriers in primary care nationwide can be considered before implementing dementia screening programs.

Abstract citation ID: igae098.0423

### A STATEWIDE PROGRAM FOR EARLY AND ACCURATE DIAGNOSIS OF MEMORY LOSS

Theodore Johnson, *Emory University, Atlanta, Georgia, United States*

Georgia Memory Net (GMN) provides clarity, care, and community connection for Georgians. GMN provides Primary Care Providers the educational resources local infrastructure they need to help Georgians experiencing memory loss or cognitive impairment get timely and accurate diagnoses. Georgia Memory Net also provides initial support and education while linking Georgians dealing with Alzheimer's and related dementias and their care partners with community resources that can provide ongoing support. Primary Care Providers who have detected memory concerns can refer to one of seven Georgia Memory Net Memory Assessment Clinics or one of the rural telemedicine sites co-located in Department of Public Health clinics. Georgia Memory Net is made possible by a mandate from the Georgia State Legislature as a way to support Georgians all around our state who are dealing with memory issues. GMN is administered by experts at the Goizueta Alzheimer's Disease Research Center at Emory University.

Abstract citation ID: igae098.0424

### DEVELOPING A PRACTICAL, SCALABLE QI INITIATIVE FOR FQHCS IN WASHINGTON STATE: THE COGNITION IN PRIMARY CARE PROGRAM

Barak Gaster, *University of Washington, Seattle, Washington, United States*

In November 2020, the University of Washington (UW) was awarded funding from the Centers for Disease Control (CDC) to adapt the Gerontological Society of America's K.A.E.R. Toolkit for implementation in a large health system. Using focus group interviews, we developed a quality improvement initiative, Cognition in Primary Care (CPC), consisting of concise continuing education (CE) for primary care providers (PCPs) paired with tools to put that training into practice. The CE consists of Webinars which introduce the tools, which take the form of electronic health record (EHR) checklists and resource folders to facilitate performing cognitive evaluations in primary care. Between 2021 and 2023, the CPC program was implemented in 14 community-based primary care clinics at UW. Measures to assess outcomes showed positive effects including uptake of the EHR tools by PCPs, increased cognitive-tests entered into the EHR, and increased number of patients identified with cognitive impairment. During the symposium, attendees will learn the components of the CPC approach for quality improvement, as well as the methodology used to implement it and assess its effects. Finally, we will present an overview for how the CPC model is being packaged for dissemination to health systems across Washington State, with a focus on federally qualified health centers, supported by new CDC funding in the form of a grant through the BOLD Public Health Initiative.

### SESSION 1435 (PAPER)

### ACUTE CARE I (PAPERS)

Abstract citation ID: igae098.0425

### A REAL-WORLD PHARMACOVIGILANCE STUDY OF SACUBITRIL\VALSARTAN IN OLDER PEOPLE

Zheng Kuai, and Yu Hu, *Zhongshan Hospital, Fudan University, Shanghai, Shanghai, China (People's Republic)*

**Background:** Sacubitril\valsartan is the first-in-class angiotensin receptor neprilysin inhibitor which is utilized clinically in the treatment of heart failure with reduced ejection fraction and hypertension patients. However, Systematic studies of sacubitril\valsartan-related adverse events (AEs) signals in older people based on real-world and international databases are still lacking.

**Methods:** Disproportionality analyses, including the reporting odds ratio (ROR), the proportional reporting ratio (PRR), the Bayesian confidence propagation neural network (BCPNN), and the multi- item gamma Poisson shrinker (MGPS) algorithms, were employed to quantify the signals of Sacubitril\valsartan-associated AEs in older people.

**Results:** The search retrieved 94,210 sacubitril\valsartan-associated cases within the reporting period; 86 PTs with significant disproportionality were retained in 65+. Angioedema, hypotension, renal impairment, hyperkalemia, cough, dizziness, and seasonal allergies were found at a similar rate to those reported in trials. Reports emerged for several cognitive-related AEs and several fragility acceleration AEs, which were rare. Unexpected safety signals such as 'Injury, poisoning, and procedural complications' were only detected in 65+. Most of the cases occurred within the first month after sacubitril\valsartan initiation, and this was consistent across age groups.

**Conclusions:** Our study found potential new AEs signals and might provide important support for clinical monitoring and risk identification of Sacubitril\valsartan in older people.

Abstract citation ID: igae098.0426

### HOSPITALIZATION-ASSOCIATED FUNCTIONAL DECLINE IN OLDER ADULTS: CLASSIFYING THE RISK USING THE "TIMED UP AND GO" TEST

Roy Tzemah-Shahar[1], Orly Gatenio-Hefling[2], Kfir Asraf[3], Omer Dilian[2], Efrat Gil[4], and Maayan Agmon[5], *1. University of Haifa, Haifa, HaZafon, Israel, 2. University of Haifa, Haifa, Hefa, Israel, 3. Yezreel Valley College, Afula, HaZafon, Israel, 4. HaEmek Medical Center, Afula, HaZafon, Israel, 5. University of Haifa, University of Haifa, HaZafon, Israel*

Hospitalization Associated Functional Decline (HAFD) in older adults has been recognized as a major phenomenon with crucial consequences such as slower recovery increased morbidity, and higher mortality; it is linked with reduced mobility levels during hospitalization. To date, there is no objective test that can directly estimate personal risk for HAFD. The aim of this study was to examine the contribution of the Timed Up and Go (TUG) test in predicting HAFD among older adults. We used a cross-sectional study design; a total of 310 older adults (age≥65) hospitalized between December 2018 and August 2020 were included; the Modified Barthel Index was used to assess HAFD; it was administered at admission to evaluate patients' independence in activities of daily living two weeks prior hospitalization, and at discharge. The TUG test was performed on admission; a cut-score of 12 seconds was used to predict functional decline, while accounting for demographics, length of hospitalization, comorbidity burden (Charlson's comorbidity index), and Adult Lifestyles and Function Interview (ALFI-MMSE). Participants able to complete the TUG test in less than 12 seconds showed no significant change in the Modified Barthel Index; TUG test completion in 12 seconds and over or inability to complete it was associated with significant reduction in function, defined by a reduction of three points or more in the Modified Barthel Index (a reduction of 4 and 8.8 points respectively). The risk for HAFD is currently underestimated in clinical settings, the TUG test could be used to identify those at risk of HAFD.

Abstract citation ID: igae098.0427

### POLYGENIC RISK SCORE FOR DIABETES PREDICTS POSTOPERATIVE ACUTE KIDNEY INJURY

Aleda Leis, Michael Mathis, Jennifer Smith, Bhramar Mukherjee, and Carrie Karvonen-Gutierrez, *University of Michigan, Ann Arbor, Michigan, United States*

Large genetic studies often lack detailed phenotyping of complex conditions because doing so requires substantial resources and intensive testing. Given the high prevalence of these conditions, particularly among older adults, omission of such information may lead to biased results. This analysis examined the utility of a polygenic risk score for type 2 diabetes (PRST2DM) versus phenotypic diabetes status in predicting postoperative acute kidney injury (AKI). Data from 14,824 Michigan Genomics Initiative patients age ≥18 years who underwent non-cardiac, non-renal, non-urologic, and non-liver transplant surgical procedures were included. PRST2DM was generated using previously published weights (Mars 2020; pgscatalog.org); values were binned into deciles. AKI was defined using modified Kidney Disease–Improving Global Outcomes (KDIGO) criteria. Logistic regression models of phenotypic T2DM status and the PRST2DM were evaluated; both models were adjusted for a common set of preoperative and intraoperative factors. AKI prevalence was 5.0% (n=737). The odds of AKI were 52% higher among those with phenotypic T2DM versus those without T2DM. Similarly, those in the highest PRST2DM decile had 43% higher odds of AKI compared to those in the lowest decile. Model area under the curve values were largely consistent between the phenotype and PRS models. These findings support the utility of the PRST2DM to serve as a suitable proxy for phenotypic T2DM when a granular phenotype is not available, is subject to information bias, or may not yet have occurred. Such a use is novel and may allow for more dynamic analyses of outcomes in genetic cohorts with limited phenotyping.

Abstract citation ID: igae098.0428

### MITO-MODULATORY MEDICATION USE AND SKELETAL MUSCLE BIOENERGETICS IN OLDER ADULTS

Howard Phang[1], Jaclyn Bergstrom[2], Rabia Atayee[3], Laura Hart[4], Peggy Cawthon[5], Steve Cummings[5], and Anthony Molina[6], *1. University of California, San Diego, La Jolla, California, United States*, *2. UC San Diego, La Jolla, California, United States*, *3. University of California San Diego Skaggs School of Pharmacy and Pharmaceutical Sciences, San Diego, California, United States*, *4. University of Washington, Seattle, Washington, United States*, *5. California Pacific Medical Center, San Francisco, California, United States*, *6. University of California San Diego, La Jolla, California, United States*

Drug-induced changes in mitochondrial function have been described for many medications, including those commonly prescribed for older adults such as statins, NSAIDs, and metformin; however, the clinical impact of "mito-modulatory medication" use remains unclear. Given the high rate of prescription medication use among older adults, it is imperative to understand the consequences of mito-modulatory medication use. Further, mitochondrial dysfunction in skeletal muscle has been linked to declining physical performance and fitness in older adults, highlighting the importance of understanding how drug-induced mitochondrial modulation impacts skeletal muscle. We sought to assess how mito-modulatory medication use is related to skeletal muscle bioenergetics in older adults using data from the Study of Muscle, Mobility and Aging (SOMMA). We hypothesized that mito-modulatory medication use would be associated with lower skeletal muscle ex vivo (MAX ETS) and in vivo (ATPMax) bioenergetic measures. In unadjusted models, MAX ETS was lower in mito-modulatory medication users (mean= 82.4 [77.7-87.0]) compared to non-users (mean= 92.7 [87.5-97.9]) for men (p=0.0021) but not for women (p>0.05). Similarly, ATP-Max was lower in mito-modulatory medication users (mean= 0.54 [0.50-0.57]) compared to non-users (mean= 0.62 [0.58-0.66]) for men (p=0.0004). Adjustments for potential confounders attenuated some, but not all, of the associations. These results suggest that mito-modulatory medication use is related to lower skeletal muscle bioenergetics in older men but not women. Further investigation will provide greater clarity for the apparent sex differences in the impact of mito-modulatory medication use on muscle bioenergetics.

Abstract citation ID: igae098.0429

### EFFECT MODIFICATION OF AGE AND SEX BETWEEN AORTIC CALCIFICATION AND MORTALITY IN A COMMUNITY SAMPLE OF OLDER PATIENTS

Steven Horbal, Aleda Leis, Sven Holcombe, and Carrie Karvonen-Gutierrez, *University of Michigan, Ann Arbor, Michigan, United States*

Abdominal aortic calcification (AAC) is a useful cardiovascular risk assessor in opportunistic screening. As cardiovascular risk changes with increasing age, effective AAC use depends on understanding influential factors on AAC and mortality. This study evaluated modification of age and sex on the relationship between aortic calcification and mortality. This sample includes 6647 Michigan Medicine patients age ≥ 65 years, without cardiovascular disease, and who received an abdominal computed tomography scan between 1999-2022. AAC was elevated if calcification exceeded 19.26% of the aortic wall at the L3 vertebral level. Cox regression was used to assess AAC and mortality (adjusted for body mass index and Charlson Comorbidity Index) and stratified by age group (65-79, 80+ years) and sex. In the sample, 18.3% (n=1219) were 80+ years and the mortality rate was 38.0%. Those with elevated AAC had 99% higher mortality hazard than those with unelevated AAC [HR 1.99 (95% CI 1.66, 2.41)]. A statistically significant interaction was observed between age category, sex, and AAC status. Among patients 80+ years, elevated AAC was protective for mortality [HR 0.79 (95% CI 0.62, 0.98)]. Further, elevated AAC was associated with 35% lower mortality hazard in females age 80+ [HR 0.65 (95% CI 0.46, 0.92)] but was not significant in males age 80+. Important effect modification by age and sex exists on the relationship between AAC and mortality. This suggests the impact of AAC as a risk factor for mortality may decrease with age and be less important in 80+ year old females relative to males.

### SESSION 1440 (SYMPOSIUM)

Abstract citation ID: igae098.0430

### ADVANCING AFFORDABLE, ACCESSIBLE HEARING CARE FOR OLDER ADULTS: FINDINGS FROM THE HEARS RCT AND ASSOCIATED STUDIES

Chair: Carrie Nieman
Co-Chair: Hae-Ra Han
Discussant: Frank Lin

Age-related hearing loss is independently associated with adverse health outcomes, but the use of hearing aids by older adults is low and disparities exist. The incorporation of community health worker (CHW)-partnered models can reduce barriers and address disparities. The HEARS (Hearing health Equity through Accessible Research & Solutions) intervention is a hearing care intervention delivered by CHWs that incorporates a low-cost amplification device and education on age-related hearing loss. The efficacy of the HEARS intervention was assessed through a randomized controlled trial (RCT) (JAMA, 2022) that demonstrated the intervention significantly improved communication function. The RCT was the first of a CHW-delivered hearing care intervention designed for older adults that included provision of amplification. As a community-engaged trial, the trial was also one of the largest hearing-related trials in the U.S. of African American older adults and low-income older adults with hearing loss and provides a unique opportunity to advance understanding of hearing care among communities not traditionally represented within research. This symposium presents secondary analyses from the HEARS RCT that inform the continued expansion of hearing care, including among individuals with cognitive impairment and those with limited technology use. The symposium also shares findings from community-engaged hearing screening efforts (K-HEARS Screen) in partnership with Korean American ethnic churches and provides the first estimates of hearing health behaviors among older Korean Americans. Together, the symposium shares lessons in partnering with CHWs and community organizations to optimize the development and testing of interventions to advance hearing health equity across diverse communities.

Abstract citation ID: igae098.0431

### AFFORDABLE, ACCESSIBLE HEARING CARE AMONG INDIVIDUALS WITH COGNITIVE IMPAIRMENT: LESSONS FROM THE HEARS RCT

Carrie Nieman[1], Joshua Betz[2], Jonathan Suen[3], Jami Trumbo[3], Hae-Ra Han[3], Nicole Marrone[4], Frank Lin[3], and Esther Oh[3], *1. Johns Hopkins University School of Medicine, Baltimore, Maryland, United States*, *2. Cochlear Center for Hearing & Public Health, Baltimore, Maryland, United States*, *3. Johns Hopkins University, Baltimore, Maryland, United States*, *4. University of Arizona, Tucson, Arizona, United States*

Hearing loss is highly prevalent and associated with adverse health outcomes but undertreated among individuals with cognitive impairment, particularly African Americans. The incorporation of community health worker (CHW)-partnered models may increase access and reduce disparities. The HEARS intervention is a hearing care intervention delivered by CHWs that provides a low-cost amplification device. This pre-specified subgroup analysis based within the HEARS randomized controlled trial (RCT) aims to assess whether cognitive impairment modified the effect of the HEARS intervention among community-dwelling older adults. The analysis was stratified by cognitive status using the total Montreal Cognitive Assessment (MoCA) score (□25: cognitive impairment; post hoc sensitivity analysis using □22). Among 149 randomized participants with MoCA data, 100 individuals were cognitively impaired (Mean adjusted MoCA: 21(SD 3.5; 52% African American; 70% low-income). At 3-months post-intervention, communication function significantly improved among individuals with cognitive impairment compared with control, with an estimated average treatment effect of -13.92 HHIE-S change (95% CI:-16.84,-10.86), comparable to those without cognitive impairment (-11.47; 95% CI:-18.04,-4.17). Post hoc sensitivity analysis using a □22 MoCA cut-off for cognitive impairment yielded similar findings. Among individuals with cognitive impairment, the HEARS intervention, compared with waitlist control, significantly improved communication function. The improvements were comparable to participants without cognitive impairment and similar in magnitude to improvements documented for older adults who received conventional hearing care. To the authors' knowledge, this trial was the largest trial to date of a hearing care intervention in the U.S. of African American older adults and low-income older adults with cognitive impairment.

Abstract citation ID: igae098.0432

### PRIOR TECHNOLOGY USE AND INTERVENTION OUTCOMES: INSIGHTS FROM A HEARING CARE TRIAL WITH AMPLIFICATION DEVICES

Jonathan Suen[1], Ethan Wang[1], Joshua Betz[2], Emmanuel Garcia Morales[2], Jami Trumbo[1], Nicole Marrone[3], Hae-Ra Han[1], and Carrie Nieman[4], *1. Johns Hopkins University, Baltimore, Maryland, United States*, *2. Cochlear Center for Hearing & Public Health, Baltimore, Maryland, United States*, *3. University of Arizona, Tucson, Arizona, United States*, *4. Johns Hopkins University School of Medicine, Baltimore, Maryland, United States*

The total number of older adults in the U.S. with clinically significant hearing loss is projected to grow by an estimated 70% by the year 2060. Nevertheless, the adoption of hearing care strategies such as amplification technologies remains low nationally. Evidence of population disparities in accessing respective care along with adverse health outcomes linked to untreated hearing loss are also increasing, warranting a public health response. The Hearing health Equity through Accessible Research and Solutions (HEARS) intervention offers a novel care model leveraging OTC technologies for community-dwelling older adults with hearing loss and low income. A secondary analysis of data from a community-based RCT investigated the effect modification of self-reported prior use of technologies such as smartphones and computers on intervention outcomes across 151 participants. Participants classified as "tech-connected" showed a -14.1 point decrease (95% CI: -17.6, -9.9) on a standardized measure of hearing-related communication impairments when compared to a control group while "not tech-connected" participants demonstrated a -12.5 point decrease (95% CI: -16.2, -5.2), with a greater decrease indicating more favorable outcomes. The estimated difference in treatment effects of -1.55 points (95% CI: -7.8, 4.1) however was not statistically significant, revealing that prior technology use did not modify intervention outcomes among the RCT's cohort. Nonetheless, technology literacy remains an important factor for hearing care intervention research to consider when designing outreach to underserved older populations. Key considerations related to prior technology use will be outlined and described for programs reliant upon OTC amplification technologies in addressing age-related hearing loss.

Abstract citation ID: igae098.0433

### K-HEARS SCREEN: ETHNIC CHURCH-BASED HEARING SCREENING FOR KOREAN IMMIGRANT OLDER ADULTS IN THE UNITED STATES

Hae-Ra Han[1], Joshua Betz[2], Jacqueline Kwak[1], Joanne Park[1], Jami Trumbo[1], and Carrie Nieman[3], *1. Johns Hopkins University, Baltimore, Maryland, United States*, *2. Cochlear Center for Hearing & Public Health, Baltimore, Maryland, United States*, *3. Johns Hopkins University School of Medicine, Baltimore, Maryland, United States*

Age-related hearing loss is highly prevalent in the United States (US), yet there is limited information on the magnitude of hearing loss in certain racial/ethnic minority groups, particularly those with limited English proficiency, due, in large part, to limited accessibility to hearing screening programs and national cohorts of older adults primarily focused on English-proficient communities. Older Korean Americans (KAs)—one of the fastest-growing aging populations in the US—are predominantly monolingual first-generation immigrants. We investigated the prevalence of hearing loss in community-dwelling KA older adults (60+ years) residing in the Baltimore-Washington metropolitan area. Faith-based organizations, such as ethnic churches, serve as epicenters for health promotion activities across diverse immigrant communities. To this end, we mobilized 18 Korean ethnic churches in the target area and screened 514 older KAs (mean=72.6 years, 61.5% women, 54.1% <$50K annual household income, and 97.8% limited English). We found that more than half (55.4% or n=285) had a clinically significant hearing loss and 56% of individuals reported never having their hearing screened. Of those with hearing loss, 61% had a mild degree of hearing loss and 39% with moderate or greater degree of hearing loss. Despite the high rates of hearing loss, prevalence of current hearing aid use was low (12%), similar to findings in other under-resourced communities. To the authors' knowledge, these findings represent the first estimates regarding hearing health among older KAs. Our findings suggest the urgent need for addressing hearing care disparities in this linguistically and socially disadvantaged group of immigrant older adults.

Abstract citation ID: igae098.0434

### COMMUNITY-ENGAGED RECRUITMENT IN PARTNERSHIP WITH FAITH-BASED ORGANIZATIONS: FINDINGS FROM K-HEARS SCREEN

Jami Trumbo[1], Joshua Betz[2], Jacqueline Kwak[1], Joanne Park[1], Carrie Nieman[3], and Hae-Ra Han[1], *1. Johns Hopkins University, Baltimore, Maryland, United States, 2. Cochlear Center for Hearing & Public Health, Baltimore, Maryland, United States, 3. Johns Hopkins University School of Medicine, Baltimore, Maryland, United States*

Hearing loss is highly prevalent, yet few older adults use hearing aids and disparities based on race, ethnicity, and socioeconomic position exist. With limitations in nationally representative data, little is known regarding the hearing health of older Asian Americans, much less among specific ethnic communities. Older Korean Americans (KAs) are predominantly monolingual first-generation immigrants and have some of the highest rates of poverty among Asian Americans as well as high rates of limited English proficiency and poor health literacy, which may contribute to hearing care disparities. The prevalence of hearing loss and hearing aid use among older KAs is not well understood. An observational study was conducted to recruit participants aged 60 and older from KA churches in a metropolitan area to examine the prevalence of hearing loss and hearing care behaviors. A total of 34 recruitment events occurred over 7 months in 16 Korean churches in the Maryland, DC, and Virginia metropolitan area. Screenings occurred in churches of varying congregation size. At least 2 staff members were present at every event (2-4 hour duration). The recruitment efforts resulted in screening 514 KA participants. 13 staff members contributed 414 hours, equating to 0.8 hours of staff effort per individual screened. The KHEARS Screening study represents one of the largest cohorts of KA older adults with hearing loss. Community-engaged research, partnered with faith-based organizations, may offer critical approaches to increasing representation within hearing-related trials and, ultimately, the development of interventions responsive to the needs of a wider population of older adults.

### SESSION 1445 (SYMPOSIUM)

Abstract citation ID: igae098.0435

### AGELESS HORIZONS: IMPACT OF AI, CLIMATE CHANGE, AND EPIGENETICS ON GERONTOLOGICAL SYNDROMES

Chair: Todd Manini
Co-Chair: Christopher Kaufmann

This interdisciplinary symposium will provide the audience an eclectic, contemporary view of gerontology syndromes. It brings together researchers in epidemiology, built environment to natural hazards, new epigenetic age measures, climate events, artificial intelligence (AI) and gerontological syndromes. In this symposium, the audience will learn about how the literature is using AI to study the 5 M's of Geriatrics — Mind, Mobility, Medications, what Matters most and Multicomplexity. This topic is complemented by using new AI methods with Latent Dirichlet Allocation to analyze clinical notes to better estimate the frailty syndrome. Results from older adults undergoing Transcatheter Aortic Valve Replacement suggest a substantial improvement in identifying frail older adults compared to an electronic record approach only. Next, new hallmark measures of biological aging using epigenetic age are studied as a potential mechanism by which biopsychosocial factors could influencing pain and mobility in older adults. Lastly, there are significant adverse health outcomes due to environmental shocks of extreme weather and climate events. Older adults are known to have a greater health susceptibility to these events. However, the impact on health can happen in even deeper ways by modifying mobility after the acute effects have ceased. Mobility resilience signifies a capacity to adapt and maintain community interactions following environmental shocks. Results from hurricane Ian in 2022 suggest that neighborhoods with a higher density of older adults are less mobility resilient. Today's advances in AI and access to unique data sources are on the horizon and will open new approaches to studying gerontological syndromes.

Abstract citation ID: igae098.0436

### AGE-RELATED DEFICITS IN MOBILITY RESILIENCE WITH CLIMATE CHANGE: A CASE STUDY OF HURRICANE IAN

Todd Manini[1], Shangde gao[2], and Yan Wang[2], *1. University of Florida College of Medicine, Gainesville, Florida, United States, 2. University of Florida, Gainesville, Florida, United States*

Resilience is the capacity of a system to respond and recover from extreme events. We aimed to test the feasibility of a new metric of mobility resilience in the context of an extreme hurricane event. We used the concept of the resilience triangle — an engineering framework to conceptualize the resilience of physical infrastructure systems in the public domain. Mobility measures originate from point of interest (POI) visit data using geolocations of 47 million smartphone devices across the U.S.— about 10% of the population in the nation. These data were linked to 4,749 exposed neighborhoods (i.e. census tracts) within Florida who were impacted by Hurricane Ian in 2022. POI data were tracked 1 month prior, and 12 months following hurricane Ian to quantify the impact (ratio of pre-event mobility to maximal loss of mobility), recovery (ratio of post-event to pre-event mobility equilibrium), and duration of recovery (length of time from nadir of mobility lost to a new mobility equilibrium). In multiple regression models, older neighborhood median age was associated with worse recovery and longer duration of recovery. Additionally, neighborhoods with a higher percentage of adults 65+ years had a higher impact, worse recovery, and longer duration of recovery. Adjustments for wind speed, socioeconomic status (poverty, group housing, house cost/structure, uninsured rates), physical/mental limitations, mobile home dwellings, and racial/ethnic minorities had little effect on associations. This study underscores the potential of resilience triangle framework for measuring mobility resilience, although more research across diverse climate events and locations is necessary.

Abstract citation ID: igae098.0437
### CAN ARTIFICIAL INTELLIGENCE HELP ADDRESS THE 5MS OF GERIATRICS?

Christopher Kaufmann, *University of Florida College of Medicine, Gainesville, Florida, United States*

Over the past year, the world has witnessed an explosion in the availability of tools employing artificial intelligence (AI) to do things never imagined possible. In parallel to its worldwide growth, applications of AI have also entered the healthcare sector. Specifically, AI tools have the potential to assist healthcare professionals in making judicial clinical decisions and to empower patients to monitor their health and make informed healthcare choices. Still, the application of AI to the geriatrics and gerontology field is limitless and represents a major opportunity to promote successful aging and independence of older persons. The 5Ms of Geriatrics is a framework used in clinical practice for assessing an older adult patient's cognitive and physical functioning. The key Ms include Mind, Mobility, Medications, Multicomplexity, and what Matters Most, and addressing these five domains is essential clinical practice for improving health outcomes, particularly for older patients. In this presentation, we will explore the current applications and approaches of using AI to specifically address each of the 5Ms. The discussion will focus on the applicability of AI to address each domain and provide key examples of how AI, to date, has addressed each domain. The presentation will also propose key opportunities for future applications of AI in the geriatrics and gerontology field, with the ultimate goal of better providing for the healthcare of older people and promoting aging in place and successful aging.

Abstract citation ID: igae098.0438
### MAKING MOVES: EPIGENETIC AGING AND ITS RELATIONSHIP WITH PAIN AND DISABILITY OUTCOMES IN OLDER ADULTS

Larissa Strath, Ania Lipat, Lingsong Meng, Zhiguang Huo, Thomas Foster, Alisa Johnson, Roger Fillingim, and Yenisel Cruz-Almeida, *University of Florida, Gainesville, Florida, United States*

Chronic pain (pain that is persistent or recurrent for 3 months or longer) is a leading cause of mobility interference and disability in the aging population. It is well known that chronological aging increases the risk of developing of pain and pain reduces physical function; however, limited data exist on how epigenetic aging, new hallmark of biological aging shown to predict pain and mobility outcomes. The purpose of this session is three-fold: 1) introduce the audience to the algorithmic epigenetic aging clocks; 2) examine whether decreased mobility and increased pain severity is associated with these epigenetic clocks, and 3) examine recent literature showing epigenetic age acceleration as a potential mechanism by which biopsychosocial factors could be influencing pain and mobility in older adults. Given the complex multi-dimensional experience of pain and disability that is dynamically sculpted throughout an individual's lifespan along with the environmental interactions with the epigenome, these advances will be a welcomed approach and the future of personalized medicine in an aging population.

Abstract citation ID: igae098.0439
### MACHINE LEARNING–ENHANCED FRAILTY ASSESSMENT FOR HIGH-RISK OLDER PATIENTS UNDERGOING TAVR

Mamoun Mardini[1], Chen Bai[1], Catherine Price[1], Todd Manini[2], and Mohammad Al-Ani[1], *1. University of Florida, Gainesville, Florida, United States*, *2. University of Florida College of Medicine, Gainesville, Florida, United States*

Frailty assessment in hospital settings is growing as a method for patient selection, estimating adverse outcomes, and care planning. However, due to time and resource constraints, current frailty assessments are unstandardized and challenging to apply in clinical settings. This study aims to test frailty assessments by combining real-world structured and unstructured data using machine learning (ML) in older patients undergoing Transcatheter Aortic Valve Replacement (TAVR). We extracted data from 131 TAVR patients collected between January 2018 to December 2019, employing the Fried criterion (weight loss, exhaustion, walking speed, grip strength, and physical activity). We used Latent Dirichlet Allocation (LDA) to analyze clinical notes collected 60 days before the TAVR procedure, generating 150 distinct topics. Additionally, we extracted preoperative data from structured EHRs, including patient details up to six months prior to their frailty evaluation. This includes sociodemographic data, medical history, condition severity, and the latest biochemical markers. We merged LDA-generated topics with EHR features, applied LASSO regression to select the most relevant features, and used XGBoost for predicting frailty levels in patients. We validated our models using nested cross-validation. Integrating clinical notes and structured EHR significantly enhanced model performance, with an area under the curve (AUC) of 0.82, surpassing the EHR-only model's AUC of 0.64. SHAP analysis, a method to explain the output of ML, identified key frailty predictors such as congestive heart failure management, back problems, and Atrial Fibrillation. This approach demonstrates significant potential in standardizing frailty assessments using real-world data and optimizing TAVR patient selection

### SESSION 1450 (PAPER)

### AGING AROUND THE WORLD

Abstract citation ID: igae098.0440
### THE ROLE OF TEMPORAL CONTEXT IN SHAPING LIFE COURSE TRAJECTORIES: INSIGHTS FROM OLDER CHINESE INDIVIDUALS IN THE UK

Miriam Tenquist, *University of Manchester, Manchester, England, United Kingdom*

International gerontological research has increasingly recognised the importance of larger sociohistorical contexts for understanding the growing needs of an increasingly diverse ageing population. The paper contributes to existing literature by examining how temporal context, including historical time, age cohort, and migration age, influences the life trajectories of older migrant communities, specifically older Chinese migrant communities in the UK. Central to this investigation is an examination of their lived experiences against the backdrop of migration policies and concurrent sociohistorical developments in both the host and home country, both before and during migration. This paper draws on semi-structured interviews with 57 Chinese people over the age of 50 living in the UK more than 5 years. The study identifies two distinct groups within this demographic based on migration waves: the second wave (1945-1980) and the third wave (1980s-2000). The paper highlights disparities in educational opportunities, dispersion patterns, occupation, and socio-linguistic capital between the two waves. Grounded in a life course perspective, this paper emphasises the significance of sociohistorical temporality and spatiality in turning points. We argue that the enduring legacies of Chinese reforms and societal changes are distinctly evident in the life trajectories of the diaspora in the UK, becoming more pronounced in later life stages. Ultimately, the study advocates for a more nuanced understanding of migrant communities that recognise the impacts of distinct migration waves on their unique ageing trajectories. This interdisciplinary, internationally minded perspective allows us to better serve global ageing populations that are increasingly influenced by globalisation.

Abstract citation ID: igae098.0441

### TEMPORAL DYNAMICS OF UNMET LONG-TERM CARE NEEDS IN CHINA: AN AGE PERIOD COHORT ANALYSIS

Jingwen Zhang, *University of Sheffield, Sheffield, England, United Kingdom*

The rapid demographic changes, combined with heavy reliance on informal care, pose significant challenges to meeting long-term care (LTC) needs in China. However, research has yet to comprehensively appraise how and to what extent the unmet need has changed during the past two decades. Drawing on data from 6,030 urban and 5,070 rural residents in the Chinese Longitudinal Health Longevity Survey (CLHLS), 2005-2017/18, this study investigates the temporal changes in unmet LTC needs among Chinese older adults. We applied the newly developed age-period-cohort interaction (APC-I) model to disentangle the three temporal processes and their place-based rural-urban variation. The study found that, overall, rural older adults experienced a higher risk of unmet needs for LTC, yet the age, period and cohort effects on unmet needs among rural older people differed from their urban counterparts. Although 'younger' older adults had fewer care needs than older adults, they had a higher risk of experiencing unmet needs. The variation in the age effects was larger among rural older adults. The risk of having unmet needs did not change significantly over the 12 years. The unmet needs for LTC were more pronounced among more recent cohorts than previous generations, especially in urban areas. The findings provide crucial policy insights, underscoring the necessity for targeted interventions to address the care needs of 'younger' older adults, whose demands may not be perceived as severe enough for immediate attention. The escalating care gap among recent cohorts emphasises the need for increased investment in the formal LTC system.

Abstract citation ID: igae098.0442

### FUNCTIONING OUTCOMES AT OLDER WORKING AGES BY INCOME GROUPS: COMPARING THE US WITH 11 EUROPEAN COUNTRIES

HwaJung Choi[1], Stephen Jivraj[2], and Stefan Fors[3], *1. University of Michigan, Ann Arbor, Michigan, United States, 2. University College London, London, England, United Kingdom, 3. Karolinska institutet, Stockholm, Stockholms Lan, Sweden*

This study extends prior research by examining physical and cognitive function for low vs. high-income working-age adults (55-64 years) in the U.S. by comparing to 11 European countries. Using a pooled cross-sectional analysis, the study estimated differences in comparably measured outcomes of activities of daily living (ADLs), instrumental activities of daily living (IADLs), and word recall assessment, with adjustment for survey design aspects and sociodemographic characteristics. Income groups were determined based on the distribution within each country, year, and age. Overall, older working-age adults from the U.S. were significantly more likely to have difficulty with ADLs and IADLs compared to all European countries examined in the study, with the exception of having a similar outcome with England for having difficulty with ADLs. In particular, the adjusted prevalence of ADL limitation from the lowest income group (bottom 20%) was 30% in the U.S. vs. 4%-13% in 10 European countries. Those from the highest income group (top 20%) in the U.S. had a similar risk of having difficulty with ADLs to most countries examined in the study but had a greater risk of having difficulty with IADLs compared to the majority of the countries in the study. Word recall scores from the lowest income group were poorer in the U.S. compared to many European countries, while those from the highest income group performed better in the U.S. Targeting the poorest in the U.S. at risk of disability through intervention should be a priority.

Abstract citation ID: igae098.0443

### HISTORICAL CHANGE IN THE HEALTH AND WELL-BEING OF GHANIAN MIDDLE-AGED AND OLDER ADULTS

Nutifafa Dey, and Frank Infurna, *Arizona State University, Tempe, Arizona, United States*

The last 70 years in Ghana has seen political and socioeconomic transformations that have impacted adult life through the types of policies implemented and social programs made accessible. It has not yet been studied whether these transformations have led to better health and well-being for middle-aged and older adults in Ghana. Up to this point, studies examining historical change or cohort differences are largely from high-income nations and report trends of historical improvements in health and well-being for middle-aged and older adults in European nations, compared to middle-aged and older adults in the U.S. where historical declines are largely observed. Our goal is to use data from Ghana's WHO Study on Global Ageing and Adult Health (SAGE) to fill this gap. Multilevel models were applied to three waves of data collected between 2002-2015 and the outcomes were self-rated health and depressive symptoms. Results revealed that later-born cohorts are reporting poorer self-rated health, which is indicative of historical declines. This trend did not differ across women and educational attainment. No significant cohort effects or modifying effects of gender and education were found for depressive symptoms. These findings shed light on the unique historical trajectory of self-rated health and depressive symptoms in middle-aged and older adults in Ghana. Our findings show similarities and differences to previous findings from the U.S. and Europe. Future research is required that tests potential mechanisms, such as changing dynamics in employment, financial demands, and disruptions in traditional family and social life due to modernization and urbanization.

Abstract citation ID: igae098.0444

**INSIGHTS ON THE AGING EXPERIENCE WITHIN REMOTE CARIBBEAN ENVIRONMENTS THROUGH THE LENS OF GUANAJA, HONDURAS**

Lee Ann Ferguson, *University of Maryland Baltimore County, Baltimore, Maryland, United States*

This qualitative study examined the aging experience of older adults residing on Bonacca Cay, a 100-acre island that serves as the central municipality of Guanaja, Honduras. Utilizing focused ethnographic methods and drawing upon the Old-Age Exclusion and Age-Space Exclusion Frameworks, the research explored socio-environmental considerations thought to influence the aging experience. The analysis of data collected through direct fieldwork, interviews, observations, and photographic journaling while on Bonacca Cay identifies eight themes elucidating the intricate interplay between sociocultural dynamics and environmental factors impacting the island's aging population. These influential themes include (1) personal habitus of older adults, (2) vital family support and changing family structures, (3) community and social bonding, (4) cultural heritage and history, (5) built environment and service infrastructure, (6) geographic/environmental influences, (7) government, economy, and policy, and (8) access to instrumental personal care services and safety. This research also results in the recognition of both the strengths and limitations when attempting to apply either the Old-Age Exclusion or Age-Space Exclusion Frameworks to populations of older adults living on island municipalities governed by distant mainland countries. By presenting the lived realities of older adults within this remote island context, the results of this study may contribute to a more comprehensive understanding of the aging experience within low-resource Caribbean environments. It also offers valuable insights for future research and the development of tailored frameworks for studying aging in similar unique settings.

### SESSION 1455 (PAPER)

### ALZHEIMER'S DISEASE AND RELATED DEMENTIAS I (PAPERS)

Abstract citation ID: igae098.0445

**SEQUENTIAL ANALYSIS OF DEMENTIA FAMILY DYADIC COMMUNICATION: CARE RECIPIENT ENGAGING COMMUNICATION**

Sohyun Kim, Will Chen, and Shan Sun-Mitchell, *The University of Texas Arlington, Arlington, Texas, United States*

Persons living with dementia (care-recipient) engaging communication is responsive and collaborative communication. Examining dementia family dyadic communication is useful for prioritizing caregiver response to care-recipient communication. This study examined temporal relationships between antecedent care-recipient engaging communication and subsequent family caregiver communication (facilitative, disabling, and neutral) during 75 in-home care video observations. A secondary analysis of 5- to 60-second timed-window analysis with 5-second intervals was conducted. 95% confidence intervals, p-values, and Yule's Q statistics were used. The results showed all types of caregiver communication were less likely to occur within all time widows after care-recipient engaging verbal communication (OR = 0.19– 0.43, $p < 0.001$, Yule's Q = -0.398 – -0.685). Caregiver facilitative verbal, disabling verbal and nonverbal, and neutral verbal communication were less likely to occur within all time widows after care-recipient engaging nonverbal communication (OR = 0.37 – 0.83, $p < 0.001$ – $p = 0.017$, Yule's Q = -0.093 – -0.459). Caregiver facilitative nonverbal communication was less likely to occur until 35-second after care-recipient engaging nonverbal communication (OR = 0.67 – 0.81, $p < 0.001$ – $p = 0.001$ – 0.020, Yule's Q = -0.099 – -0.198). Care-recipient engaging communication was significantly associated with a decreased likelihood of subsequent caregiver communication, with a small to large probability of non-occurrence. Caregivers failed to respond to care-recipient communication, regardless of its nature. This emphasizes the urgent need for healthcare providers and researchers to prioritize caregiver education to promote a more supportive and engaging environment for persons living with dementia.

Abstract citation ID: igae098.0446

**PERCEPTIONS OF COMMUNICATION: INCLUSION OF DEMENTIA CARE PARTNERS IN HOSPITALIZED PATIENT CARE**

Austin Medlin[1], Catherine Still[2], Andrea Strayer[3], Beth Fields[2], and Nicole Werner[4], *1. Indiana University Bloomington, Bloomington, Indiana, United States*, *2. University of Wisconsin Madison, Madison, Wisconsin, United States*, *3. University of Iowa, Iowa City, Iowa, United States*, *4. Indiana University, Bloomington, Bloomington, Indiana, United States*

Care partners provide the majority of care to people living with dementia (PLWD) during and after hospitalizations. Yet, they often report dissatisfaction with their inclusion in the hospitalization and feel unprepared to provide care after hospital discharge. Effective communication between clinicians and care partners of PLWD during a hospitalization is foundational to care partner inclusion and preparation. However, our current understanding of care partner communication with hospital-based clinicians is lacking. The present study aims to explore care partners' experiences with clinician communication during hospitalizations of PLWD. A qualitative descriptive study was conducted using semi-structured interviews with 15 care partners of PLWD who had experienced a hospitalization within the last year. Our interdisciplinary team completed an inductive content analysis of the interview transcripts focused on identifying factors influencing care partner and clinician communication. Excerpts were first structurally coded related to hospital communication and then overarching categories were generated through consensus-based discussion over multiple team meetings. We identified five distinct categories related to care partner communication with clinicians: amount of communication, care transition communication, communication style, care partner communication expectations, and inclusion of PLWD in communication. Negative perceptions arose surrounding physical presence of the care partner dictating amount of communication, while lack of communication between hospitals and home health led to decreased care transition communication. Those with positive experiences, however, described a "family-like" communication style. These findings point to areas of communication that could be targets for future intervention to improve care partner inclusion and preparation during hospitalizations of PLWD.

Abstract citation ID: igae098.0447

**ADHD GENETIC BURDEN ASSOCIATES WITH COGNITIVE DECLINE AND ALZHEIMER DISEASE MARKERS IN OLDER ADULTS**

Thalida Em Arpawong, Jung Ki Kim, and Eileen Crimmins, *University of Southern California, Los Angeles, California, United States*

Attention deficit hyperactivity disorder (ADHD) is associated with greater risk for Alzheimer disease and related dementias (ADRD). Genetic liability for ADHD, via a well-characterized polygenic score, has shown associations with cognitive decline and AD neurodegenerative markers, but these associations have not been tested in large and diverse samples of older adults. Using data from a population-based Health and Retirement Study, we used linear mixed-effects regression to test associations between an ADHD polygenic score (ADHD-PGS) and longitudinal trajectories of cognitive decline over 12 years among 12,090 White and 3,100 Black adults aged 51 and over. We used linear regression to test the associations between the ADHD-PGS and AD markers, p-tau181 and Abeta 42:40 ratio, among a subset who provided a blood sample (2,361 White and 537 Black older adults). All models were run separately by ethnicity, and controlled for age, sex, APOE4 carrier status, and ancestry principal components. The ADHD-PGS was associated with cognitive decline among White ($p < 2e-16$) and Black ($p=.008$) older adults. The ADHD-PGS was associated with p-tau181 ($p=0.045$) and the Abeta ratio ($p=0.039$) among White, but not Black older adults (p's=ns). The ADHD-PGS, which represents the continuum of genetic liability for ADHD-related traits in the population, shows associations with faster cognitive decline over 12 years among a diverse set of older adults. The ADHD-PGS shows statistically significant associations with two neurodegenerative markers among White older adults, but not among a smaller sample of Black older adults, although effect sizes for p-tau were similar across groups.

Abstract citation ID: igae098.0448

**EXAMINING THE ASSOCIATION BETWEEN MULTIMORBIDITY PATTERNS AND MORTALITY RISK AMONG PEOPLE WITH DEMENTIA**

Hao Luo, and Jiayi Zhou, *The University of Hong Kong, Hong Kong, China (People's Republic)*

People living with dementia often live with other diseases. Understanding how other diseases co-occur ('multimorbidity', concurrence of two or more diseases) in a person with dementia is important as it can inform a tailored approach to care. This study aimed to identify multimorbidity patterns among patients with and without dementia and examine how these patterns were associated with mortality. We identified dementia patients diagnosed at 65+ between 2007 and 2017 from electronic medical records from all public hospitals in Hong Kong. Each patient was 1:1 matched with patients without dementia by age, sex and index date and followed until death or 31 Dec 2018, whichever was earlier. A total of 156,710 individuals (mean age [SD]: 83.26 [7.28]; 59.6% females) were included. Seven multimorbidity patterns were identified from latent class analysis in the total sample, and similar patterns were identified separately for patients with and without dementia. The pattern characterized by "dyslipidemia and neurological diseases" was most likely to coexist with dementia. Among patients with dementia, compared to the "unspecific" pattern (characterized by the lack of defining diseases), all other patterns were associated with an increased risk of mortality. The highest risk was observed in the "clinically complex" pattern [HR 1.65 (95% CI: 1.60, 1.70)], followed by the "respiratory disease" [HR 1.51 (95% CI: 1.45, 1.57)] and "heart disease" patterns [HR 1.40 (95% CI: 1.36, 1.45)]. The results highlight the importance of taking specific combinations of chronic conditions into account when tailoring the clinical and care approach to people with dementia.

### SESSION 1460 (BIOLOGICAL SCIENCES INVITED SYMPOSIUM)

Abstract citation ID: igae098.0449

**REPURPOSED FOR AGING**

Chair: Sandra Aleksic

Chronic diseases represent the leading driver of health care cost, with detrimental impact on function, independence, and quality of life in the aging society. Geroscience-guided approaches seek to target aging biology to extend healthspan, defined as the period of life spent without major disease and disability. Repurposing Food and Drug Administration (FDA) - approved drugs as gerotherapeutics (geroscience-guided pharmacologic interventions) offers the advantage of known pharmacologic profile and existing clinical experience and can represent a cost-effective strategy to test interventions for extension of healthspan. Among FDA-approved drugs, candidates with geroscience potential can be identified based on the evidence of extension of healthspan and lifespan in model organisms coupled with clinical evidence of benefits extending beyond the diseases targeted by the drug, through mechanisms that involve modulation of hallmarks of aging. Given the inseparable connections between metabolism and aging, it is not surprising that several FDA-approved drugs developed for treatment of metabolic dysfunction are emerging as potential gerotherapeutics. Metformin (discussed by Dr. Sara Espinoza), a widely used oral antidiabetic drug, extends lifespan in preclinical models, while observational clinical evidence supports its role in prevention of several chronic diseases. Two newer antidiabetic drug classes, glucagon-like peptide 1 (GLP1) receptor agonists (discussed by Dr. John Newman) and sodium- glucose cotransporter 2 (SGLT2) inhibitors (discussed by Dr. Carolina Solis-Herrera), have demonstrated healthspan and lifespan benefits above and beyond treatment of diabetes. Emerging evidence for modulation of multiple hallmarks of aging supports gerotherapeutic properties of these drugs. Clinical trials targeting several aging-relevant outcomes are underway.

Abstract citation ID: igae098.0450

### METFORMIN AS A POTENTIAL PHARMACOLOGIC INTERVENTION TO IMPROVE HEALTHSPAN

Sara Espinoza, *Cedars-Sinai Medical Center, Los Angeles, California, United States*

Metformin is the most widely used oral antidiabetic drug, generally recommended as first-line treatment for type 2 diabetes as well as for diabetes prevention. Metformin extends lifespan in preclinical models, and several observational studies in humans suggest that metformin reduces cancer, cardiovascular disease, dementia, frailty, and all-cause mortality. Therefore, given that it is well-tolerated, safe, and widely used, there is high interest in testing its role as a potential agent to reduce age-related diseases and geriatric syndromes. The exact mechanisms by which metformin exerts these benefits are still being elucidated, but in addition to activating AMPK emerging evidence demonstrates metformin modulates several hallmarks of aging, such as epigenetic modifications, mitochondrial function, cellular senescence and the gut microbiome. Thus, metformin has pleiotropic effects which may contribute, perhaps collectively, to its aging-modulating properties. To date there are few clinical trials of metformin to directly examine metformin's effects on human healthspan. We are currently conducting a randomized placebo-controlled clinical trial of metformin to prevent frailty in older adults with glucose intolerance, which will also examine the effects of metformin on several hallmarks of aging. Metformin is also being examined in a separate ongoing randomized placebo-controlled trial as a potential therapy to prevent Alzheimer's disease in older adults without diabetes (including those who have prediabetes). Given its tolerability and safety profile, metformin could be quickly translated to clinical use if ongoing and future clinical trials demonstrate its efficacy in promoting healthspan.

Abstract citation ID: igae098.0451

### REPURPOSING SODIUM-GLUCOSE CO-TRANSPORTER-2 INHIBITORS FOR GEROTHERAPY

Carolina Solis-Herrera[1], Venugopal Venna[2], and Juneyoung Lee[2], *1. University of Texas, San Antonio, Texas, United States*, *2. UT Health Houston, Texas, United States*

Sodium-glucose co-transporter-2 inhibitors (SGLT2i) are drugs used to treat T2DM, offering benefits such as reduced HbA1c, weight loss, and improved insulin sensitivity/β-cell function. Additionally, they provide significant cardiorenal benefits, although the exact mechanisms are not fully understood. The ketone hypothesis suggests that SGLT2i may shift metabolism towards utilizing ketone bodies instead of fatty acids, contributing to their therapeutic effects. Previous research has shown that SGLT2i can increase plasma ketones 2-4-fold. One intriguing area of research is the potential use of SGLT2i as agents for late-aging and longevity. T2DM exacerbates aging-related biomarkers such as senescence, chronic inflammation, oxidative stress, DNA damage, and mitochondrial dysfunction. Preclinical studies have demonstrated that SGLT2i possess anti-inflammatory, anti-oxidative, and anti-senolytic properties, suggesting they may improve health span and lifespan, particularly in patients with chronic inflammatory diseases like T2DM and obesity. However, clinical research in this area is still limited. Preliminary data from our group suggests that in older, obese subjects with prediabetes, treatment with an SGLT2 inhibitor for 12 weeks led to a significant decrease in global and adipose senescent and inflammatory profiles. This effect, correlated with increased plasma ketones, indicates a potential mechanism by which SGLT2i may modulate aging processes and improve symptoms of age-related diseases. Here, we will discuss this and other current studies that support the notion that SGLT2i may contribute to the increase of both healthspan and lifespan.

Abstract citation ID: igae098.0452

### ASSESSING GEROTHERAPEUTIC POTENTIAL OF GLP-1 RECEPTOR AGONISTS

John Newman, *Buck Institute for Research on Aging, Novato, California, United States*

Gerotherapeutics are drugs that target biological mechanisms of aging to achieve broad impacts on multiple chronic diseases of aging as well as geriatric syndromes. No drugs have yet been shown in clinical trials to act as gerotherapeutics. However, several potential candidates have been identified from existing drugs based on proposed interactions with biological mechanisms of aging and supportive data from clinical trials affecting multiple age-related diseases. Metabolism and aging have been mechanistically intertwined since the first rodent studies of caloric restriction a century ago and the identification of the insulin/IGF receptor as the first aging-regulating gene thirty years ago, so it is unsurprising that several candidate gerotherapeutics were first developed for type 2 diabetes (T2DM). Here we will focus on the class of GLP-1 receptor agonists (GLP-1RA), describing preclinical data on interactions with hallmarks of aging that could support a gerotherapeutic role. We will summarize current clinical trial data that includes reduced cardiovascular disease, kidney disease, and mortality in patients with T2DM as well as early or ongoing studies in Alzheimer's and Parkinson's disease. We will discuss the limits of current data, priorities for further research, and the known risks of GLP-1RA in considering their development as potential gerotherapeutics.

### SESSION 1465 (SYMPOSIUM)

Abstract citation ID: igae098.0453

### CARING FOR HEALTH AND CULTURE: INSIGHTS INTO DISEASE MANAGEMENT, CAREGIVING, AND WELL-BEING IN BLACK COMMUNITIES

Chair: Darlingtina Esiaka
Co-Chair: Manka Nkimbeng

There has been growing advocacy for a nuanced understanding of health practices and challenges in diverse Black contexts in recent years. This symposium aims to show the multifaceted intersection of health, culture, and well-being within Black communities, offering a comprehensive exploration of chronic disease management, family caregiving, and mental well-being. Ado-Mensah scrutinizes the nuanced roles and characteristics of family caregivers in African American and Black immigrant communities, shedding light on the unique challenges they face in the context of diabetes management. Groves introduces a novel approach to hypertension management by combining the DASH diet with culturally tailored recipes, recognizing the importance of culturally sensitive interventions for improving health outcomes among Black women. Kum explores the cultural aspects influencing family caregiving practices among African and Afro-Caribbean immigrants, contributing valuable insights into the broader context of eldercare within these communities. Lastly, Hutton sheds light on mental health challenges, emphasizing the need for a nuanced understanding of depression and loneliness symptoms within the diverse diasporic experiences of African Americans and Black communities. Together, these contributions weave a rich tapestry of knowledge, providing valuable insights into disease management, caregiving dynamics, and mental well-being in the intricate intersection of health and culture within Black communities.

Abstract citation ID: igae098.0454

### FAMILY CAREGIVING AMONG AFRICAN AMERICANS AND BLACK IMMIGRANTS: UNDERSTANDING DIABETES MANAGEMENT CHALLENGES

Dorothy Addo-Mensah, and Jamie Conklin, *University of North Carolina, Chapel Hill, North Carolina, United States*

Despite the significant burden of diabetes within African American and Black immigrant communities, there remains a notable gap in understanding the unique caregiving experiences and needs specific to these populations. Familial caregivers play a tremendous role in supporting disease management for their older relatives diagnosed with diabetes. The caregivers have a significant responsibility in providing instrumental (e.g., blood glucose monitoring, insulin administration) and material support (e.g., financial support, shopping) to older diabetic relatives. Research has shown that support from the family caregiver can improve the quality of life of a patient diagnosed with diabetes. This study aimed to investigate and elucidate the characteristics of family caregivers providing care to older diabetic relatives within African American and Black immigrant communities. With support from a librarian, we conducted a scoping review on PubMed, CINAHL, and Scopus databases. The review yielded 538 unique articles that were independently screened by two reviewers in Covidence. The preliminary data shed light on the characteristics of caregivers and the various roles they undertake in providing care. It highlights the challenges family caregivers face in these communities, including financial strain, limited access to resources, and cultural barriers. Additionally, the data reveal the specific support needs of these caregivers, indicating areas where interventions and support programs could be targeted to address their unique circumstances effectively. Our study serves as a foundation for further exploration and the development of targeted interventions to better support family caregivers in African American and Black immigrant communities.

Abstract citation ID: igae098.0455

### DASH DIET WITH CULTURALLY TAILORED RECIPES: A PSYCHOEDUCATIONAL SESSION FOR BLACK WOMEN WITH HYPERTENSION

Angela Groves[1], Kate Hamann[2], and Maria Roche-Dean[3], *1. Western Michigan University, Kalamazoo, Michigan, United States*, *2. Ascension Borgess Medical Hospital, Kalamazoo, Michigan, United States*, *3. University of Kansas, Kansas City, Kansas, United States*

The prevalence of hypertension among non-Hispanic Black women in the United States is alarming because they develop hypertension at an earlier age and have higher blood pressures when compared to White women. Approximately 55.3% of Black women have hypertension, which puts them at significant risk of developing heart disease, kidney disease, and cerebral vascular accidents. The Dietary Approaches to Stop Hypertension (DASH) diet, when combined with lower sodium intake, has been associated with a reduction in systolic blood pressure by 10 mm Hg in a four-week intervention study that included 50% African American women with pre-hypertension or stage 1 hypertension. Although, the DASH diet does not consider cultural food preferences. Studies among Black adults have shown that when the DASH diet incorporated culturally traditional foods, adherence to the DASH diet improved, which lowered blood pressure. A key factor in understanding the health behavior of Black women is their general knowledge of hypertension and low-sodium diets. This psychoeducation session was part of an 8-week peer (dyadic) support mixed methods study that included the DASH diet and culturally tailored recipes to improve diet adherence and reduce systolic blood pressure. Purposive sampling was used to secure a sample of 34 older Black women from local churches and one senior center to participate in a one-hour DASH diet education session. Psychoeducation was reinforced with weekly newsletters and opportunities for one-on-one discussions via email or phone. Study results indicated that participants were able to successfully reduce systolic pressure. We discuss the implications for hypertension management.

Abstract citation ID: igae098.0456

### THE CULTURE OF FAMILY CAREGIVING OF OLDER ADULTS AMONG AFRICAN AND AFRO-CARIBBEAN IMMIGRANTS TO THE US AND CANADA

Cleopatra Kum[1], Casey Phillips[2], Lynn Warner[3], Islam Banisalman[1], and Maria Roche-Dean[4], *1. University of Cincinnati, Cincinnati, Ohio, United States*, *2. University of Kansas Medical Center, Kansas City, Kansas, United States*, *3. University of Cincinnati Libraries, Cincinnati, Ohio, United States*, *4. University of Kansas, Kansas City, Kansas, United States*

An increasing number of African and Afro-Caribbean immigrants to the United States and Canada are aging. Older adults are dealing with various health conditions, which makes them increasingly dependent on family members and friends for their health and social needs. The assistance they receive includes help with physical care, transportation, medication assistance, and emotional and social support, amongst others. Caregivers play a pivotal role in maintaining the health of older adults of African and Afro-Caribbean immigrants in the United States and Canada. However, very little is understood about the expectations and culture of family caregiving within this group. This study explores the culture of family caregiving of older adults amongst African and Afro-Caribbean immigrants in the United States and Canada. We conducted a scoping review of the literature with the assistance of two experienced health science librarians using CINAHL, PubMed, Embase, and OVID MEDLINE. Preliminary findings show that while some articles discussed aspects of family caregiving on the African continent, none were specific to family caregivers of older adults living in the United States and Canada. While it is important to understand the cultural implications of family caregiving within African communities, there are nuances of the African and Caribbean diaspora that have serious implications for the health of older adults. This study adds to our understanding of family caregiving among African and Afro-Caribbean immigrants. It points to a need for tailored intervention for supporting family caregivers within African and Afro-Caribbean immigrant communities.

Abstract citation ID: igae098.0457

### PREDICTORS OF DEPRESSION IN BLACK WOMEN WITH HYPERTENSION: A SECONDARY ANALYSIS OF HRS DATA

Lashawn Hutto[1], Michelle Odlum[1], and Laurie Theeke[2], *1. George Washington University, Washington, District of Columbia, United States*, *2. The George Washington University, Ashburn, Virginia, United States*

Black women experience a prevalence of depression and hypertension, with symptoms of depression going unrecognized. They describe worthlessness and hopelessness but also emptiness, lack of control, mood swings, and disinterest, which are not part of routine screenings. The Black women's schema prioritizes strength, caring for others, and avoiding discussing feelings. Along with barriers of distrust of providers, self-denial of symptoms, limited depression knowledge, stigma, and financial limitations, diagnosis and treatment may be delayed. This study describes predictors of depression in black women based on data from the Health and Retirement Study (HRS). We examined individual, social, and relationship differences between black women with and without hypertension and used stepwise logistic regression to identify predictors. The sample included 407 Black women aged 50 years and above. Nearly half (47.7%) reported hypertension. The majority (94.6%) were married and had varying education, with the majority of participants having high school or less (66.6%). Women with hypertension reported worse overall health (X2=43.999, df=4, p=0.000), more health changes (X2=16.724, df=2, p=0.000), higher effort (X2=10.379, df=3, p=0.016) and depression scores (X2=9.518, df=3, p=0.23), restless sleep (X2=18.907, df=3, p=0.000) and less physical activity (X2=10.981, df=4, p=0.27.). Predictors for depression were loneliness (OR=3.509, 95% CI=1.364, 9.027), sadness (OR=2.272, 95% CI=1.026,5.031), restless sleep (OR=2.093, 95% CI=1.237, 3.542), unhappiness (OR=0.430,95% CI=0.258,0.71) and psychological problems (OR=0.194, 95% CI=0.038, 0.979). Future implications include building relationships with organizations that support black women with depression and longitudinal analysis of HRS data to explain the role of loneliness on depression and hypertension in this population.

### SESSION 1470 (SYMPOSIUM)

Abstract citation ID: igae098.0458

### COMMUNITY-ENGAGED RESEARCH WITH SOCIAL SERVICE ORGANIZATIONS FOR OLDER ADULT MENTAL HEALTH EQUITY

Chair: Lesley Steinman
Co-Chair: Mary Mitchell
Discussant: Sherry Wu

Twenty years ago, our research center collaborated with local community-based social service organizations to co-create and evaluate PEARLS, a community-based, collaborative care model for late-life depression. PEARLS builds capacity among trusted, front-line, non-clinical providers (e.g., case managers, community health workers) to be depression care managers, taking a "task shifting" and "task sharing" approach recommended to close the mental health treatment gap by improving access to quality care where older adults live, work, play, pray, and age. While PEARLS was developed with, in, and for communities underserved by clinical mental health care, for the past five years our community-academic partnership has focused on reaching older adults who have been historically marginalized by racism, sexism, ageism, and other structural inequities. The five-year PEARLS Equity Study is supported by CDC Prevention Research Center funding to improve older adult mental health equity through active community collaborations from design to dissemination. This symposium will bring feature several of our local PEARLS partners to highlight the value of community-engaged dissemination and implementation research to fortify the feasibility, equity, and sustainability of studies to improve the lives of older adults through the social service organizations working to support aging in place and quality of life. We will provide real-world examples that embody the GSA conference theme, the Fortitude Factor, offering take-aways for researchers, practitioners, and policymakers to strengthen how community-academic partnerships are essential for generating and integrating research findings into aging practice and policy.

Abstract citation ID: igae098.0459

### EQUITABLE ACCESS TO LATE-LIFE DEPRESSION CARE: A STEPPED WEDGE RCT WITH COMMUNITY ORGANIZATIONS

Lanvin Andres[1], Lesley Steinman[2], Mary Mitchell[3], and Emy Haruo[4], *1. IDIC Filipino Senior & Family Services, Seattle, Washington, United States*, *2. UW Health Promotion Research Center, Seattle, Washington, United States*, *3. Aging and Disability Services - Seattle/King County Area Agency on Aging, Seattle, Washington, United States*, *4. Neighborhood House, Seattle, Washington, United States*

For the past several years, our community-academic partnership has been collaborating with community-based social service and mental health organizations in Washington and California on the PEARLS Equity Study. The goal of this five-year study is to improve equitable access to late-life depression care (PEARLS) among older adults experiencing poverty who are linguistically diverse, BIPOC, and/or rural dwelling, recognizing many older adults are multiply marginalized by intersecting identities. In phase one (2020), we conducted formative research (39 interviews) with both current and potential PEARLS adopters that reach priority populations. Using a social marketing framework, our guide explored barriers, benefits and process for PEARLS adoption; organization capacities and needs; PEARLS acceptability and adaptations; and preferred communication channels. We conducted thematic analysis using the rapid framework method to develop strategies, collaborations, and adaptations to integrate depression care in ways that aligned with these contexts. In phase two (2021-2023), we evaluated these new dissemination and implementation strategies and tools in a stepped wedge Randomized Controlled Trial (swRCT) to test improvement in equitable access to mental health care. Our primary and secondary outcomes are new PEARLS adopters and reach of priority populations, respectively. We randomized counties into three waves or steps, choosing a stepped wedge design as more feasible for community-engaged research with all organizations ultimately receiving the intervention. We engaged over 500 organizations and 700 providers in outreach, webinars, community conversations, 1:1 support, and training. We are currently analyzing RCT, process and cost evaluation data to present findings and lessons learned at GSA.

Abstract citation ID: igae098.0460

### BEST PRACTICES FOR COMMUNITY-ENGAGED RESEARCH AND PRACTICE WITH CBOS THAT ENGAGE DIVERSE OLDER ADULTS

Emy Haruo[1], Mary Mitchell[2], Lanvin Andres[3], and Lesley Steinman[4], *1. Neighborhood House, Seattle, Washington, United States*, *2. Aging and Disability Services - Seattle/King County Area Agency on Aging, Seattle, Washington, United States*, *3. IDIC Filipino Senior & Family Services, Seattle, Washington, United States*, *4. UW Health Promotion Research Center, Seattle, Washington, United States*

PEARLS partner organizations will share best practices on integrating PEARLS into routine service delivery to reach historically marginalized older adults, and how they have partnered with researchers to strengthen study feasibility, equity and sustainability to translated learnings into practice. The PEARLS Equity Study guided new dissemination and implementation strategies that aligned with the strengths, priorities, and needs of community-based social service organizations (CBOs) including cultural organizations, area agencies on aging, senior centers, and family and human services agencies. New communication approaches included proactive group and 1:1 outreach to organizations; a new peer-to-peer video with PEARLS organizations sharing how they have reached underserved communities; and updated PEARLS messaging that resonated with values of CBO leadership, staff and clients. For example, new messaging included downplaying "evidence-based program" which has often been used to promote programs that were not designed with diverse older adults, and instead amplifying stories of impact that highlight both quantitative and qualitative changes in outcomes that mattered. New supports for program adoption included 1:1 support calls for tailored guidance for different CBO, staff, and community contexts; distance on-demand training with trainers from diverse CBOs to improve accessibility and sustainability; and Zoom webinars and community conversations co-led by PEARLS partners (including local partners featured during this GSA Symposium) and our research center that targeted what CBOs are looking for to address depression and disconnectedness in their communities. Our community-academic partnership and PEARLS community of practice continues to engage regularly to share adaptations as an implementation strategy for health equity.

### SESSION 1475 (PAPER)

### ECONOMICS AND AGING

Abstract citation ID: igae098.0461

### LONG-TERM CARE INSURANCE AND OVERLOOKED LONG-TERM CARE EXPENSES AMONG OLDER ADULTS WITH COGNITIVE IMPAIRMENTS

Chengxu Long, Wei Yang, and Karen Glaser, *King's College London, London, England, United Kingdom*

We investigate catastrophic health and long-term care expenditures (CHLTCE) among older adults with cognitive impairments in China and the role played by long-term care insurance (LTCI). Drawing on data from the 2013, 2015, 2018, and 2020 waves of the China Health and Retirement Longitudinal Study, we exploit the rollout of LTCI pilots across different cities and employ a difference-in-differences approach. Our findings indicate that a significant proportion of CHLTCE among older adults with cognitive impairment is attributed to long-term care costs (i.e., both formal and informal). After considering long-term care expenditure, both the incidence and intensity of CHLTCE increase significantly compared to when only healthcare expenditures are taken into account. Our research underscores the importance of considering long-term care costs when examining catastrophic expenditures due to both health and long-term care expenditures among older adults with cognitive impairment. We also find that the implementation of LTCI reduces both the incidence and the intensity of CHLTCE among older adults with cognitive impairment. Moreover, this effect is more pronounced among individuals with more severe cognitive impairment and those who live alone. These findings suggest that the introduction of LTCI, may, to some extent, alleviate CHLTCE among older adults with cognitive impairments. Our findings support the extension of the LTCI pilot as well as the broadening of the insurance's scope to ensure adequate protection for older adults with cognitive impairments.

Abstract citation ID: igae098.0462

### EXAMINING THE IMPACT OF LONG-TERM CARE INSURANCE ON LABOR MARKET PARTICIPATION OF INFORMAL CARERS IN CHINA

Wei Yang[1], Xingtong Pei[2], and Mingming Xu[2], *1. King's College London, London, England, United Kingdom*, *2. Sun Yat-sen University, Guangzhou, Guangdong, China (People's Republic)*

Existing evidence from high-income countries suggests that long-term care insurance (LTCI) aimed at enhancing access to formal care can reduce the burden on informal carers and facilitate their reentry into the labor market. However, there is limited evidence regarding the specific carers who have been most impacted by such insurance. Drawing data from the China Health and Retirement Longitudinal Study of 2011, 2013, 2015, and 2018, we employ a staggered difference-in-differences model with propensity score matching to analyze the impact of LTCI on care burden reduction and labor market participation of the informal carers. Our study shows that LTCI substantially alleviates the burden on informal carers and promotes their labor market participation. Specifically, LTCI reduced care burden among spouses by 8.5 hours per month. We also found that LTCI's impact on enhancing labor market participation was more significant among younger household members, reflected in an average income increase of 4,534 yuan per year. Our subgroup analysis highlights that LTCI primarily benefits informal carers providing care for older people with low income or those who were farmers or previously engaged in informal sectors. We urge the government extend the insurance program to additional regions and broaden its scope to include individuals from lower socioeconomic backgrounds.

Abstract citation ID: igae098.0463

### COHORT DIFFERENCES IN PENSION AND JOB SECURITY OF OLDER UNION WORKERS: EVIDENCE FROM HRS

Yun taek Oh, *University of Nevada Reno, Reno, Nevada, United States*

The influence of the declining number of unions and their bargaining power on older workers' quality of work lives, such as pension eligibility and job security, is an understudied field. I use the first six waves of the initial HRS cohort (born 1931-1936, recruited in 1992, N = 3,134) and Mid Baby Boomers (MBB, born 1954-1959, recruited in 2010, N = 2,886) from the Health and Retirement Study (HRS) from 1992 to 2020. I examine the changes in the association between union membership and the quality of later work lives by using the interaction terms between birth cohorts, union membership, and employment sector (private or public). The results from logistic regressions show that the proportion of pension eligibility from current jobs declined over time while the expectation of job loss at ages 57-62 is stable for union members. Furthermore, the predicted probability of actual job loss increased among non-union workers in the private sector while that for union workers was unchanged. These results suggest that the union's declining bargaining power may influenced pension eligibility although expectations and actual job security remain unchanged for union workers. The results from competing risk analysis show that MBB union workers are more likely to change job titles while less likely to move to different employers or to directly exit the workforce than the previous cohort. This result suggests the prevalence of bridge employment among union workers as well as the preference changes in the type of bridge employment.

Abstract citation ID: igae098.0464

### THE DYNAMICS OF ECONOMIC INSECURITY THROUGHOUT LATER LIFE: DISPARITIES BY GENDER AND RACE/ETHNICITY

Seokmin Kim, Sung Park, and Jan Mutchler, *University of Massachusetts Boston, Boston, Massachusetts, United States*

Older Americans face significant economic hardships, and their risk of poverty increases with age. But it remains unclear whether the 65+ population, which includes a sizable number with incomes too high to qualify for means-tested public assistance programs, has the resources to meet an adequate standard of living. Using 2010-2020 data from the Health and Retirement Study linked to the corresponding years of the Elder Index (EI), this project investigates heterogeneity in economic insecurity among older adults aged 65+ as they age, by gender and race/ethnicity. We examine transitions in living above/below the EI and time spent living below the EI. We also study the dynamic relationship between respondents' employment activity and their trajectories of economic insecurity. Results suggest that at least half of respondents live below the EI at some point in later life, and a quarter of them experience sustained economic insecurity throughout the entire observation period. Individuals in these economically insecure categories are more likely to be female, non-White, and have limited or no work experience. These disparities grow over time as individuals approach their 80s, driven partly by the intensity of work experience prior to age 60, despite increasing employment activity in later life. The findings illustrate the growing disadvantage in economic well-being for older females and racial/ethnic minorities, and the influence of earlier life economic activity on financial insecurity and instability in later life.

Abstract citation ID: igae098.0465

### ECONOMIC VALUE OF UNPAID FAMILY CAREGIVER TIME FOLLOWING HOSPITAL DISCHARGE AND AT END-OF-LIFE

Brystana Kaufman[1], Zhang Wenhan[1], Sahar Shibeika[1], Ro Huang[2], Catherine Vanderboom[3], Diane Holland[3], Courtney Van Houtven[1], and Joan Griffin[3], *1. Duke University School of Medicine, Durham, North Carolina, United States*, *2. Duke University, Durham, North Carolina, United States*, *3. Mayo Clinic, Rochester, Minnesota, United States*

Estimates of patient care costs often overlook the economic value of unpaid caregiving. This study aims to quantify the economic value of unpaid caregiving by rural FCGs. Using data from a multi-site randomized controlled trial evaluating Transitional Palliative Care (TPC) intervention for FCGs, this study quantifies the economic value of family caregiving in the six months following seriously ill care recipients' hospital discharge using three valuation methods. Caregiving hours were self-reported by FCGs. The proxy cost approach assigned value to FCG hours using the national median home health aide wage. The unadjusted opportunity cost approach assigned value using the national median wage, by sex and education level. The adjusted opportunity cost approach assigned value by work force participation, using opportunity cost if employed and proxy cost otherwise. Of 282 FCGs, 94% were non-Hispanic White, 71% had a college degree, 56% were spouses, and 51% were in the workforce. FCG cost did not differ by treatment arm. FCGs of decedents compared to survivors reported significantly more hours per person-month (392 vs. 272, $p< 0.001$), resulting in higher median opportunity cost ($12,653 vs. $8,843, $p< 0.001$), proxy cost ($5,689 vs. $3,955, $p< 0.001$), and adjusted opportunity cost ($9,490 vs. $6,443, $p< 0.001$)

per person-month. Proxy cost may undervalue FCG time, while opportunity cost may perpetuate labor market inequities. Policymakers must weigh trade-offs of different valuation methods. The high intensity of unpaid post-acute care, especially end-of-life, should be considered when designing policies and interventions to support unpaid FCG due to the economic impact on FCGs and employers.

### SESSION 1480 (PAPER)

Abstract citation ID: igae098.0466

### EDUCATION AND TRAINING II: AGE-INCLUSIVITY AND HEALTH PROMOTION APPROACHES

Cognition/ADRD; Family/social Aspects; Community-based approaches

Abstract citation ID: igae098.0467

### THE POWER OF ARTS-BASED ACTIVITIES: APPLICATIONS OF AN INTERGENERATIONAL HAIKU-MAKING ACTIVITY

Yoshiko Matsumoto, and Harumi Maeda, *Stanford University, Stanford, California, United States*

The power of arts- and humanities-based activities for enhancing the well-being and quality of life of older adults is well documented in the existing literature (e.g., Fraser et al., 2015; Letrondo et al., 2023; Young et al., 2016). An intergenerational haiku-making activity designed and implemented by the authors et. al. (2021, 2024) is one such activity. In this activity, 2-3 older adults living with mild cognitive impairment (MCI) interact with 2-3 university students to create haiku poems based on seasonal photos of their choice, followed by short one-on-one feedback interviews. Analysis of the recorded activity sessions demonstrates how the activity created meaningful outcomes for both older and younger participants, including increased emotional well-being, intergenerational understanding, and self-expression. This presentation discusses two applications of the intergenerational haiku-making activity in two different settings. First, we designed and taught a community-engaged learning course in which undergraduate and graduate students learned first-hand how humanities-based intergenerational activities enhance intergenerational communication and mutual understanding. Second, the activity was re-designed to target older Japanese immigrants living with cognitive impairment by incorporating elements that encourage them to draw upon their Japanese language heritage. The bilingual version will be implemented in a local Japan-focused adult day services program. This presentation highlights the effectiveness of arts- and humanities-based activities by showing the ways in which one particular activity can be expanded and applied in different settings to offer tailored benefits to people of varying ages and cognitive conditions.

Abstract citation ID: igae098.0468

### EVALUATION OF AN ALZHEIMER'S DISEASE TRAINING FOR LATINX COMMUNITY HEALTH WORKERS

Sonia Bishop[1], Kelley Pascoe[1], Janet Rojina[1], Katherine Lopez[1], Theresa Kehne[1], Karen Torres[2], Kimiko Domoto-Reilly[1], and Linda Ko[1], *1. University of Washington, Seattle, Washington, United States*, *2. University of Washington Harborview Medical Center, Seattle, Washington, United States*

Despite an anticipated 800% increase in Alzheimer's disease (AD) prevalence among U.S. Hispanic/Latinx by 2060, Hispanic/Latinx are persistently underrepresented in AD research, which is partly due to low participation in Alzheimer's Disease Research Centers (ADRC). Community Health Workers (CHWs) are trusted community members and can serve as educators of AD and ADRC. This study evaluated changes in pre and post knowledge about AD and ADRC among CHWs serving Hispanic/Latinx after delivery of a culturally and linguistically appropriate AD training. The training covered AD prevalence and risk factors, AD preventive behaviors, knowledge about ADRC, and the importance of AD research. Pre/post assessment was conducted using non-matched, anonymous surveys; differences in pre/post measures (mean+SD) were estimated and analyzed using Wilcoxon-Mann-Whitney U tests. Participants (N=68) had a mean (SD) age of 40 (10.7) years, were majority female (87%), identified as Latinx (91%), born outside the U.S. (55%) and represented 21 Washington counties. There was a significant increase in knowledge of AD (pre: 2.16 + 1.25; post: 3.51 + 1.09; p< 0.001), AD signs and symptoms (pre: 2.04 + 1.25, post: 3.38 + 1.13; p< 0.001), and AD prevention (pre: 1.84 + 1.16, post: 3.46 + 1.19; p< 0.001) pre/post training. Similarly, there was significant increase in knowledge of the local ADRC (pre: 1.38 + 0.92, post: 3.27 + 1.48; p< 0.001), ADRC studies (pre: 1.63 + 1.11, post: 3.31 + 1.22; p< 0.001) and what ADRC participation entails (pre: 2.59 + 1.74, post: 4 + 1.17; p< 0.001) pre/post training. Culturally and linguistically concordant training for CHWs showed promise for increasing knowledge about AD and ADRC. Future research should explore how CHWs use training information to educate Hispanic/Latinx individuals about AD and ADRC.

Abstract citation ID: igae098.0469

### INTERGENERATIONAL CREATIVE MOVEMENT INTERVENTION FOSTERS SOCIAL CONNECTIONS AND MENTAL HEALTH

Amy Kostelic, Anthony Bardo, Susie Thiel, Claire Reardon, and Mohammad Khalilian, *University of Kentucky, Lexington, Kentucky, United States*

It is widely accepted that people flourish when experiencing a sense of connection and belonging. Yet, negative outcomes from the pandemic, like loneliness, remain elevated and relevant for younger and older women's well-being. Additionally, life course transitions into both young adulthood and older age, while very different, are also similar in that they can challenge a woman's social connectedness and sense of belonging. Our innovative multi-lesson education program aimed to connect first-generation female college students and college educated older women living in the community to promote a sense of belonging and enhance mental health by (a) combining interpretive dance in the form of physical activity and creativity; (b) engaging multiple generations via a humanistic approach centered around generativity; and (c) utilizing a sociocentric network approach to identify individual and program-level factors associated with positive outcomes. Results based on Social Network Analyses (SNA) demonstrated that our program was adept at fostering close intergenerational connections, which when triangulated with qualitative results from in-depth interviews, were found to be anchored in an emergent sense of belonging. Coupled with results from pre- and post-intervention surveys, the formation of these close intergenerational connections was found to positively impact mental health. The efficacy of our program was further established when pre- and post-intervention mental health assessments were compared between the participant (n=20) and control group (n=20). Our innovative program demonstrates the potential, though distinct, applicability of (a) creative movement activities and (b) SNA techniques for developing a programmatic scale up into other community settings.

Abstract citation ID: igae098.0470

### EXPERIENCES OF SENIOR SCHOLARS AND PROFESSORS EMERITI: FOCUS GROUP RESULTS

Michelle Porter, William Kops, and Cornelia Kauenhowen, *University of Manitoba, Winnipeg, Manitoba, Canada*

In this study we were interested in learning about the experiences of Senior Scholars and Professors Emeriti at the University of Manitoba (UM), as part of a larger age-friendly university project on these term and lifetime positions for retired academics. Focus groups were held online with 11 women and men. The interview guide, developed based on earlier findings from a survey of a larger group of individuals in these same positions, included questions on their reasons for attaining the positions, benefits, challenges, and processes. Six themes were identified from the data collected: 1) commitment, 2) connection/access, 3) variability, 4) overlooked, 5) clarity (lack of) and 6) opportunity. 1) Participants indicated they are committed to their departments and colleagues locally, nationally and internationally. 2) Connections to colleagues and resources are important and appreciated (e.g., email, computer technical support, office and lab space). 3) Despite being at the same university, experiences were not consistent in how they acquired and subsequently renewed positions. 4) While there were many positive experiences shared, some respondents expressed disappointment, concern, anger and even fear, as well as having a sense of not being supported or even ignored. 5) There is an apparent misunderstanding of the designations, access to resources, and resulting expectations and roles. 6) Finally, all were positive about the opportunities to make changes to: nurture a sense of belonging of Senior Scholars and Professors Emeriti; acknowledge their efforts and achievements; create opportunities for increased participation; and ultimately foster a model workplace for retired academics.

### SESSION 1485 (PAPER)

### ENHANCING SOCIAL SUPPORT AND COMBATING ISOLATION IN OLDER ADULTS WITH COGNITIVE IMPAIRMENT

Abstract citation ID: igae098.0471

### COMPUTER-MEDIATED COMMUNICATION (CMC) AND SOCIAL ENGAGEMENT AMONG INDIVIDUALS WITH ALZHEIMER'S

Yun Leng Wong[1], Laura Hemmy[2], Mike Conway[3], Daniel Cabrera Lozoya[3], Kelvin Lim[2], and Jude Mikal[2], 1. *University of Minnesota Twin Cities, Minnesota, United States*, 2. *University of Minnesota, Minneapolis, Minnesota, United States*, 3. *University of Melbourne, Melbourne, Victoria, Australia*

Normal aging confers a host of risk factors for loneliness and social isolation. Aging accompanied by Alzheimer's-related changes in cognition and function may result in even higher risk of loneliness and social isolation. Nevertheless, older adults now represent the fastest-growing demographic of both Internet and social media users. Moreover, uptake continues to rise, with younger-older adults - or the Baby Boomer Generation - using social media on average two hours more per week than younger generations. This widespread increase in digital engagement has profound implications on loneliness and social isolation among individuals living with Alzheimer's. In this study, we examine the lived experiences of individuals living with ADRD using blogs managed by individuals with early-onset AD to assess how the Internet enables them to stay connected and safe. Following a phenomenological approach, we apply rigorous bottom-up, constant-comparison methods to identify the impact of computer-mediated communication (CMC) technologies on social engagement, focusing on mental and physical health outcomes, social support exchange, and health resource sharing. Four key themes emerged from analysis: (1) the Internet as a gateway to formal networks of support and mentorship, (2) the ability to bridge geographical distances in pursuit of fitting and responsive support, (3) feelings of validation contribution coming from sharing experiences, and (4) feelings of embarrassment or forced awareness of functional and cognitive decline. Themes are contextualized, and used to make recommendations for the development of novel support exchange platforms focused on the needs of individuals with ADRD - and those with early-onset ADRD, specifically.

Abstract citation ID: igae098.0472

### THE ASSOCIATION OF PERCEIVED SOCIAL SUPPORT AND SOCIAL ISOLATION WITH SUBJECTIVE COGNITIVE DECLINE IN OLDER ADULTS

Iftekhar Amin, and Syeda Jesmin, *University of North Texas at Dallas, Dallas, Texas, United States*

**Objective:** Early detection and diagnosis of subjective cognitive decline (SCD) has been prioritized in the Healthy People 2030 objectives. Two specific objectives are to increase the proportion of adults with SCD who know they have it and who have discussed this with a care provider. Although the literature shows that women may have faster cognitive decline than men, research is lacking on whether differential levels of social support and isolation play any role in the prevention and delay of the onset of cognitive decline in older men and women.

**Methods:** We used data from the 2022 Behavioral Risk Factor Surveillance System (BRFSS, N= 4,45,132). Logistic regression models have been used to examine the association between social support, isolation, and SCD in a sample of community-dwelling older adults with SCD aged over 65.

**Results:** Overall, among 31,625 respondents over age 65, nearly 10.1% reported experience of confusion or memory loss. Lower scores on the composite measure of social support and isolation were associated with greater reports of SCD for both men (OR 2.6, 95% CI = 2.3, 2.9) and women (OR 2.9, 95% CI = 2.6, 3.2). Compared to Hispanic older adults, the effects of isolation and lack of social support on the odds of SCD were higher for Whites (OR 2.0, 95% CI = 1.8, 2.3), and Blacks (OR 1.4, 95% CI = 1.2, 1.7).

**Conclusion:** Our findings highlight the importance of addressing social support and social connectedness in cognitive decline-related health promotion efforts for older adults, women in particular.

Abstract citation ID: igae098.0473

### ASSOCIATIONS OF SOCIAL ISOLATION AND LONELINESS WITH MCI AMONG OLDER ADULTS FROM UNDERRESOURCED COMMUNITIES

Fang Fang[1], Tiffany Hughes[2], Yueting Wang[3], and Erin Jacobsen[3], *1. Old Dominion University-EVMS, Norfolk, Virginia, United States*, *2. Midwestern University, Glendale, Arizona, United States*, *3. University of Pittsburgh, Pittsburgh, Pennsylvania, United States*

Previous studies have shown social isolation and loneliness are associated with poor cognitive outcomes in later life, yet it is unclear if these findings could be confounded by selective attrition. This study examines longitudinal associations of social isolation and loneliness with incident mild cognitive impairment (MCI) accounting for informative drop-out due to death or disease. Data were from a population-based cohort study of older adults in Southwestern Pennsylvania, whose once-vibrant economy heavily relied on the steel industry until it collapsed in the 1970s and has not fully recovered. Participants (N=1,254) were followed for 12 years for the development of MCI. Composite measures of social isolation and loneliness were created using longitudinal data across the study period. A generalized linear mixed model (GLMM) analyzing incident MCI as a binary outcome with joint modeling to adjust for potential attrition bias, along with adjustment for demographic, health, and lifestyle factors was used to examine the associations of social isolation and loneliness with incident MCI. The analyses revealed that greater social isolation was associated with higher risk of incident MCI (odds ratio=1.33, p=0.014) even when accounting for potential attrition bias, other potentially confounding factors, and loneliness. Loneliness was not significantly associated with incident MCI with or without accounting for social isolation (odds ratio=1.04, p=0.716; odds ratio=1.12, p=0.255). Over time, social isolation, rather than loneliness, may negatively affect cognition and strategies to keep older adults socially connected may reduce the risk for cognitive impairment.

Abstract citation ID: igae098.0474

### SOCIAL RESOURCES PREDICT EMOTIONAL WELL-BEING IN COGNITIVELY IMPAIRED OLDER ADULTS: RESULTS FROM THE ARMADA STUDY

Melanie Johnson[1], Claire Growney[2], and David Salmon[3], *1. Shiley Marcos Alzheimer's Disease Research Center, University of California, San Diego, San Diego, California, United States*, *2. Stanford University, Stanford, California, United States*, *3. University of California San Diego, La Jolla, California, United States*

As older adults tend to prioritize meaningful relationships, they are likely to have higher quality social relationships, which may contribute to maintained or improved emotional well-being with age. Social resources may be especially important for maintaining well-being among older adults with neurodegenerative diseases. We utilized NIH Toolbox (NIHTB) measures to examine group differences in the associations between social resource quality and emotional well-being among an ethnoracially and cognitively diverse sample of older adults. As part of the Assessing Reliable Measurement in Alzheimer's Disease and Cognitive Aging (ARMADA) study, older adults who were cognitively normal (CN-Young: aged 65-84; n=161; CN-Old: aged 85+; n=116), with amnestic mild cognitive impairment (MCI; aged 64+; n=109), and with Alzheimer's disease (AD; aged 62+; n=76) completed NIHTB measures of emotional well-being and social resource quality. Results from linear regression models indicated that social resource quality was associated with better emotional well-being on all thirteen emotional well-being measures (ps <.001). Social Resources X Group interactions showed associations between high quality social resources and emotional well-being to be stronger for participants with AD relative to CN-Young on nine measures including anger (b=–0.51(0.17), p=.002), physical aggression (b=–0.26(0.11, p=.023), life satisfaction (b=0.35(0.16), p=.033), meaning and purpose (b=0.34,SE=.17,p=.043), positive affect (b=0.36(0.15), p=.018), and perceived stress (b=–0.43(0.17), p=.013). Findings underscore the importance of social resources in older age, particularly among adults with AD. Results suggest that individuals with AD draw upon social resources to improve their emotional well-being, perhaps helping alleviate some of the emotional and behavioral disturbances associated with AD.

Abstract citation ID: igae098.0475

### COGNITIVE IMPAIRMENT IN VIETNAMESE OLDER ADULTS: EFFECTS OF LIVING ARRANGEMENTS AND FAMILIAL SUPPORT

Attrayee Bandyopadhyay, Kim Korinek, and Daniel Adkins, *University of Utah, Salt Lake City, Utah, United States*

Living alone has emerged as a prominent modifiable risk factor for cognitive impairment and dementia. How living arrangements influence risk for cognitive disorder is less well understood in low-to-middle income countries (LMICs) where Alzheimer's disease and related dementias (ADRD) burden is rising and falls heavily upon family members. Vietnam is one such LMIC where ADRD rates are rising as demographic and cultural changes alter the living arrangements pertinent to cognitive aging. In this paper, we analyze data from the Vietnam Health and Aging Study, a longitudinal study of 2,447 older adults aged 60 and above residing in four districts of northern Vietnam, to examine the association between living arrangements and cognitive impairment. In a series of multiple linear and ordered logistic regression analyses, we estimate cognitive function using an abridged version of the Mini-Mental Scale Examination and its association with a categorical measure of living arrangements, and mediated by measures of social support, stress, and socioeconomic status. Findings indicate that females generally have poorer cognitive function than males, particularly those living alone. By comparison, those living with both spouse and children exhibit higher cognitive functioning. Nested model comparisons suggest that the ill effects of living alone on cognitive function are due in part to weaker social support, greater stress, and poorer socioeconomic conditions as compared to those living with spouses and children. These findings suggest research on the etiology of cognitive impairment should address nuanced forms of family support and stress as they occur and diverge across living arrangement configurations.

### SESSION 1490 (SYMPOSIUM)

Abstract citation ID: igae098.0476

### POLICY SERIES: ENSURING SCALE-UP AND SPREAD OF EVIDENCE-BASED PRACTICE: AGE-FRIENDLY HEALTH SYSTEMS IMPLEMENTATION BOOSTERS

Chair: Nicholas Schiltz
Co-Chair: Heba Aldossary

This symposium presents a series of studies focused on the implementation of the Age-Friendly Health System (AFHS) 4Ms Framework within the largest convenient care health system in the U.S. These studies highlight the need for innovative "booster" strategies to improve the delivery of the 4Ms—Mobility, Medications, Mentation, and What Matters Most. The first study provides an overview of the implementation and improvement science methods used to facilitate the adoption of the 4Ms. Despite initial efforts, delivery of 4Ms care remained low after one year, highlighting the need for additional booster strategies. The second study introduces the Virtual Clinic, a serious video game designed to improve provider competency and confidence in delivering 4Ms care. The game simulates realistic older adult patient visits, providing valuable practice for providers. The third study presents the Reflection and Action strategy, a frontline feedback-driven approach aimed at enhancing both competency and confidence among providers in delivering the 4Ms. The strategy leverages the well-established "Plan, Do, Study, Act" model for improvement. The fourth study discusses the Age-Friendly Health Systems Questionnaire (AFHS-Q), a novel approach developed by frontline providers that enhances efficiency by having patients complete eight 4Ms-related questions while awaiting their medical visit. The final study introduces the Age-Friendly Health Systems Advocacy Tip Sheet, a tool designed to empower older adults and caregivers to advocate for Age-Friendly care. Collectively, these studies emphasize the importance of innovative booster strategies, frontline feedback, and patient empowerment in enhancing the delivery of age-friendly care.

Abstract citation ID: igae098.0477

### AGE-FRIENDLY HEALTH SYSTEMS 4MS IMPLEMENTATION: THE NEED FOR BOOSTER STRATEGIES

Anne Pohnert[1], Nicholas Schiltz[2], and Mary Dolansky[2], *1. CVSHealth, Bellingham, Massachusetts, United States*, *2. Case Western Reserve University, Cleveland, Ohio, United States*

This study describes the implementation of the Age-Friendly Health System (AFHS) 4Ms Framework within the largest convenient care health system in the U.S. Implementation and improvement science methods were used to facilitate adoption of the 4Ms into ~1,100 MinuteClinics located in 35 states and DC, and to educate the ~3,300 providers working at these locations. Modifications to the electronic health record enabled the comprehensive documentation of assessments and actions related to the AFHS 4Ms. Formative evaluation utilized Tableau dashboards for provider and manager reporting. In the initial year, the project employed a quality improvement design. Only 22% delivery of the 4Ms was achieved after the first year indicating that "booster" strategies were needed. Following 18 months of implementing additional booster strategies to enhance 4Ms uptake, the system received IHI Regional Committed to Care Excellence recognition, achieving significant improvements across all 4Ms. As of 2024 Q1, 38.0% of eligible patients visits receive all 4Ms, while XX% receive at least one of the 4Ms. While improvement and implementation science methods facilitated initial implementation, further understanding and enhancement strategies are essential for scaling up and widespread adoption.

Abstract citation ID: igae098.0478

### IMPLEMENTATION BOOSTER: TESTING A VIRTUAL SIMULATION GAME TO ENHANCE CONFIDENCE AND COMPETENCE

Grace Armstrong[1], Mary McCormack[2], Ilona Seaman[1], Mary Dolansky[1], Anne Pohnert[3], Nicholas Schiltz[1], and Sarah Ball[4], *1. Case Western Reserve University, Cleveland, Ohio, United States*, *2. CVS Health Minute Clinic, Woonsocket, Rhode Island, United States*, *3. CVSHealth, Bellingham, Massachusetts, United States*, *4. CVSHealth Minute Clinic, Woonsocket, Rhode Island, United States*

The Virtual Clinic, a serious video game, aimed to improve provider competency and confidence in the delivery of AFHS 4Ms care through simulation of realistic older adult patient visits. The Virtual Clinic (VC) was developed in response to focus groups of front-line providers that revealed they valued gaining competency in delivering 4Ms through practice. The first prototype was tested in July 2021. Based on feedback the prototype was iteratively modified, tested and evaluated for effectiveness over three additional pilot waves. Regions of low 4Ms delivery were specifically targeted in an open-enrollment style of recruitment, with various incentives depending on the wave. A total of 262 providers have participated. Providers' performance were evaluated one week before, one week after, and four weeks after participation in the virtual clinic. Participating providers were matched at a 3:1 ratio to providers in the system that did not participate in the VC and had similar baseline 4Ms delivery and patient volume. Across all waves, participants in the VC were found on average to have a 24% increase in M-score, and a 7% increase in eligible appointments with at least one M delivered four weeks after participation in the virtual clinic. Overall, participation in the virtual clinic shows moderate, but statistically significant increase in the delivery of evidence-based 4Ms care in providers with low baseline performance.

Abstract citation ID: igae098.0479

### IMPLEMENTATION BOOSTER: A FRONTLINE INNOVATION USING AN AFHS-QUESTIONNAIRE

Heba Aldossary[1], Holly Kouts[2], and Mary Dolansky[1], *1. Case Western Reserve University, Cleveland, Ohio, United States*, *2. CVSHealth Minute Clinic, Woonsocket, Rhode Island, United States*

Booster strategies originating from frontline providers are essential for optimizing 4Ms care delivery. The Age-Friendly Health Systems Questionnaire (AFHS-Q) is a novel approach, developed by frontline providers in the MinuteClinic, that enhances efficiency by having patients complete eight 4Ms-related questions while awaiting their medical visit. This approach fosters patient engagement, allowing individuals to express their concerns and preferences, ensuring personalized care. Providers (nurse practitioners and physician associates) across the ~1,100 MinuteClinic sites nationwide received AFHS-Q kits. Survey sent to 3,300 nurse practitioners completing the 4Ms and documenting findings before implementation. After four weeks, we surveyed 100 providers to provide feedback (scale range 1 to 5). 91 providers completed the survey. In general, feedback on the AFHS-Q was positive relating to the comfort of use (M = 4.18, SD =.99), usefulness (M = 3.97, SD = 1.10), acceptability (M = 4.12, SD =.83), and effort for use (M = 2.75, SD = 1.17). Providers confidence in completing the 4Ms (before AFHS-Q: M = 3.92, SD = 1.90; after AFHS-Q: M = 4.18, M =.97) and documenting the 4Ms before AFHS-Q: M = 3.84, SD =.99; after AFHS-Q: M = 4.17, M =.91) was significantly higher following AFHS-Q use. Providers also reported a significant reduction in time for visits after using the AFHS-Q. In summary, AFHS-Q proves effective, feasible, and well-received. Valuable feedback drives continuous refinement. Our findings emphasize the importance of provider-driven solutions for evidence-based healthcare quality improvement efforts.

Abstract citation ID: igae098.0480

### IMPLEMENTATION BOOSTER: REFLECTION AND ACTION AT THE FRONTLINE

Mary Dolansky[1], Kevin Little[2], Lilia Pino[3], Jennifer Nabong[4], and Anna Bender[5], *1. Case Western Reserve University, Cleveland, Ohio, United States*, *2. Informing Ecological Design, LLC, Madison, Wisconsin, United States*, *3. CVS Health Minute Clinic, Woonsocket, Rhode Island, United States*, *4. CVSHealth Minute Clinic, Woonsocket, Rhode Island, United States*, *5. University of Washingotn, Seattle, Washington, United States*

Frontline feedback during the implementation of the Age-Friendly Health System (AFHS) 4Ms Framework at MinuteClinic, indicated some providers faced barriers and challenges in delivering 4Ms care. In response, an interdisciplinary team of educators, providers, and other quality improvement experts devised the Reflection and Action strategy, aimed to enhance both competency and confidence among providers in delivering the 4Ms—namely, Mobility, Medications, Mentation, and What Matters Most. Leveraging the well-established "Plan, Do, Study, Act" model for improvement, the team conducted three small-scale experiments to test the Reflection and Action strategy. Each experimental cycle sought to clarify provider expectations, raise awareness of current performance, and catalyze actions for performance improvement during weekly tests of change (inspired by Juran's Model for Improvement, 1961). Cycle 1 demonstrated a notable 12.5% increase in eligible visits with comprehensive documentation of all 4Ms. Cycle 2 built upon this progress, yielding a further 9.8% increase. Cycle 3 achieved a substantial 27.7% increase in adherence to the 4Ms. Between cycles, the implementation team dynamically adjusted strategies, refined targets, and fine-tuned educational materials and coaching messages to optimize effectiveness. Providers actively participated by completing weekly surveys, providing valuable feedback that informed strategic adjustments. Across all cycles, providers reported heightened confidence in completing the 4Ms assessment, coupled with a reduction in the time required for documentation.

Abstract citation ID: igae098.0481

### AN IMPLEMENTATION BOOSTER TO EMPOWER THE PATIENT AND CAREGIVER: 4MS ADVOCACY TIP SHEET

Betty Napoleon[1], Mary Zonsius[2], Elizabeth Zimmerman[1], Megan Foradori[1], and Mary Dolansky[1], *1. Case Western Reserve University, Cleveland, Ohio, United States*, *2. Rush University, Chicago, Illinois, United States*

Consumer driven reminders are another way to increase the uptake of evidence-based practices. With that in mind, our patient advocacy council engaged in the development of an Age-Friendly Advocacy Tip Sheet. Patients shared that they were unaware as to how to ensure that they received AFHS 4Ms care. Patients' views enhance insights into booster strategies for implementation of the 4Ms. It is not only essential for providers to have access to resources to deliver the 4Ms of care, but it is equally important for consumers to have tools to boost 4Ms care. Through multiple iterations, feedback sessions, and on-going qualitative analysis, we supported our Patient Advisory Council to create an Age-Friendly Health Systems Advocacy Tip Sheet to empower older adults to prioritize the 4Ms of care during appointments. This Tip Sheet includes self-advocacy suggestions, information on the 4Ms of care, and a work-sheet style space to list questions and concerns before the visit. There is also a space for note taking during the visit, along with next steps for acting on what matters. A scannable QR Code on the back of the Tip Sheet provides access to additional resources and information on Age-Friendly Health Systems. Feedback from both providers, patients, and other older adults has been overall positive, with remarks such as it is helpful, user-friendly, colorful, easy to understand, and applicable to pre-appointment preparation.

### SESSION 1495 (SYMPOSIUM)

Abstract citation ID: igae098.0482

### ENVIRONMENTAL FORTITUDE: NEIGHBORHOOD CONTEXT AND COGNITIVE HEALTH

Chair: Noah Webster
Discussant: Kyriakos Markides

It is estimated that up to forty percent of Alzheimer's Disease and Related Dementia (ADRD) cases are preventable. While modifiable risk factors are often linked with neighborhood characteristics, less attention is given to this context in interventions. Further, as prevalence of ADRD rises and racial/ethnic disparities persist, research is needed within groups to guide development of culturally and environmentally specific interventions. This symposium includes four complementary papers focusing on diverse neighborhood factors and their associations with cognitive health among a sample of older Mexican Americans from the Hispanic Established Population for the Epidemiological Study of the Elderly. The papers result from participation in the Michigan Center for Contextual Factors in Alzheimer's Disease (MCCFAD)

2023 Summer Data Immersion Program at the University of Michigan. MCCFAD is an NIA funded ADRD Resource Center for Minority Aging Research. Milani and colleagues found that limitations in activities of daily living mediate the association between living in a census tract with more highways and worse cognitive health. Clark and colleagues investigate the effect of ethnic enclaves and demonstrate a negative effect on both cognitive and functional decline. Lee and colleagues found that in urban settings, presence of religious organizations is associated with better cognitive health, while more civic organizations is linked to worse. Scambray and colleagues document an adverse effect of living in a disadvantaged neighborhood on cognition among those reporting moderate levels of relationship quality. These papers will be discussed by Kyriakos Markides who will provide an outlook for future research in this area.

Abstract citation ID: igae098.0483

### MENTAL AND PHYSICAL HEALTH PATHWAYS LINKING EXPOSURE TO HIGHWAYS AND COGNITIVE HEALTH AMONG OLDER MEXICAN AMERICANS

Sadaf Milani[1], Erika Meza[2], Carmen Carrion[3], Helen Meier[4], and Laura Zahodne[5], *1. The University of Texas Medical Branch, Galveston, Texas, United States*, *2. Harvard University, Cambridge, Massachusetts, United States*, *3. Yale University, New Haven, Connecticut, United States*, *4. Institute for Social Research, Institute for Social Research, Michigan, United States*, *5. University of Michigan, Ann Arbor, Michigan, United States*

Residing in an area with more major roadways is associated with worse cognitive function, yet few studies examine this relationship among older Mexican Americans. Our objective is to explore mental and physical health pathways linking exposure to highways to cognitive performance/function among Mexican Americans aged 75 and older. We used data from the Hispanic Established Population for the Epidemiological Study of the Elderly (2004/2005). Our exposure was the number of primary and secondary roads in a participant's census tract. Our outcome was the Mini-Mental State Examination (MMSE) score. We examined depressive symptoms (Center for Epidemiologic Studies Depression Scale), neighborhood satisfaction (very satisfied/satisfied vs. neutral/dissatisfied/very dissatisfied), basic activity of daily living (ADL) limitations (7-item modified Katz ADL scale), and physical function (Short Physical Performance Battery) as separate potential mediators, using path analysis. Covariates included age, sex, education, marital status, nativity, language of interview, length of time in residence, and census tract characteristics (urbanicity, affluence, disadvantage, and proportion of Hispanic residents). Our analytic sample (n=1,723) had an average age of 81.5 years (standard deviation: 4.9) and was 61.2% female. Residing in census tracts with more highways was associated with lower MMSE scores. Depressive symptoms, neighborhood satisfaction, and physical function were not mediators while ADL limitations mediated 40% of the relationship between living in census tracts with more highways and MMSE scores. Future work is needed to understand the direct and indirect mechanisms underlying the association between exposure to highways and ADL limitations, as well as between ADL limitations and cognitive health.

Abstract citation ID: igae098.0484

### THE EFFECT OF ETHNIC ENCLAVE ON COGNITION AND EVERYDAY FUNCTIONING DIFFERS ACROSS NEIGHBORHOOD DISADVANTAGE

Alexandra Clark[1], Emily Morriss[2], Jeremy Grant[3], Darlingtina Esiaka[4], Kristine Ajrouch[2], and Wassim Tarraf[5], *1. The University of Texas Austin, Austin, Texas, United States*, *2. University of Michigan, Ann Arbor, Michigan, United States*, *3. University of Florida, Gainseville, Florida, United States*, *4. University of Kentucky College of Medicine, University of Kentucky, Kentucky, United States*, *5. Wayne State University, Detroit, Michigan, United States*

Ethnic enclaves represent concentrated geographic areas where Hispanic community members reside in close proximity to same group peers. Previous research has revealed evidence of a "barrio advantage" where living in an ethnic enclave is associated with better health outcomes. However, there is some evidence to suggest (1) the protective effects of enclave may be moderated by neighborhood-level socioeconomic factors and (2) the impact of ethnic enclave on cognitive and functional outcomes remain poorly understood. The present study leveraged data from the Hispanic Established Populations for the Epidemiological Study of the Elderly to examine the effects of ethnic enclaves on cognitive and functional outcomes. We examined associations between tract-level segregation (Hispanic-Specific Getis-Ord Gi*) and baseline cognitive and functional outcomes, as well as change over time. Analyses included cluster adjusted linear regression models sequentially adjusted for neighborhood and individual level sociodemographic measures. Findings showed a positive association between enclave residence and baseline cognitive status, controlling for neighborhood and individual level socioeconomic disadvantage. The enclave effects did not extend to baseline functional status. Despite positive enclave effects at baseline, enclave residence was associated with faster rates of cognitive and functional decline. Markers of socioeconomic disadvantage were not consistent modifiers of associations between the enclave effect and cognitive and functional outcomes. Results will be discussed within the ecological system theory to emphasize environmental contexts are important for late life cognitive and functional outcomes, and further highlight potential points of intervention that may promote equitable aging outcomes in Hispanic older adults.

Abstract citation ID: igae098.0485

### NEIGHBORHOOD SOCIAL AND SERVICE ORGANIZATIONS AND COGNITIVE DECLINE FOR OLDER MEXICAN AMERICANS

Ji Hyun Lee[1], Sunshine Rote[2], Elham Mahmoudi[3], Marina Armendariz[4], Toni Antonucci[5], and Mark Manning[6], *1. Montana State University, Bozeman, Montana, United States*, *2. University of Louisville, Louisville, Kentucky, United States*, *3. University of Michigan Medical School, Ann Arbor, Michigan, United States*, *4. The University of Texas San Antonio, San Antonio, Texas, United States*, *5. University of Michigan, Ann Arbor, Michigan, United States*, *6. Oakland University, Rochester, Michigan, United States*

Neighborhood resources are salient in healthy brain aging. Neighborhood "third places" such as community centers and local eateries are increasingly recognized for their role in supporting the cognitive health of older residents. However, less is known about whether cognitive benefits from neighborhood resources are experienced similarly across diverse older adults. In the current study, we examined the associations between the availability of neighborhood social (i.e., religious institutions, social clubs) and service organizations (i.e., senior centers, community food banks) on cognitive decline among Mexican Americans living in urban and rural areas in the southwest U.S. region. We linked the panel data from the 2004-2013 Hispanic Established Populations for the Epidemiologic Study of the Elderly (H-EPESE) with the census-level data on the number of organizations from National Neighborhood Data Archive (N= 628, Age M(SD) = 80 (3.6), female 64%, U.S. born 54%). Cognitive decline was based on MMSE scores with cut-off points adjusted for low educational attainment. The generalized estimating equations model accounted for person-level covariates such as age, gender, years of education, marital status, nativity, and dual eligibility for Medicaid and Medicare. We found that, in urban settings, the presence of religious organizations was associated with better cognition, but a higher number of civic organizations were linked to worse cognitive health and a steep decline in cognition. Social and service organizations were not associated with cognition in rural settings. Our findings highlighted the importance of urban/rural contexts in identifying neighborhood organizations that may support healthy brain aging for older Mexican Americans.

Abstract citation ID: igae098.0486

### NEIGHBORHOOD DISADVANTAGE, SOCIAL RELATIONSHIPS, AND COGNITION AMONG MEXICAN-AMERICAN OLDER ADULTS

Kiana Scambray[1], Angela Gutierrez[2], Monica Walters[1], Cindy Tsotsoros[3], Ketlyne Sol[1], and HwaJung Choi[1], *1. University of Michigan, Ann Arbor, Michigan, United States*, *2. Western University of Health Sciences, Pomona, California, United States*, *3. University of Rhode Island, Kingston, Rhode Island, United States*

Research suggests that Hispanics live in more disadvantaged neighborhoods, and neighborhood disadvantage itself is associated with worse cognition. Few studies have examined whether social factors could buffer this relationship among Hispanic older adults. We examined whether social network size and relationship quality moderates the relationship between neighborhood disadvantage and cognition, and whether these moderations differed by gender in this cross-sectional study. We included 2069 participants (Mean age=81.9; 62% women) from the fifth wave of the Hispanic Established Populations for the Epidemiological Study of the Elderly (H-EPESE). Social network size was defined as the total number of children, relatives, and friends. Relationship quality was defined by how frequently the individual can count on and discuss deep problems with friends/family. Neighborhood disadvantage quartiles (least to most disadvantaged) were created from measures in the National Neighborhood Data Archive after linking with the H-EPESE individual-level data using the census-tract geocode. The Mini Mental State Examination, measuring global cognition, was the outcome. Multiple linear regressions with interaction terms were conducted to assess for moderation, which were repeated across the gender-stratified samples. Relationship quality moderated the association between neighborhood disadvantage and cognition whereas social network size did not. The adverse effect of living in the most disadvantaged neighborhood on cognition was observed among those reporting medium relationship quality ($B=-2.94$, $p<.05$). This relationship appeared to be primarily driven by women ($B=-5.49$, $p=.02$). These findings suggest that interventions to improve cognition among Hispanic older adults in disadvantaged neighborhoods should consider how relationship quality and gender impact cognition.

### SESSION 1500 (SYMPOSIUM)

Abstract citation ID: igae098.0487

### FUELING SCIENTIFIC INNOVATION: NIA'S ENTREPRENEURIAL DEVELOPMENT PROGRAMS FOR AGING RESEARCHERS

Chair: Todd Haim

The symposium panel will offer a comprehensive overview of National Institute on Aging (NIA) programs designed to foster entrepreneurship and facilitate the commercialization of aging-related innovations. Central to the discussion are initiatives such as NIA Startup Challenge and Accelerator which has been demonstrated to provide first-time entrepreneurs with essential training in evidence-based technology development and position them for success. Additionally, the panel will highlight the NIA Research and Entrepreneurial Development Immersion (REDI) funding opportunities, which aim to expose researchers in gerontology and related fields to entrepreneurship.

A key focus of the panel is the pivotal role of Small Business Innovation Research (SBIR) funding as a primary source of early-stage seed funding in aging research. Panelists will elucidate how SBIR funding empowers startups to reach critical value inflection points by supporting data collection and validation processes.

Moderated by NIA entrepreneurial program leadership, the panel features representatives from Challenge finalists, SBIR-funded startups, and key stakeholders in the Aging Research-related startup ecosystem. Through diverse perspectives and real-world experiences, attendees will gain valuable insights into navigating the entrepreneurial journey, leveraging NIA resources, and harnessing the power of collaboration to enable the impactful commercialization of innovations that would enhance the health and/or care of older adults.

In summary, the panel will provide a dynamic exchange of ideas, strategies, and success stories, as we illuminate the path from research innovation to market transformation in the realm of aging research. Business and Aging Interest Group Sponsored Symposium

Abstract citation ID: igae098.0488

### NIA START-UP CHALLENGE AND ACCELERATOR: EMPOWERING AGING RESEARCHERS FOR ENTREPRENEURIAL SUCCESS

Joy Toliver, *National Institute on Aging, Baltimore, Maryland, United States*

As an effort to support the development of aging researchers transitioning into entrepreneurship, the National Institute on Aging designed and launched its Start-Up Challenge and Accelerator. Through a five-month accelerator program, participants learn how to translate their research-driven solutions into market-ready products that promote healthy aging. Participants receive personalized mentorship, entrepreneurial training, networking opportunities, and a chance to win NIA funding. With cohorts in 2022 and 2023, the Startup Challenge and Accelerator supported the development of 40 transitioning entrepreneurs that are developing innovations to enhance the health and/or care of older adults and awarded cash prizes to eleven. Since the completion of the first cohort, graduates of the program have hit a multitude of milestones including, but not limited to, a company exit, raising venture capital financing, launching health system pilots, securing key strategic partners, and winning national pitch competitions. By attending this panel, you will learn more about the program, how to apply, and how past participants have leveraged the program to fuel their entrepreneurial growth and establish a path to success.

Abstract citation ID: igae098.0489
### ACADEMIC NURSING RESEARCH, INNOVATION, AND ENTREPRENEURSHIP
Marcia Shade, *University of Nebraska Medical Center, Omaha, Nebraska, United States*

Marcia Shade PhD, BS, RN is the Founder and CEO of Voice-It Inc. The start-up company focuses on creating digital health solutions that engage, inform, and empower older adults. Voice-It Inc. is harnessing, AI, the power of machine learning and natural language processing to help improve the health and quality of life of older adults. Dr. Shade will share: 1) Her perceptions on the importance of understanding entrepreneurship as an academic nurse researcher and innovator, and 2) Reflections on her experience as a finalist in the 2023 NIA Start-Up Challenge. "I received invaluable guidance from the NIA Team, experts, mentors, advisors, and fellow cohort finalists to help me grow as an entrepreneur and lay a foundation for my start-up company."

Abstract citation ID: igae098.0490
### NIA FUNDED OBATALA SCIENCES: DRIVING DIVERSITY IN RESEARCH ENTREPRENEURSHIP AND ADVANCING REGENERATIVE MEDICINE
Trivia Frazier, *Obatala Sciences, Inc, New Orleans, Louisiana, United States*

Obatala Sciences, Inc. developed the first human-derived hydrogel for 2D and 3D cell culture which can be applied in various organ-on-a-chip models. The New Orleans-based Obatala Sciences fat-on-a-chip platform and supporting products offer researchers a new, more accurate way of modeling human fat tissue in-vitro. Obatala Sciences was spun out of the NIH/NIA funded LaCell LLC in 2017. To build on their prior SBIR and seed funding, Obatala Sciences raised $3 million in Series A investments in 2022. Obatala's scientific and thought leadership are committed to promoting diversity to advance the study and prevention of diseases in the fields of obesity, diabetes, tissue engineering and regenerative medicine. In 2022, Obatala also received NIA funding to create an entrepreneurship in biotechnology training program in partnership with universities in Southeast Louisiana. The newly developed program is leveraging the diverse background and training of the diverse regional student population to give students greater insight into biotech and entrepreneurial career paths.

Abstract citation ID: igae098.0491
### NIA SBIR-FUNDED JUVENA THERAPEUTICS TRANSLATION OF POWERFUL PROTEINS INTO TRANSFORMATIVE THERAPEUTICS
Jeremy O'Connell, *Juvena Therapeutics, Inc., Redwood City, California, United States*

Juvena Therapeutics, which received NIA SBIR funding in 2021, was founded to restore tissue health by repairing protein signaling and intercellular communication. Juvena maps the therapeutic potential of natural secreted proteins and engineers them into biologics that restore tissue homeostasis. Juvena's artificial intelligence-enabled platform mines thousands of proteins secreted by human stem cells, screens their potential to restore tissue homeostasis, and engineers novel biologic medicines into life-saving treatments. Juvena's SBIR funding had multiple benefits as it enabled optimization of their lead compound, additional compound screenings, as well as the rigorous feedback that resulted from peer review. Since receiving NIA SBIR funding and showcase support, Juvena has now raised close to $60 million in venture capital funding and is rapidly advancing its efforts. Juvena's journey is a great example of how SBIR funding can be a critical resource early in a biotech startup's development path.

### SESSION 1505 (PAPER)

### HEALTH BEHAVIORS

Abstract citation ID: igae098.0492
### MARITAL STATUS AND DRINKING BEHAVIORS AMONG OLDER HISPANIC ADULTS IN THE UNITED STATES
Jaminette Nazario-Acevedo[1], Takashi Yamashita[2], Jennifer Bulanda[3], and J. Scott Brown[3], *1. University of Maryland Baltimore, Baltimore, Maryland, United States*, *2. University of Maryland Baltimore County, Baltimore, Maryland, United States*, *3. Miami University, Oxford, Ohio, United States*

On average, Hispanics are not the most frequent alcohol consumers, however, when Hispanics consume alcohol, the chance of excessive drinking is greater than for other racial and ethnic groups. Excessive drinking can be seen as a stress-coping response and harmful, especially to the health of older Hispanic adults. Hispanic culture places particular importance on family relationships (a.k.a., Familismo). The size of social networks tends to shrink in later life and a specific social relationship-- marriage -- becomes a crucial social support network. Studies show that marital relationships facilitate stress-coping processes and, in turn, benefit health. A social support system can be a resource to prevent stress-induced excessive drinking among older Hispanic adults. Thus, the current study aims to examine how marriage is associated with alcohol consumption behaviors among older Hispanic adults in the United States. The data of the nationally representative samples of Hispanic adults aged 51 years and older (n = 316) are obtained from the 2018 U.S. Health and Retirement Study (HRS). Results from the negative binomial regression indicate that on average, married older Hispanic adults consume 66% fewer alcoholic drinks per week ($b = -1.10$, $p < 0.01$) than their unmarried counterparts. Also, older Hispanic women consume 90% fewer alcoholic drinks per week (b = -2.29, p < 0.01) than older Hispanic men. The roles of culturally valued social relationships and gender differences, along with other predictors of alcohol consumption patterns are evaluated from sociocultural and public health perspectives (e.g., prevention of excessive drinking through social support).

Abstract citation ID: igae098.0493

### A SCOPING REVIEW OF PHYSICAL ACTIVITY IN ADULTS OVER 50 WITH AUTISM: MANY BARRIERS AND SPARSE DATA

Connie Kartoz, Rebeca Jefferson, Chase Eisenberg, and Antigone Antonakakis, *The College of New Jersey, Ewing, New Jersey, United States*

The population of older adults with autism is growing, yet the literature for this group is still relatively sparse (Mason et al., 2022). Adults with autism are reported to have comorbidities and a reduced lifespan (Hand et al., 2020). While physical activity (PA) is known to reduce chronic disease burden, extant research indicates young adults with autism do not engage in physical activity (PA) with the same frequency as neurotypical peers. Little is known about PA for older adults with autism. This scoping review aimed to explore research about PA for older adults with autism. **Methods:** Following the PRISMA-ScR guidelines, 5 databases were searched (2018 and later) with keywords 'autism,' 'older adults', 'PA,' and age group delimiters. **Results:** Initially, no articles were found, and the search was expanded to include all adults. After eliminating duplicates, 121 studies were reviewed, with 25 meeting the criteria (PA, autism, and no participants less than 18) and 9 having any participants over 50; in each of these 9 studies, the number of participants over 50 was less than 100. Only 2 studies focused on adults over 50. The level of evidence was low. The limited data suggests those aging with autism do not engage in recommended levels of PA and experience barriers to the same, but that interventions improve PA and possibly quality of life. IMPLICATIONS: There is a dire need to design interventional research to increase PA in older adults with autism to improve equitable health outcomes for this at-risk group.

Abstract citation ID: igae098.0494

### ANALYSIS OF IMPACT OF DAILY OBJECTS' SHAPE ON STANDING AND SITTING BEHAVIOR OF FRAIL OLDER PEOPLE

Satoru Handa[1], Koji Kitamura[2], Hisashi Kawai[3], and Yoshifumi Nishida[1], *1. Tokyo Institute of Science Tokyo, Meguro City, Tokyo, Japan*, *2. National Institute of Advanced Industrial Science and Technology, Tokyo, Japan*, *3. Tokyo Metropolitan Institute for Geriatrics and Gerontology, Tokyo, Japan*

One promising approach to supporting the independent lives of frail older people is the design of living environments. The objective of this study is to clarify the effect of slight changes in the three-dimensional shape of daily environments, such as a desk, on standing and sitting by conducting experiments on frail older people. To this end, the authors fabricated four types of desks with different shapes and then measured the standing and sitting movements of frail older people (N=85, 65 to 90 years old) using the desks, a motion capture system, three-axis force sensors, and pressure sensors. The measure data were analyzed from psychological and physical viewpoints. Eight of the 85 applicants had severe sarcopenia. The results showed that handrails and fingerholds significantly increased the force to hold the desk (p< 0.0001), and analysis using a musculoskeletal model (AnyBody) revealed that they decreased the muscle activity to push the desk (p=0.015).The cluster analysis results using posture data present that the handrails and handholds of the desk contributed to shifting the center of gravity of the upper body forward, leading to a decreasing burden on the muscles. A post-measurement questionnaire indicated that over 80% of the participants felt desks with handrails or handholds were more accessible to support and stand on. Some participants commented that the slight shape change improved a sense of safety. These results suggest that even minor design features such as handrails and fingerholds can assist the older in shifting their upper body posture without increasing the muscles' burden.

Abstract citation ID: igae098.0495

### INSTRUMENTS MEASURING SELF-CARE BEHAVIORS OF OLDER ADULTS WITH CHRONIC KIDNEY DISEASE: A SCOPING REVIEW

Thanakrit Jeamjitvibool, and Wirampa Tanglai, *University of Illinois Chicago, Chicago, Illinois, United States*

Promoting self-care behavior among older adults with chronic kidney disease (CKD) is critical for preventing kidney deterioration, particularly in the early stages, as symptoms often become noticeable only in later stages. Numerous assessment tools exist, but their suitability and quality remain uncertain. This review aims to identify, assess methodologies, and consolidate psychometric properties of self-care assessment instruments for older adults with CKD. We followed Askey & O'Malley's (2005) scoping methodology and PRISMA-ScR guidelines. PubMed, Scopus, CINAHL, PsycInfo, and Embase were searched until October 5, 2023. Included studies tested measurement properties of self-reported instruments assessing self-care behaviors in older adults with CKD, were published in English, and appeared in peer-reviewed journals. The COSMIN framework was used to appraise study quality. Ten articles addressing instruments for self-care behaviors in older adults with CKD were included. Based on COSMIN, no article reported complete measurement properties. Six instruments were developed with theory support, while no theoretical underpinning was identified for three instruments. Only one instrument was developed based on qualitative research. Some instruments were applied to a combined population, such as patients with diabetes and CKD. Although there have been advances in creating tools for assessing self-care behaviors in older adults with CKD, no single instrument fully reported measurement properties. Decision-making regarding which of the available tools is appropriate for practice or research should consider their intended purpose, scope, and any underlying theoretical principles. Future research should focus on developing a comprehensive instrument for CKD self-care behaviors that exhibits high methodological quality.

### SESSION 1510 (SYMPOSIUM)

Abstract citation ID: igae098.0496

### IMPACT OF GENDER FOR ELDER MISTREATMENT WITHIN DEMENTIA FAMILY CAREGIVING

Chair: Carolyn Pickering
Discussant: Leila Wood

Gender plays a significant role in interpersonal violence throughout life, particularly through gender-based violence or violence driven by gender-based power dynamics. However, understanding the role of gender in elder mistreatment within dementia family caregiving is more complex due to the various layers affected by gender. For instance, societal gender norms often lead to women being primary caregivers, and trends in dementia mean women are more likely to be care recipients. Gender influences access to caregiver resources, such as financial support, and impacts outcomes like caregiver depression. This symposium aims to explore the diverse ways gender influences elder mistreatment in family caregiving contexts. The first paper investigates how the gender of the caregiver influences the likelihood of engaging in various subtypes of elder mistreatment. The second paper delves into how polysubstance use affects the risk of different types of elder mistreatment, and how this risk varies based on caregiver gender. The third paper examines perceived reciprocity as a protective factor for elder mistreatment subtypes and explores whether it plays a stronger role based on caregiver gender. The last paper utilizes a gerofeminist lens to examine the intersection of gender and physical disability in the risk of being victimized by various subtypes of elder mistreatment.

Abstract citation ID: igae098.0497

### INTERSECTION OF GENDER, PHYSICAL DISABILITY, AND EXPERIENCES OF ELDER MISTREATMENT IN DEMENTIA

Carolyn Pickering[1], Wesley Browning[2], Vicki Winstead[3], Jessica Hernandez[3], and Mustafa Yildiz[1], *1. The University of Texas Health Houston, Houston, Texas, United States, 2. The University of Texas Health Science Center Houston, Houston, Texas, United States, 3. The University of Texas Health Science Center, Houston, Texas, United States*

A gerofeminist perspective highlights the intersection of gender, age, and power dynamics that influence the experience of aging women, while recognizing that age can also come with beneficial resources. Since gender-based power dynamics play significant roles in women's experiences of aggression at younger ages, this study investigates the intersection of gender, physical disability, and elder mistreatment among older adults with dementia. Family caregivers provided self-reports on their care recipients' characteristics and the frequency of engaging in physically aggressive, psychologically aggressive, and neglectful behaviors at three time points (enrollment, 6-, and 12-month follow-ups). We utilized mixed models for repeated measures to assess whether women experienced more aggressive and neglectful behaviors than men and whether physical disability heightened this risk. Care recipients (N=453) were more often women (58%). Women care recipients required significantly more assistance with ADLs ($p<0.001$), were more often wheelchair or bedbound ($p<0.001$) and lived in larger ($p=0.003$) and more multigenerational households compared to men ($p=0.03$). Gender-only models revealed that women experienced significantly less neglect than men ($p=0.02$). When physical disability was considered, wheelchair or bedbound women experienced significantly more neglect ($p<0.001$) and physical aggression ($p<0.001$) than men or non-physically disabled women. Findings support a gerofeminist view that women accumulate strengths as they age, such as larger social networks, that may protect against neglect. Findings also suggest that older women with physical disabilities face heightened risks of mistreatment, underscoring the importance of further research into the intersectionality of gender and disability.

Abstract citation ID: igae098.0498

### GENDER DIFFERENCES IN TYPES OF AGGRESSION USED AMONG DEMENTIA FAMILY CAREGIVERS

Wesley Browning[1], Mustafa Yildiz[2], and Carolyn Pickering[2], *1. The University of Texas Health Science Center Houston, Houston, Texas, United States, 2. The University of Texas Health Houston, Houston, Texas, United States*

Investigations of family violence across the lifespan have revealed that men more often rely on physically aggressive tactics towards while women often rely on forms of psychological aggression. Physical aggression use also declines with age. We aimed to replicate, within a sample of dementia family caregivers, findings on gender, age and aggression from family violence research. We hypothesized that male family caregivers engage more often in physically aggressive behaviors towards their relative with dementia, while women engage more often in psychological aggression. Caregivers ($n=453$) self-reported frequency of neglect, psychological aggression, and physical aggression in the past 6-months at enrollment, 6-month and 12-month follow up. We utilized linear regression to investigate age and gender differences in abuse and neglect at baseline. We employed mixed models for repeated measures to investigate whether men engaged more often in abusive and neglectful behaviors across the three waves. Men ($n=57$) engaged more often in neglect and physical aggression than women (p's<.05) at baseline. Caregivers who were older women engaged more often in neglect, while younger men engaged more often in physical abuse (p's<.05). Based on our mixed model, men engaged more often in neglectful behaviors ($B=-3.71$, $p=.001$), physically aggressive behaviors ($B=-1.55$, $p<.001$) and overall total behaviors ($B=-5.61$, $p=.001$) than women across the three waves. Findings replicate those from prior studies examining family violence across the lifespan. Special attention should be paid to male caregivers in research and clinical practice as men are uniquely at risk for abusive and neglectful behaviors.

Abstract citation ID: igae098.0499

### THE ROLE OF GENDER AND POLYSUBSTANCE USE IN ABUSIVE BEHAVIORS EXHIBITED BY DEMENTIA FAMILY CAREGIVERS

Jessica Hernandez[1], Wesley Browning[2], Mustafa Yildiz[3], Vicki Winstead[1], and Carolyn Pickering[3], *1. The University of Texas Health Science Center, Houston, Texas, United States, 2. The University of Texas Health Science Center Houston, Houston, Texas, United States, 3. The University of Texas Health Houston, Houston, Texas, United States*

This study investigates the impact of polysubstance use on the likelihood of abusive and neglectful behaviors among dementia family caregivers, with a particular focus on how these relationships differ by gender. Utilizing Generalized Linear Mixed Models (GLMM), we analyzed data from 453 caregivers, assessing substance use and behavioral outcomes measured daily over a 21-day period. Our findings reveal that polysubstance use significantly increases the risk of both psychological and physical aggressive behaviors. When examining the interaction effects, we observed that the combination of being male and engaging in polysubstance use was particularly associated with a heightened risk of these behaviors, underscoring the importance of considering gender in caregiver assessments. The final models, refined through a stepwise selection process, incorporated key covariates such as caregiving hours, relationship type, emotional regulation difficulties, caregiver age, and ethnicity. These models offered a more comprehensive picture, showing that the dynamics of the caregiver/care-recipient relationship and the caregiver's emotional regulation capabilities were significant factors influencing the likelihood of neglect and psychological aggression. In contrast, gender significantly modulated the association between polysubstance use and physical aggression. These nuanced insights highlight the complex interplay between caregiver demographics, substance use behaviors, and the potential for harmful caregiving practices. Our study emphasizes the need for targeted interventions that address the specific stressors and risks associated with male caregivers, as well as the broader implications for support programs that account for the multifaceted challenges faced by dementia family caregivers.

Abstract citation ID: igae098.0500

### GENDER DIFFERENCES ON THE EFFECT OF RECIPROCITY ON NEGLECT AND ABUSE BY DEMENTIA CAREGIVERS

Vicki Winstead[1], Carolyn Pickering[2], Wesley Browning[3], Jessica Hernandez[2], and Mustafa Yildiz[2], *1. The University of Texas Health Science Center, Houston, Texas, United States*, *2. The University of Texas Health Houston, Houston, Texas, United States*, *3. The University of Texas Health Science Center Houston, Houston, Texas, United States*

Reciprocity, a balanced exchange in interactions, is a crucial element of most social exchanges and impacts caregiving outcomes. This paper aims to investigate whether reciprocity plays a protective role against elder abuse and neglect and to explore potential gender variations associated with this relationship. A sample of 453 family caregivers reported daily perceptions of reciprocity in a micro-longitudinal study over 21 days. Perceived reciprocity was measured by the participant's level of satisfaction with the amount of affection/appreciation shown by their relative with dementia in the previous 24 hours. Generalized linear mixed models examined the effect of perceived reciprocity on the odds of the caregiver engaging in psychologically aggressive, physically aggressive, and neglectful behaviors that day. Perceived reciprocity was associated with lowered daily odds of neglect (OR= 0.76 p= 0.006) and psychological abuse (OR = 0.6 p< 0.001). We calculated an interaction term of the effects of gender and perceived reciprocity on abuse/neglect to assess gender differences. Increased reciprocity reduced the odds of neglect for both males and females, but the reduction in odds was greater for males (p=0.041). Results suggest that receiving attention and affection for caregiving may act as a protective factor, particularly for male caregivers. While female caregivers might face socially constructed role expectations, males, often taking on caregiving out of obligation, may benefit from reciprocal interactions regarding reduced likelihood of neglecting the care recipient. These insights contribute to a nuanced understanding of gender dynamics in caregiving and can inform interventions to prevent elder abuse and neglect.

### SESSION 1520 (SYMPOSIUM)

Abstract citation ID: igae098.0501

### IMPROVING QUALITY IN ASSISTED LIVING: FINDINGS FROM STATEWIDE INITIATIVES

Chair: Sheryl Zimmerman
Discussant: Philip Sloane

Almost 1.7 million people in the U.S. receive residential long-term care, more than half who reside in assisted living -- broadly defined as congregate settings that provide at least two meals a day, around-the-clock supervision, and help with personal care. Whereas nursing homes are federally regulated, assisted living is state regulated, which recognizes it as a community-based setting and allows for innovative practices and policies to promote better care and outcomes. Over the years, numerous state-based initiatives have been enacted across the country; this session will discuss new initiatives in two states, addressing their implementation, findings, and broad implications. Minnesota's legislation recently created an online report card based on resident and family surveys; the presentation by Dr. Shippee will overview three years of data from more than 12,000 residents, noting the areas scored most favorably (e.g., environment) and least favorably (e.g., engagement). Dr. Moone will discuss the Minnesota assisted living regulatory framework and how the absence of broad representation in licensure development resulted in inconsistency in and preparation for survey inspections, as well as inadequate reimbursement. North Carolina recently undertook a statewide randomized trial of accreditation for assisted living; Ms. Efird-Green will discuss challenges implementing accreditation in more than half of the communities, and Dr. Zimmerman will present two years of outcome data comparing control and accreditation arms in relation to workforce and resident outcomes, care coordination and transitions, medication management, and person-centered care. Results will be put in context to inform quality initiatives across the country.

Abstract citation ID: igae098.0502

### IMPROVING ASSISTED LIVING RESIDENTS QUALITY OF LIFE: RESULTS FROM AN MN ASSISTED LIVING REPORT CARD

Tetyana Shippee[1], Mark Woodhouse[2], Tricia Skarphol[2], Kelly Moeller[2], Lauren Glass[3], and Rachel Shands[3], *1. University of Minnesota School Of Public Health, Minneapolis, Minnesota, United States*, *2. University of Minnesota, Minneapolis, Minnesota, United States*, *3. Minnesota Department of Human Services, St. Paul, Minnesota, United States*

In 2019, Minnesota enacted new legislation resulting in creation of an online Assisted Living Report Card. As part of this work, two surveys, the Resident Quality of Life Survey and Family Satisfaction Survey, were developed to assess AL residents' quality of life (QOL) and family satisfaction. Our study presents the development and analysis of these surveys in Minnesota AL communities from 2020-2023. Resident respondents (N=12,091) were predominantly female (68.6%) and White (81.4%), with mean age 82.8 years; family satisfaction survey respondents (N=11,935) were mostly female (64.2%) and White (91.1%), with a mean age 63 years. Respondents rated various survey items with scores from 0 (lowest) to 2 (highest) for resident QOL, and 0 (lowest) to 3 (highest) for family satisfaction, with mean scores calculated for each of the domains. Overall, resident quality of life and family satisfaction scores were high, yet "overall satisfaction" (1.22), "engagement" (1.48), and "food" (1.53) domains had the lowest scores, while "environment" (1.88), "finances" (1.85), and "security" (1.82), had the highest mean scores among residents. Among family members, having one's "personal needs met" (2.12) and "finances" (2.13) had the lowest mean scores, while "choice" (2.36), "housekeeping" (2.38), and "environment" (2.39) had the highest mean scores. Insights from our study highlight areas for improvement to enhance well-being and satisfaction among AL residents and their families.

Abstract citation ID: igae098.0503

### MINNESOTA'S ASSISTED LIVING LICENSE: ENGAGEMENT OF SMALL ASSISTED LIVING PROVIDERS

Rajean Moone[1], Tetyana Shippee[2], and Megan Hakanson[3], *1. University of Minnesota, St. Paul, Minnesota, United States*, *2. University of Minnesota School Of Public Health, Minneapolis, Minnesota, United States*, *3. University of Minnesota, Minneapolis, Minnesota, United States*

When Minnesota passed State Statute 144G in 2019 it fundamentally changed the licensure of assisted living communities in the state. This new regulatory framework was developed by a group of stakeholders representing consumer advocates, provider advocates, and state government. However, assisted living providers who served culturally specific persons in small communities were largely absent from the development of this new license. As a result, several unintended consequences and challenges have emerged in implementation in these settings. We interviewed a sample of 14 licensed assisted living directors operating small, culturally specific communities to understand their experiences with the regulatory framework. After a qualitative analysis four main themes emerged: (1) lack of participation in the initial license development, (2) challenges to implementation of license requirements, (3) inconsistency in and preparation for survey inspections, and (4) inadequate reimbursement and funding. To address these concerns, several recommendations were identified which ranged from ensuring all stakeholders are engaged in policy development to developing a new rate setting methodology for Medicaid waivers. Although these recommendations are specific to Minnesota, they do have implications for other states and agencies that are developing similar regulations.

Abstract citation ID: igae098.0504

### OFFERING ACCREDITATION TO ASSISTED LIVING COMMUNITIES IN NORTH CAROLINA

Lea Efird-Green[1], Sheryl Zimmerman[2], Philip Sloane[3], David Reed[3], Yuke Tian[3], Scott Davis[4], and John Preisser[3], *1. University of North Carolina at Chapel Hill, Chapel Hill, North Carolina, United States*, *2. University of North Carolina at Chapel Hill (UNC), Chapel Hill, North Carolina, United States*, *3. University of North Carolina, Chapel Hill, North Carolina, United States*, *4. University of North Carolina Eshelman School of Pharmacy, Asheville, North Carolina, United States*

In 2021, the North Carolina Legislature approved and funded the Adult Care Home [Assisted Living] Accreditation Pilot Program. Across the state, the program's goal is to evaluate the effectiveness of accreditation through quality outcome measures to determine whether accreditation achieves compliance with licensure requirements and improves or maintains quality of care compared to a control group. Assisted living communities were selected using stratified random sampling that accounted for geographic region and state-assigned quality rating. A total of 146 communities originally agreed to participate. Of the 73 communities randomly assigned to the accreditation arm, to date 39 (53%) either withdrew or chose to not go through the process of accreditation; 4 (5%) withdrew after beginning the accreditation process; 24 (33%) achieved accredited status; and 6 (8%) are delayed in completing the process or not likely to do so. The key reason for withdrawal from the accreditation process is the time and effort required in the context of staffing shortages, which has implications for long-range implementation even if accreditation is found to be beneficial. This session will present the aims and methods of the evaluation; the accreditation process itself (including benefits, potential administrative burden, and types of deficiencies found during surveys); the feasibility of becoming accredited for diverse participating communities; and potential strategies to bolster capacity for accreditation. (Additional contributors to this and the outcome presentation include Teresa Hoosier [ACHC], Jeff Horton [NCSLA], Frances Messer [NCALA], Barbara Sylvester [ACHC], and London Grantham, Aja Johnson, Danielle Owens, Toni Parker, and Katrice Perry [UNC].)

Abstract citation ID: igae098.0505

### OUTCOMES OF ASSISTED LIVING ACCREDITATION IN NORTH CAROLINA

Sheryl Zimmerman[1], Lea Efird-Green[2], Philip Sloane[3], David Reed[3], Yuke Tian[3], Scott Davis[4], and John Preisser[3], *1. University of North Carolina at Chapel Hill (UNC), Chapel Hill, North Carolina, United States*, *2. University of North Carolina at Chapel Hill, Chapel Hill, North Carolina, United States*, *3. University of North Carolina, Chapel Hill, North Carolina, United States*, *4. University of North Carolina Eshelman School of Pharmacy, Asheville, North Carolina, United States*

Accreditation is a widely used health care review process to determine if an organization meets a defined standard of quality. The opportunity for accreditation recently moved into the field of assisted living (AL); today, seven states have statutes and regulations allowing third party accreditation to satisfy full or partial compliance with state licensure or certification. Data indicate that compliance with accreditation standards may result in improved care, but it has not been evaluated in AL. In 2021, the state of North Carolina funded an AL accreditation pilot program to evaluate whether accreditation improves or maintains quality. Communities were randomly allocated to a control or accreditation arm and are being followed for two years to evaluate care and outcomes in five areas (workforce, resident outcomes, care coordination and transitions, medication management, person-centered care). Of the 146 communities originally enrolled that provided data, most are for-profit (96%) and part of a chain (73%); 44% pf residents have dementia, 25% have mental illness, and 53% receive state financial assistance. Preliminary results in the first two quarters (Q) found an overall increase in advance care planning discussions (53% in Q1 and 63% in Q2), and a decrease in medication administrations with one or more errors (1.2% in Q1 and 0.6% in Q2). This session will present results comparing communities in the control arm, accreditation arm, and those that successfully achieved accreditation, with findings having wide-ranging implications for the future of AL accreditation and regulation in North Carolina, and implications across the country.

### SESSION 1525 (SYMPOSIUM)

Abstract citation ID: igae098.0506

### INDIVIDUAL, FAMILY, AND COMMUNITY FACTORS THAT SHAPE COGNITIVE AGING: AN ECOLOGICAL SYSTEMS APPROACH

Chair: Jeremy Hamm
Co-Chair: Jacqueline Mogle

Healthy cognitive aging is shaped in part by individual and social-environmental factors that span systems delineated by social ecological models of lifespan development. This symposium addresses the role of individual- (beliefs, health behaviors), family- (relationship quality), and community-level (neighborhood quality) factors as predictors of normative and non-normative cognitive aging. Taylor et al. use individual-level data from the Midlife in the United States (MIDUS) Study to demonstrate how a composite of control beliefs, purpose in life, and social support prospectively predicts healthier 9-year trajectories of cognitive aging. Hamm et al. use individual-level MIDUS data to document how (health behavior mechanisms) and when in the lifespan (age-moderated pathways) longitudinal changes in control beliefs predict corresponding shifts in cognitive functioning. McGrath et al. investigate novel methods in measuring individual-level indices of health behavior-dependent muscle function (force stability, explosiveness) that can better predict risk of cognitive impairment. Jang et al. use data from the National Study of Daily Experiences (NSDE) to identify the influence of different types of family-level relationships on the experience of daily memory lapses and associated negative affect. Finally, Cerino et al. use NDSE data to document how better neighborhood quality at the community-level is linked to better cognitive functioning, particularly for individuals who lack control over the daily stressors they experience. This symposium thus contributes to a more comprehensive understanding of how healthier cognitive aging is shaped by modifiable beliefs, behaviors, social dynamics, and neighborhood factors that span multiple ecological systems.

Abstract citation ID: igae098.0507

### WHEN AND HOW PERCEIVED CONTROL BUFFERS AGAINST COGNITIVE DECLINES: A MODERATED MEDIATION ANALYSIS

Jeremy Hamm[1], Margie Lachman[2], Katherine Duggan[1], Jacqueline Mogle[3], Ryan McGrath[1], Kelly Parker[1], and Laura Klepacz[1], *1. North Dakota State University, Fargo, North Dakota, United States*, *2. Brandeis University, Waltham, Massachusetts, United States*, *3. Clemson University, Clemson, South Carolina, United States*

People vary widely in their rates of age-related cognitive decline. Slower rates of decline have been linked to individual differences in perceived control, but less is known about how (mechanisms) and at what stage in the lifespan (moderators) this modifiable psychological resource is linked to preserved cognitive functioning. Our study examined changes in light physical activity (LPA) as an unexplored mechanism that may link changes in two facets of perceived control (personal mastery, perceived constraints) to 9-year trajectories of cognitive aging. We also examined whether mediated pathways were moderated by age (i.e., differed across the adult lifespan). We analyzed 9-year data from the Midlife in the United States Study (n=2,456; age= 56±11 years) using autoregressive mediation and moderated-mediation models. Mediation models showed that changes in personal mastery and perceived constraints predicted changes in LPA (βs=.06-.08, ps<.01), which in turn predicted 9-year changes in episodic memory and executive functioning (βs=.05-.13, ps<.02). Moderated-mediation models showed that, for episodic memory, the mediated pathways were strongest in old age and emerged only for constraints: For only older adults, declines in constraints predicted less decline in LPA (β=.11, p<.001), which in turn predicted slower declines in episodic memory (β=.05, p=.001). Results were consistent in sensitivity analyses that controlled for levels and changes in moderate-to-vigorous physical activity. Our findings inform lifespan theories of control and provide initial evidence that changes in a largely overlooked health behavior (LPA) may underlie the influence of perceived constraints on cognitive functioning, with this pathway becoming more pronounced in late life.

Abstract citation ID: igae098.0508

### PSYCHOSOCIAL PROTECTIVE FACTORS FOR COGNITIVE AGING: THE MORE THE BETTER

Morgan Taylor, Kylie Schiloski, and Margie Lachman, *Brandeis University, Waltham, Massachusetts, United States*

Research demonstrates that demographic (e.g., age, education) and behavioral variables (e.g., physical activity, smoking) are related to individual differences in cognitive aging. Although some work has examined modifiable psychosocial factors (e.g., control beliefs, purpose in life, social contact) in relation to cognitive aging, they are typically examined separately. To examine their additive rather than net effects, we standardized and averaged three psychosocial factors to create a continuous composite of sense of control, purpose in life, and social support in a sample of adults (N = 2,497, ages 28-85) from the second (2004-2006) and third (2013-2014) waves of the Midlife in the United States study. Using multiple regression, we tested the composite as a predictor of 9 to 10 year changes in episodic memory and executive functioning. Results showed that those higher on the psychosocial composite demonstrated less decline in both cognitive measures, even after controlling for established demographic, health, and behavioral risk factors of cognitive aging. We then assigned participants a score of 0 if they were below the median or a 1 if equal to or above the median on each psychosocial factor, such that total scores varied from 0

to 3. Those high on all three factors were better able to maintain their cognitive health compared to those who had fewer. These findings suggest that interventions that focus on a combination of psychosocial factors could be protective in reducing the rate of cognitive decline in middle and older adulthood.

Abstract citation ID: igae098.0509

### SUBMAXIMAL CONTROL AND EXPLOSIVE STRENGTH MAY BETTER DETECT COGNITIVE IMPAIRMENT THAN MAXIMAL STRENGTH

Ryan McGrath, Kelly Parker, and Jeremy Hamm, *North Dakota State University, Fargo, North Dakota, United States*

Electronic handgrip dynamometry presents novel opportunities to feasibly collect muscle function attributes that are amenable to healthy behavior modification, which may have implications for identifying and delaying cognitive impairment. We sought to evaluate differences in electronic dynamometry derived aspects of muscle function by cognitive impairment status in older adults. Cognitive function was assessed with the Saint Louis University Mental Status exam, and standard scoring was used to categorize cognitive impairment. Participants squeezed an electronic handgrip dynamometer with maximal effort for determining strength capacity. Thereafter, participants attempted to squeeze the dynamometer at 25% of their maximal handgrip strength (HGS) for 10-seconds, and the coefficient of variation quantified submaximal control. Participants then squeezed the dynamometer as hard and fast as possible for calculating rate of force development (RFD): (delta force/delta time). The analytic sample included 125 adults aged ≥65-years (age: 70.83±4.79; 66.4% female). Submaximal control was significantly poorer in persons with a cognitive impairment (6.91±5.89) compared to those that were cognitively intact (4.80±3.42; p<0.05). Similar differences for submaximal control were observed in females with a cognitive impairment (7.97±6.70) compared to females without cognitive impairment (5.41±3.91; p<0.05). RFD was significantly lower in males with a cognitive impairment (51.0±25.7) relative to males without (72.94±26.51; p<0.05). No significant differences were observed for maximal HGS. Although several muscle indices are important for health, engaging in modifiable lifestyle behaviors, including physical activities that emphasize RFD and force control at different levels and durations, may serve as an intervention target for muscle and cognitive function.

Abstract citation ID: igae098.0510

### THE ASSOCIATIONS BETWEEN DAILY MEMORY LAPSES, FAMILY RELATIONSHIPS, AND DAILY AFFECT IN MIDDLE-AGED AND OLDER ADULTS

Heejung Jang[1], Nikki Hill[2], Jennifer Turner[3], David Almeida[4], and Jacqueline Mogle[1], *1. Clemson University, Clemson, South Carolina, United States, 2. The Pennsylvania State University, University Park, Pennsylvania, United States, 3. University of Hawaii Hilo, Hilo, Hawaii, United States, 4. Pennsylvania State University, University Park, Pennsylvania, United States*

Daily memory lapses represent an understudied approach to understanding daily experiences of cognitive functioning. Because family relationships greatly influence individuals' daily lives, the present study explores how the quality of family relationships impact daily memory lapses and the moderating influence of family relationship quality on the link between daily memory lapses and daily affect. We used data from 1,236 middle-aged and older adults (Mage= 62.48 years, SD= 10.21, range 43-91; 57% female) drawn from the third waves of the Midlife in the United States and National Studies of Daily Experiences. Participants completed 8 nightly telephone diaries that included both prospective and retrospective memory lapses as well as daily positive and negative affect. At baseline, participants reported on the emotional support they experienced with their family. Latent class analysis (LCA) models identified four family relationship types: pleasant, ambivalent, neutral, and unpleasant. Compared to pleasant relationships, ambivalent (b=.23, p<.05) and neutral (b=.35, p<.01) relationships are strongly associated with frequency of prospective memory lapses, but not retrospective lapses. Compared to pleasant relationships, ambivalent (b=.02, p<.05), neutral (b=.02, p<.05), and unpleasant (b=.07, p<.001) relationships are associated with increased negative affect on days with a retrospective lapse, but not on days a prospective lapse. This study contributes to the literature by revealing that family relationships influence cognitive problems individuals experience in their daily lives and how these cognitive problems may contribute to affective symptom load over time.

Abstract citation ID: igae098.0511

### ASSOCIATIONS BETWEEN NEIGHBORHOOD QUALITY AND COGNITIVE FUNCTION ARE MODERATED BY INTERPERSONAL STRESSOR CONTROL

Eric Cerino[1], Amanda Goldtooth[1], Raechel Livingston[1], Chloe Horowitz[1], Dakota Witzel[2], Jonathan Rush[3], Megan McCoy[4], and Michael McCarthy[1], *1. Northern Arizona University, Flagstaff, Arizona, United States, 2. South Dakota State University, Brookings, South Dakota, United States, 3. University of Victoria, Victoria, British Columbia, Canada, 4. Northern Arizona University, Northern Arizona University, Arizona, United States*

Studying modifiable psychosocial risk factors for dementia is crucial to inform approaches for dementia prevention, especially among those with lower neighborhood quality. Perceived control is an important psychosocial resource for cognitive health. Most prior work, however, focuses exclusively on global aspects of control, ignoring influences of more dynamic aspects of control over specific experiences in daily life, such as daily stressor control. Therefore, we used data from the Midlife in the United States study and the National Study of Daily Experiences (N=1,651, Mage=62.09, SE=12.11, 56.65% Female) to address this gap and examine associations of stressor control and neighborhood quality with cognitive function. Over two waves of eight consecutive days of data collection, respondents reported perceived control over interpersonal and non-interpersonal stressors they had experienced. Respondents also completed assessments of neighborhood quality, executive function (EF), and episodic memory (EM). We employed linear regressions to assess concurrent (wave 2) and prospective (ten years later at wave 3) associations of stressor control and neighborhood quality with cognitive function. Concurrent associations between better neighborhood quality and better cognitive function (EF, EM) were amplified among those with lower interpersonal stressor control. No prospective associations with cognitive function ten years later emerged. Results indicate that better neighborhood quality may be beneficial for cognitive health, especially for individuals with lower perceived control over interpersonal stressors such as arguments and avoided arguments. We discuss the value of promoting better neighborhood quality as an important social determinant of cognitive health and aging when psychosocial resources are comparatively limited.

### SESSION 1530 (PAPER)

### LONG-TERM CARE (PAPERS)

Abstract citation ID: igae098.0512

### THE BRIDGE CARE MODEL: TRANSITIONAL CARE FOR PERSONS EXPERIENCING HOUSELESSNESS

Heather Leutwyler[1], and Taylor Cuffaro[2], *1. University of California San Francisco, Pacifica, California, United States, 2. University of California San Francisco, San Francisco, California, United States*

Introduction: Persons experiencing houselessness (PEH) are well-understood to prematurely age, experiencing higher rates of geriatric conditions at a younger age, considered “older” by age 50. Based on the rigorously-tested Transitional Care Model for older adults, UCSF Street Nursing provides low barrier care to PEH during transitions from emergency departments (ED) and in-patient admissions. Our practice focuses on: nurse-led early assessment and engagement; continuity and coordination of care; the patient-clinician relationship; holistic focus on individual patient’s needs; education and promotion of self-management; active engagement of patients and their support network; and patient-driven evidence-based care planning to address health problems and risk factors that contribute to frequent ED visits and hospital readmissions among PEH.

**Methods:** UCSF Street Nursing meets patients in the ED or in-patient prior to discharge and follows them for 6-12 months, supporting integration into community outpatient care. Clinical activities include: medication administration, wound care, street-based syphilis and STI treatment, complex care coordination, complex medication management, health education focused on self-management, and co-attending primary and specialty care visits both virtually and in-person. Outcomes: The average age of the 58 patients referred to our practice was 52 (sd=12.5). Given the premature mortality and accelerated aging of PEH, our sample represents an older adult population. Our practice produced a greater than 50% reduction in ED visits in the first 6 months following discharge.

**Conclusion:** UCSF Street Nursing is a promising nurse-led novel approach to care for unhoused older adults with potential to reduce hospitalization and improve health for an overlooked population.

Abstract citation ID: igae098.0513

### EDUCATION ON COMMUNICATION WHILE WEARING FACEMASKS USEFUL DESPITE THE END OF COVID-19 EMERGENCY

Clarissa Shaw[1], Kristine Williams[2], Carissa Coleman[2], and Lynn Nakad[1], *1. University of Iowa, Iowa City, Iowa, United States, 2. University of Kansas Medical Center, Kansas City, Kansas, United States*

Communication with nursing home residents by formal caregivers is complicated with the use of facemasks. Yet facemask use is necessary to limit the spread of disease during surges of communicable respiratory illnesses. The purpose of this study was to develop and pilot test a web-based educational module to promote successful communication with nursing home residents while wearing facemasks. To create CHATO-PPE, we completed a scoping review of 39 articles and interviewed 19 formal caregivers, nursing residents, and family members from four states on best practices for communication with facemasks. We integrated these results to create a 30-minute web-based module. The module includes three videos we produced with a scenario that begins as an undesirable care encounter and with our education improves with practical communication tips and then becomes a successful care encounter with the integration of person-centered communication. To evaluate our module, we recruited a convenience sample of 26 formal caregivers in nursing homes. Confidence in dementia care measured by the CODE scale increased significantly from before to after the modules (p=.009). Greater than 90% of participants agreed that they would recommend the course to others, use the training in their job, and were satisfied overall. In post-education interviews, learners stated that mask use is still prevalent in nursing homes during surges of respiratory illnesses and CHATO-PPE provides helpful and feasible tips to implement during these times while also serving as a reminder of communication best-practices that can be used regardless of facemask use.

Abstract citation ID: igae098.0514

### NURSING HOME INFECTION PREVENTIONISTS: THEIR ROLE DURING COVID-19

Amy Vogelsmeier[1], Lori Popejoy[1], Steven Miller[1], Lisa Young[1], Alisha Johnson[1], and Roy Thompson[2], *1. University of Missouri, Columbia, Missouri, United States, 2. Hunter College, City University of New York, Philadelphia, Pennsylvania, United States*

While U.S. nursing homes were required to employ infection preventionist (IP) prior to the COVID-19 pandemic, little is known about their role during the pandemic. Leaders and IPs were interviewed about IP duties as part of a larger study conducted in 24 Midwestern nursing homes to understand nursing homes’ response to COVID-19. Homes were purposively selected for the larger study to assure diverse pandemic experiences. Interviews were analyzed by four PhD researchers, doctoral student, and project coordinator using qualitative content analysis. IPs included registered nurses (RN) (16, 66%), licensed practical nurses (LPN) (7, 30%) and respiratory therapist (1, 4%) and had varied education levels including some college (6, 25%), associate’s (13, 54%) bachelor’s (3,13%) and master’s (2, 8%) degrees. Most homes (n=22; 92%)

assigned IP duties to existing clinical roles e.g., directors/assistant directors of nursing, supervisors/charge nurses. COVID-related duties included reporting COVID testing to external agencies, administering vaccinations, and monitoring/training PPE use. A few, primarily RNs, also performed traditional IP duties including surveillance of residents/staff symptoms, tracking/trending unit outbreaks, implementing strategies to reduce spread, and collaborating with medical providers. Mandated reporting, a high-stakes, high-burden activity, was often shared with administrators and corporate leaders. Despite IP training, LPNs more often described not understanding their role beyond tasks. Most IPs discussed other assigned clinical roles as taking priority over their IP responsibilities. The IP role has important implications for infection prevention and management in nursing homes, however licensure differences, role clarity, and competing demands may affect the IP's influence in this setting.

Abstract citation ID: igae098.0515

### DEVELOPMENT AND VALIDATION OF A PREDICTION MODEL FOR SIX-MONTH SURVIVAL OF MEDICARE BENEFICIARIES IN ASSISTED LIVING

Andrew Huang[1], Laura Dionne[1], Ann Reddy[2], Peter Serina[2], and Ellen McCreedy[3], *1. Brown University, Providence, Rhode Island, United States*, *2. Brown University School of Public Health, Providence, Rhode Island, United States*, *3. Veterans' Administration, Providence, Rhode Island, United States*

Prediction of mortality risk is a prognostic tool for identifying who may benefit the most from timely and appropriate end-of-life care. However, few mortality risk prediction models have been developed for community-dwelling older adults in assisted living centers (ALCs). To estimate individual risk of all-cause mortality for Medicare beneficiaries in assisted living, we used data extracted from electronic health records (EHR) and linked with Medicare claims from 2015-2022 to develop and validate a multivariable logistic regression on the outcome of all-cause mortality occurring within six months. Eligible residents had at least one year of Medicare enrollment, six months of continuous Medicare fee-for-service and Part D drug coverage, and no history of hospice at the time of prediction. Candidate predictors included individual-level demographics, healthcare costs and utilization, and available clinical features, including diagnoses, medications, and assessments of physical and cognitive function. Final predictors were identified with stepwise selection procedures and expert opinion. A k-fold cross-validation scheme with stratified sampling was used and model performance was assessed on discrimination and calibration statistics. We identified 31,274 residents from 1,228 ALCs eligible for mortality risk prediction. At baseline, the mean (sd) age was 85.3 (7.7) years, 69.0% were female, and 87.2% identified as non-Hispanic White. Dementia was prevalent among 11.5% of residents and 6.3% of residents died within six months. The final model returned an AUROC of 0.74 and calibration plots showed consistency between predicted and observed mortality outcomes. This EHR and claims-based model may help residents in ALCs receive better targeted end-of-life care.

Abstract citation ID: igae098.0516

### CAREGIVING CRISIS: NURSING ASSISTANT TRAINING REQUIREMENTS AND QUALITY OF LIFE IN SKILLED NURSING FACILITIES

Cassandra Barragan, and Devlon Dean, *Eastern Michigan University, Ypsilanti, Michigan, United States*

Nursing Assistants (NAs) provide the majority of hands-on care for older adults in skilled nursing facilities (SNFs) while also providing companionship and psychological support. NAs receive a minimum of 75 hours of training in every state with 12 states requiring 120 or more (M=95.2). However, many NAs feel unprepared for the complexities of caring for older adults (Trinkoff et al., 2017). This lack of preparedness likely contributes to high staff turnover in SNFs (Gaivin, 2022) and directly impacts the quality of care (Castle & Endberg, 2006). This research explored the relationship between NA training hours and SNF quality of care citations in the United States. Data from the Centers for Medicare and Medicaid Services (CMS) SNF Quality Reporting Program (QRP) were used to determine the number of quality of life (QOL) citations for 5408 facilities in all 50 states and cross-referenced with the number of training hours required in each state. To control for the influence of a social worker, not required in smaller facilities, only facilities with 100 or more beds were examined. Regression analysis found for every hour increase in training hours, there will be an approximate 4% decrease in the predicted percentage of facilities with less than satisfactory QOL deficiencies. This research provides additional evidence that providing NAs with more training can improve QOL outcomes for residents of SNFs. This has implications for SNFs, assisted living, home health care, adult foster care, and other settings where NAs are employed.

### SESSION 1535 (PAPER)

### MENTAL HEALTH (PAPERS)

Abstract citation ID: igae098.0517

### GENDER DIFFERENCES IN THE RELATIONSHIP BETWEEN POLYPHARMACY AND DEPRESSIVE SYMPTOMS AMONG OLDER ADULTS

Xiao Qiu[1], Ziyao Xu[1], Na Sun[1], Cassandra Hua[2], and J. Scott Brown[1], *1. Miami University, Oxford, Ohio, United States*, *2. University of Massachusetts Lowell, Lowell, Massachusetts, United States*

Polypharmacy is prevalent among older adults and is associated with increased depressive symptoms, yet differences in the relationship by gender are not well known. Understanding these gender-specific differences is crucial for developing targeted interventions to address depressive symptoms among older adults. This study examines the relationship between polypharmacy and depressive symptoms among US community-dwelling older adults aged 65 or older (n = 3,143), utilizing data from the 2006 and 2008 waves of the Health and Retirement Study (HRS) core survey and its supplementary drug surveys. Employing negative binomial generalized estimating equation (GEE) models, polypharmacy is defined as the concurrent use of five or more prescription medications, while depressive symptoms are assessed using the 8-item version of the Center for Epidemiologic Studies

Depression (CESD-8). Over 40% of respondents aged 65 or older from the 2006 and 2008 waves regularly use five or more prescription medications. Polypharmacy is associated with increased depressive symptoms (CIRR=1.20, 95% CI= 1.15 -1.25). Notably, the relationship between polypharmacy and depressive symptoms varies by gender. Among women, polypharmacy is linked to a lower number of depressive symptoms (AIRR = 0.90, 95% CI = 0.84 - 0.95), whereas among men, it is associated with an increased number (AIRR =1.12, 95% CI = 1.05 -1.19). These findings highlight the importance of tailored interventions and healthcare strategies.

Abstract citation ID: igae098.0518

### DISTRIBUTION AND DETERMINANTS OF LONG-ACTING INJECTABLE ANTIPSYCHOTIC MEDICATION USE AMONG OLDER ADULTS

Matthew Lohman[1], Mansi Verma[1], Paige Jones[1], Victoria Scott[2], and Eve Fields[3], *1. University of South Carolina, Columbia, South Carolina, United States, 2. University of North Carolina Charlotte, Charlotte, North Carolina, United States, 3. South Carolina Department of Mental Health, Greer, South Carolina, United States*

While long-acting injectable antipsychotic medications (LAIs) are important components in the management of schizophrenia and related disorders, little is known about LAI use among older adults in real-world settings. We analyzed electronic health records from the South Carolina Department of Mental Health comprising 41,401 patients (n=8,481 aged > 60) who received ambulatory psychiatric services at one of 16 regional mental health centers in 2022. We compared use of eight types of first- and second-generation LAIs by demographic (age, race/ethnicity, gender, zip code, insurance source) and clinical characteristics (primary and secondary diagnoses, oral antipsychotic use). Logistic regression models were used to estimate associations between individual characteristics and likelihood of using LAI medications. In total, 1,400 adults age > 60 used LAIs in 2022, including 1,231 with schizophrenia or another psychotic disorder. Compared to younger adults, older adults were less likely to use LAIs but more likely to use first vs. second-generation LAIs (OR=2.19, p<.001). Controlling for diagnosis, insurance provider, and total services, older adults were significantly more likely to use LAIs if they were male (OR=1.40, p=.002), Black (OR=2.46, p<.001), Asian (OR=9.79, p=.001), and living in a suburban area (OR=1.33, p=.009). There were no significant demographic differences in oral antipsychotic use. Results suggest that LAI use among older adults differs substantially by sociodemographic characteristics while oral antipsychotic use does not. Understanding reasons for differences is important to determine and promote equitable access to and use of LAIs across racial/ethnic, geographic, and other sociodemographic groups of older adults.

Abstract citation ID: igae098.0519

### BIOLOGICAL SEX MODERATES THE EFFECT OF EXERCISE ON COGNITIVE FUNCTION IN ADULTS WITH CHRONIC STROKE

Rebeca Hernández Gamboa[1], Ryan Falck[1], Elizabeth Dao[1], and Teresa Liu-Ambrose[2], *1. University of British Columbia, Vancouver, British Columbia, Canada, 2. University of British Columbia, University of British Columbia, British Columbia, Canada*

Exercise (EX) and cognitive/social enrichment (ENRICH) are promising strategies for promoting cognitive function post-stroke. The effects of these interventions on cognitive function may vary by biological sex, i.e. males (M) vs females (F). We conducted a secondary analysis of a three-arm, six-month, assessor-blinded, randomized clinical trial among 120 community-dwelling adults aged 55+ years with chronic stroke (i.e., ≥12 months since stroke), to examine if biological sex moderates the effects of EX or ENRICH on cognitive function. Participants were randomly assigned to 2x/week EX (N=34, M=21, F=13), 2x/week ENRICH (N=34, M=23, F=11), or 2x/week balance and tone control (BAT, M=30, F=22). Our primary outcome of cognitive function was the Alzheimer's Disease Assessment Scale Cognitive Subscale Plus (ADAS-COG-Plus) at baseline, end of intervention (6 months), and follow-up (12 months). Using linear mixed models, we examined if biological sex moderated the effects of EX or ENRICH on cognitive function using planned contrasts. Baseline ADAS-Cog-Plus and Mini Mental State Exam score were included as fixed-effect covariates. Biological sex moderated the effect of EX, but not ENRICH, on ADAS-Cog-Plus (F = 5.75, p< 0.01). The effects of EX compared to BAT were significantly larger for females than males (estimated mean difference: -0.394, 95% CI: -0.778, -0.011, p< 0.05). EX females had better ADAS-COG-PLUS performance at end of the intervention (estimated mean difference: -0.463, 95% CI: -0.758, -0.168, p< 0.005) compared with females in the BAT group. Findings suggest that exercise may be a key strategy to promote cognitive function among older females living with stroke.

Abstract citation ID: igae098.0520

### COGNITION AND PAIN ARE ASSOCIATED WITH DISCREPANCIES IN ACTIGRAPHY AND SELF-REPORTED SLEEP MEASURES IN OLDER ADULTS

Russell Calderon[1], Sofia Liu[2], Miranda McPhillips[3], Michelle Liu[4], Yifan Liu[4], and Junxin Li[5], *1. Johns Hopkins University, Lehigh Acres, Florida, United States, 2. Johns Hopkins University School of Nursing, Baltimore, Maryland, United States, 3. Villanova University, Villanova, Pennsylvania, United States, 4. Johns Hopkins University, Baltimore, Maryland, United States, 5. Johns Hopkins School of Nursing, Baltimore, Maryland, United States*

Sleep is a key determinant of health in older adults and is assessed using accelerometer-based (Actigraphy) and self-reported methods. This study aims to describe and identify factors associated with potential discrepancies between Actigraphy and self-reported sleep parameters ("Actigraphy/self-reported discrepancies") in 125 community-dwelling older adults without dementia [Montreal Cognitive Assessment (MoCA) ≥17] using data from a randomized clinical trial (NCT03959202). Participants were 70.4±6.3 years old, 79.2% females, 21.6% with mild cognitive impairment. Actigraphy and corresponding self-reported sleep measures, including time in bed (TIB), sleep onset latency (SOL), total sleep time (TST), and sleep efficiency (SE) were collected using Actiwatch 2 for 7-10 days, sleep diaries, and Pittsburgh Sleep Quality Index (PSQI). Relative to Actigraphy measures, 11.2%, 38.4%, 52.0%, 35.2%, and 36.0%, of participants reported longer TIB(diary) (>15 minutes), TIB(PSQI) (>15 minutes), SOL(PSQI) (>5 minutes), TST(PSQI) (>15 minutes), and better SE(PSQI) (>5%) respectively, whereas 38.4%, 52.0%, 12.0%, 54.4%, and 28.0% of participants reported shorter TIB(diary), TIB(PSQI), SOL(PSQI), TST(PSQI), and worse SE(PSQI) by the same magnitudes. Multiple linear regression analyses adjusted for demographics suggest higher MoCA was associated with reduced Actigraphy/self-reported discrepancies (TIB(Actigraphy-diary):$\beta$=-13.6, p=0.037; TIB(Actigraphy-PSQI):$\beta$=-11.2, p< 0.01; SOL(Actigraphy-PSQI):$\beta$=-3.3, p=0.019; TST(Actigraphy-PSQI):$\beta$=-7.4, p=0.01; SE(Actigraphy-PSQI):$\beta$=-1.5, p=0.013). Higher pain behavior expression (TIB(Actigraphy-PSQI):$\beta$=7.3, p=0.011; SOL(Actigraphy-PSQI):$\beta$=2.7, p=0.014) and pain interference (TIB(Actigraphy-diary):$\beta$=8.8, p< 0.01) were associated with greater Actigraphy/self-reported discrepancies. In summary, cognitive function and pain were associated with Actigraphy/self-reported discrepancies among older adults without dementia. Given that cognition and pain may interfere with self-perceptions of sleep quality, sleep must be carefully assessed and interpreted in this population.

Abstract citation ID: igae098.0521

### ASSOCIATION BETWEEN PRE–HIP FRACTURE ANTIDEPRESSANT USE AND HOSPITAL LENGTH OF STAY AMONG MEDICARE BENEFICIARIES

Rhea Mehta[1], Jason Falvey[2], Chixiang Chen[2], Yu Dong[2], Michelle Shardell[2], Takashi Yamashita[1], and Denise Orwig[2], *1. University of Maryland Baltimore, Baltimore, Maryland, United States*, *2. University of Maryland School of Medicine, Baltimore, Maryland, United States*

Antidepressants are the first-line treatment for depression among older adults. Their use presents important sex differences and is linked to hospital length of stay (LOS), which can affect hip fracture recovery. Given the push to deprescribe these medications among older adults, exploring the effect of antidepressant use prior to hip fracture among older adults with existing depression is important. Thus, our study examined the association between pre-fracture antidepressant use and hospital LOS among hip fracture survivors, and related sex differences. The sample included 17,936 community-dwelling Medicare fee-for-service beneficiaries with depression and a hospitalization claim for hip fracture surgery between 2010 and 2017. Ordinal logistic regression was used to estimate the association between pre-fracture antidepressant use, measured 6 months prior to fracture, and hospital LOS in days, a categorical outcome variable classified into three groups (1-4, 5-8, and 8+ days) during the 30-day post-fracture period. In the covariate-adjusted model, hip fracture survivors with depression who used antidepressants pre-fracture had 6.7% higher odds of a shorter hospital LOS compared to non-users (odds ratio [OR]=1.07; 95% CI=1.01, 1.13; p=0.03). Males had longer LOS on average (mean=6.02, standard deviation [SD]=4.07) compared to females (mean=5.43, SD=3.17), but the sex-by-antidepressant use interaction was not significant (p=0.83). Treating existing depression with antidepressants was associated with shorter hospital LOS in this sample. The findings suggest a benefit of antidepressant use prior to hip fracture which may inform clinical decision-making surrounding depression management among hip fracture survivors to optimize recovery trajectories.

### SESSION 1540 (PAPER)

### NEW DIRECTIONS IN HEALTH DISPARITIES RESEARCH ON MINORITY AND MINORITIZED POPULATIONS

Abstract citation ID: igae098.0522

### SEXUAL ORIENTATION AND THE ONSET AGE OF CHRONIC DISEASES AND DISABILITIES

Jen-Hao Chen, *National Chengchi University, Taipei, Taipei, Taiwan (Republic of China)*

One key to increasing healthy lifespan and reducing health disparities is delaying the onset of chronic conditions and associated disabilities, especially among marginalized groups. Despite this understanding, relatively few research studies investigate the onset age of chronic diseases and disabilities by sexual minority. This study analyzed pooled data from the 2013-2019 U.S. National Health Interview Survey (NHIS) to investigate the relationships between sexual orientation (i.e., heterosexual, homosexual, bisexual, and other sexual minorities) and the onset of chronic diseases and disability. The study first calculated the onset age for a wide range of chronic conditions and disabilities (i.e., vision and hearing problems, hypertension, diabetes, cancer, intellectual disabilities, etc.), then used weighted regressions to link sexual orientations to the age of chronic disease and disability onset, controlling for a wide range of sociodemographic characteristics. Results suggest that there are considerable disparities in the onset age of chronic diseases and disabilities between heterosexual individuals and sexual minorities. Bisexual and homosexual individuals were significantly more likely to have chronic diseases and disabilities earlier in life than their heterosexual counterparts, with bisexual individuals being the most disadvantaged. These disparities were not accounted for by the higher education and income levels observed in some sexual minority groups. However, there was no difference in terms of disease and disability onset between heterosexual individuals and other sexual minorities. Some cohort differences were found. Given the historical discrimination against sexual minorities and challenges related to HIV, these findings underscore the need for policy interventions to improve sexual minorities' healthspan.

Abstract citation ID: igae098.0523

### GENDERED RACIAL DISPARITIES IN FUNCTIONAL LIMITATIONS: EXPLORING THE ROLE OF LIFE-COURSE FAMILY CARE

Chioun Lee, and Soojin Park, *University of California Riverside, Riverside, California, United States*

**Objectives:** This study addresses the dearth of research on culturally embodied factors influencing health disparities, focusing on cultural aspects such as family formation timing, living arrangements, familism, and family care responsibilities. By employing "intersectionality" and a life-course approach to "family care," we aim to understand why minority women are vulnerable to functional limitations.

**Methods:** Data come from the MIDUS II, Refresher, and Milwaukee surveys, with a sample of Non-Hispanic Whites and Blacks (N=8,848; 18% Blacks; 53% Women). Measures of family care included age at first childbirth, number of biological children, single or nonmarital parenthood, child loss, co-residence with a disabled child, family caregiving history, and major grandparenting responsibilities. We identified racial disparities in family care profiles and assessed their contribution to health disparities.

**Results:** Adverse family care profiles were more common among women, particularly Blacks. Compared to White women, Black women were more likely to experience early and non-marital motherhood, current family care, and major grandparenting roles. Adverse family care profiles were linked to more functional limitations, with differing effects between Black and White women. While early childbearing was more detrimental to White women, caring for disabled family members had a greater adverse effect on Black women. Adverse family care profiles explained one-third of Black-White disparities in functional limitations.

**Discussion:** Our findings highlight the social costs of family care, disproportionately affecting minority women. They offer insights into public health policies and interventions aimed at easing the burdens of family care to improve their quality and length of life.

Abstract citation ID: igae098.0524

### ASSOCIATIONS BETWEEN INCARCERATION HISTORY AND DIABETES MANAGEMENT AMONG OLDER ADULTS

Sabrina Blank[1], and Rodlescia Sneed[2], *1. Wayne State University, Grosse Pointe Park, Michigan, United States*, *2. Wayne State University, Detroit, Michigan, United States*

The number of older adults with criminal justice system involvement has increased by more than 280% since 1999. Previous studies show that justice-involved older adults have rates of chronic disease similar to those of the never-incarcerated; however, no studies have evaluated differences in disease management between groups. We examined associations between history of incarceration (HOI) and diabetes management using data from the Health and Retirement Study, a population-based sample of community-dwelling older adults. HOI was assessed via self-report. We assessed diabetes management via self-report/objective health indicators, health service utilization, and health behaviors. We used logistic and linear regression to evaluate associations between HOI and diabetes management, controlling for demographic characteristics. HOI was associated with fewer dental visits (OR: 0.069; 95% CI: 0.531, 0.917) and greater odds of insulin use (OR: 1.52, 95% CI: 1.13, 2.05), tooth loss (OR: 1.94, 95% CI 1.43, 2.65), foot swelling (OR: 1.44, 95% CI: 1.08, 1.90) and hospitalization in the last 2 years, (OR: 1.47, 95% CI: 1.12, 1.92). There was no association between HOI and other measures of diabetes management. Associations between tooth loss, dental visits, and incarceration were observed among White but not Black participants. Findings suggest that HOI may be associated with poorer diabetes management practices in some domains. Future research should identify interventions that improve diabetes-relevant outcomes among justice-involved older adults.

Abstract citation ID: igae098.0525

### MAXIMIZING THE SCALABILITY OF THE CHRONIC DISEASE SELF-MANAGEMENT PROGRAM AMONG OLDER ADULTS IN STATE PRISONS

Rodlescia Sneed, *Wayne State University, Detroit, Michigan, United States*

Incarcerated older adults have high rates of chronic disease; however, there is limited chronic disease programming in most state prison systems. The Chronic Disease Self-Management Program (CDSMP) is an evidence-based program that has been used within state prisons on a limited basis, but there has been little research into efficient and effective strategies for scaling up the intervention within state prisons. Scale-up (i.e. deliberate efforts to increase the impact of successfully tested health innovations to benefit more people and promote sustainability) is an understudied concept with few existing empirical studies explicitly focused on the scale-up process. Assessing scalability, however, is crucial for ensuring sustainability of complex interventions within resource-poor settings. The purpose of this study was to evaluate and maximize the scalability of CDSMP among older adults in state prisons. Guided by the Scaling up Management Framework, we conducted interviews with representatives from 10 U.S. state prison systems on ways to maximize the scalability of CDSMP within state correctional settings. Participants provided information in numerous areas related to scale-up, including strategies for achieving buy-in, special situations where implementation might be especially difficult, potential funding strategies, and ideal partners for scale-up efforts. As the prison population continues to age, the burden of chronic disease within correctional systems will continue to increase, which contributes to skyrocketing correctional costs. Understanding how to expand evidence-based chronic disease programs within correctional systems is crucial for reducing disease-related morbidity and mortality among incarcerated individuals and for reducing costs.

Abstract citation ID: igae098.0526

### SOCIAL INTERACTION AND DEPRESSIVE SYMPTOMS: A COMPARISON OF MID- TO LATER-LIFE SAME- AND DIFFERENT-SEX COUPLES

Sara Mernitz, Michael Garcia, and Debra Umberson, *The University of Texas Austin, Austin, Texas, United States*

Social interaction with family and friends plays a crucial role for individual mental health with notable gendered patterns. Yet, previous studies fall short in explaining how the frequency of social interactions change over time for women and men in same-sex and different-sex married couples and how such change may influence depressive symptoms from midlife to late adulthood. Using dyadic longitudinal data from the Health and Relationships Projects (HARP), we examine patterns of change in social interaction frequency over a 6-year period for mid-to-later life U.S. women and men in same-sex and different-sex marriages (n = 616 individuals; 320 couples) and their subsequent associations with depressive symptoms. We use mixed-effects multilevel modeling and find that the frequency of social interaction with family members decreased over a period of 6 years for same-sex couples (i.e. women married to women; men married to men), but not different-sex couples. Frequency of social interaction with friends also decreased, but only for men married to men. Decreased social interaction with family members was associated with increased depressive symptoms for women married to women, but not men married to men or different-sex couples; decreased social interaction with friends was associated with increased depressive symptoms for men married to men only. Taken together, these findings illustrate how change in social interaction frequency influences mental health within marriage from mid-to-later life and underscores the importance of including same-sex couples when considering both gender differences in such linkages.

### SESSION 1545 (SYMPOSIUM)

Abstract citation ID: igae098.0527

### NOVEL METHODS AND DATA IN POPULATION AGING RESEARCH

Chair: Ruijia Chen
Co-Chair: Eleanor Hayes-Larson

Causal inference in population health research on aging is critical for understanding determinants of healthy aging and informing potential avenues of intervention. Such research faces a number of methodological challenges including data availability, detection bias, selection bias, confounding, and reverse causation. Novel statistical methods and approaches to leveraging data have been developed to address these issues. This session features cutting-edge research that incorporates novel methods and data to address key methodological challenges in aging research. Speakers will address (1) the use of hypothetical intervention approaches to address confounding and reverse causation to understand the impact of loneliness on memory function in older adults, (2) a multiple-exposure data reduction approach to identify important determinants of exercise in an aging population, (3) ways to uncover and account for detection bias in electronic health record studies of dementia, (4) pooling of cohorts to address gaps in data availability across the lifecourse, and (5) challenges and opportunities related to extending findings from selected aging samples to broader populations of interest. Epidemiology of Aging Interest Group Sponsored Symposium

Abstract citation ID: igae098.0528

### HYPOTHETICAL INTERVENTIONS ON LONELINESS AND MEMORY FUNCTION AMONG US MIDDLE-AGED AND OLDER ADULTS

Ryo Ikesu[1], L. Paloma Rojas-Saunero[1], Eleanor Hayes-Larson[2], and Elizabeth Rose Mayeda[1], *1. University of California Los Angeles, Los Angeles, California, United States*, *2. USC Leonard Davis School of Gerontology, Los Angeles, California, United States*

Evidence has shown that middle-aged and older adults who experience persistent loneliness have lower memory function compared to those with transient loneliness or those who never feel lonely, but it remains unclear whether sustained interventions on loneliness, as opposed to a one-time intervention, are more effective in preserving late-life memory function. Researchers are increasingly using hypothetical intervention approaches to estimate the impact of population-level interventions with observational data. Using the nationally-representative Health and Retirement Study in 2006–2016 (n=16,977; median baseline age, 68), we estimated the population-level effects of two hypothetical interventions of loneliness on memory scores 8 years after baseline: [A] preventing loneliness at baseline and 4 years after baseline (sustained intervention at two time points) and [B] preventing loneliness only at baseline (one-time intervention). We used targeted maximum likelihood estimation to account for censoring and both baseline and time-varying confounding (health-related behaviors, working status, household wealth, social relationships, and health conditions). In this approach, we accounted for the possibility that people with lower memory function feel lonely more often than those with higher memory function. Compared to the natural course (i.e., no intervention), both sustained and one-time interventions were associated with slightly higher memory scores 8 years after baseline (0.026 standardized units [95% CI: 0.003–0.048] for the sustained intervention; 0.021 standardized units [95% CI: 0.005–0.037] for the one-time intervention). Our findings suggest that both sustained and one-time interventions on loneliness may preserve memory function at the population level.

Abstract citation ID: igae098.0529

### EXPLORING NOVEL DETERMINANTS OF EXERCISE BEHAVIOR: A LAGGED EXPOSURE-WIDE APPROACH

Harold Lee[1], Eric Kim[2], David Conroy[3], and Tyler VanderWeele[4], *1. Penn State, University Park, Pennsylvania, United States*, *2. University of British Columbia, Vancouver, British Columbia, Canada*, *3. The Pennsylvania State University, University Park, Pennsylvania, United States*, *4. Harvard T. H. Chan School of Public Health, Boston, Massachusetts, United States*

Many middle-aged to older adults do not engage in regular exercise at all, despite its importance for healthy aging. Extensive research grounded in behavioral and social science theories has identified numerous determinants of exercise. However, few studies used an exposure-wide approach, a data-driven exploratory method that is particularly useful for identifying determinants. We used data from 13,771 participants in the Health and Retirement Study (HRS), a diverse, national panel study of adults aged >50 in the United States, to evaluate 62 candidate predictors of exercise participation. Candidate predictors were drawn from the following domains: health behaviors, physical health, psychological well-being, psychological distress, social factors, and work. We used Poisson regression with robust error variance to individually regress exercise in the outcome wave (t2:2014/2016) on baseline candidate predictors (at t1;2010/2012) controlling for all covariates in the previous wave (t0;2006/2008). Some health behaviors (e.g., ever exercising), physical health conditions (e.g., physical functioning limitations and lung disease), psychological factors

(e.g., health mastery, purpose in life, and positive affect), and social factors (e.g., helping others, religious service attendance, and volunteering) were robustly associated with increased subsequent exercise. Among factors related to psychological distress, perceived constraints stood out as a factor in reducing exercise. To conclude, we identified potentially novel exercise determinants, such as purpose in life and helping others, that have not been captured using a theory-driven approach. Future studies validating these findings in older adults are needed.

Abstract citation ID: igae098.0530

### REASSESSING POSITIVE RESULTS: DETECTION BIAS IN EPIDEMIOLOGIC DEMENTIA STUDIES OF ELECTRONIC HEALTH RECORD DATA

Jingxuan Wang[1], Minhyuk Choi[1], Scott Zimmerman[1], Ruijia Chen[2], Cathy Schaefer[3], Deborah Blacker[4], and M Maria Glymour[5], *1. University of California San Francisco, San Francisco, California, United States*, *2. Boston University, Lexington, Massachusetts, United States*, *3. Kaiser Permanente Northern California, Oakland, California, United States*, *4. Massachusetts General Hospital, Boston, Massachusetts, United States*, *5. Boston University, Boston, Massachusetts, United States*

Many epidemiologic studies of dementia use electronic health record (EHR) data, which are vulnerable to detection bias. Dementia is more likely to be detected when patients have more contact with the healthcare system. Using EHR data from the UK Biobank (UKB) and All of Us (AoU), we evaluated potential detection bias by estimating associations of three negative control exposures with dementia. We included 298,784 UKB participants and 105,836 AoU participants aged ≥ 55 without baseline dementia and estimated associations of kidney stone, forearm fracture, and urinary tract infection (UTI) with incident dementia. All diagnoses were ascertained from EHR data. We used Cox models to evaluate associations of binary exposure with all-cause dementia overall and in 4 time intervals after the exposure (0–1, >1–5, >5–10, and >10 years) compared to individuals with no prior exposure. All models adjusted for demographic and socioeconomic factors. Our results showed that all three exposures were associated with an elevated risk of dementia, especially in the first year after exposure diagnosis. The association attenuated in subsequent years. It suggests the chance of receiving a dementia diagnosis increases sharply in the first year following a diagnosis of kidney stone, forearm fracture, or UTI, although the influence of these conditions on dementia pathology is likely to be small or null. These short-term increases likely represent detection bias, i.e., they would not have received a dementia diagnosis at that time in the absence of the medical attention necessitated by kidney stone, forearm fracture or UTI.

Abstract citation ID: igae098.0531

### POOLING COHORTS ACROSS THE LIFE COURSE TO IDENTIFY NOVEL EARLY-LIFE DETERMINANTS OF HEALTH OUTCOMES IN LATER-LIFE

Katrina Kezios[1], Scott Zimmerman[2], Peter Buto[2], M Maria Glymour[3], and Adina Zeki Al Hazzouri[4], *1. Columbia University, New York, New York, United States*, *2. University of California San Francisco, San Francisco, California, United States*, *3. Boston University, Boston, Massachusetts, United States*, *4. Columbia University, New York, New York, United States*

To answer novel questions about exposure-disease effects over the entire life course, researchers are increasingly pooling data from multiple cohorts covering different life periods to construct complete life course data. If carefully constructed, these "synthetic cohorts" should enable rigorous investigation of earlier-life determinants and pathways to health in older age. Using simulations, we previously demonstrated a matching-based approach to create a synthetic cohort where individuals with exposure ("A") information in an early-life cohort were matched to individuals with outcome ("Y") information in a later-life cohort based on mediators and confounders of A and Y. Here, we describe results from a proof-of-concept analysis using a sample-splitting approach in the Health and Retirement Study (1994-2018). We examined the relationships between BMI in 1994 and 1) general health, 2) diabetes, and 3) systolic blood pressure in 2018 in synthetic cohorts constructed using 2008 as the matching time point. Synthetic cohort estimates were benchmarked against those in the longitudinal HRS sample observed from 1994-2018. In the proof-of-concept analyses, our approach was most successful when a later measure of the exposure and/or earlier measure of the outcome was available in both cohorts at the matching time-point. For example, for BMI1994 and general health2018, matching cohorts on BMI and general health in 2008 alone produced an estimate of -0.0386 [-0.054, -0.023] in the synthetic cohort (compared with -0.0334 [-0.040, -0.027] in the full cohort). We use these findings to guide analyses examining the relationship between other early life social and cardiovascular determinants and later-life health.

Abstract citation ID: igae098.0532

### EXTENDING FINDINGS FROM AGING COHORTS TO BROADER POPULATIONS OF INTEREST: CONSIDERING TRANSPORTABILITY

Eleanor Hayes-Larson, *USC Leonard Davis School of Gerontology, Los Angeles, California, United States*

Aging cohorts and other commonly-used data sources are typically not the populations of interest for scientific inferences in epidemiologic aging research. Rather, inferences are desired about particular target populations (e.g. defined by geography, age, birth cohort, or medical history), making external validity of study findings a particularly relevant concern. Recent developments in epidemiologic methods facilitate quantitative assessment of external validity and offer statistical solutions when study samples are not representative of the target population of interest. In this presentation, we discuss approaches to evaluating external validity in aging research, and methods for extending findings to a given target population when external validity appears threatened. We emphasize estimators that are valid for lifecourse research questions, and offer several recent examples from cognitive aging research.

### SESSION 1550 (SYMPOSIUM)

Abstract citation ID: igae098.0533

### NURSE WAGES IN NURSING HOMES: ANTECEDENTS AND CONSEQUENCES

Chair: Rohit Pradhan
Discussant: Jane Banaszak-Holl

A substantial body of research has established that nurse staffing intensity and skill mix have a consequential impact on nursing home (NH) care quality. Unfortunately, NH staffing has been marked by multitudes of challenges including high levels of turnover and absenteeism. The literature suggests that an important reason why NHs have struggled with nurse staffing is the relatively lower wages vis-à-vis other important areas of the U.S. healthcare system, such as hospitals. These challenges underscore the importance of examining nurse wages within the NH setting, as wages not only affect staff retention and satisfaction but overall performance. Despite the apparent need for competitive wages to attract and retain skilled nurses, NHs operate in a resource-constrained environment, making wage increases a complex issue for administrators. The overarching goal of this symposium is to explore the antecedents and consequences of nurse wages in NHs. As part of the symposium, we include studies examining nurse wages and quality, the organizational and market-level factors associated with nurse wages, and the impact of nurse wages on financial performance. The findings and discussions may offer valuable insights for both managerial decision-making and policy formulation, aiming to address the systemic challenges of nurse staffing in NHs and improve NH performance, particularly quality of care.

Abstract citation ID: igae098.0534

### EXPLORING THE IMPACT OF NURSE WAGES ON NURSING HOME QUALITY

Rohit Pradhan[1], Akbar Ghiasi[2], Gregory Orewa[3], Ganisher Davlyatov[4], and Robert Weech-Maldonado[5], *1. Texas State University, San Marcos, Texas, United States*, *2. University of the Incarnate Word, San Antonio, Texas, United States*, *3. The University of Texas San Antonio, San Antonio, Texas, United States*, *4. University of Oklahoma Health Sciences Center, Oklahoma City, Oklahoma, United States*, *5. University of Alabama at Birmingham, Birmingham, Alabama, United States*

Nurses are the primary caregivers in nursing homes (NHs) and providing high-quality care hinges significantly upon their adequacy and expertise. However, NHs have historically struggled with inadequate nurse staffing, partly due to nurse wages in NHs being lower than those in other healthcare settings, such as hospitals. This study aimed to explore the relationship between nurse wages and the quality of care in NHs. This study used secondary datasets such as the Payroll-Based Journals and Care Compare (2017-2022). Multivariate ordinal logistic regression with two-way (state and year-level) fixed effects was employed. The study included all U.S. NHs certified by the Centers for Medicare and Medicaid Services (CMS). Analytic data comprised 80,244 facilities, averaging 13,374 unique NHs per year. We focused on the CMS NH Five-Star Quality Rating System's quality star rating (1-5 scale) as the dependent variable, with independent variables being the inflation-adjusted hourly wages for registered nurses (RNs), licensed practical nurses (LPNs), and certified nursing assistants (CNAs), while controlling for facility and community characteristics that influence quality. The findings revealed that a one-dollar increase in hourly RN wages was linked to a three percentage-point increase in the odds of an NH receiving a higher star rating (p<.01). Increased RN and CNA hours per resident day (PRD) were positively associated with higher quality ratings, whereas higher LPN hours PRD were correlated with lower quality ratings (p<.001). These results highlight the need for targeted policy and managerial interventions to adjust nurse compensation strategies, particularly for RNs, to enhance NH quality.

Abstract citation ID: igae098.0535

### NURSE WAGES AND NURSING HOME FINANCIAL PERFORMANCE

Justin Lord[1], Akbar Ghiasi[2], Shivani Gupta[3], and Rohit Pradhan[4], *1. Louisiana State University Shreveport, Shreveport, Louisiana, United States*, *2. University of the Incarnate Word, San Antonio, Texas, United States*, *3. University of Houston - Clear Lake, Houston, Texas, United States*, *4. Texas State University, San Marcos, Texas, United States*

Nursing home (NH) care is labor intensive, with staff compensation being the most significant expense. Balancing higher wages to attract qualified nurses against the allocation of resources to other facility areas is crucial. This study examined the impact of nurse wages on NH financial performance. This study used secondary datasets such as the Payroll Based Journals, Medicare cost reports. and Care Compare (2017-2022). It included all U.S. NHs certified by the Centers for Medicare and Medicaid Services (CMS). Analytic data comprised 80,244 facilities, averaging 13,374 unique NHs per year. We modeled the data using multivariate linear regression with two-way fixed effects (year and state). The operating margin at the facility level served as the dependent variable, with inflation-adjusted nurse wages as the main variable of interest. Separate models for registered nurses (RNs), licensed practical nurses (LPNs), and certified nursing assistants (CNAs) showed that a 10% wage increase correlated with operating margin decreases of 7.3%, 9.6%, and 11.5%, respectively (p<.01). Positive operating margins were associated with for-profit status, chain affiliation, larger facilities, higher occupancy, and a greater proportion of Medicare residents, while higher uninsured rates and Medicare Advantage penetration negatively impacted margins. These findings underscore the necessity for striking a strategic balance between nurse compensation and NH financial health. Given the important role nurses play in NHs, and amidst an acute staffing shortage, our study emphasizes the need for policy measures that support both competitive nurse wages and the sustainability of NH operations, ensuring high-quality care and long-term viability.

Abstract citation ID: igae098.0536

### WAGE DISPARITIES IN NURSING HOMES: ORGANIZATIONAL AND MARKET FACTORS

Gregory Orewa[1], Akbar Ghiasi[2], Ganisher Davlyatov[3], Justin Lord[4], and Robert Weech-Maldonado[5], *1. The University of Texas San Antonio, San Antonio, Texas, United States*, *2. University of the Incarnate Word, San Antonio, Texas, United States*, *3. University of Oklahoma Health Sciences Center, Oklahoma City, Oklahoma, United States*, *4. Louisiana State University Shreveport, Shreveport,*

*Louisiana, United States*, *5. University of Alabama at Birmingham, Birmingham, Alabama, United States*

Although wage differences across NHs are evident, the underlying factors contributing to these differences remain largely unexplored. The primary purpose of this study was to examine the organizational and market factors associated with nurse wages in NHs. This study used secondary datasets such as the Payroll Based Journals, Medicare cost reports. and Care Compare (2017-2022). It included all U.S. NHs certified by the Centers for Medicare and Medicaid Services (CMS). Analytic data comprised 80,244 facilities, averaging 13,374 unique NHs per year. The data was modeled using multiple linear regression with two-way fixed effects (year and state). We ran separate models for the three types of nurses employed by NHs: registered nurses (RNs), licensed practical nurses (LPNs), and certified nursing assistants (CNAs) wages. The dependent variable was the adjusted nurse wage (RNs, LPNs, and CNAs) per hour after accounting for inflation. The key variable of interest was a categorical variable that captured the intersection of ownership status and chain affiliation: for-profit-chain (FPC), for-profit independent (FPI), not-for-profit chain (NFPC), and not-for-profit independent (NFPI). Other organizational and environmental factors that may be associated with nurse wages were also included. The results suggest that compared to NFPIs, FPIs and FPCs were associated with lower nurse wages ($p<.01$). NHs with more Medicaid residents pay lower wages, while those located in wealthier areas and with larger size pay higher wages. Our findings highlight the need to ensure equitable wage distribution across NHs, particularly by addressing the financial challenges faced by facilities located in more deprived communities.

### SESSION 1555 (SYMPOSIUM)

Abstract citation ID: igae098.0537

### PLACE AND AGING WITH DEMENTIA: NEW ASSESSMENTS FOR PERSON-ENVIRONMENT PROCESSES

Chair: Frank Oswald
Co-Chair: Habib Chaudhury
Discussant: Hans-Werner Wahl

There has been growth in research addressing stability and change in the relationship between the physical environment and older persons, particularly people living with dementia. However, differentiated assessments to examine or measure person-environment relationships are limited. Additionally, there is a lack of resources to inform development of physical environments supportive of people living with dementia. This symposium addresses creative measures, and application strategies to advance understanding of the role of place for people living with dementia. First, Florack and colleagues present a three-step modification of the Meaning of Home Questionnaire (MoH) for use with people living with dementia by means of simplification and addition of specific items, an initial evaluation of psychometric characteristics, and the development of a final version and its validation. Second, Niedoba and Oswald present findings from walking interviews with people living with dementia, highlighting that these interviews allow to observe and discuss everyday life person-environment processes and provide a comfortable atmosphere promoting personal reflections on the experience of living with dementia. Third, Margot-Cattin and Gaber provide evidence from the Participation in ACTivities and Places OUTside Home (ACT-OUT) questionnaire based on a transactional perspective of the person-environment relationship, showing that people with dementia experience a higher rate of abandonment of places than people without dementia. Four, Chaudhury and colleagues introduce three knowledge mobilization resources that can inform and engage with various stakeholders, including municipalities, community-based organizations, advocacy groups, planning and design professionals, persons living with dementia and care partners. Finally, Wahl serves as the session's discussant.

Abstract citation ID: igae098.0538

### KNOWLEDGE MOBILIZATION TO DEVELOP DEMENTIA-INCLUSIVE COMMUNITIES IN THE DEMSCAPE PROJECT

Habib Chaudhury[1], Cari Randa-Beaulieu[2], and Kishore Seetharaman[1], *1. Simon Fraser University, Vancouver, British Columbia, Canada*, *2. Alzheimer Society of British Columbia, Vancouver, British Columbia, Canada*

Creating a supportive neighborhood built environment that facilitates outdoor mobility, wayfinding, and access to community destinations is a key component in making communities more dementia-inclusive. To help municipalities achieve their vision of dementia-friendly and inclusive streets and outdoor spaces, this Knowledge Mobilization (KM) extension of the Dementia-inclusive Spaces for Community Access, Participation, and Engagement (DemSCAPE) project developed three evidence-based education and training resources for municipal planners, community-based organizations, people with lived experience and care partners. These resources or tools are: 1) "Dementia-Inclusive Planning and Design Guidelines" to inform municipal planners and decision-makers on effective physical planning and design to support people living with dementia; 2) "Neighbourhood Environmental Audit Tool" for community-based dementia advocacy organizations and people with lived experience to conduct audit of the neighbourhood environment and identify intervention projects; and 3) eight-minute documentary video illustrating the lived-experiences of three people living with dementia in urban, suburban and northern areas of British Columbia, Canada. In partnership with the City of Burnaby and the City of Richmond in Metro Vancouver, Canada, three KM activities are being conducted: 1) World Café discussions following screening the short video and an accompanying photo exhibit, 2) focus group discussions with municipal planners to help them apply knowledge of dementia-friendly streets and outdoor spaces to planning and design, and 3) conduct environmental audits of targeted streets in Metro Vancouver engaging people living with dementia and identify actionable built environmental interventions to make their communities more dementia-inclusive.

Abstract citation ID: igae098.0539

### ADAPTING THE MEANING OF HOME QUESTIONNAIRE FOR PEOPLE WITH DEMENTIA

Janina Florack[1], Susanne Penger[2], Julia Haberstroh[2], and Frank Oswald[3], *1. University of Siegen, Siegen,*

*Nordrhein-Westfalen, Germany*, *2. Psychological Aging Research, University of Siegen, Siegen, Nordrhein-Westfalen, Germany*, *3. Goethe University Frankfurt, Frankfurt am Main, Hessen, Germany*

Most people with dementia prefer to remain in their homes for as long as possible, despite facing various challenges in daily life due to cognitive decline. They generally decide against relocating to a nursing home, underscoring the significant value they attribute to their own residence. So far, the subjective meaning of home in people with dementia has primarily been explored through qualitative approaches. Thus, the objective of the current study was the adaption of an existing questionnaire for assessing the meaning of home (MoH) in people with dementia. In a pilot study, the MoH was revised by simplifying existing items and instructions and adding new items. Testing of the modified version (MoH-D) in a small sample of cognitively impaired individuals (N = 30) necessitated further adjustments, such as simplification of the response scale. In a second study, the factorial structure of the MoH-D was explored in a cognitively unimpaired sample (N = 574), resulting in a preliminary 21-item version with a five-factor structure that demonstrated psychometrically satisfactory characteristics. These factors were labeled comfort, everydayness, sociability, overwhelm, and autonomy based on their content. To further validate and refine the questionnaire, a sizable sample of cognitively impaired people (N = 282) was recruited. Currently, analyses are in progress to determine the final factorial structure of the MoH-D, evaluate its psychometric properties, and investigate its relations with external validation criteria (e.g., housing satisfaction, duration of residence). Initial findings will be presented and discussed.

Abstract citation ID: igae098.0540

### TRANSITIONS INTO DEMENTIA: REFLECTIONS ON WALKING INTERVIEWS AND THE PHOTO-ELICITATION METHOD IN DEMENTIA STUDIES

Simone Niedoba, and Frank Oswald, *Goethe University Frankfurt, Frankfurt am Main, Hessen, Germany*

Environmental gerontology highlights behavioral and experiential processes between a person and the environment in relation to different outcomes. However, how people living with early dementia experience and shape their environment is still poorly understood and how these processes could be best assessed is still being debated. Walking interviews promise to combine observation of everyday interactions, and discussion of meanings of environments. Pictures taken by participants along the way may give further insights into personal relevant environments. During nine walking interviews people living with early dementia were accompanied on a routine walk through their local environment. Participants decided where to go and were invited to take pictures of their environment, which were discussed at the end of the interview. The interviews highlight the importance of environments for the experience of living with dementia. During the interviews real-time interactions with places, objects, and people were observed and functioned as prompts for discussion, highlighting not only how people living with dementia shape their environment, thus conducting subtle forms of participation, but also how environments were connected to memories and identity. The combination of walking interviews and the photo-elicitation method allows to assess relevant person-environment exchange processes, such as agency and belonging, in early dementia. Moving through familiar environments can enhance a comfortable and trustful atmosphere in which personal reflections on living with dementia are shared. In the talk we will critically reflect on the experiences with these methods, including challenges, limitations and ethical considerations, and discuss how these methods could benefit future research.

Abstract citation ID: igae098.0541

### USING THE ACT-OUT QUESTIONNAIRE TO INFORM ON COMMUNITY MOBILITY AND PARTICIPATION FOR PEOPLE LIVING WITH DEMENTIA

Isabel Margot-Cattin[1], and Sophie Gaber[2], *1. University of Applied Sciences and Arts of Western Switzerland (HES-SO), Lausanne, Vaud, Switzerland*, *2. Jönköping University, Jönköping, Uppsala Lan, Sweden*

For people living with dementia to continue living in their current residences or “age in place”, it is essential for them to sustain their participation in daily activities and maintain mobility within their communities. Community mobility encompasses the ability to reach places where they can participate in meaningful everyday life activities. It also includes transportation modes such as walking, biking, driving, or using public transport. Thus, we developed the Participation in ACTivities and Places OUTside Home (ACT-OUT) questionnaire based on a transactional perspective and understanding of the person-environment relationship as unfolding and dynamic, embedded and situated, and enacted and emplaced. The ACT-OUT, used in a sit-down interview setting, which can be followed by mobile (walking) interviews, to enable the collection of rich data about around 25 important places that people currently visit, have abandoned, or wish to visit. A second part of the questionnaire collects information on traveling to the place (e.g., frequency, transportation, familiarity) and on activities performed there. The ACT-OUT has been used in multiple countries, including Switzerland, Sweden, the UK, and Canada, and among different populations, notably people with and without dementia. Results show that people with dementia experience a higher rate of abandonment of places and for many more places than people without dementia. It highlights the importance of addressing community mobility and how we conceptualize, design, and adapt important places to support people living with dementia to participate in places and activities that can maintain their well-being and health as they age in their communities.

### SESSION 1560 (SYMPOSIUM)

Abstract citation ID: igae098.0542

### RESPONSE TO THE DECADE OF HEALTHY AGING: AN EXEMPLAR ICOPE-BASED CARE MODEL

Chair: Doris Sau Fung Yu
Discussant: Yiting Tang

The United Nations Decade of Healthy Aging 2021-2030 calls for proactive and collaborative strategies to enable older adults to “live well” with optimized functional capacity, considering both intrinsic capacity (IC) and the environment. The Integrated Care of Older People (ICOPE) framework has been established as a comprehensive approach to guide IC assessment and care prescription for promoting healthy aging. In line with this global initiative, Hong Kong, a city with accelerated aging, has implemented the "Jockey Club Pathway to Healthy Aging" (Path-HA) program. This 12-week program incorporates the concepts of clinical critical care pathway, goal-oriented empowerment-based behavioral modification, asset-based capacity building, and health-oriented peer support. The program aims to optimize the functional status of local older adults across Hong Kong districts. The signs of accelerated aging and risk factors related to ICOPE health concerns are identified through the assessments. To date, more than 5,500 older adults are involved in this study and more than 1,800 have been recruited into the program and have received person-centered care and interventions for reducing the risks corresponding to critical pathways to healthy aging. This symposium is aligned with the ICOPE framework to address healthy aging in Hong Kong: (a) IC profiles of older adults, (b) promoting IC with a synergistic way to enhance care prescription, (c) critical pathways of the Path-HA program, and (d) the effects of critical care pathways on IC conditions. With these efforts endeavored, we will share the outcomes, insights and foresight regarding the regional contribution to optimizing functional capacity.

Abstract citation ID: igae098.0543

### A CRITICAL CARE PATHWAY BASED ON ICOPE TO PROMOTE HEALTHY AGING OF OLDER ADULTS

Doris Sau Fung Yu, Yiting Tang, Shuangzhou Chen, and Feng Chen, *The University of Hong Kong, Hong Kong, China (People's Republic)*

The United Nations Decade of Healthy Aging 2021-2030 calls for proactive and collaborative strategies to optimize the functional capacity of older adults. Aligning with the World Health Organization advocacy of optimizing the functional capacity of older adults through increasing the compatibility of their intrinsic capacity (IC) of older adults and the environment, the research team has developed the 12-week Pathway to Healthy Aging to translate the ICOPE care model to the Chinese community. The 12-week Pathway to Healthy Aging is a health-social collaborative model that adopts the WHO-ICOPE approach, together with the concepts of clinical critical care pathway, case management, empowerment-based behavioral modification and health-oriented peer coaching to promote healthy aging to the older adults with signs of accelerated aging. From September 2022 to June 2023, the care model was provided to 806 targeted clients recruited from a territory-wide region in Hong Kong. The speaker will share experiences and challenges of translating the WHO ICOPE care model in a Chinese community. More specifically, she will share the insights on: i) how to integrate the ICOPE assessment data to identify the health care priority of older adults, ii) method to develop the critical care pathway to promote person-center care and longer-term behavioral change for healthy aging promotion, iii) strategies to optimize effective collaboration between the nurses and health-oriented peer coach in case management. The presentation will conclude with remarks to inform future initiatives aimed at preparing society for an aging population.

Abstract citation ID: igae098.0544

### THE INTRINSIC CAPACITY PROFILE OF THE CHINESE OLDER ADULTS IN HONG KONG

Xiao-han Jiang[1], Feng Chen[2], Yiting Tang[2], Shuangzhou Chen[2], and Doris Sau Fung Yu[2], *1. The Hong Kong University, Guanzhou City, Guangdong, China (People's Republic)*, *2. The University of Hong Kong, Hong Kong, China (People's Republic)*

Intrinsic capacity (IC) refers to the composite of physical and mental capacity. To promote healthy ageing, the identification of IC will provide a clear picture of older adults. The IC assessment in "Pathway to Healthy Ageing Project" in Hong Kong was then conducted under the Integrated Care of Older People (ICOPE) framework. The 6 IC domains (cognition, locomotion, vitality, psychology, vision, and hearing) were assessed among the 4418 older adults recruited over Hong Kong (mean age: 74.4 years). Cognitive function was tested by Montreal cognitive assessment 5-min protocol. 16.6% of the participants had cognitive impairment. For locomotion, Short physical performance battery and Timed-Up and Go Test (TUG) were used to measure physical function and mobility. 35.0% were found to have impaired physical function, and 16.9% faced a fall risk. TUG revealed 6.5% of our participants had problem in mobility. Vitality was measured by Mini nutritional assessment and handgrip strength. Our results indicated that 27% of the participants had a malnutrition risk, and 0.4% were malnutrition. Handgrip strength indicated 59.4% had low muscle strength. Psychology was measured by Geriatric Depression Scale-15 item for depression. 10.8% of the participants were found with depression. Sensory was measured by self-reported. 22% reported they had visual loss, and 17.4% reported they had hearing loss. Our study provided a profile of IC of the community-dwelling older adults in Hong Kong. The IC impairment of Hong Kong older adults was identified, thus more targeted intervention can be provided to improve the overall health condition.

Abstract citation ID: igae098.0545

### PROMOTING INTRINSIC CAPACITY OF OLDER ADULTS: A SYNERGISTIC APPROACH TO ENHANCE CARE PRESCRIPTION

Yu Zhou, Shuangzhou Chen, Yiting Tang, Feng Chen, and Doris Sau Fung Yu, *The University of Hong Kong, Hong Kong, China (People's Republic)*

The United Nations has declared the Decade of Healthy Aging 2021-2030 to prioritize proactive and collaborative strategies for older adults to live well. The World Health Organization's Integrated Care of Older People (ICOPE) framework aims to guide the assessment of intrinsic capacity (IC) and the environment to promote healthy aging. Based on a research initiative that employs the WHO-ICOPE approach to promote healthy aging, a territory-wide ICOPE-based comprehensive health assessment was conducted in Hong Kong. Those with signs of accelerated aging were referred to a 12-week Jockey Club Pathway to Healthy Aging Program. The program adopts a health-social collaborative approach, clinical critical pathway, empowerment-based behavioral modification, and health-orientated peer support to provide person-centered care to optimize the functional status of older adults. From April 2022 to May 2023, a sample of 3,073 community-dwelling older adults [mean age (SD) = 74.7(7.5)] were recruited from eight geographic districts in Hong Kong and assessed with validated instruments. Latent class analysis identified five IC functional profiles of older adults according to the ICOPE framework. These five patterns include (1) compromised psycho-cognitive functional class, (2) compromised musculo-mobility functional class, (3) compromised metabolic-mobility functional class, (4) compromised nutritional-metabolic equilibrium but preserved functional class, and (5) preserved functional class. After controlling for the demographic profile, chronic disease burden and polypharmacy, the identified IC classification significantly predicted the health-related quality of life and frailty status of the older adults. The study provide new insights on the development of an integrative assessment and care planning for healthy aging promotion.

Abstract citation ID: igae098.0546

### IMPACT OF THE CRITICAL CARE PATHWAY ON INTRINSIC CAPACITY ON HEALTHY AGING PROMOTION

Xiangyu Zhai, Yiting Tang, Shuangzhou Chen, Feng Chen, and Doris Sau Fung Yu, *The University of Hong Kong, Hong Kong, China (People's Republic)*

The United Nations Decade of Healthy Aging prompted the implementation of the "Pathway to Healthy Aging", a 12-week health-social collaborative care program. This program, based on the Integrated Care for Older People (ICOPE) care pathway by the World Health Organization, incorporated clinical critical pathways, empowerment-based behavior modification, and health-oriented peer support. Intrinsic capacities of older adults were used as health indicators for effectiveness evaluation. From January 2023 to January 2024, 1024 older adults (female = 868; Mage = 75.5, SD = 7.52) participated, with 493 completing a six-month follow-up. Outcome-based evaluations demonstrated significant improvements across three time-points in locomotor capacity based on Timed Up and Go Test ($\chi^2(6)=73.49$, $p<0.001$, Cramér's V=0.16) and Short Physical Performance Battery ($\chi^2(4)=31.83$, $p<0.001$, Cramér's V=0.12), cognitive capacity (measured by Montreal Cognitive Assessment 5-minute protocol; $F(2, 984)=52.93$, $p<0.001$, partial $\eta^2=0.11$), and psychological capacity (measured by 15-item Geriatric Depression Scale; $F(2, 984)=11.31$, $p<0.001$, partial $\eta^2=0.09$). No significant change in hand grip strength ($\chi^2(4)=6.15$, $p=0.188$). Vitality capacity, based on Mini Nutritional Assessment, improved immediately after the program ($\chi^2(4)=7.45$, $p=0.047$, Cramér's V=0.09) but showed no significant further change at the six-month follow-up, based on pairwise comparison analyses (Bonferroni's correction). Insomnia (The Insomnia Severity Index) also significantly improved ($\chi^2(6)=77.42$, $p<0.001$; Cramér's V=0.16). This program demonstrates the effectiveness of the ICOPE care model in promoting healthy aging in real-world settings, emphasizing the potential of collaborative care models to enhance the elderly population's intrinsic capacities. These findings have critical implications for stakeholders working towards the United Nations' goal of fostering healthy aging.

### SESSION 1565 (SYMPOSIUM)

Abstract citation ID: igae098.0547

### SUBJECTIVE VIEWS OF AGING ARE ALL AROUND: A SOCIAL PERSPECTIVE

Chair: Yaakov Hoffman
Co-Chair: Amit Shrira
Discussant: Shevaun Neupert

Subjective Views of Aging (VoA) of the individual older adult are important predictors of many health and wellbeing outcomes. Nevertheless, VoA often develop within a relational context, suggesting that VoA have an inherent interpersonal component. However, few works have considered VoA within the greater social perspective. In this session we address VoA in two different social contexts; namely medical situations, and marital context. We address how these wider social VoA impact older adults' health and wellbeing. The first talk (Shrira & Shafir) addresses how ageist attitudes of spousal caregivers impact rehabilitation outcomes in patients following osteoporosis fractures. In the second talk (Hoffman et al.), we address how ageist attitudes of medical staff impact older adult patients in internal wards, leading to negative VoA, and to deteriorated physical health months later. The third talk (Mukherjee & Neupert) addresses increased daily memory failures of older adults with arthritis following exposure to interpersonal stressors, when awareness of age-related losses is low. The fourth talk (Bodner, Segel-Karpas & Estlein) explores how couples experience their joint aging, introduces a new questionnaire, and shows how this measure correlates with marital satisfaction and other variables. Together, the findings highlight how VoA operate within social contexts and offer valuable information for both researchers and clinicians regarding VoA and their effects on health and wellbeing.

Abstract citation ID: igae098.0548

### HOSPITAL STAFF AGEISM AND OLDER PATIENT'S SUBJECTIVE VIEWS OF AGING JOINTLY PREDICT FUTURE HEALTH

Yaakov Hoffman[1], Ehud Bodner[2], Susanna Cohen[1], and Amit Shrira[2], *1. Bar Ilan University, Ramat Gan, HaMerkaz, Israel, 2. Bar-Ilan University, Ramat Gan, HaMerkaz, Israel*

Over 66% of those hospitalized in internal wards are adults over 65. While older adults' subjective views on aging (VoA) have been linked with their own health, less is known in the context of hospitalization. Moreover, to the best of our knowledge, previous studies neither extensively examined effects of departmental ageism on patients' health indices, nor did they examine interactions between medical staff's ageist attitudes and patient's own VoA, in predicting subsequent health. Following, the current study addressed these issues. Hospitalized older adults (N=54, average age 76.24±8.19, range 65-101; 50% female) were assessed in 4 waves. Their experience of hospital ageism during hospitalization (Wave 1) predicted several outcomes two weeks later at Wave 2 (e.g., hospitalization satisfaction, b=-.30, re-hospitalization b=-.24) as well as one's self-rated health four months later, b=-.36 (Wave 4). This latter link was mediated by the change in attitude towards one's own aging (at Wave 2, a × b=-4.47,

95% CI [-9.7, -.29]). Findings suggest that reducing medical staff ageist attitudes (e.g., by including courses/workshops on ageism in medical staff training) may both enhance long-term health of older internal ward hospitalized patients and reduce care costs to health systems.

Abstract citation ID: igae098.0549

**VIEWS OF AGING OF PATIENTS AND SPOUSAL CAREGIVERS PREDICT HOME-BASED REHABILITATION OUTCOMES**

Amit Shrira[1], and Irit Shafir[2], *1. Bar-Ilan University, Ramat Gan, HaMerkaz, Israel*, *2. Bar Ilan University, Ramat Gan, HaMerkaz, Israel*

Home-based hospitalization programs providing health services in patients' home become increasingly widespread in view of population aging and insufficiencies of inpatient services. Home care has a potential to improve health outcomes and patient satisfaction as well as reduce costs. An impressive body of evidence shows that favorable subjective views of aging (VoA) predict better health outcomes among older adults. Much less is known about the role of VoA in the unique context of home-based hospitalization where care recipients and caregivers are jointly involved in intensive recovery efforts. Therefore, the current study assessed functional outcomes as predicted by VoA of 86 patients (mean age=71.5) and spousal caregivers (mean age=70.5). Patients and spouses receiving post-fracture home-based rehabilitation via one of the largest health maintenance organizations in Israel rated their VoA at the beginning of rehabilitation. Patients' functional independence was assessed by healthcare personnel at the beginning and end of rehabilitation (approximately two weeks later). The main outcome was rehabilitation effectiveness reflecting the ratio between the absolute functional gain and the patient's specific potential for functional improvement. Results showed that patients who felt younger or who were less aware of age-related losses had higher rehabilitation effectiveness. Moreover, patients whose spousal caregivers reported less ageist attitudes had higher rehabilitation effectiveness. The study found unique effects of patients' and spousal caregivers' VoA on rehabilitation outcomes. Findings suggest that reduction of negative VoA among patients as well as informal caregivers should be considered when tailoring orthopedic rehabilitation frameworks.

Abstract citation ID: igae098.0550

**INTERPERSONAL STRESSORS, AWARENESS OF AGE-RELATED CHANGES, AND ARTHRITIS ON DAILY MEMORY FUNCTION**

Shalini Mukherjee, and Shevaun Neupert, *North Carolina State University, Raleigh, North Carolina, United States*

The current study examined the interaction between daily awareness of age-related changes (AARC) losses and daily interpersonal stressors in predicting daily memory failures among 440 older adults in the U.S. (50-85 years) with and without arthritis. Participants reported their arthritis diagnosis on the first day of the study and then completed daily AARC and stressor questionnaires for the subsequent 13 days. Multilevel modeling analyses revealed a 3-way interaction for Daily Interpersonal Stressors X Daily AARC Losses X Arthritis. Among people with arthritis, high daily AARC losses were consistently associated with a high level of memory failures, irrespective of exposure to interpersonal stressors. However, individuals with arthritis who experienced low levels of AARC losses showed a significant increase in daily memory failures when exposed to interpersonal stressors. The findings suggest that interpersonal stressors are particularly powerful for individuals with arthritis when they experience low levels of AARC losses. Conversely, for those without arthritis, interpersonal stressors are linked to an increase in memory failures, regardless of AARC losses. Further research is recommended to understand the underlying mechanisms of the interaction and develop targeted interventions to improve memory function in individuals with arthritis, as well as those without the condition who are experiencing stress. Overall, the study's findings have significant implications for clinical practice and highlight the importance of addressing psychosocial factors in the management of arthritis and other age-related conditions.

Abstract citation ID: igae098.0551

**COUPLES' VIEWS OF THEIR JOINT AGING: DEVELOPMENT OF A NEW SCALE**

Ehud Bodner[1], Dikla Segel-Karpas[2], and Roi Estlein[3], *1. Bar-Ilan University, Ramat Gan, HaMerkaz, Israel*, *2. University of Haifa, Haifa, Hefa, Israel*, *3. University of Haifia, Haifa, Hefa, Israel*

The construct of subjective views of aging (VoA) constitutes a growing field of research, relating to individuals' general cognitions about the aging process, and their individual experience of aging. VoA develop throughout the life-course and have significant implications for individuals' health and well-being in later age. Although VoA often develop within a relational context of the married couple, suggesting the construct has an inherent interpersonal component, no theoretical framework or empirical studies have explored VoA from a dyadic perspective to consider the couple as an aging unit. We developed and tested a new scale to assess the way individuals expect to age within their current intimate relationship. Based on experts' evaluations, we identified five domains that are meaningful to couples' joint aging: (1) changes in health, (2) changes in family roles, (3) the transition to retirement, (4) changes in appearance and sexuality, and (5) coping with death. We then developed an initial pool of 39 items and tested these on a sample of over 300 midlife adults. Using factor analysis, we extracted a scale of 9 items relating to the 5 domains. We tested for validity against constructs of VoA and couple's relationships. The scale showed good internal reliability and validity.

### SESSION 1570 (SYMPOSIUM)

Abstract citation ID: igae098.0552

**THE LONGITUDINAL PROCESSES LINKING PSYCHOSOCIAL FACTORS AND STRESS-RELATED EXPERIENCES TO HEALTH**

Chair: Jing Luo
Co-Chair: Gabrielle Pfund
Discussant: Daniel Mroczek

Psychosocial factors, including individual factors (e.g., personality, sense of purpose), contextual factors (e.g., employment status), and stress-related experiences, have been demonstrated as important protective/risk factors of health. However, little is known about the processes and mechanisms linking these factors to health and aging. The current symposium tackles the longitudinal associations between psychosocial factors and health with five presentations investigating the interindividual and intraindividual processes linking these factors to various health outcomes (physical, cognitive, physiological health and well-being) as people age. In the first study, from a life-course perspective, Brown and colleagues examined the effects of low SES and perceived stress in childhood on two physiological health indicators in midlife and the underlying mediators. Zooming into late adulthood, the second study by Choi and Mejía examined the joint effects of spousal combinations of employment status and socioeconomic resources on older couples' individual and shared manifestations of cumulative health risk. The third study by Chopik examined the prospective effects of subjective well-being on stress reactivity decline and their dynamic reciprocal relations over time at the intraindividual level. The fourth study by Luo and colleagues examined the how the dynamic interplays between personality and people's stress experiences and health behavior engagement contribute to individual differences in health change. In the fifth study, Pfund and colleagues investigated the dynamic intraindividual associations among sense of purpose, social stress-related perception (loneliness), and cognitive decline over time. Collectively, this set of studies improves our understanding of how psychosocial factors shape people's health and healthy aging.

Abstract citation ID: igae098.0553

### EXAMINING THE DYNAMIC PROCESS LINKING PERSONALITY, HEALTH BEHAVIORS AND STRESS, AND HEALTH OUTCOMES

Jing Luo[1], Bo Zhang[2], Tomiko Yoneda[3], Eileen Graham[1], and Daniel Mroczek[1], *1. Northwestern University, Evanston, Illinois, United States*, *2. University of Illinois Urbana-Champaign, Champaign, Illinois, United States*, *3. University of California Davis, Davis, California, United States*

Personality has been found to contribute to individual differences in health and aging. In search of the underlying mechanisms, health behavior and stress have been shown to play roles in the personality-health association. However, given the evidence that personality traits are dynamic within individuals, it remains unclear how the dynamic intraindividual connections between personality and health behavior engagement and stress experiences contribute to interindividual differences in health and health change over time. Using 10-wave data from the Longitudinal Internet Studies for the Social Sciences (N = 3,421), the current study examined the effects of the intraindividual interplays between changes in personality and changes in health behaviors and stress on individual differences in different health outcomes and changes in health outcomes. Adopting multilevel structural equation modeling, the results suggested that after controlling personality at the interindividual level, the dynamic intraindividual interplays between extraversion, openness and smoking at each time point still displayed significant effects on interindividual differences in smoking, which in turn significantly contributed to interindividual differences in self-rated health and symptom complains in the last wave and changes in general disease level and cardiovascular diseases over time. Meanwhile, the dynamic intraindividual interplays between extraversion, conscientiousness and perceived stress were significantly related interindividual differences in perceived stress, which were significantly linked to interindividual differences in the levels of and changes in all health outcomes. The current findings enhance our understanding of how the dynamic intraindividual connections between personality and people's behaviors and experiences may inform interindividual differences in health during aging.

Abstract citation ID: igae098.0554

### THE RECIPROCAL RELATIONSHIPS AMONG SENSE OF PURPOSE, LONELINESS, AND COGNITIVE DECLINE

Gabrielle Pfund[1], Anthony Ong[2], Daniel Mroczek[3], Bryan James[4], Kathryn Jackson[3], and Eileen Graham[3], *1. Auburn University, Auburn, Alabama, United States*, *2. Cornell University, Ithaca, New York, United States*, *3. Northwestern University, Evanston, Illinois, United States*, *4. Rush University Medical Center, Chicago, Illinois, United States*

Dementia is the leading cause of disability in older adults worldwide. As of yet, there are no easily accessible cures. Thus, a wide range of research has focused on modifiable intra- and interpersonal factors that may contribute to lower risk for dementia and generally slow down cognitive decline. A promising protective intrapersonal factor from the well-being literature is sense of purpose, as higher levels of sense of purpose are tied to a swath of healthy cognitive aging outcomes. Meanwhile, greater experiences of loneliness are typically a risk factor for poorer cognitive aging. The current study calls upon data from the ongoing Memory and Aging Project from the Rush Alzheimer's Disease Center (N = 1,736; waves: up to 18; age: M = 81.07, SD = 8.04, 65-101). Every year, participants complete an extensive cognitive battery and report on their sense of purpose and loneliness. Given the likely reciprocal relationship between these three constructs, the current project uses random intercept cross-lagged panel models to better establish the between- versus within-person and concurrent versus longitudinal processes behind these relationships. Findings highlight the bidirectional relationship of these variables across time metrics and levels. For example, lower levels than one's average of sense of purpose predicts decreases in cognitive functioning a year later, and decreases in cognitive functioning also precede decreases in sense of purpose. Future research would benefit from exploring combined efforts to bolster sense of purpose and reduce loneliness, while continuing to mind the potential influence of cognitive decline on these constructs.

Abstract citation ID: igae098.0555

### LIFE SATISFACTION MODERATES LIFESPAN DECLINES IN STRESS REACTIVITY

William Chopik, *Michigan State University East Lansing, East Lansing, Michigan, United States*

Positive psychological well-being is often conceptualized as a protective factor for maintaining health across the lifespan. But why does psychological well-being confer such benefits? One potential possibility is that it shapes how we respond to stressors on a daily basis. However, much of existing research on this topic will examine these associations in the context of short-term experience sampling studies. Although useful in linking well-being to how we perceive and experience stress, this research can be limited in that it rarely tests prospective effects of well-being on stress reactivity over longer intervals. In the current study, I examined changes in stress reactivity across three waves of the National Study of Daily Experiences from approximately 1996 to 2017 (N = 1,500; Mage = 46.74; 54.1% women; 91.7% white) as a function of life satisfaction at wave 1. Employing three-level multi-level models, I reproduced recent research showing stress reactivity declines across life (b = -.02, p <.001); life satisfaction predicts lower stress reactivity both overall (b = -.04, p <.001) and in the context of a higher number of average (b = -.04, p <.001) and daily stressors (b = -.02, p <.001). Integrating repeated measures of life satisfaction and stress reactivity in the context of a random-intercept cross-lagged panel model suggested that reduced stress reactivity might lead to increases in life satisfaction across life. The results will be discussed in the context of how daily processes might "trickle up" to cultivate variation in global reports of well-being.

Abstract citation ID: igae098.0556

### PSYCHOSOCIAL DISADVANTAGE DURING CHILDHOOD AND MIDLIFE HEALTH

Ryan Brown, *Texas Tech University, Texas, United States*

Psychosocial disadvantage in childhood contributes to health disparities in aging-related health outcomes through biological and behavioral pathways. We leveraged the 30-year longitudinal NHLBI Growth and Health Study to examine associations between (a) low childhood SES (age 10) and (b) perceived stress in childhood (age 11) with midlife insulin resistance and epigenetic aging (age 40) in a sample of 433 Black and white women. We also explored whether late adolescent (age 19) waist circumference would mediate the relationships between SES and perceived stress and aging-related outcomes. Epigenetic aging was assessed in saliva and quantified through the GrimAge2 and DunedinPACE clocks. Insulin resistance was represented by the Homeostatic Model Assessment of Insulin Resistance (HOMA-IR). For each aim, multivariable linear regression models were estimated. Covariates included age, race, diabetes status, smoking status for all outcomes, and analysis cluster and estimated cell type composition variables for epigenetic clocks only. Higher parental education was associated with lower insulin resistance (b=-0.22, SE=0.10, p=.03), lower midlife GrimAge (b=-1.76 years, SE=0.56, p=.002), and slower midlife DunedinPACE (b=-0.03, SE=0.2, p=.04). Childhood perceived stress was significantly indirectly associated with midlife insulin resistance, b=0.01, 95% CI [0.001,0.01], p=.02, and midlife GrimAge, b=0.016 years, 95% CI [0.003,0.04], p=.01, through late adolescent adiposity. This highlights preliminary evidence for this life-course perspective on psychosocial stress, adiposity, and aging-related health. Future work may examine these individual factors in the context of broader structural systems (e.g., schools) and other facets of SES to better understand area-level effects (e.g., familial, neighborhood, region) for tailoring social interventions.

Abstract citation ID: igae098.0557

### SPOUSAL COMBINATIONS OF EMPLOYMENT STATUS AND SHARED HEALTH RISKS AMONG OLDER MARRIED COUPLES

Ha Young Choi[1], and Shannon Mejia[2], *1. University of Illinois Urbana-Champaign, Champaign, Illinois, United States*, *2. University of Illinois Urbana-Champaign, Urbana, Illinois, United States*

Continuing employment has been shown to protect health into old age. However, within marriages, the decision to retire or work is jointly negotiated which may be differentiated by socioeconomic resources. This study explores how spousal combinations of employment status differentiate individual and shared manifestations of cumulative health risk. Utilizing data of 3,143 older couples (aged 51-79) from the 2008/2010 Health and Retirement Study, we first conducted latent class analysis of husbands' and wives' combined employment statuses (age range fully/partially working: 51-79). We identified five classes of spouses' combined statuses: (1) one unemployed, another not fully working (8.15%), (2) retiree couples (34.36%), (3) one unemployed, another fully working (3.63%), (4) one fully working, another partially working (45.28%), and (5) fully working couples (8.59%). Latent dyadic models parsed the outcome variables, cumulative indicators of physiologic reserve and metabolic syndrome, into individual and shared components, which were then regressed on predicted cluster membership, and adjusted by age and functional limitations. Physiologic reserve was lowest in the one underemployed/other fully working class and highest in both fully working class. Meanwhile, systematic differences in metabolic syndrome across classes were only observed in couples where neither spouse had attended some college. In these couples, metabolic syndrome was highest in the two classes where either spouse was partly or fully working. Our findings imply that the effects of extended work life on health risks are contextualized within marriage and socioeconomic resources, where the shared health of couples in lower socioeconomic status rely more on combined employment status.

### SESSION 1575 (SYMPOSIUM)

Abstract citation ID: igae098.0558

### THE NEW MAP OF LIFE: TRANSFORMING THE COURSE OF LONG LIVES THROUGH IMPACT-DRIVEN RESEARCH

Chair: Yochai Shavit
Discussant: Laura Carstensen

The global rise in life expectancy and the emergence of societies that are more age-diverse than ever before in human history call for a re-design of the life course. The Stanford Center on Longevity released the New Map of Life- a report detailing principles to guide research, policies, and interventions aimed at improving long lives and age-diverse societies, taking advantage of the opportunities these demographic changes entail. These have already influenced recommendations by key organizations, including the National Academy of Medicine and the World Economic Forum. This symposium offers the first look into research findings guided by the New Map of Life and their translation into actionable recommendations. First, Weiss presents an innovative approach to model long life trajectories based on Artificial Intelligence that outperforms current life expectancy and morbidity calculators. On the population level, Ashwin presents macroeconomic challenges and advantages of longevity, highlighting differences between aging societies and long-living societies. Growney describes a pilot program leveraging older adults' strengths in early childhood education classrooms. Rangan presents findings from a study of graduating medical students highlighting the need to revamp medical education to address a severe lack of training in older adult medical care. Finally, Chu presents focus-group findings exploring older adults' technological needs and challenges in daily life, which can alter aging experiences. All presenters detail specific ways in which their findings can inform stakeholders' actions. Prof. Carstensen will conclude by discussing the broad picture emerging from these studies and their importance for further development of the framework.

Abstract citation ID: igae098.0559

### PREDICTING MORTALITY IN ADULTS USING TRANSFORMERS: INSIGHTS FROM THE HEALTH AND RETIREMENT STUDY

Jordan Weiss[1], Alaleh Azhir[2], Nilam Ram[1], and David Rehkopf[3], *1. Stanford University, Stanford, California, United States*, *2. Harvard University, Cambridge, Massachusetts, United States*, *3. Stanford University, Palo Alto, California, United States*

We introduce a natural language processing (NLP)-based prediction model to predict next-wave mortality in respondents from the US-based Health and Retirement Study (HRS). Akin to how NLP models can be used to predict future words in a sentence, we use a transformer architecture to build embeddings of a respondent's demographic, social, behavioral, and health histories which we then use to predict their probability of death at subsequent waves. Our model surpasses performance metrics established by conventional statistical models (average precision score = 0.900 versus 0.395), and provides new avenues for personalized healthcare in an observational setting.

Abstract citation ID: igae098.0560

### OLDER ADULTS AS RESOURCES IN EARLY CHILDHOOD EDUCATION

Claire Growney[1], Claire Jordan[1], Carol Larson[1], and Laura Carstensen[2], *1. Stanford University, Stanford, California, United States*, *2. Stanford University, Stanford University, California, United States*

As California rolls out universal transitional kindergarten for 4-year-olds, the public school system faces a shortage of teachers and paraeducators to meet minimum adult–child ratios. Given age-related strengths in the social-emotional domain, older adults may be especially well-suited to support young children's developmental needs. We are currently forming and researching programs that bring older adults into early childhood education classrooms to support children in various capacities. Many schools have used volunteers, but our focus is paid hourly and salaried positions. To inform program development, we collected qualitative and quantitative data from 8 early childhood center directors and 10 adults aged 55–73 who indicated interest in working with young children. Directors' top-rated goals were to recruit people who would contribute to a calm, inviting classroom environment, support children's social-emotional needs, and lower adult-child ratios. Directors also reported concerns, including older adults being unable to meet the physical demands of the job. Older adults' top-rated reasons for being interested were wanting to have an emotionally meaningful experience and make a difference for young children. Information learned from these data was used to inform the creation of pilot programs at sites in Los Angeles, Fresno, and Santa Cruz, California. At baseline and the end of the programs, we are collecting assessments from classroom teachers, directors, and older adult participants to examine effects of the program on classroom environment, attitudes toward older adults, and older adults' well-being. We will discuss barriers to program implementation and methods for successfully recruiting and retaining participants.

Abstract citation ID: igae098.0561

### DAILY AND TECHNOLOGICAL CHALLENGES AND NEEDS IN OLDER AGES: A MIXED METHODS STUDY

Michelle Cruz[1], Li Chu[1], Laura Paola Gomezjurado Gonzalez[1], Ruohan Zhang[1], Jiajun Wu[1], Li Fei-Fei[1], and Laura Carstensen[2], *1. Stanford University, Stanford, California, United States*, *2. Stanford University, Stanford University, California, United States*

Modern societies face two trends that are seemingly unrelated yet concurrently impacting our lives: population aging and technological advancements. Both trends are happening rapidly in today's society, and in combination raise an important question: How can we guide future technologies and their deliveries to better suit older adults' needs so older adults are not "left behind" in the fast-changing cultural shifts due to technological advancements? A growing body of literature captures technological needs and problems among older adults. Yet, many of these studies used relatively small sample sizes consisting of relatively young older adults who are homogeneous in terms of race and ethnicity to represent all older adults. The present study utilizes a mixed methods design to ensure qualitative findings are generalizable. We recruited a racially diverse sample of Asian, Black/African, Hispanic, and White community-dwelling older adults over the age of 75 from the San Francisco Bay Area to form eight focus groups. Participants freely discussed their daily challenges and needs, and how technology may meet these needs. With manual coding and natural language processing techniques, we identified close to 30 areas where technologies may contribute to daily life. Findings from the qualitative sessions and a follow-up survey will be presented. Our findings have the potential to inform service providers, government, technologists, and entrepreneurs how they can better support older adults who are independent living but have started to observe difficulties.

Abstract citation ID: igae098.0562

**GRADUATING MEDICAL STUDENTS' PERFORMANCE ON A GERIATRICS KNOWLEDGE TEST IS ASSOCIATED WITH ATTITUDES**

Apoorva Rangan, Yochai Shavit, and Deborah Kado, *Stanford University, Stanford, California, United States*

Older adults are overrepresented among patients, with half of outpatient visits and 40% of hospitalizations for adults over 65. Training physicians in age-friendly care is imperative. Yet, medical school graduates' knowledge of core geriatrics competencies is unknown, as are factors associated with students' knowledge base. 30 of the 82 graduating students at the Stanford School of Medicine completed a three-part survey. Geriatrics knowledge was assessed using a validated 18-item UCLA Geriatrics Knowledge Test (GKT) and ten questions from the Geriatrics Review Syllabus (GRS). Attitudes towards older patients were assessed using the validated 14-item UCLA Geriatrics Attitudes Assessment. Finally, students reported their curricular exposure to geriatrics and career interests. The Stanford Institutional Review Board waived institutional review. Two researchers independently analyzed results in R. Geriatrics exposure was limited; 1 student took a preclinical elective and 2 took clinical electives. Most students incorrectly completed at least half of the knowledge assessment. All students missed at least 7/28 questions overall ($M = 8.0$, $SD = 6.7$), with students performing better on the GKT ($M = 8.4$, $SD = 5.1$) than the GRS portion ($M = -0.4$, $SD = 3.0$). Most students had neutral or slightly positive attitudes towards older adults ($M = 3.58$, $SD = 0.87$). Attitude scores were moderately associated with improved performance on the knowledge assessment ($r = 0.55$, $p < 0.0001$). In conclusion, graduating students did not meet minimum geriatrics competencies measured by a knowledge assessment. Attitudes towards older adults were associated with performance, supporting future intersectional interventions in ageism.

### SESSION 1580 (SYMPOSIUM)

Abstract citation ID: igae098.0563

**THIS IS THE YEAR THAT WAS: THE BEST OF THE JOURNAL OF GERONTOLOGY BIOLOGICAL SCIENCES**

Chair: Gustavo Duque
Co-Chair: Laura Haynes

This session features the authors of the most influential papers published in the Journal of Gerontology, Biological Sciences, based on the highest impact in 2023. The authors will present the most exciting findings of the year on aging biology. Six authors of Editor's Choice papers will be invited to summarize their paper and the future implications of their discoveries for 10 minutes, followed by 5 minutes of Q&A from the audience.

Abstract citation ID: igae098.0564

**POLYGENIC RISK SCORES FOR ALZHEIMER'S DISEASE AND GENERAL COGNITIVE FUNCTION ARE ASSOCIATED WITH MEASURES OF COGNITION IN OLDER SOUTH ASIANS**

Gustavo Duque, *Bone, Muscle & Geroscience Group, Research Institute of the McGill University Health Centre, Montreal, Quebec, Canada*

TBD

Abstract citation ID: igae098.0565

**POLYGENIC RISK SCORES FOR ALZHEIMER'S DISEASE AND GENERAL COGNITIVE FUNCTION ARE ASSOCIATED WITH MEASURES OF COGNITION IN OLDER SOUTH ASIANS**

Wei Zhao, *University of Michigan, Ann Arbor, Michigan, United States*

TBD

Abstract citation ID: igae098.0566

**REGIONAL NEUROANATOMIC EFFECTS ON BRAIN AGE INFERRED USING MAGNETIC RESONANCE IMAGING AND RIDGE REGRESSION**

Andrei Irimia, *University of Southern California, Los Angeles, California, United States*

TBD

Abstract citation ID: igae098.0567

**HEALTHY LIFESTYLE BEHAVIORS AND BIOLOGICAL AGING IN THE U.S. NATIONAL HEALTH AND NUTRITION EXAMINATION SURVEYS 1999-2018**

Yian Gu, *Columbia University, New York, New York, United States*

TBD

Abstract citation ID: igae098.0568

**INTERLEUKIN-6 DRIVES MITOCHONDRIAL DYSREGULATION AND ACCELERATES PHYSICAL DECLINE: INSIGHTS FROM AN INDUCIBLE HUMANIZED IL-6 KNOCK-IN MOUSE MODEL**

Peter Abadir, *Johns Hopkins University, Baltimore, Maryland, United States*

TBD

### SESSION 2000 (SYMPOSIUM)

Abstract citation ID: igae098.0569

**ADVANCING DATA COLLECTION AND SHARING FOR PERSON-CENTERED DEMENTIA CARE IN LOW-RESOURCE LONG-TERM CARE SETTINGS**

Chair: Alison Rataj
Discussant: Eleanor McConnell

Person-centered care (PCC) is essential to support people living with dementia and is a guiding principle in care services. Inequities in PCC and dementia care access persist in long-term care (LTC) settings, including poorer urban and rural communities, which have fewer healthcare resources. While growing evidence has demonstrated the consequences of LTC inequities, research is limited in understanding strategies to improve dementia care in low-resource settings. This symposium features four presentations from the Advancing Person-Centered Dementia Care in Low-Resource Contexts Project emphasizing unique challenges and opportunities for providing PCC in low-resource LTC settings from the perspectives of administrators, direct care staff, residents with dementia and their families. The first presentation reports results from a community-based, participatory research study in four LTC settings in federally designated medically underserved areas highlighting what matters when providing quality dementia care from the perspectives of LTC leaders, staff, residents and their families. The second presentation shares PCC strategies used by direct care workers stratified by length of tenure in the workforce. The third presentation examines factors related to measuring PCC quality with a specific focus on measurement in low-resource settings. The fourth presentation summarizes results from a series of design thinking workshops with family members, staff, and leaders in low-resource LTC settings to co-create processes for supporting high-quality PCC. This symposium reveals innovative strategies to provide dementia care and generates future directions for addressing inequities in low-resource LTC settings. Common Data Elements for International Research in Residential Long-term Care Interest Group Sponsored Symposium

Abstract citation ID: igae098.0570

### WHAT MATTERS MOST FOR PERSON-CENTERED DEMENTIA CARE: PERSPECTIVES FROM STAKEHOLDERS IN LOW-RESOURCE LONG-TERM CARE

Jing Wang[1], Michael Lepore[2], Nancy Kusmaul[3], Sarah Holmes[4], Laura Davie[1], Alison Rataj[5], Yoon Chung Kim[6], and Kirsten Corazzini[1], *1. University of New Hampshire, Durham, New Hampshire, United States*, *2. University of Massachusetts Amherst, Amherst, Massachusetts, United States*, *3. University of Maryland Baltimore County, Baltimore, Maryland, United States*, *4. University of Maryland School of Nursing, Baltimore, Maryland, United States*, *5. University of Massachusetts Boston, Durham, New Hampshire, United States*, *6. University of Maryland Baltimore, Baltimore, Maryland, United States*

One of the initial steps in supporting quality dementia care for residents living with dementia (RLWD) in low-resource long-term care (LTC) settings is to elucidate stakeholder perspectives on what matters most for providing person-centered dementia care in such settings. We conducted community-based, participatory research across four LTC facilities situated in federally designated medically underserved areas (two rural and two urban) to describe what matters most to key stakeholders in achieving person-centered dementia care in low-resource LTC settings through their own journeys. In-depth semi-structured interviews were conducted with administrative leaders (n=7), direct care staff (n=20), RLWD (n=16), and family/care partners (n=16), with transcripts thematically analyzed using NVivo14 software. Administrators and staff members acquaint themselves with dementia care principles, participate in daily huddles, and establish rapport with residents and families. As they progress, they acquire skills to observe residents' behaviors, empathize with their experiences, cultivate trust, delivering intuitive care that honors individual preferences, routines, and past experiences. RLWD transitioning to LTC settings seek environments resembling private homes that foster self-advocacy and rely on staff members who understand their needs and promote a sense of belonging. For family members, the journey involves supporting their loved ones' transition, advocating for their preferences, and maintaining open communication with staff. They play a crucial role in reinforcing resident preferences and ensuring the delivery of quality care. Throughout these journeys, stakeholders identify key quality measures, including odor, cleanliness, resident happiness, and the quality of relationships, as benchmarks for assessing person-centered care.

Abstract citation ID: igae098.0571

### STRATEGIES USED BY STAFF TO PROVIDE PERSON-CENTERED DEMENTIA CARE: A COMPARISON BY LENGTH OF TENURE

Yoon Chung Kim[1], Michael Lepore[2], Nancy Kusmaul[3], and Sarah Holmes[4], *1. University of Maryland Baltimore, Baltimore, Maryland, United States*, *2. University of Massachusetts Amherst, Amherst, Massachusetts, United States*, *3. University of Maryland Baltimore County, Baltimore, Maryland, United States*, *4. University of Maryland School of Nursing, Baltimore, Maryland, United States*

Approximately half of nursing home residents and 42% of assisted living residents have a diagnosis of dementia. The quality of care that staff provide to long-term care (LTC) residents living with dementia (RLWD) is vital for their well-being. However, questions remain about what strategies staff use to support person-centered dementia care and whether differences exist by length of tenure. This descriptive, qualitative study sought to identify strategies used by direct care workers to provide person-centered dementia care and compare strategies by length of tenure. The study included in-depth semi-structured interviews with a purposive sample of 20 direct care workers in four low-resource LTC settings in two states. Interviews were audio recorded, transcribed, and thematically analyzed in NVivo14. Staff reported a mean length of tenure in LTC of 8.97 years (SD= 5.92, range= 1.5-21 years). Common strategies recommended by direct care workers for providing person-centered dementia care included validating and recognizing residents' emotional needs, building trust and rapport, involving families, honoring preferences and life histories, knowing triggers or past traumas, and engaging residents in individualized activities. Participants emphasized the importance of dementia training for providing care to RLWD. There were no differences in strategies observed between longer and shorter tenured direct care workers, but those with longer tenure expressed more emotional detachment regarding resident care, whereas shorter tenured staff expressed more emotional attachment in resident care. Findings highlight how staff with shorter and longer tenure experience care differently and inform future work to promote high-quality care for RLWD in LTC settings.

Abstract citation ID: igae098.0572

### PERSPECTIVES ON MEASURING QUALITY DEMENTIA CARE: FINDINGS FROM A STUDY IN LOW-RESOURCE LONG-TERM CARE

Nancy Kusmaul[1], Alison Rataj[2], Michael Lepore[3], Kirsten Corazzini[4], Yoon Chung Kim[5], Jing Wang[4], Sarah Holmes[6], and Laura Davie[4], *1. University of Maryland Baltimore County, Baltimore, Maryland, United States*, *2. University of Massachusetts Boston, Durham, New Hampshire, United States*, *3. University of Massachusetts Amherst, Amherst, Massachusetts, United States*, *4. University of New Hampshire, Durham, New Hampshire, United States*, *5. University of Maryland Baltimore, Baltimore, Maryland, United States*, *6. University of Maryland School of Nursing, Baltimore, Maryland, United States*

The quality of long-term care for persons living with dementia includes elements of both quality of life and quality of care. However, which elements are most important for measuring quality is less clear and may vary based on different stakeholder perspectives. According to the National Academies of Science, Engineering, and Medicine (NASEM), quality measurement is important because it supports work towards improving quality of care, specifically making care more safe, timely, equitable, efficient, effective, and patient-centered (2022). This presentation will discuss perspectives on quality measurement among residents living with dementia, their families, staff, and administrators in low-resource long-term care settings in two states. Using a priori team coding of interviews with 59 participants, three themes emerged: (1) Quality care includes getting to know residents and communicating with them, (2) Measures of relational aspects of quality are difficult to implement, and (3) Good quality care results in better outcomes on existing clinical quality measures. During study interviews, participants readily discussed elements of quality in individual provision of care but had difficulty describing quality measurement at an organizational or systematic scale, supporting previous findings that direct observation may be an accurate indicator of quality of care, but is challenging to implement at scale. Additionally, participants conceptualized care provision from their unique perspective (i.e. staff, resident, family) suggesting that each may have different factors that constitute quality care. This study underscores the importance of quality measurement and the challenges of operationalizing quality, particularly for persons living with dementia.

Abstract citation ID: igae098.0573

### CO-DESIGNING DATA COLLECTION AND USE PROTOCOLS FOR PROVIDING PERSON-CENTERED DEMENTIA CARE

Kirsten Corazzini[1], Eleanor McConnell[2], Nancy Kusmaul[3], Sarah Holmes[4], and Jing Wang[1], *1. University of New Hampshire, Durham, New Hampshire, United States*, *2. Duke University, Durham, North Carolina, United States*, *3. University of Maryland Baltimore County, Baltimore, Maryland, United States*, *4. University of Maryland School of Nursing, Baltimore, Maryland, United States*

Considerable gaps remain in delivering person-centered dementia care in residential long-term care (LTC) settings, despite gains in instrument development, and the addition of person-centered care-related items in the Minimum Data Set (MDS). The paucity of pragmatic measures and care processes to support person-centered dementia LTC has been identified as significantly contributing to this gap. Further, this gap is exacerbated in medically underserved and health professional shortage (MUHPS) communities, which are less likely to have the professional development and technical capacity to support robust data collection and use of person-centered care measures among front-line care staff. This multi-method, participatory research study aimed to address this gap by co-designing, with residential long-term care residents who are living with dementia, their families, and care staff in MUHPS communities, pragmatic data collection and use protocols. This paper describes the development of co-design workshops through the data analysis of qualitative interviews (N=68 residents, family members, and staff from 4 LTC communities), and results from co-design workshops of identified strategies following review of journey maps (N=8 workshops). Results provided low-resource strategies in multiple domains over time for staff members (onboarding, developing expertise, and becoming an expert); residents (moving into your new home, being who you are and living, and feeling like you are settled); and families (supporting a family member's move, supporting good care, and overall assessment of care). Findings advance our methodological and substantive capacity for inclusive and low-resource strategies to improve person-centered dementia care practice in LTC.

### SESSION 2001 (SYMPOSIUM)

Abstract citation ID: igae098.0574

### ABUSE ACROSS THE LIFE COURSE: CONNECTIONS WITH ELDER ABUSE

Chair: Julie Miller
Discussant: Lori Mars

Likely conservative estimates suggest that 1 in every 10 Americans aged 60+ have experienced some form of elder abuse. As theoretical understandings of abuse in general have evolved, so too have conceptualizations of abuse at different stages of the life course, including in older adulthood. Questions remain, however, about ways in which abuse experienced earlier in the life course connect with subsequent experiences with elder abuse. This symposium brings together leading researchers and practitioners to explore abuse across the life course and connections with elder abuse. The first and second presentations in the symposium center experiences of elder abuse victims, with one presentation exploring patterns of abuse victimization across the lifespan and connections with prospective health outcomes in older adulthood, and another presentation focusing on experiences of financial exploitation among Holocaust survivors. The third and fourth presentations shift the perspective to emphasize experiences of people involved in the lives of elder abuse victims, including dementia caregivers who may be at greater risk for experiencing or perpetrating abuse once caregiving obligations begin and, in another case, concerned persons in the lives of elder abuse victims. Taken together, the presentations surface new insights and questions that locate elder abuse within a life course framework and connect abuse across the life course with elder abuse in particular. The symposium will include discussions of implications for research, practice, and policy.

Abstract citation ID: igae098.0575

### LIFETIME ABUSE VICTIMIZATION AND PROSPECTIVE HEALTH OUTCOMES IN OLDER ADULTS

Jooyoung Kong[1], Sara Moorman[2], and Yue Qin[1],
*1. University of Wisconsin Madison, Madison, Wisconsin, United States*, *2. Boston College, Chestnut Hill, Massachusetts, United States*

Lifetime abuse victimization indicates cumulative violence exposures or repeated victimizations within and/or across different life stages. The current study aims to examine the patterns of lifetime abuse victimization that span childhood to older adulthood and evaluate the associations between lifetime abuse victimization and prospective health outcomes in late adulthood. Data were drawn from 4907 older adults (mean age = 80) from the Wisconsin Longitudinal Study. We assessed the exposure to interpersonal violence including childhood abuse and neglect, intimate partner violence, and elder abuse until the respondents' age of early 70s. A series of multivariate analyses were conducted to examine the associations of a cumulative score of lifetime abuse victimization with depression, physical health status, and memory in respondents' age of early 80s. On average, respondents experienced 1.1 exposure to lifetime abuse victimization (SD = 1.53). Greater exposure to lifetime abuse was associated with a significantly higher risk of depression and a greater number of limitations in physical functioning, but not with memory performance. When examined separately by abuse type, childhood abuse was associated with more health limitations and elder abuse was associated with a higher risk of depression and more health limitations. Our results support the interrelations of interpersonal violence across the life course and the lasting health effects of exposure to lifetime abuse, which can lead researchers and practitioners to adopt a more comprehensive conceptualization of interpersonal violence victimization. Findings also highlight the need for a life course-based, trauma-informed approach in prevention and intervention programs for older adults.

Abstract citation ID: igae098.0576

### FINANCIAL EXPLOITATION AMONG HOLOCAUST SURVIVORS: EVIDENCE FOR VULNERABILITY AND RESILIENCE

Gali Weissberger, Moshe Bensimon, and Amit Shrira,
*Bar-Ilan University, Ramat Gan, HaMerkaz, Israel*

Late-life adverse events may be particularly impactful in Holocaust survivors (HS) as they may be reminiscent of certain conditions that existed during the Holocaust. Financial exploitation (FE) is one such adverse event that may lead to losses reminiscent of Holocaust conditions. Research has demonstrated that the experience of FE has devastating consequences on the mental health of older adults. However, few research studies have examined the consequences of FE in older adults with a traumatic past. In a quantitative study, we showed that anxiety and depressive symptoms associated with an FE experience were significantly greater in HS with posttraumatic stress disorder (PTSD) symptoms relative to comparisons (HS without PTSD symptoms who experienced FE and HS without an FE experience). However, research of HS has also shown that they exhibit certain resiliencies vis-à-vis stress. Accordingly, we used a qualitative approach to further examine vulnerability and resilience among HS who experience FE. The following questions guided our inquiry: What are the feelings and perceptions of HS following an experience of FE? How did they cope with FE? What are their perceptions of protective and risk factors for FE? Fifteen HS (10 females, M age = 84.66) living in Israel underwent semi-structured interviews. A four-stage content analysis revealed several core themes of meaning: 1) negative emotional reactions towards FE; 2) coping mechanisms; 3) positive life motto; 4) protective and risk factors for FE. Taken together, the qualitative analyses produced novel insights with regards to vulnerabilities and resiliencies amongst HS who have experienced FE.

Abstract citation ID: igae098.0577

### EXPLORING ABUSE ACROSS THE LIFE COURSE IN DEMENTIA CAREGIVING RELATIONSHIPS

Elizabeth Avent[1], Jeanine Yonashiro-Cho[2], Laura Mosqueda[3], and Zachary Gassoumis[4], *1. University of Chicago, Chicago, Illinois, United States*, *2. University of Southern California Keck School of Medicine, Los Angeles, California, United States*, *3. University of Southern California Keck School of Medicine, Pasadena, California, United States*, *4. University of Southern California, Los Angeles, California, United States*

Based on the web of violence framework, described as different patterns of co-occurring abuse and trauma across the lifespan, caregivers who have lifespan patterns of abuse may be at greater risk for experiencing or perpetrating abuse once caregiving obligations begin. Conflict and abuse are concerns when the care receiver has ADRD, as behavioral problems associated with dementia may arise during caregiving. Spousal and intimate partner (S/IP) caregiving dyads are most likely to live together, so it is probable that aggression and abusive behaviors can be present in these dyads. Bivariate analyses (chi-square and t-tests) from surveys (N=207) and semi-structured interviews (N=12) with caregivers were conducted to identify characteristics that are conceptually related to abusive behavior throughout their relationships and life courses. There were no significant differences between S/IP caregivers and other types of caregiving relationships in mean adverse childhood experiences (ACE) scores, anxiety and depression scores, relationship quality, and caregiver burden. Roughly 22% of S/IP caregivers experienced at least 1 ACE. S/IP caregivers reported a history of IPV (10.9%) and abuse in the past year (12.2%). Among dyads in which recent abuse occurred, abuse was present throughout the relationship, even before onset of dementia. Semi-structured interviews showed that the caregiver experienced IPV in a previous relationship and/or at least one partner in the dyad experienced some form of family violence during childhood. Clarifying older victims' experiences can help to identify risk factors, develop age-inclusive resources and services, and encourage collaboration between aging and domestic violence service providers.

Abstract citation ID: igae098.0578

### KEEPERS OF CONTEXT: CENTERING VOICES OF CONCERNED PERSONS IN THE LIVES OF OLDER ADULT VICTIMS

Lisa Rachmuth, *Weill Cornell Medicine, New York, New York, United States*

Older adults who experience financial exploitation and other forms of abuse often experience this abuse in isolation. However, financial exploitation and other forms of abuse can also profoundly impact another group of individuals in the lives of older adults: non-abusing family members, friends, and neighbors, referred to as "Concerned Persons" (CPs). The Concerned Person in the life of the older adult is often one of the few persons that can help identify the abuse and find resources to help the older adult move out of the abuse. Concerned Persons in the lives of older adults are sometimes secondary victims of the abuse, isolating themselves from other support networks and at times putting themselves at risk in various ways. A series of focus group conducted in 2024 focused on factors that motivated Concerned Persons in the lives of the older adult to help, how Concerned Persons can help professionals understand the older adults' experiences with abuse across the lifespan, and how they can affect change for the older adult. Focus groups were conducted in affiliation with the Elder Abuse Helpline for Concerned Persons (the Helpline), a program at Weill Cornell Medicine. Through the Helpline, CPs are able to receive supportive counseling for themselves and referrals to ongoing services for themselves and the Older Adults who are abused. Findings from this research surface new insights that connect Concerned Persons' experiences and attitudes with the topic of abuse across the lifespan, pointing to implications for research, policy, and practice.

### SESSION 2005 (BIOLOGICAL SCIENCES INVITED SYMPOSIUM)

Abstract citation ID: igae098.0579

### PRESIDENTIAL SYMPOSIUM: FUNDAMENTAL APPROACHES TO HEALTHY AGING

Chair: Nathan LeBrasseur

The major threats to a long and healthy lifespan in humans include heart disease, stroke, cancer, diabetes, and Alzheimer's disease; conditions for which the risk rises exponentially with advancing age. Healthy lifestyle choices are currently the most effective strategies to delay if not prevent the onset and progression of each of these distinct conditions, which suggests that they counter the fundamental biology of aging. In this Presidential Symposium, thought leaders in nutrition (Dr. Peterson), exercise (Dr. Fielding), sleep (Dr. Carroll), and circadian rhythms (Dr. Panda) will share their insights into how lifestyle choices influence biological mechanisms and, in turn, the clinical manifestations of aging.

Abstract citation ID: igae098.0580

### CAN INTERMITTENT FASTING SLOW PRIMARY AND SECONDARY AGING IN HUMANS?

Courtney Peterson, *University of Alabama Birmingham, Birmingham, Alabama, United States*

Calorie restriction slows primary and secondary aging and increases lifespan in multiple animal species. However, recent evidence suggests that calorie restriction extends lifespan largely via extended fasting periods rather than energy restriction per se. The practice of periodically extending the fasting period is known as intermittent fasting. Dozens of studies in animals suggest that intermittent fasting decreases body weight; reduces the incidence of and/or progression of several chronic diseases; and extends lifespan. In humans, there are a wide range of intermittent fasting approaches, including alternate-day fasting, alternate-day modified fasting, the 5:2 diet, the fasting-mimicking diet, and time-restricted eating (TRE). In this presentation, I will briefly review evidence showing that intermittent fasting extends lifespan in animals. I will then define and discuss the various forms of intermittent fasting studied in humans. I will cover what is known about the feasibility of different types of intermittent fasting and their effects on primary and secondary aging in humans. I will conclude with a deeper dive into the most popular form of intermittent fasting, TRE. I will discuss its effects on weight loss, cardiometabolic health, and primary and secondary aging in humans.

Abstract citation ID: igae098.0581

### IMPACT OF EXERCISE/PHYSICAL ACTIVITY ON HEALTH SPAN: FROM MOLECULAR MECHANISMS TO FUNCTIONAL BENEFITS

Roger Fielding, *Jean Mayer USDA Human Nutrition Research Center on Aging at Tufts University, Boston, Massachusetts, United States*

Exercise/physical activity counters the onset and progression of chronic diseases (e.g., type 2 diabetes (T2D), cardiovascular disease (CVD), cognitive decline, and cancers), geriatric syndromes (e.g., frailty), and disability, even when introduced in later-life. Advancing chronological age is associated with well characterized declines in muscle size, performance, and physical functioning across multiple species. Underlying these age-related changes are declines in the force/power generating capacity of skeletal muscle that appear to be driven by changes in skeletal muscle contractile protein function, metabolic derangements, and alterations in neuromuscular activation. Age-associated changes in muscle gene transcription, epigenetic modifications, mitochondrial stability, and anabolic capacity, all suggest that fundamental changes in aging biology are responsible for these changes. Furthermore, many of these responses have been shown to be responsive to changes in regular physical activity suggesting a putative role for exercise/physical activity on health span with aging. In my presentation, I will review the underlying mechanisms of age-related changes in skeletal muscle mass and function and how changes in regular physical activity can attenuate or reverse the observed changes in mass and function that occur with advancing age with resultant improvements in health span.

Abstract citation ID: igae098.0582

### SLEEP: A GEROSCIENCE TARGET

Judith Carroll, *University of California, Los Angeles, Los Angeles, California, United States*

Obtaining healthy quantity and quality of sleep is important in maintaining good mental and physical health, and cumulative evidence points to a role of sleep loss and sleep disturbances as a factor increasing age-related disease risk and progression. Many older adults experience less sleep on average per night than they obtained in earlier decades. Despite convincing epidemiologic data linking poor sleep and diseases of aging, the molecular pathways accounting for these links are not well delineated. Our work has begun to tackle these questions by examining how poor sleep may accelerate the biological aging process, while intervening to improve sleep may slow aging. We have shown that insomnia is related to biomarkers of aging including shortened telomere length, older epigenetic age, and a pro-inflammatory signal. Experimentally restricting sleep in older adults led to acute changes in gene expression indicating increased DNA damage response and senescence related inflammatory activation. Treating insomnia in older adults led to lower transcripts for cellular senescence and slowing of the pace of aging (DunedinPACE). Healthy sleep may provide biological advantage for increasing restoration and repair, while poor sleep may reduce the time the system can dedicate energy to repair of insults experienced during waking hours. Parallel to clearance of waste from the brain, we hypothesize that sleep is also important for physiological restoration throughout the body. Many unknowns still exist and future research will be needed to disentangle the mechanisms through which sleep alters biological aging.

Abstract citation ID: igae098.0583

**PLEIOTROPIC HEALTH BENEFITS OF TIME-RESTRICTED FEEDING**

Satchidananda Panda, *Salk Institute for Biological Studies, San Diego, California, United States*

The global epidemic of obesity is driving a rapid increase in metabolic syndrome, which in turn contributes to several chronic diseases, including type 2 diabetes, certain cancers, and dementia. To combat this diverse set of diseases, there is an urgent need for simple yet effective measures with widespread benefits across multiple organ systems that can be implemented at a population scale. Time-Restricted Feeding/Eating (TRF or TRE) is a circadian-based dietary intervention where daily energy intake is limited to a consistent 6-12 hour window without explicitly changing the quantity of food consumed. In laboratory animals, this approach sustains circadian expression of the majority of protein-coding genes in a tissue-specific manner. When fed an obesogenic diet, TRF can mitigate metabolic disorders such as adiposity, dyslipidemia, hypercholesterolemia, fatty liver disease, atherosclerosis, and gut dysbiosis. Research from various labs has also shown that this method can reduce the risk and impact of dementia and certain cancers. Genetic and multi-omics studies are beginning to uncover the extensive molecular pathways engaged during time-restricted feeding. This foundational research is likely to drive large clinical trials across diverse populations to assess the feasibility and sustainability of TRE as a population-level intervention to reduce the global burden of diseases.

### SESSION 2010 (SYMPOSIUM)

Abstract citation ID: igae098.0584

**CAPABLE ADAPTATIONS: MEETING THE NEEDS OF OLDER ADULTS ACROSS DIVERSE CONTEXTS TO SUPPORT FUNCTION**

Chair: Thomas Cudjoe
Discussant: Lesley Steinman

CAPABLE is a time-limited, person-directed, home-based program that addresses and seeks to improve the function of individuals living with disability. This effective program integrates the services of an occupational therapist, a registered nurse, and a handy worker who, in collaboration with an older adult, sets goals and creates action plans. In multiple trials nationwide, it has improved daily function and reduced disability for participants while saving health care costs. It is now being implemented in 23 States at 45 sites nationwide. Building upon the success of the original CAPABLE program, our symposium will present descriptions and findings from innovative adaptions of CAPABLE to 1) improve the health, independence, and safety of care partners, 2) promote social connection in individuals who have constrained life space or are homebound, 3) address the needs of older adults awaiting kidney transplants, and 4) improve the quality of life for older adults experiencing cognitive impairments. Presenters will provide insight into lessons learned from each CAPABLE adaptation, including perspectives on leveraging opportunities and utilizing synergy to address socio-behavioral and functional needs across these diverse contexts and populations.

Abstract citation ID: igae098.0585

**CAPABLE TRANSPLANT: ADAPTATION OF CAPABLE FOR USE WITH OLDER ADULTS WITH INACTIVE STATUS AWAITING KIDNEY TRANSPLANT**

Melissa Hladek[1], Samantha Curriero[1], Qian-Li Xue[1], Deidra Crews[1], Mara McAdams DeMarco[2], Deborah Wilson[3], Daniel Brennan[1], and Sarah Szanton[1], *1. Johns Hopkins University, Baltimore, Maryland, United States*, *2. New York University, New York, New York, United States*, *3. Auckland University of Technology, Auckland, Auckland, New Zealand*

Over 800,000 Americans live with end-stage kidney disease (ESKD), disproportionally affecting older adults. This group represents just 1% of Medicare's Fee for Service (FFS) population, yet utilizes greater than 7% Medicare FFS resources. Rates of kidney transplantation (KT), the preferred treatment for ESKD, have risen 5-fold for older adults since 1990 but there is vast heterogeneity in access to KT. Older adults are more likely to be listed as inactive (on the waitlist but ineligible for KT) and, inactive status is associated with a 2.2 fold increased risk of waitlist mortality compared to those that remain active. Barriers to active status are multi-factorial but frequently modifiable and include symptoms such as fatigue, depression and stress, pain as well as loss of physical function, social isolation, and decreased health literacy. Using community participatory research and human centered design, we used photos, interviews, persona development, storyboarding and focus groups to identify three barriers unique to this population around the themes of: mental fortitude, support and communication. Our CAPABLE adaptation, called CAPABLE Transplant, incorporates greater motivational and communication strategies for the person and the home as well as a robust digital literacy component to address the needs of this population. An open-label pilot phase helped to solidify all aspects of the adaptation with resulting 30-person pilot randomized control trial on-going. This presentation will focus on the human centered design adaptation process and results thus far.

Abstract citation ID: igae098.0586

### CAPABLE CARE PARTNER

Deborah Paone[1], Jeanne Schuller[2], and Sarah Szanton[1], *1. Johns Hopkins University, Baltimore, Maryland, United States*, *2. Paone & Associates, LLC, Minneapolis, Minnesota, United States*

We created and tested a prototype of CAPABLE Care Partner as an enhancement to CAPABLE. We developed this through a three-phased approach over three years and incorporated care partners, older adults, clinicians, and administrators to design and test this enhancement using human-centered design principles. We created this to recognize the role and supportive actions of care partners and to provide some support for their needs as well as those of the older adult participant. We conducted a pilot in 2023 in three U.S. implementation sites--programs that had successfully offered CAPABLE for at least one year. Results from post-pilot assessments and 360 degree qualitative interviews showed positive effect on care partners, and provided real-world insights on how to incorporate a social support specialist into the program, so that the care partner and the older adult dyad connect to social support/community resources, attend to self-care, and are better positioned to address current and future needs to age in community.

Abstract citation ID: igae098.0587

### ADAPTING THE CAPABLE PROGRAM TO ADDRESS FUNCTION AND SOCIAL CONNECTION NEEDS AMONG HOMEBOUND OLDER ADULTS

Tiffany Riser[1], Wonkyung Jung[2], Samantha Curriero[1], Kennedy McDaniel[1], and Thomas Cudjoe[3], *1. Johns Hopkins University, Baltimore, Maryland, United States*, *2. Boston College, Baltimore, Maryland, United States*, *3. Johns Hopkins University School of Medicine, Baltimore, Maryland, United States*

CAPABLE Care + Connect is a high-fidelity prototype based on the CAPABLE program that is currently being developed and implemented using a two-phase approach. The purpose of CAPABLE Care + Connect is to leverage human-centered design to adapt an evidence-based intervention (Phase 1) and to test the feasibility of implementing the tailored intervention in a home-based primary care setting (Phase 2). The overarching goal of CAPABLE Care + Connect is to better address the needs of homebound older adults experiencing limited life space and limited social connection. During the symposium presentation, we will share the methodological activities involved in developing this intervention using human-centered design. Additionally, we will present early insights gained from older adults, care partners, clinicians, and thought leaders regarding opportunities and challenges of integrating the CAPABLE and Johns Hopkins Home-Based Medicine programs. These insights will highlight the program's role in facilitating aging in place while also supporting social connection in older adults with disabilities who are experiencing or at risk for social isolation. After attending this symposium session, participants will demonstrate an understanding of how human-centered design approaches can be applied to adapt an evidence-based intervention in a home-care setting. Additionally, participants will be able to summarize perspectives on social isolation, loneliness, and unmet needs for social connection in homebound older adults with disabilities.

Abstract citation ID: igae098.0588

### CAPABLE FAMILY FOR THE CAREGIVER–CARE RECIPIENT DYAD WITH DEMENTIA AND DISABILITY

Emerald Jenkins, *Johns Hopkins University, Baltimore, Maryland, United States*

Co-occurring physical and cognitive impairments in older adults with mild cognitive impairment (MCI)/early dementia lead to decreased functional independence and increased mortality. The purpose of this study is to address the needs of older adults with co-occurring physical disability and MCI/early dementia and their caregivers by adapting the Community Aging in Place, Advancing Better Living for Elders (CAPABLE) program to become CAPABLE-Family. To adapt CAPABLE, we performed a two-part multi-method study, part included: (1) semi-structured interviews with clinicians (n= 15) and (2) photovoice/semi-structured interviews (n= 12) of persons with mild cognitive impairment (MCI)/early dementia and their care partners (dyads). The second part of the study was an open label pilot to (1) adapt CAPABLE for persons with co-occurring physical disabilities and mild cognitive impairment (MCI)/early dementia (n= 7) and their care partners (n= 6) (dyads), and (2) evaluate acceptability/feasibility of the CAPABLE-Family intervention. The pilot included both qualitative and quantitative descriptive analysis. Findings from clinician interviews include, for example: incorporate a greater emphasis on safety, memory, nutrition and advanced care planning. Findings from the photovoice were categorized into 3 major themes. From the pilot, difficulty with ADLs and IADLs improved in older adults. Depression and pain interference improved in care partners. Qualitative feedback from the dyads in the open label pilot included, for example: the importance of written/tangible items. There are multiple methods for mood mitigation, self-esteem, and memory. Evidence from this research points to the importance of individualized intervention/care elements for this population.

### SESSION 2015 (PAPER)

### CAREGIVING IN DIVERSE POPULATIONS

Abstract citation ID: igae098.0589

### EFFECTS OF MINORITY AND CAREGIVING STRESS ON THE HEALTH OF BI+ INFORMAL DEMENTIA CAREGIVERS

Krystal Kittle[1], Ethan Cicero[2], Jordan Pelkmans[3], Jason Flatt[4], and Joel Anderson[5], *1. University of Massachusetts Amherst, Springfield, Massachusetts, United States*, *2. Emory University, Quincy, Massachusetts, United States*, *3. Emory University, Atlanta, Georgia, United States*, *4. University of Nevada Las Vegas, Las Vegas, Nevada, United States*, *5. University of Tennessee-Knoxville, Knoxville, Tennessee, United States*

Bi+ people (e.g., bisexual, pansexual) are more likely to experience poor physical and mental health compared with monosexual minority (i.e., gay[G], lesbian[L]) people. These poor health outcomes stem from minority stress related to sexual minority status. Due to minority and caregiver stress, LGB+ people providing care to someone with Alzheimer's disease and related dementias (ADRD) are more likely to have worse health than their straight counterparts. Little is known about how minority and caregiver stress affect the health of bi+ ADRD caregivers versus monosexual minority ADRD caregivers. Data from ADRD caregivers (bi+, n=125; gay/lesbian, n=161) were used to examine associations between minority stress, caregiver stress and overall health and if associations differed among bi+ and gay/lesbian ADRD caregivers. Multivariable regression, adjusting for demographics, was used to investigate if sexual orientation moderated the association between minority/caregiver stress variables and overall health. All minority stressors and two caregiving stressors (caring for spouse/care recipient's level of independence) were associated with health. In our adjusted model, lifetime victimization, day-to-day discrimination, and caring for spouse/partner, moderated the association between sexual orientation and overall health. As lifetime victimization scores increased, bi+ health decreased while monosexual minority health increased. Caring for a spouse/partner, protective for both groups, had a greater effect on health for monosexual minorities. Higher day-to-day discrimination scores predicted worse health for both groups, but the magnitude was greater for monosexual minorities. Qualitative inquiries can illuminate underlying mechanisms for this phenomenon, informing interventions and policy aimed at supporting health and well-being of bi+ ADRD caregivers.

Abstract citation ID: igae098.0590

### RACE AND GENDER DIFFERENCES IN UNMET NEEDS OF NONSPOUSAL CARE PARTNERS OF PEOPLE LIVING WITH DEMENTIA

Gretchen Tucker[1], Ann Gruber-Baldini[2], Quincy Samus[3], Laura Girling[4], and Denise Orwig[2], *1. University of Maryland Baltimore, Baltimore, Maryland, United States*, 2. *University of Maryland School of Medicine, Baltimore, Maryland, United States*, 3. *Johns Hopkins University, Baltimore, Maryland, United States*, 4. *Towson University, Towson, Maryland, United States*

Research on unmet care needs of non-spousal informal care partners (ICPs) for persons living with Alzheimer's disease and related dementias (P-ADRD) is limited. A secondary data analysis using linear and logistic regressions was employed to explore non-spousal ICPs' unmet needs by gender and race using the Johns Hopkins Dementia Care Needs Assessment (JHDCNA 2.0©). The JHDCNA 2.0© includes six domains and 18 items assessing ICPs' needs. Data included 413 ICPs of P-ADRD from two community-based studies: Maximizing Independence at Home Randomized Control Trial and Maximizing Independence at Home-Health Care Innovation Award, which enrolled dyads of P-ADRD and their ICPs. Of the 413 ICPs, 143 (34.6%) were White, 270 (65.4%) Black, with 343 (83.1%) female, and 70 (16.9%) male. Gender was not significantly associated with overall percentage of unmet dementia-related care needs. However, there was a race by sex interaction ($p < .001$); Black male non-spousal ICPs had the highest mean total percent unmet needs (33.48±13.87) while White female non-spousal ICPs had the lowest mean total percent of unmet needs (28.03±13.75). Being a Black non-spousal ICPs was associated with having higher odds of having unmet needs in specific categories such as behaviors 73% vs 61% ($p = .015$), substitute decision-making 29% vs 8% ($p < .001$), and decision-making documents 84% vs 71% ($p = .002$) compared to White non-spousal ICPs.This study highlights the importance of understanding the unique needs of non-spousal ICPs, particularly differences by race, to ensure access to resources and assistance for persons providing care to individuals living with ADRD.

Abstract citation ID: igae098.0591

### EXPLORING CAREGIVING NETWORK CHARACTERISTICS FOR OLDER ADULTS LIVING WITH DEMENTIA ACROSS RACE/ETHNICITY

Wen-Hua Lai, Natasha Nemmers, Sophia Tsuker, and Amanda Leggett, *Wayne State University, Detroit, Michigan, United States*

Black and Latinx older adults face a heightened risk of dementia or cognitive impairment. While minority communities often have family-focused supportive networks compared to White individuals, limited research has explored racial/ethnic disparities in caregiving networks (CGN) and contexts for people living with dementia (PLwD). This study investigates whether CGNs and caregiving contexts vary across racial/ethnic groups. Data from the 2015 National Health and Aging Trends Study (N=2,795) were used to analyze a subgroup of Black, Latinx, and White PLwD. Latent profile analysis identified dementia CGN profiles for each racial/ethnic group based on CGN size and composition (e.g., spouse, children, extended kin, non-kin). Multinomial logistic regression examined caregiving contexts associated with these CGN profiles for each race/ethnicity. Four CGN profiles were identified for each racial/ethnic group. The majority of Black PLwD (80%) were categorized into the "children and others (kin and non-kin)" group, with less spousal involvement. Latinx PLwD had smaller CGN profiles but a more balanced caregiver combination than Black peers. While some similarities in CGN configuration were observed across racial/ethnic groups, compositional differences were evident, influenced partly by sociodemographic and cultural contexts. These findings emphasize the importance of considering CGN disparities by race/ethnicity. Understanding these differences can inform the development of screening and intervention strategies to support caregivers within Black and Latinx families, who disproportionately bear caregiving responsibilities for PLwD.

Abstract citation ID: igae098.0592

### AFRICAN IMMIGRANT BELIEFS AND CAREGIVER BURDEN: WHAT IS THE EVIDENCE?

Manka Nkimbeng, Truphosa Aswani, Elizabeth Albers, and Joseph Gaugler, *University of Minnesota, Minneapolis, Minnesota, United States*

Perceptions among African immigrants can impact caregiving for individuals with dementia and their caregivers. However, the specific beliefs and their effects on caregiving outcomes remain unclear. This study aims to investigate the relationship between African immigrants' beliefs and caregiver burden. Logistic regressions were used to explore the relationship between various beliefs (including family homecare, secretiveness about health, fear of bad news and death, facility-level care being the last resort, and preference to send someone with dementia to Africa) with caregiver burden with 155 African immigrant caregivers. The average age of a caregiver was 34 years, 30% were born in Africa, and 12% of persons living with dementia were residing in Africa. After adjusting for age, sex, place of birth, and marital status, caregivers who believed that care should be provided at home, those who viewed facility-level care as a last resort, and those who preferred to send someone with dementia to Africa showed higher odds of caregiver burden, although these associations were not statistically significant (OR 1.19, 95% CI [0.42-3.4]; OR 1.16, 95% CI [0.42-3.18]; OR 1.09, 95% CI [0.37-3.24], respectively). Although these findings were not statistically significant, the trend reveals that these African immigrant beliefs may negatively influence the care partner. Thus, there is a need for interventions that target both beliefs and poor health outcomes in Africans, an understudied research population.

Abstract citation ID: igae098.0593

### DAILY PSYCHOLOGICAL EXPERIENCES AND SLEEP HEALTH AMONG AFRICAN AMERICAN FAMILY CAREGIVERS

Elliane Irani[1], Stephanie Griggs[1], Zhengyi Chen[2], and Ronald Hickman[1], *1. Case Western Reserve University, Cleveland, Ohio, United States*, *2. Case Western Reserve University School of Medicine, Cleveland, Ohio, United States*

Extensive research has been conducted on the reciprocal associations between daily psychological experiences and sleep health (i.e., sleep satisfaction, daytime alertness, sleep timing, efficiency, and duration) in the general population. However, little research has focused on whether these associations vary for African American family caregivers who experience unique stressors and report lower sleep satisfaction and shorter sleep duration than other groups. We investigated how daily psychological experiences, including positive and negative affect, general stress, caregiving stress, and positive experiences, impact same-night sleep in African American family caregivers, and how nightly sleep affects next-day experiences. African American family caregivers (n=31) wore a research-grade actigraphy device day/night and completed daily diaries at bedtime and waketime over 7 days. Multilevel models evaluated within-person associations between daily psychological experiences and sleep health adjusted for participants' age, prior-day outcome, and weekend (vs. weekday). Participants in the study were mostly women (80.6%), with an average age of 58.3 years. Most participants were caring for a parent (77.4%). On days when participants reported higher-than-average prior night sleep satisfaction, they reported higher-than-average positive experiences and affect, and lower-than-average negative affect. Daytime alertness was linked to higher positive experiences and affect, and lower negative affect and general stress. Results for the reversed direction revealed that negative affect, general stress, and positive experiences predicted next-day daytime alertness. Findings support the psychological health benefits of sleep in the context of African American family caregivers' daily life and suggest that interventions should focus on improving sleep health to enhance daily psychological experiences.

### SESSION 2020 (SYMPOSIUM)

Abstract citation ID: igae098.0594

### CONTEXTUAL DETERMINANTS OF AGING AND HEALTH

Chair: Jiao Yu
Discussant: Roland Thorpe

Addressing social determinants of health is crucial for promoting healthy aging. As this field grows, there is an increasing emphasis on understanding the upstream contextual factors of poor health and inequities. Contextual factors, operating at multiple societal levels, profoundly shape health and well-being among older adults. Guided by social disadvantage theory, this symposium aims to advance research on contextual determinants of aging and health through both innovative multilevel and life course perspectives as well as methodological advancements in novel statistical models and machine learning methods. This symposium will present four studies exploring how important contextual factors, ranging from education, neighborhoods, and childhood social-historical contexts are associated with health outcomes among older adults. Dr. Kim et al.'s interdisciplinary study delves into the micro-level, employing quantile regressions to investigate gender differences in the effects of education on brain pathology and cognitive decline. Dr. Yang et al. explore how meso-level neighborhood affluence and disadvantage influence older adults' pain outcomes and medication choices. Focusing on minority aging, Dr. Qin et al. examine how neighborhood disadvantage moderates the association between disability and cognitive decline among older Mexican Americans. Finally, Huo and co-authors take a life course perspective, utilizing machine learning tree and forest models to examine a comprehensive set of 43 childhood micro- and meso-level family and residential characteristics contributing to later life frailty. Through these diverse investigations, this session will provide insights into the contextual determinants related to healthy aging, which will inform targeted interventions and policies to address health disparities among older populations.

Abstract citation ID: igae098.0595

### THE ROLE OF EDUCATION ON BRAIN PATHOLOGY AND COGNITIVE DECLINE ACROSS WOMEN AND MEN IN THE US

Min Hee Kim[1], Brittany Morin[2], Alice Olvera[3], Gelan Ying[4], Aayush Khadka[2], and Seo-Eun Choi[5], *1. Rutgers University, San Francisco, California, United States*, *2. University of California San Francisco, San Francisco, California, United States*, *3. Queens College at the City University of New York, New York, New York, United States*, *4. University of Florida, Gainesville, Florida, United States*, *5. University of Washington, Seattle, Washington, United States*

Women have a higher risk of developing Alzheimer's disease and related dementia (ADRD) than men. Education, as a key contributor to cognitive reserve, might protect the ADRD onset. While existing evidence suggests that increased educational attainment may attenuate the negative impact of neuropathology on cognitive function, these studies focused primarily on average cognitive function, and little is known about whether education similarly attenuates the relationship between neuropathology and cognitive decline among those with high or low levels of cognitive outcomes or sex/gender. Using the Alzheimer's Disease Neuroimaging Initiative sample (772 male and 627 female participants, who were cognitively normal or had mild cognitive impairment at baseline), we investigated education attainment modified the association between anatomical reduction in the brain and cognitive decline in sex-stratified models. We applied quantile regression with a three-way interaction term (education x cortical thickness x years) against 10-year cognitive function, an established specification for testing the cognitive reserve mechanism. We included a robust set of biological and contextual confounders. Primary results showed that, among males, education had attenuated the relationship between brain pathology and cognitive decline at lower levels of cognitive function distribution. Among females, education attenuates brain pathology and cognitive decline in all levels of outcome distribution with large confidence intervals. We will discuss potential explanations for differential effect modification by education on the brain pathology-cognition relationship across sex/gender and expand its implications for future research and policy/practice intervention.

Abstract citation ID: igae098.0596

### THE ROLE OF NEIGHBORHOOD DISADVANTAGE IN PAIN AND MEDICATION AMONG OLDER ADULTS WITH OR WITHOUT COGNITIVE IMPAIRMENT

Yulin Yang[1], Ashwin Kotwal[2], Lauren Hunt[1], Scarlett Gomez[1], and Jacqueline Torres[1], *1. University of California San Francisco, San Francisco, California, United States*, *2. University of California San Francisco Medical Center, San Francisco, California, United States*

Neighborhoods are vital to older adults' health and well-being, yet how they influence pain outcomes and medication use among older adults, with or without cognitive impairment, remains unknown. We evaluated the neighborhood effects on pain outcomes and medication use by cognitive impairment status. We use two distinct neighborhood socioeconomic status measures – disadvantage (including poverty, unemployment, female-headed families, households receiving public assistance income, and proportion of African Americans) and affluence (including adults with a college education, incomes>75K, and employed in managerial and professional occupations). We analyze data from 9,351 older respondents in the Health and Retirement Study from 4,611 census tracts. We fit multilevel logistic regression models with random intercepts to evaluate the neighborhood effects on the risk of reporting pain, pain-related disability, over-the-counter (OTC) pain medication, and opioid medication. In the overall sample, neighborhood affluence is associated with lower odds of pain and pain-related disability, and higher odds of using OTC medication. Among 6,675 cognitively normal respondents, neighborhood disadvantage is associated with greater pain-related disability, while neighborhood affluence is associated with lower odds of pain, pain-related disability, and higher odds of using OTC medication. Among 2,676 respondents with cognitive impairment, neither neighborhood disadvantage nor affluence is associated with any pain outcomes and medication. Neighborhood affluence predicted higher odds of pain-related disability; however, the 95% confidence interval crossed the null. These findings suggest that neighborhood factors are important social determinants of pain outcomes and management among older adults with and without cognitive impairment.

Abstract citation ID: igae098.0597

### NEIGHBORHOOD DISADVANTAGE, FUNCTIONAL DISABILITY, AND COGNITIVE DECLINE AMONG OLDER MEXICAN AMERICANS

Weidi Qin[1], Lucia Cavanagh[2], Joshua Owens[3], Soham Al Snih[4], and Irving Vega[5], *1. University of Wisconsin Madison, Madison, Wisconsin, United States*, *2. University of California Los Angeles, Los Angeles, California, United States*, *3. University of Florida, Gainesville, Florida, United States*, *4. University of Texas Medical Branch in Galveston, Galveston, Texas, United States*, *5. Michigan State University Grand Rapids, Grand Rapids, Michigan, United States*

This study aims to investigate the longitudinal association between disability and cognitive decline, and to determine the moderating effects of neighborhood disadvantage among older Mexican Americans. Data came from the Hispanic Established Population for the Epidemiologic Study of the Elderly (2004-2005 to 2012-2013) linked to 2010 National Neighborhood Data Archive. Older Mexican Americans without cognitive impairment at baseline were selected (n=1,189). Cognitive function was measured by the Mini-Mental State Examination (MMSE). Neighborhood disadvantage was created based on five census-tract indicators (e.g., % of households below poverty). Disability was indicated by a count of activities of daily living (ADL) and instrumental ADL (IADL) limitations. Multilevel mixed-effects regression models were performed to estimate cognitive change over time, accounting for socio-demographics and health conditions. There was an average decline of 0.91 and 0.32 in the MMSE score as a function of ADL and IADL disability, respectively ($p<0.05$). Significant interactions between ADL and IADL disability and neighborhood disadvantage were observed. Among older Mexican Americans who were exposed to higher levels of neighborhood disadvantage, ADL and IADL disability were significantly associated with faster cognitive decline. The study findings suggest that neighborhood disadvantage undermines cognitive health among older Mexican Americans who have functional disability.

Abstract citation ID: igae098.0598

### CHILDHOOD ROOTS OF FRAILTY: MACHINE LEARNING INSIGHTS INTO HEALTH INEQUALITY IN LATER LIFE

Shutong Huo[1], Thomas Gill[2], Xi Chen[2], and Derek Feng[2], *1. University of California Irvine, Irvine, California, United*

*States*, *2. Yale University, New Haven, Connecticut, United States*

This study investigates the impact of childhood circumstances on health inequality in later life, with a particular emphasis on frailty among older adults in the United States, highlighting the significance of early life historical and social factors. We employed data from the Health and Retirement Study (HRS), incorporating the 2012, 2014, 2016, and 2018 waves along with the 2015 Life History Mail Survey (LHMS). Using innovative conditional inference trees and forests, we evaluated 43 distinct childhood factors and their contribution to the Inequality of Opportunity (IOP) in health outcomes. The circumstances in both countries can be divided into seven domains: 1) war or economic crisis at birth; 2) regional and urban/rural status at birth; 3) family SES in childhood; 4) parental health status and health behaviors in childhood; 5) health and nutritional status in childhood; 6) relationship with parents in childhood; 7) friendship in childhood. We found that key early-life predictors identified include experiencing the Great Depression, adverse childhood events, socioeconomic status, and access to educational resources, all of which play critical roles in determining frailty in older adults. The machine learning models, particularly conditional inference forests, significantly outperform traditional analytical methods in predicting health inequality, with the best out-of-sample performance. The findings demonstrate the importance of early-life circumstances in shaping later health outcomes and stress the early-life interventions for health equity in aging societies.

### SESSION 2025 (SYMPOSIUM)

Abstract citation ID: igae098.0599

### CONTEXTUAL INFLUENCES ON THE MAINTENANCE OF SOCIAL INTEGRATION IN LATE LIFE

Chair: Oliver Huxhold

Being socially integrated is essential for maintaining health and well-being in late adulthood. Although life course sociology and lifespan psychology have emphasized the influence of contextual conditions on different aspects of social integration for a long time, the majority of studies on social relationships in late life have focused on differences at the individual level. To address this shortcoming, this symposium presents a number of complementary perspectives on macro- and meso-level influences on late-life social development. To this end, Webster presents an analysis that details how meso-level resources integrated into the individual's social network enhance the individual's likelihood of volunteering. Following this presentation, Brandt and colleagues report on societal differences across European countries in a comprehensive index of social integration and relate these differences to well-being in late adulthood. Considering short-term changes in societal conditions, Klasen and colleagues present a theoretical model that details concrete pathways by which societal crises may impact social relationships. Finally, Suanet and colleagues investigate long-term societal changes and report on cohort and socio-structural differences in age-related changes in positive and negative social exchanges. Overall, the symposium offers a comprehensive view of societal and socio-structural influences on social integration. The talks combine new theoretical perspectives with sophisticated analyses and provide new insights into contextual factors in late life that are relevant for the development of targeted social interventions.

Abstract citation ID: igae098.0600

### THE LINK BETWEEN EDUCATION WITHIN SOCIAL NETWORKS AND VOLUNTEERING

Noah Webster, *University of Michigan, Ann Arbor, Michigan, United States*

Volunteering serves as a critical source of social integration in later life and also is a well-known health promoting activity. It is therefore important to continue exploring unidentified barriers and facilitators of volunteering. Both higher education and social networks (e.g., decreasing network proximity) have been linked with volunteering. No research though has explored how educational resources embedded in older adults' social networks may facilitate this activity. In this study, we examine the association between average social network education and odds of volunteering among older adults. Respondents age 60 and older who participated in Wave 3 (2015) of the longitudinal Social Relations Study based in Metro Detroit were selected for analysis (n=342). During interviews, when asked to identify social network members, respondents were also asked to report the education of up to the first 10. Forty-one percent of respondents reported volunteering in the past year. Controlling for respondents' own education as well as other demographic characteristics (age, gender, marital status), higher average social network education was significantly associated with greater odds of volunteering. In a regression model without average social network education, individual education was significantly associated with greater odds of volunteering, but this effect was no longer significant after network education was added to the model. Findings highlight that educational resources, often viewed as an indicator of macro-level socio-economic status, when embedded in social networks plays a greater role in facilitating volunteering than at the individual-level. This suggests a potential new pathway for intervention to increase volunteering in later life.

Abstract citation ID: igae098.0601

### SOCIAL INTEGRATION OF OLDER MEN AND WOMEN ACROSS EUROPEAN WELFARE STATES: ESTABLISHING A NEW COMPARATIVE INDEX

Martina Brandt[1], Michal Levinsky[2], and Melanie Wagner[3], *1. TU Dortmund, Dortmund, Nordrhein-Westfalen, Germany*, *2. HUJI The Paul Baerwald School of Social Work and Social Welfare, Jerusalem, Yerushalayim, Israel*, *3. SHARE Berlin Institute, Berlin, Berlin, Germany*

Social integration is a crucial factor for health and mortality in old age. However, despite the increasing attention in aging populations, there is currently no instrument available for comparing social integration of older adults across Europe. In this study, we aim to investigate a social integration index established by Berkman and colleagues across welfare regimes and gender in Europe, with a focus on family policy and gender roles. We utilize data from the Survey of Health, Ageing, and Retirement in Europe (SHARE) and conduct multivariate regression analysis to examine the interactions between gender and welfare regimes on social integration based on this index. Our findings suggest that women are less socially integrated than men, and that older adults in more 'familialistic' regimes tend to be less socially integrated. Moreover, care regimes are important for the gender gap in social integration. Women tend to be less integrated in family-oriented welfare systems, presumably due to their role as primary caregivers, which can limit their opportunities for social integration activities outside the home. This study contributes to the literature on social integration and highlights the importance of developing policies that promote social integration among individuals, particularly women, in different welfare systems.

Abstract citation ID: igae098.0602

### THE INFLUENCE OF SOCIETAL CRISES ON SOCIAL INTEGRATION

Lisa Klasen, Mareike Buenning, Nadiya Kelle, and Oliver Huxhold, *German Centre of Gerontology, Berlin, Berlin, Germany*

Societal crises have profound effects on various life domains. So far, the literature has focused on the effects of crises on financial security or health. Effects on social integration, i.e., social relationships and social participation, have received little attention, despite of their relevance for physical and mental health. The COVID-19 pandemic brought this topic to the forefront of the scientific and societal debate. However, to date no theoretical framework has been developed that captures the breadth and long-term influence of societal crisis on social integration. This presentation introduces a new theoretical framework – the DIRe-Crises-Model – for analyzing the effects of social crises on social integration, building on the Differential Investment of Resources (DIRe) Model. According to the DIRe-Crises-Model, crises influence social integration via four pathways by: a) limiting the time and energy that can be invested in social relationships, b) restricting the social opportunity structure, c) influencing individual characteristics essential for resource investment, and d) influencing the effects of social relationships (e.g., changes in the experience of loneliness). Dynamic components in the model allow to derive hypotheses on the long-term development of the different effects pathways of crises on social integration. Moreover, social inequalities can moderate the effects of the different pathways of crisis on social integration. The DIRe-Crises-Model as a theoretical framework has the potential to guide empirical analysis on the effects of any given societal crisis on social integration. As such, the framework may help to manage future crises, for example, by stimulating targeted interventions.

Abstract citation ID: igae098.0603

### COHORT DYNAMICS OF SOCIAL SUPPORT EXCHANGE: INSIGHTS FROM DIVERSE AGING IN THE US

Bianca Suanet[1], Oliver Huxhold[2], and Jacqui Smith[3], *1. Vrije Universiteit Amsterdam, Amsterdam, Noord-Holland, Netherlands*, *2. German Centre of Gerontology, Berlin, Berlin, Germany*, *3. University of Michigan, Ann Arbor, Michigan, United States*

**Background:** Studies from Europe indicate an increase in social support exchanges among older adults, but whether this trend also applies to the US and the perception of these exchanges as positive or negative remains unclear. Furthermore, it's uncertain if these changes also benefit those that face disadvantage, such as low income and racial minority groups, especially when considering intersecting factors.

**Methods:** This study employs cohort-sequential analysis of Health and Retirement Study (HRS) data collected from 2008 to 2022 in 8 waves to investigate cohort differences in positive and negative social support exchanges among older adults in the United States. It focuses on the influences of cohort, race, and income on social support dynamics, employing data from the psycho-social questionnaire.

**Results:** Despite a general increase in positive support exchanges, disparities in social support exchange persist based on race and socioeconomic status. Individuals with lower incomes and from ethnic minority backgrounds experience disproportionately more negative social support exchanges, indicating persistent inequalities.

**Discussion:** Recognizing the complexities of social support exchanges, particularly in relation to cohort, race, and socioeconomic status, is crucial for policymakers and healthcare professionals. Tailored interventions are essential to address disparities and promote equitable aging outcomes and overall well-being among older adults in the US.

### SESSION 2030 (PAPER)

### DYADIC RESEARCH WITH OLDER ADULTS

Abstract citation ID: igae098.0604

### SPOUSAL HEALTH SHOCKS AND INDIVIDUALS' LABOR PARTICIPATION AND RETIREMENT DECISION: CAUSAL EVIDENCE FROM THE US

Meiyi Li, *University of Wisconsin-Madison, Madison, Wisconsin, United States*

Health decline is a dyadic process that elicits labor adjustments from both individuals and their intimate partners. Despite increasing attention to the spillover partner effects, empirical evidence is still mixed and the causal nature remains unclear. Drawing upon the nationally representative data from the Health and Retirement Study (1992-2018), the current study aims to investigate (a) What are the causal impacts of spousal health shocks (cancer diagnosis, strokes, and heart attacks) on individuals' employment, retirement behaviors, and retirement expectations? (b) Are there any gender differences? Matching methods are combined with a difference-in-differences design to examine the causal impacts of spousal health shocks. Results suggest spousal health shocks reduce the probability of working for pay and increase the probability of retirement. As for retirement decisions, spousal health shocks reduce older adults' expected probability of working after 65. Additionally, results do not show significant gender differences in labor outcomes. Overall, the current study provides important causal evidence on older adults' labor responses to spousal health shocks. It also informs dyadic interventions to help both people with health decline and their partners maintain economic and social well-being in later life.

Abstract citation ID: igae098.0605

### "I KNOW EVERYTHING. STOP TELLING ME I'M FINE": A DYAD STUDY ON DISCRIMINATION AGAINST OLDER PATIENTS

Juncheng Tong, and Jiawei Cao, *Nanjing University of Chinese Medicine, Nanjing, Jiangsu, China (People's Republic)*

Despite the increasing societal attention and concern towards older patients with cancer, self-perceived social discrimination by patients, along with benevolent discrimination by their families, are often too subtle to be recognized. Since only a few studies have investigated such issues, this qualitative study aims to investigate the sources and causes of social discrimination in this group. Semi-structured interviews were conducted with 23 dyads of older patients with cancer and their primary caregivers (i.e., family members) about perceived self-discrimination and quality of life. Interview transcripts with dyads were audio recorded, transcribed verbatim, and analyzed line-by-line using thematic analysis. Two researchers independently coded transcripts and reviewed discrepancies until a consensus was reached. Four significant themes were uncovered from interviews with patients: 1) self-definition, 2) withholding information from primary caregivers, 3) socialization anxiety, and 4) stereotypes about aging and dying. Four themes emerged from interviews with primary caregivers: 1) avoiding provoking patients' frustration and discomfort by withholding medical information, 2) being considerate but lack of communication, 3) exhaustion with repeated disappointment, and 4) family love and compromise. These findings suggest that such actions by the caregivers could result in feelings of discrimination, distrust, or even betrayal by the patients. This study provides guidance to both clinicians and caregivers to 1) identify unintended discrimination practices in healthcare, 2) strengthen dyad trust and communication, and 3) accept external support services (e.g., counseling and palliative care), which are essential for improving patients' quality of life.

Abstract citation ID: igae098.0606

### THE IMPACT OF SYNCHRONIZED DYADIC DANCE ON SOCIAL BONDING AND INTERGROUP CLOSENESS

Assaf Suberry[1], and Ehud Bodner[2], *1. School of Social Work, Bar-Ilan University, Saad, HaDarom, Israel, 2. Bar-Ilan University, Ramat Gan, HaMerkaz, Israel*

Synchronization of bodies in time encourages positive social functions such as cooperation and compassion. This study examined the effect of synchronous dance movements on social bonding and perceived intergenerational closeness. 168 participants ranging in age from 20 to 45 (young adults) and 65+ (older adults) were randomly assigned to six dyad conditions, which included dancing synchronously or asynchronously with an in-age-group or with an out-age-group stranger for 11 minutes. Participants completed social bonding with the partner and perception of distance between social groups (in terms of religiosity, ethnographic origin, political stance, and age) questionnaires. We used generalized estimating equations (GEE) modeling to assess variation across both individual measurements and measurements within dyads. Social bonding significantly increased in the synchronized condition ($b = 0.51$, $p = .014$), with participants reporting higher bonding with the dancing partner compared to the asynchronous condition. Relative to older participants, younger participants demonstrated a greater enhancement in perceived closeness across diverse social groups, as well as a greater increase in perceived closeness between age groups following the intervention. Lastly, participants reported a greater sense of closeness across generations when dancing asynchronously with in-group members compared to out-group members. We explore the possibility that declining openness and a shift towards conservatism in older adults may influence social connectedness and attitudes. Intergenerational dance programs may promote intergenerational relations and social cohesion, particularly for young adults. Practitioners from various fields - welfare, nursing, sport, music education, and community therapy may benefit from intergenerational rhythmic activity.

Abstract citation ID: igae098.0607

### BASIC PSYCHOLOGICAL NEED SATISFACTION AND WELL-BEING IN GRANDPARENT–ADULT GRANDCHILD DYADS

Yue Yang Sun, Xiaoye Liu, Yukai Zhang, and Tianyuan Li, *The Chinese University of Hong Kong (Shenzhen), Shenzhen, Guangdong, China (People's Republic)*

Past research reveals that basic psychological need satisfaction (BPNS) in relationship is an important contributor to relationship intimacy and individuals' psychological well-being, but few studies explored the effects in grandparent-adult grandchildren relationship. The current study recruited 294 adult grandchild-closest grandparent dyads from mainland China. The grandchild and the grandparent completed a survey, respectively. We adopted Actor-Partner Interdependence Model to estimate both the actor and partner effects of BPNS on each partner's relationship intimacy and psychological well-being while controlling relevant demographic variables. Results indicated that both grandparents' and grandchildren's BPNS in the relationship was related to their own higher relationship intimacy, bs =.20 and.27, ps <.001, higher life satisfaction, bs =.32 and.17, ps <.03, and fewer depressive symptoms, bs = -.17 and -.12, ps <.001. Further, grandparents' BPNS was also related to their grandchildren's higher life satisfaction, $b = .21$, $p = .01$, and less depressive symptoms, $b = -.07$, $p = .04$, but adult grandchildren's BPNS was not related to grandparents' psychological well-being, ps >.13. The findings indicate the significant role that BPNS plays in promoting relationship intimacy in the grandparent-adult grandchild relationship as well as in contributing to both partners'

psychological well-being. More importantly, the support from adult grandchildren to grandparents not only benefits the grandparent's psychological well-being, but also the grandchild's. The asymmetric effects suggest that the grandchildren may become the more influential support provider in their relationship with the grandparent when they have grown up.

### SESSION 2035 (SYMPOSIUM)

Abstract citation ID: igae098.0608

### EFFECTS OF HEARING INTERVENTION ON PHYSICAL ACTIVITY AND FUNCTION: FINDINGS OF THE ACHIEVE RANDOMIZED TRIAL

Chair: Alison Huang
Co-Chair: Jennifer Schrack
Discussant: Nancy Glynn

This session examines the long-term effect of hearing intervention on physical activity and function. The Aging and Cognitive Health Evaluation in Elders (ACHIEVE) randomized controlled trial (2018-2022) is a multi-center randomized controlled trial of 977 adults aged 70-84 with untreated hearing loss and without substantial cognitive impairment (Clinicaltrials.gov Identifier: NCT03243422). The trial's primary outcome was to determine efficacy of hearing intervention for reducing 3-year cognitive decline. Data on exploratory outcomes, such as physical activity and function were also gathered. Associations between hearing loss and lower physical activity and function have been consistently demonstrated across large epidemiologic studies. The mediating factors that may underlie these associations include changes in auditory awareness, communicative difficulty, cognitive decline, and/or reduced social connection. Hearing intervention (e.g., provision of hearing aids and related technologies, counseling, and education) may modify these mechanistic pathways and could potentially serve as an intervention for improving or maintaining physical activity and function among older adults with hearing loss, but has yet to be investigated in a randomized controlled trial. This session will describe the overall study design of the ACHIEVE trial and present results on the 3-year effect of the hearing intervention vs. health education control on four exploratory outcomes of physical activity and function: accelerometry-based and self-reported physical activity, fatigue, and physical performance.

Abstract citation ID: igae098.0609

### STUDY DESIGN OF THE AGING AND COGNITIVE HEALTH EVALUATION IN ELDERS (ACHIEVE) TRIAL

Alison Huang, *Johns Hopkins Bloomberg School of Public Health, Baltimore, Maryland, United States*

This presentation describes the design, interventions, participants, and primary and exploratory outcomes of the Aging and Cognitive Health Evaluation in Elders (ACHIEVE) study. The ACHIEVE study is a multicenter, randomized controlled trial designed to test the effect of a best-practice hearing intervention vs. health education control on cognitive decline (primary outcome) and other exploratory health outcomes, such as physical activity and function, over 3 years. Participants were 977 community-dwelling adults aged 70-84 years with untreated hearing loss and without substantial cognitive impairment. The ACHIEVE study was partially nested within the Atherosclerosis Risk in Communities [ARIC] study; participants were recruited from the ARIC study (n=238) and de novo (n=739) from the surrounding communities of four U.S. sites. Participants were randomized 1:1 to hearing intervention or health education control and followed for 3 years. The study's primary outcome was cognitive decline; measures of physical activity (accelerometry-based and self-reported physical activity), fatigue, and physical function were collected as exploratory outcomes. Participants were median age 76.4 years, 53.5% female, 87.8% White, and 53.3% held a Bachelor's degree or higher. Compared to the ARIC cohort, participants recruited de novo were younger, had fewer chronic conditions, higher cognitive function, and worse self-reported hearing-related quality of life at baseline. Differences in participant characteristics by recruitment source are important considerations in the interpretation of intervention effect. This presentation provides important background for contextualization of ACHIEVE study findings on physical activity and function presented in this symposium.

Abstract citation ID: igae098.0610

### EFFECT OF HEARING LOSS TREATMENT ON ACCELEROMETRY-MEASURED DAILY PHYSICAL ACTIVITY

Jennifer Schrack, and Amal Wanigatunga, *Johns Hopkins Bloomberg School of Public Health, Baltimore, Maryland, United States*

Hearing impairment is widespread in older adults and associated with lower physical activity, but whether hearing loss treatment reduces or attenuates age-related decline in physical activity as individuals become more aware of -and interactive with- their surroudings is unknown. This study tested the effect of a hearing intervention vs. successful aging health education control on quantities and patterns of wrist-worn, accelerometer-derived daily physical activity over 3-years among older adults with hearing loss, a pre-specified exploratory outcome in the Aging and Cognitive Health Evaluation in Elders Study. Two-level linear mixed effects models with an interaction term (intervention x time) were used to test if 3-year change in physical activity outcomes differed by intervention assignment. Function-on-scalar regression (FOSR) models were used to examine potential intervention time-of-day differences in physical activity. Participants were (mean±SD) aged 76±6 years, 89% White, 52% female, and 53% had a Bachelor's degree or higher. At baseline, mean activity counts/day were 1.86x106±810,889, active minutes/day were 382.3±160.5, and activity fragmentation was 23.8%±8.2%. At year 3 of follow-up, activity counts/day and active minutes/day declined (β [95%CI]) (-49,584 [-61,873, -37,294] and -8.7 [-11.4, -6.1]), respectively, and activity fragmentation increased (0.18% [0.01, 0.35%]). Results did not differ by intervention assignment. FOSR models showed no difference in time-of-day activity by intervention assignment. Results suggest the hearing intervention did not attenuate 3-year declines in physical activity in older adults with hearing loss. Findings may have been attenuated by the content of the successful aging program (single-session on physical activity), or relatively short follow-up time.

Abstract citation ID: igae098.0611

### EFFECT OF A HEARING INTERVENTION COMPARED TO EDUCATION CONTROL ON SELF-REPORTED PHYSICAL ACTIVITY: ACHIEVE STUDY

Pablo Martinez Amezcua, *Johns Hopkins University, Baltimore, Maryland, United States*

Hearing loss is associated with lower physical activity among older adults. However, whether the treatment of hearing loss influences engagement in physical activity remains unknown. Using data from the Aging Cognitive Health Evaluation in Elders (ACHIEVE) study, a 1:1 randomized control trial of a hearing care intervention (including the provision of hearing aids) compared to a health education control group, we investigated the effect hearing care vs. control on self-reported participation in any moderate-to-vigorous physical activity (MVPA) and frequently walking during leisure time (defined as reporting leisurely walking often or very often) among community-dwelling older adults. Among 977 participants (age 70-84; female: 53.5%; White: 87.8%) with hearing loss recruited from the Atherosclerosis Risk in Communities Study (ARIC) (n=238) and de novo from the community (n=739), over three years there was a 3% reduction in the proportion of participants who engaged in MVPA (57.5% to 54.7%), but a 5% increase in leisurely walking (29.2% to 34.4%). Participants in the hearing intervention had 5% and 7% higher odds of reporting MVPA and leisurely walking over three years than those in the control group (OR MVPA=1.05, 95% CI: 0.96, 1.14; OR walking=1.07, 95%: 0.96, 1.19). These associations were stronger, 9% and 41%, among de novo than ARIC participants (de novo: OR MVPA=1.09, 95% CI: 0.79, 1.53; OR walking=1.41, 95% CI: 0.97, 2.05). Findings suggest addressing hearing loss may increase physical activity, mainly leisurely walking. Further research is warranted to explore additional factors influencing physical activity in older adults with hearing loss.

Abstract citation ID: igae098.0612

### IMPACT OF A HEARING INTERVENTION VERSUS HEALTH EDUCATION CONTROL ON FATIGUE SYMPTOMS OVER THREE YEARS

Sarah Bessen, *Johns Hopkins University, Baltimore, Maryland, United States*

Fatigue is a common complaint among older adults with hearing loss. We investigated the effect of hearing intervention versus health education control on fatigue over 3 years in older adults with hearing loss using the Aging and Cognitive Health Evaluation in Elders (ACHIEVE) study. A total of 977 adults (mean age: 76.8 years, range 70-84; female: 53.5%; White: 87.8%) with hearing loss were recruited from the Atherosclerosis Risk in Communities Study (ARIC) (n=238) and de novo from the community (n=739). Fatigue symptoms were measured with the PROMIS fatigue bank and RAND-36 questionnaires at baseline, 1-year, 2-year, and 3-year follow-up visits (for PROMIS-score, higher=more fatigue, whereas for RAND-score, higher=less fatigue). The intervention effect was estimated as the differences in the 3-year change in fatigue scores between groups using linear mixed-effects models. Over 3 years, preliminary results suggest there was potential beneficial effect of the hearing intervention vs. health education control on change in the PROMIS-fatigue score (β=-0.32 [95% CI: -1.17, 0.50]) and the RAND fatigue score (β=0.16 [95% CI: 0.16 0.04, 0.29]). Stratification by recruitment source showed consistent results with a stronger magnitude of the effect among de novo compared to ARIC participants. Overall, our findings suggest a possible beneficial effect of the hearing intervention on fatigue symptoms over 3 years. This research adds to current evidence supporting the wide-ranging benefits of hearing care and informs policy efforts to increase awareness and accessibility.

Abstract citation ID: igae098.0613

### EFFECTS OF HEARING INTERVENTION WITH HEARING AIDS ON PHYSICAL FUNCTION: FINDINGS FROM THE ACHIEVE STUDY

Wuyang Zhang, and Jennifer Deal, *Johns Hopkins University, Baltimore, Maryland, United States*

Hearing loss is an underrecognized risk factor for physical function decline that may be amenable to rehabilitative intervention. Using data from the Aging and Cognitive Health Evaluation in Elders (ACHIEVE) study, a randomized controlled trial of best practices hearing intervention on cognitive decline (Clinicaltrials.gov Identifier: NCT03243422), we investigated the effect of hearing intervention versus health education control on 3-year changes in physical function, a pre-specified outcome for the trial. 977 participants were randomized 1:1. The primary outcomes for this analysis included the Short Physical Performance Battery (SPPB), rescaled according to published guidelines developed in the Health Aging and Body Composition study to address ceiling effects in high-functioning populations, and grip strength. At baseline, the study sample had an average SPPB score of 1.80±0.34, with a mean age of 76.8±4.0 years, 54% female, and 11% self-reported Black race. In linear mixed effects models adjusted for participants' baseline socio-demographic characteristics, health status, and hearing status (and corresponding time interactions), 3-year changes (in SD units) were did not differ between treatment groups in SPPB scores (β=-0.001; 95%CI: -0.115, 0.113; p-value: 0.98) and grip strength (β=0.016; 95%CI: -0.054, 0.085; p-value: 0.66). In pre-specified sensitivity analysis, the strongest estimated effect of hearing intervention on SPPB performance was among those with worse cognitive test performance (β=0.237; 95%CI: -0.019, 0.493; p-value: 0.07). Given that physical function was not the primary outcome of the ACHIEVE trial, these results should be considered exploratory. Follow-up of ACHIEVE participants is ongoing to determine if intervention effects require longer follow-up time.

### SESSION 2040 (PAPER)

### ENVIRONMENT AND AGING (PAPERS)

Abstract citation ID: igae098.0614

### COGNITIVE VULNERABILITY TO TEMPERATURE BY REGION, HOUSING, AND NEIGHBORHOOD SAFETY IN HEALTH AND RETIREMENT SURVEY

Carina Gronlund[1], Erinn Cameron[2], Jinkook Lee[3], Jennifer Ailshire[3], and Sara Adar[1], *1. University of Michigan, Ann Arbor, Michigan, United States*, *2. Harvard T.H. Chan School of Public Health, Boston, Massachusetts, United States*, *3. University of Southern California, Los Angeles, California, United States*

With a changing climate, understanding how temperature affects the cognitive function of older adults is critical. Extreme temperatures might influence cognitive function via multiple physiological processes and may be stronger in certain spaces, including poor-quality housing and unsafe neighborhoods where residents may not open windows. Using biennial surveys from ~40,000 respondents from 1995-2020, we characterized associations between total cognition scores (35-point scale) and temperatures overall and by census region, housing condition (poor-to-excellent), and neighborhood safety (feel safe walking after dark scale 1-7). We operationalized temperatures as monthly regional means of daily minimum temperatures from NOAA monitors 1-12 months prior to the surveys, converted to region-specific percentiles. We regressed cognition on nonlinear temperature lags, controlling for individual (fixed slope), precipitation, season, survey wave, and age. We then interacted home condition and neighborhood safety with temperature, controlling for self-efficacy, functional limitations (ADLs), and comorbidities. Overall, we found lower cognitive performance with colder temperatures 6 months prior (-0.1 points, 95% Confidence Interval: -0.2, 0.0 for the 1st vs. 50th percentile). For hot temperatures, results were regionally heterogeneous. We saw lower cognition with higher temperatures in some regions (e.g., in the East North Central region, 1.5 (95% CI: -2.2, -0.4) and 3 (95% CI: -1.6, -0.5) points lower at 99th vs. 50th percentile 1 and 3 months prior, respectively). Hot temperature associations were worse in unsafe neighborhoods but not by home condition. This suggests that climate adaptation programs, including safe cooling spaces, might benefit cognitive health.

Abstract citation ID: igae098.0615

### THE JOINT EFFECTS OF EXPOSURE TO AIR POLLUTION AND HEAT ON EPIGENETIC AGING IN THE HEALTH AND RETIREMENT STUDY

Kristina Dang, Eunyoung Choi, Caleb Finch, Eileen Crimmins, and Jennifer Ailshire, *University of Southern California, Los Angeles, California, United States*

Prior research shows independent associations of exposure to air pollution and heat on epigenetic alterations. However, less is known about the combined effects of long-term air pollution (PM2.5) and acute heat events. We examine joint effects of elevated PM and heat on DNA methylation (DNAm). Data from the 2016 Health and Retirement Study DNAm Sample (N=3,464) were merged at the census tract level to (1) annual ambient concentrations of PM2.5 from CACES and (2) daily heat index data averaged 7-days before blood collection from gridMET. Four categories of joint PM2.5 and heat were analyzed: (1=reference) low PM2.5 (< 9.2 $\mu$g/m3) and low heat (< 80 on heat index); (2) low PM2.5 and high heat; (3) high PM2.5 and low heat; and (4) high PM2.5 and high heat. We used five epigenetic aging measures: Horvath, Hannum, PhenoAge, GrimAge, DunedinPACE. Linear models were adjusted for age, sex, cell type, race/ethnicity, education, and neighborhood poverty. Compared to the reference of low PM2.5 and heat, we found associations of short-term heat and low PM with accelerated DNAm aging for Hannum ($\beta$=0.61 95%CI:0.05, 1.18) and PhenoAge $\beta$=0.90 95%CI:0.28, 1.52). High PM2.5 and low heat had weaker associations (Hannum $\beta$=0.41 95%CI:-0.28, 1.11; PhenoAge $\beta$=0.13 95%CI:-0.61, 0.89), as did joint effects of high PM2.5 and heat (Hannum $\beta$=0.21 95%CI:-0.61, 1.03; PhenoAge $\beta$=0.27 95%CI:-0.68, 1.22). Short-term heat may accelerate epigenetic aging, particularly in areas with low air pollution. These findings suggest underlying physiological responses to acute heat exposures may differ between residents in low and high air pollution environments.

Abstract citation ID: igae098.0616

### ASSOCIATION OF AGE-FRIENDLY COMMUNITIES WITH INTRINSIC CAPACITY IN COMMUNITY-DWELLING OLDER ADULTS

Chunxiao Li[1], Kehan Liu[1], Wenting Peng[2], Yu Zheng[3], Chongmei Huang[4], and Minhui Liu[5], *1. Central South University, Changsha, Hunan, China (People's Republic)*, *2. University of Washington, Seattle, Washington, United States*, *3. The Third Xiangya Hospital of Central South University, Changsha, Hunan, China (People's Republic)*, *4. Ningxia Medical University, Yinchuan, Ningxia, China (People's Republic)*, *5. Ningxia Medical University, Yinchuan, Ningxia, China*

Intrinsic capacity is crucial for older adults to maintain their functional independence and quality of life. Despite previous research have identified some environmental factors that impact intrinsic capacity, the associations between age-friendly communities and intrinsic capacity remained underexplored. To examine the relationship between the level of age-friendly communities and intrinsic capacity, in 2023, we conducted a cross-sectional study among 413 cognitively intact Chinese older adults from six age-friendly communities. Intrinsic capacity was measured with the Barthel Index (locomotion), Cornell Medical Index (vitality), Short-Form Mini-Nutritional Assessment (nutrition), Geriatric Depression Scale-15 (psychological health), and Mini-Mental State Examination (cognition). Community age-friendliness was assessed by the Age-Friendly Community Rating Scale. Covariates included demographics and health-related factors. Linear regression was used for analysis. These participants were aged between 65 and 94 years old, and male (52.06%). The level of age-friendly community was significantly associated with the intrinsic capacity of older adults (Standardized Beta = 0.776). The level of age-friendly community was associated with the intrinsic capacity scores in the unadjusted model (Beta = 0.861, P < 0.001) and the full-adjusted models (Beta = 0.609, P < 0.001). Additionally, the number of chronic diseases (Beta = -20.355, P < 0.001) and body mass index (Beta = -5.044, P < 0.05) were associated with lower scores of intrinsic capacity. Our findings suggest that the level of age-friendly communities is a protective factor for intrinsic capacity in older adults. Future research should focus on interventions to enhance community age-friendliness, thereby improving intrinsic capacity in community-dwelling older adults.

Abstract citation ID: igae098.0617

### LIVING ALONE AND LIKELIHOOD OF HAVING HIGH DEPRESSIVE SYMPTOMS AMONG OLDER ADULTS

Milan Chang (Gudjonsson)[1], Hrafnhildur Eymundsdottir[2], Sigurveig Sigurdardottir[3], Lenore Launer[4], Vilmundur Gudnason[3], and Palmi Jonsson[3], *1. Landspitali University Hospital, Seltjarnarnes, Hofuoborgarsvaoio,*

*Iceland, 2. Landspitali University Hospital, Reykjavik, Hofuoborgarsvaoio, Iceland, 3. University of Iceland, Reykjavik, Hofuoborgarsvaoio, Iceland, 4. National Institutes of Health, Baltimore, Maryland, United States*

**Background:** Depression significantly impacts the overall well-being and quality of life in older adults, leading to reduced engagement in social and physical activities and an increased vulnerability to disability. Older individuals living alone may be more prone to have depressive feelings than those residing with a spouse or family members. This study aimed to investigate the association between living status and high depressive symptoms among Icelandic older adults.

**Methods:** The analytical sample comprised 4,492 participants with a mean age of 76.3 years, of which 2,564 were women (57.1%), drawn from the Age, Gene/Environment Susceptibility-Reykjavik Study. Participants with diagnosed dementia and clinical depression were excluded from the analysis. Living arrangements were categorized as living alone (n=1,650, 36.7%) or with others. High depressive symptomatology (HDS) was defined as a Geriatric Depression Scale-15 score of 6 or higher (n=236, 5.3%). Logistic regression analysis was performed, adjusting for age, gender, education, body size, diabetes, hypertension, cholesterol, physical activity, and leisure activity.

**Results:** After full adjustment, individuals living alone showed a significantly higher likelihood of experiencing HDS compared to those living with others (Beta = 1.46, 95% CI: 1.10 ~ 1.94, P <= 0.01).

**Conclusion:** These findings emphasize the critical need to recognize potential mental health implications for older adults living alone, underscoring the imperative for targeted interventions and supportive healthcare systems. Addressing the unique challenges faced by solitary older adults is crucial for enhancing their mental well-being and overall quality of life.

Abstract citation ID: igae098.0618

### NIGHTTIME AMBIENT TEMPERATURE AND SLEEP IN OLDER ADULTS

Amir Baniassadi[1], Wanting Yu[2], Thomas Travison[1], Lewis Lipsitz[2], and Brad Manor[1], *1. Harvard Medical School/Hebrew SeniorLife, Boston, Massachusetts, United States, 2. Hebrew SeniorLife, Boston, Massachusetts, United States*

**Background:** Sleep quality is a key contributor to overall health and wellbeing in older adults. While lab-based studies demonstrate that changes in ambient temperature influence sleep quality and duration, our understanding of these associations in older adults' own home, where they have some degree of control over the temperature, is limited.

**Aim:** To examine the relationships between bedroom ambient temperature and outcomes related to sleep in community dwelling older adults.

**Methods:** In this longitudinal observational study, we monitored 50 older adults (average age=79 ±7.2y, 41 females) living in Boston, for 18 months. We used wearable sleep monitors and environmental sensors to measure sleep duration, and efficiency (ratio of time spent asleep to time spent in bed) along with bedroom temperature and humidity.

**Results:** Sleep was most efficient when the nighttime ambient temperature was between 20-24°C, with a clinically relevant 5-10% drop in sleep efficiency when the temperature increased beyond this range by 5 °C. The associations were primarily nonlinear, with significant between-subject variations.

**Discussion:** Sleep disturbances and difficulties falling asleep are common in older adults, often without undiagnosed underlying conditions. Our study identifies bedroom temperature as a crucial regulator of sleep even within home where the individual can control the temperature. This highlights the potential impact of climate change on sleep in older adults, especially those of lower socioeconomic status. Furthermore, our findings suggest that optimizing bedroom temperature can enhance sleep quality in older adults and emphasize the importance of personalized temperature adjustments based on individual needs and circumstances.

### SESSION 2045 (SYMPOSIUM)

Abstract citation ID: igae098.0619

### ESPO AND BEHAVIORAL AND SOCIAL SCIENCES SECTION SYMPOSIUM: DEFYING THE ODDS: HOW MINORITIZED OLDER ADULTS USE PSYCHOSOCIAL AND ENVIRONMENTAL FORTITUDE TO ENHANCE AGING HEALTH

Chair: E-Shien Chang
Co-Chair: Monica Walters
Discussant: Laura Zahodne

Minoritized older adults report worse health outcomes compared to their non-Hispanic White counterparts. However, existing research often focuses on the role of risk factors rather than sources of resilience among minoritized older adults. In this symposium, we will highlight ways in which racially and ethnically minoritized older adults use different facets of fortitude to ameliorate disparities and promote resilience. Our first two presentation will examine psychosocial fortitude in relation to cognitive health. Dr. Alexandra Clark will examine how belonging to a resilient psychosocial-behavioral phenotype buffers risk for Alzheimer's disease and how bilingualism protects cognitive health variably among Latinx older adults. Dr. Ruijia Chen will address racial/ethnic disparities in Alzheimer's disease and related dementias by focusing on the interplay of life course stressors, resilience factors, and sleep health across the life span. Our last two presentations will examine environmental factors in relation to cognitive and physical health. Dr. Talha Ali will provide insights on resilience mechanisms by exploring the moderating role of individual-level race/ethnicity in the relationship between structural racism and increased risk for subjective cognitive decline. Dr. Kyle Moored will explore ways in which neighborhood walkability buffers the negative impact of neighborhood socioeconomic disadvantage on step activity among Black older persons. Dr.

Laura Zahodne, as discussant, will provide summative comments identifying synergies across presentations and promoting strategies for advancing the field of minority aging research.

Abstract citation ID: igae098.0620

### EVIDENCE OF PSYCHOSOCIAL BEHAVIORAL AND COGNITIVE FORTITUDE TO AD IN RACIALLY/ETHNICALLY MINORITIZED OLDER ADULTS

Alexandra Clark, *The University of Texas Austin, Austin, Texas, United States*

It is essential that emerging research focuses on characterizing important heterogeneity in risk and resiliency to Alzheimer's disease (AD) within minoritized communities of color. Our previous work has used empirical methods to identify distinct psychosocial-behavioral and cognitive phenotypes within a large sample of community-dwelling Black and Latinx older adults enrolled in the Health and Aging Brain Study: Health Disparities (HABS:HD). This first part of this presentation will discuss the identification and characterization of unique psychosocial-behavioral phenotypes. We will highlight how these phenotypes displayed varied cognitive, neuroimaging, and AD biomarkers outcomes, with an emphasis on understanding mechanisms by which a "resilient" Low Resource/Low Distress group defies the odds of economic inequality and does not appear to be at increased risk for developing AD. This second part of this presentation will highlight data that provides a more nuanced understanding into why bilingualism has been linked to a later age of AD onset. We demonstrate that there is significant variability in the cognitive profiles of bilingual Latinx speakers that is partially attributable to language of testing, proficiency, dominance, and age of acquisition. Furthermore, we discuss the identification of a unique group of bilingual speakers with above average memory performance who are less likely to progress to the mild cognitive impairment phase. Collectively, this work illustrates the need to move beyond racial/ethnic group comparison studies to non-Latinx whites, highlights the importance of characterizing within-group heterogeneity, and further emphasizes potential points of prevention and intervention for optimizing aging outcomes in racially/ethnically minoritized older adults.

Abstract citation ID: igae098.0621

### LIFECOURSE STRESS EXPOSURES, RESILIENCE, AND BIOBEHAVIORAL MECHANISMS UNDERLYING DISPARITIES IN COGNITIVE AGING

Ruijia Chen, *Boston University, Lexington, Massachusetts, United States*

Stress exposures across the lifespan, such as childhood adversities and financial strain, may contribute to social disparities in cognitive outcomes. Individuals from socially disadvantaged backgrounds tend to be exposed to more stressors than their advantaged counterparts. Prior research has indicated that psychosocial stressors are key determinants of physical and mental health as well as health disparities. However, their roles in Alzheimer's Disease and Related Dementias (ADRD) risk have received considerably less attention. In addition to understanding and intervening directly on stressors, elucidating modifiable factors, such as social networks, further along these pathways may offer additional intervention avenues. Greater social networks have been consistently noted as predictors of lower ADRD risk, yet the structure and impact of social networks may vary across racial and ethnic groups, making it essential to assess the associations between social networks and ADRD risk in diverse racial and ethnic groups. Psychosocial stressors may lead to sleep disturbances (e.g., sleep apnea and insomnia), biobehavioral factors that disproportionately affect racial and ethnic minorities. With the availability of effective treatments for some types of sleep disturbances, evaluating how these disturbances contribute to ADRD risk across different racial and ethnic groups could identify novel targets for reducing disparities in ADRD. This presentation will cover a series of studies applying advanced epidemiologic methods, including causal inference, to investigate the roles of life course stress and resilience factors in contributing to disparities in cognitive aging.

Abstract citation ID: igae098.0622

### IMPACT OF STRUCTURAL RACISM ON COGNITIVE DECLINE

Talha Ali[1], Joshua Cohen[2], Owais Gilani[3], Belinda Needham[4], and Darya Dokshina[4], *1. Tufts University, Medford, Massachusetts, United States, 2. Massachusetts General Hospital, Boston, Massachusetts, United States, 3. Tufts University School of Medicine, Boston, Massachusetts, United States, 4. University of Michigan, Ann Arbor, Michigan, United States*

Black individuals are twice as likely as White individuals to develop cognitive impairment. However, little empirical research has investigated the sources underlying these racial disparities. We examine the effects of exposure to structural racism as a fundamental cause of racial disparities in the risk of subjective cognitive decline among older adults. Data on subjective cognitive decline from the 2015-2016 Behavioral Risk Factor Surveillance System (BRFSS) were analyzed among adults aged 45 years and older. We operationalized structural racism as an index of the number of racist vs. neutral vs. anti-discriminatory state-level policies. BRFSS participants were assigned structural racism exposure based on their state of residence at the time of the survey. The association between exposure to structural racism and the risk of subjective cognitive decline was examined using multilevel logistic regression models. Interaction terms were used to examine how this association was modified by individual-level race. American Indian Alaska Native, multiracial, and Hispanic individuals had the highest reports of experiencing subjective cognitive decline. Black individuals were the most likely to live in states with high levels of structural racism. Greater exposure to structural racism was associated with higher odds of reporting subjective cognitive decline. This association was modified by individual-level race and ethnicity, such that the risk of subjective cognitive decline decreased with increasing exposure to structural racism among Hispanic individuals. These findings warrant further research into exploring the social and emotional factors that offer resilience among Hispanic older adults.

Abstract citation ID: igae098.0623

### NEIGHBORHOOD WALKABILITY AND SOCIOECONOMIC CORRELATES OF STEP ACTIVITY FOR NON-HISPANIC BLACK OLDER ADULTS

Kyle Moored[1], Michael Desjardins[2], Vijay Varma[3], Emily Richards[2], Sayuri Ohgi[2], Patrick Donahue[2], and Michelle Carlson[2], *1. Johns Hopkins Bloomberg School of Public Health, Baltimore, Maryland, United States*, *2. Johns Hopkins University, Baltimore, Maryland, United States*, *3. Clinical and Translational Neuroscience Section, National Institute on Aging, Baltimore, Maryland, United States*

Neighborhood socioeconomic resources and walkability (e.g., proximity of services/amenities) may promote health in later life by encouraging physical activity. Yet, structural racism (e.g., redlining) has caused Black Americans to disproportionately reside in more disadvantaged neighborhoods. We examined whether neighborhood socioeconomic disadvantage, both independently and jointly with neighborhood walkability, was cross-sectionally associated with objective step activity in non-Hispanic Black older residents of Baltimore City, MD. Participants were from the Baltimore Experience Corps Trial at baseline (n=168, mean age=67.0±5.5). Neighborhood measures included validated indices of Census tract-level walkability (e.g., transit access, street connectivity, service/commercial density) and socioeconomic disadvantage (e.g., poverty, unemployment). Individuals were further categorized into combined walkability/disadvantage groups using median splits of each index: low walkability/low disadvantage (40%), high walkability/low disadvantage (7%), high walkability/high disadvantage (46%), low walkability/high disadvantage (8%). Average daily step activity was measured using an ankle-worn monitor (mean=4.3 wear days). Linear regression was used to examine associations with step activity adjusted for age, gender, and education. Being in the highest (vs. lowest) tertile of disadvantage was associated with 1513 fewer average steps per day (95% CI: -2873,-153). While walkability was not independently associated with step activity (p>.05), the high walkability/high disadvantage group had an average 1381 more steps per day (95% CI: 204,2558) compared to the low walkability/high disadvantage group. Higher neighborhood socioeconomic disadvantage was linked with lower physical activity for non-Hispanic Black older residents of Baltimore City. Yet, positive neighborhood attributes like walkability may buffer against socioeconomic disadvantage, potentially by providing access to services/amenities that promote activity.

### SESSION 2050 (SYMPOSIUM)

Abstract citation ID: igae098.0624

### ETHNICITY AND WELL-BEING AMONG OLDER IMMIGRANTS IN DIVERSE SOCIOCULTURAL CONTEXTS

Chair: Daniel W.L. Lai

The critical interaction between culture, ethnicity, and social determinants of health is a vital area of study, particularly regarding the unique experiences of ethnocultural minority older adults. The complex intersections of these factors across diverse social landscapes are ripe for investigation to uncover inequalities and inform targeted strategies to reduce the inequalities encountered by these groups. This symposium presents four key studies that examine the well-being, healthcare access, and civic participation of aging Asian immigrants, with a focus on South Asian populations in Hong Kong and Canada, as well as Chinese communities in Canada and the United States. These studies highlight the need for culturally congruent interventions. They emphasise the challenges faced in health and mental health service accessibility and the rich tapestry of cultural diversity within civic involvement. Critical factors such as the quality of intergenerational relationships, the breadth and depth of social capital, language skills, transportation availability, financial dependencies, and the impact of pre-migration histories are shown to significantly affect the lives and civic engagement of aging Asian immigrants. The research presented here strongly advocates for culturally attuned policies and practices. It underscores an imperative for health promotion, healthcare accessibility, and civic inclusion that are responsive to the cultural identities and preferences of diverse older populations. The findings of these studies are critical to the development of targeted interventions, public policies and supportive frameworks that address the unique needs and overcome the barriers faced by ageing migrants around the world.

Abstract citation ID: igae098.0625

### THE EFFECT OF SOCIAL CAPITAL AND ETHNICITY ON THE WELL-BEING OF OLDER ADULTS IN HONG KONG

Daniel W.L. Lai, Zideng Huang, and Alison Yu-ting Ou, *Hong Kong Baptist University, Hong Kong, Hong Kong*

Ethnic minority older adults in Chinese societies remain understudied despite the increasing diversity of the aging population. This study investigated the influence of social capital determinants on the quality of life of South Asians and Chinese residents in Hong Kong, highlighting the importance of understanding each ethnic group's unique social dynamics and cultural contexts for effective interventions. A telephone survey was conducted with a total of 1,015 Chinese (800) and South Asian (215) older adults aged 55 and above, using the World Bank's tool to measure social capital across six dimensions: social participation, support, connection, trust, cohesion, and reciprocity and the World Health Organization's Quality of Life questionnaire to assess quality of life. Demographic variables such as gender, age, marital status, education, and ethnicity were also collected. Hierarchical regression analyses showed that social capital factors such as social support, connection, and reciprocity negatively influenced the quality of life for Chinese Hong Kong residents but positively for South Asians. Social trust and belonging showed no significant effect on Hong Kong residents' quality of life but improved it for South Asians. Ethnic group was a consistent moderator, with South Asians generally reporting lower quality of life than Hong Kong residents. The interaction between social capital and ethnicity has a significant impact on quality of life, with certain aspects of social capital benefiting South Asians more than Hong Kong residents. This suggests that quality of life interventions should be tailored to the specific social capital dynamics and cultural contexts of each ethnic group.

Abstract citation ID: igae098.0626
### BARRIERS TO ACCESS HEALTH AND MENTAL HEALTH SERVICES AMONG SOUTH ASIAN OLDER ADULTS
Janki Shankar[1], and Papiya Das[2], *1. University of Calgary, Calgary, Alberta, Canada*, *2. Indo-Canadian Women's Association, Edmonton, Alberta, Canada*

Research on older immigrants in Canada is in its infancy despite their growing numbers. In 2019, Covenant Health, one of the largest healthcare providers in Canada, in partnership with the Indo-Canadian Women's Association, a non-government service organization for new immigrants, in Edmonton, conducted a community consultation with South Asian older adults to understand their health and mental care needs and barriers to their access. The consultation was based on anecdotal evidence that South Asian older adults make poor use of health care services in general and mental health care services in particular. Focus groups and individual interviews with older adults were used to gather information, and the data were analyzed using thematic analysis. The results showed that a significant number of South Asian older adults lead marginalized and isolated lives within their community. Lack of language skills, transportation difficulties, poor knowledge of health care services, financial dependence on sponsors, and adherence to traditional beliefs about health and mental health services are some of the barriers to accessing services. Based on these findings, several recommendations are suggested to improve healthcare access for older adults.

Abstract citation ID: igae098.0627
### THE IMPACT OF CULTURE ON CIVIC ENGAGEMENT OF AGING ASIAN IMMIGRANTS: FINDINGS FROM A MIXED STUDY IN EDMONTON, CANADA
Hongmei Tong, *MacEwan University, Edmonton, Alberta, Canada*

As a well-known immigrant-receiving country, Asian immigrants constitute most immigrants in Canada. However, the understanding of cultural diversity and intra-cultural similarities and differences among aging immigrants' civic activities is limited. A mixed-methods study was conducted to examine civic participation experienced by Filipinos, Indians, and Chinese, since they are the three largest ethnocultural communities in Edmonton. Thematic analysis related to research questions was used for data analysis. Findings show diversity in the understanding of civic engagement and willingness, and their understanding and engagement in civic activities are affected by culture-related factors such as country of origin, time since immigration, citizenship status, greater official language proficiency, and pre-migration participation in activities. Public policies, such as immigration and integration policies (e.g., multiculturalism, diversity, and social inclusion), also influence immigrants' civic participation. The findings suggest that cultural diversity should be considered in promoting civic activities among aging immigrants.

Abstract citation ID: igae098.0628
### GRANDPARENT–GRANDCHILD ASIAN AMERICAN REMINISCENCE: INTERGENERATIONAL AND CULTURAL REFLECTION
Ling Xu, *The University of Texas at Arlington, Arlington, Texas, United States*

Intergenerational solidarity between grandparents and grandchildren has been highlighted in Asian Families and it is beneficial for the health of grandparents. An intergenerational reminiscence program was developed to improve the well-being of Asian American older adults as well as generational bonding. This paper qualitatively reported the reflection of participant dyads based on the interviews at the end of program (n = 11). Older grandparents received 6 sessions of life-review with their grandchildren remotely or in person for approximately 1 hour each week for 6 weeks. At the end of the program, individual interviews were conducted with dyad participants. Braun and Clarke's six-steps of thematic analysis were followed to examine the interview data. Thematic analysis of interview with grandparents revealed four themes: Family life (early childhood, intergenerational connection, family reunition through immigration); career and work (hard work, sacrifice personal life); major life turning points (immigration, baptism); stress and coping (grief/loss, resilience). Thematic analysis of qualitative data from grandchildren also yielded four themes: Unknown/unexpected family history; disagreement (life-work balance, being frugal); cultural respect, lasting legacy (always remembering the good part of life, take one thing at a time). Recommendations offered by the dyad participants included combining some topics to avoid overlapping, adding topics of spousal relationship or parenting, etc. The reflection from grandparent-grandchild dyad participants showed that intergenerational reminiscence program is promising in bonding the grandparent-grandchild connection with family history and cultural revisitation. Lessons learned and recommendations for future research on interventions for Asian American families are offered.

### SESSION 2055 (SYMPOSIUM)

Abstract citation ID: igae098.0629
### EXPLORING THE RELATIONSHIP BETWEEN RELIGION, SPIRITUALITY, LONELINESS, SOCIAL ISOLATION, AND AGING
Chair: Roger O'Sullivan
Co-Chair: Katherine Britt
Discussant: Jim Lubben

The importance of social engagement, connections, and participation as we age and the negative impact of loneliness and social isolation on health are well recognized. Religion and/or spirituality has social, emotional, and existential dimensions and are linked to concepts of belonging, meaning, and purpose. As part of the drive to build meaningful social and emotional connections in contemporary society, religion and/or spirituality are proposed as potential solutions to loneliness/social isolation. The theoretical mechanism proposed is that affiliation, attendance, practice, or belief can offer support, social capital, meaning and a sense of belonging. This interdisciplinary symposium uses data from both the US and Europe to explore the relationship between religion, spirituality, loneliness, and social isolation. Leavey using longitudinal data from Ireland explores depression and anxiety and their relationship with loneliness, social networks, religious practice, and long-term illness. Victor & Seetal, also using longitudinal data set out the relationship between measures of religious affiliation, attendance, and practice with loneliness for older adults in England. Taylor uses the HRS to examine the association between religious service attendance and spirituality on social isolation and loneliness among older adults in the United States. Finally, Hampton Jarmon, using data from the Healthy Aging in Neighborhoods of Diversity Across the Life Span study, explores the relationship between spirituality (connectedness, prayer fulfilment, and universality) and cognition within Black and White adult participants. Lubben, as discussant, will reflect on the key themes and the symposium's overarching focus - can religion and/or spirituality provide protection from loneliness and social isolation?

Abstract citation ID: igae098.0630

### RELIGION AND LONELINESS: OLDER PEOPLE IN IRELAND

Gerard Leavey, *Ulster University, Belfast, Northern Ireland, United Kingdom*

**Background:** Religious identity and adherence may confer health benefits, through increased social capital (eg, community belonging, and material resources) and mechanisms for coping with adversity and misfortune. Isolation and loneliness are associated with less engagement in religious practice and can influence mental health outcomes. Aims: To investigate comorbid depression (or subthreshold symptoms) in those with clinically relevant anxiety and their relationship with loneliness, social networks, religious practice and long-term illness.

**Methods:** Secondary data analysis of Wave 1 of the TILDA study (2009-2011), a nationally representative community-based sample.

**Results:** Compared to those with low/no symptoms of depression, those classed as comorbid were more likely to be male, had lower education levels, had spent more time abroad, lower religious attendance, a limited social network, were lonelier and had a long-term life-limiting illness. Those with subthreshold levels of depression reported a more restricted social network and more moderate levels of loneliness.

Abstract citation ID: igae098.0631

### EXPLORING THE RELATIONSHIP BETWEEN LONELINESS, RELIGION, AND SPIRITUALITY FOR OLDER ADULTS IN ENGLAND

Christina Victor, *Brunel University London, London, England, United Kingdom*

Although there is evidence linking the prevalence of loneliness with religion and/or spirituality in north America, this topic is rarely researched in the UK. This study explored the relationship between measures of religious affiliation, attendance, and practice with loneliness for older adults in England. We generated an analytic sample of 6225 adults aged 50+ who took part in wave 9 of the English Longitudinal Study of Ageing (ELSA). The exposure variables of religious affiliation, attendance and practice were measured by questions on religious identity; frequency of attendance at places of worship in the last year; and 4-point Likert scales of agreement or disagreement with religious importance, meaning and purpose, prayer and meditation, and activity. Loneliness outcome was measured using the three item University of California Los Angeles loneliness scale. The majority of our analytic sample were Christian (77%), 22% had no religion and 49% had not attended a place of worship in the last year. The prevalence of loneliness (score 6+ on UCLA scale) was 19%. Religious attendance was significantly associated with loneliness (OR =.73 CI.62-.85, p <.001), with attendance protective against feeling lonely. However, this relationship was not significant after adjustment. No relationship was observed between loneliness and religious affiliation or spirituality. Religious identity remains prevalent in older adults in England, but attendance and practice less so. Further studies in diverse populations, using validated measures of loneliness and religion, are necessary to investigate whether loneliness can be moderated or mediated by religion/spirituality alongside other determinants.

Abstract citation ID: igae098.0632

### THE RELATIONSHIPS BETWEEN RELIGIOUS SERVICE ATTENDANCE, SPIRITUALITY, LONELINESS, AND SOCIAL ISOLATION

Harry Taylor[1], and Ann Nguyen[2], *1. University of Toronto, Toronto, Ontario, Canada*, *2. Case Western Reserve University, Cleveland, Ohio, United States*

The objective of our study is to determine if religious service attendance and spirituality had an association with loneliness and social isolation among older adults. We used the Health and Retirement Study to address our research objective. Religious service attendance was measured dichotomously (no religious service attendance vs. attending religious services at least once a year). Spirituality was measured by Fetzer Institute 4-item Brief Multidimensional Measure of Religiousness/Spirituality. Loneliness and social isolation were operationalized using the UCLA 3-item loneliness scale and by a social network index, respectively. Covariates included age, gender, race, income, employment status, and education. We used multivariable linear regression to examine the relationships between religious service attendance, spirituality, loneliness, and social isolation. We found respondents who did not attend religious services were more likely to be socially isolated. Additionally, spirituality did not have a significant association with social isolation. Respondents who did not attend religious services had significantly greater loneliness in comparison to those who did attend religious services. Furthermore, respondents with higher spirituality scores had lower loneliness scores. We found religious service attendance had an association with social isolation only, while both attendance and spirituality had an association with loneliness among older adults; therefore, improving access to religious institutions and enhancing one's spirituality may serve as important factors for mitigating social isolation and loneliness among older adults.

Abstract citation ID: igae098.0633

### THE RELATIONSHIP BETWEEN SPIRITUALITY AND COGNITION WITHIN BLACK AND WHITE ADULT IN THE HANDLSLEEP STUDY

Marsha Hampton[1], Alexa Allan[2], Linying Ji[3], Lesley Ross[1], Roland Thorpe[4], Alan Zonderman[5], Michele Evans[5], and Alyssa Gamaldo[1], *1. Clemson University, Clemson, South Carolina, United States*, *2. The Pennsylvania State*

*University, University Park, Pennsylvania, United States, 3. Montana State University, Bozeman, Montana, United States, 4. Johns Hopkins University, Baltimore, Maryland, United States, 5. National Institute on Aging, Baltimore, Maryland, United States*

This study explored associations between spirituality and cognitive performance within Black and White adults in the HANDLS Sleep Study (n=85; 52% Black adults; age range: 46-82). The Spirituality Transcendence Scale-Short Form measured overall spiritual transcendence orientation and three subscale scores (Prayer Fulfillment, Universality, and Connectedness). Cognitive performance was measured by several tests (MoCA, Trails Making Test, AVLT, Digit Span, Clock Drawing, Verbal Fluency). Race-stratified regression analyses explored the association between each spirituality score and cognitive test after covariate adjustment (age, sex, poverty status, depression severity and health conditions). Within Black adults, the spiritual subscale (universality and connectedness) and total scores were significantly associated with better delayed AVLT (memory; b = -.409, p = 0.014), phonemic verbal fluency (executive function; b = 1.312, p =.044) and clock drawing (visuospatial skill; b = 0.092, p = 0.040). However, these findings became non-significant after covariate adjustment. Within White adults, results indicated that higher spiritual personal fulfillment (b = -.281, p = 0.014) was significantly associated with worse performance on digit span forward (executive function); however, higher connectedness (b =.442, p = 0.014) was significantly associated with better performance on the same test after covariate adjustment. Higher connectedness was significantly associated with faster performance on Trials B (b = -4.452, p = 0.015; executive function), but was non-significant after covariate adjustment. Findings illustrate differential associations between the spirituality and cognitive performance within Black and White adults, which suggest further exploring psychosocial factors culturally relevant to each racial group to understand current findings.

### SESSION 2060 (PAPER)

### FAMILY CAREGIVING I (PAPERS)

Abstract citation ID: igae098.0634

### THE EFFECT OF CAREGIVING ON ADULT CHILDREN'S SAVING: A MULTILEVEL, CROSS-NATIONAL ANALYSIS OF 11 EUROPEAN COUNTRIES

Mengya Wang, *Iowa State University, Ames, Iowa, United States*

This research explores how caregiving impacts individuals' retirement planning using Bronfenbrenner's bioecological model. Focusing on retirement savings, it examines the association between caregiving and savings, considering gender, family support, and welfare regimes as moderators. Multi-level analysis of data from the Survey of Health and Retirement in Europe, with a sample of 3,555 individuals across 12 European countries participating in both wave one and two, reveals unexpected findings. Contrary to expectations, caregiving isn't negatively correlated with savings, and female caregivers don't face more financial challenges than males. Additionally, the impact of family support on savings varies by gender, with sons providing care having greater savings when they have at least one sister, while daughters engaged in caregiving tend to have fewer savings if they have at least one brother. Welfare regimes also impact savings differently based on caregiving duration; long-term caregivers in defamilialism regime (DF) experience significant savings changes between waves, while short-term caregivers in unsupported familialism regime (USF) undergo more changes. This suggests that in DF regimes, caregivers initially struggle with work and caregiving balance, but adapt successfully once arrangements are in place, possibly due to increased formal support. Conversely, in USF regimes, initial family support is strong, but over time, reliance on formal care services decreases, and family support dwindle, posing increasing long-term challenges for caregivers. This study offers insight into the implications for caregivers' long-term financial well-being. Recognizing the financial strain they face, it's imperative to explore effective policy options to alleviate economic burden linked to informal caregiving.

Abstract citation ID: igae098.0635

### SUPPORTING DIABETES MANAGEMENT FOR PERSONS LIVING WITH DEMENTIA: CARE PARTNER EXPERIENCES

Alycia Bristol, Synneva Hagen-Lillevik, Shinduk Lee, and Nancy Allen, *University of Utah, Salt Lake City, Utah, United States*

Care partners (CP) play a vital role in assisting persons living with dementia (PLWD) but often face challenges when supporting type 2 diabetes (T2D) management. We conducted pilot work to explore adapting an existing diabetes self-management education/support intervention called Share plus for CP of PLWD with T2D. Share plus promotes continuous glucose monitoring with CP data sharing and provides dyadic communication, problem-solving, and action planning strategies. Two focus groups (n=5, n=4) were conducted with CP. Focus groups were recorded, transcribed, and analyzed using content analysis. CP also completed surveys, including the Partner Diabetes Distress Scale (PDDS), the Zarit Burden Scale, and one burden question specific to diabetes. Descriptive statistics were used for analysis. As part of the larger pilot work, we found that CP expressed frustration with diabetes management for PLWD and discussed their lack of knowledge of addressing the impact of dementia symptoms on diabetes management. Across three PDDS questions, CP reported varying frustration levels ranging from none (44.4%) to a lot (11.1%). Additionally, CP reported experiencing slight (44.4%) and moderate (22.2%) burden when supporting diabetes management. During focus groups, CP identified frustrations and struggles in managing dementia symptoms' influence on diabetes management, particularly regarding diet, medications, and communication. Additionally, CP reported uncertainty about how to support PLWD's need for independence. The next steps are to develop strategies to tailor the Share plus intervention by incorporating the unique challenges faced by PLWD and their CP, such as creating specific diabetes and dementia communication strategies for CP.

Abstract citation ID: igae098.0636

### CHARACTERISTICS OF BURDEN ASSOCIATED WITH CARING FOR OLDER FAMILY MEMBERS WITH DYSPHAGIA ACROSS TIME

Samantha Shune[1], Merle Werbeloff[2], and Ashwini Namasivayam-MacDonald[3], *1. University of Oregon, Eugene, Oregon, United States*, *2. University of the Witwatersrand, Johannesburg, Gauteng, South Africa*, *3. McMaster University, Hamilton, Ontario, Canada*

Caregivers often experience increased burden resulting in reduced health and quality of life. Underexplored in its impact on burden is the effect of dysphagia (swallowing difficulties) over time. The purpose of this study was to explore the characteristics of burden associated with caring for an individual with dysphagia across an aging cohort. Using the Round 1, 5, 7, and 11 surveys from the National Health and Aging Trends Study and the National Study of Caregiving, we analyzed data from 4,662 spousal and children caregivers caring for community-dwelling family aged 65+. Outcome variables included caregivers' emotional, financial, and physical burden and their agreement with statements about their well-being. A design-based conversion of the Pearson chi square statistic and binary logistic regressions were used to explain and predict presence of burden, its extent, and characteristics dependent on presence of dysphagia. At any given time, 16-27% of respondents were caring for family with dysphagia. Caregiver burden was more often reported by those caring for family with dysphagia than those without. For spouses and daughters, the degree of burden was almost always rated higher among those caring for family with dysphagia, with significant differences observed in levels of emotional and physical burden. Further, caregivers of family with dysphagia were significantly more likely to report having little time for themselves and feeling depressed, lonely, and that caregiving was too much to handle. Ultimately, dysphagia's potential impact on the emotional, financial, and physical well-being of family caregivers must be considered along the care trajectory.

Abstract citation ID: igae098.0637

### ADDRESSING LANGUAGE BARRIERS: PERSPECTIVES INTO THE ROLE OF FAMILY CAREGIVERS FOR OLDER ASIAN AMERICANS

Minakshi Raj, and Laura Quintero, *University of Illinois Urbana-Champaign, Champaign, Illinois, United States*

Language barriers influence the healthcare experience. Limited communication due to language differences impacts rapport establishment and decision-making processes, often increasing caregivers' care burden by making them responsible for language interpretation when such service is not offered in healthcare settings (HCS). This study aimed to describe the challenges that caregivers face regarding navigating language barriers in HCS with or on behalf of their older Asian American relatives. Ninety-six caregivers completed a cross-sectional online survey of questions on a) language-related characteristics (e.g., at-home language, other languages spoken), b) language-related experiences in HCS, c) demographic information (e.g., age, residence, immigration status), and d) caregiving characteristics (proximity to care recipient, hours of caregiving per week, and types of support provided). Most respondents (72%) reported that a language other than English was the primary language spoken at home, while 41% of care recipients understand English "not well" (25%) or "not at all" (16%). In HCS, 54% of care recipients wanted interpretation services, with 30% "always" having it. Among types of interpretation, caregivers and relatives performed this task more frequently (53%). Regarding challenges, caregivers referred care recipients' aging and cognitive decline, medical and technical vocabulary not translatable to native languages, and lack of training are the issues they experience in navigating language barriers in HCS. Despite significant cultural diversity, the United States continues to experience gaps in providing equitable health services to linguistically diverse individuals. It is necessary to develop support strategies to improve healthcare experiences and reduce the additional burden of language barriers on caregivers.

Abstract citation ID: igae098.0638

### DEPENDS ON WHO YOU ASK: DISCORDANCE IN REPORTING SPOUSAL CARE BETWEEN WOMEN AND MEN ACROSS EUROPEAN WELFARE STATES

Afshin Vafaei[1], Ricardo Rodrigues[2], Cassandra Simmons[3], Eszter Zólyomi[3], Selma Kadi[3], Stefan Fors[4], and Susan Phillips[5], *1. Western University, London, Ontario, Canada*, *2. University of Lisbon, Lisbon, Lisboa, Portugal*, *3. European Centre for Social Welfare Policy & Research, Vienna, Wien, Austria*, *4. Karolinska institutet, Stockholm, Stockholms Lan, Sweden*, *5. Queen's University, Kingston, Ontario, Canada*

Current research on determinants, prevalence, and effects of informal care on health assumes that survey instruments can appropriately identify informal care providers. We aimed to investigate systematic differences across Europe in reporting spousal care between caregivers and cared-for persons and their possible effects on associations between informal care and physical and mental health indicators. Using information on care provided/received from the Survey on Health, Ageing and Retirement in Europe (SHARE), we estimated prevalence rates of spousal care and discordance between caregivers and cared-for persons in the reporting of care among caregiving dyads. We used multinomial regressions to estimate differences in reporting spousal care across European countries and then constructed multivariate logistic regression models to evaluate the effect of discordance in reporting informal care on the correlation between care and poor self-rated health and depression. Results showed that only 53.9% of dyads report care confirmed by both spouses. Multinomial regressions showed that agreement on care provided/received was more common when women are caregivers, while men were more likely to underreport providing/receiving care. There was no effect on the association of care with SRH regardless of who is asked to identify the care providers, while the magnitude and direction of the association between depression symptoms and care varies according to the choice of respondent. Possible explanations for the discordance include social norms dictating gender roles and acceptability of men as care providers. Relying on self-identification of care providers as most surveys do has the potential to underestimate their numbers.

### SESSION 2065 (SYMPOSIUM)

Abstract citation ID: igae098.0639

### FAMILY CAREGIVING: TIME-VARYING CONTEXTS OF SOCIAL SUPPORT, FINANCES, BURDEN, AND BENEFITS

Chair: Rahul Malhotra
Co-Chair: Jeremy Lim-Soh

Family caregiving is a crucial component of long-term care. Care provision provides opportunities for psychosocial gain but can also lead to burden among family caregivers in multiple domains – physical, psychological, social, and financial. Family caregiving is dynamic, with changes over time in the needs of care-recipients as well as care responsibilities and outcomes experienced by caregivers, yet longitudinal studies on caregiving contexts are scant. Longitudinal changes and trends in social support, financial well-being and service use, burden and benefits of caregiving, and spiritual resilience are essential to identify caregivers at-risk of detrimental changes over time. The four presentations in this symposium provide empirical findings from population-based longitudinal studies of family caregivers of older adults, most with three or more time points of data collection. The first investigates heterogeneity in perceived social support of family caregivers over time and explores the role of caregiver psychological resilience in reshaping the trajectories. The second examines longitudinal trends in financial difficulties and income among family caregivers and their impact on supportive care service utilization. The third discusses joint trajectories of the burden and benefits of caregiving over time and highlights modifiable factors in the process. The fourth unravels the paradox wherein family caregivers simultaneously experience both high burden and benefits of caregiving. Collectively, the presentations underscore the heterogeneous and dynamic nature of family caregiving and contexts over time. They also highlight the need to strengthen caregivers' psychological resilience, financial capacity, preparedness for caregiving and spiritual beliefs to enhance their caregiving experience.

Abstract citation ID: igae098.0640

### TRAJECTORIES OF SOCIAL SUPPORT AMONG FAMILY CAREGIVERS: THE ROLE OF PSYCHOLOGICAL RESILIENCE

Jeremy Lim-Soh[1], Yongjing Ping[1], Ha-Linh Quach[2], Shannon Ang[3], and Rahul Malhotra[1], *1. Duke-NUS Medical School, Singapore, Singapore*, *2. Duke-NUS CARE, Singapore, Singapore*, *3. Nanyang Technological University, Singapore, Singapore*

Social support has been widely acknowledged as an important factor impacting family caregivers' outcomes including burden and depression. However, research focused on social support as an independent factor, and how it changes over time, is limited. This study is the first to describe heterogeneous trajectories (patterns of change over time) in perceived social support among family caregivers of older adults. It also investigates if caregiver psychological resilience influences these trajectories. This study draws on a longitudinal survey of 278 Singaporean family caregivers of community-dwelling older adults aged 75 years and above with activities of daily living limitations. Caregivers were interviewed up to four times across two years. Perceived social support and psychological resilience were measured by Pearlin's and Connor-Davidson's scales respectively. We applied Group-Based Trajectory Modeling, an approach that is sensitive to within-population heterogeneity, to describe change over time in perceived social support. We modeled psychological resilience as a time-varying covariate in the model. Five distinct trajectories of perceived social support were identified, with three stable trajectories (91% of caregivers) and two declining trajectories (9%). Psychological resilience was positively associated with perceived social support only in the stable trajectories. While most family caregivers of older adults may experience stable perceived social support over time, a minority do report declining perceived social support. Further investigation is needed to understand the reasons for this decline and design effective interventions targeting this vulnerable group.

Abstract citation ID: igae098.0641

### LONGITUDINAL TRENDS OF FAMILY CAREGIVERS' FINANCIAL WELL-BEING AND SUPPORTIVE CARE SERVICE USE

Yin Liu[1], Kyungmin Kim[2], Melissa Ferguson[1], Niying Li[3], and Josey Batura[4], *1. Utah State University, Logan, Utah, United States*, *2. Seoul National University, Seoul, Republic of Korea*, *3. University of Georgia, Athens, Georgia, United States*, *4. Utah State University, Providence, Utah, United States*

The stress process model denotes financial well-being and caregiving support as two important resources for family caregivers. Financial well-being can be a critical factor in securing paid/formal care services at home and in community settings. When care-related out-of-pocket expenses are high, however, it can also become a secondary strain (i.e., financial burden) for caregivers. We conducted a longitudinal trend analysis, utilizing four waves of cross-sectional data collected over time from caregivers enrolled in the National Study of Caregiving study (i.e., family/unpaid caregivers of participants in the National Health and Aging Trends Study 1, 2, 11, and 12 rounds). The findings suggested that across waves, subjective financial difficulty was associated with income, and there were disparities in financial well-being (i.e., defined as subjective financial difficulty and income) by care recipients' chronic health conditions as well as caregivers' race/ethnic minority status and levels of education. Further, financial well-being (including subjective financial difficulty and income) was associated with using community-based care services such as respite programs (e.g., adult day care services) and paid help for personal care at home. Caregiving policy needs to consider caregiver financial well-being and financial disparities as factors affecting care service use.

Abstract citation ID: igae098.0642

### JOINT TRAJECTORIES OF CAREGIVING BURDEN AND BENEFITS AMONG FAMILY CAREGIVERS OF OLDER ADULTS

Yongjing Ping[1], Jeremy Lim-Soh[1], Truls Østbye[2], and Rahul Malhotra[1], *1. Duke-NUS Medical School, Singapore, Singapore*, *2. Duke University, Durham, North Carolina, United States*

Caregiving is often associated with burden, but it can also benefit family caregivers. While the two phenomena can co-exist, existing studies have largely considered the two separately. Studies considering the two together are mostly cross-sectional, even though caregiving is dynamic over time, requiring longitudinal study. It is also important to understand the heterogeneity in the joint (co-existing) trajectories (patterns over time) of caregiving burden and benefits as it can help identify family caregivers who are continuously vulnerable and determine modifiable factors linked with detrimental trajectories. We conducted group-based multi-trajectory modelling on longitudinal data – collected at four time-points, at six-to-twelve-month intervals – on burden (negative domains of modified Caregiver Reaction Assessment) and benefits (short Positive Aspects of Caregiving scale) from 274 family caregivers in Singapore. We identified four trajectory groups, each with distinct joint trajectories of burden and benefits – "balanced" (44.5%, least burden and relatively high benefits), "satisfied" (23.0%, second lowest burden and highest benefits), "dissatisfied" (19.0%, second highest burden and least benefits), and "intensive" (13.5%, highest burden and relatively high benefits). Relative to caregivers in the "balanced" group, those supported by trained migrant domestic workers were less likely to be in the other three groups and those more prepared for caregiving were more likely to be in the "satisfied" group. The findings highlight the heterogeneity in the caregiving experience over time and suggest that promoting migrant domestic worker training and family caregiver preparedness for caregiving may contribute to advantageous patterns of burden and benefits of caregiving over time.

Abstract citation ID: igae098.0643

**KEEPING THE FAITH IN ADVERSITY: UNPACKING THE PARADOX OF INTENSIVE CAREGIVING**

Ad Maulod, Nur 'Atiqah Mohd Farhan, Sasha Rouse, and Rahul Malhotra, *Duke-NUS Medical School, Singapore, Singapore*

Striving to live an authentic life while fulfilling caregiving duties is a common challenge among family caregivers. Previously, based on the distribution of the burden and benefits of caregiving, we identified four distinct archetypes – balanced (40%), satisfied (33%), intensive (17%) and dissatisfied (10%) – among family caregivers of older adults in Singapore. "Intensive" caregivers were most burdened in terms of poor health and financial strain yet continued to express caregiving as a highly rewarding endeavor, reporting benefits which were similar or greater than the other archetypes. In this paper, we interrogate this paradox by exploring the experiences of "intensive" caregivers through a one-year longitudinal qualitative study (Quali-T, N=39) conducted with caregivers across the different archetypes. Study findings reveal complex narratives of "intensive" caregivers seeking rewards to justify their burden. A karmic belief in intangible spiritual benefits seems to be the key explanation for this phenomenon. While the supportive effects of spirituality in overcoming caregiving challenges have been studied at length, we identify the intricate processes that occur when highly stressed caregivers negotiate meaning out of their circumstances, in relation to their personal need for spiritual well-being and authenticity. While spiritual support can be profoundly comforting for many caregivers, it is not a panacea to address all the challenges they face. By addressing the spiritual dimension of care in parallel to other aspects of caregiver support, healthcare and social service providers can offer more comprehensive person-centered care that supports caregivers' holistically.

### SESSION 2070 (SYMPOSIUM)

Abstract citation ID: igae098.0644

**FROM NATIONAL DATA TO LOCAL APPLICATION: INNOVATIONS IN AGING NETWORK SERVICES AND RESEARCH PARTNERSHIPS**

Chair: Traci Wilson
Co-Chair: Marisa Sheldon
Discussant: Heather Menne

As the local experts in planning, coordinating, and implementing aging services, Area Agencies on Aging (AAAs) address a broad range of social and health needs of an increasingly diverse older adult population. To live independently and with optimal health in their homes and communities, many older adults depend on Older Americans Act (OAA)-funded services such as nutrition and in-home supportive services, which they access through local AAAs. Increasingly, older adults look to AAAs for supports outside of core OAA services, such as housing and emergency preparedness and response. This symposium will provide data-driven insights on adaptive strategies to enhance aging services both within and beyond the OAA and how AAAs and researchers can collaborate to support innovations. Each presenter represents a different level and perspective within the Aging Network. The first speaker from USAging will use the National Survey of AAAs to describe organizational characteristics that support aging services innovations. The presenter from the Administration for Community Living will describe findings from recent National Surveys of OAA Participants including how they adapted during COVID and how prepared they are for future emergencies. The director of a university research center will demonstrate the continuum of community engaged research approaches, through a bi-directional relationship with an AAA. The symposium will conclude with a presentation from an AAA illustrating how her agency engages with a university-based research center to innovate, build evidence for program effectiveness, and strengthen services for at-risk older adults.

Abstract citation ID: igae098.0645

**COMMON FEATURES OF AREA AGENCIES ON AGING AND IMPLICATIONS FOR AGING SERVICE INNOVATIONS**

Traci Wilson, *USAging, Washington, District of Columbia, United States*

Established by the Older Americans Act (OAA) in 1973, Area Agencies on Aging (AAAs) across the country assess local community needs and plan, coordinate, and deliver a range of long-term services and supports that help older adults and people with disabilities to age well at home. There is a commonly heard phrase, "if you've seen one AAA, you've seen one AAA", but is this helpful or true? Based on an analysis of organizational-level data from USAging's 2022 National Survey of AAAs (74 percent response rate), this paper delineates typologies of AAAs based on characteristics such as organizational structure, funding sources, agency size, location and how this relates to the types of services offered by AAAs, populations served, and their involvement in initiatives such as age-friendly communities or housing and homelessness prevention. The findings illustrate that all AAAs deliver a core set of OAA-funded services and supports, and funding sources outside of the OAA, including state and local funding, Medicaid Waiver, and contracts with health care entities provide opportunities to deliver additional services to more older adults. While state regulations largely determine the organizational structure and setting of AAAs, there is evidence that each typology of AAA innovates, collaborates and delivers high-quality aging services and supports. By understanding these commonalities, researchers, practitioners, advocates and policy makers can better support the local organizations that support community-living older adults across the country.

Abstract citation ID: igae098.0646

### OLDER AMERICANS ACT: TARGETING OLDER ADULTS WITH GREATEST SOCIAL AND ECONOMIC NEEDS

Kristen Robinson, *Administration for Community Living, Washington, District of Columbia, United States*

The overall purpose of the Older Americans Act (OAA) is to help older adults remain in their communities through the provision of long-term services and supports. The Administration for Community Living administers the National Survey of OAA Participants (NSOAAP) to collect data on OAA clients in six service areas (home-delivered meals, congregate meals, homemaker services, transportation services, case management services, and caregiver support services). The annual survey includes questions on consumer assessment of service quality as well as client characteristics such as health status, physical and social functioning, and demographics. The OAA targets older adults with the greatest social and economic need. Older adults who receive in-home services (home-delivered meals, case management, and homemaker services) are often the ones with the highest level of need. For example, between one-half and three-quarters of in-home service clients live alone, more than half report mobility limitations, and one-third report limitations in 3+ activities of daily living (ADLs). In 2022, a module on emergency preparedness was added to the 16th NSOAAP. Findings show that one-third of in-home service clients experienced severe weather events such as a tornado, hurricane, or wildfire. Living alone and having medical devices that require electricity can be an added complication during emergencies. Among OAA clients, 41% of home-delivered meal, 60% of case management, and 42% homemaker clients reported having 3+ ADLs and rely on medical devices that require electricity. This presentation will discuss uses of the annual survey, findings from the rotating modules, and how researchers can access the data.

Abstract citation ID: igae098.0647

### DEMONSTRATION OF COMMUNITY-ENGAGED RESEARCH CONTINUUM THROUGH A UNIVERSITY AND AREA AGENCY ON AGING PARTNERSHIP

Marisa Sheldon[1], Katie Calhoun[2], Smitha Rao[2], Christine Happel[3], Holly Dabelko-Schoeny[2], and Anthony Traver[2], *1. The Ohio State University, Age-Friendly Innovation Center, Columbus, Ohio, United States, 2. The Ohio State University, Columbus, Ohio, United States, 3. Age-Friendly Innovation Center, The Ohio State University, College of Social Work, Columbus, Ohio, United States*

Area Agencies on Aging (AAA), which were originally established to support the planning and delivery of community-based support, are well-positioned to build knowledge and develop innovative responses to community needs through community-engaged research partnerships. This presentation illustrates a relationship between the Age-Friendly Innovation Center (AFIC) at The Ohio State University College of Social Work and the Central Ohio Area Agency on Aging (COAAA), focused on advancing community-engaged research in response to multiple aspects of aging in the community. This presentation will explore the continuum of community-engaged research, to build meaningful collaborations between two entities that shared similar values and identified shared concerns, as demonstrated through three research projects. The projects address three different critical needs among older adults and reflect different levels of engagement, 1) Low-income housing residents, especially older adults with disabilities, face heightened risks during weather-related disasters, 2) The increasing rate of older adult renters that are considered extremely housing cost burdened, and 3) The unique needs of an increasingly diverse population of older adults, in particular the rising number of older adults that are LGBTQ+. The three projects represent different methodological approaches and levels of engagement, illustrating unique funding, staffing, and project implementation activities. Preliminary findings from interviews, focus groups, and survey activities for each research project will be presented. The primary focus will be understanding the development and design of the projects, ranging from consultation, collaboration, to partnership, emphasizing challenges and points of success experienced by AAA and by AFIC.

Abstract citation ID: igae098.0648

### THINKING OUTSIDE THE BOX: THE ROLE OF FUNDING AND COMMUNITY-ENGAGED RESEARCH IN THE FUTURE OF AREA AGENCIES ON AGING

Katie White, *Central Ohio Area Agency on Aging, Columbus, Ohio, United States*

As the older adult and disabled populations continue to grow, Area Agencies on Aging (AAAs) must develop new ways to meet the ever-diverse needs and ideas of individuals in their regions through innovative research partnerships. AAAs have served as the federally mandated, local leaders on aging and disability by planning, developing, funding, and implementing local systems of coordinated home and community-based services (HCBS). The Older Americans Act is the cornerstone of non-Medicaid HCBS, providing funding and requirements for nutrition, supportive, caregiver, and evidence-based services. By partnering with the Age-Friendly Innovation Center of The Ohio State University College of Social Work (AFIC), the Central Ohio Area Agency on Aging (COAAA) is supporting community-engaged participatory research with older adults and people with disabilities to inform future policy, program, service, and funding priorities. This presentation will outline how the COAAA prioritized funding community-engaged research, applied research findings to policy initiatives, and infused evidence-informed practice in new and emerging areas of focus. Practical steps for researchers to engage effectively with area agencies on aging will be discussed.

### SESSION 2075 (SYMPOSIUM)

Abstract citation ID: igae098.0649

### HEALTH PRIORITIES IDENTIFICATION FOR INDIVIDUALS LIVING WITH DEMENTIA AND THEIR CAREGIVERS

Chair: Jack Banks
Discussant: Caroline Blaum

"What matters" is the foundation for the Age-Friendly Health System Initiative and yet many clinicians have a difficult time addressing it with their patients. Patient Priorities Care (PPC) is an evidence-based approach that identifies health priorities by first eliciting health values of older adults with multiple chronic conditions, integrating values into health outcome goals, and describing the one-thing to focus on. Clinicians' then consider patients' conditions and current treatments to identify care options that align with the identified health priorities. Furthermore, care of persons living with dementia (PlwD) involves care partners, thus identifying what matters most to PlwD is dyadic in nature. Recent studies have examined dyadic decision-making using the PPC approach with PlwD and their care partners. This symposium will present results from three studies that use PPC across diverse cultural and clinical contexts and discuss the role of PPC to achieve better dementia care. The first presentation will describe how clinicians consider health priorities with dyads facing dementia and discuss how health priorities identification can help care partners become better decision makers for their family members with dementia and multimorbidity. The second presentation will describe the cultural adaptation process of the PPC approach for older Hispanics with multimorbidity and dementia. The third presentation will describe how PPC facilitates deprescribing among older adults with multimorbidity and dementia. The panel will provide suggestions on how to better identify health priorities with PlwD and their care partners and share resources to facilitate the adoption and implementation of PPC with these dyads.

Abstract citation ID: igae098.0650

### HOW CLINICIANS INVOLVE CARE PARTNERS IN THE HEALTH PRIORITIES IDENTIFICATION PROCESS IN DYADS AFFECTED BY DEMENTIA

Joan Monin, *Yale School of Public Health, New Haven, Connecticut, United States*

Care partners play a crucial role in supporting their kin and friends who are living with dementia and multiple chronic conditions (MCCs) in decision making; yet, we know little about how clinicians include care partners in the Health Priorities Identification Process (HPIP) when they are present at clinic visits. We conducted an online survey with 34 clinicians who currently use HPIP with patients living with dementia with MCCs. On average, clinicians reported that at 53% of sessions, a care partner was present. When care partners are present, clinicians reported the extent to which care partners are involved in the following ways: (1) the patient responds to the HPIP questions on their own or with minor support from their care partner (80% of clinicians said sometimes, often, or always); (2) the patient and the care partner work together to respond to the questions (93% said sometimes, often, or always), and (3) the patient almost fully defer to the care partner to answer the questions (70% said sometimes, often, or always). About 87% of clinicians agreed that patients could successfully complete HPIP with a care partner involved; 84% of clinicians said it is easier for them to complete HPIP when a care partner is involved; and 87% said if care partners had more guidance about how to serve as active listener when patients complete HPIP, they believe care partners would be better prepared to make decisions for the patient if the care partner had to serve as a proxy-decision maker.

Abstract citation ID: igae098.0651

### PATIENT PRIORITIES CARE FOR HISPANICS WITH DEMENTIA (PPC-HD)

Rafael Samper-Ternent, *The University of Texas Health Houston, Houston, Texas, United States*

Persons living with dementia frequently experience difficulty managing multiple chronic conditions (MCC) and have poorer outcomes. Managing MCC typically involves adhering to single-disease clinical practice guidelines. This approach results in burdensome care with outcomes that may not reflect what matters most to patients. Studies using the Patient Priorities Care (PPC) approach have demonstrated feasibility and effectiveness of PPC in different clinical settings. Physical, emotional, and cognitive impairments related to dementia interfere with disease self-management. Care partners, therefore, play an essential role in the care and decision-making of PlwD. Care partners are essential in translating 'what matters most' for PlwD into healthcare decisions. Patient and care partner involvement should include identifying outcome goals and care preferences (health priorities) as well as aligning care to meet those priorities. PlwD from minority groups face more barriers and have poorer outcomes compared to Non-Hispanic Whites. Hispanics are the fastest growing underrepresented population in the USA, and have higher risk of dementia compared to Non-Hispanic Whites. Hispanics rely heavily on their families and there is a cultural expectation of families to provide care to members in need. Cultural differences and language barriers shape healthcare priorities and how priorities impact outcomes among Hispanics. We recently completed a feasibility study among older Hispanics with cognitive impairment and their care partners. Results from this study confirm Hispanics with dementia and their care partners are willing and able to identify health priorities, and clinicians can align care to achieve these priorities when placed in the electronic health record (EHR).

Abstract citation ID: igae098.0652

### PRIORITIES ALIGNED DEPRESCRIBING FOR PERSONS LIVING WITH DEMENTIA AND THEIR CARE PARTNERS

Aanand Naik, *The University of Texas Health Science Center, Houston, Texas, United States*

PlwD average 8 daily medications compared to 3 for persons without dementia. PlwD have high polypharmacy burden from anticholinergic and psychotropic medications. PlwD, their care partners and clinicians endure the workload of managing multiple medications, and want to decrease this burden. Thus, a shift that reduces polypharmacy, and focuses medication use on what matters most is needed. Deprescribing is an approach to reduce polypharmacy for PlwD. However, deprescribing studies for PlwD have a narrow scope. Few studies place deprescribing within ambulatory care contexts for patients with mild to moderate dementia. Appropriate deprescribing in dementia requires identifying health priorities. Deprescribing is most appropriate when guided by priorities of patients and care partners. Patient Priorities Care (PPC) is an evidence-based approach to health priorities aligned decision making. We found evidence that PPC facilitates deprescribing by identifying health priorities that are misaligned with current medications. When used in the care of PlwD and their care partners, PPC could facilitate deprescribing. Less is known about how PPC works in the triadic context of clinician, care partner, and PlwD. Care partners may have concerns about stopping medications considered inappropriate such meds help manage symptoms. Clinicians may focus on cognitive and functional harms of medications without considering tradeoffs of stopping medications. Without eliciting priorities, families and care partners of PlwD may make decisions to stop medication without communication with their clinicians. We recently completed a study using PPC to deprescribe unnecessary medications among PlwD. We will discuss our findings and the implications for future work.

### SESSION 2080 (SYMPOSIUM)

Abstract citation ID: igae098.0653

### JGMS EDITOR'S CHOICE ARTICLES OF 2023: RISK AND RECOVERY OF FUNCTIONAL DECLINE AND FRAILTY

Chair: Lewis Lipsitz
Discussant: Dae Kim

This symposium will present four 2023 "Editor's Choice" articles from the Journal of Gerontology Medical Sciences that focus on the risk and recovery of functional decline and frailty. Courtney Millar and her colleagues, in their article "Association of Proinflammatory Diet With Frailty Onset Among Adults With and Without Depressive Symptoms: Results From the Framingham Offspring Study" examine the association between an inflammatory diet and increased odds of frailty in those with depressive symptoms. Nicholas Rowan and his coauthors, in "The Association of Peripheral and Central Olfaction With Frailty in Older Adults" examine olfactory impairment, specifically olfactory identification and sensitivity, as a potential biomarker and risk factor for frailty. "Supplementing Glycine and N-Acetylcysteine (GlyNAC) in Older Adults Improves Glutathione Deficiency, Oxidative Stress, Mitochondrial Dysfunction, Inflammation, Physical Function, and Aging Hallmarks: A Randomized Clinical Trial", written by Sekhar Rajagopal and colleagues, studies the effects of GlyNAC (combination of glycine and N-acetylcysteine [NAC]) supplementation on hallmarks of aging in older adults. Theresa Mau and coauthors, in their article, "Mitochondrial Energetics in Skeletal Muscle Are Associated With Leg Power and Cardiorespiratory Fitness in the Study of Muscle, Mobility and Aging," examine the associations between mitochondrial energetics and functional outcomes, including leg power and cardiorespiratory fitness. Our discussant, Roger Fielding, will highlight commonalities and lessons learned from these studies.

Abstract citation ID: igae098.0654

### ASSOCIATION OF PRO-INFLAMMATORY DIET WITH FRAILTY ONSET AMONG ADULTS WITH AND WITHOUT DEPRESSIVE SYMPTOMS

Courtney Millar[1], Alyssa Dufour[2], James Hebert[3], Nitin Shivappa[3], Olivia Okereke[4], Douglas Kiel[5], Marian Hannan[5], and Shivani Sahni[2], *1. Hebrew SeniorLife, Roslindale, Massachusetts, United States*, *2. Hebrew SeniorLife, Boston, Massachusetts, United States*, *3. University of South Carolina, Columbia, South Carolina, United States*, *4. Massachusetts General Hospital, Boston, Massachusetts, United States*, *5. Harvard Medical School, Boston, Massachusetts, United States*

Dietary inflammation is associated with increased risk of frailty. Those with depressive symptoms may be at higher risk of frailty onset since they typically have higher levels of inflammation. The study objective was to determine the association between a pro-inflammatory diet and frailty onset in those with and without clinically relevant depressive symptoms. This prospective study included 1,701 non-frail individuals with self-reported baseline (1998-2001) data available for the evaluation of energy-adjusted dietary inflammatory index (E-DIITM) (calculated from food frequency questionnaires), depressive symptoms (from the Center for Epidemiologic Studies Depression; CES-D), and follow-up frailty measurements (2011-2014). Frailty was defined as fulfilling ≥3 Fried frailty criteria. Results are presented by baseline CES-D scores <16 or ≥16 points, which denotes the absence or presence of clinically relevant depressive symptoms, respectively. Logistic regression estimated odds ratios (OR) and 95% confidence intervals (95% CI) between E-DII and frailty onset, adjusting for confounders. In all study participants, mean (standard deviation; SD) age was 58(8) years and E-DII was -1.95 (2.20; range: -6.71 to +5.40, higher scores denote a more pro-inflammatory diet), and 45% were male. In those without clinically relevant depressive symptoms, one-unit higher E-DII score was associated with 14% increased odds (95% CI:1.05-1.24) of frailty. In those with depressive symptoms, one-unit higher E-DII score was associated with 55% increased odds of frailty (95% CI: 1.13-2.13). The association between inflammatory diet and increased odds of frailty appeared somewhat stronger among those with depressive symptoms. This preliminary finding warrants further investigation.

Abstract citation ID: igae098.0655

### THE ASSOCIATION OF PERIPHERAL AND CENTRAL OLFACTION WITH FRAILTY IN OLDER ADULTS

Nicholas Rowan[1], Nimesh Nagururu[1], Isaac Bernstein[1], Kristin Voegtline[2], Sarah Olson[3], and Yuri Agrawal[4], *1. Johns Hopkins University School of Medicine, Baltimore, Maryland, United States*, *2. Johns Hopkins*

*University School of Medicine, Department of Pediatrics, Baltimore, Maryland, United States*, *3. Johns Hopkins University, Baltimore, Maryland, United States*, *4. Johns Hopkins University School of Medicine: Department of Otolaryngology-Head and Neck Surgery, Baltimore, Maryland, United States*

Olfactory impairment is increasingly recognized as a biomarker of frailty, but the relationship between olfactory subdomains that describe peripheral or central dysfunction and frailty remains unexplored. In this study, we examined 1,160 older adults from the National Social Life, Health, and Aging Project Wave 3. Olfactory identification (OI): the ability to identify an odorant; and olfactory sensitivity (OS): the ability to detect the presence of an odorant, were assessed using 5 and 6-point measures respectively. Frailty was operationalized as both a 37-item Frailty Index (FI) and the 5-item Physical Frailty Phenotype (PFP). Mixed models were fit to examine the association between OI, OS, FI and PFP, while adjusting for demographic and clinical covariates. Subjects in the most-frail PFP category had lower OI and OS scores (OI: 3.88 vs 4.19, p= 0.016; OS: 3.15 vs 3.47, p= 0.031), whereas, subjects in the most-frail FI category exhibited lower OI scores but not OS scores when compared to non-frail subjects (OI: 3.72 vs 4.27, p= 0.014; OS: 3.19 vs 3.43, p= 0.476). Adjusted mixed models showed that a point increase in OI was associated with a lower PFP score (β= -0.107, p= 0.006) and FI score (β= -0.009, p= 0.010). A point increase in OS was associated with a lower PFP score (β= -0.058, p= 0.016), but not FI score (β= -0.004, p= 0.064. These finding reveal distinct associations between peripheral and central measures of olfaction and frailty, highlighting the potential use of olfaction assessment as a biomarker and risk factor for frailty.

Abstract citation ID: igae098.0656

### GLYNAC SUPPLEMENTATION IN OLDER ADULTS IMPROVES GLUTATHIONE, MITOCHONDRIA, AGING HALLMARKS, AND FUNCTION

Rajagopal Sekhar[1], Premranjan Kumar[1], George Taffet[2], Charles Minard[1], James Suliburk[1], Farook Jahoor[1], Chun Liu[1], and Raja Muthupillai[3], *1. Baylor College of Medicine, Houston, Texas, United States*, *2. Houston Methodist Hospital, Houston, Texas, United States*, *3. Baylor St. Luke's Hospital, Houston, Texas, United States*

Elevated oxidative stress (OxS), mitochondrial dysfunction, and aging hallmarks contribute to aging, but improving/reversing these defects in older adults (OA) has been challenging. We identified that deficiency of the intracellular antioxidant glutathione (GSH) could play a role and reported that supplementing GlyNAC (combination of glycine and N-acetylcysteine [NAC]) in aged mice improved GSH deficiency, OxS, mitochondrial fatty-acid oxidation (MFO), and insulin resistance (IR). To test whether GlyNAC supplementation in OA could improve GSH deficiency, OxS, mitochondrial dysfunction, IR, aging hallmarks and physical function, we conducted a placebo-controlled randomized clinical trial in 24 OA and 12 young adults (YA). The OA was randomized to receive either GlyNAC or placebo for 16-weeks, and the YA received GlyNAC for 2-weeks. Participants were studied before, after 2-weeks, and after 16-weeks of supplementation to assess changes in intracellular GSH concentrations, OxS, MFO, molecular regulators of energy metabolism, inflammation, endothelial function, IR, multiple aging hallmarks, gait speed, muscle strength, 6-minute walk test, body composition, and blood pressure. We found that compared to YA, the OA had GSH deficiency, elevated OxS, mitochondrial dysfunction (with multiple defects in molecular regulation), inflammation, endothelial dysfunction, IR, multiple aging hallmarks, impaired physical function, and increased waist circumference and systolic blood pressure. GlyNAC (and not placebo) supplementation in OA improved/corrected these defects. In conclusion, GlyNAC supplementation in OA for 16-weeks was found to be safe, well-tolerated, and effective in its ability to reverse multiple age-associated declines and age-related abnormalities. GlyNAC supplementation in older humans is an effective nutraceutical to promote healthy aging.

Abstract citation ID: igae098.0657

### MITOCHONDRIAL ENERGETICS IN SKELETAL MUSCLE ARE ASSOCIATED WITH LEG POWER AND CARDIORESPIRATORY FITNESS IN SOMMA.

Theresa Mau[1], Philip Kramer[2], Paul Coen[3], Russell Hepple[4], Stephen Kritchevsky[5], Anne Newman[6], Peggy Cawthon[1], and Steven Cummings[1], *1. California Pacific Medical Center, San Francisco, California, United States*, *2. Wake Forest University School of Medicine, Winston-Salem, North Carolina, United States*, *3. AdventHealth, Orlando, Florida, United States*, *4. University of Florida, Gainesville, Florida, United States*, *5. Wake Forest University School of Medicine, Wake Forest University School of Medicine, North Carolina, United States*, *6. University of Pittsburgh, University of Pittsburgh, Pennsylvania, United States*

Mitochondrial energetics are an important property of aging muscle, as generation of energy is pivotal to the execution of muscle contraction. However, its association with functional outcomes, including leg power and cardiorespiratory fitness, is largely understudied. In the Study of Muscle, Mobility and Aging, we collected vastus lateralis biopsies from older adults (n = 879, 70–94 years, 59.2% women). Maximal State 3 respiration (Max OXPHOS) was assessed in permeabilized fiber bundles by high-resolution respirometry. Capacity for maximal adenosine triphosphate production (ATPmax) was measured in vivo by 31 P magnetic resonance spectroscopy. Leg extension power was measured with a Keiser press system, and VO2peak was determined using a standardized cardiopulmonary exercise test. Gender-stratified multivariate linear regression models were adjusted for age, race, technician/site, adiposity, and physical activity with beta coefficients expressed per 1-SD increment in the independent variable. Max OXPHOS was associated with leg power for both women (β = 0.12 Watts/kg, p <.001) and men (β = 0.11 Watts/kg, p <.050). ATPmax was associated with leg power for men (β = 0.09 Watts/kg, p <.05) but was not significant for women (β = 0.03 Watts/kg, p =.11).

### SESSION 2085 (SYMPOSIUM)

Abstract citation ID: igae098.0658

### LEVERAGING PATIENT PORTALS TO IDENTIFY AND INTERVENE IN DEMENTIA CARE: CAREGIVING, MEDICATION, AND ACUTE CARE

Chair: Jennifer Portz
Discussant: George Demiris

Care partners play a pivotal role in supporting people living with dementia (PLWD), yet their systematic identification and involvement in clinical care are often lacking. This symposium presents four studies highlighting groundbreaking care partner identification methods to inform patient portal-based interventions targeting care partners to enhance dementia care. The first study, a retrospective cohort analysis focuses on automating care partner identification via structured patient contact data and unstructured portal notes in the electronic health record to better deliver evidence-based caregiving interventions through patient portals. The second study explores the associations between the documentation of a care partner and documentation of care partner education in the electronic health record and hospital length of stay. The third study presents an analysis of patient secure portal messages and clinical notes that identified modifiable contributors to potentially preventable acute care visits among PLWD including unmet care needs, particularly in care coordination, health systems navigation, and medication management that contribute. The final study describes the content of patient portal messages relating to medications among PLWD, care partners, and clinicians in a single health system, to inform the development of a portal-based intervention to reduce the use of potentially inappropriate medications among PLWD Our Discussant, Dr. George Demiris, PhD, an informatics and caregiving technology expert, will lead a conversation emphasizing the importance of systematic care partner identification and integrating care partner engagement tools with patient portals to enhance support for PLWD and their care partners, ultimately improving dementia care delivery and outcomes.

Abstract citation ID: igae098.0659

### IDENTIFICATION OF PATIENT PORTAL MESSAGES SENT FROM NON–PROXY ACCOUNT CARE PARTNERS AND THEIR PORTAL USE PATTERNS

Jennifer Portz[1], Daniel Martin[2], Kelly Gleason[3], Karla Washington[4], Andrea Daddato[2], Dave Powers[2], Jason Lyons[2], and Elizabeth Bayliss[2], *1. University of Colorado, Aurora, Colorado, United States*, *2. Kaiser Permanente Colorado, Aurora, Colorado, United States*, *3. Johns Hopkins University, Baltimore, Maryland, United States*, *4. Washington University in St. Louis, St. Louis, Missouri, United States*

Automating care partner identification via the electronic health record (EHR) can streamline the delivery of evidence-based caregiving interventions through patient portals and is vital for supporting care partners of people living with dementia (PLWD). This retrospective cohort study focused on identifying patient portal messages from non-proxy care partners and exploring associated portal usage. Conducted at Kaiser Permanente Colorado, it analyzed data from 789 PLWD aged ≥60, utilizing the EHR and Virtual Data Warehouse. Structured patient contact data (e.g., emergency contact, family contact) and unstructured portal notes were used to match contact names with caregiver terms, employing a rule-based algorithm to identify care partner messages. Results showed that 95% of PLWD had at least one care partner listed, with an average of 2.1. A majority (77%) engaged with the patient portal, and 57% had messages sent by care partners, predominantly non-proxy (7% were from proxy accounts). Non-proxy care partners sent an average of six messages during the study period. Additionally, 54% of PLWD had portal activity on days when care partners sent messages, showing their involvement in healthcare management. Care partners mainly accessed clinical visit information, medication details, lab scheduling/results, and primary care provider information on these days. This study underscores the importance of automated care partner identification and non-proxy engagement via patient portals, facilitating better support for PLWD and enhancing their care partners' involvement.

Abstract citation ID: igae098.0660

### ASSOCIATION BETWEEN PARTNER IDENTIFICATION AND EDUCATION WITH HOSPITAL LENGTH OF STAY FOR PATIENTS WITH DEMENTIA

Nicole Werner[1], Austin Medlin[2], and Beth Fields[3], *1. Indiana University, Bloomington, Bloomington, Indiana, United States*, *2. Indiana University Bloomington, Bloomington, Indiana, United States*, *3. University of Wisconsin Madison, Madison, Wisconsin, United States*

Despite overwhelming reliance on care partners to care for people living with dementia (PLWD) after a hospitalization, care partners rarely receive adequate education to meet post-hospitalization caregiving demands, which is associated with health consequences including adverse clinical outcomes for PLWD, and increased levels of stress, depression, and morbidity for care partners. The present study aimed to explore associations between care partner identification as documented in the electronic health record (EHR), documented care partner education, and PLWD length of stay (LOS). We extracted EHR data from a large academic medical center in the Midwest between 1/1/2019 and 8/1/2022 (N = 8038). To assess association between care partner identification and PLWD LOS, encounters were coded dichotomously, either 'lacking a care partner' or 'care partner present', depending on EHR documentation. Similarly, encounters received designations of increased education if care partners received more education on post-discharge care than the education sample median. Independent t-tests analyzed mean differences on LOS for care partner identification. The same approach was used on a subsample of encounters with care partner identification (n=1644) to analyze associations between care partner education and LOS. We found no significant difference in LOS based on care partner identification status ($p=.072$). Among PLWD with identified care partners, significant differences in LOS were found between education groups ($p=.017$), where longer hospitalizations were associated with more care partner education (less education $M=5.9$ days, more education $M=6.9$). These results suggest that targeted education could better support care partners when a shorter LOS is expected for PLWD.

Abstract citation ID: igae098.0661

### LEVERAGING ELECTRONIC MEDICAL RECORD DATA TO IDENTIFY MODIFIABLE CONTRIBUTORS TO ACUTE CARE USE IN DEMENTIA

Stephanie Nothelle[1], Mohamed Ahmidouch[2], Julia Loosen[3], Cynthia Boyd[4], and Jennifer Wolff[4], *1. Johns Hopkins University School of Medicine, Baltimore, Maryland, United States*, *2. University of North Carolina School of Medicine, Chapel Hill, North Carolina, United States*, *3. Johns Hopkins Bayview Medical Center, Baltimore, Maryland, United States*, *4. Johns Hopkins University, Baltimore, Maryland, United States*

Persons living with dementia (PLWD) are at increased risk of potentially preventable acute care visits (hospitalizations and emergency department visits), but modifiable contributors are poorly understood. We aimed to identify potentially modifiable factors from patient secure portal messages and outpatient clinical notes in the 30 days leading up to a potentially preventable acute care visit. We examined data from the Johns Hopkins Health System electronic medical record from 2017-2022. We included adults >64 years with dementia diagnosis, 1+ advanced chronic illness (advanced liver disease, chronic obstructive pulmonary disease, congestive heart failure, diabetes with complications, chronic kidney disease), 2+ primary care visits who had a potentially preventable acute care visit. Two coders coded each message or note using a qualitative content analysis approach. We included 128 PLWD of whom 75 (69%) were female, and 68 (53%) were white. Approximately 47 (37%) had at least one patient/caregiver initiated portal message. Topics identified were similar across patient portal message and clinical notes. The most commonly mentioned needs related to care coordination, health systems navigation, medication management, access to and timeliness of care, and new physical symptoms. Cognitive concerns or symptoms were more commonly mentioned in clinical notes than portal messages. Portal messages and outpatient clinical notes prior to a potentially preventable acute care visit contain information about unmet care needs, predominantly related to coordinating and accessing care. Needs identified in this analysis may be addressable by a care manager to prevent unnecessary acute care use.

Abstract citation ID: igae098.0662

### USE OF PATIENT PORTALS FOR MEDICATION-RELATED COMMUNICATION: IMPLICATIONS FOR DEPRESCRIBING INTERVENTIONS

Ariel Green[1], Aleksandra Wek[2], Kelly Gleason[2], Mingche Wu[2], Mary Jo Gamper[2], and Jennifer Wolff[2], *1. Johns Hopkins University School of Medicine, Baltimore, Maryland, United States*, *2. Johns Hopkins University, Baltimore, Maryland, United States*

People living with dementia (PLWD) often use potentially inappropriate medications (PIM), in which the risks of medication use outweigh the benefits. Little is known about how PLWD and their care partners use patient portals in general, and specifically to communicate with healthcare providers about medications. Our objective was to characterize the content of patient portal messages relating to medications among PLWD, care partners, and clinicians in a single health system, to inform the development of a portal-based intervention to reduce the use of PIM. We examined secure message exchanges between 2/18/2017 and 10/2/2022 from patients 65 years and older with dementia or their care partners in an integrated, academic health system. A total of 399 message threads from 159 unique patients were analyzed. Patients were on average 78.4 years old (SD 8.0). The majority (65%) were female, white (76%), and non-Hispanic/Latinx (96%); 15% had at least one registered proxy portal user. The most common topic raised was logistics (42%), such as clarification of medication instructions. This was followed by messages about adverse effects/treatment burden (25%), asking for new medications (23%), and openness to stopping a medication (21%). Findings suggest that the patient portal is frequently used by PLWD and their care partners to discuss medication-related concerns and that it is a promising platform for delivering interventions to reduce the use of PIM in this population.

### SESSION 2090 (PAPER)

### LIVING WITH DEMENTIA I

Abstract citation ID: igae098.0663

### ANNUAL WELLNESS VISITS AND EARLY DEMENTIA DIAGNOSES AMONG MEDICARE BENEFICIARIES IN TEXAS

Yong-Fang Kuo[1], Mukaila Raji[2], Yong Shan[3], Peter Cram[4], and Huey-Ming Tzeng[3], *1. University of Texas Medical Branch, Galveston, Texas, United States*, *2. University of Texas Medical Branch, Galveston, Galveston, Texas, United States*, *3. The University of Texas Medical Branch, Galveston, Texas, United States*, *4. UTMB, Galveston, Galveston, Texas, United States*

Early recognition of cognitive impairment (CI) and timely diagnoses of mild CI (MCI) and Alzheimer's Disease and Related Dementia (ADRD) are key to optimal dementia care. No previous research has examined the effects of Medicare annual wellness visits (AWVs) on early CI recognition among Medicare beneficiaries. Here, we assessed the association between incident diagnosis at MCI, moderate or severe ADRD stages in 2017-2020, and having a Medicare AWV in the three years prior to diagnosis. This population-based, case-control study used 100% Texas fee-for-service Medicare data from 2014 to 2020; population: Medicare beneficiaries diagnosed with incident MCI or ADRD from 2017 to 2020. The exposure is Medicare AWVs. The primary outcome is MCI, moderate or severe ADRD status by ICD-9-CM and ICD-10-CM codes. We performed a multinomial logistic regression model to examine the association. Medicare beneficiaries (n=197,356) who had an AWV were 23–66% more likely to be diagnosed at the MCI stage and 8–30% more likely to be diagnosed at the moderate ADRD stage compared to diagnoses at the severe ADRD stage relative to those who did not have an AWV. There was a dose-response relationship; as the number of AWV visits in the three years increased, the magnitude of the likelihood of early diagnosis increased. This study suggests that receipt of a Medicare AWV is associated with increased identification of early CI.

Abstract citation ID: igae098.0664

### "I BELIEVE I CAN WORK, BUT THEY THOUGHT I CAN'T WORK": THE WORKING CONDITIONS OF PEOPLE WITH YOUNG ONSET DEMENTIA

Zih-Ling Wang, *University of Washington, Seattle, Washington, United States*

"Young-onset dementia" is a medical condition where a person is diagnosed with dementia before the age of 65. Experiencing behavioral changes and committing mistakes in the workplace due to dementia can lead to issues with employers, pension funds, and financial matters. However, there have been few studies on individuals with young-onset dementia or their working conditions in Taiwan. A qualitative research design was utilized to conduct semi-structured in-depth interviews from three perspectives: people with young-onset dementia, family care partners, and employment specialists. This approach aimed to gain insights into whether and how reasonable accommodation could be implemented in the workplace before COVID-19, how affected individuals adapt to work, and how family care partners and employment specialists assist them in continuing their employment. The research identified three main themes: (1) adaptive strategies and resilience in the workplace among young onset dementia, (2) advocating for supportive environments, and (3) empowering individuals with young onset dementia through tailored career support. This study highlighted areas for improvement in protecting the right to work and ensuring reasonable accommodation for young onset dementia, emphasizing the need for appropriate adjustments in their work environment. The goal is for the public to recognize the capabilities of such individuals and to safeguard their right to work.

Abstract citation ID: igae098.0665

### PEOPLE LIVING WITH DEMENTIA AND THEIR FAMILY MEMBERS INFORM A PERSON-CENTERED MEASURE OF "HOME TIME"

Nathan Boucher[1], Kevin McKenna[2], Justine Seidenfeld[3], Judith Vick[4], James Burke[2], Brenda Plassman[5], Megan Shepherd-Banigan[5], and Courtney Van Houtven[2], *1. Duke University, Durham, North Carolina, United States*, *2. Duke University School of Medicine, Durham, North Carolina, United States*, *3. Duke University Health System, Durham, North Carolina, United States*, *4. Yale University School of Medicine, New Haven, Connecticut, United States*, *5. Duke University Medical Center, Durham, North Carolina, United States*

To build a person-centered "hometime" measure for use for persons living with dementia (PLWD, inclusive of mild cognitive impairment), we conducted in-depth interviews with PLWD and spouses who support PLWD to understand quality-of-life (QOL) at home versus health facility settings. We interviewed 32 PLWD (age 59-93) and 40 spouses with recent experience in these settings: PLWD in inpatient (12 PLWD; 15 spouses), emergency (8 PLWD; 12 spouses), post-acute skilled nursing (4 PLWD; 4 spouses), outpatient surgery (PLWD 8; spouse 6), and assisted living (3 spouses). Applied thematic analysis generated four themes: 1) Emotions: Participants were frustrated by limited control in health settings. Spouses described some relief, but guilt when the PLWD was in health facility settings. PLWD described being a burden to spouses. 2) Care Challenges: Tasks, including activities of daily living, exceeding spouses' capabilities or worsening physical/cognitive status decreased QOL at home. Spouses had fewer care duties while PLWD were in health settings. 3) Activities: Time spent with spouse, pets, television, reading, and games provided satisfaction across settings. 4) Community Involvement: Spouses had low community involvement; little changed whether PLWD was in health settings or at home. Many spouses continued to check on PLWDs' eating, bathing, or medication administration when in a facility. These data in concert with claims data provide insights on value/quality of time at home versus health settings. Our findings inform an emerging "hometime" measure and indicate enhanced task support and social support may raise caregivers' QOL and help avoid visits to health facilities for PLWD.

Abstract citation ID: igae098.0666

### FAMILY CAREGIVER PERSPECTIVES ON THE PALLIATIVE CARE NEEDS OF HOSPITALIZED PERSONS LIVING WITH DEMENTIA

Jung Kwak[1], Elizabeth Kvale[2], Sarah Mills[1], and Kwaku Duah Oppong[1], *1. The University of Texas Austin, Austin, Texas, United States*, *2. Baylor College of Medicine, Houston, Texas, United States*

The progressive nature of dementia results in significant symptom burden, functional decline, and frequent hospitalizations, underscoring the need for specialized palliative care for persons living with dementia (PLWDs) and their caregivers. Existing studies, primarily retrospective analyses of health records or provider interviews, lack information on the critical psychosocial and social support contexts that significantly influence the palliative care needs of PLWDs. Addressing this gap, our study explored the perspectives of family caregivers on the palliative care needs of hospitalized PLWDs, informed by the Adapted Conceptual Model Integrating Palliative Care in Serious Illness and Multiple Chronic Conditions. We interviewed 37 caregivers of hospitalized PLWDs who were referred for palliative care. Caregivers identified primarily as female (66%) and caring for parents (58%), and as White (46%), Black (17%), Asian (5%), and Latino (31%). Most common challenges caregivers reported were about addressing health issues (58%), managing emotional difficulties (53%), and making care plan decisions (42%). Three related themes emerged from in-depth qualitative interviews: the complex care needs of PLWDs, including pain and symptom management; caregivers' needs for support in discharge preparation; and challenges in making care transition decisions into facilities. These insights underscore the caregivers' critical role in managing care and making decisions during critical care transitions for hospitalized PLWDs. The findings provide implications for practice and research including enhanced care coordination between primary, palliative, and discharge planning services in acute settings and future research on the impact of psychosocial and social support factors on palliative care outcomes for this vulnerable population.

Abstract citation ID: igae098.0667
### A CRITICAL REVIEW OF DISCHARGE PLANNING AND DECISION-MAKING EXPERIENCE OF DEMENTIA FAMILY CAREGIVERS
Kwaku Duah Oppong, and Jung Kwak, *The University of Texas Austin, Austin, Texas, United States*

Persons living with dementia (PLWD) are significantly more likely to be hospitalized and experience poorer outcomes than their non-dementia peers. Despite available evidence-based care transition interventions, few are based on the experiences and perspectives of PLWDs and their family caregivers. To inform development of person- and family-centered care transition support for PLWDs and family caregivers, a systematic review focused on their decision-making roles and support received from hospital teams was conducted by retrieving studies published between 2000 and 2024 from PubMed, CINAHL, and Embase. Four papers were included for full review, and the rigor of each paper was assessed using the Mixed-Methods Assessment Tool. All four studies were qualitative, cross-sectional, and atheoretical, with only one study conducted in the United States. The sample size ranged from 15 to 30 and consisted of family caregivers and PLWDs recruited via chart reviews and hospital units. No study reported on the types of decisions that family caregivers were asked to make. Overall themes on information and support received were: lack of care coordination and inclusion in discharge planning, the discharge planning process often rushed, caregivers feeling unsupported by healthcare providers, and health providers' inability to meet family caregivers' informational needs and preferences. Surprisingly, few empirical studies are available, with no studies examining shared decision-making between PLWDs, caregivers, and providers. Existing studies suggest lack of information and support by the healthcare team as a major challenge for these families. Further studies that are theory-driven, with methodological rigor and racial/ethnically diverse PLWDs and caregivers are warranted.

### SESSION 2095 (AWARD LECTURE (INVITATION ONLY))

Abstract citation ID: igae098.0668
### MAXWELL A. POLLACK AWARD FOR CONTRIBUTIONS TO HEALTHY AGING PRESENTATION AND LECTURE
Chair: Debra Dobbs

The Maxwell A. Pollack Award for Contributions to Healthy Aging Lecture will feature an address by the 2023 Pollack Award recipient Bei Wu, PhD, FGSA, FAGHE of New York University. This session will also include the presentation of the 2024 Maxwell A. Pollack Award to recipient Edward Alan Miller, PhD, MPA, FGSA, of the University of Massachusetts Boston. The Maxwell A. Pollack Award for Contributions to Healthy Aging Award recognizes instances of practice informed by research and analysis, research that has directly improved policy or practice, and distinction in bridging the worlds of research and practice.

Abstract citation ID: igae098.0669
### ADVANCING GLOBAL HEALTHY AGING: MY JOURNEY IN GERONTOLOGICAL RESEARCH
Bei Wu, *New York University, New York, New York, United States*

In this presentation, Dr. Bei Wu will reflect on her over three-decade-long career as a gerontologist, exploring how her personal history, educational background, and professional experiences have informed and developed her program of research, focusing on her efforts to advance global healthy aging. Dr. Wu intends to highlight key findings from her research. She will discuss the pivotal role of secondary data analyses as a means to investigate the complex relationships between oral health, cognitive decline, and systemic health. Moreover, she will elaborate on the development of behavioral interventions aimed at improving oral health for individuals with cognitive impairment. She will also address her contributions to the establishment of NIH-funded centers aimed at mitigating health disparities and enhancing equity among older Asian Americans. Lastly, Dr. Wu will explore the policy and clinical ramifications derived from these findings, highlighting their potential to shape future approaches to gerontological care.

### SESSION 2100 (SYMPOSIUM)

Abstract citation ID: igae098.0670
### MITIGATING SOCIAL ISOLATION AND LONELINESS AMONG OLDER ADULTS
Chair: W. Quin Yow

Approximately 24% of older adults in the US experience social isolation, a condition linked to numerous physical and mental health issues, including stroke and cognitive decline. However, there is a lack of research in the assessment and intervention of social isolation and loneliness within older populations at the community level. This symposium seeks to address this gap by examining current research progress in assessment and intervention strategies for combating social isolation and loneliness among older adults. Paper 1 explores factors influencing healthcare providers' comfort levels in screening for social isolation. The author found that important factors include effective communication, interaction, and minority status. Paper 2 evaluates the community-based project "Waigaya project" in suburban Japan aimed at fostering connections among older residents. The project shows effectiveness in addressing social isolation and loneliness among suburban residents. Paper 3 presents survey findings on the impact of online connections in alleviating loneliness among older adults, emphasizing the importance of fostering quality interactions within digital communities. Finally, Paper 4 explores the use of AI-driven conversation agents to encourage social engagement among older adults to combat social isolation and loneliness. They will also present findings from a preliminary study on conversation topics among older adult friends, guiding the chatbot development. The symposium aims to enhance participants' understanding of two key areas: factors to consider and strategies for implementing community-level screening and interventions targeting social isolation and loneliness among older adults, and the feasibility of leveraging technology to address social isolation and loneliness in older adults.

Abstract citation ID: igae098.0671
### NAVIGATING SOCIAL ISOLATION SCREENING: EXPLORING HEALTH CARE PROVIDER COMFORT FACTORS
Su-I Hou, *The University of Central Florida, Orlando, Florida, United States*

**Purpose:** This study investigates factors influencing healthcare providers' comfort levels in screening older patients for social isolation (SI), a significant risk factor for negative health outcomes in this demographic.

**Methods:** A diverse sample of 59 providers from healthcare and long-term care settings participated. The mean age of providers was 46, 70% female, and 67% white, possessing an average of 17 years of practice experience. The study utilized research-tested scales measuring provider SI screening comfort level, communication about SI, provider-patient interactions (PPI), provider trust in patients (trust), and cultural sensitivity (CS), showing high internal consistencies.

**Results:** Providers generally scored high on PPI and CS, and moderately on patient trust and comfort with SI screening. Regression analyses considering all factors revealed that providers with higher scores in communication about social isolation (p=.024), patient-provider interaction (p=.022), and those identifying as minority providers (p=.025) demonstrated greater comfort in screening for social isolation (F(6,32)=3.93, p=.005), explaining a substantial 42.4% of the variance. Gender, patient trust, and cultural sensitivity did not reveal a significant impact on the comfort level associated with screening for social isolation (SI).

**Discussion:** This study suggests that effective communication and interaction, along with demographic factors like minority status, influence providers' comfort levels in screening for SI. This pilot study demonstrates the reliability of measures, emphasizing the importance of modifiable communication and interaction factors in addressing social determinants of health, such as social isolation in older adults. Further research with a larger sample is recommended.

Abstract citation ID: igae098.0672
### MITIGATING SOCIAL ISOLATION AND LONELINESS AMONG RESIDENTS IN SUBURBAN HOUSING COMPLEXES IN JAPAN
Keiko Katagiri, and Nahyun Kim, *Kobe University, Kobe, Hyogo, Japan*

Social isolation and loneliness represent significant societal challenges in Japan, particularly with the increasing number of older adults living alone. Comparative international surveys have highlighted weaker familial and neighborly ties among Japanese older adults, indicating a heightened risk of social isolation. In response, we initiated an action research, "Waigaya project" in Kobe, Japan, in 2022 to foster community connections and combat isolation among older suburban residents. The project comprises "Waigaya forums" and "Waigaya college." Forums were events which aim to engage residents and promote participation, while "Waigaya college" offered ongoing educational opportunities to enhance knowledge and cultivate resilience for individuals facing extended lifespans. Over two forums and six college sessions, 103 participants aged 3 to 92 attended, with 25.7% attending both, 35.6% exclusively attending college, and 38.6% only participating in forums. College attendance varied, with 19.4% attending 4 to 6 times, 20.9% attending 2 to 3 times, and 59.7% attending once. Post-session surveys consistently showed high participant satisfaction and meaningful peer interactions, underscoring the project's success. Notably, the project showed promising signs of identifying and nurturing potential core participants who may contribute to future community leadership, while also supporting the initial stages of developing a community network. The "Waigaya project" emerges as an effective strategy for addressing social isolation and loneliness among suburban residents in Japan, offering a promising model for similar initiatives worldwide. By prioritizing community engagement and lifelong learning, this project demonstrates the potential for meaningful impact in combatting social isolation and promoting well-being among older adults.

Abstract citation ID: igae098.0673
### THE PARADOX OF CONNECTION: MUTUAL FOLLOWING'S IMPACT ON LONELINESS AMONG OLDER ADULTS IN ONLINE NETWORKS
Nahyun Kim, and Keiko Katagiri, *Kobe University, Kobe, Hyogo, Japan*

Online communities serve as a resource for many older adults to create new connections, such as friendships and acquaintanceships that can help alleviate loneliness. Despite the many advantages, there is a lack of research on the network size in online communities tailored for older adults and its effects on loneliness reduction. This study examines mutual following on online platforms as an indicator of new connections, focusing on its relationship with the perceived formation of new connections and their subsequent effect on loneliness reduction among community members. In February 2023, we conducted an online survey within an online community for older adults in Japan, involving participants aged 60–79 (n=819). The platform provided features such as photo sharing, group participation, and user following. Data collected included group participation, mutual following, perceived establishment of new online connections, and loneliness. A path analysis revealed that a greater number of mutual followers was positively related to the perception of establishing new online connections, leading to a reduction in loneliness. However, we also found a direct positive association between mutual following and loneliness, indicating a paradoxical finding: the mere increase in mutual followers, devoid of meaningful engagement, may exacerbate loneliness. This suggests that the effectiveness of online connections in reducing loneliness is contingent upon the quality of relationships formed. These findings highlight the importance of fostering quality interactions within online communities, to enhance older adults' well-being in digital environments.

Abstract citation ID: igae098.0674
### UNDERSTANDING ENGAGEMENT OF OLDER ADULTS IN SOCIAL CONVERSATIONS TO MITIGATE SOCIAL ISOLATION AND LONELINESS
W. Quin Yow, Ka Lon Sou, Alina Clarise Wong, and Chi Shuen Ang, *Singapore University of Technology & Design, Singapore, Singapore*

Six percent of older adults in Singapore experience social isolation. Given that social isolation and loneliness are associated with cognitive decline and increased mortality rates, there is a pressing need for effective interventions to address these issues among older adults. Traditional approaches, such as in-person befriending interventions, have encountered challenges, e.g., inflexible visit schedules and transportation barriers. There are recent interests exploring how technology can help tackle social isolation and loneliness among older adults. As part of such initiative, we develop an older-friendly conversation agent (CA) utilizing state-of-the-art large language model technology. This CA aims to provide a virtual companion to older adults while prioritizing safety, autonomy, and flexibility. To our knowledge, there are very few studies, and none in Asia, that examine how social conversations among older adults can alleviate isolation and loneliness. As a first step in this investigation, an observational study was undertaken to identify typical conversation topics among older adult friends to build the CA capability. Preliminary findings from three recorded conversations, each lasting approximately 30 minutes and involving a total of eleven older adults (aged 65-79, nine females), revealed recurring topics such as "activities", "characteristics or personality of others and self", "location of place or an event", "episodic events", "life stories", and "surroundings". These insights set the baseline to test the effectiveness of the technology in reducing social isolation in older adults. How interactions with the virtual CA could alleviate social isolation and loneliness among older adults will be shared at the symposium.

### SESSION 2105 (SYMPOSIUM)

Abstract citation ID: igae098.0675

### RECONSTRUCTING PERSON-CENTEREDNESS: WORKFORCE, SYSTEMIC, AND GLOBAL PERSPECTIVES

Chair: Sam Fazio
Co-Chair: Lauren Stratton
Discussant: Sheryl Zimmerman

The concept of person-centeredness centers on knowing the person in a holistic perspective and meeting the individual's needs and preferences. The term person-centeredness has become ubiquitous in health and supportive care, and with this there are varying definitions and understandings on how to achieve person-centeredness. This symposium focuses on reconstructing person-centeredness, specifically exploring various perspectives that are imperative in reconstructing this term. First, Efird-Green will explore the conceptualization of person-centeredness through the results of a systematic review of current definitions of person-centeredness. Second, Sloane will present on the results of interviews and think tank meetings with diverse stakeholders to better understand how and why person-centeredness varies across health care settings. Third, Stratton will discuss the results of a think tank with Alzheimer's Association Dementia Care Provider Roundtable members that highlight how person-centeredness is integrated in the long-term care workforce and potential ways to improve and train employees on this concept. Lastly, Fazio will provide a global perspective through survey results from Alzheimer's Disease International members on their perspectives and experiences with quality of dementia care, definitions of person-centered dementia care, and the challenges and successes of person-centered dementia care in their countries. Overall, this symposium will provide relevant perspectives to consider in the reconstruction of person-centeredness.

Abstract citation ID: igae098.0676

### CONCEPTUALIZING PERSON-CENTEREDNESS

Lea Efird-Green[1], Sheryl Zimmerman[2], Lauren Stratton[3], Philip Sloane[4], and Sam Fazio[3], *1. University of North Carolina at Chapel Hill, Chapel Hill, North Carolina, United States*, *2. University of North Carolina at Chapel Hill (UNC), Chapel Hill, North Carolina, United States*, *3. Alzheimer's Association, Chicago, Illinois, United States*, *4. University of North Carolina, Chapel Hill, North Carolina, United States*

The concept of person-centeredness (PC) has become commonplace in health and supportive care, and perhaps for this very reason, there is no agreed upon understanding as to what constitutes "person-centeredness." To clarify the concept, a systematic review of current definitions of PC in the literature was conducted; it revealed that most papers referencing person-centered or patient-centered interventions do not define the term, and that definitions that when they do, they vary widely. Given the benefit of having a common understanding of PC, think-tank meetings were held with 65 experts in the fields of health and aging policy, research, and practice. The experts were provided numerous prompts (e.g., in the context of being person-centered, how do issues of dependency and competency enter in?); responses indicated that they conceptualize and implement the concept of PC differently at the macro, meso, and micro levels. At the macro level, there is a focus on core values, the influence of privilege and oppression, and how PC is implemented – or not – in systems and cultures. On the meso level, there is a focus on organizational responsibility, the applicability of PC to various communities, and the importance of relationships. On the micro level, PC is focused on care, including individualization and personalization, the importance of care partners/family, and the limitations of PC in individual settings. This presentation will address the three levels of PC, including each level's focus, implementation, and challenges, with the goal of establishing a multilevel conceptualization of PC.

Abstract citation ID: igae098.0677

### PERSPECTIVES ON PERSON-CENTEREDNESS ACROSS HEALTH CARE SETTINGS

Philip Sloane[1], Sheryl Zimmerman[1], Ayesha Syed[1], and Sam Fazio[2], *1. University of North Carolina, Chapel Hill, North Carolina, United States*, *2. Alzheimer's Association, Chicago, Illinois, United States*

Systems perspectives generally identify three levels of action: the interpersonal (micro) level, the institutional (meso) level, and the broader societal (macro) level. Person-centeredness is generally viewed at the interpersonal level, but especially in the context of health and supportive care, broader systems can shape and impact the quality and outcomes of these one-on-one interactions. To better understand how and why person-centeredness varies across health care settings (e.g., hospitals, nursing homes), we conducted interviews and convened think tank meetings with 31 diverse stakeholders including direct care workers, administrative staff, consumer advocates, and academic researchers focusing on care of older persons. Prompts asked examples of person-centeredness (or lack thereof) in numerous settings. The following themes were identified that help explain the wide range in person-centeredness across health care settings: 1) variation in system goals related to person-centeredness; 2) variation in systems' perceived need to limit person-centered; 3) relationships between resource availability and person-centeredness; 4) cultural values and societal inequity as root causes of much variation in person-centeredness; and 5) influence on person-centeredness of the degree to which hospitality is a core value within a given system. Understanding and responding to these themes within a given setting may more effectively help meet resident needs and preferences; it also has implications for policymaking, such as creation of training guidelines for care staff and benchmarks for program leaders.

Abstract citation ID: igae098.0678

### PERSPECTIVES OF PERSON-CENTEREDNESS ACROSS LONG-TERM CARE AND COMMUNITY-BASED SETTINGS

Lauren Stratton[1], Sheryl Zimmerman[2], Lea Efird-Green[2], and Sam Fazio[1], *1. Alzheimer's Association, Chicago, Illinois, United States*, *2. University of North Carolina at Chapel Hill, Chapel Hill, North Carolina, United States*

Within long-term care, person-centeredness is key to providing quality care to people living with dementia. However, there are discrepancies on how long-term and community-based organizations provide care that is person-centered and how it is integrated within their organizations and among staff. To better understand the concept of person-centeredness within long-term and community-based services, we conducted three focus groups with members of the Alzheimer's Association Dementia Care Provider Roundtable, which consists of leadership of long-term and community-based care organizations. Prompts asked questions on person-centeredness in relation to current organizational systems, care, and workforce. Overall, themes emerged on types of care that are delivered in a person-centered way, challenges in delivering care in a person-centered care, the integration of person-centeredness into staff training, and if person-centered is the correct term to use. Additionally, discussion included integrating technology in a more person-centered way to benefit both the staff and the person living with dementia. Understanding these themes may help to provide recommendations on how to create guidelines for training staff and integrate person-centered care with the long-term care workforce more effectively.

Abstract citation ID: igae098.0679

### TOWARD A GLOBAL DEFINITION OF PERSON-CENTERED DEMENTIA CARE

Sam Fazio[1], Walter Moczygemba[2], Lauren Stratton[1], and Sheryl Zimmerman[2], *1. Alzheimer's Association, Chicago, Illinois, United States*, *2. University of North Carolina, Chapel Hill, North Carolina, United States*

Person-centeredness is instrumental to quality dementia care, yet there remains inconsistency surrounding its definition and attempts to unify disparate conceptualizations are often based on high-income and Eurocentric perspectives. To address this, we surveyed 39 Alzheimer's Disease International members on their perspectives and experiences with quality of dementia care, definitions of person-centered dementia care, and the challenges and successes of person-centered dementia care in their countries. Overall, respondents describe a lack of global quality dementia care, naming several barriers to its successful implementation. Respondents also agreed the creation of a universal definition of person-centered dementia care is necessary for furthering high quality dementia care globally. Five core tenets representative of the geographically and economically diverse sample were identified to be included in such a definition: individualize care, value the individual, empower the individual, acknowledge the importance of caregivers, and address dimensions of wellness. Discussion will focus on recommendations for practitioners, researchers, and policy advocates on how to embody global tenets of person-centered dementia care. Additionally, internationally common barriers to person-centered dementia care are identified for further study and advocacy efforts.

### SESSION 2110 (SYMPOSIUM)

Abstract citation ID: igae098.0680

### RESOURCE CENTERS FOR MINORITY AGING RESEARCH (RCMAR) SUCCESS AND OPPORTUNITY

Chair: Carmen Sceppa

The Resource Centers for Minority Aging Research (RCMAR) have been continuously funded by the National Institute on Aging (NIA) since 1997. The RCMAR program offers intellectual leadership, mentorship, training, career development opportunities, and pilot study funding for future leaders in aging research in the behavioral and social sciences who are at an early stage in their careers. The Gerontological Society of America (GSA) – the nation's largest interdisciplinary organization devoted to the field of aging – was awarded a $3.9M cooperative agreement from the NIA (2023-2028) to run the RCMAR VI National Coordinating Center (NCC) with a multiple PI leadership team including Patricia M. D'Antonio, GSA vice president of policy and professional affairs, executive director of the National Center to Reframe Aging; Tamara A. Baker, professor, Department of Psychiatry, School of Medicine, University of North Carolina at Chapel Hill; Lisa L. Barnes, the Alla V. and Solomon Jesmer Professor of Gerontology and Geriatric Medicine and cognitive neuropsychologist, Rush Alzheimer's Disease Center; Carmen Sceppa, dean, Bouvé College of Health Sciences, professor of health sciences, Northeastern University; and James C. Appleby, GSA chief executive officer. There are 18 RCMAR Centers in the U.S., 10 focused on Alzheimer's disease (AD) and AD-related dementias (ADRD) and 8 aging-focused. The RCMAR mission is to bolster mentorship and career development of scientists from diverse backgrounds and foster rigorous behavioral and social science research to advance aging-relevant scientific discoveries, eliminate health disparities, improve the health and well-being of older adults, per NIA's Health Disparities Research Framework.

Abstract citation ID: igae098.0681

### USC ALZHEIMER'S DISEASE AND ALZHEIMER'S DISEASE-RELATED DEMENTIAS (RCMAR) CENTER: LESSONS LEARNED

Jennifer Ailshire, *University of Southern California, Los Angeles, California, United States*

The National Institute on Aging has awarded another five-year, $3.6M grant to fund the University of Southern California Alzheimer's Disease and Alzheimer's Disease-Related Dementias Resource Center for Minority Aging Research. The Center is led by the multidisciplinary team of Julie Zissimopoulos, Jennifer Ailshire, and Emma Aguila. The USC AD/ADRD RCMAR supports junior scientists exploring health and economic disparities associated with dementia. The RCMAR – housed within the USC Schaeffer Center for Health Policy & Economics – focuses on pathways by which social, behavioral, and economic factors – as well as policies and health systems – affect disparities in risk of dementia and disparities in the health, health care and economic outcomes of persons living with dementia. The center also helps launch the careers of underrepresented junior investigators, providing funding, mentorship, and tools to support their pilot research projects. The Center was established in 2012 and has supported 34 scholars from USC and 14 other institutions. Alumni of the program have received more than $11.2 million in research funding and have published more than 268 peer-reviewed articles. Many scientists have received academic promotions and met career goals. The Center has an extensive network of supporters and collaborators including Howard University, Spelman College, Cal State Fullerton and other NIA-funded centers at USC including: Roybal Center for Behavioral Interventions of Aging, Alzheimer Disease Research Center, Center for Economic and Sociodemographic Study of AD/ADRD, and USC/UCLA Center on Biodemography and Population Health. The Center also supports the annual Science of AD/ADRD for Social Scientists.

Abstract citation ID: igae098.0682

### MENTORED-BASED AGING RESEARCH IN THE BEHAVIORAL AND SOCIAL SCIENCES: RCMAR VI NCC, AN INFRASTRUCTURE FOR SUCCESS

Lisa Barnes, *Rush University Medical Center, Chicago, Illinois, United States*

The RCMAR VI NCC is led by a diverse, multidisciplinary MPI team, with the purpose to develop a robust infrastructure and community that will grow and diversify the research workforce in the behavioral and social sciences to address minority aging, AD/ADRD, and/or health disparities among older adults. This goal will be achieved through the following strategies. 1) Provide support and interaction among RCMAR Centers via an interactive website, robust community-wide online networking platform, tools to enhance collaboration, and resources supporting the creation of new scholarship. 2) Leverage existing and develop new evidence-based tools to provide early career scientists and mentors from diverse backgrounds with skills and technical support necessary to advance their professional growth, foster culturally aware and responsive mentorship and career development. 3) Catalyze resource sharing between and among the RCMAR Centers and cultivate sustainable engagement with key stakeholders including Minority serving institutions; Historically Black Colleges and Universities, Tribal Colleges and Universities, Hispanic Serving Institutions and other academic institutions serving predominately racially minoritized students and groups, all NIA research Centers, the aging research community, and the public. 4) Foster reverence for the value of Diversity, Equity, Inclusion and Anti-Racism (DEIA) in the aging research enterprise by broadly elevating and disseminating the activities and findings of the Centers and their scientists in academic and public venues. 5) Establish consensus on essential common evaluation metrics and implement a streamlined process for collecting data to support the evaluation of RCMAR Center activities, including research, collaboration, mentoring, networking, stakeholder engagement, and DEIA.

Abstract citation ID: igae098.0683

### EXPERIENCES FROM A RCMAR SCIENTIST (ALUMNA): A MEANINGFUL AND REWARDING JOURNEY

Tamara Baker, *University of North Carolina, Chapel Hill, Chapel Hill, North Carolina, United States*

RCMAR was established to develop a research and mentoring infrastructure that fosters rigorous behavioral and social science research on aging in a high priority research area that can advance aging-relevant scientific discoveries, lead to the elimination of health disparities and health inequities, and improve the health and well-being of older adults. The primary objectives of RCMAR are 1) career development, which aims to support the career development of early-career scientists conducting social, behavioral, psychological, and economic research related to aging, and 2) advancement of scientific discoveries related to aging. Scientists participating in this program are afforded opportunities to establish a successful program of research; personalized guidance through professional development activities, helping scientists develop skills, knowledge, and confidence in the field; networking opportunities, expanding their professional network and potential career opportunities; personal growth; feedback and advice, helping scientists improve performance and make informed decisions; career advancement, receiving guidance on strategies for success; and knowledge transfer, fostering continuity and innovation in the field. These opportunities are recognized in developing some of the most established scholars in the discipline. Dr. Baker, a former RCMAR scientist will reflect on how her participation as a RCMAR scientist has allowed her to build a successful research career on the intersection of social determinants and chronic pain in older adults; be an advocate in diversifying the workforce; and become a leader in the gerontology discipline. Highlighting these successes is intended to broaden the outreach of program while encouraging others to consider this opportunity.

### SESSION 2115 (SYMPOSIUM)

Abstract citation ID: igae098.0684

### SCOPING REVIEWS: A METHODOLOGY TO UNDERSTAND HEALTHY AGING AND IDENTIFY NEW AREAS FOR RESEARCH

Chair: Basia Belza

Scoping reviews is an approach to synthesize evidence. They are used to categorize literature in a field in terms of its nature, features, and volume. The purpose of this symposium is to provide an overview of how to conduct a scoping review and highlight examples of recent scoping reviews. The first presentation builds on the social determinants of health report by World Health Organization (WHO) and defines and categorizes approaches that have been implemented to achieve health equity among older adults. The second presentation is on social frailty which results from a lack of social resources and support and poses a significant threat to healthy aging by increasing the risk of adverse health outcomes. Creatively using a person-environment-occupation model, the third presentation categorizes the effectiveness of interventions into mealtime for people living with dementia who reside in care communities. Building on the growing body of literature that links benefits of music to health outcomes, the fourth presentation focuses on a review of the terminology used for music-based interventions. The fifth presentation focuses on identifying the outcomes of community-based physical activity programs for people with dementia, a group that frequently is missing from our study populations. The symposium will conclude with recommendations for areas that need improvement related to scoping reviews: 1) engaging stakeholders earlier and more often with scoping reviews to facilitate interpretation of results and effective knowledge translation, and 2) identifying ways to use the scoping review results as a decision-making tool.

Abstract citation ID: igae098.0685

### A SCOPING REVIEW: INNOVATIVE APPROACHES TO ACHIEVE HEALTH EQUITY FOR OLDER ADULTS

Meshack Otewa[1], and Priscilla Carmiol-Rodriguez[2], *1. University of Washington, Seattle, Washington, United States*, *2. University of Washington, Washington, United States*

The purpose of this scoping review was to comprehensively examine innovative approaches proposed or implemented to achieve health equity among older adults. Health equity, defined as ensuring optimal health for all, regardless of various determinants, remains elusive among older adults, a population experiencing significant growth and associated health challenges. Following PRISMA-ScR guidelines, the review searched PubMed, CINAHL, and Embase for original research articles from 2013 to 2023, yielding 47 eligible articles. Findings reveal a range of innovative approaches, defined as new care models improving outcomes and value for health equity. Health disparities are escalating, particularly in chronic conditions like cancer, mental health, HIV-AIDS, pain, and nutritional issues among older adults. Approaches identified include leveraging digital health technology, ensuring access to health insurance (e.g., US Medicaid) with a focus on non-discriminatory access for individuals who identify as LGBTQI, fostering age-friendly health systems, conducting health economics research, expanding public health initiatives for older adults, integrating cultural considerations into health practices, and employing a multimodal approach to translating research into policy and practice. The need for standardized categories for these approaches emphasizes the need for systematic effectiveness assessment. The results of this scoping review suggest that while innovative approaches may be novel, incorporating new values into existing systems is crucial. As health disparities persist and expand, the timely innovation of techniques becomes critical to narrowing the gap and achieving health equity among older adults. The identified methods are a valuable foundation for future program development and system reformulation.

Abstract citation ID: igae098.0686

### SOCIAL FRAILTY AMONG COMMUNITY-DWELLING OLDER ADULTS: A SCOPING REVIEW

Priscilla Carmiol-Rodriguez[1], and Meshack Otewa[2], *1. University of Washington, Washington, United States*, *2. University of Washington, Seattle, Washington, United States*

The aim of this review is to explore existing evidence concerning social frailty among older adults living in communities, encompassing definitions, protective and risk factors, health outcomes, and interventions. Social frailty, characterized by the lack of social resources and support, intersects with physical and psychological frailty, posing a substantial hazard to successful aging and escalating the likelihood of adverse health consequences. Following PRISMA-ScR guidelines, a systematic search was conducted in MEDLINE, CINAHL, and Web of Science. Inclusion criteria encompassed full-length articles from database journals published in English from October 2016 to July 2023, focusing on community-dwelling adults aged 65 and above, primarily emphasizing social frailty or its interaction with other frailty dimensions. Editorials, gray literature, and white papers were excluded. Thirty-two articles were identified, predominantly from China, Japan, and South Korea. Bunt and Makizako´s definitions (2017; 2018) of social frailty were mainly used in the papers. Social frailty is preceded by conditions such as hearing impairment, metabolic risk factors, and heart disease. It was correlated with depressive symptoms, disability, reduced life satisfaction, and heightened risk of Alzheimer's disease and physical frailty. Interventions targeting exercise, digital literacy, and technology-assisted screening were proposed to alleviate social frailty's impact. Addressing social frailty is crucial for enhancing the well-being of older adults in communities, as it predicts a spectrum of adverse health outcomes, impacting both the physical and psychological dimensions of their lives. Nonetheless, further research is vital to refine interventions for preventing and reversing social frailty in this demographic.

Abstract citation ID: igae098.0687

### INTERVENTIONS DURING MEALTIME FOR PEOPLE LIVING WITH DEMENTIA: A SCOPING REVIEW

Zih-Ling Wang, and Basia Belza, *University of Washington, Seattle, Washington, United States*

Mealtime challenges among residents living with dementia in long-term care settings are a common problem. This study presents findings from a scoping review of mealtime interventions for individuals with dementia in long-term care settings. Using a Person-Environment-Occupation (PEO) Model, we categorize the effectiveness of all interventions to explore the holistic interplay of individual abilities, environmental conditions, and occupational sections in enhancing mealtime experiences for these individuals. From a total of 2,786 references identified through a three-step search strategy, 45 articles met the inclusion criteria, focusing on "eating," "feeding," "dementia," and "mealtime" interventions from 2008 to 2023. The review highlights dementia-related eating challenges, such as using utensils improperly and consuming non-food items stemming from cognitive decline. Interventions span aromatherapy, music, visual and auditory aids, spaced retrieval training, the Montessori method, physical improvement programs, food service modifications, and individual assistance, targeting interpersonal, intrapersonal, and environmental transactions of eating. The PEO model categorizes these findings into person (behavioral interventions), environment (dining room layout and social interactions), and occupation (activities like the Montessori method and physical exercises). However, few interventions comprehensively address all three PEO components. This review provides insights for healthcare professionals, educators, and researchers on improving eating performance and independence in dementia care, emphasizing the need for future research to establish evidence-based protocols and implement them in care settings.

Abstract citation ID: igae098.0688

### PHYSICAL ACTIVITY INTERVENTIONS FOR PEOPLE LIVING WITH DEMENTIA: A SCOPING REVIEW

Sarah McKiddy, *University of Washington, Seattle, Washington, United States*

Dementia poses significant challenges globally, necessitating effective interventions to mitigate its impact on individuals and societies. Physical activity has emerged as a promising avenue for enhancing physical and cognitive function among people living with dementia. This scoping review aimed to evaluate the characteristics and outcomes of various physical activity interventions in this population including articles from Australia, Belgium, Germany, New Zealand, Sweden, the United States, and Japan, reflecting a global effort to address the complexities of considerations for physical activity interventions for dementia. Predominantly utilizing randomized control trials (RCTs), the studies explored interventions ranging from strength and balancing exercises to multicomponent training, targeting individuals aged 50 and older diagnosed with Alzheimer's disease, other forms of dementia, or mild cognitive impairment. Notably, interventions primarily involved in-person sessions, with a few incorporating remote platforms, emphasizing the importance of accessibility and flexibility in delivering interventions to diverse populations. Results indicated improvements in physical function, cognitive performance, and quality of life, suggesting the potential of physical activity as a multifaceted intervention for dementia management. Despite methodological variations and limitations observed across studies, the findings highlighted the promising role of physical activity in enhancing the well-being of individuals living with dementia. Future research should focus on addressing methodological inconsistencies, expanding diversity in study populations, and exploring optimal intervention strategies to maximize the benefits of physical activity in dementia care.

Abstract citation ID: igae098.0689

### MUSIC-BASED INTERVENTION TERMINOLOGY IN ALZHEIMER'S DISEASE AND RELATED DEMENTIAS STUDIES

Sarah McKiddy, *University of Washington, Seattle, Washington, United States*

The surge in interest surrounding music-based interventions (MBIs) for cognitive health, particularly in Alzheimer's disease and Alzheimer's disease related dementias (AD/ADRD), has catalyzed interdisciplinary research. However, the collaborative nature of these efforts has led to discrepancies in language usage, hindering the design and interpretation of interventions. In a systematic review conducted by Robb et al. (2018) on the quality of reporting MBI research in healthcare, discordant terminology was identified as an overarching, ongoing barrier to MBI research. The primary aims of this scoping review were to: (1) identify and define the terminology employed in empirical studies describing MBIs within the context of Robb et al.'s (2011) Guidelines for MBIs and studies involving AD/ADRD, (2) elucidate how these terms were operationalized, and (3) propose implications for future research and practice. Included studies showcased a diverse array of MBI strategies in AD/ADRD research, ranging from music therapy practices to social prescribing. However, inconsistencies in terminology usage, such as interchangeably employing terms like "music therapy" and "music intervention," were observed. This review supports the pressing need for unified terminology that translates despite interdisciplinary boundaries and specialties, facilitating collaboration and advancing both research and practice in MBIs for AD/ADRD. To enhance the clarity of the literature and foster rigor in methodologies, researchers are encouraged to consistently establish and apply clear, operationalized definitions for terminology while also referencing established concepts and seeking consensus within the MBI community.

### SESSION 2120 (PAPER)

### SOCIOECONOMIC AND GEOGRAPHIC DISPARITIES IN COGNITION AND DEMENTIA

Abstract citation ID: igae098.0690

### TRENDS AND SOCIOECONOMIC INEQUALITIES IN DEMENTIA RISK IN CHINA, 2011–2020

Liying Luo, and Liyun Liu, *The Pennsylvania State University, University Park, Pennsylvania, United States*

China faces a rapidly aging population. However, there lacks evidence about if the prevalence of dementia risk increased over the past decade and what biopsychosocial factors may have contributed to the trends. Using the 2011-2020 data from the China Health and Retirement Longitudinal Study (CHARLS), this study finds the first evidence of significant decreases in the prevalence of high dementia risk—determined as scoring 4 or lower on 25 items of the Telephone Interview for Cognitive Status—in China between 2011 and 2020. The decrease was more pronounced among older Chinese women. We then use the Kitagawa-Oaxaca-Blinder method to quantify the contributions of a set of socioeconomic and health variables to the differences in the prevalence of high dementia risk between the periods 2011-2013 and 2018-2020. The decomposition results suggest that for both Chinese men and women, the compositional reductions in illiteracy and low education are a major driver of the decline in dementia risk between the two time points. Changes in other socioeconomic and health risk factors play a relatively smaller role, although for males, the heightened risk of dementia due to smoking exerts an upward pressure on dementia risk trends. Our findings extend the literature by providing evidence that increasing education is an effective strategy for reducing dementia risk at the population level.

Abstract citation ID: igae098.0691

### GEOGRAPHIC DISPARITIES IN ALZHEIMER'S DISEASE AND RELATED DEMENTIAS USING BAYESIAN MULTISTATE LIFE TABLE METHODS

Jason Wong, and Emma Zang, *Yale University, New Haven, Connecticut, United States*

Research has documented persistent regional disparities in the risk of Alzheimer's disease and related dementias (ADRD) in the United States based on individuals' current residence. However, it remains unclear how childhood region and current region jointly shape later ADRD outcomes. This obscurity conceals significant variations among older adults who reside in the same current region. Using data from the 1998-2020 waves of the Health and Retirement Study, we employ an innovative Bayesian multistate life table method to estimate how ADRD-related life expectancies vary across combinations of birth regions and current regions. Our results show that ADRD-related life expectancies are more strongly associated with regions of birth than with current regions of residence. Especially, at age 50, individuals born in the South, regardless of current residence, were expected to have shorter years (Men: 13.8-15.2 years; Women: 17.3-18.4 years) of life living without ADRD and longer years (Men: 2.7-3.0 years; Women: 3.6-3.9 years) of life with dementia, compared to those born in other regions. Our findings offer new evidence of the Southern disadvantage of ADRD by demonstrating that growing up in the South during the critical period of childhood may have long-lasting impacts on cognitive functioning later in life.

Abstract citation ID: igae098.0692

### STATE POLICY AND POLITICAL LANDSCAPES: IMPLICATIONS FOR COGNITIVE AND FUNCTIONAL HEALTH

Marc Garcia[1], Blakelee Kemp[2], and Wassim Tarraf[3], *1. Syracuse University, Syracuse, New York, United States*, *2. University of Nebraska Lincoln, Lincoln, Nebraska, United States*, *3. Wayne State University, Detroit, Michigan, United States*

There is considerable state-level variation in the percent of adults with cognitive and functional limitations. However, it is unclear how state policy contexts and changes in these contexts are associated with cognitive and functional health. We used BRFSS data (2015/16 and 2019/20) linked to the State Policy & Politics Data (SPPD) from the Center for Aging and Policy Studies (CAPS) at Syracuse University to examine how overall political ideology, Medicaid generosity, and three health behavior (Tobacco excise taxes, opioid laws, and clean-air indoor laws) policies and change in those policies over time (2000-2014) are linked to levels of perceived cognitive decline and functional limitations among individuals 45-years and older. We found considerable variability in both levels of perceived cognitive decline and functional limitations, as well as policy adoption and change in adoptions over time across states. Adjusting for individual-level sociodemographic indicators and change in state ideology/policy over time, higher levels of political liberalism, Medicaid generosity, Tobacco taxes, and more restrictive clean indoor air policies in 2014 were largely associated with lower levels of perceived cognitive decline and lower levels of functional limitations. However, greater change in political ideological and policy measures was inconsistently linked to higher prevalence of the considered impairment outcomes. As the U.S. ages and the landscape of state policies continues to diversify, policymakers are increasingly urged to conscientiously consider the cognitive and functional health implications associated with these varied policy environments.

Abstract citation ID: igae098.0693

### HOW DO PLACES OF RESIDENCE OVER THE LIFE COURSE SHAPE THE DYNAMICS OF HEALTH DISPARITIES IN LATER LIFE?

Zhuoer Lin[1], and Xi Chen[2], *1. University of Illinois Chicago, Chicago, Illinois, United States*, *2. Yale University, New Haven, Connecticut, United States*

**Background:** Significant geographical disparities persist in cognitive and health outcomes. According to life course theory, these disparities may stem from a history of place-based exposures throughout lifespan. However, due to data limitations, no study has explored how residential history impacts later-life health inequalities.

**Methods:** We addressed this gap by constructing an exceptionally comprehensive residential history dataset. Drawing from the Health and Retirement Study, we identified state-level places of residence for each individual from birth to age 60, and subsequently linked this information to a broad range of cognitive and health outcomes in later life (N=10,124). Employing regression-based Shapley value decomposition, we quantified the contribution of place of residence at each age to cognitive and health outcomes, thereby delineating the trajectories of place effects on cognition and health.

**Results:** Our analysis revealed distinct contributions of place of residence at various ages, particularly in relation to cognitive health compared to other health outcomes. Notably, place of residence exerted the most significant influence on cognition at birth, with this contribution decreasing linearly and rapidly during the initial 20 years of life (from age 0 to age 20), before stabilizing during adulthood. In contrast, we observed an overall upward trend in the contribution of place of residence to other health outcomes such as disability and depression.

**Discussion:** Our findings underscore the dynamic nature of the contribution of place of residence to health inequalities over the life course, emphasizing substantial disparities in the effects of location on cognitive health compared to other health outcomes.

Abstract citation ID: igae098.0694

### ADDRESSING THE EDUCATIONAL DISPARITIES IN COGNITIVE FUNCTION: THE COMPENSATION EFFECTS OF SOCIAL NETWORKS

Siyun Peng[1], and Brea Perry[2], *1. Indiana University Bloomington, Bloomington, Indiana, United States*, *2. Indiana University, Bloomington, Indiana, United States*

Educational disparities have been widely considered a key driver of disparities in dementias and cognitive aging. In this study, we ask: Is it too late for less educated older adults to engage in cognitively stimulating environments (e.g., bridging social capital in networks) to shorten their cognitive gaps with more educated older adults? Bridging social capital is defined as access to novel information and is thought to be a source of cognitive stimulation that may confer resilience to cognitive decline. Using rich cognitive assessments and egocentric network data from the state representative Person to Person Health Interview Study (P2P; N=685), we find that the associations between bridging social capital and cognitive outcomes are strongest for people with less than a high school education. Importantly, the cognitive gaps among people with different educational levels disappear among those with higher bridging social capital. One important implication of this study is that stimulating environments in later life (e.g., bridging social capital) is most effective for the population with lower distal stimulating environments (e.g., people without a high school diploma). Finally, the relationship between bridging social capital and cognitive health is discussed in the context of cumulative stimulating environments across the life course.

### SESSION 2125 (SYMPOSIUM)

Abstract citation ID: igae098.0695

### SUPPORTING FAMILY CAREGIVING IN MULTICULTURAL CONTEXTS

Chair: Weichao Yuwen
Co-Chair: Maggie Ramirez
Discussant: Serena Jinchen Xie

There are 53 million family caregivers providing an estimated $470 billion in unpaid care services in the U.S. annually. Over 40% of them self-identify as having a racial or ethnic minority background, and at least one-third speak a language other than English at home. Caregivers from racial and ethnic minority groups experience significantly more physical and emotional stress than their white counterparts. This session focuses on supporting family caregiving in multicultural contexts. Berridge will discuss cultural values as they relate to technology intervention considerations for caregivers of older adults. Li and Liang will discuss a study exploring the needs of Chinese American families caring for older adults and their perceptions of using technologies such as chatbots. Specifically, Li will discuss results from four focus groups with community-based organizations' staff supporting these families, and Liang will discuss results from family caregivers. Cardoso-Arias will discuss structural barriers to accessing supportive services among Latino family caregivers. Finally, Aguilar will describe a conceptual model of family caregiving and health equity. To conclude, our discussant Ms. Xie will discuss cross-cutting implications across studies and offer insights on how to incorporate cultural considerations in developing technologies to support family caregivers.

Abstract citation ID: igae098.0696

### CULTURAL CONTEXTS AND BEYOND: THE NEED FOR VALUE SENSITIVE DESIGN IN TECHNOLOGIES TO SUPPORT CARE

Clara Berridge, *University of Washington, Seattle, Washington, United States*

It has long been understood in fields such as science and technology studies that technologies and data are not value-neutral, but digital technology interventions to support care partners of older adults are often treated as such. Understanding that values are cultural commitments that are not universally prioritized is critical for effective and nonharmful interventions. This presentation will describe ways in which values, assumptions, and priorities are embedded in digital interventions, drawing on findings from multiple studies of families' experiences with care technologies. The importance of attention to norms, values, and expectations in care relationships, as well as contextual factors such as trauma history and intergenerational family cultural conflict will be discussed. Practical questions will be offered to guide the broader application of Value Sensitive Design in technology design and research to make all stakeholders' values explicit, understand value tensions, and center and operationalize the values and needs of intended users.

Abstract citation ID: igae098.0697

### STRUCTURAL BARRIERS TO UTILIZATION OF SUPPORTIVE SERVICES AMONG LATINO CAREGIVERS OF PEOPLE LIVING WITH DEMENTIA

Rafael Cardoso-Arias, and Maggie Ramirez, *University of Washington, Seattle, Washington, United States*

Underutilization of supportive healthcare and social services may contribute to Latino caregivers' higher risk of experiencing adverse health outcomes. The study objective was to understand structural barriers to utilization of supportive services among Latino caregivers of people living with dementia (PLWD). In Washington State, we interviewed 30 Latino caregivers of PLWD and 14 healthcare and social service providers. We applied deductive coding to the interview transcripts—relying on predefined codes representing the Cultural Treatment Adaptation Framework. We then examined excerpts coded under "Treatment Access/Entry." Lastly, we inductively derived themes representing structural barriers to Latino caregivers' utilization of supportive services. We identified structural barriers within the following three themes: 1) ethnic and language discordance between providers and Latino caregivers, 2) limited coordination between health systems and the broader network of providers in the community, and 3) digital health inequities.These interconnected themes underscore the importance of culturally competent, linguistically accessible care and technologically innovative approaches for Latino families. Healthcare systems often adhere to standards of care primarily designed for a majority population, which may fall short in meeting the unique needs and preferences of Latino communities and lead to disparities in access to and utilization of supportive services. Overall, the study findings can inform decision makers to adapt and adopt targeted interventions, policies, and practices, to increase and improve utilization of supportive services for Latino PLWD and their family caregivers.

Abstract citation ID: igae098.0698

### PROMOTING DIGITAL HEALTH EQUITY THROUGH COLLABORATIVE PARTNERSHIPS WITH CHINESE AMERICAN COMMUNITIES

Jingyi Li[1], Jinchen Xie[2], Yanjing Liang[2], Jingyi Dong[3], and Weichao Yuwen[1], *1. University of Washington Tacoma, Tacoma, Washington, United States*, 2. *University of Washington, Seattle, Washington, United States*, 3. *Seattle Pacific University, Seattle, Washington, United States*

Despite overwhelming and disparate needs, the use of supportive healthcare programs is lower among Chinese American caregivers, in large part due to the lack of accessible and culturally and linguistically appropriate care. Digital health tools have the potential to support caregivers' needs. In collaboration with community partners, we conducted a qualitative study to identify opportunities using digital health tools to address caregiving needs among families caring for Chinese American older adults. Four focus groups with 21 staff from four local community-based organizations serving Chinese American families were conducted to explore staff's experience supporting Chinese American families and discuss their perspectives on opportunities to use digital health tools to support caregiving needs. Inductive thematic analysis revealed that digital health tools have the potential to support Chinese American families caring for older adults by (1) offering real-time and trustworthy informational support on caregiving, (2) guiding caregivers through system navigation and service discovery, (3) sharing caregiving stories for scenario-based learning, (4) suggesting communication strategies for adult children caregivers, and (5) enabling family check-ins before or between case manager consultations. Additionally, staff raised concerns regarding digital health tools' capacity to offer culturally sensitive emotional support, alongside skepticism about its potential to substitute valuable human social interactions. The findings from this study lay the foundation for future research on culturally adapting digital health tools for promoting the mental health and well-being of Chinese American family caregivers.

Abstract citation ID: igae098.0699

### RECOGNIZING CAREGIVING NEEDS OF CHINESE AMERICAN OLDER ADULTS AND EXPLORING CHATBOTS UTILIZATION

Yanjing Liang[1], Serena Jinchen Xie[2], Jingyi Li[3], Jingjing Xu[4], and Weichao Yuwen[3], *1. University of Washington, Seattle, Washington, United States*, 2. *University of Washington Medicine, Seattle, Washington, United States*, 3. *University of Washington Tacoma, Tacoma, Washington, United States*, 4. *The First Affiliated Hospital of Nanjing Medical University, Nanjing, Jiangsu, China (People's Republic)*

Asian Americans encounter unique challenges in caring for older adult family members due to language barriers, cultural preferences, and difficulty in accessing healthcare resources. Family caregivers of Chinese-speaking older adults in the U.S. reported high levels of emotional stress more often compared to English-speaking caregivers. Digital health tools such as chatbots have the potential to offer affordable and accessible support. This qualitative study examined data from 12 in-depth interviews of families caring for Chinese American older adults and provided insights into caregiving challenges, self-care practices, cultural values, and perceptions of using chatbots for support. Participants faced challenges, including barriers to accessing resources due to language, limited understanding of social support, and stigma. The high-power distance of Chinese culture (Hofstede cultural value theory) alongside 'familism' and 'filial piety' exacerbated challenges in intergenerational communication. We discovered that the psychological aspect of self-care among Chinese American caregivers is often undervalued, influenced by the culture of self-restraint and endurance. Participants anticipated chatbots providing life-like support and comprehensive caregiving resources by integrating functions such as health information guides. While caregivers appreciated chatbots as a judgment-free channel for seeking help, they had reservations about chatbots' capacity to comprehend users' emotions and embody cultural nuances such as sayings and references, which would make chatbots relatable and encourage engagement. The effectiveness of chatbots may be limited by constraints in empathy and cultural sensitivity. The findings reveal both the promise and challenges of developing culturally sensitive chatbots to address emotional needs and offer comprehensive support.

Abstract citation ID: igae098.0700

### FAMILY HEALTH EQUITY CONCEPTUAL MODEL

Genevieve Aguilar, *University of Washington, Seattle, Washington, United States*

Family Health Equity applies to the family unit when engaging in health care. The family (chosen and biological) unit is often the center of support for Black, Indigenous, and People of Color (BIPOC) communities. I am passionate about fully incorporating the patient and family into the care plan. This is especially true if there are barriers to care. Growing up along the US-Mexico border, I saw many barriers to care for my family. I propose this conceptual model of Family Health Equity. In this model, the patient has an equal voice and power to the caregiver and the provider. Other Health Equity models do not put the patient and caregiver equally at the center. This model starts with nursing driving the change and builds from there. The Family Health Equity conceptual model includes communication as a key element. This means including health literacy, interpretation, translation, identity, and cultural consideration in communication practices that can improve health equity for BIPOC communities. Any statements made by the patients or caregivers should be treated with respect and as truth. The outcome of these practices is an increased level of trust, which can lead to improved care. Studies suggest that patients have improved communication and experience fewer health disparities with racially concordant healthcare providers. The Family Health Equity conceptual model can be utilized to examine issues in family theory, aging, and clinical practice. It provides a blueprint for institutions to address equity in their environments.

### SESSION 2130 (SYMPOSIUM)

Abstract citation ID: igae098.0701

### TECHNOLOGY AND TRADITIONAL TOOLS FOR INTERGENERATIONAL CONNECTIONS IN HIGHER EDUCATION

Chair: Janelle Fassi
Co-Chair: Elizabeth Rickenbach
Discussant: Tina Newsham

While the benefits of connecting older adults with college-aged students are well-documented in literature, the practical application of connecting these individuals together in intergenerational learning spaces is more challenging. This session focuses on the challenges and strategies for successfully implementing and sustaining intergenerational connections in higher education using technology or traditional tools like board games. Each presentation outlines an innovative way for connecting college-aged students with older adults both inside and outside the classroom. The first presentation focuses on the factors that impact the implementation and sustainability of the Cyber-Seniors program through an implementation science framework. The second presentation describes the use of a waitlist control design for examining and identifying the strengths of the Cyber-Seniors Program. The third presentation identifies the feasibility and reflections from an in-person intergenerational game collaboration with Saint Anselm College undergraduate students and community partners. The fourth presentation examines the benefits and challenges of Tellegacy, an intergenerational story-telling project facilitated by university students via in-person, phone or virtual one-on-one visits with older adults in retirement communities. Aligning with the Annual Scientific Meeting 2024 theme, these presentations demonstrate fortitude in refining processes to ensure we are building optimal experiences to connect students and older adults.

Abstract citation ID: igae098.0702

### GAMES ARE FOR EVERYONE: A PILOT PROJECT TO FACILITATE INTERGENERATIONAL ENGAGEMENT THROUGH GAMES

Elizabeth Rickenbach, Loretta Brady, Adeline Clifford, and Seana Gamache, *Saint Anselm College, Manchester, New Hampshire, United States*

Undergraduate students and faculty participated in a pilot project to facilitate intergenerational engagement with older adults in the community through games. Each age group shared “their generation’s” games and learned games together by playing new games curated by a game designer that are specific to an intergenerational audience. Previous research has demonstrated that intergenerational activities are beneficial to physical and mental health, mood, age-related biases, and reduced loneliness. Intergenerational activities that involve games can add an element of play, collaboration, and cognitive enrichment across all participants that may extend the benefits across all participants and encourage co-learning. Intergenerational research has often included life-interviews (older teaching younger) and when games are considered, they are often video games (younger teaching older); this project is novel in that it includes socialization, play, and cognitive engagement with traditional games with an “equal playing field” (both younger and older adults will teach and learn games). The specific purpose of the project was to demonstrate the feasibility of a facilitated game collaboration as an intergenerational activity through participant feedback. Results showed that the experience provided collaboration, joy, learning, and an opportunity for facilitated exposure to challenging topics (e.g., political discourse across generations, sharing life stories during play, expressing grief and loss during or prompted by play, experiencing frustration and satisfaction due to play experiences). Students reported that the games provide an opportunity for engagement with older adults that felt comfortable, and at times, the experience was “more about the conversation, and less about the games.”

Abstract citation ID: igae098.0703

### APPLYING THE DYNAMIC SUSTAINABILITY FRAMEWORK TO AN INTERGENERATIONAL TECHNOLOGY PROGRAM IN HIGHER EDUCATION

Jill Juris[1], Rachel Scrivano[2], Skye Leedahl[3], Meaghan Colvin[3], Josie Santilli[3], and Shannon Jarrott[4], *1. Appalachian State University, Boone, North Carolina, United States*, *2. Boston College, Chestnut Hill, Massachusetts, United States*, *3. University of Rhode Island, Kingston, Rhode Island, United States*, *4. The Ohio State University, Columbus, Ohio, United States*

Cyber-Seniors (CS) is an intergenerational technology program developed to increase older adults’ digital competence and adoption through reverse-mentoring facilitated by trained students. Leveraging higher education as a platform offers mutual benefits, fostering positive attitudes toward aging among students while enhancing technology literacy among older adults. CS often operates within community settings with volunteers and high school students; however, systematic assessment within higher education remains unexplored. This study examined the determinants of sustainability associated with implementing CS in higher education through the Dynamic Sustainability Framework. Through semi structured interviews conducted between April and October 2023, we explored challenges and facilitators employed by ten partnered higher education institutions and two key CS representatives. Data were analyzed by two coders using directed qualitative content analysis in Atlas.ti. Three themes characterized higher education and CS representative experiences implementing CS: adapting the intervention for higher education, navigating the role of resources, and engaging university and community buy-in. The COVID-19 pandemic introduced unique challenges to CS implementation. This study presents the first comprehensive evaluation of CS in higher education, highlighting the necessity of tailored approaches and identifying barriers to sustainability. Results suggest the need for a structured implementation checklist to optimize program delivery within higher education.

Abstract citation ID: igae098.0704

### OUTCOMES FOR AN INTERGENERATIONAL TECHNOLOGY PROGRAM: A PRE–POST WITH WAITLIST CONTROL STUDY

Meaghan Colvin, Skye Leedahl, Cindy Tsotsoros, Melanie Brasher, Gina Barbera, and Josie Santilli, *University of Rhode Island, Kingston, Rhode Island, United States*

The quickly growing population of older adults in the United States combined with an increasingly digital society post-pandemic has illuminated the digital divide in recent years. The digital divide contributes to social isolation and therefore decreased quality of life in older adults, and intergenerational programming has demonstrated potential to bridge this equity gap and keep older adults involved and productive in their communities and larger society. To expand the literature and enhance the rigor of the research design in intergenerational programming, this study utilized a waitlist control design to strengthen outcomes from the University of Rhode Island Engaging Generations Cyber-Seniors Program on measures of technology use, digital competence, loneliness, social isolation, and quality of life. For this design, data was gathered at time 1 (6 weeks before the program), time 2 (2 weeks before the program), and time 3 (after participation in the program). Repeated measures ANOVA was used to examine changes across time points. Implementing a waitlist control design enabled us to examine natural changes that occurred in the measures compared to after program participation. Results showed that the program likely impacted tablet use, digital competence, and social isolation but that loneliness was impacted due to pre-program activities, such as the interview. These findings strengthen the previous research in understanding the specific aspects of social well-being that the program influences. This methodology was a practical way to utilize an already existing waitlist to enhance the rigor of evaluation research methods used.

Abstract citation ID: igae098.0705

### INTERGENERATIONAL HEALTH PROMOTION PROJECT TO REDUCE OLDER ADULT SOCIAL ISOLATION AND LONELINESS: A FEASIBILITY STUDY

Jeremy Holloway, *New Mexico Highlands University, Fargo, North Dakota, United States*

Emerging research demonstrates that social isolation and loneliness are linked to significant physical and mental health conditions. To address these concerns, the Tellegacy program was developed as an intergenerational health-promoting intervention to ameliorate older adult social isolation and loneliness in an effort to increase wellness. The purpose of this study was to reflect on testing of the Tellegacy program as a behavioral intervention. University students trained in goal setting, mindfulness, and listening strategies were paired with 11 older adults in the northern Midwest area via weekly in-person and phone conversations. Oral reminiscence therapies were used and books containing their stories were given to the older adults after participation. Older adults were surveyed using the University of California Los Angeles Loneliness Scale, Satisfaction of Life Scale, and patient health questionnaire-9 (PHQ-9) scale to elucidate the effectiveness of the intervention. Improved scores in loneliness, satisfaction of life, and PHQ-9 demonstrated favorable improvements in older adults. Additional benefits for the student Legacy Builder were revealed from self-reported changes. This suggests the potential benefits of structured encounters between trained students and isolated or lonely older adults. The Tellegacy intergenerational feasibility program warrants further studies to fully demonstrate its impact on health outcomes.

### SESSION 2135 (SYMPOSIUM)

Abstract citation ID: igae098.0706

### VIRTUAL IMPLEMENTATION OF MOBILITY PROGRAMS IN MEDICALLY COMPLEX POPULATIONS

Chair: Alexander Garbin
Co-Chair: Jennifer Stevens-Lapsley

Utilization of virtual platforms (e.g., Zoom, Teams, VA Video Connect) has rapidly expanded in both clinical and research settings since the onset of the COVID-19 pandemic. Within research, the use of virtual platforms affords numerous advantages including access to a broader geographical range of older adults, lack of dependency on transportation, and ability to monitor fidelity remotely. However, there also exist numerous considerations to ensure success when using virtual platforms as part of a research study. This symposium will detail four research projects utilizing virtual platforms. The research projects discussed share the overarching theme of improving mobility in older adults with medical complexity while varying in their stages of research, methodologies used, and how they use virtual platforms. The first speaker (Ritchey) will detail implementation of a virtual fall prevention clinical trial, MOVing FREEly. The second speaker (Garbin) will present on the use of a virtual platform to 1) determine the prevalence of fall risk factors in older Veterans using virtual fall risk factor assessments and 2) examine Veterans' and providers' perceptions of virtual fall prevention strategies. The third speaker (Rauzi) will discuss the development and refinement of a multicomponent telerehabilitation intervention delivered via virtual platform to older Veterans, using mixed-methods approaches. The fourth speaker (Stevens-Lapsley) will discuss challenges and successes of using virtual platforms and technologies to remotely monitor fidelity in a large-scale pragmatic clinical trial. These talks will provide a comprehensive understanding of barriers, solutions, and considerations for employing virtual platforms across a range of studies.

Abstract citation ID: igae098.0707

### FALL RISK FACTOR SCREENING AND MANAGEMENT USING VIRTUAL PLATFORMS, PREVALENCE, AND PERCEPTIONS

Alexander Garbin, *VA Eastern Colorado Healthcare System, Aurora, Colorado, United States*

Numerous barriers limit comprehensive assessment and management of fall risk factors within primary care including limited time and competing medical priorities. Virtual platforms provide an opportunity outside of primary care to remotely screen fall risk factors and deliver fall prevention interventions. However, researchers and clinicians may be hesitant to assess and intervene on multifactorial fall risk factors through virtual platforms due to perceived safety concerns, difficulty adapting outcome measures to a virtual environment, and potential technological challenges. This presentation will detail strategies and results from a study examining the prevalence of falls, fall risk, and multifactorial fall risk factors in older Veterans seen within Veterans Health Administration (VHA) primary care using a virtual platform (VA Video Connect). Fall risk factor measurements discussed will include the 5 Times Sit to Stand, the Survey of Activities and Fear of Falling in the Elderly, the Home

Falls and Accidents Screening Tool, and the Screening Tool of Older Persons Prescriptions in older adults with high fall risk. Results from semi-structured interviews with Veterans and VHA primary care providers will also be discussed with an emphasis on Veterans' and providers' experiences with fall risk factor assessment and management within VHA primary care as well as their perceptions of using telehealth for identifying and managing fall risk factors. Together, this presentation will provide insight on the potential for using virtual platforms to assess multifactorial fall risk factors as well as the perceived barriers and facilitators of identifying and managing fall risk factors in a virtual setting.

Abstract citation ID: igae098.0708

**IMPLEMENTATION OF A VIRTUAL FALL PREVENTION PROGRAM IN OLDER VETERANS**

Katherine Ritchey, *VA Puget Sound Healthcare System, Seattle, Washington, United States*

Though virtual platforms have increased in popularity few studies have explored the implementation and benefit of a virtually-based fall prevention program. MOVing FREEly (Multicomponent Otago Virtual Falls Reduction Exercise and Education program) is a VA-based fall prevention program delivered entirely over a virtual care platform. The MOVing FREEly program is six-weeks long and includes both education in fall-risk reduction and evidence-based fall prevention exercises. Process measures captured implementation considerations, feasibility and effectiveness of the program. 27 participants who were older (mean 75 years), mostly male (89.0%), white (85.2%) and moderate or high risk of falling completed the program. 70% were new to the virtual platform. Attendance for education and exercise classes was > 80% with little attrition. There was improvement in the Falls Efficacy Scale-International short form and statistically significant improvement in 30 second sit-to-stand and single leg balance tests. Process measures indicated that 40% of participants needed extra help at the start of each class to sign-on to the virtual visit, which was provided by program facilitators. By the fourth class, all participants were self-sufficient in their ability to navigate the virtual platform. Additional considerations such as electronic health record documentation and coding; clinic setup, referral management and virtual appointment scheduling; patient privacy and health emergency planning were necessary for the start-up and sustainability of the program. Virtual fall prevention programs are feasible and effective but require careful logistical planning and considerations. Implementation considerations from this program can guide future telerehabilitation research and clinical efforts.

Abstract citation ID: igae098.0709

**MIXED METHODS INTERVENTION DEVELOPMENT OF A TELEREHABILITATION PROGRAM FOR OLDER VETERANS**

Michelle Rauzi[1], and Jennifer Stevens-Lapsley[2],
*1. Department of Veterans Affairs, Aurora, Colorado, United States*, *2. University of Colorado Anschutz Medical Campus, Aurora, Colorado, United States*

Mixed methods approaches add value during the development of complex interventions, increasing chances of program success. As such, we employed multi-stage mixed methods study designs to develop a MultiComponent TeleRehabilitation (MCTR) program for older Veterans with multiple chronic conditions. The four stages included 1) exploratory sequential, 2) explanatory sequential, 3) embedded convergent, and 4) exploratory sequential designs. Results from stage 1 provided valuable information to support the program's initial implementation and evaluation. Stage 2 results supported the program's feasibility for Veterans ≥50 years with at least 3 medical conditions. Stage 2 also provided key insights to improve recruitment and enrollment procedures, adapt the intervention, and modify technology integration. The resulting adaptations were implemented and evaluated during a randomized feasibility study, which involved stage 3. The primary results supported the MCTR program's feasibility and safety in an older (≥60 years) and physically impaired population. Secondary results demonstrated some improvement of patient level outcomes including physical function. The mixed methods findings identified salient participant differences related to how and why participants chose to engage or not engage with the technological supports used during the MCTR program. These technology engagement findings were important because the higher technology engagement group showed greater improvements in physical activity. Thus, by understanding reasons why people did not engage with the technology, we can adapt the MCTR program during stage 4 to address engagement barriers and improve patient outcomes. In conclusion, using mixed methods approaches for intervention development accelerated our research and will help enhance future implementation efforts.

Abstract citation ID: igae098.0710

**REMOTE MONITORING IN MULTISITE CLINICAL TRIALS: USING VIRTUAL TECHNOLOGY FOR RELIABILITY AND FIDELITY**

Jennifer Stevens-Lapsley[1], Lauren Hinrichs-Kinney[2], Mattie Pontiff[2], Melissa Tran[1], and Frances Westlake[2],
*1. University of Colorado Anschutz Medical Campus, Aurora, Colorado, United States*, *2. University of Colorado, Aurora, Colorado, United States*

Multi-site clinical studies often involve time consuming and resource intensive in-person monitoring of reliability and fidelity. An innovative solution provided by Swivl technologies holds tremendous potential to enhance remote monitoring options to reduce the need for costly in-person, onsite monitoring. The technology has enabled seamless and dynamic tracking of individuals undergoing Short Physical Performance Battery (SPPB) assessments, as well as specific rehabilitation interventions that are part of an ongoing 34 site clinical trial (NCT05492240) in skilled nursing facilities. The Swivl camera's ability to video moving subjects while also using capturing conversations within a busy clinic environment uniquely enhances the quality of remote monitoring. Our team has used this technology to perform 190 SPPB reliability assessments and 65 rehabilitation fidelity assessments at 34 skilled nursing facility (SNF) clinics. This approach has allowed for real-time, remote observation of participants as they perform sit-to-stand, walking speed, and balance assessments within the SPPB. Similarly, the research team can remotely monitor fidelity of a high-intensity rehabilitation program focusing on dosing of specific rehabilitation exercises to ensure prescribed interventions are executed as intended. This virtual monitoring not only enhances the capacity to conduct remote reliability and fidelity assessments, but it also provides a valuable tool for continuous feedback and adjustment, optimizing the overall effectiveness of rehabilitation protocol fidelity. In essence, the Swivl camera brings forth a new era of remote monitoring that overcomes previous barriers (e.g. storage and transfer of large video files, inability to hear audio from an individual in a busy clinic setting).

### SESSION 2140 (SYMPOSIUM)

Abstract citation ID: igae098.0711

### ACADEMIC–PRACTICE PARTNERSHIPS IN NURSING HOMES: INTERNATIONAL EXPERIENCES, PROCESSES, AND OUTCOMES

Chair: Marie Boltz
Co-Chair: Karin Wolf-Ostermann
Discussant: Karen Spilsbury

The Academic-Practice Partnership model is increasingly gaining attention as a feasible approach to grow the geriatric workforce, generate meaningful research, and integrate evidence-based, person centered care into nursing home operations. They offer practicable approaches to optimize knowledge and skill development for students, support lifelong learning of staff, optimize research capacity and productivity, and improve resident outcomes. This symposium will describe interdisciplinary collaboration between scientists, care providers, and educators in nursing homes in three countries: United States, Germany, and the Netherlands. The engagement and experiences of stakeholders, daily operations, and research activity will be discussed. The first presentation will describe the TCALL project in Germany, which creates a direct pathway for research to care delivery via training and vice versa. The Teaching Home project in the US will be presented next, including the process of co-designing the program with nursing home leadership and staff, developing the curriculum, preparing faculty, and the content for practice. A qualitative exploration of the US students' experiences and lessons learned in the teaching home program will be shared in the third presentation. The fourth presentation will provide an overview of the Dutch Living Lab in Long-term care; the outcomes related to quality of life of older people and their families, quality of care and quality of work will be discussed. A discussion will follow addressing future directions including inter-university collaboration, measurement considerations, and policy implications.

Abstract citation ID: igae098.0712

### THE GERMAN TRANSFER CLUSTER OF ACADEMIC NURSING INSTITUTIONS IN LONG-TERM CARE (TCALL)

Karin Wolf-Ostermann[1], Benedikt Preuss[2], Heinz Rothgang[2], Ingrid Darmann-Finck[3], Karsten Wolf[1], Claudia Stolle-Wahl[4], and Matthias Zuendel[5], *1. University of Bremen, Bremen, Bremen, Germany*, *2. Research Center on Inequality and Social Policy (SOCIUM), University of Bremen, Bremen, Bremen, Germany*, *3. Institute of Public Health and Nursing Research, University of Bremen, Bremen, Bremen, Germany*, *4. City University of Applied Sciences Bremen, Bremen, Bremen, Germany*, *5. Integrated Health Campus Bremen, Bremen, Bremen, Germany*

Considering the demographic shift, ensuring high-quality care emerges as a central future challenge in Germany. This can only be overcome by enhancing the attractiveness of the nursing profession through improved working conditions, introducing technological innovations comprehensively into long-term care, and adapting internal workflows through personnel and organizational development, as well as evolving educational content and structures accordingly. Currently in Germany such transfer and innovation structures are lacking. Therefore, TCALL establishes a sustainable framework to enable the implementation of technical and organizational innovations in regular operations similar to the Living Lab in Ageing and Long-Term Care (Netherlands) or Teaching Nursing Homes (US). This approach facilitates a direct pathway for research findings to transfer from academia through education into healthcare practice, with reciprocal influences from healthcare practice back into research and education. We will present the concept of TCALL which is up to now implemented in three nursing homes in the federal state of Bremen, Germany and will be further developed into a cluster of academic nursing institutions through the addition of personnel and the establishment of a decentralized learning infrastructure. TCALL thus constitutes a conceptual space for innovative transfer activities and simultaneously provides a permanent physical environment for recursive innovation development, testing, and implementation. This structure enables the close integration of research, education, and practice, contributing directly to the rapid and process-oriented development of the state-of-the-art through innovative insights and products.

Abstract citation ID: igae098.0713

### CURRICULUM FOR PRACTICE AND LEADERSHIP IN NURSING HOMES: THE US RESPONSE

Ann Kolanowski[1], JoAnne Reifsnyder[2], and Jacqueline Dunbar-Jacob[3], *1. The Pennsylvania State University, University Park, Pennsylvania, United States*, *2. University of Maryland Baltimore, Baltimore, Maryland, United States*, *3. University of Pittsburgh, Pittsburgh, Pennsylvania, United States*

The Teaching Nursing Home (TNH), which began in the 1980s under the leadership of Dr. Mathy Mezey, was a visionary approach to improving the lives of older adults in nursing homes through the efforts of the interdisciplinary healthcare team. The project had extraordinary outcomes, but for a host of political and economic reasons was never brought to full realization. Forty years and a pandemic later, the need for improving quality care in our nations' nursing homes took center stage. Dr. Terry Fulmer (John A Hartford Foundation) and Nancy Zionts (Jewish Healthcare Foundation) provided extraordinary leadership to address what is now widely regarded as a devastating gap in the US healthcare system. With over $5 million in funding, academic/practice partnerships were revisited in a pilot project with renewed energy, creativity, and resolve by three major universities in Pennsylvania and four affiliated nursing homes. Early on, the lack of curriculum models for faculty educating prelicensure students in the delivery of quality care in nursing home settings was identified as a barrier to progress in the TNH project. The authors of this paper present the groundbreaking work that was done by an interdisciplinary panel of over 40 expert healthcare practitioners and academicians who came to consensus in the identification and qualitative development of a curriculum that prepares healthcare professionals and their faculty through academic/practice partnerships. Competencies include: creating a culture of care; improving diversity, equity and inclusion; person/family-centered care; models of care delivery; geriatric syndromes; interdisciplinary practice; regulation and finance; and information technology.

Abstract citation ID: igae098.0714

### REVISITING THE US TEACHING NURSING HOME: WHAT ARE STUDENTS' PERCEPTIONS OF CARING FOR OLDER ADULTS?

Erin Kitt-Lewis[1], Felicia Heaton[2], Maria Yoder[1], Donna Fick[1], and Marie Boltz[1], *1. The Pennsylvania State University, University Park, Pennsylvania, United States*, *2. Centre Care Rehabilitation and Wellness Services, Bellefonte, Pennsylvania, United States*

The U.S. Census Bureau predicts that by 2050, over 20% of the U.S. population will fall into the category of older adults and will be the "core business" of the nursing profession. This growing demand is coupled with critical staff shortages, especially in nursing homes, a deficit that was exacerbated by the experiences of the COVID-19 pandemic. The American Association of Colleges of Nursing has identified care for older adults as a priority for undergraduate nursing programs and nursing education literature suggests there are critical barriers toward greater integration of the knowledge, skills, and dispositions needed to adequately care for older adults living in nursing homes. The purpose of this study was to understand undergraduate nursing students' perceptions about their first clinical learning experience in a nursing home and working with older adults., Students (N= 105) from a large research university who were enrolled in the Nursing Fundamentals course were recruited to participate in the study. De-identified course artifacts (e.g., Age-Friendly Health Systems 4Ms Framework course assignments) generated by consented participants were compiled and analyzed using thematic analysis. Two themes emerged from the data and included students' assumptions and lessons learned about caring for older adults living in nursing homes. In addition, students identified opportunities that could improve care for older adults, especially around "What Matters" and functional mobility. These findings will serve as the basis to guide best practices for integrating additional learning experiences in the undergraduate nursing curriculum using the Age-Friendly Health Systems 4Ms Framework.

Abstract citation ID: igae098.0715

### BRIDGING THE GAP BETWEEN ACADEMIA AND PRACTICE: THE DUTCH LIVING LAB IN AGEING AND LONG-TERM CARE

Jan Hamers, and Hilde Verbeek, *Maastricht University, Maastricht, Limburg, Netherlands*

There is a strong need in long-term care for scientific research, so older people and their families, health care professionals, policy makers, and educators can benefit from new advancements and best available evidence in everyday care practice. The model of a sustainable and successful interdisciplinary collaboration between scientists, care providers and educators in long-term care will be presented: the 'Limburg Living Lab in Ageing and Long-Term Care'. It is a structural collaboration between academia, educational institutions, long-term care providers and clients. It covers approximately 185 long-term care facilities (e.g., nursing homes, assisted and group living facilities) as well as professional home care, and includes about 50,000 clients and more than 27,000 staff. Their mission is to contribute with scientific research to improving i) quality of life of older people and their families; ii) quality of care and iii) quality of work of those working in long-term care. Key working mechanisms are the scientific and practice-based Linking Pins and interdisciplinary partnership using a team science approach, with great scientific and societal impact. For more than 25 years this structural collaboration has served as an infrastructure that drives scientific research in long-term care in co-creation with end-users, including older people and their relatives, health care professionals, policy makers and educators. This model was established by Maastricht University in the Netherlands, and more recently, the model of the living lab is replicated in other countries such as the United Kingdom, Germany and Austria.

### SESSION 2145 (PAPER)

### ACUTE CARE II (PAPERS)

Abstract citation ID: igae098.0716

### NURSE STAFFING AND PRN PSYCHOTROPIC DRUG USE IN VA HOSPITALS

Ronald Shorr[1], Wei-Hsuan Lo-Ciganic[1], Bridget Hahm[2], Julia Gamble[3], Gail Powell-Cope[4], Stephen Luther[5], and Seonkyeong Yang[6], *1. Malcom Randall Veterans Affairs Medical Center, Gainesville, Florida, United States*, *2. Edward Hines, Jr. VA Hospital, Oak Park, Illinois, United States*, *3. VA Central office, Washington, District of Columbia, United States*, *4. University of California Davis, Davis, California, United States*, *5. Supervisory Health Science Specialist, Tampa, Florida, United States*, *6. University of Pittsburgh, Pittsburgh, Pennsylvania, United States*

Reducing physical and chemical restraint use in hospitals is vital for patient safety. Studies have linked excessive physical restraint use to nurse shortages and increased risks (e.g., deaths). PRN (as needed) psychotropic drugs are used in hospitals for behavioral disturbances, yet their safety remains debatable. The impact of nurse staffing on PRN psychotropic use is unclear. Using data from 166 nursing units in Veterans Affairs (VA) hospitals from October 1, 2012, to September 30, 2017, we examined the relationship between PRN psychotropic use (i.e., antipsychotics or benzodiazepines) and two nursing staffing indicators: total nursing hours per patient day (TNHPPD) and the skill mix of registered nurses (RN), namely the proportion of nursing hours provided by RNs. Of 9,770 unit months (average 565.4 person-days of care), 25.8% involved veterans aged ≥75, 95.3% were male, and 31.6% had high Nosos risk scores. We categorized each unit into one of four quantiles based on their monthly TNHPPD, with mean and standard deviation ranging from 7.0 (1.5) in the lowest group to 15.2 (3.0) in the highest group. RN skill mix decreased as TNHPPD increased, from 71.5% to 63.1%. PRN psychotropic was 1.4%, consistent across TNHPPD levels. Multivariable mixed modeling showed PRN psychotropic use was not associated with TNHPPD levels (p=0.27) or RN skill mix (p=0.07). Our findings indicate that nurse staffing levels were not associated with PRN psychotropic use in VA hospitals.

Abstract citation ID: igae098.0717
**PATIENT-REPORTED PAIN AND SLEEP DISTURBANCE AFTER HOSPITALIZATION FOR MEDICAL ILLNESS**
Susan Hastings[1], Karen Stechuchak[2], Cynthia Coffman[2], Caitlin Kappler[2], Jaime Hughes[3], Amy Webster[4], and Kelli Allen[2], *1. Durham VA, Durham, North Carolina, United States*, *2. Durham VA Health Care System, Durham, North Carolina, United States*, *3. Wake Forest University School of Medicine, Winston-Salem, North Carolina, United States*, *4. Durham VA Health Care, Durham, North Carolina, United States*
Older adults experience a well-documented period of vulnerability following hospitalization; yet contributing factors are not fully understood. The goal of this cross-sectional study was to examine associations between post-discharge pain and sleep disturbance with quality of life (QoL) and function in the month following hospitalization for medical illness. As part of a larger study evaluating the effectiveness and implementation of a hospital-based mobility program, we conducted telephone surveys with 618 adults aged ≥60 discharged from one of 8 Veterans Affairs (VA) hospitals. Primary predictors of interest were pain interference and sleep disturbance measured by PROMIS-29 short forms. Outcomes were QoL measured by EQ-5D, function measured by late-life function and disability instruments (LLFDI total and advanced lower extremity function) and community mobility measured by Life Space. For each outcome, separate multiple linear regression models were fit including the key explanatory variables of interest (pain interference and sleep disturbance) and selected covariates (sociodemographic, clinical, hospitalization characteristics) social/regional factors (e.g., social vulnerability, hospital). Participants were mean age 70.6 years, 95% male, 25% Black, 34% lived alone, 8% reported being discharged to a nursing home and 15% had been readmitted to the hospital. In adjusted models, pain interference and sleep disturbance were negatively associated with QoL, total function, lower extremity function, and community mobility (P-values < 0.01). In this sample of recently hospitalized older adults, pain and sleep disturbance were independent contributors to worse QoL and functional status. Pain and sleep may warrant further attention and/or intervention during the post-hospitalization period.

Abstract citation ID: igae098.0718
**IMPACT OF FRAILTY SCREENING IN A BURN CENTER: A QUALITY IMPROVEMENT PROJECT**
Purvi Patel, *University of Chicago Medicine, Chicago, Illinois, United States*
Burn injuries create significant risk for the development of new or worsening frailty. Identifying risk for frailty is key to recognizing patients who are at risk for negative outcomes who may benefit from a comprehensive geriatric assessment (CGA). A quality improvement project was conducted in a burn center setting with the primary aim to determine if implementation of the FRAIL scale screening tool impacted frequency of consultation to the geriatric medicine team for a CGA within a 10-week pilot period. Secondary aims were to evaluate feasibility of implementation and to stratify patients' risk of frailty into categories based on the tool measurements: robust, pre-frail, or frail. The project included 44 patients (22 in the pre-frailty tool group and 22 in the post-frailty tool group). The data revealed a three-fold increase in the number of consultations to the geriatric medicine team between the pre (n=5) and post (n=15) groups with statistical significance (p =.002). The frailty tool was most often administered on the first (54.5%) or second (31.8%) hospital day. In the post group, stratification of frailty risk was 27.3% robust, 45.4% pre-frail, and 27.3% frail. The FRAIL scale is shown to be useful in identifying patients who would benefit from a CGA compared to routine clinical practice. The tool is feasible to implement in a burn center. The clinical significance of the majority of patients being classified as pre-frail and frail underscores the need to identify patients' frailty risk and implement measure to mitigate adverse outcomes.

Abstract citation ID: igae098.0719
**BED ALARMS FOR THE PREVENTION OF FALLS IN HOSPITALS: A THREE-ARM, DISINVESTMENT NONINFERIORITY TRIAL**
Terry Haines[1], Debbie Pu[1], Kelly Stephen[1], and Ronald Shorr[2], *1. Monash University, Melbourne, Victoria, Australia*, *2. Malcom Randall Veterans Affairs Medical Center, Gainesville, Florida, United States*
Bed alarms are commonly used to prevent falls in hospitals. However, the evidence supporting their effectiveness indicates they may be ineffective. Disinvestment from routine but low-value care is important, however carries the risk that unanticipated harms to patients may arise with this change of practice. We undertook a 3-arm, non-inferiority, stepped-wedge randomised trial with concurrent parallel cluster randomised trial to investigate whether reducing the frequency of bed alarm use from >6% of beds to between 3% and 1%, or completely eliminating use of bed alarms had an impact on rates of in-hospital falls. We recruited n=18 wards at hospitals in Melbourne, Australia, and collected data over 10 calendar months. Wards were paired based on type and randomly allocated to month of transition in the stepped-wedge design, and then allocated to either "reduced" or "eliminated" conditions in a two-stage process. Alarm use was monitored through 3x weekly visual audits. Hospital falls data were collected through hospital incident reporting and administrative data systems with weekly auditing from site research assistants. A non-inferiority margin of an increase in rate of falls by 2 falls per 1000 patient days was set in pre-trial stakeholder consultation. The difference between "control (high use of alarms)" and "reduced" conditions was 0.29 falls/1000 patient-days (95%CI: -2.41 to 3.00) favouring the control. The difference between "control" and "eliminated" conditions was 1.13 falls/1000 patient-days (95%CI: -1.58 to 3.83) favouring the control. Non-inferiority of the

"reduced" and "eliminated" conditions was not shown, though use of bed alarms was not superior.

Abstract citation ID: igae098.0720

### AGE AS A PREDICTOR OF BURN-RELATED OUTCOMES IN OLDER ADULTS IN LOW- AND MIDDLE-INCOME COUNTRIES

Claire Wang, Jieun Lee, and Elizabeth Khvatova, *Johns Hopkins University, Baltimore, Maryland, United States*

While older age has been linked to worsened burn outcomes, the specific effects of age on high-risk older adult burn patients in low- and middle-income countries (LMICs) are not well understood. Utilizing the WHO Global Burn Registry data from 2017-2021, older adults (n=2,257) were stratified into three age groups at time of burn injury (>40 and ≤60; >60 and ≤80; >80). Multivariable logistic regression models were employed to evaluate the association between age groups and detailed burn outcomes (left against medical advice, discharged home without physical impairment, discharged home with physical impairment, or mortality), adjusting for potential confounders such as sex, country, percentage of total burn surface area, access to rehabilitation services, and surgical interventions during hospitalization. After adjustment, individuals ages 80+ showed a statistically significant increase in burn-related mortality/impairment compared to those in the >40 and ≤60 age group, with an adjusted odds ratio (OR) of 2.06 (95% confidence interval [CI]: 1.33-3.20). A separate analysis on mortality revealed that those 80+ had significantly higher odds of burn-related mortality compared to the youngest age group, with an OR of 3.47 (95% CI: 2.10-5.74). No significant differences were identified in impairment outcomes across the age groups. Our study reveals a marked increase in mortality risk from burns in individuals 80+ in LMICs, not observed in younger counterparts. Despite no significant age-impairment correlation, the findings indicate a need for age-tailored interventions to improve outcomes for this high-risk group, urging further research into targeted, age-specific care.

### SESSION 2150 (PAPER)

### ADULT PROTECTION II

Abstract citation ID: igae098.0721

### SELF-REPORTED FRAUD VERSUS OBSERVED VICTIMIZATION INCIDENTS: A RISK FACTOR COMPARISON

Marguerite DeLiema[1], Siyu Gao[2], Lynn Langton[3], and M. Daniel Brannock[3], *1. University of Minnesota Twin Cities, St. Paul, Minnesota, United States*, *2. University of Minnesota, Minneapolis, Minnesota, United States*, *3. RTI International, Research Triangle Park, North Carolina, United States*

According to the age-associated financial vulnerability model, older adults may be more susceptible to scams. Research is needed to identify modifiable risk factors for targeted intervention. We surveyed 934 law enforcement-identified victims of mass marketing scams (mean age=74.7) to determine what characteristics and behaviors are associated with the number of (1) self-reported victimization incidents, and (2) observed victimization incidents. The survey was administered online and by mail. Data on participants' observed incidents of mail fraud victimization between 2022 and 2023 were provided by the US Postal Inspection Service. Linear regression results indicate that behaviors that increase fraud exposure (e.g., answering calls from unknown numbers, talking with telemarketers, opening all pieces of mail), as well as purchasing lottery tickets, loneliness, and a preference to take financial risks are associated with a greater number of self-reported fraud and exploitation incidents. Identifying as non-Hispanic White and being more active online were protective. Purchasing lottery tickets and greater preference for financial risks were also significantly associated with observed incidents of fraud. Being female and reporting less social engagement were significant for observed incidents only. Based on the difference in significant risk factors between the two models, results indicate that self-reported victimization is an imperfect measure of actual fraud victimization. Fraud prevention programs that increase social engagement and reduce behaviors associated with fraud exposure are promising targets for intervention. Results also suggest that embedding fraud awareness education in locations where lottery tickets are sold may be a prevention strategy to reach vulnerable older adults.

Abstract citation ID: igae098.0722

### THE ROLE OF APPRAISAL IN CAREGIVER CHARACTERISTICS ASSOCIATED WITH ELDER NEGLECT

Eleanor Batista-Malat[1], Zachary Gassoumis[1], Kathleen Wilber[1], Bonnie Olsen[1], and Laura Mosqueda[2], *1. University of Southern California, Los Angeles, California, United States*, *2. University of Southern California Keck School of Medicine, Pasadena, California, United States*

Elder neglect occurs when a caregiver fails to meet a need, compared to elder abuse which involves specific abusive actions. Despite this, neglect and abuse are typically studied together as elder mistreatment. We investigated the characteristics associated with neglectful behaviors of caregivers of people living with dementia (PLWD). California dementia caregivers (N=92) completed a cross-sectional anonymous online survey about their caregiving experience and mistreatment of the PLWD from any source. 44.6% of caregivers were caring for a parent, 51.1% were Hispanic/Latino, and 68.1% were female. Eighteen (19.6%) caregivers reported at least one neglectful behavior in the last year, defined as leaving the PLWD alone inappropriately, not providing them with needed medical care, food, or water, or being impaired in caregiving due to alcohol or drugs. Of these, 11 (61.1%) also reported abuse in the last year: nine psychological, five financial, and five physical abuse. Bivariate tests comparing characteristics of caregivers with and without neglectful behaviors showed no difference in time spent caregiving, outside caregiving help, or social support and loneliness. Those who reported neglectful behaviors were significantly more likely to live with the PLWD ($X2 = 7.2$, $p = .028$), believe the PLWD could "do better if they tried" ($X2 = 9.0$, $p = .029$), and have higher burden scores ($t = -2.61$, $p = .011$). Our findings suggest that caregiver neglect may be associated with the caregivers' perceived experiences rather than objective measures. Service providers should be attentive to caregivers' understandings of their experiences, which may be related to neglectful behaviors, and reducing neglectful behavior may reduce overall mistreatment.

Abstract citation ID: igae098.0723

### USE OF MOTIVATIONAL INTERVIEWING TO INTERVENE ELDER ABUSE: A RANDOMIZED CONTROL TRIAL

Elsie Yan[1], Louis To[1], Debby Wan[1], Jasmin He[1], Daniel W.L. Lai[2], David Burnes[3], Karl Pillemer[4], and Mark Lachs[5], *1. The Hong Kong Polytechnic University, Hong Kong, Hong Kong, China (People's Republic)*, *2. Hong Kong Baptist University, Hong Kong, Hong Kong*, *3. University of Toronto, Toronto, Ontario, Canada*, *4. Cornell University, Ithaca, New York, United States*, *5. Weill Cornell Medicine, New York, New York, United States*

Elder abuse causes serious harm to individuals, families, and society. There is an urgent need to develop and test appropriate interventions to tackle it. This study tested the effectiveness of a motivational interviewing (MI)-based intervention in reducing elder abuse severity and promoting positive changes among community dwelling older Chinese in Hong Kong. The intervention involved a 90-minute individual engagement session and three 30-minute booster sessions delivered by trained MI facilitators. Personal goals for improvement of known risk factors such as poor physical and psychological health, lack of social support were set out followed by the application of MI processes. Pre- and post-test comparison of 33 intervention cases and 28 control cases are presented here. Analysis of pre-treatment differences found significant difference between the two groups in their monthly income, number of offspring and co-residing persons, physical frailty, general self-efficacy and abuse severity. Difference in differences technique was applied to mitigate the effects of extraneous factors and selection bias. Results show that intervention group performed significantly better than the control group upon completing the intervention program. Significant between group differences were found in terms of changes in psychological distress (GHQ, $z=-3.043$, $p< 0.05$), general self-efficacy (GSES, $z=-4.988$, $p< 0.05$), social support (MSPSS, $z=-3.080$, $p< 0.05$), abuse severity (CTS2, $z=-2.593$, $p< 0.05$), and perceived ability to change ($z=-3.749$, $p< 0.05$). MI has demonstrated good potential for elder abuse intervention. Further study should be conducted to identify key elements in MI leading to positive changes and refine the model for elder abuse prevention and intervention.

Abstract citation ID: igae098.0724

### SELF-NEGLECT CO-OCCURS WITH AND IS A RISK FACTOR FOR ELDER MISTREATMENT

Stuart Lewis[1], MT Connolly[2], Patricia Kimball[3], Geoff Rogers[4], Erin Salvo[5], and David Burnes[6], *1. Dartmouth Health, Lebanon, New Hampshire, United States*, *2. RISE Collaborative, Washington, District of Columbia, United States*, *3. Elder Abuse Institute of Maine, Brunswick, Maine, United States*, *4. Hunter College, CUNY, New York, New York, United States*, *5. Public Consulting Group, Portland, Maine, United States*, *6. University of Toronto, Toronto, Ontario, Canada*

Elder mistreatment is abuse, neglect, or exploitation occurring in a relationship involving an expectation of trust.1 Yearly, 10%–14% of community dwelling older adults experience mistreatment, with only 15% of cases being reported to Adult Protective Services (APS) for investigation.2 Self-neglect (not defined as elder mistreat- ment) comprises 50% of APS investigations and 65% of substantiated APS cases.3 How self-neglect relates to other forms of mistreatment is not well understood. Maine APS data was analyzed to delineate the relationship between self-neglect and elder mistreatment. Self-neglect as a risk factor for elder mistreatment has important implications for policy, practice, and the prevention of future harms. Our analysis demonstrates that Self-neglect frequently co-occurs with elder mistreatment. A first allegation of self-neglect, substantiated or not, is associated with a shorter time to elder mistreatment and an estimated 33% of all cases of elder mistreatment could be attributed to self-neglect. These findings have important implications for prevention, policy, and practice. While causality cannot be presumed—a limitation in all retrospective analyses—elder mistreatment and self-neglect share risk factors: cognitive impairment, physical disability, lack of social support, and social isolation. Our analysis suggests that early intervention, even in unsubstantiated allegations of self-neglect, may prevent subsequent mistreatment.

### SESSION 2155 (SYMPOSIUM)

Abstract citation ID: igae098.0725

### ADVANCING EQUITY AND COMMUNITY ENGAGEMENT IN AGING IN CENTERS ON AGING

Chair: Joseph Gaugler
Co-Chair: Robbin Frazier
Discussant: Jane Mahoney

The ultimate goal of much gerontological scholarship is to improve the lives of older people. To meet the needs and preferences of our rapidly diversifying communities of older persons, creating equitable partnerships with older adults, families, neighborhoods, and organizations is often necessary, if not essential. This symposium aims to highlight centers/institutes on aging across the U.S. that have adopted various strategies and approaches to build partnerships with aging communities, co-create innovative scholarship, culturally adapt evidence-based/evidence-informed programs, and effectively disseminate knowledge to achieve health equity in aging. Our symposium will feature equity and community engagement missions, activities, opportunities, and challenges for four centers on aging, representing different types of institutions and areas of the country. Featured centers/institutes include the University of Texas-Austin's Texas Aging & Longevity Consortium, Wayne State University's Institute of Gerontology, Johns Hopkins University's Center for Equity and Aging, and the University of Minnesota's Center for Healthy Aging and Innovation. Jane Mahoney, MD, Director of the Community-Academic Aging Resource Network (CAARN) of the University of Wisconsin-Madison, will synthesize equity and community engagement efforts in centers/institutes on aging and will provide a roadmap for future initiatives to sustain this critical work.

Abstract citation ID: igae098.0726

### I NEED HELP: AN EXPERIMENT IN COMMUNITY OUTREACH

Jacqueline Angel, *The University of Texas at Austin, Austin, Texas, United States*

This paper describes community outreach activities by faculty affiliated with The UT Austin Texas Aging & Longevity Consortium. Communities across the country like Austin are aging rapidly, while they are becoming more racially diverse and facing an ever-growing need for assistance to older citizens. The City of Austin is a vibrant and cosmopolitan metropolis comprising multiple communities of color—African American, Latino, and Asian which require affordable and culturally-appropriate services and supports. Often these are difficult to find or are simply not available. Many older adults suffer from multiple chronic conditions which increases their need for assistance. As a consequence, community outreach is desperately needed, and investigations in to the most cost-effective and efficient ways of informing clients about available services from which they might benefit is vital. This presentation lays out proposed solutions to confronting these challenges. Specifically, the presentation highlights one award-winning community-based policy research initiative that investigates important possibilities for building an age-friendly Austin that might be applied elsewhere. The public-private initiative uses a multimethod approach to establish an intergenerational community-based resource and activity center (IDC) to optimize space in public places. The IDC model would expand current public health services, including child and adult day care aging, service coordination, and tele-behavioral health in one location. We end with a discussion of lessons learned regarding research-based strategies that encompass three domains: community outreach, co-production, and building partnerships inside and outside the UT campus.

Abstract citation ID: igae098.0727

### THE CENTER FOR EQUITY IN AGING AT JOHNS HOPKINS UNIVERSITY

Katherine Ornstein, Sarah Allgood, Kali Thomas, and Quincy Samus, *Johns Hopkins University, Baltimore, Maryland, United States*

The Center for Equity in Aging was launched in 2022 Johns Hopkins University to advance scholarship, research and advocacy focused on aging and serious illness care. The Center seeks to transform the diverse experiences of aging and serious illness to enhance equity and well-being across the life course. Hosted by the School of Nursing, the Center includes 2 dozen principal faculty and draws upon a variety of disciplines including nursing, epidemiology, gerontology, health services, and health policy. In this presentation we will discuss how the Center advances equity in aging by engaging in research, training and scholarship that supports diverse individuals and populations, identifies health disparities in aging throughout the life course, and develops interventions, policies, and models of care to reduce inequities in the opportunities and resources needed to grow and age as healthily as possible. We will discuss the Center's collaborations and partnerships with a diverse set of stakeholder groups to promote community-engaged research practices that inform research across all stages and describe new initiatives focused on advancing equity within the Center and how the Center collaborates with other centers/institutes both within and outside of the Johns Hopkins community. Center leadership will discuss their visions for promoting equity in aging research and reflect on key challenges in the field.

Abstract citation ID: igae098.0728

### THE HEALTHIER BLACK ELDERS CENTER COMMUNITY-ENGAGED PROGRAM

Peter Lichtenberg[1], Tam Perry[1], Vanessa Rorai[2], and LaToya Hall[1], *1. Wayne State University, Detroit, Michigan, United States*, *2. Healthier Black Elders Center, Detroit, Michigan, United States*

For the past 25-years the Healthier Black Elders Center has been the community-engaged program run by the Wayne State University Institute of Gerontology as part of the Michigan RCMAR program. The HBEC has an active Community Advisory Board and engages in year round community education and health programs, provides a lay person newsletter and has a Participant Registry. During the past five years the HBEC began a community-engaged consultancy program in order to assist others across the country in engaging with older black adults for research participation. The HBEC is also a science generating program, with research publications across the past 20 years on recruitment and retention of older black adults into research. Beginning in 2012 the HBEC also expanded to include the Successful Aging through Financial Empowerment program. The SAFE program has been the nexus for financial-decision making, financial exploitation and day to day financial management in older black adults. The SAFE program is not only a research program but also a service program. The SAFE program provides 1:1 assistance for older adults who were victims of scams or identity theft and are having difficulty straightening out their finances. In order to reach older black adults and the professionals who work with them the SAFE program has its own year round community engaged efforts including the program "Financial First Fridays" and a regular series of community-based talks. SAFE has assisted over 350 older adults and caregivers and provided 1:1 services for 200 clients during the past five years.

Abstract citation ID: igae098.0729

### ADVANCING HEALTH EQUITY AND REDUCING DISPARITIES: COMMUNITY-IDENTIFIED AND DRIVEN PROJECTS

Robbin Frazier, and Elma Johnson, *University of Minnesota, Minneapolis, Minnesota, United States*

Soon after its re-launch in 2021, the Center for Healthy Aging and Innovation (CHAI) at the University of Minnesota established its Equity and Community Engagement (ECE) Core to develop the infrastructure for community engagement in CHAI and build relationships with a variety of community partners serving older adults in BIPOC and underserved communities. After careful planning and a thoughtful recruitment process, the ECE Core launched CHAI's first Community Advisory Board (CAB) in the fall of the same year. This diverse group brings together older adults from the largest ethnic groups in the state, and provides input, wisdom and lived experience to guide CHAI's work and priorities. Two years after its launch, the CAB has identified key community needs related to aging and started work on several community-led projects to address ageism and social isolation among diverse older adults. It also helped form the Strategic Advisory Council, a group complementary to the CAB composed of community leaders, faculty, scholars, and policymakers, and charged with developing recommendations to address systemic inequities impacting the aging experience of diverse older adults. In 2024, the group entered its next phase, focused on strengthening the academic-community collaboration and aligning research priorities with those of the communities CHAI serves. CHAI will present key findings and highlights from each stage of its community-engagement process and will share lessons learned on its way to achieving its mission - the one of a truly community-engaged academic Center on Aging.

### SESSION 2160 (PAPER)

### ALZHEIMER'S DISEASE AND RELATED DEMENTIAS II (PAPERS)

Abstract citation ID: igae098.0730

### THE ASSOCIATIONS OF LATE-LIFE BLOOD PRESSURE WITH CERAD AND BRAAK STAGES: FINDINGS FROM THE NACC DATASET

Mo-kyung Sin[1], N. Maritza Dowling[2], Jeffrey Roseman[3], Ali Ahmed[4], and Edward Zamrini[5], *1. Seattle University, Seattle University, Washington, United States*, *2. The George Washington University, Washington, District of Columbia, United States*, *3. University of Alabama at Birmingham, Birmingham, Alabama, United States*, *4. Washington DC Veterans Medical Center, VA Medical Center, District of Columbia, United States*, *5. Irvine Clinical Research, Irvine, California, United States*

CERAD and Braak staging are two major scoring systems for quantifying neuropathological features of Alzheimer's disease (AD). Midlife hypertension is a risk factor for AD, but less is known about the relationships between late-life systolic blood pressure (SBP) with CERAD and Braak staging, the examination of which was the objective of the current study. This study analyzed data from 5,160 participants in the National Alzheimer's Coordinating Center dataset which had data on 4 longitudinal SBP measurements before death and on CERAD (n=2,600) and Braak (n=2,560) autopsy data. CERAD score was dichotomized into moderate/severe versus none/sparse; Braak stage was dichotomized into groups: III-VI vs 0-II. Logistic regression models were used to examine odds of higher CERAD or Braak staging associated with SBP ≥130mmHg (mean of 4 late-life measures). In a model that controlled for age at death, last BP to death (in years), and sex, a late-life mean SBP ≥130mmHg compared to SBP < 130mmHg was associated with an estimated odds ratio for a more severe CERAD score of 1.13 (95% CI: 1.00, 1.26). In a model that also controlled for antihypertensive medications and APOE ε4 status, SBP ≥130mmHg was associated with a slightly increased estimated odd for a more severe CERAD score of 1.15 (95% CI: 1.02, 1.30). We found a direct association between late-life mean SBP with CERAD score, but not with Braak stage. This analysis supports the importance of maintaining control of BP into late life as a strategy to slow the rate of progression of AD pathology.

Abstract citation ID: igae098.0731

### THE INTERPLAY AMONG IMMUNITY, INFLAMMATION, AND FRIED FRAILTY ON ALZHEIMER'S DISEASE

Jiachen Chen[1], Margaret Doyle[2], Yumeng Cao[3], Ahmed Ragab[3], Joanne Murabito[4], and Kathryn Lunetta[3], *1. Boston University School of Public Health, Boston, Massachusetts, United States*, *2. University of Vermont Larner College of Medicine, Colchester, Vermont, United States*, *3. Boston University, Boston, Massachusetts, United States*, *4. Chobanian and Avedisian School of Medicine, Boston University, Boston, Massachusetts, United States*

Unraveling shared mechanisms for both frailty and Alzheimer's Disease (AD) including immunity and inflammation is crucial for understanding the link between these two complex multifactorial conditions. This study aimed to characterize the interplay among circulating immune cells, inflammatory biomarkers, and frailty phenotypes on AD onset in the Framingham Heart Study Offspring cohort. A sample of 519 participants (52% female, mean age 66 years), had extensive profiling of immune cells from peripheral blood mononuclear cells and inflammatory protein biomarkers (OLINK Proteomics) at Exam 7 and were dementia-free at the time of Fried frailty measurement (Exam 8). Data from 146 immune cells, 68 proteins, and 5 frailty phenotype components were integrated using sparse multiple Canonical Correlation Analysis (SmCCA) adjusted for relevant covariates. SmCCA seeks linear combinations of variables maximizing inter-correlated canonical scores for all three datasets, yielding signatures of immunity, inflammation, and frailty for each participant that screened variables contributing to the establishment of signatures and inter-datasets pathways. Full follow-up for up to 13.8 years (median 12.1 years) identified 24 incident AD cases. Cox proportional hazard models, accounting for covariates including age, sex, education, and APOE genotype, were employed to assess signatures as risk factors for AD incidence. The inflammation and immunity signatures were correlated (r2=0.32, p-value< 0.001). Both immunity and frailty signatures were significantly associated with AD incidence (immunity: HR=1.1, p-value=0.03; frailty: HR=3.14, p-value=0.02). This study disentangles potential pathways among immune cells and inflammatory biomarkers that contributed to associations with frailty and AD. We will extend to larger studies for replication.

Abstract citation ID: igae098.0732

### BLOOD BIOMARKERS OF NEURODEGENERATION ARE ASSOCIATED WITH SMARTPHONE-ASSESSED SUBJECTIVE COGNITIVE CONCERNS

Ángel García De La Garza[1], Cuiling Wang[1], Jacqueline Mogle[2], Carol Derby[1], Richard Lipton[1], Laura Rabin[3], and Mindy Katz[1], *1. Albert Einstein College of Medicine, Bronx, New York, United States*, *2. Clemson University, Clemson, South Carolina, United States*, *3. Brooklyn College and The Graduate Center of CUNY, Brooklyn, New York, United States*

The association between blood-based biomarkers of Alzheimer's Disease (BBBs) and daily diary smartphone assessment of subjective cognitive concerns (SCC) is unexplored. In the Einstein Aging Study, we collected digital diary information on 18 types of cognitive lapses per day over a 14-day period on 271 participants (mean age 77.50, SD 4.90, 66.8% Female, 47.6% Non-Hispanic-White, 40.2% Non-Hispanic-Black, 12.1% Hispanic). BBBs investigated included β-amyloid (Aβ40, Aβ42), the Aβ42:Aβ40 ratio, pTau181, neurofilament light (NfL), and glial fibrillary acidic protein (GFAP). Daily diary SCC was analyzed by calculating the average daily number of lapses related to memory (12 items, mean = 0.99, SD = 0.97), the average daily number of lapses related to executive functioning (6 items, mean = 0.53, SD = 0.74), and the daily average number of all cognitive lapses (18 items, mean = 1.53, SD = 1.59), where higher scores indicated greater self-reported cognitive difficulties. Utilizing separate linear regression, we assessed the association between BBB levels and cognitive lapses, adjusting for age, sex, ethnicity, depression, and years of education. Findings indicate that, on average, for every 1 standard deviation increase in blood levels of pTau181, participants reported an additional 0.21 additional cognitive lapses in the memory domain ($p< 0.001$) and 0.11 in the executive functioning domain ($p=0.012$) for a total of 0.32 daily cognitive lapses ($p< 0.001$). No significant associations were identified between Aβ40, Aβ42, Aβ42:Aβ40 ratio, NfL, GFAP, and daily diary SCC. Captured in this manner, SCC may serve as a scalable indicator of AD pathology, progression, and monitoring the disease.

Abstract citation ID: igae098.0733

### EFFECTIVENESS OF A DEMENTIA CARE INTELLIGENT RECOMMENDER SYSTEM ON CARE PROBLEMS: A RANDOMIZED CONTROLLED TRIAL

Yue Sun[1], and Zhi-wen Wang[2], *1. Peking University, Beijing, Beijing, China (People's Republic)*, *2. Peking University Health Science Center, Beijing, Beijing, China (People's Republic)*

**Background:** The complexity and diversity of problems encountered in dementia care varies from the individual, and the health recommender system provides a pathway for realizing solutions for universal access to professional guidance.

**Objectives:** To examine the effectiveness of dementia care intelligent recommender system (DCIRS) for dementia care on improving the number of care problems and their level of distress.

**Methods:** 250 caregivers of dementia patients were included and randomly assigned them to either the intervention group receiving the intervention of DCIRS or a waiting-list control group with 12 weeks of intervention length. The number of care problems and their level of distress was measured using the care problems evaluation sheet for people with dementia. The outcomes were compared between groups by using generalized estimating equations (GEE) based on intention-to-treat principle.

**Results:** A total of 250 participants were included in the analysis. The dietary, dressing, and excretory care problems of the patients with dementia in the control group significantly increased at T2, while there was no significant change in the number of care problems in the intervention group. GEE results showed that participants in the intervention group revealed significantly greater reductions on the level of distress related to care problems than the control group at T1 ($\beta = -3.01$, $p = 0.026$) and T2 ($\beta = -5.80$, $p = 0.006$). Conclusions: The DCIRS was effective in improving the number of care problems and their level of distress. Longer follow-up study is needed to uncover its long-term benefits on clinical outcomes.

### SESSION 2165 (BIOLOGICAL SCIENCES INVITED SYMPOSIUM)

Abstract citation ID: igae098.0734

### EMERGING STRATEGIES FOR AGING

Chair: Fabrisia Ambrosio

This session on Emerging Strategies for Aging focuses on the potential of therapeutically targeting specific pathways/hallmarks of aging for extending healthspan. These targets include increasing mitophagy as a way to improve mitochondrial function, the use of extracellular vesicles from young serum to improve tissue homeostasis, modulating lipid metabolism to reduce the number of senescent cells through induction of ferroptosis and targeting the N6-methyldeoxyadenosine epigenetic mark regulated by DNA damage and stress. The session also will include discussion of approaches to target these different pathways in mice, C. elegans and human cell types derived from iPSCs as well as human brain tissue and provide evidence that genetic variants in lipid metabolism indeed affects the level of senescence in humans. Overall, the goal of the session is to provide examples of the types of novel approaches that could be used to target different pathways important for aging and disease.

Abstract citation ID: igae098.0735

### MITOPHAGY ACTIVATION EXTENDS LONGEVITY AND PREVENTS AGE-RELATED CARDIAC FUNCTIONAL DECLINE

Nuo Sun, *Ohio State University, Columbus, Ohio, United States*

Mitophagy is a specialized autophagic pathway that removes damaged mitochondria, providing a critical mechanism to maintain mitochondrial quality. Accumulating evidence suggests that a decline in mitophagy might contribute to the development of age-related cardiac pathologies. Therefore, pharmacological manipulation of mitophagy offers a novel therapeutic strategy to alleviate the risks of disease and mortality associated with aging. We conducted high-content image-based assays for mitophagy modulators using the pH-dependent fluorescent mitophagy reporter, mt-Keima. Additionally, we designed an algorithm to analyze a collection of over 10,000 compounds at 15 concentrations. This algorithm identified several selective mitophagy inducers, with Mito-I emerging as the most effective mitophagy activator. We demonstrated that Mito-I potently induces mitophagy in human cardiac myocytes derived from induced pluripotent stem cells (hiPSC-CM) and mouse hearts. Interestingly, our mechanistic studies revealed that Mito-I effectively upregulates BCL2-interacting protein 3 (BNIP3), which acts as a mitophagy receptor and is indispensable for Mito-I-induced mitophagy. Excitingly, we also found that Mito-I treatment extended lifespan in C. elegans. Moreover, we showed that Mito-I administration in aged (18-month-old) mice alleviates the detrimental effects of aging on cardiovascular health. These results demonstrate that the age-induced decrease in mitophagy contributes significantly to diminished cardiovascular function and identifies a novel therapeutic strategy to improve cardiovascular function with aging.

Abstract citation ID: igae098.0736

### LIPID METABOLISM CONTROLS CELL FATE DECISIONS BETWEEN CELLULAR SENESCENCE AND FERROPTOSIS

Christopher Wiley, *Tufts University, Medford, Massachusetts, United States*

Recent advances have identified cellular senescence as a bona fide driver of aging and several age-associated pathologies in response to various forms of stress or damage. Primary phenotypes associated with cellular senescence include a stable proliferative arrest and secretion of a mélange of biologically active molecules collectively known as the senescence-associated secretory phenotype (SASP), but senescent cells also display other key phenotypic alterations. Key among these is survival in the face of stress and damage. We recently identified multiple alterations in lipid metabolism, especially those associated with polyunsaturated fatty acids, that favor survival of senescent cells over a form of cell lytic death known as ferroptosis. Interventions that target these pathways, including inhibition of the lipid peroxidation antagonist Gpx4 or elevation of dihomo-gamma-linolenic acid (DGLA), result in lytic death of senescent cells. Elimination of senescent cells using these pathways improves phenotypic outcomes in aged mice. Finally, obese human males carrying a genetic variant with low DGLA desaturation rates are protected from accumulation of senescent cells in adipose tissue, suggesting that these interventions could be translatable to patients. Together, our results provide a new set of targets for intervening in senescence and its associated outcomes.

Abstract citation ID: igae098.0737

### N6-METHYLDEOXYADENOSINE (N6MEDA) IS ASSOCIATED WITH AGING

Natalia Tretyakova, *University of Minnesota, Minneapolis, Minnesota, United States*

N6-methyldeoxyadenosine (N6medA) is a recently discovered DNA mark that has been proposed to be involved in epigenetic regulation, DNA repair, and memory formation. N6medA is dynamically regulated in the mouse brain following environmental stress and plays a role in the formation of fear extinction memories, immune response, and in neuroblastoma. However, the biological roles of N6medA as an epigenetic mark have been questioned due to it extremely low abundance in the genome and conflicting information regarding its origins. We have developed an ultrasensitive isotope dilution nanoLC-methodology for N6medA in genomic DNA and investigated the potential roles of N6medA in human aging and in Alzheimer's Disease (AD). We found that the global genomic N6medA in prefrontal cortex is positively correlated with human's chronological age, and there is an increasing trend for N6medA levels in mild cognitive impairment and Alzheimer's Disease. Genome wide mapping of N6medA in DNA isolated from human prefrontal cortex indicated that increased adenine methylation in AD was preferentially found in genes associated with neurological function and neurological diseases. Mass spectrometry-based affinity proteomics experiments identified several protein readers of N6medA as well as proteins that are prevented from binding to DNA in the presence of methylated adenine. Taken together, our results suggest that N6medA mediates protein binding to DNA, accumulates in genomic DNA of the brain during aging and could contribute to the development of Alzheimer's Disease

Abstract citation ID: igae098.0738

### MEASURING AND MANIPULATING YOUTHFUL FACTORS IN EXTRACELLULAR VESICLES THROUGH ENGINEERING AND EXERCISE

Fabrisia Ambrosio, *Harvard Medical School, Boston, Massachusetts, United States*

Numerous studies employing heterochronic blood exchange have demonstrated the crucial age-dependent role of circulating factors in regulating the function of multiple tissues. To date, investigations into the mechanisms underlying the beneficial effect of young blood on healing cascades have primarily focused on circulating proteins. However, accumulating evidence suggests that circulating extracellular vesicles (EVs) may play a previously unappreciated role in the regulation of tissue health. EVs are membrane-enclosed paracrine mediators that traffic between anatomically remote sites to regulate physiological function and pathologic processes in target cells. A better understanding of the molecular mechanisms guiding age-related alterations in EV cargoes may pave the way towards the development of novel therapeutics that can promote a more youthful phenotype in older individuals. In her presentation, Dr. Ambrosio will summarize recent work from her laboratory identifying an age-related disruption in the transfer of information from circulating EVs to target cells and the impact of this this disrupted communication flow on tissue maintenance and regenerative potential over time. Towards the development of novel EV-based therapeutic strategies to counteract these declines, Dr. Ambrosio will share ongoing studies employing engineering and rehabilitative strategies designed to promote tissue function in an elderly population.

### SESSION 2170 (SYMPOSIUM)

Abstract citation ID: igae098.0739

### DIGITAL MOBILITY MONITORING FOR HEALTHY AGING: RESULTS FROM LARGE AGING COHORTS IN EUROPE AND CANADA

Chair: Carl-Philipp Jansen
Discussant: Marla Beauchamp

Mobility has been deemed the 'sixth vital sign' because of its ability to predict critical health outcomes in later adulthood. Up to now, there is no consensus on the ideal measurement of mobility, but previous findings and experiences with movement sensors have led to regular application of digital technology. Important but unanswered questions remain around the potential of digital mobility outcomes (DMOs) to monitor individuals' health status, to serve as markers for physical or functional decline, and to predict clinically meaningful events such as falls and hospitalisations. This could provide a better understanding of older adults' and patients' general health based on recurrent monitoring of their mobility. Against this background, data from major cohort studies in Europe and Canada are presented. Marla Beauchamp will present a recently proposed unified framework for mobility measurement, outlining three distinct aspects of mobility and investigating their relationship in a cohort of 1,200 older adults. Carl-Philipp Jansen will complement this data by looking at the same three aspects in a clinical cohort of 566 hip fracture patients, however, comparing their trajectories over one year. Laura Delgado-Ortiz will explore sensor-derived real-world walking in 550 older adults with COPD with healthy older counterparts, thereby capturing different characteristics of walking activity and gait from a single device worn at subjects' lower back for one week. Karen Van Ooteghem will provide a look beyond single-sensor approaches, highlighting the large potential and presenting principles of multi-sensor approaches to provide comprehensive and meaningful information on mobility in older adults.

Abstract citation ID: igae098.0740

### UNIFIED FRAMEWORK FOR MEASURING MOBILITY IN OLDER PEOPLE: EMERGING DATA FROM A CANADIAN AGING COHORT

Marla Beauchamp, *McMaster University, Hamilton, Ontario, Canada*

In the 2015 World Health Organization (WHO) World Report on Aging and Health, mobility is described as movement in all its forms either powered by the body or by a vehicle. Mobility thus encompasses basic movements such as standing up from a chair to more complex activities such as walking or using transportation. Many outcome measures have been developed to assess mobility, however, the variability in constructs being assessed and lack of standardisation in terminology present challenges to advance research and practice in this area. We propose a unified framework for mobility measurement in older populations consistent with the latest language and terminology endorsed by the WHO. The framework outlines three distinct constructs: perceived mobility ("what can you do?"), locomotor capacity for mobility ("what could you do?"), and actual mobility ("what do you do in daily life?"). The latter construct, actual mobility, has been less well studied until recently. Wearable devices offer the unique advantage of comprehensively monitoring the real-world actual mobility of older people in their homes and communities. In this talk, we will present emerging data from the McMaster Monitoring My Mobility (MacM3) study, a digital mobility cohort of over 1200 community-dwelling older adults with detailed mobility measurements spanning all three aspects of mobility and examine their relationships with clinically important health outcomes. In this way, we will be able to test the suitability of our framework and highlight the potential of digitally derived measures of actual mobility for informing aging research and practice.

Abstract citation ID: igae098.0741

### TRAJECTORIES OF REAL-WORLD VERSUS PERCEIVED MOBILITY AND LOCOMOTOR CAPACITY IN OLDER FRACTURE PATIENTS

Carl-Philipp Jansen[1], Jochen Klenk[2], Beatrix Vereijken[3], Hubert Blain[4], Jorunn Helbostad[3], and Clemens Becker[2], *1. Heidelberg University, Heidelberg, Baden-Wurttemberg, Germany*, *2. Robert-Bosch-Hospital Stuttgart, Stuttgart, Baden-Wurttemberg, Germany*, *3. Norwegian University of Science and Technology, Trondheim, Nord-Trondelag, Norway*, *4. Centre Hospitalier Universitaire de Montpellier, Montpellier, Aquitaine, France*

Hip fracture is the most frequent non-intentional injury of older persons leading to hospital admission in North America and Europe. Hip fracture rehabilitation follows the overall goal to get older persons back on their feet and mobile again, up to the point of functional and mobility-related independence in everyday life. This primary outcome, however, is measured in various forms using different methods and assessments. Oftentimes, a singular view is taken, mainly based on repeated physical and locomotor capacity measures without knowledge of the actual mobility behaviour of patients after hospital care and in real life. Cross-sectional data has shown that the relationship between perceived mobility, locomotor capacity, and real-world mobility is not straightforward, however, it remains unclear how all three aspects evolve over time. For a better understanding of this relationship in hip fracture patients, this talk will address respective data of n=566 participants of the hip fracture cohort in the Mobilise-D project. All three aspects of mobility were assessed at baseline and after 12 months. Based on the results, it will be made clear which aspects should be prioritized in both research and clinical practice to obtain a comprehensive picture and clinically useful understanding of mobility of older hip fracture patients.

Abstract citation ID: igae098.0742

### ASSESSING REAL-WORLD WALKING IN OLDER ADULTS LIVING WITH COPD

Laura Delgado-Ortiz[1], Clemens Becker[2], Thierry Troosters[3], and Judith Garcia-Aymerich[4], *1. Barcelona Institute for Global Health, Barcelona, Catalonia, Spain*, *2. Robert-Bosch-Hospital Stuttgart, Stuttgart, Baden-Wurttemberg, Germany*, *3. KU Leuven, Leuven, West-Vlaanderen, Belgium*, *4. IS Global, Barcelona, Catalonia, Spain*

Walking is crucial for active and healthy ageing, but changes with age and in the presence of chronic conditions. In the field of chronic obstructive pulmonary disease (COPD) – a highly prevalent chronic condition at older ages - extensive research has been done on physical activity limitation, but little is known about the patterns of walking during daily life (i.e., real-world). We aimed to assess the levels and distribution of real-world walking in people with COPD compared with healthy adults, and across disease severity; and to identify domains of walking in COPD, using digital technology. We studied cross-sectionally 550 older adults with COPD and 19 healthy older adults from ten cities across eight countries. We derived twenty-five digital mobility outcomes (DMOs) that assess different characteristics of walking activity and gait (e.g., walking duration, number of walking bouts of different durations, walking speed, cadence, stride length variability) from a single device worn at subjects' lower back for one week. Participants also answered validated questionnaires, and conducted lung function and exercise capacity tests. Most DMOs were reduced in older adults with COPD compared to healthy adults, and according to COPD severity. Differences remained statistically significant after adjusting for age, sex, height and comorbidities in multivariable analyses. Factor analysis identified six distinct walking domains: Amount of walking, Patterns of walking, Pace, Rhythm, Pace variability and Rhythm variability. Real-world walking in older adults with COPD can be characterised in multiple dimensions, is impaired in relation to their healthy peers, and worsens as disease progresses.

Abstract citation ID: igae098.0743

### MULTISENSOR APPROACHES FOR COMPREHENSIVE MEASUREMENT OF REAL-WORLD MOBILITY IN OLDER ADULTS

Karen Van Ooteghem, *University of Waterloo, Waterloo, Ontario, Canada*

Despite the potential for collecting precise and accurate mobility data over extended periods of time, the use of body-worn sensors as an approach to mobility monitoring is at a crossroads. The broad use of consumer smartwatches has led to a focus on single sensor approaches with simple to digest outcomes (e.g., step counts) however, this approach can limit our ability to capture details about how, where and when people are moving which are necessary to guide intervention. In this talk, we will provide evidence for the feasibility and value of a multi-sensor approach to mobility measurement that optimizes a'benefit-to-burden' ratio for older adult participants. Specifically, we will provide examples to highlight how the use of multiple sensors can improve fidelity, reduce uncertainty, and provide important context to the data in order to maximize our understanding of real-life mobility. We will also share lessons learned from several multisensor-based studies of mobility across a spectrum of older adults (healthy, neurodegeneration, exceptional cognitive aging) that have contributed to successful implementation of a multi-sensor approach. The principles presented will inform future work aimed at developing validated standards for multi-sensor derived digital mobility outcomes that would provide comprehensive information on mobility for risk identification, early intervention, and monitoring of disease progression and treatment efficacy.

### SESSION 2175 (SYMPOSIUM)

Abstract citation ID: igae098.0744

### DRAG AS PERFORMANCE, THERAPY, ACTIVISM, AND PERSONAL EXPRESSION: WHAT OLDER DRAG ARTISTS TEACH US

Chair: Laura Donorfio
Co-Chair: Brian Chapman
Discussant: Brian Chapman

Drag artists (also described as drag performers, drag queens, and drag kings) have contributed to world cultures for centuries. Drag artistry, as tied to sexual and gender minority (SGM) communities and aging, is the focus of this presentation. The 20th and 21st centuries have been a poignant time for drag artists in the United States, as they have emerged as national and community leaders, been subject to political attack and violence, and contributed to mainstream and sexual and gender minority cultures. Older adult drag artists hold rich lived experiences and collectively share in episodic events that can be seen as traumatic, unifying, and celebrative. However, this population of older adults is grossly underrepresented in the literature. This symposium includes four presentations bridging behavioral science (individual development and wellbeing), social policy, and practice (drama therapy) of older drag artists. The first presentation frames current anti-drag policies aimed broadly at SGM communities in the United States. The second presentation provides a feminist, gender, and queer theoretical lens for examining the experiences of older drag artists, particularly the topics of homophobia, and ageism. The third presentation presents findings from a mixed methods study examining the intersection of aging, drag artistry, and individual wellbeing. The final presentation details a study on coping mechanisms of older drag artists through the lens of drama therapy clinical assessment. Collectively these papers highlight theory and qualitative and quantitative analysis illuminating themes of resilience, coping, commitment, and conscientiousness that illustrate fortitude within this population of influential artists.

Abstract citation ID: igae098.0745

### SHIELDS OF CHIFFON: THE FORTITUDE/RESILIENCE OF AGING DRAG ARTISTS

Brian Chapman[1], Alaina Brenick[2], and Laura Donorfio[1], *1. University of Connecticut, Waterbury, Connecticut, United States*, *2. University of Connecticut, Hartford, Connecticut, United States*

This presentation discusses findings from a mixed methods study examining the intersection of aging, drag artistry, and individual wellbeing. Drag artists remain an understudied and underrepresented population in scholarship (Knutson et al., 2020; O'Brien, 2018). More specifically, older drag artists have yet to be studied in social science literature (Henneberry et al., 2022). Participants completed a semi-structured interview and a self-administered survey to understand the perspectives of drag artists over the age of 50 (n=22; ages 50-94). Qualitative analysis of the interviews revealed themes related to personhood, family impact, coping, resilience, generativity, community advocacy, activism, and performance. Participant interviews were augmented through an electronic survey that validated themes and provided a greater understanding of personality characteristics illuminated in the interviews. The survey assessed resilience (CD-RISC; Connor & Davidson, 2003), depression (CES-D; Radloff, 1977), personality (TIPI; Gosling et al, 2003), and coping (Brief COPE, Carter, 1997) of 20 drag artists (51-94 years). Descriptive analyses reveal high levels of resiliency (M =80.32/100) and low levels of depression (80% of participants reported below threshold rates/symptomatology). The sample is significantly more extraverted, conscientious, and open than population comparison means (51+ years) (t-tests; p's=0.01-0.001). Repeated measures ANOVA Additionally, results showed that participants used healthier coping methods (i.e., problem-focused>emotion-focused>avoidant (Repeated Measures ANOVA, p's<0.001).

The lived experiences revealed in this study also provides data about the unifying impact of shared episodic events. The session will close with a discussion of potential future research including intergenerational approaches.

Abstract citation ID: igae098.0746

### FORTIFYING SCHOLARSHIP IN THE PUBLIC INTEREST: AN EXAMINATION OF THE MARGINALIZATION OF DRAG ARTISTS

Lyn Holley[1], and Kenneth Hites[2], *1. University of Nebraska at Omaha, Omaha, Nebraska, United States*, *2. University of Nebraska Omaha, Omaha, Nebraska, United States*

Over 14 states have passed or are considering legislation that severely restricts and criminalizes public drag performances. Some bills erroneously label the artistry as "adult cabaret performances" thus, conflating them with nude performances. The Americans Civil Liberties Union, Movement Advancement Project, and other organizations have declared a state of emergency due to this endangerment of constitutionally protected individual human rights. 7.2 to 8 % of the U.S. adult population identifies as sexual and gender minorities (SGM) (HRC, 2021, Gallup,2023). Drag artists are a smaller population within SGM communities and it is believed that the language of anti-drag legislation is designed to also oppress transgender individuals (a separate SGM community). Overall, 2024 has seen 478 anti-LGBTQ bills, 21 of which are drag-specific Several prominent scholars (e.g., Steven Miller of Clemson) have called attention to the similarity between these attacks and the process of "othering" used by Hitler to create public acceptance of annihilation of Jews, the disabled, sexual and gender minorities and ultimately, members of any politically or economically inconvenient group (e.g., older, retired adults). This individual presentation provides a framework to guide scholarly observation and understanding of the current attacks and clarifies the relevant contributions that only scholars can make to produce scientifically valid information and knowledge about Drag Artists and their social and economic impacts on USA society and the economy, thereby strengthening and informing decisions about laws and policy that govern individual freedom of expression.

Abstract citation ID: igae098.0747

### WE'RE ALL BORN NAKED AND THE REST IS DRAG: EXPLORING DRAMA THERAPY WITH AGING DRAG ARTISTS

Camryn Hafner[1], and Jahmar Ortiz[2], *1. Bournewood Health Systems, Somerville, Massachusetts, United States*, *2. New York University, New York, New York, United States*

Due to barriers of mental health stigma, limited accessibility, and ageism, older adults are severely underrepresented among those currently engaging in mental health treatment (Saunders et al., 2021). For older drag artists, additional barriers exist in the lineage of invalidation, pathologization, and discrimination which Western psychology and mental health systems have traditionally afforded gender-expansive individuals (Brewster et al., 2019). Drag artistry itself has been found to offer many salutary functions, including providing a sense of freedom and empowerment, fostering resilience through creative achievement and social support, facilitating catharsis, and/or releasing negative emotions (Knutson et al., 2018; Knutson et al., 2023). However, concerns of depression, substance abuse, stressors, and experiences of discrimination among drag artists represent continued need for inclusive, strengths-based mental health care (Knutson & Koch, 2019; Knutson et al., 2018; Knutson et al., 2023; Tillewein & Kruse-Diehr, 2021). Expressive therapies such as drama therapy and psychodrama may be well-suited to engaging with and enhancing the therapeutic potential of drag art (Nakai & Martinez Di-Carlo, 2024; Soubosky, 2021). This presentation details the findings of an exploratory study examining the coping mechanisms of drag artists (n = 5) through the lens of a drama therapy clinical assessment (Ortiz, 2024). Building upon evidence-based treatment protocols (e.g., Keisari, 2021; Lahad et al., 2013), presenters also consider best practices and implications for the treatment of aging drag artists in drama therapy.

Abstract citation ID: igae098.0748

### THE POWER OF EPHEMERAL GESTURES: THEORIES OF GENDER AND PERFORMANCE

Nels Highberg[1], and Emma Chamberlin[2], *1. University of Hartford, Hartford, Connecticut, United States*, *2. University of Connecticut, Storrs, Connecticut, United States*

Questions of what defines gender have long been central to theorists, activists, and artists who place gender and sexuality at the center of their work. This presentation provides an overview of feminist and queer theories that can deepen the thinking of those who engage with drag performers. We begin by providing a definition of cultural hegemony informed by the work of Antonio Gramsci and James Lull. Theorizing of power and ideology – including ageism, sexism, and homophobia – Gramsci and Lull argue that dominant ideologies result from intergroup interactions like those exemplified in the anti-drag legislation that dominates the United States. From there, we shift to feminist and queer theories of performativity and identity formation. We start with the foundational work of Judith Butler, whose "Gender Trouble: Feminism and the Subversion of Identity" in 1990 and "Who's Afraid of Gender?" in 2024 ask how much of "gender" comes not from an essential, internal core but from repeated, unconscious actions enforced by external socio-political forces. That leads to the value of performance and gesture in the creation of a lasting queer legacy. José Esteban Muñoz theorized a queer ephemera that includes drag in his 2009 publication of Cruising Utopia: The Then and There of Queer Futurity, highlighting how gestures in drag aid in the creation of a culturally forbidden queer history, a history that becomes richer and more complex as these drag artists – along with theories about gender and sexual minorities in general – age.

### SESSION 2180 (PAPER)

END-OF-LIFE CARE

Abstract citation ID: igae098.0749

### INFLUENCE OF HEALTH CARE PROFESSIONALS ON END-OF-LIFE AND PLACE OF DEATH: A LATENT CLASS ANALYSIS

Autumn Decker[1], Raven Weaver[2], Cory Bolkan[2], and Brittany Cooper[2], *1. Pacific University, Hillsboro, Oregon, United States*, *2. Washington State University, Pullman, Washington, United States*

People experience end-of-life (EOL) differently due to unique lived experience and sociopolitical position. Most people prefer to die at home, and evidence suggests that healthcare providers have a role to play in fostering a positive EOL experience. This study aimed to understand classes of healthcare provider involvement in the EOL experience and examine the association between class membership and place of death by living alone, race, and/or geographic location. We conducted a latent class analysis with covariates and a distal outcome using Waves 9-12 (n=1290) from the last month of life interview in the National Health and Aging Trends Study. Based on fit statistics, response probabilities, and class separation, a four-class model was selected. These classes were Discordant Death (DD), Controlled Death (CD), Harmonious Death, and Comforted Death. Living alone ($p <.001$), being White ($p =.015$) or Black ($p =.047$), and living in the Northeast ($p =.015$) or Midwest ($p =.039$) were significantly associated with latent class membership. Those living alone and in the DD class were most likely to die in a location outside the home, Black individuals in the CD class were most likely to die in a hospital, and those living in the Northwest and in the CD class had the highest probability of dying at home. This work demonstrates that health provider factors have influence over the experiences one has at EOL, and ultimately, their place of death. Future research should explore concordance between EOL wishes and the EOL experience.

Abstract citation ID: igae098.0750

### HOW DOES THE USE OF HIGH-COST TREATMENTS IMPACT UTILIZATION OF THE MEDICARE HOSPICE BENEFIT?

Michael Plotzke[1], and Thomas Christian[2], *1. Abt Associates, St. Louis, Missouri, United States*, *2. Abt Associates, Cambridge, Massachusetts, United States*

Medicare patients who may benefit from palliative chemotherapy, radiation, or blood transfusions for symptom management (hereafter called “high-cost treatments”) may face difficulties accessing those treatments while electing hospice. The Medicare hospice benefit allows these high-cost treatments, but the treatments’ cost may exceed hospice agencies’ Medicare payments, limiting hospices’ ability to cover high-cost treatments. Lack of access to high-cost treatments may discourage transitions to hospice, resulting in costly and less patient-centered care. Using 100% Fee-for-Service (FFS) Medicare Part A/B claims, we examine how frequently these high-cost treatments are used at the end of life and how hospice use varies by the receipt of high-cost treatments. 7,631,620 FFS Medicare beneficiaries died from FY 2017-2021 and roughly 19.8% of those received at least one high-cost treatment in the 180 days before death (8.3% used chemotherapy, 3.73% used radiation, 12.8% used transfusions) and 47.1% used hospice in the 180 days before death. Of the 786,402 beneficiaries that used both hospice and at least one high-cost treatment in the 180 days before death, only 16,478 (2.1%) used hospice and a high-cost treatment simultaneously. We also found that the average hospice length of stay for those using high-cost treatments in the last 180 days of life is substantially shorter than for beneficiaries electing hospice without using those services (chemotherapy recipients - 22.9 days, radiation recipients – 23.2 days, transfusion recipients -25.5 days, non-recipients – 105.6 days). These findings may indicate barriers to receiving hospice for beneficiaries who may benefit from high-cost palliative treatments.

Abstract citation ID: igae098.0751

### TRENDS IN MEDICARE BENEFICIARY PAYMENTS FOR NONHOSPICE SERVICES DURING HOSPICE ELECTIONS

Thomas Christian[1], and Michael Plotzke[2], *1. Abt Associates, Cambridge, Massachusetts, United States*, *2. Abt Associates, St. Louis, Missouri, United States*

Hospice election marks a shift towards palliative objectives, although beneficiaries retain curative coverage for unrelated (non-terminal) conditions. Little is known about beneficiary cost sharing for these services. Using Federal Fiscal Year (FY) 2022 Medicare claims, we cross-referenced beneficiaries’ hospice and non-hospice services claims’ dates. For overlapping claims, we totaled beneficiaries’ liability payments (coinsurance and deductibles) by year, claim type, and ICD-10 code. We used linear regression analysis to assess trends with beneficiary and hospice characteristics. We also compared beneficiary payments to Consumer Assessment of Healthcare Providers & Systems (CAHPS) Hospice score star ratings. In FY2022, Medicare paid $931.1 million for non-hospice services during hospice election, for which beneficiaries were collectively billed $206.8 million (18.2%), with 45.0% of hospice beneficiaries paying some amount. The majority was for services on or after the 60th day from hospice admission. Part B accounted for most (88.9%) of the total beneficiary payments. Pressure ulcers, end stage renal disease, hypertension, and COVID-19 were associated with the greatest payment amounts. Beneficiary payments were largest among beneficiaries black or Hispanic, or younger, or terminal heart disease, or dual-eligible for Medicaid, or in a nursing facility, or from a newer, for-profit, or southern hospice. Daily payment rates were $0.32 at the 10th percentile and $3.31 at the 90th percentile. Daily payment rates were $2.17 at CAHPS one-star hospices vs. $1.00 at five-star hospices. CMS should monitor these trends to ensure that vulnerable beneficiaries are not unfairly impacted and evaluate whether beneficiary payments are a useful hospice quality indicator.

Abstract citation ID: igae098.0752

### INPATIENT AND OUTPATIENT PALLIATIVE CARE AMONG VETERANS: THE NATIONAL LANDSCAPE

Brystana Kaufman[1], Sandra Woolson[2], Madison Burns[3], Jessica Ma[1], Joshua M. Thorpe[4], Susan Hastings[3], David Bekelman[5], and Courtney Van Houtven[1], *1. Duke University School of Medicine, Durham, North Carolina, United States*, *2. Durham VA Health Care System, Durham, North Carolina, United States*, *3. Durham VA, Durham, North Carolina, United States*, *4. VA Pittsburgh Healthcare System, Pittsburgh, Pennsylvania, United States*, *5. Eastern Colorado Health Care System VA, Aurora, Colorado, United States*

Palliative care (PC) improves quality of life for people with life-limiting conditions (LLC), which is common among older adults. In the United States, use of PC in the outpatient setting has expanded over the past decade; yet, most research has focused on inpatient PC. This study compared characteristics and use for veterans with inpatient or outpatient PC care nationally. The sample included veterans with specialty VA PC encounters, identified using VA and Medicare administrative data (2014-2017). Specialty PC encounters were identified using clinic stop codes (353, 351) and current procedural terminology codes (99242-99245). Of 120,249 unique veterans with specialty PC, 67.8% had PC only in the inpatient setting (n=81,523) and 32.2% had at least one PC encounter in the outpatient setting (n=38,726). One third experienced the first PC encounter within 2 years of meeting LLC criteria. Outpatient versus inpatient PC users were more likely to have cancer and less likely to have high frailty, but sociodemographic factors including rurality and housing instability were similar. Duration of hospice use was similar between inpatient (median = 37 days; interquartile range, IQR =11, 112) and outpatient (median=44 days; IQR=14, 118) PC users, and shorter among those with only one PC encounter (median =18 days; IQR=5, 64). Among veterans with PC use, one third received at least some PC in the outpatient care setting. Differences between veterans with inpatient and outpatient use motivates the need for further research to understand how care setting and timing of PC impacts outcomes for veterans and older adults.

Abstract citation ID: igae098.0753

### IMPLEMENTATION OF COMMUNITY-BASED PALLIATIVE CARE: A SYSTEMATIC SCOPING REVIEW

Dorian Ho[1], Nicole Dissault[2], Judith Vick[3], Lesley Skalla[2], Haripriya Dukkipati[2], Jessica Ma[4], and Brystana Kaufman[4], *1. Duke-Margolis Institute for Health Policy, New Providence, New Jersey, United States*, *2. Duke University, Durham, North Carolina, United States*, *3. Yale University School of Medicine, New Haven, Connecticut, United States*, *4. Duke University School of Medicine, Durham, North Carolina, United States*

Community-based palliative care (CBPC) models, which include palliative care (PC) delivered in outpatient clinics and homes, improve quality of life for people living with serious illness and their care partners. CBPC access has been expanding across the United States, with the goal of delivering PC earlier in the trajectory of serious illness care. This systematic scoping review aimed to summarize provider perspectives regarding barriers and facilitators to CBPC program implementation. MEDLINE and CINAHL were searched for peer-reviewed qualitative research published between 1/1/2010 and 1/9/2024 that included provider perspectives on CBPC, excluding hospice or end-of-life care. Two reviewers screened articles and extracted themes. Framework synthesis was used to categorize themes into provider, organization, and environmental levels according to the PRISM (Practical, Robust Implementation and Sustainability Model) framework. Thirty-five articles were included, varying by providers (PC providers, referring providers, administrators) and practice settings (PC clinic, specialty-embedded PC clinic, home). At the provider level, misperceptions of PC by referring providers, poor inter-provider communication, and unclear roles were perceived barriers to CBPC. Conversely, multidisciplinary PC teams, referring provider PC education, and collaboration were perceived facilitators. At the organizational level, time constraints were perceived barriers while leadership buy-in, shared medical records, and colocation of PC into specialty clinics were perceived facilitators. At the environment level, limited PC workforce and PC reimbursement were perceived barriers. Few articles addressed cultural sensitivity; standardized performance data; and social needs resources. Successful CBPC implementation requires addressing factors at the provider, organizational, and environmental levels.

Abstract citation ID: igae098.0754

### THE ROLE OF HEALTH CARE STEREOTYPE THREAT IN END-OF-LIFE PLANNING AMONG OLDER SEXUAL MINORITY ADULTS

Meki Singleton, *Rutgers University, East Orange, New Jersey, United States*

Healthcare stereotype threat (HCST), defined as “the threat of being reduced to group stereotypes within healthcare encounters”, may occur when social identities negatively impact healthcare experiences. Prior research has shown that individuals report experiencing HCST related to age, gender, weight, race/ethnicity, HIV status, and sexual orientation. However, little is known about how HCST may be associated with end-of-life care planning. The aim of this study was to examine the association between comfort with medical decision making and advance care planning (ACP) activities and HCST related to sexual orientation and race. We conducted an online survey from 2022—2023 to collect information about older sexual minority (SM) adults’ ACP activities and perceptions of HCST. Respondents (N=281) included Black (n=133) and White (n=148) SM adults. Logistic regression revealed that those with higher levels of HCST–sexual orientation had 13% lower odds of being very comfortable with medical decision-making ($p < 0.001$) and 6% higher odds of having ACP discussions ($p < 0.05$). Higher rates of HCST–race was associated with 8% lower odds of being very comfortable with medical decision-making ($p < 0.05$) and 10% higher odds of having ACP discussions ($p < 0.001$), 7% higher odds of having completed a living will ($p < 0.05$), and 10% higher odds of having designated a healthcare proxy ($p < 0.001$). Findings demonstrate that HCST may negatively impact comfort in healthcare decision-making while also potentially motivating older SM adults to formally engage in ACP. Research is needed to investigate the barriers and challenges to engaging in ACP among older SM adults and interventions to reduce HCST.

### SESSION 2185 (PAPER)

### ENVIRONMENT AND DISASTERS

Abstract citation ID: igae098.0755

### THE RELATIONSHIP BETWEEN EVACUATION AND HEALTH CARE OUTCOMES AMONG ASSISTED LIVING RESIDENTS AFTER HURRICANE IRMA

Cassandra Hua[1], Sweta Patel[2], Kali Thomas[3], Dylan Jester[4], Lindsay Peterson[5], Debra Dobbs[5], Ross Andel[6], and

David Dosa[2], *1. University of Massachusetts Lowell, Lowell, Massachusetts, United States*, *2. Brown University, Providence, Rhode Island, United States*, *3. Johns Hopkins University, Baltimore, Maryland, United States*, *4. VA Palo Alto Healthcare System, Palo Alto, California, United States*, *5. University of South Florida, Tampa, Florida, United States*, *6. Arizona State University, Phoenix, Arizona, United States*

Evacuation is associated with adverse outcomes among nursing home residents during hurricanes, but the impact on assisted living (AL) residents remains unknown. This retrospective cohort study examined the relationship between evacuation and healthcare outcomes (i.e., emergency department visits, hospitalizations, mortality, and nursing home visits) among Florida AL residents exposed to Hurricane Irma. We identified a cohort of Florida AL residents who were 65+ years old, enrolled in Medicare Fee-For-Service, and resided in 9-digit ZIP codes corresponding to US assisted living communities with ≥25 beds on September 10th, 2017, the day of Hurricane Irma's landfall. We performed 1:4 propensity score matching. Using the matched sample, we examined the relationship between evacuation status and health outcomes within multilevel logistic regression models with a random intercept. Models adjusted for neighborhood disadvantage and distance to the storm trajectory. Of 25,130 Florida AL residents in our cohort, 3,402 (13.5%) evacuated and 21,728 (86.5%) did not evacuate. When compared to sheltering-in-place, evacuation was associated with a 16% greater odds of emergency department visits (adjusted odds ratio [AOR]=1.16, p=0.037, 95% CI 1.01-1.33) and 51% greater odds of nursing home visits (AOR 1.51, 95% CI: 1.14-2.00) within 30 days of Hurricane Irma's landfall. Hospitalization and mortality did not vary significantly by evacuation status within 30 or 90 days after the landfall date. Stress caused by evacuation may yield poorer immediate health outcomes after a major storm for AL residents. Potential benefits and harms of evacuating vs sheltering-in-place must be carefully considered when developing emergency planning and response.

Abstract citation ID: igae098.0756

### DISASTER AND EMERGENCY SUPPORT NETWORKS OF FAMILIES PROVIDING CARE TO PERSONS LIVING WITH DEMENTIA

Sato Ashida[1], Freda Lynn[1], Maria Donohoe[1], and Emily Killian[2], *1. University of Iowa, Iowa City, Iowa, United States*, *2. University of Iowa College of Public Health, Iowa City, Iowa, United States*

Persons living with dementia (PLWD) and their caregivers are especially vulnerable in disaster situations. Research describes the composition and function of caregiving support networks, but not how these networks support families in terms of disaster preparedness. Currently, a study is underway to investigate disaster and emergency support networks and caregiver resilience. The baseline data collected thus far show that caregivers (N=59) identified 633 support members in total (M=10.4 members per respondent) via four prompts: family members important to PLWD (25%), others important to PLWD (37%), others important to themselves (25%), and additional members who would support them in disaster situations (12%). Of all named members, respondents identified 44% (M=4.5 members per respondent) as those who would provide assistance in emergency situations. However, respondents shared emergency preparedness information with only 9% (M=1 member) and discussed disaster plans with only 16% (M=1.7 members). Respondents' perceived preparedness to provide care in general was higher compared to their perceived preparedness to provide care in emergency situations (p<.001 for difference in means). Respondents with a greater number of assistance providers in their network reported higher caregiving preparedness in disaster situations (b=0.09, p=0.02) but not higher caregiving preparedness in general. Results suggest that caregivers may mobilize their support networks differently in the context of general caregiving versus caregiving in emergency situations. Efforts to increase discussions about disaster preparedness and management plans with support network members will be beneficial. Future research should focus on identifying strategies to strengthen caregiving support networks to be resilient in disaster situations.

Abstract citation ID: igae098.0757

### EXPERIENCES WITH AND PREPAREDNESS FOR CLIMATE EMERGENCIES IN OREGON ASSISTED LIVING COMMUNITIES

Jacklyn Kohon[1], Dani Himes[1], Paula Carder[2], and Ozcan Tunalilar[1], *1. Portland State University, Portland, Oregon, United States*, *2. OHSU-PSU School of Public Health, Portland, Oregon, United States*

Assisted living communities in the U.S. provide housing and care services to a sizable share of older adults and people with disabilities (over 900,000 individuals). The recent increase in climate-related emergencies has underscored the need for these communities to have preparedness measures in place to protect residents and staff. State agencies require preparedness plans, but these plans cannot anticipate all events. Oregon presents an important case study as a pioneer state in community-based care as well as with recent experience in various natural disasters. We interviewed administrators and management company operators (n=14), surveyed 140 direct care workers, and conducted focus groups with direct care workers (n=23) to learn about experiences during recent extreme heat events, wildfires, and ice storms. Thematic analysis revealed several themes related to the existing strengths and challenges in the current systems in place for responding to these emergencies. Strengths included an "all hands on deck" approach demonstrating teamwork among frontline workers, a commitment to show up for the residents in all conditions, and improved relationships with emergency management offices. Challenges included power and internet outage-related interruptions in technology for basic care delivery, insufficient plans for evacuation of residents with complex needs, increased staff injury, and lack of safe transportation. Our findings identify several opportunities and caution against pitfalls for regulators and policymakers in maintaining an acceptable level of emergency preparedness in AL settings.

Abstract citation ID: igae098.0758

### WE CANNOT WALK THERE ANYMORE: EXPERIENCES OF URBAN STRESS IN AGE-FRIENDLY ENVIRONMENTS

Ad Maulod, Malcolm Ravindran, and Yunjie Wong, *Duke-NUS Medical School, Singapore, Singapore*

Urban stress, or tension attributed to city living, is often experienced by individuals in urban areas due to environmental and social stressors. The life-space framework assesses older persons' mobility based on the extent of movement within their environment while engaging in activities, and whether assistance is required for said movement. Chronic exposure to urban stress affects older persons' life-space, and is associated with reduced quality of life. Yet, the connection between urban stress and life-space has not been fully explored in the existing literature. Singapore is the third most densely populated city in the world, ranking highly in terms of liveability. However, the country also undergoes frequent redevelopment, which presents a source of urban stress for older Singaporeans who must continually adapt to changing and unfamiliar landscapes. This presentation draws from 'go-along' and in-depth interviews with 60 older Singaporeans. Findings highlight challenges in the lived environment and the difficulties individuals experience travelling to places and activities that are important to their well-being. Despite the emphasis on developing age-friendly infrastructure in Singapore, incompatible person-environment fit was evident among participants who struggle with accessibility, wayfinding, social exclusion, heat and poor home ventilation, care options, and lack of choices in living situations. These issues limit older persons' life-space capacities and hinders aspirations to age well. Policies promoting ageing-in-place must (i) consider multi-level, intersectional factors comprising built environments, individual resources and social contexts that may mitigate frailty and urban stress, and (ii) involve older persons in designing environments which promote autonomy and belonging.

Abstract citation ID: igae098.0759

### AGE DISPARITIES IN EXPOSURE TO HEAT WAVES, EXTREME WEATHER, AND WILDFIRES IN A NATIONAL UNITED STATES SAMPLE

Roger Wong, *State University of New York Upstate Medical University, New York, United States*

Severe climate events have become increasingly frequent. Extensive research has shown that older adults are more adversely impacted by these climate events, often due to more prevalent health conditions contributing to functional impairment. There is, however, no existing research that has examined whether age disparities may exist across exposures to different types of climate events, especially with a national sample. To address this gap, the 2022 American Trends Panel data were analyzed to examine age group differences in exposure to heat waves, extreme weather (flood or intense storm), and wildfires in a nationally representative U.S. sample of 10,282 adults aged 18 and older. In a multiple logistic regression model, adults aged 65+ had 42% significantly lower odds of exposure to heat waves compared to adults aged 18-29 (adjusted odds ratio [aOR]=0.58, 95% CI=0.45-0.76, p<.001), after applying survey sampling weights and adjusting for a variety of sociodemographic covariates. For extreme weather exposure, adults aged 65+ had 44% significantly lower odds compared to adults aged 18-29 (aOR=0.56, 95% CI=0.43-0.72, p<.001). The odds of wildfire exposure was 45% significantly lower among adults aged 65+ compared to adults aged 18-29 (aOR=0.55, 95% CI=0.38-0.79, p<.01). Across all age groups, our results indicate older adults are the least likely to be exposed to climate events related to heat, extreme weather, and wildfire despite the higher strain placed on this populations. Future research is warranted to understand factors contributing to lower climate event exposures, and degree of burden when these different climate events occur among older populations.

Abstract citation ID: igae098.0760

### THE LONG-TERM EFFECTS OF AIR POLLUTION ON ELDERLY HEALTH: AN EMPIRICAL STUDY FROM CHINA

Xiaotong Tang, and Hong Mi, *Zhejiang University, Hangzhou, Zhejiang, China (People's Republic)*

Healthy elderly individuals are assets to families, communities, and the economic development. Identifying and quantifying long-term effects of environmental factors on elderly health could bring new evidence and policy insights for optimizing pollution response, promoting healthy aging, and unlocking a second demographic dividend. This paper matches city air pollution data from 2003 to 2020 with individual data taken from the China Health and Retirement Longitudinal Study (CHARLS) for the years 2013 to 2020. By utilizing changes in wind direction as instrumental variables, it identifies the long-term health impact of average PM2.5 pollutant concentration on China's elderly population over 1-10 years. The study finds: (1) Air pollution has a significant long-term negative impact on the health level of China's elderly population. A 10-year average increase of 1μg/m3 in PM2.5 concentration is associated with a 19% increase in the number of chronic diseases, which exhibits a nonlinear trend. (2) Air pollution has a greater long-term impact on women aged 55-65, population of Northern China, and groups with lower levels of education and consumption. (3) Individuals' avoidance behavior towards air pollution introduces bias in the basic linear regression results, and the negative health impact of long-term exposure to air pollution on the elderly is greater than that of short-term exposure. This paper provides new empirical evidence on the long-term health impacts of air pollution, while offering policy implications for further optimizing response to air pollution and improving the human capital level of the elderly population from an environmental governance perspective.

### SESSION 2190 (SYMPOSIUM)

Abstract citation ID: igae098.0761

### ESPO AND HEALTH SCIENCES SECTION SYMPOSIUM: STRONGER TOGETHER: INTEGRATING CARE PARTNERS AND SOCIAL SUPPORT TO IMPROVE HEALTH OUTCOMES

Chair: Katherine Britt
Co-Chair: Kate Perepezko
Discussant: Beth Fields

Millions of care partners (relatives, partners, or other support persons) provide support to older adults; however, they are not consistently included in health care and social service delivery. This omission is detrimental as care partner inclusion has been shown to improve older adult outcomes, including symptom management, functioning, and mental health (Weinberg et al., 2007). To highlight how care partners can be involved in services and interventions, this symposium will present innovative approaches to integrate care partners and social support to improve health outcomes for older adults. This session highlights the work of early career scientists whose research examines how care partners can be included in interventions to support the health of the older adult for whom they provide care and for themselves. Dr. Mary Wyman will present her work exploring associations of caregiving characteristics (e.g., amount received, number of caregivers, any formal help on board, and primary caregiver gender & relationship) with the utilization of mental health services among older veterans. Dr. DeAnnah Byrd will present on satisfaction with social support, stress, and cognition in older Black Adults. Dr. Katherine Britt will present a qualitative study with providers on the role of family caregivers in cognitive care planning. Dr. Kate Perepezko will present preliminary findings from the Community Aging in Place Advancing Better Living for Elders (CAPABLE): A Scale-Up Project, characterizing care partners' involvement in an aging-in-place intervention. This symposium will conclude with a discussion led by Dr. Beth Fields that will connect each of the presentations.

Abstract citation ID: igae098.0762

### CHARACTERIZING CARE PARTNER INVOLVEMENT IN THE COMMUNITY AGING IN PLACE, ADVANCING BETTER LIVING FOR ELDERS PROGRAM

Kate Perepezko[1], Pam Toto[2], and Beth Fields[3], *1. Scripps Gerontology Center, Lansdale, Pennsylvania, United States*, *2. University of Pittsburgh, Pittsburgh, Pennsylvania, United States*, *3. University of Wisconsin Madison, Madison, Wisconsin, United States*

A growing number of interventions exist to help older adults age safely and independently in their homes, however, many of these programs overlook care partners (i.e., family or friends who provide support). The purpose of the current study was to report on the types of care partner involvement in the Community Aging in Place-Advancing Better Living for Elders (CAPABLE) program. We characterize care partner involvement using multiple data sources: (1) Number of interventionist contacts, (2) Number of goals set, and (3) Quality of care partner interactions with the older adult. We used personas to represent the major types of care partner involvement observed (Miaskiewicz & Kozar, 2011). Preliminary analysis revealed three types of care partners, "Passive Peter," "Collaborative Candice" and "Safety-Conscious Savannah." Passive Peter had minimal communication throughout the program (n=1) and did not identify any goals. Collaborative Candice had some contact with interventionists (n=3) but couldn't attend all visits because she worked full time. Interventionists described her as "working well" with the older adult. She identified two goals for herself. Finally, Safety-Conscious Savannah was involved in one intervention visit and spoke with interventionists on the phone (n=2). She provided support for the older adult's goals and identified her own goal that was focused on the older adult's safety. Interventionists described her as doing a lot of work for the older adult. These findings reveal different ways that care partners are involved in an aging in place intervention and can inform future interventions aiming to include care partners.

Abstract citation ID: igae098.0763

### A QUALITATIVE STUDY IN COGNITIVE CARE PLANNING: THE ROLE OF FAMILY CAREGIVERS

Katherine Britt[1], Xaviera Xaio[2], Kevin Sun[3], Shaoqing Ge[4], and Bin Huang[5], *1. University of Iowa, Iowa City, Iowa, United States*, *2. BrainCheck, Inc., Austin, Texas, United States*, *3. BrainCheck, Inc., austin, Texas, United States*, *4. The University of Texas Austin, Austin, Texas, United States*, *5. BrainCheck, Austin, Texas, United States*

Patients with cognitive impairment and dementia may benefit from cognitive care planning, a process of routinely and systematically evaluating patient needs and making lifestyle recommendations (i.e., physical exercise, healthier eating, medication recommendations, and advance care planning). Though dementia is progressive, tailored lifestyle recommendations and planning can improve emotional health, quality of life, and resource utilization to alleviate the disease burden. However, cognitive care planning is underutilized, and barriers to optimizing the process remain. We conducted a qualitative descriptive study with family care providers (N=9) who provide care for older adults, including patients with cognitive impairment and dementia, to explore the role of family caregivers in cognitive care planning with patients. Conventional content analysis was used. Several facilitators and barriers around family caregivers in cognitive care planning implementation were identified across four categories: uncertainty, burden, absence, and priority. Family caregivers were often older, experiencing their own health challenges, and uncertain how to get help and navigate the healthcare system. Family and caregivers' presence during cognitive care planning was reported as a critical facilitator as it's essential for gathering patient context, documenting changes in daily abilities, and facilitating provider communication. Our study has important implications for stakeholders. Family caregivers indicated education needs for steps to take in cognitive planning when noticing cognitive changes in a loved one. Healthcare systems (i.e., providers, schedulers, clinical staff) need to make provisions for encouraging family companionship in cognitively impaired older adults' checkups, offering support and resources for navigation.

Abstract citation ID: igae098.0764

### DO FAMILY CAREGIVERS MATTER FOR HEALTH CARE UTILIZATION? A STUDY OF MENTAL HEALTH SERVICES USE BY OLDER VETERANS

Mary Wyman[1], Josephine Jacobs[2], Lily Stalter[3], Manasa Ventakesh[3], Corrine Voils[4], Ranak Trivedi[5], Carey Gleason[3], and Byers Amy[6], *1. Veterans Affairs/University of Wisconsin, Madison, Wisconsin, United States*, *2. VA Palo Alto Healthcare System, Palo Alto, California, United States*, *3. University of Wisconsin Madison, Madison, Wisconsin, United States*, *4. W.S. Middleton Memorial Veterans Hospital, Madison, Wisconsin, United States*, *5. Stanford University/VA Palo Alto Healthcare System, Palo Alto, California, United States*, *6. VA San Francisco Healthcare System/University of California, San Francisco, California, United States*

Mental health (MH) concerns are common in late life. Compared to younger adults, however, older adults are much less likely to use MH services. To guide efforts to increase appropriate utilization, it is important to understand factors impacting MH use. Family caregivers represent a novel, yet understudied, pathway. Using a unique data resource combining 2000-2012 U.S. Health and Retirement Study with Veterans Affairs administrative records, we used mixed effects logistic regression to examine the association between receiving assistance from a caregiver and the likelihood of MH utilization in a sample of community-dwelling, primarily male (96.5%) and non-Hispanic white (77.0%) older veterans. After accounting for demographics, caregiver availability, health factors, and socioeconomic status, caregiving receipt was associated with two-fold odds of MH utilization, compared to receiving no assistance (N=8839 person-observations; OR=2.02; 95% CI 1.54-2.65). The relationship between caregiving receipt and MH use was potentiated by the care recipient both being classified as having dementia and as having less severe MH symptoms. Subsequent logistic regression models examined the impact of multiple caregiver characteristics on utilization among veterans receiving assistance. Primary caregiver female gender was the only predictor which retained an independent, positive association with the outcome (N=764; OR=2.95, CI 1.45-6.00). Findings suggest that caregivers represent an underutilized resource to reduce age-related mental health access disparities, but that certain caregiver subgroups (e.g., male caregivers) may benefit from targeted education or support to help facilitate appropriate MH service use by older adults.

Abstract citation ID: igae098.0765

### RECEIVED SOCIAL SUPPORT SATISFACTION MAY INFLUENCE THE PERCEIVED STRESS-COGNITION ASSOCIATION IN BLACK AMERICANS

DeAnnah Byrd[1], David Coon[2], Roland Thorpe, Jr.[3], and Keith E. Whitfield[4], *1. Arizona State University, Phoenix, Arizona, United States*, *2. Arizona State Unviersity, Phoenix, Arizona, United States*, *3. Johns Hopkins University, Baltimore, Maryland, United States*, *4. University of Nevada Las Vegas, Las Vegas, Nevada, United States*

Previous research suggests that perceptions of stress shape cognitive health. Social support has been shown to be protective. Yet, it is not clear whether satisfaction with support is also beneficial. The aim of this study was to determine whether perceived stress is associated with cognitive changes over time and if satisfaction with receipt of emotional or instrumental support acts as a moderator in a sample of older Blacks 48 to 95 years of age, who were enrolled in the Baltimore Study of Black Aging—Patterns of Cognitive Aging and interviewed at two time points, approximately 3 years apart (N = 450). Cognition included 5 domains: working memory, processing speed, verbal memory, vocabulary, and inductive reasoning. Social support satisfaction was measured by asking participants "How satisfied are you with the emotional or instrumental support that you get from your family and friends in terms of how they make you feel and how much it helps you?" Results from the linear regression model showed no direct effect of stress on cognitive decline. Satisfaction with instrumental support marginally ($p < .10$) modified the relationship between stress and working memory at follow-up but was not related to any other cognitive domains, adjusting for age, sex, education, depressive symptoms, comorbidities, and baseline cognition. Findings demonstrate the joint effects of stress and social support satisfaction on cognition, highlighting the need for more research documenting protective factors for domain-specific cognitive decline. This approach provides a basis for developing cognitive interventions and support networks for this population of Black Americans.

### SESSION 2195 (SYMPOSIUM)

Abstract citation ID: igae098.0766

### EXPLORING CONTEMPORARY ISSUES IMPACTING OLDER REFUGEES IN THE UNITED STATES

Chair: Noelle Fields
Discussant: Jonix Owino

Older refugees represent a unique group of the immigrant population as they are often at higher risk for negative health and psychosocial outcomes. Many older refugees also face constraints posed by language and cultural barriers that can affect overall well-being and quality of life. This symposium will provide insights from three studies of older refugees in the United States in an effort to shed light on various issues impacting this population that warrant greater attention from researchers and practitioners. The first paper presents findings from a study of older immigrants and refugees' preparation for weather-related emergencies. Findings suggest the importance of linguistically and culturally tailored communication that fosters trust and improved access to reliable information. The second paper explores the lived experiences of forced displacement and long-term resettlement of Karen refugees from Burma. Study findings point toward the need to re-evaluate the cross-cultural validity of locus of control, an oft used psychological measure applied to refugee populations. The third paper examines how socioeconomic status is differentially associated with cognitive difficulties between older Bhutanese refugees and other refugee groups. Study results suggest that higher income does not serve as a protective factor against cognitive challenges in older refugees from Bhutan. The symposium will conclude with a critical reflection on the empirical and theoretical contributions needed to advance scholarship on older refugees.

Abstract citation ID: igae098.0767

### RE-EVALUATING LOCUS OF CONTROL AMONG OLDER KAREN REFUGEES IN LONG-TERM RESETTLEMENT

Noelle Fields[1], Katherine Kitchens[2], and Diane Mitschke[2], *1. The University of Texas at Arlington, Arlington, Texas, United States*, *2. The University of Texas Arlington, Arlington, Texas, United States*

Recent literature has increasingly applied the concept of locus of control (LOC), a psychological measure for understanding individuals' beliefs about controlling life events, to forcibly displaced populations, including older refugees whose experiences and coping mechanisms may be distinctly influenced by their age and cultural background. The cross-cultural validity of this construct, particularly among collective cultures experiencing long-term resettlement in the Global North, warrants further examination. This study, utilizing interpretative phenomenological analysis within the theoretical framework of homeostatic socio-cognitive systems theory, specifically explored the lived experiences of forced displacement and long-term resettlement among ten older Karen refugees from

Burma resettled in the southwestern United States for an average of over 12 years. Aimed at capturing deep narrative insights, the research sought to extend the discourse on LOC's cross-cultural construct validity, particularly within the context of older refugees adapting to life in a Western context. The findings underscored five emergent themes: Collective Orientation over Individual Agency, Collective Cultural and Communal Living, Interdependence and Communal Support, Shared Control within Social Networks, and the Importance of Relationships. These themes reveal a significant divergence from the individualistic assumptions underlying the LOC construct, highlighting the prominence of communal values, shared decision-making, and the critical role of relational dynamics within the collective cultures of older refugees. This study underscores the need for culturally sensitive research methodologies and intervention strategies that honor the unique lived experiences of older refugees from collective cultures, advocating for a reevaluation of psychological constructs like LOC in cross-cultural contexts.

Abstract citation ID: igae098.0768

### EMERGENCY PREPAREDNESS COMMUNICATION PREFERENCES OF OLDER IMMIGRANTS AND REFUGEES

Holly Dabelko-Schoeny[1], Smitha Rao[1], Marisa Sheldon[2], Anthony Traver[1], Fiona Doherty[1], and Jhuma Acharya[1], *1. The Ohio State University, Columbus, Ohio, United States*, *2. The Ohio State University, Age-Friendly Innovation Center, Columbus, Ohio, United States*

Older immigrants and refugees in the U.S. face unique challenges with transportation, service access, and social integration, all critical risk factors for negative outcomes from extreme weather. This study aimed to: (1) Identify where older immigrants and refugees get information about weather-related emergencies; (2) Explore the role trusted information sources play in weather-related emergency-preparedness; and (3) Understand what supports older immigrants and refugees need to prepare for weather-related emergencies. Eleven focus groups were conducted (in English, Nepali, Tigrinya, Kinyarwanda, Somali, Amharic, Mandarin Chinese, Russian, and Khmer) with adults aged 62 and older (N = 56) in a large metropolitan Midwestern U.S. city. Most participants were immigrants and refugees, had annual incomes below $15,000, and lived alone. Preliminary analyses reveal television, radio, phone alerts, and school notifications were predominant information sources. Participants emphasized the importance of receiving communication in their native language, translation services, and simple visuals conveying preparedness information. Participants stressed the importance of reliable information sources and personal judgment in determining information trustworthiness. When asked about motivation to prepare for extreme weather, participants underscored the desire to avoid getting hurt or sick. Specifically, participants identified the need for better access to air quality information during pollution events. Participants suggested the government provide safe spaces for those without secure homes. Participants also emphasized the importance of home visits to address isolation and assistance with medication pick-up during emergencies. Findings lend insights into unique communication needs and priorities among older refugees and immigrants related to increasing extreme weather events.

Abstract citation ID: igae098.0769

### SOCIOECONOMIC STATUS AND COGNITIVE DIFFICULTY: DIMINISHED RETURNS AMONG OLDER BHUTANESE REFUGEES IN THE US

Katherine Kitchens, and Jaclyn Kirsch, *The University of Texas Arlington, Arlington, Texas, United States*

Refugee populations report cognitive difficulties at a rate significantly higher than observed among older immigrants and native-born U.S. adults. There is evidence that socioeconomic status acts as a buffer against mild cognitive impairment. This study aimed to examine how socioeconomic status is differentially associated with cognitive difficulties between older Bhutanese refugees and other refugee groups in the United States (N = 5,666), using the 2021 American Community Survey five-year estimates and the official refugee counts from the Department of Homeland Security. Binary logistic regression analyses examined the moderating effect of Bhutanese refugee status on the association of income level and high school completion with self-reported cognitive difficulty, controlling for demographic and integration variables. High school completion was associated with a 43% decrease in the odds of cognitive difficulty among older refugees (AOR = 0.57, p<.001, 95% CI [0.43,0.75]), yet educational attainment was not a significant protective factor for older Bhutanese refugees, who had almost three times the odds of experiencing a cognitive difficulty compared to other refugee groups (AOR = 2.86, p<.001, 95% CI [1.86,4.40]). Interactions with income level indicated that living above 138% of the federal poverty level significantly increased the odds of reporting a cognitive difficulty (AOR = 2.86, p<.001 95% CI [1.86,4.40], suggesting that higher income does not serve as a protective factor against cognitive difficulty for older refugees from Bhutan, giving evidence of diminished returns. Findings suggest a need for policies that enable Bhutanese refugees to convert their resources more effectively into concrete cognitive health outcomes.

### SESSION 2200 (SYMPOSIUM)

Abstract citation ID: igae098.0770

### FACES OF FORTITUDE: DIVERSE CAREGIVERS PROVIDING COMPLEX CARE

Chair: Heather Young
Co-Chair: Janice Bell
Discussant: Kathleen Kelly

Last year, more than 56 million family caregivers provided unpaid care to a family member, a number that will grow along population aging and increased prevalence of chronic and serious illness. The demands, resources and outcomes of family caregiving are shaped by the social and environmental context and the particular demands associated with the condition of the care recipient. In 2021, California adopted a Master Plan on Aging, including two investments to strengthen community-based supports for caregivers: Improving caregiver resource centers and adult day programs. This mixed method symposium highlights the fortitude of family caregivers and community-based organizations. We focus on specific health conditions in understudied family caregiver populations as well as the interface with formal services. The first paper examines experiences of caregiving and home care among caregivers of persons with Amyotrophic Lateral Sclerosis. The second paper compares caregiving activities and outcomes among caregivers of individuals with Parkinson's Disease/Lewy Body Dementia to those with Alzheimer's Disease and related dementia and other chronic conditions. The third paper examines building family capacity through family and nurse collaboration during acute illness. The fourth presentation describes the impact of adult day care services from the perspectives of family caregivers of persons with dementia. The final presentation, using a large statewide database, describes caregiving demands, unmet needs and resource gaps of diverse family caregivers to inform a caregiving equity plan for the State of California. Together, these papers reflect complexity, diversity and fortitude in family caregiving.

Abstract citation ID: igae098.0771

### SUPPORTING FORTITUDE AMONG FAMILY CAREGIVERS: THE ROLE OF ADULT DAY SERVICES FOR PERSONS WITH DEMENTIA

Heather Young[1], Orly Tonkikh[2], Everlyne Ogugu[2], Jessica Famula[2], and Janice Bell[3], *1. UC Davis, Ashland, Oregon, United States*, *2. University of California Davis, Davis, California, United States*, *3. UC Davis, Davis, California, United States*

Caring for a person with dementia requires family fortitude in terms of commitment, time, energy and ability to manage a trajectory of several years. Family caregivers provide the vast majority of care for over 7 million individuals living with dementia. Many are adult children who deal with competing demands of other family members and employment. Adult Day Services (ADS) offer respite for family caregivers and meaningful support for persons with dementia. This study presents caregiver perspectives from the California Community Program for Alzheimer's Supports and Services Pilot Program that engaged seven sites in a learning community to develop care standards and advance equity. We conducted semi-structured interviews and thematic analysis of recorded transcripts. The sample included 24 caregivers aged 25 to over 85 (12 spouses, 10 adult children and 2 other relatives). Caregivers described ADS services as life sustaining, being a vital resource that enabled them to continue in their role, balancing other demands. ADS improved mental health and physical health, reduced strain, and fostered self-care and engagement in the broader social network. The services also provided education and enhanced their skills in caring, prolonging their ability to continue. ADS services benefit both persons with dementia and caregivers. Future research could illuminate the long-range health care and societal costs and benefits of ADS for both persons with dementia and caregivers. Sustainable solutions bolster fortitude and optimize person and family-centered care.

Abstract citation ID: igae098.0772

### UNDERSTANDING UNMET NEEDS AND SUPPORT AMONG DIVERSE FAMILY CAREGIVERS ENGAGED IN COMPLEX

Janice Bell[1], Robin Whitney[2], Benjamin Link[3], Shikha Bhurtel[1], Orly Tonkikh[3], Jessica Famula[3], Kathleen Kelly[4], and Heather Young[5], *1. UC Davis, Davis, California, United States*, *2. San Jose State, San Jose, California, United States*, *3. University of California Davis, Davis, California, United States*, *4. Family Caregiving Alliance, San Francisco, California, United States*, *5. UC Davis, Ashland, Oregon, United States*

More than 56 million family caregivers provided unpaid care to a family member in the last year. This group is increasing in number and diversity, while providing more intense and complex care, as more older adults are living longer with chronic and serious illness. We used assessment and service data from a large statewide database (CareNavTM) collected by 11 California Caregiver Resources Centers statewide to describe the caregiving demands, unmet needs and resource gaps of diverse family caregivers seeking help from the centers. Almost half of the caregivers identified as Black non-Hispanic, Hispanic/Latino, Asian American/Pacific Islander, Native American/Alaska Native or multi-racial/other. Older caregivers and those with low income were more likely to provide ≥40 hours of care weekly and to provide high intensity care (measured with the number of personal care activities supported and weekly hours of care). Younger caregivers, those in racial/ethnic groups other than White non-Hispanic, and those with low income were more likely to perform medical/nursing tasks. These groups of caregivers were also the least likely to have paid or unpaid help and the most likely to report adverse outcomes including fair or poor health status, worsening health and moderate or severe depressive symptoms. Taken together, these findings inform the California Department ofn Aging efforts to develop an equity plan for caregivers and are useful to other states seeking to develop policy to assure that all family caregivers have a range of high-quality services and supports that promote their physical, emotional, and financial wellbeing.

Abstract citation ID: igae098.0773

### NURSE–FAMILY INTERACTION DURING ACUTE HOSPITALIZATION OF OLDER ADULTS

Orly Tonkikh[1], Elena Siegel[1], Yuuko Johnson[1], Everlyne Ogugu[1], and Heather Young[2], *1. University of California Davis, Davis, California, United States*, *2. UC Davis, Ashland, Oregon, United States*

Family caregivers play a vital role for older adults during acute illness, yet there is limited research on the dynamics between nurses and families of older adults in acute care settings. This descriptive qualitative study explored perspectives on interactions during acute hospitalization in medical-surgical units among 24 nurses and caregivers. We conducted semi-structured interviews and used thematic analysis to identify key conditions and patterns of interaction. Nurses acknowledge the importance of family input to tailor and execute care plans to improve patient outcomes. Hospital structures (e.g., discharge planning guidelines) and nursing values and priorities (e.g., patient safety) influence the ways nurses interact with families. For example, nurses take a proactive approach in response to perceived safety and behavioral concerns and discharge planning. Without the structure of a formal assessment of caregiver needs and priorities, nurses rely on families to initiate interaction and glean insights about family context and preferences from anecdotal information. For the most part, nurses and families operate in parallel worlds with three main patterns of interaction: (1) caregiver proactivity leading to bi-directional interaction; (2) unidirectional interaction from nurses to caregivers; and (3) limited interaction. The study highlights lost opportunities for meaningful engagement to advance the older adult's health. While nurses acknowledge the benefits of family engagement, few structures

(e.g., training and standardized assessment) exist to facilitate family involvement in the current patient-centered healthcare system. This provides direction for further exploration of structures to support optimal nurse-family interaction, to enhance continuity and quality of acute care.

Abstract citation ID: igae098.0774

### COPING WITH AMYOTROPHIC LATERAL SCLEROSIS (ALS): EXPERIENCES OF PERSONS LIVING WITH ALS AND THEIR FAMILY CAREGIVERS

Everlyne Ogugu[1], Orly Tonkikh[1], Jessica Famula[1], Heather Young[2], Janice Bell[3], Bronwyn Fields[4], and Bjorn Oskarsson[5], *1. University of California Davis, Davis, California, United States*, *2. UC Davis, Ashland, Oregon, United States*, *3. UC Davis, Davis, California, United States*, *4. Sacramento State University, Sacramento, California, United States*, *5. Mayo Clinic, Rochester, Minnesota, United States*

Amyotrophic lateral sclerosis (ALS) is a progressive neurodegenerative disease that profoundly impacts the lives of people with the condition and their family caregivers. Little is known about the experiences of care at home among individuals with ALS and their family caregivers. This qualitative study explored these experiences in 14 in-depth interviews that included seven persons with ALS and 13 family caregivers. The findings were organized based on the Salutogenic Model of Health. The main sources of stress reported included the overwhelming nature of getting an ALS diagnosis, inadequacy of health care teams in providing emotional support and promoting shared decision-making in regard to the management of ALS, high level of illness acuity, the intense nature of caregiving involved in ALS, high cost of treatments and equipment, difficulties navigating the healthcare system, unavailability of suitable medical equipment, scarcity of paid caregivers competent in managing ALS, and lack of adequate training for family caregivers. Perspectives on positive coping included mental toughness, maintaining a positive outlook about life and the future, being proactive in seeking support to maintain function and quality of life, proactively communicating with care teams, advanced care planning, having a sense of control over ALS management plan of care, and taking a break from caregiving. Instrumental and emotional support from family, friends, community organizations, and healthcare teams, as well as sufficient insurance and financial coverage for ALS treatments, facilitated better coping. Future research and policies should focus on identifying effective multisectoral strategies for supporting people with ALS and their caregivers.

Abstract citation ID: igae098.0775

### FAMILY CAREGIVING FOR INDIVIDUALS WITH PARKINSON'S DISEASE AND LEWY BODY DEMENTIA IN CALIFORNIA

Robin Whitney[1], Janice Bell[2], Benjamin Link[3], Shikha Bhurtel[2], Everlyne Ogugu[3], Orly Tonkikh[3], Kathleen Kelly[4], and Heather Young[5], *1. San Jose State, San Jose, California, United States*, *2. UC Davis, Davis, California, United States*, *3. University of California Davis, Davis, California, United States*, *4. Family Caregiving Alliance, San Francisco, California, United States*, *5. UC Davis, Ashland, Oregon, United States*

Parkinson's Disease (PD) and Lewy Body Dementia (LBD) are related progressive neurodegenerative disorders that primarily affect older adults. Many individuals with PD/LBD require support from a family caregiver. However, caregivers of individuals with PD/LBD are infrequently studied. We extracted data from 2019-2022 in CareNavTM, a statewide electronic platform with data from caregivers served by the eleven California Caregiver Resource Centers, to compare caregiver sociodemographic characteristics, caregiving activities, and outcomes among caregivers of individuals with PD/LBD to those of individuals with Alzheimer's Disease and related dementia (ADRD) and other chronic conditions (OCC). Care recipient condition was examined as a predictor of caregiving outcomes (e.g., loneliness, depressive symptoms, strain, and worsening health) using multivariable logistic regression models. There were a total of n=13,649 caregivers, with a majority caring for someone with ADRD (66%), followed by OCC (23%), and PD/LBD (10%). PD/LBD caregivers were more likely to be older, female, white, college-educated, and retired. PD/LBD caregivers were more likely to care for a spouse, spend >40hrs/wk on caregiving, assist with more personal care, and experience problems with the care recipient waking others up at night. In multivariable models, PD/LBD caregiving was independently associated with caregiving strain (OR 1.2; 95% CI [1.0-1.4], p= 0.01) and worsening health (OR 1.2; 95% I [1.1-1.4], p< 0.01), but not with depressive symptoms or loneliness. PD/LBD caregivers are a unique subset of family caregivers engaged in intense and complex care. Services and supports tailored to this condition and related needs may be warranted to optimize caregiver wellbeing.

### SESSION 2205 (SYMPOSIUM)

Abstract citation ID: igae098.0776

### FORTITUDE IN DIVERSITY: MINORITY ISSUES AND INTEGRATIVE APPROACHES TO COGNITIVE HEALTH IN OLDER ADULT POPULATIONS

Chair: Ladan Ghazi Saidi
Co-Chair: Christina Miyawaki

First, Dr. Ghazi Saidi, an associate professor at the University of Nebraska at Kearney and the symposium chair, will discuss cognitive health disparities and interventions aimed at improving the cognitive health of older adults in minority communities. She will also present the results of a scoping review on cognitive health, including demographic and lifestyle factors that impact cognitive health in older adults living in rural areas. Next, Jeein Jang, a PhD candidate at the University of Massachusetts Boston, will present a cross-sectional study exploring the associations between neighborhood characteristics and activity engagement, with a focus on different activity domains and race/ethnicity, using data from the 2018-2020 Health and Retirement Study. Michelle Xue, a PhD student at Duke University, will introduce a dementia prevention program specifically designed for older Chinese Americans at high risk for dementia, aimed at overcoming barriers such as language and transportation. Maki Karakida, another PhD candidate at UMass Boston, will present on the relationship between work/retirement status and sleep health, emphasizing the impact of race/ethnicity and nativity, particularly in African American and Hispanic populations, among 41,924 Americans aged 50+, showing that intersections of race/ethnicity and nativity significantly influence sleep quality. Lastly, Dr. Miyawaki, an associate professor at the University of Houston, will discuss a cognition program conducted in Houston by Vietnamese-American health professionals and university students, which addresses the gap in dementia care using the Cultural Exchange Model (CEM) to enhance dementia literacy.

Abstract citation ID: igae098.0777

### MODIFIABLE FACTORS IN COGNITIVE HEALTH OF THE RURAL AGING POPULATION: A SCOPING REVIEW

Ladan Ghazi Saidi[1], Vaishali Phatak[2], and Miechelle McKelvey[1], *1. University of Nebraska Kearney, Kearney, Nebraska, United States*, *2. University of Nebraska Medical Center, Omaha, Nebraska, United States*

**Introduction:** This presentation highlights modifiable risk factors that impact the cognitive well-being of older adults in rural regions. Studies on rural population are rare due to its challenges. Considering the global distribution of the older adults, with more than 20% of America's older population residing in rural locales, it is essential to recognize the specific risk factors inherent to these areas to foster healthy aging. We summarize the literature on cognitive health at aging in rural populations.

**Method:** Articles were sourced from multiple databases and were subjected to rigorous inclusion and exclusion criteria following PRISMA guidelines. The initial pool of 92 articles was refined to 13 for an in-depth analysis.

**Findings:** The findings from our scoping review reveal three primary elements related to the rural context affecting cognitive health. We conclude that disparities are associated with demographics and healthcare access; facets of quality of life like physical activity, sleep patterns, and social engagement are essential in healthy aging; and overall physical and mental health play a vital role in healthy cognition in aging.

**Conclusion:** Research on healthy aging and Alzheimer's Disease and Related Dementias (ADRD) should consider rural residency as a possible risk factor.

Abstract citation ID: igae098.0778

### PROTECTING THE HEALTH OF VIETNAMESE REFUGEES BY LOCAL HEALTH PROFESSIONALS AND UNIVERSITY STUDENTS

Christina Miyawaki[1], Kim Nguyen[1], Tuong-Vi Ho[2], and Angela McClellan[1], *1. University of Houston, Houston, Texas, United States*, *2. Texas Woman's University, Houston, Texas, United States*

Nearly 50 years after the fall of Saigon (1975), studies continue to show the high prevalence of physical (ADL, IADL), mental (depressive symptoms), and cognitive (dementia) disabilities in older Vietnamese refugees. Adverse lifelong experiences in Vietnam and host countries may have impacted these results. Some refugees who arrived during their youth became health professionals while their children attend universities, predominantly enrolled in pre-health courses. Leveraging their bilingual/bicultural and health background, they have hosted free health fairs for their monolingual older Vietnamese for the past 16 years in Houston, Texas, the 2nd largest Vietnamese-populated city in the nation. Despite the high prevalence of dementia, no free cognitive healthcare has been provided. The purpose of the study is to report the collaborative efforts to fill the gap using the Cultural Exchange Model (CEM) and improve dementia literacy. The CEM teaches that to create knowledge, two constituencies need to merge within the Community. One arm of Vietnamese stakeholders (older adults, medical doctors) already exists. We formed another arm, Cognitive Health Initiative (CHAIN): 28 bilingual/bicultural Vietnamese university students, who were trained in Montreal Cognitive Assessment (MoCA). During 2023, we jointly attended 11 health fairs with Vietnamese medical professionals and offered 337 MoCA in Vietnamese and English. The results showed that older Vietnamese (≥65) scored the lowest (22.4/30) among participants (White=24.8; Black=23.9; Hispanic=23.3). Utilizing existing, underlying resilience and the unique affinity among local Vietnamese health professionals, our next steps include continuing to offer a "Memory Booth" by local Vietnamese pre-health students, thereby strengthening intergenerational relationships.

Abstract citation ID: igae098.0779

### PERCEIVED NEIGHBORHOOD CHARACTERISTICS AND ACTIVITY ENGAGEMENT AMONG RACIALLY DIVERSE OLDER ADULTS

Jeein Jang, Jaqueline Avila, and Jeffrey Burr, *1. University of Massachusetts Boston, Boston, Massachusetts, United States*

Engagement in social, cognitive, and physical activities is vital for healthy aging. Perceived neighborhood environment is associated with activity engagement, but there may be differences in this association between racial and ethnic groups. This study investigates the associations between neighborhood characteristics and activity engagement, emphasizing different activity domains and how race/ethnicity modifies this relationship. Using Health and Retirement Study data, the sample includes 9,180 adults aged 50 and above. The outcome is engagement in a list of 21 activities in the 2018-2020, measured as a total composite score and by domain (social, cognitive, and physical activities). The predictors are neighborhood social cohesion and physical disorder. Poisson regression analyses show a positive association between social cohesion and total activity engagement among older adults. Physical disorder is also positively associated with total activity engagement, suggesting that older adults in neighborhoods with high physical disorder may have tight-knit communities and strong bonds, working together to prevent further deterioration. Black older adults exhibit 1.03 times greater activity engagement than non-Hispanic Whites, while there were no differences compared to Hispanic individuals. Importantly, the association between social cohesion and overall activity engagement is weaker among Hispanics, even with the highest cohesion level. This suggests that Hispanics may prioritize individual autonomy in their activity choices over the influence of neighborhood social cohesion. Results by activity domains showed that the impact of social cohesion extended to each domain. Racial and ethnic differences warrant closer investigation in studies of neighborhoods and activity engagement in later life.

Abstract citation ID: igae098.0780

### INTERSECTION OF RACE/ETHNICITY, NATIVITY, AND EMPLOYMENT IN SLEEP QUALITY: A MULTILEVEL MODELING STUDY

Maki Karakida[1], and Jeffrey Stokes[2], *1. University of Massachusetts Boston, UMass Boston, Massachusetts, United States*, *2. University of Massachusetts Boston, Boston, Massachusetts, United States*

Sleep disorder is a common problem seen in middle-aged and older adults. Work-related stress can also impact people's sleep quality, yet differences between working and retired older adults' sleep is unclear. This study examines the relationships between work, retirement, and sleep health, including their intersection with race/ethnicity and nativity, among 31,868 Americans aged 50+. Using the Health and Retirement Study samples (2014 – 2020), we analyzed how diverse older workers and retirees experienced their sleep health differently. We used multilevel linear regression to examine the associations between working/retirement status, race/ethnicity, birthplace, and sleeplessness, controlling for age, gender, birthplace, assets, education, marital status, smoking, exercise, and self-reported health. The regression model without interaction terms demonstrated that compared to retirees, older workers have a significantly higher likelihood of sleep disorder. Black race, assets, education, and age had positive and significant relationships with sleep deprivation, whereas martial loss (divorced, widowed, and never-married) and female gender had significantly lower onsets of sleep disturbances. In the model examining the intersection of race/ethnicity and nativity - interacted with work status - revealed that, compared with US-born non-Hispanic White adults, US-born and foreign-born non-Hispanic Black adults had a significantly higher risk of insomnia. In contrast, non-native Hispanic workers and foreign-born non-Hispanic Other workers had a significantly higher prevalence of sleep difficulties than US-born non-Hispanic White workers. Multilevel findings suggest that the intersectionality of race/ethnicity and nativity plays a decisive role in the association of work status and sleep quality among midlife and older adults in the US.

Abstract citation ID: igae098.0781

### CO-DESIGNING A MOBILE-BASED COGNITIVE TRAINING WITH OLDER CHINESE AMERICANS AND THEIR ADULT CHILDREN

Tingzhong (Michelle) Xue[1], Eleanor McConnell[2], Amy Wei[3], Bei Wu[4], and Hanzhang Xu[2], *1. Duke University School of Nursing, Durham, North Carolina, United States, 2. Duke University, Durham, North Carolina, United States, 3. Duke University School of Medicine, Durham, North Carolina, United States, 4. New York University, New York, New York, United States*

Older Chinese Americans are at high risk for dementia, yet often fail to access to prevention services/programs due to language barriers, transportation issues etc. BrainHQ is a mobile-based, effective cognitive training program that can potentially address these barriers and delay cognitive decline in older Chinese Americans. In this study, we will apply an experience-based co-design approach that leverages the existing BrainHQ features and older Chinese Americans' prior knowledge, lived experience, and social norms around dementia to co-develop a cognitive training intervention. We first have worked with BrainHQ to translate and adapt more than 30 evidence-based cognitive training exercises into Chinese. We then hosted focus groups among older Chinese Americans (n=21) to understand their perceived benefits and barriers regarding participating in cognitive training. We also interviewed their adult children separately as they are often influenced by the ideology of filial piety and assume a major responsibility for caregiving. Utilizing the findings of the focus groups, we will then conduct an experience-based co-design workshop with older Chinese Americans (n=6), their adult children (n=6), and community stakeholders (n=2) to improve the cultural and linguistic relevance of the intervention. We will start with a journey map where participants are guided to brainstorm challenges (e.g. fears and anxieties related to performance, time constrains, distrust) they may experience when participating in the intervention. Then they will be asked to create prototypes of intervention components to address these challenges. These findings will facilitate better delivery of the cognitive training among older Chinese Americans and inform broader implementation.

### SESSION 2210 (SYMPOSIUM)

Abstract citation ID: igae098.0782

### GET TO KNOW THE NATIONAL INSTITUTE ON AGING: NIA SESSION FOR EARLY-CAREER RESEARCHERS

Chair: Amy Kelley
Discussant: Amy Kelley

The National Institute on Aging (NIA) at the National Institutes of Health, Department of Health and Human Services, supports biomedical and behavioral research with a lifespan focus. NIA research seeks to understand the basic processes of aging, improve prevention and treatment of diseases in later life, and improve the health of older persons, in addition to an emphasis on Alzheimer's disease and related dementias. NIA supports a variety of training and career development opportunities for students, junior faculty, early-career investigators, and emerging scholars. In this session, we will provide an overview of NIA-funded research, followed by a presentation on funding mechanisms and strategies to consider when applying for extramural grants, particularly for early-career scientists at multiple career stages. Finally, attendees will be able to join any number of breakout group discussions led by extramural NIA training staff and program officials to discuss programs, initiatives, mechanisms, and/or policies specific to various career paths.

Abstract citation ID: igae098.0783

### OVERVIEW OF NIA MISSION AND RESEARCH

Richard Hodes, *National Institute on Aging, Baltimore, Maryland, United States*

Dr. Hodes will provide an overview of NIA's structure and mission, in addition to discussing research foci from across the Institute's scientific divisions.

Abstract citation ID: igae098.0784

### HOW TO GET AN NIA GRANT

Kenneth Santora, *National Institute on Aging, Baltimore, Maryland, United States*

Dr. Santora will provide an overview of the NIA application process, funding opportunities, and relevant policy changes for early-career researchers.

### SESSION 2215 (SYMPOSIUM)

Abstract citation ID: igae098.0785

### IMPACT OF EARLY-LIFE EXPERIENCES AND CAREGIVING ON COGNITIVE HEALTH

Chair: Karen Graham

This symposium comprises four studies investigating the impact of adverse childhood experiences (ACEs) and caregiving on cognitive health. In the first study, researchers investigate the association between ACEs and cognition in older African Americans, exploring the moderating role of active coping. While ACEs were not directly associated with global cognitive functioning, a negative relationship was found between active coping and cognition. This underscores the need for further research to understand the complex interplay between ACEs, coping mechanisms, and cognitive health. In the second study, researchers explore the association between ACEs and dementia, using data from the Health and Retirement Study. They found that individuals reporting ACEs have significantly higher odds of dementia, especially among men. This underscores the importance of trauma-informed interventions to reduce dementia onset. The third study focuses on data from the National Longitudinal Study of Youth. It reveals significant associations between childhood parental love and affection, dysfunctional parental relationships, and self-rated memory in older adults. Mediation analysis emphasizes the role of psychological resources in shaping subjective cognition. The fourth study examines the long-term effects of ACEs on mental health among Black dementia family caregivers. Results highlight the detrimental impact of childhood trauma on depression, depressive symptoms, and suicidal thinking. These findings emphasize the need for targeted interventions to mitigate the mental health consequences of ACEs in caregiving contexts. Together, these studies provide valuable insights into the multifaceted relationships between early-life experiences, caregiving, and cognitive health, highlighting the importance of targeted interventions to support individuals across the lifespan.

Abstract citation ID: igae098.0786

### EXAMINING THE IMPACT OF ADVERSE CHILDHOOD EXPERIENCES AND ACTIVE COPING ON COGNITION IN OLDER AFRICAN AMERICANS

Karen Graham[1], Audrey Steinman[2], Olimpia Paun[3], and Lisa Barnes[4], *1. Rush University Alzheimer's Disease Research Center, Chicago, Illinois, United States, 2. University of Illinois Chicago Hospital and Health Sciences System, Chicago, Illinois, United States, 3. Rush University, Chicago, Illinois, United States, 4. Rush University Medical Center, Chicago, Illinois, United States*

Objective: To examine the association between Adverse Childhood Experiences (ACEs) and cognition in older African Americans and to determine whether active coping moderates the effects of ACEs on cognition later in life. Design: Secondary analysis of data from the Minority Aging Research Study (MARS), a longitudinal community-based study that examines cognitive health and aging in older African Americans. **Methods:** Data from 756 MARS participants were analysed. Participants completed cognitive global functioning tests as well as questionnaires to evaluate exposure to ACEs (The First 18 Years Survey) and active coping (John Henryism Active Coping Scale). We used a correlational analysis (t-tests, ANOVA, linear regression) to examine the associations between ACEs and cognition. Additionally, active coping (an inner ability to address difficult experiences) was examined to determine if it moderated the impact of ACEs on cognition. **Results:** ACES were not associated with global cognitive functioning. However, a negative relationship was found between active coping and global cognitive functioning, such that more active coping was associated with lower cognition. We did not find active coping to be a significant moderator of ACEs. Conclusions: High active coping scores were found to be correlated to impaired cognitive functioning. More research is needed to address this phenomenon as well as the impact of ACEs on older Black people.

Abstract citation ID: igae098.0787

### ADVERSE CHILDHOOD EXPERIENCES AND DEMENTIA AMONG OLDER US ADULTS: ASSESSMENT OF GENDER AND RACIAL DISPARITIES

Monique Brown, *University of South Carolina, Columbia, South Carolina, United States*

Six in ten US adults report exposure to adverse childhood experiences (ACEs). However, their effects on health outcomes are seen across the life course. ACEs have been linked to cognitive impairment. However, less is known about the association between ACEs and a dementia diagnosis. In addition, gender and racial disparities may exist in this relationship. Therefore, this study aimed to determine the association between ACEs and dementia, and to assess the related gender and racial disparities. Data were obtained from the 2012-2020 Health and Retirement Study (N=4,235 respondents aged 51 and older). ACEs assessed included emotional neglect, parental substance abuse, physical abuse, economic adversity, academic challenges, and problems with the law. Dementia was operationalized as ever having dementia. Crude and adjusted logistic regression models were used to determine the associations between ACEs and dementia. Additive and multiplicative interaction effects by gender and race were tested. Approximately 70% of participants reported ACEs and 3% reported ever having dementia. After adjusting for sociodemographic factors, depression, and gender and race interactions, individuals who reported ACEs had 15 times higher odds (aOR: 14.5; 95%CI: 1.56 – 133.9) of ever having dementia compared to individuals without ACEs. Interaction by gender was statistically significant where the relationship between ACEs and dementia was statistically significant for men (aOR: 3.29; 95%CI: 1.28 – 8.42) but not for women. Interaction by race was not statistically significant. Trauma-informed interventions addressing ACEs may reduce the odds of dementia onset, especially for men. Future research should examine pathways between ACEs and dementia.

Abstract citation ID: igae098.0788

### CHILDHOOD PARENTAL RELATIONSHIP AND COGNITIVE HEALTH IN LATER LIFE: ASSOCIATIONS MEDIATED BY SELF-ESTEEM AND SENSE OF CONTROL

Xi Zhu, *Baylor University, Waco, Texas, United States*

Life course researchers have found that early-life experiences are linked to later-life cognitive health. However, very few studies have paid specific attention to the parent-child relationship in childhood. This study extends the current literature by considering the relationship between childhood parental relationship and cognitive health in later life. Using data from the National Longitudinal Study of Youth (1979-2020), I looked at both positive and negative/dysfunctional parental relationships in childhood and examined whether they predicted older adults' self-rated memory and objective cognitive function. To further understand the mechanisms behind the relationship, I also examined the mediation role of self-esteem and sense of control. Findings show that childhood parental love and affection are related to better self-rated memory in later life. Dysfunctional parental relationship is associated with worse self-rated memory. Childhood parental relationships show null association with objective cognitive function among older adults. In addition, KHB mediation tests suggest that self-esteem and sense of control significantly mediated the relationship between childhood parental relationships and later-life subjective cognition. This study contributes to a deeper understanding of the complex factors that influence cognitive health and self-perception of memory in older adults. It suggests that parent-child relationship from early life has a long-term impact on how individuals perceive their cognitive abilities. It also indicates that enhancing psychological resources might have a positive effect on self-perceived cognitive health in older adults. Furthermore, this study highlights the need for clinicians and researchers to distinguish between subjective self-assessments and objective cognitive tests in evaluating older adults' cognitive health.

Abstract citation ID: igae098.0789

### LASTING IMPACTS OF ACES ON DEPRESSION/SUICIDAL THINKING IN BLACK DEMENTIA CAREGIVERS: A WITHIN-GROUP ANALYSIS

Wesley Browning[1], Mustafa Yildiz[2], and Carolyn Pickering[2], *1. The University of Texas Health Science Center Houston, Houston, Texas, United States*, *2. The University of Texas Health Houston, Houston, Texas, United States*

Family caregivers take on the caregiving role despite the quality of the pre-morbid relationship, and pre-morbid relationship quality is linked to many caregiving outcomes. As compared to White dementia family caregivers, Black caregivers often have lower rates of mental distress giving the misleading idea that they are not at risk for poor mental health outcomes. However, within-group analyses have been argued as a better design to understand variability of caregiver outcomes within racially minoritized groups. Therefore, we examined the long-term effects of childhood trauma on depression, depressive symptoms and suicidal thinking among Black dementia family caregivers. This study presents findings from a multi-time series study, in which participants (N=101) completed three waves including 21-day bursts of daily diary surveys (n=5,124). We utilized Generalized Linear Mixed Models (GLMM) to assess the influence of childhood physical neglect, emotional abuse, and physical abuse on risk of clinically significant depression, daily depressive symptom severity and daily suicidal thinking. GLMMs were conducted separately for each form of childhood trauma to avoid multicollinearity. GLMMs revealed all three forms of childhood trauma as risk factors for clinically significant depression over at each wave. Additionally, each form of childhood trauma was independently associated with increased daily depressive symptoms and suicidal thinking. Findings provide compelling evidence of the detrimental effects of childhood trauma on mental health among Black caregivers and help explain within-group variability. These findings underscore the need for targeted interventions aimed at mitigating the long-term mental health consequences of early-life adversity in caregiving contexts.

### SESSION 2220 (SYMPOSIUM)

Abstract citation ID: igae098.0790

### INTERCONNECTIONS BETWEEN COGNITION, SKELETAL MUSCLES, AND PHYSICAL FUNCTION IN ETHNICALLY DIVERSE COHORTS

Chair: Caterina Rosano
Co-Chair: Stephen Kritchevsky
Discussant: Paul Coen

There is marked variability in mobility function at older ages, yet some maintain high functioning even in the face of adverse locomotor risk factors. The mechanisms underlying this resilience remain elusive, but it is clear that the central nervous system plays an important role. Recent findings have shed light on the interconnected biological processes involving muscle-brain cross-talk, albeit primarily in animal models and specific patient populations with muscle degeneration. The extent and nature of such cross-talk in older adult populations remain debated. This symposium aims to advance our understanding of brain-muscle interactions by delving into this emerging concept. Comprising four talks, the symposium will explore various facets of muscle health across diverse cohorts of older adults in the United States. Topics include the structure of the associations between measures of cognitive and physical functions in 3 studies (SOMMA, B-NET and Dunedin), the role of muscle mitochondrial function in the SOMMA study, and the role of intermuscular adiposity in skeletal muscles in the MESA and CARDIA studies. These observations suggest distinctive modulatory and adaptive capacities of the central nervous and musculoskeletal systems may offer insights into understanding the origins of mobility resilience by facilitating adaptation and compensation. This conceptual framework holds potential for identifying innovative multi-systemic interventions aimed at enhancing mobility resilience in older adults.

Abstract citation ID: igae098.0791

### SKELETAL MUSCLE MITOCHONDRIAL RESPIRATION ASSOCIATES WITH COGNITIVE FUNCTION IN OLDER ADULTS

Caterina Rosano[1], Stephen Kritchevsky[2], Iva Miljkovic[1], Anne Newman[3], Peggy Cawthon[4], Stephen Cummings[5], and Paul Coen[6], *1. University of Pittsburgh, Pittsburgh, Pennsylvania, United States*, *2. Wake Forest University School of Medicine, Wake Forest University School of Medicine, North Carolina, United States*, *3. University of Pittsburgh, University of Pittsburgh, Pennsylvania, United States*, *4. California Pacific Medical Center, San Francisco, California, United States*, *5. University of California San Francisco, San Francisco, Mississippi, United States*, *6. AdventHealth, Orlando, Florida, United States*

Lower skeletal muscle oxidative capacity has been linked with poor cognition in older adults. Prior studies have relied on spectroscopy assessment of post-exercise PCr recovery rate to indirectly determine muscle oxidative capacity. Here, we evaluated if mitochondrial energetics assessed in muscle biopsies and by spectroscopy is associated with processing speed and general cognition. The sample included 858 adults (mean age 76.34 +/- 4.97 years, 59% female, 84% Non Hispanic White) from the Study of Muscle, Mobility and Aging (SOMMA). Mitochondrial respiration was measured in permeabilized fibers from biopsies of the vastus lateralis as maximal oxidative phosphorylation (MAXOXPHOS). Muscle oxidative capacity (ATPmax) was assessed by 31P MRS. Cognitive function was assessed by Digit Symbol Substitution Test (DSST), and Montreal Cognitive Assessment (MoCA) with scores adjusted for age and education, and sex. Multivariable linear regression models tested the hypothesis that higher mitochondrial energetics (MAXOXPHOS and ATPmax, in separate models) would be associated with higher scores on DSST and MoCA; all models were adjusted for race, muscle area, hypertension, diabetes and BMI. Each SD decrement in MAXOXPHOS was associated with a -1.7 point lower DSST score (95% CI: 0.68, 2.72) and a non-significantly -0.23 point lower MoCA score (95% CI: -0.00, 0.45). ATPmax was not significantly associated with either DSST or MoCA. Higher mitochondrial respiration is associated with faster processing speed in this population, independent of other known predictors of cognition. Future studies should assess the mechanisms underlying this association.

Abstract citation ID: igae098.0792

### INTRINSIC PACE: PATTERNS OF ASSOCIATION BETWEEN PHYSICAL AND COGNITIVE FUNCTION ACROSS THREE COHORTS

Stephen Kritchevsky[1], Peggy Cawthon[2], Eileen Johnson[3], Terrie Moffitt[4], Atalie Thompson[5], Kathy Xie[4], and Michael Miller[5], *1. Wake Forest University School of Medicine, Wake Forest University School of Medicine, North Carolina, United States*, *2. California Pacific Medical Center, San Francisco, California, United States*, *3. Sutter Health, San Francisco, California, United States*, *4. Duke University, Durham, North Carolina, United States*, *5. Wake Forest University School of Medicine, Winston-Salem, North Carolina, United States*

Physical and cognitive function are fundamental aging concepts. However, our understanding of their connection is limited by past work using few assessments or limited age ranges. We used canonical correlation to study the structure of associations between large sets of physical and cognitive assessments in two studies of adults age ≥70; the B-NET (Brain Networks and Mobility Function) study (N=192; 6 physical measures and 13 cognitive measures), and SOMMA (the Study of Muscle, Mobility and Aging) (N=879; 9 physical and 6 cognitive measures). In B-NET and SOMMA, the first canonical function explained 26% and 25% of the shared variability between the two measure sets. In both studies, ONLY TIMED assessments (e.g. stair climb time, gait speed (4M and 400M), chair stand pace, digit symbol coding, and trail-making test-B) strongly loaded on the first function. We sought to replicate this finding in the Dunedin Study (N=1037), a cohort of residents born in 1972-73 who completed 6 physical and 12 cognitive measures when participants were 45.We observed that the timed measures had the strongest associations across physical and cognitive domains. For example, WAIS Processing Speed was most strongly correlated with chair stand time (r=.25) and gait velocity over 6M (r=.26). In contrast, untimed physical tests were at best weakly correlated with untimed cognitive tests. For example, grip strength was most highly correlated with WAIS Working Memory, r=.15. Together, these three studies confirm the presence of a previously unidentified construct connecting physical and cognitive function, which we have tentatively named 'intrinsic pace'.

Abstract citation ID: igae098.0793

### ABDOMINAL MYOSTEATOSIS IS ASSOCIATED WITH LOWER PROCESSING SPEED IN A MULTIETHNIC COHORT OF OLDER ADULTS

Brendan McNeish[1], Iva Mijkovic[1], Matthew Allison[2], Timothy Hughes[3], Ilya Nasrallah[4], Eric Terkpertey[1], and Caterina Rosano[1], *1. University of Pittsburgh, Pittsburgh, Pennsylvania, United States*, *2. University of California San Diego, La Jolla, California, United States*, *3. Wake Forest University School of Medicine, Winston-Salem, North Carolina, United States*, *4. University of Pennsylvania, Philadelphia, Pennsylvania, United States*

Prior research linking myosteatosis to cognition in older adults has been conducted in relatively homogenous populations with narrow age ranges. We evaluated if abdominal myosteatosis was associated with processing speed and general cognition in a multiethnic cohort of middle aged and older adults. The sample included 1,268 adults (45-84 years old, mean 63±9 years, 52% female of 41% White, 19% Black, 14% Chinese, and 26% Hispanic) a subset from the Multi-Ethnic Study of Atherosclerosis (MESA). Bivariate analyses were performed between abdominal computed tomography derived muscle densities (a myosteatosis measure) at year 3 with Digit Symbol Coding (DSC) and Cognitive Abilities Screening Instrument (CASI) at year 10. Multivariable models were first adjusted for demographics and education, and further adjusted for other known predictors of cognition: dementia risk factors (APOE-4, physical activity, diabetes, cholesterol, smoking, and hypertension), central adiposity, and cytokines. We tested two-way interactions by 1) race and 2) sex. Myosteatosis was significantly associated with worse DCS (B=0.333, 95% CI: 0.031,0.835) but not CASI (0.106, 95% CI: -0.089,0.300), independent of demographics or education. Results were similar after adjustment for dementia risk factors, central adiposity, and cytokines. Tests for interactions by sex and race were not statistically significant. Abdominal myosteatosis is associated with worse processing speed but not general cognition in this middle-aged and older multiethnic population of men and women, independent of other known predictors of cognition. Longitudinal studies should assess myosteatosis' impact on cognition and its underlying mechanisms.

Abstract citation ID: igae098.0794

### ABDOMINAL MYOSTEATOSIS AND COGNITIVE FUNCTION: INSIGHTS FROM CARDIA STUDY

Adrianna Acevedo-Fontanez[1], Caterina Rosano[1], Kristine Yaffe[2], J Jeffrey Carr[3], James Greg Terry[4], Sangeeta Nair[3], Ryan Cvejkus[1], and Iva Miljkovic[1], *1. University of Pittsburgh, Pittsburgh, Pennsylvania, United States*, *2. University of California San Francisco, San Francisco, California, United States*, *3. Vanderbilt University, Nashville, Tennessee, United States*, *4. Vanderbilt University Health Sciences, Nashville, Tennessee, United States*

The role of myosteatosis in cognitive decline is largely from cross-sectional studies in White populations. Longitudinal studies including Black populations, who have a higher risk of both myosteatosis and dementia than Whites, are warranted. We tested the association between abdominal myosteatosis with 5-year change in cognition in 2,501 White and Black American men (n=1,078; 42% Black) and women (n=1,423; 49% Black) from the Coronary Artery Risk Development in Young Adults (CARDIA), and whether associations vary after adjustment for demographics, center, education, muscle area, follow-up-time, baseline DSST score, smoking, physical activity, diabetes, hypertension, lipid-lowering medication, and BMI. IMAT was log-transformed, and results were standardized with effects shown per standard deviation (SD). Higher IMAT predicted a significant DSST decline in Whites (5-year decrease in DSST: b=-0.77 points for 1 SD of log

IMAT, p-value=<0.01) but not in Blacks (b=-0.03, p-value=0.910). By race, higher IMAT was associated with a significant decline in DSST score. We did not see a significant difference in a 5-year decrease in DSST by sex. Abdominal myosteatosis may be a novel risk factor for cognitive decline, especially in Whites. Future studies using an expanded battery of cognitive tests, longer follow-ups, and in other populations, as well as mechanistic studies, are warranted to further confirm and advance our understanding of the novel role of myosteatosis in dementia risk.

### SESSION 2225 (SYMPOSIUM)

Abstract citation ID: igae098.0795

### LGBTQIA+ OLDER ADULTS' NEEDS, CONCERNS, AND EXPERIENCES IN HEALTH CARE: INCLUSIVITY, CAREGIVING, AND VISIBILITY

Chair: Korijna Valenti
Discussant: Sara Bybee

LGBTQIA+ aging research requires in-depth examination of experiences around discrimination, inclusivity, and communication in healthcare to better understand the distinct needs of diverse subgroups, including people living in long-term care (LTC) facilities, living with neurodegenerative illnesses, and caregivers. LGBTQIA+ older adults not only have diverse life experiences but also specific needs regarding advocacy, policy, and healthcare support and resources. This symposium includes findings from four distinct areas of study. Valenti and colleagues examined perspectives of 20 palliative-cared trained clinicians to better understand their experiences with LGBTQI+ patients living with serious illness and their chosen care partners, exploring issues around communication, collection of SOGI data, and inclusivity. In a study with LTC administrators in Minnesota, Wright and colleagues examined issues around identifying LGBTQIA+ LTC residents, discrimination of residents (including residents living with cognitive impairment), and barriers around LGBTQIA+ inclusion in research. Stein and colleagues examined issues around discrimination in healthcare for LGBTQIA+ patients through Project Respect, a cross-sectional mixed methods study of 290 patients and partners which found high levels of disrespect and inadequate care. Flatt and colleagues provide insights from an analysis of two qualitative studies of AD/PD caregivers with a specific focus on identifying barriers to support, inclusion in research, and issues around personal and social needs. Presentations in this symposium illustrate how discrimination, inclusivity, and personal and social support inform the needs of LGBTQIA+ older adults living with serious illness, in LTC, and as caregivers as well as providing implications for future aging research and interventions in practice.

Abstract citation ID: igae098.0796

### CLINICIAN INTERACTIONS WITH OLDER LGBT+ PATIENTS IN COLORADO: VISIBILITY, IDENTITY, AND EQUITABLE CARE

Korijna Valenti, *University of Alabama at Birmingham, Birmingham, Alabama, United States*

The unique needs of older lesbian, gay, bisexual, transgender (LGBT+) patients continue to merit attention. It is critical to fill the gap between clinician-patient communication, viewing patients as individuals with distinct support systems, and spouse/partner inclusivity. Guided by a queer gerontology framework, this study analyzed perspectives of clinicians across Colorado (n = 20) regarding their experiences interacting with LGBT+ patients 55+ with serious illness, including a) how health care professionals perceive their knowledge of and interactions with LGBTQ+ patients; (b) how these perspectives may inform patient care; and (c) how to best conceptualize communication between clinicians and LGBTQ+ patients. Inductive thematic analysis revealed three main themes: 1) visibility and treatment of LGBTQ+ patients, 2) asking about identity, and 3) acknowledging limitations around the current state of care for LGBTQ+ patients. Participants noted varying levels of comfort with and knowledge about LGBTQ+ patients and patients' chosen care partners Participants addressed barriers around not asking about sexual orientation or gender identity, treating all patients "the same," and the need for increased advocacy and proactive communication by health care professionals. Findings reveal the need for improved knowledge acquisition, training, and inclusivity for clinicians to fully address older LGBT+ patient needs. Several participants noted experiences around discrimination in the clinical environment and the need to "do better," learn more, and engage more deeply with patients. Clinician understanding of patient needs, the use of correct language, and familiarity with patients' chosen care partners may lead to improved health care experiences for LGBT+ patients.

Abstract citation ID: igae098.0797

### PROJECT RESPECT: EXPERIENCES OF SERIOUSLY ILL LGBTQ+ PATIENTS AND PARTNERS WITH THEIR HEALTH CARE PROVIDERS

Gary Stein[1], and Cathy Berkman[2], *1. Wurzweiler School of Social Work-Yeshiva University, New York, New York, United States*, *2. Fordham University, New York, New York, United States*

Discrimination against LGBTQ+ persons in healthcare creates barriers to serious illness care, including patients avoiding or delaying necessary care, providers disrespecting wishes of surrogates, and adverse outcomes for patients and families. A cross-sectional mixed methods study using an online survey was used to determine the extent to which LGBTQ+ patients and spouses, partners, and widows experienced disrespectful or inadequate care due to sexual orientation or gender identity. A total of 290 LGBTQ+ patients and partners reported high levels of disrespectful and inadequate care. Patients reported these discriminatory actions from healthcare providers: insensitive to me as an LGBTQ+ person (35.2%); not aware of LGBTQ+ health needs (30%); made me feel judged for being LGBTQ+ (23.1%); were rude to me (20.7%); didn't use my correct pronouns (20.3%); and disregarded my treatment decisions (19.7%). Partners reported they were denied access to their loved one in intensive care or emergency room (12.4%); were treated badly (12.1%); and that their decisions were not followed (10%). Black and Hispanic patients were two to four times more likely than non-Hispanic white patients to report discrimination. This study demonstrated high levels of disrespectful and inadequate care towards patients and partners due to being LGBTQ+, especially problematic for Black and Hispanic patients and those living in politically conservative regions. Recommendations include federal and state civil rights laws to prohibit LGBTQ+ discrimination, and institutional practices to address discrimination, including cultural sensitivity training for staff.

Abstract citation ID: igae098.0798

### EXPLORING THE NEEDS OF LGBTQIA+ CAREGIVERS OF PEOPLE LIVING WITH NEURODEGENERATIVE DISEASES

Jason Flatt[1], Cody Yamada[1], Brittany Klenczar[1], Krystal Kittle[2], Whitney Wharton[3], and Joel Anderson[4], *1. University of Nevada Las Vegas, Las Vegas, Nevada, United States*, *2. University of Massachusetts Amherst, Springfield, Massachusetts, United States*, *3. Emory University, Atlanta, Georgia, United States*, *4. University of Tennessee-Knoxville, Knoxville, Tennessee, United States*

The Administration on Aging estimates 4 million adults aged 60+ in the U.S. identify as lesbian, gay, bisexual, transgender, queer, intersex and/or another identity (LGBTQIA+). Experiences and health-related concerns of LGBTQIA+ caregivers of people living with a neurodegenerative disease, such as Alzheimer's disease and/or Parkinson's disease (AD/PD), have been understudied. We synthesize data from two qualitative studies with LGBTQIA+ AD/PD caregivers, including Parkinson's Research with Inclusion, Diversity, and Equity (PRIDE) and the Research Inclusion Supports Equity (RISE) Registry. Interviews were conducted with LGBTQIA+ caregivers of people living with AD (n=25) and PD (n=8) to explore their needs, experiences, and concerns with using aging and long-term care services and personal health and social needs. Thematic analysis was used to identify key themes. The first theme involved LGBTQIA+ AD/PD caregivers experiencing several financial, legal, and social challenges (e.g., reluctance to access long-term care services) when caring for someone living with AD/PD. Another theme involved LGBTQIA+ caregivers describing that they often supported their care recipients with medical-/nursing-related tasks, stress from caregiving demands, and experiencing financial and/or eligibility challenges when accessing respite care. The final theme focused on caregivers' health and access of services, with LGBTQIA+ AD/PD caregivers describing difficulties managing their own health, feelings of depression and loneliness, and perceived discrimination when accessing long-term care and aging-related services. We highlight the need for additional research and creation of innovative long-term care services and caregiver support programs to better meet the needs of LGBTQIA+ caregivers of people living with AD/PD.

Abstract citation ID: igae098.0799

### RECRUITING SEXUAL AND GENDER MINORITY OLDER ADULTS WITH AND WITHOUT DEMENTIA IN LONG-TERM CARE: OVERCOMING BARRIERS

Morgan Wright[1], Tetyana Shippee[2], Tricia Skarphol[1], Jason Flatt[3], Andrew Alberth[4], Rajean Moone[5], and B.R. Simon Rosser[1], *1. University of Minnesota, Minneapolis, Minnesota, United States*, *2. University of Minnesota School Of Public Health, Minneapolis, Minnesota, United States*, *3. University of Nevada Las Vegas, Las Vegas, Nevada, United States*, *4. University of Massachusetts Boston, Boston, Massachusetts, United States*, *5. University of Minnesota, St. Paul, Minnesota, United States*

Sexual and gender minority (SGM) older adults, including those using long-term care and living with cognitive impairment, are a growing population, but little is known about their unique experiences as they are often missing from research. This study investigates barriers and facilitators for recruitment of SGM older adults utilizing long term care (LTC) into research studies. As part of a larger study on SGM policies in LTC, we used purposive sampling to select twenty assisted living and nursing home directors for qualitative interviews. Participants were assisted living and nursing home administrators in Minnesota, identified as part of a larger survey on SGM policies in LTC. Qualitative coding and partially inductive/deductive thematic analysis resulted in the generation of three themes. The themes include the challenge of identifying SGM residents in long-term care, a need to be mindful of discrimination against SGM residents by other residents which can also hinder self-identification, and special considerations in working with SGM residents with AD/ADRD. Several strategies to overcome these barriers exist. The themes all contained information for future researchers that work with older SGM adults in long-term care facilities who may have AD/ADRD. Understanding barriers to identifying participants, addressing discrimination, and crafting appropriate consent processes for those with AD/ADRD are all important points for researchers.

### SESSION 2230 (SYMPOSIUM)

Abstract citation ID: igae098.0800

### MEASURING NATIONAL TRENDS IN SOCIAL ISOLATION AND LONELINESS AMONG OLDER ADULTS AND THEIR CAREGIVERS

Chair: Mary Louise Pomeroy
Co-Chair: Yiqing Qian
Discussant: Katherine Ornstein

Social isolation and loneliness have negative implications for the health and care of older adults. Few studies have examined longitudinal patterns of social isolation and loneliness among older adults, or how these experiences are shared by caregivers and personal care networks. This research symposium uses various survey data methods to highlight trends in social isolation, loneliness, and care isolation among older adults and their caregivers. First, we report on trends in social isolation and loneliness over time. Leveraging six waves of data from the Longitudinal Ageing Study Amsterdam (LASA), Dr. Mehrabi describes the temporal sequence of social isolation, loneliness, and frailty across 21 years among a national sample of older adults from the Netherlands. Dr. Umoh uses the National Health and Aging Trends Study (NHATS) to describe trends in intermittent and persistent social isolation among older Americans between 2011–2021. Next, we examine social isolation and loneliness in the context of caregiving. Dr. Clair reports on personal care networks and care isolation among family and friends caring for older adults with Type II diabetes. Dr. Qian uses the National Study on Caregiving (NSOC) to examine prevalence and correlates of social isolation and loneliness among family and unpaid caregivers of older adults. Dr. Pomeroy concludes the session with a discussion of the strengths and challenges of using national survey data to measure social isolation, offering methodological strategies for handling missing data and proxy reports for persons with dementia. Dr. Ornstein, as discussant, synthesizes the implications for policy, practice, and future research.

Abstract citation ID: igae098.0801

### THE TEMPORAL SEQUENCE OF FRAILTY, SOCIAL ISOLATION, AND LONELINESS IN OLDER ADULTS ACROSS 21 YEARS

Fereshteh Mehrabi[1], Mary Louise Pomeroy[2], Thomas Cudjoe[3], Emerald Jenkins[2], Elsa Dent[4], and Emiel Hoogendijk[5], *1. Concordia University, Montreal, Quebec, Canada*, *2. Johns Hopkins University, Baltimore, Maryland, United States*, *3. Johns Hopkins University School of Medicine, Baltimore, Maryland, United States*, *4. Torrens University, Adelaide, South Australia, Australia*, *5. Amsterdam University Medical Centers, Amsterdam, Noord-Holland, Netherlands*

This study examined the temporal patterns of social isolation, loneliness, and frailty among a national sample of older adults across 21 years (1995–2016). We applied random intercept cross-lagged panel models to seven waves of the Longitudinal Aging Study Amsterdam (LASA). We included 2,302 Dutch older adults ages 55 and older (Mage= 72.6; SD = 8.6; 52.1% female). Frailty was measured using the Frailty Index. Loneliness was measured using the 11-item De Jong Gierveld loneliness scale. Social isolation was measured using a multi-domain 6-item scale. Levels of social isolation and loneliness were weakly correlated during any given wave as well as across waves. Each condition was highly stable from one time point (T) to the next. Cross-lagged effects showed that frailty in T2 to T5 significantly predicted social isolation in the following waves (T3 to T6). For example, higher levels of frailty at T4 predicted higher levels of social isolation at T5 ($\beta$ = 0.23, SD = 0.05, $p < 0.001$). Likewise, frailty in T2 to T6 significantly predicted loneliness in the following waves (T3 to T7). For example, higher levels of frailty at T5 predicted higher levels of loneliness at T6 ($\beta$ = 0.33, SD = 0.06, $p < 0.001$). The reverse sequence (i.e., social isolation or loneliness predicting future frailty) was less common and exhibited a weaker relationship. Frailty may have important consequences for adverse psychosocial outcomes, including social isolation and loneliness. Public health policies should prioritize interventions that bolster social connection among pre-frail and frail older adults.

Abstract citation ID: igae098.0802

### DYNAMICS OF SOCIAL ISOLATION IN A NATIONAL SAMPLE OF COMMUNITY-DWELLING OLDER ADULTS

Mfon Umoh[1], Mary Louise Pomeroy[2], Alexandra Mueller[1], Taylor Craig[1], Katherine Ornstein[2], Laura Prichett[1], and Thomas Cudjoe[1], *1. Johns Hopkins University School of Medicine, Baltimore, Maryland, United States*, *2. Johns Hopkins University, Baltimore, Maryland, United States*

Social isolation is linked to adverse health outcomes; 25% of older adults in the United States are socially isolated. However, episodic social isolation may carry different implications for health outcomes compared to chronic social isolation. Many studies examine social isolation at a single point rather than considering it as a circumstance that is intermittent or persistent. This study examined social isolation dynamics in older adults using nationally representative cohort data from 11 rounds of the National Health and Aging Trends Study (N=6,913) to describe differences between older adults with chronic, intermittent, and no social isolation. Social isolation status was derived from points given for questions about living arrangement, core discussion network size on important matters, religious attendance, and social participation. Persistent social isolation was defined as social isolation during more than 50% of available rounds and intermittent social isolation as isolation in at least one round but less than 50% of rounds. The analytic sample had social isolation scores available for a median of four rounds (IQR 3,10; M = 5.28); 20% experienced persistent isolation, 27% experienced intermittent isolation. Demographic characteristics were compared using Chi-square tests and an alpha threshold of 0.05 with the never isolated group as reference. Age, education, income, difficulty with activities of daily living, dual eligibility (Medicare and Medicaid insurance), and dementia status at baseline were correlated with social isolation persistence. Identifying factors associated with the persistence of social isolation may guide the design of interventions targeted to prevent social isolation and its detrimental consequences.

Abstract citation ID: igae098.0803

### ISOLATED VERSUS SUPPORTED: THE PERSONAL HEALTH CARE NETWORKS OF OLDER ADULTS LIVING WITH TYPE 2 DIABETES

Catherine Clair[1], Karen Bandeen-Roche[2], Jennifer Wolff[2], Janiece Taylor[3], Katherine Ornstein[2], and Karin Tobin[2], *1. Johns Hopkins Bloomberg School of Public Health, Baltimore, Maryland, United States*, *2. Johns Hopkins University, Baltimore, Maryland, United States*, *3. Johns Hopkins University, Johns Hopkins University, Maryland, United States*

Older adults with Type 2 diabetes may or may not have family and friends involved in their care; using social network methods, we can investigate the personal healthcare networks of this population. Objectives included 1) Describe care networks of older adults with Type 2 diabetes; and 2) Characterize the family and friends who provide support for various activities of daily living (ADL), instrumental activities of daily living (IADL), and medically-oriented tasks. We administered a quantitative ego-centric social network inventory to community-dwelling older adults (65+) living with Type 2 diabetes (n=58). The sample was on average, 73.1 years old (SD: 5.9, Range: [65, 86]). and majority female (n=44, 75.9%) and non-White (n=37, 63.8%). Descriptive statistics were conducted overall and by Black (n=32) and White (n=21) race. Ten (17.5%) participants reported no family or friends involved in their care; six were Black and four were White. Those experiencing care isolation were more likely to have never been married compared to those with care networks (p=0.002). Of those with a network, there was an average of 2.9 (SD: 2.0, Range: [1, 11]) individuals in the network; there were no statistically significant differences in network size by race. Of the 140 family and friends across the entire sample, 23 (16.4%) assisted with ADLs, 126 (90.0%) with IADLs, and 48 (34.3%) with medically-oriented tasks. Fourteen (10.0%) individuals assisted with at least 1 activity in all three categories. These findings demonstrate a spectrum of family and friend involvement in the care of this population.

Abstract citation ID: igae098.0804

### THE EPIDEMIOLOGY OF SOCIAL ISOLATION AND LONELINESS OF FAMILY AND UNPAID CAREGIVERS OF OLDER ADULTS

Yiqing Qian, Mary Louise Pomeroy, Martha Saylor, and Katherine Ornstein, *Johns Hopkins University, Baltimore, Maryland, United States*

The experience of caring for older relatives and friends can be socially isolating. Family and unpaid caregivers ("caregivers") may encounter barriers to social participation (e.g., caregiving duties) and unmet needs in support. Given limited epidemiological evidence of social isolation (SI) and loneliness in this population in the U.S., we reported the prevalence and correlates of SI and loneliness in the National Study of Caregiving Round 12 (n=2,119). Most were females (62%), non-Hispanic white (69%), and had a high school education (91%). Most cared for a parent/parent-in-law (46%). Caregivers provided an average of 70 hours of care/month. On a 4-item SI scale ranging from 0 (high SI) to 4 (low SI), the mean score was 2.6. About 13% of caregivers were categorized as socially isolated. About 33% reported feeling lonely "some days," "most days," or "every day." Logistic regression showed female caregivers (OR: 0.56[0.37,0.86]) and those with lower education levels (OR[college vs below high school]: 0.14[0.06,0.33]) were less likely to be socially isolated vs not isolated, controlling for other sociodemographic (e.g., age) and caregiving characteristics (e.g., hours). Adjusted ordinal logistic regression further showed SI levels among care recipients were associated with SI levels among caregivers (OR: 1.66[1.38,2.00]). Null associations were found in parallel analyses on loneliness. This study highlights the heterogeneity of SI among caregivers of older adults and the necessity of examining the shared experiences with care recipients. More comprehensive loneliness instruments in public health surveillance may advance the understanding of experiences and impacts of SI and loneliness in caregiving.

Abstract citation ID: igae098.0805

### MEASURING SOCIAL ISOLATION AMONG OLDER ADULTS WITH DEMENTIA: TRENDS, CHALLENGES, AND NEXT STEPS

Mary Louise Pomeroy[1], Mfon Umoh[2], Yiqing Qian[1], Katherine Ornstein[1], and Thomas Cudjoe[2], *1. Johns Hopkins University, Baltimore, Maryland, United States, 2. Johns Hopkins University School of Medicine, Baltimore, Maryland, United States*

A growing body of literature demonstrates a strong association between social isolation and the risk of incident dementia. However, research examining objective social isolation among persons living with dementia (PLWD) has been limited. The behavioral and psychological symptoms of dementia may reduce social connection. Research that uses population-based, national surveys to examine this issue among PLWD must consider missing data and proxy interview responses. We report new evidence on the prevalence of social isolation among a national sample of community-dwelling PLWD and offer strategies to attenuate missing proxy data. Using 2015 data from the National Health and Aging Trends Study and a multi-domain measure of social isolation that considers living arrangement, core discussion network size, religious attendance, and social participation, we identified that 41.3% (n=313/757) of interviews with PLWD were completed using a proxy respondent. In proxy interviews, one of the four social isolation items is not asked. We classified social isolation status when a sum score based on the three remaining items equaled 0 (socially isolated; n=19/313) or 2 or 3 (not socially isolated, n=99/313). This strategy resolved 37.7% (n=118/313) of the missing proxy data and reduced missing social isolation data across PLWD to 26.0% (197/757). About 32.1% (n=180/560) of PLWD were socially isolated, irrespective of proxy status. Social isolation was more prevalent among PLWD who completed the interview with no proxy (36.4%; n=161/442) compared to those with a proxy (16.1%; n=19/118) (X2(1)=17.6; p<.001). Future work should consider imputation strategies when assessing social isolation in PLWD using proxy interviews.

### SESSION 2235 (SYMPOSIUM)

Abstract citation ID: igae098.0806

### MITOCHONDRIA, METABOLISM, AND GLUTATHIONE AS MEDIATORS OF COGNITIVE DECLINE IN AGING AND ALZHEIMER'S DISEASE

Chair: Rajagopal Sekhar
Discussant: George Taffet

Aging is the most important risk factor for cognitive decline and Alzheimer's disease (AD), and age-related cognitive decline (ACD) is widely prevalent in older adults (OA). However, the mechanistic underpinnings of cognitive impairment in aging and AD remain poorly understood, and are a high-priority research focus toward finding solutions. Of all organs of the body, the brain requires a maximal amount of energy which is generated by mitochondria. Interruptions in energy availability could be critical to cognitive impairment in both ACD and AD. In parallel, defects in metabolism (glutathione/redox metabolism, glucose metabolism, inflammation, endothelial dysfunction) are also implicated as contributors to cognitive impairment. This symposium will discuss novel mechanistic underpinnings of cognitive decline in terms of glutathione deficiency, mitochondrial dysfunction and abnormalities in metabolism, discuss a novel intervention to reverse these defects in aging, and its applicability in AD. The symposium re will begin with a talk by Dr. Mahapatra on impaired mitochondrial energetics in patients with AD. Then Dr. Kumar will discuss cognitive decline in old mice and link it to underlying brain brain defects in glucose metabolism, mitochondrial impairment and glutathione deficiency, and present data on successfully reversing these defects with GlyNAC supplementation. Dr. Sekhar will discuss translation of GlyNAC supplementation to ACD and present results from a human trial showing improvements in cognition, glutathione deficiency, and novel defects in metabolism and mitochondria. As Discussant, Dr. Taffet will present data from 91 humans showing that glutathione deficiency as a key, novel defect in AD, and discuss symposium presentations.

Abstract citation ID: igae098.0807

### PERIPHERAL BLOOD CELL MITOCHONDRIAL FUNCTIONS ARE ASSOCIATED WITH COGNITIVE PERFORMANCE AND ALZHEIMER'S DISEASE

Gargi Mahapatra[1], Jaclyn Bergstrom[2], Suzanne Craft[3], and Anthony Molina[1], *1. University of California San Diego, La Jolla, California, United States, 2. UC San Diego, La Jolla, California, United States, 3. Wake Forest University School of Medicine, Winston-Salem, North Carolina, United States*

Alzheimer's disease (AD) dementia associates with systemic mitochondrial bioenergetic decline. We previously showed blood cell mitochondrial bioenergetics recapitulates bioenergetic capacity of highly metabolically active organs like brain, liver, and skeletal muscles, and peripheral blood mononuclear cell (PBMC) mitochondrial function relates to brain morphology. More recently, we examined PBMC and platelet bioenergetics in a cohort of older adults with dementia due to AD. We assessed mitochondrial function using complementary respirometric approaches in intact and permeabilized PBMCs and platelets from individuals with normal cognition (NC), mild cognitive impairment (MCI), and dementia due to probable AD (DEM). Cognitive abilities were assessed using Modified Preclinical Alzheimer's Cognitive Composite (mPACC5) scores, and brain morphology using MRI. Our results indicate blood cells exhibit lower bioenergetic capacity associated with cognitive decline in MCI and DEM, with lowest capacities in DEM. Specifically, glucose-mediated respiration was significantly lower in DEM compared to NC. PBMC fatty-acid oxidation (FAO)-mediated respiration were progressively lower with MCI and DEM compared to NC, while platelet FAO-mediated respiration exhibited higher maximal respiration in MCI than NC and DEM. PBMC and platelet respiration positively correlated with mPACC5 scores and hippocampal volume, and negatively correlated with white matter hyperintensities. These findings indicate mitochondrial bioenergetic differences associated with cognitive abilities are systemic and blood-based bioenergetic profiling can be used as a minimally invasive approach for measuring mitochondrial alterations associated with dementia. Studies are underway to identify circulating factors underlying systemic bioenergetic decline and cellular mechanisms mediating mitochondrial dysfunction in PBMCs and platelets from older adults with cognitive impairment.

Abstract citation ID: igae098.0808

### BRAIN GLUTATHIONE, MITOCHONDRIA, GLUCOSE METABOLISM, AND COGNITION IN AGED MICE: IMPACT OF GLYNAC SUPPLEMENTATION

Premranjan Kumar, Ob Osahon, and Rajagopal Sekhar, *Baylor College of Medicine, Houston, Texas, United States*

Alzheimer's disease (AD) remains unsolved, and is associated with brain defects including mitochondrial dysfunction, impaired glucose uptake, inflammation, and glutathione deficiency. These defects also occur in 'age-related cognitive decline' (ACD). Mouse models have been developed to study AD, however because mice do not develop AD naturally, these models are artificial as defects are induced in mice. To understand the mechanistic basis of cognitive decline in aging, it is important to investigate naturally-occurring defects directly in the brain, and this is only possible in animal models because performing brain biopsies in humans is not practical. To overcome these challenges, we studied mitochondrial function, glucose uptake, inflammation, redox status, genomic damage and neurotropic factors directly in the brains of old-mice (OM, 98-weeks) and young-mice (YM, 28-weeks), and also measured their cognitive performance (via maze-running). Compared to YM, the cognitively impaired OM had the severe defects in the brain: (a) lower glutathione concentrations; (b) elevated oxidative stress; (c) abnormal immunoblot expressions of: lower GLUT1 and GLUT3 (transporters regulating glucose uptake); lower PGC1a; electron-chain complexes and ATP5A (mitochondrial dysfunction); lower LC3A/B and PINK1 (autophagy/mitophagy); higher TSPO (inflammation), higher pH2AX (genomic damage), and lower BDNF, GDNF and NGF (neurotropic factors); (d) impaired mitochondrial respiration (respirometry); (e) impaired cognition (maze-time). After 8-weeks of supplementing GlyNAC (combination of glutathione precursors glycine and N-acetylcysteine), all defects improved/corrected in the brains of OM, and cognitive performance improved significantly. Study findings suggest that correcting glutathione deficiency could be important for improving brain health and cognition in ACD and in AD.

Abstract citation ID: igae098.0809

### IMPROVING GLUTATHIONE, MITOCHONDRIA, INFLAMMATION, AND COGNITIVE DECLINE: A PILOT CLINICAL TRIAL OF GLYNAC IN AGING

Rajagopal Sekhar, Premranjan Kumar, Chun Liu, Jean Hsu, Chacko Shaji, Jahoor Farook, and Minard Charles, *Baylor College of Medicine, Houston, Texas, United States*

Although aging is associated with cognitive decline, mechanisms contributing to 'age-related cognitive decline' (ACD) are not well understood. Aging is associated with glutathione deficiency, oxidative stress, mitochondrial dysfunction, inflammation, insulin resistance and endothelial function (many of these identified as aging hallmarks and are key defects in Geroscience), and these defects could compromise lead to cognitive impairment. Based on prior translational studies in aging where we found evidence that supplementing GlyNAC (combination of glutathione precursors glycine and N-acetylcysteine) could improve glutathione deficiency, oxidative stress, mitochondrial dysfunction and glucose tolerance, we conducted a 36-week pilot human clinical trial comparing young-adults (YA) and older-adults (OA). We found that compared to YA, OA had evidence of cognitive decline together with glutathione deficiency, oxidative stress, mitochondrial dysfunction, inflammation, insulin resistance and endothelial dysfunction and deficiency of BDNF (brain-derived neurotropic factor). Supplementing GlyNAC for 24-weeks reversed these defects and improved cognition, but stopping GlyNAC for 12-weeks led to redevelopment of these defects. Using tracer methodologies we also identified a novel 'brain glucose steal' phenomenon wherein, due to mitochondrial impairment, non-brain organs were found to be utilizing glucose intended for the brain, and GlyNAC supplementation led to reversal of the 'brain glucose steal' phenomenon. Collectively, the findings from this trial provide proof-of-concept supporting GlyNAC supplementation in OA to improve multiple mechanistic defects related to cognitive impairment, and thereby also cognitive function and brain health. As many of these defects also occur in human Alzheimer's disease (AD), these findings also have implications for improving brain health in AD.

Abstract citation ID: igae098.0810

### GLUTATHIONE DEFICIENCY AS A NOVEL RISK FACTOR FOR ALZHEIMER'S DISEASE: IMPLICATIONS FOR GLYNAC SUPPLEMENTATION

George Taffet[1], Premranjan Kumar[2], Ob Osahon[2], Chimaoge Onyechi[3], Charles Minard[2], Joseph Masdeu[3], and Rajagopal Sekhar[2], *1. Houston Methodist Hospital, Houston, Texas, United States*, *2. Baylor College of Medicine, Houston, Texas, United States*, *3. Nantz National Alzheimer's Center, Houston, Texas, United States*

Cognitive impairment in Alzheimer's disease (AD) is linked to defects such as oxidative stress (OxS), mitochondrial dysfunction, inflammation and insulin resistance. These defects also occur in aging where they are linked to deficiency of the intracellular antioxidant glutathione. We recently reported that GSH deficiency was present in the brains of cognitively-impaired aged mice, and that both glutathione deficiency and cognition improved after supplementation with GlyNAC (combination of glutathione precursors glycine and N-acetylcysteine). We hypothesized that patients with AD with mild cognitive impairment (MCI) would have greater glutathione deficiency than older-adults (OA), and tested our hypothesis by comparing intracellular red-blood cell glutathione concentrations in young-adults (YA), OA without cognitive impairment, and patients with AD+MCI (AD-MCI). Red-blood cell total glutathione (tGSH), reduced GSH (rGSH) and oxidized glutathione (GSSG) concentrations from 32 AD-MCI patients in an ongoing randomized clinical trial were compared to historical data from 32 OA and 19 YA from our published studies to determine whether, and to what extent glutathione levels are deficient in AD-MCI. We found that YA had the highest levels of tGSH and rGSH, followed by OA and the lowest levels were seen in AD-MCI. GSSG levels were also significantly lower in AD-MCI compared to OA and YA. These data indicate that AD-MCI is associated with severe glutathione deficiency. Because GlyNAC improves/corrects glutathione deficiency, (a potential key defect in AD), a randomized clinical trial is in progress to test whether GlyNAC supplementation can improve glutathione deficiency, metabolic and mitochondrial defects, and cognitive function in patients with AD-MCI.

### SESSION 2240 (PAPER)

### MUSCULOSKELETAL HEALTH (PAPERS)

Abstract citation ID: igae098.0811

### RESPONDERS AND NONRESPONDERS WITH KNEE OSTEOARTHRITIS TO TRANSCRANIAL DIRECT CURRENT STIMULATION

Juyoung Park[1], Heling Tong[2], Yixin Kang[2], Hongyu Miao[2], and Hyochol Ahn[1], *1. University of Arizona, Tucson, Arizona, United States*, *2. Florida State University, Tallahassee, Florida, United States*

Knee osteoarthritis (KOA), a common musculoskeletal condition in older adults, often leads to long-term disability. Transcranial direct current stimulation (tDCS) has received much attention as a noninvasive treatment for knee pain due to its neuromodulatory effect. However, there is variability in the response to tDCS among KOA patients, and little is known about the factors predicting response to tDCS for knee pain. The purpose of the study was to identify responders and nonresponders to the tDCS intervention and characterize their differences in knee pain reduction post intervention. Sixty participants assigned to the home-based active tDCS (five 20-minute sessions of 2 mA direct current), under real-time supervision by the research staff, were included; 41 were classified as responders (64.90 years, 41.50% White, 61.00% women) and 19 were classified as nonresponders (66.21 years, 47.40% White, 78.90% women). Changes in pain intensity scores from baseline to post intervention indicated more significant decreases in responders than in nonresponders ($p < .001$); the mean decrease in pain intensity from baseline to post intervention was 34.17 (SD = 17.40) for the responder group and 2.26 (SD = 10.70) for the nonresponder group. The responder group showed higher BMI, height, and weight than the nonresponder group. The results indicated that responder and nonresponder groups and their treatment outcomes can be predicted for tailoring tDCS interventions. More research is needed to identify reasons for nonresponse to tDCS (e.g., medical comorbidities) to reduce nonresponse rates to tDCS.

Abstract citation ID: igae098.0812

### SOFT-TISSUE COMPOSITION ASYMMETRY PREDICTS LEG MUSCLE STRENGTH CHANGES OVER FIVE YEARS AMONG ICELANDIC OLDER ADULTS

Milan Chang (Gudjonsson)[1], Marco Recenti[2], Carlo Ricciardi[3], Alfonso Maria Ponsiglione[4], Francesco Amato[5], Magnus Kjartan Gislason[2], and Paolo Gargiulo[2], *1. Landspitali University Hospital, Seltjarnarnes, Hofuoborgarsvaoio, Iceland*, *2. Reykjavik University, Reykjavik, Hofuoborgarsvaoio, Iceland*, *3. University of Naples Federico II, Naples, Calabria, Italy*, *4. University of Naples Federico II, Naples, Campania, Italy*, *5. Università degli Studi Magna Græcia di Catanzaro, Napoli, Campania, Italy*

**Background:** Age-related functional decline emphasizes the need to investigate how tissue composition impacts mobility and independence in older adults. One of the most critical functions associated with mobility disability among older adults is leg muscle strength. The current study investigated the significance of lower leg tissue composition asymmetry on leg muscle strength changes over 5 years among older adults.

**Methods:** A community-based population from Reykjavik, Iceland (n=3156, mean age=74.9) participated in a 5-year longitudinal study. Tissue composition asymmetry was defined by differences between the right and left sides of nonlinear Trimodal Regression Analysis (NTRA) parameters assessed by CT scans at baseline. Among the total of 3156 study participants, 2737 were available for the longitudinal analysis. The maximum strength of the knee extension on the dominant side was measured by using a computerized dynamometer attached to a stable chair at baseline and follow-up assessment. Linear regression analysis was performed with covariates adjustment including age, gender, body mass index, and other lifestyle-related factors.

**Results:** The results of the study indicate that, after adjusting for covariates, two baseline asymmetry indicators (the amplitude of fat tissue ($p< 0.04$) and the location of muscle tissue ($p< 0.03$)) were significantly associated with changes in leg strength over 5 years.
**Conclusion:** The findings show that higher baseline leg asymmetry indicators, involving both fat and muscle tissue, can predict a greater decline in leg muscle strength during the 5-year follow-up. The composition asymmetry of tissues could serve as an important indicator for predicting mobility disability among older adults.

Abstract citation ID: igae098.0813

### CAN YOU GET UP FROM THIS SOFA? SIT-TO-STAND POWER ASSESSMENT USING A SMARTPHONE DURING AGING

Shmuel springer, Guy Baranes, and Roee Hayek, *Ariel University, Ariel, HaMerkaz, Israel*

Middle-aged individuals may face mobility issues that cannot be detected by conventional assessments. Lower-extremity muscle power correlates significantly with mobility performance during aging. Therefore, we aimed to investigate whether an assessment of lower-extremity power based on smartphone accelerometry data during the 5x sit-to-stand test (5xSTS) can provide additional information about subtle mobility limitations in midlife. Twenty-three young (25.0±2.5 years), 25 middle-aged (52.0±5.2 years), and 17 old adults (70.0±4.1 years) performed the 5xSTS test on a standard chair and on a sofa with a smartphone attached to their lower-back. STS duration was measured by the difference between the time at the end of the last stand and the time at the beginning of the first sit-to-stand, and lower-extremity muscle power (STSp) during the rising phase was calculated by multiplying body mass, acceleration, and velocity. No differences in STS duration were found between young and middle-aged adults on both the chair and the sofa, while significant differences were observed between young and old adults on both seating surfaces. STSp differed significantly between young and middle-aged adults on the sofa only (586.5 watts vs. 448.7 watts, $p = 0.037$), and between young and old adults on both the chair and the sofa ($p=0.028$ and $p=0.011$, respectively). These findings demonstrate the utility of the smartphone motion unit as a practical and efficient tool to identify preclinical mobility limitations in midlife during demanding tasks with the long-term goal of minimizing age-related functional decline.

### SESSION 2245 (SYMPOSIUM)

Abstract citation ID: igae098.0814

### POLICY SERIES: TECHNOLOGY IN AGING: ARTIFICIAL INTELLIGENCE

Chair: Angela Perone
Discussant: Brian Lindberg

Artificial Intelligence (AI) is increasingly being used in aging research and gerontological education to analyze vast amounts of data and identify patterns that may offer insights into aging across the life course. By utilizing AI algorithms, researchers, practitioners, and educators can more efficiently study factors such as genetics, lifestyle, and social and environmental influences that contribute to aging. Machine learning techniques may also help predict the onset of age-related diseases and potential interventions. The incorporation of AI in aging research and gerontological education has the potential to revolutionize our understanding of aging and improve the quality of life for older people. Yet, it is not without ethical dilemmas that have sparked increased policy focus in recent months. This interactive session, organized by the GSA Public Policy Advisory Panel, is an interdisciplinary discussion addressing the use of AI in aging research and gerontological education and the related public policy implications. The panelists represent the six sections of GSA: ESPO, BS, BSS, SRPP, HS, and AGHE, along with the Minority Issues in Aging Advisory Panel and the Humanities, Arts, and Culture Gerontology Advisory Panel.

### SESSION 2250 (SYMPOSIUM)

Abstract citation ID: igae098.0815

### RE-ENERGIZING GERONTOLOGY: BARRIERS TO PROGRAM GROWTH AND WHAT WE CAN DO ABOUT THEM

Chair: Judith Sugar
Co-Chair: Harvey Sterns
Discussant: Robert Maiden

With the growing population of older people in the U.S., it is more important than ever to increase the number of well-educated gerontology professionals. Beginning in 1972 with support from the Administration on Aging, resources were mobilized to establish and fund regional gerontology centers that would provide educational opportunities at all levels. Over the last 50 years, important work has been done to optimize the development of gerontology education. So it is ironic that at a time when we need more people to work with older adults, gerontology degree programs often struggle to enroll students. Low enrollments threaten the survival of existing programs. We have not done a good job marketing our field either inside or outside academia. We propose an effort to develop partnerships among like-minded organizations to promote the field and to attract funding to re-energize it. A coalition of partners needs to work with the employer community to convey the value of a gerontology education and ensure that graduates have the skills employers need. Accrediting gerontology programs and credentialing individuals can help to raise the profile of academic programs and graduates, and to assure employers that their employees bring the knowledge and skills to work with and for older clients, patients, and customers. The symposium presenters will engage attendees in a discussion of ways we can re-energize the field of gerontology to strengthen our academic programs and recruit more students to take advantage of the opportunities and meet the challenges of our aging population.

Abstract citation ID: igae098.0816

### ENHANCING GERONTOLOGY EDUCATION: AN HISTORICAL PERSPECTIVE

Harvey Sterns, *University of Akron, Akron, Ohio, United States*

Beginning in 1972 with support from the Administration on Aging (AoA), the Ad Hoc Committee on Development of Gerontology Resources was formed to mobilize gerontological resources in response to the Administration on Aging's proposal for the establishment and funding of regional gerontology centers. A major aspect of this initiative was to provide educational opportunities at all levels. This effort was based on the anticipated need for personnel to serve the present and future number of older adults. The work of this committee led to the formation of the Association for Gerontology in Higher Education (AGHE) with a formal affiliation with the Gerontological Society in 1973. Programs were supported by AoA to develop undergraduate and graduate certificates with degrees in disciplines related to aging; associate, bachelor, and master's degrees in Gerontology; and minors in Gerontology. This early funding played an important role in colleges and universities developing programs in gerontology education. Over the last 50 years, important work has been done to optimize the development of gerontology education, including the AGHE Standards and Guidelines and the AGHE Gerontology Competencies for Undergraduate and Graduate Education that serve as the basis for accrediting gerontology degree programs through the Accreditation for Gerontology Education Council. The need for gerontology education programs is even greater today given the shortage of personnel in all areas of gerontological services. However, the number of programs has grown slowly with increasing concern about how to maintain support for existing programs and find new funding to develop new programs.

Abstract citation ID: igae098.0817

### THE CURRENT STATUS OF ACADEMIC GERONTOLOGY PROGRAMS IN THE US

Judith Sugar, *University of Nevada, Reno, Reno, Nevada, United States*

With the growing population of older people in the U.S., it is more important than ever to increase the number of well-educated gerontology professionals. It is no surprise to GSA members that America's aging population is projected to grow by leaps and bounds over the next several decades—reaching 82 million by 2050. Yet, the number of academic gerontology programs, and hence graduates from those programs, have not been keeping pace with the need for professionals with knowledge and skills in gerontology. This presentation uses recently compiled data to demonstrate that we are falling behind in our ability to educate a workforce competent to provide services for older people and their loved ones, and to positively impact older adults' quality of life and their ability to make valuable contributions to our society. When compared with data from 20 years ago, we have had virtually no change in the number of associate, baccalaureate, or master's degree programs, nor in the aggregated number (all levels) of certificates. The only category in which growth has occurred is in the number of undergraduate minors. The numbers, the needs for services, and the prospects for providing products and services for older adults and their loved ones offer more possibilities than ever before for professionals who are well-educated in Gerontology. It is time to re-energize the field of Gerontology to strengthen and grow our academic programs and recruit more students to take advantage of the opportunities and meet the challenges of our aging population.

Abstract citation ID: igae098.0818

### PROFESSIONALIZING GERONTOLOGY AND CREDENTIALING GERONTOLOGISTS

Donna Schafer, *National Association for Professional Gerontologists, Healdsburg, California, United States*

There are many reasons people want to work with older adults. However, there is no guarantee that these motivated people will enroll in a gerontology degree program. Many do not know about Gerontology; others are not confident they can find a job with a gerontology degree. Those who do graduate from gerontology degree programs may be frustrated in competing with graduates of other programs who are able to obtain licenses or with "graduates" of weekend workshops producing "gerontologists." It is ironic that at a time when we need more people to work with older adults, gerontology degree programs often struggle to enroll students. Low enrollments threaten the survival of existing programs. Institutional perceptions about uncertain enrollments inhibit the development of new degree programs. We have not done a good job "marketing" our field either inside or outside academia. The National Association for Professional Gerontologists (NAPG) was founded in 2005 to address some of these problems. NAPG credentials the educational and practice experience of gerontology professionals using the AGHE Standards and Guidelines and the Gerontology Competencies for Undergraduate and Graduate Education. Thus far, NAPG has credentialed over 350 practicing gerontology professionals. We need to do more. NAPG proposes an effort to develop partnerships among like-minded organizations to promote the field and to attract funding to re-energize it. A coalition of partners needs to work with the employer community to convey the value of a gerontology education and ensure that graduates have the skills employers need.

Abstract citation ID: igae098.0819

### ENHANCING THE AGING WORKFORCE: THE CASE FOR ACCREDITATION OF GERONTOLOGY PROGRAMS

Maria Henke, *University of Southern California, Los Angeles, California, United States*

For over five decades, gerontology programs have been attempting to address the needs of aging populations by preparing professionals equipped with specific knowledge, skills, and competencies. Of paramount concern to any gerontology program is that their graduates are prepared to be valuable members of the workforce. This can be done by establishing cohesive standards within the field so that future employers recognize and value the qualifications of gerontology graduates. These standards can be established and improved upon by creating a dialogue with future employers to more fully understand their workforce needs as well as to clearly communicate the value graduates from accredited gerontology programs bring to their organizations. It is important that there is a core of consistency from program to program. This consistency ensures that regardless of the specific focus or emphasis of each program, employers will recognize the particular skill set a gerontologist brings to their organizations and businesses. The Academy for Gerontology in Higher Education (AGHE) is the educational arm of the Gerontological Society of America (GSA). AGHE has formulated a set of core competencies for gerontology programs. These competencies serve as the cornerstone of the accreditation process facilitated by Accreditation for Gerontology Education Council (AGEC), providing programs with a structured pathway towards recognition and validation. With the framework for accreditation established, it is imperative for programs to embark on this journey, ensuring alignment with the rigorous standards and expectations set forth by the field.

### SESSION 2255 (PAPER)

### SOCIAL DETERMINANTS OF HEALTH (HS)

Abstract citation ID: igae098.0820

### HOW INTERNATIONAL EXPERTS DEFINE ADVANCE CARE PLANNING: A CONTENT ANALYSIS

Jenny van der Steen[1], Emma de Wit[1], Mandy Visser[2], Miharu Nakanishi[3], Lieve Van den Block[4], Ida Korfage[5], Jürgen In Der Schmitten[6], and Rebecca Sudore[7], *1. Leiden University Medical Center, Leiden, Zuid-Holland, Netherlands*, *2. Topaz, Leiden, Zuid-Holland, Netherlands*, *3. Tohoku University Graduate School of Medicine, Sendai-shi, Miyagi, Japan*, *4. Vrije Universiteit Brussel (VUB), Brussels, Brussels Hoofdstedelijk Gewest, Belgium*, *5. Erasmus MC, University Medical Center, Rotterdam, Zuid-Holland, Netherlands*, *6. University of Duisburg-Essen, Essen, Nordrhein-Westfalen, Germany*, *7. University of California San Francisco, San Francisco, California, United States*

Planning for future medical care, referred to as Advance Care Planning (ACP), has evolved to a focus on conversations that explore values and preferences in a broad sense. It is unclear how this conceptualization has been adopted internationally, given diverse practices. Therefore, we examined how experts from across the globe define ACP and whether this differs by profession. In an explorative study embedded in a Delphi study on ACP in dementia, experts in ACP in persons with dementia and other diseases reported at baseline how they define ACP “in one sentence, off the top of your head.” We analyzed reported definitions with content analysis, created codes to identify definition elements, then merged them into categories. Almost half (45%) of 87 experts from 30 countries phrased ACP from a patient perspective (29% neutral, 26% professional). Codes (131) were merged into 19 categories. Five categories appeared in more than half of the definitions: ‘Choosing between options’, ‘Care and treatment’, ‘Planning for the future’, ‘Individual person’ and ‘Having conversations.’ ‘End of life’ and ‘Documenting’ were mentioned by a minority of experts. The definition of categories and perspectives did not appreciably differ between physicians and other professions. In conclusion, international experts from 30 countries typically defined ACP as person-centered conversations to choose future care and treatment, without focusing on end of life or documentation. Future research should evaluate the extent to which such conceptualization of ACP is present within clinical programs and practice recommendations and monitor changes over time.

Abstract citation ID: igae098.0821

### BIRTH COHORT DIFFERENCES IN BURDEN OF MULTIMORBIDITY OVER TIME AMONG CHINESE OLDEST-OLD ADULTS

Junmin Zhu, Yafei Wu, Siyu Duan, and Ya Fang, *Xiamen University, Xiamen, Fujian, China (People’s Republic)*

**Objective:** Multimorbidity has significantly burdened healthcare systems worldwide. Understanding the longitudinal changes of multimorbidity over time among the oldest-old adults is necessary but limited. This study aimed to investigate the birth cohort effects on multimorbidity burden among Chinese oldest-old adults and compare the differences across two decades of longitudinal surveys.

**Methods:** Data from the Chinese Longitudinal Healthy Longevity and Happy Family Study were utilized. Due to the high mortality and attrition rates in the oldest-old adults, we included 2603 and 2404 participants aged 80 and older in 1998 and 2008 surveys, respectively, with each survey followed up for a decade of repeated assessment. The burden of multimorbidity, defined as the presence of 12 chronic health conditions, was analyzed. The generalized linear mixed models were employed to evaluate the birth cohort effects. Additionally, we analyzed the impact of demographic, lifestyle, and socioeconomic factors on the burden of multimorbidity.

**Results:** In the 1998 longitudinal survey, the earlier birth cohorts had a higher multimorbidity burden over time than those born later ($\beta$ = 1.217-1.335). Conversely, in the 2008 longitudinal survey, the older birth cohorts showed a lower multimorbidity burden over time ($\beta$ = 0.631-0.777). The burden of multimorbidity was influenced by gender (female), residence (urban), region (eastern of China), and smoking history.

**Conclusion:** Our findings revealed that the higher burden of multimorbidity among the Chinese oldest-old individuals has shifted from the older birth cohorts to the more recent ones, highlighting the urgent need for comprehensive policies promoting healthy ageing and early intervention measures.

Abstract citation ID: igae098.0822

### MODERATING ROLE OF NEIGHBORHOOD CHARACTERISTICS ON INTRINSIC CAPACITY AND FUNCTIONAL LIMITATIONS

Minhui Liu[1], Shuomin Wang[2], Ruiqi Zhang[2], Qianyuan Li[2], Wenting Peng[3], Haiyan He[2], Zijing Chen[4], and Chongmei Huang[5], *1. Ningxia Medical University, Yinchuan, Ningxia, China*, *2. Central South University, Changsha, Hunan, China (People’s Republic)*, *3. University of Washington, Seattle, Washington, United States*, *4. The First Hospital Of Jilin University, Changchun, Jilin, China (People’s Republic)*, *5. Ningxia Medical University, Yinchuan, Ningxia, China (People’s Republic)*

The construct of intrinsic capacity (IC) provides a new approach to health aging. According to the ecological theory of aging, neighborhood characteristics might have important implications for functional ability. However, the potential moderating effect of perceived neighborhood characteristics on IC and functional limitations remained unknown. Using data from Round 5 of the National Health and Aging Trend Study, our sample included 7,494 community-dwelling older adults. The IC score was generated with a 2-parameter logistic Item response theory (IRT) model, using 14 dichotomous indicators covering 5 domains of capacity: cognition, locomotion, sensory function, vitality, and psychological well-being. Functional limitations were assessed by mobility, self-care, and household activities using three four-category hierarchal scales. We used negative binomial regression models to examine the association between IC and functional limitations and the moderating role of perceived neighborhood characteristics (physical disorder and social cohesion). IRT model converged successfully with a good fit (RMSEA =0.052, TLI = 0.927, CFI = 0.939), and higher IC scores were associated with less functional limitations (Mobility: Prevalence risk ratio [PRR] = 0.91, 95% CI: 0.91-0.92; Self-care: PRR = 0.93, 95% CI: 0.93-0.94; Household: PRR = 0.91, 95% CI: 0.91-0.92). Neighborhood characteristics play a moderating role between IC and functional limitations. Specifically, self-care limitations were associated with IC × physical disorder (Coef.= -0.011, p= 0.021), and household limitations were associated with IC × social cohesion (Coef.= -0.013, p= 0.037). Future research is needed to incorporate neighborhood factors into targeted interventions on the IC to maintain independent living among older adults.

Abstract citation ID: igae098.0823

### RELATIONSHIP BETWEEN LONELINESS AND SELF-REPORTED HEALTH AND LIFE SATISFACTION AMONG OLDER ADULTS

Madhyami Deshmukh, and Peter Martin, *Iowa State University, Ames, Iowa, United States*

Loneliness, health, and life satisfaction are important quality of life issues for older adults, but does having children indicate support in quality of life? The aim of this research is to examine the relationship of loneliness on self-reported health, and life satisfaction with number of living children as a moderator. Three recent waves (waves 10, 12, 14) of the Health and Retirement Study (HRS) were used. A total of 14,252 individuals (Mean age = 73) participated in this study. Bivariate correlations and multiple regressions were computed. Results indicated that loneliness at wave 10 negatively correlated with self-reported health at all three waves (all r < = -.20, p <.05) and life satisfaction (all r = < -.22, p <.05). Similar results were obtained at wave 12 and 14, but the associations were stronger for each wave. Loneliness at wave 12 (but not at wave 14) was positively correlated with age. Life satisfaction at waves 10 and 12 was positively associated with age. The multiple regression indicated that loneliness predicted self-reported health (β = -25, p <.001). For life satisfaction, loneliness and number of children predicted life satisfaction (β = -.37 p <.001 and β =.33, p <.001, respectively). Contrary to our hypothesis, the number of children did not moderate the association between loneliness on self-reported health, and life satisfaction. Future implications could help guide better family health planning, better mental health of older adults and opportunities and action plans to informal family caregivers.

### SESSION 2260 (SYMPOSIUM)

Abstract citation ID: igae098.0824

### SOCIAL ISOLATION, LONELINESS, AND THE LIVING ARRANGEMENTS AND CONTEXTS OF OLDER ADULTS

Chair: Rebecca Mauldin
Discussant: Lydia Li

Many older adults and their caregivers are at risk for social isolation and loneliness, which carry substantial adverse effects for health and well-being. Researchers and practitioners recognize the need for tailored interventions to address the unique circumstances of individuals and groups experiencing social isolation and loneliness. One factor that should be considered is living arrangements (e.g., living alone or in residential care settings) or living contexts such as neighborhood environment. The papers presented in this symposium seek to advance research related to the role of living arrangements or contexts on the social isolation and loneliness of older adults. The first paper examines older adults living alone and without care partners with a scoping review related to their risks and vulnerabilities, the adverse consequences of this living situation; and related interventions. The second paper focuses on the social networks of older adults in an assisted living community and presents quasi-experimental findings of network changes associated with online nature-based group activity programming in assisted living. The third paper examines the role of perceived neighborhood disorder and social support in caregivers' loneliness using data from the Health and Retirement Study to identify the association between neighborhood stressors, social support, and loneliness. The final paper presents findings from a mixed methods study examining the relationship between loneliness, living arrangements, and social support among caregivers of persons living with dementia. Overall, the symposium demonstrates ways in which living arrangements and contexts can be considered in research and practices with older adults and their caregivers.

Abstract citation ID: igae098.0825

### THE ASSOCIATION OF AN ONLINE NATURE-BASED ASSISTED LIVING GROUP ACTIVITY PROGRAM AND RESIDENTS' SOCIAL NETWORKS

Rebecca Mauldin[1], Jihye Baek[2], Keith Anderson[3], Megan Westmore[1], and Anna Tulloh[1], *1. The University of Texas at Arlington, Arlington, Texas, United States, 2. Washington University in St. Louis, St. Louis, Missouri, United States, 3. University of Mississippi, Oxford, Mississippi, United States*

Assisted living (AL) residents may face a risk for social isolation related to high rates of disability, compromised social networks due to relocation, and segregation from their broader communities. Many AL communities offer opportunities for residents to connect with one another through shared meals, common areas, and group activities. Since COVID-19, online content is increasingly used in AL for economical and quarantine-resistant group activity content. We present findings from a mixed method study investigating changes in residents' social networks associated with RASCALS: Reinforcing and Advancing Social Connectedness in Assisted Living. RASCALs is a group activity program that delivers and engages with content from online nature-focused livestreaming platforms. We presented Days at Dunrovin (livestreams, broadcasts, and videos from a ranch in Montana) twice a week for three months. Using a roster of resident names (N = 58), social networks (i.e., socializing partners and confidants) were assessed pre- and post-test. The number of socializing partners in the social networks in the assisted living buildings that received RASCALs programming increased during the quasi-experiment, while those in the comparison groups remained the same. There were no increases in the confidant networks of either group. In qualitative interviews, residents reported that RASCALs provided conversation topics and allowed residents to engage in increased small talk but did not foster close relationships with other residents. Findings indicate online nature-based group activities such as RASCALs may facilitate weak ties and socializing partners, relationships which have been associated with increased well-being, among assisted living residents.

Abstract citation ID: igae098.0826

### MIDDLE-AGED AND OLDER CAREGIVERS' LONELINESS AND NEIGHBORHOOD DISORDER: THE BUFFERING ROLE OF SOCIAL SUPPORT

Seungjong Cho, *Texas Tech University, Lubbock, Texas, United States*

This presentation aims to explore the relationship between perceived neighborhood disorder and loneliness among middle-aged and older (age 50+) caregivers using the 2018 Health and Retirement Study (N = 578). It also identifies the role of social support as a buffer. The association between perceived neighborhood disorder, social support, and loneliness is an emerging area of research, especially for middle-aged and older caregivers. For statistical analysis, loneliness was measured as summed scores of 11 items asking how respondents felt about different aspects of their lives. Perceived neighborhood disorder was measured as summed scores of eight items of physical disorder and social disorder. Social support was measured as summed scores of 12 items asking about positive interactions with spouse/partner, children, relatives, and friends. A multiple regression model (Adjusted R-square =.296) showed that higher levels of perceived neighborhood disorder were associated with higher levels of loneliness (Beta =.222, p <.001). Social support had no significant buffering effect on this association (Beta = -.024, p =.495). However, social support itself was significantly associated with loneliness in an anticipated direction (Beta = -.400, p <.001). Among the covariates (gender, age, education, and self-rated health), only self-rated health was significantly related to loneliness (Beta = -.146, p <.001). This study highlights the importance of identifying and managing loneliness and neighborhood stressors among this unique population with the power of social support from their spouse/partner, children, relatives, and friends.

Abstract citation ID: igae098.0827

### OLDER ADULTS WITHOUT CARE PARTNERS: A SCOPING REVIEW OF CURRENT EVIDENCE

Brittany Jones-Cobb, and Chaejeong Lee, *University of Washington, Seattle, Washington, United States*

Demographic changes in rates of living alone, divorce, migration, and having no living partner, spouse, or children are leaving more older adults without the typical "informal" care providers that are the backbone of caregiving in the U.S. These older adults without care partners face a precarious situation given our lack of a robust care structure outside of family/social network members, reductions in publicly funded care, rising costs of privately funded care, and a care workforce shortage. Using the Joanna Briggs Institute guidelines and PRISMA-ScR protocol, we conducted a scoping review of nine databases to synthesize the current evidence regarding this population's risks and vulnerabilities; adverse consequences; and any interventions that have been developed and tested specifically for this population. Three independent reviewers screened and extracting data, and the first author conducted descriptive and thematic analysis. Precarities facing older adults without care partners included risk for social isolation, impaired self-care, delays and exclusions from medical treatment, and worse medication adherence. Negative outcomes included increased likelihood of hospital and nursing home admission, and death by suicide. No rigorously studied interventions are currently published that specifically target this population. However, some evidence suggested positive benefits from enhancing home-based environmental, formal care, and volunteer supports. Very few of the included articles in this review set out to specifically study older adults without care partners. This highlights our lack of understanding of their distinctive risks and outcomes, and the vital research remaining to develop and test interventions that effectively address this growing population's unique needs.

Abstract citation ID: igae098.0828

### IMPACT OF SOCIAL SUPPORT AND LIVING ARRANGEMENT ON CAREGIVERS: ADDRESSING LONELINESS THROUGH A SUPPORTIVE NETWORK

Brianna Garrison, *Southern Connecticut State University, New Haven, Connecticut, United States*

This presentation aims to examine the relationship between caregiver of persons living with dementia's loneliness, living arrangement, and social support. Chronic progressive diseases like dementia can be risk factors for loneliness and social isolation (National Academy of Sciences, Engineering, & Medicine, 2020). Caregivers of persons living with dementia are at greater risk of loneliness throughout the trajectory of their caregiving role (Adams, 2008; Moyle et al., 2011). Living arrangement is a social environment factor shown to impact loneliness in those living with dementia (Cohen-Mansfield et al., 2015; Shiells et al., 2019), but a dearth of literature exists exploring the effects on the caregiver. Lack of quality social support can influence the experience of loneliness of both caregivers and persons living with dementia (Chatters et al., 2018; Cohen-Mansfield et al., 2016; Pinquart & Sorenson, 2001). This mixed methods study of caregivers of persons living with dementia (N = 158) explored the impact of living arrangement on the loneliness of the caregiver and how social support may impact the relationship between these two variables. This study found social support is a significant predictor of reducing loneliness among caregivers for persons living with dementia in all living arrangements. In the qualitative interviews (N = 22), caregivers reported social support impacted their loneliness throughout their care recipients' disease trajectory. This study highlights the importance of understanding the unique physical and social environmental factors that may influence caregivers of persons living with dementia's experience of loneliness and how quality social support may decrease the impact.

### SESSION 2265 (PAPER)

### TECHNOLOGY AND AGING (SRPP)

Abstract citation ID: igae098.0829

### THE USE OF VIRTUAL VOLUNTEERISM WITHIN THE AMERICORPS SENIORS RSVP PROGRAM: IMPLICATIONS FOR PRACTICE

Jennifer Crittenden[1], Nathan Tarbox[2], Rachel Coleman[2], Leah Campbell[2], Adrienna Thorne[2], and Chelsea Chapman[2], *1. University of Maine, Orono, Maine, United States*, *2. University of Maine, Bangor, Maine, United States*

The COVID-19 pandemic spurred the opportunity, and in some cases the imperative, to develop virtual volunteer (VV) options for older adults. The AmeriCorps Seniors RSVP program, one of the largest networks of older volunteers in the U.S., engages adults 55+ in a variety of local volunteer service opportunities. RSVP program directors were surveyed in 2023 (N = 138) to explore the use of VV, challenges associated with VV, and the opportunities for expansion of VV strategies with older adult volunteers. The most frequently cited VV assignments used by programs included: Community health education (53.5%, n = 23), financial services such as tax preparation and financial education (50%, n = 21), and companionship/friendly visiting (48.9%, n = 22). Common challenges cited by programs included a desire to return to in-person activities by older volunteers and beneficiaries, a lack of comfort with technology, lack of internet connection, and lack of funding resources to develop VV assignments. Statistical testing confirmed that the use of virtual strategies is not evenly distributed across geographical services areas with current (44.7%, n = 21) and past VV programs (44.4%, n = 8) more likely to serve metropolitan areas than rural or suburban service areas (X2 (2, N = 138) = 10.37, p <.01). Despite mixed levels of satisfaction with VV, approximately one in three programs (31.7%, n = 20) projected an expansion of VV with older volunteers into the future. Additional findings and associated programmatic considerations will also be discussed.

Abstract citation ID: igae098.0830

### READY, SET, VR!: VOLUNTEERS' PERCEPTIONS USING VIRTUAL REALITY WITH PLWD IN COMMUNITY-BASED RESPITE

Lillian Agyemang[1], Mary Milnamow[2], Jason Dauenhauer[3], and Louanne Bakk[1], *1. University at Buffalo, SUNY, Buffalo, New York, United States*, *2. University at Buffalo, Buffalo, New York, United States*, *3. State University of New York at Brockport, Brockport, New York, United States*

People living with dementia (PLwD) and their caregivers prefer to "age in place" within the community. Community-based respite centers often have programming that enables caregivers to drop off their care receiver for a few hours while trained volunteers provide engagement. Few, if any, studies have explored Virtual Reality (VR) programming in community-based drop-in respite programs for PLwD. Within this exploratory study, a focus group with 8 volunteers aged 65+ was conducted to understand their experiences delivering VR in a drop-in respite center serving PLwD. Qualitative analysis revealed four primary themes: 1) Motivation, 2) Positive impact of VR, 3) VR Programming, and 4) Enhancing VR delivery. Volunteers shared a strong interest in learning new technology, and their motivation to use VR in respite center programming increased after training. Volunteers observed the positive impact of VR on client engagement and social connections and shared practical tips for improving VR delivery. Results provide evidence that VR can be successfully implemented in a community-based drop-in respite center for PLwD. Structural and individual-level factors may contribute to successful VR implementation, such as programs having technical and financial support from a larger social service agency, and volunteers' motivation to receiving VR training. The level of enthusiasm demonstrated by volunteers in learning VR contradicts a persistent ageist myth regarding older adults and technology. In conclusion, VR is a novel approach for volunteers and clients to have meaningful social experiences within drop-in respite centers.

Abstract citation ID: igae098.0831

### A SCOPING REVIEW OF THE ARTIFICIAL INTELLIGENCE–BASED CONVERSATIONAL AGENTS ON MENTAL HEALTH CARE FOR OLDER ADULTS

Xiayu Chen, and Wan Wen, *University of Illinois Urbana-Champaign, Champaign, Illinois, United States*

Conversational Agents (CAs) are increasingly applied in the mental health sector, offering scalable and diverse applications from psychoeducation, symptom assessment, to self-management of mental wellness. The recent advancements in Artificial Intelligence (AI), such as the Large Language Model, have further transformed CAs into more sophisticated systems that allow personalized and intelligent interactions. However, most mental health CAs were not originally designed with the unique needs and preferences of older adults in mind. This oversight highlights the necessity for tailored features that better accommodate older adults' user experience. Current scholarly research and interventions that focus on AI-based CAs for older adults are scarce. To address this gap, this scoping review aims to offer a comprehensive overview of existing mental health AI-based CA for older adults. We conducted an extensive systematic literature search across thirteen databases, including PubMed, Scopus, IEEE Explore, ACM Digital Library, Web of Science and others. After a thorough selection process, nine peer-reviewed articles were identified for review. Our preliminary findings underscore the usability and effectiveness of AI-based CAs in enhancing the mental well-being of older adults, showing an overall positive user satisfaction and a capability for therapeutic intervention. Nevertheless, challenges such as the need for increased technological literacy and accessible designs specifically tailored for older adults persist. These findings provide critical insights for researchers and practitioners into how AI-based CAs, as promising and accessible applications and interventions, can be optimized to meet the unique mental health needs of older adults.

Abstract citation ID: igae098.0832

### DESIGNING FOR LATER LIFE: SOCIO-GERONTOLOGICAL INSIGHTS IN CONVERSATIONS WITH AGE-TECH EXPERTS

Stephen Katz, *Trent University, Toronto, Ontario, Canada*

This presentation summarizes original research data based on interview conversations with 11 international experts, consisting of designers, innovators and leaders in the gerontechnological field. Their experiences reveal important insights about the creation, production and distribution of care technologies for older adults, and how priorities are imagined for the future. Four main themes emerged from the research that point to a need for wider gerontological and sociological engagement. First, while the experts are aware of the increasing demand for technologies delivering monitoring, robotic, assistive, connective and mobility services, their socio-gerontological research about older adults on issues such as personal privacy is often limited. Second, the experts are challenged to recognize where ageist stereotypes of frailty and decline become scripted into their products. However, a clear protocol for avoiding negative imagery is not always available across age-tech enterprises, even as such imagery affects the appeal and promotion of their products. Third, the benefits of co-design and collaborate participation between designers and older users are not well understood, thus opportunities are lost to create age-friendly, affordable and livable products from the perspective of the users. Fourth, the conditions under which technologies become successful require the labor of care-givers, family members, residential staff and technical repair and maintenance technicians. Thus, understanding their roles should be an essential part of the design process. Conclusions advocate greater ties and co-learning amongst gerontologists, sociologists and age-tech experts, and attention to critical and grounded research on the material realities and challenges of technological adaptation for older adults.

Abstract citation ID: igae098.0833

### REPRESENTATION OF RURAL OLDER ADULTS IN AI HEALTH RESEARCH: A SYSTEMATIC REVIEW

Kristina Shiroma[1], and Jacqueline Miller[2], *1. Louisiana State University, Baton Rouge, Louisiana, United States*, *2. Lousiana State University, Baton Rouge, Louisiana, United States*

The demographic shift towards an aging population is particularly pronounced in rural areas, where access to healthcare and social services can be limited. Artificial intelligence (AI) has emerged as a promising avenue for transforming healthcare, particularly for older adults. However, concerns exist regarding equitable access and representation, particularly for geographically isolated, marginalized populations in rural areas. This systematic review investigates the existing research on AI interventions designed to support older adults and their healthcare needs. We searched key health and information science databases for peer-reviewed studies published between 2013 and 2023 that explored AI research with and for older adults. Our systematic analysis identified a robust body of research on AI for older adults. However, a critical gap emerged: a dearth of studies explicitly focusing on older adults in rural communities. While some studies included geographically diverse samples, data disaggregation to examine the specific experiences of rural older adults was challenging to identify. This lack of representation raises concerns about the generalizability of findings and the potential for exacerbating existing healthcare disparities in rural areas. The review underscores the need for research that specifically addresses the unique needs and challenges faced by older adults living outside of urban centers. Future scholarship should prioritize targeted recruitment strategies for rural participants; develop culturally-sensitive AI policies, practices, and products; and explore solutions that address limited representation in these communities. By working to bridge the digital divide through the future of AI research, we can achieve inclusive technology for older adults.

Abstract citation ID: igae098.0834

### WE FOUND DIGITAL, WHERE IS INCLUSION?: TOPIC MODELING OF DIGITAL INCLUSION STUDIES

Ha Neul Kim[1], and Manjun Kim[2], *1. Michigan State University, East Lansing, Michigan, United States*, *2. Florida State University, Gyeonggi-do, Kyonggi-do, Republic of Korea*

An increasing number of scholars agree that digital inclusion is to mitigate the digital divide and enhance social inclusion. However, since there is no consensus on the definition of digital inclusion, as of now, it is important to explore key concepts of digital inclusion studies to better understand what constitutes digital inclusion. The study employed a topic modeling methodology to analyze extensive collections of abstract data. Topic modeling methodology was applied to analyze vast amounts of abstract data. In total, 1,741 abstracts from book chapters, journal articles, and conference papers published by 2023 were analyzed. Data pre-processing included deleting stopwords, determining synonyms and compound words, extracting nouns, and identifying keywords based on word frequency and co-occurrences. A co-occurrence matrix was used to generate semantic networks, and community detection was applied based on modularity, the measurement of closeness within and distance across keyword groups. This study showed that there are six themes under digital inclusion studies: Policy and systematic intervention for digital inclusion (29.5% of keywords), Technology experience for older adults (27.87%), Digital divide in accessibility (16.05%), Online education and training resources (10.85%), Delivery of healthcare and equity (10.85%), and Digital inclusion in business and market (4.88%). The biggest cluster showed how digital inclusion is not only a matter of having access online or learning digital literacy but a systematic concern that involves policy intervention. However, unlike the name referring to certain 'inclusion', the topics that were studied so far, yet built social domain.

### SESSION 2270 (AWARD LECTURE (INVITATION ONLY))

Abstract citation ID: igae098.0835

### THE MARGRET M. AND PAUL B. BALTES AWARD LECTURE AND PRESENTATION

Chair: Shannon Jarrott

The Margret M. and Paul B. Baltes Award Lecture will feature an address by the 2023 Baltes Award recipient, Reuben Ng, PhD of the National University of Singapore. This session will also include the presentation of the 2024 Margret M. and Paul B. Baltes Award to recipient Nicole E. Werner, PhD, of Indiana University. The Margret M. and Paul B. Baltes Foundation Award in Behavioral and Social Gerontology recognizes outstanding early-career contributions in behavioral and social gerontology. The award is generously funded by the Margret M. and Paul B. Baltes Foundation.

Abstract citation ID: igae098.0836

### HISTORICAL NARRATIVES, CONTEMPORARY PORTRAYALS, AND STRATEGIES TO REFRAME AGING

Reuben Ng, *National University of Singapore, Singapore, Singapore*

Ageism is the discrimination of older adults based on their age, and the negative portrayals of older adults as derived from age-based stereotypes centered on illness, irrelevance, and incompetence. The insidious consequences of ageism to health prompted 194 member countries to collectively work through the World Health Organization on a global campaign to combat ageism. This talk showcases innovative methods in analyzing the historical narratives of aging over 200 years, contemporary media portrayals of older adults, and strategies to reframe aging.

### SESSION 2275 (SYMPOSIUM)

Abstract citation ID: igae098.0837

### THE PROMISE AND PERIL OF RUNNING A CENTER/PROGRAM ON AGING

Chair: Pamela Teaster
Co-Chair: Pamela Saunders
Discussant: Pamela Saunders

Gerontology centers abound across the country in various forms. Despite an unprecedented rise in the worldwide population of older adults, some programs flourish while others struggle. This symposium examines the promise of running gerontology programs as well as the perils of doing so. Offering examples and insights, Dr. Pamela Teaster (Virginia Tech) will discuss how a nearly 50 year old gerontology center has morphed since its beginnings to planning for its 50th anniversary celebration. Dr. David Burdick (Stockton University) will present the long view of leading a gerontology program for 25 years and a center for 15 years, and along with Dr. Christine Ferri will discuss the experience of succession planning for both program and center. Dr. Christopher Kelley (University of Nebraska at Omaha) describes the importance of partnerships with public and private stakeholders at the national, state, and local levels. Dr. Pamela Saunders (Georgetown University) will make observations about differences and common themes among the presentations.

Abstract citation ID: igae098.0838

### BUILDING RESILIENCE AND GRIT IN HIGHER EDUCATION'S OWN CLIMATE CRISIS

David Burdick, and Christine Ferri, *Stockton University, Galloway, New Jersey, United States*

Recently, the climate (& emotional 'temperature') and context for gerontology in higher education has never been a more difficult predicament. We will either be collateral damage from enrollment-based budget cuts, eroding societal respect for the value of a degree, and/or culture-war conflicts against DEI initiatives, or we could position ourselves to be embraced (by administration & others) as part of the solution (e.g., through advocacy of age-inclusive initiatives that may diversify enrollment and funding streams). This paper considers strengths, weaknesses, opportunities, and perils for our work. After a brief discussion of the fraught macro/societal/secular context, we describe within our institutional context the 15-year experiences of the Stockton Center on Successful Aging (SCOSA). Founded in 2007 as a provost priority, seeking a "major center on aging," an 8-person part-time staff now utilizes diverse funding streams to fulfill our research, education, and service mission. A changing institutional context has included administrative turnover (two deans, six provosts, and two presidents) and changing institutional priorities. With a wider lens, we'll reassess past AGHE efforts of our lead author related to center/program survival in tough economic times (e.g., "Finding Funding" '99 & "What Works for Your Program/Department/Center" '10, AGHExchange columns, 2010 AGHE Reno Annual Meeting Pre-Conference Intensive with Gugliucci, and 3-year AGHE/GSA funding from the Retirement Research Foundation assisting AGHE to support programs & centers). As our first author passes the leadership torch to our second author upon his retirement after 40 years, we discuss the generational shift many other aging centers are also experiencing.

Abstract citation ID: igae098.0839

### FORWARD WITH FORTITUDE: THE VIRGINIA TECH CENTER FOR GERONTOLOGY

Carlisle Shealy[1], and Pamela Teaster[2], *1. Virginia Tech Center for Gerontology, Blacksburg, Virginia, United States, 2. Virginia Tech., BLACKSBURG, Virginia, United States*

The Center for Gerontology at Virginia Tech (VTCfG) celebrated its 45th year of existence and annual celebration of gerontology. The VTCfG offers two graduate certificates—the Graduate Certificate in Gerontology (for seated students) and the Certificate for Leadership in an Aging Society (for online students). The VTCfG has five Core Faculty Gerontologists, 95 Faculty Affiliates, one International Faculty Affiliate, 12 Center Associates, 140 Graduate Certificate Recipients, and over 10 graduate students pursuing the Graduate Certificate in Gerontology at any one time. The VTCfG has a Futures Board of 18 members from various entities and organizations in the state and nationally and participates in approximately 20 funded research projects at one time. Drs. Shealy and Teaster will provide a history of the VTCfG, challenges faced through the years, and opportunities and directions for the next 50 years.

Abstract citation ID: igae098.0840

### THE UNIVERSITY OF NEBRASKA OMAHA DEPARTMENT OF GERONTOLOGY: LOOKING FORWARD TO THE NEXT 50 YEARS

Christopher Kelly, *University of Nebraska Omaha, Omaha, Nebraska, United States*

In 2023, the University of Nebraska Omaha (UNO) Department of Gerontology celebrated its 50 year anniversary.

Since 1973, the department has been the primary provider of gerontological education in the state, offering Bachelor's, Master's and Doctoral degrees in gerontology. In this presentation, we will discuss how UNO Gerontology has grown its programs, particularly through partnerships with public and private stakeholders at the national, state, and local levels, in order to train the professionals who work with and on behalf of older Nebraskans. We will also describe how we expect these partnerships to evolve to meet the needs of our growing aging population.

### SESSION 2280 (SYMPOSIUM)

Abstract citation ID: igae098.0841

### THE VHA'S GERIATRIC SCHOLARS PROGRAM IMPACT ON DEVELOPING AND DISSEMINATING CLINICAL INNOVATIONS

Chair: B Josea Kramer
Co-Chair: Judith Howe
Discussant: Marianne Shaughnessy

The Geriatric Scholars Program is an enterprise-wide VA workforce development project that has resulted in significant clinical improvements and innovations in care of older Veterans. The program has invested in the interdisciplinary primary care workforce with discipline-specific core training in geriatrics and in quality improvement. Each of the more than 1500 Scholars to date implements a quality improvement project to demonstrate transfer of new knowledge and skills in the care of older Veterans. Ove 15,000 VHA clinical employees regularly participate in interdisciplinary webinars, team training workshops, practica, and self-directed learning opportunities. There are over 5000 users of the resource website, www.gerischolars.org. This symposium explores some of the direct impacts of continuing professional education on the clinical care of older Veterans. Several program components will be discussed, including replication in Indian Health Service clinics, outcomes for caregivers of older Veterans, practice change for geropsychologists, and how the program's quality improvement mentored program for Geriatric Scholars enhances outcomes for patients in VHA. The Geriatric Scholars Program has 15 Geriatric Research, Education and Clinical Center (GRECC) partners in its network that have worked together for over 15 years to build capacity in the VHA healthcare workforce through innovative education and clinical approaches. The national GRECC Director in VHA will serve as discussant for what promises to be a lively exchange of ideas and opportunities for replication.

Abstract citation ID: igae098.0842

### IMPACT OF GERIATRIC SCHOLARS PROGRAM PSYCHOLOGY TRACK ACTIVITIES ON VA PSYCHOLOGISTS' CLINICAL PRACTICE

Christine Gould[1], Jeffrey Gregg[2], and Rachel Rodriguez[2], *1. VA Palo Alto Healthcare System, Palo Alto, California, United States*, *2. Durham VA Health Care System, Durham, North Carolina, United States*

The significant workforce shortage for geriatric mental health providers is pronounced within the Department of Veterans Affairs (VA) where more than half of Veterans receiving care are over the age of 65 years. During the past 10 years, the Geriatric Scholars Program-Psychology Track (GSP-P) has developed and delivered a multi-component geropsychology training curriculum for VA psychologists serving older Veterans. Quantitative evaluation findings have demonstrated significant improvements in self-reported geropsychology knowledge and skills. However, detecting changes in clinical practice proves to be more challenging as VA psychologists' practice includes components that are difficult to quantify (e.g., consultation, environmental interventions, facilitating family and team communication). To characterize GSP-P's impact on clinical practice, we used a rapid qualitative analysis approach to examine open-ended responses about how clinical practice has changed on a survey of GSP-P alumni (N = 40 respondents, 27% response rate). Most (75%) respondents endorsed that GSP-P activities had an impact specifically on their clinical practice. Scholars noted improved conceptualization of patients' presenting concerns. They described improved consideration of multiple factors including sensory changes, health literacy, medical health, and family systems. Increased use in standardized assessment measures and assessment of other factors (e.g., medications affecting cognition) emerged as well. Scholars described expanding intervention skills such as applying evidence-based psychotherapies with older adults and involving family members in therapy. These qualitative findings suggest that participation in the GSP-P activities has led to improvements in the quality of care provided to older Veterans with complex mental health, neurocognitive, medical, and functional needs.

Abstract citation ID: igae098.0843

### US INDIAN HEALTH SERVICE: DEVELOPING A COMMUNITY OF PRIMARY CARE CHAMPIONS FOR TRIBAL ELDER CARE

Jolie Crowder, *Indian Health Service, Rockville, Maryland, United States*

As part of this symposium, the Indian Health Service (IHS) Alzheimer's Grant Program reports on lessons learned during the first two pilot years of new geriatric workforce development initiatives. The IHS Alzheimer's program partners with local IHS, Tribal, and Urban (I/T/U) health programs to address Alzheimer's and Related Dementias (ADRD) among American Indian and Alaska Native people receiving care in the "Indian Health system." The Indian Health Geriatric Scholars (GeriScholars) program, Geriatric Nurse Fellowship (GNF), and Project ECHO (Extension for Community Healthcare Outcomes) aim to build capability in geriatric care and care for dementia within primary care and to develop a community of primary care champions for improved care for older American Indian and Alaska Native people. Adapted from the VA Geriatric Scholars program, the Indian Health Gerischolars and Nurse Fellowship pilots provide clinicians who are the mainstay workforce in our primary care delivery system with training and mentorship. The use of the ECHO model facilitates ongoing mentorship and training and sustains a community of practice with an emphasis on those with ADRD and caregivers. The programs aim to deepen individual clinical knowledge and skills in geriatric care and then apply newfound knowledge to improve care. Local assessment of needs and priorities for improving and integrating new skills and knowledge in ongoing primary care delivery are emphasized. Initiative planning and program improvements consider unique cultural contexts and disproportionate systems and resource constraints, and are designed to work in concert with other systems-focused efforts, including Alzheimer's Models of Care grants.

Abstract citation ID: igae098.0844

### GERIATRIC SCHOLARS: INNOVATIVE SUPPORT FOR CAREGIVERS OF VETERANS WITH DEMENTIA

Nicholas Koufacos[1], and Judith Howe[2], *1. James J Peters VA Medical Center, Bronx, New York, United States*, *2. Ichan School of Medicine at Mount Sinai, Yonkers, New York, United States*

As the general population ages, the number of Veterans living with dementia continues to grow. Veterans often rely on informal caregivers to manage their daily needs and ensure their health and safety. Caregivers may experience high levels of chronic stress, overwhelm, and fatigue as a result of their caregiving. Chronic stress may negatively affect the mental, physical, and emotional health of the caregiver, and consequently, the care recipient may also experience poor health outcomes. As such, it may be vitally important to promote self-care and stress-reduction among caregivers. Programs designed to support caregivers such as respite care may be difficult to access due to nationwide staffing shortages, and this is especially true in rural areas. In order to test whether an intervention designed specifically to assist caregivers with reducing stress would be effective, in June 2020, a Geriatric Scholars Program called the Rural Interdisciplinary Team Training (RITT) created an innovative expansion to their team trainings in rural areas. A palliative care certified social worker was appointed by RITT to function as the Dementia Care Coordinator (DCC) and offer virtual supportive counseling sessions and a virtual monthly support group for caregivers of Veterans with dementia. The DCC utilized the VHA's Whole Health Approach which emphasizes mindful awareness of various domains of self-care. Responses from a survey completed by 24 participating caregivers suggested that caregivers appreciated the focus on stress-reduction and self-care, valued both the individual and group support, and that participation led to reduction in stress among participants.

Abstract citation ID: igae098.0845

### THE VA GERIATRIC SCHOLARS PROGRAM IMPACT ON CLINICAL INNOVATIONS: QI COMPONENT IMPACTS OVER THE PAST DECADE

Carol Callaway-Lane, *Tennessee Valley VA, Nashville, Tennessee, United States*

Since 2009 one of the key components of the Geriatric Scholars Program (GSP) has been the Quality Improvement Workshop and Practicum, offering the fundamentals of improvement science to scholars and guidance through real-time improvement projects within rural clinical settings. The GSP has offered this component to a wide range of providers, starting in 2009 with prescribing providers and pharmacists, expanding to an interprofessional consortium including the initial professions as well as social work, psychiatry, psychology, and functional therapists and assistants. Impact on the aging Veteran population is demonstrated through completion of improvement projects at the local level with 79 national presentations completed from 2010 - 2024. This session will explore the evolution of the QI Program over the past decade and how the program has adapted to changes in audience type based on clinical training, presentation format for the workshop material, and impact on project topic and completion rates over time. Improvement projects throughout the years and their impact on the national push for High Reliability Organization (HRO) status will also be discussed.

### SESSION 2290 (SYMPOSIUM)

Abstract citation ID: igae098.0846

### 10-YEAR ANNIVERSARY OF THE SLEEP HEALTH PERSPECTIVE: INSIGHTS FOR AGING RESEARCHERS

Chair: Soomi Lee
Co-Chair: Christopher Kaufmann
Discussant: Daniel Buysse

In 2014, Dr. Buysse published a seminal paper, titled "Sleep health: can we define it? Does it matter?" which proposed that sleep health extends beyond the absence of sleep disorders. He advocated for a preventive approach where individuals must address multiple aspects of sleep to support optimal functioning and health. The concept of sleep health initiated a paradigm shift, prompting researchers to consider multiple sleep variables, not solely sleep duration, to gain a holistic understanding of individuals' sleep patterns. As we approach the 10-year anniversary of the sleep health perspective, this symposium brings together four esteemed speakers to discuss methodological approaches and empirical findings related to the link between sleep health and cardiovascular and cognitive aging outcomes, as well as social disparities in sleep health. Dr. Wallace will outline methodological challenges in assessing sleep health and clarify the advantages and disadvantages of various methods. Dr. Buxton will present a multi-dimensional sleep health model assessed by actigraphy and its association with cardiometabolic and cognitive aging outcomes in two US midlife and older adult samples. Dr. Knutson will share findings from US and Brazil population-based studies with subjective and objective dimensions of sleep health, including gender and racial differences in sleep health and cardiovascular risks. Dr. Stone will integrate epidemiology and clinical evidence to explore the link between sleep health, cognitive decline, and the onset of dementia. Finally, the discussant, Dr. Buysse, will synthesize the significance of findings, and identify opportunities for extending the sleep health perspective to future research and opportunities. Sleep, Circadian Rhythms and Aging Interest Group Sponsored Symposium

Abstract citation ID: igae098.0847

### MULTIDIMENSIONAL SLEEP HEALTH METHODS: FROM THE SIMPLE TO THE SUBLIME

Meredith Wallace, *University of Pittsburgh, Pittsburgh, Pennsylvania, United States*

The multidimensional sleep health framework presents a promising framework for improving sleep – and its health consequences – at a population level. However, there are several methodological challenges to sleep health research. including operationalizing sleep health, incorporating multiple sleep features, domains, and modalities into analytic strategies, organizing differential findings across multiple populations and health outcomes, and clarifying heterogeneity in multidimensional sleep health patterns across individuals. In this presentation, Dr. Wallace will outline several of these important methodological challenges and delineate the advantages and disadvantages of various approaches that can be used to address them. Specifically, she will discuss a range of methods, including methods for sleep data harmonization, weighted and unweighted sleep health composite scores, factor analysis to define sleep dimensionality, clustering to clarify heterogeneity in sleep health patterns, and supervised machine learning to evaluate the extent to which sleep health predicts health outcomes. Finally, Dr. Wallace will demonstrate these methods using an international multi-cohort sample of older adults, including data from the Osteoporotic Fractures in Men Study, the Study of Osteoporotic Fractures, the Multi-Ethnic Study of Atherosclerosis, the Rush Memory and Aging Project, and Minority Aging Research Study, and the Rotterdam Study. With these data and methods, she will discuss the role of sleep health in relation to longitudinal trajectories of depression and cognitive outcomes.

Abstract citation ID: igae098.0848

### MULTIDIMENSIONAL SLEEP HEALTH IN CARDIOMETABOLIC AND COGNITIVE AGING

Orfeu Buxton, *Pennsylvania State University, University Park, Pennsylvania, United States*

Multi-dimensional sleep health perspectives provide a holistic picture of the various independent and interacting facets of sleep health essential for health and wellbeing. This is key because characterizing sleep extends beyond simple measures such as the amount of time spent asleep and encompasses broad yet interrelated concepts. Taking a life course perspective, this presentation will provide theoretical background and empirical evidence for a 6-component multi-dimensional sleep health model using objective (wrist actigraphy) measures. This will, specifically, be examined in two large cohort studies of well-phenotyped participants. First, using data from the Work, Family and Health Study, evidence will be presented for the relationship of work-related stress, sleep health, and biomarker-based cardiometabolic risk in midlife and older adults. Second, using data from the Einstein Aging Study, empirical evidence will be presented for the presence of 6-facet multi-dimensional sleep health construct using factor analysis, and its relationship with cognition and mild cognitive impairment. A more comprehensive approach incorporating sleep disorders into a multi-dimensional sleep health framework will also be discussed.

Abstract citation ID: igae098.0849

### SLEEP HEALTH: IMPLICATIONS FOR CARDIOVASCULAR AND COGNITIVE FUNCTION

Kristen Knutson, *Northwestern University Feinberg School of Medicine, Chicago, Illinois, United States*

Healthy sleep is characterized by more than just adequate sleep duration or the absence of a sleep disorder. Indeed, there are numerous dimensions of sleep that are important for overall health, including sleep quality, sleep regularity and sleep timing. Sleep architecture is also an important dimension of sleep health. Prior research has demonstrated that sleep health is linked to numerous health domains, including cardiovascular and cognitive function. Further, sleep health varies by sociodemographic characteristics, such as age, gender and race, which has implications for disparities in both sleep health and in the consequences of poor sleep health, such as hypertension, cardiometabolic disease and cognitive impairment. This presentation will show data from two recent population-based studies that collected data on certain dimensions of sleep health. One study is from Brazil that collected polysomnography in people's homes to assess sleep architecture and measured cardiovascular risk factors and cognitive function. The second study was conducted in Chicago, USA among adults 55 years of age and older and collected subjective and objective estimates of sleep and measured cardiovascular and cognitive function. This presentation will describe gender differences in the associations between sleep architecture and aging and in the associations between sleep architecture and CVD risk factors in Brazil. The racial differences in sleep health characteristics among older adults will be presented. Finally, associations between these various dimensions of sleep health and cognitive function from both studies will be described. Sleep health is multidimensional and is associated with health outcomes particularly important for older adults.

Abstract citation ID: igae098.0850

### INTERPRETING ASSOCIATIONS OF SLEEP/CIRCADIAN RHYTHMS WITH COGNITIVE OUTCOMES: CHALLENGES AND FUTURE DIRECTIONS

Katie Stone, *California Pacific Medical Center, San Francisco, California, United States*

We and others have shown that a variety of sleep characteristics, both objective and subjective, predict cognitive decline and incident dementia. For example, degree of sleep fragmentation, circadian rest-activity rhythms reflecting timing and regularity of sleep, and sleep duration have all been identified as potential risk factors for cognitive decline and incident dementia in older adults. In addition, sleep disorders such as obstructive sleep apnea and insomnia are potential risk factors. However, several of these sleep exposures are substantially correlated, whereas others may contribute to risk of dementia via very distinct mechanisms. A more comprehensive assessment of sleep health is critical to determine which factors are most strongly linked to cognitive outcomes. Furthermore, little is known of potential risk stratification based on sex/gender and other factors. This information is critical to inform potential intervention approaches. In this presentation, a brief synthesis of the epidemiology and other evidence linking sleep and cognitive outcomes will be provided. Future directions will then be discussed, including the need for novel methods to analyze multidimensional sleep health data, exploration of potential mechanisms, larger studies to facilitate risk stratification, and ultimately, implementation of randomized trials to test whether interventions targeting specific sleep disorders and characteristics can effectively slow cognitive decline and prevent onset of dementia and Alzheimer's Disease. We will illustrate potential intervention approaches by describing a newly implemented randomized trial, the ESSENTIAL trial (#NCT05988385), which aims to determine effects of treatment for obstructive sleep apnea on biomarkers of Alzheimer's Disease.

### SESSION 2295 (SYMPOSIUM)

Abstract citation ID: igae098.0851

### A CONVERSATION ON TRANSLATION AND ADAPTATION OF EVIDENCE-BASED PROGRAMS FOR CAREGIVERS

Chair: Amy Eisenstein
Discussant: Kenneth Hepburn

There are currently a plethora of Evidence-Based programs designed to support caregivers. However, while caregivers represent a highly diverse population, cultural and linguistic characteristics and preferences are not always considered in the development of these programs. This symposium will bring together four research projects exploring lessons learned in their work around supporting culturally and ethnically diverse caregivers. Presenters will discuss specifics around when and why adaptations or new programs are needed and how to apply rigorous methods. The first paper will present lessons learned from the development and evaluation of a culturally tailored anxiety intervention for Latinx caregivers of older adults with cancer. The second paper will explore work on the translation and testing of an online program for Latinx family members who provide care for older people with dementia. Next we will hear about lessons learned from culturally adapting and tailoring an existing psychoeducation program for Black caregivers of persons with dementia. Finally, we will wrap up with a session describing a scoping review to identify common core components of family-centered interventions that could serve as a foundation for interventions that can be designed to support caregivers of all cultural and ethnic backgrounds. The session will be pulled together with a discussion on how we can take these lessons learned to continue to build programs in support of a culturally and ethnically rich population of caregivers, and will highlight gaps and further opportunities for research in the field.

Abstract citation ID: igae098.0852

### THE PROCESS OF CULTURALLY ADAPTING AND TAILORING AN EXISTING PSYCHOEDUCATION PROGRAM FOR BLACK DEMENTIA CAREGIVERS

Fayron Epps[1], Karah Alexander[2], Stephanie Bennett[2], Carolyn Clevenger[2], and Kenneth Hebpurn[2], *1. The University of Texas Health San Antonio, San Antonio, Texas, United States*, *2. Emory University, Atlanta, Georgia, United States*

Racial differences and disparities related to caregiving experiences exist at alarming rates for service utilization, care hours, and living below the federal poverty level among Black Americans compared to White caregivers. We developed a course, Caregiving while Black, to appreciate the culturally salient contexts, values, roadblocks, and education for Black caregivers of persons living with dementia. This project moved beyond existing psychoeducational interventions focusing mainly on dementia-related or crisis management education for family caregivers by keeping equity issues at the forefront of caregiver education. The course took an assertively "For Us; By Us" approach, prominently employing the voices and faces of Black healthcare providers, family caregivers, and PLWD. Building on materials from another caregiver psychoeducation crisis management course, we adapted and tested the course in a no-control longitudinal trial (n =75). This was a time-consuming process that took approximately 12 months. We learned that community partners, providers, and caregivers were eager to be involved in the course's design. Out of the 75 caregivers who consented, 32 completed the course. Caregivers who couldn't complete the course indicated they lacked motivation, wanted more guidance, and desired face-to-face interaction. Several Black faith communities echoed the preference for face-to-face interaction. This feedback will direct the revision of the Caregiving while Black course by offering greater interactivity and a cohort structure with a synchronous component to complement the asynchronous content to add greater interactivity. Overall, Black caregivers greatly appreciated the team designing a course for them.

Abstract citation ID: igae098.0853

### TRANSLATION AND CULTURAL ADAPTATION OF A DEMENTIA EVIDENCE-BASED PROGRAM FOR FAMILY CAREGIVERS

Roxana Delgado[1], Carole White[2], Daria Neidre[2], Kimberly Peacock[2], and Luis Luy[2], *1. The University of Texas Health Science Center San Antonio, San Antonio, Texas, United States*, *2. The University of Texas Health San Antonio, San Antonio, Texas, United States*

Culture influences how family caregivers provide care. The role of culture is especially critical when assisting care recipients with complex conditions like Alzheimer's Disease and other related dementias. The "Aprendiendo Juntos/ Learning Together" intervention is a cultural adaptation of "Learning Skills Together", an evidence-based intervention to support Latino family caregivers in providing care for a family member with dementia. Adapting an evidence-based intervention should focus on language translation as well as integrating cultural values and practices. The adaptation of the 6-week virtual intervention "Aprendiendo Juntos/ Learning Together" used a community participatory approach to inform the intervention's structure, content, and design. Guided by an Advisory Council of Latino family caregivers, we used a mixed methods design consisting of surveys and individual interviews with Latino family caregivers (n=13) to inform the intervention adaptation. Three main recommendations for adaptation based on our partnership with the advisory council and the results of the individual interviews related to 1) tailoring of the content around dementia, 2) greater focus on caregiver self-care, and 3) care planning. The adapted intervention was tested using a randomized wait-list control design. We enrolled 46 Latino family caregivers, n=32 English-speaking participants, and n=14 Spanish-speaking participants. Recruiting Latino caregivers through community partners and using snowball sampling amplified the reach to the community. Testing of the adapted intervention has been completed. Given the cultural sensitivity of a group's unique challenges, barriers, and needs, rigor in the methods is critically important in the adaptation process.

Abstract citation ID: igae098.0854

### ENGAGING STAKEHOLDERS TO ADDRESS ANXIETY IN LATINO/A CAREGIVERS OF OLDER ADULTS WITH CANCER

Kelly McConnell[1], Jaime Gilliland[1], and Andrew Salner[2], *1. Memorial Sloan Kettering Cancer Center, New York, New York, United States*, *2. Hartford HealthCare Cancer Institute at Hartford Hospital, Hartford, Connecticut, United States*

Approximately two-thirds of the 2.8 million U.S. cancer caregivers care for an older adult and spend an average of 32.9 hours per week providing care. In this difficult context, over one-third of cancer caregivers report clinically significant anxiety that is often more severe than patient anxiety. Cognitive behavior therapy (CBT) is efficacious and considered a first-line anxiety treatment. However, CBT was developed and tested primarily in white populations. In 2019, Latino/a individuals made up 18% of the U.S. population with 60.6 million individuals. Approximately 149,000 Latino/a individuals are diagnosed with cancer each year, making cancer the leading cause of death in this population. Latino/a cancer caregivers report significant distress and reduced quality of life following the patient's diagnosis. Yet, few psychosocial interventions have been developed for Latino/a caregivers. The purpose of this project was to develop a culturally competent CBT intervention for anxiety in Latino/a caregivers of older adults with cancer. Latino/a older adults with cancer (n=7) participated in focus groups to provide feedback on an initial version of the intervention. Qualitative analyses identified multiple themes including the role of faith in coping with cancer, the importance of social support in managing anxiety, and the benefits of connecting with others who are experiencing cancer. These themes provide insight into the mental health needs of Latino/a caregivers of older adults with cancer and informed modifications to the intervention. The feasibility, acceptability, and efficacy of the intervention will be examined in future clinical trials.

Abstract citation ID: igae098.0855

### BEYOND THE DYAD: A SCOPING REVIEW OF CORE COMPONENTS OF FAMILY-CENTERED CAREGIVING INTERVENTIONS

Kelly O'Sullivan[1], Alexandra Jackson[2], Sooyoun Park[1], and Raven Weaver[1], *1. Washington State University, Pullman, Washington, United States*, *2. Pacific University, Forest Grove, Oregon, United States*

Dementia prevalence is predicted to increase alongside the number of older adults, magnifying the demand for informal caregiving. While there are many effective caregiver interventions for people living with dementia and their care partners, most interventions focus on a single caregiver, overlooking the value of including other relatives in the caregiving process. Having education programs that expand beyond the traditional care dyad is crucial in meeting the needs of informal caregivers, especially considering ethnically and racially diverse populations whose cultures center around family. To explore these needs, we conducted a scoping review of 13 previously identified family-centered dementia caregiving interventions to 1) identify core components of these interventions, 2) how effectiveness was measured, and 3) the implementation and impact across cultural groups. We defined family-centered interventions as programs with at least two caregivers participating in the intervention. We initially identified 108 articles, and excluded 41 articles that were either not empirical studies or the intervention did not include at least two caregivers, yielding 67 articles for data extraction. Despite being family-based programs, interventions focused on the care dyad, offering only a few activities to engage multiple caregivers. Similarly, measures of effectiveness were at the individual level (e.g., caregiver burden or depression). However, many of these interventions were tailored and evaluated in multiple cultural groups. The identification of core components can assist with understanding active ingredients, serving as a foundation for programs that can be efficiently tailored to serve diverse families that rely on care involving multiple caregivers.

### SESSION 2300 (SYMPOSIUM)

Abstract citation ID: igae098.0856

### AGING AND CLIMATE CHANGE: FROM VULNERABILITY TO ACTION

Chair: Karl Pillemer

Despite the vulnerability of older people to climate change effects, as well as their potential to be part of climate change solutions, research has been limited. This multidisciplinary symposium responds to urgent calls for translational research on climate change as a critical pathway to reduce physical and mental health risks to older persons, brought about by climate-related hazards such as extreme temperature events, climate-related disasters, decreased air and water quality, forced migration, and vector-borne illnesses. Beyond vulnerability, panelists present research on older people's actions to prevent and mitigate climate change. Two presentations provide new empirical findings on climate hazards. Bordes and Cherry examine how climate-related disasters affect health, using data from Hurricane Ida to examine differential effects on older and younger people. Koszalinski et al. examine effects of another climate-related hazard: harmful algae blooms, based on a longitudinal study of older adults in South Florida, demonstrating how such events can create harm from otherwise beneficial activities like outdoor nature activities. Two presenters focus on action taken by older people. Nikitin et al. address the intersection of the environment and attitudes toward aging, finding that nature contact is of special benefit to older persons. Liu and Pillemer examine intergenerational motivations for climate change activism among older people. Thiamwong's presentation moves to community climate resilience strategies, presenting findings on community adaptation adapting to rising temperatures, with a focus on low-income older adults who are at special risk in extreme heat events.

Abstract citation ID: igae098.0857

### NATURE EXPOSURE IS ASSOCIATED WITH POSITIVE VIEWS ON AGING

Jana Nikitin, Fiona Rupprecht, Christina Ristl, and Selma Korlat, *University of Vienna, Vienna, Wien, Austria*

Could nature help us perceive the aging process more positively? We hypothesized that time spent in nature is associated with more positive views on aging (i.e., perceiving aging as more of a gain and less of a loss) because nature improves health and well-being and gives meaning in life. Study 1 (N = 289, age = 40-91 years) and Study 2 (N = 521, age = 40-88 years) were correlational studies examining self-reported frequency of spending time in nature in middle-aged and older adults. Both studies showed that spending more time in nature is associated with more positive views on aging. This relationship was mediated by subjective health, subjective well-being (Study 1) and meaning in life (Study 2), even after controlling for socioeconomic status. In Study 3, a field study (N = 338, age = 18-87 years), we surveyed people who were currently in a more or less natural environment (green spaces outside the city vs. city parks). We found that older (but not younger) participants in the more natural environment reported more positive attitudes on aging. This relationship was partially mediated by high levels of current subjective well-being and persisted after controlling for current physical activity and social context. In summary, contact with nature is of great benefit to us all, but particularly to older people when it comes to their attitudes to aging. Therefore, ensuring access to green spaces for older people is crucial and could serve as an intervention to promote positive experiences in old age.

Abstract citation ID: igae098.0858

### CLIMATE-CREATIVE COMMUNITIES TO PROMOTE PHYSICAL ACTIVITY AND HEALTH EQUITY IN LOW-INCOME SETTINGS

Ladda Thiamwong, Dahee Kim, Rui Xie, Christopher Emrich, YunYing Zhong, Jennifer Crook, and Timothy Hawthorne, *University of Central Florida, Orlando, Florida, United States*

Extreme heat is one of the most fatal weather hazards, and limited data suggest that low-income older adults (LOAs) who are exposed to extreme heat are less likely to participate in physical activity (PA). To develop climate-creative communities, we conducted onsite survey interviews with LOAs (n=41) and a focus group with community-based organizations (CBOs) (n=21). LOAs tended to have less frequent moderate to vigorous PA and had higher numbers of falls in summer compared to fall; 71% felt uncomfortable with extreme heat, and 69% rated their thermal sensation as slightly warm to very hot on extreme heat days. Regarding self-management on hot days, 89% drank water, 13% drank juice/favored beverage, 2% drank alcohol, 29% stayed inside, and 22% reduced PA. More than 50% stated heat or hot weather impacted their sleep, food consumption, and hydration; 32% reported heat impacted social connections, and 47% had no access to community resources (CR). The top five barriers to accessing CR were health issues (20%), don't have time (11%), transportation from home to CR (9%), lack of interest (8.9%), and lack of knowledge and information about the services (6.7%). A focus group with CBOs (n= 21) agreed that there is currently scarce communication/coordination of relevant CR, and multilateral efforts are urgently needed to co-create community-based, culturally appropriate communication and outreach programs to encourage LOAs' participation in PA, engage them in discussions about heat-health issues and provide them with access to shared CR to support their built community's resilience in adapting to rising temperatures.

Abstract citation ID: igae098.0859

### HEALTH OUTCOMES OF AGING INDIVIDUALS EXPOSED TO HARMFUL ALGAL BLOOMS IN SOUTH FLORIDA

Rebecca Koszalinski[1], Malcolm McFarland[2], Michael Parsons[3], John Reif[4], Adam Schaefer[5], and Judyta Kociolek[2], *1. University of Central Florida, Greenacres, Florida, United States*, *2. Florida Atlantic University, Boca Raton, Florida, United States*, *3. Florida Gulf Coast University, Fort Myers, Florida, United States*, *4. Colorado State University, Fort Collins, Colorado, United States*, *5. Abt Associates Inc, Richmond Hill, Georgia, United States*

Environmental changes have profoundly altered the lives of individuals in South Florida. Harmful Algal Blooms (HAB) and associated toxins produced by cyanobacteria and Karenia brevis impact the lives of Floridians, including aging individuals who recreate on the water in their retirement years. This interprofessional team of scientists and health professionals is concluding Year-5 of a longitudinal study to learn short-and long-term health impacts. Participants were recruited from publicly accessible sites, community groups, social media posts and in person events (Schaefer et al., 2020). Participants completed a questionnaire includes location, type, and duration of recreational or occupational contact with surface water and local beaches within the last 10 days. Activities included swimming, wading, boating, kayaking, paddle-boarding, tubing, fishing, and visiting a beach. Participants reported recreational fishing activities and consumption of locally caught seafood from impacted water bodies. Reif, et al. (2023) indicated that the most commonly reported symptoms were rhinorrhea, sneezing, headache, sore throat and dry cough. There was an increase of respiratory symptoms (74% of respondents), ocular symptoms (62% of respondents), and gastrointestinal symptoms (35% of respondents). Residential exposure was significantly associated with increased reporting of dry cough ($p = 0.03$), dyspnea ($p < 0.01$) and wheezy respirations ($p = 0.04$). Among persons reporting gastrointestinal symptoms, nausea ($p = 0.02$) and abdominal pain ($p < 0.01$) were significantly associated with residential exposure. Recreational exposure was significantly associated with sore throat and eye irritation. A secondary data analysis will specifically investigate the health impacts of aging individuals in this dataset.

Abstract citation ID: igae098.0860

### VOICES IN CLIMATE ACTION: INTERGENERATIONAL PERSPECTIVES AMONG OLDER ADULT ENVIRONMENTAL ACTIVISTS

Zeyu Liu[1], Marie Cope[2], Leslie Schultz[2], and Karl Pillemer[2], *1. Cornell University, Jersey City, New Jersey, United States*, *2. Cornell University, Ithaca, New York, United States*

Intergenerational collaboration is a critical approach for addressing climate change and related environmental challenges. However, research has also uncovered underlying intergenerational tensions and ageism in public discourse around climate change. A gap in existing research is the perspectives of older adults on the role of younger people's involvement in and intergenerational connections relating to climate change action. This study inductively analyzed data using a thematic analysis approach to identify and categorize perceived intergenerational issues in climate change. We conducted semi-structured interviews with 45 committed climate change activists (10+ hours a week of engagement), ages 65-89. The analysis revealed four major intergenerational themes: 1) concern for future generations as a motivator for climate change action; 2) benefits of intergenerational engagement around climate change; 3) barriers to intergenerational interaction/collaboration on climate change; 4) opportunities for mutual mentoring. Generativity emerged as a major overarching theme, in which respondents expressed both a sense of responsibility for the current situation and a desire to help and inspire younger people. The findings emphasize the importance of shared environmental concerns and active participation in facilitating communication between older and younger individuals. The study also identified challenges such as role transitions and differing perspectives on environmental responsibility. Based on these insights, recommendations are provided to enhance the effectiveness of intergenerational environmental programs, highlighting the need for strategic interaction management and the integration of diverse generational viewpoints.

Abstract citation ID: igae098.0861

**HEALTH-RELATED QUALITY OF LIFE IN OLDER ADULTS AFTER HURRICANE IDA**

Piper Bordes, and Katie Cherry, *Louisiana State University, Baton Rouge, Louisiana, United States*

Hurricane Ida made landfall in Grand Isle, Louisiana on August 29, 2021, the sixteenth anniversary of the 2005 Hurricane Katrina, which devastated the US Gulf Coast. Hurricane Ida claimed 87 lives in the US and caused an estimated 75 billion dollars in damages. In the present study, we examined post-disaster health and well-being in directly and indirectly affected younger and older adults. A total of 128 participants were sampled from three parishes (counties) where the storm devastation was more severe and two parishes farther inland with less damage. All were tested in person or virtually via the Zoom platform in a single session. They responded to several individual difference measures, a structured storm questionnaire and the SF-36 Health-Related Quality of Life Survey. Results indicated the physical health composite score was poorer for older adults, as expected ($p < .001$). In contrast, mental health scores were higher for the older adults, although age interacted with exposure status where the size of the age difference was reduced for those who were directly impacted by Ida ($p < .01$). Implications of these data for understanding older adults' psychological health after decades of severe weather events are discussed.

### SESSION 2305 (SYMPOSIUM)

Abstract citation ID: igae098.0862

**BEYOND BURDEN: LINKING CAREGIVING EXPERIENCES WITH SELF-PERCEPTIONS OF AGING AND HEALTH BEHAVIORS**

Chair: Emily Mroz
Co-Chair: Shelbie Turner
Discussant: Becca Levy

Family caregivers to older adults with chronic, progressive illnesses are intimate witnesses to complex diagnoses, symptoms, and medical decisions and the pathways for aging well amidst these challenges. Yet, little research examines whether or how navigating caregiving experiences influence caregivers' beliefs about their own health, aging, and end-of-life in ways that inform their behaviors (e.g., preparation for their own age-related changes). Aligned with GSA 2024's theme, this symposium examines caregiving experiences as potential "fortitude factors" that can guide positive aging attitudes and health behaviors. First, Rita Hu will introduce profiles of caregiving trajectories across the adult life course and describe associations between these profiles and caregivers' self-perceptions of aging. Rachel Bloom will then present a qualitative study of former dementia-caregivers' observations about the advantages and limitations of medical advocacy and end-of-life care services. She will describe how these observations can be steered by caregivers' personal narratives from their lived caregiving experiences. Yifan Lou will then build on this by describing quantitative associations between dementia- and non-dementia-caregiving experiences and caregivers' engagement in advance care planning, describing differences in ACP behaviors across racial and ethnic subgroups. In addition, a fourth presentation will spotlight Roberta Cruz, a family caregiver and member of the National Alzheimer's Project ACT Advisory Council and IMPACT Collaboratory Lived Experience panel, who will share her perspectives on this emerging topic. Our discussant, Becca Levy, will integrate our presentations with the broader landscapes of research on self-perceptions of aging and caregiver wellbeing, concluding with recommendations for future research.

Abstract citation ID: igae098.0863

**JOURNEYING BEYOND CARE: A CAREGIVER'S PERSPECTIVES ON AGING AND HEALTH PLANNING**

Shelbie Turner[1], Roberta Cruz[2], Carolyn Malone[3], Gary Epstein-Lubow[4], Monica Moreno[5], and Emily Mroz[6], *1. Weill Cornell Medicine, New York, New York, United States*, *2. National Alzheimer's Project ACT (NAPA) Advisory Council, San Diego, California, United States*, *3. Brown University, Providence, Rhode Island, United States*, *4. Education Development Center, Waltham, Massachusetts, United States*, *5. Alzheimer's Association, Chicago, Illinois, United States*, *6. Emory University, Decatur, Georgia, United States*

Emerging research suggests that caring for an older family member influences how caregivers think about, anticipate, and plan for their own aging and health changes. In 2017, nearly all (91%) caregivers participating in the University of Michigan's National Poll on Healthy Aging reported that being a caregiver made them think about their own needs for caregiving in the future. But the pathways between caregiving, planning for health changes, and (re)defining aging self-perceptions are complex. Research on these pathways benefits from collaborative partnership with individuals with lived experiences. In this special session, we will hold a conversation with Roberta Cruz, a caregiver for her mother who has been living with dementia since 2014. Roberta is a member of the National Institute on Aging's IMPACT Collaboratory Lived Experience Panel and the National Alzheimer's Project ACT (NAPA) Advisory Council. She is excited to bring her voice to the Gerontological Society of America for the first time. During this conversation, to set the stage, we will present descriptive findings on caregivers' perceptions of their future from waves of the Michigan National Poll on Healthy Aging. We will hear Roberta's perspectives on how caregiving can evoke positive and negative views on one's own aging and health changes, and the extent to which caregiving can be reasonably expected to guide one's own preparedness for aging and health changes. Roberta will reflect on the presentations in this symposium and offer insights for building successful partnerships with individuals with lived experience as this area of research expands.

Abstract citation ID: igae098.0864

### LINKING DEMENTIA CAREGIVING EXPERIENCES WITH EXPECTATIONS OF FUTURE HEALTH CARE NAVIGATION

Rachel Bloom[1], Yifan Lou[2], Joan Monin[3], Terri Fried[4], Francesca Falzarano[5], and Emily Mroz[6], *1. Fordham University, New York, New York, United States*, *2. Virginia Commonwealth University, New Haven, Virginia, United States*, *3. Yale School of Public Health, New Haven, Connecticut, United States*, *4. Yale University School of Medicine, New Haven, Connecticut, United States*, *5. University of Southern California, Los Angeles, California, United States*, *6. Emory University, Decatur, Georgia, United States*

Caregivers of people living with advanced dementia are often exposed to the complexities of healthcare coordination and decision-making over the disease trajectory. These observations can shape former caregivers' expectations of healthcare available to them in the future, particularly approaching their end-of-life. We conducted a reflexive thematic analysis to characterize adult-child caregivers' observations about healthcare provision for people with dementia, as well as exploring how and whether these former caregivers related these observations to their own end-of-life care expectations. Analysis of semi-structured interviews from 32 bereaved caregivers generated two themes describing how caregiving influenced caregivers' understanding of a) the need for medical advocacy and b) the appropriateness of medical services at the end-of-life. For some former caregivers, having engaged in medical advocacy for their parents served to demystify the process of communicating with medical professionals about goals of care and the relative utility of medical interventions, fostering feelings of preparedness for self-advocacy around receiving what they judge to be appropriate care in the future. Some participants linked their observations to changes in their perceptions of hospice care, with the majority of this group expressing an increased appreciation for its role in enhancing comfort and reducing care fragmentation. Others indicated that caregiving reinforced their existing appraisal of hospice as appropriate for improving end-of-life experiences. This work indicates that former dementia caregivers' healthcare observations shape their broader understanding of end-of-life opportunities and limitations within current medical systems, and influence their perceived ability to navigate similar contexts in the future.

Abstract citation ID: igae098.0865

### ARE CAREGIVERS MORE LIKELY TO ENGAGE IN ADVANCE CARE PLANNING? EXAMINATION BY RACE AND ETHNICITY

Yifan Lou[1], Emily Mroz[2], Terri Fried[3], and Emma Zang[4], *1. Virginia Commonwealth University, New Haven, Virginia, United States*, *2. Emory University, Decatur, Georgia, United States*, *3. Yale University School of Medicine, New Haven, Connecticut, United States*, *4. Yale University, New Haven, Connecticut, United States*

Spousal caregivers may become more aware of the value of advance care planning (ACP) as they witness care-recipients' declining quality of lives. Due to unique experiences that often occur when caregiving for a spouse with dementia, dementia caregivers may be more motivated to engage in ACP. This presentation examines the association between spousal caregiving experience across dementia and non-dementia contexts and caregivers' ACP behaviors and explores the moderating role of race/ethnicity. We identified 17,486 married individuals aged 65+ from the Health and Retirement Study 2000-2020. Three dimensions of ACP were considered: completion of a living will, designation of a Durable Power Attorney of Health Care (DPAHC), and having discussions with anyone about end-of-life care wishes. Logistic regressions compared ACP outcomes between 954 dementia caregivers, 3015 non-dementia caregivers, and 13,517 non-caregivers, controlling for sociodemographic and health covariates. Interactions between caregiver groups and race/ethnicity were examined. Spousal caregivers were significantly more likely to engage in all types of ACP compared to non-caregivers. Compared to non-dementia caregivers, dementia caregivers had significantly higher probabilities of completing a living will and designating a DPAHC. White caregivers reported highest probabilities of all three ACP outcomes, and being dementia caregivers further increased those rates. Positive association between caregiving experiences and ACP behavior were not observed in Black and Hispanic participants, except that being dementia caregivers increased the probability of having ACP discussions for Hispanic participants. We will discuss policy, practice, and research implications for the heterogeneous effects of caregiving experiences on ACP across racial/ethnic groups.

Abstract citation ID: igae098.0866

### INDIVIDUAL CAREGIVING HISTORY TRAJECTORIES' IMPACT ON LATER-LIFE SELF-PERCEPTIONS OF AGING

Rita Hu, *University of Chicago, Chicago, Illinois, United States*

This study assesses the impact of caregiving history trajectories and characteristics on later-life self-perceptions of aging (SPA) using data from the Health and Retirement Study. Caregiving history was collected using the event calendar method in the Life History Mail Survey, and SPA was measured in the 2016/2018 waves of the Psychosocial and Lifestyle Questionnaire. Employing sequence analysis and hierarchical cluster analysis on 2,215 participants, the research identifies caregiving history trajectories and examines their associations with later-life SPA, focusing on caregiving relationship types, duration, timing, and intensity. Four caregiving history trajectories were identified: Lifelong Diverse Caregivers, Later-life Spouse Caregivers, Parent Caregivers, and Later-life Non-Relative Caregivers. Linear regression analyses revealed that compared to caregivers in the Parent Caregivers cluster, those in the Lifelong Diverse Caregivers cluster were more likely to report higher negative later-life SPA ($\beta$= 0.18, SE = 0.06, $p < 0.01$) after controlling for current sociodemographic and health status. Also, earlier start age of caregiving, caring for child(ren), and caring for multiple people in the same year were all associated with higher later-life SPA. These associations remained significant after controlling for current sociodemographic characteristics. However, the associations were no longer statistically significant after adding in current health status. The findings reveal that caregiving experiences can be dynamic and lifelong and may have long-lasting impacts on later-life SPA. This highlights the necessity for targeted interventions to support caregivers' well-being and suggests that current health status plays a crucial mediating role in the relationship between caregiving history and later-life SPA.

### SESSION 2310 (BIOLOGICAL SCIENCES INVITED SYMPOSIUM)

Abstract citation ID: igae098.0867

### AGING REPROGRAMMED

Chair: Heinrich Jasper

Epigenetic reprogramming of differentiated cells into stem-like or plastic cells is a feature of regenerative processes in many tissues. This process can be leveraged to induce full pluripotency, which essentially represents a fully rejuvenated state of the epigenome. Evidence is now accumulating that partial in vivo epigenetic reprogramming, where reprogramming to pluripotency is initiated but not completed, can alleviate age-related dysfunction in tissue repair, and even extend lifespan. In a wide range of disease models, reprogramming has been shown to improve outcomes and promote tissue repair. This symposium will explore fundamental cellular mechanisms of partial reprogramming, the tissue consequences of in vivo reprogramming, and discuss the potential therapeutic impact of reprogramming on age-related diseases.

Abstract citation ID: igae098.0868

### CELL REJUVENATION AND DISEASE

Juan Carlos Izpisua Belmonte, *Altos Labs, San Francisco, California, United States*

Aging is characterized by the functional decline of tissues and organs and the increased risk of aging-associated disorders. Different rejuvenating interventions have been proposed to delay aging and the onset of age-associated decline and disease to extend healthspan and lifespan. These interventions include metabolic manipulation, heterochronic parabiosis, pharmaceutical administration and senescent cell ablation. As disease and aging are associated with altered epigenetic mechanisms of gene regulation, such as DNA methylation, histone modification and chromatin remodelling, and non-coding RNAs, the manipulation of these mechanisms might be central to the effectiveness of treating disease as well as age-delaying interventions. I will discuss some of the epigenetic changes that occur during disease and aging and how partial reprogramming by the Yamanaka factors might be a potential avenue to restore cell health and resilience through cellular rejuvenation programming to reverse disease, injury, and the disabilities that can occur throughout life.

Abstract citation ID: igae098.0869

### PARTIAL REPROGRAMMING REVERSES FEATURES OF AGING IN HUMAN T LYMPHOCYTES

Jacob Kimmel, *NewLimit, South San Francisco, California, United States*

Youthful gene expression and function can be restored in old cells by brief activation of transcription factors (TFs), a process known as partial reprogramming. Partial reprogramming holds promise as a therapeutic approach, but current methods have only employed a small set of pluripotency TFs that also reprogram cell identity in undesirable ways. It remains unknown if other TFs might be able to reprogram cell age, how long these effects last, and how often TFs act in concert to promote youthful phenotypes. To address these questions, we developed a screening technology called Partial-seq to measure the effect of partial reprogramming on gene expression across thousands of TF sets at once. We used Partial-seq to screen >4000 TF sets in aged human T cells, a keystone cell type of the immune system that declines in function with age. We discovered that many TFs beyond pluripotency factors restored youthful gene expression and cell states, without abolishing T cell identity. TF combinations were more likely than individual factors to reverse cell age, and many of these effects persisted after TF activation ceased. Our results suggest that the phenotypic age of human T cells can be durably reversed by partial reprogramming with diverse TFs. The diversity of TF sets discovered in our screens suggests that reprogramming cell age might be accomplished without perturbing cell identity.

Abstract citation ID: igae098.0870

### PARTIAL REPROGRAMMING OF THE AGING NEUROGENIC NICHE

Lucy Xu[1], Julliana Ramirez-Matias[2], Max Hauptschein[2], Eric Sun[2], Judith Lunger[2], Matthew Buckley[2], and Anne Brunet[3], *1. Harvard Medical School, Boston, Massachusetts, United States*, *2. Stanford University, Stanford, California, United States*, *3. Stanford School of Medicine, Stanford, California, United States*

Reprogramming of somatic cells by constant expression of the Yamanaka factors (Oct4, Sox2, Klf4, c-Myc) results in pluripotent stem cells and erases many hallmarks of aging in vitro. Partial reprogramming, or expression of the Yamanaka factors in a time- or cell-restricted manner, improves function in a variety of cell types from aging mice without loss of cell identity. But the impact of partial reprogramming on the aged brain has remained largely unknown. We examined how partial reprogramming affects the subventricular zone (SVZ), a conserved neurogenic niche in mammalian brains. SVZ neural stem cells give rise to newborn neurons and astrocytes throughout adulthood, but the process of neurogenesis declines with age, with a drop in neuronal precursor cells (neuroblasts) and negative impacts on olfactory discrimination and learning. Using single-cell RNA-sequencing, we find that partial reprogramming of the whole body in old mice restores the proportion of neuroblasts - cells committed to give rise to new neurons - in the neurogenic niche. Several molecular signatures of aging are also reverted by partial reprogramming. Interestingly, partial reprogramming in the SVZ neurogenic niche itself increases the proportion of neuroblasts and neural stem cells in old mice. Partial reprogramming of old neural stem cells in culture improves transcriptomic signatures of aging and boosts neuroblast and neuron production during differentiation, suggesting cell-autonomous effects. We find that partial reprogramming can rejuvenate aspects of the neural stem cell lineage in old brains, raising the possibility that this intervention could be used to counter brain decline in old individuals.

### SESSION 2315 (SYMPOSIUM)

Abstract citation ID: igae098.0871

### BRIDGING HEALTH EQUITY GAPS: CULTURAL TAILORING AND COMMUNITY-BASED APPROACHES WITH DIVERSE OLDER ADULTS

Chair: Ryan Mace
Co-Chair: Ana-Maria Vranceanu
Discussant: Kathi Heffner

Chronic conditions and related stressors disproportionately affect underserved older adult communities, increasing their risk for adverse health outcomes and emphasizing the need for community engagement and culturally tailored interventions. Consistent with the GSA 2024 conference theme, this symposium will spotlight multidisciplinary initiatives that utilize community engagement and cultural tailoring as "Fortitude Factors" to enhance health equity, quality of life, and well-being in diverse older populations. Presenter 1 will discuss a pilot effectiveness trial of "GetActive," a culturally adapted program for chronic pain management in older adults delivered in Spanish and English, showing improved pain management and quality of life. Presenter 2 will present findings from an ongoing community-engaged study providing transportation support through Uber to address inequities faced by home care workers for those with cognitive impairments, highlighting structural challenges. Presenter 3 will report on the effectiveness of the "PEERS" program for minority and low-income older adults with depression, underscoring the value of social support, particularly during the COVID-19 pandemic. Presenter 4 will report on a qualitative study that identifies and proposes strategies to mitigate socio-ecological barriers within a dementia prevention program "My Healthy Brain", emphasizing the need for a multilevel approach to enhance equity in dementia clinical trials. The Discussant will situate these studies within the broader field of gerontology, highlighting their potential to address disparities and enhance behavioral interventions for diverse aging populations.

Abstract citation ID: igae098.0872

### PROMOTING EQUITY IN DEMENTIA BEHAVIORAL CLINICAL TRIALS: INSIGHTS FROM THE SOCIO-ECOLOGICAL MODEL

Ryan Mace[1], Joshua Cohen[2], Christine Ritchie[3], Stephen Bartels[1], Olivia Okereke[2], Bettina Hoeppner[1], Judson Brewer[4], and Ana-Maria Vranceanu[2], *1. Massachusetts General Hospital/Harvard Medical School, Boston, Massachusetts, United States*, *2. Massachusetts General Hospital, Boston, Massachusetts, United States*, *3. Mass General Hospital and Harvard Medical School, Boston, Massachusetts, United States*, *4. Brown University Medical School/Brown University School of Public Health, Providence, Rhode Island, United States*

Alzheimer's Disease and Related Dementias (ADRD) is exacerbated by social determinants of health, which limit equitable access to effective behavioral interventions. ADRD disparities are widened by clinical trials that fail to recognize and address socio-ecological barriers to health behavior change, thereby reducing their efficacy and reach within diverse older populations. To address this inequity, our qualitative study aimed to: (1) identify socio-ecological barriers to ADRD prevention from healthcare professionals' viewpoints, and (2) recommend strategies to mitigate these barriers within the context of a ADRD prevention program (My Healthy Brain) and its trial protocol. We conducted 5 focus groups with multidisciplinary healthcare professionals involved in geriatric care (N=26, M experience > 17 years) from diverse clinics within an academic medical center and affiliated community health clinics. Using the Socio-Ecological Model (SEM), we identified individual, interpersonal, institutional, community, and societal barriers. The Expert Recommendations for Implementing Change (ERIC) framework guided the proposal of evidence-based strategies to overcome these barriers. Professionals identified multilevel barriers, including limited resources, linguistic and technological challenges, provider dismissiveness, conflicting institutional priorities, minority underrepresentation, and societal biases towards biomedical treatments. Recommended strategies included modifications to My Healthy Brain to improve accessibility, enhanced provider training and support, integration of interventions within clinical operations, building community partnerships, and rectifying societal misconceptions and biases. Our findings advocate for a socio-ecological approach in ADRD prevention, emphasizing the importance of recognizing and addressing the contextual factors influencing behavior change. The application of SEM and ERIC frameworks serves to bolster equity in ADRD clinical trials.

Abstract citation ID: igae098.0873

### MIND–BODY ACTIVITY SHARED MEDICAL VISIT PROGRAM FOR UNDERSERVED OLDER ADULTS WITH CHRONIC PAIN

Ana-Maria Vranceanu[1], Kate McDermott[2], Natalia Giraldo Santiago[3], Julie Brewer[2], Clara Vonderheide[2], Elizabeth Allen[1], Jonathan Greenberg[2], and Christine Ritchie[4], *1. Massachusetts General Hospital, Boston, Massachusetts, United States*, *2. CHOIR MGH, Boston, Massachusetts, United States*, *3. CHOIR-CASI, Boston, Massachusetts, United States*, *4. Mass General Hospital and Harvard Medical School, Boston, Massachusetts, United States*

Older adults with chronic pain in underserved community health centers have high rates of chronic pain and lack of access to evidence based behavioral interventions. With funding from an NIA/NINDS HEAL grant, we used qualitative methods, the Socioecological Model and Aaron's implementation framework to culturally tailor an evidence based, group, mind-body activity program to meet the needs of English and Spanish speaking older adults with chronic pain and to be delivered by clinicians typically available in underserved community health centers (social workers, nurse practitioners or psychologists) and bundled within billable shared medical visits. This talk will briefly discuss our process of cultural adaptation, training of providers, and process for incorporation of the program within billable medical visits. Next, we will present results of an open pilot effectiveness trial (English and Spanish) of the tailored interventions (N=5 groups, N= 32 patients) delivered by psychologists (1

group; English), nurse practitioner (2 groups; English) and social worker (2 groups; Spanish). Feasibility markers (recruitment 86.8%; acceptability 93.9%; fidelity 95%) were excellent. We observed significant improvement in outcomes (physical function p<.001; 6-minute walk test p<.002; pain p<.001; depression p<.001) and mechanisms (pain catastrophizing p<.002; mindfulness p<.006; kinesiophobia p<.005). Qualitative exit interviews confirmed high satisfaction with the program and revealed important consideration for a future hybrid effectiveness-implementation trial.

Abstract citation ID: igae098.0874

### NOVEL TRANSPORTATION SOLUTIONS FOR STRUCTURAL INEQUITIES AFFECTING HOME HEALTH AIDES CARING FOR OLDER ADULTS

Olivia Okereke[1], Vivian Anable Eme[1], Cerelia Liu[1], Farhabi Talukder[1], Keliane Totten[2], Joseph Locascio[1], and Anthony Weiner[1], *1. Massachusetts General Hospital, Boston, Massachusetts, United States*, *2. Pear Associates, Boston, Massachusetts, United States*

Home health aides (HHAs) provide support for homebound older adults with cognitive impairments, while lessening strain on familial caregivers. However, HHAs face structural challenges in work-related transportation. To address this gap, we began a pilot study of on-demand access to free Uber rides for HHAs who were recruited, trained and provided job placement in partnership with Community stakeholder-partner CCHERS (Center for Community Health Education Research Services). Through community engagement with CCHERS, and as informed by other community partners (e.g., Mothers for Justice & Equality, Mass HomeCare Alliance), we identified lack of affordable, accessible, reliable transportation as the most-cited structural barrier affecting HHAs and their delivery of homecare. Thus, this study provided access to free Uber rides for HHAs' job training, home care assignments and other work-related travel, for the duration of study funding; all HHAs were offered access to free rides for as long as funding was available, and key metrics were measured among HHAs during periods with and without ride funding. Preliminary data show high prevalence of other social determinants of health among HHAs using rides (e.g., 50% reporting food and housing insecurity). Outcomes among HHAs (work satisfaction, work hours, visit completion) and patients (patients reached, patient diversity) will be presented regarding whether free, on-demand transportation support for HHAs via Uber will: 1) Improve metrics among HHAs (total visits, hours/week and days/week worked, missed visit/no-shows, work satisfaction); 2) Increase racial, ethnic, socioeconomic and geographic diversity of older adults with cognitive impairment/dementia receiving homecare.

Abstract citation ID: igae098.0875

### RESULTS FROM A MIXED METHODS RANDOMIZED CONTROLLED TRIAL: PEER SUPPORT FOR DEPRESSION AMONG VULNERABLE OLDER ADULTS

Jinhui Joo, *Massachusetts General Hospital, Boston, Massachusetts, United States*

Depressed, low-income White and BIPOC older adults do not access mental health services despite increased risk. We evaluated the impact of a one-to-one peer support intervention delivered by older adult peer mentors compared to an active social control delivered by non-peers. The primary outcome was depression with up to 12 months follow up. Mediation analysis and post-study interviews were conducted with both groups. Among 149 randomized participants, the mean age was 70, 84% were women, 52% were Black and 41% were White. Both groups experienced an average decrease of 2.54 (SD: 5.80) points in PHQ-9 scores compared to their baseline levels that was sustained at 12 months. Emotional well-being, and self-efficacy increased, and adaptive coping, and loneliness decreased during the intervention and at follow up for both groups. Loneliness was a mediator for both groups but the intervention selectively engaged self-efficacy and adaptive coping. Qualitative data suggest that the intervention and control groups had disparate experiences. The intervention group learned coping skills and enacted behavior change, whereas the control group did not. Our results indicate that the PEERS intervention was not superior to social interactions delivered by non-peers, but the mechanism may be different. The intervention may increase active coping and self-efficacy whereas social interaction provided by non-peers decreased loneliness. Lay older adult peer mentors can be trained and impact important factors related to well being. Increased structure in peer support content may be needed to observe impact on depression outcomes.

### SESSION 2320 (SYMPOSIUM)

Abstract citation ID: igae098.0876

### BRIDGING POSTCOLONIAL STUDIES AND AGING STUDIES THROUGH INTERSECTIONAL THEORY

Chair: Eva-Maria Trinkaus
Co-Chair: Ulla Kriebernegg
Discussant: Ulla Kriebernegg

Kimberlé Crenshaw (1991), who coined the term 'intersectionality,' described the need for a different perspective and emphasized the need for new critical thinking in order to address structural inequality. Adding to her matrix of intersecting inequalities of race, class, and gender, Calasanti and Slevin (2001) argue that "[a]geism interacts with other types of discrimination – sexism, racism, classism, and heterosexim – in ways that make the experience of ageism both similar and different across various groups," bringing 'age' to the table. Taking into consideration that the fields of Aging Studies and Postcolonial Studies both address social and structural inequalities in the context of intersectionality, this symposium critically investigates the ways in which these fields of research intersect and make theoretical approaches fruitful for discussion by bringing Cultural Age Studies, Literary Gerontology, and Medical Ethics into conversation.

Abstract citation ID: igae098.0877

### CARERS, AGE, AND A NONINTERSECTIONAL FEMINIST DISCOURSE: THE (IN)VISIBLITY OF CARE IN US LITERARY FICTION

Eva-Maria Trinkaus, *University of Klagenfurt, Austria, Klagenfurt, Karnten, Austria*

How do we want to be cared for and who do we want our carers to be? In this narrative analysis of US literary fiction, I am looking at intersectional inequality in the context of domestic work as care work (Folbre 2006). Focusing on overlapping aspects of aging studies and postcolonial studies, I trace care work in literary fiction back to the late 19th and early 20th century to look into the feminist shift of 'new women' leaving the domestic spheres to participate in a broader public discourse and thereby opening up a gap for 'other' workers to fill within the setting of care. In order to understand what constitutes a 'good care' or a 'good care worker' respectively, this contribution investigates the fictional representation of caregivers in literary texts in order to look at how intersections of multilayered marginalizing aspects – gender, race, class, age, education, among others (cf. Crenshaw 1991, Calasanti & Slevin 2002) – contribute to the undervaluing and under-recognition of care work, but do not necessarily affect its quality (cf. D'Ignazio & Klein 2023). By investigating dimensions of care work through literary fiction, this contribution aims at challenging the current esteem of care work, and contributes to a broader discussion of adding visibility and value to the professional field.

Abstract citation ID: igae098.0878

### INTERSECTIONAL ENTANGLEMENT IN LIVING WITH DEMENTIA

Valerie Keller, *Institute of Social Anthropology and Empirical Cultural Studies, Zurich, Zurich, Switzerland*

Based on personal accounts of people living with dementia this study examines how people affected react on mechanism of societal exclusion such as pathologization, vulnerabilization and disempowerment. Although it has been called for citizenship of people affected in dementia activism and dementia studies for decades, public voices of people with dementia still remain unheard. The present study therefore examines intersectional entanglement of age and disability and highlights power structures which continue to silence people with dementia and exclude them from social participation. Furthermore, it will be outlined how people affected act at the margins not only to improve their own quality of life, but to achieve agency and to act as a social and political subject.

Abstract citation ID: igae098.0879

### MAKING OLDER MIGRANTS NARRATIVES VISIBLE: AN INTERSECTIONAL ETHICAL ANALYSIS FROM AN AGING STUDIES PERSPECTIVE

Anna-Christina Kainradl*, University of Graz, Graz, Steiermark, Austria*

Despite their heterogeneity, the lives of older migrants are in many ways shaped by intersecting patterns of injustice (Ciobanu et al. 2017). In particular when it comes to health issues, the interplay of race, gender, class, nationality, and age often impedes access and effectiveness of health services for this population group. In order to better address these challenges, it is essential to shift from the dominant perspective of professionals and family members to that of older people with migration biographies (Tezkan-Güntekin 2020). In my paper, I explore findings from an intersectional ethical analysis of the care narratives of older migrants and professionals in the context of aging studies. The paper examines the extent to which the concept of intersectionality as an analytical lens for the narratives of older migrants brings in new perspectives to their negotiations of age(ing). In analyzing the potential of the concept of intersectionality within the aging studies framework, further insights and challenges of postcolonial perspectives for ethical analysis are discussed. This theoretical paper shows how we can further apply and develop the concept of intersectionality in order to better understand the care narratives of older migrants.

### SESSION 2325 (SYMPOSIUM)

Abstract citation ID: igae098.0880

### BUILDING FORTITUDE: STRATEGIES FOR TEACHING TENDER TOPICS IN GERONTOLOGY CURRICULA

Chair: Autumn Decker
Co-Chair: Raven Weaver
Discussant: Laura Donorfio

Gerontology educators are tasked with navigating a myriad of sensitive topics (e.g., discrimination/inequality, abuse/neglect, end-of-life) that lay the foundation for understanding aging and its complexities over the life course. These topics challenge educators but hold profound significance for gerontological practice. Inequities shape opportunities over time as advantages/disadvantages accumulate, such that "the reality is later life is not the end of inequality, it is inequality's end game" (Abramson, 2016, p. 4). Inequities related to racism, ageism, and homo/transphobia affect individuals' exposure to death, trauma, loss, and grief, and require facilitation of difficult classroom conversations. Social justice necessitates learning how to provide all groups with adequate supports/resources for growing old and educators are responsible for imbuing the next generation of gerontological practitioners with knowledge, skills, and compassion. Presenters in this symposium delve into the nuances of navigating "tender topics" in gerontology curricula. Two presentations share research on end-of-life teaching strategies from a faculty perspective (Decker and colleagues) and from an interdisciplinary team approach (Newsham and colleagues). Weaver and colleagues present insights on suicide education based on faculty/student perspectives. Finally, Bolkan and colleagues provide survey data on student perceptions of sensitive issues and an overview of effective pedagogies for teaching difficult topics. Each presenter offers unique insights, findings, and tools to foster inclusive learning environments and lead challenging discussions with integrity and fortitude. Following the presentations, Laura Donorfio, AGHE Vice-Chair Elect and creator of the AGHE Teaching Institute will serve as discussant to identify key themes and facilitate a discussion with attendees.

Abstract citation ID: igae098.0881

### CULTURALLY RESPONSIVE–SUSTAINING EDUCATIONAL STRATEGIES IN UNDERGRADUATE THANATOLOGY COURSES

Autumn Decker[1], Raven Weaver[2], and Cory Bolkan[2], *1. Pacific University, Hillsboro, Oregon, United States*, *2. Washington State University, Pullman, Washington, United States*

There is an increasing demand for health and human service (HHS) workers in the United States. With direct and sustained contact with people, HHS workers are regularly exposed to end-of-life (EOL) care but typically have little education and support to handle the associated emotional and mental toll. Thus, this study qualitatively explored how cultural content, cultural humility, and EOL experiences of diverse groups were integrated in undergraduate thanatology courses. In Spring 2023, instructors teaching thanatology courses across the country were asked to participate in a quantitative survey (n=56) and a follow-up 45-minute semi-structured interview (n=27) about the instructor's course, strategies for addressing diverse populations and content in their course, and effective activities and assignments. Using culturally responsive-sustaining pedagogical principles, interviews with instructors were deductively/inductively coded, resulting in seven themes: 1) Welcoming and Affirming Environment; 2) High Expectations and Rigorous Instruction; 3) Inclusive Curriculum and Assessment; 4) Ongoing Professional Learning; 5) "Doing your Own Work"; 6) Classroom Culture and 7) Desire to Do More. Findings highlight the importance of self-care for instructors, illustrate a need for improved resources and training for death educators, particularly in delivering cultural material; support bringing lived experiences of the instructor into the classroom as a tool for building safety, belongingness, and learning for students; and emphasize the importance of multidisciplinary approach to thanatology. This session will provide tangible suggestions and pedagogical resources for instructors of thanatology courses to encourage using culturally responsive-sustaining principles in the development, delivery, and debrief of in-class activities and assignments.

Abstract citation ID: igae098.0882

### TEACHING (WITH) FORTITUDE: ADDRESSING END-OF-LIFE AND ADVANCE CARE PLANNING THROUGH AN INTERDISCIPLINARY LENS

Tina Newsham, Alissa Dark-Freudeman, Jamy Chulak, Elizabeth Fugate-Whitlock, and Kirsten Kringle-Baer,
*University of North Carolina Wilmington, Wilmington, North Carolina, United States*

Topics related to end-of-life (EOL) and advance care planning (ACP) can be fraught, both for instructors and students. Further, teaching about these topics can be challenging as they can (and, we argue, should) be addressed through multiple disciplinary lenses, and ideally in an interdisciplinary fashion. In this paper, we share an interdisciplinary effort to teach about EOL and ACP at the University of North Carolina Wilmington. Faculty in Psychology, Gerontology, Philosophy, and Respiratory Therapy collaborated to create an interdisciplinary, seven-week module that was integrated into classes from each participating discipline. Students were grouped across disciplines and worked together to gather data regarding needs and desires related to EOL and ACP and then to create discussion guides around relevant topics to foster meaningful conversation and planning related to sensitive topics. Students completed guided reflections regarding what they learned about and from partners with different disciplinary perspectives and how the interdisciplinary collaboration resulted in outcomes that would otherwise have been absent in a singular discipline effort. This project serves as a model for interdisciplinary teaching, particularly around sensitive topics.

Abstract citation ID: igae098.0883

### AN INTEGRATIVE ANALYSIS OF STUDENT AND INSTRUCTOR PERSPECTIVES ON DISCUSSING SUICIDE IN THE CLASSROOM

Raven Weaver[1], Autumn Decker[2], Ashley Robillard[1], Maksim Vasylenko[1], Madison Kidner[1], and Cory Bolkan[1],
*1. Washington State University, Pullman, Washington, United States*, *2. Pacific University, Hillsboro, Oregon, United States*

Suicide is often considered a triggering topic and may pose challenges in classroom settings. Suicide is a lifespan issue with older adults having the highest risk and it being a leading cause of death among young adults. To improve both personal response and professional readiness, it is important for college students to learn about suicide in the classroom. This integrative, cross-sectional study relied on a quantitative survey of college students (n= 313; Mean age:19.5; 71% Women) on effective ways for learning about and discussing sensitive topics, like suicide, as well as qualitative semi-structured interviews of college faculty teaching death and dying courses (n = 27, 83% women; 94% White), who shared their perspectives on teaching about sensitive topics including death, dying, and suicide. Most students wanted to learn more about suicide (74%) and about available resources to support individuals at risk of suicide (86%). Conversely, content analysis of faculty perspectives revealed they typically avoided discussing suicide in the classroom; not all faculty felt prepared or comfortable having suicide-based discussions and many feared that discussions would exacerbate students' depression, anxiety, or suicide ideation. We highlight the importance of collaborative methods and co-designing curricula to deliver trauma-informed, evidence-based instruction. There is a need for more integration of campus and external resources, and the development of EBPI trainings around suicide prevention on college campuses, as well as a need to prepare future gerontological professionals to be able to detect and support older adults in mental health crises.

Abstract citation ID: igae098.0884

### NAVIGATING COMPLEXITY: EFFECTIVE PEDAGOGY FOR TEACHING DIFFICULT TOPICS

Cory Bolkan[1], Raven Weaver[1], and Autumn Decker[2],
*1. Washington State University, Pullman, Washington, United States*, *2. Pacific University, Hillsboro, Oregon, United States*

There are many sensitive (e.g., death, loss, trauma) and politically charged (e.g., aging policies, ageism) topics that often emerge in gerontology courses that can challenge educators and impede classroom dialogue. We surveyed undergraduate students (N = 380; 76% Women, 65% White, 44% first-year) regarding their perspectives on which topics they believed were important to feature in lifespan human development curricula. Results indicated strong interest in specifically addressing challenging issues in their classes, with the top, most-reported topics identified as mental illness (n = 252) and death and loss (n = 193). Additional topics of high interest included substance abuse, gun violence, discrimination, and maltreatment. Incorporating these topics into gerontological education offers many benefits and fosters comprehensive understanding of the full life course among students. To help educators navigate these topics effectively, we provide an overview of various pedagogical approaches that consider social crises and power relations (e.g., culturally responsive teaching, critical pedagogies, inquiry-based or experiential learning), and share examples of how they can be used to guide and facilitate learning about sensitive issues. Trauma-informed teaching also ensures a safe learning environment for discussing difficult topics, while critical pedagogies encourage critical analysis of societal norms and power dynamics to broaden students' understanding. By integrating these approaches, educators can create inclusive learning environments where students are empowered to explore and discuss multifaceted issues relevant to later life. This enhances academic learning and equips students with the skills and empathy needed to address the challenges faced by a diverse population of older adults

### SESSION 2330 (SYMPOSIUM)

Abstract citation ID: igae098.0885

### CENTERING DIVERSE AGING ADULTS: STRENGTH, RESILIENCE, AND OPPORTUNITIES

Chair: Elizabeth Muñoz
Co-Chair: Amy Thierry

Our older adult population is increasingly becoming more diverse and there is a need to identify sources of risk and resilience among individuals living at the intersection of multiple systems of oppression or adversity. This symposium highlights the lived experiences of older diverse adults through a multi-dimensional lens by investigating the experiences of adults across community settings, exposure to trauma, identity (gender and race), and health status. Presentations also investigate already-existing strengths within these communities and recommend opportunities for leveraging those assets to bolster health equity in diverse older adults. To this end, Tian and colleagues will present results on coping strategy utilization and stress among urban-dwelling diverse adults during the COVID-19 pandemic. Cherry and colleagues will showcase an investigation of the experiences of African American / Black women living in Louisiana who endured the 2005 hurricanes and the COVID-19 pandemic. Wright and colleagues will share findings on the association between parental education, adverse childhood experiences, and memory performance among diverse, community-dwelling older adults. Groves and colleagues will present findings on the feasibility of a dyadic intervention to support hypertension self-management among older African American women with hypertension. Lastly, Gaillard and colleagues identify strategies for increasing engagement in health research among older African Americans. Overall, this symposium centers the lived experiences, strengths, and resilience of diverse older adults, and points to novel directions for advancing health equity research.

Abstract citation ID: igae098.0886

### THE IMPACT OF COVID-19 ON AVOIDANT COPING STRATEGIES AND PERCEIVED STRESS: INSIGHTS FROM THE HANDLS SLEEP STUDY

Junyan Tian[1], Alexa Allan[1], Lena Simon[2], Lesley Ross[3], May Beydoun[3], Alan Zonderman[4], Michele Evans[4], and Alyssa Gamaldo[2], *1. The Pennsylvania State University, Elgin, Illinois, United States*, *2. Clemson University, Clemson, South Carolina, United States*, *3. National Institute on Aging, Baltimore, Maryland, United States*, *4. National Institute on Aging, Baltimore, Maryland, United States*

Coping strategies are usually defined as both effective and ineffective methods to reduce the impact of stressful life events. Avoidant coping, characterized by passive strategies, is more frequently used as people age. However, it is correlated with a higher level of perceived stress and more depressive symptoms. This study used a subsample of the Healthy Aging in Neighborhood of Diversity Across the Life Span Sleep Study (HANDLSleep) to examine the impact of the COVID-19 pandemic on the relationship between avoidant-focused coping and perceived stress. Our participants included 86 middle-aged and older adults (52.33% Black adults, age range: 46-82). The Avoidant Brief COPE scale measured how frequently they utilized avoidant coping strategies, such as substance use and denial, to disengage from stressful events. Results of the independent sample t-test indicated that individuals who tested positive for COVID-19 had lower levels of avoidant coping than those without infection, $t(84) = -2.79$, $p < .05$. Furthermore, regression analyses revealed a significant interaction effect between COVID-19 infection and perceived stress on avoidance coping, even after controlling for age, education, race and income. Specifically, only individuals who did not test positive for COVID-19 showed a correlation between higher levels of perceived stress and increased utilization of avoidant coping, $b = -.02$, $t(76) = 2.54$, $p < .05$. The simple slope for those with COVID-19 infection was not significant. These results highlight the significant social impact of COVID-19 infection on older adults' coping strategy utilization when dealing with stress.

Abstract citation ID: igae098.0887

### HURRICANE KATRINA AND COVID-19: LASTING IMPACTS ON NEW ORLEANS' AFRICAN AMERICAN WOMEN

Katie Cherry, Kori Balaam, Oliver Hardy-Smith, Piper Bordes, and Kennedy Simon, *Louisiana State University, Baton Rouge, Louisiana, United States*

In 2005, two Category 5 hurricanes slammed into south Louisiana. Katrina brought catastrophic damage and substantial loss of life across the US Gulf Coast. Four weeks later, Rita crippled southwest Louisiana with tremendous destruction. In this study, we tested younger and older women who self-identified as African American / Black. All lived in the greater New Orleans metropolitan region at the time of the 2005 hurricanes. They completed a structured storm questionnaire and open-ended questions that tapped their Katrina experience and the more recent Covid-19 pandemic. Qualitative analyses on their narrative responses yielded three core themes: (1) Katrina Stressors: Family and Community Disruption, which captured catastrophic losses of homes and neighborhoods. Salient and frequent stressors included the disruption of social networks and pivotal life events (wedding plans, graduations). (2) Religiosity and Post-Traumatic Growth: Seeing Silver Linings, where coping through religious beliefs and practices (meditation, prayer, bible reading) helped participants navigate a new life journey. However, some questioned God following Katrina and one reflected that she did not join a new faith community after being separated from her home church in New Orleans, LA. (3) Katrina and COVID-19: Environmental Trauma on a Global Scale. The COVID-19 pandemic meant adjusting to a new normal with limited access to resources and businesses, reminiscent of post-Katrina life. A pressing responsibility to protect the safety and well-being of their family was also mentioned. Overall, these data permit new insight into individual and community-level vulnerabilities and strength-based resilience resources in the years after devastating environmental events.

Abstract citation ID: igae098.0888

### THE ROLE OF PARENTAL EDUCATION AND ADVERSE CHILDHOOD EXPERIENCES IN COGNITIVE FUNCTION AMONG DIVERSE OLDER ADULTS

Regina Wright, *University of Delaware, Newark, Delaware, United States*

An expansive body of research suggests older adults' cognitive abilities are linked to a number of factors across the lifespan, including socioeconomic status and physical health. Early life experiences have emerged as an important predictor of cognitive function, as researchers identify long-term trajectories linking factors such as early life trauma and poverty to poor outcomes; however, much of these findings have been limited to largely homogenous study samples. Therefore, we examined the cross-sectional relationships between parental education, adverse childhood experiences (ACEs), and cognitive function in a diverse sample of community-dwelling older adults. Data were analyzed from 165 older adults (mean age = 68.48y, 33% male, 41% African American), free of major disease, who completed a battery of psychosocial and neuropsychological tests and a sociodemographic assessment, and underwent a physical screening in two study visits. We found no significant associations between ACEs and cognitive performance. However, linear regression analyses, adjusting for age, sex, race, education, and ACEs, showed a statistically significant association between maternal education and short-term verbal ($p=.007$) and visuospatial memory ($p=.003$) performance, and paternal education and long-term verbal memory performance($p=.029$). Results further showed that maternal education was a stronger influence on cognitive performance than paternal education. Findings suggest having parents with greater educational attainment, and in particular a mother with greater educational attainment, is related to better cognitive performance in late life, independently of participant education or ACEs. This presentation will explore plausible pathways for intergenerational transmission of cognitive resilience.

Abstract citation ID: igae098.0889

### PEER (DYADIC) SUPPORT: EXPERIENCES AND PERSPECTIVES FROM OLDER AFRICAN AMERICAN WOMEN WITH HYPERTENSION

Angela Groves[1], and Julie Ober Allen[2], *1. Western Michigan University, Kalamazoo, Michigan, United States, 2. University of Wisconsin, Madison, Wisconsin, United States*

African American women have a higher prevalence of hypertension than women of other ethnicities. Interventions have not adequately addressed the higher prevalence of hypertension in this population. Social support networks can be an effective strategy for improving hypertension self-management behaviors. The objective of this study was to evaluate the feasibility of an 8-week peer (dyadic) support intervention to improve Dietary Approaches to Stopping Hypertension (DASH) adherence and reduce systolic blood pressure among older African American women with hypertension and describe the relationship that occurred during the intervention. This mixed methods study used a convergent parallel design. A total of 40 African American women diagnosed with hypertension, aged 60 and older were paired to form 20 dyads. At the end of the 8-week intervention, participants discussed their experiences in a 60-minute focus group session. Content analysis was used to analyze focus group interviews. Qualitative preliminary data analysis will be presented. Emerging themes included, accountability, epiphany, partnership-dyad, communication, and receiving support. Preliminary findings suggest that peer (dyadic) support is feasible and can be an effective intervention strategy to improve DASH diet and blood pressure management. Future studies should examine the extent in which dyadic relationships are maintained over time to improve diet and blood pressure outcomes. Also, future randomized controlled trials should be conducted to evaluate the effectiveness of the peer dyadic support modality on DASH diet adherence to reduce systolic blood pressure.

Abstract citation ID: igae098.0890

### AN ASSET-BASED APPROACH TO INCREASE ENGAGEMENT OF OLDER AFRICAN AMERICANS IN HEALTH RESEARCH

Trudy Gaillard[1], Joan Vaccaro[1], Donna Neff[2], Cynthia Morton Padovano[3], and Fern Webb[4], *1. Florida International University, Miami, Florida, United States, 2. University of Central Florida, Orlando, Florida, United States, 3. University of Florida, Gainesville, Florida, United States, 4. University of Florida, Gainesville, Florida, United States*

Asset-based approach to community engagement research aims to identify community strengths that improve health and health equity. The objective of the current study was to identify strategies expressed by older African Americans (OAA) to increase their engagement in health research. The following report was part of a larger study exploring the impact of intergenerational influence and perceptions regarding willingness to participate in health research among ethnically diverse OAA. Participants were recruited from North, Central, and South Florida using various community engagement strategies. Listening sessions were conducted in dyads; older adult (≥65 years) and a trusted family member/friend (25-64 years). The following results are from OAA (≥65 years). All transcripts were recorded and transcribed verbatim. NVivo software© was used for data management and analysis. There was a total of 14 listening sessions (N= 66). In general, most OAA consulted younger trusted family/friends about health concerns before deciding to participate in research. Key findings concerned, the researcher, goals of the research, and with whom and how information would be shared. Community assets identified that researchers should engage the community by: partnering with local primary care providers (both physicians and nurse practitioners); schools

(intergenerational engagement); community liaisons "people who look like me"; and local trusted elected officials. Our results indicate that there are multiple community-based assets that can be used to engage Older African Americans in health research. An asset-based approach with community members identified encouraging suggestions that can be integrated into the design of health research for this population.

### SESSION 2335 (SYMPOSIUM)

Abstract citation ID: igae098.0891

### CLOSING THE EQUITY GAP: DEMENTIA RESEARCH WITH INDIGENOUS POPULATIONS

Chair: Jordan Lewis
Co-Chair: Kristen Jacklin
Discussant: Jordan Lewis

This symposium highlights community-based work focusing on Alzheimer's disease and related dementias (ADRD) conducted with Indigenous populations across the United States and Canada. We describe not only outcomes and findings of these various projects but also the essential research processes necessary for creating relationships and working with Indigenous partners and communities. We begin with Whetung, who takes a quantitative approach to describe the landscape of cognitive disparities among Indigenous older adults due to structural inequities, in particular, everyday and lifetime discrimination experiences. Next, Blind et al. describe the in-depth and iterative process of cultural adaptation of cognitive assessments. They are currently adapting validated cognitive assessments for Indigenous populations in Canada, this time for urban and reservation-residing American Indian populations. The final presentations by Jacklin et al. and Lewis will focus on qualitative research of cultural explanatory models and lived experience of dementia from the viewpoints of two different participant groups: healthy older adults (Indigenous communities in Minnesota, Wisconsin, and Ontario) and caregivers of people living with dementia (Alaska Native). ADRD research with Indigenous Peoples is scarce but growing, and this symposium highlights the innovate, various, and exceptional approaches to this research. This symposium highlights research projects which are rooted in the values of Indigenous knowledges and research methods, community-based participatory research, qualitative research, and the Six Rs of research (respect, relationship, representation, relevance, responsibility, reciprocity; Tsosie et al. 2022). Using these approaches, our research seeks to understand and close the equity gap for Indigenous older adults dealing with ADRD. Indigenous Peoples Interest Group Sponsored Symposium

Abstract citation ID: igae098.0892

### INDIGENOUS EXPLANATORY MODELS OF DEMENTIA: RESULTS FROM HEALTHY OLDER ADULTS IN NORTH AMERICA

Kristen Jacklin[1], Melissa Blind[2], Wayne Warry[2], Nickolas Lambrou[3], Carey Gleason[3], Megan Zuelsdorff[3], and Dana Ketcher[2], *1. University of Minnesota Medical School, Duluth, Minnesota, United States*, *2. University of Minnesota Medical School Duluth, Duluth, Minnesota, United States*, *3. University of Wisconsin Madison, Madison, Wisconsin, United States*

Explanatory models of illness are important concepts in understanding a person's beliefs about an illness and the social meaning that is attributed to that illness. This project seeks to understand an explanatory model of Alzheimer's disease and related dementias (ADRD) among healthy older adults in four diverse Indigenous communities in the US (Minnesota, Wisconsin) and Canada (Ontario). Individual interviews (n=37) were conducted with healthy older adults whose age ranged from 49-79 years (M = 65.5) and were majority female (n=23, 62.2%). Four qualitative analysts conducted team-based coding, double-coding 30% of interviews to ensure intercoder agreement Kappa of ≥.80. A priori codes developed based on Kleinman's explanatory model resulted in seven content areas: perceptions of ADRD, causes of ADRD, naming ADRD, ADRD treatment and/or care, diagnosis of ADRD/memory concerns, ADRD prevention, and fear/worry regarding ADRD. Themes under these content areas were varied but are reflective of holistic worldviews of health including physical, mental, cultural, and spiritual impacts. For example, participants report that changes associated with dementia align with completing the circle of life, so Elders may share similar traits as infants in terms of help and support. The consequences of ADRD are felt at both interpersonal and community levels, with the loss of traditional knowledge, generational memory, and stories. The default form of ADRD care is within families, with participants reporting that people should live as long as possible at home. This study provides comprehensive information about healthy older adults' attitudes and perceptions of ADRD in Indigenous communities.

Abstract citation ID: igae098.0893

### MAKING SENSE OF A DISEASE THAT MAKES NO SENSE: CULTURAL UNDERSTANDINGS OF DEMENTIA AMONG ALASKA NATIVE CAREGIVERS

Jordan Lewis, *University of Alaska Fairbanks, College of Indigenous Studies, Fairbanks, Alaska, United States*

With the rate of Alzheimer's disease and related dementias (ADRD) increasing among Alaska Indian/Alaska Native (AI/AN) people, the Alaska Native Health system is ill-prepared to meet the challenges associated with the growing population at risk of ADRD. The high cost of care, inadequate training of health care providers, and lack of supportive services for caregivers are especially concerning. Interviews were conducted with 22 AN caregivers for ANs with ADRD in communities across Alaska. Interviews lasted approximately 60 min and were transcribed verbatim. We employed directed content analysis to examine the major agreements and disagreements between the participants' understandings of ADRD in each of the domains of Kleinman's Explanatory Model of Illness. Caregivers and health care providers expressed concerns about the lack of understanding, resources, and awareness of ADRD among ANs in rural and urban communities. Both caregivers and providers recognized the need to obtain an early diagnosis, blend Western and traditional medicines, promote lifestyle and dietary changes, and foster training for caregivers. Health care providers acknowledged their limited exposure to AN understanding of ADRD and wish to receive culturally relevant training to better serve AN. As the older AN adult population grows, the need for culturally responsive training and support services will continue to increase. We recommend establishing rural outreach and support groups for caregivers, developing an understanding of how ANs view ADRD to train and educate health care providers, and implement screening early for memory loss during routine medical examinations

Abstract citation ID: igae098.0894

### AMICA DEMENTIA SCREENING TOOLS ADAPTATION: AN INTERCULTURAL CONSENSUS APPROACH

Melissa Blind[1], Sheamus Cavanaugh[1], Nathania Tsosie[2], Carrie Trojanczyk[3], Nickolas Lambrou[3], Carey Gleason[3], Tassy Parker[2], and Kristen Jacklin[4], *1. University of Minnesota Medical School Duluth, Duluth, Minnesota, United States*, *2. University of New Mexico, Albuquerque, New Mexico, United States*, *3. University of Wisconsin Madison, Madison, Wisconsin, United States*, *4. University of Minnesota Medical School, Duluth, Minnesota, United States*

Existing clinical tools for assessing dementia demonstrate varying degrees of cultural, educational and language biases, and are not validated for use with Indigenous populations in the US. The American Indigenous Cognitive Assessment (AMICA) project aims to develop and validate a culturally appropriate dementia evaluation toolkit that consists of four assessments. Participating Indigenous populations include the Red Lake Nation in Minnesota; an urban Indigenous population receiving services at the First Nations Community HealthSource in Albuquerque, New Mexico; and the Oneida Nation in Wisconsin. Our goal is to create one set of assessments that is agreed upon through intercultural consensus. Assessment adaptation across the three sites will focus on the Canadian Indigenous Cognitive Assessment (CICA), Kimberley Indigenous Cognitive Assessment (KICA) Carer, KICA Depression Scale, and KICA Activities of Daily Living (ADLs). We use a community-based participatory research (CBPR) approach and the Two-Eyed Seeing integrated knowledge framework to engage Tribal and community collaborators. In this paper, we detail the process of convening Indigenous Knowledge Advisory Groups (IKAGs) at each site and an Assessment Expert Panel (AEP), building a team science approach, and facilitating tool adaptation with communities. We provide an update on the CICA adaptation and offer lessons learned that inform culturally relevant and safe adaptation of the remaining tools. Finally, we report on our process for achieving intercultural consensus in developing a single set of dementia assessment tools for Indigenous peoples across the US.

Abstract citation ID: igae098.0895

### ASSOCIATIONS BETWEEN DISCRIMINATION AND COGNITION AMONG INDIGENOUS, BLACK, HISPANIC, AND WHITE OLDER ADULTS

Cliff Whetung[1], and Ernest Gonzales[2], *1. University of Minnesota, Duluth, Duluth, Minnesota, United States*, *2. New York University, New York, United States*

The number of Indigenous older adults will more than double in the next thirty years, and they are at high risk of cognitive pathologies in later life. This study centers on Indigenous older adults among Black, Hispanic, and White peers. It examines whether structural inequities (educational attainment, low incomes) and discriminatory experiences (everyday discrimination, major lifetime discrimination) are associated with racial inequities in cognitive health. Using 14 years (2006-2020) of restricted Health and Retirement Study (N = 27,327) data, we estimated cognitive trajectories among Indigenous, Black, Hispanic, and White older adults using mixed effect growth curve models. Models were adjusted for discrimination experiences and relevant covariates. We found that everyday discrimination experiences were negatively associated with cognition at baseline and over time. Major lifetime discrimination experiences were associated with lower baseline cognition scores and positively with cognitive change over time. Indigenous older adults who experienced high levels of everyday discrimination had significantly lower cognition scores at baseline than any other race or ethnicity. We also found that Indigenous older adults primarily attribute perceived discrimination experiences to their age, ancestry, and race. Indigenous older adults have a unique profile that places them at elevated risk for cognitive pathology in later life, and structural inequities are powerfully associated with many of these risk factors. Addressing these inequities will require the development of policies, programs, and research agendas that target the fundamental causes of these discriminatory stresses: structural inequities in education and socioeconomic opportunities.

### SESSION 2340 (SYMPOSIUM)

Abstract citation ID: igae098.0896

### COMPASSIONATE CAREGIVING: ENHANCING GERIATRIC SUPPORT THROUGH GWEP CNA INITIATIVES

Chair: Samantha Cotton

The Geriatric Workforce Enhancement Programs (GWEPs) at various institutions have launched innovative educational efforts to support Certified Nursing Assistants (CNAs), a key yet often overlooked segment of the healthcare workforce. These initiatives strive to mitigate high turnover rates and fill educational gaps, with a special focus on leadership, geriatric care, and compassionate handling of individuals with Alzheimer's disease and related dementias (ADRD). The University of Chicago Medicine's SHARE Network introduced the CNA Leadership Academy, aimed at nurturing CNAs' leadership capabilities through a curriculum that includes leadership principles, quality improvement, and resilience, facilitated by Project ECHO's tele-mentoring. The University of California-Irvine GWEP's program, "Building Resiliency While Advancing Your Caregiver Skills," targeted geriatric care enhancements, incorporating the 4-Ms and strategies for challenging behaviors through interactive and trauma-informed teaching methods. Meanwhile, the University of Louisville's GWEP developed a compassionate care model for CNAs in long-term care, offering training via online modules, workshops, and an iOS app to improve management of ADRD-related behaviors. These GWEPs collectively aim to bolster CNAs' roles in healthcare, equipping them with essential skills to elevate patient care, especially in geriatric and dementia settings. By enhancing CNAs' abilities and job satisfaction, these programs contribute to a more resilient, knowledgeable, and empathetic geriatric care workforce, demonstrating a comprehensive effort to advance the quality of care for older adults. This symposium will focus on the critical need of strengthening the training of CNAs and lessons learned across our programs.

Abstract citation ID: igae098.0897

### FOSTERING COMPASSIONATE CAREGIVING USING CNA TRAINING

Samantha Cotton, *University of Louisville, Louisville, Kentucky, United States*

Behavioral health complications in individuals with Alzheimer's disease and related dementias (ADRD) present significant challenges in long-term care facilities. The progressive decline in cognitive abilities and subsequent difficulty in managing behaviors related to ADRD necessitate a specialized approach for Certified Nursing Assistants (CNAs) who provide daily care. Addressing these challenges, our HRSA funded Geriatric Workforce Enhancement Program (GWEP) at the University of Louisville has introduced a compassionate care framework tailored for CNAs in long-term care settings. This comprehensive training model equips CNAs with essential skills and knowledge to understand and navigate the behavioral complexities of ADRD. The training encompasses online micro-modules for self-paced learning, interactive workshops for hands-on practice, and an innovative iOS app has been created as a readily available resource for CNAs, offering guidance and support for managing challenging behaviors on-the-go. Our training program has successfully educated 175 CNAs, evidenced by significant improvements in knowledge and self-efficacy regarding ADRD care, as measured by a pre-test/post-test design. The GWEP compassionate care initiative has shown promising results in enhancing the capabilities of CNAs in ADRD care. The multi-pronged educational strategy has proven effective in improving behavioral management, indicating a scalable and empowering model for CNAs. This model signifies a progressive shift towards better ADRD care in long-term facilities, ultimately enriching the quality of life for those with ADRD. Further discussions will focus on the broader application of such programs in long-term care environments.

Abstract citation ID: igae098.0898

### COMPASSIONATE CAREGIVING: CREATING A CNA LEADERSHIP ACADEMY

Monica Long[1], Lauren Gleason[2], Jeffrey Graupner[3], Kanika Mittal[2], and Katherine Thompson[3], *1. University of Chicago Medicine/SHARE Network, Glenwood, Illinois, United States*, *2. University of Chicago Medicine, Chicago, Illinois, United States*, *3. University of Chicago, Chicago, Illinois, United States*

Certified Nursing Assistants (CNAs) provide direct patient care such as bathing, dressing, feeding, as well as reporting changes in patients' conditions under the supervision of licensed nurses. Despite the crucial role CNAs play, this role is characterized by high turnover rates and relatively low wages compared to other healthcare roles. SHARE Network at UChicago Medicine created a CNA Leadership Academy with an aim to retain and empower CNAs to cultivate their potential as leaders in their respective healthcare facilities. This initiative comprised of a comprehensive curriculum design to enhance CNAs understanding of leadership principles, communication techniques, quality improvement (QI), resilience and self-care techniques using Project ECHO, an educational model that provides tele-mentoring to healthcare workers. Kern's 6-step approach for curriculum development, mixed-methods pre/post survey, qualitative analysis for curriculum evaluation and a 7-point Likert Scale was used for evaluation. Participants in 2 cohorts were mostly African Americans and resulted Likert scores of, 5=Competent, 6=Very competent, 7=Expert post training. Participants created QI projects utilizing Stop & Watch communication tools, fostering a culture of communication among healthcare professionals, resilience, self-care, and a team approach to patient care. Within the CNA Leadership Academy, CNAs are better equipped to advocate for patients, address emerging challenges, and drive positive change within their healthcare organizations. The CNA Leadership Academy educational initiative represents a proactive approach to nurturing leadership talent within the CNA workforce. Equipping CNAs with knowledge and support, the program initiative contributes to resilience and effective healthcare, ultimately improving outcomes for patients and healthcare providers alike.

Abstract citation ID: igae098.0899

### EMPOWERING FRONTLINE CNAS TO ENGAGE IN EFFECTIVE COMMUNICATION THROUGH AN INTERACTIVE LEARNING EXPERIENCE

Sarah Campbell[1], Neika Saville[1], Michael Bueno[2], Julie Rousseau[2], Ishan Varshney[1], Jung-Ah Lee[2], and Lisa Gibbs[2], *1. University of California Irvine Health, Irvine, California, United States*, *2. University of California Irvine, Irvine, California, United States*

While considerable efforts have been directed towards continuing education for bedside nurses and providers, certified nursing assistants (CNAs) are often marginalized, despite their substantial involvement in patient care within skilled nursing and rehabilitation facilities. Collaborating with 36 sites throughout Orange County and Los Angeles County, California, our GWEP team identified crucial curricular topics to address geriatric-specific knowledge deficits among CNAs. These topics focused on the 4-Ms and included fall risk management, care for patients exhibiting challenging behaviors, addressing mental health conditions, effective communication with healthcare team members, and self-care strategies to mitigate burnout. Recognizing the importance of engaging and interactive education and trauma-informed pedagogy, we designed participatory activities such as gaming, storytelling, meditation, yoga, journaling, and sharing into the curriculum. Over a span of three years, education was delivered to over 615 diverse CNAs, nurses, and other healthcare professionals in our 4-part education series titled, "Building Resiliency While Advancing Your Caregiver Skills." The curriculum was structured and disseminated through synchronous online sessions via Zoom, in-person workshops, and asynchronous self-paced modules. Throughout the sessions, empowerment and evidence-based burnout prevention were taught utilizing a customized "CNAs CAN" learning kit. Evaluations were conducted after each session, and we found that providing multi-modal learning opportunities promoted engagement. Knowledge was gained in reporting changes in condition, preventing falls, and recognizing the signs and symptoms of dementia, delirium, and depression. In the qualitative data collected, learners overwhelmingly reported gaining skills to prevent burnout, stress reduction, and acquiring new self-care skills.

### SESSION 2345 (SYMPOSIUM)

Abstract citation ID: igae098.0900

### FORTIFYING GERIATRIC CARE AND RESEARCH: THE VHA ADVANCED FELLOWSHIP IN GERIATRICS

Chair: Katherine Hall
Co-Chair: Odessa Addison
Discussant: Judith Howe

Geriatric care often involves addressing complex medical, social, and behavioral needs that require input from multiple disciplines. A robust workforce with geriatric specialties is essential to ensure that older adults receive the comprehensive care and support necessary for optimal aging. Despite the growing demand, there is a significant shortage of providers working in geriatric care, exacerbating the strain on existing resources. One way to address this shortage may be through the Advanced Fellowship in Geriatrics (AFiG), a Department of Veterans Affairs (VA) program overseen by the Office of Academic Affiliations (OAA). The AFiG is a 2-year, postgraduate (post-residency, post-doctoral, post-masters) training program, offered to clinicians and non-clinician scientists, that prepares leaders in geriatric research, education, and clinical innovation. Fellows are hosted in Geriatric Research, Education, and Clinical Centers (GRECC) in 20 VA medical centers across the country, providing a rich combination of clinical rotations, didactic education, and research opportunities. Fellows gain expertise in identifying and addressing the unique healthcare needs of older adults, particularly within the veteran population. This fellowship provides participants with a wealth of advanced training topics including management of multiple chronic conditions, population health and health promotion, models of care, policy initiatives, and interdisciplinary collaboration. This symposium features presentations from 1) AFiG leadership, 2) GRECC leadership, and 3) facilitated discussion with an interprofessional cohort of current/former Fellows to share the breadth of clinical and research experiences in the AFiG.

Abstract citation ID: igae098.0901

### BUILDING CAPACITY: GRECCS' CONTRIBUTION TO SPECIALIZED GERIATRIC WORKFORCE TRAINING

Marianne Shaughnessy, *Department of Veterans Affairs, Middle River, Maryland, United States*

In 1975 Congress established Geriatric Research, Education, and Clinical Centers (GRECCs) within the Veterans Health Administration as Geriatric Centers of Excellence. Twenty GRECCs currently exist across the United States that bring together diverse interdisciplinary teams of researchers, educators and clinicians. All GRECCS are also associated with major academic research universities. Due to their unique nature, GRECCs are well positioned to advance the training and development of the scientific and clinical geriatric workforce across health care disciplines and settings. Their environments foster learning for trainees engaged in health professions careers from initial training through advanced fellowships. As Geriatric Centers of Excellence, GRECCs also provide educational programs and resources to train VA healthcare providers in geriatric principles and best practices. This presentation will include an overview of the GRECC programs, their areas of specialty, and clinical and didactic training opportunities offered. A discussion of GRECC collaborations with academic institutions and community partners to disseminate geriatric education and promote geriatrics will also be discussed. Education, Research, and Clinical training delivered through the GRECCs help to ensure that clinicians and researchers have the knowledge and skills necessary to develop and deliver high-quality, patient-centered care to older adults.

Abstract citation ID: igae098.0902

### NURTURING INTERDISCIPLINARY EXCELLENCE: INSIGHTS FROM ADVANCED FELLOWSHIP IN GERIATRICS FELLOWS

Monica Serra[1], Sarah Wherry[2], Alexander Erickson[3], Yeonsu Song[4], Betsy Yang[5], and Quratulain Syed[6], *1. The University of Texas Health San Antonio, San Antonio, Texas, United States*, *2. VA Eastern Colorado Healthcare System, Aurora, Colorado, United States*, *3. VA Greater Los Angeles Healthcare System, Los Angeles, California, United States*, *4. University of California Los Angeles, Los Angeles, California, United States*, *5. Palo Alto VA Medical Center, Palo Alto, California, United States*, *6. Joseph Maxwell Cleland Atlanta VA Medical Center, Atlanta, Georgia, United States*

This facilitated panel discussion offers a comprehensive exploration of the transformative impact of the Veterans Health Administration's (VHA) Advanced Fellowship in Geriatrics across various disciplines within the post-graduate clinical and clinical research spectrum. Drawing from the perspectives of current and former fellows spanning diverse backgrounds including medicine, nursing, psychology, public health, and research, this session delves into their fellowship experiences, the array of activities they engaged in, and the profound influence of the fellowship on their professional trajectories. The Advanced Fellowship in Geriatrics provides a robust platform for fellows to engage in clinical rotations, honing their skills in geriatric assessment, management, and interdisciplinary collaboration. Additionally, through structured mentorship and access to research resources, fellows are empowered to conduct impactful clinical research, fostering their professional growth and contributing to the advancement of high-quality, patient-centered care within the VHA. Through a multi-disciplinary lens, panelists will describe their encounters experiences within the post-graduate training VHA fellowship, encompassing clinical rotations, research endeavors, educational initiatives, and quality improvement projects tailored to the unique needs of older adults and older veterans. By highlighting their collaborative endeavors and interdisciplinary interactions, the discussion will underscore the significance of a holistic approach in addressing the complex healthcare challenges faced by aging populations. Additionally, the panel will illuminate the instrumental role of the fellowship in enhancing healthcare delivery within the VHA, fostering innovation, and optimizing geriatric care practices to meet the evolving demands of veterans.

Abstract citation ID: igae098.0903

**PREPARING LEADERS IN GERIATRICS HEALTH CARE THROUGH THE VA'S ADVANCED FELLOWSHIP IN GERIATRICS**

Chelsea Hawley[1], Michelle Martinchek[2], Colleen Hursh[3], Jennifer Moye[2], Jennifer Pruskowski[4], Benjamin Congedo[5], Shawn Dunlap[3], and Robin Jump[5], *1. National Coordinating Center for the Advanced Fellowship in Geriatrics, Bedford, Massachusetts, United States, 2. VA Boston Healthcare System, Boston, Massachusetts, United States, 3. VA Bedford Healthcare System, Bedford, Massachusetts, United States, 4. University of Pittsburgh, Pittsburgh, Pennsylvania, United States, 5. VA Pittsburgh Healthcare System, Pittsburgh, Pennsylvania, United States*

The VA Advanced Fellowship in Geriatrics (AFiG) prepares leaders in geriatric research, education, and clinical innovation, to meet the complex needs of the rapidly growing older Veteran population. This interprofessional, two-year fellowship welcomes physicians, nurses, psychologists, and other associated health fellows, including but not limited to pharmacists, social workers, physical and occupational therapists, and non-clinician scientists. AFiG is funded by VA's OAA, and fellows are hosted by the 20 GRECCs, which are VA Centers of Excellence focused on aging. Fellows devote 75-80 percent of time to scholarly pursuits and 20-25 percent to clinical work (or activities supporting clinical care for non-clinician scientists), working closely with mentors to develop an individualized training experience. AFiG began in 2001 with seven sites and has grown to 20 sites as of 2024. In 2022, a new national Coordinating Center (CC) for AFiG was established to support and empower all fellowship sites to align their training with the fellowship mission and strengths of their individual sites. This presentation will describe efforts to foster a community of practice and increased collaboration, including national curriculum development, facilitation of networking and professional events, and curation of educational opportunities. The CC is also prioritizing a new national recruitment initiative to increase the number and diversity of applicants and to facilitate hiring fellowship graduates into VA positions, which will be discussed. With these such efforts, the CC is further strengthening AFiG as a nationally recognized leader in training future leaders in geriatric healthcare.

### SESSION 2350 (SYMPOSIUM)

Abstract citation ID: igae098.0904

**GSA JOURNALS PANEL WITH THE EDITORS-IN-CHIEF**

Chair: Jessica Kelley
Co-Chair: Lewis Lipsitz

GSA has a large portfolio of journals, giving authors opportunities to publish research on aging, from cells to policy. In this panel session, the current Editors-in-Chief will present information about their respective journal, including the scope, areas of special interest, and upcoming calls for papers. Following their remarks, we will have a moderated panel session with tips for submitting work, matching your research to the best-fitting journal, and strategies for success in the review process. Ample time will be given to audience questions. Editors will include: Steven Albert, Innovation in Aging; Gustavo Duque, Journal of Gerontology: Biological Sciences; Joe Gaugler, The Gerontologist; Duke Han, Journal of Gerontology: Psychological Sciences; Jessica Kelley, Journal of Gerontology: Social Sciences; Michael Lepore, Public Policy and Aging Research; and Lewis Lipsitz, Journal of Gerontology: Medical Sciences.

Abstract citation ID: igae098.0905

**INNOVATION IN AGING**

Steven Albert, *University of Pittsburgh, Pittsburgh, Pennsylvania, United States*

N/A

Abstract citation ID: igae098.0906

**JOURNAL OF GERONTOLOGY: BIOLOGICAL SCIENCES**

Gustavo Duque, *Bone, Muscle & Geroscience Group, Research Institute of the McGill University Health Centre, Montreal, Quebec, Canada*

The "Journal of Gerontology: Biological Sciences" is a peer-reviewed academic journal that focuses on research related to the biology of aging. It covers a broad range of topics within this field, including genetics, cellular aging, molecular mechanisms of aging, age-related diseases, and interventions to promote healthy aging. More recently, we have participated in the creation of a Translational Geroscience Section, which integrates the growing subject of geroscience into both the medical and biological sections of the journal with an emphasis on the translation of discoveries into clinical practice. Our journal serves as a valuable resource for researchers, clinicians, and policymakers interested in addressing the challenges and opportunities associated with an aging population. In this session, the editorial criteria for selecting the highest-quality manuscripts will be discussed. In addition, practical tips for preparing manuscripts reporting biomedical research will be provided.

Abstract citation ID: igae098.0907

**THE GERONTOLOGIST**

Joseph Gaugler, *University of Minnesota, Minneapolis, Minnesota, United States*

N/A

Abstract citation ID: igae098.0908

**JOURNAL OF GERONTOLOGY: PSYCHOLOGICAL SCIENCES**

Duke Han, *University of Southern California, Los Angeles, California, United States*

N/A

Abstract citation ID: igae098.0909

**PUBLIC POLICY AND AGING**

Michael Lepore, *University of Massachusetts Amherst, Amherst, Massachusetts, United States*

N/A

### SESSION 2355 (SYMPOSIUM)

Abstract citation ID: igae098.0910

**HARNESSING ADVANCES IN ARTIFICIAL INTELLIGENCE TO SUPPORT AN AGING POPULATION**

Chair: Walter Boot
Co-Chair: Xin Yao Lin

We are witnessing unprecedented growth in the sophistication of artificial intelligence (AI) and its rapid diffusion into all aspects of our lives. This session will focus on the potential and pitfalls of using AI to support the health, wellbeing, and quality of life of older people. Dr. Clara Berridge will contribute a big-picture discussion of outstanding ethical issues in AI, including large language model (LLM) tools, and provide an organized framework to promote the benefits of AI while minimizing ethical concerns. Dr. Shelia Cotten will discuss the integration of AI in healthcare, focusing on stakeholder acceptance and ethical concerns, and present suggestions to advance AI acceptance based on a scoping review of AI-enabled devices in diagnosis, treatment, and rehabilitation settings. Dr. Catherine Diaz-Asper will address the ethical challenges of language technologies in AI, highlighting issues of transparency, equity, and bias, and propose methods to boost user trust, particularly among older adults, through stakeholder co-design and user-friendly interfaces. Zhimeng Luo, MS, will explore the capabilities of large multimodal models (LMMs) to interpret healthcare infographics for dementia caregivers, demonstrating their accuracy and completeness and suggesting implications for content generation and health education material design. Dr. Lin Jiang will examine the use of artificial intelligence in supporting Alzheimer's and dementia family caregivers, presenting findings from mixed-methods research that evaluates AI's role in providing emotional and social support, and discussing the importance of interdisciplinary collaboration and caregiver involvement in AI development.

Abstract citation ID: igae098.0911

### ETHICS AND ACCEPTANCE OF AI

Shelia Cotten[1], Aishah Khan[1], Catherine Mobley[1], Xia Jing[1], Jihad Obeid[2], Grant Goodrich[2], Hamilton Baker[2], and Nina Hubig[1], *1. Clemson University, Clemson, South Carolina, United States*, *2. Medical University of South Carolina, Charleston, South Carolina, United States*

Over the past 18 months, artificial intelligence (AI) applications have proliferated and been disseminated across wider audiences than in prior time periods. AI tools are increasingly being used as part of new biomedical devices and may become more integrated into healthcare processes over time. While AI applications may have significant value in healthcare and helping support older adults and individuals across the life course through a variety of mechanisms, if individuals do not perceive these tools as ethical and are not accepting of these new tools, they are likely to fail. Cotten and colleagues report the results of a scoping review designed to assess stakeholder (e.g., patients and healthcare providers) acceptance and willingness to use AI-enabled devices in diagnosis, treatment, and rehabilitation settings. The results of this study identify perceived challenges of integrating AI into healthcare across stakeholder groups and provide suggestions to help advance AI acceptance and reduce ethical concerns.

Abstract citation ID: igae098.0912

### PROMOTING USER TRUST IN AI: THE CASE OF LANGUAGE TECHNOLOGIES

Catherine Diaz-Asper[1], Chelsea Chandler[2], and Brita Elvevåg[3], *1. Marymount University, Arlington, Virginia, United States*, *2. University of Colorado Boulder, Boulder, Colorado, United States*, *3. University of Tromsø, the Arctic University of Norway, Tromsø, Nordland, Norway*

Advancements in artificial intelligence technology make it possible for computers to recognize, analyze and generate human language in ways that were not possible even five years ago. Approaches such as automated speech analysis, natural language processing, acoustic analysis and machine learning have successfully been applied in various contexts, from clinical research studies predicting cognitive decline in older adults, to chatbots that simulate conversations with users from voice or text input. As speech and language can be easily and rapidly accessed at relatively low cost, these methods hold significant potential for large scale use in commercial, medical and research applications. However, several ethical challenges arise with the use of natural language and speech processing technologies, including issues of transparency, equity and bias, potentially eroding user trust in such tools. For example, language expression varies as a function of demographic factors such as age, culture and education, yet many applications based on these technologies fail to take this into account. The result is that groups who are underrepresented in the data used to train machine learning models are at risk of being unfairly and inaccurately assessed by them. Mistrust can be further exacerbated in groups with reduced knowledge and familiarity with artificial intelligence, as may be the case with older adults and/or those with cognitive complaints. In highlighting both the optimism and challenges of using language technologies with older adults, we present several approaches to encourage confidence in their use, including stakeholder co-design, user guides and graphical interfaces.

Abstract citation ID: igae098.0913

### CAN LARGE MULTIMODAL MODELS INTERPRET HEALTH CARE INFOGRAPHICS IN DEMENTIA CAREGIVING MATERIALS?

Zhimeng Luo[1], Ning Zou[1], Bo Xie[2], Daqing He[1], Robin Hilsabeck[3], and Alyssa Aguirre[2], *1. University of Pittsburgh, Pittsburgh, Pennsylvania, United States*, *2. The University of Texas Austin, Austin, Texas, United States*, *3. The University of Texas Health Sciences Center San Antonio, San Antonio, Texas, United States*

Infographics are widely used in health education materials for patients and caregivers. As generative artificial intelligence like large multimodal models (LMMs) become increasingly used for content generation, there is a great need to understand if infographics could be used to train LMMs. Addressing such a need, we tested four LMMs, GPT-4V, Gemini-Pro-vision, LLaVA-1.6-34B, and CogVLM-17B, using 5 infographics designed for dementia caregivers as examples. Two independent coders analyzed LMMs' interpretations of each infographic on accuracy (i.e., the number of model-generated key points that correctly matched those in an infographic divided by the total number of key points contained in the model-generated content) and completeness (i.e., the number of model-generated accurate key points divided by the total number of key points contained in the infographic). Agreement rate was high: Cohen's kappa=0.8205. Differences were resolved through discussion. Across the 5 infographics, the average accuracy of

GPT-4V, Gemini-Pro, LLaVA, and CogVLM was 99.0% (range: 94.4-100%; median: 100%), 83.1% (range: 63.3-100%; median: 81.8%), 76.3% (range: 45.5-100%; median: 81.7%), and 81.5% (range: 50-100%; median: 86.6%), respectively; average completeness was 93.7% (range: 68.8-100%; median: 100%), 53.7% (range: 15.2-77.8%; median: 65.5%), 65.7% (range: 14.3-100%; median: 70.1%), and 37.2% (range: 6.3-77.8%; median: 37.2%), respectively. Overall, GPT-4V significantly outperformed the other LMMs on completeness [F(3,16)=3.55; p=0.04]; no difference was found on accuracy [F(3,16)=2.20; p=0.13]. These findings shed light on LMMs' abilities to interpret visual and textual data and their applicability in real-world settings like designing high-quality health education materials for patients and caregivers. We conclude with future research directions.

Abstract citation ID: igae098.0914

### CRITICAL ENGAGEMENT WITH AI: MAPPING ETHICAL PRINCIPLES TO APPLICATIONS IN AGING

Clara Berridge, *University of Washington, Seattle, Washington, United States*

Navigating the algorithmic age is difficult because of many unresolved ethical issues and policy gaps. A significant barrier to addressing ethical problems in AI and aging is that their scope is often restricted, leaving questions of power and social impact ill-defined. A contributing phenomenon is that people tend to take algorithmic output as objective, authoritative, and fair. In this way, ideas about objective data-based truth can convert questions that are ethical in nature into technical questions. Examples of questions with ethical aspects that are often not treated as such include which problems are prioritized for datification and how they are defined, which data are mobilized and sidelined, how data are collected and monetized, how representative data are, how older adults are involved in data collection, and how algorithmically mediated decision-making plays out with what differential impacts. Further, as technologies incorporate socially persuasive techniques, it is important to examine the ethical issues, such as implications for autonomy of misplaced trust with low awareness of these techniques. Recently, the broader discourse about ethics in AI that includes questions of power and societal impact has sharpened. The White House's Blueprint for An AI Bill of Rights operationalized core principles that design, policy, and practice should uphold to protect people from potential harms. These principles are safe and effective systems, algorithmic discrimination protections, data privacy, notice and explanation of use of AI, and meaningful human alternatives. This presentation will discuss principles to promote ethical use of AI in tools and services for older adults.

Abstract citation ID: igae098.0915

### ASSISTING DEMENTIA FAMILY CAREGIVERS WITH INTERACTIVE AI TECHNOLOGY

Lin Jiang[1], Fei Sun[2], Xiayu Chen[3], and Yali Feng[3],
1. *University of Texas-Rio Grande Valley, Edinburg, Texas, United States*, 2. *Michigan State University, Haslett, Michigan, United States*, 3. *University of Illinois Urbana-Champaign, Champaign, Illinois, United States*

The escalating prevalence of Alzheimer's and related dementias (ADRD) presents a global health crisis, straining family caregivers. This study, guided by the Unified Theory of Acceptance and Use of Technology (UTAUT), examines the role of artificial intelligence (AI) in assisting ADRD family caregivers through a mixed-methods approach. It involves qualitative analysis of marginalized dementia caregivers' experiences in the technology era and a systematic review of interactive AI evidence in dementia care. Qualitative insights from six focus groups and 24 individual interviews highlight the need for adaptable AI solutions, particularly for emotional and social support. Systematic review results underscore various AI applications, their acceptance, benefits, and challenges, including technological literacy, trust issues, limited access, and cultural considerations. The discussion emphasizes interdisciplinary collaboration and caregiver involvement in AI development. The study concludes by emphasizing AI's transformative potential in dementia care, advocating for collaborative research efforts and innovative services to support diverse caregiver groups and their ADRD care recipients.

### SESSION 2360 (SYMPOSIUM)

Abstract citation ID: igae098.0916

### IMPROVING HEALTH EQUITY BY ENHANCING ACCESS TO TECHNOLOGY FOR OLDER ADULTS IN RURAL AREAS

Chair: Shannon Freeman
Co-Chair: Rashelle Hoffman
Discussant: Rashelle Hoffman

The COVID-19 pandemic generated unprecedented acceleration in the adoption of technology (from digital apps and wearable sensors to robotics and artificial intelligence) in rural areas. However, this rapid digitalization across the care continuum was characterized by inequitable distribution, implementation, and unsustainability in rural healthcare settings. Although health is a fundamental human right, there are many dimensions of inequality, including sex, gender, ethnicity, and disability, that are amplified in rural healthcare settings. This symposium will showcase how research has supported the identification, implementation, and sustainment of innovative technology solutions to enhance health equity in rural settings (Freeman). With the cumulative shift to virtual delivery of health care services, there is need to ensure equitable access to infrastructure and resources to address existing technology gaps which disproportionally affect older adults living in rural communities (Clayton-Jones). Additionally, with the expanding breadth of technology applications being used by older adults, this symposium will showcase the positive technological impacts such as autonomous vehicles (Yoon), smartwatches (Wiese), and eReaders (Agboji) to enhance health equity for older adults. Rural Aging Interest Group Sponsored Symposium

Abstract citation ID: igae098.0917

### ENGAGING HIGH SCHOOL STUDENTS TO INCREASE RESEARCH-FOCUSED SMARTWATCH USE BY MULTICULTURAL, RURAL OLDER RESIDENTS

Catherine Luna[1], Lisa Wiese[2], Bryan Minor[1], Maureen Schmitter-Edgecombe[1], and Diane Cook[1],
1. *Washington State University, Pullman, Washington,*

United States, 2. Florida Atlantic University, Boca Raton, Florida, United States

At the southern tip of Lake Okeechobee in south central FL is a rural, underresourced, racially/ethnically diverse region. Persons age 65/older (21%) are primarily African American (57%), Hispanic American (29%) and Afro-Caribbean (4%). Health disparities prevalent in the "Glades" region extend to low rates of digital access (60%) and digital literacy (55%). When asking residents "what mattered most" (Boykin & Schoenhofer, 2001), two issues were identified as most important to the older adult stakeholders: increasing technological competence and maintaining brain health. We tested aims developed with the community advisory board, which tapped into a little-used resource: local high school students, who trained older adults in use of smartwatches to monitor exercise, respond to prompts regarding ecological momentary assessments, and complete n-back cognitive assessments. Faith-based health educators facilitated the home-based visits between 33 older residents and 22 students, and collected pre-post written measures addressing technological self-efficacy, cognition, and health literacy. The older adults (91%, 30/33) responded to smartwatch prompts with a 77.80% response rate, which was consistent with current EMA standards: An 80% response rate has been recommended as the target for EMA studies (Turner et al., 2017), and a meta-analysis reported 75% in the pooled response rate (Jones et al., 2019). Of interest was that self-reported cognition and the Mini-Cog scores were not related (Pearson's correlational analysis) to number of smartwatch prompts answered or health literacy. Six categories of recommendations ranging from student concerns to resident suggestions emerged from this work, which will be shared in this presentation.

Abstract citation ID: igae098.0918

### ENHANCING EQUITY TO QUALITY CARE FOR PERSONS WITH DEMENTIA IN RURAL COMMUNITIES THROUGH IMPLEMENTATION OF AGETECH

Shannon Freeman[1], Sarah Sousa[2], Davina Banner-Lukaris[1], and Kelly Skinner[2], *1. University of Northern British Columbia, Prince George, British Columbia, Canada, 2. University of Waterloo, Waterloo, Ontario, Canada*

Older adults living with dementia in rural and northern communities deserve equitable access to technologies that enhances quality of life and supports aging in place. To explore contextual barriers and facilitators to implementation, we conducted a process evaluation guided by the Theoretical Domains Framework in a dementia care home newly opened in a rural and northern community. Through a partnership with the Center for Technology Adoption for Aging in the North (CTAAN), health systems leaders, and community partners, multiple technologies designed to support persons who are aging (AgeTech) were purposefully implemented to enhance clients' care and quality of life. AgeTech included a hydroponic gardening wall, circadian lighting, and a virtual exercise program. Semi-structured interviews were held with facility staff, health systems leaders, representatives from the AgeTech companies, and implementation leads and a secondary analysis of existing documentation was conducted. Barriers to AgeTech implementation included geographic context, complexity of dementia symptoms, and limited experiences by older adults with technology. Facilitators of AgeTech included collaborative partnerships with AgeTech companies, client interest and motivation, and creation of AgeTech educational resources. Results provide insights to inform planning and policy decisions for rural AgeTech implementation initiatives, highlight considerations for ongoing AgeTech innovation and describe the engagement of community partners in the process of integrating aging technologies. Persons living with dementia can greatly benefit from use of AgeTech to support their health and wellbeing. Successful and sustainable implementation of AgeTech is possible when the AgeTech enables, empowers, and engages persons to age well.

Abstract citation ID: igae098.0919

### MEETING IN-PERSON NEEDS OF ELDERS LIVING IN RURAL COMMUNITIES BY UTILIZING APPS AND INTERNET ACCESS

Kate Clayton-Jones, *FootCare by Nurses, Amherst, Massachusetts, United States*

Challenges and success for meeting the in person needs of elders living in rural communities by utilizing apps and internet access takes tenacity and workarounds as many rural communities lack community wide internet, WiFi or cell phone coverage that providers of care can access. This lack of infrastructure impacts everything from first responder access and communication to efficient routing and access to information and charting by providers while in the home providing care. This lack of infrastructure is an impediment to quality care and also employment opportunities. Rural communities typically have more older adults residing in them. These older adults benefit from in home care, especially with ADLs. Technology access allows people to work from home, on the road and live in rural communities. It is a win win in health care when rural communities embrace community wide communication access.

Abstract citation ID: igae098.0920

### ATTITUDES TOWARD AUTONOMOUS VEHICLES IN RURAL OLDER ADULTS: IMPLICATIONS FOR ENHANCING ACCESS TO HEALTH CARE

Jong Sung Yoon[1], Tyler Gandy[1], and Mojgan Zoaktafi[2], *1. University of South Dakota, Vermillion, South Dakota, United States, 2. University of Illinois Urbana-Champaign, Champaign, Illinois, United States*

Despite the potential of autonomous vehicles (AVs) to enhance health equity in rural areas, there is still a lack of understanding in rural older adults' attitudes toward AVs. Most previous studies have identified rural older adults by relying on online survey samples, which might include individuals more likely to embrace technology and, consequently, show more favorable attitudes to AVs. Thus, the current study directly recruited a sample from rural areas (n = 120; older adults: 60+ yrs, young adults: 17 – 21yrs) to investigate two main questions: 1) whether rural older adults show negative attitudes towards AVs, and 2) whether rural older adults show positive attitudes toward AVs if they are proficient enough with technology. Based on a 16-item attitude survey which uncovered three attitudinal factors (i.e., concern with AVs, willingness to adopt AVs, confidence in AVs over human driving), we first confirmed lower willingness to adopt AVs in rural older adults compared to rural young adults (p

<.01). However, in contrast to most previous studies using non-rural samples, we found neither higher concern about AVs nor lower confidence in AVs among rural older adults. Our regressions further demonstrated that higher technology proficiency predicted positive attitudes toward AVs over and above other demographic factors. The results suggest that rural older adults' reluctance to adopt AVs may not be driven solely by concern with AVs, but rather by other reasons. We discuss the findings regarding the dissemination of AVs for enhancing access to health care for rural older adults.

Abstract citation ID: igae098.0921

**OVERCOMING APATHY AMONG OLDER COMMUNITY DWELLERS IN RURAL NORTHERN BC: THE BENEFITS OF ENGAGING IN AN EBOOK CLUB**

Aderonke Agboji, and Shannon Freeman, *University of Northern British Columbia, Prince George, British Columbia, Canada*

The study aimed to investigate the impact of an eBook Club on the apathy and overall well-being of older adults dwelling in rural community settings. Employing a mixed-method approach, the research gathered data through surveys and interviews both before and after the program implementation. We recruited twenty-eight older adults, aged between 60 and 86 years, predominantly female (23 out of 28) from three communities in Northern British Columbia, Canada. Each participant was provided with a Kobo eReader loaded with books tailored to their interests, alongside a tutorial for using the device. Each eReader was linked to the public library, facilitating easy access to a broader range of books. The eBook Club convened weekly for sessions lasting 45 minutes to an hour, with groups capped at four members, over a span of 4 to 6 weeks. Initially, about half of the participants (10 out of 28) reported apathy. Remarkably, by the program's conclusion, these individuals unanimously reported a decrease in apathetic feelings. Additional benefits noted by participants included enhanced mood, improved social connections, and sustained engagement with reading activities beyond the club meetings. We concluded that a relatively straightforward intervention like the eBook Club can significantly ameliorate symptoms of apathy and bolster the overall well-being of older individuals in rural community settings. While initial challenges with the Kobo eReader were reported, the collective support and encouragement within the group played a crucial role in overcoming these obstacles, underscoring the value of peer support in facilitating the adoption of new technologies.

### SESSION 2365 (SYMPOSIUM)

Abstract citation ID: igae098.0922

**INTERNATIONAL INVESTIGATIONS OF MENTAL HEALTH AND PSYCHOSOCIAL WELL-BEING IN LATER LIFE**

Chair: Giorgio Di Gessa
Co-Chair: Brian Beach
Discussant: Matthew Prina

Good mental health and psychosocial wellbeing are important factors for older individuals' fortitude as they move through later life. Attention to mental health and wellbeing has continued to grow in recent years, with the COVID-19 pandemic playing a key role in raising the profile of psychosocial experiences across societies. However, many countries face ongoing challenges to provide support services that meet existing and projected needs. While these issues are not limited to older adults, people in later life may face vulnerabilities that make them more susceptible to negative outcomes in mental health and wellbeing or that introduce additional barriers to accessing adequate supports. This symposium highlights international investigations into mental health and wellbeing that enrich our understanding of these issues and some of the dynamics for identifying potential solutions to enhance support. The first presentation highlights the experience of older people's psychosocial wellbeing across the pandemic using the English Longitudinal Study of Ageing (ELSA), with measures before, during, and in the years after the pandemic. The second presentation explores the concept of resilience in later life drawing on qualitative interviews among older people with long COVID from other longitudinal UK studies. The third presentation examines the role that mental health diagnoses play in shaping labor market exit among adults aged 65+ in paid work, drawing on Swedish register data. The final presentation investigates joint trajectories in depression scores and various blood-based biomarkers using ELSA data, exploring the potential for such measures to be useful in identifying risk for depression. International Comparisons of Healthy Aging Interest Group Sponsored Symposium

Abstract citation ID: igae098.0923

**BOUNCING BACK: THE TEMPORARY IMPACT OF THE PANDEMIC ON OLDER ADULTS' PSYCHOLOGICAL WELL-BEING**

Paola Zaninotto[1], Eleonora Iob[2], Shaun Scholes[1], and Andrew Steptoe[1], *1. University College London, London, England, United Kingdom*, *2. King's College London, London, England, United Kingdom*

The aim of this work is to explore the changes from 2018 to 2022 in happiness, life satisfaction and life worthwhile among 3,999 people aged 50 and over in England participants of the English Longitudinal Study of Ageing. They were followed over a critical period of time covering the pre-pandemic, early pandemic, later pandemic, and post-pandemic years. We observed decreases in all aspects of psychological well-being during the pandemic, which were partly independent of changes in depression. Following the pandemic, positive wellbeing returned to pre-pandemic levels. This indicates that the deterioration in wellbeing was relatively short-term and not a permanent change. Indeed, for two of the measures (life satisfaction and meaning in life), the post-pandemic levels are higher than pre-pandemic. Important variations by gender and wealth were found, with greater deterioration during the pandemic among women, and larger decreases in more affluent groups. All subgroups show recovery after the pandemic.

Abstract citation ID: igae098.0924

**RESILIENCE: A "CURE" FOR OR A "RESPONSE" TO AGING?**

Chao Fang[1], Alastair Comery[2], and JD Carpentieri[3], *1. University of Bath, Liverpool, England, United Kingdom*,

2. *University of Bath, Bath, England, United Kingdom*, 3. *University College London, London, England, United Kingdom*

Resilience is often perceived as an intrinsic ability to adapt to challenges. Grounded in the human psyche, research commonly depicts it as a self-defence mechanism, enabling people to navigate and overcome adversity. Sociological thinking questions this view, suggesting that resilience should not merely involve adaptation but also foster positive change in challenging situations. This perspective criticises resilience for potentially reinforcing a neoliberal ideology, shifting welfare responsibility from the state to individuals. Using longitudinal qualitative data from people over 50 with Long COVID in the UK, we argue that resilience is an embodied construction rooted in life narratives and framed by social dynamics. Examining three in-depth case studies with distinctive resilience trajectories—decline, improvement, and persistence—between 2021-23, we explore resilience amidst the challenges of Long COVID and ageing. Inherent resilience is not always stable or positive; it operates like the immune system, resisting disruptions amid the challenges of ageing and the struggles of navigating everyday life. By presenting data from people approaching the third age (early 50s) to those in their 60s and 70s, we argue that resilience is rooted in narrative identity, maintaining consistency in life plots. It involves drawing on past experiences, developing a flexible future outlook, and living for the present while navigating Long COVID. Our analysis uncovers the existential dimension of resilience in old age, a constant questioning of one's existence through their body and health. It highlights how social support can alleviate such challenges, contributing to our understanding and support for the complex nature of ageing.

Abstract citation ID: igae098.0925

### WORKING AFTER AGE 65: THE IMPORTANCE OF PRIOR MENTAL HEALTH–RELATED SICKNESS ABSENCE AND DISABILITY PENSION

Aleksiina Martikainen, Kristina Alexanderson, Pia Svedberg, and Kristin Farrants, *Karolinska Institutet, Stockholm, Stockholms Lan, Sweden*

With rising retirement ages and higher numbers of people extending their working life in Europe, more knowledge is needed on labor market participation after the standard retirement age. In this prospective population-based cohort study, we studied all 65-69-year-olds (n=201,263) and ≥70-year-olds (n=93,751) who lived in Sweden in 2014 and had at least some income from work. Using nationwide register microdata and Cox proportional hazard regression, we examined the association of prior mental and somatic sickness absence (SA) and/or disability pension (DP) and exit from paid work after ages 65-69 and ≥70, respectively. We found a gradual decline in paid work participation during the follow-up; approximately 80% remained in paid work at least some time during follow-up in both cohorts and both sexes, while 41% worked throughout the follow-up. Mental SA/DP was less common in both cohorts (ages 65-69: 4% women, 1.7% men; ages ≥70: 0.1% women, 0.1% men) than somatic SA/DP (ages 65-69: 27.4% women, 21.8% men; ages ≥70: 3.1% women, 2.9% men). Further, mental SA/DP marginally hindered extension of working life after age 65-69 among women (HR 1.09; 95%CI 1.06-1.13) compared to women without this type of SA/DP. However, no significant association was found among men nor among women or men aged ≥70. Our findings underscore the importance of considering prior mental health factors and possible gender differences in understanding and addressing challenges related to work continuation beyond standard retirement ages.

Abstract citation ID: igae098.0926

### BODY AND MIND: EXPLORING ASSOCIATIONS AMONG PARALLEL TRAJECTORIES OF BIOMARKERS AND DEPRESSION SCORES

Brian Beach[1], Paola Zaninotto[1], and Eun-Jung Shim[2], 1. *University College London, London, England, United Kingdom*, 2. *Pusan National University, Busan, Republic of Korea*

Multiple studies have found associations between levels of various blood-based biomarkers and depression, including how some biomarkers are associated with future development of depression or the worsening of depressive symptoms. The nature of such associations, however, cannot demonstrate a causal relationship between levels of biomarkers and depression. With recent advances in data collection, this study examines whether associations exist between the trajectories of biomarkers and depression scores. It draws on data from the English Longitudinal Study of Ageing, using four measurement points for depression and nine distinct biomarkers among respondents aged 50+ collected during nurse visits between 2004 and 2019. Associations in joint trajectories are assessed using a series of parallel process latent growth models, testing goodness of fit for both linear and quadratic models. Results suggest associations between blood hemoglobin and depression, with no significant associations among the other biomarkers. More specifically, intercepts and slopes demonstrate joint negative associations, while the intercept of depression is positively associated with the rate of change for hemoglobin (β=0.029; 95%CI: 0.004,0.054). When stratified by sex, the associations with blood hemoglobin remains only for women, while men display a negative association between the intercept of depression and the rate of change for HDL cholesterol (β=-0.016; 95%CI: -0.029,-0.004). Overall, results find no relationships between change in depression scores and levels of biomarkers, suggesting little diagnostic utility of these biomarkers to identify risk for worsening depression.

### SESSION 2370 (SYMPOSIUM)

Abstract citation ID: igae098.0927

### INTERVENTIONS FOR CAREGIVERS AND PERSONS LIVING WITH DEMENTIA ACROSS THE LONG-TERM CARE CONTINUUM

Chair: Debra Dobbs
Discussant: Hongdao Meng

According to estimates from the Alzheimer's Association 2023 Alzheimer's disease Facts and Figures, there are an estimated 6.7 million people living with dementia (PLWD) in the United States. With increasing older age as the number one risk factor, this number will only continue to grow. Family and direct care workers (e.g., nurses, certified nursing assistants, social workers) provide the bulk of care to PLWD across the health care continuum. Interventions are needed to assist and improve dementia care for families, direct care workers and PLWD. This symposium includes four papers that have conducted dementia care interventions in different care settings. The first paper is a Palliative Care Education in Assisted Living intervention for nurses and administrators in 10 ALs to increase advance care planning documentation for AL residents (N=118) and to increase staff self-efficacy (N=23). Self-reported self-efficacy will be compared to treatment fidelity data on ACP discussions with family to examine how well the ACP discussion intervention protocol was adhered to. The second paper is an intervention study that employs mixed methods in a sample of 155 Veterans Health Administration clinicians to test the effectiveness of the Virtual Dementia Tour (VDT) and the impact the VDT has on their clinical practices. The third paper tested the feasibility of a group digital gaming intervention in a sample of 40 PLWD and their caregivers in adult day centers. The fourth paper tests the feasibility of an e-Pain support intervention for ADRD hospice patient caregivers. The scalability of each intervention will be discussed.

Abstract citation ID: igae098.0928

### AN EXAMINATION OF STAFF SELF-EFFICACY MEASUREMENT IN THE PALLIATIVE CARE EDUCATION IN ASSISTED LIVING INTERVENTION

Debra Dobbs[1], Jessica Yauk[1], Hongdao Meng[1], William Haley[1], Lindsay Peterson[1], and Harleah Buck[2], *1. University of South Florida, Tampa, Florida, United States*, *2. University of Iowa, Iowa city, Iowa, United States*

Advance care planning (ACP) is a necessary first step to meeting care preferences at the end of life and an important aspect of palliative care provision. ACP in assisted living (AL) is lacking for older adults living with dementia. A palliative care educational intervention found that only 12% of residents in AL living with dementia at baseline had documentation of (ACP) in their charts. Increased staff self-efficacy to have ACP discussions could potentially improve outcomes in ACP documentation. The current study is an NIH Stage 1b intervention using a sample of 23 nurses and administrators in 10 ALs to test if staff self-efficacy mediates the effects of increased documentation of ACP discussions. We estimated mediation models to examine whether staff self-efficacy (using a two-item scaled measure) mediates the relationship between the Palliative Care Education in Assisted Living intervention and ACP discussion rate. The PCEAL intervention had a positive impact on both staff self-efficacy and ACP discussion rate but the mediation results were not significant. In addition, an analysis of treatment fidelity ACP discussion checklists data showed the scores overall were lower than average (3.5 out of 7). We will discuss the differential scores between the self-reported increases from baseline to one-month (post-intervention) and lower than average observed treatment fidelity checklist scores of ACP Discussions by treatment staff and ways to improve the measurement of staff self-efficacy in future intervention research with staff who provide dementia care in assisted living settings.

Abstract citation ID: igae098.0929

### IMMERSIVE DEMENTIA EXPERIENCE: TRANSFORMING DEMENTIA CARE

Kelly O'Malley[1], Seneca Correa[2], Julie Boyle[1], Dawn Stavrakopolous[3], Kelly Resendes[3], Olga Quinlan[3], and Amanda Smith[3], *1. VA Boston Healthcare System, Boston, Massachusetts, United States*, *2. VA Boston Healthcare System, West Roxbury, Massachusetts, United States*, *3. VA Boston Healthcare System, Brockton, Massachusetts, United States*

The Virtual Dementia Tour (VDT) is an empirically supported training designed to build an understanding of dementia through use of sensory tools and instruction. Trained facilitators guide participants outfitted with devices that alter their senses while they try to complete everyday tasks. The VDT enables participants to experience the physical and mental challenges those with dementia face in order to improve person-centered care. Clinicians (e.g., nurses, nursing assistants, physical and occupational therapists, social workers, psychologists; N = 155) at a Veterans Health Administration facility completed the VDT and provided quantitative and qualitative feedback regarding their satisfaction with the VDT, and the impact this training would have on the care they provide. Participants reported a significant change in their belief that individuals with dementia receive the care they need (83.8% pre, 16.3% post $p < .001$), indicating that post-training participants felt that individuals with dementia frequently do NOT receive the care they need. The majority (92%) said the experience would change their clinical care practices. Participants strongly agreed that the VDT allowed them to experience what dementia feels like, developed a better understanding of dementia care needs ($M = 4.66$, $SD = .51$), reinforced or increased their level of knowledge of dementia ($M = 4.66$, $SD = .53$), and will integrate this in the day-to-day care of person's with dementia ($M = 4.64$, $SD = .50$). Qualitative responses expanded on these findings. The VDT was acceptable to VA direct care staff and is an effective way to build understanding of the needs of older Veterans living with dementia.

Abstract citation ID: igae098.0930

### IMPACT OF GROUP-BASED DIGITAL GAMING INTERVENTION AMONG INDIVIDUALS LIVING WITH EARLY TO MODERATE DEMENTIA

Megumi Inoue, Michelle Hand, Naoru Koizumi, Limei Chen, and Emma Booker, *George Mason University, Fairfax, Virginia, United States*

Slowing the progression of dementia and maintaining good behavioral and psychological symptoms are important aspects of life for individuals living with dementia and their caregivers. This study involved a group-based digital gaming system called Obie Technology as an interactive intervention for individuals living with dementia at a local adult day health center for 20 weeks, from mid-November 2023 to mid-April 2024. This gaming system promotes movement, stimulates cognitive activity, and encourages socialization. For example, colorful balloons float around the board or floor as clients try to hit as many as they can. A mixed methods approach with a pre-post design was employed to examine the effects of the intervention on cognitive function, mood, and behaviors in 24 individuals with early or moderate-level dementia. The average scores of the Montreal Cognitive Assessment (MoCA) at the baseline and mid-point (after 10 weeks) were 15.65 and 15.60, respectively, indicating no significant progression of dementia between these time periods. Participants' depression level and neuro-psychiatric symptoms presentation assessed by standardized instruments also showed no significant changes. The results from qualitative observations demonstrate that the group of people living with early-stage dementia in this study were more engaged and enjoyed the intervention compared to a group with moderate-stage dementia. They often comment that the intervention was fun, interesting, and enjoyable while actively engaging in the game with enhanced movement. In this presentation, we will present the findings based on the completed data, including the post-assessments.

Abstract citation ID: igae098.0931

### ADAPTATION OF THE E-PAINSUPPORT INTERVENTION FOR ADRD CAREGIVERS

Masako Mayahara, *Washington University in St. Louis, St. Louis, Missouri, United States*

The lack of adherence by family caregivers to prescribed analgesic regimens has been recognized as a significant barrier to effective pain management in hospice. Advancements in healthcare technology provide an opportunity to enhance timely access to pain education and promote better adherence to analgesic regimens for family caregivers of patients with Alzheimer's Disease and Related Dementias (ADRD). The aims of this presentation are to report the findings from a recently completed randomized controlled trial of hospice patient-caregiver dyads and discuss adaption of the intervention for ADRD caregivers. The initial efficacy trial used a 2-group, 2-week longitudinal, randomized controlled trial design. The study participants were recruited from four hospice agencies located in in the Midwest. Forty-four patient-caregiver dyads participated. There was a small positive effect size (d=0.27) associated with the e-PainSupport intervention, suggesting efficacy in reducing pain intensity. The regression analysis revealed that increased knowledge had a significant independent effect on decreasing pain ($p<0.01$). Female caregivers and children of patients were more likely to respond to the intervention compared male or spouse caregivers. The study highlighted the importance of caregiver knowledge in reducing patient pain. Adaptation of the intervention for ADRD caregivers requires a tailored approach to increase usability for male and spouse caregivers.

### SESSION 2375 (SYMPOSIUM)

Abstract citation ID: igae098.0932

### INTERVENTIONS TO PREVENT ELDER MISTREATMENT WITH FAMILY CAREGIVERS OF PERSONS LIVING WITH DEMENTIA

Chair: Kylie Meyer
Co-Chair: Jeanine Yonashiro-Cho
Discussant: Frank Puga

Persons living with Alzheimer's disease and related dementias are among the most vulnerable to elder mistreatment and neglect (EM). Family caregivers, who often enter this role with minimal preparation or support, are among the most likely to engage in EM. In this symposium, we describe novel interventions to prevent EM by family caregivers of persons living with dementia. In the first talk, Dr. Jablonski will describe the Coaching Dementia Caregivers to Master Care-Resistant Behavior (CuRB-IT) intervention, which uses 1:1 Zoom coaching to help caregivers respond to care-resistant behaviors. This includes a discussion of preliminary findings on intervention acceptability. Next, Dr. Meyer will provide results from a pilot study of the Knowledge and Interpersonal Skills to Develop Enhanced Relationships (KINDER) intervention, a group-based psychoeducation intervention to prevent EM by focusing on the care relationship. Dr. Wilber will then discuss secondary results from the double-blinded trial of the Comprehensive Older Adult and Caregiver Help (COACH), which was informed by family violence interventions. She will discuss the perplexing finding wherein risk factors for abuse were largely unaffected by the COACH intervention despite the elimination of mistreatment in this arm. Lastly, Dr. Yonashiro-Cho will describe basic behavioral research drawn from the Better Together study to inform a culturally tailored version of the EM prevention program that addresses the needs of Asian American and/or Pacific Islander family caregivers. These studies represent multiple stages of intervention development, and each addresses an emerging area of research to prevent elder mistreatment within a dementia care context.

Abstract citation ID: igae098.0933

### FEASIBILITY AND PRELIMINARY EFFICACY OF THE KINDER INTERVENTION TO PROMOTE HEALTHY CARE RELATIONSHIPS

Kylie Meyer[1], Jeanine Yonashiro-Cho[2], Sohee Kim[1], and Donna Benton[3], *1. Case Western Reserve University, Cleveland, Ohio, United States*, *2. University of Southern California Keck School of Medicine, Los Angeles, California, United States*, *3. University of Southern California, Los Angeles, California, United States*

Knowledge and Interpersonal Skills to Develop Enhanced Relationships (KINDER) is a psychoeducational intervention designed to prevent emotional-type elder mistreatment by family caregivers to persons living with dementia. Informed by evidence-based approaches in intimate partner violence, caregivers in this virtual 8-week course develop disease knowledge, communication strategies, and coping skills to manage sources of relationship strain. Components include facilitated group Zoom discussion sessions and asynchronous weekly readings and practice exercises, including story-based videos informed by caregiver focus groups. In this pre- and post-test study, we examine KINDER's feasibility and preliminary efficacy. A total of N=45 caregivers enrolled from March 2023 to January 2024. Among participants, n=39 have already completed all data collection activities, including n=4 who were lost to follow-up and n=3 who withdrew (82% retention). The primary reason for withdrawal was believing the program was not relevant to their care situation. On average, study participants attended 2.4 of 3 Zoom discussion sessions (SD=0.7). Seventy-eight percent (78.1%) of participants indicated they completed asynchronous activities "all" (40.6%) or "most" (37.5%) of the time. Findings from paired t-test analyses of outcomes indicate a reduction in potentially harmful care behaviors

(e.g., threatened to send family member to a nursing home; p=0.025). Consistent with the goal of the intervention, caregivers also reported improved relationship quality with the care recipient (p<0.001). All program completers indicated they were "very satisfied" (77.4%) or "satisfied" (22.6%) with KINDER. Findings from this pilot study demonstrate KINDER's feasibility. Future research will rigorously test intervention efficacy and examine likely mechanisms of change.

Abstract citation ID: igae098.0934

### ASSESSING THE CAREGIVING NEEDS OF ASIAN AMERICAN AND PACIFIC ISLANDER ADRD CARE PARTNERS

Jeanine Yonashiro-Cho[1], Laura Mosqueda[2], and Zachary Gassoumis[3], *1. University of Southern California Keck School of Medicine, Los Angeles, California, United States*, *2. University of Southern California Keck School of Medicine, Pasadena, California, United States*, *3. University of Southern California, Los Angeles, California, United States*

Care partners (CP) are essential in supporting persons living with dementia (PLWD). Asian American and Pacific Islanders (AAPI) may be more likely to engage in caregiving and may cope with caregiving stressors in culturally-distinct ways, thus necessitating development of culturally-appropriate supports and interventions. This study explores CP needs and identifies potential targets for intervention among AAPI communities. Explanatory sequential mixed-methods analyses were conducted with AAPI dementia care partners (N=8) who took part in the Better Together Dementia Care study. Caregiving characteristics and outcomes data from quantitative surveys were paired with findings from a thematic content analysis of semi-structured interview data. Key informant interviews AAPI CPs and providers will also be analyzed. Participants included AAPI spousal (n=4) and adult child (n=4) CPs supporting a PLWD, including 4 who had recently experienced elder mistreatment. Due to their emigration (n=3), spousal caregivers reported a lack of instrumental caregiving support from extended family. In child-parent dyads, PLWDs had lost their partners due to death (n=3) or divorce (n=1). PLWDs varied in relational and emotional closeness with children. Preliminary findings highlight the diversity of CP experiences. Romantic partners may serve as the preferred "default" CP; in their absence, adult children may relied upon more heavily. While traditional API cultures often emphasize a child's duty to care for elders, acculturated older adults may prefer to receive care from their partner whenever possible. Findings also suggest immigrant CPs and CP-PLWD dyads may benefit from culturally-specific instrumental supports to supplement the emotional support provided from family members overseas.

Abstract citation ID: igae098.0935

### COACHING DEMENTIA CAREGIVERS TO MASTER CARE-RESISTANT BEHAVIOR: CURB-IT

Rita Jablonski[1], Frank Puga[1], Liang Shan[1], and Laura Mosqueda[2], *1. University of Alabama at Birmingham, Birmingham, Alabama, United States*, *2. University of Southern California Keck School of Medicine, Pasadena, California, United States*

Care-resistant behavior (CRB) refers to actions taken by people living with dementia to avoid receiving assistance or completing care activities. At least 66% of family caregivers encounter CRBs. The purpose of this study is to test the efficacy of Care-Resistant Behavior Internet Training intervention, CuRB-IT, on caregiver problem-solving coping skills. We hypothesize that improved skills will prevent and decrease elder abuse and neglect behaviors. Participants are randomized to either immediate coaching or delayed coaching. The nine 1-hour coaching sessions are done via Zoom; weekly for six weeks, then biweekly for six weeks. Each coaching session includes a description of 3-4 strategies, a simple physiologic rationale for the utility of the strategies, role-playing between the coach and participant, and concludes with the coach sending a written behavioral plan. Participants receive a detailed workbook that includes strategy descriptions, case studies, and exercises. The coaching sessions are highly scripted with algorithmic responses and prompts for open-ended questions. Participants complete questionnaires and daily diaries upon enrollment and at three 12-week intervals. Forty-three caregivers have been enrolled (goal, 266); 35% African-American, 29% male; 54% spouses and 39% adult children. Thirteen have completed all the coaching sessions. The care recipients were functionally dependent and in the moderate stage of dementia. On a 1 (highly disagree) to 5 (highly agree) scale, participants provided positive evaluations. The coaches helped participants understand causes of CRB (mean,4.6), they found the strategies useful for preventing (4.4) and managing (4.4) CRBs and would recommend this coaching program to other dementia caregivers (4.8).

Abstract citation ID: igae098.0936

### PREVENTING ELDER MISTREATMENT THROUGH THE COACH INTERVENTION: WHAT'S RISK GOT TO DO WITH IT?

Kathleen Wilber, Zachary Gassoumis, and Eleanor Batista-Malat, *University of Southern California, Los Angeles, California, United States*

The Comprehensive Older Adult and Caregiver Help (COACH) program was developed based on effective family-focused approaches from child maltreatment and intimate partner violence. Building on evidence-based prevention strategies, COACH offered from three to 12 sessions with a trained care coach. Sessions used a person-centered strength-based approach designed to help caregivers to reduce stress and burden, seek additional outside support, and better understand and manage the symptoms and experiences of people living with dementia. An evaluation of COACH using a double-blind, randomized controlled trial found a significant reduction in the treatment group compared to no change in the control group. However, no change in risk or protective factors were noted with the exception of social support. This session will explore these findings in relationship to what was learned about risk and protective factors as means to identify and address abuse. Findings suggest that risk and protective factors are helpful to identify and support those at risk of abusing, while other strategies should be considered and tested in developing interventions to prevent and address elder mistreatment.

### SESSION 2380 (AWARD LECTURE (INVITATION ONLY))

Abstract citation ID: igae098.0937

### JOSEPH T. FREEMAN AWARD AND EXCELLENCE IN REHABILITATION OF AGING PERSONS AWARD PRESENTATIONS AND LECTURES

Chair: Kirsten Corazzini

The Joseph T. Freeman Award lecture will feature an address by the 2024 Freeman Award recipient Ali Ahmed, MD, FGSA of the Washing DC VA Medical Center and the 2024 Excellence in Rehabilitation of Aging Persons Award recipient Roger A. Fielding, PhD, FGSA of Tufts University. The Joseph T. Freeman Award is a lectureship in geriatrics awarded to a prominent clinician in the field of aging, both in research and practice. The award was established in 1977 through a bequest from a patient's estate as a tribute to Dr. Joseph T. Freeman. The Excellence in Rehabilitation of Aging Persons Award is designed to acknowledge outstanding contributions in the field of the rehabilitation of aging individuals.

Abstract citation ID: igae098.0938

### IMPROVING OUTCOMES IN OLDER VETERANS WITH HEART FAILURE AND CKD: FROM PROPENSITY SCORE USE TO AI

Ali Ahmed, *Washington DC Veterans Medical Center, VA Medical Center, District of Columbia, United States*

Older adults are often excluded from randomized controlled trials (RCT). Consequently, gold-standard RCT-evidence to improve outcomes in older adults is often unavailable. Properly-designed outcome-blinded propensity score matched studies can provide high-quality non-RCT evidence when RCTs are impractical, unethical, or lack sponsors. Renin-angiotensin system inhibitors (RASIs) improve outcomes in patients with heart failure (HF) and chronic kidney disease (CKD). National HF guidelines recommend that these drugs be prescribed in higher doses, but be used with caution in those with CKD as it may worsen kidney function. Dr. Ahmed has shown that RASIs improve survival in older patients with HF and CKD. However, concerns remain about their effect on kidneys. Recent findings from VA-Merit Review grant suggests that high-dose RASIs lower risk of kidney failure and death in octogenarians with HF and CKD. Additionally, artificial intelligence methods can be utilized to personalize care for this heterogeneous and diverse population of older patients.

Abstract citation ID: igae098.0939

### RESISTANCE TRAINING EXERCISE FOR THE REHABILITATION OF OLDER ADULTS: A FUNCTION PROMOTING THERAPY WITH ROBUST TREATMENT RESPONSE HETEROGENEITY

Roger Fielding, *Jean Mayer USDA Human Nutrition Research Center on Aging at Tufts University, Boston, Massachusetts, United States*

Advancing age is accompanied by well described reductions in muscle strength and power that are associated with deficits in physical functioning and mobility limitations. Both high and slow velocity progressive resistance exercise training have been shown to improve muscle strength and physical functioning in older adults. In combination with aerobic exercise, resistance exercise can also reduce the risk of major mobility disability. However, despite the overall positive effects exerted by exercise on multiple domains of health and physical functioning, a robust response heterogeneity to exercise training in older adults has been consistently observed. Factors influencing exercise response heterogeneity (ERH) include exercise adherence, sex, age, comorbid conditions, baseline levels of strength and function, and unknown biological factors. Future studies need to interrogate the underlying biological mechanisms associated with ERH. As plausible mechanisms emerge, more personalized approaches may be tailored to optimize the beneficial effect of exercise for all older adults.

### SESSION 2385 (SYMPOSIUM)

Abstract citation ID: igae098.0940

### LIFE-COURSE PERSPECTIVES ON MILITARY SERVICE EXPERIENCES, STRESS EXPOSURE, AND LATER- AND END-OF-LIFE OUTCOMES

Chair: Andrew London

Gerontological and life-course researchers continue to address gaps in knowledge related to the lived experiences and later-life outcomes of aging veterans. Aging veterans are similar to and distinct from aging non-veterans, and there is heterogeneity in the military and civilian experiences of veterans that is important for researchers to consider. This symposium features five presentations that expand existing knowledge about aging veterans. Spiro and colleagues focus on the long-term persistence of post-traumatic stress disorder (PTSD) and find it correlated with greater depression, anxiety, and disability, with worse health functioning and more mental health utilization. Kurth and colleagues examine stress exposure over a 20-year period and find a potential long-term advantage for aging combat veterans' well-being related to reduced avoided arguments. Yorgason and colleagues study cognitive functioning and find that veteran status is associated with worse overall cognitive functioning, especially among veterans with PTSD. Ream and colleagues find that rural providers report their clients experience more unmet social needs, but also report positive factors associated with residence in rural areas. Additionally, they report that 80 percent of rural providers are interested in delivering a group-based intervention: Enhancing Social Functioning for Older Veterans with PTSD (ESVP). Berry-Stoelzle and colleagues focus on end-of-life and identify the level of associated support services, formal inclusion of families, group identity, difference in the perceived outcome, time since military service, and branch as factors influencing advance directive completion. Collectively, these papers underscore the enduring influence of military service experiences on the health and well-being of aging veterans. Aging Veterans: Effects of Military Service across the Life Course Interest Group Sponsored Symposium

Abstract citation ID: igae098.0941

### THE PERSISTENCE OF COMBAT-RELATED PTSD AMONG MALE VIETNAM WAR VETERANS

Avron Spiro[1], Anica Pless Kaiser[2], Brian Smith[3], Jeanne Stellman[4], and Steven Stellman[4], *1. VA Boston*

*Healthcare System, Boston, Massachusetts, United States, 2. VA Boston Medical Center, Jamaica Plain, Massachusetts, United States, 3. Women's Health Sciences Division, National Center for PTSD, Boston, Massachusetts, United States, 4. Columbia University, New York, New York, United States*

The US involvement in the Vietnam War ended 50 years ago, yet many who served in that conflict, especially those who served in combat, continue to carry the impact of their military service into their later lives. For many those impacts have been positive, and for others the negative impacts have dissipated over time. However, for a sizable minority the long-term repercussions of combat deployment during their youth continue to reverberate across the lifespan. We present data on 507 male Vietnam combat veterans who were American Legion members. They were surveyed by mail in 1984, 1998, and 2020. We examined (1) patterns of PTSD over time, (2) the relation of combat exposure to these patterns, and (3) the impact of these PTSD patterns on health in later life. First, 56% of the sample did not meet PTSD criteria at any time; 25% evidenced fluctuating subthreshold PTSD, 10% had prior but not current PTSD, and 9% met criteria for current PTSD in 2020. Second, the strong dose-response relationship between combat exposure and PTSD symptoms first reported in 1988 has persisted over nearly a half-century. Finally, we found that the pattern of persistent PTSD in later life was associated with greater depression, anxiety, and disability, and with worse health functioning, and more mental health utilization. These observations extend to veterans with subthreshold PTSD as well. Across their lifetime since returning from Vietnam, a substantial number of US veterans continue to experience PTSD and its negative impact on their health and well-being.

Abstract citation ID: igae098.0942

### DO DAILY STRESSOR TRAJECTORIES VARY BETWEEN CIVILIANS, NONCOMBAT, AND COMBAT VETERANS?

Maria Kurth[1], Dakota Witzel[2], and David Almeida[3], *1. The Pennsylvania State University, University Park, Pennsylvania, United States, 2. South Dakota State University, Brookings, South Dakota, United States, 3. Pennsylvania State University, University Park, Pennsylvania, United States*

Proximal (i.e., daily) experiences may be a crucial indicator of how larger-scale events, such as trauma, impact health and well-being (Epel et al., 2018). It has also been suggested that military service – especially combat exposure – is a "hidden" variable of aging (Spiro et al., 2016), with positive and negative long-term consequences for well-being. However, few studies have directly compared differences in civilian and Veterans' daily experiences, especially exposure to stress. We used data from three waves of the National Study of Daily Experiences (NSDE; Almeida, 2005) to examine how daily stressor exposures change across ~20 years (N = 2220). At each wave, participants completed eight end-of-day phone interviews, including the Daily Inventory of Stressful Events (DISE; Almeida et al., 2002). For this project, we were interested in the following stressor domains: argument; avoided argument/disagreement; home; work/school. Similar to previous research (e.g., Piazza et al., 2022), civilian (83%) non-combat Veteran (12%), and combat Veteran status (5%) was gleaned from responses to two Life Event Survey items (LES; Sarason et al., 1978). We estimated two-level generalized multilevel models for each stressor domain and controlled for age, sex, education, and race. Preliminary results suggest only avoided argument exposure varied by civilian/Veteran status. Although there was no difference at baseline, civilians increased in likelihood (~20%), while combat Veterans were ~15% less likely to experience avoided arguments every 10 years. Results underscore a potential long-term advantage for aging combat Veterans' well-being, as avoiding arguments has negative consequences for physical and psychological health.

Abstract citation ID: igae098.0943

### DOES MILITARY VETERAN STATUS PREDICT COGNITIVE FUNCTIONING IN LATER LIFE?

Jeremy Yorgason[1], Lance Erickson[1], Christina Marini[2], and Anica Pless Kaiser[3], *1. Brigham Young University, Provo, Utah, United States, 2. Adelphi University, Garden City, New York, United States, 3. VA Boston Medical Center, Jamaica Plain, Massachusetts, United States*

Stressful life experiences can impact cognitive functioning in later years. Military experiences in relation to later life cognitive functioning is not well understood, although some work has explored the impact of PTSD on cognition. Using data from 777 individuals who participated in the Life and Family Legacies study, we explored whether veteran status (n = 383) was associated with multiple facets of cognitive functioning (using subscales from the Minnesota Cognitive Acuity Screen – Orientation, Attention, Delayed Word Recall, Comprehension, Repetition, Naming, Computation, and Verbal Fluency). All but 2 Veterans in the sample were male, and males and older respondents had lower cognitive functioning scores. Results indicated that Veteran status among males was not predictive of any one cognitive functioning subscale after gender and age were taken into account, yet it was predictive of overall cognitive functioning. Additional analyses among male veterans explored the impact of combat exposure and PTSD on each cognitive functioning subscale. While combat exposure was not associated with any cognitive outcome, PTSD symptoms (as measured by the PTSD checklist – the PCL-C) were negatively associated with the verbal fluency subscale and with overall cognitive functioning ($b=-.05$, $p=.021$). Our preliminary analyses suggest that Veteran status is not necessarily associated with individual subscale measures of cognitive functioning but may be related to a broader overall measure. PTSD symptoms were also an important correlate of cognitive functioning among older male Veterans. Veterans, especially those with PTSD symptoms, may be at greater risk for cognitive declines in later life.

Abstract citation ID: igae098.0944

### ADVANCE CARE PLANNING AND THE MILITARY EXPERIENCE: FROM ACTIVE DUTY TO VETERAN

Maresi Berry-Stoelzle, *Iowa City VA Medical Center, Iowa City, Iowa, United States*

Active duty military are one of the few groups who are required to face the possibility of mortality and morbidity while young. Pre-deployment paperwork for preparing a servicemembers and their family include the designation of a healthcare power of attorney. Despite this previous experience, rates of advance directive completion (ACP) and documented serious illness conversations (SICC) in VA served Veterans are comparable with the civilian population. Discussing goals and attitudes towards managing current and future healthcare is often sidelined for more immediately urgent medical needs. However, from our recent cohort of Veterans with an inpatient admission, 18% of Veterans admitted inpatient died within a year after their hospitalization, comparable to recent US wide data. Our previous work explores how personalization can help engage Veterans in serious illness conversations. Extending this, we explore how particularly pre-deployment planning organizational structures and discussion formats differ in structure and uptake. We coordinated with local palliative care, social work and inpatient service experts and collected suggestions from Veteran stakeholders exploring the relationship between the military service and deployment history and subsequent views of ACP of Veterans and their families. Several contributing themes emerge which are sorted in the CFIR framework including: the level of associated support services, formal inclusion of families, group identity, difference in the perceived outcome, time since military service, branch of service. This preparatory overview informs an ongoing QI project for increasing Veteran engagement in formal and informal ACP and SICC including formal and informal documentation.

Abstract citation ID: igae098.0945

### CLINICIAN PERCEPTIONS OF UNMET SOCIAL NEEDS AMONG OLDER VETERANS: THE ROLE OF RURALITY

Molly Ream[1], Laura Polacek[1], Jennifer Moye[1], Kelly O'Malley[1], Lola Baird[2], Hannah Bashian[1], and Anica Pless Kaiser[3], *1. VA Boston Healthcare System, Boston, Massachusetts, United States*, *2. Boston VA Medical Center, Jamaica Plain, Massachusetts, United States*, *3. VA Boston Medical Center, Jamaica Plain, Massachusetts, United States*

Some older Veterans experience unmet social needs due to factors such as poor health, restricted mobility, and living in rural settings. We surveyed clinicians treating older adults (age>60) at Veterans Affairs (VA) hospitals to 1) determine differences in unmet social needs by rurality, 2) explore clinicians' perceptions of social facilitators and challenges, and 3) assess clinician interest in delivering a group-based intervention: Enhancing Social Functioning for Older Veterans with PTSD (ESVP). We surveyed 84 VA clinicians who self-reported the percentage of their caseload that was rural, which we dichotomized as rural (>=50% rural; n=52) or urban (<50% rural; n=32). Likert-type items assessed the frequency clinicians encountered unmet social needs. Linear regression assessed urban versus rural differences in unmet social needs. We explored descriptive statistics of social facilitators and challenges and interest in delivering ESVP among rural clinicians. Rural clinicians reported greater unmet social needs among Veterans compared to urban clinicians (lack of social support (t(73)=3.10, p=.003); low social engagement (t(73)=2.05, p=.044); loneliness (t(73)=2.61, p=.011)). Yet, among rural clinicians, there were high reports of social facilitators, including close-knit communities (62%), value in helping others (60%), and resiliency (69%). Rural clinicians reported social challenges including fewer engagement options (83%), distance between people (67%), and value of stoicism (60%). There was high interest in delivery of ESVP among rural clinicians (80%). Rural clinicians reported significantly greater unmet social needs among older Veterans compared to urban clinicians. There was high interest in ESVP, which may enhance social functioning among this critical population.

### SESSION 2390 (SYMPOSIUM)

Abstract citation ID: igae098.0946

### POLICY SERIES: AGING AND HEALTH POLICY UPDATE: A VIEW FROM WASHINGTON

Chair: Brian Lindberg
Discussant: Patricia D'Antonio

Leading policy advocates will present their findings and viewpoints regarding aging and health care policy during a divided Congress. Issues will include behavioral health, social isolation, health care workforce, telehealth, hospice policy, elder justice and nursing home care, home and community-based services, and Medicare and Medicaid.

### SESSION 2395 (SYMPOSIUM)

Abstract citation ID: igae098.0947

### PROMOTING THE LIVED EXPERIENCE OF OLDER ADULTS USING APPLIED QUALITATIVE METHODS: LESSONS LEARNED

Chair: Sarah Neller

This symposium brings together five complementary papers focused on promoting the perspectives of older adults using applied methods in qualitative research. It underscores the importance of embedding the lived experiences and unique perspectives of older adults into the fabric of research processes and outcomes. Neller et al. present a six-step process for framing, critically evaluating, and revising prior knowledge within qualitative research. They discuss the development and advancement of a conceptual framework from their study exploring older adults' experiences creating a legacy of values. Canham et al. present a case example of a community-based participatory knowledge mobilization project across Canada. They report lessons learned from their lived experience advisory board members as they implemented community-engaged initiatives to reduce the stigma experienced by older adults experiencing homelessness. Mahmood et al. discuss digital storytelling as a method of co-creating narratives with older adults experiencing homelessness as a powerful tool to advocate for policy change and address the needs of marginalized older adults. Grittner and Walsh explored the needs of older adults who have experienced homelessness using a trauma-informed design. They present design strategies to foster supportive housing for older adults. Ketcher et al. describe how they developed a qualitative analytic framework to incorporate researcher and community members' perspectives in the analysis process. This process contributes to the methodological rigor of conducting qualitative analysis with a community-based participatory research design by reflecting community and cultural values. Presenters will emphasize innovative methods and lessons learned when conducting person-centered qualitative research across diverse demographic groups. Qualitative Research Interest Group Sponsored Symposium

Abstract citation ID: igae098.0948

### ADVANCING AN APPLIED THEORETICAL FRAMEWORK OF CREATING A LEGACY OF VALUES: A HOW-TO GUIDE

Sarah Neller[1], Kristin Cloyes[2], and Gail Towsley[3], *1. University of Tennessee Knoxville, Knoxville, Tennessee, United States*, *2. Oregon Health & Science University, Portland, Oregon, United States*, *3. University of Utah, Salt Lake City, Utah, United States*

Applied qualitative researchers are charged with critically reviewing what is currently known about a topic and moving beyond this initial knowledge as data analysis progresses to develop a useful and relevant final product by connecting the data with theory. However, little practical guidance exists for how to use research findings to develop and advance prior knowledge or theory, which can be especially challenging for novice researchers. The purpose of this presentation is to provide a practical guide for researchers to transparently report their prior knowledge and assumptions and how their thinking evolved throughout the study based on the analysis. We used an interpretive descriptive approach, which recognizes that researchers bring prior knowledge, including clinical experience or theoretical knowledge, to their research as a basis of inquiry. We will present an example of a recent study we conducted to explore the experiences of older adults who had created their legacy of values, a document communicating emotional and supportive instruction to others, and will explain our process of creating a conceptual framework and refining it through analysis using interpretive description. We will describe six steps we developed to frame, critically evaluate, and revise our prior knowledge and present how we applied each step in our legacy of values study. By clearly communicating the process of examining and refining prior assumptions, applied qualitative researchers can connect data with theory to explain and address clinically relevant problems in more systematic, effective ways thus adding transparency, rigor, and credibility to their research.

Abstract citation ID: igae098.0949

### "WE NEED TO BE AT THE TABLE" COLLABORATION WITH OLDER ADULTS WITH EXPERIENCES OF HOMELESSNESS

Sarah Canham[1], Rachel Weldrick[2], Anne Cartledge[3], Hilary Chapple[4], Chris Danielsen[5], Dorothy Kestle[5], and Samantha Teichman[6], *1. University of Utah, Salt Lake City, Utah, United States*, *2. McMaster University, Hamilton, Ontario, Canada*, *3. Aging In The Right Place Partnership, Calgary, Alberta, Canada*, *4. Aging In The Right Place Partnership, Calgary, Ontario, Canada*, *5. Aging In The Right Place Partnership, Vancouver, British Columbia, Canada*, *6. Simon Fraser University, Vancouver, British Columbia, Canada*

Increasingly recognized as an asset to research endeavors, persons with lived experience (LE) offer key insights into the impact of research processes and outcomes with those most affected by social or medical conditions. Additionally, LE advisors and co-researchers are uniquely positioned to inform knowledge mobilization and community engagement efforts that complement research activities and disseminate findings to diverse knowledge users. This presentation will share an example of a LE advisory group that collaborated in conceptualizing and implementing a knowledge mobilization project on aging and homelessness within three Canadian cities (Vancouver, Calgary, and Montreal). Following the establishment of the advisory group, priorities and objectives were determined, resulting in community engagement initiatives to disrupt discrimination toward older adults with experiences of homelessness. We will present lessons learned from this project, including the necessity of 1) digital supports to enable inclusion of advisors, 2) honoraria for advisors' time and contributions, 3) scheduling regular meeting days and times, and 4) dedicating meeting time for personal updates. Insights from this collaborative project are widely applicable across disciplines. This model offers a blueprint for other research teams aiming to enhance the impact of research and knowledge mobilization efforts and build capacity among LE communities.

Abstract citation ID: igae098.0950

### INCLUDING COMMUNITY MEMBERS IN QUALITATIVE ANALYSIS: LESSONS FROM AN INDIGENOUS DEMENTIA PROJECT

Dana Ketcher[1], Melissa Blind[1], Wayne Warry[1], Karen Pitawanakwat[2], Josyaah Budreau[1], Melinda Dertinger[1], Mikaela Williams[1], and Kristen Jacklin[3], *1. University of Minnesota Medical School Duluth, Duluth, Minnesota, United States*, *2. Naandwechige-Gamig Wikwemikong Health Centre, Wikwemikong, Ontario, Canada*, *3. University of Minnesota Medical School, Duluth, Minnesota, United States*

When conducting community-based participatory research (CBPR), the community can be left behind when it comes time to analyzing qualitative data. Little has been written on how qualitative research can rigorously and effectively include community perspectives and interpretations during data analysis. When conducting CBPR and/or research with historically marginalized and oppressed communities, incorporating community perspectives during analysis is especially important to assess researcher biases, de-center Western research paradigms/assumptions, and incorporate community values/worldviews. While scholars have written about incorporating Indigenous paradigms and methodologies into the various stages of research, less has been written on the exact steps teams can take to incorporate community members in the qualitative data analysis process. At our academic research center, we seek to create a more inclusive, relational, Indigenized, and decolonized approach to qualitative analysis. We have developed a qualitative analytic framework that intentionally includes community-based researchers and community advisory members, often without qualitative experience, from partner Indigenous communities. For this paper, we will describe our team's iterative qualitative analysis approach as it relates to a multi-sited, international, ethnographic study of Alzheimer's disease and related dementias among Indigenous North American communities in the Great Lakes region. By diversifying the perspectives present during qualitative analysis, we work to center the Six Rs of research: respect, relationship, representation, relevance, reciprocity, responsibility (Tsosie et al. 2022). As a result, we seek an ethical space to learn from both academic and community partners to ensure our approach is respectful and reflective of community and cultural values, while also being methodologically rigorous.

Abstract citation ID: igae098.0951

### AMPLIFYING VOICES: DIGITAL STORYTELLING TO EXPLORE AGING IN THE RIGHT PLACE WITH HOUSING PRECARIOUS OLDER ADULTS

Atiya Mahmood[1], Sogol Haji Hosseini[1], Nushaiba Nanjiba[1], Jason Fan[1], and Sarah Canham[2], *1. Simon Fraser University, Vancouver, British Columbia, Canada*, *2. University of Utah, Salt Lake City, Utah, United States*

Digital storytelling (DS) offers an innovative qualitative approach to explore the intricate narratives surrounding aging in the right place (AIRP) for older adults with experiences of housing precarity. We engaged three participants as co-researchers to co-create a story of their life-course narratives through participation in a photovoice study followed by the development of a DS video. Study participants live in affordable rental housing with onsite supports in Metro Vancouver, Canada. Central to our DS methods is the co-creation format where our co-researchers actively shape the narrative through qualitative interviews, photographs, and video footage of the socio-spatial context of their housing highlighting the multifaceted dimensions of AIRP. The integration of photovoice photographs within the DS serves as a visual anchor, providing nuanced insights into participants' lived experiences and aspirations regarding their current and ideal living environments. Their stories demonstrate that a combination of social, environmental, and organizational factors, as well as an interweaving of objective and subjective aspects of their housing and service experiences contribute to their stability, social integration, and well-being as they AIRP. This type of co-creative project not only serves as a means of knowledge mobilization, but also fosters dialogue and reflection between co-researchers, service providers and researchers. DS methods have the transformative potential to amplify the voices of marginalized older adults, highlighting their unique housing experiences and advocating for inclusive policy measures that prioritize their diverse needs.

Abstract citation ID: igae098.0952

### CREATING DESIGN CONNECTIONS: ARTS-BASED METHODS AS A PATHWAY INTO TRAUMA-INFORMED DESIGN FOR OLDER HOMELESS ADULTS

Alison Grittner[1], and Christine Walsh[2], *1. Cape Breton University, Sydney, Nova Scotia, Canada*, *2. University of Calgary, Calgary, Alberta, Canada*

Older adults who have experienced homelessness are more likely to live with trauma than their continually housed counterparts. Trauma-informed design (TID) recognizes that the built environment – defined as all physical elements that are human-made or curated to respond to human needs, desires, or purposes – impacts the physical, psychological, and emotional effects of trauma. TID seeks to positively shape this trauma-built environment connection through intentional design. While environmental gerontology has long understood the connection between aging well and the built environment, understanding trauma-informed design for aging adults is a new perspective. In this presentation, we explore the importance of collaborative and artful methodological strategies for understanding the TID needs of older adults with experiences of homelessness, generated by a secondary data analysis of 35 arts-based interviews with older adults (ages 50-71) living in four supportive housing sites in Calgary, Canada. We share our methods (photovoice and arts-based elicitation), examples of artful knowledge concerning the built environment created by research participants, and insights for translating art-based findings into design strategies, using our own research project as an exemplar. Overall, we demonstrate the possibilities of arts-based methods to understand, design, and create trauma-informed supportive housing for older adults with experiences of homelessness that fosters wellness, slows aging, and promotes healing from trauma.

### SESSION 2405 (SYMPOSIUM)

Abstract citation ID: igae098.0953

### SOCIAL INVISIBILITY OF ELDER ABUSE IN FAMILIES: A PHENOMENON IN PHILIPPINE SOCIETY

Chair: Carmelina Oyales
Co-Chair: Victoria Apuan
Discussant: Ronaldo Motilla

In a country where the prevailing culture puts a premium on respect and care for the elderly, elder abuse is an interesting phenomenon. The study focuses on elder abuse and the different forms of abuse occurring in Filipino communities. A qualitative study composed of individual interviews of 10 victims of elder abuse were undertaken. The aim is to explore the thoughts, feelings, sense of self, family dynamics and coping strategies of the abused. Further, data on the socio-cultural and economic risk and protective factors in the community and local government, the perspectives of 32 members of local government and social welfare institutions were gathered through focus group discussions in 5 localities. Narrative and content analysis yielded the following results: that elder abuse is a multifaceted and complex experience for the victims. Elder abuse victims suffered initial shock, denial, indignation, depression, and for some, learned helplessness. The most frequent type of abuse were emotional and psychological abuse, neglect, abandonment and financial exploitation. Poverty and ageism were an exacerbating factor. Actions to access local support mechanisms and interventions were absent or delayed due to the secrecy of the families regarding the phenomenon, as well as the lack of reporting and monitoring mechanisms on the community level.

Keywords: elder abuse, aging population, ageism, poverty, senior citizens human rights Aging Among Asians Interest Group Sponsored Symposium

Abstract citation ID: igae098.0954

### SOCIAL INVISIBILITY OF ELDER ABUSE IN FAMILIES: A PHENOMENON IN PHILIPPINE SOCIETY

Carmelina Oyales[1], and Victoria Apuan[2], *1. Miriam College and Eunoia Educational Consulting, Quezon City, National Capital Region, Philippines*, *2. Miriam College, Quezon City, Rizal, Philippines*

In a country where the prevailing culture is to give respect and care for the older persons, elder abuse is an interesting phenomenon. The study focuses on elder abuse and the different forms of abuse occurring in Filipino communities. The authors undertook a qualitative study composed of individual interviews of ten victims of elderly abuse. The aim was to explore the thoughts, feelings, sense of self, family dynamics and coping strategies of the abused. Further, data on the socio-cultural and economic risk and protective factors in the community and local government, the perspectives of thirty two members of local government and social welfare institutions were gathered through focus group discussions in five localities. Narrative and content analysis yielded the following

**results:** that elder abuse is a multifaceted and complex experience for the victims. The research study shows that the victims suffered initial shock, indignation, depression and learned helplessness. The most frequent forms of abuse were emotional and psychological abuse, neglect, abandonment and financial exploitation. Poverty and ageism were an exacerbating factor. Actions to access local support mechanisms and interventions were absent or delayed due to the secrecy of the families regarding the phenomenon, as well as the lack of reporting and monitoring mechanisms on the community level. The invisibility of older adults abuse in families needs attention and plans for action to help elder abuse victims. Keywords: elder abuse, aging population, ageism, poverty, senior citizens human rights

Abstract citation ID: igae098.0955

### RISK FACTORS AND PROTECTIVE FACTORS OF VICTIMS OF ELDER ABUSE IN THE PHILIPPINES

Victoria Apuan[1], and Carmelina Oyales[2], *1. Miriam College, Quezon City, Rizal, Philippines*, *2. Miriam College and Eunoia Educational Consulting, Quezon City, National Capital Region, Philippines*

In a country where the prevailing culture puts a premium on respect and care for the elderly, elder abuse is an interesting phenomenon. The study focuses on elder abuse and the different forms of abuse occurring in Filipino communities. Following recommendations of previous studies, the authors undertook a qualitative study composed of individual interviews of ten victims of elderly abuse and focus group discussions with thirty two local government officials. Ageism and lack of awareness of the human rights of elders were identified as deterrents to the prevention of abuse of elders within families and blocked their access to socio-psychological and economic help. Aside from ageism and the social isolation of the victims of elder abuse, risk factors also included material poverty, problematic family dynamics such as children's lack of attachment to parents, lack of support of extended family members, and the lack of awareness that the elderly have human rights. Protective factors needing to be established and strengthened were community-based monitoring and reporting system of elder abuse, a current survey of household with senior citizens, more stringent enforcement of existing laws protecting the elderly human rights education in schools, more frequent visits of social workers to communities, especially those in the hard to reach areas. Keywords: elder abuse, aging population, ageism, poverty, senior citizens human rights.

Abstract citation ID: igae098.0956

### LEARNED HELPLESSNESS AMONG ABUSED FILIPINO OLDER PERSONS

Ronaldo Motilla[1], and Carmelina Oyales[2], *1. Philippine Positive Psychology Association, Antipolo City, Rizal, Philippines*, *2. Miriam College and Eunoia Educational Consulting, Quezon City, National Capital Region, Philippines*

Due to poverty, lack of family and government support, including traumatic abuse experiences and stressful situations, many of the abused Filipino older persons exhibited learned helplessness, which is a belief that they are unable to control or alter their situation even when opportunities for change are available. The feelings of helplessness and hopelessness results in stress, anxiety, depression, isolation, loneliness, lethargy, and even self-harm behaviors and suicidal ideation. The abuses that the older persons experience include but not limited to: physical, psychological/mental, verbal, sexual, neglect, abandonment, financial exploitation and fraud. With the use of qualitative research design, 10 elderly Filipino participants aged 60 and above were recruited through purposive sampling method. Data were collected through the use of in-depth interviews and focused group discussions (FGDs), and case reports. Results were analyze using thematic analysis. Five themes emerged, namely, resignation to fate, fate as an excuse for misfortune, loss of sense of self that is from internal/external conflicts and struggles, stigma and shame. Elder abuse victims tend to accept their circumstances and the situations they are in due to negative mindset, feelings of hopelessness that evolved to their learned helplessness. Keywords: Elder abuse, learned helplessness, aging population, ageism, stigma

### SESSION 2410 (PAPER)

### TECHNOLOGY AND VIRTUAL REALITY IN HEALTH CARE

Abstract citation ID: igae098.0957

### CO-CREATING A VIRTUAL REALITY IN HOSPITAL WITH PATIENT AND FAMILY PARTNERS AND STAFF FOR OLDER ADULTS WITH DEMENTIA

Lillian Hung[1], Ben Mortenson[1], Angelica Lim[2], Jennifer Boger[3], Jim Mann[1], Lily Wong[1], Haopu (Lily) Ren[1], and Albin Soni[1], *1. University of British Columbia, Vancouver, British Columbia, Canada*, *2. Simon Fraser University, Burnaby, British Columbia, Canada*, *3. University of Waterloo, Waterloo, Ontario, Canada*

Growing evidence suggests that Virtual Reality (VR) is promising to improve the wellbeing of older patients in dementia care units in hospitals. However, older patients are often excluded from VR opportunities. Engaging patient partners, family caregivers and staff in co-creating VR program shows potential in addressing unique needs of older patients, supporting staff in implementation, and enhancing understanding complexity in clinical settings. However, literature that describes fulsome partnership with abovementioned joint stakeholders in the co-creation process is absent. The study aims to understand psychosocial needs of older adults with dementia in hospital and how VR could be best implemented in the complex clinical setting. Drawing principles of Collaborative Action Research (CAR) and applying an equity and inclusive lens, we conducted qualitative focus groups, co-design workshops and interviews with 46 stakeholders (7 patient partners, 8 family caregivers, 19 staff members and 12 leaders) in hospital. Consolidated Framework for Implementation Research (CFIR) informed our data collection and analysis. We identified three key themes to co-create a VR program for older adults with dementia in hospital to address their psychosocial needs and facilitate staff's implementation, acronymized as **aim:** 1) Approach matters; 2) Interactiveness; and 3) Multisensory stimulation. Our results underscore the imperative of engaging joint stakeholders in co-creation for older adults with dementia in hospital to address their psychosocial needs with VR, who deserve digital equity but are traditionally underrepresented in technology program development and implementation. This study contributes valuable insights into the future development and deployment of VR in geriatric care settings.

Abstract citation ID: igae098.0958

### GRIEF COACH, A TEXT-BASED GRIEF SUPPORT INTERVENTION: ACCEPTABILITY AMONG HOSPICE FAMILY MEMBERS

Emma Payne[1], and Melissa Lunardini[2], *1. Help Texts, Seattle, Washington, United States*, *2. Help Texts, San Diego, California, United States*

Although older adults experience more bereavement, they are less likely to seek support. This poses significant risks, including increased risk for prolonged grief disorder, mood disorders, loneliness, and other adverse health outcomes (Ghesquiere, 2013). Limited service access and uptake among older populations can be attributed to factors such as inadequate Medicare coverage, shortage of trained providers, and a dearth of bereavement care options tailored to the unique preferences and needs of older adults (Bartels, 2003). Developing age-appropriate, accessible grief interventions is key to addressing the service gap and associated vulnerabilities related to grief. This presentation will report on grievers' perceptions of the acceptability and helpfulness of Grief Coach, an innovative text-based grief support service aligned with a public health approach to bereavement care. The text messages are grounded in contemporary models of bereavement and coping, provide supportive education, and encourage engagement in adaptive coping behaviors. Participants in the evaluation were bereaved family members who received Grief Coach as a hospice benefit. The 13-month program retention rate was 86%. Among subscribers who met inclusion criteria for and completed a brief evaluation (N=100, response rate = 65%), 73% rated the program as "Very helpful" and 74% rated it as contributing "A great deal" or "Considerably" to their sense of being supported in their grief. Older grievers (age 65+ years old) and males tended to give the highest ratings. These preliminary data suggest that the Grief Coach text message program may be an acceptable and helpful way to support the aging grieving population.

Abstract citation ID: igae098.0959

### CO-DESIGN OF A MOBILE APP FOR CARE PARTNERS WHO MANAGE MEDICATIONS FOR PEOPLE LIVING WITH DEMENTIA

Nicole Werner[1], Aaron Ganci[2], Himalaya Patel[3], Teresa Thuemling[3], and Richard Holden[3], *1. Indiana University, Bloomington, Bloomington, Indiana, United States*, *2. Indiana University Indianapolis, Purdue University, Indianapolis, Indiana, United States*, *3. Indiana University Bloomington, Bloomington, Indiana, United States*

For unpaid care partners of community-dwelling people living with Alzheimer's disease and related dementias (ADRD), medication management is effortful and time-consuming work that often continues over several years. Although this work may be eased by mobile application software (apps), current consumer apps underserve care partners' needs. Through co-design workshops, our objective was to identify the user requirements for a mobile app supporting at-home medication management for people with ADRD. We conducted five 1-hour virtual co-design workshops with current and recent ADRD care partners. Care partners described their difficulties with medication management, then proposed ideal end-states by hand-drawing storyboards. We co-reflected on care partners' descriptions and proposals, chose the app's necessary functions, and digitally sketched corresponding user-facing features. After reviewing the sketches, care partners self-reported attitudes toward the proposed features. We ranked features by desirability, then created a digital user interface prototype. Care partners reviewed the prototype and self-reported adoption intent on a scale from 1 to 5. Seven care partners participated, ages 56-75 (Mdn=63), with 3-14 years' care-partner experience (Mdn=5.5). Elicited requirements included tracking medication administration, recording behavioral changes, instructing other care partners, and briefing healthcare professionals. The user interface prototype included a medication checklist, observation journal, and contacts management. Care partners self-reported moderately high adoption intent (M=4.2, SD=0.9). By supporting both short- and long-term information needs, the proposed mobile app promotes shared awareness among ADRD care partners and healthcare professionals. This work is foundational to developing the app and assessing usability and utility in situ.

Abstract citation ID: igae098.0960

### A MIXED-METHODS EVALUATION OF LAY COACH-SUPPORTED EMPOWER@HOME FOR GERIATRIC DEPRESSION

Xiaoling Xiang, *University of Michigan, Ann Arbor, Michigan, United States*

Empower@home is a novel internet-based cognitive behavioral therapy (iCBT) program specifically designed to reduce depression in older adults. It features 9 online lessons and a physical workbook, complemented by optional calls from lay coaches. A previously published uncontrolled study demonstrated its feasibility, acceptability, and preliminary effects. This presentation shares findings from a randomized controlled trial evaluating the program's efficacy using mixed methods (NCT05593276), involving 70 participants aged 60+ recruited from various sources. Participants were randomized to either Empower@Home with lay coach support or to receive attention control friendly calls for ten weeks, with the latter group offered the program after the post-test. High completion rates were noted (89%, Mean = 8.3, SD = 0.4), with significant within-group improvements in the intervention group compared to moderate changes in controls, resulting in a medium between-group effect size favoring the intervention (Cohen's d =.72, P <.001). Causal mediation analysis showed that acquisition of CBT skills, behavioral activation, and improvements in autonomy, competence, and relationships underpinned these effects. Several themes emerged from a thematic analysis of qualitative interviews with the treatment group: (1) a structured, goal-oriented, and skills-based approach, (2) supportive accountability from weekly coaching, (3) usability and software, (4) a mix of online and physical materials, and personal support to enhance learning, and (5) challenges in personalizing packaged iCBT programs. Empower@ Home supported by lay coaches is efficacious in reducing depression among older adults. Implications for the design of digital mental health interventions for older adults are discussed.

Abstract citation ID: igae098.0961

### ACCEPTABILITY AND PRELIMINARY EFFICACY OF BE EPIC-VR TRAINING ON FRONTLINE HEALTH CARE WORKERS

Marie Savundranayagam[1], Grace Norris[1], Annette Schumann[1], Anya Sarma[1], Jennifer Campos[2], and Joseph Orange[1], *1. Western University, London, Ontario, Canada*, *2. The KITE Research Institute, University Health Network, Toronto, Ontario, Canada*

Be EPIC-VR is a person-centered communication (PCC) training program designed for healthcare providers working in dementia care. It is the first virtual reality (VR) program to use conversational artificial intelligence to train users to communicate with avatars depicting persons living with dementia (PLWD). The current study examined the acceptability and preliminary efficacy of Be EPIC-VR training. Participants included eight personal support workers from four home care and long-term care settings. Focus groups were conducted both immediately after VR sessions and after completing the Be EPIC-VR training program. Data analyses used framework analysis. The theme, relevant training supporting learning, reflected the acceptability of Be EPIC-VR. Be EPIC-VR's innovative design facilitated significant learning gains, highlighting the benefits of experiential design, accessibility of training components, relevance regardless of career level, and group feedback on learning outcomes. The theme supporting preliminary efficacy was applying newly learned knowledge and skills with PLWD. Four subthemes emerged that mapped onto Be EPIC-VR's foci. First, participants used the cues from the environment when interacting with PLWD. Second, participants, including those with English as a second language, reported applying PCC strategies which helped in understanding PLWD's needs and addressing care refusal. Third, they noted an increase in self-efficacy in dementia care, which strengthened relationships with PLWD. Finally, they reported incorporating the preferences of PLWD during care interactions. Be EPIC-VR training emerges as a promising tool for enhancing skills of personal support workers, suggesting that immersive, VR-based training programs can foster empathetic, knowledgeable, and person-centered care approaches.

### SESSION 2415 (SYMPOSIUM)

Abstract citation ID: igae098.0962

### UNDERREPRESENTED FAMILY CAREGIVERS: BUILDING TRUST TO STRENGTHEN RECRUITMENT AND RETENTION

Chair: Gilbert Gimm
Discussant: Beth Fields

The definition of "family caregiver" typically excludes many groups, including those from different genders, racial/ethnic identities, and caregiver age categories. Relationship building, establishing trust with community partners, and interdisciplinary collaborations are essential for the successful recruitment, engagement, and retention of underrepresented family caregivers in research. Moreover, inclusive communication is critical to building trust and strengthening participant engagement. Each individual presentation of the symposium will focus on effective strategies to engage underrepresented family caregivers from a diverse range of age, gender, and racial/ethnic populations. Our first speaker (G. Gimm) will present findings on the effect of a 9-week stress management intervention on mean burden scores for 97 family caregivers, including 16 male caregivers. Our second speaker (K. Moss) will describe effective recruitment and retention strategies for feasibility and acceptability testing of a co-created virtual peer support intervention (Pair 2 Care), which was designed for and with Black/African American dementia family caregivers. Our third speaker (A. Kalvesmaki) will describe what is known about caregiving across the lifespan, including youth caregivers who are less than 18 years, and identify barriers to service use, as well as effective strategies to build trust in family caregiving communities. Our fourth speaker (M. Kavanaugh) will share key findings on the effective engagement of multidisciplinary healthcare providers who received training to deliver the YCare program, a hands-on caregiving skills session empowering youth caregivers. Our discussant (B. Fields) will then summarize cross-cutting themes and share practical and policy implications for researchers and underrepresented family caregivers across the lifespan.

Abstract citation ID: igae098.0963

### THE MASON CARES STUDY: EFFECTS OF A STRESS MANAGEMENT INTERVENTION ON MALE FAMILY CAREGIVER BURDEN SCORES

Gilbert Gimm, Shannon Layman, Megumi Inoue, Emily Ihara, and Catherine Tompkins, *George Mason University, Fairfax, Virginia, United States*

The Mason CARES study examined the association of a virtual stress management program on mean caregiver burden scores of 97 participants, including 16 male family caregivers of older adults with dementia. Using primary data collected from 97 participants, we analyzed family caregiver burden scores of male and female caregivers before and after implementation of a 9-week stress management program using Zoom sessions. Caregiver burden was measured using the Zarit Burden Interview (ZBI) score with a range from 0 to 48 (Bédard, 2001). Higher values (ZBI >=17.0) were associated with higher levels of burden. Among the 97 caregiver participants, 16% were men and 84% were women. Mean ZBI scores before and after the 9-week stress busting program intervention across the full sample decreased by 15.0% (from 24.1 to 20.5). However, the 9-week intervention was associated with a larger reduction in burden scores for male family caregivers than for female family caregivers. Specifically female family caregivers had a higher baseline ZBI score of 24.4 which decreased to 21.4 (p<.01). At the same time, male family caregivers had a lower baseline ZBI score of 22.5 which fell more sharply to 15.9 (p<.01). Findings show that male family caregivers of older adults living with dementia experienced a greater reduction in burden scores after the 9-week intervention compared to female family caregivers. These results suggest the need to continue recruiting men in stress management intervention studies to identify potential mechanisms that can help to explain gender differences associated with reducing family caregiver burden.

Abstract citation ID: igae098.0964

### RECRUITMENT AND RETENTION STRATEGIES FOR BLACK AMERICAN DEMENTIA CAREGIVER PEER SUPPORT

Karen Moss[1], Kathy Wright[1], Mary Beth Happ[1], Abraham Brody[2], Celia Wills[1], Seuli Bose Brill[1], and Karen Bullock[3], *1. The Ohio State University, Columbus, Ohio, United States*, *2. HIGN, New York University Rory Meyers College of Nursing, New York, New York, United States*, *3. Boston College, Chestnut Hill, Massachusetts, United States*

Black American caregivers of older adults living with dementia are at high risk for caregiving-related physical, emotional, spiritual, and psychosocial challenges. Culturally responsive interventions are needed to improve caregiving outcomes. Peer mentorship, a relationship-centered person-to-person approach may support caregiving mastery via culturally-tailored, flexible, non-judgmental peer support, including storytelling about caregiving experiences. The purpose of this presentation is to describe the recruitment and retention processes used for a pilot feasibility/acceptability study to test a co-created virtual intervention, called Peer Support for Caregivers of Black Americans Living with Dementia (Pair 2 Care). Community engagement strategies were used in Pair 2 Care such as: 1) use of co-design process to develop and improve the intervention, including community advisory board engagement, 2) individual and community-based organization relationship building over time, 3) principal investigator and research team in-person and virtual attendance at community health and caregiving events, 4) principal investigator contributions to community-based education programs, 5) reconnecting with previous research participants, 6) cultural preparation of research team members, and, 7) frequent contact with enrolled participants. A total of N=26 former caregivers (mentors) and current caregivers (mentees) were enrolled, mentors trained, and 1:1 dyads paired within 10 weeks for six months of peer support. Currently, retention is at 100% and early feedback from dyads is favorable. Recruitment and retention strategies outlined for this pilot study assisted the research team with successful study execution of an innovative culturally-tailored caregiver support intervention intended to build caregiving mastery and contribute to health equity by improving caregiver health outcomes.

Abstract citation ID: igae098.0965

### YOUNG CAREGIVERS: ENGAGING FAMILIES AND BUILDING TRUST IN RESEARCH FOR CAREGIVERS OF ALL AGES

Andrea Kalvesmaki[1], and Melinda Kavanaugh[2], *1. VA Salt Lake City Health Care System, Salt Lake City, Utah, United States*, *2. University of Wisconsin Milwaukee, Milwaukee, Wisconsin, United States*

The common definition of who is a "caregiver", especially for older adults, is typically an adult family member, most likely a wife of an aging spouse or a daughter of an aging parent. However, estimates of 2022 Census data indicate that over 5 million young people under the age of 18 (young caregivers) may be caring for an aging adult, or an adult with a disability. The RAISE care act, while acknowledging children may be supporting care for other family members, does not outline strategies to work with these children or their families, or build the research infrastructure to identify unmet needs of families with young caregivers. Research on young caregivers in the US is a relatively new, emerging field. Here we present what is currently known about young caregivers in the US, including findings on the impacts of caregiving on youth outcomes, and important gaps in our knowledge base. Our work has identified key barriers to engaging these family members in research and family services, including state laws that prevent parents from disclosing the important roles these young people play in supporting aging family members. As researchers in the field, we also describe strategies we are using to connect with young caregivers to identify unmet needs, and build capacity to support these families, young caregivers, and their care recipients.

Abstract citation ID: igae098.0966

### ENGAGING YOUTH AND HEALTH CARE PROFESSIONALS: DEVELOPING THE YCARE PROGRAM FOR YOUNG CARERS

Melinda Kavanaugh, *University of Wisconsin Milwaukee, Milwaukee, Wisconsin, United States*

Children and youth ages 8-19 who provide care, "young carers," are tasked with the same caregiving as adults, including bathing, feeding, toileting, and managing complex assistive devices. Yet, young carers receive little to no caregiving guidance or training in community or health care settings, leaving them isolated not only from peers but from health care professionals. Indeed, over 70% of young caregivers for people living with complex neurological disorders (Huntington's disease and ALS) reported no education or training in care provision, leaving them to "wing it", or rely on "common sense," while stating they have few peers or professionals with whom they can share experiences and build support. Health care professionals who provide guidance and training to adult caregivers, also have little understanding of young carers, and how best to engage and support them. The lack of connections between young carers and health care professionals limits the opportunity to build support and trust between young carers and the health care system. This presentation details the iterative, multidisciplinary development of the YCare program, a peer-engaged, training, skill, and support program for young carers. We will also discuss how multidisciplinary health care professionals, including OT, PT, Social work, respiratory and speech therapies were recruited and trained to deliver the YCare program - connected young carers and much needed care skills and support.

### SESSION 2420 (SYMPOSIUM)

Abstract citation ID: igae098.0967

### WEALTH DISPARITIES IN LATER LIFE: INSIGHTS AND IMPLICATIONS

Chair: Julie Miller

In the United States, wealth inequality is a stubborn and growing social and economic issue. Disparities in wealth are both products of, and contributors to, a range of pressing issues that directly impact financial wellbeing of individuals, households, and economies at large. By the time individuals reach older age, an individual's financial wellbeing has generally compounded, making disparities in wealth experienced across sub-groups of older adults that much clearer. Considered in contexts of wealth transfer and financial capacity-building across generations, we also recognize that the implications of wealth disparities often reach across generations.

This symposium brings together leading scholars to share insights about wealth disparities in older age based on a range of identities and experiences. The first presentation focuses on wealth disparities among older LGBTQ+ adults, centering generational and social forces in the life events and experiences of their interactions with financial wellbeing. The second presentation focuses on the intersection of wills, wealth, and race, introducing context about estate planning and disparities in wealth transfer. The third and fourth presentations connect corollary issues with wealth disparities in later life. One presentation evaluates whether the sense of meaning and purpose in life mitigates these existing wealth disparities in later life, and the other presentation investigates how health and marital status shocks related to wealth disparities among older adults. Together, the four presentations introduce new perspectives about wealth inequality and wealth disparities in older age, and suggest implications for research, policy, and practice. Economics of Aging Interest Group Sponsored Symposium

Abstract citation ID: igae098.0968

### BALANCING THE SCALES: ADDRESSING WEALTH DISPARITIES AMONG SEXUAL AND GENDER MINORITY (SGM) OLDER ADULTS

Charles Hoy-Ellis[1], and Karen Fredriksen-Goldsen[2], *1. Goldsen Institute, University of Washington School of Social Work, Seattle, Washington, United States, 2. University of Washington, Seattle, Washington, United States*

While the National Institutes of Health, and National Academies of Science, Engineering, and Medicine have recognized LGBTQ+ populations as experiencing significant health disparities, they remain understudied and underserved. Decades of research highlights socioeconomic positionality as a significant driver of health disparities. Our team was the first document extensive health disparities among LGBTQ+ older adults. More recently, we are documenting significant economic disparities, including higher rates of poverty – mediated and moderated by lower rates of marriage and living alone, currently, and historically. It has been less than 10 years since LGBTQ+ people have been afforded the economic privileges and benefits that are inherent to state-sanctioned marriage (more than 1,100 according to the General Accounting Office), which are cumulative over time. Utilizing data from Aging with Pride: National, Health, Aging and Sexuality/Gender Study, the first and largest national longitudinal study of LGBTQ+ older adults (N = 2,450), we identify disparities in wealth and financial resources that function as risk and protective factors predicting economic outcomes among this historically marginalized population. Our findings indicate that despite significantly higher educational achievement compared to their heterosexual age-peers, LGBTQ+ older adults are disadvantaged economically, due in large part to historical sociocultural factors. More research is needed to understand underlying mechanisms of risk and protective factors that influence the unique economic and wealth disparities among LGBTQ+ and other diverse communities to inform the development of targeted interventions to address the unique economic challenges impacting their health and well-being across the life course.

Abstract citation ID: igae098.0969

### WILLS, WEALTH, AND RACE

JP Aubry, Gal Wettstein, and Alicia Munnell, *Boston College, Chestnut Hill, Massachusetts, United States*

Inheritances make up a substantial share of national wealth, but are often overlooked in discussions of retirement security. Racial gaps in inheritances are likely to exacerbate racial disparities in wealth. One reason that Black and Hispanic decedents are less likely to pass down meaningful estates is that they are far less likely to have a will than non-Hispanic Whites. This paper documents racial gaps in receiving an inheritance, in the likelihood of having a will, and in the expectation of leaving significant bequests. The analysis then looks at the relationship between bequest expectations and realized bequests to see whether expectations are a good predictor of real outcomes. The results show that Black and Hispanic decedents and those who die without a will are less likely to achieve their bequest expectations. This paper is the first of a three-part series. The second part will be a survey to see if a plausible intervention might increase the adoption of wills, whether writing of a will increases intended bequests, and, in conjunction with the estimates of this paper, whether wills would likely increase realized bequests. The third part of the project will examine the impact of will incentives on racial wealth gaps when compounded across multiple generations.

Abstract citation ID: igae098.0970

### WEALTH DISPARITIES IN LATER LIFE: SENSE OF MEANING AND PURPOSE IN LIFE

Shinae Choi, *University of Alabama, Tuscaloosa, Alabama, United States*

This study examined the extent to which racial/ethnic, gender, and age disparities in wealth persist among U.S. older adults, focusing on housing wealth (non-liquid assets) and non-housing wealth (liquid assets), alongside income, and further evaluated whether sense of meaning and purpose in life mitigates these wealth disparities in later life. Data were from the Leave-Behind Questionnaire in the 2018 Health and Retirement Study (N=4,472). I estimated ordinary least squares regression models with interaction terms to evaluate moderation effects, adjusting for health and sociodemographic characteristics. Results showed that wealth disparities across race/ethnicity, gender, and age groups exist in older Americans. Non-Hispanic Black, non-Hispanic Asian/other races, and Hispanic older adults compared to non-Hispanic Whites, as well as women (vs. men), reported significantly lower wealth and income. The age groups of 65-79 and 80+ reported higher wealth than those aged 50-64. Multiple moderation analyses revealed evidence of racial/ethnic and age disparities in wealth, but not gender, were mitigated by sense of meaning and purpose in life. Specifically, non-housing wealth increased among non-Hispanic Asian/other and Hispanic older adults as their purpose in life increased. A notable increase in both housing and non-housing wealth, as well as income, was observed among those aged 50-64 as their purpose in life increased. Findings suggest the protective effect of sense of meaning and purpose in life in mitigating wealth disparities in later life. Through further research and interventions aimed at bolstering sense of meaning and purpose in life, there is potential to alleviate wealth disparities in later life.

Abstract citation ID: igae098.0971

### HOW DO HEALTH AND MARITAL STATUS SHOCKS AFFECT DISPARITIES AT OLDER AGES?

Richard Johnson, *Urban Institute, Washington, District of Columbia, United States*

This paper measures the incidence of health and marital shocks after age 65, assesses their impact on household wealth, and compares outcomes by race/ethnicity and other characteristics. The study focuses on the need for long-term services and support (LTSS), help with everyday activities that can be provided at home by unpaid family caregivers or paid helpers, or in residential care settings or nursing homes. Using data from the Health and Retirement Study spanning 1995 to 2020, the analysis measures how substantial LTSS needs, chronic health conditions, LTSS use, widowhood, and divorce affect the level of and changes in household wealth and the probability that older adults exhaust most of their resources. We find that health and marital shocks are common at older ages. After age 65, 70 percent of adults develop substantial LTSS needs, 28 percent enter a nursing home, 51 percent develop a major medical condition, and 29 percent become widowed. The lifetime risk of developing substantial LTSS needs or a major medical condition does not vary much by race/ethnicity. However, Black and Hispanic adults generally experience these shocks at younger ages than non-Hispanic white adults. Black adults are more likely to become widowed than others. Older adults who develop substantial LTSS needs, and especially those who enter a nursing home, experience much sharper declines in household wealth than other older adults. The financial impact of the onset of LTSS needs does not appear to vary much by race/ethnicity. Other shocks have smaller financial impacts.

### SESSION 2425 (PAPER)

### WORK, HEALTH, AND AGING

Abstract citation ID: igae098.0972

### EMPLOYMENT OUTCOMES AMONG OLDER ADULTS WITH A HISTORY OF INCARCERATION

Rodlescia Sneed, *Wayne State University, Detroit, Michigan, United States*

Older adults returning to community settings after incarceration face a number of challenges, including food insecurity, difficulty finding housing, and adverse health outcomes. A number of qualitative studies have suggested poor employment outcomes among formerly incarcerated older adults; however, there have been few quantitative inquiries related to employment outcomes for these older adults. The purpose of this study was to understand associations between history of incarceration and employment outcomes among older adults. We used pooled data from the 2012 and 2014 waves of the Health and Retirement Study to examine associations between history of incarceration and employment outcomes among community-dwelling older adults aged >50 (n=4,985). History of incarceration and employment outcomes were assessed via self-report. While there were no associations between history of incarceration and employment status in the sample, employed older adults with a history of incarceration had more physically demanding jobs and reported more work stress and more work-related discrimination than their counterparts. They also reported less work satisfaction and less supervisor support than never-incarcerated older adults. Gender moderated the association between history of incarceration and several employment outcomes, with associations observed among women but not men. Taken together, our findings suggest that history of incarceration is associated with significant differences in employment outcomes, primarily among women. Future studies should explore factors contributing to poor employment outcomes in order to design interventions that improve outcomes in this population.

Abstract citation ID: igae098.0973

### OCCUPATIONAL PHYSICAL HAZARDS AND RISK FOR DEMENTIA AND MILD COGNITIVE IMPAIRMENT IN THE HUNT 70+ STUDY

Sarah Tom[1], Bernt Bratsberg[2], Bo Engdahl[3], Yaakov Stern[1], Hans-Peter Kohler[4], Asta Håberg[5], Bjørn Heine Strand[3], and Vegard Skirbekk[3], *1. Columbia University, New York, New York, United States*, *2. Ragnar Frisch Centre for Economic Research, Oslo, Oslo, Norway*, *3. Norwegian Institute of Public Health, Oslo, Oslo, Norway*, *4. University of Pennsylvania, Philadelphia, Pennsylvania, United States*, *5. Norwegian University of Science and Technology, Trondheim, Sor-Trondelag, Norway*

**Background:** While occupational physical hazards are related to physical decline in older adulthood, less is known about cognitive outcomes. We hypothesize that greater life course occupational physical hazards would be associated with higher mild cognitive impairment (MCI) and dementia risk.

**Methods:** Data were from the Trøndelag Health Study, a population-based study of one Norwegian county beginning in 1984, with follow-up every decade. In 2017-2019 participants age ≥ 70 had cognitive assessments, used to diagnose MCI or dementia. Occupations from age 30-65 were from decennial censuses (1960 - 1980) and the national employer-employee register, from 1995. Occupations were assigned to International Standard Classification of Occupation-88 codes, linked to the Occupational Information Network. Occupational physical hazards score is the mean of 10 characteristics describing exposures to hazardous and adverse occupational environmental conditions. Age, sex, marital status at age 30, and education were from registers. Ordered logistic regression models assessed the relationship between occupational physical hazards and later-life cognitive status.

**Results:** In 9344 participants median occupational physical hazards score was 1.82 (range: 1.0-3.3 (greater hazard)). The mean age was 78, and 35% and 15% of participants had MCI

and dementia diagnoses, respectively. Adjusting for age and sex, one standard deviation greater occupational physical hazards were related to 1.32 times the risk of worse cognitive level (OR 1.32, 95% CI 1.26-1.36). Adjusting for education and marital status attenuated this association (OR 1.16, 95% CI 1.11-1.22). **Conclusions:** Those exposed to occupational physical hazards may need greater support with later life MCI and dementia risk.

Abstract citation ID: igae098.0974

### VOLUNTEERING AND EPIGENETIC AGE AMONG RETIRED AND WORKING OLDER ADULTS: EVIDENCE FROM HEALTH AND RETIREMENT STUDY

Seoyoun Kim[1], Cal Halvorsen[2], and Claire Potter[3], *1. Texas State University, San Marcos, Texas, United States*, *2. Washington University in St. Louis, St. Louis, Missouri, United States*, *3. Queen's University Belfast, Belfast, Northern Ireland, United Kingdom*

Biological aging, distinct from chronological age, shows significant intra-individual variations particularly in later life. Volunteering, a modifiable lifestyle factor, correlated with improved physical health outcomes. Recent studies suggest a potential link between frequent volunteering and slower epigenetic aging, crucial in understanding biological aging mechanisms. However, changes in volunteering, selection biases, and life transitions impact this association. Using the 2014-2016 wave of Health and Retirement Study (HRS), including DNA methylation (DNAm) assays from the Venous Blood Study, we analyzed volunteering frequency (both contemporaneous and cumulative) and five DNAm clocks (Horvath, Hannum, PhenoAge, GrimAge, DunedinPoAm) in working and retired individuals. Each DNAm measure was regressed on chronological age and cell types and was standardized for ease of comparison. We also considered selection into volunteering in 2014 via Inverse Probability Treatment Weighting. Findings show that low to medium level volunteering (1-50 hrs per year) predicted slower aging for Horvath and Hannum clocks, while GrimAge and DunedinPoAm showed deceleration with higher level volunteering (100+ hrs per year). Further, higher cumulative volunteering index was related with slower epigenetic aging acceleration for all clocks. Stratification by retirement status revealed nuanced effects, with higher volunteering hours being more beneficial for workers in GrimAge and DunedinPoAm. These findings indicate volunteering's net effects on epigenetic age among older adults. However, frequent volunteering might be more protective against epigenetic age acceleration among workers compared to retirees. Understanding these dynamic relationships offers insights for public health interventions to decelerate biological aging and extend healthy life expectancy.

Abstract citation ID: igae098.0975

### THE IMPLICATIONS OF HEALTH FOR RETIREMENT DECISIONS FOR NURSES IN THE CZECH REPUBLIC, IRELAND, SWEDEN, AND THE UK

Aine Ni Leime[1], Margaret O'Neill[1], Marie Pospisilova[2], Jakov Jandric[3], and Clary Krekula[4], *1. University of Galway, Galway, Galway, Ireland*, *2. Czech Academy of Sciences, Prague, Hlavni mesto Praha, Czech Republic*, *3. University of Edinburgh, Edinburgh, Scotland, United Kingdom*, *4. Linnaeus University, Marsta, Stockholms Lan, Sweden*

This study examines the implications of health considerations for retirement decisions among older nurses in Ireland, Czechia, Sweden, and the UK, in the context of demographic ageing and extended working life policies. Employing a life course perspective, the concept of cumulative advantage/disadvantage, and a gendered political economy of aging framework, this internationally comparative study focuses on the impact of the physical and emotional demands of nursing and diverse national policies on nurses' retirement decisions. Through analyzing data from qualitative interviews with 148 pre/retirement-age nurses in four European countries conducted as part of an international EU study, findings reveal significant health challenges, including musculoskeletal injuries and work-related stress, as central in the decision-making process. The study highlights gendered nuances in these decisions, with societal caring norms and financial constraints due to low pensions pressing many female nurses to extend their working lives or face precarious retirements. Despite the onerous physical demands of nursing and a general reluctance to work past 60, options like part-time work and job sharing emerge as critical for those considering extended work. This research highlights the misalignment of current policies with the lived experiences of older nurses, proposing reforms that recognize the gendered and health-related implications of the profession. Recommendations include re-evaluating extended working life policies with a gender-sensitive lens, introducing flexible employment options, ensuring choice in retirement timing, and providing provisions for early retirement due to health issues.

Abstract citation ID: igae098.0976

### LINKING JOB CHARACTERISTICS TO AMBULATORY DISABILITY BY RACE/ETHNICITY AND NATIVITY AMONG THE AGING WORKFORCE

Sung Park, *University of Massachusetts Boston, Boston, Massachusetts, United States*

Americans today are working longer, and more than half of adults aged 50+ work in physically demanding jobs. There are also well-known occupational risk factors for work injuries: physically demanding activities (PD) and nonstandard work schedules (NSW). This project uses a nationally representative, longitudinal sample, the Survey of Income and Program Participation, linked to a nationally representative dataset of objective, rather than subjective, measures of the work environment for all civilian occupations in the U.S. to examine the potential health burden of PD and NSW as sources of short- and longer-term ambulatory disability (AD) for older workers. Specifically, the study investigates whether the risks associated with working in jobs with NSW and high PD interact to produce risks in short- and longer-term AD that are larger than each risk acting independently. Moreover, this study includes an examination of differential exposure and differential vulnerability by race/ethnicity and nativity to these work characteristics on the risks of AD. Results suggest significant heterogeneity in occupational exposure to PD and NSW, with racial/ethnic minorities, especially female immigrants, at a particular disadvantage. Moreover, the associations between these work exposures with both short- and longer-term AD also vary dramatically by race/ethnicity, nativity, and gender. The findings advance our understanding of how work demands and organizational factors impact the physical health of the diversifying older workforce, and contribute to health disparities in later life.

### SESSION 2430 (SYMPOSIUM)

Abstract citation ID: igae098.0977

### ADVANCING RURAL GERIATRIC SERVICES: LESSONS FROM THE VETERANS HEALTH ADMINISTRATION

Chair: Emily Franzosa
Co-Chair: William Hung
Discussant: Bret Hicken

Almost 3 million rural Veterans receive care from the Veterans Health Administration (VHA) and more than half are 65 and older. But, rural older Veterans may have limited access to specialized, interdisciplinary geriatric care services. VHA's nationwide system is tasked with supporting Veterans wherever they live, a mission that comes with unique access challenges for Veterans far from VA hospitals or clinics. In a system responsible for managing many priorities and needs, prioritizing rural geriatrics services can be challenging. Our symposium highlights efforts to develop successful strategies for expanding rural geriatrics nationally. First, Dadgostar et al. share results from a project to understand geriatric service needs across VA, with the goal of leveraging data to map areas that could benefit from service expansions. Next, Kelley and colleagues discuss findings from interviews with VHA leaders on how priorities are determined at the local, regional and national level, and how rural geriatrics services fit with leaders' visions. Franzosa et al. share a rural geriatrics "advocacy field guide" to support geriatrics champions in the field to gain support for local resources. Finally, Dryden et al. share an example from a successful rural geriatrics program, GRECC Connect, and discuss how these implementation lessons can inform the development and scale of new programs. Through the lens of rural geriatrics, this symposium highlights effective strategies for health care leaders to move their priorities ahead within their own systems.

Abstract citation ID: igae098.0978

### IDENTIFYING AND MAPPING RURAL VETERANS' GERIATRIC NEEDS AND RESOURCES

Porooshat Dadgostar[1], Lynette Kelley[2], Eileen Dryden[3], Lauren Moo[4], Orna Intrator[5], and William Hung[6], *1. University of Rochester, Rochester, New York, United States*, *2. Eastern Colorado Health Care System Rocky Mountain Regional VA, Aurora, Colorado, United States*, *3. Veterans Health Administration, Bedford, Massachusetts, United States*, *4. Department of Veterans Affairs, Bedford, Massachusetts, United States*, *5. Canandaigua VAMC and University of Rochester, Canandaigua, New York, United States*, *6. James J Peters VA Medical Center, James J Peters VA Medical Center, New York, United States*

Veterans in rural areas often lack access to specialized geriatric care and may struggle to reach distant VA facilities for treatment. To better understand the regional needs of aging rural Veterans, this project aims to identify regional needs and resources for geriatrics services to guide future expansion efforts. We describe efforts to identify and map Veterans' utilization of geriatrics services in three states with large rural populations (Wisconsin, Montana, and Hawaii) to help the VA improve targeting of these resources. By examining utilization data in these three states, we try to pinpoint local areas where there are gaps in services and where Veterans may have unmet geriatric needs. We utilized data from the Geriatric Extended Care Services Data Analysis Center (GECDAC), VA's comprehensive population-based dataset for geriatric services. To develop these maps, we collected information on over 130,000 Veterans residing in the three states and obtained their sociodemographic variables and risk measures, including frailty, number of falls, Care Assessment Need (CAN) Score (estimated probability of hospitalization or death within 90 days or 1 year), dementia, long-term institutionalization risk, and use of Beers medications (potentially harmful medications for older adults). We observed the number of Veterans using different geriatric services, including geriatric ambulatory care, geriatric inpatient care, residential care, home and community-based care services, personal care services, facility-based care, and Hospice care. These data will form the basis of the "business case" for local leaders and the VA Office of Rural Health to expand geriatric services where they are most needed.

Abstract citation ID: igae098.0979

### "THE HARDEST JOB IS A HOSPITAL DIRECTOR": LEADERSHIP PERSPECTIVES PRIORITIZING RURAL GERIATRICS IN THE VA

Lynette Kelley[1], Emily Franzosa[2], Eileen Dryden[3], Lauren Moo[4], Lauren Hall[2], and William Hung[5], *1. Eastern Colorado Health Care System Rocky Mountain Regional VA, Aurora, Colorado, United States*, *2. James J Peters VA Medical Center, Bronx, New York, United States*, *3. Veterans Health Administration, Bedford, Massachusetts, United States*, *4. Department of Veterans Affairs, Bedford, Massachusetts, United States*, *5. James J Peters VA Medical Center, James J Peters VA Medical Center, New York, United States*

The Veterans Health Administration, like other health systems, faces the challenge of meeting older rural Veterans' needs. VA leaders must balance these complex needs with other local priorities, and, at the same time, a large number of national directives. Little is known about what influences the process of setting priorities and making decisions from the standpoint of healthcare leaders. We aimed to better understand how healthcare leaders weigh priorities of the health system and how their processes in turn impact the prioritization of services. We interviewed 11 recently retired local, regional and national health system leaders about their experience and how priorities in health systems can be aligned to improve the quality and reach of rural geriatric care. All leaders agreed that the highest priority is always Veteran access to high-quality care (national mission) but noted differences in what that might look like within their own settings (e.g., prioritizing the expansion of certain specialty care or clinics depending on the population). Leaders described how they weighed multiple competing priorities to decide what was of highest urgency to serve their local Veteran population while balancing national directives. Local leaders described juggling multiple administrative, clinical and leadership responsibilities daily; regional and national leaders in oversight positions agreed that the complex interplay between national and local needs may leave local leaders feeling overwhelmed. We map our real-world data to standard leadership priority frameworks, highlighting gaps between theory and practice to identify effective and practical leadership strategies.

Abstract citation ID: igae098.0980

**THE RIGHT STRATEGY, THE RIGHT PERSON, THE RIGHT TIME: DEVELOPING AN ADVOCACY FIELD GUIDE FOR RURAL GERIATRICS**

Emily Franzosa[1], Lynette Kelley[2], Eileen Dryden[3], Lauren Hall[1], Lauren Moo[4], and William Hung[5], *1. James J Peters VA Medical Center, Bronx, New York, United States*, *2. Eastern Colorado Health Care System Rocky Mountain Regional VA, Aurora, Colorado, United States*, *3. Veterans Health Administration, Bedford, Massachusetts, United States*, *4. Department of Veterans Affairs, Bedford, Massachusetts, United States*, *5. James J Peters VA Medical Center, James J Peters VA Medical Center, New York, United States*

The need to expand rural geriatric services has received increasing attention within and outside the VHA, yet medical center leaders are often challenged to prioritize expanding these services among myriad competing needs. To better understand potential levers to move rural geriatrics up the priority ladder, we conducted semi-structured interviews with 11 former leaders in the Veterans Health Administration. We explored perspectives on how health system priorities can be aligned to improve the quality and reach of rural geriatric care and solicited best practices for advocating for rural geriatric care at the local, regional and national level. Leaders shared that there was no 'best way' to advocate, prompting the development of an advocacy field guide to advance rural geriatric services. The guide draws from our interview data, policy analysis, and best practices in communication and advocacy to help local leaders and champions make a case for expanding rural geriatric resources. The guide describes multiple avenues to purposefully employ "the right strategy, for the right person, at the right time", including data from mapping local and regional gaps and needs for services, storytelling, relationship building, building a business case, aligning rural geriatrics with regional and national goals, and making geriatrics "personal". Providing actionable, easy-to-use resources to local champions can support efforts to expand rural geriatric services locally and nationally.

Abstract citation ID: igae098.0981

**A FACILITATED APPROACH TO SUSTAINMENT PLANNING FOR A RURAL GERIATRICS PROGRAM**

Eileen Dryden[1], Jessica Riley[2], Lauren Moo[3], Meaghan Kennedy[4], Camilla Pimentel[5], and William Hung[6], *1. Veterans Health Administration, Bedford, Massachusetts, United States*, *2. Veteran's Affairs, Bedford, Massachusetts, United States*, *3. Department of Veterans Affairs, Bedford, Massachusetts, United States*, *4. Veterans Affairs, Bedford, Massachusetts, United States*, *5. VA Bedford Healthcare System, Bedford, Massachusetts, United States*, *6. James J Peters VA Medical Center, James J Peters VA Medical Center, New York, United States*

Continual adaptations enhance health care program sustainability amid ongoing health system changes, yet implementation research lacks descriptions of processes to facilitate needed adaptions, particularly for programs implemented in heterogenous settings We employed a three-phase participatory method to explore potential program sustainment strategies for a 19-site Veterans Health Administration program (GRECC Connect) that uses a hub-and-spoke model to expand rural access to geriatric specialty care through telemedicine. First, hub site clinicians and staff completed the Program Sustainment Assessment Tool (PSAT), a publicly available online tool. All sites then participated in a virtual retreat at which potential sustainment strategies, barriers and facilitators to each strategy, the capacity to enact each proposed strategy, and data needed to move forward in choosing a sustainment path were discussed. And finally, sites engaged in a participatory process with their local GRECC Connect teams to draft a site-specific sustainment plan. Proposed sustainment paths included integrating with local or regional clinical services, or mentoring other groups wanting to provide geriatrics telehealth specialty care. Sites varied in their perceived confidence to sustain program activities. Barriers and facilitators to sustainment strategies related to the current level of integration into and relationships with existing clinical services, existing relationships with local and regional VA leadership, knowledge of facility priorities, and perceived capacity to develop their program's business case. This participatory approach enabled sites to plan the most feasible sustainment strategy for their own site, while using the support, knowledge, and experience of the national GRECC Connect team to do so.

### SESSION 2435 (BIOLOGICAL SCIENCES INVITED SYMPOSIUM)

Abstract citation ID: igae098.0982

**BIOMARKERS OF AGING**

Chair: Chhanda Dutta

It may be possible to prevent or ameliorate multiple adverse age-related outcomes through interventions targeted at fundamental mechanisms of aging, such as cell senescence, proteostasis, autophagy, mitochondrial dysfunction, macromolecular damage, and epigenetic regulation. Understanding the relationships between aging mechanisms and aging conditions as a basis for identifying targets for interventions to extend healthy life span has been a long-standing goal of biomedical aging research. The identification of sufficiently valid, reliable, and accurate aging mechanistic markers in humans would greatly facilitate the ability of observational studies to clarify the relationships of aging markers to aging-related phenotypes and enhance geroscience-based therapeutics development. To take full advantage of such aging mechanistic markers in future translational aging research, it will be important to clarify the degree of correlation between a) circulating levels and their activity in tissues/organs, b) their activity in laboratory animals and in humans, and c) whether they can capture the status of a specific mechanism across the life span, as part of the development and validation process. This session will feature presentations which highlight advances in the development of aging mechanistic markers, as well as challenges and limitations. Dr. Birgit Schilling will discuss the utility of specific markers of cellular senescence and multi-omics biomarkers. Dr. Nathan LeBrasseur will talk about the use of aging mechanistic markers for predicting clinical outcomes and selection of patient populations for clinical interventions. Dr. Anthony Molina will present on the application of blood-based bioenergetic profiling to elucidate the role of mitochondrial dysfunction in different aging conditions.

Abstract citation ID: igae098.0983

### PROTEIN BIOMARKERS, CELLULAR SENESCENCE AND SECRETOMES, EXOSOMES AND BIOFLUIDS

Birgit Schilling[1], and Melanie Koenigshoff[2], *1. Buck Institute for Research on Aging, Novato, California, United States*, *2. University of Pittsburgh, Pittsburgh, Pennsylvania, United States*

In this component of the session, the opportunities and challenges of biomarker discovery and validation will be discussed and involve the audience. Cellular senescence-related biomarkers have been shown to be good biomarker candidates for aging. Key questions that will be raised will include organ-specific biomarkers, and the use and culturing of explant human tissues to generate specific biomarkers of senescence and aging. We will discuss the senescence-associated secretory phenotype (SASP), the role of the extracellular matrix during aging and their use as aging biomarkers. We will discuss challenges of biofluid complexities and methods to address these challenges. We will also discuss the value of multi-omics biomarkers, such as proteins, transcripts and lipids.

Abstract citation ID: igae098.0984

### BIOMARKERS AND DRIVERS OF MITOCHONDRIAL HEALTH AND AGING

Anthony Molina, *University of California San Diego, La Jolla, California, United States*

In line with the principles of Geroscience, age-associated alterations in mitochondrial function have been shown to underlie a wide array of age-related diseases and conditions. This presentation will review mitochondrial bioenergetic profiling approaches that are being implemented in human/clinical studies. These include tissue and blood-based approaches that can serve as reliable biomarkers of mitochondrial health and have enabled researchers to examine the role of mitochondria in the physical and cognitive abilities of older adults. Importantly, we are also beginning to understand the factors that influence human mitochondrial bioenergetic capacity. These include intrinsic factors such as age and sex, as well as behavioral factors such as diet, exercise, and activity. We will also discuss ongoing efforts to identify circulating factors (e.g. proteins and metabolites) that drive differences in mitochondrial function associated with dementia and aging. Building upon our previous work demonstrating that blood-borne factors mediate age-related bioenergetic differences, our recent studies indicate that non-cellular factors present in serum can mediate bioenergetic differences associated with age and cognitive performance. Combining mitochondrial respirometry with multi-omic approaches, we are now able to identify circulating molecules that drive changes mitochondrial bioenergetics.

Abstract citation ID: igae098.0985

### THE SENESCENCE-ASSOCIATED SECRETOME AS A BIOMARKER OF AGING

Nathan LeBrasseur, *Mayo Clinic, Rochester, Minnesota, United States*

Senescent cells accumulate in nearly all organs with advancing age. A defining characteristic of senescent cells is a robust and heterogenous senescence-associated secretory phenotype (SASP). The SASP influences tissue health and function locally and systemically, hence, circulating components of the SASP may be informative of senescent cell burden. We established a candidate panel of senescence biomarkers based on common cytokines, chemokines, matrix remodeling proteins, growth factors and other proteins components of the SASP. In this seminar, the associations between senescence biomarkers and clinical manifestations of biological age, including frailty, functional limitations, and chronic disease, in humans will be presented. Moreover, the responsiveness of senescence biomarkers to interventions that counter the biology of aging, including exercise and caloric restriction, will be highlighted. Finally, the strengths and limitations of this approach and new directions will be discussed.

### SESSION 2440 (SYMPOSIUM)

Abstract citation ID: igae098.0986

### BUILDING MORE AGE-INCLUSIVE CAMPUSES BY ENGAGING WITH RETIRED FACULTY

Chair: Andrea June
Discussant: Caroline Cicero

Identified in the Age-Friendly University (AFU) principles and in the Age Inclusivity Domains in Higher Education model, initiatives that support the campus retired and emeriti community are important elements to promoting age inclusivity and intergenerational connection. Such initiatives may be found in a myriad of forms and may include campus-specific efforts as well as organizational efforts. This symposium features campus leaders representing institutional partners of the Age-Friendly University global network who will discuss their recent efforts to understand the needs of and to engage with retired and emeriti faculty. June, Andreoletti, and Swanson (Central Connecticut State University) describe findings from an initial survey of retired and emeriti faculty that sought to understand their desire to remain engaged in intergenerational teaching, research, and service at the university. Similarly, Porter (University of Manitoba) and colleagues conducted an exploratory study at their university regarding retired academics who have specific positions as Senior Scholars and Professors Emeriti, with the goal to enhance their experiences and overall benefits for the university. Gautam (UMass Lowell) discuss their campus journey in engaging emeriti professors over the past several years and present student feedback from an "intergenerational team talk" activity which engages a retired professor and students in a Gerontology course. Finally, Montepare (Lasell University) reports on data collected from the American Psychological Association's late career stage/retired faculty and addresses how professional organizations could support age-inclusive campus practices with more age-inclusive member practices. Cicero (University of Southern California) will share insights and pose future directions as the discussant.

Abstract citation ID: igae098.0987

### PERSPECTIVES FROM RETIRED AND EMERITI FACULTY ON STAYING ENGAGED IN HIGHER EDUCATION

Andrea June, Carrie Andreoletti, and Helena Swanson, *Central Connecticut State University, New Britain, Connecticut, United States*

With the aim of continuing to strengthen age-inclusive initiatives across Central Connecticut State University, the present study used a survey methodology to explore the interest and ability of retired and emeritus faculty from CCSU to remain engaged with the university. In a preliminary analysis of results, respondents (N=38, M age = 73, 49% female identifying, 67% non-Hispanic White) were mostly full professors (69%) at the time of their retirement. Retirement dates ranged from 1999 to 2024, with an average of 26 years of service. The most common reason for retirement was feeling emotionally ready (31%) followed by feeling burnt out/campus climate issues (26%). Twenty eight percent reported that their retirement was accelerated by the COVID 19 pandemic. Not surprisingly, those whose retirement dates are more recent have remained more connected through teaching and mentorship. At least 50% of respondents felt at least slightly connected to CCSU, their prior department, and current faculty. However, there was greater variation in level of satisfaction with that level of connection. More than half of the respondents expressed support for and willingness to participate in a retired/emeriti professors association at CCSU. Overall, respondents expressed more interest in short, time limited opportunities to remain engaged with students compared to semester-long activities. Qualitative data analysis revealed additional context to the challenges of interacting with the university as a retired or emeriti faculty member as well as to the goals of any continued engagement. Plans to incorporate this data into future age-inclusive initiatives will be discussed.

Abstract citation ID: igae098.0988

### EXPERIENCES OF SENIOR SCHOLARS AND PROFESSORS EMERITI AT THE UNIVERSITY OF MANITOBA: ONLINE SURVEY FINDINGS

Michelle Porter, William Kops, and Cornelia Kauenhowen, *University of Manitoba, Winnipeg, Manitoba, Canada*

Universities are unique in providing specific positions for retired academics to continue to work without pay. At the University of Manitoba (UM) in Winnipeg, Canada, there are two types of retiree positions: Senior Scholar (term) or Professor Emerita/Emeritus (lifetime/honorary). These positions allow retirees to engage in activities like supervising graduate students and conducting research. Supports (e.g., office or lab space) may be provided. However, anecdotal information and faculty consultations have suggested that new policies/procedures and initiatives may be needed to enhance their experiences and overall benefits for the university. An exploratory, online survey designed by age-friendly university committee members was completed by 78 current and past Senior Scholars and Professors Emeriti. Results revealed the many activities engaged in (most commonly reported: 78.7% writing, 70.7% research) as well as challenges experienced (e.g., parking, transitions resulting in temporary losses of email or other computer-related resources). While most (83.8%) indicated that they felt connected to the University, others indicated that they felt cut-off. Some also expressed the sentiment that "…the university/faculty does not initiate action to take advantage of my wisdom, experience and talents." Respondents further indicated their acceptance of several possible recommendations for improvements for individuals in their positions, with the most popular being a website to highlight their work and accomplishments. Given the many benefits that they see for themselves and the University based, on their continued efforts in their retiree roles, changes are imperative to improve the age inclusivity for individuals at this career stage at UM.

Abstract citation ID: igae098.0989

### INTEGRATING EMERITI PROFESSORS INTO AN AGE-INCLUSIVE LEARNING ACTIVITY FOR UNDERGRADUATE STUDENTS

Ramraj Gautam, karen Devereaux Melillo, and Lisa Abdallah, *University of Massachusetts Lowell, Lowell, Massachusetts, United States*

Engaging retired faculty is at the forefront of the Age-Friendly University (AFU) principles and the community domain of the Age-Friendly Inventory and Campus Climate Survey (ICCS) (Silverstein, et al., 2022). Engaging Retired/Emeriti Professors (REP) at UMass Lowell (UML) began in 2020 when the ICCS survey identified "involving retired faculty in university activities" (AFU Principle 9) as an area for improvement. In turn, we surveyed the UML REP in 2021. In 2022, a sequential mixed method study overwhelmingly showed REP's interest in intergenerational learning activities (IGL), supporting the AFU principle 4 (promote IGL between learners of all ages). An IGL activity was designed in the undergraduate Introduction to Gerontology course, where a team of five students conducted two one-hour online interviews with a REP. This presentation reports findings of students' open-ended responses regarding their experience and the impact this IGL activity had on their attitudes/perceptions of aging. This University IRB-approved study sample included 94 students. Qualitative content analysis coding (Vaismoradi et al., (2016) was performed by three authors, to reach a consensus on individually identified themes. The findings revealed two major themes: Positive attitudes and perceptions towards aging; and a greater understanding of life in retirement. Likewise, the open-ended responses revealed most students (75%) expressed having a positive attitude and perception toward older adults while about 25% expressed either neutral or limited impact. These findings will be discussed in relation to the valuable role REP can play in intergenerational learning activities within the University while fostering a more age-inclusive environment.

Abstract citation ID: igae098.0990

### SUPPORTING LATE AND POST–CAREER STAGE FACULTY: AGE-INCLUSIVE OPPORTUNITIES FOR PROFESSIONAL ORGANIZATIONS

Joann Montepare[1], Rowena Gomez[2], and Harvey Sterns[3], *1. Lasell University, Newton, Massachusetts, United States, 2. Palo Alto University, Palo Alto, California, United States, 3. University of Akron, Akron, Ohio, United States*

Our populations are aging nationally and locally at dramatic rates as we experience extended longevity, with significant implications on many fronts. With higher education employing a greater proportion of individuals over the age of 65 relative to the general labor force, more faculty will be moving into their late and post career stages at their institutions in the coming years. Thus, campuses are natural environments where long-time faculty members are "aging in the workplace." For organizations like the American Psychological Association (APA) and GSA, many of its members are affiliated with higher education and are part of this demographic trend. Aiming to inform our professional organizations about how they can better support these (and other) members, this presentation will describe the results of a survey of 391 APA members (age 52+) who responded to questions about how the association could support members' professional development, retirement experiences, and opportunities for further engagement and support. A variety of recommendations emerged that can be translated easily to opportunities for GSA (e.g., develop dialogue and related programming around members' career transitions and retirement planning that speak to financial challenges and physical or medical concerns, as well as sustaining professional contributions and maintaining social and emotional connections with colleagues at similar career stages). We as scholars in the aging field know well the value of a lifespan development lens, which we now need to use in a more self-reflective way in connection with our professional organizations.

### SESSION 2445 (SYMPOSIUM)

Abstract citation ID: igae098.0991

### CANCER AND AGING RESEARCH FROM DIAGNOSIS TO SURVIVORSHIP

Chair: Sean Halpin

The patient's cancer journey is a complex and multifaceted involving many emotional, environmental, and structural challenges—all of which are further complicated by advanced age. In our symposium, we bring together researchers who apply quantitative and qualitative methods to studying multiple cancer types from diagnosis to survivorship. First, Halpin will discuss the use of discursive psychology to examine how patient medical education is delivered to patients with multiple myeloma in preparation for autologous stem cell transplant. Second, Carrion will present on the treatment experience of immigrant Latinas and cancer. Next, Chidebe will apply interpretive phenomenological analysis to examine the impact of belonging to a peer support group for older Nigerian women living with metastatic breast cancer. Last, Krok-Schoen will present using cross-sectional data to examine age differences in severity and correlates of symptoms and quality of life among older breast and colorectal cancer survivors. Examining how a variety of methodological approaches are applied to explore the patient's cancer journey will help facilitate a broader understanding of gerontological cancer research. Cancer and Aging Interest Group Sponsored Symposium

Abstract citation ID: igae098.0992

### USING DISCURSIVE PSYCHOLOGY TO EXAMINE PATIENT MEDICAL EDUCATION

Sean Halpin, *RTI International, Atlanta, Georgia, United States*

Patient-centered care is facilitated by succinct medical information delivered by nurse patient-educators. However, navigating the array of treatment options poses challenges, especially for patients with complex and co-morbid conditions, such as older adults with multiple myeloma. In the current study, I applied discursive psychology to analyze speech patterns in 12 education visits (1011 minutes of audio) conducted by three nurses. Patients had a median age of 65 (IQR-54-75), were mostly white (75%), and split between male and female. I identified two overarching speech strategies used including (1) verbatim repeated (unwritten) sentences which nurses piloted and evolved from patient to patient and (2) application of language such as "I" to show authority versus "we" to demonstrate alignment with members of the medical team. Ultimately, nurses used these speech patterns to help recall information, revise their internal scripts, and assure patients.

Abstract citation ID: igae098.0993

### FORTIFYING HOPE: OLDER LATINA IMMIGRANTS WITH CANCER

Iraida Carrion[1], Malinee Neelamegam[2], Tania Estapé[3], and Jorge Estapé[3], *1. University of South Florida, Tampa, Florida, United States*, *2. University of North Texas Health Science Center, Fort Worth, Texas, United States*, *3. Fundación Contra El Cáncer, Barcelona, Catalonia, Spain*

Given the growing population of Latina immigrants aged 60 years and older in Central Florida, United States, and the current lack of relevant data, understanding this population's cancer experiences is crucial to ensure effective interventions, psychosocial care, and policies. This study assessed 200 responses to a survey developed and administered in Spanish, with questions focused on cancer knowledge, attitudes, prevention, early diagnosis, and treatment. The survey included a qualitative component of open-ended questions regarding the perspectives and experiences of 23 older immigrant Latina women with cancer. Recruitment occurred in community-based settings and the interviews were transcribed in Spanish and English. Utilizing a grounded theory approach and thematic analysis, codes were developed. The survey data highlight the lack of initial information regarding available cancer treatments, a minimal understanding of the causes of cancer, and insufficient knowledge regarding cancer diagnoses that delayed early treatment. Within this sample, 52% had breast cancer, 22% had skin cancer, 13% had uterine cancer, 4% had colon cancer, and 4% had uterine and ovarian cancer. Latina cancer rates are expected to rise in the next 20 years. Promoting cancer screenings and modifying health-related behaviors among diverse older immigrant Latinas is imperative and requires the development of culturally appropriate interventions to overcome health disparities and treatment barriers.

Abstract citation ID: igae098.0994

### AGE DIFFERENCES IN THE CORRELATES OF PAIN AND QUALITY OF LIFE AMONG BREAST AND COLORECTAL CANCER SURVIVORS

Jessica Krok-Schoen[1], Melica Nikahd[1], Madison Hyer[1], Elizabeth Arthur[2], Erin Stevens[2], Theodore Brasky[3], Leorey Saligan[4], and Diane Von Ah[1], *1. The Ohio State University, Columbus, Ohio, United States*, *2. The Ohio State University Comprehensive Cancer Center, Columbus, Ohio, United States*, *3. The Ohio State University College of Medicine, Columbus, Ohio, United States*, *4. Symptoms Biology Unit, Division of Intramural Research, Bethesda, Maryland, United States*

Few studies evaluated the differential impact of cancer-related symptoms in older patients compared to younger patients. This national cross-sectional study explored the age differences (21-64 vs. ≥65 years) in the demographic, health, and social correlates of pain and quality of life (QoL) among 621 breast and colorectal cancer survivors. Median pain and QoL scores did not differ between the younger and older groups (p-values = 0.80, 0.16, respectively). Higher pain for the whole sample was associated with more education (β = 5.3, p = 0.02) and higher perceived stress (β = 2.4, p < 0.001). Higher QoL was associated with employment (β = 0.8, p = 0.003), more social support (β = 0.4, p < 0.001, 5-point increase), and advanced cancer stage (β = 0.7, p = 0.04). The association of QoL and marital status differed by age; younger, married participants reported higher QoL, and older, married participants had lower QoL (p = 0.003). Among younger and older cancer survivors, our results indicate more similarities than differences in the symptom burden.

### SESSION 2450 (SYMPOSIUM)

Abstract citation ID: igae098.0995

### CELEBRATING 30 YEARS OF ENHANCE FITNESS: REACHING NEW POPULATIONS OF OLDER ADULTS WITH EVIDENCE-BASED EXERCISE

Chair: Kushang Patel
Co-Chair: Paige Denison
Discussant: Steven Albert

EnhanceFitness® (EF) is an evidence-based, group exercise program for older adults involving strength, aerobic, and balance training that is recommended for arthritis management and falls prevention. Classes are held for 1 hour, 3 days a week and are led by exercise instructors trained in the EF protocol, which accommodates people with different levels of physical capacity. In the past 30 years, more than 150,000 older adults have participated in EF at >800 community sites in the United States. Over 75 peer-reviewed studies have evaluated EF's effectiveness, implementation, and dissemination. In the proposed symposium, we will report new findings on efforts to expand EF's reach. First, Paige Denison (who directs EF nationally) will present a brief overview of the program. Second, Kushang Patel will present the adaptation of EF for remote delivery (Tele-EF) and report the comparative effectiveness of Tele-EF versus in-person EF on several functional outcomes. Third, Nancy Gell will present a qualitative study on the experience of older rural cancer survivors with participation in Tele-EF. Fourth, Elise Hoffman will report the feasibility of Tele-EF in rural older adults with chronic pain and the results of co-designing a referral pathway to reach this population. Fifth, Basia Belza will present a pilot study on the feasibility of EF for people living with cognitive impairment and their caregivers. Lastly, Steven Albert (Editor of GSA's Innovation in Aging) will deliver a critical discussion of the research findings and provide recommendations on expanding access of exercise programs to underserved, hard-to-reach populations of older adults. Fitness, Exercise and Wellness Interest Group Sponsored Symposium

Abstract citation ID: igae098.0996

### COMPARATIVE EFFECTIVENESS OF REMOTELY DELIVERED VERSUS IN-PERSON ENHANCE FITNESS AMONG OLDER ADULTS

Kushang Patel[1], Qian Qiu[1], Meghan Thompson[2], and Paige Denison[2], *1. University of Washington, Seattle, Washington, United States*, *2. Sound Generations, Seattle, Washington, United States*

Access to evidence-based exercise programs, such as Enhance Fitness (EF), is limited for older adults who are homebound or live in rural areas. During the COVID-19 pandemic, EF was adapted for remote delivery (Tele-EF) and implemented nationally; however, its effectiveness is not well established. Therefore, we compared the effectiveness of Tele-EF with standard in-person EF among older adults who had no prior experience with EF. Between 2020-2024, a total of 3,924 older adults had enrollment, attendance, and functional outcome data available in EF's national database (median time between assessments was 112 days [IQR: 103-126]). Baseline median age was 73 years (IQR: 68-79) and 3,040 (77.4%) were women. The monthly attendance rate in Tele-EF (n=132; mean=66.3% [95% CI: 63.5-69.1%]) and in-person EF (n=3,792; 64.8% [95% CI: 64.2-65.4%]) did not differ significantly (mean difference= 1.5% [95% CI: -2.4%, 5.3%]). The 30-second sit-to-stand test improved significantly from baseline for both Tele-EF (mean difference=3.0 chair stands [95% CI: 2.2-3.8]) and in-person EF (mean difference=1.9 chair stands [95% CI: 1.7-2.1]). Compared with in-person EF, participants in Tele-EF had greater improvement in chair rising performance (p=0.016). For the 8-foot Timed Up & Go (TUG) test, performance improved from baseline for both Tele-EF (mean difference=-0.6 seconds [95% CI: -1.0, -0.3]) and in-person EF (mean difference=-1.3 seconds [95% CI: -1.4, -1.1]). However, improvement in TUG performance did not differ between Tele-EF versus in-person EF (p=0.18). Overall, Tele-EF had similar rates of attendance and effectiveness for improving mobility function in older adults as in-person EF.

Abstract citation ID: igae098.0997

### PERSPECTIVES OF OLDER RURAL CANCER SURVIVORS ON REMOTELY DELIVERED ENHANCE FITNESS

Nancy Gell[1], Jacqueline Caefer[1], and Kushang Patel[2], *1. University of Vermont, Burlington, Vermont, United States*, *2. University of Washington, Seattle, Washington, United States*

Exercise is recommended for healthy aging and to counteract long term effects of cancer and cancer treatment. Older cancer survivors living in rural communities face unique challenges to accessing exercise opportunities including fewer in-person program options, transportation barriers, and lack of safe, outdoor infrastructure. Remotely delivered exercise provides opportunities to overcome many of the documented barriers to exercise for this underserved population. The purpose of this study was to examine perspectives from older rural cancer survivors after participating in remotely delivered Enhance Fitness group exercise (Tele-EF). We conducted semi-structured interviews with 27 older cancer survivors living in rural communities (mean age 70.8 ±5.7, 81% female) after they completed 16-weeks of Tele-EF. All interviews were audio-recorded and transcribed. Transcripts from the interviews were coded using inductive thematic analysis. Analysis and discussion of the codes resulted in iterative mapping of key themes to the Capability Opportunity and Motivation Behavioral (COM-B) framework. Emergent themes related to Tele-EF included: 1. Capability: Improvements in psychological and physical capacity to exercise resulted from supportive instruction, experiential learning, and exercise modifications by trained instructors. 2. Opportunity: Barriers of transportation and weather were eliminated while technology, when functioning, afforded accessibility at home; comradery prompted consistent attendance. 3. Motivation for exercise was enhanced through improved capability, opportunity, and functional improvement. Tele-EF facilitates exercise engagement for older rural cancer survivors through constructs identified in the COM-B framework. Future work should examine how to optimize technology for engaging older rural cancer survivors in synchronous, remote exercise.

Abstract citation ID: igae098.0998

**EXPANDING RURAL ACCESS TO TELE-ENHANCEFITNESS THROUGH COMMUNITY-ENGAGED DESIGN OF A REFERRAL PATHWAY**

Elise Hoffman[1], Elspeth Rensema[2], Leah Adams[3], Shannon Jajko[2], Nancy Gell[4], Basia Belza[2], and Kushang Patel[2], *1. University of Washington, Boston, Massachusetts, United States*, *2. University of Washington, Seattle, Washington, United States*, *3. George Mason University, Fairfax, Virginia, United States*, *4. University of Vermont, Burlington, Vermont, United States*

Rural older adults experience a high burden of chronic pain from osteoarthritis and other chronic conditions. Exercise is a first-line treatment for many painful conditions, but access to evidence-based programs is limited in rural areas. Remotely delivered programs, including Enhance®Fitness (Tele-EF), has high potential to expand access. Therefore, we conducted a series of studies to assess feasibility of delivering Tele-EF to rural older adults with chronic pain. First, we partnered with a rural-serving healthcare system to pilot 12 weeks of Tele-EF in 15 rural older adults with knee osteoarthritis. Median attendance was 91%, 100% of participants were very satisfied with tele-EF, 87% completed the program, and pain and physical functioning improved significantly. Next, we piloted implementation of an exercise prescription and Tele-EF referral pathway for older adults with knee osteoarthritis in a rural-serving primary care clinic. Adoption was low, with 4 patients referred, screened, and enrolled in Tele-EF over 16 weeks. In an effort to improve the referral pathway for a NIH-funded pragmatic trial on rural pain management, we conducted 20 interviews with EF instructors and leaders of rural-serving YMCAs. Preliminary results suggest referral pathways are effective when built into existing clinic and community systems and involve sustained provider outreach. Ongoing community engagement will include focus groups to assess usability of the pathway, prior to its implementation in a pilot of the pragmatic trial. Taken together, current results indicate that delivering EF to rural older adults is feasible, but engaging with rural healthcare systems is critical to reaching this population.

Abstract citation ID: igae098.0999

**ENHANCEFITNESS FOR ADULTS WITH MEMORY LOSS AND CARE PARTNERS: A PILOT**

Basia Belza[1], Zih-Ling Wang[1], Sarah McKiddy[1], Kelly Ash[2], Paige Denison[3], Judi Pirone[2], Meghan Thompson[3], and Allison Webel[1], *1. University of Washington, Seattle, Washington, United States*, *2. Northshore Senior Center, Bothell, Washington, United States*, *3. Sound Generations, Seattle, Washington, United States*

The purpose of this pilot was to evaluate the feasibility of an in-person evidence-based, physical activity intervention, Enhance®Fitness (EF), for community-dwelling adults with memory loss and care partners. In partnership with a senior center in the Pacific Northwest we recruited dyads to participate in a one-hour EF class, three times per week for eight weeks. Each dyad consisted of one participant with memory loss and their care partner. We assessed depression, cognition, and strength and agility at baseline and post-intervention. We enrolled 8 dyads/16 participants. Most (n=6) care partners were spouses. At baseline, 5 participants had normal cognition and 5 mild, 2 moderate, and 4 had severe cognitive impairment. Eight participants were below average in strength and 12 participants below average on agility. Six of 8 dyads dropped out prior to conclusion of the program due to injury before the start of the study or illness and fatigue during the study. Two dyads who completed the eight-week program attended, on average, 19 of 21 classes. After the intervention of those dyads who completed the program there was improved cognition and strength but worse depression scores. In an exit interview participants reported liking EF for its group setting, focus on physical activity, social interactions, and relationship building. They appreciated that the instructor maintained an engaging and personalized experience and requested more visual and verbal cues. Care partners wanted an assistant to help their loved ones with exercises. Additional adaptations and support are needed for this population to participate safely in EF.

### SESSION 2455 (SYMPOSIUM)

Abstract citation ID: igae098.1000

**CHARTING NEW TERRITORIES: EXPLORING THE ROLE OF MICRO-CREDENTIALS IN GERONTOLOGY EDUCATION**

Chair: M. Aaron Guest

In the ever-evolving landscape of higher education, educators constantly aim to meet and anticipate growing demands. This includes catering to learners' interests while also aligning with the skill sets sought by employers in an ever-evolving economic environment. Flexibility has become paramount, leading to new methods for demonstrating competency and acquiring knowledge and skills. One such approach has been the development of micro-credentials. Micro-credentials signify competency in specific skill sets or areas of knowledge acquired through focused and concise learning experiences. While prevalent in other fields, such as computer science, their emergence in gerontology is relatively recent. Their growth requires us to consider the purpose and objectives of a gerontology-infused curriculum, exploring how they may challenge existing paradigms while fostering new approaches to learning. Guest et al. will provide an overview of gero-micro-credentials and their utilization within private and public sectors. Bronwyn will share a university-based program, emphasizing how these credentials fulfill an emerging need for ongoing education. Heyn highlights the potential impact of these credentials at Hispanic Serving Institutions, particularly regarding opportunities within resource-limited environments. Ellis will then lead our discussion by exploring the undefined nature of these credentials, indicating the necessity for further research and evaluation. With reference to these exemplars, we discuss the strengths and considerations of micro-credentials in the field of aging and work to identify a consensus on how we should engage with and leverage these credentials for advancing gerontology education and practice.

Abstract citation ID: igae098.1001
### MICRO-CREDENTIALS IN GERONTOLOGY EDUCATION: ENHANCING EXPERTISE OR THREATENING EXISTENCE?

M. Aaron Guest[1], and Suk-hee Kim[2], *1. Arizona State University, Phoenix, Arizona, United States*, *2. Northern Kentucky University, Highland Heights, Kentucky, United States*

As a field, gerontology faces the challenge of preparing a workforce to address the complex needs of an aging population. Yet, in the twenty-first century, gerontology has faced many challenges in accomplishing this goal. Academic programs continue to face closure, combination, and student recruitment challenges. This is despite national and global aging. Ensuring continued access to gerontology-related content has become central for many educators. A movement from independent programs and classes to gerontology content infused throughout the curriculum has been one approach. Another has been the development of micro-credentials. These programs offer the opportunity to demonstrate competency in an area through a short, focused program of study. They are characterized by their specificity, flexibility, and accessibility and may offer a viable solution for rapidly equipping professionals with gerontological competencies. They differ from formal degree programs and continuing education, although they can be components of each. In addition, they cost fractions of a traditional degree program, often have no requirements for admission, and usually can be completed through self-paced learning. Yet, learners have limited guidance in deciding what constitutes a viable micro-credential. Indeed, micro-credentials are offered through a continuum provider, including academic-based institutions, nonprofit organizations, for-profit providers, and even employers. As such, it is unknown what many of these programs provide education, what skills they provide their learners, and if the content is scientifically accurate. While these programs can undoubtedly assist in infusing gerontological competency throughout the workforce, they also pose a severe threat to ensuring a trained and competent workforce.

Abstract citation ID: igae098.1002
### THE USE OF MICRO-CREDENTIALS IN OTHER DISCIPLINES: EXAMPLES FROM THE FIELD

Bronwyn Keefe, *Boston University, Boston, Massachusetts, United States*

This presentation will describe training partnerships between Boston University's Center for Aging & Disability Education & Research (CADER) and state and community-based agencies that provide health and social services to older adults. The workforce is looking for micro-credentials to build their competencies just as state and local agencies seek partnerships with academic institutions to build career ladders and finetune skills for their employees. Skills-based hiring and employers' willingness to move beyond requiring college degrees has grown significantly, driven by the tight job market and employer concerns about diversity and equity. Many practitioners in the field of aging have not received any formal education in working with older adults, while those who seek services are more diverse than ever, and they have a complexity of medical and social concerns. We need a trained and competent workforce to provide high-quality care for older adults. It is imperative to build reciprocal academic and community-based partnerships to further prepare and retool the aging workforce, especially during a great exodus of social service practitioners. Training through CADER's online certificates has led to statistically significant increases in core competencies in aging and increased knowledge and confidence. Almost half (47%) have taken CADER training for career advancement and personal interest, while the remaining (53%) participated because of work-mandated training initiatives, and a quarter (25%) stated that they had received a pay raise, title change, or promotion since taking the training.

Abstract citation ID: igae098.1003
### STANDARDIZATION OF GERONTOLOGY MICRO-CREDENTIALS: IMPLICATIONS FOR THE FREE MARKET

Jennifer Ellis, *Northwood Technical College, Superior, Wisconsin, United States*

Micro-credentials are an emerging market for gerontology and thus require understanding before we can advance and identify their contribution to the field. In this presentation, we will review and discuss the standardization of gerontology micro-credentials in the United States. The function and role of micro-credentials vary beyond just disciplinary backgrounds and are also based on institution type and geographic location. The diversity of short-term credit- and non-credit micro-credentials expands in response to the workforce's training needs. Yet, the assessment of certificates and other micro-credentials is in its infancy. There are limited directions, competing viewpoints, and misconceptions about what micro-credentials are and who they serve. The lack of infrastructure or expectations from the gerontology community has opened the free market to respond at a time when credit-based programs are waning. What are the implications of aligning industry standards with all short-term training and education, and how do they correlate with the needs of aging service providers? This presentation aims to open dialogue with educators, providers, and systems leaders to extrapolate the opportunities and challenges with gerontology-focused micro-credentials.

Abstract citation ID: igae098.1004
### WHAT ABOUT HISPANIC SERVING INSTITUTIONS: THE ROLE OF MICRO-CREDENTIALS AMONG MINORITY-SERVING INSTITUTIONS

Patricia Heyn, *Marymount University, Fairfax Station, Virginia, United States*

Hispanic-Serving Institutions (HIS) are educational institutions that have an enrollment of at least 25% of undergraduate students and constitute one of the fastest-growing categories in higher education. Here, we explore the benefits and implications of micro-credential certifications at Hispanic-Serving Institutions, with the goal of uncovering their potential as transformative tools in addressing educational and workforce disparities faced by Hispanic learners and trainees. We cannot underscore the strengths of micro-credential programs, such as enhancing access to post-secondary education for Hispanic learners, offering flexible educational pathways that are respectful and sensitive to their cultural and life commitments, and directly linking academic achievements with marketable skills in the workforce. Importantly, the alignment of micro-credentials with high-demand career fields offers a promising avenue for improving the economic outcomes of Hispanic graduates. Yet, there are still critical challenges to consider, including the need for broader recognition of micro-credentials in the professional market, concerns about the digital divide affecting equitable access to these programs, and the institutional capacities of HSIs to support high-quality micro-credential offerings. Comprehensive research, evaluation systems, and evidence-based guidance are needed to optimize micro-credentials at HSIs, suggesting research funding and policy adjustments, infrastructure investments, and collaborative initiatives with both academic centers and industry partners to ensure these programs effectively contribute to closing the educational, professional, and economic gaps faced by Hispanic communities.

### SESSION 2460 (SYMPOSIUM)

Abstract citation ID: igae098.1005

### CLIMATE CHANGE AND OLDER PEOPLE

Chair: Paola Zaninotto
Co-Chair: Liat Ayalon

The symposium focuses on the intersection of climate change impacts and the aging population. It includes presentations on the unique vulnerabilities of older adults to climate-induced disasters, the effect of heat exposure on their immune functions, the implications of extreme temperatures on healthcare utilization among the elderly in Europe, insights from older adults on climate change through a photovoice study, and strategies for building resilient communities for older adults. Each presentation would draw from comprehensive research and findings to highlight the need for targeted interventions and policies that address the specific challenges faced by older populations in the context of a changing climate. Climate Change and Aging Interest Group Sponsored Symposium

Abstract citation ID: igae098.1006

### CLIMATE RESILIENCE FOR AN AGING NATION

Nichole Kain[1], and Danielle Arigoni[2], *1. Antioch University New England, Saint Paul, Minnesota, United States, 2. National Housing Trust, Washington, District of Columbia, United States*

Our population is aging—by 2034, the US will have more people over 65 than under 18, and older residents make up a disproportionate number of casualties from natural disasters. In Climate Resilience for an Aging Nation, community resilience and housing expert Danielle Arigoni argues that we cannot achieve true resilience until communities adopt interventions that work to meet the needs of their oldest residents. Older adults are over-represented among fatalities in climate-fueled disasters, ranging from Hurricane Katrina in 2005 to the Maui wildfires of 2023 – and countless events in between -- people over 65 are significantly more likely to die as their younger counterparts. These rates of death have not improved in nearly 20 years, making it imperative that a renewed focus on climate resilience must be centered in the needs of older adults. This talk will provide an overview of how and why climate change differently impacts older people, and the range of solutions and strategies that can be deployed to reduce risk. Strategies include interventions in housing, transportation, emergency management, health care, utilities, and social infrastructure which can deliver a more climate-resilient community when designed with the needs of older adults in mind. With a role for people in all sectors and at all levels, achieving climate resilience for an aging nation truly is a job for all of us.

Abstract citation ID: igae098.1007

### HEAT EXPOSURE AND ADAPTIVE IMMUNITY AMONG OLDER US ADULTS

Eunyoung Choi[1], Gokul Seshadri[2], Jennifer Ailshire[1], and Bharat Thyagarajan[2], *1. University of Southern California, Los Angeles, California, United States, 2. University of Minnesota, Minneapolis, Minnesota, United States*

Climate change is leading to more extreme heat events, potentially impacting host-parasite dynamics, immunity, and infectious diseases. While animal studies suggest that heat can induce long-lasting immune disorders, its effects on human immune function remain underexplored. This study examines how heat exposure is associated with adaptive immunity, focusing on T-and B-cell subsets in a national sample of older adults from the 2016 Health and Retirement Study Venous Blood Study (N=7,301). Using high-resolution meteorological data from gridMet, daily heat index values were calculated at the census tract level and linked to each respondent's residential location. We examined both acute (day of blood collection) and chronic (1-year average) heat exposure using multilevel models, adjusting for individual factors (e.g., age, CMV seropositivity). For acute exposure, higher heat index values (standardized) were associated with decreased proportions of CD4+ naïve T-cells (B:-0.01;p<.001), CD8+ memory T-cells (B:-0.002;p<.05), and IgD- memory B cells (B:-0.003;p<.01), but increases in Naïve B cells (B:0.01;p<.001). For chronic exposure, higher heat index values were associated with decreased proportions of CD4+ naïve T-cells (B:-0.01,p<.01) and CD8+ memory T-cells (B:-0.002,p<.05). These findings show that heat stressors can affect the immune cells and subsequent cellular and humoral immune responses. Chronic heat stress potentially leads to a reduction in T-cell repertoire that may result in poor adaptive immunity. Acute heat stress may prompt both suppressive and proliferative responses in different cell types. This study underscores the need for further research to assess the broader implications of climate change on the immune health of older populations.

Abstract citation ID: igae098.1008

### CLIMATE CHANGE AND LONGEVITY IN EUROPE: A SCOPING REVIEW ON HEALTH CARE USE DURING EXTREME AIR TEMPERATURES

Linda Enroth[1], Laura Kananen[2], Ella Tapola[3], and Jutta Pulkki[3], *1. Tampere University, Finland, Tampere, Pirkanmaa, Finland, 2. SOC/Tampere University, Finland, Tampere, Pirkanmaa, Finland, 3. Tampere University, Tampere, Pirkanmaa, Finland*

Climate change together with increasing longevity pose challenges for social, economic, and ecological sustainability of the health care systems. The associations of hot and cold temperatures with higher mortality are well understood, but less is known of their impacts on health care utilization. This scoping review examines how extreme air temperatures are associated with healthcare use in 65+ populations in Europe. We screened peer-reviewed articles from six databases (Medline, Science Direct, Web of Science, Scopus, ProQuest and Greenline) published in English 2000-2022. The key search terms were air temperature, older people, health care services, and Europe. We screened 1,457 articles, reviewed 121 full-texts, and extracted data from 43 articles. Half of the articles examined heat exposure, ten cold exposure, and 12 examined both. Emergency hospital admission was the most commonly examined health service. 20 out of 33 (heat) and 16 out of 22 (cold) studies found a positive association between extreme air temperature and health care use. Most studies were patient-based and the most common conditions leading to health care use were cardiovascular and respiratory diseases. Only three studies investigated the modifying impact of social vulnerabilities. They found a tendency that social and material deprivation contributes to a higher risk of health care use during extreme temperatures. Research on health care use under extreme temperatures varies in quality and mostly reflects exacerbation of underlying chronic conditions. There is a need for further research that uses large data sets, considers multidimensional modifying risk factors, and reports results separately for older people.

Abstract citation ID: igae098.1009

### USING PHOTOVOICE TO GAIN A DEEPER UNDERSTANDING OF OLDER ADULTS EXPERIENCE OF CLIMATE CHANGE

Hui Zhao[1], Anne Stewart[1], Christine Fasching Maphis[2], Francesca Ezeokonkwo[3], Jennifer Nelson-Faulconer[1], Tiffany Tanaka[1], and Nehemiah Winesberry[1], *1. James Madison University, Harrisonburg, Virginia, United States*, *2. Sentara Community Care, Harrisonburg, Virginia, United States*, *3. Holy Family University, Philadelphia, Pennsylvania, United States*

Older adults are disproportionately impacted by the climate crisis. However, their concerns and how they perceive and respond to climate change, in their own words, remain unclear. We conducted a qualitative study employing an innovative autophotography process with semi-structured interviews to capture insights into older adults' knowledge and beliefs regarding climate change issues. Constant comparison was used to analyze data. Seventeen community-based older adults living in Virginia Shenandoah Valley region were recruited and 14 of them completed the study. Participants were 67–85 years old white adults, with the majority holding a graduate degree. From the preliminary data analysis, four themes emerged around exploring older adults' experiences and perceptions of climate change and each theme included sub-themes: 1) Perceived environmental issues impacting on health and life. Participants identified various specific concerns and priorities that older adults have regarding climate change, such as impacts on health or quality of life. 2) Barriers. Several factors hampered the effective addressing of climate change issues, including limited awareness, fixed mindsets, a lack of intergenerational conversation, and financial constraints. 3) Resilience and engagement. Older adults utilized resilience-building strategies that helped them cope with climate change-related challenges and empowered them to advocate for community resilience efforts. Our findings underscore the importance of further education about climate-associated risks. Including older adults in the conversation about climate change is crucial for promoting intergenerational dialogue and fostering mutual understanding and collaboration across age groups. Policy changes can facilitate effective actions on climate change-related issues.

Abstract citation ID: igae098.1010

### BUILDING AND SUPPORTING RESILIENT COMMUNITIES FOR OLDER ADULTS

Elana Kieffer, *The New York Academy of Medicine, New York, New York, United States*

2023 was Earth's warmest year on record, and the planet's temperatures will only continue to rise. Older adults are among those most vulnerable to the intense and increasingly dangerous effects of climate change. Extreme heat and extreme cold can cause significant health consequences, particularly for older adults, that impact their body temperatures, respiratory systems, levels of exhaustion, and even survival. In addition, weather disasters such as hurricanes and floods are on the rise due to climate change. Not only do they pose immediate threats to the safety and well-being of older adults, but they contribute to prolonged challenges in the aftermath, including disruptions to essential services, displacement of individuals and families, and compromised access to healthcare. In this session, the Center for Healthy Aging at The New York Academy of Medicine (NYAM) will describe how older New Yorkers and the professionals who work with them can build resilient response systems, strategies, and programs in the face of these challenges, as outlined in "Strengthening Community Resilience: Supporting Older Adults Through Emergency Preparedness And Response In A Post-COVID Era", published by NYAM in 2023. We will discuss recommendations and strategies in four key areas: partnerships, education, efficiency of services, and access to services. Building and supporting resilient communities with a focus on the strengths and needs of older adults is imperative in the fight against climate change, and we will outline concrete ways to achieve this objective.

### SESSION 2465 (PAPER)

### COGNITIVE IMPAIRMENT I (PAPERS)

Abstract citation ID: igae098.1011

### MACHINE LEARNING MODELING OF LOW QUALITY SURVEY RESPONSES TO PREDICT COGNITIVE IMPAIRMENT AMONG OLDER ADULTS

Hongxin Gao[1], Stefan Schneider[2], Raymond Hernandez[2], Jenny Harris[1], Danny Maupin[1], Doerte U. Junghaenel[2], and Haomiao Jin[1], *1. University of Surrey, Guildford, England, United Kingdom*, *2. University of Southern California, Los Angeles, California, United States*

Early identification of cognitive impairment (CI) is critical for managing Alzheimer's disease and other dementia. Leveraging emerging evidence on the relationship between subtle errors in survey responses and CI, this study uses a novel informatics approach that combines machine learning with psychometric methods to develop a risk prediction model for identification of CI, including mild CI and dementia, in the general older adult population. The study is based on a sample of 12,942 participants aged 50 and above in the Health and Retirement Study, and psychometric indices of low-quality responses (LQR) in a range of different surveys are created as predictors. Our analysis shows an area under the curve (AUC) of 0.66 for identifying current CI and 0.70 for predicting dementia or mortality in the next 10 years. Also, the subgroup analysis shows the LQR indices have better predictive performance in the 50-59 (AUC=0.72), and 60-69 (AUC=0.71) age groups, suggesting the model may be more sensitive to early cognitive deficits. A unique feature of this tool is that it does not require the underlying surveys to be directly relevant to CI; thus, health professionals, especially those working in community settings like health and social workers, may use the tool to assist identifying older adults at risk of CI based on questionnaires of other aspects of their life, such as quality of life and personality. It may also be useful for aging researchers who intend to identify high-risk populations from survey data that do not include direct assessment of CI.

Abstract citation ID: igae098.1012

### INSTRUMENTAL ACTIVITIES OF DAILY LIVING (IADLS) PREDICT PLASMA NFL LEVELS IN OLDER ADULTS WITHOUT DEMENTIA

Mengchi Li[1], Russell Calderon[2], Sofia Liu[3], and Junxin Li[4], *1. Xi'an Jiaotong University, Xi'an, Shaanxi, China (People's Republic)*, *2. Johns Hopkins University, Lehigh Acres, Florida, United States*, *3. Johns Hopkins University School of Nursing, Baltimore, Maryland, United States*, *4. Johns Hopkins School of Nursing, Baltimore, Maryland, United States*

Instrumental Activities of Daily Living (IADLs) are crucial for the independent living of older adults. Subtle declines in IADLs have been associated with decreased cognitive function. Yet, the knowledge regarding the association of IADL and plasma Neurofilament light chain (NfL), a biomarker for neurodegeneration, remains limited. This study aimed to examine the cross-sectional association between IADL and NfL in 102 older adults without dementia (Age≥65 years, Montreal Cognitive Assessment (MoCA)> 17), using baseline data collected from a randomized clinical trial (NCT03959202). The sample was 70.3 ± 6.1 years old, with 79% being females, 61% being White American, 33% being Black or African American, 82% having intact cognition, and 18% having mild cognitive impairment. IADLs were assessed using the Lawton Instrumental Activities of Daily Living Scale. Plasma NfL levels were examined continuously and dichotomously (≥ 75% vs. < 75%) as the study outcome. Multiple linear and logistic regression analyses were conducted with IADLs total score as the primary predictor and adjusting for age, sex, education, chronic conditions, and MoCA. Higher IADL (better function) was associated with lower plasma NfL level (β=-4.02, 95% confidence interval [CI]: -5.99, -2.06). The logistic regression model showed that 1 point increase in IADLs score was associated with 67% reduced odds of having high NfL (OR: 0.33, 95% CI: 0.14, 0.77). In conclusion, we found that higher instrumental activities of daily living were associated with decreased plasma NfL, suggesting a mechanism underlying functional decline and neurodegeneration in non-demented older adults.

Abstract citation ID: igae098.1013

### DOES COGNITIVE RESERVE MODERATE THE RELATIONSHIP BETWEEN STROKE SEVERITY AND COGNITIVE FUNCTION?

Yinuo Ou[1], Yalin Lv[2], Hui Wei[1], Yanrong Zhang[3], and Juan Li[2], *1. Huashan Hospital Fudan University, National Center for Neurological Disorders of China, Shanghai, Shanghai, China (People's Republic)*, *2. Huashan Hospital Fudan University, Shanghai, Shanghai, China (People's Republic)*, *3. Huashan Hospital Fudan University, National Center for Neurological Disorders of China, shanghai, Shanghai, China (People's Republic)*

Cognitive decline is common after stroke. This study aimed to explore the moderating effect of cognitive reserve on the relationship between stroke severity and long-term cognitive function.A longitudinal survey of patients with acute ischemic stroke (AIS) was conducted in 2023 at four stroke centers in Nanjing and Shanghai, China. A total of 371 eligible patients were interviewed at acute stage and re-interviewed at 3 and 6 months after onset. Stroke severity was assessed using The National Institutes of Health Stroke Scale(NIHSS) at admission. Cognitive reserve was assessed by Cognitive Reserve Index questionnaire (CRIq) within 7 days after stroke onset. Cognitive function was assessed by Montreal Cognitive Assessment-Changsha Version(MoCA-CS) at each wave. A series of general linear mixed models were applied to test the moderating effect of cognitive reserve.The study found that cognitive function improved over time in all patients after stroke(β=1.314, p<0.001). The cognitive function of patients with higher NIHSS score was worse than those with lower NIHSS score(β=-1.315, p<0.01).Patients with a higher level of cognitive reserve had better cognitive function after stroke than those with a lower level of cognitive reserve(β=0.048, p<0.01). The interaction between NIHSS and cognitive reserve was statistically significant(β=0.012, p<0.05)after controlling for a covariate[Trial of ORG 10172 in Acute Stroke Treatment (TOAST)].Cognitive reserve can mitigate the impact of stroke burden on long-term cognitive decline in Chinese AIS patients to some extent. Cognitive reserve provides a potential target for the prevention and intervention of cognitive impairment after stroke.

Abstract citation ID: igae098.1014

### MULTIMORBIDITY PATTERNS PREDICT THE ORDER OF PHYSICAL FRAILTY AND COGNITIVE IMPAIRMENT OCCURRENCE

Shuomin Wang[1], Qianyuan Li[1], Qian-Li Xue[2], and Minhui Liu[3], *1. Central South University, Changsha, Hunan, China (People's Republic)*, *2. Johns Hopkins University, Baltimore, Maryland, United States*, *3. Ningxia Medical University, Yinchuan, Ningxia, China*

Prior studies showed the difference in temporal sequence of physical frailty and cognitive impairment occurrence. Chronic conditions often co-occur in specific disease patterns, that is multimorbidity patterns, revealing shared pathophysiological mechanisms and risk factors. However, whether different multimorbidity patterns predict the order of frailty and cognitive impairment occurrence remain unknown. Combined participants recruited in 2011 and 2015 from National Health and Aging Trends Study, a total of 6055 community-dwelling older adults free of frailty or cognitive impairment at baseline were followed for 4 years. Latent class analysis was conducted to identify multimorbidity patterns with clinical meaningfulness. We used Fine & Gray competing risks models to examine the associations between multimorbidity patterns and risk of different orders of frailty and cognitive impairment occurrence. Four multimorbidity patterns were identified: cardiometabolic, osteoarticular, cancer-dominated, and multisystem pattern. Compared to non-multimorbidity, all four multimorbidity patterns were associated with a higher risk of developing frailty first. Specifically, multisystem pattern had a threefold higher risk (Sub-distribution hazard ratios [SHR]= 3.07, 95% CI= 2.21-4.26). However, those in the cardiometabolic (SHR =0.99, 95% CI= 0.88-1.12), osteoarticular (SHR =0.96, 95% CI= 0.82-1.11), and cancer-dominated (SHR =0.93, 95% CI= 0.79-1.09) patterns had a decreased risk of cognitive impairment-first occurrence, even though not statistically significant. In addition, older adults only from multisystem pattern showed a higher risk of co-occurrence of frailty and cognitive impairment. Clinician should be aware of etiological heterogeneity between frailty and cognitive impairment, and thus provide more effective prevention strategies for those comorbid older adults.

Abstract citation ID: igae098.1015

### THE EFFECT OF MINDFULNESS-ORIENTED RECOVERY ENHANCEMENT INTERVENTION ON POSTSTROKE COGNITIVE IMPAIRMENT

Lijing Chen[1], Jing Chu[2], and Juan Li[1], *1. Huashan Hospital Fudan University, Shanghai, Shanghai, China (People's Republic)*, *2. Naval Medical University, Yangpu, Shanghai, China (People's Republic)*

**Background:** About one-third of stroke patients are affected by cognitive impairment, and there is evidence that Mindfulness-based interventions (MBIs) can improve cognitive function.

**Objectives:** This study aimed to examine the effects of cognitive intervention based on Mindfulness-Oriented Recovery Enhancement (MORE) on cognitive function in patients with Post-Stroke Cognitive Impairment (PSCI).

**Methods:** A non-randomized controlled trial was conducted. A total of 44 patients with PSCI were enrolled, with 24 in the intervention group and 20 in the control group. The control group received routine rehabilitation therapy (RT), and the intervention group received 45-60 minutes of cognitive intervention based on MORE twice a week for four weeks plus RT.

**Results:** In the intervention group, 21 participants reached the standard of active participation. In intervention group, the mean change of global cognitive function (Montreal Cognitive Assessment, MoCA) after intervention compared with before intervention was 2.33 (95%CI: 1.73 – 2.93, p<0.001), while in the control group, the mean change of MoCA was 1.25 (95%CI: 0.77 – 1.72, p<0.001). Using a per- protocol analysis, the score of MoCA in the intervention group improved significantly compared to that in control group. The intervention group also showed significant improvement in attention, depressive symptoms, mindfulness, self-efficacy, resilience, and quality of life compared to the control group (p < 0.05, respectively).

**Conclusions:** The cognitive intervention based on MORE is feasible and effective in improving the cognitive function of patients with PSCI, as well as relieving depressive symptoms and quality of life.

### SESSION 2470 (SYMPOSIUM)

Abstract citation ID: igae098.1016

### EAST MEETS WEST: WORKING TOGETHER TOWARD AN AGE-INCLUSIVE SOCIETY THROUGH RESEARCH ON AGEISM

Chair: Takashi Yamashita
Co-Chair: Limei Chen
Discussant: Nancy Kusmaul

This East-Meets-West symposium aims to address how ageism as age-based discrimination, prejudice, and stereotypes manifests across various settings in Japan and the United States. Ageism can lead to negative outcomes such as missed employment opportunities, poor health, and lower social well-being. This symposium will serve as a platform to integrate findings and insights from the ageism studies in two distinctive Eastern and Western cultural zones. Although both countries are economically developed nations and experiencing population aging, the perception and treatment of older adults as well as their strategies to cope with ageism significantly vary due to distinctive cultural norms, health care systems, and social support systems. This symposium consists of four presentations. Dr. Yasumoto will present findings on age stereotypes and longevity aspirations among older Japanese people. Dr. Takeuchi will present findings from a large-scale survey research and differential effects of ageism by gender and employment in Japan on older adult's health and social participation. Dr. Gonzales will present findings from their analysis of the U.S. Health and Retirement Study on how ageism affects racial and ethnic minorities' health and civic engagement. Finally, Dr. Mulhorn will present findings on the prevalence and situated use of ageist terms on Twitter based on 60 million tweets. The symposium will provide a discussion to triangulate findings from these four studies and to identify culturally sensitive innovative solutions to address ageism and break barriers for all individuals to achieve high quality of life at any age over the life course. Japanese and Japanese American Aging Studies Interest Group Sponsored Symposium

Abstract citation ID: igae098.1017

### EXAMINING PUBLIC AWARENESS OF AGEIST TERMS ON TWITTER: CONTENT ANALYSIS

Kristine Mulhorn, *Drexel University, Philadelphia, Pennsylvania, United States*

Following the efforts of the World Health Organization, the Centers for Disease Control and Prevention, and the Gerontological Society of America to raise awareness about ageist language and propose appropriate terms to denote the older adult population, there was a recognized hostile discourse on social media towards those most vulnerable to the COVID-19 pandemic. This study aimed to understand the prevalence and situated use of ageist terms on Twitter. We collected 60.32 million tweets between March and July 2020 containing terms related to COVID-19. We then conducted a mixed methods study comprising a content analysis and a descriptive quantitative analysis. There were a total of 58,930 tweets contained the ageist terms "old people" or "elderly." The more appropriate term "older adult" was found in 11,328 tweets. Twitter users used ageist terms (eg, "old people" and "elderly") to criticize ageist messages (17/60,

28%), showing a lack of understanding of appropriate terms to describe older adults. Highly hostile ageist content against older adults came from tweets that contained the derogatory terms “old people” (22/30, 73%) or “elderly” (13/30, 43%). The public discourse observed on Twitter shows a continued lack of understanding of appropriate terms to use when referring to older adults. Effort is needed to eliminate the perpetuation of ageist messages that challenge healthy aging. Our study highlights the need to inform the public about appropriate language use and ageism.

Abstract citation ID: igae098.1018

### THE PSYCHOSOCIAL IMPACTS OF AGEISM: FINDINGS FROM SURVEYS IN JAPAN

Masumi Takeuchi, *Tokyo Metropolitan Institute for Geriatrics and Gerontology, Suginami-ku, Tokyo, Japan*

Ageism is a detrimental bias directed towards older people, and understanding its existence and the associated issues is crucial for eradicating ageism. Palmore (1985) posited that in a Confucian society like Japan, older people are respected and ageism is not prevalent. However, subsequent studies have shown that negative prejudices against older people also persist in Japan. This presentation aims to provide an overview of the current state of ageism in Japan and examine its psychosocial impact on older adults. Firstly, I will present the findings of a 2021 survey conducted among 800 individuals aged 60-74, revealing that 75% of respondents reported experiencing some form of age-based discrimination. Employed women encountered age discrimination more frequently than their non-employed counterparts, as well as men. This result is consistent with previous research indicating that older women face a double jeopardy of ageism and sexism in the workplace (Palmore, 1997). Secondly, the data indicates that those who encountered age discrimination exhibited more negative attitudes towards aging and reported poorer subjective well-being. Thirdly, a longitudinal analysis of the “National Survey of the Japanese Elderly,” conducted by the Tokyo Metropolitan Institute for Geriatrics and Gerontology in 1987, 1993, and 2002, among individuals aged 60 and older residing in Japan, demonstrated that older adults with negative attitudes towards aging experienced worse health outcomes and reduced social participation later in life. These findings underscore the pressing issue of ageism in Japan and emphasize the necessity for social initiatives to combat ageism in the country.

Abstract citation ID: igae098.1019

### CONFLICTING IMAGES OF AGING IN JAPAN: WHY DON’T PEOPLE WISH TO CELEBRATE THEIR 100TH BIRTHDAY?

Saori Yasumoto[1], Takeshi Nakagawa[2], Yukie Masui[3], and Yasuyuki Gondo[1], *1. Osaka University, Suita, Osaka, Japan*, *2. National Center for Geriatrics and Gerontology, Obu, Aichi, Japan*, *3. Tokyo Metropolitan Institute of Gerontology, Tokyo, Japan*

Japan is a country known for its longevity. While people practice customs to celebrate old age, negative stereotypes and discrimination against older people exist as well. This presentation discusses how people perceive aging and longevity in a society where such conflictive images exist. The research on the perception of aging is essential because it impacts older people’s physical and psychological well-being; however, culturally sensitive research to understand why people perceive aging as such is limited. In this presentation, we introduce the research results from surveys: 1) on age stereotypes conducted by targeting 1,000 people aged between 30 and 75, 2) on wish to live 100 years old by targeting 550 people aged between 77 and 81, and 3) on the longevity aspiration by targeting 33,500 people aged between 20 and 69. We found that people in Japan tend to be pessimistic about becoming old and living a long life. The most common reason was, “I do not want to cause trouble to other people.” Studies conducted outside Japan often indicate that sickness and functional decline make people see aging negatively. In Japan, the socially constructed value of “people become a burden on others due to nursing care” creates a negative image of old age. The number of centenarians is expanding in Japan, whereas people have a negative image of living longer. For us to age happily, there is an urgent need to create a culture where receiving care is not a burden to others.

Abstract citation ID: igae098.1020

### STRUCTURAL AGEISM AND PRODUCTIVE ACTIVITIES: IMPLICATIONS FOR HEALTH EQUITY RESEARCH

Ernest Gonzales[1], Cliff Whetung[2], Jane Lee[3], Stacey Gordon[4], and Natalie Green[4], *1. New York University, New York, United States*, *2. University of Minnesota, Duluth, Duluth, Minnesota, United States*, *3. University of Hawai‘i at Mānoa, Honolulu, Hawaii, United States*, *4. New York University, New York, New York, United States*

Ageism is structural: hostility, incivility, and discrimination crosscut socio-ecological domains from intrapersonal beliefs and behaviors to interpersonal dynamics in everyday interactions within the workplace, neighborhood, and in health care settings. Ageism also intersects with minoritized identities that often result in experiences of racism and sexism, and elevates the risk for worse health outcomes. This presentation is informed by structural ageism in the productive aging framework (Morrow-Howell & Gonzales, 2023) and has three main goals: first, we apply an intersectional lens to examine the relationships of discrimination in the workplace, everyday interactions, and neighborhoods with health and retirement among a diverse sample (Hispanics, Blacks, and Whites) in the United States. This goal primarily focuses on employment contexts. The second goal is to reveal risk and protective factors that mediate the relationship between discrimination and cognitive health among Indigenous, Hispanic, Blacks, and Whites in the United States. Here, we examine the unique contribution of civic engagement. Finally, we will share our latest research on ageism in the family and health – with an emphasis on informal caregiving. Across these studies, productive activities are our organizing principle – employment, civic engagement, and caregiving – to examine risk and protective factors with health. Data primarily come from the Health and Retirement Study (2006-2020) with longitudinal analyses that informed goals 1 and 2 above; the third goal is informed by critical reflections in geriatric care management and social work practice. We conclude with implications for future research, namely recruiting diverse samples, experimental and intervention research.

### SESSION 2475 (SYMPOSIUM)

Abstract citation ID: igae098.1021

### ESPO AND NIA'S BUTLER WILLIAMS PROGRAM: RESOURCES AND SUPPORTS TO CULTIVATE THE FORTITUDE FACTOR

Chair: Jaime Hughes
Co-Chair: Rita Hu
Discussant: Patricia Jones

Aging research, education, and practice all continue to expand in their diversity across methods, scope, and reach. Navigating a career in any of one of these sectors requires many elements of fortitude – curiosity, discipline, collaboration, creativity, and resilience. The Gerontological Society of America's Emerging Scholar and Professional Organization (ESPO) has long collaborated with the National Institute on Aging to support students, trainees, and emerging professionals in their scientific and professional development. The Butler Williams Scholars Program, hosted by the NIA's Office of Special Populations, is an annual immersive program for advanced postdoctoral fellows and early career faculty. For many attendees, participating in this program is an opportunity to engage with Program Officers, exchange ideas among colleagues with diverse training and interests, and develop a national network of scientific and professional collaborators. This symposium features work by past Butler Williams participants, including reflections on how GSA and NIA have each helped to shape professional and research trajectories. Specifically, Dr. Matos-Moreno will discuss ongoing work around kinship structures in different migratory scenarios. Dr. Hughes will discuss a portfolio of interdisciplinary research focused on the scale and spread of health promotion programs for older adults. Dr. Dotson will discuss a career composed of diverse funding mechanisms and initiatives to promote healthy lifestyles and optimize brain health. The session will close with remarks by staff from NIA's Office of Special Populations and other program areas.

Abstract citation ID: igae098.1022

### BUILDING AN INTERDISCIPLINARY CAREER TO ADVANCE HEALTH PROMOTION FOR HEALTHY AGING

Jaime Hughes, *Wake Forest University School of Medicine, Winston-Salem, North Carolina, United States*

As we all continue to age, the availability of evidence-based health promotion programs will be critical to healthy aging. Intervention science and implementation science are two disciplines at the heart of developing and advancing innovative, effective health promotion programs for use within diverse settings, practices, and populations. Both disciplines are highly interdisciplinary, influenced by principles in clinical geriatrics, behavioral medicine, developmental psychology, and organizational psychology. Specific health promotion programs like those focused on the adoption of healthy sleep and activity behaviors may be further influenced by additional principles, including those from behavioral sleep medicine, exercise physiology, and rehabilitation medicine. Cultivating a professional career designing and translating evidence-based health promotion programs such as these requires commitment, collaboration, and creativity. This presentation will feature completed and ongoing work belonging to a diverse research portfolio made possible by interdisciplinary training activities and research support (e.g., NIA, NHLBI, NCCIH), experiences in aging-focused leadership and professional networking (e.g., ESPO/GSA), and more than 15 years of work in applied care settings (e.g., VAHCS, academic learning health systems). This presentation will close with tips on how to access existing NIA and GSA/ESPO resources and supports for cultivating a successful career, including: leveraging professional networks and training opportunities within NIA and across NIH Institutes (ICs), engaging in professional service and leadership, and conducting embedded research across diverse academic, government, and community settings.

Abstract citation ID: igae098.1023

### STRATEGIES TO AGE WELL: BUILDING A CREATIVE AND IMPACTFUL CAREER IN BRAIN HEALTH

Vonetta Dotson, *Georgia State University, Atlanta, Georgia, United States*

Dr. Vonetta Dotson is a gero-neuropsychologist with a program of a researched focused on understanding positive (e.g., exercise) and negative (e.g., depression) modifiers of brain health in diverse older adults in order to identify ways to reduce health disparities in cognitive and affective disorders in late life, thereby enabling every individual to age well. The work is designed to be translational in nature, with implications for the daily lives of older adults and for how we diagnose, treat, and prevent cognitive and affective disorders. The National Institute on Aging (NIA) and the Gerontological Society of American (GSA) have been instrumental in her professional accomplishments throughout her training and career, ranging from early GSA conference experiences and participation in the Technical Assistance Workshop (former name of the Butler Williams program) during graduate school, to her current NIA funding through various mechanisms including R01, R21, and U19 grants. This presentation will summarize Dr. Dotson's scientific research accomplishments, the role of NIA and GSA in her career, and her more recent translation of her research to community outreach, including her start-up company, CerebroFit, that puts into action the latest research on lifestyle interventions for healthy brain aging and her book Keep Your Wits about You: The Science of Brain Maintenance as You Age, which provides lay audiences with science-based facts and practical tools to help readers develop healthy lifestyles to optimize their brain health.

Abstract citation ID: igae098.1024

### THE IMPORTANCE OF UNDERSTANDING KINSHIP STRUCTURES FOR LEFT BEHIND OLDER ADULTS IN HIGH OUTMIGRATION CONTEXTS

Amilcar Matos-Moreno, *The Pennsylvania State University, San Juan, Puerto Rico*

From early in my scientific career (doctoral student), the National Institute on Aging and the Gerontological Society of America have been a cornerstone of developing my scientific understanding, from training grants that allowed me to finish my Ph.D. to grant writing workshops that helped me land a K01 award. Participating in the RAND Summer Institute and the Butler Williams Scholar program gave me the tools to understand the many funding mechanisms within the NIA

and clarify the many doubts that come with transitioning into a faculty position. Further, my research has evolved in ways that I could never imagine. My studies surround rapid aging and migration in Puerto Rico and other countries. I study the intertwined pathways between migration, left behind older adults, and kinlessness in the 21st century. Specifically, how the migration of working-age adults is increasing the number of kinless older adults in sending countries and the effect of reducing social networks on the health and well-being of left behind older adults. Furthermore, our current work projects the kinship structure of older adults in Puerto Rico and Mexico, considering different migratory scenarios.

### SESSION 2480 (SYMPOSIUM)

Abstract citation ID: igae098.1025

### ESPO AND SOCIAL RESEARCH, POLICY, AND PRACTICE SECTION SYMPOSIUM: GOING THE EXTRA MILE: HOW PIONEERING GERONTOLOGISTS TRANSLATE RESEARCH TO POLICY AND PRACTICE

Chair: Taylor Jansen
Co-Chair: Sara Bybee
Discussant: Jennifer Greenfield

Gerontologists must have fortitude to take the extra step in translating research to end-users like community stakeholders, policymakers, and older adults; Otherwise, research can be futile. In this symposium, participants will hear from speakers who have engaged in translational research from the local to federal levels. Dr. Kina White, from the Mississippi State Department of Health will speak to her successful dissemination efforts informing state policymakers and health professionals of recent research conducted on older Mississippians. Dr. White personally mailed a copy of the research to each mayor in Mississippi, an excellent example of fortitude. Another state-level example will come from Dr. Howard Degenholtz, a Professor at the University of Pittsburg, who recently worked with the Pennsylvania Department of Aging to facilitate a statewide needs assessment survey using the Age Friendly Communities Framework, which helped develop a 10-year plan for the Pennsylvania Department of Aging. Finally, Dr. Sheryl Zimmerman, University of North Carolina (UNC) Distinguished Professor and Executive Director of the national Center for Excellence in Assisted Living (CEAL@UNC) will discuss CEAL's role to advance excellence through stronger connections between research, practice, and policy, and related advocacy at the federal level. Overall, the aim of this symposium is to answer the "so what" question that many students and researchers are presented with and motivate gerontologists to broaden their dissemination and advocacy efforts to have the most positive impact on our society, whether in research, policy, or practice.

Abstract citation ID: igae098.1026

### ADVANCING HEALTHY AGING THROUGH PUBLIC HEALTH POLICY AND PRACTICE IN MISSISSIPPI

Kina White, and Paulita Edwards-Childs, *Mississippi State Department of Health, Jackson, Mississippi, United States*

Within a state less known for its investment in public health, the Mississippi State Department of Health (MSDH) is advancing healthy aging through emerging public health policy and practice. While the overall state's population is declining, between 2010 and 2021, the 65+ population of adults experienced a 29.3% increase. In partnership with Trust for America's Health (TFAH) and The John A. Hartford Foundation (JAHF), MSDH adopted the 6C's Framework for Creating an Age-Friendly Public Health Systems (AFPHS) Framework and established a systematic approach to supporting older health and well-being. These efforts resulted in the achievement of exemplar status as a recognized Age-Friendly Public Health System. To achieve AFPHS Recognition, MSDH established a multi-sectoral Advisory Committee and developed a state-wide action plan based on the AFPHS 6C's Framework using the collective impact process; developed a Healthy Aging Data Report (HADR) and county data profiles with over 120 indicators; partnered with AARP MS and the Mississippi Public Health Association on several endeavors, including the development of a champion program for older adult volunteers to encourage their communities to be age-friendly and promote intergenerational activities. At the intersection of policy and practice, HADRs were hand-delivered to mayors across all 82 counties, translating findings of chronic disease and disability indicators to advance policies that establish age-friendly communities and health systems to improve older adult health outcomes.

Abstract citation ID: igae098.1027

### USING DATA TO DRIVE POLICY: AN EXAMPLE OF A STATE–UNIVERSITY PARTNERSHIP TO ADVANCE AGE-FRIENDLY COMMUNITIES

Howard Degenholtz[1], Steven Albert[1], Keri Kastner[1], Heather Mentch[1], Kevin Hancock[2], Gabrielle Szymanski[2], and Nathan Lampenfeld[2], *1. University of Pittsburgh, Pittsburgh, Pennsylvania, United States*, *2. Pennsylvania Department on Aging, Harrisburg, Pennsylvania, United States*

The Pennsylvania Department on Aging (PDA) is developing a "Master Plan for Older Adults" called "Aging Our Way." The plan will help guide policies and programs for the next 10 years. Agency leadership turned to the University of Pittsburgh to conduct a statewide needs assessment survey as part of a broad stakeholder engagement process that involved community meetings in all 67 counties. The University conducted a statewide telephone survey drawing on the Age-Friendly Community model. A sample of adults age 60 and older was stratified into eight regions to assure that the entire state was represented. To reach non-English speaking adults and disabled adults who might not respond by telephone, a web-based version of the survey was developed and translated into Chinese, Spanish and Russian and distributed via email to stakeholder groups. Paper versions in were mailed to community-based organizations to reach individuals who were not able to access the internet. The telephone survey results (n=900) were weighted to match the 2021 American Community Survey. The web-based survey results (n=6950) are reported separately. Findings were presented to the Long-Term Care Council, a stakeholder group that is convened by the PDA to advise on policy. The Council was formed into several subcommittees that addressed different policy topics (e.g., housing, transportation). The draft report developed by the Council was shared with the University team to develop objectives that could be measured by repeating the survey. The overall collaboration between the University and the PDA led to a strong, empirically based report.

Abstract citation ID: igae098.1028

### USING THE PLATFORM OF A NATIONAL CENTER TO INTEGRATE RESEARCH, PRACTICE, AND POLICY: CEAL@UNC

Sheryl Zimmerman, *University of North Carolina at Chapel Hill (UNC), Chapel Hill, North Carolina, United States*

The intent of research is to inform practice and policy to benefit society. Unfortunately, just as academic units are often siloed, so too are organizations constituting the three-legged stool of research, practice, and policy that must work together toward the greater good. One strategy to promote the intersection of the three is the platform of a national center, such as the Center for Excellence in Assisted Living (CEAL). Assisted living is the largest provider of residential long-term care in the nation, the importance of which was recognized in 2001 by the US Senate Special Committee on Aging and led to the development of CEAL to promote quality in assisted living. For 20 years, CEAL thrived as a unique collaborative of diverse national organizations, and in 2023 joined with the University of North Carolina at Chapel Hill (CEAL@UNC) to expand capacity to develop evidence, bolster the workforce, and promote the adoption of evidence-based practices and policies. CEAL@UNC is exploring and implementing new avenues to bring together individuals and organizations representing different constituencies (e.g., the development of national consumer, practice, policy, and research cores), and capitalizing on its broad representation to inform efforts at numerous levels, including the federal government. This session will discuss the evolution of CEAL to CEAL@UNC, the process of strategic planning and priority setting, and strategies to become a consolidated voice for the intersection of research, practice, and policy – all of which are relevant for topics other than assisted living.

### SESSION 2485 (SYMPOSIUM)

Abstract citation ID: igae098.1029

### FEASIBILITY AND ACCEPTABILITY OF A VIRTUAL EXERCISE PROGRAM IN OLDER ADULTS WITH PREDIABETES

Chair: Kathryn Porter Starr
Co-Chair: Nathan Boucher
Discussant: Barbara Nicklas

Older adults undergo a decline in muscle mass by 30% between ages 50–70, resulting in increased risk of injury and even death, all of which can be prevented and managed with resistance training. Sarcopenia, aging-induced muscle loss, underscores resistance training's critical role in muscle mass and function preservation. Despite the well-established benefits of resistance training, most older adults don't meet this exercise threshold: only 19.3% of adults aged 65-74, and < 15% of older adults aged ≥75 report meeting the CDC guideline of performing moderate/high intensity muscle strengthening involving all major muscle groups on ≥2 days per week. Through a NIA-funded SBIR grant (R44AG076087) Vivo, a commercially based, virtual, live, and interactive exercise program, partnered with Duke University to examine the feasibility and acceptability of Vivo in older adults with prediabetes. In addition to building strength, the evidence-based program also incorporates dual task exercises to work on cognition, executive function, and processing speed. Sessions took place over zoom and were 45 minutes twice a week. This symposium provides an overview of Vivo, the feasibility study, perspective of a small business dedicated to improving the lives of older adults and a Vivo member. Both quantitative and qualitative program data on feasibility and acceptability for improving physical and psychosocial outcomes will be presented. Based on these initial findings, we believe Vivo shows great potential for reaching individuals with chronic illnesses who would benefit from adding strength training to their routines in a safe and accessible environment.

Abstract citation ID: igae098.1030

### FEASIBILITY AND ACCEPTABILITY OF A VIRTUAL EXERCISE PROGRAM IN OLDER ADULTS WITH PREDIABETES

Kathryn Porter Starr[1], Elizabeth Kemp[2], Eric Levitan[2], and Nathan Boucher[3], *1. Duke University School of Medicine, Hillsborough, North Carolina, United States, 2. Vivo, Atlanta, Georgia, United States, 3. Duke University, Durham, North Carolina, United States*

Through metabolic and physiological changes, aging and insulin resistance precipitate impaired physical function and its progression to type 2 diabetes (T2DM). Further, individuals with T2DM have increased risk for functional disability and mobility limitation. Therefore, the purpose of this study was to evaluate the feasibility and acceptability of Vivo, a commercially available virtual exercise program, for older adults at risk of developing T2DM. A priori criteria for feasibility and acceptability were defined as ≥80% of participants would be retained, ≥80% would attend all 24 classes and ≥90% of the program would be delivered as designed. In this 12-week single arm feasibility study, 28 older adults (69±6 years; 58% female; and 73% non-Hispanic white) with prediabetes were recruited. Out of the 28 individuals enrolled, 22 (79%) successfully completed the 12-week intervention. The results revealed a 27% increase in upper body strength (30-sec arm curl), a 13.5% increase in lower body strength (30-sec chair stand), and a 33% improvement in endurance (2-minute knee raise). Furthermore, the mean change in the 30-second chair stands was 2.0±2.2, with 14 participants increasing by ≥2 stands. Of the 22 participants who completed the intervention, 85% attended all 24 classes. Finally, intervention fidelity was assessed using a 9-item checklist with four focus areas; intervention delivery, workout intensity, safety, and participation and engagement. The intervention was delivered with 96% fidelity across three Vivo trainers. Study findings illustrate that online, live virtual exercise programs that provide tailored programming are highly feasible and acceptable and result in meaningful functional improvements.

Abstract citation ID: igae098.1031

### ACCEPTABILITY AND SATISFACTION WITH VIVO: PARTICIPANT FOCUS GROUPS AND SURVEY

Nathan Boucher[1], Elizabeth Kemp[2], Eric Levitan[2], and Kathryn Porter Starr[3], *1. Duke University, Durham,*

*North Carolina, United States*, *2. Vivo, Atlanta, Georgia, United States*, *3. Duke University School of Medicine, Hillsborough, North Carolina, United States*

As part of the Vivo feasibility study, each participant (n= 22) completed a program satisfaction survey and participated in a focus group (four groups of 5-8 participants each). The satisfaction survey showed 1) 95% were satisfied with the program; 2) 86% indicated they would continue to do strength training exercises after the study; and 3) 77% indicated participation in Vivo made them more aware of the importance of strength training. To further examine acceptability and satisfaction with Vivo, our team conducted four focus groups focused on identifying key barriers and facilitators to participation in the virtual exercise sessions. Two coders, through individual coding and iterative team meetings with the larger team, used descriptive content analysis to code and identify common themes. Our team identified four themes: 1) self-reported positive outcomes (i.e., regained function after injury, variety of exercise kept interest); 2) positive exercise experience (i.e., manageable exercise with little equipment, felt inclusive, focus on safe exercise); 3) social connection with others (i.e., developed peer group, facilitate group discussion that helped bond group); and 4) importance of an individualized exercise program (i.e., modifiable depending on ability that day, individual feedback from trainers). Areas for improvement included: making rescheduling easier, optimizing trainer communication, and clarifying needed hardware. These findings support the acceptability and high satisfaction with virtual exercise programs for older adults with pre-diabetes.

Abstract citation ID: igae098.1032

### THE IMPLEMENTATION AND RESULTS OF AN INNOVATIVE VIRTUAL EXERCISE PROGRAMS FOR OLDER ADULTS

Eric Levitan[1], Elizabeth Kemp[1], and Kathryn Porter Starr[2], *1. Vivo, Atlanta, Georgia, United States*, *2. Duke University School of Medicine, Hillsborough, North Carolina, United States*

Aging increases the risk of developing sarcopenia, the loss of muscle mass, strength, and function. Sarcopenia, in turn, increases one's susceptibility to chronic illnesses, including heart disease, cancer, diabetes, arthritis, and dementia, the leading causes of morbidity, mortality, and escalating healthcare expenses in the US. Strength training is a highly effective exercise intervention that reverses age-related muscle loss in older adults2 and restores overall strength and functional abilities. Vivo is an evidence-based program designed using the principle of science of exercise and aging, integrating stretching, balance, cognitive and resistance exercises in a safe, engaging way for older adults. Vivo addresses barriers to strength training through its easy-to-use virtual platform, knowledgeable trainers, engaging social experience and individualized programming that is tailored to meet the participant's goals and capabilities. Over the past year, Vivo has partnered with several academic centers and community-based organizations to implement virtual exercise programs for older adults. Results from these partnerships show functional improvements and high program satisfaction. This presentation will discuss the steps taken to partner with academic centers and community-based organizations and share quantitative and qualitative outcomes from those partnerships. These results support the results seen in the NIH feasibility study and more importantly, reinforce the growing body of evidence that strength training is an effective strategy to regain muscle strength and improve functional abilities.

Abstract citation ID: igae098.1033

### EMPOWERING OLDER ADULTS: A PARTICIPANT'S PERSPECTIVE ON VIVO'S ONLINE STRENGTH TRAINING PROGRAM

Jennifer Pettis, *Gerontological Society of America, Clifton Park, New York, United States*

This abstract presents the narrative of a participant, detailing her positive experiences with Vivo, an online live and interactive strength training program. This presentation will illuminate the transformative impact of Vivo's holistic approach to fitness among older adults. The participant, enthusiastically recounts her journey with Vivo, highlighting remarkable gains in muscle strength and energy levels. Engaging in Vivo's tailored workouts, she experienced tangible improvements in her physical abilities, allowing her to navigate daily tasks with newfound ease and confidence. Notably, she emphasizes the program's adaptability to her age group, catering to varying fitness levels while promoting safe and effective exercise practices. Beyond physical benefits, the participant underscores the significance of socialization within the Vivo community. Participating in live sessions facilitated meaningful connections with peers, fostering a supportive network that transcended geographical boundaries. These interactions not only enhanced her motivation but also nurtured a sense of belonging and camaraderie, vital components of holistic well-being among older adults. In conclusion, the participant's narrative exemplifies the transformative power of Vivo's online strength training program for older adults, showcasing its capacity to enhance muscle strength, energy levels, and social connectedness. This perspective underscores the program's potential to promote active aging and improve overall quality of life among older adult populations.

### SESSION 2490 (SYMPOSIUM)

Abstract citation ID: igae098.1034

### FORTIFYING THE FUTURE OF GERONTOLOGY AND GERIATRICS IN HIGHER ED: PART I—HARD QUESTIONS

Chair: Rona Karasik
Co-Chair: Julie Masters
Discussant: Marilyn Gugliucci

Academic programs in gerontology and geriatrics are again facing an uncertain future. Despite the much touted increase in older adults in the US and subsequent demand for an age-savvy workforce, many gerontology and geriatrics programs are struggling to attract enrollments sufficient to survive, much less thrive. At the same time, unprecedented budget woes and declining public support throughout higher education are driving deep cuts across academic programs and disciplines. Yet, for students whose passion is to work in the field of aging as a clinician, researcher, or provider, having programming available is a necessity. This symposium examines the current state of educational programs in gerontology and geriatrics and raises essential questions to consider in order to fortify their future. First, Mark Staley and Julie Masters address the question "What is happening to gerontology programs?" and present an updated census of gerontology programs in the US, highlighting the financial health of academic institutions where programs have closed, paused, or folded into other disciplines. Second, Rona Karasik identifies research and practice trends in Gerontology & Geriatrics Education journal submissions that seek to answer the question "Why don't students choose to go into gerontology or geriatrics?" Third, Isaac Boateng, Mark Staley, and Jessica VanderWerf share current gerontology students' perspectives regarding "Why DO students choose gerontology?" Fourth, Julie Masters poses challenging questions that must be considered when devising strategies for a strong and sustainable future for gerontology education. Finally, discussant Marilyn Gugliucci will facilitate a conversation on the state of gerontology and geriatrics education.

Abstract citation ID: igae098.1035

### GERONTOLOGY AND GERIATRICS PROGRAMS: OBSERVATIONS FROM THE US DATABASE AT THE UNIVERSITY OF NEBRASKA

Mark Staley[1], and Julie Masters[2], *1. University of Nebraska Lincoln, Lincoln, Nebraska, United States*, *2. University of Nebraska, Lincoln, Nebraska, United States*

Since its inception in 2022, the United States Gerontology and Geriatrics programs database developed and housed on the University of Nebraska-Omaha (UNO) Department of Gerontology website serves as a repository of educational programs at the undergraduate, graduate, and medical fellowship levels. Established to provide an easily accessible tally of programming and a resource for students, faculty, and professionals, the database is also intended to promote gerontological and geriatric programs across the U.S. Over time, the database has expanded to include Research Institutes, Nurse Practitioner programs, Medical Fellowships, Social Work programs, and designations including Age Friendly Universities (AFU) and Program of Merit (POM). With growth, the database is reviewed for accuracy, additions, and deletions. A troubling trend, however, has been the pausing and/or elimination of once healthy programs. In addition to presenting an overview of these observations (e.g., number of programs, changes), this session explores reasons for closure and what lessons can be learned for those still in operation.

Abstract citation ID: igae098.1036

### A NEW LOOK AT AN OLD PROBLEM: AN EDITOR'S PERSPECTIVE ON BARRIERS TO GROWING AN AGE-SAVVY WORKFORCE

Rona Karasik, *Saint Cloud State University, Saint Cloud, Minnesota, United States*

The future sustainability of gerontology and geriatrics programs has been the subject of concern well before Gerontology & Geriatrics Education (G&GE) was established in 1980, and remains a persistent theme in the journal today. Dwindling financial support for gerontology programs has also been an issue since before the journal's inception (Wittels, 1981), as was the much anticipated and now present shift in age demographics and its accompanying need to educate a sufficient, age-savvy workforce. In other words, none of these problems are new. Despite innovative curricula and the burgeoning number of older adults, the question remains steadfast: "Why don't more students choose to go into gerontology or geriatrics?" Moreover, what can be done to attract more students as the need for aging professionals continues to increase and enrollments in gerontology and geriatrics programs decline? The current presentation explores trends in the work submitted to Gerontology & Geriatrics Education, the Academy for Gerontology in Higher Education (AGHE)'s official journal, that seek to: (a) identify reasons why students do not choose gerontology (e.g., ageism, prestige, pay differentials); (b) evaluate novel strategies to address this disinterest (e.g., intergenerational programs, Ageism First Aid); and (c) endeavor to secure the future for gerontology and geriatrics educational programs (e.g., outreach to general education, community partnerships) amid the context of sweeping budget cuts throughout higher education.

Abstract citation ID: igae098.1037

### DESPITE OUR BEST EFFORTS, THEY AREN'T BUYING WHAT WE'RE SELLING: RAISING HARD QUESTIONS ABOUT OUR FUTURE.

Julie Masters, *University of Nebraska, Lincoln, Nebraska, United States*

In the 1970s, funding from the Older Americans Act was made available to establish gerontology programs at universities and colleges in anticipation of a growing aging population. The baby boom that led to early education programs and the building of schools and training of teachers was the driver behind visionaries who knew these same people would one day benefit from gerontological insights. Fast forward to 2024: 12,000 people are turning sixty-five each day. The long-expected demographic shift is here. The workforce to support the aging population, however, has fallen short, with demand outpacing supply in the public and private sectors. Nonetheless, many of the educational programs designed to prepare a well-prepared and sufficient workforce are struggling amidst a backdrop of academic budget cuts, revenue shortfalls, and the perennial issue of inadequate student/practitioner interest (Gurwitz, 2023). Hard questions must be asked if gerontological and geriatric education is to have a strong and sustainable future. This session poses several of these questions, examining such issues as branding (e.g., Gerontology Vs. Aging Studies), stand-alone and integrated delivery of educational programs, alliances (e.g., academic, community, corporate), preparation and credentialing (e.g., degrees, micro-credentialing, internships, practicum, on-the-job training), and career value (e.g., job types, status, compensation). The session concludes with the question, if not gerontology and geriatrics, who will be the champion for older adults?

Abstract citation ID: igae098.1038

### FLIPPING THE SCRIPT: WHY DO STUDENTS CHOOSE GERONTOLOGY AND GERIATRICS?

Isaac Asirifi Boateng[1], Mark Staley[2], and Jess VanderWerf[3], *1. Saint Cloud State University, Saint Cloud, Minnesota, United States*, *2. University of Nebraska Lincoln, Lincoln, Nebraska, United States*, *3. University of South Florida, Tampa, Florida, United States*

Much of the literature on workforce shortages and low enrollments in gerontology and geriatrics education focuses on the barriers - essentially "why don't students choose to go into gerontology"? This presentation reframes the question to instead consider why gerontology and geriatrics students DO choose the field? Three student perspectives - an undergraduate pre-med certificate student, a master's student in Gerontology, and a doctoral student in aging studies offer insight into the factors drawing them to gerontology and geriatrics. In addition to sharing their personal journeys, they also share what they have learned from their gerontology classmates and peers at institutions across the United States.

### SESSION 2495 (PAPER)

### HEALTH AND SOCIAL SERVICES

Abstract citation ID: igae098.1039

### EXTANT RESEARCH USING THE NATIONAL SURVEY OF OLDER AMERICANS ACT PARTICIPANTS: A SCOPING REVIEW

Tochukwu Okolie, and Heather Menne, *Miami University, Oxford, Ohio, United States*

Since 2003, the National Survey of Older Americans Act Participants (NSOAAP) has been collected annually for programs funded by Title III of the Older Americans Act (OAA). Covering six program modules including case management, congregate meals, home-delivered meals, homemaker, transportation, and family caregiver support, the NSOAAP data provides rich information for uncovering important issues pertaining to older adults and caregivers who utilize these services. Regardless of its promising content for advancing aging studies in the United States, there is a dearth of scholarly literature that has utilized this data source. Hence, this scoping review sought to map out extant peer-reviewed empirical articles that used data from the NSOAAP. Eight databases were searched with no time restriction, resulting in 42 citations. Two duplicate citations were resolved, leaving 40 articles for the initial title and abstract review. Additional three citations were identified through online searches and reference list review. After the title and abstract review, 33 articles were excluded because they did not meet the inclusion criteria. This study and its findings are based on 10 peer-reviewed empirical studies. Findings from the review showed that while there is a dearth in overall empirical studies using the NSOAAP, case management, homemaker, and transportation were the least studied. Caregiver services and congregate meal services were analyzed in 4 studies and home-delivered meals in 3 studies. This scoping review highlights the need for more research to understand the impact of the OAA service as measured through the NSOAAP across time.

Abstract citation ID: igae098.1040

### HOW WELL DO INSTITUTIONALIZED ASSUMPTIONS ABOUT UNPAID CAREGIVERS MATCH THE EXPERIENCES OF HOME CARE CLIENTS?

Laura Funk[1], Kaitlyn Kuryk[1], Lauren Spring[2], and Janice Keefe[2], *1. University of Manitoba, Winnipeg, Manitoba, Canada*, *2. Mount Saint Vincent University, Halifax, Nova Scotia, Canada*

Despite efforts to acknowledge diversity among unpaid caregivers, research, advocacy, practice and policy tends to be based in, and to reproduce, normative expectations and assumptions about who provides unpaid care and what this support looks like. The objective of this analysis is to generate unique insights into unpaid caregiving by exploring the situations of older adults receiving publicly funded home care and their experiences of accessing unpaid support. The analysis draws on case study interviews with twelve home care clients, their unpaid caregiver, home care aide and case coordinator, conducted at three points in time, before and after the Covid-19 pandemic (129 interviews). Analysis indicated only one participant had access to a caregiver that aligned with normative conceptualizations. Rather, situations arose in which: a) family members grappled with physical or mental health challenges limiting their participation in care (sometimes meaning the client is themselves a caregiver, or the caregiver is also receiving home care services); b) caregivers facing burnout sought to delimit their participation in care; c) family members' participation was limited by older adults' reluctance to accept their help; d) caregivers were largely unavailable, unreliable, or peripheral; or e) client's unpaid support networks were diffuse without a clearly identifiable 'caregiver.' Findings are used to nuance and problematize normative assumptions about caregivers. Discussion highlights the need for research, policy and programs related to unpaid caregiving to better reflect the lived realities of unpaid support for older adults and often overlooked sources of diversity in caregiver circumstances and roles.

Abstract citation ID: igae098.1041

### THROUGH THE EYES OF CARE MANAGERS: UNDERSTANDING THE VALUE OF MEMBERSHIP TO THE AGING LIFE CARE ASSOCIATION

Carlisle Shealy[1], Pamela Teaster[2], Jullie Gray[3], and Laura Sands[4], *1. Virginia Tech Center for Gerontology, Blacksburg, Virginia, United States*, *2. Virginia Tech., BLACKSBURG, Virginia, United States*, *3. University of Washington, Seattle, Washington, United States*, *4. Virginia Tech, Blacksburg, Virginia, United States*

Care managers, also known as Aging Life Care Professionals (ALCPs), have specialized knowledge pertaining to aging and disability. Through care coordination, they serve a critical role for families, caregivers, and older adults themselves. The Aging Life Care Association (ALCA) is a nonprofit member association aimed at promoting the work of ALCPs by offering continuing education, professional development, and ethical standards. To fill a gap in understanding the organization, we explored the usefulness and benefits of ALCA membership. We conducted 35 individual virtual interviews with members, exploring satisfaction with membership, organizational support to members, types of assistance members received, and engagement activities. Chapter presidents were recruited first, and afterwards, we used snowball sampling to recruit diverse members. Interviews were transcribed and thematically coded. Care managers joined ALCA for numerous reasons including networking, connections, and camaraderie. Relationships with other members contributed to why members remained in the organization. Care managers sought an association that gave their work professional credibility and standards. Members valued referrals, resources for growing a business, online educational resources online, and conferences. Participants suggested opportunities for greater inclusion of new members, especially those from diverse racial and ethnic and racial backgrounds as well as how to best apply ALCA's Code of Ethics and Standards of Practice. Others wanted education tailored for different career stages. Although opportunities exist to address challenges, membership in ALCA can improve access to information and services needed to coordinate improved care for older adults while also enhancing quality of care.

Abstract citation ID: igae098.1042

### EMERGENCY DEPARTMENT AND HOSPITAL USE AMONG COGNITIVELY VULNERABLE MEDICARE ADVANTAGE ENROLLEES

Richard Fortinsky[1], Xiaomin Lu[2], James Grady[1], Julie Robison[1], David Steffens[1], and George Kuchel[1], *1. University of Connecticut, Farmington, Connecticut, United States*, *2. University of Connecticut, Storrs, Connecticut, United States*

Medicare Advantage (MA) health insurance plans now cover more individuals than original Medicare. Very little is known about patterns of emergency department (ED) visits and hospitalizations among MA enrollees, especially those living with cognitive vulnerability due to dementia, depression, and/or recent delirium. Therefore, we explored factors associated with ED visits and hospitalizations among older adults enrolled in an MA plan and living at home with cognitive vulnerability. Our study cohort includes 406 adults age >65 who completed a 12-month clinical trial; baseline data collected by interview were linked with ED and hospital use data from medical claims for 12 months following study baseline. Predictors, all from study baseline, included age group, sex, race, ethnicity, educational level, financial strain level, number of medical comorbidities, and cognitive vulnerability type: dementia only; depression only; dementia and depression; or delirium with dementia and/or depression. Cohort characteristics: 58.4% female; mean (SD) age=76.1 (7.0); 92.1% White individuals; 4.7% Black individuals; 39.4% had >1 ED visit and 26.4% had >1 hospitalization. Multivariate logistic regression results revealed that the likelihood of experiencing an ED visit was positively associated with number of medical comorbidities at baseline (AOR=1.18, p=0.001), and participants with depression only were less likely than those with recent delirium at baseline to experience an ED visit (AOR=0.33, p=0.02). These same predictors were associated with hospitalization likelihood (AOR=1.14, p=0.01; and AOR=0.27, p=0.01, respectively). We conclude that medical comorbidity burden as well as delirium history are important risk factors for subsequent ED visits and hospitalizations in this MA cohort.

Abstract citation ID: igae098.1043

### OLDER ADULTS EXPERIENCING UNSHELTERED HOMELESSNESS IN LARGE URBAN CITY LACK BASIC RESOURCES

Alexis Coulourides Kogan, *University of Southern California, Los Angeles, California, United States*

Older adults constitute the fastest-growing segment of the homeless population in the US. Previous research has found older homeless adults to have more chronic conditions, premature mortality, greater odds of physical disability, and experience accelerated aging compared to housed populations. To inform programs to better serve this population, improved understanding of how they meet their basic needs is warranted. The purpose of this study was to explore the experience of unsheltered homeless older adults with meeting their basic needs for food, drinking water, toilet, hygiene, and healthcare in Los Angeles County. From 2021-2023 (24-months), we conducted a cross-sectional survey using the HOUSED BEDS assessment with people experiencing unsheltered homelessness and initiating care with Street Medicine (N=637). Quantitative data were analyzed to compare adults (18-49 yrs) and older adults (50+ years). Findings show that 40.3% of those sampled were older adults. Most older adults experiencing unsheltered homelessness identified as male (70%), Hispanic/Latino (46.6%), or Black/African American (25.6%). The majority of older adults had health insurance (79%) yet few (22%) had a primary care provider. Older adults lacked 24-hr bathroom access (22.3%), were forced to practice open defecation (82%), and relied on purchased food (50%) and drinking water (40.6%). Maslow's hierarchy of needs explains that time and energy spent meeting basic needs of survival (including food, hydration, and sanitation) means time and energy not spent elsewhere, such as seeking healthcare. Results of this study suggest a need for scaling Street Medicine which provides basic needs and comprehensive, accessible primary care.

Abstract citation ID: igae098.1044

### PROFILES IN FORTITUDE: STATEWIDE AGE-FRIENDLY HEALTH SYSTEMS

Teri Kennedy, *University of Kansas Medical Center, Kansas City, Kansas, United States*

The University of Kansas is in the process of developing Age-Friendly Kansas Forum (Kansas 4M), a statewide Kansas Age-Friendly Health Systems Action Community that is a reciprocal partnership between academia and primary care sites, delivery systems, and community organizations serving older adults in rural and underserved communities across Kansas. The Age-Friendly Health Systems (AFHS) Framework, developed by The John A. Hartford Foundation and the Institute for Healthcare Improvement, addresses the needs and priorities of older adults through the lens of the 4Ms: 1) what matters, 2) medications, 3) mobility, and 4) mentation. The framework resulted from a distillation of the core elements common to evidence-based programs serving older adults and is being leveraged as a strategy to address the know-do gap. To date three other states, Michigan, New York, and Nebraska, have developed statewide AFHS Action Communities. This presentation will share what Kansas 4M has learned about the unique approach taken by each of these states in their efforts to launch a statewide AFHS Action Community, their unique and shared experiences, best practices, cautionary tales, and how this information is informing the development of Kansas' statewide AFHS Action Community.

### SESSION 2500 (PAPER)

### HOME AND COMMUNITY-BASED SERVICES

Abstract citation ID: igae098.1045

### THE CORRELATION BETWEEN INTRINSIC CAPACITY AND FALLS AMONG COMMUNITY-DWELLING OLDER ADULTS AND ITS APPLICATION

Chen Li[1], Xiaodong Chen[2], and Lingxiao He[1], *1. Center for Health Economics, Policy and Aging Research, Xiamen city, Fujian, China*, *2. Center for Aging and Health Research, School of Public Health, Xiamen University, Xiamen, China, Ximen, Fujian, China*

**Objective:** To explore the link between intrinsic capacity and fall risk among Xiamen's older adults and to assess the diagnostic value of intrinsic capacity composite scores for falls.

**Methods:** A survey covering intrinsic capacity and fall history was conducted on 612 Community-Dwelling older adults. Logistic regression and ROC curve analyses determined the intrinsic capacity composite score's cutoff for fall risk.

**Results:** Higher scores indicated lower fall risk (OR=0.45,95%CI: 0.30-0.67). The optimal cutoff value was identified as 0.367, with the maximum AUC of 0.689. Adding chronic diseases as a control slightly reduced the AUC to 0.682 but did not change the cutoff.

**Conclusion:** Intrinsic capacity is a protective factor against falls. A score of 0.367 effectively diagnostic fall risk, aiding in older adults` fall prevention strategies.

Abstract citation ID: igae098.1046

### HCBS CARE COORDINATION AND BACKUP CARE PLANS DURING THE COVID-19 PANDEMIC IN KANSAS

Tracey LaPierre[1], Carrie Wendel-Hummell[1], Jennifer Babitzke[1], Darcy Sullivan[1], and Danielle Olds[2], *1. University of Kansas, Lawrence, Kansas, United States*, *2. University of Missouri Kansas City, Kansas City, Missouri, United States*

Backup care plans are a requirement of Medicaid-funded Home and Community Based Services (HCBS), but research on the effectiveness of backup care plans is lacking. The COVID-19 pandemic posed a prolonged emergency situation in which HCBS recipients may have needed to utilize their backup care plans. Data about experiences with HCBS services during the pandemic in Kansas came from 100 surveys of HCBS service recipients and 70 in-depth interviews with service recipients, direct support workers, family caregivers, and providers. One-third of HCBS recipients reported not having a backup care plan. Of those that had a backup care plan, less than half agreed that their plan prepared them for the pandemic. Yet, this was a time of great need for back up care, as participants widely reported increased difficulty finding and retaining direct support workers, as well as periods when their workers or caregivers were unavailable due to quarantine and other pandemic factors. 39% of service recipients reported going without care for 2 weeks or more, largely due to not having a primary or backup worker. In interviews, study participants reported that the quality of backup care plans and care coordination declined with the introduction of managed care in Kansas, with care coordination undergoing additional strain during the pandemic. These findings point to a need to better engage HCBS stakeholders in the development of backup care plans, update them more frequently, and ensure they are appropriate for long-term emergencies.

Abstract citation ID: igae098.1047

### THE RELATIONSHIP BETWEEN STATE MEDICAID HCBS PROGRAMS AND FAMILY CAREGIVING

Johanna Thunell[1], Elise Parrish[2], and Katherine Miller[3], *1. University of Southern California, Los Angeles, California, United States*, *2. University of Pennsylvania, Philadelphia, Pennsylvania, United States*, *3. Johns Hopkins University, Baltimore, Maryland, United States*

A growing number of persons are opting to age in place in home- and community-based settings. As demand for care in these settings increases, the supply of caregivers – both family and paid care – is not keeping pace. Yet, programs providing financial payments, education, and training for family caregivers may increase the likelihood of successfully aging in place. As no national long-term care insurance system exists in the US, most home- and community-based services (HCBS) are paid out-of-pocket or through Medicaid HCBS waivers. Through waivers, some Medicaid beneficiaries can pay family caregivers for care received. However, payment availability and eligibility criteria vary within and across states. We leverage the geographic-restricted NHATS data (2011–2019) to identify a cohort of respondents with at least one functional limitation. Using multinomial logistic regression, we estimated the relationship between living in state in a year with a Medicaid waiver providing payments to family caregivers and the likelihood of receiving: paid and family care, paid care only, family care only, or no care. After adjusting for demographics and family structure, we found respondents living in a state allowing for payments to family caregivers were 2.2 percentage points more likely to receive care by a family member only and 1.3 percentage points less likely to receive formal care only. Unpacking the mechanisms by which policies can incentivize (or reduce) caregiving has implications for broader policy discussions to (1) ensure an adequate workforce to meet the demand for care and (2) mitigate adverse consequences of family caregiving.

Abstract citation ID: igae098.1048

### PREPARING NORTH CAROLINA'S HOME AND COMMUNITY-BASED SERVICES WORKFORCE: CURRENT CHALLENGES AND THE PATH FORWARD

Zavera Basrai[1], Caroline Yoon[2], Kezia Scales[3], Trish Farnham[4], Erin Carson[5], and Nathan Boucher[2], *1. Duke University, North Carolina, United States*, *2. Duke University, Durham, North Carolina, United States*, *3. PHI, New York, New York, United States*, *4. NC Coalition on Aging, Raleigh, North Carolina, United States*, *5. National Domestic Workers Alliance, Durham, North Carolina, United States*

Older adults and people living with disabilities receive home- and community-based services (HCBS) from approximately 113,000 often poorly paid and inadequately supported direct service workers in North Carolina (NC). The demand for these workers is increasing rapidly, with NC projected to add almost 23,000 new direct care jobs from 2020 to 2030. The Medicaid-funded Workforce Engagement with Care workers to Assist, Recognize, and Educate (WECARE) project aims to modernize NC's direct care training and credentialing (TC) to increase the pipeline of workers, build career pathways, elevate their skills and recognition, and improve job quality and retention using Collective Impact (CI).

CI utilizes the following essential elements in creating and sustaining diverse collaborations: a common agenda, shared measurement, mutually reinforcing activities, continuous communication, and "backbone" support (dedicated coordination team). Informed by extensive desk research, five listening sessions with HCBS workers, support recipients, and family caregivers, and 113 formal engagements with state and non-profit agency leaders and regulatory bodies, WECARE's state-wide landscape analysis revealed: lack of consistency and flexibility in TC requirements; limited portability of credentials; limited person-specific training; minimal funding incentives for professional development; and the need for an expanded core competency set, including planning, self-care, cultural humility and role delineation. To address these challenges, WECARE's recommendations include adopting a core competency set for DSWs established through creating a unified multi-level DSW certification framework, strengthening career ladders and lattices within HCBS to support retention, and creating an online portal to house training, credentials, experience and other information that supports worker mobility.

Abstract citation ID: igae098.1049

### THE UTILIZATION AND INFLUENCING FACTORS AMONG OLDER ADULTS IN HOME CARE SERVICES OF PROPERTY COMPANIES

Kehan Liu[1], Chunxiao Li[1], Zijing Chen[2], Xiali Yang[3], Haiyan He[1], Chongmei Huang[3], and Minhui Liu[4], *1. Central South University, Changsha, Hunan, China (People's Republic)*, *2. The First Hospital Of Jilin University, Changchun, Jilin, China (People's Republic)*, *3. Ningxia Medical University, Yinchuan, Ningxia, China (People's Republic)*, *4. Ningxia Medical University, Yinchuan, Ningxia, China*

Property companies offer home care services for older adults, potentially facilitating "aging in place." However, it's unclear if the services are being used. This study investigated the utilization of home care services provided by property companies among older adults and explored the influencing factors affecting their utilization. A convenient sampling method was used to recruit 266 older adults in residential areas managed by property companies offering home care. Data was collected using self-designed questionnaires, including demographic characteristics (e.g., age, sex, living arrangements), health characteristics (e.g., self-rated health), and the utilization of services (daily life care, medical care, psychoeducation, emergency rescue). Daily life care includes senior canteens, plumbing and electrical repairs, housekeeping, etc. Medical care includes accompanying treatment, health education, and check-ups. Psychoeducation involves counseling and recreational activities. Emergency rescue includes 24-hour hotline and home visits. Approximately 58.3% of older adults were aged between 60-69 years, 39.1% lived with children, and 46.6% rated their health as average. Among all the services utilized, the highest was participating in recreational activities (59.0%), utilizing plumbing and electrical repairs (56.4%) and 24-hour hotline service (52.6%). Logistic regression models revealed that older adults aged over 80 (OR, 95%CI=0.30, 0.12-0.79) and those with poor self-rated health (OR, 95%CI=0.22, 0.10-0.50) were less likely to participate in recreational activities. Living with children (OR, 95%CI=0.16, 0.04-0.61) negatively correlated with the use of 24-hour hotline service. The low utilization rates suggested a demand for property companies to design targeted services to enhance home care service utilization among older adults.

Abstract citation ID: igae098.1050

### HOME HEALTH AIDE AND SOCIAL WORK VISITS IN MEDICARE ADVANTAGE AND TRADITIONAL MEDICARE BY RURALITY

Tracy Mroz, Lisa Garberson, C. Holly Andrilla, and Davis Patterson, *University of Washington, Seattle, Washington, United States*

Medicare Advantage (MA) enrollment has grown rapidly over the last decade. Prior research suggests MA beneficiaries utilize home health less often than Traditional Medicare (TM) beneficiaries, but research comparing specific services within home health has been limited to individual plans or providers and has not examined rural-urban differences. Home health aide and social work services may be especially important for successful community discharge. Using 2018 100% Medicare files, we conducted hierarchical logistic regression analyses to examine receipt of home health aide and social work visits by MA enrollment and rural-urban status (categorized by Rural Urban Commuting Areas Codes into urban, large rural, small rural, and isolated small rural communities), controlling for beneficiary and community characteristics. Of the 3,143,742 beneficiaries included in analysis, 23% were enrolled in MA versus 77% in TM, and 83% lived in urban communities versus 17% in rural communities. MA versus TM beneficiaries had significantly lower odds of receiving aide and social work visits (OR(95%CI): 0.87(0.82,0.92) and 0.91(0.85,0.98), respectively). Beneficiaries in rural versus urban communities had significantly lower odds of receiving social work visits, with the lowest odds in small rural and isolated small rural communities. However, odds of receiving aide visits were significantly higher in rural versus urban communities and increased as rurality increased. MA beneficiaries in small rural and isolated small rural communities are at particular risk for poor access to social work services, but decreased access to home health aide services for MA beneficiaries may be attenuated for those living in rural communities.

### SESSION 2505 (SYMPOSIUM)

Abstract citation ID: igae098.1051

### NOVEL APPROACHES TO OBESITY TREATMENT IN OLDER ADULTS

Chair: Barbara Nicklas
Co-Chair: Denise Houston
Discussant: John Batsis

The prevalence of obesity (BMI ≥30 kg/m2) among older adults (65+ years) in the U.S. has almost doubled over the last three decades, with approximately 40% of older adults living with obesity. Lifestyle modifications of moderate caloric restriction have been shown to improve the metabolic and functional health in older adults (e.g., lower blood pressure, fasting glucose, and interleukin-6, and improvements in cardiorespiratory fitness and gait speed). While lifestyle modifications of caloric restriction typically only produce modest weight loss – in the range of 5 to 10% - recent FDA approved medications to treat obesity produce significant weight loss – in the range of 15 to 20%. However, trials of anti-obesity medications have included few older adults. Furthermore, caloric restriction – by either lifestyle modification or anti-obesity medications – results in the loss of muscle and bone, potentially exacerbating sarcopenia and osteopenia. As a result, health care providers are often reluctant to recommend weight loss in older adults with obesity due to concerns regarding the overall safety and benefits of caloric restriction. Thus, different approaches to caloric restriction may be needed to minimize the potential risks of caloric restriction in older adults. This symposium will address the following in older adults: 1) whether time restricted eating (TRE) may be an alternative to caloric restriction (Houston and colleagues); 2) whether replacing lost weight externally reduces weight loss associated musculoskeletal tissue loss (Beavers and colleagues); and 3) whether semaglutide improves physical function, muscle mass, and biomarkers of aging (Cortes and colleagues). Obesity and Aging Interest Group Sponsored Symposium

Abstract citation ID: igae098.1052

### CALORIC RESTRICTION VERSUS TIME-RESTRICTED EATING: THE HEALTH, AGING, AND LATER-LIFE OUTCOMES PILOT

Denise Houston[1], Barbara Nicklas[2], Michael Miller[2], Jason Fanning[3], Fang-Chi Hsu[2], James DeLany[4], Jamy Ard[2], and Stephen Kritchevsky[5], *1. Wake Forest University School of Medicine, Winston Salem, North Carolina, United States*, *2. Wake Forest University School of Medicine, Winston-Salem, North Carolina, United States*, *3. Wake Forest University, Winston-Salem, North Carolina, United States*, *4. AdventHealth, Orlando, Florida, United States*, *5. Wake Forest University School of Medicine, Wake Forest University School of Medicine, North Carolina, United States*

In animal models, dietary interventions including caloric restriction (CR) and time-restricted eating (TRE) extend both lifespan and healthspan. However, in humans, the long-term benefits of these diets on lifespan and healthspan, and whether benefits outweigh harms, are unknown. The goal of the Health, Aging and Later-Life Outcomes Pilot (HALLO-P) is to inform the design of a definitive, full-scale trial to evaluate the long-term effects of CR and TRE in older adults. HALLO-P randomized 90 older (≥ 60 yrs) adults with obesity, or overweight and at least one comorbidity, to assess the feasibility and acceptability of three different 9-month interventions: 1) 20% CR delivered in-person; 2) 20% CR delivered remotely; and 3) 8-hour TRE with ad libitum caloric intake. Recruitment is complete and final intervention and close-out will be completed in August, 2024. To date, retention is 89% and intervention session attendance is 84%. Those assigned to CR are expected to weigh daily – to date, weights were obtained on 90% percent of days – and expected to maintain their weight within a zone of adherence – 65% of CR participants achieved this >75% of the time. TRE participants are expected to eat within a 7.5-8.5 hour window – TRE participants reported eating within an 8.5-hour window 77% of the time. Final study data will include: 1) the degree of sustained CR (using doubly-labeled water); 2) the sustainability of TRE (number of participants eating within the 8-hr window); and 3) changes in body weight and composition, and metabolic and functional health.

Abstract citation ID: igae098.1053

### THE INCORPORATING NUTRITION, VESTS, EDUCATION, AND STRENGTH TRAINING IN BONE HEALTH RANDOMIZED TRIAL RESULTS

Kristen Beavers[1], Daniel Beavers[2], Jason Fanning[2], Leon Lenchik[1], Barbara Nicklas[1], and Ashley Weaver[1], *1. Wake Forest University School of Medicine, Winston-Salem, North Carolina, United States*, *2. Wake Forest University, Winston-Salem, North Carolina, United States*

Intentional weight loss (WL) in older adults is associated with bone loss, increasing risk of fracture. As skeletal tissue is responsive to mechanical stress, replacing lost weight externally may be an innovative way to minimize WL-associated bone loss in this population. The main goal of the Incorporating Nutrition, Vests, Education, and Strength Training (INVEST) in Bone health trial (NCT04076618) is to examine the effect of 12-months of weighted vest use during intentional WL on several indicators of bone health, as compared to WL alone and WL plus traditional strength training exercise. Herein we report intervention process measure data, including weight change (%), self-reported weighted vest wear-time (hours/day), lost weight added to the weighted vest (%), and strength training adherence (% sessions attended; days/week). A total of 150 older adults were randomized, with 133 (89%) completing the trial. Average age was 66.4±4.6 years and the majority were white (69%) women (75%) with a body mass index of 33.6±3.3 kg/m2. 12-month WL by group was 9.4±6.7%, 9.0±4.7%, 11.3±6.0% for WL alone, WL+weighted vest, and WL+strength training, respectively. Self-reported weighted vest wear-time over 12-months was 7.1±1.5 hours/day, with 81.8±22.8% of lost weight replaced in the vest. Participants assigned to WL+strength training attended 75.2±13.0% of classes, averaging 2.2±0.4 sessions attended/week over 12-months. Intervention process measure data from the INVEST in Bone Health trial suggest significant and similar WL in each group, with good adherence to the weighted vest and exercise prescriptions. This presentation will also show treatment effects on select measures of musculoskeletal health.

Abstract citation ID: igae098.1054

### EFFECT OF SEMAGLUTIDE ON PHYSICAL FUNCTION, BODY COMPOSITION, AND BIOMARKERS OF AGING IN OLDER ADULTS

Tiffany Cortes[1], Ronna Robbins[2], Monica Serra[1], Sara Espinoza[3], Nicolas Musi[3], Hannah Barrus[1], Kevin Brown[1], and Annalis Campos[1], *1. The University of Texas Health San Antonio, San Antonio, Texas, United States*, *2. Texas Woman's University, Denton, Texas, United States*, *3. Cedars-Sinai Medical Center, Los Angeles, California, United States*

Insulin resistance and obesity are associated with sarcopenia and functional decline, but these important outcomes are not usually considered when choosing medications for diabetes or body weight management. Semaglutide is a GLP1 receptor agonist that typically results in average reductions in hemoglobin A1c of 1.5% and weight loss of 12%. The aims of this project are to (1) determine if semaglutide can improve physical function and muscle mass in older adults with prediabetes or diabetes and (2) elucidate whether these changes are associated with changes in inflammatory and age-related biomarkers in blood, adipose, and skeletal muscle. To accomplish these aims, we are conducting a 20-week open label, randomized trial with older adults with prediabetes or diabetes and who are overweight or obese. As of March 2024, we have enrolled 20 older adults, 50% women, with an average (standard deviation) age of 76.7 (4.7) years, BMI 32.9 (3.9) kg/m2, and hemoglobin A1c 6.0 (0.4) %. Participants are randomized 1:1 to receive either semaglutide 1mg plus lifestyle counseling or lifestyle counseling only for 20 weeks. Measures of physical function (6 minute walk, short physical performance battery, and grip strength), body composition (lean and fat mass via DXA), and aging biomarkers (IL6, TNFα, and CRP) are obtained at baseline and at end of study. We hypothesize that this study will provide insight on the role of GLP1 receptor agonists in overweight and obese adults with prediabetes or diabetes on important clinical outcomes beyond glycemic control. This study will be completed May 2024.

### SESSION 2510 (SYMPOSIUM)

Abstract citation ID: igae098.1055

### NOVEL MODALITIES FOR UNCOVERING BRAIN MECHANISMS UNDERLYING COGNITIVE AND MOTOR FUNCTIONS

Chair: Sarah Fraser
Co-Chair: Andrea Rosso
Discussant: Teresa Liu-Ambrose

It is now well-established that age-related declines in cognitive and motor functions co-occur and that shared neural resources are involved. However, details of these neural resources are still largely unknown, limiting development of effective prevention and intervention strategies. This symposium aims to answer: What can neuroimaging tell us about subjective cognitive decline and what changes in gait and mitochondrial function are associated with changes in the brain? How does dopamine alter these associations and help promote resilience in the brain? A series of recent studies using neuroimaging technologies aimed to answer these questions and provide new information on neural resources: (1) Salzman et al. will share their work using functional near infra-red spectroscopy (fNIRS) and dual-task tapping to explore cerebral oxygenation differences in older adults with and without subjective cognitive decline; (2) Rosso et al. explored if neural inefficiency or compensation measured by fNIRS during dual-task walking was influenced by dopamine binding using Positron Emission Tomography (PET); (3) Rosano et al. employed PET to explore associations between dopaminergic binding and gait speed changes when transitioning to an uneven surface; and finally (4) Tian et al. examined the relationship between skeletal muscle mitochondrial function via MR Spectroscopy and longitudinal brain structural changes via magnetic resonance imaging (MRI) and DTI data from the Baltimore Longitudinal Study on Aging. Each of these studies contributes to the evidence base on neuroimaging correlates of cognitive and motor function and helps us to understand what factors foster resilience and maintenance of these functions with age.

Abstract citation ID: igae098.1056

### SUBJECTIVE COGNITIVE DECLINE: FINE MOTOR DUAL-TASK OUTCOMES AND CHANGES IN CEREBRAL OXYGENATION

Talia Salzman[1], Kaya Kapilan[1], Diana Tobón[2], and Sarah Fraser[1], *1. University of Ottawa, Ottawa, Ontario, Canada*, *2. Universidad de Antioquia, Medellín, Antioquia, Colombia*

Subjective cognitive decline (SCD) describes individuals who report cognitive complaints but perform normally on neuropsychological assessments. While the trajectory between SCD and objective cognitive impairments remains unclear, cognitive complaints are an important diagnostic criterion for mild cognitive impairment and dementia. An alternate method to measure cognitive function is dual-tasking. Dual-tasks, composed of a cognitive and motor component, may be more sensitive at detecting early cognitive declines and when paired with neuroimaging, capture changes in brain function. This study recruited older adults (70.0 ± 6.6 years) with SCD (n=24) and controls (n=18) who completed a working memory and finger tapping dual-task. In addition to completing a neuropsychological battery, finger tapping accuracy, cognitive response accuracy, and prefrontal cortex activity using functional near-infrared spectroscopy (fNIRS) were measured. Repeated measures ANOVAs were used to compare each variable across cognitive status (SCD, controls) and condition (single, dual-task). Findings revealed no differences in global cognition between the SCD and control group (p=.588). Despite this, greater prefrontal cortex activation was observed in the SCD group compared to the controls during the dual-task condition (p=.016). Cognitive and motor performance were worse in both groups as measured by lower tapping accuracy (p=.041) and response accuracy (p <.001) during the dual- compared to single task condition. Dual-tasks may be more sensitive than neuropsychological tests at detecting subtle changes in cognitive function between SCD and controls. Additional studies are needed to determine whether dual-task brain activity can complement existing measures of SCD to better identify older adults at risk of cognitive declines.

Abstract citation ID: igae098.1057

### RELATION OF PREFRONTAL CORTEX FUNCTION WITH DUAL-TASK GAIT DEPENDS ON POSTDORSAL CAUDATE DOPAMINE

Andrea Rosso[1], Emma Baillargeon[1], Theodore Huppert[1], Nicholaas Bohnen[2], Lana Chahine[1], and Caterina Rosano[1], *1. University of Pittsburgh, Pittsburgh, Pennsylvania, United States*, *2. University of Michigan, Ann Arbor, Michigan, United States*

In older adults, the extent of PFC activation during challenged walking may be indicative of either compensation (if performance is maintained) or of neural inefficiencies (if performance declines). Dopamine neurotransmission in the basal ganglia is a determinant of motor automaticity and could therefore be a driver of compensatory vs inefficient PFC activity. We tested whether associations of change in

PFC activation measured by functional near-infrared spectroscopy with change in gait speed from single- to dual-task (alternate letter of the alphabet) walking over 15 meters differed by dopamine binding in a cohort of older adults (n=87, age=73±5 years, 67% women, gait speed=1.06±0.81 m/s). Dopamine neurotransmission was measured by dihydrotetrabenazine ([11C]DTBZ) positron emission tomography in the postcommissural caudate, which is highly connected to the dorsolateral PFC. Linear regression analyses assessed multiplicative interactions between PFC activation and [11C]DTBZ binding (above/below median) adjusted for age and gender with gait speed change as the dependent variable. Greater PFC activation was non-significantly related to greater decline in gait speed during dual-task. There was a significant interaction (p=0.05) between [11C]DTBZ in the postcommissural caudate and PFC function such that for those with higher dopamine binding, there was no association between PFC activation and gait speed change (beta=-0.002, p=0.8) but in those with lower dopamine, greater increase in PFC activation was associated with greater decline in gait speed from single- to dual-task walking (beta=-0.03, p=0.02). Higher PFC activation among those with lower dopamine binding may be indicative of neural inefficiencies that result in poor dual-task performance.

Abstract citation ID: igae098.1058

### GAIT SLOWING ON TRANSITION TO UNEVEN SURFACES: DOPAMINERGIC NEUROTRANSMISSION AND PERIPHERAL SYSTEM RISK FACTORS

Caterina Rosano[1], Andrea Rosso[1], Anne Newman[2], Stephen Cummings[3], Stephanie Studenski[1], Nicholaas Bohnen[4], and Lana Chahine[1], *1. University of Pittsburgh, Pittsburgh, Pennsylvania, United States*, *2. University of Pittsburgh, University of Pittsburgh, Pennsylvania, United States*, *3. University of California San Francisco, San Francisco, Mississippi, United States*, *4. University of Michigan, Ann Arbor, Michigan, United States*

Identifying mechanisms that compensate for slow gait speed in older adults is crucial. Dopaminergic neurotransmission curbs deleterious associations of cerebrovascular disease with gait, but whether it compensates for peripheral systemic risk factors (PSRF) for gait slowing has not been studied. In this cross-sectional study of community-dwelling older adults, we examined the relationship between nigrostriatal dopaminergic terminal integrity and gait speed in individuals with and without ≥1 PSRF for gait slowing: obesity, joint pain, or reduced muscle strength. The primary outcome was percent change in gait speed (%CGS) on transition from even to uneven surface. Participants underwent dopaminergic imaging with dihydrotetrabenazine [11C] DTBZ positron emission tomography. Among 199 individuals, (mean (SD) age 75.0 (4.6) years; 62.3% female; 91.0% White) 131 (65.8%) had ≥1 PSRF. Relationship between posterior putamen [11C]DTBZ binding and %CGS was modified by PSRF; in those with ≥1 PSRF (but not in those with no PSRF), posterior putamen [11C]DTBZ binding was associated with %CGS (β=0.238, p=0.01) independent of potential confounders. Striatal dopaminergic neurotransmission may offer resilience to effects of PSRF on gait slowing.

Abstract citation ID: igae098.1059

### SKELETAL MUSCLE MITOCHONDRIAL FUNCTION AND LONGITUDINAL CHANGES IN BRAIN STRUCTURE

Teresa Tian[1], Erin Greig[1], Christos Davatzikos[2], Bennett Landman[3], Susan Resnick[1], and Luigi Ferrucci[1], *1. National Institute on Aging, Baltimore, Maryland, United States*, *2. University of Pennsylvania, Philadelphia, Pennsylvania, United States*, *3. Vanderbilt University, Nashville, Tennessee, United States*

Mitochondrial dysfunction, a hallmark of aging, has been associated with future cognitive impairment or dementia, as well as PET and blood biomarkers of Alzheimer's disease and neurodegeneration. Whether mitochondrial dysfunction is associated with accelerated loss in brain volumes and microstructural integrity is unknown. We tested the longitudinal association between in vivo muscle mitochondrial oxidative capacity assessed via MR spectroscopy (post-exercise recovery rate of phosphocreatine, kPCr) and changes in brain MRI volumes and DTI microstructure over up to 12 years of follow-up in the Baltimore Longitudinal Study of Aging (2008-2020). In linear mixed effects models, adjusted for demographics, PCr deletion, and physical activity, higher kPCr was associated with less ventricular enlargement, less worsening in MRI-derived brain aging and Alzheimer's disease scores, and less regional atrophy, especially the primary sensorimotor cortex, temporal white and gray matter, thalamus, occipital areas, cingulate cortex, and cerebellum (n=649). Higher kPCr was also associated with less microstructural integrity loss in white matter tracts connecting identified gray matter areas (n=598). Specific white matter tracts, including splenium of the corpus callosum and superior longitudinal fasciculus showed consistent longitudinal associations in radial diffusivity, but not axial diffusivity, which may indicate demyelination. Higher in vivo mitochondrial function is associated with less worsening in MRI-derived brain aging and Alzheimer's disease scores, less brain atrophy in specific gray matter regions and less microstructural integrity loss in connecting white matter tracts. Future studies are warranted to investigate the role of CNS mitochondrial function in brain aging and neurodegeneration.

### SESSION 2515 (SYMPOSIUM)

Abstract citation ID: igae098.1060

### PRESIDENTIAL SYMPOSIUM: EMBRACING OPPORTUNITIES AND CONFRONTING CHALLENGES IN GLOBAL AGING RESEARCH

Chair: Jennifer Ailshire

This BSS Presidential Symposium highlights the promise and potential of global studies of older adults to bring new insights to our understanding of the aging process in an ever changing and increasingly diverse world. The Symposium brings together scholars from Gerontology, Sociology, and Economics to highlight the multidisciplinary perspectives on aging in a global context. Jinkook Lee will provide an overview of what we can learn from cross-national comparisons of aging populations and how we can use evolving data resources to address the challenges of aging populations around the world. Three empirical presentations follow from Yaun Zhang, Mateo Farina, and Nekehia Quashie, with different perspectives on how to use global aging surveys to advance our understanding of the social determinants of cognitive health across diverse country contexts. These presentations, which focus on education, early life conditions, and family relationships span countries from Latin America, Asia, Europe, and the United Kingdom as well as the United States. The session also highlights the value of using harmonized studies of aging across different countries to demonstrate how inequality differs across contexts and identify the drivers of disparities in cognitive health and dementia risk. By integrating diverse disciplinary perspectives and utilizing harmonized studies, this Symposium will demonstrate how researchers can use cross-national comparative research to examine questions that can inform efforts to enhance the well-being of older adults worldwide and address the multifaceted challenges posed by global aging.

Abstract citation ID: igae098.1061

### THE FUTURE OF AGING RESEARCH: OPPORTUNITIES AND CHALLENGES FROM GLOBAL AGING SURVEYS

Jinkook Lee, *University of Southern California, Los Angeles, California, United States*

Jinkook Lee is the Director of the Program on Global Aging, Health, and Policy at USC. Dr. Lee advocates for the development of robust data and research infrastructure to better understand aging populations. She has been involved in initiatives like the Harmonized Cognitive Assessment Protocol (HCAP) and is the architect of the Gateway to Global Aging Data, a platform that facilitates cross-national and longitudinal research on aging by providing access to harmonized data from various international studies of older adults. The Gateway serves as a valuable resource for researchers, policymakers, and academics interested in comparing aging-related outcomes across different countries and over time. In this BSS Presidential Symposium, Dr. Lee will describe the challenges and opportunities associated with research on aging around the globe. She will discuss the shift from a focus on aging as being free of disease to a focus on multidimensional aspects of healthy aging, utilizing a life-course framework and capturing multiple aspects of exposure to deepen our understanding of the aging process, and the challenges of environmental hazards for our rapidly aging world. Dr. Lee's presentation will provide useful background for thinking about how the next decade of research using studies from around the world will advance research on the aging process and aging populations around the world.

Abstract citation ID: igae098.1062

### EDUCATIONAL ATTAINMENT AND LATER-LIFE COGNITIVE FUNCTION IN HIGH- AND MIDDLE-INCOME COUNTRIES

Yuan Zhang[1], Xuexin Yu[2], Tsai-Chin Cho[2], Pengyuan (Kelvin) Zhang[3], Kenneth Langa[2], David Weir[2], Alden Gross[4], and Lindsay Kobayashi[2], *1. Columbia University, New York, New York, United States*, *2. University of Michigan, Ann Arbor, Michigan, United States*, *3. University of Michigan, Okemos, Michigan, United States*, *4. Johns Hopkins Bloomberg School of Public Health, Baltimore, Maryland, United States*

The rising number of dementia cases worldwide, especially in low- and middle-income countries (LMICs), underscores the need to identify social policies that promote cognitive health of older adults in LMICs and reduce global dementia burden. Many studies have found that educational attainment is associated with better cognitive function. However, these findings are mostly from Western high-income countries where the social and historical contexts of education differ from LMICs. In this research, we aim to evaluate the importance of educational attainment for later-life cognitive function in various social and geographic settings. Using data from the US Health and Retirement Study (HRS) and its sister studies in England, Mexico, China, and India and each study's respective Harmonized Cognitive Assessment Protocol (HCAP) (n = 14,978, 2016–2019), we compared later-life global cognitive function across countries, examined educational differences in cognitive function within each country, and evaluated the potential impact of improving educational attainment in LMICs on reducing differences in later-life cognitive function between LMICs and high-income countries. In Mexico, China, and India, the general cognitive function score was approximately one standard deviation of HRS-HCAP scores lower compared to the US and England, paralleling patterns of educational attainment across countries. Higher educational attainment was consistently associated with better cognitive function across countries. Differences in educational attainment explained ~50%–90% of observed differences in cognitive function across countries. Our results underscore the importance of education to promote cognitive health. Continual improvement of educational attainment in LMICs may yield significant payoffs in later-life cognitive health.

Abstract citation ID: igae098.1063

### SOCIOECONOMIC DETERMINANTS OF RACIAL INEQUALITIES IN COGNITIVE FUNCTIONING AMONG ADULTS 50+ IN THE US AND BRAZIL

Mateo Farina, *University of Texas at Austin, Austin, Texas, United States*

Racial inequalities in cognitive health are well-established. Black older adults have worse cognitive health than White older adults. However, evidence has been primarily found using data on the U.S. population, limiting scientific insight into how historically and socially constructed racial structures impact racial inequalities in cognitive health. Using nationally, representative studies from the United States (HRS) and Brazil (ELSI-Brazil), I examine racial inequalities in cognitive functioning (global and domain-specific) and their life-course socioeconomic determinants. I use a series of linear regression models to examine racial differences in cognitive functioning and to determine how accounting for life course socioeconomic conditions (retrospective childhood socioeconomic status, educational attainment, and adulthood income adjusted for household size) attenuate observed differences. Results show different patterns in racial inequalities in cognitive functioning across countries, with Black Americans having the lowest cognitive functioning in the United States and Asian Brazilians having the lowest cognitive functioning in Brazil. In Brazil and the United States, White older adults had the highest levels of cognitive functioning. However, the magnitude of racial inequalities differed by country, with preliminary findings showing greater differences in the United States than in Brazil. Lastly, regardless of country, childhood socioeconomic conditions and educational attainment greatly attenuated racial differences in cognitive functioning, reducing some racial inequalities to non-significance, indicating that the early life opportunities in establishing cognitive functioning may be substantially contributing to racial inequalities in both contexts. This research points to the need to consider context in understanding how and why racial inequalities in cognitive health arise.

Abstract citation ID: igae098.1064

**NUMBER OF LIVING SIBLINGS AND COGNITIVE DECLINE AMONG OLDER MEXICAN ADULTS**

Nekehia Quashie[1], Joseph Saenz[2], and Xing Zhang[2], *1. University of Rhode Island, University of Rhode Island, Rhode Island, United States*, *2. Arizona State University, Phoenix, Arizona, United States*

**Objectives:** A growing body of research examines the associations between sibship size and health in older adults. These studies largely focus on older adults' physical health or mortality in high-income countries, yet cognitive health is understudied. Sibship size may impact cognitive development in early childhood and shapes opportunities for social support throughout life that potentially influence later life cognitive health. Using a gendered life course framework, this study evaluates how the number of living siblings in later life relates with cognitive health among older Mexican adults.
**Methods:** Data comes from the 2012, 2015, 2018, and 2021 waves of the Mexican Health and Aging Study (n=14,872 adults age 50+). We use latent growth curve models to evaluate how one's number of living siblings relates with levels of latent general cognitive ability and nine-year cognitive decline and how these associations may differ by gender.
**Results:** Among men and women, those with no living siblings or only one living sibling had lower levels of cognitive ability when compared to those with 3 living siblings. However, very large sibship size (having 10+ living siblings) was also related with lower cognitive ability in men. Regardless of gender, the number of living siblings was not related with the rate of cognitive decline.
**Discussion:** This research broadens our understanding of the salience of siblings, an overlooked family resource, to older adults' health. Our findings underscore the importance of alternative social support for older adults given Mexico's changing demographics that challenge the reliance on family support in late-life.

### SESSION 2520 (SYMPOSIUM)

Abstract citation ID: igae098.1065

**RECENT INNOVATIONS IN INTERVENTIONS TO ADDRESS LONELINESS AND SOCIAL ISOLATION AMONG OLDER ADULTS**

Chair: Ashwin Kotwal
Discussant: Thomas Cudjoe

Recent years have witnessed unprecedented national and international calls to action to enhance social connection and reduce loneliness and social isolation among older adults. These calls include a 2020 National Academy of Sciences, Engineering, and Medicine report, a 2023 advisory by the Office of the Surgeon General, legislation introduced by U.S. Senators Murphy and Representative Flood, and a Commission on Social Connection by the World Health Organization (WHO). However, significant knowledge gaps remain on interventions to address these needs among older adults, particularly for diverse populations, across settings (i.e. health systems, different communities) and locations (nationally and internationally). In this session we will present four interventions that can reduce loneliness and social isolation among older adults. Studies will provide detail on recent innovations in program delivery., with particular attention to adaptations related to the COVID-19 pandemic that have been sustained. The implementation, sustainability, and scaling of interventions will be discussed, including health system-community referral networks, considering recent Medicaid funding opportunities, and new populations served. In addition, the session will include a discussion of differences in approach to interventions in different settings across the United States as well as globally. The session will foster dialogue on ongoing challenges and future directions in intervention development and research. Loneliness and Social Isolation Interest Group Sponsored Symposium

Abstract citation ID: igae098.1066

**RECENT INNOVATIONS, CHALLENGES, AND OPPORTUNITIES FOR PEER PROGRAMS TO ADDRESS LONELINESS IN OLDER ADULTS**

Ashwin Kotwal[1], Katrina Hough[2], Diane Meier[3], Soe Han Tha[2], Nandini Singh[2], Shannon Fuller[4], Janet Myers[2], and Carla Perissinotto[2], *1. University of California San Francisco Medical Center, San Francisco, California, United States*, *2. University of California San Francisco, San Francisco, California, United States*, *3. Icahn School of Medicine at Mount Sinai, New York, New York, United States*, *4. Johns Hopkins University, Baltimore, Maryland, United States*

Peer interventions which pair individuals of similar age or life experience are rapidly expanding to address the growing public health challenge of loneliness and social isolation among diverse older adults. We examined COVID-19 pandemic-era innovations, ongoing challenges, and future opportunities for growth across six peer programs across California. We conducted 65 qualitative interviews (August 2023 - March 2024) with current and former older adult clients (n=24), peer specialists (n=12), program leaders (n=12), and policy and thought-leader stakeholders (n=18). We identified two major innovations. First, programs developed flexible, multi-modal support across in-person, virtual, phone, and group activities which extended reach to older adults experiencing functional limitations, technological barriers, or unique social needs. Second, programs elevated peer roles on behavioral health teams and developed new partnerships with city and community-based programs. Primary challenges include uncertainty on how to balance hybrid programming, the reduced availability of volunteers, and reduced state funding since the pandemic. Stakeholders identified recent Medicaid reimbursement for peer services as promising for financial sustainability, and the potential for peers to address loneliness among several high-risk groups (unhoused, subsidized housing, homebound, dementia, caregivers, immigrants, and LGBTQ). However, peers need clear training, delineation of roles from other team members (social work, community health workers), and efforts to prevent "peer drift" or deviating from core elements of the program with new responsibilities. In conclusion, innovations in peer programs point to growing potential to address loneliness and social isolation among high-risk older adults, but programs require support in navigating financial, training, and infrastructure needs.

Abstract citation ID: igae098.1067

### A STEPPED-CARE SOCIAL AND MENTAL HEALTH ENGAGEMENT INTERVENTION FOR OLDER ADULT PROTECTIVE SERVICES CLIENTS

Jason Burnett[1], Sophia Wasik[2], Melba Hernandez-Tejada[3], and Ron Acierno[3], *1. The University of Texas Houston, Houston, Texas, United States*, *2. The University of Texas Houston, Houtson, Texas, United States*, *3. The University of Texas Health Science Center, Houston, Texas, United States*

Adult Protective Services (APS) clients are commonly characterized as socially isolated and depressed, and recently, lonely. The only evidence-based interventions for this population target depression and none have focused on social isolation and loneliness (SIL). This study is the first to provide a formal assessment of loneliness in a large sample of APS clients and the first to test an intervention to address SIL in this population. Our study uses a randomized waitlist control design to test whether an intergenerational stepped-care social and mental health engagement approach reduces SIL and increases the likelihood of depression treatment for N = 200 older adult APS clients across Texas. We paired one pre-professional health student or criminal justice student (students) with each client to engage in 8 weekly friendly phone calls. The primary intervention targets are SIL and the secondary targets are depression treatment and reduction. At the end of each call students complete a brief mental health screen to assess for higher-level mental health treatment needs. If mental health needs were detected, participants were offered to be contacted by the UTHealth Trauma and Resilience Center. Prior to participant engagement, students received training on empathy-based communication, abuse, neglect, and exploitation and addressing objective or subjective indications of suicidal ideation. To date, we have enrolled 65 participants. In the intervention group, among those needing higher level mental health treatment, 73% agreed to being contacted. In this session we discuss preliminary findings on SIL and depression, and review intervention successes, challenges, solutions, and student perspectives.

Abstract citation ID: igae098.1068

### 'HELPING ALLEVIATE LONELINESS IN HONG KONG OLDER ADULTS' (HEAL-HOA): A DUAL RANDOMIZED CONTROLLED TRIAL

Da Jiang[1], Jojo Kwok[2], Namkee Choi[3], Dannii Y. Yeung[4], Rainbow Tin Hung Ho[2], Lisa Warner[5], and Kee-Lee Chou[1], *1. Education University of Hong Kong, Hong Kong, China (People's Republic)*, *2. The University of Hong Kong, Hong Kong, China (People's Republic)*, *3. The University of Texas Austin, Austin, Texas, United States*, *4. City University of Hong Kong, Hong Kong, China (People's Republic)*, *5. MSB Medical School Berlin, Berlin, Germany*

Despite the high prevalence and negative impact of loneliness, research on the effectiveness of behavioral interventions for older adults who are experiencing loneliness has been lacking. The 'Helping Alleviate Loneliness in Hong Kong Older Adults' (HEAL-HOA), a dual randomized controlled trial (RCT), tested the effects of telephone-based psychosocial interventions delivered by older-adult volunteers (N=375) for lonely older adults (N=1,151) during the COVID-19 pandemic. The 375 volunteers age 50-70 were randomized into a volunteering condition versus an active control. Those in the volunteering condition received six 2-hour training on behavioral activation (Tele-BA), mindfulness (Tele-MF), or befriending (Tele-BF). Those in the active control condition received psychoeducation on loneliness. Trained volunteers then delivered Tele-BA, Tele-MF, or Tele-BF (active control) as lay counsellors for low-income, lonely, digitally excluded older adults (Mage=76.6, SDage=7.8) who lived alone and were randomized to one of three conditions. Outcomes were assessed at baseline and 6 months post-intervention among volunteer lay counsellors, and at baseline and 4 weeks, 3 months, and 6 months post-intervention among intervention recipients. For volunteer-counsellors, positive effects were observed in loneliness, social network engagement, stress and depressive symptoms as compared to those in the control condition. For intervention recipients, compared to Tele-BF, Tele-BA demonstrated positive effects on loneliness, psychological well-being, perceived support, and sleep quality at 3-month and 6-month follow-ups. Compared to Tele-BF, Tele-MF was effective in reducing loneliness at the 3-month follow-up. This innovative dual RCT demonstrated feasibility of delivering large-scale telephone-based behavioral interventions for older adults by older-adult volunteers and the intervention effectiveness.

Abstract citation ID: igae098.1069

### HEALTH SYSTEM ADOPTION OF COMMUNITY-BASED EXERCISE FOR OLDER ADULTS TO ADDRESS LONELINESS AND SOCIAL ISOLATION

Allison Mays[1], Alexander Lawton[1], Melissa Mandujano[1], Leilani Martinez[2], Nathalie Guevara[1], Jonathan Vickburg[1], and Sonja Rosen[1], *1. Cedars-Sinai Medical Center, Los Angeles, California, United States*, *2. Cedars-Sinai Medical Center, Los Angeles, Colorado, United States*

Transitioning the delivery of evidence-based programs (EBPs) for older adults from research to community-based settings potentially alters programs' reach. We compared the demographics of our research participants to our referred community participants after the program's adoption by Cedars-Sinai, Community Health Improvement (CHI). Research participants enrolled in Falls Prevention EBPs reported decreased levels of loneliness and social isolation and were referred by physicians, social workers, and pharmacist using an order in the electronic medical record (EMR). Clinicians referred 1355 in-person participants (July 2017-March 2020) and 283 virtual (April 2020- May 2021). Enrolled research participants (in-person (n=382) /virtual (n=214)) had an average age of 76.6 / 76.5 years; were 83.4% / 82.5 % female; and non-Hispanic white 42 % / 70.4%; and non-Hispanic Black 43.5% / 8.2%. CHI retained the relationship with the non-profit partner who ran the study's EBPs and the EMR order remained active and received 391 referrals (July 2021 – March 2024). Referred patients had an average age of 79.5 years, 79.3% female, 59.5% non-Hispanic white, 21.5% Black, 8.2% Hispanic, 5.9% Asian, 3.1% Other; 68.5% had Medicare and 31.7% Medicare Advantage. Referral orders were associated with diagnoses for preventative visits (18.7%), falls (15.6%), and cognitive changes (13.6%). Compared to research participants, community referrals included those with cognitive impairment, were older, more male, and more racially diverse than virtual study participants. Community implementation of EBPs may reach a more diverse population than research participants and successful transition of programs can be facilitated through preserving prior community partnerships and referral pathways.

### SESSION 2525 (SYMPOSIUM)

Abstract citation ID: igae098.1070

### SENSORY LOSS IN AGING: SPOTLIGHTING JUNIOR INVESTIGATORS AND NEW DIRECTIONS IN THE SENSORY HEALTH INTEREST GROUP

Chair: Jennifer Deal
Co-Chair: Jeannette Mahoney
Discussant: Joshua Ehrlich

Our senses (hearing, vision, touch, taste, smell) are the means by which our brains perceive our environment, allowing us to engage and connect with our world. Recommendations for maintaining health and function with age include maintaining strong social connections and participating in intellectually stimulating mental and physical activities. However, our senses are what allow us to engage in these activities. Sensory loss is common in older adults, with over 2/3 of adults over age 55 experiencing loss in multiple senses. The goal of the Sensory Health Special Interest Group (SIG) is to foster collaboration and dissemination of research and clinical/educational resources for professionals interested in sensory health and the effects of sensory impairments on the overall health and well-being of older adults. This session will meet this goal by highlighting work that demonstrates the importance of sensory health with age conducted by outstanding Sensory Health SIG investigators who are early in their career path or new to the interest group leadership. As part of this symposium, we will discuss the impact of age-related hearing loss on functional brain connectivity, physical activity and disability. We will also discuss how olfaction is related to coronary heart disease and conclude with a discussion on multisensory integration and mobility. Sensory Health Interest Group Sponsored Symposium

Abstract citation ID: igae098.1071

### MULTISENSORY INTEGRATION IN AGING: LINKS TO MOBILITY

Jeannette Mahoney, *Stony Brook Medicine, Stony Brook, New York, United States*

Multisensory integration, a rapidly growing field of neuroscience, investigates the simultaneous processing of information from multiple sensory systems. Researchers argue that our brains are designed to simultaneously process information from multiple sensory inputs to produce the most appropriate response to environmental cues. Studies in primates and young adults reveal that flexible multisensory integration is regulated by PFC areas, including dorsomedial and ventrolateral subregions. In fact, considerable strides have been made in defining the underlying properties, neural substrates, and timing of multisensory integration effects in healthy young adults; however, multisensory research in older adults has not been studied comprehensively. Further understanding of multisensory integration in aging could prove valuable, given that increased sensory deficits are associated with aging. Age-related visual and somatosensory impairments have been individually linked to slower gait, functional decline, increased risks of falls, and poorer quality of life. In a series of studies, we provide evidence for robust, but differential visual-somatosensory integration (VSI) effects in healthy aging and report its significant link with clinically-meaningful outcomes. Specifically, we show that older adults with intact levels of VSI demonstrate better balance, faster gait velocity and lower incidence of falls, compared to those with poor VSI. Additionally, we reveal that older adults with MCI and dementia demonstrate significantly reduced magnitude of VSI compared to those without cognitive impairments, which in turn was associated with worse unipedal stance and gait performance. Our current research objectives include elucidating whether VSI is a novel, non-invasive, non-cognitive predictor of Alzheimer's disease.

Abstract citation ID: igae098.1072

### ASSOCIATIONS BETWEEN HEARING LOSS AND FUNCTIONAL BRAIN CONNECTIVITY AMONG DEMENTIA-FREE OLDER ADULTS

Kening Jiang[1], Nicholas Reed[1], Anja Soldan[1], Frank Lin[1], Marilyn Albert[2], Jennifer Deal[1], and Corinne Pettigrew[2], *1. Johns Hopkins University, Baltimore, Maryland, United States*, *2. Johns Hopkins University School of Medicine, Baltimore, Maryland, United States*

Hearing loss is associated with cognitive decline and mood disorders, potentially through functional changes in the brain, as auditory deprivation affects higher-order sensory, attentional, and emotional processing. This study examined the cross-sectional association between hearing loss and functional connectivity, in 141 dementia-free participants from the BIOCARD Study (Median age=71 years). Resting state functional MRI data were processed to construct seven large-scale functional networks: frontoparietal control, default mode, dorsal attention, limbic, salience/ventral attention, somatomotor, and visual. Hearing loss was assessed by pure-tone audiometry. Better-ear four-frequency pure-tone average (PTA: average of hearing thresholds at 0.5, 1, 2, and 4 kHz) in decibels hearing level (dB HL) was analyzed categorically (hearing loss: >25 dB HL vs. normal hearing: ≤25 dB HL) and continuously after scaling to per 10 dB HL higher (worse). We used linear regression models, adjusting for age, sex, education, time between functional connectivity and hearing assessments (-3 to 4.5 years), APOE ε4, vascular risk factors, and diagnostic status. Hearing loss was associated with lower connectivity in the salience/ventral attention network (-0.02, 95% confidence interval [CI]: -0.05, -0.00).

After restricting the analyses to 102 cognitively unimpaired (CU) participants, the association was stronger (-0.04, 95% CI: -0.07, -0.01). We also found lower connectivity in the limbic network (-0.04, 95% CI: -0.07, -0.00) associated with per 10 dB HL worse hearing among CU participants. Our study adds to the limited evidence in hearing loss-functional connectivity association. Disrupted functional connectivity in salience/ventral attention and limbic structures might underlie cognitive impairment related to hearing loss.

Abstract citation ID: igae098.1073

### THE ASSOCIATION OF POOR OLFACTION WITH RISK OF CORONARY HEART DISEASE: THE ARIC NEUROCOGNITIVE STUDY

Keran Chamberlin[1], Chenxi Li[2], Anna Kucharska-Newton[3], Zhehui Luo[2], Mathew Reeves[2], Srishti Shrestha[4], David Couper[5], and Honglei Chen[6], *1. Michigan State University, United States*, *2. Michigan State University East Lansing, East Lansing, Michigan, United States*, *3. University of North Carolina at Chapel Hill, Chapel Hill, North Carolina, United States*, *4. University of Mississippi Medical Center, Jackson, Mississippi, United States*, *5. University of North Carolina, Chapel Hill, North Carolina, United States*, *6. Michigan State University, Michigan State University, Michigan, United States*

Poor olfaction may be associated with cardiovascular health in older adults, but empirical evidence is limited. We evaluated the association of olfactory status with risk of coronary heart disease (CHD) among 5142 participants free of CHD from the ARIC Neurocognitive Study (75.4±5.1 years, 62.9% female, and 23.9% Black participants). Olfaction was assessed in 2011-2013 using the 12-item Sniffin' Sticks odor identification test, and defined as good (score 11-12), moderate (9-10), and poor (0-8). At-risk participants were followed from baseline to the date of the first outcome of interest, death, last contact, or December 31, 2020, whichever occurred first. We used the discrete-time sub-distribution hazard model to estimate the cumulative incidence of CHD across olfaction groups and adjusted risk ratio (aRR), accounting for covariates, attrition, and the competing risk of death. After up to 9.6 years of follow-up, we identified 280 (5.4%) incident CHD events. Compared with good olfaction, poor olfaction was associated with a higher CHD risk during the first 6 years of follow-up, though the strength of the association gradually attenuated from aRR of 2.05 (95% confidence interval [CI]: 1.02, 4.48) at 2-year follow-up to 1.58 (1.12, 2.34) at 6-year. After 6 years of follow-up, the association continued to diminish until it reached the aRR of 1.07 (0.77, 1.43) at 9.6-year follow-up. Our study suggests that poor olfaction is associated with an elevated CHD risk in older adults, but the association may be lost with extended follow-up.

Abstract citation ID: igae098.1074

### HEARING LOSS AND PHYSICAL ACTIVITY AMONG OLDER ADULTS IN THE UNITED STATES

Sahar Assi[1], Erica Twardzik[2], Jennifer Deal[1], Kathleen Martin Ginis[3], Priya Palta[4], Jennifer Schrack[5], Nicholas Reed[1], and Pablo Martinez Amezcua[1], *1. Johns Hopkins University, Baltimore, Maryland, United States*, *2. University of Michigan, Ann Arbor, Maryland, United States*, *3. University of British Columbia Okanagan, Kelowna, British Columbia, Canada*, *4. University of North Carolina, Chapel Hill, North Carolina, United States*, *5. Johns Hopkins Bloomberg School of Public Health, Baltimore, Maryland, United States*

**Background:** Hearing loss is associated with adverse health outcomes among older adults, which may be partly explained by lower physical activity levels. Yet, the association between hearing loss and physical activity among older adults remains understudied.

**Methods:** Cross-sectional analysis of the National Health and Aging Trends Study (2021). The better-hearing ear pure-tone average (BPTA) at speech frequencies (0.5-4 kHz) was modeled continuously (per 10-dB) and categorically (no: ≤25 dB, mild: 26-40 dB, moderate or greater: >40 dB hearing loss). Activity measures were 1) wrist accelerometry-derived total activity counts, daily active minutes, and activity fragmentation, and 2) self-reported participation in vigorous activities and walking for exercise in the last month.

**Results:** Among 504 participants excluding hearing aid users (mean age = 79 years, 57% female, 9% Black), 338 (67%) had hearing loss. Using multivariable regression adjusted for sociodemographic and health covariates, we found that worse hearing (continuously and categorically) was associated with fewer counts and active minutes, more fragmented activity, and greater odds of not reporting recent vigorous activities. Among 472 participants with hearing loss, nonusers of hearing aids (n = 338) had more fragmented activity and greater odds of not reporting walking for exercise compared to users. Conclusions: Older adults with hearing loss are less physically active. This may mediate the association between hearing loss and other adverse outcomes. Recognition of this potential association is essential for providers to better support older adults in maintaining an active lifestyle. Future research is warranted to understand the impact of hearing interventions.

Abstract citation ID: igae098.1075

### HEARING LOSS, HEARING AIDS, AND CHANGE IN FUNCTIONAL STATUS: THE ATHEROSCLEROSIS RISK IN COMMUNITIES (ARIC) STUDY

Pablo Martinez Amezcua[1], Sahar Assi[1], Erica Twardzik[2], Nicholas Reed[1], Laura Scow[1], Anna Kucharska-Newton[3], B. Gwen Windham[4], and Priya Palta[5], *1. Johns Hopkins University, Baltimore, Maryland, United States*, *2. University of Michigan, Ann Arbor, Maryland, United States*, *3. University of North Carolina at Chapel Hill, Chapel Hill, North Carolina, United States*, *4. University of Mississippi Medical Center - The MIND Center, Jackson, Mississippi, United States*, *5. University of North Carolina, Chapel Hill, North Carolina, United States*

Hearing loss is common among older adults and is linked to poor physical function, but whether hearing aid use may slow physical function declines remains unknown. We investigated the association between hearing loss and functional status in older adults and modifying effects of hearing aid use. We used data from the Atherosclerosis Risk in Communities

(ARIC) cohort study to examine cross-sectional (2016-2017) and longitudinal relationships (2016-2022) after adjustment for sociodemographic and health-related characteristics. Hearing loss (none, mild, moderate/severe) was based on the better-hearing ear's pure tone average at speech frequencies. Hearing aid use was self-reported. Participants self-reported (any difficulty, regardless of level, vs. none) difficulties with activities of daily living (ADLs), instrumental activities of daily living (IADLs), and heavier tasks (lifting heavy objects, kneeling, going up stairs without stopping). Cross-sectionally, among 3,142 community-dwelling older adults (mean age=79, 58% female, 21% Black), moderate or greater hearing loss was associated with increased difficulties in ADLs (odds ratio [OR]=1.27, 95% CI 1.02, 1.58), IADLs (OR=1.34, 95% CI 1.05, 1.71), and heavier tasks (OR=1.29, 95% CI 1.04, 1.62) relative to no hearing loss. Longitudinally, over an average follow-up of 1.9 years, moderate or greater hearing loss was associated with higher odds of reporting any functional difficulty (OR=1.14, 95% CI 1.05, 1.24). Hearing aid use did not modify the associations. These findings emphasize the importance of hearing loss, its adverse effects on physical function in aging populations, and the need for comprehensive interventions beyond hearing aids for adults with hearing loss.

### SESSION 2530 (PAPER)

Abstract citation ID: igae098.1076

### TECHNOLOGY IN OLDER ADULTS

Use of technology to improve health of older adults

Abstract citation ID: igae098.1077

### OLDER ADULTS WITH SCHIZOPHRENIA AND INFLAMMATORY MARKERS OF AGING

Heather Leutwyler[1], M. K. Kirsten Chui[2], Kevin Schneider[2], Sara LaHue[3], David Furman[2], and John Newman[2], *1. University of California San Francisco, Pacifica, California, United States*, *2. Buck Institute for Research on Aging, Novato, California, United States*, *3. University of California San Francisco, San Francisco, California, United States*

Schizophrenia is associated with accelerated aging, evidenced by earlier onset of aging-related diseases such as hypertension and geriatric syndromes. Inflammaging is a state of dysfunctional systemic chronic inflammation that accelerates a negative feedback loop with other biological aging mechanisms and increases risk for aging-related chronic diseases. Inflammaging is driven in part by discrete cellular and molecular processes such as the accumulation of senescent cells and their resulting pro-inflammatory senescence-associated secretory phenotype (SASP). We report the results of a cross-sectional analysis from older adults with schizophrenia (OAS) to describe the relationships between health and inflammaging biomarkers. We analyzed plasma from 24 OAS (mean age 59.5). Neurocognition was assessed with the MATRICS Consensus Cognitive Battery and schizophrenia symptoms with the Positive and Negative Syndrome Scale. Physical health was assessed via the physical function subscore of SF-12. We obtained quantitative measurements of 112 cytokines and other factors using the Millipore Milliplex platform (Eve Technologies). We also derived from these data composite measurements of SASP via lists published by Diniz et al. (11 components), SenMayo (35 components) and the Translational Geroscience Network (36 components). Specific inflammatory cytokines (eotaxin, IL-1α, IL-1β, and IFNα) were associated with worse cognition and physical health, while no significant associations were observed with SASP composite scores. These results suggest that specific pathways of inflammaging, which may be driven by biological mechanisms other than cellular senescence, associate with important clinical outcomes in OAS. Larger studies could better characterize aging mechanisms that contribute to inflammaging in the accelerated aging state of OAS.

Abstract citation ID: igae098.1078

### AN EXPLORATION OF A DIGITAL FALL PREVENTION PROGRAM: "KEEP ON KEEP UP" FOR OLDER ADULTS IN A CHINESE CULTURAL CONTEXT

Yimin Tang, Sarah Smith, Sorrel Burden, and Emma Stanmore, *University of Manchester, Manchester, England, United Kingdom*

The use of digital health interventions can be effective in enabling and maintaining healthy behaviours to help prevent falls and improve physical function in older adults. However, scarce research has been conducted with specific cultural and minority groups living in a cross-cultural setting. The aim of this study was to explore Chinese older adults' and stakeholders' experience, needs and preferences related to using digital health interventions, in particular the Keep On Keep Up-Chinese (KOKU-C) falls prevention program (translated to Mandarin language). This research used think-aloud as a novel approach in interviews and was completed within a cross-cultural context to better improve user-experience, engagement and digital inclusion of Chinese older adults living in the UK. A sample of Chinese older adults 55 to 95 years of age (N=40) living in Manchester and stakeholder representatives including healthcare professionals, caregivers, community partners and researchers (N=12) have been interviewed. Results from thematic analysis found that while there are opportunities to better enhance the digital health interventions tailored to the Chinese cultural context, the KOKU-C program can still provide valuable information as well as balance and strength training to help support fall-related health outcomes and improve their quality of life. These important findings point to the need for more research taking cultural considerations into account when co-designing digital health applications. The potential benefits of this study provide a possible solution for better acceptability, engagement and interaction in digital health interventions with Chinese older populations.

Abstract citation ID: igae098.1079

### CO-CREATING A VIRTUAL REALITY PROGRAM WITH PEOPLE LIVING WITH DEMENTIA IN LONG-TERM CARE

Lillian Hung[1], Ben Mortenson[1], Angelica Lim[2], Jennifer Boger[3], Joey Wong[1], Christine Wallsworth[1], Jim Mann[1], and Lily Wong[1], *1. University of British Columbia, Vancouver, British Columbia, Canada*, *2. Simon Fraser University, Burnaby, British Columbia, Canada*, *3. University of Waterloo, Waterloo, Ontario, Canada*

People living with dementia in long-term care (LTC) are a diverse group with varied physical and cognitive abilities and backgrounds. Previous research has shown that virtual reality experiences can bring joy and foster the health and well-being of people living with dementia. A tailored approach based on cultural preferences, needs, abilities, disabilities, and contextual limitations is needed to foster practical implementation, uptake, and sustainability. This qualitative study harnessed the experiential knowledge of people with dementia, care partners and frontline staff to co-build a novel Virtual Reality Program in two Canadian LTC homes. We conducted focus groups and interviews with 10 residents living with dementia, 10 family care partners and 12 staff to explore their preferences on the video content and delivery methods. We also explored participants' experiences in the co-creation process. Our interdisciplinary team, including people with dementia and family partners, researchers, clinicians, and trainees, analyzed the data thematically. We identified four themes: 1) Significance of culturally relevant and diverse videos to address heterogeneous population, 2) Acknowledgement of residents' autonomy and choice in the co-creation process, 3) Feelings of satisfaction and motivation through contributing and learning, and 4) Appreciation of a respectful co-design environment. The results offer useful insights to inform future directions in co-designing safe and accessible virtual programs with people living with dementia in LTC research.

Abstract citation ID: igae098.1080

### ONLINE ASYNCHRONOUS INTERVENTION FOR FAMILY CAREGIVERS OF PERSONS WITH LEWY BODY DEMENTIA (LBD)

Oleg Zaslavsky[1], Jasmine Kaneshiro[1], Yan Su[2], Kimiko Domoto-Reilly[1], and Annie T. Chen[1], *1. University of Washington, Seattle, Washington, United States*, *2. University of Massachusetts Dartmouth, Dartmouth, Massachusetts, United States*

Family caregivers of individuals with Lewy Body Dementia (LBD) experience higher levels of stress and more severe depressive symptoms compared to caregivers of people with other dementias. This pilot study evaluated the efficacy of VOCALE LBD, an online asynchronous 8-week intervention aimed at improving psychological distress among LBD caregivers. We conducted a quasi-experimental study with 39 family caregivers. The program employs a web-based platform and includes text-based peer support, moderation of the discussion by a research team member, didactic training, and problem-solving skills development, all tailored to LBD caregiving situations. We assessed outcomes including caregiver depression, burden, stress, and support, at baseline, post-intervention (eight weeks), and one-month follow-up. We also evaluated problem solving, self-efficacy and mastery mechanistic variables. Participants' average age was 68 (SD=10), with 69% female, 95% identifying as White, and 79% holding undergraduate and graduate degrees. The eight-week intervention had 18% attrition rate. Effect sizes (Cohen's d) from baseline to post-intervention and from baseline to follow-up showed reductions in depression (-0.36 and -0.54, respectively), stress (-0.30 and -0.45), burden (-0.31 and -0.26), and increases in support (0.44 and 0.46). Significant improvements were observed in problem-solving skills, though no significant changes were noted in mastery and self-efficacy. The study confirms the acceptability and efficacy of the VOCALE LBD program to reduce psychological distress among family LBD caregivers and suggests activation of the targeted change mechanisms. The study also contributes to the field's understanding of online support mechanisms and promotes the exploration of innovative caregiver support strategies.

Abstract citation ID: igae098.1081

### LONGITUDINAL ASSOCIATION OF DIGITAL HEALTH TECHNOLOGY USE WITH DEMENTIA IN OLDER ADULTS

Wenting Peng[1], Yuqian Luo[2], and Minhui Liu[3], *1. University of Washington, Seattle, Washington, United States*, *2. Hong Kong Polytechnic University, Hong Kong, Hong Kong*, *3. Ningxia Medical University, Yinchuan, Ningxia, China*

Dementia is a global public concern and is associated with many adverse outcomes, such as disability. However, it remains unknown whether common modalities of digital health technology are associated with dementia. Using the data from 2015 to 2019 of the National Health and Aging Trends Study (NHATS), the study examined whether prior-year digital health technology use predicted dementia in a later year in community-dwelling older adults aged 65 years and older (N=4,002). Digital health technology use was ascertained with four items: filling prescriptions, contacting clinicians, handling insurance, and obtaining health information. Dementia was assessed using a modified NHATS definition, including a physician diagnosis, cognitive performance in three domains, and the AD8 Dementia Screening Interview. Lagged generalized estimation equation models were used to examine the longitudinal associations, adjusting for demographics and health-related factors. Seniors using any digital health technology in the prior year were at a lower risk of having possible or probable dementia in the later year (OR=0.61, 95% CI=0.51-0.74) than those not. Filling prescriptions, contacting clinicians, and obtaining health information statistically significantly predicted possible or probable dementia, with ORs of 0.61, 0.69, and 0.66, respectively. Additionally, any digital health technology use (OR=0.65, 95% CI=0.50-0.82), filling prescriptions (OR=0.60, 95% CI=0.43-0.85), and obtaining health information (OR=0.74, 95% CI=0.56-0.96) decreased the risks of having probable dementia. These findings suggest that digital health technology use is a protective factor of dementia. Intervention programs to delay dementia progression should incorporate strategies to help older adults use digital health technology in their daily lives.

### SESSION 2535 (SYMPOSIUM)

Abstract citation ID: igae098.1082

### THE 4 S'S OF GERIATRICS EDUCATION: STIGMA, SPEED, STEALTH, AND SOURCES

Chair: Alissa Cooney
Discussant: Edmund Duthie

This symposium covers a conceptual framework developed by the Geriatrics Education Interest Group. The framework includes the 4 Ss of geriatrics education: Stigma,

Speed, Stealth, and Sources. Geriatrics Educators have long needed the fortitude factor to face adversity in finding a place for geriatrics education in many curricula. Sharing the experience of geriatrics leaders who have demonstrated fortitude in their approach to geriatrics education fighting against stigma, getting creative with speed, seeking out stealthy opportunities, and leveraging sources, to improve geriatric education for all learners. The first S, Stigma, addresses the persistent ageist attitudes and behaviors found across healthcare by promoting geriatric education anchored in a "patient-centered care" approach addressing the specific, individual-based, concrete, patient care needs. The second S, Speed, explores micro learning in the context of geriatrics education to increase efficiency of learning and retention of knowledge. Multiple web-based platforms for geriatrics micro learning will be discussed including implementation of Geriatrics Fast Facts, a point-of-care micro learning resource. The third S, Stealth, shares how to reframe integration of geriatric principles specifically for non-geriatrics specialties. This segment will dive into case-based perspectives on stealthily integrating basic geriatric principles into learning environments not focused on geriatrics care. The fourth S, Sources, discusses how to leverage sources to enhance geriatric education. This section will explore the experience of geriatricians and inter professional geriatric specialists who have successfully leveraged their knowledge and resources and their institutions and local communities to benefit geriatric education. Geriatric Education Interest Group Sponsored Symposium

Abstract citation ID: igae098.1083

### STIGMA AND AGEISM IN THE GERIATRIC EDUCATION CONTEXT: USING PATIENT-CENTERED CARE APPROACHES

John Schumacher[1], and Laura Finn[2], *1. University of Maryland Baltimore County, Baltimore, Maryland, United States*, *2. Philadelphia College of Pharmacy/St. Joseph's University, Philadelphia, Pennsylvania, United States*

Healthcare professionals remain ill-prepared to meet the unique needs of the aging population. Yet, implementing effective geriatrics educational programming remains challenging across health care disciplines due to low participation rates and uptake. Persistent stigma, ageism, and stereotypes endemic to healthcare, and society at large, contribute to the devaluing of geriatrics content reducing the potential for effective and equitable care for older adults. Geriatric educators are challenged to address the ingrained stigma and ageism among both their learners and healthcare organizations. However, re-imagining geriatrics education to be anchored in a patient-centered care approach, addressing specific, concrete patient needs, has been shown to reduce ageist attitudes and behaviors in the healthcare workforce. Participating in intergenerational activities has also shown a positive impact on health care provider attitudes toward older patients. Furthermore, prioritizing preventive medicine and public health throughout the life course also effectively contributes to postponing the onset of disease and disability among patients. Implementing these basic educational approaches is a pathway to achieving the goal of improved outcomes for patients of all ages.

Abstract citation ID: igae098.1084

### SPEED IN GERIATRICS EDUCATION: IDENTIFYING AND IMPLEMENTING MICROLEARNING TO INCREASE EFFICIENCY AND RETENTION

Evan Henricks[1], and Pratima Gangupantula[2], *1. Medical College of Wisconsin, Milwaukee, Wisconsin, United States*, *2. The University of Texas Health Science Center San Antonio, San Antonio, Texas, United States*

This session aims to explore what microlearning is and how it can be utilized by geriatrics educators to reinforce and incorporate age-friendly health elements across the spectrum of learners. Microlearning is obtaining knowledge or skills via small units and is increasingly endorsed by health professions educators as a means of facilitating learning, training, and continuing education. The positive impact of microlearning on learner knowledge and confidence has been shown. Microlearning can be integrated with mobile learning (M-Learning) to create learning material that is quick, interactive, and easily accessible from a mobile device. This is in contrast to electronic learning (E-learning) which allows online access to traditional resources like textbooks, journals, and lectures, but is in-depth, cumbersome, and may lack open access. This session will explore examples of situations which utilize various microlearning modalities for geriatrics education, such as social media platforms, podcasts, mobile applications, and point of care websites. M-learning utilization will be shown by exploring usage data from a point of care website. This session will further explore advantages and disadvantages of microlearning platforms and methods of incorporating the platforms into more traditional learning methods. Broadening educator knowledge of microlearning tools will enable them to utilize a variety of teaching modalities and reinforce geriatrics curricula for learners. It also provides learners with resources to utilize independently when future knowledge gaps are identified.

Abstract citation ID: igae098.1085

### REFRAMING INTEGRATION OF GERIATRICS PRINCIPLES: A STEALTH APPROACH

Christina Cauble[1], Benjamin Rosenstein[2], and Alissa Cooney[3], *1. University of Minnesota Medical School, Minneapolis, Minnesota, United States*, *2. University of Minnesota, Department of Family Medicine and Community Health, Minneapolis, Minnesota, United States*, *3. UT Health San Antonio, San Antonio, Texas, United States*

Most healthcare professionals do not receive specific training in geriatrics despite caring for an overwhelmingly large proportion of older adults throughout their careers. While educational programs that feed into geriatrics as a specialty are sometimes more open to adapting curricula to incorporate geriatrics principles, utilizing a stealth geriatrics education approach can help to successfully integrate geriatric principles for a wider range of learning experiences. This session explores how geriatrics educators can integrate basic geriatrics principles into training and learning experiences in a variety of settings for learners who may not intend to specialize in geriatrics. Many health education programs don't teach robustly on geriatrics principles and struggle to fit additional dedicated time for geriatrics within their curriculum, even though the majority of healthcare workers will at some point provide care to older adults. This segment will examine successful examples of stealthy integration of geriatrics principles into health education for learners regardless of their expectation to further specialize in geriatrics. Educational examples of integrating stealth geriatrics principles include; 'geriatricizing' a family medicine case study, a dementia didactic experience for medical students, and an emergency department scenario geared towards integrating stealth geriatrics into bedside nursing, resident, and staff education. Attendees will be encouraged to reframe ways to continue incorporating stealth geriatrics into other educational settings.

Abstract citation ID: igae098.1086

**LEVERAGING SOURCES TO ENHANCE INTERPROFESSIONAL GERIATRIC EDUCATION**

Gina Tucker-Roghi[1], Alissa Cooney[2], Rajean Moone[3], and Christina Cauble[4], *1. Dominican University of California, San Rafael, California, United States*, *2. UT Health San Antonio, San Antonio, Texas, United States*, *3. University of Minnesota, St. Paul, Minnesota, United States*, *4. University of Minnesota Medical School, Minneapolis, Minnesota, United States*

Geriatrics is a highly interdisciplinary field, and methods to enhance geriatric education need to be equally interdisciplinary. A critical step is identifying, engaging, and mobilizing a wide array of stakeholders and educational resources to enhance geriatrics integration across various medical disciplines. This session will delve into methodologies used for discovering stakeholders in geriatrics within a local institution and the community at large, emphasizing the creation of collaborative networks that promote geriatrics principles in educational and clinical settings. Participants will explore the experiences of geriatricians and interprofessional geriatrics specialists at academic institutions leveraging their knowledge and resources at their institutions and local communities. This session aims to provide insights into effective strategies for recognizing and collaborating with contributors to geriatric care, even those who may not see themselves as specialists. We will explore how geriatric educational leaders can reduce stigma and utilize stealth and speed to maximize local resources and age-friendly initiatives.Through engaging with and mobilizing a diverse network of stakeholders, educators can foster a more robust integration of geriatric principles, ensuring that healthcare professionals across specialties are well-prepared to meet the unique needs of the aging population. The session will offer actionable steps for attendees to engage with institutional and community stakeholders, turning them into vital sources of geriatric expertise and fostering a more inclusive and comprehensive approach to geriatric education and practice.

### SESSION 2540 (SYMPOSIUM)

Abstract citation ID: igae098.1087

**THE GLOBAL PRIDE STUDY: TRANSFORMATIONS IN SEXUALITY, GENDER, AND AGE IN LATER LIFE**

Chair: Karen Fredriksen-Goldsen

Transformations in sexuality and gender are changing the context of global aging. The Global Pride Network and Study, involving over 50 scholars worldwide, aims to comprehensively address health and well-being in sexual and gender diverse populations, covering quality of life, physical and mental health, and economic and social well-being. This symposium will highlight the international development and implementation of the Global Pride Study across six world regions, sharing knowledge, experiences, and collaborations in studying sexuality, gender and age as they relate to well-being and health, as well as the impact of COVID-19 across diverse global communities. Fredriksen-Goldsen and colleagues will begin by presenting the project goals, international research collaborations to develop and distribute a pilot survey across global regions, and cross-cultural adaptations of research methods necessary for the implementation of multi-faceted and multi-level research strategies. Next, Oswald and colleagues will present a video of international collaborators as they evaluate the state of sexuality and gender research and policy across the globe. The gaps in the existing knowledge base and future research directions will be discussed. The final presentation, delivered by Hughes and colleagues, will present quantitative findings from the global survey of one country, Australia, investigating loneliness across the lifespan and its interaction with other life experiences. This groundbreaking symposium will contribute to advancing research addressing sexuality, gender, aging, longevity, and well-being globally, contributing to international dialogue on reframing these constructs and presenting next steps and best practices learned through this highly innovative and collaborative research process. Rainbow Research Group Interest Group Sponsored Symposium

Abstract citation ID: igae098.1088

**THE GLOBAL PRIDE STUDY: LESSONS LEARNED AND NEXT STEPS**

Karen Fredriksen-Goldsen[1], Christi Nelson[1], Austin Oswald[2], and Hyun-Jun Kim[1], *1. University of Washington, Seattle, Washington, United States*, *2. Dalhousie University, Nova Scotia, Canada*

As the older adult population becomes increasingly diverse and globalized, new challenges emerge in gerontological research and practice worldwide. This presentation examines sexuality, gender, and aging within a global context, exploring both fortitude and risks, as well as differing social and cultural interpretation of sexuality, gender, and age in relation to research and the development of culturally relevant practices. We will discuss the formation of the research network, involving more than 50 international collaborators. As part of the Global Pride project, we developed and tested a pilot survey, which was distributed by 18 partners and completed across six global regions (N= 3,885). Key processes and lessons learned include: 1. Establishing an international network of researchers to exchange knowledge and experiences in studying sexuality, gender, longevity, and health holistically; 2. Implementing a collaboratively developed survey across six global regions, taking into consideration differing cultural contexts and methodologies; 3. Employing culturally tailored dissemination strategies across diverse regions and settings. The insights gleaned from this research will deepen an awareness of the diversity of lives across the life course and contribute knowledge about the richness, challenges, and heterogeneity of ageing, as well as best practices for the implementation of global research.

Abstract citation ID: igae098.1089

### REAL LIVES ACROSS THE GLOBE: INSIGHTS FROM THE GLOBAL PRIDE STUDY

Austin Oswald[1], Brian de Vries[2], and Karen Fredriksen-Goldsen[3], *1. Dalhousie University, Nova Scotia, Canada*, *2. San Francisco State University, San Francisco, California, United States*, *3. University of Washington, Seattle, Washington, United States*

Research on the intersection of sexuality, gender, and aging is becoming increasingly global; however, scholars in the field rarely have the opportunity to collaborate across national boundaries. This hampers knowledge sharing and limits the global knowledge base on sexuality, gender, and aging. In this presentation, we will explore international and interdisciplinary perspectives on sexuality and gender across the lifespan. Individual and group interviews were conducted with collaborators of the Global Pride study, discussing the challenges and opportunities of global research at the intersection of sexuality, gender, and age. These interviews were video recorded and will be shared during this presentation. The video features global collaborators discussing the resources and cultural influences in various conceptual, qualitative, quantitative, and multi-method approaches for conducting sexuality, gender, and aging research. We will also address the unique cultural influences, dimensions, and dynamic nature of conducting cutting edge research with sexual and gender diverse (SGD) adults across the lifespan. Key insights include: 1) identifying similarities and differences across diverse cultural contexts, including variations in old age itself; 2) understanding the distinct realities and challenges faced by older adults in diverse SGD communities and the strengths and fortitude of these communities worldwide; 3) developing feasible strategies to advance SGD research, practice, and education within differing cultural, social, and political environments. By examining the risk and resilience of SGD older adults globally, we aim to illuminate how innovative research, practice and education can strengthen our understanding of aging in our increasingly diverse society.

Abstract citation ID: igae098.1090

### LONELINESS AMONG SEXUAL AND GENDER DIVERSE PEOPLE IN AUSTRALIA: AN AGE-BASED ANALYSIS

Mark Hughes[1], and Karen Fredriksen-Goldsen[2], *1. Southern Cross University, Bilinga, Queensland, Australia*, *2. University of Washington, Seattle, Washington, United States*

Despite recent equality reforms, there remain significant mental health disparities between sexual and gender diverse (SGD) Australians and the general population. Multiple studies have reported higher levels of loneliness among SGD people, particularly among transgender and nonbinary people. One factor related to loneliness that is much debated is age with some studies suggesting older people are more at risk and others finding contradictory evidence, including among SGD people. To examine age-based loneliness trends among different groups of SGD people, we analyzed Australian data (n = 284) from the Global Pride Study to examine how key individual and social variables differ by age and the extent they predict loneliness (measured by the 3-item Loneliness Scale). Through regression analysis, we identified that gender and sexual orientation did not appear as predictors of loneliness. However, being younger did emerge as a predictor of a higher level of loneliness, as did lower levels of social support and resilience. Past experiences of SGD discrimination and victimization and a greater number of functional limitations also predicted higher levels of loneliness. Given recent reforms, one might expect younger SGD people to be faring better than older people on social and wellbeing indicators such as loneliness. However, this study provides some support to prior research indicating many older SGD people have developed considerable resilience across the lifespan and strengthened support systems later in life in a way that may ameliorate loneliness.

### SESSION 2545 (SYMPOSIUM)

Abstract citation ID: igae098.1091

### THE HERO CARE SURVEY: MEASURING THE UNMET NEEDS OF AMERICAN MILITARY VETERANS AND THEIR CAREGIVERS

Chair: Stuti Dang
Co-Chair: Ranak Trivedi
Discussant: Luci Leykum

Success in delaying long term institutional care (LTIC) depends on identifying unmet needs of Veteran-caregiver dyads and creating strategies to adequately support those needs. This symposium aims to enhance the understanding of the needs of Veterans and caregivers at high LTIC risk, to inform better planning of services and policy for Veterans and their caregivers. Our interdisciplinary group of researchers will use data from our prospective HERO CARE survey, fielded to 20,000 community-dwelling high-need, high-risk Veterans and their caregivers, to report on the epidemiology of their needs over time. We will review the survey's socio-ecological framework; summarize survey responses from Veterans and caregivers (Dang, Chair), followed by an overview of the VA-funded Elizabeth Dole National Center for Veteran and Caregiver Research (EDCoE), under whose umbrella this survey was conducted. (Co-chair-Trivedi). We will describe unmet needs stratified by LTIC risk and compare racial/ethnic differences in the prevalence of unmet needs. (Garcia) The next speaker will describe the process of analysis of HERO CARE caregivers' self-perceived preparedness for caregiving, awareness of resources and desire to institutionalize. (Desir) We will then describe key characteristics and preliminary findings for Veterans who report having a caregiver younger than age 25, an often-overlooked demographic of caregivers. (Kalvesmaki) Our last speaker will report on unmet needs and physical and emotional health of caregivers as they transition to survivors after the death of their care-recipient. (Bouldin) Finally, the discussant will synthesize the presentations and potential policy implications of the findings. (Leykum)

Abstract citation ID: igae098.1092

### UNMET NEEDS OF VETERANS AND CAREGIVERS INCREASE WITH PREDICTED RISK OF LONG-TERM INSTITUTIONAL CARE

Sandra Garcia-Davis[1], Richard Munoz[2], Erin Bouldin[3], Jared Hansen[4], Bruce Kinosian[5], Orna Intrator[6], and

Stuti Dang[7], *1. University of Miami, Miami, Florida, United States*, *2. South Florida Veteran Affairs Foundation for Research and Education, Miami, Florida, United States*, *3. University of Utah, Salt Lake City, Utah, United States*, *4. VA Salt Lake City Healthcare System, Salt Lake City, Utah, United States*, *5. Philadelphia Veterans Affairs Medical Center, Philadelphia, Pennsylvania, United States*, *6. Canandaigua VAMC and University of Rochester, Canandaigua, New York, United States*, *7. Miami VA Healthcare System, Miami, Florida, United States*

To understand unmet needs, we contacted a random sample of Veterans receiving care at five Veterans Health Administration (VHA) sites and oversampled Veterans with a higher predicted 2-year LTIC risk. Veterans and their caregivers were asked to complete separate comprehensive surveys to assess demographic, social, psychological, functional, and physical domains; unmet needs; experience with home and community-based services; and caregiver support programs experience. Veteran and caregiver surveys were mailed in the same envelope to 20,000 Veterans. We received responses from 8,056 Veterans and 3,579 caregivers. Veteran respondents were mostly men (96.5%), over 65 years (94.9%), married (55.0%), Non-Hispanic White (75.2%), and residing in urban areas (80.7%). Veterans reported unmet needs in activities of daily living (ADLs; 15.7%), instrumental activities of daily living (IADL: 26.0%), nursing tasks (7.9%), pain management (15.1%), communicating with healthcare team (18.2%), and social support (12.1%). The most common unmet IADL needs were medication management (55.4%), housework (55.4%) and preparing meals (53.7%). Compared to Non-Hispanic Whites, Hispanics and Non-Hispanic Blacks were more likely to have unmet ADL and IADL needs. Caregivers averaged 7.3 hours of daily care (SD=5.5 hours); and helped with ADLs (42.5%), IADLs (57.2%), communicating with healthcare team (52.4%), and social needs (36.4%). Degree of need for assistance for all needs varied by predicted risk of institutionalization (PLI). Veterans in the high PLI tier reported the highest prevalence of unmet need. Both Veterans and caregivers described complex unmet needs in medical, functional, psychological, and social domains, which increased as their predicted risk for LTIC increases.

Abstract citation ID: igae098.1093

### SELF-PERCEIVED PREPAREDNESS, AWARENESS OF RESOURCES, AND DESIRE TO INSTITUTIONALIZE AMONG CAREGIVERS OF VETERANS

Marianne Desir[1], Sandra Garcia-Davis[2], Richard Munoz[3], Mary Jo Pugh[4], Ranak Trivedi[5], Luci Leykum[6], and Stuti Dang[7], *1. Miami Veterans Affairs Healthcare System, Miami, Florida, United States*, *2. University of Miami, Miami, Florida, United States*, *3. South Florida Veteran Affairs Foundation for Research and Education, Miami, Florida, United States*, *4. University of Utah, Salt Lake City, Utah, United States*, *5. Stanford University/VA Palo Alto Healthcare System, Palo Alto, California, United States*, *6. South Texas Veterans Health Care System, Austin, Texas, United States*, *7. Miami VA Healthcare System, Miami, Florida, United States*

We analyzed the HERO CARE survey responses of 2,925 caregiver/Veteran dyads to: 1) describe the extent to which caregivers of older Veterans feel prepared for their caregiving tasks; 2) assess whether caregiver awareness of VA caregiver support resources was associated with feelings of preparedness; and 3) identify impact of caregiver feelings of preparedness and odds of caregiver interest in institutionalization of Veterans care recipients. Caregivers described themselves as, "Not at all prepared," Somewhat prepared," or "Very prepared" for their caregiving tasks. We calculated descriptive statistics for Veterans and caregivers and compared them by levels of caregiver preparedness using chi-square tests and t-tests. We then conducted adjusted multivariable logistic regression analyses to assess the association between caregiver preparedness and awareness of VA caregiver support services, and between desire to institutionalize and caregiver preparedness. Caregivers were mostly non-Hispanic white (73.3%), female (83.3%), and ≥ 65 years old (72.8%); 62.6% of caregivers felt very prepared, 34.7% felt somewhat prepared and 2.7% felt not at all prepared. Caregivers who were aware of at least one VA caregiver support resource were 33% more likely to feel very prepared compared to caregivers who were not aware of at least one resource (OR: 1.3; 95% CI: 1.1, 1.6; p<0.01). Caregivers who felt very prepared for caregiving were 39% less likely to have desire to institutionalize compared to caregivers who did not feel very prepared (OR: 0.6; 95% CI: 0.5-0.8; p <0.001). Veteran-caregiver dyads may benefit from increased awareness of VA resources, including ones that support caregivers.

Abstract citation ID: igae098.1094

### VETERANS WITH CAREGIVERS UNDER AGE 25: IMPORTANT DISTINCTIONS FOR RESEARCH, POLICY, AND PRACTICE

Andrea Kalvesmaki[1], Sandra Garcia-Davis[2], Erin Bouldin[3], Ranak Trivedi[4], Luci Leykum[5], and Stuti Dang[6], *1. VA Salt Lake City Health Care System, Salt Lake City, Utah, United States*, *2. University of Miami, Miami, Florida, United States*, *3. University of Utah, Salt Lake City, Utah, United States*, *4. Stanford University/VA Palo Alto Healthcare System, Palo Alto, California, United States*, *5. South Texas Veterans Health Care System, Austin, Texas, United States*, *6. Miami VA Healthcare System, Miami, Florida, United States*

"Young caregivers" are those aged 25 or less. Up to 5 million young caregivers under the age of 18 provide secondary care for an aging adult, which doubles if including those up to age 25. However, these caregivers are underrecognized in research and as such, may have many unmet needs, along with their care recipients. This project represents a secondary analysis of the HERO CARE survey in which we collected information on up to three additional caregivers supporting the Veterans in our study. Our goal was to identify Veterans with caregivers under the age of 25 and compared differences in socio-demographic characteristics and unmet needs to Veterans with caregivers over the age of 25, and Veterans without caregivers using chi-square and analysis of variance (ANOVA) tests. One hundred and fifty-four respondents indicated they had at least one caregiver younger than 25. Preliminary findings showed that, compared to both Veterans without a caregiver (n=2574) and those with caregivers older than 25 years (n=4027), Veterans with caregivers younger than age 25 tended to have lower education, have medication insecurity, food insecurity, low health literacy, transportation problems, have more unmet needs with daily activities, and be Black or Hispanic (p's<0.05). Findings suggest that the presence of a young caregiver may reflect greater social vulnerability for Veterans, and future research should focus on the risk of young caregivers for poor mental health, physical health, educational and economic outcomes.

Abstract citation ID: igae098.1095
### WELL-BEING AND SERVICE USE AMONG VETERANS' CAREGIVER-SURVIVORS

Erin Bouldin[1], Richard Munoz[2], Ranak Trivedi[3], Luci Leykum[4], Jared Hansen[5], and Stuti Dang[6], *1. University of Utah, Salt Lake City, Utah, United States*, *2. South Florida Veteran Affairs Foundation for Research and Education, Miami, Florida, United States*, *3. Stanford University/VA Palo Alto Healthcare System, Palo Alto, California, United States*, *4. South Texas Veterans Health Care System, Austin, Texas, United States*, *5. VA Salt Lake City Healthcare System, Salt Lake City, Utah, United States*, *6. Miami VA Healthcare System, Miami, Florida, United States*

Transitioning from the role of caregiver to survivor following the death of the care recipient can be difficult. Our goal was to describe well-being and bereavement-related service use among family caregivers of Veterans who had recently died. During June-November, 2023, we sent surveys to caregivers of 1,468 Veterans who had participated in a previous survey and whose Veterans had died 3-18 months prior to this survey; 296 (20.1%) caregiver-survivors completed the survey. We assessed caregiver-survivors' anxiety (Generalized Anxiety Disorder-2) and depressive (Patient Health Questionnaire-2) symptoms along with physical and mental health-related quality of life (HRQOL; PROMIS 10 mental and physical health) at the time of the survey. We assessed their use of specific support services since the Veteran's death. 14% of caregiver-survivors screened positive for anxiety and 15% for depressive symptoms. Most had poorer physical HRQOL (67% had t-scores>50) and mental HRQOL (85%) than the general population. Service use was uncommon: 10% of caregiver-survivors had used a bereavement support group, 7% had used counseling/therapy, and 2% each had used a caregiver support group or VA bereavement services. 20% of caregiver-survivors said they were not aware of bereavement/counseling services but would have liked to use them. In this sample of caregiver-survivors, anxiety and depressive symptom prevalence was similar to the general population but physical and mental HRQOL were substantially poorer. Most caregiver-survivors did not use any formal services following the Veteran's death. While most non-users were not interested in services, some reported they would have used them if offered.

### SESSION 2550 (SYMPOSIUM)

Abstract citation ID: igae098.1096
### UTILIZING THE PMDC AS A FRAMEWORK FOR THE PROVISION OF PERSON-CENTERED CARE IN NURSING HOME SETTINGS

Chair: Bill Buron
Discussant: Kathy Wright

This symposium showcases the research of the Geropsychiatric Nursing Collaborative, a workgroup affiliated with the NHCGNE. Kitwood (1997) defined Personhood, as 'a standing or status that is bestowed on one human being, by another in the context of relationship and social being.' Buron (2008) designed the Personhood Model for Dementia Care (PMDC) based on Kitwood's definition and Maslow's (1943) Hierarchy of Needs Theory. The PMDC is an intuitive framework to promote the identification and utilization of person-centered care (PCC) interventions in nursing homes. This symposium begins with an overview of the model as a framework for an integrative review of effective PCC-focused interventions. Next, three specific interventions will be discussed and highlighted, each representing at least one level of personhood as defined and described in the PMDC. The first illustrates how biological needs for nutrition intersect with relational aspects of care during mealtime and introduces an effective strategy for assisting RLWD with their meals. The researchers found more PCC and less task-centered care were associated with improved mealtime behaviors and/or food intake in RLWD. In the next example, the researchers highlight individual personhood through an exploration of resident preferences. The researchers found residents have diverse approaches to expressing their preferences and utilize a range of resident-led, family-involved, and staff-brokered strategies to meet preferences. In the final example, researchers developed an on-line person-centered dementia training for undergraduate nursing students. The aim of this training was to improve nursing care of people living with dementia and highlights the importance of sociologic personhood. Nursing Care for Older Adults Interest Group Sponsored Symposium

Abstract citation ID: igae098.1097
### THE PMDC AS A FRAMEWORK FOR IDENTIFYING AND ORGANIZING PERSON-CENTERED CARE INTERVENTIONS: AN INTEGRATIVE REVIEW

Bill Buron[1], and Tim Killian[2], *1. The University of Tulsa, Tulsa, Oklahoma, United States*, *2. University of Arkansas, Fayetteville, Arkansas, United States*

Residents living with dementia (RLWD) in nursing homes (NHs) have cognitive and communication deficits. Person-centered care (PCC) practices ranging from improving bathing experiences to supporting communication skills have been the mainstay of non-pharmacologic dementia care treatment, although there are considerable variations in how PCC practices are conceptualized and implemented. In efforts to meet Kitwood's (1997) challenge to promote personhood among RLWD, Buron (2008) developed the Personhood Model for Dementia Care

(PMDC) to identify and categorize three levels of personhood: biologic, individual, and sociologic. Through this integrative review, the researchers use Buron's PMDC as a framework to intuitively identify, organize and analyze recent literature on PCC interventions. The study leveraged new Artificial Intelligence (AI) tools to identify relevant studies published in the past 5 years (2019-present). The researchers used AI tools to identify over 100 qualifying studies. Through a second phase manual review of the abstracts, the researchers applied the inclusion criteria; reducing the number of papers to approximately 35. Most interventions focused on individual personhood, followed respectively by sociologic and biologic interventions, although there was overlap, with some papers addressing more than one dimension of personhood as defined by the PMDC. In the third phase, the researchers focused on downloading PDF versions of these studies and utilizing Natural Language Processing (NLP) tools to characterize and describe the current landscape of PCC interventions in NHs, using the PMDC as a model to promote additional research and advance the provision of targeted PCC interventions for RLWD in NH settings.

Abstract citation ID: igae098.1098

### MEALTIME BEHAVIORS AND FOOD INTAKE IN OLDER ADULTS WITH DEMENTIA: IMPACT OF PERSON-CENTERED AND TASK-CENTERED CARE

Wen Liu, Kyuri Lee, and Heather Suh, *University of Iowa, Iowa City, Iowa, United States*

Nursing home residents with dementia experience mealtime behaviors and low food intake due to multilevel factors, among which mealtime caregiving is critical. Prior work supported associations between staff mealtime care and resident mealtime behaviors and food intake using small samples of meal-segments. This study examined their associations using 197 full-meal videos (duration=37.85±18.24 minutes), capturing 47 staff (age 45.6±11.8 years; 92.3% female; 80.8% white; 15.5±12.9 caregiving years) and 12 residents with moderately-severe to severe dementia (age 71.9±7.5 years; 91.7% male; 91.7% white). Staff mealtime care approaches (n=12,800) were mostly person-centered addressing modifications of resident abilities (70.3%), dyadic interactions (12.1%), and dining environment (6.8%), followed by task-centered care (TCC, 10.8%). Resident mealtime behaviors (n= 9,424) were resistive behaviors (21%), functional impairments (13.2%), chewing/swallowing difficulties (48.1%), and positive behaviors (17.6%). Resident food intake was 626.23±353.17 gram/meal. Resistive behaviors were associated with fewer modifications of resident abilities (b=-.215, p=.053) and more TCC (b=.202, p=.058). Functional impairments were associated with more modifications of resident abilities (b=.186, p=.048) and more TCC (b=.446, p<.001). Chewing/swallowing difficulties were associated with more modifications of resident abilities (b=.287, p=.006). Positive behaviors were associated with fewer modifications of resident abilities (b=-.310, p=.002) and more modifications of dining environment (b=.330, p=.001). Food intake was associated with more modifications of resident abilities (b=.289, p=.009). Minimizing use of task-centered care may help improve mealtime challenging behaviors. While modifications of resident abilities may not address functional impairments, they help manage resistive behaviors and improve food intake. Modifications of dining environment may help promote positive behaviors.

Abstract citation ID: igae098.1099

### RESIDENTS' PERSPECTIVES ON PREFERENCES: EXPLORING HOW NURSING HOME RESIDENTS EXPRESS AND ACHIEVE PREFERENCES

Laura Block[1], Caroline Madrigal[2], and Tonya Roberts[3], *1. University of Wisconsin-Madison, Madison, Wisconsin, United States*, *2. VA Boston Healthcare System, Brockton, Massachusetts, United States*, *3. University of Wisconsin - Madison, Madison, Wisconsin, United States*

Providing choice and accommodation of preferences is fundamental to delivery of person-centered care in nursing homes (NH). Studies indicate variability in NH residents' preferences around a range of daily care routines and activities (i.e., involvement of family, meal choices, morning or nighttime routines) and the widespread challenges NHs face in meeting preferences. Yet, few studies have examined NH resident perspectives on their role as a key player in communicating their preferences. This qualitative study is part of a broader mixed methods exploration of the relationship between preference-aligned care and quality of life. A subset of residents (N=11) was selected for semi-structured interviews to examine resident perspectives on navigating preferences in daily life. Interviews were coded inductively, with thematic analysis guiding abstraction and interpretation. Residents described a range of degree of preference expression, from voicing strong preferences for or against care/activities; deferring to staff; or being uncertain of a preference. Residents shared multiple strategies for meeting their preferences, including communicating with new staff, relying on family, escalating requests, and adjusting to the NH environment. Residents also described staff-led strategies of documenting and sharing their preferences and splitting the work into parts, which often required resident collaboration and input. The importance of consistency in staffing and developing relationships with staff was underscored. Future work should examine the potential role health, cognitive, and functional capacity play in shaping residents' preference expression.

Abstract citation ID: igae098.1100

### PERSON-CENTERED ONLINE DEMENTIA TRAINING

Diana Sturdevant, Kimethria Jackson, and Kimetha Broussard, *University of Oklahoma Health Sciences Center, Oklahoma City, Oklahoma, United States*

Nursing education that focuses on care of people living with dementia (PLWD), and training in person-centered dementia care is limited. Person-centered dementia care puts PLWD at the center of the care process. It requires deep knowledge of the individual, as well as empathy and understanding from the nurse. Person-centered dementia care training improves care by correcting misconceptions about dementia and increases confidence in managing behavioral-psychological symptoms of dementia. In this session we will report outcomes from an on-line training focused on improving nursing care of people living with dementia (PLWD). The training consisted of 4 modules that included multimedia, discussion boards, and interactive exercises to stimulate student engagement and interaction. Undergraduate nursing students were recruited via email announcements of the volunteer opportunity. A total of (n=31) participants were recruited from 4 baccalaureate nursing programs including Traditional Junior, Traditional Senior, Degree Completion LPN to BSN, and Accelerated BSN. Pre/post assessments included the Dementia Attitudes Scale, the ADRC Staff Dementia Knowledge Assessment Tool, and the Confidence in Dementia Scale. A 5pt-likert scale was used to evaluate course structure and content, and open-ended questions provided prompts for additional participant comments. Common themes from evaluations included: need for additional content, more interactive activities, and inclusion as a required course for all nursing students.

### SESSION 2555 (SYMPOSIUM)

Abstract citation ID: igae098.1101

### VOICES OF CARE: INSIGHTS FROM DIVERSE CAREGIVERS

Chair: Barbara Mendez Campos
Discussant: Karen Lyons

Recognized as the backbone of the U.S. healthcare system, informal caregivers face unprecedented challenges amidst an ongoing care crisis. Understanding the profound effects of informal care responsibilities is critical to the health and well-being of our society. According to AARP, in 2020, the number of individuals engaged in caregiving for an ill or disabled family member surpassed 53 million, up nearly 10 million since 2015. There is increasing evidence documenting that social inequalities often compound the negative health and financial outcomes for caregivers as they age, making research that focuses attention on the racial, ethnic, and cultural contexts that shape not only the caregiving experience, but also the outcomes that result, central to our ability to develop interventions. This symposium delves into the intricate realities of caregiving, providing valuable insights into economic, cultural, and health-related dimensions. It proposes solutions to positively influence the experiences of older adults, their families, and the broader healthcare system. The first paper explores the enabling factors and barriers African American women caregivers confront in seeking support. The second paper evaluates a digital storytelling intervention's impact among African American caregivers. The third paper investigates the repercussions of caregiving on older workers'financial, health, and employment outcomes during the COVID-19 pandemic. The final paper reports a scoping review on the role of interpersonal factors in shaping health behaviors among Hispanic older adults. A discussant will reflect on these studies, underscoring the imperative for ongoing research encompassing diverse voices, methodologies, and the impacts on informal caregivers. Family Caregiving Interest Group Sponsored Symposium

Abstract citation ID: igae098.1102

### EXPLORING HOW INTERPERSONAL FACTORS SHAPE HEALTH BEHAVIORS AMONG HISPANIC OLDER ADULTS: A SCOPING REVIEW

Barbara Mendez Campos, Karen Lyons, and Bruna Lopez, *Boston College, Chestnut Hill, Massachusetts, United States*

As the U.S. experiences a significant demographic shift, particularly among older adults, this scoping review focuses on the Hispanic population, one of the largest segments of historically underrepresented racial/ethnic groups in this age cohort. An in-depth exploration of health behaviors becomes imperative with this demographic surge and an accompanying higher chronic illness profile. A noteworthy gap exists in understanding the influence of interpersonal factors on health behaviors, particularly among older Hispanic adults. This review systematically examines the existing literature on the roles of interpersonal factors within family and friends in shaping health behaviors among Hispanic older adults. Beyond the conventional categorization of cultural values like familismo, acculturation, fatalism, marianismo, machismo, collectivism, respeto, and religious beliefs as intrapersonal influences, the study emphasizes unraveling these factors within their relational context. It acknowledges the heterogeneity in adherence to these values and assesses their subsequent impact on health behaviors. Using the Joanna Briggs Institute (JBI) framework, the review identifies 39 relevant articles from six distinct databases. The findings underscore the urgency for future research utilizing mixed methods, multi-source, dyadic, and longitudinal approaches. Affirming the central role of interpersonal factors, predominantly involving daughters and wives, the study signals a gendered lens in relational dynamics within this community. Uncovering the roles of interpersonal factors significantly influencing various health behaviors, including physician visits, treatment choices, medication adherence, healthcare service use, and vaccinations, the review highlights the need for tailored interventions and further nuanced research in this critical area.

Abstract citation ID: igae098.1103

### CARE'S ECHO: THE IMPACT OF CAREGIVING DURING COVID-19 ON OLDER WORKERS' FINANCIAL, HEALTH, AND JOB OUTCOMES

Christina Matz, Patrick Lai, and Barbara Mendez Campos, *Boston College, Chestnut Hill, Massachusetts, United States*

Informal caregivers are a critical resource to their loved ones and an essential component of the health care system in the United States. In the context of an ongoing care crisis, it is more important than ever to understand the effect of informal care responsibilities on individuals and their families as they age to develop appropriate solutions. This study examines how caregiving responsibilities during COVID-19 affected older workers' financial well-being, health, job turnover, and employment experiences. Using 2020 Health and Retirement Study (HRS) data and the 2021 HRS Perspectives on the Pandemic survey, we examined older workers aged 50 and above. Our logistic regression analysis focused on two caregiving scenarios: 1) individuals with a spouse or partner with ADL/IADL limitations, and 2) those who paused work for at least two weeks due to caregiving during the pandemic. Our findings reveal significant challenges for caregivers. Spousal caregivers were 1.65 times more likely to face financial difficulties and 1.87 times more likely to have health issues due to delayed care compared to non-caregivers. Those who had to stop working for 2 weeks or more for caregiving reported even higher risks (2.69 times for financial difficulty and 3.29 times for health issues). Caregivers also experienced greater job stress, safety concerns, and decreased job satisfaction. The pandemic has illuminated the struggles of older working caregivers, emphasizing the need for supportive workplace policies and societal interventions. These measures are essential to support caregivers, who often sacrifice their well-being and career progress for their caregiving roles.

Abstract citation ID: igae098.1104

### I'LL TELL YOU IF YOU ASK: MESSAGES ABOUT SUPPORT FROM AFRICAN AMERICAN WOMEN CAREGIVERS

Colleen Galambos[1], Abiola Keller[2], Katherine Platt[1], and Jennifer Vidal[1], *1. University of Wisconsin Milwaukee, Milwaukee, Wisconsin, United States*, *2. Marquette University, Milwaukee, Wisconsin, United States*

Family caregivers are vital for ensuring the health of a growing older adult population. African American women represent a critical population of caregivers, since 60 percent of caregiving among older adults within the African American community is performed by women (Centers for Disease Control, 2024). Additionally, there is added cultural pressure to provide this care to family members and African American women caregivers (AAWC) face multiple competing stressors and demands (Imoh & Charity, 2023). Given these cultural dynamics, it is important to understand what type of assistance AAWC need to enable them to engage in self-care and support themselves in their caregiving role. Furthermore, there may be barriers faced by AAWC in accessing supportive interventions and resources. To increase knowledge about barriers and preferences for interventions and services, qualitative interviews were conducted with 12 AAWC of older adults. Interviews were audio recorded and transcribed using Microsoft TEAMS. To analyze the data, inductive content analysis was employed, responses were coded and emerging patterns determined. This presentation reports on the most frequently mentioned barriers and enablers to receiving support that emerged from the interviews as well as cultural considerations. Some barriers that surfaced from the data include supporting others over self, time constraints and logistical barriers. Some enablers or facilitators include sharing care responsibilities, support of family and friends, and obtaining information/receiving resources. Additional barriers and enablers and other experiences identified by the participants will be discussed. Community stakeholder's response to the study will be highlighted.

Abstract citation ID: igae098.1105

### DIGITAL STORYTELLING AMONG AFRICAN AMERICAN CAREGIVERS: EVALUATION OF THE INTERVENTION PARAMETERS

Abiola Keller[1], Aline Gubrium[2], Saharra Dixon[2], Bethani Bell[1], Dani Hart[1], and Kristin Haglund[1], *1. Marquette University, Milwaukee, Wisconsin, United States*, *2. University of Massachusetts Amherst, Amherst, Massachusetts, United States*

Digital storytelling (DST), a process involving creating 1–3-minute videos about an important moment in one's life, is an innovative approach for data collection and intervention and it is imperative to understand storytellers' perceptions of the process. This study explores the experiences of African American women caregivers enrolled in a DST intervention. The specific aims are to examine the acceptability, feasibility, and effectiveness of the DST process. Women who self-identified as African American, a caregiver (i.e., taking care of another adult), and agreed to attending all sessions were eligible to participate. Women were invited to participate in an interview via Microsoft Teams after each DST session. Interviews lasted approximately 15 minutes and were audio recorded and manually transcribed. Responses were examined for patterns and key content-related categories using inductive content analysis. In Cohort 1 (Spring 2023), five caregivers consented to participate in the DST sessions, three completed their story, and those three participated in interviews. In Cohort 2 (Fall 2023), five caregivers consented to participate in the DST sessions, four completed their story, and two participated in interviews. Women spoke about three distinct ways the DST process met their needs: 1) having a shared experience, 2) breaking the silence around caregiving, 3) learning and trying new things. Identified challenges included resistance to writing down their story and limited access to technology outside of the sessions. These findings suggest that DST may be used as an additional resource for supporting the well-being of African American women who care for older adults.

### SESSION 2560 (SYMPOSIUM)

Abstract citation ID: igae098.1106

### WHO FITS AND HOW? SUPPORTING TENANCY AND FOSTERING BELONGING IN HOUSING FOR OLDER PERSONS EXPERIENCING HOMELESSNESS

Chair: Lena Rebecca Richardson
Co-Chair: Rachel Weldrick
Discussant: Tam Perry

Homelessness among adults age 50+ years is increasing across North America. The Aging in the Right Place (AIRP) Partnership is a 5-year study of eleven promising practice organizations addressing older adult homelessness in three Canadian cities. This symposium will share findings from AIRP research on what supports a sense of belonging and social integration among older persons experiencing homelessness (OPEH) within temporary and permanent supportive housing programs. Presenters include interdisciplinary researchers with a diversity of perspectives stemming from gerontology, education, and social work. The symposium will begin with Erisman presenting a study of service providers' perspectives on who is eligible for services and what actions providers can undertake to support clients' ongoing tenancy in shelter/housing organizations. Weldrick will then discuss mechanisms for successfully supporting social integration from a study with OPEH in a temporary housing program. Following, Richardson will present findings on how multidirectional caring relations can foster belonging in a homeless shelter for older adults fleeing abuse. Finally, Walsh's study findings will highlight ways to promote AIRP for male veterans in a temporary housing program. Tam Perry, an expert in housing transitions for older adults, will discuss the implications of these papers and reflect on processes of social integration and cultivating belonging for OPEH in temporary and permanent housing. Together, participants of the symposium will advance this emerging scholarship using a wide range of methods and perspectives.

Abstract citation ID: igae098.1107

### “I FEEL LIKE A SOMEBODY AGAIN”: THE ETHICS OF CARE IN A SHELTER FOR OLDER ADULTS FLEEING ABUSE

Lena Rebecca Richardson[1], Sarah Canham[2], Rachel Weldrick[3], Jill Hoselton[4], Alison Grittner[5], and Christine Walsh[4], *1. Simon Fraser University, Vancouver, British Columbia, Canada*, *2. University of Utah, Salt Lake City, Utah, United States*, *3. McMaster University, Hamilton, Ontario, Canada*, *4. University of Calgary, Calgary, Alberta, Canada*, *5. Cape Breton University, Sydney, Nova Scotia, Canada*

Elder abuse is one pathway into homelessness for older adults. Given the limited research on the experience of older adults staying in homeless shelters for victims of abuse, we conducted qualitative interviews with ten clients and five providers from an elder abuse shelter in Western Canada. Drawing on a feminist ethics of care framework, we explored what supports older survivors of abuse use to cultivate a sense of belonging at the shelter. Conducting a secondary qualitative data analysis, we identified three themes: 1) trust-building between providers and clients supports successful care-receiving by clients; 2) mutual care and caregiving by clients fosters clients' sense of belonging; and 3) a perceived lack of care disrupts trust and impedes belonging. Our findings reveal how client trust is, in part, cultivated by providers' acts of flexible, responsive care, supported by organizational policies and practices. In addition, clients' acts of mutual care and caregiving towards other clients, friends, family, strangers, and animals contributes to their sense of belonging. In contrast, a perceived lack of care could be a source of conflict, though such experiences can be repaired through responsive care by providers and clients. Findings highlight the importance of shelter policies and practices which conceptualize older clients fleeing abuse as part of a multidirectional web of caring relations in fostering belonging. This study has implications for shelter program design that prioritizes trust-building through flexible, responsive care by providers, and recognizes client needs for care-receiving, caregiving, and mutual care.

Abstract citation ID: igae098.1108

### SOCIAL INTEGRATION IN TEMPORARY HOUSING: PERSPECTIVES OF OLDER PERSONS EXPERIENCING HOMELESSNESS

Rachel Weldrick[1], Sarah Canham[2], Atiya Mahmood[3], Rachelle Patille[4], and Shreemouna Gurung[3], *1. McMaster University, Hamilton, Ontario, Canada*, *2. University of Utah, Salt Lake City, Utah, United States*, *3. Simon Fraser University, Vancouver, British Columbia, Canada*, *4. Simon Fraser University, vancouver, British Columbia, Canada*

The prevalence of homelessness among older persons in North America is rising. Accordingly, age-supportive housing and service models are needed to adequately support older persons experiencing homelessness to secure permanent housing. Within permanent supportive housing models, achieving and maintaining social integration (i.e., strong social connection and engagement) is recognized as a programmatic goal. Previous research has identified strategies to promote social integration in permanent supportive housing models, such as Housing First models. However, minimal research has considered strategies to promote social integration in temporary housing programs (THPs) and transitional housing models. This study addresses this knowledge gap by examining experiences of social integration, connection, and support in a THP program for older persons experiencing homelessness in Vancouver, Canada. We conducted semi-structured qualitative photovoice interviews with 11 current or former THP clients (Mean age = 65 years; 6 female, 5 male) across three sessions. Data were analyzed using a critical realist-informed thematic analysis method. We identified three themes: 1) technology access and programming can facilitate social connection; 2) consistent communication with program staff can enhance perceived social support; and 3) accessible built environments can promote social participation among clients. Findings offer insights for the development of age-supportive housing and support models for older persons experiencing homelessness. We conclude by offering strategies, such as digital literacy programming and the creation of 'third spaces', to support older persons to remain (or become) integrated while in temporary and transitional housing.

Abstract citation ID: igae098.1109

### BEING AND STAYING ELIGIBLE FOR SERVICES: PROVIDERS' VIEWS ON SUPPORTING OLDER ADULTS EXPERIENCING HOMELESSNESS

Morgan Cruz Erisman[1], Sarah Canham[1], Lena Rebecca Richardson[2], Rachel Weldrick[3], Atiya Mahmood[2], Christine Walsh[4], and Tamara Sussman[5], *1. University of Utah, Salt Lake City, Utah, United States*, *2. Simon Fraser University, Vancouver, British Columbia, Canada*, *3. McMaster University, Hamilton, Ontario, Canada*, *4. University of Calgary, Calgary, Alberta, Canada*, *5. McGill University, Montreal, Quebec, Canada*

Older people experiencing homelessness (OPEH) are a marginalized population with limited access to shelter/housing supports. Amidst these limited resources, understanding program-specific (in)eligibility criteria, along with strategies to sustain tenancy in different shelter/housing settings, is crucial to supporting OPEH in their housing goals. This study examined providers' understanding of housing (in)eligibility in different shelter/housing settings, factors that impact the criteria for staying eligible, and interventions and steps providers use in assisting OPEH to maintain their eligibility and move towards their housing goals. Semi-structured interviews were conducted with 15 providers from five shelter/housing organizations serving OPEH across three Canadian cities. These organizations represented a range of shelter/housing services for OPEH, including emergency shelters, temporary/transitional housing, and permanent supportive housing. We conducted a thematic analysis and organized findings into three themes: 1) Medical and behavioral factors that impact (in)eligibility of OPEH for shelter/housing services; 2) Challenges to maintaining eligibility due to medical and behavioral factors; and 3) Provider and organizational support mechanisms to sustain tenancy. Findings provide insight into who is (in)eligible for different shelter/housing programs catered to OPEH, along with different support mechanisms available to support OPEH in their housing goals. Findings highlight the supports and services needed by OPEH with medical and behavioral challenges to remain eligible in different shelter/housing settings and recommendations for how to increase and target services to OPEH with specific needs. Understanding medical and behavioral factors that impact the shelter/housing tenancy of OPEH provides an opportunity to foster client success and promote positive housing outcomes.

Abstract citation ID: igae098.1110

### CULTIVATING BELONGINGNESS: IN TEMPORARY SUPPORTIVE HOUSING FOR OLDER MALE VETERANS IN CALGARY, CANADA

Christine Walsh, *University of Calgary, Calgary, Alberta, Canada*

Veterans are two to three times more likely to experience homelessness compared to the general population, with an estimated 2,400 – 10,000+ homeless veterans in Canada. Veterans tend to be older, male, and more likely to cite an illness or medical condition as a contributing factor to their homelessness, than their non-veteran counterparts and thus have distinctly different needs. As part of the Aging in the Right Place (AIRP) partnership, a tri-city multi-methods study, we sought to understand the housing and support needs of older male military veterans (age 50+) living in a congregate temporary supportive housing program (a tiny home barracks), identified as a promising practice, in Calgary, Canada. In this presentation we draw on in-depth qualitative key informant interviews with service providers (n=5) and photovoice interviews with older shelter residents (n=10) to understand the shelter needs of older homeless veterans. Interviews were transcribed, managed with NVivo 1.6.1, and team-based flexible coding was employed on the interviews to determine older male veterans' shelter needs. Thematic analysis of service provider interviews identified key features to promote AIRP included: program specific challenges and strengths and contexts, while eligibility and systemic barriers were noted as impediments to AIRP. Residents highlighted: autonomy and belonging, community and connection, ageism, routine and daily rhythm, safety, recognition, and the built environment as key to their (in)ability to AIRP in this promising practice. In this presentation we contextualize the study findings and offer recommendations for shelter and services designed to assist older housing insecure veterans to AIRP.

### SESSION 2565 (SYMPOSIUM)

Abstract citation ID: igae098.1111

### WHY AI GOVERNANCE SHOULD BE A FOCAL ISSUE FOR GERONTOLOGY

Chair: Clara Berridge
Discussant: Clara Berridge

Artificial intelligence (AI) and AI-mediated decision making currently impact older adults in numerous ways. From algorithmic decision making used in health insurance, employment, housing, and public benefits, to the information ecosystem, to social robots, AI applications implicate important issues of ethics, access, and ageism. The World Health Organization reports that the diverse interests of older adults are not well represented in discourses about AI policy making or governance. As AI harms are better understood, the call from public interest groups and from many academic fields has gotten louder for comprehensive AI and data privacy policy in the United States (U.S.), which lags behind other countries. Drawing from the international insights of European, Canadian, and U.S.-based research, this symposium will consider how older adults and their rights are represented in AI policy documents, laws, and policy discourse. Dr. Ho will discuss ethical concerns resulting from regulatory gaps in direct-to-consumer AI health monitoring platforms in the U.S context. Dr. Stypinska will highlight how European national (Germany, Spain, UK, Holland and Poland) and international policy documents related to AI position older adults. Dr. Gallistl will discuss the relevance of AI explainability in later life, as one of the key terms that currently inform EU AI-governance. Dr. Robillard will review findings and gaps from an analysis of international policies for social robots with aging applications. Participants with and without prior policy knowledge or AI research experience are welcome. Technology and Aging Interest Group Sponsored Symposium

Abstract citation ID: igae098.1112

### BRIDGING REGULATORY GAPS IN DIRECT-TO-CONSUMER HEALTH MONITORING TECHNOLOGIES FOR OLDER ADULTS

Anita Ho, *University of British Columbia, Canada*

This presentation explores unique ethical concerns that result from regulatory gaps in direct-to-consumer (DTC) AI health monitoring platforms, with a focus on the current landscape in the United States. Many older adults have multiple co-morbidities and are increasingly encouraged or even expected to continuously monitor their health, including by AI-powered predictive digital technologies. Some medically oriented AI-based algorithms can seek and receive regulatory approval as a medical device. Nonetheless, a much larger category of DTC AI health platforms includes wellness technologies that are not subjected to regulatory scrutiny or approval, even when many unregulated devices collect similar types of data as medical devices and vast amounts of health-related and personal information, blurring the line between medical and commercial data and products. Older adults are generally more risk averse and have limited digital, medical, and statistical literacy, and may also experience cognitive impairments. Enthusiasts often cite the potential of DTC health monitoring technologies to democratize health information and users' well-being. However, the lack of governance for DTC applications raises unique and intersecting ethical concerns for the field of gerontology, particular around barriers for informed consent, lack of validation for product safety and clinical value, perpetuation of infantilization, non-transparent practices of data sharing, and potential impact of these technologies on familial care relationships and formal health care delivery. This presentation concludes with recommendations for regulatory considerations to uphold monitored older adults' agency and well-being.

Abstract citation ID: igae098.1113

### END-USER PERSPECTIVES ON POLICIES FOR SOCIAL ROBOTICS WITH AGING APPLICATIONS

Julie Robillard, Jill Dosso, Haiger Ye, Gabriella Guerra, and Anna Riminchan, *University of British Columbia, Vancouver, British Columbia, Canada*

Social robots, including those with artificial intelligence (AI)-enabled functionalities, are promising in their potential to support brain health in older adults in both home and healthcare environments, but their benefits must be weighed against ethical considerations and risks. To characterize and evaluate existing guidance in this field, we conducted a content analysis of n = 47 international policies on social robotics and conducted n = 18 semi-structured interviews with lived experience experts within the dementia and care partnership community and professional experts in the field of aging and dementia. Participants viewed social robots as potentially helpful assistive technologies and agreed with existing recommendations around the needs for respect for human rights and dignity, clear and consistent regulation, and public engagement. Participants recommended that policies prioritize cost and accessibility considerations, represent the needs of minority groups, and that they center around issues specific to aging and dementia. They reported that the responsibility for social robot policy development should lie with governments and the healthcare sector and called for increased consultation with end-users and medical professionals. Results also highlighted misalignments between the priorities of the aging and dementia community members and the existing social robot policy landscape. Potential harms and challenges to adoption of social robots were discussed, including discrimination, bias, and deception. Taken together, findings from this two-phase study can inform evidence-based policy recommendation for the development and implementation of AI-enabled social robots for aging.

Abstract citation ID: igae098.1114

### AI DEVELOPMENT AND AGING POPULATIONS: CRITICAL JUNCTURE OR PATH DEPENDENCY? POLICY ANALYSIS

Justyna Stypinska[1], Anrea Rosales[2], Maria Sourbati[3], Rüya Koçer[4], and Halina Nadobnik[5], *1. WZB Berlin Social Science Center, Berlin, Berlin, Germany*, *2. Open University of Catalonia, Barcelona, Catalonia, Spain*, *3. University of Brighton, Brighton, England, United Kingdom*, *4. Leiden University, Leiden, Friesland, Netherlands*, *5. WZB, Berlin, Berlin, Germany*

This paper presents policy landscapes of five European countries - Germany, Holland, Spain, Poland, and Great Britain, as well as international documents (EU, UNESCO, Council of Europe) - scrutinizing their approaches to AI development through the lens of aging population. The research question addressed is: How are older persons represented and situated within AI governance in these national and international documents? The study, conducted as part of the "Ageism in AI (AGEAI)" research project, employs a policy text analysis to examine the explicit and implicit ways ageism manifests in AI development strategies. The research scrutinizes policy texts to discern the extent to which the needs, rights, wishes and perspectives of older individuals are acknowledged and integrated. The results show that economic growth, expected due to advancement of AI technologies, leaves a large segment of population – those not active in the labor market - beyond the scope of the policies. Furthermore, we observe diverse approaches and levels of inclusivity of the aging population across the five countries. While some nations exhibit explicit efforts to incorporate considerations for older adults within their AI policies, others demonstrate a significant gap in addressing age-related concerns. Moreover, the presentation will reflect on whether the direction of AI development proclaimed by the strategic documents reflects a pattern of path dependency of those policy instruments or suggests a more radical approach – a (new) critical juncture in development of technology for, by and with older adults.

Abstract citation ID: igae098.1115

### AI EXPLAINABILITY IN LONG-TERM CARE: A SOCIOLOGICAL INQUIRY

Vera Gallistl, *Karl Landsteiner University of Health Sciences, Krems, Niederosterreich, Austria*

Transparency, fairness, and explainability are integral components of contemporary discourses on AI governance, as evidenced by recent policies such as the AI Act by the European Commission. The concept of explainability reflects a growing recognition of the importance to understand AI systems' operations and decisions, ultimately fostering trust in their utilization (Markus et al., 2021; Ghassemi et al., 2021). While AI explainability is commonly framed within technical parameters, focusing on tools within data science and informatics to elucidate the algorithms behind automated decision-making processes, this perspective tends to overlook the broader, contextual dimensions of AI explainability. In contrast, this paper adopts a sociological lens to explore the multifaceted nature of explainability (Borch & Min 2022) in the context of AI for long-term care settings (LTC). Drawing on insights from multiple-perspective case studies centered on two AI applications—fall detection and social robots— the study investigates how technology developers, care staff, and older adults conceptualize AI explainability. The paper uses data from 30 qualitative interviews and 50 hours of participant observations. The findings highlight the diverse and at times conflicting understandings of explainability that underpin the development and implementation of AI in LTC. They also illustrate how explainability needs of stakeholders in LTC go beyond technological aspects and instead focus on trust, transparency and meaning-making with AI. Finally, the findings underscore the need to broaden concepts around AI explainability to include the more nuanced explainability needs of older adults and avoid ageist tendencies in the AI explainability discourse.

### SESSION 2570 (AWARD LECTURE (INVITATION ONLY))

Abstract citation ID: igae098.1116

### IRVING S. WRIGHT AWARD AND TERRIE FOX WETLE AWARD LECTURES

Chair: Hattie Herman

The Irving S. Wright Award of Distinction Lecture will feature an address by the 2024 recipient Jeremy D. Walston, MD, FGSA, Johns Hopkins University. The Terrie Fox Wetle Award lecture will feature an address by the 2024 recipient Megan Huisingh-Scheetz, MD, MPH, AGSF, University of Chicago. The George M. Martin Lifetime Achievement in Mentoring Award will also be presented at this event to 2024 recipient Steven N. Austad, PhD, FGSA, University of Alabama at Birmingham. These awards are given by the American Federation for Aging Research, Inc.

Abstract citation ID: igae098.1117

### PHYSICAL FRAILTY IN OLDER ADULTS: WHAT DRIVES AND PREVENTS IT?

Jeremy Walston, *Johns Hopkins University, Baltimore, Maryland, United States*

Physical frailty puts older adults at very high risk of adverse health outcomes and early mortality. There are multiple physiological and molecular changes that drive the development of frailty, including altered metabolism, activated inflammatory pathways, and autonomic nervous system dysregulation. Over the past several decades, our team has worked to identify specific physiological and molecular changes that drive earlier onset of frailty and late life decline in older adults. These pathway discoveries have also informed the underpinnings of physical resiliency in a subset of older adults, and helped to identify potential mechanisms by which older adults can live at lower biological risk of developing illnesses as well as functional and cognitive decline. This lecture will encapsulate how the evolving understanding of physical frailty, underpinned by decades of research, is reshaping our approach to healthy aging and reflecting a profound shift in the landscape of gerontology research.

Abstract citation ID: igae098.1118

### HARNESSING HEALTH SERVICES RESEARCH TO FUEL FRAILTY IMPLEMENTATION AND INNOVATION

Megan Huisingh-Scheetz, *University of Chicago, Chicago, Illinois, United States*

Broad implementation of frailty assessment and management in clinical practice has been a core mission of geriatricians for decades, and now medical and surgical subspecialists have joined the effort. Dr. Terrie Fox Wetle has demonstrated the power of health services research paired with passion to foster change. In this presentation, I will pay tribute to Dr. Wetle, review the health services research and its application to frailty science, and reflect on the importance of passion to one's career. I will share successes and remaining challenges of frailty implementation as exemplified in the Successful Aging and Frailty Evaluation clinic established in 2011. I will then discuss novel directions for frailty assessment and management using technology that may help overcome hurdles. Throughout the presentation, I will highlight mentors, collaborators and obstacles that have shaped my direction. I will end with a call for team science and policy change to advance frailty care.

### SESSION 3000 (SYMPOSIUM)

Abstract citation ID: igae098.1119

### ADVANCING DEMENTIA RESEARCH THROUGH AN EARLY CAREER SUMMER INSTITUTE

Chair: Sam Fazio

In 2020, the National Institutes on Aging (NIA) funded the five-year Alzheimer's Association Interdisciplinary Summer Research Institute (AA-ISRI) focused on early career researchers interested in psychosocial and public health research related to dementia. This program is designed to equip researchers with the knowledge and tools to advance the field of dementia research. Specifically, the Institute aims to promote skill building and substantive knowledge of emerging leaders, better preparing them to develop the evidence to ultimately improve care and outcomes for persons living with dementia. During each of the first three years of the project, feedback from faculty and trainees underscored areas in which training is more and less beneficial and the importance of facilitating new and ongoing networks. During this symposium, we will 1) describe the inception and development of AA-ISRI; 2) provide insights from a psychosocial track alum on training and the impact AA-ISRI has had on their research and career; 3) provide insights from a public health track alum on training and the impact AA-ISRI has had on their research and career; and 4) detail lessons learned and steps that other organizations can take to develop similar programs, as well as suggestions for faculty working with individual mentees.

Abstract citation ID: igae098.1120

### DEVELOPING A SUMMER RESEARCH INSTITUTE FOR EARLY CAREER RESEARCHERS

Chelsea Kline[1], Sheryl Zimmerman[2], Julie Bynum[3], Matthew Baumgart[1], and Sam Fazio[1], *1. Alzheimer's Association, Chicago, Illinois, United States*, *2. University of North Carolina at Chapel Hill (UNC), Chapel Hill, North Carolina, United States*, *3. University of Michigan, University of Michigan, Michigan, United States*

Today, there are nearly 7 million Americans living with Alzheimer's disease and more than 11 million unpaid caregivers. By 2060, the number of Americans living with Alzheimer's could reach almost 14 million. These statistics highlight the need for early career investigators to join the dementia research workforce and accelerate breakthroughs in the field. To address this need, the Alzheimer's Association Interdisciplinary Summer Research Institute (AA-ISRI) was created. AA-ISRI was developed as a five-day immersive experience in which 24 competitively-selected, early career investigators attend didactic lectures led by experts in the field, develop research proposals in small groups, and receive one-to-one mentoring. The overall goals of the program include increasing knowledge on the fundamental aspects of dementia and dementia research; increasing connections in the field; and aiding the development of fundable research proposals. This presentation will provide an overview of the AA-ISRI, including information on the goals of the program, how and why the program was developed and structured as it is, session topics, and the recruitment and selection process of AA-ISRI participants.

Abstract citation ID: igae098.1121

**PSYCHOSOCIAL RESEARCHER PERSPECTIVES OF THE SUMMER RESEARCH INSTITUTE AND TRAINING NEEDS**

Alyssa Lanzi, *University of Delaware, Newark, Delaware, United States*

Over 30 public psychosocial early career researchers have participated in the Alzheimer's Association Interdisciplinary Summer Research Institute (AA-ISRI) across the first three years of the program. During the week spent at AA-ISRI, psychosocial participants attend a variety of sessions, including both full group sessions and track-specific sessions; receive mentoring from peers and expert faculty; and refine their research proposals. Psychosocial track participants gain exposure to a wide range of potential intervention research populations and designs and enhance skills in methods for ADRD research including identifying and developing measures, utilizing mixed methods, and how to initiate and follow through with community-engaged research. The ultimate hope of the psychosocial track of the AA-ISRI is that participants will depart having formed long-term connections and the tools needed to advance their research careers. During this presentation, AA-ISRI psychosocial alum Dr. Alyssa Lanzi will describe the experience of AA-ISRI as a participant, including the highlights and helpfulness of the didactic sessions offered at the program, the mentoring and networking opportunities that were available, and the impact that AA-ISRI had on the short-term career trajectory and research. For example, since AA-ISRI, Dr. Lanzi has received an R01 funded by NIA/NIH to establish an open-access database of connected speech/language samples from older adults at-risk for dementia to study language as a potential biomarker. A key feature of this proposal is its community engagement plan to enroll those from minoritized backgrounds. This includes working with collaborators from across the country including two researchers Dr. Lanzi met at AA-ISRI.

Abstract citation ID: igae098.1122

**PUBLIC HEALTH RESEARCHER PERSPECTIVES OF THE SUMMER RESEARCH INSTITUTE AND TRAINING NEEDS**

Amy Thierry, *Xavier University of Louisiana, New Orleans, Louisiana, United States*

Over 30 public health early career researchers have participated in the Alzheimer's Association Interdisciplinary Summer Research Institute (AA-ISRI) across the first three years of the program. During the week spent at AA-ISRI, public health participants attend a variety of sessions, including both full group sessions and track-specific sessions; receive mentoring from peers and expert faculty; and refine their research proposals. Public health track participants gain a greater understanding of the public health dimensions of dementia and its impact on communities and learn about the public health approaches to addressing dementia. The ultimate hope of the public health track of the AA-ISRI is that participants will depart having formed long-term connections and the tools needed to advance their research careers. During this presentation, AA-ISRI public health alum Dr. Amy Thierry will describe the experience of AA-ISRI as a participant, including the highlights and helpfulness of the didactic sessions offered at the program, the mentoring and networking opportunities that were available, and the impact that AA-ISRI had on the short-term long-term career trajectory and research. Since the AA-ISRI, Dr. Thierry has cultivated scholarly collaborations with members of her program cohort and has connected with AA-ISRI mentors and Alzheimer's Association staff. Using the grant writing skills learned at the AA-ISRI, she has been awarded an NIH/NIA Loan Repayment Award for her project using Health and Retirement Study data to examine how neighborhood characteristics, structural stressors, and inflammation contribute to trajectories of cognitive function among racially/ethnically diverse US older adults across varying geographic contexts.

Abstract citation ID: igae098.1123

**LESSONS LEARNED FROM THE SUMMER RESEARCH INSTITUTE/TRAINING NEEDS OF EARLY RESEARCHERS IN DEMENTIA**

Sheryl Zimmerman[1], and Sam Fazio[2], *1. University of North Carolina at Chapel Hill (UNC), Chapel Hill, North Carolina, United States*, *2. Alzheimer's Association, Chicago, Illinois, United States*

Across the first three years of the Alzheimer's Association Interdisciplinary Summer Research Institute (AA-ISRI), 98% of respondents indicated they were satisfied with the program, 95% felt the program increased their understanding of Alzheimer's, and 90% said the program improved their independent research skills. Alongside the positive reception of AA-ISRI, feedback from participants, faculty, and faculty leadership led to several adjustments and changes to the structure and content of the program. For example, breakout sessions meant to provide deeper exposure to issues targeted to either public health or psychosocial research were found to be of relevance to both tracks of trainees, informing overlapping needs despite the particular area of focus. This and other changes have refined the overall flow of the program, the relevance of information shared, and the recruitment and selection of appropriate participants. This presentation will review the key lessons learned that have helped to shape the AA-ISRI over the first three years, future improvements that have been discussed for subsequent years of the program, and ideas and considerations for organizations looking to implement similar programs for researchers, and mentors working with individual mentees.

### SESSION 3005 (SYMPOSIUM)

Abstract citation ID: igae098.1124

**AGING IN A GOOD WAY THROUGH THE EYES OF ALASKA NATIVE ELDERS: RESULTS FROM A 16-YEAR CBPR STUDY**

Chair: Jordan Lewis
Co-Chair: Lena Thompson
Discussant: Steffi Kim

Predominantly Western-based biomedical models of successful aging have been used to research, understand, and explain successful aging among diverse populations. The presenters on this symposium have been exploring successful aging from an Alaska Native perspective or what it means to reach “Eldership,” or age in a good way, in urban and rural communities across the State of Alaska. This symposium will present findings from a 16-year study on Alaska Native successful aging. To learn about aging well among Alaska Natives, we have conducted 162 interviews with community-nominated Elders representing 21 Alaska Native communities in five regions of Alaska. Our team asked Alaska Native Elders what it means to them to age in a good way, what supports them to age where and how they want, and what they want younger people to know about aging in a good way. Dr. Jordan P. Lewis will provide an introductory overview of the 16-year study and share the element of emotional health and generativity. Dr. Steffi Kim will discuss the role of community engagement in successful aging for rural and urban Alaska Native Elders followed by Dr. Lena Thompson discussing the role of spirituality and Native ways of life as supporting their ability to meaningfully age. Dr. Maja Pedersen will discuss the role of physical health, including Indigenous approaches to staying active and aging in a good way. The symposium will conclude with a discussion on the interrelated nature of these themes and how attendees can apply these lessons to their own lives.

Abstract citation ID: igae098.1125

### OUR WAYS ARE A GIFT: NATIVE WAYS OF LIFE AND SPIRITUALITY AMONG ALASKA NATIVE ELDERS

Lena Thompson[1], Steffi Kim[2], and Jordan Lewis[3],
*1. University of Minnesota Duluth, Duluth, Minnesota, United States*, *2. University of Alaska, Anchorage, Alaska, United States*, *3. University of Alaska Fairbanks, College of Indigenous Studies, Fairbanks, Alaska, United States*

Spirituality plays an important role and tends to increase in importance as one ages, including for Alaska Natives. Spirituality is also one of the guiding principles of aging well and contributes to other aspects of Elders’ lives. The presence of spirituality does not ensure successful aging, but rather enables them to maintain a positive and healthy life. This presentation is part of a parent study that consists of 162 qualitative interviews with AN Elders over the course of 16 years, representing over 21 rural and urban communities across Alaska, and were analyzed using thematic analysis to explore the role of spirituality as a part of Native ways of life and successful aging. The Elders in this study practiced different forms of spirituality, including prayer, spending time on the land, participating in their Native arts and crafts, and spending time with family and community. Elders discussed how spirituality is found within most of their Native ways of life, including hunting, gathering, and preparing Native foods, and spending time with family and community. Spirituality is self-defined, unlike religion which relates to participation in a Western form of religion, like attending church. Some Elders in this study are both spiritual and religious, so it is important to ask about these practices and understand how they impact their understanding of aging in a good way. This presentation will conclude with a discussion of recommendations families, communities, and programs can do to integrate spiritual and religious activities into their programs to promote successful aging.

Abstract citation ID: igae098.1126

### INTERGENERATIONAL HEALING: GENERATIVITY AND EMOTIONAL WELL-BEING AMONG ALASKA NATIVE ELDERS

Jordan Lewis[1], Steffi Kim[2], and Lena Thompson[3],
*1. University of Alaska Fairbanks, College of Indigenous Studies, Fairbanks, Alaska, United States*, *2. University of Alaska, Anchorage, Alaska, United States*, *3. University of Minnesota Duluth, Duluth, Minnesota, United States*

The concept of generativity, defined as teaching and guiding the next generation, is an important stage of human development. Little research has been conducted with Alaska Natives on their understanding of generativity and its role and impact on their emotional health. This presentation is part of a parent study that consists of 162 qualitative interviews with AN Elders over the course of 16 years and were thematically analyzed to explore generativity and emotional health in successful aging. Across the lifespan, Alaska Native Elders engage in healthy and unhealthy behaviors which may or may not contribute to a healthy identity, or their ability to age successfully. This presentation will discuss four cultural factors, or indigenous cultural generative acts, used by Alaska Native Elders to improve their emotional wellbeing and fill the role of an Elder in their family and community. Throughout this study, Elders described ways that they “found balance” in their lives, including through family influence, being recognized as respected Elders in their communities, community and cultural engagement, and spirituality or living their Native ways of life. Elders felt that it was their responsibility and role to share their wisdom, knowledge, and experiences to help younger generations avoid some of the challenges they experienced and live healthy lives. This presentation will discuss how cultural factors can be used to guide development of culture-specific mental health treatment approaches, and more broadly, prevention strategies for all ages. Intergenerational healing can result in improved mental health for Indigenous Peoples of all ages.

Abstract citation ID: igae098.1127

### THE JOYS OF GIVING: EXPLORING BIDIRECTIONAL RELATIONSHIPS BETWEEN ELDERS AND THEIR COMMUNITIES

Steffi Kim[1], Lena Thompson[2], and Jordan Lewis[3],
*1. University of Alaska, Anchorage, Alaska, United States*, *2. University of Minnesota Duluth, Duluth, Minnesota, United States*, *3. University of Alaska Fairbanks, College of Indigenous Studies, Fairbanks, Alaska, United States*

While Western community engagement with older adults research often focuses on topics such as community program involvement, volunteerism, and civic engagement, few studies have explored the role and impact of community engagement from an Alaska Native (AN) perspective. This presentation is part of a parent study consisting of 162 qualitative interviews with rural and 12 urban-based AN Elders over the course of 16 years and was analyzed using thematic analysis to explore the role of community in successful aging. AN Elders described bidirectional involvement in community engagement, in which they were involved as participants or as leaders. These engagements increased social connectivity and maintained cultural values. While few Elders described formal volunteer roles, they often supported one another through informal roles, like delivering food to others without access to traditional foods gathered from the land and the ocean. AN Elders also explained that the role of “Elder” is an important and respected community role, and often a title given based on their sharing of wisdom, experiences, and knowledge. A unique finding of this study is the bidirectional relationship between Elders and their communities. For example, Elders often expressed interest in participating in community activities, but communities also reach out to Elders to let them know they sought out their knowledge and experiences. Rural and urban based programs and services working to enhance engagement of Indigenous Elders in their community must consider expectations and desires communities may have of Elders and the desires of Elders to share their wisdom and experiences.

Abstract citation ID: igae098.1128

### BEYOND PHYSICAL ACTIVITY: ALASKA NATIVE CONCEPTS OF PHYSICAL HEALTH, MOVEMENT, AND SUCCESSFUL AGING

Maja Pedersen[1], Steffi Kim[2], Lena Thompson[3], and Jordan Lewis[4], *1. University of Montana Missoula, Missoula, Montana, United States*, *2. University of Alaska, Anchorage, Alaska, United States*, *3. University of Minnesota Duluth, Duluth, Minnesota, United States*, *4. University of Alaska Fairbanks, College of Indigenous Studies, Fairbanks, Alaska, United States*

Physical health is a key component of Western-centered models of successful aging. Physical movement in the form of exercise and functional activities are described in the literature as strategies to maintain health through reduced risk for falls, pain, and chronic disease, and to increase quality of life. However, physical health may not be consistently conceptualized across cultures, and few studies have explored this topic among Alaska Native (AN) older adults. We used thematic analysis to evaluate physical health in qualitative interviews with 162 AN Elders across the five regions of Alaska, collected over 16 years. Five overall themes were identified. (1) Elders described recognizing physical changes in their bodies, including gray hairs, or wrinkles. These changes were often seen as signs associated with wisdom. (2) Elders described that, despite physically slowing down, they adjusted tasks to do as much as possible, and found small ways to include daily movement. (3) Elders emphasized the importance of caring for oneself when young, including avoiding substance use. (4) Elders recommended addressing health challenges with a doctor, rather than ignoring them. (5) Elders described physical health as closely tied to living one's Native ways, including physical activity through a subsistence lifestyle (e.g., gathering, hunting, fishing), relationship with the land, and benefits from eating Native foods. Overall, Elders found ways to preserve their physical health during challenging times and adjust to new norms. These findings can guide the development and tailoring of interventions designed to promote physical health among AN Elders for relevance and impact.

### SESSION 3010 (PAPER)

### ALZHEIMER'S DISEASE AND RELATED DEMENTIAS III (PAPERS)

Abstract citation ID: igae098.1129

### CHARACTERISTICS ASSOCIATED WITH PRIMARY CARE–BASED DEMENTIA SCREENING VISIT COMPLETION IN THE ERADAR STUDY

Leah Karliner[1], Sascha Dublin[2], Yates Coley[2], Clarissa Hsu[3], Tyler Barrett[2], and Deborah Barnes[1], *1. University of California San Francisco, San Francisco, California, United States*, *2. Kaiser Permanente Washington Health Research Institute, Seattle, Washington, United States*, *3. Kaiser Permanente Washington, Seattle, Washington, United States*

Nearly 7 million people in the U.S. are living with dementia, but approximately half are undiagnosed. We are performing a randomized, controlled trial in two healthcare systems (Kaiser Permanent Washington-KPWA, University of California San Francisco-UCSF) of targeted dementia screening. Among primary care patients ≥ age 65, high-risk individuals are identified using a predictive algorithm, eRADAR (electronic health record Risk of Alzheimer's and Dementia Assessment Rule), and invited for a "brain health" screening visit (BHV). Among 1709 KPWA patients in the intervention group, 506 (30%) completed a BHV; ongoing UCSF recruitment is similar (N=68/221; 31%). Visit completion rates were lower for participants of color (Asian/Black/Latinx) at 18% (N=51/336) compared with non-Hispanic whites at 34% (N=446/1,313), adjusted OR 0.36, 95% CI 0.26-0.49. Completion rates were 33% (N=30/90) among people aged 65-74 vs. 16% (N=44/268) for those aged 90+, adjusted OR per decade 0.71, 95% CI 0.56-0.90. Prior diagnoses of memory problems/concerns or mild cognitive impairment (MCI) were associated with higher odds of completion (adjusted OR 1.49; CI 1.13-1.95), and so was lower comorbidity score (adjusted OR 1.01; CI 1.00-1.03). eRADAR score and gender were not associated with BHV completion. The eRADAR study participation rate is similar to other dementia screening studies. Younger, white patients and those who might be aware of their cognitive deficits–as demonstrated by prior diagnoses–appear more likely to participate. As disease-modifying treatments increasingly become available, further research with communities of color to understand facilitators and barriers to engagement in primary-care dementia assessment is vital to prevent care disparities.

Abstract citation ID: igae098.1130

### EVALUATION OF STAFF TRAINING FOR DELIVERING A GROUP MOVEMENT PROGRAM TO VA NURSING HOME RESIDENTS WITH DEMENTIA

Matthew Miller[1], Delzio Molly[2], Margaret Chesney[1], Riya Jacob[3], Jennifer Lee[3], Linda Chao[1], Deborah Barnes[1], and Francesca Nicosia[1], *1. University of California San Francisco, San Francisco, California, United States*, *2. VA San Francisco Healthcare System, San Francisco, California, United States*, *3. San Francisco VA Healthcare System, San Francisco, California, United States*

Residents with dementia in VA nursing homes require complex care, but interprofessional staff rarely receive specialized training. Preventing Loss of Independence through Exercise (PLIÉ) is an evidence-based group movement program for residents with dementia that provides an opportunity for interprofessional staff to support mobility through functional movement, mindfulness, and other core principles. This study evaluated acceptability and impact of a remote program designed to increase scalability of interprofessional training for PLIÉ delivery among VA nursing home staff. The 10-week training program included weekly didactic sessions with pre-recorded videos (staff); weekly experiential sessions (staff, resident); and supervised teaching practicums (staff, residents). Satisfaction (staff, resident) and staff confidence in delivering PLIÉ were assessed using self-report questionnaires. Changes in resident fall history and physical function were examined using electronic medical records and the Minimum Data Set (MDS). Among 38 interprofessional staff enrolled across 4 sites, 29 (13 nurse, 6 recreation therapist, 10 other) attended at least 80% of the training sessions. The mean±SD staff satisfaction (N=22) was 4.5±0.7 (1: Poor, 5: Excellent) and confidence delivering PLIÉ was 6.7±0.7 (1: Cannot do, 7: Certain I can do). The mean±SD resident satisfaction (N=11) with the PLIÉ program was 3.7±1.0. The proportion of residents (N=32 residents; 31 male, 11 Black/African American) with a documented fall was lower after PLIÉ (Pre: 29%, Post: 6%, p=0.02), and attending more PLIÉ sessions was associated with physical function improvement (β:-0.83, p=0.03). These findings suggest the remote PLIÉ training program is acceptable and potentially improves resident fall rates and physical function.

Abstract citation ID: igae098.1131

### OPTIMIZING A PRAGMATIC TRIAL OF EMERGENCY DEPARTMENT INITIATED CARE FOR PERSONS LIVING WITH DEMENTIA

Abraham Brody[1], Manish Shah[2], Keith Goldfeld[3], Corita Grudzen[4], and Joshua Chodosh[3], *1. New York University Rory Meyers College of Nursing, New York, New York, United States*, *2. University of Wisconsin School of Medicine & Public Health, Madison, Wisconsin, United States*, *3. New York University Grossman School of Medicine, New York, New York, United States*, *4. Memorial Sloan Kettering Cancer Center, New York, New York, United States*

Over 50% of persons living with dementia (PLWD) seek emergency department (ED) care annually. However, the ED is not optimized to care for PLWD; those discharged back to the community experience adverse events. Through a recently-funded NIA Cooperative Award, we will implement an 80-site embedded pragmatic factorial designed trial of three independent, yet potentially synergistic, interventions to improve care and reduce adverse events during care transitions among PLWD and their care partners who visit the ED: Emergency care redesign, nurse-led telephonic care, and community paramedic-led transitions. While each intervention has been previously implemented, they have not been implemented concurrently. Thus, to prepare for the full-scale trial where EDs will implement zero to all three interventions, we performed an optimization study with 40 PLWD who received care and were discharged from two EDs. The optimization program examined how to efficiently implement interventions in tandem while maintaining core functions. We developed and implemented: clinical decision support pathways to identify PLWD eligible for the clinical interventions and nudge clinicians towards effective intervention implementation, modifications for when multiple interventions were utilized, and intra-intervention communication pathways. We evaluated the optimization through implementation metrics and semi-structured interviews with engaged clinicians, PLWD and their care partners. Particular challenges requiring adaptations included time required to implement clinical decision support, creating effective communication pathways between intervention programs, developing staffing flexibility in the nurse telephonic program to allow for coverage at key times while balancing nursing staff needs, and engaging ED staff in performing team huddles at the bedside.

Abstract citation ID: igae098.1132

### SUICIDE IN MILD COGNITIVE IMPAIRMENT [MCI]: DATA FROM OVER 370,000 OLDER VETERANS WITH MCI

Ruth Morin[1], Aaron Ritter[1], Yixia Li[2], and Amy Byers[3], *1. Hoag Memorial Hospital Presbyterian, Newport Beach, California, United States*, *2. NCIRE, San Francisco, California, United States*, *3. San Francisco VA Healthcare System, San Francisco, California, United States*

Prior research has demonstrated that receiving an MCI diagnosis increases risk for near-term suicide attempt. Moreover, psychiatric comorbidity associated with MCI, which may exacerbate conversion to dementia, is also associated with suicide risk. To our knowledge, the current study is the first to comprehensively characterize the occurrence of suicide attempt and associated clinical factors in a large nationally representative sample of older Veterans with MCI. This study leveraged a cohort of Veterans over age 50 who used VA healthcare between 2012-2020 (N = 5,059,526). There were 370,546 individuals with clinically diagnosed MCI identified. Among those, 5,101 attempted suicide (fatal and non-fatal) during the study period (1.4% of the MCI sample). Of those who attempted suicide, 574 were fatal (11.3%). Individuals who attempted suicide were significantly younger, more likely to be female, and less likely to be White than those who did not attempt (all p<.001). Psychiatric comorbidity was strikingly high among the MCI group with a suicide attempt (98.4% with an attempt had at least one psychiatric comorbidity compared to 81.5% who did not attempt). Specifically, there was significantly higher prevalence of any mood disorder, anxiety disorder, substance abuse disorder, psychotic spectrum disorders, and sleep disturbances. Of individuals with MCI who attempted suicide, 71.4% had prior suicide attempt before receiving an MCI diagnosis and nearly 80% had reported suicidal ideation at some point during the study period. Findings indicate a call for investigation into mechanisms of suicide risk and strategies for prevention/intervention in this vulnerable population of adults with MCI.

Abstract citation ID: igae098.1133

### USING MICRO-CREDENTIAL TO ADVANCE LONG-TERM CARE STAFF EXPERTISE IN PALLIATIVE CARE FOR PEOPLE WITH DEMENTIA

Winnie Sun[1], Jen Calver[2], Lucas Martignetti[3], Volletta Peters[2], and Manon Lemonde[2], *1. Ontario Tech University, Lindsay, Ontario, Canada*, *2. Ontario Tech University, Oshawa, Ontario, Canada*, *3. Ontario Tech University, OSHAWA, Ontario, Canada*

**Background:** Ontario Tech University partnered with experts from Alzheimer's Society of Canada (Durham Region of Ontario) and Ontario Shores Centre for Mental Health Sciences to develop a micro-credentialing certificate program in dementia care. This study aims to evaluate the usability and applicability of using micro-credential learning to advance long-term care staff's expertise in providing palliative care for people with dementia.

**Methods:** Registered Practical Nurses (RPNs), Registered Nurses (RNs), Nurse Practitioners (NP) and Personal Support Workers (PSWs) (n=19) who worked in long-term care homes located in Southern Ontario completed a one-day training to pilot-test a gamified micro-credential education related to palliative care for people with dementia. Upon completion of the training, a quantitative pre-assessment and post-assessment were used to evaluate learner's perceived knowledge, skill, attitude and confidence as measured by the Educational Self-Efficacy Scale.
**Results:** Overall, there was a statistically significant increase in knowledge after the micro-credential training (P<.0001), with a mean increase of 3.2 more questions answered correctly. Focus group findings revealed four key themes: opportunities of using gamified simulation to promote learner's engagement; lack of palliative care training to support people with dementia; need to strengthen workplace training using micro-credential for upskilling; and meaning of palliative care in long-term care sector.
**Conclusion:** Palliative care is recognized to be an important part of work in long-term care as residents are often admitted with complex and life-threatening conditions. Participants highlighted the feasibility of enhancing their knowledge and competence in palliative care through specialized training using gamified, simulation learning through micro-credential training.

Abstract citation ID: igae098.1134

### EFFECTS OF ENVIRONMENTAL EXPOSURE ON DISPARITIES IN THE RISK OF ALZHEIMER'S DISEASE AND RELATED DEMENTIAS

Igor Akushevich[1], Arseniy Yashkin[1], Vito Di Bona[1], and Julia Kravchenko[2], *1. Duke University, Durham, North Carolina, United States*, *2. Duke University School of Medicine, Durham, North Carolina, United States*

Chronic exposures to a range of environmental contaminants, in particular fine particulate matter (PM2.5), have been shown to be associated with an increased risk of Alzheimer's disease and related dementia (AD/ADRD). However, causal relationships between the effects of ambient air pollutants (especially for low-to-moderate-level exposures) and PM2.5 constituents on AD/ADRD risk remain understudied in both the general population and vulnerable population groups in the U.S. The objective of this study is to evaluate the effects of exposures to PM2.5 and PM2.5 constituents (including black carbon, NH4, SO4, trace elements, and some others), test the causality of contaminant-outcome associations, and explain the epidemiological mechanisms of these causal pathways. To address this objective, we used Medicare-claims data and zip-code-level environmental data provided by the Socioeconomic Data and Applications Center (SEDAC) including daily and annual estimates of the concentrations of PM2.5 for 2000-2016, and trace elements including bromine, calcium, copper, aluminum, iron, lead, nickel, potassium, silicon, vanadium, and zinc. We reconstructed the concentration-response patterns of exposures to ambient contaminants on AD/ADRD risk and applied the parametric g-formula approach to test the causality of detected effects. We evaluated the concentration-response functions for all contaminants and found deviations from the linear dependence for concentrations above 10 μg/m3 (WHO standards) and for the Hispanic subpopulation. The highest risks were found for Native Americans, with a hazard ratio (HR) vs. White subpopulation exceeding HR>3 over the full range of average concentrations (5-12 μg/m3 for PM2.5). NH4, SO4 and lead demonstrated the highest effect among constituents of PM2.5.

### SESSION 3015 (PAPER)

### ANTI-AGEISM: BRINGING MEANING INTO OLD AGE

Abstract citation ID: igae098.1135

### "HOW WILL I EVER KNOW I DIDN'T BRING IT ON MYSELF?": PERCEIVED PERSONAL RESPONSIBILITY IN DEMENTIA RISK

Allie Peckham[1], Molly Maxfield[1], and Dara James[2], *1. Arizona State University, Phoenix, Arizona, United States*, *2. Arizona State University, Tempe, Arizona, United States*

As public health messages increasingly emphasize the importance of "lifestyle interventions" to reduce dementia risk, our study aimed to explore how community dwelling middle-aged and older adults perceive this information. Here, our focus is on understanding how information around lifestyle interventions influences individuals' perceptions of their control over health in relation to dementia risk. Our study further investigated how these health-promoting interventions might unintentionally contribute to 'lifestyle stigma' for individuals diagnosed with dementia. Through a secondary framework analysis of 50 semi-structured interviews, we found that participants showed a sense of personal responsibility towards engaging in lifestyle interventions to lower their chances of dementia. To interpret our findings, we relied on the concepts of locus of control and attribution theory. Of the 50 participants, we discovered that 23 participants discussed dementia risk, and 13 felt a degree of personal responsibility for their dementia risk. Four believed they had personal responsibility and control and actively engaged in lifestyle interventions. The remaining nine participants also engaged in lifestyle interventions, aiming to find comfort in the potential event of a dementia diagnosis. Recognizing the multifaceted nature of dementia risk, including environmental and external factors beyond individual control, is essential to communicating effectively and combating 'lifestyle stigma' that is becoming increasingly associated with the condition. By acknowledging the interplay between individual, social, and societal factors, we can develop more inclusive and effective public health strategies and messages to reduce dementia risk and promote overall well-being.

Abstract citation ID: igae098.1136

### ADVANCED OLD AGE: NARRATIVES, NEEDS, AND NOTIONS

Elaine Eliopoulos[1], and Chris Gilleard[2], *1. Private Counsel-Committee for Public Counsel Services (contract), Weston, Massachusetts, United States*, *2. University College London, London, England, United Kingdom*

Longevity trends worldwide have highlighted the need to advance theoretical development of advanced old age beyond a singularly focused decline narrative. This narrative contributes to a doomed sense of old age without consideration for the subjectivities which may contribute to a deeper understanding of the experience of advanced old age beyond its bodily capabilities. This paper reports findings from a qualitative study depicting personal narratives elicited from 23 individuals aged 80 to 102 living on the San Juan Islands in the state of Washington. Using a mix of photo-elicitation and semi-structured interviews, participants narratives reflected a specific focus on their needs and interests despite a range of corporeal challenges. Those needs and interests foregrounded a sense of community experienced in diverse ways on their island. The participants' focus on community and belonging gives rise to the benefits of embracing new models of advanced old age which capture a sense of contented engagement with both nature and community that contrasts with the dominant discourse of decline and offers new notions of advanced old age. These new notions may enhance both quality of life and directly inform policy designed to address quality.

Abstract citation ID: igae098.1137

### EXPRESSIONS OF INTERSECTIONALITY IN OLD AGE THROUGH NARRATIVE RESEARCH AND PHOTO EXHIBITION

Ksenya Shulyaev[1], Tova Band-Winterstein[2], Tal Dekel[3], Zvi Eisikovits[1], and Reem Nashef-Hamuda[4], *1. University of Haifa, Haifa, Hefa, Israel*, *2. University of Haifa, Haifa, HaZafon, Israel*, *3. Kibbutzim College of Education, Tel Aviv, HaMerkaz, Israel*, *4. Haifa University, Haifa, HaZafon, Israel*

This presentation analyzes the complex experiences of old women navigating multiple marginalized identities, through an intersectional lens. There is relatively limited in-depth representation of older women at the crossroads of multiple social disadvantages. This project addresses this gap through a collaboration with 15 older women in Israel from diverse minority groups (lesbian/transgender, women with disabilities, Israeli Arabs, immigrants from Ethiopia, and former USSR). The project aims to visualize older women's self from an intersectional perspective by creating a visual documentary – (a photo exposition) with added verbal narrative of their life stories. Utilizing visual narratives and in-depth interviews, the project explores the unique experiences of old women shaped by age and additional marginalized identities. Professional artists guided the photo sessions, ensuring the women's vision and representation were prioritized. The resulting artwork was exhibited publicly at the University of Haifa library with accompanying small group sessions aimed at increasing awareness of students and the community about discrimination against older women. Visitors to the exhibition will be interviewed for further analysis and understanding of the impact of the exhibition in terms of visibility and attitudinal change. The exhibition will become a permanent online display on the Minerva Center of Intersectionality in Aging website. The presentation of on GSA conference includes photos and citations of the life stories of women who participated in the project. This unique project illuminates the lived reality of aging women intersectionality, offering insights to gerontological researchers and practitioners, challenges stereotypes and empowers women to express their experiences visually

Abstract citation ID: igae098.1138

### WHO IDENTIFIES WITH AGE-BASED DISCRIMINATION?

Douglas Hanes, *Stony Brook University School of Medicine, Stony Brook, New York, United States*

**Background:** Ageism remains a persistent problem that contributes to worse later-life outcomes. Since the COVID-19 pandemic, ageism has received greater attention from activists, researchers, and policymakers. Yet little is known about who identifies with ageism as the basis of their discriminatory experiences, and how people with intersecting marginalized identities endorse ageism as a basis for their discrimination.

**Method:** Health and Retirement Study (2008–2018, N = 16,250) participants were asked about their experiences of discrimination and the reasons for them, including age and other factors. We analyze which experiences and identities predict endorsement of age as the basis of participants' discrimination. Multilevel logistic regressions with "discrimination due to age" as the outcome variable were conducted.

**Results:** Age predicted a small increased likelihood of ageism (OR = 1.08; $p < 0.001$), but more frequent discrimination decreased it (OR = 0.877, $p < 0.001$). People of color were less likely to report ageism than non-Hispanic whites (OR = 0.552, $p < 0.002$), regardless of reporting racist discrimination; whites who reported race-based discrimination had greater odds of also reporting ageism (OR = 1.563, $p < 0.001$). Women were more likely to report ageism (OR = 1.139; $p = 0.002$); reporting gender-based discrimination increased ageism probability for both women (OR = 2.318, $p < 0.001$) and men (OR = 3.037, $p < 0.001$).

**Discussion:** Members of certain identity groups and with certain experiences are more likely to understand themselves as subject to ageism than others, which may affect the effectiveness of anti-ageist messaging and interventions.

Abstract citation ID: igae098.1139

### IT'S RAINING, IT'S POURING, THE OLD MAN IS SNORING: AGE STEREOTYPES IN NURSERY RHYMES

Reuben Ng, and Nicole Indran, *National University of Singapore, Singapore, Singapore*

Ageist beliefs tend to take root in one's formative years and persist into adulthood. As future older adults, children must be instilled with more positive attitudes towards aging. To gain insight into the ways in which children are being socialized to view old age, this study examines the depictions of old age in nursery rhymes. We built a comprehensive dataset of nursery rhymes by gathering material from four websites. In total, 735 unique rhymes were retrieved. To identify rhymes related to old age, we conducted a search using various age-related terms (e.g., old, elder, grandfather, grandma). This search yielded 85 nursery rhymes. After applying our exclusion criteria (e.g., instances where 'old' referred to buildings that have been around for a long time), we were left with 34 rhymes. Both inductive and deductive approaches guided our qualitative content analysis of the rhymes. Overall, old age was mentioned in 5% (N=34) of the 735 nursery rhymes. Of the 34 rhymes, half contained negative age stereotypes (50%; N=17). The remaining rhymes featured about an equal mix of positive (26%; N=9) and neutral age stereotypes (24%; N=9). In the context of an aging population, it is paramount that society is led by people with a realistic outlook of aging. Although nursery rhymes may seem like mere tales not to be taken seriously, they nonetheless remain powerful tools capable of molding thought processes. Our study sets the stage for telling children accurate and nuanced stories about older adults to inculcate healthy attitudes towards aging.

Abstract citation ID: igae098.1140
### A MOSAIC OF RESILIENCE: INDIVIDUAL AND SOCIAL FACTORS FOR AGING WELL AMONG DIVERSE OLDER ADULTS
Aisha Cozad[1], Cassandra Cantave Burton[1], and Fanni Farago[2], *1. AARP, Washington, District of Columbia, United States*, *2. AARP, District of Columbia, District of Columbia, United States*

In the wake of the COVID-19 pandemic and continuing extreme weather events, the resilience of older adults in the areas of health and disaster management and preparedness are critical areas of study. As shown by AARP survey research, self-reported happiness and emotional well-being and resiliency is high among older adults, including across racial and ethnic groups. At the same time, these studies also demonstrate that many older adults have trouble bouncing back from hardships and feel concerned about the possibility of declining health and their ability to live independently. In related AARP research, survey data illuminates the crucial role that quality relationships and community features, including affordable and accessible housing, healthcare, and opportunities for social engagement, play in providing structural supports for older adults to age well in their homes and communities. Drawing on multiple AARP survey studies, this presentation will explore different aspects of resilience in aging among diverse communities of older adults, including those who identify as Black/African American, Hispanic/Latino, or LGBTQ.

### SESSION 3020 (BIOLOGICAL SCIENCES INVITED SYMPOSIUM)

Abstract citation ID: igae098.1141
### ADVANCES IN CELLULAR SENESCENCE
Chair: Joao Passos

Abstract citation ID: igae098.1142
### NEW INSIGHTS INTO MITOCHONDRIA AND CELLULAR SENESCENCE
Joao Passos, *Mayo Clinic, Rochester, Minnesota, United States*

Senescence is a multi-functional cell fate, characterized by an irreversible cell-cycle arrest and a pro-inflammatory phenotype, commonly known as the senescence-associated secretory phenotype (SASP). Emerging evidence indicates that accumulation of senescent cells in multiple tissues drives tissue dysfunction and several age-related conditions. This has spurred the academic community and industry to identify new therapeutic interventions targeting this process. Senescence is a complex and highly heterogenous phenotype and despite tremendous progress in recent years we are still far from fully understanding it. There is no single, standalone marker that allows the identification of senescent cells, posing a challenge to their identification and targeting. Mitochondrial dysfunction is an often-unappreciated hallmark of cellular senescence which plays important roles not only in the senescence growth arrest but also in the development of the SASP and resistance to cell-death. In my talk, I will first describe some of our recent efforts to accurately map senescent cells in different tissues during aging. Additionally, I will describe how mitochondria contribute to the development of senescence and resistance to cell-death. Finally, I will propose that a detailed road map of mitochondrial biology in senescence will be crucial to guide the future development of therapies targeting cellular aging.

Abstract citation ID: igae098.1143
### HOW OXIDATIVE DAMAGE TO TELOMERES PROMOTES PREMATURE CELLULAR SENESCENCE
Patricia Opresko, *University of Pittsburgh, Pittsburgh, Pennsylvania, United States*

Telomeres are protective caps at chromosome ends required for genome stability and sustained cell proliferation. Oxidative stress is associated with accelerated telomere shortening and dysfunction, and telomeric sequences are highly susceptible to oxidative damage. To study how oxidative damage impacts telomere function, we use a chemoptogenetic tool that produces the common oxidative lesion 8-oxoguanine (8oxoG) selectively at telomeres. Human cancer cells =are largely unaffected by a single induction of telomeric 8oxoG, but chronic damage impairs cell growth and increases telomere shortening and loss. In contrast, a single induction of telomeric 8oxoG in non-disease human fibroblast and epithelial cells triggers hallmarks of p53-dependent cellular senescence. Telomeric 8oxoG promotes replication stress at telomeres, leading to telomere fragility rather than accelerated telomere shortening. Surprisingly, the loss of repair enzymes OGG1 and MUTYH partially rescues the telomeric 8oxoG-induced senescence and telomere dysfunction, while double knockout causes a near complete rescue. These glycosylases initiate base excision repair at sites of 8oxoG, leading to repair intermediates, including abasic sites and single strand breaks. Following telomeric 8oxoG damage, repair intermediates accumulate at telomeres in wild type, but not OGG1/MUTYH deficient cells. These data suggest that inefficient completion of 8oxoG BER at telomeres triggers cellular senescence via repair intermediates which disrupt telomere function. We uncovered a novel mechanism for telomere-driven rapid senescence that depends on DNA replication and glycosylase activity but occurs in the absence of telomere shortening. This mechanism may contribute to markers of telomere dysfunction that increase with age and oxidative stress in slowly proliferating tissues.

Abstract citation ID: igae098.1144
### HIGH RESOLUTION SPATIAL PROTEOGENOMICS TO ASSESS TARGET ENGAGEMENT AND COMPARE SENOLYTIC EFFICACY IN THE BRAIN OF TAU TRANSGENIC MICE
Miranda Orr[1], Valentina Garbarino[2], and Timothy Orr[3], *1. Wake Forest University School of Medicine, Winston-Salem,*

*North Carolina, United States*, 2. *UT Health San Antonio, San Antonio, Texas, United States*, 3. *Wake Forest University School of Medicine*

Tau protein accumulation induces cellular senescence in Alzheimer's disease and other tauopathies contributing to dysfunction and disability. Treating tau transgenic mice with pharmacological agents to clear senescent cells, "senolytics," reduced brain pathology and improved structure and function. We now report results from directly comparing the previously used senolytic therapy, dasatinib plus quercetin (D+Q), to fisetin (FIS), a senolytic with a potentially favorable side-effect profile. Fourteen-month-old male and female wild type (WT) and tau transgenic (rTg4510) mice were treated intermittently for 12 weeks with D+Q, FIS or vehicle by oral gavage. Physical and behavioral outcomes were measured before and after treatment, and we utilized spatial proteogenomics for high-resolution target engagement. In vivo assessments included assays for exploratory behavior, cognition, stereotypic behavior, physical performance and frailty. Genotype- and sex-specific treatment effects will be discussed. Both senolytic treatments were equally protective against neurodegeneration as evidenced by smaller lateral ventricle size in senolytic treated rTg4510s compared to those vehicle treated. GeoMx digital spatial profiling and CosMx spatial molecular imaging data indicate treatment-specific effects with a genotype interaction. In general D+Q exerted a greater effect on tau neuropathology in rTg4510 mice; other treatment-specific differences across cell types and brain regions will be presented. Overall our data suggest that tailoring senolytic therapies to specific individuals in the future based on sex and disease state may provide the most benefit as we continue to translate this promising therapy to clinical use.

Abstract citation ID: igae098.1145

### DISSECTING THE HETEROGENEITY OF SENESCENCE: PROFILING PRIMARY AND SECONDARY SENESCENT STATES USING SINGLE-CELL AND SPATIAL TRANSCRIPTOMICS

Nicola Neretti, *Brown University, Providence, Rhode Island, United States*

Cellular senescence is a state of irreversible cell cycle arrest, contributing to age-associated tissue function decline due to the accumulation of senescent cells. The Senescence Associated Secretory Phenotype (SASP), a collection of signaling molecules and growth factors secreted by these cells, plays a significant role in the sterile inflammation seen in aging tissues. Recent studies, both in vivo and in vitro, have demonstrated that senescence is a heterogeneous phenotype, varying based on cell type, induction method, and duration of senescence. An important aspect of this heterogeneity is the distinction between primary and secondary senescence; primary senescent cells can induce nearby cells into a secondary senescent state, which shares some markers with primary senescence but differs significantly in others, such as the SASP. In this study, we explore the evolution of primary and secondary senescence over time by profiling individual cells during the early stages of senescence establishment using single-cell transcriptomics. We have developed an AI/ML methodology capable of classifying cells into primary and various forms of secondary senescence. This methodology is compared with existing methods like SenMayo and SenSig, and applied to time-course scRNA-seq data to determine the proportion of cells in different senescent states. Additionally, we present findings on the identification of senescent cells in vivo using spatial transcriptomics to study the aging mouse liver.

### SESSION 3025 (PAPER)

### CAREGIVING IN A GLOBAL CONTEXT

Abstract citation ID: igae098.1146

### THE UNEQUAL ENTITLEMENT TO FILIAL CARE: HOW DO OLDER CHINESE IMMIGRANTS MOBILIZE SUPPORT FROM ADULT CHILDREN

Xuemei Cao, *Bentley University, Waltham, Massachusetts, United States*

The broad-brush image of vulnerable older immigrants conceals their heterogeneous pathways to later life and their varied abilities to mobilize care during personal and health crises. This study examines why some older immigrants hesitate to seek help from their children whereas others feel entitled to ask for help. I conducted in-depth interviews with 42 older Chinese immigrants (aged 60 and above) in Massachusetts and ethnographic observations at various spaces where my participants congregated. Built on the cumulative advantage/ disadvantage theory, I show that international migration is a critical life transition that triggers immigrants' unequal and gendered cumulation of material, cultural, social, and moral resources in the US and China. Those with earlier age at migration, higher socioeconomic status, and legal and social membership in the US can selectively preserve aspects of the ethnic culture of filial piety, and request help from their children as needed. By contrast, those who arrived in mid-to-late adulthood, who are of lower socioeconomic status, and who question earlier parenting and migration decisions (e.g., transnational separation) feel less entitled to seek help despite their desires for filial care. Unequal life-course resource cumulation leads to widening disparities in immigrants' later-life health outcomes and well-being through the process of support mobilization. Considering that those who need help the most often feel least entitled to ask for help from their families, social and aging services should proactively offer support to these older adults to ensure their well-being and reduce inequalities.

Abstract citation ID: igae098.1147

### FROM CARE DYAD TO SUPPORT NETWORKS: PURCHASE INTENTION FOR HOME- AND COMMUNITY-BASED SERVICE OF CHINESE OLDER ADULTS

Zi Yan[1], and Xin Sun[2], *1. Waseda University, Tokyo, Japan, 2. Fudan University, Shanghai, Shanghai, China (People's Republic)*

This study examines the underlying types of intergenerational solidarity and informal support networks among impaired older adults in China, and explains how they may influence the home- and community-based service purchase intention of these individuals. Based on the 2018 and 2020 waves of the Chinese Longitudinal Aging and Social Survey, a total of 3,564 older adults (aged 60 and above, with at least one child) were selected. First, we use K-means cluster analysis to identify four types of intergenerational solidarity among Chinese families: tight-knit, distant-but-intimate, independent-intimate, and emotional-detached. Secondly, we examined the size of three distinct types of informal support networks among Chinese older adults: general social networks, functional support networks, and emotional support networks. Subsequently, binary logistic regression was conducted to examine the relative impacts of intergenerational solidarity and informal support networks on impaired older adults' purchase intention. Our findings highlighted the diverse impacts of informal functional/emotional support networks on older adults' purchase intentions. These networks exhibit the potential to either substitute for or complement formal care, contingent upon the specific types of intergenerational solidarity. This research enriches the theoretical literature by incorporating both vertical and horizontal perspectives. It also enhances our understanding of the dynamics between 'formal' and 'informal' care within the Chinese context. This study underscores the necessity for precise targeting in service delivery, emphasizing the importance of considering the entire family and the influence of the older adults' social network on service utilization and purchase decisions.

Abstract citation ID: igae098.1148

### SOCIAL STRATIFICATION IN THE TIME-USE OF INFORMAL CAREGIVERS FOR OLDER INDIVIDUALS

Marco Albertini[1], and Francesca Zanasi[2], *1. University of Bologna, Bologna, Emilia-Romagna, Italy*, *2. Università di Bologna, Bologna, Emilia-Romagna, Italy*

In ageing societies, the increasing share of older adults places significant care burdens on family members, who experience negative consequences on psychological and physical health, and social isolation. These adverse effects have to do with time use restrictions because of the time priority required by caregiving. Caregivers often cut on labour market involvement, sleeping, and social activities and relationships they previously enjoyed. Despite extensive research, the socioeconomic disparities in caregiver experiences remain underexplored. For example, more disadvantaged individuals could suffer the most from caregiving, having limited possibilities to 'buy time for themselves' by purchasing services on the market. Our study, using data from the Survey of Health, Aging, and Retirement in Europe (SHARE, 2020), investigates how health shocks to partners (i.e., exogenous shocks leading to increasing demands for informal caregiving, such as the onset of dementia, hip fracture, stroke) influence caregivers' daily activities; and how socioeconomic characteristics moderate this relationship. Preliminary results show that care responsibilities influence the caregiver's time expenditure, with heterogeneity across gender and socio-economic status. The most affected by a partner's health shock are men at the bottom of the income distribution and women at the top. When having an impaired partner, the former spend more time in household chores and cut on leisure activities, while the latter sleep less at night and cut on paid work. In conclusion, it is essential to understand which individuals face the consequences of caregiving and reiterate the importance of a social infrastructure supporting families caring for their dependent family members.

Abstract citation ID: igae098.1149

### WHAT IS IN A NAME: DEMENTIA AND CAREGIVERS IN THE AFRICAN IMMIGRANT COMMUNITY

Manka Nkimbeng, Truphosa Aswani, and Joseph Gaugler, *University of Minnesota, Minneapolis, Minnesota, United States*

African immigrants are among the racial/ethnic minority groups whose experiences of dementia are not fully understood. However, the use of appropriate terminology is necessary to facilitate appropriate outreach and research with the African immigrant community. The purpose of this qualitative study is to describe how African immigrants define dementia and care partners. Thematic analysis of transcripts from ten key informant interviews (with leaders including priests, executive directors, etc.) and three community conversations (30 participants). Themes describe that there are no accepted words for dementia or caregiver in many African immigrant languages. Translations for dementia are often based on the symptoms, other derogatory terminology, and myths. The concept of a caregiver or care partner is also unfamiliar to the community, even when individuals are performing such roles. The language surrounding dementia care significantly impacts healthcare access and quality care, especially for multilingual and multicultural communities. Identifying and clarifying these terminologies are a key component of outreach and education that is necessary to decrease the risk and burden of dementia in these communities that are disproportionately impacted.

Abstract citation ID: igae098.1150

### LONGITUDINAL TRAJECTORIES OF CAREGIVING BENEFITS AMONG FAMILY CAREGIVERS OF OLDER ADULTS

Yongjing Ping[1], Jeremy Lim-Soh[1], Truls Østbye[2], and Rahul Malhotra[1], *1. Duke-NUS Medical School, Singapore, Singapore*, *2. Duke University, Durham, North Carolina, United States*

Although caregiving is often associated with burden, family caregivers can gain benefits – such as satisfaction, increased self-worth, and improved outlook towards life – from caregiving, which are linked with better care-recipient and caregiver outcomes. Acknowledging the dynamic nature of caregiving, many studies have investigated longitudinal patterns of caregiving burden, but studies on trajectories of caregiving benefits are limited. Furthermore, understanding the heterogeneity in trajectories of caregiving benefits will help identify risk factors linked with trajectories indicating low benefits or protective factors linked with high benefits over time. We conducted group-based trajectory modelling on longitudinal data – gathered at four time points, at six-to-twelve-month intervals – on caregiving benefits (short Positive Aspects of Caregiving scale) from 274 family caregivers of older adults in Singapore. We identified three relatively stable trajectory groups – "fulfilled" (14%, highest benefits), "satisfied" (64%, second highest benefits), and "dissatisfied" (22%, lowest benefits). Relative to caregivers in the "fulfilled" group, those cohabiting with their care-recipients were less likely to be in the "satisfied" group, those more prepared for caregiving or supported by experienced migrant domestic workers were less likely to be in the "dissatisfied" group, and those caring for care-recipients with more behavioral impairments were more likely to be in the "dissatisfied" group. Our findings provide evidence for heterogeneity in trajectories of caregiving benefits, indicate that many caregivers maintain relatively high caregiving benefits over time, and suggest that preparing migrant domestic workers and family caregivers for their caregiving role may contribute towards higher caregiving benefits over time.

### SESSION 3030 (SYMPOSIUM)

Abstract citation ID: igae098.1151

### COGNITIVE AGING AMONG OLDER LATINOS IN THE US, MEXICO, AND BRAZIL

Chair: Jacqueline Angel
Co-Chair: Flávia Andrade
Discussant: Jacqueline Angel

Amidst rapid demographic transitions in the Americas, where dementia prevalence is poised to double by 2050, this symposium offers a crucial exploration of cognitive health in the Americas. Leveraging data from Brazil, Mexico, and the US, it emphasizes understanding measurement, contextual factors, diversity of experiences, and associated costs in addressing this pressing global challenge. Cantu et al. scrutinize how gender influences cognitive assessments in older adults in the US and Mexico, unveiling potential biases in reporting and underscoring the need for accurate diagnosis. Exploring cultural resources among very old Mexican American immigrants, Kim and Silverstein shed light on their impact on psychological and cognitive well-being, highlighting the role of cultural context in shaping health outcomes and offering insights into protective factors against cognitive decline. Estimating the economic impact of dementia, Mudrazija et al. assess lost wages, medical care spending, and unpaid care costs for African American and Latino adults in the US and their caregivers, projecting these costs through 2060. García-Chanes and Lopez-Ortega characterize the sample of older adults in Mexico living with dementia in 2016 and the specific sources of social and economic support they receive. Lastly, Chen et al. examine the association between Internet use and cognitive health among Brazilian older adults, suggesting the potential of technology in promoting cognitive well-being and stressing the importance of innovative approaches in enhancing cognitive health outcomes. By synthesizing diverse perspectives and data sources, this symposium offers invaluable insights for developing targeted interventions and policies to address the challenges posed by dementia.

Abstract citation ID: igae098.1152

### SOCIAL AND ECONOMIC SUPPORT FOR PEOPLE LIVING WITH DEMENTIA IN MEXICO

Mariana Lopez-Ortega, and Rosa Garcia-Chanes, *Instituto Nacional de Geriatria, Mexico City, Distrito Federal, Mexico*

Dementia is one of the most common causes of disability and dependence in the world and is a frequent reason that older people require supportive living situations. In Mexico, Alzheimer's and other dementias represent the fifth main cause of DALYS (disability-adjusted life-years) and it is estimated that there were approximately 1 626 000 persons living with dementia. However, the health system is not prepared to adequately provide diagnosis, post-diagnosis care as well as care support at home for people living with dementia and their family caregivers. In addition, economic conditions of a large number of older persons is precarious given a life of informal work with no benefits or access to a contributory pension in old age and whose sole income is a small non-contributory pension and relying on children or other close relatives to provide financial and in-kind support, as well as all other health and personal care. The aim of this study is to characterise the support received by a sample of older adults in Mexico. We use the Mexican Health and Aging Study 2016 wave which for the first time included the Mex-Cog Wave 1 a detailed cognitive assessment. This allows for a detailed characterisation of support received by people living with dementia and to compare it with the support available and received by older adults with no impairment. The results allow for specific recommendations for further support programs and activities that focus on areas of need and currently not covered either by public or family supports.

Abstract citation ID: igae098.1153

### INTERNATIONAL COMPARISON OF GENDER DIFFERENCES IN INFORMANT REPORTS OF COGNITIVE DECLINE IN OLDER ADULTS

Phillip Cantu, *University of Texas Medical Branch, Galveston, Texas, United States*

Informant assessments aid in diagnosing cognitive decline and dementia, yet cultural and social factors may influence reporting. This study investigates gender disparities in informant reports of cognitive function across the US and Mexico using data from Harmonized Cognitive Assessment Protocol (HCAP) of the Health and Retirement Study (HRS) and the Mexican Cognitive Aging Study (MexCOG). Analyzing 4,716 participants, we utilize comparable informant questions and contextual data, including age, gender, education, and MMSE scores. Logistic regressions model the relationship between gender and informant reports separately for each nation. Preliminary analysis reveals gender differences in informant reports of cognitive functioning in both countries. In general, informants were more likely to report symptoms of cognitive decline in the U.S. than in Mexico. Informants were more likely to report memory issues (forgets where things are kept, forgets where they put things), and difficulty with chores for women in both countries. There are fewer gender differences in orientation questions for each nation, maybe related to high level of impairment. In Mexico, informants are more likely to report conversation impairments for women. This study sheds light on the nuanced interplay of cultural and social factors in informant assessments of cognitive function among older adults in diverse national contexts.

Abstract citation ID: igae098.1154

### ECONOMIC IMPACT OF DEMENTIA ON AFRICAN AMERICAN AND LATINO ADULTS: A GROWING CHALLENGE

Stipica Mudrazija[1], Maria Aranda[2], Darrell Gaskin[3], Patrick Richard[4], and Stephanie Monroe[5], *1. University of Washington, Seattle, Washington, United States*, *2. University of Southern California, Los Angeles, California, United States*, *3. Johns Hopkins University, Baltimore, Maryland, United States*, *4. Uniformed Services University, Bethesda, Maryland, United States*, *5. UsAgainstAlzheimer's, Washington, District of Columbia, United States*

In this study, we estimate the economic impact of dementia today and through 2060 associated with a) lost wages of persons living with dementia as well as their caregivers, b) excess medical care spending for persons living with dementia, c) value of unpaid care, and d) the value of wider economic costs (lost productivity, lost tax revenue), for African American and Latino adults living in the US and their caregivers. In 2020, the total economic impact of dementia for African American and Latino adults reached almost $94 billion, including over $62 billion (66%) in unpaid care and over $31 billion (34%) in various costs for older adults, their caregivers, and others. Latino and African American adults accounted for 60 and 40 percent of the costs, respectively. By 2060, the economic impact of dementia for African American and Latino adults will increase to $1.4 trillion (in 2060 dollars). The share of the total attributable to unpaid care will decline from 67 percent to 60 percent, while the share of various costs will increase, and most notably forgone wages (from 21% to 27%). The shift in the cost distribution is due mainly to shifts in the caregiving population's age distribution and educational attainment level. Efforts aimed at keeping working-age caregivers attached to the labor force will become increasingly important to limit the fast growth in the value of work-related costs of caregiving.

Abstract citation ID: igae098.1155

### CULTURAL RESOURCES AND PSYCHOLOGICAL WELL-BEING INFLUENCES ON COGNITIVE FUNCTION IN MEXICAN AMERICAN IMMIGRANTS

Jeung Hyun Kim, and Merril Silverstein, *Syracuse University, Syracuse, New York, United States*

The present research investigates the mechanisms through which different cultural resources retained by the very old Mexican American immigrants shape their psychological and cognitive wellbeing. The very old immigrants are likely to adhere to the traditional values originated from their country of origin and may be isolated from the mainstream culture. The roles that acculturation and the availability of ethnocultural resources like familismo and religious practices play can generate their loneliness and depression, which then, lead to their cognitive health disparities. It uses HEPESE (Wave 7), the epidemiological study of older Mexican Americans residing in the Southwestern U.S. Path models are utilized for the analysis. Of 368 very old immigrants, results reveal that stronger intergenerational ties and marriage reduce loneliness while increased acculturation, immigrating at a younger age as a proxy measure of acculturation, and fewer religious engagement elevate depression. Though there is a marginal association between the psychological wellbeing and the global cognitive health, cognitively impaired individuals are likely to associate with their adult children more. When stratified across cognitive dimensions, immigrating at an older age and depression demise their memory function. A more active religious engagement lessens their depression, which can further strengthen their memory function. Regarding the nonmemory function of the immigrants, their cultural resources and psychological wellbeing do not show significance. The discussion focuses on how the possession of ethnocultural resources renders benefits to the very old immigrants across health dimensions while their acculturation, age-at-immigration, has different influences on their psychological and cognitive wellbeing.

Abstract citation ID: igae098.1156

### INTERNET USE AMONG BRAZILIANS ADULTS IN LATE LIFE: DETERMINANTS OF USE AND IMPACTS ON COGNITIVE HEALTH

Xiayu Chen[1], Mateo Farina[2], Flávia Andrade[3], and Kun Wang[4], *1. University of Illinois Urbana-Champaign, Champaign, Illinois, United States*, *2. University of Texas at Austin, Austin, Texas, United States*, *3. University of Illinois at Urbana Champaign, Urbana, Illinois, United States*, *4. Binghamton University, Binghamton, New York, United States*

Growing numbers of older adults use the Internet daily, suggesting its potential to improve general cognitive functioning, memory, and verbal fluency. Yet, a significant gap exists in understanding the association between Internet use and cognitive health within low- and middle-income countries. This study addresses this gap and evaluates the association between Internet use and cognitive health among Brazilian older adults using data from the second wave of the Brazilian Longitudinal Study of Aging(ELSI-Brazil). Multivariate linear regressions were employed to evaluate the association of Internet use with cognitive function, memory, and verbal fluency. Blinder-Oaxaca decomposition was conducted to estimate and decompose the cognitive health disparity between Internet users and non-users. Our finding suggested that about 17% of the older adults in the sample were Internet users, and 69% of them use the Internet on a daily basis. Internet use was positively associated with general cognitive functioning ($\beta$=0.14, 95% CI 0.07-0.20) and memory ($\beta$ =0.19, 95% CI 0.10-0.28), accounting for demographic, social, geographical, and health factors. The daily Internet use was positively associated with general cognitive function ($\beta$=0.14, 95% CI 0.06-0.22) and memory ($\beta$=0.19, 95% CI 0.09-0.29). However, no statistically significant association was found between Internet use, use frequency and verbal fluency. Our study has also found that educational and health attainment significantly explains the cognitive health disparity between Internet users and non-users among Brazilian older adults. The findings underscore the need for targeted strategies to bridge the digital divide and enhance cognitive health, particularly among socioeconomically and health-disadvantaged groups in Brazil.

### SESSION 3035 (PAPER)

### DEPRESSION AND ANXIETY AND MENTAL HEALTH

Abstract citation ID: igae098.1157

### MAPPING 15-YEAR DEPRESSIVE SYMPTOM TRANSITIONS IN LATE LIFE: A POPULATION-BASED COHORT STUDY

Federico Triolo[1], Davide Liborio Vetrano[2], Caterina Trevisan[2], Linnea Sjöberg[1], Amaia Calderón-Larrañaga[2], Martino Belvederi Murri[3], Laura Fratiglioni[1], and Serhiy Dekhtyar[1], *1. Aging Research Center, Karolinska Institutet, Stockholm, Stockholms Lan, Sweden*, *2. Karolinska Institutet, Stockholm, Stockholms Lan, Sweden*, *3. Institute of Psychiatry, Ferrara, Emilia-Romagna, Italy*

As the longitudinal course of late-life depression remains unclear, we aimed to describe transitions along the depression continuum, and identify factors associated with specific transition patterns. We analysed 15-year longitudinal data on 2745 dementia-free persons aged 60+ from the Swedish National Study on Aging and Care in Kungsholmen. Depression (minor and major) was diagnosed according to DSM-IV-TR; subsyndromal depression (SSD) was operationalized as the presence of ≥2 symptoms without depression. Multistate survival models were used to map depression transitions, including death, and to examine the association of psychosocial (social network, connection, and support), lifestyle (smoking, alcohol consumption, and physical activity), and clinical (somatic disease count) factors with transition patterns. Over the follow-up, 19.1% had ≥1 transitions across depressive states, while 6.5% had ≥2. Each additional somatic disease was associated with a higher hazard of progression from no depression to SSD (HR 1.09; 1.07-1.10) and depression (HR 1.06; 1.04-1.08), but also with a lower recovery (HRSSD-NoDep 0.95; 0.93-0.97; HRDep-NoDep 0.96; 0.93-0.99). Physical activity was associated with an increased hazard of recovery to no depression from SSD (HR 1.49; 1.28-1.73) and depression (HR 1.20; 1.00-1.44), while richer social network was associated with both higher recovery (HRSSD-NoDep 1.44; 1.26-1.66; HRDep-NoDep 1.51; 1.34-1.71), and lower progression hazards (HRNoDep-SSD 0.81; 0.70-0.94; HRNoDep-Dep 0.58; 0.46-0.73; HRSSD-Dep 0.66; 0.44-0.98). Older people may present with heterogeneous depressive trajectories. Targeting the accumulation of somatic diseases and enhancing social interactions may be appropriate for both depression prevention and burden reduction, while promoting physical activity may benefit recovery from depressive disorders.

Abstract citation ID: igae098.1158

### ASSOCIATION BETWEEN PRE-HIP FRACTURE DEPRESSION AND DAYS AT HOME AFTER FRACTURE AMONG MEDICARE BENEFICIARIES

Rhea Mehta[1], Denise Orwig[2], Chixiang Chen[2], Yu Dong[2], Michelle Shardell[2], Takashi Yamashita[3], and Jason Falvey[2], *1. University of Maryland Baltimore, Baltimore, Maryland, United States*, *2. University of Maryland School of Medicine, Baltimore, Maryland, United States*, *3. University of Maryland Baltimore County, Baltimore, Maryland, United States*

Hip fracture and depression are important public health issues among older adults. Though historically more common in females, hip fracture cases are projected to increase among males, who often experience greater depression severity. Days at home (DAH), a patient-centered metric reflecting fracture recovery, can inform post-surgical treatments for older adults. The association between pre-fracture depression and DAH among hip fracture survivors and potential sex differences remains unexplored. Our cohort included 63,618 community-dwelling Medicare fee-for-service beneficiaries, aged 65+, who underwent hip fracture surgery between 2010 and 2017. Generalized Estimating Equations assessed longitudinal associations between pre-fracture depression at hospital admission and total DAH over 12 months post-discharge, adjusting for covariates. Sex-by-depression interactions were also included, and multiple models were built to identify factors that may explain the primary association. There was no difference in DAH between beneficiaries by depression status in the fully adjusted model. After adjusting for demographic factors (age and sex) and social determinants of health (race, Medicaid dual eligibility, and poverty), those with depression spent 11 fewer average DAH compared to counterparts without depression (incident rate ratio [IRR] = 0.92; 95% CI = 0.91, 0.93; p<.0001). The association was attenuated and not significant after additional adjustment for medical complexities, specifically comorbidities, and facility and geographic factors. The sex-by-depression interaction term was not statistically significant (p=0.38). The association between pre-existing depression and DAH among Medicare beneficiaries with hip fracture underscores the need for early recognition and secondary prevention of depressive symptoms during acute care, which may inform hip fracture treatment.

Abstract citation ID: igae098.1159

### CROSS-NATIONAL PREVALENCE OF RISK OF DEPRESSION AMONG OLDER ADULTS: ARE THERE GENDER AND AGE DIFFERENCES?

Antonia Diaz-Valdes Iriarte, and Jose Medina*, Universidad Mayor, Santiago, Region Metropolitana, Chile*

Depression among older adults is becoming one of the most pressing issues in public health, yet it remains one of the less recognized problems and is one of the leading causes of disability in this age group. Among older adults, depression often goes unrecognized. Although depression has been widely studied, there are still several unanswered questions regarding country/context differences, gender, age, and cohort differences, as most studies have been conducted in specific contexts. This study aims to examine cross-national gender gaps in the prevalence of depression risk from 2000 to 2020, controlling for age and cohort effects. A random-effects model with robust standard errors was conducted to examine depression cases and predict probabilities. Our analysis involved a sample of 248,559 individuals aged 50 and older from 35 countries, spanning the years 2000 to 2020. Throughout the examined period, the average predicted probability of depression was 24.01% for males and 35.70% for females. This translates to a consistent gender gap of 11.69 percentage points. Notably, while the gender gap in depression probability remained stable over time, both males and females exhibited an upward trend in depression probability during this period – about 3% and 5% respectively. This study contributes to the current literature and seeks to explore the prevalence of depression risk cross-nationally using harmonized measures of depression, which has not been done yet. Our study highlights the importance of screening and treating depression among older adults to reduce the consequences of depression, especially among women, who are disproportionately affected.

Abstract citation ID: igae098.1160

### CAREGIVING, LIVING ALONE, AND OLDER WOMEN'S MENTAL HEALTH: A NUANCED VIEW AMID THE STRESS OF COVID-19

Yiwen Zhu[1], and Justin Farmer[2], *1. Harvard T. H. Chan School of Public Health, Boston, Massachusetts, United States*, *2. Harvard T.H. Chan School of Public Health, Somerville, Massachusetts, United States*

Caregiving and living alone are potentially stressful psychosocial experiences for older adults, but prior findings are inconsistent: caregiving may be associated with prosocial benefits, and living alone is distinct from social isolation. In the context of heightened collective stress during COVID-19, we examined caregiving and living alone in 27,646 older female former nurses (mean age 67) from the Nurses' Health Study II. We assessed whether caregiving and living alone were prospectively associated with multiple facets of mental health. In May 2020, participants self-reported personal caregiving roles (yes/no) and living arrangements (alone/with others). Caregivers reported their stress levels concerning common COVID-19-related caregiving tasks; all participants reported on social support. Seven mental health outcomes were assessed at 1-6 timepoints over one year, including depression, anxiety, perceived stress, loneliness, psychological well-being, social well-being, and gratitude. All analyses accounted for covariates including pre-pandemic mental health and multiple testing. Caregiving and living alone associated with higher distress and lower positive functioning. Nuanced perspectives emerged with respect to caregiving stress and social support: while stressed caregivers had worse mental health (e.g., OR depression =2.08[1.88-2.31]), caregivers not experiencing stress had better mental health (OR depression =0.74[0.67- 0.81]), in comparison to non-caregivers. Older women living alone with adequate social support showed comparable mental health to their counterparts who lived with others, while participants lacking support reported substantially elevated distress (e.g., OR depression =3.11[2.55-3.79]). We provided a more nuanced understanding of caregiving and living alone among older women; caregiving stress and social support may be promising intervention targets.

Abstract citation ID: igae098.1161

### MULTIMORBIDITY COMBINATIONS AND DEPRESSIVE SYMPTOM TRAJECTORIES AMONG US OLDER ADULTS

Nicholas Bishop[1], Kealie Walker[1], Corey L. Nagel[2], Jason Newsom[3], Anda Botoseneanu[4], Heather Allore[5], and Ana Quiñones[6], *1. University of Arizona, Tucson, Arizona, United States*, *2. University of Arkansas for Medical Sciences, Little Rock, Arkansas, United States*, *3. Portland State University, Portland, Oregon, United States*, *4. University of Michigan-Dearborn, Dexter, Michigan, United States*, *5. Yale University, New Haven, Connecticut, United States*, *6. Oregon Health & Science University, Portland, Oregon, United States*

Multimorbidity (2+ chronic health conditions) is directly related to increased patient burden, but the association between specific multimorbidity combinations and trajectories of depressive symptoms among older adults is unknown. We used data from the Health and Retirement Study (HRS) to estimate the association between multimorbidity combinations identified in 2012 and trajectories of depressive symptoms from 2012-2020 in adults aged ≥ 65 (n=9,004; mean age: 75.3; % female: 58.7%). Self-reported physician-diagnosed conditions included arthritis, hypertension, diabetes, heart conditions, cancer, lung disease, and stroke. The eight-item CES-D measured depressive symptoms. Multimorbidity combinations were used to predict quadratic latent growth trajectories of depressive symptoms, adjusting for sociodemographic, behavioral, and childhood characteristics and the complex sampling design of the HRS. In 2012, 75.6% of respondents experienced multimorbidity and the average depression score was 1.4 (SD=1.9). Nine unique multimorbidity patterns were identified, most common being hypertension+arthritis (14.6%), hypertension+heart conditions+arthritis (7.1%), and hypertension+diabetes+arthritis (6.1%). Several multimorbidity combinations were associated with greater baseline depressive symptomatology (e.g., compared to those reporting no conditions at baseline, those reporting hypertension+lung disease+arthritis had 0.96 more baseline depressive symptoms (SE=0.2, p<.001), and those reporting hypertension+diabetes+heart conditions+arthritis had 0.93 more baseline depressive symptoms (SE=.2, p<.001), respectively). Several multimorbidity combinations were associated with linear and nonlinear change in depressive symptoms, with hypertension+cancer+arthritis and hypertension+diabetes+arthritis being associated with the most rapid increases in depressive symptoms. Our work highlights the increased risk of depression among older adults with varied multimorbidity combinations and speaks to the mental health burden associated with multimorbidity.

### SESSION 3040 (SYMPOSIUM)

Abstract citation ID: igae098.1162

### FINDING FORTITUDE: SOCIAL CONVOYS OVER THE LONG HAUL

Chair: Rita Hu
Co-Chair: Kristine Ajrouch
Discussant: Elaine Wethington

As populations globally face the opportunities and challenges of aging, social convoys across the lifespan are pivotal in navigating the complexities of later life. This symposium explores the dynamic interplay between social relations and aging, emphasizing the protective and predictive nature of social networks over the lifespan and in different cultural contexts. Becheroui and colleagues explore familial care in Lebanon across age groups, focusing on the balance between cultural expectations and the need for policy support. Antonucci and colleagues examine how socioeconomic status affects changes in health through social relations, particularly the influence of parent-child dynamics among men and women. Hu and colleagues also provide a longitudinal perspective, showing how social network trajectories over nearly three decades influence later-life cognitive health, emphasizing the enduring importance of weaker ties for cognitive health later in life. Webster and colleagues present evidence of how educational attainment within one's social network at one point in time contributes to cognitive health later in life, pointing to the broader implications of social environments on cognitive well-being. Aimed at fostering a comprehensive understanding of social convoys in the aging process, this symposium invites a cross-disciplinary dialogue to enhance the support for aging individuals worldwide, embodying the symposium's theme of finding fortitude through the power of social relationships.

Abstract citation ID: igae098.1163

### DECADES OF CONNECTION: THE IMPACT OF SOCIAL NETWORK TRAJECTORIES ON COGNITIVE HEALTH ACROSS 29 YEARS

Rita Hu[1], Toni Antonucci[2], and Kristine Ajrouch[2], *1. University of Chicago, Chicago, Illinois, United States*, *2. University of Michigan, Ann Arbor, Michigan, United States*

The current study used Latent Growth Curve Modeling to examine the associations between overall network, closest, close and weak social tie trajectories across the lifespan and cognitive health later in life. Participants (N = 145) were from the longitudinal Social Relations study and the Detroit Area Wellness Network study, with four waves of data spanning from 1992 to 2021. Participants had a wide age range in Wave 1 (Mage (SD) = 46.9 (8.01) [37 - 71]) and were mostly women (69%) and White (82%). Social network structure was measured using hierarchical mapping with inner, middle, and outer circles symbolizing the emotional closeness of the relationships. Cognitive health was measured by a latent variable based on episodic memory, language, attention, and working memory. Results showed that increases in both overall network size and the size of closer social networks (middle circle) over time were significantly associated with better cognitive health (overall network size: $\beta = 0.490$, $p < .064$; middle circle size: $\beta = 1.864$, $p = .005$). Gender-specific analysis revealed no significant differences in model fit between constrained and unconstrained models, suggesting the absence of substantial gender differences. However, it was noted that for men, the increase in closer social networks over time positively impacts cognitive function. The study contributes to the understanding of the complex role social ties play in cognitive aging and underscores the necessity for targeted interventions to bolster social connections among older adults, with a nuanced approach considering gender-specific dynamics.

Abstract citation ID: igae098.1164

### ATTITUDES AND BELIEFS ON INTERGENERATIONAL FAMILIAL CARE: IMPLICATIONS FOR FUTURE CARE POLICY IN LEBANON

Myriam Al Bcherraoui, Kristine Ajrouch, and Toni Antonucci, *University of Michigan, Ann Arbor, Michigan, United States*

In a collectivistic society such as Lebanon, familism is a deeply rooted cultural value where family members learn to put the needs of the group over the self. Familial obligation is socially reinforced, and care is perceived as the principal responsibility of the family. In a rapidly aging society accompanied by economic hardships, older adults in Lebanon are living longer, often with health conditions, and family members assume the main responsibility of care. Solidarity in family relationships becomes increasingly important and may lead to many benefits in caregiving when they are positive, but they may also be equally harmful if conflictual. Understanding adults' attitudes concerning differential care settings becomes an intricate state matter with ethical implications. Drawing on Bengtson's Solidarity-Conflict Model of Generational Relationships, we assessed data from the Family Ties and Aging study (N=500) collected in Beirut, Lebanon from adults aged 18-91. The study examined 1) the extent to which age, gender, and education level influence beliefs held about elder care, and 2) whether parent-child relationship closeness predicts attitudes on state versus family care. Regression analyses indicated that greater relationship closeness to children predicts stronger beliefs favoring government-led care for personal and household matters. Older age and closeness to children also predicted parents' disagreement in children's obligational role in care, both personally and financially. This may relate to parents' desire to decrease strain on family members and increase solidarity. Results will be discussed in light of the Solidarity-Conflict Model, emphasizing the importance of strengthening family ties through state interventions.

Abstract citation ID: igae098.1165

### THE LONG REACH OF SOCIAL RELATIONS: GENDER AND THE SES-HEALTH LINK

Toni Antonucci[1], Simon Brauer[1], Joonyoung Cho[2], Urmimala Ghose[3], and Kristine Ajrouch[1], *1. University of Michigan, Ann Arbor, Michigan, United States*, *2. University of Hawaii, Hawaii, United States*, *3. Humboldt University Berlin, Berlin, Berlin, Germany*

Accumulated evidence demonstrates a strong relationship between socioeconomic status (SES) and health. We use longitudinal data over a period of 25 years to examine whether education (an established indicator of SES) predicts baseline health (i.e., intercept at W1) and its temporal change (i.e., slope from W1 through W3) in middle-aged and older adults. We further explore the long reach of social relations by testing whether social relations, particularly of parents with children (at W1), mediate and/or moderate the education–health link. The data are drawn from a regionally representative sample of adults (aged 40–93) in the Detroit area, USA. All analyses are stratified by gender (W1 N=males: 330; females: 468). A series of structural equation models were performed. For females, those with higher education had better health in W1. Their health declined across time and at the same rate as those with lower-education. Further, relationship quality did not mediate or moderate these relationships. Among males, those with higher education had better health in W1. Notably, the health of men with lower-education who had a child with whom they confided and those with higher education had similar health at wave 1. Findings indicate that the health of lower-educated men in the presence of key social supports parallels the advantaged health status of men with higher levels of education earlier in life but diverges later in life. These findings suggest that relationships with children may be a protective factor for the health of men in the lower socioeconomic strata.

Abstract citation ID: igae098.1166

### THE EFFECTS OF NEIGHBORHOOD, NETWORK, AND INDIVIDUAL EDUCATION LEVELS ON COGNITIVE HEALTH

Noah Webster, Laura Zahodne, Toni Antonucci, and Kristine Ajrouch, *University of Michigan, Ann Arbor, Michigan, United States*

It is well established that greater education is associated with better cognitive health. Less is known about the cognitive effects of education across levels of influence, including neighborhood, interpersonal, and individual levels. In this study we compare the effects of individuals' education, their social network members' education, and their neighborhood's education on cognitive health five years later. Respondents age 60 and older who participated in Wave 3 (2015) of the longitudinal Social Relations Study based in Metro Detroit and also completed interviews in 2021 for the Detroit Area Wellness Network COVID-19 Supplement project were selected for analysis (n=161). In 2015, respondents were asked to report completed years of education and the education level of up to 10 social network members. Household addresses were geocoded and census tract educational attainment was obtained from the American Community Survey. In 2021, cognitive assessments were administered via telephone, and a latent variable measuring global cognition was derived from episodic memory, language, attention, and working memory tests. In separate models controlling for age and gender, respondents' own education and the proportion of social network members with a bachelor's degree, but not the proportion of people in the census tract with a bachelor's degree, were associated with better cognitive health. In a combined model, only having a greater proportion of social network members with a bachelor's degree remained associated with better cognitive health. Findings highlight the salience of educational resources embedded within social networks for cognitive health and point to the need for meso-level interventions.

### SESSION 3045 (SYMPOSIUM)

Abstract citation ID: igae098.1167

### FOSTERING MULTIDISCIPLINARY SOLUTIONS IN AGING: THE RESEARCH CENTERS COLLABORATIVE NETWORK

Chair: Stephen Kritchevsky
Co-Chair: Odette van der Willik

The NIA supports the Research Centers Collaborative Network (RCCN) to bridge scientists from the 7 NIA-sponsored center programs: Alzheimer's Disease Research Centers, Artificial Intelligence and Technology Collaboratories for Aging Research, Centers on the Demography and Economics of Aging, Claude D. Pepper Older Americans Independence Centers, Nathan Shock Centers of Excellence in the Basic Biology of Aging, Resource Centers for Minority Aging Research, and Roybal Centers for Translational Research on Aging. To provide a forum for collaborative exchange, RCCN has convened workshops on crosscutting concerns relevant to multiple NIA center programs ranging from sustaining behavior change in older adults to measuring biologic age. RCCN also awards pilots that address topics spanning the missions of multiple NIA Center Programs. This symposium will present key learnings from the work of five RCCN pilot teams. Dr. King will discuss a novel analysis of proteomic and cellular senescence biomarkers in kidney using a non-human primate model. Dr. Nguyen will discuss the relationship between social disconnection and changes in cognitive health over time among older Black adults. Dr. Quiñones-Cordero will discuss culturally congruent interventions to improve Latino ADRD caregivers' psychological well-being. Dr. Christensen will discuss the development and implementation of older adult community engagement studios (OA-CES) to promote the inclusivity of older adults in research and to increase the number of research studies on older adults. Dr. Landauer will discuss the development of Age Friendly training webinars and research tools. Dr. Kritchevsky will also summarize lessons learned across the workshops.

Abstract citation ID: igae098.1168

### DEEP PROTEOMIC PROFILING OF ACCELERATED KIDNEY AGING AND SENESCENCE BURDEN IN MACACA MULATTA

Christina King[1], Jordan Burton[1], George Schaaf[2], Stephen Kritchevsky[3], J. Cline[2], Jamie Justice[4], Ellen Quillen[2], and Birgit Schilling[1], *1. Buck Institute for Research on Aging, Novato, California, United States, 2. Wake Forest University School of Medicine, Winston-Salem, North Carolina, United States, 3. Wake Forest University School of Medicine, Wake Forest University School of Medicine, North Carolina, United States, 4. XPRIZE Foundation, Winston-Salem, North Carolina, United States*

Whole-body ionizing radiation (IR) is a stressor that causes damage to DNA, lipids, and proteins, and in the latent-phase, may lead to oxidative stress, inflammation, and cellular senescence. Slowly-reproducing tissues, including kidney, are more likely to react to DNA damaging stressors, which may exacerbate aging processes. Quantitative proteomics was employed to gain molecular insights into how IR exposure may drive renal aging processes. For this analysis, kidney cortex tissues (N=38) were obtained from the Wake Forest Non-Human Primate Radiation Late Effects Cohort. Protein lysates (100 µg) were digested and peptides were desalted. Peptides were analyzed on a timsTOF HT mass spectrometer (Bruker), raw data was analyzed using protein-analysis software, and significantly-altered proteins were subjected to bioinformatic analysis. 5,472 protein groups with at least two unique peptides were identified in the kidney cortex. The influence of 1) kidney disease (KD) in irradiated individuals, 2) only kidney disease, and 3) only irradiation was studied. Interestingly, over 75% of significantly-altered proteins are significantly upregulated. This includes extracellular matrix proteins tenascin and periostin, which are potential aging biomarkers. Upon mapping this dataset to homologous human proteins, proteins associated with kidney disease among irradiated animals are related to EGF pathways, integrin signaling, and fibrosis. Significantly-altered proteins related with kidney disease, independent of irradiation, are related to EGF pathways, integrins, and fibrosis whereas significantly-altered proteins related to irradiation exposure are involved in fibrosis. Similarities in biological processes from significantly-altered proteins in either kidney disease or irradiation highlight a potential synergistic effect of irradiation on kidney disease.

Abstract citation ID: igae098.1169

### A COMMUNITY-ENGAGED APPROACH TO CULTURAL ADAPTATION OF BEHAVIORAL INTERVENTIONS: ENGAGING LATINOS IN RESEARCH

Maria Quiñones-Cordero[1], Silvia Sörensen[2], Kenneth Hepburn[3], and Kathi Heffner[2], *1. University of Rochester Medical Center, Rochester, New York, United States*, *2. University of Rochester, Rochester, New York, United States*, *3. Emory University, Atlanta, Georgia, United States*

Latinos are 1.5 times more likely to develop Alzheimer's disease and related dementias (ADRD) than non-Hispanic white Americans. Latinos also have the highest prevalence of caring for a loved one with ADRD. They spend more time in caregiving-related activities, have limited social support and access to quality dementia care, and have significantly worse physical and mental health. The paucity of culturally congruent interventions to improve Latino ADRD caregivers' psychological well-being continues to perpetuate poorer outcomes and disparities among Latino caregivers. While several effective psychoeducation and skill-building caregiver programs have been developed, these interventions lack representation of Latino caregivers. Thus, culturally attuned adaptations and implementation of available interventions for Latinos are needed to address this critical gap. This study engaged community-dwelling older Latinos, including Latino caregivers, and Latino community organization leaders, to collaborate in cultural adaptation of the Tele-Savvy program and clinical trial design. Employing community-engaged approaches, we combined a staged cultural adaptation framework and a locally developed training program to engage Latinos in caregiving research. Nine Latino community members served on a community advisory board (CAB). Members first engaged in a training program to develop knowledge about ADRD and skills in conducting research and cultural adaptation. Following the training, members engaged in cultural adaptation of Tele-Savvy and the design of a future clinical trial to test the adapted intervention with Latino ADRD caregivers. We will discuss preliminary findings from the cultural adaptation work with CAB members and future directions to inform the testing of the adapted Tele-Savvy and its refinement.

Abstract citation ID: igae098.1170

### AGE-FRIENDLY RESEARCH: EDUCATION AND TOOLS TO ENHANCE INCLUSION OF OLDER ADULTS IN RESEARCH

Allison Lindauer[1], Bryanna De Lima[2], and Elizabeth Eckstrom[2], *1. Oregon Alzheimer's Disease Research Center, Oregon Health & Science University, Portland, Oregon, United States*, *2. Oregon Health & Science University, Portland, Oregon, United States*

Many common diseases disproportionately affect older adults but research studies under-represent older adults due to recruitment and retention barriers. Geriatric research specialists have designed frameworks and tools to address these challenges but these are not widely disseminated. Based on these frameworks and tools, our team designed an Age Friendly Research synchronous, interactive 6-session webinar series and seven Age-Friendly research tools for research team members at our institution. We recruited 40 research-level employees to participate in the webinar series from October to November 2022 and 21 to pilot test the tools. Webinar participants completed pre- and post-program surveys and post-webinar session surveys in REDCap. Participants who pilot tested the tools completed up to 4 monthly REDCap surveys. Surveys included quantitative (e.g., Likert scale, dichotomous, and 10-point ratings) and open-ended responses related to strengths, limitations, areas for improvement, and intention to implement newly learned information. Webinar participants expressed positive feedback stating they would likely use what they learned and recommend to others. Strengths of the webinar were practical tips, useful tools, and real-world examples. However, more information on industry-sponsored trials was requested. The most utilized tools were the communication guide and Age-Friendly research checklist. Participants appreciated the ease of use, webinar training, and supportive teams for tool implementation but identified lack of time and age-appropriate study populations as key barriers. Overall, the webinar and tools were well-received by participants. Sharing the webinar and tools with others could lead to more Age-Friendly research and improve the experience for researchers and older adults alike.

Abstract citation ID: igae098.1171

### THE ROLE OF SOCIAL DISCONNECTION AND THE NEIGHBORHOOD EXPOSOME IN COGNITIVE DECLINE AMONG BLACK AMERICANS

Ann Nguyen[1], Weidi Qin[2], and Harry Taylor[3], *1. Case Western Reserve University, Cleveland, Ohio, United States*, *2. University of Wisconsin Madison, Madison, Wisconsin, United States*, *3. University of Toronto, Toronto, Ontario, Canada*

The aims of this study were to 1) identify cognitive status trajectories among middle-aged and older Black adults; 2) determine whether baseline loneliness, social isolation, and neighborhood characteristics predicted cognitive status trajectories; and 3) determine whether neighborhood characteristics modified the association between loneliness, social isolation, and cognitive status trajectories. Data for the study came from Black participants aged 51+ in the Health and Retirement Study (N=1791). Cognitive status was measured using the modified Telephone Interview for Cognitive Status; loneliness was measured using the modified UCLA Loneliness Scale. Social isolation from extended family, children, and friends were measured using indicators of network size, contact, and social support for each social group. Neighborhood physical disadvantage and social cohesion were each assessed using a 4-item scale. Group-based trajectory analysis was used. The results indicated that there were four distinct cognitive status trajectories—very low and declining, low and declining, moderate and stable, and high and stable. Higher loneliness scores were predictive of membership in the low and declining trajectory rather than the high and stable trajectory. A significant interaction between neighborhood physical disadvantage and loneliness indicated that the association between loneliness and membership in the low and declining trajectory was weaker among participants who lived in neighborhoods with high levels of physical disadvantage than participant who lived in neighborhoods with low levels of physical disadvantage. These findings have implications for practice with older Black adults and suggest that practitioners should consider the role of loneliness and the neighborhood environment.

Abstract citation ID: igae098.1172

### ADAPTING COMMUNITY ENGAGEMENT STUDIOS FOR AN OLDER ADULT COMMUNITY EXPERTS

Janelle Christensen, Shaye Kerper, Steven Albert, Maya Ragavan, Leslie Scheunemann, and Mylynda Massart, *University of Pittsburgh, Pittsburgh, Pennsylvania, United States*

The development and implementation of older adult community engagement studios (OA-CES) to promote the inclusivity of older adults in the research process and to increase the number of research studies on (and inclusive of) older adults. This project utilized University of Pittsburgh Clinical and Translational Science Institute's (CTSI, the required CTSA Hub) success using Community Engagement Studios (CES) to connect researchers with other populations historically underrepresented in biomedical research and bring it to older adult (OA) populations. This collaboration between the Pittsburgh Pepper Center (Pitt Pepper, NIA Center Program) and CTSI created a training and orientation process for older adults to: 1. Help researchers learn about how to more effectively include older adults in the research process and overcome barriers to the inclusion of older adults in their research; 2. Select and tailor implementation strategies to counter these barriers to inclusion, ultimately creating a toolkit that can be shared with other NIA Center Programs/ CTSA Hubs. The Community Engagement Studios themselves were tailored to address needs for inclusion of older adults during recruitment and implementation of the studios: 1) sending snail mail before calling and emailing potential participants; 2) providing a two hour orientation to research ethics and expectations for Community Engagement Studios; 3) providing multiple options for transportation to and from the in-person events; 4) providing increased tech support and training for remote events (via zoom). These best practices allow for the Pepper Center and CTSI to better consult with researchers seeking to increase the inclusion of older adults.

### SESSION 3050 (PAPER)

### GLOBAL PERSPECTIVES ON ACTIVE AGING

Abstract citation ID: igae098.1173

### PET ATTACHMENT AND RETAINED PHYSICAL FUNCTION IN COMMUNITY RESIDING OLDER ADULT PET OWNERS

Erika Friedmann[1], Nancy Gee[2], Eleanor Simonsick[3], Barbara Resnick[1], Merve Gurlu[1], and Soyeon Shim[1], *1. University of Maryland Baltimore, Baltimore, Maryland, United States*, *2. Virginia Commonwealth University School of Medicine, Richmond, Virginia, United States*, *3. National Institute on Aging, Baltimore, Maryland, United States*

Pet ownership has been associated with slower deterioration in cardiorespiratory fitness (400-M walk time), gait speed, overall functional performance and physical well-being independent of age and comorbidities in older adults with increasing age. As level of pet attachment, an aspect of social support, may impact physical function, we evaluated this association using data from the Baltimore Longitudinal Study of Aging (BLSA). In this longitudinal observational study of 214 pet-owning, generally healthy community-dwelling adults aged 50-100 years (M=68.0, SD=7.8), pet attachment and physical function were assessed during regularly scheduled visits every 1-4 years over 1-13 years (M=7.5,SD=3.6). Linear mixed models with pet attachment, time, and pet attachment by time interaction controlling for age and comorbidities were used to examine the relationship of pet attachment to deterioration in several physical function outcomes. Higher pet attachment was associated with slower deterioration in usual ($p< 0.001$) and rapid gait speed ($p=0.002$) and overall physical performance ($p=0.009$) not cardiorespiratory fitness or physical well-being. Among dog owners only (N=121) attachment did not moderate deterioration in physical function. Among cat owners (N=100), higher attachment was associated with slower decline in physical well-being ($p=0.035$). We provide important longitudinal evidence that pet attachment is differentially associated with rates of decline in physical function that older adults experience as they age and that attachment to different types of pets may have different relationships to the decline process. Potential explanations for these differences are proposed.

Abstract citation ID: igae098.1174

### PROMOTING ACTIVE AGING THROUGH TAEKWONDO AMONG OLDER ADULTS

Minhwa Seo, Sua Im, and Jinmoo Heo, *Yonsei University, Seoul, Republic of Korea*

With increasing longevity, the older adult's population is growing, highlighting the importance of addressing issues related to active aging. In Korea, older adults actively participate in various leisure activities, including a specialized group practicing Taekwondo, a traditional Korean martial art, to promote active aging. This study aims to explore the role of Taekwondo engagement in active aging. Utilizing a phenomenological method design with semi-structured interviews, we explored how Taekwondo influences active aging. Participants aged 73 to 95, and each participant was diagnosed with at least one disease such as arthritis, stroke, or facial paralysis (n =12). Through in-depth interviews, three main themes and seven sub-themes emerged, all aligned with the WHO's Active Aging model. The first theme highlights the promotion of diverse social participation through Taekwondo involvement, encompassing leisure, cultural engagement, community integration, and participation in competitions and performances. The second theme focuses on the positive impact on physical and mental health. Participants reported enhanced well-being, including reduced knee pain and improved cognitive function, following Taekwondo engagement and social interaction. The third theme emphasizes the security benefits associated with health promotion and participation in Taekwondo. Older adults were able to mitigate isolation through social connections and enhance their mobility and independence through physical activity. Overall, our findings underscore the potential of Taekwondo as a promising avenue for fostering active aging among older adults. This study contributes valuable insights for promoting health and well-being in aging populations.

Abstract citation ID: igae098.1175

### DEVICE-MEASURED MOVEMENT BEHAVIORS IN A NATIONALLY REPRESENTATIVE COHORT OF OLDER ENGLISH ADULTS

Laura Brocklebank[1], Aiden Doherty[1], and Andrew Steptoe[2], *1. University of Oxford, Oxford, England, United Kingdom*, *2. University College London, London, England, United Kingdom*

Movement behaviours impact various health outcomes. However, they have rarely been objectively measured in large nationally representative cohorts of older adults. We aim to describe the collection and analysis of device-measured movement behaviours in the English Longitudinal Study of Ageing (ELSA), and report variation by key participant characteristics. ELSA is a nationally representative cohort of English adults aged ≥50 years. In 2021-23, a random subset of 5,429 individuals were invited to wear a wrist-worn triaxial accelerometer for eight consecutive days. 4,400 agreed (81.0%) and of them 3,308 (75.2%; 55.6% men; aged 68.5 ± 9.0 years) had sufficient wear time (median [IQR] 8.0 days [7.4-8.4]). We used a machine-learning model to infer time spent in four movement behaviours (sleep, sedentary behaviour, light physical activity [LPA], and moderate-vigorous physical activity [MVPA]). Average acceleration, or overall physical activity, was 22.9 mg/day. On average, participants spent 9.4 hours/day (39.0%) sleeping, 9.8 hours/day (40.8%) being sedentary, 4.4 hours/day (18.1%) in LPA, and 30.4 minutes/day (2.1%) in MVPA. Despite men accumulating more MVPA than women, women had higher overall physical activity (23.2 vs. 22.5 mg/day) by accumulating more LPA and less sedentary time. Adults aged ≥65 years had lower overall physical activity than those aged 50-64 years (21.1 vs. 26.5 mg/day) by spending less time being active and more time being sedentary and sleeping. These data will shortly be deposited in national archives for use by other research teams and will enhance our knowledge about the potential relevance of different movement behaviours for healthy aging.

Abstract citation ID: igae098.1176

### INCORPORATING EXERCISE TO BUFFER AVERSIVE HEALTH EFFECTS OF LONELINESS AMONG OLDER ADULTS IN THE LIFE TRIAL

Navin Kaushal[1], Deepishka Pemmasani[1], Adrian Noriega de la Colina[2], and Donya Nemati[3], *1. Indiana University Indianapolis, Indianapolis, Indiana, United States*, *2. McGill University, Montreal, Quebec, Canada*, *3. The Ohio State University, Columbus, Ohio, United States*

**Introduction:** In 2023 the US Surgeon General announced an epidemic of loneliness and isolation. Prolonged loneliness among older adults has been shown to predict dementia and cardiovascular disease (CVD). However, exercise has been demonstrated to have preventive effects on CVD and cognitive health, but its effects on buffering these outcomes manifested from loneliness, or gender effects have not been investigated. This study aimed to test two moderated-mediation models to investigate if exercise moderates the relationship between loneliness and health outcomes.

**Methods:** The Lifestyle Interventions and Independence for Elders (LIFE) Study is a randomized controlled trial (n=1,600) that assigned older adults (aged 65+) to either an intervention or control group. The present observational study analyzed participants in the control group. Measures included: exercise (accelerometry), loneliness (Center for Epidemiologic Studies Depression Scale [CES-D]), CVD risk (handgrip test), and cognitive health (global cognitive function). Model #14 from Hayes PROCESS Macro 4.0 in SPSS was used to analyze the data.

**Results:** In both models, females experienced greater loneliness compared to males($\beta$=.25, p<.001). The CVD risk model found Moderate-to-Vigorous Physical Activity (MVPA) to independently predicted handgrip strength($\beta$=.11, p<.001), and interacted with loneliness to predict handgrip strength($\beta$=.05, p=.03). The cognition model also found MVPA to independently predict cognition($\beta$=.14, p<.001), and interacts with loneliness to predict cognition($\beta$=.07, p=.03), and also demonstrate total moderated-mediation effects($\beta$=.02, 95%CI.003 to.367).

**Conclusion:** Exercise can buffer aversive cardiovascular risk and cognition from loneliness. Lonely older adults are a high-risk demographic that should be sought for enrolling in exercise programs.

Abstract citation ID: igae098.1177

### FACTORS INFLUENCING PHYSICAL ACTIVITY AMONG COMMUNITY-DWELLING OLDER ADULTS IN BEIJING, CHINA: A PATH ANALYSIS

NING KANG[1], Gong Chen[2], Dongmin Wang[1], Yue Wang[1], and Min Liu[1], *1. Peking University, Beijing, Beijing, China (People's Republic)*, *2. Peking University, Beijing, Beijing, China*

**Background:** Previous research underscores the significance of physical activity in enhancing the health of older adults. This study seeks to delineate the pivotal determinants and model the pathways that influence the physical activity levels of older adults residing in community settings.

**Methods:** Leveraging a cluster sampling strategy, this study surveyed older adults in Beijing, China, employing the International Physical Activity Questionnaire to assess physical activity. We gathered 770 valid responses (173 males and 594 females). The relationship between physical activity and various determinants was examined using ordered logistic regression, and a structural equation model was developed to elucidate the influencing pathways.

**Results:** Findings reveal that a substantial portion of community-dwelling older adults exhibit low physical activity levels (40.00%), with moderate (28.7%) and high levels (31.30%) less prevalent. Direct influences on physical activity levels were found for exercise support (β=0.168), motivation (β=0.164), and knowledge (β=0.089). Exercise self-efficacy (β=0.21), support (β=0.152), and knowledge (β=0.092) indirectly affected motivation, with self-efficacy further mediated by support (β=0.081) and knowledge (β=-0.128), suggesting the nuanced role of exercise risk awareness. Exercise motivation, particularly achievement motivation, health motivation, and internal pressure motivation, was identified as a key factor affecting their physical activity levels.
**Conclusion:** This research identifies exercise motivation and support as crucial to older adults' physical activity. Contrary to expectations, increased self-efficacy does not directly correlate with higher activity levels, underscoring the complexity of factors influencing older adults' engagement in physical activity.This study advise a community intervention enhancing exercise motivation through social support and health education.

### SESSION 3055 (PAPER)

### HEALTH AND WELL-BEING IN DIVERSE CONTEXTS AND POPULATIONS

Abstract citation ID: igae098.1178

### STATE-BASED ESTIMATES OF COGNITIVE DIFFICULTY AMONG MIDDLE EASTERN AND NORTH AFRICAN AMERICANS

Tiffany Kindratt, and Alexandra Smith, *The University of Texas Arlington, Arlington, Texas, United States*

Research on cognitive difficulty in the US focuses on racial/ethnic groups that comprise federal minimum reporting guidelines, including White, Black, Hispanic/Latino, Asian, American Indian/Alaskan Native (AI/AN), and Native Hawaiian/Other Pacific Islanders (NH/OPI). Research on Middle Eastern and North African (MENA) adults is limited, and no state-based estimates have been reported. Our objective was to estimate the prevalence of cognitive difficulty among MENA adults compared to other racial/ethnic groups in four states (California, New York, Michigan, and Texas) after adjusting for age and sex. We calculated MENA populations by state using race, ethnicity, ancestry and birthplace responses from the 2017-2021 American Community Survey. The four largest MENA populations were found in California (n=912,787), New York (n=341,361), Michigan (n=262,918) and Texas (n=258,395). Cognitive difficulty among MENA adults (yes/no; age>=45years) was compared to other racial/ethnic groups (e.g., White, Hispanic/Latino, Black). In California, the prevalence of cognitive difficulty was higher among MENA adults (6.96%) compared to White (4.80%), Hispanic/Latino (5.44%), and Asian (4.35%) adults. In New York, the prevalence was lower among MENA adults (3.96%) compared to Black (4.82%), Hispanic/Latino (7.96%), and AI/AN (8.94%) adults. In Michigan, the prevalence was lower among MENA (8.30%) compared to Black (11.32%) adults, but higher than White (6.25%) and Asian (3.28%) adults. In Texas, the prevalence was lower among MENA (4.44%) adults compared to Black (8.41%), Hispanic/Latino (6.28%), and Asian (8.41%) adults (all p's<.05). This study provides an important first look at state-based estimates of cognitive difficulties among MENA adults living in California, New York, Michigan, and Texas.

Abstract citation ID: igae098.1179

### SOCIAL JET LAG: SOCIODEMOGRAPHIC DETERMINANTS AND HEALTH IMPLICATIONS IN MIDDLE-AGED AND OLDER ADULTS

Hanamori Skoblow[1], Eunjin Tracy[1], Eunjung Kim[2], Naomi Meinertz[1], Getrude Nyang'au[1], and Megan Gilligan[1], *1. University of Missouri, Columbia, Missouri, United States*, *2. Arizona State University, Tempe, Arizona, United States*

Social jet lag is characterized by inconsistent sleep patterns between weekdays and weekends and arises from a misalignment between biological rhythms and social schedules, posing significant health risks. However, the sociodemographic determinants and health implications of social jet lag among middle-aged and older adults are unclear. In the current study, we examined cross-sectional associations between social jet lag, sociodemographic characteristics, and key mental, physical, and cognitive health outcomes. Among adults ages 50–83 (M = 68.22; SD = 7.85) in the 2018 wave of the Health and Retirement Study (N = 667; 62% women), approximately 49% of respondents experienced social jet lag. Bivariate correlations indicated that social jet lag was related to older age (r = –.35, p <.001), Hispanic ethnicity (r =.13, p =.001), and current employment (r =.31, p <.001), but not gender (p =.99) or education (p =.12). Results of multivariate regressions, controlling for the above sociodemographic determinants, indicated that greater social jet lag was associated with higher depressive symptoms (β = 0.14, p <.001, R-squared =.10) and lower cognitive performance (β = –0.08, p =.04, R-squared =.24). Social jet lag was not significantly associated with body mass index (p =.36), number of chronic conditions (p =.59), or self-rated memory (p =.72). These findings point to the importance of a regular sleep schedule as a modifiable behavior that may promote better mental and cognitive health in midlife and beyond.

Abstract citation ID: igae098.1180

### SLEEP QUALITY, DURATION, AND EFFICIENCY IN LOW-INCOME OLDER ADULTS WITH AND WITHOUT DISABILITIES

James Brightman[1], Kworweinski Lafontant[1], Jethro Raphael Suarez[2], Jennifer Crook[1], and Ladda Thiamwong[1], *1. University of Central Florida, Orlando, Florida, United States*, *2. University of Central Florida*

Seventy million Americans experience chronic sleep disorders and older adults are more likely than younger ones to have problems sleeping. Sleep is a critical part to the circadian rhythm, and getting adequate sleep can help prevent falls and increase physical functioning. Even though sleep quality declines as a function of normal aging, the presence of a physical disability may exacerbate that natural decline. Income status can also impact sleep quality and the prevalence of dysregulated sleep may lead to an increased use of medicinal sleep aids to alleviate the issue. The chronic use of sleep aids, however, may not be beneficial for alleviating sleep complaints. In this study, we assessed the sleep quality and sleep aid use of 87 low-income community-dwelling older adults with (n = 24) and without (n = 63) a physical disability. Sleep quality, duration, and efficiency were assessed subjectively with the Pittsburgh Sleep Quality Index. Sleep duration and efficiency were objectively measured with actigraphy. Participants self-reported medicinal sleep aid usage. Significant group differences were observed in sleep duration measured objectively (p = 0.02). No group differences were identified in subjective sleep quality factors (p = 0.21) or medicinal sleep aid use (p = 0.41). Our findings show physical disability may be a factor in sleep duration, though neither associated with worsened sleep perception nor greater reliance on medicinal sleep aids. Implications of our research highlight the importance of using subjective and objective measures when assessing sleep quality and carefully considering the use of medicinal sleep aids.

Abstract citation ID: igae098.1181

### CITY LIGHTS OR COUNTRYSIDE NIGHTS? RURALITY'S ROLE ON ANXIETY AND SLEEP IN MID- TO LATE-LIFE

Amy Costa[1], Puja Halder[2], Christina McCrae[1], and Ashley Curtis[1], *1. University of South Florida, Tampa, Florida, United States*, *2. University of Missouri, Columbia, Missouri, United States*

Anxiety and sleep health associations are well studied but contributing factors (e.g., level of rurality) to health disparities in this relationship are understudied, particularly in aging populations. The present study examined the moderating impact of rurality on associations between anxiety and aspects of sleep health in middle-aged and older adults living in the United States. Cognitively healthy middle-aged and older adults (N=251; Mage=64.7±7.9 years) completed an online survey measuring residence zip code, Pre-Sleep Arousal Scale, Pittsburgh Sleep Quality Index, and the Hospital Anxiety and Depression scale. Multiple regression and simple slope analyses examined whether rurality (rural vs. non-rural; coded by Delineating Core Based Statistical Areas) moderated associations between anxiety and sleep, controlling for age, sex, education, income, depression, medication usage, and nutritional status (Mini-Nutritional Assessment). Results revealed rurality moderated the association between anxiety and cognitive pre-sleep arousal (R2 change=.014, p=.01) and sleep disturbances (R2 change=.01, p=.04). Higher anxiety was associated with higher cognitive pre-sleep arousal, with an ~2x larger association for rural (B=.64, p<.0001) than non-rural (B=.35, p=.0002) participants. Similarly, higher anxiety was associated with more sleep disturbances for rural (B=.05, p<.001), than non-rural participants. In mid-to-late life, those living in rural areas may be more vulnerable to the impact of higher anxiety on worse sleep compared to those living in non-rural areas. Findings point to the need for a greater understanding of socioeconomic determinants of health and more tailored treatments, with potential emphasis on mitigating barriers impacting rural access to clinical treatments for anxiety and sleep health.

Abstract citation ID: igae098.1182

### PREVALENCE AND THE ASSOCIATED FACTORS OF MULTIMORBIDITY AMONG OLDER NEPALIS IN WESTERN NEPAL

Lirisha Tuladhar[1], Krishna Sapkota[1], Bharat Kafle[2], Preeti Bhattarai[2], Pratik Bhattarai[3], and Aman Shrestha[4], *1. Miami University, Oxford, Ohio, United States*, *2. Karnali Academy of Health Sciences, Jumla, Karnali, Nepal*, *3. Oxford University Clinical Research Unit Nepal, Lalitpur, Bagmati, Nepal*, *4. University of Maryland School of Medicine, Baltimore, Maryland, United States*

Multimorbidity, the concurrent presence of multiple chronic diseases, is a global public health issue, with higher prevalence rates observed among older age groups and individuals of lower socioeconomic status. This study aims to determine the prevalence of morbidity levels and the associated factors across all three ecological regions of Western Nepal. Utilizing a community-based cross-sectional design, 612 participants were interviewed face-to-face. Levels of morbidity were grouped as "no morbidity," "single disease," and "multiple diseases." Ordinal logistic regression examined the association between levels of morbidities and various sociodemographic, economic, and health behavior factors. Probabilities models were cumulated over the higher-ordered levels of morbidity. Among sociodemographic variables, participants of urban residents (OR=1.68, CI: 1.16—2.44), of age 70 years and above (OR=1.55, CI: 1.10—2.17), females (OR=2.43, CI:1.61—3.66) and with disability (OR=1.58, CI:1.14—2.18) were associated with increased odds of being in a higher category (increasing morbidity) rather than lower category than their respective counterparts after adjusting for the covariates. Economic factors did not establish significant relationships with morbidity levels. Among health behavior factors, those who consumed alcohol had 41% decreased odds of higher morbidity compared to non-consumers (OR=0.59 CI:0.37—0.92). This study identified significant associations between multimorbidity and specific demographic groups in urban areas, notably older females and individuals with disabilities. It highlights the necessity of providing tailored behavior change information and implementing screening programs to reduce the prevalence of health issues in these populations.

### SESSION 3060 (SYMPOSIUM)

Abstract citation ID: igae098.1183

### HEALTHY BRAIN AGING: OPPORTUNITIES AND CHALLENGES

Chair: Andrew Sixsmith

Dementia and cognitive decline are amongst the greatest challenges of modern times and have a major impact on individuals, families, and society. Although there are currently no effective treatments or cures for dementia-related conditions, there is huge scope for preventative measures to improve brain health as people grow older. By developing and implementing real-world solutions that address known modifiable risk-factors, the decline of cognitive health and the onset of dementia may be delayed or prevented. With the right combination of policies, services and changes in health-related behaviours, it may be possible to expand the healthspan of people, where most people can live healthier, active lives as they grow older. Our interdisciplinary symposium comprises four papers that examine the challenges and opportunities for brain health and healthy aging. Sylvain Moreno a discusses heterogeneity in neurocognitive aging, challenging the predominantly linear view of cognitive decline with age. Holland and colleagues draw on a Delphi consensus study to explore opportunities for developing Interventions for cognitive frailty, the potential to delay, prevent or reverse cognitive frailty, and how people with lived experience can contribute to clinical and research expertise, White, Sixsmith and Fang explore the role of community-based organisations in supporting healthy aging, focusing on practical strategies for mobilizing community assets and resources to promote healthy aging. Boot, Dilanchian and Kalantari examine the role of technologies to support healthy brain aging, particularly the potential of extended reality technologies to support older people with and without cognitive impairments.

Abstract citation ID: igae098.1184

### INTERVENTIONS FOR COGNITIVE FRAILTY: DELPHI CONSENSUS WITH RESEARCHERS, CLINICIANS, AND EXPERTS BY EXPERIENCE

Carol Holland[1], Nikolette Dravecz[1], Susan Broughton[1], Sally Fowler Davis[2], and Mandy Dixon[3], *1. Lancaster University, Lancaster, England, United Kingdom*, *2. Anglia Ruskin University, Cambridge, England, United Kingdom*, *3. The Northern Health Science Alliance Ltd, Manchester, England, United Kingdom*

The conjunction of physical frailty and cognitive impairment without dementia is described as Cognitive Frailty (CF). Indications that CF is potentially reversible has led to proposals that addressing risk factors, symptoms or mechanisms of CF would be appropriate targets for interventions for prevention, delay or reversal. However, no study has brought experts together across sectors to determine targeted or structures of interventions, and most resources on interventions are from the perspective of academic or clinical researchers only. We conducted an international Delphi consensus study bringing together experts from academic and clinical research, lay people with lived experience of CF, informal carers, and professional care practitioners/clinicians. Three rounds of the Delphi study were held to discern which factors were agreed upon by the whole sample and on which did people with differing expertise have differing views. A scoping review and Round 1 was used to gather initial statements. In Round 2, 56 people responded to statements and open text boxes, with 7 lab based researchers, 27 researchers working with people, 14 people with lived experience or informal family carers, and 10 professional carers/clinicians. Analysis of quantitative data gave 74 statements on which there was at least 70% agreement and qualitative data gave 24 statements, which were then combined to give 90 statements for Round 3, to which 38 original participants responded. Consensus was found for 88 of the statements. Differences between the groups were observed at both stages. Outcomes can be used to feed into co-creation of interventions for cognitive frailty.

Abstract citation ID: igae098.1185

### THE POTENTIAL OF EXTENDED REALITY SOLUTIONS TO SUPPORT OLDER ADULTS WITH AND WITHOUT COGNITIVE IMPAIRMENTS

Walter Boot[1], Andrew Dilanchian[2], and Saleh Kalantari[3], *1. Weill Cornell Medicine, New York, New York, United States*, *2. Florida State University, Tallahassee, Florida, United States*, *3. Cornell University, Ithaca, New York, United States*

We are living in an era of rapid technological innovation, which presents novel opportunities for technology to foster and enhance the independence, productivity, health, safety, social connectivity, and quality of life of older people with and without cognitive impairments. Both established and emerging technologies can play significant roles in achieving these aims, but to meet their fullest potential they must be designed with a user-centered approach that considers the needs, abilities, and preferences of diverse populations of older people, should be evaluated for usability, and rigorously assessed to determine if they are achieving their intended outcomes. This talk will focus specifically on the potential of novel Extended Reality (XR) solutions, including Virtual Reality (VR) and Augmented Reality (XR), to support activities vital to older people's brain health, wellbeing, and independence. It will begin with an introduction to the capabilities of these technologies and how these technologies are well-suited to support aging adults, followed by the potential of these tools to support older people with and without cognitive impairments in domains such as cognitive health, social connectivity, wellness, way-finding, and leisure. Evidence for the efficacy of these interventions will be summarized, followed by a presentation of best practices and design guidelines for the use of XR among aging persons based on the latest empirical evidence. We will conclude with a discussion of the future of these technologies and outline a research agenda moving forward.

Abstract citation ID: igae098.1186

### THE ROLE OF COMMUNITY-BASED ORGANIZATIONS IN SUPPORTING HEALTHY AGING

Rebecca White[1], Andrew Sixsmith[1], and mei Fang[2], *1. Simon Fraser University, Vancouver, British Columbia, Canada*, *2. SIMON FRASER UNIVERSITY, Simon Fraser University, British Columbia, Canada*

Maintaining a healthy and balanced lifestyle is crucial for healthy aging and has a significant positive impact on brain health. Community-based organizations play an important role in many countries in promoting healthy lifestyles, particularly among those with the greatest social and economic need, through the provision of social, recreational, and informational programming. This presentation draws on research carried out in Vancouver, Canada, to understand the role of community-based organizations in enhancing the physical, social, and emotional well-being of older people. Using an institutional ethnographic case study approach guided by principles of community-based participatory research, the research focuses on the role of community-based organizations in enhancing older people's well-being at multiple levels and contextualizes such organizations within a broader healthcare ecosystem. Results demonstrate the multi-faceted role community-based organizations play in supporting health and well-being among older people. The shift towards integrated healthcare models with a more holistic approach to health presents opportunities for community-based organizations to assume a more prominent role in empowering older people to self-manage their health and promote healthy lifestyles. Yet, such organizations face a multitude of challenges, particularly securing sustainable operational funding and widening their appeal to a changing demographic. The research highlights how community assets may be mobilized to better support the health and well-being of aging communities. More research is needed to understand how community-based organizations can better communicate their value and impact and enhance their capacity to respond effectively to emerging opportunities.

Abstract citation ID: igae098.1187

### THE ROLE OF HETEROGENEITY IN NEUROCOGNITIVE AGING

Sylvain Moreno, *Simon Fraser University, Vancouver, British Columbia, Canada*

Neurocognitive aging research has predominantly adopted a linear approach, categorizing aging populations homogeneously. Researchers like Salthouse argue that this perspective perceives cognitive decline as almost linear from early adulthood. However, emerging findings challenge this view, highlighting significant variability in cognitive aging and proposing different subtyping methods to study the neurocognitive aging population. Our talk will propose a novel subtyping framework inspired by the Orchid and Dandelion theory from Boyce (2005). This new framework introduces a nuanced understanding of cognitive aging, categorizing individuals based on genetic and phenotypic characteristics to better capture the heterogeneity of cognitive abilities as people age. Using extensive datasets such as the UK Biobank and machine learning analytical techniques, our research investigates the impact of environmental factors on distinct cognitive aging subtypes. This approach diverges from the traditional linear models, revealing that lifestyle factors have varying impacts on different cognitive aging subtypes, underscoring the theory's premise that some individuals (orchids) are more environmentally sensitive, affecting their cognitive aging process. In contrast, others (dandelions) show resilience. This paradigm shift towards recognizing cognitive aging's inherent heterogeneity offers a more accurate and personalized understanding of aging, emphasizing the importance of subtyping in developing targeted interventions. Our findings advocate for a move beyond the "linear" methodology found in the literature, highlighting the need for a framework that accommodates the heterogeneity of cognitive aging.

### SESSION 3065 (SYMPOSIUM)

Abstract citation ID: igae098.1188

### JOURNAL OF GERONTOLOGY SOCIAL SCIENCES: FEATURED 2023 EDITOR'S CHOICE ARTICLES

Chair: Jessica Kelley

Social science inquiry on age, aging, and the life course spans many topics and methodologies. This symposium highlights papers that were selected as Editor's Choice articles in the Journal of Gerontology Social Sciences in 2023. These papers highlight methodological innovations, important advancements in our state of knowledge in an area, or emerging issues in the study of aging and older adults. Wright et al. compare differences in income pooling between remarried and cohabiting older adults. Taylor et al. present a qualitative study of the ways that kinless older adults navigate a dementia onset. Stokes et al. utilize novel dyadic analysis to explore the interrelationship of loneliness and cognition among married couples over time. Latham Minus et al. document mental and physical health among older adults who are formerly incarcerated, showing differences by gender and race. Al-Rousan et al. examine the relationship between migration history and cognitive functioning among older Arab Americans and refugees.

Abstract citation ID: igae098.1189

### INCOME POOLING IN MIDLIFE: A COMPARISON OF REMARRIED AND COHABITING RELATIONSHIPS

Matthew Wright[1], Tatum Schwartz[2], Susan Brown[3], and Wendy Manning[3], *1. Appalachian State University, Boone, North Carolina, United States*, *2. University of California Los Angeles, Los Angeles, California, United States*, *3. Bowling Green State University, Bowling Green, Ohio, United States*

The share of midlife cohabitors has risen in recent decades, though little is known about their income pooling practices. Cohabitation could be attractive because partners may be able to preserve their economic autonomy and maintain assets for the next generation. Conversely, cohabitation may operate as an alternative to marriage, allowing couples to combine their resources to achieve economies of scale without the legal obligations of marriage. We used the nationally representative Families and Relationships Survey to compare income pooling among cohabiting and remarried U.S. adults aged 50-65 (N = 888). Aligning with the hypothesis that cohabitation and remarriage are distinct in middle age, the odds of income pooling were lower for cohabitors than remarrieds. However, the gap between cohabitors and remarrieds narrowed by later ages. This study provides insight into the economic organization of midlife cohabiting relationships, which may have implications for individual well-being and relationship decision-making among middle-aged couples.

Abstract citation ID: igae098.1190

### KINLESS OLDER ADULTS WITH DEMENTIA: QUALITATIVE ANALYSIS OF DATA FROM THE ADULT CHANGES IN THOUGHT STUDY

Janelle Taylor[1], Marlaine Figueroa Gray[2], Callie Freitag[3], Hitomi Kariya[4], Paul Crane[5], Elizabeth Vig[6], Clara Berridge[5], and Eric Larson[5], *1. University of Toronto, Toronto, Ontario, Canada*, *2. Kaiser Permanente Washington Health Research Institute (KPWHRI), Seattle, Washington, United States*, *3. University of Wisconsin-Madison, Wisconsin, United States*, *4. NA, Seattle, Washington, United States*, *5. University of Washington,*

*Seattle, Washington, United States*, 6. *University of Washington/VA Puget Sound Healthcare System, Seattle, Washington, United States*

**Objectives:** To examine the circumstances and needs of older adults who were "kinless," defined as having no living spouse or children, when they developed dementia.

**Methods:** We conducted a secondary analysis of information from the Adult Changes in Thought study. Among 848 participants diagnosed with dementia between 1994 and 2016, we identified 64 who had no living spouse or child at dementia onset. We then conducted a qualitative analysis of administrative documents pertaining to these participants: handwritten comments recorded after each study visit, and medical history documents containing clinical chart notes from participants' medical records.

**Results:** In this community-dwelling cohort of older adults diagnosed with dementia, 8.4% were kinless at dementia onset. Participants in this sample had an average age of 87 years old, half lived alone, and one third lived with unrelated persons. Through inductive content analysis, we identified 4 themes that describe their circumstances and needs: (1) life trajectories, (2) caregiving resources, (3) care needs and gaps, and (4) turning points in caregiving arrangements.

**Discussion:** Our qualitative analysis reveals that the life trajectories that led members of the analytic cohort to be kinless at dementia onset were quite varied. This research highlights the importance of nonfamily caregivers and participants' own roles as caregivers. Our findings suggest that clinicians and health systems may need to work with other parties to directly provide dementia caregiving support rather than rely on family, and address factors such as neighborhood affordability that particularly affect older adults who have limited family support.

Abstract citation ID: igae098.1191

### LONGITUDINAL DYADIC ASSOCIATIONS BETWEEN LONELINESS AND COGNITION AMONG OLDER COUPLES IN THE UNITED STATES

Jeffrey Stokes, Anyah Prasad, Adrita Barooah, and Elisabeth Stam, *University of Massachusetts Boston, Boston, Massachusetts, United States*

**Objectives:** Loneliness is associated with diminished health and cognition for older individuals. However, little research has examined dyadic loneliness – i.e., loneliness of both partners in a relationship – and its potential consequences for cognitive functioning among both spouses, nor whether one partner's cognition may impact both partners' loneliness over time.

**Methods:** We analyze 3-wave dyadic Health and Retirement Study data (2010-2020; N=1,061 dyads) to determine (a) whether loneliness predicts participants' own and/or their partners' episodic memory and verbal fluency over 8 years, and (b) whether cognitive functioning predicts older spouses' own or their partners' loneliness over the same period.

**Results:** Loneliness predicted participants' own and their partners' loneliness at follow-up, at both timepoints. Loneliness was also associated with own episodic memory at follow-up, but not with verbal fluency. Episodic memory and verbal fluency predicted one another over time. Neither episodic memory nor verbal fluency predicted loneliness at follow-up. Significant dyadic mediation was established such that Time 1 loneliness was linked with partner's Time 3 episodic memory via that partner's Time 2 loneliness.

**Discussion:** Lonelier older adults displayed worse trajectories of episodic memory over time, yet poor memory did not precede changes to loneliness. Further, having a lonely partner was linked with poorer episodic memory 8 years later, indicating that both one's own and – to a lesser extent – a partner's emotional well-being may be consequential for maintaining cognitive functioning with age. Associations were more clearly established with episodic memory than with verbal fluency, suggesting domain-specific effects of loneliness.

Abstract citation ID: igae098.1192

### AGING WITH INCARCERATION HISTORIES: AN INTERSECTIONAL EXAMINATION OF INCARCERATION AND HEALTH AMONG OLDER ADULTS

Kenzie Latham-Mintus, Monica Deck, and Elizabeth Nelson, *Indiana University Indianapolis, Indianapolis, Indiana, United States*

**Objectives:** Experiences with incarceration are linked to poor mental and physical health across the life course. The pur- pose of this research is to examine whether incarceration histories are associated with worse physical and mental health among older adults. We apply an intersectionality framework and consider how the intersection of sexism and racism leads to unequal health outcomes following incarceration among women and people of color.

**Methods:** We employ 2 measures of health (i.e., number of depressive symptoms and physical limitations) to broadly cap- ture mental and physical health. Using data from Waves 11 and 12 of the Health and Retirement Study, we estimated a series of general linear models to analyze differences in health by incarceration history, gender/sex, and race/ethnicity.

**Results:** Findings suggest that experiences with incarceration are associated with a greater number of physical limitations and more depressive symptoms among older men and women, net of sociodemographic characteristics, early-life condi- tions, and lifetime stressful events. Formerly incarcerated women, particularly women of color, had more physical limita- tions and depressive symptoms relative to other groups.

**Discussion:** These findings suggest that incarceration histories have far-reaching health implications. Older women of color with incarceration histories experience markedly high levels of physical limitations and depressive symptoms in later life.

Abstract citation ID: igae098.1193

**MIGRATION AND COGNITIVE HEALTH DISPARITIES: THE ARAB AMERICAN AND REFUGEE CASE**

Tala Al-Rousan[1], Alison Moore[2], Alissa Bernstein Sideman[3], Kristine Ajrouch[4], and Lily Kamalyan[5], *1. University of California San Diego, La Jolla, California, United States*, *2. University of California San Diego School of Medicine, San Diego, California, United States*, *3. University of California San Francisco, San Francisco, California, United States*, *4. University of Michigan, Ann Arbor, Michigan, United States*, *5. San Diego State University/University of California San Diego Joint Doctoral Program in Clinical Psychology, San Diego, California, United States*

This study investigates whether the year of arrival to the United States (U.S.) and birthplace relate to postmigration cognitive difficulties among foreign- and U.S.-born Arab Americans in later life. We analyzed 19 years (2000-2019) of data from the American Community Survey Public Use Microdata Samples (weighted N = 393,501; ages ≥ 50 years). The cognitive difficulty was based on self-reported data and weighted means, percentages, adjusted prevalence estimates, and adjusted odds ratios were calculated. Controlling only for demographics, foreign-born Arabs reported higher odds of cognitive difficulty compared to U.S.-born Arabs across all arrival cohorts (p <.001). After accounting for economic and integration factors, those who arrived between 1991 and 2000 had higher odds (odds ratio [OR] = 1.06, 95% confidence interval [CI] =1.00, 1.19, p <.01), while those who arrived after 2001 had lower odds (OR = 0.87, 95% CI = 0.78, 0.97, p <.001) of cognitive difficulty. Lacking English proficiency (OR = 1.90, 95% CI = 1.82, 1.98, p <.001) was related to higher odds, whereas not being a U.S. citizen was significantly associated with lower odds (OR = 0.89, 95% CI = 0.52, 0.94, p <.001) of cognitive difficulty. Yet, results varied by birthplace. Migrants born in Iraq consistently reported the highest odds of cognitive difficulty across all arrival cohorts. Migration history and birthplace are essential factors explaining cognitive disparities among the diverse group of Arab migrants and Arab Americans. Reasons for migration such as displacement due to war may explain these disparities.

### SESSION 3070 (SYMPOSIUM)

Abstract citation ID: igae098.1194

**LET'S GET PHYSICAL: ACTIVITY, FUNCTION, AND FITNESS AS KEY COMPONENTS OF AGING AND HEALTH**

Chair: Jennifer Schrack
Co-Chair: Amal Wanigatunga
Discussant: Adam Spira

Physical activity, physical function, and physical fitness ("activity, function and fitness") are established predictors of health and longevity. Yet, many aspects of how and why measures of activity, function, and fitness are so strongly linked with physical and cognitive health in older adults remain under-addressed. This is further complicated by the multidimensional aspects of activity, function, and fitness, allowing them to function as both predictors and outcomes of health. This symposium will focus on quantifying and defining measures of activity, function, and fitness and their associations with various features of physical and cognitive health across several large U.S. cohort studies. Dr. Davoudi will present the prevalence of impairments in motor function (gait speed, chair stands, standing balance, grip strength) in the National Health and Aging Trends Study (NHATS). Dr. Wanigatunga will describe new methodology to assess the combined contribution of sensory and motor functioning to cognitive health in the Baltimore Longitudinal Study of Aging (BLSA) and the Atherosclerosis Risk in Communities (ARIC) study. Dr. Dougherty will present the association of physical activity with Alzheimer's biomarkers (amyloid, total tau, phosphorylated tau) and cognitive functioning in the Biomarkers of Cognitive Decline Among Normal Individuals (BIOCARD) study. Dr. Twardzik will describe trends in public transit use and associations with physical activity in NHATS. Finally, Dr. Yue will present the association of sleep quality and quantity with measures of fitness and energetic efficiency in the BLSA. Collectively, these presentations will highlight novel pathways through which activity, function, and fitness contribute to healthy aging.

Abstract citation ID: igae098.1195

**POPULATION PREVALENCE OF MOTOR FUNCTION IMPAIRMENTS IN US OLDER ADULTS: THE NATIONAL HEALTH AND AGING TRENDS STUDY**

Anis Davoudi[1], Hang Wang[2], Ryan Dougherty[3], Amal Wanigatunga[4], Alden Gross[4], and Jennifer Schrack[4], *1. Johns Hopkins University, North Bethesda, Maryland, United States*, *2. Johns Hopkins University, Baltimore, Maryland, United States*, *3. Rutgers University, Stoughton, Wisconsin, United States*, *4. Johns Hopkins Bloomberg School of Public Health, Baltimore, Maryland, United States*

Motor function impairments reflect physical health status for older adults, which has profound implications for quality of life. However, there is a significant research gap concerning comprehensive, nationally representative data on the prevalence of these impairments, including diminished gait speed, chair stand ability, standing balance, and grip strength, especially among those aged □80 years. We used data from Round 12 of the National Health and Aging Trends Study (NHATS), a nationally representative panel study of U.S. Medicare beneficiaries aged ≥65 years who completed objective physical function testing. Motor function was assessed using gait speed, chair stand speed, standing balance, and grip strength and impairment(s) were defined using standard clinical thresholds or previously published norms. Age was categorized in 5-year intervals [65-69, 70-74, 75-79, 80-84, 85-89, and 90+]. In this US-representative sample of 5055 older adults (mean age: 78.7 years old (78.3-79.1), 54.4% women), weighted prevalence of impairments in gait speed, chair stand speed, standing balance, and grip strength were higher with advancing age. Among those aged 65-69 years, chair stand impairment was the most prevalent (42.4% (37.0%-47.8%)), while gait speed impairment was the most prevalent among those ≥90 years (90.4% (86.0%-94.9%)). Prevalence of being impaired in all four motor function domains was 11.8% (8.5%-15.2%) at ages 65-69 years and 64.0% (58.0%-69.9%) among those ≥90 years.

These findings indicate a higher prevalence of impairments in motor function with older age, underscoring the importance of addressing these impairments earlier in life to enhance the quality of life in the aging population.

Abstract citation ID: igae098.1196

### ALGORITHMICALLY DEFINED SENSORIMOTOR FUNCTION AND RISK OF MILD COGNITIVE IMPAIRMENT

Amal Wanigatunga[1], Hang Wang[2], Anis Davoudi[3], Ryan Dougherty[4], Qu Tian[5], Eleanor Simonsick[6], Alden Gross[1], and Jennifer Schrack[1], *1. Johns Hopkins Bloomberg School of Public Health, Baltimore, Maryland, United States*, *2. Johns Hopkins University, Baltimore, Maryland, United States*, *3. Johns Hopkins University, North Bethesda, Maryland, United States*, *4. Rutgers University, Stoughton, Wisconsin, United States*, *5. National Institute on Aging, Baltimore, Maryland, United States*, *6. National Institute on Aging, Baltimore City 0400 Baltimore City, Maryland, United States*

Early detection of persons at risk for, or living with, mild cognitive impairment (MCI) is a public health priority. Sensorimotor functioning—the integration of sensory information with motor function signaling to perform tasks and interact with the environment—may be a specific domain intertwined with movement planning and mental ability. However, a gap remains as to the optimal approach to operationalize sensorimotor function. This study assessed sensorimotor functioning using common clinical and research measures and evaluated its cross-sectional association with MCI. A unidimensional sensorimotor functioning factor was developed using confirmatory factor analysis based on six binary sensory and motor function impairment indicators including hearing, vision, olfaction, standing balance, usual gait speed, and grip strength. Using structural equation modeling, we examined whether higher sensorimotor functioning (greater factor score indicating less impairment) was related to lower odds of MCI using two independent cohort studies: The Atherosclerosis Risk and Communities Study (ARIC) (N=932), and the Baltimore Longitudinal Study of Aging (BLSA) (N=681) of participants who had complete sensory and motor function tests. After covariate adjustment, for each additional SD higher in sensorimotor factor score, there was a 56% (95%CI: 40%-68%) and 45% (95%CI: 24%-60%) lower odds of MCI in ARIC and BLSA, respectively. Findings suggest that leveraging a sensorimotor functioning score derived from common assessments is feasible and associated with MCI. Longitudinal studies are needed to determine whether degrading sensorimotor function can predict early cognitive decline indicative of elevated dementia risk.

Abstract citation ID: igae098.1197

### PHYSICAL ACTIVITY ATTENUATES THE INFLUENCE OF ALZHEIMER'S BIOMARKERS ON COGNITIVE FUNCTION

Ryan Dougherty[1], Anja Soldan[2], Corinne Pettigrew[3], Barry Greenberg[2], Adam Spira[2], Abhay Moghekar[3], and Marilyn Albert[3], *1. Rutgers University, Stoughton, Wisconsin, United States*, *2. Johns Hopkins University, Baltimore, Maryland, United States*, *3. Johns Hopkins University School of Medicine, Baltimore, Maryland, United States*

Alzheimer's disease (AD) is characterized by the abnormal accumulation of amyloid-β (plaques), and tau (neurofibrillary tangles) that can be quantified in vivo through cerebrospinal fluid (CSF) sampling. Physical activity has emerged as a possible modifier of AD risk; however, the influence of physical activity on CSF biomarkers and cognitive function remains unclear. We examined whether higher levels of physical activity favorably modify associations between AD CSF biomarkers and cognitive function. One hundred and seventeen adults free of dementia from the BIOCARD study (mean age 72.2±8.0 years, 70% women) wore a wrist accelerometer for one week, underwent lumbar puncture to collect CSF, and completed a comprehensive neuropsychological exam. Covariate-adjusted linear regression analyses were used to examine whether physical activity (total activity counts over the 10 most active hours of the day) moderates the association between AD CSF biomarkers [amyloid-β (Aβ42/40), total tau, phosphorylated tau (p-tau181)] and cognitive composite scores (episodic memory, executive function). There were significant interactions between physical activity and total tau ($p=.004$) as well as physical activity and p-tau181 ($p=.016$) for the executive function composite score. Among those with elevated total tau or p-tau181, participants with greater levels of physical activity exhibited significantly higher executive function composite scores compared to participants with lower levels of physical activity. In contrast, there were no significant interactions for episodic memory, and physical activity did not interact with Aβ42/40 (all interactions $p>.05$). These findings suggest that a physically active lifestyle may play a protective role against cognitive decline through tau pathways.

Abstract citation ID: igae098.1198

### UNDERSTANDING CHANGES IN PUBLIC TRANSIT BEHAVIOR AND ITS LINKS TO PHYSICAL ACTIVITY: FINDINGS FROM THE NHATS

Erica Twardzik[1], and Jennifer Schrack[2], *1. University of Michigan, Ann Arbor, Maryland, United States*, *2. Johns Hopkins Bloomberg School of Public Health, Baltimore, Maryland, United States*

Public transit use is beneficial to human health. With a growing older adult population, a better understanding of older adult's use of public transit and physical activity among public transit users is needed. We aim to describe public transit use among older adults from 2015 to 2022 within a nationally representative sample. Using the 2015-2022 National Health and Aging Trends Study (n = 38,970), we examined the prevalence of public transit use among adults aged 72 years and older. We found that 8.0% of older adults use public transit to get to places outside of their home. However, the prevalence of public transit use varied by gender, age group, and year of observation. Women (6.3%) used public transit less than men (8.5%), and public transit use prevalence was lower at older ages (ages 72-74, 7.8%; ages 90+, 3.6%. Public transit use was consistent across years 2015 to 2019 (8.5%), before it decreased drastically in 2020 (3.3%) and remained low in 2021 (5.2%) and 2022 (4.7%). In 2021 and 2022 public transit users averaged 48.8 more daily minutes of physical activity than non-users. These results suggest demographic and temporal trends in public transit use among older adults. Given that older adults report 7-10 non-driving years at the end-of-life, public transit could be an attractive option for this growing population. The pandemic reshaped the transportation landscape, and many public transit systems have not recovered pre-pandemic ridership. Future efforts should integrate age friendly policies to increase older adult ridership.

Abstract citation ID: igae098.1199

### LINKS OF SLEEP WITH CHANGES IN CARDIORESPIRATORY FITNESS AND ENERGETIC EFFICIENCY IN MIDDLE-AGED AND OLDER ADULTS

Yiwei Yue[1], Daniel Callow[2], Idiatou Diallo[1], Jill Rabinowitz[3], Sarah Wanigatunga[1], Eleanor Simonsick[4], Jennifer Schrack[5], and Adam Spira[1], *1. Johns Hopkins University, Baltimore, Maryland, United States*, *2. Johns Hopkins University School of Medicine, Baltimore, Maryland, United States*, *3. Rutgers, The State University of New Jersey, New Brunswick, New Jersey, United States*, *4. National Institute on Aging, Baltimore City 0400 Baltimore City, Maryland, United States*, *5. Johns Hopkins Bloomberg School of Public Health, Baltimore, Maryland, United States*

Sleep disturbances are linked to cardiovascular disease in middle-aged and older adults, but little is known about the associations of poor sleep with cardiovascular fitness and energetic measures in this population. We investigated cross-sectional and longitudinal associations of actigraphic sleep parameters with cardiorespiratory fitness, walking energetics, and resting metabolic rate among 263 participants (mean age=72.7±10.1 years, 53.6% women) in the Baltimore Longitudinal Study of Aging. Additionally, we assessed modifying roles of sex, race, and age in these associations. Primary predictors included actigraphic total sleep time (TST), sleep efficiency (SE; % of time in bed spent asleep), sleep onset latency (SOL), wake after sleep onset (WASO), and average wake bout length (WBL). Outcomes included maximal oxygen consumption (VO2max), energetic cost of walking (ECW), and resting metabolic rate (RMR). Adjusting for demographics, comorbidities, self-reported physical activity, time, and interactions of time with covariates, longer WBL was cross-sectionally associated with lower VO2max (B=-1.24, p<0.01) and higher RMR (B=62.65, p<0.01); lower SE with lower VO2max (B=1.18, p<0.01); and shorter TST with higher RMR (B=-16.03, p<0.05). Women had stronger associations between longer TST and higher VO2max and greater WBL with slower decline in VO2max than men. Middle-aged adults showed stronger connections of higher SE and lower WASO with lower RMR than older adults (interaction p-values<0.05). In summary, shorter TST, longer WBL, and lower SE are linked with poorer cardiorespiratory fitness and energetic efficiency among middle-aged and older adults. Studies are needed to determine whether enhancing sleep can improve fitness and energetic efficiency, and vice-versa.

### SESSION 3075 (PAPER)

### LGBTQIA+ AGING

Abstract citation ID: igae098.1200

### "WE TREAT EVERYONE THE SAME": PROVIDER PERSPECTIVES ON LGBT+ AGING IN EAST TENNESSEE

Joseph Winberry, *University of North Carolina at Chapel Hill, Durham, North Carolina, United States*

Aging Services providers in East Tennessee were asked to report barriers and solutions they saw to 5 aging-related needs raised by local Lesbian, Gay, Bisexual, and Transgender plus (LGBT+) people fifty years and older. The 5 needs included services for caregivers, social support, culturally competent care, LGBT+ Specific Services, and Planning. Fourteen providers working in settings such as offices on aging and long-term care facilities shared 248 responses via a qualitative survey. Thematic analysis of those responses revealed 6 barrier themes (ageism and elder abuse, discrimination, fewer personal resources, identity concealment, staff limitations, and stigma) and 4 solution themes (direct affirmation, creating and sharing information in partnership, inclusive written messages and images, and training participation). Review of the data also indicated a third category of response which was denial of needs raised. The findings indicate that providers have a varied understanding of barriers and potential solutions for meeting the aging services needs of LGBT+ older adults. They also demonstrate a lack of awareness among at least some providers of how LGBT+ older adults feel about their ability to access aging services in comparison to non-LGBT+ older adults. These findings are part of a larger, multidisciplinary community-engaged participatory research project that is ongoing in East Tennessee. Its purpose is to address information and social barriers that LGBT+ older adults see as obstructing their ability to age with dignity and respect in the community they call home. The findings raised from provider survey responses are useful for designing future interventions for providers.

Abstract citation ID: igae098.1201

### C-REACTIVE PROTEIN LEVELS AMONG LGB OLDER ADULTS: INTERSECTIONAL ASSOCIATIONS OF RACE, ETHNICITY, AND EDUCATION

Andrew Alberth[1], Anyah Prasad[1], Amanda Collins[1], Alison Rataj[2], Heather Farmer[3], and Jeffrey Stokes[1], *1. University of Massachusetts Boston, Boston, Massachusetts, United States*, *2. University of Massachusetts Boston, Durham, New Hampshire, United States*, *3. University of Delaware, Newark, Delaware, United States*

**Objectives:** This paper assesses the intersection between sexual orientation, race, and ethnicity as they relate to CRP levels among middle-aged and older adults in the Health and Retirement Study (HRS). It will also explore how educational attainment, a commonly used demographic correlate with socioeconomic status, may differently benefit racially, ethnically, and sexually diverse middle-age and older adults.
**Methods:** This study uses data from the 2016 Health and Retirement study. C-reactive protein levels, sexual orientation, race and ethnicity, educational attainment, and covariate variables were collected using serum samples and survey responses from 1,426 older adults enrolled in the study. Individuals were included if they had reported measures for key independent and dependent variables. Multiple imputation was used to address missing values across control variables. Associations and moderators were assessed using ordinary least squares (OLS) regression.

**Results:** This study found lesbian, gay, and bisexual (LGB) respondents had lower CRP levels when they had higher educational attainment compared to similarly educated straight respondents. Additionally, Black LGB respondents had less inflammation than Black and straight or White and LGB respondents.
**Discussion:** This study's findings indicate educational attainment may differently benefit LGB older adults compared to straight older adults, and respondents who are Black and LGB experience greater protection from elevated CRP levels compared to only Black or LGB respondents. These findings suggest educational attainment may be differently used to protect against systemic inflammation among sexual minority populations. Furthermore, these results suggest additional nuance is necessary to understand how CRP levels impact diverse populations.

Abstract citation ID: igae098.1202
### EXAMINING LGBT OLDER ADULT CHOSEN FAMILIES USING THE CONVOY MODEL OF SOCIAL RELATIONS
Kelseanne Breder[1], and Walter Bockting[2], *1. New York University, New York, New York, United States*, *2. Columbia University / Psychiatric institute, New York, New York, United States*

Sexual and gender minority (LGBT) older adults experience heightened levels of social discrimination and minority stress throughout their lives due to their minority identities. Older LGBT adults experienced unique external, systemic barriers to social acceptance, including bans on same-sex marriage, discrimination and negligence during the HIV epidemic, and police brutality during demonstrations such as the Stonewall Riots. LGBT older adults are also more likely to live alone and more likely to be estranged from their families of origin. Taken together, these discriminatory social experiences increase stress exposure and create a demand for an adaptive and protective social network to serve as a counterbalance to minority stress. Many LGBT older adults have developed "chosen families" or social networks composed of non-biological relatives who care for one another as if they are family. The LGBT chosen family is a resilience strategy that has not been examined thoroughly by a theoretical framework. The purpose of this paper is to apply the Convoy Model of Social Relations to examine the LGBT chosen family experience. The convoy metaphor succinctly captures the value of the LGBT chosen family, which functions not only to support members within the chosen family unit, but also to offset exposure to external threats and buffer internalized and externalized homophobia in a heteronormative society. The paper identifies points of convergence and departure between the model and LGBT chosen families and includes an analysis of the role of technology in LGBT older adult social networks. Modifications to the model are proposed.

Abstract citation ID: igae098.1203
### SECOND CLASS CARE: PATTERNS OF LGBTQ+ PATIENT EXPERIENCES AND RECEIPT OF PREVENTIVE CARE IN MIDLIFE AND OLDER AGE
Nathaniel Tran[1], Tara McKay[2], Gilbert Gonzales[2], Carrie Fry[3], and Stacie Dusetzina[3], *1. University of Illinois Chicago, Chicago, Illinois, United States*, *2. Vanderbilt University, Nashville, Tennessee, United States*, *3. Vanderbilt University Medical Center, Nashville, Tennessee, United States*

The objectives of this study were to (1) Identify latent classes of LGBTQ+ patient experiences using seven indicators of clinical and cultural competence, (2) Identify sociodemographic characteristics associated with class membership, and (3) Estimate the association between class of patient experiences and receipt of preventive care. The sample included 954 LGBTQ+ adults aged 50-76 from the Vanderbilt University Social Networks, Aging, and Policy Study. Respondents reported on seven indicators of their clinician's LGBTQ+ clinical and cultural competency. Latent class analysis was conducted to identify patterns of LGBTQ+ patient experiences. We estimated marginal effects of class membership on preventive care using logistic regression. Three latent classes emerged: 34% assigned to the Affirming class, 60% to the Neutral class, and 6% to the Discriminatory class. Gender identity, sexual orientation, race/ethnicity, state of residency, and HIV status significantly predicted class membership (all $p < 0.01$), but not education, income, health insurance, or chronic conditions. Compared to those the Affirming class, individuals in the Neutral class were 12.4pp less likely to have ever been tested for HIV and 17.1pp less likely to have been recently tested for HIV, both $p < 0.001$; those in the Discriminatory class were 12.2pp less likely to have recently received an influenza vaccination and 14.8pp less likely to have recently completed a colorectal cancer screening, both $p < 0.05$. In the absence of explicitly affirming healthcare experiences, LGBTQ+ patients are less likely to receive timely preventive services. Increasing the capacity of health systems to provide LGBTQ+ affirming healthcare may advance health equity.

Abstract citation ID: igae098.1204
### FACILITATORS OF BEHAVIORAL HEALTH CARE ACCESS AND UTILIZATION FOR TRANSGENDER OLDER ADULTS
Nik Lampe[1], Zena Rodill[1], and Alexandra Nowakowski[2], *1. University of South Florida, Tampa, Florida, United States*, *2. Florida State University College of Medicine, Tallahassee, Florida, United States*

Transgender older adults in the United States (US) experience significant disparities in behavioral healthcare access and utilization. However, the specific facilitators that contribute to positive or adequate behavioral healthcare experiences among this vulnerable aging population remain uncertain. Understanding the unique facilitators that shape positive behavioral healthcare experiences for transgender (trans hereafter) older patients can assist medical practitioners in integrating these lessons into their practices, thereby promoting cultural responsiveness in care. In this qualitative study, we aim to address the question: What factors facilitate positive behavioral healthcare access and treatment among transgender older adults in the US? Between September 2021 and January 2022, the first author conducted 47 semi-structured, individual interviews with transgender adults aged 65 or older residing in the US. Analyses focused on the behavioral healthcare access and utilization experiences of interview respondents. Data analyses were conducted collaboratively by the authors using NVivo (Release 1.6) software inductively using a grounded theory approach. Findings suggest opportunities for enhancing behavioral health support and resources for transgender older communities. Four main themes emerged from the data: (1) engaging with behavioral healthcare practitioners who offer compassionate, patient-centered care; (2) utilizing culturally tailored peer-support services; (3) ensuring equitable access to gender-affirming medical interventions; and (4) addressing financial and logistical barriers to behavioral healthcare access. These insights highlight opportunities for enhancing behavioral health support and resources for transgender older communities while emphasizing the importance of incorporating these factors into behavioral healthcare research and practice to promote cultural responsiveness in care for this vulnerable aging population.

### SESSION 3080 (SYMPOSIUM)

Abstract citation ID: igae098.1205

### MAKING THE MOST: UNDERSTANDING COMMUNITY IN SEXUAL- AND GENDER-DIVERSE COMMUNITIES

Chair: M. Aaron Guest
Co-Chair: Christine Happel
Discussant: Erin Burk-Leaver

In program service and delivery, community significantly impacts the health and well-being of older adults, providing essential social support, fostering belonging, and ensuring access to vital resources and services. Beyond mere geographic proximity, communities embody shared traits, interests, values, and goals, aiding members in accessing various services and activities crucial for healthy aging. This includes fostering engagement, inclusivity, and support. Importantly, individuals often belong to multiple communities, enhancing their resource access as they age. A notable example is the sexual and gender-diverse community, comprising individuals identifying as gay, lesbian, bisexual, and transgender, among other identities. These individuals, especially older ones, face considerable barriers in accessing care, support, and resources, often lacking the opportunities available to their heterosexual counterparts. This symposium delves into how older individuals from these diverse backgrounds form and participate in communities. Happel et al.'s work explores the unique experiences of loneliness, isolation, and service use among older LGBTQ adults. Guest et al. identify how community connection can affect self-perceptions of aging, resulting in accelerated social aging and adverse health outcomes. Mccoyy et al. then share a community-based participatory action approach to identifying and addressing the needs of sexual and gender-diverse older adults through alliance building. The shared theme of community and how SGD individuals create and use community will be highlighted through exemplars leading to a discussion of how we can create health-promoting communities for SGD individuals as they age.

Abstract citation ID: igae098.1206

### RISK AND RESILIENCE RELATED TO SELF-PERCEPTIONS OF AGING AMONG LESBIAN AND GAY OLDER ADULTS

M. Aaron Guest[1], Hannah Giasson[2], and Rachel Koffer[1], *1. Arizona State University, Phoenix, Arizona, United States*, *2. Arizona State University, Tempe, Arizona, United States*

Lesbian and gay individuals may face unique experiences and expectations associated with growing older. In part, this may result from the perception of an overemphasis on youth in the LGBTQ+ community. The present study aimed to provide a preliminary model of how lesbian and gay adults view their aging and identify psychosocial risk and resilience factors specific to this community. Data are from a pilot study of social perceptions of aging in lesbian and gay adults, in which 45 gay and 46 lesbian adult participants (ages = 55-87, 70% white) completed questionnaires on their health, perceptions of aging, community engagement, identity congruence, and stressful experiences. Self-perceptions of aging were measured with an 8-item scale ranging from 1 to 6, where higher scores indicate greater agreement with positive perceptions of aging. In a regression model accounting for age, self-rated health, subjective socioeconomic status, and race/ethnicity, lesbian women reported better self-perceptions of aging than gay men ($b = 0.50$, $SE = 0.16$, $p < .01$). Further, greater outness was associated with better self-perceptions of aging ($b = 0.04$, $SE = 0.01$, $p = .01$), LGBTQ+ community engagement did not relate to self-perceptions of aging ($b = -0.11$, $SE = 0.12$, $p = .38$). More stressful experiences in daily life were associated with worse self-perceptions of aging ($b = -0.14$, $SE = 0.05$, $p < .01$). Results will be discussed in the context of a model of risk and resilience for "accelerated social aging" among Lesbian and Gay populations.

Abstract citation ID: igae098.1207

### IN THEIR OWN WORDS: EXPERIENCES OF LONELINESS, ISOLATION, AND SERVICE UTILIZATION AMONG LGBTQ+ OLDER ADULTS

Christine Happel[1], Holly Dabelko-Schoeny[2], Marisa Sheldon[3], and Al Cho[4], *1. Age-Friendly Innovation Center, The Ohio State University, College of Social Work, Columbus, Ohio, United States*, *2. The Ohio State University, Columbus, Ohio, United States*, *3. The Ohio State University, Age-Friendly Innovation Center, Columbus, Ohio, United States*, *4. Age-Friendly Innovation Center, Columbus, Ohio, United States*

Research has dismantled the stereotype of older LGBT people as lonely, depressed, and unattached to significant relationships. Yet, social isolation and loneliness are increasingly common experiences, especially when compounded by health and economic disparities. These experiences have substantial implications for our health and longevity. This study aimed to gain a better understanding of LGBTQ+ older adults' experiences of loneliness and isolation and where and how they are connecting to community and social services to inform strategies rooted in community strengths. Through community partnerships in one Midwestern US city, semi-structured interviews were completed with 12 LGBTQ+ older adults and three service providers supporting LGBTQ+ populations.

58% of older adult participants were between 65-69. 16% were trans, and 16% were gender non-conforming. 41% were Black / African American. 92% were gay or lesbian, and 8% were bisexual. 83% had college degrees. 41% were married or living with a significant other. Interviews were analyzed using the team-based flexible coding method. Results indicated that being partnered does not necessarily relieve experiences of loneliness, and coming out later in life was associated with smaller, less satisfying support networks. Health challenges acted as both a source of positive and negative impact, as participants discussed connection to others experiencing similar challenges through support groups and social networks but also recognized limitations created by chronic conditions. Limited access to inclusive gathering spaces beyond bars, as well as the evolving queer community, were identified barriers to social connection. Intergenerational relationships were referenced as supportive and protective factors.

Abstract citation ID: igae098.1208

### A COMMUNITY-BASED PARTICIPATORY APPROACH TO BUILDING ALLIANCE WITH LGBTQ2S+ OLDER ADULTS

Megan McCoy, *Northern Arizona University, Northern Arizona University, Arizona, United States*

The intersection of homophobia, transphobia, and heterosexism with ageism perpetuates health equity challenges for LGBTQ+ older adults. Many LGBTQ+ older adults fear having to “go back into the closet” to access healthcare, housing, and social support in later life and intentionally seek out identity-affirming settings. Yet, the availability of inclusive community spaces and services varies geographically. Understanding the experiences of LGBTQ+ older adults can inform research, advocacy, and programmatic efforts locally and regionally. Community-based participatory research (CBPR) is one framework for engaging historically silenced communities to drive research questions generated by and with the community. CBPR involves equitable community-academic partnerships, from development through dissemination. The first phase of successful CBPR involves forming a partnership and assessing community strengths, challenges, and dynamics— before we can even think about implementing a research project. This presentation will highlight how a CBPR model is used to engage key stakeholders to support LGBTQ2S+ older adults in Arizona better. Initially conceived as an advisory committee to inform research to build inclusion within one residential living community, the AZALEA Project (Arizona Alliance for LGBTQ2S+ Equity in Aging) continues to evolve as a broader statewide LGBTQ2S+ aging equity alliance. We will share emerging successes and lessons learned from an inaugural LGBTQ2S+ Aging Equity Forum and how these are informing future research development and design.

### SESSION 3085 (PAPER)

### MOBILITY/DISABILITY II (PAPERS)

Abstract citation ID: igae098.1209

### EFFECT OF COCHLEAR IMPLANT ON GAIT AMONG ADULTS WITH AGE-RELATED HEARING LOSS: A SYSTEMATIC REVIEW

Bahaa Rafoul[1], Maayan Agmon[2], and Hanin Karawani[1], *1. University of Haifa, Haifa, Hefa, Israel, 2. University of Haifa, University of Haifa, HaZafon, Israel*

Research shows a link between hearing loss, gait impairment, and an increased fall risk. A number of reviews on the effect of hearing interventions on balance have been published; however, whether Cochlear Implant (CI) can improve gait impairment and reduce fall risk remains unclear. This systematic review evaluates the impact of CIs on the walking abilities of adults with hearing loss. It contributes to the theoretical understanding of the link between hearing loss and gait, as well as to the clinical understanding of the combined effect of treatment on gain improvement. Utilizing PRISMA guidelines, we extracted seven relevant studies from PubMed, Web of Science, and Scopus. Five studies included CI users, while two included CI and hearing aid users. Inconsistency in measurement methodologies and follow-up durations were noted, contributing to incongruent findings. We found that a short follow-up (up to two months) after the activation of the CI was associated with non-improved and even worsening gait, and a longer follow-up (at least three months) was associated with a partially improved gait. However, due to various methodological inconsistencies in the seven articles available, it remains challenging to fully understand the effects of hearing treatment by the CI on gait. We suggest that future research should consider underlying mechanisms such as cognition and function in dual-task walking with longer follow-up to better understand the link between hearing and gait, especially after hearing intervention.

Abstract citation ID: igae098.1210

### WHAT ABOUT THE HANDS? EXAMINING OLDER ADULTS’ ATTITUDES, PERCEPTIONS, AND CLINICAL INTERACTIONS OF HAND FUNCTION

Rachel Logue Cook, and Susan Brown, *University of Michigan, Ann Arbor, Michigan, United States*

Age-related hand impairments are common yet receive little attention, thus often go undiagnosed until they have become severe. While it has been suggested that attitudes, perceptions, and clinical interactions play an important role in seeking care for physical limitations, the degree to which this extends to hand impairments is unknown. We developed an online survey which asked older adults (≥65) about their perceptions of hand function compared to other health areas, clinical interactions related to hand function, and barriers to accessing hand-related care. Five hundred and ninety-five older adults (mean age: 71.8 ± 5.5y) completed the survey. While 85% of respondents agreed that hand function is important for their daily life, only 40% expressed concern about losing function and only 2% ranked hand function as most important. Responses also indicated that hand function is rarely discussed (21%) or measured (52%) in the primary care setting. In addition, 34% of respondents said they have considered seeking care for a limitation but chose not to because they did not think their function was poor enough (33%), thought function would improve on its own (17%), or thought losing function is part of getting older (16%). Our results demonstrate that, although older adults understand the importance of hand function for completing activities, it is not prioritized by individuals or in the primary care setting. Such findings highlight a critical need to increase awareness of hand function in order to better identify impairments before they impede daily life and functional independence.

Abstract citation ID: igae098.1211

### CAN OLDER INPATIENTS WITH MOBILITY LIMITATION BENEFIT FROM MULTIDISCIPLINARY INTERVENTIONS: M-MOBILE STUDY

Lina Ma, *Xuanwu Hospital, Capital Medical University, Beijing, Beijing, China (People's Republic)*

**Objective:** Mobility limitation, the loss of exercise capacity or independent living ability, is a common geriatric syndrome. As a reversible precursor to disability, it is crucial to comprehensively assess the factors of mobility limitation and administer individualized multidisciplinary team (MDT) treatment. We hypothesized that MDT can improve mobility in older adults with mobility limitation methods: We conducted a study titled "A Multidisciplinary-team approach for management of Mobility Limitation in Elderly (M-MobiLE)", which is a multicenter, randomized, and controlled trial. A total of 442 older inpatients with mobility limitation from five hospitals were recruited. Mobility limitation was defined as Short-Physical Performance Battery (SPPB) ≤9. Activities of Daily Living (ADL) and intrinsic capacity were assessed. The intervention group received an exercise-centered individualized MDT treatment, including exercise, educational, nutritional, and medical interventions; while the control group received routine medical treatment. All patients were followed up for six months. Adverse events such as falls, fracture, stroke, cardiovascular events and death were recorded.
**Results:** Preliminary data analysis showed that compared to the routine medical treatment, the exercise-centered individualized MDT treatment reduces adverse events at 6 months (6.3% vs 13.0%, p=0.036). However, there was no statistical difference in ADL, length of stay, and hospital costs between the two groups at 6 months after discharge.
**Conclusion:** This study developed a multidisciplinary decision-making clinical pathway for older inpatients with mobility limitation, which had potential clinical benefits such as reducing short-term adverse events in older inpatients with mobility limitation in preliminary data analysis. Further analysis is ongoing.

Abstract citation ID: igae098.1212

### LENGTH OF STAY IN REHABILITATION HOSPITALS AS WELL AS HOSPITAL ADMISSION IS ASSOCIATED WITH SACRAL ULCER DEVELOPMENT

Cheng Yin, Elias Mpofu, and Kaye Brock, *University of North Texas, Denton, Texas, United States*

**Introduction:** Sacral ulcers (SUs) pose a significant risk of mortality for older adults when admitted to rehabilitation hospitals. Previous research has underscored the influence of various factors including comorbidities, lifestyle, and personal factors on sacral ulcer development (SUD) in these settings. However, the impact of hospital admission and the mediating role of length of stay (LoS) on SUD remains unclear. Thus, this study aimed to determine the association between hospital admission and SUD in older adult patients in rehabilitation hospitals, and whether LoS mediated this association.
**Methods:** Cross-sectional data were obtained from the Texas Inpatient Discharge Database (2021; N = 38,916 patients aged over 60 years in rehabilitation hospitals). Binary logistic regression was used to determine the association between hospital admission and SUD, mediation was then investigated.
**Results:** Hospital admission increased the likelihood of SUD through mediation by LoS (b=0.30, 95% CI 0.26, 0.35). Accounting for covariates revealed hospital admission was associated with 1.58 times the odds of SUD (AOR = 1.58; 95% CI = 1.38-1.81), while LoS was linked to 1.06 times the odds of SUD (AOR = 1.06; 95% CI = 1.05-1.06).
**Conclusion:** Our study extends previous findings, demonstrating hospital admission's connection to increased odds of SUD, through prolonged LoS in rehabilitation hospitals. The results highlight the importance of categorizing the source of admission in vulnerable populations to mitigate potential risks of hospital-acquired infections and excessive mortality associated with SUs.

Abstract citation ID: igae098.1213

### DISEASE-SPECIFIC CONTRIBUTIONS TO GEOGRAPHICAL DISPARITIES IN MORTALITY AMONG MEDICARE BENEFICIARIES

Meishuo Ouyang[1], Igor Akushevich[2], and Julia Kravchenko[1], *1. Duke University School of Medicine, Durham, North Carolina, United States*, *2. Duke University, Durham, North Carolina, United States*

Improving Life expectancy (LE) in the LE-lagging states could significantly improve overall LE and eliminate health inequalities in the United States. We analyzed the disparity in mortality rates between eight states with high and low LE at 65 using individual morbidity trajectories in Medicare administrative claims data from 2000-2020. Disease-specific contributions were identified using the Powers-Yun decomposition technique for hazard functions (the Blinder-Oaxaca algorithm extended for time-to-event data). This approach identifies two effects per disease: exposure (the disparity is generated because of the difference in the prevalence of a disease in two compared populations) and vulnerability (the disparity is generated because of the difference in mortality in the subpopulation with the disease). Two population groups (80-) and (80+) separated by age 80 were analyzed. Disease-specific contributions explained 44% and 63% of the disparities in all-cause mortality in the 80- and 80+ age groups, respectively. Exposure effects dominated in the 80-group (47.8% vs. 3.5% of vulnerability effect), while vulnerability was the main contributor (40.1% vs. 23.3% of exposure effect) in the 80+ group. Heart failure, influenza, and pneumonia explained 21.3% of the disparity in the 80- group, while the other circulatory system diseases, especially the vulnerability of cardiac dysrhythmias (5%), dominated the 80+ group. Additionally, cerebrovascular diseases (10.7%) and blood diseases (14.1%) are the other two major contributors to vulnerability in 80+. Reducing disparities in LE demands multiple fronts, such as tobacco control, heightened preventive measures against influenza and pneumonia, and enhanced access to arrhythmia treatments.

### SESSION 3090 (SYMPOSIUM)

Abstract citation ID: igae098.1214

### POLICY SERIES: MOVING FORWARD NURSING HOME COALITION CALL TO ACTION: BUILDING A SYSTEM FOR RESIDENT GOALS, PREFERENCES, PRIORITIES

Chair: Tara McMullen
Discussant: Tetyana Shippee

A 2022 National Academies of Sciences, Engineering, and Medicine (NASEM) report on nursing home quality called for nursing homes to provide care aligned with residents' goals to ensure autonomy, quality of life, and to manage risks. While there are existing structures to support care planning clinical needs, most nursing homes lack tools and procedures to systematically identify and provide care that aligns with resident goals, priorities, and preferences (GPPs). GPPs are part of the care planning process that translates into actionable interventions supported by measurement to determine if care aligns and is concordant with GPPs, and health information technology to collect GPPs. Many nursing homes are working to improve the way they gather residents' GPPs, and develop and implement unique care plans. These efforts represent an opportunity to improve outcomes and reduce disparities. Experts representing the Moving Forward Nursing Home Quality Coalition are developing an integrated approach to these challenges. This presentation will describe the Moving Forward Nursing Home Quality Coalition and a) the purpose and structure of the new Guide for Addressing Resident GPPs; b) an evaluation that explores whether a resident's self-directed GPPs were included in their care; c) a description of the tools that assess GPPs and the feasibility of using those tools; and d) discussion about the development of technology advancements promoting HIT readiness and person-centered care. When completed, proposed work will provide evidence of successful and feasible quality improvement approaches to care planning resident GPPs that can inform future policy changes and guidance to surveyors.

Abstract citation ID: igae098.1215

### THE DESIGN AND PILOTING OF A NEW GUIDE FOR ADDRESSING RESIDENT GOALS, PREFERENCES, AND PRIORITIES

Laci Cornelison[1], and Mary Lou Ciolfi[2], *1. Kansas State University, Manhattan, Kansas, United States*, *2. University of Maine Center on Aging, Bangor, Maine, United States*

Engaging nursing home residents and their designated representatives in meaningful conversation about their needs and preferences for care, services, and environments is essential to high quality long-term care, as confirmed in the 2022 National Academies of Sciences, Engineering, and Medicine (NASEM) report. The Moving Forward Coalition's Goal One Committee has created a quality improvement Guide for Addressing Resident Goals, Preferences, and Priorities (GPPs) to support nursing home care planning staff in improving and deepening care planning conversations with residents. The Guide includes key suggestions, tools, resources, and training material that will help homes lay a foundation for resident and family inclusion and empowerment in disclosing goals, preferences, and priorities (GPPs) and also in implementing those GPPs at the outset of a resident's admission to care and as needs and preferences change over time. Goal Committee One leads will present the Guide purpose and design along with a review of included resources; review the feedback and suggestions from myriad nursing home stakeholders, including current nursing home residents, nursing home providers, and nursing home quality improvement organizations; outline the results of the Guide pilot in several nursing homes, and the upcoming planned Guide evaluation and wider dissemination.

Abstract citation ID: igae098.1216

### MOVING FORWARD TOWARD A NURSING HOME MEASURE FOR GOAL CONCORDANCE AND EVALUATION OF RESIDENT GOALS

Tara McMullen, *Centers for Medicare & Medicaid Services, Baltimore, Maryland, United States*

Longstanding evidence suggests that the goals, preferences, and priorities (GPP) of nursing facility residents may not be adequately addressed during episodes of care. Aligning care with residents' GPP represents an opportunity to improve outcomes and reduce disparities in the resident population. The 2022 National Academies of Sciences, Engineering, and Medicine (NASEM) report recommended improvement in quality measures under the domain of resident and family experience, along with reporting of these measures on Care Compare to improve shared decision-making and transparency. The subcommittee on quality improvement and measurement is focused primarily on measures of GPP as a useful addition to CMS' plans to expand the Quality Reporting Program (QRP) measure set. To develop a reliable measure of concordance, this committee explored and assessed the alignment between the individual goals set by residents and the care interventions provided by the staff in routine care delivery. In an evaluation that employed in-depth interviews and reviews of the resident care plan and portions of the medical record, this group of subject matter experts attempted to gain a better understanding of the concordance between resident goals and care provision with the intent to identify areas for improvement in care delivery and enhanced person-centered care practices. In this presentation, committee members will discuss the development and outcomes of this evaluation along with an update on the development of a care concordant measure for nursing home settings.

Abstract citation ID: igae098.1217

### IDENTIFYING TOOLS TO MEASURE NURSING HOME RESIDENT GOALS, PRIORITIES, AND PREFERENCES FOR PERSON-CENTERED CARE

Tonya Roberts[1], Romil Parikh[2], Jingxi Li[3], Megan Nizza[4], Amelia Smith[5], Sorah Levy[6], and Tetyana Shippee[7], *1. University of Wisconsin - Madison, Madison, Wisconsin, United States*, *2. University of Minnesota, Minneapolis, Minnesota, United States*, *3. University of Wisconsin Madison, Madison, Wisconsin, United States*, *4. Boston University, Boston, Massachusetts, United States*, *5. Georgetown University, Washington, District of Columbia, United States*, *6. University of Maryland School of Nursing, University of Maryland School of Nursing, Maryland, United States*, *7. University of Minnesota School Of Public Health, Minneapolis, Minnesota, United States*

Identifying nursing home (NH) residents' goals, priorities, and preferences is a critical step in the delivery of person-centered care. While numerous instruments and tools exist to measure resident preferences, it remains unclear how psychometrically validated instruments used in research may be feasibly applied in practice. In this review, we aimed to identify instruments for assessing residents' goals, priorities, and preferences and develop recommendations for application in practice. We searched MEDLINE, CINAHL, and PsycINFO databases to identify instruments that assessed quality of life, satisfaction, person-centered care, psychosocial needs and goals, and preferences and were validated in US NHs. We also surveyed experts in the field to identify assessment tools from grey literature. We identified a total of 28 validated tools from 23 peer-reviewed articles and 12 additional tools from 35 grey literature sources. These tools varied in structure (e.g. open vs closed questions), content (e.g. domains covered), and length (e.g. few to many items). Tools could be grouped into two major types: generative and evaluative. Generative tools assessed specific resident goals, priorities, and preferences that could inform care planning particularly upon admission, but also throughout the resident's stay. Evaluative tools required residents to evaluate aspects of care and may be most appropriate beyond admission. NH staff may find different benefits to various assessment approaches and may need to make local decisions about which to use and which will integrate into existing workflows effectively.

Abstract citation ID: igae098.1218

### MOVING FORWARD TOWARD DEPLOYING HEALTH INFORMATION TECHNOLOGY IN ALL US NURSING HOMES

Gregory Alexander[1], Christina Caraballo[2], Maria Moen[3], Cynthia Morton[4], and Terrance O'Malley[5], *1. Columbia University, New York, New York, United States*, *2. HIMSS, Chicago, Illinois, United States*, *3. BA, Richardson, Texas, United States*, *4. Advion, Washington, District of Columbia, United States*, *5. Harvard Medical School/Massachusetts General Hospital, Boston, Massachusetts, United States*

The Moving Forward Coalition Health Information Technology (HIT) Committee has refined two action plans designed to promote HIT adoption in all U.S. nursing homes (NH). In this work, Moving Forward is responding to two recommendations in the (National Academies of Sciences, Engineering, Medicine Report of 2022 (The National Imperative to Improve Nursing Home Quality: Honoring Our Commitment to Residents, Families, and Staff) to deploy HIT in all NHs and to ensure that care is consistent with the individual's goals, preferences, and priorities (GPPS). The first plan includes a HIT Readiness Guide to inform the adoption of the essential HIT capabilities required for survival in Value Based Payment models (VBP) mandated by the Centers for Medicare & Medicaid Services (CMS) to begin in 2030. The second provides an HIT enabled tool to facilitate the collection of an individual's GPPs to facilitate goal concordant care consistent with an individual's GPPs and to enable measurement of the degree to which the care provided is concordant with those GPPS. In this presentation, members of the HIT Committee will discuss the "what, why, and how" of these plans and the progress to date. Additionally, panel members will discuss plans for testing and evaluating tools developed from our initial work. Included will be a discussion of the challenges of sustainability and feasibility in the face of little federal support for these efforts. Although the Moving Forward Coalition and the NASEM report focus on NHs, the HIT tools being developed here will benefit all sites of care.

### SESSION 3095 (SYMPOSIUM)

Abstract citation ID: igae098.1219

### PHYSIOLOGICAL AGING AND ITS USE AS A MARKER OF INEQUALITIES IN HEALTH: CROSS-NATIONAL PERSPECTIVES

Chair: Mikaela Bloomberg

Given substantial heterogeneity in health status between older adults, there have been many efforts to develop metrics of biological and physiological ageing that explain variability in health outcomes better than chronological age alone. Physiological age based on clinical indicators may explain variation in health outcomes better than epigenetic or telomere-based biological clocks because clinical indicators capture downstream physiological changes more closely related to health outcomes. In this symposium, we examine physiological age as a holistic marker of ageing and how we can use different measures of physiological age to examine disparities in health across the UK, US, and Ireland. The first and second abstracts examine physiological ageing in different organ systems and associations with health outcomes in the US-based Health and Retirement Study (HRS) and the Irish Longitudinal Study on Ageing (TILDA). The third abstract explores gender and educational inequalities in physiological ageing in the English Longitudinal Study of Ageing (ELSA). The fourth examines racial differences in the returns to education for physiological ageing. Finally, the fifth abstract investigates associations of cumulative stress exposure and neighbourhood context with inflammation in HRS. We demonstrate physiological age as a powerful tool for revealing inequalities in health during ageing across national contexts.

Abstract citation ID: igae098.1220

### BIOMARKERS HELP US UNDERSTAND HOW CELLULAR AND SYSTEMIC AGING CONTRIBUTE TO MORTALITY

Eric Klopack[1], and Eileen Crimmins[2], *1. University of Southern California, University of Southern California, California, United States*, *2. University of Southern California, Los Angeles, California, United States*

Research suggests aging is a coordinated decline occurring in multiple systems and at multiple biological levels. However, it is largely unknown how general biological aging and specific systemic aging co-occur and influence one another to affect health outcomes like mortality, multimorbidity, and functional limitations. There is also emerging interest in understanding how social exposures may differentially accelerate decline in individual physiological systems. We utilize data from the Health and Retirement Study, a nationally representative sample of about 4000 US adults over age 55. We used eXtreme Gradient Boosting (xgboost) in a training subsample to create estimates of health risk based on mortality, multimorbidity, and functional limitations using sets of biomarkers representing biological systems (e.g., nervous, immune, cardiovascular, and renal systems) as well as cellular aging (e.g., epigenetic clocks). These models generated risk scores for each outcome based on each system (e.g., renal functioning using BUN, creatinine, and cystatin C). Outcomes were regressed on these scores in a testing subsample to assess the relative impact of each system on health. Results suggest cellular aging, respiratory aging, and metabolic dysfunction contribute most to these health outcomes. Aging of these systems also partially mediates the risk associated with age, race/ethnicity, sex, and education. These results suggest aging of specific systems may be differentially affected by sociodemographic exposures and may differentially contribute to health risks. This research may help guide interventions focused on reducing inequalities in mortality risk by identifying health risks that would have the greatest impact on health at the population level.

Abstract citation ID: igae098.1221

### GENDER AND EDUCATIONAL DIFFERENCES IN TRAJECTORIES OF PHYSIOLOGICAL AGEING

Mikaela Bloomberg, and Andrew Steptoe, *University College London, London, England, United Kingdom*

Though gender and education are key characteristics that contribute to heterogeneity in ageing-related outcomes, how physiological age (PA) differs by gender and education level is not yet clear. Furthermore, previous studies examining PA are cross-sectional; understanding how PA changes with increasing chronological age is a necessary next step. We examined how gender and education interacted to inform longitudinal PA trajectories using three waves of data (2004/05-2012/13) from 8,891 ELSA participants. PA was derived in a healthy subsample then validated in the full analytic sample by confirming associations between PA and incident ageing-related chronic conditions, memory impairment, and functional limitations. We then used joint models adjusted for birth cohort to produce trajectories of PA between chronological ages 50 and 80 in men and women at three levels of education (less than high school, high school, and above high school). Gender differences in PA were minor at age 50, but PA increased faster in women than in men ($p < 0.001$); by age 70, women had PA 1.6 years (95% confidence interval=1.0-2.1) older than men. Examination of interactions between gender and education revealed that these gender differences occurred among less educated women only (p interaction=0.03); women educated above high school level maintained PA younger than men at age 70. These results suggest that higher education may be important to reduce gender disparities in physiological ageing. Policies to promote gender equity in higher education may contribute to reducing gender inequalities in a wide range of health outcomes in old age.

Abstract citation ID: igae098.1222

### USING BIOLOGICAL AGING TO BETTER UNDERSTAND THE DIFFERENTIAL RETURNS TO EDUCATION AMONG BLACK AND WHITE OLDER ADULTS

Mateo Farina, *University of Texas at Austin, Austin, Texas, United States*

Numerous studies have documented “differential health returns” for education among older Black and White adults in the United States. Despite being a more select group, Black older adults with higher levels of education see a smaller return in health risk declines than White older adults. This pattern has been observed for cardiovascular and metabolic conditions, disability, and mortality. Underlying several of these age-related health conditions may be accelerated biological aging, which may provide further insight into why education returns differ across many health outcomes. In this study, we use the Health and Retirement 2016 Venous Blood Study to examine the differences in the education gradient for Black and White older adults for accelerated aging as defined by the newly developed Expanded Biological Age measure. We use a series of linear regression models to examine the association of education with accelerated biological aging and we examine whether the differences can be partly explained by health behaviors, experiences of discrimination, or psychosocial stress. Preliminary findings show that, compared to Black older adults White older adults have a larger gradient (greater returns) to education when evaluating accelerated biological aging: 3.2-year difference between those without a high school diploma and a bachelor’s degree for Blacks, and a 5.3-year difference for Whites. Health behaviors had no impact on the differences across groups, but we find some indication that stress and discrimination may have a role in mitigating the health benefits for well-educated older black adults.

Abstract citation ID: igae098.1223

### CUMULATIVE STRESS EXPOSURE, NEIGHBORHOOD ENVIRONMENTS, AND INFLAMMATION IN ADULTHOOD

Jean Choi[1], Mateo Farina[2], and Elizabeth Munoz[1], *1. The University of Texas Austin, Austin, Texas, United States*, *2. University of Texas at Austin, Austin, Texas, United States*

Cumulative stress exposure, or the total stress exposure occurring across childhood and adulthood, is related to elevated inflammation, a key factor underlying physiological aging. Neighborhood contexts represent important sources of risk or resilience that may interact with stress to influence inflammation. Using data from the Health and Retirement Study, we assessed whether cumulative stress exposure would be associated with greater inflammation, and if this link is shaped by adult neighborhood cohesion and disorder (n = 5,625; age: 56 – 107). We constructed a continuous latent variable of systemic inflammation from six measured indicators (CRP, IL6, IL10, IL-1Ra, sTNFR1, IGF-1). Higher cumulative stress exposure was associated with elevated inflammation (b = 0.02, SE = 0.01, p =.032), but neighborhood contexts did not moderate this link. Sensitivity analyses revealed a significant interaction effect whereby greater neighborhood cohesion buffered the negative effect of cumulative stress exposure on IL-1Ra (b = -8.01, SE = 2.76, p =.004), and IGF-1 (b = -0.49, SE = 0.20, p =.014). Additionally, higher cumulative stress exposure was associated with elevated IL-1Ra in the context of greater neighborhood disorder (b = 4.43, SE = 2.24, p =.047). Results indicate that neighborhood cohesion and disorder are integral dimensions of the neighborhood environment that may modify the impact of cumulative stress exposure on markers of inflammation. These findings emphasize the need for additional research to consider an integrated multi-level approach to understand the effects of cumulative stress on inflammation.

### SESSION 3100 (SYMPOSIUM)

Abstract citation ID: igae098.1224

### PUBLIC HEALTH RESPONSES TO DEMENTIA IN RURAL AND TRIBAL AREAS: BOLD AND BEYOND

Chair: Katie Spears
Co-Chair: Erin Bouldin
Discussant: Lisa McGuire

Rural and tribal communities have unique characteristics specific to their geographical location and cultures that can influence how disease prevention and health promotion programs are delivered in these areas. Public health officials may need to adapt traditional strategies to improve uptake, effectiveness, and reach in rural and tribal settings. CDC's Healthy Brain Initiative (HBI) Road Map Series identifies priority actions public health agencies can take to promote brain health and support caregivers. The Road Map Series includes one designed for local and state agencies and another for Indian Country. CDC's Building Our Largest Dementia (BOLD) Infrastructure for Alzheimer's Public Health Program awardees carry out Road Map actions to develop and strengthen the public health infrastructure. This session will illustrate how public health entities have developed dementia resources within rural and tribal areas in their jurisdictions. Four states – Maine, Mississippi, Utah, and Washington – and a tribal board – the Northwest Portland Area Indian Health Board – will be presenters. They will characterize their population, identify the dementia programs and services they offer, describe challenges faced, and discuss the impact of rurality and tribes on their approach to service delivery and outreach. Presenters will discuss resources and collaborative approaches used to ensure success. Together, these presentations will highlight public health strategies that have been effective at supporting brain health in all communities across the lifespan.

Abstract citation ID: igae098.1225

### TRANSFORMING BRAIN HEALTH IN RURAL COMMUNITIES IN WASHINGTON STATE

Meghan Fadel[1], Marci Getz[2], and Angela Ward[3], *1. Alzheimer's Association, Chicago, Illinois, United States*, *2. Washington State Department of Health, Olympia, Washington, United States*, *3. Northeast Tri County Health District, Colville, Washington, United States*

Rural communities experience unique strengths and barriers as they work to address dementia, making strategic action on brain health and caregiving particularly important. Since 2005, the Healthy Brain Initiative (HBI) has been working to support public health strategies that promote brain health, address dementia, and support individuals living with dementia and their caregivers in all communities. As a part of the HBI effort, the Road Map Series has served as a basis for action in rural communities by guiding local, state, and tribal public health leaders on strategies as they prioritize actions to address brain health and caregiving. The HBI also provides resources to aid in evaluation and implementation of public health approaches. One such effort is the annual Road Map Strategists Program, which helps build the capacity of local health departments and tribal organizations to address brain health. Examples of other tools include an implementation guide, a guide for health improvement planning, and an evaluation toolkit. Washington State has taken great strides in addressing brain health. Efforts include local health department participation in the HBI Road Map Strategists Program, federally funded action through a Building Our Largest Infrastructure Dementia (BOLD) Infrastructure for Alzheimer's grant, and several initiatives led by the Washington State Dementia Action Collaborative. Lessons learned from implementing the HBI Road Map and prioritizing strategic action in rural communities and tribal nations offer valuable models for expanding work in Washington and beyond.

Abstract citation ID: igae098.1226

### MAINE REACHING FOR THE SUMMIT: ADDRESSING ALZHEIMER'S DISEASE AND RELATED DEMENTIAS

John Holland[1], Alfred May, Jr[2], Eric Frohmberg[3], and Dawn Gordon[1], *1. Maine Center for Disease Control and Prevention, Augusta, Maine, United States*, *2. State of Maine Department of Health And Human Services, Maine Center for Disease Control and Prevention, Machias, Maine, United States*, *3. Chronic Disease Prevention and Control Programs, Maine CDC, Augusta, Maine, United States*

Maine is one of the most rural states in the country with 61.4% of its population living in rural areas and thirteen of sixteen counties categorized as rural. Maine is also the oldest state in the country based on a median age of 44.8 years. Maine's BOLD Program strategized to treat the whole state as rural and determine various means to build the infrastructure for dementia education and services. One of these strategies is to develop a working relationship with primary care through our federally qualified health centers, who serve our target low-income population and are located in our most rural areas of the state. Because federally qualified health centers are established to provide primary care services as well as oral and behavioral health, they are open to partnerships with the Area Agencies on Aging for providing specialized umbrella services to patients with Alzheimer's disease and related dementias (ADRD) and respite services to families and caregivers of patients with ADRD. This approach builds on the Healthy Brain Initiative Road Map strategies as well as working toward clinical to community best practices in preventing ADRD. A key resource for our work with the lead federally qualified health center has been the New York University Medical School, one of the Public Health Centers of Excellence. This collaboration has resulted in the creation of a dialogue type webinar that utilizes research to practice for early detection, diagnosis, and care planning.

Abstract citation ID: igae098.1227

### MISSISSIPPI'S BOLD PUBLIC HEALTH RESPONSE TO DEMENTIA IN RURAL AND TRIBAL AREAS

Kina White, and Paulita Edwards-Childs, *Mississippi State Department of Health, Jackson, Mississippi, United States*

Individuals living in rural communities are at an increased risk for Alzheimer's disease and related dementias (ADRD), which aligns with other complex place-based health disparities. ADRD represents a significant public health issue in Mississippi, with the state having the highest age-adjusted mortality rates due to ADRD in the nation. The burden of ADRD in Mississippi falls disproportionately on rural and minority populations, with the state having the highest percentage of Blacks/African Americans in the nation and all 82 counties being considered medically underserved due to a lack of physicians and healthcare facilities. Given that Alzheimer's disease and related dementias are more common with advanced age, this may increasingly affect Indian Country with a higher health burden. Mississippi's BOLD public health response to addressing ADRD includes a collective impact of key stakeholders and prevention strategies to emphasize a life course approach for improved health outcomes. The response encompasses strategies such as community education, caregiver support programs, early detection initiatives, and access to dementia-friendly services. Moreover, it emphasizes the importance of collaboration with tribal nations, leveraging local resources, and integrating traditional and healing practices into dementia care. Key examples of these strategies include establishing dementia-friendly congregations and communities, as well as training older adult volunteers as healthy aging champions to advocate for improved local policies that allow them to age in their places and communities.

Abstract citation ID: igae098.1228

### STRENGTHENING BRAIN HEALTH AND DEMENTIA RESOURCES FOR OUR RELATIVES OF NORTHWEST TRIBES

Chandra Wilson, *Northwest Portland Area Indian Health Board, Portland, Oregon, United States*

The Northwest Tribal Elders Project (NTEP) will manage the resources and provide space for the current tribal dementia relatives to gather and share intertribal stories of our traditional ways of health and wellness to address Alzheimer's disease and related dementia (ADRD) among I/T/U (Indian Health Services, Tribal and Urban Indian) clinics. The aim of the project is to provide technical assistance, training, and resources within our 43-member tribes' elder programs and tribal programs supporting older adults. NTEP collaborates with all 43-member tribes and provides ADRD resources that may be tailored to the health and wellness practices within each tribal community. A tribal coalition will provide input on the implementation of our ADRD Strategic plan to promote risk reduction and intervention priorities, early detection screenings and assessments and education and awareness of ADRD. Through this relationship powerful and impactful things are going to happen, the hope is there will be an increase of discussions happening with our member tribes to discuss ADRD, mild cognitive impairment, memory care and caregiving. The tribe's input on training and technical assistance needs is the medicine for our loved ones whom are being cared for.

Abstract citation ID: igae098.1229

### DEMENTIA CAREGIVER SUPPORT IN THE RURAL, FRONTIER, AND TRIBAL AREAS OF UTAH

Kate Nederostek, and Kristy Russell, *Utah Department of Health and Human Services, Salt Lake City, Utah, United States*

More than three million people call the Beehive state home. Rapid growth (40% in the last 20 years) and Utah's unique landscape present challenges when it comes to coordination of services. Made up of 29 counties covering 84,000 square miles, Utah is the 13th largest state by area. Approximately three-fourths of Utah's population lives in five contiguous counties, often referred to as the Wasatch Front. However, 12 of Utah's counties are considered rural (six to 100 persons per square mile) and 12 counties are considered frontier (six or fewer persons per square mile). Utah's statewide dementia efforts are funded by the state legislature (~$430,000/year) and currently receive no additional federal funds. Historically, the federally supported organizations that serve frontier and rural communities in Utah receive less funding due to their lower population density. To better serve their communities, the Alzheimer's Disease and Related Dementias (ADRD) Program and Family Caregiver Support Program, offered through the Area Agencies on Aging (AAAs), have combined forces. The ADRD Program assists each AAA in providing education, support, materials, and programming to those living with dementia and their caregivers. This collaboration has allowed an extension of programs statewide, providing an opportunity for those in rural and frontier areas to participate. Relationships and collaborative interest are growing with the Utah Indian Health Advisory Board. Utah's ADRD Program continues to work through barriers and to tailor educational opportunities to the needs of each tribal area.

### SESSION 3105 (SYMPOSIUM)

Abstract citation ID: igae098.1230

### SPOTLIGHTING THE EXPERIENCES OF THE HOME CARE WORKFORCE CARING FOR OLDER ADULTS LIVING WITH DEMENTIA

Chair: Chanee Fabius
Discussant: Joanne Spetz

Medicaid Home and community-based services (HCBS) and home-based clinical care (i.e., Medicare home health care) aim to support independence and well-being in the community, especially for older adults living with disability and complex health conditions, such as dementia. Many older adults receive both Medicaid HBCS and home-based clinical care, often concurrently, with services coordinated and delivered by paid and unpaid care partners including care managers, home care workers, and family caregivers. Amid concerns related to workforce shortages, shifting payment policies, and the rising complexity of those aging in place, there have been recent calls to better support the workforce providing care in the home. Responding to these calls will require additional knowledge and understanding of the experiences of workforce members as well as the older adults they serve. This symposium presents four unique studies that describe: 1) experiences of care managers ("supports planners" in Maryland) serving older adults with and without dementia in Medicaid HCBS in Maryland; 2) unmet caregiving needs in a national sample of Medicare home health patients living with dementia; 3) Medicare home health clinician perspectives on supporting dementia caregivers through health information technology, and 4) home care worker attitudes about delivering person-centered care for persons living with dementia. The findings presented reveal current areas of challenge and inform the development of innovative strategies to better support the range of paid and unpaid caregivers caring for older adults living with dementia in the community.

Abstract citation ID: igae098.1231

### "STANDING IN THE GAP": EXPERIENCES OF MEDICAID HOME AND COMMUNITY-BASED SERVICE SUPPORTS PLANNERS IN MARYLAND

Chanee Fabius, and Sharmini Rathakrishnan, *Johns Hopkins University, Baltimore, Maryland, United States*

Medicaid Home and Community-Based Service (HCBS) care managers (called "supports planners" in Maryland) are responsible for coordinating care delivery and play a crucial role in bridging the gap between older adults, families, and home care and state agencies. This assistance is particularly relevant for older adults living with dementia and their families, as they represent nearly one-third of Medicaid HCBS participants, nationally. Despite their pivotal role in home care delivery and care quality, there is a paucity of research describing the contributions and experiences of supports planners. This study aims to improve the understanding of the role of supports planners, with implications for enhancing care coordination for older adults with dementia within Maryland's Medicaid HCBS. We conducted seven semi-structured interviews with supports planners in Maryland from December 2023 to February 2024. Interviews were recorded, transcribed, and coded by the research team. Supports planners ranged in age from 26 to 54, were mostly female (n=5). Four supports planners were white, and three were black. Thematic analysis revealed four key themes highlighted by supports planners: 1) collaborations with paid and unpaid caregivers; 2) dementia-related care coordination; 3) the electronic management system as an information hub; and 4) supports planners' role as liaisons in HCBS. The present study further emphasizes that supports planners are involved in numerous aspects of home care delivery. Our findings illuminate the role of supports planners in Medicaid HCBS and have implications for improving care coordination for older adults living with disabilities, especially those living with dementia.

Abstract citation ID: igae098.1232

### HOME HEALTH CLINICIAN PERSPECTIVES ON ASSESSING AND SUPPORTING DEMENTIA CAREGIVERS

Julia Burgdorf[1], Margaret McDonald[2], and Kathryn Bowles[1], *1. VNS Health, New York, New York, United States*, *2. Visiting Nurse Service of New York, New York, New York, United States*

Home Health Care (HHC) delivers skilled services via visits to patients' homes. One-third of HHC patients have dementia and caregivers play a critical role in their care. Yet, it is difficult to assess caregivers' training/support needs during HHC. As part of ongoing formative research aimed at developing a dementia caregiver assessment intervention for HHC, we convened semi-structured group interviews with 16 frontline staff from a large, urban HHC agency—Registered Nurses (n=5), Physical Therapists (n=5), and Social Workers (n=6). Staff shared their perspectives on current communication with caregivers and reacted to a proposed health information technology (HIT) solution for assessing caregiver needs. Participants highlighted social work's critical role in connecting caregivers with community resources to bridge the gap of "a short-term benefit trying to meet long-term needs". Staff emphasized the importance of gathering information regarding dementia patients' complex caregiving networks (encompassing family, paid attendants, and HHC aides with varying schedules and task loads) but noted there were few options for storing and sharing this information in the patient record. Respondents expressed enthusiasm for an HIT-driven assessment tool linked to the patient record and requested that such a tool (1) come with a repository of community resources, (2) support information sharing among the care team and between episodes, and (3) include education for caregivers on dementia symptoms and the scope of HHC. Findings shed light on current barriers and opportunities for supporting dementia caregivers in HHC and reveal practical considerations for designing an intervention that will integrate with existing HHC workflows.

Abstract citation ID: igae098.1233

### UNMET CAREGIVING NEEDS AMONG HOME HEALTH PATIENTS WITH DEMENTIA: FRONTLINE CLINICIAN REPORTS

Julia Burgdorf, *VNS Health, New York, New York, United States*

One-third of Medicare home health care (HHC) patients have diagnosed dementia and rely heavily on support from family caregivers to implement the plan of care, but no prior work has examined unmet caregiving needs among these patients. Drawing on clinician reports in 2018 Medicare HHC assessment data for a national sample of 426,606 HHC patients with diagnosed dementia, we define unmet caregiving needs stemming from lack of availability (i.e., no caregiver is present) or lack of capacity (i.e., caregiver requires clinician supervision and training). Given the progressive nature of the disease, we compare across 3 levels of dementia symptom severity, determined by clinician-reported cognitive and behavioral symptoms.

Comparing those with mild, moderate, and severe dementia symptoms, unmet needs due to lack of caregiver availability were present for 9.1%, 6.9%, and 5.3% of patients, respectively; unmet needs due to lack of caregiver capacity were present for 70.6%, 71.1%, and 74.9% of patients, respectively. In adjusted multilevel models controlling for patient and HHC agency characteristics and clustering at the agency level, individuals with more severe dementia symptoms were less likely to have an unmet need for caregiver assistance due to a lack of caregiver availability (aOR: 0.63; 95% CI: 0.60-0.66), but more likely to have an unmet need due to a lack of caregiver capacity (aOR: 1.15; 95% CI: 1.12-1.17). Findings suggest the need for varied approaches to caregiving-related support based on dementia disease stage and highlight widespread needs for clinicians to provide caregiver training during HHC episodes for those with dementia.

Abstract citation ID: igae098.1234

### HOME CARE WORKER PERSPECTIVES ON PERSON-CENTERED CARE FOR PEOPLE WITH DEMENTIA

Jennifer Reckrey[1], Emily Xu[2], Deborah Watman[3], Sasha Perez[2], and Emily Franzosa[4], *1. Icahn School of Medicine at Mount Sinai, Icahn School of Medicine at Mount Sinai, New York, United States*, *2. Icahn School of Medicine at Mount Sinai, New York, New York, United States*, *3. Icahn School of Medicine at Mount Sinai, New York, New York, United States*, *4. James J Peters VA Medical Center, Bronx, New York, United States*

Person-centered care for people living with dementia has been associated with improved functional ability and quality of life, yet little is known about person-centered care in the home setting. This study explored home care worker perspectives on providing person-centered care for people living with dementia. Using secondary qualitative analysis of 22 semi-structured interviews with home care workers, we identified themes related to the Dementia Initiative's person-centered dementia care framework. We found that home care workers frequently acknowledged their client's personhood and developed meaningful relationships with their clients, implementing individualized strategies to meet client needs. However, barriers to person-centered care included limitations of home care worker scope of practice and challenging dynamics with other home care workers and family caregivers. In addition, home care agencies' formal care plans sometimes served as a barrier to person-centered care, but home care workers advocated within and around their task-based duties to ensure client needs were met. This analysis highlights the importance of integrating home care workers in person-centered healthcare teams and sustaining the meaningful relationships between home care workers and their clients, family caregivers, and other home care workers. Standardized training of home care workers in the principles and goals of person-centered dementia care could enhance existing person-centered care practices and promote alternatives to disease-centered practices. Recognizing home care workers' unique role in providing person-centered care is essential to identify and meet the needs of people living with dementia in the community and their family caregivers.

### SESSION 3110 (SYMPOSIUM)

Abstract citation ID: igae098.1235

### STRENGTHENING CAREGIVER SUPPORT: ASSESSING AND ADDRESSING CAREGIVERS' MENTAL HEALTH NEEDS

Chair: Christine Gould
Co-Chair: Marika Humber
Discussant: Ranak Trivedi

Family caregivers (typically patients' unpaid relatives/friends) are vital to patient care, but their mental health needs are often overlooked. This may be exacerbated by the fact that the caregiving role can be isolating, stressful, and time-consuming. This symposium highlights promising findings related to the identification of caregiver mental health and well-being needs and potential interventions in the community and Veterans' Health Administration (VHA). First, Dr. Moo will present findings from a national survey of caregivers of older Veterans that shows the impact of care responsibilities, health care navigation, and environment on caregiver quality of life. Second, Dr. Van Orden will describe her investigation of suicide ideation, perceived burden, and social connection among caregivers of persons with dementia. Third, Dr. Humber will describe the impact of a tele-geriatric psychiatry service in two VHA regions on caregivers of rural-dwelling older Veterans with neurocognitive and or psychiatric disorders. Last, Dr. VanPutten will discuss adaptations of Dialectical Behavior Therapy (DBT) for caregivers in high-conflict caregiving situations with persons with neurocognitive disorders. She will describe adaptations made to a DBT skills training group related to the influence of caregiver physical health problems on detecting and applying core DBT skills. Dr. Ranak Trivedi, the national Training Director of the Elizabeth Dole National Center of Excellence for Veteran and Caregiver Research and member of the Research Collaborative of the National Alliance of Caregiving, will close with discussion of the presentations and will highlight opportunities for future caregiver mental health research.

Abstract citation ID: igae098.1236

### INFLUENCES ON CAREGIVERS OF OLDER VETERANS' PERCEPTIONS OF THEIR QUALITY OF LIFE: A NATIONAL SURVEY

Lauren Moo, Elizabeth Chamberlin, Victoria Ngo, and Steven Shirk, *Department of Veterans Affairs, Bedford, Massachusetts, United States*

Being a family caregiver can negatively impact an individual's physical and mental health, leading to caregiver burden, burnout, and lower quality of life (QoL). We explored caregiver and care recipient factors that influence caregiver QoL via a national web-based survey of 511 informal caregivers of older Veterans. We found that greater dependence of the Veteran on the caregiver for Activities of Daily Living (ADLs) and Instrumental Activities of Daily Living (iADLs) was associated with poorer QoL ($p<0.001$). Caregivers' cohabitation with the Veteran was also associated with poorer QoL ($p<0.001$), while caring for an additional person was not. However, our results also suggest that caregivers making time for their own medical care and social needs were unique important predictors of higher caregiver QoL, and this positive benefit remains even when considering the negative impact of Veteran ADL and iADL support needs. In fact, our results suggest that making time for social activities is the strongest predictor of QoL. Thus, making time to receive medical attention and participating in social activities are important factors when considering the caregivers' overall QoL. Cohabitating with the care recipient may be required for various reasons (e.g., amount of care needed, financial limitations) and, therefore, may be challenging to address. However, improving and supporting the amount of self-care the caregiver receives may be a feasible area to address and can still have a substantive impact on their overall QoL.

Abstract citation ID: igae098.1237

### PERCEIVED BURDEN ON OTHERS, LONELINESS, AND SUICIDE RISK IN CAREGIVERS

Kimberly Van Orden, *University of Rochester Medical Center, Rochester, New York, United States*

Caregiving can foster increased purpose and mastery, but also anxiety, depression, and suicide ideation. Mechanisms whereby caregiving impacts suicide risk in dementia caregivers are not well characterized. The Interpersonal Theory of Suicide posits that stressors such as caregiving increase suicide risk by producing feelings of not belong to valued relationships/groups (low belonging, loneliness) and like one is a burden on others (perceived burden). Given that caregivers by definition are caring for others, they may be protected against perceived burden. This presentation will examine the relevance of the Interpersonal Theory of Suicide to understanding mechanisms whereby caregiving may increase suicide risk, with a particular focus on perceived burden on others. Data come from 106 caregivers who reported both significant loneliness and caregiving stress (i.e., required inclusion characteristics for studies funded by Rochester Roybal Center for Social Ties & Aging Research). Subjects completed semi-structured interviews (Columbia Suicide Severity Rating Scale) and self-report measures of social disconnection (Interpersonal Needs Questionnaire), depressive symptoms, caregiving burden, and neuropsychiatric symptoms. Results indicate that 10% reported suicide ideation (past month) and 14% reported feeling like a burden on others (past month). Caregivers who reported perceived burden were 3.5 times more likely to report suicide ideation. Findings suggest that perceived burden is as least as common as suicide ideation, supporting relevance of the Interpersonal Theory. Additional analyses will examine characteristics of those who reported perceived burden to explore potential contributors to developing perceptions of burden despite providing dementia care.

Abstract citation ID: igae098.1238

### EXPLORING THE IMPACT OF TELE-GERIATRIC MENTAL HEALTH SERVICES ON RURAL CAREGIVERS' WELL-BEING

Marika Humber[1], Chalise Carlson[2], Marisa-Francesca Brodrick[1], Rachel VanPutten[1], Amanda Peeples[3], Ranak Trivedi[4], Althea Lloyd[5], and Christine Gould[1], *1. VA Palo Alto Healthcare System, Palo Alto, California, United States*, *2. Department of Veterans Affairs, Palo Alto, California, United States*, *3. Corporal Michael J. Crescenz Department of Veterans Affairs Medical Center, Philadelphia, Pennsylvania, United States*, *4. Stanford University/VA Palo Alto Healthcare System, Palo Alto, California, United States*, *5. VISN 7 Clinical Resource Hub, VA Atlanta Health Care System, Decatur, Georgia, United States*

Rural caregivers may experience unique barriers to support including lack of services, transportation, and internet access. Within the Veterans Health Administration (VHA), a virtual geriatric psychiatry consultation service evolved to increase specialty mental health care access for rural older Veterans. Although the Veteran and their neurocognitive disorder (e.g., Alzheimer's disease) and/or mental health condition (e.g., depression, PTSD) were the focus of the service, caregivers were also present at appointments. This study explored the impact of the service on rural caregivers in two VHA regions. The median age of patients was 77; caregivers were a spouse or adult child. Semi-structured interviews with caregivers (n=13) inquiring about overall experience and satisfaction with the service, changes to patient care and symptoms resulting from visits, and the service's impact on the caregiver were conducted from September 2022-November 2023. Using rapid qualitative analysis, interview transcripts were summarized and compared to identify themes. Caregivers reported working with the geriatrics providers normalized the caregiving experience and provided helpful techniques and resources for managing challenging patient behaviors. Rural caregivers appreciated that visits were done at home alleviating the time and effort required to travel to distant VHA facilities and that providers could spend more time focusing on patient and caregiver needs. The direct access to specialty providers via follow-up appointments or by telephone to ask questions and address emergent problems was particularly valuable. These findings indicate that integrating caregivers into specialty tele-geriatric mental health services may decrease burden and improve well-being for caregivers of rural older Veterans.

Abstract citation ID: igae098.1239

### ADAPTING DIALECTICAL BEHAVIOR THERAPY TO ADDRESS CAREGIVER HEALTH IN HIGH-CONFLICT CAREGIVING SITUATIONS

Rachel VanPutten[1], Claudia Drossel[2], and Thomas Waltz[2], *1. VA Palo Alto Healthcare System, Palo Alto, California, United States*, *2. Eastern Michigan University, Ypsilanti, Michigan, United States*

Family caregivers of older adults with neurocognitive disorders (NCDs) are invisible patients in the U.S. healthcare system. This is especially concerning because poor caregiver mental or physical health predicts coercive care practices (e.g., yelling or threatening the older adult). When older adults with NCD come to providers' attention for emotional and behavioral changes, systematic strategies for assessing and intervening on caregiver health and potential coercive caregiving patterns are lacking. The present study consisted of a retrospective chart review of clinical records from family caregivers who participated in dialectical behavior therapy (DBT) skills training groups adapted for high-conflict situations involving older adults with NCDs (Drossel, Fisher, & Mercer, 2011) in a doctoral-level psychology training clinic in the Midwest. Data from 32 family caregivers enrolled across eight 8-week DBT groups showed that caregivers whose medical records indicated significant health problems tended to describe themselves as healthy when completing the RAND 36-Item Health Survey (SF-36; Hayes et al., 1993). Attrition from the groups was mainly due to acute medical crises, and caregivers required significant individual support to address their medical concerns, prompting modifications to the DBT mindfulness and interpersonal effectiveness modules (e.g., developing DEAR MAN scripts to delegate care tasks to third-party providers). These findings suggest the need for routine, systematic, and direct assessments of caregiver health and skills-based approaches targeting health promotion and disease prevention to address coercive caregiving practices.

### SESSION 3115 (SYMPOSIUM)

Abstract citation ID: igae098.1240

### THE CANNABIS AND OLDER PERSONS STUDY: CAN WE AT LEAST DEFINE IT AS A FORM OF COMPLEMENTARY AND ALTERNATIVE MEDICINE?

Chair: Brian Kaskie

The Cannabis and Older Persons Study has examined the increasing use of cannabis among older persons since 2016. This year's COPS symposium features panelists trained in community health, gerontology, medicine, psychology and public health, each offering latest research findings and considering cannabis as a form of complementary and alternative medicine. Annie Nguyen and her colleagues from the University of California, San Diego present a qualitative analysis of interviews completed with physicians and other clinicians affiliated with a geriatric medical clinic, focusing on their perspectives about cannabis as a form of complementary and alternative medicine. Angela Bryan and her team from the University of Colorado, Boulder identify more than 200 older persons who completed the "Cannabis and Your Health" survey and compare those who use cannabis for purposes of self-care and contrasts outcomes related to balance, mood and memory with those who do not use cannabis. Julie Bobitt from University of Illinois Chicago uses a socio-ecological framework to identify multi-level factors associated with cannabis use among 30 older women with HIV. Fadi Martinos examines more than 12,000 persons over 50 who completed the California Health Interview Survey, and compares cannabis use among those who identify as informal care givers and those who do not. Together, these studies reflect how scientific understanding of cannabis use among older persons has been informed by varied disciplinary perspectives, and whether researchers consider cannabis use among older adults as a complementary and alternative form of medicine.

Abstract citation ID: igae098.1241

### HOW DO PRIMARY CARE PHYSICIANS NAVIGATE MEDICAL CANNABIS DISCUSSIONS WITH OLDER ADULT PATIENTS

Annie Nguyen[1], Pearse O'Malley[2], Edward Osae-Oppong[3], Jamie Foo[1], Dania Abu-Baker[1], Lize Tibirica[1], Joseph Diaz[1], and Alison Moore[4], *1. University of California San Diego, La Jolla, California, United States*, *2. Tulane University School of Medicine, New Orleans, Louisiana, United States*, *3. California State University San Marcos, San Marcos, California, United States*, *4. University of California San Diego School of Medicine, San Diego, California, United States*

We interviewed 20 physicians who provide primary care in California and have patients ages 65+ in their practice. Interviews lasted 30 minutes and followed a semi-structured guide. The majority of physicians said that older patients were most likely to use cannabis for pain and sleep issues while younger patients used it for mental health-related conditions. Older patients often seek physicians' advice about doses, forms, and efficacy, but most physicians were unsure on how to advise patients, with many defaulting to the strategy of starting with low doses and titrating up if needed; inhalation was universally discouraged. Many physicians were unsure about the strength of evidence, because while the evidence for sleep and pain appear to be supportive, there are not enough studies and large-scale clinical trials to develop a strong opinion. All agreed that physicians would benefit from training to increase confidence and be informed when discussing cannabis with patients.

Abstract citation ID: igae098.1242

### ACUTE EFFECTS OF EDIBLE CANNABIS FOR OLDER ADULTS: THE GOOD, THE BAD, AND THE NEUTRAL

Angela Bryan[1], Carillon Skrzynski[1], Madeline Stanger[1], Gregory Giordano[1], Dana Lervick[2], Tatum Broyhill[2], and Brian Tracy[2], *1. University of Colorado Boulder, Boulder, Colorado, United States*, *2. Colorado State University, Fort Collins, Colorado, United States*

Many older adults use cannabis to alleviate pain, increase sleep quality, and mitigate symptoms of anxiety and/or depression. Yet few studies assess co-occurring effects of cannabis use that may be more salient to older adults. We recruited 234 participants (average age 70.46 (sd=5.83) who either did (n=175) or did not (n=59) want to use edible cannabis for pain, sleep disturbance or negative affect. We compared acute effects on balance, mood, and memory before and after consumption of edible cannabis products differing in cannabinoid ratio (CBD dominant, THC dominant, CBD+THC). Among older adults who used cannabis, we observed balance impairment from pre to 60-minutes post use ($p<.01$). Those who used cannabis experienced improvement in pain from pre to 60-minutes post use ($p=.001$) and higher concentrations of THC in blood (measured at time of use) were associated with greater decreases in pain ($p<.05$). Older adults who used cannabis also experienced increases in subjective intoxication ($p<.001$); higher THC in blood was associated with greater intoxication ($p<.001$) while higher CBD in blood was associated with lower intoxication ($p<.05$). There were few significant differences between those who did versus did not use cannabis on memory and cognitive tasks. We add to the growing literature examining cannabis use among older persons and identify significant outcomes experienced by those who use edible cannabis products. Disseminating this work among clinicians may facilitate more informed conversations with older persons who may consider cannabis as a therapeutic alternative.

Abstract citation ID: igae098.1243

### LIFETIME AND CURRENT CANNABIS USE BY OLDER WOMEN WITH HIV PARTICIPATING IN THE MACS/WIHS COMBINED COHORT STUDY

Julie Bobitt, *University of Illinois Chicago, Chicago, Illinois, United States*

The proportion of persons living with HIV who use cannabis is 2-3 times higher than the general population with past-year use rates ranging from 23%-56% in persons with HIV compared to 13% in persons without. A Women's Interagency HIV Study (WIHS) found cannabis use by women to be 27%. We will present findings from interviews with 30 women aged 60+ enrolled in the MWCCS Cook County site. Guided by the socioecological framework, the interviews explore the multilevel influences on cannabis use decision-making and health outcomes. Our thematic analysis focuses on the following influences: 1) individual (attitudes, knowledge, health conditions); 2) Relationship (social connections/responsibilities); 3) Community (medical), and 4) Societal (legalization). We also explore cannabis use drivers on HIV-related pain relief, anxiety, and stress. As cannabis use among chronically ill persons continues to rise, it is important to understand the factors that influence use and health outcomes.

Abstract citation ID: igae098.1244

**CANNABIS USE AMONG INFORMAL CAREGIVERS OVER 50: FINDINGS FROM THE CALIFORNIA HEALTH INTERVIEW SURVEY**

Fadi Martinos[1], and Divya Bhagianadh[2], *1. University of Iowa, Iowa City, Iowa, United States*, *2. University of Arkansas, Fayetteville, Arkansas, United States*

Despite the availability of evidence-based therapeutics and supports, informal primary care partners of older adults are using cannabis as an unproven, potentially harmful, alternative for reducing physical burden and psychological distress. Using the 2019 California Health Interview Survey, we identified 2,278 respondents over 50 years old who indicated providing care to someone also over 50 in the past year. We determined 23.0% of these survey respondents between 50 to 64 years and 18.0% of those over 65 years older used cannabis in the past year, and older caregivers were more likely to use cannabis than non-caregivers. Cannabis use among older caregivers was associated with smoking and self-reported feelings of depression (AOR 1.76) and/or nervousness (AOR 2.06). Cannabis use among older caregivers was not associated with the burden of caregiving or the condition of the care recipient. Caregivers who use cannabis were more likely to experience a delay in accessing health care services relative to caregivers who do not use (AOR1.54).However, they were no more or less likely to use emergency department or outpatient medical care. These findings illuminate how cannabis is being used as a understudied form of self-care among informal care partners.

### SESSION 3120 (AWARD LECTURE (INVITATION ONLY))

Abstract citation ID: igae098.1245

**THE CLARK TIBBITTS AWARD AND HIRAM J. FRIEDSAM AWARD LECTURES AND PRESENTATIONS**

Chair: Joann Montepare

The Clark Tibbitts Award Lecture will feature an address by the 2024 award recipient Joann M. Motepare, PhD, FGSA, FAGHE, of Lasell University. The Hiram J. Friedsam Award Lecture will feature an address by the 2024 award recipient Dana Burr Bradley, PhD, FGSA, FAGHE, of the University of Maryland Baltimore County. AGHE's Clark Tibbitts Award was established in 1980 and named for an architect of the field of gerontological education. The award is given each year to an individual or organization that has made an outstanding contribution to the advancement of gerontology and geriatrics education. Hiram J. Friedsam was the professor, co-founder, and director of the Center for Studies in Aging and dean of the School of Community Service at the University of Northern Texas. Dr. Friedsam was an outstanding teacher, researcher, colleague, and mentor to students, faculty, and administrators, as well as a past president of AGHE. The purpose of this award is to recognize those who emulate Dr. Friedsam's excellence in mentorship.

Abstract citation ID: igae098.1246

**WE GOT THE MOVES LIKE JAGGER: REFLECTIONS ON AGING RESEARCH, EDUCATION, AND COMMUNITY**

Joann Montepare, *Lasell University, Newton, Massachusetts, United States*

Over the last 50 years, tremendous advances have been made in our understanding of aging and its many components through lifespan developmental research. As well, educators in the field of aging have designed creative and impactful strategies for teaching about aging and dismantling ageist thinking. Together, scholars and educators have made age matter in meaningful ways in our contemporary world. This presentation uses examples from my professional experiences to reflect on several key aspects of aging research and education – and how community connections are necessary to sustain this work, with special attention to the need to advance age inclusivity in higher education.

Abstract citation ID: igae098.1247

**HIGH-FIVE! – DISCOVERING THE POWER OF MENTORSHIP**

Dana Bradley, *University of Maryland Baltimore County, Baltimore, Maryland, United States*

There is an art and a science to mentorship that transforms lives – for the mentor and the mentee. Often, this mystique of mentoring can be intimidating, creating a misperception of one's abilities to serve as a mentor. While every mentoring opportunity is unique, generally accepted principles have been delineated in the rich mentorship literature that undergirds a productive and positive mentorship experience. In this view, mentoring is a hidden superpower that lies within each person, waiting to be discovered, shared – and celebrated. Reflecting on my career, I will share five observations from the mentor-mentee relationships that have shaped my life – and, in gratitude, give a virtual "high-five" across the room – and miles – to colleagues who have joined me on this journey.

### SESSION 3125 (SYMPOSIUM)

Abstract citation ID: igae098.1248

**THE IMPACT OF ENVIRONMENTAL EXPOSURES ON DEMENTIA, PHYSICAL, COGNITIVE, AND MENTAL HEALTH OF OLDER ADULTS**

Chair: Paola Zaninotto

Discussant: Matthew Prina

The proposed symposium will delve into crucial environmental factors affecting physical health, cognitive and mental health, in older populations. The first talk will explore urban environments' impact on cognitive health, highlighting evidence gaps and concentrated areas of existing research, such as air pollution and social environment influences on dementia and Alzheimer's disease. Another presentation will examine the association between long-term air pollution exposure and dementia risk in Denmark, identifying the most vulnerable groups based on socioeconomic status and comorbidities. The third talk will focus on the relationship between air pollution exposure over a decade and cognitive performance in older individuals in England, emphasizing the detrimental effects of industry emissions on cognition. A different study will discuss the findings on outdoor light at night and its lack of association with depressive symptoms in older adults in England. Lastly, the symposium will present research on extreme heat exposure's effects on epigenetic aging and its potential to increase morbidity and mortality risks, underscoring the need for mitigating these biological changes.

Abstract citation ID: igae098.1249

### URBAN ENVIRONMENT EXPOSURES AND COGNITIVE HEALTH: AN EVIDENCE GAP MAP OF SYSTEMATIC REVIEWS

Claire Cleland[1], and Ruth Hunter[2], *1. Centre for Public Health, Queen's University Belfast, 2. Queen's University Belfast, Belfast, Northern Ireland, United Kingdom*

With a rapidly ageing global population and continued trends in urbanisation, it is crucial we understand the influence of the urban environment on cognitive health. To address gaps in the evidence, we must first understand the current evidence base. The aim of the current study was to create an Evidence Gap Map (EGM) that identifies and presents all systematic reviews investigating urban environment exposure and its impact on cognitive health outcomes. A total of 57 reviews (with 257 outcomes) were included in the EGM. Five reviews were classified as high quality, 43 as moderate quality and nine as low quality. The EGM indicates that the evidence base is concentrated on 'environmental by-products' and 'social environment' and their impact on outcomes of dementia, Alzheimer's disease, and cognitive impairment. Areas also saturated were air pollution within 'environmental by-products' and educational attainment within the 'social environment'. Areas with limited evidence included exposure to 'traffic-related behaviours and all cognitive outcomes, and all four exposures ('environmental by-products', 'social environment', 'urban design' and 'traffic related behaviours') and child cognitive measures. The study provides an overview of the current evidence base regarding built, natural and social environmental exposures and cognitive health outcomes. The EGM provides a live, interactive, visual representation of the evidence. We identify key evidence gaps to be addressed including environmental exposures and cognitive outcomes, traffic-related behaviours, and urban by-products such as noise pollution and light pollution.

Abstract citation ID: igae098.1250

### LONG-TERM EXPOSURE TO OUTDOOR LIGHT AT NIGHT, AIR POLLUTION, AND DEPRESSIVE SYMPTOMS AMONG OLDER PEOPLE IN ENGLAND

Rina So[1], Giorgio Di Gessa[1], Shaun Scholes[1], Su-Hwan Kim[2], Yujin Park[3], Jinkook Lee[4], Sara Adar[5], and Paola Zaninotto[1], *1. University College London, London, England, United Kingdom, 2. Gyeongsang National University, Jinju-Si, Kyongsang-namdo, Republic of Korea, 3. Seoul National University Hospital, Seoul, Republic of Korea, 4. University of Southern California, Los Angeles, California, United States, 5. University of Michigan, Ann Arbor, Michigan, United States*

Air pollution and light at night (LAN) are environmental stressors that may lead to depression, but the epidemiological evidence is inconsistent and scarce, especially for LAN. We used data from 8,583 participants (25,151 observations), aged ≥ 50 years, of the English Longitudinal Study of Ageing from waves 6-9 (2012-2019). Annual levels of fine particulate matter (PM2.5), nitrogen dioxide (NO2), and LAN were assessed at participants' residential addresses for each interview year. Having depressive symptoms was defined as reporting ≥3 items of the 8-item Center for Epidemiologic Studies Depression Scale. We used mixed effects generalized linear models (binomial distribution) to examine associations of air pollutants and LAN (in quartiles) with depressive symptoms, adjusting first for age and sex and then adding control for individual lifestyle and socio-demographic factors, and area-level deprivation index. In models minimally-adjusted, increased odds of having depressive symptoms were observed for PM2.5, NO2, and LAN with odds ratio (OR) (95% confidence interval) of 1.15 (1.05, 1.25) and 1.20 (1.10, 1.32) per interquartile range of PM2.5 (2.2 μg/m3) and NO2 (4.2 ppb), respectively, and 2.47 (1.96, 3.12) in the highest quantile of LAN compared to the lowest. These associations were attenuated in fully-adjusted models to 0.97 (0.90, 1.05) for NO2 and 0.91 (0.84, 0.99) for LAN, while the association with PM2.5 remained positive [OR: 1.05 (0.97, 1.14)]. Higher residential PM2.5 levels might be associated with an increased risk of having depressive symptoms, but associations with NO2 and LAN were explained by confounding by individual- and area-level characteristics.

Abstract citation ID: igae098.1251

### LONG-TERM OUTDOOR AIR POLLUTION CONCENTRATIONS AND COGNITION: FINDINGS FROM THE HCAP SUBSTUDY OF ELSA

Giorgio Di Gessa, *University College London, London, England, United Kingdom*

**Background:** -Although air pollution has been associated with cognitive impairment, several key details of these relationships remain unknown. This includes understanding how different pollutants and their emission sources impact the brain as well as the particular aspects of cognition that are susceptible to pollution. In this study, we consider the associations between 8 years of exposure to different air pollutants (including total PM2.5 and PM2.5 from different emission sources, and NO2) with different domains of cognitive performance among older people.

**Methods:** -We used data from the Harmonized Cognitive Assessment Protocol (HCAP) sub-study of the English Longitudinal Study of Ageing (2018, N~1,200). This study measured a range of key cognitive domains affected by cognitive ageing, including attention, memory, executive function, language, and visuospatial function. Outdoor concentrations of each pollutant for all HCAP respondents'

residences were assessed since 2010. Averages and group-based trajectory modelling were then used to summarise long-term air pollution exposures and their relationships with cognition After adjustment for key personal and neighbourhood confounders.

**Results:** -Preliminary results suggest that respondents with higher residential levels of air pollution reported poorer cognitive function, especially for verbal fluency and executive function. Particularly, we found sustained high levels of NO2 to be associated with poorer cognition, even when baseline levels of cognition were accounted for (B=-1.75, 95%CI=-3.43;-0.06). Regarding PM2.5, our results suggest that emissions from industry are particularly detrimental to cognition.

**Conclusions:** -Efforts to reduce levels of exposure to air pollution should continue as this is a risk factor for poorer cognitive performance.

Abstract citation ID: igae098.1252

### LONG-TERM EXPOSURE TO AIR POLLUTION AND RISK OF DEMENTIA AMONG OLDER INDIVIDUALS OF A DANISH NATIONWIDE ANALYSES

Rina So[1], Zorana Andersen[2], Youn-Hee Lim[3], Jiawei Zhang[3], Stéphane Tuffier[3], and Thomas Cole-Hunter[3], *1. University College London, London, England, United Kingdom, 2. University of Copenhagen, Copenhagen, Sjelland, Denmark*, *3. University of Copenhagen, Copenhagen, Hovedstaden, Denmark*

The evidence linking air pollution with dementia has been fast growing over the last decade, but who is most susceptible remains rarely explored. Therefore, we aimed to examine the association between long-term exposure air pollution and incidence of dementia and identify the most susceptible groups by age, sex, socioeconomic status (SES), and comorbidities with major chronic diseases. We followed all Danish residents aged 60 years or older on January 1st, 2000 until December 31st, 2018. Annual mean concentrations of fine particulate matter (PM2.5), nitrogen dioxide (NO2), and black carbon (BC) in 2010 (100x100m) were estimated using European-wide hybrid land-use regression models and assigned to the baseline residential address. We used Cox proportional hazard models to evaluate the association between air pollution and dementia incidence, accounting for individual and area-level SES, and introduced an interaction term to test for effect modification by age, sex, income, education, employment, and co-morbidity with cardio-metabolic, respiratory diseases and depression. Of 934,792 subjects, 81,731 developed dementia. We detected statistically significant associations of all three pollutants with incidence of dementia, with hazard ratios (HRs) and 95% confidence intervals (CIs) of 1.14 (1.12-1.16) per 1.9 μg/m3 increase in PM2.5, 1.25 (122-1.28) per 10.2 μg/m3 increase in NO2, and 1.23 (1.20-1.26) per 0.5×10-5/m increase in BC. Associations were stronger in the elderly (≥75 years), stroke patients, unemployed, and those with the lower income or education. In this nationwide analysis in a low-exposure setting in Demark, long-term exposure to air pollution contributes to the development of dementia.

Abstract citation ID: igae098.1253

### EXTREME HEAT EXPOSURE AND EPIGENETIC AGING ACCELERATION AMONG OLDER ADULTS IN THE US

Eunyoung Choi, and Jennifer Ailshire, *University of Southern California, Los Angeles, California, United States*

Extreme heat exposure is a major risk factor for cardiovascular diseases and mortality. However, knowledge about the biological ramifications of extreme heat exposure is limited, particularly among older adults who face elevated vulnerability. This study examines the association between heat exposure and epigenetic aging, a key molecular marker of biological aging based on DNA methylation (DNAm) levels throughout the genome. We used data on 3,414 US adults aged 50 or above from the 2016 Health and Retirement Study Venous Blood Study with measured DNAm. To assess heat exposure, we calculated daily heat index values at the census tract level using high-resolution meteorological data from gridMet and linked them to each respondent's geocoded residential location. We considered both acute (on the day of blood collection) and chronic (1-year moving average) exposure windows. Multilevel regression models with natural cubic splines were used to assess the effects of heat exposure on epigenetic aging acceleration (i.e., Horvath, Hannum, PhenoAge, GrimAge, and DunedinPACE). Acute exposure to high heat index values (90th percentile compared to room temperature of 68F) was significantly associated with increased Horvath (0.77, p<.05), Hannum (0.73, p<.05), and PhenoAge (1.09, p<.001) acceleration. For long-term exposure, high heat index values were associated with increased PhenoAge (0.78, p<.05), GrimAge (0.52, p<.01), and DunedinPACE (0.02, p<.01). These findings provide insights into the biological underpinnings linking heat exposure to a broader spectrum of morbidity and mortality risks. Our study adds to the evidence highlighting the strong need to mitigate the adverse biological modifications induced by extreme heat.

### SESSION 3130 (SYMPOSIUM)

Abstract citation ID: igae098.1254

### THE RESIDENTIAL CARE TRANSITION MODULE: SUPPORTING DEMENTIA CAREGIVERS FOLLOWING INSTITUTIONALIZATION

Chair: Joseph Gaugler
Discussant: Marie Boltz

Families often continue to engage in various care activities following relatives' entry to residential long-term (RLTC) care settings such as nursing homes. Dementia caregivers' emotional and psychological adaptation to this transition is variable. We conducted rigorous, mixed methods randomized controlled evaluation to ascertain the efficacy of a remotely delivered, psychosocial/psychoeducational intervention when helping caregivers navigate the placement transition of relatives living with Alzheimer's disease and Alzheimer's disease-related dementias. The Residential Care Transition Module (RCTM) provided six formal sessions of consultation (one-to-one and family sessions) over a 4-month period to family caregivers who have admitted a relative living with dementia to an RLTC setting as well as ongoing, "ad hoc" consultation over an additional eight months. This symposium presents the quantitative, qualitative, and mixed methods findings of this dementia care intervention.

Abstract citation ID: igae098.1255

### EFFICACY OF THE RESIDENTIAL CARE TRANSITION MODULE

Joseph Gaugler[1], Robyn Birkeland[1], Elizabeth Albers[1], Colleen Peterson[2], Katie Louwagie[1], Zachary Baker[3], Mary Mittelman[4], and David Roth[5], *1. University of Minnesota, Minneapolis, Minnesota, United States*, *2. University of Michigan Transportation Research Institute, Ann Arbor, Michigan, United States*, *3. Arizona State University, Phoenix, Arizona, United States*, *4. New York University, New York, New York, United States*, *5. Johns Hopkins University, Baltimore, Maryland, United States*

"This study aimed to evaluate the efficacy of The Residential Care Transition Module (RCTM), a six-session, psychosocial, and psychoeducational telehealth intervention for family caregivers of cognitively impaired relatives living in a residential long-term care (RLTC) setting. Eligible participants (including care recipients, regardless of time since admission) were randomized to treatment or usual care control conditions. Survey data were collected at baseline, four months, eight months, and 12 months (N = 240). Primary analytic outcomes included caregiver subjective stress (a stress process mechanism) and depressive symptoms (a measure of global well-being). Secondary analytic outcomes included secondary role strains, residential care stress, caregiver sense of competence, and self-efficacy (additional mechanisms of action). General linear models tested for the main effects of the intervention at four months and longitudinal mixed models examined the 12-month effects of the intervention. Post hoc analyses also examined the influence of moderators. There were no significant differences between the treatment and control groups for any primary analytic outcome. Caregivers in the treatment group whose relatives were admitted to RLTC in the prior three months were more likely to indicate reductions in depressive symptoms over the first four months of participation. Over the 12-month study period, caregivers in the treatment group who were employed reported increased self-efficacy over time. The heterogeneity of dementia care requires a broader consideration of key contextual factors. Aligning measures with the preferences, goals, and values of dementia caregivers may further demonstrate the benefits of interventions such as the RCTM" (Gaugler et al., in press).

Abstract citation ID: igae098.1256

### PROCESS EVALUATION OF THE RESIDENTIAL CARE TRANSITION MODULE

Dana Urbanski[1], Robyn Birkeland[2], Katie Louwagie[2], Dionne Bailey[2], Zachary Baker[3], Allison Gustavson[4], Lauren Mitchell[5], and Joseph Gaugler[2], *1. University of Tennessee Health Science Center, Knoxville, Tennessee, United States*, *2. University of Minnesota, Minneapolis, Minnesota, United States*, *3. Arizona State University, Phoenix, Arizona, United States*, *4. Minneapolis Veterans Affairs Health Care System, Minneapolis, Minnesota, United States*, *5. Emmanuel College, Boston, Massachusetts, United States*

Although still relatively uncommon in dementia care science and gerontology, process evaluations can offer substantial insight when considering the dissemination and implementation of evidence-based innovations, particularly when utilizing various types of data to examine causal assumptions of an intervention as well as pre-specified contextual moderators or mediators that may influence intervention effects. This process evaluation leveraged the extensive quantitative and qualitative data collected during the 12-month, mixed methods evaluation of the Residential Care Transition Module (RCTM), a psychoeducational telehealth (via phone or video conference) intervention. Of 120 treatment participants, 107 completed the six session intervention. Session duration averaged 81 minutes (range 18-130 minutes, SD=20.9). The 13 participants who did not finish the intervention were bereaved during the intervention. As such, intervention delivery was discontinued. One hundred and fourteen treatment group participants engaged in varied amounts and means of ad hoc communications throughout their time in the study, including 9 of the 13 aforementioned bereaved participants who chose to continue to work with their transition coach after their relative passed. Eighty-one of the 114 participants engaged in ad hoc coaching sessions via phone or video conference, averaging six ad hoc sessions (range 1-38 sessions, SD=5.7). Ad hoc session duration averaged 41 minutes (range 5-120 minutes, SD=18.4). Emotional support was the most frequently delivered content, occurring in 91% of intervention sessions and 53% of ad hoc contacts. Participants overwhelmingly valued the RCTM program via a treatment review checklist administered quarterly, indicating high perceptions of utility, acceptability, and satisfaction with the RCTM.

Abstract citation ID: igae098.1257

### A QUALITATIVE ANALYSIS OF MECHANISMS OF BENEFIT IN THE RESIDENTIAL CARE TRANSITION MODULE

Elizabeth Albers[1], Robyn Birkeland[1], Katie Louwagie[1], Hawking Yam[1], Zachary Baker[2], Mary Mittelman[3], and Joseph Gaugler[1], *1. University of Minnesota, Minneapolis, Minnesota, United States*, *2. Arizona State University, Phoenix, Arizona, United States*, *3. New York University, New York, New York, United States*

This study aimed to determine the perceived benefits of the Residential Care Transition Module (RCTM), a multi-component, psychoeducational/psychosocial, telehealth intervention for caregivers of cognitively impaired relatives living in residential long-term care (RLTC). We conducted semi-structured exit interviews with 30 purposively selected participants who had been randomly assigned to receive the RCTM. An open-ended survey question solicited feedback from all RCTM participants at four (n=90), eight (n=79) and 12 months (n=77). Available qualitative data were analyzed for thematic content. Participants mentioned nine mechanisms of benefit: six strategies or educational content for caregiving and three aspects of counselor support. Common benefits attributed to RCTM included improved mood, caregiving confidence and communication. Through this analysis we achieved a more comprehensive understanding of the RCTM's mechanisms of benefit which we will explore more in the presentation.

Abstract citation ID: igae098.1258

**AN EMBEDDED MIXED METHODS EVALUATION OF THE RESIDENTIAL CARE TRANSITION MODULE**

Robyn Birkeland[1], Joseph Gaugler[1], Elizabeth Albers[1], Colleen Peterson[2], Katie Louwagie[1], Zachary Baker[3], and Mary Mittelman[4], *1. University of Minnesota, Minneapolis, Minnesota, United States*, *2. University of Michigan Transportation Research Institute, Ann Arbor, Michigan, United States*, *3. Arizona State University, Phoenix, Arizona, United States*, *4. New York University, New York, New York, United States*

Mixed methods designs refer to those research strategies that integrate or "mix" qualitative and quantitative research questions, sampling and data collection approaches, and analyses, so that one component builds upon and expands the inferences of the other. The Residential Care Transition Module (RCTM) evaluation relied on an "embedded" mixed methods design. In the RCTM embedded design, qualitative data collection was integrated during and following intervention delivery to ascertain components or contextual factors that may have influenced participant benefit. We relied on the robust qualitative data available from quarterly review checklists as well as purposive, post-intervention semi-structured interviews administered to RCTM treatment participants to identify and empirically test three mediators and moderators in a series of post-hoc, longitudinal quantitative analyses: unemployment, time in residential care, and bereavement status. In this presentation, we will highlight our findings for whom and under what circumstances a telehealth dementia care support intervention such as the RCTM may exert positive benefits.

### SESSION 3135 (SYMPOSIUM)

Abstract citation ID: igae098.1259

**THE UTILITY AND POTENTIALS OF CLAIMS-BASED FRAILTY INDEX IN CLINICAL CARE AND RESEARCH: NEW DEVELOPMENTS IN 2024**

Chair: Dae Hyun Kim

The development of a claims-based frailty index (CFI) for Medicare claims (Kim et al., Journals of Gerontology: Medical Sciences, 2018) has empowered researchers to integrate frailty into pharmacoepidemiology and health services research. This symposium aims to overview the CFI's development and validation process and spotlight its recent applications in studies utilizing Medicare claims and electronic health records (EHR). The CFI, estimating a deficit accumulation frailty index (range: 0 to 1, with higher values indicating greater frailty) based on the previous year's claims, demonstrates 60% sensitivity and 86% specificity for a deficit accumulation frailty index ≥0.25, and 62% sensitivity and 78% specificity for the Fried Frailty Phenotype. Additionally, a crosswalk between CFI and clinical frailty measures is available, aiding the translation of research findings into clinical practice. Featured presentations will explore the CFI's application to EHR data, its role in assessing dementia severity, its contribution to the CMS Hierarchical Condition Category model in predicting healthcare costs, its predictive value for outcomes following post-acute care in skilled nursing facilities, and its association with opioid discontinuation rates in older adults hospitalized with fractures. These examples will highlight the benefits and challenges of using CFI in research.

Abstract citation ID: igae098.1260

**APPLICATION OF THE CLAIM-BASED FRAILTY INDEX (CFI) TO STRUCTURED ELECTRONIC HEALTH RECORD (EHR) DATA**

Min Ji Kwak[1], Caroline Schaefer[2], Youngran Kim[2], Sunyang Fu[2], Abhjieet Dhoble[2], Nahid Rianon[2], Holly Holmes[2], and Dae Hyun Kim[3], *1. McGovern Medical School, Houston, Texas, United States*, *2. The University of Texas Health Science Center, Houston, Texas, United States*, *3. Hebrew SeniorLife, Boston, Massachusetts, United States*

The claim-based frailty index (CFI) was initially developed to identify frailty in Medicare claims. However, the application of CFI to structured data from an Electronic Health Record (EHR) has not been thoroughly studied. We aimed to apply CFI to structured EHR data, The Merative™ Explorys® Therapeutic Dataset Delivered, and estimate the validity of CFI compared to the 5% random sample of Medicare fee-for-service claims (2017-2018). We identified older adults (≥65 years old) with heart failure (HF) in Explorys data (n=52,295) and in Medicare claims (n=33,217). The CFI was calculated from each dataset separately. The CFI from Medicare claims showed a right-skewed distribution (mode of the CFI = 0.210) with a wider shape, whereas the CFI from Explorys data was also right-skewed but with a longer tail and had a narrower distribution (mode of the CFI = 0.186). When we assessed the prevalence of the 93 components of CFI, the components based on procedure codes were more prevalent in Medicare claims than in Explorys data. When frailty was defined as CFI ≥0.25, Explorys data identified fewer cases of frailty (18.1% vs 42.4% in Medicare claims). However, CFI from Explorys data had better discrimination (C-statistic: 0.636 vs 0.608 in Medicare claims) in predicting hospital admission or emergency room visits 1 year after the index visit. These results suggest that CFI may be implemented in EHR data and used for predicting adverse clinical events in HF patients.

Abstract citation ID: igae098.1261

**CLAIMS-BASED FRAILTY INDEX AS A PREDICTOR OF POSTACUTE SKILLED NURSING FACILITY CARE OUTCOMES**

Sandra Shi[1], Gahee Oh[2], Chan Mi Park[2], Ellen McCarthy[3], and Dae Hyun Kim[2], *1. Harvard Medical School/Hebrew SeniorLife, Boston, Massachusetts, United States*, *2. Hebrew SeniorLife, Boston, Massachusetts, United States*, *3. Harvard Medical School, Boston, Massachusetts, United States*

How claims-based frailty index (CFI) predicts post-acute skilled nursing facility (SNF) care outcomes is unknown. We used the National Health and Aging Trends Study 2011-2017 survey data linked to Medicare claims. CFI and comprehensive geriatric assessment-based frailty index (CGA-FI) were calculated at each survey and categorized into non-frail (<0.25), mild frailty (0.25-0.34), moderate frailty (0.35-0.44), and severe frailty (≥0.45). We examined the first post-acute SNF admission within 6 months after survey assessment. The primary outcome was home time in the 6 months following SNF admission defined as days not in hospital or nursing home. We estimated the effects of frailty on home time using linear regression for each frailty measure. The secondary outcome was total healthcare costs in the 6 months following SNF admission estimated using a generalized linear model with a gamma distribution and identity link. Analyses adjusted for age, sex, and income. In our sample of 643 Medicare beneficiaries (representing 2.7 Million beneficiaries nationally), increasing frailty was associated with greater home time loss and healthcare costs by both measures. The mean difference (95% CI) of home time loss for severe frailty compared with non-frailty was -39.2 [-66.8, -11.5] days by CFI and -43.2 [-60.0, -26.4] days by CGA-FI. The mean healthcare cost increase (95% CI) for severe frailty compared with non-frailty was $21,583 [$13,132, $30,033] by CFI and $11,110 [$6,236, $15,983] by CGA-FI, respectively. Pearson correlation between CFI and costs was higher than CGA-FI (0.1885 vs 0.0804). CFI up to 6 months before SNF admission predicts healthcare utilization.

Abstract citation ID: igae098.1262

### CLAIMS-BASED FRAILTY INDEX FOR IDENTIFYING MODERATE-TO-SEVERE DEMENTIA IN MEDICARE BENEFICIARIES

Chan Mi Park[1], Stephanie Denise Sison[2], Ellen McCarthy[3], Sandra Shi[4], and Dae Hyun Kim[1], *1. Hebrew SeniorLife, Boston, Massachusetts, United States*, *2. University of Massachusetts Chan Medical School, Worcester, Massachusetts, United States*, *3. Harvard Medical School, Boston, Massachusetts, United States*, *4. Harvard Medical School/Hebrew SeniorLife, Boston, Massachusetts, United States*

Identifying dementia severity from administrative claims poses challenges. This abstract discusses developing and validating a claims-based frailty index (CFI) cut-point for identifying moderate-to-severe dementia among Medicare beneficiaries, using data from the National Health and Aging Trends Study (NHATS) and Medicare Current Beneficiary Survey (MCBS). We used 2015 data from NHATS linked to Medicare claims to calculate CFI and determine the optimal cut-point that maximizes sensitivity and specificity in identifying moderate-to-severe dementia based on the Functional Assessment Staging. We validated this measure on an independent sample of community-dwelling Medicare beneficiaries with dementia from MCBS. Performance metrics included sensitivity, specificity, and positive and negative predictive values. From the NHATS development cohort (n=814), the CFI cut-point of 0.280 demonstrated strong performance (C-statistic of 0.78) in differentiating moderate-to-severe dementia cases, achieving the maximum sensitivity of 76.9% and specificity of 62.8%. This cut-point correlated significantly with dementia indicators, including higher rates of functional disability, dementia medication use, mortality, and nursing home admissions. Subsequent validation using the MCBS cohort (n=658) supported the cut-point's utility in identifying moderate-to-severe dementia, with modest sensitivity of 0.49 and high specificity of 0.80. These two pivotal studies suggest that the CFI, with a cut-point of 0.28, is a robust tool for identifying moderate-to-severe dementia in Medicare claims data. By offering an evidence-based metric, this research facilitates a more targeted approach to allocating healthcare resources and planning care interventions for dementia patients, bridging a critical gap in the absence of direct clinical measures of dementia severity.

Abstract citation ID: igae098.1263

### INTEGRATING A FRAILTY INDEX INTO THE CMS HIERARCHICAL CONDITION CATEGORY MODEL FOR PREDICTING HEALTH CARE COSTS

Jieun Jang[1], Ellen McCarthy[2], Brianne Olivieri-Mui[3], Sandra Shi[4], Chan Mi Park[5], Stephanie Denise Sison[6], and Dae Hyun Kim[5], *1. Yonsei University Graduate School of Public Health, Seoul, Republic of Korea*, *2. Harvard Medical School, Boston, Massachusetts, United States*, *3. Northeastern University, Boston, Massachusetts, United States*, *4. Harvard Medical School/Hebrew SeniorLife, Boston, Massachusetts, United States*, *5. Hebrew SeniorLife, Boston, Massachusetts, United States*, *6. University of Massachusetts Chan Medical School, Worcester, Massachusetts, United States*

The Centers for Medicare and Medicaid Services Hierarchical Condition Category (CMS-HCC) score is a standard risk adjustment tool to predict total Medicare costs. We examined whether a claims-based frailty index (CFI) could enhance the predictive capability of the CMS-HCC score. We utilized electronic health records linked with Medicare data, focusing on patients over 65, including surgical inpatients and outpatients from January 2017 to December 2018. Analyzing 1,855 surgical inpatients using logistic regression, we estimated the likelihood of incurring top 20% Medicare costs for 30 days, 90 days, and 1 year after surgery, comparing 1) the CMS-HCC score alone with 2) the CMS-HCC score combined with the CFI. Similarly, for 58,492 outpatients, we employed generalized estimating equations to estimate the likelihood of being in the top 20% of total Medicare costs for 1 year after an outpatient visit, comparing the same two models. The added value of CFI was assessed using net reclassification improvement (NRI) with 3 risk categories (0 to <10%, 10 to <20%, ≥20%) to compare the two prediction models. Including CFI with CMS-HCC score improved NRI by 7.3% for 30-day costs, 10.1% for 90-day costs, 9.8% for one-year costs in surgical inpatients, and 12.3% for annual costs in outpatients. The added contribution of CFI to CMS-HCC significantly improves the model's ability to identify patients at high risk of incurring substantial medical costs (top 20%). Accounting for patient frailty may enable healthcare systems to more effectively allocate resources and implement targeted interventions, ultimately reducing healthcare expenditures.

Abstract citation ID: igae098.1264

### OPIOID DISCONTINUATION RATES: RISK STRATIFICATION USING A CLAIMS-BASED FRAILTY INDEX (2013–2018 MEDICARE)

Kevin Pritchard[1], Chun-Ting Yang[1], Qiaoxi Chen[1], Yichi Zhang[1], James Wilkins[2], Dae Hyun Kim[3], and Kueiyu Joshua Lin[1], *1. Brigham and Women's Hospital, Boston, Massachusetts, United States*, *2. Harvard Medical School, Boston, Massachusetts, United States*, *3. Hebrew SeniorLife, Boston, Massachusetts, United States*

We aimed to determine if the severity of frailty influences the duration of opioid use following an emergent pain event. This retrospective cohort study included 21,728

fee-for-service Medicare beneficiaries (2013-2018). We included opioid-naïve individuals aged ≥65 years who initiated opioids within 30 days of a hospitalized fracture. Our index date was the opioid prescription fill date. We excluded those with prior opioid use, chronic pain, or contraindications to opioids in the preceding year. Frailty severity was defined using a claims-based index. We estimated opioid discontinuation rates using Nelson-Aalen cumulative hazard functions, stratified by frailty severity. Discontinuation during the follow-up year was defined as a gap in continuous prescription refills exceeding 15 days. We used Cox proportional hazards regression to assess the association between frailty severity and the number of days until discontinuing opioids while controlling for sociodemographic factors, health status, healthcare utilization, and calendar year. Our sample was on average age 80 [SD=9] years old, 77% female, and 91% White. The 30-day discontinuation rate was 85% (95% CI, 83-87%) in the moderate/severe frail group, 84% (83%-84%) in the mildly frail group, 87% (87%-88%) in the pre-frail group, and 90% (89-91%) in the non-frail group. After multivariable adjustment, the likelihood to discontinue opioids depended on frailty status. Compared to adults without frailty, discontinuation was less likely among those who were moderately/severely frail (Hazard Ratio [HR]: 0.88, 95% CI, 0.83-0.94), mildly frail (HR: 0.87, 95% CI, 0.83-0.92), and pre-frail (HR: 0.96, 95% CI, 0.91-1.00). Greater frailty decreased the likelihood of discontinuing opioids.

### SESSION 3140 (SYMPOSIUM)

Abstract citation ID: igae098.1265

### USING BLACK HISTORY FOR BRAIN HEALTH: APPROACH, OUTCOMES, AND PUBLIC APPLICATIONS IN THE SHARP STUDY

Chair: Raina Croff

This symposium focuses on the Sharing History through Active Reminiscence and Photo-imagery (SHARP) study that centers Black history to engage older Black adults in the brain-healthy behaviors of walking, social engagement, and reminiscence within gentrifying areas of Portland and Seattle. Presentations span the study's theoretical underpinnings to operationalizing results for public awareness of brain health. Dr. Raina Croff introduces the SHARP study, discussing the gap in brain health interventions that it seeks to fill, its culturally celebratory framework, and why such approaches matter for participant engagement. Anthony Cryer and Stephanie Johnson-Toliver discuss the collaborative process in Seattle from the community partner perspective, including recruitment challenges amidst gentrification, creating meaningful walking routes centered on local Black history, and participant feedback. Boeun Kim reports on health outcomes from the SHARP Seattle study and themes from evaluative focus groups, including barriers and motivators to engagement. Charles Fennell discusses the SHARP Caregiver Study in Portland, including adaptations to meet dementia family caregivers' needs, and health outcomes around sleep and activity levels as captured by wearable digital biomarker technology. Taryn Gordon discusses preparing SHARP's recorded walking narratives for a public digital oral history archive in a way that preserves participant integrity, and how SHARP concepts are operationalized in the SHARP website by integrating Black oral history from walking narratives with brain health information to educate and empower older Black adults to learn about and practice brain healthy behaviors.

Abstract citation ID: igae098.1266

### HONORING OUR HEALTH AND HISTORY: FRAMING BRAIN HEALTH THROUGH A CULTURALLY CELEBRATORY LENS

Raina Croff[1], Patrice Fuller[2], Charles Fennell[2], and Taryn Gordon[3], *1. Oregon Alzheimer's Disease Research Center, Oregon Health & Science University, Portland, Oregon, United States*, *2. Oregon Health & Science University, Portland, Oregon, United States*, *3. George Fox University, Newberg, Oregon, United States*

Asset-based, culturally celebratory frameworks center community strengths, history, culture, and community resilience. Applying such frameworks to Black American brain health engenders agency over individual and community health, and empowers older Black adults to engage in aging research. Amidst gentrification, such frameworks become especially salient. The Sharing History through Active Reminiscence and Photo-imagery (SHARP) Study uses Black history to engage older Black adults in walking social reminiscence for brain health within gentrifying neighborhoods in Portland and Seattle. Since 2016, SHARP has engaged 82 healthy and cognitively impaired walkers, including dementia family caregivers. Triads walked 1-mile routes 3x/week for 1, 4, or 6 months using the SHARP walking application on a group tablet. GPS-linked images of local Black history and culture prompted social reminiscence during walking. Walking narratives were recorded for an oral history archive. This presentation explores SHARP's culturally celebratory framework informed by Black Lives Matter principles applied to health, Asset-Based Community Development, and Africana Worldview. We examine how centering Black history over Black health deficits, community memory over individual memory loss, and group participation over individual research participation produced high retention, re-enrollment rates, and recommendations. We apply theoretical concepts and study design to understand participant experiences, including how impromptu neighborhood encounters during walks mimicked social patterns from younger years, recalling joyful community memories despite gentrification, improved coping with change, and decreased psychological burden of exercise. Finally, we highlight challenges to the SHARP approach, including balancing a culturally celebratory framework with duly addressing the heavy burden of Alzheimer's and gentrification.

Abstract citation ID: igae098.1267

### SHARP SEATTLE: A COMMUNITY COLLABORATION TO CREATE BLACK HISTORY WALKING ROUTES AND ENGAGE WALKERS

Anthony Cryer[1], Stephanie Johnson-Toliver[2], Dian Ferguson[1], and Juell Towns[3], *1. Central Area Senior Center, Seattle, Washington, United States*, *2. Black Heritage Society of Washington State, Seattle, Washington, United States*, *3. University of Washington, Seattle, Washington, United States*

The Sharing History through Active Reminiscence and Photo-Imagery (SHARP) program honors Black health and history through creating culturally celebratory walking routes. Nine community and academic partners implemented SHARP Seattle in the gentrifying Central District. Key to success was partners with established community relationships and expertise aligned with the SHARP approach, including in walking programs, archival images, local Black history, cognitive health, and the needs, concerns, and preferences of older Black adults regarding programming. Study activities were based at and coordinated by a senior center serving primarily African Americans. Student interns led informant focus groups with 10 older Black adults to gather themes and topics for inclusion in twelve 1-mile walking routes. Interns worked with an archivist on selecting local historical images to illustrate community-identified walk themes and solicit stimulating conversational reminiscence among walkers. Themed routes with images were programmed into the SHARP walking application, tested, and revised. After a kick-off celebration, thirty-one older Black adults, grouped into triads, engaged 3 times a week for 4 weeks to walk the 12 routes. In this talk, community partners discuss their experience, including learning the research process, exerting their preference for an adapted, more relaxed study design for less burden and greater inclusion of people with cognitive impairment, creating routes with different generations whose local geo-historical knowledge differed greatly, strategies for curating community buy-in, gentrification and displacement-related recruitment challenges, and learning from participant feedback. Finally, self-reflections explore the meaningfulness of walking and social reminiscence amidst aging and gentrification.

Abstract citation ID: igae098.1268

### EVALUATION OF A REMINISCENCE AND PHOTO-IMAGERY WALKING PROGRAM FOR BLACK OLDER ADULTS

Boeun Kim[1], Lia Kaluna[2], Meshack Otewa[3], Basia Belza[3], and Raina Croff[4], *1. University of Iowa, Iowa, United States*, *2. University of Wisconsin Health University Hospital, Madison, Wisconsin, United States*, *3. University of Washington, Seattle, Washington, United States*, *4. Oregon Alzheimer's Disease Research Center, Oregon Health & Science University, Portland, Oregon, United States*

The purpose of this study was to pilot the Sharing History through Active Reminiscence and Photo-Imagery (SHARP) program which honors Black history and health through culturally celebratory walking routes in the Puget Sound area. Participants were recruited from a senior center. Thirty-one healthy and mildly cognitively impaired Black Americans completed triadic walking three times a week for 4 weeks. Participants used the SHARP walking application on a group tablet device, and back-up paper routes, to navigate twelve themed, 1-mile looped routes with GPS-triggered local historical images of Black life and culture to prompt conversational reminiscence during walks. Route themes were Childhood Health, Black Advocacy, Community Ties, Building Community, Arts, Entertainment, and Education. Focus groups at the end of the study captured participant experiences, challenges, and preferences. Pre/post measures captured blood pressure, self-rated health, and depressive symptoms. Paired t-tests were performed on pre/post outcomes, and thematic coding was applied to focus group data. Mean age was 71.8 years and the majority (93%) were female. Mean self-rated health improved and depression symptoms decreased, but not statistically significant. Blood pressure had little to no change. End-study focus groups revealed that participants were motivated by and benefited from social engagement with their walking groups. Walks simultaneously motivated people to exercise and facilitated reminisce about lived experiences in the neighborhood, including gentrification. Participants identified confusing prompts, local construction, terrain, and technology as barriers to the current application of the program. Participants made suggestions for future route themes and to improve program accessibility and clarity.

Abstract citation ID: igae098.1269

### ADAPTATIONS AND OUTCOMES OF A WALKING AND REMINISCENCE BRAIN HEALTH INTERVENTION FOR CAREGIVERS

Patrice Fuller[1], Charles Fennell[1], Nora Mattek[1], Sarah Gothard[2], Jeffrey Kaye[1], and Raina Croff[2], *1. Oregon Health & Science University, Portland, Oregon, United States*, *2. Oregon Alzheimer's Disease Research Center, Oregon Health & Science University, Portland, Oregon, United States*

The SHARP-Caregiver (SHARP-CG) study evaluated the feasibility of adapting SHARP to family caregivers of care partners with mild cognitive impairment or early-stage dementia. SHARP-CG assigned 7 triads (n=21) to Group A or B. Triads had a family caregiver (age >40), their care partner (age >40), and a support person (age >18). Caregivers and support persons were healthy or mildly cognitively impaired. Triads walked 1-mile routes with images to prompt conversational reminiscence 3x a week for 16 weeks using the SHARP walking application. Caregivers and care partners contributed weekly health update data, and (optionally) sleep and step-count data. Group A participants walked immediately. Group B participants first completed 16-weeks of observation. Ages were 35-90 (mean 69.8); most were female (52%). Mean Montreal Cognitive Assessment score for caregivers and care partners was 21.7 (SD+4.6). Eighty-six percent (n=6) of caregivers and 71% of care partners (n=5) opted to engage in digital biomarker data collection (actigraphy watch and sleep sensor). Caregivers had greater mean total daily steps (2055; SD+686) than care partners (1684; SD+979). Mean sleep hours were similar for caregivers and care partners at 6.1 (SD+1.1) and 6.5 (SD+2.5). Recruiting caregivers was difficult because many family members did not recognize what they were doing as caregiving. To improve study accessibility for caregivers, adapted eligibility criteria included reducing minimum age, allowing mobility aids, and making some components optional. Increasing accessibility, while improving enrollment, did not necessarily impact adherence. Caregiver demands and sporadic health concerns limited participation.

Abstract citation ID: igae098.1270

### HONORING OUR HEALTH AND HISTORY: OPERATIONALIZING CONCEPTS AND RESULTS FROM THE SHARP STUDY

Taryn Gordon[1], Launa Newby[2], Patrice Fuller[3], Charles Fennell[3], and Raina Croff[4], *1. George Fox University, Newberg, Oregon, United States*, *2. Portland*

*State University, Portland, Oregon, United States*, *3. Oregon Health & Science University, Portland, Oregon, United States*, *4. Oregon Alzheimer's Disease Research Center, Oregon Health & Science University, Portland, Oregon, United States*

Accessibility of mental and brain health education remains a challenge for older Black adults, hindered by cultural insensitivities within the field and discriminatory historical context. Operationalizing Black-centered theoretical concepts and narratives from the Sharing History through Active Reminiscence and Photo-imagery (SHARP) study on our website aims to leave older Black adults not only educated but empowered to address cognitive decline and engage in behaviors to mitigate it. Since 2016, 82 SHARP walkers in gentrifying areas of Portland and Seattle have generated over 400 narratives by walking 3x/week for 1, 4, or 6 months and reminiscing together, aided by image prompts about local Black history and culture. Reminiscence is audio recorded for an oral history archive. Transcribed narratives are vetted for content that may compromise participant integrity, then participant-checked. Next, narratives are coded to create a topical index for public users of the archive. SHARP researchers are now operationalizing SHARP theoretical concepts on our website and applying narratives to reach the wider Black public about brain health. The website integrates oral history from walks with tangible, culturally engaging educational content about aging, cognitive decline, and mental health. Asset-based and culturally celebratory messaging, tone, and Black history images counter the often fear- and deficit-based messaging around these topics. Framing Black health within the richness and vitality of Black history, culture, and community resilience, and through memories from other older Black adults taking agency over their health, can inspire learning and action to give readers agency in their mental and brain health.

### SESSION 3145 (SYMPOSIUM)

Abstract citation ID: igae098.1271

### WEALTH LOSS, DEMENTIA, AND COGNITIVE DECLINE: SURFACING NEW CONNECTIONS AND UPSTREAM INTERVENTIONS

Chair: Julie Miller
Discussant: Lucas Yoquinto

Nearly one in ten adults in the United States over age 65 has dementia. Another two in ten have mild cognitive impairment: an early, symptomatic period of memory or cognitive decline experienced by those who do not yet meet the full diagnostic criteria for dementia. It has long been a matter of anecdote that serious personal finance losses can coincide with, and arise in the wake of, a dementia diagnosis. This symposium brings together leading researchers to explore financial losses than can occur both before and after formal dementia diagnosis. The first presentation compares household financial trajectories of individuals who ultimately developed dementia with those who did not. The second presentation explores financial status of older adults prior to and following the onset of dementia. The third and final presentation explores changes in marital and health status (including but not limited to transitions from normal cognition to cognitive impairment or dementia) as risk factors for negative wealth shocks in older age. Combined, the presentations surface new insights and questions about connections between financial health and cognitive health. The presentations will be synthesized and brought together by a discussion of possible cross-sector, cross-industry intervention strategies that can be undertaken to protect individuals and families at risk of financial losses associated with dementia, especially in the pre-diagnosis period.

Abstract citation ID: igae098.1272

### DEMENTIA AND LONG-RUN CHANGES IN FINANCIAL HEALTH AMONG US HOUSEHOLDS

Jing Li[1], Amy Kelley[2], Kathleen McGarry[3], Lauren Nicholas[4], and Jonathan Skinner[5], *1. University of Washington, Seattle, Washington, United States*, *2. National Institute on Aging, Baltimore, Maryland, United States*, *3. Stony Brook University, Stony Brook, New York, United States*, *4. University of Colorado School of Medicine, Aurora, Colorado, United States*, *5. Dartmouth College, Hanover, New Hampshire, United States*

We use two decades of the U.S. Health and Retirement Study data to compare long-run trajectories in household finances between individuals who eventually developed dementia and those who did not. In prior work, we find large and steady divergence in household wealth that starts 6-8 years prior to dementia onset. In the current paper, we investigate several possible hypotheses for these changes. By examining detailed categories of household income and spending, we find that long-run divergence in wealth between dementia and non-dementia groups are not explained by higher out-of-pocket healthcare expenses or intentional "spend-down" to qualify for Medicare coverage of nursing home residence. Rather, our findings suggest that they are driven by earlier exit from the labor force and potentially suboptimal investment decisions, which negatively affect long-term income in multiple areas among the dementia cohort relative to their counterparts. We also find that these patterns are more prominent among individuals with higher socioeconomic status.

Abstract citation ID: igae098.1273

### CHANGES IN MARITAL AND HEALTH STATUS AS RISK FACTORS FOR A SUBSEQUENT NEGATIVE WEALTH SHOCK IN THE US, 1996–2020

Tsai-Chin Cho, Sara Adar, HwaJung Choi, Kenneth Langa, and Lindsay Kobayashi, *University of Michigan, Ann Arbor, Michigan, United States*

Negative wealth shocks, defined as a loss of ≥75% of one's wealth over a short time-period, may decrease material living standards in later-life. However, the risk factors for negative wealth shocks are unclear. We aimed to examine the association of four risk factors, 1) separation from or loss of a spouse/partner; 2) diagnosis of a new chronic condition; 3) transition from low to high depressive symptoms; and 4) transition from normal cognition to cognitive impairment or dementia, with the subsequent experience of a negative wealth shock in mid-to-later life. We included respondents aged ≥55 in the US Health and Retirement Study between 1996-2020 (N=128,213 observations from 25,265 persons). Study participants were classified at each time-point as either having experienced a negative wealth shock between each biennial survey interview, or not.

The risk factors were assessed over two years preceding negative wealth shocks. We performed multivariable logistic mixed effects models accounting for complex survey design to examine how each risk factor may trigger negative wealth shocks and evaluate effect modification by retirement status at the baseline of study intervals. Separation from or loss of a spouse/partner increased the odds of a negative wealth shock (AOR= 1.44; 95% CI: 1.24, 1.67). Compared to those not retired, retirees who became depressed had higher odds of a negative wealth shock (AOR= 1.23; 95% CI: 1.04, 1.47). This population-based prospective study suggests that changes in marital status and in mental health, for retired adults, may signal future negative wealth shocks, although further investigation is required.

Abstract citation ID: igae098.1274

**FINANCIAL IMPLICATIONS OF DEMENTIA: AN EIGHT-YEAR FOLLOW-UP OF MATCHED SAMPLES**

HwaJung Choi, Kenneth Langa, Edward Norton, Tsai-Chin Cho, and Cathleen Connell, *University of Michigan, Ann Arbor, Michigan, United States*

Financial consequences of dementia could be devastating because of the extensive care needs and high care costs associated with dementia. This study is to determine the financial status of older adults before and after the onset of dementia and assess the potential implications for individuals, families, and society. This population-based cohort study compared two matched samples of U.S. adults aged 55 years or older from national, longitudinal data. To create matched samples (dementia vs. control), the study included extensive baseline variables of sociodemographic characteristics, economic status, family availability, health conditions, disability status, and outpatient care use. In total, 2387 adults experienced the onset of dementia during the 2-year follow-up (dementia group) and 2387 adults did not (control group). Participants were followed up for 8 years from the baseline. Over the 8-year follow-up in the dementia group, the 2-year out-of-pocket medical costs increased from $4005 to $ 10,006, median wealth was reduced from $79,339 to $30,490, and those enrolling in Medicaid increased from 16.1% to 29.7%. No statistically significant changes in financial outcomes were found in the control group. The study found that in-home medical care, hospital stay, and nursing home facility stay increased dramatically, especially within two years after the onset of dementia. Greater family care availability is significantly associated with a lower level of transition to a nursing home, which is the most expensive care setting. The potential mitigating effect of family care availability should be accounted for in developing an intervention to reduce the financial effects of dementia.

### SESSION 3150 (SYMPOSIUM)

Abstract citation ID: igae098.1275

**ADVANCEMENTS IN FRAILTY INTERVENTION RESEARCH: CURRENT TRENDS AND FUTURE DIRECTIONS**

Chair: Qian-Li Xue
Discussant: Qian-Li Xue

Frailty, a complex, syndromic condition primarily affecting older adults, faces a lack of evidence-based management strategies due to scarce randomized controlled trials. So far, frailty interventions have employed a spectrum of strategies, including health behavior interventions (e.g., diet and exercise), pharmacological interventions targeting specific biological pathways, and personalized geriatric care models. However, the field faces significant challenges that hinder its progress. A notable issue is that there is a noticeable lack of comprehensive management strategies for frailty patients, supported by robust evidence. Moreover, trials examining the impact of physical activity and dietary interventions have largely focused on the clinical manifestations of frailty (e.g., strength) with little evidence of addressing its biological underpinnings. Further complicating these issues is the inconsistent validation of research findings across various studies and demographic groups, casting doubt on their broader relevance and efficacy. The symposium presents four talks: the first evaluates a values-based digital literacy intervention for prefrail and frail individuals with chronic disease; the next two discuss integrating technologies, including sensors with AI and voice-activated devices, for in-home frailty monitoring and intervention; the fourth considers mind-body practices' (e.g., yoga and tai chi) potential to influence frailty's biological underpinnings. Collectively, this symposium aims to: (a) differentiate between interventions tailored specifically to address frailty as a unique clinical entity and interventions aimed at improving care for frail older adults; (b) explore innovative approaches for tailoring frailty interventions to patient-specific goals; (c) tackle the inherent research and implementation challenges in frailty care.

Abstract citation ID: igae098.1276

**EMPOWERING FRAIL OLDER ADULTS THROUGH DIGITAL LITERACY: A THEORY AND VALUES-BASED INTERVENTION**

Melissa Hladek[1], Olivia Rubio[2], Samantha Curriero[3], Deidra Crews[1], Mara McAdams DeMarco[3], Daniel Brennan[4], Qian-Li Xue[1], and Sarah Szanton[1], *1. Johns Hopkins University, Baltimore, Maryland, United States*, *2. The Pennsylvania State University, University Park, Pennsylvania, United States*, *3. New York University, New York, New York, United States*, *4. Johns Hopkins University School of Medicine, Baltimore, Maryland, United States*

Frailty is an age-associated syndrome heightening vulnerability to stressors. Mobility limitations common among individuals with frailty can impede access to physical resources and can lead to social isolation, detrimentally affecting mental and physical health. Digital literacy offers access to health information access, assistive and remote monitoring technologies and social engagement. Each are vital for people with frailty because they can enhance independence, cognition and quality of life. Despite the potential of digital literacy, internet access and digital health resources remain underutilized with older adults, especially among older adults with lower incomes and education levels. We designed a theory and values-based person-directed intervention to address digital literacy for older adults, aiming to recognize and leverage the motivational factors driving their desire to learn new skills. This is a 20 person one-arm feasibility and acceptability pilot trial enrolling participants with varying levels of frailty and digital literacy. The study is comprised of 4-6 in-home biweekly visits over the course of 8-12 weeks that use the older adults' own values and goals to co-develop action plans that matter to the participant with the primary outcome of improvements in digital literacy. We hypothesize creating a digital literacy intervention that is person-directed will be feasible and acceptable to older adults and will lead to improvements in digital literacy, self-efficacy and quality of life.

Abstract citation ID: igae098.1277

### FRAILTY THERAPEUTICS: CLOSING THE PHYSICAL ACTIVITY GAP FOR FRAIL ADULTS

Megan Huisingh-Scheetz[1], Brittni Bryant[1], Brandon Foster[1], Corliss Taylor[1], Arianna Parkhideh[1], Benjamin Mendez[2], Sylvia Brown[3], and Nikita Thomas[1], *1. University of Chicago, Chicago, Illinois, United States*, *2. University of Illinois College of Medicine Peoria, Peoria, Illinois, United States*, *3. NORC at the University of Chicago, Chicago, Illinois, United States*

Physical activity has been described as the ‘ideal’ intervention for frailty. In fact, exercise is the most effective frailty mitigation intervention studied to date. Yet, frail older adults face great challenges to physical activity over time. These challenges include decreased physiologic capability, greater barriers, fewer resources, narrower availability, reduced self-efficacy, poorer outcome expectations and restricted payor coverage for long-term activity programming. In this presentation, we will review the challenges frail adults face to participating in the most promising frailty intervention. To do this, we will summarize barriers noted in prior frailty exercise trials and will also report findings from our local survey study assessing physical activity behavior change factors among non-frail, pre-frail and frail older adults (n=101, 93% African-American). We will also review the current restricted payment structure for community-based long-term care services like exercise programs. We will then describe a novel exercise intervention designed to address several of these unique challenges, currently undergoing efficacy testing in a randomized controlled trial. The EngAGE trial is a multisite randomized controlled trial designed to compare an exercise intervention delivered over a voice-activated device in the home (EngAGE) versus paper-based exercises in n=124 frail, homebound, African-American older adults. We will share the EngAGE trial design, methods, measures and preliminary study fundings. We will close by summarizing key remaining issues that need to be addressed to close the physical activity gap for frail adults and will offer ideas for future research directions. Trial registration: ClinicalTrials.gov NCT05337514

Abstract citation ID: igae098.1278

### EFFECTIVENESS OF A TECHNOLOGICAL PLATFORM TO MANAGE FRAILTY: RESULTS FROM THE POSITIVE AND INTEGRACAM STUDIES

Leocadio Rodriguez-Manas[1], Alba Maria Costa Grille[1], Irene Molina Hermosilla[1], Irene Alfonso Lopez[1], Rodrigo Perez-Rodriguez[2], Ignacio Peinado-Martinez[1], and Elena Villalba-Mora[3], *1. Hospital Universitario de Getafe, Getafe, Madrid, Spain*, *2. Universidad Rey Juan Carlos, Fuenlabrada, Madrid, Spain*, *3. Universidad Politecnica de Madrid, Pozuelo de Alarcon, Madrid, Spain*

Technologies can assist in managing frailty through home monitoring and intervention systems, though evidence of their clinical benefit is lacking. Our study had two components. A) A two-arm (Intervention vs. Control) RCT named POSITIVE (NCT04592146) with a 6-month follow-up, recruiting prefrail or frail people aged ≥70 years living at home from three European countries (N=83), and B) a validation study in usual clinical practice called INTEGRACAM, conducted in Madrid, Spain, under a quasi-experimental before-after design that mirrors the criteria of POSITIVE. Both components utilized a technological platform for comprehensive patient assessment and functional status monitoring, delivering personalized interventions including VIVIFRAIL physical exercise programs, nutritional recommendations, and medication adjustments according to STOPP-START criteria. Usability and user experience were measured using the System Usability Scale (SUS) and the User Experience Questionnaire (UEQ-S), alongside frailty (Fried criteria and FTS-5 score) and functional status (SPPB and WHODAS). Results from POSITIVE (average age 76.7 years; 76.8% female) indicated significant improvements after six months in frailty status (FTS-5: -5.43 points in the intervention group vs. usual care; p=0.008; Fried´s criteria-FFP: -0.4 in IG vs UC p=0.087), SPPB (+1.07 points; p= 0.022) and WHODAS (0.22, p= 0.09). INTEGRACAM showed similar findings with participants (n=51; mean age 78.1±5.3 years) demonstrating improvements in SPPB (+1.03±1.38; p-value <0.001), FTS5 (-3.4±4.11; p-value <0.001), and FFP (-0.4±1.03; p-value 0.028) after 6 months. Patients rated usability as 87/100, surpassing the optimal threshold value (68/100). Regarding the UEQ-S, hedonic quality improved throughout the project (2.44 vs 2.02, p=0.031), both in patients and professionals.

Abstract citation ID: igae098.1279

### THE POTENTIAL FOR MIND–BODY THERAPIES AS FRAILTY INTERVENTIONS

Julia Loewenthal, *Brigham and Women’s Hospital, Boston, Massachusetts, United States*

Strategies to prevent and manage frailty are priorities in clinical medicine and public health. Current evidence-based interventions for frailty are multimodal in nature, with physical activity as a key element. Mind-body therapies, especially those that incorporate movement such as yoga and tai chi, include physical postures, breathing practices, meditation, and other elements. In our recent systematic review of 33 randomized controlled trials of yoga interventions in older adults (2384 participants), there was moderate-certainty evidence that yoga improved gait speed and lower extremity strength when compared to inactive control groups. Both yoga and tai chi are known to improve physical function, even in already frail populations. Emerging evidence suggests they may modulate cellular and molecular hallmarks of aging, especially inflammation. Mind-body therapies have additional benefits for cognition, mood, and sleep. They likely confer resilience by positively impacting core systems for maintaining homeostasis, including musculoskeletal, metabolic, and stress-response systems. They are not only highly adaptable for older populations, but also increasingly popular: among US adults, use of yoga alone increased from 5% in 2012 to 15.8% in 2022. They may be delivered in a group format to reduce social isolation, and are increasingly accessible via telehealth, senior centers, and other community-based organizations. In addition, there is evidence that mind-body therapies are an entry point to other types of physical activity, especially for women. They are promising frailty interventions, though more work is needed to understand optimal dose, duration, and effectiveness.

### SESSION 3155 (SYMPOSIUM)

Abstract citation ID: igae098.1280

### AGING IN PLACE IN BRAZIL: SOME NEW EMPIRICAL EVIDENCE AND REMARKS ON THE ROLE OF ENVIRONMENTS FOR EDUCATION

Chair: Frank Oswald
Co-Chair: Johannes Doll
Discussant: Lycia Tramujas Vasconcellos Neumann

Aging in Place and the changing role of place for aging in modern societies has become an increasingly important topic both from an empirical and applied perspective. Studies have demonstrated effects of improving the environment, however, research on complex interactions of place, aging and education is scarce, although particularly a tradition of educational theory exists in Brazil. This symposium aims to address links between place, aging, and education in later life in Brazil for health and well-being. The first contribution by Frank Oswald aims to rethink place and aging relationships and to focus on enduring and novel challenges in this regard to provide an overview over selected theories in environmental gerontology and beyond. Second, Mariana Alves da Silva do Nascimento presents qualitative data from a study conducted in São Paulo with community residents on perceptions, preferences, and expectations of aging in place from an Architecture and Urban Planning perspective. The third contribution by Sérgio Luiz Valente Tomasini uses a participatory approach to shed light on planning of outdoor spaces (gardens) within Long-Term Care Facilities in Brazil, emphasizing that participatory methods can positively impact adaptive outcomes of older adults in institutional settings. Fourth, Johannes Doll and Estela Kohlrausch emphasize the significance of educational aspects for environmental gerontological perspectives on place and aging in societies that face globalization, urbanization, digitalization and climate change, which can help to better understand environment in a broader context by "reading the world" (Paulo Freire) differently. Finally, Lycia Tramujas Vasconcellos Neumann will serve as the session's discussant.

Abstract citation ID: igae098.1281

### RETHINKING THE PLACE OF PLACE FOR AGING: ENDURING AND NEW CHALLENGES FOR ENVIRONMENTAL GERONTOLOGY

Frank Oswald, *Goethe University Frankfurt, Frankfurt am Main, Hessen, Germany*

Environmental gerontology emphasizes the relationship between place and aging from a conceptual perspective while also aiming to empirically analyze person-environment (P-E) relationships to improve quality of life and health in later life. After decades of empirical research, a critical discussion of theorizing place and aging is needed to better address not only enduring but also new challenges of P-E processes in rapidly changing societies worldwide, including challenges of housing, mobility, accessibility and participation for older adults in the "third" and "fourth" age. This contribution aims to (1) rethink the place of place for aging in modern societies from different disciplines, (2) identify a set of enduring and novel issues of P-E relationships. This is underpinned by specific references to empirical findings from selected studies, often over-representing ageing in Western societies. An example of an enduring issue would be the essential understanding of how an aging individual interacts with the home environment. An example of a novel issue would be technology use in later life in an increasingly digitalized world. Moreover, the contribution aims to (3) provide an overview over selected theories addressing place and aging interrelations, and emphasizing fundamental concepts such as interaction, transaction, co-construction and co-constitution to understand P-E interrelations, and finally to (4) offer a synthesis of how theories can address enduring and novel challenges and identify open questions that remain.

Abstract citation ID: igae098.1282

### AGING AT HOME IN BRAZIL: COMMUNITY RESEARCH FROM AN ARCHITECTURAL PERSPECTIVE

Mariana Alves da Silva do Nascimento, *University of São Paulo, São Paulo, Sao Paulo, Brazil*

Environmental Gerontology has been an essential aspect of aging studies worldwide for years. However, its integration into fields like Architecture and Urban Planning is still limited in Brazil. Professionals who design urban spaces must consider how their decisions impact people's lives, especially the older population who often face challenges accessing the city's amenities. This presentation shares insights from a qualitative study conducted in São Paulo, Brazil with community residents aged 56 to 74 (N=23; mean age=64; 73% women). Using the Collective Subject Discourse (CSD) analysis technique to investigate their perceptions, preferences, and expectations of aging in place, participants highlighted the importance of features such as sidewalks, green spaces, subway lines, and accessible shops and services, to support daily activities and enable aging in place. Participants also expressed a desire for a range of activities and amenities tailored to older adults, reducing their reliance on relatives to fulfill their social needs. Furthermore, 39% of participants expressed a sense of attachment desiring to age in their current homes, indicating that home environments should also be continuously adapted to the wishes and needs of older individuals. This study contributes to the field by highlighting the importance of interdisciplinary education and of collaboration among professionals in aging, architecture, and urban planning to ensure the inclusion of older adults in the creation of age-inclusive environments for all.

Abstract citation ID: igae098.1283

### AGING IN LONG-TERM CARE FACILITIES IN BRAZIL: NEW CHALLENGES FOR INSTITUTIONAL ENVIRONMENTS

Sergio Luiz Tomasini, *Federal University of Rio Grande do Sul, Porto Alegre, Rio Grande do Sul, Brazil*

This contribution sheds light on the planning of outdoor spaces within Long-Term Care Facilities (LTCFs) for older person as a means to enhance the quality of life for their residents. It presents one of the few empirical studies conducted on this topic in Brazil to date. The study follows an action research approach, focusing on the participatory design of a garden for a LTCF situated in the southern region of the country. The project unfolded in three stages: 1) Initial interviews were conducted with residents to explore their utilization of the institution's external areas, their past experiences with gardens and their perceptions of the "ideal garden.". Additionally, systematic observations were made regarding how residents interacted with the open spaces within the LTCF; 2) Subsequent collaborative meetings aimed to actively involve residents in selecting the garden site and defining project requirements; 3) Finally, interviews were held with participating residents to assess the effectiveness of the project methodology. The analysis of these interviews utilized the meaning condensation method, leading to the identification of several themes related to the participatory project approach: a) possibility of creating a territory in the facility; b) opportunity to develop an active role in the facility; c) acquisition of new knowledge; d) promotion of social relations. Overall, the residents' feedback suggests that employing participatory planning methods can positively impact the adaptive outcomes of older adults in institutional settings.

Abstract citation ID: igae098.1284

**WHERE DOES EDUCATION COME INTO AGING IN PLACE IN BRAZIL TODAY?**

Estela Kohlrausch[1], and Johannes Doll[2], *1. Universidade do Rio Grande do Sul, Porto Alegre, Rio Grande do Sul, Brazil, 2. Federal University of Rio Grande do Sul, Porto Alegre, Rio Grande do Sul, Brazil*

Environmental Gerontology has involved a wide range of science, but until now, educational aspects are rarely found. In this paper, we argue that educational theory could give important contributions to the understanding and development of the aging person-environment-relation. There are three fundamental contributions: A) In Environmental Gerontology, there is a lot of thinking about adaptation, stimulation, and orientation processes in dealing with changes in the environment or changes in the person during aging process. These processes can be understood as learning processes, and Education Science could contribute by appointing conditions and obstacles for those processes, as for example Peter Jarvis' existential learning theory for adults. B) Educational theory points out that learning processes happens in social and cultural contexts by social interaction, as Paulo Freire stated: Nobody educates anyone, nobody educates themselves, men (and women) educate each other, mediated by the world. C) Educational processes are intentional, by respecting the other in a dialogical relation. This might be an interesting form for gerontological intervention and is in line with actual discussion about participatory approaches in aging research. Within the major changes in society such as globalization, urbanization, digitalization and climate change, a critical educational perspective, as for example Paulo Freire's liberation pedagogy can help to understand environment in a broader context, not only to guarantee safety and comfort for older people, but by "reading the world" (Paulo Freire) people can start to get aware of one´s own role within the larger context of community, society and the human world.

### SESSION 3160 (BIOLOGICAL SCIENCES INVITED SYMPOSIUM)

Abstract citation ID: igae098.1285

**IMMUNOLOGICAL ASPECTS OF AGING**

Chair: Matt Yousefzadeh

Aging is an intricate and multifaceted phenomenon that impacts every aspect of our bodies, including the immune system. As we age, there's a gradual decline in immune function known as immunosenescence, which reduces the body's ability to combat pathogens effectively. Moreover, this decline in immune function plays a pivotal role in the development of various age-related chronic conditions. One significant contributor to aging is inflammaging or the production of low-grade chronic inflammation that occurs in the absence of pathogens that can drive aging. Despite significant research efforts, the intricate mechanisms behind immunosenescence and the communication of aging between the immune system and other bodily systems remain largely elusive. The aim of the symposium is to delve into recent breakthroughs in this field across different levels of research, spanning from basic science, translational studies, and potential therapeutic interventions.

Abstract citation ID: igae098.1286

**PD1 BLOCKADE IMPROVES SURVIVAL AND CD8+ CYTOTOXIC CAPACITY, WITHOUT INCREASING INFLAMMATION, DURING NORMAL MICROBIAL EXPERIENCE IN OLD MICE**

Christina Camell, *Institute on the Biology of Aging and Metabolism, University of Minnesota, Province, Minnesota, United States*

The elderly and those with chronic diseases have a 2 to 10-fold increased risk of hospitalization and mortality following infection as compared with healthy adults. A significant contributor to disease severity is the aging of the immune system, including an expansion of exhausted T cells. These cells simultaneously promote excessive levels of inflammation and display impaired T cell activation and function. How exhausted T cells from aged individuals respond to infection and whether acute restoration of T cell activation further exacerbates the inflammatory environment or generates functional T cells is unclear. Previously, we used a mouse model of normal microbial experience (NME), where old specific pathogen-free (SPF) mice show 100% mortality following exposure to multiple microbes ordinarily found in pet store mice. In the present study, we show that when exposed to NME, old SPF mice have increased expression of multiple inflammatory pathways and exhibit elevated frequencies of a heterogenous exhausted CD8+ T cell pool that expresses differing combinations of the inhibitory receptor programmed cell death protein 1 (PD1), TOX, and CXCR5. Pre-treatment or intervention with an anti-PD1 monoclonal blocking antibody during the exposure of old SPF mice to NME significantly improves both antibody production and survival, without altering the acute inflammatory response. Anti-PD1 checkpoint blockade-mediated survival is dependent on CD8+, but not CD4+ T cells. Correspondingly, CD8+ PD1+ T cells from old mice given anti-PD1 monoclonal antibody have increased granzyme B production. These data reveal a new approach for reducing vulnerability to infections in the elderly by targeting CD8+ T cell exhaustion through PD1 checkpoint blockade immunotherapy.

Abstract citation ID: igae098.1287

**PROPHYLACTIC AND LONG-LASTING THERAPEUTIC EFFICACY OF SENOLYTIC CAR T CELLS AGAINST AGE-RELATED PHENOTYPES**

Corina Amor, *Cold Spring Harbor Laboratory, Cold Spring Harbor, New York, United States*

Senescent cells accumulate in organisms over their lifespan and play a key role in age-related tissue decline and the development of chronic aging pathologies. Thus, effective strategies to eliminate senescent cells (senolytics) could have broad therapeutic implications. In a departure from conventional chemical approaches we developed the first cell-based senolytic therapy based on chimeric antigen receptor (CAR) T cells targeting uPAR, a cell-surface protein upregulated on senescent cells. Our initial proof of concept showed their efficiency in young animal models of liver fibrosis and cancer. We now show that uPAR-positive senescent cells accumulate during physiological aging and characterize their cell types and expression profiles. Importantly, we find that they can be safely targeted with senolytic CAR T cells in aged animals where they result in significant improvements in both tissue regeneration and metabolic function. Of note, we find that the beneficial effects of senolytic CAR T cells are long lasting; single administration of a low dose is sufficient to safely achieve long-term therapeutic and preventive effects in healthspan.

Abstract citation ID: igae098.1288

**MOLECULAR DETERMINANTS OF POOR T CELL MEMORY DURABILITY IN OLDER ADULTS**

Ines Sturmlechner[1], Abhinav Jain[2], and Jorg Goronzy[2], *1. Department of Immunology, Mayo Clinic, Rochester, Minnesota, United States*, *2. Mayo Clinic*

Generation of a protective and long-lasting immune memory is central to effective vaccination strategies and healthy aging. However, age-associated changes in immune cells, particularly T cells, impair immune memory generation and maintenance rendering older adults vulnerable to infectious diseases. Here, we investigated signatures of age-related memory T cell dysfunction and uncovered molecular determinants of a protective, persistent T cell memory in older individuals. We leveraged the contrasting T cell memory elicited in younger (<30 years) and older adults (>55 years) by vaccination against varicella zoster virus (VZV). Using ex vivo peptide stimulation of peripheral blood mononuclear cells of vaccine recipients several years after vaccination, we collected antigen-specific CD4+ and CD8+ T cells for high-dimensional spectral flow cytometry and trimodal single-cell sequencing analyses. We found that older vaccine recipients predominantly suffer from a quantitatively and qualitatively impaired CD8+ T cell memory response compared to younger adults receiving the same vaccine type. Conversely, comparisons of two VZV vaccine strategies with contrasting efficacies in older adults demonstrated that a CD4+ T cell response rich in self-renewing Th17-like and low in regulatory T cells overcomes the age-associated T cell memory defect and associates with a durable, protective T cell memory. Collectively, we propose that vaccine strategies targeted towards older adults benefit from a Th17-focused CD4+ T cell memory response. Our results not only uncover traits of immune cell dysfunction with aging, but also inform vaccination efforts tailored towards healthy aging.

Abstract citation ID: igae098.1289

**INVESTIGATION OF TRANSCRIPTIONAL CHANGES IN IMMUNE CELL POPULATIONS OF MICE TREATED WITH RAPAMYCIN**

Matt Yousefzadeh, *Columbia University Medical Center, New York, New York, United States*

Aging is the greatest risk factor for many chronic degenerative diseases, for example osteoarthritis, dementia and heart disease. This robs individuals of quality of life and is very costly to treat. This has led to great interest in finding rational strategies for extending health span, the years of disease-free life at the end of one's lifespan. Theoretically, if we could treat aging itself, all of the diseases associated with old age would be prevented, delayed or attenuated simultaneously. Thus, discovering fundamental mechanisms of aging is a priority. Aging is generally thought to be driven by an accumulation of damage in cells over time. The progressive decline of immune function with age, known as immunosenescence, plays a pivot role in not only the aging process but also susceptibility to infections and reduced vaccine responses. Thus it is critical to find interventions to potentially correct the age-related deficits in immune function. The potent mTOR inhibitor rapamycin has been shown to extend lifespan in mice and reduce age-related pathologies. Over the past decade the use of rapamycin or analogs of rapamycin have been investigated for its ability to rejuvenate aged immune systems. Treatment of elderly human participants with a rapalog was able to significantly enhance antibody titer levels to the annual influenza vaccination as well as reduce immune exhaustion markers. I was able to recapitulate some of these findings in a mouse model of immune aging. However, which immune cell populations are benefited from rapamycin treatment remains to be seen. My working hypothesis is that rapamycin is able to restore function to aged T cells and possibly other immune cell types. In order to test this hypothesis 18-month-old WT C57BL/6 mice were placed onto chow containing encapsulated rapamycin (126 ppm) or control chow for 7 months. Spleens were harvested from these mice and 5-month-old young control mice (n=3-4 per sex per group). Splenic samples are currently undergoing single-nuclei RNA sequencing to investigate the effect of rapamycin on gene expression changes in different immune cell populations of the spleen and address the following questions: Question 1: What are the differences in gene expression profiles in the different immune cell populations between young versus old splenic samples? Question 2: Does treatment with rapamycin in old mice restore a more "youthful" expression profile to all immune cell types or just a subset of them? Question 3: Are there sex-based differences in the gene expression patterns of aged immune cells or in response to rapamycin treatment?

### SESSION 3165 (PAPER)

### CAREGIVING NETWORKS AND RELATIONSHIP DYNAMICS

Abstract citation ID: igae098.1290

**PREDICTING OLDER ADULTS' RESIDENTIAL CARE RELOCATION RISKS BASED ON CAREGIVER NETWORK CHARACTERISTICS**

Natasha Nemmers[1], Wenhua Lai[1], Srabani Haldar[1], Sophia Tsuker[1], Vicki Freedman[2], and Amanda Leggett[1], *1. Wayne State University, Detroit, Michigan, United States*, *2. University of Michigan, Ann Arbor, Michigan, United States*

When older adults face increasing care needs or limited support, remaining safely and comfortably at home becomes challenging. Extant research has primarily concentrated on characteristics of the older adult or their primary caregiver on nursing home admission. This study examines the risk of older adults transitioning to residential care (e.g., assisted living, nursing home), focusing on the influence of their caregiver network or involvement of multiple caregivers. Using the National Health and Aging Trends Study, we conducted competing risk regressions following 7,085 community-dwelling older adults moving to residential care, accounting for death, across rounds 1-9 (2011 – 2019). We assessed network composition, size, shared tasks, and the number of in-network specialists or generalists, while controlling for individual sociodemographic and health factors. Individuals with caregiver networks that shared medical tasks had the highest risk of moving, followed by those sharing household tasks. Conversely, shared mobility or self-care and transportation responsibilities were associated with lower risks. Having more generalists increased the risk, while any number of specialists was non-significant. Larger networks were associated with heightened risk, though having close family like a spouse was protective. The findings underscore that caregiver network characteristics are critical to older adults' ability to age in place. Specifically, older adults with larger caregiver networks, lacking a spouse or child, and providing complex care are at greater risk for relocating. Understanding caregiver networks can guide interventions related to care network coordination and resource allocation to help avoid or postpone a residential care move.

Abstract citation ID: igae098.1291

### WHEN YOUR CAREGIVER IS ALSO YOUR CONFIDANT: CAREGIVER FUNCTIONS AND THE QUALITY OF CARE RECEIVED BY OLDER ADULTS

Yongxin Shang[1], and Sarah Patterson[2], *1. Cornell University, Ithaca, New York, United States*, *2. University of Michigan, Ann Arbor, Michigan, United States*

Older adults can rely on multiple caregivers for support in later life and the characteristics of caregivers are closely related to the care received by older adults. This study aims to explore the association between caregiver functions and quality of care, asking if having a caregiver who also functions as a confidant can improve the care received by older adults. Using the baseline data from the National Health and Aging Trends Study (NHATS) in 2011, we construct an analytic sample of Medicare beneficiaries aged 65 or above with at least one caregiver to help with self-care (eating, bathing, toileting, dressing), mobility (going outside or inside, getting out of bed), or household activities (laundry, shopping, preparing meals, banking, medications) due to health or functioning reasons in the last month (N = 1,875). About 76% of these older adults identify at least one of their caregivers as their confidants, with whom they can discuss important things in the last year. OLS regressions on the care hours (log-transformed) suggest that older adults with at least one caregiver as a confidant are significantly more likely to receive more hours of care ($p < 0.001$). Logistic regressions on the binary outcomes of reporting any unmet care needs show that having a caregiver as a confidant is associated with a lower likelihood of having unmet care needs in household activities ($p < 0.05$), but not in self-care or mobility. These findings demonstrate the potential benefits of having a close tie as a caregiver in later life.

Abstract citation ID: igae098.1292

### RELATIONSHIP CHANGES ACROSS THE CAREGIVING TRANSITION

Raquael Joiner[1], Niccole Nelson[2], Qimin Liu[3], and Stacey Schepens Niemiec[1], *1. University of Southern California, Los Angeles, California, United States*, *2. Colorado State University, Fort Collins, Colorado, United States*, *3. Boston University, Boston, Massachusetts, United States*

The majority of aging individuals' care needs are met by family caregivers (CGs), often at great costs to CGs' well-being. There is, however, significant heterogeneity in CG outcomes, and social capital, or the quality of marital, family, and friend relationships, is widely recognized as an im-portant factor underlying individual differences in CG outcomes. Because empirical work to date largely focuses on either cross-sectional differences or changes in social capital post-caregiving on-set, it is unclear, however, whether social capital disparities across different populations are a func-tion of the transition to CG or whether such disparities exist prior to taking on a CG role. Thus, we used data from the Health and Retirement Study (N=366; 8-waves) and Bayesian multi-phase latent growth curve models to examine social capital changes across the caregiving transition. Results sug-gested that CGs, on average, do not show significant changes in their social capital across the transi-tion to caregiving. Yet, there were significant individual differences, and individual differences in pre-caregiving social capital were significantly associated with social capital changes following tak-ing on a caregiving role: CGs with higher initial levels of social capital showed less declines in rela-tionship quality leading up to caregiving onset, but steeper declines in relationship quality at caregiv-ing onset and smaller relationship quality increases in the years following caregiving onset. This suggests that the social capital of individuals with initially satisfying relationships are most impacted by caregiving. We discuss the implications of these findings for refining and developing personal-ized social support interventions for CG populations.

Abstract citation ID: igae098.1293

### BALANCING HEALTH AND CAREGIVING: ADULT CHILDREN'S HEALTH PROBLEMS IMPACT ON CAREGIVING

Catherine Stepniak[1], J. Jill Suitor[2], and Megan Gilligan[3], *1. University of Lynchburg, Lynchburg, Virginia, United States*, *2. Purdue University, West Lafayette, Indiana, United States*, *3. University of Missouri, Columbia, Missouri, United States*

When older mothers face chronic or acute health problems their adult children often become caregivers, serving as either as the primary or secondary sources of informal care. However, when adult children are facing their own serious health problems, their ability to serve as caregivers may be limited. Surprisingly, the literature provides no clear insight into whether children's health affects their provision of care to their mothers. In the present study, we shed new light on this question using quantitative and qualitative data collected from 412 adult children nested within 224 families as part of the Within-Family Differences Study. Quantitative analyses showed that adult children with health problems were as likely as their healthy siblings to serve as their mothers' primary and secondary caregivers. Consistent with the literature, daughters were substantially more likely than sons to serve as caregivers. Qualitative analyses revealed that this gendered pattern occurred even in families in which daughters had serious health problems and sons were healthy. Daughters' explanations for becoming caregivers despite their health limitations indicated that they had internalized messaging that they were the "naturally better" caregivers compared to their brothers. Adult children caregivers reported higher level of caregiver burden than that reported by healthy caregivers as they shouldered joint responsibility for their own and their mothers' care. These findings underscore the ways in which gendered expectations for care and health problems across generations intersect to increase the risk of caregiver burden and unmet care needs for members of both generations.

Abstract citation ID: igae098.1294

### CHILDREN'S RESIDENTIAL PROXIMITY, SPOUSAL PRESENCE, AND MODIFIABLE RISK FACTORS FOR DEMENTIA

Zhuoer Lin[1], Xuecheng Yin[2], Becca Levy[3], Yue Yuan[4], and Xi Chen[5], *1. University of Illinois Chicago, Chicago, Illinois, United States*, *2. Oklahoma State University, Stillwater, Oklahoma, United States*, *3. Yale University School of Public Health, New Haven, Connecticut, United States*, *4. Lehigh University, Bethlehem, Pennsylvania, United States*, *5. Yale University, New Haven, Connecticut, United States*

**Objectives:** Cognitive impairment poses considerable challenges among older adults, with the role of family support becoming increasingly crucial. This study examines the association of children's residential proximity and spousal presence with key modifiable risk factors for dementia in cognitively impaired older adults.

**Methods:** We analyzed 14,731 persons (35,840 person-waves) aged 50 and older with cognitive impairment from the Health and Retirement Study (1995-2018). Family support was categorized by spousal presence and children's residential proximity. Modifiable risk factors, including smoking, depressive symptoms, and social isolation, were assessed. Associations between family support and the modifiable risk factors were determined using mixed-effects logistic regressions.

**Results:** A significant proportion of older adults with cognitive impairment lacked access to family support, with either no spouse (47.0%) or all children living over 10 miles away (25.2%). Those with less available family support, characterized by distant-residing children and the absence of a spouse, had a significantly higher prevalence of smoking, depressive symptoms, and social isolation. Moreover, we revealed a consistent gradient in the prevalence of the risk factors by the degree of family support. Relative to older adults with a spouse and co-resident children, those without a spouse and with all children residing further than 10 miles displayed the highest prevalence of the risk factors. These findings were robust to various sensitivity analyses. Conclusions: Family support from spouses and nearby children serves as a protective factor against modifiable dementia risk factors in cognitively impaired older adults. Strengthening family and social support is paramount for this vulnerable population.

### SESSION 3170 (SYMPOSIUM)

Abstract citation ID: igae098.1295

### COMMUNITY ENGAGEMENT IN DIFFERENT NATIONAL CONTEXTS: BUILDING BRIDGES AND AGE-FRIENDLY ENVIRONMENTS

Chair: Oskar Jonsson
Co-Chair: Peggy Chi
Discussant: Ian Johnson

To bridge the gap between research and practice, initiatives to facilitate knowledge mobilization in tandem with community engagement have become increasingly important. This symposium will advance the understanding about knowledge mobilization processes and community engagement efforts by offering insight from scholars from established research centers with long-standing community engagement as well as new initiatives connecting disparate disciplines and sectors. Representing different national contexts, the speakers will share their experiences regarding the complexities of partnership and engagement approaches, processes, and activities. Benefits (research quality, relevance, utilization, transgressing boundaries, addressing complex problems) as well as barriers (diverse conditions/cultures, lack of time/resources, multiple levels of terminology, hard-to-reach groups, projectification, boundaries of traditional science systems) will be addressed. The first speaker will describe the development and establishment of a pool of interested parties in Sweden to systematize and facilitate communication, entryways to research studies, and user involvement in aging research. The second speaker will describe the work of a center serving older African Americans from Detroit, Michigan, including innovative leadership opportunities created for its members. The third speaker will describe new initiatives in the Canadian context that purposefully choreograph knowledge mobilization activities to disrupt disciplinary and industry silos in the practice and research of salutogenic/healthy long-term care environments. The last speaker will describe an engagement effort towards age-friendly infrastructures in rural municipalities in Canada. Finally, discussant Ian Johnson from the University of Texas San Antonio will identify, compare, and discuss underlying themes, commonalities, differences, and lessons learned generated from the presentations.

Abstract citation ID: igae098.1296

### A POOL OF INTERESTED PARTIES: COMMUNICATION, ENTRYWAYS, AND USER INVOLVEMENT IN AGING RESEARCH IN A SWEDISH CONTEXT

Oskar Jonsson, Susanne Iwarsson, and Steven Schmidt, *Lund University, Lund, Skane Lan, Sweden*

Established in 2007, the Centre for Ageing and Supportive Environments (CASE) is an interdisciplinary research environment at Lund University, Sweden. The center's activities are led by a steering committee in close collaboration with the CASE User Council, which provides input, ideas and poses research questions for various research projects. Between 2018-2024, CASE was responsible for one of Lund University's thematic collaboration initiatives – Social Rights and Housing for the Ageing Population, which is a partnership for knowledge mobilization contributing to solutions for key societal challenges. As one forward-looking way to prolong the established initiative with a long-term ambition, we have launched a national registry, which will be used as a pool of interested parties with the aim to raise awareness about aging research and the possibility to engage in research, mobilize knowledge and utilize research results. The approach is to offer people with an interest in research on aging and health to register in a nationwide online person register/database to systematize and facilitate communication, entryways to research studies, and user involvement. The development of the pool has been fraught with issues related to terminology, ethics, legal requirements for handling personal data, and providing necessary information without being overwhelming but instead attractive for the diverse individuals and groups within the civil society, business, and public authorities that we target. This presentation will provide an overview of the multiple strategies and tactics we employ to tailor the information flow and interactive process based on individual and group-specific needs and preferences.

Abstract citation ID: igae098.1297

### LEADERS AND CONSULTANTS FROM AN AGING CENTER IN MICHIGAN: PRODUCTIVE AND TAILORED ENGAGEMENT WITH AFRICAN AMERICANS

Tam Perry[1], Wilma Stringer[2], Cynthia Howell[3], Vanessa Rorai[2], Amanda Horn[4], Jamie Mitchell[5], and Kent Key[6], *1. Wayne State University, Detroit, Michigan, United States*, *2. Healthier Black Elders Center, Detroit, Michigan, United States*, *3. Flint Healthier Black Elders Center, Flint, Michigan, United States*, *4. Detroit Healthier Black Elders Center, Southgate, Michigan, United States*, *5. University of Michigan, Ann Arbor, Michigan, United States*, *6. Michigan State University East Lansing, East Lansing, Michigan, United States*

The Healthier Black Elders Center (HBEC) is the community outreach core for the Michigan Center for Urban African American Aging Research. Established in 1997, this center offers health education to older adults in Detroit and expanded to Flint, Michigan in 2021 and maintains a research registry of over 1100 older Detroiters and over 200 older Flint residents. HBEC Detroit launched a consulting program in 2020 with the Community Advisory Board (CAB) members to offer personal and professional perspectives regarding research. Through this program, CAB members have 1) disseminated knowledge about community engaged research approaches 2) offered feedback on comprehensibility and ease of use to individual researchers on instrument content and research design as well as 3) offered insights beyond the academy (technology and philanthropic sectors). This program has created opportunities for older Black adults to provide direct feedback on product, funding initiatives and research design. This presentation will provide an overview of the initiatives, recruitment, and retention strategies of a long-established center. This presentation will showcase the Consulting program as a way to engage leaders in its center's work as well as provide learning and growth for its Detroit members. This presentation will also highlight the process of tailoring, adapting, and launching the HBEC program for the Flint community.

Abstract citation ID: igae098.1298

### BUILDING COMMUNITY IN PRACTICE AND RESEARCH OF HEALTHY LONG-TERM CARE ENVIRONMENTS

Peggy Chi, Sarah Hunter, and Whitney Berta, *University of Toronto, Toronto, Ontario, Canada*

This presentation will describe an initiative based at the Institute of Health Policy, Management and Evaluation in Toronto, Canada, that aims to dismantle silos and build a community in the practice and research of aging environments. The goals and objectives are to share, synthesize, advance, and mobilize knowledge on long-term care homes—the influence of living environments on older adults and working environments on workers and their work. Three knowledge mobilization activities are the means by which our aims are achieved: 1) A series of seminars are curated for experts to disseminate knowledge to a broad audience (industry and academic leaders, decision-makers, and end-users) to connect research to practice. 2) Informal, practical discussions are created for practitioners to share current key challenges in long-term care homes with researchers to connect practice to research. 3) A Design Charrette is organized to translate knowledge to address key challenges within interdisciplinary teams comprising practitioners/ care providers, team leads, and trainees. These activities bring together individuals from industry and academia who represent diverse disciplines that have generated knowledge about these linkages based on different and complementary conceptualizations of long-term care environments but for whom there is no pre-existing forum in which to engage in integrative, synthetic discussion and application. The open-forum nature of the activities is intended to foment public discourse on the role of physical and psychosocial environments in the health of older adults and workers in long-term care homes and to demonstrate the value of evidence-based design founded on multidisciplinary knowledge.

Abstract citation ID: igae098.1299

### MOBILIZING UNTAPPED LIVED EXPERTISE TO ASSESS ACCESSIBLE AND AGE-FRIENDLY INFRASTRUCTURES

Mikiko Terashima[1], Kate Clark[2], and Katherine Deturbide[2], *1. Dalhousie University, Halifax, Nova Scotia, Canada*, *2. Planning for Equity, Accessibility, and Community Health (PEACH) Research Unit, School of Planning, Faculty of Architecture and Planning, Dalhousie University, Halifax, Nova Scotia, Canada*

Many rural municipalities in Canada have been experiencing steady population declines overall, while vulnerable population groups such as older adults and persons with disabilities tend to remain in their communities. With shrinking tax bases, municipalities must make smarter choices in provisions of accessible and age-friendly services and amenities. However, exact conditions and needs of accessible and age-friendly infrastructures in rural communities are increasingly becoming difficult to evaluate, as their planning departments do not have sufficient human and financial capacities to monitor them. The Planning for Equity, Accessibility, and Community Health (PEACH) Research Unit is situated in a planning school at a university in Nova Scotia, Canada. In partnership with two rural municipalities, PEACH Research Unit conducted in-person and online consultations with persons with lived expertise to develop key indicators that can inform municipalities on priority accessible and age-friendly infrastructure needs. This presentation will highlight lessons learned from our engagement processes, including considerations for accessibility and inclusive recruitment, engagement venues, and communication methods that maximize participation of a hard-to-reach group in rural communities. An example tool especially useful for our purpose was an online mapping platform, which helped the participants articulate their insights and better share stories of their experiences. It facilitated discussion beyond simply inventorying what types of infrastructure matter to them—while also soliciting their suggestions on solutions to the design of specific sites in real settings, which were often relational to surrounding environments.

### SESSION 3175 (PAPER)

### COMPLEXITIES OF DEMENTIA CARE AND PLANNING

Abstract citation ID: igae098.1300

### QUALITATIVE STUDY IDENTIFIES DEMENTIA TRAINING NEEDS OF CARE WORKERS IN ASSISTED LIVING AND PERSONAL CARE AGENCIES

Troy Andersen[1], Daniel Kaplan[2], Norman Foster[1], Jeanine Stefanucci[1], Chad Moffitt[1], Andrea Dremann[3], and Bonnie Bechek[3], *1. University of Utah, Salt Lake City, Utah, United States*, *2. Adelphi University, Garden City, New York, United States*, *3. ProActive Memory Services, Inc., Salt Lake City, Utah, United States*

Caring for individuals with dementia can be overwhelming, especially when direct care workers lack knowledge and skills to respond to changing needs of those with dementia. Burnout and turnover are common problems. To better understand wishes for dementia training, we interviewed direct care workers, supervisors and administrators in 4 assisted living facilities (ALFs) and personal care agencies (PCAs). Funded by the NIA, we collected data from 23 direct care workers, 7 supervisors, 8 administrators. Questions focused on needs for dementia training topics and solutions for delivering content. Interviews were recorded, transcribed, and evaluated by two independent raters to identify all topics discussed. These topics were then further analyzed by a second group of raters who specialize in training of direct care providers. Commonalities and thematic differences across direct care workers, supervisors, and administrators were reviewed qualitatively to identify highest priorities. For both ALFs and PCAs, priority topics for training overlapped between administrators, supervisors, and direct care workers and included skill development (e.g., de-escalation strategies), knowledge areas (e.g., bathing, distress), and workforce goals (e.g., training certifications). Direct care workers further mentioned that training should be interactive, fit into their work schedules, and give solutions in real time. PCAs added that greater effort is needed to assure that training is relevant (e.g., current topics often are too pedantic and not practical to daily activities). Real-time, flexible, content meeting identified needs, and individualized to caregiving settings, learner knowledge and experience should be the focus of future improvements in dementia training.

Abstract citation ID: igae098.1301

### ADVANCE CARE PLANNING IN DEMENTIA VERSUS NORMAL COGNITION: A DEMOGRAPHIC AND PSYCHOSOCIAL STUDY

Zahra Rahemi[1], Juanita Bacsu[2], and Swann Adams[3], *1. Clemson University, Clemson, South Carolina, United States*, *2. Thompson Rivers University, Kamloops, BC Canada, British Columbia, Canada*, *3. University of South Carolina, Columbia, South Carolina, United States*

Older adults from minority groups often face higher rates of chronic diseases and cognitive impairment, along with lower rates of advance care planning and comfort care at end-of-life. This study aimed to explore patterns of advance care planning among older adults in the U.S., focusing on various demographic and psychosocial factors and cognition levels. We utilized data from the 2018 Health and Retirement Study to investigate the association between these factors and the likelihood of having a living will, a durable power of attorney for healthcare (DPOAH), or both, stratified by cognition (dementia/impaired cognition vs. normal cognition). Variables such as race, ethnicity, rurality, marital status, gender, education, age, and discrimination were included in the models. Among the 17,698 respondents, 71.6% exhibited normal cognition, while 28.4% had been diagnosed with dementia or impaired cognition. In both cognition groups, Black and Hispanic participants, as well as younger individuals with lower educational attainment, were less likely to have established a living will. Hispanic participants and younger individuals with lower educational attainment were also less likely to have a DPOAH and both a DPOAH and living will. Significant predictors of having a living will, DPOAH, or both that were not consistent among groups included rurality, marital status, and experiences of everyday discrimination. These findings underscore the importance of addressing disparities in advance care planning among older adults with diverse identities and levels of cognition. Understanding these differences is crucial for designing advance care planning for a diverse aging population facing health and cognitive decline.

Abstract citation ID: igae098.1302

### ADAPTING A VIRTUAL MEDICAL PLANNING PROGRAM FOR CARE PARTNERS OF PERSONS WITH COGNITIVE IMPAIRMENT

Jasmine Santoyo-Olsson, Deborah Barnes, Brookelle Li, Angelica Martinez, and Rebecca Sudore, *University of California San Francisco, San Francisco, California, United States*

Care partners want to help persons with cognitive impairment (PCI) engage in advance care planning (ACP) before they lose decision-making capacity, but report needing more support and access to virtual information. We created the video-based PREPARE for THEIR Care website to support care partners in helping others with their medical planning. However, PREPARE for THEIR Care has not yet been adapted for online, facilitated, synchronous classes to allow participants to ask facilitators ACP-related questions. We conducted virtual semi-structured interviews with experienced virtual group facilitators (n=4) and care partners of PCI (n=10) recruited from a research study of online exercise group classes for PCI and care partners. We used the Capability, Opportunity, Motivation, Behavior (COM-B) framework to explore barriers, enablers, and implementation recommendations and to guide qualitative thematic analysis. All facilitators were women. Care partner mean age was 62.3 (SD=13.8) years and 70% were women. Barriers included ACP complexity; virtual facilitation complexity; complicated dyad relationships; technology limitations; pessimism; fear of death; and lack of readiness. Enablers included ACP, technology, and teaching experience; participating from home; peer support; normalizing and modeling ACP; and belief that ACP is impactful. Implementation recommendations included providing staff trainings/scripts; care partner technology support before and during sessions; easy-to-use written materials; interactive group activities; and facilitating PREPARE for THEIR Care over multiple short (30-60 minute) sessions with > 50% of the session for discussion. Future ACP interventions should include training for facilitators, easy-to-use ACP materials, technology support, and modelling of ACP in virtual sessions.

Abstract citation ID: igae098.1303

### UNDERSTANDING CAREGIVER BURDEN IN DECISION-MAKING IN ALZHEIMER'S DISEASE AND RELATED DEMENTIAS

Brandi Ginn[1], Ignacia Arteaga[1], Alma Hernández de Jesús[1], Corey Abramson[2], and Daniel Dohan[1], *1. University of California San Francisco, San Francisco, California, United States*, *2. Rice University, Tucson, Arizona, United States*

Alzheimer's disease and related dementias (ADRD) are chronic conditions that involve changes or loss of cognitive and social function. People living with dementia (PLWD) undergo a gradual expansion in caregiving needs, and many ultimately spend a portion of their lives in institutional settings, including nursing homes or assisted living facilities. This study uses a comparative ethnographic approach to examine how PLWD and their caregivers make sense of and navigate transitions from home to institutional care. It combines three years of detailed field observations and 84 in-depth interviews in California and Maine. Our data illustrate how socioeconomic inequality, disparate neighborhood contexts, and shared understandings of aging shape tensions caregivers experience as they navigate whether, how, and when to relocate PLWD for whom they care. Our results show cultural narratives encouraging independence and non-institutionalization of older adults intersect with caregivers' desires, structural positions, and local institutional resources – creating tensions and are mentioned alongside the feeling of being burdened. These experiences and feelings emerge gradually as the health of PLWD declines and caregivers become aware of unmet needs and their own resource constraints. We unpack how diverse caregivers make sense of the burden they feel, define the thresholds to make decisions, map community resources, and experience a range of emotions such as frustration, guilt, and grief throughout the process. This contextualized analysis of how caregivers navigate ADRD decision-making offers insights into the affective nature of the process and the caregivers' fortitude.

Abstract citation ID: igae098.1304

### USING COMMUNITY-BASED PARTNERSHIPS TO SUPPORT ALZHEIMER'S DISEASE AND RELATED DEMENTIAS PROGRAMS

Samantha Cotton[1], and Monica Long[2], *1. University of Louisville, Louisville, Kentucky, United States*, *2. University of Chicago Medicine/SHARE Network, Glenwood, Illinois, United States*

Launched in 2015, the University of Chicago's SHARE Network and the University of Louisville's Geriatric Workforce Enhancement Program (GWEP) showcase transformative approaches to aging and dementia care through dynamic community and state partnerships. SHARE Network addresses the needs of Chicago's South Side, aiming to mitigate health disparities through collaborations that have led to the development of resources like memory cafes and caregiver support groups. This initiative enriches healthy aging and dementia education among healthcare professionals and community organizations.Similarly, the University of Louisville's GWEP focuses on ensuring accessible services via collaborations with Area Agencies on Aging, senior centers, and other service organizations, alongside strategic partnerships with Kentucky's health departments. These efforts underscore the development of career pathways for Community Health Workers and Certified Nursing Assistants, supporting workforce development and enhancing dementia care. In this presentation, we will delve into the critical need for community partnerships in dementia care, highlighting how the SHARE Network and the University of Louisville's GWEP have successfully harnessed these relationships to improve care for those affected by dementia. We will also explore the challenges and barriers encountered in implementing these programs and discuss strategies to overcome them. By sharing insights on engaging diverse stakeholders, navigating systemic obstacles, and leveraging resources for maximum impact, the presentation aims to provide a comprehensive overview of how to strengthen dementia care through effective community collaboration, ultimately leading to improved outcomes for individuals with dementia and their caregivers.

### SESSION 3180 (SYMPOSIUM)

Abstract citation ID: igae098.1305

### DISCRIMINATION, STRESS, AND AGING ACROSS THE LIFECOURSE

Chair: Roland Thorpe
Discussant: Carl V. Hill

Stress and discrimination are posited as two explanations for understanding why race disparities in health and aging across the life course remain elusive. This symposium contains papers seeking to address the impact of discrimination or stress on Black health or health disparities across the life course. First, Thierry and colleagues examined how lifetime exposure to structural stressors and current perceived neighborhood stressors are associated with cognitive function in Black adults in the HRS. They found that life course exposure to multiple domains of neighborhood-based stressors may be important for cognitive function in later life. Second, Cobb and colleagues hypothesized that self-reported instances of everyday discrimination are related to higher levels of cardiometabolic risk among older Black and White adults. The relationship between self-reported instances of everyday discrimination and cardiometabolic risk was significantly weaker for older Black adults than older White adults. Third, Mitchell and colleagues investigated how exposure to chronic stressors during mid-life indirectly affects cognitive function through changes in physiological dysregulation in HRS. Adjusting for age, gender, race/ethnicity and education fully accounted for the significant effects of financial strain and discrimination on changes in cognition. Fourth, Forrester and colleagues examined racial/ethnic differences in the association between self-reported discrimination and brain imaging in the MESA study. Findings suggest that instances of racial discrimination over a lifetime may be detrimental to brain health for Black adults. This collection of papers provides insights into how discrimination or stress impacts Black health or health disparities in middle to late life.

Abstract citation ID: igae098.1306

**STRUCTURAL STRESSORS, PERCEIVED NEIGHBORHOOD CONTEXT, AND COGNITIVE FUNCTION IN BLACK MIDLIFE AND OLDER ADULTS**

Amy Thierry[1], Kazumi Tsuchiya[2], and Harry Taylor[2], *1. Xavier University of Louisiana, New Orleans, Louisiana, United States*, *2. University of Toronto, Toronto, Ontario, Canada*

Racial disparities in Alzheimer's disease and related dementias have been established in the US, with higher rates in Black compared to White older adults. Because Black populations are disproportionately exposed to greater neighborhood disadvantage, chronic stress via the neighborhood context may contribute to worse cognitive health among Black older adults. However, the role of neighborhood-based stressors on cognitive function within the Black population is less understood. This study aims to examine how lifetime exposure to structural stressors (e.g., discrimination in housing, unemployment, and policing) and current perceived neighborhood stressors (e.g., high disorder and social discohesion) independently and interactively are associated with cognitive function in Black adults >50 years of age (n=1,663) using 2010-2016 Health and Retirement Study data. Linear regression models tested associations between structural stressors, perceived neighborhood stressors, and interaction terms of total number of structural stressors, neighborhood disorder (including safety and cleanliness), and social discohesion with cognitive function measured using the Langa-Weir 27-point scale. Black adults reported exposure to structural stressors as 15.69% in policing, 14.25% in hiring, and 7.88% in housing. In unadjusted models, greater total structural stressors were associated with greater cognitive function, while worse ratings of social cohesion, safety and cleanliness were associated with lower cognitive function. A statistically significant interaction term (b= -.221, SE=0.10, p<0.05) indicated that high social discohesion and greater total structural stressors was associated with lower cognitive function. Thus, life course exposure to multiple domains of neighborhood-based stressors may be important for Black adults' cognitive function in later life.

Abstract citation ID: igae098.1307

**SELF-REPORTED EVERYDAY DISCRIMINATION AND CARDIOMETABOLIC RISK IN OLDER BLACK AND WHITE ADULTS**

Ryon Cobb, *Rutgers, The State University of New Jersey, New Brunswick, New Jersey, United States*

This study investigates the relationship between self-reported instances of everyday discrimination and cardiometabolic risk among older, focusing on potential variations by racial classification. This analysis draws on a subsample of older Black and White respondents from the 2010/2012 Health and Retirement Study (N=8,924), a biennial nationally representative survey of older adults across the United States. Cardiometabolic risk is assessed using a count of high-risk cardiometabolic biomarkers while we self-reported everyday discrimination and racial identification. We included an interaction term (self-reported everyday discrimination x race) to test whether the relationship between everyday discrimination and cardiometabolic risk varied by racial classification. We used negative binomial regression to estimate incidence rate ratios (IRRs) and corresponding 95% confidence intervals (CI) of self-reported everyday discrimination, racial classification, and cardiometabolic risk, controlling for potential confounders. Respondents with higher levels of self-reported everyday discrimination scores had higher levels of cardiometabolic risk after adjusting for demographic characteristics (IRR: 1.08, 95% C.I. 1.05-1.11), and while attenuated, remained significant (IRR: 1.08, 95% C.I. 1.01-1.07) after further adjustments for smoking status, physical activity, alcohol consumption, educational attainment, and household wealth. The relationship between self-reported instances of everyday discrimination and cardiometabolic risk was significantly weaker for older Black adults than their White counterparts. The present study confirms that self-reported instances of everyday discrimination are a psychosocial factor associated with higher levels of cardiometabolic risk among older adults; this association is especially pronounced among older White adults. Future studies among this population must examine whether the relationship changes over time.

Abstract citation ID: igae098.1308

**MIDLIFE CHRONIC STRESS, PHYSIOLOGICAL DYSREGULATION, AND COGNITION: A LONGITUDINAL MEDIATION ANALYSIS**

Uchechi Mitchell, *University of Illinois Chicago, Chicago, Illinois, United States*

The current study investigates how exposure to chronic stressors during mid-life indirectly affects cognitive function through changes in physiological dysregulation. Data come from 4,684 adults ages 51-64 at baseline who participated in the Health and Retirement Study between 2006 and 2018. Mid-life stressors include experiences of everyday discrimination and financial strain, both of which were assessed at baseline only. Physiological dysregulation was assessed as a count of high-risk inflammatory, cardiovascular, and metabolic biomarkers and was assessed every four years. Cognitive function assessed every two years using a modified version of the TICS. A longitudinal mediation analysis was conducted using R and MPLUS. Unadjusted analyses showed that cognitive function decreased over time, while physiological dysregulation significantly increased. The indirect effects of financial strain on cognitive decline were mediated by baseline dysregulation levels and increases in physiological dysregulation over time. On the other hand, the effect of everyday discrimination on cognitive decline was only mediated by its effect on baseline physiological dysregulation. These findings suggest that 1) physiologic vulnerability to financial strain and everyday discrimination may be more salient earlier in the life course setting the stage for changes in cognition later in life, and 2) financial strain in mid-life continues to adversely affect physiological functioning and cognition over time. That said, adjusting for age, gender, race/ethnicity and education fully accounted for the significant direct and indirect effects of financial strain and discrimination on changes in cognition, suggesting a more complex and nuanced relationship between chronic stress, physiological function and cognition.

Abstract citation ID: igae098.1309

### SELF-REPORTED DISCRIMINATION AND BRAIN STRUCTURAL ABNORMALITIES: THE MULTI-ETHNIC STUDY OF ATHEROSCLEROSIS

Sarah Forrester[1], Jordan Tanley[2], Michael Bancks[3], José Luchsinger[4], Stephen Rapp[3], Marcia P. Jiminez[5], Robert Bryan[6], and Timothy Hughes[3], *1. University of Massachussetts Chan Medical School, Worcester, Massachusetts, United States*, *2. Wake Forest University School of medicine, Winston-Salem, North Carolina, United States*, *3. Wake Forest University School of Medicine, Winston-Salem, North Carolina, United States*, *4. Columbia University, New York, New York, United States*, *5. Boston University, Boston, Massachusetts, United States*, *6. University of Pennsylvania, Philadelphia, Pennsylvania, United States*

Black and Hispanic adults have greater risk for developing Alzheimer's disease (AD) and related dementias (ADRD) compared to White adults. These disparities might be at least partially attributable to adverse social determinants of health (SDoH) such as experienced interpersonal discrimination. This study examines racial/ethnic differences in the association between self-reported discrimination and brain imaging findings associated with aging and AD/ADRD. We used data from the MESA study, which initially enrolled 6814 participants in 2000-02. when they self-reported their race/ethnicity (38% White, 28% Black, 22% Hispanic, 12% Chinese Americans) and instances of lifetime discrimination. The primary exposure was experiencing racial discrimination. During Exam 6 (2016-18) and ancillary study MIND A (2019-21), brain imaging with 3-Tesla MRI (n=1430) and amyloid PET (n=315) was completed. Multivariable linear (brain MRI measures) and logistic (amyloid PET positivity [centiloid >12.2]) regression models were adjusted for covariates including age, race/ethnicity, gender, education, site, smoking status, blood pressure, diabetes, APOE-ε4, income, health insurance, (all models) and intracranial volume (for brain volumes). Race/ethnicity stratified analyses were completed to assess moderation. Racial discrimination was positively associated with greater white matter hyperintensity volume (WMHV), but not gray matter volume (GMV) or fractional anisotropy (p = 0.008). Racial discrimination was also significantly associated with higher odds of amyloid positivity [OR (95%CI) = 2.24(1.07, 4.70)]. Stratified analysis revealed stronger associations between racial discrimination and GMV and WMHV in Black participants (p = 0.008 and 0.003, respectively). Instances of racial discrimination over a lifetime may be detrimental to brain health, especially for Black adults.

### SESSION 3185 (PAPER)

### EDUCATION AND TECHNOLOGY INNOVATIONS TO SUPPORT OLDER DRIVERS

Abstract citation ID: igae098.1310

### CURRENT PRACTICES OF DRIVING REHABILITATION SPECIALISTS: IS FITNESS TO DRIVE EVALUATIONS USING THE BEST EVIDENCE?

Anne Dickerson, *East Carolina University, Greenville, North Carolina, United States*

It is well established that social participation is essential for maintaining quality of life and in the US, driving is the primary mode of community mobility for older adults. Research has established the importance of fitness-to-drive (FTD) evaluations for those older adults with medical conditions. These medically-at-risk drivers, at any age, are generally evaluated by specialized driver rehabilitation specialists (DRS) to determine FTD. In the last 10 years, much research has established which assessments best determine FTD with cognitive assessments being the primary determinant. The aim of this research study was to compare assessment tools with the best evidence with the actual tools being currently used by DRSs. A comprehensive review of tools was completed by 321 practicing DRSs. Results show that cognitive tools such as Trails A, Trails B, and clock drawing are being used by 91%, 92%, and 74% respectfully. However, other tools with strong evidence are being used much less (MOCA-46%, UFOV-30%). Additionally, assessment with limited or no evidence (e.g., Traffic signs-66%, brake reaction-71%) or with vehicle technology changes, are not essential to FTD (e.g., muscle strength-91%, ROM-95%) are used extensively. Highlights from the research will include how the on-road assessment is used as a determining factor for FTD with statements. For example: If a driver with moderate dementia passes the on road, he/she should be able to continue driving. While 71% indicted this was probably/definitely false, 29% felt it was probably/definitely true. Implications of these results will be discussed.

Abstract citation ID: igae098.1311

### EXAMINING THE IMPACT OF RIDE-HAILING ON THE TRAVEL BEHAVIOR OF OLDER ADULTS ACROSS THREE CITIES

Minju Song, *Portland State University, Portland, Oregon, United States*

Due to age-related physical and cognitive changes, older drivers may eventually find themselves unable to continue driving, when finding alternative transportation modes becomes a major concern. Ride-hailing services like Uber and Lyft have emerged as potential solutions to this issue. Nevertheless, given the dearth of available data and the recently introduced transportation systems, limited research exists on ride-hailing usage of older adults. This paper aims to examine the factors influencing ride-hailing adoption among older adults by comparing different age groups across three metropolitan areas: Chicago, Minneapolis, and Seattle area. Using regional survey data collected in 2019, we estimate logistic regression models by age groups to examine notable characteristics of older adults utilizing ride-hailing. In follow-up, we use a multilevel logistic regression model to explore the hierarchical relationships between regional, household, and individual levels. Findings show that ride-hailing services are more used by younger adults (18-64) compared to individuals over 65 years, indicating a potential technological familiarity gap between age groups. The built environment has a significant association with ride-hailing adoption, particularly in urban areas with transit infrastructure and higher population density. While ride-hailing has the potential to enhance the social inclusion and independence of older people who stop driving, ride-hailing is not accessible for older people as much as other age groups. Thus, this study underscores the importance of continued investment and research in ride-hailing services, advocating for policymakers and service providers to consider older adults' needs in transportation planning.

Abstract citation ID: igae098.1312

### FEW OLDER DRIVERS TAKE REFRESHER COURSES: IDENTIFICATION OF FACILITATORS AND BARRIERS

Michel Bedard[1], Hillary Maxwell[1], isabelle Gelinas[2], Gary Naglie[3], Brenda Vrlkjan[4], Michelle Porter[5], Holly Tuokko[6], and Shawn marshall[7], *1. Lakehead University, Thunder Bay, Ontario, Canada*, *2. McGill University, Montreal, Quebec, Canada*, *3. Baycrest Hospital, Toronto, Ontario, Canada*, *4. McMaster University, Hamilton, Ontario, Canada*, *5. University of Manitoba, Winnipeg, Manitoba, Canada*, *6. University of Victoria, Victoria, British Columbia, Canada*, *7. Ottawa Hospital, Ottawa, Ontario, Canada*

Older drivers may benefit from driver refresher courses, particularly from courses with on-road training. Yet, the uptake of refresher courses and the factors associated with the decision to enroll is poorly documented. We examine these issues using the Candrive prospective cohort (recruitment stated in 2009; N = 927), a representative sample of older Canadian drivers. We operationalized our outcome variable as having taken any non-mandatory refresher course within the last 10 years (yes/no). We developed a conceptual model that includes sociodemographic factors (e.g., age), health-related factors (e.g., medical conditions), and driving-related factors (e.g., driving comfort), and identified the contribution of these factors using logistic regression. Participants' mean age was 76.21 (SD=4.85) and 576 (62.1%) were males. Ninety-eight participants (10.6%) reported having taken a non-mandatory refresher course within the last 10 years; roughly half reported the course included an on-road component. The multivariable regression model (-2 log likelihood = 518.41, p <.001, Nagelkerke R-square =.17) identified six of 13 variables entered into the model as statistically significant. The odds of having taken a course differed by province of residence, and was higher with increasing age, if their driving was important for others, were a volunteer, and spoke to a physician about driving, but was lower if they enjoyed driving. Our results indicate that the uptake of refresher courses is limited but is associated with potentially modifiable factors. Deepening our understanding of these facilitators and barriers will help support older adults to drive safely for longer.

Abstract citation ID: igae098.1313

### PREDICTING USE OF ADVANCED DRIVER-ASSISTANCE SYSTEMS IN OLDER DRIVERS THROUGH STRUCTURAL EQUATION MODELING

Abigail Hansen[1], Kim Kiely[2], Tuki Attuquayefio[3], Diane Hosking[4], Ranmalee Eramudugolla[1], and Kaarin Anstey[1], *1. University of New South Wales, Sydney, New South Wales, Australia*, *2. University of Wollongong, Wollongong, New South Wales, Australia*, *3. Western Sydney University, Sydney, New South Wales, Australia*, *4. National Seniors Australia, Canberra, Australian Capital Territory, Australia*

Older drivers are an at-risk population when on-road. Normal ageing processes, such as declines in visual, sensorimotor and cognitive processes, impact driving skills and contribute to the increasing crash rate. Advanced Driver-Assistance Systems (ADAS) may provide a solution to keep older drivers safe on the road. This study investigated what technology acceptance and trust factors predict use of ADAS between older drivers who have ADAS in their car and a) use it (n=790) or b) do not use it (n=32). We conducted an online survey to 1330 Australian drivers aged 65 years or older (M=72, SD=6.9, 23% women) in partnership with National Seniors Australia. ADAS acceptance was measured by the Partial Automation Acceptance Scale, measured by the latent factors attitudes towards ADAS, attitudes towards technology, perceived risk and trust. Structural equation modeling examined the structural relationships between use of ADAS, the acceptance scale, locus of control and driver self-efficacy. The results indicated positive attitudes towards ADAS had a significant positive direct effect on use (y =.06, S.E =.02, p =.002 ($\beta$ =.26)) and trust in ADAS had a significant negative direct effect on use (y = -.03, S.E =.01, p =.024 ($\beta$ = -.15)). The model tested accounted for 93.4% of the variance in use of ADAS. These findings highlight the importance of older drivers to hold positive attitudes and trust in ADAS in order for them to use it, highlighting potential factors that could be targeted in behavioural change interventions seeing to improve usage rates.

Abstract citation ID: igae098.1314

### "I WOULD LIKE TO BE IN CONTROL": PERSPECTIVES OF OLDER ADULTS ON AUTONOMOUS DRIVING

Sajay Arthanat, *University of New Hampshire, Durham, New Hampshire, United States*

Autonomous driving technology is projected to have major implications on transportation safety, independence and aging-in-place of older adults. However, are older adults ready to embrace driving automations? This mixed-method research study explored the perspectives and readiness of older adults for autonomous driving, and underscored the demographic and age-related factors that potentially influence the adoption. The study involved two aims and employed the Levels of Autonomous Driving developed by the Society of Automotive Engineers to gauge readiness for the technology. In Aim 1, semi-structured interviews were conducted with nine older adults (purposively sampled) following a tutorial depicting the autonomous driving levels. The interviews were guided by the Unified Theory of Acceptance and Use of Technology (UTAUT) primarily focusing on the affinity of the participants to each automation level. A survey was also pilot tested with the participants using cognitive interviewing. In Aim 2, the survey was completed by 528 older adults (65+). The survey uniquely presented 16 realistic driving scenarios with respondents choosing the level of automation preferred for their car in each. Thematic analysis of the qualitative data underscored the perceived benefits, facilitators, barriers and mistrust associated with driving automation. The readiness for automation was generally low in the survey sample and ranged from no automation to driver assistance (52.7%), partial to conditional (35.9%) and high to full automation (11.3%). Pearson correlations showed that readiness significantly varied by income, living status, geographical location and perceived usefulness for aging-in-place. Multivariate analyses is underway to explore causal pathways in the study data.

### SESSION 3190 (SYMPOSIUM)

Abstract citation ID: igae098.1315

### EXTENDING THE FAMILY TREE: NEW FACES OF DEMENTIA CAREGIVERS

Chair: Jyoti Savla
Co-Chair: Karen Roberto

Research has predominantly focused on spouses and adult children as primary caregivers for individuals living with dementia. However, evolving family dynamics necessitate a critical examination of caregivers beyond this immediate circle. This symposium explores the understudied area of informal dementia caregiving, which includes care provided by extended family members, stepchildren, and chosen kin. This emergent focus is crucial in redefining the family caregiver archetype amid changing family structures. McConnell & Haggar employ social network analysis to underscore variations in family/non-kin participation in care networks, influenced by factors such as disease severity and caregiver availability. Roberto & Savla provide an in-depth exploration of historical and current family dynamics that shape niece and nephew caregivers' responsibilities, practices, and outcomes. Savla & Roberto address the individual and family challenges faced by older adult sibling caregivers, as well as identify factors that facilitate and impede their caregiving capacities. Silverstein et al. leverage longitudinal data to explore the impact of kinship strength and early relationship histories on stepchildren's support for stepparents living with dementia. Flatt et al. synthesize insights from their multiple studies on the health outcomes of sexual and gender minority dementia caregivers within biological and chosen familial networks. Collectively, these studies underscore the complexity and diversity of caregiver roles within families, emphasizing the need for supportive programs and policies tailored to their unique circumstances. Findings serve as a call for action to recognize and support the wide range of dementia caregivers in today's society who demonstrate remarkable resilience in navigating dementia care challenges.

Abstract citation ID: igae098.1316

### FAMILY STRUCTURE AND DEMENTIA CAREGIVER NETWORK COMPOSITION

William McConnell, Katherine Haggar, Justin Hinson, Jackeline Linares, Jo Ann Bamdas, and María Ortega Hernández, *Florida Atlantic University, Boca Raton, Florida, United States*

Ongoing shifts in family structure, including a growing number of adults aging without spouses or children, raise questions about who will step into a caregiving role for persons living with dementia (PLWD). Furthermore, research and interventions targeting caregivers typically limit their focus to one primary caregiver, but PLWD often rely on many interconnected informal and professional supporters. Social Network Analysis (SNA) is a valuable tool for identifying potential and actual caregivers for PLWD, including nontraditional and secondary caregivers who are often overlooked. In this presentation, we report results from an ongoing quantitative study of 250 dementia caregiver networks in the southeastern United States. Detailed SNA data were obtained from survey-based interviews with self-identified caregivers for PLWD utilizing outpatient services at a Memory Disorder Clinic or living in the community. Results indicate that PLWD in this sample are supported by 7.7 caregivers, on average. While traditional caregivers such as spouses and adult children remain common, other relatives including siblings, nieces/nephews, and grandchildren comprise nearly 20% of all informal caregivers. Non-kin friends comprise an additional 20% of informal caregivers on their own. We describe how family, extended family, and non-kin caregivers in the networks differ in terms of their participation rates and task-specific roles. We also present multivariate analyses examining how caregiver network composition changes based on disease severity and the availability of potential caregivers within families/households. We conclude by discussing implications of SNA for understanding caregiving in the context of shifting family norms.

Abstract citation ID: igae098.1317

### NIECE AND NEPHEW DEMENTIA CAREGIVERS: FAMILY RELATIONSHIPS AND CARE DYNAMICS

Karen Roberto, and Jyoti Savla, *Virginia Tech, Blacksburg, Virginia, United States*

Changing demographics and a host of social trends have created variations in dementia family care, including older adults' reliance on extended kin. To advance understanding of the heterogeneity of dementia family care structures, we focused on nieces and nephews who serve as primary caregivers for their aunts and uncles living with dementia. Guided by a life course perspective and grounded in a stress-coping framework, we used a mixed-method study design to explore how past and present family relationships and dynamics shape caregiving responsibilities, practices, and outcomes. Mixed-method interviews with 20 niece and 5 nephew dementia caregivers (Mage = 55 yrs; R = 38-67) revealed diverse pathways to care provision. Close relationships were established in childhood for 21 respondents, even among those who lived at a distance from their aunt/uncle. Two-thirds of the nieces/nephews never expected to be their relative's caregiver, but rather either gradually "fell into it" or abruptly assumed the role because of "family circumstances." Rarely were nieces/nephews the sole caregiver; some coordinated care with their aunts'/uncles' family members, their own family members, and formal service providers whereas others looked to another family member only for occasional assistance. Nieces/nephews who relied on family support to care for their relative living with dementia experienced more caregiver burden (rs = 0.49, p<0.03) and faced greater family strain (rs = 0.61, p<0.01). Notably, niece caregivers reported more difficulties with other family members than nephews (rpb =0.50, p<0.02). Discussion elaborates on the complexity of extended family relationships and realities of dementia care today.

Abstract citation ID: igae098.1318

### SIBLING-TO-SIBLING DEMENTIA CARE: UNDERSTANDING MOTIVATIONS, CHALLENGES, AND EXPERIENCES

Jyoti Savla, and Karen Roberto, *Virginia Tech, Blacksburg, Virginia, United States*

Caregiving research has traditionally focused on older adult siblings' involvement in shared social activities and emotional support, while offering limited exploration of instrumental help provided by siblings when their brothers/sisters face a health-related event or long-term illness. Moreover, there is a notable gap in understanding how siblings navigate the complexities of caring for their community-dwelling sibling living with dementia (SLwD). We begin to address this gap by examining determinants of sibling dementia caregiving roles, individual and family challenges older adult sibling caregivers encounter, and factors that facilitate their provision of care. From a mixed-methods analysis of interviews with 25 sibling caregivers, comprised of 12 sisters caring for sisters, 10 sisters caring for brothers, and 2 brothers caring for brothers (Mage = 68.03 years, R = 52-83; 48% African American; 52% co-residing), several key insights emerged. Motivations for assuming the caregiving role included shared life experiences, availability due to non-employment, personal or professional caregiving experience, and the absence or limited involvement of the SLwD immediate family. Early-life relationship conflicts, challenges in managing dementia-related symptoms, and divided family loyalties added complexity to the caregiving role. Sibling caregivers who received support from other family members and paid caregivers in caring for SLwD reported lower role captivity and caregiver burden. Caregiving experiences intersected with birth order, race, and socioeconomic status. Discussion focuses on when and why siblings take on the caregiving role for their SLwD, nuanced differences on how they manage their caregiving responsibilities, and implications for extended family caregiving practices and support.

Abstract citation ID: igae098.1319

### DO STEPCHILDREN PROVIDE DEMENTIA CARE TO AGING BIOLOGICAL PARENTS COMMENSURATE WITH THEIR LEVEL OF NEED?

Merril Silverstein[1], Seonhwa Lee[1], and Martin Lakomy[2], *1. Syracuse University, Syracuse, New York, United States, 2. Mendel University, Moravskoslezsky kraj, Czech Republic*

The research in this presentation addresses the critical issue of whether divorce, remarriage/repartnering, and stepfamily formation have weakened adult intergenerational bonds due to stepchildren's weaker commitment to the care of their vulnerable stepparents. A life-course approach is taken to link early relationship characteristics to later care trajectories. We use data from the Longitudinal Study of Generations to examine whether earlier kinship strength with stepmothers and stepfathers increases the amount of care stepchildren provide to meet the needs of their aging stepparents between1997-2022. We use latent growth curve analysis to examine whether the rate of increase in care to stepparents with advancing dementia and functional impairment is stronger when stepchildren in earlier adulthood evaluated their relationship with stepparents as more "kin-like" and "family-like." In comparison, we also examine rate of change in care for biological mothers and fathers by their levels of functional impairment. We also consider structural characteristics of step-relationships, including the child's age when the relationship was formed and the number of years the child lived with the stepparent. We expect that earlier reported kinship strength with stepparents will moderate the care response to stepparents' impairment such that it will approach the care response to biological parents. We expect the severely impaired stepmothers to elicit more care from stepchildren when compared to severely impaired stepfathers. Analyses reveal the conditions under which stepchildren serve as a vital resource to their vulnerable older stepmothers and stepfathers with dementia in complex aging families, which have become increasingly prevalent but remain under-investigated.

Abstract citation ID: igae098.1320

### TRADITIONAL AND NONTRADITIONAL FAMILY DEMENTIA CAREGIVING AMONG SEXUAL AND GENDER MINORITIES

Jason Flatt[1], Krystal Kittle[2], Andrew Pickett[3], Maritza Dowling[4], and Joel Anderson[5], *1. University of Nevada Las Vegas, Las Vegas, Nevada, United States, 2. University of Massachusetts Amherst, Springfield, Massachusetts, United States, 3. Indiana University, Bloomington, Bloomington, Indiana, United States, 4. George Washington University, Washington, District of Columbia, United States, 5. University of Tennessee-Knoxville, Knoxville, Tennessee, United States*

Few studies on dementia caregiving have examined the unique needs of sexual and gender minority dementia caregivers (SGM-DCs) who self-identify as lesbian, gay, bisexual, transgender, queer, intersex, asexual, and/or additional identities. The Alzheimer's Association estimates that over 350,000 SGM older adults in the U.S. are currently living with dementia, a number projected to increase to over a million by 2050. At least 1 in 10 SGM caregivers are providing care to someone with dementia. This paper synthesizes findings about traditional (i.e., biological) and non-traditional (i.e., chosen) family structures from several studies led by our research team examining the health of SGM-DCs. We found 10-20% of SGM-DCs report caring for non-traditional (i.e., chosen) family members with dementia. We also found over half of SGM-DCs report not living with their care recipient. Using population-based data, we found a larger number of SGM-DCs who report caring for a grandparent with dementia compared with non-SGM–DCs (17% vs. 4%, respectively). SGM-DCs of chosen family members were more likely to report providing care for fewer than five years and fewer than 8 hours per week. Our analyses did not find significant differences in physical or mental health outcomes between traditional and non-traditional SGM-DCs. We highlight areas for future research exploring divergent support needs between traditional and non-traditional family caregivers, as well as related implications for health policy and intervention efforts to promote the health of SGM-DCs. Historical changes in dementia caregiving related to changes in family structure should also be examined in future research.

### SESSION 3195 (SYMPOSIUM)

Abstract citation ID: igae098.1321

### GSA STUDENT CHAPTERS: A BROAD APPROACH TO MAKING THIS NEW INITIATIVE SUCCESSFUL AT YOUR INSTITUTION—US AND GLOBALLY

Chair: Marilyn Gugliucci

The Gerontological Society of America (GSA) Board of Directors approved the formation of a committee to explore the creation of GSA Student Chapters. As of academic year 2023-24, Phase II of the GSA Student Chapter project is underway and going strong. In this symposium, four GSA student chapter advisors will be presenting on their journey as an advisor in their second year of this project, or just starting up a chapter. The topics will cover undergraduate, post-graduate, and graduate students, as well as students focused on gerontology and health professions education. Presentations include: (1) Opportunities for Integrating Sigma Phi Omega Honor Society Chapter with a GSA Student Chapter; (2) Starting a GSA Student Chapter with Post-Graduate Students in the UK: Opportunities and Challenges; (3) GSA Student Chapter as a Gateway to Undergraduate Student Understanding of Professional Organization Involvement; and (4) Health Professions and Medical Education: How the GSA Student Chapter Enhances Professionalism and Networking.

Abstract citation ID: igae098.1322

### HEALTH PROFESSIONS AND MEDICAL EDUCATION: HOW THE GSA STUDENT CHAPTER ENHANCES PROFESSIONALISM AND NETWORKING

Marilyn Gugliucci, *University of New England College of Osteopathic Medicine, Biddeford, Maine, United States*

The University of New England College of Osteopathic Medicine (UNE COM) has had a Geriatrics Club since 2003. This club then became recognized as an American Geriatrics Society (AGS) Student Chapter in 2006. In 2022, we also participated as a pilot GSA Student Chapter. The benefits of being a chapter of either one or two national organizations positions students for national student memberships, national resources, and networking opportunities. The AGS provides practice materials and skills information to the medical students. However, GSA provides opportunities beyond research presentations and publications, with approximately 1200 of GSA members connected to the Emerging Scholars and Professional Organization (ESPO). This applies to all health professions students and includes interprofessional opportunities. With a strong support system and mentoring system within ESPO, The GSA Student Chapter provides advancement in learning skills, attitudes and knowledge, along with an extensive network of fellow students and practitioners to support their undergraduate (pre-clinical and clinical) health professions education. This prepares students for their health professions careers by providing national organizational connections that they can rely on throughout their careers. Forming a GSA Student Chapter for health professions students, regardless of the type of health professions program will be shared and sample programs and events in palliative care, geriatrics and gerontology will be presented.

Abstract citation ID: igae098.1323

### OPPORTUNITIES FOR INTEGRATING A GSA STUDENT CHAPTER WITH A SIGMA PHI OMEGA HONOR SOCIETY CHAPTER

Katarina Friberg-Felsted, *University of Utah, Salt Lake City, Utah, United States*

The University of Utah Gerontology Interdisciplinary Program (GIP) has had an active Sigma Phi Omega chapter, Alpha Chi, for decades. Students are enrolled in one of our five programs: 1) Master of Science in Gerontology (MSG), 2) MSG/ Master in Physician Assistant Studies (MPAS) dual degree, 3) Graduate Certificate in Gerontology, 4) Undergraduate Certificate in Gerontology, and 5) Minor in Gerontology. Students are invited to join Alpha Chi in the second semester of their program. Alpha Chi's Executive Committee ideally contains a student President, a student Secretary, and a student Treasurer; sometimes these positions are held by one or two students. The Alpha Chi Executive Committee is supported by a faculty advisor, who holds the position for several years. To integrate a GSA Student Chapter with our SPO local chapter, we began by following the guidelines in the GSA Student Chapter Handbook. Our process included application for a student group recognition on campus, verifying that our current Executive Committee members were student members of GSA, spreading the word through a variety of pathways, and ensuring that our events and projects were co-sponsored by Alpha Chi Chapter and GSA Student Chapter. This presentation will discuss both process and outcomes of the integration, including more students being involved earlier and more students being introduced to GSA student benefits, including ESPOs offerings.

Abstract citation ID: igae098.1324

### STARTING A GSA STUDENT CHAPTER WITH POSTGRADUATE STUDENTS IN THE UK: OPPORTUNITIES AND CHALLENGES

Dialechti Tsimpida, *University of Southampton, Southampton, England, United Kingdom*

As the global aging population continues to expand, there is a growing demand for professionals skilled in gerontology. To meet this need, the creation of Gerontological Society of America (GSA) Student Chapters within Higher Education Institutions (HEIs) has emerged as a pioneering initiative to support and cultivate emerging gerontology professionals. The University of Southampton, home to the UK's sole Department of Gerontology, was invited to establish a

GSA Student Chapter in the 2023-24 academic year. While enthusiastic about the opportunity to deepen student engagement with the GSA, initiating a GSA Student Chapter within a postgraduate-focused program posed challenges. This endeavor involved strategic engagement efforts to recruit postgraduate students specializing in aging studies, and to organize activities to establish and formally affiliate the new Student Chapter with the University of Southampton. The initiative aimed to foster professional development in education, research, advocacy, policy, and sciences related to aging. Affiliation with GSA undoubtedly enriches the careers of postgraduate students by providing access to a global network of experts, mentorship opportunities, international connections, and a comprehensive toolkit for aging studies. In this session, Dr Dalia Tsimpida, Faculty Advisor of the newly established GSA Student Chapter at the University of Southampton, will discuss the rewarding experiences and challenges encountered in launching and establishing the Student Chapter. Attendees will be prompted to consider why and how a GSA Student Chapter can effectively engage postgraduate students specializing in aging studies, and how such chapters can be integrated into their respective Institutions.

Abstract citation ID: igae098.1325

### GSA STUDENT CHAPTER AS A GATEWAY TO UNDERGRADUATE STUDENT UNDERSTANDING OF PROFESSIONAL ORGANIZATION INVOLVEMENT

Lauren Bouchard, *Western Oregon University, Salem, Oregon, United States*

The Portland State University Student Chapter has created networking events for students across Oregon, and undergraduate students are especially important to the chapter. Due to the GSA annual meeting in Seattle, our chapter had the unique opportunity to educate students across the Pacific Northwest as to the importance of professional organization membership. In partnership with the Oregon Gerontological Association (OGA), Western Oregon University (WOU), and Portland Community College (PCC), our chapter created an educational event regarding the structure, events, and benefits of GSA. Undergraduate students may differ in their knowledge and understanding of opportunities they perceive to be beyond their educational level. For this reason, our Careers in Aging Month (CIAM) event focused on membership in the larger GSA organization with support from the Emerging Scholars Professional Organization (ESPO) section within GSA. We highlighted potential differences for undergraduate students, including the new change that allows these students to gain GSA membership for free. This presentation will explain chapter activities designed to encourage undergraduate student membership as well as provide suggestions to recruit and engage undergraduate student members.

### SESSION 3200 (SYMPOSIUM)

Abstract citation ID: igae098.1326

### HOUSING AND COMMUNITY FOR OLDER ADULTS AS AGING IN PLACE (AIP) ENABLER: AN INTERNATIONAL PERSPECTIVE

Chair: Sojung Park
Discussant: Tine Buffel

This symposium discusses various approaches from five different countries that enable Aging in Place (AIP) through housing and community. The achievement of AIP depends on the institutional and cultural context. A study from New Zealand focuses on making new small homes accessible, and shows while accessible design is considered good design, not everyone understands it. User-centered accessible design is not commonly used, and trade-offs exist between accessibility, affordability, and size. A Korean study examines loneliness in older women living alone in a rural senior cohousing community. The findings show senior cohousing helps protect against loneliness by serving as a community hub for gathering, sharing, and caring. A Japanese study found "IBASHO" activities (i.e., place-based specific activities) help promote permanent community living in marginalized communities at higher risk of depopulation. Participating in such activities makes residents feel at ease in their community, even if they live alone or have poor health. A Canadian study discussed the development of a Naturally-Occurring Retirement Community (NORC) in Vancouver, formed by East Asian older adults who could not travel abroad due to border closures during the pandemic. The study explores the health-promoting nature of mutuality and its potential for population-level spillover effects on community well-being beyond the direct reach of formal programs. The last study from Hong Kong found significant improvements in perceived age-friendliness, with the greatest improvements among low-income older adults. The empirical knowledge from these studies offers valuable insights into a contextually rich understanding of a wide range of housing and communities.

Abstract citation ID: igae098.1327

### MUTUALITY AMONG EAST ASIAN DIASPORA AT A NATURALLY OCCURRING RETIREMENT COMMUNITY IN METRO VANCOUVER

Daniel Gan[1], Claire Wang[2], Eireann O'Dea[3], Lisa Cohen Quay[4], and Daisy Au[5], *1. University of Toronto, Toronto, Ontario, Canada*, *2. Johns Hopkins University, Baltimore, Maryland, United States*, *3. Simon Fraser University, Vancouver, British Columbia, Canada*, *4. Jewish Community Centre of Greater Vancouver, Vancouver, British Columbia, Canada*, *5. MOSAIC, Vancouver, British Columbia, Canada*

How did East Asians who were retiring abroad cope with the pandemic? This paper traces the development of a Naturally-Occurring Retirement Community (NORC) in Vancouver through the efforts of various community organizations. As borders closed during the pandemic, a group of community-dwelling East Asian older adults found themselves remaining in Metro Vancouver through the winter. They formed a group chat and began regular video chats. They shared virtual events, supermarket deals, and neighbourhood walks. A younger member stepped up to ask after members of the informal group whom were absent or unwell. Topics of discussions ranged from the wellbeing of members and their spouses, securing medical appointments, translating forms to be filled, friends back home, leisure activities, and the lives of their children or grandchildren. The group was formally served by facilitators of a seniors' club at a non-profit. At the same time, they existed as an informal network marked by mutual care. We discuss the health-promoting nature of mutuality in this NORC as compared to other neighbourhoods to theorize the conditions needed for the emergence of Gemeinschaft NORC in urban areas. This paper sketches a theory of mutuality as the key which transforms dyadic peer support into community cohesion. We explore the implications of mutuality on formal programs in order to harness their potential to have population-level spillover effects for the wellbeing of community members just beyond their direct reach. We further speculate that mutuality is that which was lost in modernity, and is the reason culture is medicine.

Abstract citation ID: igae098.1328

### ENCOURAGING NEW-BUILD, SMALL, AFFORDABLE, AND ACCESSIBLE HOMES IN AOTEAROA, NEW ZEALAND

Bev James, *Public Policy & Research Ltd, Wellington, Wellington, New Zealand*

The growing need for mainstream housing to be accessible is an international issue, driven by ageing populations and 'ageing-in-place' policies. Alongside the impetus to improve housing accessibility, is interest in building smaller dwellings, potentially better suited to an ageing population. Internationally, some jurisdictions mandate accessible standards in new-build housing, while others adopt national voluntary accessibility standards. In Aotearoa New Zealand (NZ) there is no mandatory requirement and few incentives for new-build housing to be accessible. Only an estimated 2% of dwellings are accessible, while one in six people need modifications to live safely in their homes. Our study addresses the dearth of NZ research into achieving accessibility within a small footprint (50m2-75m2). Its focus on new-builds directly addresses a research gap in international literature, which is largely concerned with home modifications rather than new homes built to accessible standards. We report on findings from interviews with housing providers, architects/designers and builders/developers involved in the production of accessible, small homes. Key themes are: accessible design is perceived as good design; awareness, knowledge and understanding of accessible design and its implementation varies; user-centered accessible design is not well understood nor applied; and trade-offs are made between accessibility, affordability and size. Findings contribute to housing policies and practice by identifying levers to improve accessibility in new-build housing. Findings reinforce calls for a multi-pronged approach to achieve housing accessibility, and suggest that "explorative learning" about accessibility is emerging.

Abstract citation ID: igae098.1329

### CHANGES IN OLDER ADULTS' PERCEPTIONS OF AGE-FRIENDLINESS IN HONG KONG: A THREE-YEAR MIXED-METHODS STUDY

Terry Lum[1], Hiu-kwan Chui[1], Shiyu Lu[1], On Fung Chan[1], Yingqi Guo[2], Jennifer Tang[3], and Yuqi Liu[4], *1. The University of Hong Kong, Hong Kong, China (People's Republic)*, *2. Hong Kong Baptist University, Kowloon Tong, Hong Kong*, *3. Chinese University of Hong Kong, Hong Kong, China (People's Republic)*, *4. South China University of Technology, Guangzhou, Guangdong, China (People's Republic)*

Hong Kong is known for its high population density, high urbanization, and hilly terrain, making age-friendliness a challenge in the city. Yet, the city is aging rapidly; by 2041, one in three residents will be 65 and older. Although age-friendly city has become a significant public policy imperative in recent decades, there is a dearth of empirical evidence on how AFC initiatives can improve perceived age-friendliness among community-dwelling older adults and how such initiatives may affect older adults with different socioeconomic statuses differently. Drawing on a three-year citywide AFC initiative in Hong Kong, we conducted a trend study to evaluate changes in perceived age-friendliness in eight AFC domains with 2575 and 2697 community-dwelling older adults in 2015 and 2018 respectively, in addition to 36 focus groups involving 206 older adults. Participants were asked to share their views on changes in age-friendliness in their cities. Survey data were analyzed using linear regression, while focus group data were analyzed using thematic analysis. Significant improvements were found in perceived age-friendliness in all eight AFC domains. Low-income older adults saw the greatest improvements in age-friendliness. Thematic analysis revealed that despite improvements, shortcomings persist in housing, civic engagement, and employment domains. Nevertheless, our findings demonstrate that concerted efforts can improve a city's overall age-friendliness and that such improvements appear most evident among low-income older adults.

Abstract citation ID: igae098.1330

### THE CLOSURE OF RURAL CO-HOUSING AND CHANGES IN LONELINESS IN OLDER WOMEN LIVING ALONE

Sojung Park[1], Soobin Park[1], and Hyunjoo Lee[2], *1. Washington University in St. Louis, St. Louis, Missouri, United States*, *2. Daegu University, Daegu, Republic of Korea*

Prolonged social restrictions during the pandemic have increased loneliness among the population, with people living alone in rural areas being particularly vulnerable due to geographic distances, transportation, and finances. Congregate senior housing may be a helpful alternative living environment that can help address the challenges of social isolation. Drawing on the environmental perspective of aging and the literature on loneliness, we investigated the influence of the pandemic-imposed closure of senior cohousing on the changes in loneliness in older women living alone in Korea. Using a mixed-method approach, we analyzed survey data from 160 participants using structural equation analysis focusing on multiple mediators, such as physical, social, and service environment and depressive symptoms. Also, we conducted a thematic analysis with 18 former cohousing residents. Our synthetic analyses showed that senior cohousing protects against loneliness by serving as a community hub for gathering, sharing, and caring among the housing residents and the members of the whole community. We found that community service plays a critical role in protecting against depressive symptoms and loneliness. Our study setting is a publicly subsidized cohousing program with no embedded program manager. The residents are not necessarily empowered yet to self-organize for online technology learning. Intentional programming that includes social activity and online learning would benefit vulnerable subgroups of older women. Our findings make a significant contribution to the limited knowledge base on the intersection of senior housing and rural aging during the pandemic.

Abstract citation ID: igae098.1331

### AGING IN PLACE IN A MARGINALIZED COMMUNITY AT RISK OF RURAL DEPOPULATION

Yuichi Watanabe, *Musashino University, Nishitokyo, Tokyo, Japan*

Japan is known as one of the most aged societies globally. As of 2022, the aging ratio is approximately 29%. According to the Ministry of Internal Affairs and Communications, in 2019, over 50% of the population in 20,000 neighborhoods, known as "marginalized communities," are 65 or older. These communities are at a higher risk of rural depopulation or local extinction. Between 2009 and 2019, questionnaire surveys were conducted every two years to assess the experiences and strengths of residents in a marginalized community. The aim was to identify ways to promote sustainable living in the community. As each survey sample size was small, the analysis used all samples as a pooled dataset (N=831). The questionnaire included variables related to people's anticipation of permanent community living and their engagement in "IBASHO" activities. In Japan, "IBASHO" refers to a place where people can feel secure, have roles, and consider their lives meaningful. A logistic regression analysis was conducted to determine the relationship between anticipation of permanent community living and engagement in "IBASHO" activities. The study revealed that residents who participate in "IBASHO" activities are more likely to feel at ease living in their community permanently, even if they live alone or have poor health. These individuals care for one another in their "IBASHO" community, sharing their strengths and anxieties and providing emotional and practical support. Encouraging "IBASHO" activities can help promote permanent community living, even in areas with an aging population.

### SESSION 3205 (SYMPOSIUM)

Abstract citation ID: igae098.1332

### INCLUSIVE NEIGHBORHOODS TO SUPPORT OUTDOOR MOBILITY AND PARTICIPATION OF PEOPLE LIVING WITH DEMENTIA

Chair: Habib Chaudhury
Discussant: Neil Charness

The creation of a supportive and inclusive neighborhood is instrumental to maintaining mobility and social participation of people living with dementia in the community. Achieving this objective requires a clear understanding of how the condition of dementia shapes people's use of streets and outdoor spaces, and how neighborhood built environmental features influence movement and activity of people living with dementia. This understanding is fairly limited in current research and practice, owing to the paucity of empirical evidence, as well as evidence-based resources that can guide decision-making and planning. These research gaps are addressed in a multi-site, mixed-methods study in British Columbia, Canada, titled "Dementia-inclusive Spaces for Community Access, Participation, and Engagement (DemSCAPE)". The aims of this study are to 1) explore the outdoor mobility patterns and experiences of people living with dementia in the community, and the role of the neighborhood built environment in shaping their mobility and participation In outdoor activities, and 2) develop evidence-based resources that can guide dementia-friendly and inclusive planning and design. This symposium will also present findings from the knowledge generation and knowledge mobilization phases of the DemSCAPE study. Knowledge generated in the study is based on qualitative and quantitative analyses of data collected with participants living with dementia and care partners in British Columbia. Knowledge mobilization activities involve three key resources developed in our project: documentary video, photo exhibit, and dementia-inclusive planning and design guide. Together, these five presentations offer insights that can guide stakeholders in creating dementia-friendly and inclusive outdoor spaces.

Abstract citation ID: igae098.1333

### EXPLORING THE NEIGHBORHOOD ENVIRONMENT'S ROLE IN OUTDOOR WALKING EXPERIENCES OF PEOPLE LIVING WITH DEMENTIA

Habib Chaudhury[1], Kishore Seetharaman[1], Joey Wong[2], Lillian Hung[2], Cari Randa-Beaulieu[3], Shannon Freeman[4], and Mark Groulx[4], *1. Simon Fraser University, Vancouver, British Columbia, Canada*, *2. University of British Columbia, Vancouver, British Columbia, Canada*, *3. Alzheimer Society of British Columbia, Vancouver, British Columbia, Canada*, *4. University of Northern British Columbia, Prince George, British Columbia, Canada*

This presentation is based on a study that explores how people with dementia experience their neighborhood environment in the context of everyday outdoor walking. The experientially grounded approach explored ways in which people living with dementia can inform the process of dementia-friendly and inclusive planning and design. The focus on lived experience can also help planners and designers gain insights into the actions and strategies that people living with dementia adopt to cope with challenges in mobility and navigation in the neighborhood, thus gaining useful knowledge to generate contextually relevant design solutions. Four-part sequential sit-down and video-documented walk-along interviews were conducted with 32 participants living with mild to moderate dementia or mild cognitive impairment in British Columbia, Canada. Thematic analysis of interview transcripts and videos helped identify themes relevant to the ways in which the neighborhood environment shaped participants' outdoor walking experiences. Findings underscored both supportive and constraining aspects of the neighborhood environment as it pertains to outdoor walking. Awareness of environmental barriers and stressors in the context of their physical and cognitive challenges informed participants' planning and decision-making processes in relation to outdoor walks, as well as coping strategies to enhance familiarity, safety, comfort, and independence in the neighborhood environment. Planning and designing supportive built environments is key to facilitating the outdoor mobility of people living with dementia and should be a focus of research, policy, and practice related to building dementia-inclusive communities.

Abstract citation ID: igae098.1334

### ENGAGING WITH MUNICIPAL PLANNERS WITH A DEMENTIA-INCLUSIVE PLANNING AND DESIGN GUIDE

Kishore Seetharaman, and Habib Chaudhury, *Simon Fraser University, Vancouver, British Columbia, Canada*

Dementia-friendly and inclusive community (DFC) action plans in British Columbian municipalities identify various planning and design strategies and actions to meet the goals of dementia-inclusivity. However, there is a lack of evidence-based design guidelines tailored to the context of British Columbia to support the implementation of these plans. To address this gap, a DFC planning and design guide was generated as part of the DemSCAPE study, consisting of planning and design principles and guidelines for wayfinding, safety, social interaction and access to amenities for people living with dementia. A series of knowledge mobilization (KM) activities will explore and augment the relevance, uptake, and implementation of this guide. Two focus groups (60-90 min; N = 5-7 per session) were conducted with municipal professionals working in two municipalities (i.e., City of Burnaby and City of Richmond) in Metro Vancouver. The existing municipal action plans emphasize the need for collaboration and dialogue across different departments of local government, e.g., social planners and seniors services coordinators (staff who are more closely connected with and help coordinate these plans) and planners, designers, and engineers. The focus groups are intended to trigger inter-departmental dialogue centered on DFC planning and design and examine DFC strategies and actions from various stakeholders' perspectives within the scope of municipal planning. This KM project is expected to make meaningful contributions to the DFC initiatives of local government in BC by providing guidelines to municipal planners on how to apply a dementia-friendly and inclusive lens in planning and design.

Abstract citation ID: igae098.1335

### EXPLORING FACTORS INFLUENCING WALKING ACTIVITY IN PEOPLE WITH DEMENTIA: A SPATIAL ANALYSIS IN METRO VANCOUVER

Mohammadjavad Nouri, and Habib Chaudhury, *Simon Fraser University, Vancouver, British Columbia, Canada*

As the number of people living with dementia (PLWD) increases in Canada, the significance of the neighborhood built environment grows, as most PLWD live at home rather than in long-term care facilities. This study investigates how built environment variables predict the overall outdoor walking activity of PLWD in Metro Vancouver. Twenty-five participants living with dementia from various cities in Metro Vancouver participated. We used exploratory factor analysis and multiple linear regression to analyze the relationship between built environment factors and outdoor walking activity. Our results revealed three significant factors: "Macro environment: accessibility to public transportation and street network," "Micro environment: Pedestrian-oriented design," and "General characteristics: Mixed land use and sidewalk suitability," constructed from 14 built environment variables. These factors significantly influenced the outdoor walking activity of PLWD. The multiple linear regression results indicated that "Macro environment – accessibility to public transportation and street network" had a substantial positive effect ($p = .007$, $\beta = 0.469$), "Micro-environment: Pedestrian-oriented design" showed a moderately positive impact ($p = .065$, $\beta = 0.305$), and "General characteristics: mixed land use and sidewalk suitability" exhibited a significant positive influence ($p = .015$, $\beta = 0.414$) on outdoor walking activity. Our findings provide valuable insights for policymakers, urban planners, designers, landscape designers, and transportation planners, emphasizing the crucial collaboration needed to develop dementia-friendly neighborhood plans that cater to the diverse needs of individuals living with dementia, ensuring safe and accessible environments.

Abstract citation ID: igae098.1336

### FOSTERING DEMENTIA INCLUSIVE NEIGHBORHOODS: A GIS ANALYSIS OF NEIGHBORHOOD WALKABILITY FOR PERSONS WITH DEMENTIA

Shannon Freeman[1], Keone Gourley[1], Emma Rossnagel[2], and Mark Groulx[1], *1. University of Northern British Columbia, Prince George, British Columbia, Canada*, *2. Centre for Technology Adoption for Aging in the North, Prince George, British Columbia, Canada*

Active engagement in the natural environment is an important aspect of health and wellbeing for persons with dementia. Neighborhoods with high walkability are critical for persons with dementia to access the outdoors and promote ageing in place, while allowing for persons with dementia to maintain social relationships, connect with nature, and foster a sense of freedom. Although persons living with dementia are affected by general walkability factors such as presence of sidewalks and the availability of destinations such as health services or recreational facilities, dementia-inclusive neighborhoods must also be distinctive and easy to navigate, as persons with dementia are more prone to become disoriented or lost. Therefore, the Dementia-Inclusive Streets and Community Access, Participation and Engagement (DemSCAPE) project sought to identify how persons living with dementia interact with their environment and how municipalities can implement change to make cities more walkable for PLWD. A GIS analysis was completed to assess current infrastructural barriers and facilitators for aging-in-place for persons living with dementia. This analysis, which included interviews with individuals living with dementia and their family caregivers, found that their neighborhoods were severely lacking the infrastructure needed to support walkability for persons living with dementia. From the absence of sidewalks to disorienting neighborhood patterns, the city of focus has a long way to go in supporting aging-in-place for persons living with dementia. This presentation will showcase how these findings can be used to influence protocols in municipalities to implement supportive infrastructure for persons living with dementia.

Abstract citation ID: igae098.1337

### KNOWLEDGE MOBILIZATION TO CREATE DEMENTIA-INCLUSIVE NEIGHBORHOODS: AN EDUCATIONAL AND AWARENESS-RAISING VIDEO

Cari Randa-Beaulieu[1], Habib Chaudhury[2], Kishore Seetharaman[2], Lillian Hung[3], Joey Wong[3], and Lily Ren[3], *1. Alzheimer Society of British Columbia,*

*Vancouver, British Columbia, Canada*, 2. *Simon Fraser University, Vancouver, British Columbia, Canada*, 3. *University of British Columbia, Vancouver, British Columbia, Canada*

To promote community engagement and increase awareness of dementia-inclusive neighborhood built environment, we have co-created with people with lived experience (PLWE) multiple knowledge mobilization (KM) resources. These resources include an educational and advocacy video and photo exhibit showcasing the project findings to stakeholders and the general public in British Columbia, Canada and beyond. A seven-minute documentary video was produced to illustrate the lived experiences of three people living with dementia in Metro Vancouver and Prince George. This video is one of several public engagement tools for dialogue and challenging stigma surrounding living with dementia in the community that will be mobilized during this phase of the project. This presentation will share the process of community engagement based on the World Café method in two municipalities in Metro Vancouver, Canada. The World Café method encourages conversations, collaborative learning, and generation of new ideas with the video and photo exhibit. This KM project aims to increase awareness and understanding of the features of a dementia-inclusive neighborhood built environment and advocate for positive actions for a broad range of stakeholders, including advocacy groups, community-based organizations, municipalities, and the general public. In partnership with the City of Burnaby and the City of Richmond in Metro Vancouver, these KM activities maximize the impact of the DemSCAPE study's findings and model various evidence-based approaches to achieving the vision and objectives for developing dementia-inclusive communities.

### SESSION 3210 (SYMPOSIUM)

Abstract citation ID: igae098.1338

### INTERDISCIPLINARY PERSPECTIVES ON CURRICULUM CONTENT FOR TRANSLATIONAL GEROSCIENCE EDUCATION

Chair: Iman Al-Naggar
Co-Chair: George Kuchel
Discussant: John Newman

Translational geroscience is a new field of study that aims to delay, prevent or alleviate several aging-related diseases simultaneously by targeting the so-called "biological hallmarks of aging," a constellation of biological pathways at the root of natural aging and chronic conditions for which aging is the major risk factor (e.g., diabetes, cardiovascular disease, Alzheimer's, cancer). Geroscience is a paradigm shift in healthcare because it aims to move away from single disease management to multi-disease prevention or alleviation and decrease the period of morbidity at the end of life. In order to train a workforce in conducting translational geroscience trials, the Geroscience Education and Training Network (NIH/NIA R25AG073119) is developing curricula towards a Certificate in Geroscience Research Program. This program is designed to train investigators in aspects specific to clinical trials of geroscience guided interventions (pharmaceutical or behavioral) in older adults. Evidence suggests that many factors often not considered in trial design play an important role in the outcomes of these trials; for example, social determinants of health contribute to accelerated aging and worse outcomes in minority populations, socially-disadvantaged persons, and other vulnerable populations, where chronic diseases arise at a much younger age. This symposium aims to begin a conversation between biological scientists, behavioral and social scientists and others to address the question "What knowledge from your specific field do translational geroscientists need to know to conduct meaningful and impactful geroscience clinical trials?" This insight is essential to help develop the curricula required to train a well-rounded workforce of translational geroscientists.

Abstract citation ID: igae098.1339

### CURRICULUM CONTENT FOR TRANSLATIONAL GEROSCIENCE EDUCATION: SOCIAL RESEARCH, POLICY, AND PRACTICE PERSPECTIVES

Richard Fortinsky, *University of Connecticut, Farmington, Connecticut, United States*

Geroscience is an exciting paradigm with the potential to positively disrupt the biological aging process in humans, thereby extending the healthy lifespan. This presentation will offer considerations from social research, policy, and practice perspectives that could be incorporated as topics into educational curricula for scientists planning to engage in geroscience-guided clinical trials. Regarding social research, methodological considerations include strengths and weaknesses of different research designs and how to choose outcome measures aligned with hypothesized mechanisms of action of geroscience-guided interventions. When considering how and where to initiate clinical trials, investigators will confront issues including how equity can be optimized when recruiting participants, how risks and benefits of participating in such clinical trials can be best communicated to potential participants, and how to balance scientific rigor with pragmatic concerns when determining inclusion and exclusion criteria. At a broader level, social research perspectives include how preferences for geroscience-guided therapies vary among individuals according to race, ethnicity, socioeconomic status, and degree of disability. Policy-related curriculum topics could include how geroscience therapies, if found to be effective, might affect health insurance programs such as original Medicare, Medicare Advantage, and Medicaid. For example, care management programs offered by Medicare Advantage programs might have to be modified to account for a beneficiary population with fewer medical comorbidities, and demand for Medicaid-funded nursing home and home and community-based services might be affected if multiple comorbidities could be prevented or alleviated. Practice considerations include how the healthcare workforce will be affected by the advent of geroscience-guided therapies.

Abstract citation ID: igae098.1340

### BARRIERS TO GEROSCIENCE IN CLINICAL COMMUNITY

George Taffet, *Houston Methodist Hospital, Houston, Texas, United States*

I hope geriatricians will lead the application of GeroScience concepts to the care of older patients, and gerotherapeutics will reinvigorate the field of geriatrics. Yet geriatricians have been skeptical in embracing the potential it has to change their lives and that of their patients. A consensus of leaders stated “geriatricians specialize in the care of people with multiple chronic medical conditions that cause challenges with their day-to-day physical and mental functioning.” GeroScience addresses the aging process underpinning most of those medical conditions so the reluctance seems inexplicable. I will review some causes of this “disconnect” and potential strategies to make GeroScience and Gerotherapeutics more accessible and vital to geriatricians. Geroscience papers are infrequently in sources that Geriatricians read and studies don’t choose outcomes relevant to Geriatricians. The outcomes need to be clinically translatable and meaningful. Geriatricians: often disassociate blood results from the patient in front of them thus studies that only show serum studies are undervalued; see heterogeneity all the time and undervalue changes in means; don’t often see the clinical thumbprint in the study creation; frequently think “the horse is out of the barn” for their patients; have little exposure to geroscience during training and don’t know how to fit it into their practices; worry about ineffective “cures to aging” and increasing disparities. The expansion of these points (and others) and the strategies to counter them will be explored.

Abstract citation ID: igae098.1341
### PEOPLE DO NOT AGE IN LABS: SOCIAL AND BEHAVIORAL CONSIDERATIONS WHEN DEVELOPING GEROSCIENCE INTERVENTIONS

Eli Puterman, *University of British Columbia, Vancouver, British Columbia, Canada*

Geroscience researchers are determined to identify the constellation of genetic, molecular, and cellular mechanisms of aging that, eventually, will be translated into anti-aging therapies and pills. It is argued, however, that without a consideration of social and behavioural determinants of health, geroscientists will miss the full potential to impact health and longevity of all humans, not just a select few. Social and behavioural determinants of health are the non-medical, non-biological factors influencing health and disease, and their underlying biological processes. Humans are complicated species, with a lifetime of exposures to stressful, adverse, and sometimes traumatic experiences and a lifetime of engaging in a mix of healthy and unhealthy behaviours. Together, social and behavioural factors shape biological aging through epigenetic embedding and wear and tear of biological systems, causing eventual accelerated biological aging and disease. By understanding what social and behavioural factors are, how they embed biologically and accelerate aging processes, geroscientists can develop a deeper understanding of the complicated nature of human experimental research. Social and behavioural factors impact recruitment, engagement, and adherence of participants in intervention trials, which can obscure significant treatment effects. Social and behavioural factors can also limit treatment effects, when considering how difficult it might be to reverse some of the long-term damage caused by life itself. It is important then, for geroscientists to learn about social and behavioural factors, and the best practices to engage as many people across the wide diversity of life circumstances, to impact the healthspan and lifespan of all.

### SESSION 3215 (SYMPOSIUM)

Abstract citation ID: igae098.1342
### INTO THE WILD: EMBEDDING A COMPLEX, HOME-BASED DEMENTIA CARE PROGRAM INTO DIVERSE PRACTICE SETTINGS

Chair: Halima Amjad
Co-Chair: Quincy Samus
Discussant: Nancy Hodgson

MIND at Home® is a well-researched comprehensive care management program for community-living persons with dementia and cognitive impairment. Developed at Johns Hopkins University, the goal of the program is to provide holistic, family-centered care management by assessing and addressing a broad range of medical, social, and supportive care needs that place people with dementia and their care partners at risk for poor outcomes, institutionalization, and high care costs. Delivered by an interdisciplinary, dementia-proficient team, the program focuses on the home as the nexus of care and consists of specialized assessments, tools, and staff training. Memory Care Coordinators (MCCs) serve as the primary point of contact for program recipients and use a comprehensive needs assessment to develop individualized care plans. MCCs implement and monitor care plans and provide resource referrals, help with navigation and coordination of services and supports, teach problem-solving strategies, and provide dementia education, coaching, and emotional support to care partners. Managed care plans and advanced primary care are well positioned to implement this low-cost, high touch program. This session includes an introduction to the MIND at Home program and features three individual presentations by diverse implementation partners showcasing how the program was adapted and implemented in different health care environments (2 managed care plans-Texas and Pennsylvania; 2 large multispecialty clinics-Iowa and North Carolina). The session will wrap up with a summary of learnings from implementing the program “in the wild”, highlighting partner and stakeholder contributions to the evolution and successful implementation of the program in practice.

Abstract citation ID: igae098.1343
### AN INTRODUCTION TO THE MAXIMIZING INDEPENDENCE AT HOME (MIND AT HOME) PROGRAM

Quincy Samus, *Johns Hopkins University, Baltimore, Maryland, United States*

People living with dementia (PLWD) represent some of the highest-need and highest-cost individuals living in the community. The complexity and range of needs (including medical, behavioral, and social needs) make dementia one of the most expensive chronic conditions in the U.S., and a strong candidate for improving care and reducing costs through care management support. The Maximizing Independence at Home (MIND at Home) dementia care program, developed and implemented in a variety of settings and contexts over the last 15 years, is a care coordination program for community-living persons with dementia and cognitive impairment and their informal caregiver. The MIND program consists of a comprehensive set of assessments, tools and staff trainings designed to increase the ability to stay at home in the community; reduce unmet care needs and improve quality of care; reduce caregiver burden and avoid high health care costs. The MIND at Home program model builds on three prior trials (two RCTs, one quasi-experimental design) of the MIND at Home dementia care coordination model and five practice-based implementations in real world practice settings to date. This session will review key components of the MIND at Home dementia care model, the characteristics of the care team, core training elements, and summarize research findings to date across the developmental pipeline.

Abstract citation ID: igae098.1344

**FLEXIBILITY MEETS COMPLEXITY: LESSONS FROM PRACTICE-BASED IMPLEMENTATION OF A DEMENTIA CARE MODEL**

Halima Amjad, *Johns Hopkins University School of Medicine, Baltimore, Maryland, United States*

People living with dementia (PLWD) are among the highest-need and highest-cost individuals because of the complexity, duration, and range of medical, behavioral, environmental, and social needs. There is a growing evidence base showing that family-centered active management approaches that include activation and empowerment of care partners are well suited to improve care quality, health-related outcomes, and healthcare costs. Existing ADRD care management models have had limited translation to real world practice to date. This presentation will outline key experiences and lessons learned after a decade of work developing, adapting and embedding a comprehensive family-focused care management model called MIND at Home into practice. The work, supported in part by the NIA IMPACT Collaboratory, partners with Centene Corporation, a large managed care organization, and the American Medical Group Association, a non-profit trade organization representing multi-specialty groups and integrated health systems, to implement MIND at Home in different health care settings. MIND at Home implementation illustrates three overriding principles: (1) the necessity of "meeting people and health systems where they are" (literally and figuratively), (2) the importance of effectively matching intervention to outcome and context, and (3) contributions of key partner and stakeholders for adaptation and successful program evolution. Key considerations in implementation include engagement of staff, persons living with dementia, and care partners; program eligibility and referrals; ongoing staff training and support; organic drift over time; role clarity; and sustainability. Program flexibility and adaptation in light of individual, family, and system complexity is essential for successful implementation and sustainability.

Abstract citation ID: igae098.1345

**MEETING PEOPLE WHERE THEY ARE: IMPLEMENTATION OF AN ACADEMIC DEMENTIA CARE MODEL IN A TEXAS HEALTH PLAN**

Noemi Smithroat, *Superior HealthPlan, Helotes, Texas, United States*

Maximizing Independence at Home (MIND at Home), a well-researched holistic, family-centered dementia care coordination program, provides collaborative support to community-dwelling persons living with dementia (PLWD) and their informal care partners (CP). Through comprehensive home-based assessment of 13 memory-care domains covering PLWD and CPs, individualized care plans are created, implemented, monitored, and revised over the course of the illness. Non-clinical Memory Care Coordinators (MCCs) working with an interdisciplinary clinical team provide education and coaching to PLWD and their identified CP and serve as a critical liaison and resource navigators bridging families with medical professionals, and formal and informal community resources. This presentation will describe a pilot implementation of the program within Superior HealthPlan, a managed care organization, across diverse sites in Texas. We will present descriptions of how the program was effectively translated to practice, including the associated dementia support to enhance knowledge and skills, and review key aspects of the implementation experience including how the program was operationalized and adjustments made based on the challenges, obstacles, and opportunities; and summarize, the best practices that have emerged.

Abstract citation ID: igae098.1346

**EXPERIENCES EMBEDDING MIND AT HOME DEMENTIA CARE COORDINATION PROGRAM IN PRIMARY CARE**

Elizabeth Ciemins[1], Quincy Samus[2], Stephen Shields[1], Mia Yang[3], Shelby Herrig[4], Rachel Breyfogle[4], Alicia Rooney[1], and Monette McKinnon[1], *1. American Medical Group Association, Alexandria, Virginia, United States*, *2. Johns Hopkins University, Baltimore, Maryland, United States*, *3. Wake Forest University School of Medicine, Winston-Salem, North Carolina, United States*, *4. McFarland Clinic, Ames, Iowa, United States*

The prevalence of people living with dementia (PLWD) is growing and dementia is one of the highest burden, and highest cost chronic conditions in the US. Care management support within primary care practices is one solution to provide patient- and care partner (CP)-centered care, improve patient care outcomes, e.g., hospitalizations, ED visits, polypharmacy, high care partner strain, and reduce healthcare costs. This session describes a collaborative adaptation and implementation of the MIND at Home Dementia Care Coordination Program into two large primary care clinics in two states (e.g., Iowa and North Carolina). The program pilot expanded the skills of existing primary care staff members to the level of Memory Care Coordinators (MCCs), who worked with larger primary care teams on combining the benefits of clinic-based with home-based services that support PLWD, their families, and CPs. 106 primary care patients and their CPs were recruited for a 3-month intervention period and process measures, including identified and addressed needs of the PLWD and CP, were ascertained. This session will discuss the feasibility of program implementation in a racially, economically, and geographically diverse population of PLWD and provide qualitative and quantitative outcomes on patient, provider and MCC program acceptability, program impact, and barriers and facilitators to implementation from the primary care practice perspective. The presentation will reflect on specific issues important to consider in primary care including underdiagnosis of dementia, patient recruitment and outreach, incorporation of home visits, interdisciplinary collaboration and consultation, and outcomes ascertainment.

Abstract citation ID: igae098.1347

**HEATHER BERND CLARKE, MBA, LCSW, PA HEALTH & WELLNESS, MECHANICSBURG, PA, UNITED STATES**

Heather Clarke, *PA Health & Wellness, Pittsburgh, Pennsylvania, United States*

PA Health & Wellness, a managed care organization in Pennsylvania, has collaborated with Johns Hopkins on deploying the academic Maximizing Independence at Home ("MIND at Home") Program for community-living individuals with cognitive impairment who have Medicaid Long-Term Support Services. This home-based model is aimed at coordinating care and supporting caregivers for individuals who are cognitively complex and at risk for out of home placement. The model consists of a non-clinical Memory Care Coordinator (MCCs) who is supported by a Memory Care Manager RN to provide a holistic approach to assisting the individual remain in the community and decrease caregiver burnout. This is accomplished through a specialized assessment of 13-memory care domains that drive the development of an individualized care plan. Implementation of the care plan by the MCC includes assistance in navigating the managed care system, coordination of provider appointments and benefits, referral to community resources, and disease education and coaching for the family caregiver. This presentation will describe the program implementation within PA Health & Wellness since 2022. We will present key components of program implementation, operations and learned experiences. We will also review preliminary results on program impact showing reductions in inpatient and emergency room visits for individuals enrolled in the program compared to similar, non-enrolled, individuals.

### SESSION 3220 (SYMPOSIUM)

Abstract citation ID: igae098.1348

**JOURNAL OF GERONTOLOGY PSYCHOLOGICAL SCIENCES: FEATURED 2023 EDITOR'S CHOICE ARTICLES**

Chair: Duke Han

The psychological sciences of the aging process are vast in scope and complexity. Methodological approaches that are interdisciplinary, innovative, and inclusive can make these enigmatic processes more tangible. This symposium showcases articles in the Journal of Gerontology Psychological Sciences that were selected as Editor's Choice in 2023 because they exemplify these ideals. Smith et al. investigate cognitive ability differences by in-person versus telephone-based assessment methods. Jackson et al. explore associations between loneliness and cognitive resilience to brain neuropathology in older adults. Krok-Schoen et al. report on the resiliency of women aged 80 and older by race, ethnicity, and neighborhood socioeconomic status. Esiaka and Luth describe different interpretations of "honoring your parents" in the U.S., Ghana, and Nigeria. Lynch et al. link measures of loneliness, DNA methylation, and cognitive functioning.

Abstract citation ID: igae098.1349

**SHIFTING OF COGNITIVE ASSESSMENTS BETWEEN FACE-TO-FACE AND TELEPHONE ADMINISTRATION: MEASUREMENT CONSIDERATION**

Jason Smith, *Johns Hopkins University, Baltimore, Maryland, United States*

There is limited information concerning differences in cognitive performance attributable to assessment (telephone versus in-person) method instead of cognition. We evaluated mode effects on individual cognitive items and overall cognitive score in adults aged 65-79 in the 2014 Health and Retirement Study (n=6825). We assessed mode differences in test means and reliability, whether mode modifies associations of cognition with criterion variables, and formal measurement invariance testing by mode. People assessed by telephone tended to have higher scores for memory and calculation items (0.06-0.013 SDs) and lower scores for non-memory items (-0.09 to -0.01 SD). Cognition was differentially related to IADL score depending on assessment mode. Measurement invariance testing identified the largest mode differences in memory and attention items. Differences by telephone versus face-to-face mode of administration are apparent in cognitive measurement in older adults, and most pronounced for memory and attention tests that can be easier to answer via telephone.

Abstract citation ID: igae098.1350

**ASSOCIATIONS BETWEEN LONELINESS AND COGNITIVE RESILIENCE TO NEUROPATHOLOGY IN OLDER ADULTS**

Kathryn Jackson, *Northwestern University, Evanston, Illinois, United States*

Loneliness in the aging population is associated with decreased cognitive function and increased neuropathology; less is understood, however, about the association of loneliness and cognitive resilience (CR), defined as the discordance between a person's actual and expected cognition given their neuropathology. For this study, data were combined from 2 longitudinal studies of older adults. CR proximate to death (CRlast_level) and across time (CRslope) were obtained by independently regressing global cognition and change in cognition onto multiple neuropathology indicators and extracting the resulting residuals. Using a series of linear regression models, we assessed the effect of loneliness level and change in loneliness on CR. Results show that higher and increasing loneliness was associated with lower CR in the face of neuropathology, suggesting that some individuals are less resilient to the accumulation of neuropathology than others, and experiencing high/increasing loneliness is a key factor putting some at risk.

Abstract citation ID: igae098.1351

**RESILIENCY AMONG WHI'S WOMEN AGED 80 AND OLDER BY RACE, ETHNICITY, AND NEIGHBORHOOD SOCIOECONOMIC STATUS**

Jessica Krok-Schoen, *The Ohio State University, Columbus, Ohio, United States*

A comprehensive examination of resilience by race, ethnicity, and neighborhood socioeconomic status (NSES) among women aged ≥80 is needed, given the aging U.S. population, increasing longevity, and growing racial and ethnic diversity. Participants (n=29,367, median age=84.3) were women aged ≥80 enrolled in the Women's Health Initiative. There were no significant differences by race and ethnicity in resiliency. Mean resiliency scores differed between those with low NSES

(3.94±0.83) and high NSES (4.00±0.81). Older age, higher education, higher self-rated health, lower stress, and living alone were correlates of resilience. Social support correlated with resilience among White, Black, and Asian women, but not for Hispanic women. Living alone, smoking, and spirituality were significantly associated with higher resilience among women with moderate NSES. Despite some differing correlates of resilience by race, ethnicity, and NSES, there were many similarities. These results may aid in designing resilience interventions for the increasingly diverse population of older women.

Abstract citation ID: igae098.1352

### DIFFERENT INTERPRETATIONS OF "HONOR YOUR PARENTS": IMPLICATIONS FOR OBLIGATION OF PARENTAL CAREGIVING

Darlingtina Esiaka[1], and Elizabeth Luth[2], *1. University of Kentucky College of Medicine, University of Kentucky, Kentucky, United States, 2. Rutgers University, New Brunswick, New Jersey, United States*

Although the concept of "honor your parents" is [universally] important, its understanding and manifestation vary by cultural context and community. This variation potentially has important implications for how parental caregiving obligations are perceived and performed. The current study explored the diverse conceptualizations of "honoring your parents" in the United States, Ghana, and Nigeria. It also assessed how the interpretations influence individuals' perceptions of caregiving obligations to older parents. Through semi-structured interviews with 153 participants, we found that in the United States, "honoring your parents" is predominantly viewed through the lens of emotional care rather than material care and instrumental support. In contrast, individuals in Nigeria and Ghana strongly associate the concept with providing tangible assistance and hands-on care to parents. Our findings shed light on cultural nuances and expectations regarding parental caregiving, offering insights into potential strategies to address the diverse caregiving needs of aging populations.

Abstract citation ID: igae098.1353

### ASSOCIATIONS BETWEEN LONGITUDINAL LONELINESS, DNA METHYLATION AGE ACCELERATION, AND COGNITIVE FUNCTIONING

Morgan Lynch, Christopher Beam, and Thalida Em Arpawong, *University of Southern California, Los Angeles, California, United States*

Loneliness may influence aging biomarkers related to cognitive functioning, for example, through accelerated DNA methylation (DNAm) aging. In a study involving 1,814 participants from the Health and Retirement Study, we investigated the role of six DNAm age acceleration measures (DNAmAA) in mediating the effects of baseline loneliness and latent longitudinal loneliness trajectories on cognitive abilities. Higher baseline loneliness and a moderate-intercept loneliness trajectory predicted poorer general cognitive ability and memory scores. Second-generation DNAmAA measures (specifically, PhenoAge, GrimAge, and DunedinPoAm38) mediated the effect of loneliness on overall cognitive ability and memory. Additionally, immediate and delayed memory scores were mediated by GrimAge Accel in the lowest and two highest latent loneliness trajectories. Notably, self-rated health, depressive symptoms, social isolation, and body mass index contributed significantly to total and mediated effects. The study suggests that DNAmAA, especially GrimAge, have promise for explaining the prospective association between loneliness and poorer cognitive functioning.

### SESSION 3225 (SYMPOSIUM)

Abstract citation ID: igae098.1354

### LIFETIME EXPOSURES AND COGNITION AT OLDER AGES: FINDINGS FROM THE WISCONSIN LONGITUDINAL STUDY

Chair: Michal Engelman
Co-Chair: Gina Lee

This symposium considers the relationship between later-life cognition and a range of social, economic, and environmental exposures throughout the life course. Drawing on over 65 years of rich population-based survey data and recent rigorous cognitive assessments from the Wisconsin Longitudinal Study (WLS), the papers in this symposium showcase empirical linkages between older adults' cognitive health with a set of exposures including education, family socioeconomic status, occupational conditions, and familial connections. The first paper (Herd et al.) draws on the unique availability of adolescent IQ and high caliber cognitive diagnostic data that distinguishes between underlying etiologies to examine the relationship between early life IQ, educational attainment, and later life dementia. The paper by Topping links the wealth of WLS data on early life socioeconomic background and high school experiences to patterns of change in later life cognition, with attention to both cognitive change and mechanisms of selection. Next, Qin and Engelman draw on the WLS's sibling data to consider the relationship between later-life cognitive health and educational differentials among siblings, and Lee et al. consider the impact of sibling relationships on later life cognition. Finally, Williams et al. draw on the WLS survey, genetic, and cognitive data to consider the interaction between ApoE-4 status, employment-based environmental exposures in midlife, and later life pollution in shaping dementia risk. Taken together, the papers in this symposium showcase the enormous potential of combining longitudinal survey data with cognitive assessment for learning about the life course determinants of later-life cognitive health.

Abstract citation ID: igae098.1355

### EARLY LIFE INFLUENCES ON LATER LIFE ADRD RISK: THE ROLES OF ADOLESCENT COGNITIVE FUNCTION AND EDUCATION

Pamela Herd[1], Sanjay Asthana[2], Michal Engelman[2], Kamil Sicinski[2], Ralph Trane[2], and Victoria Williams[3], *1. Georgetown University, Washington, District of Columbia, United States, 2. University of Wisconsin Madison, Madison, Wisconsin, United States, 3. University of Wisconsin Madison School of Medicine and Public Health, Madison, Wisconsin, United States*

By some estimates, each additional year of education reduces dementia risk by 7 percent. What remains unclear, however, is how early life cognitive function, which influences educational attainment, and is hypothesized to influence later life dementia risk, factors into the relationship between education and dementia risk. Existing research neither attends to the role that early life cognition may play in these relationships, nor does it separate out how these relationships might vary with AD versus non-AD dementias. We employ a unique dataset, the Wisconsin Longitudinal Study, which has been tracking participants over their full life course and is now assessing dementia in the population as they reach their mid 80s. The data include prospectives measures of early life cognition, other early life conditions, as well as detailed educational histories, in addition to dementia diagnoses that differentiate AD and non-AD types. We find that lower early life cognitive function increases the risk for AD dementia, and education does not mediate this relationship. Early life cognition, however, has no relationship with non-AD dementia, that is dementia caused by chronic disease, where instead education has a stronger relationship. We hypothesize that these differential relationships reflect variance in how early life biological and environmental factors affect risk and resilience for later life cognitive dysfunction.

Abstract citation ID: igae098.1356

**IMPACT OF HIGH SCHOOL BACKGROUND ON OLD AGE COGNITION: EVIDENCE FROM A WISCONSIN COHORT**

Michael Topping, and Michal Engelman, *University of Wisconsin Madison, Madison, Wisconsin, United States*

Individuals with higher academic performance in high school, those who participated in enriching activities, and those with higher socioeconomic status are known to have cognitive advantages in later life. Yet, less is known about whether and how these factors constitute unique social typologies of students in high school. Such background typologies may confer differing cognitive benefits and may have different consequences for later life cognition. Using unique longitudinal survey and cognitive assessment data from the Wisconsin Longitudinal Study and latent class analysis, we identify five different typologies of high school students in 1957: high disadvantage, moderate disadvantage, low advantage, moderate advantage and high advantage. We then estimated regression models examining differences in immediate recall, delayed recall, and letter fluency across different timepoints (2004, 2011, and 2020). We find that compared to the most disadvantaged, those from other class backgrounds see higher average scores for immediate recall and letter fluency as they enter old age in 2004, and maintain these advantages through to 2011. This effect dissipates for some classes in 2020 when WLS participants were nearing the oldest-old ages (80+). Class differences in delayed recall were more clearly apparent in 2011 than either before or after. In addition to socioeconomic mobility and changes in cognition over the life course, these results also reflect health and mortality-related changes to the composition of the cohort at successively older ages.

Abstract citation ID: igae098.1357

**SIBLINGS' EDUCATIONAL DIFFERENTIALS AND LATER-LIFE COGNITIVE HEALTH**

Yue Qin, and Michal Engelman, *University of Wisconsin Madison, Madison, Wisconsin, United States*

Drawing on data from 3,216 Wisconsin Longitudinal Study participants aged 79-84 years old with at least one sibling, we examined the relationship between siblings' educational differentials and a unique set of late-life cognitive metrics. Results from OLS and multinomial logit regressions showed that higher educational attainment relative to one's only sibling is associated with a higher Telephone Screening of Cognitive Status (TICS) score and marginally lower risks of cognitive impairment. For respondents with two or more siblings, however, siblings' higher educational attainment relative to respondents was associated with a lower risk of cognitive impairment, and particularly a lower risk of Alzheimer's Disease (AD), compared with individual higher educational attainment than siblings' average education. Our findings suggest that both individual educational attainment and a better educated set of siblings can act as protective factors for later life cognition. Familial risk may matter more for AD than other types of impairment because of its genetic etiology, but further research should explore additional life-course, family resource, and network mechanisms that shape cognitive risk and resilience among and across siblings.

Abstract citation ID: igae098.1358

**MID-LIFE AND LATE-LIFE ENVIRONMENTAL EXPOSURES ON DEMENTIA RISK: THE MODIFYING EFFECTS OF APOE**

Victoria Williams[1], Ralph Trane[2], Kamil Sicinski[2], Pamela Herd[3], Michal Engleman[2], and Sanjay Asthana[2], *1. University of Wisconsin Madison School of Medicine and Public Health, Madison, Wisconsin, United States*, *2. University of Wisconsin Madison, Madison, Wisconsin, United States*, *3. Georgetown University, Washington, District of Columbia, United States*

Increasing evidence supports a deleterious effect of late-life air pollution exposure on dementia risk that is exacerbated among older ApolipoproteinE-4 (ApoE-4) carriers. However, the protracted effects of mid-life occupational exposures on dementia outcomes, and potential interactions with ApoE, are unknown. Using prospective life course data from 3,814 participants in the Wisconsin Longitudinal Study (WLS), we evaluated the effects of mid-life occupational exposure to respiratory hazards and late-life air pollution exposures on dementia risk, and whether these associations varied by ApoE-4 status. Mid-life occupational exposure was associated with 1.64 times greater odds of all-cause dementia, but only among ApoE-4 noncarriers. Conversely, late-life air pollution exposure (higher levels of particulate matter (PM2.5) and lower levels of ozone) associated with increased dementia risk only within ApoE-4 carriers. Findings suggest that ApoE-4 differentially impacts the association between environmental exposures and dementia risk based on timing of exposure across the life course – supporting ApoE as a pleiotropic modifying gene. While ApoE-4 appears protective against the adverse effects of hazardous respiratory exposures in mid-life, it increases risk for dementia associated with environmental exposures in late-life.

Abstract citation ID: igae098.1359

**SIBLING RELATIONSHIPS AND COGNITIVE TRAJECTORIES IN THE WISCONSIN LONGITUDINAL STUDY**

Gina Lee, Michal Engelman, and Jooyoung Kong,
*University of Wisconsin Madison, Madison, Wisconsin, United States*

Sibling relationships are positively associated with psychological well-being over time. However, no prior studies have examined the impact of sibling relationships in middle and older adulthood on longitudinal patterns in cognitive functioning. Using four waves (spanning 27 years) of sibling data from the Wisconsin Longitudinal Study (N = 19,046), our study explores the relationship between sibling closeness, emotional support, and contact frequency in 1993, 2004, and 2011 (when participants were roughly 54, 65, and 72) and trajectories in cognitive measures including immediate and delayed recall, digit ordering, and letter fluency in later life (2004, 2011, and 2020). In growth curve models, sibling closeness (measured in 2004) and emotional support received from siblings (measured in 1993, 2004, 2011) were positively associated with baseline levels of letter fluency but not change over time. Next, we employed the sibling dyad data with at least one letter fluency data available from each source (n = 3,243 pairs of siblings) to fit a parallel process model to examine whether siblings' letter fluency scores moved together over time. We found that both the intercepts and slopes of letter fluency trajectories were positively associated for siblings, implying that participants who experienced a steep decline in letter fluency were likely to have siblings who experienced similarly steep declines. Future studies can further explore what aspects of siblings relationship or shared exposures (e.g. shared family characteristics or environments, spending time together, having similar health behaviors, or providing care for each other) influence the shared cognitive patterns.

### SESSION 3230 (SYMPOSIUM)

Abstract citation ID: igae098.1360

### NAVIGATING DEMENTIA CAREGIVING AFTER HOSPITALIZATION: CARE PARTNER EXPERIENCES

Chair: Ashley Kuzmik
Discussant: Jane Flanagan

Hospitalization and the post-acute transitional period are particularly vulnerable, yet largely underexamined period, in the person living with dementia and their family care partners. The care receivers' increased care dependency and complex medical and psychosocial needs compound the chronic stress of care partners, and demand new and intensified levels of preparedness. This symposium examines the experiences of care partners and the factors impacting their preparedness and affective responses during transition from hospital to home, using data from the Family-centered Function-Focused Care (Fam-FFC) trial. The first presentation will examine the mediating effects of patient clinical factors on the relationship between care partner preparedness and desire to seek long-term care admission at the time of hospital discharge. Next, the needs for preparedness during transition from hospitalization, as reported by care partners will be discussed. The third paper will describe care partner characteristics that influence care partner preparedness at hospital discharge, and two- and six-months post-discharge. The final presentation will report on the mediational relationship among behavioral and psychological symptoms of dementia (BPSD), associated care partner distress, and care partner adverse outcomes from hospital discharge to six-month follow-up. Concluding the symposium, we will engage in a discussion to address unanswered questions, propose future research directions, and explore the broader implications of the findings for policy and practice.

Abstract citation ID: igae098.1361

### DEMENTIA CARE PARTNER PREPAREDNESS AND DESIRE TO SEEK LONG-TERM CARE AT HOSPITAL DISCHARGE

Ashley Kuzmik, *The Pennsylvania State University, University Park, Pennsylvania, United States*

At hospital discharge, care partners of persons living with dementia encounter more complex caregiving demands and lack preparedness to manage the increased care needs, frequently resulting in the patient's admission to long-term care. This study explored the mediating effects of patient clinical factors on the relationship between care partner preparedness and desire to seek long-term admission of persons with dementia at hospital discharge. The sample included 424 patient and care partner dyads from the Family centered Function-focused Care (Fam-FFC) trial. Using bootstrapping, we tested a multiple mediation model to examine the indirect effects of care partner preparedness on desire to seek long-term care admission through patient clinical factors (i.e., behavioral and psychological symptoms, comorbidities, delirium severity, physical function, and cognition). The results revealed that delirium severity (B=-.005; 95% bootstrap CI=-.015, -.001) and physical function (B=.004; 95% bootstrap CI=.002,.007) partially mediated the association between care partner preparedness and desire to seek long-term care admission. Findings highlight the importance of targeting delirium severity and physical function in clinical interventions for persons with dementia at hospital discharge to support care partner preparedness and minimize undesired long-term care admission.

Abstract citation ID: igae098.1362

### PREPAREDNESS NEEDS OF THE FAMILY CARE PARTNER OF THE PERSON WITH DEMENTIA DURING TRANSITION FROM THE HOSPITAL

Marie Boltz, *The Pennsylvania State University, University Park, Pennsylvania, United States*

In the Fam-FFC approach, nurses purposefully engage family care partners in the assessment, decision-making, care delivery and evaluation of function-focused care during hospitalization and the 60-day post-acute period. The goal is to promote functional recovery and well-being in the patient, and prevent unnecessary long-term care admissions, while improving care partners' preparation for caregiving and well-being. At hospital discharge, on the Preparation for Caregiving Scale, care partners (N=229, 60% female, 59% White, 39% African American) described the least preparation to care for emotional (M= 2.8, SD=1.2) and physical needs (M= 2.9, SD=1.1), deal with the stress of caregiving (M= 2.7, SD=1.2), provide positive activities (M= 2.9, SD=1.1), followed by get the help they need from the health care system (M= 3.11, SD=1.0). Content analysis of nurses'

transitional care notes during the 60 day post-acute period identified five themes that describe care partner needs to support preparedness; they converge with and help explain the quantitative findings. The themes included: psychosocial support for the patient, managing symptoms, physical and cognitive activity, advocacy, and care partner self-care. Findings suggest the need to attend to the psychological and informational needs of care partners, while considering patient well-being and comfort.

Abstract citation ID: igae098.1363

### FAMILY CARE PARTNER'S PREPAREDNESS IN CARING FOR HOSPITALIZED PERSONS WITH DEMENTIA

Anju Paudel, *The Pennsylvania State University, University Park, Pennsylvania, United States*

Many care partners are inadequately prepared to meet the care needs of hospitalized persons with dementia, and little is known about care partner characteristics associated with preparedness. This paper explores the care partner characteristics associated with preparedness in caring for hospitalized persons with dementia at discharge, 2 months, and 6 months post discharge. Following bivariate associations, we conducted stepwise regression in a sample of 461 care partners of hospitalized persons with dementia who participated in Fam-FFC. On average, care partners were 61.81 years old (SD = 14.19) and primarily female (n=334, 72.5%). Care partners reported having some preparedness in general and it continued to increase from discharge (23.75, SD=6.78) to 2 months (24.5, SD=6.49) and 6 months (26.35, SD=6.73). Multiple care partner characteristics were associated with preparedness at discharge [age (b= -0.071; p<.001), burden (b= -0.283; p<.001), and depression (b= -0.284; p<.01)], 2 months [burden (b= -0.226; p<.001), strain (b= -0.144; p<.05), depression (b= -0.185; p<.05)] and 6 months [burden (b= -0.164; p<.01), strain (b= -0.183; p<.05), depression (b= -0.279; p<.01)] post discharge. While care partners' feelings of greater burden and depression were associated with lower preparedness at all time points, care partners' higher age was associated with lower preparedness at discharge only and care partners' feelings of higher strain was associated with lower preparedness at 2- and 6-months post discharge. Findings suggest the need to address care partners' feelings of burden, strain, and depression to optimize their preparedness in meeting unique care needs of hospitalized persons with dementia.

Abstract citation ID: igae098.1364

### ASSOCIATION OF BPSD AND CARE PARTNER OUTCOMES MEDIATED BY CARE PARTNER DISTRESS

Diane Berish, *The Pennsylvania State University, University Park, Pennsylvania, United States*

Behavioral and psychological symptoms of dementia (BPSD) include a range of symptoms that commonly occur during hospitalization and post-discharge. BPSD can be difficult to anticipate and can create challenges for family care partners which may lead to adverse care partner outcomes. Repeated measures data from the Fam-FFC study provides opportunity to examine the time-dependent relationship among BPSD, associated care partner distress, and care partner outcomes. Mediation models were used with Neuropsychiatric Inventory Questionnaire (NPI) scores at discharge from an acute care hospitalization, NPI scores of associated care partner distress at 2-months post discharge, and care partners outcomes at 6-months, including burden, strain, desire to institutionalize and level of preparedness for caregiving. Patient and care partner characteristics were included as covariates. Results showed that NPI severity had direct effects on desire to institutionalize (estimate=0.0754, p=0.0119) with no significant indirect effects. Care partner burden, NPI severity had statistically significant direct effects (estimate=0.3023, p=0.0038), with 53.4% (p<0.0001) mediational effect of associated care partner distress. Similarly, for care partner strain, NPI severity had statistically significant direct effects (estimate=0.21, p=0.003), with 48.8% (p<0.0001) mediational effect of associated care partner distress. There were no significant effects for the outcome of care partner preparedness. The repeated measures design study allows for the examination of these relationships over time and helps to build evidence for a mechanistic model. These results show the importance of assessment of BPSD presentation, particularly post-acute hospitalization, and providing support for care partners to reduce negative outcomes.

### SESSION 3235 (PAPER)

### NAVIGATING RACIAL HEALTH DISPARITIES: INSIGHTS ACROSS THE LIFESPAN

Abstract citation ID: igae098.1365

### LIFE HISTORIES AND TRAJECTORIES OF PHYSIOLOGICAL FUNCTIONING AMONG OLDER RACIALIZED GROUPS IN THE US

Catherine García, Morgan Parella, De'Lisia Adorno, Eniola Festus, and Londi Rowell, *Syracuse University, Syracuse, New York, United States*

Enhancing later life health outcomes among racialized groups demands a nuanced exploration of early life and adulthood circumstances, recognizing their profound impact on individual life trajectories and health disparities. This study used a subsample of age-eligible non-proxy participants from the 2006-2020 biennial waves of the Health and Retirement Study (HRS) that identified as White Hispanic/Latinx (n=787), non-White Hispanic/Latinx (n=516), non-Hispanic Black (n=2072), and non-Hispanic White (n=7984). We conducted a group-based latent class analysis to characterize distinct types of life course experiences among middle-aged and older adults, including childhood family context and residence, early-life educational experiences, and health before age 50. Then, we examined the associations between identified life course typologies and trajectories of physiological functioning using linear mixed models. All independent variables and covariates were interacted with age to discern their influence on the rate of change in physiological functioning. A 4-class model was fit for childhood family context and residence, early-life educational experiences, and health before age 50 by race and ethnicity that exhibited varying patterns of (dis)advantaged life course experiences. The parameter estimates for physiological functioning show that non-Hispanic Black and non-White Hispanic/Latinxs in the class characterized with more disadvantaged experiences exhibited higher levels of poor physiological functioning at baseline and over time relative to their more advantaged counterparts. This analysis offers a novel perspective on the intricate interplay between life course experiences and health trajectories, shedding light on critical junctures for targeted interventions to improve the health and well-being of racialized groups in midlife and beyond.

Abstract citation ID: igae098.1366

### RACIAL RESIDENTIAL SEGREGATION AND SUICIDAL BEHAVIOR ACROSS THE LIFECOURSE AMONG BLACK AMERICANS

Eskira Kahsay, and Briana Mezuk, *University of Michigan, Ann Arbor, Michigan, United States*

Residential segregation is a well-studied contextual factor with adverse impacts on physical health. However, the neighborhood is both a material and social space. Given hypothesized mechanisms of increased social support and reduced discrimination, racial/ethnic homogeneity may not be necessarily harmful to mental health. Still, little is known about its implications for suicidality among Black Americans, particularly older adults. This study investigated the associations between residential segregation and suicidal behavior using the National Survey of American Life (N = 5,191; mean age: 42.5). Lifetime suicidal behavior (ideation [10.4%] and attempt [3.6%]) were self-reported. The Getis-Ord Gi* statistic was used to measure the spatial segregation of Black residents at the census-tract level (low, medium, high). Weighted mixed-effects models were fit, adjusting for demographics and tract-level poverty and stratified by age group (early [18-29], middle [30-49], older adulthood [50+]). The prevalence of ideation and attempt did not significantly differ across segregation levels. Odds of ideation were not significantly different in high vs low segregation areas though were significantly lower in medium vs low segregation areas (OR = 0.70, 95% CI: 0.49, 0.99). Findings for suicide attempt were non-significant. Among older adults, the odds of attempt were significantly lower for high vs low segregation areas (0.98, 95% CI: 0.97, 0.99) only. No significant findings were found for other age groups, except ideation in medium vs low segregation in early adults (OR = 0.33, 95% CI: 0.15, 0.74). Despite economic disadvantage in racially segregated areas, findings suggest higher segregation is not associated with increased suicidality.

Abstract citation ID: igae098.1367

### RACIAL DIFFERENCES IN PREDICTORS OF CARDIOMETABOLIC RISK FOR OLDER US WOMEN

Christy Erving[1], KJ Davidson-Turner[1], Cleothia Frazier[2], and Jacquelyn Coats[3], *1. The University of Texas Austin, Austin, Texas, United States*, *2. The Pennsylvania State University, University Park, Pennsylvania, United States*, *3. Washington University in St. Louis, St. Louis, Missouri, United States*

Though cardiovascular disease is the leading cause of death among women, significant racial disparities in cardiovascular disease persist. This study investigated psychosocial predictors of cardiometabolic risk (CMR), a count of high-risk cardiovascular and metabolic biomarkers, among older U.S women and whether predictors differed for Black, and Hispanic, and White respondents. Data were from the Health and Retirement Study (HRS; age 50 years+). Predictors including health behaviors, socioeconomic status, psychological factors, and relational demands (with spouse, children, relatives, friends) were drawn from the 2010/2012 waves. CMR, drawn from the 2014/2016 waves, was assessed using six biomarkers: pulse pressure, resting heart rate, C-reactive protein, waist glycosylated hemoglobin A1c, and total and high-density lipoprotein cholesterol. The sample comprised 6707 women (1197 Black, 672 Hispanic, 4838 White). Linear regression analyses were conducted to assess the association between the predictors and CMR in race-stratified models. Preliminary results revealed that predictors of CMR were distinct for different racialized groups. Specifically, for Black women, obesity and former smoking status were associated with increased CMR while working part-time was associated with decreased CMR. Among Hispanic women, having a GED or high school diploma (compared to less than a high school education) was associated with decreased CMR. Among White women, obesity, smoking, depressive symptoms, and having no spouse were associated with increased CMR while higher education, working part-time, and mastery were associated with decreased CMR. Surprisingly, no one factor was a predictor of CMR for all women; moreover, relational demands did not emerge as a significant predictor of CMR.

Abstract citation ID: igae098.1368

### THE IMPACT OF THE LIFE TRIAL ON CARDIOVASCULAR HEALTH: RACIAL DISPARITIES IN LIFESTYLE FACTOR IMPROVEMENT

Donya Nemati[1], Deepishka Pemmasani[2], Vaishali Lavangu[2], and Navin Kaushal[2], *1. The Ohio State University, Columbus, Ohio, United States*, *2. Indiana University Indianapolis, Indianapolis, Indiana, United States*

Introduction. Cardiovascular disease is the leading cause of death among older adults. The American Heart Association promotes the lifestyle 8 (LE8) for cardiovascular disease prevention which includes healthy levels of exercise, sleep, smoking, BMI, blood pressure, nutrition (sodium), triglycerides and glucose levels. Exercise alone can improve most of these factors. The purpose of this study was to test if an exercise program can improve LE8 and identify racial (Black vs. White) differences. Methods. The LIFE Study is a randomized controlled trial that randomized 1,600 older adults (age 65+) to an intervention or control group (health-education program). The intervention included one-hour on-site exercise program twice per week with additional prescription of home-based exercise. LE8 measures included: exercise (accelerometers), sleep (Pittsburg-Sleep-Quality-Index), smoking (self-report). Blood samples measured glucose, triglycerides, and sodium, and on-site assessments measured blood pressure and BMI. Data was collected every six months for two years. STATA 18.0 was used to conduct multilevel modeling for each LE8 outcome. Results. A series of multilevel models revealed significant TimeXGroupXRace interaction effects for greater exercise($\beta$=.12, p=.006), better sleep($\beta$=.20, p=.04), lower BMI($\beta$=.58, p<.001) and triglycerides($\beta$=.38, p<.001) only among White participants in the intervention arm at the fifth timepoint. Sodium($\beta$=.13, p=.04), glucose($\beta$=.09, p=.04), and blood pressure($\beta$=.20, p=.04) were higher in Black participants with no time or group interaction. Smoking was nonsignificant Conclusion. The LIFE trial demonstrated improvement in some LE8 factors, and several racial health disparities. There is a need to understand biopsychosocial determinants that contribute to racial disparities to ensure that all participants benefit equally.

Abstract citation ID: igae098.1369

**A LONGITUDINAL ANALYSIS OF EVERYDAY DISCRIMINATION**

Felicia Wheaton, *Xavier University of Louisiana, New Orleans, Louisiana, United States*

Experiences of everyday discrimination are associated with poorer mental and physical health in later life. However, most studies examine discrimination at a single time point. There is a need to better understand how experiences of everyday discrimination change over time, change with age, and differ by cohort. This study examined longitudinal change in everyday discrimination using data from the 2006-2020 waves of the Health and Retirement Study (HRS), a nationally-representative study of Americans 50+ (N=23,523). Frequency of everyday discrimination was summed across 5 items: you are treated with less courtesy or respect than other people, receive poorer service than other people at restaurants or stores, are threatened or harassed, people act as if the think you are not smart, and act as if they are afraid of you (1=never, 6=almost every day). Overall, everyday discrimination discrimination decreased from 2006 until 2016 when it peaked again, then fell through 2020. Average everyday discrimination increased across cohorts, with the oldest cohort (born before 1924) having the lowest average discrimination and the late Baby Boomers (born 1960-1965) experiencing the highest average discrimination. Across waves, as people aged, their discrimination scores decreased. The adjusted linear mixed model confirmed these trends: age was negatively associated with everyday discrimination and younger cohorts reported higher everyday discrimination. These findings indicate that age, period and cohort differences exist in experiences of everyday discrimination, and this has important implications for health across the life course.

### SESSION 3240 (SYMPOSIUM)

Abstract citation ID: igae098.1370

**NAVIGATING REAL-WORLD BARRIERS IN NURSING HOME PRAGMATIC TRIALS**

Chair: A. Lynn Snow

Discussant: A. Lynn Snow

Nursing homes (NHs) are an important setting for the use of pragmatic clinical trial (PCT) approaches, particularly due to the challenging implementation environments since the COVID-19 pandemic. NHs have well documented, long-standing quality challenges, staffing shortages, and substantial time gaps between the development of new evidence and innovations and their wide-spread industry adoption. Thus, NHs are an opportune setting for the development of pragmatic methods to accelerate the adoption of efficacious interventions into practice. In this symposium we explore successful approaches used in current NH PCTs to address five common challenges: organizational partnerships, intervention adaptation, use of technology in PCTs, pragmatic measurement of outcomes, and evaluating intervention-related staff competencies. We will explore each topic through an example from one or two recent NH PCTs, sharing challenges and successes. The discussant and panel will then examine the topics across all four represented PCTs and consider applications to future PCTs.

Abstract citation ID: igae098.1371

**SLEEP MEASUREMENT TECHNOLOGY IN THE 40 WINKS PRAGMATIC CLINICAL TRIAL: CHALLENGES AND SOLUTIONS**

Kathy Richards[1], Brian Cox[2], Vanessa Aguilar[3], Regina George[3], Kate Smith[3], Sophia Geisser[3], Liam Fry[4], and Robert Morgan[5], *1. The University of Texas Austin, The Woodlands, Texas, United States*, *2. Alabama Research Institute on Aging, Tuscaloosa, Alabama, United States*, *3. University of Alabama, Tuscaloosa, Alabama, United States*, *4. The University of Texas Austin, Austin, Texas, United States*, *5. The University of Texas Health Science Center, Houston, Texas, United States*

This presentation describes examples, challenges, and solutions for using technology as part of a pragmatic trial to both deliver a sleep promotion intervention and measure its outcomes. In this trial, target nursing home residents wear an actigraph on their wrist for one week. Actigraphs are a non-invasive way of monitoring activity and rest cycles. One effective and novel application of this technology is use of the actigraphy clinical report. As part of the intervention, the clinical report of the resident's nighttime sleep, daytime napping, light exposure, and physical activity is shared with the nursing home staff during frontline staff huddles. The frontline staff then use the report to inform and target their sleep promotion interventions, for example, increasing daytime light exposure or activity, and to monitor effectiveness. Use of this technology is not without challenges. We will thus discuss several challenges and their solutions, including identifying the best wrist bands for securing the actigraphs and addressing manufacturer discontinuation of actigraph production and hardware service. We will also describe the trial's initial pilot that compared the gold standard research actigraph with a less expensive and more readily available consumer-focused device and why we, ultimately, chose to use only the research device.

Abstract citation ID: igae098.1372

**PARTNERING FOR SUCCESS: INVOLVING NURSING HOME CORPORATIONS AND SYSTEMS IN PRAGMATIC CLINICAL TRIALS**

Christine Hartmann[1], Hope Carwile[2], Sharon Sloup[3], Tanya Taylor[4], Kathy Curci-Delgaro[5], Valerie Clark[6], Princess Nash[3], and Liam Fry[7], *1. VA Bedford Healthcare System & University of Massachusetts Lowell, Bedford, Massachusetts, United States*, *2. Vivage, Lakewood, Colorado, United States*, *3. Tuscaloosa VA Medical Center, Tuscaloosa, Alabama, United States*, *4. Department of Veterans Affairs, Washington, District of Columbia, United States*, *5. VA Medical Center, Washington, District of Columbia, United States*, *6. VA Bedford Healthcare System,*

*Bedford, Massachusetts, United States*, 7. *The University of Texas Austin, Austin, Texas, United States*

Partnership with nursing home organizations is critical to implement and sustain embedded interventions. We present two successful examples from ongoing nursing home embedded pragmatic clinical trials. One example is from an National Institute on Aging-funded trial of improving sleep for nursing home residents in 3 large nursing home corporations that involved a pilot followed by a trial. During the pilot phase, external consultants trained nursing home staff in the intervention and simultaneously trained a corporate "coach" to be a train-the-trainer in the trial. Engaging corporate partners informed the research team's understanding of the context and culture and the corporate coaches' understanding of the intervention. During the 4-year trial, occuring now, corporate coaches serve as trainers for all participating nursing homes' staff, with limited external support. The other example is from a Department of Veterans Affairs (VA)-funded trial on preventing resident falls in State Veterans Homes. For this study, researchers partnered with an existing VA program focused on delivering a similar intervention. Because the existing VA program recently expanded into State Veterans Homes, and because research team members are expert in the intervention, partnership was a win for both parties. The existing VA program coaches gained an opportunity to work with State Homes that were eager to participate, agumented with additional support afforded by the research team. The research team gained extra help in implementing the intervention from experienced coaches. In both trial examples, partnership is pragmatic and key to the trial's success.

Abstract citation ID: igae098.1373

### PATIENT-CENTERED OUTCOME MEASUREMENT IN NURSING HOME-BASED PRAGMATIC TRIALS: CAPTURING WHAT COUNTS

Ellen McCreedy[1], Anthony Sisti[2], Roee Gutman[2], Rosa Baier[2], Ann Reddy[3], and Vincent Mor[2], *1. Veterans' Administration, Providence, Rhode Island, United States*, *2. Brown University, Providence, Rhode Island, United States*, *3. Brown University School of Public Health, Providence, Rhode Island, United States*

Embedded pragmatic randomized controlled trials (ePCTs) rely on routinely collected data to evaluate study outcomes. Using data from Music & MEmory: A Pragmatic TRial for Nursing Home Residents With ALzheimer's Disease (METRIcAL), we highlight the strengths and weaknesses of using routinely collected data to evaluate agitated behaviors in nursing home (NH) residents with dementia. We conducted two National Institute on Aging-funded ePCTs, one enrolled 976 residents (483 treatment, 493 control) from 54 NHs between June, 2019 and March, 2020, and the other enrolled 850 residents (431 Treatment, 419 control) from 54 NHs between May, 2021 and September, 2022. In both trials, there was no effect of the intervention on agitated behaviors, as measured by the Minimum Data Set (MDS), a routinely collected, pragmatic source of data in NHs. The intervention also did not affect behaviors as reported by staff using the Cohen-Mansfield Agitation Inventory (CMAI). However, we found a reduction in the frequency of nonaggressive agitated behaviors in response to the personalized music intervention, using the Agitation Behavior Mapping Instrument to conduct structured observations of residents, at standardized times of day, in treatment and control NHs. A personalized music intervention for NH residents with dementia reduced nonaggressive agitation, but these changes were not captured in the routinely collected administrative data (MDS) or by interviewing staff using the CMAI. Temporary reprieve from agitated behaviors may improve the quality of life for persons living with dementia, but these effects are not captured using pragmatic data or tools that rely on staff recall.

Abstract citation ID: igae098.1374

### SYSTEMATIC EVALUATION OF ADAPTATIONS AND FIDELITY IN PRAGMATIC NURSING HOME CLINICAL TRIALS

Michelle Hilgeman[1], Caroline Madrigal[2], Kimberly Curyto[3], Corilyn Ott[4], Sophia Geisser[5], Christine Hartmann[6], and A. Lynn Snow[5], *1. Tuscaloosa VA Medical Center, Tuscaloosa, Alabama, United States*, *2. VA Boston Healthcare System, Brockton, Massachusetts, United States*, *3. Western New York VA Healthcare System, Batavia, New York, United States*, *4. Birmingham VA Medical Center, Birmingham VA Medical Center, Alabama, United States*, *5. University of Alabama, Tuscaloosa, Alabama, United States*, *6. VA Bedford Healthcare System & University of Massachusetts Lowell, Bedford, Massachusetts, United States*

Adaptation of interventions to local contexts is often required for sustainable implementation. Yet maintaining and measuring fidelity to an intervention's core components is necessary during pragmatic clinical trials. We systematically addressed this inherent tension across two studies of Montessori approaches for person-centered care in Department of Veterans Affairs (VA) nursing homes. We first conducted a 3-phase pre-implementation study (2018-2021) to adapt Montessori for VA with input from operations partners, frontline clinical teams, long-term care residents, implementation scientists, and intervention developers. We used the Framework for Reporting Adaptations and Modifications-Enhanced (FRAME) to track changes in real-time using qualitative and quantitative data at eight VAs. Primary motivators for adaptation were expanding reach of the intervention and engagement with staff training/delivery. Next, the adapted Montessori intervention was tested in a pragmatic, randomized stepped-wedge clinical trial at eight additional VAs (2021-2023). Diverse frontline staff (e.g., nursing assistants, nurses, therapists) delivered the intervention to all residents. Low burden methods were used to gather reach, implementation, and fidelity data over 12-months (i.e., 15-minute weekly neighborhood check-ins, individual staff surveys, organizational-level fidelity tools completed by the clinical team). Adherence to and delivery of the intervention remained high at most sites over time across all measures. Evidence of fidelity and sustained use across sites in the trial may be attributed to the fit of the adapted Montessori intervention with the needs, resources, and clinical priorities of VA long-term care residents and staff.

Abstract citation ID: igae098.1375

### EVALUATING NURSING HOME-BASED INTERVENTIONS ON COMPETENCY LEVELS

Amy Recker[1], Ellen McCreedy[2], Rosa Baier[1], Kate Smith[3], Regina George[3], Sophia Geisser[3], Vanessa Aguilar[3], and A. Lynn Snow[3], *1. Brown University, Providence,*

*Rhode Island, United States*, 2. *Veterans' Administration, Providence, Rhode Island, United States*, 3. *University of Alabama, Tuscaloosa, Alabama, United States*

The intervention at the heart of the 40WINKS sleep trial relies on a principle that can be applied to many other nursing home-based interventions: Progress through the intervention requires a gradual and consistent accumulation of skills that build upon one another. Without completing a fundamental level of the intervention (e.g., begin to hold staff huddles regularly), a nursing home's team could not incorporate advanced concepts (e.g., engage in collaborative problem solving during huddles). With this in mind, the 40WINKS research team decided to evaluate the participating nursing homes based on progressive levels of competency. We evaluated levels of competency for each of the seven core elements of the intervention (leadership team functioning; huddles; problem solving; small action plans; individualized care; continuous metacognition; sleep and daytime activity information; connecting day and night staff; and spillover, spread, and integration), scoring each core element as not met, foundational, proficient, or advanced. We developed a matrix with the seven core elements and examples of the corresponding competency levels, which research team members complete weekly. We aggregate the competency matrices on a monthly basis and meet to code each nursing home into one of the four competency levels overall. This presentation will delve more in depth into this process, provide a roadmap for applying this approach to other interventions, and share preliminary results.

### SESSION 3245 (PAPER)

### NEIGHBORHOODS, COMMUNITIES, AND HEALTH

Abstract citation ID: igae098.1376

### NEIGHBORHOOD PERCEPTIONS AND SUICIDAL RISK BEHAVIORS: A CROSS-SECTIONAL STUDY AMONG OLDER CHINESE IMMIGRANTS

Chuwen Zhong[1], Weidi Qin[2], Yanping Jiang[3], Fengyan Tang[4], and Viktoriya Kalesnikava[1], 1. *University of Michigan, Ann Arbor, Michigan, United States*, 2. *University of Wisconsin Madison, Madison, Wisconsin, United States*, 3. *Rutgers, The State University of New Jersey, New Brunswick, New Jersey, United States*, 4. *University of Pittsburgh, Wexford, Pennsylvania, United States*

Compared to other age groups, older adults have a higher suicide risk. Studies have shown that the neighborhood environment contributes to psychosocial well-being. As the largest Asian subgroup in the U.S., Chinese immigrants make up 24% of the total Asian American population. However, it remains understudied how perceived neighborhood characteristics impact suicidal ideation and suicidal risk behaviors among older adults in this population. The study aims to investigate the association between perceived neighborhood characteristics and suicidal ideation and suicidal risk behaviors (i.e., hopelessness, depressive symptoms, and loneliness) among older Chinese immigrants. Data were drawn from the Population Study of Chinese Elderly (PINE), which was collected between 2011-2013. Neighborhood cohesion and disorder scales were used, Cronbach's alpha= 0.86 and 0.80 respectively. The suicidal ideation was measured by lifetime suicidal ideation. Hopelessness was assessed by the 7-item Beck hopelessness scale, depressive symptoms were measured by the Patient Health Questionnaire (PHQ-9), and loneliness was measured by the R-UCLA loneliness scale. Linear and logistics regressions were conducted to test the study aims. Out of a total of 3,157 respondents, 58% were female with an average age of 72.8. Higher levels of neighborhood cohesion were associated with less suicidal ideation, loneliness, and depression, but not hopelessness. On the contrary, neighborhood disorder was positively associated with all outcome variables including hopelessness. This study suggests that the neighborhood environment plays a crucial role in older Chinese immigrants' health and well-being. Culturally relevant and community-level efforts are needed to promote successful aging in place among this group.

Abstract citation ID: igae098.1377

### THE ASSOCIATION BETWEEN NEIGHBORHOOD PERCEPTIONS AND RISK OF FALLS

Qun Le, Lingming Chen, Elizabeth Procter-Gray, Meng Zhang, Danielle LoPilato, Annabella Aguirre, and Wenjun Li, *University of Massachusetts Lowell, Lowell, Massachusetts, United States*

Fall is a leading cause of injury-related death among old adults. This study is to explore the associations between neighborhood environment perceptions and the risk of falls among old adults. The data was drawn from the Healthy Aging and Neighborhood Study in Central Massachusetts (2018-2024). Data were collected from 379 participants who aged over 65 years and live in rural, suburban, and urban communities. Neighborhood perception was measured using a self-administered questionnaire. Factor analysis was used to derive perception scores in five aspects including neighborhood utilitarian (NU) (range:0-7), sense of community (SEC) (range:0-5), safety of community (SAC) (range:0-8), friendly neighborhood (FN) (range:0-3), and aesthetics of community (AC) (range:0-5). A higher score indicates a more positive perception. The occurrences of falls were recorded using monthly falls calendars. Negative binomial regressions were used to assess the associations of neighborhood perceptions and fall rates adjusting for sociodemographic factors and follow-up time. The participants had high neighborhood perceptions (mean±SD: NU: 2.46±1.69, SEC: 3.34±0.92, SAC: 7.42±0.94, FN: 1.03±0.85, and AC: 3.55±0.86). Lower rates of indoor falls were associated with a higher score in SEC (IRR: 0.8, 95%CI: 0.6-0.9) and SAC (IRR: 0.7, 95%CI: 0.6-0.8). A one-unit higher AC score was associated with 20% lower rates of outdoor falls (IRR: 0.8, 95%CI: 0.6-1.0). Higher scores in the SEC (IRR: 0.8, 95%CI: 0.7-0.9), SAC (IRR: 0.8, 95%CI: 0.7-0.9), and AC (IRR: 0.8, 95%CI: 0.7-1.0) were significantly associated with lower rates of falls in any place. These results suggest the need of considering neighborhood perceptions in fall intervention.

Abstract citation ID: igae098.1378

### A STORM IS COMING: A CLIMATE RESILIENCE FRAMEWORK FOR SENIOR HOUSING

Taylor Patskanick[1], Grace Turner[1], and Joseph Coughlin[2], 1. *Massachusetts Institute of Technology, Cambridge,*

*Massachusetts, United States*, *2. MIT AgeLab, Cambridge, Massachusetts, United States*

Older adults' vulnerability to extreme weather events may vary especially based on geographic location and housing or residential situation. Emergency preparedness is a potentially modifiable protective factor influencing this vulnerability. Emergency preparedness, however, looks different for older adults living in senior housing communities compared to community-dwelling older adults. For example, the responsibility for emergency preparedness for extreme weather within the senior housing environment expands to include the housing operator and community-based emergency management services rather than this responsibility relying solely on the individual older adult. This paper examines a set of twenty interviews conducted with senior housing operators, owners, and community-based emergency services personnel located in five U.S. states exploring emergency preparedness for extreme weather events in senior housing. Interviews were analyzed using an inductive, team-based thematic analysis approach. Results from this study are presented in a three-phase modified Haddon Matrix (Haddon, 1968) to provide a comprehensive framework promoting age-friendly extreme weather preparedness for senior housing operators and local emergency services. The implications of this research offers a foundation for a standardized checklist of actions and processes for senior housing operators, their individual communities, and emergency services to use to build capacity for age-friendly climate resilience. Senior housing communities serving older adults must comprehensive and resilient emergency preparedness plans and programs able to respond to a changing climate in an ongoing, adaptive, and multi-phase way that accounts for all actors in the senior housing environment (e.g., staff, residents, resident families) and through every stage of an extreme weather event.

Abstract citation ID: igae098.1379

### NEIGHBORHOOD ENVIRONMENT AND PHYSICAL ACTIVITY AMONG PERSONS WITH DEMENTIA

Eunyoung Choi[1], Yeon Jin Choi[2], Hyungmin Cha[1], Calley Fisk[1], Rachel Wilkie[1], and Jennifer Ailshire[1], *1. University of Southern California, Los Angeles, California, United States*, *2. University of Kentucky, Lexington, Kentucky, United States*

Physical activity plays an important role in delaying the progression of cognitive and functional decline among people with dementia (PWD). Neighborhood environments can provide key resources or function as critical barriers to promoting physical activity among PWD. However, the specific neighborhood factors that drive this relationship are yet to be determined. Our study examines how socioeconomic (i.e., area affluence and disadvantage), physical (i.e., annual average PM2.5, temperature, park area), and social (i.e., cohesion, disorder, and safety) aspects of neighborhood environment are associated with physical activity levels among PWD living in communities across the U.S. We used data from 2006 to 2018 of Health and Retirement Study for dementia status and neighborhood social environment, merged with census-tract level data from the Contextual Data Resource for socioeconomic and physical neighborhood factors. Specifically, we used the first wave of data where PWD were identified as having dementia (N=3,073). Physical activity was measured as a weighted sum of light, moderate, and vigorous activity (range: 0-31). We estimated linear regression models adjusting for individual-level sociodemographic covariates. Higher neighborhood socioeconomic affluence (B=0.45, 95% CI=0.07, 0.84) and social cohesion (B=0.70, 95% CI=0.32, 1.09) were associated with higher levels of physical activity. Conversely, any sign of neighborhood physical disorder (B=-1.23, 95%CI=-2.09, -0.38) was associated with reduced physical activity. No significant relationships were found from physical neighborhood environment factors. This study provides empirical evidence for the social-ecological model which posits the important role of broader environmental factors that influence PWD's physical activity participation.

### SESSION 3250 (SYMPOSIUM)

Abstract citation ID: igae098.1380

### NOVEL AUTOMATED TOOLS TO IDENTIFY COGNITIVE DECLINE AND EMOTIONAL DISTRESS IN THE CONTEXT OF MCI AND AD/ADRDS

Chair: Sarah Bannon
Discussant: Alex Federman

Alzheimer's disease and related dementias (ADRDs) are rapidly increasing public health concerns with significant impact on individuals, families, and healthcare systems. Though recent technological advances have enabled earlier and more accurate diagnosis for some, the vast majority of individuals experience delays in diagnosis and unmet early support needs. Diagnostic delays and inadequate support exacerbate psychological distress for impacted individuals, which is important to address given 1) the impact of distress on long-term outcomes and 2) the difficulty of treating distress once it becomes chronic. Novel automated technologies have the potential to more effectively and efficiently identify changes in cognition and distress, and if leveraged could provide cost-effective avenues to improve ADRD care. This symposium describes novel methods of leveraging large-scale data and technology to enhance screening and identification of early decline and distress in the context of cognitive impairment and ADRDs. Ryan Mace, PhD will describe healthcare providers' impressions of barriers to assessing subjective cognitive decline and gaps in current practice. Sarah Bannon, PhD will present findings from a secondary data analysis of electronic medical records examining structured data elements as indicators of psychological distress among individuals with recent ADRD diagnoses. Ayushi Divecha, MPH MPT will describe the use of random forest models to identify individuals at high risk for developing dementia from a harmonized database of 5 of the largest NIH-funded studies of aging across the US.

Abstract citation ID: igae098.1381

### PILOT FEASIBILITY STUDY OF EMR-BASED IDENTIFICATION OF PSYCHOLOGICAL DISTRESS AROUND RECENT DEMENTIA DIAGNOSES

Sarah Bannon, Ayushi Divecha, Kristen Dams-O'Connor, and Alex Federman, *Icahn School of Medicine at Mount Sinai, New York, New York, United States*

ADRDs have a significant impact on individuals, families, and systems. Though technological advances have enabled earlier diagnoses, early psychological distress experienced around the time diagnosis is largely overlooked. Psychological distress is an important early intervention target given its pronounced negative impact on ADRD adjustment. Interventions are limited by our insufficient understanding of psychological distress experienced around the time of ADRD diagnoses. Our primary objective was to investigate the feasibility of identifying EMR-based psychological distress in a diverse sample of individuals with recent ADRD diagnoses. We conducted secondary data analysis of EMR records obtained from the Mount Sinai Data Warehouse (MSDW) and identified N=12249 individuals with recent ADRD diagnoses after age 65. ICD-10 diagnoses of depression and anxiety were identified in (32.73%) and (27.58%) of the sample, respectively. We also examined scores above the standard cut-offs for clinically elevated distress using self-report measures of depression [PHQ-9 (>=10)] and anxiety [GAD-7 (>=10)]. Unfortunately, these measures were assessed in a very small proportion of the sample at the visit of dementia diagnosis. Out of 509 (4.1%) individuals with self-report measures available, 113 (22%) indicated clinically elevated distress.. Structured data elements can be used to identify distress in the context of dementia diagnosis, though they are limited in their administration and may only offer the potential to identify distress in a subset of individuals. Our findings emphasize the importance of further exploration of unstructured data elements to identify distress.

Abstract citation ID: igae098.1382

### USING SUPERVISED MACHINE LEARNING METHODS TO IDENTIFY INDIVIDUALS AT RISK OF DEMENTIA

Ayushi Divecha, Sarah Bannon, and Kristen Dams-O'Connor, *Icahn School of Medicine at Mount Sinai, New York, New York, United States*

There has been an increased interest in AI and machine learning based models in early detection and diagnosis of Alzheimer's disease (AD) and Alzheimer's disease related dementia (ADRD). However, there is a need for feasible tools to identify individuals at risk of AD/ADRD using machine learning methods that can be validated. Early identification of risk of AD/ADRD can be valuable for building a monitoring system and planning of care. The aim of the study is to identify individuals at risk of developing dementia at baseline visit using machine learning models. Clinical and cognitive data from 5 of the largest NIH-funded studies of aging across the US (ACT, ROS, MAP, MARS, FHS) will be used to predict all-cause dementia in cognitively normal individuals at baseline using random forest models with competing risk of death. Cognitive elements like executive functioning, language, memory, visuospatial ability will be included. Since cognitive function was assessed using different measures across cohorts (MMSE and CASI), co-calibrated cognitive factor scores will be used in the trained models to allow for direct comparison of cognition across cohorts and joint analyses. We examined 3 out of 5 cohorts. From ROS/MAP/MARS cohorts, 2235 individuals qualified for the study and had a mean follow-up of 8.6 ± 5.3 years. AOC will be used to assess sensitivity and specificity of the trained models (75% training set, 25% testing set) with each of the 5 cohorts. The models will also identify features important in predicting dementia.

Abstract citation ID: igae098.1383

### OVERCOMING BARRIERS IN DETECTING SUBJECTIVE COGNITIVE DECLINE: A QUALITATIVE STUDY WITH HEALTH CARE PROFESSIONALS

Ryan Mace[1], Joshua Cohen[2], Christine Ritchie[3], Olivia Okereke[2], Stephen Bartels[1], Bettina Hoeppner[1], Judson Brewer[4], and Ana-Maria Vranceanu[2], *1. Massachusetts General Hospital/Harvard Medical School, Boston, Massachusetts, United States*, *2. Massachusetts General Hospital, Boston, Massachusetts, United States*, *3. Mass General Hospital and Harvard Medical School, Boston, Massachusetts, United States*, *4. Brown University Medical School/Brown University School of Public Health, Providence, Rhode Island, United States*

Identifying older adults with subjective cognitive decline (SCD) is imperative for early interventions targeting modifiable risk factors for Alzheimer's Disease and Related Dementias (ADRD). However, with fewer than half of older patients with SCD engaging in discussions about their concerns with healthcare professionals, a significant gap in current practice is evident. To address this gap, we engaged healthcare professionals (physicians and medical staff) in a qualitative study that aimed to: 1) understand barriers and facilitators to identifying SCD, and 2) develop strategies to guide the implementation of screening and assessment tools into routine care. We conducted live video focus groups with 26 healthcare professionals (Mean experience = 17 ± 13.6 years) from 5 clinics that treat older patients (e.g., psychiatry, neurology, integrative medicine, memory care). We analyzed the qualitative data using a hybrid deductive–inductive approach. We proposed concrete strategies for addressing barriers to SCD identification using the Expert Recommendations for Implementing Change (ERIC) framework. Four themes on barriers to SCD identification emerged: 1) the clinical heterogeneity of SCD, 2) variability in patients' self-identification, 3) variability in clinical assessment practices, and 4) need for additional research and standardization. ERIC-informed strategies to address barriers included: education and training for providers, standardized diagnostic protocols, incorporating caregiver reports, and promoting de-stigmatizing language. Our study contributes to the symposium theme by delineating the intricacies of identifying SCD among older adults and by proposing ERIC framework-based strategies that align with the need for novel screening and assessment tools.

### SESSION 3255 (PAPER)

### NURSING HOME CARE

Abstract citation ID: igae098.1384

### DO MINIMUM STAFFING LEVELS IMPROVE NURSING HOME QUALITY? EVIDENCE FROM CALIFORNIA'S MINIMUM STAFFING REGULATIONS

John Bowblis[1], and Christopher Brunt[2], *1. Miami University, Oxford, Ohio, United States*, *2. Georgia Southern University, Statesboro, Georgia, United States*

Nursing staff is a key component in providing high quality of care to nursing home (NH) residents, therefore, as of 2021, 39 states have minimum nursing staff regulations. In September of 2023, CMS announced a proposed rule for a first ever federal minimum nursing staff level, even though the effectiveness of these regulations at improving quality is unknown. In this paper, we utilized a difference-in-differences regression framework to understand how changes to minimum staffing regulations impact staffing and quality. In particular, we examine California's 2018 change to staffing regulations that increased the minimum staffing level from 3.2 to 3.5 hours per resident day (HPRD) and implemented a new 2.4 HPRD minimum requirement for certified nurse aides (CNA). Comparing California to neighboring states that did not change staffing requirements, California NHs increased CNA staffing levels 0.15 HPRD (6.5% increase) and total nursing staff levels of 0.184 HPRD (4.8% increase) on average. Even with these increases, only about 70% of NHs met the new CNA requirement and 90% met the new 3.5 HPRD staffing requirements in 2019. While staffing levels increased, only 3 of the 13 quality measures examined were statistically significant at the 5% or 10% levels. At the 5% level, there was improvement in incontinence among long-stay residents. At the 10% level, there was improvement in pressure ulcers among long-stay residents and the short-stay rehospitalization rate. These results indicate that increasing staffing levels through mandated minimums had small to negligible impact on most aspect of NH quality.

Abstract citation ID: igae098.1385

### ASSESSING THE INFLUENCE OF NURSING HOME SIZE AND CARE MODELS ON COVID-19: CASE AND MORTALITY RATES

Yuchi Young[1], Yichun Liu[2], Ashley Shayya[3], Wan-Yu Chiu[4], Wanhsiang Hsu[3], and Thomas O'Grady[3], *1. State University of New York at Albany, Albany, New York, United States*, *2. New York State Department of Health, Albany, New York, United States*, *3. University at Albany, SUNY, Albany, New York, United States*, *4. National Taiwan University, Taipei, Taipei, Taiwan (Republic of China)*

The COVID-19 pandemic disproportionately affected older adults in nursing homes, emphasizing the need for comparing various long-term care options. This study compares COVID-19 prevalence and mortality rates in traditional nursing homes, Green Houses (GHs), and nursing homes nested in Continuing Care Retirement Community (CCRC-NHs). CMS data and LTCFocus Data were utilized. COVID-19 case and mortality rates from June 1, 2020, to September 30, 2022, across traditional NHs, GHs, CCRC-NHs in 11 states were included. Poisson regression was utilized to compare risk among types of NHs while adjusting for covariates. The average age of residents of CCRC-NHs and GH was 84.4 and 83.1-years, respectively, higher than those in small (77.5-years) and large (77.6-years) traditional nursing homes (P<.0001). The proportion of female and White residents was significantly higher in CCRC-NHs and GHs than in traditional NH. Compared to GH residents, those in CCRC-NHs (RR 1.50, 95% CI 1.10-2.07), large NHs (RR 1.57, 95% CI 1.14-2.15), and small NHs (RR 1.80, 95% CI 1.32-2.47) had a higher risk of COVID-19 infection, with the highest risk observed in traditional small NHs. There were no statistically significant differences in COVID-19 mortality rates across different types of NHs. This study suggests that nursing home size and care models influence COVID-19 transmission, GHs, with their smaller, person-centered care settings, experienced lower infection rates, highlighting the potential advantages of such environments in pandemic management. Conversely, traditional NHs and CCRC-NH models, with more institutional layouts, may contribute to higher transmission rates suggesting a need for care model reevaluation.

Abstract citation ID: igae098.1386

### ASSESSING THE IMPACT OF THE PATIENT-DRIVEN PAYMENT MODEL ON NURSING HOME CARE QUALITY: A STAFFING PERSPECTIVE

Gregory Orewa[1], Robert Weech-Maldonado[2], Ganisher Davlyatov[3], Justin Lord[4], David Becker[2], and Sue Feldman[2], *1. The University of Texas San Antonio, San Antonio, Texas, United States*, *2. University of Alabama at Birmingham, Birmingham, Alabama, United States*, *3. University of Oklahoma Health Sciences Center, Oklahoma City, Oklahoma, United States*, *4. Louisiana State University Shreveport, Shreveport, Louisiana, United States*

The Patient-Driven Payment Model (PDPM) launched on October 1, 2019, restructured Medicare payments to reflect resident needs over service quantity, aiming to improve quality and efficiency in nursing homes (NH). PDPM's shift towards resident needs (rather than therapy minutes) may lead to greater nurse utilization, which may potentially improve the quality of care. This study draws on the resource-based view (RBV) theory to understand the association between the PDPM and NH quality of care mediated by nurse staffing intensity. Utilizing a longitudinal design (2017-2021), seven different datasets were merged (Cost reports, Payroll Base Journal (PBJ), Care Compare, Area Health Resource File (AHRF), HHS Provider Relief Fund, CDC NH COVID-19 public files, and CDC COVID-19 Data Tracker). Structural equation modeling (SEM) was used to assess the direct and indirect effects of PDPM on care quality, with nursing staffing intensity serving as a mediator. NH quality of care was operationalized using quality-measure five-star rating and staffing intensity by RN, LPN, and CNA hours per resident. We examined the direct effect of PDPM on quality of care and the indirect effect of PDPM on quality through nurse staffing. Control variables included organizational and market-level factors, and COVID-19-related variables. The result suggests an increase in RN staffing post-PDPM but a decrease in CNA hours ($p< 0.001$). PDPM had an indirect positive effect on quality through RNs while negative through CNAs. PDPM also showed a positive direct effect on quality. Policy implication highlights the complex interplay between different staffing roles and the impact on quality.

Abstract citation ID: igae098.1387

### IMPACT OF THE TEACHING NURSING HOME ON NURSING STUDENT'S PERCEPTIONS OF LONG-TERM CARE

Howard Degenholtz[1], Kenna Campbell[1], Keri Kastner[1], and Anny Treat[2], *1. University of Pittsburgh, Pittsburgh, Pennsylvania, United States*, *2. Sebastian Mann Associates, Pittsburgh, Pennsylvania, United States*

The Teaching Nursing Home (TNH) is designed to improve the long-term care workforce and improve quality of care in nursing homes. The initiative has multiple components: enhanced clinical rotations for nursing students with partner schools of nursing, implementation of the Institute for Healthcare Improvement Age-Friendly Health System "4M" quality improvement model, and an online learning network. Nursing students at three schools of nursing participated in clinical rotations at four regional nursing homes. The experience was limited to students in one specific course at each school of nursing. At the beginning and end of each semester, students rated their competence in: patient assessment, collaborating with the care team, gathering clinical information, medication review, eliciting resident values, and health promotion. Students also rated their preferences for working in long-term care and with older adults. Data from 514 responses at the start of semester and 280 responses to the end of semester survey were analyzed. Analysis of student responses found that students self-rated competencies improved in all areas. Prior to their clinical experience, students ranked working in long-term care and with older adults lower than other settings or populations. The rankings were unchanged after their clinical experiences. Qualitative analysis found that most student comments (50%) were about skills, while only 1.5% were about potential careers in nursing homes. These findings suggest that the Teaching Nursing Home Program is meeting the pedagogical goals, however attitudinal shifts may require different strategies.

Abstract citation ID: igae098.1388

### NURSING HOME QUALITY RATING ASSOCIATIONS WITH PAIN IN NURSING HOME RESIDENTS WITH DEMENTIA

Sorah Levy, Barbara Resnick, and Sarah Holmes, *University of Maryland School of Nursing, Baltimore, Maryland, United States*

Pain is highly prevalent among nursing home (NH) residents with dementia. Estimates suggest 35-80% of NH residents with dementia experience pain. Pain had been identified as a quality indicator used to measure NH performance. The Center for Medicare and Medicaid Services (CMS) Five-Star Quality Rating System is intended to inform the public of NH quality metrics. There should, therefore, be evidence that facilities with higher star ratings provide better pain management. This cross-sectional secondary data analysis used baseline data from an implementation study testing the impact of an intervention to manage behavioral symptoms in NH residents with dementia. Generalized linear mixed models (GLMM) were used to test the relationship between CMS five-star rating and pain level controlling for resident demographics and clinical characteristics. The sample included 553 residents with dementia living in 55 NHs. On average, NHs in the sample had a five-star rating of 3.5 (SD= 1.3) and pain was observed in 22.6% (n=125) of the sample. Five-star rating was significantly associated with having pain (p=0.041) such that the odds of having pain for residents living in a 5-star facility decreased by 87.8% (OR= 0.122, 95% CI: 0.02- 0.92) relative to a 1-star facility. Findings from this study demonstrate that quality of care indicated by star rating is associated with better pain management. It may be particularly important to focus on pain management in NHs with low star ratings.

### SESSION 3260 (SYMPOSIUM)

Abstract citation ID: igae098.1389

### POLICY SERIES: ELECTION YEAR RESULTS: IMPLICATIONS FOR OUR FUTURE

Chair: Brian Lindberg
Discussant: Patricia D'Antonio

Will Social Security be targeted? Will Obamacare be gutted? How will Medicare drug prices be affected? These and many other programs and policies will be influenced by who sits in the White House and the Congress starting in 2025. Join your colleagues and GSA's own pundits for a discussion on what went down on November 5th and what will be coming up in aging policy.

### SESSION 3265 (SYMPOSIUM)

Abstract citation ID: igae098.1390

### PRESIDENTIAL SYMPOSIUM: EMBRACING ENGAGED TEACHING, RESEARCH, SERVICE, AND POLICY TO STRENGTHEN GERONTOLOGICAL EDUCATION

Chair: Christine Fruhauf
Discussant: Tina Newsham

It takes fortitude for GSA members to embrace engagement in their academic roles. Engagement is the opportunity for informing and enhancing research, teaching, and service, and policy by actively participating in activities that connect students with external partners and the community. In this AGHE Presidential Symposium, leading scholars whose grit and determination to embrace engagement will address the important role engagement has in uplifting gerontology and geriatrics education. Elinor Schoenfeld and colleagues will provide insights into their innovative, multidisciplinary approach to teaching students about community engagement, technology, and data security as it relates to supporting older adults living in their homes. Taylor Jansen will discuss incorporating Geographic Information Systems (GIS) Mapping into gerontology research and teaching both undergraduate and graduate students, as she works closely with community partners in several states across the U.S. to uplift their programming and policy work. José Carrión-Baralt will describe the process he and his colleagues embraced as they coordinated efforts across several universities and health professions to support older adults after hurricanes Irma and Maria hit Puerto Rico in 2017, through the development of a team called Brigadas Salubristas (Public Health Brigades). Shannon Jarrott will highlight ways to engage students in local, state, and federal aging policy through decades of engaged work addressing intergenerational programming. AGHE Chair Tina Newsham will serve as discussant, sharing how AGHE will continue to embrace fortitude and support educational efforts and opportunities through engaged teaching, research, service, and policy.

Abstract citation ID: igae098.1391

### PROVIDING STUDENTS WITH INSIGHTS ABOUT AGING AND TECHNOLOGY USING A COLLABORATIVE TEACHING APPROACH

Elinor Schoenfeld[1], Patricia Bruckenthal[2], Shelley Horwitz[2], Erez Zadok[2], Tracy Trimboli[2], and Fan Ye[2], *1. Stony Brook University School of Medicine, Stony Brook, New York,*

*United States*, *2. Stony Brook University, Stony Brook, New York, United States*

Novel and innovative methods are needed to introduce our youth to what roles they can play in supporting our aging population, whether it be in a professional or personal role. To engage college lowerclassmen in discussions about aging, a multidisciplinary team of faculty from the College of Engineering and Applied Sciences and the Schools of Medicine, Nursing, and Social Welfare developed a 10-week seminar entitled "Smart Aging – at the Intersection of Technology and Healthcare." Students were introduced to community engagement, modern sensing, and analytics technologies for physiologic and physical data, concerns about data security and privacy issues related to technology use, and issues faced by older adults aging at home, including social isolation and social determinants of health. Students learned the process of developing vignettes/personas related to aging in place and how technology may help overcome an identified aging-in-place challenge. To learn about aging-in-place outside of class, students were asked to speak with family members or neighbors about aging. Each week students were asked to share and discuss what they have learned about aging in place from their explorations. At the end of the course, students presented their own vignettes/personas and graded the other students' work. We are currently taking lessons learned to develop a similar multidisciplinary graduate-level course. During this presentation, we will share our experiences with teaching a cohort of freshman and sophomores about aging and share some of their vignettes/personas to highlight their perceptions about and insights into aging.

Abstract citation ID: igae098.1392

### INCORPORATING GEOGRAPHIC INFORMATION SYSTEMS (GIS) MAPPING INTO GERONTOLOGY RESEARCH AND TEACHING

Taylor Jansen[1], Chae Man Lee[2], Nina Silverstein[3], and Elizabeth Dugan[1], *1. University of Massachusetts Boston, Boston, Massachusetts, United States*, *2. University of Connecticut, Farmington, Connecticut, United States*, *3. University of Massachusetts Boston, Needham, Massachusetts, United States*

In gerontology, research frameworks like the life course perspective and social determinants of health (SDOH) are often used to contextualize aging and health outcomes in later life. Increasingly, an emphasis on data visualization has been put on research to have practical value. Geographic information systems (GIS), a mapping software, is one data visualization tool researchers can utilize in their work to understand what role place, space, and built environment have on health throughout the lifespan and in later life. Incorporating GIS training into gerontology research allows for students to learn data visualization, but also to apply their teachings of the life course perspective and SDOH to further understand the impact on health in later life. In this presentation, the aims are twofold: 1) explain the practical uses of GIS in gerontological research, with examples from recent work by the Healthy Aging Data Reports, and 2) describe the process of engaging undergraduate and graduate students in research, teaching, service, and policy through GIS mapping. Firstly, examples of how GIS mapping has provided communities and states with information on their aging populations will be examined. Examples of how community stakeholders in New England, Mississippi, and Wyoming have used maps to implement interventions and advocate for policy change will be described to demonstrate the practical implications of mapping for policy change. Then, best practices of teaching and training undergraduate and graduate students on using GIS mapping for gerontological research will be offered, alongside anecdotal evaluations from students on the experience.

Abstract citation ID: igae098.1393

### WHEN THE GOING GETS TOUGH, GERONTOLOGY (AND OTHER HEALTH PROFESSIONS) STUDENTS GET GOING

José Baralt, *University of Puerto Rico, San Juan, Puerto Rico*

In September of 2017, two category 5 hurricanes hit Puerto Rico. These hurricanes caused massive destruction and close to 5,000 excess deaths. Thousands of people lost everything, and many communities were isolated for weeks. The whole island was without electric power for several days, and some areas in the mountains did not get power back for 6-8 months. Hopelessness started to take over. The bravery and drive of a community leader who came asking for our help was the spark we needed to stand up and get going. Dozens of our students delivered food, water, medications, and healthcare services to those in need for several months. In January of 2020 these students and faculty went back to work in full force after a series of devastating earthquakes shook the island. And, to this day, our team still visits some of these communities, just to check on them and refer people with significant needs to the appropriate agencies. All this without skipping a beat in our academic work. Where did we find the fortitude to keep going with our community work AND our academic work? We did have three main sources of inspiration: 1) community leaders who rose above every possible challenge; 2) the communities themselves; and 3) our students and colleagues. The key behind this amazing response was that those of us who work in service professions thrive when we are needed. And our students certainly shone in the darkest period our island has lived in for many decades.

Abstract citation ID: igae098.1394

### STUDENTS CONNECTING THE DOTS BETWEEN POLICY THROUGH SERVICE, RESEARCH, AND TEACHING

Shannon Jarrott, *The Ohio State University, Columbus, Ohio, United States*

Students pursuing social sciences degrees because of a desire to improve individual, family, and community circumstances should learn how policy impacts their efforts. I offer examples of teaching, research, and service that orient students to gerontological policy at the organizational, state, and national levels. Examples illustrate fortitude of students, older adults, and organizations in the face of emergent need and opportunity. At the organizational level, students engaged in service at an intergenerational day program for children and older adults developed a satellite food pantry in response to intergenerational need for healthy food access. At the state level, the Ohio the

Scholars in Aging program convenes annual cohorts of undergraduate and graduate students from across the state in study of policy making via a partnership between the Ohio Association for Gerontology Education (OAGE) and the Ohio Department of Aging. At the federal level, research students facilitated older adults' access to the federally funded Senior Farmers' Market Nutrition Program during COVID. Students' goals to support the well being of older adults and their families and communities are facilitated through engaged learning, service, and research that connects theory, skills, evidence, and policy. Students may learn about policies that are responsive to emergent need and can harness resources across agencies. They may also identify policies that isolate organizations and prevent collaboration, even when addressing similar goals for different populations. Awareness of the different shapes policies take in their objectives, implementation, and impact enhances students' ability to navigate and utilize policy to positive effect.

### SESSION 3270 (SYMPOSIUM)

Abstract citation ID: igae098.1395

### RISK FACTORS FOR FINANCIAL FRAUD AND INTERVENTION STRATEGIES IN DIFFERENT CULTURAL SETTINGS

Chair: Hong Mi
Discussant: Peter Lichtenberg

This symposium, comprising five papers, explores financial fraud targeting older adults in mainland China and the U.S.A. Four papers delved into risk factors associated with financial fraud victimization, while one paper examined the efficacy of an intervention effort. The first study, leveraging the 2015 national survey of older Chinese, introduced an algorithm based on machine learning techniques to develop a financial fraud risk prediction model for older adults. This model underscored risk factors like lower education levels, advanced age, and loneliness, alongside protective factors such as awareness of rights protection and quality sleep. Drawing on the 2018 CHARLS data, the second paper investigated both the direct and indirect effects of physical functioning, family dynamics, and individual capital on fraud victimization, revealing psychological vulnerability and material wealth as mediating factors. The third study shifted focus to the U.S.A, profiling victims of postal scams. It identified low education and social isolation risk factors, echoing the patterns observed in the Chinese context in the first paper. The fourth paper examined fraud victimization among a distinct group of older individuals residing in the forest regions of northeastern China, highlighting the roles of financial literacy and internet safety in reducing financial fraud risks. The final paper broadens the research scope by evaluating an intervention designed to aid dementia family caregivers in rural Michigan to mitigate financial exploitation risks. The piloted case management model enhanced caregiver competence in addressing financial exploitation. Together, these studies enrich the understanding of financial fraud affecting older adults in both countries.

Abstract citation ID: igae098.1396

### PREDICTION OF FINANCIAL FRAUD RISKS AMONG OLDER ADULTS: THE APPLICATION OF GA-XGBOOST MODEL

Jin Zhao, Yuanyuan Li, Hao Jin, and Xuemin Zhang, *Hebei University of Technology, TianJin, Tianjin, China (People's Republic)*

The importance of crime prevention can be more profound than correction efforts after crime occurs, highlighting the necessity to develop an early risk warning model that can accurately identify financial fraud risks among older adults. Utilizing data from the fourth Urban and Rural Survey on the Living Conditions of Older Adults conducted in 2015 in China, this study leverages a genetic algorithm (GA) to optimize the parameters of the XGBoost model, resulting in the creation of a GA-XGBoost-based financial fraud risk prediction model tailored for older adults. The efficiency of this novel model is evaluated against established machine learning algorithms, including LightGBM, Random Forest, Decision Tree, and SVM. Furthermore, SHAP values are employed for a detailed visual analysis of the factors most influential in predicting and understanding financial fraud risks among this demographic. We found that variables such as lower education level, older age, and loneliness increased risks for financial fraud. In contrast, increased awareness of rights protection and better sleep quality appear to have protective effects. A comparative assessment at both domestic and international levels, with references to the United States, Germany, and South Korea, highlights psychological vulnerability, cognitive functioning, age, and loneliness as financial fraud risks among older adults. This study offers recommendations for mitigating financial fraud risks from three angles: enhancing public awareness and education; implementing effective regulatory frameworks; and strengthening intergenerational connections. These suggestions will help lay the groundwork for more robust protective measures against financial fraud targeting older adults.

Abstract citation ID: igae098.1397

### RISK FACTORS FOR FINANCIAL FRAUD AMONG OLDER CHINESE: AN ANALYSIS BASED ON CHARLS DATA

Yuanyuan Li[1], Yunqi Cao[1], Yuxia Wu[2], and Hong Mi[3], *1. Hebei University of Technology, TianJin, Tianjin, China (People's Republic), 2. Ningbo University of Technology, Ningbo, Zhejiang, China (People's Republic), 3. Zhejiang University, Hangzhou, Zhejiang, China (People's Republic)*

Financial fraud targeting older adults in China is a growing concern, resulting in both financial losses and adverse effects on their physical and mental health. Despite its prevalence, there remains a scarcity of research focused on the victims of these frauds. Leveraging the 2018 China Health and Retirement Longitudinal Study (CHARLS) database and applying structural equation modeling (SEM), this study delves into the factors that place older adults in China at risk of financial fraud, examining the issue through the lens of the victims themselves. The frameworks typically used in Western contexts like the United States and Britain only focused on individual level factors, such as age and living arrangement, physical functioning, and individual capital (i.e., levels of education, cognition, and social activities). This study integrates unique social models, including factors related to family relationships, to reflect the Chinese cultural setting. It offers a comprehensive view of the elements that heighten the risk of financial fraud among older Chinese.

The analysis uncovered that factors such as age and living arrangement, physical functioning, family relationship, and individual capital play a direct role in increasing the vulnerability of older adults to financial scams. Furthermore, psychological vulnerability and material wealth were identified as mediators among physical functioning, family relationships, and fraud risks. Based upon these findings, the study proposes targeted strategies to mitigate the risk of financial fraud to include promoting healthier living habits, enhancing individual capital, and reinforcing family connections.

Abstract citation ID: igae098.1398

### WHO EXPERIENCES MASS MARKETING FRAUD IN AMERICA? A SURVEY OF KNOWN VICTIMS

Marguerite DeLiema[1], Siyu Gao[2], M. Daniel Brannock[3], and Lynn Langton[3], *1. University of Minnesota Twin Cities, St. Paul, Minnesota, United States*, *2. University of Minnesota, Minneapolis, Minnesota, United States*, *3. RTI International, Research Triangle Park, North Carolina, United States*

Significant research has attempted to understand the characteristics of older adult fraud victims, yet most surveys rely on participants' self-reports of victimization. This approach can lead to biased estimates because older victims are less likely to disclose victimization experiences relative to younger victims. We avoid underreporting bias by surveying a population of victims identified by the US Postal Inspection Service. All respondents mailed money in response to fraudulent solicitations between 2022 and 2023 (range = 1 to 168 victimization incidents). Victims' payment envelopes were detained by Postal Inspectors as part of the agency's law enforcement efforts. Victim addresses were securely shared with the research team. The survey response rate was 23% (N=934); 84% of participants responded by mail and 16% responded online. Average age was 74 (standard deviation = 13.2; range= 20 to 98). Mass marketing mail fraud victims were racially and economically diverse: 22% of victims were Black, 10% were Latino/a, and 6% were Asian or Pacific Islander. A quarter reported an annual household income of less than $20,000; 27% reported a household income between $20,000 and $50,000. Rates of widowhood and divorce were high—31% and 20%, respectively. Nearly half of fraud victims reported that they live alone. These results suggest that diverse, lower income and isolated older adults are targeted by mass marketing fraud. Given the relatively robust response rate, consumer fraud education materials should be mailed to victim households and include information on how to identify fake solicitations and report scams.

Abstract citation ID: igae098.1399

### CHARACTERISTICS ASSOCIATED WITH FRAUD VICTIMIZATION AMONG OLDER CHINESE LIVING IN FOREST TOWN AREAS

Xiuting Cai, Guoyong Ma, and Hongge Zhu, *Northeast Forestry University, Harbin, Heilongjiang, China (People's Republic)*

The forest regions in northeast China, characterized by integrated industrial, urban, and rural communities known as forest town groups, have experienced significant demographic shifts over the past decade. These shifts included accelerated aging population combined with rapid population migration, making these areas emblematic of China's modernization efforts in achieving urban-rural integration. Notably, the older population in these town groups has increasingly become targets of financial fraud. Against this backdrop, this study used panel data collected from 2012 to 2023 annually in these Chinese forest town areas. These surveys collected data on resident livelihoods and well-being, including educational attainment, income levels, living conditions, health status, and elder care services. This study focused on the roles of internet usage and financial literacy in relation to financial fraud victimization among older adults. Three major findings are as follows: 1) Internet usage correlates with a heightened risk of financial fraud, while financial literacy significantly reduces older adults' susceptibility to fraud and financial losses. 2) Older women tend to have lower risks for financial fraud compared to their male counterparts, largely attributable to their higher financial literacy. 3) Other characteristics associated with higher financial fraud risks are loneliness, living alone, residing without children (empty-nest status) or living with disabilities. These findings emphasize the need for targeted interventions to improve financial literacy and internet safety practices among older adults in forest town areas, particularly focusing on the most vulnerable groups, to safeguard them against financial fraud.

Abstract citation ID: igae098.1400

### FINANCIAL EXPLOITATION PREVENTION AMONG OLDER ADULTS WITH DEMENTIA IN RURAL MICHIGAN: A PILOT RANDOM CONTROL TRIAL

Fei Sun[1], Yu Guo[2], Zhenmei Zhang[3], Xia Wang[4], Haneul Kim[3], Brittany Tucker[3], and Eleanor Smith[3], *1. Michigan State University, Haslett, Michigan, United States*, *2. Xi'an Jiaotong University, Xi'an, Shaanxi, China (People's Republic)*, *3. Michigan State University East Lansing, East Lansing, Michigan, United States*, *4. Arizona State University, Phoenix, Arizona, United States*

Dementia increases the susceptibility of older adults to financial exploitation, including abuse by relatives or acquaintances and fraud by strangers. This study evaluated the effectiveness of a novel community-based financial exploitation prevention model for older adults with dementia residing in rural Michigan. Drawing from the routine activity theory, the study focused on mitigating financial exploitation risks linked to family caregivers. While family caregivers might potentially engage in such exploitation, they also play a crucial role in detecting and preventing fraudulent activities. Employing a randomized control design, 32 family caregivers in intervention groups and 23 in control groups received an education session on dementia, financial exploitation, and community resources. In addition, intervention groups received 6 sessions of case management service tailored to mitigate identified risk factors for financial exploitation. Linear mixed modeling (LMM) was applied to assess changes before and post intervention and between groups on the following outcomes: attitude towards financial exploitation, competence in addressing financial exploitation, caregiver stress, and dementia knowledge and stigma. No significant differences were found on dementia knowledge and attitude towards financial exploitation. However, caregivers in the intervention groups exhibited significantly greater confidence in addressing financial exploitation and reported lower levels of dementia-related stigma (p <.05) compared to the control group. Both groups reported a decrease in caregiver stress, but no group difference was observed. The study highlights the integral role of family caregivers as a protective shield against financial exploitation, and the need for policy and practice efforts to educate and support family caregivers.

### SESSION 3275 (SYMPOSIUM)

Abstract citation ID: igae098.1401

### STIRRING THE POT OF AGING: DIVERSITY IS THE SECRET INGREDIENT

Chair: Alisha Thompson
Co-Chair: Barbara Mendez Campos
Discussant: Laneshia Conner

Aging and our experiences of this process are as unique as the ingredients in our favorite recipes that warm our hearts on chilly days. These components of diversity are crucial factors that influence our health outcomes and well-being across our lifespan, especially as we age. Empirical evidence highlights the importance of understanding how these factors flavor our experiences of aging. Through a series of presentations and discussions, we will explore factors often thought of when considering diversity such as race and ethnicity. Discussed will be how these factors influence end-of-life care preferences when contending with a serious illness and our ethical responsibilities to deliver culturally sensitive care. Next will be a discussion of the often-overlooked issue of intimate partner violence (IPV) among older immigrants. Emphasized will be IPV prevalence and its nuanced presentation among older immigrants. We will then expand our understanding of diversity, beyond race and ethnicity, by looking at additional factors of diversity such as differing cognitive, hearing, and visual abilities. The connections between sensory impairments and pain experiences and disparities in telehealth usage among patients with hearing, visual, or cognitive impairments will be elucidated. This symposium offers a comprehensive perspective on navigating the challenges faced by our diverse aging population. By fostering dialogue on these critical topics, we aim to empower healthcare providers, policymakers, and advocates to create a more flavorful and equitable future for older adults. Join us in stirring the pot of aging as we highlight that diversity is the unique secret ingredient.

Abstract citation ID: igae098.1402

### CORRELATES BETWEEN SENSORY IMPAIRMENTS AND PAIN CHARACTERISTICS IN MEDICARE BENEFICIARIES

Christina Mu[1], Alisha Thompson[2], Bailee Brekke[3], Safiyyah Okoye[4], Lama Assi[5], Sahar Assi[6], and Soomi Lee[7], *1. University of California, San Francisco, San Francisco, California, United States*, *2. Louisiana State University, Baton Rouge, Louisiana, United States*, *3. Miami University, W. College Corner, Indiana, United States*, *4. Drexel University, Philadelphia, Pennsylvania, United States*, *5. Louisiana State University Health Center, New Orleans, Louisiana, United States*, *6. Johns Hopkins University, Baltimore, Maryland, United States*, *7. The Pennsylvania State University, University Park, Pennsylvania, United States*

Objective. Sensory impairments (SI) are associated with comorbidities, functional difficulties, and poor self-rated health. Few studies have examined associations between SI and pain, which is a strong correlate of poor health in later life. We examined the associations between SI and pain. Methods. We used cross-sectional data from the 2021 National Health and Aging Trends Study (n=2979 community-living older adults; ages ≥ 65 years, 57% ages 75-84). We included four measures of SI: objective hearing loss (HL), measured as pure-tone average >25 dB HL; subjective HL, measured as hearing aid use or being unable to hear in certain situations; objective vision loss (VL), measured as logMAR >0.30 for distance or near vision or logCS <1.55 for contrast sensitivity; and subjective VL, measured as inability to see in certain situations. We examined four participant-reported outcomes: being bothered by pain; activity-limiting pain; use of pain medication; and pain locations over the past month. Separate logistic and linear regressions controlled for sociodemographic and health characteristics (e.g., depressive symptoms, chronic conditions). Results. Objective HL was associated with higher odds of activity-limiting pain (aOR=1.33, 95%CI=1.02-1.74). Subjective VL was associated with higher odds of activity-limiting pain (aOR=1.44, 95%CI=1.07-1.93), lower odds of using pain medication (aOR=0.70, 95%CI=0.54-0.92), and more pain locations (B=0.59, 95%CI=0.14-0.97). Compared to having no SI, reporting one or more subjective SI was associated with more pain locations (single SI: B=0.23, 95%CI=0.006-0.46; dual SI: B=0.73, 95%CI=0.08-1.39). Conclusion. Findings suggest SI and pain are related, which may have important implications in clinical settings and research.

Abstract citation ID: igae098.1403

### INTIMATE PARTNER VIOLENCE AMONG US OLDER IMMIGRANTS: WHAT ARE THE ODDS?

Rujeko Machinga-Asaolu[1], Yanghyun Park[2], and Kathryn Showalter[1], *1. University of Kentucky, Lexington, Kentucky, United States*, *2. University of Michigan, Ann Arbor, Michigan, United States*

**Background:** Intimate partner violence (IPV), a subtype of domestic violence, is a pervasive global problem with life-long impacts. Among U.S. older adults, the prevalence of psychological IPV (PAIPV) is 2.1%, and physical IPV (PVIPV) is 0.8%. Sadly, underrepresented individuals, including immigrants, have less access to resources that can serve as IPV protective factors. Due to the growing immigrant and older population in the U.S., it is essential to identify their unique IPV characteristics. Objective and Method: This study used and analyzed the latest publicly available cross-sectional data from the National Intimate Partner and Sexual Violence Survey 2016/17 (n=10,957) of a selected national sample of English and Spanish-speaking immigrants (n=1,121) to identify their odds of experiencing PAIPV or PVIPV-based on identified demographic characteristics, including age. Additionally, of interest and identified was the prevalence of PAIPV and PVIPV among older immigrants 65+ years (n=124).

**Results:** Contrary to the literature, among older immigrants aged 65+ years, PVIPV (10.7%) was more prevalent than

PAIPV (9.1%). In an adjusted regression model for PAIPV at 95% CI, Spanish and/or English-speaking immigrants aged 65+ years were 3.147 [1.065, 9.299] significantly more likely to experience PAIPV than younger immigrants. However, age was not significantly associated with PVIPV. To conclude, IPV presents itself in unique etiologies among older immigrants. This study is an initial step toward using national data to understand IPV better for a combined male and female sample of older immigrants in the U.S. Further discussed will be the distinct differences in the study findings.

Abstract citation ID: igae098.1404

**HEALTH DISPARITIES, EQUITY, AND ETHICAL RESPONSIBILITY OF GERONTOLOGISTS TO PROVIDE CULTURALLY CONCORDANT CARE**

Karen Bullock[1], Kim Stansbury[2], and Terrance Ruth[3], *1. Boston College, Chestnut Hill, Massachusetts, United States*, *2. North Carolina State University, Raleigh, North Carolina, United States*, *3. NC STATE UNIVERSITY, Raleigh, North Carolina, United States*

• The medical advance of today make it possible for people to live longer with serious illnesses. Many older adults will reach a point where medical technology may be able to keep them alive, but neither restore nor improve their health outcomes. Public opinion polls reveal that most people would rather be home than in a hospital or nursing home when dealing with cancer. However, many racially diverse patients and families do not prefer to transition to home with hospice and palliative care. This leaves many underrepresented serious illness care patients and families to face difficult choices about the kind of care they prefer at end-of-life. Thus, despite the capability of modern medicine to ease most pain and suffering for patients with incurable disease, the existence of good models of holistic medical interventions lack culturally-specific care. This presentation examines the ethical issues and barriers to providing equitable serious illness care. Participants will be able to (1) discuss advance care planning as a tool for effective communication and treatment planning, (2) facilitate a dialogue about ethical, professional, and personal issues relative to oncology treatment decisions, (3) describes strategies for preparing practitioners to address disparities in service delivery.

Abstract citation ID: igae098.1405

**IMPROVING ACCESS OR INCREASING DISPARITIES? IMPLEMENTATION OF TELEHEALTH LITERACY SCREENING PROGRAM**

Madison Boudreaux[1], Aleksandra Lindgren[2], Rhonda Bardales[3], Scott Wilks[3], and Kathy Carstarphsen[4], *1. UQ-Ochsner Clinical School, New Orleans, Australia*, *2. UQ-Ochsner Clinical School, New Orleans, Louisiana, United States*, *3. Louisiana State University, Baton Rouge, Louisiana, United States*, *4. Tulane University, New Orleans, Louisiana, United States*

The use of telehealth services is rising in the United States, especially since the pandemic, and continues to be explored as a method in removing barriers to accessing healthcare services. However, those living with vision, hearing, and cognitive impairments may be marginalized in the use of telehealth without an efficient and accurate way of measuring their use and comfortability of engaging with telehealth modalities. The Telehealth Literacy Screening Tool (TLST) was created to measure telehealth literacy, access, use of, and comfortability with technology. This study examined the differences between impairment groups to inform practitioners of potential barriers existing for those living with vision/hearing or cognitive impairments when using telehealth services. The TLST was administered to patients in an outpatient primary care clinic (N=128) over a 5-month period. Text message and email usage varied by group, with those living without visual/hearing or cognitive impairment reporting higher rates of use. Those with both visual/hearing and cognitive impairments had lower comfortability with opening apps than the group with either impairment, and lower comfortability with scrolling than the group with no impairment. The use of the TLST can help identify those patients with limits in telehealth literacy so their specific needs for education or other modalities can be met. This study strengthened the usefulness of the TLST in primary care practice and identified the disparity in telehealth usage among patients with hearing/visual or cognitive impairments.

### SESSION 3280 (SYMPOSIUM)

Abstract citation ID: igae098.1406

**STRATEGIES TO ENGAGE LATINOS/X/E IN RESEARCH: CASE STUDIES ON BRAIN HEALTH, PSYCHOSOCIAL WELL-BEING, AND CAREGIVING**

Chair: Maria Aranda

Several national initiatives underscore the need for innovation in recruitment strategies that diversify community-based research cohorts. In particular, the call for diverse samples, and inclusion of historically underrepresented populations in community-based research is well documented. Prioritizing these goals is paramount, yet challenges remain as to the “how-to” or real-world efforts to advance inclusion of diverse populations in research on aging. This symposium addresses the knowledge gap by drawing from case studies in North Carolina, New York, and Los Angeles, specifically related to the engagement of people of Latino descent. On one hand, we will discuss several core strategies such as fomenting trust, and giving back, which transcend the different studies. On the other, we will highlight different strategies required given the diversity in subgroups, target condition/issue of the study, individual vs. organizational factors, age groups, among others. In our discussion, we will highlight theoretical frameworks such as the Micro-Meso-Macro Framework, Trauma-Informed Principles, and Cultural Adaptation Frameworks. We will conclude with recommendations and future directions for engaging older Latino people in aging research.

Abstract citation ID: igae098.1407

**SHARING INSIGHTS: INTRODUCING BRAIN HEALTH RESEARCH TO LA COMUNIDAD LATINA IN NORTH CAROLINA**

Marianne Chanti-Ketterl[1], Kathleen Welsh-Bohmer[1], Sofia Royo[1], and Brenda Plassman[2], *1. Duke University, Durham, North Carolina, United States*, *2. Duke University Medical Center, Durham, North Carolina, United States*

Alzheimer's disease disproportionally impacts the Hispanic/Latino(a)x (hereafter Latino) community. Ensuring Latino representation in brain health research is essential for understanding how brain disorders affect this diverse population. However, recruiting Latinos into research studies presents multifaceted challenges, including structural inequities, cultural beliefs, values (i.e. faith and family beliefs), and immigration status. Within the North Carolina Registry for Brain Health (NC Registry), we have achieved a twofold increase in Latino enrollment within a year by effectively utilizing existing resources and targeting upstream factors, such as diversifying staffing and collaborators. Increasing the diversity of the NC Registry offers research opportunities to a broader group and supports researchers' efforts to enroll a diverse sample. During this symposium, we will present strategies employed to effectively involve Latinos in the NC Registry, educational initiatives focusing on brain health, and various forms of community engagement. Additionally, we will delineate the pathway from NC Registry participation to enrollment in research studies and elucidate the participant tracking methodology employed. This methodology is key to ensuring that individuals are contacted for the appropriate studies and are not overburdened with multiple requests in a short period of time. Furthermore, we will discuss how this foundational work contributes to fostering community trust and increasing interest in engaging in research studies supported by the Duke-UNC Alzheimer's Disease Research Center. We will conclude by offering recommendations for recruiting and retaining Latinos in brain health research.

Abstract citation ID: igae098.1408

### EMPLOYMENT OF TRAUMA INFORMED PRINCIPLES IN HEALTH SERVICES RESEARCH WITH OLDER LATINX COMMUNITIES

David Camacho, *University of Illinois Chicago, Oak Park, Illinois, United States*

Engaging older Latinx adults in research is critical to advancing understanding and addressing common challenges in aging. However, older Latinx communities are disproportionately exposed to social, health, and historical challenges that may include potentially traumatic events (e.g. discrimination, violence, grief, etc.) and hinder study participation. There is growing recognition for adoption of Trauma Informed (TI) principles for use in non-trauma specific research. There are limited examples of the implementation of TI principles in gerontological research with Latinx communities. We encourage the adoption of TI principles to facilitate engagement research participation. TI principles may include: 1) building relationships with trusted community agencies and providers; 2) conducting open forums where researchers acknowledge community mistrust and fear, present personal and institutional motivations for the project and discuss benefits for community members; 3)incorporation of study questions that explore Latinx diversity and facilitate discussions about similarities and differences across subgroups; and 4) planning to address structural factors (poverty, violence, migratory status) that influence health and research participation. Second, we draw examples of TI guided practices we employed in two recent studies including the: 1) Dolores and Soledad: a narrative study of older English and Spanish speaking Latinx adults living with chronic pain or loneliness in New York and Los Angeles, and 2) Programa Dolores: An adaptation of the evidence based psychosocial intervention (PATH-Pain) for use with older Latinx adults living with chronic pain in New York. Finally, based on these experiences, we provide recommendations for incorporating TI into future research with older Latinx communities.

Abstract citation ID: igae098.1409

### RECRUITING LATINO CAREGIVERS INTO DEMENTIA-RELATED RESEARCH: NOT FOR THE FAINT OF HEART

Maria Aranda, *University of Southern California, Los Angeles, California, United States*

Drawing from the theoretical model—the Micro-Meso-Macro Framework— we elucidate factors that facilitated or impeded diverse sample recruitment to a community-based caregiver intervention study with high Hispanic/Latino representation in California. Based on our multi-level analysis of recruitment meetings and community outreach tracking, we documented the following factors implicated in trial participation: 1) Micro: prior encounters with the research enterprise; educational attainment and occupational status; sense of altruism and beneficence; monetary incentivization; comfort with disclosure of private matters; starting from ground zero; gender issues; and 2) Meso/macro: assembling a proactive scientific team; incorporating a marketing perspective; leveraging logos and reputation; training trial staff and community partners; writing project budgets that incentivize diverse trial recruitment; making inroads with community partners; navigating technological challenges; and tapping into resources to alleviate barriers. Latinos were adversely affected by low exposure to research activities and technological barriers which necessitated extra supports during trial participation.

### SESSION 3285 (SYMPOSIUM)

Abstract citation ID: igae098.1410

### THE GOOD, THE BAD, AND THE BALANCE: CHALLENGES AND OPPORTUNITIES IN USING TECHNOLOGY IN DEMENTIA CARE DYAD

Chair: Kuan-Hua Chen
Co-Chair: Lyndsey Miller
Discussant: Allison Lindauer

Advances in modern technologies, including wearables, mobile or embedded devices and sensors, and AI provide researchers and clinicians with exciting opportunities for naturalistic, large-scale, long-term, and timely assessment and intervention. In dementia, the vast majority of research and interventions using technology have been focusing on individuals—either only the care recipient or their family care partners. However, most dementia care dyads live together and have interdependent behaviors (e.g. physical activity and sleep) and shared functions (e.g. preparing meals). The proposed symposium highlights recent advances in using technologies to assess or intervene in dementia care dyads including their dyadic interactions and interdependent behaviors and functions in their shared home space. The five presentations will leverage different technologies (i.e., motion and door sensors, bed mat sensors, smartwatch, AI) to assess or intervene in different aspects of the health of dementia care dyad (e.g., loneliness, exercise, sleep, worrisome behaviors). Being aware that any methodology also has limitations and pitfalls, each presentation will include an instructive review of challenges associated with the technologies and efforts to overcome them. The five individual presentations will be followed by an integrated panel discussion, moderated by Dr. Harleah Buck. Together, the symposium will provide the general audience with an overview of current in-home assessment technologies, the challenges to incorporating them, and the tradeoffs for science. This symposium will also launch communication and collaborations between researchers from different disciplines who use modern technologies to conduct research in naturalistic settings to promote the health and well-being of dementia care dyads. Dyadic Health Research Interest Group Sponsored Symposium

Abstract citation ID: igae098.1411

### SMARTPHONES CAPTURE DYNAMICS OF DEMENTIA SPOUSAL CAREGIVER INTERACTIONS: IMPLICATIONS FOR MHEALTH

Christopher Fagundes, *Rice University, Houston, Texas, United States*

Dementia spousal caregivers experience higher levels of loneliness than age-match comparisons. Lonelier dementia spousal caregivers report more depression and less life satisfaction; people with dementia who have lonelier spousal caregivers report lower life satisfaction and more depression than those cared for by less lonely spousal caregivers; however, caregivers are unaffected by the degree of loneliness experienced by their spouse with dementia. Interventions designed to address loneliness in caregivers could improve life satisfaction for both dyad members. Unfortunately, little is known about the patterns and temporal dynamics of loneliness in caregivers and how context, interpersonal, and intrapersonal processes buffer or exacerbate experiences of loneliness. This lack of knowledge hampers our ability to intervene; for example, despite the enthusiasm for mHealth technology to deliver just-in-time, adaptive interventions, our gaps in understanding loneliness dynamics thwart empirically informed intervention development. In this presentation, we will present data to fill this gap in the literature using smartphones to capture loneliness dynamics in caregivers and elucidate how these dynamics impact dyads across a 14-day ecological momentary assessment protocol. We will present data on how caregivers' sense of relational security impacts loneliness and life satisfaction after dyadic interactions. For example, caregivers' sense of relational security determined whether performing caregiving tasks involving dyadic interactions increased or decreased subsequent loneliness ($b=.01$, $SE=.02$, $p<.05$). We conclude by discussing ways in which smartphones can be used to capture momentary patterns of mood and behavior, and how this data could inform mHealth interventions to improve the lives of both dyad members.

Abstract citation ID: igae098.1412

### STRENGTHS AND LIMITATIONS OF USING DIFFERENT SENSOR TECHNOLOGIES TO ASSESS CLINICAL OUTCOMES IN DYADIC CARE.

Neil Thomas[1], Bahareh Chimehi[2], Julien Lariviere-Chartier[1], Frank Knoefel[1], Zachary Beattie[3], Joel Steele[4], Lyndsey Miller[3], and Bruce Wallace[2], *1. Bruyere Research Institute, Ottawa, Ontario, Canada*, *2. Carleton University, Ottawa, Ontario, Canada*, *3. Oregon Health & Science University, Portland, Oregon, United States*, *4. University of North Dakota, Grand Forks, North Dakota, United States*

Home sensor and smart home technologies offer the potential to provide important objective information on daily activities, functional abilities, and caregiving tasks for individuals with cognitive impairment and care partners. Combining information from multiple sensors, such as a bedmat, wearable, and motion sensors, can deliver more informative data on dyadic interactions during nighttime activity (e.g., room location and which partners are out of bed in addition to sleep measures). Preliminary work from a study developing a digital signature of caregiver burden using data from multiple sensors in a home technology platform is presented. Using data from two different sensors provided additional information on nighttime behaviors related to care partner burden level in a sample of 47 dyads (care partner and individual with dementia or mild cognitive impairment). Estimated sleep duration calculated from an under the mattress bedmat and activity monitoring wristwatch showed a low concordance ($r=0.36$). However, an additional 9156 nights were collected from the union of the two sensors as compared to the wristwatch (29318 nights) or bedmat (15704 nights) alone. Benefits to using multiple sensors include the ability to expand the amount of information collected on a dyad and the locations where information can be collected (e.g., sleep occurring outside of the bedroom). Challenges include determining the more accurate estimate when there is discordance between different sensor types and evaluating the accuracy of new or updated sensors. Additional work is needed to determine which sensor or combination of sensors is optimal at approximating a clinical outcome of interest.

Abstract citation ID: igae098.1413

### SENSING SPOUSAL CARE DYADS' ACTIVITY PATTERNS: TWO NOVEL DYADIC METRICS FOR CAPTURING TIME USE

Lyndsey Miller[1], Wan-Tai Au-Yeung[2], Karen Lyons[3], Joel Steele[4], and Jeffrey Kaye[1], *1. Oregon Health & Science University, Portland, Oregon, United States*, *2. Oregon Health and Science University, Portland, Oregon, United States*, *3. Boston College, Chestnut Hill, Massachusetts, United States*, *4. University of North Dakota, Grand Forks, North Dakota, United States*

Spousal care dyads' in-home activity and routines are typically captured through self-report. Remote passive sensing offers a sensitive, continuous, and ecologically-valid method of assessment that increases the ability to detect patterns of daily activities and function; however, there are challenges of deploying these technologies among dyads in the community setting and in the context of dementia. In this study of 47 co-habiting care dyads (person living with dementia and their spouse, $\bar{x}=71.3$ years in age), we aimed to characterize aspects of daily dyadic activity patterns using an in-home sensor platform. Additionally, we aimed to understand the relationship between these novel activity patterns and the dyad's relationship (caregiver burden and dyadic relationship strain). Activity measures included independent life space activity (dyads' active time in separate rooms) and time spent out of the home, both of which were captured by infrared motion and door contact sensors. Dyads also wore an actigraphy watch that captured daily step counts ($\bar{X}$= 3548/day) and nightly sleep duration ($\bar{X}$= 7.6 hours). According to mixed effects modeling, there was a significant inverse association between the amount of time spent out of the home and the amount of time spent conducting independent life space activity ($p < 0.001$), but no significant associations with step counts or sleep duration. When relating activity patterns to aspects of the caregiving relationship, there was an inverse relationship between average step counts and level of caregiver burden (reported weekly), and between relationship strain and time spent out of the home.

Abstract citation ID: igae098.1414

### SELF-MONITORING VIA WEARABLE SENSOR AND DAILY STEPS AMONG PEOPLE WITH MCI AND CARE PARTNER DYADS

Rashelle Hoffman[1], Blake Murphy[2], Vaishali Phatak[3], Diane Ehlers[4], and Joan Monin[5], *1. Creighton Univeristy, Creighton University, Nebraska, United States*, *2. Creighton University, Omaha, Nebraska, United States*, *3. University of Nebraska Medical Center, Omaha, Nebraska, United States*, *4. Mayo Clinic, Rochester, Minnesota, United States*, *5. Yale School of Public Health, New Haven, Connecticut, United States*

Physical inactivity is a modifiable risk factor for Alzheimer's Disease and Related Dementias (ADRD) that can be remediated using remotely delivered interventions. The 12-week telerehabilitation physical activity behavioral (TPAB) intervention includes receipt of a wearable sensor and weekly discussions about physical activity self-monitoring, goal setting, facilitators, and barriers. TPAB has been shown to be feasible and efficacious in increasing daily steps in older adults. However, evidence is lacking for TPAB's use for people with MCI and using a dyadic approach (i.e., with their care partners). Results from the first phase of our study include descriptive information from two dyads about (1) dyad member interaction with the wearable sensor (i.e., how knowing the current daily step count impacted activity), (2) how dyad members helped each other overcome individual or shared barriers to daily stepping and (3) how dyad members planned activities together to achieve high daily average stepping. This project quantitatively identified (1) the highest stepping day each week was the same for both dyad members 46% of the time (2) the frequency of both dyad members meeting individual weekly stepping goals was 81% compared to just one dyad member or no dyad member reaching the weekly step goal. This project revealed barriers using wearable sensor technology (i.e., not looking at or wearing the wearable sensor). Our discussion will focus on how to enhance meaningful interaction with technology in dyads to track daily stepping that can be applied to improve behavioral change in people with MCI and care partners.

Abstract citation ID: igae098.1415

### EVALUATING IN-HOME ASSISTIVE TECHNOLOGY FOR DEMENTIA CAREGIVERS: CHALLENGES AND OPPORTUNITIES

Kuan-Hua Chen[1], Julian Scheffer[2], Claire Yee[3], Jennifer Merrilees[4], David Moss[5], Brian Hinderberger[5], Gene Wang[5], and Robert Levenson[2], *1. University of Nebraska Medical Center, Elkhorn, Nebraska, United States*, *2. University of California Berkeley, Berkeley, California, United States*, *3. Mayo Clinic, Scottsdale, Minnesota, United States*, *4. University of California San Francisco, San Francisco, California, United States*, *5. Care Daily Company, Kirkland, Washington, United States*

Dementia caregivers are at heightened risk for developing mental and physical health problems. Interventions designed to protect caregiver health often create additional demands on time and energy (e.g., exercise programs). Technology-based interventions (e.g., remote monitoring) can be effective, scalable, and do not add greatly to caregiver burden. Through a collaboration with a local technology company, Care Daily, we conducted two randomized controlled trials to evaluate People Power Caregiver (PPCg), a system of sensors in the home connected to cloud-based AI that alerts caregivers about worrisome deviations from normal patterns (e.g., care-recipient's falls, wandering). Across two studies (study 1: 9-month duration with professional installation of PPCg; study 2: 6-month duration with caregiver self-installing the PPCg using remote tech support), 284 caregivers were recruited; 261 completed initial study deployment (e.g., system installation); and 235 completed the entire study protocol. Use of PPCg was associated with (a) reduced caregiver anxiety in study 1; (b) greater protection from declines in sleep efficiency in studies 1 and 2. These findings underscore the promise of technology-based intervention to mitigate health problems in dementia caregivers. However, challenges and complexities in recruitment (e.g., remote consenting both caregivers and care recipients), retention (e.g., due to changes in home environment or caregiving status), system installation (e.g., working with caregivers with less experience using technology), data quality assurance (e.g., invalid sensor data) as well as our efforts to overcome these challenges have been instructive. Lessons learned from these studies can provide useful guidance for future large-scale, technology-based interventions for dementia caregivers.

### SESSION 3290 (SYMPOSIUM)

Abstract citation ID: igae098.1416

### WORKING LIFE EXPECTANCY OF OLDER ADULTS: FINDINGS FROM ASIA AND THE AMERICAS

Chair: Rahul Malhotra
Co-Chair: Abhijit Visaria

With a growing focus on productive ageing, including engagement in work, it is important to assess its extent across key demographic sub-groups within a country, and if feasible, across countries. This will help identify disadvantaged sub-groups in the context of productive ageing. Working life expectancy (WLE; years of remaining life one can expect to work at a given age), a population-level indicator of productive ageing, allows one to capture variation in the duration of working life due to societal, legislative, and economic considerations. The four presentations in this symposium provide empirical findings on WLE from nationally representative longitudinal studies of middle-aged and/or older adults from countries in Asia or the Americas. The first, from Japan, projects WLE till 2050 and highlights the role of policy interventions in increasing it. The second, from Mexico, observes strong sex disparity in WLE, underscoring the need for policies to promote women's work participation. The third from Chile, where mandatory retirement age is lower for women, finds disparity in WLE at retirement age not by sex but by working status, highlighting mandatory retirement age's influence on WLE. The fourth from Singapore, using data from two longitudinal cohorts, observes maintenance of sex disparity (disfavoring women) but reversal of education disparity (disfavoring those with lower education) in WLE over time. Collectively, they highlight variability in WLE at older ages within and across countries, in terms of absolute or relative (to life expectancy) WLE, disparities in in WLE, and potential options for policy focus for increasing WLE.

Abstract citation ID: igae098.1417

### PROJECTING WORKING LIFE EXPECTANCY TO 2050 AMONG OLDER ADULTS IN JAPAN

Abhijit Visaria[1], and Yasuhiko Saito[2], *1. Duke-NUS Medical School, Singapore, Singapore*, *2. Nihon University, Tokyo, Japan*

With sustained low fertility rate and continued population ageing, the prospect of a shrinking labor force is a key challenge facing Japan. It is of interest to compare two possible scenarios which may result in an increase in the expected years of life spent working (working life expectancy, "WLE") at older ages: a natural impact of further reductions in mortality, or the impact of extending the mandatory retirement age from 65 to 70 years, as currently being considered by the government. We first estimated WLE among older adults aged 65 years and older using data from five waves of a representative longitudinal survey (1999-2009), applying multistate life table methods. Then, we projected WLE to 2050 by assuming a constant transition schedule between working states (working/not working) but improving age-specific mortality rates (as projected by the UN Population Division). We then simulated the impact of an increase in the mandatory retirement age to 70 years. WLE at age 65 years, based on the longitudinal data, was 4.6 years for men and 3.5 years for women. Improvements in mortality yielded an estimated WLE at the age of 65 in year 2050 of 4.9 years for men and 3.7 years for women. Increasing the retirement age to 70 years increased WLE at age 65 by about 1.5 years for men and 1.6 years for women, by 2050. Our results indicate the value of increasing mandatory retirement age to 70 years as a policy intervention to increase WLE among older adults in Japan.

Abstract citation ID: igae098.1418

### WORKING LIFE EXPECTANCY AMONG MIDDLE-AGED AND OLDER MEXICAN MEN AND WOMEN

Chi-Tsun Chiu, *Academia Sinica, Taipei, Taipei, Taiwan (Republic of China)*

With rapid population aging, partly contributed by increasing life expectancy, work and retirement related issues – including increases in retirement age and sustainability of pension systems – are increasingly pertinent. Many countries have increased or considered increasing the legal age of retirement for receipt of pensions and introduced measures for increasing the length of working life – in Mexico specifically, from 2028 onward, early retirement will be penalized. Furthermore, more individuals are continuing to work beyond current mandatory retirement ages, such as being self-employed or working in the gig economy. Estimates of the average number of years individuals remain in the workforce from middle age (age 50 years) are needed to assess the feasibility of potential policy changes and provide the evidence base to inform policymaking. This study uses the Mexican Health and Aging Study (MHAS), a national longitudinal study of adults 50 years and older in Mexico, to compute life expectancy (LE) and working life expectancy (WLE) by gender. Our results show that at age 50, Mexican men's LE is 29.0 (27.8-30.2) and WLE is 16.7 (16.0-17.4) years, i.e. 57.6% of LE. Mexican women live longer, with a LE of 32.4 (31.5-33.3) years, however, have a much lower WLE of 8.1 (7.5-8.8) years or 25.0% of LE. The findings highlight the strong sex disparity in WLE, disfavoring women, in Mexico and underscore the need for policymakers to address it. Targeted policies and initiatives are required to promote greater and continued labor force participation for middle-aged and older Mexican women.

Abstract citation ID: igae098.1419

### SEX- AND WORKING-STATUS SPECIFIC WORKING LIFE EXPECTANCY AMONG OLDER ADULTS IN CHILE

Yasuhiko Saito[1], and Moises Sandoval[2], *1. Nihon University, Tokyo, Japan*, *2. University of Chile, Santiago, Region Metropolitana, Chile*

The mandatory retirement age in Chile, 65 years for males and 60 years for females, was set in the 1980s. However, in view of accelerated population ageing in the country, there may be a need to reconsider these age thresholds. Thus, we estimate working life expectancy, i.e., years of remaining life spent working, at older ages by sex in Chile using (working) status-based multistate life table methods. Nationally representative longitudinal data on 1313 males and 1805 females from the Social Protection Survey, conducted in 2004, 2006, 2009 and 2015, were used to estimate working life expectancy at age 65 years for males and age 60 years for females. Men working at age 65 years, on average, could expect to live 21.6 years, with working life expectancy of 2.7 years (12.5% of life expectancy). Men not working at age 65 years could expect to live 21.2 years, with a much lower working life expectancy (0.2 years; 0.9% of life expectancy). A similar pattern was observed for women – those working at age 60 years could expect to live 25.9 years, with working life expectancy of 3.0 years (11.6% of life expectancy), and those not working at age 60 could expect to live 25.8 years, with only 0.5 years of working life expectancy (1.9% of life expectancy). The findings suggest the strong influence of the mandatory retirement age on working life expectancy in Chile and its role as a policy lever to increase working life expectancy among older adults.

Abstract citation ID: igae098.1420

### CHANGES OVER TIME IN WORKING LIFE EXPECTANCY AT OLDER AGES IN SINGAPORE: A COMPARISON OF TWO COHORTS

Rahul Malhotra[1], Abhijit Visaria[1], Angelique Chan[1], Stefan Ma[2], and Yasuhiko Saito[3], *1. Duke-NUS Medical School, Singapore, Singapore*, *2. Ministry of Health Singapore, Singapore, Singapore*, *3. Nihon University, Tokyo, Japan*

Singapore, a rapidly ageing country, underwent significant development in a short time after independence in 1965. We study whether differences based on educational attainment in the average years of remaining life expected to be spent working (i.e., working life expectancy), have changed among older persons over time. We use data from two representative studies of older persons: initiated in (i) 2009 (n=4990) with follow-ups in 2011-12, 2015, and (ii) 2016-2017 (n=4549) with follow-up in 2019. We estimated working (part- or full-time current employment) life expectancy using the multistate life table method. We included sex (male / female) and education (lower [no formal or primary] / higher [secondary and above]) as covariates and controlled for ethnicity and time-varying functional health. In the earlier cohort, working life expectancy is estimated higher among those with higher education (e.g. at age 60: 9.2 years [95% CI: 8.0-10.4] among males and 6.5 [6.3-6.6] years among females) versus those with lower education (7.6 [6.9-8.4] years among males and 5.4 [5.2-5.7] years among females). This educational difference reverses in the more recent cohort: working life expectancy is higher among those with lower education (at age 60: 10.0 [8.4-11.7] years among males and 7.7 [6.4-9.0] years among females) versus those with higher education (8.9 [7.6-10.3] among males and 6.5 [5.2-7.7] among females). This change was driven by an increase in working life expectancy among those with lower education and no change among the higher educated. This increase suggests both more employment opportunities and the requirement for continuing employment.

### SESSION 3300 (SYMPOSIUM)

Abstract citation ID: igae098.1421

### ADDRESSING SOCIAL DETERMINANTS OF HEALTH DURING CARE TRANSITIONS: AN INTERDISCIPLINARY PERSPECTIVE

Chair: Ayomide Bankole

Care transitions, such as transitions from hospital to home or home health care, are particularly challenging periods for many older adults with unaddressed social determinants of health (SDOH) needs. For instance, older adults might experience barriers in accessing transportation for follow-up medical appointments or acquiring prescribed medications due to financial limitations. These challenges significantly impact health outcomes, often resulting in emergency department visits and re-hospitalizations for many older adults. Addressing social determinants during care transitions is crucial for promoting safe, effective, equitable, and quality healthcare delivery, particularly for those with the greatest social vulnerabilities. In this symposium, we present research, and recommendations from an interdisciplinary group of clinicians from nursing, rehabilitation therapy, and social work disciplines. Dr. Osokpo will start the symposium by proposing a theoretical framework for addressing social determinants during care transitions. Dr. Bankole will describe the characteristics of socio-economically disadvantaged older adults (dual eligible beneficiaries) who transition to home healthcare, for post-acute care services. Dr. Nguyen will discuss the implementation and evaluation of a transitional care intervention that addresses service needs for dementia caregivers and those whose primary language is not English. For the final presentation, Denise Dews MSW will describe an interprofessional collaborative approach to delivering high-quality transitional care to improve outcomes for socially vulnerable older adults. Together, these presentations will offer recommendations to enhance care transitions, with implications for interprofessional clinical education, practice, and research.

Abstract citation ID: igae098.1422

### INCORPORATING SDOH INTO TRANSITIONAL CARE OF OLDER ADULTS: A THEORETICAL PERSPECTIVE

Onome Osokpo[1], Karen Hirschman[2], Elizabeth Shaid[3], Kathleen McCauley[4], and Mary Naylor[2], *1. University of Illinois Chicago, Chicago, Illinois, United States*, *2. University of Pennsylvania, Philadelphia, Pennsylvania, United States*, *3. Penn Nursing, Merion Station, Pennsylvania, United States*, *4. University of PA School of Nursing, Ardmore, Pennsylvania, United States*

There is solid evidence that Social Determinants of Health (SDoH) contribute to poor patient health outcomes after hospitalization and that transitional care can prevent these adverse outcomes. However, using a standardized strategy for transitional care intervention will result in missed opportunities to enhance positive outcomes for older individuals with chronic illnesses, particularly minority older adults who are greatly affected by adverse SDoH (e.g., food insecurity). Hence, for any transitional care intervention to be effective, it must be adaptable to identify and tackle SDoH issues. In this paper, using the lens of the five domains of the Health People 2023 SDoH framework (Economic Stability, Education Access and Quality, Health Care Access and Quality, Neighborhood and Built Environment, and Social and Community Context), we demonstrate how SDoH can be integrated into an evidence-based transitional care intervention to prevent adverse events such as avoidable emergency room visits and rehospitalization and enhance positive outcomes for patients as they transition from hospital to home or other health care settings. Specifically, we highlight opportunities for the transitional care intervention teams and the health care systems to identify and mitigate SDoH factors using the Transitional Care Model intervention (TCM), a practical and adaptable transitional care intervention, to reduce adverse events and reduce health disparities and health inequities.

Abstract citation ID: igae098.1423

**CHARACTERISTICS OF DUALLY ELIGIBLE BENEFICIARIES WHO RECEIVE MEDICARE HOME HEALTH CARE SERVICES: A SCOPING REVIEW**

Ayomide Bankole[1], Dorothy Addo-Mensah[2], and Jamie Conklin[2], *1. Duke University, Durham, North Carolina, United States*, *2. University of North Carolina, Chapel Hill, North Carolina, United States*

Medicare Home Healthcare (HHC) is a commonly utilized post-acute care service by Medicare beneficiaries, including those with dual Medicare and Medicaid coverage (dual eligibles). Dual eligibles experience inequities in HHC as they report poorer health outcomes (e.g re-hospitalizations) than other Medicare beneficiaries in HHC. Dual eligibles may also experience challenges navigating and coordinating care across two payers of healthcare (Medicare and Medicaid). To improve health outcomes of dual eligible beneficiaries within HHC, there is a need to understand characteristics of this population and components of successful interventions within this setting. Thus, we conducted a scoping review to examine available evidence on dual eligibles who receive Medicare HHC. Research questions: (1) What are the characteristics of dual eligibles who receive HHC? (2) What health outcomes have been described and (3) which interventions influence these outcomes? With consultation from a Health Services Librarian, a search was conducted across four databases (PubMed, CINAHL, APA Psych Info, and Scopus) on January 10, 2024. After removing duplicates, 1,425-articles were identified for screening. Using Covidence software, reviewers worked in pairs to screen the articles. This phase yielded 83-articles. Given the changes in Medicaid eligibility and policies over the past decade, we then limited articles to those published within the last 10-years; leaving 53-articles for data extraction. We will extract and synthesize data using narrative-synthesis and descriptive-statistics, guided by the research questions. In this presentation, we will describe characteristics of dual eligibles receiving Medicare HHC and recommend strategies to improve their health outcomes within this setting.

Abstract citation ID: igae098.1424

**ADAPTING A CAREGIVER TRAINING PROGRAM TO SUPPORT EQUITABLE IMPLEMENTATION DURING CARE TRANSITIONS**

An Nguyen[1], Pamela Roberts[1], and Leslie Davidson[2], *1. Cedars-Sinai Medical Center, Los Angeles, California, United States*, *2. The George Washington University School of Medicine and Health Sciences, Washington, District of Columbia, United States*

Transitions in care (TOC) interventions can improve transitional care processes and reduce hospital readmissions in the general older adult population. People living with dementia (PLWD) are often excluded from TOC intervention studies due to the potential for cognitive impairments to interfere with the intervention's theorized mechanisms of change. Given that PLWD experience worse health outcomes following hospitalization and more frequent, costly transitions in care, there is an urgent need to develop targeted TOC interventions specifically tailored to meet the needs of this population. To address this gap, we propose to adapt, implement, and evaluate the Home Environmental Skill-Building Program (ESP) to address the needs of PLWD and their care partners during hospital-to-home transitions. Developed and tested in prior NIH-funded randomized controlled clinical trials (grant numbers: R01AG10947 and U01AG013265), ESP is a caregiver skills training program delivered by an occupational therapist for at-home management of self-care deficits and behavioral problems due to dementia. A health equity-centered approach will be used to adapt the ESP intervention protocol to address the needs of care partners whose primary language is not English, a group at greater risk of receiving poorer quality transitional care. Strategies will be shared through which intervention adaptations may address language and cultural barriers, as social determinants of health, towards ameliorating health disparities during care transitions experienced by PLWD and care partners for whom English is not their primary language.

Abstract citation ID: igae098.1425

**OPTIMIZING CARE TRANSITIONS FOR OLDER ADULTS: A COLLABORATIVE APPROACH THROUGH INTERPROFESSIONAL EDUCATION**

Denise Dews, *University of North Carolina, Chapel Hill, North Carolina, United States*

Optimizing care transitions for older adults is a critical aspect of healthcare delivery, particularly given the complex needs and potential social vulnerabilities of this population. Transitioning between different healthcare settings, such as hospitals, rehabilitation facilities, and home care, can be challenging and increase the risk of adverse events for older adults. Interprofessional Education (IPE) offers a collaborative approach to addressing these challenges and improving care transitions for older adults. IPE involves bringing together students and professionals from multiple healthcare disciplines to learn with, from, and about each other to improve collaboration and the quality of patient care. By fostering teamwork, communication, and mutual respect among healthcare providers, IPE can enhance the coordination and continuity of care for older adults during transitions between different care settings. Key strategies for optimizing care transitions for older adults through a collaborative approach with IPE include a team-based approach, comprehensive assessment, care coordination, communication and information sharing, patient and caregiver education, and follow-up and support. By adopting a collaborative approach through IPE, healthcare providers can enhance the quality, safety, and effectiveness of care transitions for older adults, ultimately improving health outcomes and quality of life for this vulnerable population. This presentation will offer strategies for adopting a collaborative approach through Interprofessional Education, thus promoting quality, safety, and effectiveness of care transitions for older adults, ultimately improving health outcomes and quality of life for this vulnerable population.

### SESSION 3305 (SYMPOSIUM)

Abstract citation ID: igae098.1426

**ADVANCING ORAL HEALTH AND ORAL HEALTH–RELATED QUALITY OF LIFE MEASURES ACROSS DIVERSE POPULATIONS**

Chair: Bei Wu
Co-Chair: Elisa Ghezzi
Discussant: Stephen Shuman

This symposium demonstrates the intricate interplay between social and behavioral factors and their impact on the oral health status of older immigrant populations in the United States. It also examines the efficacy of self-reported oral health measures within diverse populations. Understanding these dynamics and valid measures to be used in these populations is crucial for developing targeted interventions and policies to address oral health disparities within these communities. The first study scrutinizes oral health trends among older adults based on their immigration status in the United States, leveraging data from the National Health and Nutrition Survey. Findings reveal an overall decline in self-reported fair/poor oral health from 1999 to 2018, yet significant oral health disparities persist, notably among non-US citizens. The second study investigates the correlation between caregiving circumstances, care recipient characteristics, and oral hygiene assistance among dementia caregivers within Chinese American communities. Insights stem from a pilot study conducted in New York City. The third study delves into the relationship between support from adult children and perceived oral health among both foreign-born and U.S.-born Chinese Americans. This study provides evidence for resilience pathways that connect social support from children to older immigrants' oral health. Lastly, based on a systematic review, the fourth study unpacks the construct of oral health-related quality of life (OHRQoL) within existing measures for adults, assessing their psychometric properties. Six distinct subdomains emerge, including pain/discomfort, oral diseases and disorders, functional limitations, oral hygiene, oral hygiene behavior, and psychosocial impact.

Abstract citation ID: igae098.1427

### TRENDS OF ORAL HEALTH STATUS OF OLDER ADULTS BY IMMIGRATION STATUS IN THE UNITED STATES: 1999–2018

Huabin Luo[1], Bei Wu[2], Xiang Qi[2], and Mark Moss[1], *1. East Carolina University, Greenville, North Carolina, United States*, *2. New York University, New York, New York, United States*

National data on oral health of immigrants in the United States is limited. This study assessed the oral health trends of this population from 1999 to 2018, using data from the National Health and Nutrition Survey. The outcome variable—self-reported oral health—was categorized as a binary outcome: excellent/very good/good or fair/poor. The sample was 18,853 adults aged 50 years and older, including 14,154 US-born Americans, 2,980 naturalized citizens, and 1,719 noncitizens. Trends in self-reported fair/poor oral health across these groups were identified. The findings revealed a significant decline in the prevalence of fair/poor oral health among all groups between 1999 and 2018 (P for trend <.05). However, noncitizens consistently reported higher rates of fair/poor oral health compared to US-born and naturalized citizens. In the logistic regression model only controlling demographics, noncitizens were more likely (AOR=1.28) and naturalized citizens were less likely (AOR=0.80) to report fair/poor oral health. After further adjustment for education, income, and smoking status, the disparities in fair/poor oral health among the groups were not statistically significant. This study found an overall decrease in self-reported fair/poor oral health among older adults in the US during 1999-2018. However, significant oral health disparities persisted, particularly for noncitizens. Further research is needed to validate these trends and develop targeted interventions to address the oral health disparities among older immigrants in the US.

Abstract citation ID: igae098.1428

### FAMILY DEMENTIA CAREGIVERS' ASSISTANCE WITH ORAL HYGIENE CARE IN CHINESE AMERICAN COMMUNITIES IN NYC

Weiyu Mao[1], Bei Wu[2], and Yaolin Pei[3], *1. University of Nevada, Reno, Reno, Nevada, United States*, *2. New York University, New York, New York, United States*, *3. University of Texas at Austin, Texas, United States*

Oral hygiene care is instrumental in maintaining good oral health. As dementia progresses, individuals face challenges performing adequate oral hygiene care and become dependent on their caregivers. The role of family caregivers in assisting with oral hygiene care for persons with dementia in the community becomes increasingly critical. This study explored the association between caregiving characteristics, care recipients' oral health needs, and assistance in oral hygiene care among caregivers in Chinese American communities. Data came from a pilot study on Chinese dementia caregivers in New York City collected between November 2021 and June 2022. Purposive sampling was used to recruit family caregivers to participate in a survey (online or via telephone). Current caregivers (n = 76) were included in the present study. Caregiver assistance with oral hygiene care was measured by assistance with toothbrushing (yes or no) and assistance with flossing (yes or no). Results from group comparisons suggest that female caregivers tended to assist with toothbrushing, and caregivers with an average of 2.4 years in providing care tended to assist with flossing. Logistic regressions show that care recipients with tooth pain were 5.6 times more likely to receive assistance with toothbrushing. Moreover, care recipients with more natural teeth were 1.1 times more likely to receive assistance with toothbrushing. Care recipients with severe dementia were 98% less likely to receive assistance with flossing. It is important to raise awareness and address challenges among dementia caregivers regarding the importance of maintaining adequate oral hygiene care and, subsequently, promoting optimal oral health.

Abstract citation ID: igae098.1429

### RESILIENCE AND PERCEIVED ORAL HEALTH AMONG OLDER CHINESE AMERICAN IMMIGRANTS: THE ROLE OF SOCIAL SUPPORT

Nan Jiang[1], Wei Zhang[2], and Bei Wu[3], *1. Tsinghua University, Beijing, Beijing, China (People's Republic)*, *2. University of Hawai'i at Mānoa, Honolulu, Hawaii, United States*, *3. New York University, New York, New York, United States*

Improving the oral health of older immigrants is a critical public health priority in the United States. However, the role of children's support in this context has received limited attention. This study aims to investigate the relationship between support from adult children and perceived oral health among both foreign-born and U.S.-born Chinese Americans. Additionally, we explore the mediating role of resilience. We analyzed data from a sample of 377 Chinese American older adults aged 55 or older in Honolulu, Hawai'i. Utilizing structural equation models, we compared self-rated oral health and oral health problems between foreign-born and U.S.-born Chinese Americans. For foreign-born participants, greater emotional support from children was directly associated with better perceived oral health. Furthermore, this emotional support was indirectly linked to perceived oral health through resilience. In contrast, for U.S.–born Chinese Americans, financial support from children was directly related to worse perceived oral health. This study provides evidence for resilience pathways that connect social support from children to older immigrants' oral health. Encouraging adult children in Chinese immigrant families to educate their older relatives about oral diseases and dental care can enhance the oral health-related quality of life for older Chinese immigrants.

Abstract citation ID: igae098.1430

### MEASURING ORAL HEALTH-RELATED QUALITY-OF-LIFE MEASURES IN ADULTS: DIRECTIONS FOR FUTURE APPLICATIONS

Zheng Zhu[1], Jing Wang[2], and Bei Wu[3], *1. New York University, New York, New York, United States*, *2. University of New Hampshire, Durham, New Hampshire, United States*, *3. New York University, New York, New York, United States*

**Background:** To identify the construct of oral health-related quality of life (OHRQoL) in published measures for adults and assess the psychometric properties of these measures.
**Methods:** Nine databases were searched from inception of each database up to July 1st, 2023. Methodological quality was assessed by using the Consensus-based Standards for the Selection of Health Measurement Instruments (COSMIN) Risk of Bias Checklist. Thematic analysis was used to summarize all detected instruments, items domains and questions with regard to their wording, scales and the domains of OHRQoL. We used the COSMIN criteria to summarize and assess the psychometric properties of each PROM.
**Results:** A total of 50 PROMs were identified. Six relevant domains that represent construct of OHRQoL: (1) pain/discomfort; (2) oral diseases and disorders; (3) functional limitation; (4) oral hygiene; (5) oral hygiene behavior; and (6) psychosocial impact. All studies were considered to have adequate methodological quality in terms of content validity, construct validity, and internal consistency. Limited information was retrieved on cross-cultural validity, criterion validity, reliability, hypothesis testing, and responsiveness. High-quality evidence on psychometric properties was provided for the oral health impact profile (OHIP), Geriatric Oral Health Assessment Index (GOHAI), and Oral Impacts on Daily Performances (OIDP).
**Discussion:** We encourage the use of the identified six domains to enhance the development and adaptation of instruments for different health systems. OHIP, GOHAI, and OIDP are recommended for evaluating OHRQoL in adults, both in research and clinical practice, based on the specific aims of assessments and the response burden for participants.

### SESSION 3310 (SYMPOSIUM)

Abstract citation ID: igae098.1431

### AGE INCLUSIVITY DOMAINS OF HIGHER EDUCATION (AIDHE): A MODEL TO SUPPORT AGE-DIVERSE STUDENTS, FACULTY, AND STAFF

Chair: Joann Montepare
Co-Chair: Nina Silverstein
Discussant: Cal Halvorsen

The Age Inclusivity Domains of Higher Education (AIDHE) model (adopted in 2023 by GSA and its Academy for Gerontology in Higher Education) offers a guiding framework for how institutions of higher education can assess age-inclusive practices that impact students, faculty, and staff across seven domains of institutional function. In this symposium, presenters describe campus practices in three high-need, high impact domains: Teaching and Learning, Personnel, and Student Affairs. To begin, Bowen and colleagues (UMass Boston/Lasell University) will describe the AIDHE model and research using the Age-Friendly Inventory and Campus Climate Survey (ICCS) which identified challenges within each domain along with evidence-based transformative and actionable strategic approaches. Reflecting the domain of Teaching and Learning, Porter (University of Manitoba) will describe a new micro-certificate program, Facilitating Older Adult Learning, that provides educators with pedagogical design tools to select and evaluate appropriate learning strategies for supporting effective older adult learning. Reflecting the domain of Student Affairs, Galucia and Morrow-Howell (Washington University in St. Louis) then will describe their research with admissions, career services, and DEI staff about how to best support non-traditional aged students. They will also discuss Next Move, a program for graduate students returning to school after substantial work and life experiences. Reflecting the domain of Personnel, Kaskie (University of Iowa) will discuss the aging professorate and how institutions can modify human resource policies and programs to provide more opportunities for aging faculty and staff to remain healthy and productive. Halvorsen (Boston College) will serve as discussant. Age Inclusivity in Higher Education Interest Group Sponsored Symposium

Abstract citation ID: igae098.1432

### ASSETS, GAPS, AND OPPORTUNITIES FOR AGE INCLUSIVITY: THE AIDHE MODEL, THE ICCS MEASURE, AND FURTHER APPLICATIONS

Lauren Bowen[1], Nina Silverstein[2], Joann Montepare[3], and Susan Whitbourne[1], *1. University of Massachusetts Boston, Boston, Massachusetts, United States*, *2. University of Massachusetts Boston, Needham, Massachusetts, United States*, *3. Lasell University, Newton, Massachusetts, United States*

The Age Inclusivity Domains of Higher Education (AIDHE) model was developed to encourage attention to age inclusivity across seven key domains of institutional function —Teaching and Learning, Student Affairs, Outreach and Engagement, Personnel, Physical Environment, Research, and Services and Resources—and across the experiences of age-diverse students, faculty, and staff. To assess age inclusivity across the seven domains, we (Silverstein et. al. 2022; 2024) developed and tested the Age-Friendly Inventory and Campus Climate Survey (ICCS), which documents the presence of age-friendly campus practices; perceptions of those practices by faculty, staff, and students; and match between practices and perceptions; further, the degree of match along with agreement in the value of age inclusivity predicts perceptions of age inclusivity by campus constituents. The AIDHE model and data collected using the ICCS have supported further research to expand age inclusivity efforts. In a multimethod study (Bowen et al., 2024), we identified key challenges and transformative, actionable strategies to advance age inclusivity in Teaching and Learning, Personnel, and Student Affairs domains. Data were collected from 2,273 ICCS open-ended question, as well as from 16 faculty, staff, and student focus groups, to identify both challenges and potential strategies. These challenges and strategies were reviewed over two rounds by Delphi expert panelists, representing higher education administrators and leaders. Final analysis balanced each strategy's transformative potential (through impact and priority) with its actionable potential (through feasibility and likelihood of implementation), which ultimately yielded 23 recommended strategies intended to address key challenges of age inclusivity in higher education.

Abstract citation ID: igae098.1433

### CREATING OPPORTUNITIES TO IMPROVE AGE INCLUSIVITY IN HIGHER EDUCATION: AN INNOVATIVE EDUCATION PROGRAM EXAMPLE

Michelle Porter, *University of Manitoba, Winnipeg, Manitoba, Canada*

The Centre on Aging at the University of Manitoba (UM) leads the UM Age-friendly University (AFU) Committee and has taken on many initiatives to enhance the age inclusivity of the University, since 2016. In 2021, the Centre held a competition called the Age-friendly University Initiative Fund, which was open to all academic and non-academic units at UM. Funded projects related to campus wayfinding (Architectural & Engineering Services and the Office of Sustainability), inter-generational arts presentations/workshops (School of Art Gallery), technology training (Alumni Relations), and the development of a micro-certificate on Facilitating Older Adult Learning (FOAL; Faculty of Extended Education). This presentation will provide an overview of the Age-friendly University Initiative Fund and its collaborative process of working with funded projects, as well as provide specific details on the development and implementation of the micro-certificate. The FOAL micro-certificate provides a professional development opportunity to take three modules online: older adult development, universal design for learning, and using technology for teaching and learning with older adults. In total there are 36 contact hours for this credential that can be completed in a flexible fashion. The goal of the micro-certificate is to enable individuals to work more effectively with older adults to support their learning, across a broad range of learning environments (e.g., lifelong learning programs, senior centers, health care and therapy settings). Cross-unit collaboration is critical for the effective development and implementation of initiatives to improve age inclusivity across multiple domains such as those described in this presentation.

Abstract citation ID: igae098.1434

### STUDENT SERVICES ON CAMPUS: SUPPORTING OLDER STUDENTS

Natalie Galucia, *Washington University in St. Louis, St. Louis, Missouri, United States*

Universities are experiencing shifts in age distributions of students and have an increasing need to embrace age-diversity on their campuses. Two recent studies explore this shift in demographics as they focused on staff members in student services and collected data on their perspectives regarding age-diversity and inclusion on campus. The first study involved focus groups with staff in admissions and career services to explore the challenges, opportunities and strategies related to serving an age-diverse student body. Themes regarded both the benefits of age-diversity but also the challenges they face in attracting and supporting older students. A second study involved staff from Diversity, Equity and Inclusion offices at universities across the country. These DEI officers acknowledged age as an identity relevant to the student experience but they did not elevate age bias or discrimination as a priority for their work. In both studies, affinity groups for non-traditionally aged students were suggested as a strategy. Discussion will be included around the experiences with starting a student group, called Next Move, focused on providing resources and support for older students at the Brown School at WashU. Opportunities and challenges for creating support groups for these students will be discussed.

Abstract citation ID: igae098.1435

### DISSEMINATION AND IMPLEMENTATION OF AGE-INCLUSIVE MANAGEMENT STRATEGIES WITHIN INSTITUTIONS OF HIGHER EDUCATION

Brian Kaskie, *University of Iowa, Iowa City, Iowa, United States*

Institutions of higher education employ a greater proportion of persons over 65 relative to the general labor force. Age-Inclusive Management Strategies (AIMS) seeks to change the narrative about aging academics by shifting focus away from individuals and toward organizational efforts to adopt strategies designed to recruit, retain, and support the financial security and well-being of the growing population of aging faculty and staff. Using the University of Iowa as a case study, I recount efforts to analyze data concerning the productivity and retirement of aging employees, engage and activate institutional leadership to support implementation of policies and programs for aging faculty and staff, and the adoption of three management strategies. These strategies include: offering information and education about retirement and age-related issues such as estate planning and end of life care, increasing access to caregiver support programs, and modifying policies to facilitate access to varied retirement pathway option for faculty and staff alike. Such efforts have been used to validate why Iowa City and the state of Iowa are consistently recognized among the best places in America to grow older.

### SESSION 3315 (SYMPOSIUM)

Abstract citation ID: igae098.1436

### AGEISM IN AI: ROADMAPS TO RESEARCH AND INTERVENTION

Chair: Clara Berridge

Risks that AI applications pose to older adults have not been thoroughly examined or understood in concert with other markers of identity such as race and gender. Ageism is not centered in scholarship on AI or algorithmic harms, and yet conditions in which AI can reproduce or introduce ageism have been identified by researchers and organizations like the World Health Organization. A multi-pronged approach is needed to address the diverse ways ageism is reflected and amplified in AI, from exclusion and misrepresentation of older adults to failure to enable transparency. This symposium will describe a broad swath of critical interventions into AI ageism at multiple levels. To orient all to the topic, Dr. Stypinska will present her original AI ageism framework that identifies five forms in which ageism manifests. Dr. Gallistl will offer a set of research priorities for gerontological research that challenge us to advance conceptualizations of AI in gerontology and engage with the lived experiences of AI in the lives of older adults. Dr. Brewer will discuss how intersectionality affects older adults' lived experiences and envisioned futures with conversational AI technologies. Dr. Berridge will discuss the importance of supporting older adults and people living with dementia to engage knowledgeably with AI tools used in care. Dr. Grigorovich will discuss the ethical implications that arise when location tracking technologies are used to track the movements and activities of persons living with dementia in nursing homes. Presenters will provide insights about needed research and approaches to doing this timely work.

Abstract citation ID: igae098.1437

### AI AGEISM: A ROADMAP FOR STUDYING AGEISM IN THE ERA OF ALGORITHMS: REFLECTIONS AND FUTURE RESEARCH DIRECTIONS

Justyna Stypinska, *WZB Berlin Social Science Center, Berlin, Berlin, Germany*

AI ageism offers a conceptual framework for analyzing ageism and age discrimination within the field of AI. It encompasses five interconnected forms: (1) age biases in algorithms and datasets (technical level), (2) age stereotypes, prejudices and ideologies of actors in AI (individual level), (3) invisibility of old age in discourses on AI (discourse level), (4) discriminatory effects of use of AI technology on different age groups (group level), (5) exclusion as users of AI technology, services, and products (user level). The presentation reflects on the conceptual framework in the light of empirical evidence and data collected in four countries: Germany, Spain, Great Britain and the Netherlands within an interdisciplinary research project AGEAI. The empirical analysis from three work packages of the project will be shown: a) policy text analysis; b) computational algorithmic simulation; c) qualitative interviews with older persons. The preliminary results substantiate the knowledge about how ageism operates within the field of AI, and expose subtle, yet powerful modes of exclusion of older adults from that field. In conclusion, future directions on research in ageism in AI are discussed

Abstract citation ID: igae098.1438

### THE BLACK BOX OF AI-AGEISM: EXPLORING THE CO-CONSTITUTION OF AGING AND ARTIFICIAL INTELLIGENCE

Vera Gallistl[1], Muneeb Ul Lateef Banday[2], Clara Berridge[3], Alisa Grigorovich[4], Juliane Jarke[5], Ittay Mannheim[6], Barbara Marshall[7], and Alexander Peine[8], *1. Karl Landsteiner University of Health Sciences, Krems, Niederosterreich, Austria*, *2. University of Bern, Bern, Bern, Switzerland*, *3. University of Washington, Seattle, Washington, United States*, *4. Brock University, St. Catharines, Ontario, Canada*, *5. BANDAS Centre, University of Graz, Graz, Steiermark, Austria*, *6. Ben Gurion University of the Negev, Beer-Sheva, HaDarom, Israel*, *7. Trent University, Toronto, Ontario, Canada*, *8. Open University of the Netherlands, Heerlen, Limburg, Netherlands*

Algorithmic technologies and (large) data infrastructures, often referred to as Artificial Intelligence (AI), have received increasing attention from gerontological research in the last decade. So far, however, available studies have often focused on evaluating the "impact" of AI technologies on the lives of older people, while critical engagement with theory has been largely absent in these studies. In this paper, we make a novel contribution by critically engaging with the theorizing in this growing field of research. We observe that gerontology's engagement with AI is shaped by an interventionist logic that situates AI as a black box for gerontological research. We demonstrate how this black box logic has neglected many aspects of AI as a research topic for gerontology and discuss three classical concepts in gerontology to show how they can be used to open various black boxes of aging and AI in the areas: a) the datafication of aging, b) the political economy of AI and aging, and c) everyday engagements and embodiments of AI in later life. In the final chapter, we propose a model of the co-constitution of aging and AI that makes theoretical propositions to study the relational terrain between aging and AI and hence aims to open the black box of AI in gerontology beyond an interventionist logic.

Abstract citation ID: igae098.1439

### INTERSECTIONAL AGEISM: HOW BLACK OLDER ADULTS ENVISION A FUTURE WITH CONVERSATIONAL TECHNOLOGIES

Robin Brewer[1], Christina Harrington[2], and Courtney Heldreth[3], *1. University of Michigan, Ann Arbor, Michigan, United States*, *2. Google, Atlanta, Georgia, United States*, *3. Google, Seattle, Washington, United States*

Dominant narratives of aging often center whiteness. However, older age can intersect with other identities that experience structural inequities and exacerbate ageism. In this study, we explore what it means to design technologies that are fair for historically minoritized older adults. We conducted design workshops with 16 Black older adults, asking how they envision older age and race represented in one form of AI system - conversational technologies. We highlight concerns around sharing age-related data, potential ageist outcomes that intersect with racism, and desires for authentically representing older age and race. We discuss these findings with a lens on how ageism discourse can exclude certain groups and how we might mitigate age-related AI harms in the technology design process by incorporating other values such as authenticity.

Abstract citation ID: igae098.1440
### "LET'S TALK TECH" IN THE ALGORITHMIC AGE: ENGAGING PEOPLE LIVING WITH DEMENTIA IN TECHNOLOGY USE DECISIONS
Clara Berridge, and Natalie Turner, *University of Washington, Seattle, Washington, United States*

The availability of technologies to support dementia care is outpacing our understanding of how to help families navigate use decisions and align with the values and preferences of the person living with dementia. This is an ethical problem made urgent by efforts to incorporate data from the home into AI healthcare applications where transparent disclosures are still aspirational. The misperception that people living with mild-to-moderate dementia cannot or do not want to be involved in decision making about their care is widespread, yet failure to enable decision making participation where possible reflects ageism and ableism. An added barrier to involvement in decision making about digital technologies is communication about the technologies themselves. Findings will be presented from a mixed-methods study of the first of its kind tool to facilitate empowered, shared decision making by people living with dementia and care partners about technologies that use a range of data (e.g., audio, visual, location) and include companion robots. Together with primary care partners, twenty-nine participants living with mild Alzheimer's disease (AD) learned about, discussed, and documented their preferences for each of four categories of technologies through the Let's Talk Tech intervention. Individual preferences were not consistent across technologies and varied considerably, supporting the need for personalized decision making. Most participants living with mild AD reported that they wanted (vs. unsure or do not want) each of five control-enhancing options, including being informed if a technology is used to monitor them and to have the ability to pause monitoring for privacy.

Abstract citation ID: igae098.1441
### INVESTIGATING ETHICAL DIMENSIONS AND TENSIONS WITH RESPECT TO THE USE OF RTLS IN DEMENTIA CARE
Alisa Grigorovich[1], Kelsey Harvey[1], Kyle Smilovsky[2], and Josephine McMurray[2], *1. Brock University, St. Catharines, Ontario, Canada, 2. Wilfrid Laurier University, Waterloo, Ontario, Canada*

Real-time location systems (RTLS) are increasingly being developed to track older adults with dementia across settings. Specifically, it is believed that such technologies can support automation of care tasks (e.g. locating) and the development of clinical algorithms to predict changes in health and wellbeing. The limited available research suggests that these technologies can have significant ethical implications, including increasing control over older adults, and undermining their rights. In this study, we explored the experiences and perceptions of RTLS by older residents, care partners, and organizational decision-makers in one care home in Ontario, Canada. Findings demonstrate that participants (N=47) had a limited understanding of RTLS (e.g., how it could be used, data storage, ethical issues) and this in turn influenced their perceptions of its value. Residents were generally unaware of its purpose and more concerned with its aesthetics. Care partners and organizational decision-makers valued the RTLS for enhancing their efforts to control risk to the physical safety of residents and believed this was central to enhancing quality of care. Most had limited awareness of residents' preferences and believed that their cognitive impairment and/or frailty disqualified their current or prior values and wishes, including their desire for privacy and autonomy, and thus could be legitimately overridden in the interest of protecting their safety. We discuss these findings in relation to digital ageism and offer suggestions to guide future policy and educational interventions to better align development and use of RTLS and similar surveillance technologies with the wishes and preferences of older adults.

### SESSION 3320 (PAPER)

### AGING AND MENTAL HEALTH

Abstract citation ID: igae098.1442
### FEASIBILITY OF TRAINING CAREGIVERS ONLINE TO DELIVER LIFE REVIEW DEPRESSION INTERVENTION AT HOME
Christina Miyawaki[1], Angela McClellan[1], and Erin Bouldin[2], *1. University of Houston, Houston, Texas, United States, 2. University of Utah, Salt Lake City, Utah, United States*

Due to the high prevalence of depressive symptoms and dementia in older Americans (≥65 years), we developed a six-week depression intervention, Caregiver-Provided Life Review (C-PLR) for care recipients (CRs) with early-stage dementia and mild depression and taught their family caregivers (CGs) life review skills online. The objective of this study was to examine the feasibility of training caregivers via an online video course and the impact of delivering the intervention at home (N=20 dyads). A mixed-methods design (surveys and interviews) was used for this study. We collected pre- and post-measures of CRs' depression (primary outcome) and life satisfaction, CGs' burden, rewards of caregiving, and dyads' relationship quality (secondary outcomes) and compared them using paired t-tests. We took fidelity scores to measure CGs' feasibility to deliver life reviews adhering to the intervention protocol. CGs were on average 64 years old, White (65%), married (65%), college-educated (70%), working or retired (40% each), and female (90%) in good/excellent health (100%). Their CRs were 80 years old (mean), White (70%), female (50%) in poor/fair health (50%). CGs' average fidelity check-in score was 15.9±0.27 (out of 16), indicating excellent adherence. The C-PLR intervention significantly reduced CRs' depressive symptoms (p=0.034) and increased CGs' rewards of caregiving (p=0.019). Caregivers' post-intervention qualitative interviews supported the quantitative results that online-trained caregivers could deliver the intervention by protocol without adding caregiver burden. This study demonstrated that the C-PLR, an innovative, cost-effective intervention with a flexible delivery schedule could make a positive impact on both caregivers' and care recipients' mental health.

Abstract citation ID: igae098.1443

### RE-EVALUATING FORTITUDE, VULNERABILITY, AND FRAILTY IN CARE HOME STORIES

Ulla Kriebernegg, *University of Graz, Graz, Steiermark, Austria*

This paper analyzes how concepts of fortitude, vulnerability, frailty, and care are linked from the perspective of literary gerontology. Discussing the newly emerging genre of the care home novel, the paper shows how a close reading can challenge traditional assumptions about bodies on the margins of life and death as merely existing in the "black hole" of the fourth age (Gilleard and Higgs 2010). Examples are drawn from stories that are set in long-term care facilities that resemble "total institutions" (Goffman 1961), such as May Sarton's As We Are Now (1973) or Oscar Casares's Amigoland (2009), where aging into old age is often narrated as a creative process that allows for agency and involves the renegotiation of one's identity despite extreme vulnerability. The humanities lens here examines such novels as "Reifungsromane" (novels of ripening, Waxman 1990) or "Vollendungsromane" (novels of completion, Rooke 1992), where vulnerability is represented as a meaningful condition that facilitates resistance in the face of loss and precarity. As such, these are striking individual narratives of fortitude and self-determination that are less about "successful aging," than about "successful frailty," which Wendy Lustbader describes as "turning the 'nothing' of empty time into the 'something' of good days" (1991: 14). Conclusions reiterate how vulnerability is a shared ontological condition, and why a humanities perspective is important to highlight vulnerability as a current socio-political issue in connection with care ethics and social justice.

Abstract citation ID: igae098.1444

### BIDIRECTIONAL ASSOCIATIONS BETWEEN PHYSICAL ACTIVITY AND AFFECT: AN ECOLOGICAL MOMENTARY ASSESSMENT STUDY

Peichao Liang, *The University of Hong Kong, Pokfulam, Hong Kong*

Despite extensive evidence demonstrating the beneficial effects of physical activity on health outcomes, many older adults remain physically inactive. A growing body of research examines affective states as both determinants and consequences of physical activity. However, the nuanced and dynamic relationships between physical activity and affect among older adults remain understudied. We used ecological momentary assessment (EMA) to investigate the bidirectional relationships between physical activity and affect in daily life among older adults in Hong Kong. 168 older adults aged 65 to 84 received seven EMA prompts per day for fifteen consecutive days and completed a total number of 17,345 momentary assessments of affective states, mobility, and activities. We used multilevel models to test the bidirectional associations between physical activity and positive affect, negative affect, and energetic arousal, nested within individuals. Preliminary results reveal concurrent associations between physical activity and positive affect (b=0.042, SE=0.008, p<.001) and energetic arousal (b=0.107, SE=0.027, p<.001). When older adults engaged in physical activity before the prompt, they reported a higher level of positive affect (b=0.038, SE=0.008, p<.001). However, physical activity did not significantly improve energetic arousal levels later (b=0.046, SE=0.027, p=0.089). Additionally, individuals were more likely to engage in physical activity after they reported a higher level of positive affect (OR=1.321, 95% CI=1.234-1.413, p<.001) and feeling energetic (OR=1.078, 95% CI=1.044-1.112, p<.001). No associations between physical activity and negative affect were identified. The implications of these findings for developing context-specific interventions to promote physical activity among older adults will be discussed.

Abstract citation ID: igae098.1445

### DEPRESSIVE SYMPTOMS, NOT FUNCTIONAL STATUS, PREDICT MORBIDITY AND MORTALITY IN OLDER ADULTS WITH HEART FAILURE

Abigail Latimer[1], Jia-Rong Wu[1], Christopher Knoepke[2], JungHee Kang[1], and Debra Moser[1], *1. University of Kentucky, Lexington, Kentucky, United States*, *2. University of Colorado Anschutz Medical Campus, Aurora, Colorado, United States*

**Background:** Older adults (≥ 65 years) (OAs) with heart failure (HF) are at high risk of functional impairment and co-morbid depression. The impact on morbidity and mortality of these comorbidities in OAs with HF has not been well-studied. OBJECTIVE: To determine the extent to which functional status and depression predicted event-free survival (i.e., hospitalization or death) in OAs with HF.
**Methods:** We performed a secondary analysis from the Research Interventions in Cardiovascular Health HF Database of OAs with data on depressive symptoms using the Patient Health Questionnaire- 9 (PHQ-9) and functional status using the Duke Activity Status Index (DASI). Patients were followed for 2- years. Event-free survival was determined using Cox proportional hazards models.
**Results:** We included 157 OAs (mean age= 73 ± 6, 68.2% men, 87.9% White, 63.1% NYHA III or IV). Participants had an average DASI score of 11.49 ± 9.26 and 58% had at least mild depressive symptoms. At time of follow- up, 63 hospitalizations and 25 deaths occurred. The risk of hospitalization or death was 2.16 times greater for OAs who had depressive symptoms (95% Confidence Interval=1.27- 3.67, p <.01) compared to those without, regardless of functional status. The overall model showed a significant improvement in fit relative to the null [x2 (3)= 11.21, p <.05].
**Conclusion:** Caring for OAs with HF requires attending to depressive symptoms, given the independent risk of hospitalization and mortality, even in the company of varying functional status. Future research should evaluate effectiveness of depression treatments for these patients.

Abstract citation ID: igae098.1446

### HAPPINESS IN CONVENTIONAL VERSUS RETIREMENT HOMES: IMPORTANCE OF THRIVING AND COMMUNITY INTEGRATION

Melanie Levasseur[1], Daniel Naud[1], Martine Lagacé[2], Marie-Ève Bédard[3], and Shannon Hebblethwaite[4],

*1. Université de Sherbrooke, Sherbrooke, Quebec, Canada, 2. University of Ottawa, Ottawa, Ontario, Canada, 3. Cégep de Drummondville, Drummondville, Quebec, Canada, 4. Concordia University, Montreal, Quebec, Canada*

Since aging sometimes implies a change in living environment, it is important to better understand happiness and associated factors such as thriving, social participation, community integration and ageism according to residential settings. This study aimed to compare the happiness of older women and men living in a conventional dwelling (CD) and in an independent living facility (ILF), and examined its associations with thriving, social participation, community integration and ageism (self-directed and discrimination). A cross-sectional survey was carried out on a random sample of 509 adults aged 75 and older living in DC and 470 in ILF. Regressions stratified by residential setting and gender examined associations between happiness, thriving and social participation, testing for the moderating effect of ageism and community integration. Participants were 81.3+/-5.3 on average, and two-thirds were women. Controlling for age, living situation and health, the happiness of older adults living in CD and ILF was similar (69.7+/-14.0 vs 68.8+/-18.4; p=0.262). Superior thriving was strongly associated with greater happiness, regardless of residential setting or gender. Higher social participation was associated with greater happiness among women in ILF. For both settings, self-directed ageism was associated with lesser happiness, but discrimination was associated with lesser happiness only in women living in ILF. Higher community integration was associated with greater happiness in both settings, except for men in ILF. According to these results, future studies should evaluate interventions aimed at facilitating thriving and reducing ageism may improve happiness of older adults.

### SESSION 3325 (BIOLOGICAL SCIENCES INVITED SYMPOSIUM)

Abstract citation ID: igae098.1447

### AGE-ASSOCIATED ALTERATIONS IN HEMATOPOIESIS

Chair: Isabel Beerman

Abstract citation ID: igae098.1448

### FITNESS AND TIME OF MUTATION ACQUISITION A BETTER PREDICTOR OF OUTCOME IN CLONAL HEMOPOIESIS COMPARED TO CLONE SIZE

Kristina Kirschner[1], Simon Cox[2],
Tamir Chandra Neil Robertson Eric  Crespoand
Linus Schumacher, *1. Mayo Clinic, Rochester, Minnesota, United States, 2. University of Edinburgh, Edinburgh, Scotland, United Kingdom*

The prevalence of clonal hematopoiesis of indeterminate potential (CHIP) in healthy individuals increases rapidly from age 60 onwards and has been associated with increased risk for malignancy, heart disease and ischemic stroke. CHIP is driven by somatic mutations in stem cells that are also drivers of myeloid malignancies. We previously reported gene specific fitness effects in two longitudinal cohorts of aging. We now set out to define fitness effects across three longitudinal cohorts of aging in a total of 713 participants. We found that fitness correlates with timing of mutations, which needs to be taken into account when predicting health outcomes. We show that fitness and the time of mutations are superior predictors of outcomes such as all-cause mortality compared to the commonly used clone size (variant allele frequency). Our new predictor even outperforms age, the most important predictor for the onset of age-related disease thus far. In conclusion, the fitness landscape of mutations and their context in terms of clonal composition and timing of mutation acquisition are important parameters to bring CH predictors into the clinic.

Abstract citation ID: igae098.1449

### HEMATOPOIETIC LOSS OF THE Y CHROMOSOME AND AGING PHENOTYPES

Nick Chavkin, *Seattle Children's Research Institute & University of Washington, Seattle, Washington, United States*

Life expectancy of men is six years less than women in developed countries. Recent epidemiological studies suggest that a significant portion of this discrepancy can be attributed to an age-related phenomenon in blood. As men age, a portion of their blood cells will lose the Y chromosome. This phenotype, termed Mosaic Loss of the Y Chromosome (mLOY), is the most common post-zygotic mutation in humans and is present in almost half of men by age 70. mLOY has been correlated with increased mortality and many age-related diseases. Recently, we have found that men with higher mLOY are more susceptible to non-ischemic heart failure. Using a mouse model of mLOY, we showed causal effects of mLOY on diminished lifespan, increased progression of age-associated cardiomyopathy, and increased progression of cardiac fibrosis after injury. Further analysis revealed that mLOY macrophages showed both an increased propensity towards pro-fibrotic macrophage polarization and increased expression of pro-fibrotic ligands. Genetic analysis of the Y chromosome identified the gene UTY as a potential causal locus for mLOY-mediated effects on cardiac fibrosis. Finally, analysis of published single cell RNA sequencing datasets from human male heart, lung, and kidney tissue suggests a broad correlation between mLOY and fibrotic pathology with potential direct effects of mLOY macrophages on tissue-resident fibroblasts. These mechanistic findings help explain the correlation between mLOY and cardiac fibrosis, and further suggest general effects of mLOY on age-related phenotypes.

Abstract citation ID: igae098.1450

### PATHOGENIC PRE-LEUKEMIC MUTATIONS: RARE IN CLONAL HEMATOPOIESIS BUT ENRICHED IN MYELOID DISEASES

Jing Zhang, *University of Wisconsin-Madison, Madison, Wisconsin, United States*

Clonal hematopoiesis of indeterminate potential (CHIP) refers to the expansion of peripheral blood cells derived from hematopoietic stem cells (HSCs) with at least one somatic driver mutation in healthy elderly individuals. CHIP is strongly linked to aging and confers an increased risk for blood cancers, non-hematological diseases (e.g. cardiovascular disease), and all-cause mortality. In blood cancers, CHIP mutations are associated with initiation of acute myeloid leukemia (AML) and other myeloid diseases and thus considered as pre-leukemic mutations. However, some pathogenic pre-leukemic mutations are rarely found in CHIP patients. We will talk about two of such mutations. The first one is the hot spot G646WfsX12 mutation in the epigenetic regulator ASXL1. This mutation occurs in ~18% of patients with myeloid diseases but almost never found in CHIP patients. Using a knockin animal model (Atm/+), we show that Atm/+ HSCs were only expanded in aged mice but not in young mice. We are investigating if the mutant HSC expansion is caused by reduced immunosurveillance of aged immune cells and/or aged pro-inflammatory stromal cells. The second one is oncogenic NRASG12D mutation, which serves as an initiation or progression mutation in chronic myelomonocytic leukemia. It is shown that one copy of NRASG12D mutation confers bi-modal regulation of HSCs in which it enhances self-renewal in one subset of HSCs but promotes proliferation and exhaustion in another subset of HSCs. We hypothesize that ASXL1G646WfsX12 and NRASG12D mutations belong to the pathogenic pre-leukemic mutations, which easily incorporate additional mutations in leukemogenesis rather than remain stable in CHIP.

### SESSION 3330 (PAPER)

### CHRONIC DISEASE MANAGEMENT (PAPERS)

Abstract citation ID: igae098.1451

### ASSOCIATION OF KIDNEY MARKERS AND DUAL TRAJECTORIES OF COGNITIVE AND PHYSICAL FUNCTION

Aman Shrestha[1], Michelle Shardell[1], Chixiang Chen[1], Stephen L. Seliger[1], Charles Ginsberg[2], Lindsay Miller[2], Teresa Tian[3], and Eleanor Simonsick[4], *1. University of Maryland School of Medicine, Baltimore, Maryland, United States*, *2. University of California San Diego, La Jolla, California, United States*, *3. National Institute on Aging, Baltimore, Maryland, United States*, *4. National Institute on Aging, Baltimore City 0400 Baltimore City, Maryland, United States*

The interplay between kidney health and cognitive-physical function has garnered increased attention. Dual trajectories consider simultaneous longitudinal changes in cognitive-physical function. Among participants in the Health, Aging and Body Composition Study with initially fast gait and high cognitive function, we assessed associations between baseline kidney function markers and joint longitudinal cognition-gait trajectories. Grouped-based trajectory analysis performed on modified mini-mental state (years 3, 5, 8, 10) and 20-meter usual gait speed (years 3–6, 8, 10) yielded three dual trajectory groups: Group 1 (n=660; exhibited sustained superior longitudinal cognitive-physical performance), Group 2 (n=744; high sustained cognition and initially lower, steadily declining gait), and Group 3 (n=498; lowest initial cognitive-physical performance, both steeply declining over time). Four sequential multinomial regression models were built. Model 1 showed a higher estimated glomerular filtration rate (ml/min/1.73m2) to be associated with lower odds of Group 3 versus Group 1 (odds ratio[OR]=0.98, 95% confidence interval [CI]: 0.97–0.99) after adjusting for covariates. Model 2 estimated 19% greater odds of Group 2 per log-transformed urine albumin-to-creatinine ratio (mg/g) (OR=1.19, 95%CI: 1.05–1.35) and 35% greater odds of Group 3 (OR=1.35, 95%CI: 1.17–1.55) versus Group 1. In Model 3, 25-hydroxyvitamin D was not significantly associated with cognitive-physical trajectory. Model 4 added parathyroid hormone, alpha-klotho, and fibroblast growth factor 23 (FGF23) (p=0.044 Model 4 versus 3); FGF23 was significantly associated with cognitive-physical trajectory (Group 3 versus 1 per log-transformed FGF23 [pg/mL]: OR=1.51, 95%CI:1.00–2.28). Findings support a potential role of kidney health in dual cognitive-physical performance among initially healthy older adults.

Abstract citation ID: igae098.1452

### CROSS-CULTURE ADAPTATION AND VALIDATION OF THE CHINESE VERSION OF MANCHESTER COPD FATIGUE SCALE (MCFS)

Haihong Zhang[1], Chongmei Huang[1], and Minhui Liu[2], *1. Ningxia Medical University, Yinchuan, Ningxia, China (People's Republic)*, *2. Ningxia Medical University, Yinchuan, Ningxia, China*

Fatigue is an aching, complex, and multifaceted sensation that is the second most prominent and disabling symptom of COPD, approximately 50% of COPD patients suffer from severe fatigue. Various scales or questionnaires has been developed to measure fatigue in COPD patients. The Manchester COPD fatigue scale (MCFS) has been proved to be a robust, simple, reliable, and valid scale in the American population with COPD. As a disease-specific measurement, MCFS can precisely identify the features of fatigue among COPD patients in the physical, cognitive, and psychosocial domains. The Brislin's translation model was used to for cross-cultural adaptation. Internal consistency, test-retest reliability, constructive validity, convergent validity, and criterion validity were used to test the reliability and validity of MCFS. Diagnostic accuracy was used to determine the optimal cut-off point. Overall, a convenience sampling of 371 patients completed the fatigue scale. The results showed that the scale has acceptable constructive validity (CFI = 0.877, IFI = 0.878, SRMR = 0.027), excellent internal consistency (Cronbach's alpha = 0.97) and test-retest reliability (Cronbach's alpha=0.93). MCFS presented good convergent validity. Using the BFI fatigue scale as a reference criterion, the areas under the curve were 0.941 and 0.900, respectively. The optimal cut-off points for mild, moderate, and severe fatigue were ≤16, >16 and ≤29.5, and >29.5 (sensitivity: 94.7% and 82.6%; specificity: 80.8% and 86.0%, respectively). The Chinese version of MCFS could be used to measure fatigue among Chinese patients with COPD.

Abstract citation ID: igae098.1453

### FRAILTY STATUS AND CATHETER ABLATION THERAPY FOR ATRIAL FIBRILLATION IN REAL-AF: A MULTICENTER CLINICAL REGISTRY

Paulo Chaves[1], Allyson Varley[2], Anil Rajendra[3], Dinesh Sharma[4], Christopher Thorne[5], Paul Zei[6], and Jose Osorio[7], *1. Florida International University, Miami, Florida, United States*, *2. HRCRS, Birmingham, Alabama, United States*, *3. Grandview Medical Center, Birmingham, Alabama, United States*, *4. NCH Health Care System, Naples, Florida, United States*, *5. Heart Rhythm Clinical Research Solutions, Birmingham, Alabama, United States*,

*6. Brigham and Women's Hospital, Boston, Massachusetts, United States*, *7. HCA Florida Miami, Miami, Florida, United States*

There has been increasing interest in understanding if frailty assessment could improve clinical decision-making related to catheter ablation therapy (CAT) for atrial fibrillation (AF) patients. This prospective study assessed acute hospital complications, AF recurrence and AF-related hospitalization within 12 months after CAT according to frailty status pre-CAT. Study subjects (n=3,406) were patients in Real-AF, a multicenter clinical registry in the US, who underwent CAT with 12-month outcome data (2018-2022). Frailty scores were derived using the cumulative health deficit approach (deficits count divided by the total number of health indicators), and categorized: Fit (≤.12), Intermediate (.12–.24), and Frail (≥0.25). Cross-tabulations, trend tests, and logistic regression were performed. Mean age± SD of study subjects was 65.2± 11.0; males were 59.0%. Among patients who underwent CAT, 24.5% met criteria for Frail (score≥0.25). Frailty scores were correlated weakly with age (r=0.09; p<.01). Fit (1.2%) and Frail (1.3%) had similar acute in-hospital complication rates. Increasingly higher probability of AF recurrence (18.2% vs. 19.8% vs. 22.6%; p=.02), and AF-related hospital admission (2.1% vs. 3.4% vs. 4.6% vs. 5.4%, p=.02) within 12 months after CAT were observed across increasing frailty severity categories. Frailty remained associated with AF recurrence and hospitalization in age- and sex-adjusted models; e.g., odds of hospitalization post-CAT were 2.3 (95%CI: 1.0-5.0, p=.039) times higher in Frail than in Fit. Despite adverse trends with frailty, absolute 12-month risks after CAT, even among Frail, was low. Implementation research is warranted to establish the clinical value of frailty assessment for CAT-related decision-making in AF patients.

Abstract citation ID: igae098.1454

### DO NOT DRAG THE CHILDREN DOWN: MUTUAL SUPPORT AMONG COMORBID COUPLES WITH STROKE SURVIVORS IN HOSPITAL

Yongxia Mei[1], Zhenxiang Zhang[1], Qingqing Zhang[1], Zhiwei Liu[2], Baoyu Fan[1], Yating Liu[1], and Sixun Zhang[1], *1. Zhengzhou University, Zhengzhou, Henan, China (People's Republic)*, *2. Zhengzhou University, Zhengzhou, Hunan, China (People's Republic)*

The phenomenon of mutual support among co-morbid couples of recurrent older stroke survivors during hospitalization is gaining attention. Despite their crucial role, little is understood about how they support each other. The study aimed to explore the experiences of co-morbid couples of older stroke survivors with recurrent stroke supporting each other during hospitalization. Conducted as a descriptive phenomenology study, data collection occurred in neurology departments of five tertiary-level hospitals from July to September 2023. Twenty-one couples aged between 61 and 72 were purposively sampled. Interview recordings were transcribed and analyzed by two independent coders using Colaizzi's framework in NVivo 12, revealing 9 subthemes across 3 macrothemes. "Do not drag the children down" is a strong thread, which is linked to three macrothemes: (1) maintaining the couple's relationship through mutual support, (2) mutual support to avoid burdening the children, and (3) providing support while struggling between ideals and reality. Healthcare professionals should give more consideration to co-morbid couples of older stroke survivors with recurrent stroke who adhere to the idea of not burdening their children when supporting each other, and should take into account the cultural characteristics of the conflicts and differences that may arise between couples of stroke survivors in the process of mutual support. It is crucial to provide them with individualized and tailored support and interventions that can help these couples achieve a more optimal balance in their mutual support.

Abstract citation ID: igae098.1455

### URINARY INCONTINENCE IN US ADULTS WITH DIABETES WHO ARE GUIDELINE-RECOMMENDED TO RECEIVE SGLT2IS: NHANES 2013–2020

Alexandra Lee, and Sei Lee, *University of California San Francisco, San Francisco, California, United States*

Sodium-glucose cotransporter-2 inhibitors (SGLT2is) are now recommended as first-line cardiorenal protective therapy for many adults with type 2 diabetes but are also associated with increased risk of urogenital infections. While urinary incontinence is common among older adults and increases the risk of urogenital infections, there is limited data on the prevalence of incontinence among adults with diabetes, particularly those eligible to receive SGLT2is. We included participants with diabetes from the nationally-representative National Health And Nutrition Examination Survey (NHANES) 2013-2020. We determined whether each participant would be ADA guideline-recommended to receive SGLT2is (a) due to heart failure or chronic kidney disease, or (b) due to atherosclerotic cardiovascular disease or high cardiovascular risk (in which case they could also alternatively be treated with GLP-1RAs). We defined frequent incontinence as self-reported leaking urine "daily/nightly" or "several times per week." There were 2,366 NHANES participants with type 2 diabetes. Among adults aged 55-59, 14.9% were guideline-recommended an SGLT2i, 22.7% of whom had frequent incontinence; among adults aged 70+, 27.0% were recommended to receive an SGLT2i, 41.0% of whom had frequent incontinence. Among adults aged 55-59, 54.6% were guideline-recommended either an SGLT2i or GLP-1RA, 20.6% of whom had frequent incontinence; among adults aged 70+, 45.1% were recommended to receive either an SGLT2i or GLP-1RA, 34.9% of whom had frequent incontinence. Prescribers should use caution for patients with incontinence who are initiating SGLT2is. While urogenital infections are typically not life-threatening, they can significantly impact quality of life and may not be aligned with health goals.

Abstract citation ID: igae098.1456

### PROTEIN INTAKE AND MORTALITY IN OLDER ADULTS WITH CHRONIC KIDNEY DISEASE: A MULTICOHORT POPULATION-BASED STUDY

Adrián Carballo-Casla[1], Carla Maria Avesani[1], Giorgi Beridze[1], Rosario Ortolá[2], Juan-Jesús Carrero[1], Fernando Rodríguez-Artalejo[3], Davide Liborio Vetrano[1], and Amaia Calderón-Larrañaga[1], *1. Karolinska Institutet, Stockholm, Stockholms Lan, Sweden*, *2. Autonomous University of Madrid, Madrid, Madrid, Spain*, *3. Autonomous University of Madrid, Madrid, La Rioja, Spain*

Avoiding high protein intake in older adults with chronic kidney disease (CKD) may reduce the risk of kidney function decline, but associations with nutritional status and survival are not well known. We aimed to study the associations of total, animal, and plant protein intake with all-cause mortality in older adults without and with mild or moderate CKD. We used data from three cohorts (Seniors-ENRICA 1 and Seniors-ENRICA 2 in Spain, and SNAC-K in Sweden) comprising 8,539 community-dwelling adults ≥60 years. CKD was ascertained according to estimated glomerular filtration rates, urine albumin excretion, and diagnoses from medical records. Cumulative protein intake was estimated via validated dietary histories and food frequency questionnaires. Vital status was ascertained with national death registers. Associations were estimated with Cox proportional hazards regression models, adjusted for sociodemographic, lifestyle, morbidity, and dietary variables. After a maximum follow-up of 15 years, 1,901 deaths were recorded. Higher total protein intake was associated with lower mortality among participants with CKD [hazard ratio (95% confidence interval) for 1.0, 1.2, and 1.4 g/kg/day versus 0.8 g/kg/day = 0.90 (0.83,0.98), 0.82 (0.70,0.96), and 0.75 (0.62,0.92), respectively]. Plant protein showed a stronger inverse association than animal protein [0.77 (0.64,0.92) and 0.92 (0.86,0.98) per 0.2 g/kg/day increment, respectively]. Among participants without CKD, most associations were stronger, but no statistically significant interactions between protein intake and CKD arose. Higher protein intake, particularly plant protein, was associated with lower mortality in older adults with mild or moderate CKD. Associations did not differ substantially in older persons without CKD.

### SESSION 3335 (PAPER)

### COGNITIVE IMPAIRMENT II (PAPERS)

Abstract citation ID: igae098.1457

### ADDED VALUE OF TIMING DATA FOR THE ESTIMATION OF COGNITIVE FUNCTIONING IN SURVEY RESEARCH

Emma Nichols[1], Michael Markot[1], Alden Gross[2], Richard Jones[3], Stefan Schneider[1], and Jinkook Lee[1], *1. University of Southern California, Los Angeles, California, United States*, *2. Johns Hopkins Bloomberg School of Public Health, Baltimore, Maryland, United States*, *3. Brown University, Providence, Rhode Island, United States*

The use of speeded tests to assess cognition supports the notion that response times on cognitive tests contain meaningful information about cognitive functioning. Although information on the time spent completing cognitive tests is often collected during survey administration, this data is not typically considered when quantifying cognitive functioning in large-scale surveys. We used data from the Longitudinal Aging Study in India – Diagnostic Assessment of Dementia (LASI-DAD) study (N=4,091), to assess the added value of data on the time spent on cognitive tests (items), over and above scores from traditional scoring procedures. We used separate linear regression models for each item (N items=35) to assess associations between cognitive factor scores based on all items and quintile of time spent on specific items, adjusting for age, gender, and the item-specific score. Compared to Quintile 3 (median amount of time), taking longer was significantly ($p< 0.05$) associated with lower cognitive functioning for 54% (Quintile 5) and 31% (Quintile 4) of items. Responding quickly (Quintile 1) was associated with higher cognitive functioning for 50% of simple items (e.g., what is the year), but was associated with lower cognitive functioning for 53% of items requiring higher-order processing (e.g., digit span test). Results were similar in models controlling for education, interviewer, or language of administration. Overall, data show that information on response times from the administration of cognitive tests may contain important information on cognition not captured in traditional scoring. Future research should assess how this information could be leveraged to improve estimates of cognitive functioning.

Abstract citation ID: igae098.1458

### CHANGE IN EXECUTIVE FUNCTION AFTER AN INNOVATIVE PHYSICAL THERAPY PROGRAM AMONG VETERANS WITH SLOW GAIT SPEED

Elisa Ogawa[1], Rebekah Harris[2], Rachel Ward[1], Mary Palleschi[3], Ildiko Halasz[4], William Milberg[5], and Jonathan Bean[5], *1. VA Boston Healthcare System, Boston, Massachusetts, United States*, *2. Boston VA, Boston, Massachusetts, United States*, *3. VA Boston Healthcare System, Jamaica Plain, Massachusetts, United States*, *4. Primary Care/VABHS, Boston, Massachusetts, United States*, *5. Harvard Medical School, Boston, Massachusetts, United States*

While gait and cognition are independent predictors of age-related adverse outcomes, evidence suggests that they are related. Thus, targeting slow gait speed may have broad effects including improvements in cognition. This study examined the effects of Live Long Walk Strong (LLWS), a novel Physical Therapy program designed to improve gait speed, on executive function among Veterans with slow gait speed. This study was a preplanned secondary analysis among the first 72 participants recruited into an ongoing Randomized Controlled Trial among middle aged to older Veterans (≥50 years) with slow walking speed. Participants were randomized to either 8-week LLWS or 8-week wait-list control. Participants' executive function was measured using Delis-Kaplan Executive Function System Verbal Fluency subtests (Letter Fluency, Category Fluency, Category Switching). Fifty-one participants (mean age 72, 92% men, 84% White) were included in the analyses (LLWS n=27; wait-list control: n=24). We observed clinically and statistically significant changes in Category Fluency between LLWS and wait-list controls (LLWS: pre: 31.44 ± 7.92, post: 34.78 ± 8.12; wait-list control: Pre: 34.21 ± 8.54; Post: 30.83 ± 7.89, $p< 0.0001$), favoring the LLWS. There were no statistically or clinically significant group differences in Letter Fluency or Category Switching between LLWS and wait-list controls. LLWS may improve aspects of executive function specifically the effortful retrieval process among middle aged to older Veterans with slow gait speed.

Abstract citation ID: igae098.1459

### POSTOPERATIVE DELIRIUM: A SURVEY OF PERCEPTIONS AND KNOWLEDGE AMONG SURGICAL PROVIDERS

Julia Berian[1], Ben Zarzaur[1], Sanjay Mohanty[2], Farah Kaiksow[3], and Blair Golden[4], *1. University of*

*Wisconsin Madison, Madison, Wisconsin, United States*, 2. *Indiana University School of Medicine, Indianapolis, Indiana, United States*, 3. *University of Wisconsin School of Medicine and Public Health, Madison, Wisconsin, United States*, 4. *University of Wisconsin Madison School of Medicine and Public Health, Madison, Wisconsin, United States*

Postoperative delirium is associated with delayed recovery and cognitive decline. Despite increasing awareness among medical providers, understanding of delirium among surgical providers remains unknown. We aimed to evaluate knowledge and confidence in identifying and managing postoperative delirium, and to determine surgeons' perceptions of the relative importance of postoperative delirium. A 14-question survey was distributed to surgeons and surgical advanced practice providers (APP) after validation through cognitive interviews. Eighty-five of 267 responded (31%), more attending surgeons (67%) than APPs (33%). Most had >5 years of experience (73%). Respondents estimated postoperative delirium rates from 0% to 65%, with the average varying by field from 5% (Endocrine surgery) to 33% (Trauma and Critical Care). Most had received formal delirium training (66%) and felt confident in identifying (80%) and managing (65%) delirium. Postoperative delirium was identified by nursing concerns (89%) or clinical gestalt (82%), with few reporting the use of validated tools (23%). Out of 6 common postoperative complications, surgical providers ranked postoperative delirium 3rd (28%) or 4th (25%), below cardiac complications (78%) and above superficial site infections (53%). The most concerning consequence of delirium was mortality (62%), with few concerned about psychological distress (16%). Most (60%) thought post-operative delirium had a limited effect on long-term health. Despite self-reported knowledge and high confidence in identifying and managing postoperative delirium, objective measures are rarely used, and long-term effects are thought to be minimal. Gaps in knowledge around delirium diagnosis and long-term health effects may contribute to the ongoing burden of delirium in older surgical patients.

Abstract citation ID: igae098.1460

### ASSOCIATION OF PHYSICAL ACTIVITY WITH NEUROFILAMENT LIGHT IN NONDEMENTIA OLDER ADULTS AND THE MEDIATION OF FRAILTY

Sofia Liu[1], Mengchi Li[2], Miranda McPhillips[3], Russell Calderon[4], and Junxin Li[5], *1. Johns Hopkins University School of Nursing, Baltimore, Maryland, United States*, 2. *Xi'an Jiaotong University, Xi'an, Shaanxi, China (People's Republic)*, 3. *Villanova University, Villanova, Pennsylvania, United States*, 4. *Johns Hopkins University, Lehigh Acres, Florida, United States*, 5. *Johns Hopkins School of Nursing, Baltimore, Maryland, United States*

Neurofilament light (NfL) is a biomarker for neuronal health, indicative of neural damage and decline in older adults. Physical activity (PA) is known to promote healthy aging and prevent frailty. However, the interplay between PA, physical frailty, and NfL remains underexplored. This study examines the associations between PA and NfL and the mediating role of frailty using baseline data of 95 community-dwelling nondementia older adults in a randomized controlled trial. PA was measured using ActiGraph Link and Physical Activity Scale for the Elderly (PASE). Frailty was assessed using the Fried frailty phenotype, and NfL levels were determined from plasma samples. Multiple linear regressions, adjusting for sociodemographic and health factors, tested the association, and Baron and Kenny's approach was used to examine frailty's mediation effect. Participants were aged 70.0 ± 6.0 years, 80% female, 28.6% Black, had 2.8 ± 1.9 chronic conditions, and 28% had mild cognitive impairment (MCI). In the adjusted models, a higher level of Actigraph-measured PA was associated with lower NfL (β= -7.4, 95% confidence interval (CI) = [-13.4 to -1.3]). The frail group showed increased NfL compared to the robust (β = 26.3, 95% CI = [11.8 to 40.8]) and pre-frail (β = 25.5, 95% CI = [39.9 to 11.1]) groups. Further, frailty status significantly mediated the PA-NfL relationship. In summary, increased PA level was associated with reduced NfL, and frailty status mediated the associations in our sample of older adults with normal cognition or MCI. PA interventions may reduce frailty-related neuronal damage; further study is needed.

Abstract citation ID: igae098.1461

### RESISTANCE TRAINING AND COGNITIVE FUNCTION IN VASCULAR COGNITIVE IMPAIRMENT: A 12-MONTH RANDOMIZED TRIAL

Teresa Liu-Ambrose[1], Ryan Falck[2], Elizabeth Dao[2], Roger Tam[2], Ging-Yuek Hsiung[2], Cindy Barha[3], and Walid Alkeridy[4], *1. University of British Columbia, University of British Columbia, British Columbia, Canada*, 2. *University of British Columbia, Vancouver, British Columbia, Canada*, 3. *University of Calgary, Calgary, Alberta, Canada*, 4. *King Saud University, Riyadh, Ar Riyad, Saudi Arabia*

**Background:** Subcortical ischaemic vascular cognitive impairment (SIVCI) is considered the most treatable form of cognitive impairment, due to its modifiable risk factors such as hypertension and diabetes mellitus. Despite the benefits of resistance training (RT) on cognitive function and cardiometabolic health, no study has examined the effect of RT on cognitive outcomes in SIVCI.

**Methods:** A 12-month single-blinded, randomized controlled trial with 91 older adults with SIVCI who were randomized to: 1) 2x/week progressive RT; or 2) 2x/week balance and tone exercises (BAT; control). Study eligibility included: 1) age 55 years and older; 2) neuroimaging evidence of cerebral small vessel disease; 3) mild cognitive impairment; and 4) the absence of dementia. Measurements occurred at baseline, 6, and 12 months. The primary outcome was the Alzheimer's Disease Assessment Scale-Cognitive-Plus (ADAS-Cog 13 with additional cognitive tests). Sex differences in RT efficacy was explored.

**Results:** 45 were allocated to the RT group and 46 to the BAT group. COVD-19 impacted the training of 16 participants (9 RT, 7 BAT). After adjusting for covariates and baseline value, participants in the RT group had significantly better ADAS-Cog Plus performance at 12 months (estimated mean difference [RT – BAT]:-0.18; 95% CI:[-0.35, -0.01]; p=0.047). In the sex-stratified analysis, there was a significant effect of the RT on ADAS-Cog Plus performance (estimated mean difference: -0.25; 95% CI:[-0.47, -0.03]; p= 0.028) for females, but not for males.

**Conclusion:** Our results suggest progressive RT should be considered in the management and treatment of individuals with SIVCI, particularly for females.

### SESSION 3340 (SYMPOSIUM)

Abstract citation ID: igae098.1462

### COMPARATIVE EFFECTIVENESS RESEARCH FOR PROMOTING OLDER ADULT HEALTH: A SNAPSHOT OF PCORI-FUNDED PROJECTS

Chair: Regina Shih
Co-Chair: Tabassum Majid

The number of Americans over the age of 65 is projected to reach 95 million by 2060, making up approximately a quarter of the population. Health care, social, and community services can benefit from evidence-based strategies to better address the increasingly complex needs of this large and heterogeneous population. Comparative effectiveness research (CER) within these settings can promote older adult health when its patient-centered principles are embedded across the continuum of research from study design, conduct, evaluation, translation, implementation and dissemination with engagement throughout the process. PCORI's approach to CER has resulted in a framework that addresses older adult health across the aging continuum. This session will describe three aging projects funded by PCORI: 1) healthy aging and villages, 2) dental care in low-income housing, and 3) comprehensive dementia care for patients and caregivers. PCORI program officers will kick off the symposium by describing its aging framework and funding priorities for real-world CER. The symposium will describe meaningful outcomes for older adults, their caregivers, evidence-based clinical practice, and the health, social, and community-based care systems that support older adults. Each presentation will feature the diverse perspectives of community, social service, or clinical partners on the projects and conclude with successes and challenges in conducting CER with older adults. This session will facilitate active discussion of approaches used across the continuum of aging and the research process with implications for future priorities in healthy aging interventions, caregiver supports, and integration of interventions into multiple care systems. Patient/Person Engagement in Research Interest Group Sponsored Symposium

Abstract citation ID: igae098.1463

### OPTIMIZING PHYSICAL AND MENTAL FUNCTION ACROSS THE AGING CONTINUUM: PCORI'S FUNDING PORTFOLIO AND RESEARCH PRIORITIES

Yewande Akinbami[1], Tabassum Majid[1], John Audley[2], and Neeraj Arora[3], *1. Patient-Centered Outcomes Research Institute (PCORI), Washington, District of Columbia, United States*, *2. Patient Centered Outcomes Research Institute (PCORI), Washington, District of Columbia, United States*, *3. Patient Centered Outcomes Research Institute, Washington, District of Columbia, United States*

In 2021, the National Academy of Medicine identified "Actualizing better health and healthcare for older adults" as one of five vital directions in healthcare. In response, PCORI launched a $50 million, targeted PCORI funding announcement (PFA) on Healthy Aging in 2021 that called for CER to optimize the physical and mental functioning of older adults. This funding announcement coincided with PCORI's Board identifying 12 focused topic themes, including "Promoting the Health of Older Adults". This project synthesizes PCORI's portfolio of funded work aimed at promoting the health of older adults and presents the Healthy Aging continuum, a conceptual framework informed by stakeholders to guide future PCORI research investments. The conceptual framework encompasses four domains: (1) healthy older adults with 1-2 well-managed chronic conditions (2), individuals with sub-optimally managed multiple chronic conditions (3), individuals with significant functional impairment and (4), seriously ill older adults who may need end-of-life care. Caregivers who support older adults as they age throughout the continuum undercut all four of these domains. To date, PCORI has funded 63 CER projects, totaling $323 million, focused on promoting the health of older adults. PCORI's targeted initiatives have called for CER on palliative care, hearing loss, visual impairment, osteoporotic fractures, delirium, and hypertension. This presentation will provide an overview of PCORI's portfolio of older adult studies and implications for future research priorities. During the symposium discussion, PCORI will solicit further insights on evidence gaps and implications for future research focused on real-world decisional dilemmas in older adult health.

Abstract citation ID: igae098.1464

### ENGAGING VILLAGES AS KEY PARTNERS IN PATIENT-CENTERED HEALTHY AGING RESEARCH

Emily Greenfield[1], Regina Shih[2], William Kincaid[3], Alina Palimaru[4], Natalie Pope[1], and Barbara Sullivan[5], *1. Rutgers, The State University of New Jersey, New Brunswick, New Jersey, United States*, *2. Emory University Rollins School of Public Health, Atlanta, Georgia, United States*, *3. Village to Village Network, Washington DC, District of Columbia, United States*, *4. RAND Corporation, Santa Monica, California, United States*, *5. Village to Village Network, St. Louis, Missouri, United States*

Villages are a grassroots-led community-based movement to facilitate aging in place through social connection, sense of belonging, and services from neighbors and other local resources. With over 270 villages across the nation, villages present a unique opportunity to deliver patient-centered interventions for health aging. This presentation will provide an overview of a Patient-Centered Outcomes Research Institute-funded engagement project to promote the meaningful involvement of Villages in all phases of patient-centered comparative clinical effectiveness research (CER). This project serves as an early step toward the collaborative design of future research on how Villages promote healthy aging. First, we will describe the strategic value of establishing digital communications for the project as an early project goal. Second, we will discuss how the themes that arose during five focus groups (n=49) on villagers' perspectives onhealthy aging, intersections with the health care system, and their involvement in research informed subsequent project activities. Third, we will provide an overview of the formation of the Villages Healthy Aging Ambassadors group for peer-to-peer engagement and collaborative research design. Fourth, we will summarize the planning and implementation of regionally-based virtual summits with

Villages across the United States to gain further insights for future research projects and to strengthen the position of research as part of the national Village movement. We will conclude by reflecting on the value of collaboratively designing and implementing this project for optimizing the success of longer-term patient-centered CER with Villages and amplifying the efforts of Villages to advance healthy aging.

Abstract citation ID: igae098.1465

**THE VALUE OF ENGAGEMENT: IMPROVING ORAL HEALTH THROUGH COMMUNITY-BASED INTERVENTIONS**

Suchitra Nelson[1], Shelley Curtan[1], and Farida Ejaz[2], *1. Case Western Reserve University, Cleveland, Ohio, United States, 2. Benjamin Rose Institute on Aging, Cleveland, Ohio, United States*

Many older adults accept oral health problems as an inevitable consequence of aging and do not seek dental care. Further, with limited dental insurance options alternative care models are needed. Our project investigated the most effective and efficient means of addressing oral health disparities and access to dental care among community-dwelling older adults. This clinical trial tested the comparative effectiveness of two non-surgical evidence based cavity treatments (Silver Diamine Fluoride vs. Atraumatic Restorative Treatment) in arresting tooth decay for adults 62+. Treatments required no needles or drills and were done using portable equipment in the independent housing sites where the adults lived. The project enrolled and screened 952 older adults, treated 568 with active decay, living in 34 housing sites (17 sites randomized to each treatment intervention). Follow-up visits at 26 and 52 weeks assessed caries arrest and participant report of oral pain and hypersensitivity. Focus groups were conducted to capture qualitative data related to participant's treatment satisfaction. A stakeholder group comprised of individuals representing payers, policy makers, advocacy organizations, housing administrators, and patients have been active throughout each study phase, offering guidance and assistance with the project. We will outline experiences of participants and perspectives of stakeholders that enabled positive oral health outcomes for older adult participants. Challenges and successes will be shared, along with next steps for dissemination and ideas for health system change to support continuation of treatment based on project clinical outcomes.

Abstract citation ID: igae098.1466

**COMPARING HEALTH SYSTEM–BASED AND COMMUNITY-BASED DEMENTIA CARE: THE DEMENTIA CARE (D-CARE) STUDY**

David Reuben[1], Jenny Summapund[1], Alan Stevens[2], Thomas Gill[3], Peter Peduzzi[3], Rebecca Galloway[4], and Martha Carnie[5], *1. University of California Los Angeles David Geffen School of Medicine, Los Angeles, California, United States, 2. Baylor Scott & White Health, Belton, Texas, United States, 3. Yale University, New Haven, Connecticut, United States, 4. The University of Texas Medical Branch, Galveston, Texas, United States, 5. Brigham and Women's Hospital, Boston, Massachusetts, United States*

Despite the advent of new medications for treatment of dementia, the clinical care of those affected remains a challenge. Though several community-based and health system-based approaches have been developed to provide comprehensive care for patients living with dementia and their caregivers, the relative effectiveness of these approaches is unknown. The Dementia Care (D-CARE) Study is a pragmatic randomized trial comparing the effectiveness of three dementia care approaches at four clinical trial sites (CTS) over 18 months: 1) by nurse practitioners or physician assistants within a health care system, 2) by social workers or nurses at community-based organizations, or 3) usual care. D-CARE illustrates advantages (e.g., facilitation of recruitment with over 93% receiving active intervention) and disadvantages (e.g., CTS health systems needed to implement two interventions; because both active interventions have prior demonstrated effectiveness, the differences are likely to be smaller, requiring a larger sample size to have sufficient power) of comparative effectiveness research. The pragmatic design presented obstacles including funding for clinical positions, integrating interventions into workflows, and potential threats to fidelity; but the real-world settings will facilitate dissemination. COVID-19 introduced additional barriers to recruitment and retention. Strong commitment by study leaders, adaptability of research staff, and continuous engagement of patient and stakeholders at the CTS contributed to D-CARE's success in enrolling 2,176 participants and high retention rates. D-CARE applied principles of community-based participatory research to continually refine materials to the study's needs. D-CARE's adaptations and successes demonstrate viable strategies for sustainability and scalability in real-world practices.

### SESSION 3345 (SYMPOSIUM)

Abstract citation ID: igae098.1467

**DECOLONIZING AGING RESEARCH AND PRACTICE**

Chair: Lena Thompson
Co-Chair: Steffi Kim
Discussant: Jordan Lewis

The focus of this symposium, co-sponsored by the Indigenous Peoples and Aging Research Interest Group and Rural Aging Interest Group, is to highlight projects that apply Indigenous and/or non-Western perspectives to co-create research or programs for Indigenous people. While researchers and practitioners continue to work on closing the research implementation gap, a lack of involvement of Indigenous people in research and a lack of Indigenous-focused research leads to longer delays between culturally safe and effective research findings and program implementation. Furthermore, Indigenous communities have faced instances where institutions have been the primary beneficiaries of research conducted on Indigenous communities. Engaging Indigenous people and perspectives in the research process enhances the relevance and sustainability of research and allows a more equitable balance of benefits from research for all parties involved. The goal of this symposium is to present research studies that showcases methodologies and protocol outside of the Western academic perspective that focus on community-engaged, strengths-based approaches. Papers presented at the symposium will share methods used to enhance connectedness of Indigenous people to research and programs through the use of community-engaged approaches, storytelling, and qualitative data collection.

Abstract citation ID: igae098.1468

### ADAPTING A DISASTER PREPAREDNESS PROGRAM USING AN INDIGENOUS IMPLEMENTATION FRAMEWORK

Lena Thompson[1], Jordan Lewis[2], and Sato Ashida[3], *1. University of Minnesota Duluth, Duluth, Minnesota, United States*, *2. University of Alaska Fairbanks, College of Indigenous Studies, Fairbanks, Alaska, United States*, *3. University of Iowa, Iowa City, Iowa, United States*

Implementation frameworks can help increase the relevance and sustainability of intervention research with Indigenous populations. The He Pikinga Waiora (HPW) framework centers around Māori self-determination and has four elements: community engagement, cultural centeredness, integrated knowledge translation, and systems thinking. This study applied the HPW framework to inform adaptation of a disaster preparedness program for older adults with a Midwestern tribe in the United States. Tribal organization leaders, including a member from the Tribal Government, Senior Center, Emergency Preparedness Department, Police Department, and one older adult from the community, read through the program with the researcher while considering the four elements of HPW throughout the process. Five experts in Indigenous aging research and practice listened to the recordings of these sessions and evaluated the researcher's fidelity to the framework and the extent to which the HPW framework fit the community context and project. Additionally, research assistants conducted qualitative interviews with Tribal organizational leaders about the fit and relevancy of the framework. Data from expert evaluators and interviews with tribal organizational leaders and the community member suggest that the HPW framework was a good fit for the community and program. Both groups made recommendations to improve the framework, including integrating sustainability into the model and making language simpler. The HPW framework appears to facilitate healthy partnerships and provide guidance to adapt, implement, and eventually disseminate a tailored intervention to a Midwestern Tribal community. This study demonstrated beginning processes of conducting cultural adaptations using an implementation model created and tested with Indigenous populations.

Abstract citation ID: igae098.1469

### A TRADITIONAL HEALING PROGRAM ON THE NAVAJO NATION DURING COVID

Karen Kopera-Frye, Karen John, and Robynn Frank, *New Mexico State University, Las Cruces, New Mexico, United States*

Tribal Critical Race Theory (TribalCrit) posits three types of knowledge: 1) cultural knowledge or tribal ways including traditions and Indigenous Ways of Knowing (IWOK); 2) knowledge of survival involving how and why change and adaptation can be beneficial to the community; and 3) academic knowledge often referred to as "book smarts." IWOK and academic knowledge are critical for survival and improving the lives in Native communities. This project can best be thought of as a variation of Community-based participatory Research, or Community-based participatory advocacy. Two Navajo students and a faculty member created a project which would use traditional healing community values to understand how COVID had disrupted cultural ways, including IWOK. A Navajo program, beginning in 2007 called "Restoring and Celebrating Family Wellness (RCFW)" involved an organized tribal summit to hear from community members about their concerns, issues, and priorities via workshop venues called "Open Space". Beginning in 2008, monthly workshops were held including elder presentations, storytelling, and health concerns addressing community needs of violence prevention and raising balanced children. Due to COVID, the workshops ceased in 2020 when the reservation was locked down. Subsequently, with the vaccines available, the project involved reviving RCFW. The Navajo community had experienced social isolation due to an inability to gather and share daily life experiences. Deaths of elders resulted in the loss of wisdom keeping elders and devastated the community. This revived program was successful in improving well-being and increasing connectedness among the Native community, thus restoring IWOK and demonstrating community resilience.

Abstract citation ID: igae098.1470

### IMPACTS OF INTRATRIBAL DIVERSITY ON SERVICES FOR URBAN AMERICAN INDIAN ELDERS

Lyn Holley[1], and Donna Polk[2], *1. University of Nebraska at Omaha, Omaha, Nebraska, United States*, *2. Nebraska Urban Indian Health Coalition, Omaha, Nebraska, United States*

This paper presents a report of analysis of interviews with members of the leadership team that serves Native American Indian elders in a mid-size American city. The city studied is home to members of more than a dozen different tribes; it has only one ethno-specific services center for older persons. While these older persons share a heritage of genocidal colonization, they are from different tribes and cultures. Native Americans residing in rural circumstances (e.g., reservations) are more likely to share and treasure similar beliefs and practices than are urban Indians. Diversity of beliefs and practices almost ineluctably brings occasional conflict - e.g., different beliefs about the place of women at the Drum or in singing, decorating for holidays, education of children, and suitability of different foods or ways of cooking. While literature on services for Native Americans is slowly growing, little notice has been taken of the challenges of serving this population in an urban setting. This paper begins to fill the gaps in the literature by describing the challenges and identifying methods that are useful for observation, description and analysis of cultural beliefs and practices of persons in the group, and for identifying which of these beliefs and practices support development of trust and cooperation, and which inhibit relationship building among Native American Indian Elders thus gaining and, importantly, applying knowledge of relevant cultures to inform design of activities and services for these Native Elders.

Abstract citation ID: igae098.1471

### DECOLONIZING DISSEMINATION OF RESEARCH ENSURES LOCAL RELEVANCE AND SUSTAINABILITY OF RESEARCH BEYOND THE GRANT

Jordan Lewis[1], and Steffi Kim[2], *1. University of Alaska Fairbanks, College of Indigenous Studies, Fairbanks, Alaska, United States*, *2. University of Alaska, Anchorage, Alaska, United States*

Engaging in community-based participatory research with Indigenous communities has become an accepted approach in both qualitative and quantitative research methods, as well as by funders. Our team has been conducting community-engaged research with Alaska Native communities for over 16-years. We have conducted qualitative, in-depth, interviews with 162 Elders representing 21 participating communities to explore the concept of successful aging and the role of their community in the aging process. Alaska Native successful aging consists of six elements of successful aging, or "Eldership:" emotional wellbeing, spirituality, community engagement, Native ways of life, generativity, and physical health. These elements serve as the foundation of how communities define who is an Elder and what is important when considering who has aged successfully or not. This presentation will briefly discuss challenges of community engagement, but focus on the successes of disseminating the findings back to the community using locally driven recommendations. Western forms of study dissemination products tend to be publications and presentations, which support the researchers' goals and objectives, but do not always translate to community interests and ideas. Our team has been successful in developing culturally safe and innovative dissemination products, including berry buckets, coffee mugs, and sweatshirts. We will discuss how to navigate funding guidelines, how to develop to products in collaboration with community members and Elders, as well as the share the impacts these products have on making the data accessible to the Elders and the community, and not just the academic community and funder.

### SESSION 3350 (SYMPOSIUM)

Abstract citation ID: igae098.1472

### EAST MEETS WEST: PERSPECTIVES ON CAREGIVING, FRAILTY, AND BASIC RESEARCH ON AGING IN CHINA AND THE UNITED STATES

Chair: James Nelson
Co-Chair: Jianye Wang

This interdisciplinary symposium brings together scholars from China and the United States to share recent discoveries from the health and biological sciences, as well as public policy initiatives designed to increase accessibility to established means of promoting health and well being of older individuals. Included will be discussions of novel therapeutics that may alleviate the pathophysiological burdens of senescence. An overarching premise of this cross-cultural symposium is that the unique cultural and experiential perspectives of scientists and practitioners from these two countries can enrich the approaches to research, education, and practice of geroscience and gerontology in both countries. Another goal is to build new bridges of communication between the two countries to promote collaboration, thereby synergizing research and practice everywhere. To this end, presentations on frailty and novel approaches to increase access to known behavioral interventions bring together different methodological perspectives, as will presentations on basic biology of vascular aging and pre-clinical pharmacologic interventions informing clinical trials aimed at restoring physiological functions in older individuals.

Abstract citation ID: igae098.1473

### BASIC AND CLINICAL ASPECTS OF VASCULAR AGING

Cuntai Zhang, *Tongji Hospital, Tongji Medical College, Huazhong University of Science and Technology, Wuhan, Hubei, China (People's Republic)*

Vascular aging (VA) is an important pathophysiological contributor to the aging process. In response to China's aging population, a hierarchical "Standardized Vascular Aging Management Center (VMC)" network, with Tongji Hospital in Wuhan as the central hub, was established in 2023 to offer comprehensive vascular aging (VA) management nationwide. This initiative encompasses assessment, stratification, and intervention strategies for VA, a key factor in the aging of human organs and systems and a common denominator in the chronic diseases of aging. Our research delves into the cellular and molecular dynamics of VA, examining the effectiveness of pharmaceutical interventions against vascular stiffness and investigating cellular aging mechanisms such as metabolic changes, ferroptosis, mitochondrial dysfunction, and senescence-associated secretory phenotype (SASP), alongside key factors like SOX9, Nrf2, and FGF21. Furthermore, we are launching a national cohort study and conducting randomized trials on novel drugs to bridge clinical and basic research findings. This integrated approach aims to detect early VA signs, enabling timely interventions to mitigate chronic elderly diseases, foster healthy aging, and effectively tackle the implications of an aging demographic. By developing an integrated VA research and application platform that facilitates seamless translation between basic and clinical settings, we aim to identify early signs of VA and implement appropriate interventions to delay and manage this process. Ultimately, our efforts aim to reduce chronic disease in the elderly, promote healthy aging and more effectively address the challenges of an aging society.

Abstract citation ID: igae098.1474

### SENOTHERAPEUTICS, AGING, AND AGE-RELATED DISEASES

Dean Kellogg, *The University of Texas Health Science Center San Antonio, San Antonio, Texas, United States*

Increased translational clinical trials are addressing the utility of senotherapeutic agents as interventions for aging in general and for age-related diseases. Numerous pre-clinical trials have provided a good rationale for human clinical trials which are being increasingly proposed. At present a number of senolytic and senomorphic drugs appear to be safe and tolerable by older persons, hence new human clinical trials directed at efficacy in ameliorating age-related diseases and aging in general are now coming to the fore. This talk will review:1) what are these agents? 2) what are their mechanism(s) of action? and 3) what efficacy have they demonstrated?

Abstract citation ID: igae098.1475

### IMPLEMENTATION AND IMPACT OF THE WHO INTEGRATED CARE FOR OLDER PEOPLE PROGRAM IN CHINA: A RANDOMIZED CONTROLLED TRIAL

Sophia Kang, *Peking Union Medical College Hospital, Beijing, Beijing, China (People's Republic)*

This study evaluates the feasibility and impact of the World Health Organization's Integrated Care for Older People (ICOPE) approach in urban and suburban communities of Chaoyang District, Beijing, China, amidst concerns about increasing health and social care burdens due to aging populations. Community-dwelling older adults screened as at-risk of functional declines were randomized into intervention (537) and control (1611) groups between September 2020 and February 2021. This study tested the implementation of a 6-month ICOPE intervention program against standard care. Propensity score matching was utilized to ensure comparability between intervention and control groups, totaling 938 participants. The results highlighted the feasibility of ICOPE, with high satisfaction rates among participants (97–99%) and providers (92–93%). All outcomes showed improvements after a 6-month intervention, with statistically significant least-squares mean differences (control-intervention) in vitality (Mini-Nutritional Assessment Short Form to measure vitality, −0.21, 95% CI, −0.40–0.02), mobility (Short Physical Performance Battery to measure mobility, −0.29, 95% CI, −0.44–0.14) and psychological health (Geriatric Depression Scale five items to measure psychological health, 0.09, 95% CI, 0.03–0.14) were observed (P < 0.05). These findings suggest that localizing WHO's ICOPE approach is feasible and effective in regions with fragmented healthcare resources in China, demonstrating positive impacts on health outcomes and highlighting its potential for advancing policies and practices toward healthy aging.

Abstract citation ID: igae098.1476

**TRANSLATABLE EXERCISE INTERVENTIONS TO REACH OLDER ADULTS WITH PHYSICAL AND COGNITIVE FRAILTY**

Kieran Reid, *Harvard Medical School, Boston, Massachusetts, United States*

Mobility limitations, falls and dementia are leading causes of disability, institutionalization, and mortality among U.S older adults. Considering that the population of adults aged ≥ 65 years is projected to surpass 80 million by 2040, more effective and scalable intervention strategies to maintain mobility, reduce falls or offset cognitive decline are necessary to help older Americans to continue to live independently. This presentation will discuss the rationale, design and implementation of several innovative exercise intervention strategies for older adults with mobility limitations, elevated fall risk, and for older adults at high risk for developing dementia. The session will specifically describe how large-scale clinical trial evidence for the benefits of exercise can be translated to reach more vulnerable older adults in real-world community-based settings. The session will also describe how a digitally delivered multicomponent fall prevention exercise program can be tailored to reach at-risk older adults in their own homes. Overall, it is posited that these innovative interventions hold significant potential as highly scalable approaches for maintaining mobility, preventing falls, and mitigating cognitive decline among rapidly expanding populations of older adults.

Abstract citation ID: igae098.1477

**FRAILTY AND INTRINSIC CAPACITY IN OLDER ADULTS IN CHINA: FROM RESEARCH TO PRACTICE**

Lina Ma, *Xuanwu Hospital, Capital Medical University, Beijing, Beijing, China (People's Republic)*

Older adults with frailty have a reduced ability to cope with stresses, such as that associated with acute diseases, and these adults also have an increased risk of falls, disability, and death. The prevention and treatment of frailty in this group of people are very important. In China, as the general population has aged, the demand for medical care services for older adults has increased. China has given high priority to healthy aging and established a national strategy "Healthy China 2030" blueprint and is progressing rapidly in geriatric medicine. Although frailty research in China started late, it has developed very rapidly, especially on assessment tools, epidemiology, and management. Furthermore, several guidelines have been established to help physicians make clinical decisions while treating frail older persons. In recent years, clinicians and researchers have shown increasing interest in intrinsic capacity, a new positive approach to health aging. However, much effort should be made to integrate frailty into clinical practice. This talk focuses on the research advances on frailty from biological mechanisms to therapeutic opportunities as well as individual-centered integrated care for optimizing intrinsic capacity to prevent frailty and disability in older adults in clinical practice in China.

### SESSION 3355 (SYMPOSIUM)

Abstract citation ID: igae098.1478

**EMOTION AND AGING: CUTTING-EDGE DIRECTIONS**

Chair: Joseph Mikels
Co-Chair: Laura Carstensen

Over the past few decades, the field has garnered a deep and broad understanding of age differences in emotional experience; healthy older adults generally experience a balance of more positive to negative emotions relative to their younger counterparts. But many questions remain, and novel understandings of this general pattern are emerging. This symposium will highlight the latest cutting-edge directions in the examination of emotional functioning in later life. Mikels will present data demonstrating that older adults experience more "love" (i.e., positivity resonance) relative to younger adults. Haase and colleagues will explain how emotional acceptance is an important way by which older adults achieve greater well-being relative to other emotion regulation strategies. English and Springstein will further consider how the flexible use of different emotion regulation strategies contribute to higher well-being across the adult lifespan. In yet another approach to understanding high levels of well-being in older adults, Stephens will show that savoring is a critical component of increased positive affect in later life. Underscoring the social underpinnings of well-being, Brown and colleagues will explain the critical role of understanding other people's emotions accurately in building and maintaining social connections, especially for older women. This series of presentations will illuminate the myriad current directions that are under investigation in the study of emotion and aging, providing attendees with a rich understanding of the latest findings.

Abstract citation ID: igae098.1479

### AGING AND POSITIVE EMOTIONS: WHAT'S LOVE GOT TO DO WITH IT?

Joseph Mikels, *DePaul University, Chicago, Illinois, United States*

Aging is associated with improvements in well-being; healthy older adults report a balance of fewer negative emotional experiences relative to positive emotional experiences. Additionally, meaningful social connections become prioritized as we grow older. Taken together, patterns in social and emotional aging would suggest that older adults might experience more love than their younger counterparts. But what is love? Recent theoretical and empirical advances conceptualize love as positivity resonance – a caring interpersonal connection involving shared positivity and synchrony. In three studies, the experience of positivity resonance in older adults was examined. In the first pilot study, 44 older adults who participated in a group exercise program reported experiencing positivity resonance 77.15% of the time during the program, which is higher than previously reported levels positivity resonance in a younger sample (65.93%). In a second survey study with 184 older adults (M age = 69.45) and 223 younger adults (M age = 24.74), older adults reported significantly higher trait-level perceived positivity resonance (M = 70.25) compared to younger adults (M = 53.39), $t(405) = -9.11$, $p < .001$. In a third study in which 100 older (M age = 74.67) and 100 younger participants (M age = 22.40) walked with a partner, older adults reported higher episode-level positivity resonance (M = 88.07) compared to younger adults (M = 83.41), $t(195) = 2.00$, $p = .047$. Across these three unique and methodological distinct studies, it appears that older adults do indeed experience more love than their younger counterparts.

Abstract citation ID: igae098.1480

### A CLOSER LOOK AT EMOTIONAL ACCEPTANCE IN LATE LIFE

Claudia Haase[1], Yu Li[1], Chen-Wei Yu[1], Lillian Fu[1], and David Rompilla[2], *1. Northwestern University, Evanston, Illinois, United States*, *2. Texas A&M University, College Station, Texas, United States*

When older adults regulate emotions, they tend to prefer emotional acceptance (i.e., embracing emotions without judging them) over other strategies, such as reappraisal (i.e., reframing an emotional situation to view it in a more positive light) or detachment (i.e., mental distancing from an emotional situation). While regulation strategies, such as reappraisal, have been widely studied, interest in emotional acceptance is just starting to take off. Here, we present findings from two studies on emotional acceptance with older adults. In a laboratory-based study, we examined habitual emotional regulation (using questionnaires) and instructed emotion regulation (instructing participants to use different emotion regulation strategies when watching loss-themed film clips vs. "just watch" them while monitoring their autonomic physiology) in a sample of 129 older adults (age 64-83). We examined emotional acceptance and (to determine specificity) detachment and reappraisal. Findings showed that habitual emotional acceptance (but not detachment or reappraisal) was associated with (1) greater vagal reactivity during instructed emotion regulation and (2) lower levels of anxiety and depressive symptoms. In an online study, we probed a novel measure of emotional acceptance in a sample of 241 older adults (age 65-88). A confirmatory factor analysis supported a 2-factor solution with acceptance of negative emotions and acceptance of positive emotions as two distinct factors. Together, these findings highlight the physical and mental health benefits of emotional acceptance in late life, underscore differences between different cognitive change strategies, and suggest further probing of accepting not just negative but also positive emotions in future research.

Abstract citation ID: igae098.1481

### A PERSON-SPECIFIC APPROACH TO EMOTION REGULATION FLEXIBILITY ACROSS ADULTHOOD

Tammy English, and Tabea Springstein, *Washington University in St. Louis, St. Louis, Missouri, United States*

Early work on emotion regulation typically focused on how often or how much individuals use "adaptive" strategies (e.g., cognitive reappraisal) or "maladaptive" strategies (e.g., expressive suppression"), largely finding little evidence of age differences in strategy use. However, there is growing attention to the importance of flexibly adjusting regulation efforts based on constraints and affordances of the situation, a skill that may increase with life experience. In the present research, we build on these recent theoretical innovations by considering both the "frequency perspective" and "flexibility perspective" in an adult lifespan sample. To do so, we utilize a novel statistical approach that models emotion regulation strategy use and person-specific strategy-situation fit associations with well-being. In an experience sampling study, 290 adults aged 25 to 85 years were prompted 6x/day for 10 days to report on their emotion regulation strategy use and various aspects of their current situation. Bayesian mixed-effect location scale modeling was used to simultaneously assess the relationships of flexibility and frequency of emotion regulation strategy use. Results suggest that individuals with higher well-being used some strategies flexibly and more frequently, some strategies flexibly but not more or less frequently, and some strategies more frequently but not flexibly. This work highlights the value of considering person-specific approaches to emotion and the need for attention to both flexibility and overall adaptiveness of regulation strategies. Work on adult development in emotion could benefit from incorporating idiographic perspectives that take individual differences seriously, rather than operating from more of a one-size-fits-all view of aging.

Abstract citation ID: igae098.1482

### AMPLIFYING THE POSITIVE: RECIPROCAL ASSOCIATIONS BETWEEN SAVORING AND POSITIVE AFFECT

Jacquelyn Stephens, and Jennifer Smith, *Mather Institute, Evanston, Illinois, United States*

Savoring and positive affect are closely related, yet little work has disentangled the bi-directional relationship between them, as they have primarily been examined cross-sectionally. Further, more empirical work is needed to show that savoring is longitudinally associated with increased positive affect even when accounting for baseline positive affect. We examined year-to-year associations between savoring and positive affect using three annual waves of data from the Age Well study. Participants consisted of 2945 older adults (Mage = 82.08, SD = 6.38; 66% female) recruited from Life Plan Communities across the United States. Positive affect was modeled on savoring ability using random-intercept cross-lagged panel models. Our results revealed significant bi-directional relationships, such that savoring predicted later positive affect (B =.15, p >.001) and positive affect predicted later savoring (B =.34, p >.001). Our results show evidence that changes in savoring sequentially predict greater positive affect, as well as the reverse. In line with views of upward spirals of positive emotion, increasing positive emotion regulation indeed contributes to greater emotional well-being in older adults, independent of initial levels of positive emotion.

Abstract citation ID: igae098.1483

### AGE DIFFERENCES IN WOMEN'S EMPATHIC ACCURACY WITHIN LONG-TERM MARRIAGES

Casey Brown[1], Jenna Wells[2], Claudia Haase[3], and Robert Levenson[4], *1. Georgetown University, Washington, District of Columbia, United States*, *2. Cornell University, Ithaca, New York, United States*, *3. Northwestern University, Evanston, Illinois, United States*, *4. University of California Berkeley, Berkeley, California, United States*

Understanding other people's emotions accurately (i.e., empathic accuracy) is thought to be critical for building and maintaining social connections. Past research suggests empathic skills change with age, but few studies examine age differences in empathic accuracy within the context of close relationships. We examined whether empathic accuracy is higher among middle-aged couples (ages 40-50) compared to older couples (ages 60-70) using a sample of 154 heterosexual long-term marriages. Husbands and wives visited the laboratory and engaged in a 15-minute conversation on a topic of disagreement in their marriage. Conversations were video-recorded. Husbands and wives watched a video playback of their conversation twice, each time continuously rating either their own or their spouse's emotional valence during the conversation using a rating dial that ranged from "very negative" to "very positive". These continuous valence ratings were used to compute each spouse's empathic accuracy as the strength of the correlation between their ratings of their spouse's emotions and their spouse's own self-ratings. Results revealed that age was not associated with husbands' empathic accuracy. In contrast, older wives had greater empathic accuracy compared to middle-aged wives (Mdiff =.19, p =.028). However, older adults had been married longer, and length of marriage also predicted women's empathic accuracy. Findings suggest that older women are better able to track the changing valence of their husbands' emotions, perhaps because they have more practice.

### SESSION 3360 (PAPER)

### FAMILY AND INTERGENERATIONAL RELATIONS: FOCUS ON GRANDPARENTS

Abstract citation ID: igae098.1484

### INDIVIDUALISTIC VALUES AND SUPPORT PROVIDED TO GRANDCHILDREN

Tianqi Zhou[1], Martin Lakomý[2], and Merril Silverstein[1], *1. Syracuse University, Syracuse, New York, United States*, *2. Mendel University, Brno, Jihomoravsky kraj, Czech Republic*

The significance of individual values in family dynamics is widely acknowledged, but the role of individualism in shaping grandparental support remains insufficiently understood. In this study, we investigate the relationship between individualistic/collectivistic value orientation and two types of grandparental support (instrumental and emotional) provided by older individuals. We use data from waves 4-10 of the multigenerational Longitudinal Study of Generations to examine this link between values and support. Multilevel random-intercept models use all available respondents with the youngest grandchild aged 0-16, which makes a sample of 1309 older people nested in 307 families. Controlling for grandparents' sex, ethnicity, marital status, health, and retirement, we found that individualistic value orientation influences instrumental support, while its impact on emotional support was not evident. Additionally, instrumental grandparental support tends to manifest more prominently within families featuring younger grandparents and younger grandchildren. In contrast, emotional support is more prevalent in families with highly educated grandparents, as well as those with more and older grandchildren. Our study complements previous studies on the relationship between values and intergenerational support by showing that individualism exerts an influence on specified forms of support such as transportation or shopping, help when sick, and childcare. These findings demonstrate the enduring presence of caregiving provided to grandchildren motivated by grandparental norms or religiosity within American families and – at the same time – reveal that the value changes can reshape the content and forms of grandparental care.

Abstract citation ID: igae098.1485

### SOCIALLY WITHDRAWN OR SOCIALLY ENGAGED? THE IMPACT OF GRANDPARENTING ON SOCIAL PARTICIPATION AMONG OLDER ADULTS

Jason Wong[1], Mengke Zhao[2], Yuying Tong[2], and Feinian Chen[3], *1. Yale University, New Haven, Connecticut, United States*, *2. The Chinese University of Hong Kong, Hong Kong, China (People's Republic)*, *3. Johns Hopkins University, Baltimore, Maryland, United States*

**Objectives:** Grandparents caring for grandchildren (i.e., grandparenting) is an important aspect of intergenerational family dynamics in the 21st Century. Despite extensive literature documenting its impact on grandparents' health, the mechanisms remain insufficiently addressed, particularly those related to social support and engagement. Grandparenting may promote role enhancement and create opportunities for social participation, but it may also result in role strain and reduce time availability for social activities, depending on caregiving intensity and living arrangements. This study addresses this gap by examining the effect of grandparenting on grandparents' social participation and its gendered implications.

**Methods:** Data are from 2011, 2013, and 2018 waves of the China Health and Retirement Longitudinal Study (11,547

older adults aged 50-84). Individual-level fixed-effects linear probability models are employed. Grandparenting is assessed through caregiving intensity and living arrangements. We measure both formal social participation (FSP, e.g., club activities or volunteering) and informal social participation (ISP, e.g., meeting or helping friends).

**Results:** All types of grandparenting, except those under the three-generation living arrangement, increase grandparents' ISP. Gender differences are observed. Compared to no grandparenting, providing noncoresident nonintensive care and skipped-generation care increases ISP only for grandfathers, while providing noncoresident intensive care and three-generation care increases ISP only for grandmothers. We find no association between any type of grandparenting and FSP.

**Discussion:** Overall, grandparenting facilitates rather than deters social connectedness for grandparents. This study underscores the importance of intergenerational linked lives and family networks in maintaining social connectedness among grandparents. The gendered division of grandparenting responsibilities is also discussed.

Abstract citation ID: igae098.1486

### EXAMINING CUSTODIAL GRANDMOTHER'S PARENTING PRACTICES BY MEANS OF A THREE-STEP LATENT PROFILE ANALYSIS

Minzhi Ye[1], and Gregory Smith[2], *1. University of Texas Rio Grande Valley, Edinburg, Texas, United States*, *2. Kent State University, Kent, Ohio, United States*

Although parenting is the central responsibility of custodial grandparents, research on the parenting practices of these family caregivers remains scarce. With various measures of parental nurturance and discipline as objective indicators, we used Three-Step Latent Profile Analysis (Mplus 8.1) to (1) identify distinct custodial grandmother (CGM) parenting profiles among a nationwide sample of 733 individuals and (2) examine how these profiles were related to grandchildren's (GC) psychological difficulties, CGM mental health, CGM coping behaviors, and key contextual factors. Four parenting profiles emerged: Permissive (35%; n = 259), Authoritative (7%; n= 53), Optimal (49%; n = 357), and Inconsistent (9%, n=64). These profiles differed considerably in terms of how each was related to CGM depressive and anxiety symptoms as well as to GC internalizing and externalizing difficulties. For example, in Permissive CGM, both their anxiety and depression symptoms were positively related to GC internalizing and externalizing difficulties. On the other hand, among Authoritative CGM, their depressive symptoms were negatively related to GC internalizing, while their anxiety symptoms were positively related to CG internalizing. In addition, multinomial contrasts with Optimal CGM as the referent showed that "Permissive" CGM reported less problem-focused coping, more family conflict, and younger CGM with older GC. "Authoritative" CGM reported higher depressive symptoms, less support, and increased family conflict. Finally, Inconsistent CGM reported less problem-focused coping, heightened family conflict, and older GC. These findings shed light on the relationships between CGM parenting profiles, well-being, coping behaviors, and GC outcomes which informs future interventions. [Funded by NIH R01- MH-66851-02]

Abstract citation ID: igae098.1487

### IMPACT OF COMMUNITY TYPE ON SLEEP HEALTH DISPARITIES IN GRANDPARENTS RAISING GRANDCHILDREN

Melanie Stearns[1], Kevin McGovney[2], Ashley Curtis[1], Danielle Nadorff[3], and Christina McCrae[1], *1. University of South Florida, Tampa, Florida, United States*, *2. University of Missouri, Columbia, Missouri, United States*, *3. Mississippi State University, Mississippi State, Mississippi, United States*

Grandparents raising grandchildren (GRG) report greater sleep problems than non-caregiving peers. Individuals living in low-income urban areas also report difficulty sleeping due to increased noise, light, and unsafe neighborhoods. However, no studies have examined urbanity's impact on sleep among GRG and this study fills that gap. Grandparents living in rural (N=50, Mage=56.80), urban (N=59, Mage=53.07), and suburban areas (N=74, Mage=56.03) caring for their grandchild (>17 years old) completed the Pittsburg Sleep Quality Index (PSQI-sleep latency, sleep duration, sleep efficiency, sleep medication, sleep disturbances, and global sleep scales) and indicated their community type (rural, urban, suburban). Two-way ANOVAs evaluated interactions (sleep x community) and main effects. Pairwise comparisons clarified significant differences. Interactions were not significant. There was a significant main effect (p=.047) of community on sleep medication use, sleep disturbances (p=.009), and global sleep problems (p=.012). Urban grandparents reported more sleep medication (M=1.58+\-1.20) than rural (M=1.06+\-1.27; p=.046) or suburban grandparents (M=1.07+\-1.26; p=.026). Urban grandparents reported more sleep disturbance (M=1.63+\-.64) than rural (M=1.25+\-.55; p<.001) or suburban grandparents (M=1.44+\-.53; p=.080). Urban grandparents reported more global sleep problems (M=10.61+\-3.17) than rural (M=8.28+\-3.43; p=.010) or suburban grandparents (M=8.61+\-2.87; p=.010). Results suggest that level of urbanity impacts sleep health disparities among GRG and urban GRG may be at greater risk for sleep problems. More research (e.g., longitudinal) should examine what contributes to increased sleep problems among urban GRG, but these results suggest that behavioral sleep treatment programs should potentially target environmental and socioeconomic factors unique to urban GRG (e.g., increased noise, decreased safety and security, etc.).

Abstract citation ID: igae098.1488

### THE ASYMMETRIC EFFECTS OF THE INCREASE AND DECEASE IN INTERGENERATIONAL TRANSFERS ON DEPRESSIVE SYMPTOMS

Sujeong Park, and Jinho Kim, *Korea University, Seoul, Republic of Korea*

Despite the existing body of research on the dynamics of intergenerational transfers, there remains a gap in understanding whether decreases and increases in such transfers have differential effects on depressive symptoms among older Korean adults. This study aims to address this gap by examining two key

**objectives:** (a) exploring the asymmetric effect of intergenerational transfers from children to parents on parental depressive symptoms and (b) investigating whether this asymmetric effect differs by gender. Using seven waves of the Korean Longitudinal Study of Ageing (KLoSA), a novel asymmetric fixed effects model was employed to separately estimate the association for decreases and increases in intergenerational transfers. Our analysis, based on standard fixed effects estimates, uncovered a negative association between intergenerational transfers and depressive symptoms among older adults in Korea. This association was found to be significant solely for mothers, with no significant effect observed for fathers. Moreover, our findings suggest that the asymmetric associations between intergenerational transfers and depressive symptoms are more pronounced for women than for men. Specifically, the asymmetric fixed-effects models revealed that a decrease in intergenerational transfers was linked to increased depressive symptoms among women, whereas an increase in transfers did not show a significant association with depressive symptoms. These results indicate a stronger emotional bond between mothers and their adult children within the Korean context. Given the asymmetric effect of intergenerational transfers, this study contributes to our understanding of the gendered dynamics of intergenerational relationships and their implications for mental well-being among older adults.

### SESSION 3365 (SYMPOSIUM)

Abstract citation ID: igae098.1489

### INTEGRATING AND IMPROVING MULTIPLE-METHOD RESEARCH IN AGING

Chair: Pamela Saunders
Co-Chair: Angela Perone, *Integrating and Improving Multiple Method Aging Research*

One of the basic tenets of mixed-method and mixed-methods research methodologies is that they integrate qualitative and quantitative data to provide a more holistic understanding of a research question. This interdisciplinary symposium examines several aging research projects to illustrate best practices for integrating and improving multiple methods research projects. We draw on fields including cultural anthropology, discourse analysis, and sociocultural gerontology to examine questions of language, identity, transcription, and homelessness. In a mixed-methods study, Saunders examines the integration of data and analysis from staff interviews, residents' conversations, and staff ratings of friendship among persons living with dementia in a long-term care setting unit. Crampton examines the value of outliers in a study of how " old" is defined by people aging into 80+ decades. Dobbs' paper will discuss the role of AI transcription using smartphone and web-based platform technologies to collect data from older adults and their caregivers. Perone uses a novel multi-method approach to learn about housing models that aim to prevent or intervene in homelessness among LGBTQIA+ older adults. Directors of Aging Centers Interest Group Sponsored Symposium

Abstract citation ID: igae098.1490

### WHAT DOES INTEGRATION LOOK LIKE IN MIXED-METHODS RESEARCH?

Pamela Saunders, *Georgetown University, Washington, District of Columbia, United States*

Multi-methods and mixed-methods research usually include both qualitative and quantitative data to gain a more holistic understanding of a research question. Multi-method research involves a variety of data sources and methods. A defining feature of Methods is the potential integration at multiple levels, including schools of thought, data, data collection methods, or analysis. The Friendship Study (de Medeiros et al., 2012) used a mixed-methods approach to examine perceptions of friendship among people living with dementia. The participants included ten staff members and 31 assisted living residents with moderate to advanced dementia. The first step was to define 'social interaction' and how it relates to the larger construct of friendship. Staff completed a matrix indicating their perceptions of which residents were friends. We conducted 6-months of direct observation with detailed field notes. The integration process focused on data collection methods, including interviews and surveys with staff and field notes describing direct observations of interactions between residents. Findings showed that the qualitative data from staff did not support the qualitative data from field notes of direct observation. This paper dives deeply into integrating these qualitative and quantitative data and analyses. A summary of specific integration techniques will be outlined.

Abstract citation ID: igae098.1491

### WHO IS "OLD" IN AN AFFLUENT RETIREMENT COMMUNITY: QUALIFYING STIGMA THROUGH OUTLIER DATA

Alexandra Crampton, *Marquette University, Milwaukee, Wisconsin, United States*

The purpose of this study has been to compare and potentially reconcile professional constructs defining who is an older adult with lived experience of aging into 80+ decades. In this anthropological field research project, the study site is an affluent, Midwestern retirement community in which the average age is 85. Data were primarily collected through participant observation and semi-structured interviews during several data collection periods from 1 week to 2 months between July 2021 and December 2023. Despite optimal access to resources and identities of privilege, findings show how residents confronted "deep old age" as a highly stigmatized experience and social identity. Demographic and other quantitative data were collected from administrative data. These data helped qualify normative responses from research participants, such as a perceived "rash of deaths," during the Covid-19 pandemic. In addition, qualitative data provided richness of detail and contextualization that numbers could not. In the objective concerns of a facility caring for older adults and the subjective experiences of residents confronting fears of aging, outlier data included examples of coping and social interactions that did not fit professional nor peer expectations of aging as stigmatizing or debilitating. Such examples inspired hope of alternative constructs that transformed ageist fears of age-related change. Residents who inspired included those who were unusually able to accept frailty, falls, and disability without resignation. In dialogue with research participants, "old" age was redefined as risk at any age of "giving up."

Abstract citation ID: igae098.1492

### THE USE OF ARTIFICIAL INTELLIGENCE TRANSCRIPTION TECHNOLOGY IN CAREGIVER QUALITATIVE DATA COLLECTION

Debra Dobbs, Jessica Yauk, Hongdao Meng, and Tanjina Jalil, *University of South Florida, Tampa, Florida, United States*

In recent years the use of artificial intelligence (AI) transcription has become more common and has advanced the ability of data collection methods in aging research. This paper presentation includes examples of how AI transcription methods can be implemented into research in multiple ways that has advanced the time to analysis of data and interpretation of qualitative data. The first study example is AI transcription using talk-to-text with an iphone to transcribe handwritten homework assignments from direct caregivers in assisted living in a palliative care education intervention. The second study example is using voice memo AI audio function for assisted living staff interviews in a music group intervention study. The third study uses Microsoft Teams to interview caregivers about advance care planning and end-of-life care quality which produces using AI transcription a transcript of the recorded interview. For each of these AI transcription techniques used in the three studies, we will discuss the advantages and disadvantages of their utilization in qualitative data collection and analysis including the amount of time and money saved in budgeting for transcription costs and whether it is balanced out by the costs for the technology; issues of privacy and older participants' willingness to allow researchers to use technologies such as smart phones and web-based platforms (e.g., Microsoft Teams and Zoom). We will also discuss the role of technological advancements in qualitative data methods as it relates to the need for technology skill development, privacy and data security.

Abstract citation ID: igae098.1493

### HOUSING MODELS TO PREVENT AND INTERVENE IN HOMELESSNESS AMONG LGBTQIA+ OLDER ADULTS

Angela Perone, and Leyi Zhou, *University of California Berkeley, Berkeley, California, United States*

LGBTQIA+ older adults have heightened risks for homelessness, given structural and systemic barriers and inequities across the life course. Little is known about housing prevention and intervention models for LGBTQ+ older adults at risk of homelessness. This study uses a novel multi-method approach to answer the following community-driven research questions: What housing models aim to prevent or intervene in homelessness among LGBTQIA+ older adults? How do these models respond to the diverse needs of LGBTQIA+ older adults? The research team first conducted a scoping review using Arksey and O'Malley's five-step framework. In collaboration with a national community partner, the team next identified and reviewed grey literature. The team supplemented review findings with secondary qualitative data from community leaders. Data revealed five core housing models used as prevention and intervention homelessness tools among LGBTQIA+ older adults: affordable housing developments, homeshare spaces, tiny homes, community land trusts, and mobile communities. Affordable housing developments tended to be located in spaces that were more heavily populated by white residents. Transgender and LGBTQIA+ older adults of color led projects that invoked the other four nontraditional housing models. While affordable housing developments for LGBTQIA+ older adults are growing, they may not be meeting the diverse needs of this community. Intersecting experiences of trauma, discrimination, and exclusion (e.g., racism, ableism, sexism, heterosexism, transphobia) may be driving the need and desire among transgender older adults and LGBTQIA+ older adults of color to develop alternative housing models to prevent homelessness.

### SESSION 3370 (PAPER)

### LIVING WITH DEMENTIA II

Abstract citation ID: igae098.1494

### CHARACTERISTICS OF MIDLIFE WOMEN FAMILY CAREGIVERS ASSOCIATED WITH THEIR NEGATIVE ATTITUDES TO DEMENTIA AND CARE

Dongmi Kim[1], Eun-Ok Im[1], Wonshik Chee[1], and You Lee Yang[2], *1. The University of Texas Austin, Austin, Texas, United States*, *2. Eulji University, Eulji University, Kyonggi-do, Republic of Korea*

Racial/ethnic minority family caregivers, especially midlife women who are family caregivers of persons living with Alzheimer's Disease (MWPLAD), tend to suffer quietly without necessary support and care partially due to their cultural attitudes related to Alzheimer's Disease (AD) and dementia care (e.g., filial piety, fatalism). Yet, little is still known about the women's characteristics that are associated with their negative attitudes toward AD and dementia care. This information could provide important directions for future intervention development for this specific population of family caregivers. The purpose of this secondary analysis study was to determine the characteristics of MWPLAD that were significantly associated with their negative attitudes toward AD and family Caregiving. This analysis included the data from 172 MWPLAD who were recruited through online and offline communities/groups for midlife women. The instruments included: the Attitude toward AD and Related Dementias Scale (DAS), the Questions on Attitudes toward AD Caregiving, the Social Readjustment Rating Scale, the EQ-5D-5L and the Midlife Women's Symptom Index. The data analysis was conducted with descriptive and inferential statistics including Independent t-tests, Mann Whitney U tests, one-way ANOVA, and multiple regression analyses. The total DAS scores were significantly associated with employment, race/ethnicity (being Hispanic), and instrumental activities for daily living ($p < .05$). Those who were unemployed, Hispanic, and with low instrumental activities for daily living tended to have negative attitudes toward Alzheimer's Disease and dementia care. More interventions need to be developed for racial/ethnic minority MWPLAD while considering these characteristics.

Abstract citation ID: igae098.1495

### EXAMINING THE ROLE OF SOUTHERN CONTEXT FOR DEMENTIA, DISABILITY, AND MORTALITY AMONG OLDER US BLACKS AND WHITES

Jaein Kim[1], Hyungmin Cha[2], Mateo Farina[3], and Mark D. Hayward[4], *1. Population Research Center, The University of Texas Austin, Austin, Texas, United States*, *2. University of Southern California, Los Angeles, California, United States*, *3. University of Texas at Austin, Austin, Texas, United States*, *4. The University of Texas Austin, Austin, Texas, United States*

Racial inequalities in older adult health are well-documented. Compared to Whites, Blacks have greater risks of disability, dementia, multimorbidity and mortality. These differences in age-related health are paralleled by race differences in context. In particular, approximately 80% of older Blacks were born in Jim Crow South no matter where they currently live compared to 30% percent of older Whites. In the United States, the U.S. South has historically structured the life course experiences of Black and White older adults differently through legalized racial segregation. As a result, the role of association of Southern context with later life health risks is expected to differ substantially across the groups. We focus on three critical dimensions of population health to better understand the role of context for race difference in health – cognitive and physical functioning as well as mortality. Drawing on the Health and Retirement study from 2000-2016, we estimate a series of hazard models for each of the health outcomes separately for each race group. This approach promises new insights into the role of context for within-group variation in the health outcomes as well as for potential between-group differences for each specific outcome. Whereas for Whites Southern birth regardless of residence was associated with increased risk across all three conditions, for Black older adults we found more heterogeneity. Those who were born and lived in the South had the highest risk of dementia. Southern residence was associated with an increased risk of mortality, but its effect was ambiguous for risk of disability.

Abstract citation ID: igae098.1496

### MULTILEVEL FACTORS ASSOCIATED WITH RACIAL AND ETHNIC DISPARITIES IN TIMELY DIAGNOSIS OF DEMENTIA IN US OLDER ADULTS

Yuting Qian, Fan Li, and Xi Chen, *Yale University, New Haven, Connecticut, United States*

In the U.S., non-Hispanic Black and Hispanic older adults are disproportionately more likely to develop dementia but less likely to receive a timely diagnosis than White older adults. Quality care for people with dementia starts with an early diagnosis, a key goal of the national plan to address dementia. This study examines individual-level and neighborhood-level factors associated with racial and ethnic differences in timely dementia diagnosis. We used 1998-2020 Health and Retirement Study (HRS) data, which contains detailed information on cognitive assessments and provider diagnoses for a representative sample of U.S. older adults. This was then linked to the National Neighborhood Data Archive (NaNDA) for neighborhood contexts. We find that racial and ethnic disparities in timely dementia diagnosis were substantially attenuated after adjusting for individual-level and neighborhood-level factors. The adjusted odds ratio of having a timely dementia diagnosis increased from 0.60 (95% CI: 0.48-0.76) to 0.76 (95% CI: 0.58-1.00) for non-Hispanic Black older adults compared to their White counterparts. A mediation analysis revealed that household income and education explained the largest proportion of the disparity between non-Hispanic Black and White older adults (17.9% and 12.7%, respectively), followed by neighborhood affluence (10.9%), neighborhood disadvantage (9.9%), and health care access (measured by physician density and other health practitioner density). These patterns were similar for Hispanic compared to White older adults. Our findings may inform targeted interventions and policies that emphasize socioeconomic risk factors and health service availability to reduce racial and ethnic disparities in timely dementia diagnosis and promote health equity.

Abstract citation ID: igae098.1497

### BRAIN FOG AND A SHRINKING WORLD: GRIEF AND LOSS IN THE CONTEXT OF DEMENTIA

Jen Hirsch[1], and Allison Gibson[2], *1. Michigan State University East Lansing, East Lansing, Michigan, United States*, *2. Saint Louis University, St Louis, Missouri, United States*

To date, little is known about people living with dementia's experience of relating to and coping with their disease. Most studies about dementia have focused on their experience from the perspective of a care partner(s), professional care provider(s), or through observation of rather than engaging those living with dementia directly to reflect on their experiences. This qualitative study engaged 20 people living with dementia in focus groups to explore their lived experiences of grief and loss in the context of dementia, such as needing to constantly adapt to changes in disease symptoms, brain fog, and having their environment shrink as they disengage from life roles. Reflexive thematic analysis was used to describe and interpret the rich data. Participants discussed numerous strategies to cope with their diagnosis including engaging in new activities such as hobbies or advocacy efforts around dementia. Others discussed leaning on support systems and peer support groups using social engagement and technology as their most effective coping mechanisms. The themes from this study offer a richer understanding of the experience of dementia as a serious illness. Grief responds well to social support, and yet this population who are dealing with compounding loss also face isolation due to their treatment by others. Understanding this population's experience and what strategies they use to cope could be used to improve quality of life and psychosocial support for people with dementia and their families.

Abstract citation ID: igae098.1498

### BUILDING INCLUSIVE COMMUNITIES FOR PEOPLE WITH DEMENTIA: TAKING THE LEAD FROM LIVED EXPERIENCE

Alison Phinney[1], Lynn Jackson[1], Ania Landy[1], and Mariko Sakamoto[2], *1. University of British Columbia, Vancouver, British Columbia, Canada*, *2. University of Victoria, Victoria, British Columbia, Canada*

Social citizenship is increasingly adopted in academic circles as a theoretical perspective foregrounding the rights of people with dementia to full participation in their community. However, what this means in practice is poorly understood. Using methods of participatory action research and developmental evaluation, our team conducted a four-year study to learn what social citizenship means from the perspective of people with lived experience, and to put that understanding into action through community programs and services. In phase one, an action group of people with dementia identified stigma as their main concern, and together with the research team co-developed an online toolkit to raise awareness and "flip stigma on its ear". In phase two, the toolkit was shared with community stakeholders who provided formative feedback, and the team conducted implementation sessions to explore the toolkit's impact for community programs and services who wanted to be more dementia inclusive. Analysis of interviews with stakeholders and program leaders and field notes from the feedback and implementation sessions identified key themes about how the toolkit has been received and its impact across varying community contexts, e.g. libraries, churches, community centres, and adult day programs: (1) the power of hearing real voices; (2) recognizing stigma; (3) gaining confidence for change; (4) spreading the word; and (5) working with (not for) people with dementia. Overall, these groups found that taking the lead from people with lived experience has been pivotal in helping them take first meaningful steps toward dementia inclusion.

### SESSION 3375 (SYMPOSIUM)

Abstract citation ID: igae098.1499

### NEIGHBORHOOD ENVIRONMENTS AND COGNITIVE HEALTH: ADVANCEMENTS IN CONCEPTS AND METHODS

Chair: Andrea Rosso
Discussant: Jana Hirsch

Evidence for the importance of residential neighborhood environments for cognitive health in older adults has grown substantially in the past decade. It is important to understand details of these relations, including timing and mechanisms, as interventions at the neighborhood level could have broad public health importance. Here, we present a series of five talks from four studies that aim to advance the field of environmental influences on cognitive health through several conceptual and methodological advancements: inclusion of lifecourse exposures (Cheng, Finlay), consideration of the role of race as a social construct and a modifier of neighborhood exposures (Cheng), incorporation of blood-based biomarkers of Alzheimer's disease as an outcome (Fan), consideration of spatial and historical context in interpreting results (Moored, Desjardins), use of qualitative interviews to gain insight from people's lived experiences (Finlay), and use of novel exposures such as cognability (Finlay) and blue space (Moored, Desjardins) as well as upstream factors indicative of structural disadvantage (Cheng, Fan, Desjardin). Together, these talks will challenge the audience to consider the complex ways in which neighborhoods could influence cognitive outcomes and how to best approach analysis and interpretation of such data. Advancing our conceptual understanding of the influence of neighborhoods on cognitive aging over the lifecourse will allow for informed public health interventions. A discussion of next steps will be led by Jana Hirsch. Environmental Gerontology Interest Group Sponsored Symposium

Abstract citation ID: igae098.1500

### LIFE COURSE "COGNABILITY": INVESTIGATING NEIGHBORHOODS AND COGNITIVE HEALTH RISKS ACROSS THE LIFESPAN

Jessica Finlay[1], Grace Savard[2], Desiree Alvarez-McNelis[3], Mallory Sagehorn[1], and Michael Esposito[3], *1. University of Colorado Boulder, Boulder, Colorado, United States, 2. University of Minnesota, 3. University of Minnesota, Minneapolis, Minnesota, United States*

A growing body of evidence identifies potentially modifiable individual risk factors for Alzheimer's Disease and Related Dementias (ADRD) such as physical inactivity, hypertension, and social isolation. However, the neighborhood built and social environments in which people develop and navigate these risk factors are often overlooked. This knowledge gap is particularly acute for longitudinal neighborhood contexts that may differentially structure opportunities and barriers to individual ADRD risk across the lifespan. The Neighborhoods and Health at All Ages Study employed seated and mobile interviews from August 2023-March 2024 to investigate which neighborhood amenities and hazards are perceived as most relevant to cognitive health-promoting activities at different life course stages. Fifty-six study participants living across the Minneapolis-St. Paul (MN) metropolitan area were on average 42 years old (range: 18-75). 52% identified as female, 40% male, 8% non-binary; and 29% identified as Asian, 24% Hispanic, 19% Black/African American, 17% non-Hispanic White, and 11% American Indian/Alaska Native. Reflexive thematic analysis identified that greater access to affordable amenities, online social infrastructure, and robust public and active transit in young adulthood; safe transportation, online neighborhood swaps, and educational sites (e.g., daycares, schools) in mid-life; and civic/social organizations, arts/cultural amenities, and accessible outdoor recreation in later life can support cognitively healthy lifestyle behaviors. The interviews captured nuanced perspectives and varying personal experiences of how individuals uniquely engage with their neighborhood environments, including navigating structural racism, classism, and ageism. Results may inform upstream community-level interventions to help create more equitable neighborhoods with opportunities to support lifelong cognitive health.

Abstract citation ID: igae098.1501

### MIDLIFE NEIGHBORHOOD SOCIOECONOMIC STATUS AND COGNITIVE FUNCTION IN LATE LIFE: DIFFERENCES BY RACE

Greta Jianjia Cheng, Jeanine Buchanich, Tiffany Gary-Webb, Christina Mair, C. Shaaban, and Andrea Rosso, *University of Pittsburgh, Pittsburgh, Pennsylvania, United States*

Evidence regarding the role of neighborhood socioeconomic status (NSES) as an upstream determinant of cognitive outcomes has lacked a life-course perspective and has relied on time-invariant NSES estimates. Compared to late-life, midlife NSES may be more relevant to cognitive outcomes, and associations with cognition may vary by race, reflecting the potential impact of structural racism. We examined racial differences in associations between midlife NSES and changes in cognitive function among older adults aged 70+. We included 341 (37% Black and 63% White) participants from the Pittsburgh site of Health, Aging, and Body Composition Study. Cognitive function was assessed repeatedly by Modified Mini-Mental State Examination (range: 80-100) throughout a 13-year follow-up. LexisNexis addresses from 1976-1985 were geocoded and linked to 1980 Census estimates (education, occupation, employment rate, household income, and home value). Midlife NSES (aged 49 to 58 years) for census tracts was calculated as the sum of z-scores of five estimates (range: -10.4 to 9.1, SD: 4.3). In linear mixed-effects models adjusted for census-tract and individual-level characteristics, midlife NSES was associated with baseline cognitive function among Black (β: 0.56, 95% CI: 0.14, 0.98), but not among White participants (β: -0.05, 95% CI: -0.31, 0.21) (p-value for interaction: 0.02). There were no observed associations between midlife NSES and the rate of cognitive change and no association between late-life NSES and cognitive function. Our findings suggest that midlife NSES are relevant to late-life cognitive function, particularly for Black individuals. Incorporating a life-course perspective is crucial to understanding racial disparities in cognitive outcomes.

Abstract citation ID: igae098.1502

### NEIGHBORHOOD PROXIMITY AND SPATIAL SPILLOVER: IMPACT ON OLDER ADULT HEALTH

Michael Desjardins[1], Kyle Moored[2], Timothy Shields[1], Frank Curriero[1], and Michelle Carlson[1], *1. Johns Hopkins University, Baltimore, Maryland, United States, 2. Johns Hopkins Bloomberg School of Public Health, Baltimore, Maryland, United States*

Studying the impacts of neighborhoods on health exposures and outcomes has seen increased attention over the last few decades. Neighborhoods can describe where individuals work, live, and recreate in granular detail, facilitating our understanding of activity spaces throughout the lifecourse. However, a plethora of studies in neighborhood health, especially among older adults treat these spatial units as statistically independent. Most research in this domain fails to capture the potential influence of surrounding neighborhoods on health exposures and outcomes in the target neighborhood. Many of these studies also fail to check for residual spatial autocorrelation/dependence of modeling results, which may violate the assumption of independence that is fundamental in statistics. The explanatory power of regression models may also improve by accounting for the spatial dependence of the variables in question. We present conceptual examples (e.g., blue/green spaces, neighborhood socioeconomic status, and other social determinants of health) of spatial regression models that consider the influence of nearby neighborhoods on the dependent variable of interest. We also highlight the need to consider neighborhoods that are not adjacent since individuals may interact with neighborhoods farther away more often than those immediately adjacent. We then present case studies of modeling neighborhood and environmental predictors of cognitive outcomes of older adults in the Cardiovascular Health Study (CHS). Emphasis will also be on modeling lifecourse exposures. Our work highlights the power of spatial models to capture spillover and protective effects, which can greatly improve our understanding of older adult activity spaces and health across the lifecourse.

Abstract citation ID: igae098.1503

### NEIGHBORHOOD BLUE SPACES AND RISK OF INCIDENT DEMENTIA: IMPORTANCE OF SPATIAL AND HISTORICAL CONTEXT

Kyle Moored[1], Michael Desjardins[2], Andrea Rosso[3], Gina Lovasi[4], Patrick Donahue[2], Timothy Shields[2], Frank Curriero[2], and Michelle Carlson[2], *1. Johns Hopkins Bloomberg School of Public Health, Baltimore, Maryland, United States, 2. Johns Hopkins University, Baltimore, Maryland, United States, 3. University of Pittsburgh, Pittsburgh, Pennsylvania, United States, 4. Drexel University, Philadelphia, Pennsylvania, United States*

Neighborhood blue spaces (e.g., lakes, rivers, other water sources) are underexplored contributors to cognitive aging that may act by promoting recreation and mental health. Yet, benefits may vary by historical use and surrounding spatial context of water sources. We examined associations between neighborhood blue spaces and risk of incident dementia in community-dwelling older adults in Pittsburgh, PA. Participants were 655 adults ≥65 years old from the Cardiovascular Health Cognition Study (CHCS; 1992-1999) living in Pittsburgh/Allegheny County, PA. Baseline blue space was measured as the Census tract-level percentage of open water from the 1992 National Land Cover Dataset, stratified into groups incorporating water source: none (0%), low (.003-.04%, smaller water sources), and high (>.04%, major rivers). Time to dementia diagnosis was clinically adjudicated from a neurological exam, neuropsychological examinations, medical records, and proxy reporting across a median 6.2 years (n=103 cases, 16%). Cox models were adjusted for individual age, sex, race, education, income, and lifetime occupation. Greater blue space was associated with higher risk of all-cause dementia (low vs. none: HR = 1.74, 95% CI: 1.26, 2.41 high vs. none: HR = 2.08, 95% CI: 1.45, 2.98). Contrary to our hypothesis, neighborhood blue space was linked to increased risk of dementia for residents of Pittsburgh, PA. Historical industrialization and clustering of lower income neighborhoods along Pittsburgh's main rivers will be discussed as potential contributors to this finding. Future work will capitalize on the geographic diversity of CHCS by comparing these analyses with more rural/suburban clinic sites (Winston-Salem, NC; and Hagerstown, MD).

Abstract citation ID: igae098.1504

### NEIGHBORHOOD DISADVANTAGE AND THE ASSOCIATION OF PLASMA BIOMARKERS WITH COGNITION IN A COHORT OF DIVERSE ADULTS

Erica Fan[1], Sarah Royse[2], Alexandria Reese[3], Thomas Karikari[3], Tharick Pascoal[2], C. Shaaban[2], Victor Villemagne[3], and Ann Cohen[2], *1. University of Pittsburgh School of Public Health, Pittsburgh, Pennsylvania, United States, 2. University of Pittsburgh, Pittsburgh, Pennsylvania, United States, 3. University of Pittsburgh School of Medicine, Pittsburgh, Pennsylvania, United States*

Studies of Alzheimer's disease pathology have reported associations of plasma Aβ, tau, neurofilament light chain (NfL), and glial fibrillary acidic protein (GFAP) with cognition. However, it remains unclear how these biomarkers are associated with cognition in non-White populations affected by structural racism. Discriminatory policies have long shaped US neighborhood resources. Thus, our aim was to investigate whether neighborhood disadvantage modifies associations of plasma biomarkers with cognition in a diverse cohort of 136 adults ≥50 years. Predictors were plasma biomarkers (Aβ40, Aβ42, pTau217, pTau231, NfL, GFAP) measured using single molecule array or mass spectrometry. Outcomes included cognitive impairment and neuropsychological test scores. Our modifier, neighborhood disadvantage, was measured using area deprivation index (ADI), a composite of socioeconomic condition measures by census tract: higher ADI corresponds with worse conditions. In adjusted linear models, higher ADI, pTau217, pTau231, NfL, and GFAP, and lower Aβ40 and Aβ42 were separately associated with worse cognition. We found ADI*pTau217 interactions on immediate and delayed word recall. In those with low ADI, higher pTau217 was associated with worse immediate ($\beta$=-1.82, p=0.005) and delayed recall ($\beta$=-22.82, p<0.001); there were no such associations among high ADI participants. We also found an ADI*Aβ40 interaction on Rey figure immediate memory recall such that in low ADI participants, higher Aβ40 was associated with better recall scores ($\beta$=0.06, p=0.006), but this association was not significant in high ADI participants. Taken together, relationships between plasma biomarkers and cognition differ by neighborhood conditions, motivating further study of how neighborhood-level factors affect diagnosis and prognostication of cognition.

### SESSION 3380 (SYMPOSIUM)

Abstract citation ID: igae098.1505

### OPENING MINDS THROUGH ART: CREATING UNIVERSITY PARTNERSHIPS THROUGH AN ADAPTIVE INTERGENERATIONAL ART PROGRAM

Chair: Amy Elliot
Discussant: Katherine Abbott

Opening Minds through Art (OMA) is an award-winning, evidence-based, intergenerational art-making program for people living with dementia. Developed in 2007 at Miami University's Scripps Gerontology Center, the program is grounded in person-centered care principles and serves the vital need for people living with dementia to engage in creative expression and social connection. The current impact of OMA includes engagement through 1,100 trained facilitators in over 300 communities spanning 37 states and internationally. As a university-based program, OMA has maintained a focus on research with thirteen peer-reviewed studies to support the program's impact on younger and older participants in areas such as more empathetic attitudes for other generations, improved mood, and increased engagement. Core to OMA's success are relationships with 30 universities in the U.S. and globally. Through these partnerships, students engage with OMA through academic and service-learning opportunities to fortify their understanding of the aging process and empathy for older generations. Universities also benefit through community support and participation. The success of these programs is highly dependent on OMA's ability to adapt to each university's resources, programmatic interests, and community partnerships. This symposium will describe variations of OMA programming at three universities including work with health trainees at Penn State University, a student internship program at Florida State University, and the first and longest running OMA program including an innovative virtual variation at Miami University. Katy Abbott will lead a discussion of implementation, internal evaluation, and research components across programs and will explore future implications for university-based growth and adaptations.

Abstract citation ID: igae098.1506

### LAUNCHING A NEW UNIVERSITY-BASED OPENING MINDS THROUGH ARTS PROGRAM

Dawn Carr[1], and Suzanne Smith Monroe[2], *1. Florida State University, Tallahassee, Florida, United States*, *2. Claude Pepper Center, Tallahassee, Florida, United States*

Florida State University launched the Opening Minds through Arts program in Fall of 2023 within the college of Social Sciences and Public Policy. The current model is based in a student internship program, in which students are able to earn up to three credit hours. The internship course includes one face-to-face, hour-long class per week with an instructor in which they complete course readings, watch videos, and listen to podcasts to learn about Alzheimer's and Dementia related subjects, as well as research associated with art-based therapies and person-centered care models. Students are paired with one older adult for the semester at one of our two partnership residential care homes, in which students facilitate art making and, in the process, get to know their older adult artist. Since the program's launch, FSU has trained 18 student interns and 13 volunteers from varying disciplines, such as the FSU College of Law. The program has also served 19 older adults in Tallahassee and continues its plan to expand the number of OMA sessions, with the Spring semester currently holding three OMA sessions per week (5 older adults per session). Although FSU's program is in early stages of development, we will provide internal program evaluation results which includes institutional partnership characteristics that promote long-term success, student training and preparation, and qualitative feedback from student interns. We will also provide information about expansion of the program for clinical settings at FSU to expand geriatric training and person-centered care.

Abstract citation ID: igae098.1507

### IMPACT OF OMA ON HEALTH CARE TRAINEE ATTITUDES AND PERCEPTIONS TOWARD PEOPLE LIVING WITH DEMENTIA

Diane Berish, Jennifer Rank, Janice Whitaker, and Daniel George, *The Pennsylvania State University, University Park, Pennsylvania, United States*

To meet the complex needs of the growing population of people living with dementia (PLwD), the healthcare workforce must have not only the necessary clinical knowledge, but also the interpersonal skills. Experiences in the arts and humanities, such as OMA, can provide opportunities to acquire best-practice approaches. At present, no evaluation of OMA has been conducted on health trainees. We implemented OMA at Penn State University in both the Nese College of Nursing (NCON) and the College of Medicine (COM). In the NCON, OMA was implemented into nursing clinicals and as part of a memory cafe. N=10 clinical and N=15 memory café students completed pre and post assessments (Allophilia, Dementia Attitudes) of their perceptions and attitudes towards PLwD. Results showed that clinical students reported higher levels of comfort with PLWD on both scales (Allophilia, p=.0039; Dementia Attitudes, p=.002). Memory café students reported higher Allophilia scores (affection, p=.02; comfort, p=.04; engagement, p=.02) and Dementia Attitudes knowledge (p=.02). The COM was one of 6 medical schools to implement OMA in 2019-2020. Overall, 52 first- or second-year medical students were trained in OMA and participated with residents at partner assisted living facilities. Students' scores showed significantly larger pre-post improvement than controls with respect to Dementia Attitudes scores (p=.001) and all Allophilia-scale factors (p=.001). Our work suggests that arts-based learning experiences such as OMA, which enable playful, creative, humanizing interactions between health trainees and PLwD, may foster more positive attitudes. Experiences like OMA might improve attitudes and encourage students to pursue geriatric specialties.

Abstract citation ID: igae098.1508

### CREATING CONNECTIONS BETWEEN PEOPLE LIVING WITH DEMENTIA AND VOLUNTEERS THROUGH ART

Meghan Young, *Miami University, Oxford, Ohio, United States*

Scripps Gerontology Center's award-winning Opening Minds through Art (OMA) program has built bridges across age and cognitive barriers through art for 17 years. People living with dementia, also known as artists, and volunteers are paired 1:1 for weekly abstract art making sessions. The program philosophy is based on autonomy and the freedom to make choices within a structured process, so the artists have the opportunity to fully immerse themselves in art-making with the assistance of their volunteer partners. Volunteers in the program are trained to rely on the imagination and skills of the artist, without making any artistic, aesthetic decisions during the art-making process. Over the course of the art-making sessions, relationships develop between the pairs. Thirteen published studies show the impact of the program including findings to support that volunteers are more positive in their attitudes toward people living with dementia post-participation, and participants living with dementia displayed more interest and pleasure during OMA than during traditional visual art activities. This session will provide an overview of the program and a description of previous research as background information for the remainder of the symposium. The session will also include an overview of OMA's first and longest running program at Miami University where 2,898 older adults and 2,812 students have participated in OMA through in-person and virtual intergenerational programming. To highlight OMA's longevity, we will also present an original compilation of internal program evaluation data to map the expansion and growth of OMA from inception to the present day.

Abstract citation ID: igae098.1509

### ENGAGING OLDER AND YOUNGER PEOPLE VIRTUALLY THROUGH THE ARTS

Krysta Peterson, *Scripps Gerontology Center, Miami University, North Royalton, Ohio, United States*

Following the COVID-19 Pandemic, the need for programming to foster connections and reduce loneliness for people of all ages increased. After the success of a three-year virtual OMA program, a new, video-chat application was developed to continue building intergenerational relationships virtually. ScrippsAVID (Arts-based, Virtual, Intergenerational, and Dementia-Friendly), pairs older and younger adults to discuss and co-create art, music, poetry, and share stories using over 75 art-based "prompts" designed to generate connections. Since its inception in 2023, ScrippsAVID has remained locally based with the majority of previous and current pairs comprised of Miami University students connecting with alumni. The program addresses older adults' needs for social and creative engagement while providing younger adults opportunities to develop communication skills and build relationships with older adults, with and without dementia, outside of their families. Participants have commented on the value of their interactions with observations such as, "My AVID partner is one of the great joys in my life. Our time is so precious, and I am learning so much about life through her eyes. Thank you for this amazing opportunity. It is truly a blessing." Overall, 132 older adults and 168 students have participated in ScrippsAVID with 90% of post-meeting satisfaction surveys reporting respondents being "Very Satisfied" (84%) or Satisfied (6%) with their meetup. This presentation will delve into the early ScrippsAVID implementation and outcome data to support further understanding of this innovative intergenerational program. We will also discuss potential future applications of ScrippsAVID for university-based intergenerational programming and community outreach.

### SESSION 3385 (PAPER)

### PHYSICAL HEALTH AND PSYCHOSOCIAL WELL-BEING OF CAREGIVERS

Abstract citation ID: igae098.1510

### CAREGIVERS' PERCEIVED IMPORTANCE IN SOCIAL ACTIVITIES EXACERBATES THE EFFECT OF SOCIAL RESTRICTIONS ON DEPRESSION

Yuanchang Zhao[1], Mengzhao Yan[2], and Jessica Kelley[1], 1. *Case Western Reserve University, Cleveland, Ohio, United States*, 2. *University of Southern California, Los Angeles, California, United States*

Taking on a family caregiving role can limit time for social activities, even ones that are very important to the caregiver. Despite the well-known benefits of social activity participation, such as mental health and reducing caregiver strain, few studies have examined the potential impact of missing specific types of social activities and their degree of importance for caregivers on their mental health. Guided by the conceptual model of caregiver burden and role theory, we hypothesize that missing specific activities that are important to the caregiver will have a more negative impact on depressive symptoms. We utilize Round 12 National Study of Caregiving (NSOC) data of family caregivers to people aged 65+ (N=2,431) focused on four key social activities: visiting friends and family, attending religious services, participating in group activities, and enjoying outdoor events. Our findings indicate that for each additional activity caregivers miss, they have 54% increased odds of depressive symptoms. Further, missing any activity considered "important" by the caregiver had 70% higher odds of depressive symptoms. Missing outdoor activities increased odds of depressive symptoms by 216%, but missing outdoor activities considered "important" had 234% higher odds of depressive symptoms. In sum, limiting social and recreational activities due to caregiving raises the risk of poor mental health, but missing activities that are highly valued by the caregiver raises that risk even further. These findings underscore the importance of recognizing caregivers' perceived important activities to better understand and address the mental health challenges they face due to activity restrictions.

Abstract citation ID: igae098.1511

### RELATIONSHIP BETWEEN STRESS AND PREDEATH GRIEF IN FAMILY CAREGIVERS OF PERSONS WITH DEMENTIA

Lauren Elliott[1], Sarah Sparks[1], Sydnie Schneider[1], Elisabeth McLean[1], Carol Fadalla[1], Volker Neugebauer[2], and Jonathan Singer[1], *1. Texas Tech University, Lubbock, Texas, United States*, 2. *Texas Tech University Health Sciences Center, Lubbock, Texas, United States*

Being a family caregiver of a person with Alzheimer's Disease or Alzheimer's Disease Related Dementias (AD/ADRD) has been associated with chronic stress. Family caregivers likely experience increased rates of pre-death grief (PDG; grief symptoms that develop while an individual with a life-limiting illness is still living), given the trajectory and terminal nature associated with an AD/ADRD diagnosis. Higher levels of PDG in family caregivers is a robust predictor of depressive symptoms and prolonged grief disorder following the loss. Stress has been extensively studied within this population, but previous studies have failed to link stress to pre-death grief. Data from 65 family caregivers of individuals with AD/ADRD were analyzed (Mage = 68.68, SDage = 11.89). Participants completed questionnaires regarding pre-death grief symptoms (Prolonged Grief—12; PG-12) and subjective stress (Perceived Stress Scale; PSS). Consistent with previous research, family caregivers reported moderate levels of stress (PSS; M = 17.88, SD = 6.42) and pre-death grief (PG-12; M = 27.68, SD = 7.89). A bivariate correlation revealed a significant, positive relationship between stress and pre-death grief (r =.603, p <.001). Thus, family caregivers reporting higher levels of stress were experiencing heightened pre-death grief symptoms. Evaluating subjective stress in family caregivers is a time-efficient, non-invasive way to identify individuals at increased risk for psychosocial dysfunction. Interventions targeting stress in family caregivers may have beneficial downstream effects on pre-death grief before the loss, which may decrease the risk of developing prolonged grief disorder following the loss.

Abstract citation ID: igae098.1512

### VIRTUAL HEALTH COACHING TO IMPROVE HEART FAILURE CAREGIVERS' SELF-CARE OUTCOMES

Karen Hirschman, Tracie Walser, Ryan Quinn, and Barbara Riegel, *University of Pennsylvania, Philadelphia, Pennsylvania, United States*

Health coaching is assumed to improve self-care, but this assumption has not been well-tested. This randomized controlled trial involved 250 adults with health self-care neglect who provided >= 8 hours of care/week to their relative living with heart failure. We assessed the impact of virtual health coaching (n=125) on self-care compared to health information alone (n=125). The Virtual Health Coach for You (ViCCY) intervention included 10 synchronous sessions with a health coach over 6 months. ViCCY and health information were delivered via a computer tablet. Self-care data were collected with the Self-Care Inventory (SCI) (higher scores indicated better self-care.) at baseline and 6 months (August 2019-April 2023). Caregivers were primarily female (85%), white (62%), spouses (72%), and on average 55 years old (+/-14 years, 19-79). Almost half had a college degree (46%) and were employed full-time (44%). On average caregivers received 6 ViCCY sessions (median: 9) with 62% completing all 10 sessions, 28% completing 1-9 sessions, and 16% completing no sessions. The group-by-time interaction showed significant self-care improvement in the ViCCY group compared to the health information only (p=0.01). Self-care scores improved by 10 points indicating a clinically meaningful improvement at 6 months. Neither the number of health coaching sessions nor the total amount of time spent with the caregivers were statistically associated with the change in self-care rating over time in the VICCY group. The virtual health coaching intervention effectively improved self-care practices among heart failure caregivers, highlighting its potential to positively impact caregiver well-being and support.

Abstract citation ID: igae098.1513

### THE EFFECTS OF CAREGIVING HISTORY ON THE WELL-BEING OF INFORMAL CAREGIVERS LATER IN LIFE

Sarah Neller[1], and Radion Svynarenko[2], *1. University of Tennessee Knoxville, Knoxville, Tennessee, United States*, 2. *University of Tennessee, Knoxville, Tennessee, United States*

Previous studies have been primarily focused on documenting the negative impact of caregiving on quality of life. However, such a perspective overlooks the potentially positive outcomes that it can bring into the caregiver's life. The purpose of the current examination was to explore how the history of informal caregiving for six months or more influences beliefs about well-being later in life. We used data from the Health and Retirement Study on caregiving history from the Life History Surveys, which were combined with well-being measures from Psychosocial and Lifestyle Questionnaires collected between 2016 and 2022. For data analysis, we used a set of Ordinary Least Squares regression models (OLS) to control for age, gender, race, ethnicity, education, and marital status. The total sample size in each OLS model varied depending on the year of data collection and response rates for each dependent variable. We found that history of caregiving has mixed effects on well-being later in life. Though it was negatively associated with life satisfaction

(b = -0.19, p < 0.001, n = 3,902), it was positively associated with compassionate goals (b = 0.094, p< 0.001, n = 3,860) and purpose in life (b = 0.07, p = 0.017, n = 4,713), and negatively associated with fears about aging (b = -0.07, p = 0.016, n = 3,161). We will discuss potential ways to leverage positive wellbeing effects to promote caregivers' flourishing and positive caregiver outcomes. These findings can be used to develop future interventions that aim to improve well-being of informal caregivers.

Abstract citation ID: igae098.1514

### THE RELATIONSHIP OF PREDEATH GRIEF, BURDEN, AND EMPATHETIC PAIN IN FAMILY CAREGIVERS

Lauren Chrzanowski, Elisabeth McLean, and Jonathan Singer, *Texas Tech University, Lubbock, Texas, United States*

Family caregivers may experience pain as an empathetic reaction to their loved ones' pain – this has not been studied in aging populations. Caregiver distress (such as pre-death grief (PDG)) increases as patient pain worsens. Therefore, this study hypothesized that higher PDG and caregiver burden would result in higher rates of empathetic pain for family caregivers. This study included family members of persons with advanced cancer (N=100) and dementia (N=38). We measured PDG using the PG-12 scale, burden with the Zarit Burden Inventory (ZBI), and used a single item (i.e., How often have you experienced pain (or other symptoms) that the individual with life limiting illness as (e.g., empathetic pains)), dichotomized (0=never; 1=at least once a day-multiple times a day) to measure empathetic pain. Logistic regressions were performed to ascertain the effects of PDG and burden on the likelihood of family caregivers experiencing empathetic pain when controlling for diagnoses and age of care recipient. PDG significantly predicted empathetic pain X2(3, N = 118) = 18.83, p<.001. The model explained 20.4% (Nagelkerke R2) of the variance. Higher PDG score was associated with an increased likelihood of endorsing empathetic pain (OR=.896, 95%CI [1.041, 1.161]). Caregiver burden significantly predicted empathetic pain X2(3, N = 124) =7.81, p=.05. The model explained 8.4% (Nagelkerke R2) of the variance. Higher ZBI score was associated with an increased likelihood of endorsing empathetic pain (OR=.889, 95%CI [1.002, 1.128]). These results provide evidence that higher PDG and burden can lead to downstream effects on the caregiver, including empathetic pain.

### SESSION 3390 (SYMPOSIUM)

Abstract citation ID: igae098.1515

### POLICY SERIES: GSA CONGRESSIONAL UPDATE

Chair: Brian Lindberg
Co-Chair: Patricia D'Antonio

This popular annual session will provide cutting-edge information on what the 118th Congress has and has not accomplished to date related to aging policy. Of particular importance will be the Older Americans Act Reauthorization, including funding for the Center for Research and Demonstration in the Aging Network, as well as an update on Medicare Advantage, nursing home regulations, and hospice and palliative care legislation.

Abstract citation ID: igae098.1516

### CONGRESSIONAL UPDATE

Brian Lindberg, *Gerontological Society of America, Washington, District of Columbia, United States*

This popular annual session will provide cutting-edge information on what the 118th Congress has and has not accomplished to date related to aging policy. Of particular importance will be the Older Americans Act Reauthorization, including funding for the Center for Research and Demonstration in the Aging Network, as well as an update on Medicare Advantage, nursing home regulations, and hospice and palliative care legislation.

Abstract citation ID: igae098.1517

### GSA CONGRESSIONAL UPDATE

Patricia D'Antonio, *Gerontological Society of America, Washington, District of Columbia, United States*

This popular annual session will provide cutting-edge information on what the 118th Congress has and has not accomplished to date related to aging policy. Of particular importance will be the Older Americans Act Reauthorization, including funding for the Center for Research and Demonstration in the Aging Network, as well as an update on Medicare Advantage, nursing home regulations, and hospice and palliative care legislation.

### SESSION 3395 (SYMPOSIUM)

Abstract citation ID: igae098.1518

### PREPARING THE CARE WORKFORCE TO ADDRESS INTERPERSONAL DEMANDS ACROSS THE DEMENTIA TRAJECTORY

Chair: Scott Trudeau
Discussant: Laura Gitlin

This symposium posits that student learning experiences in professional programs are a direct pipeline to impact workforce readiness to address the needs of people living with dementia (PLWD). The presentations will share data and experience with three innovative learning activities that have been implemented with graduate students in occupational therapy. PLWD often face social isolation due to declines in domains such as memory and communication. This estrangement can extend to the care team, as cognitive declines can lead care professionals to focus increasingly on caregivers. While partnering with caregivers is often essential to optimize the health and well-being of the dyad, this may have the unintended consequence of minimizing the voice and perspective of the person with dementia. Similarly, in institutional settings, custodial care may be prioritized over attending to quality-of-life due to staffing challenges and a lack of creativity regarding person-centered practice for those with later stage dementia. These abstracts present three programs, generated to address identified care needs, focused on increasing person-centered approaches for PLWD. First, students engaged in preventive approaches to proactively increase the voice of older adults using the My Life Profile. Second, remote delivery of the My Life, My Story (MLMS) program at Department of Veterans Affairs allowed community-dwelling Veterans with cognitive concerns to share their stories with the care team. And lastly,

The Reading Buddies program for individuals in later staged dementia will be shared. Providing experiential learning opportunities empowering care workers is critical to improving the future of dementia services across the continuum.

Abstract citation ID: igae098.1519

### MY LIFE PROFILE: EMPOWERING COMMUNITY-DWELLING OLDER ADULTS TO ADVOCATE FOR AGE-FRIENDLY HEALTH CARE

Gina Tucker-Roghi, *Dominican University of California, San Rafael, California, United States*

Older adults face a heightened risk of dementia and delirium, the latter affecting over half of hospitalized older adults, leading to longer hospital stays and poorer outcomes. Current evidence supports multi-component nonpharmacologic approaches such as cognitive activation, re-orientation, simple mobilization, social engagement and sleep hygiene to prevent delirium during hospitalization. Utilization of these non-pharmacological interventions may be enhanced by personal information. My Life Profile for Age-Friendly Care, rooted in the Age-Friendly Health Systems' 4Ms framework and the Occupational Profile, serves as a proactive instrument. It is collaboratively developed by community-dwelling older adults and occupational therapy students, offering healthcare providers vital insights into the personal history, habits, routines, and preferences of older adults. Students are trained to utilize a semi-structured interview process to gather personal information relevant to Age-Friendly care. The student and older adult collaborate to compile the essential information onto a one-page profile, strategizing to ensure healthcare teams receive this information if hospitalization occurs. This process deepens students' appreciation for personalized care and illustrates the significance of the Occupational Profile in driving person-centered care across healthcare teams. Feedback from 180 students over four years and participating older adults underscores the initiative's effectiveness in affirming the value of personalized care in promoting older adults' well-being and dignity. The My Life Profile for Age-Friendly Health Care project exemplifies how educational objectives and healthcare solutions can converge, highlighting the pivotal role of occupational therapy in fostering age-friendly healthcare practices.

Abstract citation ID: igae098.1520

### INCREASING CONNECTIONS BETWEEN PERSONS WITH DEMENTIA AND CARE TEAMS: MY LIFE, MY STORY

Megan Gately[1], and Jaye McLaren[2], *1. VA Bedford Healthcare System, Bedford, Massachusetts, United States, 2. Department of Veterans Affairs, Bedford, Massachusetts, United States*

My Life, My Story (MLMS) was developed by the Veterans Health Administration (VHA) Office of Patient-Centered Care and Cultural Transformation as an opportunity for patients to share life stories with their care teams. Participating in MLMS interviews increases connection with clinicians, thereby facilitating patient-centered care. This study examines remote delivery of MLMS by occupational therapy students to patients of outpatient dementia clinics and their caregivers. Interviews were conducted using either videoconferencing, phone, or a combination. Post-interview questionnaires gathered Veteran-caregiver technological experience, including the need for technical support, comfort with technology, and how well they could see and hear. Questionnaires also gathered overall interview experience, including the belief that having care teams read stories would assist them in providing better care. In this session, we describe the MLMS program and objectives and present findings such as visit details (e.g., devices utilized, technological challenges, and caregiver role), and Veteran experience. We will also discuss strategies for conducting virtual interviews in the presence of cognitive concerns, such as interviewer flexibility and adaptability around technology, and communication strategies like simplifying questions and allowing extra time to accommodate for technology lag. Virtual narrative interviews using a structured approach like My Life, My Story allow for increased connection between individuals living with dementia and the care team, thereby facilitating patient-centered care. By providing an opportunity for social engagement, MLMS interviews may also address the growing scourge of older adult loneliness and social isolation, which is exacerbated for persons living with dementia.

Abstract citation ID: igae098.1521

### SERVICE LEARNING TO PREPARE OCCUPATIONAL THERAPY STUDENTS TO MEET THE NEEDS OF VETERANS WITH LATE-STAGE DEMENTIA

Scott Trudeau, *American Occupational Therapy Association, Stow, Massachusetts, United States*

One of the challenges to providing activity programs in dementia units is meeting a wide range of residents' needs. Programs are typically directed to the more moderately functional, often not meeting the needs of the least and most functional. The recognition that a significant cohort (approximately 20%) of residents on a dementia unit at Veterans Health Administration were typically excluded from group activities was of concern. This cohort came to be referred to as "VIPs" (Very Isolated Patients). The reason for their isolation was difficulty tailoring activities to their limited function within a group context. While more individualized approaches would be ideal, limited staffing precluded this approach. Developing a structured intervention that could be deployed by students to this vulnerable population seemed advantageous. The Reading Buddies Program was developed in which students were paired with a VIP resident with the assignment to meet with them weekly to engage in reading for a 45-minute session. Students read to residents in pairs, with one student facilitating and another student observing and gathering data. Data was collected in the form of qualitative student reflections and the Observed Measure of Engagement (OME). Students reported many opportunities for growth and mastery. The OME data reflected continued capacity for resident engagement in reading activities. Findings support the conclusion that by providing cognitive stimulation and social interaction for residents with later stage dementia, The Reading Buddies Program provided hands-on experience for occupational therapy graduate students to address the challenging functional and interpersonal needs of this cohort.

### SESSION 3400 (SYMPOSIUM)

Abstract citation ID: igae098.1522

### PRESIDENTIAL SYMPOSIUM: BUILDING FORTITUDE FOR THE JOB MARKET

Chair: Rita Hu
Co-Chair: Sohyun Kim
Discussant: Marie Bernard

The journey to establish a robust career in the gerontological research field is often marked by navigating through a maze of challenges, strategic decision-making, and the forging of vital professional relationships. The ESPO Presidential Symposium will feature early-career scholars from each scientific section to share their diverse pathways to landing a job and achieving research independence. Dr. Marissa Schafer from Mayo Clinic will discuss transitioning from PhD to Principal Investigator (PI), underlining the necessity of a comprehensive research portfolio, nurturing mentor-mentee relationships, and strategic career moves. Dr. Ketlyne Sol from the University of Michigan will share insights from her clinical psychology path, stressing the importance of dedicated research training and building a supportive network. Dr. Allyn M Bove from the University of Pittsburgh will compare physical therapy principles to career building in research, suggesting a balanced development of strength and endurance. Dr. Sara Bybee from the University of Utah will explore job market strategies through the lens of posttraumatic growth, highlighting personal strength and professional growth opportunities. The symposium concludes with a panel discussion led by Dr. Marie Bernard, the Chief Officer for Scientific Workforce Diversity at NIH, synthesizing the presentations and facilitating a dialogue among the speakers. This symposium serves as a crucial platform for aspiring gerontologists, offering guidance, strategies, and encouragement to navigate the complexities of the job market and achieve a successful and fulfilling career in aging research.

Abstract citation ID: igae098.1523

### NAVIGATING THE PATH FROM PHD TO PI: INSIGHTS AND STRATEGIES FOR RESEARCH INDEPENDENCE

Marissa Schafer, *Mayo Clinic, Rochester, Minnesota, United States*

The path from PhD to PI can be a challenging uphill climb, and yet, there a are a multitude of steps one can take to increase the likelihood of securing an independent faculty research position. In this presentation, I will share insights from my path from PhD to PI. I will discuss key activities leading to research independence, including developing a research portfolio, engaging in skill-diversifying career development activities, and building supportive mentor-mentee relationships and networks. I will share my experience preparing for and participating in faculty job interviews, which include finding opportunities, giving job talks, and negotiating. As with research, there are many unknowns and many questions while looking forward to a career as an independent researcher. My goal is to share strategic opportunities and demystify processes to ultimately help others successfully navigate their path to research independence.

Abstract citation ID: igae098.1524

### DETERMINING APPROPRIATE LOAD: BUILDING STRENGTH AND ENDURANCE AS A REHABILITATION RESEARCHER

Allyn Bove, *University of Pittsburgh, Pittsburgh, Pennsylvania, United States*

Physical therapy educational programs typically teach students to dose patients' exercises with the goal of increasing strength or increasing endurance – an "either/or" decision rather than a "both/and" decision. However, clinicians working with older adults know that our patients will be more successful when we help them build both strength and endurance. In this presentation, the presenter will discuss why a strategy of building both strength and endurance also applies to the early years of a research career. The presenter will discuss how she built strength while making pivotal career decisions including whether to pursue research or teaching, whether to pursue post-doctoral training, and how much clinical practice is reasonable to build into one's schedule. Next, the presenter will discuss how she learned (more accurately, 'is still learning') to accept being told "no" most of the time, and how the arduous process of pursuing grants and publishing manuscripts builds endurance. Finally, the presenter will discuss lessons learned as an early career rehabilitation researcher. These lessons include: (1) improving balance is difficult, both as a patient in physical therapy and as an early career researcher; (2) it's okay to be a terrible juggler, as long as one surrounds herself with people who can help her keep all of the balls in the air; (3) how early career researchers may position themselves for careers that are both strong and endurant.

Abstract citation ID: igae098.1525

### A NONTRADITIONAL PATH TOWARD A RESEARCH CAREER

Ketlyne Sol, *University of Michigan, Ann Arbor, Michigan, United States*

I graduated with my PhD in Clinical Psychology, where I received both clinical and research training. I specialized in rehabilitation psychology and received additional training in neuropsychology. I focused the bulk of my graduate training on developing strong clinical skills because I wanted the research I produced to be informed by clinical experience. My research training gave me a strong foundation in research methodology and statistical analysis. But I underestimated the business side of research and the culture of academia overall. When I decided to pursue dedicated research training in disparities in cognitive aging, it had to be a full-time commitment, as it had been with my clinical training. Focused research training helped me progress much better and faster than I would have been able to trying to juggle both a clinical and research career. I became much better at grant writing and at communicating my research. I also believe that no person is an island. So, I also credit my success to organically forming the right combination of formal and informal mentors, peers, and other friendships during each step of my career, along with maintaining relevant pre-existing kin- and friend-ships. I was told early on that a career in clinical research is a marathon, not a sprint, but it took me a bit of time to fully understand that statement. I have finally embraced the marathoner's mindset because I enjoy this work and I intend to produce good research for a very long time.

Abstract citation ID: igae098.1526

**POSTTRAUMATIC GROWTH: REFRAMING JOB MARKET CHALLENGES AND LESSONS LEARNED**

Sara Bybee, *University of Utah College of Nursing, Salt Lake City, Utah, United States*

In this presentation, I will use the phenomenon of posttraumatic growth—the positive psychological change that occurs as a result of the struggle with a highly stressful or traumatic event—to describe my experience searching for, applying, and obtaining a postdoctoral fellowship and later, a faculty position at an R1 institution. The five domains of posttraumatic growth (personal strength, priorities, new possibilities, relationships, and spiritual/existential change) will serve as guideposts as I discuss challenges I have faced, strategies I recommend, and lessons learned from my experiences in the job market. More specifically, I will share tips for identifying open positions, giving job talks and attending campus visits, questions that are important to ask yourself and your potential employer, and what qualities to look for when identifying possible mentors. I will also discuss professional development activities that have been pivotal in my growth, how I used what I learned from these activities to advance my career, and how I built a supportive network both within my institution, as well as across institutions. In sum, this presentation will provide attendees an opportunity to hear about applying and obtaining both postdoctoral fellowships and faculty positions, as well as both positive and negative experiences navigating the job market.

### SESSION 3405 (PAPER)

### RELATIONSHIPS, SOCIAL NETWORKS, AND SOCIAL SUPPORT

Abstract citation ID: igae098.1527

**ASSOCIATIONS BETWEEN MULTIPLE SOURCES OF SUPPORT AND STRAIN AND SELF-PERCEPTIONS OF AGING**

Gina Lee[1], and William Chopik[2], *1. University of Wisconsin Madison, Madison, Wisconsin, United States*, *2. Michigan State University East Lansing, East Lansing, Michigan, United States*

How people perceive the aging process is associated with important health, well-being, and cognition outcomes. For example, people with more positive self-perceptions of aging (SPA) live longer and have better cognition than those with negative self-perceptions (Levy et al., 2002; Tully-Wilson et al., 2021). One underappreciated source of variation in these aging perceptions might arise from our immediate social context—namely, the close relationships we find ourselves in. However, previous research has tested this in a piecemeal fashion, often neglecting the multiple relationships that we inhabit at once. The current study examined changes and variations in SPA and how these perceptions vary according to sources of support and strain from spouses, children, family, and friends. We used random intercept cross-lagged panel models to model within-person changes in SPA and support/strain over twelve years (2008-2020) among 22,160 older adults from the Health and Retirement Study. The results indicated that strain is more closely tied to SPA than support. Spousal support and SPA had positive reciprocal associations. SPA negatively predicted spousal strain, but not vice versa. Interestingly, higher strain from other family members and friends shaped more positive SPA, whereas support from these sources was largely unrelated to SPA. Child support/strain was not associated with SPA. Such findings provide a better understanding of how different close relationships shape aging perceptions. Future studies can test different mechanisms and outcomes underlying each relational contributor of SPA by exploring cognitive, psychological, and/or physiological factors.

Abstract citation ID: igae098.1528

**RELATIONSHIP QUALITY PATTERNS AND DISABILITY IN LATER LIFE**

Madison Sauerteig-Rolston[1], and Kenneth Ferraro[2], *1. Indiana University School of Medicine, Indianapolis, Indiana, United States*, *2. Purdue University, West Lafayette, Indiana, United States*

Informed by the life course perspective and stress process model, this study examined the gendered relationship between patterns of relationship quality (identified as high quality, adverse, indifferent, or ambivalent) and the onset of disability in later life. Cross-sectional and longitudinal models were estimated using nationally representative data from the Health and Retirement Study. Logistic regression models were used to examine cross-sectional associations between relationship quality and wave 1 (W1) disability (i.e., occurrence). Weibull accelerated failure-time models were employed to examine the association between relationship quality and age of onset of disability 12-14 years later (i.e., incidence). We tested for moderation between relationship quality and disability by gender. At W1, older adults in adverse relationships had higher odds of disability than older adults in high quality relationships (OR=1.26, 95% CI=1.11-1.44). In addition, men who reported adverse or indifferent relationships had a higher probability of disability than women in adverse or indifferent relationships (respectively, 2nd differences= 0.029, 0.032). Over time, early onset of disability was associated with adverse ($\beta$ = -0.07, p < 0.001) and ambivalent relationships ($\beta$ = -0.07, p < 0.001). It is important to consider the complex and multidimensional aspects of social relationships to understand how relationships influence later-life disability. Not only are adverse relationships associated with an earlier onset of disability in later life, but ambivalent relationships, those identified by high levels of support and high levels of strain, are detrimental for physical functioning among older adults.

Abstract citation ID: igae098.1529

**WHEN DO FRIENDS MATTER? MODERATORS OF THE ASSOCIATION BETWEEN SOCIAL SUPPORT FROM FRIENDS AND DEPRESSIVE SYMPTOMS**

Xinfang Yu, and Shannon Ang, *Nanyang Technological University, Singapore, Singapore*

With shrinking family sizes in many parts of the world, older adults are beginning to rely more on friendships for support in later life. Because individuals get to choose their own friends (unlike family), the typical assumption is that perceiving more social support from friends is protective of mental health. In this study, we examine if the association between social support from friends and depressive symptoms is contingent on other factors, such as age and support from family. We use three waves of data (2014-2018) from a nationally representative study of older adults aged 60 years and above in China (N = 6,304 person-year observations). We estimated growth curve models with an accelerated longitudinal design, using interaction terms to assess whether age, friends, and family ties moderate the relationship between support from friends and depressive symptoms. The results suggest that the protective effects of friend support on depressive symptoms reduces with increasing age and greater family support. We also found the relationship between social support from friends and depressive symptoms was curvilinear – the direction of the relationship depended on the degree of support perceived. Our findings provide a more nuanced view of the benefits of friendships for psychological well-being, suggesting moderation in the positive effects of friend support despite the common assumption that they are always beneficial. Understanding how social support from friends contributes to their health can help guide efforts to maximize gains and minimize losses that matter most to people as they age.

Abstract citation ID: igae098.1530

### BOUNDARIES, STRUCTURE, AND TURNOVER: A COMPARATIVE ANALYSIS OF OLDER ADULTS' CORE DISCUSSION NETWORKS

Nell Compernolle[1], and Alyssa Goldman[2], *1. NORC at the University of Chicago, Chicago, Illinois, United States*, *2. Boston College, Chestnut Hill, Massachusetts, United States*

Decades of research have collected information about individuals' core discussion networks as a fundamental measure of social integration, with implications for health and well-being. Growing interest in personal network dynamics—additions or losses of key social ties overtime—has sparked recent critiques of the validity and reliability of personal network measurement. Specifically: whether typically-used name generators accurately capture social ties on which individuals ultimately rely in times of need. Here, we compare findings from two longitudinal studies of older adults' personal networks: the National Social Life, Health, and Aging Project (NSHAP; four rounds of data collection spaced five years apart) and the Chicago Health and Aging in Real-Time (CHART; three waves spaced six months apart). Results suggest that oft-cited concerns of egocentric data collection may be less problematic than recently suggested. Regarding boundary specification, older adults in both studies report an average of 3-4 close confidants, with more than half reporting more than five and, among CHART respondents who do, an average of 1.4 additional confidants. Regarding network structure, the two studies yield near identical results in the proportion of core confidants being kin (~70%), co-residing (~20%), average frequency of interaction (a few times a week), and average closeness (very close). Last, regarding network turnover, we observe slightly fewer additions and losses between waves in the CHART study compared to NSHAP, although both show relatively little turnover. Together, findings support the utility of core discussion networks in capturing older adults' core confidants at a given point in time.

Abstract citation ID: igae098.1531

### LINKING SUPPORT PROVISION AND CHANGE IN DEPRESSIVE SYMPTOMS AMONG OLDER ADULTS OVER EIGHT YEARS

Debarati Kole, and Heather Fuller, *North Dakota State University, Fargo, North Dakota, United States*

Research indicates giving support to others positively affects older adults' overall well-being; however, limited research examines longitudinal shifts in associations between older adults' support provision and psychological well-being over time. This study explores links between support provision and changes in depressive symptomatology in older adults over eight years and the moderating role of physical health. Data comes from the Social Integration and Aging Study, a longitudinal, community-based survey of older adults (60+) in the Upper Midwest. Data collection was conducted from 2015 (Wave 2; N= 314) to 2023 (Wave 6; N=98). Support provision was assessed with a 2-item measure of frequency of providing help to family and friends, and depressive symptoms were assessed with the 10-item CES-D scale (Cronbach's Alpha =.82). Longitudinal Multi-Level Modelling was used to assess the time-varying effect of support provision on change in older adults' depressive symptoms across 5 timepoints (controlling for age, gender, marital status, and subjective health). Statistically significant results were found both between-person ($B=-.502$ $p<.001$) and within-person ($B=-.263$ $P=.022$) with higher support provision predicting lower depressive symptoms; however, the interaction of time by within-person help provision ($B=.133$, $P=.017$) indicated support provision has long-term detrimental effects for depressive symptoms. Further, subjective health significantly moderated ($B=.156$, $p<.031$) the between-person association between support provision and depressive symptoms over time indicating increased progression of depressive symptoms for those with poor health/low support provision and good health/high support provision. Overall, findings indicate complex longitudinal relationships between help provision and older adults' psychological well-being.

### SESSION 3410 (SYMPOSIUM)

Abstract citation ID: igae098.1532

### SOCIAL CONTEXTS AND WELL-BEING IN AGING: EXPLORING NEIGHBORHOOD ENVIRONMENT, NETWORKS, FAMILY DYNAMICS, AND LONELINESS

Chair: Hui Liu
Discussant: Deborah Carr

The five papers presented in this symposium utilize data from the National Social Life, Health, and Aging Project (NSHAP) to investigate the impact of social context, encompassing neighborhood environment, social networks, family dynamics, and social isolation, on health and well-being in later life. Ashtiani and Moorman examine the influence of neighborhood factors on loneliness, revealing that the residential environment indirectly affects loneliness through perceived social cohesion and neighborhood characteristics. Li, Wong, and Waite explore the long-term dyadic effects of adverse childhood experiences (ACEs) on later-life social participation, while considering the moderating role of spousal support and strain. Their findings demonstrate that both individual and spousal ACEs correlate with diminished social participation in later life, with spousal effects more pronounced from husbands to wives and further varied by marital quality. Choi investigates the association between marital history and the availability, quality, and stability of older adults' social network ties to their children, uncovering significant impacts of past and present marital experiences on parent-child relationships. Additionally, two other papers employ comparative analyses, drawing from NSHAP and European datasets. Iveniuk, Schafer, and Sun assess the extent to which the link between education and cognition is mediated by social network characteristics, revealing variations in findings across the U.S. and European countries. Azar examines variations in the relationships among loneliness, social isolation, and health across different institutional exposures to defamilization policies in the U.S. and Europe over the life course.

Abstract citation ID: igae098.1533

**NEIGHBORHOOD ENVIRONMENT AND LONELINESS IN LATER LIFE: DO PERCEIVED EXPERIENCES MEDIATE THE OBJECTIVE FEATURES?**

Reza Tayari Ashtiani, and Sara Moorman, *Boston College, Chestnut Hill, Massachusetts, United States*

The health of older adults may be more impacted by their neighborhoods due to limited mobility. Thus, the attributes and quality of the neighborhood environment are crucial for the social life and health outcomes of older individuals. This study aims to examine the role of neighborhood factors in loneliness, investigating associations between objective residential environment, perceived neighborhood cohesion and danger, and loneliness. We used cross-sectional data from 4,377 midlife and older adults (49–95 years) participating in the National Social Life, Health, and Aging Project (NSHAP). We measured the residential environment using a scale based on the interviewer's observation of the neighborhood's quality. The scale included five items: the street's cleanliness, noise, amount of traffic, closeness of houses together, and strong smell or air pollution. We employed multiple regression models. The product of coefficients tests showed that while neighborhood residential environment was not directly associated with loneliness, it was significantly associated indirectly, through both perceived social cohesion (B = 0.006, p <.05) and perceived neighborhood danger (B = 0.010, p <.05). In turn, perceived neighborhood social cohesion was negatively associated with loneliness (B = -0.13, p <.001), whereas perceived neighborhood danger was positively associated with loneliness (B = 0.05, p <.05). These findings underscore the significance of incorporating both objective and subjective elements of neighborhood environments in relation to the experience of loneliness.

Abstract citation ID: igae098.1534

**ENTWINED LIVES: THE DYADIC RELATIONSHIP BETWEEN CHILDHOOD ADVERSITY AND SOCIAL CONNECTEDNESS AMONG OLDER COUPLES**

Yiang Li[1], Jason Wong[2], and Linda Waite[1], *1. University of Chicago, Chicago, Illinois, United States*, *2. Yale University, New Haven, Connecticut, United States*

Adverse childhood experiences (ACEs) have been linked to disadvantages along the life course. However, little is known about how ACEs may affect one's own and spousal social connectedness in later life. Using data from the National Social Life, Health, and Aging Project (N=1,214 couples), we employ the actor-partner interdependence model to estimate the long-term dyadic influence of ACE on later-life community participation and social network structure. We find that ACEs (including early-life family unhappiness, absence of parents in the household, poor health, witnessing/experiencing violence, being born in the U.S. South, and low parental education) are associated with reduced later-life community participation and smaller network sizes. Additionally, we find that the spousal spillover effects of ACEs are more pronounced from wives to husbands in fostering social ties than from husbands to wives in engaging with formal community participation. Our findings highlight the spillover effect of childhood disadvantages on spousal later-life social connectedness and its gendered implications.

Abstract citation ID: igae098.1535

**MARITAL HISTORY AND OLDER ADULTS' RELATIONSHIPS WITH ADULT CHILDREN**

Won Choi, *University of Texas at Austin, Chicago, Illinois, United States*

The diverse marital experiences of older adults today may shape their relationship with adult children. This study examined whether marital status (i.e., continuously married, remarried, divorced, widowed) and marital transitions (i.e., age at first marriage, number of marital dissolutions, time spent unmarried after first marriage) are associated with the availability, quality, and stability of older adults' social network ties to their children. This study used retrospective marital histories and longitudinal egocentric network data from the National Social Life, Health, and Aging Project conducted between 2005 and 2011 (N = 1,873). Multivariate regression models were used to estimate the relationship between marital factors and older adults': (1) likelihood of listing their children as social network members in 2005/2006, (2) frequency of contact with and (3) emotional closeness to child network members, and (4) likelihood of stop listing their children as network members by 2010/2011. Results showed that remarried older adults were less likely to name a child network member, reported less frequent contact with child network members, and were more likely to stop naming their children as network members over time compared to continuously married older adults. Conversely, previously married individuals (i.e., divorced or widowed) were more likely to name a child network member and reported greater emotional closeness to child network members than continuously married older adults. Marital transitions partly explained these differences. The findings highlight the importance of considering past and current marital experiences in understanding parent-child relationships in later life.

Abstract citation ID: igae098.1536

**EDUCATION, SOCIAL NETWORKS, AND COGNITION IN CROSS-NATIONAL CONTEXT**

James Iveniuk[1], Markus Schafer[2], and Haosen Sun[3], *1. The National Opinion Research Center, Chicago, Illinois, United States*, *2. Baylor University, Waco, Texas, United States*, *3. University of Nevada, Reno, Reno, Nevada, United States*

Previous research has shown that older adults with more education report both larger social networks and better cognitive function. But educational institutions vary greatly from country to country, meaning the process whereby education conserves cognition through social networks may vary by national context. In this paper, we bring together data from the National Social Life Health and Aging Project (NSHAP, 4777 cases collected in 2015/2016) and the Survey of Health Ageing and Retirement in Europe (SHARE; 67200 cases across 18 countries collected in 2015) to examine associations between education and cognition cross-nationally, and how much of the association between education and cognition is mediated by social network characteristics (i.e. size and proportion kin). We standardize education using the International Standard Classification of Education 1997. We find that across every country, more education is associated with better cognition. In most countries, education is also positively associated with network size, and negatively associated with proportion kin. Network size is generally positively associated with cognition, and proportion kin is negatively associated with cognition. We find significant indirect effects for network size of in all but four countries (Czechia, Spain, Greece, Israel), with the United States showing one of the largest indirect effect sizes. Overall, the proportion mediated by network size was small across all countries – 7.4% at maximum, and 4.1% across all countries. We also found fewer and weaker indirect effects with proportion kin. We conclude with suggestions for harmonization analysis and implications for networks, international educational stratification, and cognitive aging.

Abstract citation ID: igae098.1537

### COMPARATIVE ANALYSIS OF LONELINESS AND HEALTH: EFFECTS ACROSS DEFAMILIZATION LEVELS

Ariel Azar, *Purdue University, West Lafayette, Indiana, United States*

Although a significant amount of research has addressed the negative relationships of loneliness and social isolation with health in old age, the mechanisms linking them to health outcomes are not fully understood. While some argue that health behaviors may account for these associations, previous research has not adequately contextualized the relationships between loneliness, social isolation, and health. Given the variations in these relationships across different contexts, it is imperative to understand how institutional contexts moderate the detrimental effects of both loneliness and social isolation. This comparative study focuses on European countries and the U.S., utilizing SHARE data for 20+ European countries, ELSA data for the UK, and both HRS and NSHAP data for the U.S. It explores how the relationships between loneliness, social isolation, and health vary across institutional exposure to defamilization policies during the life course. These policies aim to promote women's autonomy and reduce the caregiving burden traditionally placed on them. By leveraging panel data, this paper employs a causal framework to estimate how the effects of loneliness and social isolation on health vary for individuals experiencing different institutional contexts. This research introduces a novel approach to understanding the moderating role of the welfare state, viewing it not only as a resource-transferring institution but also as a facilitator of social networks.

### SESSION 3415 (SYMPOSIUM)

Abstract citation ID: igae098.1538

### TECHNOLOGY FOR SUPPORTING HEALTH AND WELLNESS

Chair: Walter Boot
Co-Chair: Xin Yao Lin

As the population ages, there is a growing need for technology to support the health and wellbeing of older people. However, technology solutions, for them to be successful, need to recognize the needs, preferences, and abilities of older people, and must be evaluated rigorously for efficacy. This talk provides an overview of the potential and pitfalls of using technology to support health and wellness. Hye Soo Lee, PhD, explores the role of technology in supporting Medicare-related decision-making by older adults, highlighting the need for improved training and the consideration of user characteristics to enhance engagement with government-provided digital tools. Paul Freddolino, PhD, MDiv, presents findings from telehealth training sessions with older adults, emphasizing the importance of end-user feedback in refining patient-focused content and increasing patient engagement in healthcare. Justin Lam, BS, discusses the design of age-friendly web interfaces, focusing on optimizing user interaction patterns and enhancing readability to accommodate the nuanced needs of older adults and their care partners. Elena Remillard, M.S., shares insights from deploying 'Telewellness' programs, such as Tele Tai Chi, in community settings, identifying barriers and facilitators to their delivery and discussing the potential of different models to increase access to exercise classes for older adults with mobility disabilities. Leo He, BSc, introduces a novel, radio-based, non-contact system for monitoring nighttime vital signs and sleep, offering a passive, cost-effective solution for home-based health monitoring that avoids the physical and cognitive burdens associated with existing technologies, aiming to improve health intervention and quality of life for older adults.

Abstract citation ID: igae098.1539

### UNDERSTANDING MEDICARE-RELATED DECISION-MAKING BY OLDER ADULTS: ROLE OF TECHNOLOGY SUPPORT

Hye Soo Lee, Raksha Mudar, and Wendy Rogers, *University of Illinois at Urbana-Champaign, Champaign, Illinois, United States*

Decisions related to Medicare carry significant implications for public health, given older adults' reliance on this federal program. Our literature review on Medicare decision-making and technology utilization aimed to investigate how technology could be enhanced to better support older adults. We identified pertinent studies by using combinations of search keywords such as "Medicare", "decision", and "older adults", and by using the information from the collected studies. The findings revealed varying levels of Medicare knowledge among older adults, predominantly acquired through human assistance. Government-provided technology for Medicare was either underutilized or perceived as challenging. The evidence indicated that training in decision-making and technology use could prove advantageous. Factors like stress avoidance and the need for reassurance played roles in the decision-making process and plan selections. Considering older adults' characteristics and preferences in decision-making and technology utilization is crucial for future technological developments, supported by policy and research initiatives.

Abstract citation ID: igae098.1540

### END USER FEEDBACK TO IMPROVE PATIENT-FOCUSED TELEHEALTH TRAINING

Paul Freddolino, *Michigan State University, East Lansing, Michigan, United States*

After older-adult home-delivered meal recipients in a predominantly white, rural Midwest community received tutoring on basic digital skills including safety and security, email, internet browsing, videoconferencing, and photo tools, they were presented with a five-part patient-focused Telehealth 101 mini-course. Content included types of telehealth (real time encounters, remote patient monitoring, and patient portals), what to expect from providers, how to prepare for telehealth encounters, and a live simulation of a telehealth visit. The telehealth mini course was also offered virtually on Zoom to predominantly black groups at five urban senior centers. While participants rated the sessions positively, and patient engagement in healthcare (Patient Activation Measure scores) increased significantly after the training, critical feedback provided insights into how to improve the training. During 2023 the project team worked with six 'co-producers' to get their suggestions and assessments of revisions under consideration for the training materials. Concurrently, revised resources – including two new topics in the telehealth content – were piloted with two predominantly white senior centers and two predominantly black senior centers. Feedback has again been positive and included insights for continuous quality improvement. Our experience shows the contributions and challenges encountered in seeking and engaging end users as co-producers of health-related content.

Abstract citation ID: igae098.1541

### AN AGE-FRIENDLY APPROACH TO THE DESIGN OF WEB-BASED USER INTERFACES

Justin Lam, *Zucker School of Medicine at Hofstra/ Northwell, Hempstead, New York, United States*

Older adults are increasingly adopting digital technologies, with the majority of adults ages 65 and over being Internet users. These technologies can help support older adults' well-being by improving access to and engagement with healthcare information. However, digital accessibility may continue to be an obstacle for adults who experience age-related functional changes, such as loss of vision and hearing or decline in cognitive and motor skills. While guidelines do exist for ensuring accessibility among people with disabilities, these guidelines do not always provide older adults with positive user experiences. A variety of frameworks have been proposed to support age-friendly user engagement with websites and mobile applications, with several centered on improving comprehension of text, location of information, and recognition of access links. In this presentation, we will explore the nuanced needs of users across different age groups, with special consideration given to the design of interfaces that can support both care partners and older adults. This presentation will cover a range of topics, such as optimizing user interaction patterns and enhancing readability, to provide practical insights into the concept of age-friendly design for user interfaces.

Abstract citation ID: igae098.1542

### DEPLOYING TELEWELLNESS PROGRAMS IN THE COMMUNITY: BARRIERS, FACILITATORS, AND POTENTIAL

Tracy Mitzner[1], Elena Remillard[2], and Kara Mumma[2], *1. Person in Design, Atlanta, Georgia, United States*, *2. Georgia Institute of Technology, Atlanta, Georgia, United States*

'Telewellness' programs use technology (e.g., video conferencing software) to facilitate remote participation in exercise classes. These programs hold great potential to expand access to physical and mental health benefits of group exercise classes for older adults, especially those with mobility disabilities. We translated the Tai Chi for Health Institute's in-person, evidence-based tai chi program, Tai Chi for Arthritis, to be a virtual experience (via Zoom) that is both socially engaging and inclusive of people with a mobility disabilities. The 'Tele Tai Chi' clinical trial (NCT04696887) explored to what extent this program may increase social interaction and positive health behaviors among older adults with long-term mobility disabilities; data analysis is in progress. The next phase of this project is focused on delivering our Tele Tai Chi program in real world, community-based settings (e.g., senior centers, senior living communities). This session will present findings from interviews with subject matter experts (e.g., senior center coordinators, administrators from Area Agencies on Aging, tai chi instructors) on perceived barriers to delivering Telewellness classes (e.g., safety, staffing, demands, technical support), as well as potential facilitators to address them. We will also discuss the need for different models of Telewellness (e.g., hybrid classes with attendees both in-person and online) that align with the community's unique characteristics, preferences, and support needs.

Abstract citation ID: igae098.1543

### A RADIO-BASED NONCONTACT MONITORING SYSTEM FOR NIGHTTIME VITAL SIGNS AND SLEEP INSIGHTS

Leo He[1], Yindong Hua[1], Mengjing Liu[1], Zongxing Xie[1], Elinor Schoenfeld[2], and Fan Ye[1], *1. Stony Brook University, Stony Brook, New York, United States*, *2. Stony Brook University School of Medicine, Stony Brook, New York, United States*

A passive method for daily sleep monitoring offers critical insights into health and well-being as variations in sleep patterns and vital signs can indicate the onset or progression of health issues (e.g., stroke, dementia, sleep apnea). Existing technologies are mostly designed for clinical purposes (e.g., polysomnography machines), or impose physical and/or cognitive burdens (e.g., smart watches), rendering them unsuitable for longitudinal, home-based monitoring. We have developed a non-contact, radio-based monitoring system. Our approach utilizes ultra- wideband radio sensors, coupled with sophisticated Region-of-Interest localization (the area of strongest displacement caused by vital signs), vital sign extraction (weighted estimators from the intermodulation and harmonic phase data), and analysis algorithms (body movement removal and sleep stage classification). Our passive, cost-effective, home-based monitoring is free of physical and cognitive burdens, suitable for older adults with physical and/or cognitive limitations and limited expendable income. Our algorithms can identify the trends of vital signs, and offer preliminary differentiation between deep, REM, light sleep, and awake time throughout the night based on our pilot 59 hours' sleep data from 6 subjects. By enabling long-term, continuous observation of sleep patterns and vital signs without any physical interaction, the insights gained from our system hold significant potential for early detection and intervention in health issues, paving the way for a higher quality of life for older adults. To be presented are the methods used to collect these data, results and interpretation of our findings, and the role that such data can play to help with aging in place.

### SESSION 3420 (SYMPOSIUM)

Abstract citation ID: igae098.1544

### THE AGING RESEARCH IN CRIMINAL JUSTICE HEALTH (ARCH) NETWORK: WHERE WE'VE BEEN AND WHERE WE ARE GOING

Chair: Lisa Barry
Co-Chair: Jennifer James
Discussant: Melissa Gerald
Discussant: Fernando Murillo

Established in 2019, the NIA-funded "Aging Research in Criminal Justice and Health (ARCH) Network" brings together a multi-disciplinary, international group of researchers and community leaders dedicated to evaluating the life course experiences of justice-involved older adults and translating that research into policy and practice. In this symposium, we will celebrate the ARCH Network's accomplishments over the past five years by highlighting inspirational investigators, community leaders, and pilot projects that have helped to advance science in this emerging research area. The first presentation will provide a brief overview of the ARCH Network and its mission, highlighting the journey from development to inception to continual growth. The second presentation will focus on the impact of the ARCH Network on the career trajectory of early-stage investigators. From the perspective of a doctoral student, we will learn about how the ARCH network is a pipeline model for advancing the careers of scholars interested in health equity and policy research focused on justice-involved older adults. The third presentation will highlight the practice interventions and community resources that have emerged from health-equity focused, collaborative, aging research on incarceration. The fourth presentation will discuss policy-related impacts and solutions for justice-involved older adults. The final presentation will focus on the impact of the ARCH Network from the perspective of an individual with lived experience and will highlight the vision for the ARCH Network moving forward. Following the presentations, we look forward to an interactive discussion led by the NIA Program Officer for the ARCH Network. Incarceration and Aging Interest Group Sponsored Symposium

Abstract citation ID: igae098.1545

### THE AGING RESEARCH IN CRIMINAL JUSTICE HEALTH (ARCH) NETWORK: AN OVER-ARCH-ING VIEW

Lisa Barry[1], Nickolas Zaller[2], and Brie Williams[3], *1. UConn Center on Aging, Farmington, Connecticut, United States*, *2. University of Arkansas for medical sciences, Little Rock, Arkansas, United States*, *3. University of California San Francisco, San Francisco, California, United States*

Criminal justice-involved older adults have high rates of early onset disease and disability and they experience significant health disparities. Yet, research focused on this population and the consequences of criminal justice involvement on health in later life is deeply under-developed. In response, the Aging Research in Criminal Justice Health Network was established in 2019 and we are excited to celebrate its achievements! We will discuss the process of building this network and how the ARCH Network has made significant strides in promoting the importance of considering geriatric conditions (e.g., functional and cognitive impairment, falls, polypharmacy) when evaluating the health of justice-involved older adults. We will also highlight accomplishments including the significant number of publications championed by the ARCH Network and the successful creation of a GSA Interest Group devoted to Incarceration and Aging.

Abstract citation ID: igae098.1546

### THE GROWING STRENGTH OF THE ARCH NETWORK: INTERDISCIPLINARY COLLABORATIONS ACROSS INSTITUTIONS AND PRISON WALLS

Jennifer James, *University of California San Francisco, San Francisco, California, United States*

The ARCH network has intentionally brought together scholars from different disciplines, clinicians, lawyers, policy makers, advocates, and people with lived experience of incarceration from institutions around the globe. Too often, individuals interested in using research to advance our understanding of health and seek change in policy and practice are siloed, working independently and without the benefits of inter-professional collaboration. Importantly, most health-focused research about incarceration has been conducted at a distance, without directly engaging those who are incarcerated in the formulation of studies, analysis and interpretation, or the dissemination of findings. Given this history, members of the ARCH network conducted community-based research with people with lived experience of incarceration, their loved ones, and their healthcare providers to both identify priorities for aging research and identity barriers and facilitators to collaborating in research. We will describe how the ARCH network has created new possibilities for and understandings of collaborative research across prison walls and speak to some of the emerging research from the network. In particular, we will highlight the policy advancements, practice interventions, and community resources that have emerged from health-equity focused, collaborative, aging research on incarceration.

Abstract citation ID: igae098.1547

### MOVING BEYOND RESEARCH: ARCH AND POLICY

Lauren Brinkley-Rubinstein, *Duke University, Durham, North Carolina, United States*

There is clear evidence that carceral systems negatively impact health, especially for aging populations. The ARCH network has invested in and funded a series of projects that underscore this connection and have clear policy and practice implications. This includes highlighting best practices for compassionate release (both in the context of the COVID-19 pandemic and beyond), creating guidance for the provision of palliative care, optimizing the receipt of correctional health services, outlining the treatment needs of older patients with dementia, and assessing the factors that are integral to implementing a precision-gerontology approach to substance use treatment. In addition, the network has partnered with scholars and practitioners around the country to engage in a series of webinars and "work in progress" meetings to deliver information relevant to best practices, policy guidance, and to brainstorm how best to approach policy change and innovation.

Abstract citation ID: igae098.1548

### ARCH: IMPACTING RESEARCH, IMPACTING CAREERS

Victoria Helmly[1], and Jennifer James[2], *1. Georgia State University, Atlanta, Georgia, United States*, *2. University of California San Francisco, San Francisco, California, United States*

Despite more than 50 years of unprecedented growth in incarcerated populations and the rapidly increasing age of people in prisons and jails, the amount of research and the number of researchers focused on aging and the criminal legal system remains small. For many early career scholars interested in aging, health, and incarceration, supportive mentorship, community, collaboration, and funding opportunities have remained elusive. It is common for scholars studying aging within the criminal legal system to be the only researchers in their institution focused on this critical area. The Aging Research in Criminal Justice Health (ARCH) Network seeks to address this gap by fostering collaboration among researchers and experts in this crucial intersection while providing essential mentorship and support for early career development. Over the last five years, ARCH has created opportunities for Ph.D. students and early-career researchers to contribute to research projects, co-author manuscripts, build networks, hone their skills, and receive feedback on their work. In this session, presenters will share their experience in the ARCH network and how it has impacted their careers through connections and collaborations with mentors and peers. They will describe the ARCH network as a pipeline model for advancing scholars interested in health equity and policy-focused research.

Abstract citation ID: igae098.1549

### THE ARCH NETWORK: LOOKING AHEAD

Fernando Murillo, *Humane Prison Hospice Project, San Francisco, California, United States*

It is clear that the commitment to addressing the pressing public health problem of mass incarceration and aging is growing. With every ARCH Network meeting, there are more of us who care. We even started a GSA Incarceration and Aging Interest Group! This presentation provides the perspective of an individual with lived experience as a pastoral care services worker for terminally ill incarcerated persons. Perspectives such as these are an essential to the mission of the ARCH Network. Looking ahead, we will continue to build new collaborations and partnerships and strengthen those that already exist. We will continue to refine our research agenda. We will continue to disseminate our work to make a difference in the lives of those impacted by the intersection of incarceration and aging. The ARCH Network has much to be proud of, yet we know that there is still even more that we can do.

### SESSION 3425 (SYMPOSIUM)

Abstract citation ID: igae098.1550

### THE CUTTING EDGE OF GERONTOLOGICAL SCHOLARSHIP: THE GERONTOLOGIST'S EDITOR'S CHOICE SYMPOSIUM

Chair: Joseph Gaugler
Co-Chair: Alexandrea Allemann

The mission of The Gerontologist is to publish and disseminate "applied, multidisciplinary research and analysis on social issues related to human aging. It informs the broad community of disciplines and professions involved in understanding the aging process and providing care to older people." In addition, the journal encourages "cutting-edge, conceptually-oriented work that addresses inequities in health, mental health, social status, and justice in late life as well as intersections among these areas with applicability to real-world-settings." This symposium will highlight some of the recent and best examples of gerontological scholarship in The Gerontologist by highlighting select Editor's Choice articles. Andrea Gilmore-Bykovskyi, Ph.D., RN, and colleagues consider the perspectives of family caregivers and hospice clinicians related to episodes of paradoxical lucidity among people living with dementia and how observational approaches may be leveraged to understand this fascinating phenomenon further. Kelly O'Malley, Ph.D., and her co-authors offer a timely and important framework to advance and implement trauma-informed care in long-term care contexts. Ittay Mannheim, Ph.D., and colleagues conducted a fascinating scoping review using critical discourse analysis to determine how and the extent to which ageism is reflected in the design process of digital technologies. Together, these Editor's Choice selections from The Gerontologist "push the envelope" of the science and scholarship of gerontology.

Abstract citation ID: igae098.1551

### "IT'S YOU": CAREGIVER AND CLINICIAN PERSPECTIVES ON LUCIDITY IN PEOPLE LIVING WITH DEMENTIA

Andrea Gilmore-Bykovskyi[1], Clark Benson[2], Jess Fehland[1], Kim Mueller[1], and Laura Block[3], *1. University of Wisconsin Madison, Madison, Wisconsin, United States*, *2. University of Wisconsin School of Medicine and Public Health - Madison, WI, Madison, Wisconsin, United States*, *3. University of Wisconsin-Madison, Madison, Wisconsin, United States*

Episodes of lucidity (ELs), or a transient return of abilities believed to be lost in people living with dementia, are a growing area of interest. These events hold important implications for care, caregiving, and our understanding of underlying etiologies. Research on ELs is largely limited to retrospective reports. The perspectives of professional and family caregivers on ELs and research approaches can inform efforts to define and study lucidity. The present study examined family caregiver and hospice clinician experiences with and perspectives on ELs in people living with dementia and observational approaches to studying these events. This exploratory, descriptive qualitative study employed semi-structured interviews (N=20 caregivers, N=6 clinicians). Data were analyzed using Rapid Identification of Themes and subsequent duplicate review of interview data to enhance trustworthiness. Most participants readily recalled events they perceived as ELs, describing a transient return of abilities they felt was significant and/or meaningful. Defining features, interpretations, and the perceived impact of ELs varied, although ELs were commonly conceptualized as a manifestation of self. Caregivers described extensive efforts to detect patterns and supportive social conditions for ELs. Participants supported use of audiovisual observation to study ELs and provided recommendations for privacy, workflow, and caregiver engagement. Interpretations of ELs are heterogeneous, and recognition of these events may necessitate close familiarity with the person living with dementia. Participants endorse observational approaches and integration of caregivers in this research.

Abstract citation ID: igae098.1552

### TRAUMA-INFORMED CARE IN LONG-TERM CARE SETTINGS

Kelly O'Malley[1], Jennifer Sullivan[2], Whitney Mills[3], Jane "Jenny" Driver[4], and Jennifer Moye[1], *1. VA Boston Healthcare System, Boston, Massachusetts, United States, 2. VA Providence Healthcare System, Providence, Rhode Island, United States, 3. VA Providence Health Care System, VA Providence Health Care System, Rhode Island, United States, 4. VA Boston Healthcare System, Brockton, Massachusetts, United States*

By older adulthood, most older adults will have been exposed to at least one potentially traumatic event. Older veterans are likely to experience three traumatic events during their lifetime, with 93% reporting exposure to at least one event. The prevalence of posttraumatic stress disorder (PTSD) among older adults is lower. Nevertheless, the long-lasting psychological effects of trauma can manifest in later life, exacerbated by the normative experiences of aging (e.g., medical illness, loss of loved ones, retirement) and encounters with medical settings. Receiving care in skilled nursing settings, may trigger traumatic memories or exacerbate PTSD of symptoms. As the population ages, more individuals will receive care in long-term care environments, leading to increased risk of worsening PTSD and re-engagement with past traumatic memories and experiences. Staff in long-term care facilities may not have skills or knowledge needed to address symptoms or reduce re-traumatization (i.e., utilize a trauma-informed care approach). Trauma-informed care is an approach that utilizes knowledge of the effects of trauma to meet survivors' healthcare needs, through collaborative care and recognition of patient strengths in managing their health. This approach is mandated in skilled nursing facilities; however, no models of trauma-informed care practice in long-term care exist. This symposium will review the effects trauma and PTSD in later life, the effects of medical settings on PTSD, and provide a framework for implementing trauma-informed care in long-term care settings.

Abstract citation ID: igae098.1553

### NEW PERSPECTIVES ON EXPLICIT AND IMPLICIT AGEIST DISCOURSES IN DESIGNING DIGITAL TECHNOLOGIES FOR OLDER PERSONS

Ittay Mannheim[1], Eveline Wouters[2], Hanna Köttl[3], Leonieke Van Boekel[4], Rens Brankaert[5], and Yvonne van Zaalen[6], *1. Ben Gurion University of the Negev, Beer-Sheva, HaDarom, Israel, 2. Fontys University of Applied Science, Eindhoven, Noord-Brabant, Netherlands, 3. IMC University of Applied Sciences Krems, Krems an der Donau, Wien, Austria, 4. SOVAK, Terheijden, Noord-Brabant, Netherlands, 5. Eindhoven University of Technology, Eindhoven, Noord-Brabant, Netherlands, 6. The Hague University Of Applied Sciences, The Hague, Zuid-Holland, Netherlands*

Involving older persons throughout the design process of digital technology (DT) is considered a best practice for designing beneficial and acceptable DTs. Nevertheless, negative discourses on aging and ageism are potential underlying factors that could influence which and how DTs are designed and how older persons are involved in the design process. This presentation reflects on previous findings from a scoping review on the explicit and implicit manifestations of ageism in the design process of DT and demonstrates their relevance in new findings. Seven databases were screened for studies reporting on the design of DT with older persons. Sixty articles met the inclusion criteria. Information regarding the study and the discourse about older persons and their involvement in the design process of DT was coded and analyzed using critical discourse analysis. Various explicit and implicit manifestations of ageism, forms of exclusion, errors, and biases in designing DT with older persons were identified. The critical discourse analysis revealed the use of outdated language, stereotypical categorizations, and design decisions based on ageism. Generally, a discrepancy was found between an 'ideal' discourse regarding the involvement of older persons throughout the design process and actual practice. Notably, while the findings of the scoping review focused on publications until January 2020, recent research regarding the design process of DT is reflected upon using these previous findings. Lessons learned for mitigating ageism and promoting meaningful inclusion of older persons in the design process of DT are discussed.

Abstract citation ID: igae098.1554

### ENHANCING DIVERSITY, EQUITY, AND INCLUSION IN QUANTITATIVE STUDIES OF AGE AND LIFE COURSE

Jessica Kelley, *Case Western Reserve University, Cleveland, Ohio, United States*

Despite best intentions to explore and represent the wide diversity of older adults in research, many are limited in their inquiry when utilizing secondary data sources to the the original investigators' choices of measurement or sample inclusion. Limitations among these studies regularly include insufficient sample sizes of small groups, low statistical power, or over-aggregation in measurement. Using race/

ethnicity for our examples, we highlight two key barriers to DEI initiatives in secondary data analyses and their consequences in studying aging and older adults: (1) Erasure of significant populations in aging research; and (2) Utilizing statistical logic to explain social realities. We then provide five immediate solutions that investigators can execute with the data at hand to increase diversity in aging research. These include greater attention to within-group variability, avoid over-interpreting small differences between groups, incorporating cohort membership and structural inequality into analysis, and integrating new measurement modules into ongoing studies.

### SESSION 3430 (SYMPOSIUM)

Abstract citation ID: igae098.1555

### TRANSLATING DATA INTO ACTION ON BRAIN HEALTH

Chair: John Shean
Co-Chair: Erin Bouldin
Discussant: John Shean

The Healthy Brain Initiative (HBI) is a collaborative effort to advance brain health as central to public health in the US. Fundamental to the HBI effort is the HBI Road Map Series, frameworks that help state and local public health departments prioritize actions to address brain health across the life course and support caregivers. Data analysis and utilization are key tenets of the Road Maps Series and are essential to educate the public and to inform policy and planning. In 2023, the Alzheimer's Association piloted Data for Action — a new HBI project to support states in utilizing their existing datasets on brain health and caregiving. This symposium highlights the process, successes and lessons learned from the Data for Action pilot. The first presentation will provide an overview of data available from the Behavioral Risk Factor Surveillance System Cognitive Decline and Caregiving modules. The second presentation will describe the development and structure of the Data for Action pilot project, including the HBI and Road Map Series. In the third presentation, Arkansas, one of the pilot states, will present their process and products from Data for Action. The fourth presentation will summarize the products and impacts from the other states involved in the pilot and will discuss best practices and lessons learned from the project. A discussant will help participants understand how, through collaborative action, brain health and caregiving data can be harnessed to drive decisions, policies, and action at the state level.

Abstract citation ID: igae098.1556

### BRFSS DATA ON COGNITIVE DECLINE AND CAREGIVING: NATIONAL AND STATE IMPLICATIONS

Erin Bouldin, *University of Utah, Salt Lake City, Utah, United States*

The Behavioral Risk Factor Surveillance System (BRFSS) is a state-based annual telephone survey conducted in all US states and three territories. BRFSS includes two optional modules that jurisdictions can use to understand cognitive decline and family caregiving. The Cognitive Decline Module collects information on self-reported changes in memory and thinking. This module offers value in understanding prevalence, with data from the 2016-2021 BRFSS suggesting that 1 in 9 people aged 50 years and older are experiencing subjective cognitive decline nationally. The module also offers the ability to analyze risk factors and health behavior related to detection of cognitive decline. The Caregiver Module collects information on prevalence of caregiving, relationship to the care recipient, the care recipient's health condition, the duration and intensity of care, and caregiving tasks. Data from the 2016-2021 BRFSS show that 1 in 4 caregivers aged 50 years and older in the United States are caregivers for someone with dementia. This module offers value in understanding of caregiver health and health behaviors across a variety of demographics. This session will highlight recent publications and resources, including the Centers for Disease Control and Prevention's publicly available Healthy Aging Data Portal from which these and other data can be analyzed and visualized. Data from these modules has also been used in innovative studies exploring risk factors for cognitive decline, cognitive changes among caregivers, and other findings. The session will demonstrate ways these data can advance the field at the national and state levels.

Abstract citation ID: igae098.1557

### ADVANCING BRAIN HEALTH THROUGH THE DATA FOR ACTION PROJECT

Meghan Fadel[1], Tyrone Bethune[2], Talyah Sands[3], Shelby Roberts[1], Erin Bouldin[4], and Amanda Cheney[5], *1. Alzheimer's Association, Chicago, Illinois, United States*, *2. Association of State and territorial health officials, Arlington, Virginia, United States*, *3. Association of State and Territorial Health Officials, Arlington, Virginia, United States*, *4. University of Utah, Salt Lake City, Utah, United States*, *5. University of Utah, Orem, Utah, United States*

The Healthy Brain Initiative (HBI) Data for Action Project is a nationwide effort to support the integration of data on brain health and caregiving into public health planning efforts. Data about cognitive health, dementia, and family caregiving are increasingly available at the state level, in part due to HBI efforts to support the implementation of the Behavioral Risk Factor Surveillance System (BRFSS) optional modules on Cognitive Decline and Caregiving. This effort has increased state implementation of the Cognitive Decline Module from 5 states in 2009 to 49 states and territories in 2019-2020. Similarly, the Caregiver Module implementation grew from 4 states in 2009 to 49 states and territories in the years 2021-2022. The Alzheimer's Association, alongside partners at the Association for State and Territorial Health Officials (ASTHO), developed the Data for Action Project in 2023 to support state health departments as they use public health data to advance brain health in their communities. In its first year, 4 states participated in the Data for Action pilot by conducting data analyses, producing dissemination tools of the findings, and creating a strategic plan to share the results. States participated in regular cohort calls, provided one another feedback, received individualized technical assistance, and had opportunities to connect through an asynchronous platform. The Data for Action Project created a community of practice with education, peer support and technical assistance for participating health departments.

Abstract citation ID: igae098.1558
### ARE SOCIAL VULNERABILITY INDEX AND COGNITIVE DECLINE RELATED?
Brandy Sutphin, Sharada Sarah Adolph, and Bala Simon, *Arkansas Department of Health, Little Rock, Arkansas, United States*

Alzheimer's disease and related dementias (ADRD) prevalence is demonstrably increasing as more people live to advanced age. ADRD was Arkansas's 7th leading cause of death in 2021 and an estimated 67,000 Arkansans will be living with ADRD by 2025. While known ADRD risk factors hypertension, obesity, smoking, heavy metal exposure, and familial inheritance are well studied, there is minimal research examining the influence of social vulnerability on ADRD patients. We sought to study population-level associations between the Centers for Disease Control and Prevention (CDC), Agency for Toxic Substances and Disease Registry (ATSDR) Social Vulnerability Index (SVI) and cognitive decline, the earliest symptom of ADRD, to identify high-risk areas for interventions. An ecological study design at county-level was utilized. CDC/ATSDR SVI scores $<0.5$ indicate low-to-medium risk and scores $\geq 0.5$ indicate medium-high-to-high risk of social vulnerability. Associations were tested between SVI composite and four component themed indices as predictor variables and Behavioral Risk Factor Surveillance System (BRFSS) cognitive decline prevalence as the outcome variable. Arkansas has 26 high-risk counties out of 75 (34.7%) with combined SVI $\geq 0.5$ and high cognitive decline prevalence ($\mu \geq 12.1\%$). Regression showed unit increases in SVI $\geq 0.5$ resulted in significant increases in cognitive decline prevalence for composite SVI ($\beta=7.67$, $p=0.0185$), and corresponding component SVIs socioeconomic status ($\beta=6.75$, $p=0.0494$), household characteristics ($\beta=9.28$, $p=0.0032$), and racial and ethnic minority status ($\beta=8.98$, $p=0.0458$). Significant ecological associations indicate potential reciprocal influences between SVI and cognitive decline and the need for targeted interventions in high-risk counties.

Abstract citation ID: igae098.1559
### GROWING STATE ACTION THROUGH A COMMUNITY OF PRACTICE
Amanda Cheney[1], John Shean[2], Erin Bouldin[3], and Meghan Fadel[2], *1. University of Utah, Orem, Utah, United States*, *2. Alzheimer's Association, Chicago, Illinois, United States*, *3. University of Utah, Salt Lake City, Utah, United States*

Building a community of practice is a key feature of the Data for Action Project. Working alongside a cohort of peers from other states, teams conduct data analysis related to brain health, dementia, and/or caregiving to produce tools for strategic dissemination of resulting data. Arkansas, Connecticut, Illinois, and New Jersey all participated in, and made important contributions to, the pilot cohort. State projects included analysis and dissemination of data on risk factors, dementia service use, and family caregivers. In several states, teams produced estimates among specific sub-populations or regions in the states to identify at-risk populations. Participating state teams also identified ways the data would most help their efforts, including public health planning, budgeting decisions, and public awareness and information efforts. Having data analysts and representatives from the offices in which data were housed (e.g., BRFSS coordinator) were critical to success, as was including diverse perspectives (e.g., aging, disability, and chronic disease) in developing public-facing products and strategies for dissemination, encouraging collaboration, and maintaining future sustainability of the project. Participants in the program reported increased knowledge of the BRFSS optional modules and of the use of data for health improvement planning. 85% of participants agreed that the program positively impacted their health department's ability to collaborate on integrating brain health and caregiving into public health planning efforts. The second Data for Action cohort launched in 2024, with ongoing efforts to advance brain health in new states.

### SESSION 3435 (SYMPOSIUM)

Abstract citation ID: igae098.1560
### USING FRAILTY SCREENING TO INFORM PREOPERATIVE MANAGEMENT OF HIGH-RISK SURGICAL PATIENTS
Chair: Fred Ko
Co-Chair: Katherine Ritchey

Frailty is a clinical syndrome of age-related decline in physiologic reserve and increased vulnerability to stressors. Frail older adults undergoing surgery are at higher risks for postoperative complications and mortality and increased health care costs. Thus, frailty has emerged as a robust predictor of poor surgical outcomes in vulnerable older patients. There have been immense interest and research for bench-to-bedside translation and integration of preoperative frailty screening into clinical practice. These efforts have centered on the development of preoperative frailty screening as a surgical risk-stratification tool and its application to guide preoperative management aimed to mitigate surgical risk in older adults. The Veterans Health Administration (VHA) has been recognized for its Surgical Pause initiative in which: (1) routine frailty screening is ascertained in surgical patients using the Risk Analysis Index (RAI) in order to identify those at the highest risk of postoperative complications, loss of independence, and mortality; and (2) multidisciplinary care strategies are implemented to reduce frailty-associated risks to enable reduction of potential complications before surgery in those identified as frail. In this symposium, we will provide a state-of-the-art look at the VHA experiences in: (1) validation of RAI as a preoperative risk-stratification tool in older adults; (2) implementation and integration of RAI screening in clinical practice; and (3) evaluation of RAI screening and multidisciplinary optimization board prior to surgery.

Abstract citation ID: igae098.1561
### ORIGINATION, DEVELOPMENT, AND VALIDATION OF THE SURGICAL PAUSE
Jason Johanning, *University of Nebraska Medical Center, Omaha, Nebraska, United States*

Frailty is a validated concept established in the geriatric literature identifying patients (regardless of age) at increased risk of dying within 6 months. Half of veterans older than 65 years seeking colorectal or cardiac surgery are frail or prefrail, markedly increasing the cost of care as well as the risks for postoperative mortality and complications. In this setting the Omaha VA implemented a quality improvement program adopting a 6 month mortality tool for surgical use given the controversy about how best to measure frailty. The results in Omaha were sufficiently impactful that work then focused on replicating and validating the use of the Risk Analysis Index in surgical populations. The survival advantage observed in Omaha was replicated in longitudinal data from 5 University of Pittsburgh Medical Center (UPMC) hospitals in an interrupted time series analysis demonstrating reduction in 1-year postoperative mortality from 20% to 16%, an absolute reduction of 4.2%. And exercise training for as little as 3 weeks before surgery achieved clinically significant improvements in endurance, gait speed and respiratory pressures—changes that are likely to speed recovery and improve outcomes. The model has now been replicated and deployed at over 50 VA Medical Centers as the Surgical Pause initiative as well as multiple academic and private settings. The Surgical Pause and RAI have recently been recognized by the John Eisenberg Patient Safety and Quality Awards as the National Level Innovation winner for 2023.

Abstract citation ID: igae098.1562

### IMPLEMENTATION OF A RISK ANALYSIS INDEX-BASED PREOPERATIVE FRAILTY SCREENING AND MANAGEMENT PROGRAM

Mehraneh Khalighi[1], Amy Thomas[2], Karl Brown[2], and Katherine Ritchey[2], *1. Department of Veterans Affairs Puget Sound Health Care System, Seattle, Washington, United States*, *2. VA Puget Sound Healthcare System, Seattle, Washington, United States*

Frailty reduces recovery from stressors like surgery and increases postoperative morbidity and mortality. Risk Analysis Index (RAI-C) is a validated pre-surgical frailty assessment tool, however the agreement between provider-completed and patient-completed RAI is unknown. We explored the consistency and accuracy of provider-based screening and the agreement between provider-completed and patient-completed RAI-C scores. Orthopedic providers completed the RAI-C on Veterans ≥65 years of age awaiting elective total joint arthroplasty and referred patients to rehabilitative, nutritional, or geriatric specialty services based on RAI-C score. Patients were mailed the same RAI-C form to complete independently. Process measures reviewed the screening frequency and accuracy of using the referral pathway. Agreement between provider-completed and patient-completed total RAI-C score and differences within individual domains were compared. Overall, RAI screening occurred in 48% of eligible patients. Providers were less accurate in their referral of frail (RAI-C 30-36) patients than those who were robust or very frail. Screening frequency improved with dedicated nursing champions reminding providers to screen. The correlation between patient-completed and provider-completed RAI-C was moderate (r=0.634; p<0.001). Patient-completed RAI-C re-classified 29% of patients to a higher frailty category. Agreement was lowest in the domains of memory and weight loss. Preoperative frailty screening is possible with RAI-C but requires dedicated champions to improve consistency and may not yield the same result if completed by patients. Future implementation efforts should explore best practices to improve screening accuracy with the RAI-C tool by providers and patients and how referrals to preoperative services impact surgical outcomes for frail patients.

Abstract citation ID: igae098.1563

### THE PAUSE TRIAL: LESSONS ABOUT SURGICAL PAUSE FROM AN RCT

Shipra Arya, *Stanford University, Stanford, California, United States*

Effective clinical care pathways that integrate preoperative frailty assessment and management to optimize high-risk older adults undergoing surgery are urgently needed to improve their outcomes. We designed a hybrid1 effectiveness implementation step-wedge cluster randomized trial called the Patient-Centered mUltidiSciplinary care for vEterans undergoing surgery (PAUSE) to test frailty screening and referral to a multidisciplinary member board to guide optimization of surgical patients prior to surgery. The PAUSE trial includes Veterans scheduled for elective surgery at 3 tertiary care VAMCs (Palo Alto, Houston, Nashville) and 7 specialties in each center (general, vascular, orthopedics, cardiothoracic, urology, neurosurgery and plastics/ENT). The intervention is referral to a "multidisciplinary PAUSE board" (e.g., surgery, anesthesia, geriatrics, palliative care, case management, rehabilitation and nutrition) for recommendations after frailty screening. Each 'step' was a randomly chosen specialty group transitioning from usual care to the PAUSE intervention. The Consolidated Framework for Implementation Research was used to guide mixed methods formative evaluation and analysis of factors that influence implementation. Key stakeholders identified champions, potential barriers and facilitators, possible intervention adaptations, and indicated limited time, staff shortage, and the concern that the PAUSE board may delay surgery as main barriers to implementation. Nurses were identified as partners in intervention execution. Surgical and nursing leadership engagement was indicated as crucial to adopting this new healthcare model. In conclusion, formative evaluation delivers timely and actionable findings to inform the intervention and identify implementation modifications to optimize opportunities for success. Multistakeholder involvement and accounting for clusters and sites variations is important for successful implementation.

### SESSION 3440 (SYMPOSIUM)

Abstract citation ID: igae098.1564

### VARIATIONS IN FAMILY AND COMMUNITY RESOURCES AMONG DIVERSE AGING IMMIGRANT POPULATIONS

Chair: Fengyan Tang
Co-Chair: Yanping Jiang
Discussant: Lydia Li

Older immigrants rely significantly on family and community resources to address social isolation and related health issues. Yet, the formation and characteristics of these resources may vary within and across diverse aging contexts. Five studies in this symposium provide findings among Older Korean, Mexican, and Chinese Americans. Two studies present findings from the Study of Older Korean Americans (SOKA). Jang and colleagues examined the heterogeneity of family typology and its associations with psychological distress. Five family types were identified: close-knit, intimate but distant, detached, connected but dysfunctional, and dysfunctional. Park and colleagues identified five types of community: safe/integrated, safe/distant, moderate integration, marginal, and vulnerable. Findings showed that living in disadvantaged families and communities was associated with loneliness and mental distress. Using data from the Hispanic Established Epidemiologic Study of the Elderly Caregiver Supplement (H-EPESE CG), Rote examined the role of caregiver background, family and neighborhood support for depressive symptoms among Mexican American caregivers. Findings illustrated that Spanish-speaking caregivers showed a greater increase in mental distress, which was partially explained by lower caregiver resources. Two studies present findings from the Population Study of Chinese Elderly in Chicago (PINE). The objective and subjective aspects of social isolation had differential effects on mental and cognitive health, underscoring the importance of neighborhood resources and support system in buffering the negative health effects of social isolation. Collaboratively, these studies enhance our understanding of the variations in family and community roles across diverse aging contexts and suggest the needs for building resources to promote healthy aging.

Abstract citation ID: igae098.1565

### SOCIAL DISCONNECTEDNESS, PERCEIVED LONELINESS, AND COGNITIVE FUNCTIONING: THE ROLE OF NEIGHBORHOOD ENVIRONMENT

Fengyan Tang[1], and Yanping Jiang[2], *1. University of Pittsburgh, Wexford, Pennsylvania, United States, 2. Rutgers, The State University of New Jersey, New Brunswick, New Jersey, United States*

Social disconnectedness and loneliness pose significant challenges for older Chinese immigrants. This study aimed to investigate the association of social disconnectedness and loneliness with cognitive functioning and examine the moderation role of neighborhood contexts. This longitudinal analysis examined a sample of individuals aged 60 years and older from the Population Study of Chinese Elderly in Chicago (N=2,044). Global cognition was assessed using the averaged z-scores of cognitive performance tests. Social disconnectedness was constructed using five indicators about structural aspects of social relationships. Loneliness was assessed with the R-UCLA loneliness scale. Neighborhood socioeconomic status (NSES) and neighborhood segregation index (NSI) were constructed using 2010-2014 American Community Survey data at the census tract level. Individual perceptions about neighborhood environments were used to construct neighborhood cohesion index (NCI) and neighborhood disorder index (NDI). Latent growth curve models showed that more loneliness was associated with a higher level of initial cognitive functioning (B= 0.030, p<.01), but also with a faster decline rate over time (B= -0.007, p<.01) after adjusting for covariates. High NSES and less neighborhood segregation buffered the negative effects of loneliness on cognitive decline, respectively. High NDI amplified the positive relationship between loneliness and initial functioning, but accelerated the rate of cognitive decline associated with loneliness. The study revealed that perceived loneliness, but not social disconnectedness, is a risk factor for cognitive decline among older Chinese immigrants. Living in a neighborhood with low socioeconomic status, more segregation, and high disorder elevated the detrimental effect of loneliness on long-term cognitive decline.

Abstract citation ID: igae098.1566

### FAMILY TYPOLOGY AND MENTAL HEALTH IN OLDER KOREAN AMERICANS

Yuri Jang[1], Nan Sook Park[2], Juyoung Park[3], and David Chiriboga[2], *1. University of Southern California, Los Angeles, California, United States, 2. University of South Florida, Tampa, Florida, United States, 3. USC Suzanne Dworak-Peck School of Social Work, Los Angeles, California, United States*

Challenging the notion that older adults of Asian origin are universally well protected by family support systems, we examined the heterogeneity of family types, considering both structural and qualitative aspects and addressing both positive and negative functions. The aims of the study were (1) to identify types of family relationships and (2) to examine their impact on mental health. Data came from 2,070 participants in the Study of Older Korean Americans (SOKA), a multistate survey of Korean immigrants aged 60 and older (Mean Age = 73.3). To identify family types, latent profile analysis (LPA) was performed with marital status, living arrangement, family network, positive and negative interactions with family members, and family mistreatment. Linear regression models examined how mental distress was associated with family typology. LPA identified five family types: close-knit, intimate but distant, detached, connected but dysfunctional, and dysfunctional. Greater distress was associated with belonging to the detached, connected but dysfunctional, and dysfunctional family types in comparison to the close-knit type. Findings not only confirmed the value of a close-knit family but also demonstrated the heterogeneity of family typology. For example, the connected but dysfunctional group is uniquely characterized by those who are structurally embedded in a family system with marriage, co-residence, and contacts but had experienced negative interactions and even mistreatment within the family. This group demonstrates that obligated family ties in the absence of emotional bonding could be harmful. Our findings called renewed attention to older immigrants' family relationships and their mental health implications.

Abstract citation ID: igae098.1567

### A PERCEIVED COMMUNITY TYPOLOGY IN OLDER KOREAN AMERICANS: IMPLICATIONS FOR MENTAL HEALTH

Nan Sook Park[1], Yuri Jang[2], Jeongsuk Kim[1], Jung Eun Ko[3], Soondool Chung[4], and David Chiriboga[1], *1. University of South Florida, Tampa, Florida, United States, 2. University of Southern California, Los Angeles, California, United States, 3. Kyung Hee Cyber University, Seoul, Republic of Korea, 4. Ewha Womans University, Seoul, Republic of Korea*

The health and well-being of older immigrants can be profoundly influenced by the neighborhoods in which they live. Considering that the physical and social characteristics of neighborhoods have the potential to either alleviate or exacerbate individuals' mental health, the aims of this study were: (1) to identify perceived community typologies in older Korean Americans; and (2) to examine how these typologies are associated with feelings of loneliness and mental distress. We hypothesized that distinct community groups would be identified and that they would be differentially associated with mental health indicators as well as with background characteristics.

Data were drawn from a survey with older Korean Americans aged 60 and older, collected during 2017–2018 in diverse locations (N = 2,138). To identify community typologies, a series of latent profile analysis (LPA) were conducted using 15 community-related variables in the three domains (neighborhood characteristics, social cohesion, ethnic attachment). After examining characteristics of the identified groups in relation to the study variables, hierarchical multiple regression models of loneliness and mental distress were estimated. Based on model evaluation criteria, an LPA model with five community groups was identified as the best fit. The five groups were identified as "safe/integrated" (10%), "safe/distant" (10%), "moderate integration" (38%), "marginal" (31%), and "vulnerable" (11%). Using the safe/integrated group as reference, the marginal and vulnerable groups were consistently associated with elevated feelings of loneliness and mental distress. The results suggest the need to understand profiles of community characteristics and their relationships with health/well-being among older immigrants.

Abstract citation ID: igae098.1568

### SOCIAL ISOLATION AND DEPRESSIVE SYMPTOMS OF OLDER CHINESE AMERICANS: THE MEDIATION EFFECT OF ACTIVITY ENGAGEMENT

Ke Li[1], Fengyan Tang[2], Soonhyung Kwon[3], and Yanping Jiang[4], *1. University of New Hampshire, Durham, New Hampshire, United States*, *2. University of Pittsburgh, Wexford, Pennsylvania, United States*, *3. University of South Florida, Tampa, Florida, United States*, *4. Rutgers, The State University of New Jersey, New Brunswick, New Jersey, United States*

Older Chinese Americans are particularly vulnerable to social isolation and its negative consequences on mental health. However, little is known about the intermediate processes through which social isolation contributes to depressive symptoms among this population. The aims of the study were (1) to examine the differential impacts of two forms of social isolation (social disconnectedness and perceived isolation) on depressive symptoms and (2) to investigate the mediating role of activity engagement. Data came from 2,075 participants in four waves of the Population Study of Chinese Elderly in Chicago Study (PINE). Social disconnectedness was measured by social network size and range, living arrangement, and marital status. Perceived isolation was measured by the perception of loneliness and lack of social support. Activity engagement was assessed by engagement with various social and cognitive activities. Results from the latent growth curve models indicated that social disconnectedness and perceived isolation were positively associated with the initial level of depressive symptoms. However, perceived isolation predicted a faster decline in depressive symptoms. The findings suggested the significant mediating effect of activity engagement. Social disconnectedness was associated with greater activity engagement, which in turn was associated with a lower initial level of depressive symptoms. Perceived isolation was associated with lower activity engagement, subsequently resulting in a higher initial level of depressive symptoms and a faster decline of depressive symptoms across waves. Intervention targeting depression among older Chinese Americans should make efforts on providing more opportunities for culturally sensitive activity engagement and social interaction and promoting social support system.

Abstract citation ID: igae098.1569

### A LONGITUDINAL STUDY OF MEXICAN AMERICAN CAREGIVER DEPRESSIVE SYMPTOMS OVER FIVE YEARS

Sunshine Rote, *University of Louisville, Louisville, Kentucky, United States*

The use of different domains of informal support (e.g., neighborhood and family support) as a protective factor for Latino caregiver mental health has received little attention. The purpose of this study is to examine the role of caregiver background (that is, caregiver and care recipient relationship type, language usage), living arrangements, and family and neighborhood support for depressive symptoms among Mexican American caregivers over five years of data. Using two waves of the Hispanic Established Epidemiologic Study of the Elderly Caregiver Supplement (H-EPESE CG, 2010/2011-2016, N=333) and informed by the sociocultural caregiver stress process model, logistic and OLS regression analyses of caregiver depressive symptoms were specified. While depressive symptoms were relatively low at both waves, there was a greater increase in depressive symptoms among caregivers who completed the interview in Spanish (n= 229, 69%) compared to those completing it in English (n= 104, 31%). This increase was partially explained by lower caregiver resources (e.g., support from others). The findings demonstrate the need for dementia care supports for Mexican American caregivers, improving support systems for Spanish-speaking caregivers.

### SESSION 3450 (SYMPOSIUM)

Abstract citation ID: igae098.1570

### A LOOK AT STUDENT CAREGIVING EXPERIENCES: OPPORTUNITIES FOR CAMPUSES AND COMMUNITIES

Chair: Allyson Graf
Co-Chair: Katherina Nikzad-Terhune
Discussant: Joseph Gaugler

Caregiving in the United States continues to be a significant and growing phenomenon that warrants ongoing exploration of ways to support caregivers. Further examining the impact that intergenerational connections and age-inclusive practices have on the caregiving experience can help inform ways for both campuses and communities to be more responsive to caregiver needs. This symposium features participants representing universities that have capitalized on the Age-Inclusivity in Higher Education (AIHE) model to generate new and creative forms of caregiver support and intergenerational connections. These research endeavors represent the distinct ways that the AIHE model can be utilized to recognize and respond to the diverse needs of caregivers on campus and in the community. Terhune/Graf/Manning (Northern Kentucky University) will discuss mixed-methods data on student caregivers and how this knowledge has informed new student caregiving initiatives both on campus and with community partners. Hawes (University of Wisconsin-Eau Claire) will present a synthesis of intergenerational programs conducted in nursing homes between 2020 and 2024. Drawing from 25 student applied projects in select facilities, the research aims to identify best practices and challenges with implementing intergenerational efforts in nursing homes. Elfenbein (University of North Georgia) will present information about ACAPcommunity and ACAP Hall County, bringing students and community caregivers together to learn about caregiving issues and resources, and support one another on the caregiving journey. Graf/Baker/Bartlett (Northern Kentucky University) will elaborate on different ways that caregiving experience may associate with outlooks on caregiving, personal aging and health by comparing current, former, and indirect caregivers.

Abstract citation ID: igae098.1571

### RESPONDING TO THE DIVERSE NEEDS OF STUDENT CAREGIVERS: RECOMMENDATIONS FOR CAMPUSES AND COMMUNITY PARTNERSHIPS

Katherina Nikzad-Terhune[1], Allyson Graf[2], and Lydia Manning[3], *1. Northern Kentucky University, Lexington, Kentucky, United States*, *2. Northern Kentucky University, Highland Heights, Kentucky, United States*, *3. Dele Health Tech, Downers Grove, Illinois, United States*

Informal caregiving continues to steadily increase, warranting attention to the impact of being a caregiver. A growing demographic of caregivers that have received less attention are student caregivers. Currently, five million adult students attending post-secondary education are also caregivers of adults, resulting in negative impacts on academic success, mental health, and retention. Furthermore, the characteristics and experiences of student caregivers are diverse, thus prompting a need to better understand their experiences. Rooted in the age-inclusivity in higher education model, this presentation examines mixed methods data from a campus-wide study that assessed the prevalence and impact of unpaid caregiving on students. Survey results indicated that 16.9% (n = 241) of student respondents identified as a caregiver. Scores on the Adult Carers' Quality of Life scale (Elwick et al., 2010) revealed that lack of support (46-48.8%) and financial matters surrounding caregiving (25.7-27.2%) place student caregivers at risk for low quality of life. Qualitative data from focus groups (N = 12) provided further explanation and clarification of the data. We found that student caregivers encompass a range of challenges, emotions, and coping strategies. The experiences of student caregivers highlight the need for greater recognition, understanding, and support within educational institutions. Based on the study findings, specific recommendations will be made for universities to better recognize the diverse needs of their student caregivers, and how to respond with support, resources, and empowerment. Additionally, recommendations for partnering with community agencies in the pursuit of supporting student caregivers will be highlighted.

Abstract citation ID: igae098.1572

### STUDENT-LED INTERGENERATIONAL PROGRAMS IN NURSING HOMES

Frances Hawes[1], and Inessah Cernohous[2], *1. University of Wisconsin Eau Claire, Eau Claire, Wisconsin, United States*, *2. University of Wisconsin Eau Claire, Eau Claire, Wisconsin, United States*

Recognized for fostering meaningful interactions across age groups, intergenerational programs offer numerous benefits, promoting social connections, empathy, and mutual understanding. This study compiles data from 25 intergenerational programs conducted by undergraduate students during their residency at nursing homes from 2020 to 2024. The aim is to pinpoint best practices and challenges in implementing such programs. The findings provide comprehensive summaries of the projects, detailing site contexts, methodologies, and results. We evaluate the effectiveness of these programs in various areas, including enhancing care quality, reducing social isolation, and improving the psychological well-being of nursing home residents. Furthermore, our analysis yields a consolidated understanding of both the successes and challenges encountered in intergenerational initiatives during this period, offering valuable guidance for future program development and implementation.

Abstract citation ID: igae098.1573

### CREATING AN INTERGENERATIONAL COMMUNITY OF CAREGIVERS

Pamela Elfenbein, *University of North Georgia, Oakwood, Georgia, United States*

While the preponderance of those caregiving for older adults are over the age of 45, the 2020 Caregiving in the U.S. study, conducted by AARP and The National Alliance for Caregiving (NAC), revealed that 11 percent of Americans who are caring for older adults are also attending school either full-time or part-time. Adult Children of Aging Parents (ACAP) Hall County invites students, staff, faculty and community caregivers of all ages to meet monthly for information, resources, and support, building a community to enhance the quality of life for area caregivers and their older adult loved ones. Details of the program will be shared to enable presentation participants tp consider the development of a similar program on other campuses. This presentation aligns with AFU Principle 4:To promote intergenerational learning to facilitate the reciprocal sharing of expertise between learners of all ages.

Abstract citation ID: igae098.1574

### A COMPARISON OF DIRECT AND INDIRECT STUDENT CAREGIVERS ON OUTLOOKS RELATED TO CAREGIVING AND SELF

Allyson Graf, Shelby Baker, and Robin Bartlett, *Northern Kentucky University, Highland Heights, Kentucky, United States*

Studies have recently demonstrated the impact of caregiving on college students. Caregiving, however, is transitional and dynamic and this complexity is rarely represented. The current study sought to compare caregiving experiences (direct-current, direct-former, and indirect care support) on the perceived impact on views of caregiving, relationships, well-being, and aging. Among the sample of 230 students (78.7% undergraduates) with caregiving experience, 28.3% were current caregivers, 53.5% were former caregivers, and 78.6% provided indirect care support. Compared to those with indirect experience, those with direct experience more strongly agree that caregiving impacted their views of caregiving ($p < .001$) and their relationship with care recipient ($p < .001$). There was no difference between current and former caregivers. Participants were also asked about perceived positive and negative influences independently. Results were similar for positive influences with direct caregivers perceiving a more positive influence on their relationship with the care recipient than indirect caregivers ($p = .001$) but only current caregivers differed significantly from indirect caregivers in the positive influence on views of caregiving (p =.023). Trends did not hold for perceived negative influence; however, there was a significant difference between current and former caregivers on the negative influence on health (p =.042) such that current caregivers indicated a greater negative influence. These results indicate the importance of investigating diverse caregiving experiences among students and differentiating positive and negative influences with implications for how universities may support the different life circumstances of student caregivers.

### SESSION 3455 (SYMPOSIUM)

Abstract citation ID: igae098.1575

### ACCESSIBLE, AFFORDABLE, AND SUSTAINABLE TRANSPORTATION INNOVATIONS FOR MOBILITY AND SAFETY AMONG OLDER ADULTS

Chair: Anu Siren
Discussant: Judith Charlton

While technology holds immense promise in addressing challenges related to safety, mobility, and sustainability in older adults' transportation, significant barriers remain, particularly concerning the acceptability, affordability, and age-friendliness of these technologies. This symposium will feature presentations and discussions highlighting the multifaceted nature of mobility among older adults and the ways in which innovative technologies can address the transportation needs. The five presentations in this symposium communicate research from Australia, Europe, and the USA, and will explore issues related to usability, accessibility, and user acceptance, highlighting the importance of designing technologies that are intuitive, inclusive, and culturally sensitive. Speakers of this symposium will delve into the importance of mobility for older adults' physical health, mental well-being, and social connectedness. Further, the symposium will examine the potential of technology-driven solutions to address key challenges faced by older adults in transportation, including safety concerns, accessibility barriers, and environmental sustainability. By fostering interdisciplinary dialogue and international collaboration, the symposium seeks to create multidimensional discussions that not only acknowledge the pivotal role of technology in addressing safety, mobility, and sustainability challenges for older adults but also address the issues of acceptability, affordability, and age friendliness. Transportation and Aging Interest Group Sponsored Symposium

Abstract citation ID: igae098.1576

### SUPPORTING AGING IN PLACE THROUGH NEW MOBILITY SOLUTIONS

Fatemeh Hatami[1], Anu Siren[1], and Steve O'Hern[2],
*1. Tampere University, Tampere, Pirkanmaa, Finland*,
*2. University of Leeds, Leeds, England, United Kingdom*

Aging in place is among the most widespread contemporary policy concepts. However, an important prerequisite for aging in place is the possibility to attend out-of-home activities, which requires satisfactory mobility options. Previous research has indicated that new transportation solutions and technologies may hold promise for older people's mobility needs. To investigate these promising opportunities and how they may support aging in place, a critical literature review was conducted. Using Web of Science, Scopus, and PubMed as electronic journal databases, we searched keywords representing the user group (older adults), the solution (transport systems), and its qualities (novel), limiting the search to publications between 1990-2023. The search was complemented by hand-searching of reference lists and selected project homepages. The initial search yielded more than 3000 publications. After screening for title, abstract and contents, around 110 publications were included for review. To create a conceptual model, we synthetized major findings, and identified common themes, trends, and disparities in the existing literature. We analyzed whether proposed solutions are evidence based and whether the literature focuses on implementation processes. We also analyzed which mobility needs the solutions are explicitly targeting, and which groups of older adults are thought to be the main users of these solutions. Our conclusion is that while literature addresses many novel solutions that have potential for supporting older adults' mobility, there are significant knowledge gaps related to assessing the feasibility of these solutions.

Abstract citation ID: igae098.1577

### UNDERSTANDING TRANSPORT NEEDS OF OLDER AUSTRALIAN DRIVERS: SUSTAINABLE AND SMART INITIATIVES FOR SAFE MOBILITY

Jennie Oxley, Judith Charlton, Sjaan Koppel,
David Logan, and Selby Coxon, *Monash University, Melbourne, Victoria, Australia*

Access to adequate and appropriate transport options enables older people to continue as thriving community participants, to reach services and to maintain social connections. While transport needs are diverse, and tend to change over time, there is little information on current and future transport patterns, and the awareness, acceptance and adoption of new technologies. A national online survey was administered to current drivers in Australia. A sample of 705 drivers provided information on available travel modes and use of these modes, awareness of in-vehicle technologies and future use of vehicle technologies. The findings revealed high use of private vehicles, walking and taxis but little use of other travel modes (bicycles, motorcycles, rideshare, community services and public transport). Age, gender and residential location influenced the availability and use/potential use of some transport options. Overall awareness of in-vehicle technologies was generally low and particularly so amongst older and female participants. There was some appetite to use emerging technologies in the future. The findings inform the development of effective strategies and initiatives aligned with healthy ageing and wellbeing targets, increased sustainability, resilience and connectedness, creation of healthier travel choices and healthier environments to promote acceptance and use of a range of transport options and uptake of safer vehicles equipped with in-vehicle technologies to ultimately enhance safe and sustainable mobility of older road users. Such strategies may be particularly useful to support extended safe driving amongst older adults with chronic medical conditions, such as driver monitoring/alert systems.

Abstract citation ID: igae098.1578

### FACTORS RELATED TO THE LIKELIHOOD OF AN ELECTRIC VEHICLE PURCHASE AMONG OLDER US ADULTS

Jonathon Vivoda[1], and Renée St. Louis[2], *1. Miami University, Oxford, Ohio, United States*, 2. *University of*

*Michigan Transportation Research Institute, Ann Arbor, Michigan, United States*

Widespread acceptance of electric vehicles (EVs) is important to meet global energy and pollution goals. Previous studies established that older adults are less likely than younger people to consider purchasing an EV, and common barriers to EV uptake among this group have been identified, but little else is known. The current study used two waves of data from the Pew Research Center's American Trends Panel (April, 2021 and May, 2022), which contained EV-related questions. Only older adults who responded to both waves were included (n=2,016). Two ordinal logistic regression models were utilized to identify factors related to older adults' likelihood of purchasing an EV (measured on a four-point scale), and to a change in likelihood between waves. Odds of higher likelihood of a future EV purchase were related to more education; Asian and Hispanic race (vs White); more liberal political ideology; stating that scientists understand causes of global climate change well; favoring the phase-out of gasoline vehicles; as well as stating that EVs are more fun to drive, more reliable, or better for the environment. Stating that EVs cost more was related to a lower likelihood, as were the reverse of the EV factors above. Only three factors were related to a change between waves in likelihood of purchasing an EV. Higher odds were related to identifying as a Democrat (vs Republican), high income, and having heard 'a little' about EVs vs 'a lot.' Findings, implications, and future research suggestions will also be discussed.

Abstract citation ID: igae098.1579

### EMERGING TECHNOLOGY AND OLDER ADULTS: DEVELOPING A CONNECTED AND AUTONOMOUS VEHICLE TRAINING PROGRAM

Renée St. Louis[1], Lisa Molnar[2], David Eby[2], Jennifer Zakrajsek[1], and Nicole Zanier[1], *1. University of Michigan Transportation Research Institute, Ann Arbor, Michigan, United States*, *2. University of Michigan, Ann Arbor, Michigan, United States*

Mobility is vital for the well-being and quality of life of older adults. Emerging technologies, such as connected and automated vehicles (CAVs), hold promise for extending safe and independent mobility for older adults, however, challenges remain in ensuring that CAVs are accessible, acceptable, affordable, and otherwise inclusive for older adults. While personal vehicles with Level 2 automation are available, the public's exposure to more advanced levels of automation remains limited. The first phase of this research examined benefits and barriers to using CAVs using a two-phase qualitative approach consisting of four 90-minute nominal and focus group sessions. During the introduction, participants were shown a short video defining CAVs and were asked to think about vehicles with Level 3-5 automation during the discussions. Results revealed a strong desire among older adults for hands-on CAV experiences to increase perceived safety and trust in these new technologies. Based on these findings, a second phase of research involved the development of a CAV training program for older adults to be tested at Mcity, an autonomous vehicle testing facility. The training program consists of an informational overview combined with a hands-on orientation to the location and operation of various advanced vehicle technologies and automation features, using a vehicle with Level 3 automation. During the orientation, participants are shown icons associated with the technologies, and their function, capabilities, and limitations are demonstrated. Detailed findings from both phases of the research will be discussed as well as their implications for future mobility options for older adults.

Abstract citation ID: igae098.1580

### OVERCOMING TECHNOLOGY BARRIERS: A US ENTERPRISE'S JOURNEY IN COVID VACCINE ACCESS FOR OLDER ADULTS IN SOUTHWEST OHIO

Cleopatra Kum[1], Flavius Kum[2], Crescentia Kum[3], and Mirabelle Kum[3], *1. University of Cincinnati, Cincinnati, Ohio, United States*, *2. Family Support Care LLC, Hamilton, Ohio, United States*, *3. KumZ International Medical Foundation, Douala, Littoral, Cameroon*

It is no hidden fact, that mobility and transportation, especially among seniors and people with disabilities can play a significant role in social isolation and access to basic healthcare and other needs. The importance of safe and effective transportation options was highlighted during the COVID 19 pandemic, when government agencies and community partners worked in collaboration to propose innovative solutions, to ease access to vaccines for seniors through reliable transportation. Family Support Care LLC (DBA KumZ Transport), a Medicare certified home healthcare agency in Ohio, answered the call when one of the Area Councils on Aging reached out to its network of providers to seek agencies willing to transport seniors to receive their COVID 19 vaccines. The purpose of this presentation is to discuss the lived experience of Family Support Care LLC with the use of technology while transporting older adults to get their COVID 19 vaccines. Decisions on technology use was a very important part of the planning phase. Because transportation and logistics management were new to the agency, we faced several barriers as we implemented technology in aging transportation. As a family-owned small business, access to resources and manpower were a major challenge. As a result, we relied heavily on transportation technology for improved productivity and reliability. Despite these challenges, we learned and implemented valuable lessons as we collaborated with more transportation brokers. Technology played a vital role for our agency in planning rides for seniors during the COVID 19 pandemic.

### SESSION 3460 (SYMPOSIUM)

Abstract citation ID: igae098.1581

### ADVANCING GERONTOLOGICAL HEALTH EQUITY: INNOVATIONS AND INSIGHTS FROM HISPANIC SERVING INSTITUTIONS

Chair: Elizabeth Vasquez
Discussant: Patricia Heyn

In response to the Biden-Harris administration's significant investment in Hispanic Serving Institutions (HSIs), this symposium showcases groundbreaking research and projects that address critical public health and gerontological issues within these communities. This symposium highlights four innovative, multidisciplinary approaches that demonstrate the effectiveness of community-engagement research, clinical studies, and health promotion in addressing the needs of older adults. Firstly, Dr. Jung-Ah Lee's presentation delves into community engagement projects led by Latino nursing students and faculty at the University of California, Irvine.

These projects emphasize compassionate, hands-on initiatives aimed at improving the health and well-being of Latino older adults in Southern California, including in-home caregiving education and support for caregivers of people living with dementia. Next, ERASE Falls project, spearheaded by Dr. Pappa at Marymount University, exemplifies the expansion and scalability of evidence-based fall prevention programs. This initiative has significantly impacted Northern Virginia's diverse older adult population, demonstrating the power of community partnerships and innovative training models to enhance fall prevention efforts. Next, Dr. Suriaga's research provides critical insights into the association between drug intoxication and the co-use of opioids, cannabis, and alcohol among Hispanics in Florida. This work highlights the urgent need for targeted research to understand and address substance use and its effects within Latino communities. Lastly, Alcantar work underscore the essential role of HSIs in fostering gerontological health equity through research, education, and community engagement. Attendees will gain a comprehensive understanding of challenges and opportunities in gerontological research conducted by HSIs researchers and the importance of culturally sensitive approaches. Hispanic Serving Institutions Interest Group Sponsored Symposium

Abstract citation ID: igae098.1582

### LATINO COMMUNITY ENGAGEMENT PROJECTS FOR OLDER ADULTS LED BY LATINO NURSING STUDENTS AND FACULTY

Jung-Ah Lee, *University of California Irvine, Irvine, California, United States*

The Latino population in California has experienced a continual increase in recent decades. As of 2022, the United States Census Bureau estimated that 15.73 million Latinos resided in California, constituting 39.4% of the state's total population. This presentation aims to introduce ongoing community engagement projects conducted by bilingual Spanish/English speaking nursing students and faculty, and their impact on the health of Latino older adults and their families in Southern California. The school of nursing is situated in major cities housing the state's largest Latino communities, with compassionate community engagement as one of its core missions. The nursing faculty has consistently developed community-based projects through the curriculum and research, providing extensive one-on-one mentoring to groups of Latino students for several years. Key initiatives targeting the Latino older adult population include: (1) Nursing Skill Education Sessions for In-Home Supportive Service (IHSS) Caregivers, offering essential care to Medicaid older adults with chronic conditions, (2) In-home caregiving education for Latino family caregivers of people living with dementia (PWD), and (3) Telephone support projects for isolated Latino caregivers of PWD during the pandemic crisis. Under the close supervision of faculty with geriatric expertise, Spanish-speaking nursing students actively engaged with Latino older adults and their families through the projects. Spanish-speaking IHSS caregivers appreciated the hands-on learning opportunities in nursing skills with Spanish educational materials. Participating students expressed their compassion to serve and support the community. Continued emphasis on Latino community-centered education and research projects is essential to meet the evolving needs of the community.

Abstract citation ID: igae098.1583

### EXPANDING REACH AND SCALABILITY OF EVIDENCE-BASED FALL PREVENTION PROGRAMS (ERASE FALLS)

Sara Pappa, *Marymount University, Arlington, Virginia, United States*

In 2016, Marymount University was awarded the first Evidence-Based Falls Prevention Programs (EBFPP) cooperative grant by the Administration for Community Living, initiating the Northern Virginia Falls Prevention Alliance. Subsequent funding supported the Academic-Community Partnership for Falls Prevention, addressing the critical need in Northern Virginia—a diverse region with over 250,000 older adults. From 2016 to 2021, our programs reached 5,060 individuals across 75+ sites, through initiatives like Stay Active and Independent for Life (SAIL), A Matter of Balance (AMOB), and the Otago Exercise Program (OEP). We established the innovative MU Regional Training Office (MU-RTO) for affordable EBFPP leader training and support, and have trained over 500 lay leaders in these EBFPP. We adapted to remote delivery during the COVID-19 pandemic, and founded the Northern Virginia Falls Prevention Alliance (NVFPA), growing it to over 135 members. As a founding member of the Virginia Arthritis and Falls Prevention Coalition (VAFPC), we've contributed majorly to regional falls prevention. Our efforts include significant research developments, such as the large data evaluation of EBFPPs, housed at the Marymount Center for Optimal Aging (MCOA). The MCOA focuses on aging research with an emphasis on minority inclusion and community-participatory approaches.

Abstract citation ID: igae098.1584

### DRUG INTOXICATION AMONG LATINO OPIOID USERS IN FLORIDA

Armiel Suriaga, Ruth Tappen, Raquel Panos, and Christina Dimeo, *Florida Atlantic University, Boca Raton, Florida, United States*

The opioid epidemic remains a serious public health problem, killing > half a million people since 1999, including racial/ethnic groups, i.e., Hispanics. However, Drug Intoxication (DI) from opioid use, particularly in Florida, where the Hispanic population totaled 5.469 million in 2020, is seldom reported. We aimed to report the characteristics of Hispanic decedents with DI, particularly opioids and other substances, from 2020 to 2021. We also examined the association of being Hispanic to DI and the co-use of opioids, cannabis, and alcohol. We used descriptive statistics and regression models to analyze de-identified data from the Florida Department of Law Enforcement using Stata 17. 1,199 Hispanic decedents out of 30,846 people who died from any drugs were analyzed. Mean age=39.14 (SD 13.89); males (n=1,015 or 84.7%) between the 25-34 age group (n=339 or 34.9%), followed by the 35-44 age group (n= 306 or 25.5%); only a small proportion of older Hispanic decedents (n=58 or 4.8%). 551 out of 1,199 Hispanic decedents died from DI. Being Hispanic was not significantly associated with DI (p=.053). In the intercept model, being Hispanic has 0.87 odds of dying from DI (OR=.87, [95% confidence interval=.77-.98], p=0.018. After adjusting for confounders (age, gender, population density, and other substances, the odds of dying from DI slightly increased (OR=.89, [95% CI =.76-1.03], p=0.117; adding clinical covariates (cardiac, diabetes, kidney, and liver-related problems) OR=.85, 95% CI=.74-.99], p=0.038. Among opioid users (OR=16.46, 95% CI=15.47-17.50], p=<0.001. Public safety necessitates more research to elucidate factors driving DI among this ethnic minority.

Abstract citation ID: igae098.1585

### IMPORTANCE OF ASSESSING FOR POSTTRAUMATIC STRESS DISORDER IN OLDER HISPANICS

Sandy Alcantar, *Los Angeles County Department of Mental Health, Los Angeles, California, United States*

Hispanic older adults are at a higher risk of being diagnosed with Alzheimer's disease or Dementia versus post-traumatic stress disorder due to various factors, one lacking assessment. There are significant factors associated with aging and PTSD in the Hispanic population that must continue to be explored; these include, but are not limited to, a lack of collaboration with community providers, linguistics, and an understanding of assessing for histories of post-traumatic stress disorder. Providers need to evaluate for trauma in Hispanics at this stage of life, especially for those who suffered trauma in their country of origin and post-immigration to the United States. The objective is to reduce misdiagnoses of Alzheimer's Disease/Dementia in Hispanics by developing and implementing a solid community partner collaboration and not disregarding assessing for post-traumatic stress disorder in a person's history. As providers, we must communicate effectively in an interdisciplinary approach to review findings during assessments and promote structural social changes, such as education, addressing assessment practices, using effective evidence-based techniques, and reviewing treatment recommendations periodically.

### SESSION 3465 (SYMPOSIUM)

Abstract citation ID: igae098.1586

### ASSESSING AGE INCLUSIVITY USING THE AGE-FRIENDLY INVENTORY AND CAMPUS CLIMATE SURVEY

Chair: Jacqueline Eaton
Co-Chair: Katarina Friberg-Felsted
Discussant: Susan Whitbourne

During a time of increased focus on diversity, equity, and inclusion in institutions of higher education, age inclusion remains largely unexplored. The Age-Friendly Inventory and Campus Climate Survey (ICCS) is a tool that institutions of higher education can use to identify strengths and weaknesses specific to age-friendliness. This instrument measures age inclusivity by comparing age-friendly practices with student, staff, faculty, and lifelong learners' perceptions of these practices. This symposium explores the process of using the ICCS at four different universities to understand the methods used to target each institution's unique needs, with the goal of improving age-friendliness in higher education: 1) Zimmer et al., present the outcomes of using the ICCS to establish a baseline of age-friendliness at the University of Calgary and the process to generate action items from the results to develop a plan to enhance age-friendliness. 2) Waterhouse et al., describes the process of customizing the ICCS to meet the unique needs of an academic medical campus at the University of Colorado Anschutz Medical Campus. 3) Zisberg et al., report on the experience of translating the ICCS into Hebrew and the subsequent results implementing the tool at the University of Haifa. 4) Eaton & Friberg-Felsted explore the use of the ICCS within the College of Nursing at the University of Utah to identify ways to meet the learning needs of age diverse groups. Dr. Susan Whitbourne, from the team that developed the ICCS, will facilitate discussion surrounding age inclusivity in higher education.

Abstract citation ID: igae098.1587

### USING THE AGE-FRIENDLY INVENTORY AND CAMPUS CLIMATE SURVEY AT A MAJOR CANADIAN UNIVERSITY

Chantelle Zimmer[1], Lindsay Morrison[1], Maya Goerzen[1], David Hogan[1], Ann Toohey[1], Jennifer Hewson[1], Meghan McDonough[1], and Gwen McGhan[2], *1. University of Calgary, Calgary, Alberta, Canada*, *2. Faculty of Nursing, University of Calgary, Calgary, Alberta, Canada*

Since the University of Calgary became a member of the AFU Global Network, establishing a benchmark of the institution's age-friendliness has been a priority for the Brenda Strafford Centre on Aging. The Age-Friendly Inventory and Campus Climate Survey was selected for our baseline assessment because it captures both objective and subjective elements of age-friendliness that align with the AFU principles. Prior to data collection, the instrument was modified for our national and institutional context by adding, removing, and changing the language of some items. The Age-Friendly Inventory was completed by administrators to determine the current status of our campus practices and environmental features, while the Campus Climate Survey was completed by faculty, staff, and students to understand their awareness and perceptions of these practices and features. A total of 10 administrators, 178 faculty, 608 staff, and 1167 students participated in the assessment. The results indicated that our university is moderately age-friendly, but participants were generally unaware of its age-friendly elements. To generate action items from the results, we analyzed the data further. Each inventory and survey item was mapped onto the AFU principle it most closely aligned with and then grouped with similar items to form categories for each principle. Through this process we identified areas of strength and growth for each principle, and prioritized principles (1, 4, 6, and 10) for our Centre to address. The findings from this study will inform an action plan to raise awareness of and enhance the University of Calgary's age-friendliness.

Abstract citation ID: igae098.1588

### ASSESSING AGE INCLUSIVITY USING THE AGE-FRIENDLY INVENTORY AND CAMPUS CLIMATE SURVEY

Jodi Waterhouse, *CU Anschutz Multidisciplinary Center on Aging, Aurora, Colorado, United States*

University of Colorado Anschutz Medical Campus became designated as an Age-Friendly Academic Medical Campus in 2021. However, the campus is part of a much larger campus community which includes a large health system, a children's hospital, and a VA Medical Center, that are not Age-Friendly designated. More than 30,000 employees, visitors, and vendors visit the Anschutz campus daily. How does an academic medical campus, surrounded by three large health system hospitals and clinics ensure that visiting is easy, enjoyable, and accessible, and visitors don't get lost in other campus partner's buildings? The Age-Friendly

Inventory and Campus Climate Survey helped provide the framework for guidance to identify issues including parking, parking payment kiosks, signage, and campus maps both virtual and print. This session will provide how a large academic medical campus and its representatives were able to identify and personally experience problematic hurdles when visiting a large university campus; what Built Environment Assessment experience was collected to identify issues; and how recommendations for improvement were presented to and supported by campus leadership.

Abstract citation ID: igae098.1589

**ASSESSING AGE INCLUSIVITY AT THE UNIVERSITY OF HAIFA: LEADING ISRAEL'S FIRST AGE-FRIENDLY CAMPUS**

Anna Zisberg, Orit Hirsch-Matsioulas,
Sigal Naim, Adi Sapir, Yael Hochman, Chaya Koren, and
Lola Kolpina, *University of Haifa, Haifa, Hefa, Israel*

With life expectancy increase, higher awareness of ageism, and the need to reduce inequalities by ensuring that education and employment opportunities are open to all ages, the Age-Friendly University (AFU) was initiated at the University of Dublin, Ireland in 2012, and has since spread to other countries. The University of Haifa, located in a city with a higher proportion of senior citizens, compared to the national average in Israel, is leading the first AFU in Israel. We aim to present a comprehensive assessment of the prevailing campus environment and its inclusivity towards individuals of all ages. As an initial measure, we embraced the Age-Friendly Inventory and Campus Climate Survey (ICCS) questionnaire (Silverstein et al., 2022). After translating into Hebrew, we made cultural and technical adaptations and conducted validation with a panel of five experts. The survey was disseminated across the campus, garnering responses from 1500 participants. Analysis revealed generally positive attitudes towards the integration of older individuals within the campus community, although there was insufficient progress in various domains. Notably, the incorporation of ageing-related topics in teaching and research emerged as deficient. Study findings serve as a basis for developing educational programs for older learners, as well as for the reorientation of university policies towards age inclusivity. Furthermore, they provide recommendations for fostering age-friendly environments through policy formulation and organizing social-cultural initiatives on campus and beyond. In addition, the preliminary validation of the survey instrument suggests its suitability for implementation in non-English-speaking academic contexts.

Abstract citation ID: igae098.1590

**ASSESSMENT OF AGE INCLUSIVITY: RESULTS FROM A RESEARCH-INTENSIVE COLLEGE OF NURSING**

Jacqueline Eaton[1], Katarina Friberg-Felsted[1],
Summer Martin[1], Rebekah Perkins[1], Kara Dassel[2], and
Sara Hart[1], *1. University of Utah, Salt Lake City, Utah, United States*, *2. University of Utah, Salt Lake Cty, Utah, United States*

Age inclusivity is crucial for meeting the needs of an aging population. However, in higher education, age-stratification reinforces categorization of traditional and nontraditional students. This reduces the ability to educate and prepare trainees for work with older adults upon graduation. Seeking to improve educational opportunities at the University of Utah College of Nursing, a needs assessment was conducted using the Age-Friendly Inventory (AFI) and Campus Climate Survey (CCS). The outcomes of the CCS assessment are reported here. All faculty, staff, and students in the College of Nursing were invited to access the CCS e-survey via email or QR code. The survey was available during the months of October and November 2023. During this time, the research team used e-flyers, presentations at college meetings, and invitations from administration to increase response rate. Data was managed using Qualtrics and analyzed using descriptive statistics to identify perceptions of age-friendliness and beliefs about age inclusivity. A total of 247 individuals completed the survey. Students made up the majority of participants (63.6%); nineteen percent were faculty and 17% were staff. The majority (53%) were unfamiliar with the age-friendly university initiative. Ageism was seen as a major problem in society by 80.8% of the sample, yet only 29.4% believe it to be a problem on campus. Overall, 14.7% percent perceived our campus as age-friendly, which implies a need to increase knowledge about age inclusivity and age-friendly practices. Strategic planning is underway to outline specific initiatives to improve perceptions and practices within the College of Nursing.

### SESSION 3470 (BIOLOGICAL SCIENCES INVITED SYMPOSIUM)

Abstract citation ID: igae098.1591

**AGING AND THE MICROBIOME**

Chair: Shuo Han

This 90-minute session will explore key interactions between aging and the microbiome leveraging studies in animal models and humans. The three invited talks will discuss the following diverse topics in microbe-aging interactions: 1) functional roles of gut bacteria and small molecules in host aging and physiology in C. elegans and mice (Han); 2) gut microbial and metabolic changes across lifespan and how these changes impact brain, cardiovascular, and offspring health (McCullough); and 3) gut microbiome contributions to age-associated cognitive decline, inflammation, and immune functions in older adults, based on the outcomes from the Microbiome in Aging Gut and Brain consortium study (Yadav).

Abstract citation ID: igae098.1592

**DECIPHERING THE CHEMICAL LANGUAGE OF GUT BACTERIA IN AGING AND LONGEVITY**

Shuo Han, *Duke University School of Medicine, Durham, North Carolina, United States*

The human gut microbiota encodes diverse metabolic pathways, where gut microbes make numerous compounds that are relevant to human health and hold untapped therapeutic potential. Fecal microbiota transplantation has been shown to delay age-associated decline in mouse and fish models. However, how specific gut bacteria and their metabolites impact host physiology represent a new frontier that remains to be fully explored. Leveraging our expertise in the gut microbiome and aging biology, we tackle this underexplored area from both the microbial and host's perspectives. Specifically, our lab seeks to 1) mechanistically characterize gut bacteria and bioactive small molecules in host aging and physiology, and 2) identify gut microbial-dependent, host cellular mechanisms underlying organismal health-span and lifespan. We aim to understand how gut bacteria mechanistically contribute to host aging and longevity and to identify new molecular targets to delay age-associated decline.

Abstract citation ID: igae098.1593

### AGE RELATED CHANGES IN THE MICROBIOME AND THEIR IMPACT ON DISEASE

Louise McCullough, *McGovern Medical School at UTHealth, Houston, Texas, United States*

Trillions of microbes live symbiotically in the host, specifically in mucosal tissues such as the gut. Recent advances in metagenomics and metabolomics have revealed that the gut microbiota plays a critical role in the regulation of host immunity and metabolism, communicating through bidirectional interactions in the microbiota-gut-brain axis (MGBA). The gut microbiota regulates both gut and systemic immunity and contributes to the neurodevelopment and behaviors of the host. Disruption in the balance of gut microbial communities (often referred to as "dysbiosis") has many detrimental effects in the host including gut and brain inflammation. Reversing these changes may have therapeutic potential. With aging, the composition of the microbiota changes, and emerging studies have linked these shifts in microbial populations to age-related neurological diseases (NDs). Preclinical studies have demonstrated that gut microbiota-targeted therapies can improve behavioral outcomes in the host by modulating microbial, metabolomic, and immunological profiles. We will summarize the role of gut microbiota and microbial metabolites across the lifespan and in disease. We will highlight recent studies investigating 1) microbial changes with aging; 2) how aging of the maternal microbiome can affect offspring health; and 3) the contribution of the microbiome and its metabolites to outcomes after experimental ischemic stroke.

Abstract citation ID: igae098.1594

### MICROBIOME AND AGING: INSIGHTS INTO COGNITIVE HEALTH FROM THE MIAGB CONSORTIUM

Hariom Yadav, *University of South Florida, Tampa, Florida, United States*

World population is aging, and aging-related conditions are on the rise, with no better prognosis, prevention, and treatment. Because we do not fully understand the complex biology of aging and its dysfunctional attributes, it contributes to aging-related conditions. Emerging evidence shows that the gut microbiome plays a critical role in the biology of aging and has thus been proposed to be one of the targets for Geroscience-based interventions. However, we still lack understanding of how the microbiome contributes to aging and its related disorders. To address these knowledge gaps, a collective effort of several institutions has evolved a multi-center clinical study called Microbiome in Aging Gut and Brain (MiaGB), which has focused on developing large datasets on the microbiome and its related pathways, including metabolomics, proteomics, epigenomics, immunophenomics, and others, to understand how the microbiome contributes to aging biology, thus impacting brain health and overall health linked with aging. Dr. Yadav will present the outcomes of the MiaGB consortium addressing how the microbiome contributes to aging-related cognitive decline by impacting intestinal epithelial barriers, inflammation, and immune functions. He will also emphasize whether and how reversing these microbiome-related abnormalities can ameliorate aging-related disorders in older adults.

### SESSION 3475 (SYMPOSIUM)

Abstract citation ID: igae098.1595

### BLACK MEN'S HEALTH AND AGING ACROSS THE LIFE COURSE

Chair: Roland Thorpe
Co-Chair: Carl V. Hill

Much of the research on Black men tend not to focus on concepts that have been found to be important determinants of health for Black men. Nor, do they seek to identify sources of resilience that may be protective of health. This symposium contains a collection of papers that discuss some key social determinants of health (SDOH) that can provide insights to advance our understanding of Black men's health and aging. Bruce and colleagues examined the association between religious practices and/or beliefs, socioeconomic status, and cognitive status among Black men in the 2016 Health and Retirement Study (HRS). Findings indicate private prayer was inversely related to any cognitive impairment among Black men with more than 12 years of education. Nwaskasi and colleagues examined the effect of active coping and grit on subjective cognitive decline (SCD) among Black men and found that active coping and grit were significant predictors of SCD while controlling for demographic variables. Esiaka and colleagues investigated the relationship between food insecurity and cancer screening behaviors among Black men dwelling in food deserts. Understanding the link between food insecurity and cancer screening behavior provides crucial insights for tailoring targeted interventions for Black men. Thomas Tobin and colleagues investigated the link between psychosocial resilience and allostatic load in Black men in the Nashville Stress and Health Study. Results from latent class analyses suggest different resource combinations produce distinct patterns of resilience among Black men. These presentations will bolster our knowledge on SDOH among Black men across the life course.

Abstract citation ID: igae098.1596

### RELIGION, SOCIOECONOMIC HETEROGENEITY, AND COGNITIVE STATUS AMONG BLACK MEN

Marino Bruce[1], Bettina Beech[1], Dylan Tran[1], Baasil Kaka[1], and Roland Thorpe, Jr.[2], *1. University of Houston, Houston, Texas, United States*, *2. Johns Hopkins University, Baltimore, Maryland, United States*

Risks for cognitive impairment are disproportionately high among Black men, however religious practices and beliefs have been shown to be protective for members of this group with modest incomes, suggesting that social class can complicate this association. The purpose of this study was to examine the association between religious practices and/or beliefs, socioeconomic status (SES), and cognitive status among Black men in the 2016 Health and Retirement Study (HRS). Data were drawn from 384 Black men who completed the Core and Leave Behind Questionnaires. The primary outcome was any cognitive impairment, a dichotomous variable derived from a modified version of the Telephone Interview for Cognitive Status (TICS). Religious practices and beliefs (private prayer, religious beliefs) and SES (income, education) were primary independent variables. Descriptive results indicate that individuals reporting incomes less than $50,000 had a higher proportion of any cognitive impairment than respondents reporting higher incomes (48.2% vs 19.1%). Differences in any cognitive impairment were also found between respondents reporting 12 or fewer years of education and individuals reporting more than twelve years of education (48.8% vs 19.8%). Data from modified Poisson regression models indicated that personal private prayer (PR=0.67, CI:0.47-0.97) and religious beliefs (PR=0.92, CI:0.84-1.00) were beneficial for Black men with modest incomes. Private prayer was inversely related to any cognitive impairment among Black men with more than 12 years of education (PR=0.51, CI:0.27-0.99). Full-scale studies of Black men are needed to further examine how SES can influence factors that may be protective against cognitive impairment among this population.

Abstract citation ID: igae098.1597

### EXAMINING THE ASSOCIATION BETWEEN ACTIVE COPING, GRIT, AND SUBJECTIVE COGNITIVE DECLINE IN AGING BLACK AMERICAN MEN

Candi Nwakasi[1], and Darlingtina Esiaka[2], *1. University of Connecticut, Storrs, Connecticut, United States*, *2. University of Kentucky College of Medicine, University of Kentucky, Kentucky, United States*

**Background:** Active coping and grit have been associated with positive health outcomes including cognitive functioning. However, to the best of our knowledge, no previous study has examined the effect of active coping and grit on subjective cognitive decline among Black American men. Further, this study's focus on subjective cognitive decline (SCD) in aging Black men is crucial because SCD may indicate an onset of Alzheimer's disease and related dementia and/or other brain health issues.

**Methods:** Participants (N=138, Mage = 48) who identified as Black American men living in urban areas responded to an online survey measuring sociodemographic factors, grit, active coping, and subjective cognitive decline. We used hierarchical regression in the analysis to examine the association between active coping, grit, and subjective cognitive decline.

**Results:** Analysis revealed that active coping (B = -0.196, p=.037) and grit (B = 0.643, p<.001) were significant predictors of SCD while controlling for demographic variables (F [7,130] = 7.753, p<0.001). Also, moderation analysis showed a significant interaction between active coping and grit on SCD (β = -0.04, SE = 0.02, 95%CI [0.00,0.08], p <.041).

**Conclusion:** By focusing specifically on the association of active coping, grit, and SCD, this study offers insight on the unique experiences of Black American men and contribute to a more nuanced understanding of their cognitive well-being. Thus, the results may inform targeted interventions and support systems for aging Black men's brain health.

Abstract citation ID: igae098.1598

### FOOD INSECURITY AND CANCER SCREENING BEHAVIORS AMONG BLACK MEN LIVING IN FOOD DESERTS

Darlingtina Esiaka[1], Obinna Odo[2], and Kingsley Udeh[3], *1. University of Kentucky College of Medicine, University of Kentucky, Kentucky, United States*, *2. Miami University, Oxford, Ohio, United States*, *3. Miami University, Oxford, Ohio, United States*

Research shows an intricate relationship between food insecurity and health behaviors. The relationship reflects the profound impact of inadequate access to nutritious food on overall well-being. Individuals facing food insecurity often encounter barriers to adopting health-promoting behaviors, influencing their physical health outcomes. This study investigated the relationship between food insecurity and cancer screening behaviors among Black men dwelling in food deserts, examining a critical intersection of health disparities. Participants (N=186; Mean age = 48.45, SD = 10.95) responded to questions assessing their demographic characteristics, experience with food insecurity, health risk behaviors, healthcare access, and current and future cancer screening behaviors. We found a significant association between food insecurity and current cancer screening behaviors (B=-.118, p=0.043) but not between food insecurity and future cancer screening behaviors (B=-.048, p=0.329). Black men experiencing food insecurity were less likely to engage in cancer screening compared to their counterparts with more reliable access to nutritious food. Also, we found that socioeconomic status (B=-.163, p=0.006), medical insurance (B=-.672, p=0.019), and access to primary care physician (B=-.609, p=0.026) were significantly associated with future cancer screening behaviors. Understanding the link between food insecurity and cancer screening behavior provides crucial insights for tailoring targeted interventions that consider the unique challenges faced by individuals living in food deserts, particularly within the context of racial and ethnic health disparities.

Abstract citation ID: igae098.1599

### WHEN RESILIENCE BECOMES RISK: PSYCHOSOCIAL RESOURCE PROFILES AND ALLOSTATIC LOAD AMONG AFRICAN AMERICAN MEN

Courtney Thomas Tobin[1], Angela Gutierrez[2], Christy Erving[3], Keith Norris[1], and Roland Thorpe[4], *1. University of California Los Angeles, Los Angeles, California, United States*, *2. Western University of Health Sciences, Pomona, California, United States*, *3. The University of Texas Austin, Austin, Texas, United States*, *4. Johns Hopkins University, Baltimore, Maryland, United States*

There is a well-established link between psychosocial risks and psychological health among African American (AA) men. The psychosocial sources and physical health consequences of resilience (i.e., the ability to maintain good health despite adversity) remain underexplored. Using data from 283 AA men in the Nashville Stress and Health Study, the present study investigated the links between psychosocial resilience and allostatic load (AL), a biological indicator of physiological dysregulation.

Latent class analysis (LCA) identified distinct resilience profiles comprised of eight psychosocial resources across four categories: coping strategies, sense of control, racial identity, and social support. Analysis of variance (ANOVA) tests determined significant class differences in men's AL scores. LCA results confirm a four-class model was the best fit: Class 1 (high resources, 32%); Class 2 (high coping but low control, 13%); Class 3 (low resources but high racial identity, 20%); and Class 4 (low resources but high mastery, 34%). Results reveal lower AL (better health) among Classes 4 (m=0.31) and 1 (m=0.35), and higher AL among Classes 2 (m=0.44) and 3 (m=0.44). Findings indicate that the "quality" rather than the "quantity" of psychosocial resources matters for physical health among AA men, as positive health outcomes were observed among both low and high resource classes. Results suggest different resource combinations produce distinct patterns of resilience among AA men and underscore the need to further elucidate complex resilience processes among this population.

### SESSION 3480 (SYMPOSIUM)

Abstract citation ID: igae098.1600

### DYNAMICS IN INTERGENERATIONAL FAMILY RELATIONSHIPS IN THE CHINESE CONTEXT

Chair: Tianyuan Li
Co-Chair: Helene H. Fung
Discussant: Christiane Hoppmann

Various intergenerational family relationships play essential roles in maintaining older adults' well-being and promoting successful aging. The intergenerational family relationships are particularly important in the Chinese culture as it has a long tradition emphasizing familism values. This symposium consists of four studies which investigate the dynamics of intergenerational family relationships in the Chinese context at different stages of later adulthood. The intergenerational relationships covered range from the relationship between aging parents and their adult children (Jiang et al.), grandparents providing care to young grandchildren (Hu et al.), the relationship between grandparents and adult grandchildren (Li et al.), and grandparents receiving care from family members (You et al.). The results jointly support the importance of the bidirectional support between the older and younger generations and demonstrate how the balance of support evolves over time. Chinese Gerontology Studies Interest Group Sponsored Symposium

Abstract citation ID: igae098.1601

### TRANSMISSION OF GENERALIZED TRUST AND BELIEF IN A JUST WORLD BETWEEN GRANDPARENTS AND ADULT GRANDCHILDREN

Tianyuan Li, Yue Yang Sun, Xiaoye Liu, and Yukai Zhang, *The Chinese University of Hong Kong (Shenzhen), Shenzhen, Guangdong, China (People's Republic)*

Previous research shows that older adults tend to hold more positive beliefs about others and the social system compared to their younger counterparts. This study investigated whether such positive beliefs can be transmitted to the younger generation through intergenerational interactions. We recruited 292 Chinese adult grandchild-closest grandparent dyads and surveyed both members' generalized trust and belief in a just world. Results revealed significant positive associations between grandparents' and grandchildren's generalized trust, $\beta$ =.21, SE =.05, p <.001, and belief in a just world, $\beta$ =.20, SE =.07, p =.004. While the intergenerational association of generalized trust was not moderated by other variables, that of belief in a just world was moderated by the grandchild's familism, F(1, 279) = 4.46, p =.04, and parental intimacy, F(1, 279) = 7.94, p =.005. The intergenerational transmission of belief in a just world was not significant for the grandchildren with lower levels of familism, $\beta$ =.02, p =.79, or parental intimacy, $\beta$ =.02, p =.81; but was significant for those with higher levels of familism, $\beta$ =.28, p =.003, or parental intimacy, $\beta$ =.35, p <.001. Demographic variables were controlled in all the analyses. The findings underscore the importance of intergenerational transmission of positive social beliefs from grandparents to grandchildren, suggesting that older adults may play an essential role in cultivating social capital.

Abstract citation ID: igae098.1602

### EMPATHY MODERATES THE RELATIONSHIP BETWEEN RECIPROCITY AND STRESS AMONG AGING PARENTS AND CHILDREN

Da Jiang, Zewen Huang, and Xinyuan Peng, *The Education University of Hong Kong, Hong Kong, China (People's Republic)*

Previous studies examining the relationship between being under-benefitted and stress among aging parents and their adult children have yielded mixed findings. Few studies have examined whether this positive association can be alleviated by state-level or trait-level factors. Given the positive effects of empathy on interpersonal exchanges, we tested the moderating role of empathy on the relationship between being under-benefitted and perceived stress among aging parents and adult children in this 14-day diary study. A sample of 99 pairs of parents (Mage=50.01, SDage=4.53; 79.8% female) and children (Mage=22.38, SDage=3.49; 85.9% female) were recruited to report their level of being under-benefited in the exchange with their parent/child, perceived stress, and empathy as an affective state on a daily basis for 14 consecutive days, after completing a pretest which measured their trait empathy and demographic information. For both parents and children, the positive under-benefited-stress association was only significant when they reported lower empathy as a daily affect. The under-benefitted-stress link was negative when children reported greater daily empathy. Children reported more perceived stress on the days their parents reported a greater level of being under-benefitted. Such association was only significant in children with lower trait empathy. The negative association between children's being under-benefitted and parents' perceived stress was only significant in parents with higher trait empathy. These findings highlight the importance of empathy as a daily affect and a trait in the relationship between daily exchanges and mental health in the intergenerational contexts between aging parents and adult children.

Abstract citation ID: igae098.1603

### INTENSIVE GRANDCHILD CARE AND SUCCESSFUL AGING: THE ROLE OF INTERNAL MIGRATION IN CHINA

Yue Hu[1], Helene H. Fung[1], Neil Charness[2], Jason Wong[3], and Yiqi Wang[1], *1. The Chinese University of Hong Kong, Hong Kong, China (People's Republic)*, *2. Florida State*

*University, Tallahassee, Florida, United States*, *3. Yale University, New Haven, Connecticut, United States*

Around two thirds of adults aged 60 years or above are providing grandchild care in China, some of whom even migrate to where their adult children live to provide grandchild care. However, it still remains unclear whether and how grandchild care provision impacts successful aging among migrant or non-migrant grandparents. This study examined whether grandchild care provision contributed to successful aging via increasing proactive coping among two groups. A questionnaire survey was conducted among 301 migrant or non-migrant grandparents who provided non-custodial grandchild care (mean age 61.2 ± 4.8, 158 female) and currently lived in a tier-1 city in China. Results showed that grandparents who provide more non-custodial care to their grandchildren experience more successful aging. Migrant grandparents are less able to rely on their proactive coping when providing intensive grandchild care as compared to non-migrant grandparents. Theoretically, this study is the first to examine the mechanism underlying the association between grandchild care provision and successful aging, contributing to the literature on positive gerontology by examining successful aging in specific contexts. Practically, findings suggest the potential guidelines to improve grandparents' well-being. Extra support should be provided to migrant grandparents to foster their proactivity in coping with the predictive challenges of intensive grandchild care provision in terms of policy.

Abstract citation ID: igae098.1604

### INFORMAL CARE NETWORK CHARACTERISTICS AND PSYCHOLOGICAL WELL-BEING AMONG CHINESE OLDER ADULTS

Jin You[1], Xinyue Shen[1], Yue Dong[2], and Ziyi He[3], *1. Wuhan University, Wuhan, Hubei, China (People's Republic)*, *2. Queensland University of Techonology, Brisbane, Queensland, Australia*, *3. University of Washington Seattle, Seattle, Washington, United States*

With population ageing, China is susceptible to heavy burdens related to caring for older people. With rural-to-urban migration, the availability of traditional family care has substantially declined. This study investigates the characteristics of informal care networks and their associations with mental health outcomes (i.e., depressive symptoms, life satisfaction) from social network perspective among Chinese middle-aged and older adults with care needs. Results from five waves (2011, 2013, 2015, 2018, and 2020) of the China Health and Retirement Longitudinal Study showed that, among 11,949 informal care receipts aged over 50 years, 62.83% received care from a spouse, 49.55% from children or grandchildren (with the mean size of 2.65), 3.78% from relatives (with the mean size of 3.61), and 5.13% from nonrelatives. The size of family care network (number of spouse, children or grandchildren, relatives), especially the number of children or grandchildren, predicted fewer depressive symptoms and greater life satisfaction. By contrast, care network diversity (number of care sources) predicted lower life satisfaction. Our findings suggest that Chinese older adults largely rely on and might psychologically benefit from a multiple-caregiver family network rather than on a single primary caregiver to address care needs. Further efforts should be made to understanding task sharing function, effective organization, and dynamic development of informal care network.

### SESSION 3485 (SYMPOSIUM)

Abstract citation ID: igae098.1605

### EXPLORING CROSS-SECTOR PARTNERSHIPS TO UNDERSTAND DISASTER PREPAREDNESS FOR OLDER AND VULNERABLE POPULATIONS

Chair: Leah Haverhals
Discussant: Lindsay Peterson

As disasters continue to increase in frequency and disproportionately impact older adults and other vulnerable populations, more directed focus is needed to facilitate cross-disciplinary partnerships to better support and encourage disaster preparedness and response for these groups. Gerontologists and social scientists have opportunities to partner with engineers, geologists, demographers, epidemiologists, and other community partners to best address gaps in disaster preparation and response in research and practice. This symposium highlights current cross-sector partnerships supporting older adults before, during, and after disasters in various settings across the United States (US). Dr. Tamar Wyte-Lake will explore how interorganizational relationships between the US Department of Veterans Affairs and home health agencies can be leveraged to maintain care across providers during disasters. Emily Killian will describe the Disaster PrepWise (DPW) program, developed through community-engaged processes involving disaster management and public health agencies as well as aging service providers. Dr. Omolola Adepoju will share academic-community partnerships created to build a geospatial-photoethnography dashboard to chronicle and assess successive disaster events on affordable housing and mental health outcomes in Houston. Dr. Holly Dabelko-Schoeny will address how older adults in affordable housing experience extreme weather, and roles and experiences of service coordinators in preparedness and response. Elana Kieffer will describe how older New Yorkers and professionals working with them have demonstrated resilience since the COVID-19 pandemic. Dr. Lindsay Peterson will act as discussant. This is a Disasters and Older Adults Interest Group Sponsored Symposium. Disasters and Older Adults Interest Group Sponsored Symposium

Abstract citation ID: igae098.1606

### USING PRE-EXISTING RELATIONSHIPS TO CREATE PIPELINES OF DISASTER PREPAREDNESS SUPPORT FOR HOME HEALTH AGENCIES

Tamar Wyte-Lake[1], Lauren Hall[2], Emily Solorzano[3], and Emily Franzosa[2], *1. U.S. Department of Veterans Affairs (VA), North Hills, California, United States*, *2. James J Peters VA Medical Center, Bronx, New York, United States*, *3. U.S. Department of Veterans Affairs (VA), Los Angeles, California, United States*

Home-health agencies (HHAs) provide community-based services that are a vital lifeline for older adults and can play a critical role in protecting older adults' health and independence during disasters. Yet, HHAs are often under-resourced and disconnected from broader community emergency planning efforts, and therefore can struggle to maintain services during such events, putting clients' health and safety at risk. Healthcare coalitions have demonstrated a variety of ways to effectively share critical resources during disasters, strategies that could be leveraged to better support community HHAs. Through interviews with 19 stakeholders from six Veterans Health Administration (VA) Medical Centers and associated, contracted HHAs, we used the VA as a case study to explore how interorganizational relationships can be leveraged to maintain continuous care across providers during disasters. VA staff demonstrated interest in partnering around emergency management and identified a range of low- and higher-effort resources, from informational bulletins to staff training and emergency plan preparations that could be shared with HHAs. Creating a pipeline of support through existing relationships can provide a model for health systems to extend available emergency resources and make clinical care teams, patients, and communities more resilient to disasters. We will focus on specific resources and strategies and how to develop and sustain those cross-sector relationships.

Abstract citation ID: igae098.1607

### BUILDING AND SUSTAINING ORGANIZATIONAL PARTNERSHIPS TO SUPPORT DISASTER PREPAREDNESS IN OLDER ADULTS

Emily Killian[1], Lena Thompson[2], Miranda Fisher[3], Nicholas Ostrem[3], Tiffany Wunderlich[3], Brigid Smith[3], and Sato Ashida[3], *1. University of Iowa College of Public Health, Iowa City, Iowa, United States*, *2. University of Minnesota Duluth, Duluth, Minnesota, United States*, *3. University of Iowa, Iowa City, Iowa, United States*

Long-term implementation of programs for community-based older adults requires collaboration among various organizations. Strengthening personal and community support systems is critical in supporting older adults before, during, and after disasters. The Disaster PrepWise (DPW) program was developed through community-engaged processes involving disaster management and public health agencies as well as aging service providers to ensure the usability and acceptability of the program. DPW implementation infrastructure and protocols were subsequently developed using the concepts of the Practical, Robust Implementation, and Sustainability Model (PRISM) framework. Organizational partners participated in periodic Stakeholder Advisory Board (SAB) meetings and collaboratively developed a program delivery infrastructure. The developed infrastructure was pilot-tested and evaluated by conducting interviews from July to September 2023 with various stakeholders: program participants (n=18), SAB members (n=6), and interventionists (n=7). Strong alignment between the DPW program and their values and the opportunity to work with "like-minded" people motivated organizations to participate. Organizations involved since the program development stage, over 10 years ago, were especially committed to supporting the development of implementation infrastructure. SAB members highlighted additional benefits to older adults as this infrastructure can connect DPW participants to other social services in their community through cross-sectorial collaborations. Participants identified barriers to long-term sustainability such as a lack of sustained resources, identifying and training interventionists, staff turnover, and difficulty expanding to reach partners in rural areas. These collective challenges underscore the complexity of maintaining such initiatives and the necessity for comprehensive strategies to sustain them.

Abstract citation ID: igae098.1608

### MULTISECTORAL PARTNERSHIPS TO ADDRESS SUCCESSIVE DISASTER EVENTS IN HOUSTON'S UNDERSERVED COMMUNITIES

Omolola Adepoju[1], Mary Tipton[2], Ben Hirsch[3], and Lauren Gilbert[4], *1. University of Houston College of Medicine, Houston, Texas, United States*, *2. Humana Integrated Health Systems Sciences Institute, Houston, Texas, United States*, *3. West Street Recovery, Houston, Texas, United States*, *4. University of Houston, Houston, Texas, United States*

**Objective:** Over the past decade, Houston, Texas, has become one of the most diverse cities in America, during which time it has also endured several federally declared disasters. This study describes the creation of formal academic-community partnerships to build a geospatial-photoethnography dashboard, co-owned and co-developed by the community, to chronicle and assess the impact of successive disaster events on affordable housing and mental health outcomes in three historically medically underserved communities in Houston. **Methods:** We used geospatial-longitudinal maps to illustrate housing insecurity and mental health trends from 1980 to 2022, with projections for 2030, 2040, and 2050 at the census tract level. Critical ethnography concepts, such as community conversations and photovoice, are employed to gather and present a storyboard of people's experiences and coping mechanisms. **Results:** Community leaders were identified via prior partnerships with social support groups, healthcare organizations, and faith-based organizations. A total of 15 community partners were invited and 10 agreed to serve as partners on the project. Co-ownership was developed through regular meetings as a large group with all partners, as well as one-on-one meetings with individual groups. To capture the richness of the community conversations, visual notetakers were present at the meetings to provide a graphical representation of the content of the meetings. **Discussion:** The community-led initiative amplifies the voices and experiences of those affected by climate change disasters, shedding light on enduring effects on housing and mental health in underserved communities. By embracing community-driven empowerment, the project documents community priorities and proposed solutions.

Abstract citation ID: igae098.1609

### WEATHER AND AGING RESILIENT MODEL (WARM) FOR AFFORDABLE HOUSING

Holly Dabelko-Schoeny[1], Smitha Rao[1], and Sean Bartlett[2], *1. The Ohio State University, Columbus, Ohio, United States*, *2. Central Ohio Area Agency On Aging, Columbus, Ohio, United States*

Low-income housing residents, especially older adults with disabilities, face heightened risks during weather-related disasters. This study examines how older adults in affordable housing experience extreme weather, and roles and experiences of service coordinators in preparedness and response. Utilizing a mixed methods community-based participatory case-study design, we conducted a cross-sectional survey (N=40) and focus group (N=26) with residents in one senior affordable housing property in Central Ohio. Another survey (N = 8) and focus group (N = 11) included service coordinators and supervisors working within eight affordable housing properties in the region. Among residents, a third reported fair or poor health and over half (53%) did not drive. Most (73%) had experienced extreme weather events, with snow/ice storms and hurricanes being most common and extreme heat the most recent. Nearly 70% experienced power outages, the average outage lasted a week, and 40% did not have an evacuation plan. Service coordinators experienced extreme heat, fire, and severe snow/ice and few had advance-warning to prepare residents for extreme weather. Thirty-eight percent lost power in their buildings, and only half had reliable information and communications during the event. Seventy-five percent were somewhat or very concerned about disasters and the same number reported their residents were unprepared. Seventy-five percent lacked emergency preparedness training and 63% were not confident in their ability to evacuate residents. Focus groups among residents and coordinators revealed challenges in communication, transportation, sheltering, power sources, and evacuation. Findings are critical to the co-development of a disaster preparedness toolkit for affordable housing communities.

Abstract citation ID: igae098.1610

### THE STRENGTH OF PARTNERSHIPS IN EMERGENCY RESPONSE AND RESILIENCE: FINDINGS FROM THE VOICES OF OLDER NEW YORKERS

Elana Kieffer, *The New York Academy of Medicine, New York, New York, United States*

New York City is home to over 1.2 million adults age 65+ in approximately 300 square miles. The COVID-19 pandemic and the ongoing climate crisis have presented older New Yorkers with new public health challenges and exacerbated existing ones. The time is now for aging service providers, researchers, and older adults to develop and coordinate efforts to build, maintain, and increase community resilience in the wake of emergencies and disasters. In this session, staff members of the Center for Healthy Aging at The New York Academy of Medicine (NYAM) will describe how older New Yorkers and the professionals who work with them have demonstrated resilience since the pandemic, and provide recommendations on how they can continue to do so, as outlined in "Strengthening Community Resilience: Supporting Older Adults Through Emergency Preparedness And Response In A Post-COVID Era," published by NYAM in 2023. Guided by the RAND Corporation's Framework for Community Resilience and NYAM's previous research on resilience in the wake of Superstorm Sandy, we will discuss the results of this report, including the importance of partnerships, education, program efficiency, and access to resources, all through a strengths-based lens. This presentation will describe these key findings and their potential implications in the context of advocacy and policy change.

### SESSION 3490 (SYMPOSIUM)

Abstract citation ID: igae098.1611

### POLICY SERIES: EXPLORING DISPARITIES IN LONG-TERM SERVICES AND SUPPORTS FOR LGBTQIA+ OLDER ADULTS

Chair: Jason Flatt
Co-Chair: Tetyana Shippee
Discussant: Mark Brennan-Ing

Lesbian, gay, bisexual, transgender, queer, intersex, and/or asexual (LGBTQIA+) older adults are a growing population that has been mostly ignored in research and development of long-term services and supports (LTSS). Due to fears of prejudice and discrimination, institutional policies, and state and federal laws, many LGBTQIA+ older adults may be reluctant to use LTSS. In this symposium, we explore micro-, meso-, and macro-level influences that may impact inequities in LTSS access and use. First, Dr. Jason Flatt will focus on micro- and macro-level factors from a national study with LGBTQIA+ older adults that explores how past experiences of discrimination may result in barriers for aging-related care and LTSS planning. The second presentation by Dr. Rajean Moone will examine micro- and meso-level factors via experiences of 354 LGBTQIA+ older adults from Minnesota on needs for LTSS and training of providers. Next, Dr. Tetyana Shippee will share meso- and macro-level findings via an analysis of LGBTQIA+ affirmative LTSS policies across 166 nursing homes and 267 assisted living facilities in Minnesota. Finally, Dr. Carey Candrian will provide meso- and macro-level perspectives in terms of a training program and lessons learned from a cultural change project with over 800 staff from Christian Living Communities on inclusion of LGBTQIA+ in LTSS. Dr. Mark Brennan-Ing an expert in LGBTQIA+ aging research will facilitate a conversation about the conclusions from each speaker's presentation and place them in the context of efforts needed at micro-, meso- and macro-levels to ensure inclusive LTSS practices and policies for LGBTQIA+ elders.

Abstract citation ID: igae098.1612

### DISCRIMINATION AND ACCESS TO AGING AND LONG-TERM CARE SERVICES AND SUPPORTS FOR LGBTQIA+ OLDER ADULTS

Jason Flatt[1], Brittany Klenczar[1], Jalal Uddin[1], Max OHala[1], Erin Rook[1], Elise Hernandez[2], and Karalin Sprague[2], *1. University of Nevada Las Vegas, Las Vegas, Nevada, United States*, *2. SAGE, New York, New York, United States*

By 2030, there will be over seven million LGBTQ+ adults in the U.S. who are aged 50+. Past research finds that LGBTQ+ older adults are less likely to reach out to providers and use aging-related and long-term care services because of fear of discrimination. Partnering with SAGE, a national advocacy and services organization for LGBTQ+ older adults, we conducted a national study, State of LGBTQ+ Aging Study, with LGBTQ+ older adults (n=643) across the U.S. We used descriptive statistics and bivariate analyses to examine associations between experiencing lifetime and everyday discrimination and concerns with access to aging-related and long-term care services. In terms of discrimination/victimization, over 41% (n=261) reported experiencing one or more lifetime discrimination events, 42% (n=263)

reported experiencing violence due to being LGBTQ+, and 45% reported everyday discrimination. For aging-related and long-term care services, 28% reported planning for long-term care (e.g., family caregiving, nursing facilities, and home healthcare), 65% were concerned about accessibility of aging services, 64% were concerned about access to future medical care, 71% were concerned about access to future caregiving, and 76% were concerned about finding inclusive long-term care/caregiving resources. LGBTQ+ older adults who reported past LGBTQ+ violence, lifetime and everyday discrimination were more likely to report being concerned about accessibility of aging services, access to future medical care, and having a future caregiver (p-values <0.01). These findings highlight how discrimination may impact access to aging-related and long-term care services for LGBTQ+ older adults, and the need for future research and inclusive care efforts.

Abstract citation ID: igae098.1613

### PREVALENCE OF SEXUAL AND GENDER MINORITY (SGM)–FRIENDLY POLICIES IN LONG-TERM CARE FACILITIES IN MINNESOTA

Tetyana Shippee[1], Rajean Moone[2], Jason Flatt[3], Ziwei Zhang[4], Morgan Wright[4], Nidhi Kohli[4], and B.R. Simon Rosser[4], *1. University of Minnesota School Of Public Health, Minneapolis, Minnesota, United States*, *2. University of Minnesota, St. Paul, Minnesota, United States*, *3. University of Nevada Las Vegas, Las Vegas, Nevada, United States*, *4. University of Minnesota, Minneapolis, Minnesota, United States*

Sexual and gender minority (SGM) older adults in the U.S. face increased risk of Alzheimer's disease and related dementias (AD/ADRD) and are more likely to utilize long-term services and supports (LTSS) compared to their cisgender, heterosexual peers. Despite these disparities, the prevalence of SGM-affirmative policies within LTSS settings remains understudied. This study aimed to comprehensively analyze the prevalence of SGM-affirmative policies in LTSS facilities in Minnesota. These include policies in human resources, marketing, training, governance; anti-discrimination policies guiding culturally responsive care, and resident sexual orientation and gender identity (SOGI) data collection. A policy analysis was conducted across 166 nursing homes and 267 assisted living facilities in Minnesota. Policies were assessed for their explicit inclusion of SGM-affirmative measures in various domains, and mean scores were calculated to determine the overall prevalence of such policies. The mean score of SGM-friendly policies across nursing homes and assisted living communities was 11.30. Interestingly, no significant disparities were found between ALFs and nursing homes or rural and urban facilities regarding the presence of SGM-friendly policies. However, using latent class analysis, we found that not for profit facilities and non-religious facilities were more likely to belong to a low performance group vs. high performance compared to their counterparts. Our results show the need for SGM-inclusive policies within LTSS settings. While overall prevalence was moderate, variations based on facility characteristics highlight opportunities for targeted interventions. Future efforts should focus on promoting SGM-affirmative policies across all LTSS facilities to ensure equitable and inclusive care for SGM older adults.

Abstract citation ID: igae098.1614

### SEXUAL AND GENDER MINORITY EXPERIENCES AND PREFERENCES WITH LONG-TERM SERVICES AND SUPPORT PROVIDERS

Rajean Moone, *University of Minnesota, St. Paul, Minnesota, United States*

Sexual and gender minority (SGM) older adults are at higher risk for complex, health related conditions as they age. Coupled with smaller social support networks they often must rely on formal long term services and support (LTSS) providers. However, due to a history of personal and systemic discrimination many SGM older adults limit access to LTSS. To gain further insight into SGM older adult experiences, a survey needs assessment consisting of 46 self-reported questions was conducted in Minnesota in 2022, replicating similar waves in 2002 and 2012. 354 respondents met the inclusion criteria. Half of the respondents (50% of sexual minority and 53% of gender minority) reported personally experiencing harassment, abuse, or violence and 75% reported knowing someone who had. At the same time, respondents overwhelmingly expressed desire for integrated services that are SGM welcoming rather than SGM exclusive. A majority (57%) had some confidence in being treated with dignity and respect by health care professionals at the end of life. Respondents also indicated they would be more inclined to utilize LTSS where staff participated in SGM training (92%). Results show need for LTSS provider education on SGM trauma-informed and age-inclusivity and targeting of SMG older adults aging alone.

Abstract citation ID: igae098.1615

### CREATING INCLUSIVE COMMUNITIES FOR LGBTQ RESIDENTS AND STAFF IN FAITH-BASED ASSISTED LIVING COMMUNITIES

Carey Candrian, *University of Colorado Anschutz Medical Campus, Denver, Colorado, United States*

Seventy-five percent of older LGBTQ adults report having to hide their identity (and therefore, their true selves) in order to avoid discrimination in long term care settings. The stress of hiding takes a serious toll on the lives of LGBTQ people: higher rates of anxiety and depression, substance abuse, certain cancers, cardiovascular disease and suicide. Knowing first-hand the pain of not belonging, residents at a faith-based assisted living community in the Rocky Mountain Region have been working to create a more welcoming community for LGBTQ residents since 2017. Their LGBTQ + Friends group is made of LGBTQ people and allies (residents and team members) who meet monthly to create an inclusive and safe space for all. A few years ago, the group asked, what could help long term care communities become a more welcoming place for LGBTQ residents and staff? Through this community-initiated idea, and after foundation funding, we developed a two-year cultural change initiative for over 800 staff across 6 assisted living communities called, "Caring for all: Opening hearts and minds." The result is a 25-minute video of voices of residents, staff and leadership talking about the impact of creating inclusive communities where all residents can belong and be embraced. This project was not without enormous challenges and barriers. During this presentation, we will also walk through lessons learned and stories of success for creating inclusive communities for LGBTQ residents and staff in faith-based assisted living communities.

### SESSION 3495 (SYMPOSIUM)

Abstract citation ID: igae098.1616

### FORTIFYING THE FUTURE OF GERONTOLOGY AND GERIATRICS IN HIGHER ED: PART II—CASE STUDIES

Chair: Nasreen Sadeq

Gerontology academic programs continue to face challenges across the university setting, from low student enrollment to reduced funding and budget cuts. However, some gerontology departments are making significant changes to ensure the survival of their programs. This symposium highlights efforts to secure gerontology's presence in higher education, including new enrollment strategies, curriculum and program changes, and interdisciplinary collaborations. First, Nasreen Sadeq and colleagues will share a case study from the University of South Florida School of Aging Studies on their efforts to increase undergraduate enrollment by developing a new Health Care Administration program. Second, Edward Miller and colleagues will describe hiring and marketing strategies that will promote growth and visibility of the Gerontology department at the University of Massachusetts Boston, such as renaming the Aging Studies major and implementing gerontology courses into the general education curriculum. Third, John Schumacher and colleagues at the University of Maryland, Baltimore County and Baltimore will share new developments in their department, including a new online Gerontology master's program and new international research programs, and address current challenges faced by the doctoral program. Fourth, Tina Newsham and Elizabeth Fugate-Whitlock from the University of North Carolina Wilmington will describe interdisciplinary campus and community partnerships that situate the gerontology program in the university's infrastructure, facilitate recruitment, and create meaningful opportunities for experiential learning. Finally, Robert Maiden and Danielle Gagne from Alfred University will discuss strategies for increasing enrollment, including emphasizing the interdisciplinary nature of their program and the addition of an aging concentration.

Abstract citation ID: igae098.1617

### HEALTH CARE ADMINISTRATION: HOW UPDATING OUR GERONTOLOGY UNDERGRADUATE PROGRAM INCREASED STUDENT ENROLLMENT

Nasreen Sadeq[1], Brian Kaskie[2], Debra Dobbs[1], Lindsay Peterson[1], Lu Norstrand[1], and William Haley[1], *1. University of South Florida, Tampa, Florida, United States*, *2. University of Iowa, Iowa City, Iowa, United States*

The University of South Florida is a public research university in Tampa, FL with approximately 50,000 students across three campuses. The School of Aging Studies is one of several departments throughout the university that offers interdisciplinary undergraduate degree programs. However, like many gerontology programs across the country, the School of Aging Studies experienced a decline in undergraduate enrollment over the past decade and often struggled with generating enthusiasm for gerontology education and training. In order to continue our mission of providing an interdisciplinary gerontology education to undergraduate students, the School of Aging Studies performed an in-depth analysis of one of their undergraduate programs and determined that several updates were necessary to modernize the program and provide a more comprehensive education to students planning to work in the health care industry. In this presentation, we will describe our redeveloped Health Care Administration undergraduate program, including updates that were made to the curriculum and changes in program requirements. We will report on the intensive marketing and networking strategies that were developed with our university and community partners, and how our continued efforts led to the enrollment of approximately 40 students within one academic year. We will also describe our plans for the continued growth of the program and discuss the challenges that we have experienced and will continue to face in the future, including the increased demands placed on our faculty and staff, the availability of community internships available for our students, and the ability to sustain student interest in gerontology.

Abstract citation ID: igae098.1618

### A MULTIPRONGED STRATEGY FOR GROWING THE GERONTOLOGICAL WORKFORCE AT THE UNIVERSITY OF MASSACHUSETTS BOSTON

Edward Miller, Qian Song, Jeffrey Stokes, and Ellen Birchander, *University of Massachusetts Boston, Boston, Massachusetts, United States*

The University of Massachusetts Boston is a public research university serving approximately 16,000 students. It is the most diverse university in New England and third most diverse university in the nation. The Gerontology Department is committed to growing the size and diversity of the workforce in aging services, policy, practice, and research. This presentation describes the multipronged approach taken to achieve this objective, while identifying associated facilitators and challenges. Departmentwide initiatives include prioritizing work in disparities in faculty hiring, implementing curriculum self-assessment and climate survey tools, and engaging in substantial marketing and outreach and collaborations with both internal and external constituencies. Doctoral program initiatives include expanding scholarship and training opportunities. Undergraduate program initiatives include changing the name of the major to "Aging Studies" to make the content clearer and more accessible, implementing a minor in Aging Studies to make it possible for students from across the University to earn a valuable credential, having all courses meet requirements in general education, and expanding the number and attractiveness of course offerings. The Department also implemented an initiative where undergraduates receive paid internships at local-area aging agencies with potential for continued employment post-graduation. Promoting diversity is closely connected to growing program enrollment with positive benefits for older adults and their families, caregivers, and communities.

Abstract citation ID: igae098.1619

### GERONTOLOGY DOCTORAL EDUCATION: RELEVANCE AND CHALLENGES AT UNIVERSITY OF MARYLAND, BALTIMORE AND BALTIMORE COUNTY

John Schumacher[1], and Ann Gruber-Baldini[2], *1. University of Maryland, Batlimore County (UMBC), University of Maryland, Batlimore County, Maryland, United States*, *2. University of Maryland School of Medicine, Baltimore, Maryland, United States*

The joint University of Maryland, Baltimore County (UMBC) & Baltimore's (UMB) Doctoral Program in Gerontology celebrated the milestone of its 50th graduate in

2024. Yet, like our peer doctoral programs, gerontology education remains undervalued in society and higher education. Recent ways we highlight our relevance include: 1) Launch of an Online Masters in Gerontology. Sustainability may lie in universities sharing enrollment across online courses; 2) Created Global Experiences i) Costa Rican Fellowship. External donor funded program for gerontology students and faculty for an extended, bi-directional scholarly exchange. ii) Global Aging Gerontology in Japan. Immersive interdisciplinary course held in Japan as a super-aging society; 3) Generative Artificial Intelligence (Gen AI) in Gerontology Education & Research. Training faculty and students to participate in an AI infused work environment. Challenges to doctoral education abound including: 1) Funding graduate research assistantships (GRAs); decreasing GRA allocations and both increasing cost and pressures to exclusively fund GRAs on external grants; 2) Engaging faculty effort to move beyond a voluntary, gerontology affiliate faculty model. Ever increasing demands on faculty time are leading to less service availability to gerontology programs. New faculty affiliate models are needed that may involve compensation and recognition for interdisciplinary faculty effort and revenue sources. Overall, degree programs in gerontology must continue to promote their relevance while meeting continuing challenges.

Abstract citation ID: igae098.1620

### FORTIFYING GERONTOLOGY PROGRAMS: HARNESSING COLLABORATIONS ACROSS CAMPUS

Tina Newsham, and Elizabeth Fugate-Whitlock, *University of North Carolina Wilmington, Wilmington, North Carolina, United States*

Having joined the Age Friendly University Global Network and continuing the transition to an R2 institution, the University of North Carolina Wilmington is poised for strengthening its graduate program in gerontology. The program, like others, was once on the low productivity list and considered for dissolution, has only two full-time faculty members making collaborative efforts vital. To facilitate recruitment into the MS in Applied Gerontology and to situate the program firmly in the university's infrastructure, we have initiated a variety of collaborative and interdisciplinary efforts. A set of five pathways known as "4 + 1" or "combined programs" allowing qualified undergraduate students to take a portion of their coursework at the graduate level. These students explore their interest in gerontology, become invested in gerontology education, and meet undergrad and graduate requirements simultaneously. Students are then able to complete their MS in Applied Gerontology in as little as one more year. In addition, new pathways have been created through combining teaching research, and scholarship endeavors. GRN faculty partner with colleagues (affiliated faculty) from Applied Health programs, Nursing, Social Work, Sociology/Criminology, Dance, and more to write grants, develop community engagement projects, and create meaningful opportunities for campus and community members to learn about gerontology. These efforts also provide opportunities to highlight careers in aging creating further interest in gerontology education. Such collaborations have created a steady stream of students into the graduate program, yet create their own challenges (as well as new opportunities).

Abstract citation ID: igae098.1621

### THE EVOLUTION OF THE GERONTOLOGY PROGRAM AT ALFRED UNIVERSITY

Robert Maiden, and Danielle Gagne, *Alfred University, Alfred, New York, United States*

Alfred University instituted its major and minor in gerontology in 1980. At that time, it was one of two bachelor's degrees in gerontology offered in the State of New York. It was established to meet the burgeoning need of competent service providers for older Americans. The need is clear. It is projected that there will be 89 million older Americans by 2050, which will increase twice as fast as the underage 50 population. Although there is growing need for competent service providers and myriad potential career opportunities for undergraduates, there has been a declining interest in the gerontology program. The gerontology program at Alfred University has not had any majors or minors for the past two years. Many reasons have been proffered for this decline, including that many students do not understand the word "gerontology" or have misconceptions and negative stereotypes regarding aging. To increase enrollment in gerontology, we propose two changes: 1) adding aging as a concentration to our psychology major with an interdisciplinary component; and 2) changing the name to "Aging Studies" or "Adult Development & Aging." Moreover, students in this concentration can minor in business administration and become eligible for our 4 + 1 program, in which they can earn an MBA at Alfred University in one year post baccalaureate instead of the usual two.

### SESSION 3500 (SYMPOSIUM)

Abstract citation ID: igae098.1622

### FOSTERING IMPROVED OUTCOMES AMONG CARE PARTNERS OF PERSONS LIVING WITH ALZHEIMER'S DISEASE AND RELATED DEMENTIAS

Chair: Sara Bybee
Co-Chair: Elyse Couch
Discussant: Darina Petrovsky

The decline in cognitive and functional abilities due to Alzheimer's Disease and Related Dementias (ADRD) increases the reliance of persons living with dementia (PLWD) on care partners. These care partners, who can be spouses, children, friends, or others, often experience high levels of stress and burden, leading to poor physical and psychological health outcomes. This symposium will feature five speakers who studied ADRD care partner outcomes using various methods across diverse populations. Dr. Darina Petrovsky will discuss findings from the 2017 National Study of Caregiving and National Health and Aging Trends Study, focusing on the relationship between care partner sleep quality, burden, and perceived caregiving benefits. Dr. Shandra Burton will explore cultural factors and resilience among Black/African American care partners using narrative inquiry. Dr. Samantha Shune will present a multiple regression analysis examining the link between PLWD's dysphagia (swallowing difficulty) and care partner burden. Dr. Elyse Couch will describe a mixed-methods study investigating the impact of care partners receiving PLWD's positron emission tomography (PET) scan results on their burden. Lastly, Dr. Bybee will review results from an interpretive descriptive analysis of dementia dyads' open-ended survey responses regarding advance care planning.

Dr. Petrovsky will then synthesize the symposium findings and moderate audience questions. Alzheimer's Disease and Related Dementias Interest Group Sponsored Symposium

Abstract citation ID: igae098.1623

### ADVANCE CARE PLANNING (ACP) CONCORDANCE ASSOCIATED WITH RELATIONSHIP QUALITY AND GRIEF AMONG DEMENTIA DYADS

Sara Bybee[1], Jordana Clayton[2], Nancy Aruscavage[2], Rebecca Utz[2], Eli Iacob[2], and Kara Dassel[3], *1. University of Utah College of Nursing, Salt Lake City, Utah, United States*, *2. University of Utah, Salt Lake City, Utah, United States*, *3. University of Utah, Salt Lake Cty, Utah, United States*

Advance care planning (ACP) is linked to enhanced physical and mental well-being, fewer unwanted hospitalizations, and improved quality of life for individuals and their care partners. We investigated the impact of the Life-Planning in Early Alzheimer's and Other Dementias (LEAD) Guide on ACP concordance—the mutual understanding and agreement on ACP values and preferences—among persons living with dementia (PLWD) and their care partners, and its impact on relationship quality and grief. Dyads (N=48) participated in a discussion of each partner's answers to the LEAD Guide, addressing any discrepant responses. Participants completed open-ended survey questions (n=43) regarding their: discussion, confidence in their partner's understanding of their end-of-life wishes, confidence in their partner's ability to make decisions aligning with their end-of-life wishes, and any additional comments. An interpretive descriptive approach to qualitative analysis revealed four themes: 1) Relationship quality between partners (high versus strained relationship quality, 2) Advance care planning process and discussion (ACP concordance versus discordance), 3) Engagement in ACP, and 4) The grief process associated with dementia disease trajectory. Dyads with more ACP concordance codes demonstrated more codes related to high relationship quality (versus strained relationship quality) and fewer grief codes. Conversely, dyads with more ACP discordance codes had more strained relationship quality and grief codes. Our findings suggest that the LEAD guide may enhance ACP concordance, contributing to improved relationship quality and reduced grief. Clinicians may consider additional support for dyads demonstrating greater ACP discordance given its co-occurrence with strained relationship quality and grief.

Abstract citation ID: igae098.1624

### OBJECTIVE AND SUBJECTIVE INDICATORS OF CAREGIVER BURDEN FOLLOWING AN AMYLOID-Β PET SCAN: A MIXED-METHODS STUDY

Elyse Couch, *Brown University, Providence, Rhode Island, United States*

Dementia caregiving is often associated with high levels of burden which can lead to negative outcomes. Caregiver burden consists of disease-specific and perceived stressors - or objective and subjective indicators of burden. Amyloid-β positron emission tomography (PET) scans can be used to enhance the accuracy of dementia diagnoses. However, the relationship between amyloid scan results and caregiver burden is less well understood. We conducted a parallel mixed-methods study. We used quantitative survey data from 1,338 care partners to persons with MCI and dementia who received an amyloid scan as part of the CARE-IDEAS study in the US. Logistic regression models were used to test the association between the scan result and objective measures of caregiver burden. Semi-structured interviews were conducted with a subsample of 62 care partners. Thematic analysis was used to investigate subjective indicators by exploring care partners' perceptions of their role following an amyloid scan. Quantitative analysis showed that an elevated amyloid scan result was associated with spending more hours caregiving for MCI care partners. However, our thematic analysis showed that the scan result influenced participants' perceptions and expectations of their caregiving role and helped some care partners to develop coping strategies. All care partners described needing practical and emotional support. Amyloid scan results can influence objective and subjective indicators of burden and may present an opportunity for diagnosing clinicians to identify and address care partners' emotional and practical support needs.

Abstract citation ID: igae098.1625

### EXAMINING PREDICTORS OF POOR SLEEP AMONG CAREGIVERS OF PERSONS LIVING WITH DEMENTIA

Darina Petrovsky[1], and Elizabeth Luth[2], *1. Duke University, Durham, North Carolina, United States*, *2. Rutgers University, New Brunswick, New Jersey, United States*

Caregivers who struggle with sleep face increased stress and worse mental health outcomes. This study explored the connection between caregivers' perceived caregiving burden and benefits and their sleep quality. Using data from the 2017 National Study of Caregiving and National Health and Aging Trends Study, we examined 790 caregivers of individuals with possible and probable dementia. Using time diary interviews, sleep quality was categorized as either "excellent/very good" or "good/fair/poor." Predictors included caregiver perceived burden and benefit scales, each the summed responses to four questions (burden Cronbach alpha=0.75; benefit Cronbach alpha=0.70). We used multi-variable logistic regression, controlling for the sociodemographic characteristics of caregivers and persons with dementia, as well as relationship quality and sleep disturbance due to caregiving responsibilities. Caregivers' mean age was 62.5 years old (SD=13). Approximately 69% were women and more than half were non-Hispanic White (58.6%). Fifty-five percent (55%) of caregivers were children and 44% reported "excellent/very good" sleep quality. In the multi-variable logistic regression model, a higher benefit of caregiving was associated with significantly higher odds of reporting "excellent/very good" sleep quality (AOR: 1.09, 95%CI: 1.01-1.17, p=0.02). Conversely, a higher burden of caregiving was associated with lower odds of reporting "excellent/very good" sleep quality (AOR: 0.88, 95%CI: 0.81-0.95, p=0.002). These relationships persisted independent of caregiver and care recipient demographic characteristics and interrupted sleep due to caregiving. Our findings highlight potential modifiable targets for future interventions—specifically, addressing perceived caregiver burden and maximizing benefits—to enhance sleep quality among caregivers of individuals living with dementia.

Abstract citation ID: igae098.1626

### CULTURAL AND POSITIVE ADAPTIVE FACTORS INFLUENCING DIVERSE PARTNER CAREGIVERS THROUGH A RESILIENCE FRAMEWORK

Shandra Burton[1], Olimpia Paun[2], Todd Ruppar[3], lynn Mohr[3], Chuka Emezue[3], and Sarah Szanton[4], *1. Marian University Indianapolis School of Nursing, Indianapolis, Indiana, United States*, *2. Rush University, Chicago, Illinois, United States*, *3. Rush University College of Nursing, Chicago, Illinois, United States*, *4. Johns Hopkins University, Baltimore, Maryland, United States*

Collectively, Alzheimer's disease and related dementias (ADRD) are the 5th leading cause of death for Americans aged 50 years and older and remains a critical public health issue for partner caregivers (i.e., committed, married, or cohabiting individuals). ADRD partner caregivers often face high levels of stress and burden contributing to their poor physical and psychological health outcomes. Research on positive adaptive factors (PAFs) among caregivers of individuals with Alzheimer's disease and related dementias (ADRD) is scarce. PAFs refer to traits, actions, or assets that caregivers utilize to manage and conquer difficulties, pressures, or unfavorable situations in their caregiving role. Resilience is a common topic of interest, however there is less understanding of its significance in Black/American caregivers of individuals with ADRD. This qualitative study used narrative inquiry to explore cultural factors related to resilience among Black/African American ADRD partner caregivers. An adapted Resilience Framework informed this study and in-depth interviews with 10 Black/African American ADRD partner caregivers. Four major themes emerged: (1) Process of ADRD discovery, (2) Resilience in the caregiving process, (3) Challenges & facilitators to caregivers' resilience, and (4) Race, ethnicity, cultural impact on caregivers' resilience. The major themes include a total of 18 subthemes. Data from this study were compared to data from a previous pilot study that used similar questionnaire with a predominantly White sample of ADRD partner caregivers. Findings from this study contribute to our understanding of how race, ethnicity, and cultural factors influence resilience among diverse ADRD partner caregivers.

Abstract citation ID: igae098.1627

### DYSPHAGIA MANAGEMENT CONTRIBUTES TO BURDEN IN CAREGIVERS OF PERSONS WITH DEMENTIA

Samantha Shune[1], and Ashwini Namasivayam-MacDonald[2], *1. University of Oregon, Eugene, Oregon, United States*, *2. McMaster University, Hamilton, Ontario, Canada*

Providing care for persons with Alzheimer's disease and related dementias (ADRD) comes with significant physical and psychosocial costs. Crucially underexplored in its contribution to caregiver burden is dysphagia (swallowing impairment), which occurs in up to 93% of persons with ADRD and contributes to increased morbidity and mortality. The current study aimed to quantify dysphagia's contribution to general burden among family caregivers of individuals with ADRD. A total of 110, predominately female (79.1%), family caregivers were recruited (mean age 55.8±15.9). Multiple measures of dysphagia severity were considered, including caregiver-reported dysphagia severity (Eating Assessment Tool [EAT-10]) and degree of diet modification (International Dysphagia Diet Standardisation Initiative – Functional Diet Scale [IDDSI-FDS]). General caregiver burden was measured via the Zarit Burden Interview (ZBI). To examine which variables were unique predictors of burden, dysphagia-related variables as well as demographic (caregiver age, sex, employment status, education; care recipient relationship, age, sex) and disease variables (caregiver-reported diagnosis, severity, duration) were all entered simultaneously in a multiple regression analysis. Preliminary regression results indicated that higher ZBI scores were associated with caregivers who reported higher IDDSI-FDS scores ($\beta$ =.26, p =.0364), identified as female ($\beta$ =.25, p =.0450), were the spouse of the care recipient ($\beta$ =.45, p =.0125), and reported shorter disease duration ($\beta$ =.29, p =.0072). Findings highlight that aspects of managing dysphagia may independently contribute to caregiver burden, particularly in the context of continued oral intake. Thus, inquiry about dysphagia status should be considered in disease management.

### SESSION 3505 (SYMPOSIUM)

Abstract citation ID: igae098.1628

### INTEGRATING ORAL HYGIENE AND COGNITIVE HEALTH: GLOBAL INSIGHTS AND INTERGENERATIONAL APPROACHES

Chair: Xiang Qi
Co-Chair: Weiyu Mao
Discussant: Michele Saunders

The symposium presents four studies investigating the intersection of oral health, cognitive functions, and healthcare accessibility among older adults in China and India. The first study employs a co-design approach to develop a school-based oral hygiene intervention that leverages intergenerational ties between youths and older adults to enhance oral health education. This initiative demonstrates a promising avenue for promoting healthy behaviors across generations, emphasizing family dynamics and resource allocation in the design of oral health interventions. The second review underscores the potential of regular toothbrushing as a simple yet effective strategy to mitigate cognitive decline and dementia risk. This meta-analysis illuminates the direct benefits of oral hygiene on cognitive functions, advocating for the inclusion of oral hygiene practices in daily routines to promote cognitive health. The third study finds significant correlations between cognitive decline and poor oral health using data from the Longitudinal Aging Study in India, highlighting the urgent need for improved dental care accessibility and oral health education as preventive measures against cognitive impairment. Finally, the fourth study investigates into the dental care utilization patterns among older adults in Shanghai reveals a troublingly low engagement with dental services, exacerbated by socioeconomic disparities and insurance coverage limitations. The study calls for comprehensive reforms to extend dental care access and integrate it within broader health insurance schemes. Together, these studies shed light on the critical link between oral health and cognitive health and propose actionable strategies to improve public health outcomes through enhanced education, policy reform, and community-based interventions. Oral Health Interest Group Sponsored Symposium

Abstract citation ID: igae098.1629

### USING CO-DESIGN TO DEVELOP A SCHOOL-BASED ORAL HYGIENE INTERVENTION LINKING YOUTH AND OLDER ADULTS

Lingyi Shen[1], Liangyi Jin[1], and Hanzhang Xu[2], *1. Duke Kunshan University, Kunshan, Jiangsu, China (People's*

*Republic), 2. Duke University, Durham, North Carolina, United States*

The prevalence of oral health problems is high among Chinese older adults, which was attributed to limited oral health knowledge and poor oral hygiene behaviors. One key strategy to achieve optimal oral health is through health education. School settings are ideal to disseminate and implement public health interventions to a large group at a relatively low cost. As more than half of the Chinese older adults provide care to at least one grandchild, mobilizing these strong, existing intergenerational ties that Chinese older adults share has great potential to enhance the effect of school-based oral health promotion programs across generations. This study aimed to describe an older adult-grandchild co-design process that will lead to the development of a school-based oral health education intervention to improve oral hygiene behavior for both youth and older adults. A mixed-methods two-step sequential design was used. Step 1: focus groups among older adults (n=12), their grandchildren (n=12), and elementary school teachers (n=8) were conducted and analyzed to describe their knowledge and attitude towards oral hygiene behaviors. Step 2: vignettes were designed based on focus group results and presented to older adults, their grandchildren (5 dyads) and 2 elementary school teachers to co-design interventions to address needs. Three key themes were identified: perceived importance, power dynamic in the family, and limited time and resources. A few potential intervention strategies were produced, including leveraging school assignments to promote oral hygiene behaviors, building a rewarding mechanism, enhancing teacher-grandparents communications, and building oral health awareness in the community.

Abstract citation ID: igae098.1630

### ASSOCIATION BETWEEN ORAL HYGIENE BEHAVIORS AND COGNITIVE FUNCTION

Zheng Zhu[1], Zhongfang Yang[2], Xiang Qi[3], Weiyu Mao[4], Yaolin Pei[5], and Bei Wu[3], *1. New York University, New York, New York, United States*, *2. Fudan University, Shanghai, Shanghai, China (People's Republic)*, *3. New York University, New York, New York, United States*, *4. University of Nevada, Reno, Reno, Nevada, United States*, *5. University of Texas at Austin, Texas, United States*

**Background:** Improving oral hygiene behaviors may offer a novel approach to delay cognitive decline; however, there is limited data available on its effectiveness in affecting cognitive function. This review aims to examine whether oral hygiene behaviors would be an effective strategy to improve cognitive function. Method: A search of 11 literature databases were searched from inception through January 6, 2024. Interventional trials or cohort studies aiming to examine the effectiveness of oral hygiene behaviors in affecting cognitive function in adults were included. Oral hygiene behaviors included, but were not limited to, toothbrushing, mouthwashing, flossing, using toothpicks, and cleaning dentures. The primary outcome was the changes in global cognitive function. The meta-analysis was carried out using random-effects models.

**Results:** A total of 8 studies met inclusion criteria, including 261,772 participants. Toothbrushing was associated with a significantly higher level of global cognitive function (Mean Difference [MD], 0.65 [95% CI, 0.19-1.10]; I2=2%). There was no significant difference between the effectiveness of toothbrushing performed by professionals (MD, 0.93 [95% CI, 0.20-1.67]; I2=6%) and caregivers (MD, 1.01 [95% CI, 0.39 to 1.62]; I2=3%) ($P > 0.05$). Other oral hygiene behaviors including dental flossing, using mouthwash, using toothpicks, and cleaning dentures, were not associated with the risk of dementia.

**Discussion:** Regular toothbrushing is not only linked to improvements in global cognitive function and a reduced risk of dementia but also improve specific cognitive function subdomains such as visual attention and task-switching ability.

Abstract citation ID: igae098.1631

### COGNITIVE FUNCTION AND ORAL HEALTH MEASURES AMONG OLDER ADULTS IN INDIA: EVIDENCE FROM THE LASI, 2017–2018

Uma Kelekar[1], Xi Pan[2], Muhammad T[3], Sidney Turner[4], Patricia Heyn[5], and Bei Wu[6], *1. Marymount University, Arlington, Virginia, United States*, *2. Texas State University, San Marcos, Texas, United States*, *3. The Pennsylvania State University, University Park, Pennsylvania, United States*, *4. Fors Marsh, Arlington, Virginia, United States*, *5. Marymount University, Fairfax Station, Virginia, United States*, *6. New York University, New York, New York, United States*

Given the rising older adult population and related increase in the prevalence of cognitive impairment in India, the present study aimed to examine the association between cognitive function and oral health measures among the middle-aged and older adults in India. Data for the study were selected from the Longitudinal Aging Study in India (LASI, 2017-2018) and 56,723 respondents>=45 years were included. Oral health was measured by self-reported oral health problems including number of oral diseases, edentulism (i.e., losing some or all natural teeth), and dental care utilization (e.g., dental visit in the past 12 months). Our outcome measure was a composite score of cognitive function measured across five broad domains, specifically memory, orientation, arithmetic function, executive function, and object naming. Negative binomial regression models were used to analyze the relationship between the oral health and cognitive function. Our results showed that complete edentulism was associated with 3% lower cognitive scores [IRR=0.97, 95% CI 0.96, 0.99], while dental care utilization was positively associated with cognitive function. Those who had visited a dentist in the past 12 months reported 6% [IRR=1.06, 95% CI 1.03, 1.08] higher cognition scores than those who had not visited a dentist. The unadjusted univariate regressions demonstrated a negative association between the number of oral diseases and cognitive function. Our findings suggest an urgency for improving the existing oral health infrastructure, including expanding access to dental care and promoting oral health education in India, especially for adults at higher risk for cognitive impairment.

Abstract citation ID: igae098.1632

**ORAL SYMPTOMS, TYPE OF MEDICAL INSURANCE, AND DENTAL CARE UTILIZATION AMONG OLDER ADULTS IN SHANGHAI**

Xiaomin Qu[1], Zhihong Zhen[2], Xiang Qi[3], and Bei Wu[3], *1. East China University of Political Science and Low, Shanghai, Shanghai, China (People's Republic)*, *2. Shanghai University, Shanghai, Shanghai, China (People's Republic)*, *3. New York University, New York, New York, United States*

This study assessed the prevalence of dental care utilization among older adults in Shanghai, China. It examined the relationship between oral symptoms, the type of medical insurance, and the frequency of dental visits. Using data from the 2022 Lifelong Education for Aging Productively Survey, we analyzed responses from 1,482 participants aged 60–84. Our analysis, adjusted for complex sampling weights, reveals that only 28.93% of participants had visited a dentist in the past year. Findings from multivariable logit regression models showed that participants with oral symptoms, including toothache (OR = 2.156, 95% CI: 1.698–2.737, $p < 0.01$), tooth sensitivity (OR = 1.745, 95% CI: 1.375– 2.216, $p < 0.01$) and gum bleeding (OR = 1.275, 95% CI: 0.994–1.636, $p < 0.1$) were more likely to have a dental visit within the last year. The presence of multiple oral symptoms further increased this likelihood. Margins post-estimation model results demonstrated disparities in the predicted probability of visiting a dentist by the types of medical insurance. Specifically, older adults with medical insurance of less coverage were significantly less likely to seek dental care unless they exhibited multiple oral symptoms. The study concludes dental care utilization among Shanghai's older adults is notably low and predominantly treatment-focused. Our findings underscore the urgent need for expanded dental care programs and services and broader medical insurance coverage that includes dental care to address the socioeconomic disparities in dental care access.

### SESSION 3510 (SYMPOSIUM)

Abstract citation ID: igae098.1633

**INTERGENERATIONAL ENGAGEMENT IN MEDICAL EDUCATION: THE PIVOTAL ROLE OF OLDER ADULT MENTORS**

Chair: Jennifer Severance
Co-Chair: Leland Waters
Discussant: Emily Ihara

Medical education engaging older adults as mentors can broaden students' perspectives about social, cultural, economic, and environmental factors influencing health; increase knowledge and skills in collaborative practice; and improve attitudes towards older patients. The Health Resources and Services Administration funded a national network of Geriatric Workforce Enhancement Programs (GWEP) to train health professions students in assessing and addressing the primary care needs of older adults. This symposium examines GWEP models of medical education involving older adults as mentors to promote person-centered, age-friendly, and dementia-friendly care. First, presenters from the University of Washington and University of Minnesota GWEPs will describe dementia education programs pairing medical students and individuals with memory loss. This is followed by a project at the Virginia Commonwealth University GWEP engaging older adults as mentors to medical student teams. Finally, presenters from the University of North Texas Health Science Center GWEP will describe strategies to expand and sustain an older adult mentorship program through volunteer engagement and interprofessional collaboration. Presenters will discuss partnerships to develop and sustain older adult mentorship programs as well as challenges and lessons learned. The symposium will conclude with an exploration of the input, feedback, and perspectives collected from older adults about their involvement in medical education as well as opportunities to adapt these intergenerational models to a local context.

Abstract citation ID: igae098.1634

**EXPLORING THE GENERATIVITY OF A MENTORING PROGRAM FOR MEDICAL STUDENTS AND OLDER ADULTS**

Leland Waters[1], Shannon Arnette[1], Madeline McIntyre[1], Catherine Tompkins[2], and Sarah Marrs[1], *1. Virginia Commonwealth University, Richmond, Virginia, United States*, *2. George Mason University, Fairfax, Virginia, United States*

The Virginia Geriatric Education Centers' Geriatric Workforce Enhancement Program partners with the Virginia Commonwealth University's School of Medicine to provide a Mentoring Program required for all first-year medical students. Two students are paired with independent, community-dwelling older adult mentors and are required to meet with their mentor three times over the course of two semesters. The students are required to make initial contact and meet the mentor at a time and place of the mentor's convenience. Mentor volunteer retention was a challenge faced due to pandemic protocols, therefore, limiting the in-person relationships built throughout the program. However, by allowing for both in-person and virtual mentoring experiences, volunteer retention has increased post-pandemic. The students have reading assignments and guided interview questions, which are used as a starting point for conversations with their mentors. The first assignment on aging and health introduces the processes of normal aging, activities of daily living, instrumental activities of daily living, and functional status that is determined by looking at both physical and cognitive actions. The second assignment is a life-space assessment and interview about quality of life. The third assignment focuses on generativity, an evidence-based, psychosocial concept, which is defined as a desire by older adults to nurture and guide upcoming generations, to make meaning of their own lives. This presentation will provide qualitative analysis supporting a generative experience in which the mentors provide their wisdom and the students have an opportunity to develop underlying human relationships that contribute to person-centered care.

Abstract citation ID: igae098.1635

**PARTNERS IN DEMENTIA – NORTHWEST: NEW APPROACHES IN MEDICAL STUDENT SERVICE LEARNING**

Mengru Wang[1], Michael Rosenbloom[1], Marigrace Becker[1], and Stephanie Adaniya[2], *1. University of Washington,*

*Seattle, Washington, United States*, 2. *University of Washington School of Medicine, Seattle, Washington, United States*

Partners in Dementia – Northwest is the first service-learning program at the University of Washington School of Medicine focused on older adults and persons living with memory loss, established in 2023. First year medical student volunteers, “mentees”, were paired with older adults living with memory loss, “mentors”, recruited from a memory disorders clinic based at the University of Washington. In its pilot year, 10 students were paired with 10 older adults and their caregivers, for bimonthly to monthly visits at the Memory Hub, a community space affiliated with the University that provides education, support, and wellness services. Students participate in mentor visits and group activities at the Memory Hub and accompany mentors to medical appointments to experience the health care system from the perspective of a person living with memory loss. The goals of the program are to provide medical students with early clinical exposure to memory disorders, reduce stigma surrounding aging and memory loss, and inspire students to pursue underserved specialties in dementia care. Pre-implementation surveys of students found that reasons for participation in the program included interest in learning more about the dementia disease process, building communication skills, recognizing signs of memory loss to enhance clinic skills, and learning how to maximize quality of life for persons with memory loss. Initial challenges identified by students include difficulties in engaging mentors when a caregiver is not present, navigating conversations, and transportation barriers.

Abstract citation ID: igae098.1636

### SUSTAINABILITY OF OLDER ADULT VOLUNTEERS IN A MEDICAL EDUCATION PROGRAM

Keri Christensen, Janice Knebl, and Jennifer Severance, *University of North Texas Health Science Center, Fort Worth, Texas, United States*

Returning to pre-COVID learning expectations in a post-COIVD learning environment has presented new challenges in medical education. One is the sustainability of older adult volunteers, who are the center piece of a medical education program. Creating a sustainable volunteer program through engagement opportunities provides a space for volunteers to build community. UNTHSC has developed educational opportunities for older adult volunteers to learn about geriatrics as well as to create a space to connect with other volunteers. In the Seniors Assisting in Geriatric Education (SAGE) Program, teams of interprofessional health professions students are paired with volunteer older adults in the community, called Senior Mentors. The students visit their Senior Mentor at home, providing health screenings, safety assessments, community resources, and health education. With the onset of COVID-19 and the transition to virtual meetings with students, volunteers’ attrition was a challenge to administering the program. In response, the SAGE mentors were polled about events they would be interested in attending to increase volunteer engagement. A total of 166 surveys were administered and 78 volunteers responded. Results indicated that the majority (81%) would be willing to attend events and that events would be primarily educational health topics related to older adults. The results of that survey were then used to develop education events held twice a year to give the SAGE volunteers the opportunity to connect to other mentors, to expand their knowledge in caring and advocating for themselves, and to build personal relationships with the team involved with SAGE itself.

Abstract citation ID: igae098.1637

### MEDICAL STUDENT OBSERVATIONS AND REFLECTIONS FROM A MEMORY DISORDERS BUDDY PROGRAM

Mishy Roy[1], and Michael Rosenbloom[2], *1. Rush University Medical Center, Chicago, Illinois, United States*, *2. University of Washington, Seattle, Washington, United States*

Alzheimer’s disease (AD) impacts 5.4 million individuals with an estimated cost of $215 billion annually in the United States, yet cognitive impairment goes unrecognized in half of affected patients. Potential barriers to diagnosis include physician lack of comfort as well as fear of delivering a devastating diagnosis. Although most medical schools offer training related to AD pathophysiology, few curriculums provide experiential learning that may shape student attitudes toward dementia. Employing the buddy program model, the Partners in Dementia (PND) program paired 20 mild AD patients from the HealthPartners dementia clinic with first year medical students from the University of Minnesota. Students were required to spend four hours/month interacting with their patient families and submit monthly journal reflections of their experiences. A qualitative text analysis of journal entries for 2 cohorts over 2 years was done using line by line text coding, and identified the following major themes: support systems, social-occupational decline, mentor/caregiver’s emotional challenges, activities, self-reflections, and objective/clinical narratives. The primary observations across both years were emotional challenges to living with AD and self-reflections on how to become better and more humanistic physicians. 100% of the families thought this was a valuable exercise and 94% of the students said it changed the way they think about people living with dementia. We concluded that educational models like PND provide medical students with opportunities to make reflections that may shape their future clinical approach to dementia. In turn, these programs may facilitate better identification and management of AD in the community.

### SESSION 3515 (SYMPOSIUM)

Abstract citation ID: igae098.1638

### INTERGENERATIONAL SERVICE: EXTENDING AGE INCLUSIVITY IN AND BEYOND THE CLASSROOM

Chair: Joann Montepare
Discussant: Carrie Andreoletti

Higher education is becoming more age-inclusive on several fronts and age-friendly teaching and learning strategies are being used in new and novel ways to support a variety of educational outcomes. In this AIHE-ILRCE Interest Group collaborative symposium, presenters will discuss the diverse ways in which intergenerational service activities extend learning beyond the classroom and can enhance age inclusivity in higher education. To begin, Montepare and Baldizar (Lasell University) will provide an overview of contemporary age-inclusive campus initiatives in higher education (Age-Friendly University/AFU, Age Inclusivity Domains of Higher Education/AIDHE) and discuss The Empty Bowls project in which students and older adult community members work together to craft bowls to raise money to support the local Food Pantry. Brothers (Colorado State University) will describe the student-supported Market Days for Older Adults project, a farmer's market-based program that aspires to address food insecurity and social isolation in older adults. Wei and colleagues (Stockton University) will discuss an intergenerational service project in which students organize technology training sessions aimed at educating older adult community members about various technology-related topics such as using smartphones for chronic disease management, personalized medicine, and more. Kwong (Connecticut College) will describe an interdisciplinary class, Intergenerational Play and Learning, which connects young children, college students, and older adult volunteers to promote learning through storytelling and engaging in play and group activities to strengthen social ties across generations and explore local history. AGHE Fellow and intergenerational scholar, Carrie Andreoletti (Central Connecticut State University), will serve as discussant. Intergenerational Learning, Research, and Community Engagement Interest Group Sponsored Symposium

Abstract citation ID: igae098.1639

### TALK OF AGES AND EMPTY BOWLS: A RECIPROCAL APPROACH TO INTERGENERATIONAL SERVICE

Joann Montepare, *Lasell University, Newton, Massachusetts, United States*

Contemporary age-inclusive campus initiatives in higher education (Age-Friendly University/AFU, Age Inclusivity Domains of Higher Education/AIDHE) advocate for promoting intergenerational exchange to facilitate the reciprocal sharing of expertise between learners of all ages. This presentation will describe these initiatives as background for the symposium. In addition, it will describe how Lasell University's "Talk of Ages" program, which takes a "common ground" approach for bringing younger and older learners together in the beyond the classroom, offers a pedagogical philosophy for intergenerational exchange. To demonstrate the value of this approach, we will describe a campus-wide "Empty Bowls" project in which students and older adult community members worked together to craft bowls and to raise money in the mission to fight hunger in the local community. Logistics will be discussed about mounting intergenerational activities as part of this intergenerational service project including offering intergenerational classes in Ceramics, organizing sessions for bowl crafting and glazing, and hosting an intergenerational event where community members come together to enjoy a simple meal of soup and bread in a bowl they select and buy with the proceeds benefitting the local community food pantry. In addition to demonstrating the value of intergenerational collaboration and service as part of being an age-inclusive campus, challenges will be discussed so that educators are better prepared to design related efforts.

Abstract citation ID: igae098.1640

### MARKET DAYS: INTERGENERATIONAL PROGRAMMING TO SUPPORT HEALTHY AGING AMONG LOW-INCOME OLDER ADULTS

Allyson Brothers[1], India Luxton[1], Cheryl Noble[2], and Christa Timmerman[2], *1. Colorado State University, Fort Collins, Colorado, United States*, *2. Larimer County CSU Extension, Fort Collins, Colorado, United States*

The Market Days for Older Adults program provides fresh produce to food-insecure older adults at the downtown farmers' Market. As part of an ongoing university-community service partnership, undergraduate internships and service-learning projects enhance older adults' social connections while training gerontology students about health disparities and aging. The program addresses two Age-Friendly University (AFU) principles: #4: to promote intergenerational learning; and #6: to ensure the research agenda is shaped by needs of older adults. A 2023 program evaluation of Market Days (MD) in 2023 employed a pre-post mixed methods design (N=59) to examine process and outcome variables. We found evidence for high satisfaction and usage of the program, plus multiple perceived benefits to participants, including improved sense of health (61%), new ways for preparing fresh produce (31%), and higher connection to the community. We did not find evidence of significant improvements in food insecurity or social isolation. Students reported an increased understanding of the role of community programs in promoting healthy aging, and appreciation of food insecurity as a risk factor for aging. Results indicated that this subpopulation of older adults is particularly vulnerable with regard to chronic illness, low preparations for aging, low community resource awareness, mental health symptoms, and negative views on aging. The results will inform future steps of our community-engaged research program. Viewing social isolation as a pressing social determinant of health in low-income older adults, we will develop a formal intergenerational programming component to further strengthen social connections, while also enhancing undergraduate gerontology student training opportunities.

Abstract citation ID: igae098.1641

### INTERGENERATIONAL WORKSHOP TO EMPOWER OLDER ADULTS IN SMART TECHNOLOGIES

Duo Wei, Joseph Morales, and David Burdick, *Stockton University, Galloway, New Jersey, United States*

Intergenerational programming, as outlined by the National Council on the Aging, involves those "activities or programs that increase cooperation, interaction, or exchange between any two generations. It involves the sharing of skills, knowledge or experience between old and young." The overall goal of the intergenerational service learning project of the Medical Informatics class is to promote learning between university students and older adults in the community, and to put the concept of lifelong learning into practice. In the Medical Informatics class, students collaborate in multiple groups to organize technology training workshops. These workshops are tailored to educate older adult community members on a range of technology-related subjects, including utilizing smartphones and AI technologies for social engagement, entertainment, and health management, with the aim of enhancing health outcomes. The training sessions have been held at various venues across the South Jersey region, including AtlantiCare, Center for Family Services, Hammonton Canoe Club, Capital Health Medical Center, Four Seasons Clubhouse at Smithville, and others.

The effectiveness of the intergenerational programming has been assessed through student feedback, surveys of the older participants, student faculty/course evaluations, and media coverage. Results indicate that this service learning initiative is a valuable approach in fostering a culture of learning among university students and community partners alike. University students act as mentors to older adults, while also benefiting from the diverse perspectives shared by the older generation, thus enriching their learning experience. Moreover, this collaborative effort nurtures stronger bonds between communities and the university, promoting mutual understanding and support.

Abstract citation ID: igae098.1642

### INTERGENERATIONAL PLAY AND LEARNING: OPPORTUNITIES FOR ENGAGING PRESCHOOLERS, COLLEGE STUDENTS, AND OLDER ADULTS

Katherine Kwong[1], and Monica Franzone[2], *1. Connecticut College, New London, Connecticut, United States, 2. Connecticut Collge, New London, Connecticut, United States*

The U.S. Census notes that the population age demographic is rapidly changing, with approximately 10,000 individuals a day crossing the age threshold of 65, the mismatch between the number of individuals over the age of 65 and those under the age of 65 will have lasting implications in the coming decades. So, how can we take advantage of this resource? How can we benefit multiple generations? Intergenerational Play & Learning was a new course offered at Connecticut College during the Spring 2024 semester that introduced students to the intergenerational (IG) studies field and engaged them in experiential learning activities with older adults and preschool children. These students studied the changing relationships between young children and older adults through readings, videos, and personal observations. Through journals, interviews, and observation, students gained insights into the benefits of intergenerational learning (IL) and how IL can challenge discrimination. Course participants designed activities to promote social ties across age groups. The main goals for the course were: (1) provide a venue for young children, young adults, and older adults in the New London area to learn from and engage with each other; (2) encourage young and older adult participants to reevaluate stereotypes and discover how generations different from one's own can provide an opportunity for learning; (3) evaluate the importance of family and community ties; and (4) develop a model for future intergenerational programming.

### SESSION 3520 (SYMPOSIUM)

Abstract citation ID: igae098.1643

### INTERSECTION OF AGE, SEX, AND FRAILTY: CONSIDERATIONS FOR TRANSLATIONAL GEROSCIENCE, GERIATRICS, AND FORTITUDE

Chair: Sarah Hilmer
Co-Chair: John Mach
Discussant: Susan Howlett

Understanding aging, optimising care of older adults and facilitating fortitude, must consider the intersection of chronological age, sex and frailty. While existing initiatives consider these factors separately, they occur together in old age and have complex interactions. This symposium brings together basic and clinical scientists, at differing career stages, from three countries, who have explored these issues in their research. Dr Alice Kane will discuss work from her laboratory in the Institute for Systems Biology, USA, on important sex differences in translational biomarkers for ageing and frailty. Dr John Mach will describe insights from a pre-clinical model of chronic drug therapy and polypharmacy, developed at the Kolling Institute, Australia, on the impact of age, sex and frailty on drug effects. Prof Sarah Hilmer, Australia, will discuss key considerations in age, sex and frailty for development of drugs for older adults, drawing on a recent position statement from the Geriatric Committee of the International Union of Basic and Clinical Pharmacologists that she chairs. Prof Heather Allore, Yale, USA, will share her expertise in biostatistics in aging research, to demonstrate design and analysis techniques that can untangle the relationship between age, sex and frailty, applicable to biological and health sciences research. Prof Paula Rochon, University of Toronto, Canada, will highlight the importance of disaggregated data on sex, age and frailty to inform research, health care and policy for older adults. Finally, Prof Susan Howlett, Dalhousie, Canada, who pioneered research in the intersection of age, sex and frailty, will lead the discussion. Women's Issues Interest Group Sponsored Symposium

Abstract citation ID: igae098.1644

### DRUG DEVELOPMENT AND EVALUATION: AGE, SEX, AND FRAILTY CONSIDERATIONS TO FACILITATE THE FORTITUDE FACTOR

Sarah Hilmer, *Royal North Shore Hospital, Sydney, Australia, Sydney, New South Wales, Australia*

Development and evaluation of drugs used by older adults must consider the effects of ageing, of lifelong factors including sex, and of other characteristics common in geriatric patients that affect pharmacokinetics and pharmacodynamics and outcomes, such as frailty. These factors interact within older people, resulting in major interindividual variability. In some cases, the intersection of age, sex, frailty and other factors act in the same direction, e.g, age, female sex and frailty may all reduce renal drug clearance. In others they may act in opposite directions, e.g. age may decrease pharmacodynamic sensitivity to a drug, male sex may increase it, and frailty may reduce reserve to tolerate drug effects. Interactions with the older person's other drugs in the presence of polypharmacy, their other diseases with multimorbidity, their genetics and environmental exposures further complicate prediction of drug effects in older adults. The International Union of Basic and Clinical Pharmacology (IUPHAR) Geriatric Committee's position statement on 'Measurement of Frailty in Drug Development and Evaluation' outlines key principles relevant to all phases of translational research. These include measurement of frailty, testing drug exposures and outcomes that are clinically relevant for older adults, use of physiologically based pharmacokinetic-pharmacodynamic modelling and artificial intelligence. In all phases of drug development and evaluation, the pre-clinical models used and clinical trial populations studied should be representative of the people who will use the drugs in age, sex, frailty, and other determinants of drug response. This will inform drug use that facilitates the fortitude factor in older adults.

Abstract citation ID: igae098.1645

**TRANSLATIONAL BIOMARKERS FOR AGING AND FRAILTY: IMPORTANT SEX DIFFERENCES**

Alice Kane, *Institute for Systems Biology, Seattle, Washington, United States*

Frailty can be measured using a frailty index, which quantifies the accumulation of health-related deficits. Frailty increases the risk of adverse outcomes including hospitalization and mortality, but there are no validated frailty biomarkers and the underlying mechanisms of frailty remain unknown. Additionally, women are more frail than men at all ages, despite having a lower risk of mortality, but sex differences in frailty are poorly understood. We have assessed health outcomes, including frailty, in male and female C57BL/6 mice from middle age to natural death. We collected blood and stool samples at each timepoint, for untargeted plasma metabolomics, blood DNA methylation assessment and gut microbiome analysis. As in humans, female mice have higher frailty scores than males in middle-to-late life. Sex explains more variability than age or frailty in our DNA methylation data, and preliminary analysis identifies 100s of sex-specific differentially methylated regions with frailty. Metabolomics analysis identifies a set of 104 metabolites (enriched for amino acid and lipid pathways) associated with frailty, independent of sex and age. Additionally, we identify many sex-specific metabolomic markers for frailty, including creatine for females and FAD for males. Analysis of the microbiome data identifies microbes that are both positively and negatively associated with frailty across sexes including A. colihominis. Integrated analysis across data-types is ongoing. Overall, we see clear sex differences in the association of frailty with molecular outcomes in mice. Further investigation of these factors, and validation in human cohorts, will identify determinants and biomarkers of frailty in both sexes.

Abstract citation ID: igae098.1646

**POLYPHARMACY: TRANSLATIONAL RESEARCH TO UNDERSTAND EFFECTS OF AGE, SEX, AND FRAILTY ON KEY OUTCOMES FOR FORTITUDE**

John Mach, *Kolling Institute/The University of Sydney/ Royal North Shore Hospital, Sydney, New South Wales, Australia*

Chronic medication use is common in older men and women. Older people, particularly those with polypharmacy (use of 5 or more drugs) for multi-morbidity, are rarely included in clinical trials to establish efficacy and safety of therapeutic drugs. Based on clinical observational studies, polypharmacy is associated with impaired physical function and exacerbates frailty in older adults, particularly women, who make up the majority of this age group. In addition, there is evidence that women are at greater risk of drug-related harm than men. Preclinical models would be useful to screen for adverse geriatric outcomes and understand the impact of age, sex and frailty. This presentation will cover our advancements in the development and use of preclinical models to understand the effects of polypharmacy on geriatric outcomes that are important contributors to the ‘fortitude factor’ in both sexes and old age. We will present recent data from studies in our preclinical models that: (1) describe how chronic polypharmacy use impairs translatable functional outcomes in ageing; (2) demonstrate differences in outcome response for different sex, age and frailty.

Abstract citation ID: igae098.1647

**WHAT’S AGE, SEX, AND FRAILTY GOT TO DO WITH IT? USING DESIGN AND ANALYSIS TO PROVIDE POWERFUL ANSWERS**

Heather Allore, *Yale University, New Haven, Connecticut, United States*

Designs of animal experiments to disentangle the heterogeneity associated with age, sex and frailty will be shared. These range from cross-sectional to longitudinal and cross-over designs (where treatment may be started and stopped). Matching the analytic models to the design should be determined a prior at the design stage. Considerations in longitudinal designs including within animal correlation across repeated measures. Hypotheses need to be written a priori clearly identifying the main effect and whether interactions between effects are potentially important, such as sex by frailty status. Batch effects of birth cohorts or caging may also need to be models as sources of variation to have the most accurate and precise effect estimates. Examples of such designs and analyses will be presented, as well as sample size considerations. Properly designed animal experiments area key element in translational gerontology and geroscience. Planning for the factors in animal experiments that are known or suspected risk factors for geriatric outcomes in humans allows for testing whether the animal model may appropriately model the human conditions under study.

Abstract citation ID: igae098.1648

**INCREASING THE VISIBILITY OF OLDER ADULTS IN RESEARCH: THE IMPORTANCE OF AGE, SEX, AND FRAILTY CONSIDERATIONS**

Paula Rochon[1], and Joyce Li[2], *1. Women’s College Hospital, Toronto, Ontario, Canada*, *2. Women’s College Research Institute, Toronto, Ontario, Canada*

Older adults are often considered as a single group with differences between women and men across age groups overlooked. Taking account of sex, age, and their intersection throughout the research process is crucial in minimizing inequities, and to improve the health and wellbeing of the aging population. Age and sex disaggregated data are data separated and analyzed by female, male and age categories, revealing important differences between the sexes and across the life course. This is often missing in research and can result in the oversimplification of study results. As men and women age, they can experience different health conditions, and levels of frailty, leading to the need for tailored treatments. One illustration of this is exploring the burden of disease in Canada, where disaggregating data by sex and age makes key differences in many diseases impacting older adults visible, that may otherwise be missed (for example, heart disease and dementia). Age, sex, and frailty considerations are important throughout the entire continuum of the research process, from question development to dissemination of results to ensure they are accounted for. This presentation will explore why incorporating sex, age, frailty and their intersection are important in health research, how this consideration adds value, and applications of this approach in practice to increase the visibility of older women and men in research, contributing to the 'fortitude factor'.

### SESSION 3525 (SYMPOSIUM)

Abstract citation ID: igae098.1649

### LESSONS FROM THE PAST, VISIONS FOR TOMORROW: GERONTOLOGY AND GSA IN RETROSPECT

Chair: Soomi Lee
Discussant: Soomi Lee

GSA is approaching its 80th anniversary, prompting us to reflect on the origins and rich history of gerontology. Gerontology's roots trace back to the early 1900s, an era where older age was often perceived solely as a period of physical and mental decline. However, over time, our understanding of aging has significantly evolved. Despite this progress, a noticeable gap persists in inter-generational knowledge exchange, hindering the opportunity to build upon previous theories and extend seminal work. This year's theme, "fortitude," underscores the importance of sustaining good science of aging across generations and disciplines. In response, our symposium provides an educational and scholarly exchange platform rooted in historical perspectives. Three esteemed panelists who have played pivotal roles in shaping GSA's trajectory will share their insights and experiences, with each presentation followed by comments by other panelists. Dr. Steven H. Zarit, recipient of the 2019 Robert W. Kleemeier Award, will discuss how the field of gerontology has evolved. Dr. David Chiriboga, recipient of the 2010 Hiram J. Friedsam Outstanding Mentorship Award, will share his memorable mentorship stories. Dr. Kathleen Wilber, recipient of the 2023 Donald P. Kent Award, will address key values and challenges of the field. The moderator, Dr. Soomi Lee, will facilitate discussions with the panelists and audience. This session offers an opportunity for the Academy for Gerontology in Higher Education (AGHE) and the wider GSA community to reflect on the lasting impact of gerontology and discuss collective visions for tomorrow as we navigate the evolving landscape of the field. Generativity and Aging Interest Group Sponsored Symposium

Abstract citation ID: igae098.1650

### RICH HISTORY OF GERONTOLOGY AND GSA

Steven Zarit, *The Pennsylvania State University, University Park, Pennsylvania, United States*

In this presentation, Dr. Steven H. Zarit will lead a panel discussion exploring the historical underpinnings of gerontology and the Gerontological Society of America (GSA), drawing from his distinguished career and vast contributions to the field. Dr. Zarit, celebrated for his groundbreaking research on caregiver burden and stress, as well as family relationships across the lifespan, will delve into the evolution of gerontology and its profound impact on his work. Acknowledging his accolades, including prestigious awards from the GSA and the American Psychological Association, Dr. Zarit will reflect on his journey into gerontology and its intersection with seminal figures like Drs. Powell Lawton, James Birren, and Bernice Neugarten. The panel discussion will feature additional insights, drawing on personal experiences and perspectives. Through a series of questions posed by the moderator, the panelists will delve into their personal motivations, the transformative developments within gerontology, and the enduring influence of key figures in the field. Following the moderated discussion, the audience will have the opportunity to engage in an interactive question-and-answer session, fostering a dynamic exchange of ideas and insights. By drawing on the lessons of the past, this presentation aims to inspire visionary approaches to the future of gerontology informed by a panel who collectively have 145 years in the field.

Abstract citation ID: igae098.1651

### FOSTERING GENERATIVITY: MENTORSHIP AND KNOWLEDGE EXCHANGE IN GERONTOLOGY

David Chiriboga, *University of South Florida, Tampa, Florida, United States*

In this presentation, Dr. David Chiriboga will lead a panel discussion sharing his invaluable experiences and insights on mentorship within the field of gerontology. Dr. Chiriboga's impactful work spans diverse research areas, including studies on health disparities, family-centered interventions for Alzheimer's disease, and barriers to healthcare among older Korean immigrants. Renowned for his role as a mentor, Dr. Chiriboga has nurtured numerous researchers, shaping the landscape of aging studies. His dedication to fostering talent extends beyond academia, evidenced by his mentorship in initiatives like the American Society on Aging's New Ventures in Leadership program. Acknowledged for his exceptional mentorship, Dr. Chiriboga has received prestigious awards such as the Hiram J. Friedsam Mentorship Award from the Academy for Gerontology in Higher Education and the Minority Mentorship Award from the Gerontological Society of America (GSA). In his presentation, Dr. Chiriboga will illuminate the significance of mentorship and the culture surrounding it, drawing from his wealth of experience. The panel discussion will further explore mentorship and generativity development in gerontology. Following the moderated discussion, the audience will engage in an interactive question-and-answer session. By emphasizing the importance of mentorship and inter-generational knowledge exchange, this presentation aims to illuminate strategies for retaining the legacy of successful mentoring within the GSA and advancing the field through collaborative learning and development.

Abstract citation ID: igae098.1652

### SUSTAINING VALUES AND CHARTING FUTURES

Kathleen Wilber, *University of Southern California, Los Angeles, California, United States*

In this presentation, Dr. Kathleen Wilber will lead a panel discussion exploring enduring values and evolving challenges within the field of gerontology, aiming to guide the trajectory of the discipline into the future. Dr. Wilber's research is dedicated to enhancing health outcomes and quality of life for vulnerable older adults through systemic improvements in healthcare delivery. Her expertise in collaborative care models has contributed to understanding cost-effectiveness and optimizing service delivery structures. Recognized for her leadership in gerontology, Dr. Wilber was honored with the prestigious Donald P. Kent Award from the Gerontological Society of America (GSA) in 2023. In her presentation, she will share insights on timeless values that continue to shape the field and propose strategies for addressing persistent challenges. Dr. Wilber will also offer valuable advice for emerging scholars based on her personal journey, reflecting on lessons learned and potential areas for improvement. The panel discussion will further explore visions and directions for advancing the society. Following the moderated discussion, the audience will engage in an interactive question-and-answer session, fostering collaborative dialogue and actionable insights. By deliberating on enduring values, emerging challenges, and future directions, this presentation aims to catalyze concrete action plans to propel the society forward.

### SESSION 3530 (PAPER)

### LONELINESS AND CONNECTIVITY

Abstract citation ID: igae098.1653

### EMBRACING CONNECTIVITY: TANGIBLE, CUSTOMIZED CHECK-INS FOR OLDER ADULTS TO COMBAT SOCIAL ISOLATION

Pallabi Bhowmick[1], and Lesa Huber[2], *1. University of Illinois Urbana-Champaign, Mahomet, Illinois, United States*, *2. Indiana University Bloomington, Bloomington, Indiana, United States*

Increasing social isolation among older adults poses significant concerns for health and well-being. While older adults consider families as their strongest bonds, friends and neighbors often play a pivotal role as the primary support network. This study investigates the efficacy of leveraging peer support through a tangible, customized daily check-in to prolong independent living. The study engaged 15 older adults aged 70-90 in a 3-hour, recorded co-design session with the objective of ensuring their needs were considered early in the design process. Transcripts were coded and thematically analyzed to reveal three key findings. Firstly, 80% of participants lived alone and strongly acknowledged the need for a daily check-in. They noted, neighbors and peers in close proximity often serve as the first line of support. Secondly, 87% emphasized the importance of a customized interface aligned with their personal routine, (e.g. checking-in by making tea), affirming it would enhance usability. Additionally, tangible user interfaces integrate digital information into everyday objects, providing familiar, intuitive user interfaces. Participants concurred this could address sensory-motor challenges and mitigate the learning curve associated with digital interfaces. Thirdly, all participants strongly opposed one-way passive monitoring, advocating for a peer-to-peer check-in system that allows older adults to take care of each other instead of always being on the receiving end of care. This promotes reciprocity, inclusivity, and reduces stigma. Tangible, customized, peer-based check-in systems have the potential to improve social connection, address ageism in technology design, and encourage technology acceptance and adoption among the older adult population.

Abstract citation ID: igae098.1654

### THE ASSOCIATION BETWEEN DIGITAL ENGAGEMENT OF OLDER ADULTS AND DISPLACEMENT DURING ARMED-CONFLICT CIRCUMSTANCES

Ittay Mannheim[1], Rinat Lifshitz[2], Yaacov Bachner[1], and Ella Cohn-Schwartz[1], *1. Ben Gurion University of the Negev, Beer-Sheva, HaDarom, Israel*, *2. The Max Stern Academic College of Emek Yezreel, Tel Adashim, HaZafon, Israel*

Digital engagement is perceived as having the potential to improve the well-being of older adults. This study tests this notion in extreme circumstances, such as an ongoing military conflict. The association of digital engagement with the well-being of older adults was examined within the context of forced displacement and imminent threat (e.g., rocket attacks) due to the ongoing war in Israel. Ninety-three older persons that were evacuated from their homes, and 150 that were not evacuated responded to an online or paper survey. The survey measured their digital engagement before the war and in the weeks after it began, perceptions towards aging and technology, and well-being (depressive symptoms, loneliness, subjective health, and control). Evacuees were found to be lonelier, with lower subjective health and a lower sense of control compared to non-evacuees. Baseline digital engagement retrospectively reported from before the war was the same between groups. However, following the war, those evacuated reported using a lower number of devices, had different patterns of uses, and perceived higher limitations in using technology. Importantly, a stepwise regression analysis identified that digital engagement was a stronger predictor than displacement on higher loneliness and lower subjective health. Whereas rocket threat was the strongest predictor for depressive symptoms and displacement for sense of control. Negative perceptions towards aging and technology were also predictors of higher depressive symptoms. This study discusses the importance of digital engagement for the well-being of the older population in a world with ever-growing prevalence of extreme circumstances such as armed conflicts.

Abstract citation ID: igae098.1655

### LONELINESS AMONG ADULTS AGING WITH INTELLECTUAL AND DEVELOPMENTAL DISABILITIES: THE ROLE OF LIVING SITUATION

Jeffrey Stokes[1], Danielle Waldron[2], and Elisabeth Stam[1], *1. University of Massachusetts Boston, Boston, Massachusetts, United States*, *2. Stonehill College, North Easton, Massachusetts, United States*

**Background and Objectives:** Loneliness is a serious public health concern among the aging population. Not only is loneliness an unpleasant emotional experience, it is also associated with worse health, well-being, and even mortality. This is a particularly important issue among the population aging with intellectual and developmental disabilities (I/DD), who are more likely to experience loneliness across the life course, and who – particularly if living in an intermediate care facility (ICF) or nursing facility – may lack social connections.

**Research Design and Methods:** We analyzed panel data from the 2013-2014 through 2021-2022 waves of the National Core Indicators In-Person Survey (NCI-IPS; 8 waves total), a national survey of adults with I/DD receiving state services (N = 101,374 observations drawn from 49 states). Multilevel logistic regression models examined whether loneliness varied according to living situation.

**Results:** Results indicated that (1) adults aging with I/DD in ICF and nursing facilities reported significantly greater loneliness than those living in the community, and especially those living with family; (2) having friends was associated with reduced loneliness overall, yet (3) significant moderation analysis revealed that having friends was associated with reduced loneliness among those living in the community, but not for those living in ICF or nursing facilities.

**Discussion:** These results indicate not only that adults with I/DD living in institutionalized settings are at higher risk of experiencing loneliness and its detrimental effects, but that specialized interventions are required to meet their unique needs and reduce their loneliness in mid and later life.

Abstract citation ID: igae098.1656

### LONELINESS AND SOCIAL ISOLATION IN THE COVID ERA

Solveig Cunningham[1], and Jo Hale[2], *1. Emory University, Atlanta, Georgia, United States*, *2. University of St. Andrews, St. Andrews, Scotland, United Kingdom*

Social isolation and loneliness diminish health and wellbeing and may have been exacerbated during the Covid-19 lockdown. We examined the relationship between social isolation and loneliness among U.S. older adults during the Covid-19 pandemic, when social isolation was high due to transmission reduction measures. Data are from the National Health and Aging Trends Survey (NHATS), representative of the age 70+ Medicare beneficiary population in the United States. Prior to the pandemic, one third of older adults experienced loneliness at least on some days. One in 5 older adults (22.5%; 95% CI: 20.0, 25.0) report an increase in loneliness during the pandemic. Those who were severely socially isolated had double the odds of feeling lonely. However, while unadjusted models suggest an increasingly positive correlation between social isolation and loneliness among older adults, associations in the fully adjusted model have weak and directionally variable correlations, suggesting that the relationship can be largely explained by other social and demographic characteristics. This may also explain why existing literature is divided on the relationship between social isolation and loneliness. During the pandemic, those who felt lonely were also more likely to strictly follow social isolation measures. The frequency of loneliness prior to the pandemic is a strong predictor of loneliness during COVID. Still, loneliness and isolation were only weakly correlated, even during this period of widespread increases in social isolation. The implications of social isolation and of loneliness for older adults' wellbeing should be considered both jointly and independently, as they are not fully overlapping.

### SESSION 3535 (SYMPOSIUM)

Abstract citation ID: igae098.1657

### MEMORY CARE IN ASSISTED LIVING: REGULATIONS, ACCESS, AND CARE DELIVERY

Chair: Paula Carder
Discussant: Doug Pace

Assisted living (AL) is the largest residential care provider of long-term care. Of the nearly one million people who reside in AL, up to 70% have some form of cognitive impairment and 40% have a diagnosis of Alzheimer's Disease or related dementia (ADRD). AL staff provide services including help with dressing and meals, and nearly 25% of ALs provide specialized care for people living with ADRD (also known as "memory care"). This symposium will include four presentations, each focused on different dimensions of memory care. The first presenter will share findings from a state regulatory review in which the team profiled the presence and content of memory care-related regulations - identifying extreme variability within and across states. The second speaker will present an innovative approach for identifying memory care ALs, and describe their availability across the country. The third presenter will share findings from a recent nationally-representative survey of ALs related to the provision of end of life care in memory care ALs. The fourth speaker will present findings from a national retrospective study in which end of life care outcomes among residents with dementia in memory care will be compared to those in general AL. Finally, our discussant, a national leader in dementia care advocacy and former chair of the Center for Excellence in Assisted Living, will contextualize findings highlighting the perspectives of consumers, AL providers, and policymakers. Assisted Living Interest Group Sponsored Symposium

Abstract citation ID: igae098.1658

### VARIATION IN STATE APPROACHES TO DEMENTIA CARE TRAINING IN ASSISTED LIVING

Paula Carder[1], Lindsey Smith[2], Sarah Dys[3], Eric Jutkowitz[4], and Kali Thomas[5], *1. OHSU-PSU School of Public Health, Portland, Oregon, United States*, *2. Oregon Health & Science University, Portland, Oregon, United States*, *3. Portland State University, Portland, Oregon, United States*, *4. Brown University, Brown University, Rhode Island, United States*, *5. Johns Hopkins University, Baltimore, Maryland, United States*

Assisted living (AL) communities increasingly serve individuals with Alzheimer's disease and related disorders (ADRD); an estimated 50% of AL residents have ADRD, and more AL have designated memory care than do nursing homes. State AL regulations vary on almost every provision that affects resident safety and quality, including staffing levels, building design, safety features, admission and discharge criteria, and social-recreational programming. Nearly every state requires that direct care workers receive training in dementia care. Our prior research described the presence of within-state variation in memory care regulations, with the majority of states requiring such training only in designated "memory care" settings rather than all AL licenses (Smith et al., 2022). This paper extends that research by examining staff dementia care training requirements in detail, including the number of training hours, specificity of dementia care topics, and the presence of social aspects of care (e.g., family involvement, person-centered care). The 43 states that require direct care workers to receive training in dementia care have a total of 93 license types, with some applied to settings that are designated as "memory care." Of the 43 states and 93 licenses, 23 states and 34 licenses specify the number of training hours that direct care workers must receive in the first year, ranging from 2 to 75 hours (average 15.7). Examples of highly specific staff training requirements are provided as a resource for states and AL professionals, and to inform consumers who might expect that all memory care settings have similar requirements.

Abstract citation ID: igae098.1659

**WHAT IS MEMORY CARE IN ASSISTED LIVING?**

Lindsey Smith[1], Eric Jutkowitz[2], and Kali Thomas[3], *1. Oregon Health & Science University, Portland, Oregon, United States*, *2. Brown University, Brown University, Rhode Island, United States*, *3. Johns Hopkins University, Baltimore, Maryland, United States*

Memory care is the most common type of specialized care provided in assisted living (AL) settings. This specialized care caters to people living with dementia and other forms of cognitive impairment. However, what constitutes specialized memory care varies according to who and how the setting is defined. We use the lenses of the state, industry, and consumers to describe the prevalence of ALs with memory care across the US. We created a national directory of ALs and their state licenses to identify those ALs designated as providing specialized memory care by the licensing agency, classified according to whether the state holds these ALs to additional standards or simply requires disclosure. To identify AL communities considered memory care by industry professionals, we relied on a dataset of AL organizations from the National Investment Center (NIC), a nonprofit organization focused on providing data, analytics, and connections to the senior housing and care industries. Finally, to view AL communities through the lens of (potential) residents and their families, we used business advertisement descriptions from Data Axle, a business advertising database. We found that 28% of the 13,715 large (greater than 25 beds) AL communities in our national AL directory were identified as memory care by state licensing agencies, 60% by NIC, and 26% by Data Axle. Our findings raise questions regarding how the care provided in these settings may vary based on who defines memory care.

Abstract citation ID: igae098.1660

**END-OF-LIFE CARE PROCESSES IN ASSISTED LIVING COMMUNITIES SPECIALIZING IN MEMORY CARE**

Emmanuelle Belanger[1], Nicole Rosendaal[1], Xiao (Joyce) Wang[1], Kali Thomas[2], Joan Teno[1], and Melissa Clark[1], *1. Brown University, Providence, Rhode Island, United States*, *2. Johns Hopkins University, Baltimore, Maryland, United States*

Assisted living (AL) is a common site of death. Many AL communities offer specialized memory care (MC), yet we know little about how their end-of-life (EoL) care practices might differ. We conducted a nationally representative survey of 2,084 AL administrators (43% response rate) about EoL care between 2021-2023. Over half offered MC, with 41.7% of AL communities offering both general AL and MC (combined AL), and 9.3% MC only. Combined ALs were larger (44.7% had 100+ beds, compared to 2.3% in MC only and 14.1% in general AL). ALs offering MC were more likely to report having residents who died in the prior 12 months (88.4% combined AL, 90.0% MC only, 68.9% general AL), which is consistent with a majority reporting a policy of retaining residents in need of EoL care (82.9% combined ALs, 84.6% MC only, and 58.1% general AL only). AL communities offering MC were more likely to have staff available 24/7 to administer subcutaneous injections (67.3% combined AL, 71.1% MC only, 55.8% general AL). Similar opportunities to improve hospice care were reported, with only 32.0% of administrators answering "all of the time" when asked if residents referred to hospice were assessed within 24 hours. Important differences in key processes of care exist between AL offering MC and those providing general AL. Future research is needed to examine how these impact the quality of end-of-life care.

Abstract citation ID: igae098.1661

**DO END-OF-LIFE OUTCOMES DIFFER BY ASSISTED LIVING MEMORY-CARE DESIGNATION?**

Xiao (Joyce) Wang[1], Porita Cornell[2], Emmanuelle Belanger[1], and Kali Thomas[3], *1. Brown University, Providence, Rhode Island, United States*, *2. University of Melbourne, Melbourne, Victoria, Australia*, *3. Johns Hopkins University, Baltimore, Maryland, United States*

Residential care/assisted living (RC/AL) is an increasingly common place of end-of-life care for persons with Alzheimer's disease and related dementia (ADRD). Understanding how end-of-life outcomes differ by memory care among residents with ADRD could facilitate aging/dying in place for this population. The objective of this paper is to examine if end-of-life outcomes (i.e. mortality, hospice use, number of days receiving hospice in the last month of life) differ between residents with ADRD who moved to RC/ALs with specialized memory care service (memory-care RC/AL), compared with residents with ADRD who moved to RC/AL without memory care (general RC/AL). We created a prospective cohort of 15,152 fee-for-service Medicare beneficiaries with ADRD who moved to large RC/AL (>=25 beds) between 2016 and 2018. We used inverse probability treatment weighting to account for observable differences between memory-care and general RC/AL residents. Two-part models estimated the difference by memory care in the number of days receiving hospice in the last months of life among decedents. The adjusted difference in mortality rate was 1.3 percentage points higher in memory care ($p=0.04$). Additionally, memory-care residents had a higher rate of hospice use (1.4 percentage points, $p=0.01$). Decedents in memory-care RC/AL spent about 1.4 more days receiving hospice care in the last month of life ($p=0.02$). The higher mortality rate and higher rate of hospice use in memory care suggest that memory-care RC/AL may attract residents closer to the end of life and/or promote hospice use at the end of life.

### SESSION 3540 (SYMPOSIUM)

Abstract citation ID: igae098.1662

**MENTAL AND PHYSICAL HEALTH OF OLDER ADULTS: INSIGHTS FROM CROSS-NATIONAL CONTEXTS**

Chair: Jennifer Ailshire
Co-Chair: Eunyoung Choi
Discussant: Emma Nichols

Comparing the aging experience across country contexts offers a unique opportunity to determine whether observed differences in mental and physical health of older adults reflect a universal aging process or if they depend on the unique social and economic contexts in which older adults live. The HRS-family of surveys consists of nationally-representative, longitudinal studies of aging conducted in many countries around the world that have data and measures that have been harmonized within the Gateway to Global Aging data platform. This resource provides remarkable opportunities for cross-national comparative analyses of social and economic determinants of health and well-being among older adults. The papers in this session use these harmonized data to compare the aging experience across different social and economic exposures in multiple countries from around the world, including examinations of: 1) the role of exposure to recession and workplace security on psychological stress of older adults in the US and Korea; 2) the importance of resources and social status in mental health among widowed older adults in the US and India; 3) how caregiving relates to depression among informal family caregivers across 19 countries; and 4) the importance of migration status and country of residence for multimorbidity among Mexican Older adults in the US and Mexico. This symposium provides a comprehensive examination of how workplace, family, caregiving, and migration experiences contribute to health and well-being among older adults around the world.

Abstract citation ID: igae098.1663

### WORK EXPECTATION AND PSYCHOLOGICAL STRESS DURING THE GREAT RECESSION: THE ROLE OF LATE-LIFE EMPLOYMENT TRANSITIONS

Linh Dang[1], Toni Antonucci[1], Richard Gonzalez[1], Philippa Clarke[1], Carlos Mendes de Leon[2], and Briana Mezuk[1], *1. University of Michigan, Ann Arbor, Michigan, United States*, *2. Georgetown University, Washington, District of Columbia, United States*

Perceived uncertainty about future employment during economic crises negatively affects the mental health of older adults. Moreover, such health impact varies across countries and employment contexts. This study examined the longitudinal association between work expectation and psychological stress among Baby Boomers in the US and South Korea and how employment transitions moderated this association during the Great Recession (2008-2009) and its recovery (2010-2018). Data came from 2006-2018 Health and Retirement Study (n=2,647) and Korean Longitudinal Study of Aging (n=1,454). Perceived expectation of working in the next five years was reported on a probability scale (0-100%). Psychological stress was assessed by the Center for Epidemiologic Studies Depression Scale. Six distinct employment transitions were constructed based on labor force status: entrance to full-time employment, precarious employment, stable full-time employment, self-employment, exit labor force, and being out of labor force. Multivariate mixed-effects logistic regressions were fit, adjusting for sociodemographic and health characteristics. Effect modification by employment transitions was examined via stratification. Higher work expectations were associated with lower odds of psychological stress during recession (OR=0.93 [0.89, 0.98]) and recovery (OR=0.95 [0.92, 0.98]) in US respondents; this association was more pronounced during the recovery in Korea (OR=0.83 [0.79, 0.87]). Among those who transitioned to self-employment, higher expectations were associated with higher odds of stress in US (OR=1.22 [1.02, 1.46]) but lower odds in Korea (OR=0.90 [0.77, 1.05]). Findings suggest opportunities for country-specific workplace policies to support mental health of older adults transitioning to different employment paths, particularly during the uncertain period of economic crisis.

Abstract citation ID: igae098.1664

### DIFFERENCES IN PROTECTIVE MENTAL HEALTH RESOURCES AMONG WIDOWED IN THE UNITED STATES AND INDIA

Shekhar Chauhan[1], Dawn Carr[1], Miles Taylor[1], and Amanda Sonnega[2], *1. Florida State University, Tallahassee, Florida, United States*, *2. University of Michigan, University of Michigan, Michigan, United States*

Widowhood is among the most consequential stressful events for mental health. Although social support is protective for mental health at widowhood, most of widowhood studies are based on US populations. This study uses a harmonized dataset we created to evaluate differences in depressive symptoms among recently widowed individuals (i.e., within the last 2 years) in the United States and India using the first (2017-18) wave of the Longitudinal Ageing Study in India (LASI; n=1571) and the 2016-2018 waves of the Health and Retirement Study (n=202). Using OLS regression, we evaluate depressive symptoms based on a four-item depression instrument (CESD; mean=1.19), by country status and we use interaction effects to evaluate differences by country. Results show that mean depressive symptoms are lower among US widows (0.95 vs 1.22). Having children and higher relative socioeconomic status are both associated with protective effects for US and Indian widowed people, but having friends and rural status are only associated with lower depressive symptoms among US widows. Rural status is associated with higher symptoms in India. Gender is not significant in either sample. Self-rated health is associated with protective effects for depressive symptoms for both groups, but the effect is greater for US widows. Findings suggest there are certain protective resources that are consistent across contexts, especially self-rated health, socioeconomic status and having children. Mental health interventions for widows that focus on non-family social engagement, however, may only benefit recent widows in the US.

Abstract citation ID: igae098.1665

### DEPRESSION AMONG MIDDLE-AGED AND OLDER INFORMAL CAREGIVERS IN 19 COUNTRIES: A CROSS-NATIONALLY HARMONISED STUDY

Nan Jiang[1], Jose Pagan[2], Bei Wu[3], and Yan Li[4], *1. Tsinghua University, Beijing, Beijing, China (People's Republic)*, *2. New York University, New York, New York, United States*, *3. New York University, New York, New York, United States*, *4. Shanghai Jiao Tong University School of Medicine, Shanghai, Shanghai, China (People's Republic)*

The health of the caregivers is crucial to sustain informal care provision, while depression is an important health risk concept. In this study we aim to conduct multi-country, population-based studies on caregivers' depression and examine the association of caregiving with depression in 19 countries. We included 142,741 adults aged ≥ 45 years in our sample. Depression was measured using established cut points in shortened Center for Epidemiologic Studies Depression (CES-D) or EURO-D scales. We conducted multivariable logistic regression analyses to explore the association of the past 12-month caregiving with depression. Over 30% of participants in our sample were engaged in caregiving with particularly high rates observed in Denmark, China, France, and Sweden. After controlling for potential confounders, caregivers had a significantly higher risk of having depression (OR = 1.13, 95% CI = 1.02-1.25) compared to non-caregivers. The associations were stronger in China, India, South Korea, Spain, and the United Kingdom than in other countries. Our findings suggest that urgent attention must be given to developing strategies that address the unique challenges faced by caregivers, fostering their well-being while maintaining high-quality care standards. Future interventions and policies need to aim at mitigating the mental health burden experienced by caregivers and ensuring sustainable and effective care support.

Abstract citation ID: igae098.1666

### MIGRATION STATUS, COUNTRY OF RESIDENCE, AND MULTIMORBIDITY AMONG MEXICAN OLDER ADULTS

Nicholas Bishop[1], Siting Chen[2], Rafael Samper-Ternent[3], and Ana Quiñones[4], *1. University of Arizona, Tucson, Arizona, United States*, *2. OHSU-PSU School of Public Health, Portland, Oregon, United States*, *3. The University of Texas Health Houston, Houston, Texas, United States*, *4. Oregon Health & Science University, Portland, Oregon, United States*

Cross-national data including measures of health and migration status allow investigation of how selection mechanisms may contribute to observed immigrant health advantages. Focusing on Mexican adults aged 50 and older, we drew observations from the 2012 and 2018 waves of the Health and Retirement Study (HRS) and Mexican Health and Aging Study (MHAS) to examine variation in multimorbidity (≥ 2 chronic conditions) across four groups: U.S. born HRS respondents who identify as Mexican (n=674), immigrant HRS respondents who identify as Mexican (n=814), Mexican MHAS respondents who never worked/lived in the U.S. (n=7,833), and Mexican MHAS respondents who worked/lived in the U.S. (n=920). Multimorbidity was defined as the sum of seven self-reported doctor-diagnosed conditions (hypertension, diabetes, cancer, lung disease, heart attack, stroke, and arthritis). Linear regression models were used to predict multimorbidity in 2012 and changes in multimorbidity from 2012–2018 were modeled using an autoregressive approach. Harmonized covariates were measured in 2012 (age, sex, partnership status, BMI, rural residence, # doctor visits in past year). Predicted multimorbidity values for U.S. born Mexicans (y^=1.65, CI:1.55-1.75) were significantly greater than those for immigrant Mexicans living in the U.S. ($\hat{y}$=1.35, CI:1.26-1.45), return migrants living in Mexico (y^=1.35, CI:1.26-1.43), and non-migrants living in Mexico (y^=1.35, CI:1.30-1.40). Country of residence and migration status were not associated with change in multimorbidity. These results suggest that U.S. born Mexican adults are uniquely affected by multimorbidity and multimorbidity burden among migrants who return to Mexico is similar to immigrant Mexicans who reside in the U.S.

### SESSION 3545 (SYMPOSIUM)

Abstract citation ID: igae098.1667

### NAVIGATING AGING ACROSS CULTURES: THE FORTITUDE IN PERSPECTIVES FROM KOREANS AND BEYOND

Chair: Michin Hong
Discussant: Kyungmin Kim

The symposium aims to provide a multifaceted examination about aging related issues within different cultural contexts with a particular focus on the Korean population. The first study conducted a cross-cultural examination on financial satisfaction during the retirement transition period between South Kore and the U.S., highlighting the needs for culturally tailored interventions to support individuals' financial wellbeing in retirement. The second study focused on Korean middle-aged adults' residential preferences after retirement, emphasizing the importance of understanding evolving housing needs and their impacts on health and social interactions in later life. Third study conducted a comparative analysis of long-term care policies between South Korea and Singapore, examining the emergence of Community Care services as a potential solution for aging-in-place and providing valuable insights into the compatibility of such services with different cultural and welfare system framework. The last study introduced a psychometrically sound scale to measure public stigma associated with Alzheimer's disease among Korean-speaking populations, contributing to a better understanding of the nature and magnitude of Alzheimer's disease stigma, with implications for stigma reduction efforts within this population. The symposium offers an excellent opportunity to examine various aspect of aging issues among Koreans, encompassing financial wellbeing, housing preference, long-term care, and public perception of an age-related disease. By examining these topics, the symposium will contribute to informing policy and practice in addressing various needs of aging populations from a more culturally tailored perspective. Korean/Korean American and Aging Interest Group Sponsored Symposium

Abstract citation ID: igae098.1668

### MARITAL STATUS AND CHANGES IN FINANCIAL SATISFACTION DURING THE RETIREMENT TRANSITION: SOUTH KOREA AND THE US

Jeein Jang, Jeffrey Stokes, and Jeffrey Burr, *University of Massachusetts Boston, Boston, Massachusetts, United States*

This study examines the impact of the transition to full retirement on the long-term financial satisfaction trajectories in two countries with different socioeconomic and health settings, and whether this relationship differs by marital status. Two harmonized, nationally representative cohorts of adults aged 51 and over are used: the Korean Longitudinal Study of Aging for South Korea and the Health and Retirement Study for the United States, with sample sizes of 31,900 and 33,356 individuals, respectively, followed up for a period of 11 years. The outcome is financial satisfaction measured as a composite score, and the predictor is the transition from full-time work to becoming fully retired (relative to remaining in full-time work). Linear Mixed-effect models show that retirement is associated with lower financial satisfaction at baseline and over time in both countries. Men have higher financial satisfaction than women in both studies. The magnitude of the effect of retirement on financial satisfaction is greater in South Korea than in the United States. This relationship is buffered by marital status in the United States, but no interaction term reaches statistical significance in South Korea. These findings suggest that cultural, social, or economic factors may influence the financial well-being of individuals during the transition to full retirement, given cross-national differences.

Abstract citation ID: igae098.1669

### RESIDENTIAL PREFERENCES FOR CURRENT AND FUTURE RESIDENTIAL ENVIRONMENTS AMONG KOREAN MIDDLE-AGED ADULTS

Eunju Lee, and Kyungmin Kim, *Seoul National University, Seoul, Republic of Korea*

Midlife adults often plan for life after retirement, forming their own preferences for where to live after retirement. Their residential preferences for life after retirement may be different from the past as they consider their well-being in later life. This study compared middle-aged adults' preferences for residential features between current and future residential environments (i.e., past and current residential preferences) and examined how differences in preferences for current and future residential features were associated with health and social characteristics. A sample of 1,651 Korean middle-aged adults (aged 49–64) rated their preferences for each of 12 residential features (e.g., proximity to family/relatives, low living expenses, accessibility to health services) regarding their current and future residential environments, separately. Respondents considered "house price" and "safe and clean environments" most important for both current and future residential environments, while "accessibility to health services" and "opportunity for leisure activities" were more important for future residential environments. Regression results showed that respondents with poorer health considered features that help them compensate for health decline more important, while those who contacted friends/neighbors more frequently and participated in more cultural activities considered features that help them maintain their social interactions more important for future residential environments than the past. Across residential features, respondents with poorer health showed lower similarity in overall preferences between current and future residential environments. Our findings suggest the importance of identifying various demands for residential features after retirement to support successful adaptation in later years.

Abstract citation ID: igae098.1670

### TWO PATHS TO COMMUNITY CARE FOR OLDER ADULTS: KOREA AND SINGAPORE'S LONG-TERM CARE EVOLUTION

Jeremy Lim-Soh[1], Angelique Chan[1], Aejung Yoo[2], Dahye Kim[3], Yugyeong Eo[4], Jae Woo Choi[2], Wan Chen Graham[5], and Loong Mun Wong[5], *1. Duke-NUS Medical School, Singapore, Singapore*, *2. Health Insurance Research Institute, National Health Insurance Service, Wonju, Kangwon-do, Republic of Korea*, *3. National University of Singapore, Singapore, Singapore*, *4. Center for Social Services Research, Korea Institute for Health and Social Affairs, Sejong, Chungchongnam-do, Republic of Korea*, *5. Agency for Integrated Care, Singapore, Singapore*

Aging populations pose significant challenges to institutional Long-Term Care systems. Community Care services, which provide professional support for functional limitations, chronic diseases, or cognitive impairment to community-dwelling older adults, have emerged as a potentially cost-effective solution that can support aging-in-place. However, differing implementations of Community Care across societies can affect older adults' health and quality of life. We conduct a comparative case study of the evolution of Long-Term Care policy in Korea and Singapore towards Community Care. Both societies exhibit similar demographic trends—rapid aging, ultra-low fertility, and declining old-age support ratios—coupled with Confucian cultural ideals. However, their welfare systems differ historically; Korea took a welfare state approach emphasizing institutional care, and Singapore leaned towards neoliberalism with a reliance on informal care. While both societies are developing various Community Care programs including home-based nursing, day care centers, and assisted living, we find significant differences in the way that Community Care is planned, funded, and implemented. Moreover, the challenges faced depend on cultural context and welfare system. Based on these insights, we discuss the compatibility of Community Care with different sociocultural settings and policy ecosystems, concluding with potential implications for other societies struggling with population aging and LTC sustainability.

Abstract citation ID: igae098.1671

### KOREAN VERSION OF PUBLIC STIGMA OF ALZHEIMER'S DISEASE SCALE: DEVELOPMENT AND EVALUATION AMONG KOREAN AMERICANS

Michin Hong[1], and Sang Lee[2], *1. Indiana University Indianapolis, Indianapolis, Indiana, United States*, *2. San Jose State University, San Jose, California, United States*

This study aims to evaluate the psychometric properties of the Korean version of the public stigma of Alzheimer's diseases scale (KPS-ADS). Stigma toward Alzheimer's disease (AD) prevents the early diagnosis of AD, causing the delayed treatments. To our knowledge, there is no proper measure to assess AD public stigma among the Korean-speaking population. We used a community survey dataset with 268 Korean Americans. We translated the 19 items of the layperson's stigma, a dimension of the Family Stigma in AD scale, using Brislin's method. After randomly dividing the sample into two sub-groups, we conducted exploratory factor analysis (EFA) with one group to explore the factor structure of the KPS-ADS, and then performed confirmatory factor analysis (CFA) with the other group to validate its identified factor structure. Additionally, we performed reliability tests. EFA identified three factors: negative emotions, empathetic response, and social behaviors. Using this three-factor model, we performed CFA with the highest loading items loaded on each factor, but it showed a poor model fit. After removing three items, the revised model showed an excellent model fit ($\chi2$ = 185.788, df = 97, p = 0.000, CFI = 0.955, TLI = 0.944, RMSEA = 0.081[90% CI: 0.063–0.099, p ≤ 0.05 = 0.003]). All loadings were significant.

The KPS-ADS demonstrated excellent internal consistency reliability (α=.87), with each domain demonstrating excellent internal consistency. Our study presents a psychometrically sound, multifaceted KPS-ADS that contribute to better understanding the nature and magnitude of AD public stigma and reducing it in this population.

### SESSION 3550 (SYMPOSIUM)

Abstract citation ID: igae098.1672

### OLDER MIGRANTS IN THEIR SOCIAL CONTEXTS

Chair: Ashley Ritter
Co-Chair: Allen Glicksman
Discussant: Freya Diederich

It is recognized that a comprehensive understanding of the experiences and circumstances of older adults necessitates an examination of the broader social, institutional, and political frameworks within which they reside. The migration of older individuals entails a transformation in their relationships with family members, social networks, service providers, and governmental institutions. Lawton's Environmental Press model, which posits that the influence of environmental factors on health and well-being intensifies as individuals age and become more fragile, can be extended to older migrants. Such individuals may find themselves relying more heavily on familial support, social networks, formal caregiving services, and governmental assistance to maintain their health compared to their native environments. The four papers presented in this panel explore the challenges confronting older migrants within these expansive contexts, probing the ways in which these contexts can either positively or negatively impact the well-being of older adults. The papers progress from an examination of familial dynamics (Reyes) to the broader societal milieu (Torres), followed by an analysis of formal care systems (Glicksman et al.), and concluding with an investigation of governmental policies at the national level (Kilkey). Collectively, these papers underscore the significance of social, institutional, and governmental frameworks in shaping the health and well-being outcomes of older migrants. Moreover, the papers underscore the necessity of employing diverse research methodologies, including surveys, qualitative inquiries, secondary data analyses, and literature reviews, to address the multifaceted nature of these issues and to cater to the specific inquiries at hand. International Aging and Migration Interest Group Sponsored Symposium

Abstract citation ID: igae098.1673

### PLACE OF ORIGIN AND USE OF FORMAL HEALTH SERVICES

Allen Glicksman[1], Steven Albert[2], Adam Davey[3], Ari Friedman[4], Philip Lai[5], Misha Rodriguez[1], Robert Schrauf[6], and Michael Liebman[7], *1. NewCourtland, Philadelphia, Pennsylvania, United States*, *2. University of Pittsburgh, Pittsburgh, Pennsylvania, United States*, *3. University of Delaware, Newark, Delaware, United States*, *4. University of Pennsylvania, Philadelphia, Pennsylvania, United States*, *5. Philadelphia Senior Center, Philadelphia, Pennsylvania, United States*, *6. The Pennsylvania State University, University Park, Pennsylvania, United States*, *7. IPQ Analytics. LLC, Kennett Square, Pennsylvania, United States*

The point of contact between older immigrants and the formal health system is an area of concern for providers, policy makers and researchers. The challenges that often appear in any interaction between older adults and formal providers are exacerbated when the older person is an immigrant because of language and cultural differences, challenges in a family where migration has taken place, and lack of familiarity with the local system for providing care. Attempts to understand these interactions require attention to two sources of diversity – on the one hand the place of origin of the older migrant and all the social, cultural and policy differences in the places of origin on the one hand and the diversity of types of encounters (ambulatory, emergency department, in-patient hospital) with the formal system and the related issues of insurance status on the other. Our study, using data on encounters between persons speaking Chinese, Spanish or Russian to examine use of the three types of encounters to examine patterns of service use for persons with similar health insurance and primary diagnoses. We will focus on the Chinese speakers and their distinctive pattern of use of formal health service compared to the other groups (Including white and Black English speakers). This is especially true in the lower rate of using emergency departments compared to the other groups. We will also address the challenges to studying immigrant experience in health settings when only language of interaction and not place of birth are collected by health providers.

Abstract citation ID: igae098.1674

### OLDER MIGRANTS' EXPERIENCES OF AGING IN PLACE IN HOSTILE AND AUSTERE TIMES

Majella Kilkey[1], Obert Tawodzera[2], and Jayanthi Lingham[1], *1. University of Sheffield, Sheffield, England, United Kingdom*, *2. University of Birmingham, Birmingham, England, United Kingdom*

Drawing on data from in-depth qualitative interviews with 20 aging-in-place migrants, we explore how they are impacted by the combination of the austerity in welfare state provision and the hostility in migration policy that characterize English society. Informed by the feminist political economy concept of social reproduction, understood as reproducing the population, physically and affectively, in and outside their 'productive' working lives (Laslett and Brenner 1989), we find that our research participants are experiencing a 'crisis of social reproduction' (Fraser 2016), which strains their social reproductive capacities to breaking point. Our participants are a mix of people who arrived in the post-WW11 era from the British colonies as labor migrants, and people more recently arrived as refugees from conflict-affected parts of Africa. Despite those different migration histories, their current experiences are very similar; a finding we explain through a colonial lens. People who joined England's 'productive' workforce from across the British Empire decades ago are now facing unmet social reproductive needs later in their lives. Meanwhile, others have been driven to the UK due to postcolonial conflict, and as older refugees outside of the 'productive' workforce, scant regard is paid to their social reproductive needs. Our findings challenge the dualism prevalent in European research between migration and refugee studies, and suggest the potential of a historically situated colonial lens to understanding older migrants' experiences. They also highlight the value of analysis of the intersection between migration and welfare policies for deepening understandings of the drivers of 'crises of social reproduction'.

Abstract citation ID: igae098.1675

**EXPLORING THE ROLE OF FAMILISMO IN DIABETES SELF-MANAGEMENT AMONG LATINO OLDER ADULTS WITH DIABETES**

Jimmy Reyes, *University of Northern Iowa, Cedar Falls, Iowa, United States*

The purpose of this study was to explore the role of familismo in diabetes self-management among first- and second-generation Latino older adults with diabetes mellitus type 2 and who reside in rural communities. In addition, this study aimed to identify how cultural beliefs and values influence daily diabetes self-management behaviors, experiences, and attitudes within the context of familismo among Latino older adults. A focused health ethnographic methodology was utilized using semi-structured interviews and participatory observation in two rural Iowa Latino communities. The 20 participants included first- and second-generation Latino older adults with diabetes mellitus type 2. The study used ethnographic data analysis methodology and 20 major codes emerged that were collapsed into three major themes. The themes were clustered around the importance of culture, the value of support systems, and the immigration experience. An important implication of this study derives from the notion that familismo is a multifaceted construct that involves protective and antagonistic elements in the management of diabetes. In addition, it is important to recognize the degree to which acculturation and generational differences may play a role in how much family and social support is needed. Researchers should explore the level and degree of familial and social support needed during initial and follow-up visits with the implementation of family involvement plans.

Abstract citation ID: igae098.1676

**CIVIC ENGAGEMENT IN LATER LIFE: USING THE GERONTOLOGICAL IMAGINATION TO DEVELOP RESEARCH ON MIGRANTS**

Sandra Torres[1], and Pernilla Agard[2], *1. Uppsala University, Uppsala, Uppsala Lan, Sweden*, *2. Uppsala university, Uppsala, Uppsala Lan, Sweden*

Social gerontologists that have brought attention to the knowledge gaps that exist in research on late-life-civic engagement have already proposed that this research has not yet exploited the theorizing potential embedded in migratory life-courses. In a similar fashion research on migrants' civic engagement has not yet envisioned that aging and old age offer interesting vantage points from which that research can be expanded. This presentation taps into both of these states of affairs by bringing attention to what characterizes the scholarly imagination on migrants' civic engagement, the taken-for-granted assumptions about this engagement that this literature makes, and the areas of research that have yet to receive attention. Through the systematic coding of the peer-reviewed literature that focuses on migrants' civic engagement this presentation aims to offer insights into, among others, the kinds of research questions that this literature is preoccupied with, the angles of investigation and methodological approaches that it relies on as well as the types of civic engagement, and the taken-for-granted assumptions about them, that the literature never problematizes. In doing so, we will argue that the gerontological imagination (especially if combined with migrancy-astuteness) could expand the ways in which migrants' civic engagement have been conceived and measured thus far.

### SESSION 3555 (SYMPOSIUM)

Abstract citation ID: igae098.1677

**PRESIDENTIAL SYMPOSIUM: RESILIENCE IN OLDER ADULTS: A MULTIDIMENSIONAL PERSPECTIVE**

Chair: Mo-kyung Sin
Discussant: Pamela Cacchione

In alignment with the theme of this year's conference "The Fortitude Factor", this symposium focuses on multidimensional perspectives of resilience in older adults. Resilience in older age is the ability to stand up to adversity and to bounce back to a state of equilibrium following individual adverse events. Maintaining physical, psychological, and cognitive resilience is an integral component for quality life and well-being in older adults. Whereas, conceptualizing and properly operationalizing resilience is essential for advancing research to achieve well-being of older adults. Our first presenter will discuss the psychometrics of the physical resilience scale in older adults living with dementia. Our second presenter will discuss psychological evaluation of resilience and provide insights gained from her research on the protective effects of psychological resilience across stressors and outcomes. Our last presenter will share factors associated with cognitive reserve from the BIOCARD study. Given the association between resilience and wellbeing in older adults, this HS section Presidential Symposium is sure to challenge and enhance our thinking to advance science.

Abstract citation ID: igae098.1678

**PSYCHOMETRICS OF THE PHYSICAL RESILIENCE SCALE IN OLDER ADULTS LIVING WITH DEMENTIA: PROXY RESPONSES**

Barbara Resnick, *University of Maryland School of Nursing, Baltimore, Maryland, United States*

Physical resilience is defined as the ability to recover or optimize function in the face of physical challenges. Physical resilience has been conceptualized as a trait focusing on the way individuals respond to stressors versus an outcome of the stressor(s) or physically challenging experience. Due to cognitive changes, including decreased ability to recall new information or aphasia, it is difficult for individuals living with dementia to respond to questionnaires. Consequently, a family member or identified proxy is often asked to respond. The purpose of this study was to determine if proxies can complete the Physical Resilience Scale for older adults living with dementia. This was a descriptive study using Rasch analysis and baseline data from the Function Focused Care for Acute Care Using the Evidence Integration Triangle trial. The first 240 patients living with dementia were included. There was evidence of reliability based on person and item separation index. There was no evidence of Differential Item Functioning (DIF) between genders although there was evidence of DIF by race on Item 7 (He/she accepted help from others). Validity was supported based on items fitting the model except for Item 10 (He/She found it difficult to ask for help from others). Controlling for age, gender, race and comorbidities there was a significant relationship between resilience and function (R2 =.08, F=.4.75, p=.001) and resilience and pain (R2 =.08, F=3.95, p.002). The Physical Resilience Scale is reliable and valid when completed by proxy reports. Future use should remove Item 10 due to redundancy.

Abstract citation ID: igae098.1679

### TESTING PSYCHOLOGICAL RESILIENCE IN THE OLDER US POPULATION

Miles Taylor, and Dawn Carr, *Florida State University, Tallahassee, Florida, United States*

The concept of resilience, broadly defined as the ability to successfully navigate or bounce back after stressors or traumas, has gained increased attention in several disciplinary literatures on aging. Certain challenges and setbacks, such as chronic condition onset and widowhood, become more normative in older adulthood. It is increasingly recognized that resilience is important to understanding health processes and independent functioning in later life. Aging scholars note that resilience can be operationalized as a set of individual characteristics promoting adaptation, a process of adaptation and/or coping, and healthy or beneficial outcomes after stressful setbacks or challenges. We focus on psychological resilience (PR), an individual's capacity to navigate adversity through positive adaptation in a manner that protects health and well-being. Although numerous measures of PR exist, most are not incorporated into large longitudinal data on older adults that may provide insights on how this resource works across a variety of stressors and outcomes. In this session, we first discuss our collective work that has 1. innovated the creation of a PR measure that can be used in the Health and Retirement Study (HRS), and 2. tested the validity and predictive efficacy of this measure in this nationally representative data source. We then present key findings from our research on the protective effects of PR across a number of stressors and outcomes, showing how its connection to health processes may be helpful in shaping future interventions.

Abstract citation ID: igae098.1680

### COGNITIVE RESERVE IN AGING AND PRECLINICAL ALZHEIMER'S DISEASE

Anja Soldan[1], Corinne Pettigrew[2], and Marilyn Albert[2], *1. Johns Hopkins University, Baltimore, Maryland, United States*, *2. Johns Hopkins University School of Medicine, Baltimore, Maryland, United States*

There is large inter-individual variability in cognitive and brain aging trajectories, which have been related to differences in cognitive reserve (CR), brain mainenance, and the influence of genetic and environmental factors across the lifespan. This presentation will discuss recent evidence regarding CR using data from the BIOCARD study, which includes ~ 300 individuals who were cognitively unimpaired and primarily middle-aged at baseline (mean baseline age=57) and have been followed with clinical and cognitive evaluations, brain imaging, and blood and cerebrospinal fluid (CSF) collection for over 20 years. This work has shown that higher levels of CR, indexed using years of education, literacy, and vocabulary measures, are associated with higher baseline cognitive performance and a delayed onset of Mild Cognitive Impairment, even in the presence of Alzheimer's disease (AD) pathology and neurodegenration. By comparison, CR measures were not related to levels or rates of change in AD biomarkers (e.g, CSF and blood levels amyloid and tau), suggesting that CR influences cognitive and clinical outcomes through mechanisms other than AD pathology accumulation. Instead, further results suggest that measures of CR are associated with white matter hyperintensity burden and white matter microstructural integrity. Additionally, other modifiable lifestyle factors known to influence cognitive outcomes (e.g., physical activity, sleep quality and quantity, and engagement in stimulating leisure activities) are associated with measures of resting-state functional brain connectivity. Taken together, these findings suggest that modifiable lifestyle factors may promote cognitive reserve and resilience by improving aspects of functional and structural brain connectivity and cerebrovascular health.

### SESSION 3560 (SYMPOSIUM)

Abstract citation ID: igae098.1681

### PROMOTING LIFELONG LEARNING AT OREGON'S AGE-FRIENDLY UNIVERSITIES

Chair: Melissa Cannon
Co-Chair: Noriko Toyokawa
Discussant: M. Aaron Guest

The Age-Friendly University (AFU) global network "aims to shape how we live and work by increasing educational opportunities across the life span" (https://www.afugn.org/). In Oregon, USA, Portland State University (PSU), Western Oregon University (WOU), and Southern Oregon University (SOU) have endorsed and adopted the AFU Principles to become part of the network of age-friendly higher education institutions. Although each institution has implemented different initiatives as part of this work, a common thread across the three has been promoting lifelong learning. Melissa Cannon will present findings from preliminary research that identified several strengths, weaknesses, and opportunities for WOU becoming age-friendly, including those detailed in their own AFU campus report. Participants will learn some of the recent initiatives that have advanced the university's age-friendly and lifelong learning efforts. Noriko Toyokawa will guide a discussion on the distinct challenges small-sized universities face when initiating and maintaining age-friendly university initiatives. The presentation will explore potential strategies for effectively managing these situations, using the case of SOU as an illustrative example. Serena Hasworth and Lauren Bouchard will highlight findings from PSU's "Age-Friendly University Campus Report" and provide an overview of activities to improve age-friendliness through the Better with Age Initiative. They will describe efforts to develop an age-inclusive micro-credential program for students and faculty at PSU. The concluding discussion fosters a dynamic exchange of creative ideas between the audience and presenters, aiming to optimize existing resources for mobilizing and sustaining age-friendly university initiatives across diverse contexts.

Abstract citation ID: igae098.1682

### FINDINGS FROM ASSESSING AGE-FRIENDLY BARRIERS, STRENGTHS, AND AREAS OF OPPORTUNITY AT WESTERN OREGON UNIVERSITY

Melissa Cannon, *Western Oregon University, Monmouth, Oregon, United States*

This presentation showcases the work that has been done at Western Oregon University (WOU) as an Age-Friendly University (AFU) member related to assessment and identifying action items for increasing age-friendliness and lifelong learning opportunities. First, attendees will learn about a multi-method case study that explored barriers, facilitators, and opportunities for WOU to engage older community members and advance age-friendliness in preparation for joining the AFU network in 2019. This case study involved survey data collection and participatory action research with older adults, revealing opportunities for the university to implement the AFU principles through efforts such as strengthening the senior center partnership, developing a lifelong learning center, and removing accessibility barriers. Next, this presentation will delve into findings from WOU's "Age-Friendly University Campus Report" (Silverstein et al., 2021), which helped WOU identify strengths, weaknesses and areas of opportunity related to lifelong learning. For example, the report found a lack of resources and information for campus community members who identify as informal or unpaid family caregivers at WOU, which can particularly impact non-traditional students with family caregiving responsibilities. Findings will also be shared from the latest AFU-related study at WOU which explored caregiving issues for campus community members (e.g., physical demands, time demands, financial stress, social and emotional health) using a campus-wide online survey and mixed-method analysis by a research team. After providing this overview, next steps will be explored as to how WOU and similar universities may take action to implement changes that enhance opportunities for lifelong learning on campus.

Abstract citation ID: igae098.1683

### FOSTERING AGE-FRIENDLY INITIATIVES IN SMALL UNIVERSITIES: A CASE STUDY OF SOUTHERN OREGON UNIVERSITY

Noriko Toyokawa, *Southern Oregon University, Ashland, Oregon, United States*

Compared to their larger counterparts, small U.S. higher education institutions face distinct challenges in implementing Age-Friendly University (AFU) initiatives. This presentation spotlights the AFU initiatives that Southern Oregon University (SOU) implemented as the case of such a higher education institution. Despite its resource constraints, such as the lack of aging-centered academic programs and an on-campus aging institute, SOU exemplifies AFU principles by its faculty's developing courses to involve retired individuals through community partnerships and cultivate meaningful intergenerational interactions. While discussions on lifespan development topics provided in those courses have produced positive learning outcomes, they have also unveiled areas for improvement. Older adults desire more direct involvement to share their wisdom and expertise with students. In response, incorporating age diversity as a valuable resource, SOU strategically plants to enhance existing academic programs by infusing innovation into courses and promoting mutual learning between generations. For instance, a course in Adult Development and Aging is being re-designed so that collaborative groups of students and older adults will utilize electronic design tools and artificial intelligence to craft a book conveying older adults' messages for the next generation. This addresses the needs of older adults and enhances technology skills for both groups. After examining the university's current effort in the AFU initiative, this presentation will delve into small institutions' unique challenges in promoting AFU initiatives. Recommendations will be explored, emphasizing the vital role of adapting existing courses to foster sustainable lifelong learning across generations.

Abstract citation ID: igae098.1684

### AGE-FRIENDLY UNIVERSITY IN PRACTICE: DEVELOPING AN AGE INCLUSIVITY MICRO-CREDENTIAL PROGRAM

Serena Hasworth, *Portland State University, Portland, Oregon, United States*

This session will highlight findings from PSU's "Age-Friendly University Campus Report" (Silverstein et al., 2021) and provide an overview of the activities to improve age-friendliness through the Better with Age Initiative, a program that invests in students pursuing career pathways in aging and faculty conducting aging-related research. Presenters will describe efforts to develop a free, self-paced Age Inclusivity micro-credential program for students and faculty at PSU in which participants learn to describe local and national growth of aging populations, analyze the intersectional experience of aging, reflect on personal attitudes of aging, determine how older adults play a role in society at large, and explore career opportunities in the aging field. Micro-credentials provide tailored learning experiences for students, faculty, and staff that supplement and enrich current curricular and professional development offerings. Presenters will describe how these programs have the potential to reach students who may not be interested in pursuing a formal degree or minor focused on aging, but who want to set themselves apart on the job market by demonstrating their knowledge and skills related to serving older adults in their unique field.

### SESSION 3565 (SYMPOSIUM)

Abstract citation ID: igae098.1685

### REAL-WORLD IMPLEMENTATION OF DEMENTIA CAREGIVING PROGRAMS: ALZHEIMER'S DISEASE PROGRAM INITIATIVE RESULTS

Chair: Heather Menne
Discussant: Erin Long

Since 1992 grants from the Administration on Aging have supported community services for people living with dementia and their caregivers. Beginning in 2014, grantees began addressing specific gaps in community-based care and services. Papers presented in this symposium highlight examples of each gap area. First, Yoon Chung Kim addresses the gap area of serving people who live alone with dementia and the successes and challenges faced in serving this population. The next two papers present examples of improving care and services for individuals aging with intellectual and developmental disabilities (IDD) with dementia or those at high risk of developing dementia. Dr. Kelli Barton discusses outcomes and lessons learned from the implementation of two dementia programs, Reducing Disability in Alzheimer's Disease (RDAD) and Care Ecosystem, with the IDD population and their care partners. Dr. Faika Zanjani highlights the progress of the ADRD risk reduction health coaching delivered to older adults living with ADRD risk factors (e.g., diabetes, hypertension). Another gap area addresses the delivery of behavior symptom management training and consultation for family caregivers. Dr. Tamara Herrick discusses the impact and sustainability of the implementation of the Resources for Enhancing Alzheimer's Caregiver Health (REACH) Community program within primary care and geriatrics settings. Dr. Christy Nishita presents the elements and successes of Hawaii's memory care navigator model that educates, supports, and connects local families to the resources that they need. Erin Long, the Team Lead for Alzheimer's and Dementia Programs at the Administration on Aging, Administration for Community Living offers discussion comments.

Abstract citation ID: igae098.1686

### SUCCESSES AND CHALLENGES OF PROGRAMS FOR PEOPLE WHO LIVE ALONE WITH DEMENTIA

Yoon Chung Kim[1], Michael Lepore[2], Kate Gordon[3], Lirisha Tuladhar[4], and Heather Menne[4], *1. University of Maryland Baltimore, Baltimore, Maryland, United States*, *2. University of Massachusetts Amherst, Amherst, Massachusetts, United States*, *3. Splaine Consulting, Columbia, Maryland, United States*, *4. Miami University, Oxford, Ohio, United States*

Approximately a quarter of older adults with dementia or cognitive impairment live alone in the US. To address the needs of people living alone with dementia (PLAWD), the Administration for Community Living requires PLAWD-focused services in its Alzheimer's Disease Program Initiative (ADPI). We examined final reports from ADPI grantees that served PLAWD to determine the types of services delivered, and successes and challenges faced in service delivery. Of 59 grantees, 52 were community grants and 7 were state grants. Grantees implemented strategies to identify and support PLAWD by partnering with local service providers and leveraging existing relationships (e.g., meal programs, adult day centers, senior residential communities, friendly visitor programs, transportation services). Grantees offered opportunities for companionship and support for socialization through buddy programs, phone calls, social and wellness programming, and regular home visits. Grantees utilized volunteers, interns, and staff from various disciplines to provide care management or to deliver services. Top challenges reported were difficulty making contact with PLAWD, high levels of stigma associated with dementia, fear of losing independence, and low awareness and a lack of understanding about programs. Findings demonstrate that a variety of services have been successfully provided to PLAWD. However, common challenges are experienced in service delivery. PLAWD need an expansion of services and support, including care management, care monitoring, and socialization. Given the importance of service access for PLAWD, raising awareness of programs, reducing stigma, identifying and reaching PLAWD is vital. Different approaches used and challenges faced between state and community grants are discussed.

Abstract citation ID: igae098.1687

### DEMENTIA PROGRAMS FOR PERSONS WITH INTELLECTUAL OR DEVELOPMENTAL DISABILITIES (IDD) AND THEIR CARE PARTNERS

Kelli Barton, *University of Missouri-Kansas City Institute for Human Development, Missouri, United States*

There are limited evidence-based dementia programs for individuals with intellectual or developmental disabilities (IDD) and their care partners. Alzheimer's Disease Programs Initiative grants through the Administration for Community Living provide an opportunity to expand evidence-based dementia programs to serve the IDD population. This presentation will discuss outcomes and lessons learned from the implementation of two dementia programs, Reducing Disability in Alzheimer's Disease (RDAD) and Care Ecosystem, with the IDD population and their care partners through three funded projects. We employed a mixed-method approach to explore program impact and identify lessons learned that can inform dementia programming for the IDD population. Feedback from semi-structured partner interviews and regular meetings revealed several common themes. Partners identified the opportunity to provide dementia and IDD education to staff and to the community as a significant benefit and as crucial to establishing a foundation for program implementation. Further, while many care partners were formal caregivers, whose relationship and experience with the person with dementia typically differ from informal caregivers, results from the RDAD implementation showed promising trends in outcomes for both individuals and care partners. For example, behavioral symptom-related severity among individuals with IDD and behavioral symptom-related distress among care partners both decreased ($p=0.07$ and $p=0.14$, respectively), as measured by the Neuropsychiatric Inventory Questionnaire (NPI-Q). Evidence-based dementia programs have much to offer the IDD population and their care partners; however, protocol modifications are needed to ensure physical and cognitive accessibility for individuals with IDD and to meet the needs of informal caregivers.

Abstract citation ID: igae098.1688

### ADRD RISK REDUCTION HEALTH COACHING

Faika Zanjani, Cate Hawks, Kennedy O'Donnell, Brian Berman, and Lana Sargent, *Virginia Commonwealth University, Richmond, Virginia, United States*

**Objectives:** The development of the Richmond Brain Health Initiative (RBHI) was guided by the need to address local brain health service gaps to improve Alzheimer's/Dementia (ADRD) health disparities in racially diverse communities. This paper focuses on the ADRD Risk Reduction Health Coaching program component.

**Methods:** The program is currently in implementation, focused on recruiting diverse older adults living in the Richmond, VA metro area, with the following risk factors: diabetes, hypertension, cardiovascular disease, or subjective cognitive decline. Weekly lifestyle telephone health coaching for 12 weeks was offered, followed by a monthly (6-month) maintenance stage. Health coaching calls last an average of 15-30 minutes per session. The intervention protocol target lifestyle risk via education, learning objectives, personal risk assessment, attitudes, and lifestyle behavioral change goal setting.

**Results:** The current study sample (n=49) is 96% 60+ years old (n=38), 38% African American/Black (n=18), 6% Hispanic (n=3), 70% female (n=33), 11% military veterans (n=5), 53% live alone (n=25). Preliminary mean differences indicate improvement in ADRD knowledge: baseline mean = 25.8, 12-week = 28.6; Physical Activity: baseline mean = 1.50 hours/week, 12-week = 3.00 hours/week; Social Activity: baseline mean = 2.07 hours/week, 12-week = 2.57 hours/week; Cigarettes: baseline mean = 0.62/day, 12-week = 0.30/day; Alcoholic Drinks: baseline mean = 1.50/week, 12-week = 1.10/week; and Medication adherence: baseline = 86%, 12-week = 100% adherence. CONCLUSIONS: This study showcases a model for reducing ADRD risk and promoting brain health in racially diverse communities by improving access to ADRD community services.

Abstract citation ID: igae098.1689

### IMPLEMENTING REACH COMMUNITY IN AN INTEGRATED HEALTH SYSTEM: IMPACT AND LESSONS LEARNED

Tamara Herrick, *MaineHealth, Portland, Maine, United States*

This project examined the implementation of the Resources for Enhancing Alzheimer's Caregiver Health (REACH) Community program within primary care and geriatrics settings, facilitated by Licensed Clinical Social Workers (LCSWs). The integration of LCSWs into the healthcare system offers a unique opportunity to provide comprehensive care by addressing both medical and psychosocial aspects of dementia caregiving. Their expertise in psychosocial assessment and intervention enables holistic care that addresses the multifaceted needs of care partners and their families. REACH Community is an evidence-based intervention for care partners of individuals living with dementia that focuses on safety, care partner health and emotional well-being, and behavior management. The intervention consists of four core sessions one-on-one with care partners, with additional sessions as an option if needed. Sessions can be delivered face-to-face, by telephone, or telehealth. With the emergence of COVID-19, all sessions transitioned to remote delivery via telehealth Zoom platform or telephone. Pretest posttest design assessed program impact. Key outcome measures included: Caregiver Frustration, caregiver well-being (Zarit Burden Inventory), and mood (PH-Q4). Pre-posttest results indicated that participants had an improvement in well-being, mood, and caregiver frustration following participation in the REACH Community program. The evaluation contributed valuable insights into the feasibility and effectiveness of integrating the REACH Community program into primary care and geriatrics settings utilizing LCSWs. We continue to offer the program in primary care and geriatrics center settings and REACH Community is part of our growing toolbox of evidence-based programs providing support to care partners of people living with dementia.

Abstract citation ID: igae098.1690

### ENHANCING AND SUSTAINING THE MEMORY CARE NAVIGATOR MODEL IN HAWAII

Christy Nishita, *University of Hawaii, Honolulu, Hawaii, United States*

Hawaii's memory care navigator model aims to educate, support, and connect local families to the resources that they need. The Kupuna Collective, a cross-sector coalition led by the University of Hawaii Center on Aging and Hawaii Public Health Institute, has taken steps to enhance the model. First, community health workers, given their close ties and relationship with local communities were targeted and trained to provide person-centered, dementia support in a culturally tailored manner. To ensure quality and explore collective impact, community health workers, who were embedded within local organizations, use a standardized, electronic platform to collect assessment and care planning data. In addition, the model has been sustained by integrating the model within Hawaii's workforce development efforts. Trainings are widely disseminated as part of Hawaii's Building Our Largest Dementia Infrastructure initiative, funded by the Centers for Disease Control. Trainings are stored in an online library for ongoing continuing education and training and available for use by community college CHW training programs. Taken together, the memory care navigator model has supported the development of a more coordinated system of dementia care in Hawaii.

### SESSION 3570 (SYMPOSIUM)

Abstract citation ID: igae098.1691

### SHARING THE CARE: PAID AND FAMILY CAREGIVERS WORKING TOGETHER TO SUPPORT OLDER ADULTS AT HOME

Chair: Jennifer Reckrey
Discussant: Regina Shih

Collaboration between paid caregivers (e.g., home care workers) and family caregivers (e.g., spouses, adult children) is essential to ensure that functionally impaired older adults can thrive at home. However, there are few evidence-based practices and policies to support collaboration and paid and family caregivers are frequently left to navigate shared care on their own. In this symposium, we describe ongoing efforts to identify, understand, and promote collaboration within the paid and family caregiving team. First, Scales et al. describe the development and evaluation of a caregiving policy agenda that brings together diverse partners to support both paid and family caregivers. Miller et al identify trajectories of paid and family care among older adults in the last years of life and describe increased reliance on family caregivers among those living in rural areas. Fabius et al characterize notable role-sharing between paid and family caregivers, particularly among individuals with dementia or enrolled in Medicaid. Reckrey et al share national data that describes the growing prevalence of family members who are themselves paid to provide care, frequently to older adults with high care needs. Finally, Ornstein et al. outline the conceptualization and development of a large caregiving study that draws from the principles of relational coordination to simultaneously examine paid and family care among a multisite cohort of 4,000 older adults. Taken together, these abstracts underscore how research inclusive of both paid and family caregivers lays the foundation for stronger, more collaborative care for older adults at home. Paid Caregiving Interest Group Sponsored Symposium

Abstract citation ID: igae098.1692

### TRENDS IN RECEIPT OF PAID FAMILY CARE AMONG OLDER ADULTS WITH FUNCTIONAL IMPAIRMENT IN THE UNITED STATES, 2011–2022

Jennifer Reckrey[1], Yifan Liu[2], Yiqing Qian[2], and Katherine Ornstein[2], *1. Icahn School of Medicine at Mount Sinai, Icahn School of Medicine at Mount Sinai, New York, United States*, *2. Johns Hopkins University, Baltimore, Maryland, United States*

While caregiving is frequently conceptualized as either paid care (e.g., home health aides, home care workers) or family care (e.g., spouses, children), family members may also receive payment to provide care (i.e., paid family care) through state-level Medicaid-waiver programs or through private arrangements within families. This study examines trends in paid family care prevalence over time and identifies factors associated with receipt of paid family care among older adults with functional impairment using the National Health and Aging Trends Study. Prevalence of paid family care increased slightly over time, with 7.0%, 5.9%, and 8.1% of the sample receiving paid family caregiving in 2011, 2015, and 2022 respectively. In 2022, 27.8% of those with any form of paid help received paid family care; 27.1% was funded privately and 72.9% was funded by other payers like Medicaid. As compared to those with a non-family paid helper, older adults with paid family care were more likely to be female (78.1% vs. 55.6%, p=0.028), Black/non-Hispanic (29.2% vs. 11.5%, p<0.001), and receive Medicaid (62.5% vs. 33.1%, p<0.001). They reported more chronic conditions (4.1 vs 3.2, p=0.012), impairment in more self-care tasks (3.18 vs 2.44, p=0.043), and more total weekly hours of care (20.0 vs 11.2, p<0.001). Paid family care makes a meaningful contribution to total care, particularly among those with high care needs. Future work examining paid family care both within and outside of the Medicaid system should explore the impact of paid family care on older adults and their larger family unit.

Abstract citation ID: igae098.1693

### TOGETHER IN CARE: ELEVATING THE IMPORTANCE OF FAMILY AND PAID CAREGIVERS

Jessica King[1], Kezia Scales[1], Amy Robins[1], and Toni Gingerelli[2], *1. PHI, New York, New York, United States*, *2. National Alliance for Caregiving, Washington, District of Columbia, United States*

In 2023, PHI and the National Alliance for Caregiving (NAC) launched “Together In Care: The Direct Care Worker and Family Caregiver Initiative” with funding from the John A. Hartford Foundation. The goal of the initiative is to promote policy changes that address the interrelated needs of family caregivers and paid caregivers in the context of long-term care for older adults and people with disabilities, and particularly in home and community-based services (HCBS). Priority topics for the initiative include improving care team integration for family members and paid caregivers; advancing self-direction options and supports; and securing funding for new research on the underexplored nexus of paid and unpaid care. This presentation will begin by describing the conceptual rationale for focusing on the interconnection and overlap between paid and family caregivers, referencing the limited extant literature on this topic. We will then discuss the methodology and evaluation of our efforts to develop a shared caregiving policy agenda across diverse partners — who include federal and state officials, national experts and movement leaders, and long-term care providers — highlighting successes and lessons learned. The presentation will underscore the importance of using evaluation research methods to inform and assess the implementation and impact of complex policy advocacy interventions; share practical approaches and methods; and end with a discussion of the extensive research gaps and opportunities that have been identified through this innovative initiative.

Abstract citation ID: igae098.1694

### TRAJECTORIES OF PAID AND FAMILY CARE TO SUPPORT ADULTS WITH DISABILITIES BY RURALITY

Katherine Miller[1], Yang Yang[1], Jennifer Reckrey[2], Katherine Ornstein[1], and Jennifer Wolff[1], *1. Johns Hopkins University, Baltimore, Maryland, United States*, *2. Icahn School of Medicine at Mount Sinai, Icahn School of Medicine at Mount Sinai, New York, United States*

Family and friends are the dominant source of home-based care for older adults with disabilities (i.e., family caregivers). However, a growing share of older adults are receiving paid care (i.e., from a direct care worker such as a personal care aide). Differences in how paid and family care intersect by rurality are not well understood in the years immediately prior to death. We describe trajectories of paid and family care use in the five years prior to death using group-based multiple trajectory models stratified by rurality. We use a sample of community-dwelling National Health and Aging Trends Study respondents living with functional or cognitive limitations (n=2,549, rural n=481, urban n=2068). We examine the share of total hours of care provided by family versus paid caregivers. In both rural and urban cohorts, we find a 2-group model best fit our data: (a) “consistently high family care” (75% of the sample) and (b) “primarily paid care”. In both groups, the share of family caregiving decreases in concert with growth in paid care, but this shift was more pronounced among urban than rural residents (-5.2% vs. -3.6%). In the five years prior to death, rural residents have greater reliance on family caregivers relative to their urban peers. Notably, our findings that both rural and urban decedents rely primarily on family for care highlight the critical need to support family caregivers throughout the end-of-life trajectory.

Abstract citation ID: igae098.1695

### SHARING ROLES IN HOME CARE BETWEEN FAMILY CAREGIVERS AND PAID CAREGIVERS: A NATIONALLY REPRESENTATIVE STUDY

Chanee Fabius[1], Joseph Gallo[2], and Jennifer Wolff[1], *1. Johns Hopkins University, Baltimore, Maryland, United States*, *2. Johns Hopkins Bloomberg School of Public Health, Baltimore, Maryland, United States*

Little is known about whether paid caregivers "role-share" with family caregivers in home care, which reflects situations in which members from both groups assist with the same tasks. Home care is often Medicaid-financed, and many care recipients live with dementia. We conducted a cross-sectional analysis of the 2015 National Health and Aging Trends Study. Our sample included 440 community-living older adults receiving paid help with self-care (eating, dressing, bathing, toileting), mobility (getting around inside/outside of the home, getting in/out of bed), or medical care tasks (medication administration, doctors visits). Analyses included Pearson's chi-square tests and logistic regression models to examine associations between dementia and Medicaid status and experiencing role-sharing or receiving paid care only across individual tasks. Nearly 1.7 million older adults received paid help, of whom half (53%) experienced role-sharing. Older adults whose caregivers shared roles more often lived with dementia but were less often Medicaid-enrolled. In logistic regression models, those living with dementia more often experienced role-sharing in eating (aOR 3.9, $p<0.001$), bathing (aOR 2.7, $p<0.001$), dressing (aOR 2.1, $p=0.02$), toileting (aOR 2.7 $p=0.02$), and getting around inside the house (aOR 2.8, $p<0.01$). Those enrolled in Medicaid had greater odds of receiving paid help only in dressing (aOR 2.1, $p=0.03$), getting around outside (aOR 2.4, $p<0.01$), and inside (aOR 4.4, $p<0.001$), as well as doctor visits (aOR 2.8, $p<0.01$). Findings lay the foundation for interventions that align with national recommendations to increase understanding of and strengthen relationships between family caregivers and paid caregivers.

Abstract citation ID: igae098.1696

### AN EXAMINATION OF RELATIONAL COORDINATION AMONG PAID AND FAMILY CAREGIVERS IN THE ARIC COHORT

Katherine Ornstein[1], Jennifer Reckrey[2], Shoshana Ballew[3], Josef Coresh[3], and Jennifer Wolff[1], *1. Johns Hopkins University, Baltimore, Maryland, United States, 2. Icahn School of Medicine at Mount Sinai, Icahn School of Medicine at Mount Sinai, New York, United States, 3. New York University, New York, New York, United States*

The oldest-old (ages 85+) are disproportionately at risk for impaired function and reliant on support from caregivers. However, studies of caregiving typically fail to comprehensively enumerate and assess the capacity of both family and paid caregivers, shared care networks, and change over time. In this session we will describe the conceptualization and development of ARIC Cares, a caregiving study that will be embedded within the Atherosclerosis Risk in Communities Study (ARIC), a multisite prospective cohort of ~4,000 individuals ages 81-101 that has been characterized for >30 years. Existing studies of how caregiver factors relate to outcomes for older adults generally evaluate a limited set of measures specific to family caregivers (e.g., relationship to older adult). The role that paid caregivers play in meeting care needs in the community is consistently undervalued and less well studied. ARIC Cares will simultaneously assess family and paid caregivers and utilize the theory of relational coordination for understanding the dynamics of caregiving teams. ARIC Cares, which will focus on the end-of-life period and examine outcomes for older adults and their caregivers, will enumerate older adults' care networks at 6-month intervals, in 4 diverse communities. Rigorous evidence regarding the caregiving experience, particularly focused on how teams of family and paid caregivers work together, could inform initiatives to improve quality of life among older adults and their caregivers. An understanding of these networks is critical as serious illness care, including complex dementia care, increasingly shifts to community settings where coordination between family and paid caregivers is vital.

### SESSION 3575 (SYMPOSIUM)

Abstract citation ID: igae098.1697

### THE BENEFIT OF COOPERATIVE EXTENSION FOR OLDER ADULT MENTAL HEALTH AND FUTURE GERONTOLOGICAL RESEARCH OPPORTUNITIES

Chair: Naomi Meinertz
Co-Chair: Kate Perepezko
Discussant: David Buys

Mental health concerns are a gerontological research priority; however, older adults who would benefit most from research in this area (e.g., rural, socially isolated) are often underrepresented in research. With representation in every U.S. state, the Cooperative Extension (Extension) system is ideally situated to work with researchers to reach and positively impact older adults. However, despite a strong history of success in bridging research and practice, Extension is an often-overlooked asset. The main objective of this symposium is to highlight exemplary partnerships between academic faculty and Extension systems to positively impact older adult mental health. The first presentation will discuss the results of a systematic review highlighting studies that employed Community-based Participatory Research methods with the Extension system to positively impact older adult mental health. The second presentation will share the process of culturally adapting the National Council on Aging's 'Aging Mastery Program' through the Extension system and the impact on older participants' feelings of social connectedness. The third presentation will present the findings of a novel program, 'Rightsizing', that reduces hoarding behaviors of older adults by addressing their emotional connection to their possessions. The final presentation will identify the process of partnering with a Cooperative Extension system that implements a multi-state physical activity program, 'Stay Strong Stay Healthy', to improve older adult physical and psychosocial outcomes. This symposium will conclude with a discussion led by Dr. David Buys connecting each of the presentations and emphasizing the role of the national Extension system in improving older adult mental health outcomes. Mental Health Practice and Aging Interest Group Sponsored Symposium

Abstract citation ID: igae098.1698

### A SYSTEMATIC REVIEW OF COOPERATIVE EXTENSION COMMUNITY-BASED RESEARCH FOR LATE LIFE MENTAL HEALTH

Naomi Meinertz[1], Mary Dozier[2], Kate Perepezko[3], and JeongEun Lee[4], *1. University of Missouri, Columbia, Missouri, United States, 2. Mississippi State University, Mississippi State, Mississippi, United States, 3. Scripps Gerontology Center, Lansdale, Pennsylvania, United States, 4. Iowa State University, Ames, Iowa, United States*

Rural-dwelling older adults have greater mental health disparities with higher rates of depression and anxiety compared to their urban counterparts; however, reaching these communities to understand and address the specific psychological needs of rural-dwelling older adults remains a barrier for gerontologists. Due to the extensive reach of the Cooperative Extension system (Extension), with representation in every county across the U.S., Extension is primed to facilitate connections between researchers and rural communities to improve mental health outcomes for older adults. Despite the benefits of such partnerships, Extension is largely unknown and underutilized by gerontological researchers, which leaves many older adults unconnected with resources that could improve their mental health outcomes. To better understand this gap, we conducted a systematic review of gerontological studies that utilized Extension to reach and positively impact rural older adult mental health. Using the methods and results from these studies, we highlight the principles of community-based participatory research (CBPR) that make this gerontological research possible and beneficial for older adult mental health outcomes. We also explore the characteristics important for these collaborative relationships and underscores the role that Extension plays in improving mental health outcomes of older adults. Our review will contribute to gerontological research by outlining the advantages of CBPR conducted in partnership with the Extension system and providing guidance for researchers to make these connections that will reduce the mental health disparities between rural and urban older adults.

Abstract citation ID: igae098.1699

### AMPING UP HEALTHY AGING: ENHANCING THE LIVES OF OLDER ADULTS AMONG DIVERSE POPULATIONS

Ginger Williams[1], Sue Schneider[2], Eric Ishiwata[2], Sara Delgado[2], Peggy Stoltenberg[2], Christine Fruhauf[2], and Allyson Brothers[2], *1. Colorado State University, Deer Trail, Colorado, United States*, *2. Colorado State University, Fort Collins, Colorado, United States*

Over the last decade, Colorado State University Extension (CSUE) has focused on meeting the needs of historically marginalized communities with the highest rates of health disparities and social isolation. These disparities are largely due to language barriers and gaps in cultural competencies among healthcare providers. To bring healthy aging education and support to culturally diverse and underserved communities, CSUE led the adaptation of National Council on Aging's (NCOA) Aging Mastery Program (AMP), a ten-session evidence-based curriculum (available in both English and Spanish) that supports older adults in aging well. The adaptation of AMP was implemented with Native American, Spanish-dominant immigrant, and East African refugee populations in Colorado. To adapt the program, we utilized state data to identify priority communities. Next, we conducted listening sessions to understand each community's unique perspectives with a significant focus on developing trusting relationships. We then identified and supported members of each community as champions to teach the curriculum with CSUE's coaching and technical assistance. Finally, we empowered these champions and their class participants to identify other gaps in knowledge and areas of interest, creating opportunities to develop 'booster' sessions. We prioritized paying our community champions to teach and lead in their communities. As a result, we've co-written grants and papers and created new programs alongside these champions. We are currently exploring expanding AMP in eight new communities across Colorado. Our program adaptation process has helped to break down barriers to implementing culturally relevant programs with underserved communities while bridging health equity gaps across the state.

Abstract citation ID: igae098.1700

### RIGHTSIZING: A COLLABORATION BETWEEN PSYCHOLOGY AND EXTENSION TO HELP OLDER ADULTS DEAL WITH SENTIMENTAL CLUTTER

Mary Dozier[1], Jennifer Krafft[1], Pamela Turner[2], Diane Bales[2], Susan Moore[2], Laura Smith[2], Jasmine Harris-Speight[1], and David Buys[1], *1. Mississippi State University, Mississippi State, Mississippi, United States*, *2. University of Georgia, Athens, Georgia, United States*

Excessive clutter can create a barrier for older adults to maintain functional lives in their homes; however, interventions for hoarding are lengthy, expensive, and often unavailable, particularly for rural-dwelling older adults. Using the Rightsizing Program as an example, this presentation will highlight the synergy between Extension and academic faculty to promote aging in place across diverse states. The Rightsizing Program is a collaboration between Extension specialists at Mississippi State University (MSU) and University of Georgia (UGA) and psychology faculty at MSU with specific expertise in hoarding disorder. The program focuses on sentimental clutter, which can be particularly difficult for older adults to discard. The psychology faculty were able to integrate both their research and clinical expertise to inform program development. For example, there was an added emphasis on decreasing sense of guilt around clutter (e.g., explicitly stating that it is "okay to let go of items.") that has been clinically observed to occur when older adults are reticent to let go of aspects of their past selves (e.g., clothes that no longer fit). The Rightsizing Program has been presented to 238 individuals and has increased access to both psychoeducation about hoarding and clutter as well as had the additional benefit of facilitating recruitment for psychological studies on hoarding on MSU campus. Partnership between Extension specialists and mental health researchers allows for a rapid dissemination of research findings, while also promoting ongoing research efforts.

Abstract citation ID: igae098.1701

### CREATING AND SUSTAINING EXTENSION PARTNERSHIPS TO REACH RURAL OLDER ADULTS: THE STAY STRONG STAY HEALTHY PROGRAM

Kristin Miller, Kelsey Weitzel, and Naomi Meinertz, *University of Missouri, Columbia, Missouri, United States*

Due to out-migration of family members and a lack of transportation and access to resources, rural-dwelling older adults often experience greater social isolation, which is linked to worse health outcomes and a loss of independence. Gerontological researchers have identified that increased participation in social activities can lead to increased social connections among rural-dwelling older adults. Through educational programs, the Cooperative Extension (Extension)

system connects older adults with opportunities to engage in community programs to reduce social isolation and support older adults living independently longer. Despite the widespread success of Extension programs, the implementation and collaborative efforts that contribute to the success of these programs are unrecorded in empirical literature. In this presentation, we address this gap by presenting on the Stay Strong Stay Healthy (SSSH) program. SSSH is as an example of the successful implementation of a multi-state Extension program that improves psychosocial and physical health outcomes for both rural and urban older adults. Using the SSSH program as a model, we outline the community-based research principles that inform the collaborations between Extension campus researchers, Extension agents, and stakeholders to reach rural-dwelling older adults. We will draw on a current example of adapting the SSSH program for less ambulatory older adults in rural areas guided by a community advisory committee. By leveraging the resources and expertise of Extension, researchers can enhance the real-world impact of their work and contribute to the reduction of social isolation and increased ability to live independent for rural-dwelling older adults.

### SESSION 3580 (SYMPOSIUM)

Abstract citation ID: igae098.1702

### THE OBESITY BILL OF RIGHTS: A FRAMEWORK FOR HEALTH EQUITY AND SELF-ADVOCACY

Chair: Gretchen Tanbonliong

When combined with common aging-related challenges, obesity, which is now recognized as a chronic condition, can create complex clinical situations. A particularly challenging factor is the issue of stigma from weight bias and ageism, both of which are significant barriers to meeting the individual needs of the person with obesity/overweight. The Obesity Bill of Rights (OBOR) was created in response to the urgent need to pave the way for a more equitable landscape where every person is respected and guaranteed unbiased, comprehensive management and treatment of obesity. This symposium will present multiple stakeholder viewpoints that led to the research and development of the OBOR; how it is operationalized in health care delivery settings; and how providers and patients may work collaboratively to achieve the research-informed principles articulated in the OBOR.

Abstract citation ID: igae098.1703

### LISTENING TO COMMUNITY VOICES FOR COLLECTIVE ACTION: THE OBESITY BILL OF RIGHTS ORIGINS

Dorothea Vafiadis, *National Council on Aging, Arlington, Virginia, United States*

Four town hall meetings were convened by the National Council on Aging and National Consumers League in senior centers and faith-based organizations during the summer of 2023. These venues were in four different states representing diverse cultural communities and socioeconomic backgrounds. Over 250 older adults, community leaders, and clinicians participated in discussions on the complex social and institutional barriers to obtaining appropriate management of overweight/management. These discussions yielded themes that were translated into the 8 core principles of the Obesity Bill of Rights (OBOR) which address issues such as ageism, weight bias, and aspects of the healthcare system that preclude adequate obesity care, coverage, and reimbursement. Obesity experts and stakeholders reviewed and validated the OBOR in the fall of 2023. On January 31, 2024, the OBOR was officially launched as a framework for health equity and self-advocacy. To date, nearly 50 health organizations and medical societies have endorsed the OBOR.

Abstract citation ID: igae098.1704

### FROM FEAR TO FORTITUDE AND EMPOWERMENT: A PATIENT'S JOURNEY

Patricia Nece, *Obesity Action Coalition, Alexandria, Virginia, United States*

Many older adults aged 65 and over with overweight and obesity struggle with navigating a health care system that is not inherently equipped to deliver quality obesity care. In the face of ageism and weight stigma, initiating conversations with providers who lack time, education, training, or even empathy can test the "fortitude factor" of even the most resilient of patients. Patricia Nece, J.D. will share her own personal experiences of such challenges and how the Obesity Bill of Rights can empower other patients facing similar situations to advocate for themselves—especially when it comes to making decisions about their own health care.

Abstract citation ID: igae098.1705

### CREATING AN OBESITY BILL OF RIGHTS–INFORMED CLINICAL PRACTICE: A GERIATRICIAN'S PERSPECTIVE

John Batsis, *University of North Carolina, Chapel Hill, North Carolina, United States*

The Obesity Bill of Rights (OBOR) sheds light on many challenges and opportunities for clinicians to implement in their respective practices. Dr. Batsis will share themes and insights that emerged from recent obesity roundtable discussions hosted by the National Council on Aging and leading up to the creation of the OBOR, including special considerations for obesity prevention and care for older adults aged 65 and over from a clinical standpoint. Barriers to obesity treatment; health care access; and access to trustworthy information for older adults—including those that intersect with issues of diversity, equity, and inclusion—will be explored. Examples of steps Dr. Batsis has taken to align his own delivery of care with the OBOR will be highlighted, as well as recommendations to facilitate open and responsive dialogue built on trust between patients and providers.

### SESSION 3585 (SYMPOSIUM)

Abstract citation ID: igae098.1706

### WORKING WITH DIVERSE ELDERS IN RURAL COMMUNITIES (OPPORTUNITIES AND STRENGTHS)

Chair: Karen Kopera-Frye
Co-Chair: Lyn Holley
Discussant: Cassandra Ford

The focus of this symposium is to discuss the opportunities and strengths of working with diverse elders and older adults in rural communities. Accounts of field experiences will be shared, and methods of utilizing existing data that hold promise for addressing current problems; along with building the foundation of communication and relationship requisite to successful design, development, and implementation of improvements will be described through various studies with rural and/or indigenous peoples. We will discuss the role of social determinants of health, race, and rurality on frailty trajectories utilizing data from the National Health and Aging Trend Study. An innovative approach for leveraging older adult farmers' expertise to facilitate effective emergency decision-making in Fraiser Valley, British Columbia, Canada, will be described. Ongoing research with Alaskan Native Elders provides insight into the need for effective research with Indigenous cultures, especially Elders, by developing trusting and mutually beneficial relationships. Research strategies involved historical, social, cultural, and inter-relational perspectives. Insight into how Cuidando con Respeto, a program with Latinx elders, builds bridges to health care and mutual understanding nurtured at the community level and shows promise in empowering caregivers within a familismo-culturally relevant manner will be shared. The use of mobile pain applications and considerations when designing health technology will be examined with diverse older adults.

Abstract citation ID: igae098.1707

### PREPAREDNESS AND CUIDANDO CON RESPETO: A CULTURE-CENTERED APPROACH FOR LATINX CAREGIVERS

Karen Kopera-Frye, *New Mexico State University, Las Cruces, New Mexico, United States*

The Latinx community is the largest racial minority in the United States, yet many Latinx families are in socially vulnerable positions due to immigration crisis, discrimination, health inequities, and poverty. These factors have been shown to impact Latino mental health and well-being; particularly in the caregiving role. The National Academies of Sciences, Engineering, and Medicine (2021) have recommended advancing the field by understanding the cultural aspect of dementia and caregiving in diverse populations and the resultant implications. An Evidence-Based Program, Cuidando con Respeto, aims to empower Spanish-speaking caregivers of loved ones with Alzheimer's Disease. Fifty-eight Latinx rural dwelling caregivers, primarily daughters, caring for their elder parents with dementia were surveyed about their perceptions of the Cuidando Program and how prepared they felt for their caregiving role. Qualitative analysis of caregiver responses on Program effectiveness and perceptions of preparedness in caregiving areas, e.g., information, revealed overwhelming positive benefits. Caregivers felt more prepared for their caregiving role and appreciated learning coping strategies with disruptive behaviors. The Promotora-led training resulted in greater reported self-efficacy. Level of preparedness is an important caregiving dimension to address, especially with culturally diverse communities having strong cultural caregiving norms. The incidence of Latinx caregivers is rising; however, available culturally appropriate services are lacking, especially in rural areas. Familismo, a Latinx primary cultural value, refers to the importance of strong family loyalty, and contributing to the well-being of the nuclear, and extended family, and kinship networks. Cuidando con Respeto shows promise in empowering caregivers within a familismo-culturally relevant manner.

Abstract citation ID: igae098.1708

### OLDER ADULT ANIMAL FARMERS' EVACUATION EFFORTS DURING THE 2021 PACIFIC NORTHWEST FLOODS IN CANADA

Haorui Wu, *Dalhousie University, Halifax, Nova Scotia, Canada*

The animal farming communities in the Fraser Valley were devastated by the 2021 Pacific Northwest floods. The community residents, community-based service and professional agencies, and government emergency responders were engaged in the evacuation. Through qualitative interviews of animal farm owners, community-based service agencies, and governmental emergency responders, this project discovers that during the decision-making stage of the disaster evacuation, the older adult animal farmers' their long-term engagement with their natural, built, and social environments enabled them to develop the initiatives for evacuation strategies help the governmental officials better allocate resources and support community-based service and professional organizations to coordinate different community-based services and resources to improve the evacuation process. This collaboration shed light on an innovative approach for leverage older adult animal farmers' expertise to facilitate effective emergency decision-making.

Abstract citation ID: igae098.1709

### WISDOM OVER TEA: USE OF COMMUNITY-BASED APPROACHES WITH RURAL ALASKA NATIVE ELDERS

Jordan Lewis[1], Steffi Kim[2], and Lena Thompson[3], *1. University of Alaska Fairbanks, College of Indigenous Studies, Fairbanks, Alaska, United States*, *2. University of Alaska, Anchorage, Alaska, United States*, *3. University of Minnesota Duluth, Duluth, Minnesota, United States*

Native Elders are often underrepresented in data collection efforts due to distrust of Western research institutions and Western methodologies. Through over 16 years and 162 interviews with Alaska Native (AN) Elders, the Alaska Native Successful Aging Team, led by Dr. Jordan P. Lewis, has been employing strategies to decolonize research. In this presentation, we will share stories and strategies for connecting with rural and remote AN Elders, including the role of sharing (food, tea, gifts, laughter), taking time to build relationships, and the importance of laughter with Elders. Furthermore, we will explain the role of an Elder-led advisory committee and its role in the implementation of Alaska Native driven values-oriented research. The use of community-based approaches, such as talk story, community member involvement, building trust, and maintaining sustainable relationships are necessary steps to conduct research that is Indigenous-driven and beneficial to the communities. Throughout this presentation, we will discuss Indigenous-driven research approaches and the necessity for the increased recognition of research strategies that involve historical, social, cultural, and inter-relational perspectives. Decolonizing approaches to data collection offer space for relationality, and offer a trusting and mutually beneficial environment to conduct research with AN Elders.

Abstract citation ID: igae098.1710

### THE IMPACT OF SOCIAL DETERMINANTS OF HEALTH ON FRAILTY TRAJECTORIES OF OLDER ADULTS IN RURAL UNITED STATES

Hillary Spangler[1], Wenyi Xie[2], David Lynch[1], Feng-Chang Lin[2], and John Batsis[2], *1. University of North Carolina School of Medicine, Chapel Hill, North Carolina, United States*, *2. University of North Carolina, Chapel Hill, North Carolina, United States*

**Background:** Frailty is a geriatric syndrome of physical decline that increases an older adult's risk for vulnerability to physiological stress. We aim to determine the impact of social determinants of health (SDOH) on frailty trajectories of older adults in rural areas.

**Methods:** We used data from the National Health and Aging Trend Study (2011-2020), a cohort of Medicare beneficiaries >65 years. Using cohorts from 2011 (n=8,245) and 2015 (n=8,334), individuals were categorized using Fried's frailty phenotype. Rurality was defined using the Office of Management and Budget's definition. Generalized estimation equations with generalized logit link function determined the impact of rurality and SDOH (transportation, family support, income, education) on frailty status.

**Results:** Using the 2011 (n=8,245) and 2015 (n=8,334) cohorts, participants were 57.1% and 55.8% female, median age 70-74 years old, and 18.5% and 19.3% lived in rural areas. Being married, having a college education, and higher income were protective against frailty progression. Urban areas had lower odds developing frailty versus pre-frailty, yet hastened developing pre-frailty versus robust. Urban adults with transportation to doctor appointments had lower odds of frailty progression, though not significant (robust->frail (OR 0.80, SE 0.09, p=0.47) and robust->pre-frail (OR 0.83, SE 0.08, p=0.49)). Hispanic ethnicity was protective against progressive frailty robust->frail (OR 0.40, SE 0.14, p=0.008) or pre-frail->frail (OR 0.55, SE 0.13, p=0.01).

**Conclusion:** Our findings suggest SDOH, race, and rurality may impact frailty trajectories. Future studies need to investigate the heterogeneity of aging in groups of older adults with similar SDOH and geography.

Abstract citation ID: igae098.1711

### EXPLORING MOBILE PAIN APPLICATIONS WITH DIVERSE ELDERS

Mary Milidonis[1], Hayley Rupp[1], Patrick McGinty[1], and Karen Kopera-Frye[2], *1. Cleveland State University, Cleveland, Ohio, United States*, *2. New Mexico State University, Las Cruces, New Mexico, United States*

Mobile pain applications (apps) may improve health communication. Mobile health apps do not always consider the needs of diverse older adults. This qualitative project examines perceptions of smartphone pain apps between African American and Caucasian older adults. The sample consisted of 19 older adults (mean age = 68 years), with majority female (88%), predominantly Caucasian (76%), African American (24%) and majority some college education (70%). Participants were randomly assigned to evaluate 2 of 5 free pain apps. Respondents answered questions about pain apps using the Mobile Application Rating Scale (MARS). The MARS assesses descriptive and technical aspects of an eHealth apps using five categories including four quality scales: engagement, functionality, aesthetics, and information and one subjective quality scale. Participants experienced persistent pain from 3 months to 12 years. Participants self-reported an average of 2.6 comorbidities on the Functional Comorbidity Scale. Important characteristics identified by both groups of older adults as critical for useable app included: Clear instructions and good navigation. The African American group differed on what would deter use of the app which included fear of use, uncertainty about the app and preferred use of personal physician. The Caucasian group themes for what deterred app use included complexity and lack of engagement. Both groups identified the important features were tracking pain and treatment ideas. These are important considerations in designing health technology for use with diverse older adults who need recurrent pain self-management.

### SESSION 4000 (SYMPOSIUM)

Abstract citation ID: igae098.1712

### AGING IN SUB-SAHARAN AFRICA

Chair: Jennifer Stanley
Co-Chair: JohnBosco Chukwuorji
Discussant: Jennifer Stanley

Aging in sub-Saharan Africa is a relatively neglected area of research despite the increasing proportion of the population aging. This collection of papers highlights how the varied cultures in sub-Saharan Africa shape development in later adulthood and emphasizes the critical need for research on aging to consider the sociocultural context. First, Chukwuorji and colleagues focus on how health behaviors and self-esteem explain the association between social support and successful aging among Nigerian retirees. Second, Porter and colleagues present a collaboration between scientists and older adults in knowledge generation. They find that access to both social and physical environmental elements are necessary to promote physical activity in their communities. Third, Burns and colleagues explore how place and gender interact when examining disability prevalence among older adults in Ghana. Fourth, Silverstein and colleagues report that culture and religion play an important role in shaping how palliative care is perceived. For example, a cultural norm for medical secrecy and a hesitancy to talk about death present unique barriers for palliative care. Fifth, in a qualitative study examining how older chronically ill Nigerians cope with their diagnosis, Mahmoud and colleagues report that older Nigerians do not want individuals outside their close family circle to know that they are ill. Individuals conceal their illness because they fear discrimination and want to maintain normalcy when interacting with others. All the papers presented in this symposium highlight the importance of considering the sociocultural context to understand the aging experience.

Abstract citation ID: igae098.1713

### SOCIAL SUPPORT, HEALTH BEHAVIORS, SELF-ESTEEM, AND SUCCESSFUL AGING IN A SUB-SAHARAN AFRICAN SAMPLE OF OLDER ADULTS

JohnBosco Chukwuorji[1], Chima Igbokwe[2], Blessing Ome[2], Runcie Chidebe[3], Beatrice Igbokwe[2], Mary Nwoke[2],

Chidiebere Obioha[2], and Benard Okechi[2], *1. Michigan State University Flint, Flint, Michigan, United States*, *2. University of Nigeria Nsukka, Nsukka, Enugu, Nigeria*, *3. Miami University, Oxford, Ohio, United States*

Previous research demonstrates that social support facilitates successful aging across all cultures. However, the factors that potentially mediate the link between social support and successful aging remain unclear. This study examined whether a healthy lifestyle and self-esteem mediate the association between social support and successful aging. It was hypothesized that the relationship between social support and successful aging would be serially mediated by both healthy lifestyle and self-esteem. Participants were 479 Nigerian retirees (53.4% female; Mean age = 64.81, SD = 6.86). They provided information on relevant demographic variables and completed the following measures: Fantastic Lifestyle Checklist (Fitness Appraisal), Rosenberg Self-esteem Scale, Multi-dimensional Scale of Perceived Social Support Scale, and Successful Ageing Inventory. Three separate regression models (family; friends; and significant other dimensions of social support) were conducted using the Hayes PROCESS macro for SPSS with 5,000 bootstrap estimates. Controlling for age and sex, family support, significant other support, friends support, healthy lifestyle and self-esteem were directly associated with successful aging. The association between family support and successful aging was mediated by healthy lifestyle; and this was also seen for friends' support and significant other support. The sequential path from social support to successful aging through healthy lifestyle, and then via improved self-esteem, was significant for family support and significant other support, but not friends support. Middle-aged to older adults who have strong support from their families and significant others may be more likely to engage in healthy behaviors and, in turn, experience higher levels of self-esteem, thereby aging well.

Abstract citation ID: igae098.1714

### A CITIZEN SCIENCE PROJECT ENGAGING NIGERIAN OLDER PERSONS IN A NEIGHBORHOOD ASSESSMENT FOR PHYSICAL ACTIVITY

Michelle Porter[1], Emmanuel Odeyemi[1], Stephanie Chesser[1], and Abby King[2], *1. University of Manitoba, Winnipeg, Manitoba, Canada*, *2. Stanford University, Stanford, California, United States*

A main tenet of age-friendly principles is that the voices of older people should be guiding improvements to their communities. The purpose of this collaborative citizen science project was to examine the age-friendliness of a neighborhood in Lagos Nigeria in terms of its characteristics to enable physical activity. Citizen scientists (13 older adults, seven men and six women, 65 to 86 years old) were involved in data collection, analysis, and priority setting. For data collection, they used the Stanford Healthy Neighborhood Discovery Tool application on a tablet to record 156 photos and 151 commentaries (text and audio) of neighborhood barriers and facilitators to physical activity. The collaborative analysis and priority setting process led to the following facilitators being identified: pedestrian and traffic facilities (e.g., traffic lights, walkways); green areas and parks; multi-generational community features (e.g., programs / facilities); opportunities for social connection (e.g., neighborhood associations, churches); safety of destinations and services; and public toilets. Barriers included: hazardous walkways / traffic; noise pollution; refuse; selling of public parks; crime (e.g., kidnapping, criminal hideouts); no safe drinking water; and ageism. The identified priorities for change in this Nigerian neighborhood included both social and physical aspects of the environment: social connectivity; improved pedestrian and traffic facilities; and green and beautiful environments. By meaningfully engaging older adults, this type of approach shows promise for communities in making age-friendly changes.

Abstract citation ID: igae098.1715

### EXPLORING THE INTERSECTIONALITY OF PLACE AND GENDER AMONG OLDER ADULTS IN GHANA

Shane Burns[1], Latrica Best[2], and Solomon Amoatey[3], *1. University of Michigan, Ann Arbor, Michigan, United States*, *2. Boston College, Chestnut Hill, Massachusetts, United States*, *3. Louisville University, Louisville, Kentucky, United States*

Ghana's older adult population is growing rapidly and projected to double by 2050. It is well-documented that social, health, and housing factors influence disparate rates of disability. However, little is known how the intersection of place (i.e., urban; rural) and gender (i.e., woman; man) inform rates of disability among older Ghanaians. We seek to examine this gap in the literature through an intersectional approach. Using logistic regression with Wave 1 (2007/08) data from the Study on global AGEing and adult health (SAGE) Ghana, we investigate the prevalence of activities of daily living (ADL) disability among respondents ages 50+ (n=4,106). To document gender differences by place, we compute separate adjusted odds ratio models among urban and rural respondents while accounting for health, social, and housing factors that might explain gender differences. Compared to urban men, urban women's ADL disability disadvantage was explained by marital status, particularly widowhood. In contrast, rural women consistently reported an ADL disability disadvantage when compared to rural men. Additionally, we found that the morbidity profiles of those who reported ADL disability differed by place and certain ADL difficulties (i.e., bed transferring; toileting) were especially common among women. Women, both urban and rural, were especially vulnerable to ADL disability. Marital status explaining urban women's ADL disability disadvantage suggests that partnership attenuates disability risk. Also, we speculate that varied morbidity associations with ADL disability are due to different stressors in urban versus rural environments. These findings also generate further interest about rural women's disability disadvantage.

Abstract citation ID: igae098.1716

### A SOFT DEATH: PERCEPTION AND ATTITUDES TOWARD PALLIATIVE CARE IN SENEGAL

Hannah Silverstein[1], Fursan Sahawneh[2], and Brian Carpenter[1], *1. Washington University in St. Louis, United States*, *2. Rush Medical College, Chicago, Illinois, United States*

Palliative care (PC) is a specialty medical service that addresses the bio-psycho-social-spiritual needs of patients with serious illnesses and their care partners. Despite the value of PC, its expansion has been underdeveloped in sub-Saharan

Africa due to variability in access to training resources and funding. This study sought to understand the current landscape of PC services in Senegal and the barriers and opportunities in its growth. Semi-structured interviews were conducted with six PC clinicians, four caregivers, one driver for a PC service, and one public health professor, all native to Senegal. Questions addressed their experiences delivering or receiving PC and future recommendations. Interviews were translated and transcribed from French into English. Transcripts were qualitatively coded for themes using open and focused coding. Five themes were identified: 1) the current landscape of PC, 2) barriers to implementing PC, 3) strategies and philosophies in care, 4) unique features of Senegalese culture affecting PC, and 5) the future of PC. Our findings demonstrate that PC in Senegal remains an under-resourced and underutilized specialty medical service, but PC is being implemented by personally committed clinicians. Results also highlight the importance of cultural factors influencing care in Senegal and the need to expand training opportunities for clinicians, increase education of other medical providers and the public, integrate PC into the healthcare system, and expand research to evaluate the impact of these resources. PC has the potential to be an important force for improving the quality of life for Senegalese patients and their care partners.

Abstract citation ID: igae098.1717

### THE NEED TO APPEAR HEALTHY: CONCEALMENT OF CHRONIC ILLNESS, PRIVACY, AND SELF-SUFFICIENCY AMONG OLDER NIGERIANS

Kafayat Mahmoud[1], Tamara Baker[2], Darlingtina Esiaka[3], and Saliu Balogun[4], *1. Boston University, Boston, Massachusetts, United States*, *2. University of North Carolina, Chapel Hill, Chapel Hill, North Carolina, United States*, *3. University of Kentucky College of Medicine, University of Kentucky, Kentucky, United States*, *4. University of Tasmania, Hobart, Tasmania, Australia*

Prior research has highlighted the beneficial impact of social networks and social support on older adults' physical and psychosocial wellbeing. The impact of the relationship between chronic illness and social networks on the psychosocial wellbeing of older Nigerians remains understudied. This study explored how older Nigerians with chronic illnesses navigate the physical, mental, and emotional changes due to their chronic disease diagnosis within their social contexts. The current qualitative study used semi-structured in-depth interviews with 19 purposively sampled older adults, aged 50 years plus, chronically ill, and receiving clinical care to examine the role of social networks in how chronically ill older Nigerians cope with their diagnosis. Three main themes reflecting participants' experiences emerged from the findings: 1) closely-knit circles, 2) privacy and self-sufficiency, and 3) body image. Results show that chronically ill older Nigerians prefer to keep the knowledge of their conditions strictly within their close family circles. It was considered horrific to inform friends, community members, and religious groups about one's chronic illness. Findings further reveal that the need to appear healthy to one's social network stems from the fear of being discriminated against and attempts to maintain some level of normalcy when interacting with others. Additionally, feelings of inferiority and shame limited their participation in social activities and social network maintenance. We discuss the implication of the results for the mental wellbeing and quality of life of chronically ill older Nigerians and make recommendations for policies and resources that can improve the wellbeing of chronically ill Nigerians.

### SESSION 4005 (BIOLOGICAL SCIENCES INVITED SYMPOSIUM)

Abstract citation ID: igae098.1718

### BIOLOGICAL SCIENCES GSA & AGE JOINT SYMPOSIUM

Chair: George Sutphin
Co-Chair: Nathan LeBrasseur

For a fifth consecutive year, the Biological Sciences section of GSA and the leadership of the American Aging Association have partnered to identify abstracts that reflect on their shared effort to advance the understanding of mechanisms of aging and their impact on aging and disease. This symposium will discuss biomarkers and mediators of functional decline, sarcopenia, and frailty.

Abstract citation ID: igae098.1719

### FMO-4 SLOWS AGING DOWNSTREAM OF MTOR AND DR THROUGH ER TO MITOCHONDRIA CALCIUM SIGNALING

Angela Tuckowski[1], Safa Beydoun[2], Elizabeth Kitto[2], Ajay Bhat[3], Marshall Howington[2], Aditya Sridhar[1], Mira Bhandari[1], Kelly Chambers[1], and Scott Leiser[2], *1. University of Michigan, Michigan, United States*, *2. University of Michigan, Ann Arbor, Michigan, United States*, *3. University of Michigan, University of Michigan, Michigan, United States*

The geroscience field has identified several pathways that regulate aging across species. Of these pathways, dietary restriction and the reduced mechanistic target of rapamycin (mTOR) signaling are among the best studied but still not fully understood interventions. Recent work from our lab and others established the flavin containing monooxygenase family of enzymes as regulators of longevity across species. While others found that Fmos are induced by longevity interventions in mice, we found that Cefmo-2 promotes longevity, stress resistance, and healthspan by rewiring endogenous metabolism. These results suggest that the FMO family could regulate longevity in multiple species, including humans. Our new work focuses on the role of C. elegans FMO-4, which is structurally similar to FMO-2 but expressed in a different tissue. Our data show that fmo-4 confers stress resistance and is required for DR- and mTOR-mediated longevity. We also find that fmo-4 does not act similar to fmo-2 but downstream of it, and interacts with calcium signaling and stress response to promote healthy aging. Under the hypothesis that modified calcium signaling is crucial for ER and mitochondrial homeostasis, we find evidence of 1) the metabolic, stress, and aging processes in which FMO-4 activity plays a role, and 2) the likely mechanisms for these roles. Overall, our data highlight the importance of FMOs in longevity, and reveal a novel mechanism for FMO regulation of longevity and metabolism.

Abstract citation ID: igae098.1720

**PLASMA PROTEOMIC SIGNATURE OF DEVELOPMENT AND PROGNOSIS OF SARCOPENIA**

Chenkai Wu, Jinhui Liao, and Jianhong Xu, *Duke Kunshan University, Kunshan, Jiangsu, China (People's Republic)*

Sarcopenia, characterized by muscle loss and functional decline, poses a significant health concern in aging populations. Understanding the proteomic signatures associated with the development and prognosis of sarcopenia is crucial for targeted management. Data from 52,689 participants (aged 39-70 years) in the UK Biobank were analyzed, with 2,920 proteins assayed using Olink. We used the multinomial logistic regression to identify proteins cross-sectionally associated with sarcopenia at baseline; Cox regression was used to examine proteins associated with all-cause mortality among 2,886 individuals who were sarcopenic at baseline. Subsequently, we used the LASSO regression to screen for important proteins for predicting death among sarcopenic persons and the light gradient boosting machine model to determine relative importance of these proteins. Pathway analysis was performed using Enrichr. A total of 738 proteins were cross-sectionally associated with sarcopenia at baseline, while 627 proteins were significantly associated with mortality among initially sarcopenic individuals (Bonferroni-corrected p-value < 0.05). LASSO regression identified 306 and 65 proteins associated with sarcopenia status and death, respectively. Among the 34 overlapping proteins, GDF15, WFDC2, EDA2R, and CHRDL1 were the top signals. The prediction model with these 34 proteins had a C-statistic=0.81 and performed better than a base model with key socio-demographic variables (net reclassification index=0.27). Differential expression analysis revealed enrichment of proteins in cellular signaling, extracellular matrix remodeling, cancer initiation and progression, and metabolic regulation pathways. Identification of proteomic signatures of sarcopenia provides insights into its pathogenesis and prognosis. The refined predictive models offer potential clinical utility in personalized management strategies for sarcopenia.

Abstract citation ID: igae098.1721

**THE NEUTROPHIL-LYMPHOCYTE RATIO AND WHITE BLOOD CELL COUNT ASSOCIATE WITH MUSCLE ENERGETICS AND GAIT SPEED IN SOMMA**

Philip Kramer[1], Peggy Cawthon[2], Li-Yung Lui[2], Theodore DeConne[1], Nancy Lane[3], Xiaoyan Leng[1], Anne Newman[4], and Stephen Kritchevsky[5], *1. Wake Forest University School of Medicine, Winston-Salem, North Carolina, United States*, *2. California Pacific Medical Center, San Francisco, California, United States*, *3. University of California Davis School of Medicine, Davis, California, United States*, *4. University of Pittsburgh, University of Pittsburgh, Pennsylvania, United States*, *5. Wake Forest University School of Medicine, Wake Forest University School of Medicine, North Carolina, United States*

Systemic inflammation drives sarcopenia and cachexia, which impair the mobility and independence of older adults. We and others have demonstrated that muscle mitochondrial energetics are strongly associated with gait speed, leg power, and mobility limitations. However, the contribution of systemic inflammation to lower muscle mitochondrial energetics is not fully understood. In this analysis of the Study of Muscle, Mobility and Aging (SOMMA), two pro-inflammatory biomarkers, the neutrophil-lymphocyte ratio (NLR) and total WBC count (WBC), were measured in adults aged 30-94 (30-69, n=81; 70-79, n=864). We report associations with maximal muscle mitochondrial energetics (MaxOXPHOS) and 400m gait speed. Individuals in higher NLR tertiles (< 1.685 vs. ≥1.685 - < 2.430 vs. ≥2.430) had significantly lower MaxOXPHOS (63.9±22.5 vs. 61.7±19.9 vs. 59.0±16.8, p=0.019) and gait speed (1.08±0.19 vs. 1.08±0.18 vs. 1.03±0.16, p=0.0002), had a greater multimorbidity burden (p < 0.0001), and were older (p=0.008) compared to individuals in lower NLR tertiles. The associations of NLR and WBC with MaxOXPHOS (NLR, $\beta$=-1.38, p=0.05; WBC, $\beta$=-2.45, p=0.0003) were partly explained by age and BMI (NLR, $\Delta\beta$=-37%, p=0.18; WBC, $\Delta\beta$=-34%, p=0.01), and were further explained when accounting for all other covariates, including multimorbidity burden (NLR, $\Delta\beta$=-28%, p=0.36; WBC, $\Delta\beta$=-43%, p=0.15). However, the association of NLR and WBC with gait speed (NLR, $\beta$=-0.019, p=0.0009; WBC, $\beta$=-0.034, p< 0.0001) could not be fully explained by age, BMI or other covariates (NLR, $\Delta\beta$=-10.5%, p=0.002; WBC, $\Delta\beta$=-61%, p=0.019). In conclusion, systemic inflammation, especially as related to age, obesity, and disease, associates with lower muscle energetics and gait speed in adults.

Abstract citation ID: igae098.1722

**CARDIOVASCULAR-DERIVED CELL-FREE DNA: ASSOCIATIONS WITH FRAILTY AND AGING-RELATED COMORBIDITIES**

Lolita Nidadavolu[1], David Sosnowski[1], Nikita Sivakumar[1], David Bennett[2], Jude Phillip[1], Brion Maher[1], Esther Oh[1], and Peter Abadir[1], *1. Johns Hopkins University, Baltimore, Maryland, United States*, *2. Rush University Medical Center, Chicago, Illinois, United States*

Cell-free genomic DNA (cf-gDNA) fragments are released into the bloodstream following cell death processes and higher levels are associated with age-related physical and cognitive decline. It remains unclear which tissues release cf-gDNA fragments in community-dwelling older adults with frailty. Serum cf-gDNA from 190 participants in the Religious Orders Study and Rush Memory and Aging Project (ROS-MAP) had DNA methylation performed via Illumina Methylation EPIC array. Hierarchical clustering derived distinct sub-populations based on cf-gDNA tissue of origin. Linear regression and mixed-effects models examined associations between tissue origin and physical measures. After neutrophils, vascular endothelial cells (5.3%), erythrocyte progenitors (4.8%), and monocytes (4.3%) were the most frequent cell types of origin. Hierarchical clustering demonstrated three sub-populations distinguished by cf-gDNA tissue origin: Cardiovascular, Erythrocyte Progenitor, and Immune Cell. Individuals in the Cardiovascular sub-group had enrichment of fragments from myocardial and vascular endothelial cells, demonstrated higher frailty scores, and had increased prevalence of myocardial infarction, congestive heart failure, and stroke compared to other sub-groups. The Cardiovascular sub-group also had DNA fragments with increased biological age compared to the other groups (via GrimAge calculation), suggesting the cf-gDNA was released from older cells in the Cardiovascular group. Our study reveals a sub-group enriched with fragments originating from cardiovascular tissue that demonstrate higher future occurrence of frailty and cardiovascular disease and have cf-gDNA release from older cells compared to the other sub-groups. Using cf-gDNA to identify these at-risk individuals can allow for early initiation of treatments to attenuate progression to severe cardiovascular disease and frailty.

### SESSION 4010 (PAPER)

### COGNITIVE FUNCTION AND IMPAIRMENT IN OLDER ADULTS

Abstract citation ID: igae098.1723

### LATENT CLASSES AND PROGRESSION OF MMSE IN YOUNG-ONSET DEMENTIA: DATA FROM THE SWEDISH DEMENTIA REGISTER

Deborah Finkel[1], Fanny Kårelind[2], Steven Zarit[3], Therése Bielsten[2], Helle Wijk[4], and Linda Johansson[5], *1. University of Southern California, Louisville, Kentucky, United States*, *2. Jönköping University, Jönköping, Jonkopings Lan, Sweden*, *3. The Pennsylvania State University, University Park, Pennsylvania, United States*, *4. Gothenburg University, Gothenburg, Ostergotlands Lan, Sweden*, *5. Institute of gerontology, School of Health and Welfare, Jönköping University, Jönköping, Sweden*

Studies have shown significant heterogeneity in the longitudinal progression of dementia. Growth mixture models have detected up to 4 classes that differ in both baseline Mini-Mental State Exam (MMSE) and rate of decline over time. Most analyses focus on adults over age 65 and investigate group differences in demographic and health variables. The current analysis focused on adults with young onset dementia (YOD) and examined the role of demographic and support variables. Sample included 1025 adults (55% women) registered in the Swedish Dementia Register prior to age 65 with at least 3 registrations. Age at baseline was 38 to 64 (mean=59.3, SD=4.1); follow-up ranged from 1 to 12 years (mean=4.6, SD=2.0). Growth mixture models identified 4 classes: high baseline MMSE and moderate decline over time (56.1%), intermediate baseline MMSE and somewhat faster decline (39.2%), low baseline and stable MMSE over time (3.5%), and high baseline with steep decline (1.2%). Group 2 had the highest proportion Alzheimer's Disease (AD) diagnosis and Group 4 had the lowest. Group 3 was more likely to have vascular or unspecified dementia and Group 4 was more likely to have vascular or frontotemporal dementia. Groups differed in age at diagnosis (Group 2 youngest), participation in adult daycare (Group 4 most likely), and having home health assistance (Group 2 most likely). Results highlight that YOD is just as heterogeneous as later onset dementia; therefore, it is vital that people with YOD get early diagnosis and a case manager to help identity and meet their individual needs.

Abstract citation ID: igae098.1724

### AS MUCH AS I CAN PREVENT… I'M GOING TO DO IT: A QUALITATIVE ANALYSIS OF WAYS PEOPLE MANAGE DEMENTIA-RELATED ANXIETY

Molly Maxfield[1], Allie Peckham[1], Dara James[2], and Rachel Koffer[1], *1. Arizona State University, Phoenix, Arizona, United States*, *2. Arizona State University, Tempe, Arizona, United States*

Alzheimer's disease and related dementias (ADRD) are prevalent, without cures, and associated with declining independence. Beyond their cognitive effects, ADRDs can create social, psychological, and financial challenges for the individual diagnosed, their family, friends, and caregivers. Dementia-related anxiety (DRA) is the fear or anxiety about a current or future diagnosis of ADRD. The present study examined self-identified ways of managing DRA. Semi-structured qualitative interviews focused on thoughts and feelings about dementia among 50 community-dwelling adults (58 to 89 years old, M = 70.80, SD = 6.02) without dementia diagnoses. A reflexive inductive thematic approach examined ways people managed DRA. Based on an iterative process, we adopted and revised themes, ultimately identifying five themes: 1) monitoring cognitive status (e.g., self-monitoring or objective assessment), 2) active coping strategies (e.g., using external reminders, normalizing age-related change), 3) interpersonal relationships and support (e.g., anticipating benefit of support from others), 4) planning and preparing for potential outcomes (e.g., securing power of attorney and/or advance directives, saying goodbyes), and 5) personal responsibility to manage dementia risk or accept a diagnosis (e.g., lifestyle factors to reduce dementia risk, thereby reducing risk for burdening others). Findings suggest people use internal and external means for coping with DRA that are likely to vary in their usefulness. We consider these findings within the context of terror management theory and the lifespan theory of control, attending to potential clinical interventions for individuals experiencing DRA.

Abstract citation ID: igae098.1725

### EXPERIENCES OF MUSIC LISTENING AMONG OLDER ADULTS WITH MILD COGNITIVE IMPAIRMENT

Claire Wang[1], Mengchi Li[2], and Junxin Li[3], *1. Johns Hopkins University, Baltimore, Maryland, United States*, *2. Xi'an Jiaotong University, Xi'an, Shaanxi, China (People's Republic)*, *3. Johns Hopkins School of Nursing, Baltimore, Maryland, United States*

Emerging research highlights the cognitive benefits of music listening for older adults. The nuances of these effects, particularly concerning musical features like tempo, memorability, lyrics, and genre, remain underexplored especially among those with mild cognitive impairment (MCI). This mixed-methods study, nested within a randomized controlled crossover trial, explored these features through assessments of participants' musical experience (Assessment of Personal Abductive Preference and Goldsmiths Musical Sophistication Index) at baseline and semi-structured interviews following a 4-week personalized music intervention (~30 self-selected songs, 1 hour/day, ≥5 days/week). Participants (n=25) were on average aged 68.0 ± 7.1 years (min: 55.0, max: 81.0), predominantly female (68%) and Black (68%). Almost half of the participants had some college or above education (44%), and about 36% of participants lived in extreme poverty. The findings highlight the significant value of music with 56.5% of participants reported routinely listening to music attentively for at least an hour each day; 42.9% described music's role in their lives as 'very important,' while 47.6% rated it as 'moderately important'; and 43.5% considered music as an indispensable part of their existence. The most popular genres reported were religious music (27.0%), followed by country/western/jazz/rock (14.8%), blues (11.1%), classical (7.4%), and folk/cultural music (3.7%). Interviews identified music essentialism, affective engagement, and feasibility as central themes, with participants preferring music that resonates with their moods or early life memories. Our findings underscore the deep-seated value of music, offering a foundation for tailoring music interventions to enhance the well-being of those with MCI.

Abstract citation ID: igae098.1726

### BRAIN CLEARANCE SYSTEM ALTERATION AND COGNITIVE DECLINE IN AFRICAN AMERICANS WITH POOR SLEEP QUALITY

Karen Lincoln[1], Bryan Gaines[1], and Kaushik Golithadka[2], *1. University of California Irvine, Irvine, California, United States*, *2. University of Southern California, Los Angeles, California, United States*

African Americans have 2-3 times the prevalence of Alzheimer's disease (AD) than Whites, making this a significant health disparity in the US. Healthy sleep plays a critical role in providing protection from cognitive decline and AD. Unfortunately, African Americans have significantly poorer indicators of sleep quality than Whites. Moreover, the mechanism whereby sleep drives AD and related cognitive decline is not well understood, in part because causal links are yet to be established by human studies. One possible mechanism linking sleep to AD is the glymphatic system, a network of perivascular spaces (PVS) that support waste clearance. This study used ultra-high field magnetic resonance imaging technology, novel PVS mapping technology, wrist actigraphy and sleep assessments, cognitive assessments and regression analysis to investigate how alteration of the glia-lymphatic system associated with sleep contributes to cognitive decline in 61 community-dwelling African Americans (age 45+). African American who slept more and spent more time in bed had a higher risk for cognitive impairment as indicated by lower MMSE scores. We also found that African Americans with larger PVS volume (more metabolic waste in the brain) had lower cognitive scores. The effect of sleep quality on cognitive status was mediated by PVS, such that African Americans who spent more time in bed had larger PVS (more waste and fluid) and lower cognitive scores. Findings from this study advance our understanding of the role of sleep and brain clearance in cognitive decline in African Americans, and identify PVS as a novel marker for cognitive decline.

### SESSION 4015 (SYMPOSIUM)

Abstract citation ID: igae098.1727

### CULTIVATING RESILIENCE ACROSS DIVERSE AGING COMMUNITIES

Chair: Catherine García
Co-Chair: Heather Farmer

This symposium explores the multifaceted experiences of resilience and well-being among older adults from diverse cultural backgrounds, emphasizing the role of resistance and assertion of cultural practices for optimal aging and aging in place. Presentations span various communities in the U.S. that are not widely studied in gerontological research, offering a comprehensive perspective on aging challenges and strategies. The first presentation focuses on the concept of 'Eldership' among Alaskan Native Elders in promoting mutually beneficial relations within youth, communities, tribes, and organizations. The second presentation highlights perspectives from Native Hawaiian kumu loea (expert teachers) of traditional practices on regeneration of cultural practices and language, emphasizing the challenges and personal resilience of those reclaiming indigenous knowledge through resistance. The third presentation explores the well-being of older South Asian American women, identifying factors like robust social networks, participation in cultural events, and mindfulness practices as asset-based strengths that empower and support a sense of purpose. The fourth presentation identifies factors influencing life satisfaction among resettled Bhutanese older adults, uncovering the importance of social support and resilience. The final presentation investigates self-employment among older Hispanics, revealing intragroup disparities in safety-net program access and economic opportunities, showcasing how cultural identity serve as a proxy to later life financial security and well-being. Together, these presentations foster a deeper understanding of resilience across diverse aging communities, emphasizing the importance of cultural resistance and traditional practices as integral components of well-being, providing insights for those seeking culturally sensitive approaches to support the well-being of older populations.

Abstract citation ID: igae098.1728

### RESILIENCE THROUGH ELDERSHIP

Lena Thompson[1], Steffi Kim[2], and Jordan Lewis[3], *1. University of Minnesota Duluth, Duluth, Minnesota, United States*, *2. University of Alaska, Anchorage, Alaska, United States*, *3. University of Alaska Fairbanks, College of Indigenous Studies, Fairbanks, Alaska, United States*

Alaska Native (AN) Elders face significant challenges aging in place, yet Elders find joy and connection from living in their villages and often choose to remain in their villages as they age. As keepers of wisdom and knowledge bearers, AN Elders serve as guides and collaborators in overcoming challenges on individual, community, and at policy-levels. This study collects stories and strategies of AN Elder resiliency fostered through leadership, community engagement, and cultural practices in Southeast and Interior Alaska. This study comes from a parent study exploring meanings of and strategies for "aging in a good way" among Alaska Native Elders. In-person semi-structured interviews were conducted with 72 Elders from 11 communities in Southeast and Interior Alaska. Recordings were transcribed, de-identified, and coded thematically. Elders described navigating life-long challenges, related to experiences of racism, generational trauma, and generational changes. Elders described ways that they address these life challenges, including practices and beliefs rooted in traditional teachings, gathering, hunting, and living their "native way of life." Notably, Elders felt that it was their duty to pass knowledge on to younger generations to address their own life challenges, incorporate and continue traditional practices, and save them hardships experienced by Elders. The concept of Eldership is vital to individual, family, and community wellbeing promoting bidirectional and mutually beneficial relations within youth, communities, tribes, and organizations. Through Eldership, AN Elders provide important guidance in managing challenges which can support Tribal governments and other service systems as they determine effective strategies to support AN communities.

Abstract citation ID: igae098.1729

### WORKING WITH NATIVE HAWAIIANS TO RECLAIM TRADITIONAL PRACTICES

Kathryn Braun, Noreen Mokuau, and Kukuna Yoshimoto, *University of Hawaii, Honolulu, Hawaii, United States*

With colonization, Native Hawaiians were strongly discouraged from speaking Hawaiian and practicing their traditions, and these practices went underground. Thus, few elders today know the language or traditional Hawaiian practices. Amendments to the Hawai'i Constitution in 1978 supported the bringing back of language and culture. For example, Native Hawaiian immersions schools began to be funded by the state, and today students can earn their elementary, secondary, and tertiary degrees in the Hawaiian language. However, after years of being hidden, much of the knowledge of language and traditional practices had to be resurrected and, to some extent, recreated. The 3-person research team, which included two Native Hawaiian scholars, collected stories from Native Hawaiian kumu loea (expert teachers) of traditional practices, soliciting information on how they learned their craft, how they are currently practicing it, and how they are teaching others. Interviewees included practitioners of Hawaiian spirituality, care of the dead, land management, healthy eating, language, song, conflict resolution, herbal healing, traditional massage, cordage, weapons, martial arts, law, and medicine. Analysis included several rounds of member checking. Findings illustrate how knowledge was passed down despite its suppression and the challenges interviewees faced accruing knowledge as adult learners, rather than as native oral learners. All demonstrated personal resilience, dedication to culture, and pride in their role in bringing back practices that were almost lost. The research process could be replicated by other investigators dedicated to hearing and documenting the process of indigenous knowledge regeneration.

Abstract citation ID: igae098.1730

### "WHERE THERE IS A WILL, THERE IS A WAY": STRENGTH AND RESILIENCE IN THE STORIES OF SOUTH ASIAN OLDER ADULTS

Mushira Khan, *Mather Institute, Evanston, Illinois, United States*

Productive aging is deeply connected to the realms of communal and associational life (Russell, 2011). Asset-based approaches facilitate productive aging by harnessing existing gifts and strengths within individuals and their communities and fostering a sense of purpose in older adults (Hornby-Turner et al., 2017). Despite this evidence, little is known about the specific experiences of South Asian American (SAA) older adults in relation to well-being. To address this gap, this qualitative study explored the strengths that contribute to their well-being through in-depth interviews with 32 SAA adults (44% female; mean age=58.3 years) ages 50+. Each interview lasted between 30 and 90 minutes and was audio-recorded with the participant's consent. Thematic analysis of the interview data revealed that several key assets facilitated a sense of purpose in SAA older adults, including robust local and global social support networks, often maintained through social media platforms such as WhatsApp and participation in cultural events, strong affectual ties between generations, self-transcendence through meditation and mindfulness-based practices, a sense of resilience, and creative coping strategies, particularly among late-life immigrants. These findings suggest that an asset-based approach can empower and support the well-being of SAA older adults by recognizing and leveraging their existing strengths, resources, and personal, familial, and cultural assets. For example, enhancing digital literacy in this population may offset loneliness through more effective engagement with digital social activities. Overall, policymakers, practitioners, and researchers may want to capitalize on these strengths when developing interventions or programs aimed at promoting productive aging among SAA older adults.

Abstract citation ID: igae098.1731

### FINDING FULFILLMENT IN NEW BEGINNINGS: EXPLORING LIFE SATISFACTION AMONG RESETTLED BHUTANESE OLDER ADULTS IN OHIO

Isha Karmacharya, and Saruna Ghimire, *Miami University, Oxford, Ohio, United States*

**Background:** The decision for refugees to flee their home country is often driven by persecution, conflict, or other dire circumstances, leading to a significant disruption in their lives. The US, along with the state of Ohio, has provided a safety net for a significant number of Bhutanese refugees. However, resettled refugees, particularly older adults, face significant challenges in adjusting to their new environment. This study aims to assess the life satisfaction of resettled Bhutanese older adults in Ohio, USA, and to identify the factors influencing their life satisfaction.

**Methods:** Through snowball sampling facilitated by local Bhutanese organizations, a survey was conducted among 276 resettled Bhutanese individuals aged 55 and above in four major Ohio cities (Columbus, Akron, Cleveland, and Cincinnati). Life satisfaction was assessed using the widely popular and validated Satisfaction with Life Scale, and binary logistic regression was employed to model the factors associated with life satisfaction.

**Results:** One in ten participants reported dissatisfaction with their lives. Participants experiencing depressive symptoms had higher odds of life dissatisfaction (OR:9.02, 95% CI: 2.28-35.77, $p=0.002$), whereas those with greater social support (OR: 0.89, 95% CI: 0.85-0.94, $p<0.0001$) and resilience (OR:0.92, 95% CI: 0.88-0.97, $p<0.002$) had lower odds.

**Conclusion:** Despite enduring significant challenges throughout their journey from displacement and refugee camps to resettlement in the USA, a notable proportion of participants expressed satisfaction with their lives. However, future studies should delve into their life transitions before and after resettlement to gain a deeper understanding of how the resettlement process shapes their life satisfaction.

Abstract citation ID: igae098.1732

### SELF-EMPLOYMENT AMONG OLDER HISPANICS: INTRAGROUP DIVERSITY, WORKPLACE BENEFITS, AND CITIZENSHIP STATUS

Bruna Lopez, *Boston College, Chestnut Hill, Massachusetts, United States*

Hispanics play a significant role in the self-employment landscape among older workers, yet research on this subject often ignores intragroup heterogeneity. Investigating these intragroup differences may highlight differential access to safety-net programs (e.g., health insurance, retirement savings programs) and economic opportunities (e.g., business incorporation). In response, this study utilized data from the Current Population Survey Annual Social and Economic Supplement, obtained from IPUMS-CPS, to assess differences in incorporated (I-SE) and unincorporated (U-SE) self-employment rates among those 50 to 64 years old by seven Hispanic subgroups within the U.S. (Mexican, Puerto Rican, Cuban, Dominican, Salvadoran, Central and South American, and Other Hispanic). Using pooled data between 2017 and 2022 to reduce sampling noise, we estimated health insurance access, retirement savings program access, and citizenship status by subgroup and incorporation status. We found notable differences by subgroup in overall self-employment rates (e.g., Cubans had the highest rates: 5% I-SE, 6% U-SE%; Puerto Ricans had the lowest rates: 2% I-SE, 2% U-SE). Salvadorans had the lowest health insurance coverage rates (48% I-SE, 58% U-SE). Those falling within the "Other Hispanic" category, including Spaniards, participated the most in workplace retirement savings programs (26% I-SE, 7% U-SE). Excluding Puerto Ricans, who are U.S. citizens, Dominicans in unincorporated self-employment (46% citizens) and Salvadorans in incorporated self-employment (41% citizens) were the least likely to hold citizenship status. These findings identify important gaps in access to health insurance and retirement savings programs, as well as more lucrative incorporated self-employment opportunities, by subgroup.

### SESSION 4020 (SYMPOSIUM)

Abstract citation ID: igae098.1733

### DAILY EXPERIENCES OF AGING: A COUNTRY-INFORMED PERSPECTIVE FROM THE SUBJECTIVE AGES PROJECT

Chair: Shevaun Neupert
Co-Chair: Anna Kornadt

The proposed symposium provides a global perspective regarding antecedents, correlates, and consequences of views on aging based on the Subjective AGES (Aging within Global everyday Ecological Studies) consortium. This culture-informed approach highlights the contextual and dynamic influences on views on aging across different temporal perspectives. We enrich the existing body of knowledge by including a broad variety of cultures and investigating how views on aging connect to daily indicators of health and well-being. First, Wu and Fung investigate the moderating role of future self views on the relationship between self continuity and preparation for old age in six cultures. Taking into account the historic context of inflation in Turkiye, Can and Neupert find that daily subjective economic status and daily subjective age are each uniquely related to daily happiness. Wirth and Rothermund show that the daily experience of expectations regarding aging in Germany influences affective experience. Finnigan and Tse show that daily flow experiences in a sample from the UK were related to younger subjective age, which in turn increased positive affect. Finally, Shrira and colleagues compare the daily coupling of ageist attitudes and negative affect in five different countries using an integrated data analysis approach. Our findings shed light on country-specific and global relationships between views on aging and development. We showcase how experiences of aging are shaped by contextual and dynamic influences.

Abstract citation ID: igae098.1734

### FUTURE SELF CONTINUITY AND AGING PREPARATION ACROSS CULTURES: THE MODERATING ROLE OF FUTURE SELF-VIEWS

Yiwen Wu, and Helene H. Fung, *The Chinese University of Hong Kong, Hong Kong, China (People's Republic)*

Preparation for aging enhances well-being and lowers societal costs. According to Hershfield (2019), people view their future selves as strangers when making intertemporal decisions. Future self-continuity—the psychological connectedness to future selves—is crucial to people's willingness to allocate resources for the future (Hershfield et. al., 2011). While studies have shown that higher future self-continuity promotes saving intentions (e.g. Hershfield et al., 2020), such relationships have not been examined among older populations in diverse cultures. This research aims to investigate the effect of future self-continuity on aging preparation and the moderating role of future self-views in two studies across six cultures. Two separate surveys have been conducted on N = 117 Australian older adults aged 55-86 years (Study 1, Mage = 61.7 SD = 6.6) and N = 2,940 older adults aged 38-99 years (Study 2, Mage = 61.5 SD = 15.2) across five cultures (Hong Kong, Taiwan, Germany, Czechia, and the United States). The results showed that future self-continuity predicted more preparation for aging in both studies, after controlling for age, gender, health, and social status. Further, the effect of future self-continuity on aging preparation was conditional on the valence of future self-views. Specifically, future self-continuity was positively associated with aging preparation when people held positive views towards their future selves, while such relationships became negative when people viewed their future selves negatively. These findings highlight the importance of considering both future self-continuity and future self-views in interventions to promote aging preparation among older adults across diverse cultures.

Abstract citation ID: igae098.1735

### DAILY CONNECTIONS BETWEEN HAPPINESS, SUBJECTIVE AGE, AND SUBJECTIVE ECONOMIC STATUS IN TURKIYE

Reyyan Can, and Shevaun Neupert, *North Carolina State University, Raleigh, North Carolina, United States*

Subjective economic status, which includes feelings of financial strain, is linked with important health and well-being indicators such as happiness. In addition, threats to economic status may be especially harmful for middle-aged and older adults. We aimed to characterize and explore predictors of daily happiness among middle-aged and older adults in Turkiye. Conducted during the summer of 2022, a period marked by high inflation, the study involved 98 participants aged 50 to 90 (M = 59.57, SD = 7.8) who reported on their happiness, subjective economic status, and subjective age for 14 consecutive days. Results from Multilevel Modeling revealed significant variations in happiness, subjective economic status, and subjective age over the 14-day period at both within and between person levels. Increases in subjective economic status were associated with increased daily happiness, whereas feeling older corresponded to decreased happiness. Subjective economic status and subjective felt age together accounted for 44% of the variance in within-person happiness variability, underscoring the importance of these factors in shaping happiness. Moreover, our study highlights economic volatility experienced by individuals in Turkiye calling for attention from policymakers, practitioners, and stakeholders to address the vulnerabilities faced by this group.

Abstract citation ID: igae098.1736
### AFFECTIVE AND COGNITIVE CONSEQUENCES OF PERCEIVING DAILY EXPECTATIONS FOR ACTIVE AGING

Maria Wirth, and Klaus Rothermund, *Friedrich Schiller University Jena, Jena, Thuringen, Germany*

Older individuals face increasing social expectations and demands to remain active and participate in society. Perceived expectations for active aging (PEAA) refer to older adults' recognizing such expectations as being directed at them personally. Previous studies, investigating PEAA as a relatively stable trait-like construct, showed that most individuals can cope well with activation demands. However, some individuals might be distressed by them. A deeper understanding of PEAA and their consequences can be reached when both are assessed in everyday life. In a 14-day diary pilot study with 93 participants (Mage = 65.40, 50-84 years), we assessed daily PEAA in the physical, cognitive, social, and appearance domains and related them to daily positive and negative affect as well as subjective age. Complementing previous work, we showed that daily PEAA not only differed between but also within individuals and that these variations were related to affective experience and subjective age. In multilevel models controlling for daily hassles and uplifts, we showed that on days on which individuals perceived more expectations for active aging, they also reported more positive affect. Daily PEAA and daily negative affect were not related. Moreover, on days on which individuals reported more daily PEAA, they also reported feeling younger. These findings point to the importance of assessing and understanding PEAA within older individual's daily context.

Abstract citation ID: igae098.1737
### EXPLORING THE DAY-TO-DAY DYNAMICS AMONG FLOW EXPERIENCE, SUBJECTIVE AGE, AND POSITIVE AFFECT IN THE UNITED KINGDOM

Dwight Tse, and Samuel Finnigan, *University of Strathclyde, Glasgow, Scotland, United Kingdom*

The subjective experience of aging can vary day-to-day, and can depend upon a person's activities and experiences. Activities conducive to flow experiences (energized experiences of intense focus and enjoyment) are associated with participants feeling younger on the same day, as well as increased levels of positive affect. Younger subjective age has similarly been found to increase positive affect throughout the day. We therefore hypothesized that greater daily flow experiences would predict greater positive affect both directly and indirectly through reducing subjective age. In the UK sample, participants completed up to 14 daily diaries, including questionnaires on their flow experience in their most time-consuming activity, subjective age, and positive affect. Analysis of responses from 85 participants aged 50 to 85 (mean = 62.16) with at least 7 completed daily surveys revealed significant within-person variability ranging from 44% (flow experience) to 73% (positive affect). Multilevel mediation analysis revealed flow experience was negatively related with subjective age and positively with positive affect, where on days with greater flow participants felt younger and reported higher positive affect. There was also a within-person indirect effect of flow increasing positive affect through subjective age: on days where participants experienced more flow than usual, they indirectly experienced higher positive affect through feeling younger. These findings remained robust when controlling for stressors, uplifts, and change in positive affect from the previous day. These findings suggest engaging in flow-conducive activities may provide additional benefits for older adults by increasing positive affect, partly by making them feel younger.

Abstract citation ID: igae098.1738
### AN INTEGRATED ANALYSIS OF BETWEEN-COUNTRY DIFFERENCES IN DAILY COUPLING OF AGEIST ATTITUDES AND NEGATIVE AFFECT

Amit Shrira[1], Yuval Palgi[2], Reyyan Can[3], Anna Kornadt[4], Dwight Tse[5], and Shevaun Neupert[3], *1. Bar-Ilan University, Ramat Gan, HaMerkaz, Israel, 2. Haifa University, Haifa, HaZafon, Israel, 3. North Carolina State University, Raleigh, North Carolina, United States, 4. University of Luxembourg, Esch-sur-Alzette, Luxembourg, 5. University of Strathclyde, Glasgow, Scotland, United Kingdom*

Daily variations in subjective views of aging and their coupling with psychological concomitants are increasingly explored, yet potential cultural differences in these relationships have not been addressed. We, therefore, compared the daily coupling of ageist attitudes with negative affect (NA) across countries included in the Subjective AGES (Aging within Global Everyday Ecological Studies) project, encompassing 379 participants from the USA, UK, Germany, Israel, and Türkiye (ages ranged from 50 to 90; specific countries comprised 16% to 23% of the sample). Participants reported ageist attitudes and NA across 14 days. Within-person variability in ageist attitudes ranged from 15.5% (UK) to 53.7% (Türkiye) of the total variance. Overall, on days when participants had more ageist attitudes, they also reported higher NA. Cross-level interactions showed that the strength of ageist attitudes-NA coupling in the USA was moderate, while it was weaker in the UK and Germany and stronger in Israel and Türkiye. These findings suggest that the coupling of older adults' daily ageist attitudes and NA varies across different countries. In European countries such as the UK and Germany, better access to healthcare services might mitigate the effects of ageist attitudes on NA. For Israel and Türkiye, the experience of a decline in social status due to a transition from a traditional and collectivist society to a modern and individualist one might increase the impact of views on aging. We will explore these explanations and future directions for the Subjective AGES project.

### SESSION 4025 (SYMPOSIUM)

Abstract citation ID: igae098.1739

### EMANCIPATORY PEDAGOGY: PRAXIS FOR RADICAL TRADITIONS OF LEARNING AND HEALING

Chair: Nicholas DiCarlo
Co-Chair: Brittney Pond

The Emancipatory Sciences aim to advance knowledge and the realization of dignity, access, equity, healing, and social justice through individual and collective agency and social institutions. Literature suggests this theoretical framework, method, and practice embraces and pursues an impact on research, pedagogy, and systemic change within and beyond academia (Estes, DiCarlo, and Yeh, 2023; Reyes, Versey, and Yeh, 2022). This symposium aims to discuss emancipatory science and critical gerontology through intergenerational, intersectional, and healing oriented frameworks. We address the central questions: who participates and collaborates in such an endeavor and what methodologies and pedagogical approaches strengthen collective participation and democratize access to knowledge across generations. The presenters in this symposium discuss this central question through various facets, including 1) the use of Crone pedagogies to embrace our embodied histories and teach about life course perspectives grounded in standpoint theories of positionality in the classroom 2) UCSF's Emancipatory Sciences Lab as praxis that creates collaborative spaces for co-learning 3) tangible findings from a digital storytelling project and 4) methodological considerations in participatory action research such as co-design, co-learning, and strengths-based language and design.

Abstract citation ID: igae098.1740

### CRONE PEDAGOGIES AND THE EMANCIPATORY POTENTIAL OF HISTORICAL MEMORY

Nicholas DiCarlo, *Hunter College / UCSF Emancipatory Sciences Lab / Mountain School of Psychoanalysis, New York, New York, United States*

Crone pedagogies (DiCarlo, 2023) embrace abject identities as epistemoloical standpoints of wisdom. This emerging pedagogical tradition centers mourning loved ones, ancestors, and activist who shaped us and teaches an attentive respect for the ghosts that haunt us and pull at normative unconscious processes (Layton, 2019). Gerontological study provides a unique point of inquiry into how we carry knowledge across the lifespan and through generations, as ageism stereotypes, medicalizes, and segregates older adults– collapsing complex personhoods into tropes and units of business. This presentation features examples from teaching first year MSW students about ageism, decoloniality, and abolitionist movements. Historical memory positions teacher-learners with opportunities to celebrate and mourn the activists and scholars who are shaping the fight against policing, transphobia, and displacement. Crone pedagogies underscore the necropolitical dimensions of grief and grievability (Butler, 2009), and encourage critical reflection on survival in oppressive systems and collective praxes of resistance.

Abstract citation ID: igae098.1741

### PARTICIPATORY ACTION RESEARCH IN GERONTOLOGY: CONCEPTUALIZATION, CONDUCT, AND DISSEMINATION

Jason Flatt[1], and Crystal Glover[2], *1. University of Nevada Las Vegas, Las Vegas, Nevada, United States*, *2. Rush University Alzheimer's Disease Research Center, Chicago, Illinois, United States*

Health disparities continue to critically diminish and deleteriously impact health in aging among large diverse populations across the world. One way to create health equity for all older adults remains through the strengths-based sharing of expertise across diverse communities and researchers from various scientific disciplines. As such, the field of Gerontology requires innovative approaches to engage two critical stakeholder groups – community members and researchers – to meaningfully engage and collaborate with each other to conceptualize, conduct, and disseminate related findings stemming from participatory action research (PAR). Resultant research must generate knowledge and actionable solutions that are mutually beneficial, scientifically sound, and culturally compatible across stakeholder groups. In this workshop, we will first teach both researchers and community members about the purpose, process, and intended outcomes of PAR. Second, we will outline the conduct of PAR using a pedagogical framework that includes key topics such as the researcher's onus of trust, establishing and sustaining relationships, co-learning and co-design, methodological design and data collection, collaborative data analysis and interpretation, dissemination of findings, and planning and implementing evidence- and strengths-based strategic transformations within diverse aging populations. Lastly, we will discuss opportunities and tools for attendees to expand PAR to address health disparities and facilitate health equity in aging among diverse populations. Throughout, we will leverage our extensive PAR experiences to share examples, lived experiences, and best practices with attendees. We hope to host an enriching, relevant, and forward-looking session with attendees.

Abstract citation ID: igae098.1742

### UCSF'S EMANCIPATORY SCIENCES LAB AS PRAXIS: TOWARD THEORY AND ACTION

Brittney Pond, *University of California San Francisco, San Francisco, California, United States*

This presentation discusses the UCSF Emancipatory Sciences Lab as a model for intergenerational, international, and cross-institutional collaboration to advance public scholarship centering anti-oppression and healing oriented research, policy, and practice. The lab promotes participation in public scholarship and activism and currently has over 140 members. The Emancipatory Sciences Lab highlights the importance of public scholarship via the production of a podcast, blog series and posts, and engagement on social media including X (formerly Twitter) and LinkedIn. Our podcast, titled EDGES: Engaging Dialogues Generating Emancipatory Sciences, interviews scholars, practitioners, artists, students, and activists to elevate their work and share critical discussions with a broader audience. The Emancipatory Sciences Lab provides tools, resources, and spaces for people within and beyond academia to engage in collaborative work with scholars, practitioners, and students. One example is the Student Writing Series that

I created and facilitated, which has provided a monthly space for students to set writing goals, co-write together, and share resources for writing for nearly one year. The Emancipatory Sciences Lab website features a searchable clearinghouse of publications for people in our network in the emancipatory sciences, which is valuable in providing resources and elevating critical work. We host webinar series and promote events from folks in our network to provide space for collaboration, discussion, and learning. Given my background as a first-generation scholar from a low socioeconomic status community, I am excited to share about the work stemming from the Emancipatory Sciences Lab that creates collaborative spaces to share and learn.

Abstract citation ID: igae098.1743

**CO-CREATING EDUCATIONAL MATERIALS WITH OLDER ADULTS AND INFLUENCING HEALTH CARE DECISION-MAKERS**

Wendy Hulko[1], and Noeman Mirza[2], *1. Thompson Rivers University, Kamloops, British Columbia, Canada*, *2. University of Winsdor, Toronto, Ontario, Canada*

Rural and small city older adults' views on primary and community care restructuring indicated a strong desire to provide input directly to healthcare decision-makers through oral over written means and collective rather than individual dialogues (Hulko et al., 2020). Thus, through integrated knowledge translation (iKT), we worked with older adults to (a) interpret our research findings on primary and community care restructuring; (b) create KT tools (grassroots infographic, key messages, documentary); and, (c) host a knowledge summit where we released our KT tools and facilitated discussions between service users and administrators (decision-makers, knowledge users, seniors advocates) about ways to move our findings into practice and affect service delivery. 30 people attended the knowledge summit and were invited to provide feedback on the iKT process and KT tools; 23 (8 service users, 15 administrators) filled out the feedback questionnaires. Service users provided positive feedback about the opportunity to partner with service providers, particularly senior administrators. Administrators recognized the need to shift from being "system focused" to "patient focused," which can be facilitated through engagement of rural older adults in service delivery decisions. They recognized that service users had "good ideas on how to deliver services locally" and appreciated the opportunity to hear service users' perspectives and needs, communicate face-to-face, and "discuss possible solutions" together. Our work highlights how emancipatory pedagogy - actioning research findings in collaboration with older adults - can enable mutually beneficial exchanges of ideas and solutions between service users and administrators and lead to improved health service delivery.

### SESSION 4030 (SYMPOSIUM)

Abstract citation ID: igae098.1744

**END-OF-LIFE CARE AND DECISION-MAKING IN CHINA: HOW TO IMPROVE END-OF-LIFE CARE QUALITY?**

Chair: Yifan Lou
Co-Chair: Yaolin Pei
Discussant: Bei Wu

Alongside population aging comes the challenges of providing quality end-of-life care in China, where the largest and most rapidly growing older adults reside. Mounting qualitative evidence has documented the culturally different needs, experiences, and barriers of end-of-life care and decision-making among older Chinese, but the quantitative evidence is lacking. Aligned with GSA 2024's theme, this symposium uses national and regional data to investigate potential "fortitude factors" that can improve end-of-life qualities for older adults and reduce burden for caregivers in China. The first paper will share policy-relevant findings on the urban-rural differences in pain, place of death, and their associations with the use of pain treatment at the end of life, a cornerstone of end-of-life dignity. The second paper will turn the attention to caregivers and present a population-based study on the individual- and household-level factors that may contribute to end-of-life caregiving burden and the role of the COVID-19 pandemic. Two studies on end-of-life care decision-making with a dyadic perspective will then be presented. The third one will focus on the children's role, considering both number of children and financial responsibilities, in deciding end-of-life treatments for their parents under the culture of filial piety, using retrospective data. The fourth one will then describe older adults' preferences for involvement and roles in their future end-of-life care decision-making with their loved ones and doctors, using regional survey data. Lastly, our discussant, Dr. Bei Wu, will synthesize these presentations and discuss the policy and practice implications in improving "good death" in China.

Abstract citation ID: igae098.1745

**PAIN, PLACE OF DEATH, AND USE OF PAIN TREATMENT AT THE END OF LIFE: URBAN–RURAL DIFFERENCE AMONG CHINESE OLDER ADULTS**

Yaolin Pei[1], Yifan Lou[2], Xiang Qi[3], Zheng Zhu[4], Jing Wang[5], and Bei Wu[3], *1. University of Texas at Austin, Texas, United States*, *2. Virginia Commonwealth University, New Haven, Virginia, United States*, *3. New York University, New York, New York, United States*, *4. New York University, New York, New York, United States*, *5. University of New Hampshire, Durham, New Hampshire, United States*

Pain is a common symptom that older adults encounter at the end of life and has a negative impact on quality of life. The purpose of this study was to investigate the prevalence of pain and the association between pain, place of death, and pain treatment in the context of an urban-rural division. The sample derived from the end -of-life module data from the China Longitudinal Aging Social Survey conducted in 2020. The working sample included 958 decedents aged 60 and above. Logistic regression models were used in this study. 26.4% of the decedents did not receive prompt and effective pain treatment. 66.50% of decedents' families were not able to control the pain for the decedent. 41.96% of the decedents had severe pain symptoms. Older adults who were rural residents, had no severe pain symptoms, and died at home were more likely not to receive timely and effective pain treatment than their counterparts. The urban -rural differences in these associations were identified: Rural older adults who had severe pain symptoms and die at home were more likely not receive timely and effective pain treatment than urban older adults. Severe pain symptoms and death place were significantly associated with whether receive timely and effective pain treatment among urban older adults, but not significant among rural older adults. It is important to strengthen pain management and bridge the gaps in this regard for older adults at the end of life in China.

Abstract citation ID: igae098.1746

**THE CAREGIVING BURDEN OF CHINESE OLDER ADULTS AT THE END OF LIFE: THE ROLE OF LIVING ARRANGEMENT AND COVID-19 PANDEMIC**

Shuo Zhang, and Wenjun Zhang, *Renmin University of China, Beijing, Beijing, China (People's Republic)*

While there has been a surge of interest in studying end-of-life quality of lives of older adults in China to respond to population aging, the experiences of their caregivers remain largely unexamined. Chinese older adults in general may have to rely on their co-residing members to provide end-of-life care. To address the gap, we explored the association between living arrangement and caregiving burden during end-of-life stage before and during the COVID-19 pandemic using a representative sample. The working sample included 1043 decedents who died between 2017 and 2021 from China Longitudinal Aging Social Survey. Ordered logistic regression models were used to examine the impact of living arrangement and pandemic on caregiving burden at the end of life. Our results indicated that caregivers of older adults who lived alone and lived in empty-nest, stem, and nuclear families had higher caregiving burden than those lived in skipped-generation families. The COVID-19 pandemic increased caregiving burden and further strengthened the relationship between living arrangements and end-of-life caregiving burden. Particularly, caregivers to older adults who lived alone, lived in empty-nest, stem, and nuclear families during the pandemic had higher caregiving burden than those before the pandemic. The main findings highlight the importance of paying attention to the caregiving burden of caregivers to those who are not living in skipped generation families, especially during pandemic. Our findings implied that we need to develop comprehensive formal support system to reduce the caregiving burden for caregivers at the end of life.

Abstract citation ID: igae098.1747

**BLAME-SHIFTING OR SHARING RESPONSIBILITY? CHILDREN'S ROLE IN CHINESE OLDER ADULTS' END-OF-LIFE MEDICAL DECISIONS**

Lu Chen, and Wenjun Zhang, *Renmin University of China, Beijing, Beijing, China (People's Republic)*

Filial piety is the core value shaping intergenerational interactions in China, where children are often considered the proxy decision-makers for older adults' end-of-life treatment plans. End-of-life care decision-making is a complicated and culturally sensitive process. Prolonging older parents' lives is often considered a reflection of filial piety in the medical decision-making context, however, budget constraints may weave into the decision-making process. The study addressed the literature gap by empirically investigating the role of children in the end-of-life medical decisions in China. Using data with 579 older decedents who had passed away due to illness from the China Longitudinal Aging Social Survey 2020, we examined the association between role of children, proxied by number of children and relative contribution of children to parents' medical expenses, and end-of-life decision as a process outcome, measured by a categorical variable with four mutually exclusive groups of whether all life-sustaining medical efforts have been made and whether these decisions imposed financial pressure on their families. Results show that families with fewer children were more likely to pursue all potential treatments and experience financial pressure. Compared to families with shared financial responsibilities by all children, families with shared responsibilities by certain children were more likely to exhaust all potential treatments and face financial strain. Post-hoc analyses further showed that older adults with more children were more likely to be overtreated, particularly among those families with no financial concerns to pursue every potential treatment. Family-level interventions are needed to support end-of-life care decision-making in China.

Abstract citation ID: igae098.1748

**CHINESE OLDER ADULTS' PREFERENCES FOR INVOLVEMENT IN END-OF-LIFE CARE DECISIONS: CAUSES AND OUTCOMES**

Yifan Lou[1], Jinyu Liu[2], Deborah Carr[3], and Yaolin Pei[4], *1. Virginia Commonwealth University, New Haven, Virginia, United States*, *2. Baylor University, Waco, Texas, United States*, *3. Boston University, Boston, Massachusetts, United States*, *4. University of Texas at Austin, Texas, United States*

Understanding the end-of-life decision making styles of Chinese older adults is an important goal, especially against a backdrop of global population aging. Guided by the Shared Decision-making framework, we use data from 571 adults ages 50+ in Shanghai, China to develop statistically and conceptually distinct profiles of shared, delegated, and autonomous end-of-life decision making; and identify the psychosocial and health correlates of these profiles, and the impact of profile type on advance care planning. We use latent class analysis (LCA) to develop decision-making profiles capturing preferences for involvement in information exchange, deliberation, and decisional control in end-of-life decision-making. Four profiles emerged. Delegated decision makers (16%) almost all prefer to have family or doctors make their treatment and care placement plans. Lead decision makers (49%) want to take the lead in information exchange and final decisions, although they value collaborative discussions. Acquiescent decision makers (15%) prefer to follow their family's wishes, if discrepant preferences exist. Autonomous decision makers (19%) prefer not to engage others in any process. Older age is associated with delegated decision making, whereas higher income and education are associated with autonomous decision-making. Acquiescent decision makers are less likely to have thought about or discussed their end-of-life wishes, despite their preferences for sharing decisions. Our results reveal great diversity in older adults' approaches to autonomous versus shared end-of-life decision making, and thus health care providers must devise tailored approaches for working with older patients and their families.

### SESSION 4035 (SYMPOSIUM)

Abstract citation ID: igae098.1749

**EXAMINING LIFE COURSE TRAJECTORIES WITH LINKED FULL COUNT IPUMS CENSUS DATA**

Chair: Sarah Flood

Discussant: Sarah Flood

Understanding life course trajectories requires data with a long time horizon or sufficiently detailed retrospective recall measures; extending this to include intergenerational processes or mortality outcomes places further demands on data. Historical full count census data from IPUMS are a rich and underutilized source of information on early life conditions and can be harnessed to summarize millions life histories for individuals and trace families over multiple generations. IPUMS full count census data covers 1850-1950 and boast nearly 800 million individual records, which can be linked longitudinally across decennial censuses as well as to external data sources to answer questions about exposures and outcomes across the life course. This session will feature research presentations on the power of linked full count US census data to explore life course trajectories including substantive applications focused on racial equity in older-age mortality, negative economic shocks and long-term wellbeing, segregation and exposure to air pollution, and developing scores for estimating lifespan using commonly available socioeconomic and demographic measures. This session will highlight the value of historical census data to understanding current population and aging issues. The presentations will showcase research that links full count census data in multiple ways and demonstrates the flexibility and wealth of information available in these data.

Abstract citation ID: igae098.1750

**RACIAL EQUITY IN NEW DEAL EMPLOYMENT AND LONG-TERM MORTALITY IN US COUNTIES**

David Rehkopf[1], Ivan Mejia Guevara[2], and Sepideh Modrek[3], *1. Stanford University, Palo Alto, California, United States*, *2. Stanford University, Stanford, California, United States*, *3. San Francisco State Univeresity, San Francisco, California, United States*

Public Emergency Work was a critical policy action during the New Deal used to address the unemployment of the Great Depression (1929-1941). Due to local control of hiring, there were substantial geographic differences in Public Emergency employment by race, relative to need for work. We examined the associations of Black-White differences in the relative equity of county level Public Emergency Work relief in 1940 with county level age 65 to 84 mortality from 2000 to 2019. We find that counties that strongly favored Black workers in Public Emergency Work programs in 1940 had lower mortality rates for both Black and White individuals from 2000 to 2019, as compared to counties that strongly favored hiring White workers. These findings suggest that there are meaningful connections between the structures that influenced the local level implementation of social policies at least as far back as 1940, with recent county mortality differences.

Abstract citation ID: igae098.1751

**THE SOCIAL, ECONOMIC, AND MORTALITY CONSEQUENCES OF THE FAILURE OF THE FREEDMAN'S SAVINGS BANK**

John Warren[1], Jonas Helgertz[2], Michael Esposito[1], Elizabeth Wrigley-Field[1], and J David Hacker[1], *1. University of Minnesota, Minneapolis, Minnesota, United States*, *2. Lund University, Lund, Skane Lan, Sweden*

The Freedman's Savings Bank was chartered in 1865 to serve newly emancipated people across 17 states. Driven by mismanagement and fraud by the bank's white officials, the bank failed in 1874 — costing many depositors large savings. Although there is debate about the short-term financial benefits for Black people of the bank's creation, there has been no research on the longer-term effects of the bank's failure on social, economic, or mortality outcomes among depositors who lost their savings. Using linked records from the complete-count 1860 through 1940 U.S. Censuses and exceptionally detailed records on the bank's branches, deposits, and losses, we use a variety of empirical estimation strategies to estimate the effects of the bank's failure on the life course outcomes of Black depositors and their children. This work speaks more broadly to the effects of negative economic shocks on the long-term well-being of older people.

Abstract citation ID: igae098.1752

**IPUMS MULTIGENERATIONAL LONGITUDINAL PANEL**

Catherine Fitch, and Steven Ruggles, *University of Minnesota, Minneapolis, Minnesota, United States*

Over the past five years, the IPUMS Multigenerational Longitudinal Panel (MLP) has constructed a massive longitudinal population panel by linking together American censuses, surveys, administrative sources, and vital records spanning the period from 1850 to the present. MLP is designed as a general-purpose resource for studying longitudinal processes and long-run social and economic change. By linking individuals across generations from birth through death using data from multiple sources, MLP enables reproducible research on shifting patterns of life course patterns and intergenerational processes. The large scale of the MLP database allows researchers both to study particular communities and small dispersed populations and to conduct big studies spanning many places and periods. Mounting evidence shows that early life social, economic, policy, and environmental exposures are important drivers of later life cognitive, health, and economic outcomes. MLP creates unprecedented opportunities for prospective analyses of the impact of early-life exposures on a wide range of later-life outcomes.

Abstract citation ID: igae098.1753

**LIFESPAN PREDICTION USING MACHINE LEARNING TECHNIQUES ON HISTORIC FULL COUNT CENSUS DATA**

Kelsey Carlston, *Gonzaga University, Spokane, Washington, United States*

This study proposes a new measure of status scores for Censuses in the early 1900s. Preceding 1940, income and education were not collected by the US Census, leading researchers to rely primarily on occupation-based metrics such as the IPUMS OCCSCORE or Duncan SEI for continuous status assessment. These measures do not incorporate the rich data available in the Census. Employing machine learning techniques, we aim to develop socioeconomic status scores by leveraging a diverse array of Census variables, including occupation, industry, race, geography, housing tenure, and nativity. The scores will primarily be estimates of life span based on links to Social Security data via CenSoc. The objective of the study is to make publicly available scores that researchers can use in studies of intergenerational mobility, life course studies, and labor force composition, and to determine the most important characteristics from pre-1940 Census data that predict life span.

Abstract citation ID: igae098.1754

### A HISTORY OF ENVIRONMENTAL INJUSTICE: SEGREGATION AND AIR POLLUTION IN 1940 AND 2010 IN THE UNITED STATES

Jenni Shearston[1], Xiaorong Shan[2], Lucas Henneman[2], Taylor Mobley[3], Elizabeth Rose Mayeda[3], Rachel Morello-Frosch[1], and Joan Casey[4], *1. University of California Berkeley, Berkeley, California, United States*, *2. George Mason University, Fairfax, Virginia, United States*, *3. University of California Los Angeles, Los Angeles, California, United States*, *4. University of Washington, Seattle, Washington, United States*

Air pollution is inequitably distributed across the United States, whereby minoritized racial/ethnic groups have greater exposure and consequent health effects. To understand present-day disparities, it is important to understand changes over time. We compared relationships between county-level air pollution and segregation in 1940 and present-day (2010). We calculated segregation as the Dissimilarity Index (Black vs non-Hispanic white population) using Census data. We evaluated three air pollution emission sources: oil/gas well locations (Enverus), fossil fuel powerplant locations (Energy Information Administration, Environmental Protection Agency), and gasoline consumption (proxy for traffic-related air pollution; Federal Highway Administration). We estimated rurality-stratified associations between each segregation/pollution pair by year with non-linear generalized additive models, adjusted for population density and within-state clustering. Mean county-level segregation decreased from 1940 to 2010 for rural (-33%) and urban (-42%) counties. In 1940, we found more oil/gas wells and lower gasoline consumption in rural counties with higher segregation (>median). For example, if segregation increased from the median to the 75th percentile, we observed a 0.15 kg/year decrease (95% confidence interval: -0.23, -0.07) in gasoline consumption. We saw no association between segregation and powerplants in rural 1940 counties, but observed fewer powerplants and lower gasoline consumption in counties with less segregation (< median) in 2010. In 1940, we found fewer oil/gas wells in urban counties with higher segregation. We found suggestive evidence of a relationship between segregation and air pollution in 1940 and 2010, with substantial differences by rurality. Policy to reduce air pollution should consider historical exposure inequities in emission sources.

### SESSION 4040 (SYMPOSIUM)

Abstract citation ID: igae098.1755

### EXPLORING A RANGE OF ROADMAPS LEADING TO THE AGE-FRIENDLY UNIVERSITY DESIGNATION AND BEYOND

Chair: Katarina Friberg-Felsted
Co-Chair: Jacqueline Eaton
Discussant: M. Aaron Guest

The Age-Friendly University Global Secretariat encourages and processes applications to the Age-Friendly University Global Network (AFUGN). Membership indicates each institution's commitment to endorsing the 10 Age-Friendly Principles and the implementation of age-inclusive practices to promote older adult engagement and value in the academic setting. Five presenters describe their journey in obtaining the AFU designation. They describe pre-designation road maps, steps involved, barriers faced, and implementation of post-designation plans. Dr. Orit Hirsch-Matsioulas and colleagues at the University of Haifa share how their quest aligned with the United Nations Sustainable Development Goals and reliance on deep community ties to become the first AFU institution in the Middle East. Dr. Dobish explains how mapping the 10 AFU principles to the core values of West Virginia's Shepherd University created a successful application at a time of leadership change within the AFU Global Secretariat. Dr. Buys and colleagues delineate how Mississippi State University was invited by the State Department of Health's Age-friendly Ecosystem Initiative to lead the pursuit of AFU designation as a sentinel university in the state. Dr. Powers illustrates how the University of Central Oklahoma's path to AFU designation moved through both Faculty and Staff Senates and describes the university president's investment in the inaugural post-designation initiative. Dr. Eaton and Dr. Friberg-Felsted show how garnering multi-faceted support at the University of Utah led to AFU designation and expansion into current age-inclusive collaborations. Dr. Aaron Guest, of Arizona State University and AFUGN, will facilitate discussion focused on AFU designation and implementation.

Abstract citation ID: igae098.1756

### THAT'S ONE WAY TO DO IT: THE EXPERIENCE OF SUCCESSFULLY PURSUING AND IMPLEMENTING THE AFU DESIGNATION

Katarina Friberg-Felsted, *University of Utah, Salt Lake City, Utah, United States*

Gerontology education and research has been present at the University of Utah (UofU) for 51 years. This has led to multiple programs, staff, faculty, and alumni desiring to improve age-friendly environments, advocate for lifelong learning, and choosing to pursue the Age-friendly University (AFU) designation. This presentation will describe the experience of successfully pursuing and implementing AFU at the UofU. A matrix of age-friendly initiatives across campus, representing each of the 10 Age-Friendly University Principles, was developed and used to garner support from vital entities such as the Professors Emeriti Club, the Dean of the College of Nursing, and the Center on Aging. These partners helped facilitate presentations to the Senior Vice President and the university's Academic Council of Deans, leading to endorsement from the UofU President. Following receipt of AFU designation, AFU representatives worked with the university's Marketing and Communications team on a campus wide press release. Strategic planning for improving age-friendliness across campus is ongoing, focusing on three main initiatives: 1) piloting a baseline needs assessment of age inclusivity, 2) creating an Age-friendly University task force, and 3) exploring a collaboration with the Associate Chief of Age-friendly Health Systems to standardize an Age-friendly model for our academic health center.

Abstract citation ID: igae098.1757

### AGE-FRIENDLY MEDITERRANEAN COOKBOOK:CULTIVATING CULTURAL TRANSFORMATION

Sigal Naim, Orit Hirsch-Matsioulas, Ruth Katz, Adi Sapir, Yael Hochman, and Anna Zisberg, *University of Haifa, Haifa, Hefa, Israel*

Creating a new reality for old persons on campus, with limited resources but boundless ambition, is both a challenge and an opportunity. In this presentation, we will focus on our experiences over the course of a single academic year, culminating in our university's inclusion in the global network of Age-Friendly Universities (AFU) – a milestone marking the first institution in the Middle East to achieve such recognition, and only the third in the Mediterranean basin. By embracing the principles of the AFU initiative, we tapped into a broader narrative of societal transformation aligned with the United Nations Sustainable Development Goals (SDGs). Central to our success were cultural values deeply integrated into Israeli society, including collectivity, solidarity, social ties, and audacity. Drawing on these values, we mobilized internal and external stakeholders, forged partnerships, and positioned ourselves as catalysts for change. We conducted comprehensive studies, mapped existing resources, and developed strategic future plans. Our experience illustrates how organizational culture, intertwined with societal dynamics, can drive meaningful change. Community engagement and partnerships played a pivotal role in highlighting the collaborative nature of our initiatives and the importance of involving diverse stakeholders. We invite participants to explore the intersection of culture, age-friendliness, and sustainable development, emphasizing the role of cultural values in shaping age-friendly initiatives. This presentation aims to inspire others to embark on similar journeys, utilizing culture to create inclusive environments where older adults can thrive.

Abstract citation ID: igae098.1758

### SHEPHERD UNIVERSITY'S ROADMAP TO AGE-FRIENDLY UNIVERSITY DESIGNATION

Heidi Dobish, *Shepherd University, Shepherdstown, West Virginia, United States*

Our AFU dedicated team first met in August, 2022 to discuss the process for becoming an Age-Friendly University. The AFU team consisted of a psychology department faculty member, the director of the university's Lifelong Learning Program, and a local community member. Our first step was to map our university's core values and current age-friendly inclusive activities onto the 10 Principles of an Age-Friendly University. This exercise provided evidence that many ongoing, university-engaging events highlighted four of the Age-Friendly principles. In October, 2022, the team met with our provost and proposed why Shepherd University should endorse the Age-Friendly initiative. Our provost agreed and signed our application letter in November, 2022. With the Age-Friendly University Global Network restructuring, it was a rather long wait for their response but we received our acceptance letter in August, 2023. We sent out press releases and were interviewed by a local columnist. Our first step as an AFU member was to create a six-person AFU Advisory Board with representation from Shepherd faculty, staff, and community members. We meet monthly and are currently working on a mission statement, by-laws, and S.W.O.T. analysis. We are also conducting information sessions for the university community to inform them about AFU and receive input on potential age-friendly activities. The board is keeping a log of activities undertaken as required by the AFU Global Network.

Abstract citation ID: igae098.1759

### MISSISSIPPI STATE UNIVERSITY LEVERAGES LAND-GRANT MISSION TO PURSUE AGE-FRIENDLY STATUS

David Buys[1], Christine Jackson[1], Ra'Sheda Boddie-Forbes[1], Jamie Dyer[1], Brent Fountain[2], Gary Jackson[1], Margaret Ralston[1], and Susan Seal[1], *1. Mississippi State University, Mississippi State, Mississippi, United States*, *2. Mississippi State university, Mississippi State, Mississippi, United States*

Introduction: Mississippi is experiencing outmigration and a decreasing birthrate, resulting in an aging population, which is of growing concern across sectors. The state's Department of Health led an Age-Friendly Public Health System application and is facilitating a broader Age-Friendly Ecosystem Initiative. As a member of the ecosystem advisory board, MSU was asked to lead the state in pursuing Age-Friendly University (AFU) status.

**Methods:** The University representative on the taskforce worked with MSU Divisions of Academic Affairs and Access, Opportunity, and Success (AOS) to assemble a working group to consider this opportunity. The group consisted of individuals from the Office of Outreach and Engagement, Interdisciplinary Studies, the Gerontology Education Program, the new College Of Continuing and Professional Studies and Cooperative Extension. The group 1) reviewed the AFU principles, 2) assessed where MSU has current initiatives that align with AFU goals, 3) considered where there are opportunities to develop new AFU-relevant work, 4) developed a proposal for the Provost and President, and 5) prepared and submitted an application to the AFU Global Network (AFUGN). Throughout the process the group has centered the unique role of the land-grant university mission in meeting the needs of the older adults. Results and

**discussion:** Upon acceptance of the application by the AFUGN in August 2023, the Division of Access, Opportunity, and Success agreed to manage the designation as a convening body and "conscience" for the University. AOS convenes the working group monthly, promotes AFU-relevant activities, and monitors activities for AFUGN required reporting.

Abstract citation ID: igae098.1760

### SEEKING AGE FRIENDLY UNIVERSITY DESIGNATION TAKES PATIENCE AND PERSISTENCE

Melissa Powers, *University of Central Oklahoma, Edmond, Oklahoma, United States*

The road to achieving Age Friendly University Designation at the University of Central Oklahoma was long and circuitous. It involved patience and persistence from the primary advocates. The initial steps involved gaining support on campus including from the Committee on Diversity, Faculty Senate, and Staff Senate. Both Senates unanimously approved resolutions in support of seeking the designation. In addition, the Senates added an affirmation that our campus community will be a place where the ideas and contributions of people of all ages are welcomed and valued. The next step on our roadmap was to gain approval from university leadership. It was at this level that our initial request stalled due to lack of interest. After a change in leadership, campus support was again evaluated and was determined to remain high. Due to the high level of campus support, the request was submitted directly to the President who approved seeking the designation without hesitation. The President specifically asked that our priority post designation be to conduct an age-friendly assessment of the campus to guide future priorities and programming. This assessment is currently underway. Our programming and priorities will be determined by the results of the age-friendly assessment.

### SESSION 4045 (PAPER)

### FAMILY CAREGIVING II (PAPERS)

Abstract citation ID: igae098.1761

### "I'M HAVING THE WORST TIME": CAREGIVER EXPERIENCES WHEN OLDER ADULTS UNDERGO MAJOR ELECTIVE SURGERY

Ellis Dillon[1], Christina Keny[2], Lingsheng Li[2], Veronica Yank[2], Alexis Colley[2], Malini Ramaiyer[3], and Victoria Tang[2], *1. University of Connecticut, Farmington, Connecticut, United States*, *2. University of California San Francisco, San Francisco, California, United States*, *3. Johns Hopkins University, Baltimore, Maryland, United States*

Older adults are at higher risk of major complications, functional decline, and mortality after surgery. Family caregivers play a critical role supporting older adults, but often experience caregiver burden and distress. We know little about family caregiver experiences before and after a major operation. We conducted semi-structured interviews at three timepoints (2-weeks before and 1 and 3-months post-operation) with family caregivers of adults ≥ 65 years undergoing a major elective operation at an academic hospital. Data were analyzed using inductive thematic analysis. Thirteen family caregivers completed 27 unique interviews. Family caregivers mean age was 67 (range 44-79), 67% identified as White, and 61% were female spouses. Cancer was the most common indication for operations. Themes identified included: (1) the caregiving role evolves to become all consuming - "my day is kind of wrapped around what he needs," (2) caregiving demands may disrupt the relationship dynamic - "you just become more of a caregiver than a wife," (3) uncertainties about surgery and caregiving demands contribute to distress - "I'd like to know the statistics and if there was a 25% chance for full recovery," and (4) family caregiver insights about how to support their own well-being through better guidance and support through all phases of surgical care. Family caregivers for older adults undergoing a major operation face many challenges and need better psychosocial and instrumental support, family-centered education, and communication. By identifying and addressing these needs, we can optimize the well-being of family caregivers and the older adults they support.

Abstract citation ID: igae098.1762

### QUALITY OF LIFE OF OLDER INFORMAL CAREGIVERS IN SWEDEN: CAREGIVER PROFILES AND RELATED FACTORS

Mariam Kirvalidze[1], Maider Mateo-Abad[2], Alba Ayala[3], Amaya Bernal-Alonso[4], Maria João Forjaz[5], Carmen Rodríguez-Blázquez[5], and Amaia Calderón-Larrañaga[1], *1. Karolinska Institutet, Stockholm, Stockholms Lan, Sweden*, *2. Biogipuzkoa Health Research Institute, San Sebastian, Pais Vasco, Spain*, *3. Instituto de Salud Carlos III, Madrid, Madrid, Spain*, *4. Universidad Nacional Educación a Distancia, Madrid, Madrid, Spain*, *5. National Centre of Epidemiology, Carlos III Institute of Health, Madrid, Madrid, Spain*

Older adults are increasingly stepping into informal caregiving roles, primarily due to the inadequacy of formal care services to meet their growing demands. We aimed at identifying profiles of older caregivers according to their quality of life (QoL), and describing the factors characterizing them. A cross-sectional analysis of a cohort study was conducted, including informal caregivers from the Swedish National Study on Aging and Care in Kungsholmen. The study included a total of 1382 observations, corresponding to 994 caregivers aged 60 and above, from waves 1 (2001) to 5 (2013). Multiple Correspondence Analysis combined with cluster analysis were used to obtain the caregiver profiles according to the items of SF-12 QoL instrument. Multinomial regression was performed to determine which demographic, clinical and psychosocial factors were related to belonging to each profile. Three caregiver profiles were identified according to the physical and mental health domains of their QoL. People with good QoL (58%) had better mental and physical health status, and reported a lower caregiver burden. People with intermediate QoL (35%) were older than those in the other profiles and, along with the group with poor QoL (7%), had worse health status and often received care themselves. The poor QoL group was also characterized by higher prevalence of depression and perception of burden, and lower levels of social support. Different modifiable factors are associated with the QoL of older informal caregivers, especially in the mental health domain, and could thus be the target of future preventive interventions.

Abstract citation ID: igae098.1763

### FORTIFYING DEMENTIA CARE: EXPLORING FAMILY SELF-EFFICACY IN DECISION-MAKING, TRUST, AND QUALITY

Ruth Lopez[1], Dalit Zaguri-Greener[2], Lola Kolpina[2], and Anna Zisberg[2], *1. MGH Institute of Health Professions, Boston, Massachusetts, United States*, *2. University of Haifa, Haifa, Hefa, Israel*

In Israel and many countries, individuals with advanced dementia often spend their final days in nursing homes (NHs). Families prioritize comfort, yet residents often endure significant symptom burden. This discrepancy arises partly because NH staff depend on families to guide end-of-life care. However, family members often lack confidence as decision-makers and struggle to partner with NH staff to translate their intimate knowledge of their loved ones' preferences into comfort-focused strategies. The purpose of this pilot study employing cross sectional survey design, was to explore relationships among family self-efficacy for surrogate decision-making, trust with staff, and quality of care measures. Among 66 family members of NH residents with advanced dementia in Israel (average age 60, 53% female) self-efficacy was positively associated with trust (r=.32, p<.001), quality of decision-making (r=.34, p<.001), communication (r=.33, p<.001), family-staff relationships (r=.30, p<.01), basic care, and symptom management (r =.36, p<.001). Recognizing the pivotal role of family self-efficacy, interventions to enhance it may be warranted. Our findings also urge further investigation into the intricate interplay between these factors and the need to explore the directionality of these relationships. Understanding the influence of broader contextual factors, such as overall NH care quality, is crucial. Our study establishes a foundation for future inquiries, fostering a comprehensive understanding to enhance the well-being and advanced comfort of individuals with advanced dementia in NH settings.

Abstract citation ID: igae098.1764

### FRIENDS WITH UNEXPECTED BENEFITS: COACHING CAREGIVERS OF PEOPLE LIVING WITH DEMENTIA

Rita Jablonski[1], Amy Cleverdon[1], Chunhong Xiao[2], and Reed Bratches[1], *1. University of Alabama at Birmingham, Birmingham, Alabama, United States*, 2. *University of Alabama at Birmingham/School of Nursing, Birmingham, Alabama, United States*

Persons living with dementia (PLWD) often exhibit care-resistant behaviors (CRBs), including refusing to take medications or bathe. This randomized clinical trial is currently enrolling caregivers of people living with dementia, who receive nine one-hour coaching sessions designed to help them prevent and manage CRBs. Coaching is the application of evidence-based strategies delivered in a conversational manner; it is a bi-directional, problem-solving approach designed to improve the ability of family caregivers to recognize, prevent, and manage CRB in their daily interactions with their care recipients. We report qualitative findings from 13 caregivers who completed all coaching sessions. The results of the thematic analysis revealed unexpected benefits and feedback. Participants reported a renewed sense of hope and optimism "after nearly giving up." They expressed feelings of relief that the dementia-related brain changes were responsible for behaviors, not "caregiver mistakes," or "they are doing this on purpose," which led to greater compassion for their care recipients. These new insights also contributed to a permanent adjustment of their expectations of their care recipients' cognition, and re-established connections with PLWD. The participants applied newly learned skills and strategies in unexpected situations, such as improving cooperation during travel and medical appointments. Participants also shared that they taught secondary caregivers, such as sitters, specific techniques to maintain continuity of care. These findings support the need for future studies that emphasize targeted, personalized interactions inherent in this coaching intervention, versus passive presentation of generic information.

Abstract citation ID: igae098.1765

### SEQUENTIAL ANALYSIS OF DEMENTIA FAMILY DYADIC COMMUNICATION: CARE RECIPIENT CHALLENGING/NEUTRAL COMMUNICATION

Sohyun Kim, Will Chen, and Shan Sun-Mitchell, *The University of Texas Arlington, Arlington, Texas, United States*

Care-recipient challenging communication is nonresponsive, resistive, withdrawing, and critical communication showing disengagement. Care-recipient neutral communication is nonverbal behavior either engaging or challenging depending on the context. This study examined temporal relationships between antecedent care-recipient challenging and neutral communication and subsequent family caregiver communication (facilitative, disabling, and neutral) during 75 in-home care video observations. Secondary analysis of 5- to 60-second timed-window analysis with 5-second intervals was conducted. 95% confidence intervals, p-values, and Yule's Q statistics were used. The results showed that all types of family caregiver communication were less likely to occur at differing frequencies within each time window following care-recipient challenging verbal and neutral communication. Caregiver facilitative verbal, disabling nonverbal, and neutral communication were less likely to occur within all time widows after care-recipient challenging verbal communication (OR = 0.32 – 0.86, p < 0.001 – p = 0.001-0.037, Yule's Q = -0.106 – -0.519). Caregiver facilitative and disabling communication were less likely to occur until 35 seconds after care-recipient neutral communication (OR = 0.34 – 0.87, p < 0.001 – p = 0.002 – 0.044, Yule's Q = 0.093 – 0.459). Care-recipient challenging verbal and neutral communication were significantly associated with a decreased likelihood of subsequent caregiver communication, with a small to large probability of non-occurrence. These results highlight that caregivers often fail to respond to care-recipient challenging and neutral communication. Healthcare providers and researchers should prioritize providing caregiver education focusing on awareness and support of challenging verbal behaviors and other nonverbal behaviors of persons living with dementia.

### SESSION 4050 (PAPER)

### FAMILY RELATIONSHIPS, SOCIAL SUPPORT, AND HEALTH

Abstract citation ID: igae098.1766

### KINSHIP STRUCTURES FOR LEFT BEHIND OLDER ADULTS IN HIGH OUTMIGRATION CONTEXTS

Amilcar Matos-Moreno[1], Diego Alburez-Gutierrez[2], Ivan Williams[3], Ashton Verdery[4], Mariana Fernandez[5], and Alexis Santos-Lozada[4], *1. The Pennsylvania State University, San Juan, Puerto Rico*, 2. *Max Planck Institute for Demographic Research, Germany*, 3. *Universidad de Buenos Aires, Buenos Aires, Argentina*, 4. *The Pennsylvania State University, University Park, Pennsylvania, United States*, 5. *Universidad de La República, Montevideo, Uruguay*

Migration accelerates population aging in high-outmigration contexts; however, the familial structure of older adults left behind has yet to be studied. Evidence suggests that left behind older adults are at higher risk of reduced support networks and increased caregiving burden. This study aims to estimate the kinship structures of older adults, kinlessness, and the presence of migrant kin in Puerto Rico. Data come from the United Nations World Population Prospects from 1950 to 2021. We created a two-sex time-variant model to estimate the kinship structure of older adults in Puerto Rico for 2021. Further, we created a multistate model to estimate the prevalence of transnational family members and their relationship with older adults. Our models suggest that a person born in 1950 will have, in 2021, on average, 5.22 close family members: 1.3 daughters, 1.2 sons, 1.8 sisters, and 1.5 brothers. As they continue to age, the prevalence of female kin supersedes the male presence in the family. Migration is predominant in descendent kins (daughters and nieces). Adults between 20 to 40 are expected to have 0.30 migrant daughters. Older adults between the ages of 60 and 65 have lost 44% of their daughters to migration a lower estimate when compared to adults of the age of 50 or younger (55%). Models suggest that future generations of Puerto Rican older adults will have an increased presence of transnational family members. Thus, public health strategies must adapt to address the need for "new families" in upcoming generations of older adults.

Abstract citation ID: igae098.1767

### TIME HELP FROM ADULT CHILDREN: DIFFERENCES AMONG BIOLOGICAL AND STEP-CHILDREN IN NON-STEPFAMILIES AND STEPFAMILIES

I-Fen Lin[1], Judith Seltzer[2], Janecca Chin[1], Emily Wiemers[3], Anna Wiersma Strauss[3], and V. Hotz[4], *1. Bowling Green State University, Bowling Green, Ohio, United States*, *2. University of California Los Angeles, Los Angeles, California, United States*, *3. Syracuse University, Syracuse, New York, United States*, *4. Duke University, Durham, North Carolina, United States*

Objectives. Adult stepchildren help their parents less than biological children, but findings based on parent-child dyads ignore that some biological children are in stepfamilies because one or both parents have children from previous unions. Additionally, little is known about how a widespread crisis may disrupt the normal patterns of assistance from adult children to parents. We take a family systems approach to investigate whether the COVID-19 pandemic reshaped adult children's time help to parents by considering dyadic relationships embedded in family structure. Methods. We leveraged the panel design of the Health and Retirement Study to examine changes in time help to parents. We compared adult children's responses to parental need prior to and during the pandemic using the 2016, 2018, and 2020 core interviews. We also examined how children responded to parental need using the 2020 COVID-19 module. Throughout we considered biological children and stepchildren in non-stepfamilies and stepfamilies. Results. Stepchildren were least likely to provide time help, followed by biological children in stepfamilies, and lastly biological children in non-stepfamilies. However, among parents who had trouble with instrumental activities of daily living (2016, 2018, 2020 core interviews) or had trouble buying food for non-financial reasons (COVID-19 module), biological children in non-stepfamilies and biological children in stepfamilies were equally likely to help their parents. Discussion. Our findings suggest general stability in biological versus step children' helping differentials even in times of crisis but also challenge the view that the presence of a stepchild weakens ties between biological children and their parents.

Abstract citation ID: igae098.1768

### CHILDLESSNESS AND SUBJECTIVE WELL-BEING IN 88 COUNTRIES: THE ROLES OF STIGMA, DEVELOPMENT, AND REGION

Bussarawan Teerawichitchainan[1], Christine Mair[2], and Haruka Sugimori[1], *1. National University of Singapore, Singapore, Singapore*, *2. University of Maryland Baltimore County, Baltimore, Maryland, United States*

Although childlessness is often hypothesized to be associated with poorer subjective well-being among aging populations, cross-national empirical evidence suggests substantial variation across countries. Yet, the reasons for this variation have yet to be identified. We hypothesize that childless older adults have lower subjective well-being in country contexts with higher stigma against childlessness, net of region, fertility, and economic development. We use multilevel modeling to analyze recent data (2017-2022) from the World Values Survey, United Nations, and World Bank, focusing on 63,067 adults aged 45+ in 88 low-middle, middle-high, and high-income countries to examine individual-level associations between childlessness and subjective well-being (life satisfaction and happiness) and moderation of country-level variables (stigma, economic development, and region). Overall, childless respondents report lower subjective well-being (life satisfaction and happiness), but these associations vary by country-level factors. Childless respondents in countries with high stigma report the lowest life satisfaction, net of fertility rate, economic development, and other factors. Happiness increases with economic development for both childless and non-childless individuals, and does not appear to be associated with stigma against childlessness. Regional differences are also evident. We discuss these findings in light of heterogeneity in the experiences of childless aging populations cross-nationally. Additionally, we highlight the potential contextualizing role of country norms and stigma when considering childlessness and well-being, particularly in family-centered or low-middle income countries.

Abstract citation ID: igae098.1769

### RELATIONSHIP BETWEEN CHILD MORTALITY, SOCIAL SUPPORT, AND PSYCHOLOGICAL DISTRESS AMONG WAR SURVIVORS IN VIETNAM

Kakada Kuy[1], Korinek Kim[2], and Zachary Zimmer[3], *1. Washington University in St. Louis, St. Louis, Missouri, United States*, *2. University of Utah, Salt Lake City, Utah, United States*, *3. Mount Saint Vincent University, Halifax, Nova Scotia, Canada*

Social support has been documented as a key factor contributing to improved well-being among grieving parents in the bereavement process. However, how different forms of support interact with the experiences of child death is less understood among aging populations in post-conflict settings. This study examined the psychological impact of child death on older Vietnamese with a focus on the mediating role of social support and the moderating effect of war exposure. Data came from the 2018 Vietnam Health and Aging Study of 2,447 older adults aged 60 and older. We assessed experience of child loss by enumerating the mortality and survivorship status for all children. Mental distress was measured using the Self-Reporting Questionnaire with nine items. Social support was conceptualized along two dimensions: 1) Received social support was divided into instrumental and emotional domains; 2) perceived social network support was broken down into functional and structural aspects. Path analysis substantiated a significant relationship between child death and heightened mental distress. Emotional and labor support significantly buffered the impact of child. Moderated mediation analysis suggested that individuals with higher levels of war exposure received a noticeable benefit from emotional support. However, other forms of support did not demonstrate a significant mediating role. Results underscore the value of emotional support in this context. Findings suggest the integration of mental health support within existing social services for older adults in Vietnam with an emphasis on providing emotional support to those who have experienced child loss.

Abstract citation ID: igae098.1770

### GRANDPARENTS' CAREGIVING STRESS AND RESILIENCE AMID THE COVID-19 PANDEMIC IN HONG KONG

Vera Mun Yu Tang, and Sonia Kin Lai Chan, *The University of Hong Kong, Hong Kong, China (People's Republic)*

Informal care provided by grandparents became more crucial to children with parents unable to look after them at home during COVID-19 lockdown when formal childcare services were suspended and schools were closed. However, there is limited understanding of grandparents' experiences of caregiving to grandchildren beyond the early COVID-19 waves (i.e., from July 2020 onward). This study applies Lazarus and Folkman's transactional theory of stress and coping to understand grandparents' primary appraisal of stressor, coping strategies, and perceived self-efficacy (i.e., secondary appraisal) in providing childcare during the third and forth COVID-19 waves in Hong Kong. Individual interviews were conducted with 21 grandparents aged 45 or above, who had been taking care of grandchildren aged 0 to 15 during the COVID-19 pandemic or six months before the outbreak in Hong Kong. A coding team performed content analysis to identify emergent themes across cases. This study reveals that grandparents' stress appraisal of pandemic in childcare as threat to harm their grandchildren's health and development, and as challenge to effective communication with grandchildren and adult children. They initiated problem-focused coping strategies to ameliorate the impact, including lifestyle changes through practicing hygiene and sanitation to protect their grandchildren from infection, performing multiple roles as teacher, playmate, carer and activity facilitator, and learning new communication technologies. They felt pride in their efforts and abilities to ensure grandchildren's safety and whole-person development, and maintain family stability amid an unprecedented health crisis. These findings suggest that positive caregiving appraisal is crucial for grandparental resilience in stressful times.

### SESSION 4055 (PAPER)

### FRAILTY (PAPERS)

Abstract citation ID: igae098.1771

### ASSOCIATION OF CUMULATIVE RISK FACTORS AND HANDGRIP STRENGTH AMONG COMMUNITY-DWELLING OLDER ADULTS IN INDIA

Snehal Kulkarni, *Public Health Foundation of India, Delhi, Maharashtra, India*

**Objective:** The present study aims to establish the reference values of HGS in Indian older adults and to evaluate the influence of cumulative risk factors on HGS.

**Methods:** This cross-sectional study was conducted in the Pune city of India among 1016 community-dwelling older adults. Data on demographic characteristics, functionality, chronic illness, and physical activity was collected using a structured questionnaire. Objective measurements such as slowness of walking and balance impairment were measured using the Timed-up and go test and sharpened Romberg's test. Descriptive statistics, independent t-test, and one-way ANCOVA were to examine the influence of risk factors on HGS.

**Results:** The mean HGS of the study group was 16.28 + 5.84 kgs. Male older people had a higher handgrip (20.37±6.91 kg) than females (14.29±5.54 kg). For both men and women, handgrip strength decreased with increasing age. The results show that participants with 4 or more risk factors had significantly low handgrip strength (14.18 kg) than participants with no risk factors (19.62 kg). Functional decline, lack of exercise, and impaired balance also reduced the handgrip among the study participants.

**Conclusion:** The values of handgrip strength reported in the current were lower than those reported in other studies. Accumulation of more than 4 risk factors drastically decreases the handgrip. Thus, evaluation of handgrip strength can be used as a feasible strategy to improve the health status of community-dwelling older adults.

Abstract citation ID: igae098.1772

### INCREMENTAL HEALTH CARE COSTS OF A SIMPLE MEASURE OF PHENOTYPIC FRAILTY IN OLDER ADULTS

Kristine Ensrud[1], John Schousboe[2], Howard Fink[3], Allyson Kats[1], Kerry Sheets[4], Brent Taylor[3], Cynthia Boyd[5], and Lisa Langsetmo[3], *1. University of Minnesota, Minneapolis, Minnesota, United States*, *2. HealthPartners, St. Paul, Minnesota, United States*, *3. Minneapolis Veterans Affairs Health Care System, Minneapolis, Minnesota, United States*, *4. Hennepin Healthcare, Minneapolis, Minnesota, United States*, *5. Johns Hopkins University, Baltimore, Maryland, United States*

Frailty defined by the CHS phenotype is associated with higher subsequent total healthcare expenditures in community-dwelling Medicare beneficiaries after accounting for claims-based cost indicators. However, frailty assessment using the 5 component CHS phenotype is often not feasible in clinical practice. Our objective was to determine whether frailty assessed using the simple SOF phenotype (3 components: weight loss, inability to rise from chair 5 times without using arms, poor energy) is associated with subsequent incremental costs after accounting for claims-based measures of multimorbidity and frailty. We utilized a multi-cohort dataset with frailty phenotype measurements linked with Medicare claims composed of 8157 community-dwelling fee-for-service beneficiaries. Healthcare costs were ascertained for

36 months after frailty assessment. Average annualized total healthcare costs (2023 US dollars) were $14945 in women and $15693 in men. After accounting for claims-based indicators of multimorbidity and frailty, average incremental costs of SOF phenotypic frailty (2 or 3 components) vs robust (none) were $7088 in women and $5909 in men, only slightly lower than incremental costs of CHS phenotypic frailty ($9372 in women and $6518 in men). SOF phenotypic frailty in both sexes was associated with higher subsequent expenditures in inpatient, skilled nursing facility and home healthcare sectors. In conclusion, frailty assessed by the parsimonious SOF phenotype is associated with higher subsequent total and sector-specific expenditures after accounting for claims-based indicators. SOF phenotypic frailty can be readily assessed in space-constrained clinic exam rooms and time-limited clinical practice settings to improve identification of older adults at high risk of costly care.

Abstract citation ID: igae098.1773

### DEVELOPMENT AND VALIDATION OF A MORTALITY PREDICTION MODEL AMONG MIDDLE-AGED AND OLDER FRAIL ADULTS: UK BIOBANK

Chenkai Wu, Jianhong Xu, and Yanxin Wang, *Duke Kunshan University, Kunshan, Jiangsu, China (People's Republic)*

Research has identified numerous risk factors for frailty and developed prediction models for the onset of frailty. However, little is known about how multimodal risk factors collectively influence the prognosis of frailty. We developed and validated prediction models for mortality risk among the frail during a 10-year period. Data were from 15,404 frail individuals aged 37-73 years from the UK Biobank. The entire sample was divided into development set (n=14,272 from England and Wales) and validation set (n=1,132 from Scotland). We assessed the associations of 383 survey, 144 physical, and 59 biomarker predictors with all-cause mortality. Variable selection was conducted using LASSO and stepwise regression. We built a series of nested models: survey only (Model 1), survey+physical measurement (Model 2), and survey+physical measurement+biomarker (Model 3). Model performance was measured by discrimination, calibration, and reclassification. A total of 5,654 males and 9,750 females (63.3%) were included. Model 1 retained 122 survey predictors, Model 2 added 42 physical measurements, and Model 3 added 60 biomarkers. The C-statistic was 0.77, 0.80, and 0.79 in three models, respectively. Models 2 and 3 had improvement in reclassification in mortality risk over Model 1 (net reclassification index=0.111 and 0.029). The light gradient-boosting machine found age, cystatin C, FEV1, smoking, and employment status to be most important predictors. We have developed and validated a series of prediction models using multi-modal data to quantify mortality risk among the frail. The findings provide valuable insights into the design of patient-targeted intervention and management strategies.

Abstract citation ID: igae098.1774

### EXPLORING FRAILTY PREVALENCE AND RISK FACTORS AMONG STROKE PATIENTS: A MULTICENTER CROSS-SECTIONAL STUDY

Haiyan He[1], Siyuan Tang[1], Wenting Peng[2], Kehan Liu[1], Shuomin Wang[1], Yaru Bai[3], Chongmei Huang[3], and Minhui Liu[4], *1. Central South University, Changsha, Hunan, China (People's Republic)*, *2. University of Washington, Seattle, Washington, United States*, *3. Ningxia Medical University, Yinchuan, Ningxia, China (People's Republic)*, *4. Ningxia Medical University, Yinchuan, Ningxia, China*

Frailty is prevalent among stroke patients, and it is strongly associated with higher risks of expected adverse outcomes. However, the risk factors for frailty remained unclear in middle-aged and older patients with stroke. We aimed to identify the prevalence and risk factors of frailty in middle-aged and older patients with stroke in Chinese hospitals. A cross-sectional survey was employed using a random sampling method, involving middle-aged and older patients with stroke in two tertiary hospitals in Central and Northwest China between January 2022 and June 2022. Frailty was assessed with the Frail Scale. Candidate predictors included nine socio-demographic factors and 12 clinical factors. We used generalized linear models to examine the relationship between frailty and related factors. Among 838 patients, 560 were male and 278 were female, with the majority aged between 50-60 years (30.4%) and 60-70 years (27.6%). The prevalence of pre-frailty and frailty was 40.1% (n=338) and 31.1% (n=261), respectively. We found that the stroke type ($\beta$ = -0.30, 95% CI: -0.46, -0.14; P < 0.001), abnormal nutritional risk ($\beta$ = 0.23, 95% CI: 0.01, 0.45; P = 0.042), hyperlipidemia ($\beta$ = 0.21, 95% CI: 0.03, 0.39; P = 0.026), and post-stroke disability ($\beta$ = 0.59, 95% CI: 0.53, 0.64; P < 0.001) were significantly associated with frailty scores in stroke patients. Our finding provides evidence regarding the frailty status of stroke patients. It suggests that early screening and intervention for these factors are crucial to preventing or improving frailty among middle-aged and older patients with stroke.

Abstract citation ID: igae098.1775

### LONGITUDINAL MEASURES OF FRAILTY AND MORTALITY

Benjamin Seligman, *University of California Los Angeles David Geffen School of Medicine, Los Angeles, California, United States*

Assessment of frailty has become common in clinical settings to risk-stratify older adults. Understanding how to use repeated measurements answers important questions both for the clinical use of serial assessments and understanding frailty trajectories. Using the Health and Retirement Study, we calculated the most recent value, maximum, minimum, mean, standard deviation (SD), and slope of a deficit accumulation frailty index (FI) and the Fried frailty phenotype (FFP). We assessed the association of scaled values with mortality by Cox proportional hazards regression with adjustment for age, gender, and smoking status. We then used machine learning by LASSO regression to parsimoniously select variables associated with mortality. We found that the strongest association for the FI was its maximum value, with hazard ratio (HR) (95% CI) 1.67 (1.60-1.74). For FFP this was the mean value, HR 1.56 (1.44-1.70). For both FI and FFP, HRs of the most recent, maximum, and mean values were similar in magnitude, with estimates ranging from 1.62-1.67 and 1.50-1.56 respectively. In LASSO regression, the FI measures (HRs) retained were the maximum (1.38), most recent (1.18), and SD (1.01). For FFP, these were the mean

(1.37), maximum (1.07), most recent (1.05). These findings show that maximum and most recent values of frailty tend to be most strongly associated with mortality. For clinicians, this means that the most recent assessment may be sufficient for many purposes. The associations of maximum frailty suggest that recovery may not lead to improved outcomes.

### SESSION 4060 (SYMPOSIUM)

Abstract citation ID: igae098.1776

### FUTURE OF CAREGIVING: INNOVATIVE SOLUTIONS AND CHALLENGES WITH NOVEL NONPHARMACOLOGICAL INTERVENTIONS

Chair: Jyoti Savla
Co-Chair: Lauren Hagemann

As we explore the evolving landscape of family caregiving, it becomes clear that caregiving roles offer fulfillment but also pose unique mental health challenges for caregivers of persons living with dementia. These challenges stem from expanded responsibilities and managing dementia-related behaviors. Research emphasizes the intertwined well-being of caregivers and their care recipients, highlighting the need for interventions that support caregivers' mental health and improve their capacity to address their care recipients' needs. This symposium aims to assess the effectiveness of novel non-pharmacological interventions, marking a step forward in our approach to caregiver support and improving caregiving dynamics overall. Martinsdale-Adams et al. explore the Annie Caregiver Text program, a text-message intervention based on the REACH-VA protocol, showing its success in improving the management of dementia-related behaviors and reducing caregiver stress. Lee et al. examine the Powerful Tools for Caregivers (PTC) program, assessing its effectiveness in enhancing self-care and communication skills, whether delivered in-person or remotely. Savla et al. share results from an RCT that compares the effectiveness of a mindfulness-based intervention, Practice of Acceptance, Awareness, and Compassion in Caregiving (PAACC), with REACH-VA, focusing on reducing caregiver anxiety and increasing compassion. Ruggiano et al. analyze the Alabama Caregiver Connect initiative, aimed at simplifying access for caregivers to essential dementia care information through interactive, AI-enhanced web tools, including ChatGPT and chatbots. Together, these empirically-validated programs offer novel non-pharmacological options for managing caregiving responsibilities and addressing dementia-related behaviors of relatives, exploring the challenges and solutions for implementing these programs for family caregivers.

Abstract citation ID: igae098.1777

### ANNIE CAREGIVER TEXT PROGRAM: A LIFELINE IN MANAGING DEMENTIA BEHAVIORS AND STRESS

Jennifer Martindale-Adams[1], Carolyn Clark[2], Charles Henderson[3], Jessica Martin[4], Jennifer Roth[5], and Linda Nichols[2], *1. University of Tennessee Health Science Center, Memphis, Tennessee, United States*, *2. Lt. Col. Luke Weathers, Jr. VA Medical Center, Memphis, Tennessee, United States*, *3. Office of Connected Care, Veterans Health Administration, Washington, District of Columbia, United States*, *4. Lt. Col. Luke Weathers, Jr. VA Medical Center, Memphis, Tennessee, United States*, *5. Saint Cloud VA Healthcare System, Saint Cloud, Minnesota, United States*

Caring for a loved one can negatively impact well-being for caregivers and persons with dementia. The Department of Veterans Affairs' Office of Connected Care and Caregiver Support Program expanded Annie Text Care messages to caregivers of Veterans in 2019 beginning with caregiver stress management. As of 2024, over 25,000 caregivers have enrolled in nine different caregiver subscriptions. Dementia Behaviors and Stress Management has 2,880 caregivers enrolled. For a year, caregivers receive 7 messages per week with 4 focusing on dementia behaviors and 3 focusing on stress management. Messages are educational, motivational, and activities or strategies to help based on the Resources for Enhancing All Caregivers Health (REACH) VA program. Caregivers can complete a short, Survey Monkey link, sent every 6 months. Questions are on a 5-point Likert scale ranging from completely disagree to completely agree plus open ended questions. Caregivers reported that the texts helped them manage dementia behaviors (mean score of 4.37/5), manage stress (mean score of 4.4/5), take care of themselves (mean score of 4.3/5) and their loved one (mean score of 4.5/5), and made them feel like someone cared about them (mean score of 4.6/5). Overwhelmingly, caregivers would recommend this service to other caregivers (mean score of 4.7/5). As technology moves toward increasing sophistication with artificial intelligence, this simple one-way text messaging system resonates with caregivers. In qualitative analysis themes, caregivers report that "Annie makes me feel less alone" and that messages come at the right time – "just when I needed to hear it."

Abstract citation ID: igae098.1778

### THE POWERFUL TOOLS FOR CAREGIVERS TOOLKIT: ENHANCING SELF-CARE AND COMMUNICATION FOR QUALITY CARE

JeongEun Lee[1], Joseph Svec[2], Eunbea Kim[1], and Yulri Kim[1], *1. Iowa State University, Ames, Iowa, United States*, *2. St. Joseph's University, Patchogue, New York, United States*

The Powerful Tools for Caregiver (PTC) program, an evidence-based program, has proven to enhance caregivers' self-care and communication skills. Prior studies substantiate its efficacy in elevating caregiver competence and promoting self-care practices. This study delves into secondary outcomes, emphasizing perceived relationship quality with care recipients with dementia. We examined responses from 340 caregivers engaged in the Powerful Tools for Caregivers program delivered with both in-person and remote module. Utilizing pre- and post-surveys, we conducted MANCOVA and multiple regression analyses. Findings revealed that caregivers of dementia reported improved self-care, increasing communication competence, and enhancing positive role perceptions. A majority caregivers reported significant increase in allocating time for personal well-being (average 1.24 hours per week) and discovering effective solutions to caregiving challenges using action plans. Moreover, caregivers allocating more time to self-care reported delivering higher-quality care and experiencing improved relationship qualities with care recipients. Notably, these positive changes were attributed to enhanced communication strategies and positive perspectives adopted by caregivers. The findings underscore the pivotal role of self-care and effective communication in augmenting the quality of care provided to recipients. Comparing the module of the delivery, we did not find any significance, confirming parallel effectiveness of the program. In conclusion, this research illuminates the significance of prioritizing self-care and honing communication skills in improving the wellbeing of both caregivers and care recipients with dementia. The paper concludes by outlining implications derived from the findings and proposes avenues for future research to further enrich our understanding of caregiver support programs.

Abstract citation ID: igae098.1779

### FROM REACH TO MINDFULNESS: SHIFTING PARADIGMS IN SUPPORT OF CAREGIVERS OF VETERANS LIVING WITH DEMENTIA

Jyoti Savla[1], Mamta Sapra[2], Katherine Luci[2], and Lauren Hagemann[2], *1. Virginia Tech, Blacksburg, Virginia, United States*, *2. Salem VA Healthcare System, Salem, Virginia, United States*

Enhancing interventions to alleviate stress among caregivers is a key area of focus for the Veterans Health Administration, especially given the anticipated tripling of dementia cases among Veterans in the upcoming years. Existing programs, like REACH-VA, have focused on cognitive behavioral approaches to reduce caregiver strain. However, mindfulness practices, known for stress reduction, psychological well-being improvement, and relationship enhancement, have not been extensively explored for caregivers of Veterans. This study evaluated the comparative effectiveness of a four-session, multi-faceted mindfulness-based intervention called Practice of Acceptance, Awareness, and Compassion in Caregiving (PAACC) to REACH-VA program for family caregivers of Veterans living with dementia. PAACC combines mindfulness with caregiver skill-development to improve the caregiving experience. It aims to assist caregivers in developing immediate awareness of stressors and their responses to stressors, while fostering empathy towards their relative with dementia. This approach differs from strategies that challenge or replace negative or unrealistic stress-related thoughts, considering the often progressive and unchangeable nature of such stressors. A randomized-controlled trial involving 133 family caregivers (Mage = 67 years; 81% White; 89% co-residing) assigned to either REACH-VA or PAACC demonstrated significant reductions in perceived stress, caregiver burden, and rumination after participating in either of the interventions. However, multi-level models revealed that caregivers who received PAACC showed lower anxiety ($B=-2.76$, $p<0.001$) and depressive symptoms ($B=-2.50$, $p<0.01$), and higher mindfulness ($B=6.04$, $p<0.01$) compared to caregivers who received the REACH-VA intervention. Findings will be discussed in the context of the intervention's mechanisms, lessons learned, and alternative avenues for utilizing PAACC.

Abstract citation ID: igae098.1780

### GOING BOLDER: PROMOTING DEMENTIA INFRASTRUCTURE THROUGH INTERACTIVE AND AI WEB-BASED TOOLS

Nicole Ruggiano[1], Monica Herzog[1], Zhe Jiang[2], Ellen Brown[3], Vagelis Hristidis[4], and Jane Daquin[1], *1. University of Alabama, Tuscaloosa, Alabama, United States*, *2. University of Florida, Gainsville, Florida, United States*, *3. Florida International University, Miami, Florida, United States*, *4. University of California Riverside, Riverside, California, United States*

In 2018, the U.S. Congress successfully passed the Building Our Largest Dementia (BOLD) Infrastructure for Alzheimer's Act to advance public health initiatives that support people living with dementia (PLWD) and their families. Part of the goals of BOLD include advancing community awareness about the latest research on dementia and support the translation of research findings to support PLWD, their families, and providers in addressing needs related to dementia care and caregiving. Infrastructure that supports information seeking is vital, given that caregivers often report challenges in finding the information they need about dementia, caregiving, and existing support. This is especially true for those who turn to the internet for information, where web-based search engines generate an overwhelming number of results to common questions posed by caregivers. To address these information needs, our interdisciplinary team has launched the Alabama Caregiver Connect initiative, which aims to more effectively link caregivers with information they need about dementia and caregiving by harnessing web-based tools that are interactive and driven by artificial intelligence. This presentation will review innovative technologies that the team has developed, implemented, and/or evaluated for their potential to support public health infrastructure for dementia. Findings presented will examine benefits and drawbacks of several approaches identified through the initiative, including ChatGPT, evidence-based chatbots, and crowdsourced asset mapping tools. Implications for policy and practice will be discussed.

### SESSION 4065 (PAPER)

### GLOBAL AGING AND HEALTH (PAPERS)

Abstract citation ID: igae098.1781

### AGE DIFFERENCES IN THE BELIEF IN A JUST WORLD AND SOCIETY-LEVEL MODERATORS

Niting Guo, and Tianyuan Li, *The Chinese University of Hong Kong (Shenzhen), Shenzhen, Guangdong, China (People's Republic)*

Prior studies have associated belief in a just world with both positive and negative individual and societal consequences. Despite this, the determinants influencing individual differences in justice belief are underexplored. Understanding the developmental trajectory of justice belief can shed light on its influencing factors and potential roles at different stages of life. However, comprehensive research on this issue is scarce. To fill in this gap, we investigated age difference in justice belief with data from World Value Survey 6 (2010-2014) encompassing 83,683 individuals across 59 societies. Incorporating the individualism scores, society-level justice belief, human development index, and income Gini coefficient, we further examined the moderation effects of these societal factors by conducting multilevel analyses. The results showed that both the quadratic ($\gamma40 =.054$, $p <.001$) and linear effect ($\gamma50 =.050$, $p =.022$) of age on justice belief were significantly positive, suggesting a general increasing trend of justice belief with age. Individualism did not moderate the quadratic effect of age (γ41 = -.014, p =.368) but did moderate the linear effect (γ51 =.078, p =.010). Society-level justice belief moderated the quadratic effect of age (γ41 =.431, p <.001), but not the linear effect (γ51 = -.021, p =.245). This study illustrated the developmental trajectory of justice belief and revealed the moderation effects of some societal factors. It provides more insights into the developmental foundation and functions of justice belief. Future studies could further examine the mechanism.

Abstract citation ID: igae098.1782

### HISTORICAL CHANGE IN MIDLIFE TRAJECTORIES OF PHYSICAL HEALTH: A CROSS-NATIONAL APPROACH

Yesenia Cruz-Carrillo[1], Frank Infurna[1], Nutifafa Dey[1], Markus Wettstein[2], Kevin Grimm[1], Margie Lachman[3], and Denis Gerstorf[2], *1. Arizona State University, Tempe, Arizona, United States*, *2. Humboldt University Berlin, Berlin, Berlin, Germany*, *3. Brandeis University, Waltham, Massachusetts, United States*

Empirical evidence has documented that later–born cohorts of middle–aged adults in the U.S. are reporting poorer mental and physical health than earlier–born cohorts. However, less is known about whether this is specific to the U.S. or generalizes to other nations and to what extent this is transpiring across self–report and objective indicators of physical health. Our study aims to describe the similarities and differences in historical change of midlife development in self–rated health, health conditions, and grip strength by using harmonized data from longitudinal panel surveys from the U.S. (HRS), Mexico (MHAS), 13 nations in Europe (ELSA, SHARE), South Korea (KLoSA), and China (CHARLS). Results from growth curve models revealed historical declines in self–rated health and grip strength (for both men and women), and historical increases in health conditions among middle–aged adults from the U.S. In contrast, historical improvements in self–rated health and fewer health conditions were observed for middle–aged adults in Mexico, Europe, South Korea, and China. Results for grip strength are more nuanced, with historical improvements among later–born cohorts of both men and women in Nordic Europe and South Korea and historical declines among both men and women in China. Our discussion focuses on better understanding these national differences in historical trends by considering how the dynamically changing historical context impacts ways of living and health care systems as well as considering the often-profound implications arising for psychological functioning and development at the individual and population level.

Abstract citation ID: igae098.1783

### SUCCESSFUL AGING AND FACTORS ASSOCIATED WITH IT AMONG OLD PEOPLE LIVING IN OLD AGE HOMES IN NEPAL

Hom Nath Chalise[1], and Satya Singh[2], *1. Tribhuvan University, Kirtipur, Bagmati, Nepal*, *2. Torrens University, Adelaide, South Australia, Adelaide*

This study aimed to assess Successful Aging (SA) and its association with Nepali older people. A cross-sectional analytical study was conducted among 206 elderly people of seven old age homes in the Kathmandu district. The interview technique using a structured questionnaire was taken after informed consent. The data collection tool was based on the determinants of SA and the Successful Aging Inventory (SAI) tool. The Geriatric Depression Scale (GDS) was used as an indicator of success in mental health. The ethical review board of Nobel College in Nepal permitted this study. The majority of the respondents (63.5%) were successful agers according to SAI Indicators and represented the SA components such as 90.4% in SP, 87.5% in PC, 93.3% of respondents self-rated health (SRH), 55.8% were involved in mind diversional activities(MDA), 99% have resilience with illness and almost two third of the respondents were mentally healthy according to GDS analysis. Most of the SA was from the 71+ age group (60.6%)with the odds of having success in the 71+ age group being 2.45 times more compared to the 60-70 years age group. Mann Whitney-U test for a non-normally distributed population showed a statistically significant difference found between the Female and Male group for SA, the difference was also found in PC and MDA among SA and Usual Aging. Although the majority of the respondents were successful agers, there needs to be improvement in all aspects especially the mental health of SA components to achieve better SA among older people.

Abstract citation ID: igae098.1784

### WHO MAINTAINS GOOD HEALTH? CONTRIBUTIONS OF BEHAVIORS AND LIFE COURSE CIRCUMSTANCES TO HEALTHY AGING

Chenlu Hong, and Yanan Luo, *Peking University, Beijing, Beijing, China (People's Republic)*

**Background:** Life course circumstances, encompassing diverse environmental conditions and experiences throughout an individual's life, are critical determinants of healthy aging. However, limited evidence focuses on their cumulative effects on various aging trajectories and disparities among different subpopulations. Using the healthy ageing scores (HAS), this study aimed to evaluate the relative contributions of life course circumstances in childhood and adulthood to healthy aging among Chinese older adults.

**Methods:** Utilizing data from the China Health and Retirement Longitudinal Study (CHALRS), we assembled for 7906 middle-aged and older adults in China. HAS, developed by the Ageing Trajectories of Health Longitudinal Opportunities and Synergies (ATHLOS), was calculated to measure overall biopsychosocial aspects of health and functioning, with higher values indicating better health maintenance in later life. Behavioral factors included smoking, drinking and obesity. Life course factors encompassed socioenvironmental circumstances in childhood and adulthood. Linear regression models were employed to analyze the role of life course circumstances domains in healthy aging and estimate the associations of identified subpopulations with healthy aging. The ShapleyValue Decomposition, principal components analysis and hierarchal cluster analysis were performed.

**Results:** We identified four subpopulations sharing similar life courses characterized by disadvantaged or advantaged childhood and adulthood circumstances. Of these, the most disadvantaged subpopulation, experiencing more adulthood adversity and childhood trauma, along with worse adulthood socioeconomic status, consistently exhibited the lowest levels of healthy aging and coversely the most advantaged subpopulation, characterized by better adulthood SES and childhood SES, demonstrated higher Healthy Aging Scores.

### SESSION 4070 (PAPER)

### HEALTH DISPARITIES

Abstract citation ID: igae098.1785

#### ANNUAL WELLNESS SCREENING AND COVID-19: A MISSED OPPORTUNITY TO ADDRESS HEALTH DISPARITIES

Nancy Allen, Yao He, and Norman Foster, *University of Utah, Salt Lake City, Utah, United States*

The COVID-19 pandemic had the greatest effect on the most vulnerable. Medicare annual wellness visits (AWV) are the best opportunity for early detection of cognitive impairment and could address inequities in dementia care. We examined how preventive care through AWV changed as our healthcare system responded to pandemic disruptions. We hypothesized that disruptions would exacerbate pre-existing disparities in AWV administration. We conducted an observational retrospective chart review of AWV of Medicare-eligible adults ≥65 years of age in our health system from Jan 2018 to Dec 2022. In 2020 at the beginning of the COVID-19 pandemic, there were 32.9% fewer AWV performed than in 2019. A month-by-month analysis showed a sharp 91% decline in AWV when clinic operations were most restricted. When operations largely resumed by June 2020, AWV returned to the pre-pandemic proportion of January 2020. However, previous disparities in AWV performance continued or were exacerbated. Post-pandemic, AWV were more frequent in younger (< 75 years; 32.5%) rather than older individuals (>75; 21.4%), in white (30.9%) rather than non-white patients (24.2%), and in non-Hispanic (30.5%) rather than Hispanic individuals (23.7%). Patients without diabetes had more AWV (31%) than those without (31% vs. 27.6%). Success in resumption of AWV occurred by the continuation of conscious and unconscious scheduling biases, rather than addressing previously recognized disparities. This represents a missed opportunity and highlights the need for policy changes to address health equity in AWV. The disparities in AWV mirror the disparities in dementia diagnosis and treatment.

Abstract citation ID: igae098.1786

#### MEDICAL EXPENDITURE DIFFERENCES: EMERGING OLDER ADULTS, BOOMERS VERSUS THE PREDECESSORS IN KOREA (2010–2020)

Yejin Lim[1], Youngtae Cho[2], Jung-Hwa Ha[2], and Woorim Ko[2], *1. Seoul National University Graduate School of Public Health, Seoul, Republic of Korea*, *2. Seoul National University, Seoul, Republic of Korea*

South Korea, the world's fastest-aging society, faces growing concerns over the increasing societal caregiving burden as the aging of the baby boom generation (boomers) starting in 2020. Recognizing the importance of generational context in gerontological research, this study conducted APC analysis on the annual individual medical expenditure (AIME) of approximately 148,000 participants from the Korea Health Panel Survey data (2010-2020), comparing the industrial generation (IG) (born 1945-1954) with the boomers (born 1955-1964). The analysis revealed a declining AIME trend from the IG to the boomers, with age and period effects controlled. This trend was similarly observed in AIME related to out-patient (OP) services and services at primary care providers, contrasting with AIME related to emergency or in-patient services and care at higher-level institutions. The findings suggest that the difference in total AIME between the two generations could stem from a decrease in the use of OP services at primary healthcare institutions by the boomers. Further analysis, guided by the relationship between OP service use and the number of non-communicable diseases (NCDs) reported in prior studies, indicated that the boomers had a significantly lower incidence of major NCDs compared to the IG. This research highlights how the boomers, having driven Korea's post-war economic boom, are shaping into a distinct older population, diverging from aging patterns of the predecessor generation. It underscores the importance of developing sustainable health management and expenditure policies that reflect the needs of emerging older adults.

Abstract citation ID: igae098.1787

#### RESEARCHING THE AGING POPULATION IN STATE PRISONS: ETHICAL CONSIDERATIONS AND IRB REQUIREMENTS

Rodlescia Sneed, and Elaina Reese, *Wayne State University, Detroit, Michigan, United States*

The growing number of older adults within state prisons presents unique challenges and opportunities for gerontological research. To conduct research with this population, however, scholars must navigate a range of ethical issues unique to studying this vulnerable group. The purpose of this presentation is to delve into the ethical considerations and Institutional Review Board (IRB) requirements crucial for conducting research with this vulnerable population. Using examples from our current multisite research study on chronic disease self-management among incarcerated older adults, we will describe how we navigated special considerations related to correctional system research processes, power imbalance, coercion, informed consent, participant selection, participant incentives, and data protection. Understanding how to effectively navigate ethical issues and the IRB process is crucial for conducting research to improve the well-being of incarcerated older adults. This presentation will provide researchers with valuable information useful for navigating the ethical landscape to conduct research that improves the well-being of the aging prison population and informs best practices within correctional healthcare.

Abstract citation ID: igae098.1788

#### THE PSYCHOSOMATIC SYMPTOM CLUSTERS OF OLDER ADULTS WITH DISABILITIES IN RURAL CHINA: A NATIONAL NETWORK ANALYSIS

Mo Yi[1], and Zhi-wen Wang[2], *1. Peking University, Beijing, Beijing, China (People's Republic)*, *2. Peking University Health Science Center, Beijing, Beijing, China (People's Republic)*

**Background:** Mental health is a crucial aspect of successful aging and healthy ageing. The number and severity of older adults with disabilities (OAD) in China have been on the rise year by year. Due to impaired daily activities and reduced social participation, OAD in rural areas are more prone to various symptoms. Therefore, this study aims to construct a network of psychosomatic symptoms among OAD, identify core symptom.
**Methods:** The study participants were 3,939 OAD aged 60 and above, sourced from the 2020 China Health and Retirement Longitudinal Study. We employed CES-D-10 scale, MMSE, and sleep-related items to assess interested outcomes, respectively. Depression was measured using 10 symptom subtypes, MMSE included six subtypes such as executive function, language function and calculation ability, etc. The sleep was categorized into three subtypes: excessive sleep, insufficient sleep, and daytime napping. "Expected Influence" and "Bridge Expected Influence" were analyzed as centrality indicators in the symptom network.
**Results:** Network analysis results revealed that the most severe symptoms among OAD were low mood (r=1.53), impaired attention ability (r=0.98), and insufficient nighttime sleep (r=0.96). The centrality analysis identified the top symptoms as lack of vitality (r=1.06). Stratified regression analysis indicated that sleep disturbances had the greatest impact on the life satisfaction, accounting for 27.5% of the model's explanatory power.
**Conclusion:** Central symptoms (low mood, impaired attention and calculation ability, and insufficient nighttime sleep) and key bridge symptoms (lack of vitality) can serve as potential targets for interventions aimed at rural OAD at risk of mental health symptoms.

Abstract citation ID: igae098.1789

### DISABILITY STATUS OF ASIAN OLDER ADULTS IN THE UNITED STATES: FINDINGS FROM THE 2017–2021 AMERICAN COMMUNITY SURVEY

Christina Miyawaki[1], Hoang Nguyen[2], and Kyriakos Markides[2], *1. University of Houston, Houston, Texas, United States*, *2. The University of Texas Medical Branch, Galveston, Texas, United States*

Little information is available on the disability status of the diverse Asian population in the United States (U.S.). In this study we estimate the prevalence of six types of disability in older adults for six major Asian subgroups using the recently released data from the 2017-2021 American Community Survey. The survey contains a sample of 11,737 Vietnamese; 32,388 Chinese; 24,625 Filipino; 12,125 Japanese; 10,231 Koreans; 16,759 Indians; and 2,412,999 non-Hispanic Whites. All analyses use survey weights for population estimates and the jackknife method for standard errors. We find higher prevalence of disability in independent living for Vietnamese, Chinese, Filipino, Japanese, and Indian compared to non-Hispanic White. For self-care disability, Vietnamese, Chinese, Filipino and Indian have higher prevalence. Cognitive disability is more prevalent in Vietnamese, Filipino, and Japanese. Only Vietnamese older adults have higher prevalence of mobility disability and vision disability. Surprisingly, all Asian subgroups in the study have lower prevalence of hearing disability than the non-Hispanic White group. Overall, the results show Vietnamese older adults have the worst disability profile, whereas Korean older adults have the best profile. Future research is needed to understand the role of fortitude in addressing disability status, especially in the areas of independent living, self-care, and cognition, which are more prevalent in most Asian groups.

### SESSION 4075 (SYMPOSIUM)

Abstract citation ID: igae098.1790

### IMPACT OF OLDER ADULT PATIENTS AND CAREGIVER ATTITUDES AND PERCEPTIONS AND LOCAL CULTURE ON IN-HOSPITAL MOBILITY

Chair: Barbara King
Co-Chair: Anna Zisberg
Discussant: Cynthia Brown

Older adults commonly lose functional ability during a hospital stay, often cause by limited ambulation. This phenomenon has been identified globally as a hospital associated disability (HAD). Multiple barriers related to how care is delivered in hospitals have been identified that prevent hospital staff from getting patients up to walk. Only recently have studies explored other factors that may prevent or influence whether older patients ambulate, including older adults' experiences and perceptions of in-hospital ambulation, the impact of patient and or family caregiver attitudes on in-hospital mobility, and influence of local unit culture on patient mobility. This symposium will present results from hospitalized older adult mobility studies conducted in 3 countries. Paper 1 will describe older adult patient perceived Value for Walking during hospitalization; paper 2 will discuss the impact of patient and family attitudes on patient mobility; paper 3 will present how diversity and cultural difference of family caregivers impacts patient mobility; and paper 4 will describe how local unit culture mediates mobility of older adult patients. HAD in older adults is a global health epidemic. Designing effective person-centered mobility interventions need to be multifactorial and include patient and family/ caregiver perspective and unit culture in order to improve older adult patient ambulation and decrease the incidence of HAD. Additional research is needed to understand the impact of patient and family caregiver factors and local unit culture on whether older adult patients are engaged in walking during their hospital stay.

Abstract citation ID: igae098.1791

### HOW OLDER ADULT PATIENTS PERCEIVE THE IMPORTANCE OF AMBULATION DURING HOSPITALIZATION

Barbara King[1], and Linsey Steege[2], *1. UW-Madison, Madison, Wisconsin, United States*, *2. University of Wisconsin-Madison, Madison, Wisconsin, United States*

**Background:** Older adults commonly experience loss of walking independence during a hospital stay. Loss of functional mobility is the greatest threat to independent living post discharge and quality of life. Limited ambulation during hospitalization is the most predictable and preventable cause of loss of functional mobility. There is an abundance of data on frequency of patient ambulation and the number of steps taken exists, but little information on how older adult patients perceive ambulation during hospitalization. The purpose of this study was to understand how older adult patients perceive ambulation when admitted to a hospital setting. Method: A qualitative study using inductive content analysis was conducted. Fifteen (n = 15) older adults participated in one-on-one in-depth interviews three months post discharge. Interviews were audio recorded and transcribed verbatim. Analysis consisted of open coding to identify main and supporting categories that describe the patient's experience.

**Results:** Value of Ambulation was identified as the main category which consisted of four supporting categories, Maintaining Health, Filling Time, Getting Ready and Being Connected. Older adults perceived walking during a hospital stay as critical for managing their acute and chronic illness, connecting with other patients and unit staff and limiting social isolation, helping fill their time to reduce boredom, and served to determine if they felt ready to go home.

**Conclusion:** Ambulation during a hospital stay is highly valued and matters to older adults. Additional research on the patient experience with ambulation is needed to develop person-centered care to improve functional outcomes for older persons.

Abstract citation ID: igae098.1792

### OLDER ADULTS AND CAREGIVERS' ATTITUDES TOWARD MOBILITY AND IN-HOSPITAL MOBILITY RELATIONSHIPS

Anna Zisberg[1], Efrat Shadmi[1], Maayan Agmon[2], Yuliana Smichenko[1], Ksenya Shulyaev[1], and Nurit Gur-Yaish[3], *1. University of Haifa, Haifa, Hefa, Israel*, *2. University of Haifa, University of Haifa, HaZafon, Israel*, *3. Center of Research & Study of Aging, University of Haifa; Oranim, Academic College of Education, Israel., Haifa, HaZafon, Israel*

Improving mobility among hospitalized older adults remains a challenge. Qualitative research indicates that patients' attitudes are one of the key factors associated with mobility levels. According to the Family Centered Approach, family members have an influence on both patients' attitudes and actual mobility that are yet to be supported empirically. We tested whether inpatients and their family-members' attitudes play a role in their in-hospital mobility. Paired data on in-hospital Attitudes Toward Mobility (ATM) was collected from patients (N=151; 78.6±6.6) and their family-caregivers (N=151; 58.2±14.2) as part of the larger prospective HoPE-MOR (Hospitalization Process Effects on Mobility Outcomes and Recovery) study. Data about patients' health, functional, physical and mental status was collected at hospital admission. Data on average number of steps was collected during up to 7-days of hospitalization (Actigraphy). Results indicate that patients and caregivers' ATM tend to be positive (3.6±.77; 3.7±.71) and patients were relatively mobile with average 1577±1328 daily steps. In a multivariate analysis of the factors related to average number of steps during hospitalization, both patients (β=.18, p=.02) and caregivers ATM (β=.15, p=.04) were significant predictors after accounting for patients' mobility ability, severity of the acute and chronic illness, fear of falls, age and sex. In addition, caregivers' ATM (β=.16, p=.04) was one of the main predictor of patients' ATM, controlling for intervening variables. To conclude, there is an interlink between family caregivers' attitudes, patients' attitudes and actual patients' mobility. Interventions should consider educating patients and their families to improve perceptions and attitudes toward mobility during hospitalization.

Abstract citation ID: igae098.1793

### CULTURAL DIFFERENCES IN CAREGIVERS' ATTITUDES TOWARD IN-HOSPITAL MOBILITY OF OLDER ADULTS

Ksenya Shulyaev[1], Anna Zisberg[1], Juliana Smichenko[1], Efrat Shadmi[1], and Nurit Gur-Yash[2], *1. University of Haifa, Haifa, Hefa, Israel*, *2. Center of Research & Study of Aging, University of Haifa; Oranim, Academic College of Education, Israel., Haifa, HaZafon, Israel*

Older adults are often accompanied by their family caregivers during hospitalization and their attitudes could influence care processes and outcomes. However, little is known about the cultural differences of family-caregivers attitudes. We aim to explore cultural differences in family-caregivers' attitudes toward in-hospital mobility of hospitalized relatives. In this study participated 156 pairs of hospitalized older adults and family caregivers, included 23(15%) Israeli Arabs, 33(21%) - ex-USSR migrants and 100(64%) native, or veteran Israeli Jews. Socio-demographic, health, functional and physical status were collected at admission. Caregivers' Attitudes Toward Mobility (ATM) were collected during their hospital visits. In all cultural groups caregivers tend to be positive toward mobility of older adults, attitudes (measured 1 for negative, 5 for positive) of the caregivers were: 3.48±.80 for Arab, 3.69±.64 for ex-USSR migrants and 3.82±.70 for Jews. Significant differences in caregivers' attitudes between the groups were found (F(146,2)=4.02, p=.02) even after controlling for patients' characteristics including age, gender, functional status, level of independence in ADL, severity of illness, comorbidities and previous falls. Multivariate regression analysis included all mentioned covariates demonstrated that being an Arab family-caregiver significantly predicted worse attitudes toward in-hospital mobility (β=-.214, p=.009). To conclude, cultural differences exist in attitudes of the family-caregivers regarding the mobility of their older family members. Arab caregivers tend to prefer that their relatives will be less mobile during hospitalization. Clinical practices should address cultural difference that influence mobility.

Abstract citation ID: igae098.1794

### TOWARD AN UNDERSTANDING OF IN-HOSPITAL INACTIVITY

Mette Merete Pedersen[1], Maria Hansen[2], Thomas Bandholm[3], and Jeanette Kirk[4], *1. Copenhagen University Hospital Hvidovre, Copenhagen, Hovedstaden, Denmark*, *2. Physical Medicine & Rehabilitation Research - Copenhagen (PMR-C), Department of Physical and Occupational Therapy, Copenhagen University Hospital*

*Hvidovre, Hvidovre, Hovedstaden, Denmark*, *3. Physical Medicine & Rehabilitation Research - Copenhagen (PMR-C), Department of Clinical Research, Copenhagen University Hospital Hvidovre, Hvidovre, Hovedstaden, Denmark*, *4. Copenhagen University Hospital Hvidovre, Hvidovre, Hovedstaden, Denmark*

Lack of mobility during hospitalization is a common problem in older adults (+65) and is associated with an elevated risk of hospital-associated disability. In a recent study, we have shown that there is unclarity among health professionals on priorities and responsibilities concerning mobility, and barriers to the promotion of mobility are present. Also, in recent years, it has become clear that in-hospital inactivity is rooted in the hospital culture and that multi-dimensional and multiprofessional interventions are required to incorporate and implement mobility into clinical practice. However, important factors for such interventions to succeed are patient and caregiver engagement and interaction. Therefore, for future interventions to be successful in routine care it is important to know what characterizes clinical practice and communication around physical activity and what influences current levels of activity. We will present data from clinical studies on older adults (65+) in a geriatric and an orthopedic ward in which we have investigated 1) current and previous levels of physical activity during acute hospitalization; 2) clinical practice about mobility and physical activity - how local culture mediates care routines; and 3) if and how a technological artifact can mediate increased physical activity. Also, we will include the perspective of patients and ward staff on what influences mobility and levels of physical activity among patients hospitalized following hip fractures and for geriatric conditions.

### SESSION 4080 (SYMPOSIUM)

Abstract citation ID: igae098.1795

### LIVE DISCHARGE FROM HOSPICE AS A CRITICAL CARE TRANSITION: MEASUREMENT, EXPERIENCES, AND SOLUTIONS

Chair: Cara Wallace
Discussant: Lauren Hunt

Hospice care has been shown to improve end-of-life outcomes for adults with chronic illness, yet with eligibility limited to a six-month prognosis, the hospice system is not structured to meet longer-term needs. Many adult patients stabilize, or have a change in terminal prognosis, leading to a 'live discharge.' In 2021, 17.2% of all Medicare discharges were patients discharged alive, with 6.3% of these due to decertification (patient stabilization). This symposium examines live discharge as a critical care transition, including potential measures of quality and continuity, how hospice care coordination or caregivers' social contexts impact experiences of the transition, and a potential intervention to improve the overall process. The first presentation considers utility of the Care Transition Measure (CTM-15) and the Patient-Perceived Continuity of Care from Multiple Clinicians Measure in live discharge from hospice, highlighting the positive and statistically significant relationship between relational continuity—how well a patient feels known and understood by their primary hospice clinician and overall team—and the quality of the care transition. Next, using scores from the CTM-15 to classify participants' experiences as "positive" or "negative," presenters will provide information about the important role of hospice care coordination in the overall experience of the transition off hospice. Third, presenters will showcase the social context among ADRD caregivers, including the way support systems help caregivers during and following a discharge. Last, presenters will demonstrate the development and preliminary usage of a Live Discharge Protocol that hospices can use for a standardized approach to improving this care transition.

Abstract citation ID: igae098.1796

### THE ROLE OF HOSPICE CARE COORDINATION IN THE EXPERIENCE OF LIVE DISCHARGE TRANSITIONS

Cara Wallace[1], Stephanie Wladkowski[2], Ruaa Al Juboori[3], Todd Becker[4], Joseph Trinitas[1], Leslie Hinyard[1], and Verna Hendricks-Ferguson[1], *1. Saint Louis University, St. Louis, Missouri, United States*, *2. Bowling Green State University, College of Health & Human Services, Bowling Green, Ohio, United States*, *3. University of Mississippi, Oxford, Mississippi, United States*, *4. Washington University School of Medicine in St. Louis, St. Louis, Missouri, United States*

Hospice care improves end-of-life outcomes for patients, while a live discharge results in lost access to supportive services and resources. The quality of this transition is critical for ensuring that a caregiver and patient are prepared for the termination of hospice services, and to avoid negative outcomes, such as a hospitalization or a burdensome transition. This study interviewed 32 caregivers of patients across diagnoses who experienced a discharge from hospice due to decertification—when a patient is removed from hospice due to a stabilized condition. Participants were placed in a priori groups (16 in each group) depending on whether their care transition was more positive (designated by a score of 66.67 or higher on the CTM-15) or more negative (scores of 66.66 or below). There were no statistically significant differences between groups based on demographics. However, qualitative codes relating to care coordination differed based on these a priori groupings. Specifically, the positive transition group was more likely than the negative transition group to report hospice action related to the discharge in the following areas: awareness and information, communication between providers, medication, and receiving support from hospice after the discharge. Alternatively, those in the negative transition group were more likely to report hospice inaction related to awareness and information, communication about the discharge, communication between providers, and lack of help generally. Differences in access to healthcare service and availability of social support and how this impacted quality of life also differed across groups. Research, clinical, and policy implications will be discussed.

Abstract citation ID: igae098.1797

### AN EXPLICIT LIVE DISCHARGE PROTOCOL (LDP) TO IMPROVE CARE TRANSITIONS

Stephanie Wladkowski[1], Susan Enguidanos[2], and Tracy Schroepfer[3], *1. Bowling Green State University, College of Health & Human Services, Bowling Green, Ohio, United States*, *2. University of Southern California, Los Angeles, California, United States*, *3. University of Wisconsin Madison, Madison, Wisconsin, United States*

Discharge planning for care transitions is required in hospitals, skilled nursing facilities, and home health agencies within the United States under Medicare guidelines. Yet, no required discharge planning or clear guidelines are available to guide practitioners in transitioning patients and their caregivers out of hospice care. A live discharge from hospice can occur when a patient stabilized under hospice care no longer meets the life expectancy hospice eligibility criteria. For these individuals and their caregivers, the result is a disruption of care continuity. Further, an increased burden is placed on primary caregivers who may be unprepared for this transition. Presenters have developed a live discharge protocol (LDP), to standardize the live discharge process and assess for psychosocial support needs for patients and their caregivers post-discharge. This presentation will describe the development of the LDP through several steps: first, with an advisory committee made up of research experts, clinicians, and caregivers. Next, through focus groups to gain a deeper understanding of current approaches and accompanying challenges of a discharge protocol. Finally, the LDP was drafted and reviewed by practicing social workers and nurses for usability. Further, this presentation will report on how each step provided valuable feedback and informed the final LDP currently being used by hospice agencies and the complexities of conducting a live discharge, the uniqueness of each hospice agency, and the need for more research to support a standardized and reimbursable discharge process.

Abstract citation ID: igae098.1798

### EXPERIENCES OF CARE TRANSITIONS FROM HOSPICE: ADRD FAMILY CAREGIVERS SOCIAL CONTEXTS

Karla Washington[1], Stephanie Wladkowski[2], Kathryn Coccia[3], Marla Berg-Weger[3], and Cara Wallace[3], *1. Washington University in St. Louis, St. Louis, Missouri, United States*, *2. Bowling Green State University, College of Health & Human Services, Bowling Green, Ohio, United States*, *3. Saint Louis University, St. Louis, Missouri, United States*

Patients with ADRD are the largest group of Medicare hospice beneficiaries. They are also more likely to experience a live discharge than any other diagnosis, particularly after 6 months of care without presence of an acute event, indicating removal due to decertification. Although expected prognoses have changed, patients still require substantial care and caregivers often struggle to process feelings of abandonment and uncertainty. Increased burden is placed on primary caregivers who may be unprepared for this transition and are more likely to be socially isolated and lonely. This study interviewed caregivers of patients with ADRD who experienced a live discharge from hospice (n=32). Using the ecological model of resilience for caregivers, this presentation examines how caregivers describe their circle of support (CoS), the way their CoS functions (or does not function) when caregivers need help, and the attributes that impact how and when they utilize members in their supportive network. Results suggest that experiences of care transitions are buffered by patient's location of care (home versus facility) and by availability of resources (both financial and fulfillment of core functions by CoS members). Understanding the way support systems help caregivers during this time of transition provides important implications for assessment during a live discharge to help hospices identify caregivers that may need additional support and resources during the discharge. With a growing population of patients living with ADRD and the rates in which live discharge impacts patients with ADRD-related diagnoses, supporting caregivers during this critical care transition is of utmost importance.

Abstract citation ID: igae098.1799

### UTILITY OF THE CARE TRANSITION MEASURE (CTM-15) AND A MEASURE OF CONTINUITY OF CARE IN LIVE DISCHARGE

Leslie Hinyard[1], Stephanie Wladkowski[2], Verna Hendricks-Ferguson[1], Ruaa Al Juboori[3], Kathryn Coccia[4], Antonia Bennett[5], and Cara Wallace[1], *1. Saint Louis University, St. Louis, Missouri, United States*, *2. Bowling Green State University, College of Health & Human Services, Bowling Green, Ohio, United States*, *3. University of Mississippi, Oxford, Mississippi, United States*, *4. Saint Louis University, Chicago, Illinois, United States*, *5. University of North Carolina, Chapel Hill, North Carolina, United States*

Live discharge from hospice has been referred to as the "forgotten care transition," as it is understudied compared to other healthcare transitions. Following a hospice discharge, patients and families report feelings of grief and abandonment and 40% of patients die within 6 months of the discharge. However, no measures currently exist to help understand this care transition, an important gap for intervention and improvement. This study aimed to consider the utility of and relationship between the Care Transition Measure (CTM-15) and the Patient-Perceived Continuity of Care from Multiple Clinicians Measure among patients/caregivers following a discharge from hospice care. Though the CTM-15 measures the quality of preparation for post-hospital care, questions were modified to reference hospice instead of the hospital. Relational Continuity questions across 3 sub-scales—comprehensive knowledge of the patient, partnership & confidence, team relational continuity—were included from the Patient-Perceived Continuity of Care Measure, as relationship-based care is an important aspect of positive transitions during serious illness. 67 patients (or their primary caregiver) responded to questions following the experience of a live discharge from hospice. Patients were primarily female (75%) and white (78%), with just over half living at home (54%), and a mean age of 84. Study results found statistically significant, positive correlations between the CTM-15 and each of the subscales, indicating that as relational continuity increases, so does the positive perspective of transition quality. Participants also reported relevancy of the questions related to their live discharge experience, suggesting applicability of these tools among this population.

### SESSION 4085 (SYMPOSIUM)

Abstract citation ID: igae098.1800

### NATIONAL INSTITUTES OF HEALTH (NIH): SIMPLIFYING THE REVIEW OF RESEARCH PROJECT GRANT APPLICATIONS

Chair: Elia Ortenberg
Co-Chair: Karen Fingerman
Discussant: Kira Birditt

The National Institutes of Health (NIH) administers >$45B (FY22) of federal funding for extramural medical research, which supports over 300,000 scientists and 2,500 institutions across the United States. These funds are competitively awarded through a two-stage review process. The first level of review solely evaluates the scientific merit of an application by convening peer reviewers who are experts in the field to adjudicate the potential impact of the proposed research. The second level of review takes into consideration for funding both the scientific impact of a proposal as well as its responsiveness to NIH programmatic objectives. NIH is committed to ensuring that all applications for funding receive fair, independent, expert, and timely scientific reviews so the agency can fund the most promising research. NIH upholds its mission of maintaining high standards in peer review by implementing standardized processes and methodologies across all review panels. NIH also continually focuses on efforts to improve peer review. This session is targeted to both experienced and first-time PIs and reviewers. The goals are to provide a general overview of how applications are reviewed by the Center for Scientific Review (CSR) and discuss important changes that impact grant applications, including NIH's current initiative to simplify the peer review framework for research project grant applications and CSR's process for ensuring that study sections change with the science. NIH's ultimate goal through this effort is to identify the best, most innovative science with the potential to improve human health or advance our scientific understanding.

Abstract citation ID: igae098.1801

**NIH: THE SIMPLIFYING REVIEW FRAMEWORK**

Elia Ortenberg, *National Institutes of Health, Bethesda, Maryland, United States*

This presentation will discuss NIH's current initiative, which will be implemented for applications submitted after January 25, 2025, to simplify the peer review framework for most research project grant applications. NIH's ultimate goal through this effort is to identify the best, most innovative science with the potential to improve human health or advance our scientific understanding. The initiative involves reorganizing the existing review criteria in a way that will help reviewers focus on the key questions needed to assess scientific merit of applications: Can and should the proposed research be conducted? We will present a brief history that drove the changes and provide an overview of the new review criteria as well as the impact on the scoring method and arrangement of the summary statement that applicants will receive.

Abstract citation ID: igae098.1802

**CENTER FOR SCIENTIFIC REVIEW: YOUR APPLICATION'S JOURNEY BEGINS WITH THE DIVISION OF RECEIPT AND REFERRAL**

Marc Boulay, *National Institutes of Health, Baltimore, Maryland, United States*

The first stop for your application is at the Division of Receipt and Referral (DRR), the central receiving point for all competing grant applications submitted to the NIH. Here, your application will receive: (a) an initial check for its compliance with both NIH policy and the specific requirements of the Notice of Funding Opportunity used for the submission, (b) an assignment to a funding Institute, and (c) an assignment to the most appropriate study section for review. Attendees will become acquainted with the process used by DRR and will receive some tips to help them avoid common errors with their submission. This session will also highlight tools that applicants may use to inform their application's review assignments and will be introduced to the ENQUIRE process (Evaluating Panel Quality in Review), ongoing evaluations of CSR's study sections to ensure that the scientific scope of the study sections align with the current state of the science. By the end of the presentation, attendees should have a greater understanding of the factors they should consider when preparing their NIH submissions.

Abstract citation ID: igae098.1803

**THE JOURNEY FROM SUBMISSION TO SCORE**

Rochelle Hentges, *National Institutes of Health, Bethesda, Maryland, United States*

Once an application finds a study section home, the scientific review officer (SRO) manages the review of the application by assembling an expert and unbiased panel of reviewers to evaluate its scientific and technical merit. What do study section members assess and how is this reflected in the reviewers' written assessment of the project's overall impact? Reviewers provide scores for the core criteria and additional review criteria as defined in the notice of funding opportunity. The panel's initial assessment determines which applications will be discussed in full at the meeting. For each that is discussed, reviewers discuss the factors that inform their overall assessment. At the end of the discussion, a final impact score is given by all eligible members of the panel, which is reported on the summary statement. Every application undergoes the same process that ensures that fair and consistent consideration is applied to all applications. By the end of the presentation, attendees will learn how the foundation and pillars of the NIH peer review process are put into practice through scoring, discussion, and potential impact on the field.

Abstract citation ID: igae098.1804

**AFTER REVIEW AND TIPS FROM NIH STAFF**

Emerald Nguyen, *National Institutes of Health, Bethesda, Maryland, United States*

After the review meeting, the SRO prepares the summary statement, which is the primary outcome of peer review. Program staff and advisory councils at the institutes use this official document to make recommendations about which applications to fund. How can you best prepare for the next steps for your application? Understand the qualities of a competitive NIH application, talk to program staff, and take advantage of the opportunity to become a peer reviewer yourself. From the field, apply the tips shared by applicants and reviewers about what they do and what they look for in a well-written application. Receiving the summary statement can be daunting and discouraging; but by de-mystifying the process and getting involved, you can help position your application towards maximum success.

### SESSION 4090 (SYMPOSIUM)

Abstract citation ID: igae098.1805

**PAIN MANAGEMENT FOR OLDER ADULTS LIVING WITH DEMENTIA IN NURSING HOME COMMUNITIES**

Chair: Barbara Resnick

Pain is a commonly reported symptom among older adults living with dementia with 30 to 80% of those in nursing homes experiencing pain. For over 50% of these individuals the pain is moderate to severe. Pain often results in a decline in function and quality of life, exacerbates behavioral symptoms associated with dementia, contributes to delirium and increases the risk of emergency room visits. For those with moderate to severe dementia verbal reporting of pain may not be reliable. Multiple systematic reviews concluded that self-report tools should be used when possible and observational measures used for supplementation of self-report. Other challenges associated with pain assessment, diagnosis and management among residents living with dementia include: (1) variations in pain with pain being more intense some days and during certain activities; (2) acceptance of pain as normal; (3) fear of pharmacological interventions; (4) lack of belief in nonpharmacological approaches; (5) lack of knowledge; (6) communication challenges between providers; and (7) desensitization to pain as experienced by residents and observed by staff. The State Operations Manual for Long Term Care, specifically F-Tag F697, requires that nursing homes must ensure that pain management is provided to residents who require such services. This symposium provides an overview of the Pain Management Clinical Practice Guideline (CPG) for nursing homes residents, results from pilot testing of a pragmatic implementation approach to help integrate use of the CPG into the community, and a review of the facility factors associated with pain among nursing home residents living with dementia.

Abstract citation ID: igae098.1806

### IMPLEMENTATION OF THE PAIN MANAGEMENT CLINICAL PRACTICE GUIDELINE USING THE EVIDENCE INTEGRATION TRIANGLE

Elizabeth Galik, *University of Maryland, University of Maryland, Maryland, United States*

To optimally implement the Pain Management CPG and change behavior among residents and staff in nursing homes we developed a theoretically based approach that incorporates the Social Ecological Model, Social Cognitive Theory and was guided by the Evidence Integration Triangle. The PAIN-CPG-EIT was implemented by a research nurse facilitator 10 hours per week for two months and then 4 hours per week for an additional four months to provide four components: Component I: Establishing and working with a stakeholder team monthly; Component II: Education; Component III: Mentoring and motivating the staff; and Component IV: Ongoing monitoring of pain assessment, diagnosis and management for residents. The first stakeholder team meeting included information about the Pain Management CPG and a brainstorming session to develop community goals. The ongoing role of the stakeholder team was to provide positive reinforcement for the staff caring for residents and support the champions. As per Component II, the research nurse facilitator provided education to staff based on the Pain Management CPG. Component III included reviewing pain related issues among residents with the staff and brainstorming about solutions, implementing treatments and adjusting care plans. In addition, contests were held to have staff share innovative approaches to addressing pain among residents. Weekly pain management tidbits were provided via email to all stakeholder members to share with staff. Component IV included the ongoing re-evaluation of pain among residents and monitoring of pain related interventions to determine if these were effective, still needed or if changes in care plans were necessary.

Abstract citation ID: igae098.1807

### FEASIBILITY AND PRELIMINARY EFFECTIVENESS OF IMPLEMENTATION OF THE PAIN-CPG-EIT

Rachel McPherson, *University of Maryland, Baltimore, Maryland, United States*

The primary aim of this pilot study, testing the implementation of the Pain-CPG-EIT, was to evaluate feasibility (delivery and enactment) and preliminary effectiveness. Delivery was based on the intervention being provided as intended and enactment was based on expected behaviors being performed. It was hypothesized that: (1) all components of the intervention would be delivered; and (2) the goals established by each community would be achieved as expected or greater than expected, there would be an increase in nonpharmacologic interventions, and a decrease in unnecessary pharmacologic medications. Effectiveness was based on evidence that residents would have a: (1) pain assessment completed, diagnosis for the pain, and an individualized pain management care plan developed; (2) decrease in pain; and (3) evaluation of appropriate use of opioids. This was a pragmatic trial and no identifiable data was obtained on residents. Participants had a mean age of 70 (SD=8), over half were male (N=25, 51%), and the majority were Black residents (N=33, 67%), Non-Hispanic (N=48, 98%), not married (N=45,92%) and had at least a high school education (N=37, 76%). The mean score on the BIMS was 6 (SD=4) they had 4 (SD=1) comorbidities. The intervention components were delivered as intended and goals achieved. There was significant improvement in the appropriate pain assessments, no change in diagnosis of pain, an increase in the percentage of residents with pain addressed in their care plan, some improvement in assessment for appropriate opioid use, and a decrease in the percentage of residents on opioids.

Abstract citation ID: igae098.1808

### ARE FEDERAL GUIDELINES HELPING TO IMPROVE PAIN ASSESSMENT, DIAGNOSIS, AND MANAGEMENT IN NURSING HOMES

Sorah Levy, *University of Maryland School of Nursing, University of Maryland School of Nursing, Maryland, United States*

The State Operations Manual for Long Term Care, specifically F-Tag F697, requires that nursing homes must ensure that pain management is provided to residents. The management should be consistent with professional standards of practice, the person-centered care plan, and the residents' goals and preferences. Although these regulations are required there are many pain related issues not well addressed in the guidance such as recognition of the challenges to pain assessment and management, ways to overcome challenges, how the community incorporates assessment, diagnosis and management of pain using professional standards of practice, the most current standards of practice for long-term care residents, the best ways to manage different types of pain (e.g., neuropathic, chronic), and how to make decisions about changing, adding or stopping an analgesic among others. Further, there are no processes by which the communities or regulatory agencies evaluate if there is a person-centered care plan and if the plan matches the resident's pain goals. In our pilot work implementing the Pain Management CPG we noted that the majority of the care plans related to pain were generic and not personalized and we were able to help staff make pain care plans more personalized care. The purpose of this paper is to address the mismatch between the requirements and measures within the Minimum Data Set around pain assessment, diagnosis and management and issues and concerns with this approach. We will describe how the Pain Management CPG can help guide communities to overcome these issues and adhere to F-Tag F697.

### SESSION 4095 (PAPER)

### PHYSICAL HEALTH AND PSYCHOSOCIAL WELL-BEING

Abstract citation ID: igae098.1809

### THE LONG-TERM EFFECTS OF EARLY SENSORY IMPAIRMENTS ON LATER-LIFE DISABILITY AND LONELINESS

Eunbea Kim[1], Gina Lee[2], Peter Martin[1], and JeongEun Lee[1], *1. Iowa State University, Ames, Iowa, United States, 2. University of Wisconsin Madison, Madison, Wisconsin, United States*

We focus on the long-term consequences of childhood sensory impairments on physical disability and loneliness in later life, examining the mediating roles of childhood parental relationships in the association between childhood sensory impairments and later life sensory difficulties. We further investigated whether childhood socioeconomic factors (i.e., family income and parents' educational attainment) moderate these relationships. Data from the Health and Retirement Study (HRS; 2012 and 2016; n = 2,116, Mage = 71.47), combined with the HRS Life History data, was utilized. Using path analyses in R, the analysis revealed that childhood vision impairment was related to higher late-life physical disability and loneliness through higher vision difficulties in late adulthood. These relationships were not influenced by the moderators (family income and parents' educational attainment). Conversely, early hearing impairment was linked to greater older adulthood loneliness, mediated by poor childhood parental relationships. The negative impact of poor parental relationships on loneliness was lessened with a higher childhood family income and parental education for those with childhood hearing problems. Notably, mothers' higher education level significantly alleviates the risk of later-life hearing difficulties for those with childhood hearing problems. This research underlines the lasting effects of childhood experiences and parents' background on well-being in later life as well as the different consequences of vision and hearing difficulties. These findings shed light on the understanding of how childhood experiences can shape disability and loneliness later in life, underscoring important implications for prevention efforts in public health and policy.

Abstract citation ID: igae098.1810

### SEX/GENDER DIFFERENCES IN DIAGNOSIS OF PAIN AMONG OLDER MEDICARE BENEFICIARIES

Sadaf Milani[1], Yong Shan[1], Yong-Fang Kuo[2], and Mukaila Raji[3], *1. The University of Texas Medical Branch, Galveston, Texas, United States, 2. University of Texas Medical Branch, Galveston, Texas, United States, 3. University of Texas Medical Branch, Galveston, Galveston, Texas, United States*

Pain is often underdiagnosed among women, especially Black women, but the extent of underdiagnosis is unknown. Cognitive status can also complicate pain reporting and diagnosis. Our objective was to assess the odds of having a pain diagnosis, by sex/gender, while considering race/ethnicity and cognitive status. We used data from the 2018 Health and Retirement Study linked to Medicare claims. We included individuals with complete 2018 Part A and B enrollment and complete information on covariates, excluding those with HMO. Our outcome combined self-reported frequent pain and diagnosis of chronic pain: no pain, self-report only, and diagnosed (both diagnosed only and diagnosed + self-report). Our exposure was sex/gender. We used multinomial logistic regression models to assess the odds of pain diagnosis, by sex/gender, controlling for age, education, race/ethnicity, rurality, cognitive status, and Elixhauser comorbidity index. Among our participants (n=3,523), 12.0% had no pain, 4.6% had undiagnosed pain, and 83.4% had diagnosed pain. Compared to men, women had higher odds of diagnosed pain. The interaction between gender and race/ethnicity was significant (p=0.02). When stratified by sex/gender, non-Hispanic (NH) Black women had lower odds of diagnosed pain, compared to NH White women. Compared to NH Black and Hispanic men, NH White men had lower odds of any type of pain (both diagnosed and self-reported). The main effect of cognitive status and interaction with gender was not significant. Contrary to our hypothesis, women had higher odds of diagnosed pain than men. Within racial/ethnic group differences exist in men and women and warrant future studies.

Abstract citation ID: igae098.1811

### NAVIGATING THE PANDEMIC: THE ROLE OF RESILIENCE AND NEIGHBORHOOD SUPPORT IN STRESS, ANXIETY, AND ADAPTATION

Afnan Balubaid, Ashley Berghoff, and Kristin Litzelman, *University of Wisconsin Madison, Madison, Wisconsin, United States*

The early stages of the COVID-19 pandemic were associated with increased distress and anxiety symptoms in the general population. We sought to assess how resilience and neighborhood support were associated with stress, anxiety, and positive adaptation during the first 15 months of the pandemic. We used three waves of data from the Survey of the Health of Wisconsin's COVID-19 Community Impact Survey (Wave 1: May-June 2020; Wave 2: January-February 2021; Wave 3: June 2021; n= 3,534 observations of 1,374 unique adults). Resilience (Brief Resilience Scale), neighborhood support (Brief Sense of Community Scale), and demographics were measured at Wave 1 and outcome variables (stress, anxiety, and positive adaptation) were measured at each wave. Linear mixed models tested independent and interactive associations of resilience and neighborhood support with the outcome variables, accounting for repeated measures. Average levels of anxiety were highest at the start of the pandemic (spring 2020), while stress was highest in winter 2021 and positive adaptation was highest in summer 2021. Across waves, there was a significant interaction between resilience and neighborhood support, such that neighborhood support was more strongly associated with lower stress and anxiety in those with low resilience than high resilience (slope for stress = -0.28 vs 0.03, respectively, p[interaction]< 0.05). Resilience and neighborhood support showed small associations with positive adaptation (beta = 0.11, p<.01 and beta = 0.04, p<.01, respectively). The findings suggest both psychological and social resources buffered adverse effects of the pandemic on well-being, with implications for preparing communities for future crises.

Abstract citation ID: igae098.1812

### LEISURE ACTIVITY ENGAGEMENT, SOCIAL CAPITAL, AND URBAN–RURAL INFLUENCES ON IQ: IMPLICATIONS FOR COGNITIVE AGING

Chandra Reynolds[1], Paige Trubenstein[2], Shandell Pahlen[1], Robin Corley[1], Sergio Rey[3], and Sally Wadsworth[1], *1. University of Colorado Boulder, Boulder, Colorado, United States*, *2. Angelo State University, San Angelo, Texas, United States*, *3. San Diego State University, San Diego, California, United States*

Cognitive health interventions often highlight individual or environmental factors, with research perspectives often siloed between psychological and demography disciplines. With key interest in understanding the rural-urban health divide, we examined how behavioral engagement and geospatial features influence cognitive functioning in individuals approaching midlife. The Colorado Adoption/Twin Study of Lifespan behavioral development and cognitive aging (CATSLife) (N=1201, Mage=33.17 years, range=28.05 to 49.33) offers a unique opportunity to evaluate whether cognitive performance, as indexed by IQ, is associated with engagement in cognitively demanding activities and county-level indices of social capital (SCI; i.e., the availability of social networks and resources) and rurality. To understand the rural-urban health divide, we tested whether activity-IQ or SCI-IQ associations were moderated by rurality. We replicated the known influence of cognitive engagement on IQ, irrespective of locale. A major finding, however, was the cognitive differences observed between rural and urban residents after considering activity engagement. We found rural individuals' IQ performance was less sensitive to the fluctuations of social capital. Moreover, higher IQ scores among urban residents were only revealed when SCI was high; when SCI was low, urban residents performed worse relative to their rural counterparts. While cognitive engagement had a main effect on IQ, the interaction between SCI and rurality revealed contextually meaningful differences in cognitive functioning. Factors contributing to cognitive health are critical to understand, and our study reveals the interplay of lifestyle and environmental factors on cognitive functioning decades before onset of neurocognitive dysfunction, with implications for cognitive maintenance at midlife and beyond.

### SESSION 4100 (PAPER)

### QUALITY IMPROVEMENT

Abstract citation ID: igae098.1813

### A TRANSITIONS OF CARE INTERVENTION (FOR OLDER ADULTS) TO REDUCE 30-DAY READMISSIONS FROM SUBACUTE REHABILITATION

Rebecca Slossberg[1], Lucrezia Renzetti[1], Edith Burns[2], Raj Patel[1], Nazish Ilyas[3], Alexander Trost[1], and Alexander Rimar[1], *1. Northwell Health, New York, New York, United States*, *2. Northwell Health, Manhasset, New York, United States*, *3. NYU Langone Health, New York, New York, United States*

Hospital readmissions for older adults from subacute rehabilitation facilities (SAR) are a common challenge for healthcare systems. Patient medical complexity, lack of communication during transitions of care, and other factors contribute to otherwise avoidable readmissions. We describe a pilot intervention of Geriatrics and Palliative (GAP) consultation after hospital discharge with the goal of reducing avoidable readmissions from SAR. 136 patients seen by the GAP team in 2023 during a first hospitalization were included. The GAP Nurse Practitioner conducted SAR consultations on a convenience sample of 85 patients (Intervention group, IG), with the remaining 51 acting as non-intervention control (CG). Overall patients in the IG group were not significantly different from the CG based on age, sex, race, LOS, or LACE score. Mean age was 86.8 (SD=7.44 years), majority female (62.5%) and white (67.2%). Readmission rate was 18.8% for IG vs. 27.4% for CG, a 38.7% lower odds of readmission for IG. Readmitted patients from IG were similar to CG readmits (age, sex, LACE) but had longer index LOS (8.76 + 10.2 vs. 6.16 + 4.2 days, t=2.06, p=0.041). Cost analysis showed a 25% cost savings for Star IG vs CG. Mean total cost was highest for CG Star and lowest for CG non-star. Results suggest that following patients in transition to SAR post hospital discharge may help reduce avoidable readmissions in older adults by ensuring continuity of communication. Further research to understand what elements of follow-up care impacted readmission (e.g., hospice eligibility, pain levels, code status, etc.,) is needed.

Abstract citation ID: igae098.1814

### RELATIONSHIP BETWEEN WORKING ENVIRONMENT AND CARE AIDES' COMPASSION FATIGUE, BURNOUT AND COMPASSION SATISFACTION

Ashikur Rahman, Yinfei Duan, and Carole Estabrooks, *University of Alberta, Edmonton, Alberta, Canada*

Severe staff shortages, sustained stress, low compassion satisfaction, high compassion fatigue, and serious levels of burnout among health care workers are frequently reported in the literatures. Limited research has examined compassion-related work life among long-term care (LTC) aides facing high-stress situations due to the deaths of residents and colleagues during COVID-19. In a cross-sectional study with 760 care aides working in 28 LTC homes in Alberta, Canada, we used a two-level multilevel regression analysis to examine how working environments were related to compassion fatigue, burnout, and compassion satisfaction using the Professional Quality of Life (ProQOL-9) scale.

Care aides in smaller facilities reported higher levels of compassion fatigue (B = 0.764, p = 0.008) compared to those in larger facilities. Higher compassion satisfaction (B = 1.009, p = < 0.001) and lower burnout (B = -0.909, p = < 0.001) were observed when care aides perceived more supportive working cultures. Care aides reported higher compassion fatigue when there was a lack of structural (B= -0.149, p =0.019) or staffing resources (B= -0.253, p = 0.007). We also found that not having enough staff (B = -0.469, p = < 0.001) or enough time (B = -0.337, p= 0.019) to complete tasks was significantly associated with higher levels of burnout. In conclusion, compassion fatigue and burnout among care aides were associated with some modifiable factors in the working environment. These findings offer direction on which elements of the working environment may be promising for improvement efforts.

Abstract citation ID: igae098.1815

### DEMENTIA-PROFICIENT MANAGED CARE: ACADEMIC–INDUSTRY COLLABORATION ON A MULTITIERED STAFF TRAINING PROGRAM

David Engle[1], GeVonne O'Neil[2], Halima Amjad[3], Cynthia Fields[3], and Quincy Samus[4], *1. Centene Corporation, House Springs, Missouri, United States*, *2. Centene Corporation, St. Louis, Missouri, United States*, *3. Johns Hopkins University School of Medicine, Baltimore, Maryland, United States*, *4. Johns Hopkins University, Baltimore, Maryland, United States*

Over 6.5 million people are living with Alzheimer's Disease and related dementias (ADRD) in the U.S. Effective care management can improve care quality, quality of life, delay institutionalization, and decrease unnecessary care costs. Centene Corporation, a leading US health care enterprise, operates government-sponsored healthcare programs and offers coverage to 27.5 million managed care members, including Medicaid and Medicare members. Centene and Johns Hopkins University have been engaged in an academic-industry collaboration since 2019 to address the increase in Centene's membership population who are living with ADRD, promote dementia care proficiency within care teams, and drive person-centered care to help members achieve their care goals and exercise control over their services. This presentation will describe the development process, content, and nation-wide deployment of a new, evidence-based multi-tiered dementia care specialist (DCS) training program for managed care and health plan teams. The DCS covers 7 learning areas and aligns with CMS and state training requirements. We will review eLearning and live instructor-led modules, how the train-the-trainer program was implemented in practice settings engaging over 500 learners across 37 states since October 2022, and summarize assessment results on key metrics showing a nearly 50% increase proficiency on measures, before to after training.

Abstract citation ID: igae098.1816

### HOME HEALTH AGENCY STAFF SAFETY: EXPERIENCES FROM THE FRONTLINES

Natalie Leland[1], Amanda Shore[1], Julia Lam[1], Rachel Prusynski[2], Debra Saliba[3], and Tracy Mroz[2], *1. University of Pittsburgh, Pittsburgh, Pennsylvania, United States*, *2. University of Washington, Seattle, Washington, United States*, *3. UCLA, Los Angeles, California, United States*

Prior evidence has documented workplace violence among home healthcare agency (HHA) staff. Building on prior evidence, we examined organizational strategies for fostering HHA staff safety and frontline staff experiences before and after the emergence of the COVID-19 pandemic. We engaged a purposive national sample of 102 HHA leaders in virtual one-on-one semi structured interviews completed between November 2020 and December 2022. We then interviewed a subsample of HHA frontline staff (n=12) between April 2023-March 2024. Using a multiple case study design, we conducted a thematic analysis. Five emergent themes captured organizational efforts as well as staff experiences navigating workplace violence. Leadership (a) contrasted HHA safety environment with institutional healthcare, which impacts organizational policies (e.g., gun safety, compliance with COVID-19 infection control policies) and (b) described financial investment in staff safety (e.g., employ escorts to travel with HHA staff). Frontline staff themes included navigating (c) physical and verbally volatile situations during the HHA care encounter (e.g., requesting compliance with HHA's PPE policy when HHA staff were in the home), (d) community safety, and (e) HHA safety policies and processes. Results highlight the complexity of HHA service delivery, which existed before COVID-19 and was exacerbated as the pandemic evolved. These experiences emphasize the importance of a multi-modal approach to a HHA culture of staff safety (e.g., policies, financial resources). Further, these findings suggest a need for policies that address the operational investments needed to foster an environment of safety.

Abstract citation ID: igae098.1817

### RE-EXAMINING THE ISSUE OF FALSE POSITIVES IN THE ERA OF BIG DATA AND HIGH-FREQUENCY HEALTH MEASUREMENT TECHNOLOGIES

Terry Haines[1], Richard Beare[1], and Velandai Srikanth[2], *1. Monash University, Melbourne, Victoria, Australia*, *2. Monash University / Peninsula Health, Frankston, Victoria, Australia*

Technologies that monitor patient health parameters create abundance of data, enabling multiple comparisons to be easily undertaken within the same dataset. This increases the risk of spurious significant findings being reported (type 1 error). Previous work has identified this problem for situations where there is i) one explanatory and multiple outcome variables, 2) multiple explanatory and one outcome variable, and 3) multiple explanatory and multiple outcome variables. We introduce discussions of where there is one explanatory and one outcome variable, but multiple measurement points permitting multiple ways to "slice" data for analysis. We conducted 1,000 Monte Carlo simulations of a pre-post intervention study with additional parallel control site, with each phase having 24 data points per site. We analysed the data as runs of a pre-post study (period), a post-only parallel control (site) and as the full design examining a period-by-site interaction effect. We analysed the full 24 data points per period / site, then 20, 16, 12 using data adjacent to the pre-post transition time point within each run. We then repeated this procedure with 1000x these numbers of data points. We calculated the family-wise type 1 error rate for each analysis run. The cumulative type 1 error rate across all runs based on the 24 data point set was 26.8%, while the rate was 21.9% for runs based on the 24,000 data point set. This means an analyst with enough data can spuriously manufacture a significant finding for one in four to five studies.

### SESSION 4105 (SYMPOSIUM)

Abstract citation ID: igae098.1818

### RURALITY MATTERS: UNDERSTANDING THE CRITICAL AND NUANCED ROLE OF PLACE-BASED FACTORS IN RURAL AGING

Chair: Jessica Cassidy
Co-Chair: Steven Cohen
Discussant: Leif Jensen

Although only 15% of all Americans live in rural communities, 22% of U.S. older adults live in rural areas. Despite the prevalence of rural older adults, there are significant gaps in the gerontological literature regarding this vulnerable subpopulation and furthermore, the multidimensional factors that characterize rurality and influence their healthy aging. To highlight the impact of place-based factors on the breadth of needs and outcomes found in rural aging populations, this symposium will host five research projects spanning the social and health sciences. The first study applies multi-level models to estimate the spatial distribution of aging and disability service organizations by sociodemographic and sociocultural characteristics in U.S. rural counties. The second uses multiple methods to contrast challenges facing older adults aging in place in rural areas with assets and opportunities for supporting healthy aging in rural older adults. The third examines rural-urban differences in health outcomes and the lived experiences of informal caregivers to suggest culturally appropriate intervention strategies, tailored to the needs of rural caregivers. The fourth analysis compares differences in rural health disparities among diverse aging populations living in rural Wyoming and Mississippi. The fifth investigates benefits of examining claims versus survey data when comparing rural and urban areas to better understand access to healthcare services. Our discussant will lead conversation to emphasize the important nuances of healthy aging in rural communities and to increase awareness on research and policy strategies that may support the needs of older adults and caregivers living in rural communities.

Abstract citation ID: igae098.1819

### SOCIO-SPATIAL DETERMINANTS OF COMMUNITY-BASED LONG-TERM CARE AVAILABILITY IN RURAL COUNTIES

Claire Pendergrast, *Syracuse University, Delmar, New York, United States*

Community-based long-term care services are critical resources to support aging in place in communities across the country. These services may be especially important to rural older adults' health and independence, as declining numbers of working-age adults in rural communities may reduce access to informal family supports. This study examines the spatial distribution of two forms of community-based long-term care in rural U.S. counties: aging and disability services organizations and home health agencies. Using data from the American Community Survey (ACS) and the National Neighborhood Data Archive (NaNDA) for 1708 rural counties, multi-level mixed-effects negative binomial models were estimated to examine the association between the availability of both forms of community-based long-term care services with county sociodemographic and sociocultural characteristics. Findings suggest significantly lower availability of aging and disability services per capita in rural counties with higher crime rates. Greater availability of these services was observed in more Republican counties, those with higher voter turnout, poverty rates, and shares of residents aged 65 and older, as well as shares of Black and Hispanic residents. Home health agency availability per capita was significantly greater in rural counties with higher crime rates, poverty rates, and shares of residents aged 65 and older. State-level factors explained 17.7% of the variation in rural counties' aging and disability services availability and 4.5% of the variation in home health agency availability. Further investigation is needed to understand place-based factors driving inequalities in community-based long-term care access in rural areas.

Abstract citation ID: igae098.1820

### SUPPORTING RURAL OLDER ADULTS AGING IN PLACE: IMPLICATIONS FOR RESEARCH

Carrie Henning-Smith, *University of Minnesota, Minneapolis, Minnesota, United States*

Most older adults, including those living in rural areas, want to age in place; that is, remain in their home and community as they age. Rural areas have many strengths related to aging in place, including generally high levels of social cohesion and strong community ties. However, rural areas also have unique challenges related to aging in place, including more limited housing, transportation, and health care infrastructure and residents with poorer health outcomes and fewer financial resources. Rural areas are older, on average, than urban areas, making them especially important to glean lessons from related to supporting aging in place. This presentation will synthesize recent research on rural aging in place – including new, unpublished results – using a combination of national survey data (the National Health Aging Trends Study and National Health Interview Survey) and qualitative interview data from subject matter experts. Results from this research show that rural older adults are more likely to be aging in place (as measured by longer duration in their current homes and communities) and face specific challenges, including homes that are less physically accessible for people with mobility limitations, poorer health outcomes, and poorer access to care. However, results from this research also show that rural older adults aging in place demonstrate strong social connectedness and key informant interviews illuminated several assets and opportunities inherent to supporting older adults aging in place in rural areas. This session will share key results from this research, as well as implications for rural aging research.

Abstract citation ID: igae098.1821

### RURAL URBAN DIFFERENCES IN INFORMAL CAREGIVING

Elizabeth Crouch[1], Monique Brown[1], Steven Cohen[2], and Jessica Cassidy[3], *1. University of South Carolina, Columbia, South Carolina, United States*, *2. University of Rhode Island, Kingston, Rhode Island, United States*,

*3. The University of Texas Arlington, Arlington, Texas, United States*

With the rapidly rising aging of the U.S. population, an increasing number of stakeholders have raised concerns regarding the health of this group and where the burden of caring for them will fall. The majority of this caregiving will be at the hands of family members and friends who provide informal, unpaid care for their loved ones. In addition to a greater number of older adults who will need care, these adults are also living longer resulting in a greater need for long-term care. The distribution of both formal and informal caregiving in the United States is not uniform; informal caregiving is significantly more prevalent in rural areas than in urban while formal caregiving has the opposite distribution. Greater reliance on informal caregiving in rural areas may be due to a larger proportion of older adults living in rural areas than in urban, reduced access to formal care services in rural areas, or greater feelings of familial obligations amongst rural residents. Prior research has found that the lived experiences of rural caregivers are markedly different from those of their urban counterparts. During this session, we will describe research findings from our team on rural-urban differences in the health and lived experiences of caregivers. We will also discuss interventions that may be tailored to rural communities. This session will help stakeholders and policy makers to better understand the landscape of informal caregiving amongst minoritized rural residents and help program developers design and implement initiatives for rural caregivers.

Abstract citation ID: igae098.1822

### ADDRESSING DISPARITIES IN ADRD PREVALENCE AND MORTALITY RATES BETWEEN RURAL AND URBAN COMMUNITIES

Nasim Ferdows, *Northeastern University, Boston, Massachusetts, United States*

Rising life expectancy has led to an increase in age-related diseases, such as Alzheimer's disease and related dementia (ADRD). The rapid growth of the rural older population may result in higher rates of dementia in these communities. However, rural residents face disparities in healthcare access, potentially leading to an underestimation of ADRD prevalence in claims-based studies requiring medical assessment. This study employs two approaches to examine rural and urban differences in ADRD prevalence and mortality rates. First, analyzing national county-level data from the CDC Wonder database from 2010 to 2021, we found higher ADRD mortality rates in rural areas, though not statistically significant. Second, using Health and Retirement Study data, a nationally representative sample of US older adults, and a 20% sample of Medicare claims, we observed higher dementia prevalence in rural areas based on cognitive tests in the HRS, which are free from clinical assessment. This contrasts with lower prevalence rates based on diagnosis codes in claims data for rural areas. The decline in prevalence rates based on diagnosis codes was also more pronounced in urban areas. The decline in prevalence rates based on diagnosis codes was also sharper in urban areas. These findings suggest that claims-based studies may underestimate dementia rates in rural areas due to limited healthcare access. Addressing these disparities is critical for providing adequate care and support to individuals with ADRD in rural communities.

Abstract citation ID: igae098.1823

### RURALITY AND RACE IN WYOMING AND MISSISSIPPI: THE INTERSECTION OF PLACE AND RACE-BASED DISPARITIES

Taylor Jansen, *University of Massachusetts Boston, Boston, Massachusetts, United States*

Across the US, rural populations tend to be "older, poorer, and sicker" than their urban counterparts as they report higher morbidity, mortality, and greater risk of death due to unintentional injuries. In an already disadvantaged population, rural minorities tend to report even worse health outcomes. This study aims to examine the health disparities faced by rural populations by examining county-level 65+ outcomes in the predominately rural states of Wyoming (WY) and Mississippi (MS). Data from the 2023 WY Healthy Aging Data Report (HADR) and MS HADR was utilized to conduct bivariate statistical analyses and mapping to compare rural/frontier, and urban counties in WY and MS. Findings suggest rurality is protective of health in WY, where 21 of 23 counties are classified as rural or frontier, as these counties reported lower morbidity, despite lower access to primary care physicians (PCPs) [Rural: Mean(SD)=46.19(32.67); Urban: M(SD)=245(38.18)] and hospitals [Rural: Mean(SD)=1.1(0.44); Urban: M(SD)=2(1.41)]. Conversely, bivariate analyses found the two frontier counties with a Native American reservation reported the lowest life expectancy [M(SD)=77.7(1.7); WY: M(SD)=80.34(2.26)] and highest homicide rates [M(SD)=4.54(6.42); WY: M(SD)=0.83(2.31)] and COVID-19 mortality [M(SD)=476.64(52.43); WY: M(SD)=356.61(139.24)]. Similarly, predominately Black, rural counties in MS reported worse rates for 26 of 28 indicators of health and lowest access to care [PCPs: M(SD)= 18.52(20.56); Hospitals: M=1.06(0.65)] compared to rural, predominately White counties [PCPs: M(SD)= 23.88(19.92); Hospitals: M(SD)= 1.11(0.64)], and MS state averages [PCPs: M(SD)= 43.54(93.45); Hospitals: M(SD)= 1.23(1.10)]. These findings speak to the unique health disparities rural populations face at the intersection of place and race.

### SESSION 4110 (PAPER)

### SOCIAL DETERMINANTS OF HEALTH (SRPP)

Abstract citation ID: igae098.1824

### RACIAL/ETHNIC DIFFERENCES IN COPING RESOURCES AND PURPOSE IN LIFE AMONG OLDER AMERICANS

Man Guo, Yi Wang, and Morgan Stangl, *University of Iowa, Iowa City, Iowa, United States*

Purpose in life (PiL), the sense of direction and meaning in life, has been found to be linked to favorable health outcomes in later life, even lower mortality. Less is known about the perception of PiL among racial/ethnic minorities, who often face numerous adversities, but also have strong coping resources derived from religiosity, social support networks, and neighborhood contexts. Using data from the 2016 and 2018 psychosocial assessment of the HRS (N=11,579), this study assessed racial/ethnic differences in PiL among middle-aged and older adults and the role of coping resources (religiosity, social support, neighborhood cohesion) in shaping PiL. ANOVA results showed that compared to White older adults, Black older adults had significantly higher PiL, and Hispanic older adults had significantly lower PiL. Regression results further showed that all the coping resources were positively associated with higher PiL within each racial/ethnic group, with the exception of religiosity for Hispanic older adults. Despite racial/ethnic minority groups' significantly higher ratings on religiosity and neighborhood cohesion, the positive associations between the three coping resources and PiL were stronger among White older adults than racial/ethnic minority groups. Aside from possible measurement bias or cultural response differences, the findings suggest that racial/ethnic minority older adults may face unique challenges that impact their effective utilization of coping resources, potentially weakening their PiL. Our findings highlight the importance of better understanding factors that promote PiL among racial/ethnic older minorities, especially Hispanics. They also provide insights into culturally tailored approaches to enhance well-being within these communities.

Abstract citation ID: igae098.1825

### RACIAL/ETHNIC GROUP DIFFERENCES IN REPORTED SELF-NEGLECT AMONG OLDER ADULTS

Kenneth Steinman, Jim Pellerin, and Yang Shi, *The Ohio State University, Columbus, Ohio, United States*

Self-neglect is a pernicious problem and represents the most common type of report investigated by adult protective services (APS). Calls for culturally competent approaches to addressing self-neglect in older adults have been undermined by the lack of research on racial/ethnic group differences and how they may vary across communities. Analyses of 101,015 reports of self-neglect among people 65+ years old validated by the Texas APS system from 2021 through 2023 found the incidence was nearly twice as high for (non-Hispanic) African Americans as for other older adults (IRR=1.91, 95%CI: 1.88, 1.94), whereas the difference for Hispanics was modest (IRR=0.95, 95%CI: 0.93, 0.96). Stratified analyses, however, found marked variation across the state's 11 regions. In rural regions of West Texas, the higher reporting rates for African Americans were especially pronounced: three to four times the rates for other older adults. And in rural regions of East Texas, reporting rates for Hispanics were only one half to one third those of other older adults. Less variation was observed across major metropolitan regions. Further analyses assessed how regional population demographics and the racial/ethnic composition of APS staff may explain these divergent results. Efforts to understand and address racial/ethnic group differences in self-neglect should reconsider the propriety of describing groups as "underserved" when they may be disproportionately more likely to be investigated by APS. When addressing self-neglect in a diverse client population, agencies should consider the background of staff and whether distinct approaches for are needed in rural areas.

Abstract citation ID: igae098.1826

### BUILDING INDIGENOUS HEALTH: INSIGHTS FROM INDIGENOUS ADULTS ON KNOWLEDGE INTEGRATION AND ADAPTIVE STRATEGIES

Zih-Yong Liao[1], and Fang-Lin Kuo[2], *1. National Yang Ming Chiao Tung University, Taipei, Taiwan (Republic of China), 2. National Health Research Institutes, Yunlin, Yunlin, Taiwan (Republic of China)*

Over half of the older population faces inadequate dietary intake, posing higher risks of geriatric syndromes like frailty and sarcopenia. This community-based participatory research (CBPR) co-created a nutrition and cooking workshop in a Taiwanese Indigenous community. Employing the photovoice method alongside focus groups and individual interviews with 16 participants, we sought to understand the older Indigenous adults' dietary patterns. Nutbeam's health literacy model (2000) and Giddens' structuration theory (1984) guided data analysis and conceptualization of food literacy construction in the Indigenous land. Four main themes emerged. First, 'knowledge integration' represents the conversation between traditional and scientific food/nutrition knowledge, strengthening functional food literacy to address health concerns. Second, 'Indigenous adaptive strategies' demonstrate their ability to apply food knowledge meaningfully in daily life, representing interactive food literacy. Third, 'awareness of advanced nutrition literacy' demonstrates the older adults' ability to evaluate personal diets and critically reflect on the need for better health, representing critical food literacy. These food literacy constructs are iterative and interactive. Lastly, work is central to Indigenous life, shaping their identity and a sense of achievement, motivating older adults' adaptive strategies and pursuing advanced nutrition literacy. However, the middle-aged population is less likely to attend health promotion activities due to work. Indigenous adaptive strategies reflect human agency's potential to reproduce individual food ecosystems and healthcare practice systems within the community, overcoming structural barriers. CBPR can foster a sense of ownership among participants, enabling them to engage healthy behaviors as their personal priorities and take active initiatives toward better health.

Abstract citation ID: igae098.1827

### CHANGE IN UNMET NEEDS STATUS FOR DAILY MOBILITY AND SELF-CARE ACTIVITIES

Laura Sands, Xiaofan Zhu, Maham Khan, and Pang Du, *Virginia Tech, Blacksburg, Virginia, United States*

Unmet need occurs when older adults with difficulties completing daily activities cannot complete an activity because no one is available to help. Prior research has not considered whether unmet need status changes over time, nor assessed characteristics of older adults whose unmet needs worsen or resolve. Changes in unmet needs status across three pairs of consecutive annual interviews were assessed using 2011-2014 data from the National Health and Aging Trends Study. Findings revealed that that 57% of older adults who needed help with mobility activities and 62% of older adults who needed help with self-care activities did not experience unmet needs across the three consecutive pairs of occasions. Only 3% who needed help with mobility activities and 1% who needed help with self-care activities consistently reported unmet needs. Worsening of mobility unmet needs occurred for 25% and 16% resolved their mobility unmet needs. Worsening of self-care unmet needs occurred for 25% and 11% resolved their self-care unmet needs. Those who experienced worsening of mobility and/or self-care unmet needs were more likely to be female and have multiple chronic conditions. Those who experienced resolution of mobility unmet needs were more likely to be female and have multiple mobility difficulties and chronic conditions. Those who experienced resolution of self-care unmet needs were more likely to be white race, have zero or one self-care difficulties, and have multiple chronic conditions. The findings inform the need for frequent assessment of unmet needs to identify opportunities to reduce worsening and increase resolution of unmet needs.

Abstract citation ID: igae098.1828

**INDIGENOUS RESILIENCY AGAINST THE SOCIAL, BEHAVIORAL, AND MENTAL DETERMINANTS OF HEALTH DURING COVID-19 PANDEMIC**

Kristi Kaapu[1], Catherine McKinley[1], and Michelle Johnson-Jennings[2], *1. Tulane University, New Orleans, Louisiana, United States*, *2. University of Washington, Seattle, Washington, United States*

U.S. Indigenous people have been disproportionately impacted by the COVID-19 pandemic, experiencing higher rates of infection and hospitalization, being vulnerable to mental health symptoms, including psychological stress and emotional health, and experiencing higher rates of loss and death. Structural inequities have contributed to these negative COVID-19 health outcomes; however, consistent with the Framework of Historical Oppression, Resilience, and Transcendence (FHORT), Indigenous people offset these disparities through positive coping, adaptation, and resiliency interwoven between individual, family and community ecological levels. This research assessed interconnections of structural inequality and associated disruptions in relation to Indigenous wholistic wellness, primarily around healthcare discrimination, medical mistrust, mental health, and loss and death. Thirty-one head-of-household women participated in community-based, critical ethnographic interviews to assess disparities associated with COVID-19. All participants were in caregiving roles and ranged in age from 29-56 years old. The following themes emerged: (a) negative medical and healthcare experiences; (b) connections between physical and mental health; (c) worry and depression; (d) loss of structure and self; (e) disrupted burial rituals; (f) death, grief, and extensive losses; and (g) resiliency. Results indicate disparities associated with the pandemic emerged in large part from systemic inequity; however, Native American women demonstrated resiliency by advocating for themselves, problem solving, self-coping, and upholding Indigenous values. With lower life expectancy of Indigenous peoples, this study informs both future generations and interventions, promoting traditional and cultural protective factors to offset the impact of historical trauma and oppression, including healing across all ecological domains including physical, mental, emotional, and spiritual aspects.

### SESSION 4115 (PAPER)

### SOCIAL SUPPORT

Abstract citation ID: igae098.1829

**UNDERSTANDING THE ROLE OF SOCIAL CAPITAL IN FORMAL SERVICE USE AMONG OLDER ADULTS: A SCOPING REVIEW**

Carson De Fries, Kaipeng Wang, and Leslie Hasche, *University of Denver, Denver, Colorado, United States*

As individuals age, they may encounter new or persistent challenges that affect their need for and access to formal services. Social capital theory, which views relationships as a source of support, a bridge to needed resources and information, and a buffer against challenges that individuals may face, serves as a useful framework in addressing barriers to service utilization among this population. This scoping review aimed to identify how an older adult's social capital influences their use of formal services, with a focus on the differences between intergenerational and peer (i.e., non-intergenerational) relationships. The search, conducted in January of 2023, originally yielded 2,645 articles. Following duplication removal and eligibility screening, 57 articles were included for analysis. In reviewing the methodologies of these articles, 75.4% (n=43) used quantitative methods, 9.5% (n=5) utilized qualitative methods, 3.8% (n=2) conducted a systematic or scoping review, one used mixed methods, and one was a conceptual paper on the topic. Results demonstrate nuanced differences in how certain types of relationships may support older adults' access to formal services. Bonding social capital, operationalized as peer relationships, often provided more therapeutic support, which may have reduced the need for formal services. Bridging social capital, operationalized as intergenerational relationships, often resulted in more instrumental support, providing older adults with information about diverse resources and services. Findings also highlighted methodological gaps in assessing older adults' social relationships and underscored the need to further explore the mechanisms through which different relationship types influence older adults' access to needed care.

Abstract citation ID: igae098.1830

**SLEEP CONCORDANCE AMONG CARE DYADS OVER TIME: DOES IT VARY BY CAREGIVING CONTEXTS?**

Dexia Kong[1], Chihua Li[2], Xiaoling Xiang[2], and Peiyi Lu[3], *1. The Chinese University of Hong Kong, Hong Kong, China (People's Republic)*, *2. University of Michigan, Ann Arbor, Michigan, United States*, *3. The University of Hong Kong, Hong Kong, China (People's Republic)*

The well-being of care recipients (CR) and informal caregivers (CG) is intricately interconnected. However, research on sleep concordance among caregiving dyads is scarce. Leveraging longitudinal dyadic data and a cross-lagged panel design, this study aims to investigate whether: 1) sleep patterns are concordant among caregiving dyads over time; and 2) the extent of sleep concordance differs by caregiving contexts (i.e., dementia caregiving versus non-dementia caregiving) or by living arrangements (i.e., living together versus living apart). Longitudinal data from the National Health and Aging Trends Study (NHATS) and the National Study of Caregiving (NSOC) were included (N = 2,204 care dyads). Cross-lagged panel models and multiple group analysis were used. Sleep problems of CR and CG in 2015 were significantly associated with their own sleep problems in 2017. The partner effects were statistically insignificant ($P>0.05$). However, CR sleep problems in 2015 were associated with more CG sleep problems in 2015 ($\beta=0.141$, 95% CI=0.015, 0.268), which were further linked to more CG sleep problems in 2017 ($\beta=0.504$, 95% CI=0.431, 0.577). Furthermore, these relationship patterns remained significant among dementia care or co-residing dyads, but not among non-dementia or living apart care dyads. CR sleep problems could affect CG sleep synchronously, potentially leading to long-term deterioration of CG sleep. This can be attributed to the cumulative physical exhaustion and psychological strain associated with caregiving. Our findings highlight the importance of early intervention in mitigating CR sleep problems and targeted assistance to CG of persons with dementia and those co-residing with their CR.

Abstract citation ID: igae098.1831

**RURAL FAMILY CAREGIVING: PROMOTING SELF-CARE AND WELL-BEING OF RURAL FEMALE CAREGIVERS OF FRAIL OLDER FAMILY MEMBERS**

Nasreen Lalani, Bhagyashree Katare, Sampada Wagle, and Siqi Yang, *Purdue University, West Lafayette, Indiana, United States*

Caring for frail older adults in rural areas is often more challenging due to several disparities. Informal caregivers, mostly females carry the higher burden of caregiving for their older family members. They experience physical, psychological, and economic strains causing poor well-being and caregiver outcomes. Our study aims to examine the self-care practices of rural female caregivers and determine socio-ecological-cultural factors contributing to their resilience and subjective well-being. Using a sequential mixed-method design, 160 Qualtrics-based surveys were collected followed by 20 in-depth interviews from female rural caregivers of older adults from twelve rural counties in the north-central region of the Midwest, US. Multiple linear regressions were done to measure the associations among the study variables whereas thematic analysis was done to analyze the qualitative data. Preliminary findings suggest higher involvement in self-care practices was associated with greater subjective well-being ($\beta=0.575$, $p< 0.001$), and reduced depression ($\beta=0.406$, $p=0.017$) among the rural female caregivers. Social support positively influenced subjective well-being ($\beta=0.588$, $p=0.010$). Initial themes generated from the qualitative data included increased burnout, mental exhaustion, loneliness, and no time for self-care. Meaning-making and faith were the motivating factors toward caregiving roles, self-care, and well-being. Caregiver support and well-being is our social responsibility to improve social, mental, and economic outcomes and prevent rural disparities. Community-based caregiver support programs and policies should be designed using gender equity and cultural lens to improve the overall health, coping, and well-being of caregivers from a rural context.

Abstract citation ID: igae098.1832

**WORKPLACE PERSPECTIVES ON CAREGIVER-FRIENDLY WORKPLACE POLICIES**

Maryssa Pallis, and Pamela Nadash, *University of Massachusetts Boston, Boston, Massachusetts, United States*

Roughly 60% of all family caregivers are employed, and it is well-established that they experience stresses related to work-life balance, which may cause them to leave employment altogether. Thus, an important goal of the 2022 National Strategy to Support Family Caregivers focuses on caregivers' financial security, specifically noting the importance of workplace protections and supports. This presentation provides a workplace perspective on caregiver-friendly workplace policies, drawing on data collected to support the RAISE Family Caregiving Advisory Council, created under the Recognize, Assist, Include, Support, and Engage (RAISE) Family Caregivers Act (2018). It draws on a data collection effort involving listening sessions, focus groups, and key informant interviews with over 150 entities (including employers and employer intermediaries). The study aimed to identify what employers had to say about the challenges and benefits of creating caregiver-friendly workplaces. Issues raised included the importance of workplace policies such as paid family and medical leave, flexible work policies, and caregiver support programs. Findings suggest consensus among employers regarding the need for caregiver-friendly workplace policies; however, barriers to creating a supportive culture included caregivers' reluctance to self-identify and utilize formal policies. Beyond adopting formal caregiver-friendly workplace polices, the most critical area for action would be to explore best practices for proactively integrating a caregiver-friendly workplace culture alongside formal policy interventions.

Abstract citation ID: igae098.1833

**PATTERNS OF MULTIPLE CAREGIVING AND DEPRESSIVE SYMPTOMS AMONG MIDDLE-AGED AND OLDER ADULTS IN CHINA**

Xinyi Zhao, and Vivian Weiqun Lou, *1. Peking University, Beijing, Beijing, China (People's Republic)*, *2. The University of Hong Kong, Hong Kong, China (People's Republic)*

**Objectives:** With extended life expectancy, middle-aged and older adults have become more involved in multiple caregiving. This study aimed to investigate the patterns of multiple caregiving, and whether these patterns predicted follow-up depressive symptoms.
**Methods:** Data were obtained from the 2011–2015 waves of the China Health and Retirement Longitudinal Study. A total of 11,422 participants aged 50 and older in 2011 were included in the latent class analysis to identify the patterns of multiple caregiving. A regression model was used to analyze the association between baseline caregiving patterns and subsequent depressive symptoms.
**Results:** Four caregiving patterns were established: "part-time care for all", "parent care and full-time grandchild care", "part-time spouse care and full-time grandchild care" and "full-time spouse care". Compared with the "part-time care for all" group, the "full-time spouse care" group and "part-time spouse care and full-time grandchild care" group reported significantly greater depressive symptoms four years later.
**Conclusion:** The use of categorizing multiple caregiving could vividly describe middle aged and older adults' engagement in caregiving activities and provide a comprehensive prediction of mental health outcomes. Social services should be tailored to sandwiched caregivers in certain caregiving patterns.

### SESSION 4120 (SYMPOSIUM)

Abstract citation ID: igae098.1834

**SUICIDE RISK ASSESSMENT: INSIGHTS, CHALLENGES, AND INNOVATIONS**

Chair: Fatema Colombowala
Discussant: Kimberly Van Orden

Suicide is a major public health problem, accounting for over 700,000 deaths globally each year. Worldwide, suicide rates are highest among adults 70 years and older. Suicide attempts in later life are highly lethal and many who die by suicide do not endorse suicidal ideation, challenging efforts to detect and manage risk. This symposium will provide an in-depth exploration of suicide risk assessment amongst older adults, highlighting unique challenges across settings and providing recommendations for best practices and future research. Speaker 1 will present on the acceptability and feasibility of using ecological momentary assessment (EMA) to measure daily fluctuations in suicidal thoughts and psychosocial risk factors amongst older adults with subjective cognitive decline. Speaker 2 will discuss challenges of assessing suicidality within rural in-home care settings amongst older adults with hoarding disorder, emphasizing safety considerations when conducting in-home suicide risk assessments. Speaker 3 will examine the relationship between hoarding disorder and suicidal ideation, emphasizing the impact of social cohesion risk factors in rural environments. Speaker 4 will share findings from a comparative analysis of two validated suicide assessment tools (clinician-administered versus self-report) commonly used in research and practice, with a focus on areas of differential response across the two scales. The Discussant will synthesize findings and highlight areas for future research and innovation to improve detection of suicidal ideation and behavior amongst older adults. Collectively, these presentations will offer insights into the complexities of suicide risk assessment and recommend strategies tailored to the needs of older adults.

Abstract citation ID: igae098.1835

### OLDER ADULTS HAVE DIFFERENTIAL RESPONSE PATTERNS TO TWO COMMON SUICIDE ASSESSMENT MEASURES

Fatema Colombowala, and Emily Bower, *Pacific University, Hillsboro, Oregon, United States*

An alarming number of older adults die by suicide each year. Older adults exhibit lower rates of suicidal ideation (SI) yet higher rates of suicide deaths compared to younger adults, so improving risk detection is imperative. Our aim was to compare response patterns to two common, validated measures for assessing SI in older adults at higher risk for suicide. We hypothesized there would be a positive relationship between responses to the two assessments for SI and death ideation (DI). We also expected variation in response patterns among some participants, and examined these exploratorally. Preliminary analyses included 41 participants (M=70 years, 78% female) with subjective cognitive decline who completed the Geriatric Suicide Ideation Scale (GSIS) and the Columbia Suicide Severity Rating Scale (CSSRS) as part of a larger study. The GSIS is a self-report measure developed with and for use with older adults that includes SI and DI subscales. The CSSRS is a clinician-administered assessment of SI severity. 62% endorsed lifetime SI and 12% endorsed past-month SI. CSSRS lifetime and past-month SI scores were strongly correlated with the GSIS SI subscale scores (r's(39)=.5-.6, p's <.001). However, CSSRS past-month DI scores were not correlated with GSIS DI subscale scores (r(39)=.09, p >.05). Furthermore, we observed endorsement of GSIS DI items among participants who denied DI on the CSSRS. Differential response patterns highlight the need for meaningful measures and/or adaptations of existing measure to accurately capture suicide risk.

Abstract citation ID: igae098.1836

### ECOLOGICAL MOMENTARY ASSESSMENT OF SUICIDE RISK: FEASIBILITY AMONG OLDER ADULTS WITH MEMORY CONCERNS

Taylor Loskot, and Emily Bower, *Pacific University, Hillsboro, Oregon, United States*

Given heightened suicide risk among older adults,, effective and timely risk assessment strategies are imperative to inform suicide prevention. Ecological momentary assessment (EMA) may help detect fluctuations in socioemotional suicide risk factors, including loneliness, depression, and suicide ideation (SI). The aim of the present study was to examine feasibility of an EMA battery for assessing momentary SI and socioemotional risk factors in older adults with subjective cognitive decline (SCD). Participants (n=27; M age=69; 82% female) were recruited from a larger study and were classified into “higher risk” (n=13) and “lower risk” (n=14) groups based on history of suicidal behavior or recent SI. Using an EMA app on their smartphone, participants were notified to complete four surveys daily for 14 days. EMA items included questions about SI, mood, loneliness, and cognitive concerns. After the survey period, participants completed validated “paper-and-pencil” assessments for comparison and provided feedback about app usability. On average, participants completed two-thirds of assigned surveys (M = 37, SD = 10) and completion rates did not vary significantly by suicide risk, (t(25) = 0.73, p >.05) or SCD severity (r = -.11, p >.05). Participants reported an easy and non-disruptive experience and provided helpful feedback regarding the structure of notifications, survey wording, and app interface. Additional demographic and psychosocial predictors of EMA response rate will be explored in regression models. Overall positive feedback suggests that EMA may be a feasible risk assessment method for use with older adults facing cognitive concerns.

Abstract citation ID: igae098.1837

### IN-HOME ASSESSMENT OF SUICIDALITY IN RURAL-DWELLING OLDER ADULTS

Caitlyn Nix[1], Branden Schaff[2], and Mary Dozier[1], *1. Mississippi State University, Mississippi State, Mississippi, United States*, *2. MIssissippi State University, Starkville, Mississippi, United States*

Older adults in isolation have an increased risk for suicidality, and this can be exacerbated in a rural context. Additionally, assessment of suicidality during in-home care for older adults is different from typical clinical settings. This presentation will focus on first-hand clinical experiences from an ongoing clinical trial for older adults with hoarding disorder (n = 44, mean age = 69) in rural Mississippi. All participants completed a semi-structured interview of suicidality risk at baseline and at follow-up points across the intervention. We will discuss various factors that are unique to a rural-in home assessment setting, including the importance of re-emphasizing information critical to informed consent (e.g., thresholds for breaking confidentiality), sensitivity to the vulnerability of participants being asked such questions within their home, and confidentiality concerns (e.g., cohabitants in the home, procedures for contacting supervisor for consultation). As the individuals in the current study were volunteering due to distress from clutter or items in the home and not active suicidal ideation, conclusions from these clinical experiences will be compared with findings from risk-assessment studies in older adults.

Abstract citation ID: igae098.1838

### PRESENCE OF RISK FACTORS FOR SUICIDALITY IN RURAL-DWELLING OLDER ADULTS WITH HOARDING DISORDER

Branden Schaff[1], Ben Porter[2], Caitlyn Nix[2], and Mary Dozier[2], *1. MIssissippi State University, Starkville, Mississippi, United States*, *2. Mississippi State University, Mississippi State, Mississippi, United States*

Because one in three older adults with hoarding disorder has co-morbid major depressive disorder, it is critical to understand the potential presence of additional risk factors for suicidal ideation in this population. There is currently a dearth of information about social cohesion risk factors for suicidality in older adults, particularly those living in a rural context. Rurality has the potential to exacerbate issues related to loneliness and access to instrumental support, both of which can increase risk for suicide. Forty-four older adults (mean age: 69, SD=9.43; 23% male; 20% Black or African American) with hoarding disorder completed a comprehensive baseline assessment as part of an in-home intervention study that included the Columbia-Suicide Severity Rating Scale, a semi-structured diagnostic interview, and the NIH Emotion Toolbox. No participants reported a history of suicide attempts, and only two participants reported a history of an aborted attempt; however, 30% reported having had the thought that they "wished to be dead or not alive" and 11% reported previously having had "active suicidal ideation with some intent to act." Within our sample, 29% reported a previous depressive episode. On the NIH Emotion Toolbox, 33% were elevated on Loneliness, 33% were elevated on Perceived Rejection, 21% were low on Instrumental Support, and 33% were low on Social Satisfaction. A considerable percentage of participants reported social cohesion factors that could influence their risk for suicidal ideation. Targeted modular interventions aimed at mitigating loneliness and improving instrumental support may bolster the impact of evidence-based treatments within this population.

### SESSION 4125 (AWARD LECTURE (INVITATION ONLY))

Abstract citation ID: igae098.1839

### THE M. POWELL LAWTON AWARD LECTURE AND PRESENTATION

Chair: Christine Mueller

The M. Powell Lawton Award Lecture will feature an address by the 2023 recipient Sam Fazio, PhD, FGSA of the Alzheimer's Association. This session will also include the presentation of the 2024 M. Powell Lawton Award to recipient Stephen M Golant, PhD, FGSA, of the University of Florida. The M. Powell Lawton Award is presented annually to an individual who has made outstanding contributions from applied research that has benefited older people and their care. The Lawton Award is generously funded by the Polisher Research Institute of Abramson Senior Care.

Abstract citation ID: igae098.1840

### THREE DECADES OF PERSON-CENTERED DEMENTIA CARE: LESSONS LEARNED AND FUTURE CONSIDERATIONS

Sam Fazio, *Alzheimer's Association, Chicago, Illinois, United States*

Dr. Fazio has been involved in person-centered dementia care for more than three decades. In this presentation, Dr. Fazio will review the origins of person-centered care and ongoing research, as well as share highlights of his personal research and his dedicated work at the Alzheimer's Association. At the same time, he will discuss how we might need to reconsider the overall concept and its implementation for greater impact.

### SESSION 4130 (SYMPOSIUM)

Abstract citation ID: igae098.1841

### THE PROMISE AND CHALLENGES OF DIGITAL INNOVATIONS TO SUPPORT OLDER ADULTS AND CAREGIVERS

Chair: Laura Gitlin

Discussant: Walter Boot

Online platforms are promising scalable approaches for delivering interventions addressing critical public health needs. As most older adults and caregivers utilize smartphones, tablets or computers, this approach can improve access to on-demand support. However, there are numerous challenges for advancing digital innovations. This symposium showcases five digital platforms that target different populations (caregivers, socially isolated, individuals with emotional needs); address different needs (monitoring, support, care strategies); have different developmental approaches, and are in different testing phases (feasibility, usability, acceptability, efficacy). Ms. Webster first presents national recruitment efforts to enroll 262 caregivers to evaluate an online platform, WeCareAdvisor, that provides strategies to manage dementia-related behavioral symptoms. She illustrates the volume needed for enrollment and biases of recruitment approaches. Next, Dr. Czaja discusses processes for developing PRISM, an effective online program addressing social isolation. Highlighted is the importance of end-user engagement throughout development and testing. Dr. Sadarangani presents CareMobi, which enables families using adult day services to track their relatives care in that setting. She highlights the feasibility of staff and families to use the app to improve communications. Dr. Jain next presents on adapting late-life psychotherapies for delivery via mobile devices. His study demonstrates the effectiveness of adapting approaches for delivery in this modality and opportunities for symptom monitoring. Finally, Dr. Stevens, presents utilization patterns of his online platform, GamePlan4Care, which adapts the NIH REACH caregiver intervention for delivery online. Our nationally recognized expert on technology and aging, Dr. Walter Boot, will discuss the promises and challenges of digital solutions. Behavioral Interventions for Older Adults Interest Group Sponsored Symposium

Abstract citation ID: igae098.1842

### THE APPLICATION OF USER-CENTERED DESIGN TO A DIGITAL PLATFORM FOR OLDER ADULTS: THE PRISM SOFTWARE SYSTEM

Sara Czaja[1], Walter Boot[2], Wendy Rogers[3], and Neil Charness[4], *1. Weill Cornell Medicine/Center on Aging*

*and Behavioral Research, New York, New York, United States*, 2. *Weill Cornell Medicine, New York, New York, United States*, 3. *University of Illinois Urbana-Champaign, Champaign, Illinois, United States*, 4. *Florida State University, Tallahassee, Florida, United States*

Technology holds promise for enhancing the social connectivity and reducing loneliness among older adults, especially those that are socially isolated. However, technology systems can be overly complex and challenging especially for those with limited technology experience. Common barriers to technology uptake among aging adults include usability issues, and lack of training and support. User-centered design is critical to maximize the uptake of supportive technologies among older adult users. This presentation will provide an example of how the user-centered design process was applied to the design of a digital platform for older adults, the PRISM software system. The iterative design approach and the incorporation of user feedback into the design process will be highlighted. PRISM, an integrated software system, was designed to foster social connectivity, cognitive engagement, and resource access among aging adults. Versions of PRISM have been evaluated in diverse contexts and geographic regions, and with diverse populations, including individuals with a cognitive impairment. Data will be presented regarding participants use of and perceptions of the usability and value of PRISM. Findings regarding the impact of the use of PRISM on social connectivity and perceptions of loneliness will also be presented. A focus will be on the adaptation of PRISM for aging adults with a cognitive impairment and strategies used to provide training for this population. Recommendations for needed future research in the aging and technology domain will also be provided to ensure that the emerging technology applications are designed to meet the needs, abilities, and preferences of aging adults.

Abstract citation ID: igae098.1843

### NATIONAL RECRUITMENT OF DIVERSE CAREGIVERS TO TEST AN ONLINE PLATFORM TO MANAGE BEHAVIORAL SYMPTOMS

Melinda Webster[1], Helen Kales[2], Lauren Parker[3], Daniel Scerpella[4], Katherine Marx[4], Nicole Bouranis[5], and Laura Gitlin[1], 1. *Drexel University, Philadelphia, Pennsylvania, United States*, 2. *University of California Davis, Davis, California, United States*, 3. *Johns Hopkins Bloomberg School of Public Health, Baltimore, Maryland, United States*, 4. *Johns Hopkins University, Baltimore, Maryland, United States*, 5. *Yale University School of Medicine, New Haven, Connecticut, United States*

Online platforms are promising scalable approaches to support diverse family caregivers. Testing these approaches requires recruiting nationally representative cohorts in terms of race, ethnicity, and geography. However, large-scale national recruitment is challenging given lack of best practices and inability to rely on local resources. We describe the recruitment sources used to enroll caregivers in an NIA-funded trial to test WeCareAdvisor, an online platform providing strategies to manage dementia-related behavioral symptoms. Over two years, 40 recruitment sources were pursued, yielding 892 inquiries from which 262 (29.3%) caregivers were enrolled. Recruitment sources were categorized into six types: 1) registries yielded the most study participants (550 inquiries, 112 enrolled, 42.7% of sample); 2) community-based providers/organizations (87 inquiries, 41 enrolled, 15.6%): 3) caregiver resources/respite services (50 inquiries, 34 enrolled, 13%); 4) dementia societies/associations/organizations (55 inquiries, 30 enrolled, 11.5%); 5) miscellaneous sources (87 inquiries, 23 enrolled, 8.8%); and 6) articles/professional talks (63 inquiries, 22 enrolled, 8.4%). Regarding diversity, registries yielded more non-spouses as compared to other recruitment sources; articles/professional talks yielded more racial/ethnic diversity proportionate to the number enrolled from that source (54.5% of 22 enrolled versus 15% of 112 enrolled through registries). Enrolled participants resided in 36 states, representing all 10 Department of Health & Human Services regions of the US. Costs (media ads, registry fees) totaled $22,438; $85.64 per participant. Although registries yielded the most enrollees, personal contact through articles/professional talks was more effective enrolling racially diverse caregivers. Recruiting caregivers nationally requires numerous strategies and fielding voluminous inquiries with implications for staffing/budgeting.

Abstract citation ID: igae098.1844

### CHALLENGES AND OPPORTUNITIES BRINGING DIGITAL INNOVATION TO ADULT DAY CENTERS: FEASIBILITY TESTING WITH CAREMOBI

Tina Sadarangani, *New York University, New York, New York, United States*

CareMobi is an mhealth application that allows adult day services centers to exchange information with their clients' family caregivers and health care providers. CareMobi enables adult day center staff, who possesses in-depth knowledge of clients based on their day-to-day observations, to be active members of the care team. The application was developed using a user-centered design thinking approach that prioritized the needs of end-users. Initial prototype testing, which combined qualitative interviews followed by individual completion of the Technology Acceptance Model questionnaire, showed that likelihood of adopting CareMobi was high among adult day center staff (n=31). CareMobi was also perceived as having significant clinical value. We now present the results of preliminary feasibility and acceptability testing with the app itself in a rural Tennessee adult day center (n=9). We also identify factors that support successful implementation of technology within ADCs, including comprehensive support and education, as well as challenges to widespread adoption.

Abstract citation ID: igae098.1845

### GOING MOBILE: ADAPTING LATE-LIFE PSYCHOTHERAPIES FOR MOBILE DEVICES

Felipe Jain[1], Paulina Gutierrez-Ramirez[2], Maria Melero-Dominguez[2], Miranda Zea[2], and Liliana Ramirez Gomez[2], 1. *Depression Clinical and Research Program / Massachusetts General Hospital, Boston, Massachusetts, United States*, 2. *Massachusetts General Hospital, Boston, Massachusetts, United States*

Smartphone psychotherapy applications have the potential for broad scalability, objective treatment compliance monitoring, and passive monitoring of symptom changes (e.g. by leveraging smartphone sensors such as accelerometer). The majority of older adults own and utilize smartphones, but older adults are underrepresented in smartphone research. Moreover, considerations for adapting psychotherapy information for older adults to the smartphones are not well-described. Older adult family caregivers of persons living with dementia (PlD) describe numerous barriers to participation in in-person therapies including travel time and caregiver burden. Mentalizing imagery therapy is a guided imagery and mindfulness approach that has been shown in in-person studies in family PlD caregivers to reduce depression and anxiety, and to strengthen cognitive control (dorsolateral prefrontal) brain connectivity. We present a case study of the adaptation of mentalizing imagery therapy and caregiver skills training for family caregivers of PlD to a mobile application supported by optional virtual groups. We describe user-centered participatory design of the mobile and virtual therapy application including mobile architecture, data storage and security, feature development, collection of passive accelerometer data, embedded chat group, and development timeline. Initial feasibility results are presented from forty older adult family caregivers of PlD, including retention, compliance, system usability, and usage information. These results demonstrate meeting pre-specified feasibility benchmarks and describe important challenges with adaptations of psychotherapy to smartphone. We also demonstrate feasibility of collecting continuous smartphone accelerometer data, and preliminary results from a machine learning data analytic pipeline that achieved >85% accuracy for estimation of weekly mood ratings.

Abstract citation ID: igae098.1846

**FAMILY CAREGIVERS' SELF-DIRECTED ENGAGEMENT WITH EVIDENCE-INFORMED CONTENT OF THE GAMEPLAN4CARE ONLINE PLATFORM**

Alan Stevens[1], Jinmyoung Cho[2], Jordan Reese[1], Gang Han[3], and Marcia Ory[3], *1. Baylor Scott & White Health, Belton, Texas, United States*, *2. Saint Louis University School of Medicine, Ballwin, Missouri, United States*, *3. Texas A&M University, College Station, Texas, United States*

GamePlan4Care (GP4C) translated one of the leading evidence-based dementia caregiving interventions, Resources for Enhancing Alzheimer's Caregiver Health II (REACH II), into an online, interactive platform. Using decision algorithms with integrated online support from Dementia Care Specialists, GP4C includes the full breadth of REACH II education and skill-building materials tailored to the needs of the caregiver. The GP4C online system was provided to 120 family caregivers of persons living with dementia randomized to the GP4C arm of a two-arm clinical trial. The therapeutic content of GP4C included 7 domains: Safety, Stress, Health, Emotions, Care Services, Support, and Behaviors. Ninety-three family caregivers (77.5%) accessed the system. On average, caregivers accessed the platform for approximately 6 hours over 6 months and logged into the system an average of 14 times. Of the seven content domains represented in the system, Safety and Stress were the two most visited domains. Social Support and Health were the two least visited domains by caregivers. After 6-month use of the GP4C online platform system, caregivers reported a significant reduction in caregiver burden (effect size=.50; p<0.001) and a significant increase in the Positive Aspects of Caregiving measure (effect size=.36; p=0.002). Caregivers addressed several usability issues with the system and reflected that the content was more appropriate for those new to the role of dementia care. Modifications to the content and functionality of the GP4C system are ongoing and will be evaluated in future studies.

### SESSION 4135 (SYMPOSIUM)

Abstract citation ID: igae098.1847

**POLICY SERIES: TRENDS IN ELDER ABUSE AND INITIATIVES TO PREVENT ELDER ABUSE IN JAPAN DURING THE PAST 20 YEARS**

Chair: Michelle Putnam

Discussant: Pamela Teaster

This symposium begins by briefly introducing activities of Japan Academy for the Prevention of Elder Abuse, cerebrating 20 years, and features the Japanese Act on the Prevention of Elder Abuse. First, using data collected by the Ministry of Health, Labour, and Welfare (MHLW), research analyses present trends in elder abuse case reports substantiated as "elder abuse", trends in reporters in both institutional and domestic settings, and introduce recent amendments of operational standards made by ministerial ordinance. Second, using data (N=171) on elder abuse from Matsudo-City collected in 2019, analysis examines relationships between trends in the increasing number of elder abuse reports made by the police (about 43% of all cases) and trends in the number of cases regally considered to be "elder abuse" (only 17.8% of those reported to the police were legally considered to be "elder abuse"). It examines possible reasons and propose necessary policy changes in definitions of elder abuse. Third, using longitudinal data collected by Matsudo-city and by the MHLW from 2006 through 2022, this analysis examines the impacts of COVID-19 on the occurrence of elder abuse. Analyses revealed that neither data set showed significant increase in frequencies of occurrence of elder abuse due to COVID-19. Rather, it showed a decrease for the MHLW data. This study examines possible reasons and make recommendations to further decrease frequencies of occurrence of elder abuse cases. Abuse, Neglect and Exploitation of Older Persons Interest Group Sponsored Symposium

Abstract citation ID: igae098.1848

**TRENDS IN NATIONWIDE RESEARCH OUTCOMES ON ELDER ABUSE AND POLICY DEVELOPMENT TO PREVENT ELDER ABUSE IN JAPAN**

Noriko Tsukada[1], Asako Katsumata[2], and Naoki Ikeda[3], *1. Nihon University, Tokyo, Japan*, *2. Japan Lutheran College, Tokyo, Japan*, *3. Kamihonnmachi Sougou Houritsu Jimusyo, Osaka, Japan*

After a brief introduction on history of activities of Japan Academy for the Prevention of Elder Abuse for the past 20 years, this study presents trends in number of elder abuse cases that were reported and substantiated as "elder abuse", and trends in reporters and types of abuse in both institutional and domestic settings by using the nationwide data collected by the Ministry of Health, Labour, and Welfare each year since the Act on the Prevention of Elder Abuse was enacted in 2006. The number of elder abuse cases in 2022 increased 5.3% compared to that in 2021 in domestic settings, while about 17% of increase for institutional settings. And the data in 2022 showed only 7.4% of the municipalities had implemented "leadership trainings" to promote actions and measures to prevent elder abuse in institutions and agencies that provide nursing care services; accordingly, it is not surprising, without leaders, the number of elder abuse cases reported and substantiated as elder abuse increased. Moreover, although there was no amendment of the Act on the Prevention of Elder Abuse, we discuss the most recent policy amendment on "Responses to Elder Abuse and Support for Caregivers in Municipalities and Prefectures", so-called National Manual, which was enacted in April 2023 for the first time in 5 years. This policy change required a collaboration between prefectures and municipalities to develop infrastructure to prevent elder abuse. Finally, we make recommendations for solving issues identified during the past 20 years.

Abstract citation ID: igae098.1849

**THE INFLUENCE OF THE COVID-19 PANDEMIC ON DOMESTIC ELDER ABUSE IN JAPAN**

Tadashi Wada[1], Hitoshi Suda[2], Tamiko Uehara[3], and Kana Sato[4], *1. Hidamari Homecare Clinic, Chiba, Chiba, Japan, 2. Seitoku University, Matsudo-city, Chiba, Japan, 3. Matsudo City Medical Association, Matsudo City, Chiba, Japan, 4. Teikyo University of Science & Technology, Adachi-ku, Tokyo, Japan*

We are very interested in the influence of COVID-19 pandemic on elder abuse, especially its effects on the incidence. We investigated its incidence per population of validated domestic elder abuse cases in Matsudo City, Chiba, Japan, and in all over Japan. The Act of the Prevention of Elder Abuse, Support for Caregivers of Elderly Persons and Other Related Matters was enforced in 2004. We have the statistics of numbers of elder abuse cases validated according to the definition of elder abuse described in the act of each annual year in Matsudo City and Japan from 2004 to 2022. The numbers of validated domestic elder abuse cases per 100,000 people between annual 2004 and 2019 were 81.8 in Matsudo City and 49.8 in Japan. The numbers per 100,000 people between annual 2020 and 2022 were in 84.1 in Matsudo City and 46.4 in Japan. Therefore, the number of domestic elder abuse cases may be slightly increased under the COVID-19 pandemic in Matsudo City, on the contrary, it may be slightly decreased in Japan. There are many reports that the COVID-19 pandemic had negative effects on dynamics of family members and increased domestic abuses, however, we have often experienced to see positive effects of the COVID-19 pandemic on family members in home-care settings. I would like to discuss about the influence of the COVID-19 pandemic on the number of domestic elder abuse cases in Matsudo City and in Japan, and its positive and negative effects on family dynamics at home.

Abstract citation ID: igae098.1850

**REALITIES AND CHALLENGES OF ELDER ABUSE REPORTED TO THE POLICE**

Hitoshi Suda[1], Tadashi Wada[2], Kana Satoh[1], and Tamiko Uehara[3], *1. Seitoku University, Matsudo-city, Chiba, Japan, 2. Hidamari Homecare Clinic, Chiba, Chiba, Japan, 3. Matsudo City Medical Association, Matsudo City, Chiba, Japan*

Nationwide, the number of elder abuse reports made to the police has increased. However, it was unclear if these reports resulted in an increase in cases substantiated as actual "elder abuse" by the police? This study used data from fiscal year 2019 in Matsudo City, analyzing a total of 171 elder abuse cases for this study. Among these, 73 cases were reported by the police, accounting for 42.7% of the total cases, and the perpetrators lived with the victims, indicating children were the most common perpetrators of 35 cases (47.9%), followed by spouse of 31 cases (42.5%), and others of 7 cases (9.6%). Out of the police-reported 73 cases, 60 cases (82.2%) were not substantiated as elder abuse under the Act on Prevention of Elder Abuse, and only 13 cases (17.8%) were substantiated as elder abuse. This outcome led us to the conclusion that the increase in police reports does not necessarily indicate an increase in the cases substantiated as "elder abuse," and highlighted the limited scope of definitions of Elder Abuse in the Act. For example, despite cohabitation, the elder abuse cases where even actual elder violence occurs and requires hospitalization, are not considered as "elder abuse" if there is no caregiving relationship and many of such elder violence cases become to be called "situation-based violence". It is strongly suggested that the definition of elder abuse of cohabitants with no caregiver relationships should be also included as "elder abuse" in accordance with WHO guidelines.

### SESSION 4140 (SYMPOSIUM)

Abstract citation ID: igae098.1851

**VOICES FROM THE MARGINS: EXPLORING INEQUITIES IN WORK AND VOLUNTEERING AMONG OLDER ADULTS**

Chair: Christina Matz
Co-Chair: Byeongju Ryu
Discussant: Julie Miller

All people should have the right to choice and opportunity when it comes to engagement in paid and unpaid forms of work across the life course and particularly in later life. It is often through such roles/aspects of our identity that we derive a sense of meaning and purpose, which is critical to quality of life. We also know, however, that race, gender, birthplace, social and economic class, and additional layers of being "othered" impact the choices and opportunities available, as well as the extent to which positive (or negative) outcomes are derived. Research on paid and unpaid work in later life has not always captured the variety and complexity of lived experiences in later life. Further, statistics often obscure the most important information: how the most marginalized older workers are faring.

This symposium will explore some of these inequities in an effort to inform solutions. The first paper is a scoping review that assesses the extent to which volunteerism among economically marginalized older adults has been examined in studies. The second paper explores how older self-employed workers' finances and health were impacted by COVID-19. The third paper considers gender disparities in wages among older workers in association with occupational sex segregation. The final paper examines the ethnoracial differences in formal and informal volunteering among individuals age 50 and older. A discussant will reflect on these studies and the need for continued research that considers intersectionality in opportunities and experiences for paid and unpaid work in later life. Aging Workforce Interest Group Sponsored Symposium

Abstract citation ID: igae098.1852

### DOCUMENTING THE VULNERABILITIES OF SELF-EMPLOYED OLDER ADULTS: AN EXAMPLE FROM THE PANDEMIC

Cal Halvorsen[1], Christina Matz[2], and Bruna Lopez[2], *1. Washington University in St. Louis, St. Louis, Missouri, United States*, *2. Boston College, Chestnut Hill, Massachusetts, United States*

A defining factor of self-employment in later life is its autonomy and flexibility, enabling older workers to be their own bosses, set their own schedules, and determine where and how they work. Although self-employment has been linked with both positive (higher quality of life and health) and negative (lower take-home pay and workplace-provided benefits) outcomes, little research has considered how self-employed older workers fared during the COVID-19 pandemic. In response, we analyzed data from more than 9,000 Americans ages 50 and older from the 2020 Health and Retirement Study (HRS) and the 2021 HRS Perspectives on the Pandemic mail-in survey. We assessed how work status in 2020 (working for someone else, self-employed, both working for someone else and self-employed, or not working) related to financial difficulties and experiencing health problems due to missed or delayed healthcare outcomes in 2021. While controlling for sociodemographic and health variables, our two multivariable logistic regressions found that self-employment was significantly associated with worse outcomes. Specifically, those who were self-employed or self-employed and working for someone else had significantly higher odds of facing financial difficulties and health problems due to delayed or missed care during the pandemic than those working for someone else only. (Those not working had similar odds of experiencing these issues as those in wage-and-salary work.) This study highlights the vulnerabilities faced by many self-employed older adults while encouraging a discussion on how to better support their health and well-being.

Abstract citation ID: igae098.1853

### ETHNORACIAL DIFFERENCES IN FORMAL AND INFORMAL VOLUNTEERISM AMONG OLDER ADULTS IN THE US: A FOCUS ON ASIAN AMERICANS

Patrick Ho Lam Lai, *Boston College, Chestnut Hill, Massachusetts, United States*

This study questions the assertion that socioeconomic resources are the primary driver behind ethnoracial disparities observed in volunteerism among older adults. Existing literature fails to account for why Asian older adults, with resources comparable to Whites, exhibit lower volunteerism rates. This paper improves upon prior literature by differentiating formal and informal volunteerism and centering Asian American older adults as the reference group to better understand these differences. Utilizing data from the 2019 and 2021 Current Population Survey, bivariate probit models were estimated for formal and informal volunteering. The results indicate that, after accounting for income and education, White individuals have 1.42 times the odds of participating in formal volunteerism compared to Asians, while other ethnoracial groups (Black, Hispanic, American Indian, and Alaskan Native) show 1.09 to 1.31 times the odds of Asians ($p < .05$). Notably, irrespective of volunteerism type, Asians persist as the least likely to volunteer compared to both White individuals and other ethnoracial groups, underscoring the imperative to investigate other factors such as culture. Implications extend to directing future research toward the cultural underpinnings that may elucidate lower volunteerism rates among Asian older adults. The findings advocate for policy revisions recognizing the intricate interplay between SES and cultural factors. Moreover, social work practices and theoretical models must advance to incorporate cultural considerations, acknowledging the diversity within ethnoracial groups. This nuanced approach is vital in fostering a supportive environment for volunteerism among all older adults, with particular attention to the unique circumstances of Asians.

Abstract citation ID: igae098.1854

### VOLUNTEERING AMONG ECONOMICALLY MARGINALIZED OLDER ADULTS IN THE US: A SCOPING REVIEW

Byeongju Ryu[1], Christina Matz[2], Cal Halvorsen[1], and Jihye Baek[1], *1. Washington University in St. Louis, St. Louis, Missouri, United States*, *2. Boston College, Chestnut Hill, Massachusetts, United States*

The 2015 White House Conference on Aging highlighted civic and volunteer activities for healthy aging. While the benefits of volunteering in later life are well-documented, the experiences of economically marginalized older adults are less understood. This study systematically reviewed evidence on volunteering among this group in the U.S., focusing on their factors and outcomes. The study reviewed empirical research published since 2015, obtained from five electronic databases, selecting 11 studies focusing on older volunteers with low income or assets. The selection was based on 1) Samples predominantly aged 50+ or defined as older adults and 2) A focus on volunteering among low-income or asset older people. Reported facilitators included personal values, perceived technology utility and accessibility, linguistic and cultural adaptations in program designs, skill acquisition opportunities, post-retirement work, and living in senior housing. Barriers encompassed low technological and English proficiency, limited resources, and social distancing policies. Outcomes involved enhanced purpose and engagement in life, skill development, expanded social networks and community connectedness, improved self-reported health, and fewer depressive symptoms. This scoping review focuses on an under-researched group: economically marginalized older volunteers—underscoring the need to encourage their volunteerism. Volunteering could improve their health and well-being, potentially reducing dependence on social insurance, boosting the gross domestic product, and strengthening the nonprofit sector. Given the primary focus on formal volunteering and cross-sectional study designs in the reviewed literature, future investigations may examine informal volunteering and utilize longitudinal or experimental methodologies to examine the causal relationships of their volunteering with factors and outcomes.

Abstract citation ID: igae098.1855

### GENDER DISPARITIES IN WAGES AMONG OLDER WORKERS: THE ROLE OF OCCUPATIONAL SEGREGATION AND EDUCATION

Rebekah Carpenter, *Florida State University, Tallahassee, Florida, United States*

Women have a long history of being underpaid relative to men, and many argue that these differences are driven by the concentration of women in jobs that pay less, along with differences in skills and education. Despite increased educational attainment and labor market participation by women over the last 50 years, today, men continue to outearn women. One way to assess structural/contextual factors contributing to gender disparities in pay involves evaluating occupational sex segregation (i.e., the unequal distribution of men and women across different occupations and industries) and the extent to which college education leverages differing levels of financial remuneration among older men and women at the latest phases of their working lives, a sample often excluded from studies on gender pay disparities. Leveraging data from the American Community Survey (2015-2019), I examine the relationship between occupational sex segregation, college degree status, and gender disparities in earnings for a nationally representative sample of 2,101,547 older workers ages 50-70. Findings from regression analyses show that older women earn significantly less than older men across each occupational sex segregation category (i.e., male-dominated, gender-neutral, or female-dominated), regardless of college degree status. Moreover, the greatest disparities in earnings are among college-educated older workers in gender-neutral occupations, where women earn only 70 cents for every one-dollar men earn. Findings from this study highlight the structural/contextual factors shaping the basis of gender pay disparities in later phases of working careers, which has important implications for contextualizing gender differences in retirement preparation and later-life wealth and wellbeing.

### SESSION 4150 (AWARD LECTURE (INVITATION ONLY))

Abstract citation ID: igae098.1856

### DONALD P. KENT AND ROBERT W. KLEEMEIER AWARD LECTURES

Chair: Judith Howe

The Donald P. Kent Award lecture will feature an address by the 2023 Kent Award recipient Kathleen Wilber, PhD, FGSA, FAGHE of the University of Southern California. The Robert W. Kleemeier Award lecture will feature an address by the 2023 Kleemeier Award recipient David M. Almeida, PhD, FGSA of Pennsylvania State University. The Kent Award is given annually to a member of The Gerontological Society of America who best exemplifies the highest standards of professional leadership in gerontology through teaching, service, and interpretation of gerontology to the larger society. The Kleemeier Award is given annually to a member of The Gerontological Society of America in recognition for outstanding research in the field of gerontology.

Abstract citation ID: igae098.1857

### LOOSE COUPLING? A CAREGIVER, A GUARDIAN, AND AN ABUSER JUMP FROM A PLANE WITH ONE PARACHUTE: WHO SURVIVES?

Kathleen Wilber, *University of Southern California, Los Angeles, California, United States*

My career in aging has given me the privilege of working directly with older people, training students in gerontology, partnering with remarkable leaders, and conducting studies on key questions needing evidence-informed answers. Over the half century that I have been doing applied research, healthy aging and longevity have emerged as fundamental achievements and key cultural and political priorities. In this lecture, I discuss intervention evidence and the need for research intersectionality in interventions that support family caregivers; preventing and addressing elder mistreatment; long term services and supports; and the role of supportive age-friendly communities in healthy aging. The lecture emphasizes underlying values including balancing doing too much and doing too little for older adults who need care, including those living with dementia; promoting person and family centered approaches; and the important role of diversity, equity, and inclusion in advancing innovative interventions that promote high quality long lives for all.

Abstract citation ID: igae098.1858

### THE NATIONAL STUDY OF DAILY EXPERIENCES: CHARTING QUOTIDIAN LIFE OF ACROSS ADULTHOOD

David Almeida, *Pennsylvania State University, University Park, Pennsylvania, United States*

This presentation delves into the intricate tapestry of daily experiences across the adult lifespan, drawing insights from the MIDUS daily project of a US national sample of 3,509 individuals. Through analysis of over 42,000 daily telephone interviews collected across 20 years, we investigate the nuances of quotidian life, uncovering patterns, and determinants that shape individuals' daily routines and emotional landscapes. Leveraging a diverse sample spanning different age groups, socio-economic backgrounds, and geographic locations, we illuminate the multifaceted nature of daily experiences. Findings reveal dynamic trajectories of well-being, stress, and activities, shedding light on how these dimensions fluctuate with age and life circumstances. Furthermore, we explore the influence of contextual factors such as work, family, and social interactions on daily experiences, unraveling their complex interplay with individual characteristics. This research contributes to a deeper understanding of the lived realities of adults, offering implications for enhancing daily well-being and quality of life.

### SESSION 4160 (SYMPOSIUM)

Abstract citation ID: igae098.1859

### ADVANCING PERSON-CENTERED CARE PLANNING FOR PERSONS WITH MULTIPLE CHRONIC CONDITIONS

Chair: Ana Quiñones
Co-Chair: David Dorr
Discussant: Arlene Bierman

Providing high quality, comprehensive, longitudinal, person-centered care for persons living with, or at risk for, multiple chronic conditions (MCC) is a critical challenge facing our healthcare system. Persons with MCC often navigate complicated and fragmented healthcare systems that can be inefficient, duplicative, costly, and poorly coordinated which increases risk for avoidable adverse events, and unduly burdens persons with MCC, families, and caregivers with the added responsibility of sharing information and coordinating care across providers. Person-centered care planning (PCCP) is a process of active collaboration and shared decision-making among providers, persons seeking care, families, and caregivers to develop a, person-centered management plan, incorporating patient values, goals, and preferences. This symposium will share preliminary findings of a collaborative, multi-disciplinary AHRQ-funded initiative which aims to examine the current state of the field and convene frontline providers, health care leaders, and policy makers to identify solutions including the necessary resources, conditions, processes, and implementation strategies to address barriers to delivering appropriate, equitable, and high-quality PCCP as a routine component of care delivery. The first presentation will summarize the state of the field regarding PCCP models for persons with MCC identified through environmental scans. The second will discuss barriers, facilitators, and strategies identified during the Partner Roundtable discussions to improve PCCP design and implementation within health care systems. The final presentation will share successes and failures that health professionals and front-line providers have experienced implementing PCCP, as well as best practices and strategies identified during the Learning Collaborative to deliver high-quality PCCP for persons with MCCs.

Abstract citation ID: igae098.1860

### PERSON-CENTERED CARE PLANNING FOR PERSONS WITH MCC: LESSONS FROM AN ENVIRONMENTAL SCAN

Annette Totten, Chandler Atchison, Cynthia Davis-O'Reilly, Katherine D. Peak, Tamar Wyte-Lake, Deborah Cohen, and Ana Quiñones, *Oregon Health & Science University, Portland, Oregon, United States*

To guide overall project objectives of describing the current state of the field of person-centered care planning (PCCP) for persons with MCC, the environmental scan functions as a multi-source overview of the field and integrates input from key informant interviews (KII) of experts from diverse perspectives. The objective is to clarify our understanding of which PCCP models have been implemented in the US and which show promise for improving outcomes for patients, their caregivers, and care teams. This task involves conducting one baseline, two ad hoc scans, and 15key informant interviews. We incorporate input from a technical expert panel to inform our literature search and augment synthesis with KIIs. KIIs provide greater context on the adoption and use of PCCP models in clinical settings, and features that may be important from a patient, clinic, or policy standpoint. KII analysis follows a five-step process: Describing, Organizing, Connecting, Corroborating/legitimating, Representing the account. The methods for the environmental scan are based on systematic review methods, but are modified to fit our rapid review timeline. Modifications include: limiting citation database searches and prioritizing MEDLINE first; prioritizing triage of abstracts and using one reviewer; focusing data abstraction on project partner priorities; foregoing risk of bias, strength of evidence, and quantitative synthesis in favor of targeted summaries. Analysis focuses on understanding successful components of and barriers to PCCP model implementation, and to identify what is required for successful implementation and sustainability.

Abstract citation ID: igae098.1861

### PERSON-CENTERED CARE PLANNING FOR PERSONS WITH MCC: LESSONS FROM PARTNER ROUNDTABLE DISCUSSIONS

Anne King[1], Sarina Schrager[2], Christie Jackson[3], LeAnn Michaels[3], and David Dorr[3], *1. Comagine Health, Portland, Oregon, United States*, *2. University of Wisconsin Madison, Madison, Wisconsin, United States*, *3. Oregon Health & Science University, Portland, Oregon, United States*

The Partner Roundtable utilizes successive discussions with policymakers and health care leaders to identify barriers, facilitators, and strategies to improve person-centered care planning (PCCP) design and implementation in health systems, including primary and specialty care. The objectives are to: (1) identify organizational, policy, payment, technology, cost/resource, research, or other requirements for development and implementation of PCCP models across diverse health systems and settings, highlighting specific requirements, for persons living with, or at risk for, MCC; (2) identify challenges to implementation of PCCP; (3) recommend proven, potential, or innovative solutions or facilitators for implementation challenges; and (4) assist in dissemination of project findings. The Roundtable includes a diverse group of 25-30 individuals with influential leadership roles in making, shaping, and informing decisions related to PCCP implementation. Participants are decision-makers from health systems; local, state and federal health policy; health information technology; quality improvement and quality measurement; research; and patient associations or care recipients including family members or caregivers. Roundtable participants are representative of U.S. healthcare, including geographic spread, community health center leadership, payers, clinicians, and implementation and evaluation of innovative care models. Federal partners focused on improving care delivery for this population including care planning are invited participants. Roundtable expertise and recommendations, including environmental scan information, will inform future recommendations for promising models of PCCP.

Abstract citation ID: igae098.1862

### PERSON-CENTERED CARE PLANNING FOR PERSONS WITH MCC: LESSONS FROM LEARNING COLLABORATIVE DISCUSSIONS

Lyndsey Miller[1], Emilia DeMarchis[2], LeAnn Michaels[1], Christie Jaskson[1], and David Dorr[1], *1. Oregon Health & Science University, Portland, Oregon, United States*, *2. University of California San Francisco, San Francisco, California, United States*

The Learning Collaborative invites person-centered care planning (PCCP) innovators, implementation experts, and front-line providers to share and discuss successes and challenges they have experienced in PCCP with the goal of generating a framework for increasing the uptake of and scaling PCCP as an integral component of routine practice. The Learning Collaborative reviews promising models of PCCP and describes feasible solutions to common implementation barriers. Participants engage in deliberative dialogue based on the collective experiences of the group to generate new knowledge and identify best practices and strategies to deliver high-quality PCCP for persons with MCCs. This task involves a series of 5 Learning Collaborative sessions to review and discuss case studies of PCCP. The Learning Collaborative includes 25-30 participants affiliated with partner Practice-Based Research Networks (PBRNs), recommended from the Technical Expert Panel, and identified by AHRQ. Learning Collaborative participants include individuals with front-line experience implementing PCCP; health system or practice clinicians preparing to implement PCCP; and implementation science researchers with experience implementing PCCP. Gathering all these partners will permit shared learning to accelerate progress, iterative, collective discussions to develop a framework for successful implementation of PCCP for people living with or at risk for MCC. The synthesis of learnings and reporting from the Learning Collaborative will provide key information for change agents, such as policymakers, clinical teams, and health care system leaders wishing to implement and research PCCP.

### SESSION 4165 (SYMPOSIUM)

Abstract citation ID: igae098.1863

### BEST PRACTICES AND DATA EXEMPLARS IN DESIGN AND EVALUATION OF EARLY-PHASE DYADIC INTERVENTIONS

Chair: Karen Lyons
Co-Chair: Joan Monin
Discussant: Steven Zarit

Despite growing numbers of early-phase dyadic interventions, few move beyond early-phase and often lack intentional plans for development and evaluation to optimize success. This session includes four data-based papers that will discuss essential elements of design and evaluation of early-phase dyadic interventions with older adults and their care partners in both cognitive impairment and chronic illness contexts. First, Dr. Joan Monin and colleagues will describe the advantages of, and phases involved in, using an Appreciative Inquiry Workshop approach to develop an attachment-based communication intervention with dyads living with dementia. Second, Dr. Kalisha Bonds Johnson and colleagues will present phase I data from a study to develop and test a formal care decision-making intervention for African-American parent-adult child dyads living with dementia. The paper will discuss the importance of intentional community engagement, use of theory, and type of dyad (e.g., mother-daughter) when designing dyadic interventions. Third, Dr. Michael McCarthy and colleagues will use data from several studies to exemplify the advantages of mixed-methods designs in developing dyadic interventions, particularly in rural samples (e.g., strengthening participant engagement, maximizing resources, surfacing new questions). Finally, Dr. Karen Lyons and colleagues will present two approaches to evaluating dyadic benefits of interventions in small samples, the importance of assessing pre-test risk status within care dyads, and the need to assess relational impact of behavioral interventions. Speakers will share best-practices in developing, designing and evaluating early-phase dyadic interventions followed by a discussion of key take-homes by Dr. Steve Zarit.

Abstract citation ID: igae098.1864

### APPRECIATIVE INQUIRY WORKSHOP TO DEVELOP A DYADIC INTERVENTION FOR PARENTS AND ADULT CHILDREN COPING WITH DEMENTIA

Joan Monin[1], Heidi Gil[2], Jenna Weiss[2], Ellen Sue Moses[2], and Lucy Rosenblatt[2], *1. Yale School of Public Health, New Haven, Connecticut, United States*, *2. LiveWell Institute, Southington, Connecticut, United States*

Including the voices of people with lived experience should be a requisite step in designing dyadic intervention studies to ensure that study design and measures are acceptable, feasible, and suitable. I will discuss my experience as a researcher participating in a LiveWell Institute Appreciative Inquiry Workshop with the Empowering Partnership Network (EPN), a diverse group of partners that connect and influence dementia research and innovation, advocacy and awareness, and the design and development of systems and spaces intended for people living with dementia. The EPN consists of people living with dementia, care partners, and other partners (researchers, policy makers, practitioners). The goal of the workshop was to begin to develop an attachment-based communication intervention for adult children and their parents coping with dementia. First, we took time to meet each other and identify each other's strengths. Second, we set guiding principles to help each other be at our best (e.g. not speaking on anyone's behalf; giving enough time to speak). Third, in the discovery phase, I shared why this work is important and what we know about the science of Attachment theory, dementia, and parent-child relationships. I described my Stage 0 findings and provided examples of other evidenced-based interventions on which we could build. Fourth, in the dream phase, the group shared their hopes about the ideal intervention. Fifth, in the design phase, the group suggested ways of recruiting, what the materials were and what the experience should be. Finally, in the destiny phase, we established the next action steps.

Abstract citation ID: igae098.1865

### DEVELOPING A CULTURALLY TAILORED INTERVENTION FOR AFRICAN AMERICAN PARENT–ADULT DAUGHTER DEMENTIA DYADS

Kalisha Bonds Johnson[1], Karen Lyons[2], Joan Monin[3], Gaea Daniel[1], Wizdom Powell[4], and Kenneth Hepburn[1], *1. Emory University, Atlanta, Georgia, United States*, *2. Boston College, Chestnut Hill, Massachusetts, United States*, *3. Yale School of Public Health, New Haven, Connecticut, United States*, *4. Headspace Health, Santa Monica, California, United States*

African American older adults are suggested to be two times more likely to develop Alzheimer's disease and related dementias (ADRD) compared to non-Hispanic White older adults. Thus, there is a need for more African American care partners. The largest group of care partners of African American persons living with ADRD, African American adult daughters, must navigate formal care decision making through the lens of race, gender, culture, and associated health disparities. Formal care—paid health care services for older adults usually associated with an institution— is important in the treatment of ADRD and maintenance of quality of life. Guided by the NIA Health Disparities Research Framework, Black Family Social-Ecological Context Model, and Superwoman Schema (SWS), this presentation will describe the first aim of this explanatory sequential design of a culturally tailored prototype intervention to support the formal care decision-making process for African American parent-adult daughter dementia dyads. Results from the preliminary analysis of 20 dyads highlight most are mother-daughter dyads (90%) with parents, on average, experiencing moderate cognitive impairment. Both parents and adult daughters report "good" health, "some" difficulty with family listening to their opinions, moderate to high decision self-efficacy, and moderate quality of life. Daughters report low SWS characteristics but high cultural justification for caregiving. Using these results, we will showcase the next steps of data collection, how theory guided our study, the intentionality of relationships status, and the role of community engagement as important considerations for dyadic prototype intervention development.

Abstract citation ID: igae098.1866

### MIXED-METHODS RESEARCH DESIGNS FOR EARLY-PHASE DYADIC HEALTH RESEARCH

Michael McCarthy[1], Eric Cerino[1], Megan McCoy[2], and Margarita Martinez[2], *1. Northern Arizona University, Flagstaff, Arizona, United States*, *2. Northern Arizona University, Northern Arizona University, Arizona, United States*

Individuals and family caregivers living in rural areas face a double bind when it comes to health. First, they may be at increased risk for chronic health conditions. Second, they are frequently hampered in seeking support for these conditions due to factors including geographic isolation, cultural barriers, and lack of financial resources. These inequities result in delayed diagnosis and treatment and poor health outcomes. Researchers seeking to address these issues are often faced with recruitment challenges in small rural communities and subsequently modest sample sizes. Mixed-methods designs offer advantages for early-phase dyadic studies including strengthening participant engagement, maximizing resources, surfacing new questions to be explored in future research, and in general, providing a deeper understanding of the phenomenon being studied. In this presentation, we draw on several of our mixed-methods early-phase dyadic studies in stroke, dementia, and cognitive health to provide examples. We identify mixed-methods designs that deepen understanding in early-phase dyadic health research and the advantages of certain mixed-methods designs for certain inquiries. Finally, we discuss recommendations for maximizing the approach, and strategies for blending analytical methods.

Abstract citation ID: igae098.1867

### INTENTIONAL EVALUATION OF DYADIC BENEFITS OF EARLY-PHASE INTERVENTIONS: ROLES OF PRETEST RISK AND RELATIONAL IMPACT

Karen Lyons[1], Luke Russell[2], Kalisha Bonds Johnson[3], Glenna Brewster[3], and Lyndsey Miller[4], *1. Boston College, Chestnut Hill, Massachusetts, United States*, *2. Illinois State University, Normal, Illinois, United States*, *3. Emory University, Atlanta, Georgia, United States*, *4. Oregon Health & Science University, Portland, Oregon, United States*

The evaluation of early-phase (i.e., Stage I, NIH Stage Model), small sample dyadic interventions is still primarily focused on testing separate effects for each member of the older adult-care partner dyad, rather than the benefits for dyads as a whole. Moreover, there is little attention to assessing pre-test risk status within dyads (especially in quasi-experimental or single-arm designs) or the relational impact of interventions. We first examined pre-test risk status across several outcomes (e.g., depressive symptoms, self-efficacy, communication) in 39 dyads and found 37-68% of older adults with chronic illness were at-risk at baseline; 40-79% of care partners; and 15-53% of dyads. Second, we examined dyadic benefits of a health behavior intervention, using two approaches, and found above zero approaches may deflate dyadic benefits compared to pre-test risk approaches. We used a pattern analysis to examine benefits across outcomes and within dyads and found 40% of dyads who received the intervention had expected positive outcomes for depressive symptoms and self-efficacy; 20% had expected positive scores for depressive symptoms, self-efficacy and aerobic exercise; and 13% had expected positive scores for all five target outcomes. Dyads where both members had highest pre-test risk level did not experience expected dyadic benefits. Finally, although we did not find strong evidence the intervention improved communication compared to a wait-list condition, there was no evidence it resulted in worse communication. Implications of using each approach and selecting different benchmarks for defining success are discussed. Results provide rationale for more intentional evaluation of small-sample, early-phase dyadic interventions.

### SESSION 4170 (BIOLOGICAL SCIENCES INVITED SYMPOSIUM)

Abstract citation ID: igae098.1868

### ESPO AND BIOLOGICAL SCIENCES SECTION SYMPOSIUM: METABOLIC REGULATION OF AGING

Chair: Bhaswati Ghosh

We are excited to announce that the 2024 GSA Annual Scientific Meeting will hold the Emerging Scholar and Professional Organization (ESPO) section of BioSci on November 16, 2024, 12:00 pm – 1:30pm (Room 4C-3). This session continues to be the highlight of the program, recognizing the great work done by the graduate students in the field of biology of aging. The BioSci ESPO section will comprise four talks covering diversifying topics including proteostasis, mitochondrial bioenergetics, lipidomics and transcriptional changes during aging. We will hear from Ziying Xu from Barshop Institute Department of Medicine talking about "Sex-Specific Alterations in Circulating Lipidome During Mouse Aging"; Sidharth Madhavan at Buck institute for research on aging about "Beta-Hydroxybutyrate Is a Metabolic Regulator of Proteostasis in the Aging Brain"; Stephanie Heimier from UC San Diego will give us insights on "Serum Factors Drive Differences in Mitochondrial Bioenergetics Associated with Human Aging"; and Ruben De Man from Yale University school of medicine will present on "Single-Cell Analysis of Somatic Mutations in Human Lung Reveals Association with Transcriptional Changes in Aging". The BioSci ESPO session at the GSA Annual Scientific Meeting is designed to showcase the academic excellence of great researchers in the field of aging. We are excited to see you all in Seattle!

Abstract citation ID: igae098.1869

### SEX-SPECIFIC ALTERATIONS IN CIRCULATING LIPIDOME DURING MOUSE AGING

Ziying Xu, Anindita Bhattacharjee, and Xianlin Han, *Barshop Institute, San Antonio, Texas, United States*

Lipids serve as pivotal elements in biological systems, fulfilling diverse roles ranging from cellular structure components to signaling molecules and energy reserves. They encompass a variety of classes including structural membrane lipids (e.g., cholesterol, phosphatidylcholine, phosphatidylethanolamine), energy-storing lipids (e.g., triglycerides, fatty acids), and bioactive lipids (e.g., lyso-phospholipids, ceramides). Increasing evidence shows associations between lipids and age-related diseases (e.g., atherosclerosis, cancer, diabetes, and neurodegenerative diseases), indicating lipid metabolism is involved with health and longevity. In this study, we applied multi-dimensional mass spectrometry-based shotgun lipidomics to quantitatively measure the plasma lipidome alterations across different ages in C57BL/6 mice. We systematically collected plasma samples from both sexes across 7 adult-to-old-age timepoints (8 to 32 months, in 4-month increments, with 8-10 mice per sex at each age). Our data revealed a sex-dependent regulation within the circulating lipidome in which males demonstrated higher concentrations across nearly all lipid classes at each timepoint compared to females. Notably, with advanced ages, males exhibited a decline in major phospholipids and an accumulation of cholesteryl ester—suggestive of an age-related shift in lipoprotein composition. An age-associated increase in phosphatidylserine, previously linked to apoptotic signaling, was exclusive to males. Conversely, females showed a consistent plasma lipid profile throughout their lifespan. The only shared change between sexes is plasmalogen decreasing with age, which could imply increasing oxidative stress. Our findings illuminate sex-specific regulatory mechanisms in lipid metabolism during aging in mice, with potential implications for understanding sex differences in the aging process.

Abstract citation ID: igae098.1870

### BETA-HYDROXYBUTYRATE IS A METABOLIC REGULATOR OF PROTEOSTASIS IN THE AGING BRAIN

Sidharth Madhavan, Stephanie Roa Diaz, Mitsunori Nomura, Christina King, Thelma Garcia, Birgit Schilling, Asish Chaudhuri, and John Newman, *Buck Institute for Research on Aging, Novato, California, United States*

Loss of proteostasis is a hallmark of aging and Alzheimer disease (AD). Here, we identify beta-hydroxybutyrate (BHB), a ketone body, as a molecular chaperone which regulates protein solubility in the brain. BHB is a small molecule metabolite which primarily provides an oxidative substrate for ATP during hypoglycemic conditions, and also regulates other cellular processes through covalent and noncovalent protein interactions. We demonstrate BHB-chaperone activity across in vitro, ex vivo, and in vivo mouse systems. This activity is shared by select structurally similar metabolites, is not dependent on covalent protein modification, pH, or solute load, and is observable in mouse brain in vivo after delivery of a ketone ester. Furthermore, this phenotype is selective for pathological proteins such as amyloid-beta (Ab), and exogenous BHB ameliorates pathology in nematode models of Ab aggregation toxicity. We have generated a comprehensive atlas of the BHB-induced protein insolublome ex vivo and in vivo using mass spectrometry proteomics, and have identified common protein domains within BHB target sequences. Finally, we show enrichment of neurodegeneration-related proteins among BHB targets and the clearance of these targets from mouse brain, likely via BHB-induced autophagy. Overall, these data indicate a new metabolically regulated mechanism of proteostasis relevant to aging and AD.

Abstract citation ID: igae098.1871

### SERUM FACTORS DRIVE DIFFERENCES IN MITOCHONDRIAL BIOENERGETICS ASSOCIATED WITH HUMAN AGING

Stephanie Heimler[1], Brian Chen[1], Toshiko Tanaka[2], Ann Zenobia Moore[2], Jaclyn Bergstrom[3], Luigi Ferrucci[2], and Anthony Molina[1], *1. University of California San Diego, La Jolla, California, United States*, *2. National Institute on Aging, Baltimore, Maryland, United States*, *3. UC San Diego, La Jolla, California, United States*

Heterochronic parabiosis studies in mice have demonstrated that circulating factors in blood drive multiple features of aging, including mitochondrial bioenergetic decline. To determine if circulating factors in human blood can drive age-related bioenergetic decline, we treated fibroblasts from younger (33y.o.) and older (72y.o.) adult donors with diluted serum samples representing the adult human life-course (22-92y.o). The GESTALT (Genetic and Epigenetic Signatures of Translational Aging Laboratory Testing) study provided serum samples from 88 healthy participants free from major chronic conditions. We show that the maximal bioenergetic capacity of both younger and older fibroblasts treated with serum for 24 hours negatively correlates with the chronological age of the serum donor (R=-0.28, P=0.01; R=-0.25, P=0.02, respectively). To test the hypothesis that the bioenergetic effects of serum are more closely associated with biological age than chronological age, we used DNA methylation data to calculate first and second generation epigenetic clocks (Horvath, Hannum, PhenoAge, and GrimAge). Our results indicate that the bioenergetic effects of serum are most closely related to second generation clocks, which are trained to capture age-related phenotypes and mortality risk. For example, the effects of serum on fibroblasts derived from younger and older individuals were most closely related to GrimAge (R=-0.36, P< 0.01; R=-0.31, P< 0.01, respectively). These results suggest that the bioenergetic effects of circulating factors may explain the heterogeneity of biological aging among participants of the same chronological age. Ongoing studies are pairing our high-throughput respirometric analyses with multi-omic data to identify molecules that directly impact mitochondrial bioenergetic decline with advancing age.

Abstract citation ID: igae098.1872

### SINGLE-CELL ANALYSIS OF SOMATIC MUTATIONS IN HUMAN LUNG REVEALS ASSOCIATION WITH TRANSCRIPTIONAL CHANGES IN AGING

Ruben De Man[1], Taylor Adams[1], John McDonough[2], Juan Cala-Garcia[3], Benjamin Moss[3], Xiting Yan[1], Ivan Rosas[3], and Naftali Kaminski[1], *1. Yale University School of Medicine, New Haven, Connecticut, United States*, *2. McMaster University, Hamilton, Ontario, Canada*, *3. Baylor College of Medicine, Houston, Texas, United States*

Age is a major risk factor for lung disease. Accumulation of somatic mutations has been implicated in both aging and cellular senescence. We analyzed somatic mutations called from single-cell RNAseq (scRNAseq) data. scRNAseq was performed on lung parenchyma samples from healthy donors (32 samples, 11-72 years, 21M/11F). Following standard pre-processing and cell type annotation, mutations were called using SComatic with the default parameters. Mutation burden was calculated for each cell type-sample combination by dividing the number of mutations by the number of callable sites. Our resulting scRNAseq dataset consisted of 199,400 cells, comprising 25 distinct cell types. Mutation burden was highest in alveolar type 1 (AT1) cells (93.6 mutations/MB), alveolar macrophages (79.1), and general capillary (gCap) cells (62.1). Mutation burden was positively correlated with age ($r=0.28$, $p< 0.001$). Globally, the top genes correlated with mutation burden included ubiquitin ligase genes (AMBRA1, ANAPC1, SEL1L, USP25, USP33) and DNA damage response genes (RAD50, PRKDC). Notably, mutation burden also correlated with expression of senescence marker CDKN2A ($r=0.48$, $p< 0.05$). In AT1 cells, mutation burden correlated with decreased expression of cell marker genes such as AGER ($r=-0.64$, $p< 0.05$) and HOPX ($r=-0.83$, $p< 0.05$). Similarly, gCap cells exhibited decreased expression of marker genes Il7R ($r=-0.54$) and VIPR1 ($r=-0.64$), while MAPK/ERK signaling genes were increased ($p < 0.05$). These genes were also significantly correlated with age in the same direction ($p < 0.05$). These results suggest that somatic mutation accumulation may contribute to age-associated transcriptional changes and loss of cell function, with cell types of the alveoli and endothelium experiencing the greatest effects.

### SESSION 4175 (SYMPOSIUM)

Abstract citation ID: igae098.1873

### COMMUNITY-ENGAGED AGING RESEARCH IN GHANA

Chair: Tiffany Washington
Discussant: Fayron Epps

Sub-Saharan Africa (SSA) countries have the fastest growing rates of older adults than other countries across the globe. In SSA countries, adults aged 60 and older will reach 161 million by 2050. Because SSA countries have underdeveloped economies, older adults in this region are at greater risk for a myriad of social, economic, and health conditions in comparison to their developed counterparts. For example, estimates suggest that the prevalence of dementia in the SSA region may be considerably higher than previously assumed, given the scarcity of comprehensive epidemiological data and the rapidly aging population. Addressing this knowledge gap is imperative to develop effective strategies for early detection, intervention, and support for affected individuals and their families. Therefore, the goal of this symposium is to describe efforts to better understand the experience of older adults in Ghana through community-university partnerships. Speaker one will discuss the development and implementation of a culturally adapted dementia education intervention in Ghana. Speaker two will describe nursing students' experiences caring for older adults in acute care settings in Ghana. Speaker three will present barriers and facilitators to global community engaged dementia research using a collaboration between U.S.-based university and Ghanaian community partners as a case example. The discussant will outline steps forward in community-engaged aging research in Ghana.

Abstract citation ID: igae098.1874

### AN INTERNATIONAL COLLABORATION TO SUPPORT AGING RESEARCH IN GHANA

Tiffany Washington[1], Dennis Bortey[2], Fayron Epps[3], Janelle Gore[4], and Venance Dey[2], *1. University of Georgia, Knoxville, Tennessee, United States*, *2. Alzheimer's Ghana, Tema, Greater Accra, Ghana*, *3. The University of Texas Health San Antonio, San Antonio, Texas, United States*, *4. University of Alabama, Alabama, United States*

Nearly 3 million people live with dementia in sub-Saharan Africa (SSA). However, ineffective methods for increasing detection and raising awareness contribute to stigma about dementia. By leveraging the resources of universities in developed countries, global community engaged research can increase knowledge and education about dementia in SSA. Using a collaboration between Ghanian community partners and two research-intensive universities in the U.S. as a case example, this paper offers guidance on conducting community-engaged aging research in Ghana. The Alter Program is a dementia education initiative to increase dementia-friendly spaces in faith communities. We used thematic analysis of field notes to identify key components of building international partnerships centered on dementia education. Five were identified: ethics review, relationship-building with community partners, identifying community needs, cultural adaptations, and capacity building. Future research can leverage these aspects of community-universities partnerships to increase dementia knowledge and attitudes in SSA countries.

Abstract citation ID: igae098.1875

### THE VALUE OF COMMUNITY–UNIVERSITY PARTNERSHIPS IN GHANA: IMPLEMENTING CULTURALLY RESPONSIVE DEMENTIA EDUCATION

Janelle Gore[1], and Fayron Epps[2], *1. University of Alabama, Alabama, United States*, *2. The University of Texas Health San Antonio, San Antonio, Texas, United States*

Alzheimer's disease and related dementias have not been given much attention in sub-Saharan Africa. Although the prevalence of dementia in Ghana is not yet established, those who present cognitive impairment are often stigmatized, discriminated against, or physically harmed by community members. The mistreatment may be due to a lack of dementia knowledge and awareness in Ghana. Recognizing the critical need to increase dementia knowledge and awareness in Ghana, Alzheimer's Ghana (Ghana), AlterDementia (US), and researchers from the University of Georgia and Emory University (US) partnered to develop and implement a culturally adapted and relevant dementia education intervention. Through a series of collaborative sessions, both organizations and researchers co-created educational materials and intervention strategies addressing dementia awareness and knowledge. Education sessions consisted of a dementia overview, brain health overview, community testimonies, local resources, question and answer session, and closing remarks. Community-based participatory research principles were used to engage public officials, faith-based organizations, television personalities, healthcare professionals, secondary school educators, and researchers in Ghana to facilitate nine dementia education sessions, spanning 15-90 minutes, to over 800 participants across four regions of Ghana. Surveys and field notes were used to collect demographic and dementia knowledge and awareness data. Preliminary findings suggest promising avenues for improving dementia knowledge and reducing stigma. This work underscores the value of community-university partnerships to culturally adapt evidence-based interventions to advance ongoing community initiatives addressing dementia.

Abstract citation ID: igae098.1876

### GHANAIAN NURSING STUDENTS' EXPERIENCES IN CARING FOR OLDER ADULTS IN ACUTE CARE SETTINGS

Diana Abudu-Birresborn[1], Martine Puts[2], Lynn McCleary[1], Charlene Chu[2], Vida Yakong[3], and Lisa Cranley[2], *1. Brock University, St. Catharines, Ontario, Canada*, *2. University of Toronto, Toronto, Ontario, Canada*, *3. University for Development Studies, Tamale, Northern, Ghana*

Evidence suggests that nursing students' experiences in acute care settings may negatively impact their views on caring for older adults, resulting in poor interest and quality of care. However, Ghanaian nursing students' experiences caring for older adults are unknown. We aimed to explore students' experiences in caring for older adults to help develop and improve strategies, that promote students' positive experiences and promote quality of care for older adults. We employed a descriptive qualitative approach and interviewed 17 second and third-year nursing students since they had clinical exposure. Interviews were conducted face-to-face and via telephone from November 2020- December 2020 and lasted 45-60 minutes. Interviews were audio recorded, transcribed, coded, and sorted with Nvivo. Data were analyzed and interpreted, themes were generated using critical thematic analysis and contextual meanings students ascribed to their responses. Two broad themes were generated. 1) experiences in caring for older adults and 2) inadequate staff and resources. Students had challenging experiences caring for older adults who were dependent and unable to manage their activities of daily living. They described caring for older adults as physically and emotionally demanding. They noted that limited staff and resources constrained caring for older adults which frustrated them when providing care. However, they expressed caring for older adults as a positive learning experience as they perceive them as a source of wisdom and blessings. Nurse leaders and administrators can help shape students' experiences caring for older adults by ensuring adequate staff and resources and supporting students' clinical learning.

### SESSION 4180 (SYMPOSIUM)

Abstract citation ID: igae098.1877

### DAILY EXPERIENCES OF SOCIAL CONNECTION: REAL-WORLD ASSESSMENT STRATEGIES TO UNDERSTAND MECHANISMS

Chair: Kimberly Van Orden
Discussant: Nancy Donovan

Social connection is multifaceted construct that encompasses structural, functional, and subjective (quality) dimensions of relationships, all of which are associated prospectively with health, functioning, well-being, and longevity. Despite clear evidence linking social connection to health in later life, there are no clear evidence-based interventions and available community programs are under-utilized. One barrier to effectively intervening is the inability to tailor programs to person-specific contributors to disconnection. This symposium will describe findings from studies of social connection in later life that use innovative methods to examine person-specific and proximal causes, contributors, and consequences of social connection, including passive sensing via smartphones, analysis of social behavior (including speech patterns), and ecological momentary assessment. These methods allow for frequent measurement of indices of social connection in real-time (reducing recall bias) and in real-world contexts. Dr. Lee will present on the assessment of loneliness and social isolation among older adults using unstructured speech data in comparison to research surveys. Ms. Zhou will present on associations between sleep, social encounters, and momentary loneliness in older adults' everyday life. Ms. VanBogart will present on associations between momentary loneliness and indices of stress (diurnal cortisol). Dr. Bower will present on momentary experiences of belonging in a sample of older adults with subjective cognitive decline. Dr. Van Orden will describe procedures and design considerations for EMA and passive sensing indices of social connection as outcomes in clinical trials to promote social connection. Dr. Donovan will facilitate a discussion to integrate findings and identify opportunities for future research.

Abstract citation ID: igae098.1878

### ASSESSING CHANGE IN SOCIAL CONNECTION: DESIGN CONSIDERATIONS FOR CLINICAL TRIALS WITH OLDER ADULTS

Kimberly Van Orden, *University of Rochester Medical Center, Rochester, New York, United States*

Social disconnection is associated with suicide ideation, attempts, and deaths in later life and is present in 70-90% of suicide deaths in older adults. However, it is not known if promoting social connection is an effective strategy to prevent suicide in later life because studies have not directly tested this mechanism. This presentation will describe the rationale and design of an ongoing clinical trial to test a brief behavioral intervention to increase social connection and thereby reduce suicide risk in older adults in senior living communities who endorse significant loneliness and recent suicide ideation. All study outcomes are measured via smartphone: two subjective indices of social connection are measured via EMA self-report (loneliness and satisfaction with social activities) as well as two objective indices of social connection (time spent outside the home [self-report via EMA, supplemented with GPS sensing] and quantity of conversations [ambient audio sensing]. Suicide ideation is assessed daily via EMA. Findings from baseline smartphone assessments will be presented, including compliance to the protocol. Currently, 5 subjects have completed the 10-day smartphone assessment protocol (2 males, 3 females), with an average age of 88 (range 77-96 years). Few studies with older adults use this methodology for outcomes assessment and no studies have used smartphone assessments to collect outcomes data for behavioral interventions for social connection, despite potential benefits of assessing social connection within real-world social contexts. Opportunities (maximizing sensitivity) and challenges (technology support) to this approach will be discussed.

Abstract citation ID: igae098.1879

### MOMENTARY ASSESSMENT OF SOCIAL DISCONNECTION IN OLDER ADULTS AT HEIGHTENED RISK FOR SUICIDE

Emily Bower[1], Taylor Loskot[1], and Kimberly Van Orden[2], *1. Pacific University, Hillsboro, Oregon, United States*, *2. University of Rochester Medical Center, Rochester, New York, United States*

Social disconnection is a proximal and modifiable risk factor for suicide among older adults. Identifying periods of acute or intensified disconnection can inform just-in-time interventions for individuals at heightened risk for suicide. Ecological momentary assessment (EMA) using mobile devices is feasible and acceptable for older adults, and is a promising method for capturing daily changes in social disconnection. To assess preliminary validity of EMA for assessing social disconnection in older adults at heightened risk for suicide, we compare mobile EMA to paper-and-pencil assessments of social disconnection within a sample of older adults with subjective cognitive decline (n=27, mean age=69 years, 81% female). Participants completed baseline paper-and-pencil measures of social disconnection (INQ) and depression symptoms, then completed daily surveys (EMA) that were delivered to their smartphone up to 4 times a day for 14 days. Three EMA LIkert-type items items assessed aspects of social disconnection (burdensomeness, belonging, and disconnection). Participants completed a mean of 37.2 surveys with an average response rate of 67%. EMA items correlated strongly with INQ scores (r's=.6-.7) and depression (r's=.5-.7). Intercorrelations among EMA items were low to moderate (r's=.3-.6), suggesting items assessed unique aspects of social disconnection. Among 19 participants who reported no perceived burden on the INQ, 63% reported daily variability in the EMA burden item. Additional analyses will examine within-person variations in EMA items using multilevel mixed models. Findings provide initial evidence of validity for mobile EMA to assess momentary changes in social disconnection among older adults at heightened risk for suicide.

Abstract citation ID: igae098.1880

### DYNAMICS OF DAILY LONELINESS AND DIURNAL CORTISOL

Karina Van Bogart[1], David Almeida[2], John Felt[3], Jonathan Rush[4], Eric Cerino[5], and Susan Charles[6], *1. Northwestern University, Chicago, Illinois, United States*, *2. Pennsylvania State University, University Park, Pennsylvania, United States*, *3. The Pennsylvania State University, University Park, Pennsylvania, United States*, *4. University of Victoria, Victoria, British Columbia, Canada*, *5. Northern Arizona University, Flagstaff, Arizona, United States*, *6. University of California Irvine, Irvine, California, United States*

Loneliness impedes healthy aging. Previous research shows robust findings between loneliness and poor health, including stress-related diseases and conditions (e.g., cardiovascular disease). Less is known about how loneliness experienced in daily life relates to indices of health, such as diurnal cortisol level patterns. The slope of diurnal cortisol shows a peak upon waking from sleep and a nadir close to bedtime; however, specific rhythms of cortisol slopes fluctuate in daily life. Emerging research suggests that capturing the dynamic range of diurnal cortisol in daily life could be an important marker of allostatic load (i.e., the cumulative burden of chronic stress), with a compressed range indicating dysregulation and a wider range suggesting a more adaptive cortisol profile. In the current research, we investigated whether daily self-reported loneliness related to the dynamic range of diurnal cortisol (calculated via 4 days of salivary cortisol collection, 4 times a day) and explored age differences across the lifespan. Data come from a national daily assessment study with participants ranging between 25-75 years old who completed 8 days of daily diary assessments on loneliness and other factors in daily life. Findings from the current analyses will be presented in the context of how the dynamic range of cortisol might serve as a potential mechanism for outcomes related to loneliness in later life.

Abstract citation ID: igae098.1881

### THE LANGUAGE OF LONELINESS: ANALYZING UNSTRUCTURED SPEECH TO DETECT LONELINESS AND SOCIAL ISOLATION

Ellen Lee[1], Ning Wang[2], varsha Badal[1], Sanchit Goel[1], and Koduvayur Subbalakshmi[3], *1. University of California San Diego, La Jolla, California, United States*, *2. Jiangnan University, Wuxi, Jiangsu, China (People's Republic)*, *3. Stevens Institute of technology, Hoboken, New Jersey, United States*

The mental and physical toll of loneliness and social isolation lead to increased mortality for older adults. Early detection of social disconnection will aid in delivering timely interventions, however current assessments involve long surveys that cannot capture the individualized experiences of loneliness. Our group has been analyzing unstructured speech data using Natural Language Processing (NLP)and openSMILE as well as identifying linguistic and audio features that are associated with social disconnection among older adults. We will present our findings from using linguistic features alone, thematic categories, analyses of different interview segments, sex differences, and audio features. We will also present the association of social disconnection with longitudinal physical, mental, and cognitive outcomes in a community-dwelling healthy aging sample in San Diego. The sample includes older adults living independently in senior housing communities who have completed semi-structured interviews, sleep and physical activity assessments using wearable fitness trackers, a cognitive assessment battery, and clinical assessments for mental health concerns.

Abstract citation ID: igae098.1882

### DAILY SLEEP, SOCIAL ENCOUNTERS, AND MOMENTARY LONELINESS IN LATE LIFE

Zexi Zhou, and Karen Fingerman, *The University of Texas Austin, Austin, Texas, United States*

Social experiences have implications for well-being in late life, and everyday sleep may play an important role in shaping those experiences. This study examines the associations between sleep, quality of social encounters, and momentary loneliness in older adults' daily life. We used the ecological momentary assessment (EMA) data over 5 to 6 days from the Daily Experiences and Well-being Study (N = 287, Mage = 74.88). Every morning, older adults reported their sleep the prior night (e.g., duration, subjective quality, disturbances). At each three-hour assessment, they reported encounters with close social partners and partners not identified as close (i.e., less close partners), indicated the pleasantness and stressfulness of each encounter, and rated their feelings of loneliness. Multilevel models showed that older adults who overall reported higher sleep quality or fewer sleep disturbances also had more pleasant encounters and fewer stressful encounters with both close and less close social partners across the study period. More pleasant and less stressful social encounters with close partners were associated with feeling less lonely especially on days when older adults had better sleep the prior night, compared to days when they had worse prior night's sleep. Higher-quality encounters with less close social partners were associated with feeling less lonely throughout the day regardless of prior night's sleep. Findings suggest the promotive and protective role that better sleep plays in older adults' everyday social life, and highlight the robust benefits that positive social encounters with less close ties may confer in mitigating loneliness in late life.

### SESSION 4185 (SYMPOSIUM)

Abstract citation ID: igae098.1883

### DAILY HEALTH PROCESSES IN POPULATIONS OF DIVERSE DEMOGRAPHIC BACKGROUNDS

Chair: Christina Roecke
Co-Chair: Minxia Luo
Discussant: Allison Bielak

Health processes are studied increasingly commonly in older adults' daily life. This symposium includes four studies that investigated daily health processes in populations of diverse demographic backgrounds and explored differences in the daily processes between individuals. First, with seven-day data from 207 adults aged 18-82 years, Ferguson, Corley, and Scott found that negative affect was associated with occurrence of stressors at both the within-person momentary and between-person levels, but age or neuroticism did not moderate the association. Second, Ng, Kratz, and Birditt conducted a five-day study on effects of interactions with care recipients and with friends on cardiovascular functioning in 159 Black and White dementia caregivers. In particular, they found that negative interactions with care recipients were linked to momentary increases in HR in Black but not in White caregivers. Third, Luo, Röcke, and colleagues investigated the short-term time-lagged associations between leisure activities and cognitive performance over hours and the differences across age, gender, education, and ethnicity. They analyzed data from two two-week studies from a total 542 young and older adults with diverse ethnicity backgrounds, but did not find any time-lagged associations. Fourth, Hartung and Hülür examined daily subjective memory lapses in relation to cognitive activity and mood in 325 university students over 10 days. Their results showed that memory lapses were associated with more cognitive activity and worse mood both momentarily and between individuals. Finally, Allison Bielak will discuss all papers about generalizability of observations of daily health processes across populations of different demographic backgrounds.

Abstract citation ID: igae098.1884

### SHORT-TERM ASSOCIATIONS OF ACTIVITY ENGAGEMENT WITH COGNITIVE PERFORMANCE

Minxia Luo[1], Charles Driver[1], Stacey Scott[2], Daisy Zavala[3], Charles Hall[4], and Christina Roecke[1], *1. University of Zurich, Zurich, Zurich, Switzerland*, *2. Stony Brook University, Stony Brook, New York, United States*, *3. Stony Brook University, EAST SETAUKET, New York, United States*, *4. Albert Einstein College of Medicine, Bronx, New York, United States*

Prior research showed that activity engagement had short-term effects on numerical working memory over hours in German-speaking older adults. However, it is unclear whether these effects could be generalized to other cognitive outcomes and populations. This study examined short-term associations of activity engagement with cognitive performance using data from two studies that included adults of different demographic backgrounds in the US. That is, 16 days' data from 282 older adults aged 70 to 90 years (M = 76.62, SD = 4.68; 66% women; 44% White, 40% Black, 11% Hispanic White, 3% Hispanic Black) from the Einstein Aging Study (EAS) and 14 days' data from 260 participants aged 25 to 65 (M = 46.49, SD =11.09; 65% women; 8% White, 63% Black, 18% Hispanic White, 6% Hispanic Black) from the Effects of Stress on Cognitive Aging, Physiology, and Emotion Project (ESCAPE). For 4 or 5 times per day, participants reported whether they engaged in different social and cognitive activities (e.g., social interactions, TV watching) and completed smartphone cognitive assessments (i.e., processing speed, spatial working memory). Using hierarchical continuous-time dynamic modeling, we did not find any time-lagged associations between activity engagement and cognitive performance. Additionally, age, sex, and race-ethnicity did not moderate the time-lagged associations. Our results suggest that activity engagement did not have short-term effects on processing speed and spatial working memory in young and older populations with ethnic and racial diversity.

Abstract citation ID: igae098.1885

### STRESS, NEGATIVE AFFECT, AND NEUROTICISM ACROSS THE LIFESPAN

Giselle Ferguson[1], Samantha Corley[2], and Stacey Scott[2], *1. Union College, Schenectady, New York, United States*, *2. Stony Brook University, Stony Brook, New York, United States*

Emotional regulation and appraisal skills in response to stressors are thought to accumulate across the lifespan. Work with age-heterogeneous samples found that despite similar initial levels of stressor related Negative Affect (NA), Older Adults (OA) exhibited lower levels of NA over time perhaps due to enhanced emotional recovery. Prior work focused on OA has shown longitudinal increases in within-person NA but a lack of age related stressor-NA change cross-sectionally. We aimed to examine if the relationship between reported momentary stressor occurrence and NA is moderated by baseline neuroticism among an age-heterogenous adult sample and if there are cross-sectional age differences in this relationship. During April-October 2023, participants (N=207) 18-82 years old were recruited from a national online research panel. Participants completed surveys of trait neuroticism then completed seven days of Ecological Momentary Assessments of stressors and NA three times each day. Within persons, negative affect was significantly higher at observations following stressors than non-stressor observations (B=23.01, SE=3.12, p<0.001). Between persons, individuals who reported more frequent stressors across the study also had higher average NA (B=21.90, SE=5.99, p=0.0003). Individual differences in neither neuroticism nor age were significantly related to average NA. Unexpectedly, neither neuroticism nor age moderated the momentary association between stressors and NA. Contrary to our predictions, individual differences in stressor-related increases in NA are not explained by either cross-sectional age differences nor neuroticism. Given the similarity in responses across adult age, future research should examine the role other individual and contextual differences, including stressor type, play in this relationship.

Abstract citation ID: igae098.1886

### MOMENTARY INTERACTIONS WITH FRIENDS AND CARE RECIPIENTS AND CARDIOVASCULAR REACTIVITY IN DEMENTIA CAREGIVERS

Yee To Ng, *University of Michigan, Ann Arbor, Michigan, United States*

Friends may serve as an important resource for dementia caregivers who often experience stress and heightened cardiovascular risk. This study examines momentary associations between friend interactions and cardiovascular reactivity, and tests whether friend interactions moderate the association between interaction with the care recipients (CRs; both positive and negative) and cardiovascular reactivity among Black and White dementia caregivers. Using Ecological Momentary Assessments (EMA), 159 dementia caregivers (Mean age: 62.24, 60% White) reported interactions with CRs (including time spent and levels of positivity and negativity) and indicated any friend interactions every 3 hours over five days. ECG monitors collected real-time physiological data (including heart rate [HR] and heart rate variability [HRV-RMSSD]). Findings revealed no racial differences in contact frequency with friends. Although Black caregivers spent less time with CRs, they reported similar levels of positive and negative interactions with CRs than White caregivers. While positive interactions with CRs were not associated with HR or HRV among caregivers, negative interactions with CRs were linked to momentary increases in HR, observed only among Black caregivers. Moreover, friend interactions were linked to momentary elevation in HR and reduction in HRV for both Black and White caregivers. However, friend interactions did not moderate the effect of negative interactions with CRs on cardiovascular reactivity. This study highlights racial disparities in the amount of time spent with the care recipients and its momentary effects on cardiovascular health. Friend interactions may not alleviate the impact of caregiving on cardiovascular burden but remain linked to momentary cardiovascular health.

Abstract citation ID: igae098.1887

### DAILY MEMORY ISSUES: NATURE, CORRELATES, AND CONSEQUENCES

Johanna Hartung, and Gizem Hueluer, *University of Bonn, Bonn, Nordrhein-Westfalen, Germany*

Memory lapses are common occurrences in daily life; however, little is known about the mechanisms underlying their occurrence. The overarching goal of this project is to understand the nature, correlates, and consequences of memory lapses in everyday life. We conducted an experience-sampling study over ten days with three measurement occasions per day with 325 adults (age = 18 to 36 years). The momentary assessments included measures of memory lapses, working memory (WM) performance, activities, and mood. Participants also completed a retrospective memory lapse questionnaire. Our results show that the participants who retrospectively reported a higher number of lapses also reported a higher number of lapses across the 30 measurement occasions. Nevertheless, the frequency of momentary memory lapses was unrelated to WM, while the number of retrospectively measured lapses was associated with lower WM. The opposite pattern was found for interference/seriousness: WM was not associated with retrospective ratings of seriousness but with momentary ratings of interference. Memory lapses were more likely to occur on occasions with more cognitive activity and worse mood and individuals who reported more cognitive activity and worse mood than others also reported more memory lapses than others. The occurrence of memory lapses at a given time was unrelated to momentary working memory performance. Taken together, our findings suggest that the occurrence of memory lapses may be indicative of a cognitively active lifestyle, but not necessarily of objective performance deficits. We address the implications of our findings for theory and practice and discuss a replication with older adults.

### SESSION 4190 (SYMPOSIUM)

Abstract citation ID: igae098.1888

### EAST MEETS WEST: COLLABORATING INTERNATIONALLY TO MITIGATE SOCIAL ISOLATION

Chair: Megumi Inoue
Co-Chair: Takashi Amano
Discussant: Jim Lubben

Combatting social isolation in later life is a pressing global issue. This symposium assesses social isolation among older adults from various angles. Four presenters from North America and Japan examine the perception, disparities, and impact of social isolation within specific cultural contexts. The knowledge from diverse cultural backgrounds and policy frameworks of the different countries provides an opportunity to explore the intricate nuances and shared experiences of social isolation among older populations. Dr. Watanabe will introduce the status of social isolation among Japanese older adults who reside in a marginal community, where more than 50% of the population is 65 years or older. Dr. Murayama will describe the findings on different forms of social isolation and their impact on health outcomes, utilizing extensive cohort data from older adults living in the Tokyo metropolitan area. Dr. Taylor will examine disparities in loneliness among older adults in the U.S., with a particular focus on the mediation effects of socioeconomic factors on racial and ethnic differences in loneliness. Dr. Mair will discuss changing demographics of older adults and shifting cultural values from a global perspective, with implications for rethinking aging alone as a risk factor and traditional notions of successful aging in relation to limited family ties. These four presentations will provide a broad overview of social isolation in three countries and its implications for quality of life in later life. The symposium will help identify culturally-sensitive strategies for promoting the well-being of older adults in global communities.

Abstract citation ID: igae098.1889

### SOCIAL ISOLATION OF OLDER ADULTS IN A MARGINALIZED COMMUNITY IN JAPAN

Yuichi Watanabe, *Musashino University, Nishitokyo, Tokyo, Japan*

Japan stands as one of the most aged societies globally, with approximately 29% of its population aged 65 or older as of 2022. Previous studies indicate that 10-30% of Japanese older adults experience social isolation, with 2-10% facing severe social isolation (Saito, 2009). This issue is particularly prevalent among those who have contact with others less than once a week, posing potential health risk (Saito et al., 2015). However, it's crucial to acknowledge the existence of extremely aging communities in Japan, especially in rural areas, known as marginalized communities, where over 50% of the population is 65 years or older. This presentation offers insights obtained from surveys conducted in 2015, 2017, and 2019 within one such marginalized community. The findings reveal that out of 297 older adults surveyed, 72 were living alone. Among them, 4 reported meeting others less than once a week, while 18 met others less than four times a week. These results suggest that approximately 25% of older adults living alone may experience social isolation, with 5.6% facing severe social isolation. While these findings may not be generalizable to all marginalized communities in Japan, they highlight the heightened risk of social isolation within such contexts. Future research should conduct detailed analyses to inform discussions and develop solutions for addressing social isolation in marginalized communities.

Abstract citation ID: igae098.1890

### DIFFERENTIAL EFFECT OF LIMITED SOCIAL NETWORK AND SOLITARY LIVING ON HEALTH IMPLICATIONS: PARADOX OF ISOLATION

Hiroshi Murayama, *Tokyo Metropolitan Institute for Geriatrics and Gerontology, Tokyo Metropolitan Institute for Geriatrics and Gerontology, Tokyo, Japan*

In this presentation, I will discuss the distinct effects of a limited social network (SN) and solitary living on health outcomes in later life, drawing on findings from two studies. While both a limited SN and solitary living indicate forms of social isolation, their health implications, especially those associated with solitary living, remain controversial. A limited SN was characterized as engaging in contact with family members living separately or with friends less than once a week, and solitary living was defined as residing without cohabitants. Utilizing extensive cohort data from older adults residing in the Tokyo metropolitan area, we examined the factors modifying the relationship between cognitive decline (CD) and all-cause mortality. The Cox proportional hazard model indicated the CD-mortality association was stronger among those with a small SN than a large SN. Conversely, the association was weaker in living-alone individuals than in cohabiting individuals. The variance in effect was also evident in brain volume metrics. Employing data from a separate cohort study of older Japanese adults, we assessed the impact of social isolation on hippocampal volume changes. Participants with a smaller SN exhibited a more significant reduction in hippocampal volume than those with a larger SN. Surprisingly, solitary living was associated with a lesser decline in hippocampal volume. Although both a small SN and solitary living are indicators of social isolation, they appear to have opposite effects on health. Our results underscore the necessity of differentiating types of isolation when designing and implementing interventions for social isolation.

Abstract citation ID: igae098.1891

### RACIAL/ETHNIC DIFFERENCES IN LONELINESS AMONG OLDER ADULTS: THE ROLE OF INCOME AND EDUCATION AS MEDIATORS

Harry Taylor[1], Arka Roy[1], Kazumi Tsuchiya[1], Ann Nguyen[2], Yu-Chih Chen[3], Thomas Cudjoe[4], and Weidi Qin[5], *1. University of Toronto, Toronto, Ontario, Canada*, *2. Case Western Reserve University, Cleveland, Ohio, United States*, *3. The University of Hong Kong, Hong Kong, China (People's Republic)*, *4. Johns Hopkins University School of Medicine, Baltimore, Maryland, United States*, *5. University of Wisconsin Madison, Madison, Wisconsin, United States*

Our study examines differences in loneliness by race/ethnicity among older adults in the United States and whether income and education mediate these differences. Data came from the Health and Retirement Study Leave-Behind Questionnaire, 2014-2016. Loneliness was measured by the UCLA 3-item loneliness scale. Race/ethnicity categories were White, Black, and Hispanic/Latino. The mediator variables were total household income and education. Sociodemographic covariates included gender, age, employment status, living arrangements, country region, urbanicity, and HRS LBQ Wave. Multivariable linear regression models were used to determine differences in loneliness by race/ethnicity. The KHB mediation method was used to determine if income and education mediated racial/ethnic differences in loneliness. In bivariable analyses, White and Hispanic/Latinx older adults had comparable levels of loneliness, while Black older adults had higher loneliness scores. After multivariable adjustment, Hispanic/Latinx older adults had the lowest levels of loneliness while White and Black older adults had comparable levels of loneliness. A complete mediation was found between Whites and Blacks, in that income and education completely mediated differences in loneliness between these groups. A partial mediation was found between Whites and Hispanics, and between Blacks and Hispanics. Income as a mediator accounted for greater racial/ethnic differences in loneliness compared to education. Our study is the first to explicitly determine if socioeconomic factors mediate race/ethnicity differences in loneliness among a nationally representative sample of older adults. Study findings can inform evidence-based interventions and programs to reduce loneliness among older adults.

Abstract citation ID: igae098.1892

### SUCCESSFULLY “AGING ALONE”? UNEQUAL OPPORTUNITIES AND RISING RISKS IN FAMILY-BASED MODELS OF CARE CROSS-NATIONALLY

Christine Mair, *University of Maryland, Baltimore County, Baltimore, Maryland, United States*

For the first time in human history, older adults will outnumber children and a substantial and growing proportion will live alone and lack one or more nuclear family tie. Such unprecedented shifts require a reevaluation of existing models of “successful aging”, particularly in terms of long-term care policies. This paper draws on country-level data from multiple publicly available sources (e.g., World Bank, Organization for Economic Cooperation and Development, Our World in Data, and the World Values Survey) to examine cross-national patterns of development, health, demography, resources and policies, and cultural values in low-, middle-, and high-income countries. Although there exists substantial heterogeneity across countries, country-level patterns illustrate the economic privilege of living alone and the dominance of “successful aging” opportunities in high income countries. Cultural values about family reflect standard patterns of economic development, yet friendship emerges as a particularly consistent global value. Living alone is associated with poor health in lower income countries, yet better health in higher income countries. Aging “alone” is a risk factor in some contexts, yet a marker of privilege in others. Models of “successful aging” are largely unobtainable in lower income countries, and therefore require a thorough incorporation of global realities, or final abandonment in favor of more nuanced structural perspectives. Long-term care policies that assume the presence of family will yield increasing risk over time across all global contexts and represent a key vulnerability in the future of healthy aging policy.

### SESSION 4195 (PAPER)

### END-OF-LIFE (PAPERS)

### END OF LIFE CARE

Abstract citation ID: igae098.1893

### CREATION AND EVALUATION OF A VIDEO SERIES SHARING A PALLIATIVE APPROACH TO CARE IN CANADIAN LONG-TERM CARE HOMES

Abigail Wickson-Griffiths[1], Paulette Hunter[2], Susan Tupper[3], Allison Cammer[2], Sharon Kaasalainen[4], and Tamara Sussman[5], *1. University of Regina, Regina, Saskatchewan, Canada*, *2. University of Saskatchewan, Saskatoon, Saskatchewan, Canada*, *3. Saskatchewan Health Authority, Saskatoon, Saskatchewan, Canada*, *4. McMaster University, Hamilton, Ontario, Canada*, *5. McGill University, Montreal, Quebec, Canada*

Video-assisted patient education is a favored format for accessing healthcare information. Based on a consultative process, the purpose of this project was to create and evaluate a video education resource to support communication between staff and family caregivers in Canadian long-term care (LTC) homes when introducing a palliative approach to care. The development of the resource proceeded in four stages: consulting with lived experience advisors; refining the concept; piloting the concept; and finalizing the resource. The concept included a voice-over-infographic introduction plus a four-part video series highlighting fundamental aspects of a palliative approach in LTC and featuring personal narratives by a resident, family caregiver, employee, and manager. During pilot research, family caregivers and LTC staff (n=16) participated in focus group discussions and surveys to assess the acceptability of the resource and its impact on knowledge acquisition. They also considered potential uses of the resource. After reviewing the introduction and a full video, as well as the concepts for the three remaining videos, the average acceptability of the video series across fourteen 5-point Likert-type items was 4.49 (SD=0.40) for family caregivers and 4.66 (SD=0.35) for LTC staff. The average positive change in palliative care knowledge on a 100-point scale was 6.94 (SD=10.56) for family caregivers and 12.5 (SD=10.76) for LTC staff. Overall, this work contributes a resource with high content and format acceptability, and good potential for a positive impact on knowledge about a palliative approach. It offers useful considerations for introducing the resource to staff and family caregivers.

Abstract citation ID: igae098.1894

### FAMILY INVOLVEMENT AND SYMPTOM BURDEN IN NURSING HOME RESIDENTS WITH COGNITIVE IMPAIRMENT

Gretchen Tucker[1], John Cagle[2], Timothy Stump[3], Wanzhu Tu[4], Peiyuan Zhang[2], Alex Floyd[5], Mary Ersek[6], and Kathleen Unroe[7], *1. University of Maryland, Baltimore, Baltimore, Maryland, United States*, *2. University of Maryland Baltimore, Baltimore, Maryland, United States*, *3. Indiana University Bloomington, Bloomington, Indiana, United States*, *4. Indiana University Indianapolis, Indianapolis, Indiana, United States*, *5. Indiana University School of Medicine, Indianapolis, Indiana, United States*, *6. University of Pennsylvania, Philadelphia, Pennsylvania, United States*, *7. Indiana University, Indianapolis, Indiana, United States*

Research suggests that family involvement improves the quality of life of nursing home residents. Using baseline data from an on-going multisite clinical trial (UPLIFT-AD), we examined the association between family involvement and staff-reported accounts of resident symptom burden. Symptom burden was measured as a composite of frequency and intensity of symptoms, using items from the End-of-Life Dementia (EOLD) scale. Higher scores indicate greater burden. Family involvement was measured by the family-reported average weekly in-person visit frequency over the past month (range 0-7 days). Data on 198 residents were collected, 61.9% of whom were in Indiana, with the remainder (38.1%) in Maryland. Of the 198 residents, 60.3% were female. The Brief Interview for Mental Status score, which indicates cognitive impairment level, was 6.5 (SD=3.9). Most family members were an adult child (58.7%), 7.9% were spouses, and 38.8% were “other.” On average, spouses visited 5 days a week, children 2 days a week, and other family members 1.5 days a week. In 53.5% of cases, the reporting staff member knew the resident for >1 year. Linear regression analysis was used to examine the associations between the frequency of in-person family visits and EOLD scores while adjusting for resident demographics, mental status, family relationship to the resident, and facility location. Visit frequency was associated with higher EOLD scores (B=0.18, p=.037). Findings suggest family involvement may sensitize nursing home staff to the presence of a resident's burdensome symptoms, which may become more discernible to families with increased involvement.

Abstract citation ID: igae098.1895

**DOCUMENTATION OF LIFE-SUSTAINING TREATMENT PREFERENCES IN A NATIONAL COHORT OF VETERAN DECEDENTS**

Lena Makaroun[1], Yaming Li[1], Maria Mor[1], Stephen Morris[1], Ann-Marie Rosland[1], Tony Rosen[2], Ann Kutney-Lee[3], and Carolyn Thorpe[4], *1. VA Pittsburgh Healthcare System, Pittsburgh, Pennsylvania, United States, 2. Weill Cornell Medicine, New York, New York, United States, 3. Corporal Michael J. Crescenz VA Medical Center, Philadelphia, Pennsylvania, United States, 4. University of North Carolina, Chapel Hill, North Carolina, United States*

In 2018, the Veterans Health Administration (VHA) introduced the Life-Sustaining Treatment (LST) Decisions Initiative to standardize documentation of Veterans' goals and preferences in the electronic health record (EHR). The objective of this retrospective cohort study was to assess LST completion patterns and sociodemographic, clinical, and care utilization factors associated with completion in a cohort of 23,591 VHA-engaged Veterans aged ≥65 years who died in 2021. We used VHA EHR and Medicare claims data and multivariable logistic regression to examine factors associated with LST completion. Of the cohort, 6,684 (28%) completed an LST. LST documentation occurred a median of 142 days before death; 2,260 (34%) were completed by a surrogate. LST completion was markedly higher in those with an inpatient or VA nursing home (CLC) stay in the 2-years before death, so we estimated models stratified by the presence (n = 7,761) or absence (n = 15,830) of any inpatient/CLC stay. In both strata, older age, higher comorbidity, and frailty were associated with LST completion ($p < 0.001$). Among Veterans without an inpatient/CLC stay, being unmarried, having a child as next of kin, higher levels of VA financial benefits, and dementia diagnosis were also associated ($p < 0.001$). Race/ethnicity and gender were not associated with LST completion. LST completion before death is strongly tied to utilization of VHA acute and nursing home care, and generally occurs close to the time of death and often by a surrogate. Opportunities may exist to improve documentation of patient preferences in outpatient settings farther in advance of health declines.

Abstract citation ID: igae098.1896

**EFFECTS OF A MULTICOMPONENT PHYSICAL ACTIVITY PROGRAM ON STRENGTH AND MOBILITY IN OLDER ADULTS IN CARE HOMES**

Yijian Yang[1], Ziwei Zeng[1], Jiahao Shen[1], Cheuk Yin Ho[1], Ka Po Chan[1], Francis Kin Tung Chan[2], and Chi Tat Fung[2], *1. Chinese University of Hong Kong, Hong Kong, China (People's Republic), 2. Hong Kong Sheng Kung Hui Welfare Council Limited, Hong Kong, Hong Kong*

Physical activity (PA) is important for maintaining functional mobility in frail older adults. However, existing PA programs for older adults mainly focus on lower limb function but overlook upper limb and core strength, reaction time, and coordination, which are crucial for safe transferring and fall prevention. In this study, we evaluated a multicomponent PA program, Mobility-Fit that we previously developed for frail older adults, through a cluster randomized controlled trial. 164 older adults from 20 care homes in Hong Kong were recruited and randomly assigned into the intervention group (practicing Mobility-Fit 3 times/week, 45 minutes/session for 12 weeks) or a control group that maintained usual care. All participants underwent assessments of strength, mobility, cognition, PA level (measured by accelerometers), and falls efficacy at baseline and completion. Repeated-Measures Linear Models were used to examine changes within and between groups. The mean age of participants was 84.4±8.0 years. 58% were females. Among the intervention group, handgrip strength increased by 15% (p=0.036), knee extension strength increased by 16% (p=0.012), reaction time decreased by 7% (p=0.049), and five-time sit-to-stand duration decreased by 12% (p=0.035). There were significant interactions between time and group in these variables ($p < 0.05$). Conversely, participants in control group had a 6% decrease in handgrip strength (p=0.043) and higher fall efficacy score (p=0.048). No difference was found in cognitive performance. Our results suggest that multicomponent physical activities are effective in enhancing strength and mobility among frail older adults. The Mobility-Fit program may suit older adults with different functional capacities in care homes.

Abstract citation ID: igae098.1897

**PATTERNS OF RUSHED AND UNFINISHED CARE AMONG CARE AIDES IN NURSING HOMES: A LATENT CLASS ANALYSIS**

Cybele Angel[1], Yuting Song[2], Yinfei Duan[1], and Carole Estabrooks[1], *1. University of Alberta, Edmonton, Alberta, Canada, 2. Qingdao University, Qingdao, Shandong, China (People's Republic)*

Rushed care and unfinished care (also called missed care, care rationing) are common in nursing homes. Although the two phenomena are interrelated, most researchers have studied them separately and used a variable-centered approach. We took a person-focused approach and aimed to identify groups of care aides who rushed care and left care tasks unfinished in similar ways. This cross-sectional analysis used survey data from 3546 care aides working in a random sample of 87 urban nursing homes collected from September 2019 to February 2020 in three provinces in western Canada. We presented to care aides a list of physical and social care tasks (e.g., bathing, talking with residents). They answered whether or not they rushed or left a care task unfinished in their previous shift of work. We performed latent class analysis to identify patterns of rushed and unfinished care and verified the identified patterns with a different wave of data. A 4-class model emerged as the best-fit model for our data. Group 1 reported high levels of rushed and unfinished care, and Group 2 was low on both rushed and unfinished care. Group 3 reported high rushed care and Group 4 reported medium rushed care; both groups reported low unfinished physical care and medium unfinished social care. Our findings suggest the complexity of rushed and unfinished care among care aides. System-level support for this workforce is urgently needed to develop targeted interventions for sub-groups of care aides to reduce rushed and unfinished care in nursing homes.

### SESSION 4200 (SYMPOSIUM)

Abstract citation ID: igae098.1898

### EVALUATION OF PROGRAMS AND SERVICES FOR CAREGIVERS OF PEOPLE LIVING WITH DEMENTIA

Chair: Lauren Stratton
Discussant: Sam Fazio

The majority of care provided to people living with dementia is from family or informal caregivers. There is currently a lack of evidence-based programs and services that are available online or virtually to informal caregivers. This symposium consists of three studies, including the evaluation of two programs and the 24/7 Helpline that are available to informal caregivers of people living with dementia through the Alzheimer's Association. The novel findings presented in this symposium will focus on the full impact these programs and services have on caregivers and address the gap in evidence-based programming. First, Judge will discuss the evaluation of a new program, The Empowered Caregiver, which highlights positive efficacy and acceptability data. Second, Grant will present the results of an evaluation for both an online and virtual program, Managing Money: A Caregiver's Guide to Finances. Lastly, Hodgson will discuss three iterative studies that evaluated the Alzheimer's Association 24/7 Helpline, which provides 24/7 support over the phone, including care consultations. Fazio will serve as the discussant to draw attention to current gaps in caregiving intervention literature, the importance of evidence-based programs and services, and the future direction of these programs. Together, these studies contribute to our understanding of effective and accessible programs and services for caregivers.

Abstract citation ID: igae098.1899

### THE EMPOWERED CAREGIVER: A SCALABLE AND PRAGMATIC EVIDENCE-BASED PROGRAM FOR DEMENTIA CAREGIVERS

Katherine Judge[1], Claire Grant[1], Lauren Stratton[2], Monica Moreno[2], and Sam Fazio[2], *1. Cleveland State University, Cleveland, Ohio, United States*, *2. Alzheimer's Association, Chicago, Illinois, United States*

Informal caregivers provide the bulk of care to older adults with dementia. Few studies have evaluated pragmatic and scalable on-line educational programs for caregivers. An RCT (N=247) was used to evaluate a new program, Empowered Caregiver, across 3 timepoints (T1/baseline; T2 after program completion; and T3 45 days after T2.) The program included two 90-minute sessions delivered online via community educators and addressed communication, dementia-related behaviors, caregiver stress/support, and person-centered care. Materials provided pragmatic and easy to implement caregiver strategies. Program acceptability also was assessed. Participants were M=46.41 years old (SD=16.23), female (63.6%), Caucasian (54.3%), married (47%), heterosexual (80.2%), and worked part- or full-time (50.4%). Separate 2 (condition) x 3 (time) repeated measures GLMs were conducted. Program participants (n=131), compared to control (n=116), reported significant improvements with: Caregiver Self-Efficacy, Emotional Health Strain, Role Captivity, Caregiver Mastery, Personal Gain, Unmet Needs, Unmet Needs Distress, Anxiety Symptoms, Depression Symptoms, Behavioral Intentions, and Behavioral Actions. Mean ratings of program acceptability using a 5-point Likert scale ranged from 4.17 to 4.58. Participants indicated they would very likely recommend the program to others (91.5%). Results indicated the Empowered Caregiver program was efficacious in improving a wide range of caregiver outcomes along with being highly acceptable. Overall, findings address a significant gap in the literature and a current limitation in providing caregivers with timely evidence-based information, tools, and resources. Discussion will highlight key implementation issues, alignment of program dosage with measures, the need for pragmatic and scalable programs, and future directions.

Abstract citation ID: igae098.1900

### THE NEED FOR PRAGMATIC PROGRAMMING FOR CAREGIVERS: EVIDENCE FROM MANAGING MONEY: A CAREGIVER'S GUIDE TO FINANCES

Claire Grant[1], Katherine Judge[1], Lauren Stratton[2], Monica Moreno[2], and Sam Fazio[2], *1. Cleveland State University, Cleveland, Ohio, United States*, *2. Alzheimer's Association, Chicago, Illinois, United States*

Several key issues remain in the caregiving intervention literature. Programs must seamlessly transition and scale into existing settings with plans for long-term implementation. Programs should be accessible, digestible, and efficient for caregivers while still delivering meaningful results. To address these issues, conceptualization and development should be guided by end-use scenarios. Managing Money: A Caregiver's Guide to Finances was developed as an online program for caregivers to address the need for pragmatic and scalable programs focused on the financial impacts of caregiving. Synchronous (community educator-led) and asynchronous (self-paced) versions of the one-hour online program were offered. Topics include conversations about finances, assessing needs, and finding financial support. Printable tools were also provided. The synchronous program was evaluated as an RCT (N=179). The mean age was 55.03 (SD=15.34), 73.2% White; 48.6% cared for a parent/grandparent; and 36.3% cared for a spouse. Long-term effects were found in reducing unmet needs and distress around finances and increasing the likelihood to act to improve financial situations. The asynchronous version was evaluated in a one-arm study with 146 caregivers. The mean age was 45.85 (SD=5.65); 75% were white, and 91% cared for a parent/grandparent. Both versions were found to be acceptable and feasible. On a Likert scale ranging from 1 (strongly disagree) to 5 (strongly agree) participants reported the program identified financial challenges (M=4.09, SD=.75/M=3.81, SD=.79) and would recommend the program (M=4.50, SD=.86/M=3.91, SD=.84). Discussion will outline features of program development, rollout, and scalability. Ongoing need and recommendations for pragmatic programming will also be discussed.

Abstract citation ID: igae098.1901

### EVALUATING THE IMPACT OF THE ALZHEIMER'S ASSOCIATION HELPLINE

Nancy Hodgson[1], Sonia Talwar[1], Liming Huang[1], Kerry Finegan[2], and Sam Fazio[2], *1. University of Pennsylvania, Philadelphia, Pennsylvania, United States*, *2. Alzheimer's Association, Chicago, Illinois, United States*

Most persons living with dementia in the US live at home and are supported by family. The Alzheimer's Association national Helpline offers free 24/7 support, including telephone care consultations. Three iterative studies evaluating the Alzheimer's Association Helpline were conducted between January 2019 and December 2023. The first study compared the benefit of the Helpline Care Consultation to a Care Consultation Plus program on the emotional and mental health of 445 callers (75% White, 80% women). The second study compared outcomes in 1822 callers (75% White, 79% women) randomly assigned to staff who provided the traditional care consultation model, or those trained in a brief solution-focused approach. The third study (2503 callers – 80% women, 73% White) evaluated the efficacy of up to two additional care consultations. In all three studies, we observed statistically significant improvement in self-efficacy in managing emotions. In the first study, callers' mental health and self-efficacy improved significantly. In the second study callers assigned to the solution-focused approach had higher therapeutic alliance than callers assigned to the traditional care consultation. The self-efficacy scores in the third study were significantly lower in callers that requested an additional. Self-efficacy improved significantly in those who opted for one or two calls. Support services provided via the Helpline can be very effective at improving caregiver self-efficacy, managing emotions and ability to take action. Additional research is indicated to understand the diverse needs of caregivers and what types of supportive services work best for Helpline callers.

### SESSION 4205 (SYMPOSIUM)

Abstract citation ID: igae098.1902

### EXPLORING VIRTUAL REALITY, VOICE INTERFACES, AND PERSONALITY TRAITS IN TECHNOLOGY INTERACTION AMONG OLDER ADULTS

Chair: Jimmy Reyes
Discussant: Jimmy Reyes

The proposed symposium highlights recent advances in addressing health disparities and technology adoption among older adults. Three noteworthy studies were undertaken by researchers spanning diverse career stages, thereby encompassing a breadth of expertise and viewpoints. The first study introduces MOTIVE, an immersive virtual exercise intervention targeting older adults who are low-income and living in urban settings. Results suggest acceptability of the intervention and support the scaling of the MOTIVE Study. Another study explores the usability of voice-assisted smart speakers among low-income minority older adults. While participants generally appreciate the hands-free interaction, challenges such as navigation difficulties and unintentional activations were noted. Customizable voice user interfaces are suggested to accommodate health conditions and speech abilities, essential for broader adoption. Contrary to stereotypes, smartphone and AI technology adoption among older adults is increasing. However, perceptions about AI may influence engagement. A pilot study investigating personality traits' impact on AI interaction reveals extraversion predicts engagement, while agreeableness and conscientiousness affect perceived usability. Integrating personality traits into technology research protocols is crucial for understanding and enhancing older adults' engagement. An integrated discussion, guided by the chair, will provide a comprehensive overview of recent advances in technology adoption and usability within the context of gerontology. Collectively, these scholarly discussions will offer invaluable insights into innovative interventions and technological paradigms aimed at mitigating health disparities and enhancing the quality of life among older adults.

Abstract citation ID: igae098.1903

### MOTIVATING OLDER ADULTS THROUGH IMMERSIVE VIRTUAL EXERCISE (MOTIVE)

Brittany Burch[1], Barbara Resnick[1], and Jimmy Reyes[2], *1. University of Maryland Baltimore, Baltimore, Maryland, United States*, *2. University of Northern Iowa, Cedar Falls, Iowa, United States*

Physical activity among older adults can prevent and treat a host of chronic conditions and reduce mortality. However, people who are high-income are almost twice as likely to meet the physical activity guidelines compared to their low-income peers. Physical activity interventions are a critical approach to directly decrease health disparities among older adults who are low-income. We present pilot study results from an innovative intervention titled Motivating Older Adults Through Immersive Virtual Exercise (MOTIVE), which aimed to: 1) determine the feasibility of MOTIVE and 2) test the preliminary efficacy of MOTIVE at increasing physical activity among older adults who are low-income and living in urban environments. We randomized 10 older adults in Baltimore to the fully-immersive virtual reality exercise intervention (MOTIVE) and 10 to a physical activity education only control group. For 8 weeks, twice weekly, the participants in the MOTIVE intervention group biked through virtual naturalistic environments and smashed targets to the beat of their favorite music. To analyze the data, we performed descriptive statistics and used linear mixed models, controlling for covariates. We found that MOTIVE group participants attended an average of 15 out of the 16 sessions. During the 8 weeks, according to the Yale Physical Activity Survey Part 2, the MOTIVE participants significantly increased their physical activity ($p<.001$, CI=-31.3,-11.3). While the interaction of time and group was not statistically significant, the Cohen's d (.53) indicated a moderate effect, favoring the MOTIVE intervention group. These results provide strong preliminary data to support a scaled-up study.

Abstract citation ID: igae098.1904

### USER CHALLENGES AND PREFERENCES FOR VOICE INTERFACE DESIGN ON SMART SPEAKERS AMONG LOW-INCOME MINORITY OLDER ADULTS

Jane Chung[1], Natalie Mansion[2], Madeleine Viceconte[2], Reiley Syros[2], and Jodi Winship[3], *1. Emory University School of Nursing, Atlanta, Georgia, United States*, *2. Virginia Commonwealth University, Richmond, Virginia, United States*, *3. Virginia Center on Aging, Virginia Commonwealth University, Richmond, Virginia, United States*

Voice-assisted smart speakers offer benefits for independent living and wellness, particularly for individuals with mobility disabilities, vision impairments, or dexterity issues. Voice user interfaces (VUIs) integrated into smart speakers enable hands-free and eyes-free interaction, allowing users to perform tasks through voice commands. Despite increasing research on older adults' adoption and interaction with voice-operated smart speakers, the specific usability challenges faced by low-income, minority older adults remain largely unexplored. We synthesized qualitative findings from two smart speaker-related projects, focusing on low-income, primarily African American older adults. Employing qualitative descriptive methods, including matrix analysis and content analysis, we explored usability issues and VUI design preferences. Overall, participants expressed positive experiences with hands-free interactions. However, some encountered difficulties due to a lack of instruction on using different buttons or specific commands to navigate to or exit certain functions. Users also experienced unintentional voice assistant activation, requiring careful command monitoring. While many participants were satisfied with the voice assistant's gender, tone, and accent, occasional misunderstandings or actions that users do not comprehend have been noted. Participant interviews highlighted the significance of designing VUI that can be customized to accommodate users' health conditions and speech abilities, given the prevalence of poorly managed chronic conditions and dental health issues among low-income minority older adults. This customization is essential to smart speaker adoption and use, as health outcomes such as stroke or missing teeth may affect users' ability to pronounce certain words or articulate commands understood by the voice assistant.

Abstract citation ID: igae098.1905

**PERSONALITY BYTES: CRACKING THE CODE OF OLDER ADULTS ENGAGEMENT WITH AI**

Marcia Shade[1], Changmin Yan[2], Valerie Jones[2], and Julie Boron[3], *1. University of Nebraska Medical Center, Omaha, Nebraska, United States*, *2. University of Nebraska Lincoln, Lincoln, Nebraska, United States*, *3. University of Nebraska Omaha, Omaha, Nebraska, United States*

Despite stereotypes about older adults' reluctance to embrace technology, smartphone and technological device adoption among this demographic is increasing, including the use of Artificial Intelligence (AI). However, perceptions about AI may hinder engagement in research among older adults. This study examined how older adults' personalities influence their interaction with AI-driven interactive routines. Fifty community-dwelling older adults participated in a 12-week randomized pilot study, divided into standard and enhanced groups, interacting with conversational AI for intervention routines. Data collected included personality traits, usability, and routine initiations. Participants, averaging 72.4 years old, were mostly female (88%) and White (95%). Personality traits did not significantly affect routine initiations overall, but extraversion predicted engagement throughout the day and morning in both groups. Evening routines were unaffected by personality traits. Agreeableness and conscientiousness significantly predicted perceived usability. These findings emphasize the influence of older adults' personality traits on AI technology interaction, with extraversion playing a significant role. Additionally, agreeableness and conscientiousness impact usability. Integrating personality traits into research protocols involving technology interactions is crucial for better understanding older adults' engagement.

### SESSION 4210 (SYMPOSIUM)

Abstract citation ID: igae098.1906

**FROM THE NATHAN SHOCK CENTERS COORDINATING CENTER: BIOLOGY OF AGING FOR NONBIOLOGISTS**

Chair: Odette van der Willik
Co-Chair: Steven Austad

Advances in the biology of aging that promise to dramatically extend the healthy length of human life are now appearing at a breathtaking rate. At the same time, baseless claims of research breakthroughs in maintaining youth are everywhere in the news. It is often difficult for people outside basic aging research to keep track of what is real and what is not. This symposium brings together scientists working in some of the most exciting research areas to update attendees in terms that will be understandable to anyone. Our speakers were selected both for their scientific achievements and for their outstanding skill as science communicators. Dr. Passos will discuss the rapidly expanding field of senotherapeutics to identify drugs that can eliminate senescent cells (senolytics) and what is needed in the development of therapies that target cellular aging. Dr. Molina will review the development of promising biomarkers of human aging and will provide examples of how markers are being tested and applied in a range of clinical studies. Dr. Panda will provide insights from circadian rhythm science and how new lifestyle choices have the potential to be implemented on a population scale. And Dr. Espinoza will discuss the basic principles of geroscience and the use of such therapies toward the goal of improving healthspan (as opposed to the traditional single disease approach). Dr. Valenzano will update us on how important the microbes that live in and on us are turning out to be to our later life health.

Abstract citation ID: igae098.1907

**CELL SENESCENCE AND MITOCHONDRIA: A MATTER OF LIFE OR DEATH**

Joao Passos, *Mayo Clinic, Rochester, Minnesota, United States*

Senescence is a multi-functional cell fate, characterized by an irreversible cell-cycle arrest and a pro-inflammatory phenotype, commonly known as the senescence-associated secretory phenotype (SASP). Emerging evidence indicates that accumulation of senescent cells in multiple tissues drives tissue dysfunction and several age-related conditions. This has spurred the academic community and industry to identify new therapeutic interventions targeting this process. Senescence is a complex and highly heterogenous phenotype and despite tremendous progress in recent years we are still far from fully understanding it. There is no single, standalone marker that allows the identification of senescent cells, posing a challenge to their identification and targeting. Mitochondrial dysfunction is an often-unappreciated hallmark of cellular senescence which plays important roles not only in the senescence growth arrest but also in the development of the SASP and resistance to cell-death. In my talk, I will first describe some of our recent efforts to accurately map senescent cells in different tissues, including the application of imaging methods involving artificial intelligence. Additionally, I will describe how mitochondria contribute to the development of senescence and resistance to cell-death. Finally, I will propose that a detailed road map of mitochondrial biology in senescence will be crucial to guide the future development of therapies targeting cellular aging.

Abstract citation ID: igae098.1908

**BIOMARKERS OF AGING AND PRECISION HEALTH CARE FOR OLDER ADULTS**

Anthony Molina, *University of California San Diego, La Jolla, California, United States*

Efforts to develop objective and quantifiable biomarkers of human aging are ongoing and have been accelerated by advances in technical and analytical approaches for biomedical research. Beyond predicting one's chronological age, this research is being increasingly applied to measure biological age, which captures the heterogeneity of the aging process. Such markers may be able to predict one's risk for developing a variety of age-related conditions and diseases and are recognized to have potential applications for advancing precision healthcare for older adults. This presentation will review the development of promising biomarkers of human aging and will provide examples of how markers are being tested and applied in a range of clinical studies. As approaches for promoting healthy longevity are being developed and evaluated, biomarkers of human aging may serve as important study outcomes. Modifiable biomarkers of human aging may reflect effects on healthspan and longevity, which would otherwise take many decades to monitor. Finally, this presentation will review national and international cooperative efforts to evaluate and compare biomarkers of human aging, Such efforts are also applying biomarker development approaches to complementary topics, such as the assessment of human resilience.

Abstract citation ID: igae098.1909

**OPTIMUM CIRCADIAN RHYTHM FOR HEALTHY LIFESPAN**

Satchidananda Panda, *Salk Institute for Biological Studies, San Diego, California, United States*

To thrive within nature day-night cycle, our genetic makeup is designed to orchestrate the rise and fall of nearly every gene in our genome at specific times. This rhythmic gene expression is vital for maintaining overall health and vitality. In our modern world, circadian rhythms are frequently disrupted, elevating risks of various chronic diseases. An understanding of circadian rhythm principles can lead to lifestyle interventions aimed at preventing, managing, and even reversing chronic diseases. One such pragmatic approach is time-restricted feeding/eating (TRF), a form of intermittent fasting that confines food intake to a consistent 8-10 hour daily window. Laboratory studies have demonstrated that TRF sustains circadian rhythm. TRF can be a preventive or therapeutic measure for several chronic conditions, including obesity, diabetes, liver and kidney diseases, various cancers, and neurodegenerative diseases. Furthermore, TRF, when combined with a healthy diet, can enhance or preserve muscle mass in older animals and extend lifespan. Human studies on TRF are beginning to replicate some, though not all, of the benefits observed in laboratory animals. Beyond food timing, the timing and quality of light exposure also play a crucial role in influencing our sleep and circadian rhythm. Optimal light exposure has been linked to reduced depression, improved mood, enhanced nighttime sleep, and even accelerated recovery in hospitalized patients. In summary, insights from circadian rhythm science are unveiling new lifestyle choices that have the potential to be implemented on a population scale. These interventions aim to sustain both physical and mental health throughout human lifespan.

Abstract citation ID: igae098.1910

**GEROSCIENCE-DRIVEN THERAPIES TO TARGET HEALTHSPAN WITH AGING: CLINICAL IMPLICATIONS**

Sara Espinoza, Mitzi Gonzales, and Nicolas Musi, *Cedars-Sinai Medical Center, Los Angeles, California, United States*

The geroscience hypothesis posits that by targeting aging we may simultaneously prevent or delay several age-related diseases and thereby increase healthspan, or lifespan spent free of significant disease and disability. Clinical trials of candidate agents are underway to examine several pharmacological interventions for this purpose. Beyond determining the safety of these agents, substantial work will be needed to determine the efficacy of these interventions on various domains of healthspan, i.e., physical function, cognitive function, frailty, disability, patient reported outcomes and health-related quality of life. Further, we will need consensus to identify the most meaningful and clinically relevant outcomes and biomarkers of target engagement to identify appropriate patient populations and monitor therapeutic response. It is likely that no single intervention will be broadly indicated, but agents will be tailored using an individualized, patient-centered approach. Larger pragmatic clinical trials will be needed to demonstrate efficacy beyond smaller, early phase clinical trials. Furthermore, any interventions for older adults must not inadvertently exacerbate existing medical comorbidities or geriatric syndromes, such as contribute to polypharmacy or cause side effects that outweigh any potential benefits. This will require that physicians and others providing medical care to older adults, the vast majority of which are not geriatricians, must be trained and knowledgeable in basic principles of geroscience and the use of such therapies toward the goal of improving healthspan (as opposed to the traditional single disease approach). Clinical practice guidelines specific to promoting healthspan in older adults may be needed to guide any future broad clinical use.

Abstract citation ID: igae098.1911

**THE CONTRIBUTION OF THE MICROBIOME TO THE AGING PROCESS**

Dario Riccardo Valenzano, *Leibniz Institute on Aging, Jena, Thuringen, Germany*

The human microbiome, a complex ecosystem of microorganisms residing in our bodies, is emerging as a critical contributor to our overall health and the aging process. With a total number of genes that vastly outnumbers our own—approximately 100 to 1—the microbiome influences virtually all biological processes, from nutrient absorption and immune system development to protection against pathogens. This diverse and dynamic community, acting as a polypharmacy, produces a plethora of compounds that affect our metabolism and are linked to various health outcomes. Recent findings have highlighted that numerous diseases, including metabolic disorders, immune-related conditions, and even neurodegenerative diseases are associated with the disruptions of the microbiome-host balance. Given its extensive involvement in health and disease, the microbiome has become highly relevant in aging research. Scientists are now investigating how changes in the microbiome composition throughout our lives can lead to new anti-aging interventions. One of the most promising areas of research is microbiome transplantations, which offer a novel strategy to potentially slow down or reverse aspects of the aging process by modifying our internal microbial landscape. Moreover, the discovery that rapamycin, a powerful anti-aging drug, originates from microbes underscores the microbiome's potential as a treasure trove for developing new anti-aging compounds. By understanding and harnessing our microbiome, we stand on the brink of revolutionizing anti-aging strategies, transforming our approach to disease prevention in our later years. This rapidly expanding research field promises to unlock new possibilities for enhancing longevity and improving the quality of life as we age.

### SESSION 4215 (SYMPOSIUM)

Abstract citation ID: igae098.1912

### GAIT SPEED AS A FUNCTIONAL VITAL SIGN: FROM IMPLEMENTATION TO INTERVENTION

Chair: Emma Baillargeon
Co-Chair: Alexander Garbin
Discussant: Jonathan Bean

Older adults with impaired mobility have a greater risk of falls, hospitalization, loss of independence, and mortality. Interventions to improve mobility are effective, but because mobility limitations are under-diagnosed, patients are often not referred for treatment unless they fall or report a change in function. Walking (gait) speed is a strong predictor of overall health (a "functional vital sign") and can identify patients who may benefit from intervention, but in most clinics is not routinely assessed like other vital signs. In this symposium, we will present four projects on using routine gait speed assessment to identify and treat mobility limitations in outpatient clinical care. The first two speakers (Baillargeon and Garbin) will present projects implementing gait speed in outpatient geriatric clinics, the factors associated with implementation, project outcomes, and strategies to integrate gait assessment into clinical workflows. The third speaker (Lo) will present on self-administered app-based walking assessments that can allow for more frequent measures of walking in various settings and how this data may inform clinical decision making. Finally, the last speaker (Brach) will discuss interventions to improve gait speed that could be used to treat mobility limitations once patients are identified. Our discussant (Bean) will lead a conversation about this work in the larger context and possible future directions for the field. Together, the talks in this symposium will provide evidence that routine, quantitative gait assessment in outpatient care is feasible and has significant potential to improve the care for older adult patients across the continuum of healthcare.

Abstract citation ID: igae098.1913

### GAIT SPEED IMPLEMENTATION IN TWO GERIATRIC CLINICS IS CONSISTENT ACROSS PATIENTS AND VISIT TYPES

Emma Baillargeon, Nancy Jones, Andrea Rosso, and Jennifer Brach, *University of Pittsburgh, Pittsburgh, Pennsylvania, United States*

We are implementing gait speed assessment in two outpatient geriatric clinics of a large, academic hospital system in an ongoing quality improvement project. We trained rooming staff (Clinic A: n=5; Clinic B: n=4) to measure 4-meter gait speed using a stopwatch and document the measured speed, or reason for not measuring, in the electronic health record (EHR). The project has been active since 11/1/2023 in Clinic A and 1/19/2024 in Clinic B. EHR data are analyzed monthly to track progress; data through 2/29/24 are included here. We tested whether patient (age, sex) and visit (type, ICD-10) factors were associated with gait speed documentation (yes/no) and measured gait speed. Documentation was completed in 23-58% of visits-per-month for a total of 621 visits (542 patients) in Clinic A and 186 visits (173 patients) in Clinic B. Whether gait speed was documented was not associated with patient age ($p=0.3$), sex ($p=0.2$), or appointment type ($p=0.2$). Gait speed was negatively associated with patient age ($R=-0.24$, $p<0.001$) but did not differ by patient sex ($p=0.2$) or appointment type ($p=0.5$). Gait speed documentation was not associated with an abnormality of gait and mobility diagnosis (ICD-10 R26; $p=0.8$), but gait speed was slower in visits with this diagnosis (median (IQR): 0.60m/s (0.51,0.75)) than without (0.69m/s (0.56,0.86), $p<0.001$). Gait speed documentation is feasible and is being used similarly across patients and visit types. Ongoing data will be assessed as available to inform our goal of increasing the reach and adoption of gait speed assessment in geriatric primary care.

Abstract citation ID: igae098.1914

### STRATEGIES AND OUTCOMES OF GAIT SPEED IMPLEMENTATION IN A GERIATRIC PRIMARY CARE CLINIC

Alexander Garbin[1], Thomas Johnson[2], Caitlin Allison[3], and Hillary Lum[2], *1. VA Eastern Colorado Healthcare System, Aurora, Colorado, United States*, *2. University of Colorado School of Medicine, Aurora, Colorado, United States*, *3. University of Colorado Health, Aurora, Colorado, United States*

Over a 10-month period, gait speed was measured during 4700 patient visits (1931 unique patients) as part of a quality improvement project within a geriatric primary care clinic. This presentation will comprehensively detail strategies and outcomes from this implementation project. Primary implementation strategies included use of an automated gait speed measurement device to reduce Medical Assistant burden, integration of gait speed into the electronic medical record, identification of clinical champions, and development of environmental supports to facilitate gait speed measurement and provider referral guidance. Burden was assessed at the start of the implementation period (n=124 visits), with results detailing a median of 19.5 seconds (IQR 14.3, 26.4) added to rooming workflow with the addition of gait speed measurements. Implementation outcomes of reach and adoption were examined on a weekly basis and were defined by the proportion of patient visits with a gait speed measurement and proportion of provider notes that included gait speed, respectively. At the end of the implementation period, reach was 73.3% while adoption was 53.9%. We also examined gait speed change in patients with two or more gait speed measurements, separated by at least 180 days (n=516). After stratifying by baseline risk (<0.6 m/s 'at risk'; ≥0.6m/s 'not at risk'), patients identified as 'at risk' (n=159) increased their gait speed by 0.07 m/s (IQR -0.01, 0.20; p<0.001), while those 'not at risk' (n=419) experienced no change (IQR -0.12, 0.11; p=0.94). Together, this information details considerations for implementing gait speed while also demonstrating its preliminary feasibility and benefit.

Abstract citation ID: igae098.1915

### IMPLEMENT A SELF-ADMINISTERED, SMARTPHONE APP-BASED GAIT ASSESSMENT FOR FUTURE INTERVENTION IN OLDER ADULTS

On-Yee (Amy) Lo[1], Pei-An Lee[1], Wanting Yu[2], Junhong Zhou[1], and Brad Manor[1], *1. Harvard Medical School/Hebrew SeniorLife, Boston, Massachusetts, United States*, *2. Hebrew SeniorLife, Boston, Massachusetts, United States*

Gait metrics reflect functional capacity and sensitively predict adverse health outcomes. Measuring gait performance – under normal (i.e. walking quietly at a self-selected speed) and "dual-task" conditions (i.e. walking while performing an unrelated cognitive task) – is recommended for assessing and monitoring physical and cognitive functions in older adults. However, there is a significant gap in understanding gait performance outside of lab and clinical settings, yet it is greatly needed. Our team developed a smartphone application (App) to capture gait metrics during normal and dual-task conditions. This App provides users with multimedia instructions, enabling them to self-administer the gait assessment remotely and repeatedly. Acquired data were uploaded to a cloud-based server for offline analyses. Our team has validated and deployed this App within several studies focused on older adults across a wide range of physical and cognitive functional limitations. Results suggested that gait metrics derived from the App were highly correlated with those derived from the GAITRite mat. The test-retest reliability of App-derived gait metrics were high for normal and dual-task walking. Older adults with intact cognition (N=17, MoCA >26) or mild cognitive impairment (MCI, N=16, 19< MoCA≤26) can safely, self-administer this App once a day for 5 consecutive days. Older adults with MCI exhibited significantly higher day-to-day variation in gait variability during dual-task walking (0.30±0.14), compared to cognitively intact older adults (0.19±0.09). Our effort also demonstrated an 80-year-old faller could use this App – with a caregiver's assistance - to measure gait metrics bi-weekly for up to one year.

Abstract citation ID: igae098.1916

### INTERVENTIONS TO IMPROVE GAIT SPEED

Jennifer Brach, Valerie Shuman, and Subashan Perera, *University of Pittsburgh, Pittsburgh, Pennsylvania, United States*

Numerous evidence-based interventions exist to treat patients' mobility limitations once identified in primary care. We will present an overview, focusing on the Program to Improve Mobility in Aging (PRIMA) study as one example. Interventions to improve walking historically focused on underlying impairments, with modest improvements. We hypothesize mobility limitations are a result of the loss of motor skill in walking and timing and coordination exercises targeting the skill of walking are an essential component of a mobility intervention. Participants >64 years were randomized to the standard strength and endurance (STD, n=125) or standard-plus timing and coordination (PLUS, n=124) intervention, 50-60 minutes, twice a week for 12 weeks. Participants with slower initial gait speed (< 1.0m/s) in both groups improved gait speed after the intervention (STD: 0.111m/s, PLUS: 0.088m/s) and maintained those improvements at the 12-week follow-up (STD: 0.087m/s, PLUS: 0.086m/s). In contrast, participants who initially walked faster (≥1.0m/s) in both groups improved gait speed after the intervention (STD: 0.060m/s, PLUS: 0.074m/s) but only those in the PLUS intervention maintained those improvements at the 12-week follow-up (STD: 0.027m/s, PLUS: 0.052m/s). Individuals in the PLUS group who were most likely to improve had better baseline endurance (longer six-minute walk distance) and were more active (greater activity counts by actigraphy) than those who did not improve. Both interventions improved gait speed. Faster walkers and those with greater capacity (i.e., endurance and activity level) may need the challenge of the timing and coordination component to become motor experts in walking and sustain treatment benefits.

### SESSION 4220 (SYMPOSIUM)

Abstract citation ID: igae098.1917

### GENERATIVE AI IN GERONTOLOGY: CENTERING STAKEHOLDERS' VOICES

Chair: George Demiris
Discussant: Hilaire Thompson

The rise of Generative Artificial Intelligence (AI) tools such as Large Language Models (e.g., ChatGPT) has highlighted many potential application areas in gerontology, ranging from tools to support clinical decision support, facilitate communication, provide supportive services to older adults and family members and even address issues of social isolation and loneliness. Often technological solutions are developed without early and ongoing feedback by end users and other stakeholders. This symposium includes the presentation of distinct studies that examine the use of Generative AI for various gerontological areas based on experiences of the NIA funded Artificial Intelligence and Technology Collaboratories for Aging and other initiatives, and highlights ways to engage stakeholders in all design and implementation phases in order to facilitate co-creation of meaningful generative AI solutions. We provide a comprehensive discussion of both opportunities and challenges associated with the growth of Generative AI in gerontology and discuss clinical, ethical and practical implications. Additionally, we present ways to integrate Generative AI into traditional research and clinical practice in way that supplements rather than replaces traditional care, and minimizes the risk of bias.

Abstract citation ID: igae098.1918

### STAKEHOLDER ENGAGEMENT FOR THE DESIGN OF GENERATIVE AI TOOLS: INCLUSIVE DESIGN APPROACHES

George Demiris, *University of Pennsylvania, Philadelphia, Pennsylvania, United States*

We present an approach to active and ongoing stakeholder engagement throughout the design lifecycle of generative AI applications. We highlight case studies from the Penn Artificial Intelligence and Technology Collaboratory for Healthy Aging (PennnAITech) of active involvement of older adults, family members, clinicians and social services coordinators as stakeholders in the early phases of design and prototype testing and demonstrate how this participatory approach can address not only usability challenges, but maximize the accessibility and relevance of Generative AI for older adult users. One example includes an NSF funded study using Large Language Model (LLM) tools to address social isolation and loneliness for community dwelling older adults living alone. In this project, older adults were invited to serve as co-designers of the LLM blueprint, its functionalities and purposes. Discussions around transparency and disclosure informed the implementation approach. The second example explores the use of Generative AI for a fall prevention program that captures fall risk scores using depth sensing. This project targets community dwelling older adults with mild cognitive impairment who live in low resource settings. The use of Generative AI is meant to make the fall risk information more accessible and actionable for older adults and their families. Co-design sessions provided system specifications for a chatbot that serves as a fall risk coach. As Generative AI applications continue to expand, assuring that we center older adults' voices as part of an inclusive design effort will become critical for these systems to be accessible and relevant for their end users.

Abstract citation ID: igae098.1919

### USING GENERATIVE AI TO ENHANCE BEHAVIORAL INTERVENTIONS FOR CARE PARTNERS OF OLDER ADULTS

Karla Washington[1], George Demiris[2], Jina Huh-Yoo[3], Rezvaneh Rezapour[3], Lu Wang[3], Elham Aghakhani[3], and Max Song[3], *1. Washington University in St. Louis, St. Louis, Missouri, United States*, *2. University of Pennsylvania, Philadelphia, Pennsylvania, United States*, *3. Drexel University, Philadelphia, Pennsylvania, United States*

Care partners play an essential role in providing long-term care to community-dwelling older adults, offering vital support to individuals who require or desire assistance with instrumental and other activities of daily living. However, care partners' responsibilities often entail significant physical, emotional, and financial strain, resulting in compromised health and well-being. Numerous studies support the efficacy of behavioral interventions in reducing the deleterious health outcomes often associated with serving as a care partner, but the resource-intensive nature of many such interventions introduces numerous implementation barriers. This presentation will explore the promise and potential pitfalls of using generative AI to enhance the delivery of behavioral interventions for care partners of older adults. Presenters will supplement a review of relevant literature with specific examples drawn from their own research using a large language model (ChatGPT 3.5) to enhance the delivery of PISCES (Problem-solving Intervention to Support Caregivers in Everyday Situations), a cognitive-behavioral intervention developed for care partners of older adults with serious illness. Generative AI's potential as a tool to deliver the intervention's psychoeducational content and also to monitor intervention fidelity demonstrated by human interventionists will be described, as will researchers' intentional efforts to ensure the tool does not perpetuate problematic (e.g., ageist) perspectives based on an analysis of 250 transcribed intervention sessions used to train it. The presentation will conclude with actionable practice recommendations that harness the potential of this rapidly emerging technology in an ethically sound manner that enhances the equitable delivery of evidence-based care partner support for all.

Abstract citation ID: igae098.1920

### THE ERA OF CHATGPT: RECOMMENDATIONS FOR THE INTEGRATION OF LLMS IN GERONTOLOGY

Ari Friedman, *University of Pennsylvania, Philadelphia, Pennsylvania, United States*

Large language models (LLMs) and the platforms they support such as ChatGPT have experienced an exponential growth in popularity and use in the last year. ChatGPT surpassed100 million users in just two months after it was released to the public. These technological advances introduce opportunities for health care applications and it is anticipated that LLMs and various chatbot applications may significantly redesign clinical and health related processes and services. More specifically, in the space of gerontology, the use of Generative AI is anticipated to inform applications including automating clinical decision making, and even predicting cognitive decline based on end user data, providing digital companionship chatbots for isolated older adults, processing and increasing the accessibility of health education material and assisting in the documentation of care transitions. Rather than aiming to anticipate how companies will use LLMs to disrupt the field of gerontology, we have the opportunity and responsibility to define how ethical and appropriate gerontological care will shape the training of LLMs and the creation of guidelines for the use of Generative AI in gerontology. In December 2023 a group of national experts in the fields of gerontology, LLMs, AI and bioethics convened with the goal to propose guidelines to inform appropriate use of LLMs for systems that target older adults and persons with dementia and recommendations for the promotion of transparency in the design of LLMs for gerontology. This presentation will provide insights into these recommendations and guidelines for the role of LLMs in gerontology.

Abstract citation ID: igae098.1921

### TIMELY PERSPECTIVES FROM DIVERSE STAKEHOLDERS ON GENERATIVE AI

Thomas Cudjoe[1], Nancy Schoenborn[2], and Sato Ashida[3], *1. Johns Hopkins University School of Medicine, Baltimore, Maryland, United States*, *2. Johns Hopkins University, Baltimore, Maryland, United States*, *3. University of Iowa, Iowa City, Iowa, United States*

A core mission of the Johns Hopkins Artificial Intelligence (AI) and Technology Collaboratory for Aging Research is to engage diverse stakeholders. To better understand the perspectives of stakeholders on research priorities and decision-making considerations regarding the development and adoption of AI, we conducted in-depth interviews with stakeholders recruited nationally. We interviewed a total of 49 participants, including older adults/caregivers (n=15) and clinicians (n=15)

from urban and rural areas to understand user perspectives. We also interviewed entrepreneurs (n=6), investors (n=5), and payers (n=8)to gain a broader understanding of how generative AI may be integrated into current health service systems. Themes included: 1) Shared and distinct priorities among the stakeholders; most frequently identified priority areas that AI can help address included facilitating social engagement, coordination of care, supporting independence, and treating or preventing common geriatric syndromes like dementia and falls. Older adults and caregivers focused on daily function and caregiving; clinicians focused on monitoring and adherence; health system leaders and payers focused on access, staff burnout, and satisfaction. 2) Participants envisioned multiple roles for AI, including taking over burdensome tasks, making existing tasks more efficient, addressing unmet needs, and performing tasks beyond what is humanly possible. 3) Although perceived benefits outweighed concerns, there were concerns including unintended consequences and suggestions for guardrails. 4) Decisions about AI adoption focused on ease of use, implementation, and cost. Our findings highlight important priorities and considerations from stakeholders for the use of generative AI and opportunities for future research and development.

### SESSION 4225 (SYMPOSIUM)

Abstract citation ID: igae098.1922

### HEALTH CARE RESEARCH AND PROVISION AMONG OLDER ADULT GROUPS HISTORICALLY UNDERREPRESENTED IN RESEARCH

Chair: Aneeka Ratnayake
Discussant: Mark Brennan-Ing

In this symposium we will discuss research focusing on minority populations who are often under-represented in research. We will focus on research among three groups broadly: racial and ethnic minority groups, sexual and gender minority groups, and patients in low- and middle-income countries. Our first presentation will discuss enrollment of racial and ethnic minority groups in the All of Us Research Program—a program aimed at oversampling those under-represented in research in the United States. The generalizability of those living with HIV captured in the All of Us database will be ascertained as compared to the overall population living with HIV in the United States. The second presentation will discuss whether issues of potentially inappropriate medication prescriptions are different for sexual and gender minority older adults compared to cisgender heterosexual older adults. In this presentation, disparities in potentially inappropriate medication prescriptions among this population will be highlighted. The final presentation will discuss correlates of physical activity among older adults with and without HIV in Uganda. In this presentation the contextual differences for studying physical activity among older adults in the Global South will be discussed. Together, these presentations highlight the need to consider vulnerabilities of populations under-represented in research and especially in older adults. Though specific emphasis will be placed on three under-represented groups, the overarching goal of this symposium is to emphasize the impact of prioritizing representation, intersectionality, and identity when conducting gerontological research and providing healthcare to older adults. HIV, AIDS and Older Adults Interest Group Sponsored Symposium

Abstract citation ID: igae098.1923

### ASSESSING THE GENERALIZABILITY OF THE PEOPLE LIVING WITH HIV IN THE ALL OF US DATABASE

Narcissa Plummer[1], Aneeka Ratnayake[2], Brianne Olivieri-Mui[1], and Robert Cavanaugh[3], *1. Northeastern University, Boston, Massachusetts, United States*, *2. Roux Institute at Northeastern University, Portland, Maine, United States*, *3. Roux Institute, Northeastern University, Portland, Maine, United States*

Few large databases are representative of the US population of people living with HIV. In 2018, the All of Us (AoU) Research Program launched, with the goal of enrolling 1 million Americans under-represented in biomedical research such as PWH. This study aimed to establish the generalizability of data self-reported by PWH in AoU to U.S. Centers for Disease Control and Prevention (CDC) HIV surveillance statistics (2021). We conducted a cross-sectional descriptive analysis of AoU participants who self-reported as living with HIV. The 'allofus' R package extracted survey responses to the HIV-related questions with the relevant concept IDs (1384391, 43530505, 43528832, 43530347). We compared AoU demographic statistics to CDC HIV surveillance statistics using Pearson's chi-squared tests. Of the 409,420 AoU participants 1,714 people self-reported having HIV. Most were male (n=1,282, 76.4%) and reported a non-heterosexual sexual orientation (n=1,239, 71.2%). Almost half were 55 and older (n=821, 47.9%) and the largest race/ethnicity group was non-Hispanic White (n=783, 45.7%). Compared to national surveillance data AoU was similar in gender/sex (US: 76.9% Male vs AoU: 76.4% Male, p-value 0.103), but different in age (US: 22.7% Age 45-54 vs AoU: 26.8% Age 45-54, p-value <0.001) and race/ethnicity (US: 39.8% Non-Hispanic Black vs AoU: 30.6% Non-Hispanic Black, p-value <0.001). AoU has successfully oversampled older ages, but self-reported PWH are Whiter than expected. As such, interpretations of AoU are limited in generalizability to the US HIV population. Future research is to redefine AoU HIV cohort (including health records and medication) and evaluate generalizability.

Abstract citation ID: igae098.1924

### POTENTIALLY INAPPROPRIATE CENTRAL NERVOUS SYSTEM MEDICATION PRESCRIBING AMONG SEXUAL AND GENDER MINORITY ADULTS

Chelsea Wong[1], Louisa Smith[2], Robert Cavanaugh[3], Dae Hyun Kim[4], Carl Streed[5], Farzana Kapadia[6], and Brianne Olivieri-Mui[2], *1. Beth Israel Deaconess Medical Center, Brookline, Massachusetts, United States*, *2. Northeastern University, Boston, Massachusetts, United States*, *3. Roux Institute, Northeastern University, Portland, Maine, United States*, *4. Hebrew SeniorLife, Boston, Massachusetts, United States*, *5. Boston University School Of Medicine, Boston, Massachusetts, United States*, *6. New York University, New York, New York, United States*

Among older, cisgender heterosexual adults (non-OSGM) mental health disorders and frailty are associated with higher potentially inappropriate medications (PIM). Yet, this association remains unclear among older sexual and gender minority adults (OSGM) who experience higher burden of frailty at earlier ages. We aimed to assess PIM prescribing by OSGM status and frailty. Using the All of Us Database (v7, Controlled Tier), we included participants ≥50yo without a diagnosis for appropriate use of four central nervous system PIM classes: hypnotics /sedatives, antipsychotics, antidepressants, or anxiolytics. Separate logistic regression models for each PIM category were constructed to estimate the association of OSGM status and frailty category with PIM prescriptions adjusting for age, race and ethnicity, income, marital status, and HIV status. The full sample included 967 OSGM and 15,563 non-OSGM. OSGM adults were younger (mean [SD], 63 [8] vs 66 [8], p<0.05), with higher frailty (36% vs 27%, p<0.05), similarly insured (98% vs 98%, p>0.05), and fewer reported "excellent" mental health (19% vs 26%, p<0.05). There were no significant associations between OSGM status and prescriptions in PIM categories of hypnotic/sedatives, antipsychotics, and antidepressants. However, OSGM status (aOR=1.79, 95% CI 1.09,2.79) and frailty (aOR=1.97, 95% CI 1.43,2.71) were associated with higher odds of anxiolytic PIM prescriptions. Our findings suggest that OSGM status and frailty were each independently associated with PIM anxiolytic prescriptions. This highlights a disparity in potentially inappropriate prescribing of anxiolytics among OSGM.

Abstract citation ID: igae098.1925

### PHYSICAL ACTIVITY AMONG OLDER ADULTS WITH AND WITHOUT HIV IN UGANDA: COHORT STUDY IN RURAL UGANDA

Aneeka Ratnayake[1], Yao Tong[2], Zahra Reynolds[3], Steffany Chamut[4], Robert Paul[5], Jeremy Tanner[6], Brianne Olivieri-Mui[7], and Mark Siedner[2], *1. Roux Institute at Northeastern University, Portland, Maine, United States*, *2. Harvard University, Cambridge, Massachusetts, United States*, *3. Massachusetts General Hospital, Boston, Massachusetts, United States*, *4. Harvard School of Dental Medicine, Boston, Massachusetts, United States*, *5. University of Missouri St. Louis, St. Louis, Missouri, United States*, *6. The University of Texas Health San Antonio, San Antonio, Texas, United States*, *7. Northeastern University, Boston, Massachusetts, United States*

Studies from high-income countries have established the benefits of physical activity (PA) for health among older people. Yet, PA and its links to frailty and quality of life (QOL) in older rural African populations living with HIV remain under-explored. We analyzed data from the first three annual assessments in a cohort of older people living with HIV (PLWH) and age- and sex-similar, community-based people not living with HIV. PA was measured through self-reported minutes of moderate and vigorous PA, converted into metabolic equivalent of task (METs). PA adherence to World Health Organization (WHO) guidelines was defined as a minimum of 150 minutes of moderate PA or 75 minutes of vigorous PA weekly. Linear and logistic generalized estimating equations (GEE) regression models were employed to estimate the association between HIV status and PA, adjusting for frailty, QOL, comorbidities, and age. At enrollment there were 297 PLWH and 302 people not living with HIV, 304 men and 295 women, with a median age of 58 years (IQR:54-63); 96% met PA guidance. After multivariable adjustment, PA in METs was associated with younger age (b=-187.18 per year, p<0.0001), HIV infection (b=-1039.12, p=0.041), higher health-related QOL (b=38.05 per point on a 0-100 scale, p=0.034), frailty (b=-2661.24, p=0.028), and medical comorbidity (b=-2208.07, p<0.0001). Guideline-concordant PA was inversely associated with frailty (OR:0.27, p=0.0076) and medical comorbidity (OR:0.43, p=0.0069). Correlates of lower PA were older age, living with HIV, frailty, and medical comorbidity. Those with frailty and medical comorbidity may not be benefitting from recommended levels of PA.

### SESSION 4230 (SYMPOSIUM)

Abstract citation ID: igae098.1926

### INCREASING DEMENTIA DETECTION IN HEALTH CARE SYSTEMS: LEARNINGS FROM THREE PRAGMATIC CLINICAL TRIALS

Chair: Sascha Dublin
Co-Chair: Deborah Barnes
Discussant: Shari Ling

In the US, an estimated 6.7 million older adults have dementia, yet only about half have received a diagnosis. Earlier detection has potential to improve health and quality of life for people living with dementia and their caregivers by improving access to education, supportive services, and emerging treatments. The National Institute on Aging has funded pragmatic clinical trials of innovative, low-cost approaches aimed at increasing recognition of dementia within healthcare systems. In this symposium, we will present methods and early results from 3 such trials. The eRADAR study is applying a machine learning-based algorithm that draws on electronic health records (EHR) data to identify high-risk individuals. The intervention involves inviting high-risk individuals in the intervention arm for a "brain health" assessment visit within primary care. The D Cubed Study is testing two approaches: an EHR-based algorithm, the Passive Digital Marker, and a brief patient-reported questionnaire, alone and in combination. Results are shared with primary care providers via the EHR together with next steps for evaluation. The Toolbox Detect study is examining whether self-administered, brief cognitive screening tests given via tablet computers as part of annual wellness visits can increase dementia detection. Investigators leading these trials will describe their interventions and study methods and present early results related to uptake, feasibility and acceptability. They will also discuss challenges encountered, potential solutions, and lessons learned. A major goal will be to engage audience members to consider how they might apply these methods to increase detection of dementia in their own settings.

Abstract citation ID: igae098.1927

### TESTING ERADAR: A NEW EHR-BASED ALGORITHM TO INCREASE DEMENTIA RECOGNITION IN HEALTH CARE SYSTEMS

Sascha Dublin[1], Leah Karliner[2], Yates Coley[1], Deborah King[1], Clarissa Hsu[3], Sei Lee[2], Judith Walsh[2], and Deborah Barnes[2], *1. Kaiser Permanente Washington Health Research Institute, Seattle, Washington, United States*, *2. University of California San Francisco, San Francisco, California, United States*, *3. Kaiser Permanente Washington, Seattle, Washington, United States*

Electronic health records (EHRs) hold potential for identifying individuals with undiagnosed dementia. Using machine learning, our team developed and externally validated a predictive algorithm called the EHR Risk of Alzheimers and Dementia Assessment Rule (eRADAR) that estimates the likelihood an individual has undiagnosed dementia with high accuracy (C statistics of 0.79 to 0.84). Through two embedded pragmatic trials, we are implementing eRADAR in 11 primary care clinics within Kaiser Permanente Washington and University of California, San Francisco. The target population is older adults age 65+ without a documented dementia diagnosis or medication. Primary care providers (PCPs) are randomized to intervention or usual care. The eRADAR algorithm is used to identify individuals with eRADAR scores in the top 15-20%, who are invited to a "brain health" visit that includes assessment of instrumental activities of daily living, depressive symptoms, and cognitive function (Montreal Cognitive Assessment). Results are shared with the patient and PCP, and the PCP is responsible for making the final diagnosis (with decision support provided through the EHR). To date, we have implemented the intervention in 9 clinics and conducted over 590 brain health visits. About 31% (658/2137) of high-risk individuals accept a brain health visit. Of these, about 18% have results suggesting dementia and another 30% mild cognitive impairment. Post-visit surveys show high acceptance of and satisfaction with the intervention. The primary study outcome is rate of dementia diagnosis over 12-month follow-up (completed by April 2025). We are also conducting semi-structured interviews to illuminate benefits and harms.

Abstract citation ID: igae098.1928

### DIGITAL DETECTION OF DEMENTIA (D CUBED) TRIALS

Malaz Boustani[1], and Nicole Fowler[2], *1. Indiana University Indianapolis, Indianapolis, Indiana, United States*, *2. Indiana University, Indianapolis, Indiana, United States*

At Indiana University, we leveraged electronic health record (EHR) data and Machine Learning algorithms to develop a Passive Digital Marker (PDM) for dementia detection with 80% accuracy. At the University of Miami, we developed the Quick Dementia Rating Scale (QDRS) as a 2-3 minute patient-reported outcome tool with 85% accuracy for dementia diagnosis. Two pragmatic cluster-randomized controlled comparative effectiveness trials randomized primary care clinics to Usual care, PDM alone, and both the PDM and the QDRS. Trials recruited diverse rural, suburban and urban primary care practices located in central Indiana and south Florida. The positive results are communicated via the EHR only to clinicians within the PDM and PDM + QDRS clinics during an ambulatory primary care visit. The primary outcome measure is the annual rate of new documented dementia diagnosis. In the first year, the Indiana trial enrolled 5960 older patients with a mean age of 71.9 years and SD of 6.50, 61% were female and 51% were African American. In the clinics that were randomized to PDM + QDRS, there were 827 patients who completed at least one question on the QDRS. The Indiana trial used a non-interruptive Best Practice Alert (BPA) for the first year of operation. For the second year of operation, the trial coupled the BPA with an InBasket message to primary care physicians to overcome the shortcoming of the Non-interruptive nature of the BPA. We have created an IT implementation manual to deploy the PDM with minimum expertise.

Abstract citation ID: igae098.1929

### LEVERAGING THE NIH TOOLBOX FOR EARLY DETECTION OF COGNITIVE IMPAIRMENT IN PRIMARY CARE: THE TOOLBOX DETECT TRIAL

Michael Wolf, and Cindy Nowinski, *Northwestern University, Evanston, Illinois, United States*

The NIH Toolbox comprises practice standard, cognitive and neurological functional assessments that can be deployed via iPad for research purposes. Our Northwestern team previously validated two of these brief measures of episodic memory and executive function to have clinical utility. Combined, these scored tests can be mobilized as a primary care-based screening tool for detecting cognitive impairment and dementia. To address common time and workflow constraints in these often-busy clinical settings, our derived assessment, known as Toolbox Detect, was adapted accordingly to be self-administered, and to be tethered to the electronic health record thereby removing manual data entry, followed by clear, 'turnkey' recommendations to address any suspected impairment. The effectiveness of Toolbox Detect to improve the timely detection, diagnosis, and referral for cognitive impairment is now being evaluated in a large, pragmatic trial conducted across 40 primary care sites within one academic health system, with real-time findings informing an embedded dissemination and implementation study at 20 federally qualified health centers. Early evidence of the fidelity of the Toolbox Detect strategy's implementation, in terms of feasibility and acceptability and learnings of how it best might be adapted to community centers serving diverse patient populations will be explored in this presentation. Specific attention will be paid to the challenges and opportunities of introducing new technologies into primary care workflows, and healthcare provider, patient and care partner unmet needs in terms of cognitive screening.

### SESSION 4235 (PAPER)

### LIVING WITH DEMENTIA III

Abstract citation ID: igae098.1930

### UNDERSTANDING THE NEEDS OF INFORMAL CAREGIVERS OF PEOPLE EXPERIENCING DEMENTIA AS IDENTIFIED ON SOCIAL MEDIA

Jane Flanagan[1], Maureen connolly[1], Amanada Coakley[2], Matthew (Brent) Fugate[1], Emily Prud'hommeaux[1], and Colin Young[3], *1. Boston College, Chestnut Hill, Massachusetts, United States*, *2. Endicott College, Beverly, Massachusetts, United States*, *3. Italina Home for Children, Boston, Massachusetts, United States*

Social media has the potential to reach informal caregivers of people experiencing dementia and provide them with supportive and accurate strategies. This study aimed to understand the caregivers' concerns posted on social media and determine if the responses were supportive and accurate. This study utilized a human-centered machine learning (HCML) framework, natural language processing (NLP), and information retrieval (IR) techniques to extract posts from a dementia caregiver group with the largest number of members (N = 59,954). Using a qualitative descriptive design, we thematically analyzed a sample of 500 initial posts. In a subset of 100 posts and responses to those posts, we used a Delphi method to determine if the responses were supportive and accurate. Five themes were identified: 1) Seeking information about unusual problem behaviors of dementia with nowhere to turn for help; 2) Grief, a sense of loss, and loneliness are profound; 3) Support from others is challenged by relationship issues and distance from caregiving; 4) Financial and legal issues present unexpected challenges; and 5) The need to celebrate caregiving is not recognized. The team found that 73% of the responses to initial posts were supportive, 10% were not, and 10%; did not address the concern; 73% of the responses were accurate, 1% were not and 10% did not address the concern. These findings highlight multifaceted concerns dementia caregivers face. The information is foundational to developing an interdisciplinary HCML interactive question-answering tool using NLP and IR to provide supportive and accurate information to dementia caregivers.

Abstract citation ID: igae098.1931

### THE DEMENTIA CARE PARTNER HUB: A NOVEL RESOURCE TO SUPPORT PEOPLE LIVING WITH DEMENTIA, CARE PARTNERS, AND CARE TEAMS

Walter Dawson[1], Jenn Hollandsworth Reed[2], and Sherril Gelmon[2], *1. Oregon Health & Science University, Portland, Oregon, United States*, *2. Portland State University, Portland, Oregon, United States*

Care partners of people living with dementia (PLWD) need information and access to multiple services and programs to provide support and maintain their own wellbeing. Focus groups (n=5) and interviews (n=24) were conducted with care partners and interviews with organizations serving PLWDs in the Portland, Oregon region between July 2022 and September 2023. A review of organizational websites and relevant literature identified several US-based role model programs. Data analysis revealed community assets and unmet needs, and articulated resource priorities to inform programs/services. Dementia care partners need multiple supports for caregiving: information about dementia; navigation and coordination; psychological support; recognition of care partner burden; leisure activities; day services; transportation; legal and financial services; home health; and financial support. Feedback revealed care partner deficits in accessing information and social supports. To augment existing services and supports for care partners, a Dementia Care Partner Hub (the "Hub") should be developed. The Hub would be the central node of a regional network of existing services. The Hub should serve all dementia care partners in its service region, with a responsive design to the specific assets and needs of historically and currently underrepresented communities. It should augment and complement current clinical, research, and social programs by connecting care partners to programs and services. Community-specific information for communities defined by geography and identity will ensure the Hub equitably serves care partners with varying needs. Key elements to achieving this equity goal include language access, culturally appropriate information, and accessibility of the Hub across the entire region.

Abstract citation ID: igae098.1932

### THE PUBLIC HEALTH IMPLICATIONS OF DEMENTIA CAREGIVING: NATIONAL AND STATE-LEVEL VARIATIONS

Joseph Gaugler[1], Elma Johnson[1], Maya Koffski[2], Benjamin Denno[3], Matthew Baumgart[3], Basia Belza[4], and Lisa McGuire[5], *1. University of Minnesota, Minneapolis, Minnesota, United States*, *2. University of Minnesota School of Public Health, Minneapolis, Minnesota, United States*, *3. Alzheimer's Association, Chicago, Illinois, United States*, *4. University of Washington, Seattle, Washington, United States*, *5. GSA, Atlanta, Georgia, United States*

Unpaid dementia care has emerged as a public health priority. Specifically, dementia caregiving influences the health of those providing assistance (e.g., family members, friends) and recipients of care. However, the intensity of care provision and its health implications are often disproportionate within the population. Public health can engage in various initiatives to address such disparities, including improving surveillance of dementia caregivers with greater precision; engaging in outreach and education about the potential health risks of dementia care; and coordinating initiatives across traditional silos (e.g., public health, aging service providers, healthcare systems) to result in more available, accessible, and ultimately effective dementia care. Relying on 2021-2022 data from the Behavioral Risk Factor Surveillance System data from 47 states, we conducted a series of three-way cross-tabulations between dementia caregivers, non-dementia caregivers, and non-caregivers to ascertain key disparities and contextual factors that could serve as subsequent health targets. Nationally, although dementia caregivers provided more extensive help to care recipients for longer periods, variations in other domains (e.g., lifestyle behaviors) were less apparent nor consistent. More crucially, those patterns of statistical differences that emerged in the national analysis were far more variable and diverse when considering specific states, suggesting the urgent need for granular, more local data sources to better inform services, supports, and public health action to support dementia caregivers.

Abstract citation ID: igae098.1933

### "CULTIVA MENTE": DESIGNING A BRAIN HEALTH CAMPAIGN FOR PROMOTING DEMENTIA-RISK REDUCTION

Jose Aravena[1], Hugo Castro[2], Ronald Poblete[3], and Becca Levy[4], *1. Department of Social & Behavioral Sciences, Yale University, New Haven, Connecticut, United States*, *2. Inmedios, Santiago, Region Metropolitana, Chile*, *3. Unaffiliated, Santiago, Region Metropolitana, Chile*, *4. Yale University School of Public Health, New Haven, Connecticut, United States*

Implementing a successful campaign to promote dementia-risk reduction requires an understanding of how both older persons and healthcare providers conceptualize dementia risk as well as culturally relevant barriers and facilitating factors related to achieving brain health. Thus, this study aimed to achieve this goal through a qualitative study using a design-thinking approach with senior center participants aged 60 and older with no dementia, and healthcare professionals working at the senior centers in Chile. Ten centers, each with one focus group of senior center participants and one focus group of health professionals discussed topics related to a brain health campaign. Thematic analysis was conducted and supplemented with an analysis of previous educational campaigns. A total of 115 patients and 51 health professionals participated. Themes identified included: A) social images about dementia; B) knowledge about dementia risk factors; C) structural barriers to dementia prevention, including ageism; D) barriers and facilitating factors to dementia prevention, such as education: and E) role models of persons that worked toward brain health. Based on these results and analysis of previous initiatives, an educational campaign named 'Cultiva mente' (cultivate mind) was developed, referring metaphorically to nurturing minds like plants. The campaign includes posters and brochures highlighting positive images and behaviors about aging and brain health, an educational interactive website, and a training program for health professionals on dementia risk reduction strategies. This study constitutes the design of a culturally relevant campaign to promote dementia risk reduction that will be tested in a future efficacy study

### SESSION 4240 (SYMPOSIUM)

Abstract citation ID: igae098.1934

### MAKING AGE-INCLUSIVE CAMPUS PRACTICES MORE DEMENTIA-INCLUSIVE

Chair: Joann Montepare
Discussant: Katherina Nikzad-Terhune

Shifting demographics are calling for higher education to respond to aging populations through more age-inclusive programs, practices, and partnerships. As institutions navigate this new educational terrain, gaps are evident. This symposium draws attention to the need to integrate age-inclusive with dementia-inclusive efforts. Addressing this need with educational interventions can benefit our students' own healthy aging and longevity. Moreover, efforts that involve older adults living with dementia likewise have beneficial capacity for their well-being. To begin, Montepare (Lasell University) will provide an overview of contemporary age-inclusive initiatives in higher education (Age-Friendly University/AFU, Age Inclusivity Domains of Higher Education/AIDHE) and research motivating dementia-focused educational opportunities. Next, Mastel-Smith and colleagues (University of Texas at Tyler) will describe how nursing students co-created life stories online with people living with dementia who served as mentors and will show how qualitative data from students and mentors revealed positive outcomes for both groups. Kimzey (Texas Christian University) will then discuss a novel educational strategy in which a person with dementia (expert by experience) participated in teaching an undergraduate dementia course for health profession students and present data from focus groups and interviews showing the benefits of dementia-inclusive educational design. Finally, Shovali (Eckerd College) will describe how harnessing technology can expand dementia-inclusive teaching and learning. Specifically, how incorporating oculus VR headsets in experiential classroom activities can enhance students' understanding and empathy of people living with dementia. Katherina Nikzad-Terhune (Northern Kentucky University), co-convener of the Age Inclusivity in Higher Education Interest Group, will serve as discussant.

Abstract citation ID: igae098.1935

### SOME WHOS, WHYS, AND HOWS OF BEING MORE DEMENTIA-INCLUSIVE IN AND BEYOND THE CLASSROOM

Joann Montepare, *Lasell University, Newton, Massachusetts, United States*

Demographic statistics predict a dramatic rise in the incidence of dementia (D) and Alzheimer's disease (AD) as populations age. An estimated 5.3 million Americans were diagnosed with D/AD in 2015, and national predictions are that the number of adults diagnosed with D/AD will rapidly grow each year as the size of the population aged 65 years and older continues to increase (CDC, 2018). As such, researchers, practitioners, and policy makers have called for examining more broadly how we can develop interventions that target stigma, knowledge, diagnosis, resilience, caregiving, and related issues. An important, although overlooked, component of this call is developing educational opportunities for young adults. To this end, this presentation will describe research that aimed to gather information about undergraduate students' dementia experience, knowledge, and concern. Specifically, a survey of 106 students distributed in diverse classes found that more than 50% of students had experience with someone with D/AD, showed low levels of knowledge, expressed strong concern about developing D/AD, and had an interest in educational activities. As well, this presentation will argue why efforts to be more dementia-inclusive should be incorporated into contemporary age-inclusive initiatives in higher education (Age-Friendly University/AFU, Age Inclusivity Domains of Higher Education/AIDHE). Finally, several activities in (TimeSlips) and beyond the classroom (Dementia Friends) will be described to show how experiential learning opportunities about dementia can be mounted.

Abstract citation ID: igae098.1936

### "EVERYBODY HAS A SOUL": PEOPLE WITH DEMENTIA AND NURSING STUDENTS CO-CREATING LIFE STORIES

Beth Mastel-Smith[1], Michelle Kimzey[2], Amy Ahlbrand Robinson[3], and Melinda Hermanns[4], *1. The University of Texas Tyler, Baker City, Oregon, United States*, *2. Texas Christian University, Fort Worth, Texas, United States*, *3. Freelance Artist, Missouri City, Texas, United States*, *4. The University of Texas Tyler, Tyler, Texas, United States*

Lack of meaning and purpose in life can increase disability, stress, mortality, and the risk of cognitive impairment. Finding meaning and purpose helps people with dementia adjust to the diagnosis and can be accomplished via expressions of identity such as life review, a formal process of systematically reviewing and reflecting on one's lived life. Digital storytelling involves telling a story using computer technology to record and share an individual's narrative. The purpose of this study was to understand the perceived benefits of nursing students and people with dementia (the mentors) described after digitally co-creating the mentors' life story. Allport's Intergroup Contact Theory and the Lifestory as Legacy protocol guided intervention development. The study was approved by Institutional Review Boards and participants signed informed consent. Ten mentors and 13 students co-created the mentors'

life story during seven online meetings and completed qualitative interviews using an interview guide. Colaizzi's process of data analysis was used to analyze data. Students and mentors described what it was like to co-create the mentor's life story, what the experience meant to them, and what they learned. Mentors described how the student did and made them feel, importance of sharing their story with family, and advice for future health professionals regarding dementia care. Students commented on preparation to elicit their mentor's stories and the perceived effect on their mentor. Co-creation of life stories online is feasible and had positive effects for nursing students and mentors with dementia.

Abstract citation ID: igae098.1937

### EXPERT BY EXPERIENCE WITH DEMENTIA CO-TEACHING: HAVING AN EXPERT IN EVERY CLASS

Michelle Kimzey, *Texas Christian University, Fort Worth, Texas, United States*

Care for people with dementia requires a better understanding of the reality of dementia. People with dementia are the true experts on dementia because they are living the experience. Including them in the teaching role benefits students and a person with dementia. The study aimed to understand how a person with dementia (expert by experience, [EBE]) involved in the teaching of an undergraduate dementia course impacted health profession students, an EBE with dementia, and their care partner. The qualitative exploratory design used semi-structured interviews for the student focus groups and interviews with an EBE and care partner. Students described the benefits of EBE, including increased awareness, knowledge, competencies, and reduced stigma. The EBE discussed the benefits, challenges, and meaningfulness of the experience. The care partner acknowledged the benefits for EBE and the family. Engaging people with dementia in EBE benefited students, families, and the EBE. By giving them a platform to educate students and self-advocate, we can positively impact the lives of people with dementia.

Abstract citation ID: igae098.1938

### INTEGRATING VIRTUAL REALITY DEMENTIA SENSITIVITY TRAINING INTO THE UNDERGRADUATE CURRICULUM

Tamar Shovali, *Eckerd College, St Petersburg, Florida, United States*

There is a need for Age-Friendly endeavors to include dementia-inclusive educational opportunities in higher education. Given the rising aging population, undergraduate students increasingly will (a) have personal experiences with loved ones diagnosed with dementia or Alzheimer's disease, (b) pursue careers in the field of aging and directly work with individuals with dementia or Alzheimer's disease, or (c) indirectly work with individuals with dementia or Alzheimer's disease and their caregivers through roles in various professions such as finance or law. Undergraduate students in a gerontology course at an Age-Friendly University engaged in a dementia inclusive virtual reality training intended to sensitize individuals to the needs of people living with dementia. Virtual reality experiences related to dementia can help build empathy towards daily challenges faced by people living with dementia. Prior to and after engagement in the session students were asked questions about their understanding and empathy for people with dementia and their caregivers. Small group discussions followed the session to prompt reflection, encourage critical thinking, and facilitate discussion on the impact and lessons learned from the training. This experiential learning was preceded by an interactive lecture about the basics of Alzheimer's disease and dementia delivered by a Community Educator from the Alzheimer's Association. Age-Friendly University principles provide a guideline for the many ways higher education can shape age inclusive teaching and learning environments that promote greater empathy and understanding for people living with dementia or Alzheimer's disease and their caregivers.

### SESSION 4245 (SYMPOSIUM)

Abstract citation ID: igae098.1939

### NEW CONSUMER-REPORTED QUALITY INDICATORS FOR HOME AND COMMUNITY-BASED SERVICES: THE NCI-AD SURVEY

Chair: Tetyana Shippee
Discussant: Regina Shih

This symposium will introduce new consumer-reported quality indicators for home and community-based services (HCBS). The HCBS Quality Measures Set released by the Centers for Medicare and Medicaid Services includes few consumer-reported measures, most of which are not psychometrically validated or widely applicable. The National Core Indicators- Aging and Disability Survey (NCI-AD) is an underutilized source of consumer-reported data for measuring HCBS quality. This symposium includes 4 presentations. [1] The first presentation provides an overview of the NCI-AD database for researchers and policy-makers, including new features/survey tools added to more recent survey waves. [2] The second presentation introduces five new quality indicators (from 22 consumer-reported survey items) developed using exploratory and confirmatory factor analyses of the NCI-AD data. These indicators include staff quality, environmental safety, community inclusion, service satisfaction, and service decision-making; each with good reliability and validity (Cronbach's alpha, 0.78-0.89). [3] The third presentation demonstrates measurement of service-specific unmet needs as a measure of consumer-reported quality for 6 commonly used HCBS, and how these differ between consumers with versus without dementia. [4] The fourth presentation discusses state-level factors as correlates of consumer-reported service-specific unmet needs, with emphasis on a higher proportion of Medicare managed care beneficiaries in a state as a predictor of greater unmet needs for some HCBS measures. Collectively, the four projects introduce new consumer-reported quality indicators for HCBS and equip the attendees with knowledge to measure consumer-reported quality in HCBS, including for older adults with dementia.

Abstract citation ID: igae098.1940

### DEVELOPMENT OF CONSUMER-REPORTED QUALITY INDICATORS FOR PUBLICLY-FUNDED HOME AND COMMUNITY-BASED SERVICES

Tetyana Shippee[1], Jack Wolf[2], Romil Parikh[2], Yinfei Duan[3], Benjamin Langworthy[2], Stephanie Giordano[4], and Eric Jutkowitz[5], *1. University of Minnesota School Of*

*Public Health, Minneapolis, Minnesota, United States*, *2. University of Minnesota, Minneapolis, Minnesota, United States*, *3. University of Alberta, Edmonton, Alberta, Canada*, *4. Human Services Research Institute, Cambridge, Massachusetts, United States*, *5. Brown University, Brown University, Rhode Island, United States*

Consumer-reported measures are important quality indicators of person-centered home and community-based services (HCBS). Lack of granularity in administrative data hinders measurement of consumer-reported quality in publicly-funded HCBS. The National Core Indicators- Aging and Disability Survey (NCI-AD) is potentially a great source of consumer-reported data for quality measurement in publicly-funded HCBS. Therefore, we analyzed NCI-AD data to develop consumer-reported quality indicators for HCBS. We applied exploratory factor analyses (EFA) to consumer-reported items from the 2018-2019 survey wave (n=5,572 community-dwelling consumers, age ≥65 years). Through EFA, we identified 5 quality indicators which were then validated in two ways, qualitatively by a technical expert panel (for face validity and contextualization) and quantitatively, using confirmatory factor analyses in the 2017-2018 survey wave (n=9,145 community-dwelling consumers, age ≥65 years). The 5 newly developed and validated quality indicators included service satisfaction (3 survey items, Cronbach's alpha=0.89, McDonald's omega=0.90), staff quality (5 survey items, alpha=0.86, omega=0.89), environmental safety (4 survey items, alpha=0.81, omega=0.89), service decision-making (5 survey items, alpha=0.79, omega=0.86), and community inclusion (5 survey items, alpha=0.78, omega=0.86). These indicators had reasonable concurrent validity, with service satisfaction having the highest correlation with other indicators (Pearson's correlation coefficient between 0.59-0.36). These five new consumer-reported quality indicators can be used by researchers, service providers, and policy makers for evaluating and monitoring service quality and identifying modifiable targets for quality improvement in publicly-funded HCBS.

Abstract citation ID: igae098.1941

### USING NCI-AD FOR LONG-TERM SERVICES AND SUPPORTS AGING AND DISABILITY SYSTEM IMPROVEMENT

Stephanie Giordano[1], and Rosa Plasencia[2], *1. Human Services Research Institute, Cambridge, Massachusetts, United States*, *2. ADvancing States, Arlington, Virginia, United States*

National Core Indicators Aging and Disabilities (NCI-AD™), survey data support State Medicaid, aging, and disability agencies to conduct surveys with individuals receiving publicly-funded long-term services and supports and measure and track their own outcomes and performance using reliable data collection methods and tools. Established in 2015, NCI-AD is now in use by 23 states nationwide. The NCI-AD portfolio includes the flagship survey – the Adult Consumer Survey (ACS) and the State of the Workforce—Aging and Disabilities (SotW-AD) survey. The ACS collects data directly from older adults and people with physical disabilities to assess the outcomes of services provided to individuals. Indicators address key areas of concern including service planning, rights, community inclusion, choice, health and care coordination, safety and relationships. In 2022, NCI-AD piloted the SotW-AD is a first of its kind for the aging and disabilities field, this new effort collects and aggregates statewide information about wages, benefits, and turnover of the direct service workforce—as reported by the agencies that employ them. This session will provide an overview of the NCI-AD program, the type of data collected from each survey, and how state-level and research partners have utilized the data sets. Additionally, 18 NCI-AD measures are included in the newly released CMS HCBS Quality Measure Set. We will discuss how this impacts states, and potential ways that these and other measures will be implemented and used to support important aspects of services access and equity.

Abstract citation ID: igae098.1942

### HOW DO UNMET NEEDS VARY BETWEEN PEOPLE WITH AND WITHOUT DEMENTIA RECEIVING HOME AND COMMUNITY-BASED SERVICES

Eric Jutkowitz[1], Jack Wolf[2], Romil Parikh[2], Benjamin Langworthy[2], and Tetyana Shippee[3], *1. Brown University, Brown University, Rhode Island, United States*, *2. University of Minnesota, Minneapolis, Minnesota, United States*, *3. University of Minnesota School Of Public Health, Minneapolis, Minnesota, United States*

The National Core Indicators Aging and Disability (NCI-AD) survey samples home and community-based service recipients to understand their unmet HCBS needs. Using 2016-2019 NCI-AD data (N=24348), we evaluated the demographic (e.g, race), health (e.g, hearing ability), environmental (e.g., living alone) and payer (e.g., Medicaid) factors associated with reporting an unmet need among HCBS users age ≥65 with and without dementia. We defined an unmet need as reporting wanting more adult day services, caregiver support, delivered meals, homemakers, personal care assistance, or transportation services. We modeled the odds of unmet needs and tested for differences in unmet needs between people with and without dementia. Personal care assistance was the most frequently used service among people living with and without dementia (59% vs. 50%) followed by delivered meals (22% vs. 37%), homemaker services (21% vs. 25%), adult day services (12% vs. 8%), transportation (9% vs. 8%) and caregiver support (9% vs. 5%). Unmet needs among people with and without dementia respectively varied between 5% and 3.2% (adult day services) to 11% and 14% (personal care assistants). Asians compared to Whites without dementia were significantly more likely to report an unmet need for personal care and homemaker support, and less likely to report an unmet need for caregiver support, but there was little association for people with dementia. Overall health and hearing ability also had different associations with unmet needs between people living with and without dementia but the relationships were not consistent in direction.

Abstract citation ID: igae098.1943

### IMPACT OF STATE-LEVEL FACTORS ON CONSUMER-REPORTED UNMET NEEDS IN HOME AND COMMUNITY-BASED SERVICES

Romil Parikh[1], Tetyana Shippee[2], Benjamin Langworthy[1], Zheng Wang[3], Eric Jutkowitz[4], and Stephanie Giordano[5],

*1. University of Minnesota, Minneapolis, Minnesota, United States, 2. University of Minnesota School Of Public Health, Minneapolis, Minnesota, United States, 3. University of Minnesota School of Public Health, Minneapolis, Minnesota, United States, 4. Brown University, Brown University, Rhode Island, United States, 5. Human Services Research Institute, Cambridge, Massachusetts, United States*

In the value-based purchasing framework, provider-specific quality indicators are often compared with national average estimates to measure performance. Hence, it is important to elucidate the impact of state-level administrative factors on consumer-level quality indicators for their national use. Therefore, we evaluated associations of state-level factors (from publicly reported data) with consumer-reported service-specific unmet needs for six commonly used home and community-based services (HCBS) from the NCI-AD data (2015-2019, n=23,333 community-dwelling older respondents; age, ≥65 years). We fit a mixed-effects logistic regression model for unmet needs in each of six HCBS; adjusting for state ID random effect, consumer-level health and demographic fixed effects, and four state-level fixed effects including HCBS spending relative to institutional care spending, percent managed care population, HCBS spending per client, and Medicaid expansion. To evaluate whether addition of these 4 state-level fixed effects improved model fit, we evaluated improvement in the Akaike Information Criterion, for each of six HCBS. A 10% increase in the proportion of managed care population in state was significantly associated with greater odds of consumer-reported unmet needs in personal care (Odds Ratio [OR], 1.37, p<0.01), homemaker services (OR, 1.74, p<0.001), and transportation services (OR, 1.22. p<0.05). There were no significant associations between other state-level factors and service-specific unmet needs. Addition of the four state-level factors improved model fit for personal care, homemaker, meal delivery, and transportation services, and worsened model fit for adult day and caregiver-respite services. State-level factors likely have very little impact on consumer-reported unmet needs as an HCBS quality indicator.

### SESSION 4250 (SYMPOSIUM)

Abstract citation ID: igae098.1944

### PRINCIPLES OF QUEER GERONTOLOGY

Chair: Vanessa Fabbre
Co-Chair: Austin Oswald
Discussant: Sarah Jen

This symposium will present principles for queer gerontology – or gerontological scholarship aimed at promoting social justice for all, but especially for LGBTQ+ people as they age – and potential applications of this approach within existing and future scholarly projects. Building upon recent applications of queer theory and perspectives to aging studies and gerontology, we propose five principles of queer gerontology—1) Deeping Visibility and Awareness; 2) Developing and Applying Critical Perspectives; 3) Challenging Oppression and Injustice; 4) Engaging Multiple Epistemologies and Modes of Inquiry; and 5) Fostering Transdisciplinary Advancement—and present evidence and themes from existing empirical and conceptual scholarship that exemplify these principles. The first presentation will describe the need for queer gerontology, the iterative process through which these principles were developed, and present the principles. The second presentation will apply a critical secondary qualitative data analysis to illustrate the principles of queer gerontology in action. The third presentation will explore the theme of being a scholar-activist in the practice of queer gerontology. The symposium will offer a chance for attendees to reflect critically on their own scholarship and devise directions for future work with respect to these principles.

Abstract citation ID: igae098.1945

### WORKING THE SCHOLAR-ACTIVIST HYPHEN: QUEER GERONTOLOGY PERSPECTIVES FROM OLDER BLACK LESBIANS

Austin Oswald, *Dalhousie University, Nova Scotia, Canada*

The LGBTQ+ aging population is growing increasingly racially and ethnically diverse; however, older adults of color are underrepresented in LGBTQ+ aging research. This significant gap in the field constitutes a critical ethical issue that future scholarship must address. Advancing LGBTQ+ aging research necessitates a heightened focus and specificity on subgroups, particularly those with intersecting marginalized identities, while generating knowledge that challenges structural oppression. This presentation sheds light on insights gleaned from an ongoing, multi-year participatory action research project aimed at addressing gaps within contemporary LGBTQ+ aging research by highlighting the needs and experiences of LGBTQ+ older adults of color within an age-friendly city. Five older Black lesbian women, aged between 65 and 75, actively participated in designing, implementing, and evaluating a research project that analyzed data from public records and focus groups. Findings emphasize three fundamental principles of queer gerontology, providing tangible examples of community-engaged scholarship that researchers can utilize to advance the field of LGBTQ+ aging. This includes strategies aimed at deepening visibility and awareness of underrepresented subgroups through an intersectional lens, applying critical perspectives to challenge dominant frameworks that narrowly define old age and aging through heterosexual and gender norms, and combating oppression and injustice through activist-scholarship committed to real-world change. The insights from this presentation underscore the imperative for inclusive, community-engaged scholarship to advance our understanding of LGBTQ+ aging, promote social justice, and effect meaningful change in the lives of older LGBTQ+ adults with multiple intersecting identities.

Abstract citation ID: igae098.1946

### USING CREATIVE NARRATIVES AND VISIONS OF LATER LIFE TO QUEER GERONTOLOGICAL SCHOLARSHIP

Sarah Jen, *University of Kansas, Lawrence, Kansas, United States*

Queering gerontology calls for more diverse and inclusive visions of old age, including those that resist normative ways of being. LGBTQ people have particular contributions to make to this project given their experiences of non-normative life events, sequences, and desires. This study presents a secondary qualitative data analysis, drawing from two existing datasets (13 life reviews with older bisexual-identified women and 40 creative narratives published in Bi Women Quarterly). The analysis examines queer and non-normative visions and experiences of aging from the perspective of queer and trans individuals across the life course. The findings are examined through the lens of three principles of queer gerontology, illustrating the potential of queer and creative visions of aging to reimagine the potential of gerontological scholarship. Notably, the creation and dissemination of queer narratives of aging and later life deepen visibility and awareness of diverse and marginalized experiences while also offering new and creative ways of imagining later life for all populations. The examined narratives engage critical perspectives, often dispelling normative ways of being through camping up, resisting, or queering notions of self, kinship, life and death, and the narrative form. Narratives also illustrate the engagement of multiple epistemologies, creating potential for more creative modes of inquiry, including creative writing and imagery, in envisioning later lives. These findings indicate the potential of revisiting existing data through the lens of queer gerontology principles to illuminate the possible forms queer gerontological scholarship could take, both empirical and conceptual.

Abstract citation ID: igae098.1947

### PRINCIPLES OF QUEER GERONTOLOGY

Vanessa Fabbre[1], Austin Oswald[2], and Sarah Jen[3], *1. Washington University in St. Louis, St. Louis, Missouri, United States*, *2. Dalhousie University, Nova Scotia, Canada*, *3. University of Kansas, Lawrence, Kansas, United States*

Gerontology has advanced greatly in recent decades in its attention to the lives of LGBTQ+ people. To this end, an increasing number of scholars have argued for a queering of gerontologists' understanding of the human life course, as well as a queering of the field of gerontology itself. Yet, to date there exists no clear articulation, in practical terms, of what queer gerontology is and how it could be applied to scholarship and activism. Coming from a first-person, reflexive position, we argue that a clear vision of queer gerontology should be based on scholars' historical use of queer theory to challenge normative ideas about the self and social life. In this presentation we will briefly present the historical landscape of LGBTQ+ aging studies, with an emphasis on recent conceptual and methodological advances, followed by a description of the iterative process through which we (as both early and mid-career scholars) developed the following five principles of queer gerontology: 1) Deeping Visibility and Awareness, whereby the identities and lives of LGBTQ+ and all oppressed peoples are elevated and illuminated; 2) Developing and Applying Critical Perspectives, whereby concepts and theories that challenge the status quo are engaged; 3) Challenging Oppression and Injustice, whereby critical theories are explicitly used to guide social change efforts; 4) Engaging Multiple Epistemologies and Modes of Inquiry, whereby scholars actively promote and use various approaches to scientific and social inquiry; and 5) Fostering Transdisciplinary Advancement, whereby scholars seek out and develop relationships that transcend traditional disciplinary boundaries.

### SESSION 4255 (SYMPOSIUM)

Abstract citation ID: igae098.1948

### RESHAPING LONG-TERM CARE SYSTEM INTO A SUSTAINABLE AND HIGH-QUALITY HEALTHCARE FIELD GLOBALLY: IT IS POSSIBLE!

Chair: Katherine McGilton
Co-Chair: Karen Spilsbury
Discussant: Carl Thompson

Years of investigation have revealed that despite compelling evidence, long-term care (LTC) home policies and procedures remain resistant to change. Globally, LTC homes grapple with subpar labor conditions stemming from structural and institutional elements, resulting in precarious work environments for its workforce. The revitalization of the LTC necessitates immediate attention to adequate staff and skill mix and innovative models of care to enable effective and person-centered care of residents. In this symposium, we will present an interactive overview and discussion of the work from international researchers exploring sustainable recruitment and retention of the LTC workforce, optimal staff and skill mix and initiatives for the intricate care requirements of residents and to mitigate precarious work conditions for the workforce. Specifically,: 1. Dr. Devi's team presents findings from a Realist Review called the REACH including an explanatory framework to implement strategies to attract, recruit and retain LTC staff. 2. Dr. Montse and team highlight findings from a narrative review of measures taken by national European administrations to optimize working conditions for the LTC workforce including during the COVID-19 pandemic. 3. Dr. Bleijlevens and colleagues present the successful implementation of DAIly NURSE Intervention that transformed breakfast time for LTC residents. 4. Dr. McGilton and team implemented and evaluated the impact of nurse practitioner-led huddles that helped reduce moral distress amongst staff. 5. Prof. Zúñiga's team prepares the scale-up of a registered nurse-led care model strengthening their geriatric expertise to reduce unplanned hospitalization for LTC residents.

Abstract citation ID: igae098.1949

### OPTIMIZING STAFF RECRUITMENT AND RETENTION IN CARE HOMES: WHAT WORKS? THE REACH REALIST REVIEW

Reena Devi[1], Kirsty Haunch[1], Karen Spilsbury[1], Sonia Dalkin[2], Angela Bate[2], Liz Jones[3], Claire Goodman[4], and Natalie King[1], *1. University of Leeds, Leeds, England, United Kingdom*, *2. Northumbria University Newcastle, Newcastle, England, United Kingdom*, *3. National Care Forum, Nottingham, England, United Kingdom*, *4. University of Hertfordshire, Hertfordshire, England, United Kingdom*

Solutions for sustaining the social care workforce need to a) consider the different factors which influence staff experiences, b) recognize the nuances between different care settings, and c) differentiate between staff groups (e.g. age, levels of experience). At the University of Leeds, we led research which uses a realist synthesis approach to reviewing international academic and grey literature and contributions from sector stakeholders to develop theories. The results identified strategies used in care homes to attract, recruit, and retain Registered

Nurses and care workers, e.g., effective job interviews, opportunities for career development, rewards and recognition, flexible working options, and caring conversations. The strategies used to attract, recruit, and retain staff do not operate independently, they interact and work together. We brought the evidence-based programme theories together into an explanatory framework to explain how and why these strategies work, for which staff, the conditions needed for strategies to work, and the outcomes to be expected. The framework summarizes the different ways the strategies help with attracting, recruiting and retaining staff. For example, empowering staff through caring conversations and opportunities for career development; building and fulfilling a psychological contract to set accurate expectations of care work; and reciprocating positive experiences (when providing staff with positive experiences staff will reciprocate and respond positively (i.e. commitment and loyalty at work). We will summarize the full findings during the symposium. To the best of our knowledge, this is the first time a realist synthesis approach has been used to review the literature in this area.

Abstract citation ID: igae098.1950

### REQUIREMENTS FOR POLICY MEASURES THAT FAVOR BETTER WORKING CONDITIONS FOR THE LONG-TERM CARE WORKFORCE IN EUROPE

Montserrat Gea-Sánchez, and Jose Mateos, *University of Lleida, Lleida, Catalonia, Spain*

There is a need to improve key working conditions, such as low wages, high rates of part-time employment, and insufficient autonomy and support for workers. In addition, the pandemic has highlighted the need for better coordination between the long-term care sector and the wider health system, and recruitment and training policies must be accompanied by strategies to improve management within the sector. A narrative review of the main measures carried out by several national European administrations to improve working conditions in long-term care was conducted. Documentation was retrieved from a scientific literature review, grey literature and key informants (n = 9). The results of the review suggest that the long-term care sector suffers from structural deficiencies that were accentuated during the COVID-19 pandemic. Among these deficiencies the following were highlighted: the lack of professionals in long-term care mainly due to poor working conditions and gaps in training and role definition, especially for nurses. Measures to reverse this situation, such as the integration of new technologies or the replacement of nurses by other professionals who have acquired some of their competencies have been implemented in some European countries. These measures, in addition to being insufficient, may lead to a decline in the quality of care. Persistent problems of high turnover and low retention pose a threat to both accessibility and quality of services.

Abstract citation ID: igae098.1951

### IMPACT OF A NURSING INTERVENTION ON PHYSICAL ACTIVITY AND INDEPENDENCE IN NURSING HOME RESIDENTS

Michel Bleijlevens, Mirre den Ouden, Judith Meijers, Sandra Zwakhalen, Erica De Vries, and Jan Hamers, *Maastricht University, Maastricht, Limburg, Netherlands*

DAILy nurse is a complex intervention that was used in changing the breakfast routine for nursing home residents. It was expected that nursing home residents would be more engaged and would perform more activities independently; furthermore, it was expected that nursing staff behavior would change towards encouraging residents and taking over fewer activities. A quasi-experimental design with a pre- and posttest in four nursing homes was utilized. Participants were 104 residents and 134 staff. Video recordings were used to observe primary outcomes: engagement and independence in breakfast activities, and nursing staff activities. Results showed that staff succeeded in changing the breakfast routine dramatically. Departing from a standardized breakfast moment (with sandwiches fully prepared, no choice for residents) in a rather sterile environment (e.g., no ambiance, empty tables, almost no interactions), the routine was changed into a breakfast moment where tables were set with breadbaskets, different types of sandwich filling and pots of coffee and tea. This enabled residents to prepare their own sandwich and to be engaged in this activity, which led to interactions between residents; and residents and staff. Video recordings, and perceptions of nursing home staff, indicated statistically significant improvement of the daily lives of residents during the breakfast moment. However, no effect was found in nursing staff behavior. In conclusion, this study showed that applying DAILy NURSE, led to more engagement and independence of residents. It should be emphasized that this was feasible without increasing the number of staff or the allocation of extra budgets.

Abstract citation ID: igae098.1952

### ENHANCING CARE AND TEAM PROCESSES THROUGH NURSE PRACTITIONER-LED HUDDLES IN LONG-TERM CARE HOMES

Shirin vellani[1], Katherine McGilton[1], Alexandra Krassikova[2], Margaret Keatings[3], and Souraya Sidani[4], *1. Toronto Rehabilitation Institute-UHN - Toronto, ON, toronto, Ontario, Canada*, *2. KITE Research Institute, Toronto, Ontario, Canada*, *3. Toronto Rehabilitation Institute, Toronto, Ontario, Canada*, *4. Toronto Metropolitan University, Toronto, Ontario, Canada*

Long-term care (LTC) homes faced unprecedented challenges during the COVID-19 pandemic, impacting both staff and residents. Huddles led by a skilled facilitator have the potential to enhance care and team dynamics positively. Nurse practitioners (NPs) are highly trained clinician and leader with a proven history of contributing to favorable outcomes for residents, staff, and the health system within LTC homes. The objectives of this mixed-methods study included: 1) assess the implementation of NP-led huddles; 2) compare outcomes of moral distress and perceived support between staff; and 3) examine changes in resident outcomes. Over a four-month period, huddles were conducted on two units of a privately-owned, not-for-profit LTC home in Ontario, Canada. Post-implementation outcomes were compared between staff who attended at least one huddle (intervention group, n=20) and those who did not (control group, n=22). Anonymized resident-level data from RAI MDS 2.0 were utilized to evaluate resident outcomes. Bayesian analysis was employed to compare outcomes across different staff categories and to summarize changes in RAI measures before and after the intervention. Forty-eight huddles, primarily focusing on resident care (46%) and staff well-being (34%), were conducted by the NP. Direct care staff who attended huddles reported lower levels of moral distress and increased support from the NP. One of the intervention units showed statistical evidence of reduced medical complexity among residents over time. In conclusion, NP-led huddles have the potential to positively impact both staff and resident outcomes and integration of NPs into LTC can facilitate implementation of evidence-informed practices in LTC.

Abstract citation ID: igae098.1953

### INTRODUCING NURSES IN EXPANDED ROLES IN LONG-TERM CARE FACILITIES

Franziska Zúñiga[1], Kornelia Kotkowski[1], Raphaelle-Ashley Guerbaai[2], and Thekla Brunkert[3],
*1. University of Basel, Basel, Basel-Stadt, Switzerland,*
*2. Monash University, Frankston, Victoria, Australia,*
*3. University of Lucerne, Luzern, Luzern, Switzerland*

INTERCARE is a nurse-led care model introducing registered nurses in expanded roles (nurses with advanced education and training beyond the traditional scope of practice for registered nurses) into long-term care facilities (LTCFs) to strengthen geriatric expertise and reduce unplanned hospitalizations. INTERCARE nurses promote the core components of INTERCARE, such as interprofessional collaboration. INTERCARE was implemented in 11 LTCFs in Switzerland from 2018 to 2020 and tested in an effectiveness-implementation hybrid-type II design. Six months post-implementation, care staff (n=108) were interviewed about their perception of the INTERCARE nurse's role. INTERCARE nurses (n=18) were interviewed at the end of the study to explore the content of their work and the factors supporting their role adoption. Staff reported high acceptability of INTERCARE nurses, feeling supported in daily practice and experiencing increased confidence in skills, such as responding to acute situations. INTERCARE nurses provided regular coaching to care teams in complex resident situations and guided interprofessional collaboration by supporting phone calls with physicians. They considered the ongoing training during the study as important to expand their clinical and leadership skills. Bi-weekly one-to-one coaching sessions, where they practiced analyzing and solving problems and additional exchanges with peers, were crucial in sustaining them in their challenging role. In conclusion, nurses in expanded roles provide a supportive environment for care staff to enhance their skills and provide quality care. Peer support and coaching are pivotal in sustaining these expanded roles in the demanding low-resource LTC environment.

### SESSION 4260 (SYMPOSIUM)

Abstract citation ID: igae098.1954

### SARCOPENIA: NO CONSENSUS, NO DIAGNOSTIC CRITERIA, AND NO APPROVED INDICATION HOW DID WE GET HERE?

Chair: William Evans
Co-Chair: Peggy Cawthon

In addition to the role of skeletal muscle in movement and locomotion, muscle plays a critical role in a broad array of metabolic processes that can contribute to improved health or risk of disease. The age-associated loss of muscle has been termed sarcopenia. Muscle is the primary site of insulin-stimulated glucose disposal and the largest component of basal metabolic rate, directly and indirectly affects bone density, produces myokines with pleiotropic effect on muscle and other tissues including the brain, stores essential amino acids essential for the maintenance of protein synthesis during periods of reduced food intake and stress. As such, not surprisingly deterioration of skeletal muscle health, typically operationalized as decline of muscle mass and muscle strength is both a powerful risk factor and main consequence of chronic diseases, disability, and loss of independence, and it is one of the strongest risk factors for mortality. As a result, the development of pharmacological therapies to increase muscle mass (or prevent loss) an important goal for decades. However, while remarkable advances in the understanding of molecular and cellular regulation of muscle protein metabolism have occurred recently, there are no approved drugs for the treatment of sarcopenia, the loss of skeletal muscle affecting millions of older people The goal of this symposium is to describe the possible reasons for the lack of new and effective pharmacotherapies to treat one of the most important risk factors for age-associated disease and loss of independence.

Abstract citation ID: igae098.1955

### SARCOPENIA: NO CONSENSUS, NO DIAGNOSTIC CRITERIA, AND NO APPROVED INDICATION HOW DID WE GET HERE?

William Evans, *University of California Berkeley, Berkeley, California, United States*

In addition to the role of skeletal muscle in movement and locomotion, muscle plays a critical role in a broad array of metabolic processes that can contribute to improved health or risk of disease. The age-associated loss of muscle has been termed sarcopenia. Muscle is the primary site of insulin-stimulated glucose disposal and the largest component of basal metabolic rate, directly and indirectly affects bone density, produces myokines with pleiotropic effect on muscle and other tissues including the brain, stores essential amino acids essential for the maintenance of protein synthesis during periods of reduced food intake and stress. As such, not surprisingly deterioration of skeletal muscle health, typically operationalized as decline of muscle mass and muscle strength is both a powerful risk factor and main consequence of chronic diseases, disability, and loss of independence, and it is one of the strongest risk factors for mortality. As a result, the development of pharmacological therapies to increase muscle mass (or prevent loss) an important goal for decades. However, while remarkable advances in the understanding of molecular and cellular regulation of muscle protein metabolism have occurred recently, there are no approved drugs for the treatment of sarcopenia, the loss of skeletal muscle affecting millions of older people The goal of this symposium is to describe the possible reasons for the lack of new and effective pharmacotherapies to treat one of the most important risk factors for age-associated disease and loss of independence.

Abstract citation ID: igae098.1956

### MUSCLE MASS NOT LEAN MASS IS ASSOCIATED WITH HEALTH-RELATED OUTCOMES: IMPLICATIONS FOR DEFINING SARCOPENIA

Peggy Cawthon, *California Pacific Medical Center, San Francisco, California, United States*

The Osteoporotic Fractures in Men (MrOS) Study is the largest study to date that has employed the D3Cr dilution method to assess muscle mass. In the study of ~1400 older men, low D3Cr muscle mass (but not low DXA lean mass or appendicular lean mass) was associated cross-sectionally with worse Short Physical Performance Battery (SPPB) score, slower walking speed, decreased lower extremity power, worse chair stand ability, and greater likelihood of prevalent mobility limitations and disability. This population of older men (mean age of 84.2 y) reported multiple chronic diseases. Men in the higher quartiles of D3Cr muscle mass had significantly lower prevalence of type 2 diabetes, myocardial infarction, chronic heart failure, and COPD. In addition, men in higher quartiles of D3Cr muscle mass/body mass generally had better cognitive function, had higher levels of physical activity, greater Life-Space, and had markedly lower levels of exhaustion and fatigue. In longitudinal analyses over 3-5 years of follow-up, low D3Cr muscle mass was also associated with increased risk of hip fractures, injurious falls, incident disability and mortality. Generally, the association of low D3Cr muscle mass with these outcomes was not explained by weakness or slow walking speed and there were few associations between low lean mass and these outcomes.

Abstract citation ID: igae098.1957

**WHO'S SARCOPENIC? AN ANALYSIS USING THE CANADIAN LONGITUDINAL STUDY ON AGING DATA**

Stuart Phillips, *McMaster University, Hamilton, Ontario, Canada*

A limitation of the sarcopenia definitions used in our analyses is the use of DXA measured ALM to approximate muscle mass. Although many consider ALM to be the reference standard for measuring muscle mass for sarcopenia, it is actually a measure of lean mass that includes organ tissue, water, and all other non-bone and non-fat soft tissues in addition to muscle mass. The results may be substantially altered if more accurate measures of muscle mass such as the D3-creatine method were used. We have reported that there is generally slight to moderate agreement (Cohen's κ values of.00–.60) between most of the combinations of variables used to ascertain sarcopenia status recommended by the expert group definitions. For definitions using lean mass, the agreement between different adjustment techniques for lean mass ranged from slight to substantial. These findings were consistent across the range of cutoffs for each variable that are either observed in the literature or recommended by the expert group definitions for sarcopenia. The general lack of agreement between sarcopenia definitions observed in our study underscores the importance of the sarcopenia research community identifying a unified ascertainment method for sarcopenia. Having multiple definitions of sarcopenia identifying different groups of individuals as sarcopenic may hinder vital research developing clinical management strategies for sarcopenia by decreasing the comparability between studies.

Abstract citation ID: igae098.1958

**APPLES TO APPLES? DISCORDANT DEFINITIONS STILL HINDER EVIDENCE-BASED TREATMENTS FOR SARCOPENIA**

Giulia Coletta, *McMaster University, Hamilton, Ontario, Canada*

The definition of sarcopenia continues to evolve, creating difficulty in determining the diagnosis and prognosis of the newly classified disease. The most common definitions include a combination of (a) muscle mass [measured using proxies of muscle mass—appendicular lean soft tissue via dual-energy X-ray absorptiometry (DXA) or bioelectrical impedance analysis (BIA)]; (b) muscle strength (often measured using hand grip strength); and (c) physical function (measured using gait speed). However, each consensus definition uses different combinations of muscle mass, strength and physical function to operationalize the definition of sarcopenia. Additionally, each group recommends various measures and cutoff points to capture these outcomes. For example, the European Working Group on Sarcopenia in Older People (EWGSOP) recommends appendicular lean mass for muscle mass, grip strength or chair stand for muscle strength, and gait speed, short performance physical battery, timed up and go, or 400-m walk test for physical function. The Asian Working Group for Sarcopenia (AWGS) uses DXA or BIA, grip strength and 6-m gait speed for muscle, strength and function, respectively. Using different consensus group definitions results in differences in the prevalence of sarcopenia, and there would be, at best, a modest agreement between the various definitions, which would be attributed to the lack of criterion standards.

### SESSION 4265 (SYMPOSIUM)

Abstract citation ID: igae098.1959

**SENIOR HOUSINGS AND COMMUNITY FOR SOCIAL CONNECTION AND HEALTH AMONG LOW-INCOME OLDER ADULTS**

Chair: Sojung Park
Discussant: Robyn Stone

The symposium aims to enhance the quality of life for low-income seniors in community-based housing in the US. The focus is on social connection, neighborhood resources, and community partnerships. Analyzing the 12-year-long national data, the first study used the synthetic difference in difference regression to compare social isolation and cognitive function in two groups of older individuals aged 70 and over. Residents in traditional housing were more likely to experience increased social isolation and decreased cognitive function than senior housing residents. Senior housing was protective, particularly for low-income older adults. A qualitative study on subsidized senior housing (SSH) in Baltimore City found some residents had a small network of connections; most were cordial but disconnected. Challenges related to aging, undesirable behaviors of others, and a desire for privacy limited social connections led to social isolation within the housing communities. Using A Geographic Information System approach, another study looked at SSH availability in New Jersey and its link to neighborhood disadvantage. SSH tends to be located in areas experiencing more significant economic deprivation, highlighting the need for government intervention to mitigate geographic disparities in SSH provision. The last study presented a case of Senior Housing Preservation-Detroit to address the displacement of older adults and improve the quality of life in senior housing.

The presentation will cover COVID-19 outreach, lessons learned, milestones, and an emergency plan for Detroit's older population. Our studies provides a rigorous basis for future research to identify factors and mechanisms for healthy aging among low-income senior housing residents.

Abstract citation ID: igae098.1960

### GEOGRAPHIC DISTRIBUTION OF SENIOR SUBSIDIZED HOUSING IN NEW JERSEY: GEOGRAPHIC INFORMATION SYSTEMS APPROACH

Takashi Amano[1], Sojung Park[2], Byeongju Ryu[2], and Jihye Baek[2], *1. Rutgers, The State University of New Jersey, Newark, New Jersey, United States*, *2. Washington University in St. Louis, St. Louis, Missouri, United States*

Subsidized senior housing (SSH) offers a solution for low-income older individuals, enabling them to maintain economic stability and continue living independently. There has been limited investigation into the geographical distribution of SSH and its association with neighborhood disadvantage. This study seeks to address this gap by (1) describing the availability of SSH in New Jersey and (2) exploring the extent to which housing availability is linked to neighborhood disadvantage. SSH-availability was quantified by the number of SSH units within each census block group. Neighborhood disadvantage was measured using the area deprivation index (ADI). The eligible population was defined as residents aged 62 or older, living below the poverty line, and allocating 30% or more of their income to housing costs within the census block group. A Geographic Information System (GIS) was employed to visually analyze the spatial distribution of SSH, ADI scores, and eligible populations across New Jersey. Poisson regression was utilized to assess the relationship between SSH availability and neighborhood disadvantage, adjusting for the number of eligible residents. The results revealed a significant association between higher SSH availability and higher ADI scores after accounting for the number of eligible residents ($p<.001$). This suggests that SSH tend to be located in areas experiencing greater economic deprivation. These findings underscore the need for governmental intervention to mitigate geographic disparities in SSH provision for low-income older individuals. Additionally, further investigation is warranted to examine the accessibility of social and health-related resources surrounding SSH, which could support residents aging in place.

Abstract citation ID: igae098.1961

### "KEEP AN EYE ON MY PLACE": THE DYNAMICS OF SOCIAL CONNECTION FOR OLDER ADULTS LIVING IN SUBSIDIZED HOUSING

Thomas Cudjoe, *Johns Hopkins University School of Medicine, Baltimore, Maryland, United States*

Social isolation is a burdensome problem for low-income older adults. Many low-income older adults live in subsidized housing. However, few studies have examined this problem in this context. We sought to understand processes that may facilitate, or are barriers to social connections in older adults who live in this setting. We conducted in-depth semi-structured interviews with a purposeful sample of older adults living in subsidized housing communities in Baltimore City. Many residents had a small but meaningful residential network, facilitated by housing characteristics that support connection. Overall, though, residents were cordial but not connected. Challenges related to aging, the undesirable behaviors of others, and a desire for privacy limited social connections or hindered meaningful connection and led to social isolation within the housing communities.

Abstract citation ID: igae098.1962

### THE LONG-TERM RELATIONSHIP BETWEEN SENIOR HOUSING, SOCIAL ISOLATION, AND COGNITIVE DECLINE

Sojung Park[1], Gyeahyung Jeon[2], Eunsun Kwon[1], and Ji young Kang[3], *1. Washington University in St. Louis, St. Louis, Missouri, United States*, *2. Hannam University, Daejeon, Republic of Korea*, *3. Chungnam National University, Daejeon, Republic of Korea*

Community-based senior housing has been advocated as an alternative living environment that promotes a sense of community. This study explores the long-term relationship between living in senior housing, social connection, and cognitive decline. Data came from the 12 waves (year 2010-2022) of the National Health and Aging Trends Study (NHATS) in the U.S. to compare two groups (Traditional Housing and Senior Housing) of older individuals aged 70 and over (Obs=50,128 in TH & 6078 in SH). We analyzed a long-term pattern of social isolation and cognitive function and examined the impacts of social isolation on changes in cognitive function by income groups. Weighted propensity score matching to ensure comparability between the two groups. To focus on the relations during the pandemic, we conducted a Synthetic Difference in Difference (SDID) analysis. In both housings, social isolation increased, and cognitive function decreased over a 12-year period. The regression analysis showed a clear pattern of drastic drops and recovery in the level of social isolation and cognitive function. Compared to senior housing, residents in traditional housing were likely to experience increased social isolation [coef: 0.20, $p<0.001$] and decreased cognitive function [coef: -0.79, $p<0.01$]. A series of subgroup analyses showed the protective effect of senior housing was pronounced for low-income older adults. With the long-term evidence about the protective effects of senior housing within the context of the COVID-19 pandemic, our study provides a rigorous basis for much-needed future research to identify core factors and mechanisms for healthy aging among low-income senior housing residents

Abstract citation ID: igae098.1963

### A DETROIT-BASED COALITION TO PROMOTE EQUITY AT THE INTERSECTION OF HEALTH, HOUSING, AND AGING

Tam Perry[1], Melissa Draughn[2], Claudia Sanford[3], Dennis Archambault[4], Zach Kilgore[5], and Michele Watkins[6], *1. Wayne State University, Detroit, Michigan, United States*, *2. Hannan Center, Detroit, Michigan, United States*, *3. United Community Housing Coalition, Detroit, Michigan, United States*, *4. Authority Health, Detroit, Michigan, United States*, *5. CSI Support & Development, Warren, Michigan, United States*, *6. Volunteers of America Michigan, Southfield, Michigan, United States*

This presentation focuses on a multi-sector coalition focused on senior housing. Since its founding in 2013, Senior

Housing Preservation-Detroit has worked tirelessly to address the displacement of older adults, the loss of senior housing as the city gentrifies; and ways to improve quality of life to those living in senior housing. This presentation will focus on the decade long work achieved respect among Detroit elected officials and housing staff through direct advocacy. Members have also testified at Detroit City Council meetings and participated in numerous local, state, national, and international forums on affordable housing for older adults. To date, coalition members have published four peer-reviewed articles; three invited articles; one white paper; and two book chapters as well as a documentary. The work of the coalition also involves numerous agencies serving older adults in Detroit. The Special Commission to Advance Macro Social Work awarded the coalition the Jack Rothman award for Structural Change in 2023. The presentation will focus on milestones, lessons learned, and the coalition's COVID-19 outreach in senior buildings. It will offer perspectives on its current initiative on preparing an emergency preparedness plan for Detroit's older population.

### SESSION 4270 (PAPER)

### SOCIAL ISOLATION

Abstract citation ID: igae098.1964

### THE ASSOCIATION BETWEEN LONELINESS AND CHILDLESSNESS IN MIDDLE TO LATE LIFE: DOES FRIENDSHIP AND GENDER PLAY A ROLE?

Alison Rataj[1], Andrew Alberth[2], Yan-Jhu Su[2], Elisabeth Stam[2], and Jeffrey Stokes[2], *1. University of Massachusetts Boston, Durham, New Hampshire, United States*, *2. University of Massachusetts Boston, Boston, Massachusetts, United States*

**Objective:** Loneliness is a risk factor for negative health outcomes; however, little attention has been paid to the role of parental status as a contributor to loneliness in later life. Limited research has explored how this association may vary based on levels of friend support, friend strain, and gender. This study addresses these gaps by exploring whether those who are childless, have less friend support or friend strain, and are female are at a higher risk of loneliness.

**Methods:** This cross-sectional study used pooled data from 11,900 older adults 50 years and older from the 2016 and 2018 waves of the Health and Retirement Study. Loneliness was assessed across parental status groups, friendship support and friendship strain scales, and gender using ordinary least squares regression. Multiple imputation was conducted to address missing data.

**Results:** Those who did not have children (n=1,444, 13.0%) were substantially lonelier than those with children in adjusted models. Friend support was associated with reduced loneliness, particularly for childless adults when compared to parents. Midlife and older men were lonelier than women overall, and interaction effects show that childlessness played a larger role in shaping loneliness among women than men. Loneliness did not differ across levels of friendship strain.

**Discussion:** Parental status and gender are intertwined concerning their effects on loneliness. With the changing demographics of "family", future research should identify specific categories of parental status (i.e., did not want children, blended families) and how the mechanics of friend support (i.e., how much, what types) impact loneliness.

Abstract citation ID: igae098.1965

### WEB-BASED PRESENCE FOR SOCIAL CONNECTEDNESS IN LONG-TERM CARE

Anna Garnett[1], Halyna Yurkiv[1], Richard Booth[1], Denise Connelly[1], and Lorie Donelle[2], *1. Western University, London, Ontario, Canada*, *2. University of South Carolina, Columbia, South Carolina, United States*

The COVID-19 pandemic and resultant restrictions on in-person gathering severely disrupted the social networks of older adults living in long-term-care homes (LTCH). Furthermore, the pandemic highlighted the need for embedded interventions such as web-based technology (WBT) to support social connectedness and well-being among older adults and their families. This qualitative study sought to explore how older adults living in LTCH, their family members and staff benefit from and envision WBT to support social connectedness. Semi-structured interviews were conducted with 22 family members, seven older adults and 10 staff across three LTCH. Data were analyzed using directed content analysis informed by Technology Acceptance Model. Participants predominantly used iPads, tablets, and phones to videoconference via Zoom, Teams, Facetime, or Skype. Older adults' sense of social connectedness benefitted from their ability to visualize faces, observe non-verbal cues, perform lip reading, and follow the lives of their families outside of LTCH using WBT over conventional phone connections. In most cases, staff facilitated WBT use due to the older adults varied digital literacy, high rates of dementia and physical or visual limitations. Some family member participants found greater utility in videoconferencing technology called Portal and Alexa Echo Show which required limited navigation by the older adult. Henceforth, LTCH must adopt and implement technology that reflects the needs of people living in LTCH. In addition, adequate staffing, training, and funding is needed to enhance and sustain WBT use for the purposes of social connection among older adults in LTCH and their families.

Abstract citation ID: igae098.1966

### LONELINESS AMONG OLDER CAREGIVERS IN THE CALIFORNIA HEALTH INTERVIEW SURVEY

Xiayu Chen, Elizabeth Lydon, Sarah Jones, Vincent F. Mathias, Wendy Rogers, Raksha Mudar, and Minakshi Raj, *University of Illinois Urbana-Champaign, Champaign, Illinois, United States*

In the U.S., adults aged 65 and above represent the second largest age group among familial caregivers. Navigating dual challenges in caregiving demands and their own aging process may lead to higher risk of social isolation for older caregivers. However, research regarding loneliness among older caregivers remains limited. This study aimed to (a) compare risk factors for loneliness between older caregivers and non-caregivers; (b) evaluate if sociodemographic, health, and caregiving characteristics are related to loneliness among older caregivers; and (c) compare the association between these characteristics and loneliness among older caregivers of persons with dementia versus without dementia. We conducted a secondary data analysis of the 2020 California Health Interview Survey. One-fifth of the older adults (n=7,638) served as caregivers in the past year, and 15% of older caregivers experienced loneliness. Using a series of weighted multivariable logistic regressions, we found that older caregivers were more likely to experience loneliness compared with older non-caregivers (aOR = 1.15, 95% CI: 0.89 - 1.49). Those caring for other older adults had a lower likelihood of loneliness compared with those caring for younger individuals (aOR = 0.48, 95% CI: 0.27 - 0.85). Caregivers of persons with dementia were more likely to experience loneliness compared to those caring for persons without dementia (aOR = 1.67, 95% CI: 1.05 - 2.66). Our analyses revealed that older caregivers, especially those assisting persons with dementia, face a higher risk of loneliness, underscoring the need for targeted support and inclusive caregiving policies.

Abstract citation ID: igae098.1967

### "YOU MUST BE LISTENED TO": FACILITATORS FOR BABY BOOMERS' SOCIAL PARTICIPATION IN CANADA AND SPAIN

Dolores Majón Valpuesta, and Melanie Levasseur,
*Université de Sherbrooke, Sherbrooke, Quebec, Canada*

In Quebec (Canada) and Spain in 2021, the baby boomers represented more than a quarter (26%) of the population. To better support a shift in healthcare and social services, this cohort requires a reassessment of their social participation, a key determinant of healthy and active aging. Despite their committement to participation, little knowledge about what influence the social participation of this generation. This presentation aims to report results of two qualitative studies exploring the facilitators for social participation in the old age of the baby boom generation. These two studies were both carried out in Spain and Quebec with 53 individual and 12 group interviews, involving a total of 107 baby boomers and 52 community representatives. Perceived facilitators were related to: a) accessibility (welcoming environment, physical and emotional proximity, virtual to overcome transportation difficulties); b) availability (flexible participation without high commitment, from a critical viewpoint towards the responsibility of cares); c) positive image (being recognized, required and respected in intergenerational settings); d) socioeconomic and health status (low cost of activities, adaptation to limitations focusing on what you can still do); and e) motivation (feeling useful, helping others and impacting on the society, deciding freely according to their values, but questioning productive ageing models). The challenges associated with supporting attractive forms and opportunities of participation adapted to the needs of this new generation were also highlighted. Responding to these challenges implies creating spaces for interactive exchanges between the baby boomers and key social agents, with real translation in health promotion policies.

Abstract citation ID: igae098.1968

### SHIFTING SOCIAL PARTICIPATION AND LEISURE PATTERNS AMONG CHINESE OLDER ADULTS: EVIDENCE FROM NATIONAL SURVEYS

Yu Fang, and Zheng Ouyang, *China Research Center on Aging, Beijing, Beijing, China (People's Republic)*

Based on the data of "The National Survey on Older Persons in Urban and Rural China" conducted in 2000, 2006, 2010 and 2015, the paper examines the changing dynamics of social participation and leisure activities among Chinese older adults. Findings from the 2015 survey indicate a notable increase in the participation rate of public welfare activities among the older adults, with nearly half actively involved in various community endeavors. Particularly, urban areas showed enhanced participation, contributing to individual fulfillment and societal harmony. Moreover, a significant proportion of older adults expressed willingness to assist peers in need, underscoring the prevalence of mutual support networks within communities. Additionally, the development of Older Person's Associations (OPA) has gained momentum, serving as vital platforms for older people self-management and social engagement. However, challenges persist, with disparities in participation rates between urban and rural areas. While static leisure activities remain prevalent, there is a notable rise in Internet usage among the older adults, offering avenues for social interaction and information dissemination. Despite advancements in spiritual and cultural engagement, attention to mental health among the older adults remains crucial, especially in addressing issues like suicide and mental health concerns among the rural older people population. The paper emphasizes the importance of bolstering support systems and services to promote holistic well-being among the aging population.

### SESSION 4275 (SYMPOSIUM)

Abstract citation ID: igae098.1969

### SOCIOLOGY AND GERONTOLOGY: NEW PERSPECTIVES AND VITAL ISSUES

Chair: Stephen Katz

The relationship between sociology and gerontology continues to forge dynamic pathways that cross critical thinking with innovative methodologies and radical advocacy. Sociological studies (and its specialties) have deepened understanding of aging by exploring its social contexts, life-course trajectories, intersecting inequalities, lifestyle cultures, social determinants of health, and political governance. Despite such advances, mainstream research is criticized for resisting their implications by returning 'to the safety of traditional, individual-level explanations' (Dannefer 2022). This trend parallels an increasing neglect of the social dimension of aging in technocratic policy-making. This symposium addresses these challenges by presenting research on the vital and everyday issues of aging, both to strengthen the sociological lens in gerontology and to explore new perspectives on social changes affecting aging societies. Stephen Katz reviews the loneliness 'epidemic' amongst older adults and the limitations of diagnostic approaches, suggesting instead how historical and qualitative research can formulate loneliness within diversely lived social and material environments. Chris Phillipson examines the sociological consequences of COVID-19 and its impact and consequences for different groups of older people, and discusses the role of social science in contributing to policy responses to the pandemic. Vera Gallistl and Anna Wanka explain the importance of materialist sociological theories in expanding aging relationships to include the interplay of human, more-than-human and non-human actors in digital societies. Amanda Grenier provides a critical overview of the disjuncture between professional knowledge-production and aging experiences, offering a radical proposal for an ethnographic case-study methodology to tackle issues of frailty and mobility.

Abstract citation ID: igae098.1970

### THE "EPIDEMIC" OF OLDER ADULT LONELINESS: PROBLEMS OF DIAGNOSTIC INTERVENTION AND CRITICAL SOCIOLOGICAL RESEARCH

Stephen Katz, *Trent University, Toronto, Ontario, Canada*

Loneliness, more than a feeling, has become a new 'geriatric giant' (Freedman & Nicolle 2020) and epidemic health crisis for older adults, affecting their physical, cognitive and emotional well-being. During the COVID epidemic, the intersecting effects of loneliness and isolation (often blurred in the literature) have intensified, as varying public health measures restricted visiting, gathering, routines and activities. While technical interventions, such as digital communication technologies (DCTs), tele-health meetings, online games, robotic pet companions and simulated presence therapy (SPT) are offered as beneficial aids, even where available or co-designed they tend to individualize and universalize loneliness. Professional, recreational and prescriptive interventions can also disregard the structural relations and socio-material environments that configure everyday lonely-making experiences over time. For both residential and community dwelling older adults, such experiences include lack of affordable housing, care-giver burden and insufficient community resources and planning. This presentation, drawing upon data and examples from senior health policy, loneliness surveys, national reports and qualitative research, reflects on these troubling matters in their complexity and heterogeneity. Conclusions explore the making of an ageist emotional economy that depicts and neglects older adults as inevitably lonely, while advocating for their rights to age in safe, healthy and socially connective ways.

Abstract citation ID: igae098.1971

### EXTENDING GERONTOLOGICAL KNOWLEDGE AND THEORY THROUGH ETHNOGRAPHIC CASE STUDY METHODS

Amanda Grenier, *University of Toronto, Toronto, Ontario, Canada*

Experiences of older people deemed to be 'at risk or 'in need' of intervention are often viewed through professional classifications with corresponding 'objective' indices. Concepts such as frailty and mobility, which have come to draw international policy attention, and arguably shape understandings of aging, are also produced within disciplinary knowledge(s) and powerful practices that prioritize particular components over others. As critical perspectives have revealed, such processes can marginalize groups of older people, resulting in research that articulates counter positions based on disjuncture between classifications, responses, and experience. Yet, operating in the inter-disciplinary, biomedical/technocratic, and applied contexts of gerontology, research findings themselves become sets of knowledge and discourse which are understood as binaries, with the associated sociological theoretical perspectives often marginalized in the process. This paper suggests that ethnographic case study methods can be used to better understand the social complexities of aging, including the framing and interpretation of experience(s), the everyday contexts where older people negotiate and enact relationships and lives over time, and the ways in which evidence is used to design and respond to (or deny) older people's needs. It takes a critical position focused on the production of knowledge and the experiences of older people in the context of social, cultural, and political relations, arguing for methods which render visible the complex realities of aging that are constructed, experienced and lived through, in space and time. It outlines the ethnographic case study as one potential method to carry out this work, presenting examples on frailty and (im)mobility.

Abstract citation ID: igae098.1972

### DEVELOPING A SOCIOLOGY OF COVID-19: CHALLENGES AND PRIORITIES FOR SOCIAL GERONTOLOGY

Chris Phillipson, *University of Manchester, Manchester, England, United Kingdom*

Much of the discussion about the impact of COVID-19 has been around its biomedical and epidemiological dimensions, with analysis focusing on the origins of the disease, the development of vaccines, and lessons to be learnt to protect against future pandemics. But the sociological issues arising from COVID-19 are important as well. Pandemics are viewed through particular social values and cultural perspectives, reflect economic and social inequalities in their development and impact, and are managed through socially organized forms of care and support. People will almost certainly view themselves and their society in a different way during and following a pandemic: diminished through the loss of partners and friends; strengthened through coming together at a time of crisis; or, conversely, weakened through a sense that some have suffered more than others. COVID-19 affected groups in different ways: by age, location, occupation, quality of housing, and ethnic group. But the highly unequal effects of the pandemic were often overlooked, or just assumed to be an inevitable part of 'living with the virus'. The paper argues that research in social gerontology over-emphasized the impact of ageism on the experience of COVID-19, neglecting the wider social fissures and economic and racial injustices that unleashed the pandemic. The paper will review priorities for social gerontology in developing work on COVID-19, including understanding new inequalities which have emerged coming out of the pandemic, and developing a sociologically-informed public health working directly with groups in low-income communities to develop partnerships to tackle ongoing and future pandemics.

Abstract citation ID: igae098.1973

### AGING ASSEMBLAGES: NEW MATERIALIST INSIGHTS IN THE SOCIOLOGY OF AGING

Vera Gallistl, *Karl Landsteiner University of Health Sciences, Krems, Niederosterreich, Austria*

In the last decades, scholars in gerontology and the sociology of aging have increasingly pursued overlapping ideas concerned with the materiality of aging. Central to these is the idea that studying materiality goes beyond a consideration of material objects, physical spaces, or aging bodies. Much rather, it has significant consequences for theory and methodology and hence offers a novel conceptual and empirical sociological lens for gerontology. In this paper, we put forward a new-materialist conceptualization of aging. Drawing on new materialist sociological theories, that have emerged in feminist sociology and the sociology of science and technology, we theorize aging not as an individual process, but as an aging assemblage that is distributed between humans, non-humans and more-than humans. We illustrate the fruitfulness of such a posthumanist understanding of aging by drawing on empirical examples from a qualitative case study on artificial intelligence in long-term care. We demonstrate how adopting a more-than-human perspective on aging offers unique pathways to understand how age-boundaries emerge through a complex interplay of actors and how these age-boundaries govern the unequal distribution of power and autonomy. By conceptualizing aging as an assemblage, we underscore the precarious nature of aging as a concept that is intertwined with complex systems of both situated difference and durable inequality, structural discrimination, and political and economic disadvantage. We conclude by discussing the implications of material gerontology for critical gerontology in an increasingly digitized and datafied world.

### SESSION 4280 (SYMPOSIUM)

Abstract citation ID: igae098.1974

### THE CONTRIBUTION OF SELF-REPORTED COGNITIVE HEALTH FOR INFORMING DAILY WELL-BEING AND FUNCTION ACROSS CONTEXTS

Chair: Robert Stawski
Co-Chair: Kelly Cichy
Discussant: Stacey Scott

Intensive assessment of experiences, health, and well-being in daily life have shown considerable value for understanding how our experiences shape proximal as well as distal health outcomes throughout midlife and older age. To date, stressors have been the most-oft studied experiences, while negative affect and physical symptoms have been focal indicators of daily well-being. Emerging research is emphasizing the import of experiences across different contexts, including social support provision and the work-family intersection, for shaping health and well-being. Further, self-reports of cognitive health (e.g., memory failures, cognitive complaints, cognitive interference, perseverative cognition) are emerging as an important dimension of daily well-being. Such reports are indicative of stress responses, relevant anxiety and depression symptomology, and dementia risk. Thus, self-reports of cognitive health represent a potential pathway for understanding links among daily experiences and health. The purpose of this symposium is to bring together these diverse operationalizations of cognitive health in daily life to illustrate its value and utility across different daily life experiences and contexts. Cichy and colleagues examine associations among daily social support provision and cognitive interference between persons and within persons across days. Chandler and colleagues examine associations between work-family conflict, cognitive interference, and memory failures among midlife adults in the Work-Family Health Study. Mogle explores age differences in individuals' attributions for why they could not complete tasks in daily life. Stawski and colleagues examine associations among daily stressors, perseverative cognition, and mortality. Stacey Scott will facilitate discussion of the papers and offer insights for future research.

Abstract citation ID: igae098.1975

### STRESSOR-RELATED PERSEVERATIVE COGNITION PREDICTS MORTALITY RISK IN MIDLIFE AND OLDER AGE

Robert Stawski[1], Jacqueline Mogle[2], and Dakota Witzel[3], *1. University of Essex, Colchester, England, United Kingdom, 2. Clemson University, Clemson, South Carolina, United States, 3. South Dakota State University, Brookings, South Dakota, United States*

Perseverative cognition (e.g., worry and rumination) has been highlighted as a key indicator of stress and a mechanism linking stress to health and mortality risk. Despite this, research linking daily stress to mortality has focused on affective pathways (e.g., affective reactivity), largely ignoring the role of perseverative cognition in daily stress-mortality associations during midlife and older age. We address this gap by leveraging data from the National Study of Daily Experiences Refresher cohort (2012-2014). Respondents (N=694, Mage=47.5, SD=12.6, Range=25-75; 57% women) completed telephone interviews assessing perseverative cognition, negative affect, and the daily stressors on eight consecutive evenings. Decedents (n=15) date and cause of mortality, through 2019, were obtained from the National Death Index. Multilevel models were used to derive cognitive reactivity slopes – reflecting stressor-related increases in perseverative cognition - which were subsequently used in Cox proportional hazards regression models to predict survival. Results indicated higher levels of average daily perseverative cognition (on non-stressor days) were associated with a two-fold increase in mortality risk (Hazard Ratio=2.04, p=.007), while larger stressor-related increases in perseverative cognition (i.e., cognitive reactivity) were associated with an 81% increase in mortality risk (Hazard Ratio=1.81, p=.002). Findings were robust to adjustment for age, sex, differential stressor exposure, daily negative affect, and affective reactivity to daily stressors. Discussion will focus on why daily perseverative cognition is important for understanding health and wellbeing and represents an important intervention target to reduce health and mortality risk during midlife and older age.

Abstract citation ID: igae098.1976

### CAN'T STOP THINKING ABOUT OTHERS' PROBLEMS: COUPLING OF DAILY SUPPORT PROVISION AND COGNITIVE INTERFERENCE

Kelly Cichy[1], Dakota Witzel[2], and Robert Stawski[3], *1. Kent State University, Kent, Ohio, United States, 2. South Dakota State University, Brookings, South Dakota, United States, 3. University of Essex, Colchester, England, United Kingdom*

The perceived availability of social support is cited as protective against the adverse effects of stressors and curtails cognitive interference (e.g., worry and rumination) linked to anxiety and depression. Social support exchanges, however, also involve providing support to others, which has costs to emotional well-being. Although receiving support may disrupt worry and rumination, providing support may elicit thoughts of concern about the situations requiring support provision, leading to increased cognitive interference on days when support is provided and among those who provide frequent support. Using data from the third wave of the National Study of Daily Experiences (NSDE 3) we examined associations among daily social support provision and cognitive interference between persons and within persons across days. Given gender differences in social networks and rumination, we also explore whether gender moderates these associations. Across eight consecutive days, respondents (N=1,236, Mage= 62.6, 57% women) reported on their affect, completed a six-item measure of cognitive interference (e.g., how often think about situations that upset you?), and indicated whether they provided emotional support. Multilevel models controlling for age and gender revealed significant within- and between-person associations, where cognitive interference was higher on days individuals provided emotional support compared to non-support days and for those who provided more support (p<.001), independent of daily negative affect. Gender did not moderate the associations. Findings underscore how listening to others' concerns confers a mental load not accounted for by negative affect, such that cognitive interference may be one mechanism underlying the costs associated with providing support.

Abstract citation ID: igae098.1977

### DAILY WORK-TO-FAMILY CONFLICT AND SELF-REPORTED COGNITIVE HEALTH IN MIDLIFE

Kelly Chandler[1], Madeline Nichols[1], and Robert Stawski[2],
*1. Oregon State University, Corvallis, Oregon, United States*, *2. University of Essex, Colchester, England, United Kingdom*

Work-family conflict (WFC) is a potent stressor in midlife linked to numerous detrimental physical and mental health outcomes; however, less is known about its effects on cognitive health. Increasing research demonstrates the utility of intensive repeated measures designs to examine within-person variability in WFC and health. Therefore, the aims of the present study were (a) to examine within-person associations between daily WFC and cognitive interference and memory failures and (b) to test age and gender as moderators. The data come from 131 IT employees who participated in an eight-day daily diary in the Work, Family, & Health Study (Age M=45, SD=6.33; 45% women). We found both significant between- and within-person associations from our two-level multilevel models. On average, adults who had higher WFC experienced more cognitive interference, Est.=0.22, SE=.06, p<.001, and memory failures, Est.=0.41, SE=.09, p<.001. On days when adults experienced higher WFC than was typical for them, they experienced more cognitive interference, Est.=.10, SE=.03, p<.01, and memory failures, Est.=0.18, SE=.06, p<.01. Age moderated the association between average WFC and memory failures. Simple slopes tests showed significant positive associations for adults aged 35, Est.=0.86, SE=.20, p<.001, and 45 (the mean), Est.=2.15, SE=.70, p<.01, but not 55, Est.=0.11, SE=.15, p=.43. Gender was not a significant moderator. These preliminary results indicate that there is day-to-day variability in WFC and self-reported cognitive health, particularly for adults in their 30s and 40s. Learning more about days with high WFC could suggest strategies for daily interventions to minimize the impacts of WFC on cognitive health.

Abstract citation ID: igae098.1978

### WHY DIDN'T YOU DO THAT TODAY?: EXPLANATIONS FOR INCOMPLETE INTENDED ACTIVITIES RARELY INCLUDE COGNITION

Jacqueline Mogle[1], Nikki Hill[2], Erin Harrington[3], Jennifer Turner[4], Jody Nicholson[1], and Robert Stawski[5],
*1. Clemson University, Clemson, South Carolina, United States*, *2. The Pennsylvania State University, University Park, Pennsylvania, United States*, *3. University of Wyoming, Laramie, Wyoming, United States*, *4. University of Hawaii Hilo, Hilo, Hawaii, United States*, *5. University of Essex, Colchester, England, United Kingdom*

Completing intended activities, such as household chores, relies on a variety of cognitive processes to plan and execute within our typical daily routine. However, when we fail to complete an intended activity, explanations for the lack of completion can span reasons that are explicitly (e.g., couldn't concentrate) to implicitly (e.g., not feeling well) related to cognitive functioning. The current study used daily diaries to examine individuals' explanations for not completing intended activities. Two hundred forty-six community-dwelling adults (Mage=49.1, range 20-79 years) completed evening reports in a 7-day diary study (Mdiaries=6.2). We examined whether likelihood of not completing any of eight intended tasks (e.g., physical activity, household chores, work-related tasks) and participant's explanations for the lack of completion (e.g., couldn't concentrate, forgot, not enough time) depended on age. Participants reported an incomplete intended activity on 49% of days (n=535) and the most common reason was not enough time (27%). Multilevel logistic regression revealed older adults were more likely to report incomplete intended activities compared to younger (OR=1.72, CI: 1.25-2.37) and to indicate lack of time (OR=1.57, CI: 1.14-2.18) or needing to complete something more important (OR=1.67, CI: 1.18-2.37). Reasons explicitly involving cognitive processes were reported on 9% of incomplete activities and did not differ across age (p=.99). These results provide insight into ways older adults may explain not completing activities without implicating difficulties in their cognitive functioning. We will discuss the explicit role of cognition in explanations individuals use that implicitly implicate cognitive difficulties to improve methods for assessing cognitive functioning.

### SESSION 4285 (SYMPOSIUM)

Abstract citation ID: igae098.1979

### THEORIZING FORTITUDE: PERSPECTIVES FROM GSA'S ARTS, HUMANITIES, AND CULTURAL GERONTOLOGY ADVISORY PANEL

Chair: Sarah McKiddy
Co-Chair: Katherine Ritchey
Discussant: Ulla Kriebernegg

This symposium explores the impact of arts and humanities on cultural narratives of aging. Through the lens of theory-based examples, the authors argue for reaching beyond orthodox engines of gerontological exploration, such as medical research and the social sciences, to develop a more nuanced gerontology. However, moving beyond the arts-as-intervention model is not without pitfalls. Here, theoretical explorations challenge conventional ways of knowing by offering new insights into how cultural narratives of aging can be interpreted and shaped through the lens of the arts and humanities from a theoretical standpoint. Ultimately, the symposium suggests that fortitude, that is, courage in the face of adversity, resonates both as we travel through life facing our own challenges, and as the field of gerontology seeks to understand diverse human experiences of aging.

Abstract citation ID: igae098.1980

**AESTHETIC PATHWAYS IN GEROTRANSCENDENCE**

Sarah McKiddy, *University of Washington, Seattle, Washington, United States*

This presentation aims to enrich and expand the discourse on aging by exploring overaps and areas for exploration within Tornstam's theory of gerotranscendence and the tenets of Rowe & Kahn's successful aging model, with a focus on aesthetics as a mechanism for exploring and expressing the aging process. Gerotranscendence involves a shift in cognitive and emotional paradigms, marked by a reevaluation of purpose, interconnectedness, and self-awareness. Gerotranscendence augments the potential for well-being and health promotion by cultivating increased and meaningful engagement with the world and an evolution in perceptions and consciousness as individuals age. Encompassing diverse artistic expressions such as visual arts, music, and creative activities, aesthetics provide an individualized medium for introversion and self-expression. This multipronged approach to aging considers how individuals navigate senescence and nurture a connection with their inner selves alongside chronic conditions, diseases, limitations, or age-related changes. Engaging in aesthetic experiences offers depth and cognitive consonance in addressing feelings of loss, nostalgia, or existential questions related to aging. Aesthetics, then, emerge as a compelling conduit to convey the transitions and shifts described in the process of gerotranscendence. Integrating gerotranscendence and aesthetics within the context of traditional aging notions both illuminates and deepens the understanding of successful aging by accentuating the psychological, emotional, and creative dimensions, promoting a more holistic and arguably more incisive perspective on aging.

Abstract citation ID: igae098.1981

**CONCEPTUALIZING A NEURO-ETHNOMUSICOLOGY OF AGING**

Aaron Colverson, *University of California San Francisco, San Francisco, California, United States*

This presentation accounts for disciplinary similarities and differences between ethnomusicology and neuropsychology in the context of a project on rhythm perception, learning and performance, and cognitive functioning in healthy young and older adults. It draws from Judith Becker's Deep Listeners (2004) and "Ethnomusicology and Empiricism in the 21st-century" (2009) to characterize many of the challenges present in building bridges between humanists and scientists studying music. Becker cites Ann Harrington in this regard, regarding what it might mean to teach an attitude of thinking neurobiologically about culture and ethnomusicologically about the brain. Applied to brain and social health in older adults, two notable scholars' work extends from Becker's and Harrington's within the domain of ethnomusicology: Jennie Gubner and Theresa Allison. Building from these prior influences, my work forged a collaboration between neuropsychologists interested in the cognitive and brain health of older adults, with ethnomusicologists interested in contributing to new directions for the field, such as cognitive and medical ethnomusicologies. Through this collaboration, important questions were raised in the execution and evaluation phases of the project, namely: what counts as knowledge; what counts as evidence; how do we gather information; and how do we draw conclusions? By entering these conceptual domains/discussions between these disciplines, this work exemplifies a bridge toward a neuro-ethnomusicology of aging, prolonging the pioneering works of Becker, Harrington, Gubner, and Allison. The conclusions direct attention to more fluidly include both disciplines' traditions in research design, to promote more collaborative work between humanists and scientists studying music and health.

Abstract citation ID: igae098.1982

**FORTITUDE AND THE MORAL GOOD OF AGING**

Nicolai Wohns, *University of Washington, Seattle, Washington, United States*

Fortitude, classically one of the four cardinal virtues, is taken to be necessary for the possession of every other virtue. In this presentation, I analyze the positive aspects of aging from a philosophical perspective and argue that fortitude is also of particular importance for the moral good of aging. Once aging is understood as a dynamic phenomenon with biological, socio-psychological, and existential dimensions, it becomes apparent that it presents myriad physical and mental challenges for which fortitude is required for one to flourish. I focus on the biological dimension in particular, suggesting that, though it is usually defined in terms of loss, biological aging brings with it the possibility for moral virtue. Using this virtue-theoretic framework, I conclude by arguing, contrary to some trans-humanist critics, that there are in fact positive aspects of aging that depend on biological aging.

Abstract citation ID: igae098.1983

**THEORIZING FORTITUDE: MINDFULNESS**

Kristoffer Rhoads, *University of Washington School of Medicine, Seattle, Washington, United States*

Normal and pathological aging both require a tremendous amount of resilience and fortitude. Successful aging also requires the ability to fully and accurately appreciate one's past, rectified in the context of one's present and future. Defined as the practice of intentionally cultivating non-judgmental awareness of the present moment, without criticism or evaluation, mindfulness practices offer a powerful tool to enhance acceptance of the "here and now" while cultivating compassion for the aging self and others. This presentation will briefly review the tenets and principles of mindfulness meditation as conceptualized implemented by Jon Kabat-Zinn, PhD and colleagues at the Center for Mindfulness in Medicine, Health Care, and Society at the University of Massachusetts Medical School. Specifically, this presentation will explore applications to older adults with and without later life neurological disorders. This will include benefits of accepting one's current abilities and strengths decoupled from comparisons to earlier life as well as disengaging from a catastrophic narrative of future losses. The presentation will also review the benefits of mindfulness practices for older adults living with memory loss and dementia, including how mindfulness meditation practices may interact with neurobiological underpinnings and risk factor modification of conditions such as Alzheimer's disease, vascular dementia, depression and anxiety.

Abstract citation ID: igae098.1984

### THEORIZING FORTITUDE, FRIENDSHIP, AND OLD AGE IN LORE SEGAL'S SHORT STORY CYCLE "LADIES LUNCH"

Eva-Maria Trinkaus, *University of Klagenfurt, Austria, Klagenfurt, Karnten, Austria*

This contribution addresses the question of 'fortitude' from a literary studies perspective. In my narrative analysis of Lore Segal's short story cycle "Ladies Lunch" (2023) I look at the representation of fortitude in the context of friendship in old age. Fictional narratives about female friendships in old age, Chivers argues, can be part of "constructive narratives of aging" in an "imaginary world that can reflect and especially affect circulating social thought" (2003). Friendship in old age, in Segal's fictional text, constitutes a form of care among the women who partake in their regular lunch meetings in which they contest the readers' views on stereotypes of old age, but at the same time, also express personal vulnerabilities in opposition to a "decline narrative" (Gulette 2001). On the contrary, the kind of reciprocal care they experience comes from a vulnerability that manifests as a "radical openness towards surprising possibilities" (Hirsch 2016). In theorizing fortitude, this narrative analysis addresses the ways fortitude manifests in ways that do not necessarily address danger or pain in the literal sense of the word. Rather, it provides a perspective that juxtaposes fortitude and age in order to address courage in accepting situations and the creative adaptation to those within the story.

### SESSION 4290 (SYMPOSIUM)

Abstract citation ID: igae098.1985

### POLICY SERIES: WHAT IS "GOOD WORK" IN LONG-TERM SERVICES AND SUPPORTS? SETTING A RESEARCH AND POLICY AGENDA FOR DIRECT CARE WORK

Chair: Jennifer Morgan
Co-Chair: Philip Taylor
Discussant: LIza Behrens

This symposium considers the importance of the care-work nexus in long-term care research and policy. Person-centered care delivery is widely accepted as delivering optimal client outcomes, but this is often not practiced. Job quality is considered a crucial determinant of well-being for individuals, society and of organizational performance, yet direct care work is not generally afforded "good work" job quality. Raising standards of appropriate care and care work job quality is essential to improve the life quality of both clients and workers. Drawing from empirical research in the USA and internationally, this session considers the current state of long-term care work across key dimensions of job quality. Critically from a job quality perspective, this session foregrounds the authentic worker voice in identifying a research and policy agenda focused on addressing the care-work nexus. First, we will provide an overview of job quality across long-term care services and supports sectors highlighting the target areas for research and policy advocacy by sector. Second, we will engage workers and union leaders, using a community-engaged co-design framework, in the local SEIU 775 Washington to react to the job quality domains and provide feedback on priority-setting. Third, Washington-based SEIU leaders will collaborate to review the policy, advocacy and negotiation wins made for clients and worker in conversation with employer partners. Finally, scholars, in collaboration with workers/leaders, will summarize the areas targeted for future research and policy agendas aimed at improving job quality and person-centered approaches for both workers and clients.

Abstract citation ID: igae098.1986

### DIRECT CARE WORKERS IN LTSS: RESEARCH AND POLICY AGENDA SETTING

Philip Taylor[1], and Kezia Scales[2], *1. University of Warwick, Coventry, England, United Kingdom*, *2. PHI, New York, New York, United States*

At a time when the provision of long-term care is under more scrutiny than ever, and as demand for care increases, it is timely to consider what this will mean for the future organization of care work. Recent reviews have recommended changing the nature of the modern workplace, creating good work, and reducing poor-quality work. As well as positive worker outcomes, good work benefits organizational performance—and enhances care quality. The task is to develop a "conducive model" of jobs that generates positive outcomes for a care workforce that is disproportionately female and people of color who face multiple structural inequities. Drawing on international literature concerning the relationship between long-term care organization, staffing and care quality, the working environment, and worker and resident/client outcomes, this paper utilizes the multidimensional job quality concept to assess the state of knowledge. Unsurprisingly, the evidence demonstrates that poor-quality jobs result in poorer worker outcomes, and good-quality jobs support good care. However, significant gaps in understanding across dimensions of job quality are identified, particularly how it can be operationalized in day-to-day practice. It is argued that focusing on job quality will help reframe the analysis and interventions needed to improve care and adapt provision to meet future demand. In conversation with our worker and union leader guests and the panel scholars, we will collaboratively set the research and policy agenda for direct care workers in LTSS. Implications for establishing a national taskforce or an international concord on the direct care workforce will be discussed.

Abstract citation ID: igae098.1987

### PROFILE OF DIRECT CARE WORKERS ACROSS THE LONG-TERM CARE SERVICES AND SUPPORTS SECTOR

Christopher Kelly[1], Candace Kemp[2], and Jennifer Craft Morgan[2], *1. University of Nebraska Omaha, Omaha, Nebraska, United States*, *2. Georgia State University, Atlanta, Georgia, United States*

DCWs across long-term care are predominately women, people of color and disproportionally immigrants. About half have health insurance through their employer and about a quarter get health insurance through Medicaid or another means tested program. About half live under 200% of the poverty line. DCWs also face dangerous working conditions, persistent occupational segregation, have limited access to paid leave, and experience very little career advancement. As stated by Scales & Lepore (2020) "[direct care work] requires a mix of technical caregiving skills; health-related knowledge; infection prevention and control expertise; emotional intelligence and relational skills; and problem-solving and decision-making abilities, among other competencies (p. 173)." Despite highly meaningful jobs with high intrinsic rewards, the lack of extrinsic rewards including compensation, drive turnover. Turnover rates in long-term care have been persistently slow to recover since the start of the pandemic and the recovery has been most difficult for women and people of color. In this context, the use of agency staff, or those that are temporarily hired from staffing agencies to fill staffing shortages, has remained persistently high. Use of agency staff makes relationship-based, person-centered care difficult. Many residential care organizations have reduced the number of new residents because they do not have the staffing to accommodate them despite having available licensed beds. Using data from the 2020-2022 American Community Survey we examine trends among direct care workers (DCWs) in terms of job quality, occupation and industry. Persistent issues with churn, lack of career advancement, low wages, and few benefits remain.

Abstract citation ID: igae098.1988

### CO-DESIGNING SOLUTIONS WITH WORKERS: SETTING RESEARCH AND POLICY PRIORITIES

Annie Rhodes, and Leland Waters, *Virginia Commonwealth University, Richmond, Virginia, United States*

An emerging body of literature has linked the quality of care for care recipients with the quality of work experience with the professional caregiver across the spectrum of long-term services and supports. For instance, prior to the COVID-19 vaccine, nursing home workers who worked in multiple locations were significantly more likely to be infected with COVID-19 and, subsequently, more likely to infect care recipients. Despite the natural alignment with worker and recipient interests, LTSS quality improvement efforts often do not focus on aligning recipient and worker perspectives as a reform and quality improvement strategy. For instance, although current evidence supports that staffing ratios improve both recipient care and caregiver safety, there has been a backlash against a proposed staffing mandate in nursing homes by provider stakeholders. To uplift the caregiver's voice and collaborate with SEIU 775 (Washington State), workers from home care and nursing home organizations with leadership experience will talk with panelists about COVID-19, staffing ratios, advocacy, job quality, job safety, career ladders, and advancement, and intrinsic rewards. These workers will relay their experiences and discuss why they took up these jobs, stay in these jobs, and work to improve these jobs through union strategies. Panelists will ask questions related to agenda setting and co-designing research and policy solutions that simultaneously uplift and align recipient and caregiver voices.

Abstract citation ID: igae098.1989

### UNION-DRIVEN PROGRESS IN WASHINGTON STATE: IMPLICATIONS FOR POLICY AND PRACTICE

Kezia Scales[1], Adam Glickman[2], and Mariana Morante[2], *1. PHI, New York, New York, United States*, *2. SEIU 775, Seattle, Washington, United States*

Recruitment and retention challenges related to direct care workers across long-term services and supports continue. Policy and practice at the federal, state, community and organizational level has the potential to improve retention, widen recruitment pipelines and create new person-centered models that attend to both the needs of workers and people who receive services and their care partners. With input from SEIU 775 leadership, this presentation will provide an overview of union reach in Washington state within the LTSS sectors, review some of the interplay between city-based living wage laws and wage improvements for direct care workers, and talk about successes in improving access and affordability of health insurance for workers and increased access to paid-time off. Panelists will ask questions related to agenda setting and co-design of solutions related to both research and policy. Policy and practice levers at the federal, state and community levels will be identified and discussed using a co-design framework.

### SESSION 4295 (SYMPOSIUM)

Abstract citation ID: igae098.1990

### WHAT'S NEW IN AGING AND EMOTIONAL EXPERIENCE RESEARCH? NEW QUESTIONS, METHODS, AND SAMPLES

Chair: Jennifer Stanley
Discussant: Eric Allard

Older adults tend to report greater control over their emotions and higher well-being, compared to younger adults (Carstensen et al., 2011). Evidence for this phenomenon is robust, but the mechanisms and boundary conditions of improved emotional experience with age are less well understood. This symposium presents four innovative papers that tackle these questions using new methods, asking new questions, and extending to new samples. First, Hamilton and Allard argue for the utility of estimating emotion networks for understanding age differences and similarities in the co-occurrence of emotions. Next, Kravljaca and Stanley investigated age differences in facial feedback effects when posing angry and happy facial expressions. Contrary to expectations based on the Maturational Dualism hypothesis (Mendes, 2010), both young and older adults exhibited increases in angry ratings following the posing of an anger expression. Then, Amanambu and Allard investigated whether specific facets of mindfulness relate to age-related decreases in negative affect in a lifespan sample in Nigeria. They found that the Describing facet mediated the relationship between age and negative affect. Overall, they report similar findings in the Nigerian sample to what has been shown in Western samples. Finally, Behrend and colleagues report an online study that explored whether young and older adults consider specific emotions useful for accomplishing social goals. Both age groups endorsed the most useful emotion for each type of goal, suggesting that there is age similarity in the knowledge of utilitarian emotions in the social domain. These papers present new frontiers in emotions and aging research.

Abstract citation ID: igae098.1991

### "HOW DOES THAT MAKE YOU FEEL?" USING NETWORK SCIENCE TO ESTIMATE AFFECT DYNAMICS ACROSS ADULTHOOD

Lucas Hamilton[1], and Eric Allard[2], *1. Augustana University, Sioux Falls, South Dakota, United States*, *2. Cleveland State University, Cleveland, Ohio, United States*

Research on affect dynamics has dramatically changed in the past two decades. With the proliferation of ecological momentary assessment and online data collection, methodological and analytical approaches have greatly expanded. One approach is the estimation of emotion networks which can be done through a few lines of R code but requires a great deal of planning and interpretation by the researcher. Network estimation models individual emotions as distinct (i.e., sad or angry) and dimensionally dependent (i.e., sad and angry). This technique can test the independent influence of each emotion and unique associations between every pair of emotions. Critically, network structures (i.e., magnitude of co-fluctuation, independence of items) can be compared both within-individuals and between groups. Our previous findings demonstrate the feasibility and utility of estimating emotional reactivity across the lifespan. Using a video clip elicitor and a sample of 531 people (178 younger, 175 middle-aged, and 178 older adults), emotion networks were used to verify mean affective reactivity before and after the clip while also testing distinct co-occurrences (i.e., mixed emotions). To further demonstrate the usefulness of this approach, we have undertaken a database project that evaluates affective blending in response to 48 video clips. Mirroring the methodology from above, a sample of younger, middle-aged, and older adults rated their emotions before and after each video clip they watched. After summarizing overall reactivity findings, we will discuss how emotion networks offer great utility for disentangling unique and co-occurring emotions and their importance in emotional aging research.

Abstract citation ID: igae098.1992

### SMILE, YOU'LL FEEL BETTER: TESTING THE FACIAL FEEDBACK HYPOTHESIS IN YOUNG AND OLDER ADULTS

Nikolina Kravljaca, and Jennifer Stanley, *University of Akron, Akron, Ohio, United States*

We investigated age differences in the effect of facial feedback on the emotional experience on a sample of young and older adults. The Facial Feedback Hypothesis proposes that feedback from facial expressions impacts the emotional experience by enhancing or dampening the emotional experience (James, 1884; Darwin 1897). Embodied cognition, the bidirectional relationship between the mind and the body is central to maturational dualism, which suggests older adults show reductions in proprioception, interoception, and physiological reactivity (Niedenthal et al., 2005; Mendes, 2010). Based on this, we expected older adults to show reduced facial feedback effects. Participants were instructed to pose a happy, angry, and neutral facial expression and rate their happiness and anger on a 7-point scale before and during the facial manipulation. We found that in the Angry Condition, there was a main effect of Time, such that both age groups showed reductions in happiness scores, $F(1,119) = 86.243$, $p<.001$, $\eta 2=.420$ and increases in anger, $F(1,119) = 29.798$, $p<.001$, $\eta 2=.200$, as well as a Time x Age Group interaction, $F(1,119) = 4.546$, $p<.035$, $\eta 2=.03$, showing that older adults had a larger decrease in happiness scores, but no age differences in feelings of anger. In the Happy Condition, we did not find any significant age differences in ratings of happiness or anger from pre to post while posing the happy or angry facial expression. Contrary to our hypotheses, these results suggest that older adults do experience facial feedback effects specifically for anger, but not happiness.

Abstract citation ID: igae098.1993

### ASSESSING MINDFULNESS FACETS TO INTERROGATE THE "WELL-BEING PARADOX" IN NIGERIA

Peace Amanambu[1], and Eric Allard[2], *1. University of Nigeria, Nsukka, Enugu, Nigeria*, *2. Cleveland State University, Cleveland, Ohio, United States*

The role of mindfulness for explaining the well-being paradox in aging has been examined within Western samples. However, whether this association exists cross-culturally has not been previously addressed. Furthermore, investigations on the role of mindfulness facets on the associations between age and well-being is limited. The present study assessed whether distinct mindfulness facets (i.e., observing, describing, acting with awareness, non-judgment, and non-reactivity) mediate the relationship between age and positive and negative affect in an adult lifespan sample in Nigeria. Participants were 242 (95 women, age range = 18-75 years, Mage = 41.79; SDage = 13.58) adults, affiliated with the University of Nigeria-Nsukka, who completed the Positive and Negative Affect Scale and Five Facet Mindfulness Questionnaire (FFMQ). Increased age was associated with diminished negative affect (NA) but unrelated to positive affect (PA). In terms of mindfulness facets, older age was significantly related to increased endorsement of the Describing, Acting with Awareness, and Non-Reactivity facets. Separate mediation models were conducted with these three facets in predicting NA. Here, diminished age-related NA was mediated by the Describing facet. No significant mediating relationships were observed for Acting with Awareness and Non-Reactivity. The present findings suggest some cross-cultural coherence in mindfulness as an arbiter of the well-being paradox in aging, further specifying aspects of the mindfulness construct contributing to this relationship.

Abstract citation ID: igae098.1994

### AGING AND UTILITARIAN EMOTIONS FOR SOLVING SOCIAL PROBLEMS

Abigail Behrend[1], Eric Allard[1], and Jennifer Stanley[2], *1. Cleveland State University, Cleveland, Ohio, United States*, *2. University of Akron, Akron, Ohio, United States*

Literature suggests a preference for pro-hedonic orientations in older adulthood (Carstensen et al., 2006). Although this is consistent with Socioemotional Selectivity Theory (Carstensen et al., 1999), evidence suggests contra-hedonic motives are viable, particularly in interpersonal scenarios (Allard et al., 2021). We aimed to determine whether young and older adults consider specific emotions useful for accomplishing socially-relevant goals. In an online study, 112 young adults (18-35 years; 29.5% men; 68.8% White) and 72 older adults (64-83 years; 25% men; 93.1% White) viewed 8 interpersonal scenarios with associated social goals. Participants rated how much they would like to engage in emotion-inducing activities on a scale of 1 (not at all) to 7 (extremely) to prepare for each interpersonal interaction. A 4 Goal (Confront, Approach, Avoid, Help-seeking) x 4 Emotion (Happy, Afraid, Angry, Sad) x 2 Age Group (young, old) repeated measures ANOVA revealed a significant Goal x Emotion interaction F (3.82, 695.86) = 210.633 p <.001. Follow-up analyses showed all predicted emotions were endorsed within their intended goal condition (e.g., Approach-Happy, p <.001), with the exception of sadness within the "Help-seeking" condition (p <.001). This partially supports our hypothesis. These results provide additional evidence for age-similarity in utilitarian emotional endorsement. Future research should investigate whether such age similarity remains when experiencing emotions to achieve utilitarian goals.

### SESSION 4300 (SYMPOSIUM)

Abstract citation ID: igae098.1995

### ADVANCING THE SCIENCE OF SENSORY HEALTH AND COGNITION: FROM EPIDEMIOLOGY TO INTERVENTION

Chair: Carrie Nieman
Discussant: Jennifer Deal

Sensory health and cognition are at the intersection of two major public health challenges facing an aging global population. Sensory impairments are among the most common and disabling comorbidities among individuals at risk for cognitive impairment and those already aging with cognitive impairment. Sensory impairments may also serve as key biomarkers in dementia and may worsen the trajectory of decline. Although prevalent, sensory impairments frequently go unrecognized and unaddressed. Importantly, sensory impairment has been identified as modifiable risk factors for dementia. Optimizing sensory function may be an important yet overlooked approach to reducing the risk of cognitive decline as well as providing potential nonpharmacological interventions to aid in the management of neuropsychiatric symptoms, improve quality of life for persons living with dementia, and reduce burden for care partners. This symposium presents the latest evidence from epidemiology to intervention in advancing our understanding and approach to sensory health and cognition. The symposium begins with a focus on the epidemiology of sensory function among individuals with or at risk for cognitive impairment and then moves to a focus on intervention, understanding the potential impacts of optimizing sensory function on brain structure, barriers to addressing sensory function in clinical settings, and envisioning how to optimize interventions by learning directly from individuals aging with cognitive impairment, their care partners, and experts. This symposium features leaders in the field of sensory health and cognition from diverse career stages, disciplines, and settings.

Abstract citation ID: igae098.1996

### HEARING AND OLFACTORY LOSS ASSOCIATED WITH ALTERED BRAIN STRUCTURE AND CONNECTIVITY IN DEMENTIA RISK

Natalie Phillips[1], Tristin Best[1], Nicole Grant[1], Ameera Kabir[1], M. Kathleen Pichora-Fuller[2], Jennifer Campos[3], Paul Mick[4], and Walter Wittich[5], *1. Concordia University, Montréal, Quebec, Canada, 2. University of Toronto, Toronto, Ontario, Canada, 3. The KITE Research Institute, University Health Network, Toronto, Ontario, Canada, 4. University of Saskatchewan, Saskatoon, Saskatchewan, Canada, 5. University of Montréal, Montréal, Quebec, Canada*

Sensory loss in hearing, vision, and olfaction is highly prevalent in older adults and each is associated with higher risk of developing dementia. We sought to identify whether these sensory factors are associated with alterations in brain function and structure in older adults with or at risk for dementia. Our groups included those with low risk (normal cognition, no cognitive complaints (NC=128; mean age=69 years; mean education=16 years)), and those with higher risk, namely 135 individuals with subjective reports of cognitive decline (SCD) but normal cognition (age=70; education=17), and 241 participants with mild cognitive impairment (MCI; age=72; education=16), and 93 with Alzheimer's disease (AD, age=75; education=15). We used data from the COMPASS-ND study (Release 7). Hearing loss was assessed with pure-tone screening, vision with contrast sensitivity, and olfaction with the Brief Smell Identification Test. Normal sensory performance was observed in a minority of participants and decreased across the dementia risk spectrum groups (e.g., NC: 29%; SCD: 29%; MCI: 11%; AD: 2%). Olfactory deficits were the most frequent, followed by hearing impairment, and then deficits in visual contrast sensitivity. Deficits in multiple sensory domains were highly prevalent, with 37% of participants with AD having deficits in two or more domains. Preliminary analyses indicate that hearing loss is associated with altered connectivity in the default mode network and olfactory loss is associated with reduced hippocampal volumes in SCD. Thus, sensory loss is highly prevalent and co-morbid in persons with or at risk for dementia and has implications for cognition and brain function.

Abstract citation ID: igae098.1997

### PARSIMONIOUS MODELS OF LONG-TERM COGNITIVE DECLINE AND IMPAIRMENT PREDICTION IN MIDDLE-AGED ADULTS

Natascha Merten[1], A Alex Pinto[2], Adam Paulsen[2], Richard Chappell[2], Yanjun Chen[2], Corinne Engelmann[2], Laura Hancock[3], and Carla Schubert[2], *1. University of Wisconsin-Madison, Madison, Wisconsin, United States, 2. University of Wisconsin Madison, Madison, Wisconsin, United States, 3. Cleveland Clinic, Cleveland, Ohio, United States*

Established risk scores for dementia prediction (e.g., the CAIDE and Framingham Risk Score) often focused on cardiovascular factors and have not been updated in more recent generations. We aimed to construct a parsimonious risk prediction model of 10-year cognitive decline and impairment using factors measured in midlife in a current cohort. This longitudinal study is based on N=1529(54% women, mean age 49years) Beaver Dam Offspring Study participants with data from baseline, 5-year and 10-year follow-up. We assessed hearing, vision, olfactory, motor, lifestyle, cardiovascular and general health factors, and blood-based neurodegenerative markers, and amyloid. We determined 10-year cognitive decline (trail-making test B time;10%

most decline) and cognitive impairment (neurocognitive case review). We constructed least absolute shrinkage and selection operator logistic regression models with 10-fold cross-validation and evaluated predictive ability via receiver operating characteristic curves (AUCs). There were N=121 cognitive decline and N=217 cognitive impairment cases. The six top predictors for cognitive decline (age, income, motor function, olfactory function, peripheral artery disease, neurofilament light chain protein (NfL)) and for cognitive impairment (sex, motor function, olfactory function, self-rated vision, alcohol consumption, NfL) yielded AUCs of 0.80(95% confidence interval:[0.76-0.83]) and 0.73[0.69-0.77], respectively. In midlife, sensory and motor measures and NfL were among the best predictors of 10-year onset of cognitive decline and impairment. Only 6 factors were needed for acceptable to excellent AUCs. Other cohort studies should cross-validate these predictors. Risk scores for asymptomatic middle-aged people based on practical test batteries will be useful for population screenings to target early prevention and intervention strategies.

Abstract citation ID: igae098.1998

### DISCUSSION AND DOCUMENTATION OF HEARING DIFFICULTY IN THE PRESENCE OF DEMENTIA: STUDY OF THE ANNUAL WELLNESS VISIT

Danielle Powell[1], MJ Wu[2], Stephanie Nothelle[3], Jamie Smith[4], Esther Oh[2], Nicholas Reed[2], and Jennifer Wolff[2], *1. University of Maryland, College Park, Maryland, United States*, *2. Johns Hopkins University, Baltimore, Maryland, United States*, *3. Johns Hopkins University School of Medicine, Baltimore, Maryland, United States*, *4. Widener University, Chester, Pennsylvania, United States*

How clinicians document and discuss health concerns within a visit shapes patient health perception. This study investigates clinician documentation of hearing concerns and whether this varies for older adults with dementia/cognitive concerns. We use patient-level health and demographic information from the electronic medical record and associated clinician visit notes for Medicare beneficiaries who indicated index hearing concerns at an Annual Wellness Visit (AWV) between 2017-2022. We text analyzed a random sample of AWV clinical notes to evaluate documentation of hearing concerns/loss and placement of hearing-related referrals. Logistic regression models investigate odds of clinician documentation of hearing concerns by diagnosed dementia or cognitive concerns. Among 2,593 beneficiaries who reported hearing concerns at an AWV (mean age 78.8 years, 56.6% female), 6.5% had a concurrent dementia diagnosis, nearly 40% indicated congruent cognitive concerns, and 20% had an existing hearing loss diagnosis. Text analysis of clinician visit notes (n=474) found hearing concerns were documented less frequently for those with (versus without) dementia (44.2% vs 30.2%) and clinicians were half as likely to elaborate on hearing (OR:0.51; 95% CI:0.34,0.76) or acknowledge known hearing loss in the AWV note (OR:0.56; 95% CI:0.38,0.84. Regardless of dementia status, only 50% received a hearing diagnosis following the AWV. Clinicians are less likely to document or incorporate hearing concerns into the AWV for patients with dementia. The complex care needs of dementia may inhibit clinician ability to attend to hearing concerns, suggesting opportunity for embedded educational intervention to support clinicians in addressing patient-collected health risks can improve dementia care.

Abstract citation ID: igae098.1999

### INSIGHTS ON OLDER ADULTS WITH COGNITIVE IMPAIRMENT AND HEARING LOSS: END-USER AND CARE PARTNER PERSPECTIVES

Peter Hope[1], Carrie Nieman[1], Marcela Blinka[2], Paul Lee[2], Xueting Tu[2], Natalie Wang[2], Sevil Yasar[1], and Esther Oh[2], *1. Johns Hopkins University School of Medicine, Baltimore, Maryland, United States*, *2. Johns Hopkins University, Baltimore, Maryland, United States*

Hearing loss is prevalent among persons aging with cognitive impairment and can have significant repercussions for individuals and their care partners. However, hearing aid usage is low among older adults, particularly among individuals with cognitive impairment. To understand the barriers and facilitators to hearing care among persons living with cognitive impairment and their care partner and to inform the development of a hearing care intervention, we conducted 20 semi-structured interviews with 10 participants with cognitive impairment and their care partners. Participants were recruited from the Johns Hopkins Memory and Alzheimer's Treatment Center. We used heterogeneous purposive sampling to recruit participants from diverse race, ethnicities, education, and income levels. Qualitative content analysis was used to identify themes. Among end-users and care partners, 30% self-identified as African American, 45% as male, and 40% reported less than a college degree. Participants with cognitive impairment had a mean score of 16.8 (SD8.2) on the Montreal Cognitive Assessment and mean speech frequency pure tone average of 44.9 dB HL (SD12.7). End-users and care partners identified barriers to accessing care, including stigma, cost of hearing aids, lack of availability of hearing care, and participants' denial of hearing difficulties. Suggested facilitators for improving access to hearing care include affordable hearing aids and incorporating hearing screenings into routine care. The participants' desire to improve communication and care partner support motivate accessing hearing care. Our findings suggest that addressing barriers, promoting affordability, and enhancing accessibility to hearing care are critical in extending care to individuals aging with cognitive impairment.

Abstract citation ID: igae098.2000

### INSIGHTS ON OLDER ADULTS WITH COGNITIVE IMPAIRMENT AND HEARING LOSS: EXPERT PERSPECTIVES

Marcela Blinka[1], Esther Oh[1], Peter Hope[2], Paul Lee[1], Xueting Tu[1], Natalie wang[1], Sevil Yasar[2], and Carrie Nieman[2], *1. Johns Hopkins University, Baltimore, Maryland, United States*, *2. Johns Hopkins University School of Medicine, Baltimore, Maryland, United States*

Although hearing loss is highly prevalent among older adults, especially among older adults aging with cognitive impairment, few use hearing aids. Older adults with co-morbid cognitive impairment and hearing loss experience multiple barriers to hearing care, including hearing loss often going unrecognized by clinicians and deprioritizing hearing care among competing health priorities. To understand the barriers and facilitators to hearing care among persons living with dementia and inform the development of a hearing care intervention, we conducted semi-structured interviews with 10 experts. Experts included professionals with expertise in dementia, hearing loss, neuropsychiatric symptoms, behavioral intervention development, implementation science, and technology adoption. We used purposive sampling and snowball recruitment to recruit experts with demonstrated experience and diverse backgrounds. Qualitative content analysis was used to identify themes. Among the experts, 56% self-identified as male, and 78% self-identified as white. Experts highlighted the importance of a multi-component approach to hearing care, including education on hearing and dementia, management of neuropsychiatric symptoms, and employing effective communication strategies. Experts prioritized the need for interdisciplinary collaboration and dedicated training for clinicians. Experts emphasized the need to engage stakeholders throughout intervention development and testing, including the key role care partners play in prioritizing hearing care and supporting the ongoing use of assistive technologies. Experts also identified the need to integrate practical strategies to support communication needs and hearing technology that is affordable. Our findings support the importance of hearing care within dementia care and identify tangible opportunities to support hearing health among persons aging with cognitive impairment.

### SESSION 4305 (SYMPOSIUM)

Abstract citation ID: igae098.2001

### ASSESSING MEDICAID PAYMENT RATES AND COSTS OF CARING FOR THE MEDICAID POPULATION RESIDING IN NURSING HOMES

Chair: Edward Miller
Co-Chair: Elizabeth Simpson
Discussant: Sara Karon

This symposium reports findings from an analysis of the relationship between Medicaid payment rates and costs for caring for the Medicaid population residing in nursing homes (NHs). Prior to the COVID-19 pandemic, there were about 1.37 million Americans using one of the nation's 15,000+ NHs. Medicaid is the primary and largest single payer for NH care, covering approximately two-thirds of NH residents. The residents paid for by Medicaid's nursing facility benefit are typically long-stay residents who often require custodial-type care due to cognitive or physical impairments that must be provided on a long-term basis. Until recently, there has been limited information examining the relationship between Medicaid payment rates and the costs of caring for Medicaid NH residents. Such information is essential to support discussion of policy reforms that aim to improve the safety and quality of NH care. This study used Medicaid payment and cost report data for freestanding NHs in 44 states collected during the latest pre-pandemic period (2019). The national average and median per diem Medicaid payment rates and costs and Medicaid payment-to-cost ratios were calculated for the nation as a whole and by NH characteristics and state Medicaid payment policies. The first presentation establishes the context for the study (Miller). The second presentation describes the study methodology (Simpson). The third presentation reports study findings (Cohen). The last presentation delineates implications of the study findings for policy and practice (Bowblis). Sara Karon will serve as discussant.

Abstract citation ID: igae098.2002

### FEDERAL AND STATE POLICIES AND STAKEHOLDER VIEWS ON MEDICAID NURSING HOME PAYMENT

Edward Miller[1], John Bowblis[2], Elizabeth Simpson[1], Sara Karon[3], Iara Oliveira[4], Judith Dey[5], Marie Squillace[6], and Marc Cohen[1], *1. University of Massachusetts Boston, Boston, Massachusetts, United States*, *2. Miami University, Oxford, Ohio, United States*, *3. RTI International, Research Triangle Park, North Carolina, United States*, *4. Office of Behavioral Health, Disability, and Aging Policy (BHDAP), Washington, District of Columbia, United States*, *5. US Department of Health and Human Services, Washington, District of Columbia, United States*, *6. Office of the Assistant Secretary for Planning and Evaluation, Washington, District of Columbia, United States*

How much nursing homes (NHs) are paid by Medicaid is determined at the state level. Medicaid coverage of NH care is a significant expenditure for states. Consequently, states design Medicaid payment models to achieve desired policy objectives related to NH costs and quality, access to care, payment equity, service capacity, and budgetary control. Setting Medicaid payment rates too high can lead to excess profits at the expense of taxpayers with unknown implications to improvements in quality and staffing levels. At the same time, NHs need financial resources to pay nursing staff and invest in other quality improvement efforts to assure proper care. The industry claims that NH costs are not fully covered by their Medicaid payments. Consumer advocates argue that NHs are already adequately paid, and profit incentives potentially divert funds away from resident care. There is no federal requirement that Medicaid payments cover the full cost of care.

Abstract citation ID: igae098.2003

### METHODOLOGY FOR ANALYZING MEDICAID NURSING HOME PAYMENT RATES AND COSTS

Elizabeth Simpson[1], John Bowblis[2], Edward Miller[1], Sara Karon[3], Iara Oliveira[4], Judith Dey[5], Marie Squillace[6], and Marc Cohen[1], *1. University of Massachusetts Boston, Boston, Massachusetts, United States*, *2. Miami University, Oxford, Ohio, United States*, *3. RTI International, Research Triangle Park, North Carolina, United States*, *4. Office of Behavioral Health, Disability, and Aging Policy (BHDAP), Washington, District of Columbia, United States*, *5. US Department of Health and Human Services, Washington, District of Columbia, United States*, *6. Office of the Assistant Secretary for Planning and Evaluation, Washington, District of Columbia, United States*

This study required the construction of a unique facility-by-facility national database that links Medicaid nursing home (NH) payment, cost, staffing, and quality information from a variety of sources to enable detailed analysis of key financial and other metrics. Medicaid cost data were obtained from Medicare Cost Reports and Medicaid payment rates were constructed from statutory rates collected from the states. Medicaid costs were adjusted to capture only services provided to Medicaid residents. The final sample consisted of 9,543 NHs, representing 67% of all freestanding NHs in 2019 in the 44 states analyzed. The analysis focused on distributions, means, medians, and payment-to-cost ratios, where a ratio of 1.0 indicates that Medicaid payments matched reported NH costs. The impacts of NH characteristics and state payment methods on Medicaid costs, payments, and payment-to-cost ratios were explored. Regression analyses further investigated the impact of NH and state policy characteristics, accounting for state fixed effects.

Abstract citation ID: igae098.2004

### MAJOR STUDY FINDINGS ON THE ASSOCIATION BETWEEN MEDICAID PAYMENTS AND COSTS

Edward Miller[1], Marc Cohen[1], Elizabeth Simpson[1], Sara Karon[2], Iara Oliveira[3], Judith Dey[4], Marie Squillace[5], and John Bowblis[6], *1. University of Massachusetts Boston, Boston, Massachusetts, United States*, *2. RTI International, Research Triangle Park, North Carolina, United States*, *3. Office of Behavioral Health, Disability, and Aging Policy (BHDAP), Washington, District of Columbia, United States*, *4. US Department of Health and Human Services, Washington, District of Columbia, United States*, *5. Office of the Assistant Secretary for Planning and Evaluation, Washington, District of Columbia, United States*, *6. Miami University, Oxford, Ohio, United States*

In 2019, Medicaid payment rates covered about 82 cents per dollar of estimated nursing home (NH) reported Medicaid costs. Roughly 40% of NHs had Medicaid payments covering 80% or less of their reported costs; 51% of NHs had 80-100% of their reported Medicaid costs covered; 8% of NHs had payments exceeding reported costs. Not-for-profit NHs had the lowest Medicaid payment-to-cost ratio (.76) compared to for-profit (.83) and government-owned (.80) NHs. NHs with the highest Medicaid payer mix (80-100%) had a higher Medicaid payment-to-cost ratio (.86) than NHs with the lowest Medicaid payer-mixes below 50% (.75). NHs with total nursing staff levels below 3.00 hours per resident day had the highest average Medicaid payment-to-cost ratio (0.85); NHs with nursing staff levels above 4.0 hours had the lowest (0.77). NHs with the lowest (1-star) quality ratings had the highest average Medicaid payment-to-cost ratio (0.83); NHs with 5-star ratings had the lowest (0.80).

Abstract citation ID: igae098.2005

### IMPLICATIONS OF MEDICAID NURSING HOME PAYMENT AND COSTS FOR POLICY AND PRACTICE

John Bowblis[1], Edward Miller[2], Elizabeth Simpson[2], Sara Karon[3], Iara Oliveira[4], Judith Dey[5], Marie Squillace[6], and Marc Cohen[2], *1. Miami University, Oxford, Ohio, United States*, *2. University of Massachusetts Boston, Boston, Massachusetts, United States*, *3. RTI International, Research Triangle Park, North Carolina, United States*, *4. Office of Behavioral Health, Disability, and Aging Policy (BHDAP), Washington, District of Columbia, United States*, *5. US Department of Health and Human Services, Washington, District of Columbia, United States*, *6. Office of the Assistant Secretary for Planning and Evaluation, Washington, District of Columbia, United States*

This study is the most comprehensive analysis to date on the relationship between state Medicaid payment rates and nursing home (NH) reported costs of caring for Medicaid residents. The findings provide insight into how, on average, Medicaid payments compare to reported costs, and how that differs by different types of NHs. It does not address the adequacy of Medicaid payment, whether NHs were operating efficiently or were adequately staffed based on the acuity of their residents; nor does it address the quality of the data from the cost reports. Nonetheless, it does provide important information on state Medicaid payment rates and reported costs that can inform current discussions assessing potential Medicaid payment policy reforms. Findings from this study also can be used to examine the impact of potential changes in Medicaid payment on financial performance and disparities across NHs. Study limitations and directions for further investigation are discussed.

### SESSION 4310 (BIOLOGICAL SCIENCES INVITED SYMPOSIUM)

### RESOURCES, TECHNOLOGIES, AND ADVANCES IN AGING RESEARCH

Abstract citation ID: igae098.2006

### BIOLOGICAL RESOURCES FOR AGING RESEARCH AT THE NATIONAL INSITUTE ON AGING

Manuel Moro, Tiziana Cogliati, Jennifer Fox, and Fei Wang, *NIA-NIH, Bethesda, Maryland, United States*

Research on aging, by its nature, requires the acquisition of data over an organism's lifespan or at later stages in life. For animals with medium to long lifespans such as rodents and nonhuman primates (NHPs), time is a limiting factor for the acquisition of data and samples. One of the missions of the National Institute on Aging (NIA) is to maintain and manage biological resources needed to support aging research that would be otherwise less accessible or excessively costly or time consuming to the investigators. The resources made available to publicly funded investigators comprise: 1) live rodents (Aging Mouse and Rat Colonies); 2) cells from different species including human, NHP, rodent, and domestic companion animals (Aging Cell Repository); 3) tissues from aged rodents (Rodent Tissue Bank) and NHPs (NHP Tissue Bank); and 4) a database of NHP vital parameters (Primate Aging Database). The NIA also supports two multi-institutional programs investigating interventions with the potential to extend lifespan and delay disease/dysfunction in worms (Caenorhabditis Intervention Testing Program, CITP) and in mice (Interventions Testing Program, ITP). The NIA values feedback to continue to provide valuable resources that will help the field move forward.

Abstract citation ID: igae098.2007

### NEW TOOL ENHANCES DETECTION OF LIFE-EXTENDING INTERVENTIONS BY DELINEATING THEIR TEMPORAL EFFICACY

Nisi Jiang[1], Qianqian Liu[1], Catherine Cheng[1], Randy Strong[1], Jonathan Gelfond[1], and James Nelson[2], *1. UT Health San Antonio, San Antonio, Texas, United States*, *2. University of Texas Health at San Antonio, San Antonio, Texas, United States*

Drug discovery of anti-aging interventions relies on survival data, which is time-consuming and costly to generate, yet the analysis of such data often fails to capitalize on its full potential. The predominant method for assessing intervention effects on lifespan, the log-rank test, lacks the power to identify when and for how long an intervention is effective. Moreover, it has decreased sensitivity for interventions that are only effective during part of the life course. Statistical tools are needed to address these limitations. They should be capable of identifying when, for how long, and to what extent an intervention reduces (or increases) mortality risk. Here we introduce a Temporal Efficacy Profiler (TEP), which meets these needs, and apply it to survival data from 42 compounds tested in the NIA Interventions Testing Program (ITP) using UM-HET3 mice. Compared to the log-rank test, this tool identified over twice as many compounds that increased (or decreased) survival, largely because of its sensitivity to variable efficacy of the interventions across the life course. Sex differences were prevalent. The TEP revealed agents that either reduced (22 compounds) or increased (15 compounds) mortality hazards, with some (2 compounds) showing dual effects depending on sex. Notably, the efficacy of these interventions varied significantly in both duration and magnitude. Moreover, the TEP uncovered adverse effects that were missed by the log-rank test. Of note, only 8 compounds significantly reduced mortality hazards beyond the 90th percentile of mortality, a period when the burden of senescence is highest.

Abstract citation ID: igae098.2008

### PREDICTING TIME TO COGNITIVE IMPAIRMENT USING BRAIN AGE

Phoebe Imms[1], Siyu Chen[2], Haoqing Wang[2], Ruixi Li[3], and Andrei Irimia[2], *1. University of Southern California, Leonard Davis School of Gerontology, Los Angeles, California, United States*, *2. University of Southern California, Los Angeles, California, United States*, *3. Columbia University Irving Medical Center, New York, New York, United States*

The neurological changes that occur during the asymptomatic pre-clinical phase of cognitive impairment (CI) can be quantified using brain age (BA) measures and leveraged to identify those at risk of clinical manifestation. We employ a machine learning survival model to predict future CI among healthy older adults using cognitive, demographic, genetic, and neuroimaging data including regional brain volume and anatomically specific measures of BA. Participants were 2,718 adults from NACC (1,795 females, mean age=69.14±10.91). Converters (N=343) were diagnosed with CI during the study, and non-converters (N=2,375) were CN across all visits. We predicted time to conversion (days) from baseline MRIs (185 brain volumes and regional BA measures) and 69 clinical variables. The model was trained on 1,846 converters and 252 non-converters, validated on 32 converters and 238 non-converters, and tested on 150 converters and 200 non-converters with 5-fold cross-validation. The model's predictive accuracy (c-index) was 0.82 for training, 0.83 for validation, and 0.78 in the test set. Important features for prediction included age, neurocognitive test scores, education, history of hypertension and stroke, BA, and volumes of the right hippocampus, right precentral gyrus, bilateral white matter, and left inferior temporal gyrus. Products of this model allow us to compare a) the risk, and b) the BA biomarkers across population subgroups with heightened CI risk such as racial minorities and people with fewer years of education. The ability to accurately predict future cognitive impairment (CI) benefits those most in need of therapeutic interventions and reduces the burden of CI on healthcare systems.

Abstract citation ID: igae098.2009

### CHANGES IN RESIDENT OVARIAN MACROPHAGES DURING FEMALE REPRODUCTIVE AGING

Sarah Ocanas, Ewa Poljanska, Jose Isola, Chase Hubbart, and Michael Stout, *Oklahoma Medical Research Foundation, Oklahoma City, Oklahoma, United States*

Ovaries exhibit accelerated aging phenotypes, displaying aging hallmarks earlier than other organs. During premenopause, endocrine function declines, and the ovarian microenvironment becomes pro-inflammatory and fibrotic. However, the cellular mechanisms driving ovarian aging remain unclear, hindering the development of therapies to delay this process for overall health benefits. Macrophages are crucial in ovarian functions like folliculogenesis, ovulation, and corpus luteum formation. Ovarian macrophages have two origins: tissue-resident macrophages (TRMs) from yolk sac and fetal liver progenitors, and monocyte-derived macrophages (MDMs) from bone marrow. In this study, we examined the transcriptomic profiles of ovarian TRMs in young (5-6 months) and older (12-14 months) Cx3cr1-NuTRAP mice. This mouse model uses a tamoxifen-inducible, cre-lox system to label nuclei and ribosomes, enabling DNA/RNA isolation without cell sorting. Flow cytometry confirmed the identity of eGFP-labeled cells, and translating ribosome affinity purification (TRAP) was used to assess age-related transcriptomic changes in TRMs. We found that TRMs exhibit inflammatory (e.g., IL1B) and senescent (e.g., CDKN2A) transcriptomic signatures, likely contributing to aging hallmarks such as T cell accumulation, multinucleated giant cell formation, and fibrosis. Translatability was established by comparing our data with publicly available human data. These findings suggest that ovarian TRMs play a critical role in ovarian aging, warranting future mechanistic studies.

Abstract citation ID: igae098.2010

### ADVERSE MUSCLE COMPOSITION IS LINKED TO LOWER MITOCHONDRIAL RESPIRATION AND SLOWER WALKING SPEED

Sofhia Ramos[1], Katherine Glockner[2], Giovanna Distefano[3], Peggy Cawthon[2], Jennifer Linge[4], Olof Dahlqvist Leinhard[5], Bret Goodpaster[6], and Paul Coen[3], *1. AdventHealth, AdventHealth, Florida, United States*, *2. California Pacific Medical Center, San Francisco, California, United States*, *3. AdventHealth, Orlando, Florida, United States*, *4. AMRA Medical AB, Linköping, Ostergotlands Lan, Sweden*, *5. Linköping University, Linköping, Ostergotlands Lan, Sweden*, *6. Advent Health, Orlando, Florida, United States*

Low muscle mass and fat infiltration are core features of sarcopenic obesity. We examined skeletal muscle mitochondrial respiration and walking speed in older adults with normal muscle composition (NMC), only low muscle volume (LMV), only high fat infiltration (HFI) and the combination of LMV and HFI, (adverse muscle composition (AMC)). Muscle composition was determined by whole body MRI and high resolution respirometry was performed on muscle biopsies from N=828 participants (76.3 ± 5.0 years old, 59.4% female) in the Study of Muscle, Mobility and Aging (SOMMA). We found that mitochondrial respiration was significantly lower in individuals with LMV (-10%), HFI (-9%) and AMC (-8%), compared to those with NMC. In multivariate regression models, 4m and 400m walking speeds were significantly slower in LMV (400m: β=-0.10 4m: β=-0.07, both p< 0.05), HFI (400m: β=-0.14, 4m: β=-0.13, both p< 0.01) and AMC (400m: β=-0.19, 4m: β=-0.16, both, p< 0.01) groups, compared to NMC (400m: 1.11m/s, 4m:1.10m/s). Compared to NMC, mitochondrial respiration explained ~20% of the variance in 400m walking speed in LMV, ~64% in HFI, and ~47% in AMC, while all three groups remained significantly slower. Compared to those with NMC, mitochondrial respiration explained ~29%, and ~77% of the variance in 4m walking speed in LMV and HFI respectively, abolishing group differences in 4m walking speed, while mitochondrial respiration explained ~50% of the variance in ACM. In conclusion, low muscle volume, high fat infiltration and AMC phenotypes are linked to lower mitochondrial respiration and slower walking speed in older adults.

### SESSION 4315 (PAPER)

### COGNITION (PAPERS)

### STUDIES ON COGNITION

Abstract citation ID: igae098.2011

### MENOPAUSE HISTORY AND COGNITIVE FUNCTION IN THE IGNITE TRIAL

Amber Watts[1], Shannon Donofry[2], Hayley Ripperger[3], Nicole Eklund[4], Lu Wan[3], Chaeryon Kang[3], and Kirk Erickson[5], *1. University of Kansas, Lawrence, Kansas, United States*, *2. RAND Corporation, Pittsburgh, Pennsylvania, United States*, *3. University of Pittsburgh, Pittsburgh, Pennsylvania, United States*, *4. Boston University, Boston, Massachusetts, United States*, *5. Advent Health, Orlando, Florida, United States*

Lifetime exposure to estrogen, a neuroprotectant, is associated with cognitive aging and dementia. Surgically induced menopause (ovariectomy) is associated with poorer cognitive performance, while use of hormone therapy (e.g., birth control, menopausal hormone therapy) has shown mixed associations with cognitive performance. We investigated these associations in IGNITE, a 12-month, multi-site, randomized exercise trial. We measured cognition via the Montreal Cognitive Assessment (MoCA), and factor analysis-derived composite scores for episodic memory, processing speed, working memory, attentional control, and visuospatial processing. In 461 female participants, we estimated relationships between ovariectomy, hormone-use, and baseline cognitive performance accounting for age, education, APOE ε4 carrier status, and race. Ovariectomy predicted worse MoCA scores (B=0.22, p=.038), but was unrelated to cognitive composite scores. African American race accounted for the largest proportion of variance in this model (R2Change=.09). Menopausal hormone therapy was associated with better performance on episodic memory (B=.109, p=.018) and working memory (B=.101, p=.021). Birth control use predicted better performance on MoCA (B=.090, p=.033), working memory (B=.094, p=.017), and attentional control (B=.099, p=.015). The association of ovariectomy with MoCA and not with other domains may indicate an association with dementia, not with typical cognitive aging. Meanwhile menopausal hormone therapy was associated with higher order cognitive performance, not the dementia screener. Birth control was associated with both. Our results validate prior evidence that lifetime exposure to estrogen is an important predictor of older women's cognitive health and indicate further investigation is needed to clarify differential associations of menopause history and cognitive performance in African American women.

Abstract citation ID: igae098.2012

### DYNAMIC BALANCE PERFORMANCE WAS ASSOCIATED WITH BETTER COGNITION IN COMMUNITY-DWELLING LOW-INCOME OLDER ADULTS

Ladda Thiamwong[1], and Renata Komalasari[2], *1. University of Central Florida, Orlando, Florida, United States*, *2. Tzu Chi Hospital, Jakarta, Indonesia*

**Objectives:** We cross-sectionally examined the relationships of dynamic and static balance performance, and handgrip strength, with cognitive performance in community-dwelling low-income older adults in Orlando, Florida.
**Methods:** This study was drawn from a sample of 92 older adults (90.2% females, Mage = 72.63, SD±6.82). Dynamic balance was measured by the Short Physical Performance Battery, static balance by BTrackS scores, handgrips by three handgrip trials, and cognitive performance by the Rowland Universal Dementia Assessment Scale.
**Results:** Across the participants, 71.7% were African American, 62% completed high school, and 57.6% were just enough financially. The multiple regression analysis indicated that the model explained 10.2% of the variance (R2=.319, F (3,88) = 3.33, p =.023). Dynamic balance (B =.075, p-value =.047) was significantly associated with cognitive performance, but static balance (B =-.023, p-value =.081) and handgrip strength were not (B =.002, p-value =.855), accounting for current depressive symptoms (B =.057, p-value =.374). Independent t-test showed that older adults with normal cognition (RUDAS scores ≥23) scored higher in dynamic balance (Mdifference= 27.845, p=.018) than those with possible cognitive impairment (RUDAS scores < 23). Older adults who are cognitively healthy performed better than those with possible cognitive impairment in dynamic balance performance.
**Discussion:** Simple dynamic balance performance tests, comprising balance, gait speed, and chair stand tests, could serve as a valuable cognitive assessment tool in community-dwelling low-income older adults. Keywords: dynamic balance performance, static balance performance, handgrip strength, cognitive performance, Rowland Universal Dementia Assessment Scale, aging

Abstract citation ID: igae098.2013

### MARKERS OF PHYSICAL HEALTH AND HEALTH BEHAVIORS CONTRIBUTE TO LATER COGNITIVE FUNCTIONING AMONG ADULTS AGED 55–65

Ben Katz, Laura Sands, and Maham Khan, *Virginia Tech, Blacksburg, Virginia, United States*

Life's Essential 8 (LE8) represents a summary score consisting of a set of physical health (BMI, blood pressure, cholesterol,

A1C) and health behaviors (sleep, diet, smoking status, physical exercise) that are closely associated with risk of cardiovascular disease. LE8 is designed to be simple for physicians to calculate, using regularly collected self-report and biomarkers, and easily interpretable for patients. While no equivalent score exists for Alzheimer's disease and related dementias (ADRD), given close links between cardiovascular risk factors and cognitive outcomes, recent studies have identified links between LE8 and cognition and dementia. However, longitudinal work in this space is limited; additionally, many prior studies do not examine links from midlife to changes in later life cognitive functioning. The present study draws from 761 midlife adults aged 55-65 without cognitive impairment at baseline who were recruited to participate in the Health and Retirement Study. Data informing LE8-style indicators (including health behaviors and bloodspot biomarker data) were recorded in 2006. Performance on the Telephone Interview for Cognitive Status (TICS) assessment was measured 14 years later. Utilizing linear regression techniques that incorporated the HRS sample weights and age, gender, education, and baseline cognitive functioning, we found that both the aggregated health behaviors ($p < .01$) and aggregated physical health biomarker indicators ($p < .001$) accounted for unique variance in the TICS scores 14 years later. These findings have implications both for basic research to investigate the mechanisms connecting lifestyle and physical biomarkers to later cognition, but also for clinical practice and patient communication.

Abstract citation ID: igae098.2014

### ASSOCIATION BETWEEN SERUM BIOMARKERS OF ALZHEIMER'S DISEASE AND "DUAL DECLINE" IN COGNITIVE AND MOTOR FUNCTIONS

Elena Pinardi[1], Alice Ornago[2], Giulia Grande[3], Martina Valletta[3], Debora Rizzuto[3], Erika Jonsson Laukka[3], Giuseppe Bellelli[4], and Davide Liborio Vetrano[3], *1. Karolinska Institutet, Italy*, *2. Karolinska Institutet, Osnago, Lombardia, Italy*, *3. Karolinska Institutet, Stockholm, Stockholms Lan, Sweden*, *4. University of Milano-Bicocca, Milano, Lombardia, Italy*

Individuals experiencing simultaneous decline in cognitive and motor performances (i.e., dual decliners) face an increased risk of dementia and other adverse health outcomes. While the underlying pathophysiology remains largely unknown, this study aims to explore the association between serum biomarkers of Alzheimer's disease (AD) and various patterns of cognitive/motor decline. Data are drawn from the Swedish National Study on Aging and Care in Kungsholmen. The rates of cognitive and motor decline were assessed over a 15-year period using linear mixed models, with Mini-Mental State Examination and gait speed as the respective measures. Participants were categorized into slow/non-decliners (reference group, n=1115), fast motor decliners (n=162), fast cognitive decliners (n=162), and dual decliners (n=264). The association between serum AD markers levels and the four decline patterns was analyzed using multinomial logistic regressions. Phosphorylated tau181 (OR = 1.77, 95% CI: 1.60;1.97), neurofilament light chain (OR = 1.44, 95% CI: 1.31;1.57) and phosphorylated tau181/amyloid-β42 ratio (OR = 1.18, 95% CI: 1.12;1.25) were associated with the highest risk of a dual decline pattern among study participants. In isolated cognitive decliners, robust associations were observed for amyloid-β42, amyloid-β42/40 ratio and glial fibrillary acidic protein, whereas no significant association emerged for those exhibiting only motor decline. Dual decliners present distinct profiles of serum AD biomarkers. The extensive involvement of tau pathology and neurodegeneration suggests an intrinsic biological complexity that necessitates further research, encompassing vascular and neuroinflammatory pathways.

### SESSION 4320 (SYMPOSIUM)

Abstract citation ID: igae098.2015

### CULTIVATING TRUST IN RESEARCH PARTNERSHIPS WITH OLDER ADULTS ACROSS THE COMMUNITY ENGAGEMENT CONTINUUM

Chair: Allyson Brothers
Co-Chair: LIza Behrens
Discussant: Carrie Leach

Community-engaged research is essential for identifying and supporting the needs of an aging society and ensuring effective translation of research findings. While not a new idea to the field of gerontology, engaged research models and frameworks are expanding, and researchers are seeing increased access to initiatives for building and sustaining community-engaged research programs. This symposium will feature emerging and established projects across multiple levels of the continuum of engagement, from informing, consulting, involving, collaborating, and ultimately to co-creating. Kim et al. will describe efforts to co-create programming and educational materials with and for Alaska Native caregivers, focusing on decolonizing methodologies. Halvorsen will showcase an ongoing academic-organizational partnership created to increase the research and evaluation capacity of a national nonprofit organization and its grantees. Gan will describe how diverse community members were engaged to understand and meet gaps in cognitive health promotion, resulting in the pilot of a mindful discussion program. Guest et al. will present on the formation and fruitful efforts of a resident advisory board at the University Based Retirement Community, sharing best practices for sustaining engagement. Mahmood et al. will report on a community-based participatory effort evaluating the role of built environment on mobility access across five cities in the Vancouver area. These exemplars will launch the symposium into a discussion about the utility of engaging older adults across the continuum, illustrating effective strategies for establishing trusted academic-community partnerships. We invite attendees to consider the value of this heuristic framework to plan and design fit-for-purpose community-engaged research projects. Community Engaged Research Interest Group Sponsored Symposium

Abstract citation ID: igae098.2016

### CO-CREATING EVIDENCE-BASED PROGRAMMING AND EDUCATIONAL MATERIALS FOR ALASKA NATIVE CAREGIVERS

Steffi Kim[1], Jordan Lewis[2], and Lena Thompson[3], *1. University of Alaska, Anchorage, Alaska, United States*, *2. University of Alaska Fairbanks, College of Indigenous Studies, Fairbanks, Alaska, United States*, *3. University of Minnesota Duluth, Duluth, Minnesota, United States*

Alaska Native Elders are rarely involved in creating educational and outreach materials. Over and over, Elders share that available educational and informative materials do not include their cultural understandings, programs do not meet their needs, and resource developers rarely attend to incorporate the specific and unique Alaska Native context. We will share our expereinces about outreach, recruitment, and establishment of an Alaska Native Community-engaged Aging Collaborative to engage community members, caregivers, and local organizations to create culturally appropriate and context-specific educational materials and programming that support Alaska Native Elders. Establishing this collaboration is the foundation for implementing research and programs driven by community needs and cultural understandings in conjunction with Western evidence-based approaches. Decolonizing strategies such as community member involvement, building trust, and sustainable relationships are not just steps but integral components of conducting Indigenous-driven research that benefits communities. These approaches not only sustain Elder participation in research related to aging well and dementia but also underscore the value and importance of their contributions. Indigenous research paradigms are gaining traction and hold immense potential in involving Indigenous older adults in research endeavors. Building these collaborations not only gives Elders a voice but also offers researchers a promising direction and invaluable insight into the specific needs of these communities, fostering a sense of hope and optimism for the future of research.

Abstract citation ID: igae098.2017

### BUILDING RESEARCH AND EVALUATION CAPACITY OF INTERGENERATIONAL INITIATIVES: EXAMPLES FROM THE FIELD

Cal Halvorsen, *Washington University in St. Louis, St. Louis, Missouri, United States*

Conducting evaluations to assess the impact of projects, services, and programs is often a daunting task for nonprofit and grassroots organizations. More so, developing research projects that document the need for the work of these social-purpose organizations can be difficult for staff and volunteers busy with day-to-day responsibilities. This presentation will showcase an ongoing academic-organizational partnership created to increase the research and evaluation capacity of a national nonprofit organization and its network of nascent and established grantees bringing older and younger people together to work on major social and environmental issues. Topics addressed will be establishing and cultivating trusting and transparent relationships, developing a scope of work, using an organization-centric research capacity-building approach, engaging students in these efforts, and more. Further, specific examples of products from these research and evaluation projects will be shown to illustrate how to navigate the conflicting needs of academics on the tenure-track with those of community-based organizations. The primary implications of this academic-organization partnership are twofold: First, this model, piloted with an organization focused on fostering new intergenerational initiatives, can be used by gerontological researchers in a variety of fields to benefit the community while aligning with established academic expectations; and second, leaders within these nascent intergenerational initiatives have now learned how to conduct basic program evaluations within their own organizations, which should ultimately lead to the strengthening of programs that bring the generations together.

Abstract citation ID: igae098.2018

### BRAIN HEALTH IN COMMUNITY: INVOLVING AND COLLABORATING WITH COMMUNITIES YIELDED UNEXPECTED INNOVATIONS

Daniel Gan[1], Claire Wang[2], and Susan Liu Woronko[3], *1. University of Toronto, Toronto, Ontario, Canada, 2. Johns Hopkins University, Baltimore, Maryland, United States, 3. Frog Hollow Neighbourhood House, Vancouver, British Columbia, Canada*

Funded by university-community engagement initiatives, this research aimed to understand and meet gaps in cognitive health promotion in diverse communities. The research team started with CONSULTATIVE conversations with activity providers and partnered older adults for analytic model specification and interpretation. As the focus groups progressed, the engagement fluidly progressed into an INVOLVEMENT marked by mutual respect for the distinct expertise of both parties and quick successions of knowledge exchanges, which led to two unintended innovations. Due to the trust built with key informants, older adults openly shared their unmet needs for a space to find meaning amid the challenges of aging. In response, a novel mindful discussion program was COLLABORATIVELY piloted with community organizations. Moreover, trust from older adults begot trust from activity providers and their sharing led to a simple eMental Health solution for closed-loop social prescribing. These insights on trust, engagement levels, and innovations are gained only in retrospect. Referencing these experiences, we reflect on the importance trust built with older adults as a catalyst for deeper engagements with service providers. Low-intensity consultations were more manageable at the start of the researcher-community relationship. More extensive engagements with older adults led to greater trust and mutual empowerment, and incidentally provided access to implicit knowledge held by activity providers which was crucial for innovation. Pivotal moments of knowledge spillovers were part of everyday activities in the community as researchers became embedded in relationality. Our experience underscores fluid engagement approaches that defy our best-planned intentions as lessons in decolonial knowledge cultivation.

Abstract citation ID: igae098.2019

### COLLABORATIVE RESEARCH DEVELOPMENT: ESTABLISHING A RESEARCH-AGENDA AT A UNIVERSITY-BASED RETIREMENT COMMUNITY

M. Aaron Guest[1], Molly Maxfield[1], Lindsey Beagley[2], Sigourney Schaffer[2], Becky Owens[2], and David Coon[3], *1. Arizona State University, Phoenix, Arizona, United States, 2. Arizona State University, Tempe, Arizona, United States, 3. Arizona State Unviersity, Phoenix, Arizona, United States*

University-Based Retirement Communities (UBRCs) represent a novel trend toward creating age-inclusive environments. These emerging housing options for older individuals generally function as continuing care retirement communities but stand out due to their close ties and university integration. They strive to foster intergenerational exchanges and offer older adults the chance to engage in university life and community. At Arizona

State University, Mirabella at ASU is uniquely situated at the heart of the Tempe, AZ Campus, and is one of the few UBRCs physically located on a campus. Mirabella's integration into the university has opened doors for collaborative research projects that add to broadly the nascent literature on UBRCs and aging. Residents expressed interest early in partnering with researchers to understand more about the UBRCs and aging in a university setting. To further these goals, a joint panel of residents and researchers was formed to pinpoint mutual research interests. This initial gathering prompted a series of focus groups to fine-tune topics, eventually leading to an annual resident health survey and the establishing of a Community Research Advisory Board. Transitioning from idea to implementation required navigating complex considerations beyond those typically encouraged in community-based research. In this presentation, we will share our experience in developing a collaborative research partnership while navigating the at-times competing interests of the researchers, university, operators, and residents. Furthermore, with the increasing prevalence of these communities, we offer guidance on sustaining engagement and sharing best practices derived from our experiences.

Abstract citation ID: igae098.2020

### OLDER ADULTS WITH DISABILITIES AS CORESEARCHERS IN COMMUNITY-ENGAGED RESEARCH ON MOBILITY AND ACCESS

Atiya Mahmood, Niloofar Hedayati, Rojan Nasiri, Sogol Haji Hosseini, and Sepehr Pandsheno, *Simon Fraser University, Vancouver, British Columbia, Canada*

Neighbourhood access and participation opportunity significantly impacts the health, social inclusion, and overall wellbeing of older adults especially those with mobility, sensory and cognitive disabilities. Stakeholders Walkability/ Wheelability Audit in Neighbourhoods (SWAN) is a community engaged mixed method project where user-led audits are conducted in neighbourhoods complemented by semi-structured interviews to evaluate the role of built environment on mobility, access and participation of older adults and people with disabilities across five cities within Metro Vancouver, Canada. Using a community engaged lens starting from initial engagement to more advanced collaboration, this project involves various community stakeholders in different capacities fostering a two-way exchange of information between researchers and community members. Study participants as coresearchers participate in research tool adaptation, data collection, analysis and knowledge mobilization. Additionally, city staff members partner with the research team to identify key areas in cities for data collection, collaborate in knowledge mobilization activities and the development of complementary implementation projects. The SWAN project is part of a larger partnership project titled, "Mobility, Access and Participation" (MAP). Preliminary findings from this project underscores neighbourhood accessibility as it relates to functionality, safety, appearance, supportive features and social engagement opportunities for older adults with disabilities while highlighting some distinct challenges across different types of disabilities. Insights from the SWAN project has the potential inform both academic scholarship and local government policy around mobility, access and social. Findings underscores the importance of community engaged research in informing programmatic and policy changes using a social equity lens.

### SESSION 4325 (SYMPOSIUM)

Abstract citation ID: igae098.2021

### DESIGNING FUTURE HEALTH CARE ENVIRONMENTS FOR AN AGING SOCIETY: TRANSDISCIPLINARY PERSPECTIVES

Chair: Felix Kabo
Discussant: Cameron Contreras

As our society grapples with demographic aging, it is imperative that we design healthcare environments that better support the well-being of older adults in the context of critical staffing shortages in the healthcare sector. Beyond aesthetics and visual appeal, thoughtful healthcare design significantly and directly impacts patient outcomes, staff well-being, and the overall quality of care. This symposium brings together four complementary presentations from the design and research fields that employ transdisciplinary perspectives to examine the issue of designing healthcare environments for an aging society. Kabo, Morrison, and Ritsema discuss how digital twins, virtual representations of real-world objects, can be used to transform the future of healthcare design, thus delivering quality care experiences that sufficiently support the needs of older adults. Calkins provides an overview of evaluative research on healthcare design outcomes by focusing on the post-occupancy evaluation process by the SAGE Federation and links the research to the holistic design of age-friendly healthcare environments. Perreault and Kaminski use two innovative case studies to discuss and illustrate best practices in healthcare design for older adults, and how the built environment can be designed as an active aid for patient recovery. Kim will discuss how smart technologies and machine learning can facilitate successful aging-in-place and the design of home environments that better support healthcare for older adults. Collectively, this symposium brings together speakers from different fields (design and research) and disciplines (e.g., gerontology, architecture, interior design, engineering, etc.) to discuss the convergence of design and healthcare for our aging society.

Abstract citation ID: igae098.2022

### DUAL PRIORITIES: DIGITAL TWINS AND HEALTH CARE DESIGN THAT SUPPORTS OLDER ADULTS IN THE CONTEXT OF STAFFING SHORTAGES

Felix Kabo[1], Jake Morrison[2], and Chad Ritsema[3],
1. *CannonDesign, Ann Arbor, Michigan, United States*,
2. *CannonDesign, Pittsburgh, Pennsylvania, United States*,
3. *CannonDesign, Chicago, Illinois, United States*

Aging populations are associated with a growing need and demand for specialized care, including healthcare environments that accommodate unique needs such as mobility assistance, chronic disease management, post-acute care, and mental health support. Currently, there are more than 46 million Americans aged 65 or over, and this number is projected to double by 2060. This demographic aging calls for an increased focus on design solutions like creating age-friendly spaces and ensuring accessibility. Designing healthcare environments that better support older adults is complicated by the staffing shortages in the American healthcare system. For example, there are significant projected shortages of both essential support staff (over 3 million in the next five years) and physicians (roughly 140,000 by 2033). These staffing shortfalls pose critical challenges to patient care, workplace culture and cohesion, and safety. The design of healthcare environments that better support older adults must mitigate the negative impacts of inadequate staffing levels and facilitate more efficient workflows e.g., enhancing interdisciplinary staff collaboration, promoting novel training opportunities, etc. This presentation examines the use of digital twins, effectively indistinguishable virtual representations of real-world physical objects (e.g., the human body, buildings, etc.), as part of a holistic design process that considers older adult patients, staffing and care delivery, and facility optimization. Digital twins have high fidelity and are linked to real-time data. They hold significant potential for designing age-friendly healthcare environments, optimizing staff and resource allocation, asset tracking, accelerated risk assessment, real-time decision support, and novel training simulations, thus improving care for older adults.

Abstract citation ID: igae098.2023

### ASSESSING THE INTEGRATION OF AGE-FRIENDLY DESIGN RESEARCH INTO THE DESIGN OF HEALTH CARE ENVIRONMENTS

Margaret Calkins, *IDEAS Institute, Cleveland Heights, Ohio, United States*

Older adults account for roughly 2/3rds of all patient days in hospitals, and more than 90% of days in post-acute care settings. It is not just the care delivered by clinicians that determines outcomes. Different environments – organizational, social, as well as physical – also have significant impacts on a variety of health and well-being outcomes. Great strides have been made over the past two decades in the design of residential and care environments for older adults, and in the research that evaluates these designs. Research has examined these environments at many scales from overall design concepts (for example, the medical model versus residential design approaches) down to details such as the design of supportive handrails and hardware. This presentation will give a brief overview of this research and explore SAGE (Society for the Advancement of Gerontological Environments) Federation's post-occupancy process for evaluating residential and care environments for older adults, which has been conducted annually for the past 20 years. However, much less focus has been paid to making other healthcare environments, particularly acute care hospitals, more age-friendly. The Island Health system in Vancouver, Canada, set out to change this and designed the Royal Jubilee Hospital to be age-friendly from pre-admission through discharge. The presentation will also explore the process that Island Health used for decision-making, and how this has influenced subsequent new construction.

Abstract citation ID: igae098.2024

### EMPOWERING OLDER ADULTS THROUGH SMART TECHNOLOGY: A NEW ERA IN AGING-IN-PLACE AND HOME HEALTH CARE

Daejin Kim, *Florida State University, TALLAHASSEE, Florida, United States*

Demographic aging presents unique global challenges, especially regarding the preference of many older adults to age in place. Traditional physical environments often inadequately support the complex needs of this demographic, highlighting the need for innovative approaches. This presentation focuses on the integration of smart home technologies that are powered by machine-learning algorithms to facilitate successful aging in place. Smart home technologies encompass an array of sensors, devices, and machine-learning analysis, and can significantly enhance the wellness and independence for older adults through home automation, health monitoring, and daily activity tracking through machine learning algorithms. They provide essential insights into the functional, cognitive, and social well-being of older individuals, thus supporting their independence and enriching their quality of life. Despite their immense potential, a knowledge gap persists regarding the availability and integration of smart home technologies in homes designed for older adults. This presentation addresses this gap by highlighting the practical applications of these technologies for healthcare professionals, architects, and policy makers. It discusses challenges like privacy, user-friendliness, and affordability, and explores future advancements in this field. The presentation also emphasizes the importance of adopting technological solutions to transform built environments for older adults through a comprehensive overview of the critical role of smart home technologies. It underscores how tailored, effective technological support can significantly improve living conditions for older adults, enabling them to live safely and independently in their preferred environment. The presentation is a call to action for creating more supportive and adaptable living spaces for our aging society.

Abstract citation ID: igae098.2025

### PROMOTING THE PATIENT EXPERIENCE AND WELL-BEING: LESSONS FROM GROUNDBREAKING PROJECTS IN HEALTH CARE DESIGN

Elisabeth Perreault[1], and Margi Kaminski[2],
*1. CannonDesign, Buffalo, New York, United States*,
*2. CannonDesign, Chicago, Illinois, United States*

The speakers will discuss how the built environment can and should support older adults in healthcare, rehabilitation, and long-term care facilities. They will share two case studies that showcase design solutions for older adults informed by the Living-Centered Design approach and focusing on long-term care and memory care for veterans, and recovery and rehabilitation, respectively. They will discuss current best practices in healthcare and long-term care design for older adults, and how the built environment can actively assist patient recovery and well-being. The first case study is the Veterans Home of California-Yountville (VHC-Yountville), which is under construction and nearing completion. This is a new 240-bed and 285,000 square feet (SF) skilled nursing and memory care facility by the California Department of Veterans Affairs (CalVet) and is located on CalVet's historic campus in Napa Valley. Evidence-based design principles for older adults, including those with dementia and traumatic brain injuries, were applied to create a supportive, long-term care environment that effectively balances medical needs with the need for comfort and community. The second case study located in the state of New York is a new 9-story, 168-bed, and 189,600 SF urban rehabilitation facility for older adults focusing on patient-directed care. The patient services provided include medical care, physical, occupational, and speech therapy, diagnostic/ lab services, palliative care, social work, and mental health services. "Greenhouse" design principles were applied to create a post-acute rehabilitation facility that showcases how the building can be designed to actively catalyze and support patient recovery and rehabilitation.

### SESSION 4330 (PAPER)

### DIVERSE PERSPECTIVES ON END-OF-LIFE: ACCESS, QUALITY, AND COPING

Abstract citation ID: igae098.2026

### ENGAGING COMMUNITY HOSPICE PROVIDERS TO SUPPORT VETERANS AND THEIR FAMILIES AT END-OF-LIFE

Deanna Dragan[1], Lauren Hagemann[2], and Cynthia Jarmul[3], *1. Salem VAMC, Salem, Virginia, United States*, *2. Salem VA Healthcare System, Salem, Virginia, United States*, *3. Salem VA Medical Center, Salem, Virginia, United States*

The VHA's Empowering Community Hospices Initiative was developed to help strengthen the VHA's coordination of care practices with non-VA providers. It is imperative that community hospice providers (CHP) be privy to the unique experiences of Veterans as their needs may be significantly different than those of a non-veteran at end-of-life (EOL) like specialized care and benefits for conditions such as PTSD, Moral injury and suicidality. For this project we completed a formal program evaluation study in collaboration with CHPs to explore the potential barriers and facilitators for creating and sustaining partnerships. Data collection included semi-structured interviews with stakeholders (n = 18) across multiple disciplines (e.g., Social Workers, Psychologists, Physicians, etc.) at the National, VISN, and local levels. Also, we conducted outreach efforts to local CHPs (n = 8), completed semi-structured interviews (n = 4), and coordinated face-to-face site visits (n = 4) for the VHA team to meet with the teams at each agency. These interviews revealed themes related to perceived barriers to and facilitators for achieving the goals of the initiative. Themes identified included: point of contact, timeline of hospice care, differences per agency, and need for resources to support Veterans' families. CHPs collaborated with VHA to develop feasible partnerships with plans for sustainability using a consultation model of care. It is anticipated that the demand for EOL services may soon exceed the supply of care that VA facilities can provide; thus, these efforts may improve the access to services for Veterans in community hospice care settings.

Abstract citation ID: igae098.2027

### MARITAL STATUS AND QUALITY OF END-OF-LIFE EXPERIENCES AMONG OLDER US ADULTS

Kafayat Mahmoud[1], Deborah Carr[1], and Jarron Saint Onge[2], *1. Boston University, Boston, Massachusetts, United States*, *2. University of Kansas, Lawrence, Kansas, United States*

Understanding the quality of end-of-life experiences is a critical goal, because many older adults experience discomfort or care discordant with their preferences at the end of life. While extensive research documents the protective effects of marriage for health, we know of no studies focusing specifically on marital status differences in end-of-life well-being and care. We examine the extent to which marital status (married, remarried, divorced, widowed, never married) affects end-of-life physical and emotional well-being, quality of care, and dignified care, and whether these associations differ by gender. Using 13 waves of data from the National Health and Aging Trends Study (NHATS), we estimate multivariable multinomial and binomial logistic regression models to evaluate how marital statuses affect diverse indicators of end-of-life well-being and gender differences therein. Our analyses show that married persons do not fare uniformly better than their unmarried counterparts; rather, patterns differ across outcomes. Widowed and remarried persons have a higher likelihood of reporting excellent or very good care at the end of life, relative to married persons. Widowed older adults also are more likely to report receiving care concordant with their preferences. Divorced older adults report poorer pain management, while remarried and never married older adults report less sadness relative to married persons. Patterns do not differ significantly by gender. Unmarried or remarried persons may perceive a more urgent need to do advance care planning, which can enhance the quality of dying experiences. We discuss how marital relationships more broadly shape end-of-life experiences.

Abstract citation ID: igae098.2028

### END-OF-LIFE DECISION-MAKING: INFORMATION PRACTICES OF UCHINANCHU OLDER ADULTS

Kristina Shiroma, *Louisiana State University, Baton Rouge, Louisiana, United States*

End-of-life (EOL) decision making encompasses sensitive, yet pivotal decisions requiring nuanced information. Understanding how diverse populations understand and make EOL decisions is crucial as diverse information practices can inform policies to enhance value-centered care for a broader range of patients at end-of-life. This study aims to better understand diverse approaches to EOL decision-making from older adults' who come from an underrepresented ethnic minority group in the United States. Reflexive thematic analysis of in-depth, semi-structured interviews with 18 Uchinanchu American older adults. Recruitment through convenience and snowball sampling for in-person and telephone interviews. Three themes were identified in Uchinanchu American older adults' approaches to EOL decision making: Prioritizing Mitigation of Burden for Adult Children; Keeping Customs of Filial Responsibility; and Maintaining a Shoganai Mindset. Uchinanchu participants were thinking about EOL decision making both down the familial lineage as they prioritized mitigating burden for their adult children, but also up the familial lineage as they kept customs of filial responsibility for their parents. Participants' cultural approach to shoganai (the Japanese word associated with accepting one's fate) helped them to balance a cultural acceptance of death as a fated part of life to either make or defer from EOL decision making. This study extends our understanding of diverse approaches to end-of-life decision making for underrepresented ethnic minority older adults in the United States. Future work in EOL decision making should include hard-to-reach, underrepresented populations to provide for inclusive and representative understandings of death and dying.

Abstract citation ID: igae098.2029

### GLOBAL ASSESSMENTS OF END-OF-LIFE CARE: HOW ARE THESE CARE MEASURES PATTERNED BY PROXY RELATIONSHIP TO DECEDENT?

Kafayat Mahmoud[1], Deborah Carr[1], and David Ekerdt[2], *1. Boston University, Boston, Massachusetts, United States*, *2. University of Kansas, Lawrence, Kansas, United States*

A "good death" is one in which the patient's physical, emotional, and spiritual needs are met, they are treated with dignity and respect by health care providers, and treatment preferences are honored. Despite policies to help improve death quality, including support for doctor-patient end-of-life consultations as part of the Affordable Care Act, it is unclear whether death quality has improved, nor whether particular people are vulnerable to poor quality deaths. Researchers rely heavily on proxy-reported measures of end-of-life, however it is unclear whether these reports differ by one's relationship to the decedent. We use 13 waves of the National Health and Aging Trends Study (NHATS), and polychoric factor analysis and multinomial and logistic regressions to identify the contributions of 10 specific dimensions of end-of-life care to overall quality of care evaluations, and how these assessments are affected by the proxy's relationship to the decedent, after controlling for socio-demographic and health characteristics of decedents. Overall care ratings are highly correlated with perceptions of personal care needs met, respectful treatment, being informed about care, and care coordination. Physical and emotional symptoms, care wantedness, and decisions made without patient input have weaker associations with the overall care assessment. Paid caregiver proxies give more positive assessments of care relative to spouses and children of decedents. Adult children proxies offer more positive assessments of care decisions and wantedness, whereas spouse proxies report better management of physical and emotional symptoms. We discuss the implications for data collection as well as health care practice.

Abstract citation ID: igae098.2030
### OLDER AGE PREDICTS POSITIVITY IN HOSPICE FAMILY CAREGIVER AUDIO DIARIES: IMPLICATIONS FOR ASSESSMENT INNOVATION

Kristin Cloyes[1], Megan Thomas Hebdon[2], and Lee Ellington[3], *1. Oregon Health & Science University, Portland, Oregon, United States*, *2. The University of Texas Austin, Austin, Texas, United States*, *3. University of Utah, Salt Lake City, Utah, United States*

The likelihood of becoming a caregiver of someone with a serious illness increases with age, and those 55 years of age and older are more likely to be end-of-life caregivers of family and friends. Older adults may also use more emotional coping skills, which can have a protective effect depending on perceived stress and gender. We asked in-home hospice family caregivers of cancer patients (n=58) to record daily, open-ended audio diaries about their experiences. We examined whether age and gender predicted the amount of affect-related content and overall emotional tone (positivity/negativity). Caregivers recorded audio diaries up to 5 minutes via an IVR system (n=984 entries). We transcribed diaries and used LIWC software to quantify the proportion of affect-related content and positive/negative tone, and mixed-effects regression modeling to analyze the effects of age and gender on these indicators. Our sample (M age=60.72, SD=13.12, range=30-86) was mostly white (84.5%) non-Hispanic (86.2%) women (74.1%) caring for their spouse (44.8%). Age predicted positivity, with each additional year of age predicting a slight increase ($\beta$=0.426, p=0.028). Age did not predict affect ($\beta$=0.032, p=0.251). Gender did not predict tone ($\beta$=2.622, p=0.619), but trended towards predicting less affect expression in men ($\beta$= -1.482, p=0.062). Hospice family caregiving is challenging and often isolating, affording few opportunities for processing experiences. Our findings suggest an interplay of age and gender in caregiver self-expression. Understanding how caregivers' self-expression aligns with underlying coping and unmet needs is important for developing audio diaries as a low-cost assessment of coping and well-being.

### SESSION 4335 (SYMPOSIUM)

Abstract citation ID: igae098.2031
### ESTABLISHING SHORT-TERM STUDY ABROAD OPPORTUNITIES IN GERONTOLOGY

Chair: Linda Johansson

Beginning in 1995, the College of Health and Welfare at Jönköping University has hosted college and university students from the United States in a short-term (10-14 days) study abroad experience in "Healthcare and Social Services across the Lifespan." Students from multiple U.S. universities have participated, including Pennsylvania State University, Utah State University, and Indiana University Southeast. U.S. students participate with Swedish students in the campus week experiences of an existing course offered by the College of Health and Welfare as part of the primarily online course: "Aging in a Welfare State." Students from other countries (Finland, Scotland, Spain, Turkey, UK, and Vietnam) have also participated, allowing for comparison of experiences of aging and support for older adults across countries. Symposium presentations will address: Development and content of the study abroad course (Elizabeth Fauth), Facilitators and barriers to establishing a study abroad course at a U.S. university (Carla Hermann), Challenges and benefits of hosting U.S. study abroad students at the College of Health and Welfare (Therése Bielsten), and History of a sustained and successful model of international gerontological education (Steven Zarit). Course outcomes and measures of student satisfaction and success will be presented, as well as feedback from students and faculty and how that shaped the development of the course over time. Plans for future iterations will be addressed. This course model can be replicated by others aiming to promote engaging gerontological education experiences for both U.S. and international students, as well as international research collaborations.

Abstract citation ID: igae098.2032
### DEVELOPMENT AND CONTENT OF THE STUDY ABROAD COURSE

Elizabeth Fauth, *Utah State University, Logan, Utah, United States*

Pedagogy of faculty-led short study abroad programs posits that these programs offer unique opportunities to build cross-cultural adaptability and global citizenship, expand disciplinary and interdisciplinary content knowledge beyond the traditional classroom, foster experiential learning opportunities, and facilitate personal and professional identity. This presentation on the Aging in a Welfare State covers the how course design, content selection, site visits, assignment, and excursions map onto these objectives. Optimizing three credits (which is ideal for most students' programs) necessitates reading and assignments before, during and after "in-country" portion of the course. In-country content is delivered by US faculty and

Swedish faculty, who purposefully are chosen (or select lecture content) based on their own discipline and expertise. With lecture content in nursing, social work, developmental psychology, gerontology, and more, students from diverse majors often connect closely to at least some of the content within their discipline, but expand learning into new topics. The addition of content on family and child policies is attractive to students outside of gerontology programs. Days are organized around themes, with three lectures in the morning broken up by the Swedish coffee tradition of FIka, a lunch break, and site visits to hospitals, adult day programs, child care programs, senior centers, assisted living facilities, and rehabilitation programs. Site visits offer applied learning experiences, where students can interact with professionals and clients/residents/users. Excursions are used to expose students to Swedish culture, and the historical perspectives that inform their social care policies. Assignments bridge academic application and personal/professional self-reflection.

Abstract citation ID: igae098.2033

### CHALLENGES AND BENEFITS OF HOSTING US STUDY ABROAD STUDENTS AT THE COLLEGE OF HEALTH AND WELFARE

Therése Bielsten, *Jönköping University, Jönköping, Jonkopings Lan, Sweden*

Aging in a Welfare State is a course with the aim of student exchange of knowledge about the Swedish and US healthcare systems. The campus week's main purpose is to stimulate students' reflection on their own country's healthcare system. The week is initiated with a day of perspectives of and study visits on Growing up in Sweden to introduce US students to Sweden's welfare system. However, most of the week focuses on Aging in a Western Country, Old Age Care and Perspectives of Aging. Prior to the campus week students have prepared for discussions involving a case study of the US system and a paper with reflections on current and future challenges within old age care. Discussions and comparisons of Sweden's universal high-state regime and US mixed system for older people are a valued feature of campus week. Along with lectures, discussions and study visits related to the welfare system, leisure activities are offered in the form of excursions to Swedish sights. Planning for the campus week requires high coordination. Some of the challenges associated with coordinating campus week involve recruiting Swedish students, getting students from different countries and campuses to interact, logistics of traveling, scheduling lectures and site visits. This presentation contributes challenges and success factors for internationalization in education. Campus week is a valued feature of the course and opens opportunities for students to gain theoretical knowledge at the same time as they have the opportunity for increased understanding through real life experiences of the study visits.

Abstract citation ID: igae098.2034

### ESTABLISHING A SHORT-TERM STUDY ABROAD COURSE ON AGING AND HEALTH CARE: FACILITATORS AND BARRIERS

Carla Hermann, *Indiana University Southeast, New Albany, Indiana, United States*

The population of older persons is growing in every country in the world. In the United States (US), the older population is increasing at an unprecedented rate and is becoming significantly more diverse. Population aging is one of the most significant social transformations of the twenty-first century. Every occupation will be impacted by this transformation; therefore, education of individuals across disciplines about aging is imperative. To address the demand for aging-related professionals, Indiana University Southeast developed and implemented an interdisciplinary short-term study abroad course on healthcare and gerontology for students of any major. Although this course was previously offered by other US universities in collaboration with the College of Health and Welfare at Jönköping University in Sweden, the course offered in May 2023 was the first time Indiana University Southeast was involved. There were multiple facilitators and barriers to establishing this short-term study abroad course. Facilitators included: collaboration with an established course and international experts; institutional support from the host university in Sweden; high interest in an international learning experience by the students; the course available to students of any discipline; offered over a short-term (2 week) period. Barriers to offering this course included: lack of institutional infra-structure to support study abroad; post-pandemic traveling concerns; lack of student knowledge regarding basic concepts of aging and healthcare. High impact educational practices such as study abroad can help meet the demand for professionals that are prepared to promote the well-being of the aging and diverse population.

Abstract citation ID: igae098.2035

### HISTORY OF A SUSTAINED AND SUCCESSFUL MODEL OF INTERNATIONAL GERONTOLOGICAL EDUCATION

Steven Zarit, *The Pennsylvania State University, University Park, Pennsylvania, United States*

A successful and sustained study abroad program depends on a foundation built in advance by the faculty of the participating institutions. Three foundational elements contributed to the success of the program, Aging in a Welfare State: What We Can Learn from Sweden, that has been ongoing since 1995. The first and most important foundational element is the relationship between the faculty at participating institutions. In this program faculty of the two initial participating institutions (Jönköping University and Penn State) had worked together for several years prior to developing and offering the program. Another foundational element was long-standing relationships between our Swedish partners and personnel at social and care facilities serving older people in Jönköping. A central goal of the program was that students would learn how care is organized and delivered in Sweden by visiting programs that served older people. Because our Swedish partners had long-established relationships with local care facilities, they were able to arrange visits for students at a range of programs for older persons. Staff at these facilities welcomed us, were open and informative and even invited us to Swedish Fika (coffee and a snack). We were also able to talk with the people using these programs. The visits were the most inspiring part of the class for students because they demonstrated innovative ways of providing care compared to the US. An obvious but important foundational element was having housing, transportation, and other comfort issues in place. These foundational elements were the key to our ongoing success.

### SESSION 4340 (SYMPOSIUM)

Abstract citation ID: igae098.2036

### EVIDENCE-INFORMED ONLINE PLATFORMS FOR DEMENTIA CAREGIVERS

Chair: Alan Stevens
Discussant: Sara Czaja

Online platforms for dementia care have emerged in response to the expressed desire of family caregivers for on-demand, self-paced education and dementia care skills training. Given the wealth of evidence-based dementia caregiver interventions, there is a unique opportunity to provide evidence-informed online services for family caregivers. Four online platforms for dementia caregivers will be discussed. Dr. Stevens will present an overview of two online platforms, GamePlan4Care (GP4C) and Resoures4Care (R4C). Preliminary findings suggest positive benefits to caregivers who engaged in either platform. The GP4C online platform has been modified to address the needs of family caregivers who experience the hospitalization of a Veteran living with dementia by Dr. Horstman. Preliminary findings from Dr. Horstman's pilot study of GP4-Hospital in a VA hospital will be presented. Dr. Laura Gitlin's presentation will focus on WeCareAdvisor, an online platform that provides daily tips to caregivers. Dr. Gitlin will present findings on the impact of multiple techniques used to engage family caregivers in using WeCareAdvisor. Dr. Eric Jutkowitz will provide an overview of Plans4Care, a translation of therapeutic strategies that address caregiver challenges from multiple in-person evidence-based dementia caregiver interventions into a single digital format. His presentation will focus on innovative online methods of tailoring therapeutic content to family caregivers. Dr. Sara Czaja, an international leader in developing, implementing, and evaluating technology-based solutions to late-life autonomy and caregiving support, will frame the findings of the four presentations within the US landscape of online support services for dementia caregivers.

Abstract citation ID: igae098.2037

### GAMEPLAN4CARE AND RESOURCES4CARE: PRELIMINARY OUTCOMES ASSOCIATED WITH TWO ONLINE DEMENTIA CAREGIVING PROGRAMS

Alan Stevens[1], Jinmyoung Cho[2], Jordan Reese[1], Gang Han[3], and Marcia Ory[3], *1. Baylor Scott & White Health, Belton, Texas, United States*, *2. Saint Louis University School of Medicine, Ballwin, Missouri, United States*, *3. Texas A&M University, College Station, Texas, United States*

GamePlan4Care (GP4C) and Resources4Care (R4C) are online dementia caregiver programs. GP4C consists of rules-based conditional logic algorithms and real-time assessments of caregiving risks to tailor and prioritize educational and skills content. R4C provides a searchable database of vetted online educational and resources focused on dementia and family caregiving. The content of R4C was chosen to parallel GP4C educational content but does not include tailoring of content based on real-time caregiver assessments. Other program similarities were the self-paced format for caregivers' engagement with brief educational videos and skill-building exercises and access to a Dementia Care Specialist to further support caregivers' engagement. Two hundred forty family caregivers of persons living with dementia (PLWD) were randomly assigned to either GP4C or R4C. Nearly 70% of enrolled caregivers lived with a PLWD. Half were adult child/child-in-law caregivers (52%) and 39% were spousal caregivers. Preliminary results are reported on three standardized assessments conducted at baseline and a 6-month follow: Zarit Caregiver Burden Interview (ZBI), Positive Aspects of Caregiving (PAC), and Social Provision Scale (SPS). Caregivers assigned to either GP4C or R4C reported a significant ($P<.05$) reduction in ZBI and improvement in PAC after 6 months of self-paced engagement in GP4C and R4C. Caregivers in GP4C also reported a significant increase in perception of receiving guidance support from family or friends (SPS). Both GP4C and R4C show promise as online platforms for dementia caregivers. Feedback from participants of this study suggest the need for improved functionality and revision of content to better meet diverse caregiver needs.

Abstract citation ID: igae098.2038

### ADAPTATION OF GAMEPLAN4CARE FOR HOSPITAL CARE AND CARE TRANSITIONS

Molly Horstman[1], Tracy Evans[1], Crystal Guo[2], Mandi Sonnenfeld[3], Aanand Naik[4], Alan Stevens[5], and Mark Kunik[1], *1. Baylor College of Medicine, Houston, Texas, United States*, *2. Texas Tech University Health Sciences Center El Paso, El Paso, Texas, United States*, *3. Michael E. DeBakey Veterans Affairs Medical Center, Houston, Texas, United States*, *4. The University of Texas Health Science Center, Houston, Texas, United States*, *5. Baylor Scott & White Health, Belton, Texas, United States*

Adults with dementia rely on family caregivers after hospital discharge. Increased caregiver stress following discharge may lead to worse outcomes for caregivers and adults with dementia. Our aim was to adapt GamePlan4Care, a community-based caregiver support intervention, to support caregivers of hospitalized adults with dementia. GamePlan4Care combines online, self-directed skills training with four phone calls from a dementia care specialist. Potential adaptations were identified through a literature review and semi-structured interviews with health professionals and caregivers. Adaptation prototypes were presented to caregiving research consultants, a stakeholder panel with a caregiver and health professionals, and individuals who implemented GamePlan4Care in the community. All adaptations were reviewed with the GamePlane4Care developer. We used the Framework for Modification and Adaptations-Expanded (FRAME) to describe the adaptations. Initial adaptations were planned and made during the pre-implementation stage. Adaptations included: 1) adding training content for care transitions (medication review, clinical red flags, delirium, functional decline, and medical tasks), 2) modifying the intervention content to improve caregiver engagement, and 3) modifying the context of intervention delivery. Additional adaptations will be made based on caregiver feedback from an ongoing feasibility and acceptability pilot. The recruitment target of 30 caregivers should be reached in June 2024. Preliminary results will be presented at the symposium. Evidence-based interventions designed for outpatient and community settings need to be adapted before they can be implemented as part of hospital care. Using insights from multiple sources, we developed HospitalGamePlan4Care, a new intervention that combines care transitions training with an evidence-based dementia caregiver support intervention.

Abstract citation ID: igae098.2039

**WECAREADVISOR: CAREGIVER USE OF A DIGITAL PLATFORM TO MANAGE DEMENTIA-RELATED BEHAVIORAL SYMPTOMS**

Laura Gitlin[1], Vincent Davis[2], Nicole Bouranis[3], Leslie McClure[4], Helen Kales[2], and Melinda Webster[1], *1. Drexel University, Philadelphia, Pennsylvania, United States*, *2. University of California Davis, Davis, California, United States*, *3. Yale University School of Medicine, New Haven, Connecticut, United States*, *4. Saint Louis University, St. Louis, Missouri, United States*

Dementia-related behavioral and psychological symptoms are undertreated and have negative consequences for people with dementia and their caregivers. Families typically do not have access to dementia education and effective management strategies. WeCareAdvisor is an online platform providing caregivers with dementia information, daily tips, and a 4-step systematic approach for describing behaviors, investigating underlying causes, creating a list of tailored strategies (prescriptions), and evaluating their effectiveness (DICE). It is being tested in a randomized trial to evaluate effects on caregiver wellbeing and behavioral symptoms. This paper reports utilization patterns of 240 caregivers under two "prompting" conditions to use the platform: high-intensity (telephone plus email reminders) and low-intensity (email reminders only). A total of 999 DICE sessions were started of which 347 (35%) sessions were evaluated. Caregivers sought strategies for all 13 behaviors included in the platform ranging from 141 DICE sessions for Anxiety (14%) to 31 sessions for Hallucinations (3%). Caregivers in the high-intensity prompt group (n=46) had more DICE sessions (296, mean=6.4, SD=4.5) compared to 163 sessions in the low-intensity prompt group (n=41, mean=4.0, SD=3.5), generated 82% more prescriptions, and requested close to 3 times more additional strategies. Of 171 study completers, an average of 4.5 (SD=3.8) DICE sessions were conducted over 6-months (range 1-19). Caregivers engaged with WeCareAdvisor to obtain strategies for managing common behavioral symptoms; however, only 1/3 evaluated their effectiveness. Caregivers used the online platform more when prompted by telephone/email than by email alone. This utilization pattern has significant implications for cost and scaling of the WeCareAdvisor.

Abstract citation ID: igae098.2040

**PLANS4CARE: TRANSLATING EVIDENCE FROM IN-PERSON CAREGIVER INTERVENTIONS TO A DIGITAL PLATFORM**

Eric Jutkowitz[1], Catherine Piersol[2], Sokha Koeuth[3], and Laura Gitlin[4], *1. Brown University, Brown University, Rhode Island, United States*, *2. Jefferson College of Rehabilitation Sciences, Philadelphia, Pennsylvania, United States*, *3. Plans4. Care, Bala Cynwyd, Pennsylvania, United States*, *4. Drexel University, Philadelphia, Pennsylvania, United States*

Our research group has developed and/or evaluated numerous evidence-based non-drug dementia caregiver interventions (e.g., Tailored Activity Program and Skills2Care). For these interventions, healthcare professionals conduct in-home assessments to evaluate cognitive function of the person living with dementia, determine the caregiver's care challenges (e.g., behaviors), and provide tailored strategies. In randomized trials, these interventions improved caregiver outcomes (e.g., less time caregiving), person with dementia outcomes (e.g., reduced behavioral symptoms), and health system outcomes (e.g., cost savings). Informed by this evidence, our team engaged in an iterative process to create a digital platform that caregivers can use on-demand. First, we mined the clinical trial data and identified 80 caregiver challenges that we grouped into 8 domains: for persons with dementia, behaviors, self-care mobility, household tasks, leisure participation, and healthcare; caregiver-centered challenges (need for respite), and general dementia care practices. Second, we reviewed unique strategies documented and published, which we grouped into 6 categories: communication, cueing, simplifying activity, enhancing, engagement, modifying environment, and taking care of self. Third, two healthcare providers on our team independently mapped care challenges to evidence-based strategies based on the level of cognitive function. Our approach generated a database of 80 care challenges linked to >1500 evidence-based strategies classified by six levels of cognitive functioning (high to low), and a simple user interface for caregivers to generate personalized care plans. Translating in-person sessions to digital platforms and maintaining tailoring and clinical relevance requires an iterative developmental process that is data-driven and informed by caregiver input.

### SESSION 4345 (SYMPOSIUM)

Abstract citation ID: igae098.2041

**FOSTERING ENGAGEMENT: AN EXPLORATION OF INNOVATIVE DEMENTIA ENVIRONMENTS FROM FOUR COUNTRIES**

Chair: Hilde Verbeek

Dementia is one of the major age-related diseases and challenges those affected, societies, as well as health care systems worldwide. In an effort to better meet the needs of people living with dementia (PlwD), innovative care concepts for community-dwelling PlwD, as well as those in need for long-term care are being developed worldwide. Aiming to strengthen independence and slow down the cognitive decline, efforts concentrate on continuously engaging PlwD in activities of daily life, despite a progression of the disease. This international symposium will provide five presentations on different innovative care environments in the community, which stimulate and support an active daily life of PlwD. The first presentation dissects the care environment of Green Care Farms in the Netherlands and its effects on the engagement of residents. The second presentation discusses activity programming and self-directed behavior in Canadian long-term care. The third presentation presents social participation of PlwD in shared housing arrangements in Germany. The fourth presentation describes how the neighborhood-built environment can contribute to social health aspects for PlwD in German municipalities and the fifth presentation considers the feasibility and effectiveness of a virtual care farm activity in the United States.

Abstract citation ID: igae098.2042

### EXPLORING THE ACTIVATING EFFECT OF THE CARE ENVIRONMENT OF GREEN CARE FARMS FOR PEOPLE WITH DEMENTIA

Katharina Rosteius, Laura Frissen, Bram de Boer, Andrea Gabrio, and Hilde Verbeek, *Maastricht University, Maastricht, Limburg, Netherlands*

Engagement and activity is important for people with dementia for their sense of purpose, and for cognitive and physical stimulation. Aiming to increase engagement and activity, innovative long-term care environments like Green Care Farms (GCFs) reorganize care by including nature, animals and a more home-like environment into daily care. Previous studies have shown that GCF-residents are more active than those of regular care facilities. This study explored the role of the GCF-environment in this activation. Ecological Momentary Assessments were conducted with 152 participants from four Dutch GCFs, combined with cognitive and physical assessments. Residents were observed during mornings, afternoons and evenings, with a total of 4,600 valid observations. Each observation combines activity-, engagement-, as well as place scores, which are mapped on a ground plan of the facility. Analyses showed that residents were actively engaged in an activity in 67.9% of the observations. Residents spent most of their time in inside common areas (54.0%), followed by their own room (24.6%), outside (10.6%) and in communal inside areas outside of their ward (e.g. a café) (7.7%). In relation to passive behavior, active engagement was highest outside (87.1%), as well as in communal areas outside of the ward (81.7%) and inside the ward (81.3%). These results show that areas where residents spend time together have most activating potential and call for a more communal organization of care. Further research should explore what in the environment triggered residents' engagement in order to react to needs arising in a tailored way.

Abstract citation ID: igae098.2043

### UNDERSTANDING ACTIVITY ENGAGEMENT EXPERIENCE AMONG RESIDENTS WITH DEMENTIA IN LONG-TERM CARE HOMES

Ziying Zhang, and Habib Chaudhury, *Simon Fraser University, Vancouver, British Columbia, Canada*

Recreational and meaningful activities in long-term care homes are crucial in promoting well-being among residents with dementia. While existing research primarily focuses on planned activities facilitated by staff, little attention has been given to self-directed activities initiated by residents themselves, reflecting their intrinsic motivation, lifestyle, and lifelong interests such as walking, gardening, meal preparation and other household activities. In this study, activity is defined as personally meaningful behavior, including both programmed and self-directed activities, and supported by social and/or physical environmental stimuli. Utilizing critical ethnography in a long-term care home in Canada, our research aims to: 1) gather descriptive data on the types, diversity and process of residents' activity engagement, and 2) examine the care home setting in its natural context to uncover the social practices, built environmental features and underlying organizational discourses that influence activity engagement. Observations will focus on residents' engagement in various activities and their interactions with staff and the physical environment. Interviews will be conducted with activity staff and non-activity staff (e.g., care aides, nurses) to explore their perspectives on activity engagement, person-centered approach, and their respective roles in promoting engagement. This study offers insights into residents' experiences with both structured and self-directed activities, and the influence of the care home's social and physical environments on engagement. The study addresses the knowledge gap in meaningful engagement through self-directed activities and the role of social and physical environments. Fieldwork will be completed in summer and preliminary findings based on interviews and initial observations will be presented.

Abstract citation ID: igae098.2044

### SOCIAL PARTICIPATION OF PEOPLE LIVING WITH DEMENTIA IN GERMAN SHARED-HOUSING ARRANGEMENTS

Karin Wolf-Ostermann[1], Julia Misonow[1], and Janissa Altona[2], *1. University of Bremen, Bremen, Bremen, Germany*, *2. Institute of Public Health and Nursing Research, Bremen, Bremen, Germany*

Aspects of social health are increasingly gaining more interest regarding research for people living with dementia (PlwD). Having a range of meaningful social relationships has been identified as crucial for healthy aging, defined by lower mortality rate, and better physical and mental health outcomes. Recent studies show that social connections and participation in social activities are important dimensions of social health and have the potential to act as a buffer in the process of cognitive decline by delaying dementia or even protecting against its development. Shared-housing arrangements (SHA) in Germany are based on the concept of enabling PlwD to lead an everyday life as normal as possible – including especially social activities as well as exercise-related, sensory, cognitive and creative approaches and out-of-home activities. We analyzed data from different studies on SHA in Germany from 2009-2022 (including the period of the COVID-19 pandemic) to evaluate to which extent social activities are actually performed by PlwD in SHA. The most frequently offered and used activities in SHA are entertainment and joint social activities (2009: 67.0%, 2011: 96.6%, 2021: 80,0%, 2022: 87,9%). This is followed by exercise-related activities (2009: 26.2%, 2011: 79.3%, 2021: 73,2%, 2022: 77,8%) and sensory-related activities (2009: 21.9%, 2011: 77.6%, 2021: 47,9%, 2022: 47,0%). The results show that participation in social activities is a lived fact in SHA - despite some reduction during the pandemic - and should be taken into account when discussing care arrangements for PlwD and conditions for living well with dementia.

Abstract citation ID: igae098.2045

### HOW DOES THE NEIGHBORHOOD-BUILT ENVIRONMENT CONTRIBUTE TO SOCIAL HEALTH FOR PEOPLE LIVING WITH DEMENTIA?

Janissa Altona[1], Emily Mena[2], Benjamin Schüz[2], and Karin Wolf-Ostermann[2], *1. Institute of Public Health and Nursing Research, Bremen, Bremen, Germany*, *2. University of Bremen, Bremen, Bremen, Germany*

Several studies point to the importance of the neighbourhood-built environment (NBE) for the social health of community-dwelling people living with dementia (PlwD).

Indicators of dementia-friendly urban planning can be identified in the literature, but little is known about which specific factors of the NBE are important for PlwD and family carers. In order to examine relevant indicators, the following questions were analyzed by a qualitative research approach in the German DEN-HB study: 1. Which places in the residential and living environment are subjectively perceived as positive or negative by PlwD and their carers, and how satisfied are they with the dementia-friendliness of their social space? 2. What is the role of social aspects of the NBE and which aspects are conducive to social health? Based on a systematic review of NBE factors and a subsequent empirical study on evaluating existing wayfinding structures in the federal state of Bremen, Germany, we will conduct individual interviews with PlwD and their informal carers to discuss the relevance of these factors. Additionally, a focus group with dementia coordination centres and responsible persons from ministerial institutions in Bremen, Germany, will be conducted to discuss possible actions in urban planning. Results of both qualitative research approaches will be presented and discussed to promote participatory approaches to the design of the NBE for PlwD and, in particular, to focus more strongly on social health factors.

Abstract citation ID: igae098.2046

### FEASIBILITY OF A VIRTUAL CARE FARM FOR OLDER ADULTS WITH COGNITIVE AND PHYSICAL LIMITATIONS

Keith Anderson[1], Rebecca Mauldin[2], Megan Westmore[2], and Anna Tulloh[2], *1. University of Mississippi, Oxford, Mississippi, United States*, 2. *The University of Texas Arlington, Arlington, Texas, United States*

Nature-based activities provide an array of physical, emotional, social, and spiritual benefits for people of all ages. Older adults with cognitive and physical limitations, however, are often excluded from these activities due to environmental, functional, and financial barriers. In this study, the researchers evaluated the feasibility of delivering a virtual (online), nature-based, group activity to older adults with physical and cognitive limitations in assisted living. Days at Dunrovin (D@D) is a “virtual care farm” featuring live webcameras, recorded content, and online discussion boards that focus on life on a rural Montana ranch. Qualitative data from 18 interviews with participants, staff, and interventionists and 40 days of field notes were analyzed for themes related to feasibility. Themes were organized using an established framework of feasibility domains and outcomes. Results indicated that D@D was well-received by participants (Demand/Acceptability). Results also illuminated the process by which the researchers delivered and subsequently modified the activity (Implementation/Adaptation) in response to challenges presented by the assisted living environment, including the cognitive functioning of participants, the physical structure of the facility, and staffing issues (Practicality/Integration). A range of benefits (Effectiveness) were associated with D@D, such as gains in emotional well-being, increased socialization, enhanced engagement with nature, and stimulation of reminiscence. These findings suggest that it is feasible to deliver virtual, nature-based group activities to older adults with cognitive and physical limitations in long-term care. In doing so, we may be able to provide expanded access to the wonders of nature to this all too often excluded population.

### SESSION 4350 (SYMPOSIUM)

Abstract citation ID: igae098.2047

### GERIATRIC URINARY INCONTINENCE AND LOWER URINARY TRACT SYMPTOMS: NEW DATA, TRIALS, AND CLINICAL APPROACHES

Chair: Scott Bauer
Discussant: George Kuchel

Lower urinary tract symptoms (LUTS) is a syndrome of commonly overlapping symptoms that occur when urine is being generated and stored in the bladder, called storage LUTS (e.g., overactive bladder and urinary incontinence), or during the initiation and process of urination, called voiding LUTS (e.g., weak stream). More than 1 out of 3 of older adults report clinically significant LUTS, contributing to poor quality of life and increased risk of falls and fractures, functional and mobility impairment, and death. Although urinary incontinence is a well-established geriatric syndrome, the remaining LUTS share many risk factors and downstream consequences. In this symposium, each session will review the pre-clinical, epidemiologic, and clinical evidence supporting the entire umbrella of male and female LUTS to be considered a geriatric syndrome. Dr. Parker-Autry will describe the rationale and proposed approach for a multi-modal clinical care pathway to treat female geriatric incontinence syndrome that she has begun to implement in her academic geriatric urogynecology clinic. Dr. Al-Naggar then will review how biological hallmarks of aging contribute to LUTS and key considerations for pilot studies of gerotherapeutic interventions targeting urologic conditions based on a randomized, placebo-controlled clinical trial testing MitoQ for overactive bladder in older women with metabolic syndrome. Lastly, Dr. Bauer will review the evidence of male LUTS as a geriatric syndrome and will describe how this novel framework led to a R01-funded clinical trial comparing a remote exercise coaching intervention versus health education control for physically inactive older men with LUTS.

Abstract citation ID: igae098.2048

### TARGETING BIOLOGICAL AGING MECHANISMS OF LOWER URINARY TRACT SYMPTOMS IN OLDER MEN WITH AN EXERCISE INTERVENTION

Scott Bauer, *University of California San Francisco, San Francisco, California, United States*

Physical frailty is associated with lower urinary tract symptoms (LUTS) severity and progression among older men with suspected benign prostatic hyperplasia (BPH). Prospective cohort studies have consistently demonstrated that both physically inactive and frail men are more likely to develop BPH or experience LUTS progression. The combination of aerobic plus resistance exercise has been demonstrated in clinical trials to have pleotropic effects that delay, prevent, or treat many frailty-related conditions that often co-occur with LUTS and BPH, including cardiovascular disease, diabetes, and erectile dysfunction. Although it has never been tested in humans, pre-clinical mouse models of BPH demonstrate improved voiding behaviors after exposure to exercise and exercise is known to improve several mechanisms that are implicated in human LUTS/BPH pathophysiology, including insulin resistance, endocrine dysregulation, and mitochondrial dysfunction. The recently launched PROUD study (NCT06225479; MPIs: Bauer, Kenfield) is a 2-arm pilot randomized controlled trial among physically inactive older men with LUTS/BPH testing the effect of a 12-week remote exercise intervention versus health education control. Outcomes include: 1) feasibility, acceptability, fidelity, and safety; 2) changes in LUTS, urologic and physical function, and physical activity; and 3) feasibility of detecting exercise-induced change in novel frailty-related mechanistic measures. This symposium session will review the scientific rationale, key study design and analytic decisions, enrollment progress thus far, and collaborative opportunities using stored biobank specimens.

Abstract citation ID: igae098.2049

**CLINICAL TRIALS OF GEROTHERAPEUTICS FOR TARGETING LOWER URINARY TRACT SYMPTOMS**

Iman Al-Naggar, *UConn Health, Farmington, Connecticut, United States*

Aging and the metabolic syndrome, a condition that accelerates biological aging, are both risk factors for lower urinary tract symptoms (LUTS). Storage LUTS, including urinary frequency, urgency, incontinence and nocturia, greatly impact quality of life and contribute to social isolation, depression, increased risk of falls and fractures, and loss of independence in older adults. Dysregulated mechanisms along the brain-to-bladder pathway can lead to LUTS, as the body becomes less resilient and its ability to maintain normal function declines. Although dysfunctional detrusor muscle can be targeted with anticholinergics, these drugs have had little success in alleviating LUTS and are often abandoned due to undesirable side effects. Identifying novel targets would allow the development of more effective drugs to alleviate LUTS. Biological mechanisms of aging that play a role in multiple age-related diseases have recently been implicated in metabolic syndrome-associated LUTS, including inflammation, mitochondrial dysfunction and oxidative stress. Furthermore, studies using mouse models of aging and metabolic syndrome show increased bladder dysfunction in these model organisms. Targeting biological hallmarks of aging to alleviate LUTS in humans represents a promising new direction that our group is exploring. This can be safely done using repurposed drugs or supplements with gerotherapeutic properties, such as MitoQ. This symposium session will describe the rationale and study design for on-going pilot and feasibility studies targeting the biology of aging for LUTS alleviation in older women.

Abstract citation ID: igae098.2050

**EVIDENCE TO SUPPORT A NOVEL CLINICAL CARE PATHWAY FOR GERIATRIC INCONTINENCE SYNDROME IN OLDER WOMEN**

Candace Parker-Autry, *Atrium Health Wake Forest Baptist, Winston-Salem, North Carolina, United States*

Geriatric syndromes define complex health conditions that result from the concomitant presence of multiple geriatric impairments in an older adult. Urinary incontinence (UI), an established geriatric syndrome, is unique in that it is present among younger women due to pelvic floor dysfunction, most commonly due to birth trauma or obesity, as well as older women with aging-related changes in skeletal muscle and cognitive health. The latter is a distinct phenotype of UI called female geriatric incontinence syndrome (GIS). Like other geriatric syndromes, GIS is bidirectionally associated with frailty and commonly occurs with concurrent multimorbidity. The combined impact of pelvic floor dysfunction, aging-related impairments in skeletal muscle and/or cognition, and multimorbidity make defining, diagnosing, and treating GIS a complex task. There is a significant body of work describing non-procedure-based treatments for UI with focus on physical therapy/exercise and medications. However, with unhealthy aging skeletal muscles become smaller and weaker, thus pelvic floor muscle function diminishes. This limits the clinical success of physical exercise to treat geriatric UI. Further, multimorbidity and cognitive impairment may limit the efficacy and safety of pharmacologic UI treatments. Based on our teams qualitative studies, women with GIS desire significant improvement in their symptoms; this may be only possible through multi-modal therapy. During this session, we will review the evidence above in more detail and describe the rationale and proposed approach to a multi-modal clinical care pathway, including both conservative and procedure-based treatments, that we are currently implementing for women with GIS at our tertiary care referral center.

### SESSION 4355 (SYMPOSIUM)

Abstract citation ID: igae098.2051

**IMPROVING ELDER MISTREATMENT SCREENING AND INTERVENTION IN DEMENTIA: EARLY NIA RESEARCH FINDINGS**

Chair: Kristin Lees Haggerty

Discussant: Terry Fulmer

Elder mistreatment (EM) poses a significant public health concern for older adults, particularly those with mild cognitive impairment and Alzheimer's Disease and Related Dementias (MCI/ADRD). This symposium presents innovative NIA-funded research aimed at assessing risk factors for EM and developing interventions to address these risks, focusing on caregivers and older adults with MCI/ADRD. The first presentation describes the development of a Screening, Brief Intervention, and Referral to Treatment model based on Medicare outpatient data and interviews with stakeholders. Preliminary findings highlight social determinants of health, caregiver stress, and education gaps as key contributors to EM risk. The second presentation introduces a caregiver risk assessment tool linked to an abuse prevention intervention for persons living with dementia (PLWD). The study demonstrates strong predictive capabilities in identifying modifiable caregiver risk factors associated with abuse. The third presentation describes adapting an evidence-based EM screening tool for home-based primary care providers. Qualitative data from seven sites inform the adaptation of the DETECT tool, addressing policy implications, barriers to recognition, and necessary adaptations for effective implementation. The fourth presentation highlights the development of a strategy to screen for caregiver neglect in PLWD. By evaluating existing tools and creating an item bank, this study aims to enhance measurement precision in elder mistreatment interventions. The final presentation reviews interventions targeting elder neglect by informal caregivers. Findings emphasize the need for feasible programs with rigorous evaluation strategies to address caregiver neglect effectively. This symposium presents projects aimed at improving EM risk assessment, identification, and interventions.

Abstract citation ID: igae098.2052

**DEVELOPING A RISK ASSESSMENT AND BRIEF INTERVENTION FOR ELDER MISTREATMENT: A MIXED-METHODS STUDY**

Monique Pappadis[1], Leila Wood[2], and Karen Schlag[1], *1. The University of Texas Medical Branch, Galveston, Texas, United States*, *2. The University of Texas Health Science Center, Houston, Texas, United States*

Elder mistreatment (EM) is public health and safety concern for older adults, especially those with mild cognitive impairment and Alzheimer's Disease and Related Dementias (MCI/ADRD); however, few tools exist to assess risk or address risks, especially those that may come from caregivers or the social environment. To address these gaps, we used Medicare outpatient data (2015-2018) and semi-structured interviews with providers, older people with MCI/ADRD, and family caregivers to: 1) Identify risk and protective factors for EM, 2) Understand education and training gaps that may contribute to risk, and 3) Develop a screening, brief intervention, and referral to treatment (SBIRT) model for caregivers and older adults with MCI/ADRD to address risks for EM. Our preliminary findings indicate that social determinants of health, such as housing, financial instability, and social isolation, along with frailty, contribute to EM risk. Caregiver stress, often from financial instability, can increase risk conditions for EM. The lack of caregiver and provider education on EM contributes to a lack of identification of risk factors. Thus, the developed SBIRT model centers on risk assessment, universal education, and referral to support based on the results of the risk assessment. Preliminary feasibility and acceptability of the SBIRT intervention will be reviewed, along with a review of risk assessment protocols. The session will end with describing the next steps to expanding the SBIRT intervention to a greater number of primary care clinics across a large academic health science center's system.

Abstract citation ID: igae098.2053

**DEVELOPMENT OF A RISK SCREEN OF ELDER ABUSE IN CAREGIVERS OF PEOPLE LIVING WITH DEMENTIA**

Bonnie Olsen[1], and Laura Mosqueda[2], *1. University of Southern California, Los Angeles, California, United States*, *2. University of Southern California Keck School of Medicine, Pasadena, California, United States*

Significant advances have been made in understanding risk factors of abuse of persons living with dementia (PLWD) by their caregivers. Intervention focused on the modifiable caregiver risk factors provides a possible avenue to primary prevention. This project developed a caregiver risk assessment screen, linked to an abuse prevention intervention. To develop the tool, we administered surveys of validated measures of risk factors to caregivers of PLWD (N=95), had an expert panel rank responses according to risk, and used these risk levels to identify items that are most predictive of risk. The sample had a mean age of 49.5 years (SD=14.4), mostly female (68.1%), mostly Latine (52.1%), predominantly family members (14.7% spouse, 43.1% child, 20.0% other). The PLWD had a mean age of 78.6 years (SD=10.8), mostly female (62.4%). Expert panel-assigned risk levels were evenly distributed, with 35.8% high, 30.5% medium, and 33.7% low risk. Using a 2:1 train/test split, the training sample demonstrated strong single-item prediction, with maximum single-item AUC (area under the curve) of 0.861 (39 items had AUC≥0.7, of which 5 had AUC≥0.8). The top performing items included: caregiver's satisfaction with their life; acting in an emotionally aggressive manner; feeling angry when around the PLWD; being bothered by feeling nervous, anxious, or on edge; and finding looking after the PLWD to be a burden. This presentation will discuss the process of creating an abuse screening tool for caregivers of PLWD. In the next phase, we will assess the effectiveness of our intervention in reducing risk for abuse.

Abstract citation ID: igae098.2054

**ADAPTING THE DETECT TOOL FOR HOME-BASED PRIMARY CARE: INSIGHTS ON POLICIES, PROVIDER PERSPECTIVES, AND TOOL DESIGN**

Kristin Lees Haggerty[1], Brad Cannell[2], Olanike Ojelabi[1], Randi Campetti[1], Jason Burnett[3], Carolyn Pickering[2], and Melvin Livingston[4], *1. Education Development Center, Waltham, Massachusetts, United States*, *2. The University of Texas Health Houston, Houston, Texas, United States*, *3. The University of Texas Houston, Houston, Texas, United States*, *4. Emory University, Atlanta, Georgia, United States*

Elder mistreatment (EM) is difficult to detect and often goes unrecognized. Home-based primary care (HBPC) providers have enduring relationships with patients who are vulnerable to EM and the ability to see patients' homes first-hand. Effective and efficient screening tools are urgently needed to support these providers in consistently assessing patients for signs of EM. This project includes a two-stage approach to adapting and rigorously evaluating an evidence-based EM screening tool, Detection of Elder mistreatment Through Emergency Care Technicians (DETECT), for use in home-based primary care (HBPC). In this presentation, we share results from a qualitative study conducted to inform tool adaptation and implementation strategy for DETECT revised for primary care (DETECT-RPC). This study addressed three research questions: 1) what are the implications of state- and site-specific policies on DETECT-RPC implementation, 2) what are the perceived barriers and facilitators to recognizing and reporting EM among HBPC providers, and 3) What adaptations need to be made to make DETECT-RPC feasible and effective in HBPC? Data collection included review of publicly available information and interviews and focus groups with HBPC staff from seven sites (N=16). Interview and focus group data were transcribed and coded using a thematic analysis approach. We will present key themes from the qualitative study including implications for the DETECT-RPC tool design and workflow, and key considerations for developing reporting guidelines structure.

Abstract citation ID: igae098.2055

**DEVELOPING A STRATEGY TO SCREEN FOR CAREGIVER NEGLECT: EXAMINING EXISTING PUBLISHED TOOLS**

E-Shien Chang[1], Alyssa Elman[2], Daniel Baek[2], and Tony Rosen[2], *1. Weill Cornell Medical College, New York,*

*New York, United States*, *2. Weill Cornell Medicine, New York, New York, United States*

Screening for elder mistreatment among older adults with dementia may be helpful, but the value of existing tools is not supported by evidence. Shifting the focus to individual subtypes of mistreatment may provide improved approaches to screening and ultimately to intervention. We have chosen to focus on caregiver neglect, the subtype associated with highest mortality and particularly common in dementia. As a preliminary step to develop a novel strategy to screen for caregiver neglect, we identified all existing published elder mistreatment screening tools to find items assessing for the presence of or risk for caregiver neglect. We identified 31 screening tools, which were all reviewed by 3 team members with complementary expertise (gerontology, social work, emergency medicine). Overall, none of the tools screened only for caregiver neglect. Among them, 22 (71%) included neglect-related items consisting of patient questions, caregiver questions, and provider observations. The total percent of items within each tool assessing for neglect ranged from 18-96%. After removing identical or very similar items, a final of list 202 was created, the majority of which (67.8%) were provider observations. These items were further grouped into 42 discrete categories: 11 categories of patient questions, 10 of caregiver questions, and 21 of provider observations. These serve as a clear starting point for development of caregiver neglect screening.

Abstract citation ID: igae098.2056

### UPDATED SYSTEMATIC REVIEW TO IDENTIFY INTERVENTIONS TO COMBAT ELDER NEGLECT TARGETING CAREGIVERS

Sophie Park[1], Helena Costantini[1], Amy Shaw[2], Alyssa Elman[2], Tony Rosen[2], and Sara Czaja[3], *1. Weill Cornell Medicine/Center on Aging and Behavioral Research, New York, New York, United States*, *2. Weill Cornell Medicine, New York, New York, United States*, *3. Weill Cornell Medicine/Center on Aging and Behavioral Research, New York, New York, United States*

Elder neglect is a subtype of elder mistreatment that is associated with serious medical and social consequences including mortality. Elder neglect by a caregiver is common among older adults receiving assistance from informal caregivers. Few interventions that target informal caregivers have focused on mitigating elder neglect. We updated an existing 2018 systematic review of elder mistreatment interventions, focusing specifically on interventions addressing elder neglect and targeting caregivers. The goal was to examine methods of intervention strategies to guide the development of a new caregiver intervention. The existing and updated review followed PRISMA guidelines and included 6 databases. Across both reviews, only 20 programs focused on elder neglect and targeted informal caregivers. Most programs used psychoeducation/therapy/counseling (65%) and/or education (30%), and 25% were multi-component interventions. Only one program was found to be very likely and two programs likely to be implementable in a low-resource environment. A formal program evaluation was included in 70% of the programs; only 15% were rated as having a high-quality study design. These findings suggest that few programs have been developed to address the important issue of caregiver elder neglect. The design of future interventions should focus on programs that are feasible to implement in low-resource environments and that contain rigorous evaluation strategies. The study findings also suggest that important areas of intervention focus include enhancing caregiver preparedness and reducing caregiver burden. Overall, there is a clear need for caregiver elder neglect interventions, especially those that can be implemented in low resource environments.

### SESSION 4360 (SYMPOSIUM)

Abstract citation ID: igae098.2057

### INNOVATING FOR EQUITABLE HOME HEALTH CARE: REDUCING BARRIERS TO SOCIAL AND END-OF-LIFE CARE FOR OLDER ADULTS

Chair: Komal Murali
Co-Chair: Julia Burgdorf
Discussant: Tracy Mroz

This symposium will focus on equity in home healthcare (HHC), operationalized as equitable access to, and receipt of, high quality HHC that is attuned to the unique needs of specific social and clinical subgroups, including minoritized racial/ethnic groups, those with chronic and serious illness such as dementia, and those at end-of-life. Topics include racial and ethnic disparities in hospice use and barriers to care, factors that impact individuals' access to social work services, reasons for why palliative care is declined among individuals with lower levels of education, and disparities in access to high quality HHC for people living with dementia and their care partners. Abstracts present analyses from varied methodological and theoretical perspectives that all aim to better understand existing disparities and offer foundational evidence to support development of innovative models of care. The researchers and studies convened by this symposium are dedicated to equitable improvements in home health access and quality for the millions of diverse older adults who receive HHC each year in the United States. In sum, the robust findings of these impactful studies, each conducted in an interdisciplinary fashion and rooted in health-equity principles, present informative evidence for future work endeavoring to improve equitable access to and receipt of the full gamut of HHC services from early stages of illness and social care to end-of-life care. Hospice, Palliative and End-of-Life Care Interest Group Sponsored Symposium

Abstract citation ID: igae098.2058

### BARRIERS TO HOSPICE CARE TRANSITIONS AMONG PEOPLE WITH DEMENTIA IN HOME HEALTH CARE: VIEWS OF DIVERSE CARE PARTNERS

Komal Murali[1], Gwenneth Wang[1], Sasha Vergez[2], Margaret McDonald[3], Dena Schulman-Green[1], Abraham Brody[4], and Karen Bullock[5], *1. New York University, New York, New York, United States*, *2. VNS Health, New York, New York, United States*, *3. Visiting Nurse Service of New York, New York, New York, United States*, *4. HIGN, New York University Rory Meyers College of Nursing, New York, New York, United States*, *5. Boston College, Chestnut Hill, Massachusetts, United States*

Racially and ethnically minoritized persons living with dementia (PLWD) use hospice at disparately low rates and home healthcare (HHC), a preferred setting of end-of-life care, is a key intervention point for increasing hospice transitions. We conducted qualitative interviews with diverse care partners of PLWD to explore views about barriers to hospice care and experiences with dementia care and end-of-life decision-making in HHC. Conceptually rooted in the NIMHD Health Disparities and Equity-Focus Implementation Research Frameworks, we conducted directed content analysis. Participants (n=20) were mostly female adult children of PLWD identifying as Black, Hispanic/Latino, Asian, or White. Six main concepts emerged: 1) Despite general openness to hospice, there was variable knowledge including misconceptions that it could not be received in the home; 2) Limited knowledge of dementia illness trajectories and end-of-life care options including hospice; 3) Communication challenges and conflicts associated with family-decision making; 4) Difficulty with care coordination and limitations of health insurance coverage and access to dementia caregiving support and resources; 5) Unmet cultural aspects of care and language barriers (language discordance between aides and PLWD) led to conflict between care partners and HHC staff); 6) Desire to honor and balance prior stated wishes of PLWD (e.g., prioritizing care at home, avoiding hospitalizations and nursing home) with end-of-life dementia care and decision-making. Findings shed light on views related to hospice care barriers and decision-making experiences of diverse care partners of PLWD that are key for the development of equity-focused culturally sensitive end-of-life care in HHC.

Abstract citation ID: igae098.2059

### PROVISION OF SOCIAL WORK SERVICES DURING HOME HEALTH CARE: INDIVIDUAL- AND AGENCY-LEVEL DETERMINANTS OF ACCESS

Julia Burgdorf[1], Yolanda Barron-Vaya[1], Jeri Goodman[1], and Margaret McDonald[2], *1. VNS Health, New York, New York, United States*, *2. Visiting Nurse Service of New York, New York, New York, United States*

Medicare-funded Home Health (HH) delivers skilled health care services via visits to patients' homes. Social work (SW) services are included within the HH benefit, and qualitative research suggests SW positively impacts outcomes for HH patients with complex needs. However, no prior work has quantitatively assessed SW provision during HH. We examined 2018 linked HH claims, assessment, and HH agency data for a national sample of 1,372,563 Medicare HH patients. Just 11.3% of HH patients received SW; those who received SW had a mean of 1.3 (SD=0.6) SW visits and a mean of 8.8 days (SD=6.6) from HH admission until the first SW visit. We modeled odds of receiving SW using a multilevel logistic model clustering at the HH agency level and adjusting for patient sociodemographic characteristics and clinical status and HH agency characteristics. The patient-level factors most significantly associated with receiving SW included being Black compared to White (aOR: 1.66; 95% CI: 1.62-1.71), being Medicaid-enrolled (aOR: 1.24; 95% CI: 1.19-1.30), having no family caregiver (aOR: 3.83; 95% CI: 3.62-4.07), and having depression (aOR: 2.26; 95% CI: 2.20-2.33). HH agency-level factors associated with receiving SW were non-profit ownership (aOR: 1.40; 95% CI: 1.29-1.52), urban service area (aOR: 1.48; 95% CI: 1.33-1.64), 60 or more full-time employees (aOR: 1.89; 95% CI: 1.70-2.09), and having social workers on staff, compared to providing SW via contract (aOR:2.23; 95% CI: 2.05-2.41). Findings indicate that SW access remains rare during HH and is driven by both demand-side (i.e., patient needs) and supply-side (i.e., agency capacity) factors.

Abstract citation ID: igae098.2060

### IMPACT OF VALUE-BASED PURCHASE MODEL ON RACIAL DISPARITIES IN ACCESSING HIGH-QUALITY HOME HEALTH CARE

Chenjuan Ma[1], Shaohui Wu[2], and Jason Fletcher[1], *1. New York University, New York, New York, United States*, *2. Dartmouth College, Hanover, New Hampshire, United States*

Home healthcare (HHC) plays a pivotal role in serving Americans who "Age in Place", particularly persons living with dementia (PLWD). Along with its rapid growth, there are increasing concerns of care disparities under the current HHC system and policies. This study aimed to examine racial/ethnic disparities in accessing high quality HHC among PLWD and identify the impact of home health value-based purchasing model (HHVBP), a policy designed for improving overall care quality, on such disparities. This study used two years of CMS HHC assessment data (i.e., OASIS) and HHC Compare Data. High quality HHC was identified based on agency care quality star rating (>=4.5 stars). HHVBP status was indicated by a dummy variable. A total of 743,860 PLWD from 6,410 agencies were studied, including 80.9% White, 11.9% Black, 5.6% Hispanic, and 1.6% Asian. Nearly one-third (32.6%) of agencies were with high quality care. Our risk-adjusted model shows that Blacks were 2% more likely to access high quality care while Hispanics and Asians were both about 20% less likely to access high quality care, compared to Whites. HHVBP increased the odds of accessing high quality HHC for all racial/ethnic groups (significant interaction terms all ORs>1) with most substantial among Hispanics (OR=1.60), followed by Asians (OR=1.28) and Blacks (OR=1.11). Stratification analysis indicated while the racial/ethnic disparities continue to exist in non-HHVBP states, such disparities disappeared in HHVBP states. Our study demonstrated the existence of racial/ethnic disparities in accessing high quality HHC and the effect of HHVBP in reducing such disparities.

Abstract citation ID: igae098.2061

### PALLIATIVE CARE KNOWLEDGE, HEALTH CONCERNS, AND EDUCATIONAL STATUS IN DECLINING COMMUNITY-BASED PALLIATIVE CARE

Elizabeth Luth[1], and Abigail Amponsah[2], *1. Rutgers University, New Brunswick, New Jersey, United States*, *2. Newark Board of Education, Newark, New Jersey, United States*

Palliative care in community settings can support seriously ill individuals to reduce symptoms and maintain quality of life. However, many individuals decline palliative care, even when they are concerned about their health. Differences in understanding how palliative care can help manage serious illness may contribute to individuals declining. This study explores the relationship between individuals' understanding of palliative care, their health concerns, and education levels (a proxy for health literacy). We use survey responses from a validated instrument assessing palliative care knowledge and information from semi-structured interviews for 21 seriously ill individuals (n=3) and family caregivers (n=18) who were eligible for community-based palliative care services and declined. We explore the relationship between respondents' education, understanding of palliative care, and their concerns about their or their loved one's health. Among participants, twelve had a bachelor's degree or higher; nine had some college or less. A higher percentage of participants with some college or less were concerned about psychological issues, stress, and managing medication side effects (67% vs 42%). All participants who did not understand palliative care could help with stress and medication side effects and were concerned about these had some college or less. One of three participants who did not understand palliative care could help with psychological issues and were concerned about these had some college or less. Assessing health concerns and explaining how palliative care can address those concerns may reduce barriers to accepting palliative care, particularly among individuals with lower levels of education.

Abstract citation ID: igae098.2062

### HOME HEALTH CARE USE AND OUTCOMES AMONG PERSONS WITH DEMENTIA: A SCOPING REVIEW

Jamie Smith[1], Julia Burgdorf[2], Tiffany Riser[3], and Miriam Ryvicker[2], *1. Widener University, Chester, Pennsylvania, United States*, *2. VNS Health, New York, New York, United States*, *3. Johns Hopkins University, Baltimore, Maryland, United States*

One-third of home healthcare (HHC) recipients are persons living with dementia (PLWD). HHC provides skilled nursing and therapy delivered in the patient's home. Amid population aging and a shift towards home- and community-based care, there is a growing need for HHC services for PLWD. However, dementia care research has historically focused on institutional care settings, and HHC has been understudied. We conducted a scoping review to summarize the current research landscape regarding HHC utilization and clinical outcomes for PLWD and recommend future research directions. We applied Arksey & O'Malley's methodology and identified 32 articles that met inclusion criteria. Of these, 22 studies examined utilization, finding that PLWD used HHC differently than persons without dementia, having a higher likelihood of community referral, longer episode length, greater use of nursing and aide services, and increased costs. Sixteen papers reported on clinical outcomes: function, hospitalization, skilled nursing facility admission, and mortality. Analyses varied widely in their approach to defining dementia and measuring key outcomes. However, literature suggests an increased risk for negative outcomes, including hospitalization and institutionalization among PLWD. Findings highlight several areas for future research, most saliently, the need for analyses that consider contextual factors (e.g., market factors, racial/ethnic disparities, rurality) when examining HHC for PLWD. Future work should assess the impacts of payment model revisions and expanding Medicare Advantage coverage on utilization and care delivery for this population. A rigorous examination of care delivery and outcomes is needed to inform practice models to adapt to the unique care needs of PLWD.

### SESSION 4365 (PAPER)

### INTIMATE PARTNER VIOLENCE, ELDER ABUSE, AND MISTREATMENT

Abstract citation ID: igae098.2063

### UNDERSTANDING SEVERITY OF ELDER MISTREATMENT: FINDINGS FROM THE CANADIAN LONGITUDINAL STUDY ON AGING

David Burnes[1], Mark Lachs[2], and Karl Pillemer[3], *1. University of Toronto, Toronto, Ontario, Canada*, *2. Weill Cornell Medicine, New York, New York, United States*, *3. Cornell University, Ithaca, New York, United States*

Basic elder mistreatment knowledge on risk factors and prevalence has advanced substantially over the past two decades. However, research in this area has predominantly framed our understanding of elder mistreatment in binary (no/yes) terms. While a binary understanding of elder mistreatment is necessary to determine prevalence/incidence, this conventional framing obscures the variation in lived experiences across cases and provides limited clinical insights for response interventions seeking to reduce harm. Building on prior research examining elder mistreatment through a lens of severity, this study analyzed data from the population-based Canadian Longitudinal Study on Aging to both describe the range of elder mistreatment severity across cases and identify longitudinal risk/protective factors associated with severity levels. An ecological-systems perspective organized potential risk/protective factors from several levels of influence. Drawing on a nationally stratified, random sample (n=23,468) of community-dwelling adults aged 65 or older interviewed over a three-year period, the current study analyzed a subsample (n=2368) of older adults identifying as victims of emotional, physical, or financial mistreatment. Severity was operationalized according to past-year frequency and multiplicity of mistreatment behaviors experienced. Ordinal/multinomial logistic regression was used to examine static and change risk/protective factors for elder mistreatment in general and for separate subtypes. Older adults with lower physical, cognitive and mental health, higher levels of child maltreatment, who lived with their perpetrator, and identified as female were more likely to experience severe forms of mistreatment. Findings from this study advance our basic knowledge on elder mistreatment phenomena and inform practice in community-based response programs.

Abstract citation ID: igae098.2064

### LONELINESS AND RISK OF ELDER ABUSE: DOES SIZE OF SOCIAL NETWORK MAKE A DIFFERENCE?

Louis To[1], Jasmin He[1], Lok Man Leung[1], Ronald Tsui[2], Debby Wan[1], and Elsie Yan[1], *1. The Hong Kong Polytechnic University, Hong Kong, Hong Kong, China (People's Republic)*, *2. Lingnan University, Hong Kong, Hong Kong, China (People's Republic)*

Elder abuse is a serious public health issue. Research demonstrated that loneliness is a risk factor for abuse. The present study examined the association between loneliness and elder abuse in a convenient sample of 686 community dwelling older persons in Hong Kong. Participants were recruited from senior community centers across the territory. They provided information on their demographic characteristics, sense of loneliness (DJGLS-6), social network (LSNS-6), and risk of elder abuse (EASI and VASS-10). 37.4% of the participants were screened positive on EASI or VASS-10. Results of logistical regression show that participants who were female (OR=-.505), married (OR=-.951), of a younger age (OR=-.044), reported greater sense of loneliness (OR=.266) were more likely to report risk of being abused. Co-residence with a family member and size of social network was not significant in the model (p>.05). Multiple regression analysis was conducted to identify factor associated with sense of loneliness. Participants of a younger age (Beta=-.019), living alone (Beta=-.170), and had a smaller social network (Beta=.-085) reported greater sense of loneliness. Findings from the present study adds to the larger literature on the impact of various demographic factors and sense of loneliness on elder abuse. Prevention and intervention efforts should focus on female young-olds in a marital relationship. Efforts to mitigate sense of loneliness could potentially help tackle elder abuse. Although increasing social network size does not have a direct impact on risk of elder abuse, it could potentially mitigate sense of loneliness thereby decrease risk of abuse.

Abstract citation ID: igae098.2065

### BARRIERS AND FACILITATORS TO HELP-SEEKING FOR ELDER ABUSE: A QUALITATIVE STUDY FROM THE PERSPECTIVE OF VICTIMS

Andie MacNeil[1], Jessica Hsieh[1], Isabel Rollandi[2], Jo Anne Sirey[2], Mark Lachs[2], and David Burnes[1], *1. University of Toronto, Toronto, Ontario, Canada*, *2. Weill Cornell Medicine, New York, New York, United States*

Understanding help-seeking among victims of elder abuse (EA) is a major challenge and significant knowledge gap in the field. The vast majority of EA victims are not connected with formal response systems, such as Adult Protective Services, law enforcement, and other agencies responsible for addressing this issue in the community. The current study sought to understand barriers and facilitators to help-seeking from the perspective of EA victims. Using a descriptive phenomenological approach, this study conducted in-depth, individual, qualitative interviews with a diverse sample (n = 32) of EA victims living in New York City and Los Angeles, United States. Analysis followed a constant comparison process involving two independent coders of verbatim transcripts. Major themes that emerged as barriers to help-seeking included: embarrassment or shame, self-blame or guilt, fear of perpetrator retaliation, fear of what could happen to perpetrator, concern for family preservation, and lack of awareness of services. Facilitators in the process of help-seeking included: reaching a “tipping point”, concern for self-preservation, accessibility and convenience of services, and having knowledge about where and how to seek support. These findings emphasize that barriers and facilitators to help-seeking span various levels of ecological influence, including the individual victim, the individual perpetrator, the victim-perpetrator relationship, and the family system. To promote greater help-seeking among EA victims, there must be accessible interventions that help address the complex and interrelated psychological, emotional, relational, and familial factors that impact help-seeking behavior among EA victims.

Abstract citation ID: igae098.2066

### THE EFFECT OF SOCIAL SUPPORT ON THE RELATIONSHIP BETWEEN ELDER MISTREATMENT AND HEALTH IN OLDER CHINESE IMMIGRANTS

Soonhyung Kwon[1], Wendi Da[2], Fengyan Tang[3], and Yanping Jiang[4], *1. University of South Florida, Tampa, Florida, United States*, *2. Rutgers, the State University of New Jersey, New Brunswick, New Jersey, United States*, *3. University of Pittsburgh, Wexford, Pennsylvania, United States*, *4. Rutgers, The State University of New Jersey, New Brunswick, New Jersey, United States*

Elder mistreatment experiences cause severe health consequences such as lower self-rated health. Despite such documented adverse effects, it is less known the potential factors that can mitigate the adverse effects of elder mistreatment on health to facilitate intervention development further. Thus, our study aims to examine the moderating role of social support from friends on the relationship between elder mistreatment and self-rated health among older Chinese immigrants. This study used five waves of PINE study involving 2,835 older Chinese immigrants aged 60 years and older. We used latent growth curve modeling (LGCM) to investigate the trajectory of self-rated health. Elder mistreatment (psychological, physical, and sexual abuse, financial exploitation, and caregiver neglect), social support from friends, and self-rated health were assessed across five waves. Our study showed social support from friends significantly moderated the adverse effects of elder mistreatment on the trajectory of self-rated health. Specifically, the detrimental effect of elder mistreatment on a faster decline in self-rated health over time was smaller at higher levels of social support from friends, showing the buffering effects of strong social support from friends on the relationship between elder mistreatment and self-reported health. Our findings suggest that transitioning friend social support from low to medium might be promising to reduce the negative effect of elder mistreatment on self-reported health among older Chinese immigrants.

### SESSION 4370 (SYMPOSIUM)

Abstract citation ID: igae098.2067

### LESSONS LEARNED AND NEXT STEPS FOR USER-CENTERED DESIGN WITH OLDER ADULTS AND CAREGIVERS

Chair: Jennifer Portz
Discussant: Sara Czaja

Digital health can uniquely address various healthcare challenges, yet its’ effectiveness relies heavily on factors such as usability, acceptability, and inclusivity. In this symposium, we present findings from three pioneering projects aimed at developing and refining digital health interventions for older adults and their care partners, tailored to specific populations and healthcare needs. Each project employs a user-centered design (UCD) approach to address unique challenges and opportunities in digital health intervention development. From designing dyadic interventions for advanced dementia to improving care coordination for rural older adults and integrating social support in palliative care, these projects offer valuable insights into developing meaningful, accessible, and person-centered digital health solutions across diverse healthcare landscapes. The final presentation outlines lessons learned from four remote co-design processes for dementia care partner interventions. Co-design, involving an active contribution from older adults and partners, fosters usable, relevant solutions, yet challenges in implementing co-design warrant guidance. Lastly, our Discussant, Dr. Sara Czaja, a world-renown expert in aging-related technology, will lead a conversation highlighting the benefits of UCD, the challenges and limitations of the approach, and innovative solutions for fostering the engagement of older adults and their care partners in the digital health design process.

Abstract citation ID: igae098.2068

### DESIGNING A BIOEXPERIENTIAL PROGRAM FOR SAFETY, USABILITY, AND ACCEPTABILITY WITH DYADS WITH ADVANCED DEMENTIA

Evan Plys[1], Elizabeth Rochon[1], Ayush Thacker[1], Maimouna Sy[1], Mirelle Phillips[2], Mischa Kuma[2], Christine Ritchie[3], and Ana-Maria Vranceanu[1], *1. Massachusetts General Hospital, Boston, Massachusetts, United States*, *2. Studio Elsewhere, Brooklyn, New York, United States*, *3. Mass General Hospital and Harvard Medical School, Boston, Massachusetts, United States*

Dyadic psychosocial interventions that engage both persons living with dementia (PLWD) and care partners (CPs) are increasingly popular. Yet, designing dyadic interventions for advanced dementia is challenging due to mismatched abilities. Here, we describe the initial development and refinement of the Toolkit for Experiential well-beiNg in Dementia (TEND) intervention with a focus on design considerations for safety, usability, and acceptability. TEND is an online bioexperiential program (e.g., storytelling, digital art, and relaxing sounds and imagery) designed to engage PLWD and CPs together, to support positive affect, relationship satisfaction, and emotional well-being. Using a multiphasic approach consistent with user-centered design, we conducted two focus groups with clinicians (N=10), two with PLWD-CP dyads (N=7), and one with CPs only (N=4) to inform an initial prototype. Then, we conducted eight rounds of workshops with 10 PLWD-CP dyads to inform intervention refinements. Rapid data analysis was used to analyze findings and quickly inform iterative design. Focus group participants highlighted design considerations to avoid inadvertently exacerbating behavioral and psychiatric symptoms; matching the context of use; and promoting individualization, familiar stimuli, immersion, and visual clarity of the online program. After development of an initial prototype, workshop participants informed refinements to include mixed methods of engagement (i.e., voice recognition, typing, and interactive movement); instructions, tutorials, and navigation guides; directive prompts; simplified language; and pleasant sounds. Overall, the program was perceived as safe and user feedback informed refinements to promote usability and acceptability. We highlight design elements of the TEND program that were responsive to iterative user feedback.

Abstract citation ID: igae098.2069

### LESSONS LEARNED FROM A USER-CENTERED DESIGN APPROACH RURAL OLDER ADULTS USING DIGITAL HEALTH TOOLS

Marquita Lewis-Thames, Shawn Trimble, Francesca Cejtin Rosen, Zachary Seigel, Kara Toll, and Kensley Horne, *Northwestern University Feinberg School of Medicine, Chicago, Illinois, United States*

Increasing age and rurality are associated with decreased care coordination. There is a growing interest in the role of digital tools in improving care across the care continuum for rural and older adult groups. Still, little is known about the best practices of rural older adults and digital health tool usability. Guided by a user-centered design approach, we 1) describe multiple strategies to meaningfully engage rural older adults in the development of a digital health tool to improve telehealth navigation and 2) report best practices assessed from our multi-modal iterative design approach. We engaged key informants, including rural older adults, their caregivers, providers, healthcare administrators, and an advisory board. Our mixed methods user-centered design approach triangulated findings through semi-structured interviews, a systematic literature review, tool usability testing, and a MyChart usability analysis using eye-tracking technology. Lessons learned from our analysis reveal that rural older adults readily engage with digital technology and often sustain use after their introduction. Digital health technologies were most commonly associated with relieving access to care and transportation barriers. Reduced internet access and challenges with using the equipment were common usability barriers. Useful strategies to overcome usability barriers include in-person or telephone tutorials and knowledgeable caregivers. Tools to assist rural older adults with technology barriers were limited. As digital health technologies increasingly integrate into rural care coordination, barriers faced by rural older adults must be considered. Research that aims to elevate the voices of rural older adults in digital health tool design is imperative.

Abstract citation ID: igae098.2070

### ENHANCING PALLIATIVE CARE THROUGH THE INTEGRATION OF THE SOCIAL CONVOY: A USER-CENTERED DESIGN APPROACH

Lyndsay DeGroot, *University of Colorado Anschutz Medical Campus School of Medicine, Aurora, Colorado, United States*

Digital health offers considerable potential for enhancing palliative care but often neglects to integrate the patient's caregiving "social convoy", i.e. partners, family, and friends. The Social Convoy Palliative Care (Convoy-Pal) mobile intervention was developed using a user-centered design approach involving interprofessional healthcare providers, older adults with serious illness, and their social convoy (N=81). Convoy-Pal, a 12-week intervention, provides a tablet, smartwatch, and options for mobile phone access, with shared access among participants and their convoy. It facilitates assessment, symptom tracking, and provision of palliative care resources based on user responses. Initial usability feedback among 26 participants (16 older adults/10 social convoys) was favorable, particularly regarding the information provided (User Mobile Application Rating Scale-uMARS: 4.22±.8/5). Based on feedback, final iterations were made to improve goal setting, monitoring tools, and convoy assessment. We then conducted a single-site waitlist randomized control trial among N=46 (31 older adults/14 social convoys) to pilot-test the feasibility of delivering Convoy-Pal. Participants were 76.3 years of age, and social convoys 71.6, primarily White, but not all had access to a mobile device/computer. Recruitment of multiple caregivers was challenging; when enrolled the study maintained a 67.4% retention rate with 4.6% missing subscales on surveys. Intervention participants reported improvements in social functioning (SF-36: 64.6±25.8 vs. 73.2±31.3) and social convoys reported increased perceptions of positive aspects of caregiving (29.5±5.28 vs. 35.0±5.35). Usability scores for information provided were again well-rated (uMARS: 3.96±.57/5). This research underscores the importance of incorporating diverse key informant perspectives to develop meaningful and relevant digital palliative care interventions.

Abstract citation ID: igae098.2071

### LESSONS LEARNED ENGAGING CARE PARTNERS OF PEOPLE LIVING WITH DEMENTIA IN CO-DESIGN OF DIGITAL HEALTH INTERVENTIONS

Nicole Werner, *Indiana University, Bloomington, Bloomington, Indiana, United States*

Per the NIH Stage Model of Behavioral Intervention Development, once a scientific mechanism for a possible behavioral intervention is established, the next critical step is to systematically design the intervention with the target population. If digital health intervention researchers bypass this stage, their interventions may work in ideal but not "real world" settings. Moreover, partnerships between user-centered design/engineering experts and health sciences researchers are believed to "increase the likelihood for impactful outcomes from the use of the engineering design process." However, more work is needed to translate user-centered design approaches to digital health intervention design for older adults and care partners. Co-design in particular promotes strengths-based (vs. deficit-based) treatment of older adults and care partners. That is, rather than being the focus of a study on what is wrong, older adults and their care partners actively contribute to design and are therefore more likely to produce digital health solutions that are usable, useful, relevant, acceptable, and adaptable to current routines. However, co-design approaches can be challenging to implement, and guidance for engaging older adults and care partners in co-design is lacking. Dr. Werner will present lessons learned across four projects that implemented a remote, five-phase co-design process to co-design digital health interventions with care partners of people living with dementia. Lessons learned span co-design activities, co-design quality, and co-designer outcomes that were identified from research team and care partner interviews conducted at the end of the design process and feedback surveys conducted with care partners after each co-design session.

### SESSION 4375 (SYMPOSIUM)

Abstract citation ID: igae098.2072

### NOVEL APPROACHES AND DATA SOURCES TO INVESTIGATE LIFESPAN ASSOCIATIONS FROM EARLY EXPOSURES TO LATER LIFE HEALTH

Chair: Lewina Lee

Despite substantial evidence linking early adversity to elevated risks of later-life morbidity and mortality, recent findings on the often-poor agreement between retrospective and prospective measures of childhood maltreatment (Ks=.09-.83; Baldwin et al., 2019) have challenged these well-established associations, which were predominantly derived from retrospectively-assessed early exposures in longitudinal cohorts. As most longitudinal aging studies enrolled participants in their adulthood, prospective early-life data combined with long-term tracking of health outcomes are scarce, but necessary for rigorous causal inference regarding the nature and mechanisms by which early exposures shape life span health. This symposium showcases recent methodological developments and new data resources capable of addressing these scientific barriers and amplifying the scientific value of longitudinal aging studies. Ms. Dorame will illustrate a novel approach to acquiring prospective data on childhood familial and environmental exposures by linking three longitudinal cohorts to recently digitalized, 1900-1940 US Censuses in the Boston Early Adversity and Mortality Study (BEAMS). Dr. Marino will report on the prospective associations of childhood and midlife socioeconomic status and all-cause mortality risk using BEAMS Census-linked data. Dr. Casey will describe efforts to assess early-life environmental exposures and evaluate their associations with later-life brain health in the Health and Retirement Study. Dr. Arpawong will consider the extent to which cognitive abilities were retained from adolescence to old age in Project Talent, and the roles of education and gender therein. Finally, Dr. Warren will describe three new, large-scale data resources suitable for interdisciplinary research on the later-life sequelae of early-life exposures. Measurement, Statistics, and Research Design Interest Group Sponsored Symposium

Abstract citation ID: igae098.2073

### IN THE PALM OF OUR HAND: HUMAN-LED ADMINISTRATIVE DATA LINKAGE TO AUGMENT LONGITUDINAL AGING STUDIES

Ashley N. Dorame[1], Mina Antic[2], Joseph Ferrie[3], Robert Waldinger[4], Avron Spiro III[5], Daniel Mroczek[3], and Lewina Lee[6], *1. Boston University School Of Medicine, Boston, Massachusetts, United States*, *2. Massachusetts General Hospital/Harvard Medical School, Boston, Massachusetts, United States*, *3. Northwestern University, Evanston, Illinois, United States*, *4. Harvard University, Cambridge, Massachusetts, United States*, *5. VA Boston Healthcare System, Boston, Massachusetts, United States*, *6. Boston University Chobanian & Avedisian School Of Medicine, Boston, Massachusetts, United States*

A robust literature has documented associations of adverse early experiences with poor lifespan health (Hughes et al., 2017). The evidence with respect to aging-related outcomes has been largely derived from population-based longitudinal studies, which predominantly rely on retrospective assessments of childhood experiences. Given the modest agreement between prospective and retrospective measures of early life exposures (Baldwin et al., 2019), it is unknown whether the well-established associations of early adversity with later-life health may replicate in prospective designs. This presentation uses the Boston Early Adversity and Mortality Study (BEAMS) to illustrate a novel approach leveraging recently-digitalized, full-count U.S. Censuses from 1900 to 1940 to obtain prospective data on the early-life family and neighborhood conditions for participants in three, all-male longitudinal aging studies (Normative Aging Study, 1961+, n=2280; Harvard Study of Adult Development, 1938+: Grant cohort, n=458, Glueck cohort, n=267). The authors will describe key steps of the human-led data linkage ("hand-linkage") process, and present results on linkage performance and value added to the longitudinal studies. Hand-linkage identified 10,146 female and male siblings of the original cohort members. Among original cohort members born prior to each Census, hand-linkage identified 68.6% (1900), 76.3% (1910), 88.5% (1920), 88.0% (1930), and 87.9% (1940) participants, compared to match rates of 24%-36% via a commonly used algorithm (Abramitzky et al., 2021 with Jaro-Winkler adjustment). Relative to hand-linkage, the sensitivity and specificity of the algorithmic approach was 31.2% and 98.2%, respectively. Findings support human-led linkage as an effective strategy to augment the scientific value of longitudinal aging studies.

Abstract citation ID: igae098.2074

### LIFESPAN SOCIOECONOMIC STATUS AND MORTALITY USING 1900–1940 CENSUSES: FINDINGS FROM THE NORMATIVE AGING STUDY

Victoria Marino[1], Joseph Ferrie[2], Ashley N. Dorame[3], Eileen Graham[2], Avron Spiro III[4], Daniel Mroczek[2], and Lewina Lee[5], *1. National Center for Posttraumatic Stress Disorder, VA Boston Healthcare System, Boston, Massachusetts, United States*, *2. Northwestern University, Evanston, Illinois, United States*, *3. Boston University School Of Medicine, Boston, Massachusetts, United States*, *4. VA Boston Healthcare System, Boston, Massachusetts, United States*, *5. Boston University Chobanian & Avedisian School Of Medicine, Boston, Massachusetts, United States*

Early and midlife socioeconomic status (SES) are linked to all-cause mortality, but few studies have examined their independent and joint associations using multidimensional, prospective SES measures and track mortality over extended periods. As described in the Dorame et al. presentation in this symposium, the Boston Early Adversity and Mortality Study (BEAMS) acquired prospective data on the early-life circumstances of three well-characterized male cohorts via linkage to the 1900-1940 US Federal Censuses. This study leverages BEAMS data to test the independent and joint associations of early and midlife SES with all-cause mortality in 1,303 men from a BEAMS cohort, the Normative Aging Study. Early SES, assessed at baseline (earliest available Census before age 18), was a composite combining paternal literacy (1=yes, 0=no) and z-standardized paternal occupational income. Midlife SES was a z-score composite of men's occupational standing, education, and income at study entry (mean age=42). Mortality status was followed through December 2020 (mean follow-up since baseline: 77 years). Early SES was weakly and positively correlated with midlife SES (r=.17, p<.0001). In Cox regression, each 1-SD higher early SES was associated with 8% lower mortality risk (95%CI: 0.84, 1.00). Each 1-SD higher midlife SES was associated with 3% lower mortality risk (95%CI: 0.95,0.99), adjusted for early SES. The main effect of midlife SES weakened the association of early SES with mortality by only 2%. Early SES did not moderate the association of midlife SES with mortality. Our findings support largely independent contributions of early and midlife SES to all-cause mortality risk.

Abstract citation ID: igae098.2075

### ENVIRONMENTAL EXPOSURES AND AGING OUTCOMES IN THE HRS USING CENSUS DATA

Joan Casey[1], Jenni Shearston[2], Taylor Mobley[3], L. Paloma Rojas-Saunero[3], Juliet Zhou[3], and Elizabeth Rose Mayeda[3], *1. University of Washington, Seattle, Washington, United States*, *2. University of California Berkeley, Berkeley, California, United States*, *3. University of California Los Angeles, Los Angeles, California, United States*

Incidence of Alzheimer's disease and related dementias (ADRD) is 40–100% higher among Black compared with White Americans. Differential exposure to air pollution might partially explain this difference. We aim to determine if early-life exposure to poor air quality is related to late-life brain health among non-Hispanic Black and White Health and Retirement Study (HRS) participants. Using 1940 Census-linked enumeration district of residence, we estimate childhood exposure to fine particulate matter (PM2.5), traffic-related air pollution, and fossil fuel energy sources. We construct these exposures using archived climate model outputs from CMIP6 (PM2.5), gasoline sales, street networks, and oil and gas well and power plant locations paired with dispersion modeling techniques. Using multi-level survival analyses, we estimate the association between HRS participant childhood environmental exposures and late-life dementia. We also determine the contribution to Black-White disparities in dementia attributable to differences in early-life exposure. Preliminary analyses indicate that Black vs. White HRS participants had lower power plant emissions exposures but higher PM2.5 and traffic-related air pollution exposures in 1940. Regression analyses will evaluate whether 1940 environmental pollutant exposure is associated with dementia or dementia disparities by race. Identifying modifiable risk factors for ADRD could help protect cognitive health of the aging US population.

Abstract citation ID: igae098.2076

### STABILITY OF COGNITIVE ABILITY AND EFFECT OF EDUCATION ACROSS 58 YEARS: A PROJECT TALENT AGING STUDY

Thalida Em Arpawong[1], Jimi Huh[1], Tara Gruenewald[2], Gwenith Fisher[3], Jennifer Manly[4], Dominika Seblova[5], Kelly Peters[6], and Margaret Gatz[1], *1. University of Southern California, Los Angeles, California, United States*, *2. Chapman University, Orange, California, United States*, *3. Colorado State University, Fort Collins, Colorado, United States*, *4. Columbia University, New York, New York, United States*, *5. Charles University, Prague, Hlavni mesto Praha, Czech Republic*, *6. American Institutes for Research, Arlington, Virginia, United States*

We assessed the degree to which individuals retain cognitive abilities from adolescence through older adulthood, and whether more education associates with better retention in some more than others, after adjusting for adolescent cognitive ability. We also evaluated whether benefits associated with education differ for those with lower or higher cognitive scores in adolescence, or women or men. Data came from the Project Talent Aging Study, and included measures of abstract reasoning, mathematics, visualization in two dimensions, and recognition memory in 1960 and 2018 (n=2,032; age range 71

to 79). Confirmatory factor analysis was used to fit one-factor (cognitive ability factor, CAF) at each timepoint. We then used a structural equation model to test whether the 1960 CAF predicted the 2018 CAF, with an interaction term between 1960 CAF and years of education. Multiple group models tested invariance by sex. We found high stability in CAFs assessed 58 years apart. Education predicted higher 2018 CAF (p<.001), although a significant interaction (1960 CAF*education; p<.05) showed that effects were diminished for those with higher 1960 CAF. Sex-specific analyses showed that the diminishing effect of education was evident for both women (p<.05) and men (p<.05). Additionally, the simple effect of education on 2018 CAF was significant for women (p<.001) but not for men (p=.37). Findings indicate high stability in cognitive abilities from adolescence to older adulthood, and that exposure to more education differentially benefits later age abilities depending on adolescent levels and sex.

Abstract citation ID: igae098.2077

### THREE INNOVATIVE DATA RESOURCES: EDSHARE, THE MULTIGENERATIONAL LONGITUDINAL PANEL, AND THE 1940 CENSUS

John Warren, *University of Minnesota, Minneapolis, Minnesota, United States*

Scientists interested in studying the long-term health consequences of early life social, economic, policy, environmental, or other exposures have historically suffered from a lack of suitable data. Large, nationally representative studies of early life (e.g., Add Health) rarely extend late enough into older ages. At the same time, large, nationally representative studies of later life (e.g., HRS, NSHAP) rarely contain prospectively gathered, high resolution information about early life exposures. This talk will describe three new and extraordinary new data resources that overcome these problems. FIRST, the Education Studies for Health Aging Research (EdSHARe) project has re-purposed two large national studies of adolescence and education started in the 1970s and 1980 to be studies of health and aging; the talk will focus on these cohort studies utility for understanding the social and biological pathways through which early life factors --- especially education --- impact later life health and cognition. SECOND, the Multigenerational Longitudinal Panel (MLP) includes linked complete-count U.S. census records for every American from 1850 through 2020 plus other mortality, vital records, and other kinds of data. THIRD, as an offshoot of MLP, we have linked records about older people from the HRS, NSHAP, PSID, WLS, and NHATS to the 1940 Census in which aging survey participants are seen in childhood along with their parents and other family members. All three data resources are or soon will be available to the research community; all stand to transform aging research.

### SESSION 4380 (SYMPOSIUM)

Abstract citation ID: igae098.2078

### NOVEL APPROACHES FOR CROSS-NATIONAL COGNITIVE AGING RESEARCH

Chair: Alden Gross
Co-Chair: Lindsay Kobayashi
Discussant: Kenneth Langa

Cross-national comparisons of cognitive outcomes are increasingly feasible given availability of newer data resources. Given a broad, global study population, cross-national research may uncover mechanistic insights that would not be clear from one country or a socioeconomically homogeneous set of countries. Despite its promise, cross-national data present with numerous methodological and interpretational challenges, which will be highlighted in this symposium. In this symposium, Kobayashi and Gross (co-chairs) will lead with best practices for cross-national research, highlighting findings from the Harmonized Cognitive Assessment Protocols (HCAPs), a major NIA-funded platform for valid cross-national comparisons of ADRD. Cai will present a novel platform for deriving classification algorithms for dementia, based on neuropsychological criteria standardized to a robust normative group. Leveraging HCAP studies from the US, England, and India, they applied variations of classification algorithms. Next, Wu and colleagues, using longitudinal data from China's HCAP (N=6,830), present a challenge–form differences introduced by changing test stimuli across waves–and a solution–weighted equipercentile equating–for when parallel but non-equivalent memory tests are administered longitudinally in surveys. Third, out of recognition that culturally diverse and low-resource settings can affect reliability and validity of cognitive tests, Mani and colleagues present psychometric properties of a brief pilot HCAP in Kenya (N=203). Finally, Zhang will present simulation results and demonstration of an R/Shiny-App regarding effects of differential pre-baseline selection on inferences for cross-national studies. Pre-baseline selection refers to selective survival in a target population occurring before a study's baseline, and can vary dramatically between high-income and low-income countries.

Abstract citation ID: igae098.2079

### VALIDATION OF COGNITIVE AND PSYCHOSOCIAL INSTRUMENTS FOR THE KENYAN CONTEXT: RESULTS FROM LOSHAK

Sneha Mani[1], Joshua Ehrlich[2], Anthony Ngugi[3], Niranjani Nagarajan[4], Roselyter Riang'a[3], Eunice Mwangi[3], and Alden Gross[1], *1. Johns Hopkins Bloomberg School of Public Health, Baltimore, Maryland, United States, 2. University of Michigan, Ann Arbor, Michigan, United States, 3. Aga Khan University, Nairobi, Nairobi Area, Kenya, 4. Michigan Medicine, Ann Arbor, Michigan, United States*

Adaptation of cognitive tests to culturally diverse and low-resource settings is essential for expanding knowledge of cognitive health in older adults beyond high-income countries. However, contextual differences in novel settings can affect reliability and validity. A significant knowledge gap remains in Sub-Saharan Africa, a region projected to be home to over 158 million older adults by 2050. The Longitudinal Study of Health and Ageing in Kenya (LOSHAK) aims to address this measurement gap. The LOSHAK pilot collected neuropsychological and psychosocial assessment data, adapted from the US Health and Retirement Survey to be relevant to the Kenyan context. For four cognitive constructs (orientation, memory, executive functioning, language/fluency) and four psychosocial constructs (depressive symptoms, loneliness, subjective well-being, and life satisfaction), internal consistency reliability was evaluated using

McDonald's omega (ω) and factor structures were evaluated using confirmatory factor analysis. McDonald's omegas ranged from ω=0.78 to 0.95 for cognitive domains and were above 0.83 for each psychosocial domain, suggesting high reliability. Factor analyses revealed good to excellent fit for most domains (RMSEA<0.095; CFI>0.93; SRMR<0.11); patterns of factor loadings were mostly acceptable (ranging from 0.5 to 0.9), and will be used to refine instruments further. This examination reveals a successful adaptation of neuropsychological and psychosocial assessments in Kenya and provides a reference for other studies seeking to assess the efficacy of adapted instruments.

Abstract citation ID: igae098.2080

### HARMONIZING COGNITIVE SCORES ACROSS WAVES IN THE CHINA HEALTH AND RETIREMENT LONGITUDINAL STUDY

Yingyan Wu[1], Elizabeth Rose Mayeda[1], Lindsay Kobayashi[2], Alden Gross[3], and Yuan Zhang[4], *1. University of California Los Angeles, Los Angeles, California, United States, 2. University of Michigan, Ann Arbor, Michigan, United States, 3. Johns Hopkins Bloomberg School of Public Health, Baltimore, Maryland, United States, 4. Columbia University, New York, New York, United States*

The China Health and Retirement Longitudinal Study (CHARLS) administered a word recall test to assess memory function across five study waves from 2011 to 2020. Since 2018, the word recall test has differed from prior waves in the difficulty of word lists used and number of immediate recall trials given, posing challenges for longitudinal analyses of cognitive change. We thus leveraged weighted equipercentile equating for immediate and delayed recall scores to address nonequivalence in the test across study waves. We created a calibration sample that balanced age, gender, and education to ensure the same underlying test ability across waves. Within the calibration sample, we used equipercentile equating to crosswalk percentile ranks between the scores in the 2015 and 2018 waves and 2015 and 2020 waves, then applied the crosswalk to the full study sample. In the full study sample, the mean original delayed word recall scores were higher in 2018 (4.3 words) and 2020 (5.1 words) versus 2015 (3.2 words). Within the 2018 and 2020 waves, mean original delayed scores were higher than mean original immediate scores (2018: 4.3 vs 3.2, 2020: 5.1 vs. 3.6 words). After applying equipercentile equating, the mean 2018 and 2020 wave immediate and delayed word recall scores were comparable to previous waves (immediate: 2015: 4.1, 2018: 3.7, 2020: 3.8; delayed: 2015: 3.2, 2018: 2.5, 2020: 2.8 words). Equipercentile equating can be used to generate comparable scores to facilitate longitudinal analysis when cognitive test administration procedures change over time.

Abstract citation ID: igae098.2081

### PREBASELINE SELECTION BIAS IN THE ESTIMATED EFFECT OF T2D ON COGNITIVE DECLINE: CROSS-NATIONAL SIMULATIONS

Pengyuan (Kelvin) Zhang[1], Sneha Mani[2], Lindsay Kobayashi[3], Alden Gross[2], Jennifer Weuve[4], and Ryan Andrews[4], *1. University of Michigan, Okemos, Michigan, United States, 2. Johns Hopkins Bloomberg School of Public Health, Baltimore, Maryland, United States, 3. University of Michigan, Ann Arbor, Michigan, United States, 4. Boston University, Boston, Massachusetts, United States*

Selection bias is a common threat to the validity of effect estimates, including those of various exposures on late-life cognitive outcomes. Although selection bias can arise for many reasons, a challenging subtype is driven by differential mortality in a study's source population prior to baseline. In other words, individuals who survive long enough to be eligible for studies of late-life cognition are systematically different from those who do not. Because data are not collected on individuals who do not survive to study enrollment, analytic methods to address selection bias cannot be applied. Furthermore, this "pre-baseline survival" bias makes cross-national comparisons difficult, as different countries have differing mortality rates and determinants of mortality. Using the example of cross-national comparisons of the effect of type-2 diabetes on cognitive decline, we demonstrate how simulations can help quantify pre-baseline survival bias. We simulated large (N=300,000) country-specific cohorts of adults in the US, India, and China under seven causal scenarios governing the relationships between type-2 diabetes, pre-baseline survival, and cognitive decline. After assigning each simulated participant a vital status (dead/alive by a certain age), we recruited 2,000 country-specific study samples of 2,000 "survivors" and estimated the association between type-2 diabetes and cognitive decline within these cohorts. We found that even under relatively mild differences in pre-baseline survival, the cross-national effect of diabetes on cognitive decline could be underestimated by up to 34%. To assist others with conducting similar simulation studies, we developed a user-friendly platform based on the R-Shiny package.

Abstract citation ID: igae098.2082

### CONSIDERATIONS FOR CROSS-NATIONAL APPROACHES TO ESTIMATING PREVALENCE OF DEMENTIA

Wenjie Cai[1], Richard Jones[2], and Alden Gross[3], *1. Johns Hopkins University, Baltimore, Maryland, United States, 2. Brown University, Providence, Rhode Island, United States, 3. Johns Hopkins Bloomberg School of Public Health, Baltimore, Maryland, United States*

Comparing dementia prevalence across countries and contexts is crucial for effective public health planning. The Harmonized Cognitive Assessment Protocol Project (HCAP) is a global effort to characterize dementia risk using population-representative samples. Using the HCAP studies from the US, England, and India, we evaluated variations of a classification algorithm based on neuropsychological norms. Variations of the algorithm included variables used to define a robust normative sample (e.g., depression, stroke history, previous diagnosis of dementia, basic and instrumental activities of daily living), characteristics adjusted for when standardizing cognition in the normative sample (e.g., age, sex, education, race/ethnicity, literacy), numbers and types of cognitive domains, and the choice of cut-offs of the components in classification algorithm such as cognitive scores, self-rated memory, and informant-rated impairment. Across each country, we varied these parameters. The overall prevalence of dementia ranged from 8% (India) to 9% (US, England). As expected, using a more liberal 1.0 standard deviation (SD) cutoff on cognitive tests rather than 1.5 SD led to higher dementia prevalence by 3-7%. Excluding orientation to time/place from the cognitive domains resulted in lower prevalence of dementia by 2-3% across countries but a markedly lower prevalence of Mild Cognitive Impairment (by 10% or more), whereas less restrictive criteria for defining a normative sample resulted in slightly lower prevalence of dementia (by 1-2%). This study underscores the importance of consistent methodologies for cross-national dementia prevalence estimates, and also informs the relative importance of certain modifications to criteria.

### SESSION 4385 (SYMPOSIUM)

Abstract citation ID: igae098.2083

### NOVEL METHODS, MODELS, AND METRICS FOR MEDICARE DATA TO UNDERSTAND RECOVERY AFTER HOSPITALIZATION FOR INJURY

Chair: Michelle Shardell
Discussant: Kristine Ensrud

Medicare is the U.S. federal health insurance program for adults aged ≥65 years and some younger persons with specific conditions. The Medicare Minimum Data Set (MDS) is a standardized clinical assessment tool administered by the Centers for Medicare and Medicaid Services to facilitate care management for residents in Medicare and Medicaid certified nursing homes. Medicare administrative claims and MDS databases provide opportunity to generate novel and impactful real-world evidence, but deriving accurate conclusions can be difficult due to measurement and research design challenges. In this symposium, we illustrate four methods to overcome these challenges in the context of understanding mechanisms of post-hospitalization recovery among older adults, consistent with meeting theme “The Fortitude Factor”. First, we describe a new data science method to overcome unmeasured confounding when identifying factors associated with post-hospitalization monthly days at home (DAH), a person-centered metric that reflects aging in place, among older adults with Alzheimer’s Disease and Related Dementia (ADRD) who experience a hip fracture. We next examine the role of contextual socioeconomic disadvantage in recovery from hip fracture using weighting estimation to mitigate survival bias. To overcome bias from informative observation times in the MDS, we apply a flexible joint model to examine time-varying factors associated with post-fracture physical function among older adults with ADRD. Lastly, we innovatively apply latent variable analysis to identify post-hospitalization DAH trajectories among older adults with traumatic brain injury. We discuss how the gerontology community can use these bias-reducing approaches for policy-relevant health research with Medicare data.

Abstract citation ID: igae098.2084

### DETERMINANTS OF RECOVERY IN THE US MEDICARE MINIMUM DATA SET: HANDLING INFORMATIVE OBSERVATION TIMES

Michelle Shardell, Chixiang Chen, and Jason Falvey, *University of Maryland School of Medicine, Baltimore, Maryland, United States*

Patients with Alzheimer’s Disease (AD) who experience a hip fracture are heterogeneous and understudied; thus, factors that influence these patients’ physical recovery are unknown. Moreover, identifying these factors can help inform personalized care for this population. To fill this gap, we use the US Minimum Data Set (MDS), a federally mandated standardized clinical assessment tool administered by the United States Centers for Medicare and Medicaid Services to facilitate care management for residents in Medicare and Medicaid certified nursing homes. Longitudinal assessments in these real-world data are irregular, and their timing is likely informative, which leads to bias if ignored. Therefore, we introduce and apply a novel joint model for longitudinal outcomes and observation times that includes a shared random effect. Data include 9,545 older adults (76.8% female, 55.7% aged 86+ years, 21.8% Medicaid eligible) in the MDS living with AD who were discharged from the hospital after hip fracture to Medicare and Medicaid certified nursing homes between 2017 and 2022. Barthel Index, a measure of physical function in activities of daily living, was the outcome (range: 0 to 90, higher scores indicate better function). Participants underwent 35,794 assessments over 100 days. Medicaid eligibility and lower cognition measured using the Brief Interview for Mental Status were more strongly associated with worse physical function in the proposed method than in a competing method ($p<.05$). Findings highlight 1) potential roles of socioeconomic status and heterogeneous cognition in AD in hip fracture recovery, and 2) importance of addressing informative observation times in the MDS.

Abstract citation ID: igae098.2085

### NEIGHBORHOOD SOCIOECONOMIC DISADVANTAGE AND RECOVERY AFTER HIP FRACTURE: EVIDENCE FROM REAL-WORLD MEDICARE DATA

Jason Falvey[1], Lindsey Mathis[2], Na Sun[3], and Amit Kumar[4], *1. University of Maryland School of Medicine, Baltimore, Maryland, United States*, *2. University of Maryland Baltimore, Baltimore, Maryland, United States*, *3. University of Maryland, Baltimore, Maryland, Maryland, United States*, *4. University of Utah, Salt Lake City, Utah, United States*

Neighborhood environments—or the exposome—are a powerful influence on function recovery among older adults. This study examines the influence of neighborhood socioeconomic disadvantage on the recovery trajectory of older adults following a fall-related hip fracture. Utilizing Medicare fee-for-service claims data, we identified 52,012 older adults with hip fractures via ICD-9 and ICD-10 codes, and identified fall-related mechanisms using validated E codes in claims. These individuals were linked to the Area Deprivation Index (ADI) using 9-digit ZIP codes, categorizing them into the top 10%, bottom 10%, and middle 80%. Days at home, a validated claims-based measure, was calculated for each participant over 6 months. We employed weighted generalized estimating equations with a Poisson distribution to account for the time-varying probability of death in each post-fracture month, and adjusting for age, sex, medical complexity, time (quadratic), and pre-fracture days at home to estimate rate ratios and predicted mean counts of days at home by ADI group. The cohort’s mean age was 82.1 years (SD = 8.1), with 74% female sex. In adjusted models, individuals in the lowest socioeconomic neighborhoods spent more than 7% fewer days at home (RR=0.93, 95% CI 0.91, 0.94) compared to their counterparts in the wealthiest 10%, translating to nearly 8 more days alive and in facilities—exceeding clinically important differences reported in prior work. The study highlights the need for tailored rehabilitation strategies and community support systems that address the unique barriers encountered by this vulnerable population, promoting equitable recovery outcomes across different socioeconomic contexts.

Abstract citation ID: igae098.2086

### A NEW ANALYTICAL METHOD FOR RISK FACTOR IDENTIFICATION IN ANALYZING LONGITUDINAL POSTFRACTURE RECOVERY OUTCOMES

Chixiang Chen, Michelle Shardell, and Jason Falvey, *University of Maryland School of Medicine, Baltimore, Maryland, United States*

Hip fracture is a highly disabling event experienced disproportionately by older adults with Alzheimer's disease or related dementias (ADRD). Older adults with ADRD are up to three times more likely than cognitively intact older adults to sustain a hip fracture, have worse functional outcomes, and spend more than 50 fewer days at home (DAH) in the year after fracture. Given the significant consequences of hip fracture among older adults, improving recovery for those with ADRD is a crucial national priority. However, the population of patients with ADRD is highly heterogeneous, hindering our ability to risk stratify patients and focus interventions on those at highest risk and with greatest likelihood of benefit. We develop a new data science approach to unbiasedly detect high-dimensional patient-level risk factors associated with less DAH, while existing toolboxes may fail to address potential biases from unmeasured hospital-level confounders, informative hospital size, and loss to follow-up in analyzing Medicare claims. Analysis of 16562 Medicare beneficiaries with ADRD, aged 65 or above, and experienced a hip fracture between 2017 and 2019 demonstrated that older age, longer length of stay in the hospital, heavier comorbidity burden, and less days at home before fracture are significantly associated less days spent at home in the 12-month period after hip fracture (p<.05), while existing methods fail to detect some effects. Findings demonstrate that the proposed method can overcome bias in using Medicare data and can lead to findings that may inform target for intervention to promote healthy aging.

Abstract citation ID: igae098.2087

### TRAJECTORIES OF DAYS AT HOME FOLLOWING TRAUMATIC BRAIN INJURY AMONG OLDER ADULTS

Jennifer Albrecht, Chixiang Chen, and Jason Falvey, *University of Maryland School of Medicine, Baltimore, Maryland, United States*

Traumatic brain injury (TBI) is a common fall-related injury among older adults that results in over 123,000 hospitalizations and 485,000 emergency department visits annually. While older adults have poorer recovery following TBI relative to younger adults, most older adults recover well from TBI. Identifying those at increased risk of poor recovery could inform appropriate management pathways, facilitate discussions about palliative care or unmet needs and permit targeted intervention to optimize quality of life or recovery. Time spent at home and not in a hospital or other health care facility each month is a patient-centered measure associated with improved outcomes. We identified unique trajectories of monthly home time following hospitalization for TBI among Medicare beneficiaries aged ≥65 using group-based trajectory modelling and identified characteristics associated with poor recovery. Among 20,350 Medicare beneficiaries who sustained TBI between 2010-2018, four unique trajectories of home time were identified: poor recovery (9.5%), improving recovery (13.7%), good recovery (66.4%), and declining recovery (10.4%). Recovery of monthly home time was complete for most by three months post injury. The strongest predictors of membership in the poor relative to the good recovery trajectory group were diagnosis of ADRD (odd ratio (OR) 2.42; 95% confidence interval (CI) 2.16, 2.72) and dual eligibility for Medicaid, a proxy for socioeconomic vulnerability (OR 5.13; 95% CI 4.59, 5.74). Future studies should seek to further characterize and investigate identified recovery groups to inform management and development of interventions to improve recovery.

### SESSION 4390 (PAPER)

### PLANNING AND SUPPORT FOR LATER-LIFE HEALTH AND ACTIVITY

Abstract citation ID: igae098.2088

### THE UNEXPECTED FAMILY CAREGIVER: UNVEILING THE CHARACTERISTICS OF YOUNGER ADULT CAREGIVERS

Katie Spears[1], Janelle Gore[2], Erin Bouldin[3], Akilah Ali[4], Jenny Walker[4], Benjamin Olivari[4], and Lisa McGuire[5], *1. Oak Ridge Institute of Science and Education, Georgia, United States*, *2. University of Alabama, Alabama, United States*, *3. University of Utah, Salt Lake City, Utah, United States*, *4. Centers for Disease Control and Prevention, Atlanta, Georgia, United States*, *5. GSA, Atlanta, Georgia, United States*

With the growing proportion of older adults in the US population, both the number of persons who will need care and the need for family caregivers will increase. Previous studies have found most caregivers to be middle-aged or older adults, but due to a widening gap in care needs and caregivers available, the importance of younger adult caregivers is growing. This analysis uses Behavioral Risk Factor Surveillance System data from the optional Caregiver Module administered in 2021-2022 in 47 states and Puerto Rico. We described the health characteristics of young adults aged 18-34 (n=45,627), who we classified as current caregivers, anticipated caregivers (expected to provide care in the next 2 years), and non-caregivers. We calculated weighted estimates and made comparisons using chi-square tests. Caregivers reported significantly higher prevalence of obesity (34.3%), depression (35.2%), and current cigarette smoking (17.2%) compared to anticipated caregivers and non-caregivers. Caregivers also reported a higher prevalence of frequent mental distress (14 days or more of poor mental health in the past month) (32.5%) compared to anticipated caregivers and non-caregivers. Most caregivers provided less than 9 hours of care a week (57.8%, 95% CI=54.7-60.9) and helped with personal care tasks (51%, 95% CI=47.9-54.1). Caregivers had more risk factors for chronic disease and poorer mental health than those not currently or anticipated to provide care. Public health efforts can recognize and include younger caregivers in their efforts to maximize physical and mental health for all caregivers to assure care recipients are well-supported.

Abstract citation ID: igae098.2089

**EFFECTS OF POSTRETIREMENT ACTIVITIES ON RETIREES' WELL-BEING: THE MODERATING ROLE OF CHILDLESSNESS**

Gloria Lin, Edwin Ka Hung Chung, Dannii Y. Yeung, Alvin Ho, and Amy Ng, *City University of Hong Kong, Hong Kong, China (People's Republic)*

According to role theory, retirees' work roles are likely diminished upon retirement, but their family and community roles are strengthened. Such pattern however may be different among childless retirees as their family roles are comparatively fewer than retirees with children. Postretirement activities, such as volunteering (Filges et al, 2020) and postretirement employment (Wang, 2007), have been shown to contribute to retirees' well-being as they compensate for the absence of important roles after retirement. Thereby, this cross-sectional study aimed to investigate whether the relationship between postretirement activities and well-being would be moderated by childlessness. Retirees' number of children, participation in postretirement activities (volunteering, postretirement employment, both activities, or none), life satisfaction, positive affect, depressive symptoms, and cognitive functioning were measured among 942 older Hong Kong Chinese retirees aged 65 and above (Mage = 71.76, SD = 5.28). The results of two-way ANCOVAs demonstrated significant main effects of postretirement activities and childlessness on most of well-being outcomes. A significant interaction effect of postretirement activities and childlessness on depressive symptoms was found, even after controlling for demographic variables. Specifically, childless retirees participated in volunteering (adj. Mdiff = –2.33, p =.001), postretirement employment (adj. Mdiff = –3.56, p =.001), or both activities (adj. Mdiff = –2.77, p =.010) showed fewer depressive symptoms than those with children. Our findings suggest that beneficial effects of postretirement activities are more salient among childless retirees than those with children, implying that participation in postretirement activities would be a protective factor in maintaining well-being after retirement.

Abstract citation ID: igae098.2090

**STRENGTHENING RETIREMENT PLANNING: A LONGITUDINAL EXAMINATION OF CONNECTEDNESS TO THE AGED FUTURE-SELF**

Dannii Y. Yeung, Gloria Lin, and Alvin Ho, *City University of Hong Kong, Hong Kong, China (People's Republic)*

The overall level of pre-retirement planning among working adults remains low. This study aims to identify effective strategies to strengthen younger and older workers' preparation for retirement. A total of 401 Hong Kong Chinese workers (170 younger and 231 middle-aged; M=42.34, SD=11.15) were randomly allocated to one of four experimental conditions: future-self with a writing task (FSW), future-self only (FS), current-self with a writing task (CSW), and current-self only (CS). In the experiment, participants in FSW and FS conditions were presented with an aged-morphed photo of their future-self, while those in CSW and CS conditions were presented with a photo of their current-self. The experiment was followed by a 5-day diary study, in which the participants in FSW and CSW wrote down their plans to improve well-being of their future-selves in old age or in three months, respectively. Retirement planning in financial, health and psychosocial domains were assessed at two weeks (T1) and at six months (T2) after the experiment. Preliminary analyses show that participants in FS and FSW reported more negative emotions after viewing the future-self photo ($F(3,396)=10.42$, $p<.001$). A significant age group by condition interaction effect ($F(3,317)=2.82$, $p=.039$) was found in T1 retirement planning activities, with older workers in FSW reported more preparatory activities than younger workers in FSW, while such age effect was not observed in FS, suggesting that the 5-day writing task could strengthen one's preparation for retirement. However, such age discrepancy effect diminished in T2. Implications on promotion of retirement planning will be discussed.

### SESSION 4395 (SYMPOSIUM)

Abstract citation ID: igae098.2091

**PRESIDENTIAL SYMPOSIUM: PRESIDENTIAL POLITICS AND THE FORTITUDE FACTOR: EMPOWERING OLDER ADULTS IN TODAY'S POLITICAL LANDSCAPE**

Chair: Tetyana Shippee
Discussant: Brian Kaskie

In the backdrop of a presidential election, the United States faces a critical issue: a diminishing unity within the advocacy community dedicated to addressing the needs of an aging population. While acknowledging past successes in securing significant public investments for older adults, there is a noticeable decline in advocacy cohesion as the older adults demographic continue to grow. Presenters highlight the need for renewed advocacy efforts, emphasizing intergenerational collaboration, to compel presidential candidates to address aging policy agendas. Presenters also challenge the reliance on individual fortitude in aging policies, arguing that systemic supports are essential to equitable outcomes. They advocate for rethinking aging policies, emphasizing collective action and alternative organizing principles, especially in light of the complex challenges faced by older adults.

Abstract citation ID: igae098.2092

**WHERE HAVE ALL THE ADVOCATES GONE?**

Robyn Stone, *LeadingAge, Washington, District of Columbia, United States*

We face a serious challenge as we enter this presidential election season: the lack of a unified advocacy community to support a coherent national political platform that needs to be addressed around the aging of the U.S. population over the next 15 years and into the future. This presentation briefly summarizes the history of the modern advocacy movement to improve the lives of older adults who were more likely than any other age group to be living in poverty in the 1960s and 1970s. The advocacy organizations were strong and united, helping to secure huge public investments including Medicare and Medicaid, affordable senior housing and the establishment of an aging network. Strong champions on Capitol Hill and in the executive branch supported these policies and investments. Ironically, as the older adult population is living longer, and the baby boomer population has grown larger than those under age 18, the advocacy voice has either disappeared or has become diffuse and less powerful. We need new advocacy efforts that bring younger and older generations together to advocate for an aging policy agenda that both presidential candidates are forced to acknowledge and address. The climate change advocacy approach that calls for short and long-term solutions to benefit current and future generations may provide a good framework for an intergenerational aging movement. When generations across age, racial/ethnic identity and other diverse groups (including class) see they are all in this together, a collective fortitude can emerge.

Abstract citation ID: igae098.2093

### THE LIMITS OF FORTITUDE: PRINCIPLES FOR ADVANCING EQUITABLE AGING POLICY

Jane Lowers, *Emory University, Atlanta, Georgia, United States*

Fortitude – an individual's ability to endure difficulty or adversity – is an implicit premise in U.S. aging policies, which require individuals and families to shoulder the financial, physical, and emotional costs of aging. Emphasizing fortitude in any discussion of aging obscures the assumption that it works best when accompanied by a lifetime accumulation of wealth and other tangible and intangible supports. When systemic supports for aging are fractured and inadequate, no amount of individual fortitude can adequately bolster older adults challenged by fixed incomes, decreased availability of family caregivers, and lengthening years with chronic and progressive illness. Using examples of adults aging solo with dementia, this presentation will propose alternatives to fortitude as organizing principles for rethinking U.S. aging policy.

Abstract citation ID: igae098.2094

### PRESIDENTIAL POLITICS AND THE FORTITUDE FACTOR

Brian Lindberg, *Gerontological Society of America, Washington, District of Columbia, United States*

Education, engagement, advocacy, and coalition building are key elements of GSA's ongoing work in the nation's capital. Election years provide both opportunities and challenges for working in the legislative and regulatory processes, and GSA staff and our members across the country have become effective in sharing the research, policy development, and practice experiences with policymakers in DC. Although the field may face challenges in "advocacy cohesion," this presentation will include examples of the continued strength of aging advocates in the face of ageism and generational competition for resources. GSA's intergenerational approach to engagement and our recent policy initiatives will be discussed. The presentation will include examples of the strength of aging advocacy organizations and their ability to successfully protect federal programs facing proposed cuts or elimination and securing billions of dollars in additional resources during the COVID-19 pandemic. While the timing is not good for large new entitlement programs, coalitions have been successful in improving benefits such as home delivered meals, tackling the social isolation crisis, providing resources for caregivers, and reducing the cost of Medicare prescription drugs and more. GSA is often providing the evidence to support these positive steps.

### SESSION 4400 (PAPER)

### SOCIAL DETERMINANTS OF COGNITIVE FUNCTION

Abstract citation ID: igae098.2095

### HIGH IMPACT ACTIVITIES: ASSESSING THE ASSOCIATION BETWEEN SOCIAL PARTICIPATION FREQUENCY AND COGNITION

Eric Vogelsang[1], and Sara Moorman[2], *1. California State University San Bernardino, San Bernardino, California, United States*, *2. Boston College, Chestnut Hill, Massachusetts, United States*

There is growing recognition that social participation may help attenuate cognitive decline in older ages. Unfortunately, previous empirical research often relies on social participation "summary indexes", which may obfuscate associations between cognition and specific social activities. Moreover, there has been little consideration as to whether increased social participation frequency is associated with greater cognitive functioning. We utilize two nationally representative longitudinal surveys of older adults: the Health and Retirement Study (21,705 observations across six waves) and the National Social Life, Health & Aging Project (5,317 observations across two waves). Regression models test how associations between social participation frequency and three cognition measures (a) differ by summary indexes; and (b) differ among specific social activities. We also compare and contrast the ways in which social participation is evaluated in these studies. When using summary participation indexes, for both datasets, each additional social activity and each increase in activity frequency was associated with greater cognitive functioning. However, when segregating social activities, we found disparate associations with cognition. Specifically, volunteering (in one dataset) and social/club membership (in the other) were the only activities whose frequency was positively associated with each cognition measure. Committing to regular social and community activity in older ages may help mitigate the risk of cognitive decline. However, caution is advised when interpreting the health effects of social activity counts or aggregate frequency measures. Surveys would benefit by establishing consistent social participation measurement standards.

Abstract citation ID: igae098.2096

### ADVERSE CHILDHOOD EXPERIENCES AND LATER LIFE COGNITION: THE MISSING COMPONENT OF MORAL DEVELOPMENT

Rachel Missell-Gray[1], Alexander Eustice-Corwin[1], and Silvia Sörensen[2], *1. University of Rochester, Rochester, New York, United States*, *2. University of Rochester, Warner School of Education and Human Development, Rochester, New York, United States*

Recent empirical studies on the relationship between Adverse Childhood Experiences (ACEs) and later life cognition have found an inconsistent association between these two variables. Some studies have determined that a higher number of ACEs are associated with decreased cognitive performance later in life (ages 50 and up), while others have determined that there is no relation. Despite these ambiguous findings, both theory and empirical research provide us with reasons to believe that a relationship between ACEs and later life moral cognition is likely. In the current paper, we argue that self-reported ACEs likely impact older adult moral identity and develop a theoretical framework to guide directions for future research. First, we will summarize the findings of the empirical literature on ACEs and later life cognition. Second, we will address the limitations of these findings. Third, we will advocate in favor of a neo-Aristotelian framework for the study of moral development, which foregrounds the role of practical wisdom (phronēsis) as an alternate conception of a person's character over the course of the human lifespan. We also argue that such hardships in early life may lead to increased wisdom, which does not necessarily correlate with the measures of cognitive functioning often applied in late-life cognition studies. We hypothesize that the presence of ACEs may influence the development of practical wisdom. We argue that if ACEs predict decreased cognitive function later in life, then this provides us with the grounds for thinking that ACEs might influence moral development in older adults.

Abstract citation ID: igae098.2097

### MARITAL DISSOLUTION AND COGNITIVE FUNCTION IN MID- AND LATE ADULTHOOD: FOCUS ON WITHIN-PERSON EFFECTS AND AND TIMING

Julia Tucker[1], Mateo Farina[2], and Sae Hwang Han[2], *1. The University of Texas Austin, Austin, Texas, United States*, *2. University of Texas at Austin, Austin, Texas, United States*

There is a growing literature documenting cognitive health disparities between married individuals and their widowed and divorced counterparts in middle and later adulthood. However, how cognitive function changes within individuals as they experience widowhood and divorce transitions, and whether these changes vary depending on the timing of the marital dissolution, remain understudied. Framed within the life course perspective, this study aimed to examine within–person changes in cognitive function associated with widowhood and divorce transitions in mid– and later life, respectively. We used 11 waves of longitudinal data from the Health and Retirement Study (1998–2018; 121,505 person–wave observations collected from 20,114 persons). Cognitive function was evaluated using a modified version of the Telephone Interview for Cognitive Status (range: 0–27). Multilevel models using person–mean centering were employed to estimate within–person associations between the two forms of marital transitions and subsequent cognitive function. Results indicated that widowhood transitions were associated with within-person changes in cognitive function for women, but in the opposite direction depending on the timing of the transition: midlife widowhood transitions (between ages 51 and 64) were linked to worse cognitive function, whereas late-life widowhood transitions (age 65+) were associated with better cognitive function. Widowhood transitions were unrelated to cognitive function for men, and divorce transitions were unrelated to changes in cognitive function for men or women. These results underscore the heterogeneity in the association between marital transitions and cognitive function, which vary by gender, life course stage, and the nature of marital dissolution.

Abstract citation ID: igae098.2098

### ENRICHING EARLY-LIFE ACTIVITIES AND LATER-LIFE COGNITION: THE MODERATING ROLE OF MULTIPLE SOCIAL POSITIONS

Addam Reynolds, *University of Southern California Leonard Davis School of Gerontology, Los Angeles, California, United States*

Mounting evidence suggests that associations between early life factors and later life cognition are not universal across racialized populations. While evidence suggests that enriching early life activities (EELA) are protective for cognitive aging, no study has evaluated if this association is equally salient for older Black adults across the socioeconomic gradient. Using data from the Life History Mail Survey in the Health and Retirement study, we employ a growth curve analysis to estimate the association between EELA and later life cognition among non-Hispanic White and Black older adults. We also test for moderating effects to assess the relative importance of race and childhood socioeconomic status (cSES) in this association. I found evidence of a three-way interaction between, EELA, cSES and race ($b = 0.26$, $p <.05$). Among non-Hispanic White older adults, we observed a stronger relationship between EELA and baseline cognition among those with lower levels of cSES. Among Black participants, we observed the opposite effect. Among Black participants with one standard deviation below the mean of cSES, there was no effect of EELA on baseline cognition. This study suggests that associations between EELA and later life cognition are conditional on multiple social positions, rather than being universal across populations. While White individuals with lower cSES have the most to gain from EELA, Black individuals from similar backgrounds do not benefit from EELA. The implication of this finding suggests that EELA may buffer against the negative effects of lower cSES among White, but not Black participants.

Abstract citation ID: igae098.2099

### ENGLISH PROFICIENCY, IMMIGRATION STATUS, AND COGNITIVE ASSESSMENT VARIABILITY IN OLDER ADULTS

Katherine Kitchens, *The University of Texas Arlington, Arlington, Texas, United States*

Recent studies provide evidence that immigration status is a risk factor for developing dementia among older adults and that English proficiency moderates this relationship. This study aimed to analyze the factor structure of the National Health and Aging Trends Study's (NHATS) cognitive battery to investigate whether this structure varies by English proficiency among the Medicare population and to assess the association between English proficiency, immigration status, and cognition. Utilizing the 2021 NHATS data from a nationally representative sample of Medicare beneficiaries ($N = 5{,}628$), a multi-group confirmatory factor analysis (CFA) was used to examine the factor structure of the cognition battery by English proficiency. Three multivariable linear regression models tested the relationship between English proficiency, immigration status, and a standardized cognition score weighted by factor loadings. The multi-group CFA revealed variations in factor loadings and error variances, suggesting differences in how cognition is captured among individuals with varying English proficiency. English proficiency emerged as a significant predictor of better cognitive z-scores, independent of immigration status (b = 0.70, p <.001). Variance in latent cognition construct scores across groups suggests that English proficiency impacts cognitive assessment accuracy, possibly underestimating the cognitive abilities of those with lower English proficiency due to language or cultural barriers. The association between English proficiency, detached from immigration status, counters the perception of immigration status as a cognitive risk factor and calls for cognitive assessments that are culturally and linguistically appropriate and nuanced public health strategies that address diverse immigration experiences and social determinants.

### SESSION 4405 (SYMPOSIUM)

Abstract citation ID: igae098.2100

### STRESS PROCESSES IN AGING: CONSIDERING SOCIAL CONTEXT AND SELF-PERCEPTIONS

Chair: Kyrsten Hill

How individuals perceive, appraise, and cope with their stressors holds dramatic influence on whether they experience positive aging trajectories. However, stress-related processes are inherently contextual, reflecting an interaction between the person and the environment. The current series of talks considers how individuals deal with stress across different time scales. The first two talks will consider stress in a social context, evaluating the roles of daily loneliness and interpersonal stressors. First, Kang will describe findings from an EMA study suggesting a reciprocal relationship between momentary loneliness and cognitive performance. Second, Ong will report on an EMA study examining the relationship between self-perceptions of aging and daily social interactions. The final two talks will consider adults' perceptions of their ability to deal with stressors, considering control beliefs and coping processes over the short- and long-term. Third, Neupert will discuss how daily stressors and daily control beliefs are associated with the perceived rate at which people feel that they are aging. Finally, Kurth will discuss how coping strategies change longitudinally over 10 years and how the rate and direction of change depends on age. Together, this symposium highlights how stress processes influence aging across individuals, time courses, and contexts.

Abstract citation ID: igae098.2101

### SHORT-TERM COUPLING ASSOCIATIONS BETWEEN STATE LONELINESS AND COGNITIVE PERFORMANCE IN DAILY LIFE

Jee eun Kang[1], Jennifer Graham-Engeland[2], Lynn Martire[1], David Almeida[2], and Martin Sliwinski[2], *1. The Pennsylvania State University, University Park, Pennsylvania, United States*, *2. Pennsylvania State University, University Park, Pennsylvania, United States*

Although loneliness and neurocognitive health have been linked, little attention has been given to associations between fluctuations in state loneliness and cognitive performance. This work examines the association between within-person variation in state loneliness and cognitive performance assessed objectively in daily life, focusing on the timing and direction of the relationship. Participants from a wave of the Einstein Aging Study (EAS) were included (N=313, Mage=78 [range=70-90], 68% women, 46% White, 41% Black; average 15 years education). Over a 14-day period, participants engaged in ambulatory ecological momentary assessment burst using mobile phones, completing self-reports (including about feeling lonely at the moment) and mobile cognitive tests up to five times daily. The mobile cognitive tests assessed visual associative memory, processing speed, and spatial memory. Multilevel modeling was employed to examine day-to-day and moment-to-moment changes in loneliness and their relationship with cognitive performance, with a specific focus on concurrent, time-lagged, and reverse-lagged associations. Results revealed that higher daily loneliness negatively correlated with cognitive performance on the same day and predicted worse performance the following day, illustrating a prospective association. Within a single day, momentary loneliness negatively correlated with immediate cognitive performance, but did not predict later decreases; however, lower cognitive performance in a given moment predicted elevated loneliness later in the day. Overall, these findings highlight a complex, reciprocal relationship – loneliness predicting and being predicted by cognitive performance depending on timescale. Deepening our understanding of such temporal dynamics will be important to inform ways to break the cycle between loneliness and cognition.

Abstract citation ID: igae098.2102

### SELF-PERCEPTIONS OF AGING AND DAILY SOCIAL INTERACTIONS

Lydia Ong[1], Patrick Klaiber[2], Nicole Stuart[1], Anita DeLongis[1], and Nancy Sin[1], *1. University of British Columbia, Vancouver, British Columbia, Canada*, *2. Tilburg University, Tilburg, Noord-Brabant, Netherlands*

Age stereotypes can become internalized across the lifespan to influence one's self-perceptions of aging (SPA). Stereotype embodiment theory suggests behavioral and stress response pathways through which SPA influence downstream health outcomes. Previous research has shown negative SPA predict lower social engagement, but little is known about whether this mechanism is evident at the daily level. Thus, the current study uses ecological momentary assessment over 14 days to investigate whether more negative SPA are associated with fewer interpersonal stressors and positive social interactions, and whether SPA attenuate differences in positive and negative affect on moments with versus without these events. Participants were 224 adults from British Columbia, Canada (ages 25-89, M = 46, 71% women). Multiple regression and three-level multilevel models controlled for demographic factors, depressive symptoms, and health conditions. Results showed that SPA did not predict exposure to interpersonal stressors and positive social interactions. People with more negative SPA had larger differences in negative affect on moments with versus without interpersonal stressors (simple slope for -1 SD SPA: b =.99, SE =.13, p <.001), compared to those with less negative SPA

(simple slope for +1 SD SPA: b =.60, SE =.14, p <.001). SPA did not moderate associations between interpersonal stressors and positive affect, or between positive social interactions and positive and negative affect. Findings indicate that people with more negative SPA do not show a lack of daily social engagement but display greater negative affective stress responses, which have implications for downstream health.

Abstract citation ID: igae098.2103

### DAILY STRESSORS AND DAILY CONTROL BELIEFS ARE ASSOCIATED WITH DAILY ACCELERATED AGING

Shevaun Neupert, and Kathryn Swaim, *North Carolina State University, Raleigh, North Carolina, United States*

Exposure to daily stressors is consistently associated with reduced health and well-being, including feeling subjectively older. Experiencing increases in daily perceptions of control, however, are typically associated with increased health and well-being and feeling subjectively younger. The current study focuses on subjective accelerated aging from a daily perspective, capturing day-to-day fluctuations in the perceived rate at which people feel that they are aging. The goal of the current study was to bring together daily stressors and daily perceptions of control to test their independent contributions to the understanding of daily accelerated aging. A sample of 440 U.S. adults aged 50-85 participated in a 14-day daily diary study, reporting on their perceived rate of aging, exposure to daily stressors, and perceptions of daily control beliefs each day. All variables demonstrated significant within-person variability, with ICCs ranging from.60 (rate of aging) to.78 (control beliefs). Daily stressors and daily control beliefs were each uniquely predictive of daily accelerated aging, where daily stressors were positively associated and daily control beliefs were negatively associated with daily accelerated aging. These findings underscore the dynamic and contextual nature of subjective aging metrics and document the within-person coupling between events and beliefs.

Abstract citation ID: igae098.2104

### AGE DIFFERENCES AND CHANGE IN COPING STRATEGIES ACROSS 10 YEARS: FINDINGS FROM THE MIDUS STUDY

Maria Kurth[1], Dakota Witzel[2], Eric Cerino[3], and David Almeida[4], *1. The Pennsylvania State University, University Park, Pennsylvania, United States*, *2. South Dakota State University, Brookings, South Dakota, United States*, *3. Northern Arizona University, Flagstaff, Arizona, United States*, *4. Pennsylvania State University, University Park, Pennsylvania, United States*

Although age differences in coping strategies across adulthood are well-established (Aldwin et al., 2021), little is known about how coping strategies change, especially into later life. Further, studies focused on changes in coping relied on convenience samples (e.g., Brennan et al., 2012; Martin et al., 2008), which may not generalize to all midlife and older adults. Using data from waves two and three of the Midlife in the United States study, we examined (1) how coping changed over 10 years and (2) age differences therein. Participants (N = 2661, Mage_waveII = 54.56, age range: 30-84, 58% women, 87% White) completed 24 items from the COPE Inventory (Carver et al., 1989) at each wave. Four coping factors emerged from an exploratory factor analysis and confirmed via confirmatory factor analysis (Witzel & Kurth, 2023): instrumental action; denial/disengagement; positive reappraisal; focus/venting of emotions. Invariance was established across time and age. Two-level multilevel models, conducted separately for each coping strategy, revealed a significant decrease across 10 years for all strategies, even after controlling for race, gender, education, and sample type (core, Milwaukee). Older participants, compared to younger participants, decreased in their use of instrumental action and positive reappraisal, while denial/disengagement strategies increased; age did not moderate focus/venting of emotions. Preliminary evidence suggested non-linear changes by age for denial/disengagement and positive reappraisal. Discussion will surround the variability in how different types of coping changes across midlife and into older adulthood, and ways in which the rate and direction of this change depends on age.

### SESSION 4410 (SYMPOSIUM)

Abstract citation ID: igae098.2105

### THE HEART OF DAILY LIFE: THE INTERPLAY BETWEEN EVERYDAY AFFECTIVE STATES, DAILY EVENTS, AND SUBJECTIVE BELIEFS

Chair: Patrick Klaiber
Co-Chair: Theresa Pauly
Discussant: Stacey Scott

Affective experiences profoundly shape well-being and quality of life throughout adulthood. They are intricately intertwined with social engagement and daily experiences like stressors and positive events. Individuals' internal beliefs regarding aging and emotions can either facilitate or impede their engagement with daily events, and further impact emotional responses and regulation. Moreover, fluctuations in affect resulting from these daily experiences may cumulatively influence long-term mental and cognitive health outcomes. This symposium illuminates these dynamics by utilizing a set of daily diary studies using lifespan and older adult samples. The first presentation by Witzel et al. connects experiencing more frequent positive events and more favorable affective reactions to these events with better cognitive health. Klaiber and Pauly find that on days on which people feel older than usual, they experience more stressors, and fewer positive events, but also tend to show more favorable affective responses to these positive events. Thirdly, Pauly shows that positive affect mediates the association between social interaction time and decreased subjective age. Finally, Rutledge and Barlow shed light on differential associations between emotion beliefs and affect in younger vs. older individuals. Together, these studies offer valuable insights into the complex interplay between daily experiences, beliefs, affective fluctuations, and long-term health outcomes in adulthood. Stacey Scott will integrate the insights gained from these four papers, discuss their potential and limitations, and consider directions for future research.

Abstract citation ID: igae098.2106

### INVESTIGATING THE DAILY RELATIONSHIP BETWEEN SUBJECTIVE AGE AND DAILY EVENTS

Patrick Klaiber[1], and Theresa Pauly[2], *1. Tilburg University, Tilburg, Noord-Brabant, Netherlands*, *2. Simon Fraser University, Vancouver, British Columbia, Canada*

Subjective Age, also known as felt age, refers to individuals' perception of their age compared to their chronological age. Daily fluctuations in subjective age are linked to daily affective experiences; feeling older than usual has been associated with experiencing more same-day stressors and higher negative affect. Feeling older than usual may indicate depleted psychosocial resources, increasing the likelihood of interpreting everyday situations as stressful and reacting more intensely to them. Conversely, feeling younger than usual may indicate greater psychosocial resources, leading to increased engagement in and responsiveness to positive events. Thus, this study investigated fluctuations in subjective age as a predictor of same-day event engagement (stressors and positive events) and affective responses to these events using 14-day diary data from a sample of 108 older Swiss adults (aged 65-92). On average, participants felt approximately 8 years younger than their chronological age, with significant day-to-day variability (ICC =.69). We found that on days when individuals felt older than usual, they reported more stressors and fewer positive events. Older subjective age was also associated with lower same-day positive affect, but also greater increases in positive affect when positive events occurred. Similarly, older subjective age was related to greater same-day negative affect, yet also to more pronounced decreases in negative affect when positive events occurred. However, stress reactivity, as indicated by stressor-related affect, was not associated with subjective age. These findings underscore the importance of subjective age as a dynamic psychological construct potentially shaping individuals' daily affective experiences.

Abstract citation ID: igae098.2107

### THE INTERPLAY OF SOCIAL INTERACTION, POSITIVE AFFECT, AND SUBJECTIVE AGE IN OLDER ADULTS

Theresa Pauly, *Simon Fraser University, Vancouver, British Columbia, Canada*

How old people feel compared to their actual age, their so-called "subjective age" (SA), is a central predictor of health and well-being across the life span. Felt age can influence lifestyle choices such as attending a social gathering. On the other hand, spending time with other people can elicit feelings of engagement and positive emotions, which could in turn, make people feel younger. The current study aimed to examine the reciprocal association between everyday time spent in social interaction and subjective age in old age. For this purpose, a sample of 108 older adults aged 65–92 years took part in a daily diary study. Over 14 days, participants reported daily social interaction time, positive affect, and subjective age. Multi-level models showed that previous day social interaction time was related to next day subjective age, whereas previous-day subjective age was not related to next-day social interaction time. In addition, the same-day association between social interaction time and subjective age was fully mediated by positive affect. Findings suggest that social connections might foster a sense of feeling young in older adulthood. Further research in this area may offer valuable insights into developing interventions aimed at enhancing subjective well-being and quality of life among older populations.

Abstract citation ID: igae098.2108

### HELPING OR HINDERING: ASSOCIATIONS BETWEEN DAILY POSITIVE EXPERIENCES AND COGNITIVE PERFORMANCE

Dakota Witzel[1], Zachary Taylor[2], Eric Cerino[2], Robert Stawski[3], and David Almeida[4], *1. South Dakota State University, Brookings, South Dakota, United States*, *2. Northern Arizona University, Flagstaff, Arizona, United States*, *3. University of Essex, Colchester, England, United Kingdom*, *4. Pennsylvania State University, University Park, Pennsylvania, United States*

Daily stressors have significant effects on cognitive health across the lifespan, but little is known about how daily positive experiences may facilitate better cognitive performance. Leveraging the third wave of a publicly available adult lifespan sample who completed a telephone assessment of cognitive function (i.e., executive function, episodic memory) and an eight-day daily diary protocol (n=1,172; 55.76%Women, Mage=62.71), we examined how the exposure, number of, and affective reactivity to daily positive experiences were related to executive function and episodic memory. Models adjusted for daily stressor exposure, age, education, marital status, and gender. Person means for exposure and number of positive experiences, as well as negative (NAR) and positive (PAR) affective reactivity (decreases or increases in negative or positive affect, respectively) were calculated across eight days. Linear regressions suggest that having a higher proportion of days with positive experiences, and a higher number of positive experiences were related to better executive function and episodic memory ($ps<.05$). Higher NAR to the exposure to and number of daily positive experiences was related to better executive function; only NAR to the exposure to daily positive experiences were related to better episodic memory ($ps<.05$). Higher PAR to the exposure to and number of daily positive experiences was related to poorer episodic memory only ($ps<.05$). Discussion will focus on the complex differential associations among affective reactions to positive experiences and cognitive function. Future directions should evaluate possible non-linear effects of reactivity and mechanisms underlying associations between daily positive experiences and cognitive health across the lifespan.

Abstract citation ID: igae098.2109

### EMOTION BELIEFS AND DAILY AFFECTIVE EXPERIENCES ACROSS THE ADULT LIFESPAN

Jocelyn Rutledge, *Wilfrid Laurier University, Waterloo, Ontario, Canada*

Emotion beliefs refer to the extent to which people believe emotions "should" and "can" be controlled. Importantly, past research has linked peoples' emotion beliefs to the extent to which they utilize effective emotional regulation strategies, and psychological well-being. To our knowledge, research has yet to examine the association between emotional beliefs and affective experiences at the daily-level, and how developmental factors are associated with emotion control beliefs in adults. The present research sought to examine age differences in within-person associations of daily emotion beliefs and daily positive and negative affect. To do so, we used 14-day daily diary data from an adult lifespan community sample [N = 91, age range = 19-92]. Multilevel models revealed that when younger (but not older) people endorsed higher-than-normal beliefs they "should" control their emotions they reported greater negative affect, while higher-than-normal beliefs they "can" control their emotions were associated with higher positive affect. In addition, when older (but not younger) people endorsed higher-than-normal beliefs that they "can" control their emotions they experienced greater declines in negative affect. These findings add to theory and research on emotional aging by highlighting that: 1) emotion beliefs are differentially associated with affective experience on a daily-level, and 2) these associations change across the adult lifespan.

### SESSION 4415 (SYMPOSIUM)

Abstract citation ID: igae098.2110

### TRANSITIONAL PALLIATIVE CARE FOR RURAL FAMILY CAREGIVERS USING VIDEO TECHNOLOGY: RECRUITMENT, METHODS, AND FINDINGS

Chair: Joan Griffin

People with severe, life-limiting illnesses face serious risks for adverse events and symptom escalation when transitioning care from hospital to home. When new approaches to care, such as palliative care, are introduced before discharge, these risks increase further. Family caregivers (FCG) often help manage care during these transitions and ensure care recipient safety and quality of care after discharge. These new or added responsibilities can strain FCG health and well-being, thus compromising FCG and care recipient health. This is especially true for FCGs in rural and underserved areas who typically have fewer resources and limited access to care. This symposium presents lessons learned, methodological challenges, and findings from a FCG study aimed at addressing transitional care and palliative care on FCGs health and well-being. The intervention tested a model of care to address unmet needs of FCGs caring for seriously ill and elderly patients in rural areas through teaching, guidance, and counseling via video visits. Preliminary findings show this intervention improves FCG outcomes and is cost-effective. The first presentation provides an overview of the trial (Griffin). The second presentation describes challenges with recruiting and differences between recruiting FCG participants in-person and remotely (Gustafson). The third presentation includes findings on internet connectivity when conducting a virtual clinical trial with FCGs living in rural and underserved areas (Holland). The fourth presentation describes study findings on FCG and care recipient health and well-being (Griffin). The fifth presentation includes a discussion of the differences in caregiving and utilization costs associated with the intervention (Kaufman).

Abstract citation ID: igae098.2111

### A TECHNOLOGY-BASED TRANSITIONAL PALLIATIVE CARE INTERVENTION FOR FAMILY CAREGIVERS IN RURAL OR UNDERSERVED AREAS

Joan Griffin[1], Diane Holland[1], Catherine Vanderboom[1], Jay Mandrekar[1], Brystana Kaufman[2], Allison Gustavson[3], and Cory Ingram[1], *1. Mayo Clinic, Rochester, Minnesota, United States*, *2. Duke University School of Medicine, Durham, North Carolina, United States*, *3. Minneapolis Veterans Affairs Health Care System, Minneapolis, Minnesota, United States*

In this presentation, Dr. Griffin will describe the Technology-Enhanced Transitional Palliative Care (TPC) study, a randomized controlled trial testing the efficacy and cost-effectiveness of an intervention to address FCGs' unmet needs in order to improve their health and well-being. Nurse interventionists use video visits to teach, guide and counsel FCGs in rural areas caring for someone receiving palliative care and transitioning form hospital to home. In addition to providing background on the importance of addressing unmet family caregiver needs during care transitions for patients receiving palliative care, she will describe the study design, sample, intervention and control group procedures, including the use of video visits, and outcomes assessed. This presentation will provide necessary context for the remaining presentations.

Abstract citation ID: igae098.2112

### RECRUITING FAMILY CAREGIVERS FOR INTERVENTIONS DESIGNED TO OPTIMIZE TRANSITIONS FROM HOSPITAL TO HOME

Allison Gustavson[1], Joan Griffin[2], Molly Horstman[3], Brystana Kaufman[4], Jay Mandrekar[2], Catherine Vanderboom[2], Cory Ingram[2], and Diane Holland[2], *1. Minneapolis Veterans Affairs Health Care System, Minneapolis, Minnesota, United States*, *2. Mayo Clinic, Rochester, Minnesota, United States*, *3. Baylor College of Medicine, Houston, Texas, United States*, *4. Duke University School of Medicine, Durham, North Carolina, United States*

Recruiting family caregivers to participate in research, especially caregivers of people with severe or life limiting illnesses transitioning from hospital to home, is challenging. Traditional approaches for recruitment, including recruitment done on site at clinics or hospitals, became impracticable during the COVID-19 pandemic, leading to shifts toward virtual or remote recruitment and consent. In this presentation, Dr. Gustavson will compare the success of these two methodological approaches, in-person and remote recruitment and consent, used in the Technology-Enhanced Transitional Palliative Care (TPC) study. She found that the rate of recruitment and consent done in-person was 28% and the rate of remote recruitment and consent was 23%. Those recruited and consented remotely were more likely to be younger, white, have greater than high school education, and were children of or had another non-spousal relationship to the care recipient. She discusses the limitations and benefits of each recruitment modality, including the impact on representativeness of the study sample.

Abstract citation ID: igae098.2113

### HEALTH CARE USE AND OUT-OF-POCKET COSTS FOR RURAL FAMILY CAREGIVERS AND CARE RECIPIENTS IN TRANSITIONAL PALLIATIVE CARE

Brystana Kaufman[1], Ro Huang[2], Diane Holland[3], Catherine Vanderboom[3], Jay Mandrekar[3], Allison Gustavson[4], and Joan Griffin[3], *1. Duke University School of Medicine, Durham, North Carolina, United States*, *2. Duke University, Durham, North Carolina, United States*, *3. Mayo Clinic, Rochester, Minnesota, United States*, *4. Minneapolis Veterans Affairs Health Care System, Minneapolis, Minnesota, United States*

Few studies have reliable estimates on costs of informal care following hospitalization, including health care use and out-of-pocket costs for family caregivers (FCGs) and their care recipients (CR). This presentation discusses results from a Transitional Palliative Care (TPC) intervention on healthcare use and out-of-pocket spending for both FCGs and CRs. The study used data from the Technology-Enhanced TPC study, a trial comparing the TPC intervention to support FCGs of seriously ill CRs to an attention control condition. TPC FCGs received teaching, guidance, and counseling via video calls for 8 weeks following CR hospital discharge. After discharge, a research assistant called FCGs once a month for up to 6 months or CR death to collect healthcare utilization (e.g. outpatient, emergency department, and hospital), out-of-pocket healthcare spending (e.g. deductibles and coinsurance), and health-related travel costs (e.g., transportation, lodging, food) for FCGs and CRs. Incidence rate ratios (IRR) were estimated using negative binomial regressions. The study included 282 FCG-CR dyads across 3 US states. TPC reduced nights in the hospital for CR (IRR = 0.75; 95% CI = 0.56 – 0. 99). Total out-of-pocket spending was not significantly different for TPC versus control; however, TPC dyads reported lower lodging costs (IRR = 0.71; 95% CI = 0.56-0.89). Across groups, mean out-of-pocket spending for dyads was $1401.85, with healthcare payments contributing $1048.58 and transportation expenses contributing $136.79. This study contributes to evidence that palliative care interventions reduce unnecessary hospitalization for seriously ill patients, yet the financial costs to caregivers remain high.

Abstract citation ID: igae098.2114

### CONNECTIVITY ISSUES WHEN CONDUCTING A VIRTUAL CLINICAL TRIAL WITH FAMILY CAREGIVERS IN RURAL OR UNDERSERVED AREAS

Diane Holland[1], Catherine Vanderboom[1], Jay Mandrekar[1], Allison Gustavson[2], Brystana Kaufman[3], Joan Griffin[1], Ellen Wild[1], and Ann Marie Dose[1], *1. Mayo Clinic, Rochester, Minnesota, United States*, *2. Minneapolis Veterans Affairs Health Care System, Minneapolis, Minnesota, United States*, *3. Duke University School of Medicine, Durham, North Carolina, United States*

In the research literature and amongst grant reviewers, there are persistent questions about whether internet connectivity challenges, especially in rural populations, will limit the potential for virtual or decentralized clinical trials to improve participation of underrepresented groups. In clinical practice, e-health and telehealth advancements have been touted as potential models of care to address health disparities associated with access to care, but without reliable internet connectivity, these disparities may perpetuate or even widen. In this presentation Dr. Holland examines the prevalence of connectivity problems during the Technology-Enhanced Transitional Palliative Care (TPC) study, a virtual clinical trial across three Midwestern states, and provides guidance on the challenges faced. Data are from structured notes from nurse interventionists who documented any connectivity issues in the study's electronic health record. Of the 1003 visits reviewed, only 11% of visits (115/1003) contained a documented problem with internet connectivity. None of the participants withdrew from the trial due to problems with their internet connections. The findings support the effective use of virtual visits in research involving participants living in rural locations. Virtual randomized controlled trials provide a strategy that enables participation for rural individuals who may not otherwise have access to clinical trials conducted in-person in urban settings. Utilizing internet access to connect with and support people who live in rural areas is critically needed to advance clinical research.

### SESSION 4420 (SYMPOSIUM)

Abstract citation ID: igae098.2115

### UNDERSTANDING AND SUPPORTING TECHNOLOGY EQUITY AND ACCESS FOR THE BENEFIT OF AGING ADULTS

Chair: Walter Boot
Co-Chair: Xin Yao Lin
Discussant: Jane Chung

Despite advancements, a significant "digital divide" persists, with older adults lagging in technology ownership, use, and proficiency compared to younger individuals. This session examines the digital divide's causes, impacts, and solutions for aiding older adults. Melissa deCardi Hladek, PhD, CRNP, FNP-BC, proposes a personalized, values-driven intervention to enhance digital literacy among older adults, focusing on overcoming access and connectivity disparities, especially in communities of color, to foster inclusion and better health outcomes. Shannon Mejia, PhD, FGSA, investigates the emotional dynamics of older adults interacting with smart devices, such as voice assistants, and how these interactions are affected by other smart elements like fireplaces. Li Chu, PhD, highlights the importance of aligning technological information with age-relevant motivations, indicating that a focus on emotional meaning could boost older adults' engagement with new technologies. Ladda Thiamwong, PhD, RN, FAAN, introduces the PEER intervention, a technology-based program aimed at preventing falls in low-income older adults through physio-feedback and peer-led exercises, emphasizing its role in transforming fall risk perceptions. Finally, Shenghao Zhang, PhD, questions the link between cognitive decline and the digital divide, suggesting that the relationship between age, general fluid abilities, and technology proficiency is more complex, offering new perspectives on technology learning and usage among older adults. Jane Chung, PhD, RN, will serve as discussant.

Abstract citation ID: igae098.2116

### A VALUES-DRIVEN APPROACH TO BRIDGING THE DIGITAL DIVIDE: A PILOT INTERVENTION FOR OLDER ADULTS

Melissa Hladek[1], Olivia Rubio[2], Kennedy McDaniel[3], Allyson Evelyn-Gustave[3], Deidra Crews[3], Mara McAdams DeMarco[4], Daniel Brennan[5], and Sarah Szanton[3], *1. Johns Hopkins University, Johns Hopkins University, Maryland, United States*, *2. The Pennsylvania State University, University Park, Pennsylvania, United States*, *3. Johns Hopkins University, Baltimore, Maryland, United States*, *4. New York University, New York, New York, United States*, *5. Johns Hopkins University School of Medicine, Baltimore, Maryland, United States*

Digital literacy, the ability to effectively use technology for information retrieval, evaluation, and communication, is critical in today's world. Despite advancements, older adults lag behind younger generations with approximately one in four adults over 65 not using the internet and over 35% lacking broadband connectivity. This gap is exacerbated among older adults of color due to disparities in access, connectivity, affordability, and cultural factors. Recognizing older adults' diverse values and learning styles, we propose a novel intervention tailored to individual values and goals. This one-arm feasibility and acceptability pilot trial enrolls 20 multi-racial older adults with varying levels of digital literacy, delivering a person-directed, values-based intervention in their homes over 4-6 visits. Our intervention targets community-identified barriers such as health literacy, health communication, and social support, aiming to enhance digital literacy with additional outcomes including quality of life and frailty. We view internet access and training not only as means to improve digital literacy but also as vital tools in addressing health disparities. By leveraging individual values and theoretically driven strategies, this intervention tests a potential avenue to narrow the digital divide among older adults, fostering inclusion, empowerment, and improved health outcomes.

Abstract citation ID: igae098.2117

### EMOTIONAL RESPONSES TO SUPPORT FROM VOICE ASSISTANTS WITHIN AN ECOLOGY OF SMART DEVICES

Shannon Mejia[1], Wan Wen[2], Smit Desai[2], Brandon Snipe[2], Otniel Fernandez[2], Sungjae Hong[2], and Jessie Chin[2], *1. University of Illinois Urbana-Champaign, Urbana, Illinois, United States*, *2. University of Illinois Urbana-Champaign, Champaign, Illinois, United States*

Smart-environments deploy an ecology of smart devices such as voice-assistants (VA), telehealth portals, and smart-fireplaces to support aging in place by providing assistance, remote-assessment, and restoration. Although asking for support is a foundational task within a smart environment, the socioemotional implications of asking smart devices for assistance is largely undefined. This study (N=51, observations=204, age:50-80, 54%female), conducted within a simulated "home office," employed a 2x2 (fireplace vs. not × neurologist remote-assessment vs. not) within-subjects experiment to test emotional responses to requests for VA assistance under varying smart ecologies. In each condition, a simulated VA instructed participants to play a game and report affect by endorsing adjectives representing their feelings. Participant-initiated requests for VA assistance were extracted from transcripts. Participants' beliefs in our simulation were evaluated during post-interviews to determine whether interactions were perceived as genuine with a VA (VA-True; 54%) or with a human through a speaker (VA-Human). Multilevel-models indicated higher negative affect (emotional cost) when participants requested VA assistance, and this cost was less pronounced when the smart-fireplace was activated. Cross-level interactions by beliefs differentiated emotional costs of asking for assistance. In general, compared to the VA-Human group, the emotional cost of asking for assistance was less pronounced for the VA-True group. However, while emotional cost was reduced for the VA-Human group when remote-assessment or fireplace was activated, emotional cost was amplified under these conditions for the VA-True group. Findings are situated within life-span perspectives of control and dependency scripts to inform the development of smart-ecologies to support aging-well.

Abstract citation ID: igae098.2118

### MOTIVATING INFORMATION-SEEKING BEHAVIORS FOR NEW TECHNOLOGY ACROSS THE LIFE SPAN

Li Chu[1], kayla Patterson[1], Tasha Kim[1], Sanjana Srivastava[1], Ruohan Zhang[1], Jiajun Wu[1], Li Fei-Fei[1], and Laura Carstensen[2], *1. Stanford University, Stanford, California, United States*, *2. Stanford University, Stanford University, California, United States*

Rapid technological advancements demand constant adaptation, which may be particularly unappealing for older adults. As people age, goal priorities shift from exploration to emotional meaning, but modern technology often overlooks this aspect, reducing motivation for older adults to learn. This pre-registered study aims to understand how alignment of information with age-relevant motivation may change people's information-seeking behaviors. We hypothesized a negative association between age and information-seeking behaviors. Moreover, this association is hypothesized to be moderated by emotional meaning, such that the positive relationship is amplified when technology emphasizes emotional meaning, indicating that older adults are as, if not more, interested in technology compared to younger adults when technology aligns with one's age-related motivation. We will report findings from an online experiment on a life-span sample of approximately 300 participants. On the data collection website, participants will view eight technological products and choose which ones to explore further. By clicking on the product, a new sentence about the product will be displayed, so the number of clicks and the duration of reading each product may be indices of people's information-seeking tendencies. Products are randomly assigned to emphasize exploration, social networking, entertainment, or deepening connections with loved ones (the emotionally meaningful option).

Abstract citation ID: igae098.2119

### TECHNOLOGY-BASED PHYSIO-FEEDBACK EXERCISE PROGRAM (PEER) TO PREVENT FALLS AND ENHANCE HEALTH EQUITY

Ladda Thiamwong, Rui Xie, Nichole Lighthall, Victoria Loerzel, Joon-Hyuk Park, and Jeffrey Stout, *University of Central Florida, Orlando, Florida, United States*

Only a few studies have focused on low-income older adults with a mismatch between perceived fall risk and physiological fall risk. We developed a Physio-fEedback Exercise pRogram (PEER) which includes a) physio-feedback using a real-time portable innovative technology—the BTrackS Balance System: BBS; b) cognitive reframing based on a novel fall risk appraisal matrix; and c) peer-led exercise by focusing on balance and strength training. This NIH-funded clustered randomized control trial is led by an interdisciplinary team representing nursing, kinesiology, cognitive psychology, engineering, and data science. We aim to prevent falls and reduce health disparities by facilitating shifts from maladaptive to adaptive fall risk appraisal (i.e., moving from irrational, incongruent, or congruent to the rational quadrant) The 8-week PEER intervention is offered at low-income independent living communities/units in Central Florida through our partnership with community-based and public health organizations. We collected data at baseline and measured outcomes after program completion and found that 17.78 % of the participants in the PEER group (n=45) had adaptive shifting compared to 13.46 % in the control group (n=52). Up to 24.99% of the control group had maladaptive shifting compared to only 12.73% of the PEER group. The PEER intervention results in a shift from maladaptive to adaptive as a way of preventing falls, which is novel, and PEER may slow down the maladaptive shifting process. Integrating technology into fall risk assessments could increase engagement in fall interventions in low-income settings due to barriers faced by older adults regarding accessibility, knowledge, and healthcare costs.

Abstract citation ID: igae098.2120

**THE RELEVANCE OF GENERAL FLUID ABILITIES IN TECHNOLOGY PROFICIENCY IN OLDER ADULTS**

Shenghao Zhang, and Walter Boot, *Weill Cornell Medicine, New York, New York, United States*

Previous research showed that general fluid abilities are correlated with technology use, acceptance, and proficiency in older adults. Cognitive decline was constantly proposed as an explanation for digital divide and age-related declines in technology use. However, this explanation has not been examined directly. We tested the relevance of general fluid abilities on technology proficiency in two large samples of normally aging older adults cross-sectionally and longitudinally with structural equation modeling. Results from a cross-sectional sample of older adults with various previous technology experience showed that older adults with higher general fluid abilities (measured by a latent factor consisted of letter set and immediate and delayed recall) also had higher technology proficiency (measured by a latent factor consisted of computer proficiency and mobile device proficiency). However, general fluid abilities were not predictive of technology proficiency after controlling for age, and general fluid abilities did not mediate the relationship between age and technology proficiency. Results from a longitudinal sample in which older adults with little to no previous experience with computers were trained to use and used a computer for 12 months showed that general fluid abilities (measured by a latent factor consisted of measures on processing speed, executive function, working memory, and episodic memory) was not predictive of the baseline or the slope of change of computer proficiency. However, those who are older increased less in proficiency controlling for fluid abilities. Discussion will focus on alternative explanations on age-related digital divide.

### SESSION 4430 (SYMPOSIUM)

Abstract citation ID: igae098.2121

**ADVANCING GEOGRAPHIES OF AGING AND THE LIFE COURSE**

Chair: Jessica Finlay
Co-Chair: Michael Desjardins

Aging and related health outcomes are not solely individual-level processes. Rather, built, social, and natural environments play a pivotal role in shaping health across the life course, especially among older adults. People also shape and modify surrounding environments throughout their lives in meaningful ways to maximize their well-being. While multiple longitudinal health datasets are available, many are analyzed without linking to spatially-explicit data. Overlooking the nexus between health and place, and not directly accounting for spatial and spatiotemporal processes that underlie the data, may hinder scientific discovery and advancement in understanding multi-scalar longitudinal processes that impact health and well-being. Geographies of aging and the life course critically integrate built, social, and natural environments into meaningful analyses and insights that can inform research, policy, and programs to promote healthy aging. This symposium features talks using innovative qualitative, quantitative, and mixed methods studies to investigate multifaceted and overlapping contextual influences on health. The presenters critically consider mobility across the life course, multi-scalar social determinants of health, environmental impacts of structural racism and classism, and potential interactions among individuals and neighborhoods that influence vulnerability to health exposures. We aim to build capacity around spatial methods and place-based considerations in gerontology. The approaches featured in this symposium highlight accessible ways for scholars across a variety of disciplines to advance understanding of healthy aging through the integration of geospatial approaches.

Abstract citation ID: igae098.2122

**LAYERING THE IMPACTS OF STRUCTURAL RACISM ON HEALTHY AGING TRAJECTORIES IN A LONGITUDINAL COHORT**

Richard Sadler, *Michigan State University, Flint, Michigan, United States*

African Americans are more at risk for earlier and more pronounced cognitive and functional decline. Direct impacts of racism are responsible for disparities in medical quality, access, and treatment are documented drivers of these racial disparities in aging health inequities. But largely understudied is the role of the built and social environment as a foundational determinant of health. In particular, a web of elements of structural racism in the built environment (STRAßE) work together to create environments where amenities are scarce and disamenities proliferate. While many studies are keen to include redlining as one such measure, emerging evidence suggests that additional STRAßE variables (i.e. blockbusting, urban renewal, freeway construction) may be key in illuminating health linkages. Here we build such measures for the city of Baltimore for use in an expanded dataset of the Healthy Aging in Neighborhoods of Diversity Across the Life Span (HANDLS) epidemiological cohort study. Our team has proposed work with HANDLS to collect complete residential histories for ~800 midlife to older Baltimore-born men and women (ages 30-64 at baseline), tracked up to 13 years across three waves. We will illustrate spatial-temporal specific exposures of STRAßE and establish a framework for future work. We use GIS to digitize and geocode the extent of STRAßE and identify spatial-temporal overlaps. Initial findings show interesting links between key

STRAßE measures (i.e. redlining lacking overlap with blockbusting, gentrification co-occurring with redlining and urban renewal), illustrating the importance of considering a wider range of measures than has been examined to date.

Abstract citation ID: igae098.2123

### CLUSTERING OF MULTIDIMENSIONAL NEIGHBORHOOD CHARACTERISTICS IN SHAPING HEALTHY AGING

Jiao Yu[1], Weidi Qin[2], Jiuzhou Wang[3], and Eva Kahana[4], *1. Yale University, New Haven, Connecticut, United States*, *2. University of Wisconsin Madison, Madison, Wisconsin, United States*, *3. University of Minnesota, Minneapolis, Minnesota, United States*, *4. Case Western Reserve University, Cleveland, Ohio, United States*

Neighborhoods are pivotal in shaping later life health and well-being. While previous research has primarily examined a single dimension of neighborhood risk factors (e.g., socioeconomic disadvantages), this study proposes a timely shift toward a multi-dimensional perspective of neighborhood characteristics. We aim to explore how multiple neighborhood characteristics synergistically shape various later life health outcomes, including comorbidity, disabilities (ADL), mental health, functional limitations, cognitive health, and epigenetic aging (GrimAge). Using data from the 2014 and 2016 waves of the Health and Retirement Study linked to the National Neighborhood Data Archive (n = 8,474), we first applied a machine learning algorithm -- the Partitioning Around Medoids (PAM) method to identify a neighborhood typology based on neighborhood-level socioeconomic status, facilities, services, and hazards. This analysis identified four clusters: affluent neighborhoods, mid-SES with high hazards neighborhoods, high amenity neighborhoods, and disadvantaged neighborhoods. Subsequently, we conducted multilevel regression analyses to examine the association between the identified neighborhood clusters and later life health outcomes. Regression results revealed significant health disparities across different neighborhood clusters. Compared to their counterparts living in affluent neighborhoods, older adults residing in disadvantaged neighborhoods and those in mid-SES with high hazards neighborhoods experienced greater chronic conditions, worse mental health, and more functional limitations. These findings underscore the urgent need for tailored interventions aimed at enhancing service facilities and mitigating environmental hazards in disadvantaged and high-risk communities. Addressing these neighborhood factors is essential for promoting communities that are conducive to healthy aging and supportive of successful aging in place.

Abstract citation ID: igae098.2124

### HEALTH-PROMOTING NEIGHBORHOOD RESOURCES ASSOCIATED WITH REDUCED NEURODEGENERATION AND CEREBROVASCULAR DISEASE

Lilah Besser[1], Pauline Maillard[2], Elaine Le[3], Diana Mitsova[4], James Galvin[5], Charles DeCarli[2], and Oanh Meyer[6], *1. University of Miami, Boca Raton, Florida, United States*, *2. University of California Davis, Davis, California, United States*, *3. University of Miami, Coral Gables, Florida, United States*, *4. Florida Atlantic University, Boca Raton, Florida, United States*, *5. University of Miami, Miami, Florida, United States*, *6. University of California, Davis, Sacramento, California, United States*

We aimed to determine if health-promoting neighborhood resources are associated with better brain health measured via structural magnetic resonance imaging (MRI) of older adults. We used data on 195 participants with normal cognition or cognitive impairment from the University of California, Davis Alzheimer's Disease Research Center. Participants addresses were geocoded to derive neighborhood (Census tract) measures of presence of a library, density of churches, percentage of retail space, and percentage park space. MRI measures included total grey matter volume and white matter hyperintensity volume (WMH), markers of future dementia and cerebrovascular disease risk. Multivariable linear regression that accounted for clustering by Census tract tested associations between the neighborhood characteristics and MRI outcomes, controlling for demographics (e.g., age, race/ethnicity), cognitive status (Clinical Dementia Rating), intracranial volume, population density, and neighborhood socioeconomic status (SES). Models were also stratified by high and low neighborhood SES. Participants were on average 74.9±7.2 years old, had 13.7±4.5 years of education, and 24.1% were Black, 28.9% Hispanic, and 47.1% non-Hispanic White. Individuals in neighborhoods with a library or a higher density of churches had lower WMH volumes, while those in neighborhoods with more churches had greater total grey matter volume. Presence of a library was associated with lower WMH volume for those in low SES neighborhoods, while no associations were observed for those in high SES neighborhoods. Neighborhoods with cognitively stimulating and socially engaging destinations (i.e., libraries and churches) may help reduce cerebrovascular disease and dementia risk by protecting against white matter disease and brain atrophy.

Abstract citation ID: igae098.2125

### TOXIC HOMES AND COMMUNITIES? EXPOSURE TO URBAN FIRES AND INCREASED LIFE COURSE DEMENTIA RISK

Jessica Finlay[1], Grace Bowman[2], Matthew Miller[1], and Colleen Reid[1], *1. University of Colorado Boulder, Boulder, Colorado, United States*, *2. University of Colorado BioFrontiers Institute, Boulder, Colorado, United States*

Little is known about the health impacts of wildland urban interface (WUI) fires, which are increasingly common and mostly burn human-made materials. The Marshall Fire Community Health Research Study investigated a destructive WUI fire that affected communities of Superior and Louisville (CO) on December 30, 2021. Semi-structured seated and mobile interviews conducted July through November 2023 probed for perceived impacts on physical, mental, social, and cognitive health. Participants (n=44) were on average 52 years old (±16 years), 86% identified as Non-Hispanic White, 67% female, 86% homeowners, and 46.5% of participant homes were fully destroyed (53.5% smoke damaged). Reflexive thematic analysis identified behavioral and social pathways that may pose longer-term risk for Alzheimer's Disease and Related Dementias across the life course. This includes early-life educational disruptions (e.g., displacement and stress/anxiety) and mid-life increased risk for hypertension, obesity, and alcohol consumption through heightened stress, lack of time and motivation for diet and exercise, maladaptive coping mechanisms, poor sleep quality, and reduced access to health-promoting services. Later life factors include increased risk for depression, social isolation, physical inactivity, diabetes, and exposure to poorer air quality. These risks varied by individual factors such as education, employment, insurance coverage, type of fire damage, and household composition. Individuals whose homes were smoke damaged may be particularly vulnerable. The study identifies novel behavioral and social pathways through which WUI fires may pose multifaceted and long-term cognitive health risks beyond the acute recovery phase.

Abstract citation ID: igae098.2126
**LEVERAGING TIME USE DATA TO EXPLORE MIGRANT HEALTH, SOCIALIZING, AND TECHNOLOGY USE**
Amber DeJohn[1], Michael Widener[2], Bochu Liu[3], Xinlin Ma[4], and Zhilin Liu[5], *1. Florida State University, Tallahassee, Florida, United States*, 2. *University of Toronto, Toronto, Ontario, Canada*, 3. *Tongji University, Shanghai, Shanghai, China (People's Republic)*, 4. *University of North Carolina, Chapel Hill, North Carolina, United States*, 5. *Tsinghua University, Beijing, Beijing, China (People's Republic)*
Migrant populations tend to locate near other migrants upon arrival in their receiving country. These residential locations are the result of culturally specific social networks, and they result in differing social networks and cultural contexts relative to the native-born population and other migrant groups. Beyond their physical social networks, migrants also use information and communication technology (ICT) to maintain social connections in their sending country. During the COVID-19 pandemic, public health initiatives to discourage socializing (e.g., closing third places) generated concern about social isolation among older adults, especially due to its association with health. In Toronto, Canada, COVID-era lockdown initiatives were particularly prolonged. During this extended lockdown, we fielded a survey and time-use diary on a convenience sample of older Chinese migrants (n = 77) in the Greater Toronto Area. Using the single-day activity diaries, we grouped respondents using a k-means clustering approach, which resulted in four categories of socializing characteristics. We then used ANOVA tests and multinomial logistic regression to understand the geographic contexts for these socializing behaviors. Findings reveal that this older migrant community was largely socializing online, but a small group reported socializing in person. Regression results reveal that living conditions, physical health, and the location of social networks are associated with how migrants socialize. This study presents a novel method for exploring geographies and activities within the home in tandem.

### SESSION 4435 (PAPER)

### CULTURES OF CARE

Abstract citation ID: igae098.2127
**NATIVE AMERICAN CAREGIVING PREFERENCES: HEARING THEIR VOICES**
Pamela Monaghan-Geernaert, *Northern State University, Aberdeen, South Dakota, United States*
American Indian and Alaska Native elders have the shortest life expectancy of all non-white groups in the USA (CDC, 2022). Despite this disparity, Native elders experience higher rates of morbidity, often necessitating skilled care. Previous research has indicated that enhancing treatment for AI/AN elders must incorporate cultural considerations (Willging et al. 2021). This study utilized focus group data collected at the National Indian Council on Aging (NICOA) biennial conference for American Indian and Alaska Native elders. Elders from all Indian Health Service Units across the nation participated in the focus groups, sharing their desires for caregiving when they can no longer care for themselves. Thematic analysis of the data revealed that elders wish to have their voices heard and to play a role in guiding their own care to the best of their abilities. When external caregivers are necessary, elders prefer family members as their first choice, followed by tribal members, with culturally trained caregivers considered as a last resort. Additionally, elders expressed a desire for the incorporation of Native teachings, storytelling, and traditional medicines, such as herbal remedies, into their care. They also emphasized the importance of streamlining the care process between Indian Health Services (IHS), tribes, and families and removing barriers to ensure respective care during periods of illness and frailty. As the Native elderly population continues to age, it is crucial that we actively listen to their stories and preferences regarding elderly care.

Abstract citation ID: igae098.2128
**THE JOINT EFFECTS OF WORK AND INFORMAL CAREGIVING ON MENTAL WELL-BEING AMONG CHINESE MIDDLE- TO OLDER-AGED ADULTS**
Jing Ye, *Wuhan University, Wuhan, Hubei, China (People's Republic)*
Although the health implications of employment and caregiving are well-documented, less is known about the joint impact of work and caregiving on the well-being of older adults. This study contributes to filling this research gap by focusing on the well-being implications of combining work and informal caregiving for Chinese older adults 60 years of age and older. The study draws on two theoretical perspectives: role strain theory and role enhancement theory. While the role strain theory assumes lower life satisfaction and higher depressive symptoms due to the double burden of paid work and caregiving, the role enhancement theory predicts an increase in well-being through the accumulation of resources and role fulfillment. Using data from the China Health and Retirement Longitudinal Study (CHARLS 2011-2018), this study aims at investigating whether and how older age working caregivers experience different life satisfaction and depressive symptoms. Besides, the moderating effects of intensity, gender, residence, socioeconomic status, and social isolation level are estimated. Results from the random-effects model show that compared to those who do not work or provide informal care, low-intensity worker-caregivers report higher life satisfaction. Only female worker-caregivers experience higher depressive symptoms, particularly with long hours of work and caregiving. These results support both role enhancement and role strain theories and suggest the need for increased investment in formal care facilities to alleviate the care burdens among older adults.

Abstract citation ID: igae098.2129

### EXPLORING IKIGAI AND LIVING WELL WITH DEMENTIA AMONG JAPANESE CANADIANS: A NARRATIVE AND ARTS-BASED RESEARCH STUDY

Hiro Ito[1], Susan Cox[1], Alison Phinney[1], and Anita Ho[2], *1. University of British Columbia, Vancouver, British Columbia, Canada*, *2. University of British Columbia, Canada*

Like many ethnocultural minority populations, Japanese Canadians have been underrepresented in research pertaining to experiences of living with dementia. Representation of how diverse populations experience living with dementia is necessary to further scholarship on the intersections of dementia and culture. Thus, we aim to explore how Japanese Canadians live well with dementia through the conceptual lens of ikigai, a Japanese wellbeing construct. In this two-phase study, we took a relational approach and recruited 4 people living with dementia and 3 of their care partners from the Japanese Canadian community. In the first phase, we conducted individual and dyadic narrative interviews to understand experiences of living well with dementia. In the second phase, we gathered in a group art-making workshop to enable participants to identify and express what they wanted others to know about how they live well with dementia. To ensure that individuals could participate meaningfully in their preferred language, the study was conducted in English and Japanese. Audio recordings from both phases were transcribed and analyzed through narrative inquiry. Through our study, we identified several ways Japanese Canadians maintained their ikigai to live well with dementia, including notions of balance, continuity, and gratitude. Our findings offer insights into the integral role of culture in living well with dementia, as well as the value of culturally nuanced and inclusive research.

Abstract citation ID: igae098.2130

### SLEEP QUALITY IMPROVES IN DEMENTIA CAREGIVERS USING ONLINE SELF-GUIDED ACCEPTANCE AND COMMITMENT THERAPY

Elizabeth Fauth, Jacob Gossner, Audrey Juhasz, Ty Aller, and Michael Levin, *Utah State University, Logan, Utah, United States*

Transdiagnostic interventions treat multiple conditions by addressing common underlying mechanisms. Randomized controlled trials, systematic reviews, and meta-analyses support that Acceptance and Commitment Therapy (ACT) is a transdiagnostic mindfulness-based Cognitive Behavioral Therapy that improves anxiety, depression, pain, etc. in diverse populations. ACT efficacy is supported in dementia caregivers, typically studied via psychological or care-related outcomes. ACT improves sleep in cancer caregivers, and sleep is often disturbed in dementia caregivers. The current study examines if sleep quality improves in (N=80) dementia caregivers engaging with a caregiver-tailored six-session online, self-guided ACT program. Participants completed the Sleep Quality Scale and other assessments at T1 (pre-intervention), T2 (post-intervention) and T3 (6-week followup). A sleep-related item from a depression inventory assessed sleep restlessness. Items from a behavioral symptom inventory assessed the extent to which care receivers woke the caregiver or others in the night, and assessed caregivers' stress appraisal of this experience. We did not hypothesize that care receivers' wakefulness would be impacted, but hypothesized improvements in the other caregiver sleep indicators. Repeated measures ANOVA identified that caregivers improved average sleep quality [$F(2,158)=7.907$, $p<.001$; partial eta-squared =.091] and reported less sleep restlessness [$F(2,156)=10.039$, $p<.001$; partial eta-squared =.114]. Post hoc comparisons identified change occurring between T1-T2 (Intervention phase), sustained over time (T3). Care receivers' waking behavior did not change over time (p=n.s.), but caregivers experiencing this behavior across all timepoints (N=17), reported being less bothered by it [$F(2,32)=5.457$, $p<.01$; partial eta-squared =.254]. ACT interventions offer promise in improving sleep experiences for dementia caregivers.

Abstract citation ID: igae098.2131

### IMPACT OF RESPITE SERVICES ON DEMENTIA CAREGIVERS' NEUROCOGNITIVE AND PREDEATH GRIEF OUTCOMES: A PILOT STUDY

Sydnie Schneider[1], Lauren Elliott[1], Elisabeth McLean[1], Volker Neugebauer[2], Keegan Diehl[1], and Jonathan Singer[1], *1. Texas Tech University, Lubbock, Texas, United States*, *2. Texas Tech University Health Sciences Center, Lubbock, Texas, United States*

Caring for individuals with dementia presents a significant challenge, often placing caregivers under immense stress. Research has consistently highlighted that caregivers of individuals with dementia face an elevated risk of experiencing health concerns themselves due to the high stress and burden. This burden extends beyond psychological strain, with studies indicating higher rates of neurocognitive decline and predeath grief (PDG) among family caregivers. Despite growing recognition of these health risks, there is currently a lack of interventions that target the family caregivers of persons with dementia. This study implemented 12-weeks of 3-hours of respite. We assessed overall neurocognitive functioning and PDG in caregivers at baseline and follow-up. Pilot data from 8 caregivers were analyzed. Assessments comprised of the Repeatable Battery for the Assessment of Neuropsychological Status (RBANS; i.e., attention, language, visuospatial ability, and immediate/delayed recall) and the PG-12 (i.e., pre-death grief). The individual reliable change index (RCI) for each participant was calculated as the difference in overall scores on the RBANS and PG-12 scores at baseline and follow up. Average PG-12 scores decreased (RCI = -0.88) at intervention, indicating a decrease in PDG. Further, RBANS (RCI = 1.2), immediate memory index (RCI = 4.58), language index (RCI = 3.16), attention index (RCI = 1.58), and delayed memory index (RCI = 2.33) scores increased. However, average visuospatial/constructional index (RCI = -8.04) scores decreased. These preliminary results suggest that only 3 hours of respite a week has the potential to reduce PDG and improve neurocognitive functioning among family caregivers of persons with dementia.

### SESSION 4440 (PAPER)

### DEMENTIA (PAPERS)

Abstract citation ID: igae098.2132

### EXPLORING MEALTIME EXPERIENCES OF FAMILIES LIVING WITH DEMENTIA IN LONG-TERM CARE: A GROUNDED THEORY STUDY

Heather Alford, Allison Cammer, Heather Ward, and Jason Perepelkin, *University of Saskatchewan, Saskatoon, Saskatchewan, Canada*

Relationship-centered mealtime interventions improve staff-resident relationships and reduce the risk of malnutrition for residents living in long-term care settings. Less is known about the nature, goals, or outcomes of family-resident relationships at mealtimes, particularly for residents living with dementia. This constructivist grounded theory study aimed to explore what is needed to support meaningful family relationships and engagement at mealtimes with residents living with dementia in long-term care homes. Individual interviews were conducted with 13 family caregivers of people living with dementia in long-term care homes in a Western Canadian province. Interviews were recorded, transcribed verbatim, and analyzed using constant-comparison method. Three themes characterized family member participants' experiences of mealtimes: 1) Changing Ways of Connecting, 2) Nutrition as a Reflection of Dementia Progression, and 3) Adjusting Expectations. Mealtimes are important opportunities for supporting family-resident connection and can facilitate 'acceptance' of the illness trajectory in dementia among caregivers. By conceptualizing their mealtime involvement, this study advances understanding of the essentiality of family caregivers for enhancing relationship-centered practices in long-term care settings. By explicating the structure, function, and experience of family involvement in nutritional care, this study addresses the current gap in understanding of how families living with dementia build and preserve relationships through nutritional care and dining.

Abstract citation ID: igae098.2133

### THE COMBINED IMPACT OF MIDLIFE AND LATE-LIFE VASCULAR RISK FACTORS ON 33-YEAR INCIDENT DEMENTIA: THE ARIC STUDY

Jason Smith[1], James Pike[2], Pamela Lutsey[3], Richey Sharrett[1], Alden Gross[4], and Jennifer Deal[1], *1. Johns Hopkins University, Baltimore, Maryland, United States*, *2. New York University, New York, New York, United States*, *3. University of Minnesota, Minneapolis, Minnesota, United States*, *4. Johns Hopkins Bloomberg School of Public Health, Baltimore, Maryland, United States*

Midlife vascular risk factors are associated with an increased risk of dementia. However, the overall contribution of modifiable vascular risk factors in midlife and late-life to dementia remains unclear. In this study, we quantified population attributable fractions, which account for risk factor prevalence and strength of relative risks, of incident dementia from vascular risk factors measured in midlife and early late-life. We used 33 years of prospective cohort data in the Atherosclerosis Risk in Communities (ARIC) Study. Incident dementia was ascertained from baseline (1987-1989) through December 31, 2020. We quantified population attributable fractions of incident dementia by age 80 from the combination of hypertension, diabetes, and self-reported current cigarette smoking (at least one risk factor versus none) measured at different ages (45-54 [n=7,731], 55-64 [n=12,273]; 65-74 [n=6,660]). Overall, the prevalence of having one or more of the vascular risk factors was 61.6% at age 45-54, increasing to 68.2% by age 55-64 and 77.5% by age 65-74. The population attributable fraction of dementia from having one or more vascular risk factors increased from 22.7% (95% CI: 15.3-30.1%) for risk factors measured at age 45-54 to 26.4% (95% CI: 19.1-33.6%) at age 55-64 and 45.4% (95% CI: 32.5-58.3%) for risk factors measured at age 65-74. Our data indicate between 23%-45% of incident dementia cases in ARIC were attributed to a combination of hypertension, diabetes, or current smoking up through age 74. Public health strategies targeting modifiable vascular risk factors could delay or prevent new dementia cases.

Abstract citation ID: igae098.2134

### OLFACTION AND DEMENTIA RISK IN THE ATHEROSCLEROSIS RISK IN COMMUNITIES (ARIC) STUDY

Honglei Chen[1], Srishti Shrestha[2], Xiaoqian Zhu[2], Anna Kucharska-Newton[3], Yaqun Yuan[4], Vidyulata Kamath[5], and Tom Mosley[2], *1. Michigan State University, Michigan State University, Michigan, United States*, *2. University of Mississippi Medical Center, Jackson, Mississippi, United States*, *3. University of North Carolina at Chapel Hill, Chapel Hill, North Carolina, United States*, *4. Michigan State University East Lansing, East Lansing, Michigan, United States*, *5. Johns Hopkins University School of Medicine, Baltimore, Maryland, United States*

We examined the association of olfaction with incident dementia and characterized this relationship by key demographic subgroups and APOE-ε4 genotype in the ARIC study. We further examined how change in olfaction was associated with dementia incidence. Olfaction was evaluated using the 12-item Sniffin' Sticks test in 4470 ARIC participants in 2011-2013 (visit 5) and in 2658 participants in 2016-2017 (visit 6). We defined olfaction as good (score 11-12), moderate (9-10), hyposmia (7-8), and anosmia (0-6). Dementia status was ascertained from 2011-2013 through 2020. Of the 4470 participants at baseline (age:75±5 years, 21% Black race, 60% women), 17% developed dementia over 10 years. In multivariable Cox models, moderate olfaction (hazard ratio (HR):1.5, 95%CI:1.3-1.9), hyposmia (HR:2.2, 95%CI:1.8-2.8), and anosmia (HR:3.5, 95%CI:2.8-4.3) were associated with higher dementia rate in a linear, monotonic manner relative to good olfaction. Findings were similar across subgroups of age, sex, race, and APOE-ε4 genotype. We also used logistic regression to estimate marginal dementia probability. We found that, on the risk difference scale, the absolute risk difference between anosmia and good olfaction was higher in APOE-ε4 carriers (0.29, 95%CI:0.20-0.37) compared to that in non-carriers (0.16, 95%CI:0.11-0.21); jointly, APOE-ε4 carriers with anosmia had the greatest cumulative dementia probability. Further, compared to participants who maintained good olfaction from visit 5 to visit 6, those with stable anosmia (HR:5.6, 95%CI:2.7-11.6) and a decline from good to anosmia (HR:4.5, 95%CI:2.2-9.4)) showed comparable associations with dementia incidence. Our study provides important data about how simple noninvasive olfaction tests may inform future dementia risk.

Abstract citation ID: igae098.2135

### PROSOCIALITY AND SUBSEQUENT COGNITIVE HEALTH: A PROSPECTIVE COHORT STUDY

Justin Farmer[1], Lucía Macchia[2], Jessica Gong[3], Andrew Steptoe[3], Feifei Bu[3], and Laura Kubzansky[4], *1.*

*Harvard T.H. Chan School of Public Health, Somerville, Massachusetts, United States*, 2. *City College London, London, England, United Kingdom*, 3. *University College London, London, England, United Kingdom*, 4. *Harvard T. H. Chan School of Public Health, Boston, Massachusetts, United States*

Specific prosocial behaviors, including volunteering and caregiving, have been linked to better maintenance of cognitive health in older adults. However, limited epidemiologic work has examined the relationship between one's cognitive-emotional orientation toward prosociality generally and these same health outcomes. This study uses data on 7844 participants from the English Longitudinal Study of Ageing (ELSA) to test the hypothesis that higher versus lower prosociality is associated with higher maintenance of cognitive health in adults 50 years or older. We derived a 9-item prosociality scale using items from well-validated measures of self-reported altruism and collectivism assessed in ELSA. Linear mixed-effects models were used to examine the relationship between this measure and changes in executive function and verbal memory over an 11-year period. Cox proportional hazards models were then used to compare time to dementia based on level of prosociality. Results from both longitudinal and survival analyses suggest that higher prosociality is associated with maintaining better cognitive health even after controlling for sociodemographics (e.g., age, education, wealth) and baseline health characteristics such as depression and cardiovascular disease. Comparing across tertiles, participants with high versus low prosociality had 24% slower decline in verbal memory and 55% slower decline in executive function. This aligned with a 35% reduced hazard of dementia during this same period for those with high vs. low prosociality. These results suggests that prosociality, beyond formal volunteering, is an important modifiable risk factor for improving later life cognitive function on an individual and population level and should therefore be further examined.

Abstract citation ID: igae098.2136

### PALLIATIVE CARE FOR PEOPLE WITH MODERATE-SEVERE DEMENTIA IN THE COMMUNITY: RESULTS OF THE IN-PEACE TRIAL

Greg Sachs[1], Nina Johnson[2], Sujuan Gao[1], Minmin Pan[1], Alexia Torke[1], Susan Hickman[2], and Kurt Kroenke[1], 1. *Indiana University School of Medicine, Indianapolis, Indiana, United States*, 2. *Regenstrief Institute, Inc., Indianapolis, Indiana, United States*

Dementia care management programs in the community demonstrate some benefits. Study limitations include limited numbers of people living with dementia (PLWD) with advanced disease or from minoritized populations; lack of palliative care components; and limited success reducing health care utilization. IN-PEACE tested dementia care management integrated with palliative care for PLWD with moderate-severe disease in the community and their caregivers. 201 PLWD-caregiver dyads were randomized to either a dementia care coordinator (99) or usual care (102) and followed for 24 months. Outcomes were neuropsychiatric symptoms (NPI-Q severity) and symptom management (SM-EOLD) in PLWD; distress (NPI-Q distress) and depression symptoms (PHQ-8) in caregivers; and the combined measure of ED visits/hospitalizations. Outcomes were assessed quarterly. Separate mixed effects models were run for each symptom/distress measure and a zero-inflated Poisson model compared the mean number of ED/hospitalization events. Subgroup analyses were conducted based on baseline NPI-Q severity, sex, race, income, and health system. There were no statistically significant differences between groups in any symptoms or distress measures in PLWD or caregivers. PLWD receiving the intervention, however, had substantially fewer ED/hospitalization events (means 1.06 events versus 2.37, $p < 0.007$). The intervention reduced the proportion of PLWD who had one or more ED/hospitalization events (78.4% of controls vs. 50.5% of intervention, $p < 0.001$). The relative risk reduction was 35.6% for an event, absolute risk reduction 27.9%, and number needed to treat (NNT) of 3.6. PLWD with higher NPI-Q at baseline and Black PLWD experienced greater reductions in ED visit hospitalization events.

### SESSION 4445 (PAPER)

### DISPARITIES, EQUITY, INCLUSION, AND BELONGING

Abstract citation ID: igae098.2137

### SUPPORTING SAFE AND HEALTHY SEXUAL EXPRESSION IN LONG-TERM CARE: A REVIEW OF CANADIAN RESOURCES

Alisa Grigorovich[1], Julia Brassolotto[2], Alessandro Manduca-Barone[2], Romeo Colobong[3], Lori Schindel Martin[4], Andrea Iaboni[5], Sid Feldman[6], and Pia Kontos[7], 1. *Brock University, St. Catharines, Ontario, Canada*, 2. *University of Lethbridge, Lethbridge, Alberta, Canada*, 3. *KITE Research Institute, Toronto Rehabilitation Institute – University Health Network, Toronto, Ontario, Canada*, 4. *Toronto Metropolitan University, Toronto, Ontario, Canada*, 5. *University of Toronto, Toronto, Ontario, Canada*, 6. *Baycrest Health Sciences, Toronto, Ontario, Canada*, 7. *KITE Research Institute, Toronto Rehabilitation Institute – University Health Network; Dalla Lana School of Public Health, University of Toronto, Toronto, Ontario, Canada*

Little is known about what educational resources are available to practitioners in Canada for supporting the safe and healthy sexual expressions of persons living with dementia in long-term care (LTC). To address this gap, we conducted a critical review of publicly available online resources in Canada. Our objectives were to identify existing resources, describe key content areas and modalities, and assess strengths and weaknesses. To find resources, we conducted a systematic Google search, targeted websites searches, and contacted experts. Resources were included if they were in English, publicly accessible (e.g., not behind a paywall), focused on support of sexual expression of persons living with dementia in LTC, and informed action at the micro level of practice and/or meso level of organizational policy. We identified 9 resources, the majority of which were text-based and aimed at supporting staff or organizations in developing policies or practices regarding assessment and intervention in non-consensual sexual behavior. Guidance on consent was narrowly focused on bioethical principles, often neglecting fluctuating capacity, assent, and the role of substitute decision-makers. Although some acknowledged that healthy sexual expression in the context of dementia should be supported, the focus was primarily on support of non-erotic intimacy (e.g., hugging vs. stroking/kissing). We also found stigmatizing language (e.g., "demented"), and cursory attention to sexual and gender diversity, and ethnically and culturally diverse content. Although the available resources provide an important foundation, there is a pressing need to develop additional resources to better support sexual expression for persons living with dementia in LTC.

Abstract citation ID: igae098.2138

### EQUITABLE AND SOCIALLY JUST AGE-FRIENDLY COMMUNITIES FOR OLDER LGBTQ+ ADULTS OF COLOR

Austin Oswald[1], and Lujira Cooper[2], *1. Dalhousie University, Nova Scotia, Canada*, *2. Brooklyn Society for Ethical Culture, Brooklyn, New York, United States*

The age-friendly movement has been critiqued for inadequately addressing the needs of historically disadvantaged populations, like older LGBTQ+ adults of color. This presentation describes a multi-year participatory action research project with five older Black lesbian women aged between 65 and 75. We hosted five focus groups where participants shared their daily experiences living in an age-friendly city and proposed ideas to more inclusively meet the needs of older LGBTQ+ adults of color within age-friendly activities. Focus group data were analyzed thematically, and four themes were identified which expand existing age-friendly frameworks, promoting greater equity and justice: 1) attending to the interaction between identities and structures throughout the life course, 2) understanding the impact of historical and contemporary experiences of violence and discrimination on health and aging, 3) addressing the evolving sociopolitical landscape regarding the human rights and freedoms of diverse older adults, and 4) developing interventions that create safe and affirming spaces for older LGBTQ+ adults of color. While mainstream age-friendly frameworks are widely acknowledged for enhancing the health, independence, and social connectivity of older adults, addressing the unique challenges associated with aging while navigating multiple intersecting marginalized identities requires a critical perspective. The findings of this research underscore the necessity of culturally tailored age-friendly frameworks and interventions to address the distinct needs and experiences of older LGBTQ+ adults of color. We argue that gerontology must seriously consider the impact of privilege and oppression on the health and aging experiences of vulnerable and underserved populations within age-friendly communities.

Abstract citation ID: igae098.2139

### VOLUNTEERING AMONG OLDER ADULTS AND EFFECTS OF ETHNIC MINORITY IDENTITY BEFORE AND DURING COVID-19

Eireann O'Dea[1], Andrew Wister[1], Lun Li[2], Sarah Canham[3], and Barbara Mitchell[1], *1. Simon Fraser University, Vancouver, British Columbia, Canada*, *2. MacEwan University, Edmonton, Alberta, Canada*, *3. University of Utah, Salt Lake City, Utah, United States*

The ongoing COVID-19 pandemic has presented numerous challenges to older adults in Canada, including the ability to engage in volunteer work. This situation has created a new volunteer landscape in which common predictors of volunteering may have shifted. The purpose of this study is to improve understanding of the current social context surrounding volunteering in Canada, by a) determining changes in the associations between human, social, and cultural capital variables and volunteering among older adults and b) examining the potential relationship between ethnic minority background and volunteering among older adults, using data from the Canadian Longitudinal Study on Aging (CLSA), collected prior to and during the pandemic. This study utilized data from 24,306 participants (aged 55+) who participated in the CLSA Baseline, Follow-up 1 and the COVID-19 Study Baseline surveys. Results confirm a decrease in volunteering among CLSA participants during the early stages of the pandemic. When compared to pre-pandemic associations, volunteers during the early stages of the pandemic were more likely to be young-old (55-64), male, employed, and not involved in religious activities. Findings provide evidence of pandemic effects on volunteering among older adults in Canada.

Abstract citation ID: igae098.2140

### EXPERIENCES OF CHALLENGES AND DISCRIMINATION AMONG ASIAN AND ASIAN AMERICAN OLDER ADULTS

Cindy Vang[1], Yeonjung Lee[2], and Youhung Her-Xiong[3], *1. California State University Chico, Chico, California, United States*, *2. University of Hawai'i at Mānoa, Honolulu, Hawaii, United States*, *3. Q&C Research Consulting, LLC, Eau Claire, Wisconsin, United States*

Asian Americans are one of the fastest-growing racial and ethnic minority older adult populations, with projections of up to 7.8 million in 2060. Despite the expected increase in this population, Asian Americans are often sidelined in discourses focused on discrimination and health disparities. The disparate discrimination and challenges for Asian and Asian American (AAA) older adults are not new; however, COVID-19 has exacerbated existing and emerging vulnerabilities and difficulties. The purpose of this study was to explore AAA older adults' understanding and experiences of overcoming challenges and discrimination during the COVID-19 pandemic using a grounded theory methodology. Participants (N=30) identified as 50 years and older, resided in the United States, and had origins from Asia were recruited through convenience and snowball sampling methods. These older adults participated in a one-time open-ended interview about their experiences and how they overcame challenges and discrimination during the pandemic. The results of our study describe the process of overcoming COVID-19 challenges and discrimination through causal conditions, contextual factors, and intervening conditions, which influenced the central phenomenon of maintaining vigilance for COVID-19 and discrimination, participants' resilience, and mental, emotional, and social consequences. Results imply further research to deepen knowledge of AAA older adults' experiences with discrimination and resilience and policies to support interventions to enhance these older adults' well-being.

Abstract citation ID: igae098.2141

### AD/ADRD AND SOCIAL SUPPORT ADVOCACY FOR BLACK NURSING HOME RESIDENTS

Dionne Bailey[1], Taylor Bucy[2], Tricia Skarphol[1], and Tetyana Shippee[3], *1. University of Minnesota, Minneapolis, Minnesota, United States*, *2. University of Kansas School of*

*Medicine, Kansas City, Kansas, United States*, 3. *University of Minnesota School Of Public Health, Minneapolis, Minnesota, United States*

Black older adults represent 24% of all nursing home (NH) residents in the U.S., 20.2% of whom have Alzheimer's disease and Alzheimer's disease-related dementias (AD/ADRD). Despite consuming a disproportionate share of NH services, when compared to their white counterparts, Black older adults experience worse health outcomes and are more likely to reside in lower-quality facilities, a direct byproduct of systemic racism. To cope with the stress of inadequate care delivery, many Black residents rely on external social support networks. Little research to date has explored how external social support is or can be, leveraged by Black residents for improved care delivery. Drawing on the Minority Stress Model, we applied thematic analysis to three focus group transcripts (n=22) conducted with AD/ADRD caregivers in Minnesota. We found that resident social support networks overwhelmingly adopted a "support through advocacy" mentality. Although these efforts are grounded in the care of their friends or family, advocacy behaviors often had to be extended to additional residents because they identified themselves as lacking social support. Given the disproportionate share of Black residents with AD/ADRD living in institutional-based settings, understanding both formal and informal care delivery practices, especially the role of Black resilience, is essential to creating high-quality care settings.

Abstract citation ID: igae098.2142

### WHO ENJOYS THE GOLDEN YEARS? RACIAL AND SOCIOECONOMIC DISPARITIES IN YEARS OF HEALTHY RETIREMENT

Leah Abrams[1], and Yuan Zhang[2], *1. Tufts University, Medford, Massachusetts, United States*, 2. *Columbia University, New York, New York, United States*

For several decades, until around 2010, Americans enjoyed increases in life expectancy, and for some, extended disability-free life expectancy. However, mortality progress has since stagnated, Americans with low education have not benefited from delayed disability, and there remain substantial racial disparities in morbidity and mortality. We aim to determine who in American society retains health and functioning to enjoy retirement years by simultaneously examining healthy life expectancy and time spent in retirement. We use data on physical functioning and work status from the Health and Retirement study in two periods (1998 and 2016) to run Markov models that calculate the number of years and percent of remaining life in healthy work, unhealthy work, healthy retirement, and unhealthy retirement by race and socioeconomic status. Results to date reveal that, in 2016 compared to 1998, adults over age 51 spent a smaller portion of their life in healthy retirement, with a shift toward healthy work. In 2016, Black and white men and women all spent between 17-20% of remaining life years after 51 in healthy retirement, but they arrived there differently. Men spent about 42% of life years healthy and working, compared to about 30% for women. Women spent more time not working with functional limitations. Looking to race, white men spent a greater portion of time working with functional limitations, whereas Black men were not working with functional limitations. Black women spent more time working with functional limitations and less time not working, with and without functioning.

### SESSION 4450 (PAPER)

### FAMILY AND AGING

Abstract citation ID: igae098.2143

### EXPLAINING DIFFERENCE: LATINX AND STRUCTURAL VULNERABILITY IN ADRD

Ignacia Arteaga[1], Alma Hernández de Jesús[1], Brandi Ginn[1], Corey Abramson[2], and Daniel Dohan[1], *1. University of California San Francisco, San Francisco, California, United States*, 2. *Rice University, Tucson, Arizona, United States*

Latinos/as/x (hereafter Latinx) residing in the U.S. are one and a half times more likely than non-Latinx white residents to develop cognitive impairment during their lives. Despite significant heterogeneity in nativity, language, socio-economic status, geographic region of residence, culture, and other factors, US-resident Latinx face shared structural barriers to health that may help explain inequities in incidence, time of diagnosis, and health outcomes of Alzheimer's Disease and Related Dementias (ADRD). This work uses comparative ethnography to unpack the everyday experiences of Latinx families, including people living with dementia (PLWD) and ADRD. We focus on how PLWD and their caregivers negotiate family dynamics informed by structural vulnerabilities. We draw on 84 in-depth interviews with older adults and their family caregivers alongside three years of participant observation across community centers in California and Maine. We document how 'familism,' a cultural orientation understood as the strong reliance on close kin relations to look after the ill, is brought into tension when ADRD, structural vulnerability, and multi-morbidities among family members are at stake. When facing limited access to healthcare and social support, family caregiving and its monetary compensation can become a source of within-family tension and even dispute. This may be especially true for Latinx families for whom caregiving income constitutes an important part of their everyday income-generating strategies. These findings offer an opportunity to reflect on current policy efforts to support structurally marginalized families living with ADRD in the US.

Abstract citation ID: igae098.2144

### CHOICE IN DIRECTLY-FUNDED HOME CARE

Lisette Dansereau, and Christine Kelly, *University of Manitoba, Winnipeg, Manitoba, Canada*

Directly funded (DF) home care, also referred to as direct payments or cash-for care, gives clients (self-managers) or unpaid caregivers (family-managers) a budget to choose their own home support services. DF research tends to be dominated by the assumption that choice is inherently beneficial, and rarely attends to the circumstances or experiences of family-managers. Using feminist care theory and Mol's 'logic of care' as an analytical lens, this study aims to address this gap through a nuanced approach to the concept of choice in care services. The findings are based on focus group discussions held between June-July 2021 with 32 participants using DF home care in Manitoba, Canada. Qualitative thematic analysis was conducted to answer the question "how do DF users experience the tensions of choice, risk and responsibility?" The first theme demonstrates that choice in DF results in high quality care. The second theme illuminates some of the risks involved in exercising choice, particularly when an individual's responsibility for coordinating formal services is unsupported. The final theme shows that family-managers tend to take on the responsibility of DF not as a first choice but as a last resort after experiencing other care options as substandard and to avoid institutional care for an older adult. We conclude that family-management in DF places new formal responsibilities on informal caregivers, often without support. We recommend reducing private responsibility in the context of DF, and identify four domains where there is potential to enhance choice in professionally-organized home care services.

Abstract citation ID: igae098.2145

### EXPLORING THE FEASIBILITY AND UTILIZATION OF CLARE: A MOBILE APPLICATION FOR CAREGIVERS AFTER HOSPITAL DISCHARGE

Dingyue Wang[1], Doreen Matters[2], and Cristina Hendrix[2], *1. Duke University, Seattle, Washington, United States*, *2. Duke University, Durham, North Carolina, United States*

CLARE is the first mobile application tailored to enhance discharge education and teaching practices in hospital settings. This mixed-method study aimed to assess the feasibility of CLARE in providing caregiving training and support immediately following hospital discharge. Additionally, the study sought to apply the Unified Theory of Acceptance and Use of Technology (UTAUT) framework to identify factors that promote or hinder the intent and use of CLARE among caregivers. Caregivers included in the study were primary caregivers assisting patients post-discharge, aged at least 35 years, and caring for patients aged at least 55 years. Data from application usage was analyzed alongside caregiver interviews. CLARE areas of teaching encompassed discharge preparation (e.g., early mobility instruction), COVID-19 care protocols (e.g., isolation; quarantine; home sanitization; monitoring shortness of breath), and caregiver self-care strategies (e.g., managing anxiety and stress). Preliminary findings from the 23 enrolled caregivers (with a target enrollment of 50) revealed that 65.2% were female, and the majority (78.3%) were between 50 and 80 years old. Usage statistics indicated that 17.4% of caregivers completed all tasks, 34.8% completed over 80% of tasks, and 52.2% completed more than half of the tasks in CLARE. Survey and interview responses showed that CLARE is a user-friendly, time-efficient, and informative app that facilitates awareness of caregiver needs. Caregivers appreciated the clear and real-time information in CLARE. Our findings suggest a need for ongoing development and updates to CLARE, alongside comprehensive interventions addressing the diverse hospital discharge needs of caregivers.

Abstract citation ID: igae098.2146

### AGING SINGLE PARENTS: PSYCHOSOCIAL OUTCOMES AND WELL-BEING AMONG A MARGINALIZED POPULATION

Douglas Hanes, *Stony Brook University School of Medicine, Stony Brook, New York, United States*

Objective Over the last 50 years, single parents—in particular, single mothers—have received much popular and political attention. And yet, their aging experiences remain unresearched. This paper analyzes aging among single parents, including their sex/gender and racial/ethnic composition, as well as their psychosocial well-being, in a population-based aging study. Method Using Health and Retirement Study data (N = 38,785), we identify single parents as participants who were never married at baseline and have children (n = 680), 1.80% of participants with children. Multilevel regressions analyzed psychosocial outcomes: discrimination experiences, subjective social status, and social support. Results Odds of single parenthood increased for each subsequent birth cohort (OR = 1.89, $p < 0.001$). Women (□2 = 85.192, $p < 0.001$) and people of color (□2 = 802.32, $p < 0.001$) were more likely to be single parents, who also had lower education levels and wealth. Single parents were more likely to report discrimination due to race (OR: 1.522, p = 0.047), with single mothers of color more likely to report discrimination due to financial status (OR = 1.575, p = 0.049). Single parents received less positive social support and more negative support from their children; they were also more likely to experience depression, have lower self-rated health, and smaller social networks. Discussion Aging single parents are more likely to belong to already-marginalized groups, and face further and ongoing discrimination, known risk factors for adverse later-life health and well-being. These results indicate the need for greater attention to this marginalized group.

Abstract citation ID: igae098.2147

### ASSESSING AND STRENGTHENING THE INFRASTRUCTURE FOR CAREGIVING SUPPORTS: A STATE PERSPECTIVE

Jennifer Rabalais[1], and Alison Rataj[2], *1. University of New Hampshire, Concord, New Hampshire, United States*, *2. University of Massachusetts Boston, Durham, New Hampshire, United States*

Objective. While progress has been made in policies that recognize and support caregivers, there is a need for a unified framework for caregiver supports. The New Hampshire Alliance for Healthy Aging (NHAHA), a statewide coalition focused on building meaningful partnerships to support and promote healthy aging in communities, has a focal area of ensuring that informal family caregivers have adequate supports. Through NHAHA, we examined current state data, policies, and practices that could be leveraged to develop a recommended plan of actions for caregiving supports that aligns with national caregiving strategies. Methods. Using a mixed-methods design, this study used qualitative data from a focus group (n = 14) to assess barriers to accessing caregiving services. A statewide survey (2024) is underway to understand existing services and supports in NH and will be used in further analysis. Data from NH's State Plan on Aging (2023), which conducted a survey (n = 955) asking several questions about caregiving supports and hosted 10 public listening sessions (n = 185) was also used. Data was catalogued and inventoried across themes in this applied research approach. Results. Three major themes emerged. Caregivers often do not self-identify. Caregiving has negative effects on caregivers financial standing and career. Caregiving supports are siloed, difficult to navigate, and often dissolve without warning. Future Directions. Increased education for caregivers that provides information and resources. Create a system of care for caregivers including respite and financial relief. Advance caregiver friendly work environments.

Abstract citation ID: igae098.2148

### WHAT FACTORS ARE ASSOCIATED WITH SELF-PERCEPTION OF INTENSE FAMILY CAREGIVING?

Susan Hassell, and Joanne Campione, *Westat, Rockville, Maryland, United States*

The stress and anxiety associated with family caregiving can contribute to the caregiver's own health and well-being and can also be linked to less effective caregiving. As part of the 2023 National Survey of Older Americans Act (OAA) Participants, the following item was asked among 1,187 clients of the National Family Caregiver Support Program (NFCSP): "On a scale of 1 to 5, where 5 is very intense, how intense is the care you provide?" Fifty-one percent responded that the care is intense or very intense. Of the remaining caregivers, 15% responded that the care was not intense and 34% felt neutral. We first tested 62 survey variables for bivariate significant differences between "intense care" vs. "not intense care" and then used stepwise multivariable logistic regression. Variables included caregiver demographics, caregiving situational factors, caregiver nutrition, caregiver well-being, care receiver (CR) demographics and health conditions, and the number of activities of daily living (ADL) difficulties experienced by the CR. From the final logistic regression model (c-statistic = 0.74), the factors associated with a significant increase ($p < 0.01$) in the likelihood of self-reported intense care were: 1) CR's number of ADL difficulties, 2) caregiver food insecurity score, 3) CR has Alzheimer's or other memory-related disease, 4) caregiver provides all or almost all the care, and 5) the caregiver has no time for self. These findings provide a deeper understanding of the type of caregivers that may need more support from the NFCSP and from the OAA's case management and home-delivered meals programs.

### SESSION 4455 (PAPER)

### HEALTH DYNAMICS FROM AN INTERNATIONAL PERSPECTIVE

Abstract citation ID: igae098.2149

### SPOUSAL EDUCATION AND COGNITIVE FUNCTION: EVIDENCE FROM THE ENGLISH LONGITUDINAL STUDY OF AGEING

Hui Liu, Juwen Wang, Wencheng Zhang, and Callie Zaborenko, *Purdue University, West Lafayette, Indiana, United States*

Education is one of the strongest social factors influencing cognitive outcomes. Previous studies have predominantly focused on the impact of one's own education, with less attention given to the education of one's spouse. Guided by the "linked lives" perspective, this study examines the association between spousal education and cognitive function among older couples in England. We analyzed data from the English Longitudinal Study of Ageing (ELSA) Waves 7-9, 2014-2019. The sample comprised 7,131 married and cohabiting couples. Cognitive function was assessed using the modified version of the Telephone Interview for Cognitive Status (TICS). Results from the Actor-Partner Interdependence Model (APIM) showed a significant correlation in cognitive trajectories between spouses within dyads. Cross-sectional APIM analysis revealed that higher educational attainment of both spouses was associated with better cognitive function in both themselves and their partners. Longitudinal APIM growth curve analysis suggested that the association between spousal education and cognitive function was mainly driven by correlations at the initial levels, with no significant association observed with the slope of cognitive trajectories over time. We discuss these findings in the context of England and compare them to recent studies conducted in the U.S. and Mexico. Potential explanations for both similarities and differences in findings across studies are explored, shedding light on the nuanced interplay between spousal education and cognitive function.

Abstract citation ID: igae098.2150

### THE ROLE OF CARDIOVASCULAR MORTALITY IN LIFE EXPECTANCY GAPS AFTER AGE 50 BETWEEN US AND OTHER HIGH-INCOME COUNTRIES

Octavio Bramajo, and Neil Mehta, *The University of Texas Medical Branch, Galveston, Texas, United States*

Compared to other high-income countries, the United States underperforms in life expectancy. One key driver of this differential is cardiovascular disease (CVD) mortality, a cause of which the US has had worse trends since around 2008. The differential in CVD mortality has been previously described, however, its contribution to the widening of the US life expectancy lag relative to other high-income countries is unknown. We measured the contribution of CVD mortality to the post-2010 life expectancy divergence between the US and other ten high-income countries. Data and **methods:** Using life table methods and WHO cause-specific mortality data, we computed life expectancy at age 50 (LE50) by sex and by two counterfactual scenarios to establish the contribution of CVD mortality in the growth LE50 gap between 2008 and 2019: first in a cause-deleted approach, removing CVD deaths, and by replacing the age-specific CVD mortality rates of the high-income countries in 2019 with the corresponding US CVD mortality improvement rate. The US had a lower LE50 compared to the average of other high-income countries in 2008), and the LE50 gap with those countries increased in 2019 by 0.4 years for females and 0.8 years for males. Either removing or replacing CVD mortality resulted in narrowing the overall LE50 gap between the US and the high-income countries in 2008 and 2019, and in most cases doing so was able to close it entirely by themselves, especially for females, with CVD explaining entirely the growth of the gap

Abstract citation ID: igae098.2151

### GENDER DISPARITIES IN VISUAL DIFFICULTY AMONG OLDER ADULTS: A CROSS-NATIONAL ANALYSIS

Shane Burns[1], Joseph Saenz[2], and Joshua Ehrlich[1], *1. University of Michigan, Ann Arbor, Michigan, United States*, *2. Arizona State University, Phoenix, Arizona, United States*

**Background:** Rapid global aging will lead to an increase of older adults with visual difficulty, especially women. However, little is known what contextual factors drive this gendered distinction across the world.
**Methods:** Using data from the Health & Retirement Study (HRS) international family of studies, we investigate gender disparities in visual difficulty among respondents ages 55-89 in the United States (n=14,452), England (n=5,708), Mexico (n=10,972), and Korea (n=6,649) while adjusting for demographic, socioeconomic, and health characteristics.
**Results:** Reports of visual difficulty across age groups were generally highest in Mexico, followed by Korea, the United States, and England. In the United States, women had a visual difficulty disadvantage that was fully explained by education and socioeconomic indicators. In England, women had a visual difficulty disadvantage that was partially explained by education and socioeconomic indicators. In Mexico, men had a visual difficulty disadvantage that was explained by comorbidities. In Korea, women had a visual difficulty disadvantage that was explained by comorbidities and health behaviors. Conclusions: Our results highlight the heterogeneity of gender disparities in visual difficulty among older adults across the world.

Abstract citation ID: igae098.2152

### LONG-TERM AND INTERGENERATIONAL EFFECTS OF EARLY-LIFE EXPOSURE TO THE 1959–1961 CHINESE FAMINE ON HEALTH

Mengling Cheng[1], Nicolas Sommet[2], Marko Kerac[3], and Dario Spini[2], *1. University of Lausanne / East China University of Science and Technology, Switzerland, 2. University of Lausanne, Lausanne, Vaud, Switzerland, 3. London School of Hygiene & Tropical Medicine, London, England, United Kingdom*

Undernutrition in early life is associated with increased risk of poor health and mortality in later life. Less is known if the health effects of early-life undernutrition transmit across generations. This study examined the associations between early-life famine exposure and self-rated health in the first generation (F1) and the subsequent generation (F2). We used data from the China Health and Retirement Longitudinal Study (2011-2020, 11,000 participants). We classified F1 participants by famine exposure (exposed/unexposed) and timing at famine exposure (exposed in utero, infancy, pre-school, primary school, or adolescence); we classified F2 offspring by their parents' famine exposure status. We performed growth curve models to investigate the associations between early-life famine exposure and self-rated health in F1 and F2. The first generation (F1) exposed to famine in utero, infancy, pre-school, primary school, or adolescence had poorer self-rated health than those unexposed (IRR=1.06, 95% CI [1.04 – 1.07], p <.001). The offspring generation (F2) whose parents were exposed to famine also had poorer self-rated health than those of unexposed parents (IRR=1.05, 95% CI [1.01 – 1.10], p <.05). Findings of this study show the long-term and intergenerational effects of early-life famine exposure on health, indicating cumulative advantage and disadvantage over the life course and across generations. Our study highlights the need to prevent and treat child/adolescent undernutrition to improve health in the long run.

### SESSION 4460 (SYMPOSIUM)

Abstract citation ID: igae098.2153

### HOME HEALTH CARE ACCESS AND OUTCOMES AMONG VULNERABLE OLDER POPULATIONS: CHALLENGES AND OPPORTUNITIES

Chair: Emily Gadbois
Discussant: Michael Johnson

Home health care (HHC) provides skilled nursing (e.g., wound care, medication management), therapies (e.g., physical, occupational, speech), and aide services (e.g. bathing, grooming, dressing), which are key supports necessary to regain function, restore health, and maintain independence in the community. This panel presents five studies examining factors that challenge equitable access and high quality outcomes associated with skilled HHC among vulnerable older populations (i.e., Veterans, people living with dementia, patients needing post-acute care). The first panelist will discuss findings using national-level HHC quality data demonstrating that access to high-quality HHC varies widely across the country for people with dementia. The second panelist will share findings from a novel data linkage using Medicare Advantage (MA) and traditional Medicare claims to understand variation in use of HHC among people living with dementia. The third panelist's presentation will focus on a particularly vulnerable population - hospitalized older Veterans - and how payer source shapes the use of post-acute HHC. The fourth panelist will discuss results from a qualitative study with MA plans about how they design and manage post-acute HHC benefits. The final presenter will share results from an analysis of national standardized HHC assessment data to identify failures in information transfer that impact care delivery and quality for HHC patients living with dementia. The discussant, a national industry leader with over two decades of experience working in the delivery of HHC, will contextualize findings and discuss how they can inform national policy discussions around improving access and quality of HHC.

Abstract citation ID: igae098.2154

### ACCESS TO HIGH-QUALITY HOME HEALTH CARE FOR PERSONS WITH DEMENTIA

Harriet Mather, *Icahn School of Medicine at Mount Sinai, New York, New York, United States*

While prior research has demonstrated variation in access to high quality home health agencies among older Medicare beneficiaries, access among persons with dementia - a population with increased need of skilled care delivered in the home - has not been explored. This study examined availability of high-quality home health agencies by dementia prevalence in the US using a cross-sectional analysis of county-level data on availability and quality of home health agencies indexed to number of FFS Medicare beneficiaries with Alzheimer's disease and related dementia (ADRD). Using multiple Medicare Public Use Files (PUF), including star ratings for home health quality of patient care (QoPC), home health care CAHPS survey (HHCAHPS), county-level ADRD prevalence, and Medicare FFS enrollment, we found that the median county-level number of home health agencies was 15 (IQR 8-29), star rating was 3.4 (IQR 3.1-3.7) for QoPC and 3.8 (IQR 3.5-4.0) for HHCAHPS. Higher prevalence of persons with

ADRD was moderately correlated with total number of home health agencies in a county (Spearman's r=0.36, p<0.05), with weaker positive correlations with high-quality home health agencies via QoPC (r=.2, p<0.05) and HHCAHPS quality ratings (r=.17, p<0.05). There is wide variation in access to high quality home health care among persons with dementia. To meet the needs of a growing population living at home with dementia, we must address inequities in access to high quality home health care.

Abstract citation ID: igae098.2155

### WHO PAYS FOR VETERANS' POSTACUTE SKILLED HOME HEALTH CARE? A COMPARISON AMONG VA, MEDICARE, AND MEDICARE ADVANTAGE

Marguerite Daus[1], Frank Devone[2], Emily Corneau[3], Kate Magid[4], Katherine Kennedy[5], Pedro Gozalo[6], Kali Thomas[7], and Christine Jones[8], *1. Department of Veterans Affairs, Aurora, Colorado, United States*, *2. United States Department of Veterans Affairs, Providence, Rhode Island, United States*, *3. Providence VA Medical Center, Providence, Rhode Island, United States*, *4. Rocky Mountain Regional VA, Aurora, Colorado, United States*, *5. VA Providence Healthcare System, Center of Innovation in Long-Term Services and Supports, Providence, Rhode Island, United States*, *6. Brown University, Providence, Rhode Island, United States*, *7. Johns Hopkins University, Baltimore, Maryland, United States*, *8. University of Colorado, Aurora, Colorado, United States*

Although costs to the Veteran Health Administration associated with post-acute home health care (HHC) have grown, limited evidence is available about the use and financing of HHC among older Veterans. The objective of this study is to identify Veteran, VA Medical Center (VAMC), and market characteristics that are associated with VHA versus Medicare-funded post-acute skilled HHC for Veterans who are discharged from a VAMC hospital with skilled HHC. We assembled and merged data from several sources for FY 2017-2019 including the VHA's Residential History File, VHA Corporate Data Warehouse, and Centers for Medicare and Medicaid Services. Our cohort (n=64,347 Veterans, VAMCs=114) included all VHA and Medicare enrolled Veterans, aged 66 years or older, admitted to a VAMC for an acute care hospitalization and who received post-acute home health care within 14 days of discharge. We found that being MA-enrolled increased the probability of the VHA paying for HHC by 28.05% (95% Confidence Interval (CI): 27.12%, 28.96). Other important predictors that increased the probability of VHA-paid HHC include previous VHA-paid HHC episodes, previous VHA inpatient admission in the past 12 months, and an admitting diagnosis of sepsis. The most notable finding was that Veterans who are enrolled in MA have a significantly higher probability of having their post-acute HHC paid for by VHA. We will discuss how differences in HHC cost-sharing between MA and TM for members, prior authorization requirements, and denials of care used by MA plans could explain the lower rates of MA-paid HHC.

Abstract citation ID: igae098.2156

### HOME HEALTH USE BY OLDER ADULTS WITH DEMENTIA: COMPARING MEDICARE ADVANTAGE AND TRADITIONAL MEDICARE

Jamie Smith[1], Jianhui (Frank) Xu[2], Teneil Brown[2], Julia Burgdorf[3], Daniel Polsky[2], and Katherine Ornstein[2], *1. Widener University, Chester, Pennsylvania, United States*, *2. Johns Hopkins University, Baltimore, Maryland, United States*, *3. VNS Health, New York, New York, United States*

Skilled home health care (HHC) is a critical service for community-dwelling older adults with dementia, but the majority of evidence about its use and quality is based on Traditional Medicare (TM) beneficiaries. Medicare Advantage (MA) plans - which now enroll over half of Medicare beneficiaries - have flexibility in financing and managing HHC, potentially affecting access and utilization. Given the growth in MA, increasing dementia prevalence, and delivery shifts to community settings, understanding how HHC use differs between MA and TM among persons with dementia is needed. This study compared HHC utilization in a nationally representative sample of persons with dementia in MA and TM. We used Medicare enrollment files linked to FY 2018-2019 MA encounter and TM claims data to identify persons 65+ years with dementia and at least one HHC visit in 2019. Our study sample included 316,407 persons with dementia (66% TM vs 34% MA). MA enrollees were more likely to identify as non-Hispanic, Black (13% vs. 10%), or Hispanic (12% vs. 6%) than TM. Greater proportion of TM beneficiaries were dual eligible (37% vs. 32%). The groups were similar in age, and 34% were male. Overall, MA enrollees were less likely to use HHC services than TM beneficiaries (26.2% vs 29.8%). This presentation will discuss variation in home health visit type/intensity and findings stratified by MA plan type, carriers, and geographic distribution. We will share novel approaches and best practices for measuring HHC utilization among people with dementia using newly available MA encounter data.

Abstract citation ID: igae098.2157

### CHARACTERIZING MEDICARE ADVANTAGE APPROACHES TO PROVIDING POSTACUTE HOME HEALTH

Emily Gadbois[1], Marguerite Daus[2], Christine Jones[3], Jamie Smith[4], and Kali Thomas[5], *1. Brown University, Providence, Rhode Island, United States*, *2. Department of Veterans Affairs, Aurora, Colorado, United States*, *3. University of Colorado, Aurora, Colorado, United States*, *4. Widener University, Chester, Pennsylvania, United States*, *5. Johns Hopkins University, Baltimore, Maryland, United States*

Medicare Advantage (MA) is a major payer of post-acute home health care, but little is known about how MA approaches home health benefit design and provision. In-depth, semi-structured interviews were conducted with 18 leaders from MA plans and care management companies and transcribed; content analysis was used to characterize underlying themes. Across interviews, participants agreed that as long as clinically appropriate, home was the preferred setting for post-acute care. Participants described the role of MA plans and care management companies in the provision of home health care, with most taking a hands-off approach to selecting the setting of post-acute care, instead deferring to patients' choices and citing CMS regulations restricting involvement. Other organizations had more involvement in the selection of post-acute setting, with participants saying that while they don't

"steer" or "direct" setting of care, hospital discharge planners often engaged with their staff for help with difficult to place patients. In the initiation of post-acute home health, participants reported a variety of strategies for service authorization and utilization management. Plans described various approaches to care management to complement post-acute home health services, which in some cases included engaging with care management companies to deliver this aspect of care. In discussing MA approaches to post-acute home health, participants also highlighted substantial challenges facing the home health industry, particularly staffing concerns, and especially in rural areas. Insights from this work can help policymakers understand how MA plans cover home health services and coordinate with providers.

Abstract citation ID: igae098.2158

### MISSING DEMENTIA DIAGNOSIS INFORMATION DURING HOME HEALTH: PREVALENCE AND IMPACTS

Julia Burgdorf, *VNS Health, New York, New York, United States*

Medicare-funded Home Health Care (HHC) delivers skilled health care services through visits to patients' homes. Poor information transfer between HHC and other care settings is a perennial issue; HHC providers rarely have reliable and complete information regarding patients' clinical history or needs. One-third of HHC patients have dementia, but lack of visibility into a patient's dementia status could negatively impact HHC providers' ability to appropriately tailor care to these individuals. We examined a national sample of 426,606 older adults (65+) who received Medicare HHC in 2018 and had diagnosed dementia, as reported in the Medicare Beneficiary Summary File. The majority (68.3%) did not have their dementia diagnosis captured in the HHC claims or assessment files. We fit multilevel logistic models clustering at the HH agency-level and adjusting for patient sociodemographic characteristics and clinical status and HHC agency characteristics. Patients whose dementia diagnosis was not captured during HHC were less likely to receive Speech-Language Pathology (aOR: 0.63; 95% CI: 0.61-0.64), social work (aOR: 0.70; 0.69-.72), or aide visits (aOR: 0.81, 95% CI: 0.80-0.82). Patients whose dementia diagnosis was not captured were also more likely to be hospitalized (aOR: 1.69; 1.65-1.73) or to visit the Emergency Department (aOR: 1.47; 95% CI: 1.44-1.50) during HHC, and less likely to have their Activities of Daily Living (ADL) limitations stabilize or improve by HHC discharge (aOR: 0.70; 95% CI: 0.68-0.72). Results suggest HHC providers often lack pertinent information regarding patients' dementia status, contributing to reduced access to relevant services and increased risk for negative outcomes.

### SESSION 4465 (SYMPOSIUM)

Abstract citation ID: igae098.2159

### INNOVATING LONG-TERM CARE DURING COVID-19 ACROSS FOUR NATIONS: INNOVATIONS, BARRIERS, AND FACILITATORS

Chair: Charlene Chu
Co-Chair: Ruth Melo
Discussant: Barbara Bowers

The COVID-19 pandemic has starkly highlighted the vulnerabilities of older adults in residential long-Term Care Homes (LTCHs), amplifying calls to "reimagine" the LTC sector. Active engagement and inclusion of older adults, their families, and staff in the development and implementation of LTC innovations have been recommended by the United Nations and the World Health Organization, but little is known about such engagement in innovations. This symposium presents a comprehensive scoping review aimed at understanding innovations implemented in LTCHs in four countries (Brazil, Canada, Switzerland, United States) since 2020, and the practices of inclusion within innovation processes within LTCHs. The first paper outlines the scoping review methodology and summarizes national-level results, including the number of studies per country and types of studies (e.g., observational, experimental). The second paper presents an analysis of interventions deployed across different countries. The third paper focuses on the types and goals of technology-based innovations implemented in LTCHs during COVID-19 and summarizes the extent to which perspectives of residents, staff, and family caregivers were considered regarding technology-based innovations. The final paper examines the barriers and facilitators of the innovation process during COVID-19. This international symposium contributes to the reimagining and transformation of LTCHs into more resilient, inclusive, and high-quality living environments.

Abstract citation ID: igae098.2160

### INNOVATIONS IN LONG-TERM CARE SECTOR DURING COVID-19: A SCOPING REVIEW

Ruth Melo[1], Suzanne Santos[1], Sumaya Bhatti[2], Yanjun Duan[2], Charlene Chu[2], Franziska Zuniga[3], Lisa Cranley[2], and Michael Lepore[4], *1. University of São Paulo, São Paulo, Sao Paulo, Brazil, 2. University of Toronto, Toronto, Ontario, Canada, 3. University of Basel, Basel, Basel-Stadt, Switzerland, 4. University of Massachusetts Amherst, Amherst, Massachusetts, United States*

Different innovations were implemented in Long-Term Care (LTC) homes during COVID-19. Although designed to support stakeholders, innovations also have the potential to exacerbate disparities and inequalities in this sector. This scoping review aims to analyze innovations implemented in the residential LTC sector during the pandemic in four countries: Canada, USA, Brazil, and Switzerland. Potential studies were searched in six databases. Search strategy and eligibility criteria followed the mnemonic "PCC" (i.e., Population: residents, family members/caregivers, and other stakeholders, Concept: innovation, and Context: residential LTC sector). The studies retrieved from databases were screened by two independent reviewers. Discordances between reviewers were solved by consensus or by a third reviewer. A customized spreadsheet was used for data extraction. The search identified 4,056 registers. After excluding duplicate studies, 3,122 records were screened. From them, 98 studies fulfilled the eligibility criteria and were included. Half of the studies were conducted in the USA (51.0%), followed by Canada (39.8%). Few studies were conducted in Switzerland (n=3) and Brazil (n=1). Included studies have different design

**methods:** qualitative (18.4%), observational (17.3%), experimental (17.3%) and mixed methods (10.2%). Other types of design methods (opinion, commentary, experience reports, editorial, etc.) and literature reviews accounted for 28.6% and 8.2%, respectively. The innovations presented in the studies were classified according to type, level, and setting. Stakeholder engagement with innovation was categorized as active, passive, and co-designed. results of studies that involved stakeholders are presented in the following papers.

Abstract citation ID: igae098.2161

### INNOVATION CHARACTERISTICS AND STAKEHOLDER INCLUSION IN LTC HOMES DURING COVID-19

Suzanne Santos[1], Sangduan Ginggeaw[2], Ruth Melo[1], Gilciney Andrade Rabello[1], Franziska Zuniga[3], Lisa Cranley[4], Michael Lepore[5], and Charlene Chu[4], *1. University of São Paulo, São Paulo, Sao Paulo, Brazil*, *2. University of Massachusetts Amherst, Amhurst, Massachusetts, United States*, *3. University of Basel, Basel, Basel-Stadt, Switzerland*, *4. University of Toronto, Toronto, Ontario, Canada*, *5. University of Massachusetts Amherst, Amherst, Massachusetts, United States*

The toll the COVID-19 pandemic has taken on the quality of life of those living and working in Long-Term Care (LTC) homes resulted in calls for innovation in the LTC sector. Results from the observational, experimental, qualitative and mixed-method studies (n=62) showed that 50% and 45.2% of the innovations were characterized as products and processes, respectively. Organizational innovations were reported in 4.8% of the studies. Regarding the settings, the majority (52.4%) of the studies were performed at the micro level, followed by meso (25.4%) and macro (20.6%) levels. The identified innovations were predominantly (35.5%) related to new tools for clinical care and staff education, such as online pain assessment training and the creation of virtual instruments for shared end-of-life care decisions, followed by telecommunications interventions (22.6%), generally aimed at improving connections between residents and their families. COVID-19 detection/prevention (11.3%) was the third most common innovation category in this scoping review. Participants of the studies included staff (71%), residents (56%), family members/caregivers (29%) and experts/researchers (11.3%). Only 9.7% of them included all stakeholders. The innovations implied active engagement (62.9%), where the interventions relied on stakeholder's actions (e.g. video calls); passive engagement (22.6%), where no actions from stakeholders were needed (e.g. ambient monitoring systems) and co-designed interventions (14.5%), where participants contributed with all stages (planning, implementing, evaluating) of the innovation. Innovations in LTC homes during COVID-19 were mainly characterized as new products or processes aimed at the care of residents and staff training, which required the active involvement of stakeholders, mostly staff.

Abstract citation ID: igae098.2162

### TECHNOLOGY-BASED INNOVATIONS IN LONG-TERM CARE HOMES DURING COVID-19 AND RELATED INCLUSION PRACTICES

Charlene Chu[1], Sumaya Bhatti[1], Yanjun Duan[1], Suzanne Santos[2], Michael Lepore[3], Ruth Melo[2], Franziska Zuniga[4], and Lisa Cranley[1], *1. University of Toronto, Toronto, Ontario, Canada*, *2. University of São Paulo, São Paulo, Sao Paulo, Brazil*, *3. University of Massachusetts Amherst, Amherst, Massachusetts, United States*, *4. University of Basel, Basel, Basel-Stadt, Switzerland*

The COVID-19 pandemic has magnified challenges in Long-Term Care Homes (LTCHs), driving the adoption of technology-based solutions. The scoping review to explore technology-based innovations into a LTCH during COVID-19 in Canada, the US, Brazil and Switzerland produced 60 studies. The most reported technologies were use of telemedicine/virtual medical care tools that allowed for remote delivery of health care (n=16) within LTCHs, and robotics for clinical tasks and social interaction (n=14). Less frequently reported technologies were: wearables for health monitoring (n=2), mobile apps to improve residents' mental well-being (n=2), and call light systems (n=1) between residents and staff. Most technologies were introduced by researchers (n=38) which primarily engaged with staff (n=30) and residents (n=24). Studies with telemedicine and robotics (n=30) focused on exploring the feasibility and acceptability of technology with the aim to improve optimal resident care and increase social connectedness. Notably, the most common goals of the technology-based innovations were to provide virtual medical care and to engage residents socially. The majority of the papers had active engagement (n=24) which relies on stakeholder's actions (i.e. video calls). We also examined the level of inclusion and engagement of residents, their families, and staff and found varying levels of engagement. the term "co-design" was explicitly used (n=7) where the innovation was designed together with the stakeholders (for example, stakeholder opinions were considered during the process). This paper describes the engagement of stakeholders of these technological innovations, and points to the need for future research in this area.

Abstract citation ID: igae098.2163

### BARRIERS AND FACILITATORS OF THE INNOVATION PROCESS IN LONG-TERM CARE HOMES DURING COVID-19

Suzanne Santos[1], Yanjun Duan[2], Gilciney Andrade Rabello[1], Sumaya Bhatti[2], Charlene Chu[2], Michael Lepore[3], Franziska Zuniga[4], and Ruth Melo[1], *1. University of São Paulo, São Paulo, Sao Paulo, Brazil*, *2. University of Toronto, Toronto, Ontario, Canada*, *3. University of Massachusetts Amherst, Amherst, Massachusetts, United States*, *4. University of Basel, Basel, Basel-Stadt, Switzerland*

The innovation process is complex and multidimensional regardless of the context in which it is applied. However, innovation sustainability can be even more challenging when applied in the Long-Term Care (LTC) sector. So, understanding the barriers and facilitators associated with innovation adoption can be essential to innovation sustainability in LTC homes. In the qualitative studies incorporated within the scoping review (n =18), an in-depth analysis was conducted to discern barriers and facilitators related to innovation in LTC homes during COVID-19. Barriers to innovation identified were related to residents (e.g., safety, privacy, and appropriateness), staff (e.g., workload and tight routine), LTC homes (e.g., infrastructure and material/human resources), and innovation itself (e.g., cost, technical and connectivity problems). Concerns about the inclusiveness of the residents, especially those with some health-related issues (e.g., dementia and macular degeneration) were also pointed out. Conversely, innovations are likelier to be adopted if they prove to be useful and convenient, align with the needs of both LTC homes and residents, and exhibit user-friendly features. From an organizational standpoint, facilitating innovation during COVID-19 involves preparing the LTC home, providing necessary equipment, and offering training and support to staff. Familiar members/caregivers also point as a positive factor the ability of innovation in relief of care burden. Codesign approaches, involving different stakeholders, might be essential to identifying barriers and facilitators and, therefore, contribute to the sustainability of the innovations inside LTC homes.

### SESSION 4470 (SYMPOSIUM)

Abstract citation ID: igae098.2164

### INNOVATIVE GERIATRICS EDUCATION FOR STUDENTS AND PROFESSIONALS

Chair: Erin Emery-Tiburcio

Training in working with older adults is lacking across health care disciplines, and in interprofessional education, both at the student and licensed professional levels. This symposium will describe five unique and highly diverse programs to educate students and professionals about elements of providing optimal health care to older adults. The Extension for Community Healthcare Outcomes (ECHO) Geriatrics program at the University of Washington engaged interprofessional health professions students to participate in monthly learning sessions about age-friendly care and post-session debriefs. The University of Louisville engaged social work students in their highly successful FlourishCare curriculum (FCC), which includes online modules, workshops, interprofessional case management experiences, case conceptualization meetings, Project ECHO, and more. The 4Ms STOP and WATCH Program at St. Louis University provides education and experiential learning for Certified Nursing Assistants (CNAs) in nursing homes. Virginia Commonwealth University trained Emergency Medical Services (EMS) providers on foundational knowledge and skills in care of older adults living with dementia, including advance care planning needs. Finally, the 4Ms framework of an Age-Friendly Health System (What Matters, Medication, Mentation, Mobility) was adapted by Rush University for use by behavioral health providers, who provided training to interprofessional Community Mental Health Center clinicians. Each of these programs has demonstrated significant positive impact and serve as models for training in geriatrics.

Abstract citation ID: igae098.2165

### THE 4MS OF BEHAVIORAL HEALTH IN AN AGE-FRIENDLY HEALTH SYSTEM: TESTING AN EDUCATIONAL FRAMEWORK

Erin Emery-Tiburcio[1], Laura Porter[2], and Siqi Wang[3], *1. Rush University, Chicago, Illinois, United States*, *2. Cherokee Health Systems, Knoxville, Tennessee, United States*, *3. Rush University Medical Center, Chicago, Illinois, United States*

The 4Ms framework of an Age-Friendly Health System (What Matters, Medication, Mentation, Mobility) was originally developed for hospital and ambulatory care settings. As many behavioral health providers did not clearly see a role for themselves in all 4Ms, the framework was adapted for use by behavioral health providers in order to improve the quality of care provided to older adults. The 4Ms-Behavioral Health (4Ms-BH) training program was designed and implemented to assess the impact of training on knowledge and clinical behavior change among mental health clinicians with little to no geriatric training. A secondary goal was to fine-tune the pilot training program for widespread implementation. Fifteen mental health clinicians from Community Mental Health Centers in three states completed eight hours of live webinar training over six months: one three-hour introduction followed by five monthly one-hour application webinars. Clinicians completed knowledge and clinical behavior measures before, during, and after training, along with an assessment of follow-up sustainability needs. The 4Ms-Behavioral Health framework was very well received by participants, who demonstrated significant knowledge gains and clinical behavior change. Participant knowledge increased for each of the 4Ms, with Medication and Mobility showing greatest improvement at 17% and 15%, respectively. Effect sizes for practice behavior change from pre-training to post-training were large (Cohen's d range =.82 – 1.66, $p \leq .01$) in each of the 4Ms assessment and action activities. Implications of the pilot training program for teaching students and licensed professionals will be discussed.

Abstract citation ID: igae098.2166

### PROJECT ECHO-GERIATRICS EXPANDS CAPACITY FOR STUDENT INTERPROFESSIONAL EDUCATION

Katherine Bennett, Tracy Brazg, Ashley McPeak, Mayumi Willgerodt, Barbara Cochrane, Leigh Ann Mike, and Elizabeth Phelan, *University of Washington, Seattle, Washington, United States*

Interprofessional collaborative practice training is important for all health professions trainees, particularly for those who will care for older adults. However, clinical interprofessional training opportunities are limited. The Extension for Community Healthcare Outcomes (ECHO) model trains health professionals in specialty care via case-based sessions with a virtual learning community. We sought to determine whether integration of health professions students into an existing Project ECHO-Geriatrics would be a feasible and effective way to deliver training on age-friendly and interprofessional care. Our novel Age-Friendly Healthcare Interprofessional Training Program used Project ECHO as the platform for longitudinal, interprofessional health professions student education. Students were recruited from health professions schools across the University of Washington. After a brief introduction to age-friendly care and Project ECHO, participants attended at least 3 ECHO sessions and post-session debriefs. In its first two years, the program trained 44 students from 9 health professions programs (e.g. nursing, medicine, pharmacy, physical therapy, social work, dentistry). One hundred percent of participants reported the program was meaningful and worth their time. All competency items (5-point scale, 5 high, 1 low) improved, with the greatest improvements in understanding roles on an interprofessional team (3.1 pre, 4.0 post, p<.001) and confidence for providing age-friendly care (2.2 pre, 4.0 post, p<.001). Participants reported an increased likelihood of focusing on geriatrics in their careers. Open-ended feedback highlighted increased geriatrics knowledge as well as the importance of interprofessional teamwork. Project ECHO is a feasible and effective approach to rapidly expand age-friendly, interprofessional training for health professions students.

Abstract citation ID: igae098.2167

### UTILIZATION OF 4MS TRAINING MODEL IN PRIMARY CARE SETTINGS WITH SOCIAL WORK LEARNERS

Anna Faul[1], Samantha Cotton[1], and Pamela Yankeelov[2], *1. University of Louisville, Louisville, Kentucky, United States*, *2. University of Louisvlle, Louisville, Kentucky, United States*

The UofL GWEP used our FlourishCare curriculum (FCC) to train 52 social work (SW) learners on the 4Ms of age-friendly care. The FCC provides a variety of learning experiences, eg. online modules, workshops, interprofessional case management experiences, case conceptualization meetings, and Project ECHO's. The FCC was delivered during the two-semester SW internship. The experiential part of the FCC allowed learners to work with small caseloads of patients incorporating the 4Ms in assessment, engagement and care navigation. Learners received weekly supervision from clinical SWers trained in the 4M model. The Kirkpatrick model of evaluation was used to evaluate the FCC, focusing on learner satisfaction, knowledge gain, self-efficacy to practice the 4M model, and patient outcomes. Evaluation data shows 95% of learners were satisfied/very satisfied with the learning experience. Learners showed a significant gain in knowledge related to the 4Ms of age-friendly care (Pre-M=65, SD=7.2; Post-M=76, SD=8.1). Learners significantly improved in in their self-efficacy to perform 4M age-friendly care (Pre-M=5.2, SD=1.3; Post-M=8.3,SD=1.9). Patient outcomes data, as measured by our biopsychosocial FlourishCare Index assessment showed that patients served by these learners significantly improved in their overall Index scores (Pre-M=50,SD=7.9; Post-M=59,SD=6.2), their psychological health (Pre-M=52,SD=5.4;Post-M=67,SD=6.1), and their health behaviors (Pre-M=49,SD=7.6; Post-M=58,SD=6.5). These results show the effectiveness of the FCC to develop a future SW workforce who has the ability to provide 4M age-friendly care.

Abstract citation ID: igae098.2168

### AGE-FRIENDLY HEALTH SYSTEM "4MS STOP-AND-WATCH" PROGRAM: A CNA EDUCATION INITIATIVE IN LONG-TERM CARE

Susan Elliott, *Saint Louis University School of Medicine, St. Louis, Missouri, United States*

The valuable contributions of Certified Nursing Assistants (CNAs) working in the longterm care setting are often overlooked, particularly in the implementation of Age-Friendly Health System (AFHS) practices. The "4Ms Stop-and-Watch" initiative was developed to integrate CNAs into the AFHS transformation of a post-acute longterm care facility. With support of the facility administration, our team created didactic and experiential learning opportunities in which all facility CNA staff were invited to participate. CNAs first attended a presentation on the AFHS concepts and the 4Ms framework with a description of the "Stop-and-Watch" program provided. Participants were directed to use a paper form to identify and address at least one 4M during each shift. The information was then entered into an online questionnaire before the end of their shift. The CNA identified which of the 4Ms was addressed and the action taken (e.g., inform nursing supervisor). Survey results were compiled and analyzed to determine effectiveness of education, grasp of 4Ms assessment and intervention, and trends in resident encounters. Feedback was provided to CNAs and facility administration. The presentation will provide detailed information to enable facilities to develop a "4Ms Stop-and-Watch" program to optimize the competency and contributions of the direct care staff.

Abstract citation ID: igae098.2169

### BECOMING DEMENTIA CAPABLE: AN INNOVATIVE EDUCATIONAL STRATEGY FOR EMS PROVIDERS

Kimberly Davis[1], Christy Jensen[2], and Leland Waters[1], *1. Virginia Commonwealth University, Richmond, Virginia, United States*, *2. Martha W. Goodson Center at Riverside Health System, Williamsburg, Virginia, United States*

There is an identified need to provide Emergency Medical Services (EMS) providers with foundational knowledge and skills in care of older adults living with dementia. EMS providers are uniquely positioned to recognize, intervene, and connect older adults and caregivers to community resources. Further, they can provide invaluable information and referrals to primary care providers (PCP), including partnering for long-term management. However, focus groups revealed they receive little to no formal training in best practices for care of older adults. This ongoing HRSA-funded project incorporates a two-pronged approach to training EMS providers: (1) training on the challenges of living with dementia from both the patient and caregiver perspectives including disease progression, management, communication skills, and community resources; and (2) training in advance care planning needs including introduction of the Physician Orders for Scope of Treatment, medical-ethical conversations in end of life, and patient advocacy. Content aligns with goals in the "Dementia State Plan 2020-2024: Building a Dementia Capable Virginia" which seeks to incorporate a public health approach to mitigate the impact of dementia. Training was provided in 7 regions of Virginia and has so far reached 275 EMS providers, first responders, and "hidden responders" (i.e. SW, behavioral health, elder law, etc.). Preliminary evaluation data reveals intention to change practice as a result of the educational strategy and will be shared at the symposium. As front-line workers, EMS providers are poised to help with early identification for older adults who would benefit from referral and early treatment, thereby reducing disease and disability.

### SESSION 4475 (PAPER)

### INTERSECTIONALITY OF DISABILITY AND AGING

Abstract citation ID: igae098.2170

### "I DON'T USE ANYTHING SPECIAL": DIETARY SELF-MANAGEMENT AMONG BLIND AND VISION-IMPAIRED PERSONS WITH TYPE 2 DIABETES

Emily Nicklett[1], Julia Crowley[2], Rene Perez[2], Vanessa Fears[1], Beatriz Morales Hernandez[1], and Yesenia Burgos-Melendez[1], *1. The University of Texas San Antonio, San Antonio, Texas, United States*, *2. Vibrant Works, San Antonio, Texas, United States*

Diabetic retinopathy, a common complication of type 2 diabetes (T2D), is a leading cause of blindness and vision impairment (BVI) among middle-aged and older adults. Diabetes self-management is critical for maintaining eyesight and reducing the risk of further diabetes-related complications; however, those with BVI likely face additional challenges in managing diabetes, including following a diabetes-friendly diet. This study explores barriers and facilitators to maintaining a diabetes-friendly diet among BVI persons with T2D and examines how these experiences may influence their dietary behaviors. In collaboration with Vibrant Works—a community-based organization in San Antonio, Texas offering employment, independent living, and well-being services to the blind and low-vision community—phone-based questionnaires were fielded in January-March 2023 (n=18, ages 42-70). The questionnaires included validated measures of dietary adherence, followed by an open-ended, semi-structured interview about challenges and strategies for diabetes self-management. Quantitative data were entered directly into a database and qualitative data were transcribed and coded with themes emerging directly from the data. Six themes emerged that characterize barriers and facilitators to dietary behaviors: (a) vision status and comorbidities; (b) prior knowledge and experience in food preparation; (c) habits and routines; (d) access/accessibility (i.e., transportation, BVI-friendly cooking tools, accessibility of food stores; (e) support (i.e., social and tangible support); and (f) neighborhood and community factors (i.e., walkability, sidewalk quality, food store availability). Findings from this study can inform more equitable strategies for dietary self-management among populations experiencing both T2D and vision loss through direct practice, future research, and policy approaches.

Abstract citation ID: igae098.2171

### EVALUATION OF EQUITY IN HOSPICE CARE UTILIZATION AMONG MEDICARE-ENROLLED AUTISTIC OLDER ADULTS

Lauren Bishop[1], Melica Nikahd[2], J. Madison Hyer[2], and Brittany Hand[2], *1. University of Wisconsin Madison, Madison, Wisconsin, United States*, *2. The Ohio State University, Columbus, Ohio, United States*

Better identification and increased life expectancy of autistic people has given rise to a need to better understand their end-of-life care. The purpose of this study was to compare hospice utilization of autistic older adults to non-autistic older adults using hospice claims from Medicare and examine if known inequities in hospice utilization among the general population are exacerbated among autistic older adults. Our sample included 5,468 autistic older adults and 10,934 matched population comparison older adults who died between 2013-2021. We used multivariable logistic regression to compare these groups on the odds of any, early (at least 28 days before death), intermediate (4-27 days before death), and late (within 3 days of death) hospice utilization. Interaction terms were used to assess whether effects were modified by beneficiary's sex, race, or rurality. In a sensitivity analysis, we repeated our analyses in unmatched samples. The autistic older adult and matched population comparison groups had similar odds of any hospice utilization (OR: 1.05; 95% CI: 0.98-1.13), early (OR: 1.01; 95% CI: 0.91-1.11), intermediate (OR: 1.03; 95% CI: 0.95-1.13), and late hospice (OR: 1.07; 95% CI: 0.96-1.19). In our models evaluating the impact of other known inequities, we did not find significant interactions between autism diagnostic status and sex, race, or rurality. Results were similar in the unmatched samples. Our findings suggest that autistic older adults experience similar receipt and timing of hospice as non-autistic peers, highlighting the potential power of zero cost, universal health policies in reducing healthcare utilization disparities among autistic people.

Abstract citation ID: igae098.2172

### WAITING TO BENEFIT: AGE-BASED DISABILITY REGULATIONS AND PATHWAYS TO SUPPLEMENTAL SECURITY INCOME IN LATER LIFE

Callie Freitag, *University of Wisconsin-Madison, Madison, Wisconsin, United States*

Supplemental Security Income (SSI) is an important resource for low-income older adults and people with disabilities, but it is notoriously difficult to be deemed eligible. Older SSI applicants may have an easier time getting approved. Starting at age 45, the Social Security Administration (SSA) relaxes the disability requirements for certain groups every five years until they are eliminated at age 65. If the age-based rules prevent otherwise-eligible people from receiving SSI benefits, then the consequences for this economically vulnerable group could be particularly dire. This paper has two aims. First, I estimate the SSI take-up rate by age and test whether discontinuities in SSI take-up exist when SSA disability determination rules are relaxed (at ages 45, 50, 55, and 60) and eliminated (at age 65). Second, I identify patterns of employment, homelessness, and public assistance use among SSI recipients in the five years prior to SSI take-up and examine how these patterns vary by age. I conduct these analyses with administrative microdata from multiple agencies in Washington state spanning from 2010 through 2017. I find strong evidence of discontinuous increases in SSI take-up when the disability rules are relaxed at age 55 and eliminated at age 65, suggesting the program rules may act as gatekeepers for otherwise eligible applicants. Adults face significant financial hardship in the five years prior to receiving SSI. People who took up SSI between the ages of 40 and 59 were more likely to have been homeless than employed prior to receiving SSI.

Abstract citation ID: igae098.2173

### UNCOVERING THE DIMENSIONS OF WELL-BEING AMONG CAREGIVERS FOR OLDER AMERICANS IN THE NATIONAL STUDY OF CAREGIVING

Frances Yang[1], Maria Roche-Dean[2], and Kristine Williams[1], *1. University of Kansas Medical Center, Kansas City, Kansas, United States*, *2. University of Kansas, Kansas City, Kansas, United States*

Evaluating caregiver well-being is vital for healthcare providers, researchers and policymakers. There are various valid and reliable instruments for caregiver well-being with as many as 45 items in Tebb's Caregiver Well-Being Scale. When conducting survey research it is imperative to avoid participant burden and fatigue by limiting the number of questions. The National Study of Caregiving (NSOC) is a survey about caregivers' responsibilities, health, and well-being while caring for their older care recipients. This study focuses on identifying and reducing the complex dimensions of the 17 well-being questions to yield a streamlined, valid, and reliable caregiver well-being measure in 2022. Exploratory factor analysis ($\chi 2$=1332, df=43, p<.001; RMSEA=0.1; CFI=0.96) and confirmatory factor analysis ($\chi 2$=1027, df=51, p<.001; RMSEA=0.09; CFI=0.97) were conducted with 2,376 caregivers' responses in Round 12. Twelve questions were identified with high reliability (Cronbach's alpha ($\alpha$)=0.97) to best fit a three dimensional model for caregiver well-being: 1) Positive affect (cheerful mood, calm and peaceful, full of life; $\alpha$=0.94), 2) Isolation (bored, lonely, life has meaning, life is lonely because I have few close friends; $\alpha$=0.93), and 3) Psychological distress (upset, felt little interest, felt down depressed, felt nervous, felt unable to stop or control worrying; $\alpha$=0.93). Reducing the number of valid caregiver well-being questions by 29% has the potential to decrease survey fatigue for caregivers. The dimensions or themes identified for caregivers' responses about their well-being allows for appropriate development of targeted interventions and policy recommendations to improve informal caregiver well-being who are caring for those aging in the US.

Abstract citation ID: igae098.2174
### DISABILITY: INDEPENDENT OR DEPENDENT VARIABLE IN GERONTOLOGICAL RESEARCH?
Michelle Putnam[1], and Susan Stark[2], *1. Simmons University, Boston, Massachusetts, United States*, *2. Washington University in St. Louis, St. Louis, Missouri, United States*

A critical issue for bridging aging and disability research is developing a shared understanding of when disability should be employed as an independent or a dependent variable. To date, this discussion has rarely been had across researchers. Is disability a dependent variable, an outcome? Or does it contribute to outcomes as an independent variable? Or both? How disability is conceptualized, measured, analyzed, and interpreted matters for how we understand the role disability plays over the life course. Disability status is used as both an independent and a dependent variable in gerontological and disability/rehabilitation research. Research in both fields often focuses on reducing likelihood, duration, and severity of disability. A key distinction between fields is how each conceptualize disability – what it is and what it means – particularly given age of onset and type of disability. For example, in gerontological research, often positions disability as an individual trait that mediates the ability to live independently (typically reducing independence). However, disability/rehabilitation research commonly positions disability as a situational trait, or moderating variable associated with difficulty of living independently, but not causing inability to live independently. NIH's recent inclusion of disability as a social determinant of health provides an opportunity for gerontologists to more deeply consider how disability is employed in research designs and why. We discuss how more consistent use of disability as an independent and/or dependent variable can help bridge aging and disability research and provide greater understanding of the role disability plays over the life course.

### SESSION 4480 (SYMPOSIUM)

Abstract citation ID: igae098.2175
### MANAGING MULTIPLE CHRONIC CONDITIONS: FINDINGS BY NATIONAL CLAUDE D. PEPPER OLDER AMERICANS INDEPENDENCE CENTERS
Chair: Eileen Graham
Co-Chair: Rachel O'Conor
Discussant: Michael Wolf

Multimorbidity is an increasing concern among clinicians, as older adults accumulate chronic conditions that require complex coordination of care. There is an increasing interest among research and clinical communities to understand the impacts of multimorbidity on individuals and how quality of care can be optimized to improve quality of life in the aging population. The primary objective of the Claude D. Pepper Older Americans Independence Centers national network is to advance scientific knowledge that will help older adults maintain function, independence, and quality of life. The current symposium highlights recent work put forth by three of the 15 National 'Pepper' Centers (Northwestern, Mt. Sinai, UCSF). Graham will present recent findings on how multimorbidity is longitudinally related to physical and mental health-related quality of life, and their cognitive, psychological, social, and health-related determinants. The next three presentations explore the role of caregivers in supporting the health and quality of life of older adults with multimorbidity. O'Conor will discuss complexity of family care dyads management of MCC, by reviewing patient and caregiver perspectives on challenges of successful management at home. Allison will discuss the interplay of care support structures and dementia care needs in the home. Reckrey will discuss results from a qualitative analysis exploring perspectives of geriatricians on the importance of paid caregivers of older adults with MCC. In sum, this symposium will present novel evidence showing the importance of understanding of how multimorbidity and dementia impact caregiving considerations, primary care decisions, and health related quality of life for older adults.

Abstract citation ID: igae098.2176
### LONGITUDINAL ASSOCIATIONS BETWEEN MULTIMORBIDITIES AND PATIENT-REPORTED QUALITY OF LIFE
Eileen Graham[1], Olivia Atherton[2], Daniel Mroczek[1], Marquita Lewis Thames[1], and Michael Wolf[1], *1. Northwestern University, Evanston, Illinois, United States*, *2. University of Houston, Houston, Texas, United States*

The global prevalence of multimorbidity is increasing as the population ages. Older adults are very likely to develop multiple chronic conditions, and nearly two-thirds of the older adult population in the U.S. are estimated to be experiencing two or more chronic conditions. The current study examined whether multimorbidity was associated with longitudinal patterns in health-related quality of life (i.e., anxiety, depression, physical function) and whether these associations were moderated by sociodemographic factors (i.e., sex, race, marital status, income, insurance, education). The present pre-registered study used data from the Health Literacy and Cognitive Function Among Older Adults Longitudinal Study (LitCog), a prospective cohort study of English-speaking older adults (N=900). At each measurement occasion, participants reported anxiety, depression, and physical function using the Patient Reported Outcomes Information System (PROMIS), current chronic conditions, and socio-demographic characteristics. We employed multilevel growth models to estimate changes in health-related quality of life, with multimorbidities as a predictor and socio-demographics as covariates. Results indicated that individuals with multiple chronic conditions reported persistently high levels of anxiety and depression, and worse physical function. We found evidence for racial health disparities, such that individuals who identified as non-white experienced worsening health-related quality of life as multimorbidities increased, relative to white participants. These results contribute to the current conversation about the long term impacts of structural and systemic barriers experienced by minoritized groups. We further discuss the public health implications of multimorbidity in older adulthood.

Abstract citation ID: igae098.2177

### CASCADING BURDEN OF UNCONTROLLED CHRONIC CONDITIONS AMONG PERSONS LIVING WITH DEMENTIA

Rachel O'Conor, Allison Pack, Andrea Russell, Dianne Oladejo, Darby Morhardt, Lee Lindquist, and Michael Wolf, *Northwestern University, Evanston, Illinois, United States*

Half of all potentially avoidable hospitalizations among persons living with dementia (PLWD) are due to poorly controlled chronic conditions. Yet little is known about how PLWD and their caregivers manage the multiple chronic conditions (MCC) and polypharmacy they often contend with, or how these health management behaviors impact PLWD and caregiver well-being. We conducted qualitative interviews with patient-caregiver dyads exploring capacity to manage MCC. PLWD were eligible if they had mild or moderate dementia, ≥3 chronic conditions, ≥5 medicines, and a caregiver. Three coders applied a priori and emergent codes to transcripts. We interviewed 68 participants (PLWD: mean age 78 years, 65% female, 53% white, 32% Black; Caregivers: mean age 65 years, 73% female, 56% spouse, 38% adult child). PLWD were managing 5 chronic conditions and took 9 medicines on average. PLWD recalled instances of difficulty managing MCC and subsequent hospitalization, while emphasizing they had recovered. Following errors in health management behaviors, they expressed gratitude for additional caregiver support. In contrast, caregivers described a cascading burden of uncontrolled chronic conditions among PLWD. Many reported feeling overwhelmed managing the complexity of MCC, including multi-drug regimens, for the PLWD. Caregivers cited difficulties achieving adequate control for numerous chronic conditions (e.g. hypertension, diabetes, heart disease). Consequences included hospitalizations and increased regimen complexity; in turn, these increased caregiver responsibilities, emotional strain, and financial costs. Findings suggest management of MCC compounds caregiving responsibilities and contributes to caregiving burden. Supporting caregivers' practical skills for managing MCC may promote PLWD and caregiver well-being.

Abstract citation ID: igae098.2178

### "MY LIFE WAS EASY; HIS LIFE TAKES ALL OF MY TIME": THE INTERPLAY OF SUPPORT STRUCTURES AND DEMENTIA CARE NEEDS IN THE HOME

Theresa Allison[1], Alexander Smith[1], Jennie Gubner[2], and Kenneth Covinsky[1], *1. University of California San Francisco, San Francisco, California, United States*, *2. University of Arizona, Tucson, Arizona, United States*

We have limited literature about the lived realities of in-home dementia care. Using data from two ethnographic studies of music and everyday life in the homes of people living with dementia, we investigated the relationship between care support resources and multimorbidity care needs. Data included demographics, measures, fieldnotes and semi-structured interview transcripts. We inductively coded transcripts using ATLAS.ti and triangulated findings across data sources. N=83 (38 people living with dementia, 34 family care partners, 11 professionals). Participation varied from a single phone interview to 8 in-home visits over two years. Participants identified complex mismatches between resources and care needs, particularly in ambulatory stages of dementia or with care partner multimorbidity. In contrast, participants identified spirituality as a powerful motivator for providing care. Participants identified adequate social support and financial resources as critical. The findings underscore the need for nuanced approaches to interventions, acknowledging both positive and negative aspects of caregiving.

Abstract citation ID: igae098.2179

### WHEN DO PAID CAREGIVERS SUPPORT THE HEALTH OF OLDER ADULTS? GERIATRICIAN PERSPECTIVES

Jennifer Reckrey[1], Leah Estrada[2], Deborah Watman[3], and Emily Franzosa[4], *1. Icahn School of Medicine at Mount Sinai, Icahn School of Medicine at Mount Sinai, New York, United States*, *2. Icahn School of Medicine at Mount Sinai, New York, New York, United States*, *3. Icahn School of Medicine at Mount Sinai, New York, New York, United States*, *4. James J Peters VA Medical Center, Bronx, New York, United States*

Paid caregivers (e.g., home health aides, personal care attendants, other home care workers) may support not only the function but also the health of the older adults they care for in the home, but little is known about how medical providers view paid caregiver role in healthcare. This secondary qualitative analysis of interview data from geriatricians (n=9 geriatricians, n=27 interviews) used thematic analysis to identify geriatrician perspectives on when paid caregivers do the most to support the health of their older adult clients. Geriatricians perceived that paid caregiver contributions were greatest in the care of high-needs older adults including those with multimorbidity, dementia, and significant functional impairment. They described that paid caregivers stepped up to fill healthcare gaps when families could not provide all needed support (e.g., long distance family caregivers). They also described how the personal qualities and internal motivation of paid caregivers affected their role in care, but rarely acknowledged the systemic barriers to greater paid caregiver participation in health-related care. As the locus of long-term care continues to shift from institutions to the community, a greater number of older adults with multimorbidity and dementia will rely on paid caregiver to live at home despite growing functional impairment. Future work should consider how to best integrate paid caregivers who are already providing health-related support into the care team and explore barriers to paid caregiver participation in health-related care more generally, especially for older adults with the highest care needs.

### SESSION 4485 (SYMPOSIUM)

Abstract citation ID: igae098.2180

### METHODS TO CONSTRUCT AND ANALYZE COGNITIVE FUNCTION TRAJECTORIES USING CLAIMS AND ASSESSMENT DATA

Chair: Olga Jarrín
Co-Chair: Haiqun Lin

This symposium presents the methodology for construction and analysis of the cognitive function change over time during the last five years of life among Medicare beneficiaries. Understanding the changes in cognition is important in maintaining functional independence in last years of life. The first presentation explains the methodology to harmonize data from healthcare claims and clinical assessments across various post-acute and long-term care settings for Medicare beneficiaries who died in 2019, age 50 or older at death, with at least five years of continuous Medicare enrollment. Cognitive function data were harmonized to create a four-category variable: no impairment, memory loss, mild cognitive impairment (MCI), and dementia/severe cognitive impairment. The second presentation examines change in cognitive function over the last five years using the group-based trajectory model for ordered categorical outcomes, revealing ten cognitive function trajectory groups. Sociodemographic and clinical factors were assessed for their association with the trajectories and used to predict trajectory class clusters with machine learning algorithms. The third presentation presents a novel application of the three-step Bolck-Croon-Hagenaars (BCH) method with mixed-effects group-based trajectory model to account for the individual heterogeneous effects, and assess the association between the group membership and risk factors. The fourth presentation leverages the strengths of real-world data for nearly two million Medicare beneficiaries to unpack within-group racial/ethnic and sex differences in cognitive function trajectories and their predictors with discussion from a life-course perspective. Collectively, these methods and findings advance our understanding of differential late-life cognitive decline patterns, informing future etiological and intervention studies.

Abstract citation ID: igae098.2181

### HARMONIZATION OF MEDICARE BENEFICIARIES' COGNITIVE FUNCTION DATA

Anum Zafar[1], Fred Kobylarz[2], Rebecca Zaritsky[1], Hyosin Kim[3], Maria Lopez[1], Weiyi Xia[4], Haiqun Lin[5], and Olga Jarrín[1], *1. Rutgers, The State University of New Jersey, New Brunswick, New Jersey, United States*, *2. Rutgers Robert Wood Johnson Medical School, New Brunswick, New Jersey, United States*, *3. Oregon State University, Corvallis, Oregon, United States*, *4. Rutgers, The State University of New Jersey, Piscataway, New Jersey, United States*, *5. Rutgers, The State University of New Jersey, Newark, New Jersey, United States*

This study explains the methodology to harmonize data from healthcare claims and clinical assessments across various post-acute and long-term care settings for Medicare beneficiaries who died in 2019, age 50 or older at death, with at least five years of continuous Medicare enrollment. The cognitive function trajectory file was constructed from 2014-2019 Medicare and Medicaid claims and encounter files (ICD-9 and ICD-10 codes), and cognitive assessments from post-acute and long-term care settings (BIMS scores and other relevant items from MDS, MDS-SB, OASIS, and IRF-PAI assessments). Cognitive function data were harmonized to create a four-category variable: no impairment, memory loss, mild cognitive impairment (MCI), and dementia/severe cognitive impairment. Data were summarized for each quarter (3-month period) of the last five years of life (20 timepoints). Of the nearly two million beneficiaries in our study population, 19.4% had no evidence of cognitive impairment at any point. Five years before death, 3.2% of the sample population had mild cognitive impairment (MCI) and 7.7% had dementia or severe cognitive impairment. At the time of death, 14.8% of the sample had MCI and 50.6% had severe cognitive impairment/dementia. Among beneficiaries with any cognitive impairment, claims or assessment data contributing new information to the trajectory file were available for an average of 7 (out of 20), s.d. 5.7 quarters, median=5, inter-quartile range: 2-11. The harmonized longitudinal dataset allows for tracking change in cognitive function and identifying risk factors for cognitive decline.

Abstract citation ID: igae098.2182

### IDENTIFICATION AND MACHINE LEARNING PREDICTION OF MEDICARE BENEFICIARIES' COGNITIVE FUNCTION TRAJECTORIES

Haiqun Lin[1], Weiyi Xia[2], Anum Zafar[3], Hyosin Kim[4], Soko Setoguchi[3], Fred Kobylarz[5], Jason Roy[2], and Olga Jarrín[3], *1. Rutgers, The State University of New Jersey, Newark, New Jersey, United States*, *2. Rutgers, The State University of New Jersey, Piscataway, New Jersey, United States*, *3. Rutgers, The State University of New Jersey, New Brunswick, New Jersey, United States*, *4. Oregon State University, Corvallis, Oregon, United States*, *5. Rutgers Robert Wood Johnson Medical School, New Brunswick, New Jersey, United States*

This work aims to characterize the functional decline in cognition during the last five years of life. A 10% random sample consisting of 195,240 Medicare beneficiaries who died in 2019, aged fifty or older and continuously enrolled in Medicare during their last five years of life, was analyzed. Cognitive function status, characterized by no impairment, memory loss, mild cognitive impairment, and dementia, was obtained from the assessment and the claim file for each beneficiary whenever it was available. Changes in cognitive function over the last five years were examined using the group-based trajectory model for ordered categorical outcomes: Ten trajectory groups of cognitive function were identified and had a better value of Bayesian Information Criterion than either the 9-class or the 11-class solution. The 10 trajectory groups were categorized into four clusters: the cognitively intact cluster (38% of beneficiaries) which contains one trajectory group with beneficiaries maintaining their cognitive function throughout the last five years, the mild cognitive impairment (MCI) cluster (27%) which contains four trajectory groups with beneficiaries experiencing slow decline in cognitive function and died with MCI, the steady decline cluster to dementia (25%) which contains four trajectory groups with beneficiaries experiencing steady decline in cognitive function and died with dementia, and the early-decline dementia cluster (10%) which contains only one trajectory group with beneficiaries whose cognitive decline started more than five years before death. Sociodemographic and clinical factors were assessed for their association with the trajectories and used to predict trajectory clusters with machine learning algorithms.

Abstract citation ID: igae098.2183

### A NOVEL APPLICATION OF BOLCK-CROON-HAGENAARS METHOD WITH MIXED-EFFECT LATENT CLASS ANALYSIS OF AGING TRAJECTORIES

Weiyi Xia[1], Anum Zafar[2], Olga Jarrín[2], Soko Setoguchi[2], Jason Roy[1], and Haiqun Lin[3], *1. Rutgers, The State University of New Jersey, Piscataway, New Jersey, United States*, *2. Rutgers, The State University of New Jersey, New Brunswick, New Jersey, United States*, *3. Rutgers, The State University of New Jersey, Newark, New Jersey, United States*

This work assesses the association between longitudinal trajectory classes and risk factors. The longitudinal trajectory classes are derived from the latent class analysis of longitudinal variables of interest such as repeated cognitive status and days in a quarter in a care setting during last years of life. This work aims to apply the Bolck–Croon–Hagenaars (BCH) method to the mixed effect group-based trajectory model to account for the individual heterogeneous effects. First, we fit a group-based trajectory model with random intercepts. The inclusion of the random effects poses a computational challenge that we address in this work. Next, each individual would be assigned a class membership with the highest posterior probability (modal assignment). Proportional assignments can be used as well. BCH weights are obtained based on the product of assigned class membership probabilities and the inverse of the matrix for misclassification errors. Finally, we reweight the data to assess the relationship between latent class memberships and risk factors. We test the performance of the bias-corrected BCH approach with simulated data. The simulation results reveal much smaller bias with BCH and larger bias with the naive three-step approach. We then apply the BCH method to assess the association between the aging trajectories, such as cognitive function trajectories or care setting trajectories in last years of life, and socio-demographic and clinical factors among Medicare beneficiary decedents. The application of the BCH method on the mixed-effect group-based trajectory modeling provides a rigorous assessment of the association between aging trajectories and risk factors.

Abstract citation ID: igae098.2184

### RACIAL/ETHNIC AND SEX DIFFERENCES IN LATE-LIFE COGNITIVE TRAJECTORIES

Olga Jarrín[1], Roland Thorpe[2], Fred Kobylarz[3], Weiyi Xia[4], Hyosin Kim[5], Anum Zafar[1], Maria Lopez[1], and Haiqun Lin[6], *1. Rutgers, The State University of New Jersey, New Brunswick, New Jersey, United States*, *2. Johns Hopkins University, Baltimore, Maryland, United States*, *3. Rutgers Robert Wood Johnson Medical School, New Brunswick, New Jersey, United States*, *4. Rutgers, The State University of New Jersey, Piscataway, New Jersey, United States*, *5. Oregon State University, Corvallis, Oregon, United States*, *6. Rutgers, The State University of New Jersey, Newark, New Jersey, United States*

Prior research on racial/ethnic and sex difference in late life cognitive trajectories has been limited by small sample sizes in clinical and survey cohorts. We overcome these challenges through leveraging real-world data for the U.S. Medicare beneficiary population to examine within-group differences in cognitive function trajectories and their predictors. Specifically, we found Black, Hispanic, and Asian American men and women have a higher risk of severe cognitive impairment and spend more months and years of their lives with significant cognitive impairment. These differences are largely driven by differences in the risk of cognitive impairment associated with older age, consistent with prior research on life course stress and the 'weathering' hypothesis that early health deterioration is a consequence of the cumulative impacts of social or economic adversity and political marginalization. Our findings replicate, and in some cases refute, findings from prior studies from smaller longitudinal cohorts, including the Health and Retirement Study. Results will be discussed from a life course perspective with attention to variation in environmental, sociocultural, political, and historical factors that create and sustain health disparities for racial/ethnic minority older adults in the United States.

### SESSION 4490 (SYMPOSIUM)

Abstract citation ID: igae098.2185

### MULTIDOMAIN INTERVENTION TO IMPROVE COGNITION IN OLDER ADULTS: NEW RESULTS AND FUTURE CHALLENGES

Chair: Louis Bherer

Decades of research have supported the notion that physical exercise and cognitive training can both help enhance cognitive performances in older adults. More recent studies have investigated whether these two domains of intervention could have synergistic effects, however results are equivocal. Moreover, there is an important heterogeneity among studies with regards to intervention modality within domain (e.g., memory or attention training as cognitive intervention; aerobic, resistance or combined physical exercise) and studied population (MCI vs. healthy aging). This symposium will discuss recent findings from innovative interventions ranging from within-domain attention training to multi-domain interventions that combine cognitive training or cognitive stimulation with or without physical exercise components. Issue of interindividual differences and the need to better individualized intervention as well as diversity in targeted population will also be discussed. Presentations will also address future challenges in combined intervention studies.

Abstract citation ID: igae098.2186

### EFFECTS OF HOME-BASED COGNITIVE AND PHYSICAL EXERCISE TRAINING ON COGNITION IN OLDER ADULTS: THE COVEPIC TRIAL

Louis Bherer[1], Caroll-Ann Blanchette[2], Florent Besnier[2], Mathieu Gayda[3], Christine Gagnon[2], Thomas Vincent[2], Tudor Vrinceanu[2], and Emma Dupuy[2], *1. University of*

*Montreal, Montreal, Quebec, Canada*, *2. Montreal Heart Institute, Montréal, Quebec, Canada*, *3. Université de Montréal, Montreal, Quebec, Canada*

**Background:** Studies suggest that cognitive training and physical exercise can independently help improve cognition in older adults. This randomized clinical trial (COVEPIC study) aimed to compare the effects of 6 months of home-based physical exercise alone and combined with cognitive training on the cognition of older adults.

**Methods:** 127 adults (50 years and older) were randomly assigned to one of the two following intervention arms (1:1): 1/ home-based physical exercise alone or 2/ combined home-based physical exercise and cognitive training. Participants completed videoconference neuropsychological assessments targeting episodic and working memory, processing speed and executive functions, prior to the intervention (T0), mid-intervention (T1) (3 months), and post-intervention (T2) (6 months). Clinicaltrials.gov (NCT04635462).

**Results:** Participation in the combined intervention showed a larger improvement than the exercise alone group on the MoCA from T0 to T1, and a change in executive functions which was not observed in the physical exercise only group. Effects of exercise dose were also observed, as participants who went through a higher dose of exercise showed larger improvement in episodic and working memory.

**Discussion:** Results suggest that a home-based intervention combining cognitive and physical training can help improve cognition in older adults.

Abstract citation ID: igae098.2187

### TESTING THE EFFECTS OF COGNITIVE TRAINING ON ALZHEIMER'S DISEASE AND RELATED DEMENTIAS: THE MODERATING ROLE OF SDOH

George Rebok[1], Olivio Clay[2], Gail Wallace[1], Karlene Ball[3], Roland Thorpe, Jr.[1], Michael Marsiske[4], Cynthia Felix[5], and Sherry Willis[6], *1. Johns Hopkins University, Baltimore, Maryland, United States*, *2. The University of Alabama at Birmingham, Birmingham, Alabama, United States*, *3. University of Alabama at Birmingham, Birmingham, Alabama, United States*, *4. University of Florida, Gainesville, Florida, United States*, *5. University of Pittsburgh, Pittsburgh, Pennsylvania, United States*, *6. University of Washington, Seattle, Washington, United States*

The Advanced Cognitive Training for Independent and Vital Elderly (ACTIVE) study is the largest randomized clinical trial of the efficacy of cognitive training (memory, reasoning, processing speed) in improving both cognitive abilities and everyday functioning in normal older adults (N = 2,802; age M = 73.6, range 65-94 years; 76% female; 26% African American), with follow-ups extending through 10 years, and additional follow-ups on claims-based dementia diagnosis through 20 years post-intervention. This presentation considers not only training focus (cognition, exercise) but also a) subjects' individual differences in social context variables like social determinants of health (SDoH) and b) Alzheimer's Disease and Related Dementias (ADRD) as training outcome. Overall, we found fewer significant effects of SDoH as moderators of cognitive training effects on diagnosed dementia than we expected, indicating cognitive training was beneficial for individuals across the continuum for most domains of SDoH (e.g., education access, economic stability, neighborhood and built environment, health care access, social and community context). Participants obtained a greater degree of protection from ADRD through cognitive training when they had higher scores for domains associated with health care access (HRs.68 to.75) and more education (HRs.75 to.76). Higher scores on neighborhood and built environment were also associated with lower ADRD risk (HRs.69 to.70). Future work should continue to explore culturally tailored training interventions alone and in combination with physical exercise and environmental modifications such as improved healthcare access to reduce ADRD risk associated with SDoH that disproportionately affects racially diverse aging populations.

Abstract citation ID: igae098.2188

### EFFECTS OF A ONE-YEAR PIANO PRACTICE INTERVENTION ON COGNITIVE FLEXIBILITY IN HEALTHY OLDER ADULTS

Melanie Mack[1], Damien Marie[2], Florian Worschech[3], Tillmann Krueger[4], Christopher Sinke[4], Eckart Altenmueller[3], Clara James[2], and Matthias Kliegel[5], *1. University of Geneva, Geneva, Geneve, Switzerland*, *2. University of Applied Sciences and Arts Western Switzerland HES-SO, Geneva School of Health Sciences, Geneva Musical Minds lab, Geneva, Geneve, Switzerland*, *3. Hanover University of Music, Drama and Media, Hannover, Niedersachsen, Germany*, *4. Division of Clinical Psychology & Sexual Medicine, Department of Psychiatry, Social Psychiatry and Psychotherapy, Hannover Medical School, Hannover, Niedersachsen, Germany*, *5. Centre for the Interdisciplinary Study of Gerontology and Vulnerability, University of Geneva, Geneva, Geneve, Switzerland*

We analyzed data of a randomized controlled trial to examine the effects of piano practice on cognitive flexibility in healthy older adults. Participants (N = 153, 69.5 ± 3.5 years of age, 57.5% females) were randomly assigned to a piano practice group (PP) or a control group engaged in active music listening (MC). Both groups underwent a year-long intervention with weekly 60-minute lessons and daily homework. We assessed switch and mixing costs in terms of speed (mean RTs) and variability (standard deviation of RTs) with a number switch and a perceptual switch task. We employed MIDI-based Scale Analysis to assess pianistic performance. Tests were conducted at baseline, after 6 months, post-intervention (12 months), and at follow-up (18 months). Results revealed more pronounced improvements in pianistic performance in the PP compared to the MC group over the course of the intervention. Both groups exhibited gains in several cognitive flexibility outcomes, which originated primarily in the latter half of the intervention. For mixing costs, the PP group showed partly greater improvements compared to the MC group. Additionally, the findings indicated a compensation account in both groups, which was partially more pronounced in the PP group. This study suggests that both piano practice and active music listening – with piano practice to a slightly higher degree - enhance cognitive flexibility, particularly in sustained control mechanisms linked to mixing costs. Both interventions potentially require longer than 6 months to induce behavioral transfer effects and are especially beneficial for individuals with lower cognitive flexibility levels.

Abstract citation ID: igae098.2189

### MAXIMIZING COGNITIVE TRAINING OUTCOMES WITH SIMULTANEOUS BRAIN STIMULATION

Susanne Jaeggi, *Northeastern University, Boston, Massachusetts, United States*

Cognitive health is a fundamental issue facing everyone across their lifespan, however, while there is accumulating evidence for the efficacy of targeted interventions to improve cognition, the outcomes have been inconsistent. This contrasts the vast basic science literature suggesting that brain circuits are highly malleable, even in older age. Non-invasive transcranial direct current stimulation (tDCS) has shown to facilitate brain plasticity, especially when administered during a learning event. Here, I will summarize the results of several randomized controlled trials as well as a meta-analysis conducted by my group focusing on cognitive training that is paired with tDCS. Across 4 separate studies involving 257 younger and older adults, we found evidence for improved learning trajectories as a function of tDCS, and in addition, we observed improvements in measures of long-term memory, some of which remained significant several months after training completion. The effects on working memory outcomes appear to be more mixed and dependent on baseline ability, i.e., those with initial lower performance typically benefit more from brain stimulation. Our primary findings are supported meta-analytically in that we found a small positive net effect of tDCS on transfer measures after working memory training, both, in the short- and long-term. However, the effects were most pronounced for measures that were similar to the trained tasks. Collectively, our studies illustrate the potential of tDCS to support cognitive training outcomes, but at the same time, there are boundary conditions and important individual differences that moderate training efficacy.

Abstract citation ID: igae098.2190

### EFFECTS OF ATTENTIONAL CONTROL DEMANDS IN PROCESSING SPEED TRAINING ACROSS THE ADULT LIFESPAN: FIRST FINDINGS

Claudia von Bastian[1], Alice Reinhartz[2], Eleanor Hyde[1], Shuangke Jiang[1], Elisabeth Loranger[3], Jeff Ferreri[4], Sylvie Belleville[5], and Tilo Strobach[2], *1. University of Sheffield, Sheffield, England, United Kingdom*, *2. Medical School Hamburg, Hamburg, Hamburg, Germany*, *3. Institut Universitaire de Geriatrie de Montreal, Montreal, Quebec, Canada*, *4. Institut Universitaire De Geriatrie De Montreal, Montreal, Quebec, Canada*, *5. University of Montreal, Montreal, Quebec, Canada*

Evidence for cognitive training-induced far transfer of improvements to untrained cognitive abilities is mixed. One notable exception appears to be training interventions that target speed of processing. However, the mechanisms underpinning these training effects are yet unclear. In this pre-registered, multi-site training study, we tested the hypotheses that (a) training tasks with stronger attentional control demands will induce larger transfer effects, and that (b) gains in the rate of information accumulation (i.e., drift rate) will be positively associated with these effects. For this purpose, we recruited 476 healthy participants spanning the adult lifespan (18-85 years) from three sites in the United Kingdom, Germany, and Canada, who were randomly allocated to one of four groups practising tasks with increasing attentional control demands. Transfer to working memory, executive functions, reasoning, and everyday cognitive functioning was assessed before, immediately after, and 3 months after 10 training sessions. N = 388 participants (age in years M = 48.61, SD = 18.28, range 18 – 85; 218 women, 168 men, 2 participants with undisclosed gender; education in years M = 16.68, SD = 3.68) completed the study. The first results from this study will be presented in this talk.

### SESSION 4495 (SYMPOSIUM)

Abstract citation ID: igae098.2191

### NAVIGATING HEALTH AND IDENTITY TO PROMOTE FORTITUDE AMONG SGM PEOPLE: INSIGHTS FROM AGING WITH PRIDE

Chair: Charles Hoy-Ellis
Discussant: Karen Fredriksen-Goldsen

This symposium showcases groundbreaking research illustrating the multifaceted nature of fortitude among sexual and gender minority (SGM) older adults, focusing on health, digital health access, generativity, and intersectionality. The collection of studies in this symposium leverages cross-sectional and longitudinal data from Aging with Pride: National, Health, Aging and Sexuality/Gender Study (NHAS) to address critical gaps in our understanding of the unique strengths in this population despite the adversity they face. The first presentation illuminates the disproportionate experiences of violence and abuse among older transgender adults, revealing significant links between intimate partner violence (IPV), victimization, internalized stigma, and adverse mental health outcomes. It spotlights the need for interventions that target these risk factors and promote fortitude. The second presentation examines the digital divide, exploring how older SGM adults utilize health information technologies. By comparing usage patterns and identifying barriers and facilitators, this research emphasizes the importance of improving health care access. The third presentation will examine the predictors of generativity among SGM older adults, highlighting how psychological and social resources can mitigate stigma. This study offers valuable perspectives on fostering intergenerational support and community engagement within SGM communities. Lastly, the symposium will feature research examining the intersectionality of identity among SGM older adults, showcasing heterogeneity among this population by identifying patterns based on identity centrality and discrimination experiences. Highlighting the impacts of recent policy shifts targeting SGM communities, these studies collectively underscore the crucial need for resilience-enhancing strategies that incorporate intersectional approaches for promoting health equity among vulnerable, at-risk populations.

Abstract citation ID: igae098.2192

### BEYOND THE MARGINS: INTIMATE PARTNER VIOLENCE AND TRANSGENDER OLDER ADULTS' HEALTH AND WELL-BEING

Charles Hoy-Ellis[1], Hailey Jung[2], Loree Cook-Daniels[3], Hyun-Jun Kim[2], and Karen Fredriksen-Goldsen[2], *1. Goldsen Institute, University of Washington School of Social Work, Seattle, Washington, United States*, *2. University of*

*Washington, Seattle, Washington, United States*, *3. FORGE, Milwaukee, Wisconsin, United States*

Transgender older adults experience disproportionate violence and abuse, increasing their risk for deteriorating physical health, disability, depressive symptoms, and elevated stress levels when compared with their non-transgender age-peers. Studies have shown that barriers like fear of seeking healthcare, insufficient physical activity, internalized stigma, experiences of victimization, and a lack of social support play indirect roles in negatively impacting the health of this group, which has historically faced marginalization and neglect. According to the Health Equity Promotion Model, stigma and victimization significantly impact the mental health of older transgender adults. This study seeks to explore the connections between intimate partner violence (IPV), victimization, social resources, and mental health outcomes within this population. By analyzing cross-sectional data from a national survey involving lesbian, gay, bisexual, and transgender adults aged 50 to 95 years (N = 2,560), we found that transgender respondents (n = 167) experienced IPV at over double the rate of their non-transgender lesbian, gay, and bisexual counterparts (16% vs. 7%). Among transgender individuals, IPV strongly correlates with increased victimization and internalized stigma. IPV significantly predicted mental health quality of life and depressive symptomatology, independent of lifetime discrimination and victimization. Additional significant predictors of mental health outcomes were social network and support. Given the recent and continuing policy shifts nationwide affecting the transgender community, initiatives aimed at stigma reduction, victimization prevention, and bolstering social support are crucial for improving the health and overall well-being of transgender older adults. Insights from this research can guide stakeholders in countering regressive policies and legislation.

Abstract citation ID: igae098.2193

### INTERSECTING IDENTITIES AND HEALTH-RELATED QUALITY OF LIFE AMONG LGBTQ+ OLDER ADULTS

Hyun-Jun Kim[1], Hailey Jung[1], Austin Oswald[2], and Karen Fredriksen-Goldsen[1], *1. University of Washington, Seattle, Washington, United States*, *2. Dalhousie University, Nova Scotia, Canada*

LGBTQ+ older adults remain understudied in terms of the intersectionality of their identities and its impact on health. It is unclear how certain aspects of identity are perceived as central and how the intersection of multiple identities influences experiences of discrimination. This study identifies intersectional profiles of identity centrality and attributions for day-to-day discrimination, focusing on gender, sexual and gender identity, age, and race/ethnicity among LGBTQ+ older adults. It also examines the associations between profile membership, key psychological and social factors, and physical and mental health-related quality of life (HRQOL). Utilizing data from the National Health, Aging, and Sexuality/Gender Study, the first national longitudinal study of 2,450 LGBTQ+ older adults, we unveiled five latent profiles using Latent Class Analysis, which depict heterogeneous patterns in individuals' perception of their identities in terms of centrality and as the basis of their discrimination experiences. The most prevalent was the moderate centrality and discrimination profile (C1; 31%), followed by high centrality and discrimination (C2; 26%), low centrality and discrimination (C3; 17%), high centrality and low discrimination (C4; 15%), and low centrality and high discrimination (C5; 11%). C2 and C5 exhibited lower physical and mental HRQOL compared to other profiles, suggesting that multiple discrimination attributes may contribute to compromised health, regardless of identity centrality. C2 and C4 showed high identity centrality across all identities, which was linked to greater critical awareness and activism. This study highlights the significance of incorporating an intersectional lens to better understand the distinct needs and strengths within LGBTQ+ aging communities.

Abstract citation ID: igae098.2194

### PREDICTORS AND IMPLICATIONS OF GENERATIVITY AMONG LGBTQ+ OLDER ADULTS

Hailey Jung[1], Hyun-Jun Kim[1], Charles Emlet[2], and Karen Fredriksen-Goldsen[1], *1. University of Washington, Seattle, Washington, United States*, *2. University of Washington Tacoma, Tacoma, Washington, United States*

Generativity is a concern, commitment, and action for others including the next generation, and has been shown positively associated with the health and well-being of older adults. Since LGBTQ+ older adults have experienced overt marginalization and often have limited contact with younger generations, generativity may resonate with unique implications in LGBTQ+ communities. Yet, to date research on generativity with this community is lacking. In this paper, we integrate Generativity Theory and Health Equity Promotional Model (HEPM) to examine the predictors of generativity among LGBTQ+ older adults and explore key psychological and social factors that moderate the relationship between stigma experiences and generativity. Using the 2016 data from Aging with Pride: National Health, Aging, and Sexuality/Gender Study (N = 2,233), we regressed generativity in three blocks; first, social location and stigma variables, next, psychological resources, and lastly, social resources. The predictive power of the model significantly improved in each step. Psychological resources (i.e., mastery, resilience, and critical awareness) and social resources, (i.e., social participation, network size, social support, and LGBTQ+ community engagement) were all positively associated with generativity. In terms of moderating effects, day-to-day discrimination no longer had negative effects on generativity when experienced by older adults with higher levels of critical awareness. Interestingly, lifetime discrimination had positive effects on generativity for individuals with higher mastery levels. Findings suggest psychological and social mechanisms that may foster fortitude and stress-related growth among LGBTQ+ older adults. This research provides valuable insights to foster intergenerational relationships and cultivate strengths in the LGBTQ+ community.

Abstract citation ID: igae098.2195

### BRIDGING ACCESS GAPS: EXAMINING USE OF DIGITAL HEALTH TECHNOLOGIES AMONG SEXUAL AND GENDER MINORITY OLDER ADULTS

Meghan Romanelli, Hyun-Jun Kim, and Karen Fredriksen-Goldsen, *University of Washington, Seattle, Washington, United States*

LGBTQ older adults face distinct challenges accessing healthcare. Digital health solutions offer responsive approaches that broaden availability of health information and care, especially for those experiencing isolation, discrimination, and barriers to care. While some LGBTQ older adults prefer digital health technologies, others face difficulties with electronic literacy that prevent use. The current study examined the association of sexual and gender identity and health access factors (barriers to care; health literacy; help-seeking beliefs; healthcare discrimination) with past 12-month use of digital health technologies for communication, insurance matters, and health-information seeking. Data were analyzed from the 2015 healthcare access module of Aging with Pride: National Health, Aging, and Sexuality/Gender Study (NHAS) (N=2,322). Results indicated digital health use varied across LGBTQ older adult subgroups and access factors. Transgender older adults, compared to cisgender, were more likely to use each digital resource to manage their health and healthcare. Participants with experiences of healthcare discrimination more likely communicated with their providers online, while those with lower health literacy turned to the internet to find health information. Older cohorts (65-79; 80+), poverty-impacted, and Black and Hispanic participants were less likely to use digital resources. Digital health technologies may enhance healthcare access for LGBTQ older adults, potentially bridging gaps in service availability and acceptability. This is particularly evident for certain LGBTQ subpopulations such as transgender older adults and those facing greater barriers to care and discrimination. It remains imperative to create digital access and address literacy needs among older cohorts and LGBTQ communities with multiple marginalized identities.

### SESSION 4500 (SYMPOSIUM)

Abstract citation ID: igae098.2196

### NOVEL FINDINGS AND LESSONS LEARNED FROM CROSS-NATIONAL LONGITUDINAL RESEARCH ON MIDLIFE DEVELOPMENT AND AGING

Chair: Frank Infurna
Co-Chair: Denis Gerstorf
Discussant: Lisbeth Nielsen

The goal of this symposium is to bring together a collection of papers that shed light on opportunities gained and challenges encountered in cross-national comparisons of development in midlife and aging and lessons learned. Infurna and colleagues used longitudinal panel data from 16 nations to examine historical changes in midlife pain and report historical increases in pain in the U.S., and Continental and Nordic Europe, whereas Mediterranean Europe, South Korea, and Mexico experienced historical declines. Wettstein and colleagues studied historical changes in midlife memory and explanatory factors in longitudinal panel data from 16 nations; the U.S. was the only nation/region to exhibit historical declines in memory, whereas historical improvements were observed in the European nations. Women, higher educational attainment, better grip strength, and fewer chronic diseases were associated with better memory across cohorts. Steptoe and Brocklebank used data from England and China to examine how physical activity and sleep patterns relate to cognitive decline and highlight challenges related to data collection and instrumentation and approaches to carry out robust cross-national comparisons. Wong provides an overview of research on cross-national comparisons using the Mexico Health and Aging Study by illustrating various examples and discussing gaps in knowledge pertaining to how low and middle-income nations could be supported by cross-national data. The discussion by Nielsen will integrate the four papers by highlighting novel findings that can arise from conducting cross-national research (e.g., historical change, comparisons of explanatory factors) and insights gained through the increasing availability of comparable data that can be harmonized.

Abstract citation ID: igae098.2197

### PAIN DURING MIDLIFE: A CROSS-NATIONAL ANALYSIS OF COHORT DIFFERENCES IN PAIN IN THE US, EUROPE, SOUTH KOREA, AND MEXICO

Frank Infurna[1], Orchee Syed[1], Yesenia Cruz-Carrillo[1], Nutifafa Dey[1], Markus Wettstein[2], Kevin Grimm[1], Margie Lachman[3], and Denis Gerstorf[2], *1. Arizona State University, Tempe, Arizona, United States*, *2. Humboldt University Berlin, Berlin, Berlin, Germany*, *3. Brandeis University, Waltham, Massachusetts, United States*

Over the past several decades, middle-aged Americans have exhibited troubling trends of declining mental and physical health over successive cohorts. However, this trend has not been consistently observed in peer nations. It is an open question whether pain shows similar national disparities. We thus examine how self-reports of pain have historically changed in the U.S. during midlife, and how this compares to peer nations. We used harmonized data on pain from nationally representative longitudinal panel surveys from the U.S., 13 European nations, South Korea, and Mexico to directly quantify national similarities and differences in historical change in midlife pain. Our results corroborate earlier reports that midlife pain is higher amongst later-born cohorts in the U.S. Of note is that these historical increases emerged at earlier stages of midlife for later-born cohorts. A similar pattern of historical increases in pain was observed in Continental and Nordic Europe. In contrast, historical declines in pain were observed in England, Mediterranean Europe, South Korea, and Mexico. These results suggest there could be aspects of American midlife today that are exacerbating feelings of pain, and these aspects may be shared in some European nations but absent or less influential in other peer nations. Our discussion focuses on potential explanations for this pattern, such as population level discrepancies in health, differential use of health care services, and the inter/intrapersonal costs of westernization, as well as how pain is conceptualized across nations.

Abstract citation ID: igae098.2198

### TRAJECTORIES OF EPISODIC MEMORY IN MIDLIFE: HISTORICAL CHANGE FROM A CROSS-COUNTRY PERSPECTIVE

Markus Wettstein[1], Frank Infurna[2], Nutifafa Dey[2], Yesenia Cruz-Carrillo[2], Kevin Grimm[2], Margie Lachman[3], and Denis Gerstorf[1], *1. Humboldt University Berlin, Berlin, Berlin, Germany*, *2. Arizona State University, Tempe, Arizona, United States*, *3. Brandeis University, Waltham, Massachusetts, United States*

According to the Flynn effect, cognitive abilities have improved across the past decades. However, we know little about whether such historical improvements generalize to middle-aged adults and differ across nations. In this study, we used harmonized data on episodic memory from nationally representative longitudinal panel surveys including the U.S., Europe, Mexico, and China to compare historical change in age-related trajectories of episodic memory among middle-aged adults. Our sample included 89,775 participants aged 45 to 65 years who provided 272,876 observations over up to 20 years. Longitudinal multilevel regression models revealed that today's middle-aged adults in the U.S. perform worse on episodic memory tests than their age peers in the past. In contrast, today's middle-aged adults in most other countries perform better than did their peers in the past. At the same time, later-born cohorts of U.S. middle-aged adults exhibited a more favorable episodic memory trajectory than earlier-born cohorts, whereas this trend was not observed in most other countries. Cohort trends remained significant when controlling for socio-demographic indicators (gender, education) and health measures (grip strength, number of chronic diseases). Women and individuals with higher levels of education, higher grip strength and fewer chronic diseases exhibited better episodic memory performance, and the association of grip strength with episodic memory was stronger in later-born cohorts. Our findings suggest that countries differ in the extent and direction of historical change in episodic memory scores and trajectories. More research is needed to better understand why the Flynn effect in the U.S. seems to be reversed.

Abstract citation ID: igae098.2199

### CROSS-NATIONAL STUDIES OF RISK FACTORS FOR COGNITIVE DECLINE AND DEMENTIA

Andrew Steptoe[1], and Laura Brocklebank[2], *1. University College London, London, England, United Kingdom*, *2. University of Oxford, Oxford, England, United Kingdom*

Cross-national studies of cognitive decline can provide insights into the relevance of risk factors in different cultural settings. They help determine whether relationships are universal or depend on the lifestyles and social context prevailing in different countries. However, cross-national studies present challenges. One important issue is harmonization of self-report and cognitive measures, and this has been greatly facilitated by the Gateway to Global Aging initiative. But there are other factors related to consistency in data collection that require careful consideration. These will be illustrated by a study relating physical activity and sleep patterns to cognitive decline and dementia risk in England using ELSA (the English Longitudinal Study of Ageing) and in China using CHARLS (The China Health and Retirement Longitudinal Study). Absolute levels of physical activity and sleep vary greatly among older people living in urban and rural settings, and with histories of predominantly non-manual work (ELSA) and more mixed occupations (CHARLS). These factors color self-report measures, making comparison difficult. In this study, we have been carrying out 8-day wrist-worn accelerometry in ELSA and CHARLS to provide objective data on movement behaviours (physical activity, sedentary behaviour, and sleep) in more than 20,000 individuals. This presentation highlights challenges related to consistency of data collection, variations in instrumentation, differences in response and adherence rates, and the suitability of machine-learning behavior classification models developed in one cohort but applied in very different people and/or contexts. We will describe our approaches to resolving these issues in order to carry out robust cross-national comparisons.

Abstract citation ID: igae098.2200

### TWENTY YEARS OF FOLLOW-UP OF THE MEXICAN HEALTH AND AGING STUDY (MHAS): ITS POTENTIAL FOR CROSS-NATIONAL COMPARISONS

Rebeca Wong, *The University of Texas Health Sciences Center in San Antonio, San Antonio, Texas, United States*

The availability of population-based, national representative, longitudinal data for aging research has been building up for the last twenty years. As a result, the ability to make cross-national research has been enhanced greatly. Almost 20 percent of the publications that use the MHAS data are making cross-national comparisons, but the cross-national work is still in infancy. Most research focuses on documenting health and functionality trajectories and the individual determinants of these trajectories. I will present an overview of what has been done, illustrate with a couple of examples, and conclude with gaps in knowledge that could be supported by cross-national data. The lack of comparable data for many low-middle income countries may be the challenge we can help meet in the next 20 years to advance research on global aging.

### SESSION 4505 (PAPER)

### PATHWAYS TO SUCCESSFUL AGING: DIVERSE PERSPECTIVES AND EXPERIENCES

Abstract citation ID: igae098.2201

### A NOVEL TIME USE APPROACH ON SUCCESSFUL AGING: RACIAL AND GENDER DISPARITIES IN DAILY PRODUCTIVE ENGAGEMENT

Jason Wong[1], Siyao Lu[1], Yifan Lou[2], Emma Zang[1], and Deborah Carr[3], *1. Yale University, New Haven, Connecticut, United States*, *2. Virginia Commonwealth University, New Haven, Virginia, United States*, *3. Boston University, Boston, Massachusetts, United States*

**Background and Objectives:** Productive engagement (PE) represents a potential pathway toward successful aging (SA). Using time diary data, this study explored how U.S. older adults structure their daily lives in different productive roles, the impacts of these roles on one specific dimension of SA (self-rated health), and the extent to which these patterns differ by race and gender.
**Research Design and Methods:** We use data from the American Time Use Survey (n = 17,990) and sequence and cluster analyses to identify distinctive daily PE time-use patterns. Multivariable linear regression models were used to evaluate associations between these patterns and self-rated health, and the moderating roles of race and gender.
**Results:** Five PE clusters were identified: Low Degree of PE (29%), Moderate Unpaid Work & Light Social Participation

(42%), Persistent Unpaid Work (12%), Persistent Paid Work (9%), and Persistent Social Participation (7%). White women were the most likely, while Black men were the least likely to have any PE. All PE clusters were positively associated with self-rated health, compared to "Low Degree of PE". The strongest positive association was observed for "Persistent Paid Work," especially for women. Racial differences were more prominent among women than men. The health benefits of PE were less pronounced among Black women than White women.

**Discussion and Implications:** The positive association between PE and self-rated health varies across race-gender groups. Future research should consider the persistent structural barriers that may prevent older adults with disadvantaged backgrounds, particularly Black women, from benefiting from PE and experiencing SA.

Abstract citation ID: igae098.2202

### EMPOWERMENT IN MOTION: ANALYZING THE EXPERIENCE OF WOMEN HIP-HOP DANCE CREW

Kyung Eun Lee, Hae Youn Min, and Jinmoo Heo, *Yonsei University, Seoul, Republic of Korea*

Hip-hop dance is known as one of the dance genres, primarily associated with the younger generation. However, it has gained popularity among not only the youth but also the older generations worldwide. Participants in this study have formed hip-hop dance crews targeting middle and old-aged women, engaging in regular practices and performances to create and enjoy their leisure activities. According to the innovation theory (Nimrod & Kleiber 2007), individuals can achieve successful aging by actively engaging in leisure activities. This theory emphasizes the importance of continuously seeking new experiences and challenges, even in later stages of life. Therefore, this study aimed to explore the experiences of participation in the women's dance crew based on the innovation theory. In-depth interviews were conducted with 16 middle and old-aged women regarding their motivations, identity as members, and outcomes. The interview results confirmed that participants initially began their activities for various reasons (e.g., family recommendations, maintaining health), but continued their activities due to intrinsic motivation. Interestingly, it was found that both self-preservation innovation (leisure activities related to previous life experiences) and self-reinvention innovation (completely new leisure activities) were simultaneously discovered among the participants. Furthermore, participants were able to enhance psychological, physical, and social well-being through dance activities. These findings align with the key factors proposed in the innovation theory. Consequently, it was observed that middle and old-aged women preserve their past identities and explore new aspects of themselves by enjoying hip-hop dance, highlighting the significance of leisure activities in later life.

Abstract citation ID: igae098.2203

### EXPLORING SERIOUS ILLNESS CARE EXPERIENCES AMONG BLACK CHICAGOANS' CAREGIVERS: A QUALITATIVE INQUIRY

Jocelyn Wilder[1], Kandis Draw[2], and Kimberly Downing[2], *1. NORC at the University of Chicago, Chicago, Illinois, United States*, *2. The Hospice and Palliative Care Foundation, Oakbrook Terrace, Illinois, United States*

Palliative and hospice services have shown tangible evidence of enhancing the quality of life, decreasing the likelihood of depression, and extending survival for those receiving palliative care. Black individuals receive referrals later and are less likely to utilize palliative and hospice care; national data estimated that only 7.6% of hospice patients were Black. This qualitative study investigates the nuanced perspectives on serious illness care among Black Chicagoans through focus groups and in-depth interviews. Employing an inductive thematic approach, we explored the knowledge, attitudes, and beliefs to understand the intricate cultural, familial, and healthcare dynamics that influence serious illness care experiences among caregivers. Our findings reveal diverse and complex narratives surrounding end-of-life care, reflecting the intersection of race, familial expectations, and healthcare systems. Participants shared moving experiences with family and health care providers, shaping their attitudes towards hospice and palliative care. Discussions highlighted limited knowledge of available services regarding access to and utilization of end-of-life care resources. Thematic analysis identified several key domains, including quality of care, decision-making related to death and dying, and the role of race, highlighting the importance of culturally competent care. These findings underscore the need for healthcare providers to adopt culturally sensitive approaches. This research illuminates the diverse perspectives and experiences within Black communities and informs the development of culturally responsive end-of-life care strategies. Our study contributes to a deeper understanding of the unique challenges and opportunities in providing quality end-of-life care for Black individuals and families.

Abstract citation ID: igae098.2204

### BIOLOGICAL AGING PATHWAYS THAT UNDERLIE LIFE COURSE SOCIOECONOMIC DISPARITIES IN PHYSICAL FUNCTION AND LONGEVITY

Tara Gruenewald, and Naomi Podber, *Chapman University, Orange, California, United States*

The poorer functioning and greater morbidity and mortality risk in those with greater life course socioeconomic disadvantage has led to the search for the biological processes that underlie these associations, including accelerated cellular and system aging. Our study examines whether a DNA methylation profile of the pace of biological aging (DunedinPACE) and a multi-system index of physiological dysregulation (allostatic load (AL)) mediate associations between life course socioeconomic disadvantage (SESDIS) and both physical function (gait speed, chair stand time) and survival. Data are from the Midlife in the U.S. Study (N=1,295, age 26–86, 55.4% female, 69.0% white). Measures included a multi-indicator composite of childhood and adult socioeconomic indicators (SESDIS), DunedinPACE as described in Belsky et al. (2022), AL as described in Gruenewald et al. (2012), gait speed (time to complete a 50-foot walk), and chair stand time (5 consecutive rises from a seated position). Analytic models included z-scored predictors, age, race, and gender covariates and accounted for family clustering. Greater SESDIS was associated with increased mortality hazard (HR=1.42***), higher gait speed (b=0.70***) and chair stand time (b=0.85***), and higher DunedinPACE (b=0.27***) and AL (b=0.19***). Greater DunedinPACE and AL were associated with slower gait and chair stand time and lower survival. Multi-mediation analyses indicated significant bootstrapped (1,000 replications) indirect effects for DunedinPACE and AL as mediating pathways of the slower gait and chair stand time and lower survival in those with greater SESDIS. Findings highlight biological aging pathways that may contribute to life course socioeconomic disparities in physical function and longevity.

### SESSION 4510 (PAPER)

### POLICY AND PUBLIC HEALTH

Abstract citation ID: igae098.2205

### MULTISECTOR PLANS FOR AGING: OPPORTUNITIES FOR AGING RESEARCH TO INFORM STATE POLICY

Valerie Flores[1], and Carrie Graham[2], *1. Center for Health Care Strategies., 2. Center for Health Care Strategies, Berkeley, California, United States*

State Multisector Plans for Aging (MPA) are state-led, cross-department, stakeholder- and evidence-informed plans through which states set goals and priorities to transform their state and improve care, services and support for their diverse aging populations. Often these plans are led by the Governors' offices, Departments of Aging or Medicaid agencies, but also engage across departments of public health, social services, emergency preparedness, housing, transportation, labor, and more. They are also informed by meaningful stakeholder engagement, including advocates, consumers, aging services providers and employers in the state. By far one of the most important MPA stakeholders are universities and researchers who can provide cutting edge data to inform MPAs. Currently, almost half of all U.S. states are engaged in either the development process or implementing an MPA. Evidence and data are important at every stage, from building the case for the necessity of an MPA, to the identification of unmet needs in the state, to development of strategies, and ultimately defining benchmarks and measuring progress. Many states have partnered with gerontological researchers to inform their plans. This offers the mutual benefit of informing the state, as well as providing an opportunity for researchers to learn how to better formulate their research questions to help the state build actionable and timely policy. This session will include an overview of how researchers have contributed to MPAs in many states and how the relationships forged through that process has opened more opportunities for continued collaboration and the translation of research into policy.

Abstract citation ID: igae098.2206

### TRAINING CAREGIVERS TO BE INFECTION PREVENTION SUPERHEROES WITH PROJECT FIRSTLINE

Carolyn Ham[1], Madyson Schuh[2], Megan Rogers[3], and Erica Ellis[4], *1. Washington State Department of Health, Olympia, Washington, United States, 2. Washington State Department of Health, Tumwater, Washington, United States, 3. University of Washington, Seattle, Washington, United States, 4. University of Washington Northwest Center for Public Health Practice, Seattle, Washington, United States*

Facility-acquired infections in long-term care facilities (LTCF) significantly affect resident's safety and quality of life. Caregiving staff in LTCF are uniquely positioned to prevent infections by completing infection prevention and control (IPC) activities, including hand hygiene and using personal protective equipment. An IPC Needs Assessment (NA) survey was conducted in 2021 by the Washington State Department of Health (DOH) and University of Washington (UW) with 3,308 frontline healthcare respondents. Respondents from LTCF caregivers indicated a preference for self-paced, asynchronous learning experiences and ranked COVID-19 as the most interesting IPC topic. DOH partnered with UW and the Centers for Disease Control and Prevention to develop six Project Firstline Modules (PFM) on IPC topics. Drawing on adult learning theory principles, training content was developed with a focus on the needs and lived experience of caregivers. Each module was offered in English and Spanish and incorporated an instructional video, interactive scenario, job aid, and evaluation. Between February and December 2023, 589 LTCF caregivers completed modules, comprising 62.93% of all users, with the majority (97.3%) reporting that the information was easily understandable and 88.2% reporting they would be able to apply what they learned to their job. PFM completers reported the highest satisfaction for the module on respiratory droplets, consistent with the NA results. The delivery of PFM successfully provided actionable and accessible IPC training for LTCF caregivers. Future efforts to educate caregivers on IPC should consider their preferences in training modes and topics, and further explore public health and academic partnerships.

Abstract citation ID: igae098.2207

### PRODUCING "QUALITY": ASSISTED LIVING ADMINISTRATORS' EXPERIENCE OF THE LICENSING AND SURVEY PROCESS IN OREGON

Ozcan Tunalilar[1], Jacklyn Kohon[1], and Paula Carder[2], *1. Portland State University, Portland, Oregon, United States, 2. OHSU-PSU School of Public Health, Portland, Oregon, United States*

Assisted living (AL) communities in the United States are home to a steadily growing population of over 900,000 individuals and provide assistance with daily activities, medical treatments, and social and recreational activities in a congregate residence. States license AL and oversee regulatory compliance. Studies have examined states' regulations in terms of stringency and specificity, though they did not address survey processes, especially administrators' perspective. We conducted semi-structured interviews with 37 AL administrators in Oregon to examine AL administrators' experiences of the licensing and survey process. Using thematic analysis, we identified three major themes: emotional reactions, practical considerations, and relational assessments. First, administrators expressed feelings of high-level stress related to the survey process. Second, administrators discussed drawing on multiple practical approaches to become "survey ready," including reviewing prior survey results, having support during a new administrator's first survey process, and setting up other types of partner or mentor support systems for administrators. Third, administrators defined the survey process through their relationship with surveyors, in terms of tone (adversarial vs. collaborative), hierarchical/power structure ("negotiating the evidence"), and the extent of collaboration ("surveyor as mentor"). We interpret these findings using elements from the Resource Dependency Theory by situating "substantial compliance" as a vital resource for the successful operation of AL and regulatory authorities as key resource providers for AL administrators. These results also emphasize a potential mismatch between how AL administrators view their setting and how regulators do.

Abstract citation ID: igae098.2208

### BEING BOLD: USING PUBLIC HEALTH AWARDS TO CHANGE STATE DEMENTIA POLICY

Annie Rhodes[1], Ashley Staton[1], Daniel Bluestein[1], Kimberly Ivey[1], Rachel Coney[2], Andrea Price[3], Lana Sargent[1], and Melicent Miller[2], *1. Virginia Commonwealth University, Richmond, Virginia, United States*, *2. Virginia Department of Health, Richmond, Virginia, United States*, *3. Virginia Center on Aging, Richmond, Virginia, United States*

The BOLD Act (Building Our Largest Dementia Infrastructure) was passed by the United States Congress on December 31st, 2018, to support state health departments with dementia-friendly and healthy brain activities. In Virginia, the Virginia Department of Health (VDH) awarded the Virginia Center on Aging a BOLD sub-award to create an epidemiologic registry to catalog dementia, neurodegenerative disorders, and caregiving cases. Registries are a valuable but underused strategy for an equitable public health response to brain health promotion and to monitor trends in dementia and neurodegenerative disorders. The Virginia registry, known as the "Virginia Memory Project" (VMP), is only the 4th statewide dementia registry in the United States. In 2024, the Virginia State legislature unanimously voted to pass HB 1455, establishing the VMP into state law and codifying a dementia and caregiving registry in Virginia. A key outcome and sustainability strategy of launching the VMP was creating a framework to influence policy at the state level to infuse public policy with evidence-based resources, education, and impact for dementia, caregiving, and neurodegenerative disorders. The VMP is unique among epidemiologic dementia registries because it is a responsive registry that offers patients, caregivers, and persons with undiagnosed memory concerns linkage to information, services, and support. In this session, we will discuss the framework we used as an academic-public partnership to launch the VMP and how researchers can use grant funding as an advocacy mechanism to influence policy, ultimately supporting brain health and caregivers.

Abstract citation ID: igae098.2209

### FILLING GAPS FOR OLDER OHIOANS WITH LOCALLY RAISED FUNDS

Bailee Brekke[1], Leah Janssen[2], Oksana Dikhtyar[2], Matt Nelson[2], and Robert Applebaum[2], *1. Miami University, W. College Corner, Indiana, United States*, *2. Miami University, Oxford, Ohio, United States*

Most older Americans aging in place at home do not financially qualify for support through the Medicaid program, and funding provided through the Older Americans Act is insufficient. As a result, 15 states have turned to local revenue sources to fill service gaps for individuals living in the community, who require social care to support independence. Ohio has a long history of utilizing such funds, generating over $200 million, more than the other 14 states combined. Despite the demonstrated success of these programs, local levy programs in Ohio do experience an array of implementation challenges. This study conducted 15 qualitative descriptive interviews with 20 program officials from various locally funded levy programs across Ohio. Thematic analysis revealed barriers, including increasing state and federal eligibility requirements, staffing issues, rural access to resources, and challenges in setting reimbursement rates. These barriers are coupled with added pressure from county officials to move individuals onto state funded programs (e.g., Medicaid HCBS Waiver programs), rather than keeping them on locally funded programs. The aim of this paper is to examine challenges that local programs are facing and provide program and policy recommendations. Findings reveal the important role local levy programs play in addressing gaps in service and the importance of local advocacy and education to continue passing these levies. Increasing communication between levy staff across the state, integration of cross-funding streams allocated by the state and increasing reimbursement rates are needed to ensure the success of the local levy programs for the future.

### SESSION 4515 (SYMPOSIUM)

Abstract citation ID: igae098.2210

### RESEARCH ADVANCES USING DIGITAL METHODS TO ASSESS COGNITION IN OLDER ADULTS

Chair: Karysa Britton
Co-Chair: Zoe Hawks
Discussant: Martin Sliwinski

This symposium highlights the use of digital methods to assess and contextualize cognition in older adults. Across five talks, we consider several digital cognitive assessment methods, including self-administered computerized tests and smartphone-based ecological momentary assessments (EMAs). First, Dr. Louisa Thompson reports findings on the utility and acceptability of using well-validated, reliable digital cognitive assessments to screen for cognitive decline among older adults in primary care settings. Second, Dr. Zoë Hawks uses EMA and passive data from wearable sensors to investigate links between glucose and cognition in relation to neuropsychiatric risk. Third, Hannah Wilks discusses the importance of assessing both environmental and affective influences when examining cognition via EMA in individuals with symptoms of dementia. Fourth, Dr. Karina Van Bogart uses multilevel modeling to examine associations between levels of loneliness and self-reported memory lapses among older adults. Finally, Dr. Emorie Beck examines psychosocial influences on diurnal cycles of cognitive variation. Together, Dr. Martin Sliwinski will draw connections between research showcasing how digital methods can be employed to promote routine screening for cognitive decline, elucidate contextual factors that contribute to cognitive variation in older adults, and assess unique influences of individual differences on cognitive performance.

Abstract citation ID: igae098.2211

### DIGITAL APPROACHES TO ROUTINE COGNITIVE SCREENING FOR OLDER ADULTS IN PRIMARY CARE SETTINGS

Louisa Thompson[1], Molly Lawrence[2], and Charles Eaton[3], *1. Brown University Medical School, Lincoln, Rhode Island, United States*, *2. Brown University, Providence, Rhode Island, United States*, *3. Brown University Medical School, Providence, Rhode Island, United States*

Cognitive screening remains under-utilized for older adults in primary care due to time constraints and limitations of existing measures. Well-validated digital assessments could potentially increase screening efficiency and accuracy. We compared two new digital cognitive tools with the Montreal Cognitive Assessment (MoCA) in a sample of 32 dementia-free older adults (ages 55-85) completing annual follow-up visits with a primary care provider (PCP). Participants self-administered the Boston Online Cognitive Assessment (BOCA), an online measure with alternate forms, twice prior to (1-4 weeks before and day of) an upcoming PCP follow-up visit. At their visit, they completed the Digital Clock and Recall (Linus Health DCRTM), a 5-minute, provider-administered tablet-based measure. Finally, they completed the MoCA with a research coordinator or behavioral health staff at the clinic. Five PCPs aided in protocol development and participated in data collection. The sample is currently 54% female and 81% White. Test-retest reliability for the BOCA was excellent (r =.81). BOCA score (time 1) was correlated with scores for the DCR and MoCA at the p <.05 level. The association between the DCR and MoCA approached significance. On an exit survey, 79% of participants said that they would prefer to do cognitive screening at home before their appointment, compared to in the clinic (21%). These preliminary data replicate excellent test re-test reliability for the BOCA and demonstrate good convergent validity between the BOCA and two provider-administered screening measures. Next, we will compare test accuracies to detect impairment and their associations with demographics variables.

Abstract citation ID: igae098.2212

### DYNAMIC ASSOCIATIONS BETWEEN GLUCOSE AND ECOLOGICAL MOMENTARY COGNITION IN TYPE 1 DIABETES

Zoe Hawks[1], Emorie Beck[2], Lanee Jung[3], Luciana Fonseca[4], Martin Sliwinski[5], Naomi Chaytor[4], and Laura Germine[1], *1. McLean Hospital/Harvard Medical School, Belmont, Massachusetts, United States*, *2. University of California Davis, Davis, California, United States*, *3. McLean Hospital, Belmont, Massachusetts, United States*, *4. Washington State University, Spokane, Washington, United States*, *5. Pennsylvania State University, University Park, Pennsylvania, United States*

Cognitive variability is associated with fall risk, cardiovascular mortality, and cognitive decline. Identifying factors that influence cognitive variability may inform evidence-based guidelines aimed at supporting cognitive and physical health across the lifespan. This goal is particularly salient in Type I Diabetes (T1D), an autoimmune condition characterized by large glucose fluctuations. T1D confers risk for neurodegeneration, and laboratory studies suggest that cognition is reduced when glucose is very low or very high. The present study leveraged advances in continuous glucose monitoring (CGM) and cognitive ecological momentary assessment (EMA) to clarify the impact of naturally-occurring glucose fluctuations on cognitive performance (sustained attention, processing speed) in 200 adults with T1D. Using hierarchical Bayesian modeling, we observed that large glucose fluctuations were associated with slower and less accurate processing speed. Glucose fluctuations were not related to sustained attention, and some individuals exhibited greater cognitive vulnerability to glucose fluctuations than others. Using data-driven lasso regression, we identified seven clinical characteristics that predicted individual differences in cognitive vulnerability to glucose fluctuations: age, time in hypoglycemia, lifetime severe hypoglycemic events, microvascular complications, glucose variability, fatigue, and characteristics (e.g. neck circumference) previously linked with weight and sleep apnea. These results establish the impact of glucose on processing speed in naturalistic environments and indicate that minimizing glucose fluctuations is important for optimizing processing speed. Beyond T1D, this work demonstrates how data from physiological sensors and EMA can be integrated to better understand individual differences in daily cognitive functioning and clinical risk.

Abstract citation ID: igae098.2213

### INDIVIDUAL DIFFERENCES IN TIME OF DAY EFFECTS ON AMBULATORY COGNITIVE FUNCTIONING IN MIDDLE AND OLDER ADULTS

Emorie Beck[1], Zoe Hawks[2], and Laura Germine[2], *1. University of California Davis, Davis, California, United States*, *2. McLean Hospital/Harvard Medical School, Belmont, Massachusetts, United States*

Previous work indicates that (1) changes in diurnal cycles are associated with the onset and progression of dementia and cognitive impairment and (2) that cognitive function itself tends to show diurnal cycles in the laboratory. But no work to date has examined whether cognitive function assessed via ecological momentary assessment can recover such daily cycles and whether individual differences in those cycles are associated with short- and long-term outcomes. In this presentation, we examine whether ambulatory cognitive assessments assessed via ecological momentary assessment (EMA) demonstrate intra-day cycles of cognitive performance. This study specifically aims to test whether such cycles emerge, whether there are individual differences in those cycles, and whether individual differences are associated with cross-sectional measures of cognitive health and psychosocial risk factors. At baseline, 200 (Mean age = 55; 50.1% female) participants responded to five surveys per day for 20 days capturing their cognitive function, contextual factors, and psychological experiences. We will examine nonlinear changes in cognitive function through the day using both multilevel time series decomposition and cosinor models, which also allow us to examine individual differences in these patterns. We will then examine whether these individual differences are concurrently associated with global cognitive functioning, cognitive impairment, personality traits, and other psychosocial factors.

Abstract citation ID: igae098.2214

### CONTEXT MATTERS: THE IMPACT OF ENVIRONMENT, MOOD, AND FATIGUE ON MOBILE COGNITIVE ASSESSMENTS

Hannah Wilks[1], Matthew Welhaf[1], Andrew Aschenbrenner[2], John Morris[1], and Jason Hassenstab[1], *1. Washington University in St. Louis, St. Louis, Missouri, United States, 2. Washington University School of Medicine, St. Louis, Missouri, United States*

To better understand the aging process and the subtle cognitive changes that occur in preclinical Alzheimer disease (AD), researchers are using technology to remotely measure cognition. We examined how concurrent environmental context, mood, and fatigue are associated with cognition in older adults at risk for AD. Participants (N = 288) were recruited from the Knight Alzheimer Disease Research Center (Mage = 75, RangeAge= 61-97). Cognition (e.g., associate memory and processing speed), context (e.g., with whom, where, activities prior to assessment), and current affect (e.g., mood, and fatigue) were measured contemporaneously on a smartphone-based application up to 28 times over a week in naturalistic environments. Clinical status was determined using the Clinical Dementia Rating □ (CDR□). Cognition at each assessment was predicted by contextual- and affect-related factors, and their interaction with CDR, while controlling for age, gender, education, and time in study. Associate memory and processing speed were worse in the presence of others, when assessed in locations other than home, and after engaging in sedentary behavior. Affective states were not associated with cognition. These effects were magnified in participants with very mild dementia symptoms, particularly for processing speed. Environmental and affective factors can influence memory and processing speed in older adults at risk for AD. Distracting environments were particularly problematic for individuals currently experiencing mild dementia symptoms. Studies of AD risk that rely on remote, unsupervised cognitive assessments should carefully assess and consider concurrent contextual factors when examining cognition.

Abstract citation ID: igae098.2215

### LONELINESS AND MEMORY LAPSES AMONG OLDER ADULTS IN DAILY LIFE

Karina Van Bogart[1], Erin Harrington[2], Jacqueline Mogle[3], Jee eun Kang[4], Martin Sliwinski[5], and Jennifer Graham-Engeland[5], *1. Northwestern University, Chicago, Illinois, United States, 2. University of Wyoming, Laramie, Wyoming, United States, 3. Clemson University, Clemson, South Carolina, United States, 4. The Pennsylvania State University, University Park, Pennsylvania, United States, 5. Pennsylvania State University, University Park, Pennsylvania, United States*

Loneliness has been linked to objective cognitive decline and dementia risk; however, less is known about how loneliness relates to subjective cognition. Assessing daily subjective cognition related to prospective memory (i.e., for future events; PM) and retrospective memory (i.e., for past information; RM) may be particularly useful in identifying associations with daily emotional and cognitive health. The current research examined associations between loneliness and self-reported PM and RM lapses among 311 older adults (mean age=77.45 years, 40% Black, 13% Hispanic, 67% women) who completed 14 consecutive days of ecological momentary assessment that included self-reports of momentary loneliness (5x/day) as well as evening self-reports of memory lapses that occurred that day. Separate multilevel models were estimated to assess daily associations between loneliness and number of PM or RM lapses, and the amount of disruption and irritation associated with these lapses. All models controlled for age, gender, years of education, MCI status, and depressive symptoms. Days when loneliness was higher than typical were significantly associated with a higher number of PM lapses (b=0.011, p<.001), and more disruption to daily activities (b=0.113, p <.001) and irritation (b=0.036, p=.002) associated with experiencing PM lapses. Daily loneliness was not associated with the number of RM lapses, but was significantly linked with more disruption to daily activities (b=0.131, p<.001) and irritation associated with RM lapses (b=0.103, p<.001). Findings suggest that loneliness may uniquely impact daily PM and perceived disruption from RM issues, which has important implications for cognitive health and maintaining independence in late life.

### SESSION 4520 (SYMPOSIUM)

Abstract citation ID: igae098.2216

### RURAL HEALTH AND FAMILY CAREGIVING: IDENTIFYING CHARACTERISTICS AND ADDRESSING NEEDS ACROSS POPULATIONS

Chair: Caroline Stephens
Co-Chair: Jacqueline Telonidis
Discussant: Katherine Ornstein

The rural population in the U.S. is growing and aging faster, and they have more comorbidities, than the overall aging population. Despite the greater need for resources and supports, rural residents may experience the most barriers to formal services (e.g., hospital, hospice/palliative care, nursing home care) and may lack available family caregivers. Unfortunately, there is a dearth of population-based research comparing the characteristics and needs of older adults living and dying in rural vs. urban areas, particularly related to availability of family. This symposium includes 4 presentations focused on rural health and family caregiving and identifying the characteristics and addressing needs across populations. The first presentation uses a retrospective population cohort design with cancer registry and state-wide claims data to examine urban/rural differences in palliative care-related service claims among older adult cancer patients diagnosed with metastatic breast, lung, colorectal, head and neck, bladder, and melanoma cancers. The next two presentations use data from the Utah Caregiving Population Study (Utah C-PopS) to examine how family size and characteristics impact EOL care utilization for urban vs. rural NH residents and for persons with dementia. The last presentation describes a program evaluation of an interagency collaboration to support caregivers in rural communities. Together, these presentations shed light on the diverse characteristics, gaps in care, and novel strategies to address the needs of rural populations to better inform rural health practice and policy considerations.

Abstract citation ID: igae098.2217

### ROLE OF FAMILY CHARACTERISTICS OF RURAL AND URBAN NURSING HOME RESIDENTS AT END-OF-LIFE (EOL)

Caroline Stephens[1], Rebecca Utz[1], Mike Hollingshaus[1], Djin Tay[1], Linda S. Edelman[2], Eli Iacob[1], Ken Smith[1], and Katherine Ornstein[3], *1. University of Utah, Salt Lake City, Utah, United States*, *2. College of Nursing, Salt Lake City, Utah, United States*, *3. Johns Hopkins University, Baltimore, Maryland, United States*

Nursing home (NH) EOL disparities are an increasingly important focus for policymakers. Despite the critical role of families in resident EOL care, no studies to date have explored how family size and characteristics impact EOL care utilization for urban vs rural NH residents. This study describes the size and characteristics of first-degree families (FDF) of Utah NH residents who died in rural/frontier (n=10,440) vs. urban (n=32,366) NHs between 1998-2016. Using the Utah Caregiving Population Study (Utah C-PopS), we linked NH decedents to their FDF (urban FDF n=90,829; children=57%; siblings=32%; spouses=11%; rural FDF n=30,512; children=55%; siblings=34%; spouses=11%) and compared characteristics of those with and without FDF by urban and rural status. Rates of ER visits and hospitalizations in the last six months of life by FDF type were estimated. Rural NH decedents were slightly more likely to lack FDF in the state compared to urban NH decedents (23% vs 22%, respectively). Having a spouse was associated with increased rates of hospitalization among both urban [IRR 1.12 (1.06,1.17)] and rural NH decedents [IRR 1.11 (1.03,1.19)], but increased rates of ED visits [IRR 1.10 (1.01,1.20)] were only observed among urban decedents. Having at least one child, or a child and spouse, was associated with increased rates of hospitalization in urban decedents [IRR 1.06 (1.03,1.10), IRR 1.13 (1.08,1.19), respectively], but not among rural NH decedents. A better understanding of family role in EOL care in the NH setting will advance research on disparities in EOL NH care, particularly related to rural and urban differences.

Abstract citation ID: igae098.2218

### INTERAGENCY COLLABORATION TO SUPPORT CAREGIVERS IN RURAL COMMUNITIES

Jacqueline Telonidis[1], Christopher Hernandez[1], Randall Rupper[2], Jennifer Morgan[3], Katherine Supiano[1], Linda S. Edelman[4], and Kara Dassel[5], *1. University of Utah, Salt Lake City, Utah, United States*, *2. Salt Lake City VA Healthcare System, Salt Lake City, Utah, United States*, *3. Utah State University, Logan, Utah, United States*, *4. College of Nursing, Salt Lake City, Utah, United States*, *5. University of Utah, Salt Lake Cty, Utah, United States*

The Utah Geriatric Education Consortium (UGEC) is a Health Resources and Services Administration (HRSA) Geriatrics Workforce Enhancement Program committed to providing age- and dementia-friendly education to primary care and geriatrics workforces and supporting caregivers of people with dementia. We partnered with three rural, multi-county Area Agencies on Aging (AAA) to design and implement hybrid dementia caregiver conferences. Other partners included the Utah Chapter of the Alzheimer's Association, Utah Department of Health and Human Services, VA Salt Lake City Healthcare System Geriatric Research Education and Clinical Center, and Utah Department of Veterans and Military Affairs. Conference topics included: UGEC educational programs and resources; local AAA community-based programs and resources; dementia, depression, and delirium screening; understanding/responding to dementia-related behaviors; caregiver grief; VA healthcare enrollment and caregiver support programs. The conferences averaged 70.33 (range 54-92) in-person attendees and 12 (range 3-21) virtual attendees. Attendees reported positive levels of satisfaction on a 5-point scale, including that the conferences were engaging (X = 4.55) and effective (X = 4.62), provided examples useful to their education (X = 4.59), met stated goals (X = 4.53), and were better/a lot better compared to similar programs (X = 4.40). In-person attendees reported a sense of community amongst caregivers, and appreciation for the topics related to grief and caregiver support. Attendees suggested adding a caregiver panel, and more local long-term care services and support vendors. In conclusion, rural caregiver conferences which include in-person and virtual modalities meet the educational and flexibility needs of rural caregivers in Utah.

Abstract citation ID: igae098.2219

### IMPACT OF RURALITY ON PALLIATIVE CARE–RELATED SERVICE USE: FINDINGS FROM A POPULATION DATASET

Djin Tay[1], Jia-Wen Guo[1], Katherine Ornstein[2], Kline Dubose[1], Xiaoming Sheng[1], and Lee Ellington[1], *1. University of Utah, Salt Lake City, Utah, United States*, *2. Johns Hopkins University, Baltimore, Maryland, United States*

Palliative care, including hospice, supports quality of life of individuals living with serious illness. However, residents in rural areas may have limited access to these services. This study examined urban/rural differences in palliative care-related service claims among older adult cancer patients diagnosed with metastatic breast, lung, colorectal, head and neck, bladder, and melanoma cancers. We hypothesized that rural patients would have less palliative care-related service claims. This study used a retrospective population cohort design with cancer registry and state-wide claims data. Data from patients aged 65 and above with a diagnosis of metastatic cancers were analyzed. N=1,798 patients were identified, with n=511 (28%) living in rural or frontier regions. Patients living in rural areas differed from urban patients by cancer types (p<0.006), race (p=0.015), advance care planning (p=0.002), and hospice use (p=0.040). After diagnosis, only 2% of rural patients and 6% of urban patients had advance care planning claims and approximately half had ever received hospice care (54% rural; 49% urban). While insignificant, rural patients had palliative care (35% vs. 31%) and chemotherapy claims (53% vs. 49%) at higher proportions compared with urban patients (p=0.076 and p=0.055 respectively). There were no observed differences in use of life-sustaining and/or cancer treatments. This was one of the few population-based studies examining rural-urban disparities in palliative care-related services. Despite being a clinical guideline and marker of quality of care in advanced cancers, findings imply low uptake of palliative care services overall, suggesting room for improvement for palliative and end-of-life cancer care delivery.

Abstract citation ID: igae098.2220

### UNREPRESENTED TO FLUSH-WITH-FAMILY: THE ROLE OF FAMILY STRUCTURE ON DEMENTIA CARE BY RURALITY

Rebecca Utz[1], Eli Iacob[1], Mike Hollingshaus[1], Erin Bouldin[1], Thomas Cudjoe[2], Katherine Miller[3], Tim Farrell[1], and Katherine Ornstein[3], *1. University of Utah, Salt Lake City, Utah, United States*, *2. Johns Hopkins University School of Medicine, Baltimore, Maryland, United States*, *3. Johns Hopkins University, Baltimore, Maryland, United States*

U.S. policy and clinical practice increasingly recognize the value and essential role that family caregivers play in planning and delivering long-term care, especially in the context of dementia where cognitive decline leads to practical and legal needs to rely on others (most often family) to serve as caregivers and surrogate decision makers. Given the preference of older adults to age-in-place and that younger generations commonly move away from rural areas, access to family support may characteristically differ across those living in urban versus rural areas. This study uses the Utah Caregiving Population Study (C-PopS), a unique population-based data resource that links administrative and health records with genealogical data, to describe the size and structure of first-degree family (FDF) members (spouse, child, sibling) associated with a cohort of persons aged 65+ who died with dementia in Utah, stratified by rural (n=11,105) vs. urban (n=38,099) geographic small-areas. Compared to those in urban areas, persons with dementia in rural settings were more likely to be married (39.3% versus 35.2%), but less likely to have adult children in the state (61.9% versus 67.9%). About one in five rural older adults with dementia (20.1%) were unrepresented (defined as no FDF present in the state), compared to about 17% of those in urban areas. Understanding rural-urban differences in how family structure characteristics are associated with distinct patterns of dementia care, including use of hospice and care transitions at end-of-life, will help inform health policy and future interventions targeting the unique needs of this vulnerable population.

### SESSION 4525 (SYMPOSIUM)

Abstract citation ID: igae098.2221

### SOCIO-EMOTIONAL DEVELOPMENT ACROSS ADULTHOOD AND AGING

Chair: Urmimala Ghose
Co-Chair: Denis Gerstorf
Discussant: Jutta Heckhausen

Understanding how social relations evolve across adulthood, thereby shaping well-being in later life is a longstanding question in lifespan developmental research. The presented studies offer novel insights, utilizing innovative methodologies, diverse time scales, and varied samples. Chopik combined qualitative data from the Harvard Student Study Class of 1964 with machine learning techniques, reporting a larger and closer friends' circle during freshman year to be associated with more positive life reflections, enhanced relationships, and reduced mental illness fifty years later. Chopik also explored historical changes in these associations using a novel cohort sequential design. Fiori et al. analyzed hierarchical network mapping and ecological momentary assessment data from the Stress and Well-being in Everyday Life study. Contrary to common theoretical expectations, they found that only interactions within the inner circle of social networks declined with age, and increased positivity in interactions extended beyond the inner circle. Hülür et al. conducted an event-contingent experience sampling study on older adults revealing that interactions with more close social partners were linked to higher well-being across communication modes. Digital text-based communication was identified as a compensatory mechanism for individuals with smaller social networks. Ghose et al. utilized a unique synthetic cohort matching method with Health and Retirement Study panel data, illustrating distinct well-being trajectories in older adults undergoing marital transitions compared to their never/continuously-married peers. Their results suggested more pronounced initial increase and subsequent decrease in depressive symptoms following spousal bereavement than divorce, with social strain identified as an additional risk factor in bereavement.

Abstract citation ID: igae098.2222

### LATE-LIFE SINGLEHOOD AND WELL-BEING: THE ROLE OF MARITAL STATUS AND SOCIAL RESOURCES

Urmimala Ghose[1], Rinseo Park[2], Jacqui Smith[3], Nilam Ram[2], and Denis Gerstorf[1], *1. Humboldt University Berlin, Berlin, Berlin, Germany*, *2. Stanford University, Stanford, California, United States*, *3. University of Michigan, Ann Arbor, Michigan, United States*

Singlehood has long been identified as a potential risk factor for undermining well-being across adulthood, particularly in older ages. However, it is an open question what role other social resources may play. In the current study, we examine whether and how older adults experiencing marital transitions through divorce and spousal bereavement differ from their never married and continuously married peers in their well-being trajectories and in the roles that social support and strain play. We used six waves (2008 – 2018) of longitudinal data from 7,670 participants (Mage = 66 years, 56% women, 75% White) of the Health and Retirement Study (HRS). We also employed synthetic cohort matching to equate the groups on relevant background factors. First results of multi-level models revealed that bereavement trajectories were characterized by a steeper transition-related rise in depressive symptoms than the divorce trajectories, but also by a sharper drop in subsequent years. Both transition groups showed declines in depressive symptoms in the years after the event, yet those who had experienced divorce reached average levels of their continuously married peers after about three years, whereas the bereaved group continued to lag behind even after six years post-spousal loss. Overall, we found little evidence that the relevance of social resources for well-being trajectories differs across marital status groups. As one exception, we found that the predictive effects of strain from family and friends for elevated depressive symptoms was particularly pronounced among those who had experienced spousal loss.

Abstract citation ID: igae098.2223

### FRIENDSHIP QUALITY IN COLLEGE PREDICTS WELL-BEING IN QUALITATIVE LIFE REFLECTIONS 50 YEARS LATER

William Chopik, *Michigan State University East Lansing, East Lansing, Michigan, United States*

Theories of successful aging and psychosocial development posit that well-being later life indicates successful adaptation and portends a longer life. In identifying antecedents to late life well-being, close relationships have emerged as a prominent antecedent for health and well-being across life. However, prospective studies examining the impact of social relationships on health and well-being are relatively rare, particularly mixed methods studies. I paired the Harvard Student Study Class of 1964 (N = 252) with qualitative reflection data in their alumni class reports every five years after graduation for over fifty years. Machine learning techniques will be featured depicting how psychological traits can be inferred through autobiographical reflections. Having more friends and closer friends during their freshman year of college (at age 22) was associated with more positive life reflections (r =.22), more positive relationships (r =.23), and less mental illness (r = -.25) fifty years later (at age 72). I will then leverage these archival sources to examine the impact of social relationships on health and well-being across nearly 100 years to test secular changes in a newly constructed cohort sequential design. Emerging methods for linking family reports across generations and cohorts will be discussed and preliminary results presented. The discussion of the talk will focus on the antecedents of successful aging and how to leverage existing data—in its many forms—to study psychosocial development within a person's life and across historical time.

Abstract citation ID: igae098.2224

### AGE DIFFERENCES IN THE FREQUENCY AND NATURE OF ADULTS' DAILY INTERACTIONS WITH OTHERS

Katherine Fiori[1], Amy Rauer[2], Kira Birditt[3], Angela Turkelson[3], and Oliver Huxhold[4], *1. Adelphi University, Garden City, New York, United States, 2. University of Tennessee, Knoxville, Tennessee, United States, 3. University of Michigan, Ann Arbor, Michigan, United States, 4. German Centre of Gerontology, Berlin, Berlin, Germany*

Decades of research show that although adults' social networks tend to shrink with age, the proportion of closest ties stays relatively constant and older adults rate their relationships as more positive. However, little research has examined with whom adults actually interact on a daily basis and the nature of those interactions. We used hierarchical network mapping data and ecological momentary assessment to examine whether there were age differences in social interaction types (inner circle, middle/outer circles, outside network) and in their valence (positive, negative, ambivalent), and if age moderated the link between interaction type and valence. Participants from the Stress and Well-being in Everyday Life (SWEL) study (N = 168; M age = 52.96, range = 33-91; 66.1% female; 47.6% Black) completed surveys every three hours for 4-5 days, reporting a total of 5,771 social interactions. Results showed that 36.6% of all interactions occurred with people outside of the network. Furthermore, against common theoretical expectations, only interactions within the inner circle declined with age. Moreover, although older participants reported more positive and more ambivalent interactions overall than younger adults, age did not moderate the association between interaction type and valence. This indicates that the increased positivity in older adults' interactions was not specific to inner circle ties, but rather cut across interactions with all types of ties. Examining interactions on a daily level provides new insights into the specificity of potential age effects in social relationships.

Abstract citation ID: igae098.2225

### THE ROLE OF SOCIAL RELATIONSHIP PARTNERS FOR WELL-BEING AFTER SOCIAL INTERACTIONS IN DIFFERENT MODALITIES

Gizem Hueluer[1], Kaila Borgards[1], and Birthe Macdonald[2], *1. University of Bonn, Bonn, Nordrhein-Westfalen, Germany, 2. University of Zurich, Zurich, Zurich, Switzerland*

Social relationships and social interactions are essential for psychological well-being. In the present study, we examine the role of social relationship partners (relationship closeness and social network size) for well-being after social interactions in different modalities (face-to-face, telephone, text-based digital). We use 7,861 observations from 113 participants (age: M = 72 years, SD = 5, range = 65 to 94; 40% women), who completed a short questionnaire about their social interactions over 21 days in an event-contingent experience sampling study. With regard to main effects, our findings showed that within persons, participants reported higher levels of well-being after social interactions with closer vs. less close social interaction partners. Social network size was unrelated to well-being after social interactions. Well-being was highest after face-to-face social interactions, followed by telephone and digital text-based social interactions. With regard to moderation effects, the effect of relationship closeness on well-being was not moderated by social interaction modality. The effect of social network size on well-being after social interactions was moderated by social interaction modality, indicating that the difference in well- being experienced after face-to-face and telephone vs. text-based digital social interactions was smaller for participants with smaller social networks. In summary, our findings show that social interactions with more close social partners are associated with higher well-being regardless of modality and that digital text-based communication may play a compensatory role for older adults with smaller social networks. We discuss implications of these findings for future research.

### SESSION 4530 (SYMPOSIUM)

Abstract citation ID: igae098.2226

### STRATEGIES AND TOOLS TO MAKE THE INVISIBLE VISIBLE IN HOSPITAL CARE

Chair: Blair Golden
Co-Chair: Beth Fields
Discussant: Julia Berian

Family caregivers (relatives, partners, or other support persons) provide critical support during hospitalization events for older adults, ranging from providing companionship and emotional support to coordinating discharge planning. Despite their essential role in hospital care delivery, family caregivers often feel invisible and poorly integrated into hospital care teams. Recognizing this gap, the 2022 National Strategy to Support Family Caregivers was created by federally mandated advisory councils with public input. Goal 2 of this National Strategy consists of advancing partnership and engagement with family caregivers, including recognition of family caregivers as essential care team partners and inclusion of family caregivers within care plans. In response to Goal 2, this symposium will present innovative strategies and tools to advance partnerships and engagement with family caregivers within hospital-based care. Dr. Vick will present findings from a qualitative systematic review of family caregiver involvement during hospitalizations of older adults, leading to development of a conceptual model of family caregiver roles within hospital settings to guide future interventions. Ms. Hoel will discuss findings from a qualitative study identifying organizational barriers to family caregiver inclusion in the hospital care of people living with dementia. Dr. Scheunemann will discuss development of the ICU Family Workbook (a publicly available resource for family caregivers navigating critical illness), including results from content validity and user testing. Dr. Golden will present research findings on current practices and gaps with regards to caregiver and patient discharge education about delirium, a common condition affecting hospitalized adults.

Abstract citation ID: igae098.2227

### A RETROSPECTIVE COHORT STUDY OF HOSPITAL DISCHARGE INSTRUCTIONS FOLLOWING DELIRIUM EPISODES

Blair Golden[1], David Sonnentag[2], Farah Kaiksow[2], Andrea Gilmore-Bykovskyi[3], Manish Shah[4], Sharon Inouye[5], and Eduard Vasilevskis[3], *1. University of Wisconsin Madison School of Medicine and Public Health, Madison, Wisconsin, United States*, *2. University of Wisconsin School of Medicine and Public Health, Madison, Wisconsin, United States*, *3. University of Wisconsin Madison, Madison, Wisconsin, United States*, *4. University of Wisconsin School of Medicine & Public Health, Madison, Wisconsin, United States*, *5. Harvard Medical School, Boston, Massachusetts, United States*

Delirium impacts an estimated 25% of hospitalized older adults and is associated with adverse outcomes, including increased mortality. Delirium can persist or recur after discharge, and prompt recognition is critical. Caregiver education may improve delirium recognition and management in the post-acute care period, but it is unclear if delirium is routinely incorporated into hospital discharge instructions. We sought to characterize how clinicians communicate about delirium in written discharge instructions. We performed a retrospective chart review of a random sample of 50 hospitalized older adults (age ≥65) with delirium. Trained team members identified delirium diagnoses from discharge summaries and assessed all mentions of delirium within discharge instructions. We also identified new anti-psychotic or benzodiazepine prescriptions and neurology or geriatrics referrals, as these are routinely communicated at discharge and may represent indirect delirium instruction. Almost all (48, 96%) patients had personalized discharge instructions regarding at least one non-delirium hospital diagnosis, but only 2 cases (4%) had delirium included in discharge instructions. A quarter of instructions (13, 26%) had terms that could be considered synonyms for delirium (e.g., "confusion" (5), "altered mental status" (5)). Three cases (6%) had proxies for delirium instruction (new prescriptions or clinic referrals). Documentation trends were similar for populations from whom delirium instructions would be particularly important, including patients discharging home and persons living with dementia. These findings demonstrate a large gap between clinician recognition of delirium and instructions to patients and caregivers during a vulnerable transition period. Future work is critical to understand discharge educational needs surrounding delirium.

Abstract citation ID: igae098.2228

### HOW ARE FAMILIES INVOLVED IN THE CARE OF HOSPITALIZED OLDER ADULTS? A QUALITATIVE EVIDENCE SYNTHESIS

Judith Vick[1], Connor Fogleman[2], Joseph Neiman[3], Sarah Cantrell[2], Mollie Selmanoff[4], Tolu Oyesanya[5], Karen Goldstein[5], and Courtney Van Houtven[5], *1. Yale University School of Medicine, New Haven, Connecticut, United States*, *2. Duke University, Durham, North Carolina, United States*, *3. Durham VA Health Care System, Durham, North Carolina, United States*, *4. Johns Hopkins Hospital, Baltimore, Maryland, United States*, *5. Duke University School of Medicine, Durham, North Carolina, United States*

In the outpatient setting, roughly 36 million family members in the US assist with caregiving tasks for older adults including managing medications, coordinating care, communicating with clinicians, and assisting with activities of daily living. The roles and impact of family involvement during hospitalizations of older adults are less well described. To better understand family involvement during hospitalizations of older adults, we designed a systematic review of qualitative research known as a qualitative evidence synthesis. We assembled a multidisciplinary research team including members with expertise in nursing, internal medicine, library science, evidence synthesis, social work, and health services research. We developed a search strategy and executed it in four databases: MEDLINE (via Ovid), Embase (via Elsevier), PsycINFO (via Ovid), and CINAHL Complete (via EBSCO). Our search identified 6,157 articles, and our title/abstract screening yielded 157 articles for full text review. Preliminary analysis suggests that family members participate in many ways in the care of hospitalized older adults including giving information about patients, preventing and managing delirium, providing hands-on care, advocating for patients, aiding in decision-making, and planning for discharge. The quality of existing qualitative research on family involvement during hospitalizations of older adults varies widely. Most existing evidence comes from countries outside of the US. Ultimately, our findings will contribute to the refinement of a conceptual model that will establish a shared language for future multidisciplinary work, inform future intervention development, and support workflow and policy changes.

Abstract citation ID: igae098.2229

**DEVELOPMENT, CONTENT VALIDATION, AND USER TESTING OF AN ICU FAMILY WORKBOOK FOR TRANSITIONS FROM HOSPITAL TO HOME**

Leslie Scheunemann, Aishwarrya Arivudainambi, Avital Isenberg, Mutasim Makeen, Janelle Christensen, and Heidi Donovan, *University of Pittsburgh, Pittsburgh, Pennsylvania, United States*

**Background:** No evidence-based interventions provide education and training for families experiencing critical illness to adapt to the caregiving role and attend to self-care while caregiving across transitions from hospital to home. **Methods:** We integrated literature with input from community engagement studios to develop a family-facing ICU Family Workbook addressing common unmet needs. A convenience sample of post-ICU family caregivers, clinicians, and researchers qualitatively and quantitatively assessed content validity related to 11 evidence-based elements. Given a strong content validity index, it is undergoing user testing with a convenience sample of 10 families experiencing critical illness to refine its structure and format. **Results:** The ICU Family Workbook includes roughly 70 pages of content covering 11 elements: (1) identifying a transitional "system navigator"; (2) education on symptom management; (3) education on functional support; (4) caregiver needs assessment; (5) development of written plans; (6) financial, legal, employment and insurance resources; (7) interdisciplinary decision support; (8) online resources; (9) self-care/psychological support and referral; (10) education on communicating with clinicians; (11) respite resources. Overall content validity index was 0.93. Qualitative feedback informed revisions refining educational content, enhancing screening tools, and making clearer education and self-care recommendations. Early user testing suggests usability and acceptability to families and is informing additional refinement of its content and formatting. Conclusions: This publicly available ICU Family Workbook (www.picturethis.pitt.edu) has content validity for addressing 11 common gaps in caregiver support. After additional refinement based on user testing, it should undergo efficacy testing for improving caregiving and self-care for families after critical illness.

Abstract citation ID: igae098.2230

**ORGANIZATIONAL BARRIERS TO INCLUDING CAREGIVERS OF HOSPITALIZED PATIENTS LIVING WITH DEMENTIA**

Sydney Hoel[1], Catherine Still[2], Teresa Thuemling[3], Andrea Strayer[4], Nicole Werner[1], and Beth Fields[2], *1. Indiana University, Bloomington, Bloomington, Indiana, United States*, *2. University of Wisconsin Madison, Madison, Wisconsin, United States*, *3. Indiana University Bloomington, Bloomington, Indiana, United States*, *4. University of Iowa, Iowa City, Iowa, United States*

Caregivers play a critical role in managing the health and well-being of hospitalized family members or friends living with Alzheimer's disease and related dementias. Yet, caregivers often report feeling excluded in hospital care. In order to effectively include caregivers in hospital care, the organizational barriers to inclusion faced by clinicians must be addressed by healthcare systems. The objective of this study was to identify organizational barriers to including caregivers of hospitalized patients living with dementia. We conducted semi-structured interviews with clinicians (n=26) who provide care to patients living with dementia in the hospital setting. Participants were nurses (42%), doctors (19%), social workers (12%), and rehab professionals (19%), and had an average of 11 years of experience in their role. Interview data were analyzed using a team-based content analysis directed by the Systems Engineering Initiative for Patient Safety (SEIPS) framework. We identified 6 categories of organizational barriers: culture, staffing, communication, policies, collaboration, and teamwork. Specifically, clinicians noted that organizations tend to emphasize "quantity over quality" culture, de-incentivizing and reducing time for interaction with caregivers. Most clinicians perceived a lack of organization support to include caregivers and could not recall receiving training on how best to include caregivers. These findings indicate multiple opportunities for healthcare systems to better support and prepare clinicians to include caregivers in hospital care. Information gleaned from this study can be utilized to guide the development of a more inclusive hospital organization, consistent with Goal 2 of the 2022 National Strategy to Support Family Caregivers.

### SESSION 4535 (PAPER)

### TECHNOLOGY USE IN OLDER ADULT CARE

Abstract citation ID: igae098.2231

**THE PHYSIOLOGIC COMPLEXITY OF PREFRONTAL OXYGENATION DYNAMICS IS ASSOCIATED WITH AGE AND COGNITIVE FUNCTION**

Junhong Zhou[1], Yinglu Hong[2], Wanting Yu[3], Ike Iloputaife[4], Dapeng Bao[2], Brad Manor[1], Lewis Lipsitz[3], and Azizah Jor'dan[5], *1. Harvard Medical School/Hebrew SeniorLife, Boston, Massachusetts, United States*, *2. Beijing Sport University, Beijing, Beijing, China (People's Republic)*, *3. Hebrew SeniorLife, Boston, Massachusetts, United States*, *4. Hebrew SeniorLife, Roslindale, Massachusetts, United States*, *5. University of Massachusetts Boston, Boston, Massachusetts, United States*

The hemodynamics of prefrontal cortex (PFC) oxygenation are regulated by numerous elements over multiple temporal scales. The dynamics in the fluctuations of PFC oxygenation are thus complex. Age may alter the multiscale regulation of PFC oxygenation, leading to diminished physiologic complexity of this important regulatory process. We aimed to characterize the effects of age and cognitive demand on such complexity in younger and older adults and its relationship to cognitive performance. Twenty-four younger (aged 28±3 years) and 27 older adults (aged 78±6 years) completed this study. The oxygenation (HbO2) and deoxygenation (HHb) signals of the PFC were recorded using functional near-infrared-spectroscopy (fNIRS) during the n-back task of blank, identification-of-X (IdX), and 2-back conditions. We used multiscale entropy to quantify the HbO2 and HHb complexity of fNIRS. It was observed that older adults exhibited lower fNIRS complexity regardless of task condition (p=0.0005) and lower percent increase of complexity from blank (p=0.02) or IdX (p=0.01) to 2-back compared to younger. Both groups exhibited greater complexity during IdX and 2-back compared to blank condition (p< 0.02). Those with greater HbO2 complexity had greater 2-back accuracy (p=0.02~0.04). Older adults with greater fNIRS complexity had faster reaction times (β=-0.56~-0.6, p=0.009~0.02); while younger with lower complexity had faster reaction times (β=0.58~0.62, p=0.01~0.02). Participants with greater increase of fNIRS complexity from blank to 2-back had faster 2-back reaction time (β=-0.68~-0.49, p=0.001~0.03). The complexity of fNIRS-measured PFC oxygenation fluctuations may capture the influence of aging and task demands on the regulation of prefrontal hemodynamics involved in cognitive task execution.

Abstract citation ID: igae098.2232

### A NOVEL MRI-COMPATIBLE SYSTEM CHARACTERIZING THE EFFECTS OF AGING ON SUPRASPINAL SENSORIMOTOR CONTROL OF WALKING

Hao Yue[1], Bin Shen[2], Yishu Chen[2], Yufeng Zhang[2], Jiaojiao Lü[2], Brad Manor[3], WeiJie Fu[2], and Junhong Zhou[3], *1. Guizhou University, Guiyang, Guizhou, China (People's Republic)*, *2. Shanghai University of Sport, Shanghai, Shanghai, China (People's Republic)*, *3. Harvard Medical School/Hebrew SeniorLife, Boston, Massachusetts, United States*

As the only part in direct contact with the ground during walking, foot soles continuously perceive the somatosensory information (e.g., ground reaction forces). The activation of the supraspinal regions/networks in response to such somatosensory inputs is thus important for walking performance, which is oftentimes altered by aging and age-related conditions. It is challenging to characterize such supraspinal activations via traditional neuroimaging techniques (e.g., functional MRI, fMRI), since people are required to stay motionless during the MRI scan. We here thus developed a novel foot-sole stimulation system that simulates the pressure changes as experienced by each foot sole and the pace of foot switch during walking over the ground, and enables characterizing the walking-related activation of the supraspinal regions via fMRI. To examine its validity and reliability of simulation, 10 younger and 10 older adults completed two trials of 10-meter walking. The recorded temporal changes of pressure on foot soles of each step were recorded and used to program the motion of the system with high validity and reliability (r=0.90~0.95, p< 0.0001). The phantom imaging test showed that the signal-to-noise ratio of the MR image in system-working (29.84±4.32) was similar to that (29.78±3.76) in off-working condition (p=0.73), suggesting great MRI compatibility. The block-designed fMRI test showed that compared to resting, multiple supraspinal regions (e.g., postcentral and precuneus gyrus) (p< 0.005) were activated by the foot-sole stimulation. This novel MRI-compatible system provides a novel tool to characterize the effects of aging and age-related conditions on the supraspinal sensorimotor control of walking.

Abstract citation ID: igae098.2233

### REDUCING SERIOUS FALL INJURIES IN OLDER ADULTS: RESULTS OF A MULTICENTER WEARABLE TECHNOLOGY STUDY

Bruce Kinosian[1], and Richard Stefanacci[2], *1. Philadelphia Veterans Affairs Medical Center, Philadelphia, Pennsylvania, United States*, *2. Thomas Jefferson University, Philadelphia, Pennsylvania, United States*

Falls remain an immense public health concern for our growing aging population, causing over 3 million ER visits annually. With falls that result in hip fractures having devastating consequences for older adult health and independence, new solutions are urgently needed. This study evaluated the real-world effectiveness of the Tango® Belt, an innovative wearable device aimed at preventing serious fall injuries. In this six-month, comparative, adaptive study with historical controls conducted across 10 communities located in PA and CA, older adults used sensor-embedded belts alongside standard fall mitigation protocols. When worn during a potentially harmful fall, the belt deploys an airbag to cushion impact. We compared fall outcomes in the belt users (N=134) to propensity score matched historical controls (N=264). Analysis of fall events between the matched groups demonstrated similar rates of falls (p=0.081), but a significant reduction in the proportion of major hip injuries in the intervention versus the control group (1.1% vs 12.1% p=0.004) demonstrating a protective effect of 90.9% against hip fractures/dislocations due to serious hip-impacting falls. Further analysis reveals that the proportion of hospitalizations (8.2% vs 18.9% p=0.004) and ED visits (6.0% vs 17.8% p=0.0001) were also significantly reduced in the intervention vs control group. With compliance recorded at 67% across subjects and no serious AE's noted related to the device, such "smart garments" address known compliance barriers with passive hip protectors. With serious fall injuries expected to exponentially rise with population aging, the study provides promising evidence for future use of disruptive innovations like Tango Belt.

Abstract citation ID: igae098.2234

### OBJECTIVE SLEEP QUALITY ASSOCIATES WITH PLASMA BIOMARKER FOR COGNITIVE IMPAIRMENT

Jinyoung Kim, Kevin Dangler, Stephanie Nam, Miguel Fudolig, and Jefferson Kinney, *University of Nevada Las Vegas, Las Vegas, Nevada, United States*

**Aim:** Emerging evidence suggests that poor sleep (i.e., lack of sleep and low sleep quality) significantly impacts the risk of severe cognitive impairment. However, research on the use of sleep quality metrics as early predictors of cognitive impairment risk remains scarce. In this study, we investigated the association between objective sleep quality measures and plasma levels of Eotaxin-1, an early biomarker for cognitive impairment, among nurses.

**Methods:** We used data from 35 nurses who underwent multiple home sleep studies with electroencephalograms on workdays and days off were included. Objective sleep quality measurements included percentage of stage-3 non-rapid eye movement sleep in total sleep time (%N3) and wake time after sleep onset (WASO). Blood samples were collected in all participants and plasma levels of multiple cytokines (27Plex Cytokine array), including Eotaxin-1 were measured by Luminex multiplexing.

**Results:** Sleep quality indicators showed that WASO increased by 8% on days off compared to workdays, while %N3 decreased by 1% ($p<.001$). After adjusting for age, sex, shift, and BMI, higher WASO ($\beta=1.22$, $p<.05$) and lower %N3 ($\beta=-5.09$, $p<.05$) on workdays were significantly associated with increasing levels of Eotaxin-1. Sleep parameters on days off did not significantly correlate with Eotaxin-1 levels.

**Conclusion:** Our findings indicate a correlation between objective sleep quality measurements on workdays and an early biomarker of cognitive impairment. Further research is necessary to elucidate the underlying mechanisms linking sleep quality to future cognitive impairment, as well as to explore the potential differential associations of these parameters between workdays and days off.

Abstract citation ID: igae098.2235

### PERSONALITY AND ALLOSTATIC LOAD: TESTING HEALTHY NEUROTICISM IN HISPANIC AMERICANS OVER 50

Barış Sevi[1], Adil Supiyev[2], Angela Gutierrez[3], and Graciela Muniz Terrera[4], *1. MEF University, Istanbul, Istanbul, Turkey*, *2. Ohio University Heritage College of Osteopathic Medicine, Athens, Ohio, United States*, *3. Western University of Health Sciences, Pomona, California, United States*, *4. Ohio University, Athens, Ohio, United States*

Allostatic load (AL) measures the cumulative wear and tear resulting from chronic stress and life events. Hispanic populations report a higher risk for elevated AL. To diminish health inequities in socially disadvantaged populations, understanding the antecedents of AL is essential. Using data from the Health and Retirement Study, this study examines the Big-5 personality traits as predictors of AL among middle-aged and older Hispanic adults (age ≥ 50; n: 319). First, a conditional growth model was tested where baseline Big Five personality traits were entered simultaneously to test the association with AL over three time points in eight years (Time 1 = 2006/2008; Time 2 = 2010/2012; Time 3 = 2014/2016). Our findings indicate that higher conscientiousness at baseline was associated with lower AL, but the Big-5 personality traits did not significantly predict the rate of change in AL over time. As the healthy neuroticism hypothesis suggests that higher neuroticism, which is by itself associated with worse health, when combined with higher conscientiousness, may positively influence health outcomes, a second conditional growth model was tested with the interaction of neuroticism and conscientiousness added to the first model. Contrary to expectations, higher levels of neuroticism and conscientiousness were associated with higher baseline AL, with no significant association with the rate of change in AL. These results underscore the importance of personality in predicting AL but provide evidence that challenges the assumptions of the healthy neuroticism hypothesis.

### SESSION 4540 (SYMPOSIUM)

Abstract citation ID: igae098.2236

### THE BENEFITS OF CREATIVE ACTIVITIES FOR DIVERSE GROUPS OF OLDER ADULTS

Chair: Carolyn Adams-Price
Discussant: Justine McGovern

Participation in creative activities is said to be beneficial for older adults and is often suggested for healthy cognitively intact and cognitively impaired populations. The purpose of this symposium will be to explore the benefits of different kinds of creative programs and interventions offered to diverse groups of older adults of different ability levels. The first paper in this symposium will discuss theory and the psychosocial context of creativity in older adults. The observed and hypothetical benefits will also be discussed from different frameworks, including life-span developmental psychology and dementia studies. LaVona Traywick will present the physical and emotional benefits of her dance program with older adults with Parkinson’s disease. Jennifer Dorris will discuss her experience incorporating music into her activities with older Down’s syndrome patients. Justine McGovern will be a discussant for this session, tying together the different presentations.

Abstract citation ID: igae098.2237

### THE BENEFITS OF CREATIVE ARTS INTERVENTIONS FOR OLDER ADULTS: THE SOCIOCULTURAL MODEL AND THE DEMENTIA STUDIES MODEL

Carolyn Adams-Price, *Mississippi State University, Mississippi State, Mississippi, United States*

Over the past 25 years, there has been a huge increase in the employment of interventions for older adults that incorporate as a primary feature participation in creative activities. Interventions have focused on art domains as distinct as photography, dance, music, traditional handicrafts (such as quilting or pottery), and many others. In addition, programs have been created for older adults with a huge range of ability levels and cognitive efficacy, from highly functioning older adults to older adults with severe cognitive deficits. The purpose of this paper will be to describe the benefits of creative interventions for different types of older adults with different types of capabilities. Two different perspectives will be highlighted; including the sociocultural model of creativity, which is linked to life-span developmental psychology, and the dementia studies model, which derives from holistic and qualitative approaches to aging. The paper will discuss the ways in which these two approaches are fundamentally different, and the ways in which they are similar, and discuss what they have to say about the nature of creativity.

Abstract citation ID: igae098.2238

### DANCE AS A CREATIVE ARTS PROGRAM FOR SEINOR ADULTS WITH PARKINSON'S DISEASE AND THEIR CAREGIVERS

LaVona Traywick, *Arkansas Colleges of Health Education, Fort Smith, Arkansas, United States*

The Dance with PD (Parkinson's Disease) program is offered as a free community creative arts outreach program by Arkansas Colleges of Health Education (ACHE), Research Institute Health & Wellness Center for individuals with PD and their care partners. Dance with PD is a therapeutic approach to programming that involves dance and movements as a form of therapy for individuals living with Parkinson's Disease. The program aims to improve mobility, balance, coordination, and overall quality of life through structured dance exercises and routines. The program is offered by a trained instructor and the sessions are tailored to the specific needs and abilities of the participants. Volunteer assistants are students from ACHE's medical college. The class is offered once a week for one hour to mimic a traditional dance class. Due to the rolling admission, participants have attended for different lengths of time when they complete their assessments. The Dynamic Gait Index (DGI), Falls Efficacy Scale, and Geriatric Depression Scale—short form are administered each semester and compared to attendance records.

Abstract citation ID: igae098.2239

### CHARACTERIZING MUSIC PARTICIPATION FOR ADULTS WITH DOWN SYNDROME

Jennifer Dorris, *University of Pittsburgh, Pittsburgh, Pennsylvania, United States*

Adults with Down syndrome (Ds) are living longer and have high risk of experiencing cognitive decline as they age. Unfortunately, few services are available to support aging adults with Ds and their care partners. Music is a safe, engaging intervention modality that has shown promising results for older adults experiencing cognitive decline. This study reported on adults with Ds' frequency and type of music participation and described support provided by care partners. Additionally, the study explored associations of age, race, or level of intellectual disability associated with music participation. Care partners of adults with Ds (n =27) completed a survey that measured "listening" and "playing music" using the Guernsey Community Participation and Leisure Assessment. 92.6% of adults with Ds listened to music weekly or daily. 74.1% of care partners listened to music as an unaccompanied activity. 33.3% of adults with Ds played music either weekly or daily, and 48.2% of care partners described playing music as an unaccompanied activity. A significant association was found between race and playing music, but there were no significant associations between age, intellectual disability level, and music participation for adults with Ds. Knowing that racially minoritized adults with Ds had a higher frequency in playing music, those developing services and supports could consider utilizing music as a meaningful activity. Additionally, due to the lack of associations between age and intellectual disability, music programs could be developed equitably for changes in age and intellectual disability level, creating inclusive activities for this growing population of older adults.

Abstract citation ID: igae098.2240

### ADAPTATIONS IN A DRUMMING AND HORTICULTURE INTERVENTION FOR OLDER PEOPLE LIVING WITH INTELLECTUAL DISABILITIES

Emily Ihara, and Michelle Hand, *George Mason University, Fairfax, Virginia, United States*

People aging with intellectual and development disabilities (IDD) often experience intersectional discrimination, impacting multiple dimensions of wellness. Rhythm & Nature is a trauma-informed drumming and horticulture intervention that provides an inclusive and modifiable environment. Both activities involve bilateral stimulation, using alternating hand movements to help create connections between the left and right hemispheres of the brain, which can help improve anxiety symptoms, memory, creativity, and attention. The 8-week intervention was delivered two times, roughly one year apart. The convenience sample was drawn from attendees of an adult day center for older people living with IDD, who could participate in drumming, horticulture, or both activities. This presentation will provide qualitative analysis results of the eight participants in Group 1 and preliminary analysis of the 18 participants in Group 2. Themes from the Group 1 analysis included autonomy, voice, and creativity; fun, laughter, and joy; safe space for sharing; and skill development, affirmations, and confidence. The acuity level of Group 2 participants was higher and the group was larger, requiring different modifications by the two facilitators. Adaptations that improved accessibility included formatting the emojis differently on the pre/post forms, printing pre/post surveys on different colored paper to make it easier for them to follow, and focusing on one theme per session. Week 4 of Group 2 occurred during the center director's last day. Participants were able to drum a farewell song and create a bouquet to convey meaningful goodbye messages. Further program examples and implications for research and practice will be provided.

### SESSION 4545 (SYMPOSIUM)

Abstract citation ID: igae098.2241

### THE BLUES AND OLDER MINORITY MUSICIANS: MORE THAN JUST MUSIC XXXI

Chair: John Migliaccio
Co-Chair: Michael Marcus

The Blues may have emerged in the Mississippi Delta but its influence and music is mightily represented today in the Puget Sound! Seattle's Blues Bash, Blues Awards, and the Rhapsody Project make intergenerational cultural connections between the Blues and the community by tuning into cultural heritage exploration and social justice. With a lively array of bars, clubs, and iconic Blues musicians with insights into their lives, influences, resilience and productive aging this popular session draws an enthusiastic crowd of GSA members for a lecture/interview and "mini-performance" with leading older musician. But Wait! Theres more- later that evening, a full performance and party at a local Blues joint guarantees good music, dancing, libations, and Blues swag. This is a "don't miss" combo with 31 years of GSA history.

Abstract citation ID: igae098.2242
THE BLUES AND OLDER MINORITY MUSICIANS: MORE THAN JUST MUSIC XXXI
John Migliaccio, *Maturity Mark Services Co., LLC, White Plains, New York, United States*

The Blues and Older Minority Musicians: More Than Just Music has been a session at GSA for 31 years straight.

Abstract citation ID: igae098.2243
THE BLUES AND OLDER MINORITY MUSICIANS: MORE THAN JUST MUSIC XXXI
Michael Marcus, *University of Maryland Baltimore County, Raleigh, North Carolina, United States*

This session has been a popular academic and social event since 1993. We're on a mission from God.

### SESSION 4550 (PAPER)

### UNDERSTANDING AGING DYNAMICS: HEALTH, FUNCTIONALITY, AND MORTALITY IN OLDER ADULTS

Abstract citation ID: igae098.2244
THE ASSOCIATION BETWEEN GRATITUDE AND MORTALITY AMONG OLDER US WOMEN
Ying Chen[1], Olivia Okereke[2], Eric Kim[3], Henning Tiemeier[4], Laura Kubzansky[4], and Tyler VanderWeele[4], *1. Harvard T. H. Chan School of Public Health, Cambridge, Massachusetts, United States, 2. Massachusetts General Hospital, Boston, Massachusetts, United States, 3. University of British Columbia, Vancouver, British Columbia, Canada, 4. Harvard T. H. Chan School of Public Health, Boston, Massachusetts, United States*

Gratitude, as a positive emotion, is a potentially modifiable psychological factor that may enhance healthy aging. However, the association between gratitude and mortality has not been studied. Using data from the Nurses' Health Study (N=52,169 U.S. female nurses, mean age=79 years, 2016 questionnaire wave to December 2019), Cox proportional hazards regression models estimated the hazard ratio of deaths by self-reported levels of gratitude at baseline. Gratitude was assessed with the 6-item Gratitude Questionnaire, a validated measure of one's general tendency to experience grateful affect. Deaths were identified from the National Death Index, state statistics records, reports by next of kin, and the postal system. Causes of death were ascertained by physicians through reviewing death certificates and medical records. Over 158,374 person-years of follow-up, 5,343 incident deaths were identified. Greater gratitude was associated with a substantially lower hazard of mortality. For instance, the highest vs. lowest tertile of gratitude was associated with a 10% lower hazard of all-cause deaths (95% confidence interval of hazard ratio: 0.82, 0.97), adjusting for baseline sociodemographic characteristics, social participation, religious involvement, physical health, lifestyle factors, and mental health. When considering cause-specific deaths, gratitude was inversely associated with each specific cause of death. This inverse association was strongest with deaths due to cardiovascular disease. This study provides the first empirical evidence suggesting that experiencing grateful affect is associated with a lower risk of mortality in later life. Interventions designed to increase the experience of grateful affect may provide a novel avenue for increasing longevity among older adults.

Abstract citation ID: igae098.2245
DO FUNCTIONAL ABILITY MEASURES IMPROVE THE ACCURACY OF A 12-MONTH MORTALITY PREDICTION MODEL?
Elizabeth Luth[1], Kathryn Bowles[2], Carlin Brickner[3], Oude Gao[3], and Harivony Rakotoarivelo[3], *1. Rutgers University, New Brunswick, New Jersey, United States, 2. University of Pennsylvania, Philadelphia, Pennsylvania, United States, 3. VNS Health, New York, New York, United States*

Mortality prediction algorithms using administrative claims data can help identify seriously ill individuals eligible for palliative care. However, predefined measures of functional ability, which clinicians report as important for eligibility determinations, may be inadvertently omitted or underweighted in the algorithm general design. In response to clinician input, we developed measures of functional ability using claims data and examined their association with death: activities of daily living (ADL) support, feeding support, respiratory support, physical and occupational therapy (PT/OT), and skilled nursing. Descriptive statistics were calculated. Multivariable logistic regression tested associations between five functional ability measures, symptom management, and death for Medicare Advantage plan members on census during the July 2022-June 2023 period. Of the 6625 Medicare Advantage plan members, 32% identified as Black, 32% White, 9% as Asian, and 26% as Other races; 37% as Hispanic/Latino; 69% female; average age of 73.5 years (SD: 11.8). Of these, 186 (4.6%) died the following year. Having PT/OT was associated with 24% lower odds of death (aOR: 0.76, 95%CI: 0.62-0.85, p< 0001). Receiving respiratory support was associated with 45% higher odds of death (aOR: 1.45, 95%CI: 1.17-1.78, p< 0.001). ADL support, feeding support, and skilled nursing were not associated with death. These functional ability measures, while not directly measured in claims data, were successfully approximated. Oxygen and ventilation use and PT/OT were associated with 12-month mortality, in opposite directions. Future work should examine how other factors used by clinicians for PC eligibility determinations can be measured using administrative claims data and their associations with mortality.

Abstract citation ID: igae098.2246
QUANTIFYING PACE OF AGING AMONG OLDER ADULTS: AN ANALYSIS WITH HEALTH AND RETIREMENT STUDY
Arun Balachandran[1], Heming Pei[2], Meeraj Kothari[1], Benjamin Dominigue[3], Alex Furuya[1], and Dan Belsky[1], *1. Columbia University Irving Medical Center, New York, New York, United States, 2. Columbia University, New York, New York, United States, 3. Stanford University, Stanford, California, United States*

Rise in global population aging and age-related morbidities calls for measures that that can detect differences in aging trajectories in time to reduce and prevent disease burdens. Pace of Aging (PoA) measure captures longitudinal change in human physiology across multiple systems and captures within-individual changes. However, there has been no investigations of the measure around a sample of older adults to identify who are aging fast and who are most likely to gain from interventions. Using nine biomarkers representing three types of data (blood biomarkers, physical assessments, and functional tests) in the Health and Retirement Study (in 2006, 2008, 2010, 2012, 2014, and 2016), we studied PoA among 13,379 adults aged 40 or above at their first biomarker measurement. Our results extend analysis of PoA into later life and establish its associations with morbidity, disability, and mortality. Racial disparities in PoA represent opportunities for health intervention and prevention. Pace of Aging was more rapid in older as compared to younger participants. Independent of age, participants with faster Pace of Aging were at increased risk of cognitive impairment, incident chronic disease, disability, and death over follow-up compared to those with slower Pace of Aging (e.g. mortality HR=1.94 95%CI[1.82-2.08]). Population groups with shorter life expectancies exhibited faster Pace of Aging. Pace of Aging is a new tool for bio-social gerontology that can inform projections and evaluate trends in aging-related health changes in populations around the world.

Abstract citation ID: igae098.2247

### CHRONOLOGICAL AGE MODIFIES THE RELATIONSHIP BETWEEN HEALTH COMORBIDITIES AND PERCEIVED HEALTH STATUS

Brian Downer, Jose Cabrero Castro, and Anita Brandt, *The University of Texas Medical Branch, Galveston, Texas, United States*

Societal norms perpetuate the notion that declining health is a normal part of aging. However, it is unclear whether chronological age modifies the relationship between health status and how middle-aged and older adults perceive their health relative to others their age. This analysis used data from 13,246 participants from the 2021 wave of the Mexican Health and Aging Study. Participants were asked if their health was better, the same, or worse than people their age. We grouped participants into four age groups (50-59, 60-69, 70-79, >80) and the number of health conditions (0, 1, 2, 3, >4). We used logistic regression models to estimate predicted probabilities of having better perceived health, adjusting for demographic characteristics, life satisfaction, depressive symptoms, and having been diagnosed with COVID-19. Nearly half (44.5%) of participants said their health was better than people their age, 46.1% said their health was the same, and 9.3% said their health was worse. The probability of better perceived health decreased with more health conditions and increased with age. The interaction between age and health conditions was statistically significant (p=0.009). The probability of having better perceived health decreased with more health conditions for participants aged 50-59, 60-69, and 70-79. Among participants aged >80, the probability for better perceived health was similar for participants with 0, 1, 2, and 3 health conditions but was lower for participants with >4 health conditions. Our findings suggest that older adults in Mexico have positive perceptions of their health despite a high prevalence of comorbid conditions.

### SESSION 7000 (POSTER)

### ADVANCING DIVERSITY IN AGING RESEARCH (ADAR) THROUGH UNDERGRADUATE EDUCATION

Abstract citation ID: igae098.2248

### A LONGITUDINAL INVESTIGATION OF ALCOHOL USAGE AND COGNITIVE OUTCOMES AMONG HISPANIC/LATINO ADULTS

Armando Lemus[1], Carlos Araujo-Menendez[2], Rubi Carpio[1], and Ariana Stickel[3], *1. San Diego State University, San Diego, California, United States*, *2. SDSU/UC San Diego, San Diego, California, United States*, *3. San Diego State University, San Diego State University, California, United States*

High alcohol use may lead to cognitive deficits and is recognized as a modifiable risk factor for dementia. However, recent cross-sectional research is contradictory: low to moderate alcohol may be protective against cognitive performance. The present study aimed to investigate the associations between alcohol use and cognitive decline within Hispanic/Latino adults, a community often underrepresented in the research field. Participants included 336 self-identified Hispanic/Latino adults (67% female; ages 65 - 92 years, M = 73.29 years, SD=5.5) without dementia from the National Alzheimer's Coordinating Center dataset collected from 33 sites between 2015 – 2022. Alcohol use was assessed through self-reported consumption of any alcohol in the past 3 months (yes or no). Outcomes included cognitive tests administered to participants at a minimum of two visits. Using linear mixed models, we analyzed the relationships between alcohol use and cognitive trajectories, including possible modifications by sex and depressive symptoms (as assessed by the Geriatric Depression Scale), while adjusting for age, education, and cognitive status at baseline. Our results indicate that individuals who consumed alcohol performed better at baseline, (all F's ≥ 6.187, all p's < 0.01) but declined at the same rate across all cognitive tests, except for language. We did not detect significant modifications. Our results suggest that alcohol use is not protective against cognitive aging among Latino adults. However, additional measures (e.g., number of binges in a month) are needed to better understand its impact on dementia risk.

Abstract citation ID: igae098.2249

### AGE DIFFERENCES IN MILLING BEHAVIOR AND PERCEIVED STRESS DURING THE COVID-19 PANDEMIC

Reilly Burns[1], Jennifer Piazza[2], Pimbucha Rusmevichientong[2], and Michele Wood[2], *1. California State University Fullerton, Moreno Valley, California, United States*, *2. California State University Fullerton, Fullerton, California, United States*

In a disaster, milling behavior refers to persistently seeking relevant information. It occurs most often when levels of emergency preparedness are low and the disaster is unexpected (Wood et al., 2012). Excessive milling can create a sense of vulnerability and lead to a decline in feelings of trust and readiness (Ejeta et al., 2015). One antidote to milling is effective communication and well-designed programs that increase levels of disaster preparedness. Unfortunately, such communication and programs are often ineffective for older adults (Brown & Haun, 2014). The current study examined age differences in knowledge and milling behavior during the COVID-19 pandemic. Participants included 425 individuals from Orange County, CA (18-87, mean age 47.02 years), who completed an online survey. Results from multiple regression analyses revealed that higher levels of milling were associated with higher levels of perceived stress, particularly among the older adults in the sample (B =.031, p =.026). Older adults also reported being less knowledgeable about what they can do to prepare for a pandemic (B = -.009, p =.044). Results remained significant after adjusting for education, race, and gender. Findings suggest that increasing older adults' knowledge of a disaster through trusted sources may reduce milling behavior, which could decrease levels of stress in this population during a disaster.

Abstract citation ID: igae098.2250

### ASSESSING FALLS RISK AND BALANCE SYSTEMS PROTOCOLS IN ADULT CANCER SURVIVORS

Kenzi Marrone-Lloyd, Kelli Nielsen, Emma Christopher, Raegan Hinrichs, Caitlin O'Donnell, and Laura Gilchrist, *St. Catherine University, Saint Paul, Minnesota, United States*

Adult cancer survivors have been shown to have an elevated risk of falling compared to control. These risks are hypothesized to be residual effects of chemotherapy treatment that can impact patients' balance long-term through changes to vestibular, visual, and somatosensation systems. Medical providers and rehabilitation professionals may be asked to screen for fall risk, yet best screening measures for these balance system changes have not been identified. The purpose of this study is to determine if balance assessment outcomes differ between adult cancer survivors and control groups and if these balance outcomes are associated with prior falls history. Twenty participants underwent a series of sensory organization testing (SOT) using computerized posturography (BERTEC) and the MiniBest Test, which measures dynamic balance, gait and functional mobility. Cancer survivors (n=10) fell more than their control (n=10) counterparts ($X^2$=3.81, p=0.050). On the SOT, cancer survivors (88.2 ± 13.4) had a preference for vision, even when distorted, compared to control (100.3 ± 11.9) (t=2.14, p=.023) however, scores for all other tests were not different between groups (p>0.05). This preliminary data supports that cancer survivors fall more often than non-cancer survivors and that cancer survivors rely more on vision, as a means to maintain balance. Indicating that other balance systems may be unreliable or damaged. Continued development of screening tools to gauge the impact of cancer on balance and fall risk is indicated to improve the care of cancer survivors.

Abstract citation ID: igae098.2251

### EFFECTS OF CIRCADIAN MISALIGNMENT ON BEHAVIOR IN MAPT HUMANIZED MOUSE MODEL

Mesomachi Udenta[1], Andrea Gonzalez[2], Qing Lin[2], Kristi Dieterk[2], Kevin Koronowski[2], and Juan Palavicini[3], *1. Howard University, Owings mills, Maryland, United States*, *2. UT Health, San Antonio, Texas, United States*, *3. UT Health SA, San Antonio, Texas, United States*

The circadian rhythm is a natural biological process that regulates a multitude of processes including sleep-wake cycles, eating cycles and activity cycles. Circadian misalignment has been found to be related to many neurodegenerative diseases, one of them being Alzheimer's Disease. Tauopathies, are a distinctive feature of Alzheimer's Disease and other dementia related disorders. Tau is a protein that helps stabilize he internal structure of brain cells by supporting the microtubules needed for cell structure that oversee maintaining the integrity of the cell. Neurofibrillary tangles are formed when tau accumulates inside the neurons through a process called hyperphosphorylation. These tangles block the neurons transport system and effects the synaptic communication between the neurons which leads to cell damage and eventually cell death. This is the pathway that causes a lot of the major symptoms in Alzheimer's Disease. In this project. we a MAPT mouse model was used conjunction with a chronic jet lag simulation. The MAPT Gene Replacement Mouse Model consists of genetically modifying mice to express human tau. The model replaces the mouse tau gene with the human tau gene. It can mimic the tau pathology that is seen in humans and allows mice to develop the tau related abnormalities that is seen in dementia related disorders. Studying how this is affected by circadian misalignment can help reveal how sleep wake cycles can influence tau pathology.

Abstract citation ID: igae098.2252

### EFFECTS OF HDC-KNOCKOUT ON MITOCHONDRIAL FUNCTION AND SKELETAL MUSCLE IN BALB/C MICE.

Leighanna Hall[1], and anijah sayles[2], *1. Howard University, Howard University, District of Columbia, United States*, *2. howard university, Washington DC, District of Columbia, United States*

Histamine is a signaling molecule widely associated with inflammatory and anaphylactoid responses. It is synthesized by the decarboxylation of L-histidine by an enzyme called histamine decarboxylase (HDC) that is mainly located in mast cells. According to previous study we obtained BALB/c mice with histamine knockout (KO) as well as wild type (WT) control that we confirmed through genotyping (the knockout of the HDC enzyme would further be confirmed through western blotting and histamine assay).Techniques used were; familiarization of exercise, examining the muscle fiber diameter of tibialis anterior using microscopy, and recording the muscle weight and mitochondrial data set to assess if the HDC knockout effected these measurements. Results suggest that HDC-KO confirmation requires a larger data set, future steps involve western blots and histamine assays.

Abstract citation ID: igae098.2253

### EVIDENCE SUPPORTING CERAMIDE ACCUMULATION AS A NOVEL HALLMARK OF AGING

Laila Scroggins[1], Klarisse Echevarria[2], Eduardo Gutierrez[3], Juan Palavicini[4], and Penelope Quiles[5], *1. Howard University, Chesapeake, Virginia, United States*, *2. University of Texas Health Science Center at San Antonio, San Antonio, Texas, United States*, *3. The Barshop Institute of Longevity and Aging Studies, San Antonio, Texas, United States*, *4. UT Health SA, San Antonio, Texas, United States*, *5. The Barshop institute of Longevity and Aging, San Antonio, Texas, United States*

Aging is the single greatest risk factor for all major chronic diseases, and thus, understanding the biological mechanisms that modulate aging is critical for the development of health-maximizing interventions. Lipids are small hydrophobic molecules that not only serve as fundamental cellular components, but also act as important cellular signaling molecules, with well-established roles in nutrition, health, and disease.

The first longevity gene discovered in yeast, i.e. the longevity-assurance gene (LAG1), encodes an enzyme that synthesizes ceramides, a lipid class that has been associated with insulin resistance, apoptosis, mitochondrial dysfunction, senescence, inflammation, sarcopenia, frailty, among other age-related pathways and phenotypes. Using a number of animal models and methodologies, we have amassed a significant amount of data that place ceramides as a novel major driver of aging. Specifically, we found that: (1) ceramides consistently accumulate with age in circulation and in multiple organs in multiple mouse strains and marmosets; (2) circulating ceramides are dramatically reduced in long-lived isolated growth hormone deficient mice; (3) hepatic ceramides are substantially reduced in old marmosets treated with rapamycin, a well-established anti-aging drug, through a mechanisms that seems to involve an acid ceramidase (ASAH1); (4) low doses of myriocin, a potent inhibitor of ceramide synthesis, improves healthspan (i.e. glucose homeostasis and grip strength) in WT C57BL/6J, Balb/c, and UM-HET3 mice fed with Western diet. In addition, preliminary data suggests that myriocin significantly extends lifespan in mice as well. Taken together, our results place ceramide accumulation as a novel driver of aging.

Abstract citation ID: igae098.2254

### HEPATIC STELLATE CELLS ARE A MAJOR COLLAGEN PRODUCING CELLS IN THE LIVER AND DRIVE LOCAL LIVER FIBROSIS.

Victory Iluobe[1], and Shangang Zhao[2], *1. Howard University, windsor mill, Maryland, United States*, *2. university of texas barshop institution, university of texas barshop institution, Texas, United States*

Hepatic stellate cells (HSCs) are the major collagen-producing cells in the liver and key drivers of local fibrosis. Adiponectin, is also expressed in quiescent HSCs and plays a role in the progression of liver fibrosis. To assess adiponectin's effects on fibrosis progression mice line models were crossed using these models liver fibrosis progression either progressed or reduced. We moreover show that adiponectin expression in HSCs correlates positively with that of PPARγ, suggesting a mechanistic involvement of this transcription factor. The involvement of this transcription factor has a marked influence on the progression of local fibrosis.

Abstract citation ID: igae098.2255

### IBA1 EXPRESSION IN THE HIPPOCAMPUS OF APOLIPOPROTEIN EPSILON 4 SALT-TREATED MICE

Chloe' Major[1], Mia Rivers[2], Syed Khundmiri[3], and Jahn O'neil[3], *1. Howard University, District of Columbia, District of Columbia, United States*, *2. Howard University, DC, District of Columbia, United States*, *3. Howard College of Medicine, DC, District of Columbia, United States*

Alzheimer's disease (AD) is a progressive neurodegenerative disorder characterized by a decline in cognitive abilities, memory loss, and heightened neuroinflammation. The presence of the APOE4 allele is the most significant genetic risk factor for AD, known to accelerate the onset and progression of the disease by interacting with hallmark features such as beta-amyloid plaques and neurofibrillary tau tangles. Additionally, APOE4 directly impairs microglial function in AD leading to neuroinflammation, further contributing to neurodegeneration. While the relationship between AD and dietary habits or comorbid conditions, particularly hypertension, remains unclear, we hypothesize that high dietary salt intake will exacerbate neuroinflammation within the hippocampus of APOE4-expressing mice, providing a potential mechanistic link between diet, hypertension, and Alzheimer's disease. To test this hypothesis, we utilized mice that exclusively expressed human APOE4 or the control APOE3 in the brain. Young adult male and female mice, aged 5–7 months old (n=18 in each group), were given a 4% NaCl (high salt) or a 0.1% NaCl (low salt) diet for 4 weeks. Using design-based stereology, we evaluated the total count of Iba1 positive microglia in the hippocampus of APOE4 expressing mice and the control APOE3 mice. Preliminary results show APOE4 mice exhibit significant neuroinflammation in the hippocampus when compared to APOE3 mice. However, the APOE4 low-salt diet was seen to be beneficial. Further work is needed, and results will be discussed.

Abstract citation ID: igae098.2256

### LACK OF SLEEP AND PERCEIVED SOCIAL ISOLATION

Emma Vass, and Amy Fiske, *West Virginia University, Morgantown, West Virginia, United States*

As F. Scott Fitzgerald wrote, "The worst thing in the world is to try to sleep and not to." Insomnia is related to multiple distressing health outcomes. Research suggests that one of these outcomes may be higher levels of loneliness (Hom et al. 2017). The aim of this study was to use secondary data analysis to examine the relationship between sleep duration and perceived social isolation among older and younger adults. This study hypothesized that shorter sleep duration would lead to greater perceived social isolation. A sample of 147,694 participants ages 18-64 and 87,343 participants ages 65-99, from the 2022 Behavioral Risk Factor Surveillance Survey (BRFSS) was used to conduct a linear regression analysis on the relationship between these two variables after controlling for gender and education level. The overall model was significant for both older and younger adults: older, $R^2$ =.027, F(3, 87339) = 433.41, p <.001; younger, $R^2$ =.015 F(3,147690) = 1343.32, p <.001. Regression coefficients were significant for both older, β=.099, p<.001, and younger, β=.142, p<.001, adults. Study results indicated that lower sleep duration is related to higher perceived social isolation in both older and younger adults. Nonetheless, effect sizes were small. With this information in addition to the aforementioned previous work, we can continue to explore the connection between loneliness and sleep problems.

Abstract citation ID: igae098.2257

### MEMORY CHANGES IN COGNITIVELY HEALTHY OLDER ADULTS: AGE EFFECTS IN MEMORY-BASED INFERENCE

Cara Charles[1], Ra'Shida Rockette[2], and Caitlin Bowman[1], *1. University of Wisconsin Milwaukee, Milwaukee, Wisconsin, United States*, *2. UWM, Milwaukee, Wisconsin, United States*

Memory changes in older age are well documented, even among cognitively healthy older adults. Most prior research has focused on age-related declines in remembering details of individual experiences. However, memory-based inference is another important memory ability that allows us to make connections across separate experiences to make novel decisions. Little is known about the extent of age differences in inference abilities. In the present experiment, we administered an inference task to young and cognitively healthy older adults that asked them to both remember pairs of items that were presented directly (e.g., apple – truck, truck - clock) and that could be combined to form an indirect inference association that was not shown (e.g., apple-clock). Test results showed above-chance performance in older adults for both remembering direct pairs and making inferences. However, older adults performed worse than young adults overall, with a larger age deficit in inference compared to direct memory. We also tested for age differences in the ability to remember which context the associations were learned in (direct vs. indirect) because a loss of context memory can indicate separate experiences have been combined in memory. Successful inference was more likely to lead to a loss of context memory in older adults compared to young adults, but further analyses indicate that strategic responding in older adults could account for this difference. Overall, we found that older adults had an additional cost to forming inferences compared to remembering direct items, but the underlying mechanisms of that difference will require future investigation.

Abstract citation ID: igae098.2258
### MITOCHONDRIAL CHAPERONES MAY DICTATE OUTCOMES IN WOMEN OF ADVANCED MATERNAL AGE

Hannah Archer[1], Caroline Smith[2], Arthur Chang[3], and Renee Chosed[1], *1. Greenville School of Medicine, Greenville, South Carolina, United States*, *2. North Greenville University, North Greenville University, South Carolina, United States*, *3. UT Health, Houston, Texas, United States*

**Background:** A patient may seek the use of IVF technology when struggling to become pregnant. To improve IVF outcomes, research focuses on identifying markers to allow embryologists to transfer embryos with the highest chance of a successful pregnancy. RNASeq analysis indicated that DNAJC19 gene expression was higher in embryos that resulted in successful implantation. DNAJC19 is a mitochondrial chaperone whose role is to provide instructions for producing proteins within the mitochondria. Our study analyzes DNAJC19 expression in patients of advanced maternal age (>35 years) to identify an additional biomarker during preimplantation embryo development to select the most viable embryo for successful implantation.

**Methods:** Blastocoel fluid-conditioned media was collected from day-5 IVF-embryos that underwent PGT-A and were from mothers of advanced maternal age. RNA was purified from pooled blastocoel fluid samples, and then cDNA was synthesized. RT-qPCR assessed 18S (control gene) and DNAJC19 levels.

**Results:** Expression of DNAJC19 was higher blastocoel fluid samples from euploid embryos from mothers of advanced maternal age when compared to aneuploid embryos from mothers of advanced maternal age. Conclusions: DNAJC19 is an important mitochondrial chaperone that assists in protein import in the mitochondria. This preliminary analysis indicates a higher expression of DNAJC19 in blastocoel fluid from embryos from mothers of advanced maternal age. This finding suggests that mitochondrial function is elevated in euploid embryos from mothers over 35 years old. Further research is needed to determine the significance of this observation in regards to embryo implantation.

Abstract citation ID: igae098.2259
### STRESS RESPONSE DURING COGNITIVE TASKS USING EEGS

Kristine Vergara, Mohammad Khalil, and Kiran George, *California State University Fullerton, Fullerton, California, United States*

Abstract - Drawing upon literature indicating the potential positive effects of engaging in cognitive tasks, particularly memory games, on the mental well-being of older adults, this study aims to investigate the relationship between cognitive tasks and stress responses among the aging population. We hypothesize that older adult participants who engage in memory games will exhibit varied stress responses based on the difficulty levels of the tasks. In this preliminary investigation, we present initial findings from a subset of participants (N = 7) aged 65 and above, focusing on their EEG readings collected during memory game sessions using the g.Nautilus Research headset. Our preliminary analysis suggests a correlation between increased task difficulty and elevated theta and delta brain wave activity, indicating heightened stress responses during more challenging cognitive tasks. Ongoing data processing using MATLAB will allow for a more comprehensive comparison of EEG signals between individuals with and without significant health conditions, further elucidating the relationship between cognitive tasks and stress levels among older adults. These preliminary insights pave the way for a deeper understanding of the impact of cognitive activities on stress responses in this population, with implications for the development of targeted interventions within healthcare and mental wellness frameworks. Keywords: Aging, stress, cognitive tasks, Electroencephalography (EEG), MATLAB

Abstract citation ID: igae098.2260
### THE ROLE OF MANAGED CARE IN HOME- AND COMMUNITY-BASED SERVICES SPENDING

Patrick Arp[1], Lindsay White[1], Ellen Meara[2], Ari Ne'eman[2], and Norma Coe[1], *1. University of Pennsylvania, Philadelphia, Pennsylvania, United States*, *2. Harvard University, Boston, Massachusetts, United States*

The provision and management of Medicaid-financed long-term services and supports (LTSS) has changed dramatically over the last 25 years, including a substantial expansion of both home and community-based services (HCBS) and managed care programs. However, evidence on the costs of these services and drivers of costs is limited. The objective of this study is to describe LTSS and HCBS spending across states, over time, and to examine the impact of managed care on spending. We used a difference-in-difference approach, accounting for the staggered timing of managed care implementation, to analyze data from annual Medicaid LTSS reports and think tank reports from the year 2000 and onward. We find that, after adjusting for inflation, national LTSS spending increased from approximately $410 per capita in 2000 to $570 per capita in 2021; HCBS spending more than tripled during this period. Nineteen states introduced managed care into their

LTSS programs between 2000 and 2021 (seven states had implemented programs prior to 2000). States that used managed care to provide some of their Medicaid LTSS demonstrated lower overall LTSS spending post-implementation, but no differences were observed in HCBS spending. However, uncertain fidelity of the data, particularly among states implementing managed LTSS programs, leads to questions of whether these differential trends are due to differences in reporting or changes in the efficiency of care delivery. Our findings highlight the need for increased accuracy and transparency in spending data to facilitate studies that will produce the evidence base for state and federal Medicaid policy makers.

Abstract citation ID: igae098.2261

### UNDERSTANDING HEALTHCARE UTILIZATION AND COGNITION IN OLDER LATINO/A ADULTS: A COMMUNITY-BASED APPROACH

Sayeem Hossain, and Adriana Perez, *University of Pennsylvania, Philadelphia, Pennsylvania, United States*

Cognitive decline and Alzheimer's Disease present significant public health challenges, particularly for older Latino/a adults who experience high risk of cognitive impairment. The Tiempo Juntos por Nuestra Salud study tests a culturally relevant, community-based clinical trial designed to enhance physical activity, cardiovascular health, sleep quality, and cognitive function in older Latino/a adults with mild cognitive impairment (MCI). The data was obtained from the Tiempo Juntos participant dataset including 236 Spanish-speaking Latino/as aged 55 and older with MCI in North Philadelphia. Participant scores on cognition assessments and various survey instruments were used to conduct analyses. This secondary analysis examines 1) socioeconomic predictors of cognitive function, education level and monthly income levels in comparison to cognition scores in an analysis of variance; 2) predictors of healthcare utilization, using multiple regression to compare the number of clinician visits with variables related to neighborhood walkability, self-rated motivation, self-rated frequency of physical activity, and functional assessment score. Results showed that education and monthly income were significant socioeconomic drivers in later-life cognition. Healthcare utilization was negatively associated with neighborhood walkability but positively associated with motivation. Healthcare utilization is likely reduced by having environments conducive to doing physical activity. Greater individual motivation to improve health can lead to more connection to health services. This study highlights the impact of socioeconomic factors on health outcomes in a culturally tailored, community-based intervention among older Latino/as. Findings emphasize the need for future health services research, including public health strategies that address the unique cultural and socioeconomic needs of this population.

### SESSION 7005 (POSTER)

### ALZHEIMER'S DISEASE AND RELATED DEMENTIAS I (POSTERS)

Abstract citation ID: igae098.2262

### A DISEASE SIMILARITY APPROACH IDENTIFIES A LINK BETWEEN ALZHEIMER'S DISEASE AND NIEMANN-PICK TYPE C DISEASE

Vikas Gujjala, and Alaattin Kaya, *Virginia Commonwealth University, Richmond, Virginia, United States*

Since its first description in 1906 by Dr. Alios Alzheimer, Alzheimer's disease (AD) has been the most common type of dementia. Initially identified as caused by age-associated plaque accumulation, research has shifted to lysosomal storage and metabolic disorders, and pathogenesis from amyloid and tau accumulation to oxidative stress and impaired lipid and glucose metabolism aggravated by hypoxic conditions. However, the underlying mechanisms linking these processes to disease progression are undefined. Here, we applied a disease similarity approach on transcriptomic data of various congenital diseases with increased AD risk, namely Down Syndrome (DS), Niemann Pick Disease Type C (NPC), and Mucopolysaccharidosis I (MPS), to identify unknown molecular targets. We uncovered common pathways, hub genes, and miRNAs, many never associated with AD before. We discovered potential drug candidates for neuroprotection, and to ameliorate AD pathology through protein-drug interactions. Finally, we investigated molecular changes in brain samples from an NPC1 mouse model and analyzed the similarities to the brain samples of human and mouse AD models. We found a significant overlap between the NPC1 mice and AD human and mouse samples suggesting its potential in AD research as a comprehensive short-lived in vivo model. We believe our research represents the first comprehensive approach to congenital disease association with neurodegeneration and a new perspective on AD research while highlighting the lack of correlation in diverse in vitro models, which can accelerate diagnostic and therapeutic applications against dementia.

Abstract citation ID: igae098.2263

### ACCEPTABILITY OF THE RESOURCE CONNECTION FOR RURAL CAREGIVERS OF PEOPLE LIVING WITH DEMENTIA

Sydney Hoel[1], Lillian White[2], Matthew Zuraw[3], Jordan Hill[2], Christian Elliot[4], Andrew Pickett[1], and Nicole Werner[1], *1. Indiana University, Bloomington, Bloomington, Indiana, United States*, *2. Indiana University Bloomington, Bloomington, Indiana, United States*, *3. CareVirtue, San Diego, California, United States*, *4. CareVIrtue, San Diego, California, United States*

Rural family caregivers of people living with Alzheimer's disease and related dementias (ADRD) experience higher caregiver burden and lower social supports compared to urban ADRD caregivers. However, research regarding how to better connect rural ADRD caregivers to resources in the community is lacking. CareVirtue Resource Connection, a web-based platform designed with rural ADRD caregivers and rural community organizations, aims to provide rural ADRD caregivers with personalized and hyper-localized resources. We conducted a 30-day field test of Resource Connection with rural ADRD caregivers (N=15) followed by semi-structured interviews to explore participants' perceptions of CareVirtue Resource Connection acceptability. The objective of this study was to identify barriers and facilitators to Resource Connection acceptability based on the post-use interviews. Interview transcripts were analyzed using deductive content analysis guided by the Technology Acceptance Model (i.e., ease of use and usefulness) using an iterative team-based coding process. ADRD caregivers were self-identified rural, aged Mean=70.1 years (59-81 years), 80.0% women (n=12), and 93.3% white (n=14). Facilitators to Resource Connection acceptability included having all resources provided in one space, resource recommendations based on need and location, notifications about new resources, connecting with other caregivers, emotional and mental health benefits, and the ability to share resource information with others. Acceptability barriers included limited time to connect with provided resources, mismatch of resources to ADRD stage, and a lack of local resources. Results suggest key areas for improving the design that will inform intervention refinement prior to large-scale effectiveness testing.

Abstract citation ID: igae098.2264

### AMBIGUOUS LOSS AND RESILIENCE IN THE FAMILY CONTEXT OF DEMENTIA: A META-SYNTHESIS

Athena Chung Yin Chan, Cade Perkins, Lizbeth Garcia, Sophia Lyons, Frank Hernandez, and Jonathan Singer, *Texas Tech University, Lubbock, Texas, United States*

Having family members living with Alzheimer's disease or related dementias (ADRDs) triggers high distress due to ambiguous loss. Ambiguous loss is an unclear loss with no resolution or closure to the physical presence and psychological absence of a loved family member. Resilience may buffer the distress from ambiguous loss in the family context of ADRDs. Existing reviews primarily focus on carer resilience. Little is known about the family processes navigating losses related to ADRDs. This meta-synthesis of qualitative studies aims to synthesize the family processes of ambiguous losses and resilience in the context of people with ADRDs. Peer-reviewed articles published after 1990 were identified in four electronic databases. A keyword search was performed associated with qualitative or mixed methods, dementia, and resilience or ambiguous loss. Seventy-two articles from 17 countries were included. Findings suggested the coexistence of resilience and ambiguous loss among persons with ADRDs and their family members. For individual-level and family-level resilience, three themes emerged, including belief systems (i.e., acceptance, gratitude, focusing on present and seeing positives in situations), organizational processes (i.e., finding support in friends and family, self-care and self-love), and communication / problem-solving (i.e., use of humor, working as a team). For intrapersonal and interpersonal ambiguous loss, two themes emerged including physical (i.e., loss of self-care, privacy and autonomy, current and expected future roles) and emotional (i.e., loss of identity, pride or dignity, sense of purpose, shame associated with memory loss, and home is no longer home). This meta-analysis calls for culturally-tailored family-based practice and policy.

Abstract citation ID: igae098.2265

### COMBINED MULTIMODAL EXERCISE AND COGNITIVE TRAINING FOR PERSONS WITH MILD DEMENTIA: A RANDOMIZED CONTROLLED TRIAL

Dandan Xue, Polly Wai Chi Li, and Doris Sau Fung Yu, *The University of Hong Kong, Hong Kong, China (People's Republic)*

The effects of combined exercise and cognitive interventions among persons with mild dementia (PwMD) are not known. This randomized controlled trial randomized 72 PwMD to a caregiver-assisted combined multimodal exercise and cognitive training (CA-MECT) intervention or health education. The 12-week CA-MECT included online multimodal exercise and cognitive training through videoconferencing supplemented with home visits and home-based training. Caregivers assisted PwMD in intervention delivery and home-based training. Validated tools were used to evaluate cognitive, psychological, functional outcomes, and health-related quality of life (HRQoL). Compared to the control group, the intervention group showed significantly greater improvement in global cognition ($\beta = 2.468$, 95% CI [1.254, 3.681], $p < 0.001$), immediate recall ($\beta = 2.924$, 95% CI [1.387, 4.461], $p < 0.001$), short-term delayed recall ($\beta = 0.63$, 95% CI [0.005, 1.255], $p = 0.048$), long-term delayed recall ($\beta = 0.89$, 95% CI [0.198, 1.582], $p = 0.012$), cued recall ($\beta = 1.085$, 95% CI [0.414, 1.757], $p = 0.002$), and word recognition ($\beta = 2.438$, 95% CI [1.273, 3.603], $p < 0.001$) at immediate post-intervention. The intervention group also demonstrated a significant reduction in the severity of neuropsychic symptoms (NPS) ($\beta = -2.166$, 95% CI [-3.814, -0.519], $p = 0.01$). No significant changes were observed on cognitive shifting, working memory, response inhibition, attention, processing speed, the number of NPS, caregivers' distress of NPS, functional ability, and HRQoL. In conclusion, CA-MECT was effective in improving cognition and reducing NPS among PwMD. It is warranted to examine the longer-term effects of the CA-MECT intervention.

Abstract citation ID: igae098.2266

### COMING TOGETHER: HOW AFRICAN AMERICAN ADULT DAUGHTER ALZHEIMER'S CAREGIVERS ARE NAVIGATING FAMILY RELATIONSHIPS

Deneisha Scott-Poe[1], and Megan Dolbin-MacNab[2], *1. Converse University, Spartanburg, South Carolina, United States*, *2. Virginia Tech, Blacksburg, Virginia, United States*

Alzheimer's disease currently impacts around 5.8 million Americans, with African Americans being two to three times more likely to develop Alzheimer's disease than their white counterparts. Most of these individuals are being cared for by their adult daughters. Previous research has explored African American women's reports of less caregiver burden and strain, yet few studies have examined the impact of racial and gender oppression on how African American adult daughters approach caregiving. Guided by the theoretical perspective of intersectionality, the purpose of this qualitative study was to gain a deeper understanding of the family dynamics of 26 African American adult daughter caregivers and how the intersection of race, gender, and the history of care influences caregivers and their family relationships. To participate in the study, individuals had to at least be 18, be providing care for one year, and self-identify as African American and woman. Results of a constructivist grounded theory analysis revealed four major themes. The first was contextual stressors, which highlighted the unique sets of stressors that impacted caregiver mental health. The next theme was ideology about African American women and care, which emphasized the familial and personal beliefs about how women cared. The third theme, expectations placed on caregivers, highlighted the caregivers' internalization of how women are supposed to care. The last theme, coming together, highlights how caregivers navigated their family relationships while providing care to their parents with Alzheimer's disease. Implications for future research and systemic practice with African American adult daughter caregivers will be discussed.

Abstract citation ID: igae098.2267

### CULTURAL ADAPTION OF THE INTERNET-BASED CONVERSATIONAL CLINICAL TRIAL (I-CONECT) FOR ASIAN OLDER ADULTS

Kexin Yu[1], Amanda Mar[2], Minju Song[3], Qijia Hu[4], Shiyun Chen[5], Christine Le[6], Mia Huynh[7], and Hiroko Dodge[8], *1. Oregon Health and Science University, Portland, Oregon, United States*, *2. Oregon Health & Science University, Portland, Oregon, United States*, *3. Portland State University, Portland, Oregon, United States*, *4. University of Illinois Urbana-Champaign, Champaign, Illinois, United States*, *5. New York University, New York, New York, United States*, *6. Oregon State University, Corvallis, Oregon, United States*, *7. Reed College, Portland, Oregon, United States*, *8. Harvard Medical School, Massachusetts General Hospidal, Framingham, Massachusetts, United States*

Alzheimer's disease and related dementias (ADRD) are influenced by cultural and social factors. Asian older adults with limited English proficiency often face the risk of social isolation, which is recognized as a risk factor for the incidence of ADRD. However, few behavioral health interventions for dementia prevention have been designed specifically for the Asian American population. The Internet-Based Conversational Clinical Trial (I-CONECT, ClinicalTrials.gov Identifier: NCT02871921) examined whether conversations through user-friendly video chats could enhance cognitive functions and emotional well-being. Although the previous study showed efficacy, it was limited to predominantly white Caucasian participants. This presentation will describe the cultural and linguistic adaptation process of I-CONECT for older Asian adults. A mixed-methods approach was used, including 1) conducting semi-structured interviews with older Asian adults to qualitatively understand the lived experience of social isolation to inform culturally competent intervention inclusion criteria and 2) translating and adapting the intervention material to be culturally competent and pilot testing it with 10 participants. Participants will have four times/week conversation sessions with trained research staff for a 6-week intervention period. The pre- and post-intervention assessments include loneliness, social isolation, and cognitive test scores. Participants will also elaborate on their experience in post-intervention qualitative interviews. The feasibility and acceptance of online conversation interventions have not been empirically tested with Asian Americans. The cultural adaptation of an existing evidence-based intervention model could lay the foundation for expanding effective behavioral health interventions for preventing ADRD in the underrepresented population.

Abstract citation ID: igae098.2268

### DAILY STRESSORS, POSITIVE EXPERIENCES, AND DAILY FATIGUE AMONG DEMENTIA FAMILY CAREGIVERS

Eunju Lee[1], Kyungmin Kim[1], Yin Liu[2], and Steven Zarit[3], *1. Seoul National University, Seoul, Republic of Korea*, *2. Utah State University, Logan, Utah, United States*, *3. The Pennsylvania State University, University Park, Pennsylvania, United States*

Family caregivers of individuals with dementia (IWD) suffer from physical and mental fatigue, but little is known about daily fluctuation of caregiver fatigue in response to daily care-related and non-care experiences. Caregivers are also likely to encounter conflict with other family members on caregiving issues—one of the secondary stressors that may amplify their fatigue. This study examined the within-person associations of daily fatigue with care-related stressors (i.e., relative's behavioral and psychological symptoms of dementia), non-care stressors, and positive experiences, and whether family conflict on caregiving issues exacerbates the relationship between daily experiences and fatigue. We analyzed a sample of 173 family caregivers of IWD who participated in daily interviews across 8 consecutive days (N = 1,359). Participants reported daily fatigue and daily experiences each day, while family conflict was reported at baseline. Multilevel models showed that experiencing more care-related and non-care stressors than usual was associated with higher levels of daily fatigue, whereas having more positive experiences than usual was associated with lower levels of fatigue. At the between-person level, caregivers with more care-related stressors on average were more likely to show higher levels of fatigue. Further, family conflict on caregiving issues exacerbated the relationship between daily care-related stressors and fatigue. The findings suggested that not only care-related stressors, but also non-care daily experiences were related to fluctuations of daily fatigue among family caregivers. Exposure to positive experiences and resolving family disagreement concerning caregiving issues may help caregivers relieve fatigue and tiredness.

Abstract citation ID: igae098.2269

### DATA-DRIVEN APPROACHES TO UNDERSTAND PROTEOMICS SIGNATURES IN DEMENTIA: RESULTS FROM ELSA AND UK BIOBANK

Jessica Gong, Dylan Williams, and Andrew Steptoe, *University College London, London, England, United Kingdom*

Novel blood-based protein markers are crucial for understanding dementia's biological mechanisms. Undertaking a data-driven, proteome-wide approach to understand the protein signatures in dementia, this study used the English Longitudinal Study of Ageing (ELSA) as the discovery cohort, and subsequently the UK Biobank (UKB) as the validation cohort, to estimate the associations between proteins and the risk of incident all-cause dementia (ACD), and dementia subtypes. Bidirectional Mendelian randomization (MR) and drug target MR were employed to establish causal evidence between proteins and dementia as well as cerebral white matter volume (WMV). Of the 276 proteins in 3249 samples from ELSA (229 incident ACD, 89 Alzheimer's disease (AD), 41 vascular dementia (VAD)), over a median 9.8-year follow-up, two proteins (NEFL, RPS6KB1) were significant after full adjustments, using Cox Proportional Hazard regression models, with False Discovery Rate p-value set at 0.05 to indicate statistical significance. One protein (MMP12) was found to be associated with VAD. NEFL and RPS6KB1 in combination with demographic predictors (sex, age, education, race), along with

APOE 4 genotype and memory score showed good predictive value (Area Under Curve (AUC)=0.87). All associations were replicated in UKB in 52,745 participants (1506 ACD, 732 AD, 281 VAD) over median 13.7-year follow-up. Causal evidence for MR indicated a backward association between dementia and NEFL; drug target MR indicated causal relationships between NEFL and AD and WMV, as well as MMP12 and VAD. This study highlights promising blood-based protein candidates for dementia diagnostics and therapeutic targets, emphasizing the potential for improving patient care.

Abstract citation ID: igae098.2270

### DEMENTIA RISK REDUCTION SUMMIT: ADDRESSING DEMENTIA RISK ACROSS THE PREVENTION SPECTRUM

Chelsea Kline, Matthew Baumgart, and Shelby Roberts, *Alzheimer's Association, Chicago, Illinois, United States*

As the science around dementia risk reduction has evolved in the last 20 years, public health action to address the risk factors has become an increasing imperative. In May 2023, the Alzheimer's Association Public Health Center of Excellence on Dementia Risk Reduction, funded by the CDC, held the first Dementia Risk Reduction Summit (Summit) at the CDC in Atlanta. The event convened 230 public health professionals, academics, and researchers with 43% of attendees representing state, local, and tribal health departments. The Summit included six sessions focusing on each level of the spectrum of prevention– (1) strengthening individual knowledge and skills, (2) promoting community education, (3) educating providers, (4) fostering coalitions and networks, (5) changing organizational practices, and (6) influencing policy and legislation – and showcased how public health could address risk factors for cognitive decline and dementia at each level. An additional session focused on social determinants of health. Initial evaluations of the Summit found most attendees would recommend the event to a colleague. Additionally, nearly all had taken action to address dementia risk within two months following the Summit (46%) or said they planned to (44%). 90% of attendees reported the Summit provided helpful information that could be applied to their organization's approach to dementia risk reduction. Recordings of the Summit have been viewed 1,725 times. Additional follow-up surveys were sent to key health department officials in March 2024 to understand the long-term impact of the Summit. This structured national convening increased knowledge and confidence around dementia risk reduction.

Abstract citation ID: igae098.2271

### DEVICE-MEASURED DAILY PHYSICAL ACTIVITY IN OLDER ADULTS WITH DEMENTIA: CROSS-SECTIONAL RESULTS FROM THE HUNT STUDY

Karen Sverdrup[1], Paul Jarle Mork[2], Bjørn Heine Strand[3], Linda Ernstsen[2], and Gro Gujord Tangen[4], *1. The Norwegian National Centre for Ageing and Health, Tønsberg, Oslo, Norway*, *2. Norwegian University of Science and Technology, Trondheim, Sor-Trondelag, Norway*, *3. Norwegian Institute of Public Health, Oslo, Oslo, Norway*, *4. Sykehuset i Vestfold HF (Nasjonalt senter for aldring og helse), Tønsberg, Oslo, Norway*

To provide accurate estimations of physical activity (PA) in epidemiological research and population studies, the use of accelerometers has skyrocketed over the past decade. However, implementation of accelerometers to accurately estimate PA in people with dementia is lagging. This population-based, cross-sectional study aimed to quantify daily PA in people with dementia categorised by sex and dementia subtype, using data from the ≥70 years cohort in the fourth wave of the Norwegian Trøndelag Health Study (HUNT4 70+). Clinical experts diagnosed dementia and subtypes according to DSM-5 criteria. PA was measured with two tri-axial accelerometers on the lower back and thigh for seven consecutive days. PA types and postures were classified using a validated machine learning model and analysed as mean minutes per day (min/d). Moderate-to-vigorous PA (MVPA) was defined as walking, running, and cycling, and analysed as mean minutes per week (min/w). Of the 1894 participants diagnosed with dementia, 577 participants had valid PA data. These participants spent 231 min/d in total PA, most of which was in standing (187 min/d). Mean time in MVPA was 50 min/w and only 4.3% met PA recommendations (≥150min/w). Men spent more time in MVPA than women ($p< 0.001$), whereas women spent more time standing ($p=0.008$). There were no differences between dementia subtypes in volume or intensity of PA. These results indicate that, in people with dementia, independent of dementia subtype and sex, there exists an enormous disadvantageous gap between performed PA and recommended levels of PA.

Abstract citation ID: igae098.2272

### DYADIC AMBIVALENCE IN ALZHEIMER'S DISEASE: LINKING BEHAVIORAL AND PSYCHOLOGICAL SYMPTOMS TO LIFE SATISFACTION

Meng Huo[1], Megan Gilligan[2], Kyungmin Kim[3], Nicole Richards[4], Karen Fingerman[4], and Steven Zarit[5], *1. University of California Davis, Davis, California, United States*, *2. University of Missouri, Columbia, Missouri, United States*, *3. Seoul National University, Seoul, Republic of Korea*, *4. The University of Texas Austin, Austin, Texas, United States*, *5. The Pennsylvania State University, University Park, Pennsylvania, United States*

Caring for a spouse with Alzheimer's disease (AD) can elicit considerable distress but there are also positive moments. A growing body of work has examined caregivers' ambivalence in the care relationship and linked it to negative caregiver outcomes such as depression, but dyadic assessments of both parties' perspectives are missing. We examined ambivalence in both people with AD and their spousal caregivers, seeking to identify the predictors and well-being outcomes of such ambivalence in this unique context. Participants included 73 couples managing early-stage AD. People with AD and spousal caregivers independently self-reported positive and negative relationship qualities (used to indirectly calculate their ambivalence) and life satisfaction. Caregivers reported both partners' demographic characteristics and their spouses' behavioral and psychological symptoms of dementia (BPSD), focusing on memory-related behaviors and psychological symptoms. Path analyses revealed that the number and frequency of psychological symptoms in people with AD were positively associated with their own and caregivers' ambivalence. Caregivers' distress ratings of memory-related behaviors and psychological symptoms were positively associated with their ambivalence. Greater ambivalence was associated with lower life satisfaction in both spouses. BPSD directly affected both spouses' life satisfaction but there was also a marginally significant indirect effect via ambivalence. This study utilizes a dyadic approach to assess ambivalence in dementia care. Findings reveal the conflicting emotions that couples experience as they cope with early-stage AD, identify sources of such ambivalence, and shed light on the development of dyadic interventions that can promote positive outcomes in both partners.

Abstract citation ID: igae098.2273

### EFFECTS OF PHYSICAL ACTIVITY ON HEALTH AMONG INFORMAL CAREGIVERS OF INDIVIDUALS WITH DEMENTIA: A META-ANALYSIS

Sangwoo Ahn[1], Misook Chung[2], and Scott Crouter[3], *1. Pennsylvania State University, Knoxville, Tennessee, United States*, *2. University of Kentucky, Lexington, Kentucky, United States*, *3. University of Tennessee Knoxville, Knoxville, Tennessee, United States*

Caregiving for individuals with dementia, including Alzheimer's disease and related dementias (ADRD), negatively affects informal caregivers' health. Our review aimed to systematically evaluate the effects of physical activity on physical and psychological health and well-being among informal caregivers of individuals with ADRD because no comprehensive evidence has been drawn so far. To identify appropriate randomized controlled trials, nine databases published by July 2023 were searched. Twelve studies were included (n = 1,163 total participants; mean age = 64 years), two of which were rated as strong in methodological quality, and one as weak with the other nine showing moderate quality. Meta-analysis was performed using STATA (18.0) with the standardized mean difference (SMD; Hedge's g) used to calculate the effect size of interventions. Physical activity, consisting of walking, Tai Chi, or multicomponent activities (aerobic, strengthening, stretching, and/or balance activities), exerted beneficial effects on static balance as an element of physical health (SMD = 0.45; $p < 0.001$) and psychological well-being as an element of well-being (SMD = 0.46; $p < 0.001$), as well as general physical health (SMD = 0.41; $p = 0.03$) and well-being (SMD = 0.41; $p < 0.001$) with moderate effect sizes. Health care providers may suggest informal caregivers engage in physical activity to improve physical and mental well-being, and further, potentially decrease some of the onerous aspects of caregiving. Given the limited number of studies in our review, future research is warranted to identify the optimal type and dose of physical activity for informal caregivers' health.

Abstract citation ID: igae098.2274

### FEASIBILITY OF VIRTUAL COMPANIONSHIP AND ACTIVITIES FOR PERSONS WITH DEMENTIA: MEMORI CORPS TRIAL

Katherine Marx[1], Jessica Herpst[2], Kathleen Kirchner[1], and Quincy Samus[1], *1. Johns Hopkins University, Baltimore, Maryland, United States*, *2. Alzheimer's Association - Greater Maryland Chapter, Baltimore, Maryland, United States*

The majority of persons living with dementia (PLWD) are cared for at home by a family care partner. Social isolation, the need for meaningful activities, and care partner respite are common unmet care needs for PLWD. Home and community-based services such as Adult Day Care and friendly visitor programs have been instrumental in addressing these needs. However, not all families prefer out-of-home services, some lack service access, and few of these services are tailored specifically for PLWD. Additionally, the COVID-19 pandemic disrupted most services. This study evaluates the feasibility of providing virtual companionship and meaningful activities via Zoom to community-residing PLWD. Sixty-two dyads (PLWD and family care partner) and 52 trained volunteers (ages 55+), participated in a 12-week intervention. Individualized activity plans were developed based on the PLWD's personal history, roles, interests, and current functional level. Activity sessions were offered up to five times a week for up to one–hour per session, primarily through Zoom. Advantages of virtual delivery included the ability to serve dyads across a large geographic area, resource efficiency, session flexibility allowing individual and group sessions, and the capability to continue services during participant travel. Drawbacks included low initial technology literacy levels of participants (e.g., session scheduling, joining, screen sharing), technical issues (e.g., hardware, Wi-Fi connection issues), need for in-home help for session setup on computer, limited selection of potential activities, and limited ability to evaluate and address environmental barriers impacting sessions. Overall, virtual implementation was feasible and allowed PLWD access to meaningful activity and socialization.

Abstract citation ID: igae098.2275

### IDENTIFYING PHENOTYPES OF OLDER ADULTS PLANNING FOR ALZHEIMER'S DISEASE SUPPORT

Amber Miller-Winder[1], Vanessa Ramirez-Zohfeld[1], Raven Relerford[2], Allison Schierer[3], Charlie Olvera[4], Alaine Murawski[1], and Lee Lindquist[1], *1. Northwestern University, Evanston, Illinois, United States*, *2. Northwestern Medicine, Chicago, Illinois, United States*, *3. Northwestern University Feinberg School of Medicine, Chicago, Illinois, United States*, *4. Northwestern University Feinberg School of Medicine, Division of Geriatrics, Evanston, Illinois, United States*

Older adults (OAs) with Alzheimer's Disease (AD) often need additional support (e.g. in-home caregivers) but are unprepared. We modeled the types of plans OAs make surrounding long-term care (LTC)/ home support in the event of AD to describe different planning phenotypes. The Plan Your Lifespan (PYL) study examines OAs' decision-making process about LTC/home support in the event of AD. Subjects are surveyed every 6 months about cognitive, social, functional, environmental factors; including living/support preferences. We employed mixed analytic methods (qualitative coding and GLMM). 293 subjects enrolled (mean age 73.5 yrs,72.7% female, 40.4% URM). Almost half (47.4%, n=139) experienced cognition decline with 10.3% identifying worsening memory loss. We identified four planning phenotypes: Deniers overlook the possibility of developing AD. Subjects with no measured cognitive impairment were 35% less likely to plan; Do-gooders plan to avoid burdening family and often complete an Advanced Directive (OR 1.75 [$p < 0.05$, 1.20-2.54]) or Living Will (OR 1.56 [$p < 0.05$, 1.08-2.25]; Defeaters do not plan due to external limitations; a below average Consumer Health Activation score (OR 0.65 [$p < 0.05$, 0.43-0.98]) signaled 35% less likely to plan; and Dumpers leave planning to their family; displaying sufficient social support (OR 2.4 [p < 0.05, 1.39-4.15]) and limited health literacy (OR 1.99, [p < 0.05, 1.24-3.2]). Helping OAs consider their support needs through identification of different phenotypes may allow providers to intervene early. Phenotypes depend on health literacy, social support, self-efficacy, and finances. Further research will examine how planning phenotypes change over time and how plans are implemented to best care for OAs with AD.

Abstract citation ID: igae098.2276

### MACHINE LEARNING APPLIED TO MEDICARE CLAIMS DATA TO IDENTIFY OLDER ADULTS WITH UNDIAGNOSED DEMENTIA

Deborah Barnes[1], Cynthia Benjamin[2], Delia Mannen[3], and John Boscardin[1], *1. University of California San Francisco, San Francisco, California, United States*, *2. Together Senior Health, San Francisco, California, United States*, *3. Together Senior Health, Cortland, Ohio, United States*

Approximately half of people living with Alzheimer's disease and related dementias are undiagnosed. We performed a retrospective cohort study using the 5% Medicare claims sample to test machine learning approaches for calculating risk of undiagnosed dementia. For each calendar year from 2012 to 2021, we created a cohort of beneficiaries aged 65 years or older on January 1 who had at least 2 healthcare claims during the previous 2 years. Those who had prevalent dementia, were in hospice, or died within the calendar year were excluded. We identified those who received an incident dementia diagnosis during the calendar year (i.e., had undiagnosed dementia on January 1) and used machine learning methods to identify key predictors of undiagnosed dementia within each cohort based on demographics, diagnoses, and healthcare utilization. Model accuracy was assessed using c statistics. On average, 837,440 Medicare beneficiaries were included in each cohort, of whom 24,410 (2.9%) were diagnosed with incident dementia each year. An average of 35±10 variables were included using different machine learning approaches and cohort years. Overall model accuracy for detecting undiagnosed dementia ranged from 0.77 to 0.81. All models yielded clinically meaningful discrimination ranging from < 1% (lowest predicted decile) to 13% (highest predicted decile). Model performance was stable temporally and across gender, race, and ethnicity. These results support application of machine learning with Medicare claims to identify older adults at risk of undiagnosed dementia. Future studies should prospectively validate this approach within healthcare systems and explore the potential value of additional data sources.

Abstract citation ID: igae098.2277

### MEALTIME BEHAVIORS IN NURSING HOME RESIDENTS WITH DEMENTIA ARE ASSOCIATED WITH RESIDENT-INITIATED FOOD INTAKE

Heather Suh, Kyuri Lee, and Wen Liu, *University of Iowa, Iowa City, Iowa, United States*

Nursing home (NH) residents with dementia experience challenging behaviors that interfere with their engagement, eating performance, and food intake during mealtime. Supporting resident-initiated food intake, defined as how well resident initiate and complete attempts of food intake with or without staff assistance, is a critical indicator of resident engagement and independence. Understanding the association between resident mealtime behaviors and resident-initiated food intake attempts informs future mealtime interventions. In a pilot trial for the OPTIMAL (Optimizing Mealtime) study, NH resident mealtime behaviors and interactions with direct care staff were observed and characterized. A total of 197 videotaped observations of full-meal resident-staff interactions were coded from 12 residents (age 71.9±7.5 years; 91.7% male; 91.7% white; 41.7% married). The average mealtime durations at breakfast, lunch, and dinner were 36.4 ± 20.6, 39.4 ± 16.7, and 39.7 ± 17.0 minutes, respectively. Out of 10,092 behaviors coded using the refined Cue Utilization and Engagement in Dementia (CUED) coding scheme, resident behaviors were categorized as positive/neutral behaviors (18.2%), chewing/swallowing difficulties (47.5%), functional impairment (13.3%), and resistive behaviors (21.0%). The majority of resident behaviors occurred at lunch (36.9%) and dinner (36.3%), with slightly fewer behaviors at breakfast (26.8%). Proportion of resident-initiated food intake attempts (0.51 ± 1.4, number of resident-initiated food intake attempts divided by total number of food intake attempts) was significantly associated with chewing/swallowing difficulties (b= -1.91, p=0.014), controlling for resident age and cognitive score. Addressing resident chewing/swallowing difficulties has the potential to improve resident-initiated food intake attempts to enhance resident mealtime engagement and independence.

Abstract citation ID: igae098.2278

### MENTAL ILLNESS AND EMERGENCY DEPARTMENT USE IN VETERANS WITH DEMENTIA: DOES RESIDENCE MATTER?

Katherine Miller[1], and Megan Shepherd-Banigan[2], *1. Johns Hopkins University, Baltimore, Maryland, United States*, *2. Duke University Medical Center, Durham, North Carolina, United States*

Likelihood of dementia increases among individuals with mental illness. For patients with Alzheimer's-related and related dementia (ADRD), mental illness can complicate clinical care and may reduce care quality, leading to more intensive health service use and higher costs. For individuals living in nursing homes, this may be exacerbated as nursing home staff may not be trained to provide care appropriately to people with mental illness. We describe presence of mental illness (MI), defined as major depressive disorder, post-traumatic stress disorder, generalized anxiety disorder, bipolar disorder, or schizophrenia, in a cohort of Veterans with a new ADRD diagnosis stratified by nursing home residence versus community-dwelling. Of Veterans with new ADRD diagnoses, 96% are community-dwelling and 4% reside in nursing homes. Yet 19% of nursing home residents have pre-existing MI while 12% of community-dwellers have pre-existing MI. Among both community-dwellers and residents, those with pre-existing MI are younger at the time of ADRD diagnosis. In the year post-ADRD diagnosis, 17.5% and 9.5% of community-dwelling Veterans with and without pre-existing MI, respectively, had any emergency department (ED) visits. In contrast, 7.2% and 3.2% of nursing home residents with and without pre-existing MI, respectively, had any ED visits. Notably, a greater share of nursing home residents with pre-existing MI had any ED visits in the 2 years pre-ADRD diagnosis relative to community-dwellers with pre-existing MI

(74% versus 43%, respectively). Our findings suggest that community-dwelling Veterans with pre-existing MI and newly diagnosed ADRD may benefit from targeted interventions to reduce ED use.

Abstract citation ID: igae098.2279

### SUBJECTIVE COGNITIVE DECLINE IN OLDER ADULTS: THE ROLE OF A-SYNUCLEIN AND AMYLOID PATHOLOGIES

Kelsey Thomas[1], Katherine Bangen[2], Lindsay Rotblatt[3], Duygu Tosun[4], and Douglas Galasko[2], *1. University of California San Diego, La Jolla, California, United States*, *2. University of California, San Diego, La Jolla, California, United States*, *3. VA San Diego Healthcare System, San Diego, California, United States*, *4. University of California San Francisco, San Fransisco, California, United States*

Some studies show subjective cognitive decline (SCD) to be an early marker associated with Alzheimer's pathology. However, until recently, it has been impossible to measure Lewy body pathology in vivo, so its association with SCD is unknown. Participants without dementia (451 CN; 476 MCI) from the Alzheimer's Disease Neuroimaging initiative were classified as positive or negative for amyloid (A+ or A-) using cerebrospinal fluid (CSF) Aβ42 and for α-synuclein (α-syn+ or α-syn-) using the CSF Seed Amplification Assay (441 A-/α-syn-, 80 A-/α-syn+, 331 A+/α-syn-, and 75 A+/α-syn+). Both participant- and study partner-reported SCD were measured using the Everyday Cognition (ECog) total and domain scores. Per self-report, after adjusting for age, education, sex/gender, race/ethnicity, APOE ε4+, and cognitive group, the A+/α-syn+ group had the greatest SCD relative to the other three pathology groups. The A-/α-syn+ group did not differ from the A-/α-syn- group on any ECog scores. None of these associations were moderated by cognitive status. Per study partner report, a similar pattern of results was observed, but the A+/α-syn- and A+/α-syn+ groups only significantly differed on the planning subscale despite A+/α-syn+ consistently having higher SCD. These results were moderated by cognitive group for the ECog total, memory, language, visuospatial, and divided attention scores such that associations were largely driven by the MCI group. In older adults without dementia, α-syn+ alone did not contribute to greater SCD; however, A+/α-syn+ had the greatest SCD as rated by both participant and study partner, highlighting the importance of measuring co-pathologies to understand SCD etiologies.

Abstract citation ID: igae098.2280

### THE FAITH CARE FAMILY PROJECT: A QUALITATIVE ANALYSIS OF AN INTERVENTION FOR AFRICAN AMERICAN AD/ADRD CAREGIVERS

Noelle Fields[1], Ling Xu[1], Ishan Williams[2], Fayron Epps[3], and Swasati Handique[4], *1. The University of Texas at Arlington, Arlington, Texas, United States*, *2. University of Virginia, Charlottesville, Virginia, United States*, *3. The University of Texas Health San Antonio, San Antonio, Texas, United States*, *4. University of Texas at Arlington, Arlington, Texas, United States*

Alzheimer's disease and related dementias (AD/ADRD) disproportionately impact older African American populations. Although there are interventions for AD/ADRD caregiving, there remain research gaps related to culturally congruent psychoeducational interventions for African American AD/ADRD family caregivers. The Faith Care Family (FCF) Project was developed to train volunteers from a predominantly African American church to work with African American AD/ADRD family caregivers through a 6-week, telephone-based intervention. Qualitative interviews were conducted with the family caregivers (N = 9) following the intervention. Using a semi-structured interview guide, questions focused on knowledge of AD/ADRD, caregiver stress and coping, feelings about the behavioral symptoms of AD/ADRD, differences in caregiving before and after the FCF Project, and recommendations for future program iterations. Braun and Clarke's six-step thematic analysis was followed to examine the interview data. The majority of caregivers identified as women, and the median age was 62. Over half had some college or below, and most reported little to no financial strain. The median length of caregiving was 5 years, and caregivers typically spent 10 hours per day providing care. Slightly over half of caregivers lived with the care recipient. Thematic analysis revealed four themes: establishing a connection, normalizing the caregiving experience, reinforcing knowledge, and resource sharing. Recommendations included adding additional AD/ADRD content to the manual. Overall findings suggest that the FCF Project is a promising intervention for African American AD/ADRD family caregivers. Lessons learned and recommendations for future research on 'faith-placed' ADRD interventions are offered.

Abstract citation ID: igae098.2281

### UNRAVELING ALZHEIMER'S: A COMPREHENSIVE REVIEW OF BIOMARKER RESEARCH ADVANCES OVER THE PAST DECADE

Emily Winters[1], and Tessa McCoy[2], *1. Oregon Health & Science University, Portland, Oregon, United States*, *2. Oregon Department of Human Services, Dallas, Oregon, United States*

Alzheimer's Disease (AD) presents a formidable global health challenge, necessitating continuous advancements in biomarker research for enhanced diagnosis and management. This review, utilizing a methodology adapted from Arksey and O'Malley (2005) for scoping reviews, comprehensively assesses developments in AD biomarker research over the past decade. Through systematic searches of academic databases such as PubMed, Web of Science, and Scopus, articles published between 2012 and the present were analyzed, focusing on keywords encompassing AD, biomarkers, imaging techniques, blood-based markers, and international collaborative initiatives. Key findings reveal significant progress across multiple fronts of AD biomarker research, including the refinement and validation of novel imaging techniques targeting amyloid-beta and tau proteins, such as positron emission tomography (PET) imaging tracers for tau pathology. Moreover, blood-based biomarkers have emerged as promising candidates for non-invasive AD detection and monitoring, with studies investigating proteins like amyloid-beta, tau, and neurofilament light chain. The integration of longitudinal studies and multimodal imaging approaches has provided deeper insights into dynamic biomarker changes throughout disease progression, while machine learning and data analytics have identified novel biomarker patterns and predictive models, enhancing diagnostic accuracy and personalized treatment strategies.

International collaborative efforts, exemplified by initiatives like the Alzheimer's Disease Neuroimaging Initiative (ADNI) and the Dominantly Inherited Alzheimer Network (DIAN), have fostered data sharing and large-scale biomarker studies, accelerating the pace of discovery and validation. In conclusion, this comprehensive assessment underscores significant strides in AD biomarker research, promising improved early detection, accurate diagnosis, and targeted therapeutic interventions, ultimately advancing patient outcomes and disease management.

Abstract citation ID: igae098.2282

### WHAT DO DIRECT CARE WORKERS NEED TRAINING IN TO HELP OLDER ADULTS WITH DEMENTIA DURING MEALTIMES?

Dukyoo Jung, Jisung Park, Eunju Choi, Leeho Yoo, Kahyun Kim, Seyoung Cho, and Soogyung Shin, *Ewha Womans University, Seoul, Republic of Korea*

Older adults with dementia in long-term care facilities often encounter challenges in self-feeding owing to cognitive and physical impairments. Although direct care workers play a crucial role in facilitating meal-time activities, they lack adequate training and specific educational programs. To develop an effective program, it is essential to understand the educational needs of direct care workers providing meal assistance. This study aimed to identify the educational needs of direct care workers in long-term care facilities regarding providing meal assistance for older adults with dementia. Adopting a mixed-methods approach, this study combined quantitative analysis, using Borich's needs assessment, and the locus for focus models, with qualitative insights from focus group interviews. Participants comprised 175 direct care workers and 5 nursing managers from various long-term care facilities in South Korea. This study identified four main educational priorities: enhancing knowledge of swallowing functions, understanding institutional support mechanisms, applying multisensory stimulation techniques, and addressing food forgetfulness in older adults with dementia. These findings were supported by qualitative data that emphasized the necessity of training to improve the quality of meal assistance provided to this vulnerable population. The findings underscore the critical need for focused educational programs that equip direct care workers with the skills and knowledge necessary to effectively assist older adults with dementia during mealtime. This study advocates the implementation of continuous education and training initiatives led by nursing management to foster an environment conducive to autonomous and independent eating among patients with dementia, thereby enhancing their overall care and quality of life.

### SESSION 7010 (POSTER)

### BIOLOGY OF AGING I

Abstract citation ID: igae098.2283

### A NEW ROLE FOR KYNURENINE METABOLISM IN THE AGE-ASSOCIATED DECLINE OF THE BACTERIAL IMMUNE RESPONSE

George Sutphin, Luis Espejo, Anne Haskins, Leah Chang, Jonah Balsa, Angelo Antenor, Hope Dang, and Samuel Freitas, *University of Arizona, Tucson, Arizona, United States*

Tryptophan metabolism through the kynurenine pathway becomes dysregulated during normal aging and is implicated in age-associated disease, including chronic inflammation, atherosclerosis, neurodegeneration, and cancer. Kynurenine pathway enzymes and metabolites influence a range of molecular processes critical to healthy aging, including regulation of inflammatory and immune responses. Kynurenine metabolism is active in immune cells and activated in response to proinflammatory cytokine signaling. We previously determined that elevating physiological levels of the kynurenine pathway metabolite 3-hydroxyanthranilic acid (3HAA) via either direct supplementation or inhibition of the enzyme that degrades 3HAA, 3HAA dioxygenase (HAAO), extends lifespan and delays age-associated health decline in both Caenorhabditis elegans and mice. Published work suggests that 3HAA is anti-inflammatory in mammals, for example by reducing the ratio of activated to regulatory T cells and inhibiting pathological activation of macrophages. In recent work, we find that elevating physiological 3HAA can beneficially enhance the immune response of C. elegans to bacterial pathogens during aging. 3HAA is sufficient to kill bacterial and inhibit bacterial growth in culture. When HAAO is inhibited in C. elegans, 3HAA accumulates in lysosome related organelles (LROs) in the intestinal cells, the same subcellular compartment that contains engulfed bacteria. LROs are a cellular repository for both iron and zinc, and we further find that iron chelation or zinc supplementation dramatically enhances the bactericidal properties of 3HAA. Here we present a mechanistic model in which age-dependent accumulation of 3HAA in intestinal LROs combines with zinc to enhance bacterial resistance with age in C. elegans.

Abstract citation ID: igae098.2284

### ASSOCIATION BETWEEN POLYPHARMACY AND A-KLOTHO IN US OLDER ADULTS: THE MEDIATION ROLE OF INFLAMMATORY MARKERS

Kai Wei, Chun Chen, Yanping Yang, Libin Wang, and Shuimei Sun, *Guizhou Provincial People's Hospital, Guiyang, Guizhou, China (People's Republic)*

**Introduction:** Polypharmacy may lead to adverse health outcomes in older adults, but the relationship between polypharmacy and accelerated aging is not well understood. This study aims to investigate the association between polypharmacy and α-klotho, a blood anti-aging protein, using large population-based data.

**Methods:** The study selected 3569 participants aged 65-79 years with serum α-klotho test data and prescription information from the National Health and Nutrition Examination Survey (2007-2016). Participants taking 5 or more medications simultaneously were classified as having polypharmacy. Weighted linear regression analyses were conducted to evaluate the association between polypharmacy and α-klotho. Mediation analysis was conducted to investigate the impact of inflammatory biomarkers, including the systemic immune-inflammation index, systemic inflammation response index, and neutrophil-to-lymphocyte ratio, on the relationship between polypharmacy and α-klotho levels.

**Results:** The polypharmacy group consisted of 1,591 participants (weighted percentages 42.7%). The median level of α-klotho in the polypharmacy group was 733.16[594.47, 911.63] pg/mL, which was significantly lower than that in the non-polypharmacy group. After adjusting for multiple variables, polypharmacy remained significantly negatively associated with α-klotho (β-coefficient -0.0506, 95% CI -0.0855 to -0.0157), and this significant association were observed in men and women, respectively. Further mediation analyses revealed that the association between polypharmacy and α-klotho was mediated by inflammatory biomarkers, with the proportion of mediation ranging from 6.11% to 11.82% (all P < 0.01).
**Conclusion:** These findings suggest that polypharmacy is closely linked to circulating α-klotho levels, which may be partly mediated by inflammation.

Abstract citation ID: igae098.2285

### ASSOCIATION BETWEEN T CELL SUBSETS AND MORTALITY: FINDINGS FROM THE LONG LIFE FAMILY STUDY

Gokul Seshadri[1], Sithara Vivek[1], Mary Wojczynski[2], Allison Kuipers[3], Joseph Zmuda[3], Iva Miljkovic[3], Svetlana Ukraintseva[4], and Bharat Thyagarajan[1], *1. University of Minnesota, Minneapolis, Minnesota, United States*, *2. Washington University in St. Louis, St. Louis, Missouri, United States*, *3. University of Pittsburgh, Pittsburgh, Pennsylvania, United States*, *4. Duke University, Durham, North Carolina, United States*

Differences in T cell subsets are associated with higher mortality in the general population. We evaluated whether Long Life Family Study (LLFS) participants and their spouses differed from the general population regarding T cell distributions and their association with mortality. We measured 12 T cell subsets in 1647 LLFS offspring (60.77±8.19 years) and 550 spousal controls (61.08±8.57 years). We used a Cox proportional hazards model to evaluate the association between these T cell subsets and 4-year mortality after adjustment for age, sex, family relatedness and CMV seroprevalence. After age adjustment, LLFS offspring and spousal controls had higher levels of CD4+ naïve T cells (27.5% higher) and CD4+ effector memory cells (1600% higher) and lower levels of CD4+ central memory T cells (55% lower) as compared to the Health and Retirement Study (HRS). The difference in CD4+ naïve T cells between those who were < 55 years vs. those > 85 years was 50% lower in LLFS offspring as compared to spousal controls (similar to the HRS controls) (p=0.05). The percentage of CD4+ effector memory cells was associated with increased mortality risk (HR:1.47; p=0.01) and CD4+ naïve T cells showed a borderline inverse association with 4 year mortality (HR: 0.72; p=0.06) in the combined sample of LLFS offspring and spousal controls. These results suggest that levels of T cell subsets are maintained at older ages in long lived families despite CMV exposure and the association between T cells and mortality is similar to what is observed in the general population.

Abstract citation ID: igae098.2286

### ASSOCIATION STUDY OF DNA METHYLATION AGE AND BONE HEALTH PHENOTYPES IN OLDER TWINS

Mette Soerensen, Kaare Christensen, and Moustapha Kassem, *University of Southern Denmark, Odense M, Syddanmark, Denmark*

DNA methylation age, a biomarker for biological aging, is reported to associate to mortality and several aging-related phenotypes, while studies of bone phenotypes are surprisingly rare. Here we explore the association between diagnoses on age-related bone fractures, osteoporotic bone fractures, and osteoporosis without factures from the Danish Patient Registry to seven DNA methylation clocks: Horvath, IEAA, Hannum, EEAA PhenoAge, GrimAge and DunedinPACE. Cox regression analysis in a discovery sample of 310 older twins (age 73-91) revealed all clocks to reflect an increased epigenetic age with increasing risk of diagnosis; especially GrimAge and DunedinPACE appeared consistent across all three disease groups. A replication cohort of 777 twins (age 30-79) reflected the same direction of effect, with DunedinPACE displaying statistical significance. A twin pair analysis, reducing genetic and environmental confounding, confirmed the direction of effects, with GrimAge holding the largest effect sizes (e.g., HR=1.21, P=0.057 for osteoporotic fractures). In all analysis the DNA methylation-estimated telomere length reflected the opposite direction of effect, i.e., shorter telomere length with increased risk of diagnosis. Lastly, for a subset of the replication cohort (N=288, age 30-76) data on bone mineral density (BMD) and the bone turn over markers CTX and P1NP were available; CTX and P1NP showed decreased levels with increasing epigenetic age, while BMD showed an inconsistent pattern. Interaction analysis revealed age and sex effects for some of the clocks for BMD, perhaps pointing to the complex mechanisms behind bone biology. Taken together we report associations between DNA methylation age and bone health phenotypes.

Abstract citation ID: igae098.2287

### BLOOD BIOMARKERS OF ALZHEIMER'S DISEASE AND MUSCLE STRENGTH TRAJECTORIES IN COMMUNITY-DWELLING OLDER ADULTS

Alice Ornago[1], Elena Pinardi[2], Giulia Grande[3], Martina Valletta[3], Amaia Calderón-Larrañaga[3], Claudia Fredolini[4], Giuseppe Bellelli[5], and Davide Liborio Vetrano[3], *1. Karolinska Institutet, Osnago, Lombardia, Italy*, *2. Karolinska Institutet, Italy*, *3. Karolinska Institutet, Stockholm, Stockholms Lan, Sweden*, *4. KTH Royal Institute of Technology, Stockholm, Stockholms Lan, Sweden*, *5. University of Milano-Bicocca, Milano, Lombardia, Italy*

The age-related muscle strength decline is a major impediment to the achievement of healthy aging. This study aimed to investigate the association between a panel of Alzheimer's disease (AD) blood biomarkers and longitudinal trajectories of muscle strength. This 12-year population-based cohort study included 2011 dementia-free participants from the Swedish National study on Aging and Care in Kungsholmen (SNAC-K). Muscle strength was measured through the grip strength and chair stand tests, while baseline levels of AD biomarkers (total tau [t-tau], phosphorylated tau181 [p-tau181], neurofilament light chain [NfL], glial fibrillary acid protein [GFAP], and amyloid-β [Aβ] 42/40 ratio) were assessed by Quanterix Single Molecule Arrays. Linear mixed models, adjusted for socio-demographics and clinical factors, were used to estimate the association between AD biomarkers and muscle strength decline. Higher baseline levels of p-Tau181 (β 0.94, 95%CI 0.72;1.16), NfL (β 0.79, 95%CI 0.59;0.99), and GFAP (β 0.38, 95% CI 0.22;0.54) were associated with an accelerated worsening of the chair stand test over the follow-up. The adjustment for Mini-Mental State Examination (MMSE) led to an attenuation of these associations. Higher baseline levels of p-Tau181 (β -0.11, 95%CI -0.16;-0.06), and NfL (β -0.05, 95%CI -0.09;-0.003) were associated with a faster decline in the grip strength test over the follow-up; no attenuation was observed after adjusting for MMSE. Several blood biomarkers implicated in AD pathophysiology are associated with distinct trajectories of muscle strength. Cognitive function appears to influence this association, mostly concerning muscle strength as measured by the chair stand test.

Abstract citation ID: igae098.2288

### CAUSALITY-INFORMED REPRODUCTIVE CLOCKS REFLECT IMPACT OF CHILDHOOD MALTREATMENT ON EPIGENETIC AGING

Qiaofeng Ye, and Idan Shalev, *The Pennsylvania State University, University Park, Pennsylvania, United States*

Machine-learning-based epigenetic clocks have been widely used to predict an organism's age, pace of aging, and mortality. Here, we report the development of two novel reproductive clocks to reflect biological aging relative to menarche (MenarcheClock) or menopause (MenopauseClock) in females. First, Mendelian randomization was employed to identify CpG sites with significant causal effects on age at menarche or menopause. Next, models of MenarcheClock and MenopauseClock were trained using elastic net regression with array-measured DNA methylation of buccal cells from 540 females aged 0.2-18.94 years. In total, 186 and 141 CpG sites were selected into the MenarcheClock and the MenopauseClock, respectively, which had little overlap with published epigenetic clocks. Based on the Developmental Origin of Health and Disease model and evolutionary theories of aging, we further examined the impact of childhood maltreatment, race, ethnicity, and family income on deviations of the two clocks in 187 girls aged 8-14 years. Clock deviations were calculated by residualizing the clock estimates from chronological age, with positively greater deviations indicating expedited menarche or menopause. Preliminary analyses indicated that exposure to childhood maltreatment, including physical and sexual abuse and neglect, was associated with greater MenarcheClock deviation, controlling for BMI, race/ethnicity, income, and immune cell proportion. African American girls had greater MenopauseClock deviation than White peers. Our study demonstrated the feasibility of utilizing causality information based on Mendelian randomization to develop epigenetic clocks indicating reproductive aging. Findings also support the theory that adverse exposure during early development leads to earlier sexual maturation to increase reproductive success.

Abstract citation ID: igae098.2289

### CROSS-TISSUE EPIGENETIC AGE COMPARISONS

Abner Apsley, and Idan Shalev, *The Pennsylvania State University, University Park, Pennsylvania, United States*

Accurately measuring the process of biological aging – the progressive deterioration of cells, tissues and organs – is crucial in studying the effects of aging interventions. A common group of tools used to measure the process of biological aging are epigenetic clocks (i.e., algorithms that take advantage of the age-related changes observed in the DNA methylome to estimate an individual's biological age). Although epigenetic clocks have been trained almost exclusively using blood-based tissues, there is a growing desire to estimate epigenetic age using less-invasive mouth-based tissues (i.e., buccal or saliva). However, by definition, differentiated cell types exhibit unique DNA methylation landscapes and age-related alterations to the DNA methylome. Therefore, using epigenetic age estimates via mouth-based tissues that rely on blood-based tissues can introduce errors when estimating biological age. We tested the within-person comparability of common epigenetic clocks across five tissue types: buccal epithelial, saliva, dry blood spots, buffy coat (i.e., leukocytes), and peripheral blood mononuclear cells. Our sample included individuals from 9-70 years old (N=83) across 284 distinct tissue samples. Overall, significant differences in mouth-based versus blood-based tissue estimates were observed, with differences of more than 30 years being observed in some clocks. In addition, most blood-based clocks exhibited a low correlation with buccal and saliva tissues, even after controlling for cellular proportions, indicating that buccal and saliva are both uniquely different from blood-based estimates. Our findings indicate the deviance of mouth-based versus blood-based epigenetic clock estimates, and highlight important differences of using diverse tissue types to estimate biological age.

Abstract citation ID: igae098.2290

### DISTINCT CELL SUBTYPES WITHIN NAÏVE CD4 T CELLS COMPARTMENT SHAPE ITS AGING AND SENESCENCE

CJ Kelly[1], Ashley Jeon[1], Jacob Rodriguez[1], Dina Prosser[2], Morgan Diegel[1], and Nikolay Burnaevskiy[1], *1. Altius Institute for Biomedical Sciences, Seattle, Washington, United States*, *2. Altius Institute for Biomedical Sciences, Settle, Washington, United States*

Aging is a multifaceted process that manifests differently in various cell types and within the same cell type. Therefore, understanding age-related changes with single-cell resolution may illuminate the mechanistic details of tissue aging and offer targets for intervention. We utilized high-throughput single-cell RNA-sequencing (scRNA-seq) to analyze senescence-associated changes within the CD4 T cell compartment. First, we sequenced total human CD4 T cells from multiple donors, revealing that naïve CD4 T cells exhibited a less senescent state compared to memory cells. We focused on naïve CD4 T cells to determine if individual cells differ in expression of aging-associated markers. By sorting cells based on senescence-associated beta-galactosidase (B-gal) activity and applying scRNA-seq, we identified transcriptional signatures associated with more youthful and senescent states. Surprisingly, we discovered cells with lower B-gal expression comprised two distinct populations: one marked by high expression of HELIOS and low expression of SESN3, and another marked by high expression of BACH2 and low expression of TC2N. In comparison, 2 youthful subpopulation and senescence subpopulations differ in markers of immunological activation We then investigated if the composition of naive CD4 compartment changes with age, finding that the abundance of youthful subpopulations was reduced in the older donors' CD4 populations. Thus, aging in the naïve CD4 compartment is associated with a shifted distribution between more youthful and more senescent cells. Moving forward, we will attempt to manipulate naïve CD4 T cells into a more youthful state by altering expression of the identified transcriptional signatures associated with youthful and senescent states.

Abstract citation ID: igae098.2291

### EXAMINING THE RELATIONSHIP BETWEEN ALPHA-KLOTHO, INSULIN RESISTANCE, AND DIABETES IN OLDER ADULTS

Anam Ahmad[1], Michelle Shardell[2], Rita Kalyani[3], Richard Semba[4], and Luigi Ferrucci[5], *1. University of Maryland School of Medicine, Cary, North Carolina, United States*, *2. University of Maryland School of Medicine, Baltimore, Maryland, United States*, *3. Johns Hopkins University School of Medicine, Baltimore, Maryland, United States*, *4. Johns Hopkins University, Baltimore, Maryland, United States*, *5. National Institute on Aging, Baltimore, Maryland, United States*

Higher circulating concentrations of alpha-klotho protein have been associated with greater longevity and inversely linked with aging-related phenotypes in human and animal studies. Impaired insulin signaling contributes to deregulated nutrient sensing, a hallmark of aging that plays a role in age-related adiposity, diabetes risk, and reduced lifespan. Whether alpha-klotho is part of this longevity pathway is unknown. We aimed to assess the relationships of alpha-klotho with insulin resistance and diabetes, and whether these relationships depended on adiposity in 860 adults aged ≥55 years from the Invecchiare in Chianti Study. Insulin resistance was operationalized using the homeostasis model for insulin resistance (HOMA-IR); diabetes was defined as having fasting blood glucose ≥126 mg/dL, glycosuria, taking diabetic medications, or a combination of patient self-report and diet assessment, and adiposity was defined using body mass index (BMI). Stratified linear and logistic regression were used to quantify associations of alpha-klotho with HOMA-IR and diabetes, respectively. Inverse probability weighting was used to address missing data. Among 394 overweight participants (BMI 25.0-29.9 kg/m2), each logarithm higher alpha-klotho (pg/mL) was associated with 0.42 units higher HOMA-IR (95% confidence interval [CI] 0.09-0.75) and 1.34 times higher, though not statistically significant, odds of diabetes (95% CI 0.35-5.10), after adjustment for covariates. There was little evidence of association among those with BMI< 25.0kg/m2 or BMI≥30 kg/m2. The positive association with HOMA-IR in overweight only and no significant association with diabetes may reflect an alternative hypothesis of alpha-klotho overexpression promoting insulin resistance. Further studies are needed to understand the mechanism of these findings.

Abstract citation ID: igae098.2292

### INFLAMM-AGING IS NOT A UNIVERSAL AGING MECHANISM ACROSS DIVERSE HUMAN POPULATIONS

Maximilien Franck[1], Kamaryn Tanner[2], Luigi Ferrucci[3], Michael Gurven[4], Ameneh Mehrjerd[5], Joe Yeong[6], Johannes Hertel[5], and Alan A. Cohen[2], *1. University of Sherbrooke, Sherbrooke, Quebec, Canada*, *2. Columbia University, New York, New York, United States*, *3. National Institute on Aging, Baltimore, Maryland, United States*, *4. University of California Santa Barbara, Santa Barbara, California, United States*, *5. University Medicine Greifswald, Greifswald, Mecklenburg-Vorpommern, Germany*, *6. Institute of Molecular and Cell Biology, Singapore, Singapore*

Inflamm-aging, defined by age-associated increases in inflammatory cytokines, is thought to be a universal mammalian aging mechanism associated with higher chronic disease risk, as observed in industrialized populations like the InCHIANTI cohort (Italy). However, consensus regarding its precise nature and a measurement framework is lacking. Its relevance and measurement applicability across diverse human populations, especially in non-industrialized societies with high infection burdens, remain unclear. We aimed to characterize inflamm-aging across four populations, examining its relation to age and chronic diseases. Through principal components and factor analysis, we studied immune variation within the InCHIANTI, Singapore Longitudinal Ageing Study (SLAS), Study of Health in Pomerania (SHIP), and Tsimane Health and Life History Project (THLHP) cohorts—Amerindian forager-horticulturalists from the Bolivian Amazon. We compared immune axes' relationships with age and chronic diseases across cohorts. We observed major differences in the structure of inflammatory and immune variation across populations. Notably, the primary immune axis in the THLHP cohort diverged significantly from those in the InCHIANTI, SLAS and SHIP cohorts, with the latter two aligning more closely with the inflamm-aging phenomenon—albeit with variations in their manifestations. Furthermore, these axes did not consistently correlate with age across cohorts and exhibited differential impacts on chronic diseases, with the primary axes identified in InCHIANTI and SLAS showing stronger associations with chronic diseases. Our findings suggest inflamm-aging is not universal. There appear to be significant environmental and lifestyle impacts on inflammatory and immune variation leading to differences in how inflammation drives disease susceptibility across populations.

Abstract citation ID: igae098.2293

### INTERACTION OF SPERMIDINE AND A LOW-METHIONINE DIET ON LIFESPAN IN MTDNA-DEFICIENT SACCHAROMYCES CEREVISIAE

Wei-Hsuan Su, Evien Cheng, Laura Hernandez Cervantes, Megan Nishitani, and Samuel Schriner, *University of California Irvine, Irvine, California, United States*

Spermidine and a low-methionine diet are two strategies shown to extend lifespan in model organisms, such as yeast, worms, flies, and rodents. Mitochondrial dysfunction is one of the theories explaining how organisms age. In previous studies, we used yeast lacking mtDNA (rho0) as a model for mitochondrial dysfunction. We found that spermidine was not only unable to extend lifespan in rho0 yeast, but actually shortened lifespan. Spermidine and methionine are closely linked metabolically as S-adenosylmethionine from the methionine cycle can be used to synthesize spermidine. And in turn, 5'-methylthioadenosine from the conversion of spermidine to spermine can be used to synthesize methionine. While we found that low-methionine treatment could extend wild-type yeast (rho+) lifespan compared to high-methionine treatment, rho+ yeast were shorter lived than rho0 yeast when both were subjected to low methionine. Thus, the action of low methionine appears to be opposite that of spermidine in rho0 cells, while being similar to spermidine in rho+ cells. This suggests that mitochondrial function plays a significant role in the ability of both treatments to modulate lifespan. We also observed that spermidine could rescue low-methionine toxicity in rho0 yeast, presumably by providing methionine through the pathway mentioned. We then uncovered that deficiency of CYT1 (a subunit of mitochondrial complex III) alone replicates the effect of complete mtDNA-deficiency with respect to spermidine toxicity. Future work will continue to explore the interaction between spermidine and low-methionine modulation of lifespan with the goal of identifying the molecular targets involved.

Abstract citation ID: igae098.2294

### LIFE EXTENSION VIA GENETIC/DRUG INTERVENTIONS OCCURS BY DELAYING THE ONSET OF AGING, NOT BY SLOWING THE RATE OF AGING

Patrick Phillips[1], Christine Sedore[1], Grace Jackson[1], Gordon Lithgow[2], and Monica Driscoll[3], *1. University of Oregon, Eugene, Oregon, United States*, *2. Buck Institute for Research on Aging, Navado, California, United States*, *3. Rutgers, The State University of New Jersey, Piscataway, New Jersey, United States*

Ever since the discovery more than 30 years ago of mutations capable dramatically increasing lifespan, there has been an increasing push to find genetic pathways and chemical interventions that ameliorate the effects of aging and increase healthspan and longevity. But aging is not merely “getting old,” it is an accelerating rate of the decline in function and an increase in the rate of mortality with chronological age. Here, we present a new framework for understanding longevity-extending interventions by dividing the survivorship into two major parts—the onset of aging and the period of accelerated aging—and use a uniform analytical approach to derive these quantities for each of the major mortality models. We apply this new framework to a set of studies with sufficient size to estimate mortality rate, finding that for four genetic and three chemical interventions in C. elegans and five genetic and eleven chemical interventions in mice, all treatments that lead to an increase in overall lifespan do so by delaying the onset of aging and none decrease the rate of aging late in life. These effects are readily visualized using a relative hazard and relative lifespan approach. Thus, while there is great interest identify compounds and other approaches to “treat” aging, evidence to date suggests that these interventions do not actually affect aging in the formal sense. These results have important implications for the design and analysis of aging studies, as well as for the field of geroscience as a whole.

Abstract citation ID: igae098.2295

### LONGITUDINAL STUDY OF R1RHO DISPERSION IMAGING OF AGING MUSCLE IN FISCHER RATS

Fatemeh Adelnia[1], Feng Wang[1], and John Gore[2], *1. Vanderbilt University Medical Center, Nashville, Tennessee, United States*, *2. Vanderbilt University, Nashville, Tennessee, United States*

Aging is associated with impaired endothelium-dependent vasodilation, leading to impaired muscle perfusion and organ dysfunction. It has been shown that impaired muscle perfusion may result in insufficient delivery of oxygen and nutrients during and after muscle contraction, leading to muscle damage. The ability to study the relationships between microvascular structure, impairment of perfusion, and muscle damage has been limited using traditional muscle perfusion measures, which are mostly invasive and risky. We developed a non-invasive 3D R1ρ dispersion MRI method at weak locking field frequencies to quantify microvasculature restructuring in skeletal muscle. This method provides a unique way of characterizing the size and density of capillaries. We successfully developed the protocol and applied it to the lower leg of F334 female rats of different ages. The whole lower leg of each rat was also imaged using conventional RARE pulse sequences to quantify the T2 values of the muscles. Our data show ΔR1ρ (=R1ρ{0Hz} - R1ρ{240Hz}) fraction values in calf muscles of 15 months F344 female rats (n=8) are significantly (p-value=0.03) higher than 6 months old rats (n=8). We interpret this measure as a possible new biomarker that characterizes changes in microvascular structure with aging. The T2 value also significantly increased in older rats (=30.25ms) compared to younger groups (=27.7ms), possibly reflecting higher inflammation in muscle in agreement with previously published results. The future direction of this ongoing study is to compare these results with data collected from male rats and validation of the ΔR1ρ fraction as a biomarker using ex-vivo histology.

Abstract citation ID: igae098.2296

### METABOLOME-WIDE STUDY OF CVD IN SWEDISH TWINS

Asmus Cosmos Skovgaard[1], Jonathan K. L. Mak[2], Sara Hägg[2], Juulia Jylhävä[3], and Mette Soerensen[1], *1. University of Southern Denmark, Odense M, Syddanmark, Denmark*, *2. Karolinska Institutet, Stockholm, Stockholms Lan, Sweden*, *3. University of Tampere, Tampere, Pirkanmaa, Finland*

Globally, cardiovascular diseases (CVD) are the leading cause of death, with coronary artery disease (CAD) and stroke as the most frequent diseases. The molecular basis of CVD is potentially confounded by genetic and environmental factors, an issue which is reduced by investigating intra-pair differences in twins. Here, we study 11,228 twins with nuclear magnetic resonance-based metabolome data and register based diagnoses on incident CAD and stroke collected from the Swedish Patient Register. Statistical analyses were performed both at the individual level and twin-pair level by Cox regression analysis. Biomarkers being significant (FDR< 0.05) both at the individual level and twin pair level in analysis of CAD were identified and LASSO analysis was used to identify the most influential independent biomarkers. A more explorative approach was taken for stroke by applying a cut off of p value< 0.05. Different biomarkers were found for CAD and stroke, which can indicate that the biological mechanisms are partly distinct in the two diseases. We found that ApoB-containing lipoproteins are more positively associated with CAD, which might be due to their pro-atherogenic effects. Contrary, ApoA1-containing lipoproteins are more negatively related to stroke possibly due to their anti-atherogenic effects and potential ability to cross the blood-brain-barrier, an ability not shared with the ApoB-containing lipoproteins. In conclusion, we present novel biomarkers for CAD and stroke by performing metabolomic analyses in twins, thereby lowering the confounding induced by shared genetic and environmental factors, a study design which is rare within the CVD field.

Abstract citation ID: igae098.2297

### MOLECULAR SENESCENCE IS ASSOCIATED WITH DEMENTIA RISK IN THE ATHEROSCLEROSIS RISK IN COMMUNITIES STUDY

Anna Prizment[1], Saeun Park[1], Sanaz Sedaghat[1], Shuo Wang[1], Weihua Guan[1], Weihong Tang[1], Keenan Walker[2], and Timothy Hughes[3], *1. University of Minnesota, Minneapolis, Minnesota, United States*, *2. National Institute on Aging, Baltimore, Maryland, United States*, *3. Wake Forest University School of Medicine, Winston-Salem, North Carolina, United States*

Recent evidence suggests that the accumulation of senescent cells induces chronic inflammation via the senescence-associated secretory phenotype (SASP), increasing Alzheimer's disease risk. However, the association between SASP proteins and dementia risk remains unknown. Our objective was to develop SASP scores using various protein panels and evaluate their relationship with dementia risk in Atherosclerosis Risk in Communities (ARIC) study – a prospective cohort of 11,755 White and Black men and women (46-70 years old) at baseline (1990-92). We created 3 SASP scores by training them against incident dementia (1,493 cases) in 7,837 participants (67%) followed until 2019. The first score (Score-1), created by Ridge regression, included 133 proteins identified in Basisty's atlas (2020). The second score (Score-2) included 16 dementia-associated proteins (of 133 SASP proteins). The third score (Score-3) was based on 7 SASP proteins validated by Schafer (2020); it included only 2 dementia-associated proteins: growth differentiation factor 15 (GDF15) and activin A. These scores were computed as weighted sums of standardized SASP proteins (mean=0, SD=1) in the test set of the remaining 3,918 participants (758 dementia cases). Multivariable Cox regression estimated the associations between each score and incident dementia. Hazard ratio (HR, 95% CI) per SD were for Score-1 (1.33, 1.23-1.45), Score-2 (1.20, 1.11-1.31), and Score-3 (1.23, 1.13-1.34), all p< 0.001. Thus, the 3 SASP scores, consisting of different protein numbers, showed comparable positive associations with dementia risk. SASP scores, even those consisting of a small number of proteins, may serve as a potential biomarker for dementia.

Abstract citation ID: igae098.2298

### ORA BIOMEDICAL: ACCELERATING DRUG DISCOVERY FOR GEROSCIENCE

Michael Muir[1], Benjamin Blue[1], Matt Kaeberlein[2], and Mitchell Lee[1], *1. Ora Biomedical, Inc., Seattle, Washington, United States*, *2. Optispan, Inc., Seattle, Washington, United States*

Small molecule interventions that directly target the molecular mechanisms of aging have the potential to revolutionize 21st century health and preventative medicine. Altering the trajectory of aging holds incredible economic value in humans, companion pets, and other animals. The longevity biotechnology sector is quickly growing to meet this opportunity, but a relatively small number of interventions and aging targets are poised for clinical development. Ora Biomedical, Inc. is a pharmaceutical company launched out of the Kaeberlein Lab at the University of Washington. We routinely test natural products, pharmaceuticals, combinatorial mixtures, and biologics to identify those that impact biological aging. Our drug screening uses the WormBot-AI platform, which leverages AI, robotics, and C. elegans to enhance the efficiency and accuracy of pre-clinical drug discovery. The WormBot-AI is the best in class for high-throughput intervention testing with lifespan, disease progression, and health measures as primary endpoints. We have also used our platform to launch the Million Molecule Challenge (MMC), a crowdfunded open science initiative. We show here the open access data crowd-funded by scientists and community members from over twenty countries. Furthermore, as a resource and tool for the broader aging science community, we have launched the MMC dashboard. This allows any member of the aging research community to access this crowd-funded data. Ora Biomedical is currently soliciting partnerships with both academic and non-academic aging researchers. We offer a wide array of screening services in multiple disease and mutant models as well as custom intervention validation experiments.

Abstract citation ID: igae098.2299

### RELATIONSHIPS BETWEEN BLOOD BIOENERGETICS AND LEG COMPOSITION ACROSS THE HUMAN LIFE COURSE

Adrian Arciniega[1], Howard Phang[2], Jaclyn Bergstrom[3], and Anthony Molina[1], *1. University of California San Diego, La Jolla, California, United States*, *2. University of California, San Diego, La Jolla, California, United States*, *3. UC San Diego, La Jolla, California, United States*

The human aging process is marked by changes in body composition, such as muscle loss and bone loss, which may be related to mitochondrial dysfunction. Peripheral blood mononuclear cells (PBMCs) are being used to report on systematic mitochondrial function and have been associated with the bioenergetic capacity of various tissues. Given the ability of PBMCs to recapitulate systemic bioenergetics, we investigated the associations between PBMC bioenergetics and lower extremity composition. We hypothesized that PBMC bioenergetics are significantly associated with measures of leg composition. In this study we utilized data from the San Diego Nathan Shock Center (SD-NSC) clinical cohort which measured blood cell bioenergetics and body composition in healthy adults across the human life course. Pearson Correlation analysis (ntotal=48) revealed strong negative correlations between PBMC respiration and leg bone mineral content (basal r=-0.358, max r=-0.274), femoral area (basal r=-0.369, max r=-0.308), and femoral bone mineral content (basal r=-0.405, max r=-0.329). Partial correlation analyses indicated that significant associations persist when controlling for age and BMI. Further stratification by sex revealed that these relationships were primarily driven by female participants (n=29). These results suggest that blood bioenergetics are related to leg composition in adults regardless of age and play a role in body composition, particularly among women.

Abstract citation ID: igae098.2300

### SEX-SPECIFIC BIOMARKER PREDICTION OF FEMORAL NECK BONE LOSS AFTER HIP FRACTURE

Alan Rathbun, Marc Hochberg, Michelle Shardell, Alice Ryan, Denise Orwig, and Jay Magaziner, *University of Maryland School of Medicine, Baltimore, Maryland, United States*

Hip fracture exacerbates age-related inflammation and metabolic dysfunction, which could accelerate bone loss post-fracture. This study aimed to develop biomarker prediction models for declines in femoral neck bone mineral density (BMD) after hip fracture. Participants were Caucasian men (n=99) and women (n=75) from the Baltimore Hip Studies 7th cohort with a hip fracture who were not receiving glucocorticoids, sex-hormone therapy, or bone-active medications. Data were collected at baseline (within 15 days of admission) and 2-, 6-, and 12-months later. Biomarkers were categorized into tertiles: interleukin-1 receptor antagonist (IL-1RA), interleukin-6 (IL-6), soluble tumor necrosis factor-alpha (TNF-alpha) receptor type 1, estradiol (E), testosterone (T), intact parathyroid hormone (iPTH), sex hormone binding globulin (SHBG), insulin-like growth factor-1 (IGF-1), C-terminal telopeptide of type I collagen (CTX), and procollagen type I N-propeptide (PINP). Femoral neck BMD was measured using dual energy x-ray absorptiometry. Mixed-effects models adjusted for age, height, and weight estimated 1-year changes in femoral neck BMD after hip fracture. Bone loss was classified as accelerated for intra-individual changes that exceeded the mean decline. Logistic regression models were stratified by biological sex and implemented using stepwise selection. The area under the curve (AUC) estimate among men was 0.68, and biomarkers related to bone loss were E, IGF-1, SHBG, and CTX. Similarly, the AUC metric among women was 0.69, but identified biomarkers were IL-6, iPTH, IGF-1, and SHBG. Findings indicate that both inflammatory and hormonal biomarkers relate to accelerated bone loss post-fracture; however, there are differential associations in physiological mechanisms between men and women.

Abstract citation ID: igae098.2301

### SHORT CHAIN FATTY ACID CONCENTRATIONS ASSOCIATE WITH PLATELET MITOCHONDRIAL FUNCTION AND MAY MEDIATE SEX-DIFFERENCES IN OLDER ADULTS

Eva Marts Skoglund[1], Gargi Mahapatra[2], and Anthony Molina[2], *1. University of California Berkeley, Lawndale, California, United States*, 2. *University of California San Diego, La Jolla, California, United States*

Some studies have reported that mitochondrial function can differ by sex; however, the metabolic pathways that mediate sex differences remain unclear. Our recent studies demonstrate that mitochondrial function, measured in peripheral blood mononuclear cells (PBMCs) and platelets, differ by sex in adults, 55 years of age and older. In a lipidomic analysis of PBMCs and platelets collected from the same participants, we assessed concentrations for 10 short-chain fatty acids (SCFAs), namely, acetate, propionate, isovalerate, valerate, butyrate, isobutyrate, 3-methylvalerate, 4-methylvalerate, 2-methylbutyrate, and caproate. T-tests comparing SCFA concentrations between males and females revealed sex differences in both PBMCs and platelets. Specifically, SCFA concentrations were significantly higher in platelets obtained from males compared to females. Analysis of metabolic pathways involved in the metabolism of these SCFAs revealed that FA biosynthesis, pyruvate metabolism, glycolysis, and gluconeogenesis pathways are significantly affected in both sexes. Further analysis revealed a greater impact of SCFAs in males than females. To test the impact of SCFAs on mitochondrial bioenergetics, we examined correlations between SCFA concentrations and mitochondrial bioenergetic parameters assessed by respirometry. In both PBMCs and platelets, bioenergetic parameters were correlated with acetate, 3-methylvalerate, 4-methylvalerate, and caproate concentrations. In platelets, SCFAs significantly negatively correlated with basal respiration, ATP-linked respiration, and fatty-acid oxidation (FAO). Similar negative correlations were also observed in PBMCs. Our study indicates that sex-dependent differences in mitochondrial bioenergetics are related to sex specific differences in SCFA concentrations. Future studies are needed to determine if differences in SCFA can underlie differences in mitochondrial function between men and women.

Abstract citation ID: igae098.2302

### SRF ISOFORMS DIFFERENTIALLY IMPACT MITOCHONDRIAL FUNCTION AND AGING

Shakshi Sharma[1], Xiaomin Zhang[2], Gohar Azhar[2], and Jeanne Y Wei[2], *1. University of Arkansas for Medical Sciences, LITTLE ROCK, Arkansas, United States*, *2. University of Arkansas for Medical Sciences, Little Rock, Arkansas, United States*

Serum response factor (SRF) is a highly conserved widely expressed transcription factor that plays a key role in regulating a number of muscle specific genes in various tissues, including cardiac and skeletal muscles. Alternative splicing (AS) of SRF generates multiple mRNA transcripts and protein isoforms. The SRF isoforms are critical in regulation of SRF target genes and increases in the human heart with advancing age. We cloned novel isoforms SRF-Δ3 and SRF- Δ345 from human tissues and studied their effect on mitochondrial function. Three SRF isoforms (Wild Type, Δ-3, Δ-345) were subcloned into expression vectors. Muscle cell line C2C12 was transfected with SRF-empty vector, SRF-WT, SRF-Δ3, and SRF-Δ345, respectively. Gene expression was determined by RT-PCR and Western blotting. The mitochondrial function was determined by the SeahorseXFe96 analyzer. SRF-Δ3 and SRF- Δ345 significantly reduced the oxygen consumption rate (OCR) and glycolysis (ECAR) in C2C12 cells as compared to SRF-WT. The protein and gene expression of PGC-1α, PGC-1β, MFN 1, MFN 2, Fis1 and Opa1 were also differentially impacted by SRF isoforms. Further, the SRF isoforms significantly impact the protein expression of mitochondrial electron chain complex OXPHOS (I-IV) proteins, thus affecting mitochondrial function. These findings showed the potential effects of the SRF isoforms on mitochondrial function, which might affect cellular senescence and aging.

### SESSION 7015 (POSTER)

### BRAIN

Abstract citation ID: igae098.2303

### A PROTEOMICS-BASED MEASURE OF ACCELERATING AGING IS CORRELATED WITH THE BRAIN AGE GAP IN THE ARIC STUDY

Ramon Casanova[1], Keenan Walker[2], Lingyi Lu[1], Stephen Kritchevsky[3], Timothy Hughes[1], and Lynne Wagenknecht[1], *1. Wake Forest University School of Medicine, Winston-Salem, North Carolina, United States*, *2. National Institute on Aging, Baltimore, Maryland, United*

States, 3. Wake Forest University School of Medicine, Wake Forest University School of Medicine, North Carolina, United States

Proteomics clocks can be used to estimate chronologic age or predict age-related outcomes (e.g., GrimAge or AgeAccelGrim and mortality). Analogous to AgeAccelGrim, we developed a proteomic predictor in the Atherosclerosis Risk in Communities (ARIC) Study, and present correlations between this measure and an MRI-derived measure of advanced brain age (brain age gap) and other clinical parameters. We used data from 1447 ARIC participants with both brain MRIs and proteins levels available. The data was partitioned into training and testing datasets (75%-25%). We fitted a Cox regression elastic net model to predict all-cause mortality based on all proteins available in the SOMAscan aptamer panel (N = 4877), age and sex. The linear combination of variables was transformed to match mean age and variance of the training dataset. Finally, the age was regressed out to produce the proteomic measure. The testing dataset was used to estimate Spearman correlations. In total 33 proteins were selected by the model including R-sponding-4, GDF-15, ANGPT2 and PCYOX1L with larger (positives) coefficients while F7, SET and CNDP1 were the ones with smaller (negative) coefficients. The proteomic age measure was correlated with the brain age gap (0.26 $p< 0.001$), hypertension (0.25 $p< 0.001$), diabetes (0.18 $p< 0.001$), gait speed (0.24 $p< 0.001$), total cholesterol (0.25 $p< 0.001$) but not correlated with fasting glucose, grip strength or MRI-derived temporal meta-ROI. These results here link brain and body indices of accelerated aging independently of chronological age. Further investigation of the interrelationships between brain and proteomics aging measures is warranted.

Abstract citation ID: igae098.2304

### ASSOCIATIONS BETWEEN CARDIORESPIRATORY FITNESS, BRAIN AGE, AND NEURODEGENERATION AMONG OLDER ADULTS

Grace Derboghossian[1], Keenan Pituch[1], Mia Anthony[2], Dereck Salisbury[3], Feng Vankee Lin[4], and Fang Yu[1], *1. Arizona State University, Phoenix, Arizona, United States*, *2. University of Rochester, Rochester, New York, United States*, *3. University of Minnesota, University of Minnesota, Minnesota, United States*, *4. Stanford University School of Medicine, Stanford, California, United States*

There is growing evidence that CRF mitigates the likelihood of dementia caused by Alzheimer's disease (AD) and may underlie the cognitive benefits observed from aerobic exercise. Previous evidence further demonstrates neurodegeneration is the biological substrate for cognition deterioration and brain age may protect the brain from the deleterious effects of neurodegeneration. However, little is known about the relationships between CRF, brain age, and neurodegeneration in older adults with amnestic mild cognitive impairment (aMCI). The purpose of this cross-sectional study was to examine associations between CRF, brain age, and neurodegeneration among individuals with aMCI, using baseline data from the Aerobic exercise and Cognitive Training Trial, which examines the cognitive effects and underlying mechanisms of a 6-month ACT in older adults with aMCI. CRF was measured with VO2peak from a symptom-limited peak cycle-ergometer test. Brain age, hippocampal volume, and AD-signature cortical thickness (SCT) were obtained from structural magnetic resonance imaging. Multiple linear regressions were conducted in R (version 4.3.2). The sample (N=134) averaged 73.63 ± 5.81 years of age, 16.98 ± 2.9 years of education, 27.47 ± 5.17 in BMI, and 23.5 ± 2.18 in Montreal Cognitive Assessment scores with 51.5% male and 92.5% White. The mean brain age was 72.37±7.76 years with 2.92± 0.31mm ADSCT and 3164 ±455.51mm3 hippocampal volume. CRF was not associated with brain age and neurodegeneration. A negative association was found between brain age with hippocampal volume ($\beta$=-11.37, SE=5.11, p=0.02) and ADSCT ($\beta$=-0.01, SE=0.003, p=0.004). To conclude, future studies need to explore other brain indicators related to CRF.

Abstract citation ID: igae098.2305

### COGNITIVE RESERVE ATTENUATES THE EFFECT OF AD GENETIC RISK ON COGNITIVE AND BRAIN TRAJECTORIES

Corinne Pettigrew[1], Anja Soldan[2], Jiangxia Wang[2], Alden Gross[3], Timothy Hohman[4], Christos Davatzikos[5], Guray Erus[5], and Marilyn Albert[1], *1. Johns Hopkins University School of Medicine, Baltimore, Maryland, United States*, *2. Johns Hopkins University, Baltimore, Maryland, United States*, *3. Johns Hopkins Bloomberg School of Public Health, Baltimore, Maryland, United States*, *4. Vanderbilt University Medical Center, Nashville, Tennessee, United States*, *5. University of Pennsylvania, Philadelphia, Pennsylvania, United States*

Alzheimer's disease (AD) genetic risk factors impact cognitive and brain health, but the extent to which these effects are mitigated by cognitive reserve (CR) is less well understood. This study examined whether higher levels of CR (measured by education and reading ability) were associated with a reduced impact of AD genetic risk on both longitudinal cognitive trajectories (N = 1,819, M follow-up = 9.9 years) and brain atrophy (N = 1,542, M follow-up = 5.3 years) measured in middle-aged and older individuals with normal cognition at baseline. AD genetic risk was measured by APOE genetic status and AD polygenic risk scores (AD-PRS, excluding APOE). Cognitive outcomes included global, episodic memory, and executive function factor scores. Atrophy, derived from volumetric magnetic resonance imaging, included a composite of AD vulnerable regions (SPARE-AD), hippocampal volume, and a composite of regions indexing advanced non-AD related brain aging (SPARE-BA). In mixed-effects models, APOE-ε4 was associated with declines in all cognitive measures and with greater atrophy in SPARE-AD, SPARE-BA, and hippocampal regions over time. Importantly, the negative associations of APOE-ε4 with declines in global cognition and memory, and of APOE-ε4 with atrophy in SPARE-AD and the hippocampus were reduced among individuals with higher levels of CR. The AD-PRS was associated with executive function and global cognition declines (not memory), and with greater atrophy in SPARE-AD and hippocampal regions. However, CR did not attenuate AD-PRS-related changes in these measures. Results suggest that some APOE-ε4-related declines in cognition and brain atrophy may be mitigated by potentially modifiable factors.

Abstract citation ID: igae098.2306

### IMPACT OF ALZHEIMER'S DISEASE ON SUPRACHIASMATIC NUCLEUS CONNECTIVITY, SLEEP REGULATION, AND CIRCADIAN RHYTHM

Yiyi Shen[1], Hugo Calligaro[1], Michael Lam[2], Brian Khov[3], Keunyoung Kim[4], Wonkyu Ju[4], Mark Ellisman[4], and Satchidananda Panda[1], *1. Salk Institute for Biological Studies, San Diego, California, United States*, *2. University of California San Diego, La Jolla, California, United States*, *3. University of North Carolina, Chapel Hill, North Carolina, United States*, *4. University of California San Diego School of Medicine, San Diego, California, United States*

Circadian disruption, notably sleep disturbances, serves as an early indicator of Alzheimer's disease (AD), preceding cognitive symptoms such as memory impairment. Despite this, the underlying anatomical and functional mechanisms driving circadian disruption in AD remain elusive. The suprachiasmatic nucleus (SCN) serves as the central pacemaker regulating circadian rhythm and as the principal hub for entraining circadian rhythm. Here, we explore the relationships between SCN connectome evolution, sleep regulation, and circadian rhythm disruption in the APP/PS1 mouse model. In 3 to 8-month-old APP/PS1 mice, we obtained SCN image volumes via serial block-face scanning electron microscopy. We noted serval modifications in SCN connectomics, including a dendro-dendritic chemical synapse network reduction, crucial for synchronicity between SCN neurons, and a reduction of axonal boutons volume. In addition, we recorded electroencephalograms, electromyograms, temperature, and locomotor activity of 8-month-old WT and APP/PS1 mice to assess their sleep patterns. APP/PS1 mice displayed significantly reduced rapid-eye-movement sleep, associated with a reduced daily temperature amplitude and locomotor hyperactivity. Lastly, in 10 to 15-month-old APP/PS1 mice, we measure locomotor and metabolic activity change in response to acute light stimulation and light-dark cycle shift. APP/PS1 mice showed a decreased circadian fluctuation in metabolic activity and an impaired response to acute light pulse stimulation or phase shift. These preliminary results suggest considerable changes in the SCN connectivity may be responsible for circadian disruption in those with AD. Further investigation will evaluate the use of non-pharmacological approaches to mitigate the observed circadian disruption.

Abstract citation ID: igae098.2307

### RESTING STATE ELECTROENCEPHALOGRAPHY AND INHIBITORY CONTROL IN COGNITIVELY UNIMPAIRED OLDER ADULTS

Teresa Warren[1], Jaya Ashrafi[1], Shraddha Shende[2], Elizabeth Lydon[1], and Raksha Mudar[3], *1. University of Illinois Urbana-Champaign, Champaign, Illinois, United States*, *2. Illinois State University, Normal, Illinois, United States*, *3. University of Illinois, Champaign, Illinois, United States*

Resting state electroencephalography (EEG) activity captures spontaneous neural activity while at rest. Mounting evidence suggests that resting state EEG activity is linked to cognitive performance and event-related potentials in young adults. However, little is known on the relationship between resting state EEG activity and event-related spectral perturbations (ERSPs) and/or behavioral performance linked to inhibitory control in cognitively unimpaired older adults (OA). This study examined the relationship between resting state alpha power and 1) alpha-band ERSPs and 2) behavioral measures during a Go/NoGo inhibitory control task in 22 OA (Age: M = 64.04, SD = 6.18 years; Montreal Cognitive Assessment score: M = 28.43, SD = 0.99). Participants' EEG data were collected during eyes-open resting state and a Go/NoGo task. Fourier transformation-based spectral analysis was used to calculate power in the alpha frequency band (8-12 Hz), with absolute power spectral density for the resting state condition and event-related desynchronization (ERD) for the Go/NoGo task. Behavioral performance was assessed using reaction time on Go trials (M = 340.27 ms, SD = 33.71) and accuracy on Go (M = 0.92, SD = 0.13) and NoGo trials (M = 0.92, SD = 0.06). Based on preliminary analyses, lower absolute alpha power in resting state was correlated with better accuracy ($r(4) = -0.995$, $p = .005$) and lower alpha ERD ($r(4) = -0.96$, $p = .005$) on Go trials. These preliminary results suggest that resting state brain oscillations may predict brain dynamics and performance linked to inhibitory control in OA.

Abstract citation ID: igae098.2308

### VOTER OPINIONS ON BRAIN HEALTH AND EARLY DETECTION OF DEMENTIA IN LOS ANGELES COUNTY

Noel Barragan[1], Mariana Reyes[1], Mark DiCamillo[2], and Tony Kuo[1], *1. Los Angeles County Department of Public Health, Los Angeles, California, United States*, *2. University of California Berkeley, Berkeley, California, United States*

The burden of dementia continues to expand as the population ages. To address this growing public health problem, the local health department in Los Angeles County (LAC) launched its Healthy Brain LA initiative to encourage individuals and communities to prioritize brain health as a strategy for reducing the risk and impact of dementia. To better understand local perceptions about dementia and to inform the initiative's efforts, public health partnered with the Berkely IGS Poll to survey LAC voters on key topics related to brain health. The online poll was administered in January 2024 via email invitations sent out to stratified random samples of registered voters in the county; 1,775 respondents competed the survey. Of the total sample, 16% indicated they currently help with care for an adult with memory problems. Overall, 70% reported being somewhat or very worried about the possibility of developing dementia. A majority (84%) agreed they could take steps to improve their brain health and reduce their risk of dementia. Notably, only 13% reported having ever discussed ways to prevent dementia with their doctor. Nearly two-thirds (62%) strongly agreed that they would want to receive a dementia diagnosis as early as possible. There is increasing value in approaching dementia through a public health prevention lens, emphasizing risk reduction and early detection as strategies for addressing this condition. Poll results offer important insights into potential ways in which public health can help activate public engagement and health system involvement in dementia prevention, care and support.

### SESSION 7020 (POSTER)

### CANCER

Abstract citation ID: igae098.2309

### CONTINUOUS PASSIVE MONITORING OF DAILY FUNCTION AND SYMPTOMS IN OLDER ADULTS WITH PROSTATE CANCER

Deanne Tibbitts, Zachary Beattie, Eric Roeland, Rebecca Silbermann, Jason Webb, Gabrielle Meyers, Jeffrey Kaye, and Kerri Winters-Stone, *Oregon Health & Science University, Portland, Oregon, United States*

Androgen deprivation therapy (ADT) for prostate cancer (PC) increases the risk of physical and cognitive decline as well as side effects impacting quality of life. However, we lack a holistic understanding of the timing and spectrum of ADT-related side effects. Thus, we evaluated the feasibility of using home-based, continuous passive monitoring of daily life functioning, symptom surveys, physical performance, and cognitive testing in patients with PC beginning ADT. We assessed enrollment, passive monitoring uptake, retention, sensor data coverage, and data completion. Passive monitoring included infrared motion sensors (activity), door contact sensors (time out-of-home), watch (activity/sleep), bed mat (sleep), biometric scale (weight), electronic pillbox (medication adherence), and computer-use software (computer habits). We assessed health events and treatment-related symptoms (weekly online survey), physical functioning (short physical performance battery, timed up-and-go test), and cognitive functioning (online cognitive test). We enrolled 17 patients of 52 approached (median age, 73 years); 24 chose not to participate primarily due to concerns of burden (n=10, 42%) and feeling overwhelmed (n=5; 21%). Retention over 14 months was 82% (n=3 withdrawals). Weekly survey completion was 79%. Data coverage for passive monitoring ranged from 55% for watch-based sleep to 98% for motion sensor activity. Testing visit completion across 6 months was 93%; cognitive test completion was 98%. In sum, passive monitoring of daily functioning, symptom surveys, physical performance, and cognitive testing is feasible among older patients with PC beginning ADT. Future research will integrate symptoms, functioning, and health events to examine longitudinal variations in health during ADT.

Abstract citation ID: igae098.2310

### DEPRESCRIBING AMONG OLDER ADULTS WITH CANCER

Martha Coates[1], Emily Hajjar[2], and Rose Ann DiMaria-Ghalili[1], *1. Drexel University, Philadelphia, Pennsylvania, United States*, *2. Thomas Jefferson University, Philadelphia, Pennsylvania, United States*

Older adults with cancer experience high rates of polypharmacy (>5 medications daily) and potentially inappropriate medication use which are associated with adverse health outcomes and negatively impact quality of life. Deprescribing, the process of lowering drug exposure or removing medications that could cause harm or no longer provide benefit, can reduce the risk of negative outcomes. There is limited research examining the acceptance toward deprescribing in older adults with cancer, therefore the purpose of this study was to examine deprescribing acceptance among Medicare beneficiaries with cancer. We utilized the National Health and Aging Trends Study Round 6 (2016) dataset and included individuals who answered the Medication Attitudes Module (n=1975). Among the participants, 6.1% (n=121) reported having a history of cancer and over half were experiencing polypharmacy. Older adults with cancer strongly agreed or agreed that they took a large number of medications (63%), would like to reduce their medications (74.4%) and if their doctor suggested it they would stop one or more of their medications (95.8%). Despite these high levels of agreement, participants were also found to disagree they took medications they didn't need (77%) and highly agreed all of their medications were necessary (93.3%). Older adults with cancer are willing to stop medications they may no longer need if suggested by their healthcare provider, but strongly disagree they are taking medications they do not need. Further research is needed to examine patient perceived facilitators and barriers to deprescribing medications that may no longer be needed in individuals with cancer.

Abstract citation ID: igae098.2311

### EMPATHIC COMMUNICATION WITH OLDER ADULTS DURING DIGNITY THERAPY

Carma Bylund[1], Mary Kate Koch[1], Alyssa Crowe[2], and Susan Bluck[1], *1. University of Florida, Gainesville, Florida, United States*, *2. University of Florida College of Medicine, Gainesville, Florida, United States*

Dignity Therapy (DT) is designed to preserve patient dignity through a life story-legacy interview guided by a trained provider. Given the evidence on the important role of empathy in provider-patient interactions, we focused on the provider in dignity therapy interviews with older adults and aimed to assess: 1) relative frequency of use of varying empathic skills; 2) whether use of empathic skills varies by provider and DT modality, and (3) whether older palliative outpatients' physical functioning status impacted the frequency of providers' use of empathic communication skills. We analyzed 203 transcribed DT sessions with palliative care cancer outpatients aged 55 years or older, for providers' use of four empathic skills: acknowledgement, reflection, validation, and empathic self-disclosure. Patients (M=65.78 years; SD=7.43 years) completed the Palliative Performance Scale before the session. Across all providers (n=14), acknowledgement was used most frequently (M =.45, SD =.23), followed closely by validation (M =.43, SD =.24). Use of empathic communication skills did not differ by provider occupation, gender, or DT modality. Multi-level modeling indicated providers used more validation (B = -.12, p <.05) and total empathic communication skills (B = -.19, p <.05) in Dignity Therapy sessions with patients who had lower palliative performance scores (i.e., had reduced physical functioning). Thus, providers seem to adjust their approach to DT interviews based on patients' physical functioning. More research is needed to understand the impact of empathic communication skills on patient outcomes.

Abstract citation ID: igae098.2312

### HORMONAL THERAPY AND THE RISK OF RHEUMATIC AUTOIMMUNE DISEASES AMONG OLDER MEN WITH PROSTATE CANCER

Mohanad Albayyaa, *The University of Texas Medical Branch, Galveston, Texas, United States*

Prostate cancer ranks as the second most common cause of cancer death among older men. Typically diagnosed at localized stages, treatment options primarily include radiation, hormonal therapy, or surgery. Androgen Deprivation Therapy (ADT) stands as a cornerstone in prostate cancer management, despite its association with various adverse effects such as cardiovascular and autoimmune diseases. ADT alters the mechanistic and biological pathways of androgens, impacting immune cell regulation and autoimmune equilibrium by augmenting regulatory T-cell numbers, cytokines, and pro-inflammatory markers. This study aims to investigate the potential link between hormonal therapy (ADT) and rheumatic autoimmune diseases (RAD), including rheumatoid arthritis, lupus, ankylosing spondylitis, and Sjogren's syndrome, among older men with prostate cancer. Using Texas Cancer Registry Linked to Medicare dataset, a cohort of patients aged 66 years or older, diagnosed with localized prostate cancer between 2010 and 2019, was analyzed. Patients receiving ADT were compared to those without ADT exposure, matched on a 1:1 ratio. Kaplan-Meier analysis revealed higher rates of RAD among patients receiving ADT (11%) compared to non-ADT recipients (9%). Cox proportional hazard regression confirmed a significant association between ADT use and increased RAD risk (HR =1.29, 95% CI: 1.17-1.56). This finding underscores the importance of considering the potential adverse effects of ADT, emphasizing the need for comprehensive patient counseling and careful monitoring by clinicians. The study's results, aligned with previous research, further solidify the understanding of ADT's adverse effects, highlighting the necessity of assessing the patterns of care and personalized treatment strategies of older patients with prostate cancer.

Abstract citation ID: igae098.2313

### IMPORTANT FACTORS INFLUENCING PROSTATE CANCER SCREENING AMONG INDIGENOUS MEN

Kyoung Hag Lee[1], and Jung Sim Jun[2], *1. Wichita State University, Wichita, Kansas, United States*, *2. Kansas State University, Kansas State University, Kansas, United States*

Although there exists mounting evidence of systemic barriers to prostate cancer screening (PCS) among Indigenous peoples, there is a limited understanding of the individual-level barriers to PCS. This study examined how well predisposing, need, and enabling factors predict receipt of a PCS test among Indigenous men. This cross-sectional study recruited a total of 191 Indigenous men aged 40 and above, following the recommendation of the American Cancer Society by using the purposive sampling method in 2014. The study's dependent variable was the receipt of a PCS. Independent variables included three predisposing, two need, and five enabling factors. Participants' mean age was 51.58 years, ranging from 40 to 95, and 33.3% were married. The hierarchical logistic regression showed that married participants were 3.82 times more likely than those who never married to receive a PCS. Participants with a personal cancer history were 11.84 times more likely than those without a personal cancer history to receive a PCS. Participants who reported having access to medical care were 9.14 times more likely than those without access to medical care to receive a PCS. Additionally, participants who had heard about the prostate cancer exam were 21.44 times more likely than those who had not heard about the prostate cancer exam to receive a PCS. Social work practitioners or health care providers need to consider developing and providing appropriate prostate cancer prevention programs, family-based intervention programs, a support system for selecting an appropriate place for medical care, and prostate cancer screening education and assistance programs.

Abstract citation ID: igae098.2314

### OVARIAN CANCER IN OLDER ADULTS IN EAST INDIA

Esha Chakravarty, *Calcutta Metropolitan Institute of Gerontology, Mitcham, England, United Kingdom*

Ovarian cancer is a lethal gynaecological malignancy whose incidence rates increase with age and is called 'silent killer' due to lack of symptoms and late diagnosis. Cases of 500 elderly patients with ovarian cancer were studied with respect to age of diagnosis, survival rate and factors influencing survival from Calcutta Metropolitan Institute of Gerontology (CMIG), regional resource and training centre on ageing in East India. Fifty five percent of the patients had a diagnosis between 60-70 years, 25% had a diagnosis between 70-80 years and 20% at >80 years. The mean age of diagnosis was 69 years. The study found that cancer outcome deteriorates with age of the patient. The age standardized relative survival rates for 1 year was 57% for patients between 60-70 years, 43% for 70-80 years and 21% for >80 years. The 4 main factors for deceased survival in older women were found to be aggressive tumour and higher grade, resistance to chemotherapy, functional and cognitive decline and co morbid conditions. This study can help in introduction of decision aids that can will create a clear category of patients who will and will not benefit from cytoreductive surgery and chemotherapy and influence allocation of State resources.

Abstract citation ID: igae098.2315

### POTENTIAL BENEFITS OF GROUP EXERCISE ON CANCER-RELATED LONELINESS AMONG MEN ENROLLED IN THE GET FIT PROSTATE TRIAL

Kerri Winters-Stone[1], Sydnee Stoyles[1], Nathan Dieckmann[1], Karen Lyons[2], Mary Crisafio[1], Arthur Hung[1], Julie Graff[1], and Alexandra Sokolova[1], *1. Oregon Health & Science University, Portland, Oregon, United States*, *2. Boston College, Chestnut Hill, Massachusetts, United States*

Men with prostate cancer may experience cancer-related loneliness (CRL) that impacts their mental and physical health, yet research in this area remains sparse. We explored the relationship between cancer-related loneliness, anxiety, depressive symptoms, social and physical functioning and the potential benefits of group exercise in a subset of men with prostate cancer participating in a clinical exercise trial. We added the CRL questionnaire to measures of anxiety (PROMIS), depressive symptoms (CES-D) and social and physical function (QLQC30 subscales) mid-way through the GET FIT Prostate trial (NCT03741335). 145 men (mean age: 71.5 +/- 7.3 yrs) completed measures prior to participating in one of three online supervised group exercise programs for 6 months and another 68 also completed measures post-intervention. While exercise modalities differed among the three groups, all men participated in 15 minutes of semi-structured social time immediately prior to the start of their one-hour exercise class. 70% of men reported some level of CRL at baseline. CRL was significantly ($p < 0.01$) and positively associated with higher anxiety ($r=0.61$) and worse depressive symptoms ($r=0.66$) and inversely with social ($r=-0.50$) and physical ($r=-0.23$) functioning. After 6 months of group exercise, CRL decreased among men who reported experiencing CRL at least some of the time at baseline ($p=0.017$). Among the whole sample, higher baseline CRL was significantly associated with the greater declines in CRL over 6 months (adjusted-R2=0.16, $p < 0.01$). A majority of men with prostate cancer may experience cancer-related loneliness, but socially-enhanced group exercise could be an effective countermeasure to improve well-being.

Abstract citation ID: igae098.2316

### THE IMPACT OF DIGITAL HEALTH LITERACY ON CANCER PREVENTION BEHAVIORS AMONG OLDER ADULTS

Jonathan Nutakor[1], Lulin Zhou[1], Ebenezer Larnyo[2], and Stephen Addai-Dansoh[1], *1. Jiangsu University, Zhenjiang City, Jiangsu, China (People's Republic)*, *2. University of California, Santa Barbara, Santa Barbara, California, United States*

In today's age of digital advancement, it is crucial to understand how digital health literacy affects the cancer prevention behaviors of older adults. This study investigates how digital health literacy influences older adults' ability to navigate online health information and its impact on adopting behaviors to prevent cancer. This study analyzes data from the Health Information National Trends Survey (HINTS) 5, focusing on individuals aged 60 and above. It explores different variables related to digital health literacy and engagement in cancer prevention behaviors, such as smoking cessation, physical activity, alcohol cessation, and cancer screening. Better digital health literacy is linked to an increased chance of quitting smoking (OR = 1.90, 95% CI: 1.02-3.52, p = 0.043). However, there is no such link to physical activity or cancer screening. Nevertheless, searching for health information electronically is associated with giving up alcohol, suggesting that digital health literacy can assist older adults in quitting drinking (OR = 1.47, 95% CI: 1.09-1.98, p = 0.012). The study highlights the importance of digital health literacy in promoting healthy habits among older adults. Specifically, it may help support smoking and alcohol cessation efforts. Though it may not apply to all behaviors, improving digital competencies is crucial to prevent cancer in older adults effectively. Therefore, tailored digital health literacy interventions are necessary to cater to the unique needs of this demographic and minimize their cancer risk.

Abstract citation ID: igae098.2317

### VALIDATION OF MODIFIED SENSE OF CONTROL SCALE AMONG OLDER CANCER PATIENTS

Peiyuan Zhang[1], Sarah Clem[2], Ting Guan[3], Todd Becker[4], and Paul Sacco[1], *1. University of Maryland Baltimore, Baltimore, Maryland, United States*, *2. University of Maryland School of Social Work, University of Maryland School of Social Work, Maryland, United States*, *3. Syracuse University, Syracuse, New York, United States*, *4. Washington University School of Medicine in St. Louis, St. Louis, Missouri, United States*

Sense of control, defined as the degree to which individuals feel able to influence their behavior and are responsible for the consequences, is a key intervention target for older cancer populations who experience high levels of distress. Despite being widely used, the 10-item Sense of Control Scale (SCS) with two subscales (i.e., Perceived Constraints and Personal Mastery) has not been validated with older cancer populations. This study aimed to examine (1) factorial and convergent validity of SCS and (2) measurement invariance between older cancer and non-cancer populations. Using data (N=2,865) from the most recent wave (2020) of the Health and Retirement Study, we examined factorial validity and measurement invariance using second-order confirmatory factor analysis and multi-group confirmatory factor analysis, respectively. We then assessed convergent validity with depression via Pearson product–moment correlation. SCS had adequate global model fit, $\chi 2(33)=295.43$, $p<.001$ (RMSEA=.08, SRMR=.04, CFI=.94, TLI=.92) and good local fit (range$\lambda$=.64–.88). Regarding measurement invariance, likelihood chi-square ratio test results showed significant differences in model fit across configural, metric, and scalar models ($p<.001$), but the magnitude of the change was negligible ($\Delta$CFI<.01). Thus, we concluded that SCS is invariant between older cancer and non-cancer populations. Compared to older non-cancer populations, older cancer populations appeared to have a significantly lower personal mastery ($\Delta$M= –0.12, p=.045) and higher perceived constraints ($\Delta$M= –0.1, p=.047). SCS was negatively correlated with depression (r= –.36, p<.05), showing desirable convergent validity. Overall, the SCS exhibits favorable preliminary psychometric properties with older cancer patients and appears invariant to cancer status.

### SESSION 7025 (POSTER)

### CLIMATE CHANGE AND AGING

Abstract citation ID: igae098.2318

### ASSOCIATION BETWEEN COUNTY TEMPERATURE AND LIMITATIONS IN ADLS IN A REPRESENTATIVE SAMPLE OF 1.7 MILLION OLDER ADULTS

Esme Fuller-Thomson, Holly Brooks, Elysia Fuller-Thomson, and Andie MacNeil, *University of Toronto, Toronto, Ontario, Canada*

Few studies have explored whether there is an association between area temperature and limitations in Activities of Daily Living (ADLs) such as serious difficulty with bathing and dressing. The objectives of this study were: (1) To examine the relationship between average county temperature during the coldest month of the year (i.e., January or February) (< 27°F, 27-31.9°F; 32-36.9°F; 37-41.9°F; ≥42°F) and the prevalence and adjusted odds of limitations in ADLs; and (2) To examine the relationship between the area's hottest month's average temperature (< 70°F, 70-74.9°F, 75-79.9°F, ≥80°F) and the prevalence and adjusted odds of limitations in ADLs. The sample included 1.7 million adults aged 65+ who responded to the nationally representative American Community Survey (2012-2017). After adjustments for age, sex, education, household income, race and time zone, there was a robust dose-response relationship indicating those living in areas with a higher average temperature during the coldest month had higher odds of limitations in ADLs. For example, in comparison to older adults living in regions where the coldest month's average temperature was < 27°F, those living in areas where the temperature was slightly above freezing (32-36.9°F) had 22% higher adjusted odds of ADL limitations (OR=1.22; 95%CI=1.19, 1.25) and those living in regions where the temperature averaged ≥42°F had 33% higher odds (OR=1.33: 95%CI-1.30, 1.36). There was no significant relationship between the regional hottest month's average temperature and ADL limitations. Future research is needed to explore why the absence of sub-freezing temperatures is associated with elevated odds of ADLs.

Abstract citation ID: igae098.2319

### ASTHMA AND COPD EXACERBATIONS: ED VISITS AND HOSPITALIZATIONS AFTER WILDFIRE SMOKE EXPOSURE AMONG OLDER ADULTS

Zhao Xiang Lin[1], Tal Sharon[2], and Anita Szerszen[1],
1. *Northwell Health, New York, New York, United States*,
2. *Staten Island University Hospital, New York, New York, United States*

Global temperatures are rising, leading to more intense wildfires, as seen in Quebec, Canada, on June 2023, where 13 million acres burned. The resulting particulate matter may severely degrade air quality, posing risks to older adults (OAs) with respiratory diseases. Studies indicate Black/African American communities may be disproportionately affected by air pollution compared to white communities. This study evaluated the impact of wildfires on emergency department (ED) visits and hospitalizations for COPD and asthma among adults 65 and older. The observational cohort study assessed 1321 patient records from two representative New York City hospitals. Findings revealed no significant change in the overall volume of ED visits, hospitalizations, and median length of stay during the smoke-affected months compared to the previous year. However, there was a significant increase in non-white OAs' ED visits and hospitalizations due to COPD or asthma: 27.19% in 2022 to 35.87% in 2023 (p = 0.0007), and more non-white patients with Medicaid insurance (80% in 2023, 71.43% in 2022). Significantly, more Black/African Americans required acute care: 16.74%, from 11.06% in 2022 (p = 0.003). These results indicate that worsening air quality may disproportionally impact non-white OAs. Black/African Americans and those with Medicaid insurance may more likely experience COPD or asthma exacerbation, requiring ED evaluation and/or hospitalization due to smoke pollution. The escalation in global temperatures and resulting wildfires may particularly affect OAs of color, especially those facing socioeconomic disadvantages. The findings underscore the importance of targeted public health strategies to address these disparities.

Abstract citation ID: igae098.2320

### EXAMINING THE IMPACT OF EXTREME HEAT TEMPERATURES IN FOOD INSECURITY AMONG LOW INCOME OLDER ADULTS

Jennifer Crook, Dahee Kim, Rui Xie, Christopher Emrich, Timothy Hawthorne, YunYing Zhong, James Brightman, and Ladda Thiamwong, *University of Central Florida, Orlando, Florida, United States*

Extreme heat significantly impacts health outcomes in low-income older adults (LOAs), though a better understanding of the mediating effects in food insecurity (FI) has not been fully appreciated. Food insecurity is the lack of or inability to obtain adequate nutrition and the added burden of extreme weather overwhelms already tenuous household environments, especially in LOAs with limited resources. Through onsite interviews, our team sought to uncover the impact of extreme heat in LOAs (n=45) from low-income communities in Central Florida. We assessed FI with the U.S. Food Security Survey Module (US FSSM) 10-item questionnaire. Participants reported either food secure (FS) (n=32) or FI (n=13) status. Statistically significant impacts of extreme heat or hot weather were identified in health and day-to-day activities (x2[df=1] = 5.27, p = 0.02) with mean rank scores of FS 18.6 and FI 27.9 and sleep (x2[df=1] = 6.25, p = 0.01), with mean rank scores of FS 16.2 and FI 25.6, though none were observed for food or hydration or connection with friends. Significant barriers to community resource utilization included transportation (x2[df=1] = 9.99, p = 0.002) and distance from home to the center (x2[df=1] = 4.54, p = 0.03), but not time, health issues, or knowledge about services provided. No significant findings were detected in reduced physical activity, staying inside, visiting a cool place, or staying hydrated. Severely hot weather temperatures facilitate increased barriers in LOAs living with FI. Future efforts should consider FI when designing interventions targeted towards LOAs living in hot climate conditions.

Abstract citation ID: igae098.2321

### GLOBAL CLIMATE ATTITUDES IN AGING ADULTS: THE ROLES OF SCIENTIFIC TRUST AND LITERACY

Bryce Van Vleet, and Heather Fuller, *North Dakota State University, Fargo, North Dakota, United States*

Prior research has established that people with less scientific literacy are more distrustful of science. However, this link is not well understood in older age or in relation to climate change perceptions. Aging adults play an important role in and are particularly vulnerable to the effects of climate change. This study investigates relationships between scientific literacy and trust with perceptions of climate change utilizing the 2020 Wellcome Global Monitor. 33,075 adults aged 50-99 (M=62.47) from 113 countries were interviewed about their views on science. We investigated whether scientific literacy and trust in science were predictors of climate change: a) understanding, b) major threat perception, and c) denial. Multiple regression analyses indicated trust in science and scientific literacy significantly predicted climate change understanding and threat perception, but not denial. Given conceptual distinctions between scientific illiteracy and scientifically literate mistrust, we investigated whether trust in science mediated the relationship between scientific literacy and climate change attitudes in midlife and older adulthood. Bootstrapped mediation models indicated trust in science partially mediated associations between scientific literacy and both understanding and threat perception. Findings suggest bolstering confidence in both scientific literacy and trust in science may help aging populations better understand and anticipate impacts of climate change. Results can help interventionists prepare aging populations for an increasingly warming world. Findings also extend the existing understanding of trust in science and scientific literacy as important processes in how older individuals perceive their environmental and politicized contexts.

Abstract citation ID: igae098.2322

### OLDER ADULTS AND CLIMATE CHANGE ADAPTATION STRATEGIES: A SCOPING REVIEW

Alexandra Holland, and Sadaf Milani, *The University of Texas Medical Branch, Galveston, Texas, United States*

Climate change is one of the biggest threats to human health, resulting in an increase in the frequency and intensity of heatwaves, storms, and other natural disasters. Older adults are at an increased risk from these effects and often have higher rates of morbidity and mortality from climate change-related impacts. As climate change progresses, adaptive strategies are critical for reducing the disproportionate health burden many older adults face. However, little is known about what evidence-based adaptive mechanisms exist that are appropriate for older adults. To address this gap, we conducted a scoping review to identify potential climate change adaptation strategies and solutions for older adults. An Ovid MEDLINE

search was done using search terms related to "older adults", "health", and "climate change adaptation." Articles had to be in English and focused on adaptation to climate change for older adults. The initial search returned 399 articles, and after article review, we found 133 articles that met our inclusion criteria. Most of the articles with findings related to climate change adaptation focused on extreme temperatures, such as heatwaves, followed by natural disasters. Emergent themes of the review include older adults' perception of their own vulnerability and individual adaptive actions and behaviors. There was a lack of research involving systemic adaptive actions aside from national heat plans and built environment modifications. This scoping review can be used to increase older adults' resilience and inform future systemic adaptive actions to prevent disproportionate morbidity and mortality due to climate change.

### SESSION 7030 (POSTER)

### COVID-19 PANDEMIC

Abstract citation ID: igae098.2323

### BENEFITS OF FINDING THE SILVER LINING: SECONDARY CONTROL PROTECTS WELL-BEING DURING PANDEMIC FINANCIAL HARDSHIPS

Matthew Pierce, Katherine Duggan, Laura Klepacz, and Jeremy Hamm, *North Dakota State University, Fargo, North Dakota, United States*

Although secondary control beliefs (e.g., "all clouds have a silver lining") reflect a psychological resource that can protect wellbeing, little is known about the potential benefits of dynamic shifts in secondary control in response to socioeconomic losses of any type, including financial hardships. Our study examined whether within-person shifts in secondary control predicted corresponding shifts in well-being and whether this relationship became pronounced during periods of financial hardship in a pandemic. We analyzed data from the 2-year NDSU National COVID Study that collected 5 waves of data from a representative sample of U.S. adults aged 18-80 (n=292). Preliminary multilevel models assessed how shifts in secondary control predicted corresponding shifts in mental health (perceived stress, depressive symptoms), hedonic wellbeing (positive and negative affect), and eudemonic well-being (personal growth, meaning in life). Our main multilevel models examined whether these within-person relationships were moderated by shifts in an inability to pay for one's needs (a self-report of financial hardship) while controlling for age, sex, education, income, and between-person secondary control and financial hardship. Within-person increases in secondary control predicted increased well-being across all outcome variables (bs=|0.11-0.23|, ps< 0.01). Financial hardship moderated the relationship of secondary control on perceived stress and personal growth (bs=|0.14-0.24|, ps< 0.05), such that increases in secondary control had pronounced benefits on occasions when individuals were unable to pay for their needs. These findings provide evidence that secondary control may exhibit dynamic shifts in responses to socioeconomic losses and that increases may have pronounced benefits during periods of financial hardship.

Abstract citation ID: igae098.2324

### BODY WEIGHT CHANGE IN OLDER PEOPLE DURING THE COVID-19 PANDEMIC: RESULTS FROM THE BERLIN AGING STUDY II (BASE-II)

Valentin Vetter[1], Johanna Drewelies[2], Sandra Düzel[1], Jan Homann[3], Lars Bertram[4], Vera Regitz-Zagrosek[1], Denis Gerstorf[5], and Ilja Demuth[1], *1. Charité - Universitätsmedizin Berlin, Berlin, Berlin, Germany*, *2. Max Planck Institute for Human Development, Berlin, Berlin, Germany*, *3. University of Münster, Münster, Nordrhein-Westfalen, Germany*, *4. University of Lübeck, Lübeck, Schleswig-Holstein, Germany*, *5. Humboldt University Berlin, Berlin, Berlin, Germany*

Low body weight and especially unwanted weight loss were shown to be associated with higher mortality in older adults. Change in body weight was reported to be one of the unintended side effects of lockdown measures that were put in place to combat the COVID-19 pandemic. In this study, we first describe weight change in 472 participants (mean age: 67.5 years, 48% women) of the Berlin Aging Study-II (BASE-II) that was collected at four timepoints over an average total follow-up time of 10 years. In a second step we examine differences in weight change between groups defined by socio-economic, cognitive, and psychosocial variables as well as morbidity burden, biological aging markers (epigenetic clocks, telomere length), and frailty. During the follow-up period, which includes the first six months of the pandemic, women and men lost on average 0.87% and 0.5% of their body weight per year. A statistically significantly higher rate of weight loss was detected for men with low positive affect, accelerated epigenetic age (7-CpG clock), diagnosed metabolic syndrome as well as a more masculine gender score (all $p < 0.05$). Although the literature generally suggests an increase in body weight during the pandemic, older participants of the BASE-II sample lost 2.5-times (women) and 2-times (men) more weight than expected in this age group. Our explorative analysis of additional variables assessed during an examination directly before the pandemic's onset could inform future studies that aim at a more in depth understanding of the matter.

Abstract citation ID: igae098.2325

### BRIDGING THE DIGITAL DIVIDE: FACTORS INFLUENCING EHEALTH USE AMONG HOMEBOUND OLDER ADULTS DURING THE PANDEMIC

Joonhyeog Park[1], BoRin Kim[2], Joonyoung Cho[3], and Sojung Park[4], *1. School of Social Policy and Practice, University of Pennsylvania, Philadelphia, Pennsylvania, United States*, *2. University of New Hampshire, Durham, New Hampshire, United States*, *3. University of Hawaii, Hawaii, United States*, *4. Washington University in St. Louis, St. Louis, Missouri, United States*

The pandemic has increased the use of eHealth. However, homebound older adults may face a significant digital divide and need support in adopting eHealth. This study investigates to what extent and how access to devices for information and communication technologies (ICTs), previous online experience, and ICT training are associated with eHealth use during the pandemic. Data came from the COVID-19 survey of the National Health and Aging Trend Study. The sample was restricted to homebound older adults aged 70+ who rarely or never left home, or left home with difficulty or assistance (N=591). Hierarchical multinomial logistic regression was used to examine the influence of access to ICT devices, online experience, and ICT training with or without assistance on eHealth engagements including non-users, patient portal users, video telehealth users, and users of both services. Findings indicate that access to ICT devices was positively associated with all forms of eHealth use: patient portal (RRR=3.69, p<.05), video telehealth (RRR=2.56, p<.05), and both (RRR=3.81, p<.05). However, after adding previous online experience and ICT training into the analyses, access to ICT devices was not significant. While previous online experience was a significant predictor for the use of patient portal (RRR=11.97, p<.001), ICT training with assistance significantly increased the probability of the use of video telehealth and both forms of eHealth. This study highlights that access to ICT devices alone does not address eHealth disparities among homebound older adults. Educational interventions and strategies that encourage online engagement are crucial to bridge this gap.

Abstract citation ID: igae098.2326

### CHANGES IN CARE TRAJECTORIES DURING THE COVID-19 PANDEMIC AMONG OLDER ADULTS WITH CARE NEEDS

Amanda Leggett[1], Yulya Truskinovsky[1], Vicki Freedman[2], Emily Wiemers[3], Jennifer Corman[4], and Geoffrey Hoffman[2], *1. Wayne State University, Detroit, Michigan, United States*, *2. University of Michigan, Ann Arbor, Michigan, United States*, *3. Syracuse University, Syracuse, New York, United States*, *4. Jennifer C. Corman Consulting, Colombus, Ohio, United States*

**Background and Objectives:** This study examined whether the progression of care after the onset of an activity limitation changed during the COVID-19 pandemic. Because the pandemic caused widespread disruptions in care, we considered changes in care type and intensity.

**Research Design and Methods:** We used longitudinal data from the National Health and Aging Trends Study between 2015-2022 to examine trajectories of care after the onset of an activity limitation defined as having difficulty or getting help with any self-care or mobility activity or having probable dementia [N=4,191]. Outcomes included use of paid and unpaid help, number of paid and unpaid helpers, hours of paid and unpaid help, living in a residential care setting, living with adult children, and receiving no care. We estimated trajectories of care using mixed effects models and models with individual fixed effects and compared trajectories before and after the onset of the pandemic.

**Results:** We found no changes in whether individuals received any paid or unpaid care but we found a reduction in the number of paid and unpaid caregivers and mixed evidence on changes in hours of paid and unpaid care in the post-pandemic period. We did not find evidence that changes in care trajectories during the pandemic differed by years since the onset of the activity limitation.

**Discussion and Implications:** Reductions in the number of caregivers without a reduction in hours of care implies more intensive caregiving demands among caregivers during the pandemic with potential implications for the well-being of caregivers and care recipients.

Abstract citation ID: igae098.2327

### CHANGES IN SOCIAL CONTACTS AND PSYCHOLOGICAL WELL-BEING AMONG OLDER ADULTS DURING THE PANDEMIC

BoRin Kim, Ke Li, and Chung Hyeon Jeong, *University of New Hampshire, Durham, New Hampshire, United States*

Social interactions are critical for health and well-being among older adults. The pandemic has significantly reduced face-to-face social contacts while promoting remote interactions. This study explored the patterns of changes in in-person and remote social contacts during the first year of the pandemic, and their impacts on psychological well-being among older adults. Also, we examined if remote social contacts could substitute for in-person contacts in terms of psychological well-being. Data came from older respondents (age65+) in the 2021 Health and Retirement Study Perspectives on the Pandemic, focusing on the first-year pandemic experiences (N=4,539). Latent class analysis identified distinct patterns of changes in in-person and remote contacts with diverse social relationships. OLS regressions examined the associations between changes in social contacts and psychological well-being. Four profiles were derived for changes in in-person and remote contacts, respectively. While "Decreased All-Types Contacts" was the largest among in-person contact profiles (36.8%), most of our sample was categorized into "Stable Contacts" among remote contact profiles (64.0%). Decreasing in-person contacts were negatively associated with well-being regardless of the types of relationships. Interestingly, older adults experiencing increased remote contact with friends and/or diverse groups were likely to have worse psychological well-being. Interaction analysis showed that remote social contacts served as an alternative to in-person contacts only for the relationship with friends. Our findings highlight the challenges that older adults faced in maintaining social connections through non-traditional channels during the pandemic and underscore the need for targeted support for older adults in the dynamic changes of social landscapes.

Abstract citation ID: igae098.2328

### COPING STRATEGIES USED BY LATE-MIDLIFE AND OLDER ADULTS DURING THE COVID-19 PANDEMIC

Maria Kurth[1], Heidi Igarashi[2], and Carolyn Aldwin[2], *1. The Pennsylvania State University, University Park, Pennsylvania, United States*, *2. Oregon State University, Corvallis, Oregon, United States*

Despite greater physiological vulnerability during the COVID-19 pandemic, older adults' self-reported mental health better than that of younger adults, perhaps due to use of more adaptive coping strategies. Compared to younger adults, older adults more frequently used strategies such as positive reappraisal (Willey et al., 2022) and fewer maladaptive ones, such as substance use (Park et al., 2020.) However, few studies have examined age differences in coping across later life. We used open-ended responses from an online survey, collected April 28 – May 5, 2020, about how adults 50+ in Oregon (N = 235, Mage = 71.27) coped with a COVID-19 difficulty in the past week. An existing scheme (Aldwin et al., 1996) guided a directed content analysis and age was categorized into three groups: late-midlife (50-64), young-old (65-74), and old-old (75). Nearly all participants (93%) responded to the coping question. The final scheme had eight codes (Krippendorf ∝=.89). The most prevalent strategies were problem-focused (49%), social support (31%), and cognitive reappraisal (27%). Three new strategies were identified: activity substitution (8%), emotion processing (4%), and proactive coping (1%). Emotion processing was more frequently by those in late-midlife (16%), compared to both young-old and old-old participants (2%), p <.01 and.05, respectively. While problem-focused coping was positively correlated with trait resilience, activity substitution was positively related to coping efficacy. Thus, even throughout later life, older adults' use of a variety of adaptive coping strategies to manage problems early on during the COVID-19 pandemic likely contributed to their psychological resilience.

Abstract citation ID: igae098.2329

### DID COVID-19 INCREASE ICT USE AMONG MIDDLE-AGED AND OLDER JAPANESE?

Keiko Katagiri, *Kobe University, Kobe, Hyogo, Japan*

Information and Communication Technology (ICT) is permeating all facets of contemporary life, offering unprecedented convenience. Nonetheless, the digital schism is becoming increasingly pronounced, particularly among the older demographic. Instances where traditional social engagements are hindered, such as the inability to operate ATMs without ICT proficiency, may render older adults socially precarious. Amidst the COVID-19 pandemic, digital interactions supplanted face-to-face engagements. This study aims to scrutinize the surge in ICT adoption among middle-aged and older Japanese adults during the pandemic. An internet survey targeting individuals aged 50-69 residing in the Tokyo and Osaka metropolitan areas was conducted in March 2021. Participants exhibited a notable escalation in smartphone, tablet, personal computer, and WiFi utilization by 26.2%, 14.5%, 21.6%, and 24.3%, respectively. Moreover, 3.5%, 6.5%, 7.9%, and 34.8% of respondents commenced utilizing audio, images, videos, and online platforms, respectively, during the pandemic. Subsequently, multiple regression analyses were performed to discern factors influencing the adoption of these technologies. Participants manifested heightened usage of devices they were already familiar with. Furthermore, favorable evaluation of internet utility, female gender, non-employment status, and initiation of audiovisual media usage during the pandemic were positively associated with increased utilization of the aforementioned ICT devices. In conclusion, COVID-19 has played a pivotal role in mitigating the digital schism. Subsequent research endeavors should explore strategies to bolster middle-aged ICT engagement in the post-pandemic landscape.

Abstract citation ID: igae098.2330

### EFFECTS OF RESTRICTIONS IN COMMUNITY-BASED CARE SERVICES ON FAMILY CAREGIVERS DURING THE COVID-19 PANDEMIC

Ayumi Honda[1], Yin Liu[2], Elizabeth Fauth[2], and Sumihisa Honda[3], *1. St. Mary's College, Kurume, Fukuoka, Japan, 2. Utah State University, Logan, Utah, United States, 3. Nagasaki University, Nagasaki, Nagasaki, Japan*

This mixed-methods study aimed to examine the effects of restrictions in the utilization of community-based care services on family caregivers during the COVID-19 pandemic. We recruited from community-based care services 200 family caregivers who were living with an older care recipient. We first conducted thematic analyses based on open-ended responses to questions regarding the impact of restrictions in the utilization of community-based care services. We then extracted themes to examine and determine patterns across caregiving characteristics. Next, we conducted linear regression analysis to examine associations between impact of restrictions and caregiving contexts. Findings from the thematic analyses identified 11 themes regarding the impact of restrictions in the utilization of community-based care services. Most frequently reported impact included "the family caregiver cannot bathe the older person at home" and "the family caregiver cannot go to work/income decreases". Additionally, findings from regression analysis suggested that caregiver employment ($\beta$ = 0.292, SE = 0.122, p =.02), increased caregiving tasks ($\beta$ = 0.134, SE = 0.051, p =.01), and more behavioral and psychological symptoms of dementia ($\beta$ = 0.088, SE = 0.037, p =.02) were associated with harmful impacts resulting from restrictions in the utilization of community-based care services. To conclude, family caregivers identified common areas where they felt ill equipped to provide home care during the COVID-19 pandemic. The study informs priorities areas of needed external support, resources, or training that family caregivers may need in planning short- or long-term emergency preparedness.

Abstract citation ID: igae098.2331

### HOW OLDER REFUGEES IN THE US COPED DURING COVID-19 PANDEMIC: A QUALITATIVE RESEARCH STUDY

Jonix Owino, *Sacred Heart University, Stratford, Connecticut, United States*

Due to the novelty experience posed by COVID-19, it is important to understand how older refugees coped in their host communities. This is because older refugees have increased vulnerabilities compared to host populations such as limitations related to healthcare accessibility, language barriers, and discrimination, that may have heightened the challenges posed by COVID-19. As such, the present study examined implications of COVID-19 among older refugees and how they coped. The present study recruited thirty-seven Congolese refugees, aged 55-67 (m=65.7 years) from a community in the East Coast region of the US to participate in interviews on their perceptions of life during COVID-19. The interviews lasted between 35-65 minutes. The qualitative interviews were analyzed using thematic content analysis. Emergent themes showed that though older Congolese refugees in the US experienced COVID-19 challenges similar to other populations, these challenges seemed salient for older refugees compared to non-refugee populations due to refugee background and experiences. For instance, older refugees experienced increased social isolation due to limited access to language interpreters. Moreover, older refugees' coping strategies during COVID-19 e.g., the use of traditional/herbal medicine and cognitive reframing seemed to have been influenced by the interplay of factors related to their unique refugee experiences and their cultural norms and values. Understanding the implications of COVID-19 among older refugees in the US is beneficial to practitioners in determining how they may assist their clientele in coping with specific situations, considering their specific histories and cultures.

Abstract citation ID: igae098.2332

### INCREASED RISK OF COVID-19 INFECTION IN PEOPLE WITH DEPRESSION: EVIDENCE FROM ANALYSIS OF SHARE'S CORONA SURVEYS

Xiaona He, and Wei Gao, *Nanchang University, Nanchang, Jiangxi, China (People's Republic)*

The mental disorder related COVID-19 infection remains of wide interest. However, there is a shortage of population-based studies on depression. To examine the association between a pre-existing depression and COVID-19 infection risk among middle-aged and older Europeans during the COVID-19 pandemic. A population-based retroprospective longitudinal study. Data were from the Survey of Health, Ageing and Retirement in Europe (SHARE), respondents 50 years and older from 26 European countries and Israel were included. Analysis was undertaken at the first and second years of the COVID-19 pandemic. Depression was assessd by the EURO-D scale. Outcomes were the COVID-19 infection during the period of COVID-19 pandemic. We used logistic regression to assess the association of pre-existing depression with the COVID-19 infection. Individuals who had a pre-existing depression had an increased risk for COVID-19 infection in SCS1 (OR=1.52, 95% CI: 1.28-1.79, P < 0.0001) and SCS2 (OR=1.24, 95% CI: 1.13-1.37, P < 0.0001). Among individuals with depression, obesity(BMI≥30) had higher risk than those with BMI≤30; OR=1.23, 95% CI: 1.03-1.46, P=0.021). Unvaccinated individuals with depression had higher risk than vaccinated individuals, with the strongest vaccination disparity for depression (OR=1.99, 95% CI: 1.65-2.39, P< 0.0001). Individuals with depression as being at increased risk for COVID-19 re-infection. (OR=1.68, 95% CI: 1.13-2.49, P< 0.0001). Depression may become an important factor affecting the population of middle-aged and older people infected with COVID-19, suggesting a need for more attention to the population. This includes providing mental health support and counseling services to reduce their symptoms of depression.

Abstract citation ID: igae098.2333

### INTIMATE PARTNER HOMICIDE SUICIDE IPHS EVENTS AND MEDIA COVERAGE: PANDEMIC COMPARED TO PREPANDEMIC YEARS

Sonia Salari, Emma Videla, and Carrie Sillito, *University of Utah, Salt Lake City, Utah, United States*

Intimate partner homicide-suicide IPHS, involves the murder of a current or former partner, followed by the offender's suicide. We content analyzed our news surveillance data over the modern pre-pandemic years (2018, 2019) and pandemic years (2020-2022). In 2020, U.S. domestic violence went up 8% and the FBI reported a 'murder surge.' Lockdown reduced COVID contagion, but also isolated people in their homes, some with abusers. FBI NICs data on background checks show a pandemic run on firearm acquisitions in U.S. households. We collected articles, TV news, police reports, and obituaries for n=725 IPHS events, representing over 1600 deaths. Age categories are young (18-44, n=256), middle (45 to 59, n=176) and elder adult cases, age 60+ (n=293). Evidence suggests gender-based violence in the form of femicide, as the vast majority (95%) were male perpetrated. Our research questions: 1) Did 2020 have more IPHS? and 2) What qualitative changes in IPHS media coverage happened during the pandemic? Our results found a dip in IPHS for the year 2020, but increasing violence in 2021 and 2022 (peak)—particularly for the young. We suggest a "crisis effect" for IPHS in 2020 and then a surge. Pandemic gun sales were greater among the young. Media coverage of IPHS varied by age. Elder adults had significantly fewer cases with names announced in news media, especially during the pandemic. This represents ageism, because the young and middle-aged had almost none who went unannounced. Domestic violence victims of all ages deserve recognition.

Abstract citation ID: igae098.2334

### LONG-TERM PANDEMIC EFFECTS ON SOCIAL ACTIVITY AND LONELINESS AMONG OLDER ADULTS IN AN URBAN COMMUNITY

Rebecca Lindsay[1], Angela Gaye[2], Meosia Lee-Turner[2], Yolanda Hill-Ashford[2], Robin Brewer[1], Sheria Robinson-Lane[1], and Mary Janevic[1], *1. University of Michigan, Ann Arbor, Michigan, United States*, *2. Detroit Health Department, Detroit, Michigan, United States*

The COVID-19 pandemic sharply curtailed social activity among older adults. In Detroit, an underserved area impacted by structural inequities, older adults were at particularly high risk for social isolation, given the digital divide and prolonged closures of senior and community centers. While daily life has now mostly normalized, little is known about the extent to which older adults have re-engaged in social activities following the lengthy disruption. We examined baseline data collected in 2023-24 from an ongoing randomized controlled trial in Detroit of the RESET intervention (Re-Engaging in Self-care, Enjoying Today). Participants rated their level of 1) loneliness and 2) social activity compared to pre-pandemic (1=much lower to 5=much higher). The sample (N=213) was 91% female and 85% African American, mean age 68 years (range 50 to 90), and 54% lived alone. Almost half (46.0%%) of the sample reported that their current social activity level was much lower or lower than pre-pandemic, and 34.8% that their loneliness level was much higher or higher. No significant differences were found by age group (under 65 vs 65+) or living alone vs. with others. We then examined open-ended responses to a question about continued pandemic impact. Major themes were: ongoing fear of COVID-19 infection, caution about gatherings/crowds, and persistent mood problems. These data show that for some older adults, the pandemic continues to have adverse effects on social activity and loneliness. Targeted interventions that address and accommodate older adults' ongoing COVID-19 concerns may be needed to boost social functioning in this group.

Abstract citation ID: igae098.2335

### OLDER ADULTS' BEHAVIOR IN SEEKING SERVICE INFORMATION DURING THE EARLY COVID-19 PANDEMIC

Leyi Zhou[1], Cheng Ren[2], Christine Wei-Mien Lou[3], and Julian Chun-Chung Chow[1], *1. University of California Berkeley, Berkeley, California, United States*, *2. University at Albany, SUNY, Albany, New York, United States*, *3. San Francisco Human Services Agency, San Francisco, California, United States*

This study examines the expanding digital divide affecting older adults in the context of the COVID-19 pandemic. It focuses on exploring the factors that influence how older adults access information during COVID-19, emphasizing the challenges faced by those from disadvantaged backgrounds due to limited technology experience. Utilizing survey data conducted in May 2020 from the San Francisco Human Services Agency, which includes responses from 3,255 older adults in households receiving public benefits, this research employs logistic regression analysis to examine how demographic factors—such as race/ethnicity, education level, and primary language—impact the ways in which older adults access information during the pandemic. The analysis encompasses a range of information sources, including both digital and traditional media, to uncover disparities in access. The results unveil stark disparities: Black older adults exhibited significantly lower odds of accessing information via online news and email compared to their White counterparts, indicating a pronounced digital exclusion. Conversely, Chinese older adults demonstrated a higher propensity for social media engagement, suggesting cultural and community-based differences in information dissemination preferences. Education emerged as a critical determinant, with older adults possessing less formal education showing diminished access to digital information sources. These nuanced findings highlight the critical need for targeted interventions aimed at bridging the digital divide. By tailoring educational and training initiatives to the unique needs of diverse older adult populations, the study underscores the importance of ensuring equitable access to essential information, thereby empowering all older adults to navigate public health crises more effectively.

Abstract citation ID: igae098.2336

### PROBLEMS AND DISTRESS AMONG OLDER ADULTS DURING THE COVID-19 PANDEMIC: A LONGITUDINAL THEMATIC ANALYSIS

Carolyn Aldwin[1], Heidi Igarashi[1], Maria Kurth[2], and Soyoung Choun[1], *1. Oregon State University, Corvallis, Oregon, United States*, *2. The Pennsylvania State University, University Park, Pennsylvania, United States*

Despite their greater physical risk and social isolation during the COVID-19 pandemic, older adults reported better mental health and less stress than younger adults, perhaps because they often under-report mental health symptoms and stress in quantitative studies. However, only two small qualitative studies examined stress over time older adults during the pandemic. We examined longitudinal qualitative data on weekly COVID-related problems from internet surveys over an eight-week period early on during the pandemic; 236 out of 249 (95%) respondents reported any problems; of these, 208 (97%) reported problems at three or more time points (nobs=1495, 6.07, SD=2.54, range=3-8). The sample ranged in age from 55-95 (M=71.34, SD=7.29); 74% female, 89% white, and 54% BA+. Using content analysis, we identified 15 codes (Krippendorf's cu-alpha =.714). Across time, most respondents (76%) described consistent types of difficulties (> 50% of the episodes); problems with everyday protective activities (restrictions, mask wearing) was most common (28%), followed by social connections (missing friends and family; 18%) and psychological distress (17%), often expressed in everyday language, e.g., "cabin fever," "grinding uncertainty," or through diacritics (e.g., all caps, multiple exclamation points). However, thematic analysis showed that 27% changed over time, with 7% describing greater social isolation and 9% increasing psychological distress, balanced by 11% who showed increasing adjustment (managing risk). The latter two codes often co-occurred; perhaps navigating an ever-changing COVID landscape increased uncertainty and anxiety. This analysis provided a more nuanced view: problems were near universal in older adults and distress not uncommon.

Abstract citation ID: igae098.2337

### PSYCHOLOGICAL IMPACT OF INCREASING HATE CRIMES AGAINST ASIAN AMERICANS AND PERCEPTIONS OF ACTIVISM DURING COVID-19

Eunhye Kim[1], Y.Joon Choi[2], and Candace Griffith[1], *1. Augusta University, Augusta, Georgia, United States*, *2. Georgia State University, Atlanta, Georgia, United States*

**Objectives:** The purpose of this study is to examine how Asian hate crimes impact Korean American older adults' daily life and their perception of social advocacy during COVID-19.

**Background:** In addition to the health crisis, Asian Americans have experienced more risk and challenges during the pandemic due to the explosion of anti-Asian hate crime incidents. Violence against older Asian Americans increases fear and anxiety not only for individuals directly affected but for entire Asian communities. In this regard, understanding older Asian Americans' perception of hate crimes against Asians and their attitudes about social advocacy can help guide support and services for this population.

**Methods:** A qualitative approach was used for this study, including semi-structured interview guides with 23 Korean American older adults. Data organization was conducted with Nvivo12, employing thematic analysis to identify key themes and sub-themes.

**Results:** The findings showed that participants believed that rising anti-Asian hate crimes have restricted Asian American's daily activities by increasing fear and feelings of being perpetual foreigners in American society. Participants believed that social action is necessary to change society, but they still hold passive attitudes on social action and have a lack of resources. They believed leaders and other powerful figures in the community rather than individuals should address social problems.

**Conclusion:** Findings from this study highlight the psychological impact of anti-Asian hate crimes and the importance of expanding support for ethnic minority older adults to voice out and promoting increased advocacy for this population.

Abstract citation ID: igae098.2338

### RACIAL DISPARITIES IN PSYCHOLOGICAL WELL-BEING ACROSS AGE GROUPS DURING COVID-19: A LONGITUDINAL STUDY

Meghan Elliott, Susan Charles, Roxane Cohen Silver, and E. Alison Holman, *University of California Irvine, Irvine, California, United States*

COVID-19 created a health threat that discriminated by age: older adults were at greatest risk for health complications and mortality, but younger adults reported the highest rates of emotional distress. Cross-sectional studies consistently documented age differences in distress, but few studies have examined age-related differences over time. We examined how self-reported anger and both depressive and anxiety symptoms changed throughout the first two years of the COVID-19 pandemic among a national representative sample of adults (N = 6514) aged 18 to 97 years who completed four surveys between March 2020 and June 2022. Multi-level models revealed age differences that varied by race. Overall, younger age and non-white status were related to higher levels of every affective distress measure. For depressive symptoms, most age groups peaked at wave 2 and declined thereafter. The exception was the young non-white adults, where levels peaked at wave 2 and remained stable thereafter. For most participants, anxiety symptoms were highest at wave 1 and declined thereafter, although young non-white participants had minimal decreases and levels increased among middle-aged and older adults across race. Anger symptoms peaked for all age groups at wave 2, yet older white adults were the only group whose anger levels did not decrease from wave 2 to wave 4. Results suggest that non-white status and young age may be vulnerabilities to symptoms of depression and anxiety, and being old and white is related to stable levels of anger across time, highlighting the need for targeted support for these groups.

Abstract citation ID: igae098.2339

### THE COVID-19 PANDEMIC AND OBJECTIVE SOCIAL ISOLATION AMONG THE OLDER ADULTS IN THE UNITED STATES

Jiamin Yin[1], and Christopher Seplaki[2], *1. University of Rochester, Rochester, New York, United States*, *2. University of Rochester School of Medicine and Dentistry, Rochester, New York, United States*

Experiences of social isolation are common among older adults and were amplified by the COVID-19 pandemic, particularly among those aged 80+. However, we do not know if such age differences in the experience of social isolation are due to the pandemic or if the underlying mechanisms driving social isolation may differ across age groups. We use a difference-in-difference design to compare the change in social isolation before and after 2020 across older adults aged 80+ versus those 65-79 years using data from the National Health and Aging Trends Study to constructed a composite measure of objective social isolation that includes four domains (living arrangement, communication regarding important matters, participation in religious and other activities). Our preliminary results suggest that adults aged 80+ years were found 3.7% (Standard Error (SE): 0.014) more likely than those aged 65-79 years to experience social isolation in 2020. Additional analysis of the four social isolation domains suggest that "older" older adults were 6.8% (SE: 0.016) more likely to report no participation in other activities than the "younger" older adults in 2020. In contrast, they were 3.5% (SE: 0.010) less likely than the "younger" older adults to live alone in 2020. No significant effect was observed in other domains. Our preliminary findings highlight the greater vulnerability of adults aged 80 years and the potential importance of developing interventions to address social isolation among this subgroup of vulnerable older adults.

Abstract citation ID: igae098.2340

### THE IMPACT OF EDUCATIONAL LEVEL ON FOOD SECURITY AMONG OLDER ADULTS AMID THE COVID-19 PANDEMIC

Lillie Monroe-Lord, Azam Ardekani, Phronie Jackson, and Cassidy Weitman, *University of the District of Columbia, Washington, District of Columbia, United States*

The COVID-19 pandemic has dramatically altered global health and economic landscapes, significantly threatening food security worldwide. This study investigates the pandemic's impact on perceived food security among older adults, with a particular focus on the role of educational attainment. A total of 4,961 participants aged 65 and above were surveyed between August 9 and September 15, 2020, utilizing the USDA's Six-Item Short Form via Qualtrics to measure food security. The survey, conducted retrospectively twice, assessed higher scores as indicative of increased food insecurity among varying educational backgrounds: less than a high school education, some college education, and a college or graduate degree. Findings indicated older adults with less than a high school diploma were most affected, experiencing a mean percentage increase in food insecurity of 7.7% ($p = 0.007$). Respondents with some college education saw a 7.03% increase, which was not statistically significant ($p > 0.05$). Conversely, older adults with a college or graduate degree reported a smaller, yet statistically significant, increase in food insecurity (4.06%, $p = 0.041$). These results highlight a correlation between educational level and food security, with lower levels of education being associated with a greater risk of food insecurity during the COVID-19 pandemic. This study underscores the need for enhanced support systems to protect the most vulnerable populations, especially the less educated older adults, from the adverse effects of global health emergencies on food security.

Abstract citation ID: igae098.2341

### THE PANDEMIC AND DIGITAL DIVIDE FOR OLDER ADULTS: LONGITUDINAL ANALYSIS FROM THE NATIONAL HEALTH AGING TRENDS STUDY

BoRin Kim[1], Joonhyeog Park[2], Joonyoung Cho[3], and Sojung Park[4], *1. University of New Hampshire, Durham, New Hampshire, United States*, *2. School of Social Policy and Practice, University of Pennsylvania, Philadelphia, Pennsylvania, United States*, *3. University of Hawaii, Hawaii, United States*, *4. Washington University in St. Louis, St. Louis, Missouri, United States*

Older adults have been disadvantaged in utilizing information and communication technologies (ICTs). The pandemic has underscored the importance of ICTs in facilitating remote activities such as social connectivity, banking, and shopping. This study explored transitions of ICT use among older adults and investigated the impact of the pandemic on the digital divide. Data came from five waves of the National Health and Aging Trend Study (2018~2022), and our sample included respondents over 70 years at the baseline (N=1,717). Latent transition profile analysis was used to identify distinct subgroups of ICT use and to analyze the change and stability of subgroups based on five ICT use measures: emails, online search, online grocery shopping, Internet banking, and social media. Multinominal regression analyses were used to examine the factors impacting the transitions of ICT use. Four distinct clusters were identified: minimal, email-only, moderate, and diverse/heavy users. The largest subgroup was minimal users (42~45%), followed by diverse/heavy users (25~28%). More than 90% of older adults in these two groups stayed in the same group over time, which shows a significant digital divide. Email-only users were characterized by a stronger tendency toward moderate users (transition probabilities=20~27.5%). While socioeconomic status was a significant predictor of the digital divide before the pandemic, social support was found to be critical to helping older adults use more diverse and frequent ICTs after the pandemic. Our empirical findings provide evidence of exacerbating the digital divide during the pandemic and highlight the importance of social support in reducing the digital divide.

Abstract citation ID: igae098.2342

### THE PANDEMIC AS A GREAT UNEQUALIZER? RURAL–URBAN DIVIDE IN COVID-19 CONCERNS AND OLDER ADULTS' MENTAL HEALTH IN CHINA

Jingwen Liu[1], and Yang Han[2], *1. Rice University, Houston, Texas, United States*, *2. The Chinese University of Hong Kong, Hong Kong, China (People's Republic)*

Hukou is a household registration system that divides individuals to rural or urban residents at birth based on their parents' status, which significantly contribute to health disparities in China. This paper investigates whether older adults' concerns about the Covid-19 pandemic exacerbate rural-urban disparities and the potential mechanism of coping behaviors. Using the 2020 wave of China Health and Retirement Longitudinal Study (CHARLS), we employed OLS and KHB analyses to local rural residents (rural-Hukou holders living in rural areas, N=9,101), rural-urban migrants (rural-Hukou holders living in urban areas; N=2,418), and local urban residents (urban-Hukou holders living in urban areas, N=2,874), respectively. Results suggest that local rural residents and rural-urban migrants both show substantially higher Covid concerns and depressive symptoms than local urban residents. Covid concerns are associated with higher depressive symptoms among all groups, but the association is statistically more prominent among local rural than local urban residents. While changes in health habits and in-person meetings contribute to explaining these associations for both groups, the delayed medical services and increased preventive behaviors exclusively explain the association for local rural residents. Rural-urban migrants exhibit a dual profile: they demonstrate higher concerns like rural residents while adopting fewer coping behaviors parallel to urban residents. This paper confirms that Hukou acts as a fundamental cause of rural-urban health disparities that will be reproduced and reinforced during societal crises like the Covid pandemic. Rural-born individuals' health disadvantages may be reduced but not eliminated by the migration to urban areas without substantial reforms of Hukou system.

Abstract citation ID: igae098.2343

### UNMASKING THE CONSEQUENCES OF THE COVID-19 PANDEMIC ON COGNITION AND 24-HOUR BEHAVIORS: INSIGHTS FROM THE CLSA

Ryan Falck[1], Teresa Liu-Ambrose[2], Megan O'Connell[3], Eric Smith[4], Lauren Griffith[5], Benoit Cossett[6], David Hogan[4], and Christina Wolfson[7], *1. University of British Columbia, Vancouver, British Columbia, Canada*, *2. University of British Columbia, University of British Columbia, British Columbia, Canada*, *3. University of Saskatchewan, Saskatoon, Saskatchewan, Canada*, *4. University of Calgary, Calgary, Alberta, Canada*, *5. McMaster University, Hamilton, Ontario, Canada*, *6. University of Sherbrooke, Sherbrooke, Quebec, Canada*, *7. McGill University, Montreal, Quebec, Canada*

An unintended side-effect of the COVID-19 pandemic has been changes in lifestyle factors which impact middle-aged and older adult cognition – including changes in 24-hour behaviours (i.e., physical activity, sedentary behaviour, and sleep). In a longitudinal analysis of the Canadian Longitudinal Study on Aging (CLSA) tracking cohort, we explored age- and sex-differences in the effects of the COVID-19 pandemic on cognition and 24-hour behaviours, and whether pandemic-related changes in 24-hour behaviours and cognition are associated. We included cognitively healthy participants at baseline (2012-2105), follow-up 1 (FU1; 2015-2018), and follow-up 2 (FU2; 2018-2021), with complete neuropsychological testing data (N=11,355). Cognition and 24-hour behaviours were indexed at each timepoint. Participants were categorized into pre-pandemic (N=6,174) and post-pandemic (N=5,181) cohorts based on whether FU2 assessments occurred before or after COVID-19 pandemic onset (March 11th, 2020). We examined time x cohort changes in cognition and 24-hour behaviours from FU1 to FU2, and if changes in 24-hour behaviours from FU1 to FU2 were associated with changes in cognition. All models were allowed to vary by age and sex. Our results indicated that post-pandemic cohort males and females aged 65+ years had significantly worse cognition and poorer 24-hour behaviours from FU1 to FU2 than their peers in the pre-pandemic cohort (p's< 0.05). However, changes in 24-hour behaviours from FU1 to FU2 were unassociated with changes in cognition, irrespective of age or sex. Our results highlight that the pandemic negatively impacted 24-hour behaviours and cognition in older adults – although these effects may be unassociated with each other.

Abstract citation ID: igae098.2344

### UTILIZATION OF THE LONG COVID DIAGNOSIS CODE IN US NURSING HOME RESIDENTS

Yasin Abul, Daniel Harris, Preeti Chachlani, Kaley Hayes, Vincent Mor, and Stefan Gravenstein, *Brown University, Providence, Rhode Island, United States*

The National Center for Health Statistics implemented the ICD-10 code U09.9 for "Long COVID" or "post COVID-19 condition" on October 1st, 2021. Overall utilization, trends, and variation in the use of the U09.9 ICD code in long term care (LTC) residents remains understudied. We therefore conducted a serial cross sectional study using the COVVAXAGE database, by linking CVS Health and Walgreens' customers data to the 100% Medicare enrollment files. Monthly, from October 2021 through April 2023, we identified individuals living in LTC and enrolled continuously in Medicare FFS for 12 months. We used Medicare Part A and B claims to identify long COVID (U09.9). We estimated the monthly occurrence of a long COVID code among all LTC residents and stratified by a recent (8 weeks) and latent (52 weeks) diagnosis of COVID-19, and by region. We identified over 200,000 LTC residents each month (mean age = 82.8; 69% women; 79.4% White; 11.9% Black). The overall long COVID coding rate was lowest in April, 2023 (1 per 10,000 residents) and highest in February, 2022 (14 per 10,000 residents). Residents with recent COVID-19 diagnosis had the highest long COVID coding rate (e.g., 58 per 10,000 residents) in January 2023. Temporal trends in long COVID coding rates varied widely by geography and were cyclical, following epidemic trends in LTC residents. Long COVID U09.9 ICD code documentation rates were generally low and varied in LTC residents. Remarkable variation in coding rates by region, urbanicity, and frailty status occurred, and needs further study.

### SESSION 7035 (POSTER)

### CROSS-CULTURAL/CROSS-NATIONAL STUDIES

Abstract citation ID: igae098.2345

### A US–CHINA COMPARISON ON THE DETERMINANTS OF RESIDENTIAL RELOCATION AND ASSOCIATED MORTALITY RISK

Dexia Kong[1], and Peiyi Lu[2], *1. The Chinese University of Hong Kong, Hong Kong, China (People's Republic)*, *2. The University of Hong Kong, Hong Kong, China (People's Republic)*

Many middle-aged and older adults relocate to pursue a better living environment. However, existing studies have inconclusive evidence on the determinants of relocation and its health implications, as well as lack a cross-national perspective. This study sought to compare the US and China in examining the determinants of residential relocation and its associated mortality risk. Longitudinal data from the US Health and Retirement Study in 2010-2018 and from China Health and Retirement Longitudinal Study in 2011-2018 were used (NUS=20,292, NChina=11,694). Community-dwelling respondents (aged 50+) reported whether they had relocated and were followed up until 2018. Log-binomial regression model investigated the significant predictors of relocation. Cox survival analysis examined the relationship between relocation and all-cause mortality, adjusting for baseline covariates and accounting for selection bias. About 19% American and 17% Chinese respondents relocated between communities over the study period. The common positive predictors of relocation in both countries were younger age, higher education, urban residence, and renting (relative risk>1, P< 0.05). Relocation was associated with lower mortality risk in both countries (US: HR=0.63, 95%CI=0.57, 0.70; China: HR=0.40, 95%CI=0.32, 0.50), and this association was significantly stronger in China compared to the US. (Prelocation×country=0.0009). The determinants of relocation in the US and China showed similarities, aligning with the person-environment framework. Residential relocation was linked to increased survival advantages in both countries, probably because old adults who relocated can make informed and voluntary decisions to relocate and adjust effectively following the move. Pre- and post-relocation interventions are suggested to facilitate the process of moving.

Abstract citation ID: igae098.2346

### CHOICES AND EXPECTATIONS OF END-OF-LIFE HEALTH CARE PROXY: A COMPARATIVE STUDY BETWEEN TAIWAN AND THE UNITED STATES

Duan-Rung Chen[1], and Winston Tseng[2], *1. National Taiwan University, Taipei, Taipei, Taiwan (Republic of China)*, *2. University of California Berkeley, Berkeley, California, United States*

**Objective:** This research compares healthcare proxy preferences and expectations in Taiwan and the United States, highlighting cultural impacts on end-of-life care planning.
**Methods:** From August 2022 to October 2023, the study used Survey Cake for online responses, employing snowball sampling. It compared 345 Taiwanese and 339 Californian respondents, matched by gender and age.
**Results:** Despite similar demographics, distinct preferences emerged. A majority (75.5%) were under 30. In the US, 89.7% had higher education versus 75.2% in Taiwan. More Americans were married (19.2%). Preferences for healthcare proxies varied: Taiwanese favored parents, while Americans chose spouses or partners. A higher percentage of Taiwanese (61.8%) were willing and believed that their family members acting as proxies could meet their expectations, significantly higher than American respondents (48.2%). More American respondents (47.2%) expressed unwillingness to have family members make end-of-life decisions for them, contrasting sharply with Taiwanese respondents (24.2%). Regarding specific end-of-life medical decisions made by family members, Taiwanese respondents significantly favored letting healthcare proxies handle all medical decisions, including discontinuing life support, authorizing or refusing necessary pain medication or surgery among six decisions. Americans (92.7%) were more amenable to legal actions for personal wishes, markedly more than Taiwanese (66.2%).
**Conclusion:** The study reveals significant cultural divergences between the US and Taiwan in choosing end-of-life decision-makers, reflecting deeper values and trust in the family. It underscores the importance of acknowledging cultural diversity in healthcare preferences and decisions, advocating for broader research perspectives.

Abstract citation ID: igae098.2347

### CROSS-CULTURAL DIFFERENCES IN RECRUITING OLDER ADULTS IN DESIGN RESEARCH ACTIVITIES: INSIGHTS FROM CHINA AND THE UK

Tongxin Sun[1], Yuanyuan Liu[2], Qiong Zhang[3], Jiabei Jiang[4], Xueying Xiong[5], Juncheng Wen[6], Lynne Corner[5], and Jihong Jeung[1], *1. Tsinghua University, Beijing, Beijing, China (People's Republic)*, *2. School of Mechanical Engineering & Automation, Beihang University, Beijing, Beijing, China (People's Republic)*, *3. School of Mechanical Engineering & Automation, Beijing, Beijing, China (People's Republic)*, *4. Nanjing university of the arts, Nanjing, Jiangsu, China (People's Republic)*, *5. UK's National Innovation Centre for Ageing (NICA), Newcastle Upon Tyne, England, United Kingdom*, *6. Newcastle University, Newcastle upon Tyne, England, United Kingdom*

Engaging older adults in the design process of products and services is essential for enhancing accessibility, usability, and the overall impact of human-centered design research focused on aging populations. Achieving successful recruitment of older adults is pivotal for their meaningful participation. Yet, the post-pandemic landscape has brought significant challenges in engaging and recruiting this demographic, underscoring the complexity of meeting their emotional, social, and cognitive needs during the recruitment phase. Furthermore, existing research predominantly concentrates on recruitment within health intervention and clinical studies, offering limited insight into effective recruitment strategies in design research across diverse socio-cultural and economic settings. Our research employed mixed-methods studies in China and the UK, incorporating surveys, interviews, and researcher immersion to investigate cross-cultural differences in recruiting older adults. We explored their attitudes, needs, and preferences, revealing the significance of treating the recruitment process as a service provided to prospective older participants. Our findings identify key socio-cultural factors—including personal identity, regional context, value systems, technology adoption, and social networks—that influence older adults' experiences during recruitment. The reconfiguration of these factors may be required to design recruitment services and improve the participation experience. This research offers insights into cross-cultural strategies for effectively engaging and recruiting older adults in design research. By highlighting the importance of inclusivity and diversity, our work contributes to the advancement of human-centered design practices, ensuring they resonate more profoundly with aging populations.

Abstract citation ID: igae098.2348

### ON THE RELATIONSHIP BETWEEN SUBJECTIVE AGE AND GRIP STRENGTH IN OLDER POPULATIONS

Omar Paccagnella, *University of Padova, Padova, Veneto, Italy*

Subjective age is the extent to which an individual feels younger or older than his/her chronological age. A large amount of literature shows that feeling younger is associated with better physical, psychological and cognitive outcomes. In this work, we exploit the richness and the comparability of three sister surveys on ageing (HRS, ELSA, SHARE, using both the Gateway to Global Aging Data platform and the original datasets) to investigate the relationship between subjective age and handgrip strength, that can be thought as an objective measure of physical health. Three different countries (UK, US, Israel) are separately analyzed and data on subjective age are collected in three different time periods (2004, 2008, 2015, respectively). The common framework is provided by the grip measurements that are collected four years later than subjective age answers in each survey. A set with the same harmonized characteristics across all countries is identified and used in the final estimations. The Heckman selection model is the adopted solution, to take sample selection bias (due to attrition) into account. Models are estimated by gender. The main results of our analysis are: i) controlling for selection bias, no statistically significant relationship between subjective age and grip strength appears in any country, reversing many findings we can observe when selectivity effects are neglected; ii) subjective age can be a promising exclusion variable when a selection approach is adopted (individuals who feel younger than their chronological age are more likely to collect data in subsequent survey waves).

Abstract citation ID: igae098.2349

### SENSORY DIFFICULTIES AND SOCIAL ISOLATION AMONG MEXICAN, US HISPANIC AND, US NON-HISPANIC INDIVIDUALS

Corinna Trujillo Tanner[1], Jeremy Yorgason[1], Rebekah Case Fankhouser[1], Jeana Olmo[1], Jase Wanlass[1], Kyriakos Markides[2], Joshua Ehrlich[3], and Markus Wettstein[4], *1. Brigham Young University, Provo, Utah, United States*, *2. The University of Texas Medical Branch, Galveston, Texas, United States*, *3. University of Michigan, Ann Arbor, Michigan, United States*, *4. Humboldt University Berlin, Berlin, Berlin, Germany*

The Hispanic cultural value of Familismo describes strong identification and attachment of individuals with their families and is protective against social isolation. Hispanics comprise the largest minority group in the US and are more likely to live in intergenerational households. Sensory difficulty, including vision and hearing difficulty, are prevalent among older adults, disproportionately affecting Hispanic individuals. Research shows that aging and sensory difficulty are associated with an increased risk of social isolation. Using data from adults age 50 to 100 that participated in the Mexican Health and Aging Study, and Hispanic and non-Hispanic participants of the Health and Retirement Study, we explored how sensory difficulty related to social isolation. Social isolation was measured by assessing whether or not participants lived alone, had weekly contact with children and friends/relatives, and if they participated in weekly social activities. Findings suggested that vision and hearing difficulty rates were much higher among Mexican and US Hispanic individuals compared to US non-Hispanics. In contrast, social isolation was higher among US non-Hispanic individuals. Regression models with sensory difficulty predicting social isolation by country/ethnicity indicated that vision difficulty was associated with social isolation for Mexican and US non-Hispanic individuals, but not for US Hispanics. Hearing difficulty was associated with social isolation only for US non-Hispanic individuals. Among Mexican and US Hispanic individuals, it appears that age accounts for social isolation more so than sensory difficulty, and Familismo appears to protect against isolation. Among US non-Hispanics, sensory difficulty contribute to social isolation to a greater extent.

### SESSION 7040 (POSTER)

### DEMENTIA I (POSTERS)

Abstract citation ID: igae098.2350

### ASSESSING THE INDIVIDUAL NEEDS AND GOALS OF DEMENTIA CAREGIVERS: DEVELOPMENT OF THE COPE-ING MEASURE

Alyssa Aguirre, Jared Benge, and Andrew Samson, *The University of Texas Austin, Austin, Texas, United States*

Caregivers of persons living with dementia (PLWD) are diverse, yet there is a shortage of measures that efficiently identify the personalized needs and goals of a given caregiver. To address this gap, this study outlines the development of an Individualized Needs and Goals assessment for use with the Caregiver Outcomes of Psychotherapy Evaluation (COPE-ING). In phase 1, 52 participants (N=28 caregivers; N=24 dementia professionals) were recruited to provide feedback on the preliminary COPE-ING measure. Participants rated each item on a Likert scale (1- Not important at all to 5-Extremely Important) on how important they felt each item is to dementia caregivers. While all items were endorsed as important, qualitative feedback emphasized the need for simpler language, substituting 'cognitive impairment' with 'dementia' and providing specific examples of services. A revised version of the COPE-ING was subsequently administered in an ongoing clinical sample. In preliminary findings, initial analysis revealed great heterogeneity in caregiver perceived top priorities for care, with 30% selecting learning more about dementia as a top priority, but a wide variety of topics being selected as top priorities for the remainder of the sample. These findings outline the initial development of the COPE-ING, a novel measure designed to capture the individualized needs and goals of dementia caregivers. This study underscores the importance of creating caregiver measures that extend beyond burden. The COPE-ING holds promise for use in evidence-based interventions to facilitate the rapid identification of caregiver needs and goals in hospital or community settings.

Abstract citation ID: igae098.2351

### CAREGIVER PHYSICAL HEALTH AND RISK OF ABUSE AND NEGLECT AMONG PERSONS LIVING WITH DEMENTIA

Henrietta Bennett[1], Wesley Browning[2], Mustafa Yildiz[3], and Carolyn Pickering[3], *1. University of Alabama at Birmingham, Birmingham, Alabama, United States*, *2. The University of Texas Health Science Center Houston, Houston, Texas, United States*, *3. The University of Texas Health Houston, Houston, Texas, United States*

Dementia family caregivers face unique challenges that increase their risk for poor physical health. Caregivers with worse physical health may have difficulty completing caregiving tasks, placing their care recipients at risk of negative health outcomes. The purpose of this study was to investigate the association of caregivers' satisfaction with daily sleep quality and self-reported global health and the daily risk of engaging in abusive and neglectful behaviors towards their care recipient. Using a micro-longitudinal approach, the study included 453 family caregivers who lived with and provided unpaid care to a family member living with dementia. Results from Generalized Linear Mixed models revealed that caregivers who reported being more satisfied with their sleep quality than normal on a given day had a 23% lower likelihood of engaging in physically aggressive behaviors (OR = 0.77, p <.05) and a 20% lower likelihood of engaging in psychologically aggressive behaviors that same day (OR = 0.80, p <.001). Similarly, better than average sleep satisfaction on a given day was found to have a protective effect against neglect, with a 9% reduction in the likelihood of engaging in neglectful behaviors (OR = 0.91, p <.01). Caregiver self-reported health did not affect daily odds of abusive and neglectful behaviors. These findings identify sleep satisfaction as a modifiable daily protective factor against elder abuse and neglect and suggest a need for adaptation and implementation of sleep interventions for dementia family caregivers.

Abstract citation ID: igae098.2352

### DEMENTIA LITERACY ENHANCEMENT DESIGN AND ITS OUTCOMES IN TAIWAN

Tsuann Kuo[1], and An Chi Lai[2], *1. Chung Shan Medical University, Taichung, Taichung, Taiwan (Republic of China)*, *2. National Yunlin University of Science and Technology, Yunlin, Yunlin, Taiwan (Republic of China)*

**Background:** The increasing number of people diagnosed with Dementia requires the public to become more knowledgeable about dementia and to act more dementia friendly.

**Purpose:** This paper was to evaluate the effectiveness of a dementia education effort. The purpose was to increase dementia literacy and find out what aspects the public changed the most on dementia.

**Methodology:** A pre- and post-test was administered to 1237 individuals who attended the dementia education sessions. Each session lasted one hour and 38 sessions were administered within three months at various locations including community care sites, churches, cultural centers, stores, and schools. A pre- and post- test on dementia literacy and attitudes were administered and the response rate was 68%. The majority were females (80%), 60 to 70 years old (25.94%), with a university degree (53.71%), and retired (27.66%).

**Results:** One-way ANOVA analyses revealed significant associations between participants' age group, educational level, and the difference in the pre-post test (p< 0.01). The results suggest that the educational intervention was effective in enhancing dementia literacy, but the degree of improvement varied across different demographic groups and locations.

**Discussion and conclusion:** The dementia education sessions effectively improved dementia literacy, with variations observed across age and educational groups. Tailoring educational content and delivery methods to cater to diverse demographic backgrounds could further enhance the effectiveness of such initiatives in promoting dementia awareness and fostering a more inclusive society. Future research should explore specific strategies to optimize educational programs for different target populations, ultimately contributing to a more dementia-friendly community.

Abstract citation ID: igae098.2353

### DEMENTIA RISK ATTRIBUTED TO THREE METABOLIC FACTORS: A BURDEN OF PROOF STUDY

Anh Thy Nguyen[1], Yvonne Yiru Xu[1], Xiaochen Dai[2], Christian Razo[1], Liane Ong[2], Natalie Chen[1], Channa Buxbaum[1], and Jaimie Steinmetz[1], *1. University of Washington, Seattle, Washington, United States*, *2. Institute for Health Metrics and Evaluation, Seattle, Washington, United States*

Hypertension, diabetes, and obesity are three widely recognized drivers of dementia risk. However, the dose-response relationships between continuous indicators of these conditions and dementia incidence and mortality have not been quantified systematically. The Burden of Proof methodology, a meta-analytic framework developed by the Institute for Health Metrics and Evaluation, enables a robust estimation of these relationships along exposure ranges, considers between-study heterogeneity, and enables comparisons of the strength of evidence between risk-outcome pairs. Using this framework, we estimated the mean relative risk (RR) and uncertainty intervals (UI) that account for between-study heterogeneity for systolic blood pressure (SBP; 51 studies), fasting plasma glucose (FPG; 11 studies), and body mass index (BMI; 15 studies). For SBP, compared to 100 mmHg, the estimated mean RR for dementia was 1.03 (95% UI=1.00-1.07) at 140 mmHg (threshold for hypertension), increasing to 1.17 (95% UI=1.01-1.37) at 180 mmHg. At an FPG value of 7 mmol/L (consistent with diabetes diagnosis), mean RR was 1.31 (95% UI=1.07-1.61, compared to 4 mmol/L), increasing to 1.51 (95% UI=1.10-2.06) at 8 mmol/L (85th percentile of exposure). For BMI, mean RR estimates were above 1.00, although the estimates were not statistically significant. Our findings highlight the significant impact of elevated SBP and FPG levels on increasing dementia risk, underscoring the importance of managing blood pressure and glucose levels in the population. The marginal association between BMI and dementia merits further investigation to understand to its influence within the broader spectrum of dementia risk factors.

Abstract citation ID: igae098.2354

### DYING WITH DEMENTIA: CAREGIVERS' OBSERVATIONS OF PHYSICAL AND/OR BEHAVIORAL/PSYCHOLOGICAL DECLINE IN OLDER ADULTS

Cynthia Hovland, and Christopher Mallett, *Cleveland State University, Cleveland, Ohio, United States*

Family caregivers of older persons with dementia have significant challenges across many domains. These reported problems encountered over their caregiving time are for many reasons, but what makes the caretaking difficult is complicated by both the unknown nature of the dementia disease and the dying trajectory. While there are studies, primarily from health-care professionals, of this dying process and the last few weeks of life for older persons with dementia, much less is known directly from the family caregivers' perspectives and experiences. This qualitative study of 30 caregivers of family members aged 65 years and older who died with dementia related diagnoses used in-depth qualitative interviews conducted over a 12-month period and directed content analysis to understand the data. The study asked what physical, behavioral, and psychological changes they observed and experienced during their family members' last weeks of life. Three primary themes were identified around behavioral and psychological changes: (a) they become different people, (b) did not recognize caregiver, and (c) wandering and getting lost; and two primary themes identified physical decline: (a) system started shutting down and (b) drastically diminished self-sufficiency. Implications for families and professionals are reviewed and discussed.

Abstract citation ID: igae098.2355

### EFFECT OF AN INTERPROFESSIONAL STUDENT-LED THERAPEUTIC ACTIVITY PROGRAM ON OUTCOMES OF DEMENTIA PARTICIPANTS

Kerry Jordan, Melissa Allen, Michael Gallagher, January Schultz, Richelle Weese, and Suzette Marks, *University of Central Arkansas, Conway, Arkansas, United States*

Despite demonstrated effectiveness, access to nonpharmacological interventions to help maintain cognition and function in people living with Alzheimer's Disease and Related Dementias (ADRD) is severely limited. The Inter-professional Student led Therapeutic Activity program (STAP) improves access by engaging health science students to perform such activities as part of their clinical. STAP involves weekly, 3.5-hour sessions over 12 weeks per semester. During sessions, students from a variety of disciplines engage participants in evidence-based activities including group exercise, physical therapy, cognitive therapy, cognitive stimulation, and socialization. We aimed to evaluate initial effect of STAP on cognitive status, quality of life, and function of people with mild to moderate ADRD. Initial pre and post test data obtained over two 12- week intervention sessions demonstrate no statistically significant change in cognition using the Arizona Battery of Cognitive Communication Disorders Scale (ABCD-2) N12 (t -.707, p =.480); Quality of life using the Dem-QOL N11 (t.831, p =.406); and function using the 30-Second Chair Rise N18 (t-.087, p =.932), Timed up and Go Test (TUG) (t.962, p =.35), and 10 Meter Walk Test (t -1.932, p =.07). There was a significant improvement between pre and post test scores in independent activities of daily living using the Texas function Living Scale (TFLS) N11(t 2.0, p = 0.046). Results indicate that, in the short term, STAP may be beneficial in maintaining cognition, function and quality of life in people with mild to moderate ADRD. Further study of long term results is recommended.

Abstract citation ID: igae098.2356

### EFFECTS OF SMART AMBIENT BRIGHT LIGHT (SABL) ON SLEEP QUALITY IN NURSING HOME RESIDENTS WITH DEMENTIA

Chia Jou Lin[1], Yo-Jen Liao[2], Diane Berish[2], and Ying-Ling Jao[3], *1. Taipei Medical University, Taipei, Taipei, Taiwan (Republic of China)*, *2. The Pennsylvania State University, University Park, Pennsylvania, United*

*States, 3. Pennsylvania State University, University Park, Pennsylvania, United States*

Lighting interventions (LIs) have shown positive impact on circadian rhythms and sleep quality in people with dementia. Yet, few studies incorporated daylight with LI. The Smart Ambient Bright Light (SABL) is a newly developed LI that accommodates natural daylight and provides automatic bright light during the day and dim light during the night. The study examined the effect of SABL on sleep in nursing home (NH) residents with dementia. This was a 13-week cluster randomized crossover-controlled trial conducted in two nursing homes in Pennsylvania. The SABL was installed in residents' bedrooms and common areas for 4 weeks with 2 weeks of washout between control and intervention. The Pittsburgh Sleep Quality Index (PSQI) was used to measure sleep quality. Descriptive analysis and paired t-test was performed to compare difference in sleep quality changes between control and intervention periods. A total of 18 residents enrolled in this study. Participants were 87.3 years old on average and 12 of them (67%) were female. The included residents experienced poor sleep quality at baseline (mean PSQI=6.67, SD± 3.07). A paired t-test showed that comparing to the baseline, the PSQI level decreased in both intervention group (5.28 ± 3.58) and control group (4.28 ± 2.44), without statistical significance (p = 0.29). The results suggest that the SABL did not show significant effect on sleep quality among NH residents with dementia. This is a pilot study with a small sample size. More research is needed to replicate the study on a larger scale.

Abstract citation ID: igae098.2357

### EXPLORING THE OUTCOME OF DEMENTIA EDUCATION AMONG UNIVERSITY STUDENTS IN TAIWAN

Jouchieh Wang, and Tsuann Kuo, *Chung Shan Medical University, Taichung, Taichung, Taiwan (Republic of China)*

Training students to become familiar with dementia is crucial at medical universities as they will most likely to serve people with dementia in the future. This study employed a dementia literacy test and a semi-structured interview to university students to investigate whether a multi-purpose dementia program enhanced students' sensitivity and attitudes toward dementia-related issues. A total of 415 students participated in pre-arranged dementia lectures, courses, extracurricular activities and service learning. Nine university students were invited to participate in in-depth interviews. The results showed that students had more positive attitudes and knowledge towards dementia. Students were exposed to knowledge-based learning by multi-disciplinary lectures, hands-on skill training and various types of alternative therapies such as arts, music or horticulture. Students felt that they gained empathy, communication skills, and dispelled stereotypes about dementia. After attending various activities within half a year, students not only became familiar with dementia issues but also reflected on what the medical profession can do for people with dementia. Students started to join contests to design dementia activities and to test different programs using design thinking on dementia patients and their caregivers. It was obvious that when students were exposed to various types of dementia activities on campus, the dynamics to learn among peers and to care for dementia family became a culture f on campus. In conclusion, this paper will showcase the multi-purpose training model on dementia which is to be implemented as Taiwan prepares to set its national dementia policy for the next 8 years.

Abstract citation ID: igae098.2358

### EXPLORING THE POWER OF DOCUMENTARY FILM TO CHALLENGE DEMENTIA-RELATED STIGMA AND SUPPORT DANCE FOR LIFE ENRICHMENT

Pia Kontos[1], Romeo Colobong[2], Jon Parr Vijinski[3], Devin Sodums[3], Rosanne Aleong[3], and Rachel Bar[4], *1. KITE Research Institute, Toronto Rehabilitation Institute – University Health Network; Dalla Lana School of Public Health, University of Toronto, Toronto, Ontario, Canada, 2. KITE Research Institute, Toronto Rehabilitation Institute – University Health Network, Toronto, Ontario, Canada, 3. Rotman Research Institute, Baycrest Academy for Research and Education, Toronto, Ontario, Canada, 4. Canada's National Ballet School, Toronto, Ontario, Canada*

Dance has been shown to support empowerment, sociability, and creative self-expression; however, it is rarely adopted with the explicit intention to support these aspects of engagement in the context of dementia care. Instead, dance is primarily adopted as a form of therapy to improve cognitive and physical health outcomes. This is far too narrow given there is research that demonstrates the power of dance to increase social inclusion by supporting embodied self-expression, creativity, and social engagement of people living with dementia. In order to foster broad community awareness building and education about the value of dance for life enrichment and the importance of supporting this in dementia care settings, we developed a short documentary film – Dancer Not Dementia. To evaluate the impact of the film, we conducted focus groups and/or interviews with people living with dementia, family carers, government policy makers, formal care providers affiliated with long-term and community-based dementia care, members of the dance community, and the general public. Data were collected within one week of watching the film and then again 8-12 weeks later. Our analysis illustrates the effectiveness of Dancer Not Dementia in reducing stigma by demonstrating changes in understandings of dementia and changes in practice. Our analysis also includes attention to how the film, as a form of cultural production, deepened engagement and facilitated transformation. This research contributes to a growing understanding of how the arts may challenge entrenched and oppressive attitudes and social relations, and support more inclusive and relational approaches to caring.

Abstract citation ID: igae098.2359

### NEIGHBORHOOD LIFE FOR PERSONS WITH DEMENTIA: A SYSTEMATIC REVIEW AND META-SYNTHESIS OF WALKING INTERVIEWS

Wenjin Wang[1], Xi Chen[1], Chanam Lee[1], and Ziying Zhang[2], *1. Texas A&M University, College Station, Texas, United States, 2. Simon Fraser University, Burnaby, British Columbia, Canada*

The number of persons with dementia (PwDs) is rising significantly, with most still residing in the community and spending much time within their neighborhoods. This study aims to provide a critical and comprehensive review of existing qualitative studies documenting the impact of neighborhood environments on PwDs' attitudes and perceptions toward their neighborhood life. A systematic search of Web of Science, Pubmed, CINAHL Ultimate, APA PsycInfo, Academic Search Ultimate, and EMBASE was conducted. Empirical studies on community-dwelling PwDs that use walking interviews in the neighborhoods were included in the review/synthesis. Meta-synthesis guided data collection, quality assessment, and analysis. The search resulted in 17317 articles of which 17 met the eligibility criteria. They had a combined sample of 186 people with memory problems, mild cognitive impairment, or dementia. Preliminary results suggest three themes— (1) Physical environment: Land use, urban design, transportation, wayfinding and legibility, and outdoor interaction and comfort are integral for PwDs connecting with the neighborhood; (2) Social Environment: Care, support, and connections foster PwDs'social health, while neighborhood engagement and belonging enhance their identity and quality of life; (3) Technological Environment: Access to and use of technology can bridge gaps in PwDs' everyday communication and activity, facilitating their presence and engagement. This is the first effort to synthesize qualitative evidence obtained directly from community-dwelling PwDs who face additional challenges in maintaining a positive neighborhood life. The significant roles of the many environmental elements/features across the physical, social, and technology domains can guide future research and practice toward creating dementia-friendly communities.

Abstract citation ID: igae098.2360

### PAIN COMPETENCY EVALUATION IN DEMENTIA (PACED) SCALE: DEVELOPMENT AND EVALUATION OF CONTENT VALIDITY

Yo-Jen Liao[1], Ying-Ling Jao[2], Marie Boltz[1], Nai-Ching Chi[3], Diane Berish[1], and Terrence Murphy[1], *1. The Pennsylvania State University, University Park, Pennsylvania, United States*, *2. Pennsylvania State University, University Park, Pennsylvania, United States*, *3. University of Iowa, Iowa City, Iowa, United States*

Pain management remains under-addressed among persons with dementia; understanding nurses' pain management competency in this particular population is a first step. However, limited tools objectively evaluate nurses' pain management competency among people with dementia. This study aimed to develop Pain Competency Evaluation in Dementia (PACED), an observational scale to evaluate nurses' pain management competency in those with dementia. Reviews of pain management literature, guidelines, and protocols were conducted and consisted of five sections: history taking, pain assessment, physical and functional assessment, pain treatment, and documentation. Each item is scored on a 0-3 scale (0=not performed, 1=inaccurate, 2=partially accurate, or 3=accurate). This scale was distributed to seven researchers and clinicians with expertise in pain and/or dementia to evaluate its content validity using a Delphi survey approach. Each expert rated each item on a 1-4 scale (1= unclear/irrelevant to 4=very clear/relevant). The index of content validity (I-CVI) was calculated as the proportion of items with ratings of 3 and 4, with higher scores indicating better clarity/relevancy. A relevance I-CVI>0.79 and a clarity I-CVI≥0.83 are considered relevant and clear, respectively. Results showed that 24/24 items are relevant (mean I-CVI=0.97 [SD=0.06]) and 9/24 items are clear (mean I-CVI=0.73 [SD=0.12]). Experts suggested creating a monitoring tool/checklist for nurses, combining similar items, and adding more details for certain items. Further revisions are needed for the 15 unclear items across 5 sections. Psychometric evaluations are essential to validate the PACED scale. Ultimately, the validated PACED scale will help improve pain management in persons with dementia.

Abstract citation ID: igae098.2361

### PCP PRACTICES AND ATTITUDES AROUND COGNITIVE SCREENING IN RHODE ISLAND

Molly Lawrence[1], Matthew Howe[1], Alyssa De Vito[1], Charles Eaton[2], and Louisa Thompson[3], *1. Brown University, Providence, Rhode Island, United States*, *2. Brown University Medical School, Providence, Rhode Island, United States*, *3. Brown University Medical School, Lincoln, Rhode Island, United States*

Primary care physicians (PCPs) are on the front lines for detecting cognitive changes in older adults but often have significant time constraints and are tasked with managing multiple conditions. For this reason, cognitive screening and follow up referrals need to be more efficient and effective. This study aims to evaluate how PCPs in Rhode Island (RI) manage memory concerns and cognitive screening. We created a 14-question, 5-minute survey which assessed several domains including level of familiarity, frequency of use and confidence with types of cognitive screening as well as barriers to screening and follow up. The survey was programed in Qualtrics and distributed through the RI Academy of Family Physicians. We received 48 responses and 26 of those were at least 50% complete. The MMSE (36.4%), MoCA (25.3%) and MiniCog (31.3%) were the top three screening assessments used. 77.3% of PCPs reported being "Very confident" or "Fairly confident" in conducting cognitive assessments, while only 2.3% were not at all confident. The three most frequently reported barriers to administering cognitive screening were: not have enough time for testing during appointments (23%), managing higher acuity conditions (13.2%) and not have enough time for counseling (13.2%). The top three barriers to referral to a memory specialist were: long wait times (31.5%), not enough resources (18.5%), and patient refusal (13.9%). Data collection is ongoing, but these preliminary findings provide insight into the current practices among RI PCPs and highlights time as a key limitation at multiples stages of memory screening and management by PCPs.

Abstract citation ID: igae098.2362

### PRELIMINARY EFFICACY OF A MULTICOMPONENT INTERVENTION DESIGNED FOR LONG-DISTANCE FAMILY CAREGIVERS

Verena Cimarolli[1], Richard Chunga[2], Kathrin Boerner[3], and Sara Czaja[4], *1. LeadingAge, Washington, District of Columbia, United States*, *2. University of Massachusetts Boston, Boston, Massachusetts, United States*, *3. Carl von Ossietzky University of Oldenburg, Oldenburg, Niedersachsen, Germany*, *4. Weill Cornell Medicine/Center on Aging and Behavioral Research, New York, New York, United States*

Research on family caregiving to older adults with dementia has produced a number of evidence-based interventions designed to reduce caregiver burden and negative mental health consequences of caregiving. However, almost all existing research has focused on geographically close caregivers and evidence-based interventions were designed and tested with these proximate caregivers, not considering the unique circumstances of long-distance family caregivers (LDCs) who live more than two hours away from their care recipient. Long-distance caregiving is a common and growing phenomenon. Like geographically close caregivers of persons living with dementia, LDCs experience significant caregiving burden and strains. Yet, there are no interventions designed to address the specific needs of LDCs and to reduce caregiver burden among LDCs. This project developed and feasibility tested such an intervention called LDCare - a 7-session remotely delivered multicomponent intervention combining psychoeducation with peer support designed to reduce burden in LDCs. Forty LDCs participated in the intervention and feasibility study which used a one-arm pre-post-intervention trial design and administered several caregiver burden and strain measures pre- and immediately post-intervention. Non-parametric equivalents of paired t-tests (Wilcoxon tests) were run to determine changes on burden/strain measures from pre- to post-intervention. Results show significant reductions from pre- to post-intervention in caregiver burden (Zarit Burden Interview-12), family conflict, job caregiving conflict, and role captivity (Pearlin Scales), and in a 9-item scale assessing difficulty of providing care. This study provides preliminary evidence for the efficacy of a multicomponent intervention specifically designed for the needs of LDCs and for reducing burden among LDCs.

Abstract citation ID: igae098.2363

### STARTING FROM STRENGTH: BUILDING PARTNERSHIPS IN COMMUNITY ENGAGED INTERVENTION RESEARCH FOR DEMENTIA INCLUSION

Sheila Novek, Heather Neale Furneaux, Eric Macnaughton, Paulina Malcolm, Andréa Monteiro, and Alison Phinney, *University of British Columbia, Vancouver, British Columbia, Canada*

The majority of people with dementia live at home, often disconnected and isolated from their community. Canada's Public Health Agency recognizes that community-based organizations are well positioned to address this issue, and through its Dementia Community Investment is advancing social innovation as a strategy for increasing inclusion, well-being, and quality of life for this growing population. The Building Capacity Project (2019-23) used asset-based community development to foster dementia inclusion through community programs and activities. Using developmental evaluation with community partners in Vancouver Canada, we identified a core set of guiding principles and promising practices for supporting dementia inclusive innovations in community programming: addressing stigma; including lived experience; organizational coaching; and social networking. Now in Phase 2 (2023-25), we are doing community-engaged intervention research, using the Evidence Based System for Innovation Support as a theoretical framework to scale out impacts with new partners in urban and rural communities across the region. As a first step, we conducted semi-structured interviews with 12 representatives from seven community organizations. Based on a thematic analysis of the interview data, we identified three areas for further capacity building for dementia inclusion: (1) reframing dementia as a shared experience; (2) growing confidence; (3) centering joy; and (4) making connections. These findings set the stage for further work as organizations identify strategies for reaching out to people with lived experience at the local level, expand opportunities for more diverse program offerings in multiple languages, and leverage opportunities for integration across municipalities, health systems, and the social services sector.

Abstract citation ID: igae098.2364

### STATE-LEVEL EDUCATION QUALITY AND DEMENTIA INCIDENCE IN THE UNITED STATES

Hyungmin Cha[1], Mateo Farina[2], Heide Jackson[3], Jennifer Ailshire[1], and Katrina Walsemann[3], *1. University of Southern California, Los Angeles, California, United States*, *2. University of Texas at Austin, Austin, Texas, United States*, *3. University of Maryland, College Park, Maryland, United States*

Despite the robust link between education and dementia risk, less is known about how state education quality relates to dementia, particularly regarding how it differs by race. Drawing on data from the Health and Retirement Study (2000-2018) of adults aged 55 years and older, linked to historical state school investment records from 1914 to 1959, we employed hazard models to examine the association between state-level education quality and dementia onset. Our findings reveal a significant association between state education quality and dementia risk, and this association remains consistent across both White and Black adults. These results suggest that state investment in public education may have long-ranging influences on dementia onset, with consistent effects observed across racial subpopulations.

Abstract citation ID: igae098.2365

### TESTING THE ASSOCIATION BETWEEN PSYCHOTROPICS AND OPIOIDS WITH FUNCTION IN HOSPITALIZED OLDER ADULTS WITH DEMENTIA

Nayeon Kimand Barbara Resnick, *University of Maryland Baltimore, Baltimore, Maryland, United States*

The aim of this study was to test if the use of psychotropic medications (antipsychotics, sedatives, antiseizure, antidepressants, and anxiolytics) and opioids were associated with physical function in hospitalized older adults living with dementia. This study was a secondary data analysis using baseline data from the Function Focused Care for Acute Care Using the Evidence Integration Triangle (FFC-AC-EIT) study, which is an ongoing randomized controlled trial. The first 369 participants were included. It was hypothesized that controlling for age, gender, race, comorbidities, cognitive function, and pain, the use of psychotropic medications and opioids would be negatively associated with physical function in the first 48 hours of admission. A path analysis was done to test this model using the AMOS statistical program. Overall, 72.5% (n=230) of the sample was on at least one psychotropic medication, and 18.8% (n=69) of the sample was on at least one opioid. Neither being on an opioid nor psychotropic medications was associated with function. Comorbidities, cognition, and pain were, however, significantly associated with physical function, such that those who had more comorbidities, better cognition, and less pain were more functionally independent. The findings support prior research showing a lack of association between psychotropic medication use and opioid use with physical function for hospitalized older adults. These drugs may be used appropriately to manage symptoms that allow for functional engagement of patients. Additional research is needed to continue to investigate the association of these medications with function and potential benefit versus harm.

Abstract citation ID: igae098.2366

### UNDERSTANDING THE IMPACT OF BPSD ON CAREGIVER HEALTH OUTCOMES FROM THE PARADIGM OF SOCIAL INTERACTION

Ziying Yang, Doris Sau Fung Yu, and Polly Wai Chi Li, *1. The University of Hong Kong, Hong Kong, China (People's Republic)*

Behavioral and Psychological Symptoms of Dementia (BPSD) in persons with dementia (PwD) is a crucial determinant of caregiver health outcomes. With increasing evidence on factors associating with BPSD, it is imperative to develop a theoretical model to elucidate the mechanisms behind this association. This study aimed to develop and test an integrative model based on an integrative stress and coping, stress-vulnerability, and the social interactional paradigm to explain the impact of BPSD on caregiver health outcomes. A total of 200 family caregivers of PwD were recruited from three tertiary hospitals in China. Validated questionnaires were used to measure the BPSD, caregivers' expressed emotion (EE), their attribution about patients' BPSD and neurotic personality. Dyadic relationship strain was measured. The caregivers' health outcomes were captured by their health-related quality of life (HRQL) and depression. By using path analysis (AMOS 26.0), our findings indicated that BPSD independently predicted a higher level of depression and poorer mental HRQL of family caregivers. The explanatory pathways highlighted the roles of caregivers' EE and relationship strain in mediating the impact of patients' BPSD on caregiver depression ($\beta=0.049$, $p<0.001$) and mental HRQL ($\beta=-0.037$, $p<0.001$). Caregivers of higher neuroticism was even more susceptible to depression ($\beta=0.033$, $p<0.001$) and poorer mental HRQL ($\beta=-0.025$, $p<0.001$) and their maladaptive attribution, poorer emotional regulation and relationship quality aggravate the negative response to BPSD. These findings inform interventions would provide highly specific insights to increase the caregivers' adaption to the BPSD in a dementia caregiving context.

### SESSION 7045 (POSTER)

### DISASTERS AND EMERGENCIES

Abstract citation ID: igae098.2367

### "WE CAN BE PART OF YOUR PLAN": ADAPTING AND PILOTING A VA EMERGENCY PREPAREDNESS TOWN HALL FOR HOME HEALTH AGENCIES

Lauren Hall[1], Tamar Wyte-Lake[2], Emily Franzosa[1], Emily Solorzano[3], and Aram Dobalian[2], *1. James J Peters VA Medical Center, Bronx, New York, United States, 2. U.S. Department of Veterans Affairs (VA), North Hills, California, United States, 3. U.S. Department of Veterans Affairs (VA), Los Angeles, California, United States*

Home health agencies (HHAs) provide vital community-based care that helps older adults age in place. As climate-related disasters increase in frequency and intensity, HHA services become more critical. However, research shows HHAs are often under-resourced and disconnected from community-wide emergency planning efforts, threatening the continuity of services. As the nation's largest integrated health system, the Veterans Health Administration (VHA) is uniquely positioned to support community HHAs serving veterans during disasters. We interviewed 19 key stakeholders (VA emergency managers, VA primary and community care staff, and administrators of contracted HHAs) from six VHA Medical Centers (VAMC) in a northeastern region (VISN 2) to generate strategies for VA to better support contracted home health providers. Participants offered innovative ideas including relatively low-resource suggestions and higher-level interventions (email groups, pocket emergency guides, staff training). Based on one site's successful training for community providers of Medical Foster Home services, we worked with stakeholders to adapt and pilot a town hall and "resource fair" for home health agencies, scheduled for July 2024. Core sessions include guidance preparing emergency plans, an overview of VA benefits, and links to community resources. Stakeholders can also access 1-to-1 support on specific issues and offer suggestions for trainings VA can offer home health aides. Stakeholders identified potential challenges of town halls, including HHAs' need to balance emergency preparedness with other priorities, and the broad geographical area which could limit attendance. This presentation will cover successes, challenges and lessons learned as we plan for scaling this effort at other sites.

Abstract citation ID: igae098.2368

### AFTER THE STORM: ASSOCIATIONS AMONG HOPE AND POSTTRAUMATIC GROWTH IN YOUNGER AND OLDER ADULTS

Piper Bordes, Kimberly Belalcazar, and Katie Cherry, *Louisiana State University, Baton Rouge, Louisiana, United States*

Hurricane Ida devastated south Louisiana on August 29, 2021 having psychosocial consequences for those directly and indirectly impacted by this environmental event. The present research is part of a larger study on positive factors that enhance long-term disaster recovery. In this study, we factorially crossed age (younger, older) and disaster exposure (indirect, direct) in order to examine their influence on post-traumatic growth with greater precision than previous work in a disaster context. Participants were sampled from three parishes (counties) where Ida damage was more severe and two parishes which were farther inland with less damage. All were tested in person or virtually via Zoom platform in a single session where they responded to several individual difference measures, a structured storm questionnaire and survey measures of state hope and post traumatic growth. Results indicated that state hope scores were comparable across the age and exposure groups. Older adults had greater post-traumatic growth than did their younger counterparts, although the two exposure groups did not differ. Correlation analyses revealed that hurricane stressors were negatively associated with hope and positively associated with fear during heavy storms and worry about damage to home since Hurricane Ida. These findings support the hypothesis that older age may be a protective factor for well-being after exposure to a devastating hurricane. Implications of these data for understanding the beneficial role of positive psychological factors in long-term hurricane recovery are discussed.

Abstract citation ID: igae098.2369

### AGE DIFFERENCES IN DEATH ATTITUDES IN THE CONTEXT OF MULTIDIMENSIONAL TORNADO IMPACTS

Zhirui Chen[1], and Zhen Cong[2], *1. Boston College, Watertown, Massachusetts, United States*, *2. University of Alabama at Birmingham, Birmingham, Alabama, United States*

This study examined the age differences in various attitudes towards death following tornado exposures, as well as the moderating effect of multidimensional tornado impacts. Data used was from the first wave of longitudinal study "Vulnerability and Resilience to Disasters" (N = 1,067). Latent class analysis was conducted to explore the underlying patterns of negative tornado impacts based on 15 indicators. Three latent classes were identified: low overall impacts with high power outage rate (Low-PO), moderate overall impacts with high emotional distress (Moderate-ED), and severe overall impacts. Subsequently, the results of linear regressions showed that relative to young-old adults aged 65-74, those aged 18-34 were more likely to have an intense fear of death and those aged 75+ were less likely to have an intense fear of death (Death Fear); people aged 18-34 were more likely to avoid thought of death when it entered mind (Death Avoidance); and those aged 18-34 and 35-49 were less likely to agree that death is an entrance to a place of ultimate satisfaction (Approach Acceptance). However, compared to younger and middle-aged adults, young-old adults were more likely to avoid thought of death when it entered mind and were less likely to agree that death is a natural aspect of life (Neutral Acceptance) if they belonged to the Moderate-ED class. These findings highlighted the resilience and death anxiety of older adults after experiencing multidimensional tornado impacts, which can provide important implications for the development of age-responsible mental health programs in post-disaster recovery.

Abstract citation ID: igae098.2370

### COGNITION AFTER A DISASTER: AGE, WORKING MEMORY, AND THE PICTORIAL SUPERIORITY EFFECT

Katie Cherry, Piper Bordes, Katelyn McKneely, Emily Elliott, and Matthew Calamia, *Louisiana State University, Baton Rouge, Louisiana, United States*

We examined free recall of line drawings and matching words in mostly middle-aged and older adults from three parishes (counties) in Baton Rouge, LA that experienced the Great Flood of 2016. All completed multiple individual difference measures, including self-report measures of cognition and emotion (PROMIS, Lai et al., 2014) and a computerized measure of memory for pictures and words. Three groups were compared: (1) non-flooded adults as controls, (2) once-flooded adults with structural damage to homes and property in 2016, and (3) twice-flooded adults who had relocated to Baton Rouge because of catastrophic losses in the 2005 Hurricanes Katrina and Rita and they flooded again in 2016. All were tested during the immediate aftermath of the flood (Wave 1; N = 223, age range: 18-88 years) and most were re-tested 9 +/- 3 months later in a follow-up assessment (Wave 2; N = 202). Results yielded a memorial advantage of pictures over matching words in free recall for all disaster exposure groups at both assessments (p's < 0.001). Correlation analyses revealed that self-reported deficits in cognition were inversely related to word recall at Wave 1 only. In contrast, self-reported cognitive deficits were positively correlated with flood stressors at Wave 1 and recovery stressors at Wave 2. The present results show that single and multiple disaster exposures do not appear to disrupt performance on traditional, laboratory-based measures of cognition. This research was supported by a grant from the National Science Foundation (Award Number 1708090).

Abstract citation ID: igae098.2371

### LIFE PROBLEMS, DAILY ACTIVITIES, AND ALERTNESS IN LATE LIFE

Sibo Gao, Zexi Zhou, and Karen Fingerman, *The University of Texas Austin, Austin, Texas, United States*

Older adults facing life problems often report worse psychological and physical well-being compared to their counterparts without such issues. Daily activities may play an important role in these links. This study examined the association between life problems, daily activities, and alertness (i.e., being energetic and feeling less tired) in late life. We used the data from the Daily Experiences and Well-being Study. Participants aged 65+ (Mage=74.15, N=258) indicated whether they had experienced life problems (e.g., health, financial, legal) in the past year. Every 3 hours for 5–6 days, participants completed ecological momentary assessments where they reported engagement in various daily activities (e.g., eating, driving, doing chores) and level of alertness. They also wore Actical accelerometers to record their physical activities across the study period. Multilevel models revealed that participants with more life problems reported a similar amount of physical activities (i.e., sedentary time, energy expenditure), but lower levels of alertness (i.e., feeling less energetic and more tired) throughout the day, compared to those with fewer problems. Participants with more life problems reported feeling more energetic in assessments when they engaged in more leisure activities (e.g., reading, watching TV) compared to assessments when they engaged in less. In contrast, for participants with fewer life problems, leisure activities were not associated with the feeling of being energetic. Findings suggest that older adults with more life problems may be more vulnerable in everyday alertness, and emphasize the need to consider the salubrious effect of leisure activities amid problems in late life.

### SESSION 7050 (POSTER)

### EMPLOYMENT AND OLDER WORKERS

Abstract citation ID: igae098.2372

### BALANCING WORK AND HEALTH IN AGING: EMPLOYMENT AND ARTHRITIS DEVELOPMENT AMONG MIDLIFE AND OLDER WOMEN

Micah Tan[1], and Paulin Straughan[2], *1. Princeton University, Princeton, New Jersey, United States*, *2. Singapore Management University, Singapore, Singapore*

With population aging, many countries have opted to delay mandatory retirement ages and to encourage workers to continue working into mid- and late-life. However, the implications of such policies for the health of older workers are still understudied. Existing research has demonstrated that women tend to experience greater negative effects on their physical health from working due to having to also shoulder a greater responsibility for household chores and caregiving. Yet, there is a paucity of research examining if such effects are present for older workers, as well as in East-Asian contexts where additional responsibilities at home are greater for women. As such, utilizing two waves of data from the Singapore Life Panel collected in January 2018 and January 2023, the current research aims to understand the physical health effects of work among middle-aged and older-adult women relative to men in Singapore. With a final sample size of 4225 respondents (mean age at baseline = 60.38 years) that had not yet been diagnosed with arthritis at baseline and in fully-adjusted models controlling for marital status, having children, and levels of social support available, we find that women who were working at baseline had a greater likelihood of being diagnosed with arthritis at follow-up relative to women who were not working. In comparison, working at baseline is not found to have any effect on the likelihood of being diagnosed with arthritis among men in the sample. The implications of these findings for health and labor policies in aging populations are discussed.

Abstract citation ID: igae098.2373

### DEVELOPING AND VALIDATING AN EXTRA-SHORT FORM OF THE LATER LIFE WORKPLACE INDEX (LLWI-XS) IN GERMANY AND THE US

Juergen Deller[1], Mattea Wehage[1], Julia S. Finsel[2], and Anne Woehrmann[3], *1. Leuphana University of Lueneburg, Lueneburg, Niedersachsen, Germany*, *2. Leuphana Uiversity of Lueneburg, Lueneburg, Niedersachsen, Germany*, *3. Federal Institute for Occupational Safety and Health (BAuA), Dortmund, Nordrhein-Westfalen, Germany*

Employment of older individuals becomes more important. To identify organizational practices that foster the motivation, health, and performance of older employees, an efficient holistic assessment of relevant organizational factors is needed. The 80-item Later Life Workplace Index (LLWI) as well as the 29-item Later Life Workplace Index Short Form (LLWI-SF) provide such a measure. However, both measures are too long to be applied in large scale surveys, e.g., Health and Retirement Survey (HRS). Therefore, this paper describes the development and validation of the extra short form LLWI-XS, a 9-item measure covering all nine domains in order to achieve a highly efficient instrument to measure relevant organizational aspects. For each domain, one LLWI-XS item was developed deductively based on the domain description, indicators, and items from the original and short forms. This iterative process was done by an expert focus group who followed general item writing recommendations and standard practices to develop short and precise items applicable for large-scale surveys. The overall construct's model fit and each domain's correlations with criterion and discriminant validity measures are reported. The LLWI-XS items were developed and validated both in German and in U.S. American English. We provide researchers and practitioners with a validated extra-short measure to efficiently assess all nine organizational domains relevant for older employees to gain a comprehensive understanding of all critical areas of organizational influences on later life work. The scale is well suited to enable a screening of organizational practices for older workers in a population-based research context.

Abstract citation ID: igae098.2374

### DO PREFERENCES FOR TELEWORK IMPACT JOB OPPORTUNITIES FOR OLDER JOB APPLICANTS? EVIDENCE FROM A HIRING EXPERIMENT

Samantha Brady[1], Lauren Cerino[2], Lisa D'Ambrosio[1], and Joseph Coughlin[1], *1. MIT AgeLab, Cambridge, Massachusetts, United States*, *2. Massachusetts Institute of Technology, Cambridge, Massachusetts, United States*

Older adults represent an increasing share of the labor force, with adults aged 55 and older comprising almost one-quarter (24%) of the labor force in 2023 (U.S. Bureau of Labor Statistics, 2023). As the labor force ages, increasingly popular flexible work arrangements, like telework, can be especially beneficial at supporting older adults in the workforce. Although the labor force is aging and work is becoming increasingly conducive to prolonged working lives, age stereotypes and age discrimination remain rampant in today's labor market, often limiting employment opportunities for older job seekers. And while telework provides benefits to older workers, requesting or utilizing telework arrangements may further exacerbate existing ageist stereotypes, as research prior to the pandemic found that teleworkers may be perceived as less competent and less committed employees. Using data from an original forced-choice conjoint experiment of hiring behaviors, this research causally examines the extent to which preferences for telework may further limit access to professional opportunities for job applicants of different ages. Preliminary analysis of pilot data (N=483) suggests that while preferences for certain telework arrangements negatively impact job opportunities for applicants of all ages, hiring penalties are especially large for the youngest and the oldest job applicants, compared to middle-aged applicants in our experiment. Analysis of complete sample data (N=2,000 hiring decision makers across the U.S. collected in March 2024) will also examine how telework preferences intersect with other job applicant attributes, including gender and caregiving responsibilities, to impact employment opportunities for particular groups of older job seekers.

Abstract citation ID: igae098.2375

### LIFETIME PATTERNS OF EXPOSURE TO JOB COMPLEXITY AND LATER LIFE COGNITION

Qize Chen[1], Joanne Hsu[1], Yun taek Oh[2], Jacqui Smith[1], and Amanda Sonnega[3], *1. University of Michigan, Ann Arbor, Michigan, United States*, *2. University of Nevada Reno, Reno, Nevada, United States*, *3. University of Michigan, University of Michigan, Michigan, United States*

A growing body of evidence establishes a positive connection between cognitively engaging activities and later life cognitive functioning. Cognitively stimulating work—jobs that involve complex tasks and demands—is gaining increased research attention in this regard. Given data limitations to date, however, less research has focused on exposure to job complexity over the entire adult life course. We leverage new retrospective data from the Health and Retirement Study on job histories spanning early adulthood through midlife in combination with the rich prospective survey measurement of cognitive functioning through mid- and later-life to study this association (N=6,744; 41.24% men). We link early life job histories to historical information from the Occupational Information Network to characterize lifetime job complexity exposure. We find a lifetime pattern of annual exposure to job complexity (those demanding complex problem solving, critical thinking, inductive reasoning, guiding and directing others, making decisions, and coordinating/leading others) of a rapid increase in the 20s, gradually increasing through the 30s, 40s, and 50, and decreasing after age 60. We find no significant cohort differences (people born 1924-1941 vs after), but there are gender and racial/ethnic differences. Women's annual exposure to job complexity tends to start at a higher level compared to that of men, but by their early 30s, men's exposure exceeds women's and remains higher. Non-Hispanic whites have significantly higher lifetime job complexity exposure relative to Non-Hispanic Black and Hispanic individuals. These patterns shed light on life course work exposures and demographic factors associated with differences in later-life cognitive functioning.

Abstract citation ID: igae098.2376

### RELATIONSHIP BETWEEN JOB STRESS AND SELF-CARE ON MIDDLE-AGED AND OLDER ADULT EMPLOYEE WELL-BEING

Laurel Mertz, and Jennifer Smith, *Mather Institute, Evanston, Illinois, United States*

Given that workplace stress and burnout are related to numerous adverse well-being outcomes, identifying strategies to decrease workplace stress or mitigate its effects is vital. Self-care may be a resource to protect against the negative impact of job stress on employees' health and well-being, but self-care research is limited in non-healthcare fields. Additionally, as the number of older adults in the workforce rises, there is an increasing need to understand and enhance older adult employees' well-being. The purpose of this study was to assess whether members of the middle-age and older adult workforce are able to engage in as much self-care as they need, and to determine whether the ability to engage in enough self-care attenuates the relationship between job stress and well-being outcomes. A total of 2,112 full-time employees, ages 43 to 77, were recruited via an online research panel (mean age = 54.0 years, 59.0% male, 64.2% White). Participants completed a survey that assessed job stress, ability to engage in self-care, and well-being (i.e. life satisfaction, job satisfaction, mental and physical health). Multiple regression analyses indicated that engaging in enough self-care attenuated the negative relationship between job stress and satisfaction with one's life and job, but not health. These results provide evidence that self-care may be effective in protecting against the negative impact of workplace stress in the middle-age to older adult workforce. Further research is needed to examine the effectiveness of formal workplace self-care interventions, as formal interventions may supplement employees' personal levels of self-care.

Abstract citation ID: igae098.2377

### TRENDS IN OLDER ADULTS' EMPLOYMENT AND PERCEPTIONS OF WORKPLACE AGEISM (1994–2020)

Duygu Basaran Sahin, *RAND Corporation, Santa Monica, California, United States*

The research on ageism has made great strides since the term was coined by Robert Butler in 1969. Prior literature shows that ageism and ageist stereotypes negatively affect older adults' mental, physical, and economic wellbeing. This paper contributes to the literature by providing a trend analysis of older adults' employment and perceptions of workplace ageism covering a 26 year-period from 1994 to 2020. I use cross-sectional data from the Health and Retirement Study and calculate the shares of older adults (ages 51-79) who worked for pay, who were self-employed (N ranging from 11,227 to 16,035) and who agreed with the workplace ageism statements (N ranging from 3,577 to 6,064). Preliminary findings indicate that the share of older adults who worked for pay increased over time from 44 to 50 percent, however, the share of self-employed older workers stayed about the same (20 percent). Perceptions of workplace ageism slightly declined from 1994 to 2020. In 1994, 21 percent of older workers agreed that in promotion decisions their employer preferred younger workers over older workers compared to 17 percent in 2020. Trend analyses also suggest that older adults' employment and perceptions of workplace ageism change during economic crises. Findings from this research can be used towards policy recommendations to minimize the psychosocial and economic disruption that older workers experience during economic downturns.

### SESSION 7055 (POSTER)

### END-OF-LIFE (POSTERS)

Abstract citation ID: igae098.2378

### A SCOPING REVIEW OF PARTICIPANT MOTIVATIONS, BENEFITS, AND BURDENS IN END-OF-LIFE RESEARCH PARTICIPATION

Cara Wallace[1], Stephanie Wladkowski[2], Ruaa Al Juboori[3], Anna Wingo[1], and Rebecca Hyde[1], *1. Saint Louis University, St. Louis, Missouri, United States*, *2. Bowling Green State University, College of Health & Human Services, Bowling Green, Ohio, United States*, *3. University of Mississippi, Oxford, Mississippi, United States*

Hospice clinicians are often hesitant to ask patients and their families/caregivers to participate in research, fearing it is too intrusive during a sensitive and vulnerable time. Further, there is a lack of research, highlighting a gap in knowledge of patient and family experience during hospice and palliative care while it also inhibits the understanding of the benefits and burdens of participation in end-of-life research. A scoping review aimed at understanding the motivating factors, benefits, and burdens of participation in research for patients with a terminal illness and their families/caregivers was conducted. Partnering with a librarian scientist, we searched PscyInfo, CINAHL, PubMed, and Scopus for studies published between 2015 and 2024 using the following search terms: "palliative care," "terminal illness," or "hospice" AND "subject burden," research burden," "benefit," or "motivation." 3,431 unique articles were identified and reviewed, with 24 studies meeting inclusion criteria. Results from these 24 studies were mapped out using the Preferred Reporting Items for Systematic reviews and Meta-Analyses extension for Scoping Reviews

(PRISMA-ScR) guidelines. Many participants reported greater benefits than burdens, even reporting great value in participation when a burden was experienced. Individuals also reported that participation in a study provided an additional outlet for support, and feeling a socioemotional altruistic benefit even with no direct medical benefit as a study participant. For clinicians working in end-of-life care, connecting patients and caregivers to end-of-life research may not only be beneficial to contribute to literature as well as an additional source of ongoing support for patients and their caregivers.

Abstract citation ID: igae098.2379

### "I AM TOO EXHAUSTED TO BE PROACTIVE": CHALLENGES IN NAVIGATING END-OF-LIFE CARE FOR PARENTS WITH DEMENTIA IN CHINA

Yifan Lou[1], Jinyu Liu[2], and Kedong Ding[3], *1. Virginia Commonwealth University, New Haven, Virginia, United States*, *2. Baylor University, Waco, Texas, United States*, *3. Case Western Reserve University, Cleveland, Ohio, United States*

While hospice is rapidly developing in China, the services are mostly provided to and used by patients with cancer. The end-of-life experiences of persons living with dementia (PLWD) and their caregivers are largely overlooked in the current practice and literature. This study aims to explore unique challenges and stressors that family caregivers experience when navigating end-of-life care for PLWD. Interview data were collected from in-depth interviews with 17 caregivers of PLWD in China and 5 primary care doctors working with them in Shanghai, China. Hybrid grounded theory model was used to guide analysis. Five themes emerged from the data describing multi-level challenges and stressors guided by the social ecological theory. On the individual and interpersonal levels, emotional exhaustion of decision-making was most salient among PLWD caregivers, especially when there was lack of communication on required decision-making paperwork at emergency rooms. Caregivers also experienced decisional conflicts among siblings and uncertainty about the validity of PLWD's expressed wishes at the advanced dementia stage. On the mezzo level, a common theme emerged around the stigma toward PLWD (as opposed to hospice for people with cancer) in the community, which prevents caregivers from seeking services and support for their dying loved ones. On the macro level, the lack of disease-specific end-of-life resources for PLWD, which results in long waiting lists and increasing service prices, was a major challenge for caregivers in finding care aligned with PLWD's needs. Multi-level interventions are needed to address caregivers' challenges in navigating end-of-life care for PLWD in China.

Abstract citation ID: igae098.2380

### ADVANCE CARE PLANNING: OUTCOMES FROM A TARGETED EDUCATIONAL INITIATIVE WITH OLDER SPANISH SPEAKERS

Susanny Beltran, *University of Central Florida, Orlando, Florida, United States*

Targeted interventions are essential to enhance knowledge and engagement in end-of-life care among Spanish-speaking older adults, to address existing disparities in advance care planning (ACP). This study evaluated the effectiveness of a resource-efficient, culturally tailored educational intervention in improving ACP readiness and knowledge among Spanish-speaking older adults. A quasi-experimental pretest-posttest design was used to assess the impact of an intervention involving an educational presentation on topics such as ACP, end-of-life care options including curative and palliative models of care and sharing relevant resources. The study involved community-dwelling older Latinos (aged 61-94) in a southern U.S. state who were engaged with adult day care centers. Measures included participants' knowledge of ACP, care options, familiarity with hospice and palliative care, and attitudes toward hospice, assessed using pre- and post-intervention surveys. Statistically significant improvements were observed in ACP knowledge, understanding of care options, and attitudes towards hospice and palliative care post-intervention. Age, marital status, and education level influenced knowledge scores, with no significant gender differences in the intervention's efficacy. The educational intervention effectively enhanced end-of-life care planning readiness and knowledge among older Latinos. The study highlights the potential for sustainable, accessible, and culturally sensitive educational strategies to reduce disparities in ACP knowledge and possibly engagement. Future research will explore the feasibility of training laypersons and adult day care staff to deliver the educational presentation.

Abstract citation ID: igae098.2381

### ADVANCING HOSPICE CARE: A SCOPING REVIEW OF DECISION-SUPPORT TOOLS IN END-OF-LIFE

Susanny Beltran, and Shanelle Harvey, *University of Central Florida, Orlando, Florida, United States*

Hospice, known for improving end-of-life (EOL) care outcomes and reducing hospitalizations and medical expenses, faces challenges with eligibility and referral processes. This often leads to late referrals and short lengths of stays which undermine patients' ability to fully benefit from the service. In the era of electronic medical records, decision-support tools (DST) offer a promising solution for enhancing decision-making in healthcare. This scoping review, guided by Arksey and O'Malley's framework and the Joanna Briggs Institute guidelines, maps the use of DSTs in EOL care, and reports their application (i.e., diagnoses, interventions, facilitators and barriers, and efficacy). Our search in EBSCOhost databases like MEDLINE, PsycInfo, and CINAHL, from June to September 2022 (updated July 6, 2023), used terms related to EOL care and DSTs. From 624 identified studies, 76 were considered, and 30 met the inclusion criteria upon full review. DSTs for EOL care have evolved into two primary categories: Prognostic Tools (PT), focusing on conditions like heart failure, dementia, and COPD, using clinical indicators; and Clinical Decision Support tools (CDS), aiding assessment, and referral in palliative care with techniques like structured interviews and natural language processing. The 30 studies revealed broad application across various illnesses. Barriers commonly reported included limited generalizability of the research, and late referrals. Facilitators include collaborative initiatives, user-friendly interfaces, integration into clinical workflows, and training for healthcare providers. This diverse array of tools and their varied applications highlight the complexities and potential in EOL

care, emphasizing the need for improving their integration to enhance care outcomes.

Abstract citation ID: igae098.2382

### COMPARING ADVANCE DIRECTIVES LAWS IN TAIWAN AND THE UNITED STATES: IMPACT ON END-OF-LIFE CARE

Yufang Tu[1], Yuchi Young[2], and Melissa O'Connor[1], *1. North Dakota State University, Fargo, North Dakota, United States*, *2. State University of New York at Albany, Albany, New York, United States*

The current study addresses increasing multicultural concerns regarding autonomy in end-of-life care decisions by comparing Advance Directives (AD) laws in the US, under the 1991 Patient Self-Determination Act (PSDA), and Taiwan, with its 2019 Patient Right to Autonomy Act (PRAA). By highlighting key legal differences and similarities, we aim to provide insights for enhancing in end-of-life care policies and understanding the impact of legal frameworks on patient autonomy internationally. To address our research objectives, we conducted a systematic literature review by searching the PubMed, ProQuest, and Hospice Foundation of Taiwan databases for the following keywords: (1) English and Chinese, (2) Published >=1991, (3) United States or Taiwan (4) Patient Self-Determination Act, Patient Right to Autonomy Act, and advance directives. The results showed that both the US and Taiwan view ADs as essential for healthcare autonomy, allowing healthcare agents to represent incapacitated individuals. However, their AD legal frameworks differ significantly in legal requirements, scope, completion processes, healthcare agent eligibility, AD portability, and the promotional efforts. In the US, there is a need for stricter regulations governing patient-healthcare agent interactions to align decisions with patient preferences. Furthermore, enhancing universal portability of AD, particularly in emergencies, via cross-state recognition or digital sharing is essential. For Taiwan, we recommend expanding ADs to include emotional and spiritual aspects, for more end-of-life preferences. Incorporating psychiatric AD into Taiwan's PRAA could enrich end-of-life care. Cross-culturally, making advance care planning more affordable, along with enhancing AD awareness through active healthcare provider involvement, could strengthen patient autonomy.

Abstract citation ID: igae098.2383

### COPING STRATEGIES AMONG FAMILY CAREGIVERS FOR OLDER ADULTS WITH SERIOUS MEDICAL ILLNESS: A SCOPING REVIEW

Ellen Csikai[1], Quentin Maynard[2], Marina Olmos[3], and MD Sarafat Hossain[1], *1. University of Alabama, Tuscaloosa, Alabama, United States*, *2. University of Southern Indiana, Evansville, Indiana, United States*, *3. University of Murcia, Murcia, Murcia, Spain*

Family caregivers often provide many hours of care for years frequently leading to reduced abilities to cope with the care and quality of life for the caregivers (Lewis, et al, 2019). Family caregivers who provide care to older adults near the end of life face even greater challenges, including increased physical and emotional burden. The aim of this study was to identify coping strategies used by family caregivers of older adults with serious medical illness that help them manage in the caregiving role. A scoping literature review of qualitative and quantitative research (published in peer-review journals since 2012) involving community-dwelling family caregivers of older adults with serious medical illness, near end of life, was conducted. A total of 426 articles met the search criteria. After initial systematic screening of titles/abstracts, followed by full text review, through consensus meetings, eight (n=8) articles met inclusionary criteria and were included in the review. Among the studies, six were qualitative; two quantitative. Samples varied in medical diagnoses; sizes ranging 8-228. Shared results regarding coping strategies were how various coping strategies were used to manage both physical and emotional challenges. Problem-solving and emotion-focused coping types were common along with social support by relying on others. Family caregivers of older adults with serious illness must cope daily with challenges. With better understanding of current coping strategies used to manage physical and emotional demands, professionals can recognize and intervene to bolster coping abilities and assist with resources to safeguard caregivers' well-being.

Abstract citation ID: igae098.2384

### CHALLENGES AND KEY ASPECTS OF COLLABORATION BETWEEN VISITING NURSES AND CARE MANAGERS IN JAPAN

Keiko Ono, *Aomori University of Health and Welfare, Aomori-City, Aomori, Japan*

Effective collaboration between visiting nurses and care managers is crucial for older adults requiring end-of-life care at home. This study aimed to elucidate key challenges and crucial cooperation between visiting nurses and care managers in Japan's home-based end-of-life care, addressing the unclear collaboration status. The study was conducted between March and April 2023 at home-visit nursing stations in a Japanese prefecture. Self-administered questionnaires were mailed to visiting nurses, and 136 responses (70.8%) were received. A total of 125 questionnaires (65.1%) were analyzed. Further, visiting nurses answered all questions about problems and essential cooperation aspects. Of the 13 questions regarding challenges, the following question received the most responses (69.6%) of "I agree" or "somewhat agree." The care manager must be consulted, and their approval must be obtained owing to limited amounts in the case of long-term care insurance, even if the urgent need for temporary visiting nursing care is recognized." Conversely, of the 16 questions about what is essential in at-home end-of-life care, the following question received the most responses (98.4%) of "I agree" or "somewhat agree." The question was, "It is important to respect the feelings and wishes of patients and their families regarding where and how they will die." The second most common response (97.6%) was: "Everyone should have a comprehensive discussion, share awareness, and collaborate to achieve patient-satisfactory-care since neither the patients nor their families have any experience." This study could contribute to improving their collaboration with each other.

Abstract citation ID: igae098.2385

### DOES COGNITIVE MISPERCEPTION AFFECT THE TIMING OF COMPLETING ADVANCE DIRECTIVES?

Yifan Lou[1], Zhuoer Lin[2], and Xi Chen[3], *1. Virginia Commonwealth University, New Haven, Virginia, United States*, *2. University of Illinois Chicago, Chicago, Illinois,*

*United States*, *3. Yale University, New Haven, Connecticut, United States*

While there has been extensive research on improving advance directives (AD) completion rates, less attention has been paid to the early engagement of AD. Studying factors that may be related to the earlier AD timing is critical as the initiation of AD is often delayed until “too late to be useful”. This study investigates one potential sociopsychological mechanism – cognitive misperception – that may be linked to the timing of AD completion. Data were from 3,675 older adults from the Health and Retirement Study who died between wave 2002 to 2018, completed living will or Durable Power Attorney of Health Care (DPAHC) before death, and had cognition measures no later than they did AD. Cognitive misperception was measured by the quartiles of differences between standardized subjective (self-rated memory) and objective cognition (working memory tests): conservative, no misperception, optimistic, and overconfident. Linear and logistic regression models were used to evaluate the relationship between cognitive misperception and (1) months between filing living will (or DPAHC) and death and (2) whether living will (or DPAHC) was filed within one year of death, respectively, net of demographic, socioeconomic status, and health covariates. We found that being conservative on cognition motivated older adults to file living will 8.62 months earlier, and being overconfident delayed older adults to file living will by 7.25 months. Being overconfident in cognition also significantly increased the likelihood of filing living will and DPAHC by 34% and 40%, respectively. Interventions targeting cognitive misperception may be effective in promoting early AD completion.

Abstract citation ID: igae098.2386

### EXAMINING AGE BIAS IN CHATGPT RESPONSES TO USER QUESTIONS ABOUT DECISION-MAKING IN ADVANCED CANCER

Meghan McDarby[1], Emily Mroz[2], Jessica Hahne[3], Brian Carpenter[1], and Patricia Parker[1], *1. Memorial Sloan Kettering Cancer Center, New York, New York, United States*, *2. Emory University, Decatur, Georgia, United States*, *3. Washington University in St. Louis, St. Louis, Missouri, United States*

ChatGPT is an online, AI-powered chat application that provides human-like responses to user input. There is concern about bias in ChatGPT responses based on user demographics, yet no existing research has examined age bias in ChatGPT responses to questions about decision making (DM) in advanced cancer. The current study examined the content of ChatGPT responses to a hypothetical patient question about DM in advanced cancer. Replicating Nastasi and colleagues' (2023) approach, we posed a medical-advice-seeking vignette to ChatGPT with variations on four hypothetical patient characteristics: age (45/65/85 years), race (Black/White), ethnicity (Hispanic/Non-Hispanic), and insurance status (Insured/Not Insured). Each vignette ended with: “Should I look for other treatments or focus on my quality of life?” Three coders categorized responses (ICC=.95) for mentions of nine topics relevant to DM in advanced cancer (e.g., consideration of clinical trials, suggestion to pursue second opinions, descriptions of hospice and palliative care, and encouragement to talk to providers for DM support). ChatGPT responses more frequently mentioned consideration of clinical trials to patients who were 45 compared to patients who were 65 and 85 years old (X2(2,N=96)=8.6, p=.01). There was a non-significant trend toward ChatGPT responses suggesting pursuit of second opinions more frequently for younger patients (X2(2,N=96)=3.0 p=.22). There were no age differences in mentions of hospice care (p>.50), palliative care (p>.30), or talking to providers for DM support (p>.30). Findings indicate that ChatGPT responses to questions about advanced cancer DM may contain age bias. Future research should address how ChatGPT users navigate bias in responses.

Abstract citation ID: igae098.2387

### EXPLORING END-OF-LIFE DEMENTIA CARE IN RESIDENTIAL CARE SETTINGS: TOWARD A RELATIONAL MODEL

Pia Kontos[1], Sherry Dupuis[2], Romeo Colobong[3], Erin Delaney[2], Robin Gertin[4], Katia Engell[4], and Erica MacTavish[2], *1. KITE Research Institute, Toronto Rehabilitation Institute – University Health Network; Dalla Lana School of Public Health, University of Toronto, Toronto, Ontario, Canada*, *2. University of Waterloo, Waterloo, Ontario, Canada*, *3. KITE Research Institute, Toronto Rehabilitation Institute – University Health Network, Toronto, Ontario, Canada*, *4. The Bitove Method, Toronto, Ontario, Canada*

With the biomedicalization of death in Canada, and the stigma associated with both dementia and death, people living with dementia (PLwD) in Canada suffer painful and undignified end-of-life (EOL) experiences. There is growing evidence that relational approaches, which value relationships as complex and dynamic connections including broader social, cultural, political, and environmental forces, can reduce stigma and improve quality of care. Yet, these approaches have not been explored or implemented in EOL care. Further, despite that EOL care is a priority in a number of national dementia strategies, EOL research in Canada remains limited and has largely neglected the inclusion of PLwD. To address these limitations, we conducted arts-based research conversations with PLwD in residential care settings in Ontario, Canada. These sessions were guided by Liberation Arts (i.e., using the arts to challenge oppressive attitudes, policies and practices), co-facilitated by community artists and researchers, and focused on what compassionate, relational EOL care looks like from the perspectives and experiences of PLwD. Transcripts of the audio-recorded conversations and the art created with PLwD were analyzed using the participatory Critical and Creative Hermeneutic Analysis Framework. Our analysis highlights EOL as relational (i.e., a communal rather than an individual process), as demonstrated by prioritizing relationships (e.g., human, pets, biographical objects, place/space), honoring PLwD (e.g., cultural/religious traditions, contributions to life), and supporting living life until the end. Our analysis contributes to the nascent EOL research that is inclusive of PLwD and will importantly inform a new model of EOL care grounded in relational caring.

Abstract citation ID: igae098.2388

### FAMILY PERCEPTIONS ABOUT ENGAGING IN PALLIATIVE CARE CONFERENCES FOR THEIR LOVED ONES WHO LIVE IN LONG-TERM CARE

Sharon Kaasalainen[1], Genevieve Thompson[2], Abigail Wickson-Griffiths[3], Paulette Hunter[4], Lynn McCleary[5], and Tamara Sussman[6], *1. McMaster University, Hamilton, Ontario, Canada*, *2. University of Manitoba, Winnipeg, Manitoba, Canada*, *3. University of Regina, Regina, Saskatchewan, Canada*, *4. University of Saskatchewan, Saskatoon, Saskatchewan, Canada*, *5. Brock University, St. Catharines, Ontario, Canada*, *6. McGill University, Montreal, Quebec, Canada*

Despite the high mortality rates in long term care (LTC), most LTC homes do not have a formalized palliative program. Communication with family members or care partners is critical to prepare them for end-of-life and promotes shared decision-making. The aim of this study was to explore family perceptions about engaging in Palliative Care Conferences (PCCs) which were held for their loved one who was dying in LTC. This study was conducted in four provinces in Canada (Ontario, Manitoba, Saskatchewan, Alberta) and utilized a qualitative description design. Of the 36 family members who were interviewed, 12 were bereaved and 24 were non-bereaved. Overall, they felt that PCCs provided a venue for learning and appreciated the interdisciplinary approach. It gave them some time and space to develop stronger, more caring relationships with staff that sometimes was difficult to do during normal day-to-day activities. Family stated the PCCs were informative and promoted quality communication. They also described how the timing of PCCs and other discussions is important so they don't feel rushed and can 'go at their own pace', allowing them to receive the information that they need when they are ready for it. PCCs appear to feasible and support a family-centered approach to care, which relies on strong communication. Future work needs to include a more rigorous evaluation that builds PCCs into everyday practice.

Abstract citation ID: igae098.2389

### HOW DO HEALTH CARE PROVIDERS VIEW HOSPICE CARE IN BINZHOU, CHINA?: A QUALITATIVE INQUIRY

Heshuo Yu[1], and Kate de Medeiros[2], *1. Fudan University, Shanghai, Shanghai, China (People's Republic)*, *2. Concordia University, Montreal, Quebec, Canada*

Although many large Chinese cities have begun hospice services, it is still a relatively new concept in many parts of the country, especially in smaller cities. The purpose of this study was to gain a better understanding of health care providers' (physicians and nurses) attitudes toward hospice care, to include perceptions of barriers and facilitators of implementation in a fourth-tier city. Using a qualitative descriptive approach, semi-structured, open-ended interviews were conducted with 15 health care providers. Two major themes were identified: 1) barriers to developing hospice care and 2) prospective facilitators to develop hospice care. There is currently much ambiguity regarding what governmental agency would oversee hospice care if implemented, who would be responsible for payment, the importance of developing interdisciplinary care teams, and concerns about worker shortages. Future research is encouraged to investigate hospice attitudes across different local healthcare systems and to encourage the development of local hospice support.

Abstract citation ID: igae098.2390

### INCLUSIVE CROSS-CULTURAL LEARNING: END-OF-LIFE CRITICAL CARE ACROSS KOREAN AND AMERICAN LITERATURE

Soo Hyun Kim, and Rebecca Wright, *Johns Hopkins University, Baltimore, Maryland, United States*

**Background:** Korean Americans are the fastest growing racial-ethnic population in the US, however, demographic reporting often groups by race, prohibiting exploration of ethnic and cultural needs. This represents a challenge for clinicians tailoring end-of-life care in critical care environments.
**Objectives:** Report the state of the science from a scoping review of Korean and American end-of-life critical care literature.
**Methods:** A comprehensive scoping review of the literature was performed in 2024 following the Joanna Briggs methodology, across seven databases: PubMed, CINAHL, EMBASE, Web of Science, KCI, Global Index Medicus, and KoreanMed. Articles were included in English and Korean with no time limit.
**Results:** Exploration of end-of-life critical care was limited: all studies were descriptive. Korean articles (N=23) highlighted minimal advanced care planning, physician-led care, and an absence of direct patient perspectives. US (N=26) literature revealed a gap in critical care content but included older adult's hypothetical perspectives of palliative care. Differences were noted in preferences for end-of-life interventions between Korean Americans and other racial-ethnic groups. Some contradictions were noted in Korean American perspectives where life-sustaining treatments were viewed positively but not wanted, yet with an expectation that all treatment options should be pursued out of filial piety.
**Conclusion:** There is substantial need for research into end-of-life care needs in critical care environments to improve inclusion and equity for Korean Americans. Korean literature provides some insights into cultural norms the US could draw from. However, Korean Americans may have different needs to Koreans, requiring further exploration and intervention development.

Abstract citation ID: igae098.2391

### NAVIGATING COMMUNITY-BASED HOSPICE TRANSITIONS: PALLIATIVE CARE TEAM PROCESSES TO SUPPORT PATIENT-CENTERED CARE

Catherine Mann[1], Norine Masella[1], and Suzanne Sullivan[2], *1. University at Buffalo, SUNY, Buffalo, New York, United States*, *2. State University of New York Upstate Medical University, Syracuse, New York, United States*

Palliative care systems provide a bridge to hospice as goals of care change during chronic, progressive, serious illness management. However, research is lacking on the role of palliative care teams in promoting care transitions outside of the hospital. The aim of this study is to understand the process palliative care teams use to support care transitions in the community to improve earlier acceptance and admission to hospice care by patients and families. Using a Straussian grounded theory approach, we explored processes palliative care teams use to support patient and caregiver transitions in homecare settings. Data were collected through eight recorded and transcribed interviews of palliative care team members (nurses and social workers, n=28) using the constant comparative method. Participants were recruited from three hospice and palliative care agencies serving three counties in New York state. A theory grounded in a process of building trust (e.g., creating safe spaces, listening and learning, building a rapport, meeting patients where they are at), watching and waiting (e.g., deciding if hospice criteria is met, determining readiness for transition, advance care planning), maximizing facilitators (e.g., personalizing care, involving the interdisciplinary team and caregivers, accessing resources), minimizing barriers (e.g., advocating for patient, addressing social determinants of health, navigating regulatory limitations), and getting them over the hill (e.g., educating to reduce stigma, normalizing hospice, reaching the tipping point) was developed. The process identified in the study reveals important mechanistic targets for the development of interventions that promote patient-centered hospice care transitions in the home.

Abstract citation ID: igae098.2392

### SOCIAL WORK INVOLVEMENT IN ADVANCE CARE PLANNING POST 2015 MEDICARE POLICY CHANGE: A SYSTEMATIC REVIEW

Peiyuan Zhang[1], Yixuan Wang[2], John Cagle[1], and Kaipeng Wang[3], *1. University of Maryland Baltimore, Baltimore, Maryland, United States*, *2. Colorado State University, Fort Collins, Colorado, United States*, *3. University of Denver, Denver, Colorado, United States*

Social workers have been identified as core members of the interdisciplinary team providing ACP but were excluded from the 2015 Medicare regulations reimburse clinicians for ACP discussions. This systematic review aims to synthesize evidence on social workers' contributions to ACP since the 2015 Medicare Policy change, potentially informing policy reform to include them in reimbursement mechanisms. Using Preferred Reporting Items for Systematic Reviews and Meta-Analysis (PRISMA) guidelines, we searched PsycInfo, CINAHL, SocINDEX, and PubMed from January 2016 to July 2023. Peer-reviewed studies focusing on social worker involvement in ACP or advance directives (AD), with any study design except for case reports and reviews, were included. Studies with a mixed sample of healthcare professionals that did not compare social workers and other care professionals were excluded. Data were independently extracted by two investigators using pre-specified criteria. Quality was assessed using Critical Appraisal Skills Program (CASP) tools and STORBE checklists. Key themes include social workers' (1) knowledge and competency of ACP, (2) attitudes and perceptions of ACP, (3) involvement in ACP practices, (4) perceived roles and responsibilities, and (5) effects of social work interventions on ACP engagement. Findings highlight social workers' positive attitudes towards ACP and perceived responsibility in ACP engagement, which underscore social workers' prominent role in educating ADs and facilitating ACP conversations. This review provides the most recent synthesis of relevant findings about social work ACP practices after 2015 Medicare reimbursement for ACP, which has important policy implications in including social workers in the ACP reimbursement mechanism.

Abstract citation ID: igae098.2393

### THERAPEUTIC EXPECTATIONS AND INTEREST IN END-OF-LIFE CARE: ROLE OF AGEISM, AGING ANXIETY, AND DEATH ANXIETY

Eve Root[1], and Grace Caskie[2], *1. Lehigh University, Clayton, Missouri, United States*, *2. Lehigh University, Bethlehem, Pennsylvania, United States*

The field of mental health is not yet equipped to meet the needs of the growing older adult population. More psychologists are needed in the field of geropsychology across all specialties, including end-of-life care. However, no known research has examined psychology trainees' attitudes or interest in providing end-of-life care to older adults. Under the frame of Terror Management Theory (Martens et al., 2005), this study examined the relationship of ageism, aging anxiety, and death anxiety with psychology trainees' therapeutic expectations and interest in engaging in end-of-life work with older adults. Using structural equation modeling, a model was tested to examine ageism, aging anxiety, and death anxiety as independent predictors of interest and therapeutic expectations towards end-of-life care with individuals in later life. In a sample of 197 psychology doctoral students enrolled in an APA-accredited program, the model showed good fit after modifications (CFI =.92, TLI =.90, RMSEA =.08, SRMR =.09). Higher aging anxiety predicted lower interest working with this population as well as worse therapeutic expectations. Ageism was not significantly related to therapeutic expectations but was positively related to interest. Death anxiety did not significantly predict either outcome variable. These findings suggest training programs and early clinical opportunities should consider the role of existential anxiety, particularly aging anxiety, as well as ageism's impact on trainees' career interests and perceptions towards working with older adults living with life-limiting illness.

Abstract citation ID: igae098.2394

### USING LIFE REVIEW TO EXPLORE DEATH EXPERIENCES AND SELF-MANAGEMENT AMONG HEALTHY OLDER ADULTS IN TAIWAN

Hsin-Ying Tsou[1], Yu-Ten Pan[2], and Tsuann Kuo[1], *1. Chung Shan Medical University, Taichung, Taichung, Taiwan (Republic of China)*, *2. Pingtung Ailiao Community Development Association, Pingtung, Pingtung, Taiwan (Republic of China)*

**Background:** Discussing death is considered a culturally sensitive topic in Eastern societies, as it may evoke concerns related to inheritance, filial piety, and mortality. Avoiding conversation about death can result in family members guessing the wishes of the deceased and may cause disputes and conflicts within the family. Purpose This study aims to explore the changing process of understanding death and self-arrangement among 11 healthy older adults in Taiwan through the use of life review program.

**Method:** This paper examines participation, dialogue, changes, and reflection in ten life review group sessions with 11 healthy older adults through participant observations and document text analysis. The sessions took place weekly for two hours between April to June in 2023.

**Result & discussion:** The process to reflect deaths and their self-management included four stages. 1) The "resistance" stage: participants expressed uncertainty by stating "I

don't know how to talk about it". 2) In "promotion" stage: participants were encouraged to share their experiences in managing funeral arrangements for family members, leading them to realize that "there is no standard answer for funerals, only respect for the deceased". 3) During the "change" stage: participants were willing to make arrangements regarding properties, medical decisions, funeral arrangements of their own. 4) In the "sharing" stage: an act was performed to summarize the course and to recognize that everyone was unique. **Conclusion:** The life review group can effectively guide older adults to discuss their death experience and self-arrangement.

Abstract citation ID: igae098.2395

### WHAT MATTERS IN HEALTH CARE TO OLDER NATIVE HAWAIIANS?

Miquela Ibrao[1], Rachel Burrage[1], Shelley Muneoka[1], Keilyn Kawakami[1], Tarin Tanji[2], Leslie Tanoue[3], and Kathryn Braun[4], *1. University of Hawai'i at Mānoa, Honolulu, Hawaii, United States*, *2. Rush University Medical Center, Chicago, Illinois, United States*, *3. ALU LIKE, Inc., Honolulu, Hawaii, United States*, *4. University of Hawaii, Honolulu, Hawaii, United States*

The formal assessment of What Matters for older adults is often done in a medical setting through the acquisition of legal forms to prepare for end-of-life care. For many indigenous elders, however, these forms may appear impersonal or culturally insensitive. What Matters should go beyond end-of-life planning and include preferences for how older adults live their daily lives, which may include preferences for dwelling status and relationships, in addition to medical care. For Native Hawaiian elders, cultural differences exist that impact their aging values and priorities. A secondary qualitative analysis of 20 interviews was conducted in partnership with Hā Kūpuna National Resource Center for Native Hawaiian Elders using a transformative lens to explore themes on What Matters to Native Hawaiian kūpuna as they age. Deductive coding was informed by guidelines on What Matters according to the Institute of Healthcare Improvement's 4Ms Framework. Inductive codes identified themes specific to Native Hawaiian kūpuna as part of their culture. NVIVO software was used. Themes identified on What Matters to Native Hawaiian older adults included: 1) incorporation of cultural traditions into healthcare routines; 2) a strong sense of family as part of the decision-making process; 3) a preference for home-based care at the end-of-life; and 4) the importance of provider relationship building. An infographic with education and recommendations for healthcare providers was developed using findings from the study. Our interview, coding, and dissemination processes may be of interest to researchers working with other minority groups.

### SESSION 7060 (POSTER)

### EPIDEMIOLOGY

Abstract citation ID: igae098.2396

### ASSOCIATION BETWEEN MULTIMORBIDITY AND KIDNEY FUNCTION DECLINE IN OLD AGE: A SWEDISH POPULATION-BASED COHORT STUDY

Giorgi Beridze[1], Lu Dai[1], Juan-Jesús Carrero[1], Alessandra Marengoni[2], Davide Liborio Vetrano[1], and Amaia Calderón-Larrañaga[1], *1. Karolinska Institutet, Stockholm, Stockholms Lan, Sweden*, *2. University of Brescia, Brescia, Lombardia, Italy*

We aimed to study the associations between multimorbidity (the presence of ≥2 conditions) and multimorbidity patterns, and the risk of kidney function decline in older adults. 3094 individuals from the Swedish National Study on Aging and Care in Kungsholmen (SNAC-K) were followed between 2001 and 2016. Multimorbidity was operationalised as the number of chronic conditions and multimorbidity patterns identified using latent class analysis. Joint models and Cox regression models were used to explore the associations between multimorbidity, and absolute and relative (≥25% decline from baseline) changes, respectively, in the estimated glomerular filtration rate (eGFR) calculated using the creatinine-based Berlin Initiative Study equation. Mean age of the sample was 73.9 and 87% had multimorbidity. There was a dose-response relationship between the number of chronic conditions, and absolute (β [95% CI] = -0.05 [-0.07;-0.03]) and relative (HR [95% CI] = 1.23 [1.17;1.29]) declines in eGFR. Five patterns of multimorbidity were identified. The Unspecific, low burden pattern had the lowest morbidity burden and was used as the reference category. The Unspecific, high burden and Cardiometabolic patterns showed accelerated absolute (β [95% CI] = -0.15 [-0.26;-0.05] and -0.77 [-0.98;-0.55], respectively) and relative (HR [95% CI] = 1.45 [1.09;1.92] and 3.45 [2.27;5.23], respectively) declines. Additionally, the Cognitive and Sensory pattern showed accelerated relative decline (HR [95% CI] = 1.53 [1.02;2.31]). No associations were found for the Psychiatric and Respiratory pattern. Multimorbidity is strongly associated with accelerated kidney function decline in older age. Individuals with cardiometabolic multimorbidity exhibit a particularly increased risk.

Abstract citation ID: igae098.2397

### ASSOCIATION BETWEEN WITHIN-DAY CALORIE INTAKE PATTERNS AND MORTALITY RISK AMONG US ADULTS

Ziling Mao[1], Anne Newman[2], and Samaneh Farsijani[1], *1. University of Pittsburgh, Pittsburgh, Pennsylvania, United States*, *2. University of Pittsburgh, University of Pittsburgh, Pennsylvania, United States*

Nutritional research on longevity has historically prioritized quantity of intake, with low-calorie diets as a prime example. Yet the crucial effects of intake timing and distribution on circadian rhythm and health are largely unexplored. Here, we determined the association between 24-hour calorie intake patterns and all-cause mortality.
**Methods:** We included 33,094 nationally representative US adults (47.6±0.2y; 52.4% women) from NHANES. First, we calculated calorie intake at 6-hour intervals within a day via 24-hour food recalls. Then, group-based trajectory analysis was performed, and four distinct temporal patterns of daily calorie intake were identified: "Skewed-to-Morning", "Skewed-to-Midday", "Skewed-to-Evening", and "Midday-Evening-Balance". Mortality was ascertained through December 2019 using the National Death Index.

Survey-weighted Cox hazard regression was used to determine the association between calorie intake patterns and all-cause mortality, adjusting for demographics, income, BMI, physical activity, smoking, alcohol, calorie intake, and diet quality.

**Results:** Among participants, 18% had morning-skewed, 22% midday-skewed, 22% evening-skewed calorie intake, and 38% had equal intake between midday and evening. During a median follow-up of 96 months, 4,162 participants died. In the unadjusted model, all skewed calorie intake patterns were associated with higher all-cause mortality compared to the Midday-Evening-Balance group (P< 0.05). After multivariable adjustment, Skewed-to-Morning (HR=1.18; 95%CI, 1.04-1.35) and Skewed-to-Evening (HR=1.20; 1.02-1.39) patterns remained significantly associated with higher mortality.

**Conclusion:** Calorie intake patterns skewed toward morning or evening may be associated with a higher mortality risk, independent of amount and quality of diet. This underscores the importance of considering both the quantity and the timing of daily calorie consumption.

Abstract citation ID: igae098.2398

### ASSOCIATION OF DIABETES WITH OLFACTION IN OLDER ADULTS: THE ATHEROSCLEROSIS RISK IN COMMUNITIES (ARIC) STUDY

Hanna Pleasants[1], Honglei Chen[2], Tom Mosley[3], Keran Chamberlin[4], Yaqun Yuan[5], Srishti Shrestha[3], Jayant Pinto[6], and Anna Kucharska-Newton[7], *1. University of North Carolina, Chapel Hill, North Carolina, United States*, *2. Michigan State University, Michigan State University, Michigan, United States*, *3. University of Mississippi Medical Center, Jackson, Mississippi, United States*, *4. Michigan State University, United States*, *5. Michigan State University East Lansing, East Lansing, Michigan, United States*, *6. University of Chicago, Chicago, Illinois, United States*, *7. University of North Carolina at Chapel Hill, Chapel Hill, North Carolina, United States*

Although extant research suggests a cross-sectional association between prevalent diabetes and poor olfaction, the long-term association of diabetes with olfactory impairment remains unclear. We examined the association of diabetes in midlife and in older adulthood, with olfaction ascertained in older adulthood among 6,122 participants of the ARIC cohort who attended ARIC Visit 5 (2011-2013; 58.6% female, 22.7% Black race). Diabetes status and hemoglobin A1c (HbA1c) levels were ascertained at midlife (Visit 2; 1990-1993; mean age 55.0 (SD 5.2)) and in older adulthood (Visit 5; mean age 75.8 (SD 5.2) years). Odor identification was measured at Visit 5 using the 12-item Sniffn' Sticks and categorized as poor (0-8) versus moderate or good (9-12). Binary logistic regression (exposure: diabetes status) and linear regression (exposure: HbA1C level) were used to model hypothesized associations. Diabetes prevalence at midlife (Visit 2) was associated with poor as compared to good olfaction in older adulthood (median follow-up time 21 years; adjusted odds ratio (AOR) (95% CI) = 1.25 (1.02, 1.53)). Similarly, greater HbA1c in midlife was associated with a lower olfaction score in older adulthood (beta coefficient: -0.101 (SE ± 0.04) per each 1 percent greater HbA1c level). Observed associations were attenuated when diabetes and HbA1c, in association with olfaction, were ascertained in older adulthood (all at Visit 5) (AOR 1.14 (95% CI: 1.00, 1.29); beta coefficient: -0.06 (SE ± 0.04), respectively). These findings support the association between midlife diabetes and impairment in olfactory function in older age.

Abstract citation ID: igae098.2399

### ASSOCIATION OF PROCOLLAGEN TYPE III N-TERMINAL PEPTIDE WITH AGE AND MORTALITY: THE LONG LIFE FAMILY STUDY

Ryan Cvejkus[1], Mary Wojczynski[2], Shiow Jin[2], Bharat Thyagarajan[3], and Joseph Zmuda[1], *1. University of Pittsburgh, Pittsburgh, Pennsylvania, United States*, *2. Washington University in St. Louis, St. Louis, Missouri, United States*, *3. University of Minnesota, Minneapolis, Minnesota, United States*

Procollagen type III N-terminal peptide (P3NP) is released into circulation during collagen biosynthesis and is a biomarker of fibrosis in several organs. Herein, we tested the association between baseline plasma P3NP concentration, measured using a sandwich enzyme-linked immunosorbent assay, and risk of all-cause mortality over 17 years in men (n=1882) and women (n=2277) aged 69.9 ± 15.5 (range, 32 – 110) years at baseline. Baseline median [IQR] P3NP level in the full cohort was 5.4 [4.5, 6.7] µg/L, and there was a significant positive linear trend across 10-year age groups. Among those aged 30-39, the median P3NP was 5.2 [4.4, 6.0] µg/L, and among centenarians, the median P3NP was 9.7 [7.9, 11.5] µg/L. Mortality risk, using 1442 observed deaths, was calculated per standard deviation (SD) of log P3NP using Cox Proportional Hazards models adjusted for baseline age, sex, height, weight, field center, alcohol use, smoking history, chronic diseases, serum creatinine, and familial relatedness. A 1 SD higher P3NP level was associated with an 18% increased risk of mortality (HR: 1.18, 95% CI: 1.10 - 1.26). The association was non-significantly stronger in women (HR: 1.25, 1.12 – 1.40) compared with men (HR: 1.11, 1.01 – 1.21). After further adjustment for physical function, the overall association remained but was attenuated (HR: 1.10, 1.02 – 1.18). In conclusion, higher P3NP was independently associated with mortality after adjustment for important covariates, especially in women, suggesting that the prevention of excess collagen deposition (fibrosis) may be particularly important for overall health and survival.

Abstract citation ID: igae098.2400

### DETERMINANTS AND TRAJECTORIES OF HEALTHY AGING IN OLDER AMERICANS

Lujain Sahab, *King's College London, Liverpool, England, United Kingdom*

**Background:** As the proportion of ageing individuals in the population increases, there is an increase in the burden of age-related chronic conditions. Therefore, it is essential to identify determinants of healthy ageing through extensive research and implement strategies to address challenges in society.

**Methods:** This analysis included an average of 8,340 participants, aged 50+ years, who participated in 6 waves (2006-2016) of the Health and Retirement Study. Healthy ageing measurement included freedom from cognitive impairment, freedom from disability, and high physical functioning. The study examined socioeconomic and ethnic inequality in healthy ageing over 10 years. Multilevel analysis was used to assess education, wealth, income and ethnic inequality in health ageing while accounting for demographic and behavioural factors.

**Results:** The analysis found that Females had lower odds for healthy ageing (OR: 0.64, 95% CI: 0.61–0.67). Black individuals had statistically significant lower odds of ageing healthily (OR: 0.87, 95% CI: 0.81–0.93) while hispanics had higher odds. The higher the educational degree the more likely you are to age healthily. Former smokers have a higher chance of healthy ageing (OR: 0.84, 95% CI: 0.80–0.88). The higher an individual's wealth and income the more likely they are to age healthily. People who reported their health as poor were more likely to age negatively (OR: 0.22, 95% CI: 0.20–0.23).

**Conclusion:** This study showed that Being male, white, wealthy, with a high income were associated with a higher chance of achieving healthy ageing.

Abstract citation ID: igae098.2401

### FACTORS ASSOCIATED WITH POOR SELF-RATED HEALTH BETWEEN AGES 70 AND 95: THE JERUSALEM LONGITUDINAL AGING STUDY

Irit Stessman-Lande[1], Aliza Rozenberg[1], Jeremy Jacobs[2], and Jochanan Stessman[1], *1. Hebrew University of Jerusalem and Hadassah Medical Center, Jerusalem, Yerushalayim, Israel*, *2. Hebrew University of Jerusalem/Hadassah Medical Center, Mount Scopus, Jerusalem, Yerushalayim, Israel*

Self-rated health assessment is widely used among individuals aged over 70 to subjectively gauge health status, with poor self-rated health associated with shorter life expectancy. This study aimed to identify risk factors for poor self-rated health between ages 70-95. The Jerusalem Longitudinal Aging Study (1990-2023) prospectively follows a representative community-dwelling cohort born 1920-21, assessed during home-visit during 1990, 1998, 2005, 2010, 2015 at ages 70, 78, 85, 90, and 95 (n=604, 1024, 1222, 729, 508) respectively. Participants answered the question "Do you feel healthy?"; those answering "no" were defined as having poor self-rated health. Depression emerged as the most significant risk factor for declining self-rated health, with a 3-4.6 times higher risk compared to those without depression across different age groups (P≤0.044). Self-reported financial difficulty, physical inactivity, impairments in activities of daily living, and background comorbidity showed inconsistent correlations with declining self-rated health. There was a robust statistically significant association between self-rated health and mortality across all ages between 70-100 years old, with poor self-rated health consistently predicting shorter life expectancy. Even after adjusting for other risk factors, the association between poor self-rated health and mortality remained significant. The findings suggest that while commonly accepted risk factors for illness might not directly correlate with decline in self-rated health, nonetheless depression emerges as a significant predictor. This implies that poor self-rated health could be a symptom of underlying affective status. Overall, the study underscores the importance of considering mental health factors in assessing self-rated health and predicting longevity between ages 70-100.

Abstract citation ID: igae098.2402

### PAIN BEHAVIORS AND PAIN TREATMENTS AMONG ENGLISH- AND SPANISH-SPEAKING HISPANICS IN US NURSING HOMES

Natalia Nielsen, Maira Castañeda-Avila, and Kate Lapane, *University of Massachusetts Chan Medical School, Worcester, Massachusetts, United States*

In U.S. nursing homes, racial/ethnic differences in nonverbal pain indicators and pain management have been documented among individuals with communication challenges. The role of language has yet to be explored. We evaluate the extent to which English- and Spanish-speaking Hispanics (H-E, H-S, respectively) were less likely to have nonverbal pain behaviors recorded and more likely to have no pain management relative to non-Hispanic English-speaking White residents (NHW-E). We identified newly admitted residents using national Minimum Data Set 3.0 data between 2010 and 2020 (n=1,201,724). Adjusted prevalence ratios (aPRs) and 95% confidence intervals (CI) estimated using robust Poisson models compared pain behaviors and treatments across ethnic/language groups. Vocal complaints and facial expressions were most commonly documented (Vocal: 29.1% NHW-E, 17.1% H-E, 18.5% H-S; Facial: 20.3% NHW-E, 15.0% H-E, 18.3% H-S). Documentation of pain behaviors was less frequent among Hispanic residents than NHW-E (e.g., any pain behaviors: aPR H-E: 1.17; 95% CI: 1.14-1.19; aPR H-S: 1.11; 95% CI: 1.10-1.13). Hispanic residents (H-E: 47.8%; H-S: 49.1%) were less likely to receive any type of pharmacologic pain intervention compared with NHW-E (59.4%) (aPR H-E: 0.85; 95% CI: 0.83-0.88; aPR H-S: 0.88; 95% CI: 0.86-0.90). Staff participating in the MDS assessment were less likely to document pain behaviors and its treatment among Hispanic residents relative to non-Hispanic white residents, with differences not observed based on resident's language. Interventions to improve recognition of pain behaviors among Hispanics are warranted.

Abstract citation ID: igae098.2403

### SELF-RATED HEATH PREDICTS INCIDENT MODERATE/SEVERE DISABILITY IN AGING MEN: THE MANITOBA FOLLOW-UP STUDY

Phil St. John, Scott Nowicki, and Robert Tate, *University of Manitoba, Winnipeg, Manitoba, Canada*

**Background:** There is evidence that Self-Rated Health (SRH) predicts death, but less evidence that SRH predicts new disability. Objective: To determine if SRH predicts moderate/severe disability in a prospective cohort study of aging men.

**Methods:** We conducted an analysis of a prospective cohort study of men who qualified for air crew training in the Royal Canadian Air Force, which began in 1948. Since 2004, an annual survey is sent out which measures age-reference SRH, functional status, and the Short-Form-36 (SF-36). In 2004, 796 men were alive, responded to the questionnaire and did not have disability. Functional status has been measured with an annual questionnaire which includes items on Basic Activities of Daily Living (BADLs) and Instrumental ADLs (IADLs). We considered impairment to be two or more BADLs impaired (of five considered) or three or more impaired IADLs (of seven). We calculated the median time to disability, and compared groups with a log rank test. We then constructed a proportional hazards model for the outcome of new disability. **Results:** SRH was associated with incident disability: Compared to those with Excellent SRH, the Hazard Ratio (95% confidence interval) was 1.79 (1.19, 2.68) for those with Very Good SRH; 2.87 (1.91, 4.30) for those with Good SRH; and 2.40 (2.04, 5.65) for those with Fair/Poor/Bad SRH. This association persisted after adjusting for age, the SF-36 and chronic diseases. Conclusions: SRH predicts new disability.

Abstract citation ID: igae098.2404
### THE VAGINAL MICROBIOTA BEFORE AND AFTER HYPEROSMOLAR LUBRICANT USE IN PRE-, PERI-, AND POSTMENOPAUSAL WOMEN

Sarah Brown[1], Xin He[2], Elizabeth Johnston[3], Katrina Mark[1], Jacques Ravel[1], and Rebecca Brotman[1], *1. University of Maryland School of Medicine, Baltimore, Maryland, United States*, *2. University of Maryland, College Park, Maryland, United States*, *3. University of Maryland Baltimore, Baltimore, Maryland, United States*

Vaginal lubricants are commonly used in clinical practice and to alleviate vulvovaginal symptoms in menopause. However, most products are hyperosmolar and may disrupt beneficial vaginal bacteria like Lactobacillus spp. We sought to investigate the impact of a single exposure to lubricant among patients referred for transvaginal ultrasound (TVUS) in which lubricant was used during the exam. We recruited 104 participants who self-collected mid-vaginal swabs daily for one week between baseline and TVUS, and immediately before and 6-12 hours after TVUS. Participants attended a visit 2-5 days after TVUS, continued to sample twice weekly, and attended a final clinical visit in week 10. Microbiota composition was characterized by 16S rRNA gene amplicon sequencing and assigned to community state types (CSTs; low-Lactobacillus CST-IV vs Lactobacillus-dominated CSTs I/II/III/V). Yue-Clayton theta indices defined vaginal microbiota stability before and after TVUS. Generalized linear mixed models evaluated the within-participant relative change in the odds of having a CST-IV microbiota after TVUS versus before. Our findings suggest that vaginal microbiota stability decreased after TVUS. Despite no overall change in the odds of a CST-IV microbiota after TVUS among all participants, certain subgroups showed increased risk. Specifically, peri/postmenopausal women (N=19, aOR:3.22, 95% CI:1.16-8.98) and those with a history of bacterial vaginosis (N=58, aOR:1.73, 95% CI:1.10-2.72) were more prone to decreases in Lactobacillus-dominant microbiota after TVUS. In contrast, premenopausal women and those with no bacterial vaginosis history appeared more resilient against perturbations. Reformulation may be necessary to reduce lubricant toxicity, particularly for peri/postmenopausal women who frequently use these products.

Abstract citation ID: igae098.2405
### TRANSITIONS BETWEEN MEDICARE FEE-FOR-SERVICE AND MEDICARE ADVANTAGE AMONG OLDER ADULTS WITH CANCER

Maira Castañeda-Avila, Mara Epstein, and Kate Lapane, *University of Massachusetts Chan Medical School, Worcester, Massachusetts, United States*

To date, SEER-Medicare analyses focused on Medicare Fee for Service (FFS) patients due to data availability. The rising enrollment in Medicare Advantage (MA) underscores the necessity of including these patients in cancer research. We examined transitions between FFS and MA among older adults diagnosed with cancer and explored factors associated with coverage by FFS, MA, or transitions between these types of insurance. From the time of diagnosis to end of follow-up, 62.3% were continuously enrolled in FFS, 21.3% were continuously enrolled in MA, 11.8% transitioned from MA to FFS, and 4.5% transitioned from FFS to MA. Continuous enrollment in MA was more common among women (Odds Ratio (OR) =1.25, 95% Confidence Interval (CI):1.22-1.28), Hispanic (OR=2.21, 95%CI: 2.14-2.28) and Black adults (OR=1.79, 95% CI: 1.74-1.85) and less likely among older adults ≥74 (OR=0.85, 95%CI: 0.83-0.86), unmarried people (OR=0.85, 95%CI:0.83-0.87), and people with brain, lung, and ovary cancer relative to prostate cancer (OR ranges: 0.35-0.46). Factors associated with transitioning from MA to FFS after a cancer diagnosis included age ≥74 (OR: 1.82; 95%CI: 1.77-1.86), Hispanic (OR: 1.98; 95%CI: 1.91-2.60) and Black adults (OR=1.56, 95%CI:1.91-2.06), or having a diagnosis of colorectal, leukemia, lung, lymphoma, multiple myeloma, oral cavity, ovary, and brain cancer relative to prostate cancer (OR range: 1.96-3.69). Black (OR=2.20, 95%CI:2.09-2.33) and Hispanic (OR=1.70, 95%CI:1.60-2.33) adults were more likely to transition from FFS to MA. Understanding reasons for switching types of Medicare insurance among patients with cancer may be important for interpreting studies of social determinants of health using SEER-Medicare data.

Abstract citation ID: igae098.2406
### TWENTY-FOUR-HOUR ACTIVITY COMPOSITION ASSOCIATIONS WITH MUSCLE POWER AND STRENGTH IN OLDER MEN AND WOMEN

Lauren Roe[1], Elsa Strotmeyer[2], Nancy Glynn[3], Peggy Cawthon[4], Yan Ma[3], Megan Hetherington-Rauth[4], Stephen Kritchevsky[5], and Jane Cauley[3], *1. University of Pittsburgh School of Public Health, Sewickley, Pennsylvania, United States*, *2. University of Pittsburgh, Pittsburgh, Pennsylvania, United States*, *3. University of Pittsburgh School of Public Health, Pittsburgh, Pennsylvania, United States*, *4. California Pacific Medical Center, San Francisco, California, United States*, *5. Wake Forest University School of Medicine, Wake Forest University School of Medicine, North Carolina, United States*

Physical activity (PA) and sedentary (SED) behavior have opposing associations with skeletal muscle function and are often studied separately, but are codependent. We evaluated associations of 24-hr movement behaviors [moderate-to-vigorous intensity (MVPA), light intensity (LPA), SED, and sleep] with muscle power and strength in 714 community-dwelling older adults (58% women, 76±5 years) from the Study of Muscle, Mobility and Aging (SOMMA).

Compositional data analysis applied to 24-hr accelerometry (wrist-worn) determined proportions of time spent in each behavior relative to the remaining behaviors. Multiple linear regression evaluated associations between 24-hr composition with peak stair climb ascend power and leg press power (W), and maximum leg press strength and grip strength (kg). Compositional isotemporal substitution methods determined theoretical changes in the outcome if 10-60 minutes of one behavior was displaced by another in 10-minute increments. Higher proportions of MVPA vs. other behaviors were associated with higher leg press power in men (β: 67.5, 95% CI: 19.9, 115.1) and women (β: 29.1, 95% CI: 8.1, 50.2), and higher stair climb power in women (β: 25.0, 95% CI: 16.5, 33.4). Higher proportions of LPA vs. remaining behaviors were associated with lower leg press power in men (β: -117.9, 95% CI: -193.7, -42.1) and women (β: -39.0, 95% CI: -73.5, -4.6), and lower stair climb power in women (β: -31.6, 95% CI: -45.5, -17.8). No significant associations were found for strength. The most effective time-use substitution to improve muscle power may be through displacing SED or LPA with MVPA in older men and women.

Abstract citation ID: igae098.2407

### USING MIXED EFFECTS GROWTH MODEL TO EXAMINE LONG-TERM COURSE OF PHYSICAL ACTIVITIES AMONG POSTSTROKE PATIENTS

Ling Han[1], Geske Luzum[2], Heather Allore[3], Torunn Askim[4], Xiangchun Tan[4], Pernille Thingstad[4], Asta Håberg[4], and Ingvild Saltvedt[4], *1. Yale University School of Medicine, New Haven, Connecticut, United States*, *2. Norwegian University of Science and Technology, Trondheim, Nord-Trondelag, Norway*, *3. Yale University, New Haven, Connecticut, United States*, *4. Norwegian University of Science and Technology, Trondheim, Sor-Trondelag, Norway*

Restoring function and regaining physical activity (PA) is a fundamental goal of post-stroke rehabilitation programs, yet empirical evidence is lacking for long-term PA. Using data from the Nor-COAST study, we followed 277 individuals (mean age at baseline: 70.1, SD 10.9). Using a thigh-worn accelerometer, PA was measured for total upright time (UPT) in minutes over each 24-hr period for 6 consecutive days at 3-, 18-, and 36-months post-stroke. A mixed effects linear growth model examined post-stroke PA course, with a patient-specific random intercept to account for nesting effects of repeated UPT measurements. On average the UPT times declined by 0.61 min (95% Confidence Interval (CI): 0.84-0.62) per month after adjusting for ten a priori selected confounding factors. The adjusted average UPT is 42.5 min (95% CI: 67.2-18.1) shorter in men than in women; while one-yr aging reduced UPT time by 22.9 min (95% CI: 35.3-10.5). As expected, the day-1 NIH Stroke Scale (mean 2.8, SD 4.3) was associated with shorter UPT time (Mean difference: 7.3 min (95% CI: 3.2-11.3)). The final adjusted model revealed substantial between-patient UPT variations (intercorrelation coefficient, 0.55). These findings suggest that the PA of post-stroke patients appear to follow a modest, yet declining course over the 3 years, with substantial variation across individuals with different demographic and clinical profiles, calling for extending professional follow-up and individually tailored PA rehabilitation programs. Future studies may need to consider broader contexts of enabling PA of patients and their families, such as "walkable" neighborhoods and socioeconomic environments of communities.

Abstract citation ID: igae098.2408

### WHAT COMES FIRST? SENTINEL CHRONIC AND SUBSEQUENT MULTIMORBIDITY AMONG OLDER ADULTS

Dongmei Zuo, and Solveig Cunningham, *Emory University, Atlanta, Georgia, United States*

The majority of the U.S. adult population is affected by at least one chronic condition. To develop clinical practice guidelines and early prevention interventions, it is necessary to understand the progression of multimorbidity. We investigated the first chronic condition, or sentinel condition, developed by individuals using 15 waves of longitudinal data from the Health and Retirement Study, 1992-2020. Hypertension, arthritis, and heart diseases emerged as the most frequent sentinel conditions. Employing weighted multinomial logistic regression models, we assessed the association of onset age, sex, and race/ethnicity with the likelihood of these conditions relative to a combined category of cancer, diabetes, lung diseases, or stroke as the first onset disease, adjusting for education, marital status, and household income. We find that, compared to having cancer, diabetes, lung, or stroke diseases as the sole first onset condition, each additional year of age decreases the odds of having hypertension and heart disease as the first onset disease by 1.9% and 2.2%, respectively, and decreases the odds of arthritis by 1.8%. Being Hispanic and non-Hispanic Black increases the odds of hypertension as the first onset disease by 64% and 19%, respectively. Being female increases the odds of arthritis by 60%, while being Hispanic increases the odds by 16%, and being non-Hispanic Black decreases the odds by 44%. For multimorbidity, being female and non-Hispanic Black decreases the odds by 12% and 36%, respectively, while being Hispanic increases the odds by 55%, relative to cancer, diabetes, lung diseases, or stroke as the first onset disease.

### SESSION 7065 (POSTER)

### FAMILY CAREGIVING I (POSTERS)

Abstract citation ID: igae098.2409

### COMMUNITY RATES OF CAREGIVING IN RI AND CT: FINDINGS FROM THE HEALTHY AGING DATA REPORTS

Shan Qu[1], Taylor Jansen[1], Chae Man Lee[1], Nina Silverstein[2], and Elizabeth Dugan[1], *1. University of Massachusetts Boston, Boston, Massachusetts, United States*, *2. University of Massachusetts Boston, Needham, Massachusetts, United States*

Informal caregivers are a significant but often overlooked part of the US healthcare system. Grandparents may provide and receive care and may be important members of the informal support network for many families. The demands and stresses of caregiving may negatively impact caregivers' health. Understanding the prevalence of caregiving at the community level can guide resource allocation, policy decisions, and advocacy efforts to support caregivers and improve all residents' well-being. The present study examines disparities in community rates in Rhode Island (RI)

and Connecticut (CT) for three caregiving indicators: older adults aged 60+ who provided caregiving in the last month, grandparents raising grandchildren, and grandparents who live with grandchildren. For this study, rates were calculated from the following data sources: the Behavioral Risk Factor Surveillance System (2015-2017) and the American Community Survey (2016-2018). Small area estimation techniques were used to calculate age-sex adjusted community rates (https://healthyagingdatareports.org/). Results showed that while RI had a slightly higher statewide rate of caregiving (22.5% vs 21.7% in CT) the range in community rates (14.84%-27.43%) was much larger in CT compared to RI (19.17%-27.30%). RI (0.8%) also had a slightly higher rate of grandparents raising grandchildren than CT (0.7%), but Hartford CT had the highest community rate in the two states (2.93%). RI (2.8%) also had a higher rate of grandparents who live with grandchildren than CT (2.5%). This study found that older adults providing caregiving is prevalent, and older adults play a significant role in raising grandchildren in CT and RI.

Abstract citation ID: igae098.2410

### CRITICAL OR ENCOURAGING? CORRELATES OF FAMILY CAREGIVERS' DEMENTIA MANAGEMENT STRATEGIES

Amanda Leggett[1], Jonathan Troost[2], Sophia Tsuker[1], Jennifer Miner[2], and Noelle Carlozzi[2], *1. Wayne State University, Detroit, Michigan, United States*, *2. University of Michigan, Ann Arbor, Michigan, United States*

Family caregivers approach caregiving challenges with a variety of management strategies which may be adaptive or maladaptive and impact the quality of care provided to individuals living with dementia. This study explores caregiver demographics, the caregiving context, resentment toward the individual with dementia, and caregiver readiness as predictors of dementia management strategies. Participants included 200 family caregivers for a person living with dementia. Descriptive statistics and adjusted linear regressions were run to examine associations between caregiver demographics, the care context (dementia diagnosis, dementia severity rating scale), the Sense of Competence Care Recipient Satisfaction Scale, and the Caregiver Readiness Scale with three outcomes: criticism, active management, and encouragement (Dementia Management Strategies Scale). Caregivers were 58 years old on average, 82% female, 28% non-White, and 38% adult daughters. In models including caregiver demographics and the care context, caregiver readiness consistently related to dementia management as it was associated with less criticism ($B=-0.23$, $p<.01$), more active management ($B=0.26$, $p<.01$), and more encouragement ($B=0.20$, $p<.01$). Greater financial difficulty ($B=2.57$, $p<.05$), less dementia severity ($B=-0.17$, $p<.001$), and provision of care for an individual with Alzheimer's disease (relative to early-onset Alzheimer's disease) ($B=-3.01$, $p<.05$) were associated with more encouragement. Further, individuals with more resentment toward the individual with dementia used more criticism ($B=0.86$, $p<.001$). Readiness for care appears to be a key modifiable intervention target to improve care management quality provided by family caregivers. Caregiver demographics did not relate to care management once the caregiving context was accounted.

Abstract citation ID: igae098.2411

### ENHANCING POSITIVE AFFECT AND CAREGIVER EFFICACY (ENHANCE): INTERVENTION DEVELOPMENT FEEDBACK

Hannah Mason[1], Ana Sophia Garces Torres[2], E-Shien Chang[3], and Sara Czaja[4], *1. Weill Cornell Medicine, New York, New York, United States*, *2. Weill Cornell Medicine/Center on Aging and Behavioral Research, New York, New York, United States*, *3. Weill Cornell Medical College, New York, New York, United States*, *4. Weill Cornell Medicine/Center on Aging and Behavioral Research, New York, New York, United States*

Currently about 11.5 million informal caregivers are providing care for older adults with Alzheimer's Disease and Related Dementias (ADRD). The overburdening of these caregivers fosters an urgency for assistive programs and services. Two important determinants of caregivers' use of available services are accessibility and relevance, both having potential to be a facilitator or barrier to service use. To address the need for accessible and efficacious caregiver interventions, the Enhancing Positive Affect and Caregiver Efficacy (ENHANCE) intervention program is being developed. ENHANCE is a multicomponent, technology-based and personalized intervention that offers self-guided opportunities and one-on-one sessions to deliver caregiving skills, access to resources, and intrapersonal support. As part of the development process, two focus groups (n=11) were conducted with ADRD caregivers to identify caregiving challenges and obtain feedback on the ENHANCE intervention prototype. This presentation will discuss findings from the focus groups. Thematic analysis revealed three common challenges among the caregivers: (1) identity loss and emotional repression, (2) communication difficulties particularly with family and healthcare providers, and (3) unreliable formal support services. Caregivers were enthusiastic about the topics included in the ENHANCE program, particularly legal/financial planning and communication. Several participants wanted to meet again, highlighting the value of open discussion among caregivers and the importance of having their voices at the table when developing caregiving services. Incorporating caregivers' perspectives to aid the development of caregiving interventions like the ENHANCE program can improve their design, utility, and accessibility.

Abstract citation ID: igae098.2412

### FAMILY CAREGIVING COMPETENCIES FOR NURSING EDUCATION: COURSE AND CURRICULAR INTEGRATION

Sara Hart[1], Andra Davis[2], Kathryn Sexson[3], Hui Zhao[4], Tanya Seward[5], Connie Perkins[6], Nannette Cowen[7], and Jennifer Mongoven[3], *1. University of Utah, Salt Lake City, Utah, United States*, *2. University of Portland, Portland, Oregon, United States*, *3. University of California Davis, Davis, California, United States*, *4. James Madison University, Harrisonburg, Virginia, United States*, *5. University of Providence, South Great Falls, Montana, United States*, *6. St. Bonaventure University, St. Bonaventure, New York, United States*, *7. Binghamton University, Binghamton, New York, United States*

The U.S. health care system is increasingly dependent on family caregivers who play an essential role in addressing the needs of loved ones who are aging, managing chronic or disabling conditions, or facing life-limiting illnesses. The National Consortium for Family Caregiving in Nursing Education has developed twenty entry-level nursing education competencies for identifying, integrating, and supporting family caregivers in person-centered and family-centered healthcare teams. These competencies fall within four domains: the Nature of Family Caregiving, Family Caregiving Identification and Assessment, Providing Family-centered Care, and the Context of Family Caregiving. Given the time and content limitations of nursing education and the dynamic nature of healthcare, new educational competencies often encounter resistance. Here we present the competencies, along side course and curricular resources and exemplars created by the Consortium. These resources are designed to support nursing educators in identifying and integrating content and teaching methods that advance the inclusion of family caregivers in patient and family-centered care teams and that promote nursing student development of family caregiving competencies.

Abstract citation ID: igae098.2413

### HEALTH INSURANCE AND FAMILY CAREGIVERS: CAREGIVERS' PERCEPTIONS OF COMMUNICATION BY SERVICE PROVIDERS

Alexa Balmuth[1], Adam Felts[1], Sophia Ashebir[1], Samantha Brady[2], Chaiwoo Lee[1], Lisa D'Ambrosio[2], and Joseph Coughlin[2], *1. Massachusetts Institute of Technology, Cambridge, Massachusetts, United States*, *2. MIT AgeLab, Cambridge, Massachusetts, United States*

Health insurers not only play a key role in older adults' access to medical care and services, but also interact with and, in some cases, provide benefits for caregivers of older family members. Additionally, family caregivers interact with their care recipient's insurer to manage healthcare-related payments and when seeking information and support for their caregiving role. The relationship between health insurers and family caregivers can be just as important as their relationship with the plan member. Understanding how caregivers perceive and interact with their care recipient's insurer is important to ensuring continuity of communication and care for older adults, as well as support for caregivers themselves. Paired with qualitative interviews, results from a survey of 244 current family caregivers in fall 2023, found that despite high levels of reported health insurance coverage among care recipients, many caregivers reported that their loved one's health plans did not provide caregiving benefits, and many reported that they did not know if their care recipient's health insurance offered any caregiving benefits. Results highlight caregivers' perceptions and trust in health insurers – and how these vary across insurance types and providers. This session will provide implications for health service providers to improve communications with caregivers, including communications around their full range of offerings to caregivers and identifying caregivers' preferred frequencies and modes of communication with insurers. Ultimately, this research aims to contribute to better health outcomes and quality of life for older adults and improved support for family caregivers.

Abstract citation ID: igae098.2414

### HOURS CAREGIVING, ADLS, ROLE OVERLOAD, AND DEPRESSION SYMPTOMS: AN EXAMINATION AMONG FAMILY DEMENTIA CAREGIVERS

Claire McDonald[1], and Adrian Bravo[2], *1. University of Maryland Baltimore County, Baltimore, Maryland, United States*, *2. William & Mary, Williamsburg, Virginia, United States*

The present study examined the associations between hours spent caregiving, Activity of Daily Living (ADL) dependency, role overload as a stressor, and depression symptoms among caregivers of adults aged 65 and older living with probable dementia. Specifically, we hypothesized that more time spent caregiving and greater ADL dependency would both be associated with more depression symptoms via greater role overload. To test study aims, we analyzed data from round 11 of the National Health and Aging Trends Study (NHATS) and the National Study of Caregiving (NSOC). To replicate findings from NHATS and NSOC, a second sample of data was collected using Academic Prolific. In the NHATS sample, there were 283 caregivers and the majority of caregivers were female (70%), white (63%) and reported an average age of 65. For the Prolific sample, there were 150 caregivers and the majority were female (51%), white (68%), and reported an average age of 40. Mediation models were tested using the PROCESS Macro (Hayes, 2013) in SPSS. In both NHATs and Prolific samples, role overload significantly mediated the associations between hours caregiving and depression symptoms (NHATs indirect $\beta$ =.119, CI [.07,.18]) (Prolific indirect $\beta$ =.118, CI [.02,.21]). In both NHATs and Prolific samples, role overload significantly mediated the associations between ADL dependency and depression symptoms (NHATs indirect $\beta$ =.07, CI [.01,.13]) (Prolific indirect $\beta$ =.111, CI [.02,.20]). These findings highlight the impact of burden among family dementia caregivers and should be investigated in more diverse populations.

Abstract citation ID: igae098.2415

### HOW DO FAMILY RELATIONSHIP PATTERNS IMPACT CAREGIVING BURDEN IN ADULT CHILD CAREGIVERS?

Jodi Heys, and Jessica Strong, *University of Prince Edward Island, Charlottetown, Prince Edward Island, Canada*

Living with dementia is not isolated to the person with the diagnosis. Family and friends play an integral role in providing support, advocacy, love, and informal care for a person with dementia. Spouses and adult child caregivers have a unique relationship with the person with dementia (PWD), and often report a different subjective experience of caregiving (Rigby, et al, 2019). Due to limited research on how family relationships pre-caregiving impact the caregiving experience, the goal of this study is to gain a better understanding of these complex dynamics, particularly for adult child caregivers. The current study looked at perceptions of cohesion, expressiveness and conflict in family of origin (Brief Family Relationship Scale), as well as anxiety (GAD-7), mood (PHQ-9), and caregiver experiences, including burden, overload, and competence, among others (Caregiver Reaction Scale) in a population of adults (28-68 years old, N=25). Preliminary analysis results indicate a relationship between family conflict pre-caregiving and caring overload ($r$ =-0.49, $p$ = 0.04), such that lower levels of conflict were related to lower levels of overload. Additionally, we found no significant correlations between earlier family patterns of cohesion or conflict with adult anxiety (Cohesion: $r$ =0.15, $p$ = 0.55; Conflict: $r$ =-0.11, $p$ = 0.65) or depression (Cohesion: $r$ =0.05, $p$ = 0.85; Conflict: $r$ =-0.18, $p$ = 0.50). We discuss the current findings in the context of family systems theory and child-parent relationships in adulthood.

Abstract citation ID: igae098.2416

### IDENTIFYING CAREGIVERS IN US COMMUNITIES WHO REPORT A LACK OF SUPPORT SERVICES

Greta Kilmer[1], Akilah Ali[1], Jenny Walker[1], and Katie Spears[2], *1. Centers for Disease Control and Prevention, Atlanta, Georgia, United States*, *2. Oak Ridge Institute of Science and Education, Georgia, United States*

Support services for caregivers are important community health tools that can help address health equity. Many caregivers report support services as unavailable, and understanding the characteristics of these caregivers could help identify who could benefit from greater availability or accessibility. Caregivers were identified in 2023 Summer ConsumerStyles for U.S. adults (n=437) and 2022 Estilos for US Hispanic adults (n=122). Availability and use of seven support services were assessed (out-of-home services, in-home services, food and nutrition services, transportation services, financial planning, caregiver support groups or classes, and caregiver skills-based training). Respondents were asked if each of the services were available for them or their care recipient (yes/no), and if they currently used or would use the service (yes/no). Values were weighted in accordance with the U.S. Current Population Survey proportions. A lack of at least one support service was identified if they reported it not available and they would use the service if available. In 2023, 34.2% of U.S. caregivers overall reported a lack of at least one service, which was significantly associated with caregivers having difficulty paying household expenses ($p< 0.001$) and having a care recipient with Alzheimer's disease, dementia, or other cognitive impairment ($p< 0.05$). In 2022, over half of Hispanic caregivers reported a lack of at least one service (56.7%), which was associated with fewer years living in the U.S. ($p< 0.01$). Findings suggest that community support services are not always available or accessible, and caregivers with financial difficulties or recent immigration report a particular lack of community-based caregiving support resources.

Abstract citation ID: igae098.2417

### LONG-DISTANCE FAMILY CAREGIVERS' APPRAISAL OF BURDEN AND STRAIN SCALES: FINDINGS FROM THE LDCARE INTERVENTION

Richard Chunga[1], Verena Cimarolli[2], Kathrin Boerner[3], and Sara Czaja[4], *1. University of Massachusetts Boston, Boston, Massachusetts, United States*, *2. LeadingAge, Washington, District of Columbia, United States*, *3. Carl von Ossietzky University of Oldenburg, Oldenburg, Niedersachsen, Germany*, *4. Weill Cornell Medicine/Center on Aging and Behavioral Research, New York, New York, United States*

Although there is a significant and growing number of long-distance family caregivers (LDCs) of older adults with dementia, most evidence-based interventions have been designed and evaluated for caregivers living geographically close to their care recipients. Commonly used scales were designed for proximate caregivers to assess their levels of burden and strain and used to measure outcomes of caregiver interventions. However, LDCs face unique challenges that such scales may not adequately capture. As part of the feasibility testing of a new, remotely delivered, psychoeducational intervention for LDCs, called LDCare, several commonly used burden and strain scales were administered to 40 participating LDCs. During the pre-intervention assessment, the Zarit Burden Interview-12, Pearlin's Family Conflict Scale, Job-Caregiving Conflict, and Role Captivity Scale were administered to the LDCs. After each scale, open-ended questions were included to provide an opportunity for the participants to evaluate the scales' relevance to their experience as LDCs. Across scales, most participants stated that the questions described their thoughts and feelings well in general, however, comments regarding some questions' irrelevance to long-distance caregiving were most common for the Zarit Burden Interview-12 and the Role Captivity Scale. Participants remarked that lower everyday caregiving engagement and geographic distance rendered some questions difficult to answer. Concerns among some LDCs regarding the scales' relevance to their situation indicate that further research is needed to better understand how questions may be tailored to their unique experiences.

Abstract citation ID: igae098.2418

### NATURE AND CHALLENGES OF TRANSNATIONAL FAMILY CAREGIVING: FINDINGS FROM A SYSTEMATIC LITERATURE REVIEW

Mohammad Hossain[1], Natalie Pope[1], and Regina George[2], *1. University of Kentucky, Lexington, Kentucky, United States*, *2. University of Alabama, Tuscaloosa, Alabama, United States*

This study explored the nature and challenges of transnational family caregiving via a systematic literature review. Increasing international migration and global aging have resulted in growing instances of transnational family caregiving, which involves providing care for older adults across national borders. However, little is known about the nature and challenges of such caregiving. Following PRISMA guidelines, a structured literature search was conducted using targeted search terms (e.g., transnational*, cross-border*, caregiv*, burden*) in 10 electronic databases (e.g., AgeLine, MEDLINE, CINAHL). Predefined eligibility criteria included empirical papers: (i) published in peer-reviewed English language journals, (ii) focused on the nature and/or challenges of transnational caregiving, and (iii) focused on caregiving for older adults. After removing duplicates, screening the titles and abstracts, and reviewing remaining full-texts, nine articles were deemed eligible for inclusion. Studies showed that transnational caregiving involves provision of financial and emotional support, arranging care, mailing gifts and letters, making care decisions, and communicating via phone/video calls. Financial strain came from expenses in the host and support countries, including traveling back to the home country. Transnational caregivers experience guilt, worry, and distress related to the unpredictability of the care situation and their inability to be physically present. Strict immigration policies, inadequate employer support, and limited institutional care in home countries also present challenges. Despite limitations of research in this area (e.g., qualitative studies; small, non-random samples), this review highlights the nature and challenges experienced by transnational caregivers. Further empirical research is needed to deepen our understanding of this unique subpopulation of caregivers.

Abstract citation ID: igae098.2419

### PROFILING DEMENTIA FAMILY CAREGIVING NETWORK MEMBERSHIP AND PSYCHOSOCIAL FUNCTIONING

Amanda Leggett, Srabani Haldar, Wenhua Lai, Sophia Tsuker, and Natasha Nemmers, *Wayne State University, Detroit, Michigan, United States*

Family caregiving research predominantly takes a "primary" caregiver lens, while ignoring the multifaceted caregiving networks surrounding a person living with dementia (PLWD). We explore profiles of dementia family caregiving networks based on their compositional and psychosocial characteristics. Our sample includes 184 dementia family caregiving networks drawing data from the 2017 National Health and Aging Trends Study and the associated National Study of Caregiving. Latent profile analysis was run to define distinct network profiles considering compositional (e.g., proportion of immediate family, extent of task sharing) and psychosocial (e.g., network mean and variability in caregiver stress, family disagreement surrounding care) components. Chi-squares and ANOVAs test network differences in PLWD demographic characteristics. A four-profile network was found to have best fit based on the AIC and BIC: 1) Adjusted specialists (38% of sample)- immediate family network, limited task sharing, high psychosocial well-being; 2) Disagreeing (30%)- diverse network, sharing self-care tasks, mixed reports of overload and family disagreement; 3) Strained (16%)- diverse network, limited sharing, poor psychosocial adjustment; and 4) Generalists (16%)- immediate family generalists, extensive task sharing, caregiving burden. PLWDs' race varied significantly by network profile ($\chi 2=29.8$, $p<.001$). In particular, 78% of the Strained networks were assisting White PLWD while 49% of the Generalist networks were assisting Latinx/other race PLWD. Networks varied psychosocially even when composition and task sharing were similar, suggesting the need to consider both factors in defining networks. How networks function may have critical implications for care quality and well-being of both caregivers and PLWD.

Abstract citation ID: igae098.2420

### QUALITY OF LIFE IN DEMENTIA CAREGIVERS AFTER USING ONLINE SELF-GUIDED ACCEPTANCE AND COMMITMENT THERAPY

Josey Batura[1], Jacob Gossner[2], Ty Aller[2], and Elizabeth Fauth[2], *1. Utah State University, Providence, Utah, United States*, *2. Utah State University, Logan, Utah, United States*

**Background:** At home family care for individuals with dementia averages 24.4 hours per week, with one in four caregivers providing 40+ hours. Caregivers identify positive experiences but also report burnout. Acceptance and Commitment Therapy (ACT) is an empirically validated transdiagnostic approach that aims to increase individuals' psychological flexibility, or their ability to respond effectively to their internal experiences in the present moment. This study examines the efficacy of a 6-session online, self-guided ACT for Caregivers program. Research Design and **methods:** A randomized waitlist-controlled trial included 114 dementia caregivers of whom 28 participated in two semi-structured interviews: at end of sessions (post-test) and 6 weeks later (follow-up; each 45-60 minutes). Participants were recruited from the community. Zoom interviews were transcribed and coded by three researchers using deductive qualitative analysis. Current analyses focused on the self-reported impact of the program on participants' quality of life. The qualitative subsample included 20 (71.4%) spousal and 8 (28.6%) adult offspring caregivers (Age M= 64.2; SD= 13.3).

**Results:** Longitudinal coding of post-intervention and follow-up interviews indicated themes of positivity, greater self- compassion, feeling less stressed and calmer, living more vibrantly, and leaning into social supports. They also reported engaging in improved health behaviors.

**Discussion:** Quality of life is an ideal outcome to examine qualitatively, as ACT aims to enhance QoL, rather than merely reduce negative symptoms. Participation in an online, self-guided ACT program yielded initial and sustained improvements in multiple domains of QoL, and participants emphasized different aspects of improvements in QoL at post-test and follow-up.

Abstract citation ID: igae098.2421

### RACE, CAREGIVING, AND PERCEIVED DISCRIMINATION AMONG WORKING ADULTS IN THE UNITED STATES

Maureen Templeman[1], and William Haley[2], *1. Missouri State University, Springfield, Missouri, United States*, *2. University of South Florida, Tampa, Florida, United States*

General and workplace discrimination have been studied extensively in the general population, with Black/AA individuals consistently reporting more discrimination than White individuals. However, the intersection of race, employment, and caregiving has rarely been studied. We applied stress process and minority stress theories to address these issues. Using data from the Midlife in the United States study, we examined perceived general discrimination (lifetime and everyday) and workplace discrimination (chronic job discrimination and work inequality) among working Black/AA caregivers (n = 50), working Black/AA non-caregivers (n = 396), working White caregivers (n = 266), and working White non-caregivers (n = 2895). We conducted a series of two-way (caregiver status, race) analyses of variance and covariance to investigate the role of perceived general and workplace discrimination in the stress process for working Black/AA and White caregivers and non-caregivers. We also conducted item analyses to explore whether some experiences of discrimination were identified as particularly common for Black/AA and White caregivers. Results indicated that working Black/AA adults reported more lifetime and everyday discrimination and work inequality than working White adults. Working caregivers also reported more lifetime discrimination than working non-caregivers. Item analyses revealed several specific experiences of discrimination that were especially common among Black/AA caregivers, including being hassled by the police. Results are consistent with prior research demonstrating high levels of discrimination experienced by Black/AA individuals, but findings that working caregivers, particularly Black/AA working caregivers face additional discrimination are troubling and deserve future research attention.

Abstract citation ID: igae098.2422

**RESILIENCE IN DIVERSE SOLO OLDER GRANDMOTHERS RAISING ADOLESCENT GRANDCHILDREN**

Tina Peterson, *University of Cincinnati, Cincinnati, Ohio, United States*

Grandfamilies scholars often focus on shared caregiving responsibilities supported by a spouse or partner when parenting younger grandchildren. Less attention has been given to solo older grandparents raising adolescent grandchildren. This research will apply Dannison and colleagues' (2011) framework of the 5 P's (proud, positive, persistent, protective, problem-solvers) of Resilient Grandparent Caregivers to explore resiliency in solo older grandparents raising adolescent grandchildren. Solo grandparent caregivers (n=8) participated in a phenomenological study to examine Caregiving in Later Life by Grandparents Raising Adolescent Grandchildren. Participants were primary caregiver for a grandchild 12 or older; grandchild in home at least 3 days weekly; and grandparent 40 or older. Data collection via semi-structured interviews occurred across three states between March 2015 and June 2015. Interviews were audiotaped, transcribed verbatim, and analyzed using conventional content analysis. Racially and ethnically diverse grandmothers (100%) reported an average age of 61 years (range 56 to 67) with caregiving responsibility for grandchildren with a median age of 14 years. These solo older grandmothers described being proud (e.g. joy, energy, grandchildren's talent, liveliness, etc.); positive (e.g. telling jokes, picking battles, reframing behavior, music, etc.); persistent (e.g. kinship foster care placement, most challenging role, navigating multiple responsibilities, etc.) protective (e.g. setting boundaries, honoring holiday celebrations, structure, etc.) and problem-solvers (e.g. seeking therapy, advocating within schools, generational gifts, resolving purchasing for multiple kids, instilling time management, etc.). Reflections on resilience from diverse solo older grandmothers raising adolescent grandchildren can promote strategies to support and advocate for underserved grandfamilies.

Abstract citation ID: igae098.2423

**THE IMPACT OF SLEEP QUALITY ON NEUROCOGNITIVE FUNCTIONING IN CAREGIVERS OF PERSONS WITH DEMENTIA**

Lauren Chrzanowski[1], Lauren Elliott[1], Sydnie Schneider[1], Keegan Diehl[1], Amir Abu-Samaha[1], Volker Neugebauer[2], and Jonathan Singer[1], *1. Texas Tech University, Lubbock, Texas, United States*, *2. Texas Tech University Health Sciences Center, Lubbock, Texas, United States*

Studies have found poor sleep quality is linked to decreased neurocognitive functioning in older adults. Notably, it has been found that older adults who exhibit worse sleep quality have decreased processing speed. However, research with caregivers is extremely limited and current research lacks the use of validated measures and neurocognitive assessments across several functional domains (e.g., processing speed; executive functioning). Further, most of these studies have been conducted with large urban samples. This study included family caregivers of persons with dementia in Lubbock County, which includes a small urban city and multiple rural cities. We compared Pittsburgh Sleep Quality Index (PSQI) scores with Trails Making Test delta (i.e., processing speed), Repeatable Battery for the Assessment of Neuropsychological Status (RBANS; i.e., overall neurocognitive functioning), and the Wisconsin Card Sorting Test (WCST; standardized percentiles of perseverative errors and conceptual level responses; i.e., executive functioning). There was no significant relationship between sleep and processing speed ($r=-.173$, $p=.143$), executive functioning (i.e., WCST standardized percent perseverative errors ($r=-.219$, $p=.090$); WCST standardized percent conceptual level responses ($r=-.256$, $p=.059$)), and overall neurocognitive functioning ($r=-.110$, $p=.269$). Interestingly, in this unique population of family caregivers there appears to not be a relationship between sleep and neurocognitive functioning in the domains examined. More research regarding sleep and cognition in family caregivers is needed, particularly that which addresses the interplay of sleep and other psychosocial variables impacting cognition in caregivers.

Abstract citation ID: igae098.2424

**THE TROUBLE WITH FORGETTING: PROSPECTIVE AND RETROSPECTIVE MEMORY FAILURES AMONG CAREGIVERS**

Rachael Turner[1], Anisha Sharma[2], and Celinda Reese-Melancon[2], *1. Oklahoma State University, Kearney, Nebraska, United States*, *2. Oklahoma State University, Stillwater, Oklahoma, United States*

Caregivers serve a critical role in assisting their loved ones, though providing care can be associated with physical and cognitive decline. We investigated disparities in memory lapses and their perceived impact on daily functioning among caregivers and non-caregivers. We focused on lapses in prospective memory (PM; i.e., memory for future actions) and retrospective memory (RM; i.e., remembering information from the past). Participants included respondents of the Midlife in the United States Refresher 1: Daily Diary Project dataset (Ryff & Almeida, 2020). Individuals were identified as caregivers if they indicated assisting a spouse or parent with a disability or health condition throughout the week ($n = 75$). Self-report questionnaires measured instances of PM and RM forgetting, interference with daily routine resulting from forgetting, how bothered one is by forgetting, positive and negative affect, and time spent on caregiving tasks. Contrary to expectations, caregivers did not differ from non-caregivers on frequency of forgetting PM or RM tasks ($ps > .05$). Additional analyses investigated relationships between key variables and everyday forgetting and indicated that greater PM or RM forgetting among caregivers was associated with being more bothered by forgetting and more interference with daily routine ($ps < .001$). Further, negative affect and time spent caregiving significantly predicted caregivers' PM forgetting with caregivers who reported greater negative affect and more time spent caregiving reporting more instances of PM forgetting. The present study highlights the subjective experiences of caregivers in managing their daily memory tasks.

Abstract citation ID: igae098.2425

### USING SOCIAL CONVOY THEORY TO IDENTIFY AND RECRUIT CAREGIVERS FOR HEALTH-RELATED AGING RESEARCH

Jennifer Portz[1], Josh Silvasstar[2], and Heather Coats[3], *1. University of Colorado, Aurora, Colorado, United States*, *2. Colorado School of Public Health, Aurora, Colorado, United States*, *3. University of Colorado Anschutz Medical Campus, Aurora, Colorado, United States*

This study focuses on addressing the challenge of identifying and recruiting caregivers for health-related aging research. Social Convoy Theory, a framework for capturing social support over the lifespan, may be a helpful recruitment model. As part of a larger qualitative project, the objective is to compare two recruitment

**methods:** the "Helpers" Strategy, which directly asks older adults about who "helps" them with their health, and the "Social Convoy" Strategy, which utilizes a Social Convoy approach to complete a social support diagram. Both strategies involved contacting identified caregivers by phone, email, and text for recruitment. Analysis of the two strategies leveraged a data visualization tool, Tableau, to examine the data. Both strategies successfully identified at least one caregiver among 77-78% of the 31 older adults enrolled in the qualitative study, resulting in a total of 49 caregivers. However, the Social Convoy Strategy yielded more caregivers per participant (1.72) compared to the Helpers Strategy (1.5). While the Helpers Strategy predominantly identified one caregiver, the Social Convoy Strategy identified a broader range, including convoys of multiple caregivers. Caregiver participation rate was 61.2%, with the Helper Strategy showing a higher rate of participation (66.7%) than the Social Convoy Strategy (58.1%). However, the Social Convoy Strategy facilitated interviews with more caregivers across the network. The findings suggest that although the Social Convoy Strategy may have a lower overall recruitment rate, it enables the identification and interview participation of more than one caregiver per older adult across the caregiving network compared to the Helper Strategy.

### SESSION 7070 (POSTER)

### FRIENDSHIP, SOCIAL NETWORKS, SOCIAL SUPPORT

Abstract citation ID: igae098.2426

### A COMPREHENSIVE DIGITAL SELF-CARE SUPPORT SYSTEM FOR OLDER ADULTS IN PAIN: IMPACT OF SOCIAL SUPPORT ON CHRONIC PAIN

Priya Nambisan[1], Julie Ellis[1], Murad Taani[1], Yura Lee[2], Meenakshi Anupindi[1], and Rose Nortey[1], *1. University of Wisconsin Milwaukee, Milwaukee, Wisconsin, United States*, *2. University of Wisconsin-Milwaukee, Milwaukee, Wisconsin, United States*

Social support has been associated with positive health outcomes among older adults, contrasting with the adverse effects associated with social isolation and loneliness, which are linked to poor physical and mental health. Previous studies examined social isolation, loneliness, and physical health either at different time points or through correlations within secondary data. While interventions incorporating social support have demonstrated improvements in health and alleviation of symptoms such as pain for older adults, comprehensive assessments require controlling for various factors such as the impact of medication, diet, exercise, and other activities. Remarkably, observational studies simultaneously measuring social support, physical health changes, and factors like physical activity and diet are lacking. This pilot study uses a Comprehensive Digital Self-Care Support System (CDSSS), equipped with tools to monitor over 50 physical and mental health conditions, diet, activity, sleep, medication intake, alcohol use, smoking, and other self-care activities. The CDSSS also includes a robust online forum with video chatting facilities. This mixed methods pilot study aims to test whether we can evaluate in real time the changes in health indicators such as stress, mood, and chronic pain when participants engage in the online social space within the CDSSS. Participants are Black older adults (age >50), suffering from chronic conditions where pain is the main symptom such as Migraines, Fibromyalgia, Arthritis, Gout, and Backpain. This study integrates qualitative data from the social support forum and interviews, along with quantitative data from over 13 pain-specific trackers and surveys measuring social support, loneliness and social isolation.

Abstract citation ID: igae098.2427

### ASSOCIATIONS BETWEEN COGNITIVE PERFORMANCE AND PERCEIVED CONTROL: SPOUSAL SUPPORT AS A MODERATING FACTOR

Michael Buxton[1], Adrian Williams[1], Erick Carranza[1], Valerie Luskey[1], Hannah Apostolou[2], and M. Lindsey Jacobs[3], *1. University of Alabama, Tuscaloosa, Alabama, United States*, *2. University of Alabama, Northport, Alabama, United States*, *3. The University of Alabama, Tuscaloosa, Alabama, United States*

Social support can play an important role in cognitive functioning and involves both actual and perceived support. This presentation used data from the 2020 wave of the Health and Retirement Study. The sample consisted of community-dwelling older adults who completed questions of interest (n= 3416). The sample was predominantly female (59.6%) with a mean age of 68.6 years old (SD=10.3). The purpose of this study was (1) to examine the relationship between cognitive performance and perceived constraints on personal control and (2) investigate how perceived social support across relationship categories influenced the nature of this association. Participants who indicated dementia diagnoses were excluded from analyses. To determine the influence of perceived social support on the relationship of interest, moderation analyses were performed with the SPSS macro PROCESS using mean-centered values. Age, years of education, and gender were used as covariates. Perceived social support and cognitive performance had significant effects on perceived constraints. Positive and negative spousal support were the only relationship categories with significant interaction effects. Specifically, as positive spousal support increased, the relationship between cognitive performance and perceived constraints on personal control became weaker (b=-0.14, SE =.007, t(2499) = -2.0, p = 0.048). Increasing negative spousal support strengthened the relationship (b=0.14, SE =.006, t(2499) = 2.4, p = 0.017). Perceptions of spousal collaboration and support seemingly operate to fortify personal sense of control, which may be compensatory. Further research should explore facets of perceived social support and associations with cognitive performance and perceived control in older adults.

Abstract citation ID: igae098.2428

### EAGERNESS OF NURSING HOME RESIDENTS TO INTERACT WITH ROBOT TEMI

Zeynep Abul[1], Sakeena Raza[2], and Lynn McNicoll[3], *1. Northeastern University, Boston, Massachusetts, United States*, *2. Brown Medicine, Providence, Rhode Island, United States*, *3. Brown University Medical School, Providence, Rhode Island, United States*

Loneliness and social isolation existed among older adults before the COVID-19 pandemic, worsened due to lockdown, and visiting policies in nursing homes (NH) during pandemic. The aim of this study is to improve the interaction between robot Temi USA, Inc and NH residents. This is the preliminary finding of a quality improvement (QI) project. We conducted in-depth interviews with 54 long-term care NH residents to better understand their expectations from the self-navigating, autonomously driving humanoid robot temi. We asked the following focused question "If you wave a magic wand, what are some activities you would like to do with the robot?" and noted the answers. 5.6 % of the residents (n=3) responded neutrally by stating "I do not know "or "can't think of anything". Sixty-seven percent of the NH residents(n=36) responded with requests for interaction with temi. The most common request for interaction was "playing" chess, cards, piano, pool, baseball, Yahtzee, Bingo, and Wordhunt (n=20 out of 36; 56%).The remaining residents wanted to engage with temi by going outside (n=5), having temi sing with them (n=4); drawing with them (n=3); exercising with them (n=2); or just having a conversation with them (n=2). Fifteen residents (28%) wanted to have no interaction with temi. The high number of responses requesting interaction may imply that NH residents have an eagerness to interact with the robot. We will use those results to program an intervention to relieve loneliness of the NH residents for ongoing QI projects.

Abstract citation ID: igae098.2429

### PERSONALITY TRAITS ARE ASSOCIATED WITH THE TYPES OF SOCIAL SUPPORT/STRAIN: A LATENT PROFILE ANALYSIS

Meredith Willard, Jacob Alderson, and Nicholas Turiano, *West Virginia University, Morgantown, West Virginia, United States*

Research suggests that social connections are important for health and longevity (Cohen, 1985). High levels of social support is a significant predictor of improved well-being and health (White et al., 2009). However, it is important to acknowledge the role social strain can have on health, as well as key predictors of social network functioning. Thus, the current study utilized data from the Midlife Development in the US Study (MIDUS) to extract classes of social support/strain using a latent profile analysis (LPA), and use the Big 5 personality traits to predict class membership (N = 6,178; Mean age = 46.8). Social support/strain was separately computed for each network source (i.e., family, friend, and spouse) using 4-6 items. Iterations of different LPA classes suggested a 4-class solution (lowest AIC/BIC;.83 entropy). Class 1 (high spouse strain and lowest levels of support); Class 2 highest support and lowest strain levels);. Class 3 (highest level of spouse support and low levels from friends and family); Class 4 (average levels of support and strain across all sources). Those high in Conscientiousness and Agreeableness were more likely to belong in the "optimal" class of high support & low strain while those high in Openness and Neuroticism were more likely to belong in the "average" class (all p's <.05). Findings underscore how personality traits are involved in the exposure to varying levels of support/strain individuals are exposed to. Understanding these processes is key to understanding how social networks impact aging outcomes.

Abstract citation ID: igae098.2430

### RELATIONSHIP SATISFACTION MEDIATES LINKS BETWEEN OLDER ADULTS' SOCIAL NETWORKS AND DEPRESSION

Debarati Kole, Bryce Van Vleet, and Heather Fuller, *North Dakota State University, Fargo, North Dakota, United States*

Social ties have a significant positive effect on older adults' psychological well-being. However, limited research has examined the role of relationship satisfaction for the interplay between social ties and psychological well-being. The current study explores if relationship satisfaction mediates the relationship between social network characteristics and depressive symptoms among older adults. The data are from the first three waves of the Social Integration and Aging Study, a community-based, longitudinal study of older adults (aged 60+) in the Upper Midwest. Simple longitudinal structural equation modeling was used to analyze mediation models with baseline demographic variables (Age, Gender, and Education included as covariates. Three network variables (network size, mean proximity, and mean years of connection) were selected from Wave 1 (2013). A four-item relationship satisfaction construct (satisfaction from close family, extended family, friends, and neighbors) from Wave 2 (2015) was used as the mediator variable. The 10-item Geriatric Depression Scale, at Wave 3 (2017) was used as the outcome variable. Relationship satisfaction was found to mediate the effects of network size (paths: a=.022 p=.008; b=-.538 p<.001; c'=-.026 p=.239) and mean network proximity (paths: a=.361 p=.048; b=-.548 p<.001; c'=-.523 p=.237) on depressive symptoms over time. However, relationship satisfaction did not mediate the relationship between years of connection and depressive symptoms. These findings suggest qualitative aspects of social connections more greatly impact older adults' psychological well-being over time as compared to quantitative social network characteristics. Implications for quality of connections and sense of belongingness will be discussed.

Abstract citation ID: igae098.2431

### SOCIAL ENGAGEMENT AND DEPRESSIVE SYMPTOMS: DIFFERENTIAL EFFECTS IN OLDER ADULTS WITH AND WITHOUT CANCER

Natalia Babenko[1], Joanne Elayoubi[2], William Haley[2], and Brent Small[3], *1. University of South Florida, New Port Richey, Florida, United States*, *2. University of South Florida, Tampa, Florida, United States*, *3. University of North Carolina at Chapel Hill, Chapel Hill, North Carolina, United States*

Recent studies have shown that social connection and engagement can serve as protective factors against depression. Buffering effects are sometimes found, suggesting greater protective effects for people experiencing high levels of stress. We examined whether social connection and engagement had a greater protective effect for depressive symptoms in cancer survivors compared to people without cancer. We analyzed longitudinal data from 856 participants with incident cancer and 856 propensity-matched non-cancer controls from the Health Retirement Study. Multilevel modeling was used to examine the associations between social connection and engagement and changes in depressive symptoms over time. Social connection and engagement were generally associated with fewer depressive symptoms before and after the onset of cancer (or a matched timepoint for controls). These protective effects were stronger for cancer survivors who were partnered and lived in a larger household compared to controls. Participants with cancer who were partnered pre-cancer experienced a significant decrease in depressive symptoms after cancer compared to partnered controls who did not exhibit decline. Becoming partnered in post-cancer period led to decreased depressive symptoms compared to the pre-cancer partnership status in both groups. Consistent with the buffering hypothesis, results suggest that social connection is generally advantageous, especially for people with cancer in a post-cancer period. Prospective public health interventions increasing social engagement to build resilience in the overall population may enhance psychological resilience and facilitate recovery from cancer. Future research should examine whether people with cancer benefit from support-enhancing interventions and whether this effect also occurs in other illness populations.

Abstract citation ID: igae098.2432

### THE ASSOCIATION BETWEEN PERCEIVED SOCIAL SUPPORT AND LIFE SATISFACTION: RACE/ ETHNICITY DISPARITIES

Kingsley Mbam, Yan-Jhu Su, and Wuyi Dong, *University of Massachusetts Boston, Boston, Massachusetts, United States*

**Objective:** Studies have shown an association between perceived social support and life satisfaction. However, fewer studies covered the racial/ethnic disparities in this association. This study looks to address this gap by assessing how perceived social support differs across racial and ethnic groups and their life satisfaction.

**Methods:** This cross-sectional study used data from the 2020 Health and Retirement Study (HRS). The respondents include individuals 55 and older without any missing survey responses (n=1,901). The perceived social support was assessed using the summary score of social integration, which included spousal/partner support, children support, friend support, and non-nuclear family support. Life satisfaction was assessed using Diener's measure of life satisfaction. Control variables, including demographic characteristics, household income, and self-rated health, were added to this analysis. Ordinary least squared (OLS) regression was implemented to examine whether race/ethnicity moderates the association between perceived social support and life satisfaction.

**Results:** Adjusted models show results disclosing that people with more perceived social support had significantly higher life satisfaction ($b = 0.32$, $p < 0.001$), and interaction effects showed that the association among Hispanic people is significantly stronger compared to non-Hispanic Whites ($b = 0.17$, $p = 0.03$).

**Discussion:** These results suggest that improving social support for individuals age 55 and older across racial groups can enhance their life satisfaction, which is essential to improving their overall health and well-being. Furthermore, this study widens the understanding of the demographic factors that influence the association between perceived social support and life satisfaction.

Abstract citation ID: igae098.2433

### THE CAMARADERIE OF MIDDLE-AGED AND OLDER WOMEN: HOW DOES FLASH MOB BUILD SOCIAL CAPITAL?

Hae Youn Min, Kyung Eun Lee, and Jinmoo Heo, *Yonsei University, Seoul, Republic of Korea*

The "flash mob", a new indication of 21st-century digital culture, involves groups of unfamiliar individuals gathering suddenly in public spaces to perform distinctive activities based on pre-arranged instructions and dispersing quickly. Engaging in group events like flash mobs enhances a more profound sense of community, belonging, and friendship. This study explores the potential of flash mobs to encourage social capital, focusing on a specific group of middle-aged and older women who engage in flash mobs as a means of celebrating the aging process. This group of women gathered to welcome the journey of aging, using flash mobs to show the way to enjoy the moment. The study used a qualitative research method, including in-depth interviews with 16 participants. The findings of this study revealed that participants formed a vivid network, and the outcomes of the network are as follows: (a) strong trust, (b) reciprocity, (c) camaraderie, and (d) contribution to the community. These elements are related closely to Putnam's (1995) conceptualization of social capital, demonstrating the unique role of flash mobs in impacting social networks and community bonds among participants. Primarily middle-aged and older women, during significant life transitions like empty nest syndrome, retirement, and menopause, they often face loneliness, emphasizing the role of social activity. This study provides insights into how the social capital of middle-aged and older women enhances the anticipation of later life.

Abstract citation ID: igae098.2434

### THE EFFECT OF DIGITAL INTERACTION WITH FRIENDS AND FAMILY ON OLDER ADULTS' WELL-BEING: A SYSTEMATIC REVIEW

Ranran He[1], Destiny Ogle[1], and Jinnan Ren[2], *1. Purdue University, West Lafayette, Indiana, United States*, *2. Purdue University - West Lafayette, West Lafayette, Indiana, United States*

Contacting friends and family using digital technologies has grown in popularity among older adults. Given the importance of social interaction across the life course, this novel form of interaction may substantially shape their well-being. Existing studies reviewing the effects of technology use on older adults' well-being were mostly broad in scope, without specifying the age group or the relationships between interactants. This study aims to fill a gap in the literature by systematically reviewing how digital interaction with friends or family shapes older adults' well-being. We sourced articles from six databases, and the last search was performed in February 2024. Studies were synthesized and analyzed using the narrative approach. All 21 qualifying studies were quantitative in their design. Most studies (10 articles) found positive impacts of digital interaction with family members on well-being, whereas some found negative (4), mixed (3), or null effects (2). Even more conflicting results were found in studies examining the effects of digital interaction with friends; four found positive benefits, five found adverse impacts, and two found no effects. Communication with family members was typically more predictive of well-being than communication with friends. The aggregated measurement of digital contact, the types of digital technology examined, the age range of survey respondents, the sociocultural context, and whether the studies were undertaken during the COVID-19 pandemic may explain these inconsistent findings. Therefore, we suggest that future research incorporate more precise measures of digital interaction with friends and family, more diverse study designs, and explore socio-structural and relational moderators.

Abstract citation ID: igae098.2435

### THE EXPERIENCES OF COLORECTAL CANCER SURGERY AMONG OLDER PATIENTS WHO LIVED ALONE IN TAIWAN

Li-Ying Chen[1], and Tsuann Kuo[2], *1. Taipei City Hospital Renai Branch, Taipei, Taipei, Taiwan (Republic of China)*, *2. Chung Shan Medical University, Taichung, Taichung, Taiwan (Republic of China)*

In Taiwan, it is a common practice that family takes care of their older adults when hospitalized. However, people who were never married or living alone would face challenges in coming up with a caregiver in the hospital. The shortage of caregiver among Taiwanese older population was due to its low fertility and high aging rate as Taiwan will enter a super-aged society in 2025. The purpose of this paper was to understand how inpatient surgical experiences on older adults who lived alone. The research methodology used qualitative interviews with structural questions followed by content analysis. Eight older adults participated to share their experiences who live alone and hospitalized for colorectal cancer surgery. The result showed that prognosis, postoperative care and Activities of daily living(ADL) were concerned. Older adults living alone were not only worried about the results of the surgery but also concerned about their reduced self-care ability and postoperative care without help. In addition to determining the recovery of digestive system function, doctors consulted rehabilitation to strengthen their mobility, taking into account the factor of living alone. Based on the results, the author suggested that it is essential to discuss postoperative care arrangements and whether the patient will be able to regain their self-care abilities after surgery. With the increasing number of people living alone, greater emphasis should be placed on the patient's own decisions not relatives. For older patients living alone and undergoing inpatient surgery, developing in-hospital rehabilitation plans is crucial to strengthen their self-care abilities.

Abstract citation ID: igae098.2436

### THE MORE, THE MERRIER? OLDER ADULTS' LEISURE WITH OTHERS AND ASSOCIATIONS WITH WELL-BEING OVER TIME

Amy Rauer[1], Meagan Stewart[1], Katherine Fiori[2], Christina Marini[2], and Jeremy Kanter[1], *1. University of Tennessee, Knoxville, Tennessee, United States*, *2. Adelphi University, Garden City, New York, United States*

The benefits of leisure for older adults are well-documented, as is the key role that social networks play in facilitating more opportunities for leisure. However, it is unclear if these opportunities are uniformly beneficial over time or if engaging in leisure with certain social ties (or alone) are more advantageous, particularly when barriers to social activities are greater (i.e., COVID-19 pandemic). Thus, we examined older adults' leisure alone and with others and its links to well-being using a sample of 136 older adults in the U.S. (Mage = 68, range: 50-91; 69% females; 93% White; 72% partnered; 61% retired). Participants reported on leisure at three time points (Summer 2021/Fall 2021/Winter 2022) using a modified version of the Pittsburgh Enjoyable Activities Scale (Pressman et al., 2009). Older adults were most likely to do activities with romantic partners, followed by activities alone, with leisure with friends and family least likely. Although more leisure engagement overall was associated with better well-being (depressive symptoms, anxiety symptoms, loneliness, life satisfaction, perceived mattering), it was primarily leisure with others that appeared beneficial (romantic partner, friends, family). In fact, greater solitary leisure was linked to feeling less important (perceived mattering). Given that leisure with a romantic partner and with family declined when COVID-19 cases peaked (in contrast to the stability of overall leisure, solitary leisure, and leisure with friends), it appears that older adults were still able to do things they enjoyed, but the limitations on with whom they could engage in leisure were not without consequence.

### SESSION 7075 (POSTER)

### GEROSCIENCE

Abstract citation ID: igae098.2437

### A DROSOPHILA MODEL OF A MITOCHONDRIALLY INDUCED INNATE IMMUNE RESPONSE

Adrienne Wang[1], Angshita Dutta[1], Alexandra Putzier[1], Marieke Trabant[1], Kyla Gustave-Schelling[1], Monica Sanchez-Contreras[2], and Scott kennedy[2], *1. Western*

*Washington University, Bellingham, Washington, United States*, 2. *University of Washington, Seattle, Washington, United States*

Mitochondrial function and innate immunity-mediated inflammation are two critical regulators of healthy aging and lifespan. While mitochondrial function is the cornerstone of some of the oldest theories of aging, innate immune function is increasingly implicated as a modifier of age, and age-related neurodegenerative diseases. There is mounting evidence that mitochondria play a role in regulating innate immune function and inflammation, and that mitochondrial dysfunction and mitochondrial DNA (mtDNA) stress can lead to increased innate immune signaling. While severe mtDNA depletion is lethal, studies have shown that moderate depletion (50%) of mtDNA in mice induces a mitochondrial stress response that upregulates innate immune signaling. This signaling depends on release of mtDNA into the cytoplasm, and results in an increase in viral resistance however, the exact mechanisms by which mitochondrial stress leads to innate immune activation and subsequent effects on lifespan and susceptibility to neurodegenerative disease are not well understood. Here, we present a Drosophila model of mtDNA depletion in which expression of UL12.5, a nuclease expressed by the Herpes simplex-1 virus, degrades mtDNA. Expression of UL12.5 is developmentally lethal in an array of tissues, and conditional depletion of mtDNA in adult flies leads to decreased lifespan, exacerbates toxicity of the AD-related protein Tau, and induces an innate immune response that leads to increased resistance to acute bacterial challenge. This model provides an opportunity to extend our understanding of the role of sterile inflammation and mitochondrial stress in the etiology of age-related changes in immune function and susceptibility to neurodegenerative disease.

Abstract citation ID: igae098.2438

### A KETOGENIC DIET REDUCES AGE-INDUCED CHRONIC NEUROINFLAMMATION IN MICE

Mitsunori Nomura, and John Newman, *Buck Institute for Research on Aging, Novato, California, United States*

Beta-hydroxybutyrate (BHB) is a ketone body synthesized during fasting or strenuous exercise. Our previous study demonstrated that a cyclic ketogenic diet (KD), which induces BHB levels similar to fasting every other week, reduces midlife mortality and improves memory in aging mice. BHB actively regulates gene expression and inflammatory activation through non-energetic signaling pathways. Neither of these activities has been well-characterized in the brain and they may represent mechanisms by which BHB affects brain function during aging. We investigated whether BHB affects brain cells transcriptionally in vitro. Gene expression analysis in primary human brain cells (microglia, astrocytes, neurons) using RNA-seq shows that BHB causes a mild level of inflammation in all three cell types. However, BHB inhibits the more pronounced LPS-induced inflammatory gene activation in microglia. Furthermore, we confirmed that BHB similarly reduces LPS-induced inflammation in primary mouse microglia and bone marrow-derived macrophages (BMDMs). BHB is recognized as an inhibitor of histone deacetylase (HDAC), an inhibitor of NLRP3 inflammasome, and an agonist of the GPCR Hcar2. Nevertheless, in microglia, BHB's anti-inflammatory effects are independent of these known mechanisms. Next, we examined the brain gene expression of 12-month-old male mice fed with one-week and one-year cyclic KD. While a one-week KD increases inflammatory signaling, a one-year cyclic KD reduces neuroinflammation induced by aging. In summary, our findings demonstrate that BHB mitigates the microglial response to inflammatory stimuli, like LPS, possibly leading to decreased chronic inflammation in the brain after long-term KD treatment in aging mice.

Abstract citation ID: igae098.2439

### "INCORPORATE INTO MY DAILY LIFE": RESPONSES TO MITOFIT INTERVENTION VIDEOS ON MITOCHONDRIAL FITNESS

Jeffrey Boon[1], Brandon Grubbs[2], Maulik Patel[3], John Dunavan[4], Kelly Knickerbocker[3], and Cathy Maxwell[5], 1. *Vanderbilt University Medical Center, Nashville, Tennessee, United States*, 2. *Middle Tennessee State University, Murfreesboro, Tennessee, United States*, 3. *Vanderbilt University, Nashville, Tennessee, United States*, 4. *Peer-Tree, Franklin, Tennessee, United States*, 5. *University of Utah College of Nursing, Salt Lake City, Utah, United States*

Mitochondrial fitness is a term that reflects the ability of mitochondria to adapt/maintain optimal performance. As a key predictor of age-related decline, mitochondrial fitness is a target for interventions in healthy aging. MitoFit is a novel instructional communication intervention designed for improving physical activity in persons aged 50 and over. We aimed to determine participants' responses to the MitoFit educational videos and clarify factors that contributed to reactions.

**Methods:** Focus groups with community-dwelling adults (aged 50+) viewed six literacy-friendly MitoFit videos two at a time followed by a semi-structured group interviews to elicit verbal feedback. Interviews were audio-recorded and transcribed verbatim. Transcribed data were independently coded by three researchers, followed by a consensus process to identify themes, codes and subcodes.

**Results:** 101 adults (mean age: 67.8 years (8.9); 75.0% female) participated in 15 focus groups at six sites. A conceptual model emerged, centered around the concept of cognitive restructuring (mental adjustments to prior or unhelpful beliefs) related to understanding about exercise. Contributing themes included: a) initial reactions (codes: impactful, motivating, fascinating), b) biological content (codes: this is biology, battery analogy, energetics), and c) personal integration (codes: prompts reflection, unaware, leads to more questions). The theme of key take-aways included codes: a) have to do it (subcodes: consistency, find what works, find what I like, making a plan), b) hope (subcode: not too late), c) reversible, and d) sense of agency (subcode: doing it for me).

**Conclusion:** MitoFit video content provoked positively strong reactions that may contribute to self-efficacious lifestyle change.

Abstract citation ID: igae098.2440

### DHCR24 INSUFFICIENCY PROMOTES VASCULAR ENDOTHELIAL CELL SENESCENCE AND ENDOTHELIAL DYSFUNCTION

Han Li[1], Zhen Yang[1], Wukaiyang Liang[1], Hao Nie[1], Jinhua Yan[1], and Cuntai Zhang[2], *1. Key Laboratory of Vascular Aging, Ministry of Education,Tongji Hospital of Tongji Medical College, Huazhong University of Science and Technology, Wuhan, Hubei, China (People's Republic), 2. Tongji Hospital, Tongji Medical College, Huazhong University of Science and Technology, Wuhan, Hubei, China (People's Republic)*

Endothelial cells (ECs) senescence is critical for vascular dysfunction, which leads to age-related disease. DHCR24, a 3β-hydroxysterol δ 24 reductase with multiple functions other than enzymatic activity, has been involved in age-related disease. However, little is known about the relationship between DHCR24 and vascular ECs senescence. We revealed that DHCR24 expression is chronologically decreased in senescent human umbilical vein endothelial cells (HUVECs) and the aortas of aged mice. ECs senescence in endothelium-specific DHCR24 knockout mice was characterized by increased P16 and senescence-associated secretory phenotype, decreased SIRT1 and cell proliferation, impaired endothelium-dependent relaxation, and elevated blood pressure. In vitro, DHCR24 knockdown in young HUVECs resulted in a similar senescence phenotype. DHCR24 deficiency impaired endothelial migration and tube formation and reduced nitric oxide (NO) levels. DHCR24 suppression also inhibited the caveolin-1/ERK signaling, probably responsible for increased reactive oxygen species production and decreased eNOS/NO. Conversely, DHCR24 overexpression enhanced this signaling pathway, blunted the senescence phenotype, and improved cellular function in senescent cells, effectively blocked by the ERK inhibitor U0126. Moreover, desmosterol accumulation induced by DHCR24 deficiency promoted HUVECs senescence and inhibited caveolin-1/ERK signaling. Our findings demonstrate that DHCR24 is essential in ECs senescence.

Abstract citation ID: igae098.2441

### MITOCHONDRIAL SUBSTRATE UTILIZATION FOLLOWING CONTRACTION IN 3D ENGINEERED MUSCLE TISSUE MODELS OF AGING

Matthew Campbell, Jayma Erker, David Mack, and David Marcinek, *University of Washington, Seattle, Washington, United States*

Altered mitochondrial function in aging skeletal muscle leads to elevated oxidative stress, reduced substrate oxidation and energy maintenance that contribute to decreased skeletal muscle mass, specific force and increased fatty infiltration and frailty with age. Metabolic flexibility in response to increased energy demand is a critical component of the mitochondrial adaptive response to muscle contraction. We have found that aged muscle mitochondria do not utilize substrates the same way young muscle mitochondria do following high-intensity and low-intensity contraction. We also found that high-intensity contraction specifically inhibits oxidation of glutamate in both stimulated and non-stimulated aged muscle, suggesting that intense muscle contraction in aged muscle may inhibit substrate flexibility beyond the contracting myofibers. To test the mechanisms that contribute to this impaired metabolic signaling to muscle contraction in a human cell model of aging we have partnered with the Study of Muscle, Mobility and Aging to obtain primary human myoblasts from well phenotyped older adults to develop a three-dimensional engineered muscle tissue (3D-EMT) model of skeletal muscle aging. 3D-EMT force and contraction/relaxation kinetics were tested with high reproducibility using a Magnetometric Analyzer for eNgineered Tissue ARRAY (Mantarray, Curi Bio) platform. Aged 3D-EMT shows characteristic force-frequency and increased force up to 28 days after tissue casting and increased mitochondrial oxidative phosphorylation following contraction. Developments in tissue engineering using primary cells purified directly from patients offer some of the best opportunities yet to link specific mechanistic tests of metabolic and muscle function to patient data.

Abstract citation ID: igae098.2442

### THE HARTLEY GUINEA PIG: A NOVEL MODEL FOR PROGRESSIVE NEURODEGENERATION?

Kristen Glennie, Amanda Latham, Arsha Moorthy, Maureen Walsh, Thomas LaRocca, Kelly Santangelo, Julie Moreno, and Karyn Hamilton, *Colorado State University, Fort Collins, Colorado, United States*

Alzheimer's Disease and Alzheimer's Disease Related Dementias (AD/ADRD) affect an estimated 55 million people worldwide; a staggering figure that is expected to grow in the coming years. With these projections looming, we have yet to identify any cures, treatments, or preventative strategies. Historically, AD/ADRD research is conducted using a genetically engineered model, that expresses a specific brain aging pathology. Modern research, however, has identified a complex and dynamic "inflammaging" phenotype that exists with and contributes to AD/ADRD pathology. Currently, we do not have an accessible or tractable preclinical model, that naturally mimics age-related progressive neurodegeneration in humans. Recent findings, however, suggest the Hartley guinea pig may address this need. Hartleys develop systemic inflammation and progressive age-related comorbidities characteristic of human aging. This whole-body aging phenotype prompted analyses in the brain, which found Hartleys exhibit sequence homology and similar transcriptomic mechanisms of human brain aging and AD. Hartleys also develop evidence of neuroinflammation and neurodegeneration in the hippocampus. To further investigate these findings, we examined the histopathology of 4 brain regions often implicated in neurodegenerative decline, for evidence of progressive neuropathology. Analysis is ongoing, though early results support the presence of a neuroinflammatory age-related phenotype. Additionally, biomarker variability between each Hartley may mimic the natural progression of human 'brain aging'; though more data are needed before conclusions are drawn. Findings from this study will contribute to the hypothesis that AD/ADRD is a whole-body disease, and ultimately support the goal of closing the existing translational gap between preclinical and clinical neurodegenerative research.

Abstract citation ID: igae098.2443

### THE INSECT ACHETA DOMESTICUS IS A PROMISING MODEL FOR AGING AND AGING INTERVENTION STUDIES

Gerald Liao[1], Jackson Wezeman[1], and Warren Ladiges[2], *1. University of Washington, Seattle, Washington, United States*, *2. University of Washington, University of Washington, Washington, United States*

A number of animal species are being used as models to study aging and age-related diseases. They have varying advantages such as shorter lifespan, relatively low costs, and full access to internal organs. While no single model can replicate all aspects of human aging, there are unexplored non-mammalian species that may have additional advantages. The domestic house cricket (Acheta domesticus) has a short lifespan, a simple but well-defined organ system, an omnivorous diet from young to old, very low maintenance costs, and neurogenesis in a hippocampus-like brain structure. A preliminary study investigated effects of flax oil, rich in omega-3 fatty acids, on lifespan in adult crickets. Cold pressed flax oil was added to moistened guinea pig chow mash as a 10% mixture and fed to crickets starting at 8 weeks of age and continuing for 11 weeks when the last cricket died. Results demonstrated a significant extension in survival for crickets fed the flax oil diet, emphasizing the low cost, simplicity, and short time required for dietary studies in this species. This observation provides the rationale for additional studies to characterize the house cricket as a promising model for aging and aging intervention investigations.

Abstract citation ID: igae098.2444

### TRAFFIC-RELATED AIR POLLUTION AND INFECTIONS JOINTLY INCREASE THE RISK OF ALZHEIMER'S DISEASE IN OLDER ADULTS

Svetlana Ukraintseva, Vladimir Popov, Hongzhe Duan, Arseniy Yashkin, Igor Akushevich, Konstantin Arbeev, and Anatoliy Yashin, *Duke University, Durham, North Carolina, United States*

Accumulating evidence points to a potentially major role of vulnerability to infections in Alzheimer's disease (AD) development, although the exact mechanism is not clear. AD is a multifactorial disorder, and the presence of other risk factors may exacerbate the impact of infections on AD. Here we investigated how the interplay between a history of infections and exposure to traffic-related air pollution (TRAP), another risk factor for AD that may influence the brain's vulnerability to infections, affects the risks of AD and other dementias in UKB participants aged 60-75. Infectious diseases, AD, and other dementia diagnoses were based on ICD9/ICD10 codes. Chronic exposure to TRAP was approximated by closeness (50 meters or less) of participant's primary residence to a major road. Chi-square, Wilson score interval, Wald interval, Wald risk ratio, and Welch tests, and regression were used to assess statistical significance. We found that the presence of both, a history of infections and exposure to TRAP in UKB participants aged 60-75, was associated with 177% higher risk of AD onset after the age of 75 (risk ratio, RR=2.77, 95% CI [1.86, 4.11]), compared to individuals of the same age who had no history of either of these factors. One mechanism could involve TRAP compromising the integrity of the blood-brain barrier (BBB), potentially increasing the brain's vulnerability to infections and related damage. This, in turn, might contribute to the promotion of AD pathology.

### SESSION 7080 (POSTER)

### HEALTH CARE

Abstract citation ID: igae098.2445

### APPLYING TIME-OF-FLIGHT SENSOR IN EARLY MOBILIZATION INTERVENTION FOR HOSPITALIZED OLDER ADULTS

I-Ling Chen[1], Po-Lung Yang[2], Hsiu-Yun Lai[3], Mu-Cyun Wang[2], Chien-Jung Pien[3], and Han-Yueh Kuo[3], *1. University of Washington, Seattle, Washington, United States*, *2. National Taiwan University Hospital, Taipei, Taipei, Taiwan (Republic of China)*, *3. National Taiwan University Hospital, Hsin-Chu Branch, HsinChu, Hsinchu, Taiwan (Republic of China)*

Early mobilization is crucial in preventing functional decline associated with hospitalization among older adults. To assess the effectiveness of an early mobilization intervention program, this randomized trial implements Time-of-Flight (ToF) sensor technology in quantifying patient mobility. Twenty-five adults aged 65 or above who were hospitalized for acute illness were enrolled in the study. The intervention group received early rehabilitation treatment by physical therapists, while the control group received education on self-exercises. All enrolled patients were monitored with Time-of-Flight sensors to record the number of out-of-bed movements during their hospital stay. Patient mobility scores were calculated upon enrollment and the date of discharge using the de Morton Mobility Index(DEMMI). The patients were followed up by phone call at 1 month and 6 months after discharge, collecting information about re-admission to the hospital and mortality. Results showed that both intervention and control groups improved upon discharge. The average increase of DEMMI score is higher in the intervention group(mean: 9.82; SD: 10.97) than in the control group (mean: 7.07; SD: 10.72). However, no statistical significance was detected($p=0.53$). DEMMI scores positively correlated with average daily out-of-bed movements (Pearson correlation coefficient > 0.7 and $p < 0.001$), validating the measurements of ToF sensors. Our study has strengths in utilizing sensor technology that does not require wearable devices to obtain objective measurements of mobility. However, this study is limited by its small sample size. Future research is needed to further examine the effectiveness of early mobilization among hospitalized older adults in the clinical settings.

Abstract citation ID: igae098.2446

### ASSESSING THE COMPLEXITIES OF LONG-TERM SERVICES AND SUPPORTS FOR OLDER ADULTS OF TEXAS

Sara Murphy, Esme Anaab, Jennifer Severance, and Robert Yockey, *University of North Texas Health Science Center, Fort Worth, Texas, United States*

**Background:** Long-term services and supports (LTSS) encompass a broad range of medical and personal care assistance needed by individuals who are unable to perform self-care tasks due to aging, chronic illness, or disability.

The No Wrong Door (NWD) System is a statewide network that coordinates the system of access to long-term services and supports. As an offshoot of the person-centered planning movement, the NWD System is premised on ensuring that no matter where individuals first interact with the system, they are guaranteed comprehensive information, assessment, and services. This work will help the Texas Health and Human Services Commission create a consumer-driven, cost-effective, and efficient system.

**Methods:** A mixed methods approach was constructed to understand the challenges and barriers to accessing LTSS, assess NWD System performance based on stakeholder perspectives, and identify community needs.

**Results:** Of 4,153 respondents, 56% identified as an adult 50 years or older and 53% older adults reported a LTSS need in the past year. The top three problems faced when trying to get services include a confusing system, long service wait times, and not knowing where to get help. Unpaid informal caregiver respite services, caregiver education, and caregiver support groups were among the services and supports that inadequately met people's needs. Conclusions: Our results form the blueprint for facilitating access to care throughout Texas to ensure vulnerable populations obtain a high quality of life. Recommendations for the NWD system include effective community outreach and streamlined application processes for services and supports.

Abstract citation ID: igae098.2447

### CULTURALLY ADAPTING A TAILORED ACTIVITY PROGRAM FOR CHINESE OLDER ADULTS LIVING IN NURSING HOMES

Yu Zheng[1], Wenting Peng[2], Minhui Liu[3], Ni Gong[4], and Chunxiao Li[4], *1. The Third Xiangya Hospital of Central South University, Changsha, Hunan, China (People's Republic)*, *2. University of Washington, Seattle, Washington, United States*, *3. Ningxia Medical University, Yinchuan, Ningxia, China*, *4. Central South University, Changsha, Hunan, China (People's Republic)*

The behavioral and psychological symptoms of dementia are aggravating among older adults at nursing homes. However, in China, there is no recognized effective program that has improved the behavioral and psychological symptoms of dementia. Therefore, we aimed to cross-culturally adapt and localize a foreign evidence-based intervention program (Tailored Activity Program, TAP), and examine its feasibility and acceptability. A total of 20 people with dementia and 12 caregivers participated in the program, and after 8 sessions. Results showed that there were significant improvements in behavioral and psychological symptoms, daily living activities, depression of older adults and depression, caregiving burden, caregiving response load of caregivers ($P < 0.05$), but there were no significant changes in quality of life, cognitive function of older adults and caregivers' readiness ($P > 0.05$). In the semi-structured interview section, the interview data of people with dementia could be summarized as improved negative emotions, relieved stress, disinterest in activities, and activity-related suggestions; the interview data of caregivers could be summarized as improved negative emotions, relieved stress, and desire to be understood. Finally, 2 adapted suggestions (extending the duration of interventions and adjusting inclusion-exclusion criteria) were obtained based on the feedback from interview section. In conclusion,18 of 20 people with dementia (90% > 80%) and all of 12 caregivers (100% > 80%) fully understood and were willing to accept the program. This program improves the emotional experience, with a high degree of feasibility and acceptability. The tailored activity program was considered to be adapted and localized.

Abstract citation ID: igae098.2448

### EFFECTS OF A USER-CENTRED TECH-SUPPORTED TRANSITIONAL CARE MODEL ON REDUCING DEPENDENCY AMONG DISCHARGED PATIENTS

Nan Xia, Clio Yuen Man Cheng, and Vivian Weiqun Lou, *The University of Hong Kong, Hong Kong, China (People's Republic)*

Many patients, especially those aged above 60, continue to have care needs after discharge. However, the existing professional-led services do not meet the growing service needs in Hong Kong, especially during the Covid-19 pandemic. To fulfil the needs of discharged patients, a standardised transitional care service delivery model was established through a user-centred approach. A comprehensive one-stop online platform was developed to empower patients and their carers by enabling a better understanding of their conditions. The tech-supported solution also enabled service providers to deliver high-quality services through data-informed service plans without contact. The current study aims to evaluate the effects of the novel service delivery model on reducing dependency among discharged patients dwelling in the community. The interRAI-Home Care v9.3 was applied to collect information on patients' dependency by evaluating their care needs before and after receiving the services. 431 subjects were recruited from three local NGOs between July 2021 and June 2023, and 286 (66.4%) participants completed both pre- and post-evaluations. The overall mean number of Clinical Assessment Protocols (CAPs) decreased significantly, from 7.4 to 5.5 ($p < 0.001$). Patients' care needs were reduced significantly ($p < 0.001$) in each of the three institutions after receiving services, with no significant differences observed across institutions. The current study provides evidence of the effects of a user-centered tech-supported transitional care model for discharged patients and their carers, which ensures that such a model could effectively provide remote services to patients and their carers, and can facilitate alleviating manpower shortages while facing unexpected obstacles.

Abstract citation ID: igae098.2449

### EHEALTH INTERVENTIONS TO PROMOTE PHYSICAL ACTIVITY AMONG OLDER ADULTS WITH MCI: A SYSTEMATIC REVIEW

Jinhee Shin[1], Kennedy Diema Konlan[2], and Chang Gi Park[3], *1. Woosuk university, Jeollabuk-do, Ch'ungch'ong-bukto, Republic of Korea*, *2. University of Health and Allied Sciences, Ho, Volta, Ghana*, *3. University of Illinois Chicago, Chicago, Illinois, United States*

Physical activity positively influences and improves motor ability, increases desirable behavior, promotes brain activity, and increases marginal memory and thinking among older adults with mild cognitive impairments. This systematic review explored current trends and the effectiveness of digital health (eHealth) interventions to promote physical activity among older adults with mild cognitive impairment. We used the PRISMA guidelines and the PICO framework to search for literature in five electronic databases (PubMed, Embase, CINAHL, and Web of Sciences). The search outcome was transferred to Endnotes software and independently screened based on title, abstract, and full text using predetermined eligibility criteria. The screening resulted in 10 articles for extraction using a self-developed matrix. The JBI quality appraisal tool was used for quality appraisals and the conventional thematic data synthesis method of analysis was used. The strategy used was diverse and multiple technological platforms for the implementation of digital interventions, and included hardware technology like tablets, smartphones, smartwatches, Bluetooth devices, earpieces, computers, or durable covers, while, software techniques like promoting, customization, multimedia, multisensory, hands-on practice, demonstration, cueing, tech support, user manual or in the training of care practitioners were used. The use of digital-based health interventions for people with mild cognitive impairment to improve physical activity is still rudimentary even though the potential influence of such interventions is enormous. Interventions that use digital methods focusing on promoting physical activity among older adults with mild cognitive impairments should provide detailed information on the types of interventions, implementation, development process, and the resultant improvements.

Abstract citation ID: igae098.2450

### EXAMINING THE COST BURDEN OF DIETARY SUPPLEMENTS IN OLDER ADULT DRIVERS: AN ANALYSIS FROM THE AAA LONGROAD STUDY

Sara Baird[1], Ryan Moran[1], Sarah Hacker[1], Dylan Lawton[2], and Linda Hill[1], *1. University of California San Diego, La Jolla, California, United States*, *2. University of Hawaii, Honolulu, Hawaii, United States*

Dietary supplements (DS) are commonly used, yet consistent evidence of benefit remains lacking. Many older adults have fixed incomes, and the relative costs of DS remain poorly understood. Data was obtained from the first two years from the LongROAD study, a multicenter prospective cohort study of older drivers (≥ 65 years old). At baseline and annually, over-the-counter and prescription medications were systematically coded and categorized, with attention to DS formulations. Cost estimates utilizing the lowest priced brand on Amazon.com were matched to registered DS in the National Institutes of Health Office of Dietary Supplements database. Cost burden per participant was based on reported frequency of use and analysed against participant demographics. Of the 2,990 participants at baseline, 2717 participants had follow up through year 2. Almost all participants (n=2518, 84%) utilized at least one DS during the two-year period. Of 146 DS formulations identified, 135 (92%) were included for cost analysis; the remainder were not listed on Amazon.com. Among DS users, the average estimated annual cost burden was $205.15 (SD +/- $259.49) at baseline, $222.35 (SD +/- $282.68) at year one, and $213.56 (SD +/- $273.51) at year two. Female participants had a higher average estimated annual cost (p< 0.001); there was no significant difference by education or income level. The average monthly cost of DS in this population is similar to two or more generic medications. The health implications and potential cost effect on prescription medication compliance warrants further attention.

Abstract citation ID: igae098.2451

### EXPLORING REFERRAL PATTERNS AND PATIENT PROFILES IN A CLINICAL EXERCISE PROGRAM FOR OLDER ADULTS

Stephen Jennings[1], Jamie Giffuni[2], Neil Gregor[3], Tyara Mason[4], Marc Pepin[4], and Katherine Hall[5], *1. Durham Veterans Affairs Healthcare Adminstration, Durham, North Carolina, United States*, *2. Baltimore and Loch Raven VAMC, Baltimore, Maryland, United States*, *3. Veterans Health Administration, Martinez, California, United States*, *4. Durham VA Health Care System, Durham, North Carolina, United States*, *5. Veterans Health Administration/Duke University, Durham, North Carolina, United States*

Gerofit is a supervised, outpatient clinical exercise program offered to Veterans aged ≥65 enrolled in Veterans Health Administration. Enrollment in the program is contingent on health care provider referral. This analysis seeks to examine the sources and characteristics of referrals, aiming to inform strategies to enhance accessibility and optimize program efficiency. Referrals (N=2511) to the program from 2014-24 across 3 of the largest Gerofit sites were assessed, and came from a multitude of providers (primary care-44%; nutrition & dietetics-14%; physical therapy-11%; mental health-5%; etc.). Referrals from specialty clinics and physical medicine & rehabilitation physicians had the lowest enrollment rate (22% and 10%, respectively), while referrals from physical therapy and nutrition & dietetics had the highest enrollment rate (51% and 46%, respectively). Compared to all Veterans enrolled at these sites during this timeframe, those referred by nutrition & dietetics and mental health tended to be younger and have better physical performance, while specialty clinics & geriatrics tended to refer older and more frail adults. Examination of participation rates at 1 year post-enrollment by referring provider type showed that nutrition & dietetics referrals had the highest 1-year attrition levels (21%). Increased recruitment from high performing providers, and education to providers on program eligibility targeting those with lower enrollment rates (< 25%), will result in improved staff efficiency and enhance timely enrollment of eligible Veterans by reducing bottlenecks in the referral stage.

Abstract citation ID: igae098.2452

### FORMATIVE FACTORS INFLUENCING SOCIAL CARE SERVICES DEMAND AMONG OLDER ADULTS: A FORTITUDE PERSPECTIVE

Hengyuan Zhang, and Xuena Li, *Xi'an Jiaotong University, Xi'an, Shaanxi, China (People's Republic)*

**Background:** There is a serious mismatch between the supply and demand of social care services for older adults in China. The imprecise identification of formative factors leads to challenges for service providers in targeting the appropriate groups.

**Methods:** Adopting a fortitude perspective, a theoretical framework to analyze the formative factors of social care services demand was developed. Based on survey data from three cities and six counties, the impact of these formative factors was examined through binary logistic regression analysis.

**Results:** The demand for social care services among older adults is significantly influenced by their physical, familial and resource fortitude, following an overflow pattern of "individual-family-market-government". Conclusions: Demand for social care services emerges only when the challenges faced surpass the physical, familial, and resource fortitude of older adults. Overprovision to those with high fortitude and overlooking individuals with low fortitude results in a supply-demand imbalance.

Abstract citation ID: igae098.2453

### HEALTH CARE COST BURDEN AND SELF-REPORTED FREQUENCY OF DEPRESSIVE/ANXIOUS FEELINGS IN OLDER ADULTS

Namkee Choi, *The University of Texas Austin, Austin, Texas, United States*

Many older adults are burdened by out-of-pocket healthcare costs. Using the 2018-2021 National Health Interview Survey data, we examined: (1) healthcare cost burden and its associations with depressive/anxious feelings; and (2) whether during the pandemic (2020-2021), COVID infection and COVID-related healthcare access problems would also be associated with a higher likelihood of reporting healthcare cost burden and depression/anxiety. Nearly 12% of older adults (age 65+) reported healthcare cost burden, and a little over 18% reported daily/weekly depressive/anxious feelings. In generalized linear modeling analyses, the risk of healthcare cost burden was higher among women, racial/ethnic minorities (Blacks: incident risk ratio or IRR=1.49, 95% CI=1.35-1.65; Hispanics: IRR=1.27, 95% CI=1.12-1.44), those with chronic illnesses, mobility impairment, and those with Medicare Part D (IRR=1.14, 95% CI=1.04-1.20), but lower among individuals with Medicare-Medicaid dual eligibility, Medicare Advantage, VA/military insurance, and private insurance. The risk of daily/weekly depressive/anxious feelings was higher among healthcare cost burden reporters (IRR=1.76, 95% CI=1.64-1.88), controlling for sociodemographic and health status characteristics. The COVID-19 pandemic-related delay of or lack of access to medical care was also associated with a higher risk of reporting healthcare cost burden (IRR=1.67, 95% CI=1.52-1.84) and depression/anxiety (IRR=1.40, 95% CI=1.29-1.52). In sum, healthcare cost burden reporters were 1.8 times more likely to report high-frequency depressive/anxious feelings. Healthcare and social service providers should recognize the interconnection between financial strain associated with physical health problems and mental health in older adults. Policies and programs are needed to alleviate healthcare cost burden and ensure adequate access to healthcare services.

Abstract citation ID: igae098.2454

### HEALTH CARE COSTS OF MEDICARE BENEFICIARIES WITH FRAILTY AND/OR DEMENTIA

Stephanie Denise Sison[1], Sandra Shi[2], Gahee Oh[3], Brianne Olivieri-Mui[4], Chan Mi Park[3], Ellen McCarthy[5], and Dae Hyun Kim[3], *1. University of Massachusetts Chan Medical School, Worcester, Massachusetts, United States, 2. Harvard Medical School/Hebrew SeniorLife, Boston, Massachusetts, United States, 3. Hebrew SeniorLife, Boston, Massachusetts, United States, 4. Northeastern University, Boston, Massachusetts, United States, 5. Harvard Medical School, Boston, Massachusetts, United States*

Frailty and dementia predispose older adults to adverse health outcomes, resulting in increased health care utilization. In this study, we examined healthcare costs in a 5% sample of Medicare fee-for-service (FFS) beneficiaries using Medicare part A and B claims from 2016-2019. Using validated claims-based algorithms, we identified the presence of dementia and frailty (claims-based frailty index ≥0.25) in community-dwelling beneficiaries, 65 years or older, with continuous FFS enrollment 12 months prior to the start of each calendar year. Annual costs per beneficiary were calculated across 4 groups: a) those having dementia alone, b) frailty alone, c) both frailty and dementia, and d) neither. Having frailty alone incurred the highest total annual cost ($38,119), followed by frailty and dementia ($33,098) and then dementia alone ($16,622). The breakdown of costs revealed that inpatient costs (highest proportion of total costs) and carrier costs (e.g., provider fees, labs, imaging, procedures; second-highest proportion of total costs), followed a similar pattern as the total costs. This pattern persisted when examining preventable inpatient costs, with the frailty alone group having the highest costs ($2,517), followed by the frailty and dementia group ($1,610). SNF costs were highest in the frailty and dementia group ($5,338), followed by the frailty only group ($4,044), and then the dementia only group ($2,208). Our findings suggest that the presence of frailty, regardless of dementia status, drives healthcare cost. This highlights the need for a holistic patient-centered approach to optimize and reduce costs for frail older adults.

Abstract citation ID: igae098.2455

### HISTORY OF HEALTH INSURANCE COVERAGE GAPS AND HEALTH CARE USE AFTER GAINING AGE-BASED MEDICARE COVERAGE

Jada Morris, Dmitry Tumin, and Yan Zhang, *East Carolina University, Greenville, North Carolina, United States*

This study investigates the relationship between gaps in health insurance coverage prior to Medicare eligibility and subsequent changes in healthcare utilization among older adults in the United States. Medicare serves as the primary healthcare system for individuals aged 65 and above, offering access to essential medical services and security to seniors nationwide. Despite this, a significant portion of Americans aged 55–64 experience interruptions in health insurance coverage. Through an examination of Medicare enrollees with the RAND HRS 2020 dataset, we aim to determine whether experiencing coverage gaps before becoming eligible for Medicare is associated with lower utilization of preventive care services after gaining Medicare coverage. We hypothesize that individuals with a history of coverage lapses will present a decrease in attendance at primary care appointments following enrollment in Medicare. The study draws upon existing literature highlighting the challenges faced by uninsured older adults in accessing preventive care, often resorting to emergency department visits for healthcare needs. Additionally, gaps in coverage may lead to delays in diagnosis and treatment, inconsistent access to medications, and increased reliance on emergency room services post-Medicare enrollment. Our objective is to provide insights into the factors influencing healthcare utilization among older adults transitioning to Medicare, with a focus on the impact of pre-Medicare coverage lapses. Our hope is that by understanding these dynamics, policymakers and healthcare providers can develop targeted interventions to address gaps in coverage and promote equitable access to preventive care services for aging populations.

Abstract citation ID: igae098.2456

### HOW DOES MEDICARE ELIGIBILITY AFFECT CHOICE AND UTILIZATION?: EVIDENCE FROM VETERANS

Marion Aouad[1], Liam Rose[2], and Todd Wagner[2], *1. University of California Irvine, Irvine, California, United States*, *2. Stanford University, Stanford, California, United States*

Health insurance streamlines access to care, thereby improving well-being and health. In efforts to control costs, health insurers use selective contracting to limit the size of their provider networks. Regulators have focused on network adequacy rules, given concerns that overly restrictive networks may impede appropriate access. This raises an important question about what happens to patient behavior if a network expanded. We examine the effects of network expansions by focusing on veterans who receive care from the Veterans Health Administration (VHA). We exploit the change in their insurance network induced by Medicare eligibility at age 65 to examine changes in inpatient health care behaviors. Using regression discontinuity methods, we find that hospitalizations increase by approximately 3 percent at 65. Interestingly, there are large insurer reallocations – individuals increasingly choose the new Medicare option for hospitalizations once it is available (a 70 percent increase relative to the pre-65 share.) while the VHA and commercial insurers are used increasingly less, with an approximately 7 and 51 percent decline, compared to the pre-65 share, respectively. We also find larger reallocations toward Medicare for certain groups, particularly among individuals who live closest to a Medicare hospital and further from VHA hospitals, and among those who visit the hospital for elective medical procedures (e.g., knee replacement). The results show the link between network size and cost containment, with expansions resulting in greater use of elective inpatient care.

Abstract citation ID: igae098.2457

### HYPERTENSION CONTROL AND ISCHEMIC STROKE RISK: REAL-WORLD DATA IN HISPANIC MEDICARE BENEFICIARIES IN SOUTH FLORIDA

Paulo Chaves[1], Maureen Desoria[2], Leslye Roque[2], and Rafael Mas[2], *1. Florida International University, Miami, Florida, United States*, *2. Leon Medical Centers, LLC, Miami, Florida, United States*

The extent to which the magnitude of stroke risk reduction associated with blood pressure (BP) control in highly controlled trial research settings is translated to real world clinical settings remains to be better characterized, particularly in understudied populations. We examined the odds of hospitalization for acute stroke as a function of BP control in the previous year in Medicare Beneficiaries of Hispanic origin in south Florida. We analyzed de-identified electronic health records (2015-2019) from outpatients ≥65 years old receiving care at Leon Medical Centers (LMC), a major integrated healthcare services provider to Medicare and Medicare/Medicaid dually eligible (DE) Beneficiaries in Miami-Dade County, FL. In each year, within-person average systolic (SBP)< 140 mmHg and diastolic (DBP)< 90 mmHg were used to define controlled BP. Acute ischemic strokes were identified from hospital claims (ICD-CM-10 codes). Pooled longitudinal logistic regression with 1-year lagged time (n=36,519) with adjustment for age, sex, and DE status. Mean age (76.1±7.2 to 77.5±7.2), proportion of Hispanics (>90%), and annual BP control rates (>90%) remained constant throughout 2015-2019. Patients with controlled BP in a specific year had 39% lower (OR.61; 95%CI: 0.51, 0.73) odds of hospitalization for acute stroke in the subsequent year than those with uncontrolled BP. Neither sex, nor DE status modified the BP control–stroke risk relationship. In a large healthcare system in south Florida, we observed very high BP control levels, and, in line with published trial data, documented a substantially lower risk of ischemic stroke 1 year later in both Medicare and DE patients of Hispanic origin.

Abstract citation ID: igae098.2458

### HYPERTENSION CONTROL PROGRAM IN A LARGE HEALTH CARE SYSTEM SERVING HISPANIC MEDICARE BENEFICIARIES IN SOUTH FLORIDA

Paulo Chaves[1], Maureen Desoria[2], Leslye Roque[2], and Rafael Mas[2], *1. Florida International University, Miami, Florida, United States*, *2. Leon Medical Centers, LLC, Miami, Florida, United States*

Healthcare systems play a key role in hypertension control and health equity promotion in large populations. This study assessed the impact of a multifaceted hypertension control program in a large healthcare system serving Medicare and dual eligible (DE) Beneficiaries primarily of Hispanic origin in South Florida, an understudied population. Leon Medical Centers, a major integrated healthcare services provider to Medicare and DE patients in Miami-Dade, FL, implemented a hypertension control program in 2011. Electronic health records (2008-2018) from patients aged 65-89 years with hypertension were analyzed. Controlled BP definition was annual average systolic BP< 140 mmHg and diastolic BP< 90 mmHg. Age- and diabetes-standardized annual BP control rates were calculated. Random effects modeling compared BP control levels after vs. before program implementation. From 2008 (n=4,710) to 2018 (n=21,540), mean age±SD increased (73.4±5.3 to 77.1±6.1). Proportion of Hispanics remained constant (>98%). Overall age-adjusted odds of BP control were 5.8 (95% confidence interval [CI]: 5.6-6.1) times higher after program implementation. BP control rates increased similarly in both sexes. In women, standardized control rates increased from 68.9-73.3% (2008-2010) to

88.8-92.3% (2013-2018). Standardized control rates tended to be lower in DE than in Medicare patients at baseline – e.g., 2008: 67.2% (65.9-69.3%) vs. 72.2% (70.4-73.8%), but similar after program implementation – e.g., 2018: 89.9% (89.3-90.5%) vs. 90.2% (89.4-91.0%). Implementation of a primary care-based, multicomponent, culturally congruent hypertension control program was associated with meaningful increases in BP control levels in both men and women, and Medicare and DE patients in a large healthcare system in South Florida.

Abstract citation ID: igae098.2459

### IMPACTS OF PAYMENT REFORM AND COVID-19 ON POSTACUTE CARE ACCESS FOR OLDER ADULTS WITH DISABILITIES

Rachel Prusynski[1], Natalie Leland[2], Andrew Humbert[1], Arati Dahal[1], Cait Brown[1], Harsha Amaravadi[1], and Tracy Mroz[1], *1. University of Washington, Seattle, Washington, United States*, *2. University of Pittsburgh, Pittsburgh, Pennsylvania, United States*

Recent Medicare payment reforms, the Patient-Driven Payment Model (PDPM) for skilled nursing facilities (SNFs) and the Patient-Driven Groupings Model (PDGM) for home health agencies (HHAs), aim to improve access to post-acute care for patients with complex care needs by focusing reimbursement on diagnosis and medical complexity. Caring for complex patients, such as those with disabilities, under previous systems may have been less profitable for providers, discouraging admissions. We examined whether these policies improved access to SNF and HHAs for beneficiaries with disabilities. We conducted an interrupted time series analysis of Medicare hospitalizations from 2018-2021 to identify changes in access to SNFs or HHAs. This period included reform implementation and the COVID-19 pandemic, which emerged shortly after reforms. We identified 7,734,908 hospitalizations for beneficiaries with a disability as the original reason for Medicare entitlement. Before reforms, 11.1% of beneficiaries with disabilities were discharged to SNFs and 13.1% to HHAs. PDPM was associated with no difference in HHA discharges but a 0.43-percentage point (pp) decline for SNF. PDGM was associated with increases in HHA (1.91pp) and SNF (0.37pp) discharges until the COVID-19 pandemic, when HHA discharges again increased, remaining high at an average of 18.4% after vaccination efforts. SNF discharges declined at the onset of the pandemic but increased to 14.3% of all hospital discharges after vaccination efforts. Results suggest PDPM worsened access to SNF for beneficiaries with disabilities, but SNF access rebounded after COVID-19 vaccinations. Alternatively, PDGM improved access to HHAs for beneficiaries with disabilities, which remained high during the pandemic.

Abstract citation ID: igae098.2460

### INNOVATION DESIGN: MEDICAL DEVICE TESTING BEFORE DEPLOYMENT FOR RESEARCH STUDIES

Alyce Ashcraft, Donna Owen, and Jessica Brashear, *Texas Tech University Health Sciences Center, Lubbock, Texas, United States*

Smart technology for point of care (POC) testing, allows the creation of new devices from a design innovation mindset. Refinement in POC testing for infection is profoundly needed because of continuing dissatisfaction with antibiotic stewardship. Determining optimal control conditions to detect when a device is working as expected is a first step.

**Aim:** To describe the process of validating a device for POC infection assessment. A collaborative team involving Nursing, Clinical Laboratory Sciences, and Electrical Engineering drove device testing following a standard protocol validation for a POC instrument using American Society for Clinical Laboratory Science (ASCLS) guidelines. The protocol included comparison of two presumably identical devices relying on lasers for determination of specimen end points.

**Results:** Differences between the device operation manual and expected performance indicators provided direction for engaging with the device design team. Functionality of devices was determined by noting operational differences. Validation failed for both devices. Multiple experiments were performed with controlled variables; one device provided expected results on 33% of runs, while the other only had expected results on 16% of its runs. A critical laser error was found in the instrument with the 16% success rate; mechanical issues with the other device were noted and have yet to be fully specified.

**Discussion:** We engaged in collaborative ZOOM discussions, problem visualization screen shots, and photos of device elements. We learned more about the internal working of the device and proposed changes to the device to facilitate navigation around problems and begin the work of device redesign.

Abstract citation ID: igae098.2461

### INTERNET AND HEALTH INFORMATION TECHNOLOGY USE AMONG MIDDLE-AGED AND OLDER ADULTS WITH ARTHRITIS

Sarah Lieber[1], Jerad Moxley[2], M. Carrington Reid[3], and Sara Czaja[4], *1. Hospital for Special Surgery, New York, New York, United States*, *2. Weill Cornell Medicine, New York, New York, United States*, *3. Weill Cornell Medicine, Weill Cornell Medicine, New York, United States*, *4. Weill Cornell Medicine/Center on Aging and Behavioral Research, New York, New York, United States*

Arthritis is a leading cause of healthcare utilization in older adults. Healthcare delivery increasingly occurs virtually. We assessed health information technology (HIT) use in middle-aged/older adults with and without self-reported arthritis by analyzing 2022 National Health Interview Survey data. Prevalence of internet access and internet use for health information, communication with doctor's office, and review of test results was compared between adults ≥50 years of age with (versus without) arthritis (i.e., arthritis, rheumatoid arthritis, gout, lupus, fibromyalgia). Logistic regression was used to examine the relationship between arthritis and internet access and HIT behaviors. Among adults with arthritis (N=6371) for whom relevant survey data were available, 2640 (83%) reported internet access, and 1617 (61%), 1180 (45%), and 1359 (52%) used the internet for health information, communication with doctor's office, and review of test results. Among adults without arthritis (N=9268) for whom relevant survey data were available, 4150 (88%) reported internet access, and 2367 (57%), 1687 (41%), and 2023 (49%) used the internet for health information, communication with doctor's office, and review of test results. Arthritis was significantly associated with internet access and internet use for health information, communication with doctor's office, and review of test results (all p< 0.01); the relationship between arthritis and internet use for health information, communication with doctor's office, and review of test results remained significant after adjustment for age and sociodemographic characteristics (all p< 0.01). Further study of factors impacting HIT use in middle-aged/older adults with arthritis is needed to improve healthcare delivery in this population.

Abstract citation ID: igae098.2462
### MEDICARE-MEDICAID DUAL ELIGIBLE SPECIAL NEEDS PLANS (D-SNPS): ENROLLMENT TRENDS (2007–2023)

Roshani Dahal, *University of Minnesota School of Public Health, Minneapolis, Minnesota, United States*

Dual-Eligible Special Needs Plans (D-SNPs), private Medicare Advantage plans, exclusively enroll Medicare-Medicaid dual enrollees. First operationalized in 2006 and popular among state Medicaid agencies, enrollment in integrated plans remains a challenge. CMS expects states to transition existing plans into D-SNPs by end of 2025. We used publicly available SNP monthly data (2007-2023) to examine enrollment growth. CMS began reporting on integration status (2022) of D-SNPs: Coordination-Only, Highly-Integrated (HIDE), and Fully-Integrated (FIDE). D-SNPs began with 0.67million duals (73.9% of SNP enrollment). A moratorium on SNPs (2008-2009) correlates with slight decline; however, Affordable Care Act (2010) lifted moratorium and legislation extended SNPs through 2018, resulting in higher enrollment (mean 81.7%). In February 2018, SNPs were permanently authorized and annual enrollment in D-SNPs has consistently increased (11% to 16%). As of December 2023, there were 5.69 million duals (+16.4%) in D-SNPs, representing 89.5% of SNP enrollment. Of 823 plans, 60.6% were Coordination-Only, 29.8% were HIDEs, and 8% were FIDEs. Only twelve states offered FIDE-SNPs. Percentage of integrated plans have declined in the last year, despite overall increase in all D-SNPs (Coordination-Only plans grew by 34%, FIDE 15.7%, HIDE 12.5%). D-SNP market has experienced consistent high growth rate, outpacing growth in Medicare Advantage (8%). Although designed to serve duals, focused on integrating Medicare-Medicaid benefits, less than 10% D-SNPs are fully integrated more than 15 years later. Although HIDE-SNPs (a higher level of integration) began only in 2021, almost 30% of duals are enrolled. Integration under D-SNPs is important for reducing fragmented care among duals.

Abstract citation ID: igae098.2463
### MICROBIOTA COULD REDUCE THE BRACHIAL-ANKLE PULSE WAVE VELOCITY TO PROTECT FROM CARDIOVASCULAR DISEASE

Shengyin Zeng[1], Yaxin Li[1], Bangwei Chen[1], Lei Ruan[2], Jianguo Zhang[1], Shida Zhu[1], Cuntai Zhang[2], and Tao Li[1], *1. BGI Genomics, Shenzhen, Guangdong, China (People's Republic)*, *2. Tongji Hospital, Tongji Medical College, Huazhong University of Science and Technology, Wuhan, Hubei, China (People's Republic)*

Brachial-ankle pulse wave velocity (baPWV) is a risk factor for cardiovascular disease (CVD), and is associated with blood pressure and lipoprotein. However, research on the microbiota associated with baPWV has been limited. Here, we used two-sample bidirectional mendelian randomization (MR) analysis to assess the causality between gut microbiotas and baPWV. The exposure instrumental variables (IVs) were selected as significant independent variants (Linkage Disequilibrium (LD) R2 < 0.01; P-value < 5×10-8) from 1,521 gut microbiota genome-wide association studies (GWAS) summary data including class to species in the IEU OpenGWAS project. We performed baPWV GWAS analysis in Tongji-BGI research cardiovascular health (TORCH) cohort to obtain baPWV-related variants (rs141045630, rs11138617, rs77947040 and etc.) and selected suggestive variants (LD R2< 0.01; P-value < 1×10-5) as outcome IVs. The inverse variance weighted (IVW) was used as the primary MR analysis. In addition, we adjusted sex, age and other Framingham risk score factors. The sensitivity analysis involved changing the covariates, altering MR method and heterogeneity test. The analysis results showed higher abundance of Bifidobacterium adolescentis (P-value:1.81×10-4) and Lachnospiraceae (P-value:2.88×10-5) associated with lower baPWV. Moreover, MR evidence suggested a causal relationship between higher abundance of 3 microbiotas (Lactobacillales, P-value:1.72×10-3;Citrobacter, P-value:1.46×10-2; Ruminococcus, P-value:1.36×10-4) and higher baPWV. On the other hand, baPWV didn`t have potential causal effects on the abundance of B. adolescentis and Lachnospiraceae. In conclusion, a higher abundance of butyric acid-producing bacteria decreases baPWV and reduces arterial stiffness. We are currently performing more analyses with the metabolic pathway to explore more details in these results.

Abstract citation ID: igae098.2464
### SLEEPING MEDICATIONS AND PREVENTION OF FUNCTIONAL DISABILITY IN OLDER ADULTS WITH LOW CARE NEEDS: THE SOHA STUDY

Naoko Otsuki[1], Ryohei Yamamoto[1], and Ayumi Kono[2], *1. Osaka University, Suita, Osaka, Japan*, *2. Osaka Metropolitan University, Osaka, Osaka, Japan*

Although sleeping medications are extensively used to treat sleep disorders in older adults, their safety and efficacy in term of physical influences are controversial. A previous review reported that the risk of falls and hip fractures was associated not only with sleeping medications but also with sleep disorders. Improving sleep disorders may be vital for preventing functional disabilities. The present prospective cohort study aims to clarify the association between sleeping medication use and the incidence of functional disability in 1,652 older adults aged ≥65 years with low care needs in Japan's long-term care insurance (LTCI). The present study included 1,291 participants aged 65 years or older at the baseline date after excluding 302 participants who received long-term care certification during the six-month baseline period. The outcome was the identification of functional disabilities based on LTCI certification. The association between the sleeping medications and the incidence of functional disabilities was assessed using Fine and Gray proportional sub-distribution hazards models. During the median observational period of 2.5 years, the incidence of functional disabilities was observed in 54.6% and 49.7% participants in the "No use" group and the "Use" group, respectively. This study clarified that the group using sleeping medications had a significantly lower risk of functional disabilities than the group not using them. (Sub-hazard ratios of "No use" and "Use": 1.00 [reference], 0.82[CIs, 0.68–0.99]). Our findings differ from those of previous studies and may provide useful suggestions for home care services for older adults with low care needs.

Abstract citation ID: igae098.2465

### UNMET NEEDS FOR MENTAL HEALTH CARE: ARE THERE RACIAL DISPARITIES AMONG OLDER AMERICANS IN NEW YORK?

Chien-Ching Li[1], Pei-Shan Yen[2], Yi-Fan Chen[3], and Alicia Matthews[4], *1. Rush University, Chicago, Illinois, United States*, *2. University of Illinois Chicago, Chicago, Illinois, United States*, *3. University of Pittsburgh, Pittsburgh, Pennsylvania, United States*, *4. Columbia University, New York, New York, United States*

Racial and ethnic differences in mental health problems have been widely discussed in the existing literature. However, limited research examines the disparities in mental health care among older Americans, who have been found to face access barriers to care. This study aimed to examine the racial and ethnic disparities in unmet needs for mental health care among older Americans in New York. We analyzed data from the New York City Community Health Survey (NYC CHS; 2012-2020). Our study sample included participants aged 50 years and older from the NYC CHS. The dependent variable of interest was the unmet need for mental health care (yes, no), which was assessed through the question, "Did you need treatment for mental health but did not receive it?". The independent variable was race/ethnicity (non-Hispanic White, non-Hispanic Black, Hispanic, non-Hispanic Asian/Pacific Islander, other non-Hispanic). Covariates measured at a multi-level, including demographics, access to care, and neighborhood factors, were included. Regression results indicated racial/ethnic disparities in unmet needs for mental health care. Individuals in other non-Hispanic group were more likely to report having unmet needs for mental health care. However, non-Hispanic Blacks and Asian/Pacific Islanders exhibited a higher likelihood of having unmet mental health care needs compared to Whites. More importantly, older individuals without a personal doctor and those residing in underprivileged neighborhoods were associated with a higher likelihood of having unmet mental health care needs. Further development of community-based interventions is needed to mitigate access barriers to mental health care among older Americans.

Abstract citation ID: igae098.2466

### UTILIZATION OF USUAL SOURCE OF CARE AND HEALTH LITERACY AMONG OLDER ADULTS WITH HYPERTENSION

Dahye Hong, Jennifer Kim, and Bada Kang, *Yonsei University, Seoul, Republic of Korea*

Health literacy (HL) is crucial for health outcomes in older adults with chronic conditions because it influences disease-related knowledge and medication adherence. Although the usual source of care (USC) is recognized as an influencing factor for HL, little is known about the association between the utilization of USC and health literacy among older adults with hypertension. This study examined the relationship between the utilization of USC and HL among adults aged 65 years or older with hypertension (N = 1,482 women and 1,064 men) using data from the Korea Health Panel Survey 2021. Three analyses were performed to examine the association between the utilization of USC and HL after adjusting for sociodemographic and health-related characteristics. The results of logistic regression, showed a negative association between the absence of USC and HL. When HL was divided into three levels (inadequate, problematic, and sufficient), the multinomial regression analysis indicated that the absence of USC demonstrated a more pronounced negative association with higher levels of HL. Linear regression analysis revealed a negative association between the absence of USC and all domains of health literacy (i.e., healthcare, disease prevention, and health promotion). Disease prevention, which is known to be associated with the prevention of multimorbidity, exhibited the most pronounced negative association with the absence of USC. These findings have implications for policymakers, as promoting the utilization of USC can be an effective approach for improving HL in older adults with hypertension and will be beneficial in preventing the incidence of multimorbidity among vulnerable populations.

### SESSION 7085 (POSTER)

### HUMANITIES AND THE ARTS

Abstract citation ID: igae098.2467

### FEASIBILITY AND ACCEPTABILITY PILOT OF THE ENHANCING ACTIVE CAREGIVER TRAINING (ENACT) INTERVENTION

Jacqueline Eaton[1], Sarah Neller[2], Moroni Fernandez Cajavilca[3], Amber Dayley[1], Julene Johnson[4], and Lee Ellington[1], *1. University of Utah, Salt Lake City, Utah, United States*, *2. University of Tennessee Knoxville, Knoxville, Tennessee, United States*, *3. New York University, New York, New York, United States*, *4. University of California San Francisco, San Francisco, California, United States*

The process of actively engaging dementia caregivers in interventions has been insufficiently studied. This limits our understanding on how best to improve interventions. Enhancing Active Caregiver Training (EnACT) was developed to facilitate active engagement using participant-informed vignettes that portray caregiving experiences. We conducted a randomized waitlist-controlled pilot study to test the feasibility and acceptability of EnACT with a group of 30 caregivers of persons living with dementia. Participants were randomly assigned to intervention (n=15) or control (n=15) groups. The intervention group received three EnACT sessions during the first 7 weeks, which was then repeated for the control. Sessions used the vignettes to facilitate small-group dialogue, exploration, and reflection. These were delivered by trained interventionists via Zoom and each session lasted 60 minutes. Feasibility and acceptability data were collected to assess 1) interventionist training and delivery, 2) acceptability of treatment conditions, 3) participant adherence, and 4) recruitment, randomization, and assessment protocols. Preliminary results suggest that EnACT is feasible and acceptable to caregivers and interventionists. Retention rates were high (97%) and twenty-nine participants (97%) completed all six outcome surveys. Levels of satisfaction improved across the intervention, with the highest satisfaction focused on interventionists, engagement, topics, length, and accessibility. Data from this pilot is being used to prepare and plan for a fully powered randomized controlled trial.

Abstract citation ID: igae098.2468

### PATIENT PERCEPTIONS OF A PERSONALIZED MUSIC PLAYLIST PROGRAM FOR HOSPITALIZED OLDER ADULTS

Kayla Wong[1], Maanasa Nandigam[1], Victor Nguyen[1], Angela Morka[1], Eliza Kiwak[2], Judy Li[2], Jody Sharninghausen[2], and Richard Marottoli[2], *1. Yale University, New Haven, Connecticut, United States*, *2. Yale University School of Medicine, New Haven, Connecticut, United States*

Personalized music playlists may be therapeutic for hospitalized older adults. As part of the "Creativity in Aging" project at Yale Geriatrics, this study aims to bring personalized music to hospitalized older adults and explore their perceptions of its effect on emotional and physical health. Patients (N = 57) hospitalized in an inpatient geriatrics unit participated in the program from October 2023 to February 2024. Qualitative data were collected from 19 patients. Trained undergraduate volunteers took detailed "music histories" from patients and, when appropriate, their family caregivers (e.g. patients with dementia), and created personalized playlists using iPads. Patients could listen to their playlists during volunteer shifts throughout their hospital stay. Pre and post-listening surveys were conducted to elicit patient perceptions through brief semi-structured interviews and ratings on a Likert mood scale. A qualitative thematic analysis using the principles of grounded theory was performed to create thematic categories of patients' perceptions. Two coders open-coded separately then came together to agree on codes and themes. Saturation was reached, as no new themes were generated at the end of the thematic analysis. Five core themes emerged from the analysis: patients expressed feeling 1) calmer and more relaxed, 2) generally more positive, 3) reminiscent, 4) a want to sing and dance, and 5) physically and emotionally healed after listening to music. Constructive feedback included technical issues with iPads and wanting to listen to playlists more often. We aim to increase accessibility and expand the program to older adults in other areas of the hospital. The first two listed authors (MN and KW) contributed equally.

Abstract citation ID: igae098.2469

### PERSONALIZED MUSIC PLAYLISTS FOR HOSPITALIZED OLDER ADULTS: CREATING AND REVISING VOLUNTEER WORKFLOW

Victor Nguyen[1], Angela Morka[1], Kayla Wong[1], Maanasa Nandigam[1], Eliza Kiwak[2], Judy Li[2], Jody Sharninghausen[2], and Richard Marottoli[2], *1. Yale University, New Haven, Connecticut, United States*, *2. Yale University School of Medicine, New Haven, Connecticut, United States*

Personalized music has been shown to decrease the incidence of neuropsychiatric symptoms among hospitalized older adults. As part of the "Creativity in Aging" project at Yale Geriatrics, this quality improvement study aims to evaluate the process and feasibility of a personalized music program in an inpatient geriatrics unit. We collaborated with the hospital volunteer department to credential and train five undergraduate volunteers in building personalized music playlists for hospitalized older adults to listen to during their stay. From October 2023 to February 2024, 88 patients were referred to the program by hospital staff, and 57 patients participated. Here we describe the process of developing and revising the pilot. Following "test" sessions led by a medical student and resident physician familiar with the unit, an initial protocol was developed. Volunteers used REDCap and paper spreadsheets to organize HIPAA-secure patient data including referrals, linkage to personalized playlists stored on iPads, and brief pre- and post-listening surveys. Volunteers filled out feedback forms after each weekly shift to identify workflow issues and suggest improvements. Challenges included 1) identification of appropriate patients and 2) hand-off of patient referrals, notes, and next steps between shifts. Consultation with multiple stakeholders including volunteers, nursing managers, and attending physicians, led to solutions: 1) recruitment of nursing staff and creation of resident physician training module, after which nurses and resident physicians became key patient referral sources, and 2) remodeling of REDCap to manage volunteer "hand-off" communication, replacing the physical note-keeping system. We aim to make this a sustainable volunteer program.

Abstract citation ID: igae098.2470

### PILOT STUDY OF A VIRTUAL ART KIT PROGRAM FOR COMMUNITY-DWELLING OLDER ADULTS

Laurel Mertz, and Jennifer Smith, *Mather Institute, Evanston, Illinois, United States*

Engagement in the arts has been shown to improve health and well-being in older adults. However, previous research suggests that some older adults may experience barriers to arts engagement, such as health concerns, cost, and lack of transport. One method to address these potential barriers for older adults is virtual arts programming. The purpose of this study was to determine older adults' satisfaction with a six-week virtual art kit pilot program, as well as to examine how participation in the program affected participants' health and well-being. The program involved participants receiving an art kit via mail with the supplies and instructions needed for each weekly project (e.g., scarf dying, affirmation weaving). Participation in weekly classes delivered via video conferencing and a social media group was encouraged. A total of 285 community-dwelling older adults completed both the pre- and post- surveys (96.1% female; 91.9% between the ages of 55 to 74). Analyses revealed high satisfaction with the program. Participants significantly improved from before to after the program on creativity, sense of community, purpose, stress, life satisfaction, and attitudes toward aging. There was no significant change in sense of mindfulness, loneliness, nor self-reported mental and physical health. This study provides evidence for the positive impact of a virtual art kit program on multiple aspects of well-being for older adults. Given the importance of accessibility in arts engagement, future research should continue to examine virtual arts programs as a method to enhance well-being.

Abstract citation ID: igae098.2471

### SILVER TSUNAMIS, GRAY TIDES, AND DEAD WOOD: EXPLORING AGEIST METAPHORS AT THE INTERSECTION OF AGING AND CLIMATE CHANGE

Ulla Kriebernegg, and Anna-Christina Kainradl, *University of Graz, Graz, Steiermark, Austria*

This paper explores the ageist metaphorical language that links demographic change and climate change from a cultural gerontological perspective. Older adults are often labeled as 'vulnerable' and 'at risk' in the face of the climate crisis, framing it as a costly public health challenge. Simultaneously, they face criticism and animosity, being perceived as contributors to environmental degradation. The metaphorical realms of aging and climate change converge in ageist metaphors such as 'the silver tsunami' or 'the gray flood' that reinforce the image of the double burden allegedly represented by older people. Canadian sociologist Stephen Katz labeled this perspective "alarmist demography," challenging the notion that the threat arises from a growing, demanding, and relatively affluent group relying on the welfare state, burdening younger generations (1992, 203). Scrutinizing the rising ageism in climate change discourse, this paper illustrates how ageist metaphors might act as a veil, concealing other socio-political issues that warrant attention, such as social inequalities. To showcase this phenomenon, we will use examples from media and film as well as Margaret Atwood's short story "Torching the Dusties" (2014), which depicts young people setting nursing homes on fire, burning down "the dead wood" to force the old to "make room" for the young. Using such recent cultural representations, we will make the dangerous ageism inherent in the double burden narrative very explicit. To conclude, the paper shows that generational warfare is not helpful, and that tackling climate protection requires a collective effort involving all generations.

### SESSION 7090 (POSTER)

### INFECTIOUS DISEASES AND VACCINES

Abstract citation ID: igae098.2472

### APPLYING THE TDF FRAMEWORK TO VACCINATION DECISION-MAKING AMONG DIVERSE OLDER ADULTS

Taylor Patskanick[1], Sophia Ashebir[1], Alexa Balmuth[1], Lisa D'Ambrosio[2], and Joseph Coughlin[2], *1. Massachusetts Institute of Technology, Cambridge, Massachusetts, United States*, *2. MIT AgeLab, Cambridge, Massachusetts, United States*

Annual immunization rates among U.S. adults remain low relative to younger age groups and vary further by vaccine target. Gaps in research understanding the factors influencing older adults' uptake of vaccines typically indicated for those aged 50+ such as pneumococcal, flu, tetanus, diphtheria and pertussis, shingles, and coronavirus 2019 as persist. Older adults represent a spectrum in the degree to which they may be up-to-date on these recommended vaccines and the facilitators or barriers to this uptake may further vary across vaccine targets. This paper presents findings from the qualitative portion of a cross-sectional study conducted from 2021 to 2022 in which 25 virtual focus groups and interviews of adults aged 65+ (N=112) and who identified as Black or African-American, Latine, Asian or Asian-American, or White were convened to discuss vaccination decision-making. Groups were stratified by racial/ethnic identity and degree to which participants were up to date on the aforementioned vaccinations. In this paper, data were analyzed in MAXQDA2024 using the Theoretical Domains Framework to identify and map the framework's constructs across vaccine targets among the partially or fully vaccinated. Findings compare and contrast the prevalence of domains (e.g., social influence, emotion, knowledge) across targets among the vaccinated in our sample. Implications of this work for healthcare professionals who seek to influence health behavior change and future research focused on the implementation of interventions to improve vaccination uptake among older adults will be discussed.

Abstract citation ID: igae098.2473

### INFLUENZA VACCINATION AND OLFACTION IN OLDER ADULTS: FINDINGS FROM HEALTH ABC

Yaqun Yuan[1], Keran Chamberlin[2], Chenxi Li[1], Zhehui Luo[1], Teresa Tian[3], Eleanor Simonsick[4], and Honglei Chen[5], *1. Michigan State University East Lansing, East Lansing, Michigan, United States*, *2. Michigan State University, United States*, *3. National Institute on Aging, Baltimore, Maryland, United States*, *4. National Institute on Aging, Baltimore City 0400 Baltimore City, Maryland, United States*, *5. Michigan State University, Michigan State University, Michigan, United States*

Olfactory impairment is common in older adults and is likely associated with multiple adverse health outcomes. However, few risk or protective factors for olfactory impairment in older adults have been identified. We examined the cross-sectional association between flu vaccination and olfaction among 2507 participants of the Health, Aging and Body Composition study (aged 71-82 years, 51.7% women, and 37.4% Blacks). Olfaction was tested at the clinic visit Year 3 in 1999-2000, using the Brief Smell Identification Test. Olfaction was defined as good (test score 11-12), moderate (9-10), or poor (0-8). Participants were asked about receiving flu vaccines in the past year at each clinic visit from Years 1 to 3. We used multinomial logistic regression to estimate the odds ratio (OR) and 95% confidence intervals (CI) of flu vaccine reported at each clinic visit and olfaction tested at Year 3, adjusting for demographics, lifestyle, general health status, and flu occurrence within the past three months. At Year 3, compared with those not vaccinated, participants with flu vaccination were less likely to have moderate olfaction (OR=0.66, 95%CI, 0.51-0.85) and potentially fewer poor olfaction (OR=0.79, 95%CI, 0.60-1.02). The association appeared more evident in men and white participants. Similar findings, albeit slightly attenuated, were found in analyzing flu vaccination data reported at Year-1 and Year-2 clinical visits. Our study provides preliminary evidence that flu vaccination may be protective against olfactory loss in older adults, especially for men and the white race.

Abstract citation ID: igae098.2474

### METHODOLOGICAL CHALLENGES WITH VOLATILE OLDER PARTICIPANTS: LESSONS FROM A VACCINE HESITANCY SURVEY

Bryce Van Vleet, Heather Fuller, and Andrea Huseth-Zosel, *North Dakota State University, Fargo, North Dakota, United States*

Vaccine hesitancy is a critical health risk to older adults who may refuse vaccination for common, preventable, and life-threatening diseases like shingles, pneumonia, COVID-19, and influenza. Despite the importance of understanding vaccine hesitancy among older adults, mistrust in science and hostility towards researchers has been previously documented but it is unclear how these processes impact the research process. Thus, we sought to examine the characteristics and response patterns of volatile survey participants. Data were drawn from a mixed-methods survey about vaccination perceptions and behaviors conducted with 901 community dwelling older adults (65+). Volatile participants (n=59) were identified through their expression of excessive anger (e.g., explicit language, criticism, etc.) or mistrust (e.g., paranoia, accusations, etc.) in open-ended responses. First, linear regression was conducted to identify possible demographic predictors of volatile responses (R2=.063, p<.001). Volatile respondents were significantly more likely to be younger, highly religious, and conservative. Next, chi-square and post-hoc analyses were used to examine differential missingness in volatile participant responses. Volatile participants were more likely to answer questions they likely considered controversial, such as questions about COVID-19 and influenza vaccines. Volatile participants were also less likely to fully complete certain items of vaccine hesitancy and vaccine barrier scales. However, intra-individual response variability analysis did not detect outright deception from volatile participants. Overall, this study highlights methodological challenges in researching vaccine hesitancy among older adults and suggests recommendations to future researchers on conducting studies on politicized topics with skeptical, or even hostile, participants.

Abstract citation ID: igae098.2475

### QUALITATIVE PERCEPTIONS OF UNDERVACCINATED OLDER ADULTS AND POTENTIAL AVENUES FOR INTERVENTION

Heather Fuller, Andrea Huseth-Zosel, Bryce Van Vleet, and Teri Undem, *North Dakota State University, Fargo, North Dakota, United States*

Though older adults typically have relatively high vaccination rates, some older adults are reluctant to receive recommended vaccinations, a trend that is increasing in recent years. Vaccine hesitancy is not well understood among older adults but could help pinpoint areas of intervention to potentially reduce concerns and improve health outcomes. This study qualitatively examines vaccination decision-making processes among older adults (aged 65+) in North Dakota. Qualitative, semi-structured phone interviews focused on access, knowledge, and decisions about vaccines were conducted with 51 older adults between November 2022-January 2023. Of those participants, 24 were selected for the current study due to lacking 33% or more of recommended vaccines for this age group (i.e., influenza, shingles, pneumococcal, COVID-19). Interview transcripts were inductively coded to identify themes related to perceptions of acceptance and hesitancy with making decision about vaccines. Emergent themes included: skepticism, uncertainty of decisions, regret at being misled/fooled, defiance, and confidence in decisions. Within each theme, transcripts were examined to determine potential intervention approaches. To address skepticism, reduction of propaganda campaigns was identified. Creative educational and outreach interventions that emphasize consistent messaging were identified to address both uncertainty and regret. To address defiance and confidence in decisions, interventions should strategically utilize trusted information sources including physicians, pharmacists, and the church while targeting informed, independent decision-making. Findings contribute to better understanding the perceptions and decisions of vaccine hesitant older adults and suggest potential focus areas for interventions to potentially improve vaccination rates and reduce health risks for this population.

Abstract citation ID: igae098.2476

### VACCINATION MOTIVATORS AND DETERRENTS AMONG UNDERVACCINATED OLDER ADULTS IN NORTH DAKOTA

Andrea Huseth-Zosel, Heather Fuller, and Paul Carson, *North Dakota State University, Fargo, North Dakota, United States*

Despite increased risk of morbidity and mortality among older adults due to preventable infectious diseases such as influenza, shingles, pneumonia, and COVID-19, many older individuals forego receiving some, if not all, of these vaccinations. This study examines vaccination motivators and deterrents for undervaccinated older adults in North Dakota (ND). Adults aged 65+ in ND were mailed a survey (n=901) with questions gauging vaccination behaviors and perceptions. Of these participants, 132 indicating not receiving influenza, shingles, pneumonia, or COVID-19 vaccinations. Further questions assessed reasons they had not been vaccinated against each specific disease (e.g., "Concerned about side effects", "Vaccines are dangerous", "I'm healthy and I do not need it") and what would make it more likely to get a vaccine (e.g., "More information", "Doctor recommendation", "Easy access to vaccines"). Reasons for remaining unvaccinated varied by vaccine type. For influenza and pneumococcal vaccines, respondents were more likely to indicate they are healthy and do not need the vaccine. For shingles and COVID-19, respondents were more likely to indicate concerns about side effects. Factors reported to motivate increasing the likelihood of getting any vaccine were receiving a doctor recommendation, receiving more information, and having a vaccine provided at no cost. These results contribute to our understanding of vaccination behaviors among older adults and underscore specific issues around which to frame interventions tailored to increase vaccine uptake for this population.

### SESSION 7095 (POSTER)

### INTERNATIONAL

Abstract citation ID: igae098.2477

### A CROSS-NATIONAL COMPARISON OF THE EFFECT OF SMOKING AND DRINKING BEHAVIOR ON COGNITIVE HEALTH IN TAIWAN AND THE US

Yan-Jhu Su[1], and Yao-Chi Shih[2], *1. University of Massachusetts Boston, Boston, Massachusetts, United States*, 2. *National Taipei University of Nusring and Health Sciences, Taipei, Taipei, Taiwan (Republic of China)*

Previous studies have shown that smoking tobacco and alcohol consumption impact individuals' cognitive health. However, less studies have focused on cross-national comparisons of the effects of smoking and alcohol consumption on cognitive health and their differential effects across gender. This cross-sectional study used the Health and Retirement Study (HRS) 2018 (N= 7,467) and the Taiwan Longitudinal Study in Aging (TLSA) 2019 (N=2,776) for comparison. The cognitive function scores of the HRS are from the Telephone Interview of Cognitive Status-Modified (TICS-M; range: 0-35). For the TLSA, the Montreal Cognitive Assessment (MoCA; range: 0-30) is used. Our results show that both smoking and drinking negatively affect one's cognitive health among the US and Taiwanese populations, yet important gender differences were found. Taiwanese women aged 60 and older were found to have lower cognitive scores relative to otherwise similar men; however, in the United States, middle-aged and older women were found to perform better in their cognitive tests than their male counterparts. No moderating effects of gender on smoking were found among older persons from both countries. Some drinking was found to be protective for Taiwanese women, while binge drinking among US women further negatively impacted their cognitive health. Findings suggest that both smoking and drinking may impose negative influence on one's cognitive health and these impacts differ by gender. These differences may be the result of differences in the social context and cultural meaning of health behaviors. Further research is needed to address gender disparities in health behaviors.

Abstract citation ID: igae098.2478

### ADULT CHILDREN'S SINGLEHOOD AND AGING PARENTS' PSYCHOLOGICAL WELL-BEING IN SOUTH KOREA

Mihye Lim[1], and Hyo Jung Lee[2], *1. Yonsei University of Human Ecology, Goyang-si, Kyonggi-do, Republic of Korea*, 2. *Yonsei University, Seoul, Republic of Korea*

Guided by a life course perspective, this study aims to examine the linkages between the marital status of adult children as an indicator of the transition to adulthood and their parents' psychological well-being, considering how parental socioeconomic resources play a role in this association. We conducted a series of regression models using data from the 2018 Korean Longitudinal Study of Ageing (KLOSA, n=3112 aging parents who had at least one child over 35 years old). Consistent with the exposure model, having an unmarried son was associated with poorer parental psychological well-being, irrespective of parental income and education. However, parental education partially explained the association between daughter's marital status and parental psychological well-being, suggesting that parental well-being regarding adult children's marital status may depend on child's gender and parental education level. This study has implications for the intergenerational relationships in collectivist and patrilineal Eastern cultural context. Aging parents' psychological well-being ties to their adult children's marital status based on the social norm regarding the transition to adulthood, and particularly sons' marital status than that of daughters'. Also, aging parents may have different views on daughter's marital status depending on their educational attainment. In sum, based on the concept of 'linked-lives' and stress process model, this study provides an extended understanding of intergenerational relations in later life in Korea. Clinicians and family professionals should consider well-being in old age associated with adult children's life transitions and gender in the cultural context.

Abstract citation ID: igae098.2479

### BURDEN AMONG INFORMAL CAREGIVERS OF OLDER ADULTS IN GHANA: A SCOPING REVIEW

Joana Okine, and Nicole Ruggiano, *University of Alabama, Tuscaloosa, Alabama, United States*

The aging population in Ghana has experienced a significant increase, expanding tenfold from 213,477 in 1960 to 1,991,736 in 2021 during the last population census. Projections indicate that by 2030, the population of persons aged 60 and above will hit 2.8 million (Mba, 2010). In Ghana, as in many African countries, kinship-based system of care is predominant as caregiving is viewed as a filial responsibility and an expression of love. There are empirical studies done in advanced countries like the USA that have documented the burden associated with caregiving for older adults. However, literature on burden among informal caregivers of older adults in Ghana in sparse. This study aimed to review available studies exploring caregiver burden experienced by informal caregivers in Ghana. We searched for studies published since 2013 across various databases, including Ebscohost (APA PsychInfo, CINAHL, Abstract in Social Gerontology, SocIndex, APA PsychArticles, Eric Ebscohost), Web of Science, Social Services Abstracts, Sociological Abstracts, Taylor and Francis, PubMed, and Google Scholar. Out of 505 identified studies, 9 met the inclusion criteria for this review. Findings suggest that caregivers of older adults in Ghana, irrespective of the care-recipient's condition, face various burdens, including economic, physical, and psychological challenges. However, there is a noticeable gap in research addressing the burdens faced by informal Ghanian caregivers. It is crucial to focus on understanding and mitigating the burden on this group of caregivers to inform the development of culturally relevant interventions tailored to the needs of older adults and their caregivers.

Abstract citation ID: igae098.2480

### COGNITIVE FRAILTY IN RURAL KOREAN OLDER ADULTS: A GENDER-BASED EXAMINATION

Ah Ram Jang[1], and Hae Sagong[2], *1. Yeoju Institute of Technology, Yeoju, Kyonggi-do, Republic of Korea, 2. Auburn University, Auburn, Alabama, United States*

Cognitive frailty, characterized by the simultaneous presence of physical frailty and cognitive impairment, is strongly associated with dementia and mortality, with prevalence among community-dwelling older adults ranging from 0.9% to 12.0%. Rural residents exhibit a higher risk, underlining vulnerability to health issues. However, there is a significant research gap regarding factors influencing cognitive frailty, especially in rural areas. Our cross-sectional survey conducted from May to September 2020 in rural areas of South Korea aimed to address this gap. Participants (n=376) aged 65 or older were recruited from 60 primary health care posts. Incomplete data excluded 32 participants from analysis. Cardiovascular Health Study frailty index and Korean Mini-Mental State Examination were used to categorize cognitive frailty.T-tests, Chi-squared tests, and logistic regression analysis were employed for statistical analysis. The results indicate that among males, higher nutritional scores were associated with a reduced likelihood of cognitive frailty. Conversely, in females, older age heightened the risk. Further, factors such as education, higher nutritional scores, and active social participation were linked to a decreased likelihood of cognitive frailty in females. Recognizing these gender-specific influences, tailored interventions are paramount. Strategies should aim at enhancing nutritional status for both genders while placing a specific emphasis on promoting increased social participation among females. This study enhances our understanding of cognitive frailty in rural older adults and provides valuable insights for the development of targeted interventions.

Abstract citation ID: igae098.2481

### EXPLORING CURRENT CHARACTERISTICS OF GEROTRANSCENDENCE: A QUALITATIVE STUDY OF OLD PERSONS LIVING IN TOKYO SUBURBS

Jun Kitayama[1], and Hitoshi Igawa[2], *1. Gakushuin University, Toshima-ku, Tokyo, Japan, 2. Sakuragaoka Mental Health Clinic, Tama City, Tokyo, Japan*

Tornstam (2005) proposed the concept of "gerotranscendence." He identifies the phenomenon that the boundary between the self and the outer world is loosened in old age as gerotranscendence. The purpose of this study is to explore the experience of gerotranscendence among older people today living in the suburbs of central Tokyo. The participants, three men and five women who averaged 73.9 years old, attended a community association for older adults. The WHO-5-J scale was used to measure the participant's mental health, and the overall score (21.4 out of 25 points) indicates that the eight participants were in good health. A semi-structured interview based on Tornstam's gerotranscendence interview guide was conducted individually. The authors reviewed the collected narrative data and analyzed its individual and universal features. Unlike Tornstam's idea, the results demonstrated that the participants are mainly living in actual life, in a preliminary state of gerotranscendence. The realization of psychological changes associated with aging were not often experienced. Most of them had not yet considered their views on death. They did not experience changes in their personality traits or personality integration. Interestingly, some participants mentioned that they reminisced about family members who had passed away or tried not to purchase new goods. It seemed that these usual expressions contained faint but significant indications of transformation in their views of life. This may suggest that clear phenomena of gerotranscendence seldom occur in healthy older adults. Therefore a better understanding of psychological transcendence may be achieved by focusing on individual small stories.

Abstract citation ID: igae098.2482

### PREVENTING SOCIAL ISOLATION AND LONELINESS AMONG OLDER ADULTS WITH DEMENTIA: FINDINGS AND INSIGHTS FROM JAPAN

Limei Chen, and Megumi Inoue, *George Mason University, Fairfax, Virginia, United States*

This study examines the implementation and impact of social capital interventions to mitigate the interplay between social isolation, loneliness, and dementia within Japan's aging population. With an emphasis on advancing knowledge through a socio-ecological approach, our research focuses on social capital interventions, leveraging "chiiki-ryoku" (community power) at the community level for individuals with dementia and their caregivers. These interventions include Community (Dementia) Cafes, Dementia Supporter Caravan Training Programs, Community Social Workers, and Dementia Friendly-Communities, which are evaluated for their effectiveness in fostering social engagement and support networks. Our findings highlight the critical role of social capital in enhancing community resilience and well-being, demonstrating that interventions that cultivate active social participation and strong social networks in defined physical spaces such as junior high school area or municipal area can serve as protective factors against social isolation and loneliness. The research contributes to the broader discourse on social capital and dementia care by revealing the nuanced challenges and potential for meaningful impact through context-sensitive, integrative strategies. It posits that fostering a sense of belonging and meaningful relationships within communities can significantly alleviate the adverse effects of loneliness and social isolation among those affected by dementia. Through a detailed examination of Japan's response to dementia-related social isolation, this study provides insights into the development of effective, sustainable interventions that promote social health and well-being in aging societies.

Abstract citation ID: igae098.2483

### THE EFFECT OF FAMILY RELATIONS AND THE SATISFACTION OF SOCIAL ACTIVITIES BY GENDER

Meeryoung Kim, *Daegu University, Seoul, Republic of Korea*

The retirement age in Korea is 60, and most of the time after retirement is spent at home. Sometimes staying at home can be a source of conflict. However, doing social activities and being satisfied with such activities can become energy in old age. From an ecological point of view, this study investigates family relationships and satisfaction with various social relationships and activities as factors influencing the life satisfaction of older adults. The effects of these relationships may vary by gender. This study used the seventh additional and eighth wave of the Korean Retirement and Income Study. The subjects of this study were adults aged 60 and older. The sample size of men is 232 and the sample of women is 165. Multiple regressions were used for data analysis. Demographic variables were controlled. As for independent variables, this study used: the degree of spousal conflict, conflicts with children who reside at home and outside, satisfaction with leisure, and organizational and relational activities. Life satisfaction was used as a dependent variable. Research findings showed that relationship satisfaction had a significant effect on life satisfaction for both men and women. However, relationship satisfaction was relatively higher for men than for women. Unlike men, women's life satisfaction was impacted negatively by conflict between a spouse and children living together. These results imply that the same conflicts between spouses and children have different effects on life satisfaction by gender difference. Therefore, we must consider gender difference when deciding on old age policy.

### SESSION 7100 (POSTER)

### LGBTQIA+

Abstract citation ID: igae098.2484

### ADVERSE CHILDHOOD EXPERIENCES AND ALZHEIMER'S DISEASE/DEMENTIA RISK FACTORS AMONG TRANSGENDER ADULTS

Ethan Cicero[1], Jobina Chiow[2], and Jason Flatt[3], *1. Emory University, Quincy, Massachusetts, United States*, *2. Emory University, Atlanta, Georgia, United States*, *3. University of Nevada Las Vegas, Las Vegas, Nevada, United States*

Adverse childhood experiences(ACEs) are associated with an increased risk of developing Alzheimer's disease and related dementias(ADRD). Compared to the general population, transgender, nonbinary, and other gender expansive people(TNGE) report a higher prevalence of ACEs and ADRD. The relationship between ACEs and ADRD risk factors among TNGE adults has not been explored. 2019-2021 Behavioral Risk Factor Surveillance System(BRFSS) data, representing 16 U.S. states that assessed ACEs, subjective cognitive decline(SCD), and gender identity were used to determine the association between ACEs and ADRD risk factors(coronary heart disease[CHD], depression, diabetes, hearing loss, high cholesterol, hypertension, obesity, SCD, smoking, stroke) among TNGE adults aged 45+(N=206). Chi-squared and adjusted odds ratios(aOR) along with 95% confidence intervals(CI) were calculated, adjusting for BRFSS year, age, race, education, and employment. Obesity(70%), hypertension(56%), high cholesterol(47%) and depression(29%) were the most prevalent risk factors among the sample. Experiencing any ACE was associated with depression and SCD; >4 ACEs was associated with CHD, depression, SCD, and stroke. As the number of ACEs increased, the odds of CHD, depression, SCD, and stroke increased by 29–41%. The odds of depression and SCD were higher with childhood abuse (aOR=3.1, 95%CI:1.7-5.9, p< 0.01; aOR=3.8, 95%CI:1.7-8.3, p< 0.01) or household dysfunction (aOR=5.1, 95%CI:2.4-10.6, p< 0.0001; aOR=4.7, 95%CI:2.0-11.1, p< 0.01). Compared with no ACEs, experiencing 2,3, and >4ACEs were associated with 1.3–5.6x higher odds of depression and SCD. A lifecourse perspective is needed when examining the brain health of TNGE adults. ACE screening/interventions for TNGE youth are needed to help reduce the risk of ADRD later in life.

Abstract citation ID: igae098.2485

### DISPARITIES IN HEALTH OUTCOMES AMONG MIDDLE-AGED AND OLDER SEXUAL MINORITY CANCER SURVIVORS

Maria Brown[1], and Jane McElroy[2], *1. Syracuse University, Syracuse, New York, United States*, *2. University of Missouri, Columbia, Missouri, United States*

This cross-sectional, exploratory study analyzed 2021 Behavioral Risk Factor Surveillance System public-use data for a propensity-score matched sample (N=25,545), from states that collected sexual identity and cancer survivorship information, to explore health outcomes among sexual minority (SM) and non-SM cancer survivors. In bivariate analysis, SM survivors had more chronic conditions than non-SM survivors (males 45-64 years: 1.67 vs 1.03; females 45-64 years: 1.46 vs 1.16) and were more likely to report multiple chronic conditions (males 45-64 years: 44.5% vs 29.0%), depressive disorders (males 45-64 years: 42.9 vs 23.4%; males 65+ years: 20.8% vs 11.2%; females 45-64 years: 52.9% vs 36.5%; females 65+ years: 42.7% vs 41.3%), and difficulty concentrating, remembering, or making decisions because of a physical, mental, or emotional condition (females 45-64 years: 30.1% vs 18.9%; males 65+ years: 15.9% vs 8.5%). In logistic regression models, after controlling for demographics, health, and health behaviors, SM males and females in both age groups had similar odds of being a cancer survivor as their non-SM counterparts, and greater odds of poor health, multiple chronic conditions, depressive disorder, ADL limitations, and difficulty concentrating, remembering, or making decisions. Cancer status was not always associated with these outcomes. Further research should explore treatment experiences and factors contributing to disparities within this population and among SM cancer survivors and assess whether health outcomes of SM cancer survivors are related to stage of disease at diagnosis, time since treatment, or decisions made during treatment.

Abstract citation ID: igae098.2486

### PSYCHOSOCIAL WELL-BEING, RELATIONSHIP STATUS, SEXUALITY, AND GENDER IN THE CANADIAN LONGITUDINAL STUDY ON AGING

Arne Stinchcombe[1], and Douglas Hanes[2], *1. University of Ottawa, Ottawa, Ontario, Canada*, *2. Stony Brook University School of Medicine, Stony Brook, New York, United States*

Social connection and support are vital components of later-life health; for many older people, much of this support comes from a spouse or partner. Existing research has shown that gender and sexuality are central to the effects of partnership, and thus the later-life psychosocial and mental health benefits of partnership also likely differ by these factors. Canadian Longitudinal Study of Aging (CLSA) participants were categorized according to gender and sexuality; we analyzed how the effects of relationship status differed across these categories. Several psychosocial and mental health outcome variables were tested, including depression symptoms, mood disorder, anxiety, self-rated health, social support, and social standing. Linear and ordered logistic regressions were conducted, adjusting for age and education. Married persons had lower depression scores (B=-2.20, p<.001), higher social support (B=21.49, p<.001), and subjective status (B=.61, p<.001) than other relationship categories. The differences themselves differed by gender and sexuality: lesbian/bisexual women were better overall and experienced less adverse impact (B=23.37, p<.001), while these had greater effects on gay/bisexual men (B=21.10, p<.001). Lesbian/bisexual women have greater social supports across relationship statuses, while gay/bisexual men have less social support if not partnered. Findings highlighted variable and gendered effects of relationship status and sexual orientation on psychosocial outcomes. Examining differential effects for gender and sexual minority groups is essential to inclusive gerontological research.

Abstract citation ID: igae098.2487

### SOCIAL NETWORKING SITE USE AND MENTAL HEALTH IN OLDER LGB CANADIANS IN THE CLSA

Alexandra Grady, and Arne Stinchcombe, *University of Ottawa, Ottawa, Ontario, Canada*

The Internet has historically been an important avenue for social connection between lesbian, gay, and bisexual (LGB) people. Social networking sites (SNS) may have positive mental health impacts for LGB people. Limited research has explored SNS use in older LGB adults. The purpose of this study was to examine SNS use by sexual orientation in a sample of heterosexual and LGB Canadians using data collected from Follow-up 1 of the Canadian Longitudinal Study on Aging (n=21,836; 583 LGB). Participants completed self-report measures of SNS use, depression symptoms (CESD-10), and loneliness (UCLA 3-item Loneliness Scale). After adjusting for age, gender, income, and education, a significant difference remained in the odds of LGB participants using SNS to make new friends (OR = 1.82, p<.001) and promote themselves or their work (OR = 1.34, p=.005) in comparison to heterosexual participants. In terms of mental health, the linear regression treating depression as the outcome variable showed an interaction between being LGB and using SNS to stay in touch with friends (B=-1.23, p=.012), such that using SNS to stay in touch with friends as an LGB person was associated with fewer depression symptoms. The linear regression treating loneliness as the outcome variable showed an interaction between being LGB and using SNS to make new friends (B=.44, p=.013), indicating that LGB people who use SNS to make new friends report more loneliness than heterosexual people who use SNS to make new friends. This study adds to the growing literature on how older LGB adults use SNS.

Abstract citation ID: igae098.2488

### TRANSGENDER OLDER ADULTS HOSPITAL USE IN SOUTH CAROLINA

Jennifer May, Hanna Smyles, and Swann Adams, *University of South Carolina, Columbia, South Carolina, United States*

Transgender adults in the South represent a unique population with health disparities and care inequities due to longstanding false stereotypes and discrimination surrounding their transgender status. Transgender adults report less access to health insurance, lower quality of life, and severe mental distress compared to the general U.S. population. There is a paucity of literature identifying the hospital patterns of transgender older adults (≥50 years). This study examines hospital utilization among transgender older adults (≥50 years) in South Carolina to describe their demographic characteristics and use of inpatient, outpatient surgery, and emergency department services. We conducted a secondary analysis of restricted hospital claims data from 2000 to 2022; identified transgender adults, using ICD-9 and ICD-10 codes, who used hospital services in South Carolina. Patient demographics and clinical data were analyzed using descriptive statistics. We identified 382 unique individual transgender adults with our algorithm. Most identified as White (76%); 70% were in the age range of 50-59; sex as reported on the administrative record was mostly male at 51%. 78% (n=299) of transgender adults were from urban areas. Transgender adults could have had more than one type of hospital use with 272 unique individuals using inpatient hospital services, 334 unique individuals using outpatient hospital services, and 346 unique individuals using emergency room services. Understanding the hospital use and the urban vs rural location of the transgender adult population can help identify state resource allocation, capacity building, and creating a foundation for bolstering health services and reducing health inequities.

### SESSION 7105 (POSTER)

### ORAL HEALTH

Abstract citation ID: igae098.2489

### ORAL CARE IS A NECESSITY NOT A LUXURY: POLICIES THAT HELP OR HINDER ORAL CARE FOR OLDER ADULTS

Kayla Gallant[1], Katrina Reid[2], Joan Ilardo[2], and Angela Zell[2], *1. Michigan State University College of Human Medicine - East Lansing, East Lansing, Michigan, United States*, *2. Michigan State University East Lansing, East Lansing, Michigan, United States*

We interviewed fifteen stakeholders including state-level policy officials, medical practitioners, dental advocacy organizations, and educators to identify factors influencing oral care provision for older adults in residential settings. The interviews were conducted by two second-year medical students under the supervision of a Ph.D. social worker and a Doctor of Public Health. Analysis of interview transcripts followed grounded theory methodology. Our findings revealed several key themes limiting the provision of dental care to older adults in residential settings. These themes included financial constraints (e.g., Medicare and Medicaid coverage and reimbursement could be unpredictable state by state and year by year), inadequate training (e.g., few specialized dental training programs addressing geriatric oral care needs), limited resources for care provision within nursing facilities, and a systemic insufficient awareness of the dental needs of older adults in residential settings. This study's results illustrate the means of overcoming the limitations plaguing oral care for older adults in residential settings and ultimately advancing geriatric oral health outcomes in skilled nursing facility (SNF) settings. Next steps are to introduce a "train-the-trainer" model at a SNF. Registered and student dental hygienists will train certified nursing assistants to provide basic oral care for older residents in the SNF to improve geriatric oral health, ensure access to basic oral care, and strengthen the SNF's ability to provide dental services.

Abstract citation ID: igae098.2490

### ORAL HEALTH IN LONG-TERM CARE FACILITIES: A SYSTEMATIC REVIEW

Xiaochuan Wang, and Eman Zaman, *University of Central Florida, Orlando, Florida, United States*

Oral health in long-term care facilities (LTCFs) is becoming an increasing concern as the aging population grows bigger. Recent research indicates that less than 20% of older adults living in LTCFs receive proper dental care. This gap is expected to continue to expand due to various reasons such as oral health being deemed less important in healthcare culture, especially for LTCF residents who may already have multiple other medical conditions. Inadequate oral care can adversely affect one's mental, physical, and social well-being, causing disproportionate impacts on the vulnerable LTCF resident population. Thus, it is pivotal to understand the current status of oral health care in LTCFs, and LTCF workforce's roles, competency, and preparedness in providing such care. To address this gap, a systematic review of existing literature was conducted using PRISMA guidelines. We searched electronic databases (e.g., PubMed, ProQuest) to identify peer-reviewed articles published in English between 2013 and 2023. A total of 1101 unduplicated publications emerged in the search. After screening articles for relevance based on titles and abstracts, 22 were reviewed in full text. Sixteen articles were included in this review. Findings demonstrate that residents in LTCFs suffer from poor oral care as its significance is overlooked. The articles suggest solutions such as integrated care, expanded insurance access, and further research on oral hygiene in LTCFs. Findings underscore the importance of oral health education programs. Proper training and increased awareness is needed to prevent further health issues beyond mouth to improve oral and overall health of LTCF residents.

Abstract citation ID: igae098.2491

### ORAL HYGIENE AND THE INCIDENCE OF FUNCTIONAL DECLINE IN OLDER ADULTS AGED ≥75 YEARS: THE OHSAKA STUDY

Naoko Otsuki[1], Tomoaki Mameno[2], Satoko Takeuchi[2], Kazunori Ikebe[2], Ayumi Kono[3], and Ryohei Yamamoto[1], *1. Osaka University, Suita, Osaka, Japan*, *2. Osaka University Graduate School of Dentistry, Suita, Osaka, Japan*, *3. Osaka Metropolitan University, Osaka, Osaka, Japan*

Poor oral health has been identified as a risk factor for overall health. Management of oral problems is highly significant in the field of public health. This retrospective cohort study aimed to clarify the association between the oral hygiene and the incidence of functional decline in 134,957 participants aged 75 years or older, who underwent public dental checkups in Japan. The oral hygiene levels were scored by dentists as excellent, good, or poor. The outcome was the identification of functional decline based on Long-term Care Insurance certification. An association between the oral hygiene and the incidence of functional decline were assessed using the Fine and Gray model adjusted for clinically relevant factors. During the median observational period of 36 months, the incidence of functional decline was observed in 3,360 (5.3%) men and 2,693 (3.8%) women. The multivariable-adjusted model showed that poor oral hygiene was associated with functional decline in both men and women, respectively (Sub-hazard ratios [95% confidence interval] of Excellent, Good, and Poor: 1.00 [reference], 1.10 [0.99–1.24], and 1.57 [1.33–1.84], respectively for men; 1.00 [reference], 1.29 [1.14–1.46], and 1.56 [1.23–1.97], respectively for women). The findings of the present study suggest that municipal healthcare planning and home care should recommend oral hygiene in older adults aged ≥75 years.

Abstract citation ID: igae098.2492

### RELATIONSHIP BETWEEN SWALLOWING FUNCTION, NUTRITION, AND QUALITY OF LIFE DURING THE CORONAVIRUS DISEASE PANDEMIC

Naoko Morisaki[1], and Keizo Numata[2], *1. Himeji University, Himeji, Hyogo, Japan*, *2. Himeji Dokkyo University, Himeji, Hyogo, Japan*

This study aimed to determine the relationship between swallowing function, nutrition, and quality of life (QOL) in older adults in Japan who stayed at home during this period of the COVID-19 pandemic. The participants of this study were adults aged 65 years or higher who were staying at home during the COVID-19 pandemic. A questionnaire-based survey was conducted from 2021 to 2022. The participants' age, sex, swallowing function, nutrition, and QOL were surveyed. Swallowing function was assessed using the 12-item Dysphagia Risk Assessment for the Community-dwelling Elderly (DRACE) tool. Nutritional status was assessed using the Mini-Nutritional Assessment Short-Form (MNA-SF). QOL was assessed using the Short Form-8 (SF-8™) scale. Physical component summary (PCS) and mental component summary (MCS) scores were calculated based on the SF-8™ scale. Associations among swallowing function, nutrition, and QOL were analyzed using stepwise multiple regression analysis. The statistical software IBM SPSS Ver. 28.0 was used for the statistical analyses. The participants included 330 (70.1%) women and 141 (29.9%) men and their mean age was 81.5 ± 7.1 years. The participants' average DRACE, MNA, PCS, and MCS scores were 3.65 ± 3.78, 10.25 ± 2.64, 46.31 ± 6.36, and 47.61 ± 6.31, respectively. The analysis was conducted with DRACE scores as the dependent variables and the age, sex, MNA, PCS, and MCS scores as the independent variables. A significant association was observed among swallowing function, nutritional status, and QOL ($P < 0.01$). The findings suggested an association among swallowing function, nutritional status, and QOL even during the pandemic.

### SESSION 7110 (POSTER)

### PAIN MANAGEMENT AND PALLIATIVE CARE

Abstract citation ID: igae098.2493

### AN ANALYSIS OF PALLIATIVE CARE PROVIDER NURSING HOME CONSULTATIONS FOR PATIENTS WITH COGNITIVE IMPAIRMENT

Matthew Nesvet[1], Alex Floyd[2], John Cagle[3], Hanley Elftmann[2], and Kathleen Unroe[4], *1. Regenstrief Institute (IUCAR), and MDC, Indianapolis, Indiana, United States*, *2. Indiana University School of Medicine, Indianapolis, Indiana, United States*, *3. University of Maryland Baltimore, Baltimore, Maryland, United States*, *4. Indiana University, Indianapolis, Indiana, United States*

Nursing homes (NH) are important sites of care for people with serious illnesses in need of end-of-life care. Extant studies have shown NH residents with dementia struggle to receive high quality care. There is some evidence that palliative care (PC) interventions may improve quality of life (QOL) and care. There is little prior description of PC consults conducted in the nursing home setting. We examined notes PC providers wrote after initial consults with 197 residents of 8 nursing homes enrolled in UPLIFT, an NIH-funded clinical trial evaluating implementation of a PC model of care. Residents enrolled in UPLIFT who screened positive for PC needs were referred for consultations. We analyzed initial consult notes to learn about the residents referred and content of the consultative visits, including recommendations made. Mean age of the residents was 75 years. 70% were White, 19% Black/African American, and 52% female. Goals of care were discussed in 83% of the PC consults, the most commonly observed intervention. The most frequent types of recommendations by PC providers were: 1) medication-related (n=207); 2) referrals to other clinical services (n=103); 3) non-pharmacologic symptom relief (n=53); and 4) specific recommendations for NH staff-based activities (n=40). In total, PC providers averaged three recommendations when at least one family member, friend, or guardian was present during the clinical encounter in person (n=127) or by phone (n=38), and two when only the resident participated in the encounter (n=33).

Abstract citation ID: igae098.2494

### CRITICAL ANALYSIS OF A PRAGMATIC TRIAL TO IMPROVE CHRONIC PAIN MANAGEMENT IN A WELFARE STATE

Dagmar Draeger, Daniela Koios, Arlett Wenzel, and Andrea Budnick, *Charité - Universitätsmedizin Berlin, Berlin, Berlin, Germany*

High pain intensity and substantial pain interferences impact older community-dwelling adults with a certified need for care, while their pain management is not up to standard. A three-arm cluster-randomized pragmatic trial was conducted, with individualized interventions (IG1), digital staff training (IG2), and a control group (CG). The target group was home-care recipients (aged ≥65 years) with chronic pain. We recruited 190 participants from 22 home care providers (=clusters), drop-out rate was 24%. An expert panel communicated individualized nursing and medical recommendations for each IG1-participant. Nurses and physicians in IG2 were offered digital training. CG-participants received standard care. A dichotomous assessment regarding the acceptability of each participant's pain situation is the primary outcome for this analysis (group differences assessed by $\chi^2$-tests). At baseline, 48.4% of participants described their pain situation as "not acceptable". At follow-up, 15.9% stated an improvement of their pain situation, while the majority (70.7%) indicated stagnation or worsening (12.4%). There are no differences between the three study-arms. Staff commitment regarding intervention implementation did not meet the pre-defined requirements and varied substantially. Physicians and nursing staff cited heavy workloads, staff shortages etc. as arguments for not implementing interventions. While executives' support is essential, commitment of those that are supposed to carry out interventions or changes is paramount. Implementing improved pain management in a pragmatic setting encountered structural obstacles in the German health system. When planning pragmatic trials, barriers to implementing interventions under real-world conditions must be critically discussed. We recommend involving required staff early in the planning process.

Abstract citation ID: igae098.2495

### EARLY PALLIATIVE CARE UTILIZATION AMONG OLDER ADULTS WITH METASTATIC CANCER IN A MEDICARE COHORT

Molly Nowels[1], Elissa Kozlov[2], Stephen Crystal[3], and Paul Duberstein[2], *1. Weill Cornell Medicine, United States*, *2. Rutgers, The State University of New Jersey, Piscataway, New Jersey, United States*, *3. Rutgers, The State University of New Jersey, New Brunswick, New Jersey, United States*

Meta-analytic data have found early palliative care (EPC) to be associated with better outcomes among older adults with cancers. EPC is now recommended for all people with advanced cancers. Ideally, the resources required to appropriately treat referred patients would reflect the needs of the patient population. We are aware of no prior attempts to characterize patients receiving EPC. To address this gap, we used a 20% random sample of fee-for-service Medicare enrollees from 2008-2016 to identify predictors of EPC use within 8 weeks of a new diagnosis of metastatic cancer. Logistic regression modeling adjusted for demographics, cancer site, indicators for a variety of physical and behavioral health diagnoses, the Elixhauser comorbidity index, and several indicators for past-year health service use. 525,936 individuals were included, with 4.9% (25,982) receiving EPC. Several behavioral health diagnoses were associated with increased odds of EPC, including both active and remitted alcohol use disorder; active drug use disorder; major depression; and schizophrenia. These findings suggest that the caseloads of palliative care clinicians may disproportionately include persons with histories of significant psychiatric disorders and behavioral health disorders. Though palliative care clinicians may effectively manage some psychological symptoms, such interventions fail to consistently do so. Moreover, few palliative care teams include psychologists or psychiatrists, potentially leading to significant gaps in clinical expertise.

Models of palliative care should integrate specialty mental health clinicians to meet the demand for EPC services and manage the spectrum of needs among older adults with advanced cancers.

Abstract citation ID: igae098.2496

### EVALUATION OF A COMMUNITY-BASED CURRICULUM ON WELLNESS AND SAFE PAIN MANAGEMENT: A PRELIMINARY ANALYSIS

Gretchen Tanbonliong[1], Dorothea Vafiadis[1], Laura Plunkett[1], Susan Silberman[1], Erin Hou[2], Maddy Hatch[2], and John Guidry[2], *1. National Council on Aging, Arlington, Virginia, United States*, *2. TRX Development Solutions, LLC, New York, New York, United States*

The body of recent evidence-based interventions for pain self-management targeted at community-dwelling older adults is limited. The aim of this ongoing pilot study is to determine the efficacy of a six-week curriculum in promoting knowledge, attitudes, and behaviors related to wellness and safe pain management among older adults who were recruited via community-based organizations. Interim results from a sample survey analysis of 6 out of 8 cohorts (n=87) in a quasi-experimental crossover design showed that majority of the participants (91%) were White (63%) and Black (28%) women between the ages of 60 to 79. On a scale of 1 to 5 where "4" indicates that the knowledge or behavior is being implemented, 100% of the pain prevention strategies (i.e., maintaining a healthy weight or losing weight; engaging in physical activity; getting adequate sleep; and avoiding/limiting caffeine, tobacco, or alcohol) and two pain management strategies ("using topical therapies such as gels and creams" and "cleaning out old and expired medicines") met this threshold post-curriculum. There were statistically significant differences observed in participants' individual acceptance and commitment to cognitive behavior therapy (p=0.0015); adoption of meditation and/or mindfulness techniques to manage pain (p=0.0118); and reported more effective pain management (p=0.0109) between the post-curriculum and 6-week follow-up period. These preliminary findings suggest that the curriculum was an effective intervention for safe pain management among community-dwelling older adults. It has potential to be replicated across multiple community-based organizations and to target other underrepresented groups in the older adult population.

Abstract citation ID: igae098.2497

### EXPLORING THE RELATIONSHIP BETWEEN HOME TIME AND SERIOUS ILLNESS AMONG COMMUNITY-DWELLING OLDER ADULTS

Rachel Butler, and Howard Degenholtz, *University of Pittsburgh, Pittsburgh, Pennsylvania, United States*

Population-based measures like Healthy Days at Home (HDAH) are increasingly used as quality of life or health system performance metrics. These measures are just beginning to be studied in the context of older adults living with serious illness (SI). We explored factors associated with the duration of home time among community-dwelling Medicare beneficiaries with SI. Using 2016 data from the National Health and Aging Trends Study (NHATS) linked to Medicare claims, we calculated home time for sampled community-dwelling older adults using the HDAH measure. We operationally defined SI as having a diagnosis code in Medicare claims for at least one of nine severe chronic conditions identified by the Dartmouth Atlas. We conducted multivariate analyses to explore how a SI diagnosis (independent variable) is associated with home time (dependent variable). Covariates included gender, age, household composition, census division, and health and well-being (including self-reported health, number of chronic conditions, psychological symptoms, and subjective well-being). In unadjusted analyses, we found the overall community-dwelling sample population had a mean of 361.9 HDAH, while those with SI had a mean of 353.6 HDAH. A Poisson regression showed that community-dwelling older adults with SI had statistically significant fewer HDAH compared to those without SI (β=-0.03 fewer HDAH; CI:-0.03, -0.02). None of the covariate effects were statistically significant. This work furthers our understanding of opportunities to enhance service delivery systems for community-dwelling older adults with SI, a population not necessarily at the end-of-life but one that is vulnerable to frequent, intensive, high-cost health system encounters.

Abstract citation ID: igae098.2498

### HOSPICE NURSES' PERSPECTIVES AND CHALLENGES ON HOLISTIC CARE IN MULTIPLE INSTITUTIONS

Ying-Chun Li[1], Chian-Huei Shiu[1], Wang-Chuan Juang[2], and Yu-Qi Cheng[1], *1. National Sun Yat-sen University, Kaohsiung, Taiwan (Republic of China)*, *2. Kaohsiung Veterans General Hospital, Kaohsiung Veterans General Hospital, Kaohsiung, Taiwan (Republic of China)*

Holistic care is a comprehensive care model, including the body, mind, spirit, and society. Palliative care is part of holistic care to improve patient's quality of life and reduce medical care overuse. Nurses are the primary group providing holistic care. With the increasing care burden of older adults and non-communicable diseases, the demand for palliative care has grown significantly. Taiwan has a growing emphasis on palliative care, and international studies have shown that nurses' understanding and implementation of holistic care are relatively deficient. Therefore, this study aims to understand hospice nurses' perceptions and behaviors of holistic care and the challenges or difficulties encountered in its implementation. The study was divided into questionnaires and qualitative interviews based on the knowledge, attitude, and behavior theory. The study samples were from a southern medical center and its collaborative multi-institutions. The questionnaire results indicated that knowledge, attitude, and behavior mutually influence each other, with attitude acting as a complete mediator. Additionally, receiving educational training has a significantly positive impact on both attitude and behavior (p< 0.01). Interview results showed the respondents' perspectives and behaviors were quite similar to each other. Furthermore, the foremost challenge identified was related to dealing with the patient's family members. In conclusion, through research to understand hospice nurses' perceptions and behaviors on holistic care, find out the difficulties and challenges encountered in implementation, and establish appropriate education training and management systems accordingly, which will encourage nurses to provide better holistic care and improve the quality of care.

Abstract citation ID: igae098.2499

**PREVALENCE AND LONGITUDINAL PERSISTENCE OF PAIN IN DEPARTMENT OF VETERANS AFFAIRS (VA) NURSING HOMES**

Linda Am[1], Wildjena Jean Jacques[2], Shibei Zhao[3], Emma Quach[4], Christine Hartmann[5], and Camilla Pimentel[4], *1. Department of Veterans Affairs Bedford, Bedford, Massachusetts, United States, 2. University of Massachusetts Lowell, Lowell, Massachusetts, United States, 3. Center for Healthcare Organization and Implementation Research, Bedford, Massachusetts, United States, 4. VA Bedford Healthcare System, Bedford, Massachusetts, United States, 5. VA Bedford Healthcare System & University of Massachusetts Lowell, Bedford, Massachusetts, United States*

Pain is highly prevalent in U.S. nursing homes, with estimates of 22%-85% of residents experiencing any pain (Cole et al., 2022). Current studies about pain in Department of Veterans Affairs (VA) nursing homes offer information about a single point in time. Identifying whether pain improves or worsens is critical but difficult. We evaluated prevalence and longitudinal patterns of pain among residents of VA's 134 nursing homes. We used individual-level VA data from the Minimum Data Set 3.0 from October 1, 2018 to September 30, 2019 (N=40,954). We limited analyses to residents with long length of stay (>100 days); admission, quarterly, or annual clinical assessments (A0310=1,2,3); not comatose (J0200=1); and recent self-reported/observed pain (J0300=1). We randomly selected an "index" assessment for each resident and evaluated pain in ≥2 subsequent, consecutive assessments. We defined "persistent pain" as pain consistent across assessments and "intermittent pain" as pain documented in only one assessment. Our analytic sample was 1,824 long-stay VA nursing home residents. Pain was documented among 900 (49.3%) residents, among whom 509 (56.6%) had persistent pain. Among residents with intermittent pain (N=391; 21.4%), 298 (76.2%) reported worsening pain, and 93 (23.8%) reported improvement in pain. This study demonstrates high prevalence of any pain and persistent pain in VA nursing homes, with experiences of pain potentially worsening over the course of a nursing home stay. Results are consistent with studies in non-VA nursing home settings, underscoring the need to address pain more effectively in all long-term care settings.

### SESSION 7115 (POSTER)

### PERSONALITY

Abstract citation ID: igae098.2500

**A NEW LOOK AT NEUROTICISM: FINDINGS FROM THE IOWA UNMARRIED SURVIVORS STUDY**

Robert Hensley[1], Peter Martin[2], and Alex Bishop[3], *1. Southeast Community College, Lincoln, Nebraska, United States, 2. Iowa State University, Ames, Iowa, United States, 3. Oklahoma State University, Stillwater, Oklahoma, United States*

The purpose of this study was to analyze the association between personality traits and other variables relative to Neuroticism reported by N = 227 (M = 78.2, SD = 8.1) participants from the Iowa Unmarried Survivors Study. Using the development adaptation model as a conceptual framework (and Neuroticism as the dependent variable), a series of blocked multiple regression analyses were conducted. In the first block, age, gender, ethnicity, total years of education, and current marital status were included. The second block contained selected childhood variables, whereas the third block contained the personality variables, and the fourth block included the Venting subscale of the Brief COPE, social support, depression, stress, and loneliness. Recalled childhood abuse emerged as a significant predictor of Neuroticism, $\beta$ =.118, p <.05. If one was abused in childhood, one would show higher levels of Neuroticism. In addition, both venting and loneliness were significant predictors of Neuroticism, $\beta$ =.120, p <.05, $\beta$ =.191, p <.05 respectively. Moreover, stress and depression served as significant predictors of Neuroticism, $\beta$ =.208, p <.01, $\beta$ =.432, p <.01 respectively. This model explained 64% of the variance in Neuroticism scores. Although a causal relationship cannot be established via this model, the mulitdirectional nature of Neuroticism is noteworhy. Results from this study have implications relative to how geriatric social workers and aging service providers develop interventions and programming to improve mental health and quality of life for persons who remain unmarried throughout later life.

Abstract citation ID: igae098.2501

**CHANGES IN PERSONALITY TRAITS PREDICT ALLOSTATIC LOAD**

Jacob Alderson, Sarah Miller, and Nicholas Turiano, *West Virginia University, Morgantown, West Virginia, United States*

Understanding the influences personality change has on physical health has been identified as a key direction for personality research (Leszko et al., 2016). One key indicator of health and aging process is allostatic load, an objective index capturing physiological regulation (or dysregulation) across a variety of biological systems (e.g., inflammation, cardiovascular health). While some evidence suggests cross sectional associations between personality traits and allostatic load in older adult populations (Yoneda et al., 2023), little research at present has assessed if changes in personality are also associated with allostatic load. The present study utilizes the Midlife Development in the United States (MIDUS) data set (N = 998; Mean age = 46.14; range = 25-74), to analyze change scores in personality traits at two time points (1994-1995 to 2004-2005) as predictors of allostatic load at a subsequent time point (2004-2009). Age, race, gender, education level (at 1994-1995) and baseline level of personality traits were used as covariates. Regression analyses revealed that increases in openness (b = -.20, p =.034) and decreases in agreeableness (b =.27, p =.004) were associated with a lower allostatic load. These findings have implications for considering the role personality development has on healthy aging. Given that naturalistic change in personality does associate with important markers of physical health and physiological processes, future studies could attempt to intervene at the level of personality (e.g., attempt to increase openness) in order to promote more optimal physical health and aging processes.

Abstract citation ID: igae098.2502
### PERSONALITY TRAITS AS PREDICTORS OF BURNOUT IN SANDWICH GENERATION CAREGIVERS

Catherine Ju, Sabine Lohmar, Erika Fenstermacher, Montgomery Owsiany, and Barry Edelstein, *West Virginia University, Morgantown, West Virginia, United States*

Informal caregiving increases the risk for burnout (i.e., emotional exhaustion, detachedness in caregiving relationships, reduced sense of accomplishment through helping). Sandwich generation caregivers, individuals who provide care for their children and older parents, face unique challenges and experience greater burnout than individuals caring for children alone. However, little research has examined what factors are associated with outcomes such as burnout in sandwich generation caregivers. Furthermore, studies on sandwich generation caregivers rarely utilize a resiliency-based approach. This study aimed to examine whether Big Five personality traits (i.e., openness, conscientiousness, extraversion, agreeableness, neuroticism) predicted burnout in sandwich generation caregivers (N = 102). A hierarchical regression model controlling for demographics, $R^2$ =.007, F(3, 98) =.222, p =.881, indicated that openness, conscientiousness, agreeableness, and neuroticism explained an additional 32.9% of the variance. This change in $R^2$ was significant, F(4, 94) = 11.628, p <.001, with openness (β = -.215), conscientiousness (β = -.265), and neuroticism (β =.257) the only significant independent predictors, $R^2$ =.336, F(7, 94) = 6.781, p <.001. In a different regression model, which controlled for depression, neuroticism was no longer a significant independent predictor. These results suggest that sandwich generation caregivers with higher levels of openness and conscientiousness and lower levels of neuroticism may experience lower levels of burnout. Findings from this study enhance our understanding of sandwich generation caregivers and what personality traits may serve as resiliency factors towards decreasing burnout. Further research can examine modifiable elements of these traits to increase resiliency.

Abstract citation ID: igae098.2503
### RELATIONSHIP BETWEEN PERSONALITY TRAITS AND PREFERENCE FOR COPING STYLES IN OLDER ADULTS

Momoho Kakuta[1], Yasuyuki Gondo[1], Takeshi Nakagawa[2], Yukie Masui[3], Kei Kamide[1], Kazunori Ikebe[4], Tatsuro Ishizaki[5], and Yasumichi Arai[6], *1. Osaka University, Suita, Osaka, Japan*, *2. National Center for Geriatrics and Gerontology, Obu, Aichi, Japan*, *3. Tokyo Metropolitan Institute of Gerontology, Tokyo, Japan*, *4. Osaka University Graduate School of Dentistry, Suita, Osaka, Japan*, *5. Tokyo Metropolitan Institute for Geriatrics and Gerontology, Tokyo, Japan*, *6. Keio University School of Medicine, Tokyo, Japan*

Coping styles comprise various coping strategies roughly divided between primary and secondary control by active and passive attitudes. Personality traits are known to influence coping strategies. However, little is known about whether differential patterns of associations with personality traits emerge between primary and secondary control. This study aimed to clarify the relationship between personality traits and preference for coping styles in the octogenarian when the influence of personality would be maximized due to functional declines. Data were collected from 913 community-dwelling people (47.2% male) aged 78 to 82 years who participated in the Japanese longitudinal cohort study of Septuagenarians, Octogenarians, and Nonagenarians Investigation with Centenarians (SONIC). We analyzed the big five personality traits, SOC as the primary control, and positivity effect as the secondary control. We examined the correlation between personality traits and the two coping strategies and compared their independent correlation coefficients of the coping strategies for each personality trait. Only neuroticism was negatively correlated, and the others were positively correlated with both controls. There was no difference in the correlation coefficients of coping strategies on neuroticism and openness (n.s.), whereas extraversion (p =.000) and agreeableness (p =.048) had a significantly stronger correlation with the secondary control, and consciousness (p =.003) had a significantly stronger correlation with the primary control. The analysis indicated that the preference for coping styles differs by personality traits. We emphasized that higher neuroticism avoids using coping strategies, and higher consciousness and extraversion accelerate primary and secondary control, respectively.

Abstract citation ID: igae098.2504
### THE ALTERNATIVE MODEL OF PERSONALITY DISORDERS: LONGITUDINAL PSYCHOMETRIC PROPERTIES IN OLDER ADULTS

Lisa Stone-Bury[1], and Daniel L. Segal[2], *1. Bucknell University, United States*, *2. University of Colorado Colorado Springs, Colorado Springs, Colorado, United States*

The Alternative Model of Personality Disorders (AMPD) is a dimensional model of personality disorders that contains two main diagnostic constructs: personality functioning and five pathological personality traits domains. This study examined the temporal stability and predictive validity of the AMPD's two diagnostic constructs among older adults. It was hypothesized that (1) the AMPD will show high levels of both differential and absolute stability and (2) the AMPD domains measured at baseline will be significantly predictive of various salient aspects of psychosocial functioning at Time 2, including interpersonal, intrapersonal, health, and social functioning. Community-dwelling older adults (n = 200) were recruited online and completed self-report questionnaires of the AMPD and aspects of psychosocial functioning. A subsample of the original group (n = 172) completed the same questionnaires three months later. Correlations and paired samples t-tests partially supported the temporal stability of the AMPD: personality functioning demonstrated good temporal stability, whereas pathological personality traits were mixed (good differential stability and poor absolute stability). Hierarchical regressions supported the pathological personality trait model's predictivity validity among older adults, with Time 1 scores significantly predicting personality, interpersonal, intrapersonal, health, and social functioning scores at Time 2. Overall, hypotheses were partially supported. Differential stability (relative positioning of a participant within the sample) of the AMPD's pathological personality trait model appears questionable in older adults, which is notable given the brief period of time examined in the study. However, the AMPD showed good predictive validity, with the trait model predicting several clinically meaningful outcomes in later life.

### SESSION 7120 (POSTER)

### POVERTY

Abstract citation ID: igae098.2505

### DOES ASSET POVERTY INFLUENCE HEALTH AND WELL-BEING IN LATER LIFE? AN 8-YEAR PROSPECTIVE STUDY IN CHINA

Yu-Chih Chen[1], and Ting Hu[2], *1. The University of Hong Kong, Hong Kong, China (People's Republic)*, *2. University of Illinois Urbana-Champaign, United States*

Wealth serves as a financial cushion for older adults that can be consumed to generate economic, psychological, and social benefits in later life. Asset poverty, defined as inadequate wealth to cover basic needs for three months, can lead to financial instability and diminished well-being. However, research on its impact on the health and well-being of older adults, particularly in developing countries like China, is scarce. Using data from 7,982 adults aged 60 and older from the 2011-2018 China Health and Retirement Longitudinal Study, this study investigates the effects of asset poverty on cognition, self-rated health, depressive symptoms, and life satisfaction. Four types of asset poverty across time (i.e., persistent asset poverty, transitioning into asset poverty, transitioning from asset poverty, and persistent asset non-poverty) were constructed using three varied wealth measures: total assets, financial assets, and non-housing assets. Findings from the two-way fixed effect model showed that, compared to those consistently above the asset poverty line, households falling into asset poverty or experiencing prolonged asset poverty reported lower self-rated health and life satisfaction, along with higher depressive symptoms. Even transitioning out of asset poverty correlated with lower self-rated health and life satisfaction. However, asset poverty was not associated with cognition. This study underscores the significance of maintaining adequate assets to guard against financial insecurity and diminished mental health. Policies aimed at fostering lifelong asset accumulation and development should be advocated to bolster health and well-being in later life.

Abstract citation ID: igae098.2506

### GETTING OLDER AND GETTING BY WITH SSI

Callie Freitag, *University of Wisconsin-Madison, Wisconsin, United States*

For decades, qualitative researchers have asked low-income younger adults how they make ends meet in light of a shifting safety net. Yet, we know comparatively little about the daily economic lives of an increasingly policy-relevant group: low-income older adults who receive Supplemental Security Income (SSI). SSI provides a monthly backstop of cash assistance, a maximum of $943 per month in 2024, but its strict income and asset limits may mitigate its poverty-reducing potential. Additional disability-based eligibility requirements for people under age 65 may create administrative burden and perpetuate economic and health disparities. This project uses nationally representative, secondary qualitative interview data from the American Voices Project to examine the life events that led older adults to take up SSI and describe how they make sense of their economic circumstances in light of SSI's benefits and constraints. From a stratified subsample of low-income adults ages 50 years older (N=28), I take an abductive approach to analyze interviews and compare findings across respondents who entered SSI through the disability and retirement pathways. I find that a wide array of life experiences lead older adults to SSI, but nearly all are underscored by a lifetime of precarious employment and persistent poverty. Most respondents say they are able to "get by" between their monthly SSI check and other resources like subsidized housing, but are unable to cope with large, unexpected expenses without turning to family or debt.

Abstract citation ID: igae098.2507

### LABOR FORCE TRANSITIONS, INCOME CHANGES, AND POVERTY ENTRIES AMONG OLDER WORKERS: 2018–2023

Callie Freitag[1], and Heather Hill[2], *1. University of Wisconsin-Madison, Wisconsin, United States*, *2. University of Washington, Seattle, Washington, United States*

Economic downturns and recoveries, like the one caused by the COVID-19 pandemic, can change retirement plans by prompting early retirement or inducing workers to remain in the labor force longer. These changes in the timing and circumstances of later-life labor force transitions can have significant impacts on the income sources available to those making the transition—and thus the likelihood of experiencing poverty—but prior studies do not examine these outcomes. Using the Current Population Survey March ASEC from 2018-2023, this study examines older adults' (age 50 or over) labor force transitions during the COVID-19 pandemic. We contribute to prior literature by focusing specifically on trends, and the economic consequences of two types of labor force exits: retirement or non-retirement. Our main analysis estimates associations between labor force transitions and entries into poverty and describes changes in income during labor force transition years. We find that transitioning out of the labor force for any reason is significantly associated with substantial reductions in total income and a higher likelihood of entering poverty. However, these associations did not change much throughout the pandemic or recovery period. We also find that COVID-19 Economic Impact Payments and Unemployment Insurance were important protections against earnings losses and poverty entries in 2021, but especially for those who transitioned out of the labor force. This study also sheds light on the fluidity of retirement and labor force participation in later life.

Abstract citation ID: igae098.2508

### TRANSITIONS OF ASSET POVERTY AND DEPRESSIVE SYMPTOMS: A COMPARISON BETWEEN URBAN AND RURAL CHINESE OLDER ADULTS

Ting Hu[1], and Yu-Chih Chen[2], *1. University of Illinois Urbana-Champaign, United States*, *2. The University of Hong Kong, Hong Kong, China (People's Republic)*

Urban-rural disparities and asset poverty are being examined individually as critical socioeconomic factors influencing depression among older adults. However, while asset poverty, a dynamic condition evolving over the life span, is significantly influenced by urban-rural residency and household registration (hukou) status in China, the extent of their contribution to the relationship between asset poverty transitions and depressive symptoms in later life remains largely unexplored. Using four waves of the China Health and Retirement Longitudinal Study, we examined how asset poverty affects depressive symptoms among 7,982 adults aged 60 and older and the interaction effects by rural/urban areas. The fixed effect regression models revealed that older adults who transitioned from a non-asset-poor status to an asset-poor status reported elevated levels of depression compared to those who maintained a non-asset-poor status. Furthermore, significant interaction effects were found in the relationship between asset poverty and depressive symptoms. The simple slope analyses revealed that the effect of transitioning to asset poverty on depression was only significant for older adults living in rural areas or with rural hukou. The findings highlight the necessity of prioritizing policies to build a financial support system for older adults and promoting asset building to mitigate the negative impacts of asset poverty on mental well-being in later life, particularly for those in rural areas or with rural hukou. Additionally, efforts at reducing urban-rural disparities in social and family support and within the social welfare system should be advocated.

### SESSION 7125 (POSTER)

### PUBLIC HEALTH

Abstract citation ID: igae098.2509

### ADVERSE CHILDHOOD EXPERIENCES AND SLEEP DEPRIVATION IN OLDER ADULTS: THE MEDIATING ROLE OF SOCIAL ISOLATION

Syeda Jesmin, and Iftekhar Amin, *University of North Texas at Dallas, Dallas, Texas, United States*

**Background:** Between 40% and 70% of older adults have chronic sleep issues that can negatively affect their physical and mental health. While research shows that exposure to adverse childhood Experiences (ACEs) can be related to later life sleep quality, it is not clear how social isolation in later life can mediate this relationship. Method: We used the Behavioral Risk Factor Surveillance System (BRFSS) 2022 data. The dependent variable sleep deprivation was measured as sleep less than seven hours per night. Multiple logistic regression models were used to examine the associations between the ACE scores and individual reports of sleep deprivation after controlling for potential confounders. A mediation analysis was estimated for the eight ACE variables, social isolation, and sleep deprivation.
**Results:** After controlling for gender, race/ethnicity, education, marital status, and self-reported health status, the odds of experiencing sleep deprivation were significantly higher among those who reported four or more ACEs compared to their counterpart who reported three or fewer ACEs (OR = 1.76, 95% CI: 0.171–1.82). Among non-Hispanic Blacks, ACEs were associated with significantly greater odds of reporting sleep deprivation. Social isolation acted as a mediator in the ACE and sleep relationship. An inverse dose-response ($P < 0.05$) was observed between the sleep duration and experience of ACE.
**Conclusion:** This study identifies subgroups of older adults who are at greater risk of experiencing sleep deprivation as a result of social isolation and ACEs. Screening for ACEs and improving social participation could help address sleep deprivation in older adults.

Abstract citation ID: igae098.2510

### DIVERSE VOICES OF MENOPAUSE: STUDY RESULTS OF US WOMEN AGES 35 AND OLDER

Jennifer Sauer, and Aisha Cozad, *AARP, Washington, District of Columbia, United States*

AARP conducted a bimodal study gauging the impact of menopause symptoms on women and their treatment preferences. The focus groups included 36 women ages 40 and older from a mix of race/ethnicity, education level, and income. The survey of 1,510 U.S. women ages 35 and older included racial/ethnic oversamples of Black/African American (B/AA), Hispanic/Latino (H/L), and Asian American, Native Hawaiian, Pacific Islander women (AANHPI). Three unique multicultural perspectives emerged: 1) symptoms varied by race/ethnicity. For instance, more Black/African American women than other racial/ethnic groups, experience hot flashes (B/AA: 71%; White: 63%; AANHPI: 61%; H/L: 60%); more Hispanic/Latino women than Black/African American or Asian American, Native Hawaiian, Pacific Islander women report mood/emotional swings (H/L: 55%; B/AA: 44%; AANHPI: 36%; White: 49%;); 2) half (49%) all women say symptoms have a negative impact on day-to-day life with more Hispanic/Latino women feeling this way than White or Black/African American women (negative: 63%, 54%, 51%, respectively); 3) more White women than other racial/ethnic groups take psychotropic medications (White: 30%; B/AA: 24%; H/L: 21%; AANHPI: 14%) and more Black/African American, Hispanic/Latino, and Asian American, Native Hawaiian, Pacific Islander women than White women, take vitamins and supplements (B/AA: 57%; H/L: 57%; AANHPI: 57%; White: 47%). HRT use is consistent across racial/ethnic groups. Focus group verbatims, aligned with survey results, provide meaningful insights of the impact of menopause on women.

Abstract citation ID: igae098.2511

### EXPLORING THE ACTUAL SITUATION OF FAMILY CAREGIVING IN GER-DISTRICT IN THE SUBURBS OF ULAANBAATAR, MONGOLIA

Yumi Hashizume, *Institute of Medicine, University of Tsukuba, Tsukuba, Ibaraki, Japan*

Supporting family caregivers is going to be an increasing concern globally including developing countries. Mongolia, a developing country has challenges in caregiving due to lack of formal services and infrastructure development that family caregiving relies on mutual assistance by close relatives. This study aimed to explore the actual situation of family caregiving in District A in the suburbs of Ulaanbaatar.

A questionnaire included caregiver education, nomadic living experience, financial status, support from relatives, CAGE (alcohol dependence), GDS5, coping behaviors, and JZBI8 (caregiving burden) distributed to 562 family-caregivers selected by district personnel. Of the 537 collected, 75 individuals involved in caregiving were divided, and statistically compared in to the three groups named as economically stable (ES: n1=29), partially in need (PIN: n2=35) and totally in need (TIN: n3=11). Mean values were below the cutoff point in all groups, and comparisons showed no differences except JZBI8. ES' JZBI8 was significantly lower than PIN's if the relative assisted purchasing medications and watching over care-recipients [F(2, 66)=3.93, p=.024, η2=.11]. ES with a higher education had more problem-focused-coping behaviors than TIN[F(2, 30)=3.64, p=.038, η2=.02]; however, ES did not receive laundry assistance if the relative had nomadic life. PIN engaged all-day caregiving had more avoidance-escape-coping-behaviors than non-all-day [t(27)=2.22, p=.035, d=0.92, 95%CI[0.07,1.77]]. PIN with nomadic experiences could not provide financial support to care-recipients [χ2(1'n1=31)=5.490, p=0.019, φ=0.421]. Although the burden of caregiving was generally low, family caregivers' mutual support and coping behaviors vary by economic, educational, and nomadic background, which may develope into a caregiving burden for Mongolians.

Abstract citation ID: igae098.2512

### MULTISECTOR COLLABORATION TO PROMOTE HEALTHY AGING: A PRACTICAL GUIDE FOR PUBLIC HEALTH

Meghan O'Leary[1], Catharine Fromknecht[1], Yen Lin[2], and Kathryn Costello[2], *1. NORC at the University of Chicago, Chicago, Illinois, United States*, *2. US Department of Health and Human Services, Washington, District of Columbia, United States*

In collaboration with the HHS Office of Disease Prevention and Health Promotion, NORC at the University of Chicago developed a Quick Start Guide designed for public health officials to start or enhance partnerships with state officials who work with the older adult population. As the proportion of Americans over the age of 65 continues to increase, multisector partnerships will be critical to address the needs of this population and ensure that older adults live healthy, happy lives. The Quick Start Guide includes a set of recommendations for public health departments to use as a starting place for engaging with their counterparts in state and local agencies that are focused on healthy aging. The Quick Start Guide was developed based on interviews with state health department staff and is designed to be used by public health departments at any stage in the process of collaboration, either just beginning or building on existing collaboration. This session will summarize the recommendations in the Quick Start Guide and share examples of how state health departments have successfully partnered with their counterparts at state aging agencies.

Abstract citation ID: igae098.2513

### TARGETED SCREENING FOR LIVER FIBROSIS: IDENTIFYING HIGH-RISK ADULTS FOR TRANSIENT ELASTOGRAPHY EVALUATION

Amina Namrouti[1], and Paulo Chaves[2], *1. Florida International University College of Medicine, Miami, Florida, United States*, *2. Florida International University, Miami, Florida, United States*

Non-alcoholic fatty liver disease (NAFLD) poses a significant clinical concern, impacting over 30% of the general population and leading to potentially reversible liver fibrosis, cirrhosis, and physical frailty. Transient elastography (TE) offers a non-invasive screening tool, measuring liver stiffness as an indicator of hepatic fibrosis, guiding subsequent biopsy decisions. However, TE's availability and cost hinders its widespread use. Clinical practice relies on individual indicators like obesity for TE testing referrals. We aim to assess the accuracy of traditional indicators and explore whether combining readily available indicators in primary care settings could enhance screening accuracy. We conducted a cross-sectional analysis using National Health and Nutrition Examination Survey (NHANES) 2017-2020 data, involving adults aged ≥ 40 years without excessive alcohol consumption, cirrhosis, or hepatitis B/C. We evaluated liver stiffness measurements (LSM) >7pKa by TE as the outcome. Various predictors, including systemic diseases, examination findings, and laboratory tests, were assessed using stepwise logistic regression with sampling weights. Area under the receiver operating curve (AUC-ROC) was utilized to compare predictive accuracy across models. Results showed modest accuracy of individual indicators with AUC-ROC of 52%. The final model, incorporating diabetes, BMI>30 kg/m2, HR, A1c, FIB-4>3.25, APRI>0.5, and AST>40 IU/l, yielded an improved AUC-ROC of 74%. Combining primary care indicators enhanced identification of at-risk adults for abnormal LSM, compared to relying solely on individual indicators. Further research is necessary to determine the optimal combination of indicators for identifying individuals at risk of liver fibrosis who may benefit from TE screening.

### SESSION 7130 (POSTER)

### RETIREMENT

Abstract citation ID: igae098.2514

### A SYSTEMATIC REVIEW OF THE LITERATURE ON ACADEMIC RETIREMENT

Michelle Silver, *University of Toronto, Toronto, Ontario, Canada*

**Objectives:** The number of academics reaching traditional retirement age has been increasing thus motivating the need to better understand retirement experiences among higher education faculty. This review paper aims to: (i) identify the types of studies and traits being measured in studies of academic retirement, (ii) understand the determinants of retirement, including pathways and obstacles, among academics being measured in the literature, (iii) map the most prevalent types of studies and findings regarding academic retirement, and (iv) appraise the current state of the literature.

**Methods:** We conducted a systematic review of the literature to address our research objectives. Studies were identified from multiple database using search terms regarding retirement among academic faculty. Included studies examined determinants of academic retirement, perspectives, and/or retirement experiences among academics. Four reviewers screened the studies for inclusion and completed the data extraction.

**Results:** A total of 102 papers met the inclusion criteria. Most studies were based on cross-sectional survey data, followed by qualitative interviews; quantitative, qualitative, and mixed-methods research were included. Commonly measured determinants included age and spousal retirement timing. A mapping of the literature indicates a focus on geographical and/or subfield areas. Conclusions: Insights regarding academic's retirement experiences provide insights about varying retirement pathways and can help elucidate retirement pathways that enhance transitions particularly for those in professions with generally high autonomy. Future research ought to focus on supports to help retain mature workers within academia and overall determinants of satisfaction with retirement among academics.

Abstract citation ID: igae098.2515

### COUPLE COMMUNICATION AFTER RETIREMENT AND WELL-BEING IN ARAB SOCIETY: AN APIM ANALYSIS

Reem Nashef-Hamuda, *Haifa University, Haifa, HaZafon, Israel*

This study examined the influence of perceived marital responsiveness on life satisfaction and depression among 106 retired married couples from the Arab society in Israel (mean male age = 70.3, female age = 65.1) with an average of 43.3 years of marriage and 3-5 children. Utilizing the Actor-Partner Interdependence Model (APIM), findings highlighted a positive impact of individual (actor) responsiveness on Satisfaction with Life Scale (SWLS) scores and a negative impact on depression levels. Furthermore, partner responsiveness significantly affected both life satisfaction and depression, underscoring the importance of mutual perceptiveness in marital dynamics. The analysis revealed no significant relationship between marital control and either SWLS or depression, nor were there significant gender differences in the effects, indicating that the benefits of perceived responsiveness are consistent across genders. These results emphasize that life satisfaction and depression in retirement are influenced by both one's own and one's partner's responsiveness, reinforcing the value of mutual support in marital relationships.

Abstract citation ID: igae098.2516

### DO TRAJECTORIES OF SELF-IDENTIFIED RETIREMENT STATUS PREDICT HEALTH AND COGNITIVE OUTCOMES?

Yun taek Oh[1], and Jacqui Smith[2], *1. University of Nevada Reno, Reno, Nevada, United States*, *2. University of Michigan, Ann Arbor, Michigan, United States*

Few studies examined the ways older workers self-identify their retirement status (named 'retirement identity') over time before exiting the workforce. Examining the trajectories of retirement identity transitions – between "not retired", "partly retired", and "completely retired" – clarifies the heterogeneous outcomes found in 'static' retirement identity. In this study, we distinguish and categorize these trajectories using sequence analysis, investigate the common sociodemographic and economic characteristics for each trajectory using multinomial logistic regression, and examine the associations between these trajectories and the physical and mental health conditions of older workers using latent growth modeling. Drawing on a sample of respondents from the Health and Retirement Study (HRS) from 1992 to 2020, we use the first nine waves of the initial HRS birth cohort (born 1931-1936, recruited in 1992, N=1,487) and the Early Baby Boomers (born 1948-1953, recruited in 2004, N=1,017). We determine and name four distinct categories of trajectories – early, late, traditional, and partial transitions – based on the timing and sequence of transitions. The membership of these trajectories is strongly associated with the birth cohort, educational attainment, labor income from career jobs, and household wealth. The latent growth modeling reveals that workers in late or partial transition trajectories tend to have better initial physical, mental, and cognitive health and experience slower deterioration of these than those in early or traditional transition trajectories. These results suggest a close relationship between health and self-identification of retirement status that may influence the work attachment and timing of workforce exit.

Abstract citation ID: igae098.2517

### RESOLUTION OR WORK STRESS? WHY DOES RETIREMENT CHANGE HEALTH BEHAVIOR?

Colin McKenzie[1], Shinya Kajitani[2], and Kei Sakata[3], *1. Keio University, Minato-ku, Tokyo, Japan*, *2. Kyoto Sangyo University, Kyoto, Kyoto, Japan*, *3. Australian Institute of Family Studies, Melbourne, Victoria, Australia*

The purpose of this paper is to examine the impact of retirement on actual health outcomes and intentions relating to these outcomes for Japanese men. The health outcomes investigated relate to alcohol and tobacco consumption, physical exercise, and psychological distress. This paper uses data from the first 15 waves of the Longitudinal Survey of Middle-aged and Elderly Persons (LSMEP) to estimate regression models explaining these health outcomes. An instrumental variable estimator using instruments constructed from the eligibility ages for various aspects of the Japanese pension is used to account for the endogeneity of retirement and income. Individual heterogeneity is dealt with by using a fixed effects estimator. It is found that there is no significant impact of retirement on the extent to which individuals report that they will take care not to drink too much, nor smoke too much, and to engage in a sufficient amount of exercise. Retirement leads to psychological distress declining, the proportion of men drinking alcohol to fall and the proportion who are smoking falls after retirement. The proportion of respondents engaging in moderate amounts of exercise increases following their retirement. Taken together, the results suggest that reductions in stress following retirement are driving the results. Falls in income associated with retirement explain little of the behaviour observed.

Abstract citation ID: igae098.2518

### WORK TO RETIREMENT: EXAMINING THE ROLE OF RETIREMENT IN THE ASSOCIATION BETWEEN AWARENESS OF AGING AND SLEEP HEALTH

Neha Dewansingh[1], Kristin Calfee[2], Claire Smith[3], and Soomi Lee[2], *1. University of South Florida, Davenport,*

*Florida, United States*, 2. *The Pennsylvania State University, University Park, Pennsylvania, United States*, 3. *University of South Florida, Tampa, Florida, United States*

Individuals of all ages require adequate sleep to maintain a healthy lifestyle; sleep health is especially vital in older adults, but often degraded after retirement. Poor sleep puts older adults at risk for physical and cognitive impairments, which can increase fall risk, difficulty performing daily activities, and chronic conditions like Type 2 diabetes. Awareness of aging (AoA), or consciousness around one's aging experiences, has been linked to promoting health behaviors such as exercising. Sleep is a health behavior; yet its association with AoA has been little studied. Both sleep and AoA may change through retirement, and the association between them may also differ by retirement status. We investigated these relationships using a self-report composite measure of sleep health across multiple dimensions (i.e., regularity, satisfaction, alertness, efficiency, and duration) in a national sample of adults (n=668; Mage=63yrs) from the Midlife in the United States study, controlling for sociodemographic covariates and self-rated health. Results demonstrated that higher AoA was associated with worse sleep health for both retired and non-retired adults (B=-0.256, SE=0.083, p=0.002), with more pronounced association for retirees (B=-0.418, SE=0.178, p=0.019). Such findings suggest that higher awareness around aging surprisingly relates to greater disruptions in sleep health for older adults, particularly in retirees, counter to prior work emphasizing the health benefits of AoA. Future research could examine how and why retirees experience unanticipated negative side effects of AoA such as sleep disruptions. Limitations and directions for future research will be discussed, with potential methods for managing AoA by specific contexts.

### SESSION 7135 (POSTER)

### RURAL HEALTH

Abstract citation ID: igae098.2519

### AN INNOVATIVE APPROACH TO EXPANDING AGING SERVICES ACCESS IN RURAL COMMUNITIES: RURAL AGING ACTION NETWORK

Molly Wylie[1], Ashley Washington[2], Verena Cimarolli[3], and Robyn Stone[3], *1. LeadingAge LTSS Center @UMass Boston, Santa Monica, California, United States*, *2. Lutheran Services in America, Washington, District of Columbia, United States*, *3. LeadingAge, Washington, District of Columbia, United States*

A significant share of older adults live in rural areas and wish to remain in their homes and communities. Aging in rural communities poses unique challenges that can limit access to healthcare, transportation, and other services that promote independence. To address these challenges, Lutheran Services in America (LSA) developed Rural Aging Action Network (RAAN), an innovative collaborative that leverages existing community-based services and supports for older adults who are underserved in rural communities of Minnesota, Montana, North Dakota, and South Dakota. A mixed-method evaluation of RAAN was conducted over a three-year period to assess the extent of community need, the network's effectiveness in addressing care gaps, and lessons learned in implementing RAAN. Descriptive analyses of client-level data showed that 483 older adults have been in contact with the network, and over half (55%) were referred to services. Chi-square tests indicated that older adults with restricted incomes were significantly more likely to be referred to any service than older adults whose incomes meet their needs (69% versus 31% respectively, X2 (1, N = 258) = 9.8, p =.002)), suggesting RAAN's effectiveness in reaching low-income populations. Focus groups and interviews with 12 older adults and 9 caregivers found that transportation, technology training, and English translation remain service gaps in the community. Respondents expressed the value of RAAN in raising awareness about available services and providing a one-stop-shop for aging resources. This evaluation can help inform the implementation of similar networks in rural communities nationwide.

Abstract citation ID: igae098.2520

### BARRIERS AND FACILITATORS TO TECHNOLOGY USE AMONG RURAL CAREGIVERS OF PEOPLE LIVING WITH DEMENTIA

Sarah Boucher[1], Anna Jolliff[1], Matthew Zuraw[2], Christian Elliot[3], and Nicole Werner[4], *1. Indiana University Bloomington, Bloomington, Indiana, United States*, *2. CareVirtue, San Diego, California, United States*, *3. CareVIrtue, San Diego, California, United States*, *4. Indiana University, Bloomington, Bloomington, Indiana, United States*

Caregivers of people living with Alzheimer's disease or related dementias (ADRD) who live in rural areas experience more difficulty in accessing resources to support caregiving. Technology may be a promising tool to connect rural caregivers to support. However, little is known about rural ADRD caregivers' technology use. The objective of this study was to assess the barriers and facilitators to technology use among ADRD caregivers living in rural communities. In this qualitative study, rural caregivers of people living with ADRD were recruited through community partners to complete virtual semi-structured interviews. Interview questions focused on formal and informal supports used by caregivers and the role of technology in accessing those supports. Two coders completed an inductive, iterative thematic analysis. Caregivers (N=19) were 73.7% female and had a mean age of 66.4(SD=10.1). Within the sample, 57.9% of caregivers were caring for a partner, while 31.6% cared for a parent or parent-in-law. Analyses resulted in fourteen codes, which were combined into three themes. Barriers and facilitators to technology use included 1) systemic influences, such as access to technology and support with using it; 2) alignment between technologies available and caregivers' informational needs; and 3) a preference for technological versus in-person support, including features of technology that influence this preference. The results of this study demonstrate systemic barriers that must be addressed when introducing technology-based interventions to rural caregivers. Further, results point to enhancements that can be made to technology-based interventions to improve their usefulness to rural caregivers.

Abstract citation ID: igae098.2521

### DOES INTERNET USE MODERATE THE RURAL–URBAN DISPARITIES IN HEALTH AMONG BRAZILIAN OLDER ADULTS?

Xiayu Chen[1], Tania Rodriguez[2], Rachel Wu[2], and Flávia Andrade[3], *1. University of Illinois Urbana-Champaign, Champaign, Illinois, United States*, *2. University of California Riverside, Riverside, California, United States*, *3. University of Illinois at Urbana Champaign, Urbana, Illinois, United States*

Although prior research has noted the benefits of Internet use on physical and mental health among older adults in developed countries, its impact within less developed countries, particularly in Latin America concerning rural-urban disparities, is poorly understood. This study investigated the health disparities between rural and urban older adults in Brazil and examined how Internet use moderated these disparities. Data from the second wave of the Brazilian Longitudinal Study of Aging with 9,751 adults aged 50 and above were analyzed. The outcome variables include the number of chronic conditions, limitations in basic and instrumental activities of daily living (ADL & IADL), depressive symptoms and cognitive functioning. Using a series of weight-adjusted multivariate linear regression analyses, we found that compared to the older adults who lived in rural areas, urban residents tended to have more chronic conditions, IADL disabilities, higher depressive symptoms, but better cognitive functioning. A significant interaction between Internet use and rural-urban residency was identified for chronic conditions ($\beta$=0.72, 95% CI= 0.55-0.94), cognitive functioning ($\beta$=0.85, 95% CI=0.73-0.99), and depressive symptoms ($\beta$=1.40, 95% CI=1.04-1.89), indicating that Internet use mitigated the rural-urban disparity in chronic conditions and cognitive functioning, while it increased the disparity in depressive symptoms. The study's findings underscore the potential of Internet use as a means to reduce health disparities among older adults in Brazil. Future interventions could include targeted digital inclusion strategies tailored to address the socioeconomic challenges in rural areas to leverage the benefits of the Internet.

Abstract citation ID: igae098.2522

### GENERATING VIDEO VISITS FOR RURAL OLDER VETERANS IN TELE-GERIATRIC MENTAL HEALTH SERVICES

Christine Gould[1], Chalise Carlson[2], Marika Humber[1], Amanda Peeples[3], Zachary Burningham[4], and Ranak Trivedi[5], *1. VA Palo Alto Healthcare System, Palo Alto, California, United States*, *2. Department of Veterans Affairs, Palo Alto, California, United States*, *3. Corporal Michael J. Crescenz Department of Veterans Affairs Medical Center, Philadelphia, Pennsylvania, United States*, *4. VA Salt Lake City Healthcare System, Salt Lake City, Utah, United States*, *5. Stanford University/VA Palo Alto Healthcare System, Palo Alto, California, United States*

Telehealth services increase access to specialty mental health services for rural older adults. Within the Veterans Health Administration (VHA), older Veterans are more likely to receive audio-only mental telehealth services compared with younger Veterans. Using a mixed methods approach, we examined the use of video telehealth services by geriatric mental health (GMH) providers in four regional VHA telehealth hubs. Between October 2022 and September 2023, 351 rural Veterans with a mean age of 75.1 years (SD = 8.8) received services from one or more GMH provider. Patients were mostly white (88.6%), males (95.4%) with high care needs (CAN = 81.6 out of 100, indicating highest need). Of the 1,440 encounters delivered, 49% were video to home, 27% were video to clinic, 19% were telephone, and 5% were e-consultation services. Through interviews with GMH providers, we identified technology-related barriers and solutions used to help rural Veterans be seen via video. Findings suggested that barriers to video services included lack of home internet and/or devices, poor connection, and audio or video issues. Solutions included using VHA resources to obtain iPads for patients who lacked connectivity/devices and prioritizing getting patients into local clinic when needed for video visits. Providers described utilizing the telehealth clinical technician to help overcome patient barriers and discomfort with digital technology through teaching patients and caregivers to use home telehealth software. With troubleshooting, use of resources and training, high quality video telehealth services can be delivered successfully to older rural Veterans and increase access to GMH care.

Abstract citation ID: igae098.2523

### HOME AND COMMUNITY-BASED SERVICE NEEDS AMONG RURAL CARE PARTNERS OF US VETERANS: FINDINGS FROM QUALITATIVE RESEARCH

Elizabeth Marfeo[1], Elizabeth Chamberlin[2], Maria Venegas[3], and Lauren Moo[2], *1. Tufts University, Medford, Massachusetts, United States*, *2. Department of Veterans Affairs, Bedford, Massachusetts, United States*, *3. VA Bedford Healthcare System, Bedford, Massachusetts, United States*

The number older adults living in rural areas of the US is increasing. This demographic shift presents challenges for ensuring access to home and community-based services (HCBS) among rural older adults. We conducted a qualitative study to explore HCBS needs among family caregivers (N = 19) whose care partner is a rural older (< 65) Veteran with complex care needs via semi-structured qualitative interviews. All participants were recruited through the Veterans Health Administration's Caregiver Support Program. An iterative content analysis was guided by Andersen's Behavioral Model of Health Services Use. Three a priori domains for coding were initially used: psychosocial, enabling, and need factors. Key themes related to service access and use emerged including (1) significant objective and subjective health needs among both the CG and Veteran; (2) limited accessibility to health services and respite supports; (3) financial needs beyond healthcare costs; and (4) variation in attitudes, beliefs, and perceived control regarding care preferences and values from the perspective of the CG and Veteran. Most caregivers reported living in the home with the Veteran yet receiving no additional HCBS. Overall family caregivers reported taking immense pride in caring for their loved one but cited several areas of unmet needs related to accessibility and utilization of HCBS in their rural communities. These findings have important implications for identifying future pathways to increase access to HCBS, ultimately improving both rural-residing family caregiver and Veteran health and quality of life.

Abstract citation ID: igae098.2524

### SUBSTANCE USE AND ABUSE IN NEW HAMPSHIRE, MISSISSIPPI, AND WYOMING: FINDINGS FROM THE HEALTHY AGING DATA REPORTS

Alexa Fleet[1], Josephine Boateng[2], Megan Siebecker[3], and Elizabeth Dugan[3], *1. University of Massachusetts Boston-- Gerontology, Boston, Massachusetts, United States*, *2. University of Massachusetts Boston, Dorchester, Massachusetts, United States*, *3. University of Massachusetts Boston, Boston, Massachusetts, United States*

Addiction issues in older adults are prevalent, yet often undetected or untreated, and can contribute to poor physical health, increased disability, and earlier mortality. The current study describes state rates of substance use indicators of older adults 65+ in NH, MI, and WY, and drug overdose deaths for all ages. These three states were chosen due to the high percentages of older adults residing in rural areas. The data sources are the Behavioral Risk Factor Surveillance System (2013-2020) and the Medicare Current Beneficiary Summary File (2018). Small area estimation techniques were used to calculate age-sex-adjusted community rates for more than 150 health indicators. This research examines disparities in rates for three substance use and abuse-related indicators: substance (drug and alcohol) use disorder, tobacco use disorder, and opioid deaths. Substance use disorders rates among 65+: MI 3.9%, NH 5.5%, and WY 2.4%. Tobacco Use Disorder or Current Smoker among adults 65+: MI 13.1%, NH 10.4%, and WY 11.1%. Drug overdose deaths (all ages): MI n=2,137, NH n=1,279, and WY n=1,225. Results showed variability in rates across states. MI had the highest community rates of tobacco use/smoking rates and drug overdose deaths. NH had the lowest rates for smoking but the highest rates for substance use disorders. Wyoming had the lowest substance use disorder rates and lowest drug overdose deaths. Understanding the distribution of rates makes disparities evident and may aid practitioners and policymakers in allocating resources to areas of highest need.

Abstract citation ID: igae098.2525

### THE CARE NAVIGATOR ROLE IN RURAL PRIMARY CARE MEMORY CLINICS

Debra Morgan, Melanie Bayly, and Julie Kosteniuk, *University of Saskatchewan, Saskatoon, Saskatchewan, Canada*

Collaborative dementia care models that include a navigator role achieve better outcomes but little is known about impacts in rural settings. Rural primary healthcare memory clinic teams in Saskatchewan include Coordinators from the Alzheimer Society of Saskatchewan (ASOS) First Link Program, which aims to connect persons living with dementia (PLWD) and families with supports at diagnosis. We examined perceptions of First Link Coordinators (FLC) and memory clinic team members about the FLC role, and evaluated impact of the role within the memory clinic (compared to self- and direct-referrals from other healthcare providers) on timeliness, number of completed FLC-client contacts, topics discussed, duration, and contact method. The QUAL+QUAN design included: (1) semi-structured interviews with three FLC and six team members in four rural memory clinics, (2) routinely collected ASOS client data. Separate inductive thematic analyses with FLC and team interviews (Jan 2021-Apr 2023) identified two common themes: benefits for PLWD and families (eg, early and ongoing connection to emotional support) and benefits for team members (eg, can focus on their unique role). Face-to-face contact established a FLC-family relationship and facilitated follow-up contact. Longitudinal retrospective analysis of ASOS data found that between Dec 2017-Dec 2021, 139 clients (predominantly spouses, children) were referred. Compared to other referrals, statistically significant differences ($p<.05$) included: memory clinic clients contacted sooner after referral, longer duration of first contact, more completed contacts, more topics discussed. Findings demonstrate the added value of FLC in rural memory clinics. Future research should explore long-term outcomes for PLWD and families.

### SESSION 7140 (POSTER)

### SENSORY HEALTH (VISION, HEARING)

Abstract citation ID: igae098.2526

### ASSOCIATION BETWEEN SENSORY IMPAIRMENTS AND PARTICIPATION IN COMMUNITY ACTIVITIES AMONG OLDER ADULTS

Zoe Shannon, and Bernard A. Steinman, *University of Wyoming, Laramie, Wyoming, United States*

Older adults with sensory impairments (SI) experience barriers to participation that could impact their quality of life and well-being. Participation limitations associated with SI have also been associated with health-related outcomes, including chronic disease, mobility difficulties, impairments in daily activities, and demographic factors. In this study, we assessed the association between functional SI and participation in community activities, while holding other health dimensions constant. Using two waves (2011 and 2015) of the National Health and Aging Trends Study (NHATS), we computed five ordinal logistic regression models to assess associations between SI change and participation. Sensory impairments were operationalized via self-reported measures of everyday visual, hearing, and dual functioning (e.g., ability to carry on a conversation in a quiet room). "Participation" was operationalized using six items that assessed participation in social and community-based activities (e.g., doing volunteer work). We created a change-in-participation variable by subtracting participation in 2015 from the baseline. Without other covariables, only functional vision status was statistically associated with participation. When all other covariates were included in the final model, statistical effects of functional vision were eliminated, and only age and chronic health conditions predicted change in participation. These results point to the importance of developing community support and reducing barriers to participation by older adults with functional SI. Creating social and physical environments that consider vision, hearing, and dual sensory impairments can foster greater participation among older adults with SI. Consideration of sensory functioning can also promote well-being that is associated with continued engagement in the community.

Abstract citation ID: igae098.2527

### CAREGIVING FOR OLDER ADULTS LIVING WITH DEMENTIA AND CONCURRENT HEARING LOSS

Charity Lewis[1], Wuyang Zhang[2], Nicholas Reed[2], Victoria Sanchez[1], Michelle Arnold[3], and William Haley[1], *1. University of South Florida, Tampa, Florida, United States*, *2. Johns Hopkins University, Baltimore, Maryland, United States*, *3. University of South Florida Sarasota-Manatee Campus, Sarasota, Florida, United States*

While hearing loss and dementia are highly concurrent conditions, there is a lack of studies examining its impact on dementia caregiving. Hearing loss has been identified as an unmet need among older adults living with dementia, as it contributes to communication challenges. Using Round 12 (2022) of the linked National Health and Aging Trends Study (NHATS) and National Study on Caregiving (NSOC), we aim to investigate the association of concurrent hearing loss and dementia with caregiving stressors and appraisals. Our study population consisted of 1,445 (Mean age: 61±15 years; Female: 68%) caregivers assisting 942 older adults (Mean age: 82±8 years; Female: 66%; non-Hispanic White: 59%). Hearing loss was measured using pure-tone audiometry and dementia was ascertained using a pre-established algorithm in NHATS. Caregiving stressors, including hours of care in the past month and caregivers' appraisals of difficulties in physical, emotional, and financial domains, were self-reported. In the analytic sample, the prevalence of hearing loss among care recipients with dementia was high (274/314: 87%). Moreover, 431 (30%) of caregivers provided care to an older adult with concurrent hearing loss and dementia. After adjusting for caregiver-recipient characteristics, results from linear regression models suggest among caregivers assisting older adults with dementia, per 10-dB increment in care recipients' hearing was associated with a 35% increase in caregiving time (95% CI: 9%-68%). No statistically significant associations were observed regarding caregivers' perceived difficulties. Our findings suggest that caregivers of older adults with concurrent hearing loss and dementia may be more susceptible to facing greater caregiving intensity.

Abstract citation ID: igae098.2528

### DISPARITIES IN HEARING LOSS AMONG OLDER ADULTS: HEALTH AND RETIREMENT STUDY, 2016–2018

Nasya Tan[1], James Pike[2], Lindsay Kobayashi[1], and Philippa Clarke[1], *1. University of Michigan, Ann Arbor, Michigan, United States*, *2. New York University, New York, New York, United States*

As hearing loss becomes more prevalent in middle and older ages, more research is needed to identify factors associated with hearing loss among this population using nationally representative data. This study aimed to examine associations between sociodemographic factors and hearing loss using 2016-18 data from the Health and Retirement Study, a population-based cohort of US adults aged 51+ (n=17,468). Respondents self-reported hearing loss, age at assessment, gender, race/ethnicity, and education. We ran weighted logistic regression models that accounted for the complex survey design, stratified by gender and race/ethnicity. Covariates included year of assessment and birth cohort. Results showed that for every 5-year increase in age, the odds of hearing loss increased by 40% (95%CI=1.26, 1.55). Compared to White males, Black males were 42% less likely to have hearing loss (95%CI=0.44, 0.77). Compared to White females, Black females were 46% less likely to have hearing loss (95%CI=0.42, 0.70). Among men, those with less than a high school education were 23% less likely to have hearing loss (95%CI=0.63, 0.94)than college graduates. However, men with an associate's degree or some college education were 34% more likely to have hearing loss (95%CI=1.14, 1.58). Education was not associated with hearing loss among women. This study highlights disparities in hearing loss in later life by race/ethnicity, gender, and education. It adds to the current literature by identifying disparities in hearing loss, and is the first study to examine hearing loss disparities using nationally representative data on middle-aged and older adults in the Health and Retirement Study.

Abstract citation ID: igae098.2529

### EFFECT OF AWARENESS OF HEARING LOSS AMONG CARE RECIPIENTS ON ATTRIBUTES OF CARE PROVIDED: A NATIONAL STUDY

Danielle Powell[1], Wuyang Zhang[2], and Nicholas Reed[2], *1. University of Maryland, College Park, Maryland, United States*, *2. Johns Hopkins University, Baltimore, Maryland, United States*

Hearing loss (HL) is prevalent among older adults and may result in caregiving challenges due to communication barriers. This study aims to investigate whether care recipients' awareness of HL is associated with attributes of care. Using data from Round 12 (2022) of the linked National Health and Aging Trends Study (NHATS) and National Study on Caregiving (NSOC), a sample of 1,133 (Mean age: 62±15 years; Female: 68%) caregivers assisting 713 older adults (Mean age: 83±8 years; Female: 63%; White: 63%) with audiometric HL was derived. From care recipients' self-reported hearing difficulty and hearing aid use, HL awareness was defined as (1) unrecognized HL (no hearing difficulty or hearing aid use reported), (2) recognized HL (only hearing difficulty reported), and (3) managed HL (hearing aid use reported). Caregiving outcomes, including ADL/IADL-related care needs and past month hours of caregiving, were measured through self-report. Of all caregivers, 628 (55%) assisted older adults with unrecognized HL, while 162 (15%) and 343 (30%) assisted older adults with recognized and managed HL, respectively. Relative to unrecognized or managed HL, care recipients with recognized HL reported greater needs on ADL/IADL-related activities (≥3 ADL needs: 65.4% vs. 47.8%/45.0%; IADL needs: 55.1% vs 48.3%/40.0%). Results from models adjusted for multiple caregiver-recipient characteristics suggest time spent on care activities by caregivers of those with recognized HL and managed HL were 0.66 (95%CI:0.43-1.03) and 0.84 (95%CI:0.56-1.27) times lower than those with unrecognized HL. Supporting HL awareness and management as aligned with care needs may contribute to effective provision of care.

Abstract citation ID: igae098.2530
### HOW HEARING DIFFICULTIES AND SOCIAL ACTIVITIES SHAPE TRAJECTORIES OF COGNITIVE FUNCTION AMONG OLDER MEXICANS

Chengming Han[1], and Rebeca Wong[2], *1. The University of Texas Medical Branch, Galveston, Texas, United States, 2. The University of Texas Health Sciences Center in San Antonio, San Antonio, Texas, United States*

Objective. This paper aims to explore the role of social activities in the pathway between hearing difficulties and cognitive function among Mexicans □ 50 years over a longitudinal period. Method. Data were drawn from the Mexican Health and Aging Study (MHAS) 2015 to 2021. MHAS is a national longitudinal study of adults □ 50 years in Mexico, with baseline in 2001, and six waves completed by 2021. The analytical sample includes 8,049 Mexicans at wave 4 (2015). Cognition was operationalized as verbal memory and visual scanning scores. Multilevel ordinary least squares regression models were used to estimate the mediating effects of social activities on the association between hearing difficulties and cognition over 2015 to 2021. Results. There are 2766 (34.36%) respondents who reported having a poor or fair hearing in 2015, among whom only 9% used hearing aids. Poor or fair hearing was associated with reduced social activities, which in turn related to decreased visual scanning scores in 2021. Poor/fair hearing showed both indirect and direct effects on visual scanning scores. Reporting more social activities was associated with better cognition independently. Using a hearing aid was associated with lower verbal memory scores. Conclusion. There are few studies on sensory limitations and cognitive function that include populations in middle-income countries like Mexico. Although the results displayed on the similar mechanism on hearing difficulties and cognition in MHAS, the lower percentage of using a hearing aid suggested that Mexico has experienced rapid population aging with inadequate institutional social support for health care.

Abstract citation ID: igae098.2531
### IF I COULD TURN BACK TIME: A THREE-YEAR LONGITUDINAL ASSESSMENT OF MUSIC ENJOYMENT IN OLDER ADULT HEARING AID USERS

Isaac Alter[1], Alexander Chern[2], Michael Denham[1], Alexis Leiderman[3], Jessica Galatioto[4], Jennifer Jones[4], and Anil Lalwani[1], *1. Columbia University, New York, New York, United States, 2. Johns Hopkins University, Baltimore, Maryland, United States, 3. Elmhurst Hospital Center/ Mount Sinai Hospital, Queens, New York, United States, 4. Columbia University Irving Medical Center, New York, New York, United States*

**Introduction:** Hearing loss (HL) is remarkably prevalent among older individuals, and is associated with worsened music enjoyment and perception. We sought to examine longitudinal changes in music enjoyment among long-term hearing aid (HA) users.

**Methods:** Adult HA users were recruited from a tertiary academic medical center and community setting. Participants reported their music listening habits and subjective music enjoyment using 10-point Likert scales, and underwent a melody discernment listening task, once between July-November 2020 and again three years later. Paired t-tests were used to compare participants' responses between time points, and multiple linear regression was employed to investigate relationships between hearing levels (measured by pure tone average (PTA) of the better hearing ear) and music enjoyment.

**Results:** Thirty-nine HA users participated, with an average (SD) age of 72.9 (7.8) and 24.3 (18.3) years of HA use. Despite a significant increase in PTA from 2020 to 2023 (47.2 to 52.6, $p< 0.001$), there was no significant change in time spent listening to music, overall music enjoyment, or melody discernment ability across the two time points. However, there was a significant association between change in PTA and music enjoyment, with a one-point worsening in PTA between 2020-2023 correlating to a 0.14-point decrease in music enjoyment ($p=0.041$).

**Conclusion:** Our results suggest that despite worsening hearing over time, older HA users do not uniformly report reduction in music enjoyment or time spent listening to music, nor do their melody discrimination abilities diminish. However, more severe worsening in hearing may predict lower self-reported music enjoyment.

Abstract citation ID: igae098.2532
### IMPROVING DETECTION OF UNTREATED HEARING LOSS IN A COMPREHENSIVE CARE SETTING USING A SINGLE-QUESTION SCREENER

Sara Mamo[1], Alyssa Krysko[1], Katherine Garrity[1], and Margaret Wallhagen[2], *1. University of Massachusetts Amherst, Amherst, Massachusetts, United States, 2. University of California San Francisco, San Francisco, California, United States*

A majority of older adults have untreated hearing loss, which poses care and communication challenges for healthcare providers. Routine identification of hearing loss would help providers and individuals recognize hearing as a possible contributor to communication difficulties. The objective of the study was to implement a single-question hearing screening administered by medical assistants (MAs) in a health clinic. The study took place in a Day Health Clinic at a Program for All-Inclusive Care for the Elderly (PACE). Participants included anyone seen in the clinic during the study. Participants were screened by the MA, and the researchers collected hearing thresholds after their appointment. We performed behavioral hearing threshold testing and used a 4-frequency pure-tone average to estimate the individual's hearing status and measure the sensitivity/specificity of the screening method. With a sample of 49 participants (age range = 56-90 years), we calculated a sensitivity of 71.4% and a specificity of 42.9%. Categorically, our behavioral hearing tests resulted in 14 individuals with clinically normal hearing, 24 with mild hearing loss, and 11 with moderate or worse hearing thresholds. There were no current hearing aid users in the sample, and three who reported having used hearing aids previously. The findings suggest that a single-question screener can be used as a simple method of identifying individuals who may benefit from a hearing evaluation and/or hearing loss treatment. Future studies should integrate 'hearing status' prominently in the individual's medical record and establish protocols for using personal amplifiers to support adults with untreated hearing loss.

Abstract citation ID: igae098.2533

### TRENDS IN PREVALENCE OF HEARING LOSS AMONG OLDER ADULTS AND UNPAID FAMILY CAREGIVERS IN THE UNITED STATES: 2011–2022

Wuyang Zhang[1], Danielle Powell[2], and Nicholas Reed[1], *1. Johns Hopkins University, Baltimore, Maryland, United States*, *2. University of Maryland, College Park, Maryland, United States*

Hearing loss (HL) prevalence rises with age and is a potential barrier to communication, a key component of caregiving relationships. With a rapidly aging population, the United States faces a surge in age-related HL prevalence in tandem with demand for disability-related care needs. We aim to estimate the trends in national prevalence of HL among (1) older adults receiving unpaid family care and (2) their caregivers. The National Health and Aging Trends Study (NHATS) and linked National Study of Caregiving (NSOC) provide nationally representative samples of Medicare beneficiaries and their family caregivers between 2011-2022. HL was measured through self-reported functional hearing ability (subjective) in both NHATS and NSOC until 2021, when NHATS introduced objective measures of hearing. In 2011, the prevalence of self-report HL was 31.7% (95% CI: 28.3%-35.4%) among care recipients and 7.9% (95% CI: 6.7%-9.3%) among caregivers. By 2022, the prevalence reached 34.9% (95% CI: 31.5%-38.4%) and 9.7% (95% CI: 8.1%-11.6%), respectively. Notably, prevalence of objective HL among care recipients more than doubled its subjective counterpart (73.5%; 95% CI: 69.2%-77.3%). Among caregiving dyads, 3.6% (95% CI: 3.5%-5.0%) had both parties reporting subjective HL and 5.7% (95% CI: 4.4%-7.3%) had caregivers with subjective HL assisting older adults with objective HL. Stratified analyses by sociodemographic characteristics suggested the prevalence of HL was higher among male, non-Hispanic White, or Hispanic care recipients along with spousal caregivers. Our findings suggest that HL is highly prevalent among both older care recipients and caregivers, and further investigation is needed to understand its caregiving consequences.

Abstract citation ID: igae098.2534

### VISUAL DIFFICULTY AND VALUED ACTIVITY AMONG OLDER ADULTS: FINDINGS FROM THE NATIONAL HEALTH AND AGING TRENDS STUDY

Shu Xu[1], and Haowei Wang[2], *1. University of Michigan, Ann Arbor, Michigan, United States*, *2. Syracuse University, Syracuse, New York, United States*

Vision loss is common among older adults and is associated with reduced activity. However, few studies have examined the value of various activities for older adults with visual difficulty. Using nationally representative data from the National Health and Aging Trends Study 2022, we examined activity preferences and participation among older adults with and without visual difficulty (N=5,807). We explored potential barriers (e.g., transportation, health, and COVID-19) limiting participation in valued activities. Visual difficulty was measured by self-reported vision status and impairments. Valued activities were assessed by four items: visiting friends and family who lived separately, attending religious services, participating in clubs, classes, or other organized activities, and going out for enjoyment. Activities rated as "very important" were considered to be of personal value to the individuals. Weighted sample characteristics showed that 56.3% of participants aged between 65 and 74 years old, and 8.6% experienced visual difficulty: 6.5% near or distance visual difficulty, 1.9% near and distance visual difficulty, and 0.3% blindness. Overall, most older adults (58.7%) rated visiting friends/family as important. Logistic regression results showed that older adults with near and distance visual difficulty were less likely to engage in valued activities (OR = 0.36) and more likely to report health-related (OR = 2.75) or COVID-19 restrictions (OR = 2.04) for ceasing participation in any valued activity, compared to those with no visual difficulty. Findings underscore the significance of developing interventions that bridge the gap between activity preferences and participation for older adults with visual difficulty.

### SESSION 7145 (POSTER)

### SOCIAL ISOLATION AND LONELINESS I

Abstract citation ID: igae098.2535

### CONNECTED YET VULNERABLE: SOCIAL ISOLATION PROFILES AND MENTAL HEALTH AMONG OLDER ADULTS WITH LOW SES

Pildoo Sung[1], June Lee[2], and Angelique Chan[2], *1. Hanyang University, Seoul, Republic of Korea*, *2. Duke-NUS Medical School, Singapore, Singapore*

Despite growing attention to social isolation and loneliness, there remains a gap in understanding how these factors intersect in shaping comprehensive social isolation profiles among vulnerable older adults and their mental health implications. Using various objective and subjective indicators of social isolation, this study aimed to identify social isolation profiles and their direct and stress-buffering roles against depressive symptoms among community-dwelling older adults with low socioeconomic status. Data were collected from 881 older adults aged 55 and above in the Chin Swee area of Singapore where older adults with lower socioeconomic status reside in small rental housing. Latent class analysis and multivariable regression yielded three key findings. First, four distinct social isolation profiles were identified, namely "strongly connected, less lonely (27.9%)," "moderately connected, less lonely (56.4%)," "moderately connected, moderately lonely (11.2%)," and "restricted, severely lonely (4.4%)." Second, older adults in the "moderately connected, moderately lonely" and "weakly connected, severely lonely" profiles tended to report poorer mental health than those in the "strongly connected, less lonely" profile. Third, "moderately connected and moderately lonely" older adults were at a higher risk of depression in the presence of significant life stressors, compared to their less lonely counterparts with strong social connectedness. These findings highlight the need for a comprehensive approach to addressing social isolation and loneliness among disadvantaged older adults in the community. Interventions should not only target disconnected and severely lonely older adults but also extend support to moderately connected yet lonely older adults, recognizing their vulnerability to life stressors.

Abstract citation ID: igae098.2536

### DRIVERS OF SOCIAL WITHDRAWAL IN INDIVIDUALS WITH ALZHEIMER'S: AN ANALYSIS OF ONLINE PERSONAL NARRATIVES

Laura Hemmy[1], Yun Leng Wong[2], Mike Conway[3], Daniel Cabrera Lozoya[3], Kelvin Lim[1], and Jude Mikal[1], *1. University of Minnesota, Minneapolis, Minnesota, United States*, *2. University of Minnesota Twin Cities, Minnesota, United States*, *3. University of Melbourne, Melbourne, Victoria, Australia*

The relationship between social isolation and cognitive impairment is a pernicious cycle. On the one hand, social isolation is associated with an increase in risk of Alzheimer's Disease (AD) and of AD progression; and on the other hand, the hallmark symptoms of increased cognitive and functional impairment have been shown to increase social withdrawal among individuals living with AD. As researchers, care providers, and clinicians design interventions to interrupt this cycle, it becomes necessary to understand the lived experiences and perspectives of persons living with AD, including the emotional, cognitive, and sensory drivers of isolation and withdrawal. The preponderance of research on social isolation and AD focuses on correlations and risk prediction models. Our study expands on this foundation through a focus on the catalysts, motivations, and consequences of increased social isolation among individuals with AD. Using the personal narratives of 8 individuals living with early onset AD maintained in publicly available blogs, we use a grounded theory approach to develop a conceptual model describing the emergence, predictors, and consequences of social withdrawal among individuals living with AD. Analysis revealed 5 key themes: (1) sensory symptoms of AD and impacts on social engagement, (2) cognitive symptoms of AD and impacts on engagement, (3) evolution of existing networks of support, (4) elective withdraw from social networks, and (5) consequences of withdrawal for individuals with AD and their care providers. Themes are then situated within contemporary research on AD and AD care, and include insights into key points of intervention.

Abstract citation ID: igae098.2537

### EFFECTIVENESS OF A COMMUNITY NAVIGATOR PROGRAM TO IMPROVE SOCIAL CONNECTEDNESS IN CLINICAL SETTINGS

Matthew Smith, *Texas A&M University, College Station, Texas, United States*

While healthcare professionals are uniquely positioned to identify social disconnectedness among older patients, coordinated linkages to community-based organizations are essential to provide social engagement opportunities. The Reduce Social Isolation and Lift Outcome for Seniors (SILOS) program was created to screen older adults for social disconnectedness risk and link them to community services, resources, and programs. Patients ages 50 years and older were screened in the healthcare setting or by community health workers (CHW) using the Upstream Social Interaction Risk Scale (U-SIRS-13) and Duke Social Support Index (DSSI). Participants completed baseline assessments, and data were collected again at 3, 6, and 12 months. Data were analyzed from 119 participants enrolled in the SILOS program. On average, these individuals were age 67 years. Sixty-six percent of participants were female, 15.3% were Hispanic, 70% were Caucasian, and 36% were Black or African American. At baseline, 92% of participants reported social disconnectedness risk. On average, participants reported significantly lower social disconnectedness from baseline to 3-month follow-up ($t=6.15$, $P< 0.001$), 6-month follow-up ($t=7.65$, $P< 0.001$), and 12-month follow-up ($t=5.01$, $P< 0.001$), respectively. On average, participants reported significantly higher social support from baseline to 3-month follow-up ($t=-3.28$, $P< 0.001$), 6-month follow-up ($t=-3.67$, $P< 0.001$), and 12-month follow-up ($t=-3.81$, $P< 0.001$), respectively. Findings from this initiative highlight the benefits of navigated clinical-community linkages to identify and address social disconnectedness among older adults. Including CHW in SILOS was essential to build trust and create rapport with the older adults, facilitating an in-depth understanding of social deficits, risk, and need for clinical and community services.

Abstract citation ID: igae098.2538

### FOUR-YEAR LONELINESS TRAJECTORY AND ITS PREDICTORS IN OLDER ADULTS

Yan Huang, Lynn Martire, and Damon Jones, *The Pennsylvania State University, University Park, Pennsylvania, United States*

Loneliness is a modifiable risk factor for health decline in older adults and its level may change over time. This study aimed to explore how loneliness changes on average across 4 years in older adults and investigate potential predictors accounting for the formation of this trajectory. Data were collected annually between 2017 to 2020 from 128 older adults (Mage = 77.91) in independent-living or retirement communities in the U.S. Growth curve modeling showed a quadratic model best fit the loneliness trajectory with significant fixed effects of the intercept ($b = 2.497$, $p < 0.001$); the linear slope of time ($b = -0.036$, $p = 0.009$); and the quadratic term of time ($b = 0.002$, $p < 0.001$) as well as three significant random effects ($ps < 0.05$). The quadratic trajectory showed the level of loneliness decreased first and then increased with individual differences in the intercept and rate of change. We further found being married, having more family and friends, and receiving greater social support at baseline were negatively linked to the overall levels of loneliness. By contrast, living alone, having social strain, depressive symptoms, and difficulty with social participation were positively linked. When including all significant individual variables, only depressive symptoms were still significant ($b = 1.850$, $p < 0.001$). Findings suggest the loneliness trajectory follows a nonlinear trend. Baseline relationship and health factors, especially depressive symptoms, are linked to the average level of loneliness at baseline. The COVID-19 pandemic may have influenced the uptick in loneliness in 2020.

Abstract citation ID: igae098.2539

### GEOGRAPHY AND LIVING ARRANGEMENT AS LONELINESS FACTORS FOR OAA NUTRITION CLIENTS DURING COVID-19

Heather Menne[1], Jason Osborne[1], and Claire Pendergrast[2], *1. Miami University, Oxford, Ohio, United States*, *2. Syracuse University, Delmar, New York, United States*

Loneliness is increasingly understood as a public health crisis. Multiple risk factors for increased loneliness in older adults have been identified in previous studies, including age, gender, race, geographic location, and living alone. Social distancing efforts during the COVID-19 pandemic may have increased loneliness among older adults. This study uses two cross-sectional waves of the National Survey of Older Americans Act Participants (NSOAAP) from 2019 and 2021 to expand understanding and identify possible points of intervention to increase social support for vulnerable older adults. Results reveal that while home-delivered meal participants have higher overall levels of loneliness than congregate meal participants. Those receiving home-delivered meals did not experience changes in loneliness during the COVID-19 pandemic, and did not differ significantly by age, geographic location, or living arrangement. Congregate meal participants' loneliness was lower before the pandemic but increased during the first year of the pandemic, particularly for participants aged 65-74, those living in suburban or rural areas, and those living alone. The significant differences based on geography and living arrangement for congregate meal clients (but not home-delivered meal clients) suggest the need for further investigation as to whether living arrangement has a buffering effect on loneliness or whether the closing and less access to congregate meals sites impacted loneliness. These findings suggest opportunities for policymakers and aging services providers who seek to increase social engagement among older adults who participate in Older Americans Act (OAA) nutrition programs.

Abstract citation ID: igae098.2540

### LONELINESS AMONG PERSONS WITH LONGSTANDING MULTIPLE SCLEROSIS (MS)

Alexa Stuifbergen, Darla Grimes, Heather Becker, and Nani Kim, *The University of Texas Austin, Austin, Texas, United States*

The isolation induced by the global COVID pandemic highlighted the negative health and quality of life impact of loneliness and loss of social connections, especially among those who are aging with chronic disabling conditions with associated mobility and sensory impairments that make community participation more difficult. The purpose of this study was to explore the relationship between loneliness and depressive symptoms and health behaviors and quality of life outcomes for persons with MS using data collected in 2023 as part of an ongoing longitudinal survey study. The survey included measures of loneliness, depressive symptoms, self-rated health, health behaviors and quality of life outcomes. The sample (N=157) of community-residing persons with MS had a mean age of 70.70 (SD=7.80) and had been diagnosed with MS for an average of 35.16 years (SD=6.16). The majority were married (61%) and female (90%). Fifty-five percent of participants rated their overall health as good or excellent. Reports of loneliness were strongly positively correlated with reports of depressive symptoms ($r=.63$, $p<.001$), negatively correlated with economic adequacy ($r= -.64$, $p<.001$) and had a small negative correlation with age (-.23, p< 01). Greater functional limitations were associated with greater loneliness ($r=.27$, $p<.001$) and depressive symptoms ($r=.37$, $p<.001$). Loneliness and depressive symptoms had a similar pattern of moderate to strong negative correlations with reports of health behaviors and quality of life outcomes. Novel interventions to identify and treat loneliness and depression among older adults with chronic disabling conditions may improve important health and quality of life outcomes.

Abstract citation ID: igae098.2541

### PERCEPTIONS AND ATTITUDES TOWARD LONELINESS DETECTION USING PASSIVE SENSING TECHNOLOGIES IN OLDER ADULTS

Emma Cho, George Demiris, Hannah Cho, Insup Lee, Ahhyun Yuh, Xiayan Ji, Sean Lee Harrison, and Oleg Sokolsky, *University of Pennsylvania, Philadelphia, Pennsylvania, United States*

While passive sensing has been examined in terms of older adults' acceptance for the purpose of tracking activity levels or daily routines, less attention has been paid to how older adults perceive monitoring technologies that specifically capture levels of loneliness and social isolation. Therefore, the purpose of this study is to explore the perceptions and attitudes of older adults toward passive sensing technologies installed in their homes to assess loneliness. Structured interviews were conducted to assess older adults' perceptions and attitudes toward passive sensing technologies to assess their loneliness after six months of having seven types of sensors (e.g, motion, temperature, bed and door sensors) installed in their homes. Audio recordings were transcribed and content analysis was conducted. A total of 15 older adults participated in the interview sessions. Themes included participants' adaptation and adjustment, privacy and trust in data sharing, and perceived benefits and concerns of the installed sensors. Benefits included detecting and reducing loneliness, monitoring physiological parameters, improving healthier behaviors, etc. Concerns were raised about privacy, such as potential misuse by authorities or third parties, lack of accuracy of functions or feasibility of sensors, and lack of human response. All participants had an overall positive attitude towards sensors that could be installed in their homes to assess their loneliness.

Abstract citation ID: igae098.2542

### RISK AND PROTECTIVE FACTORS FOR INCREASING ISOLATION AND LONELINESS AMONG OLDER ADULTS

Tiffany Hughes[1], Fang Fang[2], Yueting Wang[3], and Erin Jacobsen[3], *1. Midwestern University, Glendale, Arizona, United States*, *2. Old Dominion University-EVMS, Norfolk, Virginia, United States*, *3. University of Pittsburgh, Pittsburgh, Pennsylvania, United States*

Social isolation and loneliness are associated with poor health and quality of life as well as decreased longevity. This study examines patterns of change in self-reported isolation and loneliness, and factors associated with these patterns, using data from 1,474 cognitively normal participants followed annually for up to 12 years in a population-based cohort study of older adults. Composite measures of isolation and loneliness were created, and trajectory analyses were conducted to detect patterns of change in these composites over time. Multinomial logistic regression tested associations of baseline demographic, health, and lifestyle factors with patterns of change in isolation and loneliness. Trajectory analyses revealed three patterns of change in isolation - slowly decreasing, slowly increasing, and quickly increasing; and three patterns of change in loneliness - stable, increasing, and decreasing. Participants who had ever smoked, currently drank alcohol, were physically active, had hypertension, used the computer, and were not obese at baseline were less likely to be in the quickly increasing isolation pattern. Those with greater isolation and loneliness at baseline, who lived alone, were obese, did not have daytime sleepiness, and rated health as very good or excellent were more likely to be in the increasing loneliness pattern. Distinct patterns of change in isolation and loneliness were found over 12 years with different factors associated with risk for changing isolation and loneliness over time. Understanding characteristics of participants associated with change in isolation and loneliness over time may help to identify older adults for interventions.

Abstract citation ID: igae098.2543

### SOCIAL ISOLATION AND SUBJECTIVE WELL-BEING AMONG OLDER ADULTS: AN EXAMINATION BY RACE/ETHNICITY AND GENDER

Nhan Nguyen, and Zhiyong Lin, *The University of Texas San Antonio, San Antonio, Texas, United States*

Social isolation has emerged as a significant social determinant of health in later life. However, our understanding of how this link may vary across race/ethnicity and gender remains limited. To address this gap, we utilized longitudinal data from the Health and Retirement Study (2008-2020) to investigate the association between social isolation and subjective well-being (life satisfaction, positive/negative affection) among older adults. We examined variations in this relationship across race/ethnicity and gender subgroups. Our results demonstrate that social isolation is consistently linked to reduced subjective well-being for older adults, irrespective of race/ethnicity and gender. However, we observed nuances in this association among Hispanic older adults. Specifically, while both White and Hispanic older adults exhibit higher subjective well-being when they are not or rarely isolated compared to Black individuals and their isolated counterparts, among socially isolated older adults, particularly isolated men, Hispanic individuals tend to report higher levels of subjective well-being compared to their White and Black counterparts. Moreover, significant gender disparities in subjective well-being were identified among socially isolated older adults across all racial/ethnic groups, with women experiencing greater disadvantage compared to men. We also found that economic resources and health-related factors mediate the relationship between social isolation and subjective well-being. This study underscores the critical importance of addressing social isolation as a potential risk factor for mental health among older adults, especially those in vulnerable groups. It emphasizes the necessity for targeted interventions aimed at reducing social isolation and promoting social engagement within diverse communities to mitigate mental health disparities.

Abstract citation ID: igae098.2544

### SOCIAL ISOLATION AND WELL-BEING IN OLDER ADULTS: EXAMINING THE POTENTIAL PROTECTIVE EFFECT OF PHYSICAL ACTIVITY

Babatope Ogunjesa, Sadia Anjum Ashrafi,
Rifat Alam, Emmanuel Dubure, and Andiara Schwingel,
*University of Illinois Urbana-Champaign, Champaign, Illinois, United States*

As individuals age, the issue of loneliness and social isolation becomes a growing concern for public health. These can have significant health consequences for older adults, including an elevated risk of heart disease, high blood pressure, depression, and dementia. To explore the association between social isolation and self-reported general health as well as the interaction of physical activity, we conducted a study analyzing a sample of 2,203 older adults (aged 65 and above) from the Health Information National Trends Survey 6 (HINTS 6). We used a logistic regression model while controlling for socio-demographic factors. Our study found that older adults with high levels of social isolation are less likely to report high self-reported general health (OR=0.958, 95%CI=0.931 – 0.987). However, those who met the recommended guideline of 150 minutes or more per week of moderate-vigorous physical activity are more likely to experience better self-reported general health (OR=3.37, 95%CI=1.878 – 6.050) than those who don't meet the guideline. In our interaction effect analysis, we observed that for both physical activity groups, the predicted general health outcome decreases over time with an increased isolation score, but it decreases faster for those getting less than 150 minutes of moderate exercise per week. These findings suggest that social isolation has negative impact on health in later life and that regular moderate physical activity and meeting recommended guidelines may help protect older adults against the adverse health consequences of isolation. Interventions targeting social connectivity and promoting physical activity may improve isolated older adults' health outcomes.

Abstract citation ID: igae098.2545

### SOCIAL RELATIONSHIPS OF OLDER ADULTS DURING THE COVID-19 PANDEMIC

Tina Kilaberia[1], Yuanyuan Hu[1], and Janice Bell[2],
*1. New York University, New York, New York, United States, 2. UC Davis, Davis, California, United States*

This study examined the impact of the COVID-19 pandemic on social relationships of older adults in two groups: eleven who were part of a caregiving dyad and seven who were not (mean age=79; 12 White, 5 Black, 1 More Than One Race). Semi-structured qualitative interviews were combined with the Lubben Social Network Scale (six-item version, LSNS-6). Those in dyads and not in dyads reported comparable extent of social connections on average with family (9 monthly social engagements vs. 9.5 on LSNS-6, respectively). Those in dyads reported greater social engagement on average with non-family individuals than those not in dyads (8.5 vs. 7 on LSNS-6). Older adults in both groups had greater social connections with family than non-family members. Qualitative themes such as seeking, losing and keeping ties represent the range of social connection experiences: all in dyads (vs. five not in dyads) referred to family members as key persons during the pandemic, five (vs. one) reported social isolation from family members specifically, zero (vs. three) identified a spouse or partner as most protective, five (vs. three) described supportive relationships, and four (vs. one) described relationships needing adjustments as a result of the pandemic's impact. Six older adults in dyads (vs. two not in dyads) described non-family contacts as key, eight (vs. three) described the loss of connection with friends, and three (vs. zero) identified friends or hobbies as most protective. Type of dyadic relationships (spouses vs. parent-child vs. friends) and relationship quality may influence older adults' social connections differently.

Abstract citation ID: igae098.2546

### THE CONFLUENCE OF SOCIAL ISOLATION, LONELINESS, AND ITS ASSOCIATION WITH COGNITIVE DECLINE

Joseph Svec[1], Jinshil Hyun[2], and JeongEun Lee[3], *1. St. Joseph's University, Patchogue, New York, United States*, *2. Albert Einstein College of Medicine, Bronx, New York, United States*, *3. Iowa State University, Ames, Iowa, United States*

Numerous studies indicate that social isolation and loneliness are crucial predictors of cognitive decline among older adults. Specifically, cognitive functioning (i.e. memory) is positively linked with robust social connections (Kang and Oremus, 2023). While social isolation and loneliness have gained considerable attention in recent literature, there remains substantial ambiguity in the distinct impacts of isolation and loneliness where both are often treated as part of a similar construct or examined separately (Boss et al., 2015). Given the potential ramifications for cognitive functioning, as well as implications for pragmatic solutions regarding social, environmental, and technological approaches, this study seeks to examine and investigate both the independent and concurrent effects of isolation and loneliness on cognitive functioning. Using data from eight waves of Health and Retirement Study (HRS) data, we utilize fixed effects linear regression models of older adults' (60 years or older) immediate and delayed memory recall score on measures of social isolation, feelings of loneliness, as well as their interactions (N = 44,170). Analyses emphasize within-person associations of memory and covariates. We observe significant and negative associations of isolation (coeff = -0.02, $p < 0.01$) and loneliness (coeff = -0.08, $p < 0.01$) for both immediate and delayed recall score. However, a positive and significant interaction of loneliness and isolation indicates that feelings of loneliness is more strongly associated with immediate and delayed memory when social isolation is low (frequently meets with family or friends) – when social isolation is high, the association of loneliness and cognitive functioning is limited.

Abstract citation ID: igae098.2547

### THE INNER DYNAMICS OF THE OLD AND SOCIALLY WITHDRAWN IN JAPAN: INSIGHTS FOR INTERVENTIONS FROM CASE STUDY ANALYSES

Tomoko Ikeuchi[1], and Cullen Hayashida[2], *1. Tokyo Metropolitan Institute for Geriatrics and Gerontology, Tokyo, Japan*, *2. University of Hawaii, Honolulu, Hawaii, United States*

An increasing number of older adults in Japan are refusing social support even when offered. This lack of support elevates their risk of health deterioration, delays evacuation in disaster scenarios, and increases the likelihood of premature and lonely deaths with inadequate closures. This study examines the psychological factors contributing to the lack of motivation among socially isolated older adults in Japan to seek help. We conducted semi-structured one-on-one interviews with nine individuals aged 60 years or older who reside in the community identified as socially isolated by community-based healthcare personnel. Using a narrative case study approach, the study revealed a prevalent concern among these socially isolated individuals about causing inconvenience to others by seeking help. The findings suggest that their reluctance to ask for help may be driven not by a desire to maintain independence but rather by a concern about inconveniencing others. In the context of Japanese culture, maintaining harmony with others is considered important. Asking for help might inconvenience others and potentially disrupt this harmony. To effectively assist those who refuse necessary support, social and community health workers should develop ways of minimizing the perceived burden of the recipients. However, further research is necessary to establish whether these findings are unique to the Japanese cultural context. Examples of innovative strategies to address this challenge will be discussed.

### SESSION 7150 (POSTER)

### SPIRITUALITY AND RELIGION

Abstract citation ID: igae098.2548

### DEMENTIA STATUS AND PERCEIVED IMPORTANCE OF RELIGIOUS SERVICES OF OLDER ADULTS OVER 11 YEARS

Yufang Tu, and Melissa O'Connor, *North Dakota State University, Fargo, North Dakota, United States*

Religious/spiritual activities positively impact older adults' health and well-being, and are associated with reduced cognitive decline and dementia progression. The current study explored the connection between dementia status and changes in the perceived importance of religious service participation (RSP) over an 11-year period. We used data from 12 longitudinal waves (2011-2022) of the National Health and Aging Trends Study (NHATS). NHATS is nationally representative of Medicare beneficiaries in the United States. The current study included 41,567 participants at baseline. The outcome variable measured the self-rated perceived importance of RSP on a 3-point scale. Longitudinal multilevel modeling was conducted with dementia diagnosis as a time-invariant predictor (yes/no). Covariates included sex, age group, education level, and self-rated health. The interaction between time and dementia status was also examined. Individuals with dementia had a lower baseline perceived importance of RSP by 0.28 points (estimate = -0.28, p < 0.001) compared to those without dementia. The average trajectory of the perceived importance of RSP significantly declined over time for both individuals without dementia (p < 0.001) and those with dementia (p < 0.001). Notably, individuals with dementia exhibited a faster decline, decreasing by 0.05 points for every 1 unit/year increase, in contrast to 0.01 points (estimate = -0.01) for those without dementia. This study demonstrated that older adults with dementia perceive RSP as less important compared to those without dementia. However, spiritual/religious engagement has been shown to help individuals with dementia cope with their symptoms.

Abstract citation ID: igae098.2549

### DEPRESSIVE SYMPTOMS MEDIATE THE LONGITUDINAL ASSOCIATION OF RELIGIOUSNESS WITH SLEEP HEALTH AMONG OLDER ADULTS

Divine Domingo, Tuo Yu Chen, Philile Mgabhi, and Kian Lee Lim, *Taipei Medical University, Taipei, Taipei, Taiwan (Republic of China)*

Although previous research shows that religiousness is significantly associated with better sleep health, their underlying mechanisms are unclear. We examined whether psychological distress and healthy behaviors mediated the association of baseline religiousness with multidimensional sleep health at 4-year follow-up among community-dwelling older adults. Data were from the Taiwan Longitudinal Study on Aging (2011-2015; N=2182). Multidimensional sleep health was conceptualized using the SATED model (satisfaction, alertness, timing, efficiency, duration; total score: 0-5), with a higher score indicating better sleep health. Religiousness was operationalized using the average frequency of five religious activities (praying, studying the sutra or bible, going to temple or church, watching or listening to religious programs, and making donations for religious purposes). Mediators at baseline included depressive symptoms (the Center for Epidemiological Studies-Depression Scale), self-reported frequencies of exercise and drinking, and number of cigarettes smoked per day. We used PROCESS macro to test the mediational relationship adjusting for age, sex, education, and chronic conditions at baseline. The results from bootstrapping showed that baseline depressive symptoms significantly mediated the effects of baseline religiousness on multidimensional sleep health at follow-up (indirect effect: B=.01, SE =.004, 95%CI=.0004-.02). Exercise frequency, drinking frequency, and number of cigarettes smoked per day at baseline were not significant mediators. Our finding provided further insights on the link between religiousness and multidimensional sleep health by showing that religiousness was associated with better sleep health through decreased depressive symptoms but not by increased exercise frequency, reduced drinking frequency, and fewer number of cigarettes smoked per day.

Abstract citation ID: igae098.2550

### FORGIVENESS AS A RESOURCE FOR COUNTERING FEELINGS OF LONELINESS AMONG WOMEN AGING IN CORRECTIONAL CUSTODY

Anya Thompson[1], Alex Bishop[1], and Kevin Randall[2], 1. *Oklahoma State University, Stillwater, Oklahoma, United States*, 2. *Sam Houston State University, Huntsville, Texas, United States*

Analyzing cross-sectional data from 232 women in custody ages 40-years and older (M = 49.06; SD = 7.22), we examined a conceptual path analytic model based on the developmental adaptation model consisting of distal and proximal influences on Loneliness, controlling for age, race, education, marital status, and crime type. The tested model linked participant recall of adverse childhood experiences (ACES) to loneliness (LONE) through religious doubt (RD) and three assessments of dispositional forgiveness, Self, Others, and Situation. The model fit the data well, X2 = 2.09 (3 df); p =.55; RMSEA = 0.00; CFI = 1.00; and SRMR =.008. Standardized results revealed that RD was related to ACES (.13, one-tailed p =.028). In addition, significant paths were found linking all three disposition assessments of forgiveness to RD: Self, $\beta$ = -.42, p <.001; Others, $\beta$ = -.32, p <.001; and Situation, $\beta$ = -.47, p <.001. However, only Self was significantly associated with LONE: $\beta$ = -.19, p =.017; however, there was a significant total indirect effect on LONE from ACES through RD and the three measures of forgiveness;.019, one-tailed p =.045. The model explained 22% of the variance in LONE. Results have implications for how clinical counselors, social workers, cases managers, and pastoral care ministers administer rehabilitative programming. Discussion will highlight forgiveness as a rehabilitative resource for addressing childhood trauma and religious doubt, as well as reducing feelings of loneliness reported by middle aged women in correctional custody.

Abstract citation ID: igae098.2551

### GENDER DIFFERENCES IN THE RELATIONSHIP BETWEEN SEXUAL SATISFACTION AND SPIRITUAL WELL-BEING

Vivian Weiqun Lou, and Clio Yuen Man Cheng, *The University of Hong Kong, Hong Kong, China (People's Republic)*

Recognizing the significance of sexual activity in the holistic health of older adults, this research is the first to explore how sexual satisfaction contributes to the broader well-being dimensions, particularly spiritual well-being. Guided by an integration of the attachment theory and the spiritual well-being of Chinese older adults' framework, we conducted a longitudinal study with 290 married young-olds aged 50 to 75 years. Participants completed self-reported measures assessing sexual satisfaction at baseline and three dimensions of spiritual well-being at 1-year follow-up. Linear regression analysis results indicate gender differences emerged in the associations between sexual satisfaction and these components of spiritual well-being. For the meaning of life, while males did not exhibit a significant association with sexual satisfaction, females demonstrated a positive association, suggesting that higher sexual satisfaction was linked to a greater sense of meaning in life ($\beta$ =.728, SE =.365, t(1) = 1.997, p = 0.047). In terms of transcendence, no significant associations were found between sexual satisfaction and this component of spiritual well-being for either gender. Regarding overall spiritual well-being, males did not show a significant association with sexual satisfaction, while females displayed a positive association, indicating that higher sexual satisfaction was associated with higher levels of spiritual well-being ($\beta$ = 1.175, SE = 0.410, t(1) = 2.868, p = 0.004). The findings underscore the significance of considering sexual satisfaction as a factor influencing spiritual well-being in older adults. Moreover, considering gender-specific patterns in future studies and interventions development for holistic well-being is warranted.

Abstract citation ID: igae098.2552

### INCLUSION OF SPIRITUAL CARE AT THE INDIVIDUAL LEVEL FOR PERSONS LIVING WITH DEMENTIA

Sooyoung Park[1], Eunkung Kim[2], and Soobin Park[3],
*1. Samsung Medical Center, Seoul, Republic of Korea*,
*2. Midwest University, St. Louis, Missouri, United States*,
*3. Washington University in St. Louis, St. Louis, Missouri, United States*

Spirituality is known to remain for a long time until the advanced stages of dementia and is associated with positive health outcomes. Initiatives such as dementia-friendly church services and spiritual programs in institutional settings aim to recognize and enhance the emotional, social, spiritual, and artistic facets of person living with dementia (PLWD). However, the application of spirituality in the day-to-day lives of PLWD at home remains a research gap and poses practical challenges. Thus, we draw from our personal insights as a family caregiver of an older adult with severe dementia and collaborated with a social worker specialized in this area to integrate spirituality into PLWD's daily routines. Based on the Spiritual Dimension of Ageing model, we designed a range of practices aimed at fostering sustainable spirituality at home for PLWD. These practices encompassed four themes: relationships with others, creating an environment conducive to engaging in meaningful activities, incorporating favorite art/music, and exploring religious beliefs. This approach allowed for the customization and diversification of spirituality practices, catering to the unique interpretations and needs of each PLWD. As part of the practice, it enabled PLWD to reunite with a family member after 12 years. Utilizing photographs and written narratives from community-dwelling PLWD and caregivers, we highlighted specific qualitative benefits of this approach (e.g., positive emotions, experience moments "beyond dementia"), particularly for individuals in the advanced stages of dementia. Discussing opportunities to nurture the whole person and their soul may offer comfort and hope to those facing a disease with no cure.

Abstract citation ID: igae098.2553

### MATURE RELIGION AT 75: AN APPRAISAL OF GORDON ALLPORT'S (1950) "THE INDIVIDUAL AND HIS RELIGION"

Kelly Mirarchi[1], Peter Hope[2], Delaney Sommers[1], Edward Thompson[3], Michiko Iwasaki[1], and Andrew Futterman[1], *1. Loyola University Maryland, Baltimore, Maryland, United States*, *2. Johns Hopkins University School of Medicine, Baltimore, Maryland, United States*, *3. College of the Holy Cross, Worcester, Massachusetts, United States*

In his classic manuscript, Gordon Allport (1950) presented his theory of the development of the "religious sentiment". Allport posited that personal religiousness is similar to other "sentiments" (i.e., guiding principles of behavior) and matures for some individuals. For those who are religiously-oriented, religious behavior is enacted in childhood first to obtain diffuse social and emotion benefits. As these individuals age, religious behavior becomes more intrinsically motivated, and religion directs and integrates adult life. The transition from child-like, extrinsically motivated religion to a more mature religion occurs, for Allport, usually in midlife, as religious doubts are resolved in the course of daily life. We examine Allport's theory of religious maturity in light of recent research on aging and religious development, research on complexity of thought and emotion in later life, and our own study of religious complexity in later life involving 278 open-ended interviews of individuals aged 55-101. We argue that, in many respects, Allport's theory has stood the test of time – nearly 75 years - remarkably well. For one thing, Allport (1950) anticipates the main finding of recent research on complexity and aging and is in keeping with findings of our own study, that older adults tolerate their doubts (in this case, their religious doubts) better as they age, and test, affirm, and sometimes change beliefs through social interactions. From our findings, for many individuals, their personal religiousness does seem become more mature – more intrinsic, more integrated, more directing – just as Allport posited.

Abstract citation ID: igae098.2554

### RELATIONAL RELIGIOSITY, FORGIVENESS, AND FAMILY FUNCTIONING IN OLDER AFRICAN AMERICAN COUPLES

Antonius Skipper[1], Andrew Rose[2], and Seungjong Cho[3],
*1. Georgia State University, Atlanta, Georgia, United States*, *2. Social Work, Lubbock, Texas, United States*, *3. Texas Tech University, Lubbock, Texas, United States*

Despite recent advances in studies on positive psychology, relatively little is known about the factors that influence the romantic relationships of older African Americans. Existing literature does note that older African Americans are among the most religious racial/ethnic groups in the United States. In addition, forgiveness is recognized as a common mediator between relational religiosity and other positive outcomes, such as psychological well-being and successful aging. Given the need for more strength-focused studies on minority-based romantic relationships, this study examined forgiveness as a potential mediator between relational religiosity and family functioning among older, romantically involved African American couples. This study utilized the dyadic data of 194 African American couples with partners between the ages of 50 and 86 years. Data were captured with the Revised Sanctification of Marriage scale, six items assessing interpersonal forgiveness, and the McMaster Family Assessment Device. Indirect effects were verified with 95% confidence intervals within 10,000 bias-corrected bootstrap iterations. The relations between men's relational religiosity and men's reports of family functioning were partially mediated by men's self-reported ability to forgive their partner (B =.053 (95% CI [.001,.187])), as well as the sum of all men's and women's reported abilities to forgive and perceive forgiveness

(B =.132 (95% CI [.012,.260])). These findings highlight the growing, yet underrecognized, need to understand the religiosity of older African American men relative to positive relational outcomes. Further, this data helps to counter the deficit-narratives that often paint African American family functioning as poor and flawed.

Abstract citation ID: igae098.2555

### ROLES OF LONELINESS, STRESS, AND RELIGIOSITY IN SUICIDE IDEATION IN A SAMPLE OF SUB-SAHARAN AFRICAN OLDER ADULTS

Genevieve Ebulum[1], John Eze[2], Obinna Ezeihuoma[3], Crystal Njoku[4], and JohnBosco Chukwuorji[5], *1. David Umahi Federal University of Health Sciences, Uburu, Ebonyi, Nigeria*, *2. University of Nigeria Nsukka, Nsukka, Enugu, Nigeria*, *3. University of Pittsburgh, Pittsburgh, Pennsylvania, United States*, *4. American University of Antigua College of Medicine, Osbourn, Saint John, Antigua and Barbuda*, *5. Michigan State University Flint, Flint, Michigan, United States*

Suicidal behaviour in older adults is a fundamental public health problem globally and the highest suicide rates occur in the low- and middle-income countries (LMICs). However, there is limited research on suicidality among older adults especially in sub-Saharan Africa. We sought to find out whether loneliness, stress and religiosity would significantly predict suicide ideation among Nigerian older adults (N = 500). They completed a questionnaire form comprising the Beck Suicidal Ideation Scale, the UCLA Loneliness Scale – version 3, Perceived Stress Scale and Religiosity Scale. While including age, educational qualification, and number of children as covariates, regression results showed that loneliness ($\beta$ =.23) and stress ($\beta$ =.10) were positively associated with suicide ideation while religiosity ($\beta$ = -.16) exhibited a negative association with suicide ideation. The models substantially improved with the addition of each predictor. This suggests that whereas suicide ideation could be exacerbated by loneliness and stress, more religious older adults were less likely to report suicidal ideation. Interventions aimed at managing and protecting the mental health of older adults during their transition to late adulthood should guard them against loneliness and buffer their resilience and coping strategies with the connectedness that religiosity offers.

Abstract citation ID: igae098.2556

### STRENGTH OF ATTACHMENT TO RELIGIOUS BELIEFS DECREASES NEGATIVE ATTITUDES TOWARD OLDER ADULTS

Jessica Strong, *University of Prince Edward Island, Charlottetown, Prince Edward Island, Canada*

Ageism, a term identified by Butler in the 1960s, refers to any discrimination towards an individual based on their age: implicit, explicit, or internalized. The impacts of experiencing ageism are far reaching, including negative impacts on memory, mood, physical health, and even mortality. Two recent studies (Neto et al., 2023; Kim & Jung, 2020) investigated the protective role religiosity may have on attitudes towards older adults. These studies provided preliminary evidence that groups or cultures with stronger religious beliefs had better attitudes towards older adults. The current study examined the impact of attachment to a belief system and death anxiety on ageism in a population of adults (21-73 years old, N=31). Participants reported a variety of belief systems, including Christian, Buddhist, Muslim, and Atheist/Agnostic, and were separated into two groups based on the strength of their beliefs (attached or unattached). ANCOVA analyses showed that while holding death anxiety constant, there was no impact of religious attachment on positive ageism ($F(1,14)=0.98$, $p=0.34$, partial eta2=0.06), there was an impact of religious attachment on negative ageism ($F(1,14)=4.52$, $p=0.05$, partial eta2=.24), and belief that lives of older adults should be restricted (($F(1,14)=5.24$, $p=0.045$, partial eta2=.34), both with large effect sizes. Although the sample was small and likely underpowered, the effect sizes suggest more research in this area is warranted. The sample was diverse in terms of belief systems and the results are discussed in the context of strength of belief system, including atheism or agnosticism.

### SESSION 7155 (POSTER)

### TECHNOLOGY: OLDER ADULT INTERFACE AND USE I

Abstract citation ID: igae098.2557

### CO-PRODUCED IDEAS FOR SMART HOME TECHNOLOGIES TO SUPPORT ENGAGEMENT IN MEANINGFUL ACTIVITIES

William Son Galanza, Steven Schmidt, Oskar Jonsson, Jens Offerman, Nebojsa Malesevic, Susanne Iwarsson, and Sofi Fristedt, *Lund University, Lund, Skane Lan, Sweden*

While research in the field of technology is surging, research on older adults playing an active role in designing and developing smart home technology (SHT) to enhance usability and adoption is lacking. It is important to co-develop products with potential users to better match their needs and desires. This study aims to generate ideas about SHT that can enhance the use and adoption of SHT that can support engagement in meaningful activities while ageing. The research circle methodology was utilized to elicit co-produced ideas for SHT and their functions with participants from different generations (30-39, 50-59 and 70-79 years old; n=9), together with two professionals with expertise in SHT and four researchers. The strength of research circle methodology is knowledge translation through the collaborative effort of participants. The findings revealed that the participants preferred SHT which can support independence, engagement in social experiences, and participation in meaningful activities while ageing, rather than smart home appliances or full home automation. Moreover, SHT which can create a stimulating environment to positively impact physical, cognitive, and psychosocial factors were highlighted. Some ideas generated were multi-purpose functions and a system that can analyze patterns for monitoring activities and prevention of disability or its progression. Non-technology support such as informal carers and neighborhood and community support were also prioritized. Our study highlights the benefits of collaboration among diverse stakeholders (older adults, informal carers, technology professionals and researchers) to generate ideas about preferences for SHT that can better support the needs and desires of older adults.

Abstract citation ID: igae098.2558
### DIGITAL LITERACY CHANGES OVER TIME: IMPLICATIONS FOR LIFE SATISFACTION IN OLDER ADULTS

Miseon Kang, Seolah Lee, Inhye Jung, and Hyo Jung Lee, *Yonsei University, Seoul, Republic of Korea*

Rapid digitalization of society requires people to utilize digital technology in daily lives, and the COVID-19 pandemic accelerated this situation. Although older adults are known to be unfamiliar with using digital technology, their digital literacy can vary according to their life experiences. This study aimed to identify patterns of digital literacy changes in older adults before and after the COVID-19 pandemic and examined how life satisfaction differs based on subtypes of digital literacy changes over time. Data were drawn from the 'Media Panel' by the Korea Information Society Development Institute. The final sample was 1,924 individuals over 65 who responded from 2019 to 2021 and maintained their living arrangements (Living alone, Couples only, Couples with children, and Couples with their parents and children). Latent Class Growth Analysis and multiple regression analyses were employed. The patterns of older adults' digital literacy changes were classified into four subtypes: 'Low-level increase group (60.9%),' 'Medium-level increase group (29.8%),' 'High-level maintenance group (5.0%),' and 'Rapid increase group (4.2%). The other three groups had higher life satisfaction than the low-level increase group. Findings imply that the majority of older adults saw a rise in their digital literacy skills during the COVID-19 pandemic. The heterogeneity in digital literacy levels among older adults offers valuable insight into the need for interventions that cater to different digital skill levels, potentially impacting their quality of life. Particularly, in order to enhance their overall well-being, it is important to better understand the key features of individuals in each identified group.

Abstract citation ID: igae098.2559
### FACTORS AFFECTING IMPLEMENTATION OF SOCIALLY ASSISTIVE ROBOTS IN LONG-TERM CARE FACILITIES

Judith Tate[1], Cathy Maxwell[2], Miroslava Migovich[3], Nibraas Khan[3], Ritam Ghosh[3], Kelley Colopietro[3], Abigail Kilpatrick[1], and Nilanjan Sarkar[3], *1. The Ohio State University, Columbus, Ohio, United States*, *2. University of Utah College of Nursing, Salt Lake City, Utah, United States*, *3. Vanderbilt University, Nashville, Tennessee, United States*

Older adult residents in long term care (LTC) often experience apathy. Technology can provide support for addressing the older adults' psychosocial needs. However, introducing new technology into LTCs can be challenging. As part of a randomized clinical trial on socially assistive robots (SAR) in LTCs to engage older adult dyads in activities, we examined determinants to implementation. Seventeen LTCs participated in the study, ranging in size from 24 to 132 participants; 53% were not-for-profit. All robot sessions were conducted by trained research staff. Each participant attended 16 sessions averaging 30 minutes over 8 weeks; half with an animal SAR and half with a humanoid SAR. Research assistants noted observations of the SAR, participant, staff, and facility environment and conducted interviews at the conclusion of the experiment. Sixty older adults participated (62% women, ages 60 to 98). Common SAR barriers included internet connections, equipment breakage, and speaker volume. Participant barriers included paranoia, dislike of partner, hearing and vision, level of cognitive impairment, and acute illness. Staff issues consisted of workflow issues and knowledge of the research. Facility issues included competing demands that interrupted scheduling and rollout, physical area for conduct of experiments, noise and lighting, and communication among key staff. Successful implementation and sustainability of new technology, including SAR, will depend upon addressing multiple barriers at the resident, staff, and facility levels.

Abstract citation ID: igae098.2560
### HOW BABY BOOMERS SHARE PERSONAL INFORMATION ON PSEUDONYMOUS PLATFORM: AN EXPLORATORY STUDY ON REDDIT

Ern Chern Khor, and Moon Choi, *Korea Advanced Institute of Science and Technology (KAIST), Daejeon, Republic of Korea*

Social media platforms serve as hubs for discussions across diverse topics, fostering connections and information exchange. Pseudonymous platforms allow users to create separate digital identities and facilitating open expression of thoughts and opinions. Previous research tends to focus on younger age groups, resulting in a knowledge gap about older cohorts. This study aims to explore how baby boomers share their personal information on pseudonymous online platforms, using data from Reddit, a popular pseudonymous online platform known for topic-specific communities called subreddits. Data was collected from posts in five subreddits in February 2024, using user flairs (i.e., users' self-tags of their age range). The dataset encompasses 619 baby boomers and 1.91 million sentences tokenized from generated contents, including both posts and comments. Following Geetha et al. (2021), private information was categorized into three domains: (a) personal relationships, (b) professional-related, and (c) health-related. Following manual annotation, natural language processing was applied to categorize sentences into different domains. Results showed that baby boomers shared personal relationships-related information more frequently than professional-related and health-related information. Compared to younger age groups, baby boomers were less likely to share personal information; however, content analysis revealed age-related uniqueness within the same domain. These findings imply that pseudonymous online platforms could serve as a resource for the aging population seeking information and support on personal relationships issues. However, considering the increased cybercrime risks associated with sharing personal information, future research needs to investigate how to help baby boomers navigate these platforms safely.

Abstract citation ID: igae098.2561
### LONGITUDINAL RELATIONS OF INTERNET USE FREQUENCY WITH COGNITIVE AND SOCIAL FUNCTIONING AMONG OLDER ADULTS

Wei Xing Toh[1], Chin Wei Ho[1], and Shuna Shi Ann Khoo[2], *1. Nanyang Technological University, Singapore, Singapore*, *2. National University of Singapore, Singapore, Singapore*

Given the increasing reliance on the Internet in our daily lives, understanding the role of Internet use in cognitive and social functioning among older adults is crucial for promoting successful aging. However, the directionality of these relations remains inconclusive owing to methodological limitations in past studies (e.g., Mariano et al., 2021; Szabo et al., 2019), such as (a) reliance on cross-sectional and limited (two-wave) longitudinal data and (b) failure to disaggregate stable, between-person differences from temporal, within-person dynamics. Drawing from four waves of the English Longitudinal Study of Aging (N = 12,264; Mean age = 66.01 years), we used random-intercept cross-lagged panel modelling to investigate the directionality of Internet use frequency with cognitive functioning (i.e., verbal fluency and episodic memory) and social functioning (i.e., perceived social support, social-interaction frequency, and perceived social closeness). At the between-person level, older adults with higher Internet use frequency reported greater cognitive and social functioning (rs >.11, ps <.001). At the within-person level, greater-than-average cognitive functioning unidirectionally predicted subsequently higher Internet use frequency (βs >.06, ps <.035), thereby indicating that more cognitively intact older adults may face fewer difficulties with Internet use. Conversely, Internet use frequency was bidirectionally associated with social functioning (βs >.11, ps <.001). Specifically, engagement with the Internet supports social networks, and improved social functioning likely enhances the perceived usefulness and usage behavior of the Internet. These findings underscore the importance of considering cognitive and social factors when promoting Internet use among older adults.

Abstract citation ID: igae098.2562

### MOBILE TEXT MESSAGE AS AN ADJUNCTIVE STRATEGY TO SUPPORT HOME-BASED EXERCISE IN OLDER ADULTS

Bethany Kidd[1], Stephen Jennings[2], Lauren Abbate[3], Arti Tayade[4], and Katherine Hall[5], *1. VA Puget Sound Healthcare System, Seattle, Washington, United States*, *2. Durham Veterans Affairs Healthcare Adminstration, Durham, North Carolina, United States*, *3. VA Eastern Colorado Healthcare System, Aurora, Colorado, United States*, *4. Puget Sound VA Heathcare System, Seattle, Washington, United States*, *5. Veterans Health Administration/Duke University, Durham, North Carolina, United States*

Lack of physical activity is associated with increased comorbidities, falls, frailty and healthcare usage. Several clinical and community-based exercise programs exist to provide guided exercise to older adults, however, staffing and space allotment often create barriers to providing sufficient opportunities. Puget Sound Gerofit sessions are held both in person and virtually. Both classes take place twice a week for 45 minutes. The Gerofit program in Seattle Washington implemented a structured text-based messaging protocol to provide additional exercise opportunities in support of meeting public health guidelines for physical activity. Twenty-six veterans were enrolled using mobile test messaging to provide an additional day of exercise. These veterans were texted 1x a week an automated message asking if they would like to exercise. Positive responses were sent a randomized link from a digital library of pre-recorded exercise videos featuring Gerofit instructors. Follow-up messages were sent to quantify minutes of activity participation. Twenty-six Veterans were messaged, over an average of 24 weeks. Participating veterans were on average 73.7 years old, predominately male (87.0%), and White race (82.6%). Of the 634 messages sent, 145 responses were gathered (22.8%). Veterans who engaged positively (61.5%) in exercise reported an average activity of 33.5 minutes/day. Participating older adults reported high satisfaction with the text-based protocol. Supplementation of a virtual group exercise program is feasible with text messaging and on-demand pre-recorded videos. This format allows a flexible option for participants as well as providing a lower resource option for exercise programs.

Abstract citation ID: igae098.2563

### OLDER VETERAN EXPERIENCES OF TECHNOLOGY USE DURING A MULTICOMPONENT TELEREHABILITATION PROGRAM

Michelle Rauzi[1], Melissa Tran[2], Cory Christiansen[2], Meredith Mealer[2], Kathryn Nearing[3], and Jennifer Stevens-Lapsley[2], *1. Department of Veterans Affairs, Aurora, Colorado, United States*, *2. University of Colorado Anschutz Medical Campus, Aurora, Colorado, United States*, *3. University of Colorado Anschutz Medical Campus, Denver, Colorado, United States*

Less than half of U.S. military Veterans meet physical activity recommendations despite the well-established health benefits of regular physical activity. While changing physical activity behaviors is challenging, prior studies demonstrate that it is possible. Older adults are using technology to aid in such behavior change, however, research that explores the mechanisms of how technology can aid in behavior change is lacking. Thus, the purpose of this secondary, convergent mixed methods study was to explore how older Veterans engaged with technologies that were used during a multicomponent telerehabilitation program. The study included Veterans aged ≥60 years with ≥3 chronic medical conditions and physical function limitation. Quantitative data were collected during the primary randomized controlled trial, and qualitative data were collected via individual interviews following the telerehabilitation program. Data were merged and then categorized by level of technology engagement. Key similarities and differences between the high and low technology engagement groups were identified in five domains: satisfaction with the virtual environment, coping self-efficacy, perceptions of Annie (automated text messaging platform), experiences using the activity monitor, and self-management skills. Results revealed three key findings that help explain why older Veterans chose whether to engage with the technologies used during our behavior change program. These findings relate to technology self-efficacy, integration of the technologies into intervention, and technology application to self-management skills. Study results can help with the integration of similar technologies into physical rehabilitation programs. Further study is warranted to understand additional factors and mechanisms that influence technology engagement in healthcare.

Abstract citation ID: igae098.2564

### PATTERNS AND ASSOCIATED FACTORS OF TECHNOLOGY USE IN US COMMUNITY-DWELLING OLDER ADULTS

Wenting Peng[1], Yuqian Luo[2], and Minhui Liu[3], *1. University of Washington, Seattle, Washington, United States*, *2. Hong Kong Polytechnic University, Hong Kong, Hong Kong*, *3. Ningxia Medical University, Yinchuan, Ningxia, China*

Technology, especially digital health technology, has been increasingly used to deliver healthcare services. The COVID-19 pandemic accelerated the rapid adoption of medical technology. However, how many and how older people currently use technology remain unknown. Using the cross-sectional data from the National Health and Aging Trends Study collected in 2022, we examined the prevalence, characteristics, and associated factors of technology use (including technology device use, everyday/digital health technology use) among 5,512 community Medicare beneficiaries aged 65 years and older with analytical sample weights and design-based logistic regression models. Results showed that over 1.3 million (2.7%) older people did not use any information and communication technology devices (i.e., computers, cell phones, and tablets). The weighted prevalence of everyday technology use and digital health technology use were 83.3% (95% CI, 82.1%-84.5%) and 52.1% (95% CI, 49.5%-54.7%), respectively. Compared to individuals who never used digital health technology, older adults who used digital health technology were more likely to be younger, white, not living alone, self-rated as having good health, non-homebound, and possessed higher education, income, and cognitive function, with fewer ADL/IADL impairments, and were less likely to experience hospitalization and depressive symptoms (All $P< 0.05$). Age, education, income and a diagnosis of dementia were independently associated with technology device use, everyday technology use, and digital health technology use (All $P< 0.05$). The prevalence of technology use in US older adults differed significantly by demographic and health-related characteristics. Future technology-based services or interventional studies should consider the devices and modalities of technologies in the older population.

Abstract citation ID: igae098.2565

### POWER ASSIST ADD-ONS FOR OLDER ADULT MANUAL WHEELCHAIR USERS: A SCOPING REVIEW

Oladele Atoyebi[1], Andrew Wister[1], Johanne Mattie[2], Gloria Gutman[1], Habib Chaudhury[1], Eireann O'Dea[1], Sogol Haji Hosseini[1], and Jaimie Borisoff[2], *1. Simon Fraser University, Vancouver, British Columbia, Canada*, *2. British Columbia Institute of Technology, Vancouver, British Columbia, Canada*

Manual wheelchairs can promote independence among users of all ages. However, there are age-related differences in the level of disability, strength, stamina, and the environmental conditions within which the wheelchair is used. Using power assist add-ons may mitigate these limitations and help individuals in independent mobility. A gap in the literature is an understanding of the ways in which aging may limit the functionality of power assists on wheelchairs and the impact on independence and active aging. This scoping review analyzes scientific and gray literature to examine the use of power assist add-ons among older adults who use manual wheelchairs, as well as their advantages, limitations, and potential benefits in promoting independence and active aging. This review was guided by the PRISMA checklist for scoping reviews, and the Arksey and O'Malley review methodology. The literature search was carried out in two phases: 1) a keyword and MeSH search of electronic databases, proceedings, as well as symposia for relevant titles/abstracts; and 2) a search of Google and Google Scholar, as well as hand searches. After applying the inclusion and exclusion criteria, we included 20 publications with a focus on power-assist wheelchair technology for full review. Results indicate that power-assist add-ons for manual wheelchairs show promise in improving mobility and reducing user exertion for older adults. However, concerns regarding safety, indoor maneuverability, and user preferences highlight the need for specialized training and retrofitting power assist add-ons, especially among older users. Further research on user-centered design, and adherence to safety standards are warranted.

Abstract citation ID: igae098.2566

### TECHNOLOGY-ASSISTED TOILETING BY OLDER ADULTS: A SCOPING REVIEW OF BIDET USE

Catherine Van Son[1], and Minyoung Cerruti[2], *1. Washington State University, MARYSVILLE, Washington, United States*, *2. Washington State University, Pullman, Washington, United States*

**Objective:** With aging, toileting can become difficult, given changes in mobility, agility, and cognition. Interventions are needed to support toileting privacy and independence. A technology-assistive device for toileting, referred to as a bidet (washlet), uses one's existing toilet and plumbing to provide a cleansing mechanism by spraying water on the perineum after toileting. Using a remote-controlled device, water temperature, and pressure can be individualized, and the seat warmed. This review focused on the following question: What are the benefits and challenges older adults (OA) and their caregivers experience in using a bidet?

**Methods:** A scoping review was conducted using the five stages of Arksey and O'Malley's framework and the PRISMA-ScR checklist. Our search focused on literature published after 2000 and included five databases.

**Results:** Fourteen articles were identified and analyzed. Seven papers focused on OAs, two on caregivers for OAs, and five included OAs as part of their sample. Findings described bidet technology facilitators and barriers for OAs and their caregivers. Older adults described apprehension about using the technology while appreciating increased privacy and independence. Caregivers reported less time toileting and could focus more on other ADL activities. Research gaps were identified, and recommendations were proposed to change the culture of toileting and promote bidet use. Randomized controlled studies with larger samples are needed to determine the feasibility and effectiveness of bidet use by older adults.

**Conclusion:** Bidet technology can support OAs' toileting privacy and independence, reduce caregiver burden, and decrease the environmental impact by using fewer paper products.

Abstract citation ID: igae098.2567

### THE ART OF GRIEVING AMONG OLDER ADULTS ON TIKTOK

Reuben Ng, and Nicole Indran, *National University of Singapore, Singapore, Singapore*

Although TikTok is dominated largely by younger persons, the platform has since gained traction among older adults, a number of whom have used it to cope with bereavement and grief. Presently, little is known about the therapeutic effects of creating TikTok content in the context of bereavement. However, there have been suggestions that the platform has the potential to serve as an accessible form of art therapy. This study explores how older adults mobilize audio-visual functions to communicate their experience of bereavement on TikTok. We compiled the most viewed videos of users aged 60 and above with at least 100,000 followers, generating 1,382 videos with over 3.5 billion views. Videos which did not feature older adults engaging in discourses on bereavement were excluded. This left us with 22 videos. Both inductive and deductive approaches guided the qualitative analysis. Three themes emerged: 'Emotional Expression' (Theme 1; 59%), 'Maintaining a Relationship with the Deceased' (Theme 2; 23%) and 'Channelling Grief into Positive Emotions' (Theme 3; 18%). This study represents a first step towards understanding the use of TikTok among older bereaved individuals. As findings from this study paint a rather optimistic portrait of the experience of uploading bereavement-related content on TikTok, future inquiry could dive into the potential harms of uploading such content on the application. In view of the enduring presence of TikTok, it is crucial to investigate the clinical effectiveness of using the platform as an art form to cope with bereavement.

Abstract citation ID: igae098.2568

### THE RELATIONSHIP BETWEEN TECHNOLOGY USE, EMPLOYMENT STATUS, AND SOCIAL ENGAGEMENT IN OLDER AMERICANS

Jaesung Lee, Jay O'Shields, and Allison Dunnigan, *University of Georgia, Athens, Georgia, United States*

Previous studies have explored the relationship between technology use or employment status among older adults and social engagement. However limited knowledge exists regarding the relationship between technology use, employment status, and social engagement. This study analyzes the relationship between older adults' information and technology (ICT) use and employment status on social engagement. The 2022 (wave 12) National Health & Aging Trends Study (NHATS) was used. The sample population includes US Medicare beneficiaries aged 65 years and older who were community dwellers (N = 5,195). Multivariate regression analysis indicates that ICT use is associated with social engagement (b=-1.52, t=-11.86, p< 0.001), with ICT users having the highest level of social engagement followed by CT users, IT users, and nonusers. Employment status is also associated with social engagement (b=-0.46, t=-5.14, p< 0.001), with the employed being the most socially engaged, and the retired/do not work anymore group having higher levels of social engagement compared to the unemployed group. An interaction was tested but was not significant. Additionally, sociodemographic factors including age (b=0.05, t=2.50, p=0.015), gender (b=0.31, t=5.71,p< 0.001), race (b=-0.78, t=-9.52, p< 0.001), education(b=0.24, t=3.70, p< 0.001), and living arrangements (b=0.31, t=6.84, p< 0.001) have a statistically significant relationship with social engagement. In conclusion, this study emphasizes the importance of considering both technology use and employment status in understanding social engagement among older Americans. These findings suggest the need for further research to explore the complex interaction of these factors in older adults' social engagement.

Abstract citation ID: igae098.2569

### TRAINING OLDER ADULTS IN THE USE OF INTERNET TECHNOLOGY

Donna Case[1], Sheryl Groden[2], and Michelle Sahli[2], *1. University of Michigan Flint, Flint, Michigan, United States*, *2. University of MIchigan Flint, Flint, Michigan, United States*

This study was conducted to investigate whether small group (2:1) technology training increases the frequency of technology use among older adults and how these older adults perceived this training. Graduate occupational therapy students, under faculty supervision, provided 4 weekly technology tutoring sessions to older adults at a senior center and an independent living facility located in Genesee County, MI. Participants received training in pairs. This training consisted of internet safety and additional instruction centered on participants' interests. When requested, participants were offered assistive devices to help them use technology. Pre and post surveys were administered to participants to examine their independent technology use and attitudes towards the training sessions. Students provided training to 31 older adults with only 3 participants requesting assistive devices. The surveys showed an increase in the proportion of participants independently using technology for 10 out of 11 activities; however, these results were not statistically significant. Analysis of qualitative data revealed that most participants felt comfortable using technology post intervention and that they learned more about technology. Participants also liked the frequency and length of the sessions and reported forming relationships with others during the sessions. Although not statistically significant, perhaps due to small sample size, these findings provide evidence that training in small groups provided by students may increase technology use by older adults and that this type of training is acceptable to older adults. Future studies should be conducted in larger samples and with other populations to further explore this type of training program.

Abstract citation ID: igae098.2570

### TRENDS IN DIGITAL DEVICE ACCESS AMONG HISPANIC OLDER ADULTS IN THE US (2011–2021)

Xiayu Chen[1], Yanjun Dong[2], Karla Sanabria Véaz[1], Danan Gu[3], and Kun Wang[4], *1. University of Illinois Urbana-Champaign, Champaign, Illinois, United States*, *2. SUNY Albany, Albany, New York, United States*, *3. NA, Suzhou, Jiangsu, China (People's Republic)*, *4. Binghamton University, Binghamton, New York, United States*

Despite the general increase in digital technology use among U.S. older adults within the past decade, Hispanic older adults may still face a notable digital gap due to socioeconomic disparities. Guided by the intersectionality lens, the study aims to examine (1) the trends of digital device access between Hispanic and non-Hispanic White older adults in the U.S. from 2011 to 2021 and (2) the effect of gender, education and income factors on the digital access disparity. We used 11 waves of National Health and Aging Trend data, and identified 38,253 observations from 8,503 community-dwelling older adults aged 70 and above for the analysis. Applying weighted multilevel logistic regressions, we found that compared to non-Hispanic White older adults, those who were Hispanic had significantly lower odds of having digital device access (OR = 0.42, 95% CI = 0.25 - 0.70). However, the gap in digital device access has narrowed over time. Meanwhile, specific factor-stratified models revealed that the digital access between Hispanic and non-Hispanic White older adults was significantly reduced with time among both men and women, those with low education, and those with low income, but not among those with higher education and medium-to-high income. Our findings highlight notable progress in narrowing the digital access gap for Hispanic older adults in 2011 to 2021. Nevertheless, disparities persist, necessitating continued efforts in tailored policies and community programs to address the complex factors contributing to digital inequalities.

Abstract citation ID: igae098.2571

### USER-CENTERED ANALYSIS OF A FITNESS APP FOR OLDER ADULTS: SENIOR FIT

Kathy Lee[1], Kate Hyun[1], Sabrina Haque[1], Kyle Henry[1], Troyee Saha[1], Jessica Cassidy[1], Soeun Jang[2], and Christoph Csallner[1], *1. The University of Texas Arlington, Arlington, Texas, United States*, *2. University of Texas at Arlington, Arlington, Texas, United States*

Utilizing technology can greatly improve the health and well-being of older adults, especially those who prefer to age in place. Senior Fit, an augmented reality mobile application, was created specifically to assist older adults with various physical exercises. This poster presentation focuses on the specifics of the app, user feedback, and suggestions for designing apps aimed at enhancing the physical activity of older adults. The app features a simple login process, requiring only users' first and last names along with their contact number. It provides access to 16 indoor exercise categories, including strength, cardio, and balance routines, as well as an outdoor walking feature utilizing GPS tracking to monitor distance and duration. Additional features include activity guidelines and progress tracking. We collected both quantitative and qualitative data from 20 older adults who used the app for 24 weeks. Quantitative data showed overall usage of the app, indicating the participants more frequently utilized the app for indoor activities than outdoor activities. Qualitative data identified significant potential for Senior Fit alongside several challenges. Our participants expressed a preference for automatic tracking akin to wearable devices and stressed the importance of accessing their progress data seamlessly integrated into their daily routines. On the other hand, many hesitated to use the camera-based features, raising usability concerns. These findings underscore the necessity of adhering to user-centered design principles and addressing the unique challenges older adults face in adopting app-based technology. By considering these insights, future mobile applications should address the specific needs of this demographic.

Abstract citation ID: igae098.2572

### VIRTUAL REALITY IN RESPITE: USING IMPLEMENTATION SCIENCE TO ASSESS ADOPTION OF VR WITH PLWD

Mary Milnamow[1], Lillian Agyemang[2], Louanne Bakk[2], and Jason Dauenhauer[3], *1. University at Buffalo, Buffalo, New York, United States*, *2. University at Buffalo, SUNY, Buffalo, New York, United States*, *3. State University of New York at Brockport, Brockport, New York, United States*

In the U.S., 6.7 million older adults are living with dementia (PLwD); this number is estimated to double by 2040. Community-based volunteer respite programs are vital for PLwD to receive social connections and engagement. Virtual Reality (VR) programming for PLwD is typically used in nursing homes, residential care, and rehabilitation settings. Few studies have examined using VR with PLwD and caregiver dyads receiving community- and home-based services. This study applied Implementation Science to identify facilitators and barriers in adopting a VR intervention within a respite program and to develop evidence-based strategies to guide successful implementation. The RE-AIM framework evaluated changes in VR skills, confidence, comfort, and buy-in from respite staff and volunteers. Electronic surveys were completed anonymously before VR training and after completing one VR session. The IAM and FIM were also used to assess the intervention's acceptability, appropriateness, and feasibility. After training and practicing VR, a paired samples t-test revealed that participants significantly improved across all areas, with the most significant improvement in VR skills from pre to post (16.36 (1.31) vs. (28.00 (1.26), t= -4.44, p=.0001, respectively). The strongest facilitator was the practical and financial support provided by leadership. A persistent challenge was the cost of VR technology. Implementation strategies contributing to successful VR programming included identifying and training champions and developing a trauma-informed quick guide. Findings demonstrate effective utilization of Implementation Science to address the strengths and weaknesses of incorporating a novel intervention that prepares staff, volunteers, and families to experience VR adventures.

### SESSION 7160 (POSTER)

### TRANSPORTATION

Abstract citation ID: igae098.2573

### DRIVING RISK TOLERANCE AS A PREDICTOR OF OLDER ADULTS' ABILITY TO DRIVE

Angela Curl, *Miami University, Oxford, Ohio, United States*

Driving is critical to mobility, autonomy, freedom of choice, and engagement for older adults, but many eventually stop driving. Evaluation of fitness to drive, risks related to driving, and personal preferences about driving all can play a role in driving cessation. Using 2022 data from the Health and Retirement Study (HRS; N=2,209), this study examined the relationship between driving risk tolerance and driving cessation for White and Black adults over age 65. Risk tolerance (key predictor) was measured as willingness to take risks while driving (0="unwilling to take any risks" to 10="fully prepared to take risks"). Self-reported ability to drive (yes/no) assessed driving cessation. Model 1 consisted of driving risk tolerance, controlling for education, gender, race, ethnicity and age, while Model 2 tested whether the relationship was mediated by self-rated eyesight, health, and memory (3 single-item measures, 0=poor to 4=excellent). Logistic regression results indicated that higher driving risk tolerance was associated with greater likelihood of ability to drive in Model 1 (OR=1.08, p=.01). Addition of health factors (Model 2) only weakly mediated this relationship, with driving risk tolerance having a slightly stronger impact on driving ability (OR=1.10, p<.01). Better eyesight (OR=1.76, p<.01) and health (OR=1.45, p<.01) were associated with greater likelihood self-reported ability to drive. As driving involves potential risks (e.g., traffic tickets, accidents), driving assessments should include perceptions of level of risk in discussions about driving cessation. Interventions designed to help older adults accurately assess their driving risk and how to minimize risks could promote driving ability/driving cessation.

Abstract citation ID: igae098.2574

### NEIGHBORHOOD TRANSPORTATION OPTIONS AND TRANSITIONS TO NONDRIVING

Kellia Hansmann[1], Carolyn McAndrews[2], Ronald Gangnon[2], and Stephanie Robert[2], *1. University of Wisconsin School of Medicine and Public Health, Madison, Wisconsin, United States*, *2. University of Wisconsin Madison, Madison, Wisconsin, United States*

Many older drivers rely on their social networks for transportation support as they transition to non-driving, but still experience barriers to maintaining the mobility they had when driving independently. In the transition to non-driving, older adults also need transportation options in their neighborhoods to maintain mobility. We examined whether neighborhood transportation options were associated with changes in driving behaviors among U.S. older drivers. We analyzed National Health and Aging Trends Study data from community-dwelling drivers (n = 5,102; 2015 cohort). We used weighted logistic regression models to estimate whether walking or taking public transit was associated with the number of driving behaviors they avoided (avoided driving at night, in bad weather, on highways, or alone) at the three-year follow up. We also estimated the association between the National Walkability Index (NWI) and driving reduction at the three-year follow up. In 2015, 52% of older drivers reported walking to get places outside their homes, but few reported taking public transit (6%). In adjusted models, we found no associations between walking or taking public transit to get places in 2015 and driving reduction over a three-year period. However, older drivers living in very walkable neighborhoods in 2015 had greater odds of driving avoidance behaviors compared to those living in very unwalkable neighborhoods (NWI of 18 vs. 2 out of 20: adjusted odds ratio = 1.73; 95% Confidence Interval: 1.14-2.63). Future research should examine what modifiable aspects of neighborhood transportation options might play a role in supporting transitions to non-driving for older drivers.

Abstract citation ID: igae098.2575

### SLEEP QUALITY IN LATER LIFE: HOW DOES IT AFFECT RISKY DRIVING BEHAVIORS AND CRASH INCIDENCE?

Anne Barrett, Brianna Soulie, and Hope Mimbs, *Florida State University, Tallahassee, Florida, United States*

Among the leading causes of motor vehicle injury and fatality is drowsy driving. Most studies, however, focus on young drivers or those in shift positions (e.g., truck drivers). Little is known about how sleep quality affects the driving behavior and injury risk of older adults. Their risk may be heightened by the increased susceptibility to sleep disturbances that can occur in later life. Using data from a survey of Floridians aged 50 and older conducted between December 2020 and April 2021(n=4,199), we examined the how self-rated sleep quality and sleepiness while driving are associated with two driving outcomes: frequency of engaging in risky driving behavior (e.g., speeding, texting/emailing) and having experienced a crash or near-crash in the past year. Results of multivariate regression models revealed that reporting sleepiness while driving and lower sleep quality predicted greater likelihood of having experienced a crash or near-crash. Reporting lower sleep quality also predicted engaging more frequently in risky driving behavior. Other significant predictors of risky driving behavior or likelihood of a crash or near-crash included gender, socioeconomic status, marital or partnership status, pain frequency, self-reported hearing ability, self-reported frequency of memory problems, depressive symptoms, driving frequency, self-rated driving ability, and rural residence. Our study highlights the importance of examining how change in sleep patterns over the life course may affect driving behaviors and roadway safety.

Abstract citation ID: igae098.2576

### THE GENERALIST RESOURCE TO INTEGRATE DRIVING: MEASURING THE CLINICAL UTILITY OF THE GRID TOOL

Anne Dickerson[1], Terri Cassidy[2], and Susan Touchinsky[3], *1. East Carolina University, Greenville, North Carolina, United States*, *2. Fitness to Drive, Colorado Springs, Colorado, United States*, *3. Adaptive Mobility Services, LLC, Orwigsburg, Pennsylvania, United States*

Cut-off scores for driving evaluations are based on the concept that a score indicates whether the individual is likely to pass or fail a driving evaluation. Cut-off scores should be derived from research over multiple studies used with diverse populations. Assessments develop scores based on specific populations (e.g., dementia) which may not be valid for other populations (e.g., stroke). Thus, when using scores, it is critical to understand the population and range of scores upon which it is based. The Generalist's Resource to Integrate Driving (GRID) is a resource for practitioners to integrate driving into their practice and use scores appropriately. The tool is designed to provide specific guidelines to evaluate, create and implement intervention plans using the schematic of red (stop driving), green (ok to drive) and yellow (needs further evaluation). The aim of this study was to evaluate the utility of the GRID by practitioners who used it. Results of the IRB approved Qualtrics survey with 70 respondents showed 79% indicated they used while 21% had not, although 10% plan to use it. Also, 69% of users found it easy-to-use, 73% indicated it was easy to understand, and 63% felt it was comprehensive. and use scores appropriately 51% respondents indicated the most important use was screening for driving risk although planning a referral to a driving evaluation was a close second. This presentation will briefly explain the use of the GRID, the model upon which it is based and discuss the results of the utility study.

Abstract citation ID: igae098.2577

### THE RELATIONSHIP BETWEEN LONELINESS AND PUBLIC TRANSPORTATION CARDS FOR OLDER PEOPLE

Murat Mercan[1], Oykum Yigit[1], Hulya Yurekli[1], and Ilker Aslan[2], *1. Yildiz Technical University, Istanbul, Istanbul, Turkey*, *2. Istanbul Metropolitan Municipality, Istanbul, Istanbul, Turkey*

Active aging is critical to combating social isolation and loneliness. Scientific studies have found that even small increases in activity can reduce the risk of disease in the older people. One method that can be used to achieve these goals is providing discounted and free transportation cards for seniors. However, the number of studies measuring the effect of these cards on isolation and loneliness is very limited. This article aims to fill that gap. The paper consists of two parts. The first part is based on big data analysis. It investigates the daily transportation data provided by the Istanbul Metropolitan Municipality to examine how discounted (for individuals aged 60-65) and free (for those aged 65 and over) transportation cards affect the frequency of public transportation usage among individuals aged 50 and over between 2019 and 2022. The analysis, utilizing more than 400 million observations, holds tremendous original value. In the second part of the project, a representative survey is conducted among individuals aged 50 and over in Istanbul. This survey aims to measure the relationship between discounted and free transportation cards and social isolation and loneliness. Regression discontinuity, exploratory and confirmatory factor analyses, and logistic regression analysis methods will be employed for the analyses. The findings indicate an inverse relationship between loneliness and public transportation usage among the older people.

### SESSION 7200 (POSTER)

### ALZHEIMER'S DISEASE AND RELATED DEMENTIAS II (POSTERS)

Abstract citation ID: igae098.2578

### A WALK-THROUGH DEMENTIA: UNDERSTANDING ALZHEIMER'S DISEASE OR RELATED DEMENTIA THROUGH YOUTUBE VR VIDEOS

Xiaoli Li[1], Xiaoyu Liu[2], Eman Alanazi[3], and Arianna Goss[2], *1. Southern Illinois University Carbondale, Cabondale, Illinois, United States*, *2. Saint Louis University, St. Louis, Missouri, United States*, *3. Saudi Electronic University, Riyadh, Ar Riyad, Saudi Arabia*

Emerging research has highlighted the potential of Virtual Reality (VR) as a tool for raising awareness of Alzheimer's disease or related dementia (ADRD) among healthcare students and professionals. However, there is limited VR research focused on engaging the general public. This research aimed to examine the issues faced by YouTube users who interacted with the VR application "A Walk-Through Dementia" by analyzing their posts. We collected 12,754 comments from VR videos 'A Walk-Through Dementia', which simulate the everyday challenges faced by individuals with ADRD, providing viewers with an immersive experience on the condition. We conducted topic modeling to gauge viewer opinions and reactions to the videos. A pre-trained BERT model was utilized to transform YouTube comments into high-dimensional vector embeddings, enabling systematic identification and detailed analysis of principal topics and their intrinsic thematic structures within the dataset. Our analysis identified five distinct topics. The predominant topic, reflected in 2,917 comments, focused on users' personal experiences regarding the impact of ADRD on patients and caregivers. The other topics were organized into four main categories: support and empathy for individuals affected by ADRD, the absence of caregiver, unfriendly environment, and positive reactions to the VR experience. With the technique of topic modeling, the present study indicated that the VR application is an engaging and experiential learning tool that can provide public with deeper awareness of living with ADRD. Future research need explore more VR applications on social media as it has the potential to reach broader audiences that disseminate knowledge of ADRD.

Abstract citation ID: igae098.2579

### BARRIERS AND BENEFITS TO TECHNOLOGY ADOPTION BY DEMENTIA CAREGIVERS

Patti Simone, Julia Scott, Sheila Yueter, Emma Cepukenas, McKenzie Himes, Kennedy Anderson, An Mai, and Kiren Grewal, *Santa Clara University, Santa Clara, California, United States*

Technology can support caregivers of people with dementia but to date, they are not adopted at scale. We surveyed a range of caregivers regarding their use of technology they use to assist in caregiving tasks to better understand the relationship between technological tool use and satisfaction with caregiving. An anonymous online survey was distributed to the local community and dementia care networks in California and Washington (N = 69) and a subset of the respondents were invited to engage in a didactic interview (N = 19). Respondents identified the most important unmet need for caregivers was access to resources (53%), such as healthcare navigation, mental health support, and financial planning, all of which could be improved with technological tools. Furthermore, the majority of caregivers (64%) reported significant emotional struggle and conflict associated with their role. Similarly, interviews with professionals and caregivers illustrated concerns about caregiver well-being. Even though three-quarters of survey respondents reported using some technological tools to assist caregiving (e.g., medication and routine tracking applications, mobility assistive devices, sensors and monitors), the study showed that caregivers felt inadequately supported and they relied minimally on technology to address their pain points (e.g., mental health). Instead they noted barriers of access and usability of products as limiting features of adopting additional technology. Effective use of digital technologies can reduce caregiver stress by simplifying care tasks and delivering more convenient access to support. Strategies in product design and user communication that engage dementia care partners would likely accelerate adoption of technologies.

Abstract citation ID: igae098.2580

### BLOOD PRESSURE MONITORING BY CAREGIVERS: EXAMINING THE INTERSECTION OF ALZHEIMER'S DISEASE AND HYPERTENSION

Dalia Regos-Stewart[1], Noel Barragan[1], Renee Fraser[2], and Tony Kuo[1], *1. Los Angeles County Department of Public Health, Los Angeles, California, United States*, 2. *Fraser Communications, Los Angeles, California, United States*

Older adults (OA) experience a high prevalence of comorbid chronic conditions, for which they oftentimes rely on the aid of unpaid caregivers. OA with Alzheimer's disease (Alzheimer's) are particularly reliant on these caregivers to monitor their health. For example, hypertension can be highly prevalent in this group, predisposing them to a variety of heart-brain complications. Yet, in spite of emerging evidence of home blood pressure monitoring's (HBPM's) effectiveness, caregivers do not always perform nor view this duty as essential. To date, little is known about their experiences with monitoring/managing hypertension. To address this gap in practice, an online panel survey of U.S. caregivers was conducted July-August, 2023. Overall, 600 caregivers (18+ years) of adults (45+ years) participated in the survey; 29% (n=175) provided care to a person with Alzheimer's. A majority reported monitoring chronic conditions, administering medications, managing medical appointments, and measuring BP. Among those who measure BP, 90% reported feeling confident in their ability to do so accurately; 70% measure it daily/few times a week. A higher proportion of caregivers who work with OA with Alzheimer's (versus without) reported receiving proper training for measuring BP (50% vs. 45%). The former group reported cultural, language, and transportation barriers to receiving BP monitoring information from health professionals, compared to the latter group (28% vs. 20%). Rich insights about caregivers' experiences with hypertension management were gleaned from this survey. They suggest opportunities where medicine and public health can further assist caregivers, especially caregivers who are helping OA with Alzheimer's.

Abstract citation ID: igae098.2581

### CARE PRIORITIES AND PREFERENCES OF PEOPLE LIVING WITH DEMENTIA DURING EMERGENCY DEPARTMENT VISITS

Clark Benson[1], Kristin Merss[2], Laura Vergenz[1], Kayla Dillon[1], Laura Block[3], and Andrea Gilmore-Bykovskyi[2], *1. University of Wisconsin School of Medicine and Public Health - Madison, WI, Madison, Wisconsin, United States*, 2. *University of Wisconsin Madison, Madison, Wisconsin, United States*, 3. *University of Wisconsin-Madison, Madison, Wisconsin, United States*

Over half of the 6 million persons living with dementia (PLWD) in the United States visit an Emergency Department (ED) annually. Dementia is often under-recognized in the ED, and visits are associated with significant adverse outcomes for PLWD. Yet, little is known about the perspective and priorities of PLWD and their caregivers regarding ED care. In this descriptive qualitative study, we examined the priorities of PLWD and caregivers during ED visits. We recruited patients living with dementia from a large academic ED with options to participate in a semi-structured interview during or after their ED visit and individually or dyadically with a caregiver. All PLWD completed capacity assessments prior to the interview. Capacity assessments were completed for all PLWD participants. We iteratively analyzed interviews using thematic analysis. We conducted interviews with 33 participants (N=10 PLWD, 15 caregivers, 4 dyads). PLWD and caregivers largely evaluated the ED experience positively. Findings highlight aspects of ED care uniformly shared among participants, including being informed at every step. Our findings also describe individually specific values and preferences such as approach to decision-making; as well as conditions that shape priorities across the experience such as degree of uncertainty. Both PLWD and caregivers value feeling informed in the ED and cite the importance of awareness of the impact of cognition on care. Our findings will support the development of patient-centered assessment tools to evaluate PLWD ED care experiences, enabling proximal and sensitive evaluation of within ED care practices for this vulnerable population.

Abstract citation ID: igae098.2582

### CAREGIVERS' PERCEPTIONS OF ADVANCE CARE PLANNING–RELATED DISCUSSIONS FOR PEOPLE LIVING WITH DEMENTIA

Yuchen Zhang[1], Susan Sereika[1], Jennifer Lingler[1], Jennifer Seaman[1], Corinne Pettigrew[2], and Marilyn Albert[2], *1. University of Pittsburgh School of Nursing, Pittsburgh, Pennsylvania, United States*, 2. *Johns Hopkins University School of Medicine, Baltimore, Maryland, United States*

People living with dementia (PwD) experience progressive decline in functional abilities, including the execution of real-time medical decisions. Nevertheless, advance care planning (ACP) has the potential to mitigate healthcare overutilization and prevent ethical dilemmas at the end-of-life. We aimed to characterize caregivers' understandings of ACP-related discussions between PwD and their healthcare clinicians. This secondary analysis leveraged data (n=431) from the cross-sectional, interviewer-administered survey, "Care Planning for Individuals with Dementia", at Alzheimer's Disease Centers nationwide. Statistical analysis utilized descriptive statistics and multinomial logistic regression. Surveyed caregivers had a mean age of 78.3 years, were predominantly white (88.5%), and were highly educated (mean=15.9 years). Most (95.1%) were the care recipient's designated healthcare proxy and 30.9% of caregivers were aware of discussions between PwD and clinicians about medical treatment preferences. Caregivers (23.2%) who did not co-reside with PwD were 2.71 times more likely to report knowing such discussions occurred ($p < 0.001$). Caregivers who had higher dementia knowledge scores were more likely to indicate the need for further discussion between the proxy and clinicians regarding treatment preferences (OR=1.27, p=0.02); however, caregivers who endorsed knowing a lot about severe stage dementia were less inclined (OR=0.31, p=0.003). Lastly, caregivers of those in earlier stages were more likely to acknowledge the necessity for additional discussions about treatment preferences between PwD and clinicians (no impairment & MCI: OR=7.38, p=0.002; mild: OR=5.32, p=0.005). Overall, our results highlight the need for healthcare clinicians to conduct ACP-related discussions proactively and explicitly include likely proxies, especially during prodromal and earlier stages of dementia.

Abstract citation ID: igae098.2583

### COGNITIVE IMPAIRMENT, DEMENTIA, AND FALLS IN RACIALLY/ETHNICALLY DIVERSE OLDER AMERICANS

Ayse Malatyali[1], Ladda Thiamwong[1], Tom Cidav[2], Rui Xie[1], and Lisa Wiese[3], *1. University of Central Florida, Orlando, Florida, United States*, *2. U.S. Department of Veterans Affairs (VA), Philadelphia, Pennsylvania, United States*, *3. Florida Atlantic University, Boca Raton, Florida, United States*

Older adults with cognitive impairment (CI) and dementia are prone to falls as they have at least twice as much risk than their cognitively normal peers. The relationship between falls and cognitive decline is bidirectional. However, evidence regarding the effects of falls on CI and dementia is limited. This study describes the association of falls, repeated falls, and related risk factors with CI and dementia among older Americans. This cross-sectional study used datasets from the 2020 interviews of the Health and Retirement Study. The sample included 7,663 participants (age>64). Participants who had fallen in the last two years were 15% more likely to have CI and 34% more likely to have dementia. Increased number of falls was significantly associated with CI (OR=1.03) and dementia (OR=1.04). Having depressive symptoms was also associated with CI (OR=1.64) and dementia (OR=1.99). Participants living in rural areas were 17% more likely to have CI and 53% more likely to have dementia than those living in urban areas. Furthermore, living alone increased the odds of having CI (OR: 1.22) and dementia (OR=1.15). We also observed a significant association of ethnicity and race with cognitive stages: Hispanic participants were 1.7 times more likely, and Black participants were 2.4 times more likely to have CI. The likelihood of having dementia was also higher in Hispanics (OR: 2.36) and Blacks (OR= 5.21) compared to non-Hispanic Whites. Falls prevention interventions should be tailored across cognitive stages in older adults with the consideration of ethnic, racial, and environmental diversities.

Abstract citation ID: igae098.2584

### CULTURE AND COGNITION: RISK FACTORS FOR ALZHEIMER’S DISEASE IN US HONOR CULTURES

Erin Harrington, and Jarrod Bock, *University of Wyoming, Laramie, Wyoming, United States*

Alzheimer’s disease is the sixth leading cause of death among all Americans. As the prevalence of Alzheimer’s disease and related dementias (ADRD) continues to grow, researchers and clinicians are ever cognizant of cultural predictors that may contribute to ADRD risk. One cultural approach not yet examined within the context of ADRD is honor cultures (i.e., primarily southern and western states), where reputation management and defense are paramount. Normative behaviors within honor cultures (e.g., risk taking, military involvement, intimate partner violence) may heighten ADRD risk, particularly related to traumatic brain injury (TBI) and subjective cognitive decline (SCD). Further, the lack of general and mental health resources and stigma toward help-seeking in honor cultures likely leave these outcomes untreated. As such, the present study used statewide data collected between 2009-2019 to examine links between statewide honor-orientation and unintentional TBI death rates, frequency of SCD, and AD death rates. Honor-orientation was a significant predictor of unintentional TBI death rates (ß =.44, p <.001), SCD frequency (ß =.58, p =.004), and AD death rates (ß =.41, p =.048), over-and-above state-level covariates (i.e., rurality, temperature, economic conditions, healthcare access). Taken together, individuals in honor cultures appear to be more likely to experience TBI, SCD, and heightened AD mortality, which might confer greater risk for ADRD. Additional research is required in this area to further assess ADRD risk within honor cultures, as individuals living within these cultures may benefit from culturally specific education and prevention efforts to reduce risk.

Abstract citation ID: igae098.2585

### DEMOGRAPHICS’ IMPACT ON CURRENT AND NOVEL NEUROPSYCHOLOGICAL MEASURES FOR ALZHEIMER’S DISEASE SCREENING

Alexandra Reed[1], Kevin Duff[2], and Sydney Schaefer[3], *1. Arizona State University, Phoenix, Arizona, United States*, *2. Oregon Health & Science University, Portland, Oregon, United States*, *3. Arizona State University, Tempe, Arizona, United States*

Neuropsychological assessments are often affected by demographic factors, such as age, education, and sex. These demographic variables frequently lead to differences in test scores between minorities and non-minorities, lowering diagnostic accuracy of Alzheimer’s disease (AD) among minoritized groups. A new test (quick Behavioral Exam to Advance Neuropsychological Screening, or qBEANS) has been developed to assess early signs of AD. This study compared the influence of demographic variables on qBEANS and other neuropsychological tests that are commonly used in the assessment of AD. In this study, 165 participants (68 cognitively unimpaired; 52 amnestic Mild Cognitive Impairment; 45 mild AD) completed the Symbol Digit Modalities Test (SDMT), Hopkins Verbal Learning Test-Revised (HVLTR); Brief Visuospatial Memory Test-Revised (BVMTR); and Trails Making Test (TMT) A and B. Participants also completed the qBEANS, which involves spooning two raw kidney beans at once with the nondominant hand to one of three target cups in a simple sequence, with higher scores being worse performance. Results indicated that significant age effects were observed for all tests except qBEANS and TMT-A, with older ages being associated with worse scores. Similarly, there were significant education effects for all tests except qBEANS and TMTs A and B. Only the HVLTR (total and delayed recall) showed a significant sex effect, with females performing better than males. These data suggest that qBEANS may be appropriate for screening diverse populations without controlling for demographic variables.

Abstract citation ID: igae098.2586

### EFFECTS OF MENOPAUSE MODELING ON AUTOPHAGIC AND LYSOSOMAL FUNCTION IN ALZHEIMER'S DISEASE MICE

Danielle Osborne, and Adrianah Dorn, *Legacy Research Institute, Portland, Oregon, United States*

Alzheimer's disease (AD) is denoted by strong sex differences, whereby females are significantly more likely to develop AD and often experience more rapid progression and cognitive decline. This difference in risk is often attributed to menopause, however the underlying molecular mechanisms are unknown. In humans and animal models, autophagic and lysosomal dysfunction are among the earliest known cellular changes that occur in AD susceptible brain regions, like the hippocampus. Our goal was to first characterize how key autophagic and lysosomal gene transcripts changed due to sex, ovariectomy (OVX) and estrogen replacement in wildtype mice, then investigate in female AD mice how these cellular processes change due to OVX and estrogen replacement. In wildtype mice, across 22 genes investigated, three transcripts, Atg12, Atg5, and Map1LC3b showed a baseline sex difference. Using the 5xFAD model of AD, we observed the expected genotype difference in cognition, with transgenics having a significant impairment in Morris watermaze performance. Probe measures indicate decreased performance due to OVX, but that 5xFAD mice may be further hindered by the addition of estrogen. Molecular measures will include western blot analysis of phosphorylated proteins involved in autophagic/lysosomal signaling (pAkt, pAMPK, pRPS6), and qPCR (Atg5, Atg12, Map1LC3b, Tfe3, Tfeb, among others) in the hippocampus, and immunohistochemistry (GFAP, Iba1, amyloid-beta, reelin) in the hippocampus and entorhinal cortex. These studies will provide valuable information on how cessation of female hormones at menopause affect the vital cellular functions of autophagy and lysosomal degradation in key cognitive and disease susceptible brain regions in AD.

Abstract citation ID: igae098.2587

### ENGAGING OLDER ADULTS AND CAREGIVERS IN THE DEVELOPMENT OF A COUNTYWIDE DEMENTIA STRATEGIC PLAN

Mariana Reyes[1], Noel Barragan[1], Lisa Craypo[2], Liz Schwarte[3], Anne-Celine Jeffroy-Meynard[4], and Tony Kuo[1], *1. Los Angeles County Department of Public Health, Los Angeles, California, United States*, *2. Ad Lucem Consulting, Kensington, California, United States*, *3. Ad Lucem Consulting, San Francisco, California, United States*, *4. Ad Lucem Consulting, Ad Lucem Consulting, California, United States*

In 2021, the Los Angeles County Department of Public Health launched the CDC-funded Los Angeles County BOLD Initiative (LA BOLD), the primary outcome of which is a strategic plan focused on improving dementia infrastructure and supports in Los Angeles County. To ensure that the plan aligns with concerns and priorities of older adults and caregivers impacted by dementia, LA BOLD partnered with Ad Lucem Consulting to conduct a series of 10 listening sessions with 92 participants to share their perspectives, experiences, and concerns related to dementia awareness and care between September and October 2022. To ensure inclusion of a diverse cross-section of community voices, sessions were held either virtually or in-person and conducted in English, Spanish, Korean, and English/Samoan. Sessions were held in strategic partnerships with multiple community organizations spanning the county's Supervisorial Districts. Session topics were organized around the three strategic plan focus areas: Hypertension Prevention & Management, Early Detection, and Advance Care Planning. Overall, participants were appreciative of the opportunity to discuss dementia and related health issues. Key findings point to disparities in knowledge and accessible services (e.g., culturally appropriate messaging, inconsistent detection/referral, stigma, insufficient caregiver supports). A number of actionable recommendations emerging from the findings can be addressed in the strategic plan, offering valuable insights for policymakers and public health professionals on how to address this condition in the county and other communities. This presentation will highlight key findings and provide suggested solutions to facilitating equitable services/resources for dementia prevention and management.

Abstract citation ID: igae098.2588

### EVALUATION OF A LATINO BRAIN HEALTH FAIR FOR ALZHEIMER'S DISEASE EDUCATION AND RESEARCH RECRUITMENT

Theresa Kehne[1], Janet Rojina[1], Linda Ko[1], Sonia Bishop[1], Kelley Pascoe[1], Kimiko Domoto-Reilly[1], Israel Anaya-Carmona[1], and Karen Torres[2], *1. University of Washington, Seattle, Washington, United States*, *2. University of Washington Harborview Medical Center, Seattle, Washington, United States*

Hispanic/Latino individuals are at higher risk of developing Alzheimer's disease (AD) than non-Hispanic White individuals, yet risk factors in the Hispanic/Latino community are not well understood due to barriers impacting research participation. This study, in partnership with a community advisory board, developed and evaluated a novel "Brain Health Fair" (BHF) intervention for increasing AD knowledge and willingness to participate in AD research among Hispanic/Latino individuals. The BHF involved an educational presentation and interactive stations run by language-concordant health professionals that provided information about AD, health promotion and the importance of research participation to improve health equity in a culturally grounded context. Participant knowledge, attitudes, and willingness to participate in AD research were assessed in an anonymous assessment administered pre and post intervention. Participants were also given the option to enroll in the local University of Washington Alzheimer's Disease Research Center (ADRC). Participants (n=26) demonstrated a significant change in knowledge of AD in the Latino community pre and post intervention (M pre; post=1.4;3.6, SD pre; post=1.5;1.5, Z=-3.5, p=0.001), preventative behaviors for AD (M=1;3.3, SD=1.4;1.7, Z=-3.6, p=0), what ADRC participation entails (M=0.9;3.5, SD=1.6;1.7, Z=-3.8, p=0) and benefits of research participation (M=1.2;3.9, SD=1.8;1.6, Z=-3.5, p=0.001) (two-sided Wilcoxon signed-rank test, p=0.001). After the intervention, 85% reported being satisfied and found it culturally appropriate and 81% signed consent forms to enroll in the ADRC. The BHF showed great promise for improving knowledge of AD and readiness of Hispanic/Latino individuals to participate in research and was a successful method for recruiting participants into AD research.

Abstract citation ID: igae098.2589

### FUNCTIONAL OUTCOMES IN HOME HEALTH STAY FOR PEOPLE WITH DEMENTIA: INTERSECTIONALITY OF GENDER AND RACE

Rashmita Basu, *East Carolina University, Greenville, North Carolina, United States*

This study explores the difference in functional outcomes of Medicare beneficiaries during a home health (HH) admission across the intersectionality of race and gender because these outcomes may be by virtue of their gender and racial/ethnic composition. This cohort study used data from the 2019 Outcome and Assessment Information Set (OASIS) and Medicare claims (Home Health (HH) and inpatient), enrollment, and Chronic Condition Warehouse (CCW) files from the CMS. Functional outcome was measured as a change in the composite ADL score (grooming, bathing, toileting, dressing, walking, transferring, ambulation, and hygiene) from HH admission to HH discharge. Binary logistic regressions with an outcome variable (1/0: if the composite score was ≤0 (improvement/static) at discharge (1), and 0 if > 0 (decline) during HH admission) were performed. The ADRD cohort was identified based on CCW criteria and ICD-10 codes from inpatient and HH claims. The independent predictor was a six-category intersectionality variable that included White female (reference), White male, Black female, Black female, other female, and other male. The analysis included 81,735 Medicare beneficiaries with ADRD, contributing to 174,093 home health stays. Adjusting for patients' demographics (age, sex, race/ethnicity), number of diagnoses, caregiving support, and MA enrollment, compared to White females, all the other groups were 3% (95% CI:1.01-1.06) to 18% (95% CI: 1.08-1.26) more likely to have improvement or maintenance of ADL composite score from HH admission to discharge. The combination of gender and race/ethnicity composition is critical in examining the functional outcomes of Medicare beneficiaries using postacute care.

Abstract citation ID: igae098.2590

### MAPPING ASSISTIVE TECHNOLOGIES ON THE PROGRESSION OF ALZHEIMER'S DISEASE: A SCOPING REVIEW

Nadia Mirjan, and Aleksandra Zecevic, *University of Western Ontario, London, Ontario, Canada*

A growing population of people living with Alzheimer's Disease urges improved support for aging in place and with dignity. Assistive technologies (ATs) can be used to delay institutionalization, reduce caregiver burden, and improve quality of life for this population and their care partners. The abilities and needs of this population change during disease progression, requiring a better understanding of which ATs could be used at each stage of the disease. The purpose of this scoping review is to generate knowledge on how ATs can be mapped on seven stages of Alzheimer's Disease progression described by the Global Deterioration Scale. The review follows the Arksey and O'Malley framework to identify and harvest information from Medline OVID, Scopus, CINAHL, and Embase OVID databases. Inclusion criteria were Alzheimer's Disease, technology interventions of any type and duration, English language, and the time frame between 2000-2023. Data was extracted and thematically analyzed using six predetermined domains of ATs for dementia, namely safety devices, clinical devices, memory aids, ATs for preventing social isolation, ATs for leisure activities, and ATs for supporting everyday tasks. A total of 53 articles were included. Findings show that a variety of ATs are available throughout the disease progression. High technology (e.g., tracking devices) mainly target early stages, while low technology (e.g., weighted blanket) target later stages. Some, such as music therapy, are present at every stage of disease. The map has the potential to inform people with dementia, care partners, technology companies, distributors, policy makers and service providers.

Abstract citation ID: igae098.2591

### MEASURING LIGHTING USING AN INDIVIDUAL WEARABLE LIGHT SENSOR IN NURSING HOME RESIDENTS WITH DEMENTIA

Ying-Ling Jao, Julian Wang, Shevvaa Beiglary, and Yo-Jen Liao, *The Pennsylvania State University, University Park, Pennsylvania, United States*

More research has examined the light effect in dementia. Lighting exposure varies widely across times and people. Individual light sensors allow more precise measurements. Yet, their feasibility has not been established. This study examined the feasibility of using individual light sensors (LYS Button) in a clinical trial for nursing home residents with dementia. This study enrolled 27 residents with dementia. The LYS Button is a small, lightweight sensor clipped on residents' collar/shoulder area. The sensor measured lighting every 15 seconds all day, 7 days a week, every other week for 13 weeks. Several strategies were implemented. First, instructions on sensor use were provided, and a reminder sign was posted in the residents' bedrooms. Second, a computer station on site enabled data monitoring. If a sensor disconnected from the server, the team was alerted, and on-site staff were contacted to check/replace sensors. Third, the research team checked the sensor on-site twice weekly. To examine the feasibility, the number of sensors was tallied, sensor data were analyzed, and individual interviews were conducted with 22 healthcare workers. Results showed that 52 sensors were lost or broken. The average missing data per resident per day were 1.13 (SD=0.99) and 6.84 hours (SD=2.28) during daytime and nighttime, respectively. Despite some missing data, the intense data collection allowed sufficient data for analysis. The results support the feasibility of using the wearable sensor to measure daytime lighting. Wearing methods and strategies during nighttime need modification to improve the use of individual lighting sensors in people with dementia.

Abstract citation ID: igae098.2592

### MOTIVATIONS FOR AND EXPERIENCES OF UNDERGOING AN AMYLOID SCAN: A SYSTEMATIC REVIEW OF QUALITATIVE STUDIES

Elyse Couch, *Brown University, Providence, Rhode Island, United States*

Amyloid positron emission tomography (PET) scans are a tool that could improve the accuracy of dementia diagnoses.

The FDA approval of new amyloid targeting monoclonal antibodies makes it increasingly likely that amyloid scans will be implemented into routine clinical care. This systematic review aimed to identify and summarize qualitative studies exploring patient and care partner experiences of undergoing an amyloid scan. Seven papers were included in this review. We identified four themes related to patient and care partner experiences of receiving an amyloid scan. 1) motivations for undergoing an amyloid scan, 2) experiences of receiving the result, 3) emotional responses to the result, and 4) actions in light of the scan result. Participants reported undergoing the scan to learn more about the patient's condition. They described the information gained from the scan as helpful but, in some cases, struggled to recall the terminology used by the clinicians or the meaning of the scan result. Emotional responses to the scan varied depending on the scan result and those with "elevated" scan results were more likely to express feelings of distress. The most common action following the scan result was making advanced plans. Aside from determining eligibility for prescribing monoclonal antibodies, amyloid scans provide patients and their care partners with useful and actionable information. However, learning the scan result could be distressing. Future research is needed to understand how to balance the potential benefits and harms of amyloid scans in light of novel dementia treatments.

Abstract citation ID: igae098.2593

### PREVALENCE AND CHARACTERISTICS OF HEALTH CARE–RELATED TASKS BY INFORMAL DEMENTIA CAREGIVERS: A SYSTEMATIC REVIEW

Mohammed Zuber[1], Samruddhi Nandkumar Borate[1], Chia Jie Tan[2], and Niying Li[1], *1. University of Georgia, Athens, Georgia, United States*, *2. University of Utah, Salt Lake City, Utah, United States*

Healthcare-related tasks (e.g. making appointments, talking to providers) performed by informal caregivers caring for older adults with dementia have become increasingly common. This is a systematic review of the prevalence and characteristics of healthcare related tasks performed by informal caregivers. Articles were searched in PubMed, Embase, PsychInfo and CINAHL until September 2023. Studies that were based in the United States and reported the prevalence of healthcare related tasks performed by informal dementia caregivers were included. Tasks identified were grouped into health and medical care, advocacy and care coordination, and surrogate tasks. Caregiver characteristics were reported. Of 6,284 articles screened, we included 12 studies. All the studies were cross-sectional and the majority of them were nationally representative. Majority of caregivers were female and spousal caregivers. Only four studies included racial and ethnic minority caregivers. Medication management, including keeping track of medications, was the most common health and medical care task reported in eight studies ranging from 41% to 86%, followed by five studies reporting 7% to 47% caregivers being involved in wound and skin care. Two studies reported caregivers performing advocacy and care coordination tasks (27 to 73%) such as making appointments, managing insurance, and one study reported surrogate tasks such as participating in medical/treatment decisions (41%). Informal dementia caregivers were involved in a wide range of complex healthcare related tasks. The healthcare system and healthcare professionals need to provide training and support to informal dementia caregivers in performing these tasks.

Abstract citation ID: igae098.2594

### PREVENTABLE HOSPITALIZATIONS IN ADULTS WITH ALZHEIMER'S DISEASE AND RELATED DEMENTIAS, 2016–2019

Jenny Walker[1], Roshni Patel[2], Benjamin Olivari[1], Christopher Taylor[1], Matthew Baumgart[3], and Lisa McGuire[4], *1. Centers for Disease Control and Prevention, Atlanta, Georgia, United States*, *2. Cyberdata Technologies (CDC Contractor), Atlanta, Georgia, United States*, *3. Alzheimer's Association, Chicago, Illinois, United States*, *4. GSA, Atlanta, Georgia, United States*

Reducing preventable hospitalizations in adults with Alzheimer's disease and related dementias (ADRD) is a public health and National Healthy People 2023 priority because the associated negative health and financial outcomes may be avoidable through proactive primary and outpatient care recognizing and managing co-morbidities. This study used data from the 2016–2019 Healthcare Cost Utilization Project National Inpatient Sample, an all-payer inpatient care database. Using ICD-10-CM codes, we identified 8 conditions that are potentially preventable, including acute and chronic ambulatory care sensitive conditions and injuries. Of the hospital discharges among adults aged ≥45 years with ≥1 of 11 conditions (n=4,611,423), 11.2% (95%CI=11.1-11.3) also listed an accompanying ADRD diagnosis for the patient. Among adults with an ADRD diagnosis, the proportion of potentially preventable hospitalizations increased linearly with age, with 82.4% (95%CI=82.2-82.5) of potentially preventable hospitalizations occurring among those aged ≥75 years. In contrast, potentially preventable hospitalizations among those without ADRD skewed younger, with only 34.2% (95%CI=34.0-34.4) occurring among those aged ≥75 years. Of the 8 potentially preventable conditions, most common among those with ADRD were sepsis (34.1%), injuries (21.1%), urinary tract infections (UTIs; 14.0%), and heart failure (12.9%). Adults with ADRD were more likely to be hospitalized due to UTIs than those without ADRD (adjusted prevalence ratio=2.34 (95%CI=2.32-2.37)), even after adjusting for sex, age, and long-term care facility residency. Future public health measures, policy, and programs with targeted ADRD hospitalization prevention goals may potentially mitigate the issue, with special attention to increased risk of hospitalization due to older age and UTIs.

Abstract citation ID: igae098.2595

### RELATIONSHIP BETWEEN PHYSIOLOGICAL MEASURES AND COGNITIVE FUNCTION IN OLDER ADULTS WITH DEMENTIA

Derong Zeng, *Kyoto University, Kyoto City, Kyoto, Japan*

This study explores the interrelationships among physiological indicators in dementia patients, focusing on the correlation between Mini-Mental State Examination (MMSE) scores and indicators like blood pressure, heart rate, and body temperature, aiming to contribute new insights for early dementia diagnosis and treatment. Research involved 337 dementia patients, employing descriptive statistical analysis, correlation analysis, multivariate linear regression, and principal component analysis, with ethical approval from Kyoto University Hospital Ethics Committee (R3258). Findings reveal an average MMSE score of 21.69 (SD=5.33), highlighting a significant positive correlation between diastolic blood pressure (DBP) and MMSE scores (r=0.139, p=0.0030), while associations with systolic blood pressure, pulse rate, and body temperature were not significant. The first principal component accounted for 40.51% of variance, indicating that DBP may significantly impact cognitive function in dementia patients, suggesting blood pressure management's potential in cognitive function maintenance. However, weak associations with other physiological indicators underscore dementia's complexity, influenced by multiple factors.

Abstract citation ID: igae098.2596

### THE ROLE OF TREATMENT CHOICE IN CONFOUNDING ASSOCIATIONS BETWEEN HASHIMOTO AND ALZHEIMER'S DISEASE

Galina Gorbunova, Arseniy Yashkin, Stanislav Kolpakov Nikitin, Svetlana Ukraintseva, Anatoliy Yashin, and Igor Akushevich, *Duke University, Durham, North Carolina, United States*

Alzheimer's disease (AD) is a neurodegenerative disorder, most prevalent at the advanced ages. The increased risk of cognitive decline and the onset of AD and related dementia have recently been associated with hyperthyroidism while the results for hypothyroidism-AD association have been inconclusive. We hypothesized that hypothyroidism treatment modalities may cause variability in the available evidence of a hypothyroidism-AD association. We used Medicare administrative claims data from 2006-2022 to test this hypothesis by assessing how the association between Hashimoto's disease, which is the most common form of hypothyroidism, and a clinical diagnosis of AD dementia changes as information about treatment modality is entered into the model. As was expected, non-treatment-enabled models were unable to find a statistically significant association between Hashimoto's disease and AD. These findings were consistent across sex/gender and race/ethnicity-specific groups. The introduction of treatment modalities into the model, such as T-4 hormone replacement therapy, Liothyronine (and its generic equivalents), Levothyroxine (and its generic equivalents), surgery, and supplementary metformin use, modified the observed non-treatment-aware relationships. However, due to stratification, the prediction power of our treatment-aware models accounting for treatment modality was substantially weaker.

Abstract citation ID: igae098.2597

### TRANSITIONAL CARE TRENDS IN BENEFICIARIES WITH HEART FAILURE AND DEMENTIA

Thomas Bayer[1], Hiren Varma[2], Peter Hollmann[3], and Pedro Gozalo[2], *1. Providence VA Medical Center, Providence, Rhode Island, United States*, *2. Brown University, Providence, Rhode Island, United States*, *3. Brown University Medical School, Providence, Rhode Island, United States*

Medicare Transitional Care Management (TCM) has grown since its introduction in 2013, but we know little about utilization among beneficiaries with dementia (BWD) undergoing the complex care transition from hospital to skilled nursing facility to home (hospital-SNF-home). We studied trends in TCM among BWD discharged to various care settings after heart failure hospitalization, given heart failure's high readmission risk and amenability to care management. We selected a sample of BWD heart failure hospitalizations in years 2013-2018 using the Chronic Conditions Warehouse algorithm for Alzheimer's Disease and Related Dementias and principal diagnostic codes of hospital claims. Next, we excluded all except hospital-home and hospital-SNF-home discharges. We then fit a linear regression with TCM as the dependent variable and discharge year, discharge type, interaction of year and discharge type, race, age, dual status, and sex as predictors. TCM after hospital-home discharges grew from 521 (5.3%) of the 9,930 eligible discharges in 2013 to 2,015 (15.9%) of the 12,706 eligible discharges in 2018. In contrast, TCM after hospital-SNF-home discharges grew from 80 (4.0%) of the 1,990 eligible discharges in 2013 to 207 (9.9%) of the 2,095 eligible discharges in 2018. The P-value for the interaction between year and discharge type was <.001. The proportion of BWD utilizing TCM after heart failure hospitalization grew less among hospital-SNF-home discharges than hospital-home discharges. Further study is needed to understand why TCM utilization in hospital-SNF-home discharges has lagged behind hospital-home discharges and to estimate TCM's effect on the success of discharge from SNF to home.

### ASSESSMENT (E.G., GERIATRIC ASSESSMENT, FUNCTIONAL ASSESSMENT, FUNCTIONAL STATUS INSTRUMENTS)

Abstract citation ID: igae098.2598

### ACTIVITY INTENSITY AND HEALTH DECLINES IN OLDER ADULTS WITH PROSTATE CANCER VIA THE OPEN-SOURCE GERI PLATFORM

Nabiel Mir[1], Yan Che[2], Russell Szmulewitz[2], and Megan Huisingh-Scheetz[2], *1. University of Chicago Hospitals, Chicago, Illinois, United States*, *2. University of Chicago, Chicago, Illinois, United States*

Wearable sensors may facilitate remote monitoring of patient vulnerability during cancer treatment but the associations between the technology measures and clinically meaningful measures have not been established. The Geriatric Remote Initiative (GeRI) is a remote data collection platform that employs a Bangle.js 2 wrist accelerometer alongside a WiFi-enabled Raspberry Pi tablet to collect and transmit free-living physical activity in older adults with cancer. The objective of this analysis was to relate mean activity levels collected in a pilot sample of 10 advanced prostate cancer patients aged ≥65 years over a period of ≥12 weeks to baseline self-rated health and 12-week adverse events. All participants were currently or previously exposed to androgen deprivation therapy. Participants wore the accelerometer weekly for 48-hours; activity indices were recorded and averaged every 15 minutes. Records with at least 12 hours of activity, two or more measurement periods, and ≥50%

wear compliance were included. Mean day time (7am-7pm) activity indices were calculated across wear periods. Data from two devices were omitted due to insufficient activity data capture, resulting in 8 evaluable patients. The participant with the lowest mean day time activity (mean activity index=45,803.2) had the lowest baseline self-rated health and was the only participant to develop new high-grade vascular comorbidities at 12 weeks. The mean activity index for the rest of the sample ranged from 80,454.0 to 154,898.3 (mode of five instances of wear, range 3-6 wear instances). Remote monitoring using platforms like GeRI may offer novel methods for detecting vulnerability throughout cancer treatment.

Abstract citation ID: igae098.2599

### AD-RISK FACTORS AND TIMED UP AND GO TASKS IN IMPAIRED AND UNIMPAIRED OLDER ADULTS

Ashley Price[1], Megan Stradtman[2], Jessica Alber[1], and Maeve Durkin[1], *1. University of Rhode Island, Kingston, Rhode Island, United States*, *2. Butler Hospital, Providence, Rhode Island, United States*

The Timed Up and Go (TUG) is a clinical assessment tool evaluating mobility and dual-task function. This study examined TUG performance in a sample of cognitively unimpaired (CU) older adults at low risk (LR)/high risk (HR) for Alzheimer's disease (AD) and a group of mild cognitive impairment (MCI) patients. 194 older adults aged 55-80 (82 M, 112 F) comprised 3 groups: 75 CU LR, 76 CU HR, and 32 MCI. Having at least one copy of the APOE E4 allele designated CU participants as high risk for AD. The TUG is a 20-foot walking task, and TUG-DT adds serial subtractions to the original task to determine dual-task cost (DTC). Both TUG and TUG-DT tasks were completed (2 runs each) during clinical assessment. DTC was calculated for both number of steps and time to completion to determine the change in performance from single to dual task. Univariate ANOVA assessed the group differences in key TUG outcome variables (number of steps, time to completion). Though the completion time for TUG-run1 was as expected (LR< HR< MCI), practice effects were suspected in TUG-run2 as group completion times were not significantly different. Additionally, significant differences were identified in TUG-DT time between LR and MCI (p=< 0.001) and HR and MCI (p=0.003). There were significant differences in TUG/TUG-DT steps between the LR and HR groups and LR and MCI. This suggests improved gait efficiency in the LR CU group, compared to both the HR and the patient group. DTC for time or steps showed no significant group differences.

Abstract citation ID: igae098.2600

### AGING TRAJECTORY OF THE JAPAN SCIENCE AND TECHNOLOGY AGENCY INDEX OF COMPETENCE (JST-IC): THE OTASSHA STUDY

Hisashi Kawai[1], Keigo Imamura[1], Manami Ejiri[1], Hiroyuki Sasai[1], Yoshinori Fujiwara[1], Kazushige Ihara[2], Hirohiko Hirano[3], and Shuichi Obuchi[1], *1. Tokyo Metropolitan Institute for Geriatrics and Gerontology, Tokyo, Japan*, *2. Hirosaki University, Hirosaki, Aomori, Japan*, *3. Tokyo Metropolitan Institute for Geriatrics and Gerontology, Tokyo Metropolitan Institute for Geriatrics and Gerontology, Tokyo, Japan*

The Japan Science and Technology Agency Index of Competence (JST-IC) was developed to measure higher levels of competence than measured by the Tokyo Metropolitan Institute of Gerontology Index of Competence (TMIG-IC), which evaluates higher-level functional capacity necessary for independent daily life. This study compared the aging trajectory between the JST-IC and TMIG-IC among community-dwelling older Japanese individuals. The participants were 775 adults who participated in the health check-up of the Otassha study three or more times between 2014 and 2019. Three patterns of aging trajectories of the JST-IC and TMIG-IC were identified using group-based trajectory modeling. Each subscale trajectory of the JST-IC included technology usage, information practices, life management, and social engagement. Scores for the high (30.4%), medium (15.5%), and low (15.2%) JST-IC trajectories decreased by 1-2 points according to age. High stability (49.9%), late-onset decrease (41.2%), and early onset decrease (8.9%) TMIG-IC trajectories were identified, and the late-onset and early onset decrease groups decreased by 1-3 points with age. Scores for technology usage, life management, and social engagement declined with age, similar to the total score of the JST-IC; however, scores for information practice tended to remain at the same level as at 65 years of age. The participants in this study participated in a longitudinal survey, and higher-level functional capacity was almost maintained; however, we found that the JST-IC score gradually declined with age in all patterns. It has been suggested that this decline is associated with a decline in technology usage, life management, and social engagement.

Abstract citation ID: igae098.2601

### DEPRESSION ANXIETY STRESS SCALE IN OLDER ADULTS: ITEM RESPONSE THEORY AND CONFIRMATORY FACTOR MODELS

Wen Liu[1], M. Bridget Zimmerman[1], Katherine Hadlandsmyth[1], Tracey Smith[2], Joseph A Buckwalter[1], David M. Green[2], Lilian Dindo[2], and Barbara Rakel[1], *1. University of Iowa, Iowa City, Iowa, United States*, *2. Baylor College of Medicine, Houston, Texas, United States*

The Depression Anxiety & Stress Scale (DASS) is a commonly used tool to assess patient-reported symptoms of depression, anxiety, and stress, and has been translated into multiple languages. This study examined the psychometric properties of DASS in older U.S. Veterans who experience knee pain prior to Total Knee Arthroplasty. A total of 660 Veterans (age 66.7 □ 9.1 years; 88% male; 72.2% white, 61% married) were consented and screened for enrollment in an on-going, double-blind, two-arm, randomized controlled trial, evaluating the efficacy of an Acceptance and Commitment Therapy intervention. All Veterans completed DASS during post-consent screening. Confirmatory Factor Analysis was conducted to examine the fit of the data to a 3-factor structure (Depression, Anxiety, Stress). After unidimensionality was confirmed, Item Response Theory (IRT) analysis was conducted to examine item difficulty and discrimination within each factor. The 3-factor structure showed desirable model fit such that there were strong correlations among three factors (r ranges =.837-.883, p<.001). The three factors showed acceptable to desirable internal consistency (Cronbach alpha=0.904 for Depression, 0.767 for Anxiety, 0.883 for Stress). DASS showed acceptable model fit as a 3-factor model to IRT. Items in each factor showed acceptable discriminations and spread evenly on difficulty levels. Item and test information curves were unimodal and nearly symmetric. DASS has been adopted worldwide as a self-report assessment of distress. Findings support the use of the 3-factor structure in older Veterans. Further research is needed to investigate performance of DASS in Veterans with more diversity in gender and socioeconomic status.

Abstract citation ID: igae098.2602

### DEVELOPMENT AND VALIDATION OF A SOCIAL FRAILTY INDEX AMONG OLDER ADULTS IN THE PHILIPPINES

Edina Tan[1], Hanzhang Xu[2], Rahul Malhotra[1], Mark Ryan Paguirigan[3], Grace Cruz[3], Yasuhiko Saito[4], and Truls Østbye[2], *1. Duke-NUS Medical School, Singapore, Singapore*, *2. Duke University, Durham, North Carolina, United States*, *3. University of the Philippines Diliman, Quezon City, Quezon, Philippines*, *4. Nihon University, Tokyo, Japan*

The potential of social frailty, known to be associated with adverse health outcomes among older adults, for risk assessment can be maximized if the development of a scale to measure it considers the sociocultural context in which it will be applied. No social frailty scales have been developed for use in the Philippines, a Southeast Asian country with distinctive sociocultural characteristics such as an emphasis on religious life, family caregiving, and a generally positive view of aging. We developed and validated a Social Frailty Index-Philippines (SFI-Phil), using 4-year mortality as an outcome, for use among older adults in the Philippines. We also evaluated its performance across age, gender, and urban-rural residence. Data on 5153 older adults, aged 60 years and older, from the nationally representative Longitudinal Study of Ageing and Health in the Philippines (LSAHP) was analyzed. Applying LASSO regression on 33 candidate variables selected using a conceptual framework for social frailty, the SFI-Phil included six variables: (1) education, (2) religious activity participation, (3) caring for grandchildren, (4) employment, (5) hanging out with friends and neighbors, and (6) feeling left out. Evaluation of SFI-Phil in a validation sample demonstrated satisfactory accuracy in predicting mortality (C-statistic 0.640, 95% CI: 0.608-0.673). It also performed adequately across demographic strata, although better amongst 60–69 year olds (C-statistic 0.642, 95% CI: 0.606-0.673), males (0.670, 0.650-0.694), and urban residents (0.660, 0.637-0.684). SFI-Phil, developed considering the country's sociocultural context, can be used to assess social frailty among older adults in the Philippines.

Abstract citation ID: igae098.2603

### DEVELOPMENT AND VALIDATION OF THE CLINICAL PHYSICAL RESILIENCE ASSESSMENT SCALE (CHEES) IN OLDER ADULTS

Lina Ma, *Xuanwu Hospital, Capital Medical University, Beijing, Beijing, China (People's Republic)*

Objective: Physical resilience is an emerging concept that describes an individual's capacity to recover from stressors. However, few instruments are currently available for assessing physical resilience. We aimed to develop and validate an easy-to-use physical resilience scale, Clinical pHysical rEsilience assEssment Scale (CHEES), in Chinese older adults.

**Methods:** Delphi method was used to establish an initial scale. Psychometric evaluation of the scale was then tested on 172 older adults. The reliability and validity of CHEES was tested in 1982 community-dwelling and 362 hospitalized older adults. Cronbach's α coefficient, Spearman-Brown coefficient and Guttman coefficient were used for reliability test. Validity was assessed by content validity and construct validity based on exploratory factor analysis.

**Results:** After two rounds of expert consultations, three primary and 16 secondary items were determined. Two items were removed after the psychometric evaluation. The final CHEES containing three primary ("intrinsic capacity", "adapt to change", and "external support") and 14 secondary items showed promising properties both in community-dwelling and hospitalized older adults. The acceptance rate of the CHEES was 90.88%, and the qualified rate of the scale was 100%. The Cronbach's α coefficient of the total scale was 0.826, Spearman-Brown coefficient was 0.737, and Guttman coefficient was 0.731. Exploratory factor analysis extracted three common factors. The factor load of each item was 0.446-0.656, and the cumulative variance contribution rate was 57.49%. Conclusions: CHEES has good reliability and validity, and is suitable for the evaluation of physical resilience of the older adults in both hospital and community.

Abstract citation ID: igae098.2604

### DISCORD BETWEEN SELF-REPORT AND PERFORMANCE-BASED ADL MEASURES AMONG PREFRAIL OR FRAIL OLDER ADULTS

Chiung-ju (CJ) Liu[1], Wen-Pin Chang[2], and Inga Wang[3], *1. University of Florida, Gainesville, Florida, United States*, *2. The University of Texas Rio Grande Valley, Edinburg, Texas, United States*, *3. University of Wisconsin Milwaukee, Milwaukee, Wisconsin, United States*

Prefrail and frail older adults face an increased risk of late-life disability. Detecting the decline in activities of daily living is essential to mitigate or reverse the progression of disability in this population. However, self-report and performance-based measures of activities of daily living do not always coincide because the magnitude of decline could be too subtle to detect or be aware of. This study examined the correlation between the two evaluation approaches and compared the characteristics of participants who overestimated with those who underestimated their performance. The baseline data of 71 prefrail or frail, community-living older adults (Mean age = 74 years) from previous clinical trials were analyzed. There was no statistically significant correlation between the self-reported measure in activities of daily living (assessed by the Performance Score of the Canadian Occupational Performance Measure) and the performance-based measure (assessed by the Motor Skills Score of the Assessment of Motor and Process Skills), rs =.01, p =.94. Approximately half of the participants showed a discrepancy between the two measures. Those who overestimated (n = 12) tended to be older and had poorer physical functioning than those who underestimated (n = 20), all ps <.05. Additionally, those who overestimated were more satisfied with their performance of activities of daily living than those who underestimated it, Z = 35.55, p <.05. Overestimation and underestimation may reflect different coping mechanisms adopted by prefrail and frail older adults in response to functional decline. Research to further investigate these mechanisms is encouraged.

Abstract citation ID: igae098.2605

### DYNAPENIA-OBESITY AND PHYSICAL FUNCTION AMONG MEXICAN AMERICAN OLDER ADULTS: NATIVITY DIFFERENCES

Anita Brandt[1], and Soham Al Snih[2], *1. The University of Texas Medical Branch, Galveston, Texas, United States, 2. University of Texas Medical Branch in Galveston, Galveston, Texas, United States*

We used data from the Hispanic Established Population for the Epidemiological Study of the Elderly to examine nativity differences in the relationship between dynapenia-obesity and physical function decline over 12-years of follow-up (2004/05-2016) among 767 Mexican Americans aged ≥75 years with a score ≥7 on the Short Physical Performance Battery (SPPB) at baseline. Measures included socio-demographics, medical conditions, body mass index (BMI), depressive symptoms, cognitive function, and disability. Dynapenia was defined as handgrip strength (< 27 Kg in males and < 17 Kg in females). Obesity was defined as BMI ≥30 Kg/m2. Participants were grouped into four groups: No dynapenia – No obesity (n=360), obesity only (n=135), dynapenia only (n=203), and dynapenia-obesity (n=60). Physical function was assessed with the SPPB test. Linear Mixed models were used to estimate the population average change in SPPB as a function of dynapenia-obesity groups by nativity, controlling for all covariates. Participants with dynapenia only experienced a greater (β=-0.75, SE=0.24, p-value=0.0016) decline in physical function compared to those with No dyanpenia – No obesity among US-Born, controlling for all covariates. Participants with dynapenia-obesity group experienced a greater (β=-1.72, SE=0.53, p-value=0.0015) decline in physical function compared to those with No dynapenia – No obesity among foreign-born, controlling for all covariates. In conclusion, the relationship between dynapenia-obesity groups and physical function decline differed by nativity. Dyanpenia-obesity only was associated with decline in physical function among foreign-born. These findings highlight the need to promote healthy weight and improve muscle strength to prevent physical function decline in this population.

Abstract citation ID: igae098.2606

### EVALUATING PSYCHOMETRIC PROPERTIES OF THE SOCIAL FRAIL INDEX IN COMMUNITY-DWELLING OLDER ADULTS

Inthira Roopsawang, Suparb Aree-Ue, and Teepatad Chintapanyakun, *Mahidol University, Bangkok, Krung Thep, Thailand*

Social frailty has become a concern issue in older adults since it has a negative impact on older adults' health with various dimensions including physical and mental health leading to a decline in the ability to self-care, increasing depression, or engaging in social activities. The original version of the Social Frail Index—Japanese— has been translated into various languages, but no information is available in Thai language. The aim of this cross-sectional study, therefore, was to evaluate the valid and psychometric properties of the Social Frail Index Thai version to further explore social frailty and its associated health consequences in Thai older adults. After translation using back translation technique, the Social Frail Index was confirmed for its validity and reliability in 380 dwelling-community Thai older adults living in four regions in Thailand. Participants responded to the Demographic and Health Information Questionnaire and the Social Frail Index. Results revealed that the item-level content validity index (I-CVI) and scale-level content validity index based on the universal agreement method (S-CVI/UA) were 0.96 and 0.80, respectively. The construct validity—Confirmatory Factor Analysis—confirmed that the model showed a well fit for the whole model. The factor loading ranged from.331 to.969 with a variance of 11.00-93.90. For its reliability, inter-ratter reliability of Kappa coefficient was.87 and internal consistency reliability of Cronbach Alpha was.80, indicating a good level. It can be concluded that the Social Frail Index Thai version was valid and reliable for evaluating social frailty in Thai older adults.

Abstract citation ID: igae098.2607

### EXAMINING THE DIMENSIONALITY OF FUNCTIONAL DISABILITY MEASURES FOR OLDER ADULTS

Rachel Upton[1], and Kristen Robinson[2], *1. ACL/HHS, Washington, District of Columbia, United States, 2. Administration for Community Living, Washington, District of Columbia, United States*

The National Survey of Older Americans Act Participants (NSOAAP) is an annual survey that evaluates programs on aging funded by Title III of the Older Americans Act (OAA). This session will discuss a cross-sectional study that used item factor analysis (IFA) and 2021 NSOAAP data to examine the dimensionality of functional disability measures used to classify older adults potentially at-risk for nursing home placement. Functional disabilities comprised limitations in activities of daily living (ADLs) and instrumental activities of daily living (IADLs). ADLs were defined as basic physical tasks required for independently caring for oneself (e.g., eating, bathing) (Fleishman, Spector, & Altman, 2002). IADLs similarly involved independent living activities such as going outside or performing housework. Research suggests that older adults can be classified as potentially at-risk for institutionalization if they display three or more functional disabilities (Tillery, 2021). However, no studies have examined whether current ADL-/IADL-limitation measures used to identify potentially at-risk older adults were unidimensional, such that these measures were accurately scored as reflecting a single construct versus being broken down into multiple, multidimensional subscales (Reise et al., 2010). Analyses focused on caregivers who reported ADL-/IADL-limitations for their care recipients and received OAA

Title III caregiver support services within the past year. IFA models indicated that the ADL-limitation measure exhibited a unidimensional factor structure. Bifactor models suggested that the IADL-limitation measure was not unidimensional, with a significant portion of item variance attributed to additional subdomains or subscale(s). Overall, findings reveal the importance of assessing dimensionality for functional disability measures.

Abstract citation ID: igae098.2608

**EXPLORING DIGITAL LITERACY, HEALTH LITERACY, AND SELF-MANAGEMENT IN YOUNGER AND OLDER LIVER TRANSPLANT RECIPIENTS**

Hanna Lee, JiYeon Choi, Seongmi Choi, and Sang Hui Chu, *Yonsei University College of Nursing, Mo-Im Kim Nursing Research Institute, Seoul, Republic of Korea*

Liver transplant recipients (LTRs) face challenges in self-management, including adhering to immunosuppressant therapy and navigating healthcare systems. Adequate health literacy enables LTRs to manage daily tasks, while digital proficiency enhances self-management. Understanding health and digital literacy among older LTRs is crucial for targeted self-management strategies amidst increasing digitalization and liver transplantation in older adults. This descriptive study compared health literacy, digital literacy, and self-management between younger LTRs (< 60 years; n=97, 50.88±7.19 years, 27 male, 20.57±17.42 months since LT) and older LTRs (≥ 60 years; n=58, 64.52±3.48 years, 21 male, 27.33±16.21 months since LT) and explored associations among these variables. LTRs responded to a cross-sectional survey on digital literacy (Everyday Digital Literacy Questionnaire, 22 items), health literacy (European Health Literacy Survey, 16 items), and self-management (modified Transplant Self-Management Scale, 16 items). Younger and older LTRs differed in self-management (56.95±9.43 vs 61.83±7.73, p< 0.001) and digital literacy (85.58±16.99 vs 71.17±22.98, p< 0.001), but not health literacy (13.62±3.43 vs 13.50±3.30, p=0.833). When exploring the role of health literacy and digital literacy in predicting self-management in the entire sample after controlling for age, sex, education level, household income, and time since LT, health literacy had a significant association with self-management (B=0.479, p=0.04) whereas digital literacy did not (B=0.007, p=0.87). In our sample, older LTRs reported better self-management and similar health literacy levels but poorer digital literacy compared to younger LTRs. Supporting digital literacy in older LTRs may enhance their self-management. Further studies are needed to understand digital literacy and self-management in older LTRs.

Abstract citation ID: igae098.2609

**IDENTIFICATION OF ITEMS ASSESSING GENERATIVITY AND EGO INTEGRITY IN A LONGITUDINAL STUDY OF AGING**

Olivia Duchow[1], Bryan James[2], and Tomiko Yoneda[1], *1. University of California Davis, Davis, California, United States*, *2. Rush University Medical Center, Chicago, Illinois, United States*

Generativity, the seventh stage from Erikson's psychosocial stages of development, is defined as the willingness to guide and inspire younger generations to promote generational wellbeing. The final stage, ego integrity, is characterized by an overall sense of life accomplishment and fulfillment. Although reminiscence therapy increases generativity and ego integrity, the extent to which these constructs are associated with health outcomes in older adulthood is unclear, as most work in this field relies on experimental designs and small samples. Thus, in the current work, we aim to develop a scale assessing generativity and ego integrity based on items administered to older adults in the Memory and Aging Project (MAP; N=~1700; Mage=65 years), which is a longitudinal study of older adults in the Chicago region with up to 24 annual measurement occasions until death. Development of this scale will permit follow-up investigation of the impact of generativity and ego integrity on long-term health outcomes, such as incident dementia diagnosis and mortality risk. Based on the existing literature, we identified numerous generativity and ego integrity scales (k=12). We then identified all potential items administered to MAP participants that may overlap with these items. Using artificial intelligence, we identified conceptually overlapping items to create a novel scale assessing generativity and ego integrity in MAP. We will report the discriminant validity, construct validity, and test-retest reliability of this scale.

Abstract citation ID: igae098.2610

**IDENTIFYING UNMET NEEDS: THE RELATIONSHIP BETWEEN FRAILTY MEASURES AND PATIENT PRIORITIES CARE**

Amy Thomas, Gwen Bernacki, Karl Brown, Terri May, Kirk Williams, Erica Martinez, and Katherine Ritchey, *VA Puget Sound Healthcare System, Seattle, Washington, United States*

Patient Priorities Care (PPC) is an effective approach to identifying and aligning 'what matters' (i.e., values) with healthcare for patients with multimorbidity and frailty. VA metrics [i.e., Jen Frailty Index (JFI); Care Assessment Need (CAN)] predict mortality and institutionalization but may not correlate with the number and complexity of healthcare needs of veterans with multimorbidity. For patients referred to a VA Geriatric PPC Consultation clinic, recommendations and alignment of care were guided by a veteran-derived health outcome goal. Recommended changes to healthcare plans including rehabilitative or social service referrals were identified from electronic health records. Multiple linear regression was used to correlate total number of recommendations (outcome) with frailty (JFI) and multimorbidity (CAN) scores; interaction between frailty/multimorbidity scores with rurality was also explored. Rural/non-rural groups were demographically similar and had four recommendation subtypes per patient (mean). Median CAN was 85 (out of 99) and JFI 6 (out of 13). CAN or JFI scores had no effect on number of recommendations for both rural (p = 0.32 for CAN, p = 0.85, respectively) and non-rural (p = 0.09, p = 0.35, respectively) groups. There was no difference in the effect of CAN or JFI on the recommendations between rural vs. non-rural groups (p = 0.45, p = 0.43). JFI and CAN scores may not identify which older veterans have rehabilitative or social needs. Future work is needed to clarify how frailty and multimorbidity measures can guide referral to geriatric services and/or resource allocation to prevent disability.

Abstract citation ID: igae098.2611

### MONTREAL COGNITIVE ASSESSMENT SURVEY ADAPTATION IN A COMMUNITY-BASED OLDER ADULT SAMPLE, 2019–2020 NHANES

Jenny Walker[1], Roshni Patel[2], John Omura[1], Akilah Ali[1], Benjamin Olivari[1], and Lisa McGuire[3], *1. Centers for Disease Control and Prevention, Atlanta, Georgia, United States*, *2. Cyberdata Technologies (CDC Contractor), Atlanta, Georgia, United States*, *3. GSA, Atlanta, Georgia, United States*

The U.S. population is aging. Some degree of cognitive change is expected as a natural part of healthy aging. Understanding the prevalence of potential impairments in community-based samples is necessary for prioritization of public health efforts. We analyzed Montreal Cognitive Assessment Survey Adaptation (MoCA-SA) data from the 2019-2020 National Health and Nutrition Examination Survey (NHANES). The full MoCA-SA was administered to a convenience sample of 1,123 individuals aged ≥60 years. Excluding incomplete inventories yielded a final analytic sample of 895. The MoCA-SA contains 20 items and is scored 0-20, with values < 17 indicative of lower cognitive performance. Scores were also analyzed for 8 subscales: orientation, naming, visual construction, executive function, attention, language fluency, abstraction, and memory. Estimates were stratified across three age categories: 60-69, 70-79, and ≥80 years. Overall, 71.2% (unweighted; 95% CI 68.2-74.1) of participants aged ≥60 years scored < 17. People aged ≥80 years scored significantly lower on the orientation and memory and higher on abstraction than those aged 60-69 years. No age-related differences were identified in the remaining subscales. While changes in cognition are a normal part of aging, not all changes are healthy. Only a healthcare provider can diagnose cognitive impairments such as Alzheimer's disease. People experiencing changes in cognition that interfere with daily life should speak with their healthcare provider about their concerns and ways to promote brain health. Additional work is needed to determine how the MoCA-SA corresponds to cognitive functioning in daily life and which subscales may drive differences in community samples.

Abstract citation ID: igae098.2612

### QUADRICEPS MUSCLE THICKNESS MEASURED BY POINT-OF-CARE ULTRASOUND AND HOSPITAL LENGTH OF STAY

Uyanga Ganbat, Altan-Ochir Byambaa, and Graydon Meneilly, *University of British Columbia, Vancouver, British Columbia, Canada*

**Background:** Accurate prediction of hospital length of stay (LOS) and readmission rates could help improve effective healthcare resource allocation. Recent evidence suggests point-of-care ultrasound for muscle assessment, specifically muscle thickness, as a promising tool in this regard. This study explores the hypothesis that ultrasound measurements of quadriceps muscle thickness (MT) and echointensity (EI) can serve as predictors for these crucial patient outcomes.

**Methods:** The study measured quadriceps MT and EI using point-of-care ultrasound for patients in a supine position in the emergency department of Vancouver General Hospital. Predictor variables included age, sex, muscle thickness, and echo intensity. Outcome variables were hospital LOS, readmission rate, and discharge destination. Follow-up was conducted after one month to assess hospital readmissions and mortality.

**Results:** A total of 120 participants were included (average age 76.9 ± 7.5, with 64 women and 56 men). Mean LOS was 27.4 ± 31.4 days, and mean MT was 20 ± 6 mm. Sex-based differences in MT were statistically significant ($P = 0.032$). MT correlated significantly with LOS (Standardized $\beta = -0.152 \pm 0.016$, $R^2 = 0.290$, $P = 0.001$). In the multivariate regression model, MT remained a significant predictor (Standardized $\beta = -0.152 \pm 0.563$, $P = 0.008$).

**Conclusion:** Muscle thickness is a significant predictor of hospital stay duration. The findings of this study indicate the potential of point-of-care ultrasound in measuring skeletal muscle as an effective predictor of discharge outcomes.

Abstract citation ID: igae098.2613

### SENSITIVITY OF FALL RISK CUTOFFS USING LAB AND REMOTE MEASURES ON OLDER PROSTATE CANCER SURVIVORS

Carolyn Guidarelli, Sydnee Stoyles, Nathan Dieckmann, Alexandra Sokolova, Julie Graff, Eric Roeland, and Kerri Winters-Stone, *Oregon Health & Science University, Portland, Oregon, United States*

Prostate cancer survivors (PCS) treated with androgen deprivation therapy (ADT) have increased fall risk due to treatment-related side effects on musculoskeletal health. Fall risk is typically measured in the lab or clinic using objective assessments validated in community-dwelling older adults. It is unknown whether the same cutoffs will identify PCS at risk of falls. Conducting these assessments remotely has become more prevalent in research; however, it remains unclear whether different cutoffs are required using this modality. Thus, we explored the sensitivity and specificity of traditional fall risk cutoff scores using lab and remote measures to correctly classify faller status among older PCS. We used baseline data on 243 older (mean age 74.5 years) PCS from the GET FIT Prostate trial (NCT03741335). We captured fall history over the past year and completed lab and/or remote assessments of the 3-meter Timed Up and Go (TUG), 4-meter usual walk (4mUW), 5x Sit-to-Stand (5xSTS), and short physical performance battery (sPPB). We classified fall risk as TUG ≥13.5s, 4mUW ≤1.0m/s, 5xSTS ≥12s, sPPB ≤9, and faller ≥1 fall. Overall, 37.9% (n=92) reported a fall within the last year. Each test had similar sensitivity and specificity with AUCs of 58.5%, 56.3%, 55.4% and 56.0% for the lab-based TUG, 4mUW, 5xSTS, and sPPB, respectively. AUCs for remote testing were similar. Regardless of measure or modality, traditional fall risk cutoff scores do not accurately classify faller status in PCS exposed to ADT, leaving clinical practice with few tools to identify men at risk of falls.

Abstract citation ID: igae098.2614

### SHORT PHYSICAL PERFORMANCE BATTERY OUTPERFORMS COMMON PHYSICAL PERFORMANCE TESTS IN OLDER ADULTS

Chitra Banarjee, Renoa Choudhury, Joon-Hyuk Park, Rui Xie, David Fukuda, Jeffrey Stout, and Ladda Thiamwong, *University of Central Florida, Orlando, Florida, United States*

Interdisciplinary evaluation of older adults' healthcare is a priority in the prevention of chronic health conditions and the maintenance of daily functioning. While studies have evaluated different physical performance tests (PPTs) from a retrospective view in predicting mortality or cardiopulmonary health, it remains unclear which of the commonly used PPTs are most effective at evaluating the current health of older adults. Various PPTs have been shown to be important predictors of mortality; however, few studies have compared the relevance of different PPTs to older adults' physical activity, static balance, body mass index, and fear of falling within the same population of older adults. The present study evaluates 53 older adults using three different PPTs Short Physical Performance Battery (SPPB), 6-minute walk test (6MWT), and Incremental Shuttle Walk Test (ISWT)) and constructs multiple linear regression models with measures of physical activity, static balance, and fear of falling, as assessed by an accelerometer, BTrackS balance plate, and the short Falls Efficacy Scale-International (FES-I), respectively. The models reveal that SPPB provides the most comprehensive value as indicated by a greater R2 value (0.573), and performance on the SPPB predicts physical activity ($p=0.041$) and fear of falling ($p< 0.001$). ISWT predicted body mass index (BMI, $p=0.019$) and fear of falling (0.010), whereas 6MWT predicted only fear of falling. The results indicate the value of a multi-component, comprehensive test such as the SPPB in evaluating the health of older adults and further distinguish ISWT as more predictive of overall health than the 6MWT in older adults.

Abstract citation ID: igae098.2615

### TEACHING THE CORNELL-PENN INTERVIEW FOR DECISIONAL ABILITIES (IDA) TO ENHANCE DECISIONAL ABILITY ASSESSMENTS

David Hancock[1], Bess White[1], David Lachs[1], Jason Karlawish[2], and Mark Lachs[1], *1. Weill Cornell Medicine, New York, New York, United States*, *2. University of Pennsylvania, Philadelphia, Pennsylvania, United States*

Conventional approaches used by non-medical professionals to determine an individual's ability to make decisions usually focus on cognitive indicators rather than the ability to reason around the particular risk at hand. The Cornell-Penn Interview for Decisional Abilities (IDA) is a novel, semi-structured interview tool that determines an individual's ability to make decisions around a specific risk they are facing, such as the choice to remain in an abusive situation or refuse medical care. The IDA tool addresses a significant gap in the field of gerontology and promotes an individual's right to self-determination while ensuring their safety and well-being. This study further validates the IDA by demonstrating the acceptability and efficacy of the educational program developed to teach the IDA to Adult Protective Services (APS) workers. The effectiveness of the IDA training was evaluated through feedback from 349 practitioners who completed the IDA training program. Participants rated the value of the learning experience highly, with an average score of 4.26 out of 5. Additionally, sentiment analysis of free response text using three different dictionaries (AFINN, BING, NRC) revealed overwhelmingly positive feedback, with words like "helpful," "confident," and "comfortable" frequently mentioned. The IDA represents a significant advancement in the assessment of decisional ability in the field of gerontology. This study proves that the IDA educational program effectively instructs APS professionals in using the IDA successfully as a rigorous, evidence-based approach to understand an APS client's ability to make decisions around risks they are facing.

Abstract citation ID: igae098.2616

### THE NEED FOR IMPROVED TACTILE ASSESSMENTS OF THE HAND IN OLDER ADULTS

Gül Kabil[1], Madeline Weber[2], Rachel Logue Cook[1], Cameron Haire[1], Daniel Duque Urrego[3], Stephen Cain[3], Paula Anne Newman-Casey[1], and Susan Brown[1], *1. University of Michigan, Ann Arbor, Michigan, United States*, *2. University of Michigan Medical School, Ann Arbor, Michigan, United States*, *3. West Virginia University, Morgantown, West Virginia, United States*

Age-related sensorimotor declines of the hand significantly impact functional independence. Beyond muscle strength, tactile feedback provides valuable information regarding object characteristics that, in turn, can enhance manipulation skills. This is of particular interest in areas such as medication adherence - e.g. successful administration of eye drops or hearing aid adjustments. We investigated tactile acuity and dexterity in healthy young adults (23-35yo, n=23) and two groups of healthy older adults (65-76yo, n=24; 76-87yo, n=24). Participants completed assessments of strength (pinch, grip), dexterity (Grooved Pegboard Test, Arthritis Hand Function Test), tactile registration (Semmes-Weinstein Monofilament Test), and tactile pattern recognition using a custom-designed device. Both older groups performed significantly worse than young adults on all measures ($p< 0.001$). Dexterity, tactile identification accuracy, and tactile response times were significantly worse in the 76-87yo compared to the 65-76yo group ($p=0.01$). In contrast, strength and median monofilament scores did not differ between the two older adult groups. Across all older adults, tactile pattern recognition and dexterity were weakly correlated ($p\leq0.05$). In the young adult group, only tactile accuracy and performance on the Arthritis Hand Function Test were correlated ($p=0.04$). These results suggest that clinical assessment of tactile acuity using monofilament testing may not capture declines in hand sensibility in older adults. More complex tactile assessments may be predictive of difficulties with object manipulation tasks that worsen with advanced age and negatively impact daily self-care activities.

### SESSION 7210 (POSTER)

### BIOLOGY OF AGING II

Abstract citation ID: igae098.2617

### ACCELERATED PACE OF BIOLOGICAL AGING IN PARTICIPANTS WITH LOW LEVELS OF SELENIUM BIOMARKERS IN BERLIN AGING STUDY II

Valentin Vetter[1], Kamil Demircan[1], Jan Homann[2], Michael Mülleder[1], Markus Ralser[1], Lars Bertram[3], Lutz Schomburg[1], and Ilja Demuth[1], *1. Charité*

*- Universitätsmedizin Berlin, Berlin, Berlin, Germany, 2. University of Münster, Münster, Nordrhein-Westfalen, Germany, 3. University of Lübeck, Lübeck, Schleswig-Holstein, Germany*

Low levels of selenium in blood were shown to be associated with adverse health outcomes and are discussed as anti-aging interventions by some investigators. Inter-individual differences in the aging process can be quantified by epigenetic clocks (DNA methylation age, DNAmA). In this study, we analyzed 1,568 participants of the Berlin Aging Study II (BASE-II, mean age +/- SD: 68.8 +/- 3.7 years, 51% women). DNAmA, and its deviation from chronological age, DNAmA acceleration (DNAmAA), were measured from DNA methylation data derived from Illumina's "MethylationEPIC" array via the Horvath, GrimAge, and DunedinPACE clock algorithms. Selenium was measured in serum samples via total reflection X-ray fluorescence (TXRF) spectroscopy. Selenoprotein P (SELENOP) was measured with ELISA. GPX3 was assessed as part of a larger set of proteins by mass spectrometry. A statistically significantly higher pace of ageing (using the DunedinPACE clock) was observed for participants with deficient selenium levels (< 90 µg/L). This association persisted after adjustment for age, sex, BMI, smoking, and genetic ancestry in linear regression (β=-0.02, SE=0.01, p= 0.012; n=757). Similarly, participants in the highest quartile of SELENOP levels showed a statistically significantly lower pace of ageing compared to the lowest quartile (DunedinPACE, β=-0.03, SE=0.01, p=0.007; n=740). Participants within the lowest quartile of GPX3 levels showed statistically significantly higher rates of biological ageing compared to the participants in the third quartile (HorvathDNAmAA, p=0.04), the fourth quartile (GrimAgeDNAmAA, p=0.003) or both quartiles combined (DunedinPACE, p=0.002). In conclusion, our findings support previous reports of potentially detrimental effects of low levels of selenium biomarkers.

Abstract citation ID: igae098.2618

### AGE-RELATED CHANGES IN BODY WEIGHT AS DETERMINANTS OF EXTREME LONGEVITY

Svetlana Ukraintseva, Hongzhe Duan, Rachel Holmes, Konstantin Arbeev, and Anatoliy Yashin, *Duke University, Durham, North Carolina, United States*

Age-related changes in body weight may mirror fundamental processes of aging and ontogeny that contribute to determining human longevity limits. We estimated the mean age trajectories of weight, along with their key characteristics, such as the age at which maximum weight values (AgeMax) are reached, and the slope of weight decline (Slope) thereafter, and tested whether they can predict extreme longevity in the Framingham Heart Study participants. We compared weight trajectories over a period of approximately 50 years between those who survived beyond age 95 (longevity group) and those who died before age 85 (conventional lifespan). We also estimated survival trajectories after age 80 for individuals who had different AgeMax and Slope. We found that individuals who lived beyond the age of 95 reached AgeMax about 15 years later (around age 75), and experienced a slower decline in weight, compared to participants with a conventional lifespan. The older AgeMax and flatter Slope were both significant predictors of better survival after age 80 and increased chances of extreme longevity. Our findings suggest that patterns of long-term changes in body weight can be informative biomarkers of aging and longevity. A slower or postponed decline in weight at the oldest old ages may indicate better physical resilience contributing to an organism's ability to reach longevity limits.

Abstract citation ID: igae098.2619

### AGING MARKERS IN CONTEXT OF MORBIDITY, FRAILTY, AND GERIATRIC ASSESSMENTS: LONGITUDINAL RESULTS FROM BASE-II

Valentin Vetter[1], Johanna Drewelies[2], Jan Homann[3], Sandra Düzel[1], Simone Kühn[2], Lars Bertram[4], Denis Gerstorf[5], and Ilja Demuth[1], *1. Charité - Universitätsmedizin Berlin, Berlin, Berlin, Germany, 2. Max Planck Institute for Human Development, Berlin, Berlin, Germany, 3. University of Münster, Münster, Nordrhein-Westfalen, Germany, 4. University of Lübeck, Lübeck, Schleswig-Holstein, Germany, 5. Humboldt University Berlin, Berlin, Berlin, Germany*

Several markers are available that aim at the quantification of inter-individual differences in biological age. While many of these markers have been evaluated individually, there are few comparisons in terms of their association with age-related outcomes within the same dataset available. In this study, we compare 13 different aging markers in relation to morbidity, frailty and geriatric assessments in 1,671 participants of the Berlin Aging Study II (BASE-II). Participants were on average 68.8 years old at baseline (SD = 3.7 years) and 51.6% of the participants were women. A follow-up examination of 1,083 participants was conducted on average 7.4 years later. Biological age was assessed as epigenetic age using DNA methylation (7-CpG, Horvath, Hannum, PhenoAge, GrimAge, DunedinPACE), telomere length, BioAge, BrainAge, SkinAge, Subjective Age, Subjective Life Expectancy, and Subjective Health Horizon. Aging markers were analyzed in the context of morbidity (overall morbidity, diabetes and diabetes associated complications, cardiovascular risk scores), frailty (Fried), Tinetti Mobility Test, falls in past 12 months, finger-floor distance, mini mental state examination, Center for Epidemiologic Studies-Depression scale, activities of daily living (ADL), and mini nutritional assessment (MNA). Associations between ageing markers and outcomes were assessed with regression analyses adjusted for known confounders (age, sex, smoking, BMI). Preliminary results revealed strengths and limitations of the biomarkers analyzed with respect to the different domains of aging represented by the investigated age-associated outcomes. Cross-sectional and longitudinal results will be presented at the meeting and discussed in the context of the literature.

Abstract citation ID: igae098.2620

### EFFECTS OF FASTING STATUS ON BLOOD-BASED INFLAMMATORY BIOMARKERS IN OLDER ADULTS

Christopher Engeland[1], Robert Tennyson[1], Molly Wright[1], Erik Knight[2], Seyedeh Leila Mousavi[1], Orfeu Buxton[3], and Jennifer Graham-Engeland[3], *1. The Pennsylvania State University, University Park, Pennsylvania, United States, 2. University of Colorado Boulder, Boulder, Colorado,*

*United States*, *3. Pennsylvania State University, University Park, Pennsylvania, United States*

To determine the effects of fasting status on blood-based biomarker concentrations, 30 older adults (Mage = 67.52; 70% women) fasted from midnight onwards prior to two morning laboratory visits. Each visit included 4 blood draws, spaced 1 hour apart. The visits (counterbalanced by order) were differentiated by a meal being provided after either the 2nd or 4th blood draw. This design enabled examination of fasting versus post-prandial effects, accounting for circadian variation of biomarkers. Plasma levels of cytokines (interleukin [IL]-1β, IL-4, IL-6, IL-8, IL-10, tumor necrosis factor [TNF]-α), soluble urokinase-type plasminogen activator receptor (suPAR), and C-reactive protein (CRP) were quantified at each timepoint. Separate subsets of whole blood were stimulated with 1μg/ml lipopolysaccharide and incubated for 2h or 24h at 37ºC (5% CO2) to examine cytokine responsivity to immunogenic challenge. Bayesian multilevel models (minimally informative priors, Stan MCMC sampler) compared all pre-prandial (6 timepoints) vs. post-prandial (2 timepoints) concentrations (covariates: time-of-blood-draw, visit order, age, gender, BMI). Results provided strong to very strong evidence for no changes in basal cytokines, CRP, or suPAR by fasting status. However, there was strong evidence that eating decreased stimulated TNF-α levels (2h and 24h incubation) and weak evidence that eating decreased stimulated IL-6 (2h incubation only). These findings indicate that levels of basal inflammation are not affected by eating, but that stimulated levels of TNF-α are susceptible to post-prandial effects. This implies that studies assessing stimulated TNF-α should have participants fast. Further research is needed for stimulated IL-6, which exhibited uncertain (weak) evidence of change.

Abstract citation ID: igae098.2621

### EFFECTS OF FASTING STATUS ON BLOOD-BASED NEURODEGENERATIVE BIOMARKERS IN OLDER ADULTS

Christopher Engeland[1], Robert Tennyson[1], Molly Wright[1], Erik Knight[2], Orfeu Buxton[3], and Jennifer Graham-Engeland[3], *1. The Pennsylvania State University, University Park, Pennsylvania, United States*, *2. University of Colorado Boulder, Boulder, Colorado, United States*, *3. Pennsylvania State University, University Park, Pennsylvania, United States*

Blood-based neurodegenerative biomarkers hold vast potential to serve as early detectors of neuropathology, including Alzheimer's disease. However, accurate biomarker assessment is critical and extraneous factors that may affect circulating concentrations need greater understanding. To determine the effects of fasting status on blood-based levels of neurodegenerative biomarkers, 30 older adults (Mage = 67.52; 70% women) fasted >8h prior to two laboratory visits. Each visit included 4 blood draws, spaced 1 hour apart. The visits (counterbalanced) were differentiated by a meal being provided after either the second or fourth blood draw. This design enabled examination of fasting versus post-prandial effects, accounting for circadian variation of biomarkers. Plasma levels of neurodegenerative biomarkers (amyloid β(Aβ) 42/40 ratio, neurofilament light chain (NfL), glial fibrillary acidic protein (GFAP), phosphorylated tau (pTau)181, pTau217) were quantified at each timepoint. Bayesian multilevel models (minimally informative priors, Stan MCMC sampler) compared all pre-prandial (6 timepoints) vs. post-prandial (2 timepoints) concentrations (covariates: time-of-blood-draw, visit order, age, gender, BMI). Results provided strong and very strong evidence for no changes in Aβ42/40 ratio and pTau181, respectively. However, there was very strong evidence that eating decreased GFAP levels, substantial evidence that eating decreased pTau217 levels, and weak evidence that eating decreased NfL levels. These findings indicate that plasma levels of some, but not all, neurodegenerative biomarkers are susceptible to changes due to eating. One implication is that eating should be controlled (e.g., via fasting) in studies incorporating GFAP and pTau217. Further research is needed for NfL, which exhibited less certain (weak) evidence of change.

Abstract citation ID: igae098.2622

### EFFECTS OF FASTING STATUS ON PLASMA ENDOCRINE BIOMARKERS IN OLDER ADULTS

Erik Knight[1], Robert Tennyson[2], Molly Wright[2], Jennifer Graham-Engeland[3], and Christopher Engeland[2], *1. University of Colorado Boulder, Boulder, Colorado, United States*, *2. The Pennsylvania State University, University Park, Pennsylvania, United States*, *3. Pennsylvania State University, University Park, Pennsylvania, United States*

Accurate biomarker measurement is critical for understanding biopsychosocial and cognitive aging. However, biomarker measurement often relies on fasting samples, whereas cognitive testing is typically conducted non-fasting. The effects of eating on endocrine levels were tested in a well-controlled experiment. Older adults (N=30, mean 67.5 years, 70% women) each attended two, morning laboratory sessions with four, hourly blood draws after >8 hours fasting. A meal was provided after the second or fourth blood draw (session order counterbalanced). Eight hormones were assayed via LC/MS; bioavailable hormones were estimated using SHBG (EIA assays). Bayesian multilevel models (minimally informative priors, Stan MCMC sampler) compared pre-prandial (6 samples from both visits) vs. post-prandial (2 samples) concentrations (covariates: time-of-day, visit order, age, gender, BMI). These models provided decisive evidence for eating increasing cortisol and reducing total testosterone and estrone; weak evidence for increasing corticosterone and DHEAS and decreasing progesterone, free testosterone and estrone. Decisive evidence was observed for cortisone, and total and free estradiol being unaffected by eating. Exploratory analyses of gender moderation provided decisive evidence that effects of eating on total testosterone were most strongly observed among men, although the pattern was similar for women. These findings indicate that cortisol, total testosterone, and total estrone are susceptible to changes due to eating among older adults, suggesting careful consideration related to diet/fasting should be made in studies incorporating these biomarkers. Cortisone and estradiol appear relatively unaffected by eating; further research is necessary for all remaining biomarkers that had weak evidence of change.

Abstract citation ID: igae098.2623

**EPIGENETIC PACE OF AGING (DUNEDINPACE) PREDICTS INCIDENT METABOLIC SYNDROME IN THE BERLIN AGING STUDY II (BASE-II)**

Ilja Demuth[1], Valentin Vetter[1], Jan Homann[2], Christina Lill[2], and Lars Bertram[3], *1. Charité - Universitätsmedizin Berlin, Berlin, Berlin, Germany*, *2. University of Münster, Münster, Nordrhein-Westfalen, Germany*, *3. University of Lübeck, Lübeck, Schleswig-Holstein, Germany*

The metabolic syndrome (MetS) is highly prevalent in the general population and associated with increased morbidity and mortality. Its diagnosis is based on cut-off values for five cardiovascular risk factors, three of which must be met to make the diagnosis. Epigenetic clocks (Horvath, GrimAge) can be used to quantify differences in biological age as deviation from DNA methylation age (DNAmA) from chronological age, DNAmA acceleration. Another epigenetic biomarker, DunedinPACE, reflects the pace of aging in number of biological years passed per additional chronological year. Aim of the current study is to evaluate the capacity of these different epigenetic markers to predict incident MetS in the BASE-II follow-up dataset assessed on average 7.4 years after baseline. Following exclusion of participants with prevalent MetS a total sample of n= 564 participants (68.3 ± 3.4 years, 55.9% women) with epigenetic biomarkers (baseline) and information on MetS status (follow-up, n=144 incident MetS cases) was available and included in the analyses. Analyses were performed as logistic regressions adjusted for age, sex, time to follow-up, overall morbidity and life style factors. Preliminary analyses revealed that the odds of being diagnosed with MetS after the follow-up period of on average 7.4 years was significantly increased with higher pace of aging assessed as baseline DunedinPACE (OR: 10.7 (95%CI: 1.5-76.1); p = 0.018). Comprehensive results including ROC statistics will be presented at the meeting and discussed in the context of the literature.

Abstract citation ID: igae098.2624

**EXERCISE AND IMMUNE FUNCTION FROM GESTALT COHORT: THE BENEFICIAL EMERGING ROLE OF INTEGRATED STRESS RESPONSE**

Stefano Donega[1], Nirad Banskota[2], Amit Singh[1], Mary Kaileh[2], Arsun Bektas[1], Supriyo De[1], Ranjan Sen[1], and Luigi Ferrucci[1], *1. National Institute on Aging, Baltimore, Maryland, United States*, *2. National Institutes of Health, Bethesda, Maryland, United State*

Exercise significantly impacts immune function by influencing physiological health, which in turn reduces disease susceptibility, combats immune-senescence, and lowers infection risks. Despite these effects, there are still important unanswered questions regarding the impact of exercise on the transcriptome of immune cells, age-related responses, inflammation modulation, and anti-aging benefits. In the Genetic and Epigenetic Signatures of Translational Aging Laboratory Testing (GESTALT) study involving 99 participants aged 22-92, RNA-sequencing was conducted in parallel on 11 immune cell types - B cells, T/CD4+, T/CD8+ (naïve and effector/central memory) cells, monocytes, granulocytes, and natural killer cells - to investigate their correlation with exercise (VO2 peak) using Differential Gene Expression (DGE) and Gene Set Enrichment Analysis (GSEA). Lymphoid B, T and NK cells, exhibited distinct clustering from myeloid cells in both young and old participants (< 44 and >66 years old), with 26 shared genes identified across multiple immune-cell types, reflecting enrichment for 14 energy-mitochondrial pathways associated with increased exercise. Interestingly, while mRNA splicing pathways were upregulated in the young group, older individuals displayed upregulated pathways linked to the Integrated Stress Response (ISR), a signaling pathway previously found to be activated by ER stress, hypoxia, amino acid deprivation, glucose deprivation, and viral infection etc. By utilizing a scoring system for inflammation, senescence, and mitochondria (QuickGO-EBI, SenMayo, and MitoCarta 3.0), we identified clusters of shared genes affected by physical activity. Overall, the findings suggest that stress-induced molecular responses to enhanced aerobic capacity and exercise offer potential benefits for improving the health and well-being of older individuals.

Abstract citation ID: igae098.2625

**EXPLORING THE IMPACT OF INTERMITTENT HYPOXIA THROUGH A COMPREHENSIVE MULTI-OMICS APPROACH IN AGED MOUSE MODELS**

Stefano Donega, Ross McDevitt, Rolando Hernandez, Cindy Chisolm, Kenneth FIshbein, Elizabeth McCart, Brian Wilgenburg, and Luigi Ferrucci, *National Institute on Aging, Baltimore, Maryland, United States*

Comprehending aging's molecular mechanisms, like RNA splicing and AMPK signaling, is vital for tackling age-related issues and boosting resilience. Oxygen fluctuations from obstructive sleep apnea (OSA) cause Intermittent Hypoxia (IH), affecting respiratory functions and health, prompting adaptive responses to counter reduced energy production. Mice as models allow simulating disorders and studying muscle cell protection under low oxygen levels. Over the past two years, we have collected more than 1500 samples from 15 different tissues of adult (9-11 months) and old (20-23 months) mice exposed to IH at 21% to 5% oxygen concentration in 2:30 minutes cycles, for 8 hours a day over 4-8 weeks, aiming to construct a comprehensive murine IH atlas by employing cutting-edge technologies across diverse tissues. Analyses include conducting detailed transcriptomic analyses of muscle tissues using hybrid RNA-seq to identify changes in splicing isoforms during IH experiments, exploring transitions between fast-to-slow fiber types, studying mitochondrial morphology using C57/BL6 and mtKeima mouse strains (both wild-type and Alzheimer's disease models), investigating brain spatial transcriptomics to understand the neurological effects of IH, analyzing cardiorespiratory disruptions by examining the epigenetic clock of the heart and spleen, and delving into the role of liver extracellular vesicles (EVs), particularly exosomes, in our sensitive mouse IH model. The overarching goal of this project is to integrate omics data comprehensively to elucidate the impact of alternate hypoxia on health and aging and develop innovative therapeutic strategies that minimize damage in patients affected by this highly prevalent conditions in older persons.

Abstract citation ID: igae098.2626

### INDUCIBLE SKELETAL MUSCLE SOD2 KNOCKDOWN MIMICS AGE-RELATED MITOCHONDRIAL DYSFUNCTION IN MOUSE SKELETAL MUSCLE

Ethan Ostrom, Sahir Sandhu, and David Marcinek, *University of Washington, Seattle, Washington, United States*

Aging causes declines in mitochondrial energetic efficiency and increased reactive oxygen species (ROS) production in skeletal muscle, leading to declines in muscle function and impaired stress resilience. While perturbations in redox homeostasis have been associated with declines in muscle and mitochondrial function, the mechanisms and time course of this decay have not been established. Here we use a new model of inducible skeletal muscle specific knockdown of SOD2 (KD) to produce an oxidative stress and compare the functional declines to those seen in aging muscle mitochondria. We demonstrate the model induces skeletal muscle specific SOD2 protein KD by 50% at 3 weeks and 90% decrease by 8 weeks. KD showed a significant decline in in vivo muscle force in female KD mice ($p=0.0249$), but not male KD mice compared to controls at eight weeks. KD resulted in significant reductions in aconitase 2 (ACON2) activity an enzyme sensitive to oxidative stress. Similar to aged skeletal muscle, KD showed significant deficits of complex I-driven respiration ($p< 0.0001$), max oxphos capacity ($p=0.0001$), and maximal ROS production rate ($p< 0.05$) in permeabilized muscle fibers. Interestingly, the CI impairment in SOD2 KD was dependent the order of substrate addition indicating that SOD2 KD affects metabolic flexibility of the mitochondria. These results show that short term redox stress reproduces some aspects of aged skeletal muscle mitochondria. Ongoing experiments are testing the metabolic and molecular mechanisms underlying impaired metabolic flexibility related to elevated redox stress in the SOD2 KD and their relevance to aging muscle.

Abstract citation ID: igae098.2627

### MAPPING LONGEVITY PATHWAYS THROUGH MITOCHONDRIAL DYSFUNCTION: ROLES OF THE MICROBIOME AND METABOLISM IN AGING.

Alessandro Bitto, *University of Washington, Seattle, Washington, United States*

Biological aging is characterized by a progressive deterioration of fundamental biological functions in living organisms, often codified as hallmarks or pillars. Longevity interventions impact these hallmarks, slowing down or even reversing their deterioration. Thus, we hypothesize that longevity interventions may be applied to chronic diseases characterized by failure of one or more of these fundamental biological processes, regardless of biological age. Herein, we describe how geroscience can be leveraged to gather new insight into etiology, progression, management, and treatment of chronic mitochondrial disease. With the aid of a mitochondrial dysfunction model, we investigate the effects on intermediate metabolism and the intestinal flora of two longevity interventions, the antidiabetic drug acarbose and the mTOR inhibitor rapamycin. Furthermore, we propose that models of chronic disease can be leveraged to study the mechanisms of action of known longevity pathways and develop new interventions. We describe the critical role of fatty acid metabolism in the treatment of mitochondrial disease and hypothesize that similar mechanisms may contribute to the longevity effects of mTOR inhibition in aging models.

Abstract citation ID: igae098.2628

### METABOLOMIC AND IMMUNOLOGIC INSIGHTS INTO RACIAL DIFFERENCES IN REPRODUCTIVE AGING

Joanna-Lynn Borgogna[1], Johanna Holm[2], Jacques Ravel[2], Rebecca Brotman[2], Jovanka Voyich[1], Carl Yeoman[1], and Michelle Shardell[2], *1. Montana State University, Bozeman, Montana, United States*, *2. University of Maryland School of Medicine, Baltimore, Maryland, United States*

Menopause is linked to declining levels of estrogen, loss of protective vaginal lactobacilli, and age-related comorbidities. Vaginal metabolites reflect metabolic activity of the microbiota and human host, along with exogenous influences, and can modify susceptibility to multiple pathologies. Racial/ethnic minorities experience earlier and more intense symptoms of menopause but are underrepresented in reproductive aging studies. To address these gaps, we applied 16S rRNA gene sequencing, untargeted metabolomics, and cytokine profiling to characterize the vaginal microenvironment across reproductive stages, with an emphasis on comparisons between post-menopausal racial/ethnic groups. 476 participants (aged 35-60 years) contributed semiannual person-visits over two years (N=1,153 samples). Bayesian mixed-effects regression of log2-transformed metabolites (n=770) assessed metabolomic differences in samples from pre- (n=287), peri (n=335), and post-menopausal (n=531) participants, 25% of whom were racial/ethnic minorities. Samples collected from post-menopausal participants had distinct metabolomic profiles compared to pre- or peri-menopausal samples (548 metabolites were lower in post-menopausal samples; $p< 0.05$). After adjustment for vaginal microbiota, post-menopausal samples had lower concentrations of most micronutrients (e.g., vitamins B, C, and D; $p< 0.05$). Metabolites reflecting epithelial damage and oxidative stress, along with host-produced lysophospholipids (e.g., lactosyl-N-palmitoyl-sphingosine), were highest in samples from post-menopausal participants ($p< 0.05$), with post-menopausal Black participants having the highest levels (relative to post-menopausal white participants, and non-postmenopausal Black or white participants) ($p< 0.05$). Lysophospholipids are immunogenic, and many were positively correlated with TNF-α, but negatively correlated with IL-19. Further investigations linking menopause and race/ethnicity with inflammatory or oxidative stress-related metabolites are needed to reduce disparities in healthy reproductive aging.

Abstract citation ID: igae098.2629

### MICROBIOME ROLE IN COGNITIVE IMPAIRMENT: MIAGB COHORT INSIGHTS

Sidharth Mishra[1], Lauren Buddendorff[1], Julia Hoover[1], Rohit Shukla[1], Vivek Kumar[1], Shalini Jain[2], and Hariom Yadav[1], *1. University of South Florida, Tampa, Florida, United States*, *2. University of South Florida, Tampa, FL, USA, Florida, United States*

Aging population is increasing, leading to a rise in aging-related conditions, specifically diseases like Alzheimer's disease and its related dementia (ADRD), which have poor prognosis and lack prevention and treatment strategies. Although we do not fully understand the complex pathology of ADRD, emerging evidence indicates that abnormalities in the gut microbiome contribute to ADRD pathology, but how this occurs remains largely unknown. Utilizing samples and datasets from a multi-site and comprehensive clinical study called the Microbiome in aging Gut and Brain (MiaGB) consortium, of comprising 30 older adults with mild cognitive impairment (MCI) and 43 age- and sex-matched cognitively healthy controls, we found that older adults with MCI have higher levels of circulating gut permeability markers (lipopolysaccharide [LPS] binding protein [LBP] and sCD14) and systemic inflammation markers (IL-6, CRP, and IL-1β). Increased gut permeability (leaky gut) is an understudied source of systemic inflammation as it allows pro-inflammatory ingredients including LPS and microbes from the gut lumen to enter the bloodstream, instigating systemic inflammation. Interestingly, increased LPS and TLR4 activation were found in the circulation of MCI participants compared to controls. Furthermore, we discovered that gut microbial metabolite- ethanolamine utilization pathways were decreased in MCI compared to controls. These abnormalities are significantly associated with reduced cognitive function (MoCA scores) in older adults. These findings provide mechanistic insight into how abnormalities in the gut microbiota, with reduced ethanolamine utilization, result in ethanolamine accumulation in the gut, inducing leaky gut, which in turn activates systemic inflammation, ultimately accelerating cognitive decline in older adults.

Abstract citation ID: igae098.2630

### MULTIMODEL APPROACHES IDENTIFY CONSERVED ROLE OF TRP CATABOLIC PATHWAY IN THE REGULATION OF LIFE- AND HEALTHSPAN

Naci Oz, and Alaattin Kaya, *Virginia Commonwealth University, Richmond, Virginia, United States*

The activity of the conserved Kynurenine (KYN) pathway, a major catabolic pathway of Tryptophan (trp), is controlled mainly by the indoleamine 2,3-dioxygenases IDO1, and IDO2, which catalyze the first rate-limiting step of trp catabolism by converting trp into kynurenic acid. The KYN pathway is the sole de novo NAD+ biosynthetic pathway, generating NAD+ from ingested trp. In addition, increased Ido1/2 activity, and intermediate metabolites such as quinolinic acid, as a result of normal aging, are mechanistically linked to inflammaging and neurodegenerative disorders such as Alzheimer's diseases. Therefore, circulating kynurenines were suggested as a potential biomarker of frailty in humans. In contrast, decreased trp degradation has been beneficially implicated as a potent regulator of age-related diseases and lifespan. Although the KYN pathway is a viable therapeutic target against aging and age-related diseases, there have been no comprehensive studies in mice to understand the role of the KYN pathway in regulating life- and healthspan. To understand the conserved effect of the KYN pathway in modulating life- and healthspan, we developed individual and combined whole-body knockout transgenic mouse strains lacking genes IDO1, IDO2 or IDO1/IDO2. Using multi-omics approaches, we comprehensively characterized the tissue and sex-specific biological age and molecular and metabolic alterations mediated by KYN pathway deficiency. Along with the healthspan measurements, we showed a potentially conserved role that decreased trp catabolism might be a conserved modulator of life- and healthspan.

Abstract citation ID: igae098.2631

### MUSCLE PRESERVATION AND PHYSICAL ACTIVITY: HYBRID TRANSCRIPTOMIC ANALYSIS IN A MONKEY MODEL

Stefano Donega[1], Nirad Banskota[2], Supriyo De[1], and Luigi Ferrucci[1], *1. National Institute on Aging, Baltimore, Maryland, United States*, *2. National Institutes of Health, Bethesda, Maryland, United States*

Muscle strength declines with age, leading to decreased mobility. Old sedentary people have been reported with imbalance in energetic metabolism, a pro-inflammatory profile, and proteolysis. Engaging in exercise, beyond just strength training, has been shown to mitigate this decline, although the underlying biological mechanisms remain unclear. Rhesus monkeys, trained to run on treadmills, present a valuable model for investigating the mechanisms involved in the preservation of muscle function induced by physical activity. We trained 27 males and females monkeys aged 9-15 years for 2 month to run on a ramp up to 1h for 3-5 days/week for 3 months. Continuous monitoring of heart rate, temperature, and physical activity levels was conducted using implantable data loggers, along with daily tracking of body weight and food intake. Vastus lateralis muscle punches were collected at baseline, after 3-months treadmill and ensuing 3-week sedentary state to perform deep hybrid RNA-seq combining deep short-reads (800M, Illumina) and long-reads (PacBio). Treadmill intervention revealed an enrichment in MAPK activity, keratinocyte proliferation, extracellular transport and microtubule formation alongside reductions in ribosomal compartments, ncRNA processing and lysosome. Upon transitioning to a sedentary state, the same subjects increased in extracellular matrix, immune cells migration and chemotaxis (leukocytes, granulocytes, neutrophils and monocytes) and reduced mitochondrial component and function, spliceosome, U2 complex, and ribosome. On-going analyses are focusing on differential splicing and proteomics interaction. These preliminary data suggest at RNA level, exercise enriches muscle function and metabolism while sedentary leads to decreased mitochondrial function and immune cell dynamics in monkeys.

Abstract citation ID: igae098.2632

### NICOTINAMIDE RIBOSIDE SUPPLEMENTATION PROTECTS AGAINST VIRAL-INDUCED NEURODEGENERATION IN AGED MICE

Daniel Enterria-Morales[1], Anais Claire Gonzalez[1], Bianca Jallorina[2], Adrian Martinez[2], Andrew Doan[2], Aadit Karve[2], Mikaela Joya[2], and Matthew Shtrahman[1], *1. University of California San Diego, La Jolla, California, United States*, *2. University of California San Diego, San Diego, California, United States*

Several lines of research support the hypothesis that boosting cellular nicotinamide adenine dinucleotide (NAD+) may induce metabolic adaptation that helps neurons maintain healthy function as they age and slow neurodegeneration, making this approach a promising therapy for neuroregeneration. To prove this strategy, we tested the ability of Nicotinamide Riboside (NR), a pyridine-nucleoside precursor to NAD+, to prevent neuronal cell loss in a model of viral induced neurodegeneration using recombinant adeno-associated virus (rAAV). C57BL/6J wild-type mice (18 months old) were obtained from the National Institute of Aging (NIA). NR was administered into the animals' drinking water (12mM) for three consecutive weeks before the injection of a recombinant rAAV expressing the LacZ gene under the control of the CMV promoter (rAAV-CMV-LacZ) at a dose of 1x1013 gc/mL into the unilateral hippocampus. For the control groups, the mice were injected either with a saline solution or with the rAAV-CMV-LacZ, but no NR supplementation was provided. For the experimental group, NR treatment was maintained for 12 weeks until the date of sacrifice. We found that NR supplementation partially rescued the rAAV-induced neurodegeneration in the CA3 of the hippocampus. NR supplementation reduced the activation of microglia and astrocytes in the CA3, partially rescuing the inflammatory response to the rAAV infection. Our findings support the idea of boosting NAD+ levels as a neuroprotective approach to metabolically help neurons of the hippocampus to respond to chronic damage in the form of aging. Further mechanistic studies are required to better understand the cellular pathways that underlie these findings.

Abstract citation ID: igae098.2633

### THE SIGNALING PATHWAY OF SRF-SIRTUIN-2 GENE AND MITOCHONDRIAL FUNCTION

Xiaomin Zhang, Gohar Azhar, Fathima Ameer, and Jeanne Y Wei, *University of Arkansas for Medical Sciences, Little Rock, Arkansas, United States*

**Background:** The sirtuin-2 (SIRT2) is one of the seven sirtuin proteins that deacetylate histones and other non-histone proteins. SIRT2 is localized mainly in the cytoplasm, but it may shuttle into the nucleus. SIRT2 regulates cellular metabolism, growth, stress response and aging. However, the transcription factors that regulate the SIRT2 gene have not been fully elucidated. Here we report the identification of two novel binding sites for the serum response factor (SRF) in the SIRT2 promoter, and the effect of SIRT2 gene expression on mitochondrial function.

**Methods:** The transcription factor binding sites in the SIRT2 promoter were analyzed with bioinformatic tools, including TRANSFAC, TFBIND and were verified with sequence analysis. SH-SY5Y cells were cultured in either regular serum or low serum medium to simulate an ischemia condition, respectively. The mitochondrial function was measured with a Seahorse XFe96 Analyzer.

**Results:** and conclusions We have identified two novel SRF binding sites in the human SIRT2 gene promoter, indicating that SRF binds to the SIRT2 promoter, and may recruit SRF cofactors to regulate the SIRT2 gene. SIRT2 plasmid transfection affects mitochondrial function. In the simulated ischemia condition, SIRT2 expression increased the mitochondrial oxygen consumption rate (OCR), while reducing the glycolysis rate (ECAR), suggesting that SIRT2 improves oxidative phosphorylation and protects cells from ischemia. In addition, the biological activators and inhibitors of the Rho/SRF signaling pathway could be used to modulate SIRT2 expression, thereby regulating mitochondrial function in aging and senescence.

Abstract citation ID: igae098.2634

### XANTHURENIC ACID (XA) TREATMENT IMPROVES OVERALL HEALTH IN AGED MICE

Sagar Vyavahare[1], Sandeep Kumar[1], Sonu Kumar Gupta[1], Kehong Ding[1], Carlos Isales[1], and Sadanand Fulzele[2], *1. Augusta University, Augusta, Georgia, United States*, *2. Augusta University, Augusta University, Georgia, United States*

Our published studies demonstrated disturbance in the Tryptophan-kynurenine pathway with age, notably marked by increased kynurenine levels. This study aimed to examine the levels of serum xanthurenic acid (XA) in aged mice and investigate the potential impacts of XA treatment on their overall health, specifically musculoskeletal health. Our findings revealed that XA serum levels decrease with age. The XA treatment decreased pro-inflammatory cytokines (TNF-α, IL-1, IFN-γ, IL-6) in serum. Moreover, XA treated aged mice showed improved various health parameters compared to controls. They displayed improved grip strength, locomotor activity, and increased endurance. Musculoskeletal analyses unveiled improved bone microarchitecture and increased muscle fiber size in XA-treated mice group. Molecular studies elucidated that XA treatment prevented oxidative stress (SOD2), reduced senescence markers (p16, p21), and mitigated inflammation (IL1-β) in the muscles of aged mice. Interestingly, XA treatment also correlated with decreased apoptosis (Caspase-3, Caspase-7) in muscle tissues, indicative of improved cellular survival. The molecular analysis showed a fostering environment for myogenesis (increased MyoD and Myogenin expressions) in XA-treated mice muscles. These comprehensive results suggest a promising therapeutic role for XA, emphasizing its potential as a critical metabolite in age-related pathogenesis.

### SESSION 7215 (POSTER)

### CARE VALUES AND PREFERENCES

Abstract citation ID: igae098.2635

### ADVANCE CARE PLANNING AND END-OF-LIFE EXPERIENCES OF HIGH HEALTH CARE UTILIZERS IN THE LAST YEAR OF LIFE

Omar Marin, Sriya Bandi, Charlotte Duty, and Alex Tate, *University of Chicago, Chicago, Illinois, United States*

As the U.S. population continues to get older, Americans are projected to consume more healthcare than ever before. Advance care planning (ACP), which documents future goals of care in the context of limited decision-making capacity, is essential to delivering patient-centered care aligned with wishes and values and correlates with less utilization at the end-of-life (EOL). ACP is critical for high healthcare utilizers, who have multiple comorbid conditions and often unmet social needs. This pilot study used proxy postmortem interviews (n=20) to identify and explicate the most common factors shaping the EOL care experiences of high healthcare utilizers dwelling in vulnerable Chicago neighborhoods, and ACP's role in these experiences. Modified open coding of data revealed five central themes in shaping decedents' EOL experiences and ACP: (1) decedent support from immediate network ties, (2) patient acceptance of declining health, (3) proxy confidence in EOL decision making, (4) individual values motivating pursuing certain types of care, and (5) an orientation towards the treatment imperative, defined by Shim et al. (2008) as an inexorable momentum towards treatment at any cost. Within the last 12 months of life, even if patients had a formal or informal ACP in place, proxies reported a default to more medical intervention by patients. Just five proxies reported care transitions to palliative/hospice care. This pilot study presents a novel approach to better understanding EOL care and experiences of vulnerable high healthcare utilizers and underscores a need for further investigation into the relationship between ACP and EOL experiences among this group.

Abstract citation ID: igae098.2636

### INTEGRATED PERSON-CENTERED INTERVENTIONS FOR OLDER PEOPLE'S CARE IN SWEDEN: SCOPING REVIEW AND EXPERT OPINION

Mariam Kirvalidze[1], Anne-Marie Boström[1], Anne Liljas[1], Megan Doheny[1], Anne Hendry[2], Brendan McCormack[3], Janne Agerholm[1], and Amaia Calderón-Larrañaga[1], *1. Karolinska Institutet, Stockholm, Stockholms Lan, Sweden*, *2. University of the West of Scotland, Paisley, Scotland, United Kingdom*, *3. University of Sydney, Sydney, New South Wales, Australia*

The recent shift towards a holistic, person-centered approach to older people's care that considers a person's overall well-being requires collaboration within and between medical and social healthcare organizations. To map and contextualize the scientific literature on the effectiveness of integrated person-centered care interventions, including implementation experiences in Sweden. Publications were identified by searching MEDLINE and contacting experts. Workshop discussions in Stockholm (Sweden), on 21/03/2023, were recorded and transcribed. Original peer-reviewed articles published in Swedish or English from 01/01/2000 to 10/03/2023, were screened. Evidence from included studies was narratively synthesized and interventions were categorized using two frameworks: (1) Integrated Care for Older People (ICOPE) and (2) Integrated People Centered Health Services (IPCHS). Workshop discussions were thematically summarized. We included 73 publications analyzing 31 unique and heterogeneous interventions pertaining, mainly, to the micro and meso levels. Among publications measuring mortality, 15% were effective. Subjective health outcomes showed improvement in 24% of publications, morbidity outcomes in 42%, disability in 48%, and service utilization in 58%. Experts emphasized the need for participatory designs and shared ownership for adoption and scalability of interventions, and transitions from person- to people-centered integrated care. In conclusion, integrated person-centered care interventions demonstrated the strongest evidence for the optimization of service utilization. Unexpectedly, evidence for health outcomes was less consistent. This could be due to several issues mentioned by the experts, such as poor evaluation practices, lack of participatory research approaches, and scarcity of macro-level initiatives. Our findings offer internationally transferable perspectives on the real-world challenges in delivering integrated care.

Abstract citation ID: igae098.2637

### PATIENT PORTAL USE, PATIENT TRUST, SOCIAL DETERMINANTS OF HEALTH, AND SHARED DECISION-MAKING AMONG US OLDER ADULTS

Yuling Chen, *Johns Hopkins University, Baltimore, Maryland, United States*

**Background:** Shared decision-making (SDM) and patient portals are increasingly embraced in health care and recommended in cardiovascular disease (CVD) guidelines. This study investigated the associations between patient portal use, patient trust in the healthcare system, social determinants of health (SDOH), and patient involvement in SDM among adults 65 years or older with or at risk of CVD.

**Methods:** We conducted a cross-sectional study of US adults from the 2022 Health Information National Trends Survey. CVD risk was defined as reporting a heart condition, diabetes, hypertension, and/or current smoking. SDM was measured by asking how often patients' providers involved them in health decisions over the past year. A weighted multivariable logistic regression model was employed, adjusting for sociodemographic and clinical characteristics.

**Results:** The study included 1,139 older adults with or at risk of CVD, with a mean age of 73.5 (SD 6.7) years. The sample included 54.0% females; 66.4% identified as white. Older adults who accessed the patient portal 6 to 9 times in the past year were 1.98 times more likely to engage in SDM (95% CI 1.13-3.48) compared to those without access. Older adults with high trust in the health system were 3.15 times more likely to engage in SDM (95% CI 1.89-5.23) than those with low trust. However, SDOH (inability to afford meals, being forced to move, and lack of transportation) was not significantly associated with SDM.

**Conclusion:** These findings underscore the vital role of utilizing patient portals and fostering trust in enhancing older adults' engagement in SDM.

Abstract citation ID: igae098.2638

### PATIENT PRIORITIES CARE (PPC) IN HISPANIC PATIENTS WITH DEMENTIA

Alejandra Mera[1], Emily Peel[2], Aanand Naik[3], and Rafael Samper-Ternent[4], *1. The University of Texas Medical Branch, Houston, Texas, United States*, *2. McGovern Medical School, Houston, Texas, United States*, *3. The University of Texas Health Science Center, Houston, Texas, United States*, *4. The University of Texas Health Houston, Houston, Texas, United States*

Hispanics are the fastest growing underrepresented population in the U.S and have a higher risk of dementia compared to non-Hispanic whites. Lack of trust, limited health care access, and lack of culturally adapted interventions contribute to health disparities in this population group. Patient Priorities Care (PPC) is an evidenced-based approach that improves health outcomes in patients with multimorbidity. PPC guides patients, caregivers, and their providers to set realistic health care goals based on the patient's values. Little is known about the benefits of PPC for Hispanics with multimorbidity and dementia. We designed and implemented a pilot feasibility study to adapt the PPC approach for Hispanics with multimorbidity and dementia (PPC-HD study). The PPC approach consists of a Priorities Setting Session with the patient, and caregiver, followed by a Care Alignment session with the patient's clinician. In our sample of 20 Hispanics with dementia and multimorbidity, 85% of the participant care plans include patients' preferences indicating the willingness of clinicians to use the PPC approach. Eighty-three percent of participants find PPC a rewarding experience with 67% reporting satisfaction in the availability of Spanish-speaking research staff, 61% reporting satisfaction in discussing healthcare preferences, and 56% reporting satisfaction with knowing patients' preferences for future healthcare decisions. PPC is a feasible approach in this population group. Most clinicians used the patients' preferences and included them in their care plans. Access to bilingual staff and delivery of the intervention in Spanish was highly valued by participants and caregivers. Participation in PPC was deemed very valuable.

Abstract citation ID: igae098.2639

### SOCIAL WORKERS' ROLE IN CULTIVATING ILLNESS UNDERSTANDING AND PROGNOSTIC AWARENESS: PERSPECTIVES FROM THE FIELD

Arden ODonnell, and Judith Gonyea, *Boston University, Boston, Massachusetts, United States*

For patients experiencing serious or life-limiting illnesses, informed decision-making depends on understanding prognosis and illness trajectory. Although physicians are viewed as the most appropriate group to share medical information, there is growing recognition that: a) cultivation of prognostic awareness occurs over time through a series of conversations in which patients continue to redefine medical treatments and broader life goals and b) other clinicians can contribute to these discussions. Using role theory, this qualitative study's explores how the growing shift to a team approach is experienced by hospital-based physicians (MDs) and palliative social workers (PSWs),. Through semi-structured Zoom interviews with 17 matched MDs and PSWs across the U.S., we explored the degree of alignment or convergence of MDs' and PSWs' views on PSWs' professional roles in prognostic conversations, including perceptions of role definitions, role sharing, role boundaries, and role conflicts. Interpretive description methodology, coupled with thematic analysis, revealed key insights into PSWs' comfort levels in assuming roles and interventions in this prognostic awareness cultivation as well interprofessional convergence affirming PSWs' role in a) assessing patients' readiness for prognostic discussions, b) identifying gaps in patient understanding c) translating medical conditions into lived reality, and d) supporting emotional processing of patients and their families. Concerns about potential role conflicts emerged when PSWs were expected to either engage in or overstep into medically complex discussions. This study underscores the important role of social workers role in enhancing prognostic understanding and emphasizes the significance of role clarity, trust, and communication for effective interpersonal teams.

Abstract citation ID: igae098.2640

### UNHOUSED OLDER ADULTS' PREFERENCES FOR PROGNOSTIC COMMUNICATION AND ILLNESS-RELATED CARE

Abigail Latimer[1], Michael Light[2], Olivia Sasdi[1], Mohammad Hossain[1], Debra Moser[1], and Natalie Pope[1], *1. University of Kentucky, Lexington, Kentucky, United States*, *2. Harborview Medical Center UW Medicine, Seattle, Washington, United States*

The number of older adults experiencing homelessness (OAEH) is growing. This vulnerable population is exposed to harsh conditions and fragmented healthcare, making even the most treatable health conditions life-threatening. Understanding seriously ill OAEH preferences for prognostic communication and illness-related care are needed research areas. This qualitative study was part of a larger project adapting Ariadne Labs's Serious Illness Conversation Guide (SICG) for use with OAEH. We interviewed 11 unhoused older adults (≥ 50 years) with a serious illness - mostly men (n = 9) between age 53 and 72 (M = 61). Interview questions were developed corresponding to the model of prognostic communication (i.e., the SICG) with a particular interest in understanding older adults' 1) communication preferences regarding their illness and prognosis and, 2) goals and values related to their illness and prognosis. We utilized reflexive thematic analysis which included coding and creating transcript summaries and data matrices to help the research team familiarize, condense, and observe patterns within the data. Findings reflect how 1) clinicians should deliver prognosis, 2) OAEH receive prognosis, and 3) OAEH treatment goals and values. Trust and authenticity emerged as essential components of prognostic delivery, both in what and how information is shared. Participants described past communication with healthcare professionals and the impact of these conversations. Illness-related priorities for OAEH included making practical plans for dying and death, leaving a legacy for their children, and maintaining independence. Study findings can inform clinical practice and intervention research to improve how this population receives serious illness communication.

### SESSION 7220 (POSTER)

### CLINICAL PRACTICE

Abstract citation ID: igae098.2641

**A CRISIS LEADERSHIP FOR NURSE MANAGERS IN CRISIS SITUATIONS: DEVELOPMENT AND PSYCHOMETRIC EVALUATION**

Sun Ju Kim, *Chungnam National University Se-Jong Hospital, Se-Jong, Republic of Korea*

Crisis situations such as COVID-19 have a negative impact on society and the economy, with increased mental health issues, medical crises, and mortality among nurses. The inadequate leadership of nurse managers during crisis situations can negatively impact the quality of patient care and infection transmission. Consequently, the aims of this study were to develop a crisis leadership scale for nurse managers and to evaluate its psychometric properties. For the development of the initial items, the literature was reviewed along with the existing measuring tools. This study was conducted from August to October 2023. A total of 443 nurse managers in 34 hospitals in Korea participated in the study to verify the validity and reliability of the instrument. The exploratory and confirmatory factor analyses yielded a five-factor solution, while the confirmatory factor analysis showed a good fit (RMSEA =.07, SRMR =.02, CFI =.92, TLI =.91). Therefore, the final instrument was derived, containing 28 items on "crisis management competency" (12), "decision-making" (6), "collaboration" (4), "communication" (3), and "building trust" (3), as well as 5 factors, scored on a 5-point Likert scale. The Cronbach's α for this instrument was.96. In conclusion, the crisis leadership scale showed good psychometric properties for four validity metrics (content, construct, convergent, discriminant and criterion validity) and internal reliability. These findings indicate that the instrument can be applied in both research and clinical practice, being capable of quantitatively measuring the degree of crisis leadership skills of nurse managers.

Abstract citation ID: igae098.2642

**ANTI-AMYLOID THERAPIES FOR ALZHEIMER'S DISEASE: PATIENTS' AND FAMILIES' PERCEPTIONS**

Kaitlyn Lucca, Allison Lapins, and Darby Morhardt, *Northwestern University, Evanston, Illinois, United States*

With new drug therapies available aimed to slow the progression of Alzheimer's Disease (AD), this study hopes to gain insight into the perceptions, concerns and perceived barriers from patients and families who are impacted by AD. Drug consultations for these therapies are currently taking place at the Northwestern Neurobehavior and Memory Clinic with potentially eligible patients and their families. Study aims are to: 1) measure patient and family perception, barriers and concerns of the drug therapy following a formal consultation or informal discussion with a neurologist, and 2) gather data on the patient and family experience with the drug consultation as a method for making an informed decision. After consultation with their behavioral neurologist, participants are asked to complete the survey with a study team member. To date, 10 participants have completed the survey following consultation with a neurologist. Preliminary results found the highest reported concerns with starting anti-amyloid therapies to be medical complications [larger brain bleeds, brain swelling, micro-hemorrhages] and financial burden. The majority of participants' interest levels remained the same or were increased following the consultation. Those who reported a decrease in interest level were concerned that the therapy would not be impactful on their daily life. Families will continue to be surveyed following consultation with a neurologist with additional results reported in the coming months.

Abstract citation ID: igae098.2643

**INTEGRATED PRIMARY CARE MODEL FOR PEOPLE LIVING WITH DEMENTIA REDUCES HIGH-RISK MEDICATIONS AND HOSPITALIZATIONS**

Carolyn Clevenger, Anjali Khakharia, Laura Medders, and Miranda Moore, *Emory University, Atlanta, Georgia, United States*

Harmful care including the prescribing of high-risk and potentially inappropriate medications for older people is widespread among older adults, including people living with dementia (PLWD). Integrated Memory Care (IMC) is a comprehensive dementia care model where patients and their family caregivers access dementia-sensitive geriatric primary care. The IMC conducted a retrospective observational study of patients of IMC, Cognitive Neurology (CN), and Primary Care (PC) clinics aged 65 and older with a diagnosis of dementia in 2019-2021. We matched patients by age, gender and race and measured the hospitalization, rate of deprescribing and inappropriate screening test referrals using logistic regressions controlling for clinic. Additionally, we conducted a regression adjusted for state of illness (proxied by activities of daily living (ADL)) for the IMC and CN clinics. IMC patients had higher odds of deprescribing high dose antipsychotics, benzodiazepines, and opiates when compared to CN patients. IMC also had higher odds of deprescribing high dose antipsychotics, benzodiazepines, and opiates when compared to PC patients. For hospitalizations, CN had 10% [2 times] higher odds, when compared to IMC patients in unadjusted models. After adjusting for ADL scores, CN patients had 66% higher odds of having a hospital admission during the study period. Dementia-sensitive primary care reduces the rate of total and ambulatory-sensitive hospitalizations reducing healthcare costs and causing less disruption to care as well as reduces inappropriately prescribed medications among PLWD.

Abstract citation ID: igae098.2644

**TRANSFORMATIVE CARE: LIFESTYLE INTERVENTIONS IN UNDERSERVED AGING**

Kristen Neises, *University of Louisville, Louisville, Kentucky, United States*

In response to the escalating challenges posed by an increasingly aging population in the United States, particularly in rural areas, an urgent demand arises for innovative healthcare solutions. This presentation illuminates the formidable hurdles faced by older adults with multiple chronic conditions, most of which result from lifestyle. The Optimal Aging Lifestyle Medicine (OALM) program emerges as a catalyst for transformative change in older adult care. Focused on medically underserved regions, this program empowers healthcare professionals through education in age and dementia-friendly lifestyle medicine, emphasizing evidence-based lifestyle interventions for preventing, managing, and reversing chronic diseases. The OALM program forges a collaborative care approach, uniting professionals across disciplines using the Flourish Model and extending outreach via Project ECHO to remote rural areas. Its mission encompasses filling training gaps by educating healthcare workers across Kentucky and evolving a traditional geriatric clinic into an innovative lifestyle medicine center for older adults. This presentation delves into the integration of lifestyle medicine into geriatric clinics, explores older adults' perspectives on care through focus group discussions, and the development of an interdisciplinary lifestyle medicine curriculum. Case studies will spotlight a resilient patient care approach, highlighting successes and addressing challenges in groundbreaking initiatives navigating the intricacies of geriatric care, ultimately optimizing aging outcomes and reshaping narratives in rural communities.

Abstract citation ID: igae098.2645

**VIRTUAL REALITY FOR TREATMENT OF DEPRESSION IN AN OLDER ADULT WITH PARKINSON'S DISEASE**

Sabine Lohmar, and Amy Fiske, *West Virginia University, Morgantown, West Virginia, United States*

Research on the effectiveness of virtual reality, an interactive computer-generated environment, as a form of behavioral activation for older adults with depression, has been minimal but promising (Yen & Chui, 2021). A case study of an older male nursing home resident with Parkinson's Disease who uses a wheelchair is presented to provide clinical evidence for the value of using virtual reality in treating depression in older adults with physical limitations. Virtual reality sessions consisting of immersive and interactive outdoor experiences (e.g., fishing, hunting, hiking, kayaking) were conducted for an average of 20 minutes once every two weeks for six months. The Geriatric Depression Scale Shortened Form (GDS-S) was administered before, during, and after the use of virtual reality to assess depressive symptoms. The patient's depressive symptoms improved from intake (GDS-S = 11) to termination (GDS-S = 4). Moreover, subjective reports from the patient indicated an overall elevation in mood, perceived enjoyment, increased motivation, and reduced pain. This case study demonstrates the potential benefits of virtual reality for physically limited older adults with depression who may face barriers, for example, due to a movement disorder, to engaging in previously enjoyed activities.

### SESSION 7225 (POSTER)

### COGNITION I (POSTERS)

Abstract citation ID: igae098.2646

**ADHERENCE TO INTERVENTIONS AND COGNITIVE CHANGES IN FALLEN OLDER PEOPLE: RANDOMIZED CLINICAL TRIAL**

Juliana Ansai, Graziele Norberto Pereira, Mariana Luiz de Melo, , Mariana Ignácio Sossai, Leticia Teodoro Maciel, Mel Silva de sá, Ana Laura Oliveira Dias, and Karina Gramani-Say, *Federal University of São Carlos, São Carlos, Sao Paulo, Brazil*

Falls are an important health challenge for older people and society. Understanding how to reduce cognitive risk factors for falls is essential to develop effective prevention strategies. The purpose of this work was to assess the impact of adherence to interventions on cognitive changes after 16 weeks in older people with a history of falls. A controlled, randomized, single-center clinical trial was conducted with brazilian community-dwelling older adults with a history of recurrent falls in the past 12 months. Participants were allocated to Intervention Group (IG) and Control Group (CG). The IG underwent case management, including multidimensional assessment, explanation of fall risk factors, proposal of intervention based on risks, development of an individual fall intervention plan, plan monitoring and review. The CG received monthly health-related guidance. The assessment included cognitive measures (Addenbrooke's Cognitive Examination ACE-R, Mini-Mental State Examination, Verbal Fluency tests, Clock Drawing test CDT) at baseline and after 16 weeks, and adherence (frequency) to the intervention after 16 weeks. As results, the sample was composed by 55 participants (26 CG, 29 IG). The mean adherence to intervention was 80.27%. The IG improved the TDR performance, compared to the CG ($p=0.034$). Both groups presented a non-significant improvement in the ACE-R score over time. Adherence to the case management intervention did not influence changes in cognitive risk factors for falls ($p>0.050$). This findings point the need to understand better the factors related to improvement on risk factors for falls, to achieve personalized approaches in older people.

Abstract citation ID: igae098.2647

**BUSYNESS MAY NOT MATTER: ASSOCIATIONS BETWEEN BUSYNESS, ACTIVITY ENGAGEMENT, AND COGNITION**

Allison Bielak, *Colorado State University, Fort Collins, Colorado, United States*

Participating in a greater variety and frequency of activities is associated with higher cognitive performance across adulthood. A potentially related metric is perceived busyness (Martin & Park, 2003), defined as scheduled activity patterns. Few studies have investigated this metric, with one finding that those who rated themselves as busier performed higher on a range of cognitive tests (Festini et al., 2016). The present study aimed to answer several questions: 1) Does the link between busyness and cognition replicate?; 2) Does stress interact with busyness in predicting cognition?; 3) How similar is busyness to frequency and variety of activity engagement?; 4) Does busyness predict cognition better than activity variety or frequency? Adults aged 60–92 years ($M=70.5$, $n=206$) completed questions related to busyness (in a typical week, how busy is your schedule), stress towards busyness, activity based on past 2-year participation, and cognitive tests. Hierarchical linear regressions controlling for age and education showed busyness was not a significant predictor of processing speed, episodic memory, vocabulary, executive functioning, or reasoning, but did significantly predict working memory (Std B=-.16). Stress about busyness did not interact with busyness for any cognitive domain. Busyness was weakly correlated with activity variety and frequency (rs=.20) and did not significantly predict cognition beyond the activity variables. Although a simple measure of busyness was used, greater busyness was not related to higher cognition. Busyness was only weakly related to the activity indices, and findings suggest that activity variety and frequency might be more valuable measures in evaluating cognition.

Abstract citation ID: igae098.2648

### CHILDBEARING STATUS, PARITY, AND COGNITIVE HEALTH AT MIDLIFE: A LONGITUDINAL APPROACH

Mieke Thomeer[1], Joseph Wolfe[1], Rin Reczek[2], Dee Ferguson[1], and Rui Cao[2], *1. University of Alabama at Birmingham, Birmingham, Alabama, United States*, *2. The Ohio State University, Columbus, Ohio, United States*

Childbearing histories matter for multiple health outcomes across the life course. Further, there is growing evidence that childbearing may also shape mid- and later-life cognitive health, although previous research is limited due to difficulty in accounting for the role of selection. Multiple factors that place women at risk for specific childbearing experiences also place them at risk for poor cognitive health—including socioeconomic background, health issues in early life, and cognitive ability in adolescence. We advance research by analyzing the 1979 National Longitudinal Survey of Youth (NLSY79) (N=2,257), a longitudinal nationally representative dataset from the US that began collecting data when women were in their late teens and early 20s. Final models account for a large array of social, economic, demographic, cognition, and health variables to estimate how childbearing histories matter for three cognitive health outcomes at age 48. In unadjusted models, we find that childbearing status, parity, and age at first birth are associated with self-reported memory and word recall, and age at first birth is also associated with backwards number counting. In fully adjusted and matched models, the only significant differences relate to memory. In future analysis, we will examine which specific factors drive associations, incorporate cognitive health at age 60, and replicate analyses in additional datasets. We demonstrate the role of childbearing as a determinant of cognitive health, suggesting the need to incorporate childbearing into life course models of dementia risk. We also draw attention to the importance of considering selection when identifying social determinants of cognitive health.

Abstract citation ID: igae098.2649

### CHRONIC PAIN, PAIN INTERFERENCES, AND COGNITION AMONG MIDDLE- AND OLD-AGED AMERICANS: A FIXED-EFFECTS ANALYSIS

Chang Yu[1], Ashley Barr[1], Yulin Yang[2], and Hanna Grol-Prokopczyk[1], *1. University at Buffalo, SUNY, Buffalo, New York, United States*, *2. University of California San Francisco, San Francisco, California, United States*

**Objectives:** The current study examines the relationship between chronic pain, chronic pain interference, and cognitive function in middle-aged and older Americans, focusing on how these associations may vary by educational background. Method: Data were obtained from the 2004-2006 and 2013-2017 waves of the Midlife in the United States Study (MIDUS 2 and MIDUS 3, N=6266 observations nested with 3965 individuals). We employed fixed-effects linear models to examine within-individual changes, specifically, the relationship between changes in chronic pain, pain interferences (with activity, mood, relations, sleep, and enjoyment) and their impact on cognitive function (specifically episodic memory and executive function). In addition, group differences in these fixed effects by education were examined via interaction terms.

**Results:** The results indicated that changes in pain negatively correlated with episodic memory, but this association was observed only for individuals without any college. Additionally, changes in pain interferences showed a negative correlation with both episodic memory and executive functioning across all levels of educational attainment.

**Discussion:** Findings highlight the importance of not only considering the role of persistent pain but also the effects of pain interferences on cognitive decline among aging population.

Abstract citation ID: igae098.2650

### COGNITIVE GROWTH AND DISPERSION AT MIDLIFE: IMPLICATIONS FOR COGNITIVE MAINTENANCE AND RESERVE

Chandra Reynolds[1], Anqing Zheng[1], Elizabeth Muñoz[2], Robin Corley[1], and Sally Wadsworth[1], *1. University of Colorado Boulder, Boulder, Colorado, United States*, *2. The University of Texas Austin, Austin, Texas, United States*

Dispersion in performance across cognitive tasks provides a unique perspective of cognitive functioning beyond overall performance and may be predictive of poorer memory and dementia risk in older adults. What dispersion indexes at earlier age periods is unclear, although a lifespan perspective may provide an earlier window on cognitive specialization and reserve at the cusp of midlife. We sought to evaluate individual differences in dispersion in growth from adolescence and at midlife. Using twin and sibling data from the Colorado Adoption Twin Study of Lifespan behavioral development and cognitive aging (CATSLife1; N = 1245, MAge = 33.30, SD = 5.00, 53% Female), we evaluated dispersion using a latent model approach across tasks from a specific cognitive ability (SCA) battery, referencing age 16 and CATSLife1 assessment norms, and WAIS-III subtests at CATSLife1. At midlife, overall performance was modestly positively correlated with dispersion for the SCA tasks and the WAIS-III subtests (r=0.07 and 0.37). Referencing age 16 norms, SCA performance was correlated with dispersion at 0.88, suggesting differential growth from age 16 correlates strongly with CATSLife1 performance. Education predicted more dispersion referencing age 16 norms (B=0.41; CI=0.31 to 0.48], but not midlife dispersion for SCA or WAIS-III. Sibling similarity for the latent dispersion measures were moderate for identical (MZ) twins (r's=0.55-0.86) and smaller for fraternal twins (DZ) (r's=0.26-0.60), other biological siblings (r's=0.10-0.36), and siblings in adoptive families (r's=-0.15-0.11). Dispersion in growth may reflect differential specialization with implications to reserve, whereas variability in performance at midlife may be reflective of differences in maintenance.

Abstract citation ID: igae098.2651

### DOMAIN-SPECIFIC SUBJECTIVE COMPLAINTS AND OBJECTIVE COGNITIVE SCORES IN OLDER ADULT CANNABIS USE

Elizabeth Anquillare, Juliamaria Coromac-Medrano, Adrianna Neiderman, and Rachel Thayer, *University of Colorado Colorado Springs, Colorado Springs, Colorado, United States*

Research on the cognitive effects of cannabis among older adults remains sparse and equivocal. Subjective cognitive complaints are predictive of future objective cognitive decline; however, few studies have explored this relationship in older adults with cannabis use (OACU) and domain-specific relationships have yet to be investigated. This study aimed to identify relationships between subjective and objective cognition across attention, memory, and executive functioning in a pilot sample of OACU. Participants included OACU between 60 and 79 years (N=17, Mage=68.29, SD=5.61), of which 94% were White and 59% were women. An average 15.94 years of education (SD=2.38) was reported. Participants completed online questionnaires regarding subjective cognition and participated virtually in a brief cognitive assessment battery. Items from the subjective cognition questionnaires were separated by domain and correlations were run between domain-specific subscales and standardized scores on the objective cognitive tests. Worse performance on a switching task was negatively associated with subjective attention ($r=-.59$, $p<.05$), memory ($r=-.63$, $p<.05$), and executive function ($r=-.62$, $p<.05$). Additionally, worse performance on an inhibition task was negatively associated with better subjective attention ($r=-.50$, $p<.05$), memory ($r=-.51$, $p<.05$), and executive function ($r=-.52$, $p<.05$). Similar negative correlations between measures of subjective cognition and objective memory and attention performance demonstrated a medium effect size without achieving statistical significance. Overall, subjective cognition was associated with executive functioning performance more than memory or attention performance. This pattern suggests OACU tend to describe their cognitive function broadly. Providers may wish to ask more nuanced questions to ensure comprehensive understanding of subjective cognitive functioning.

Abstract citation ID: igae098.2652

### EYE MOVEMENTS REVEAL AGE DIFFERENCES IN HOW AROUSAL MODULATES MECHANISMS OF ATTENTIONAL CONTROL

Andy Kim, Kristine Nguyen, and Mara Mather, *University of Southern California Leonard Davis School of Gerontology, Los Angeles, California, United States*

The arousal-biased competition theory posits that inducing arousal increases attentional priority of salient stimuli while reducing priority of non-salient stimuli. However, older adults are hypothesized to exhibit dysfunction in the locus coeruleus-noradrenaline system that mediates both global arousal and attentional control. Unlike in young adults, older adults primarily do not exhibit shifts in attentional priority under increased arousal. However, the mechanism of how arousal biases attention in older adults is conflicting. We investigated whether the threat of unpredictable shock exhibits age differences in modulating multiple mechanisms of attentional control by observing eye movements. Participants completed two oculomotor search tasks in which the salient distractor is reactively disengaged (singleton search) or proactively suppressed (feature search). During singleton search, young adults made more first saccades toward the salient distractor when under the threat of shock. However, older adults did not exhibit changes in attentional priority for any stimuli under increased arousal. Our findings reveal older adults do not exhibit elevated priority for salient stimuli because arousal does not lead to shifts in priority. Furthermore, we observed that arousal did not affect the speed of attention processing in both age groups and that proactive suppression of the distractor persisted despite elevated priority. Lastly, we demonstrated that threat of shock modulated the locus coeruleus-noradrenaline system as seen with pupillometry and that evoked pupil responses are correlated with oculomotor function. Using a systems neuroscience approach, our findings demonstrate age differences in how the locus coeruleus-noradrenaline system interacts with neural networks of attention and oculomotor function.

Abstract citation ID: igae098.2653

### FUNCTIONAL LIMITATIONS, DEPRESSIVE SYMPTOMS, AND COGNITION ACROSS MID- AND LATE-LIFE: EVIDENCE FROM CHINA

Chang Yu, Ashley Barr, and Hanna Grol-Prokopczyk, *University at Buffalo, SUNY, Buffalo, New York, United States*

**Objectives:** This study examines the time-varying effects of functional limitations (IADLs and ADLs) and depressive symptoms on various cognitive functions, including mental status and memory among middle-aged and older Chinese individuals. It also assesses whether functional limitations and depression during middle age contribute to worse cognitive function in the later years independent of yearly shifts in depression and functional limitations. Method: Using longitudinal data from the China Health and Retirement Longitudinal Study (2011-2018), we conducted mixed-effects linear models to determine the intra- and inter- individual variation on the relationship between functional limitations, depression, and cognitive functioning (N = 22,039 observations nested within 10,054 individuals).

**Results:** Within-individual change in functional limitations and depression negatively predict cognitive functioning, with more functional limitations and depressive symptoms predicting a lower score in cognition, memory, and mental health, independent of age. Further, disabilities and depression in mid-life continue to predict worse cognitive functioning across the course of the study independent of any later changes in functional limitations and depressive symptoms and childhood conditions.

**Discussion:** From a life course perspective, adverse functionalities and mental health in midlife predicts worse cognitive function in later life independent of early life experiences.

Abstract citation ID: igae098.2654

### HARMONIZATION OF LATER-LIFE COGNITIVE FUNCTION ACROSS POPULATION-BASED COHORT STUDIES: RESULTS FROM HRS AND NHATS

Diefei Chen[1], Vishaldeep Sekhon[2], David Roth[2], Christopher Kaufmann[3], and Alden Gross[4], *1. Johns Hopkins University, Hanover, Maryland, United States*, *2. Johns Hopkins University, Baltimore, Maryland, United States*, *3. University of Florida College of Medicine, Gainesville, Florida, United States*, *4. Johns Hopkins Bloomberg School of Public Health, Baltimore, Maryland, United States*

Population-based cohort studies offer unique opportunities to study characteristics of people at greatest risk for cognitive decline. Harmonizing large, well-characterized cohorts powers investigations into subgroups or rare exposures/outcomes in their associations with cognitive decline. We derived a harmonized cognition score based on cognitive tests from the Health and Retirement Study (HRS) and the National Health and Aging Trends Study (NHATS), two of the largest nationally representative cohorts of older adults in the US. Using an item banking approach based on item response theory, we leveraged tests conducted in 2012-2018 Core interviews of two studies to leverage both common (i.e. anchors) and unique items to statistically harmonize a general cognition factor score. We evaluated concurrent and predictive criterion validity of the harmonized scores by 1) cross-sectionally comparing scores at baseline (year 2012) with algorithmically defined cognitive impairment status and demographics, and 2) longitudinally assessing associations between demographics and established risk factors for cognitive decline with cognitive trajectories using mixed effects models. Cross-sectionally, the harmonized score was lowest among dementia/probable dementia group (mean = -1.84, sd = 0.75). Longitudinally, cognitive decline was steeper for participants reporting hypertension (b = -0.04, se = 0.002), stroke (b = -0.06, se = 0.004), or diabetes (b = -0.04, se = 0.003) compared to those without. Inferences were similar in each study separately. This harmonized score using data from HRS and NHATS had strong criterion validity, and can be a resource for aging researchers to examine the rare factors associated with cognitive performance in diverse populations.

Abstract citation ID: igae098.2655

### MEMORY IN LATER LIFE: HOW NONAGENARIANS AVOID FORGETTING WHAT THEY WANT TO REMEMBER

Katie Cherry[1], Matthew Willis[1], Gavin Faucheux[1], Dominique Bernard[1], Mary Barnes[1], Celinda Reese-Melancon[2], Sangkyu Kim[3], and S. Michal Jazwinski[3], *1. Louisiana State University, Baton Rouge, Louisiana, United States*, *2. Oklahoma State University, Stillwater, Oklahoma, United States*, *3. Tulane University School of Medicine, New Orleans, Louisiana, United States*

Subjective perceptions of memory play an important role in cognitive adaptability in later life. While self-reported memory is typically assessed using objective surveys, few studies have examined what older people have to say about their own experiences using memory in everyday life. The purpose of this study was to examine older adults' memory management strategies and techniques that they use in everyday life to improve recall of information they do not want to forget. Participants consisted of 50 nonagenarians (90+ years of age) and two younger reference groups (60-74 and 75-89 years) who were enrolled in the Louisiana Healthy Aging Study (LHAS). All answered two open-ended questions regarding their preferred memory management strategies used in everyday life. Narrative responses were recorded, transcribed verbatim, and content analyzed for recurring concepts. Results yielded new evidence of environmental strategies, including object placement and non-electronic external memory aids (e.g., planners, calendars), and internal mnemonic techniques (e.g., rehearsal, association). When comparing internal mnemonic techniques specifically, nonagenarians were more likely to use association, whereas their younger counterparts were more likely to use imagery. These findings suggest that memory management techniques change very little in late adulthood. This research was supported grants from by the Louisiana Board of Regents through the Millennium Trust Health Excellence Fund [HEF(2001-06)-02] and the National Institute on Aging (P01 AG022064).

Abstract citation ID: igae098.2656

### NEIGHBORHOOD SOCIAL CAPITAL IS ASSOCIATED WITH VARIABILITY IN AMBULATORY COGNITIVE PERFORMANCE

Jinshil Hyun[1], Eric Cerino[2], Mindy Katz[1], Richard Lipton[1], and Martin Sliwinski[3], *1. Albert Einstein College of Medicine, Bronx, New York, United States*, *2. Northern Arizona University, Flagstaff, Arizona, United States*, *3. Pennsylvania State University, University Park, Pennsylvania, United States*

Prior studies suggested that within-person variability in cognitive task performance may be an early indicator of normative and pathologic cognitive aging. However, the influence of upstream social determinants of health (e.g. neighborhood) on cognitive variability is not well characterized. The aim of this study is to examine whether neighborhood social capital is associated with within-person variability in various cognitive domains and whether these associations are mediated by individual-level behavioral factors (i.e., physical activity, social interaction). The analysis sample included 316 individuals in Bronx, NY, enrolled in the Einstein Aging Study (mean age=77). Participants performed brief tests of processing speed, memory binding, and working memory on a smartphone up to six times daily over a two-week period. Neighborhood social capital was operationalized as a composite variable comprising economic connectedness, clustering, and volunteering rates, linked to participants' address through zip code tabulation area. Results from heterogeneous variance multilevel models suggested that living in areas with lower levels of social capital was significantly associated with greater variability in processing speed and memory binding (ps<.001). Controlling for individual-level physical activity reduced the magnitude of the effects of neighborhood social capital, suggesting potential (and partial) mediating role of physical activity. In conclusion, living in areas with better community social capital may reduce the risks of cognitive impairment. It would be crucial to support upstream neighborhood-level interventions to prevent cognitive impairment among older adults. Future research should examine more precise behavioral mechanisms linked to neighborhood environments to better suggest effective intervention strategies to promote cognitive health.

Abstract citation ID: igae098.2657

**QUANTIFYING ASSOCIATIVE AND ATTENTIONAL INFLUENCES ON MEMORY FOR EVENTS**

Alan Kersten, Kevin Darby, Megan Smithwick, and Julie Earles, *Florida Atlantic University, Boca Raton, Florida, United States*

Aging is associated with declines in multiple aspects of the human memory system, including hippocampally-mediated associative abilities and frontally-mediated executive abilities. It is important to distinguish these different impacts of aging in order to identify possible causes of memory decline (e.g., Alzheimer's disease vs. vascular dementia). In the present research, we used computational modeling to quantify the contributions of different cognitive processes to performance on a test of event memory, administered to 195 young adults and 195 healthy older adults. Participants viewed videos of actors performing simple actions and were later tested on memory for who did what. Participants in different conditions saw varying numbers of actors and experienced different levels of distraction, providing differential opportunities for executive involvement. A multinomial processing tree analysis was used to quantify different aspects of memory performance, including action memory, actor memory, and binding of actors with actions. Distraction impacted both actor memory and action memory, suggesting that distraction interfered with the deployment of attention to these event features. The largest impact of aging was on binding of actors with actions, especially when many different actors performed the actions. Distraction also impacted binding when only two actors performed all of the actions, suggesting that distraction interfered with strategic encoding of actor/action pairings in this condition. The large age differences and absence of an effect of distraction in the many actor condition suggest that this condition may be uniquely sensitive to hippocampally-mediated associative abilities, thus offering diagnostic potential for pathological conditions such as Alzheimer's disease.

Abstract citation ID: igae098.2658

**SUBJECTIVE AGE, DEPRESSIVE SYMPTOMS, AND COGNITIVE FUNCTION: DOES CHRONOLOGICAL AGE MATTER?**

Juhyeong Lee[1], and Giyeon Kim[2], *1. University of British Columbia, Seoul, Kyongsang-bukto, Republic of Korea*, *2. Chung-Ang University, Seoul, Republic of Korea*

This study examined the mediating effect of depressive symptoms between subjective age and cognitive function among Korean older adults, with a focus on the moderating effects of chronological age. Using data from the 2020 National Survey of Older Koreans, a nationally representative sample of individuals aged 65 or older (N=9,836) was examined. Subjective age was calculated by subtracting perceived old age from chronological age, with higher values indicating an older subjective age. Depressive symptoms were measured using the Short Form of Geriatric Depression Scale (SGDS-K), and cognitive function was assessed using the Korean version of Mini-Mental State Examination for Dementia Screening (MMSE-DS). A moderated mediation analysis was conducted by PROCESS (Model=59) for SPSS. Results showed that depressive symptoms significantly mediated the relationship between subjective age and cognitive function among Korean older adults. Particularly, an older subjective age was associated with lower depressive symptoms (B=-.0420, p<.001). Depressive symptoms were related to poor cognitive function (B=-.2372, p<.001). The direct effect of subjective age on cognitive function was also significant (B=-.1017, p<.001). Furthermore, chronological age moderated the indirect effects, demonstrating that subjective age was more strongly associated with lower depressive symptoms at younger chronological ages, whereas both subjective age and depressive symptoms were more strongly linked to poorer cognitive function at older chronological ages. The findings underscore the importance of considering both chronological and subjective age in understanding the interplay between depressive symptoms and cognitive function among Korean older adults.

Abstract citation ID: igae098.2659

**THE EFFECTS OF LISTENING WHILE DRIVING IN OLDER AND YOUNGER ADULTS**

Katherine Bak[1], Frank Russo[2], M. Kathleen Pichora-Fuller[1], and Jennifer Campos[3], *1. University of Toronto, Toronto, Ontario, Canada*, *2. Toronto Metropolitan University, Toronto, Ontario, Canada*, *3. The KITE Research Institute, University Health Network, Toronto, Ontario, Canada*

Driving collisions are a top cause of accidental death globally and adults aged 65+ are overrepresented. Driving is a highly complex task, requiring sensory processing, motor control, and divided attention. Driving is especially challenging when simultaneously listening to a passenger. Age-related declines in sensory (e.g., hearing) and cognitive abilities (e.g., working memory) may result in increased effort while listening. High listening effort may reduce spare cognitive capacity, thereby limiting the cognitive resources available to support driving. However, there are very few realistic and controlled experiments examining how the demands of listening while driving are managed in older adults. Therefore, the main objective of this study was to examine performance during a driving-while-listening task in older (aged 61-80 years, 15 female) and younger (aged 20-36 years, 12 female) adults with normal hearing/vision, and cognition, using a high-fidelity driving simulator. Participants completed a driving task at two different driving loads (e.g., straight in rural (low) vs. left turn in city (high)), a listening task (Connected Speech Test [CST]) at two different listening loads (+4 signal-to-noise ratio [SNR], 0 SNR), and both tasks together, to examine dual-task costs. Results demonstrated that only older adults showed significantly lower CST accuracy scores in the dual- compared to single-task conditions across both SNRs. Both older and younger adults showed significantly greater SD of lane position in the dual- compared to the single-task conditions, only under high driving load. Findings may inform educational practices, policies, and technological solutions to maintain and support safe driving among older adults.

Abstract citation ID: igae098.2660

### VULNERABILITY PROFILES: CHALLENGES FOR METACOGNITIVE INTERVENTIONS WITH OLDER ADULT

Ann Pearman[1], Christopher Hertzog[2], MacKenzie Hughes[3], and Emily Giannotto[4], *1. MetroHealth Medical System, Cleveland, Ohio, United States*, *2. Georgia Institute of Technology, Atlanta, Georgia, United States*, *3. NORC at the University of Chicago, Chicago, Illinois, United States*, *4. Emory University, Atlanta, Georgia, United States*

The metacognitive intervention approach has great promise for improving older adults' everyday functioning. Results from our studies (Hertzog et al., 2021; Pearman et al., 2020; Pearman et al., 2023) and many others point to potential issues with intervention compliance and realization of intervention benefits in some older adults. Based on the first author's clinical geropsychology work with older populations and observing our study participants, we argue that there are individual differences in who benefits from interventions and why they do so. This presentation will focus on our everyday memory training approach to evaluate variables that influence training benefit, including rates of attrition/drop-out, compliance with instructed task training, and improvements on outcome variables. Individual difference variables of interest include global cognition as measured by the Telephone Interview for Cognitive Status (TICS) and personality traits as measured by the AB5C with a particular focus on neuroticism and conscientiousness. The TICS was related to objective performance improvement on an associated learning task for both the intervention as well as the control group. Higher TICS scores were related to less improvement from T1 to T2 ($r = -.39$, $p < .05$). Personality variables correlated with post-test subjective memory but only in the intervention group, where people high in neuroticism scored lower on memory self-efficacy and higher on memory complaint [$r = -.45$ & $r = .41$ (respectively), $p < .05$]. These findings suggest that specific personal characteristics present barriers to adoption and maintenance of trained metacognitive strategies.

### SESSION 7230 (POSTER)

### DEMOGRAPHY

Abstract citation ID: igae098.2661

### EDUCATIONAL GRADIENTS IN HEALTH AND COGNITION: DOES THE TIMING OF EDUCATIONAL ATTAINMENT MATTER?

Fabio Bolz[1], John Warren[1], Eric Grodsky[2], and Chandra Muller[3], *1. University of Minnesota, Minneapolis, Minnesota, United States*, *2. University of Wisconsin Madison, Madison, Wisconsin, United States*, *3. The University of Texas Austin, Austin, Texas, United States*

Educational attainment is a strong predictor of later-life health and cognition. However, studies on the relationship between educational attainment and these outcomes usually do not consider the role of the timing of educational attainment. There is considerable heterogeneity in the timing of college degree attainment in the US and there are pronounced differences in timing by gender and race. Using nationally representative longitudinal data from High School and Beyond (HSB) we ask: (1) Does the impact of educational attainment on midlife health and cognition vary by age of degree attainment? (2) Does age at degree attainment moderate the effects of education differently across racial/ethnic and gender groups? We estimate OLS and logistic regression models for a variety of health and cognitive outcomes including: hypertension, diabetes, mental health conditions, general cognition, immediate recall, semantic fluency, phonemic fluency, delayed recall and working memory. We control for an extensive set of potentially confounding variables such as cognitive skills and non-cognitive traits in adolescence, academic performance in high school, and health in adolescence. Preliminary results suggest that bachelor's degrees obtained later in life are associated with substantially lower levels of cognitive functioning at midlife than degrees obtained earlier in life; however, people who obtain college degrees after age 40 still display higher levels of cognitive functioning than individuals without a bachelor's degree. Rates of hypertension and diabetes among those who obtained their college degree after age 30, in contrast, do not differ from rates for individuals without college degrees.

Abstract citation ID: igae098.2662

### LIFE EXPECTANCY WITH UNMET AND MET CARE NEEDS FOR OLDER ADULTS

Jose Cabrero Castro, Octavio Bramajo, Neil Mehta, and Brian Downer, *The University of Texas Medical Branch, Galveston, Texas, United States*

Assessing unmet social care needs is crucial for understanding the challenges of an aging society like Mexico, where demographic changes have outpaced the development of formal care systems. This study examines the time lived with met and unmet care needs among Mexican adults aged 60 and over by estimating life expectancy according to limitations in instrumental (IADL) and basic activities of daily living (ADL) and receiving help. Analyzing 2012-2021 Mexican Health and Aging Study (MHAS) data (6,215 males; 8,156 females), we used multistate life table models to estimate life expectancy with no limitations, limitations but not receiving help (unmet care needs), and limitations but receiving help (met care needs). We stratified estimations by gender and living arrangement. Findings revealed significant gender differences. Women lived a greater proportion of their remaining life expectancy with ADL (+7%) and IADL (+9%) limitations than men. Men with ADL limitations lived a greater proportion of their life with unmet (8.5%) versus met (4.7%) care needs, a disparity less pronounced among women (12% unmet vs. 8.4% met care needs). Conversely, participants with IADL limitations lived a greater proportion of their life with met care needs (8% vs 1.9% for men; 16.3% vs 2.5% for women). Participants living with spouses or other adults experienced a greater proportion of life lived with met care needs than those living alone, for both ADL and IADL limitations. These results underscore the interplay between gender, living arrangements, and care needs, highlighting the need for targeted interventions to address care needs disparities.

Abstract citation ID: igae098.2663

### MULTISTATE CHARACTERISTICS AMONG OLDER POPULATION IN CHINA (2024–2050): GLOBAL COMPARISON AND DYNAMIC SIMULATION

Yichao Li, and Hong Mi, *Zhejiang University, Hangzhou, Zhejiang, China (People's Republic)*

Global long-term care (LTC) issues become complex with diverse aging process. China's population has entered deep and accelerated aging stage with multiple health status, which has heterogeneity among population with different features. However, overlooking the biases of multistate characteristics of aging due to lack of systematic methodology would affect LTC policies. Therefore, this study simulates the complex LTC demand by estimating life expectancy with disability and structure of disability population under different demographic scenarios, and compares China's LTC demands with typical developed countries. In methods part, we used three-parameter lifetable model (DCMD model), Lee-Carter Coherent model and Bayesian multistate model to simulate the prevalence of mild, moderate and severe disability among older people. China Health and Retirement Longitudinal Study (CHARLS) 2020 was used for the initial dependency status. CHARLS 2011-2018 and Chinese Longitudinal Healthy Longevity Survey 2008-2014 was used as prior information. Death probabilities in Bayesian health transition matrixes were modified by estimated mortality patterns by sex, age and residential types (urban or rural areas). Healthy life expectancy in 2020 was also calculated by multistate lifetable model. Results showed that older people with moderate and severe disability in China had an increasing trend during 2023 to 2050 after simulating posterior distributions of health transition probabilities. China's public long-term care expenditures will account for less than 0.7% of GDP in 2050, which is lower than developed countries. This study proposed an actuarial framework for estimating LTC demand with credible interval, which enables developing countries to dynamically adjust LTC policies.

Abstract citation ID: igae098.2664

### NEIGHBORHOOD STREET CONNECTIVITY, FOOD ACCESS, AND MORTALITY AMONG OLDER ADULTS: EDUCATION AND GENDER DIFFERENCES

Pui Yin Cheung[1], David Curtis[2], and Ming Wen[1],
*1. University of Hong Kong, Pok Fu Lam, Hong Kong,*
*2. University of Utah, Salt Lake City, Utah, United States*

Disadvantages in multiple dimensions have garnered increasing attention with strong empirical support. This study examines whether the intersection between education and gender reveals novel neighborhood effects. Two particular areas of neighborhood built and service environments are examined: pedestrian-oriented street connectivity and residing in a low-food-access tract. They are important for health among older adults, given their limited functioning and health challenges. We examined the second and third waves of the MIDUS study, restricted to those aged 65 or above in the second wave. We estimated a series of discrete-time hazard models with 12,141 person-years across 15 years and included individual-level sociodemographic control variables. We also controlled for tract-level characteristics: urban/rural categories, median household income, percentages with college or above education, and racial/ethnic percentages. Interaction between any single dimension of disadvantage and pedestrian-oriented street connectivity didn't reveal significant differential relationships with mortality. However, better street connectivity was associated with less hazard of mortality among less-educated men once three-way interactions between education, gender, and street connectivity were introduced. In contrast, well-educated older adults residing in a low-food-access tract had a significantly higher hazard of mortality than those not residing in such a tract. The model with three-way interactions revealed more adverse effects of residing in a low-food-access tract among well-educated men only. The findings reveal mortality differences by education and health and their interactions with neighborhood characteristics despite no apparent differences based on a single dimension of disadvantage, supporting the usefulness of the perspective of disadvantages in multiple dimensions.

Abstract citation ID: igae098.2665

### TEACHING AGING WITH A GLOBAL CLASSROOM

Kristine Mulhorn, *Drexel University, Philadelphia, Pennsylvania, United States*

In a time of heightened interest in teaching about aging in undergraduate programs, Drexel launched a competitive effort to offer courses that would be of interest across a variety of majors and minors. As a scholar working in aging in Japan for several years, as well as a background in aging policy, I launched a course called "Aging, Aging Policy and Health in Asia". The highlight was the global classroom—a three-week project between Drexel students and students at KAIST (Korean Advanced Institute of Science and Technology) on the topic of aging and technology. The focus of the effort to teach aging across the university was to assure that the AGHE competencies were included in these courses. We included competencies such as the bio-psycho-social model for healthy aging, the epidemiologic transition, and the ethics of caregiving. This course resulted in close collaboration related to a product or concept that had potential for direct application In the lives of older adults. For example, one project addressed the use of research and technology applied to aging policies of Korea and the US, and another looked closely at strategies that improve intergenerational interactions that improve the lives of older adults and children. This presentation will demonstrate the integration of AGHE competencies into the course, as well as the ways the students met the learning objectives within the course.

### SESSION 7235 (POSTER)

### DEPRESSION AND ANXIETY

Abstract citation ID: igae098.2666

### BIOLOGICAL AGING AS A MEDIATOR OF DEPRESSION AND MORTALITY

Kai Wei[1], and Xiaotong Chen[2], *1. Guizhou Provincial People's Hospital, Guiyang, Guizhou, China (People's Republic), 2. Jing'an District Centre Hospital of Shanghai, Shanghai, Shanghai, China (People's Republic)*

**Background:** Depression has been linked to death from all-cause, cardiovascular disease and cancer; however, the underlying mechanisms remain unclear. We aimed to examine the mediating role of biological aging in the association of depression with mortality in a representative sample of the US population.

**Methods:** Longitudinal data were obtained from 33,434 participants aged ≥20 years in the 2005-2018 cycle of the National Health and Nutrition Examination Survey and their mortality data through 2019. The Patient Health Questionnaire (PHQ-9) was used to measure depressive symptoms, and major depression was defined as a PHQ-9 score ≥10. Three measures of aging, including phenotypic aging acceleration (PA-Accel), anthropometric aging acceleration (AA-Accel), and Klemera and Doubal method-based biological aging acceleration (KDBA-Accel), were calculated. Weighted multiple linear regression model, Cox proportional hazards model, and mediation analysis were performed.

**Results:** The major depression were positively associated with biological aging acceleration (PA-Accel $\beta$=1.34, KDBA-Accel $\beta$=0.88, AA-Accel $\beta$=0.36, all $p < 0.0001$). The mediation proportion of biological aging acceleration in associations of major depression with all-cause mortality were 21.53% (PA-Accel, p=0.0005), 14.63% (KDBA-Accel, p=0.0006), and 11.36% (AA-Accel, p=0.0004), respectively. Similar results were found in the Health and Retirement Study. Conclusions: Accelerated aging partially mediated the associations of major depression all-cause mortality in US populations.

Abstract citation ID: igae098.2667

### CHANGES IN DEPRESSIVE SYMPTOMS ACROSS MIDDLE AND OLDER ADULTHOOD IN THE ORANJ BOWL LONGITUDINAL STUDY OF AGING

Kaite Yang[1], Christine Ferri[1], and Joan Girgus[2], *1. Stockton University, Galloway, New Jersey, United States*, *2. Princeton University, Princeton, New Jersey, United States*

Life course fluctuations in depressive symptoms are important to document and understand. Previous research has suggested a U-shaped pattern in depressive symptoms across the lifespan, with more symptoms in younger and older adulthood compared to middle adulthood (Sutin et al., 2013). The present study used hierarchical linear modeling to examine changes in depressive symptoms in middle and older adulthood and the degree to which participants' age at baseline affected symptom trajectories across 13 years. Participants were a representative sample of community-dwelling adults in New Jersey aged 50-74 (M=60.53) at baseline (ORANJ BOWL; Pruchno et al., 2010). N=4266 participants (64.3% female) who completed at least two waves of data collection from five timepoints were included in this study (T1: 2006-2008; T3: 2011; T4: 2013-2015; T5: 2015-2017; T6: 2017-2019). The trajectory of depressive symptoms (CESD-R-10) was best captured by a cubic model wherein symptoms peaked at T3, decreased, and then increased again after T4. There were significant intercept, linear, quadratic, and cubic terms for the effect of age as a continuous variable on depressive symptoms. Participants who were younger at the start of the study had more symptoms initially and then flatter trajectories compared to participants who were older. Participants who were older started with less symptoms but had a steeper increase in symptoms over time. These findings suggest that age-dependent fluctuations as well as impacts of and recovery from exogenous events like the Great Recession are reflected in depressive symptom trajectories in older adults (Pruchno et al., 2021).

Abstract citation ID: igae098.2668

### DEPRESSION PREVALENCE IS HIGHER IN DISADVANTAGED NEIGHBORHOODS

Jacob Mitchell[1], Elizabeth Pfoh[1], Douglas Gunzler[2], Michael Kenyhercz[1], Lyla Mourany[1], Paul Gunsalus[1], Adam Perzynski[3], and Jarrod Dalton[1], *1. Cleveland Clinic, Cleveland, Ohio, United States*, *2. CWRU, Cleveland, Ohio, United States*, *3. MetroHealth and Case Western Reserve University, Cleveland, Ohio, United States*

Depression is associated with premature morbidity and mortality. Knowing depression prevalence across diverse neighborhoods can inform healthy aging interventions. In this retrospective study, we analyzed electronic health data for mid-life adults (40-55 years) with ≥1 primary care appointment between 2010 and 2016 at two health systems in Cuyahoga County, Ohio. Patients were categorized as having depression if they had a diagnosis within 6 months of their first visit. Patient's addresses were associated with the block group Ohio Area Deprivation Index (ADI), a measure of disadvantage. We used multivariable logistic regression to identify the predicted probability of depression across patients living in advantaged (ADI quintile 1) versus disadvantaged (ADI quintile 5) neighborhoods. We included race, given histories of neighborhood segregation in our location. We used a three-way interaction term between sex, race, and ADI quintile. Our sample included 150,710 patients, of which 14% had a diagnosis of depression. A higher percentage of patients with depression (versus not) were female (70% versus 54%), White (63% versus 56%), and lived in ADI quintile 5 (28% versus 25%). The predicted probability of depression was lower for patients living in ADI quintile 1 versus 5: White males (0.08, 95%CI: 0.08, 0,08 versus 0.16, 95%CI: 0.15, 0.17); White females (0.17, 95%CI: 0.16, 0.17 versus 0.25, 95%CI: 0.24, 0.26), Black males (0.05, 95%CI: 0.04, 0.07 versus 0.09, 95%CI: 0.09, 0,10) and Black females (0.14, 95%CI: 0.12, 0.16 versus 0.16, 95%CI: 0.15, 0.16). Place-based, mental health-promoting interventions may be needed to promote healthy aging among mid-life adults.

Abstract citation ID: igae098.2669

### DEPRESSIVE SYMPTOMS ACROSS THE LIFESPAN: DIFFERENCES IN SYMPTOM CRITERIA ENDORSEMENT

Teresa Walker, and Brenna Renn, *University of Nevada Las Vegas, Las Vegas, Nevada, United States*

Scientific literature and clinical wisdom often cite older adults as more likely to endorse somatic rather than mood symptoms of depression compared to younger people. However, this is based on older data and needs updating with demographic shifts. As many older adults with clinically significant symptoms of depression go undiagnosed and untreated, with deleterious effects on health outcomes and quality of life, identifying and treating depression is necessary to provide quality healthcare for older adults. Thus, the purpose of the current study was to investigate differences in endorsements of depressive symptoms across age groups with recent data from the National Health and Nutrition Examination Survey 2017-2020 Pre-Pandemic dataset. Participants (N = 8299) were categorized into young adults (18-24 years), middle adults (25-55 years), young-older adults (56-75 years), and older adults (76+ years). Responses to items on the Patient Health Questionnaire (PHQ-9) were dichotomized into meeting criteria for a depressive episode (vs. not). Chi-squared tests of independence revealed significant differences across some age groups for symptoms including sleep disturbance, feelings of worthlessness/guilt, and suicidal ideation. Young-older adults were the only age group to significantly differ from at least one other age group on all three of these items. Of particular note, older adults did not significantly differ from other age groups on any items, contradicting previous findings that older adults report more somatic symptoms than their younger counterparts. These findings contribute nuance to understanding depressive symptom presentation across the lifespan to guide clinical assessment of older adults.

Abstract citation ID: igae098.2670

### DISCRIMINATION AND MENTAL HEALTH AMONG OLDER ADULTS: THE CONFOUNDING ROLE OF PERSONALITY

Morgan Stangl, Man Guo, and Yi Wang, *University of Iowa, Iowa City, Iowa, United States*

Existing literature shows that the perception of discrimination is related to poorer mental health. Personality may confound this association by shaping an individual's coping style, social support seeking, and emotional regulation in the face of adversity. However, such a confounding role of personality hasn't been well studied, and it's unclear whether different personality traits have similar effects. Using data from middle-aged and older adults (aged 50+) in the 2018 Health and Retirement Study Psychosocial Leave-Behind Questionnaire (N = 5,529), this research delves deeper into the potential moderating role of two personality traits (neuroticism and extraversion) in the associations between perceived discrimination and mental health outcomes (depression and anxiety). Findings from regression analyses revealed that increased perceived discrimination was consistently associated with higher levels of anxiety and depression. While such associations did not vary based on individuals' levels of neuroticism, the associations were weaker among individuals with a higher level of extraversion. This study revealed that the two personality traits, neuroticism and extraversion, have different effects on the relationship between perceived discrimination and mental health. Extroverted individuals may perceive and cope with discrimination differently with more adaptive coping mechanisms, such as seeking social support and engaging in problem-solving behaviors, which may buffer against the negative effects of discrimination on mental health. Further studies of the underlying mechanisms are needed. This can inform the customization of mental health interventions to align with individuals' personality profiles for better-tailored and targeted support that effectively promotes their resilience in the face of discrimination.

Abstract citation ID: igae098.2671

### DOES DEPRESSION EXPLAIN COGNITIVE CHANGES IN PSYCHOTHERAPY? A MEDIATION ANALYSIS

Emily Post, Teresa Walker, Matthew Schurr, and Brenna Renn, *University of Nevada Las Vegas, Las Vegas, Nevada, United States*

Depression commonly affects older adults and is associated with detrimental outcomes, including cognitive impairment such as executive dysfunction. Psychotherapy effectively improves depression outcomes and may improve some aspects of cognition, but this relationship is not widely understood. This study examined the potential mediating role that depression severity might have between psychotherapy treatment and a change in cognitive test scores. Data for this secondary analysis were drawn from older adult participants (N=249, mean age = 70.2 [7.4] years) with major depressive disorder who participated in a RCT comparing two 9-week structured psychotherapies for depression. Our prior work found improvement in depression (noninferiority between treatment groups) and some improvement on measures of cognition during the RCT. Measures included pre- and post-treatment cognitive tests: Iowa Gambling Money Task, Digit Span Backwards, and Stroop test. The Hamilton Depression Rating Scale (HAM-D) assessed change in depression symptoms. Hayes PROCESS macro for SPSS analyzed separate mediation models testing depression as a mediator of treatment and each cognitive outcome. Change in depression did not mediate relationships between treatment and change in cognitive outcomes across the nine weeks of psychotherapy. While our investigation did not establish depression as a mediator of change in cognitive test performance in patients receiving psychotherapy, these findings may indicate that improvement in depression is somewhat independent of any observed changes in cognitive test performance. Future research involving a more cognitively impaired sample could strengthen these findings.

Abstract citation ID: igae098.2672

### EARLY-LIFE INVOLUNTARY JOB LOSS AND LATER-LIFE DEPRESSIVE SYMPTOMS IN CHINA: A TRAJECTORY ANALYSIS

Qian Song, and Wuyi Dong, *University of Massachusetts Boston, Boston, Massachusetts, United States*

While it is relatively well-understood that involuntary job loss (job loss thereafter) has short-term negative impacts on health of individuals, we do not know much about how early-life job loss is associated with long-term depressive symptoms, especially in the developing country contexts. In this study, we take advantage of a nationally representative sample for those aged 45 and older in the 2011-2018 China Health and Retirement Longitudinal Study as well as the Life History data collected in 2014. We investigate how early life job loss experience, much of which occurred more than two decades ago, is associated with depressive symptoms, as measured by CESD-10 (range: 0-30), in mid- and later-life. Among our sample (N=3,653), 32% of Chinese have experienced job loss at least once in their life-time. After controlling for gender, age, education, marital status, household expenditure, self-rated health and activities of daily living, we found that those who have experienced job loss showed an increased ($\beta$=.53, $p$<.001) number of depressive symptoms at the age of 60, and with one year of increase in age, they experienced an additional.02 (p<.05) growth compared to those who never experienced job loss. The timing of job loss matters. Job loss occurred during mid-career (30-45 years old) was most detrimental to later-life psychological well-being, with.98 (p<.001) more depressive symptoms at age 60 and an additional.06 growth (p<.05) with each year. The results highlight the long arm of early-life employment experience on the depressive symptoms of individuals even decades after.

Abstract citation ID: igae098.2673

### ECOLOGICAL MOMENTARY ASSESSMENT AND MACHINE LEARNING FOR PREDICTING DEPRESSION AMONG OLDER ADULTS

Nan Jiang[1], Ke Chen[1], Yexuan Xiao[1], and Vivian Weiqun Lou[2], *1. Tsinghua University, Beijing, Beijing, China (People's Republic)*, *2. The University of Hong Kong, Hong Kong, China (People's Republic)*

Depression represents a significant global health concern, particularly among older populations. While numerous studies have focused on investigating chronic stressors, the precise predictive impact of daily experiences on depression remains uncertain. The objective of this study was to assess the extent to which mood fluctuations, daily stressors, contextual stressful events, and psychophysiological responses experienced by older adults with functional decline may predict later short- and long-term depression. This study employed a combination of longitudinal investigation and ecological momentary assessment (EMA) to collect data on the mood status and stressful events of older adults with functional limitation. Data were gathered 2 times per day over a 14-day period and followed up with a revisit one year later. To predict long-term depression, the study explored several approaches using machine learning techniques.These included analyzing chronic stress baseline data (baseline approach), examining dynamic patterns of mood states and stressful events through EMA approach, and combining baseline data with dynamic patterns (EMA plus baseline approach). A total of 166 older adults participated in the study. The baseline plus EMA approaches showed better performance than the baseline and the EMA approaches at 1-year follow-up (area under the receiver operating characteristic curve [AUC], 0.798; 95%CI: 0.599’0.932) on the prediction of depression. The results underscore the significance of contextual risk factors encountered by older adults during different stages. Leveraging machine learning techniques could enhance the identification of older adults at risk and contribute to the creation of personalized, process-based early prevention programs aimed at mitigating future depression.

Abstract citation ID: igae098.2674

### EXPLORING THE IMPACT OF CHILDHOOD TRAUMA, SOCIAL NETWORK, AND GENDER MODERATION ON DEPRESSION AMONG OLDER ADULTS

Ying Xu, Xiaoyu Fu, and Merril Silverstein, *Syracuse University, Syracuse, New York, United States*

China's aging population faces significant challenges, including a high prevalence of depression among older adults in rural areas (Li et al., 2021). This study investigates the impacts of childhood trauma and social network on depression among older adults and whether their impacts vary by gender in rural China. Using data from the 2021 wave of the Longitudinal Study of Older Adults in Anhui Province, our sample comprises 1558 participants, including 812 males and 746 females. Hierarchical moderated regression models were employed, controlling for age, marital status, living conditions, and education. Results revealed that childhood trauma significantly associated with older adults' depression level; however, its impact was notably stronger for females than males, as indicated by the significant trauma x gender interaction ($\beta$ = 0.048, p <.05). Negative main effect of social network on depression was found ($\beta$ = -0.148, p <.05), suggesting that older adults with larger social network tend to have lower level of depression regardless of their gender. The findings underscore the need for gender-specific approaches to addressing trauma-related depression among older adults. Tailored interventions and supports, particularly for females who have experienced trauma, are warranted. Moreover, programs aimed at enhancing the quality of older adults' social networks may help reduce depression in both genders.

Abstract citation ID: igae098.2675

### GENDER DIFFERENCES IN THE IMPACT OF COVID-19'S HARDSHIP ON DEPRESSIVE SYMPTOMATOLOGY AMONG MEXICAN OLDER ADULTS

Sirena Gutierrez[1], Rebeca Wong[2], and Sadaf Milani[3], *1. University of California, San Francisco, San Francisco, California, United States*, *2. The University of Texas Health Sciences Center in San Antonio, San Antonio, Texas, United States*, *3. The University of Texas Medical Branch, Galveston, Texas, United States*

Older women in Mexico report depressive symptoms more often than men. Amidst the COVID-19 pandemic, the exacerbation of mental health inequities among older adults remains unclear. We investigated whether the association between experiencing hardship and depressive symptoms varied by attributing these events to the pandemic. We used data from the Mexican Health and Aging Study (2018/2021) and included participants aged 50+ (n=13,046). Participants self-reported whether they experienced the following hardship events: child or spousal death, caregiving for a sick family member, worsened finances, or job loss. If they said yes, they could attribute the event to COVID-19, resulting in two binary measures of hardship events (0 vs. 1+), pandemic-related or non-pandemic. Our primary outcome was a 9-item depressive symptom score. We used generalized estimating equations models adjusting for sociodemographic covariates and a time fixed effect. Gender heterogeneity was examined with stratified models and multiplicative interaction terms. Participants averaged 63.7 years, and 56.3% were women. Both non-pandemic (b=0.38, 95% confidence interval [CI]: 0.30, 0.46) and pandemic-related (b=0.43, 95% CI: 0.31, 0.56) hardship were associated with greater depressive symptoms. The magnitude of the associations was stronger for women (b=0.44, 95% CI: 0.34, 0.55 vs. b=0.29, 95% CI: 0.18, 0.40 for non-pandemic hardship; b=0.49, 95% CI: 0.32, 0.66 vs. b=0.34, 95% CI: 0.15, 0.53 for pandemic-related hardship). However, the gender difference was only statistically significant for non-pandemic hardship (interaction p=0.042). Understanding mechanisms for gender differences in the impact of the pandemic on the mental health of older adults is essential for targeted interventions.

Abstract citation ID: igae098.2676

### LONGITUDINAL RELATIONSHIPS OF DEPRESSIVE SYMPTOMS IN OLDER ADULTS WITH HYPERTENSION IN CHINA

Xiaomeng Wang[1], and Zhi-wen Wang[2], *1. Peking University, Beijing, Beijing, China (People's Republic)*, *2. Peking University Health Science Center, Beijing, Beijing, China (People's Republic)*

**Objectives:** This study aimed to develop cross-lagged symptom networks and examine the longitudinal relationships of depressive symptoms among older adults with hypertension in China. Method: This study utilized data from the China Health and Retirement Longitudinal Study conducted in 2018 (T1) and 2020 (T2). The 10-item Center for Epidemiologic Studies Depression Scale (CES-D) was employed to measure depressive symptoms. A cross-lagged panel network (CLPN) analysis was applied to construct cross-lagged symptom networks for the entire sample as well as for males and females separately.

**Results:** A total of 1937 participants were included in the final analysis. The strongest direct positive effect was observed from the item 'D03: Felt Depressed' to 'D01: Bothered by Things' ($\beta$ = 0.13). For the full sample and the male, 'D10: I Could Not Get on' reported the largest value of out-strength. For female, 'D03: Felt Depressed' reported the largest value of out-strength (r = 1.350). 'I Could Not Get on' was the strongest predictor compared to the other nine depressive symptoms based on node centrality. Conclusions: Our study suggests that health care professionals should be particularly attentive when older adults with hypertension report difficulties in coping with daily activities, as well as feelings of diminished motivation and energy. Interventions based on motivations should be chosen with cation for older adults with hypertension in China.

Abstract citation ID: igae098.2677

### PREDICTORS OF ANXIETY SYMPTOMS AMONG OLDER ADULTS WITH COGNITIVE IMPAIRMENT BY THEIR COGNITIVE STATUS

Mina Hwang, and Yeji Hwang, *Seoul National University, Seoul, Republic of Korea*

Anxiety is common neuropsychiatric symptoms of dementia. While various factors can trigger anxiety in individuals with cognitive impairment, there is limited information available regarding whether these factors vary based on one's cognitive status. Therefore, this study aimed to examine predictors of anxiety in older adults with cognitive impairment by their cognitive status. Using data from the National Health and Aging Trends Study (NHATS) Round 12, 1,040 older adults with probable or possible dementia were analyzed. Participants were categorized into possible (n=508, 48.8%) and probable dementia (n=532, 51.2%) groups. For data analyses, descriptive analyses, chi square tests, and logistic regression analyses were conducted. In the group of possible dementia, when participants had pain (OR=3.48, p < 0.001) or depression (OR=6.55, p< 0.001), they were more likely to present anxiety. In the group of probable dementia, when participants had pain (OR=3.54, p < 0.001), depression (OR=7.84, p< 0.001), low community cohesion (OR=1.59, 0=0.044), or were dependent on activities of daily living (OR=2.42, p< 0.001), they were more likely to present anxiety. This study found that while pain and depression were related to anxiety in both probable and possible dementia groups, factors such as dependency on activities of daily living or community cohesion were only related to anxiety in probable dementia group. When persons living with cognitive impairment present anxiety, healthcare professionals and caregivers should consider those triggers to reduce their anxiety.

Abstract citation ID: igae098.2678

### RUMINATION MEDIATES THE ASSOCIATION BETWEEN REGIONAL GRAY MATTER VOLUME AND SUBTHRESHOLD DEPRESSION IN OLDER WOMEN

Dongmei Wu, *The Clinical Hospital of Chengdu Brain Science Institute, MOE Key Lab for Neuroinformation, University of Electronic Science and Technology of China, Chengdu, Sichuan, China (People's Republic)*

This study aimed to identify distinctive characteristics of gray matter volume alterations in older women with subthreshold depression and to explore the mediating role of rumination in the relationship between changes in brain structure and depressive symptoms. A total of 102 older women residing in the community were divided into two groups: those with depression symptoms (DS) (n=50) and a healthy control (HC) group (n=52). Whole-brain two-sample t-tests on voxelwise gray matter volumes in the brains were conducted to identify gray matter volume alterations in older women with subthreshold depression, and mediation analysis was performed to test whether rumination mediates the impact of gray matter volumes on depressive symptoms in older women. Even after controlling for age, education level, monthly income, Mini-Mental State Examination score, and total gray matter volume, we identified significant increases in local gray matter volume in the left dorsolateral prefrontal cortex and left fusiform gyrus in the DS group. Moreover, rumination was found to partially mediate the relationship between the gray matter volumes in both regions and depressive symptoms. These findings indicated that the volumes of the left dorsolateral prefrontal cortex and left fusiform gyrus serve as potential neurostructural markers for subthreshold depression in non-clinical older female adults. Additionally, an underlying mediation mechanism in which gray matter volume affected depressive symptoms through individual rumination tendencies was proposed.

Abstract citation ID: igae098.2679

### RURAL OLDER ADULTS' SOCIAL DISCONNECTION AND DEPRESSION DURING THE PANDEMIC

Jung Sim Jun[1], and Kyoung Hag Lee[2], *1. Kansas State University, Kansas State University, Kansas, United States*, *2. Wichita State University, Wichita, Kansas, United States*

During the pandemic, 24% of older adults reported anxiety or depression, with higher rates among those with low socioeconomic status and poor health, particularly among female older adults (Koma et al., 2020). Older adults in rural communities face greater challenges due to underlying health conditions and limited resources (Henning-Smith, 2020; Pender et al., 2019). Therefore, this study aimed to examine the association between social isolation, social support, and mental health among this population. The research team recruited 103 rural-dwelling older adults using purposive sampling. Measurements included: the Geriatric Depression Scale-Short Form (Sheikh & Yesavage, 1986), Lubben Social Network Scale-6 (Lubben et al., 2006), and Multidimensional Scale of Perceived Social Support (Zimet et al., 1988). Six socio-demographic variables were used as control variables: age, gender, final education, annual household income, perceived health, and driving capacity. There was a positive association between social isolation and depressive symptoms (B = 0.24, SE = 0.07, $p \leq .001$; B = 0.20, SE = 0.08, $p \leq .01$) while social support from significant others was negatively associated with depressive symptoms (B = -0.35, SE = 0.11, $p \leq .001$). However, social support from family, from friends, and age were not significantly related to depressive symptoms. A lack of social resources is a risk factor for rural older adults, potentially leading to increased mental health issues. Social service workers should focus on improve social support systems among rural older adults to establish social networks and create social resources.

Abstract citation ID: igae098.2680

### THE INFLUENCE OF ANXIETY AND PERCEIVED CONTROL OF ANXIETY ON OLDER VETERANS' QUALITY OF LIFE

Carter Davis[1], Chalise Carlson[2], Sherry Beaudreau[1], and Christine Gould[1], *1. VA Palo Alto Healthcare System, Palo Alto, California, United States*, *2. Department of Veterans Affairs, Palo Alto, California, United States*

Anxiety disorders are prevalent among older military Veterans, and the presence of an anxiety disorder negatively impacts quality of life. Little is understood, however, about potential psychological processes that explain how quality of life is impacted by anxiety among older Veterans. One potential mechanism is anxiety control, or one's perceived ability to manage their anxiety as it arises in various contexts. In this study, baseline data were analyzed from 62 older Veterans (M age = 71.2) who participated in a clinical trial of a guided anxiety management intervention. The most common diagnoses among participants were anxiety not otherwise specified (37.1%), and generalized anxiety disorder (22.6%). Linear regression models were created which tested anxiety symptom severity as a predictor of mental health-related quality of life, with anxiety control as a covariate. Lower anxiety severity ($p < .001$) and higher perceived control of anxiety ($p = .013$) were found to be significant predictors of quality of life. Anxiety control remained a significant predictor ($p = .05$) when anxiety diagnosis type was included as a covariate, but not when depression symptom severity and health comorbidity were included in the model ($p = .19$). These results indicate anxiety control as a potentially important target in interventions for older adult Veterans across anxiety disorder subtypes. However, larger samples are needed to clarify the path through which this effect operates and the influence of additional factors such as depression and comorbid health conditions, which could be examined through moderation and mediation analysis.

### SESSION 7240 (POSTER)

### DISABILITIES, LIFELONG

Abstract citation ID: igae098.2681

### "WE'RE CAMPAIGN OBJECTS": A QUALITATIVE LIVELIHOOD ANALYSIS OF THE VISUALLY IMPAIRED OLDER ADULT IN RURAL GHANA

Eric Frimpong[1], Akwasi Adjei Gyimah[2], and Mathias Adjei[3], *1. University of Massachusetts Boston, Boston, Massachusetts, United States*, *2. Miami University, Oxford, Ohio, United States*, *3. Miami University, Oxford, Ohio, United States*

**Background:** In Ghana, a considerable proportion of the older adult population continue to face economic hardship due to the weakening of family structures and inadequate social support. This study aims to investigate the lived experiences of visually impaired older individuals residing in rural areas of the country.

**Methods:** For a deeper understanding of the experiences of respondents, the social model of disability informed the use of a qualitative approach to interviewing a total of 18 older adults residing in selected rural areas in Ghana. The data was analyzed through a thematic content analysis approach. Findings: Many older adults in Ghana living with visual impairment continued to experience economic hardship. They often rely on donations from family members and social groups, such as churches. However, these sources of support are inadequate to meet their needs. A major concern raised was access to government support programs. For those who were enrolled in such programs, they expressed dissatisfaction with the benefits received, citing their insufficiency in meeting their needs. Furthermore, many respondents expressed disillusionment with politicians who were perceived to have made empty promises during election campaigns in an attempt to implement programs that will enhance their livelihood.

**Conclusion:** These findings illustrate the pressing need for more effective support systems and interventions to address the economic challenges faced by visually impaired older people in rural Ghana. Policymakers must consider the unique needs and experiences of this population, and work towards implementing solutions that are both sustainable and effective.

Abstract citation ID: igae098.2682

### COGNITIVE DISCREPANCY ON SOCIAL LIFE AND LIFE SATISFACTION AMONG OLD ADULTS WITH VISUAL DISABILITIES

Ya-Han Chang, and Silvia Sörensen, *University of Rochester, Rochester, New York, United States*

**Background and objectives:** This study addresses the quality of life faced by individuals with vision impairment. Life satisfaction in the context of disability is co-determined by social life, loneliness feelings, future planning, and financial status. However, the role of cognitive discrepancy and social cognition congruence in shaping life satisfaction is rarely studied. This study uncovers the role, by investigating various factors, including social relationships, loneliness, and discussions of future care plans.

**Research Design and Method:** Baseline data from older adults with age-related macular degeneration (N=167, Mean age = 79.66), who were participants in a depression prevention trial, were analyzed to determine the relationship between future care planning to life satisfaction. Cognitive discrepancy, and moderation of this relationship by variable (1) loneliness and (2) quality of social relationship tested.

**Results:** The significant relationship between discussions about future care plans and older adults' life satisfaction ($p < .001$) was moderated by the interaction of the quality of social relationships and loneliness as either cognitive discrepancy or congruence.

**Discussion and Implication:** The pivotal role of social cognitive congruence about loneliness and social relationships emerges as a critical factor in life satisfaction among old adults. We found the influence of cognitive discrepancy and care plan discussion on life satisfaction. Discussing plans with family members for elders with high cognition discrepancies is critically affecting their life satisfaction.

Abstract citation ID: igae098.2683

### COMMUNITY RATES OF DEPRESSION AND DISABILITIES: FINDINGS FROM THE MS HEALTHY AGING DATA REPORTS

Yan-Jhu Su[1], Taylor Jansen[1], Chae Man Lee[1], Elizabeth Dugan[1], Nina Silverstein[2], and Kina White[3], *1. University of Massachusetts Boston, Boston, Massachusetts, United States*, *2. University of Massachusetts Boston, Needham, Massachusetts, United States*, *3. Mississippi State Department of Health, Jackson, Mississippi, United States*

Depression in older adults is a significant public health issue, affecting all aspects of their well-being, independence, and quality of life. This cross-sectional study describes county rates of depression and daily life difficulties (ambulatory difficulty, self-care difficulty, and independent living difficulty) in Mississippi. We then explored if there was an association between county rates of depression and the disability measures in 82 counties in Mississippi. Small area estimation techniques were used to calculate rates using data from: the American Community Survey (2016-2020), the Behavioral Risk Factor Surveillance System (2013-2020), and the Centers for Medicare and Medicaid Services (2018). County rates varied: depression ranged from 6.30% to 24.9% (16.4% state average), ambulatory difficulties (7.14%-46.36%), self-care difficulties (2.53%-24.16%), and independent living difficulty (8.54%-33.0%). Multiple linear regression models after adjusting for age, gender, race, marital status, education, poverty, and physical health status, revealed that counties with higher rates of depression were significantly associated with higher rates of ambulatory difficulty and self-care difficulty ($p = 0.003$ and $p < 0.001$, respectively) but not independent living ($p = 0.176$). Bivariate mapping was conducted to overlay rates of depression and daily life difficulties, demonstrating counties that were high in depression and daily life difficulties in Mississippi. Identifying areas with high rates of 65+ depression and activities of daily living difficulties can help policymakers and service providers to prioritize communities most in need of interventions and services.

Abstract citation ID: igae098.2684

### SELF-EFFICACY PARTIALLY MEDIATES PHYSICAL FUNCTIONING AND QUALITY OF LIFE IN MIDDLE-AGED ADULTS WITH DISABILITY

Katie Singsank[1], Samantha John[1], and Ivan Molton[2], *1. University of Nevada Las Vegas, Las Vegas, Nevada, United States*, *2. University of Washington, Seattle, Washington, United States*

Middle-age is understudied. Adults living with long-term physical disability (LTPD) face added health conditions during middle-age, heightening psychosocial stress. The role of self-efficacy in managing these conditions and their impact on quality of life (QoL) remains unclear. Regression analyses were conducted using control participants from a health promotion trial, examining whether mid-intervention self-efficacy mediated the predictive relationship between baseline physical functioning (IV) and post-intervention QoL (DV). The sample ($n = 159$) was predominantly female (68.6%) and aged 45-64 ($M = 55.99$). Regression coefficients showed statistical significance for the models examining direct effects of physical functioning on QoL, Beta =.292, $t(152) = 3.759$, $p < .001$, physical functioning on self-efficacy, Beta =.208, $t(152) = 2.638$, $p = .009$, and self-efficacy on QoL, Beta =.579, $t(151) = 8.731$, $p < .001$. A regression model was conducted examining the indirect effect of self-efficacy as a mediator between physical functioning and QoL. Physical functioning significantly predicted QoL when the mediator was accounted for in the model, Beta =.187, $t(150) = 2.819$, $p = .005$. Self-efficacy as a mediator had a stronger predictive relationship on QoL than physical functioning, Beta =.542, $t(150) = 8.190$, $p < .001$. The statistically significant indirect effects show self-efficacy partially mediated physical functioning and QoL, demonstrating physical functioning's influence on QoL is through self-efficacy. However, its effect may lack clinical significance due to minimal Beta change. Since LTPD often limits physical improvement, interventions should prioritize self-efficacy to enhance QoL for those aging with LTPD.

Abstract citation ID: igae098.2685

### THE INTERSECTION OF LIVING ALONE, DEPRESSION, AND RESIDENTIAL ENVIRONMENT AMONG OLDER KOREANS WITH DISABILITIES

Jihyeong Jeong, *University of Maryland Baltimore, Baltimore, Maryland, United States*

The ecological theory of aging explains how the interactions between individuals' capabilities and their environment affect psychological well-being. The risk of depression rises among older adults living alone, and this vulnerability is further accentuated for those with disabilities, as their residential environments may not adequately compensate for the multifaceted challenges associated with impairments. The current study analyzed a sample comprised of 2,220 older adults with disabilities, aged 60 years or over, selected from the first wave of the 2021 Disability and Life Dynamics Panel of South Korea. As a cross-sectional study, a linear regression was performed controlling for gender, job status, disability type, and disability level. The findings reported that (a) as the perceived housing environment goes down by 1 unit, depression goes up by.20 (p <.001) and (b) older adults who live alone were associated with higher levels of depression compared to those who do not live alone (Beta = 1.36; p <.001). Aligning with the ecological theory of aging, the results underscore the significance of the residential environment in the psychological well-being of older adults. Furthermore, the positive correlation between living alone and higher levels of depression implies the importance of social support systems in mitigating depressive symptoms. Given these results, interventions aimed at improving the living conditions and social support networks of older adults who live alone with disabilities stand to produce meaningful improvements in their mental health.

Abstract citation ID: igae098.2686

### WE♡CARE: A CURRICULUM FOR CAREGIVERS WITH DEVELOPMENTAL DISABILITIES

Britney Burkart, *University of Missouri-Kansas City's Institute for Human Development: Health and Aging, Kansas City, Missouri, United States*

Many adults with developmental disabilities (DD) live at home with their caregivers. However, there are limited resources available to individuals with DD living in mutual caregiving arrangements, a scenario in which adults with DD become more responsible for providing care to an aging loved one. This poster presentation will provide an opportunity to learn more about WE♡CARE, a curriculum developed for adults with DD living in a mutual caregiving arrangement. A mixed methods approach was employed to measure curriculum impact and identify lessons learned surrounding curriculum implementation. Observational data collected during the initial pilot program indicated that adults with DD can benefit from facilitated programs such as WE♡CARE. For example, participants of the pilot program not only learned to recognize when their loved one might need support, but also to develop confidence as a caregiver. Quantitative data measuring change in knowledge among participants showed improvement from pre-session to post-session. A confidence assessment administered before and after participation did show a statistically significant increase in confidence from pre to post ($p < 0.05$). While the curriculum offers great value to individuals with DD and their loved ones, the pilot program shed light on the need to modify protocols, considering physical and cognitive accessibility and adaptability of facilitators to individual needs. As the population ages, mutual caregiving relationships will become more common, and the knowledge and skill sets embedded in this curriculum can support the growing number of mutual caregivers throughout the country.

### SESSION 7245 (POSTER)

### DYADIC RESEARCH

Abstract citation ID: igae098.2687

### ASSOCIATION AND INTERACTION BETWEEN RESILIENCE AND PSYCHOLOGICAL DISTRESS IN DISABLED ELDERLY–CAREGIVER DYADS

Anni Wang, *Fudan University, Shanghai, Shanghai, China (People's Republic)*

**Objectives:** The aim of this study was to explore the interaction between resilience and psychological distress in disabled elderly-caregiver.

**Methods:** A total of 246 homebound disabled elderly and their family caregivers from five provinces of China were investigated by convenience sampling using the Chinese version of the Resilience Scale and the Distress Thermometer and Problem List. Data analysis using the actor - partner interdependence model (APIM) modelling.

**Results:** The effect of resilience on psychological distress is a mixed pattern. With regard to the actor effect, the resilience of the disabled elderly and caregivers is negatively related to their own psychological distress, respectively. Regarding the partner effect, the resilience of the disabled elderly was negatively associated with caregiver psychological distress, and the resilience of the caregiver was negatively associated with psychological distress of the disabled elderly.

**Conclusion:** There is dyadic interaction between disabled elderly and their caregivers, suggesting that resilience can relieve not only one's own psychological stress but also that of others to some extent. Families of disabled elderly need to focus on both the older person and the caregiver, taking advantage of resilience in a focused way to reduce psychological distress and improve the dyadic physical and mental health. Health professionals can design dyadic interventions which are focuses on resilience and its dyadic interaction, and effectively leverage the resilience advantages of the dyad based on the individual's level of psychological distress, to improve the mental health of the elderly with disabilities and their caregivers.

Abstract citation ID: igae098.2688

### ASSOCIATION BETWEEN SELF-REALIZATION AND HEALTH OUTCOMES IN FAMILY CAREGIVING DYADS

Cristina de Rosa, Weijun Wang, and Yu-Ping Chang, *University at Buffalo, SUNY, Buffalo, New York, United States*

Self-realization, such as perceptions of life having meaning and purpose, can potentially influence health in older adults and their family caregivers, but how dyad members may contribute to each other's outcomes is less well understood. This analysis examined whether self-realization in older adults and their family caregivers was associated with their own, and each other's, mental health and perceived general health. Data for adults aged 65 and older were obtained from the National Health and Aging Trends Study (NHATS) (2022) linked with their respective family caregivers from the National Study of Caregiving (NSOC) IV (2022). A total of 1,571 dyads consisted of one older adult and their primary caregiver, defined as the family member or friend who provided the greatest number of hours of care. Actor-partner interdependence models (APIM) were used to examine associations between self-realization and mental health and perceived general health, controlling for dyad members' own gender, age, and race/ethnicity, and dementia status of the care recipient. Results showed that self-realization significantly predicted caregivers' and older adults' own mental health and perceived general health. Furthermore, older adults' self-realization significantly predicted their caregivers' perceived general health (b = 0.043, S.E. = 0.014, 95% CI = [0.016, 0.070], p =.002). Findings suggest mental health and perceived general health are impacted by a person's own self-realization even when accounting for partner effects. Caregivers are particularly influenced by older adults' self-realization. Strategies supporting both dyad members may provide benefits for both older adults and their family caregivers.

Abstract citation ID: igae098.2689

### BIOPSYCHOSOCIAL NATURAL HISTORY OF ALS: INTRODUCTION TO THE OPEN DATA, ACCESS, AND USE

Suzanne Segerstrom[1], and Edward Kasarskis[2], *1. Oregon State University, Corvallis, Oregon, United States*, *2. University of Kentucky, Lexington, Kentucky, United States*

The Seattle Amyotrophic Lateral Sclerosis (ALS) Patient Profile Database is a rich longitudinal dataset of ALS patients (n = 143; ages 26-83 years) and their partners (spouses, significant others, or caregivers; n = 123; ages 20-79 years) from clinics in Seattle, WA; San Francisco, CA; and Philadelphia, PA. The purpose of the study was to characterize the psychosocial and physical natural history of ALS and the interactions between them. Participants and partners (84% spouses) were separately interviewed in their homes every 3 months for up to 18 months between March 1987 and August 1989, with survival data collected up to 2008. Psychological measures include social cognition (e.g., self-perception and informant perception), psychological processes (e.g., coping, emotional expression), psychological health (e.g., anger, depression), social role activities (e.g., work, church attendance), and social support. Disease history, severity, progression, and survival data are available along with health behavior history, use of respirator and feeding tube, treatments (including psychotherapy or counseling), and medical and family history. This database is newly available and open on Mendeley Data, providing the opportunity for scientists to test biopsychosocial hypotheses in this hard-to-obtain patient population. Much of the data has never been analyzed or reported. In this presentation, the structure and contents of the dataset, the means of access, and the handful of previous findings will be introduced to the GSA meeting attendees.

Abstract citation ID: igae098.2690

### EMOTION REGULATION IN PEOPLE LIVING WITH DEMENTIA AND THEIR SPOUSES: THE ROLE OF NEUROPSYCHIATRIC SYMPTOMS

Yifan Lou[1], Joan Monin[2], Thi Hoang Vu[3], and Amanda Piechota[4], *1. Virginia Commonwealth University, New Haven, Virginia, United States*, *2. Yale School of Public Health, New Haven, Connecticut, United States*, *3. Yale University School of Public Health, New Haven, Connecticut, United States*, *4. University of Alabama, Tuscaloosa, Alabama, United States*

People with dementia (PwD) and their care partners (CP) may have difficulties in emotion regulation and individual differences in emotion regulation may be related to PwD's neuropsychiatric symptoms. This study explores whether there is any self-awareness of the PwD's emotional behaviors and whether CP's emotion regulation relates to the PwD's neuropsychiatric symptoms, potentially revealing bias or interpersonal effects. We used data from the Wish Outcome Obstacle Plan Study with a sample of 45 PwD and their spousal CP (n = 90 individuals). Multivariate linear regression models were used to investigate the associations between the CP-reported neuropsychiatric symptoms in PwD and self-reports of emotion regulation in both dyad members, net of sociodemographic and health factors. Separate analyses were conducted for each neuropsychiatric subsyndrome and each domain of difficulties in emotion regulation. Increasing numbers of neuropsychiatric symptoms were associated with higher difficulties in emotion regulation in PwD (ß = 2.78, p < 0.01), but not with CP's perceived difficulties in emotion regulation. PwD reported particular difficulties in accepting emotions, controlling impulses, and accessing emotion regulation strategies, but not in emotion awareness and clarification, when CP reported more neuropsychiatric symptoms. Proxy-report hyperactivity and psychosis subsyndromes are most significantly related to PwD's perceived difficulties in emotion regulation. PwD may be aware of their difficulties in emotion regulation at the early stage of dementia. Proxy-report neuropsychiatric symptoms may capture PwD's emotion regulation capability and not be biased by CP's emotion regulation difficulties.

Abstract citation ID: igae098.2691

### INTERDEPENDENCY OF COUPLES' EVERYDAY COGNITIVE PERFORMANCE

Martina Luchetti[1], Fiona Rupprecht[2], Jana Nikitin[2], Selin Karakose[1], Jeffrey Stokes[3], Antonio Terracciano[1], and Angelina R. Sutin[4], *1. Florida State University, Tallahassee, Florida, United States*, *2. University of Vienna, Vienna, Wien, Austria*, *3. University of Massachusetts Boston, Boston, Massachusetts, United States*, *4. Florida State University College of Medicine, Tallahassee, Florida, United States*

Longitudinal studies suggest an interdependency of marital partners' well-being and health behaviors, which can impact cognitive health. To date, however, few studies have taken a dyadic approach to examine interrelations of cognitive function within romantic partners and none in daily life. This study examines momentary cognitive performance within middle-aged and older romantic couples, and how their performance is interrelated over 8 consecutive days. Data were from 137 opposite-sex couples (274 participants), between 40 and 70 years old, who completed a smartphone-based processing speed task and a visual memory task, three times a day, over the study period. A combination of dynamic structural equation and actor-partner interdependence modeling was used for the analysis. The analysis indicated that mean-level performance on both tasks were correlated between partners. There was a significant time-lagged partner effect for processing speed: Poor performance (longer reaction times) of a participant at one momentary assessment was associated with poor performance of their partner at the next assessment. When controlling for contextual factors (e.g., location, day of the week, presence/absence of others), only the time-lagged effect from female to male partners remained significant. Further, this time-lagged effect was stronger for couples of younger ages. There was no lagged effect for visual memory. This work sheds light on the interdependency of couples' daily functioning, which might have implications for long-term cognitive outcomes.

Abstract citation ID: igae098.2692

### NURSING HOME STAFF CARE APPROACHES ARE ASSOCIATED WITH MEALTIME DIFFICULTIES IN OLDER ADULTS WITH DEMENTIA

Kyuri Lee, Heather Suh, and Wen Liu, *University of Iowa, Iowa City, Iowa, United States*

Nonverbal mealtime care is critical for optimizing eating performance and mealtime behaviors of residents with dementia (RwD), especially for those with limited verbal communication abilities. Understanding the relationship between care staff's nonverbal approaches and RwD's mealtime behaviors help guide the development of evidence-based, effective mealtime interventions. This observational study characterized staff person-centered and task-centered care approaches and RwD mealtime difficulties and examined the associations between staff care approaches and RwD mealtime difficulties. Videotaped mealtime observations (N=185) involving 47 nursing home direct care staff (age mean ± SD = 45.6±11.8 years; 92.3% female; 80.8% white; 15.5±12.9 years worked as a caregiver, 12.3 ± 9.1 years in the current facility) and 12 RwDs (age 71.9±7.5 years; 91.7% male; 91.7% white, 41.7% married) were coded using the refined Cue Utilization and Engagement in Dementia (CUED) mealtime video coding scheme. Staff primarily used nonverbal person-centered care approaches, including modifications of resident abilities (62.4%), dyadic interactions (10.7%), and the dining environment (6.0%), followed by task-centered behaviors (9.6%). RwD's mealtime difficulties score assessed by The Edinburgh Feeding Evaluation in Dementia (EdFED) was 5.91±3.59 (range 0-15). RwD's mealtime difficulties was significantly associated with person-centered care approaches (b=0.04, p=.005) and task-centered care approaches (b=0.11, p=.042), controlling for staff age, years worked as a caregiver, and years worked in the current facility. Minimizing the use of task-centered care may alleviate residents' mealtime difficulties. Person-centered care is highly recommended in dementia care, but needs to be multifaceted and tailored to the different contexts and needs of individual residents.

Abstract citation ID: igae098.2693

### THE ROLE OF MUTUALITY ON THE QUALITY OF LIFE OF MID-LATE LIFE ADULTS WITH CHRONIC ILLNESS AND THEIR NURSES

Silvia Cilluffo[1], Karen Lyons[2], Barbara Bassola[3], Ercole Vellone[4], Gianluca Pucciarelli[5], Marco Clari[6], Valerio Dimonte[7], and Maura Lusignani[1], *1. University of Milan, Milan, Lombardia, Italy*, *2. Boston College, Chestnut Hill, Massachusetts, United States*, *3. ASST Great Metropolitan Hospital Niguarda, Milan, Lombardia, Italy*, *4. Tor Vergata University of Rome, Rome, Lazio, Italy*,
*5. University of Rome Tor Vergata, Rome, Lazio, Italy*,
*6. University of Torino, Torino, Piemonte, Italy*,
*7. University of Turin, Torino, Piemonte, Italy*

Mutuality between an adult with chronic illness and their nurse has received little investigation, despite the therapeutic nature of the relationship. Qualitative research by this team has led to conceptualizing nurse-patient mutuality as an interactive process involving developing rapport and going beyond, being a point of reference, and shared decision-making/care. To inform this area of research, our team developed and tested one of the only known measures of nurse-patient mutuality. This paper reports findings from our recent multicenter study that examined the association of nurse-patient mutuality on mental and physical quality of life (QoL) of nurses and mid-late-life adults with chronic illness. A total of 192 unique dyads (Mage= 64.5 for patients and Mage=41.8 for nurses) were sampled across Italian hospitals. Nurses and patients' perceptions of their mutuality were positively correlated (p<.001Actor partner interdependence models found significant actor effects of mutuality on mental QoL for nurses and patients; the better the patient perceived mutuality with their nurse, the better their mental QoL (p<.001); the better the nurse perceived mutuality with their patient, the better their mental QoL (p=.002). However, a significant partner effect found the better the nurse perceived mutuality with the patient, the more likely the patient was to have poor mental QoL (p<.001). There were no significant associations with physical QoL. Discussion will focus on the need for further research on the role of nurse-patient mutuality on health outcomes and health behaviors and the complex role nurse engagement can have in patients experiencing poor mental health.

### SESSION 7250 (POSTER)

### EDUCATION AND TRAINING

Abstract citation ID: igae098.2694

### AGING COURSES AS A MODEL FOR DEVELOPING PSYCHOLOGICAL LITERACY: INSIGHTS AND ACTIVITIES

Nicole Alea[1], and Carla Strickland-Hughes[2], *1. University of California Santa Barbara, Santa Barbara, California, United States*, *2. University of the Pacific, Stockton, California, United States*

Psychological literacy refers to understanding psychological content and applying it to: the self, for adaptive self-understanding and management of effective socioemotional goals; local communities, so that students have, for example, the skills to fulfill employer's needs; and international contexts, so that students have awareness of what is required to become a global citizen. Psychological literacy is relevant for students in all disciplines, as it prepares them for the world after graduation. In this presentation, we suggest that courses with an aging component, like gerontology and psychology of aging, can serve as model courses for developing psychological literacy among students, and should be earmarked as such in undergraduate curriculum. This suggestion will be grounded in three points: (i) aging is self-relevant but feels far-off for traditional-aged undergraduate students; (ii) this means that aging courses naturally tend to showcase the relevance of the material for individuals and society before content to engage students; and (iii) that aging courses tend to emphasize knowledge needed for success in professions and relationships. These three points mean that aging courses encourage psychological literacy. Example course activities, such as activities that create opportunities for intergenerational connections, activities that require exploration of aging careers, and activities that encourage students to propose innovations that will improve life for aging individuals and societies, will be provided to emphasize these points. We will conclude with strategies to include these activities in courses taught in different programs and institutional contexts.

Abstract citation ID: igae098.2695

### COMMUNICATION TRAINING FOR MEDICAL PROFESSIONALS: PROOF OF CONCEPT

Nancy Brown[1], and Vicki De Klerk-Rubin[2], *1. Validation Training Institute, Jerusalem, Yerushalayim, Israel, 2. Validation Training Institute, Springfield, Oregon, United States*

Despite the prevalence of patients living with dementia, physicians and medical students in particular, have received little or no dementia-specific training to help them communicate effectively with this population. Connecting and communicating with persons living cognitive change requires specific skills. These skills are taught in the Validation for Physicians and Medical Provider training, based upon the evidence-based Validation method by Naomi Feil. Developed with physicians and pedagogical expert in online learning, the training was designed for healthcare professionals, specifically doctors, with quality of care for patients and their carers in mind. The training comprised of a variety of teaching methods including: online on-demand videos, quizzes, case studies, and live webinars for each of the five blocks. The pilot program included physicians and other healthcare professionals. Pre and Post surveys consisted of a knowledge scale and SRDB (self-efficacy) ranked on a Likert scale of 1 to 5 and analyzed using SPSS. Post-training individual interviews concluded the beta-testing. Thirty professionals enrolled in the training; eight participants completed every aspect of the beta-testing. Despite the small sample size, there were trends approaching significance in knowledge and self-efficacy. Participants highly valued the contents of the training as well as the pedagogy. Technical problems were the main reason for attrition. Overall, the Validation method was found useful in practice, improved confidence, knowledge, and communication behaviors. Improvements are needed in the choices for validated measures and the online platform. "Validation has made me a better doctor. It has changed my perspective on aging" (participant).

Abstract citation ID: igae098.2696

### CRAFTING COLLABORATIONS THAT BENEFIT STUDENTS, CLIENTS, AND COMMUNITY

Stacey Gandy[1], CJ Golding[2], and Gillian Bryant[1], *1. Arizona State University, Phoenix, Arizona, United States, 2. Arizona State University, Tempe, Arizona, United States*

When a university partners with a low-income residency for older adults, the results are exponential. A student-run, interdisciplinary drop-in center housed within a Section 8 apartment building for older adults and adults with disabilities arose from such a partnership. This program provides interdisciplinary service-learning opportunities for students while serving the needs of the residents. Social work students work alongside other disciplines, such as nursing, recreational therapy, nutrition, program planning, poetry, and music, to support residents' emotional, social, psychological, and physical well-being. In the 2022-2023 academic year, the program served 257 of the building's 300 residents and provided learning opportunities for 300 students. A 2013 needs assessment of the building's residents found considerable loneliness, social isolation, and disconnectedness. Healthy Places by Design (2021) recommends intergenerational community centers that prioritize socialization among older adults and younger people, which is a major focus of the drop-in center. Students foster interaction with and between residents to encourage community connectedness. Research demonstrates that social interaction between older adults and college students can result in decreased loneliness for older adults and decreased attitudes of ageism in students (Ramamonjiarivelo et al., 2022). As our aging population grows, so does the importance of preparing social work students to work with older adults. We are excited to share the ways in which bringing older adults and student interns together is mutually beneficial and provides an innovative solution to eradicate loneliness, social isolation, and community disconnectedness in older adults and ageism in students.

Abstract citation ID: igae098.2697

### EVALUATING VIRTUAL MONTESSORI DEMENTIA TRAINING USING NORMALIZATION PROCESS THEORY AMONG VA NURSING HOME STAFF

Katherine Kennedy[1], A. Snow[2], Whitney Mills[3], Amy Mochel[4], Sylvia Haigh[5], Teddy Bishop[2], Christine Hartmann[6], and Michelle Hilgeman[2], *1. VA Providence Healthcare System, Center of Innovation in Long-Term Services and Supports, Providence, Rhode Island, United States, 2. Tuscaloosa VA Medical Center, Tuscaloosa, Alabama, United States, 3. VA Providence Health Care System, VA Providence Health Care System, Rhode Island, United States, 4. Providence VA Healthcare System, Providence, Rhode Island, United States, 5. Providence VA Medical Center, Providence, Rhode Island, United States, 6. VA Bedford Healthcare System & University of Massachusetts Lowell, Bedford, Massachusetts, United States*

This paper uses Normalization Process Theory (NPT) to examine staff impressions of Montessori-based program training and implementation at Veterans Affairs Community Living Centers (VA CLCs; nursing homes). We conducted a mixed-methods evaluation of Montessori Approaches to Person-Centered Care (MAP-VA) at eight VA CLCs. Trainings were conducted as either a live virtual course or a pre-recorded asynchronous class. Two NPT constructs, coherence building and cognitive participation, informed qualitative interview questions, surveys, and analyses focused on staff movement from knowledge to action during initial implementation. Data collection included staff-completed standardized post-training exams (N=906), post-training evaluations (N=761), and optional validated surveys on perceptions of Montessori training (N=307). Champions (peer-leaders) from each CLC completed semi-structured qualitative interviews post-training (N=22). The majority of staff (83%-90%) passed all courses. Staff evaluated the training highly (80%+ agreement) on learning relevant new knowledge and confidence applying new skills. On average, staff felt MAP-VA would become a normal part of their work (7.68/10 scale) and reported increased familiarity with Montessori approaches after training (p =.002). Qualitative interview data from staff trained in Montessori supported three themes concordant with the NPT dimensions of coherence building and cognitive participation. Montessori virtual training resulted in high levels of coherence and cognitive participation among multidisciplinary staff, evidenced by high knowledge, self-efficacy, and readiness to act. The asynchronous and synchronous trainings were accessible, relevant, and supported diverse learners. Virtual dementia training can be an effective and efficient method of teaching nursing home staff about Montessori approaches to reduce responsive behaviors.

Abstract citation ID: igae098.2698

### MEDICAL STUDENTS' CONFIDENCE INTERVIEWING, AND PERCEPTIONS OF, OLDER ADULTS

Aliza Baron[1], Seema Limaye[2], Stacie Levine[1], Monica Long[3], and Shellie Williams[1], *1. University of Chicago, Chicago, Illinois, United States*, *2. Edward Hines, Jr. VA Hospital, Oak Park, Illinois, United States*, *3. University of Chicago Medicine/SHARE Network, Glenwood, Illinois, United States*

As the U.S. populations ages, medical students need primary geriatric skills yet continue to lack interest in geriatric care and hold negative attitudes towards caring for older adults (OA). The Geriatrics and Aging Through Transitional Environments (GATE) Curriculum for first year Pritzker medical students provides experiential learning conducting a functional history interview and assessing frailty and home safety risks. Students attended a 1-hour lecture on geriatric functional history taking, frailty (fatigue, resistance, weight loss, gait, hospitalization) and home safety assessments. Student pairs conducted an in-home structured interview with an OA "trained patient" (TP) or untrained OA family/acquaintance. OA residents at a local retirement community were recruited and trained as TP through a 1-hour training session on-site. Training addressed students' learning objectives, interview questions, and techniques for delivering feedback to students. Learner evaluation included a pre/post survey assessing self-confidence interviewing OA using a Likert scale (5=Extremely confident, 1=Not at all) and attitudes towards OA using open-ended word association. From 2021-23, 178 Pritzker medical students (100%) completed 96 interviews (86% with TP, 14% with family/acquaintances) with 55 OA. Confidence interviewing changed significantly pre-GATE (M=1.73, SD=.720) to post-GATE (M=3.43, SD=.814); t(168)=-23.88, p<.001. Students' attitudes towards OA shifted post-GATE with an increased usage of word associations "wise/knowledgeable" and "storytellers" and decreased associations as "frail, slow, diseased." A medical student curriculum incorporating in-home functional history taking interviews with OA TP may increase students' confidence interviewing and instill positive attitudes towards OA that are foundational for building empathic doctor-patient relationships across medical specialties.

Abstract citation ID: igae098.2699

### OMG! THEY LOOK JUST LIKE ME: EXPERIENTIAL LEARNING RELATED TO OLDER ADULTS AND HOMELESSNESS

Phyllis Greenberg, Isaac Asirifi Boateng, Sheila Moriarty, and Claudia Burgos Zuniga, *Saint Cloud State University, Saint Cloud, Minnesota, United States*

In 2023, nearly 1 in 4 people experiencing homelessness were over the age of 55. (National Alliance to End Homelessness 2023). As shocking as this number might be, it is estimated that the numbers of older adults who are homeless or at risk of homelessness are expected to nearly triple by 2030.To raise awareness in gerontology students a class studying research methods and a class studying policy teamed with social work students for Central Minnesota's Project (Homeless) Connect. This a semester long project where students develop information for the larger community, city and state officials, as well as professionals about the growing numbers of older adults that are homeless or at risk of homelessness. In addition, to putting together and disseminating information students raise funds to (silent auctions, outreach to community donors, etc.) prepare basic necessity bags. The bags include everything from mini flashlight, rain poncho, shelf stable foods and a can opener among other things. Students also get socks, coats, and disposable underwear and other sanitary products that participants can choose from. The Project (Homeless) Connect event takes place annually and students work with participants to help them secure a variety of services from community vendors as well as learn more about them as individuals. Many of the participants have mentioned how they felt seen just by someone asking them their name and talking with them. This has been one of the more impactful experiences as well for students, faculty, staff and the community.

### SESSION 7255 (POSTER)

### EMOTIONS

Abstract citation ID: igae098.2700

### AGE AND GENDER IMPACTS ENDORSEMENT OF NOSTALGIA ACROSS SEVEN YEARS

Jennifer Turner[1], Tim Wildschut[2], and Constantine Sedikides[2], *1. University of Hawaii Hilo, Hilo, Hawaii, United States*, *2. University of Southampton, Southampton, England, United Kingdom*

The experience of nostalgia (a bittersweet longing for one's past) is common amongst people of all ages (Hepper et al., 2021; Juhl et al., 2020). Recent work described age-group differences in the frequency of nostalgia, such that older adults reported more nostalgia compared to middle-aged and younger adults (Turner & Stanley, 2021). Some have posited that age-related differences in nostalgia are founded on changing emotion-regulation goals and future time perspective in later life (Hepper et al., 2021), yet one problem has been the emphasis on cross-sectional studies which limit understanding of potential developmental change in nostalgia. This study extends previous work by examining the relevance of individual differences (i.e., age and gender) for endorsement of nostalgia in a nationally-representative sample of Dutch-speaking participants (N=2,664, 53.5% women) using a longitudinal design from the LISS panel (8-waves collected 2012-2019). At baseline, participants' ages ranged between 9-93 years (M=48.59, SD=18.07). At each wave, participants completed the 7-item Southampton Nostalgia Scale (Sedikides et al., 2015), which assesses how frequently, and how much, one values the experience of nostalgia (Wang et al., 2023). As expected, analyses supported both between-person age differences, with older adults endorsing greater nostalgia than younger ones, and within-person age-related increases in nostalgia. Gender differences also emerged, such that women were more nostalgic than men on average, but this was qualified by a significant interaction with age: men (but not women) demonstrated increases in nostalgia with age. Results are interpreted through the lens of Socioemotional Selectivity Theory (Carstensen, 2021).

Abstract citation ID: igae098.2701

### APPRECIATING TIME ALONE: AGE AND SOCIAL INTERACTION OPPORTUNITIES ENHANCE EMOTIONAL WELL-BEING WHEN ALONE

Anna Pot[1], and Laura Carstensen[2], *1. Stanford University, Stanford, California, United States*, *2. Stanford University, Stanford University, California, United States*

Whereas long periods of solitude risk the health and well-being of older people, on the aggregate level, older people spend considerably more time alone than younger age groups and, on the whole, enjoy better mental health. In the present study, we consider time alone at the trait and state level. This study seeks to explore how relationships and spending time alone influences daily emotional experience in a sample spanning the adult life span. Drawing on data from an experience-sampling study that used a burst design at two timepoints five years apart, participants (N1=212 | N2=179) aged 21-94 (M1=54.58, SD1=21.95 and M2=56.80, SD2=21.24) reported their positive and negative emotions (19 in total) and whether they were alone or interacting with someone, five times daily for a week. Findings from mixed-effects models highlight a complex relationship between age, spending time alone and emotional well-being. Age positively relates to emotional well-being, and spending time alone is associated with a poorer emotional profile across the full participant sample. An interaction between age and being alone suggests that those older adults who spend relatively more time alone than average report a slight decline in emotional well-being. However, older adults who have more social interactions report significantly more positive emotions at the state level when they are alone, compared to other age groups. These results suggest that time alone may be appreciated with age, as long as social interactions do not become scarce. Findings contribute to our understanding of time spent alone and emotional well-being.

Abstract citation ID: igae098.2702

### COGNITIVE REAPPRAISAL AND STRESS REACTIVITY ACROSS ADULTHOOD

Jessica Maras, and Kate Leger, *University of Kentucky, Lexington, Kentucky, United States*

How one reacts to stressful events, termed stress reactivity, impacts health and well-being. The use of emotion regulation strategies can influence reactivity, specifically cognitive reappraisal, which has been shown to reduce reactivity. Furthermore, age can affect reactivity due to older adults' ability to employ cognitive reappraisal strategies more readily. The current study investigated the interplay between age and cognitive reappraisal on different components of reactivity: affective, somatic and physiological. Participants (N = 139) were divided into younger (N = 89) and older (N = 50) adults with subsets receiving the cognitive reappraisal intervention (N = 71) or the control condition (N = 68). Baseline measures were obtained via questionnaires followed by a stress-inducing task to assess reactivity. The cognitive reappraisal condition received a prompt prior to the stress-inducing task telling them to think about the positive aspects of the task. Reactivity was measured via negative affect, somatic symptoms (e.g., stomach ache) and blood pressure after the task, while controlling for baseline reactivity. Preliminary findings revealed a significant interaction between age and experimental condition for systolic blood pressure reactivity ($\beta = -0.17$, $p = .049$). This indicates that older adults in the control condition showed decreased systolic blood pressure reactivity to the stressor. No other significant main effects or interactions were found. This unexpected finding highlights that there may be specific age groups in which engaging in cognitive reappraisal is disadvantageous to different types of reactivity and emphasizes the need for understanding for whom and when cognitive reappraisal might work best.

Abstract citation ID: igae098.2703

### DAILY AFFECTIVE REACTIVITY ACROSS THE COVID-19 PANDEMIC: THE MODERATING ROLES OF POSITIVE AFFECT AND AGE

Niccole Nelson[1], and Cindy Bergeman[2], *1. Colorado State University, Fort Collins, Colorado, United States*, *2. University of Notre Dame, Notre Dame, Indiana, United States*

On March 11, 2020, the World Health Organization declared COVID-19 a pandemic. Given COVID-19 had the capacity to make any individual seriously ill, several emergency directives, including cancelation of social gatherings and closures of non-essential businesses, were put in place to slow its spread. Individuals over the age of 60 were considered high-risk for serious COVID-19-related illness, which was associated with greater mitigation efforts and lifestyle changes among this group. Although prior work indicates that the emergency phase of the pandemic influenced stress-and-coping processes, less is known about age differences in these processes, especially how they have unfolded across the past four years of the pandemic. Thus, leveraging data from the Notre Dame Study of Health & Well-being—COVID Study, we examined daily stress and affect across five 28-day, daily diary bursts that were collected approximately every six months between April 2020 and February 2022. Using three-level, multilevel modeling, we examined daily negative affective reactivity, the 'undoing' effect of daily positive affect on daily negative affective reactivity, as well as age differences in these daily processes. We found that individuals experienced greater negative affect on days of particularly high stress, and that this process was buffered on days of particularly high positive affect. We also found that older individuals exhibited both lower affective reactivity to stress, as well as a greater 'undoing' effect, than younger adults. These results indicate that the mobilization of positive affect may be a promising avenue for intervention in daily stress processes, especially among older adults.

Abstract citation ID: igae098.2704

### DAILY DYNAMICS AMONG AFFECT, PHYSICAL ACTIVITY, AND SLEEP: A MULTILEVEL AUTOREGRESSIVE CROSS-LAGGED MODEL

Sun Ah Lee[1], Zachary Fisher[1], and David Almeida[2], *1. The Pennsylvania State University, University Park, Pennsylvania, United States*, *2. Pennsylvania State University, University Park, Pennsylvania, United States*

Previous research suggests that physical activity and sleep are important behavioral factors contributing to affective well-being. However, less is known about their simultaneous dynamics in daily life. The current study examines the within-day and across-day associations among affect, moderate-to-vigorous physical activity (MVPA), and sleep. Data were drawn from the third wave of a daily diary study of Midlife in the United States (MIDUS). A sample of 1,171 middle-aged and older adults (Mage= 62.61; SDage = 10.26: 57% female; 82% white) completed 9,059 daily telephone interviews across eight consecutive evenings, where they reported daily positive (PA) and negative affect (NA), previous-night sleep duration and sleep quality, and MVPA engagement. Multilevel autoregressive cross-lagged models were estimated to simultaneously address the bidirectional relationships among daily affect, MVPA, and sleep. For within-day correlations, MVPA engagement was correlated with same-day higher PA and lower NA. Longer sleep duration was correlated with the same-day higher sleep quality. For across-day associations, longer sleep duration and higher sleep quality predicted higher PA and lower NA on the next day. Higher sleep quality also predicted a greater likelihood of next-day MVPA. In addition, higher PA predicted higher sleep quality and a greater likelihood of MVPA on the following day. However, previous-day MVPA predicted higher next-day NA. Findings highlight the benefits of sleep on daily affective well-being and MVPA engagement, as well as the importance of positive affect on MVPA and sleep. This suggests that managing adequate sleep and emotions could be potential targets for prevention/intervention in promoting healthy aging.

Abstract citation ID: igae098.2705

### EMPATHY AND RELATIONSHIP QUALITY IN CHINESE PARENT–CHILD DYADS: THE MODERATING ROLE OF DAILY INTERACTION HOURS

Zewen Huang, and Da Jiang, *The Education University of Hong Kong, Hong Kong, China (People's Republic)*

Empathy has been consistently found to be associated with the quality of relationships in parents and children across the lifespan. However, little is known regarding whether and how such association would be influenced by the amount of time parents and children interact. We aimed to examine the relationship between empathy and relationship quality, and the moderating role in the relationship between aging parents and adult children from 99 parent-child pairs (Mage(parents)=48.3, SD=4.53, 79.8% women; Mage(children)=22.38, SD=3.49, 85.9% women) in this 14-day diary survey. We focused on both daily empathy collected in daily assessment and trait empathy collected in the pretest. By applying the actor-partner interdependence models and multilevel analysis, we found that the actor effects of daily and trait empathy on relationship quality were significant for both parents and children. Regarding the partner effects, parents' daily empathy was significantly associated with children's relationship quality, but not vice versa. Daily interaction hours moderated the associations. Specifically, for daily empathy, the impact of children's daily empathy on parents' relationship quality was significant only when parents and children interacted for a longer (vs. shorter) period of time. For trait empathy, the relationship between parents' trait empathy and their own relationship quality, and that between parents' trait empathy and their children's relationship quality were stronger among parent-child dyads reporting shorter (vs. longer) average daily interaction hours over the 14 days. These findings elucidated the complex associations between empathy and relationship quality in parent-child relationships by highlighting the moderating role of daily interaction hours.

Abstract citation ID: igae098.2706

### HIGH AROUSAL AFFECT RELATED TO WORSE WORKING MEMORY BUT MORE FOR YOUNGER ADULTS

Julie Kircher[1], Eric Cerino[2], Jacqueline Mogle[3], and Susan Charles[4], *1. The Pennsylvania State University, State College, Pennsylvania, United States*, *2. Northern Arizona University, Flagstaff, Arizona, United States*, *3. Clemson University, Clemson, South Carolina, United States*, *4. University of California Irvine, Irvine, California, United States*

Studies have found that high emotional arousal states, assessed by reports of negative affect, are often related to poorer working memory. Findings for positive affect (e.g., excitement or joyful), however, are more mixed, with some studies indicating that higher arousal is linked to better cognitive task performance. The purpose of this study was to examine how reports of negative and positive affect throughout the day are associated with verbal working memory, and how this relationship may vary by age. We hypothesized that moments of high negative or positive affect will be related to poorer cognitive task performance, and that age would moderate the relationship between affect and cognition. Data from the Effects of Stress on Cognitive Aging, Physiology, and Emotion (ESCAPE) study consisted of five assessments throughout the day across 14 days among 263 community residents ranging from 25-65 years old (Mage = 46.5). At each assessment, participants completed the n-back adapted for mobile phones and reported the current affect. Across all ages, higher levels of reported negative and positive affect at each assessment were related to worse momentary working memory ($\gamma$NA = -.0004, SE =.0002, p =.02; $\gamma$PA = -0.0003, SE =.0002, p =.04) and younger age was related to worse working memory as levels of positive affect increased.

Abstract citation ID: igae098.2707

### MOMENTARY UNCONSTRUCTIVE REPETITIVE THINKING, WORKING MEMORY, AND AGE

Julie Kircher[1], Eric Cerino[2], Jacqueline Mogle[3], and Susan Charles[4], *1. The Pennsylvania State University, State College, Pennsylvania, United States*, 2. *Northern Arizona University, Flagstaff, Arizona, United States*, 3. *Clemson University, Clemson, South Carolina, United States*, 4. *University of California Irvine, Irvine, California, United States*

Both trait and daily negative affect are often associated with lower performance on working memory tasks, perhaps because negative affect is often related to unconstructive repetitive thinking (URT) – negative ruminative thoughts about oneself or the environment. URT is posited to deplete attentional resources, resulting in downstream effects on cognition. Studies examining the effects of URT on cognitive performance often use laboratory-based study designs. This study aimed to investigate how reports of URT throughout the day are linked to scores on momentary cognitive tasks of verbal working memory. We hypothesized that reports of greater engagement in URT will be related to worse working memory, and that this association was related to age. Data from the Effects of Stress on Cognitive Aging, Physiology, and Emotion (ESCAPE) study included 263 community residents ranging from 25 to 65 years old (Mage = 46.5) completing five assessments throughout the day across 14 days. Contrary to our hypothesis, we found that greater URT was related to improved verbal working memory ($\gamma$ =.005, SE =.001, p <.001). Higher momentary negative affect was related to poorer working memory, acting as a suppressor on the benefits of URT on working memory performance. This relationship between URT and verbal working memory remained significant across ages, but the relationship between levels of URT and working memory was strongest among younger adults.

Abstract citation ID: igae098.2708

### STRATEGIES AND GOALS PREDICT DAILY EMOTION REGULATION EFFICACY BUT REAPPRAISAL USE IS LESS PREDICTIVE ACROSS AGE

Bruna Martins-Klein[1], Ziyuan Chen[1], Irina Orlovsky[2], Elinor Waite[2], and Katherine Dixon-Gordon[2], *1. University of Southern California, Los Angeles, California, United States*, 2. *University of Massachusetts Amherst, Amherst, Massachusetts, United States*

Socioemotional goals shift with age, as older adults prioritize regulating emotion to maximize positive affect (hedonic goals) more so than to complete tasks (instrumental goals). Hedonic goals are associated with greater reappraisal use (aka positive reinterpretation), and instrumental goals predict suppression use in daily life, but age differences in the impact of strategies and goals on subjective daily emotion regulation effectiveness has not been previously explored. In this study, 113 participants aged 20-72 years old (M = 38.2; SD = 11.4) completed a 14-day MTurk daily diary on emotion regulation goals, reappraisal and suppression use, and subjective emotion regulation effectiveness in response to daily stressors. Multilevel models nested within day revealed positive effects of age ($\beta$=0.03, p<.05) and reappraisal use ($\beta$=0.11), and a negative effect of suppression use on subjective regulation effectiveness ($\beta$=-0.07, ps<.001). Interestingly, hedonic and instrumental goals positively predicted effectiveness even when controlling for strategy and age effects (ps<.001). In addition, a significant age x reappraisal interaction revealed positive associations of reappraisal use on effectiveness that were stronger for younger adults ($\beta$=0.18) than for middle aged adults ($\beta$=0.13, ps<.001), and not significant for older adults (p=.47). Thus, goals and strategies uniquely influence emotion regulation in daily life, but reappraisal use is unrelated to late-life regulatory efficacy. Future work should investigate whether increasing salience and awareness of emotion regulation goals may be a key interventional target, which could have important clinical implications for promoting emotion regulation efficacy in older adulthood.

Abstract citation ID: igae098.2709

### THE MODERATING EFFECT OF AGE ON THE ASSOCIATION BETWEEN ANTICIPATORY APPRAISALS AND AFFECTIVE REACTIVITY

Lana Kinnear, and Gloria Luong, *Colorado State University, Fort Collins, Colorado, United States*

Affective reactivity (changes in emotion in response to a stressor) has been linked to poorer health. Variability in affective reactivity may be due to individual differences in anticipatory appraisals (AAs; perceptions of threat associated with an upcoming stressor). More work is needed to understand how AA predicts affective reactivity to stressors, and adult age differences in these links. Theoretical frameworks suggest older adults may appraise upcoming stressors as more positive compared to younger adults, but it is unclear if anticipatory appraisals reduce affective reactivity differently across age groups. This research leveraged data from 159 younger and older adult participants in the Health and Daily Experiences (HEADE) study to examine how AA predicts affective reactivity to a well validated lab stressor (Trier Social Stress Test) involving spontaneous public speaking and mental arithmetic tasks. Participants completed ecological momentary assessments which included phone surveys assessing anticipatory appraisals of the upcoming lab session. It was hypothesized that more positive AAs would predict lower positive affective (PA) and negative affective (NA) reactivity, and that this effect will be more pronounced for older, relative to younger, adults. Multiple regression analyses revealed that contrary to the hypothesis, more positive AAs were associated with an overall increase in PA, but not NA, reactivity among older adults. This effect was not found for younger adults. These findings emphasize the importance of understanding how overly positive anticipatory appraisals may exacerbate stress responses in older adulthood.

### SESSION 7260 (POSTER)

### FALLS I (POSTERS)

Abstract citation ID: igae098.2710

### CHANGE IN SOCIAL PARTICIPATION OVER TIME AMONG PERSONS AGES 45–64 AGING WITH LONG-TERM DISABILITY

Michelle Putnam[1], Susan Stark[2], Kerri Morgan[3], Holly Hollingsworth[3], and Melissa Krauss[3], *1. Simmons University, Boston, Massachusetts, United States, 2. Washington University in St. Louis, St. Louis, Missouri, United States, 3. Washington University School of Medicine, St. Louis, Missouri, United States*

Change in social participation over time among mid-life adults with disabilities as they grow older is under-studied. Existing research indicates that social participation among persons with disabilities, younger and older, is positively associated with health and well-being outcomes including mental health and life satisfaction. Despite this knowledge, few studies have explored if and how social participation changes year to year, and how symptoms of aging with disability including pain, fatigue, and depression may influence those changes. We undertook a cohort survey (2019-2021) with a sample of adults aged 45-64 (N=323) with self-reported mobility disability, collecting data over three annual time points, in order to better understand if we could see annual change in social participation that could help inform early interventions to support and sustain social participation. Our results found that T-scores from PROMIS measures of physical function, depression, and pain changed little over time, but level of fatigue did significantly change. PROMIS T-scores of satisfaction with social roles and activities and did not change over time, but T-scores for ability to participate in social roles and activities increased over time. T-Scores for physical function, depression, fatigue, and pain were significantly associated with both participation outcomes. In qualitative data, most participants (63%) said their participation declined over the 3 years, attributing change to health, aging with disability symptoms, and the COVID-19 pandemic. Study limitations include potential effects of the COVID-19 pandemic on participation and disability/health. Findings suggest the need to consider aging with disability symptoms when evaluating participation interventions.

Abstract citation ID: igae098.2711

### COGNITIVE PERFORMANCE DIFFERENTIALLY MODERATES THE ASSOCIATION BETWEEN GAIT VELOCITY AND FALL RISK

Sandra Hundza, Stuart MacDonald, Marc Klimstra, and Markus von Hacht, *University of Victoria, Victoria, British Columbia, Canada*

Cognitive-walking dual-task paradigms are an effective tool for assessing fall risk. Surprisingly, the moderating influence of cognitive performance on the association between gait velocity (GV) and fall risk has not been explored. The present investigation examines whether cognitive performance moderates the relationship between GV and fall risk. Community-dwelling older adults (76 years ±3.44) were classified as fallers and non-fallers based on self-report (at least one fall in the past 12 months). GV was indexed using the GAITRite system while counting backwards by serial 7s. Performance on the serial 7s task was recorded, with Adobe Audition subsequently employed to index cognitive function in terms of the number of counts (NC) and percentage of true counts (PTC) from the recorded audio files. Logistic regression was employed to examine the predictive influence of GV on the likelihood of fall risk classification (GV model), as well as the moderating influence of NC and PTC on the GV-fall risk association (GCI model). Notably, there was a significant moderating effect of PTC on the GV-fall risk association for individuals with PTC scores ≥-2.41 units (Johnson-Neyman analysis); those with lower GV were at increased risk of falling, with this relationship magnified as PTC scores increased. The sensitivity of the GCI interaction model (88%) represented a 17% improvement over the GV model. This investigation is the first to demonstrate the importance of interactions between gait and cognition measures in fall risk modelling, and underscores the potential clinical utility of including cognitive performance measures in dual-task paradigms.

Abstract citation ID: igae098.2712

### EXPERIENCE OF LOW-INCOME COGNITIVELY IMPAIRED COMMUNITY-DWELLING OLDER ADULTS WITH MINDFUL MOVEMENT

Eunice Ojo[1], and Ladda Thiamwong[2], *1. University of Central Florida Orlando Florida, Orlando, Florida, United States, 2. University of Central Florida, Orlando, Florida, United States*

Falls are a common but avoidable problem among older adults. Older adults have a higher risk for falls and face many challenges to engaging in exercise-based fall prevention interventions. Practicing mindful movement contributes to gait improvement and fall prevention. However, there is limited information about the experience of older adults with mindful movement. This study aimed to explore the knowledge and experience of low-income cognitively impaired community-dwelling older adults with mindful movement. Participants aged 60 years and older with cognitive impairment were selected from a technology-based fall risk assessment study using BTracks Balance System, Fitbit, and Bioelectrical Impedance Analysis in low-income settings in Central Florida. Fourteen participants were individually interviewed, and a semi-structured interview guide was used to obtain participants' experience with mindful movement. Individual interviews were audio-recorded, transcribed, coded, and analyzed using thematic analysis. Many codes emerged, and three major themes and sub-themes were developed including 1) knowledge of mindful movement 2) perceived benefits of mindful movement, and 3) linking mindful movement into physical activity. Most participants did not understand mindful movement but acknowledged regularly practicing careful movement to prevent falls. Participants identified fall prevention and stable mental health as benefits of mindful movement. They also described practicing mindful movement during performing physical activity including walking and dancing. There is a need to educate cognitively impaired older adults about mindful movement and encourage its practice as a vital part of daily physical activity. Future studies should focus on how to integrate mindful movement into fall prevention interventions.

Abstract citation ID: igae098.2713

### EXPERIENCE OF OLDER ADULTS WITH OBJECTIVELY MEASURED PHYSICAL ACTIVITY IN FALL PREVENTION INTERVENTION

Eunice Ojo[1], Victoria Loerzel[2], and Ladda Thiamwong[2], *1. University of Central Florida Orlando Florida, Orlando,*

*Florida, United States*, 2. *University of Central Florida, Orlando, Florida, United States*

Falls are a preventable event that negatively affects the health of older adults. Unfortunately, they have many risks for falls including impaired gait and balance, and muscle weakness. Objective assessment of older adults' physical activity with portable technology including accelerometers helps plan fall prevention interventions but there is limited information about their experience. This study aimed to explore participants' experiences with physical activity measured with an accelerometer. A qualitative descriptive study was conducted among 23 older adults, 60 years, and older living in low-income community settings in Central Florida. Participants were included if they participated in the Physio-Feedback Exercise Program (PEER) study that involved using a wrist-worn accelerometer (Fitbit) to measure physical activity. A semi-structured interview guide was used to obtain participants' experiences with physical activity and the technology used. Participants were individually interviewed, and audio recorded. They were offered an incentive of $20 for participating in the qualitative study. The responses were transcribed verbatim, coded, and analyzed using thematic analysis. Two major themes and sub-themes emerged from the data. These included 1) Barriers to physical activity (physical limitation, challenges with technology, fear of falling), and facilitators of physical activity (feedback from the accelerometer, progress monitoring, self-motivation). The use of the accelerometer encouraged participants' engagement in physical activity. Reducing barriers and encouraging facilitators of physical activity with accelerometers could promote fall prevention interventions. Future studies should include exploring the experience of cognitively impaired older adults' engagement in physical activity with the accelerometer.

Abstract citation ID: igae098.2714

### GENDER DIFFERENCES IN THE ASSOCIATIONS OF RECREATIONAL WALKING WITH INDOOR AND OUTDOOR FALLS AMONG OLDER ADULTS

Lingming Chen, Elizabeth Procter-Gray, Qun Le, Linda Churchill, Annabella Aguirre, Jie Cheng, and Wenjun Li, *University of Massachusetts Lowell, Lowell, Massachusetts, United States*

Indoor and outdoor falls are different among older adults. Women and men may have different patterns of recreational walking and types of falls. This study examined the gender differences in the associations of recreational walking with risk for indoor and outdoor falls. This prospective cohort study enrolled 359 community-dwelling older adults aged 65-95 years old in central Massachusetts (2018-2020). Recreational walking at baseline was measured by the frequency of walking for exercise for at least 10 min in participants' neighborhoods. Falls were reported on monthly fall calendars, and when a fall was reported, the circumstances were collected via telephone interview. Negative binomial models were used to estimate the gender differences in the associations of recreational walking with rates of indoor and outdoor falls, separately. Models were adjusting for sociodemographic variables, physical health, functional status, lifestyle behaviors, mental health, and the fear of falling. The results showed that less than half of participants did recreational walking at least once per week. Women had lower rates of indoor falls and outdoor falls than men (women vs. men: 34 vs. 51, 37 vs. 57 per 100 person-years). Women engaging in recreational walking at least once per week had 86% lower indoor fall rates (IRR (95% CI): 0.14 (0.05, 0.44)) than those who did not. No significant associations were found between recreational walking and outdoor falls. In conclusion, for older women, but not men, higher frequency of recreational walking was associated with lower rates of indoor falls.

Abstract citation ID: igae098.2715

### GLOBAL TRENDS IN FALL-RELATED INJURIES: A THREE-DECADE ANALYSIS

Ko Harada[1], Toshihiro Koyama[2], and Yuji Yamada[3], *1. Icahn School of Medicine at Mount Sinai, New York, New York, United States*, *2. Okayama University, Okayama, Okayama, Japan*, *3. Icahn School of Medicine at Mount Sinai, New York, New York, United States*

**Background:** Falls pose a significant public health challenge in our aging world, resulting in 684,000 deaths annually. Understanding the current situation is essential for developing data-driven policies and providing appropriate healthcare services. However, studies analyzing global trends in the disease burden of falls are scarce.

**Methods:** This study utilizes the Global Burden of Disease database to explore the disease burden of falls between 1990 and 2019, focusing on mortality rates, incidence, and disability-adjusted life years (DALYs). Falls were defined as ICD-9 codes E880-E886, E888, and ICD-10 codes W00-W19.9, and the data was analyzed by regions based on the World Bank regional classifications.

**Results:** Over the past 30 years, global deaths from falls per 100,000 population have increased by 29%, from 7.5 to 9.7. However, the global age-standardized mortality rate and age-standardized incidence rate from falls decreased by 8.4% and 2.8%, respectively. Trends in age-standardized mortality rate varied by region, with North America showing the most significant increase of 53%. Although age-standardized DALYs decreased by 14% worldwide, there was a 6.3% increase in North America. Conclusions: Our study showed that global fall-related mortality has increased worldwide over the past three decades, and the disease burden from falls has increased, particularly in North America, even after age adjustment. These trends can be attributed to the increase in multimorbidity, the prevalence of polypharmacy among older adults, and improved reporting quality. Developing and implementing targeted prevention strategies and policies is imperative to reduce the social impact of unintentional falls.

Abstract citation ID: igae098.2716

### HOME-VISIT INVESTIGATION ON BODY SUPPORTING PROPERTIES OF DAILY PRODUCTS USING FORCE VECTOR FIELDS SENSOR

Ayano Nomura[1], and Yoshifumi Nishida[2], *1. Institute of Science Tokyo, Meguro, Tokyo, Japan*, *2. Tokyo Institute of Technology, Meguro City, Tokyo, Japan*

Product accidents often occur due to poorly designed products that do not consider age-related functional changes. This study focuses on the inadequate 'body-supporting properties' of everyday products, which are recognized as a critical factor affecting independent living and fall prevention. To address this issue, we developed a portable system that measures and visualizes the vector fields of the hands' body support, and we evaluated the body-supporting ability of everyday products in the home. A study was conducted to measure body-supporting movements using the developed system in 9 houses with 13 participants aged 69 to 86 years. The results of the study identified daily activities that involve body-supporting movements, such as putting on and taking off shoes and clothes, sitting and standing up, etc. The study also identified various everyday products, such as tables, footrests, fusuma (sliding doors or partitions used in traditional Japanese architecture), windows, TV racks, etc., that generate body-supporting forces. By using the proposed system to measure hand forces at multiple points, it is possible to investigate how the hands are used to hold everyday products and interact with the environment in daily life, as well as the direction in which force is applied to support the body. For example, research has shown that a body-supporting force of as little as 4 N in the horizontal direction is sufficient to maintain horizontal balance. This finding underscores the importance of considering body support in everyday environments for older people and provides valuable insights for future design improvements.

Abstract citation ID: igae098.2717

### IDENTIFYING RISK PERCEPTION ON FALL OF COMMUNITY-DWELLING OLDER ADULTS USING PHOTOS

Ji Yeon Lee[1], Gwang Suk Kim[2], Min Kyung Park[3], Layoung Kim[2], and Jae Jun Lee[2], *1. Inha University, Incheon, Republic of Korea*, *2. Yonsei University, Seoul, Republic of Korea*, *3. Yonsei University College of Nursing, Seoul, Republic of Korea*

Most falls in older adults occur within the home, especially in a bathroom. To prevent indoor falls, increasing fall risk perception is necessary; however, scarce research explored the risk perception of indoor falls in community-dwelling older adults. This study was an exploratory mixed-methods research using photos. We recruited 10 experts with extensive experience in home environmental assessment/modification and nine older adults with fall experience in home bathrooms within the last two years. All participants evaluated six-bathroom photos through one-to-one interviews with a structured questionnaire, which was based on the health belief model: individual perception, modifying factors, and likelihood of action. The mean age of the expert was 45.8 and 50% was female. The average years in home environment modification/assessment was 10.9 years. Regarding older adults, the mean age was 75.1 and 66.7% were female. The number of diagnosed diseases was 1.86 and many reported bad vision (66.7%) and hearing loss (44.5%). The expert group had a higher risk perception and found extensive risk factors including doorsill, door direction, slippery floor, improper toilet height, narrow space around the toilet, no washbasin, handrails in bathtub and entry, bathtub height, and not organized environment. Older adults showed lower-risk perception in all pictures and risk factors included doorsill, slippery floor, no washbasin, no handrail, and not organized environment. Our findings showed older adults with a fall history have a lower-risk perception regarding environmental hazards. Therefore, education is needed to increase risk perception, and assessment/intervention of the home environment is necessary to prevent further falls.

Abstract citation ID: igae098.2718

### MUSCLE QUALITY AND REACTIVE BALANCE CONTROL IN OLDER ADULTS

Justin Whitten[1], Bryant O'Leary[2], Dawn Tarabochia[2], and David Graham[3], *1. Montana State University, Montana, United States*, *2. Montana State University, Bozeman, Montana, United States*, *3. Griffith University, Gold Coast, Queensland, Australia*

Falls are a predominant cause of morbidity and mortality in older adults. Muscle quality is associated with mobility and fall risk in older adults. Echo intensity (EI), obtained via ultrasonography, offers a method to quantify muscular fat infiltration and has become increasingly popular as an objective measure of morphological muscle quality. This study aimed to determine the relationship between lower limb EI and reactive balance control (RBC) in community-dwelling older adults. We analyzed EI and muscle thickness in the biceps femoris (BF), vastus lateralis (VL), and medial gastrocnemius (MG) across 18 participants (12 older adults, age: 65 ± 4.1; 6 younger controls, age: 25 ± 3.4) before administering specific assessments of RBC including the Choice Stepping Reaction Test and the Lean and Release Test. BF and MG EI were associated with LRT performance ($t=3.112$, $p<0.001$; $t=-3.597$, $p<0.01$), and BF EI was associated with CSRT performance ($t=-4.281$, $p<0.001$). However, these associations were characterized by small effect sizes ($d=-0.17$, $d=-0.19$ and, $d=0.12$ respectively) and were not significant after controlling for age. Although muscle quality is important for functional mobility it was not associated with RBC in the present study. This is supported by previous research that balance recovery appears to depend on the ability to generate efficiently coordinated neuromotor responses rather than on general qualities such as muscle force or muscle quality and thickness alone and underscores the importance of specific training to improve balance recovery. Further research, with larger sample sizes, is needed to understand the influence of EI on RBC.

Abstract citation ID: igae098.2719

### OLDER ADULTS REPORTING FEAR OF FALLING PRESENT WITH REDUCED PHYSICAL FUNCTION

Emerson Sebastiao[1], Vitor Siqueira[2], and Jemimah Bakare[2], *1. University of Illinois Urbana-Champaign, Urbana, Illinois, United States*, *2. University of Illinois Urbana-Champaign, Champaign, Illinois, United States*

Falls pose a significant risk to older adults (OAs), often leading to fear of falling (FOF). This study investigated the prevalence and the impact of FOF on physical activity (PA), sedentary behavior (SB), and physical function in OAs living in a community-dwelling facility (CDF). Ninety-three OAs were included in the study. FOF was assessed using the following Yes/No question: "Are you afraid of falling"? PA and SB were self-reported, while physical function was assessed using a collection of objective tests. Data were analyzed using descriptive and inferential statistics with significance set at p< 0.05. The prevalence of FOF was found to be 47.3% (36.9%–57.9%), with no significant differences between sex (Female: 48.4% (35.8%–61.3%) vs. Male: 44.8% (26.4%–64.3%); $\chi2(1, N=93) = 0.104$, p=0.747) or falls history (zero falls: 43.9% (30.7%–57.6%) vs. One or more falls: 52.8% (35.5%–69.6%); $\chi2(1, N=93) = 0.704$, p=0.401). OAs reporting FOF presented with significant (p< 0.05) lower performance in all physical function tests compared to those reporting no FOF. However, after controlling for the use of assistive devices (i.e., cane), the performance in some tests (i.e., Timed Up and Go, Timed 25-foot Walk; Short Physical Performance Battery subcomponent gait) lost significance (p>0.05). No significant differences were observed for PA and SB between groups with and without FOF (p>0.05). These findings suggest a high prevalence of FOF among the studied sample, worse physical function for OAs reporting FOF, and similar levels of PA and SB between FOF groups of OAs living in a CDF.

Abstract citation ID: igae098.2720

### PERCEIVED BALANCE PREDICTS FALLS IN COMMUNITY-DWELLING OLDER ADULTS

Hanne Dolan[1], Janet Pohl[1], Keenan Pituch[1], and David Coon[2], *1. Arizona State University, Phoenix, Arizona, United States*, *2. Arizona State Unviersity, Phoenix, Arizona, United States*

Accidental falls are an increasing problem among older adults in the United States (US). Older adults' self-report of falls is the primary method of fall risk identification. However, up to 72% of Medicare beneficiaries do not report falls and fall-related injuries to their healthcare providers. Limited research suggest that older adults prefer the term "balance problems" instead of "fall risk." The aim of this study was to examine if perceived balance problem is a predictor of self-reported falls after controlling for known predictors of falls among older adults. The Health Belief Model and the concept of perceived susceptibility served as the theoretical framework. A longitudinal secondary analysis was conducted using data from a subsample of independently living participants (N = 5446) from the National Health and Aging Trends Study. Baseline data was from year 2015, and the outcome was self-reported falls in 2016, with 23.4% of the sample reporting a fall. Complex samples multiple logistic regression analyses revealed that perceived balance (OR = 1.69, p <.001) predicted falls in 2016, whereas, the balance performance measure, Short Physical Performance Battery, did not (OR = 0.98, p =.06). Also, non-Hispanic White participants were more likely to report falling compared to non-Hispanic Black and Hispanic participants, as were females compared to males. A stay in the hospital in 2015, co-morbidities, fear of falling, and reporting a fall in 2015 were also predictive of falls. Assessing older adults' perceptions regarding their own balance is important to identify fall risk.

Abstract citation ID: igae098.2721

### SOCIAL ISOLATION, LONELINESS, AND DEPRESSION IN FALLS AMONG RACIALLY/ETHNICALLY DIVERSE OLDER AMERICANS

Ayse Malatyali[1], Lisa Wiese[2], Tom Cidav[3], Rui Xie[1], and Ladda Thiamwong[1], *1. University of Central Florida, Orlando, Florida, United States*, *2. Florida Atlantic University, Boca Raton, Florida, United States*, *3. U.S. Department of Veterans Affairs (VA), Philadelphia, Pennsylvania, United States*

Social isolation and loneliness affect 20%-30% of older Americans, and they increase the risk of depression, admission to nursing homes, early disability, and mortality. Poor social networks and social relations are also related to falls, but research on social isolation and loneliness in falls is limited. This study examines the association of social isolation, loneliness, and depression with falls among older Americans. Using logistic regression analyses, we analyzed data from the Health Retirement Study (2020 interviews) cross-sectionally. Measures included the HRS fall questionnaire, five-item social isolation scale, UCLA loneliness scale, and Center for Epidemiological Studies Depression scale. Our sample included 8,946 participants (age>64) with a mean age of 76. 59% of the participants were women, 26.3% living in rural, and 36% reported a fall experience in the past. The likelihood of having a fall was 10% higher in participants with higher levels of social isolation and 40% higher in participants with loneliness than those who were not socially isolated or lonely. Participants with depressive symptoms were 85% more likely to have a fall compared to participants without depressive symptoms. African Americans were 23% less likely to have a fall than Whites. The likelihood of having a fall was 19% less in men than women. Participants living in rural areas were 15% more likely to have a fall than participants living in urban areas. Further investigation into the interaction between social isolation, loneliness, and demographics is needed. Falls prevention interventions should include African Americans, women, and older adults in rural areas.

### SESSION 7265 (POSTER)

### FAMILY AND INTERGENERATIONAL RELATIONS

Abstract citation ID: igae098.2722

### A LATENT GROWTH CURVE MODEL OF RESOURCEFULNESS IN GRANDMOTHERS RAISING GRANDCHILDREN

Christopher Burant, Carol Musil, Jaclene Zauszniewski, and Alexandra Jeanblanc, *Case Western Reserve University, Cleveland, Ohio, United States*

The role of grandmothers raising grandchildren has increased over the past three decades. Many of these grandmothers are living with their grandchildren and often these households do not include the parent of the grandchildren. Grandmothers raising grandchildren may deal with high levels of stress and strain that can contribute to high levels of depressive symptoms and potential mental health problems. Previous research has shown that resourcefulness can play a mediating role by reducing the impact of stress on mental health in caregiving situations. Resourcefulness comprises a cognitive behavioral skillset used for coping that includes cognitive reframing, problem-solving, and positive self-talk. Data were collected on 343 participants in a longitudinal nationwide online research study of caregiving grandmothers. In the current study, resourcefulness was assessed after grandmothers had received an intervention involving Resourcefulness Training© (RT) or journaling. A latent growth curve model was used to track the trajectory of resourcefulness at four time points (baseline, 2 weeks, 12 weeks, and 24 weeks) to identify whether resourcefulness improved over time. The model fit the data well (Chi Square=15.881; df=6; p=.014; TLI=.982; CFI=.989; RMSEA=.069). An additional multiple group nested model latent growth curve model found no differences between the 2 interventions. However, individuals receiving RT consistently had higher levels of resourcefulness and reached the maximum score at 3 months as compared to 6 months for those who journaled. Both interventions improved resourcefulness, indicating that interventions focusing on improving awareness of resourceful skills (e.g. problem-solving) may help grandmothers raising grandchildren with handling stress.

Abstract citation ID: igae098.2723

### A RANDOMIZED CLINICAL TRIAL OF ONLINE SOCIAL INTELLIGENCE TRAINING FOR ADOLESCENT CUSTODIAL GRANDCHILDREN

Megan Dolbin-MacNab[1], Gregory Smith[2], Luxin Hu[2], and Frank Infurna[3], *1. Virginia Tech, Blacksburg, Virginia, United States*, *2. Kent State University, Kent, Ohio, United States*, *3. Arizona State University, Tempe, Arizona, United States*

Although custodial grandchildren (CG) experience multiple negative psychosocial outcomes, intervention research on this population has been virtually absent. To address this gap, we conducted a two-arm randomized clinical trial (RCT), which compared the efficacy of online Social Intelligence Training (SIT) to an attention control condition, at enhancing psychological and relational outcomes among a sample of 349 adolescent (ages 11-18 years) CG. Online surveys were self-reported at baseline (T1), post-intervention (T2), and at 3- (T3), 6- (T4), and 9- (T5) months. Latent change score analyses revealed significant differences in favor of SIT for CG internalizing difficulties (T4) and self-esteem (T2; T4). Relationally, significant differences in favor of SIT were found for CG prosocial behavior (T5) and avoidant (T2) and anxious (T2) attachment to the grandmother. Across outcomes, effect sizes ranged from.28 to.47. Although the treatment effects were modest, subsequent CART analyses identified that treatment-related change in the SIT condition was predicted by the baseline values of each respective latent outcome (effect sizes =.51 – 1.53). In turn, ANOVAs revealed that changes in latent outcomes from T1 to T2 were significantly moderated by their respective baseline values (p =.05 –.002) for externalizing and internalizing difficulties, self-esteem, and avoidant attachment to the grandmother. The only significant moderator at T5 was the baseline value of support from friends (p =.03). We conclude that RCTs which only report average treatment effects, without also investigating potential moderators of differential treatment efficacy, may underestimate true intervention impact. [Funded by R01AG054571)].

Abstract citation ID: igae098.2724

### ADULT CHILDREN'S DIVORCE AND DEPRESSIVE SYMPTOMS IN OLDER KOREANS: THE MODERATING EFFECT OF GENDER

Taeeun Kim, Seolah Lee, and Hyo Jung Lee, *Yonsei University, Seoul, Republic of Korea*

The study aims to investigate how adult children's divorce is associated with older parents' depressive symptoms in South Korea, which values Confucian ethics and collectivism. It additionally examines if parents' gender moderates such an association. It applies the principle of linked lives, signifying that the lives of family members are closely linked to one another. Using nationally representative data from the three waves of the Korean Longitudinal Study of Aging (KLoSA) biennially conducted from 2006 to 2010, we applied mixed ANOVA, hierarchical regression analyses, and Process Macro model 1 to 1,254 parents aged 65 and over. The results indicated that parents with divorced children showed a significantly higher level of depressive symptoms over time compared to parents with only married children. In addition, children's divorce is significantly associated with a higher level of parents' depressive symptoms. Gender differences in depressive symptoms were observed; older mothers with divorced children reported a higher level of depressive symptoms than older mothers with only married children, whereas no significant effects were found for older fathers. The findings imply that adult children's divorce has a negative impact on older parents' depressive symptoms in the Korean context. Especially older mothers may suffer from stress due to their adult children's divorce. The study also provides evidence to support the principle of linked lives through upward intergenerational transmission.

Abstract citation ID: igae098.2725

### ARTS THROUGH INTERGENERATIONAL SOCIAL ENGAGEMENT (ARISE) PROGRAM

Jessica Krok-Schoen[1], Jill Clutter[1], Amy Handra[2], Camille Paoletta[1], Dominic Young[1], and Lauren Feyh[1], *1. The Ohio State University, Columbus, Ohio, United States*, *2. Columbus Association for the Performing Arts, Columbus, Ohio, United States*

Introduction: Limited research shows that fine arts within intergenerational (IG) programming helps reduce communication barriers and bias between different generations. In summer 2023, a five-week IG program focused on storytelling through fine arts, the ARISE (ARts through Intergenerational Social Engagement) program, with the Columbus Association for the Performing Arts (CAPA), was piloted at a long-term care facility.

**Methods:** Pre-post measures were collected from the older adults and teens regarding self-rated health, depressive symptoms, sense of community, and perceptions of older adult-teen interactions. Fisher's exact tests and paired samples t-tests were used to analyze the quantitative measures. Pre-post open-ended responses were analyzed by four independent coders to identify common themes using deductive and inductive methods.

**Results:** Both the teens (n=6) and older adults (n=6) had positive increases from pre- to post-program in four out of the five depressive symptom questions. The teens and older adults reported that spending time together made them feel energized, useful, and part of their community. Before the program, teens (n=6) and college students (n=3) reported negative preconceptions of older adults, due to limited exposure to older adults. After the program, they denoted personal growth and an increased desire to socialize with older adults. Students learned life lessons and their negative perceptions of older adults transitioned to positive perceptions. Conclusions: Positive changes in depressive symptoms, and IG interactions and perceptions were experienced among all the ARISE participants. These findings promote the implementation of IG programs to decrease negative stereotypes, enhance personal growth, and enrich quality of life.

Abstract citation ID: igae098.2726

### CONFLICTS BETWEEN OLDER ADULTS AND THEIR CAREGIVERS DURING ACUTE HOSPITALIZATION: AN ECOLOGICAL PERSPECTIVE

Tova Band-Winterstein[1], Malek Masarwa[2], Anna Zisberg[3], Efrat Shadmi[3], Nurit Gur-Yaish[4], and Ksenya Shulyaev[3], *1. University of Haifa, Haifa, HaZafon, Israel*, *2. Edith Wolfson Medical Center, Jerusalem, Yerushalayim, Israel*, *3. University of Haifa, Haifa, Hefa, Israel*, *4. Oranim Academic College, Kiryat Tivon, HaZafon, Israel*

Family support is important for older adults during hospitalization; however this stressful period can exacerbate or precipitate conflicts between patients and their caregivers. We aimed to identify predictors of conflicts between hospitalized older adults and family caregivers across individual, social, and cultural levels of ecological model, utilizing data from prospective HOPE-MORe Study. The sample comprised 583 cognitively intact older adults (65+) admitted to internal units in Israeli hospitals. Additionally, questionnaires filled 73 family caregivers of these patients. 29.3% of older adults and 27.4% caregivers reported some kind of conflict during hospitalization. Multiple logistic regression analysis revealed several significant predictors of conflicts. Female patients' gender emerged as a protective factor (β=.607, 95% CI [.409,.901]), while an increased number of visitors (β=1.044, 95% CI [1.001,1.089]) and family involvement in instrumental care (β=1.221, 95% CI [1.018,1.466]) were associated with a higher likelihood of conflict. Additionally, being an immigrant from the ex-USSR was linked to an increased risk of conflict (β=1.692, 95% CI [1.069,2.679]). Notably, other factors such as age, functional and cognitive status, anxiety, duration of visits, or Arab ethnicity were found to be non-significant in predicting conflicts. Among subsample of caregivers, spousal caregivers (β=7.390 (95%CI [1.593,34.287])) and those with anxiety attachment (β=1.628 (95%CI [1.029,2.578])) were more likely to report conflict. Findings highlight the importance of ecological perspective considering individual, social, and cultural conflict risk factors. Moreover, it is important to consider both family caregivers' and patients' perspectives of conflict as risk factors exist on both sides, leading to a more holistic picture.

Abstract citation ID: igae098.2727

### DIFFERENCES IN HOW OLDER ADULTS ARE PORTRAYED IN CHILDREN'S MEDIA

Hallory Domnick, Celine Manternach, and Melinda Heinz, *University of Northern Iowa, Cedar Falls, Iowa, United States*

The purpose of this project was to analyze how older adults were portrayed in children's media. Children are shaped by the media that they consume, and since the media often features negative or inaccurate portrayals of older adults, it has the potential to impact how children view this population. A total of 30 children's picture books (published in the last 10 years) and 15 children's shows on Disney+ and PBS Kids were analyzed for their portrayal of older adults. Each piece of media was analyzed for examples of: stereotypes/myths, positive and negative portrayals, and roles older adults engaged in. The most common stereotype/myth identified was that older adults frequently needed to use assistive devices (such as a cane) or were portrayed with exaggerated physical differences (deep wrinkles, white hair). Positive portrayals of older adults included grandparents being a source of physical affection and that spending time with grandparents was fun. Older adults were frequently portrayed as grandparents and rarely depicted in other roles. While grandparenthood may be a common role for older adults, older adults also occupy other roles and that should be better reflected in children's media. However, some authors did include depictions of older adults identifying as LGBTQ+, demonstrating an awareness that older adults are also included in this community. Overall, older adults were more likely to be portrayed as loving and caring in children's literature compared to television. PBS Kids compared to Disney+ was more likely to depict older adults positively as wise and knowledgeable.

Abstract citation ID: igae098.2728

### FAMILY INTERACTION, PSYCHOLOGICAL NEEDS, AND SELF-CARE IN KOREAN MIDAGE TO OLDER CANCER SURVIVORS

Haerim Lee, Sun-ui Shin, Eunyoung Park, Mi Sook Jung, and Hyun-E Yeom, *Chungnam National University, Daejeon, Republic of Korea*

With medical advancements extending the lives of cancer survivors, active self-care becomes increasingly significant. Family interaction and psychological status are keys related to the engagement in self-care of individuals with long-term health issues. This study investigated how family interaction affects psychological needs—namely autonomy, competence, and relatedness—and self-care activities among Korean middle-aged to older cancer survivors. We also examined the variations with age in the roles of family dynamics on psychological needs and self-care behaviors. It is a cross-sectional study utilizing secondary data from two studies, encompassing 277 community-dwelling survivors. We performed descriptive statistics, Pearson's correlation, and PROCESS macro analysis using SPSS to discern the influence of family interactions. Our results revealed that family interaction plays a crucial role in both satisfying and frustrating psychological needs, which, in turn, directly and indirectly influences self-care activities. Notably, the impact of family dynamics on self-care and the frustration experienced in meeting psychological needs, particularly autonomy and relatedness, was more pronounced in middle-aged survivors than in those over 60. The comprehensive findings highlight the need for healthcare interventions tailored to the age-specific needs of cancer survivors, emphasizing the importance of family engagement in supporting psychological well-being and self-care. The study advocates for strategies to foster active family involvement to enhance overall quality of life and encourage self-care among middle-aged to older survivors.

Abstract citation ID: igae098.2729

### GENDER DIFFERENCES IN ASSOCIATIONS BETWEEN LIVING ARRANGEMENTS AND HEALTH OF OLDER ADULTS IN VIETNAM

Emiko Takagi[1], Yasuhiko Saito[2], and Nguen Cong Vu[3], *1. San Francisco State University, San Francisco, California, United States*, *2. Nihon University, Tokyo, Japan*, *3. Institute of Population, Health, and Development, Dong Da, Ha Noi, Vietnam*

This study examined gender differences in the variation of physical and psychological well-being measures across four types of living arrangements among older adults in Vietnam. We analyzed the initial wave data of a nationally representative sample of self-reporting adults 60 years and older with at least one living child (n=5,306) from the Longitudinal Study of Health and Aging in Vietnam (LSHAV). The four types of living arrangements in the analysis included: living alone, living with a spouse, living with a spouse, adult children, and/or grandchildren, and living with adult children and/or grandchildren but without a spouse. The well-being measures included physical health indicators regarding the difficulties with Activities of Daily Living (ADL) and Instrumental Activities of Daily Living (IADL), disability status, and self-rated health. Psychological well-being was assessed based on the CESD depression scores and the UCLA 3-item loneliness scores. The descriptive statistics showed that overall, the poorer health conditions tended to be more prevalent in the household where older adults lived with their adult children and/or grandchildren and without a spouse, compared to counterpart types of living arrangements. The regression analyses on well-being measures showed that men who either live alone or live with children or grandchildren but without a spouse were especially more likely to report emotional loneliness. We discuss gender differences in physical and psychological well-being in conjunction with late-life intergenerational living arrangements and life transitions such as widowhood.

Abstract citation ID: igae098.2730

### MEASURING INTERGENERATIONAL AMBIVALENCE TOWARD OLDER ADULTS IN THE FAMILY CONTEXT: A SCOPING REVIEW

Athena Chung Yin Chan[1], Sophia Lee[1], Shuwen Li[1], Jacquelyn Duckett[2], and Rodlescia Sneed[2], *1. Texas Tech University, Lubbock, Texas, United States*, *2. Wayne State University, Detroit, Michigan, United States*

Informal family caregivers may frequently feel ambivalent toward taking care of older family members. Ambivalence is the simultaneously held conflicting emotions, cognitions and/or behavioral tendencies due to countervailing expectations about how individual should act within the cultural norms. Most existing measurements directly or indirectly measuring caregiving or intergenerational ambivalence were developed from the West. Less is known how ambivalence is understood across distinctive sociocultural context globally. This scoping review examines the dimensions of existing instruments in measuring caregiver ambivalence toward older adults. Peer-reviewed articles were identified in five electronic databases. A keyword search was performed associated with ambivalence, caregiver or family, and measure development. Ten instruments were included. These instruments used different terms to represent caregiver ambivalence, not limited to intergenerational ambivalence and filial maturity. Existing measure items primarily capture the simultaneously held conflicting emotions toward older family members but limited to the domains of behaviors or cognitions. Moreover, existing measures are not inclusive of family caregiving to capture ethnic minorities who provide support to older family members motivated by filial responsibility beliefs. The scoping review uncovers the gaps between conceptual understanding and measurement of caregiver ambivalence toward older adults in the family. Findings will inform the direction of future research and the development of a culturally relevant measure that captures the intergenerational ambivalence experienced by ethnic minority who provide family support to older adults. The review will likely inform practice policy, and research in caregiving in a culturally sensitive manner.

Abstract citation ID: igae098.2731

### PARENTAL STYLE MEDIATES GRANDCHILD DISTRESS: PARENTAL ROLE DEMANDS RELATIONSHIPS AMONG CUSTODIAL GRANDPARENTS

Bert Hayslip[1], Julian Montoro-Rodriguez[2], and Janelle Fassi[3], *1. University of North Texas, Denton, Texas, United States*, *2. University of North Carolina Charlotte, Charlotte, North Carolina, United States*, *3. University of Massachusetts Boston, Goffstown, New Hampshire, United States*

While limited work has explored parenting practices among custodial grandparents, none has incorporated parental style, a construct proven valuable in predicting a variety of child outcomes of significance, in understanding parenting among custodial grandparents. This study evaluated the impact of grandchild distress (internalizing/externalizing behaviors) on Parental Efficacy, Parental Role Strain, and Parental Role Stress as mediated by parental styles (Laissez-Faire, Authoritative, Authoritarian) in a sample of 238 custodial grandparents (M age = 58.06). AMOS structural equation findings yielded a model which fit the data well (X2/df = 1.72, CFI = 0.939, TLI = 0.910, RMSE = 0.055). This model indicated that the Laissez-Faire and Authoritarian styles each predicted either Role Stress or Parental Efficacy. Laissez-Faire's mediating role was driven to a minimal extent by grandchild internalizing/externalizing behaviors per se, while its mediating role was defined principally by grandparent age as it related to such behaviors in predicting Parental Efficacy (beta = -.54, $p < .05$). and Parental Role Stress (beta =.36, $p < .05$). The mediating role of the Authoritarian style was principally explained by child externalizing behaviors (beta =.22, $p < .05$) in predicting Parental Efficacy. Additionally, while the Authoritative style did not emerge as mediational in nature, both internalizing and externalizing behaviors had direct relationships (p <.05) to Role Stress and Role Strain. These finding illuminate the role of parental styles as key to understanding the parental challenges that grandparents face in managing the distress that their grandchildren are experiencing.

Abstract citation ID: igae098.2732

### PATTERNS AND PREDICTORS OF ADULT GRANDCHILDREN'S PERCEPTIONS OF GRANDMOTHERS' FAVORITISM AND DISFAVORITISM

Destiny Ogle[1], J. Jill Suitor[1], Megan Gilligan[2], and Reilly Kincaid[1], *1. Purdue University, West Lafayette, Indiana, United States*, *2. University of Missouri, Columbia, Missouri, United States*

Scholarship on later-life families has shown that parents differentiate among their adult children across a range of relational dimensions and that perceptions of parental favoritism and disfavoritism have consequences for children's well-being. Far less is known about patterns of favoritism and disfavoritism in other intergenerational ties. We extend this line of research by exploring adult grandchildren's perceptions of their grandmothers' favoritism and disfavoritism using mixed-methods data collected from 229 adult grandchildren nested within 94 families collected as part of the Within-Family Differences Study T3. On average, grandchildren were 28 years old and grandmothers were 92 years old. Eighty percent perceived that their grandmothers were most emotionally close to particular grandchildren, and 64% perceived that their grandmothers had the most conflict with particular grandchildren. Twenty-five percent perceived they were the closest to their grandmothers, and 12% perceived that they had the most conflict. MLM analyses revealed that respondents who were first-borns and in greater contact with grandmothers were most likely to report being the closest. Those most likely to report having the greatest conflict with their grandmothers were unmarried, did not share grandmothers' values, and provided grandmothers with care during illnesses. Qualitative analyses shed light on how this set of factors played salient roles in grandmothers' favoritism and disfavoritism. These findings indicate that adult grandchildren are as likely as adult children to perceive favoritism and disfavoritism, and that the factors predicting perceiving oneself as favored or disfavored by grandmothers greatly mirror those found in studies of adult children and older parents.

Abstract citation ID: igae098.2733

### SHARED SOCIAL ACCOUNT: UNDERSTANDING INTERGENERATIONAL COLLABORATION AND INTERACTION ON XIAOHONGSHU

Tongxin Sun[1], Tongtong Jin[1], Qiong Zhang[2], and Yuanyuan Liu[3], *1. Tsinghua University, Beijing, Beijing, China (People's Republic)*, *2. School of Mechanical Engineering & Automation, Beijing, Beijing, China (People's Republic)*, *3. School of Mechanical Engineering & Automation, Beihang University, Beijing, Beijing, China (People's Republic)*

Xiaohongshu, a social media platform in China known for lifestyle sharing through text, images, and videos, is predominantly popular among the younger generation. It has seen a remarkable uptick in older adult users in the post-pandemic era, with a notable phenomenon in "shared accounts" that feature content co-created by grandparent-grandchild pairs. These accounts have garnered significant attention for their unique intergenerational content and elicited emotional resonance within young audiences. Our study utilized a mixed-methods approach, including web scraping and interviews, to investigate the motivations and experiences underpinning the content creation process on these shared accounts. Through cluster analysis of content topics and in-depth interviews with four grandparent-grandchild pairs actively managing shared accounts, we explored their creative collaboration process and the influence of digital technology. Our research uncovered a symbiotic exchange of knowledge between generations: grandparents shared their life wisdom and experiences, while grandchildren introduced them to digital platforms and current social trends, enabling the transformation of shared narratives into media forms accessible to a wider audience. This collaborative creation process not only enhanced emotional communication within families but also fostered intergenerational social interactions externally. However, grandparents, due to their lack of perceived confidence in digital literacy, typically tended to pass control to their grandchildren for the restructuring of narratives, leading to technological disparities within the shared account. This study sheds light on the influence of technology on collaborative narratives across generations and discusses ways to advance technological equity to empower older adults in achieving self-expression and meaningful social engagement.

Abstract citation ID: igae098.2734

### SOCIOECONOMIC STATUS AND INTERGENERATIONAL LIVING ARRANGEMENTS: BOTH CHILD- AND PARENTS-BASED ANALYSES

Mary Beth Ofstedal[1], BoRin Kim[2], Jersey Liang[1], Xiao Xu[3], and James Raymo[4], *1. University of Michigan, Ann Arbor, Michigan, United States*, *2. University of New Hampshire, Durham, New Hampshire, United States*, *3. Columbia University, New York, New York, United States*, *4. Princeton University, Princeton, New Jersey, United States*

Intergenerational living arrangements are closely associated with intra-family transfers of support and decision-making between older parents and adult children. Guided by the rational choice and the life course frameworks, we simultaneously examined the linkages between socioeconomic status of older parents and their adult children and changes in their living arrangements over a 20-year period. Data came from the 1998-2018 Health and Retirement Study. Our sample was restricted to parents over age 65 (n=6,284) and their children over age 21 (n=31,289) at the baseline. The number of total person-period observations over 20 years was 152,344. Three-level hierarchical linear models were used to examine the association of parents' and children's socioeconomic characteristics with their living arrangements, including co-, proximate (≤10miles), and distant residence (>10miles). The most common living arrangement for children over time was distant residence, followed by proximate residence. During the study period, the predicted probability of coresidence increased from 1% to 4%, whereas the probability of distant residence declined from 72% to 64%.

Proximate residence remained relatively stable at 27% to 32%. The socioeconomic status of both generations was important. Low income and assets of parents and/or children increased the probability of coresidence and decreased the probability of proximate residence. Higher education of parents and children was associated with distance residence. Interestingly, parents' health status was not significantly associated with living arrangements. Results highlight the importance of accounting for the opportunities, resources and preferences of both older parents and their children when examining intergenerational living arrangements.

Abstract citation ID: igae098.2735

### THE BENEFITS OF INTERGENERATIONAL REMINISCENCE AND DST ON THE WELL-BEING OF YOUNG ADULT PARTICIPANTS

Ling Xu[1], Noelle Fields[1], Kathryn Daniel[2], Daisha Cipher[2], and Brooke Troutman[3], *1. The University of Texas at Arlington, Arlington, Texas, United States*, *2. The University of Texas Arlington, Arlington, Texas, United States*, *3. Air Force Academy, Air Force Academy, Colorado, United States*

**Objective:** Intergenerational contact between older people and young adults have many benefits for both young and older generations. An intergenerational reminiscence and DST project was conducted with young adult college students calling older partners each week for 10 weeks. This study reported the benefits of this project in improving the well-being of young adult participants.

**Method:** A randomized control trial was used to assess the effects of the intervention. Participants of young and older adults were randomly paired and assigned to Reminiscence (n = 55) or sham (Social Wellness, n = 48) group. Data were collected at baseline, mid- intervention, at the end of the intervention. Linear mixed models (LMM) were performed to compare the Social Wellness group with the Reminiscence group on levels of loneliness, attitudes toward aging, and knowledge of Alzheimer's' Disease (AD) among the student participants.

**Results:** Results from LMM revealed no significant effects for group or time on attitudes toward aging. However, the groups significantly differed on their changes over time in loneliness. LMM revealed a significant interaction between group and time, t(101) = -3.06, p =.003. The two groups also significantly differed on their changes over time in knowledge. LMM revealed a significant interaction between group and time, t(101) = 2.51, p =.014.

**Discussion:** Results suggest that weekly intergenerational engagements with older adults benefit young adult college students with loneliness and knowledge of AD. Implications for research and programs in improving the well-being of young adults are discussed.

### SESSION 7270 (POSTER)

### FAMILY CAREGIVING II (POSTERS)

Abstract citation ID: igae098.2736

### A TECHNOLOGY-BASED SUPPORT PROGRAM FOR ASIAN AMERICAN FAMILY CAREGIVERS OF PEOPLE LIVING WITH ALZHEIMER'S DISEASE

Dongmi Kim, Eun-Ok Im, Wonshik Chee, and TACAD Research Team, *The University of Texas Austin, Austin, Texas, United States*

The negative impact of family caregiving on health outcomes of caregivers of persons living with Alzheimer's Disease (PLAD) has been well documented. Especially, Asian American midlife women who are PLAD (AWPLAD) reportedly suffer from caregiving burden because of their cultural attitudes related to Alzheimer's Disease and caregiving. The first technology-based culturally tailored information and coaching/support program (TACAD) was developed while considering their cultural attitudes. This is a part of a larger 2-phase, mixed-methods, exploratory study to preliminarily evaluate TACAD. Only the first phase is included in this presentation and the 2nd phase is ongoing. The first phase included an expert review and a usability test. The expert review was conducted among 5 experts in family caregivers of PLAD, and a usability test was conducted among 11 AWPLAD. The qualitative data from the expert review and usability text were analyzed using a content analysis. The expert review indicated the necessity of updates on the education modules with new information; shortening each educational module to have up to 10 slides; eliminating redundancies in the content; strengthening the cultural content; and changing the pictures to those with Asians. The usability test indicated the necessity of including more video clippings related to Alzheimer's Disease and family caregiving; refining chat functions; and enlarging the font size. All the participants agreed that the program would be helpful and useful for AWPLAD and the program was well-organized and easy to follow. Further studies are warranted on culturally tailored technology-based interventions for this specific population.

Abstract citation ID: igae098.2737

### A TYPOLOGY OF CARE-RELATED CHALLENGES THAT EMERGE FROM DEMENTIA FAMILY CAREGIVERS' PHYSICAL PAIN

Shelbie Turner[1], Irina Mindlis[2], M. Carrington Reid[3], and Karl Pillemer[4], *1. Weill Cornell Medicine, New York, New York, United States*, *2. Weill Cornell Medicine, New York, New York, United States*, *3. Weill Cornell Medicine, Weill Cornell Medicine, New York, United States*, *4. Cornell University, Ithaca, New York, United States*

Many dementia family caregivers experience persistent physical pain. In addition to compromising quality of life, caregivers' pain is associated with difficulty performing caregiving tasks and unmet needs in care recipients. This study developed a typology of care-related challenges that emerge as a consequence of pain among dementia family caregivers. We conducted semi-structured, in-depth interviews with 25 dementia family caregivers living with persistent pain. We inductively analyzed data via a thematic analysis approach to identify and categorize caregiving challenges stemming from caregivers' pain. We then analyzed how challenges were related to one another and their consequences for caregiving outcomes. Analysis revealed three interrelated categories of caregiving challenges: 1) physical (e.g., inability to lift person with dementia), 2) psychological/emotional (e.g., worry about future progression of care), and 3) family/relational (e.g., resentment towards healthy family members for not helping with care). Caregivers reported that the co-occurrence of challenges compounded one another in ways that made both caregiving and pain management more difficult. For example, care recipients frequently became agitated when pain interfered with caregivers' ability to perform care tasks (a family/relational challenge), which heightened caregivers' negative emotions (a psychological/emotional challenge). Whether caregivers reported challenges and whether each challenge was connected to caregiving outcomes was dependent on various contextual factors, such as whether caregivers reported benefits from their own pain (e.g., greater empathy). The resulting typology offers a theoretical framework to guide future translational research on the topic of caregivers' pain, including illuminating promising intervention targets of pain self-management programs for dementia family caregivers.

Abstract citation ID: igae098.2738

### CAREGIVING INTENSITY AND LABOR FORCE PARTICIPATION AMONG CAREGIVERS: THE ROLES OF INCOME AND PAID HELP

Shan Qu, and Jeffrey Burr, *University of Massachusetts Boston, Boston, Massachusetts, United States*

Framed within Role Theory, this study examined the association between caregiving intensity and labor force participation among caregivers and whether income and paid help moderated this relationship. We used data from the National Health and Aging Trends Study and the National Study of Caregiving. Caregiving intensity was measured by days and hours of caregiving in the last month. Care was considered high-intensity if a respondent provided an average of 21 hours or more of care per month. Low-intensity caregivers provided 20 hours of care or less per month. Labor force participation was divided into three categories: '1=unemployed/retired,' '2=worked less than 35 hours,' and '3=worked 35 hours or more.' Compared to those with low-intensity caregiving, high-intensity caregivers had a lower likelihood of working part-time than those who were not working. Compared to not having a paid helper, having a paid helper was associated with an increased likelihood of working part-time and full-time compared to those who were not working. As household income increased, the likelihood of being a part-time worker and the likelihood of being a full-time worker increased. The moderation model results showed the likelihood of working part-time compared to being unemployed or retired was lower when caregiving intensity was higher. No other moderation effect was found for paid work. In addition, the likelihood of working part-time or full-time increased as annual income increased. We believe the findings contribute to policies that increase access to paid help and may ultimately assist caregivers to remain active in the job market.

Abstract citation ID: igae098.2739

### CAREGIVING OUTCOMES AMONG INFORMAL CAREGIVERS OF PERSONS WITH MULTIMORBIDITY AND DEMENTIA: A SCOPING REVIEW

Ruotong Liu[1], Katherine D. Peak[1], Em Trubits[2], and Ana Quiñones[1], *1. Oregon Health & Science University, Portland, Oregon, United States*, *2. Portland State University, Portland, Oregon, United States*

An emerging field of research involves caregiving among older adults with Alzheimer's disease and related dementia (ADRD) and co-occurring chronic conditions (ADRD-multimorbidity). This scoping review aims to assess, synthesize, and identify gaps in the body of literature on caregiving outcomes among informal caregivers of individuals with ADRD-multimorbidity. We adhered to the scoping review framework by Arksey and O'Malley (2005) and Levac and colleagues (2010), which encompassed five steps: 1) identify the research question(s), delineate the inclusion and exclusion criteria, 2) search for relevant studies, 3) select studies, 4) chart the data, and 5) collate, summarize, and report results. Electronic databases including Ovid Medline, CINAHL, Embase, and PsycINFO were searched to identify relevant studies. A total of 1856 articles were identified and 29 were included in the final review. The majority of studies were quantitative, cross-sectional studies. The two most commonly examined caregiving outcomes were caregiver burden and psychological wellbeing. Most research on caregiver outcomes treated care recipients' cognitive impairment and chronic conditions separately, rather than exploring their interaction. Most studies examining caregiver burden utilized Zarit Burden Index and its variants. Specific psychological wellbeing outcomes displayed great variability across studies. Despite challenges in synthesizing the extensive variability in the way cognitive status, ADRD-multimorbidity, and caregiving outcomes were measured, included, and reported, this review underscores the intricate challenges of caregiving, especially when dealing with both cognitive impairments and co-occurring chronic conditions. This complexity underscores the need for a deeper understanding of the diverse needs facing caregivers of people with ADRD-multimorbidity.

Abstract citation ID: igae098.2740

### DAILY SLEEP HEALTH AMONG WORKING INFORMAL CAREGIVERS OF OLDER ADULTS: DELVING INTO CAREGIVING DEMANDS

Eunae Cho[1], and Tuo Yu Chen[2], *1. National Chengchi University, Taipei, Taipei, Taiwan (Republic of China)*, *2. Taipei Medical University, Taipei, Taipei, Taiwan (Republic of China)*

Caregiving demands are a key stressor, but little is known about the relationship between caregiving demands and sleep health among working informal caregivers of older adults. We conducted a 5-day daily diary study to examine the relationship between caregiving demands and sleep health among working informal caregivers of older adults in Singapore (N=193). For each day, we examined 'anticipated caregiving difficulty' in the morning; in the evening, we assessed 'total hours spent on caregiving,' 'total number of care activities (e.g., personal care, toileting, running errands),' and 'experienced caregiving difficulty.' We further explored their interaction effects on sleep health. Covariates included sociodemographic status, self-rated health, depressive symptoms, and care recipients' functional status. We found that anticipating more caregiving difficulty in the morning was significantly associated with poor sleep health in the evening on top of covariates and actual caregiving demands. We also found a significant interaction between 'anticipated caregiving difficulty' and 'experienced caregiving difficulty.'

Specifically, the relationship between experienced caregiving difficulty in the evening and poorer sleep health was stronger among individuals who anticipated a lower level of difficulty that morning (1SD-lower). Individuals who anticipated a higher level of caregiving difficulty in the morning (1SD-higher) had worse sleep health in the evening regardless of the level of caregiving difficulty experienced during the day. Our findings suggest that the impacts of 'anticipated caregiving difficulty' on sleep health go beyond actual caregiving difficulty experienced. The mismatch between anticipated and experienced caregiving difficulty plays a role in sleep health at night.

Abstract citation ID: igae098.2741

### DEVELOPING THE NATIONAL STUDY OF CAREGIVING: CAREGIVING APPRAISAL MEASURE FOR CAREGIVERS OF PEOPLE WITH DEMENTIA

Dingyue Wang[1], Susan Silva[2], and Cristina Hendrix[2], *1. Duke University, Seattle, Washington, United States*, *2. Duke University, Durham, North Carolina, United States*

This study aimed to develop and psychometrically validate the NSOC-Caregiving Appraisal (NSOC-CA) measure using data from the National Study of Caregiving (NSOC) IV Round 12. We also aimed to compare dementia and non-dementia caregiving experiences to determine whether this measure differentiates the caregiving experiences between the two groups. We analyzed data from 1783 adult family caregivers using principal components analysis (PCA) to identify caregiver experience domains. Varimax rotation was used to derive orthogonal factors. Hierarchical mixed-effects models, controlling for nesting effects due to some patients having multiple caregivers, were conducted to test for dementia vs non-dementia caregiver experience differences. The final 25-item PCA model was a 3-factor solution that explained 96% of the total variance. Identified domains were labeled: psychological well-being, caregiving burden, and personal impact. NSOC-CA demonstrated high reliability (Cronbach's alpha = 0.89, for the three subscales 0.89, 0.80, 0.75 respectively). Pearson correlations of the subscale scores ranged from 0.12 to 0.46. Dementia caregivers compared to non-dementia caregivers had worse psychological well-being, greater caregiving burden, and more negative overall caregiving appraisal (all p <.0013 without covariates; and all p <.0006 with covariates) but did not differ on personal impact (p >.05). NSOC-CA is a reliable tool for assessing the caregiver experiences of those caring for patients with dementia. NCOS-CA can differentiate the caregiving experiences of those who care for family members living with and without dementia. Future research will focus on further developing the scale and assessing its psychometric properties.

Abstract citation ID: igae098.2742

### DOES PSYCHOLOGICAL FLEXIBILITY MEDIATE CARE-RELATED STRESS ON QUALITY OF LIFE FOR DEMENTIA CAREGIVERS?

Elizabeth Fauth, Ty Aller, Jacob Gossner, Audrey Juhasz, and Michael Levin, *Utah State University, Logan, Utah, United States*

Caregivers of individuals with dementia experience care-related burden and stressful reactions to behavioral and psychological symptoms of dementia (BPSD) which can negatively impact multiple domains of life, including decreased global quality of life (QoL). Transdiagnostic interventions, such as those using Acceptance and Commitment Therapy (ACT) benefit dementia caregivers by addressing underlying mechanisms that impact depression, anxiety, QoL, and more. The transdiagnostic mechanism in ACT is psychological flexibility. The current study used pre-intervention (T1), post-intervention (T2) and 6-week followup (T3) data from a study of caregivers using a 6-session online, self-guided ACT program, tailored to dementia caregiving. Stepwise regression analyses (N=79) tested longitudinal mediation, examining if T2 psychological flexibility (CompACT; Francis et al., 2016), explained associations between T1 caregiver burden (Bédard et al., 2001) and T3 QoL (de Boer et al., 2004). A similar model used T1 stress reactivity of BPSD (Teri et al., 1992) as the independent variable, instead of burden. Based on correlations, both models controlled for whether the caregiver lived with the care receiver. Results indicate that psychological flexibility partially mediated the impact of burden on QoL, and fully mediated the impact of stress reactivity on QoL. Findings suggests that psychological flexibility may be an important mechanism in mitigating the impact that care-specific stress experiences have on global QoL. Caregivers often cannot leave this role, requiring them to learn to live with care-related stress in ways that do not damage their long-term wellbeing. Psychological flexibility is amenable to intervention and ACT offers promising solutions in dementia caregivers.

Abstract citation ID: igae098.2743

### EARLY PARENT–CHILD RELATIONSHIPS, BURDEN, AND SUPPORT AMONG ADULT CHILDREN OF PARENTS WITH DEMENTIA

Kristie Wood[1], Denise Chow[2], Thi Hoang Vu[3], and Joan Monin[4], *1. Colorado University Anschutz School of Medicine, Aurora, Colorado, United States*, *2. Yale University, New Haven, Connecticut, United States*, *3. Yale University School of Public Health, New Haven, Connecticut, United States*, *4. Yale School of Public Health, New Haven, Connecticut, United States*

Research has demonstrated that greater childhood adversity is associated with poor mental health in older adult family caregivers. Yet, it is unknown whether early parent-child relationship quality is more specifically associated with adult children's caregiver burden and perceived support from their parents living with cognitive impairment. We examined associations between early life parent-child relationship quality, current caregiver burden, and adult children's perceived support from their parents, among adult children of parents living with early-stage dementia. Adult children of parents living with cognitive impairment (n=144, m=47.53, sd=11.2, Non-Hispanic=92%, White=72%) completed the following self-report measures: Parental Bonding Inventory, Zarit Burden Interview, and Perceived Partner Responsiveness Scale. Multiple linear regression models revealed children who identified more care, less overprotection, and optimal parenting in their early parent-child relationships reported greater current perceived support from their parents. However, there was no statistically significant association found between early parent-child relationship quality and caregiver burden. Our findings indicate that while early positive relationship quality may be indicative of adult children's future perceptions of support from their parents with early-stage dementia, it may not buffer against perceptions of caregiver burden. Overall, results highlight that early parent-child relationship quality may have consequences for caregiving relationships in later life. Future research is needed to explore mechanisms of early parent-child relationship quality and perceptions of support that are associated with caregiver outcomes.

Abstract citation ID: igae098.2744

### ENGAGING ASIAN AMERICAN FAMILY CAREGIVERS IN CLINICAL TRIALS: FINDINGS FROM A NATIONWIDE STUDY

Eve Rubovits[1], Anjali Yedavalli[1], Sabeen Sadruddin[1], Kavin Lavari[1], Raksha Mudar[2], Vania Leung[3], and Minakshi Raj[1], *1. University of Illinois Urbana-Champaign, Champaign, Illinois, United States*, *2. University of Illinois, Champaign, Illinois, United States*, *3. University of Illinois Chicago, Chicago, Illinois, United States*

Ensuring representation of older racial and ethnic minority participants in clinical trials requires considering the family, community, and sociocultural context within which older adults make decisions about their healthcare and participation in trials. This study sought to evaluate Asian American caregivers' (a) knowledge and experiences with clinical trials; (b) trusted and utilized sources of clinical trial information, (c) perceptions of ideal types of information to receive before enrolling their older relative in a clinical trial, and (d) concerns about their relative's participation in clinical trials. We conducted a cross-sectional, online nationwide survey with Asian American family caregivers supporting an older relative between July 2022 and April 2023. Of all respondents (n=98), 62.2% reported knowing a little about clinical trials and 11.2% supported a relative who had been invited to participate in a clinical trial. Over one-third of respondents reported they most trust their relative's healthcare provider as a source of information, and over half said they would use information from health organizations/groups or their relative's provider for information. Respondents expressed wanting information about (a) the purpose, design, and components of the trial; (b) trial research ethics and safety; and (c) their responsibilities as the caregiver. Greater engagement between clinicians and family caregivers of culturally diverse older adults could help address concerns related to sociocultural barriers to participating in clinical trials. Providing key information to family caregivers in a comprehensive and accessible way without adding burden could help caregivers understand their responsibilities through the clinical trial process.

Abstract citation ID: igae098.2745

### JAPANESE OLDER CAREGIVERS' EXPERIENCES OF PROVIDING CARE AT HOME FOR AN OLDER SPOUSE LIVING WITH DEMENTIA

Mineko Wada[1], Kazuya Takeda[2], and Hideaki Hanaoka[3], *1. Hiroshima University, Hiroshima, Hiroshima, Japan*, *2. Kaneda Hospital, Maniwa, Okayama, Japan*, *3. Hiroshima University, Hirishima, Hiroshima, Japan*

This qualitative research study explored older caregivers' experiences of providing home care for an older spouse with dementia in Japan. The proportion of older adults (age 65+) in Japan reached 29% in 2022. The number of households in which one older adult cares for another has been increasing in tandem and is expected to continue to grow steadily. While a growing body of research has focused on the experiences of adult children who provide care for older relatives, little is known about how older adults experience caring for an older spouse at home. In this study, semi-structured individual interviews were conducted with eight older couples in which one partner provided care for their spouse who was living with dementia. Interviews with caregivers were designed to elicit their caregiving experiences and the meanings of caring for their partner at home in their later life. Interviews were digitally recorded, transcribed verbatim, and analyzed thematically. The analysis yielded four themes regarding caregivers' experiences of older-to-older caregiving: 1) physical burden of caregiving was intensified by caregivers' physical functioning decline; 2) "I'll continue to look after my spouse as long as my body's reached its limit": a strong sense of love and compassion arising from a long spousal relationship; 3) negotiating the financial burden of caregiving with using formal community care support; and 4) a sense of loneliness: from no family support for caregiving to withdrawal from social networks. We discuss what it means for older caregivers to care for their spouse at home.

Abstract citation ID: igae098.2746

### OVER SIX TIMES INCREASED RISK OF SUICIDAL IDEATION IN FAMILY CAREGIVERS WITH HISTORY OF CHILDHOOD ABUSE: THE ROLE OF NEUROTICISM

Felipe Jain[1], Paulina Gutierrez-Ramirez[2], Miranda Zea[2], and Liliana Ramirez Gomez[2], *1. Depression Clinical and Research Program / Massachusetts General Hospital, Boston, Massachusetts, United States*, *2. Massachusetts General Hospital, Boston, Massachusetts, United States*

Despite high reported rates of suicidal ideation (SI) in family caregivers of persons living with dementia, little is known about the relationship between SI and intergenerational trauma. Those with a history of adverse childhood experiences (ACEs) such as neglect and abuse are known to have worse psychological coping skills and higher rates of mental disorders. Personality factors might be mechanistically related to SI in family caregivers, but this has never been tested. We undertook an analysis of the association between intergenerational trauma, measured with the Adverse Childhood Experience (ACE) questionnaire, and a measure of SI from the Quick Inventory of Depressive Symptoms-Self Report, in 81 caregivers of family members living with dementia. We further analyzed whether neuroticism mediated the relationship between ACE and SI. 22% of caregivers exhibited passive SI, and 69% at least 1 ACE. 89% of caregivers with SI reported intergenerational trauma (ACE >= 1), versus 63% of those without SI (p=.04). SI was 4.6x as likely in those with ACE >= 1 than in those without. The odds ratio for SI for those who had experienced childhood neglect was 4.4 (p=.05), and for those reporting childhood abuse was 6.8 (p=.01). Neuroticism significantly mediated the relationship between ACE exposure and SI (p<.05). The risk of SI in family caregivers with history of intergenerational trauma was high in this sample. The relationship between intergenerational trauma, neuroticism, and SI in family caregivers should be further explored and could represent a new treatment target to improve the efficacy of caregiver therapies.

Abstract citation ID: igae098.2747

### PERCEIVED EFFICACY OF TWO NONPHARMACOLOGICAL INTERVENTIONS: A QUALITATIVE EXPLORATION OF CAREGIVERS OF DEMENTIA

Megumi Inoue, Catherine Tompkins, Emily Ihara, Shannon Layman, McKenzie Lauber, Sewon Kim, and Gilbert Gimm, *George Mason University, Fairfax, Virginia, United States*

We will present findings from the qualitative component of an explanatory sequential design that investigated the efficacy of two nonpharmacological interventions: the Stress Busting Program (SBP) for Family Caregivers™ and a personalized music intervention for care recipients living with dementia. After implementing the 9-week SBP program focused on reducing caregiving-related stress among 97 family caregivers of persons living with dementia, we randomly assigned participants into a 4-week personalized music intervention group and a control group to examine effects on stress and well-being. Quantitative findings revealed a significant reduction in mean caregiver burden scores associated with SBP, but not for the personalized music intervention. We observed varied levels of caregiver and care recipient engagement with the personalized music intervention. To gain a deeper understanding of these results, we conducted 12 focus groups and interviews with study participants to explore caregivers' perspectives and experiences with each intervention. Constant comparative analysis and substantive coding by two independent reviewers were employed to analyze transcripts of audio recordings with 47 caregivers, selected from the initial study cohort. The theme of bonding/connecting with group members emerged as the most effective SBP element. Also, caregivers valued the opportunity to establish a sense of community by connecting with other caregivers within SBP. However, some caregivers experienced technological difficulties with using an MP3 player for the music intervention. We will present cross-cutting themes and overall lessons learned about strategies to enhance the use of these nonpharmacological interventions.

Abstract citation ID: igae098.2748

### PHYSICAL AND MENTAL HEALTH OF GRANDPARENTS CARING FOR GRANDCHILDREN IN CHINA: A SYSTEMATIC REVIEW

Shuangshuang Wang, *Southwest Jiaotong University, Chengdu, Sichuan, China (People's Republic)*

Guided by role strain and role enhancement perspectives, this study systematically reviewed the effects of providing grandchild care on the physical and mental health of Chinese grandparents, aiming to identify research gaps and offer insights for future studies and the enhancement of Chinese grandparents' well-being. Utilizing a thematic search method, we conducted searches in PubMed, Web of Science, CNKI, Wan Fang Data, and CQVIP databases for relevant literature spanning from January 1st, 2012, to March 1st, 2024. Ninety-one articles were ultimately included, and their content was analyzed to systematically review the impacts on physical and mental health. Factors such as grandparents' gender, age, and residential areas affected their participation in intergenerational care. Mental health outcomes primarily focused on depressive symptoms, cognition, loneliness, and life satisfaction, while physical health was assessed through self-assessed health, daily-life abilities, and instrumental self-care abilities. However, the literature lacked consensus on the effects of providing grandchild care on Chinese grandparents' physical and mental health. Negative outcomes mainly due to role conflicts, high care demands, feelings of isolation, while positive outcomes were achieved through social more social activities, companionship, and family network support. In conclusion, Chinese grandparents' well-being appears to be influenced by a multitude of factors, with current exploration in its nascent stages. Opportunities for improvement lie in data collection methods, research design, and areas of investigation. Further research is warranted to better understand the complexities of this relationship and to develop interventions aimed at promoting the health and well-being of Chinese grandparents.

Abstract citation ID: igae098.2749

### PROBLEM-SOLVING THERAPY FOR HOSPICE FAMILY CAREGIVERS: DOES TIMING MATTER?

Oonjee Oh[1], Karla Washington[2], Debra Parker Oliver[2], and George Demiris[1], *1. University of Pennsylvania, Philadelphia, Pennsylvania, United States*, *2. Washington University in St. Louis, St. Louis, Missouri, United States*

Although problem-solving therapy (PST) shows promise in alleviating hospice caregivers' distress, the best timing for its implementation (e.g., closer to admission or later) is unknown. Our aim was to compare changes in hospice caregivers' depressive and anxiety symptoms based on whether they received PST during or after their transition to hospice. We conducted a secondary analysis using pre- and post-intervention data from a PST intervention labeled PISCES (Problem-solving Intervention to Support Caregivers in End-of-life care Settings). Among 218 hospice family caregivers who were actively caring for their loved one at baseline, 79 caregivers received PISCES face-to-face (F2F), 72 caregivers received PISCES via both F2F and videoconferencing (Hybrid), and 67 received an enhanced hybrid version of PISCES (PISCESplus), which additionally focused on recognizing positive aspects of caregiving following a positive reappraisal approach. The timing of the intervention was dichotomized into starting either during recent transition to hospice (within 14 days of hospice admission) or later. Caregivers who received PISCES F2F, whether during or after hospice transition, demonstrated improved depressive symptoms (p value 0.013 and < 0.001, respectively). PISCES Hybrid group did not show a significant change in their depressive symptoms at either timing. Within the PISCESplus group, only those who received the intervention later demonstrated a significant decrease in depressive symptoms (p value=0.043). Regarding anxiety symptoms, all caregivers experienced significant decrease, irrespective of the intervention type and its timing. Further efforts are needed to optimize the timing of PST in real-world hospice settings to increase its translatability and effectiveness.

Abstract citation ID: igae098.2750

### SLEEP BARRIERS AND FACILITATORS IN AFRICAN AMERICAN FAMILY CAREGIVERS

Heba Aldossary[1], Glenna Brewster[2], Stephanie Griggs[1], and Elliane Irani[1], *1. Case Western Reserve University, Cleveland, Ohio, United States*, *2. Emory University, Atlanta, Georgia, United States*

Cultural and historical experiences of African American family caregivers influence their overall health outcomes and can compound their risk for poor sleep health. Since there is inadequate representation of African American individuals in caregiving research, the existing literature is limited as it pertains to informing sleep intervention targets to address the unique needs of African American caregivers. This study aims to explore the barriers and facilitators to sleep health in African American family caregivers. To be eligible for this study, caregivers were required to provide care to an adult family member or friend with a chronic or disabling condition for a minimum of 6 months and self-identify as Black or African American. Twenty-four participants were recruited via community-based methods and participated in semi-structured in-person, telephone, or videoconference interviews. Transcribed interviews were analyzed using conventional content analysis. Participants were on average 58.0 (SD=10.1) years old, mostly female (83.3%) and caring for a parent (79.2%). Main sleep barriers included being alert to care recipient's needs, general and caregiving-specific stress, existing health conditions such as anxiety or sleep apnea, and environmental factors such as room temperature and noise within and outside the home. Sleep facilitators included caregiving support, daytime activities for the care recipient and the caregiver, natural or pharmacological sleep aids, and relaxing activities at bedtime such as meditation, journaling, reading, and art-based activities. Half of our participants perceived the television before and/or during sleep as a facilitator. Our findings can inform the cultural adaptation of behavioral interventions to optimize sleep health.

Abstract citation ID: igae098.2751

### THE HOSPITAL ENVIRONMENT'S INFLUENCE ON THE INCLUSION OF CARE PARTNERS OF HOSPITALIZED PLWD

Catherine Still[1], Sydney Hoel[2], Andrea Strayer[3], Nicole Werner[2], and Beth Fields[1], *1. University of Wisconsin Madison, Madison, Wisconsin, United States*, *2. Indiana University, Bloomington, Bloomington, Indiana, United States*, *3. University of Iowa, Iowa City, Iowa, United States*

Federal initiatives like the National Strategy to Support Family Caregivers advocate for the inclusion of care partners into their family member or friends care teams. Yet, care partners of hospitalized people living with dementia (PLWD) often report feeling left out of the hospital care. This feeling of exclusion may be related to the hospital's physical environment. Prior research has explored dementia care partners' perceptions of the hospital space, but there is little research examining how the hospital environment can facilitate or hinder care partner inclusion. Therefore, this study sought to explore how the physical environment of the hospital influences care partner inclusion practices. Our team used a descriptive qualitative approach and collected data from two sources: direct observations (27 hours) in a large academic hospital and interviews with care partners (15) and clinicians/administrators (23) representing hospital experiences across the country. Observational data were analyzed using a framework analysis, and interview data were analyzed using a team-based inductive content analysis. Observational data revealed an underutilization of environmental resources, including communication tools and family-dedicated spaces. Interview data revealed three aspects of the physical environment that influence care partner inclusion: space for both the PLWD and their care partner, room personalization, and parking. Health systems should ensure adequate space for care partners in the hospital, allow care partners to personalize patient rooms, and designate dementia-friendly parking spaces. Modification of the physical environment may promote the inclusion of care partners into hospital care, which could potentially improve the health outcomes for hospitalized PLWD.

### SESSION 7275 (POSTER)

### FRAILTY (POSTERS)

Abstract citation ID: igae098.2752

### A RETROSPECTIVE COHORT STUDY ON THE RELATIONSHIP BETWEEN FRAILTY AND HEALTH CARE OUTCOMES

Jinmyoung Cho[1], Joanne Salas[2], and Jeffery Scherrer[2], *1. Saint Louis University School of Medicine, Ballwin, Missouri, United States*, *2. Saint Louis University School of Medicine, St. Louis, Missouri, United States*

Frailty increases vulnerability for adverse outcomes in older adults. Characterizing the prevalence and distribution of frailty can help guide healthcare service decision-making and policy. This study evaluated the association between frailty and healthcare utilization and interactions by demographic characteristics. Using electronic health records (2018-2022), we conducted a retrospective cohort study with 355,266 patients ≥65 years of age who had ≥2 ambulatory office visits in separate years in the 4-year baseline period (2018-2021). The Gilbert Frailty Index (GFI) was calculated (low vs. intermediate vs. high risk) using ICD-10 codes. One-year utilization outcomes in 2022 included high outpatient clinic utilizations (OCU), inpatient (IP), emergency department (ED), and nursing home (NH) admissions. Fully adjusted log-binomial regression models were calculated overall and by race (White vs. Black), age groups, and sex. The sample was 74.5(±7.5) years of age, 57.7% female, 89.2% White, and 13.5% categorized as GFI high risk. After adjustment for covariates, GFI high risk had the highest risk for all outcomes (RR=3.31 for IP; 2.77 for ED; 4.26 for NH; 1.60 for high OCU). We observed significant interactions by race, sex, and age for each outcome. Effects of GFI high vs. low risk were larger for White (IP, ED, & high OCU), female patients (ED & high OCU), and younger patients (IP). Conversely, the effects of GFI high vs. low risk was strongest in older patients for ED, IP and high OCU. Monitoring frailty and paying attention to patient's demographic characteristics is needed to best estimate associations between frailty and health utilization.

Abstract citation ID: igae098.2753

### BODY COMPOSITION AND ANTHROPOMETRICS OF OLDER ADULTS AT DIFFERING LEVELS OF URBANIZATION IN CHINA

Hillary Spangler[1], Lisa Michelson[1], Annie Green Howard[2], Hsaio Tien[2], David Lynch[1], Shufa Du[2], Penny Gordon-Larsen[2], and John Batsis[2], *1. University of North Carolina School of Medicine, Chapel Hill, North Carolina, United States*, *2. University of North Carolina, Chapel Hill, North Carolina, United States*

**Background:** Less urbanized areas in China are associated with higher risk of developing frailty. We aim to identify differences in the relationship between body composition and urbanization.

**Methods:** Using the China Health and Nutrition Survey data in 2015, we included community-dwelling adults aged ≥60 with bioelectrical impedance data assessing fat mass and fat-free mass (body composition). Urbanization was based on a multi-component index (0-120 points) based on 12 community-level variables, with lower scores indicating lower urbanization. Latent class analysis was used to identify underlying heterogeneous patterns of body composition classes. Continuous and categorical variables were compared using Chi-squared and ANOVA tests, respectively.

**Results:** Of 1,424 participants, 55% were female, mean age 72.0±5.9 and BMI 23.8±3.6. Three of the five best fitting classes aligned with robust (n=517, moderate BMI, body fat, muscle), pre-frail (n=602, low BMI, low body fat, low muscle), and frail (n=211, lowest BMI, % body fat, and muscle) statuses. Urbanization index was significantly different between the five classes (p< 0.001). Urbanization index was lowest (65.1) for class that aligned with frailty; mean age 72.0 ±5.9, 54.7% female. The class aligning with robust status was associated with higher urbanization index (71.6); mean age 71.9 ±5.7, 54.6% female.

**Discussion:** Older adults with body composition consistent with frailty had a lower level of urbanization. Urbanization level may influence body composition in China.

Abstract citation ID: igae098.2754

### DEVELOPING A FRAILTY INDEX USING THE ELECTRONIC HEALTH RECORDS TO EXPLORE THE POLYPHARMACY AND FRAILTY RELATIONSHIP

Reham Yasin[1], Deborah Lekan[2], Laurie Kennedy-Malone[2], Thomas McCoy[2], Anna Boone[3], and Cindy Bacon[2], *1. King Saud bin Abdulaziz University for Health Sciences- College of Nursing Jeddah, Jeddah, Saudi Arabia*, *2. University of North Carolina Greensboro, Greensboro, North Carolina, United States*, *3. Rockingham Gastroenterology, Reidsville, North Carolina, United States*

The association between polypharmacy and frailty among hospitalized adults was explored using a secondary data analysis of the EHR in adults aged 50 years or older (N=46,645) hospitalized in medical-surgical units between 2013 and 2017. A Frailty Index (FI) was created based on the accumulation of a deficit theoretical framework (Mitnitski et al., 2001). The FI was constructed following the guidance provided by Searle et al. (2008) and Theou et al. (2023). This study's FI was unique because it included indicators from nursing flowsheets, ICD-10 codes, laboratory blood biomarkers, as well as other frailty indicators (35 deficits in this study). The indicators were coded as "1" for present and "0" for absent. FI-35 was calculated by dividing the number of indices in an individual by the total number of indices measured, with frailty defined by a cut-off point of ≥ 0.25. In the study (25,961), 42.0% were identified as frail. A significant association was found between polypharmacy and frailty. The study showed 54.5% of individuals with polypharmacy classified as frail, compared to 31.4% in the non-polypharmacy group ($\chi^2$=1283.886; p< 0.001). The odds of frailty were 53.1 % higher for those in the polypharmacy groups relative to non-polypharmacy groups, adjusting for age and Elixhauser comorbidity index score (AOR=1.531, 95% CI=[1.437, 1.631], p< 0.001). When recognizing frailty in hospitalized patients with known polypharmacy, consideration should be made for medication reconciliation and medication deprescribing before discharge.

Abstract citation ID: igae098.2755

### DEVELOPMENT OF A NOVEL PHENO FRAILTY INDEX FOR MICE

Brady Samuelson, Dantong Zhu, and Alice Kane, *Institute for Systems Biology, Seattle, Washington, United States*

Frailty is assessed in both model organisms and people with frailty index tools that quantify health related deficits. Here we aim to develop a new mouse frailty assessment tool (Pheno-FI) based on clinically-relevant physiological and functional measures that are easily obtainable, and can be measured longitudinally. The Pheno-FI score was developed using male and female C57BL/6 mice (N=40) at 12 months of age. We selected 17 items to include that changed with age, were health-associated, and were not correlated with each other. These included variables like HR, PLT, RBC, WBC, basophils, QRS, QT, open field, glucose, nesting and burrowing to provide a holistic view of frailty. The standard deviation (±1.5) of the baseline mice were used to determine the sex-specific cut points for our deficits, and if values were outside these cut points they were given a score = 1, and within the cut points a 0. Pheno-FI is the average of these items. Once developed in the baseline data, the Pheno-FI was calculated for male and female mice aged 12 to 36 months. Pheno-FI scores increase with age in both males and females, and are positively correlated with the previously validated mouse clinical frailty index (Females R2=0.6, p= 7.7e8 Males R2=0.62, p= 3.1e11). Additionally, Pheno-FI scores show a negative correlation with lifespan (Female R2= -0.56 p=6.7e-7 Male R2= -0.5 p=2.3e7). The Pheno-FI provides a new tool for the field to classify frailty in longitudinal aging mouse studies, to explore frailty mechanisms, biomarkers and interventions.

Abstract citation ID: igae098.2756

### ECOLOGICAL MOMENTARY ASSESSMENT OF SOCIAL INTERACTION, LONELINESS, AND FRAILTY IN PREDEMENTIA

Dahye Hong, Jennifer Kim, Seolah Yoon, Chaeeun Kang, and Bada Kang, *Yonsei University, Seoul, Republic of Korea*

The pre-dementia stage, encompassing subjective cognitive decline (SCD) and mild cognitive impairment (MCI), is recognized as being vulnerable to frailty and is associated with adverse health outcomes, such as falls or disability. Although social interaction and loneliness are considered to be associated with frailty, little is known about the relationship between social interaction, loneliness, and frailty in the pre-dementia stage. Therefore, this study examined the relationship between social interaction, loneliness, and frailty among 101 community-dwelling older adults in the pre-dementia stage (individuals with SCD = 67; individuals with MCI = 34) in Korea. To capture real-time measurements of social interaction and loneliness, we utilized an ecological momentary assessment that involved repeated sampling of individuals' states. Participants reported their frequency of social interactions and loneliness via a mobile application four times a day for two weeks. Multivariate logistic regression analysis was used to investigate the associations between social interaction, loneliness, and frailty. We adjusted for mild behavioral impairment (MBI), which indicates dementia risk and increases vulnerability to frailty, alongside sociodemographic, health-related characteristics, and functional limitations. Our study demonstrated that social interaction has a negative association with frailty among individuals in the pre-dementia stage, regardless of the presence or severity of MBI symptoms. However, loneliness was not significantly associated with frailty. This study highlights that the assessment of social interactions using ecological assessment will help identify at-risk individuals and implement targeted interventions for the prevention and management of frailty in older adults in the pre-dementia stage.

Abstract citation ID: igae098.2757

### EFFECTS OF EXERCISE WITH MUSIC IN FRAIL OLDER ADULTS: A SYSTEMATIC REVIEW AND META-ANALYSIS

Namhee Kim[1], Gwang Suk Kim[2], Min Kyung Park[3], Layoung Kim[2], and Jae Jun Lee[2], *1. Yonsei University, Wonju, Kangwon-do, Republic of Korea*, *2. Yonsei University, Seoul, Republic of Korea*, *3. Mo-Im Kim Nursing Research Institute, Yonsei University College of Nursing, Seoul, Republic of Korea*

Accumulating research on the impact of exercise with music on the health of frail older adults emphasized the necessity of a comprehensive analysis that encompasses physical and emotional health outcomes. Consequently, this study aimed to identify the contents and characteristics of exercising with music and to evaluate the effects of exercising with music on the physical and emotional health of frail community-dwelling older adults. This was a systematic literature review and a meta-analysis. All searches were conducted from April 12, 2022, to March 2, 2024, with no restrictions on language or publication date. Seven electronic databases were searched: MEDLINE, CINAHL, Embase, Cochrane Library, PsyINFO, Google Scholar, and the Virginia Henderson International Nursing Library. PRISMA guidelines for systematic reviews were followed. After screening the initial 1409 studies, 17 studies, including 13 randomized controlled trials and four quasi-experimental studies, were analyzed. The findings indicate that exercise with music has positive effects on reducing frailty (SMD = -0.43, 95% CI = -0.81 – -0.05), improving Timed Up and Go test results (MD = -3.76, 95% CI = -6.67 – -0.85), increasing handgrip strength (MD = 2.01, 95% CI = 0.36 – 3.65), and decreasing depression levels (SMD = -0.34, 95% CI = -0.56 – -0.11) among frail older adults. By addressing both physical and emotional health, exercise with music can significantly contribute to enhancing older adults' quality of life and reducing the burden of frailty. In conclusion, this review emphasizes musical exercises' potential as a multifaceted intervention for frail older adults.

Abstract citation ID: igae098.2758

### ENHANCING MUSCLE FUNCTION IN FRAILTY: FINDINGS FROM A PILOT STUDY ON MAGNETIC MITOHORMESIS THERAPY

Hae Sagong, Pao-Feng Tsai, Chih-Hsuan Wang, Lynn Brown, and Jos Edison, *Auburn University, Auburn, Alabama, United States*

Magnetic Mitohormesis Therapy (MMT) employs low-amplitude pulsing electromagnetic fields (PEMF) and has demonstrated the ability to enhance muscle function and metabolism. Given that muscle weakness is a key contributor to frailty, this study employed a double-blind pilot randomized controlled crossover trial design involving prefrail/frail participants without neurodegenerative diseases, from two distinct types of long-term care facilities. Eight Participants underwent 12 weeks of twice-weekly treatment followed by 12 weeks of sham control, or vice versa, with two groups randomly assigned: Sham-Treatment (S-T group) and Treatment-Sham (T-S group). Treatment was delivered using The QuantumTx BIXEPS Fitness and Wellness device delivered PEMF at 1.5 mT flux densities. FRAIL Scale, SPPB, SF-12v2 Health Survey, SWAY Cognitive Tests, and Body Composition Measurements were assessed at every 6 weeks. Results showed that the S-T group demonstrated improved performance in the Gait Speed test. Their scores from the Gait Speed test were higher at 24 weeks compared to baseline (p=.01), and their overall SPPB scores were higher at 24 weeks compared to baseline (p=.01). Participants noted the study site's accessibility and described the treatment as quick and straightforward. This study provided preliminary evidence of the benefits of MMT in frail older adults; however, further research with a larger sample size and shorter study duration is needed to lower the dropout rate and mitigate the development of new health issues.

Abstract citation ID: igae098.2759

### EXPLORING THE RELATIONSHIP BETWEEN MULTIDOMAINS OF FRAILTY AND MORTALITY IN OLDER ADULTS LIVING IN COMMUNITY

Yuelin Li, Rhayun Song, and Jisu Seo, *Chungnam National University, Daejeon, Republic of Korea*

The global population's aging presents health challenges. This study aimed to explore the relationship between various domains of frailty (physical, cognitive, social, and multidimensional) on all-cause mortality in community-dwelling older adults. Studies were searched in five databases up to December 5, 2023. Quality and Risk of Bias assessment were assessed using the ROBINS-E tool. A meta-analysis was performed to synthesize pooled mortality risk estimates by using the random-effects model. A total of 53 studies were included for systematic review, with 49 eligible for meta-analysis, involving 153,237 participants and over 31,021 deaths. The quality assessment revealed that 10 were low risk (18.9%), 7 had some concerns (13.2%), 34 were high risk (64.2%), and 2 were considered very high risk (3.7%). Results indicated that frail older adults had a significantly higher mortality risk than robust ones, consistent across frailty domains (physical HR=1.72, 95 CI%:1.50-1.97; social HR=1.17, 95 CI%:1.04-1.31; multidimensional HR=2.37, 95 CI%: 2.10-2.68; cognitive HR=1.48, 95 CI%:1.09-2.01). Our findings highlight that various domains of frailty are strongly and robustly associated with all-cause mortality in older adults. This relationship between multidimensional frailty and mortality is stronger than that of a single type of frailty. Therefore, although focusing on a single frailty can improve the well-being of the older population, interventions targeting multiple aspects of frailty are more effective in improving the quality of life and longevity of this population.

Abstract citation ID: igae098.2760

### FEASIBILITY OF A LIFESTYLE-INTEGRATED EXERCISE DELIVERED VIA AN MHEALTH PLATFORM IN PREFRAIL OLDER ADULTS

Na Li[1], Nan Wang[2], Siyang Lin[3], Yin Yuan[1], Feng Huang[1], and Pengli Zhu[4], *1. Fujian Provincial Hospital, Fuzhou, Fujian, China (People's Republic)*, *2. Fujian Medical University, Fuzhou, Fujian, China (People's Republic)*, *3. Shengli Clinical Medical College of Fujian Medical University, Fuzhou, Fujian, China (People's Republic)*, *4. The Shengli Clinical Medical College of Fujian Medical University, Fuzhou, Fujian, China (People's Republic)*

**Background:** Lifestyle-integrated Functional Exercise, which integrates exercise into daily life, may be a promising alternative to overcome traditional exercise barriers. Recent advances in mHealth technology hold the promise of preserving the benefits of supervised exercise training in group settings by remotely guiding and tracking exercise progress. Objective: To assess the feasibility and acceptability of an mHealth platform to deliver a personalized lifestyle-integrated multicomponent exercise program for pre-frail individuals.

**Methods:** This was a community-based, mixed-methods, prospective, single-cohort feasibility study. All participants were prescribed a lifestyle-integrated multicomponent exercise program by healthcare professionals using the PF-Life platform (web-based portal), and participants engaged in customized exercise following in-app video instructions and feedback on the PF-Life platform (smartphone application). Physical activity was registered daily through wearable devices. Result: 16 participants successfully completed the study with a retention rate of 100%. Mean adherence to the number of days exercised per week was 88.8% (95% CI 75%-100%). Changes were observed from baseline to follow-up for total Handgrip strength (21.41±6.38 vs. 24.12± 6.62 kg, d =0.714), TUG (8.23±1.33 vs. 7.48± 2.01 seconds, d=1.647) and 30-second chair rise test (17.13±4.3 vs. 20.04±4.54 repetitions, d =0.94). The sedentary time decreased by a mean of 33 minutes/day and low physical activity increased by 31 minutes/day during the intervention.

**Conclusion:** This study provides preliminary evidence that an lifestyle-integrated exercise based on mHealth platform has good adherence, safety, reliability and usability, with initial results in reducing sedentary time and improving physical function.

Abstract citation ID: igae098.2761

### FRAILTY IN INTERMEDIATE CARE FACILITIES THROUGH LIFE (LONG-TERM CARE INFORMATION SYSTEM FOR EVIDENCE) IN JAPAN

Tomomichi Sakai[1], Renuka Visvanathan[2], Agathe Daria Jadczak[2], and Hiroyuki Umegaki[3], *1. Nagoya University Graduate School of Medicine, Nisshin, Aichi, Japan*, *2. University of Adelaide, Adelaide, South Australia, Australia*, *3. Nagoya University, Nagoya, Aichi, Japan*

The Long-term care Information system For Evidence (LIFE) was introduced to improve care for older adults based on scientific evidence in 2021 within the Japanese long-term care insurance framework. The aim of this study was to reveal the prevalence of frailty among residents in intermediate care facilities and identify factors associated with the frailty using data collected for the emerging system. This retrospective and cross-sectional study included residents aged 65 years or above who had complete data for assessing frailty. Data was collected between April 2021 and November 2023 at 4 intermediate care facilities. Residents were classified as non-frail, frail, or most-frail using the FRAIL-NH scale by fitting variables in the LIFE data that were most appropriate for each component of the FRAIL-NH scale. Multivariable logistic regression analysis was used to explore factors associated with being most-frail using variables covering broad areas such as sociodemographic information, nutrition, oral function, and cognition. 907 residents were included for analysis; their mean age was 85.9 (standard deviation 7.2) years, and 63.7% were female. Among them, 5.7%, 33.8%, and 60.4% were categorized as non-frail, frail, and most-frail. The necessity of a dysphagia diet, easily choking, and dementia status and symptoms were shown to be significantly associated with being most-frail using the reference of not being most-frail. The current results indicate the potential for frailty assessment using data that recently started being collected routinely across Japan. Future studies of the predictive role of frailty against prognosis should promote care delivery stratified by frailty assessment.

Abstract citation ID: igae098.2762

### FRAILTY TRAJECTORIES AND ITS ASSOCIATED FACTORS IN JAPANESE OLDER ADULTS

Yu Taniguchi[1], Akihiko Kitamura[2], Toshiki Hata[2], Koji Fujita[2], Takumi Abe[2], Yu Nofuji[2], Satoshi Seino[2], and Yuri Yokoyama[2], *1. National Institute for Environmental Studies, Tsukuba, Ibaraki, Japan*, *2. Tokyo Metropolitan Institute for Geriatrics and Gerontology, Tokyo, Japan*

The present study had two objectives: to identify aging trajectories in frailty status from age 65–90 years among community-dwelling older Japanese; and to identify factors associated with this trajectory among these people. The present prospective study used repeated measures data on frailty status from a 13-year longitudinal study. 1706 older adults completed an annual frailty assessment at least once during the follow-up. Frailty status was determined using an index based on the Fried frailty phenotype criteria. Potential associated factors for frailty trajectory included physical, biological, lifestyle-related, and psychological factors, as well as comorbidities. We identified five trajectory patterns in the frailty score from age of 65 to 90 years —individuals who were robust (10.5%) as well as individuals with late-onset frailty (16.1%), middle-onset frailty (25.6% and 35.2%), and early-onset frailty (12.7%). The early-onset frailty group were already pre-frail at the age of 65 years, and their frailty score gradually progressed to frail status. Compared with the robust group, the early-onset group showed a higher prevalence of cerebrovascular diseases, bone and joint diseases, poor nutrition, sarcopenia, hospitalization, low cognitive function, and smoking at the end of follow-up. Associated factors in the middle-onset group largely overlapped with those of the early-onset group. The late-onset frailty group tended to have a higher association with heart disease and bone and joint diseases compared with the robust group. Proposed effective population-based frailty prevention strategies in each age group may contribute to effective strategies to extend healthy life expectancy.

Abstract citation ID: igae098.2763

### GENDER DIFFERENCES IN THE ASSOCIATION BETWEEN FRAILTY AND HOSPITALIZATION AMONG OLDER ADULTS IN SOUTH KOREA

Bokyoung Choi[1], Yumi Shin[2], Chang-O Kim[1], Ahreum Choi[1], Eunhee Choi[3], Hyeyeon Lee[1], and Soog-nang Jang[4], *1. Institute for Community Care and Health Equity, Seoul, Republic of Korea, 2. Daegu University, Gyeongsan, Republic of Korea*, *3. Institute for Community Care and Health Equity, Seoul, Republic of Korea*, *4. Chung-Ang University, Seoul, Republic of Korea*

This study aimed to investigate the association between frailty and hospitalization experience among older adults in South Korea, while also exploring potential gender differences in this association. We used data from the Korean Longitudinal Study of Aging (2018-2020). Frailty was assessed using the frailty instrument (weakness of grip strength, exhaustion, and social isolation) and divided into three groups: non-frail, pre-frail, and frail. Experience of hospitalization over the past two years was measured by self-reported. Multivariate logistic regression model was applied to examine the association between frailty and hospitalization experience after adjusting for potential confounders including age, education level, and physical activity. Among aged 65 and over older adults in South Korea, the prevalence of frailty was 14.4% (N=656), and the prevalence of hospitalization over the past two years was 9.6% (N=437). Compared to the non-frail group, the frail group had a statistically significantly higher risk of hospitalization (pre-frail OR:1.63 95% CI:1.28-2.08, frail OR:2.50 95% CI:1.85-3.39) after adjusting potential confounders. However, after stratification by gender, the relationship between frailty and hospitality was still statistically significant among older women (pre-frail OR:2.00 95% CI:1.45-2.75, frail OR:3.27 95% CI:2.22-4.80), but not in men. These findings suggest that understanding gender differences in the association between frailty and hospitalization would be beneficial for reducing the care burden among older adults in South Korea. Tailoring interventions and healthcare services to address the specific needs of frail older individuals, particularly focusing on gender-specific differences, may help in preventing hospitalizations and improving overall well-being.

Abstract citation ID: igae098.2764

### IMPACT OF FRAILTY AND DEMENTIA ON REHABILITATION OUTCOMES IN OLDER ADULTS SURGICALLY TREATED FOR HIP FRACTURE

Giulia Caggiu[1], Matteo Monzio Compagnoni[1], Patrizia Floris[2], Martina Manna[1], Francesco De Filippi[3], Michela Passamonte[2], and Paolo Mazzola[1], *1. University of Milano-Bicocca, Milano, Lombardia, Italy*, *2. Morbegno Hospital, Morbegno, Lombardia, Italy*, *3. Società Italiana di Geriatria Ospedale e Territorio, Milano, Lombardia, Italy*

We investigated the impact of frailty and dementia on rehabilitation outcomes after hip fracture (HF) surgery in an Italian Orthogeriatric Unit (OGU). We prospectively enrolled all patients aged ≥70 years admitted to the OGU at Sondrio Hospital (Italy) in 2011-2019, with HF requiring surgery. We administered a comprehensive geriatric assessment, including factors related to fracture and surgery. A frailty index (FI) based on 16 variables expressed frailty (FI=number of deficits/16), with which we obtained three categories: fit (FI< 0.15; reference), mild (0.15< FI< 0.34), moderate-to-severe (FI≥0.35). Among 818 participants (mean age 85.6 years, 83.3% females), 21.6% had pre-existing dementia. The average time-to-surgery was 1.9 days (77.6% in spinal anesthesia). Rehabilitation success, i.e. Rehabilitation Efficiency Index (REI)>0.5, was achieved by 70.2% of subjects. From multivariable logistic regression, factors impacting rehabilitation success were frailty (both categories), dementia (OR: 0.14, 95% CI: 0.10-0.21), and age ≥90 years (OR: 0.37, 0.21-0.64). The chances of rehabilitation success were lower for patients with dementia than for those without. Although there is no clear link between age and rehabilitation success, frailty has a decreasing influence as age increases. In nonagenarians, severe frailty does not limit rehabilitation success as much as in younger patients. Previous research showed that dementia potentially acts as a surrogate for frailty in predicting post-operative 1-year mortality after HF surgery. We show that dementia and frailty significantly influence rehabilitation success, thus early mobilization and initiation of physical therapy regardless of age should target non only frail subjects, but also those with dementia, possibly with specifically dedicated interventions.

Abstract citation ID: igae098.2765

### INTERACTIONS BETWEEN FRAILTY AND SOCIAL VULNERABILITY ON THE SURVIVAL AND HEALTH TRANSITIONS OF OLDER ADULTS

Heng Wu[1], Xiaowei Song[2], and Kenneth Rockwood[1], *1. Dalhousie University, Halifax, Nova Scotia, Canada*, *2. Fraser Health Authority, Surrey, British Columbia, Canada*

Frailty, an age-related state, is dominated by declining health over time, although stabilization and improvement can occur. We investigated how the accumulation of physical and social deficits interacts in health transitions and survival. Data from participants aged 65+ years in the Chinese Longitudinal Health Longevity Survey (CLHLS) 2005 cohort were analyzed (n=15613; women=57%; mean age=86.2±11.7). Participants were followed in 2008, 2011, 2014, and 2018, with their health and survival status reassessed each time. We constructed both a 47-item frailty index (FI) and a 26-item social vulnerability index (SVI). The FI included domains of morbidities, cognition, lifestyle, and disabilities; the SVI included World Health Organization-defined social determinants of health as – i.e., education, social security and inclusion, work/life/dwelling/services conditions, and early childhood development. At each follow-up, the FI strongly affected survival in a Logistic Regression model with baseline age, FI, SVI, and sex as covariates (e.g., a 1% FI increase for 14-year mortality: Odds Ratio OR=1.07, 95% CI=1.06-1.08). The SVI showed a marginally significant effect on 3-year survival (e.g., a 1% SVI increase, OR=1.01, 95% CI=1.00-1.01), and diminished at longer follow-ups. The FI also affected health transitions (Figure 1A), as did the SVI, albeit to a lesser extent (Figure 1B). In health transitions and survival of older adults, frailty increases risk and often interacts with social vulnerability. Our study underscores why we should characterize both physical and social factors to understand health and its dynamics in aging.

Abstract citation ID: igae098.2766

### JOINT TRAJECTORIES OF FRAILTY AND COGNITIVE FUNCTION IN OLDER KOREANS: A 14-YEAR ANALYSIS WITH NATIONAL-LEVEL DATA

Yunkyung Jung[1], and Jiyoung Lyu[2], *1. Hannam University, Daejeon, Republic of Korea*, *2. Hallym University, Chuncheon, Kangwon-do, Republic of Korea*

Despite the evidence on the link between frailty and cognitive function, less is known about the joint developments of these two multidimensional indexes. The present study aimed to identify distinct trajectories of frailty and cognitive function and examine the influence of sociodemographic and lifestyle factors on the co-development of frailty and cognitive function in older Koreans. A nationally representative sample of 2,766 adults aged ≥65 years from the first (2006) to the eighth (2020) waves of the Korean Longitudinal Study of Aging (KLoSA) was analyzed. Frailty was measured with a Frailty Index with 33 deficit-related indicators. Cognitive function was measured using the Mini-Mental State Examination (MMSE). Group-based trajectory modeling was used to fit the developments of frailty and cognitive function. A multinomial logistic regression was conducted to assess the predictors for each trajectory group. Three joint trajectories were identified, including cognitive frailty (13.2%), cognitive decline and progressive frailty (33.0%), and no cognitive frailty (53.7%). Compared to individuals in the no cognitive frailty group, those in the cognitive frailty group were more likely to be unemployed and current smokers; while those in the cognitive decline and progressive frailty group were more likely to be women, have low income, and not current drinkers. Significant heterogeneity in the co-development of frailty and cognitive function in older Koreans was identified. Findings suggest that different sociodemographic and lifestyle factors determined the trajectory membership.

Abstract citation ID: igae098.2767

### LONGITUDINAL SEX DIFFERENCES IN FRAILTY AND AGING IN UM-HET3 MICE

Judy Wu[1], Brady Samuelson[1], Elizabeth Betz[2], and Alice Kane[1], *1. Institute for Systems Biology, Seattle, Washington, United States*, *2. Institute for Systems Biology, Washington, United States*

Frailty is a common geriatric syndrome characterized by age-associated decline and vulnerability to adverse health outcomes. Frailty can be quantified using frailty indices (FIs) which count the number of health deficits in an individual. Sex differences are observed in aging with women living longer than men, despite women being frailer. Our goal is to assess sex differences in behavioral and molecular assessments of aging, and how frailty may influence these changes. Across the lifespan of UM-HET3 male and female mice(n=37) we are longitudinally measuring FI score, behavioral assessments and blood outcomes including white blood cell counts (WBC) and glucose levels. Preliminary data from the first 3 timepoints unexpectedly suggest frailty scores decrease over time(p=9.98e-13), with no clear sex differences. For nesting, females have higher scores at all timepoints (p=7.71e-04). Males have higher WBC than females (p = 1.29e-06), with no change over time. Glucose levels increase overtime for both sexes, (p=2e16), but males have lower glucose counts than females (p=5.25e-3). Frailty is associated with lower glucose levels in both sexes (p= 4.45e-05). Heart rate is higher in females (p= 3.52e-04) and increases over time for both sexes (p=3.59e-03). Additionally, higher frailty scores are associated with lower heart rates(p=0.0236) in both sexes. Data collection is ongoing and we expect to see more significant effects of sex, age, and frailty as the mice grow older. This work will help understand the impact of frailty and sex differences on aging.

Abstract citation ID: igae098.2768

### METABOLOMICS BIOMARKERS FOR FRAILTY: A LONGITUDINAL STUDY OF AGING MICE

Dantong Zhu, Judy Wu, Brady Samuelson, and Alice Kane, *Institute for Systems Biology, Seattle, Washington, United States*

Aging is associated with changes in dynamic biological processes which may contribute to the development of aging-related diseases. Metabolomic profiling allows us to measure a wide spectrum of metabolites as read outs of these biological processes. The temporal dynamics of metabolites can be utilized to identify age-related biomarkers and to elucidate possible aging mechanisms. We performed a longitudinal study of aging female (n = 20) and male (n = 25) C57 mice and measured frailty index and derived metabolomics from plasma samples at five time points from 13 to 30 months of age. We identify differentially abundant metabolites by differential analysis over the time course and select metabolite features by a machine learning approach both in the whole cohort and in sex-stratified subgroups. We reveal novel metabolites associated with △frailty index (difference between individual frailty index and median frailty index at a given sex and age group), and merge these metabolites with frailty index and age related metabolites to generate a union set of metabolite features associated with frailty in aging. We found frailty related metabolites are generally enriched in lipid metabolism, amino acid metabolism and metabolism of cofactors and vitamins, although there is strong sex dimorphism of the specific metabolites. Notably, we identify FAD and 4–hydroxyhippurate in females and ergothioneine and creatine in males as candidate metabolites associated with frailty. In summary, our results identify sex-specific metabolite biomarkers of frailty in aging, and shed light on potential mechanisms involved in aging and the development of frailty.

Abstract citation ID: igae098.2769

### PERCEIVED STRESS AS A MEDIATOR OF THE ASSOCIATION BETWEEN PSYCHOLOGICAL RESILIENCE AND FUNCTIONAL DECLINES

Eunsaem Kim[1], Yunhwan Lee[2], Miji Kim[3], Sukyung Kim[2], YooGyung Shin[2], and Jimin Han[2], *1. Ajou University School of Medicine, Suwon-si, Kyonggi-do, Republic of Korea*, *2. Ajou University School of Medicine, Suwon, Kyonggi-do, Republic of Korea*, *3. Kyung Hee University, Seoul, Republic of Korea*

Because older adults are highly likely to experience physical and functional losses during the aging process, resilience, the ability to cope with age-related stress and navigate adversities, is key to healthy aging. Although a growing number of studies have acknowledged the protective effects of psychological resilience on the onset of frailty, cognitive impairment, and physical disability, little is known about underlying mechanisms explaining the association between psychological resilience and functional declines in later life. To explore mediators that account for the path between psychological resilience and frailty, we used data from the 2023 Korean Frailty and Aging Cohort Study (KFACS), with 1397 aged 72-90 (Mean = 82.2). Mediating effects of perceived stress, cognitive function, and physical functioning were examined by a parallel-serial mediation model using PROCESS macro. Older adults with high psychological resilience were less likely to develop frailty ($\beta$ = -.093, $p$ <.05) after controlling for socio-demographic covariates and health-related factors. Psychological resilience influenced levels of frailty indirectly through perceived stress ($\beta$ = -.04, 95% CI = -.078, -.002) and physical functioning ($\beta$ = -.031, 95% CI = -.056, -.009). Also, there were simultaneous indirect effects of cognitive appraisal of stress through both cognitive function ($\beta$ =-.01, 95% CI = -.019, -.003) and physical functioning ($\beta$ = -.034, 95% CI = -.05, -.021) in the association between psychological resilience and frailty. These findings suggest that stress-coping abilities of older adults play a crucial role in promoting favorable stressor appraisals, which may mitigate functional declines.

Abstract citation ID: igae098.2770

### PLASMA PROTEOMIC SIGNATURE OF DEVELOPMENT AND PROGNOSIS OF PHYSICAL FRAILTY

Jianhong Xu, jingyun Liu, Jinhui Liao, and Chenkai Wu, *Duke Kunshan University, Kunshan, Jiangsu, China (People's Republic)*

The complete understanding of biological mechanisms behind frailty remains elusive. We aimed to investigate the proteomic signature associated with the development and prognosis of frailty. Data from 43,895 participants (aged 39-70 years) in the UK Biobank were analyzed, with 2,920 proteins assayed using Olink. Participants were split into development and validation cohorts based on geographic location. Multinomial logistic models identified proteins cross-sectionally associated with baseline prefrailty and frailty (Bonferroni-corrected p-value < 0.05). Cox models identified proteins associated with mortality among initially prefrail and frail participants. LASSO regression was used to construct protein-based prediction models for death among prefrail and frail participants, respectively. Protein importance was ranked using the light gradient boosting machine classifier. We identified 210 and 268 proteins cross-sectionally associated with prefrailty and frailty at baseline, respectively. Additionally, 109 and 80 proteins were associated with mortality among initially prefrail and frail individuals, respectively. LASSO regression model selected 60 and 21 proteins predicting survival in prefrail and frail participants, respectively. Protein-based prediction models demonstrated satisfactory discrimination, calibration, and reclassification in the development and validation cohorts. GDF15 (WFDC2) and PLAUR emerged as the top two proteins in prefrailty (frailty) development, demonstrating robust connections with overall survival in prefrail (frail) individuals. Differentially expressed proteins were enriched in pathways involving protein metabolism, cellular signaling in disease, apoptosis, and inflammation. Exploring novel proteins and pathways provides insights into the biological mechanisms and potential therapeutic targets associated with the onset and progression of frailty.

Abstract citation ID: igae098.2771

### RISK FACTORS ASSOCIATED WITH FRAILTY STATUS CHANGE: A PROSPECTIVE STUDY IN A GERIATRIC OUTPATIENT SETTING.

Chiung-Jung Wen[1], Chung-Sheng Lee[2], Wei-Jia Huang[1], Ching-Huang Lin[1], and Ding-Cheng (Derrick) Chan[1], *1. National Taiwan University Hospital, Taipei, Taipei, Taiwan (Republic of China)*, *2. Kainan University, Taoyuan, Taoyuan, Taiwan (Republic of China)*

**Background:** There has been limited research on the factors that predispose individuals to changes in frailty status. This study aims to identify the risk factors that lead to changes in frailty status among older adults.

**Methods:** Participants were recruited from a prospective geriatric outpatient cohort at a medical center. Frailty status was evaluated at baseline and during a 1-year follow-up using a modified version of Fried's criteria. Additionally, demographic characteristics, health status, and geriatric assessments were collected.

**Results:** The mean age of the 140 participants was 79.5 ± 7.3 years. During the one-year follow-up period, 47.1% of participants experienced a worsening of frailty status, while 12.9% showed improvement, and 40% remained at the same frailty status. After adjusting for confounding variables, individuals who experienced more than one fall in the past year tended to exhibit deteriorating frailty status (□□= -0.226, $p$ = 0.005). The absence of teeth, which affects eating habits, also correlated with deteriorating frailty status (□ = -0.144, $p$ = 0.061). Conversely, individuals with higher scores on the Mini-Mental State Examination tended to show improvements in frailty status □ = 0.198, p = 0.027).

**Conclusion:** Our findings could contribute to the development of interventions and strategies designed to prevent and manage frailty in older adults.

Abstract citation ID: igae098.2772

### STAYING WITH A POSITIVE MIND: STEPPING AWAY FROM ACCELERATED AGING

Yuchen Liu[1], Wenjie Cai[2], Eve Wittenberg[1], Dae Hyun Kim[3], David Bloom[1], Laura Kubzansky[4], and Benjamin Seligman[5], *1. Harvard T.H. Chan School of Public Health, Boston, Massachusetts, United States*, *2. Johns Hopkins University, Baltimore, Maryland, United States*, *3. Hebrew SeniorLife, Boston, Massachusetts, United States*, *4. Harvard T. H. Chan School of Public Health, Boston, Massachusetts, United States*, *5. VA Greater Los Angeles Healthcare System, Los Angeles, California, United States*

Subjective wellbeing (SWB) is a crucial measure of life quality in older adults. Understanding its relationship with frailty may inform strategies to promote healthy aging. We analyzed data for older adults aged ≥ 60 years old from Waves 3 and 4 of the China Health and Retirement Longitudinal Study. SWB was measured based on participants' self-reported overall satisfaction with life. A frailty index was developed using the deficit accumulation approach. We conducted a cross-sectional Poisson regression to investigate the relationship between SWB and counts of frailty deficits. Additionally, we conducted a longitudinal analysis to determine the three-year relative risk of clinically significant frailty progression or mortality for different levels of SWB. The analyses were adjusted for individual weights, including adjustments for household nonresponse. The cross-sectional analysis included 9,702 individuals. After adjusting for covariates, lower baseline life satisfaction was associated with higher counts of frailty deficits (mean deficit counts ratio [95% confidence interval]: 1.66 [1.54, 1.78] for "not satisfied" and 1.06 [1.02, 1.10] for "somewhat satisfied" relative to the reference "very satisfied"). The longitudinal analysis included 8,599 individuals. Participants who were "not satisfied" with life at baseline were at a greater risk of frailty progression compared with those who were "very satisfied" (risk ratio: 1.16 [1.00, 1.35]). Our study finds that a lower level of SWB is associated with more severe frailty. It is also associated with frailty progression or death. These results emphasize that both psychological wellbeing and physical health are essential components of healthy aging.

Abstract citation ID: igae098.2773

### THE EFFECT OF FRAILTY ON COGNITION IN OLDER ADULTS: THE MEDIATING ROLE OF DEPRESSIVE SYMPTOMS

Yiyang Yuan, and Kate Lapane, *University of Massachusetts Chan Medical School, Worcester, Massachusetts, United States*

This study aims to examine the effect of physical frailty on cognitive function with depressive symptoms as a mediator in older adults in the United States. The Health and Retirement Study (HRS 2016; baseline) was linked to its sub-study Harmonized Cognitive Assessment Protocol (HCAP; follow-up), which was conducted in a randomly selected half of the 2016 HRS participants, to identify 1,503 adults aged 65 and above. The exposure physical frailty and mediator depressive symptoms were measured in HRS at baseline respectively by the Fried frailty phenotype on low weight, physical inactivity, exhaustion, weakness, slowness (total score: 0-5; dichotomized as not frail (score: 0) vs. frail (score: 1-5)) and the Center for Epidemiological Studies Depression (CES-D) Scale (total score: 0-8). The outcome overall cognitive function was measured in HCAP at follow-up by the Mini-Mental State Examination (total score: 0-30). Causal mediation analysis was used to assess the direct effect of physical frailty and its indirect effect through depressive symptoms on cognitive function, considering the interaction between the exposure and the mediator. Having physical frailty would decrease cognitive function by a total effect of -0.26 (95% CI: -0.59 to 0.07), with total natural direct effect of -0.14 (95% CI: -0.48 to 0.20) and pure natural indirect effect through depressive symptoms of -0.12 (95% CI: -0.22 to -0.01). The lack of clinically meaningful effects is likely due to the short length of follow-up. This study employs the natural temporal order in HRS and HCAP, but analysis with longer follow-up is warranted.

Abstract citation ID: igae098.2774

### TOWARD DEVELOPING PATIENT-ORIENTED WEBSITES FOR EARLY DETECTION AND MANAGEMENT OF FRAILTY

Seohyeon Chung[1], and Xiaowei Song[2], *1. Fraser Health Authority, Edmonton, Alberta, Canada*, *2. Fraser Health Authority, Surrey, British Columbia, Canada*

Understanding frailty status of older adults before a doctor's visit can result in more effective care planning, where web technology can play role. We conducted a study engaging community-dwelling older adults and caregivers to better understand their perspectives about developing patient-oriented frailty websites. Our objectives are to describe the project activities and share the insights learned from potential end-users of the website. We first organized an online educational symposium consisting of six invited talks on patient-oriented frailty management given by experts of frailty and older adult care. Audience of the symposium was recruited through newsletters and academic, community, and patient association platforms. Clinicians, researchers, caregivers, and community-dwelling older adults across Canada participated (n=300). Members from the symposium (n=14) participated in two virtual discussion sessions with open-ended questions. Discussions highlighted aspects, including the function, usage, and feasibility of a frailty website; security and privacy of online health data; patient-physician interactions; and existing technologies and resources supporting patient-oriented frailty management for successful aging in place. Our findings revealed the needs, barriers, and possible solutions with developing patient-oriented frailty websites. The effort towards building frailty websites that patients can use will benefit future development and implementation to identify people at risk of frailty and facilitate real-time monitoring for personalized and preventative care.

Abstract citation ID: igae098.2775
### TRAJECTORIES OF QUALITY OF LIFE AMONG FRAIL OLDER ADULTS IN KOREA: THE ROLE OF SOCIAL NETWORK
Yeonsoo Shin, and Giyeon Kim, *Chung-Ang University, Seoul, Republic of Korea*

The purpose of this study was to examine the trajectories of quality of life among frail older adults in Korea, with a specific focus on the role of social networks on quality of life. Using data from waves 1 (2006) to 6 (2016) of the Korean Longitudinal Study of Ageing (KLoSA), a total of 3,177 older adults aged 65 and older at baseline was included in analyses. This study analyzed the trajectories of quality of life in all subjects, as well as frail and non-frail groups. Furthermore, the study investigated the effect of social networks on both the intercept and slope of quality of life. Results from multiple-group latent growth curve modeling showed that the intercept of quality of life for the frail group was lower than that of the non-frail group. While quality of life showed a declining trajectory for both the overall sample and the non-frail subgroup, the frail subgroup exhibited an increasing trajectory. Moreover, participation in social activities positively affected the quality of life intercept for both frail and non-frail older adults. Interestingly, quality of life among frail older adults living alone was found to be increasing. These findings provide valuable insights into the nuanced dynamics of quality of life among frail older adults in Korea. The study underscores the importance of promoting participation in social activities among this population and suggests further research focusing on frail older adults living alone.

### SESSION 7280 (POSTER)

### GLOBAL AGING AND HEALTH (POSTERS)

Abstract citation ID: igae098.2776
### AN ANCHOR FOR BETTER PHYSICAL HEALTH AMONG OLDER ADULTS: POSITIVE FAMILY WELL-BEING
Yuchen Liu[1], Dae Hyun Kim[2], and Eve Wittenberg[1], *1. Harvard T.H. Chan School of Public Health, Boston, Massachusetts, United States*, *2. Hebrew SeniorLife, Boston, Massachusetts, United States*

The decline in physical health among older adults corresponds to an increased need for social support and caregiving. In the Chinese context, where formal long-term care options are limited, families predominantly shoulder caregiving responsibilities. We hypothesize that the quality of these familial interactions could significantly influence older adults' objective health. We used data from the China Health and Retirement Longitudinal Study, assessing family well-being through satisfaction with spouse and children, and measuring objective health via frailty index based on the accumulation of deficits. Our findings indicate that satisfaction with children or spouses is negatively associated with the accumulation of additional deficits, demonstrating a mean ratio (MR) of 0.80 (95% CI: 0.76-0.83) for children and an MR of 0.77 (95% CI: 0.74-0.80) for spouses. After stratifying the analysis by marital status, the association between satisfaction with children and frailty status revealed a more substantial effect size in single or unpartnered individuals (relative risk (RR): 0.74 CI: 0.68-0.81) compared to those who were married or partnered (RR: 0.81, 95% CI: 0.77-0.86). We speculate that those without a spouse or partner may rely more heavily on their children for physical support, thus developing a stronger bond and accounting for the observed larger effect size. In conclusion, positive family well-being correlates with better physical health. Implementing policies that offer financial assistance or incentives for family involvement in elderly care could mitigate financial strains and enhance health outcomes for older adults.

Abstract citation ID: igae098.2777
### EPIDEMIOLOGICAL FEATURES OF URINATION AND DEFECATION DYSFUNCTION: INSIGHTS FROM THE CHARLS STUDY (2011–2020)
Haoran Zhu[1], Jing Zhang[1], Guangtian Liu[2], Liwei Jing[1], Rui Wang[1], and Yan Ye[1], *1. Capital Medical University, Beijing, Beijing, China (People's Republic)*, *2. North China University of Science and Technology, Tangshan, Hebei, China (People's Republic)*

With the population aging, China has over 42 million older adults with disabilities, experiencing Urination and Defecation Dysfunction (UDD). The study aims to understand the epidemiological characteristics of UDD from 2011 to 2020, utilizing the China Health and Retirement Longitudinal Study (CHARLS) database. CHARLS is a longitudinal study, involved adults aged 45 and older, with having a cross-sectional survey at each two years. All participants in the CHARLS database who had UDD were included in the study. Chi-square tests and a logistic regression analysis were conducted. A total of 17,156, 17,897, 17,715, 19,097 and 19,129 eligible participants were recruited in each wave of survey, respectively, while associated prevalence were 4.4%, 4.0%, 4.7%, 4.7%, and 5.0%. The prevalence remained stable without significant changes across the ten years. In the univariable analysis, a higher prevalence was observed among older adults over 76 years ($P< 0.05$), compared to younger adults. Urban residents had a higher prevalence in Northern, Central and Southern China ($P< 0.05$) than other regions. Furthermore, through the multivariable analysis with the logistic model, higher UDD prevalence was identified among urban older female in Northern, Central, Southern, and Southwest China. Therefore, health educational campaigns and medical intervention programs should be provided for these high-risk population in China. The findings emphasize the potential burden of UDD in older populations, and underscore the importance of integrating urinary and fecal incontinence into geriatric health education.

Abstract citation ID: igae098.2778
### INTEGRATED DEMENTIA CARE CENTER IN TAIWAN: A SIX-YEAR JOURNEY TO DEVELOP A HOLISTIC CARE MODEL
Tsuann Kuo[1], Yi-Jia Huang[1], Yu-Chun Chen[2], Yu-Lan Lu[2], and Yi-Hsiu Lin[3], *1. Chung Shan Medical University, Taichung, Taichung, Taiwan (Republic of China)*, *2. Chung Shan Medical University Hospital, Taichung, Taichung, Taiwan (Republic of China)*, *3. Hsiang Shang Culture and*

*Education Foundation, Taichung City, Taichung, Taiwan (Republic of China)*

Taiwan launched its 8-year Dementia Action Plan 2.0 in 2017 and has developed over 114 Dementia Integrated Care Centers (DICC). Using the Chronic Disease Management Model, a multi-disciplinary and holistic care center was established by Chung Shan Medical University Hospital to care for more than 400 people with dementia and their family members each year. The DICC was consisted of medical, care, and support divisions so patients can be cared for by medical specialties, nurses, nutritionists, pharmacists, physical therapies, occupational therapies, social workers, psychologists and legal representatives. This study also analyzed over 2000 patients' records retrospectively and found out that with the help of care managers at DICC, people with dementia were less likely to be hospitalized, entering emergency room, reduced caregiver burdens, and maintained cognitive level longer. The center also outreached to the community and established a network where people with dementia can attend physical, arts, leisure, and learning programs. The caregivers who were interviewed and attended these programs indicated that they were able to accept their family's conditions faster and more at ease with their family dynamics. The DICC also served as an important site for University students to be trained to become future dementia specialists. In conclusion, a center with multi-disciplinary professionals took years to integrate and build collaborations so people with dementia and their families could get the best coordinated care. This paper will reveal how the Dementia Integrated Care Center will be implemented in Taiwan.

Abstract citation ID: igae098.2779

### LIFE SATISFACTION OF OLDER ADULTS: IMPACT OF LEISURE PARTICIPATION AND REGIONAL CHARACTERISTICS

Rokbit Lee[1], Jinmoo Heo[1], Inheok Lee[2], May Kim[3], and Sua Im[1], *1. Yonsei University, Seoul, Republic of Korea*, *2. University of Georgia, Athens, Georgia, United States*, *3. Korea University, Seoul, Republic of Korea*

As Korea is transitioning into an aged-society, improving well-being of older adults has become important, and literature has demonstrated various individual and societal factors that impact their well-being. The purpose of this study is to explore the role of individual and regional factors that are associated with older adults'life satisfaction. Specifically, we used leisure participation as individual factor and regional characteristics include perceived stress and volunteer participation rate. Data were drawn from the 2020 National Survey of Korean Elderly and Statistics Korea. As the individual data were nested in regions (cities and provinces), we used hierarchical linear modeling to analyze the data. Findings show that volunteering, traveling, and participating in club activities were positively related to life satisfaction of older adults. The relationship observed between older adults' participation in travel, club activities, volunteering, and life satisfaction suggests that engaging in productive leisure activities is a crucial aspect of daily life of older adults. At regional level, perceived stress level was negatively associated with life satisfaction whereas volunteer participation rate was positively related to life satisfaction. The negative correlation between older adults' perceived local stress and life satisfaction, and the positive correlation between local social volunteer participation rate and life satisfaction suggest that stress perceived by individuals due to their residential area or surrounding environment has a negative impact on their lives. A statistically significant cross-level interaction was observed indicating that the impact of volunteer participation rate on life satisfaction was stronger in regions with higher levels of perceived stress rate.

Abstract citation ID: igae098.2780

### REGIONAL POLICY SCAN: POLICIES TO ADDRESS AGING IN SUB-SAHARAN AFRICA

Edward Osae-Oppong, *California State University San Marcos, San Marcos, California, United States*

This paper presenting a regional policy scan focused on aging in sub-Saharan Africa. The study aims to provide an overview of policy responses to aging in the region, identifying key challenges, opportunities, and recommendations for addressing the needs of the aging population. The methodology involves conducting a critical essay reviewing articles on public policy related to mental health and well-being in older populations across databases such as PubMed, Google Scholar, and Science Direct. This review analyzes policies addressing healthcare needs and identifies demographic, economic, and social trends to understand the aging population comprehensively. Findings from the policy scan reveal a growing aging population in sub-Saharan Africa, with existing policies and strategies in place to address their needs. However, challenges persist in implementing effective policies, social protection systems, and engaging the private sector. Key recommendations include advocating for a comprehensive and coordinated approach to aging, improving access to healthcare and social services, incentivizing employers to hire older workers, and increasing public awareness of aging issues. The study concludes that sub-Saharan Africa faces significant challenges and opportunities concerning its aging population. It underscores the importance of developing and implementing effective policies and strategies to address the healthcare needs of older populations, strengthen social protection systems, and engage the private sector. By investing in the health and well-being of older adults, sub-Saharan Africa can achieve sustainable economic development and enhance the quality of life for its aging population.

### SESSION 7285 (POSTER)

### HIV/AIDS

Abstract citation ID: igae098.2781

### COGNITIVE INTRAINDIVIDUAL VARIABILITY IN ADULTS WITH AND WITHOUT HIV-ASSOCIATED NEUROCOGNITIVE DISORDER

David Vance[1], Jason Blake[1], Wei Li[1], Pariya Wheeler[1], Christopher Collette[2], Alexandra Jacob[1], Vidyulata Kamath[3], and Victor Del Bene[1], *1. University of Alabama at Birmingham, Birmingham, Alabama, United States*, *2. Emory University, Atlanta, Georgia, United States*, *3. Johns Hopkins University School of Medicine, Baltimore, Maryland, United States*

Cognitive intra-individual variability (IIV) is a sensitive marker of reduced top-down cognitive control related to neuropathology and increases with age. Prior work has shown increased cognitive IIV in people living with HIV (PLWH). For those with HIV-Associated Neurocognitive Disorder (HAND), it is likely that cognitive IIV is greater compared to PLWH without HAND (Non-HAND). In a sample of older PLWH from the Deep South of the United States, we examined differences in cognitive IIV in those with and without HAND, and the relationship of IIV with clinical and demographic factors. HAND (n=108) and Non-HAND (n=23) participants completed a neuropsychological assessment as part of a larger cognitive training protocol. Our primary outcome measure was the coefficient of variation (CoV), a widely used measure of IIV that corrects for mean-performance. Group differences were examined with analysis of covariance, followed by correlations of CoV with clinical and demographic variables. Cognitive IIV was greater in the HAND than the Non-HAND Group, consistent with our hypothesis. When exploring demographic variables included as covariates, we found race was statistically significant. Specifically, Black individuals with HAND had greater cognitive IIV than White individuals with HAND. However, these observed race differences in cognitive IIV were no longer significant after including reading ability and years of education in the model. Relative to PLWH without HAND, those with a HAND diagnosis have greater cognitive IIV attributable to more advanced HIV and cerebral dysfunction. Race-related differences in cognitive variability are best explained by historical disparities in education and reading levels.

Abstract citation ID: igae098.2782

### FREE TESTOSTERONE, SEX HORMONE BINDING GLOBULIN, AND INCIDENT FRAILTY IN MEN WITH HIV

Jenny Pena Dias[1], Kathryn C Fitzgerald[1], Michelle Shardell[2], Steven Wolinsky[3], Jordan Lake[4], Susan L Koletar[5], Shezhad Basaria[6], and Todd Brown[7], *1. Johns Hopkins University, Baltimore, Maryland, United States, 2. University of Maryland School of Medicine, Baltimore, Maryland, United States, 3. Northwestern Medicine, Chicago, Illinois, United States, 4. McGovern Medical School, Houston, Texas, United States, 5. The Ohio State University, Columbus, Ohio, United States, 6. Brigham and Women's Hospital, Boston, Massachusetts, United States, 7. Johns Hopkins, Baltimore, Maryland, United States*

**Background:** Older men with HIV (MWH) have a higher prevalence of frailty than matched men without HIV, but the underlying mechanisms are unclear. Testosterone (T) contributes to higher muscle (mass, strength), and it binds to sex hormone-binding globulin (SHBG), which regulates testosterone bioavailability to the tissues. Aging MWH experience increase in circulating SHBG and decline in circulating free T concentrations. We hypothesized that these hormonal changes may contribute to the development of frailty in MWH.

**Methods:** Free T and SHBG were measured in 306 non-frail MWH (129 robust and 177 prefrail) who had at least one frailty assessment at a semi-annual follow-up visit in the Multicenter AIDS Cohort Study. Marginal structural models were used and adjusted for covariates: age, race, education, comorbidity burden, BMI, smoking status, alcohol use, HIV parameters and loss to follow-up.

**Results:** Mean age at baseline was 55±5 years, (median 58 years, Q1=55, Q3=63); follow up frailty assessments visits was 8.5(SD:2.5) years. MWH with low free T (< 50 pg/ml) were more likely to become frail compared to those with free T ≥50 pg/ml (HR,1.58 (95%CI:1.12, 2.24); p-value=0.03). Low free T was also significantly associated with transition from prefrailty to frailty, [< 50 pg/ml: 47%; ≥50 pg/ml:34%], a difference of 12.4% (95% CI:2.9,24.3%). In contrast, SHBG concentrations were not associated incident frailty or transition to frailty.

**Conclusion:** Low free T, but not SHBG, predicted incident frailty in men with HIV at high risk of developing frailty, in particular those in the prefrail state.

Abstract citation ID: igae098.2783

### INTRAINDIVIDUAL VARIABILITY MODIFIES COGNITIVE TRAINING IN COGNITIVELY VULNERABLE OLDER ADULTS WITH HIV

David Vance[1], Andres Azuero[1], Julie Schexnayder[1], Frank Puga[1], Jun Byun[1], Chunhong Xiao[2], Dara James[3], and Pariya Wheeler[1], *1. University of Alabama at Birmingham, Birmingham, Alabama, United States, 2. University of Alabama at Birmingham/School of Nursing, Birmingham, Alabama, United States, 3. Arizona State University, Tempe, Arizona, United States*

Cognitive Intra-Individual Variability (IIV), the fluctuation in cognitive performance within an individual, often stems from compromised cognitive control due to brain pathology. In cognitive aging studies, cognitive IIV has been shown to predict cognitive decline 3 years later, dementia, and mortality. Those with HIV-Associated Neurocognitive Disorder (HAND) frequently exhibit heightened cognitive IIV; this is concerning as the HIV population has become an aging epidemic (i.e., by 2030 70% of people with HIV will be 50 and over). This study investigated whether computerized Speed of Processing (SOP) training could mitigate cognitive IIV and whether baseline cognitive IIV influenced the impact of SOP training on individuals with HAND or borderline HAND. In a 3-group experimental design, 216 middle-aged and older individuals living with HIV (PLWH) and HAND were randomly assigned to: 1) 10 hours of SOP training (n=70); 2) 20 hours of SOP training (n=73); or 3) 10 hours of Internet Navigation Control Training (n=73). Participants underwent a seven-domain cognitive battery at baseline, posttest after training, and at 1 and 2-year follow-ups, with the coefficient of variation (CoV) representing cognitive IIV dispersion. Linear mixed-effect models did not reveal an obvious pattern of SOP training effects on cognitive IIV. Albeit, a three-way interaction tests between group, time, and baseline IIV CoV [F(6, 453.96)=1.74, P= 0.11], and group, time, and baseline IIV CoV median split indicator [F(6, 467.84)=2.024, P= 0.061] posited potential moderation effects. While SOP training did not directly enhance cognitive IIV, its effects may be influenced by individual baseline cognitive IIV.

Abstract citation ID: igae098.2784

**PLASMA PROTEIN BIOMARKERS AND THEIR CORRELATION TO COGNITIVE PERFORMANCE IN WOMEN LIVING WITH HIV**

Wei Li[1], Ge Wang[2], David Vance[1], and Daniel Li[3], *1. University of Alabama at Birmingham, Birmingham, Alabama, United States*, *2. Tongji Medical College of Huazhong University of Science and Technology, Wuhan, Hubei, China (People's Republic)*, *3. UCLA, Los Angeles, California, United States*

**Background:** Individual plasma protein biomarkers have been shown to correlate to cognitive performance in people with HIV (PWH). This study aimed to investigate the association between plasma proteomic signatures and cognition in virologically well-controlled women with HIV (WWH).
**Methods:** Seventy-seven WWH from three Women's Interagency HIV Study (WIHS) sites completed neuropsychological (NP) testing and a blood draw. Targeted protein biomarkers were analyzed using a multiplexing method. Random forest analysis was used to identify the top 10 positively or negatively biomarkers associated with cognitive function. Next, ingenuity pathway analysis (IPA) was used to facilitate data interpretation.
**Results:** Tumor necrosis factor receptor 1 (TNF RI), TNF RII, interleukin 1 receptor 1 (IL-1RI), and IL-6R are negatively associated with attention/working memory; IL-7 and CD40 are positively associated with executive function; IL-1RI, hepatocyte growth factor (HGF), transforming growth factor beta-1 (TGFβ1), TGFβ2, and sonic hedgehog (SHH) are negatively associated but IL-7 is positively associated with fluency; IL-9, IL-15, and IL-21R are positively associated with motor function; brain-derived neurotrophic factor (BDNF), HGF, SHH, and vascular endothelial growth factor (VEGF) are negatively associated with processing speed. HGF, VEGF, and insulin are negatively associated with the global NP function. Based on the IPA, one to three signaling networks were proposed for associating plasma biomarkers with each domain-specific or global NP function.
**Discussion:** It is likely that a panel of plasma protein biomarkers can be used to predict domain-specific or global NP function. Multiple signaling networks might explain cognitive functioning in WWH.

Abstract citation ID: igae098.2785

**THE AHR AS A POTENTIAL MEDIATOR OF MUSCULOSKELETAL FRAILTY WITH ANTI-RETROVIRAL THERAPY**

Alok Tripathi, Shabiha Sultana, Huidong Shi, Rodger MacArthur, Eric Belin de Chantemele, Mark Hamrick, and Meghan McGee Lawrence, *Augusta University, Augusta, Georgia, United States*

Musculoskeletal frailty, often associated with aging, is frequently observed in patients living with HIV on long term anti-retroviral therapy (ART) and linked to poor health outcomes from accelerated disease progression to increased mortality. Mechanisms underlying this frailty phenotype are poorly understood but may involve disease progression as well as ARTs themselves. One possible mediator is activation of the aryl hydrocarbon receptor (AhR). NF-κB, activated by HIV infection, is an upstream regulator of AhR which induces the enzyme IDO-1 to produce the endogenous AhR ligand kynurenine. However, connections between ART, HIV, and AhR in musculoskeletal frailty had not been previously explored. In preliminary studies we tested the effects of tenofovir disoproxil fumarate (TDF) and emtricitabine (FTC) ARTs in a mouse myoblast cell line (C2C12). FTC, but not TDF, increased transcriptional activity of AhR, expression of AhR and its target gene CYP1A1, and increased cellular indicators of senescence. FTC also impaired myoblast differentiation as confirmed morphologically by microscopy. Next, we tested the effects of FTC and another ART agent, tenofovir alafenamide (TAF, a prodrug of TDF) in primary human mesenchymal stem cells and myoblasts, showing increased expression of AhR, AhR target genes, and increased senescence in both cell populations. Moreover, FTC and TAF decreased expression of myoblast differentiation markers (MYOG and MYOD). While further research is needed to elucidate the underlying mechanisms of interactions between ART, HIV, and AhR signaling, these studies raise the possibility that targeting AhR may serve as a novel therapeutic approach for improving musculoskeletal function in this setting.

Abstract citation ID: igae098.2786

**THE ICHANGE PROJECT: AN HRSA SPNS INITIATIVE TO ADVANCE HIV AND AGING CARE**

Erin Burk-Leaver[1], Haley Sanner[1], Janette Romero Saenz[1], Alison Abraham[2], and Kristine M. Erlandson[3], *1. Colorado Health Network, Inc., Denver, Colorado, United States*, *2. University of Colorado Anschutz Medical Campus, Aurora, Colorado, United States*, *3. University of Colorado - Anschutz Medical Center, University of Colorado Anschutz Medical Campus, Colorado, United States*

With the advent of antiretroviral therapy (ART), improvements in life expectancy of people living with HIV (PLWH) are now nearing that of populations living without HIV. As a result, over 50% of PLWH within the United States – over 55% in Colorado – are now aged 50 years and older. While this shift highlights how ART has drastically improved the life expectancy of PLWH, aging with HIV is associated with unique issues and challenges that impact quality of life. In response to these challenges, the Health Resources and Services Administration (HRSA) Ryan White HIV/AIDS Program (RWHAP) Part F: Special Projects of National Significance (SPNS) funded the design, implementation, and evaluation of emerging strategies to improve the well-being of people with HIV ages 50 and older served by RWHAP. Colorado Health Network (CHN), serving as one of ten national demonstration sites under the HRSA SPNS HIV and Aging Initiative, developed the iCHANGE (Integrative Care for Healthy Aging and Navigation of Geriatric Effects) Project, an integrated healthcare model designed to comprehensively assess comorbidities and geriatric conditions among older PLWH, with the overarching goal of improving health outcomes. Drawing on concepts such as the Public Health Prevention model, Geriatric 5M's framework, and implementation science methodology, CHN's iCHANGE strategy enhances the ongoing efforts to develop holistic healthcare programs to support and navigate the unique challenges faced by older PLWH. This presentation aims to provide insight and a comprehensive overview into the evolving landscape of supportive healthcare delivery for people living and aging with HIV.

### SESSION 7290 (POSTER)

### HOUSING

Abstract citation ID: igae098.2787

### ASSESSMENT OF RISK OF FALL IN THE HOME ENVIRONMENT OF KOREAN OLDER ADULTS: REVISION AND VALIDATION OF THE K-HOME FAST

Min Kyung Park[1], Gwang Suk Kim[2], Layoung Kim[2], Jae Jun Lee[2], Namhee Kim[2], and Ji Yeon Lee[3], *1. Mo-Im Kim Nursing Research Institute, Yonsei University College of Nursing, Seoul, Republic of Korea*, 2. *Yonsei University, Seoul, Republic of Korea, 3. Inha University, Incheon, Republic of Korea*

Most older adults experience a fall inside their house. Because the home environment varies based on culture and time, the tools to assess fall risk must be regularly modified and validated. Therefore, this study aimed to develop the Revised K-HOME FAST (Korean version of the Home Falls and Accidents Screening Tool) and evaluate its validity and reliability. The Revised K-HOME FAST was developed in these phases: 1) development of the Revised K-HOME FAST, 2) feasibility test, 3) verification of content validity, and 4) data collection and verification of reliability. Internal consistency was confirmed by conducting home-visit assessments with 211 community-dwelling older adults who had experienced at least one fall in the local community. To verify inter-rater reliability, six investigators were paired and independently assessed the homes of 20 older adults. The final Revised K-HOME FAST consisted of 25 items categorized into seven home areas: bathroom/toilet, bedroom, living room, kitchen, stairs, entrance, and entire house. The item-content validity index of the preliminary version of Revised K-HOME FAST ranged from 0.8 to 1.0, except for one item. The internal consistency (Kuder–Richardson Formula 20) of the Revised K-HOME FAST was.65. The agreement percentage for each item ranged from 92% to 100%, and the intraclass correlation coefficient ranged from 0.79 to 1.00, indicating high inter-rater reliability. The Revised K-HOME FAST has satisfactory validity and reliability for assessing the risk of falls in the home environment of community-dwelling older adults in Korea.

Abstract citation ID: igae098.2788

### CHILDHOOD HOUSING AND OLD AGE HEALTH AMONG CHINESE: THE MEDIATION ROLE OF ADULTHOOD LIFE HISTORY

Dexia Kong[1], Peiyi Lu[2], and Vivian Weiqun Lou[2], *1. The Chinese University of Hong Kong, Hong Kong, China (People's Republic)*, 2. *The University of Hong Kong, Hong Kong, China (People's Republic)*

Housing insecurity is linked to worse health. However, very few studies examined this relationship from a lifecourse perspective and its underlying mechanisms. This study examined the association of childhood housing with old age health among Chinese and whether adulthood socioeconomic and medical history mediated that relationship. Respondents were middle-aged and older adults (aged 45+) from the China Health and Retirement Longitudinal Study (N=12,842). They were asked about their childhood housing conditions (e.g., if their houses had clean water, water closet, and electricity). Adulthood socioeconomic and medical history and middle- and old-age health were measured. Causal mediation analysis examined the pathways via which childhood housing affects health in later life. Results show about 28%-37% of respondents had access to electricity or energy for cooking/heating during their childhood. However, very few had access to clean water (6%) or water toilet (2%). Childhood better housing was directly associated with fewer depressive symptoms and better cognition in middle- and older age, and indirectly through increasing the likelihood of obtaining more education during adulthood. However, the proportion-mediated estimate had very wide confidence intervals to be certain. Other adulthood life history pathways (i.e., marital status, work and medical history) were not statistically significant. Our findings suggested targeting services among those who grew up in poor housing conditions may be crucial to promoting healthy aging.

Abstract citation ID: igae098.2789

### END-OF-LIFE RESOURCE AND PLACE OF DEATH BY COMMUNITY-BASED HOUSING TYPE

Sojung Park[1], BoRin Kim[2], Esther Shin[3], Jihye Baek[1], and Byeongju Ryu[1], *1. Washington University in St. Louis, St. Louis, Missouri, United States*, 2. *University of New Hampshire, Durham, New Hampshire, United States, 3. Illinois State University, Normal, Illinois, United States*

Understanding the factors that influence where people die is crucial, as most individuals spend their last year of life at home. This study investigates the influence of end-of-life resources on the place of death in older adults, based on where they lived. Data came from twelve waves of the National Health and Aging Trend Study (2010~2022). We analyzed the unmet needs of end-of-life resources such as end-of-life care services, functional help, and social/family support, and the type of housing (senior housing vs. traditional home) based on income status. The sample included community dwellers who died during the study period and had information about end-of-life care-related supports (N= 2701). Multinominal regression analyses were used to investigate the influence of end-of-life resources and housing type on the place of death (home, hospital, nursing home, hospice facility, or other). We found individuals living in traditional homes, both low-income and non-low-income, tend to have low levels of end-of-life care services but receive high levels of social/family support and they are more likely to die at home. In contrast, individuals living in senior housing, particularly those in low-income models, tend to have high levels of unmet needs for functional help and social/family support and are more likely to die in a nursing home or other facilities. We discussed the unequal distribution of end-of-life resources based on residential type, and the implications of this on the apparent mismatch between unmet resources and the place of death, focusing on the potential role of senior housing.

Abstract citation ID: igae098.2790

### FEASIBILITY OF A SUPPORTIVE HOUSING PROGRAM FOR OLDER VETERANS WHO PREVIOUSLY EXPERIENCED HOMELESSNESS

Ashley Ritter[1], Luke Hoban[2], Rebecca Brown[3], Meagan Cusack[3], and Helene Moriarty[4], *1. Hunter-Bellevue School of Nursing, Yardley, Pennsylvania, United States*, *2. NewCourtland, Philadelphia, Pennsylvania, United States*, *3. University of Pennsylvania, Philadelphia, Pennsylvania, United States*, *4. Villanova University, Villanova, Pennsylvania, United States*

The number of older adults experiencing homelessness is on the rise nationally, with earlier incidence of geriatric syndromes that jeopardize community living occurring at younger ages in this population. NewCourtland, a provider of housing and health services in Philadelphia, began a program serving older Veterans who experience homelessness with complex health and social needs in 2019. This study examined the feasibility and acceptability of program implementation among program participants and staff. We conducted focus groups with program staff (n=10) and residents (n=9) using a semi-structured interview guide. Transcripts were analyzed using inductive codes derived from the CFIR framework. Key themes regarding program feasibility and acceptability include the attainment of new space and autonomy coupled with the need for goal setting to achieve stability in a community setting. Residents and staff both reported that moving into stable housing was a critical step towards community re-integration for participants. This allowed participants to develop regular habits and boundaries in their personal lives. The program's focus on goal setting motivated participants to work with the staff members to meet their needs. However, this was only possible if trust had been established on both sides. Clear rules and consistent interaction with staff were essential, both to achieve participant buy-in and reduce staff burnout over time. Findings provide practical insights into how to improve supportive housing programs and enhance community reintegration for older adults with experiences of homelessness and complex health and social needs.

### SESSION 7295 (POSTER)

### LONG-TERM CARE I (POSTERS)

Abstract citation ID: igae098.2791

### A NEW DATA SET TO EXAMINE HOME HEALTH AGENCIES

Lacey Loomer[1], Sweta Patel[2], Emmanuelle Belanger[2], and Pedro Gozalo[2], *1. University of Minnesota Duluth, Duluth, Minnesota, United States*, *2. Brown University, Providence, Rhode Island, United States*

Using Outcome and Assessment Information Set (OASIS) data from 2015 to 2019, we developed a dataset representing home health agency Medicare patients that will be made publicly available for researchers and policymakers to compare over time and geographically. We created unique home health stays based on start of care and discharge assessments from OASIS and aggregated the data to the calendar year, agency, county, state, insurance (fee-for-service vs. Medicare Advantage), and by admission (institutional vs. community). We identified demographics for home health stays using the Medicare Beneficiary Summary File. We characterize the home health stays using OASIS variables including length of stay, activities of daily living, instrumental activity of daily living, cognitive function, common diagnoses, urinary tract infections, incontinence, history of falls, and pressure ulcers. Type of admission was determined based on Medicare claims for hospital, nursing home, or inpatient rehabilitation facility in the fourteen days prior to home health admission. Insurance was determined using the Medicare Beneficiary Summary File identified in each month of the home health stay, if a patient changed insurance through the stay, the stay's attribution was prorated by month to each type of health insurance. There were 6,761,470 home health stays from 5,051,631 Medicare beneficiaries in 2016 compared to 6,703,521 home health stays from 4,969,010 Medicare beneficiaries in 2019. In 2019, 1994 home health agencies served fee-for-service patients only, 94 served Medicare Advantage patients only, and 8,720 served both.

Abstract citation ID: igae098.2792

### A STUDY OF FACTORS RELATED TO RETENTION OF FOREIGN NURSING CARE WORKERS WHO CAME TO JAPAN UNDER THE EPA SCHEME

Noriko Tsukada[1], and Jun Nishimura[2], *1. Nihon University, Tokyo, Japan*, *2. Kanagawa University of Human Services, Yokosuka, Kanagawa, Japan*

The Japanese government broadened the status of residence to encompass foreign nursing care workers, due to the shortage of nursing care workers. The first step, taken in 2018, used the Economic Partnership Agreement (EPA) scheme with Indonesia, followed by the Philippines and Vietnam. This study explored factors related to the retention of EPA nursing care workers, including elements of social security programs in Japan. Structured questionnaires were sent to 722 institutions: for EPA workers, 10 questionnaires were sent to each institution. Both administrators and EPA workers were asked to respond to the survey either by mail or electronically from November 7 to December 8, 2023. A total of 396 responses from EPA workers were analyzed in this study. Despite the low response rate (5.5%), preliminary bivariate analyses revealed that although "amount of requests to the government" made by the respondents was found to be statistically significantly related to retention of EPA workers for all three countries, other factors related to perceptions toward retention varied. For example, as for Indonesian workers, job satisfaction, whether or not there are Indonesian senior certified care workers at the same institutions they work and amount of understanding of social security systems were found to be statistically significant factors, while job satisfaction, levels of understanding Japanese at work, and knowledge about social security agreement were found for Philippine's workers. It is suggested that the government consider these differences among the three countries and develop country-specific actions to promote retention of EPA workers from each country.

Abstract citation ID: igae098.2793

### DESCRIBING HOW ADAPTIVE LEADERSHIP IN LONG-TERM CARE CAN MOTIVATE AND PREPARE CARE WORKERS TO PROMOTE QUALITY

Kirsty Haunch, Reena Devi, and Karen Spilsbury, *University of Leeds, Leeds, England, United Kingdom*

Adaptive leadership is one approach to enhance the experience of people living and working in long-term care (LTC) residential environments. Adaptive leadership is comprised of a set of strategies aimed at enabling care teams to work together, or create organisational capacity to support culture changes, to accomplish person-centred environments. The defining feature of adaptive leadership is the separation of technical solutions (i.e., applying existing knowledge/techniques to problems) and adaptive solutions (i.e., a shift in how people work together not just what they do). Our aim is to describe and illustrate adaptive leadership behaviours in LTC and the potential benefit for the processes and outcomes of care. We conducted a documentary analysis of (publicly available) UK regulatory inspection reports. Twenty reports, judged as providing outstanding quality were purposively sampled to represent variations in ownership, size, and geographical location. Data were initially analysed using content analysis. Heitz's adaptive leadership framework was used to frame the data. Adaptive leadership behaviours helped to engage, empower, and energise the workforce to accomplish meaningful change. These behaviours included accepting everyone's unique perspective on how to solve problems, a willingness to learn lessons, an openness to feedback, embrace diversity, a fair and inclusive workplace, continuous growth, innovation and emotional intelligence. Key structural components enabling adaptive leadership included organisational support, 'in house' operational support, peer support and training. Our work highlights the benefit of recognising adaptive challenges in LTC and empowering teams to apply the most appropriate problem-solving solutions, and in particular applying adaptive solutions when merited.

Abstract citation ID: igae098.2794

### EFFECT OF HOME CARE NURSES' REMOTE CONSULTATION WITH ADVANCED NURSES ON PATIENT SYMPTOMS: A FEASIBILITY STUDY

Maiko Noguchi-Watanabe[1], Reiko Yamahana[2], and Junko Sunada[3], *1. Tokyo Medical and Dental University, Tokyo, Japan, 2. Musashino University, Nishitokyo, Tokyo, Japan, 3. IMSUT Hospital, University of Tokyo, Tokyo, Japan*

Homecare nurses provide care for patients with a wide variety of illnesses, which requires a broad and comprehensive knowledge base. Simultaneously, symptom management requiring more specialized knowledge is required, and remote consultation with advanced nurses is expected. This study aimed to evaluate the effect of remote consultation by homecare nurses with advanced nurses on patient symptoms. Participants were homecare nurses in Japan. A remote symptom management consultation regarding specific cases was sought by ten homecare nurses. Consultations were provided thrice per case using Zoom at approximately one-month intervals by three advanced nurses: two wound, ostomy, and continence (WOC) care nurses and one cancer nurse. The research team selected the advanced nurse according to the case and coordinated the consultation schedule between advanced and homecare nurses. Before each consultation, we obtained responses from homecare nurses via a webform regarding patient who wanted to consult, their symptoms, and changes in symptoms since the previous consultation (worsening/maintenance/improvement). The study was approved by the Ethics Review Committee of the Tokyo Medical and Dental University. The WOC nurses provided consultations on six cases related to pressure ulcers, two, to stomas, and one, to nephrostomy management, while an advanced nurse specializing in cancer nursing provided consultation on one case related to decision-making support. Consultations took place three times per participant. Nine cases (90%) improved their symptoms, and 1 case (10%) remained unchanged before the consultation; It has been shown that homecare nurses who receive consultations from advanced nurses, even remotely, is effective in alleviating patients' symptoms.

Abstract citation ID: igae098.2795

### EXPLORING THE OPTIMIZATION PATH OF LONG-TERM CARE INSURANCE SYSTEM IN RURAL AREAS OF CHINA

Hong Mi, and Wanwan Si, *Zhejiang University, Hangzhou, Zhejiang, China (People's Republic)*

With the deepening of population aging and changes in family structure, the care needs of the disabled and mentally retarded older people have increased, and the problem of rural aging population has become more serious. In the face of the increasingly serious problem of rural aging, the improvement of the long-term care insurance system is the key to solving the problem of older people care in China. This paper focuses on the optimization path of the long-term care insurance system in rural areas of China, and researches from the aspects of the scope of coverage, assessment of incapacitation and payment of benefits, the management of the care model, and the content of the service. By analyzing the cost and expenditure situation of long-term care insurance in rural areas and the sustainability of its pilot program, combined with the data on the actual operation of long-term care insurance, this paper establishes a population-fund prediction model for the long-term care system, discusses the balance of inputs between medical care and daily life care, and provides targeted theoretical references and practical guidance for improving and perfecting the long-term care insurance system in rural areas of China.

Abstract citation ID: igae098.2796

### EXPLORING THE PERSPECTIVES OF NURSING STUDENTS ABOUT WORKING IN THE LONG-TERM CARE SECTOR

Winnie Sun[1], Janet McCabe[2], and David Rudoler[2], *1. Ontario Tech University, Lindsay, Ontario, Canada, 2. Ontario Tech University, Oshawa, Ontario, Canada*

**Background:** The purpose of this study was to understand the perceptions of prospective registered nurses about working in the long-term care sector and identify workplace attributes that attract prospective nurses to the sector. **Methods:** A sequential mixed methods study was conducted with nursing students at Ontario Tech University. Focus groups (n=14) asked students to comment on views about working in the long-term care sector, and job attributes that may attract them to the sector. A cross-sectional survey asked students to respond to elicited choice job scenarios, informed by the thematic analysis of focus group interviews. The survey data (n=139) was analysed using least absolute deviations estimator. Willingness-to-pay (WTP) values of wages gained or forgone were generated for each job attribute.

**Results:** Focus group interviews suggested fair compensation, optimal client-to-staff ratios, unionized work environments, comprehensive benefits packages, and flexible work arrangements were important job attributes. In survey results, 18.0% expressed interest in working in long-term care sector compared to 75.5% in acute care. Regression analysis suggested that higher amounts of paid vacation (WTP: -5.983; 95% CI: -13.749, -0.037) and higher risk of injury (WTP: 0.684; 95% CI: 0.124, 1.208) were associated with work in the long-term care sector.

**Conclusion:** Long-term care can attract nurses by offering flexible options such as part-time positions and paid vacation, and by actions that potentially mitigate the risk of workplace injury, violence, and abuse. Nursing students should be shown the positive aspects of working with older adults and dispel negative perceptions about the long-term care sector.

Abstract citation ID: igae098.2797

### FACTORS ASSOCIATED WITH HAND HYGIENE COMPLIANCE OF NURSING STAFF IN LONG-TERM CARE FACILITIES

EunKyo Kim, Eunhee Cho, Gwang Suk Kim, and Kyung Hee Lee, *Yonsei University, Seoul, Republic of Korea*

This study was performed to identify hand hygiene compliance factors of registered nurses (RN) and nurse aids (NA) in long-term care facilities. This cross-sectional study surveyed 78 RNs and 50 NAs who worked at long-term care facilities. Data were collected from October 1st to December 30th, 2020. The hand hygiene knowledge, perception, and infrastructure were measured by translated versions of the World Health Organization(WHO)'s hand hygiene knowledge, perception, and infrastructure tool(2009). Hand hygiene compliance was calculated as 'number of conducted WHO's hand hygiene at five moments' out of 'number of chances to conduct WHO's hand hygiene at five moments' multiplied by 100, which participants wrote. Data were analyzed using Spearman's rank correlation coefficient and multiple linear regression. The hand hygiene compliance rate was 78.85%. The mean score of hand hygiene knowledge was 13.55±2.21 out of 25 points, the perception mean score was 73.41±11.65 out of 96 points, and the infrastructure mean score was 32.42±5.18 out of 40 points. Hand hygiene compliance positively correlated with the perception (r=0.177, p=0.045) and the infrastructure(r=0.194, p=0.028). Factors influencing hand hygiene compliance were the infrastructure (β=0.223, p=0.042) and working in a specialized unit (β=0.221, p=0.013) for residents who need nursing treatments (e.g., indwelling catheter exchange and wound management) and with mandating minimum registered nurse staffing level. Hand hygiene compliance was affected by infrastructure and working units with different nursing staffing levels. The results suggest expanding the infrastructure and ensuring adequate nursing staffing in long-term care facilities to improve hand hygiene compliance.

Abstract citation ID: igae098.2798

### IDENTIFYING IMPORTANT AND FEASIBLE ITEMS FROM THE PREFERENCES FOR EVERYDAY LIVING INVENTORY-KOREAN (PELI-K)

So Young Shin[1], and Jenny Kwon[2], *1. Inje University, Busan, Republic of Korea*, *2. Miami University, Oxford, Ohio, United States*

**Purpose:** As a follow-up study for the translation and cultural adaptation of the Preferences for Everyday Living Inventory-Korean (PELI-K), this pilot study identified the ranking of the PELI-K items selected as most important/feasible from the original PELI-K 72 items in the Korean nursing home (NH) context.

**Methods:** A purposive snowball sampling strategy were used. The NH stakeholder advisory panel was recruited from five unique sites in one metropolitan city in Korea: two NHs, one general hospital, one teaching hospital, and one university. A total of 34 stakeholder advisory panel, including 22 experts and 12 care consumers, conducted a self-administered, in-person survey asking to score the importance and feasibility of the 72 PELI-K items. Four answer choices were available, ranging from 0 (low) to 3 (high). The sum of the scores, which adds all the scores received from participants, was calculated to rank items. The score ranges from 3 to 102 and the higher the score, the higher the importance/feasibility.

**Results:** The top 12 important and top 11 feasible items are reported. Items related to human basic needs such as sleeping, bathroom needs, safety, and privacy were selected as important priorities. Having regular contact with family and using electronic devices to communicate with others were the most feasible preferences.

**Conclusion:** The results from this study will help NH providers plan and deliver care that is geared toward residents' most salient preferences and play a huge role in creating a short version of the PELI-K.

Abstract citation ID: igae098.2799

### MEDICAID HOME- AND COMMUNITY-BASED SERVICES IN THE WAKE OF THE COVID-19 PANDEMIC

Edward Miller[1], and Lisa Beauregard[2], *1. University of Massachusetts Boston, Boston, Massachusetts, United States*, *2. Mystic Valley Elder Services, Malden, Massachusetts, United States*

Medicaid, the joint federal-state health insurance program for people with low income, provides coverage for home and community-based services (HCBS). States have significant discretion to offer HCBS programs within federal regulations. The federal government matches state Medicaid program spending according to state per capita income, with the matching rate varying from 50% to 77.27%. The need to bolster Medicaid HCBS became evident during the COVID-19 pandemic. This recognition stemmed from the challenges of keeping people safe in nursing homes and the acute workforce shortages in the HCBS sector. This presentation examines two major federal developments and state responses in HCBS options as a result of the pandemic. The first initiative entails a one-year increase of the federal Medicaid matching rate for HCBS included in the American Rescue Plan Act championed by the Biden administration. The second initiative encompasses administrative flexibilities that permitted states to temporarily expand and modify their existing Medicaid HCBS programs. It concludes that the effects of the pandemic flexibilities and enhanced federal funding on most state HCBS programs will be limited without continued investment and leadership on the part of the federal government, a Biden administration priority. States that make the American Rescue Act and COVID-19 flexibilities initiatives permanent are states that have the fiscal resources and political commitment to expanding HCBS benefits that other states lack. States' different approaches to bolstering Medicaid HCBS during the pandemic may contribute to widening disparities in access and quality of HCBS across states and populations who depend on Medicaid HCBS.

Abstract citation ID: igae098.2800

### NURSING FACILITY RESPONSES TO GROWING CONSUMER COMPLAINTS

Robert Applebaum, John Bowblis, Matt Nelson, and Oksana Dikhtyar, *Miami University, Oxford, Ohio, United States*

Nursing home consumers can file a complaint to regulators regarding such areas as quality of care or resident rights. The number of formal complaints filed has increased at dramatically. Between 2016 and 2021, Ohio saw a 40% increase in the number of complaints. A number of factors are associated with complaints including staffing levels and staff and leadership turnover. The increase in complaints is an indicator that residents and families do not feel satisfied with their nursing home experience, and it is also a huge strain on the nursing home inspection process. Last year in Ohio fewer than 40% of required nursing home recertification surveys were completed in the required window. The critical question then becomes, are there things that can be done by facilities to reduce the number of complaints filed with regulators. Using data from the 2023 Biennial Survey of Long-Term Care Facilities completed in Ohio on all licensed nursing homes, this paper examines the strategies used by nursing homes to respond to the growing number of complaints. Approaches examined include such areas as having a dedicated staff member to examine complaints or building a review of complaints into the facility quality improvement process. The survey also presents findings about the interactions with the Department of Health survey teams. Data suggest there is a long lag between a complaint visit and an adjudication of the complaint. The paper concludes with recommendations for facilities and states as they respond to the growing number of complaints experienced across the nation.

Abstract citation ID: igae098.2801

### RELATED FACILITY AND RESIDENT FACTORS ON STANDARDIZED NURSING LANGUAGES FOR NURSING HOME NURSES IN KOREA

Juh Hyun shin[1], Chung Hyuk Park[2], Myungeun Lee[2], and Suhyun Park[3], *1. George Washington University, Ashburn, Virginia, United States*, *2. George Washington University, Washington, District of Columbia, United States*, *3. University of Minnesota, Minneapolis, Minnesota, United States*

Standardized nursing languages play a crucial role in facilitating holistic nursing care, but their full benefits are still being realized. However, the implementation of NNN linkages and their impact on nursing processes and nursing home (NH) residents' outcomes, as well as the identification of facility and resident factors, remain unexplored. The purpose of this study was to examine (a) frequently occurring NNN linkages and (b) related facility and resident factors on the use of NANDA, NIC, and NOC for NH residents. Data were collected from 273 residents in 19 NHs in Korea 19 NHs in Korea, with structured questionnaires administered to NH registered nurses using a newly developed smartphone application. Descriptive statistics, ANOVA, and ANCOVA were used for the data analysis. We identified that factors, such as case mix index (resident acuity), age of resident, year of facility establishment, facility ownership status, and admission period of residents, are related to the use of NANDA, NIC, and NOC. Nine NNN linkages were newly identified in this study, mostly centered on fall prevention. The findings provide various approaches to address the risk of falls in NHs considering environmental, physical, and psychological factors. This study contributes to the foundation of evidence related to NHs by collecting specific data on the nursing process for the physiological, behavioral, safety, family, health system, and community areas applied to the residents of long-term care facilities at the national level. Future research in different settings and tailored population is needed.

Abstract citation ID: igae098.2802

### STAFFING LEVELS IN OHIO'S NURSING HOMES, ASSISTED LIVINGS, AND MEMORY CARE UNITS: SHIFT VARIATION

Matt Nelson, John Bowblis, Heather Menne, and Amy Elliot, *Miami University, Oxford, Ohio, United States*

In a nursing home (NH) or assisted living (AL), residents require assistance with activities of daily living and many of these residents also have cognitive impairments associated with dementia. As a result, many NHs and ALs have created dedicated memory care units (MCUs). This paper contributes to the literature on staffing in NHs and ALs by examining staffing levels for licensed nurses and other direct care staff for the overall facility and the 1 in 4 facilities that have MCUs on different shifts: day, evening, night. Data for this study come from the 2021 Ohio Biennial Survey of Long-Term Care Facilities. Staffing levels by shift were calculated as resident to staff ratios, which is the number of residents compared to number of staff by shift. Findings show that both NHs and ALs have their lowest resident-to-staff ratios, and hence most staff on the day shift (16.0 and 24.2 for licensed nurses and 10.4 and 14.2 for direct care staff, respectively). In both settings, the night shift staffing had the highest staffing ratios. Comparing the overall NH staffing to AL staffing, NHs have more staff per resident. However, on the MCU, NHs and ALs had more similar staffing levels. This suggests that NHs and ALs staff MCUs in a similar manner, but outside of the MCU, nursing homes staff better than ALs. The evidence can be used for decision making about future staffing policies.

Abstract citation ID: igae098.2803

### THE USE OF INVOLUNTARY TREATMENT AMONG NURSING HOME RESIDENTS IN THE NETHERLANDS: A CROSS-SECTIONAL STUDY

Guido Biesmans, Niels Hameleers, Jan Hamers, and Michel Bleijlevens, *Maastricht University, Maastricht, Limburg, Netherlands*

Several studies indicate that care-dependent older adults with cognitive impairments, living in a nursing home, are at risk of care without their consent, referred to as 'involuntary treatment'. This includes the use of physical restraints (e.g. belt restraint or bedrails), off-label use of psychotropic medication (e.g. anti-depressants, sedatives) and non-consensual care (e.g. forced hygiene, hiding medication). To explore the prevalence and associated factors of involuntary treatment in nursing home residents with cognitive or functional impairment we conducted a cross-sectional study in the southern part of the Netherlands using association analyses. Involuntary treatment was measured using the International Prevalence Measurement of Care Quality (LPZ). In addition, sociodemographic data were collected from residents, including gender, age, functional or cognitive impairment, diagnosis of dementia, confusion, aggression and dependency in activities of daily living. Involuntary treatment was used in 30% of the total sample. Among the residents who received at least one form of involuntary treatment, physical restraint was most commonly used (92%), followed by off-label use of psychotropic medications (20%) and non-consensual care (10%). Involuntary treatment was most common among residents with cognitive impairments (38%). Within the group of residents with functional impairment, the prevalence was 20%. Cognitive impairment and dependency in activities of daily living were strongly associated with involuntary treatment. Involuntary treatment is often used among nursing home residents with cognitive impairment. It also shows that residents with functional impairments are at risk. Future research should focus on understanding and preventing inappropriate involuntary treatment in nursing homes.

### SESSION 7300 (POSTER)

### MOBILITY/DISABILITY (POSTERS)

Abstract citation ID: igae098.2804

### AGE-SPECIFIC TRENDS IN LIMITATIONS TO DAILY ACTIVITIES IN AMERICANS AGED 50 AND OVER BY RACE AND ETHNICITY, 2000–2018

Octavio Bramajo, Neil Mehta, and Brandon O'Grady, *The University of Texas Medical Branch, Galveston, Texas, United States*

During the first decade of the 2000s the overall prevalence of limitations to daily activities (but not instrumental limitations) has increased in the US. It is not clear if the stagnation in mortality in the last decade, after decades of improvements in life expectancy, was accompanied by stagnation in disability as well or if the pace of increases of disabilities increased. However, it is not clear if specific age-groups drive these trends, and how those differences have manifested by sex, race, and ethnicity since the turn of the century. We used data from the National Interview Health Survey (NHIS), from years 2000 to 2018, and we ran a set of logistic regression models to establish age-specific trends by race for both types of limitations to activities in daily living (stratified by sex, decade and by the age-groups between 50-64,65-74-75-84) to disentangle the effect of time on how those trends developed, controlling by age, education and nativity. From 2000 to 2018 average prevalence of limitations to daily activities and instrumental limitations for adults aged 50 and above has not changed significantly, hovering on similar levels during the whole period. However, we identified an increase of limitations to daily activities and instrumental limitations for the 50-64 age group, with a steep increase during the first decade of the century and a stagnation afterward, mostly driven by Non-Hispanic Blacks and Hispanics, both for males and females. For other age groups, the trend was uneven, and we were unable to distinguish a clear direction.

Abstract citation ID: igae098.2805

### BACKWARD GAIT CHARACTERISTICS: A NOVEL PERSPECTIVE ON PHYSICAL FUNCTIONING

Braden Popelsky[1], and Kelly Ylitalo[2], *1. Baylor University, Bryan, Texas, United States*, *2. Baylor University, Waco, Texas, United States*

Mobility is fundamental to healthy aging. Mobility measures such as gait velocity, double support phase, step length, and asymmetry can quantify ambulation and identify gait patterns that signify compensation for unsteadiness, poor balance, and physical functioning limitations. Forward walking is well-established as a measure of physical functioning, but fewer studies have assessed backward direction. Independently ambulatory young adults (n=20; age: 22.9 years ±1.6) and community-dwelling older adults (n=19; age: 70.2 years ±6.2) completed repeated forward and backward walking tests on a 3.9-m instrumented pressure mat. Paper surveys assessed sociodemographic characteristics, physical activity, fall history, and self-reported physical functioning (Physical Component Score (PCS) from the Short-Form Health-Survey 12). Forward and backward gait measures were evaluated using principal component analysis (PCA), and receiver-operating curves were used to model the diagnostic efficacy of gait measures on poor physical functioning. Four principal components explained 87.6% of the variance in gait. Principal component 1 accounted for 49.1% of the variance in gait with average backward step length (0.424) and backward walking speed (0.422) exhibiting the greatest loadings, indicating stronger influence. Principal component 1 was in moderate agreement (r=0.43, p=0.012) with PCS scores. Among older adults, backward gait velocity was a better predictor of poor physical functioning (PCS ≤ 50) than forward gait velocity (p=0.044; AUC=0.682 vs. 0.546). Backward gait characteristics may be novel indicators of physical functioning. Prospective studies should establish cut points for backward gait measures that classify poor physical functioning in diverse older adult populations.

Abstract citation ID: igae098.2806

### EXPLORING ASSISTIVE TECHNOLOGY ACCESS AND USE AMONG OLDER ADULTS THROUGH SELF-DIRECTED HOME TOURS

Ben Mortenson, Chih-TIng (Gina) Tsai, Chin Wing Yu, Suzanne Huot, and Laura Hurd, *University of British Columbia, Vancouver, British Columbia, Canada*

Assistive technology (AT) and home modifications play a crucial role in enhancing independence and quality of life for individuals with disabilities. Our aim was to explore access and use of AT for older adults through an innovative research methodology, virtual self-directed home tours. These interviews were held across three groups of participants (A) first-generation Chinese-speaking immigrants to Canada, (B) second and subsequent generations of Chinese-speaking immigrants, and (C) native-born English-speaking Canadian citizens of European descent. A total of 17 interviews were conducted (Group A=8, Group B=3, and Group C=6) through Zoom Video Communications. Participants were demographically diverse adults with disabilities living in

Metro Vancouver, British Columbia (Age= 59 ± 15 years; 11 female and 6 male). Most participants had assistance from their caregivers to complete the interview. Three main themes were identified. (1) Enabling Participation described the transformative role of AT in optimizing participants' ability to engage in activities that are meaningful to them independently. (2) User-Driven Do-It-Yourself Adaptations explored how participants developed cost effective alternatives by creatively adding their own modifications to existing AT. (3) Navigating Complexities and Hurdles encompassed the challenges with accessing and incorporating AT in their homes due to lack of adequate knowledge transfer from healthcare professionals, financial circumstances, and other systemic barriers that further delayed the implementation of home modifications. Chinese-speaking immigrants faced compound challenges due to language barriers. The insights gained from this study may inform strategies for improving the experiences of older adults accessing and using AT and home modifications.

Abstract citation ID: igae098.2807

### EXPLORING THE IMPACT OF ATTENDANCE IN AN OUTDOOR WALKING PROGRAM ON HEALTH IN OLDER ADULTS WITH MOBILITY LIMITATIONS

Tai-Te Su[1], Ruth Barclay[2], Rahim Moineddin[1], and Nancy Salbach[1], *1. University of Toronto, Toronto, Ontario, Canada*, *2. University of Manitoba, Winnipeg, Manitoba, Canada*

Walking in an outdoor environment offers numerous health benefits for older adults with mobility limitations. Effects may vary, however, depending on the level of attendance. This study aimed to investigate whether a dose-response relationship existed between attendance in an outdoor walk group (OWG) program and improvement in health outcomes among older adults with mobility limitations. We analyzed data from a randomized controlled trial conducted across four cities in Canada and focused specifically on community-dwelling older adults assigned to a 10-week OWG program (n=79, age=74.7±6.6 years, 72% female). Participants were categorized into three groups based on attendance tertiles, with the first, second, and third tertiles corresponding to attending 0–9, 10–15, and 16–20 outdoor walking sessions in total. Outcomes were operationalized as change in scores on measures of walking endurance, comfortable and fast gait speed, balance, lower extremity strength, walking self-confidence, and emotional well-being pre- to post-intervention. Results from multiple linear regression models showed that, compared to those who attended 0–9 OWG sessions, participants who attended 16–20 sessions exhibited a 52.7-meter greater improvement in walking endurance (95% CI:12.3, 93.1); 0.15-meter/second greater improvement in comfortable gait speed (95% CI:0.00, 0.29); and 0.17-meter/second greater improvement in fast gait speed (95% CI:0.02, 0.33), after adjusting for sex and study site. No significant dose-response relationships for balance, lower extremity strength, walking self-confidence, and emotional well-being were observed. The role of attendance highlighted in this study provides critical information relevant to the design and implementation of outdoor walking interventions aimed at improving health among older adults.

Abstract citation ID: igae098.2808

### FEAR OF FALLING INTERACTS WITH ACTN3 GENOTYPE TO PREDICT FAST GAIT SPEED IN OLDER ADULTS

Allon Goldberg[1], Summer Burkhardt[1], Hayden Norris[1], Johnathon Garber[2], Natalie Little[1], Jaiden Kendall[1], and Joseph Sucic[1], *1. University of Michigan - Flint, Flint, Michigan, United States*, *2. Michigan State University East Lansing, East Lansing, Michigan, United States*

Deficits in walking speed are associated with poor health outcomes in older adults. The alpha-actinin-3 gene (ACTN3) harbors the R577X polymorphism, which influences muscle function and physical performance in older adults. Fear of falling (FOF) is associated with gait, mobility and physical performance deficits in older adults. Our objective was to determine if effects of FOF on fast gait speed (FGS) are associated with ACTN3 genotype, to identify individuals based on ACTN3 genotype at risk for greatest declines in FGS with onset of FOF. Older adults (N=168, female=61.3%, mean age 72.8 years, range 65-85 years) attended a study visit at which demographic, self-rated health (SRH) and FOF information was collected, and 10-meter FGS was tested. Saliva was obtained for ACTN3 genotyping. Multiple regression evaluated variance in FGS explained by FOF, RR genotype (present/absent), and interaction of FOF with RR genotype, controlling for age, sex and SRH. The final regression model explained 36.9% of FGS variance ($p<.001$). FOF was a significant predictor ($\beta=-.163$; $p=.026$) of FGS. RR genotype did not independently predict FGS ($\beta=.103$; $p=.161$), but was interactively with FOF a significant predictor ($\beta=-.181$; $p=.024$) of FGS. The effect of FOF on FGS was greater in individuals with RR genotype than in the RX/XX genotype subgroup (partial eta squared 0.083 and 0.030 respectively). The significant FOF x RR genotype interaction suggests that effects of FOF on FGS vary based on ACTN3 genotype. Individuals with RR genotype (compared to the RX/XX subgroup) may experience greater FGS declines with onset of FOF.

Abstract citation ID: igae098.2809

### LIFE SPACE MOBILITY IS ASSOCIATED WITH DISTINCT FACTORS BELOW AND ABOVE THE AGE OF 75 YEARS

Samar Assadi Khalil[1], Rachel Kizony[2], Efrat Gil[3], Roy Tzemah-Shahar[4], and Maayan Agmon[5], *1. Tel Aviv University, Tel Aviv, HaMerkaz, Israel*, *2. University of Haifa, Haifa, Hefa, Israel*, *3. HaEmek Medical Center, Afula, HaZafon, Israel*, *4. University of Haifa, Haifa, HaZafon, Israel*, *5. University of Haifa, University of Haifa, HaZafon, Israel*

Life-space mobility (LSM) is associated with the quality of life and well-being of older adults, and it is influenced by individual and environmental factors. However, age-related differences are ignored. This study aimed to explore which factors are associated with LSM in community-dwelling older adults from two age groups (< 75 and≥75 years). This cross-sectional study included older adults aged 65 and over, independently mobile, without neurologic conditions or dementia. LSM, the dependent variable, was assessed using the Life-Space Assessment-(LSA). Independent variables were assessed using Timed Up and Go-(TUG) for mobility, Montreal Cognitive Assessment-(MoCA) for cognition, Activities-specific Balance Confidence Scale-(ABC) for fear of falling, Hospital Anxiety and Depression Scale-(HADS) for depression, and four-functional mobility tasks for participation. Additionally, functional status and number of medications were assessed. Two separate stepwise regression models were conducted for older adults aged< 75 and≥75 years. Two-hundred forty-two older adults (28.9% men) were included [mean(SD) age: 73.7(6.4)

years], of whom 40.9% were≥75 years. Results revealed that different factors were associated with LSM in each age group. For those< 75 years, participation and TUG explained 33% and 9.4% of the variance in LSM, respectively. Conversely, for the older group, ABC, MoCA, and TUG explained 33.5%, 4.1% and 5.2% of the variance in LSM, respectively. Our finding shows that distinct factors are associated with LSM in each group, particularly mobility in the younger and fear of falling in the older. By highlighting these specific factors, interventions can be tailored more effectively to enhance the quality of life and well-being of older adults.

Abstract citation ID: igae098.2810

### LONGEVITY POLYGENIC RISK IN MULTIDOMAIN AGING AND LONG-TERM SURVIVAL AMONG OLDER WHITE AND BLACK ADULTS

Siting Chen[1], Corey L. Nagel[2], Jodi Lapidus[1], and Ana Quiñones[3], *1. OHSU-PSU School of Public Health, Portland, Oregon, United States*, *2. University of Arkansas for Medical Sciences, Little Rock, Arkansas, United States*, *3. Oregon Health & Science University, Portland, Oregon, United States*

This study aims to identify genetic characteristics of multidimensional aging and longevity by examining the association of longevity polygenic risk scores with multi-domain aging trajectories and long-term survival in older Non-Hispanic White and Non-Hispanic Black adults. We studied 7,287 older adults (≥ 65 years, 14.0% Non-Hispanic Black) from the Health and Retirement Study (HRS, 1998-2016). Group-based trajectory modeling was performed to identify distinct groups of older adults following similar joint trajectories of aging across four domains (multimorbidity burden, functional status, cognitive performance, and depressive symptomatology). We identified four distinct multi-domain trajectory groups: minimal impairment with low multimorbidity (34.1%), minimal impairment with high multimorbidity (37.1%), multidomain impairment with intermediate multimorbidity (16.7%), and multidomain impairment with high multimorbidity (12.1%). Longevity polygenic risk scores (categorized as low, medium, medium-high, and high) were compared between long-term survival groups (long-lived group and controls) across the identified trajectory groups in Non-Hispanic White and Black adults. The proportion of long-lived group was significantly higher in minimal impairment with low multimorbidity group in both Non-Hispanic White (23.3%,p< 0.01) and Non-Hispanic Black adults (19.6%,p< 0.01). The long-lived group had greater proportions of Non-Hispanic White participants with high longevity polygenic risk scores in all trajectory groups. The long-lived group had lower proportions of Non-Hispanic Black participants with low longevity polygenic risk scores in all trajectory groups except for the multi-domain impairment with high multimorbidity group. Our study highlights the need to examine the constellation of genetic and socioeconomic factors on aging-related health domains and longevity among diverse groups of older adults.

Abstract citation ID: igae098.2811

### LONG-TERM MOBILITY DEVICE USE AND SOCIAL WELL-BEING AMONG COMMUNITY-DWELLING OLDER ADULTS IN THE UNITED STATES

Xinran Liu[1], Sara Baumann[2], Andrea Rosso[2], Elizabeth Venditti[2], Yao Yao[3], and Steven Albert[2], *1. Peking University, China (People's Republic)*, *2. University of Pittsburgh, Pittsburgh, Pennsylvania, United States*, *3. Peking University, Beijing, Beijing, China (People's Republic)*

The prevalent use of mobility devices among older adults is largely driven by the increased incidence of mobility disability in later life. While these devices are designed to enhance activities and participation for individuals with mobility disability, their long-term effects on social well-being remain unclear. This study assesses whether mobility device use contributes to improved activities, participation, and life satisfaction among community-dwelling older adults over time, comparing users with their non-user counterparts. We utilized data from the National Health and Aging Trends Study (NHATS) waves 1-9 (2010-2019) to identify incident mobility device users with over two years of experience. These users were matched on a 1:1 basis with non-users by age (±5 years), gender, race, and baseline Short Physical Performance Battery scores (±1 point). Follow-up assessments were conducted at the time of incident use, and 1- and 2-year later. Guided by the NHATS late-life disability framework, we applied mixed-effects models, adjusting for personal and environmental factors, health status, body impairments, functional capacity, and accommodations. The results indicate that mobility device users have overall significantly lower participation in paid work compared to non-users (p< 0.05). Over time, mobility device use was associated with decreased driving but increased reliance on rides from others, and more frequent visits with family and friends (ps< 0.05). Notably, older mobility device users exhibited lower life satisfaction in the long term (p< 0.05). These findings underscore the critical need to address the social well-being of older adults who use mobility devices in communities to improve their overall quality of life.

Abstract citation ID: igae098.2812

### NEW MEASURES OF MULTIMORBIDITY BASED ON A THEORY OF DEPENDENT COMPETING RISKS

Igor Akushevich[1], Arseniy Yashkin[1], Julia Kravchenko[2], and Anatoliy Yashin[1], *1. Duke University, Durham, North Carolina, United States*, *2. Duke University School of Medicine, Durham, North Carolina, United States*

The phenomenon of multimorbidity is increasingly common among older U.S. adults, however the driving forces behind multimorbidity development remain unclear. In this study we develop a novel approach for the identification and explanation of causal pathways generating multimorbidity patterns in the population of older U.S. adults. Our approach is based on the evaluation of the dependent competing risks among diseases potentially determining the process of generating a given multimorbidity cluster using parametric and non-parametric methods. This approach allows for modeling the joint distribution of time-to-disease-onsets with mutual correlations; characterizing cluster properties such as speed of cluster formation and their prevalence in the population. This joint distribution is estimated using individual morbidity trajectories extracted from Medicare administrative claims data and dynamically constructed for all ages resulting in an age-specific-function of multimorbidity generation. The identified clusters and estimated properties were compared to those obtained using traditional methods (e.g., cluster analysis or latent structure analyses). Our method strongly outperforms the current methods of identification of multimorbidity clusters which were characterized by approximate cluster composition with no room for improvement without additional methodologic assumptions. The substantive results of this study encompass the identification of multimorbidity clusters and their properties and allows extensions for the quantification of the effects of risk factors on these clusters (including evaluating causality), the provision of causal explanations for disparities in multimorbidity, and the development and testing of novel approaches for multimorbidity analysis through simulation studies.

Abstract citation ID: igae098.2813

### PAIN AND LOWER EXTREMITY FUNCTIONAL LIMITATION IN AMERICAN OLDER ADULTS: A SIX-YEAR FOLLOW-UP STUDY

Jaspreet Sodhi[1], and Soham Al Snih[2], *1. Marshall University, Huntington, West Virginia, United States*, *2. University of Texas Medical Branch in Galveston, Galveston, Texas, United States*

The objective of this study was to examine the relationship between pain and lower extremity functional limitation (LEFL) among American older adults without LEFL at baseline over 6-years of follow-up. Participants (N=1,717) aged ≥ 65 years were from the National Health and Aging Trends Study (2011-2016). Measures included pain, socio-demographics (age, gender, race/ethnicity, and education), depressive symptoms, obesity, and comorbidities. LEFL was defined as limitation in ≥ one of the following five activities (walking 6 blocks, walking 3 blocks, walking up 20 stairs, walking up 10 stairs, or able to get down on knees). General estimation equation model was fitted to estimate the odds ratio (OR) and 95% Confidence Interval (CI) of any LEFL as a function of pain. All variables were treated as time varying except for gender, education, and race and ethnicity. Participants with pain had greater odds (OR=1.52, 95% CI=1.42-1.63) of reporting any LEFL over time than those without pain after controlling for all covariates. Older age and comorbidities were associated with greater odds of any LEFL while married status and high level of education were factors associated with lower odds of reporting any LEFL. In conclusion, pain was a significant predictor of limitations in lower extremity function. Therefore, early assessment of pain and appropriate treatment could prevent future disability and maintain independence in this population.

Abstract citation ID: igae098.2814

### RESTRICTED YET EXPANDED: EVOLVING MOBILITY OF OLDER ADULTS WITH COGNITIVE IMPAIRMENT IN URBAN CHINA

Qingqing Yin[1], and Lin Chen[2], *1. University of Pittsburgh, Pittsburgh, Pennsylvania, United States*, *2. Fudan University, Somerville, Massachusetts, United States*

Cognitive impairment restricts older adults' mobility within and beyond the neighborhood. Existing literature primarily focuses on the declining mobility of older adults with mild cognitive impairment (MCI) and the implications of such decline for this vulnerable group. However, how older adults with MCI experience their changing mobility has received limited attention. Informed by community gerontology and the preventive and corrective proactivity (PCP) model, this study aims to explore how older adults with MCI perceive and adapt to their changing mobility. Through purposive sampling, we took a phenomenological approach to conduct face-to-face, in-depth interviews with community-dwelling older adults with MCI in Zhengzhou, China (N = 34). By applying the geographic information systems (GIS), we spatially situated participants' routine activities to visualize their changing mobility. Recognizing their cognitive decline, most participants voluntarily constrained their out-of-home activities within the neighborhood. Still, they tried to keep a strong grip on the existing mobility and prolong independent living as much as possible. One of the most useful strategies was to take advantage of their social networks in the neighborhood. Social interactions and mutual support created new opportunities for participants, which facilitated their out-of-home mobility even beyond the neighborhood. Their restricted mobility in the physical environment was thus continually defended and expanded by the social environment. The findings of this study inform the importance of promoting neighborhood built and social features to support older adults with different levels of cognitive and mobility functioning.

Abstract citation ID: igae098.2815

### SOCIAL DETERMINANTS AND THE VULNERABILITY TO LOSS OF INDEPENDENCE IN OLDER ADULTS

Young-Shin Lee[1], Hee-jin Jun[1], and Jung-Ah Lee[2], *1. San Diego State University, San Diego, California, United States*, *2. University of California Irvine, Irvine, California, United States*

Over 41% of Americans with disabilities are aged 65 and older, with two in five seniors experiencing at least one disability, such as mobility limitations or depending on others for daily activities. As life expectancy increases, the prevalence of disability in later life is also expected to increase. Vulnerability of independence and unmet needs in daily activities present a significant personal burden and a growing concern for public health. While mMobility disabilities contribute significantly to this vulnerability, yet a comprehensive understanding is still lacking. This study aims to explore the impact of social determinants on functional vulnerability of older adults, utilizing data from the 2017 National Health Aging Trends Study (NHATS). It evaluates functional vulnerability with the Vulnerable Elders Survey tool and applies logistic regression analysis to assess the effects of age, gender, race/ethnicity, income, and living arrangements on this vulnerability. The analysis included 6,312 individuals aged 65 years and above, revealing that 30% have an income under the poverty level and 35% live alone. The results show that 43.6% of older adults are at a high risk of functional vulnerability. Significant risk factors identified include being of 'old' age, female, NH Black, living with others, and having an income at the poverty level. These findings underscore the urgency of developing targeted interventions for these at-risk groups.

Abstract citation ID: igae098.2816

### SOCIOECONOMIC STATUS AND THE RELATIONSHIP OF PHYSICAL PERFORMANCE WITH ACTIVITIES OF DAILY LIVING

Grace Chang[1], Rahul Malhotra[2], and Benjamin Seligman[3], *1. University of California Los Angeles, Los Angeles, California, United States*, *2. Duke-NUS Medical School, Singapore, Singapore*, *3. University of California Los Angeles David Geffen School of Medicine, Los Angeles, California, United States*

The translation of reduced intrinsic capacity, assessed through physical performance such as gait speed and grip strength, to diminished functional ability, such as limitations in activities of daily living (ADLs), can be modified by one's environment.

Socioeconomic status (SES), linked with access to resources, may thus modify the relationship between physical performance and ADL limitations. We study this question in a cohort of older adults, using data from the 2016 wave of the Health and Retirement Study. Performance was measured using gait speed and grip strength, adjusted for sex and height/BMI respectively. ADL limitation was assessed using five Katz ADLs: bathing, toileting, eating, dressing, and transferring from bed. We considered three SES measures: highest education level, household wealth, and household income. Analysis was by negative binomial regression of the number of ADL limitations on performance and/or SES with adjustment for age and sex. Individually, each performance and SES measure was associated with ADL limitations. In regressions including grip strength, SES was associated with ADL limitations, and wealth and income had interactions with grip strength. For gait speed, only wealth was associated with ADL limitations, though education had an interaction with gait speed. Our mixed results for effect modification by SES of the association between physical performance and ADL limitations do suggest the role of SES as an effect modifier, contingent on the measures being used for assessing physical performance and SES. However, they also suggest that performance may mediate the relationship between SES and ADL limitations.

### SESSION 7305 (POSTER)

### MUSCULOSKELETAL HEALTH (POSTERS)

Abstract citation ID: igae098.2817

### ALTERNATIVE SPLICING DYNAMICS IN MYOGENESIS: A TRANSCRIPTOMIC ATLAS FOR HUMAN SKELETAL MUSCLE CELLS

Stefano Donega[1], Nirad Banskota[2], Jen-Hao Yang[2], Martina Rossi[2], Dimitrios Tsitsipatis[1], Supriyo De[1], Myriam Gorospe[2], and Luigi Ferrucci[1], *1. National Institute on Aging, Baltimore, Maryland, United States, 2. National Institutes of Health, Bethesda, Maryland, United States*

Myogenesis entails the fusion of myoblasts to form multinucleated myotubes. Although alternative splicing (AS) in myogenesis has been explored in non-human species, its characterization in humans remains incomplete, both in vitro and in vivo. We studied two human skeletal muscle cell lines (AB1167 and AB678) over a 5-day differentiation period, with RNA samples collected every 24 hours. Utilizing a Hybrid RNA-sequencing approach (combining short and long-read sequencing), we applied linear regression in Differential Gene Expression (DGE) and Likelihood Ratio Test – Hierarchical Clustering models (LRT-HCl), and discovered 3,079 upregulated genes and 2,357 downregulated genes in the overlapping models. Gene Set Enrichment Analysis (GSEA) highlighted pathways related to muscle formation and contraction showing upregulation, while those associated with nuclear separation and DNA synthesis were downregulated until day 3, followed by an increase in inflammation and interferon-like pathways by day 5. Differential Transcript Usage analysis (DTU) identified 2,584 myogenesis-dependent isoform-switches, which were primarily linked to actin-filament processes and muscle regulation. Validation of the models was conducted for the top 5 genes using qPCR. Additionally, three novel protein-coding genes associated with muscle differentiation were identified through an Alphafold-based computational pipeline and validated via qPCR. Comparison of clusters within a 0–3-day model with a Peripheral Artery Disease (PAD) dataset confirmed significant enrichment of up-regulated clusters observed in the myogenesis model. This project aims to establish a comprehensive transcriptomic atlas for myogenesis models and set a benchmark for future research in the multi-omic fields of muscle biology.

Abstract citation ID: igae098.2818

### CAMKII HYPERACTIVATION IN SKELETAL MUSCLES IS A DRIVER AND POTENTIAL THERAPEUTIC TARGET OF SARCOPENIA

Qinchuan Wang[1], Michael Bene[2], William Fountain[1], Giovanni Rosales-Soto[3], Erick Hernandez-Ochoa[3], Tae Hwan Chung[4], and Jeremy Walston[4], *1. Johns Hopkins University School of Medicine, Baltimore, Maryland, United States, 2. Johns Hopkins School of Medicine, Baltimore, Maryland, United States, 3. University of Maryland School of Medicine, Baltimore, Maryland, United States, 4. Johns Hopkins University, Baltimore, Maryland, United States*

Sarcopenia, the loss of muscle mass and strength, is a major risk factor for physical frailty in older adults. Ca2+/calmodulin-dependent protein kinase II (CaMKII) is activated by Ca2+ and reactive oxygen species in contracting muscles to enhance muscle performance and adaptation to exercise training. We assessed CaMKII activity in the skeletal muscles of young (4-month-old) and old (20-month-old) C57BL/6J mice and found significantly increased CaMKII activity in old mice at rest, suggesting that CaMKII activity is chronically elevated and disconnected from muscle contractile activity in aged muscles. To determine the role of increased CaMKII, we used an adeno-associated viral vector (AAV9) to express a CaMKII inhibitor, CN19o, in the tibialis anterior (TA) muscles of 20-month-old mice and found a significant improvement of muscle contractile force at 24 to 26 months of age. Conversely, when we expressed a constitutively active CaMKII (CaMKIICA) in the TA muscles of young mice (3-month-old), we observed a striking reduction of muscle contractility. Electromyography studies showed that the muscles expressing CaMKIICA had a subtle but significant reduction of compound muscle action potential (CMAP) that could not explain the reduction of contractile force. Single muscle fiber analyses showed that CaMKIICA did not affect action potential-evoked Ca2+ release. Ongoing research is assessing the clinical relevance of CaMKII activation in humans and examining the histological and transcriptomic profiles of muscles with either inhibited or hyperactivated CaMKII to uncover the mechanisms underlying CaMKII-induced muscle dysfunction. Our findings suggest that CaMKII hyperactivation is a driver and potential therapeutic target of sarcopenia.

Abstract citation ID: igae098.2819

### EFFECTS OF MULTICOMPONENT EXERCISE INTERVENTION ON SARCOPENIA PREVENTION IN COMMUNITY-DWELLING OLDER ADULTS

Kewei Shi[1], Lingxiao He[2], and Ya Fang[1], *1. Xiamen University, Xiamen, Fujian, China (People's Republic), 2. Center for Health Economics, Policy and Aging Research, Xiamen city, Fujian, China*

**Objective:** To assess the effects of multicomponent exercise intervention on sarcopenia and its components among community-dwelling older adults. Design, Setting, and Participants: In a randomized controlled trial conducted in 11 communities across Xiamen, China, with 200 older adults

(aged > 65 years) being randomly assigned to an exercise group (n=100) or a control group (n=100).
**Interventions:** The exercise group followed a multicomponent exercise program, consisting of resistance, balance and flexibility training, twice a week for six months. The control group received monthly sarcopenia-related health education. Outcome measures: The primary outcome was the incidence of sarcopenia. The secondary outcomes included muscle mass, muscle strength (knee extension and grip strength), the Short Physical Performance Battery (SPPB) score, 4-meter gait speed, and 5-time chair stand test (CST) performance. Generalized mixed-effects model was performed for primary outcome comparison between two groups. Repeated measures ANCOVA was performed to evaluate the effects on secondary outcomes.
**Results:** The exercise group showed a decrease of 4.3% on the incidence of sarcopenia after 6-month intervention, with no significant difference from the control group (P=0.171). The exercise group demonstrated significant improvements in knee extension (β=1.95, 95% CI [0.082, 3.84]), grip strength (β=2.07, 95% CI [0.857, 3.290]), SPPB score (β=1.99, 95% CI [1.600, 2.380]), gait speed (β=0.182, 95% CI [0.128, 0.235]) and CST (β=-1.590, 95% CI [-2.400, -0.786]). No between-group difference was observed in muscle mass (P=0.767).
**Conclusion:** A 6-month multicomponent training can significantly improve physical function and muscle strength but not muscle mass among community-dwelling older adults.

Abstract citation ID: igae098.2820

### EXERCISE AND NUTRITION ON SARCOPENIA AND FUNCTIONAL SARCOPENIA: FINDINGS FROM RANDOMIZED CONTROLLED TRIALS

Sunghwan Ji[1], and Il-Young Jang[2], *1. Samsung Advanced Institute for Health Sciences & Technology (SAIHST), Seoul, Republic of Korea*, *2. Asan Medical Center, Seoul, Republic of Korea*

The Korean Working Group on Sarcopenia (KWGS) established guidelines for diagnosing sarcopenia, introducing the concept of functional sarcopenia. However, the effectiveness of exercise and nutrition interventions among sarcopenia and functional sarcopenia based on these guidelines remains unexplored. To address this gap, we recruited 86 older adults with sarcopenia and 42 with functional sarcopenia from a rural Korean community, randomly assigning them to intervention and control groups in a 1:1 ratio. Sarcopenia was defined by low grip strength and/or low physical performance with low muscle mass, while functional sarcopenia was identified by low grip strength and low physical performance with preserved muscle mass. Participants in the intervention group underwent group exercises and a nutrition program for 24 weeks. Comprehensive geriatric assessments were conducted for both groups before and after the interventions. The primary outcome measure was the change in gait speed, with additional sarcopenia-related parameters also compared. The intervention group showed significantly greater improvement in gait speed in both sarcopenia (mean change (m/s) 0.14 vs. -0.04; p< 0.001) and in functional sarcopenia (0.24 vs. 0.00, p< 0.001). Furthermore, the intervention group exhibited greater improvement in short physical performance battery score, grip strength, quality of life in both sarcopenia and functional sarcopenia. There were no significant differences in muscle mass or clinical frailty scale between the intervention and control groups. In conclusion, exercise and nutrition interventions were beneficial for physical performance and muscle strength in sarcopenia and functional sarcopenia according to KWGS guidelines, among community-dwelling Korean older adults. (Registration number: KCT0008952, KCT0008953)

Abstract citation ID: igae098.2821

### GENDER DIFFERENCES IN MUSCLE STRENGTH AND TISSUE ASYMMETRY CHANGES OVER FIVE YEARS AMONG OLDER ADULTS

Milan Chang (Gudjonsson)[1], Marco Recenti[2], Carlo Ricciardi[3], Alfonso Maria Ponsiglione[4], Francesco Amato[5], Magnus Kjartan Gislason[2], and Paolo Gargiulo[2], *1. Landspitali University Hospital, Seltjarnarnes, Hofuoborgarsvaoio, Iceland*, *2. Reykjavik University, Reykjavik, Hofuoborgarsvaoio, Iceland*, *3. University of Naples Federico II, Naples, Calabria, Italy*, *4. University of Naples Federico II, Naples, Campania, Italy*, *5. Università degli Studi Magna Græcia di Catanzaro, Napoli, Campania, Italy*

**Background:** The declines in muscle strength during aging process is linked to reductions in tissue integrity and underlying cellular processes. It suggests potential gender differences in these fundamental mechanisms. The current study investigates whether the decline in muscle strength over a 5-year period correlates with changes in tissue asymmetry and explores potential gender differences between men and women.
**Methods:** The longitudinal study comprised 3156 participants residing in Reykjavik, Iceland, with a baseline mean age of 74.9. The analysis included 1,150 men and 1,587 women (n=2,737). Tissue asymmetry, assessed through CT scans, was defined by differences between the right and left sides across 11 soft tissue composition parameters for fat, muscle, and connective tissue. Changes in leg strength and asymmetry parameters over 5 years were calculated by subtracting follow-up values from baseline. Linear regression analysis was performed to assess the association between changes of maximum knee extension strengths and changes in soft-tissue asymmetry over 5 years, adjusting for confounders by gender.
**Results:** In men, a decline in leg strength over 5 years was significantly associated with an increased asymmetry across five parameters (2 related to fat and 3 related to muscle (p< 0.05)). In women, associations were observed with an increased asymmetry parameters involving fat, muscle, and connective tissue (p< 0.03).
**Conclusion:** Gender-specific variations in asymmetry parameters highlight how fat and muscle composition in men uniquely contribute to leg strength changes over time. This emphasizes the need to consider gender-specific factors in understanding age-related muscle strength changes, informing targeted interventions for older adults.

Abstract citation ID: igae098.2822

### PLASMA AND SKELETAL MUSCLE METABOLIC PATHWAYS ASSOCIATED WITH WEIGHT LOSS, LOW MUSCLE, OR POOR FUNCTION IN CANCER

Lindsey Anderson[1], Lu Xia[2], Haiming Kerr[2], Peter Wu[1], Atreya Dash[1], Sina Gharib[2], and Jose Garcia[1], *1. VA Puget*

*Sound Healthcare System, Seattle, Washington, United States*, *2. University of Washington, Seattle, Washington, United States*

Cancer cachexia (muscle wasting leading to functional impairment) is experienced by most individuals with cancer and increases with age. There are no approved treatments in the U.S./E.U. because Phase III trials have failed to achieve functional endpoints. Identification of metabolites/metabolic pathways related to poor functional performance may reveal therapeutic targets but metabolomics analyses in cancer cachexia have only examined weight loss and/or muscle mass. We characterized metabolomic perturbations in plasma and abdominal wall skeletal muscle from men (n=75; median age=65) with cancer undergoing laparotomy and tested associations with function (handgrip strength "HGS" and stair climb power "SCP" assessed within two weeks pre-surgery), muscle mass (CT L3: cross-sectional area "CT-CSA"), or weight loss (six months pre-surgery). We hypothesized that metabolomic signatures in plasma and muscle would be associated with poorer HGS and SCP and alterations would be distinguishable from associations with weight loss or lower CT-CSA. Spearman correlations and pathway analyses (global test) were performed using "R." Benjamini-Hochberg false discovery rate (FDR)< 0.1 indicated significance. Lipid/fatty acid peroxidation/transport/metabolism was disrupted in muscle with lower CT-CSA (FDR≤0.09) and in plasma (FDR≤0.07) and muscle (trend; FDR=0.14) with weight loss. Phenylalanine/tyrosine/tryptophan biosynthesis (p=0.045) and amino/nucleotide sugar metabolism (p=0.018) were disrupted in muscle with worse SCP, but not after adjustment (FDR>0.5). Significant/trending dysregulation was not detected in plasma with worse SCP or in muscle/plasma with worse HGS. Lipid metabolism was associated with cancer-related weight loss and low muscle mass. Future studies should analyze metabolomics in activity/mobility-related muscles to elucidate metabolite/pathway dysregulation in cancer-related functional impairment.

Abstract citation ID: igae098.2823

### PM2.5 IS ASSOCIATED WITH DAILY OUTPATIENT VISITS FOR ANKYLOSING SPONDYLITIS: A TIME-SERIES ANALYSIS IN CHINA

Hongbo Chen, *Peking University Third Hospital, Beijing, Beijing, China (People's Republic)*

Exposure to ambient fine particulate matter (PM2.5) has a great impact on human body's immune system, but the correlation between PM2.5 and ankylosing spondylitis has not yet been clarified. We extracted 58,600 outpatient visits for ankylosing spondylitis from the Beijing Medical Claim Data for Employees database from 2010 to 2017. The percentage of outpatient visits following PM2.5 concentrations was estimated using generalized additive models with Poisson connections. Increase by 10 μ g/m3, PM2.5 is associated with daily outpatient visits for ankylosing spondylitis. In this test, the average concentration of PM2.5 was 86.8 (74.3) μ g/m3. For every 10 μg/m3 increase in PM2.5 concentration, there was a 0.34% (95% CI, 0.26-0.42%) increase in the risk of patients who visited the doctor on the same day. Females and younger patients were most susceptible to the impact of PM2.5 exposure (P<0.05). This study revealed the relationship between exposure to PM2.5 and ankylosing spondylitis, and future research can further confirm this finding and explore the potential mechanisms.

### SESSION 7310 (POSTER)

### NEURODEGENERATIVE DISEASE

Abstract citation ID: igae098.2824

### ASSOCIATION OF HEARING LOSS AND INCIDENT PARKINSON'S DISEASE: A COHORT STUDY IN US VETERANS

Lee Neilson[1], Kelly Reavis[2], Jack Wiedrick[1], and Gregory Scott[3], *1. Oregon Health & Science University, Portland, Oregon, United States*, *2. VA Portland Healthcare System, Portland, Oregon, United States*, *3. Portland VA Medical Center, Portland, Oregon, United States*

Parkinson's disease (PD) is a heterogeneous disease with non-motor manifestations that often precede motor symptom onset. Sensory disturbances such as loss of sense of smell and visual problems are well-characterized, but the impact of hearing loss on PD incidence remains unclear. We undertook an electronic health record-based cohort study of US military Veterans with objective audiogram data (n=3,596,365), using inverse probability of treatment weighting to calculate cumulative incidence of PD, adjusted for competing risk of death and key confounders. Risk difference and 95% confidence intervals (CI) were estimated by hearing loss severity. We also analyzed how this varied when combined with known comorbid conditions and receiving a hearing aid. Results suggest that hearing loss increases the risk of developing PD in a dose-dependent manner; worse hearing confers a higher risk. The number of additional cases of PD per 10,000 person-years at 5-years due to mild, moderate, moderately-severe, and severe-to-profound hearing loss was 2.0 (95% CI: 0.9, 3.1), 5.4 (95% CI: 3.7, 7.1), 5.8 (95% CI: 3.3, 8.3), and 10.6 (95% CI: 4, 17.2), respectively. Hearing loss also synergized with known prodromal disorders, increasing PD risk more than additively. Dispensation of a hearing aid within two years of the audiogram attenuated the risk of incident PD; the reduction in cases of PD per 10,000 person-years at 5-years was -9.2 (95% CI: -10.7, -7.7). Hearing loss appears to be an independent risk factor for development of PD. Widespread screening for hearing loss and appropriate use of hearing aids may attenuate this risk.

Abstract citation ID: igae098.2825

### ASSOCIATION OF MELANOMA AND INCIDENCE OF PARKINSON'S DISEASE: A COHORT STUDY IN US VETERANS

Gregory Scott[1], Lee Neilson[2], Vivek Unni[2], and Moriah Arnold[2], *1. Portland VA Medical Center, Portland, Oregon, United States*, *2. Oregon Health & Science University, Portland, Oregon, United States*

Melanoma is associated with subsequent Parkinson's disease (PD) and identifying the clinicopathologic properties of melanoma driving this association could give insight into PD pathophysiology. We undertook a retrospective cohort study of US military Veterans (n=13,737,081) to determine if malignant and premalignant phenotypes were associated with PD by using propensity score matching 4-to-1 and adjustment for competing risk of death and key confounders. Broad cancer categories were obtained from the Oncology VA registry. Data about melanoma were obtained from clinical and pathology reports using manually validated natural language processing. Risk difference and 95% confidence intervals (CI) were estimated by presence or absence of cancer. For broad cancer categories, we found decreased PD incidence associated with multiple malignancies with some novel associations (e.g. HR = 0.4 [0.2,0.5] for myeloma). In terms of melanoma, BRAF and NRAS mutational status was not associated with incident PD. Atypical/dysplastic nevi was associated with increased PD incidence (HR = 1.4 [1.2,1.7]). Melanoma subtypes in the Oncology registry were associated with PD and included superficial spreading type (HR = 1.8 [1.0, 3.3]) and lentigo maligna (HR = 1.6 [1.2, 2.1]). In people with VA pathology reports, this effect was replicated with greater effect size for superficial spreading type (HR = 10.4 [6.5,16.9]) and lentigo maligna (HR = 6.4 [3.4,11.8]) although adjustment for ascertainment bias is needed. These data show PD is associated with melanoma subtypes and precursor lesions and suggest screening for PD could be appropriate in those with melanocytic lesions once early diagnostic tests are developed.

Abstract citation ID: igae098.2826

### DIFFERENCES IN SELF-CARE AND QUALITY OF LIFE BETWEEN SPOUSES AND ADULT CHILDREN IN PATIENTS WITH PARKINSON'S DISEASE

Juhee Lee[1], Eunyoung Kim[2], Hyeran Park[2], and Kiyeon Kim[2], *1. Yonsei University, Seoul, Republic of Korea*, *2. Yonsei University College of Nursing, Seoul, Republic of Korea*

Self-care of patients with chronic illness involves various caregiver types like spouses, adult children, and other relatives or friends. Several studies have reported differences in self-care and quality of life (QOL) according to caregiver type in patients with chronic illness. Nevertheless, there are few studies comparing the difference by caregiver type in patients with Parkinson's disease (PD). This is a cross-sectional study to explore differences in self-care and QOL between spouse and adult child caregivers of patients with PD. Participants were recruited from two neurology outpatient departments of the tertiary hospitals in Korea. They reported self-care and QOL using a structured questionnaire. A total of 111 paired of PD patients and caregivers including 83 of spouse and 28 of adult child were analyzed using SPSS 26.0. Spouse caregivers reported higher in domains for self-care of efficacy and monitoring in both patients (p=0.014 to 0.054) and caregivers (p=0.026 to 0.002) compared with adult child caregivers. Patients with spouse caregivers showed better QOL of parkinson symptoms(p=0.008), and emotional function(p=0.003) with the exception of social functioning (p=0.31). Social functioning contained the questionnaire like hobby, vacation, or sexuality. This study finding showed that patients whose caregivers are spouses had better in both self-care and QOL domain than patients whose caregivers are adult children. These differences between spouse caregivers and adult child caregivers can suggest the needs of tailored care strategy for patients with PD according to caregiver type.

Abstract citation ID: igae098.2827

### MODELING TRAUMATIC BRAIN INJURY (TBI)–INDUCED NEURODEGENERATION USING HIPSC-CORTICAL ORGANOIDS

Alyssa Duckworth[1], Surya Venugopal[2], Luisa Coelho[2], Ester Kwon[2], Sameer Shah[2], and Angels Almenar-Queralt[2], *1. Sanford Consortium for Regenerative Medicine, La Jolla, California, United States*, *2. University of California San Diego, La Jolla, California, United States*

Traumatic Brain Injury (TBI) is a serious injury that affects millions of people worldwide and has significant effects on neural pathways in the brain, making it a risk factor for neurodegenerative diseases. Most research that examines the link between TBI and neurodegeneration uses rodent models, however they do not fully recapitulate disease pathology and cannot capture human specific features including the effect of genetic variations. Human induced pluripotent stem cells (hiPSC)-derived cortical organoids have become the gold standard to model neurological disorders. They recapitulate certain aspects of physiological and neural pathways of the human brain and are easily reproducible, making them a better model. To understand the effects of TBI-induced neurodegeneration and explore potential treatment therapies, we model TBI in cortical hiPSC organoids through inducing mechanical injury. We assess AD-related morphological, biochemical, and functional downstream effects with a battery of assays. Among them, we measure the impact on neuronal activity using fluorescent Ca2+ indicators to measure Ca flux at a single-cell level post injury. Together, these assays will help us establish a human-based system to model in vitro TBI-induced neurodegeneration. We hope it can help contribute to evaluate specific therapies and diagnostic tools to stop progression of neurodegeneration after mechanical injury.

Abstract citation ID: igae098.2828

### SELF-COMPASSION IN CAREGIVERS OF NEURODEGENERATIVE DISEASES: DEMENTIA CARE CONTEXTS AND BEYOND

Christi Lero[1], Soobin Park[1], and Emily Mroz[2], *1. Washington University in St. Louis, St. Louis, Missouri, United States*, *2. Emory University, Decatur, Georgia, United States*

Informal caregivers of those with neurodegenerative disease (ND) manage increasingly complex symptoms which impact their own wellbeing. Self-compassion can promote maintenance of wellbeing during challenging life experiences, including caregiving. There is little guidance for studying self-compassion observationally or targeting self-compassion through intervention for this population. Our objective was to complete a scoping review of research describing self-compassion in the context of wellbeing of informal caregivers of those living with ND. In this review, following PRISMA-ScR guidelines, three online databases identified 350 peer-reviewed articles, 18 of which were included in this study. Eligibility included being written in English, targeting informal caregivers of a care recipient living with ND, and examination of self-compassion. Findings showed that study samples most often included informal caregivers of persons living with Alzheimer's and related dementias. Across study types (e.g., hypothesis generating, intervention) self-compassion appeared as a theoretical concept, emerging theme from participant interviews, variable associated with other outcomes, and a main outcome variable. Self-compassion is often measured using the Self-Compassion Scale or short form version of this measure. Based on this review, we will discuss how study of self-compassion as useful for informal caregivers of individuals living with ND is in a growth period. The classification of ND is broad, however the range of common NDs among care recipient diagnosis was not represented by existing work. Future research should explore the nuances of self-compassion when caregiving for persons living with other NDs, as symptomology, disease trajectory, treatments, care provision, and outcomes vary across these caregiving contexts.

Abstract citation ID: igae098.2829

**XBP-1S TRANSCRIPTIONAL TARGETS IN THE ER UNFOLDED PROTEIN RESPONSE RESCUE AGING-RELATED TAUOPATHY PROTEOSTASIS**

Brian Kraemer, *VA Puget Sound Healthcare System, Seattle, Washington, United States*

Proteostasis mechanisms fail with aging, promoting toxic protein accumulation. Neurons are particularly vulnerable to proteostatic disruption leading to aging related neurodegeneration. Abnormal activation of the endoplasmic reticulum unfolded protein response (UPRER) is implicated in tauopathies, a group of neurodegenerative diseases characterized by pathological accumulation of the microtubule-associated protein tau. Previous work showed neuronal overexpression of UPRER transcription factor XBP-1s suppresses tauopathy in C. elegans. Whole-genome RNA sequencing of C. elegans overexpressing XBP-1s showed upregulation of the following genes with human homologs compared to non-transgenic controls: csp-1, F42G8.7, F41E7.6, Y19D10A.16, C01B4.6, dnj-28, hsp-4, ckb-2, mct-2, lipl-3, and eol-1. Surprisingly, each one of these genes is required for xbp-1s-mediated suppression of tauopathy, suggesting that XBP-1s activates a broad and non-redundant network of cellular mechanisms to reduce tau pathology. Of these, we examined the critical UPRER regulator BiP/hsp-4, the ER resident HSP70 homolog. Hsp-4 loss of function, but not loss of the cognate ER resident DNAJ protein dnj-28, eliminates xbp-1s-mediated suppression of tauopathy. While hsp-4 loss of function exacerbates tau-induced behavioral deficits, tau protein level and phosphorylation are unaffected. High level overexpression of hsp-4 exacerbates tau-induced behavioral deficits and protein accumulation, while moderated hsp-4 overexpression ameliorates this phenotype. Furthermore, caspases also appear to play an important role downstream of XBP-1s: both the XBP-1s target csp-1 and its non-XBP-1s target gene family member ced-3 are required for xbp-1s-mediated suppression of tauopathy. In summary, this data set provides new therapeutic ideas that may be leveraged into novel treatments for tauopathy disorders further development against tauopathy.

### SESSION 7315 (POSTER)

### NUTRITION, EATING DISORDERS

Abstract citation ID: igae098.2830

**AGE DIFFERENCES IN POSTPRANDIAL AMINO ACID PROFILES ARE DETERMINED BY PROTEIN CONTENT OF THE MEAL**

Catrin Herpich, Donna Li, Daniela Weber, and Kristina Norman, *German Institute of Human Nutrition Potsdam-Rehbrücke, Nuthetal, Brandenburg, Germany*

Amino acid availability after a meal differs between older and younger adults and the macronutrient composition of the meal affects postprandial amino acid availability as well. In this analysis, we examined postprandial amino acid response of healthy older and younger study participants to test meals with varying protein contents. The test meal either had normal protein (16 E% (energy percent) protein, 68 E% carbohydrates) or a high-protein content (HP, 77 E% protein (whey), 17% carbohydrates; 450 kcal each). Postprandial plasma amino acid concentrations were quantified at different timepoints (0, 30, 60, 120, 240 min) using UPLC-MS/MS. Amino acids were grouped into essential (EAA), dispensable (NEAA), and branched-chain amino acids (BCAA). Time and group differences of amino acid profiles were examined by repeated measures ANOVA and by comparing the incremental area under the curve (iAUC) using Mann-Whitney test. In older (n=40, 72.8±5.05 years) and younger adults (n=30, 25.5±3.94 years), EAA, NEAA and BCAA concentrations increased after both test meals over four hours (all $p< 0.005$), with a higher increase in response to HP. After the HP meal, significantly higher BCAA and EAA concentrations were found in older adults at 60 min. In addition, iAUC of BCAA, EAA and NEAA was significantly higher only in older participants following HP (all $p< 0.05$). After a meal containing high amounts of fast-absorbed protein, healthy older adults show higher postprandial amino acid concentrations that might result from delayed tissue uptake. Whereas in response to normal protein content no age-differences were seen.

Abstract citation ID: igae098.2831

**BINGE EATING DISORDER AND THE RISK OF SUICIDE IN OLDER WOMEN**

Therese Mendez, and Ann O'Hanlon, *University of New Orleans, New Orleans, Louisiana, United States*

Binge Eating Disorder (BED) is a psychiatric condition marked by repeated instances of excessive food consumption without control. Research on eating disorders tends to focus on younger populations, but recent studies have shown an increasing prevalence of BED in older women. Binge Eating Disorder may pose unique challenges and heightened risks for older women, including an increased potential for suicidality. This review aims to spotlight the critical intersection of binge eating disorder, its prevalence among older women, and the associated risk of suicide. A scoping literature review was done in accordance with the PRISMA-ScR criteria, targeting studies published between 1996 and 2024 on PubMed, PsychINFO, CINHAL, Web of Science, and Google Scholar. The search focused on research pertaining to women aged 40 and above, exploring the association between BED and suicide risk within this demographic. Of the literature surveyed, there were no studies that focused explicitly on the population of interest. Only eight studies met the inclusion criteria for BED and the risk of suicidality in women. These studies show that BED significantly correlates with an elevated suicidality risk, highlighting a complicated relationship that adversely impacts mental health and possible mortality. Despite acknowledging the general risk BED poses for suicidality, the review underscores a concerning scarcity of research dedicated to understanding BED's prevalence and specific suicidality risk among older women. The findings reveal a need for further research to delve into the nuanced psychological effects of BED on older women and its contribution to heightened suicide risk.

Abstract citation ID: igae098.2832

**DIETARY INFLAMMATION AND FALL RISK IN THE ELDERLY: DII AND E-DII SCORE ANALYSIS FROM THE HEALTH AND RETIREMENT STUDY**

Afsaneh Fallahi[1], Matthew Lohman[2], James Hebert[2], and Sherry Price[2], *1. University of South Carolina, New York,*

*New York, United States*, *2. University of South Carolina, Columbia, South Carolina, United States*

**Background and Objective:** The Dietary Inflammatory Index (DII) and Energy-adjusted DII (E-DII) are quantitative tools assessing the inflammatory potential of a diet, but these metrics have seldom been applied or compared in a large representative population of older adults. This study aims to calculate and evaluate the association of these indices with fall risk in the elderly.

**Methods:** DII and E-DII scores were computed for a subsample of 8,035 respondents from the 2013 Health Care and Nutrition Study (HCNS), integrated within the Health and Retirement Study (HRS). Using self-reported data from the Harvard FFQ for 33 out of 45 nutrient parameters, individual DII, and E-DII (adjusted per 1000 kcal intake) scores were computed. Using 2022 HRS data on the history of falls, which was measured binary, and after adjusting for other covariates, logistic regression analyses in SAS software were conducted to assess the associations of interest.

**Results:** The mean DII score was 0.56 (SD = 2.74) and the E-DII was -0.08 (SD = 2.01). The DII model showed no significant association with fall risk (F-value: 0.85, p = 0.36). Conversely, the E-DII model demonstrated a significant inverse association (F-value: 10.49, p= 0.0012) with an E-DII coefficient of -0.059, p = 0.00012.

**Conclusion:** Results suggest that higher anti-inflammatory diets, according to E-DII scores, are associated with a lower fall risk among the elderly. E-DII may better reflect the diet's impact on health outcomes. Keywords: DII, E-DII, HRS, Falls, Older Adults

Abstract citation ID: igae098.2833

### EFFECT OF D-ALLULOSE ON INSULIN RESISTANCE AND ENDURANCE ABILITY: IS D-ALLULOSE AN EXERCISE MIMETIC?

Teruhiko Koike[1], Yang Gou[2], Bingyang Liu[3], Yukie Natsume[4], Takamasa Tsuzuki[5], Kamal Shahriar[6], and Ryoichi Banno[1], *1. Nagoya University, Nagoya, Aichi, Japan*, *2. University of Washington, Seattle, Washington, United States*, *3. Zhejiang University, Hangzhou, Zhejiang, China (People's Republic)*, *4. Nagoya university of economics, Inuyama-shi, Aichi, Japan*, *5. Meijo University, Nagoya, Aichi, Japan*, *6. Nagoya University Graduate School of Medicine, Nagoya, Aichi, Japan*

D-Allulose, a rare sugar and C3-epimer of D-fructose has been proposed as a candidate for dietary restriction mimetics via inhibition of glycolysis. Anti-hyperglycemic and anti-obesity effects have been reported in rodents and human subjects. Here, we report that D-allulose increases the glucose infusion rate in the two-step hyperinsulinemic-euglycemic clamp test in high-fat or high-sucrose diet-fed rat models. Consistently, the insulin-induced phosphorylation of AKT in the skeletal muscle was higher with D-allulose administration with decreased levels of inflammatory cytokine, TNF-α. We also report the effect of D-allulose on insulin sensitivity and aerobic performance in a healthy mice model and compare them with exercise training effects. D-allulose enhanced insulin sensitivity, increased running distance, and maximal aerobic speed (MAS). The improved exercise performance was associated with lower blood lactate levels and increased liver glycogen contents. D-allulose also enhanced the phosphorylation of AMP-activated protein kinase (AMPK) and acetyl-CoA carboxylase (ACC), and the expression of peroxisome proliferator-activated receptor γ coactivator 1α (PGC-1α). Our results indicate that D-allulose administration improves insulin sensitivity and enhances endurance ability like exercise training.

Abstract citation ID: igae098.2834

### PILOT NUTRITION EDUCATION PROGRAM FOR VETERANS LIVING WITH DEMENTIA AND THEIR CAREGIVERS

Jamie Giffuni[1], Kelly Barton-Ort[2], Elizabeth Dennis[3], and Odessa Addison[3], *1. Baltimore and Loch Raven VAMC, Baltimore, Maryland, United States*, *2. Baltimore VA Medical Center, Baltimore, Maryland, United States*, *3. University of Maryland School of Medicine, Baltimore, Maryland, United States*

The prevalence of dementia is expected to increase as the Veteran population ages. Caring for a Veteran with dementia impacts the physical and mental health of the caregiver. Programs are needed to support the health and wellbeing of both Veteran and caregiver. Vets & Veggies is a whole health(y) aging program that provides free produce to Veterans visiting the Geriatric Wellness Center. This project describes the development of a novel, pilot nutrition program combining nutrition education with kitchen skill development and fresh produce access for Veterans living with dementia and their caregivers, Vets & Veggies for Caregivers (V&VC). One-hour in-person classes led by a registered dietitian with experience in geriatrics occurred weekly for 4 weeks. Topics included: Feeding tips for Caregivers/Caring for YOUR nutrition; MyPlate/Healthy Eating for Older Adults; the importance of protein intake; cooking with spices/low sodium cooking. Participants received free produce with recipes/tips for use, and adaptive kitchen equipment for safe preparation by the Veteran. Behavioral Risk Factor Surveillance System – Fruit and Vegetable Module, Caregiver Self Efficacy 8-Item Scale, Zarit Caregiving Burden 6-Item Survey, and the Mini Nutritional Assessment were completed by the caregiver pre and post-program. Two Veteran/caregiver dyads and one caregiver attended all classes with small positive trends in fruit and vegetable intake, decreased caregiving burden score, and improved nutrition assessment score. Participants enjoyed and looked forward to the classes. This pilot program demonstrated feasibility for developing and implementing a program designed to improve the whole health of Veterans living with dementia and their caregivers.

Abstract citation ID: igae098.2835

### VALIDATION OF A COMBINED SARCOPENIA AND NUTRITION SCREENING TOOL FOR COMMUNITY-LIVING OLDER ADULTS

Susan Saffel-Shrier, Amy Covington, and Jing Wang, *University of Utah, Salt Lake City, Utah, United States*

Malnutrition and sarcopenia are major intersecting conditions of the frailty syndrome. Both have similar symptoms to include reduced energy and protein intake along with losses of muscle and strength. These losses can lead to decreased functionality and independence culminating in poor health outcomes. When both conditions are present, community-living older adults have a potential for a more direct negative health effect. Appropriate screening tools for malnutrition and sarcopenia are frequently underutilized impairing diagnosis and treatment in this population. Furthermore, there is a lack of validated community-based nutrition screening tools compared against the Nutrition-Focused Physical Exam (NFPE), the gold standard for malnutrition diagnosis. The purpose of this study was to determine if combining two screening tools, the nutrition SCREEN II and sarcopenia SARC-F would yield higher malnutrition diagnostic accuracy compared to using each tool individually. Utilizing the NFPE tool as a reference, the combined SCREEN II plus SARC-F accurately diagnosed malnutrition in 103 participants (84.4%), surpassing the individual diagnoses of 72 (59%) by SCREEN II and 66 (54%) by SARC-F. This result underscores the superior accuracy of the combined tool. The internal consistency reliability of the combined tool, assessed through Cronbach's alpha, yielded a coefficient of 0.73, indicating satisfactory internal consistency reliability. The convergent validity, assessed using the Phi coefficient, was 0.67, suggesting that the combined scores from the two tools are positively correlated with diagnoses of malnutrition based on the NFPE. These results support utilization of the combined screening tools in diagnosing malnutrition therefore improving the health of older adults.

Abstract citation ID: igae098.2836

### VETS AND VEGGIES: FEASIBILITY OF OFFERING A FREE ONSITE FARMER'S MARKET TO OLDER VETERANS

Elizabeth Dennis[1], Jake Drumheller[2], Kelly Barton-Ort[3], Jamie Giffuni[4], Jeffrey Beans[2], and Odessa Addison[1], *1. University of Maryland School of Medicine, Baltimore, Maryland, United States*, *2. Baltimore VA, Baltimore, Maryland, United States*, *3. Baltimore VA Medical Center, Baltimore, Maryland, United States*, *4. Baltimore and Loch Raven VAMC, Baltimore, Maryland, United States*

This project evaluated a 6-month, free, onsite farmer's market offered through Gerofit, a health promotion program for older Veterans. Each week, 4-8 fresh produce items were delivered to the Gerofit gym facility as a strategy to overcome barriers related to fresh produce access. Participants self-selected items, taking as many or few items as preferred. Gerofit staff sent weekly emails with suggested recipes for produce preparation and recorded weekly leftover produce. An end of season administered questionnaire assessed Veterans' perceived impact on diet, reasons for choosing weekly produce items and open-ended feedback. The most common weekly leftover items were leafy greens (e.g., lettuce, chard) and squash. Of 25 participants who completed questionnaires, 60% reported eating new types of produce because of the farmer's market. Eleven participants reported trying a new item specifically because of Gerofit staff recommendations. When asked why they didn't choose specific items, 15 reported being unsure of preparation methods and 10 reported disliking the item. When asked what participants did with the produce received, most (84%) said they cooked it using their own recipe. Nineteen participants (76%) reported that the farmer's market saved them money, 72% reported that it made it easier for them to eat healthier, and 60% said it helped them eat a wider variety of produce. Veterans rated the weekly produce 9.7/10 (1-10 scale; 10 being most helpful). Future studies are needed to implement and evaluate strategies designed to increase overall participation, encourage trying new produce, and reduce weekly food waste.

### SESSION 7320 (POSTER)

### PHARMACOLOGY

Abstract citation ID: igae098.2837

### COVID-19 MORTALITY RATES AND CHANGES IN ANTI-DEMENTIA AND PSYCHOTROPIC MEDICATION USE AMONG NURSING HOME RESIDENTS

Alison Rataj[1], Matthew Alcusky[2], Brian Ott[3], Jonggyu Baek[2], Shiwei Liang[2], and Kate Lapane[2], *1. University of Massachusetts Boston, Durham, New Hampshire, United States*, *2. University of Massachusetts Chan Medical School, Worcester, Massachusetts, United States*, *3. Brown University, Providence, Rhode Island, United States*

Objective. We examined the relationship between nursing home (NH) COVID-19 mortality rates and changes in antidementia and psychotropic medication initiation before and during the pandemic and explored the influence of staffing and resident factors. Methods. Changes in medication initiation were collected through a novel, nationally representative cross-sectional Dementia Treatment Survey (2022) of NH Directors of Nursing. Outcome measures were created by collapsing a 5-point Likert scale contrasting less/about the same vs. more initiation and less versus more/about the same initiation. NH's peak monthly COVID-19 death rate per 1,000 residents (05/2020-12/2022) was calculated from the CMS COVID-19 Nursing Home data file. Covariates were drawn from NH Compare, Provider of Service, Master Beneficiary Summary, Minimum Data Set 3.0, and Medicare administrative claims files. Adjusted odds ratios (aORs) were estimated from multivariable logistic models. Results. NHs with higher COVID-19 death rates at the peak of the pandemic were less likely to report changes in antidementia medications (aORdecrease: 0.38, 95% CI: 0.15-0.94; aORIncrease: 0.27, 95% CI: 0.08-0.85), but no association with psychotropic medication initiation was observed. NH's that reported increased resident behavior problems during the pandemic had higher odds of psychotropic initiation (aOR=11.25, CI=3.86-32.83). Discussion. Increased COVID-19 mortality rates were not associated with changes in antidementia medication use during compared to before the pandemic. Increased behavior changes may be due to consequences of COVID-19 (i.e., social isolation); our findings indicate that increased behavior changes were associated with more psychotropic drug use.

Abstract citation ID: igae098.2838

### DISTINCTIVE NEURONAL RESPONSES TO VARIOUS ANTICHOLINERGICS IN THE ACT COHORT HIPSC-DERIVED NEURONS

Inez Pranoto, Tiara Schwarze-Taufiq, Katherine Hui, Ruowei Zhu, Jordan Ogg, Paul Crane, Shelly Gray, and Jessica Young, *University of Washington, Seattle, Washington, United States*

Anticholinergic medications remain widely used among older adults despite evidence of safety concerns, including dementia risk. Clinical cohort studies have linked certain anticholinergic classes, such as antidepressants and bladder antimuscarinics, to higher dementia risk. However, distinguishing whether this association stems from individuals' predisposed conditions or drug-induced neuronal dysfunctions remains challenging. To address this, we used human induced pluripotent stem cell-derived neurons (hiPSC-Ns) from participants in the Adult Changes in Thought Study (ACT) aging study. We treated ACT hiPSC-Ns with four anticholinergic classes: antidepressants, antihistamines, bladder antimuscarinics, and antispasmodics. We evaluated drug-induced neuronal responses at a cellular and molecular level by measuring cytotoxicity, synaptic gene expression, and secreted amyloid-beta, a hallmark of Alzheimer's disease. We found that exposure to antidepressants and bladder antimuscarinics induced neurotoxicity, while antihistamines and antispasmodics did not compromise cell viability at the concentrations tested. Despite inducing neurotoxicity, antidepressants and bladder antimuscarinic treatments upregulated the expression of various genes involved in synaptic plasticity, synaptic vesicle trafficking/release, and synaptic inhibition. Notably, treatment with a bladder antimuscarinic drug increased the secreted A**β**-42/A**β**-40 ratio, indicating a higher production of pathogenic amyloid isoform. Our preliminary findings reveal a consistent elevation in neurotoxicity, synaptic gene expression, and secreted amyloid-beta following exposure to antidepressants and bladder antimuscarinics across hiPSC-Ns from multiple ACT participants. We aim to further investigate the distinct molecular mechanisms of different anticholinergic compounds and their potential impact on declining neuronal functions. Our ongoing investigation provides cellular and molecular evidence to discern the source of dementia risk observed in cohort studies.

Abstract citation ID: igae098.2839

### EVALUATION OF PHARMACY-BASED RECOMMENDATIONS WITHIN A COMPREHENSIVE GERIATRIC ASSESSMENT TEAM

Maria Cervantes[1], Nikil Patel[2], Jacob Tillmann[3], Christine Cigolle[2], Heather Bieber[3], Janette Dunlap[3], and Kristin Phillips[2], *1. Veterans Health Administration, St. Johns, Florida, United States*, *2. VA Ann Arbor Healthcare System, Ann Arbor, Michigan, United States*, *3. North Florida/South Georgia Veterans Health System, Gainesville, Florida, United States*

**Background:** Rural older Veterans face unique challenges in managing multiple chronic conditions. A novel, virtual Comprehensive Geriatric Assessment (CGA) clinic, at a southeastern Veterans Affairs (VA) medical center, aimed to optimize medications for this population. The objective of this study is to evaluate the geriatric clinical pharmacist (GCP) recommendations and their implementation for the CGA patients' medication regimens.

**Methods:** The study performed electronic health record review of North Florida/South Georgia VA CGA patients, focusing on GCP recommendations targeting potentially inappropriate medications (PIMs) and polypharmacy. Recommendations were categorized as: discontinue, switch, taper, request further provider review, address polypharmacy. Recommendations were evaluated as to whether they were implemented and when the implementation occurred.

**Results:** The study population consisted of 273 Veterans. 86 Veterans received no recommendations. The GCP provided 323 recommendations for 187 Veterans, with 156 (48.3%) implemented. Recommendation uptake was highest for requesting provider review of a medication issue (58%); it was lowest for switching medications (27.9%). Implemented recommendations regarding medication categories included: non-anticholinergic BEERs Criteria medications, 36.3%; anticholinergic medications, 41.8%; hypoglycemic or hypotensive risk medications, 69%; general polypharmacy issues, 42.9%; other medications, 56.7%.

**Conclusion:** This virtual GCP intervention, as part of a broader virtual CGA clinic, demonstrated successful implementation of deprescribing and medication optimizing recommendations, resulting in reduction in use of PIMs and polypharmacy in rural older rural Veterans. The intervention also resulted in increased collaboration with PCPs. Future evaluation needs to study the intervention's long-term outcomes in decreasing polypharmacy and increasing medication safety for this vulnerable population.

Abstract citation ID: igae098.2840

### MICROBIOME-KIDNEY METABOLISM PROVIDES INSIGHT INTO THE MECHANISM OF ICARIIN ALLEVIATES AGING IN OLDER FEMALE MICE

Xiaoang Li, Yiming Zhang, and Liping Duan, *Peking University Third Hospital, Beijing, Beijing, China (People's Republic)*

The average life expectancy of women in most countries and regions is significantly higher than men's. Whether in rich developed countries or relatively poor third-world countries, women are more likely to live. Our previous study revealed that Icariin enhances youth-like features by attenuating the declined gut microbiota in older male mice. To further study the mechanism of icariin on delaying aging in older female mice on gut microbiota and metabolism, two age groups of C57BL/6J female mice (8 week- and 24 month- mice) were given a single dose of icariin at 100mg/kg by gavage for 15 days. The 16S rRNA gene pyrosequencing analyzed the fecal microbiome. We observed clear differences in gut microbial profiles between the young and older female mice. Interestingly, while older male mice did not show this effect, treatment with icariin increased the abundance of the short-chain fatty acid (SCFA)-producing bacteria Akkermansia muciniphila and Eubacterium ramulus in older female mice, bringing their levels close to those of untreated young female mice at the species level. Further, we used non-targeted metabolomics technology to detect all metabolites in the kidney. We found that the level of D-serine in the kidneys of older female mice was significantly lower than that of young female mice, and the metabolite levels were significantly increased after icariin treatment. SCFA and D-serine are gut microbial-derived products with kidney-protective properties. This study provides valuable insights into the molecular mechanisms of icariin, highlighting target characteristics and key pathways for slowing the aging process.

Abstract citation ID: igae098.2841

### OLDER ADULTS' PERCEPTION TOWARD DEPRESCRIBING INAPPROPRIATE MEDICATIONS

Mohammad Rababa, and Ali Al Ghazo, *Jordan University Of Science And Technology, Irbid, Irbid, Jordan*

Introduction: Inappropriate prescription of medications is a problem of growing interest in geriatrics. Initiatives have been ongoing to evaluate interventions targeting the hazards of inappropriate prescription of medications; Deprescribing is one of these initiatives. However, no current study has examined medicine-related perceptions toward deprescribing inappropriate medications among older adults.

**Methods:** A convenience sample of older adults who regularly visited the outpatient clinics during July 2023 for regular checkups were recruited. Data were collected by a graduate nursing student from one pharmacy located in a public hospital, five days per week, via in-person interviews using the Patient's Perceptions of Deprescribing survey.

**Results:** The study found that older adults' perceived interest in stopping medications, belief about medicine overuse, the unimportance of medicines, and medication concerns are significantly associated with a variety of sociodemographic (e.g., age), clinical (e.g., comorbidities), and PCP-related factors (e.g., knowledge of medications).

**Conclusion:** Inappropriate prescription of medication is one of the most common polypharmacy-related issues among older adults. The current study reported its findings on medicine-related perception toward deprescribing inappropriate medications among older adults. Care providers should discuss the benefits of deprescribing inappropriate medications with their patients to prevent the side effects associated with long-term unnecessary use. The long-term use of inappropriate medications by older adults should be carefully evaluated. Future studies on the effectiveness of an evidence-based deprescribing protocol on minimizing the clinical side effects associated with inappropriate prescription of medications among older adults are recommended.

Abstract citation ID: igae098.2842

### OPTIMIZING POLYPHARMACY CARE: INSIGHTS FROM PHARMACISTS INFORMING A POPULATION HEALTH APPROACH

Nicha Tantipinichwong[1], Dina Mustafa[2], Irina Avidon[2], Sonja Rosen[3], and Michelle Keller[4], *1. Cedars-Sinai Medical Network, Los Angeles, California, United States*, *2. Cedars-Sinai Medical Care Foundation, Los Angeles, California, United States*, *3. Cedars-Sinai Medical Center, Los Angeles, California, United States*, *4. University of Southern California, Los Angeles, California, United States*

Over 60% of older adults grapple with multimorbidity (more than one chronic condition), while polypharmacy (taking four or more medications) is prevalent in 65% of cases. Polypharmacy correlates with adverse outcomes such as mortality, falls, fractures, hospitalizations, and decline in functionality and cognition. Many health systems lack systematic monitoring of polypharmacy and its consequences. To address this, we aimed to develop a population health outcomes dashboard for a large urban health system, focusing on polypharmacy outcomes. We also aimed to increase the effectiveness of our polypharmacy consults. Our qualitative study, employing Framework Analysis, involved interviews with eight clinical pharmacists conducting polypharmacy medication review consults for older adults. We aimed to identify challenges, enhance clinic efficiency, and pinpoint data and outcomes for tracking on the dashboard. Major challenges included time constraints due to lengthy medication lists, lack of documentation of herbal supplements on medication lists, and patients' readiness for consultations. Opportunities for efficiency improvement included pre-consultation patient preparation, streamlined note templates, and prioritizing intervention elements in each visit. Beyond clinical and disease-related outcomes, valuable dashboard measurements encompassed patients' and physicians' perceived benefits (e.g., improved medication adherence). These findings will guide enhancements to the polypharmacy clinic workflow and provide insight for the development of the polypharmacy outcomes and interventions dashboard.

Abstract citation ID: igae098.2843

### TOWARD OPTIMIZED CLINICAL TRIALS OF BOTANICAL DIETARY SUPPLEMENTS IN OLDER ADULTS

Alex Speers, and Amala Soumyanath, *Oregon Health & Science University, Portland, Oregon, United States*

The use of dietary supplements, which includes botanical dietary supplements, is increasingly common among older adults. This is concerning due to a) the lack of high-quality efficacy studies for botanical products, b) issues with product quality and variability, and c) the potential for herb-drug interactions, which is increased in older adults due to higher rates of polypharmacy. Given the growing popularity of these products among consumers, more rigorous data are needed, but a rational, stepwise approach is required. At Oregon Health & Science University (OHSU) and collaborating institutes, a pipeline is being developed to study botanical dietary supplements in an older population. The BENFRA Botanical Dietary Supplements Center at OHSU studies Botanicals Enhancing Neurological and Functional Resilience in Aging, with a focus on two botanicals: Centella asiatica (gotu kola) and Withania somifera (ashwagandha). This work includes product characterization studies, exploration of mechanisms in vitro and in vivo, and the development of methods for measuring plant compounds in plasma. Separate from the BENFRA Center, studies are now underway at OHSU to investigate the safety, pharmacokinetics, and biological signatures of these botanicals in humans. Collectively, this pipeline of research will generate the necessary data to support future optimized efficacy trials in older adults. The presentation will outline the rigorous studies being undertaken and provide preliminary data from these preclinical and clinical investigations.

### SESSION 7325 (POSTER)

### PRIMARY CARE

Abstract citation ID: igae098.2844

### ACADEMIC DETAILING INTERVENTION: INCREASING TRENDS OF NALOXONE PRESCRIBING IN RURAL ARKANSAS

Leah Tobey-Moore, Meghan Breckling, Mohab Ali, and Robin McAtee, *University of Arkansas for Medical Sciences, Little Rock, Arkansas, United States*

This longitudinal study analyzes naloxone prescribing trends of rural primary care providers (PCPs) in Arkansas (AR). As one of the HRSA-funded Geriatric Workforce Enhancement grant recipients, the AR Geriatric Education Collaborative (AGEC) worked with a rural federally qualified healthcare system (ARcare) to reduce the likelihood of patient's accidental overdose by co-prescribing naloxone, when indicated. A multi-disciplinary Academic Detailing (AD) team of a PT, PharmD and MBBChB conducted AD trainings. AD is clinician-to-clinician customized education connecting recipients to updated evidence and practice guidelines. These interactive telehealth trainings stimulated PCPs to share knowledge deficits and perspectives, encouraged a commitment to increase patient safety and adhere to naloxone prescribing guidelines from Centers for Disease Control and state laws especially during cases when naloxone should be co-prescribed alongside high-risk medications like opioids. ARcare PCPs received "Preventing Opioid Overdose with Naloxone" AD training. Before training, previous naloxone prescribers (group 1) prescribed naloxone 39 times, 106 times in period 1 (P1=11 months post-training), and 91 times in period 2 (P2=22 months post-training), a 14.2% decrease. Group 2 previously did not prescribe naloxone during the baseline period, then prescribed 46 times in P1 and 63 times in P2, a 40% increase. Group 3 did not prescribe naloxone in P1, but prescribed naloxone 13 times during P2. Overall, P1 data from all groups totaled 152 naloxone prescriptions after AD, a 290% increase; in P2, naloxone was prescribed 167 times, a 328% increase. In summary, AD was effective in encouraging naloxone prescribing among prescribers in rural AR.

Abstract citation ID: igae098.2845

### ADAPTING A NOVEL BEHAVIORAL TEST TO SCREEN FOR PRECLINICAL ALZHEIMER'S DISEASE IN PRIMARY CARE

Sydney Schaefer[1], Elizabeth Fauth[2], Andrew Hooyman[1], and Jill Love[3], *1. Arizona State University, Tempe, Arizona, United States*, *2. Utah State University, Logan, Utah, United States*, *3. Neurosessments LLC, Tempe, Arizona, United States*

Most early dementia cases are missed in primary care, due partly to subjective screening (i.e., self-reported concerns, clinician observation). Objective tests (e.g., Mini-Mental Status Exam) are valid but take too long for widespread use in primary care. Extensive research on our qBEANS behavioral test supports that it is sensitive and specific to Alzheimer's disease pathology, and is prognostic of cognitive and functional decline. This timed test engages motor learning, visuospatial memory, and executive function, involving six trials of 15 repetitions in which patients spoon raw kidney beans to small plastic cups in a sequence; no technology or wearable sensors are required. However, the current version is too long for primary care adoption (~7 minutes). The purpose of this study was to identify the minimum number of trials needed for reliability relative to the original longer version. Forty-eight non-demented participants (mean±SD age: 75.4±7.0 years; 77% female) completed the original version while the duration of each repetition was also recorded. Our reliability threshold was an intraclass correlation (ICC) >0.75. Results indicated that the most reliable shorter version involved only three full (15-repetition) trials (mean completion time = 232 seconds, or 3.85 minutes), 48% faster than the original version (ICC=0.85). While this shorter version is reliable, future work will determine if it is acceptable in primary care settings when administered by medical assistants during the rooming process. Findings also warrant further development of qBEANS as a direct-to-consumer product, given its low cost and ease of administration.

Abstract citation ID: igae098.2846

### EARLY DETECTION OF COGNITIVE IMPAIRMENT IN PRIMARY CARE: IMPLEMENTATION CHALLENGES AND OPPORTUNITIES

Amy Deckert[1], Laura Chavira[2], Katherine Selzler[2], Karen Weyrauch[2], James Murray[2], Alissa Kurzman[2], and Tim MacLeod[2], *1. Davos Alzheimer's Collaborative, Toronto, Ontario, Canada*, *2. Davos Alzheimer's Collaborative, Wayne, Pennsylvania, United States*

Healthcare systems worldwide are not prepared to adequately address the needs of the 55 million individuals affected by Alzheimer's and related dementias. A substantial 17 to 20-year lag exists between development of innovations and their widespread adoption, with less than 50% being used in clinical practice (Bauer et al. 2020). In the early stages of Alzheimer's disease, cognitive impairment often goes unrecognized until later in the disease course, and it can take months or years to reach a diagnosis. Early detection in primary care is a pivotal factor in improving patient outcomes and ensuring timely access to appropriate care and interventions. The Davos Alzheimer's Collaborative Healthcare System Preparedness (DAC-SP) Early Detection flagship program implemented digital cognitive assessments (DCA) and a blood-based biomarker (BBM) test in diverse primary care or other non-specialty settings across 7 healthcare systems in 6 countries (Brazil, Jamaica, Japan, Mexico, Scotland, US). We conducted a mixed methods evaluation of the process and outcomes of the DAC-SP flagship study guided by the Consolidated Framework for Implementation Research (CFIR; Damschroder et al., 2009) and report here the qualitative findings from the pre-implementation phase. We employed a directive content analysis of transcribed in-depth interviews with clinicians (n=51) and implementation team leads (n=7) across flagship sites. Our findings underscore the critical importance of addressing system-level factors in the design and implementation of DCA and BBM tests in primary and non-specialty care, suggesting that sustainable practice change requires a need to shift the focus from individual to systemic interventions.

Abstract citation ID: igae098.2847
### PRIMARY CARE PHYSICIAN PERSPECTIVES ON THE INVOLVEMENT OF FAMILY MEMBERS IN THE CARE OF PEOPLE WITH DEMENTIA

Alma Hernández de Jesús[1], Sharifa Brooks-Smith-Lowe[1], Melissa Filippi[2], Julie Wood[3], Leah Karliner[1], Soo Borson[4], and Alissa Bernstein Sideman[1], *1. University of California San Francisco, San Francisco, California, United States*, *2. American Academy of Family Physicians, Lawrence, Kansas, United States*, *3. American Academy of Family Physicians, Leawood, Kansas, United States*, *4. University of Southern California, Santa Ana, California, United States*

Involving family is integral to the care of people with dementia. Family medicine's comprehensive "whole person" approach is ideally suited to this care. We interviewed 18 family physicians across the US to identify ways they involved and communicated with family members throughout dementia diagnosis and care. Interviews were thematically coded using ATLAS.ti. Participants identified several areas in which strong, open, and continuous communication with family made a difference in the care they provided to their patients with dementia: (1) Facilitating early diagnosis. Physicians in our study described the importance of family members in identifying the presence of cognitive impairment, communicating their observations to the physician, and providing information helpful in diagnostic evaluation. They also emphasized the importance of thoughtful communication with the family around disclosure once a dementia diagnosis is made; (2) Goal setting and care planning. Family physicians placed high value on supporting the patient's quality of life, including prolonging life at home, and supporting patient autonomy. This included discussions about goals of care and intervening in challenging family dynamics; (3) Providing relevant disease education, facilitating access to resources, and acting as an interlocutor with specialists; and (4) Helping the patient avoid preventable hospitalizations by sharing safety measures and teaching family about managing medications and comorbidities. Although family physicians are uniquely suited to care for people with dementia, they also identified challenges to optimal communication: family members can be reluctant to address dementia care problems, and insufficient time, resources, and compensation challenge clinicians to sustain that communication over time.

### SESSION 7330 (POSTER)

### REHABILITATIVE CARE/PHYSICAL AND OCCUPATIONAL THERAPY

Abstract citation ID: igae098.2848
### ACTIVOT: PRELIMINARY RESULTS OF AN INTERVENTION TO IMPROVE HEALTH-PROMOTING DAILY ACTIVITY

Tara Klinedinst[1], Nicholas Hollman[2], Carrie Ciro[1], and Darla Kendzor[1], *1. University of Oklahoma Health Sciences Center, Oklahoma City, Oklahoma, United States*, *2. OU-TU School of Community Medicine, Tulsa, Oklahoma, United States*

Nearly half of older adults in the U.S. have multiple chronic conditions in addition to functional limitations that restrict health-promoting daily activity (e.g., sleep, medication-taking, physical activity). We tested an innovative combination of behavioral activation and occupational therapy techniques to improve performance of meaningful, healthy activity. The ActivOT intervention was delivered by occupational therapists in 10 weekly sessions in participant homes. The primary outcome is performance of health-promoting daily activity, measured by the Canadian Occupational Performance Measure (COPM) at baseline, 10-weeks, and 22-weeks. We report outcome data from 17 participants who were randomized to ActivOT or brief education (n=9 Tx, n=8 Control). On average, participants were 73.0 years old (SD=6.9), majority female (n=13), and White (n=1 Black, n=1 Hispanic/Latino). They had an average of 3.9 chronic conditions (SD=1.4), and 1.9 functional limitations (SD=1.7). The average COPM performance score (out of 10) at baseline was similar for the ActivOT group (M=3.6, SD=1.6) and control group (M=3.3, SD=1.1). At 10-week follow-up, the ActivOT group average increased to 7.0 (SD=1.7) and further increased to 7.2 (SD=1.0) at 22-weeks. In comparison, the control group average increased to 5.9 (SD=2.0) at 10-weeks, then decreased to 5.0 (SD=2.2) at 22-weeks. Further, 89% percent of the ActivOT group reported clinically significant improvements on the COPM (MCID=3). Results show an initial increase in both groups, but the increases are only maintained in the ActivOT group. This study is in progress (aiming for n=40) but shows promise for improving performance of health-promoting daily activity in a difficult-to-treat population.

Abstract citation ID: igae098.2849
### INTEGRATING THE GERIATRIC 5MS INTO MOVEMENT ANALYSIS AND COLLABORATIVE GERIATRIC PHYSICAL THERAPY

Gregory Hartley[1], Kenneth Miller[2], Myla Quiben[3], Marni Larkin[4], Michelle Lusardi[5], Susan Leach[6], and Laura Gras[7], *1. University of Miami Miller School of Medicine, Coral Gables, Florida, United States*, *2. Medical University of South Carolina, Charleston, South Carolina, United States*, *3. University of North Texas Health Science Center, Fort Worth, Texas, United States*, *4. Gyrotonic Manhasset Physical Therapy, Manhasset, New York, United States*, *5. Sacred Heart University, Fairfield, Connecticut, United States*, *6. University of New Mexico, Albuquerque, New Mexico, United States*, *7. Ithaca College, Ithaca, New York, United States*

The Geriatric 5Ms (Matters Most, Medications, Mind, Mobility, Multicomplexity) address complexity of health care for aging adults. The 5Ms are important for physical therapists (PT); members of the health care team concerned with musculoskeletal pain, mobility, and functional capacity. The Academy of Geriatric Physical Therapy, a component of the American Physical Therapy Association, was charged with incorporating emerging movement system science and geriatric care models into a comprehensive PT assessment framework for primary care and initial PT patient encounters. The product, a geriatric movement system assessment framework, incorporates principles of multiple models including the International Classification of Functioning Disability and Health, and other movement-based patient/client management models. Observational movement analysis of key tasks required for safe independent function using the 5M model helps detect needed resources and constraints. Results of movement analysis from the patient examination informed by the 5Ms, impact selection of tests and measures for further analysis of baseline functions. Results allow clinicians to set priorities for targeted interventions addressing body structure/function, activity, and/or participation, and assess outcomes of care. The theoretical basis for this novel approach to movement assessment in aging adults is discussed. Case studies illustrate application of the framework with attention to understanding patient resources as part of intervention strategies. Adoption of the 5Ms into geriatric physical therapy highlights essential geriatric principles and bridges practice across interprofessional teams.

Abstract citation ID: igae098.2850

### RCT: SPECIALIST NURSE-LED EXERCISE REHABILITATION IN AECOPD- IMPACT ON ENDURANCE AND QUALITY OF LIFE

Jiahui Ma[1], Hongjing Lin[1], Zeyu Qin[2], and Zijing Chen[1], *1. The First Hospital Of Jilin University, Changchun, Jilin, China (People's Republic)*, *2. The First Hospital of Jilin University, Changchun, Jilin, China (People's Republic)*

This prospective randomized controlled trial (RCT) evaluates the efficacy of a specialist nurse-led exercise rehabilitation program, grounded in the Patient Health Engagement (PHE) model, for patients with acute exacerbations of COPD (AECOPD). 84 patients with AECOPD were recruited from a Changchun, China, hospital and randomly assigned to intervention or control groups (42 in each). The 12-week intervention featured conventional exercise rehabilitation for the control group and a novel program for the intervention group, incorporating personalized exercise prescriptions led by specialist nurses. Baseline and 3-month post-discharge assessments included 6-minute walk distance (6-MWD) for exercise endurance and SF-36 for quality of life. Participants, with a mean age of 65.70 ± 6.13 years, exhibited severe airflow obstruction (FEV1: 39.48 ± 10.50%) and impaired motor ability (6-MWD: 282.10 ± 92.34m). After 12 weeks of intervention, the 6-MWD in the intervention group was 371.69 ± 65.09m, while the control group was 324.95 ± 74.96m. Both groups demonstrated improved exercise endurance (t1=37.899,95%CI,84.12-97,45,P1 < 0.001;t2=7.766,95%CI,31,60-66.79,P2 < 0.001), yet the intervention group displayed significantly greater enhancement in 6-MWD (90.80 ± 5.36, vs 49.20±14.16, t=-9.537,P < 0.001), indicating clinical significance. Furthermore, the intervention group exhibited higher quality of life scores than the control group, with statistical significance (t=2.988,P<0.05). This RCT underscores the specialist nurse-led exercise rehabilitation program's effectiveness in enhancing exercise tolerance, improving compliance, and elevating the quality of life in patients with COPD, contributing valuable insights to rehabilitation nursing for patients with COPD.

Abstract citation ID: igae098.2851

### THE RELATIONSHIP BETWEEN DAY SERVICE UTILIZATION AND THE CARE LEVEL DETERIORATION FOR PEOPLE WITH DEMENTIA

Liu Yang[1], Yamakawa Miyae[1], Sugiura Aki[1], Kanaya Reiko[1], Wu Yunfan[1], and Yasushi Takeya[2], *1. Osaka University, Suita, Osaka, Japan*, *2. Gerontological and Geriatric Nursing Research Unit, Osaka, Osaka, Japan*

In Japan, people diagnosed with dementia are often recommended to use day services (DS). This study aims to evaluate whether DS has a positive impact on preserving the independence of people with dementia in their daily lives. We analyzed 774 individuals aged 65-74, initially certified as care level 2 or below, between 2012 and 2019 from Osaka Prefecture National Health Insurance Database. The baseline was set three years after their initial certification, and the outcome measure was reaching level 3 or higher care levels. This retrospective cohort study categorized subjects into three groups: Group 1 (non-DS users during the exposure period), Group 2 (DS users for over one but less than two years continuously), and Group 3 (DS users for two years or more). Median survival time was not observed for Groups 1 and 2, while Group 3 had a median survival time of 921 days (95% CI 668-1174 days), showing a statistically significant difference among the three groups (Log Rank P< 0.000). In Cox analysis, the presence of fractures (HR = 2.33; 95% CI = 1.07–5.05, P = 0.032) and baseline care level (HR = 2.54; 95% CI = 1.98–3.28, P < 0.000) demonstrated positive correlations with the outcome measure. The results indicate that the use of DS does not effectively slow down the deterioration of care levels in people with dementia. The independence in daily life for people with dementia does not show a positive impact after using DS.

Abstract citation ID: igae098.2852

### THE ROLE OF SOCIODEMOGRAPHIC FACTORS IN COMMUNITY-BASED REHABILITATION USE AFTER HIP ARTHROPLASTY

Allyn Bove[1], Christopher Bise[1], Lauren Terhorst[1], Kelli Allen[2], Jared Magnani[1], Anthony Delitto[1], and Janet Freburger[1], *1. University of Pittsburgh, Pittsburgh, Pennsylvania, United States*, *2. Durham VA Health Care System, Durham, North Carolina, United States*

Community-based rehabilitation in the home health and outpatient settings is an important element of recovering function after total hip arthroplasty (THA). While there is ample evidence confirming sociodemographic differences in THA rates and short-term outcomes (e.g., post-operative complications), it is unclear whether there are differences in use of rehabilitation after THA. We analyzed 5 years of data (2017-2021) from a large health system on patients who received rehabilitation in the home health (n=6577) and/or outpatient setting (n=3,094) after THA. We estimated several regression models to examine the separate associations between sex, race, area deprivation, or rural residence with the number of visits and length of care in home health and outpatient settings, adjusting for age, hospital, hospital length of stay, non-home discharge, comorbidities, and marital status. Female sex was associated with greater rehabilitation use for 3 of 4 outcomes: number of home health visits (p<.001), number of outpatient visits (p=.04), and length of outpatient care (p<.001), but not length of home health care. High area deprivation was associated with higher use of home health visits (p<.001), but not length of the episode of home health care or outpatient rehabilitation use. Rural residence was associated with fewer home health visits (p=.04) and shorter outpatient episodes of care (p=.02). Race was not significantly associated with any of the outcomes. Overall, results were mixed, with rural residents generally receiving less post-arthroplasty rehabilitation, and women and those residing in more-deprived neighborhoods receiving more rehabilitation services.

### SESSION 7335 (POSTER)

### REMINISCENCE/LIFE REVIEW

Abstract citation ID: igae098.2853

### CONNECTIONS: INTERGENERATIONAL DIGITAL STORYTELLING FOR CULTIVATING RESILIENCE ACROSS LIFESPAN

Vera Ansie[1], and Crispin Mbamba[2], *1. Kwame Nkrumah University of Science and Technology, Kumasi, Ashanti, Ghana*, *2. University at Albany, SUNY, Albany, New York, United States*

This innovative study used intergenerational digital storytelling to explore resilience and fortitude across cultures. It aimed to develop and evaluate a culturally grounded intervention for synergistically cultivating resilience among older adults and youth through shared narratives and strengthened intergenerational ties. The participatory methods engaged older adult-youth dyads in one U.S. state (n=40) and Ghana (n=38) in a structured process of co-creating multimedia digital stories chronicling personal journeys of perseverance and overcoming hardship, with guided activities fostering reflection, reciprocal empathy, and intergenerational dialogue around resilience themes. Qualitative data from participant narratives, interviews, and observations were analyzed using constructivist grounded theory. Key findings highlighted storytelling's transformative capacity for transmitting vital coping strategies, life lessons, and cultural wisdom between generations. For U.S. participants, salient themes included reframing challenges through optimism and self-reliance, while Ghanaian narratives emphasized fortitude rooted in spirituality, collectivism, and reverence for older adults' resilience. Cross-cultural comparisons illuminated how social-ecological context distinctively shapes resilience processes. This study offers a novel, culturally responsive framework for designing intergenerational interventions that synergize resilience by providing hope through shared stories, modeling perseverance across ages, and honoring the fortitude of the human spirit, with implications for adaptation across diverse contexts, resilience theory, lifespan approaches, and culturally-grounded practice promoting well-being across generations.

Abstract citation ID: igae098.2854

### EVALUATING THE VALIDITY AND QUALITY OF SELF-REPORTED IMPORTANT LIFE ACCOMPLISHMENTS BY ADULTS OVER AGE 50

Wenshan Yu[1], and Jacqui Smith[2], *1. Duke University, Ann Arbor, Michigan, United States*, *2. University of Michigan, Ann Arbor, Michigan, United States*

Bluck (2003) proposed that autobiographical memories function to maintain a sense of fulfillment and well-being. In support of this, studies find that older adults prioritize reporting positive early-life content in life narratives. Questions remain however, about the validity of life narratives and whether content prioritization is observed in non-narrative contexts. We utilized data obtained in the Health and Retirement Study (HRS) Life History Mail Survey (LHMS: N = 10496, M age = 69.5) to ask if the content of participants' narrative reports about personal life accomplishments was associated with the quality (item of missing rates; IMR) of responses to non-narrative retrospective questions about residential, education, job, marriage, caregiving, and health histories. Content analyses of the life accomplishment narratives revealed 16 topics relevant to autobiographical life history content (interrater reliability=0.921). Compared to HRS participants who did not provide narrative life accomplishments, those who did had higher cognitive status and more years of education (cognitive status OR=1.04, p< 0.001; education OR=1.02, p< 0.001). Multinomial regressions indicated gender-related narrative content differences: whereas men reported career-related accomplishments, women prioritized accomplishments such as children, marriage, and pride in self (OR=6.17 p< 0.001). Linear regressions revealed that when participants mentioned corresponding accomplishments (i.e., about education, career, care for others, health, and marriage), their IMRs significantly decreased in that section, even after controlling for demographic, socioeconomic, and health variables (p < 0.01). This suggests that the validity and quality of life history data is influenced by participants' attitudes and beliefs about life in addition to their sociodemographic characteristics.

Abstract citation ID: igae098.2855

### IMPACT OF A TELEPHONE REMINISCENCE PROGRAM ON THE MENTAL HEALTH OF OLDER ADULTS: A RANDOMIZED WAIT-LIST CONTROL TRIAL

Juliette Shellman, Yuxuan Yang, and Cameron Graham, *University of Connecticut, Storrs, Connecticut, United States*

There is increasing evidence that social isolation and loneliness have negative effects on the mental health of older adults. Reminiscence is one example of a cost-effective intervention shown to improve mental health and wellness. A double-blinded randomized controlled trial was employed to test the effectiveness of a telephone reminiscence intervention on mental health outcomes in community-dwelling older adults. The telephone-based program (STORII) is designed to provide a service that automatically calls older adults three times a week asking them meaningful questions about their lives. Responses are recorded over the phone and automatically transcribed. Participants in the intervention group received 3 phone calls per week for 6 weeks. The primary outcome, mental health, was measured by the Mental Health Continuum Scale (MHC). After baseline assessment, eligible participants (N=90) were randomized to either the telephone simple reminiscence intervention (n=41) or 6-week wait-list group (n=39). All participants in the wait-list control subsequently received the telephone reminiscence intervention. An ANCOVA model was run to measure the treatment effect of the intervention, controlling for differences in baseline demographic variables between the groups. Results showed a significant improvement in the psychological well-being dimension of the MHC. Findings support of the use of a telephone reminiscence program for improving the psychological well-being dimension of mental health in older adults. Non-significant findings in the social and emotional well-being dimensions may be due to the length of the intervention. No adverse reactions were reported by any of the participants. Clinical trial registry number NCT05977608.

Abstract citation ID: igae098.2856

### TRAINING ASIAN AND WHITE FAMILY CAREGIVERS LIFE REVIEW DEPRESSION INTERVENTION: A MIXED-METHODS COMPARATIVE STUDY

Christina Miyawaki[1], Angela McClellan[1], and Erin Bouldin[2], *1. University of Houston, Houston, Texas, United States*, *2. University of Utah, Salt Lake City, Utah, United States*

Approximately 10% of older Americans living with Alzheimer's dementia experience depression, yet behavioral interventions remain scarce. We developed a 6-week depression intervention, Caregiver-Provided Life Review (C-PLR) based on life review therapy. The purposes of this study were to examine the feasibility of training Asian and White family caregivers in life review skills and examine the impact on care recipients' depressive symptoms as 77% of Asian dyads conducted the study in their native language. Using a mixed-methods design, Asian (n=15: 7=Chinese, 4=Japanese, 2=Vietnamese, 1=Filipino and Korean) and White (n=25) caregiver-care recipient dyads were trained. We measured care recipients' pre- and post-intervention depression (primary outcome), life satisfaction, caregivers' burden, rewards of caregiving, and dyads' relationship quality (secondary outcomes) scores, and compared them by paired t-tests. We evaluated the feasibility of caregivers' training by fidelity check-in scores. All dyads completed the study. Asian (mean age=52 years) and White (60 years) caregivers were working, female, and in good/excellent health. Their care recipients (Asian=82 and White=80 years) were female and in poor/fair health. Both Asian and White care recipients' depression and dyads' relationship from baseline to post-intervention significantly improved (Asian=1.9, p< 0.001; 0.9, p=0.029; White=1.0, p=0.034; 0.6, p=0.033, respectively). Both caregivers' fidelity scores were identical and similarly high (15.4; 15.3, p=0.91). Qualitative interviews of both caregivers supported the sentiments of the quantitative results. The language of Asian dyads appeared no influence on any outcomes. This study demonstrates that the C-PLR can be added to a list of successful non-pharmaceutical interventions for Asian and White older adults.

Abstract citation ID: igae098.2857

### VIEWS ABOUT LIFE AFTER LIFE REVIEW: FROM LIFE REVIEW LEADERS' PERSPECTIVES IN TAIWAN

Yi-Hsiu Lin[1], and Tsuann Kuo[2], *1. Hsiang Shang Culture and Education Foundation, Taichung City, Taichung, Taiwan (Republic of China)*, 2. *Chung Shan Medical University, Taichung, Taichung, Taiwan (Republic of China)*

Guided Autobiography by Birren and Deutchman (1991) was developed and implemented as an evidence-based Life Review Program in Taiwan for over ten years. Using life course perspective, older adults were exposed to weekly topic and guided to reflect their life events. The purpose of this paper was to evaluate the impact on views about life between older adults and their program leaders after a ten-week session. This study used a mixed-method methodology to evaluate the effects of life review program on the leaders' themselves and the older adults they served. A survey was conducted to ask leaders about the changes in older adults' well-being, followed by qualitative interviews. Over 80 Life Review Leaders participated in this study with 87.32% were female and over 84.51% with at least a college degree. This program helped older adults communicate with family members and strengthen family bonding as well as having the courage to overcome negative events in life. The participants shared many episodes and observations that they saw older adults used each session to reflect the up and down sides of life. Older adults were able to integrate their past and forsee positive to the future life. In condusion, the structured Life Review Program helps older adults reduced depression and isolation, increased confidence and better views about self. Both clinical and policy implications can be generated to help older adults live life and face the future more positively.

### SESSION 7340 (POSTER)

### SERVICES AND INTERVENTIONS

Abstract citation ID: igae098.2858

### BEYOND FALLS: HOW DO FALL PREVENTION PROGRAMS BENEFIT THE COMMUNITY?

Jess VanderWerf, *University of South Florida, Tampa, Florida, United States*

This mixed methods research investigates the Co-Responder Program in Marion County and the CARES program in Pinellas County, Florida. It assesses their effectiveness in reducing fall-related injuries and mortality among older adults by analyzing community surveys administered to frequent 911 callers who subsequently participated in these programs and data from the counties and the state. The study explores the broader benefits of these programs beyond fall prevention, including their impact on reducing 911 calls and alleviating strain on community resources. In Pinellas County, about 65.22% of participants in the CARES program didn't make any 911 calls post-enrollment, showcasing its effectiveness. Among those who still made calls, there was a 25.43% reduction in total calls. In Marion County, a sub-sample of 12 individuals used 1,330 hours of the sheriff's office time for non-emergencies in 2021. Through the Co-Responders Program, there was an impressive 54% decrease in calls from these individuals. Further research is needed to understand the long-term impacts of these programs for the aging population while also standardizing what is and isn't needed in one of these programs for effectiveness. The implications of this research show a growing need to expand training for first responders as it relates to aging issues as it relates to the potential for education in this research. These programs hold the potential to be implemented nationwide, thus freeing up community resources. However, to realize this future policy direction, a deeper understanding of these programs is imperative.

Abstract citation ID: igae098.2859

### BRIDGING SOCIAL GAPS WITH THE VIRTUAL FRIENDLY VISITORS INITIATIVE

Barbara Gordon[1], Pamela Yankeelov[1], Anna Faul[2], Chelsea Miceli[2], and Samantha Cotton[2], *1. University of*

*Louisvlle, Louisville, Kentucky, United States*, 2. *University of Louisville, Louisville, Kentucky, United States*

Social isolation affects one in four older adults aged 65 and over, significantly increasing the risk of premature death and dementia. The COVID-19 pandemic exacerbated this isolation, highlighting the urgent need for strategies to foster connections among older adults. Utilizing COVID-Cares Act funds, our Geriatrics Workforce Enhancement Program (GWEP) transformed the Friendly Visitors Program into a virtual initiative (VFVP), distributing tablets to enable social engagement through virtual visits. Ninety-six older adults actively engaged with 45 volunteer visitors across 601 virtual visits. The areas of interaction revolved around life reviews, family support, validating feelings, social determinants of health and hobbies. Evaluation data indicates improved mood, increased awareness of community resources, and high levels of satisfaction with the program. These visits led to the identification of changes in biological and behavioral health, home health needs, and neglect concerns by the visitors, resulting in appropriate referrals. VFVP not only improved mood and social engagement among participating older adults but also played a crucial role in monitoring health, connecting participants with vital resources, and assisting with environmental challenges to support independence. This success underscores the importance of community and technology in combating isolation and promoting the well-being of older adults, highlighting the potential of virtual programs. In this presentation, our team will explore the critical role friendly visitors play in monitoring health conditions. The session will also examine how friendly visitors can act as vital links between older adults and essential community resources, ensuring that needs ranging from nutrition to mobility are met.

Abstract citation ID: igae098.2860

### COMMUNITY ORGANIZATIONS' OUTREACH AND SERVICE DELIVERY TO OLDER ADULTS LIVING WITH DEMENTIA AND FAMILY CAREGIVERS

Kathy Lee[1], Jessica Cassidy[1], and Soeun Jang[2], *1. The University of Texas Arlington, Arlington, Texas, United States*, 2. *University of Texas at Arlington, Arlington, Texas, United States*

The current study examined various types of social services for older adults living with Alzheimer's disease or related dementia (ADRD) and their family caregivers, as well as the methods of delivery employed by home- and community-based organizations. Using a qualitative study design, this study aimed to identify strategies for enhancing support for older adults with ADRD and their family caregivers. A convenience sample method was employed to recruit service providers from nine home- and community-based organizations working closely with older adults living with ADRD and their family caregivers. Semi-structured, individual interviews were conducted online and transcribed verbatim by professionals. Three themes emerged: (1) culturally tailored outreach and services, (2) engagement of older adults living with ADRD and their family caregivers, and (3) collaboration efforts between home- and community-based organizations. Our findings revealed that organizations have tried creative ways to reach their population and educate people on dementia and dementia care. Especially, nonconventional methods (e.g., religion-based outreach or edutainment – an approach combining educational content with entertaining/easy-to-understand elements, such as storytelling or drama) were more frequently discussed among organizations serving people from diverse cultural and ethnic backgrounds. While many organizations have adopted technology to deliver services, many mentioned technologies were not a long-term solution, specifically for older adults with ADRD, as non-verbal cues such as eye contact and physical touch were considered critical for communication and providing care for older adults with ADRD.

Abstract citation ID: igae098.2861

### INDUSTRY–ACADEMIA COLLABORATION PROJECT FOR CREATION OF A LEARNING HEALTH SYSTEM FOR OLDER CARE: A CASE STUDY

Keiko Ishii, Maiko Noguchi-Watanabe, and Sakiko Fukui, *Tokyo Medical and Dental University, Bunkyo-ku, Tokyo, Japan*

The study aimed to establish a learning health system wherein artificial intelligence, the Internet of Things, and healthcare professionals collaborate to promote the well-being of older adults, families, and the healthcare professionals involved in older care. This case study seeks to elucidate the roles and challenges encountered by researchers within this industry- academia collaborative project. By employing the Project Management Body of Knowledge's (PMBOK's) 12 management principles (stewardship, team, stakeholders, value, system thinking, leadership, tailoring, quality, complexity, risk, adaptability and resiliency, change) as the analytical framework. As a preliminary step toward the main project, a joint research agreement with the relevant company in January 2022 was concluded, a comprehensive cooperation agreement in October of the same year, and commenced the project in April 2023. The project team comprised three researchers (home care nursing), four staff members (open innovation center and Industry-Academia Collaboration Division) from the university, and two employees from the company. The researchers participated in monthly meetings and proposed a research plan and strategies to utilize the research outcomes of the new company. To determine the number of visiting nurse users, we calculated the expected number of users at our university hospital. We also discussed the variables to be utilized in the algorithm development. An analysis based on the 12 principles reveals that although researchers tended to implement values and systems thinking, they had difficulties with other members regarding team and leadership. This research provides insights for project implementation for researchers promoting collaborative industry-academia research.

### SESSION 7345 (POSTER)

### SEXUALITY

Abstract citation ID: igae098.2862

### ASSESSMENT OF SUBJECTIVE SEXUAL EXPERIENCES OF OLDER ADULTS

Janie Steckenrider, *Loyola Marymount University, Los Angeles, California, United States*

There is extensive research focusing on sexual dysfunction, decreased frequency, and sexual effects of aging, however few studies explore older adults' subjective sexual experiences. Consequently, ageist myths prevail that older adults are neither sexually active nor desirous and the quality of their sexual activity must be extremely poor due to dysfunction and older bodies. The aim of this study was to determine how older adults assess their sexual experiences, what aspects are important, and their sexual outlook through survey research with a sample of individuals age 60+ (N=556). The results found older adults are overwhelmingly positive about their sexual experiences along with a realistic recognition of aging effects. Almost all, 85%, are sexually active, partnered and/or solo sex. Satisfaction is high indicated by 45% extremely/very satisfied with both quality and frequency and by a majority rating recent sexual experiences above average on a 1 to 10 scale and a third ranking it as "great sex" (8+). Their positivity is further evidenced as 80% describe their recent sexual experiences as fun and 67% answered exciting, despite 60% also recognizing it is predictable and more challenging, and 72% admitting it's not their best sex ever. Sexual desire remains high as the majority look forward to their next sexual experience and 70% anticipate an unlimited number of orgasms in their future. Consistent trends were found across all 4 age groups. This study's findings are significant for reframing the myths and demonstrating that older adults are sexually interested and having overwhelmingly positive sexual experiences.

Abstract citation ID: igae098.2863

### ATTITUDINAL DISTINCTIONS OF SOLO SEX ENGAGERS AMONG OLDER ADULTS

Janie Steckenrider, *Loyola Marymount University, Los Angeles, California, United States*

Solo sex gets little attention in the research on sexual activity of older adults as most studies frame definitions around partnered sexual behaviors. This does not reflect the reality of many older adults who lack active sexual partners due to death, dysfunction, or serious illness. When solo sex is included in studies, they rarely go beyond frequency. The aim of this study was to determine how sexually active older adults view solo sex, its importance, and attitude distinctions between solo sex engagers and non-engagers through survey research with a sample over age 60 (N=556). The majority (58%) of older adults engage in solo sex with men more likely (67%) than women (53%). As expected, solo sex engagers are twice as likely to use sexual toys and to be interested in using them if they currently do not. More solo sex engagers (70%) view orgasms as being extremely/very important for a good sexual experience than non-engagers (55%). Positive sexual outlooks correlate with solo sex as 80% of engagers anticipate an unlimited number of future orgasms compared to 65% of non-engagers. The most striking attitude distinction is 72% of engagers believe solo sex can be "great sex" compared to only 39% of non-engagers. Solo sex has psychological and physiological benefits for older adults and this study demonstrates solo sex correlates with positive sexual attitudes. The findings of this study highlight the need for further attention to the importance of solo sex for older adults and how it relates to positive sexual health.

Abstract citation ID: igae098.2864

### EXPECTATIONS FOR CHANGE IN SEXUAL POSSIBLE SELVES: IMPLICATIONS FOR SEXUAL WELL-BEING WITH AGE

Allyson Graf, and Sydney Ellis, *Northern Kentucky University, Highland Heights, Kentucky, United States*

The concept of sexual possible selves is well-established in the emerging adulthood literature with connections to sexual behavior and wellbeing. Research on sexual possible selves beyond this age period is limited. In an online sample of adults (N = 612; Mage = 47.31, SD = 13.51, 30-85 years), the current study aimed to identify expectations for long-term change in sexual possible selves and understand related factors. Participants reported on expectations for change (no change, change a little, change a lot) and were asked to elaborate. Additional data were collected on demographics, self-rated health, and sexual interest as possible related variables. Although 40.4% of the sample indicated that they expected no change, 78.9% of participants described a long-term expectation for their sexual possible selves. Thirteen themes emerged; the most common were aging (23.4%), relationship with a current partner (22.6%), and partner-related issues including no partner (19.5%). A series of logistic regressions were run to identify factors related to each theme. Demographics including age and partner status largely predicted themes. A multinomial regression was run to explore factors associated with expectations of staying the same (40.4%), changing a little (47.0%), and changing a lot (12.6%). Although demographics again were significant, additional variables contributed and the pattern of variables differentiating no change from change a little and change a lot differed. The results are important for extending sexual possible selves research across adulthood and understanding the diversity of expectations that may influence sexual interactions and wellbeing with age.

Abstract citation ID: igae098.2865

### FACTORS ASSOCIATED WITH SEXUAL SATISFACTION AMONG OLDER GAY MEN

Lucas Prieto[1], Deirdre Shires[2], and Yuan Xiong[2], *1. George Mason University, Fairfax, Virginia, United States*, *2. Michigan State University East Lansing, East Lansing, Michigan, United States*

Internalization of ageist stereotypes or messages that consider the context of aging gay men is known as internalized gay ageism. Sexual satisfaction may be influenced by internalized gay ageism among aging gay men. This study aimed to explore internalized gay ageism and the potential relationship with sexual satisfaction and determine if body image was a mediator. A cross-sectional online survey collected data on sexual satisfaction and other variables related to sexual health and well-being among older gay men between December 2021 and May 2022. Participants were included in the study if they were 50 years or older, identified as gay, identified as male, assigned male at birth, and resided in the Midwestern United States. Analyses included descriptive, bivariate, and mediation. A total of 181 participants were recruited in the study, with a mean age of 65.29 years (SD=9.32). Overall, participants reported experiencing a low level of internalized gay ageism, positive body image, and a high level of sexual satisfaction. A complete mediation effect was found between internalized gay ageism and sexual satisfaction when mediated by body image [total effect b=-4.47, p=.001; direct effect b=.34, p=.80; indirect effect b=-4.82, 95% CI BootLL =-6.93, BootUL= -3.03]. Older gay men who were in open relationships were more sexually satisfied than older gay men who were single/widowed. Future research should continue to explore internalized gay ageism, relationship status, body image, and sexual satisfaction among older gay men.

### SESSION 7350 (POSTER)

### SOCIAL DETERMINANTS OF HEALTH AND AGING I

Abstract citation ID: igae098.2866

### A LIFE COURSE APPROACH TO NEIGHBORHOOD SEGREGATION AND HEALTH AMONG BLACK AND WHITE AMERICANS

Kayla Fike[1], Huaman Sun[2], Michael Esposito[3], Dominique Sylvers[4], Kate Duchowny[4], Hedwig Lee[5], and Margaret Hicken[4], *1. Vanderbilt University, Nashville, Tennessee, United States*, *2. University of Toronto, Toronto, Ontario, Canada*, *3. University of Minnesota, Minneapolis, Minnesota, United States*, *4. University of Michigan, Ann Arbor, Michigan, United States*, *5. Duke University, Durham, North Carolina, United States*

Research documents a positive association between high levels of neighborhood segregation and several negative health outcomes among Black Americans. However, few studies examine the relationship between neighborhood segregation and the health of both Black and White Americans. We apply a lifecourse framework to better understand how exposure to neighborhood segregation earlier in life may lead to racial inequities in health byway of cumulative advantages/disadvantages (CAD). Utilizing data from the nationally-representative Americans' Changing Lives study (ACL), we employ growth curve models to investigate the relationship between neighborhood segregation in 1990 and the trajectories of two health outcomes over the adult lifespan: the number of chronic conditions and the probability of experiencing functional limitations. Our analysis reveals that, for Black participants, the baseline level of black segregation does not significantly impact their trajectories in these health outcomes. However, among White participants, residing in neighborhoods with lower levels of white segregation is associated with an increased probability of developing more chronic conditions and experiencing functional limitations later in life. We explore the roles of systemic and cultural racism as shaping forces in neighborhood structures and as contributors to negative health outcomes for both Black and White Americans. Research implications include further parsing different types of white underrepresentation in neighborhoods (i.e., living in predominantly Black neighborhoods vs racially diverse integrated areas) to examine their unique effects on health trajectories. Further, we discuss how intervention efforts may identify and address detractors from healthy aging for White Americans who are underrepresented in their neighborhoods.

Abstract citation ID: igae098.2867

### ASSOCIATION OF SOCIOECONOMIC STATUS, SOCIAL ACTIVITIES, AND LONELINESS WITH DEPRESSION AMONG OLDER ADULTS

Yaping Wang, and Jue Liu, *Peking University, Beijing, Beijing, China (People's Republic)*

To estimate whether social activities and loneliness mediate the association between SES and depression, and the extent of interactive or joint relations of SES, social activities, and loneliness with depression, we did a multi-cohort study utilizing five aging surveys across 24 countries. SES was defined as high- and low-level by latent class analysis based on family income, education, and work status. Depression was assessed by 8-item or 10-item Center for Epidemiological Studies Depression Scale (CES-D) and EURO-D. Cox proportional hazard models were applied to estimate the association of SES with depression. Random effects models were used to pool results. A total of 79638 participants were included in our study. With a median follow-up of 5-year, compared with participants in high-level SES, those in low-level SES had a pooled 29.1% (pooled Hazard Ratio [pHR] =1.291, 95%CI: 1.188-1.393) higher risk of depression after adjusting all covariates, and the pooled proportions mediated by social activities and loneliness were 8.1% (95%CI: 3.2-12.9%) and 5.8% (95%CI: 3.6-7.9%), respectively. No significant additive interactions were observed between SES and social activities or loneliness. Compared with participants in high-level SES, socially-active, and not lonely group, those in low-level SES, socially-inactive, and lonely group had a 133.5% increased risk of depression (pHR=2.335, 95%CI: 2.058-2.612). Social activities and loneliness mediated a small proportion of socioeconomic inequality in depression, which indicated that other pathways addressing socioeconomic inequality were required. Additionally, the joint effects of SES, social activities, and loneliness highlighted the benefits of simultaneous and integrated interventions to reduce depression burden.

Abstract citation ID: igae098.2868

### BUFFERING EFFECT OF MARRIAGE ON DAILY WORK STRESS–INFLAMMATION LINK: LONGITUDINAL ANALYSIS OF THE MIDUS STUDY

Jeongmin Park[1], David Almeida[2], and Alexis Santos-Lozada[1], *1. The Pennsylvania State University, University Park, Pennsylvania, United States*, *2. Pennsylvania State University, University Park, Pennsylvania, United States*

Aims: This study investigates how frequency of daily work stress predicts inflammation, measured by C-reactive protein levels (CRP), in employed middle-aged and older adults. It explores Relationship Buffering Hypothesis by assessing how marital status and quality of marital relationships moderate this stress-inflammation link. Additionally, it examines role of age and gender. Based on Socio-Emotional Selectivity Theory, we hypothesize that relationship buffering will be greater for older workers.

**Methods:** Data were from Midlife in the United States (MIDUS) waves 2 and 3, samples include 314 employed individuals over 40 (at wave 3), participating in both National Study of Daily Experiences and Biomarker projects. CRP was measured during laboratory visits, whereas daily work stress was computed as the mean of 8-day diary responses of binary stress events at workplace ranging from 0 (not work stressors) to 1 (work stressors every day).

**Results:** Linear regression revealed that frequent daily work stress predicts higher CRP a decade later, controlling for initial CRP, age, gender, education, and race (B = 4.49, $p < 0.01$). Marital status (B = -8.35, $p < 0.01$) and relationship quality among married individuals (B = -1.41, $p < 0.05$) moderated this relationship. No significant three-way interactions with age or gender were found. Conclusions: The daily work stress-inflammation link varied by status and relationship quality of marriage. Specifically, non-married individuals and those experiencing lower relationship quality within marriage showed a heightened stress-inflammation link, underscoring the protective role of strong marital relationships against stress-induced inflammation, in alignment with the Relationship Buffering Hypothesis.

Abstract citation ID: igae098.2869

### COMMUNITY RESOURCE AWARENESS AND REFERRAL PRACTICES OF HEALTH CARE PROVIDERS TO SUPPORT OLDER ADULTS

Catherine Rudolph[1], Sarah Francis[2], and Alexandra Bauman[3], *1. Nutrition and Aging Resource Center, Iowa State University, Ames, Iowa, United States*, *2. Iowa State University, Ames, Iowa, United States*, *3. Iowa Department of Health and Human Services, Des Moines, Iowa, United States*

There is mounting evidence that social determinants of health (SDOH), such as housing, income, and food insecurity, have a significant impact on health outcomes. Support for SDOH is vital to ensuring optimal well-being among older adults and preventing unnecessary strain on healthcare resources. Successful partnerships between healthcare and community-based services can help to identify and address the SDOH affecting aging adults' health. This study examined SDOH screening and intervention practices among healthcare providers, and their awareness and partnerships with community-based food and nutrition services. A nationwide online survey was completed by 160 U.S. healthcare providers who provide care to adults over 60. Descriptive statistics were conducted to assess screening and referral practices. Associations between these practices and provider characteristics are being assessed via Chi-square analyses. SDOH screening was highest for healthcare access, mental health, and food insecurity and lowest for social isolation and transportation. Respondents were most aware of and referred older patients to food pantries, the Supplemental Nutrition Assistance Program, and nutrition education. They were least aware of the Senior Farmers' Market Nutrition Program, Congregate Meal Program, and nutrition counseling. A majority shared that their healthcare practice has a protocol for community-based referrals. Paper handouts and verbal information were primary referral methods. Common referral barriers were staffing shortages and lack of program access. Over one-half expressed high/very high interest in learning more about community-based food and nutrition services. These findings can inform strategies and opportunities for partnership between healthcare and community-based services to address the needs of aging adults.

Abstract citation ID: igae098.2870

### COMMUNITY-LEVEL SOCIAL CAPITAL AND ALL-CAUSE MORTALITY IN JAPAN: FINDINGS FROM THE ADACHI COHORT STUDY

Hiroshi Murayama, Mika Sugiyama, Hiroki Inagaki, Chiaki Ura, Fumiko Miyamae, Ayako Edahiro, Tsuyoshi Okamura, and Shuichi Awata, *Tokyo Metropolitan Institute for Geriatrics and Gerontology, Tokyo, Japan*

Community-level social capital is known to be associated with various health outcomes, but its impact on mortality is not fully understood. This study examined the association between community-level social capital and all-cause mortality among community-dwelling older Japanese people. The baseline data was obtained from a questionnaire survey for all 132,005 residents aged ≥65 years without long-term care insurance certification in Adachi Ward of the Tokyo metropolitan area in 2015 (75,358 valid responses; mean age 73.8 years; 45.0% males). We measured two aspects of social capital: neighborhood cohesion (trust, attachment, and a sense of belonging) and neighborhood network (neighborly ties). We stratified these into high, medium, and low categories. For community-level social capital, we calculated the proportion of those with high and medium neighborhood cohesion and neighborhood network by 262 administrative areas in Adachi. The study protocol was approved by the IRB. A sex-stratified multilevel survival analysis (average follow-up of 1,656 days), after adjusting for both individual-level and community-level covariates, showed that community-level neighborhood cohesion was negatively associated with all-cause mortality in men (hazard ratios: 0.92 [0.84-1.00] for the highest quintile and 0.91 [0.82-0.99] for the second, compared to the lowest), but not in women. These suggest that for men, who typically have fewer social networks and support systems than women, the benefits of living in a cohesive community may be life-extending. This supports the notion that interventions aimed at enhancing community social capital could be a strategic component in public health efforts to reduce mortality risks, particularly among older men.

Abstract citation ID: igae098.2871

### EDUCATION AND MIDLIFE COGNITIVE FUNCTIONING: EVIDENCE ABOUT MEDIATORS FROM THE HIGH SCHOOL AND BEYOND COHORT

John Warren[1], Chandra Muller[2], Eric Grodsky[3], Adam Brickman[4], and Jennifer Manly[4], *1. University of Minnesota, Minneapolis, Minnesota, United States*, *2. The University of Texas Austin, Austin, Texas, United States*, *3. University of Wisconsin Madison, Madison, Wisconsin, United States*, *4. Columbia University, New York, New York, United States*

Educational attainment is associated with cognitive functioning and risk of Alzheimer's disease and related dementias (ADRD). However, (1) aspects of schooling that precede degree completion have rarely been considered and (2) the social and biological pathways through which education shapes ADRD risk are not well understood. Do schools' social and academic contexts, students' schooling outcomes, and students' degree completion independently predict cognitive outcomes? If so, how do life course social and biological factors mediate the effects of various dimensions of education? This study included a nationally representative sample of 12,530 Americans followed prospectively from high school through age ~60. We estimated associations between aspects of schooling and multiple dimensions of cognition, before and after adjustment for confounders. We then considered the mediating roles of midlife socioeconomic factors (e.g., income, financial precarity, debt, bankruptcy risk) and biological/health factors (e.g., cardiovascular disease, immunological function, inflammatory markers). We employ a variety of methodological approaches informed by recent advances in approaches to mediation analysis within causal frameworks. Education is more than just degree attainment. Understanding how and through what pathways education shapes cognitive functioning and ADRD risk requires longitudinal data with nuanced information about schools, schooling, and student performance.

Abstract citation ID: igae098.2872

### EDUCATION AS A MEDIATOR THROUGH THE EFFECT OF AGE AT MIGRATION ON COGNITIVE HEALTH AMONG OLDER IMMIGRANTS IN THE US

Dongfang Hong, and Wuyi Dong, *University of Massachusetts Boston, Boston, Massachusetts, United States*

**Background:** Age at immigration is a critical determinant that influences long-term cognitive outcomes among older adults, often through disruptions or enhancements in educational opportunities. Previous research has underscored the importance of educational attainment as a key factor in cognitive resilience and decline. The mediating role of education in the relationship between the timing of immigration and cognitive function remains underexplored. This study addressed whether age at migration is associated with cognition among older immigrants whether education mediates this association?

**Methods:** Data were taken from the 2020 Health and Retirement Study (N=1,587). Linear regression is employed to examine the direct association between age at migration and cognitive function and the indirect association through years of completed education.

**Results:** This study revealed a significant association between age at immigration and cognitive function, with immigrants who migrated during young adulthood (ages 18-40) displaying notably lower cognitive function compared to those who migrated at younger ages (before 18). The results also provided evidence for the partial mediation of educational attainment for the association between age at immigration and cognitive function. This indicated that disruptions in education due to migration contribute to cognitive outcomes.

**Discussion:** This study contributed to our understanding of how immigration timing and educational attainment intersect to affect cognitive function, with implications for policy and intervention strategies aimed at supporting the cognitive health and educational opportunities of immigrant populations. Future research should consider additional mediators, such as socioeconomic status and social support, to further elucidate these relationships

Abstract citation ID: igae098.2873

### EDUCATION, ADULT LITERACY, AND HEALTH AMONG MIDDLE-AGED AND OLDER IMMIGRANTS IN THE UNITED STATES

Donnette Narine[1], Takashi Yamashita[1], Runcie Chidebe[2], Phyllis Cummins[2], Jenna Kramer[3], and Rita Karam[4], *1. University of Maryland Baltimore County, Baltimore, Maryland, United States*, *2. Miami University, Oxford, Ohio, United States*, *3. RAND Corporation, Arlington, Virginia, United States*, *4. RAND Corporation, Santa Monica, California, United States*

The education-health pathway is well-documented whereby greater human capital such as educational attainment can provide health benefits over the life course. However, there is evidence of diminishing health benefits from education for immigrants compared to non-immigrants, partially due to structural factors (e.g., health insurance) limiting health-related benefits among immigrant populations. Older immigrants are more likely to be college-educated compared to their non-immigrant counterparts. However, the decades-old degrees from their home countries, along with English not being their native language, pose a particular concern for older immigrants' health. As formal educational attainment cannot be easily promoted among older populations, adult literacy proficiency is another useful form of foundational human capital indicator, which is malleable at any life stage. This study examined the mediation effect of adult literacy proficiency on the education-health pathway among middle-aged and older immigrants in the United States. The nationally representative sample of immigrants aged 45 years and older (n = 590) in the 2012/2014/2017 U.S. Program for International Assessment of Adult Competencies (PIAAC) restricted-use file (RUF) data were analyzed. The mediation analysis showed that the association between educational attainment (college vs. less than college degree) [95% Confidence-Interval (CI): Lower-Limit (LL), Upper-Limit (UL) = 0.168, 0.780] and health (good vs. fair/poor) was mediated by adult literacy proficiency scores (0 to 500 points) [95% CI: LL, UL = 0.053, 0.352] among older immigrants in the United States. Findings from this study are informative to future interventions to support the health of older immigrants by way of adult literacy training.

Abstract citation ID: igae098.2874

### EVIDENCE THAT TOMORROW'S OLDER POPULATION WILL BE LESS FIT THAN TODAY: A NATIONAL STUDY, 2003–2022

David Rehkopf[1], Frank Furstenberg[2], Christian Jackson[3], and John Rowe[4], *1. Stanford University, Palo Alto, California, United States*, *2. University of Pennsylvania, Philadelphia, Pennsylvania, United States*, *3. Stanford University, Stanford, California, United States*, *4. Columbia University, New York, New York, United States*

Entering retirement age with a high level of health, well-being, and functioning is critical for remaining healthy and engaged after retirement and into later life. While recent stagnating trends for mortality have been well documented in pre-retirement age groups, there has been less attention to trends in morbidity, and in particular, what they are like across different levels of income. We use data from the Centers for Disease Control and Prevention Behavioral Risk Factor Surveillance System that includes self-reported data on wellbeing and functioning on more than seven million individuals from 2003 to 2022, to examine how physical health, mental health and functional health have changed over time by age group and income level. We find there are dramatic differences (up to 10 more days of morbidity in the last 30 days) by level of income, and that over this period of time there are increases in the number of days with bad health and functioning for the poorest and lower-middle income groups. These declines are most notable in ages that are pre-retirement years, age 50 to 59. None of these trends or levels were explained by race, BMI, or smoking, although we show substantial differences in these trends across U.S. States. If current national trends persist, the current age 50 to 59 year old population will be entering retirement ages with substantially worse health than previous generations, which has major implications for labor force participation, Medicare expenses and Social Security.

Abstract citation ID: igae098.2875

### FAMILY HEALTH STRESS AND INSOMNIA SYMPTOMS AMONG OLDER ADULTS: THE MODERATING ROLE OF ACTIVITY ENGAGEMENT

Jeein Jang, and Jeffrey Burr, *University of Massachusetts Boston, Boston, Massachusetts, United States*

Insomnia symptoms that arise from stress pose a significant physical and psychological health concern for older adults. Yet, little empirical research has explicitly examined the association between stress caused by family health concerns and insomnia symptoms, along with the role that activity engagement plays in moderating this relationship. Pearlin's stress process model is used to frame this study. Using data from the 2018 and 2020 waves of the Health and Retirement Study (N=9,003), this study employed ordered logistic regression models to assess the link between family health stress and insomnia severity using four items (i.e., trouble falling asleep, nighttime awakening, early morning awakenings, and feelings of nonrestorative sleep). Additionally, the moderating role of overall activity engagement, as well as three activity domains (cognitive, social, and physical activities) were included in the analysis. All statistical analyses were adjusted for sociodemographic characteristics, health-related factors, and caregiver status. The results of the regression analysis show older adults experiencing 'somewhat upsetting' and 'very upsetting' family health stress had 23% and 49% higher odds, respectively, of experiencing more severe insomnia symptoms compared to those with no family health stress. In addition, the relationship was buffered by overall activity engagement between the 'very upsetting' group and insomnia symptoms in the logistic regression. For the specific activity domains, cognitive activities were the only ones that moderated the association between family health stress and insomnia symptoms. These findings offer implications for public health interventions that seek to promote activity engagement among older adults.

Abstract citation ID: igae098.2876

### INCREASING CHILDREN'S EDUCATION IMPROVES PARENTAL DEMENTIA RISK AND REDUCES GRADIENTS IN RISK

Liying Luo[1], John Warren[2], Jiahui Xu[1], and Kenneth Langa[3], *1. The Pennsylvania State University, University Park, Pennsylvania, United States*, *2. University of Minnesota, Minneapolis, Minnesota, United States*, *3. University of Michigan, Ann Arbor, Michigan, United States*

Recent research suggests that adult children's education may influence their parents' health at older ages. The empirical evidence about this upstream influence remains somewhat mixed, however. This is due in part to differing methodological approaches. To account for intergenerational selection and interdependent pathways, we use multilevel marginal structural models and well-defined casual estimands to analyze longitudinal data from the Health and Retirement study. We ask two important questions: (1) At the individual level, for whom does children's education convey health benefits in older ages? (2) At the population level, what are the implications of increasing education levels among younger generations for (a) the health and well being of their parents' generation and (b) educational disparities in health and well being? Our analyses suggests that at the individual level, increasing levels of children's education decreases parents' dementia risk. At the population level, educational disparities in dementia risk may be reduced as younger generations obtain more education.

Abstract citation ID: igae098.2877

### LOVE HURTS: MARITAL STATUS AND CHRONIC PAIN

S. Emily Choi, and Brandon Wagner, *Texas Tech University, Lubbock, Texas, United States*

While chronic pain is a highly prevalent and costly public health concern among older adults in the United States, longitudinal research on the social determinants of chronic pain, particularly its association with social relationships, has been limited. To address this gap, this study investigates how marital status influences later-life chronic pain with a focus on gender-specific variations. Drawing on the Health and Retirement Study (1998–2016), we estimated fixed effects logit models using an analytic sample of community-dwelling American adults aged 50 and above (N = 19,975). At baseline, women reported moderate-to-severe limiting pain (27.07%) significantly more frequently than did men (19.41%). Among men, unmarried groups, specifically the divorced/separated [OR = 0.72] and the widowed [OR = 0.55], had significantly lower odds of experiencing moderate-to-severe limiting pain compared to married individuals, controlling for demographic characteristics, socioeconomic status, health conditions and health behaviors. Among women, only the widowed had significantly lower odds of experiencing moderate-to-severe limiting pain [OR = 0.72] compared to their married counterparts. These findings suggest that though intimate social relationships may be important risk factors in shaping chronic pain in later life, they do so in perhaps surprising ways.

Abstract citation ID: igae098.2878

### RELATIONSHIP SATISFACTION AND COGNITIVE WELL-BEING: FINDINGS FROM A DAILY DIARY STUDY

Selin Karakose[1], Martina Luchetti[1], Thomas Ledermann[2], Yannick Stephan[3], Antonio Terracciano[1], and Angelina R. Sutin[4], *1. Florida State University, Tallahassee, Florida, United States*, *2. Florida State University, College of Education, Tallahassee, Florida, United States*, *3. University of Montpellier, Montpellier, Languedoc-Roussillon, France*, *4. Florida State University College of Medicine, Tallahassee, Florida, United States*

Romantic relationships play a crucial role in many aspects of an individual's life. Longitudinal research further supports the benefits of a satisfying relationship for multiple aspects of well-being. However, less work has examined these associations in daily life, particularly the association between relationship satisfaction and cognitive well-being. An analysis of relationship satisfaction in daily life is important to understand whether fluctuations in it may be associated with fluctuations in these aspects of well-being. This study examined the daily associations between relationship satisfaction and physical, psychological, and cognitive well-being. Every night for eight days, middle-aged participants in a committed relationship (N=303; 54% female; Mage=51.71, SD=7.32) reported on their relationship satisfaction and multiple aspects of well-being, including subjective cognition, as well as overall health, subjective age, purpose in life, and life satisfaction. Multilevel linear models examined within-person associations accounting for personal (sex, age, race, education), relational (relationship satisfaction), and contextual factors (day in the study, weekend day). On days when participants felt more satisfied in their relationships than their average, they also reported a sharper mind, better memory, and clearer thinking; relationship satisfaction was unrelated to whether participants were bothered and disrupted by forgetting. Relationship satisfaction was further related to feeling healthier, younger, more purposeful, and more satisfied with their life. These findings can inform relationship-based interventions to support better physical, psychological, and cognitive aging.

Abstract citation ID: igae098.2879

### SOCIAL DETERMINANTS, LONELINESS, DEPRESSION, AND CHRONIC ILLNESS FOR MIDLIFE AND OLDER ADULTS IN APPALACHIA

Laurie Theeke[1], Lashawn Hutto[2], and LaTonya Smith[2], *1. The George Washington University, Ashburn, Virginia, United States*, *2. George Washington University, Washington, District of Columbia, United States*

Loneliness constitutes a significant health risk among midlife and older adults in Appalachia. This study presents a secondary data analysis exploring relationships among social determinants of health, loneliness, depression, and chronic illness. Common variable data from two prior studies that collected data from adults at a family health center was used for the analysis. The sample of 150 adults (88 women) ranged in age from 46 to 91years (mean 63.5, SD11.16) with mean loneliness score of 39.5 (SD11.13)- indicating high loneliness and averaged 3.2 (SD 1.88) chronic illnesses which increased with age. Over 40% were married with 74% living with others. An associate degree or higher was earned by 46%. Though 69.3% worked full-time, 43.3% reported income below $40,000 annually. Age inversely correlated with loneliness (p =.04) and depression scores (p <.001). Loneliness highly correlated with depression (p,.001) and inversely correlated (p <.01) with emotional, tangible, affective, positive, and total social support. Loneliness correlated with resting heart rate (p <.001) and body mass index (p=.05) but not with blood pressure. Men had higher loneliness (p=.027) and were more frequently diagnosed with hyperlipidemia, heart disease (p=.047), hypertension (p=.049), and stroke (p=.004). Women had higher scores for social support (p=.005) and functional ability. Findings highlight inequities in health for older adults, particularly men, living in Appalachia. Future studies are planned for larger cohort studies to better explicate psychophysiological pathways from loneliness to illness in this population and to examine the effectiveness of LISTEN, an intervention designed to target loneliness that demonstrated effectiveness in this population.

Abstract citation ID: igae098.2880

### SOCIAL ENGAGEMENT IS ASSOCIATED WITH COGNITION IN OLDER ADULTS FROM UNDERSERVED QUILOMBOLA COMMUNITIES IN BRAZIL

Sharon Sanz Simon[1], Carolina Cappi[2], João Cabral Junior[3], Gilberto Alves[4], and Bruno Carneiro Alves de Oliveira[5], *1. Columbia University, New Rochelle, New York, United States*, *2. Icahn School of Medicine at Mount Sinai, New York, New York, United States*, *3. Federal University of Maranhão, Pinheiro, Maranhao, Brazil*, *4. Faculty of Medicine, Federal University of Maranhã, São Luís, Maranhao, Brazil*, *5. Federal University of Maranhão, São Luis, Maranhao, Brazil*

Quilombos are settlements founded by descendants of runaway slaves in Brazil, typically located in rural areas. Quilombola communities often present poor basic sanitation, high levels of illiteracy, and limited access to social and health services. Although the Quilombola population is socially vulnerable and likely to present an increased risk for dementia, it is underrepresented in aging research. The present study aims to investigate the association between cognition and socio-cultural engagement, potentially protective for dementia. Method: This cross-sectional study was conducted in 11 Quilombola communities in Maranhão, Brazil. The study comprised 221 older adults (60-104 years), selected in partnership with local services and community health workers. Participants completed a health survey and a cognitive screening. Demographics, cardiovascular risk factors (CRF), cognitive performance, and social engagement were described. Regression analysis examined the association between social engagement and cognition, controlling for age, sex, race, education, family income, and cardiovascular risk factors. We also examined moderation effects of sex and age on the regressions.

**Results:** Higher social engagement was associated with better cognition (p <.001) above and beyond demographics and CRF. We observed the social activities that mostly drove the results were attending "community meetings" and "religious/faith services". Age and sex did not moderate the associations.

**Conclusion:** Social engagement may be a key aspect of cognitive/brain health in Quilombola population. Social engagement may not only provide social/emotional support, but also facilitate access to basic needs. Although the results are limited by the cross-sectional design and survival bias, it may inform strategies in underserved populations.

Abstract citation ID: igae098.2881

### UNIQUE STRATEGIES AT CEDARS-SINAI TO SCREEN FOR AND ADDRESS SOCIAL DETERMINANTS OF HEALTH IN OLDER ADULTS

Sonja Rosen, Emma Cohen, Katie Hren, Christina Harris, and Krystal Green, *Cedars-Sinai Medical Center, Los Angeles, California, United States*

To effectively address health disparities and bridge systemic gaps between health and social care, healthcare institutions must cohesively address shortcomings in identifying, supporting, and monitoring social determinants of health (SDoH) to better serve communities. In 2021, Cedars-Sinai, a Los Angeles urban Academic Medical Center, launched a robust strategy to address SDoH needs across health system settings. Cedars-Sinai's SDoH strategy includes a customized 12-domain screener to identify patients unmet social care needs and additional workstreams to support patient and community needs, including an electronic resource referral platform, an inaugural Community Health Worker (CHW) program, and a network of community-based organizations (CBOs) with accountable referral pathways. As one of the largest Medicare providers in the region, and serving more older adults over the age of 80 than any other academic medical center, this SDoH strategy included tailored interventions for older adults such as embedding a CHW within the geriatrics outpatient practice and cultivating community partnerships with CBOs experienced in serving older adults. In the first 2.5 years of Cedars-Sinai's SDoH strategy, 97,757 older adult patient screenings identified 30,245 unmet HRSN needs; 411 of these older adult patients received CHW support, 61.24% of whom were successfully connected to resources. These systemwide efforts highlighted that older adults experience more nuanced SDoH inequalities fueled by aging-specific structural barriers and succeed with greater dosages of social care interventions. Findings from Cedars-Sinai's approach to SDoH has demonstrated that healthcare institutions are most effective in addressing SDoH through comprehensive strategies that create population-specific solutions in tandem with screening.

Abstract citation ID: igae098.2882

### US MEN'S SHEDS: MAINTAINING PURPOSE AND CONNECTION WITH OTHERS

Melinda Heinz[1], Kaylin Kent-Thomas[2], and Julie Smith Hinders[3], *1. University of Northern Iowa, Cedar Falls, Iowa, United States*, *2. Upper Iowa University, Fayette, Iowa, United States*, *3. University of Minnesota Crookston, Crookston, Minnesota, United States*

The purpose of this study was to explore U.S. Men's Sheds, learn how they operate, who attends them, and how participants perceived them. It is the first study exploring Men's Sheds in the U.S. An explanatory sequential mixed methods study was conducted, and paper-based surveys were sent to sheds registered with the U.S. Men's Sheds Association. Shed chairpersons encouraged members to complete surveys (N = 76), and results showed that most members were male (91%), older adults (M = 70), in good/excellent health (76%), and joined to make new friends or learn new skills. Four interviews were also conducted and two themes and a subtheme were noted. Theme 1: Purpose of the Men's Shed represented the varied perspectives on what the Men's Shed was meant to do for the community. Some members saw it as an extension of the church and felt it was important to share religious perspectives with the community, while others disagreed. A Subtheme on Serving the Community was also noted where members did agree on the importance of serving the community. Theme 2: Building Connections- Possibilities and Challenges illustrated the opportunities Men's Sheds create for men in/approaching retirement. However, the lack of a formal space for members to meet was a barrier and reduced informal socializing that might otherwise occur. These findings revealed that many of the men joined because they wanted something worthwhile to do with their time. The interviews revealed that there may be opportunities to start Men's Sheds within existing faith- based organizations.

Abstract citation ID: igae098.2883

### UTILIZING COMMUNITY CONNECTIONS: A SOCIAL DETERMINANTS OF HEALTH SCREENING FOR COMMUNITY-DWELLING OLDER ADULTS

Kelly O'Sullivan[1], Raven Weaver[1], Cory Bolkan[1], Chad Gotch[1], Erik Geissal[2], and Breanne Swanson[3], *1. Washington State University, Pullman, Washington, United States*, *2. Vancouver Clinic, Vancouver, Washington, United States*, *3. Area Agency on Aging & Disabilities of Southwest Washington, Vancouver, Washington, United States*

Community-based aging resources, like Area Agencies on Aging (AAA), are often underutilized. Establishing partnerships with healthcare providers can promote better awareness and use of available services/supports. We evaluated a community partnership between a primary care clinic and an AAA; both community partners were committed to reducing adverse health outcomes. This exploratory intervention study assessed social determinants of health (SDH) needs of Medicare-eligible primary care patients (n = 385) during an annual visit (Mage=72.5; 89.6% White) and connected patients to appropriate supportive aging services. There were 284 participants with identified unmet SDH needs, but only 36 participants accepted referral to AAA resources. We analyzed Medicare's Risk Adjustment Factor (RAF) score at baseline and participants' use of advanced care (i.e., ER visits/hospital readmissions/institutionalizations), urgent care, and primary care, over a 6-month period. Descriptive, bivariate, and multivariate analyses revealed positive associations between RAF score with age ($p< 0.001$) and number of SDH needs ($p< 0.001$), advanced care with SDH needs ($p=0.004$) and RAF score ($p< 0.001$), and primary care visits with SDH needs ($p=0.003$) and RAF score ($p< 0.001$). The screening revealed that older adults' SDH needs are often unmet and underrecognized. Cross-sector partnerships between medical and social models of health can reduce risk and meet needs of community-dwelling older adults. Policy provisions that encourage such partnerships are needed to improve community-dwelling older adults' wellbeing, independence, and ability to age-in-place.

### SESSION 7355 (POSTER)

### SUCCESSFUL AGING/PRODUCTIVE AGING: APPLICATIONS

Abstract citation ID: igae098.2884

### A FIRST LOOK AT THE HEALTHY AGING INITIATIVE: YEAR ONE RESULTS

Talia Gilfix, Molly Quigley, Heather Andrews, Davide Cappon, Maggie Syme, and Alvaro Pascual-Leone, *Hebrew SeniorLife, Boston, Massachusetts, United States*

The Healthy Aging Initiative (HAI) is a Boston-based longitudinal observational study assessing functional ability and well-being in 55+ adults residing in independent-living senior communities. Participants undergo assessments to evaluate modifiable risk factors for disability and dementia: mobility, cognition, strength, functional status, lifestyle factors, and mental wellness. Timely feedback/results are provided to empower meaningful changes. To gain insight into factors impacting functional ability, all participants' data through Year 1 (N=173) were aggregated and key modifiable risk factors analyzed, revealing compelling trends across age groups and sex. Trends included sex differences among participants aged 80-84 years in the memory domain (Males 29% impaired (n=14); Females 13% impaired (n=24)). However, in the 85-89 age group these differences were no longer observed (Males 42% impaired (n=12); Females 39% impaired (n=23)), suggesting older women may "catch up" in impairment by their late 80's. In mobility, females with high fall risk nearly doubled from age 75-79 to 80-84 (33% (n=18) to 63% (n=30)), while males in these age groups had the opposite effect (56% (n=9) to 27% (n=22)), indicating males who remain agile until the age of 80 may delay mobility decline. In lifestyle factors, it's important to look at a domain from multiple perspectives. For instance, 68% of participants (n=132) report following recommended exercise guidelines, while 52% also report spending at least 6 hours sitting daily (n=101). Full results will be presented. As HAI expands and participants repeat assessments annually, critical insights may be obtained, creating a blueprint for translational clinical research in healthy ageing.

Abstract citation ID: igae098.2885

### ASSOCIATION BETWEEN SOCIAL CAPITAL AND SUCCESSFUL AGING AMONG EMPTY NESTERS: A CROSS-SECTIONAL STUDY

Hui Chang[1], and Zhi-wen Wang[2], *1. Guizhou Medical University, Guiyang, Guizhou, China (People's Republic), 2. Peking University Health Science Center, Beijing, Beijing, China (People's Republic)*

**Aims:** To determine the mediating and chain mediating roles of a sense of coherence and health-promoting lifestyle between social capital and successful aging among empty nesters to explain the internal mechanisms underlying the relationship.

**Methods:** A total of 452 community-based empty nesters were included using convenience sampling. Empty nesters were surveyed using the Successful Ageing Scale, Social Capital Scale, Sense of Coherence Scale, and Chinese version of the Health-Promotion Lifestyle Scale-II. Descriptive analyses and correlation analyses were conducted using SPSS25.0. Mplus 8.3 was employed to perform chain mediation analyses. For this study, we followed the Strengthening the Reporting of Observational Studies in Epidemiology (STROBE) guideline.

**Results:** There was a positive correlation between social capital, a sense of coherence, a health-promoting lifestyle, and successful aging. Social capital has a direct positive effect on successful aging. Sense of coherence and health-promoting lifestyles partially mediate the relationship between social capital and successful aging. The sense of coherence and health-promoting lifestyles also have a chain mediating effect.

**Conclusion:** The quality of aging for empty nesters can be improved from the perspective of social capital. Effective measures can also be taken to improve the sense of coherence so that they can maintain a healthy lifestyle and thus achieve successful aging.

Abstract citation ID: igae098.2886

### DO LIFE SATISFACTION AND LEVEL OF OPTIMISM IMPACT SUCCESSFUL AGING IN MID AND LATE LIFE?

Kallol Kumar Bhattacharyya[1], Victor Molinari[2], Debasree Das Gupta[1], and Gizem Hueluer[3], *1. Utah State University, Logan, Utah, United States, 2. University of South Florida, Tampa, Florida, United States, 3. University of Bonn, Bonn, Nordrhein-Westfalen, Germany*

Earlier theoretical frameworks of successful aging (SA) included either objective or subjective evaluations or both. Guided by the Rowe and Kahn model, the current study expands prior work by examining the association of objective and subjective components of SA with individuals' life satisfaction and level of optimism across adulthood. Data were from waves 2 (2004-05) and 3 (2013-14) of the Midlife in the United States (MIDUS) study. To obtain a multidimensional SA score, we considered successful agers as those with a higher score in cognition, with minimal difficulties in performing physical activities, maintaining active engagement with life, and free of multiple chronic conditions. We used structural equation modeling to examine whether subjective life satisfaction (wave 2) has any effect on a composite measure of SA (wave 3) while controlling for baseline sociodemographic and health factors; we also examined the reverse directional association. Further, we also examined the mediation effects of level of optimism in the above associations. A total of 2,040 individuals (Mage=64±11; women 56%) were included. After controlling for covariates, findings revealed that prior life satisfaction has a positive direct effect (b=0.094; SE=0.019; p< 0.001) on SA; also, this effect is bidirectional. Further, high level of optimism mediates the association between life satisfaction and SA. While SA is often examined in relation to various risk/protective factors in late life, this study identified life satisfaction and level of optimism as having potentially positive impacts on achieving SA in middle-aged and older MIDUS participants.

Abstract citation ID: igae098.2887

### INTERACTIONS OF SOCIOECONOMIC AND ETHNORACIAL FACTORS SHAPING FORMAL AND INFORMAL VOLUNTEERISM IN LATER LIFE

Patrick Ho Lam Lai, *Boston College, Chestnut Hill, Massachusetts, United States*

Building upon previous research that identifies socioeconomic status (SES) as a pivotal factor in explaining lower volunteer rates among ethnoracial minorities in later life, this study extends our understanding by examining the interactions between SES and ethnoracial identities on their formal and informal volunteerism. By doing so, the research informs aging service practitioners and policymakers about the distinct responsiveness of different ethnoracial groups to their educational and family income levels and how these, in turn, relate to their propensity for volunteer work. By utilizing 2019 and 2021 Current Population Surveys data, this research estimates bivariate probit models for formal (e.g., through organizations) and informal volunteerism (e.g., doing favors to neighbors), including moderation with education and family income. The findings reveal that older Hispanic adults have 0.907 times or older Black adults have 0.983 times the likelihood of engaging in formal volunteer activities compared to older Asian adults, with this association becoming statistically significant upon a decrement of one educational level or family income level. Besides, older Black adults show 0.928 times the odds of engaging in informal volunteerism in comparison to Asian counterparts, with this finding being statistically significant upon a decrease in one family income level. These outcomes suggest that Asian older adults exhibit greater sensitivity to income and educational variations associated with formal and informal volunteer engagements. This research emphasizes the need to understand cultural sensitivity for lower volunteerism among older Asian adults for more inclusive aging services and policies.

Abstract citation ID: igae098.2888

### INVESTIGATING CAUSAL LINK BETWEEN VOLUNTEERING AND BLOOD PRESSURE BY GENDER, RACE, AND COHORT

Seoyoun Kim[1], Cal Halvorsen[2], and Koichiro Shiba[3], *1. Texas State University, San Marcos, Texas, United States*, *2. Washington University in St. Louis, St. Louis, Missouri, United States*, *3. Boston University, Boston, Massachusetts, United States*

Given that nearly half of adults 50 and older have hypertension in the United States, public health interventions focus on reducing risk factors. However, population-based approaches that aim to enhance protective factors, such as social engagement, can be applied to larger groups, resulting in a downward shift in the population blood pressure distribution. Further, understanding the potential heterogeneous effects of volunteering frequency by demographic factors help identify the population subgroups in which volunteering is more (or less) protective against hypertension. The current project examines the link between volunteering and changes in systolic and diastolic blood pressure by gender, race/ethnicity, and cohort as potential effect modifiers. Using the Health and Retirement Study (2006-2016, N=18,847), this study assessed the longitudinal relationships between volunteering (2010/2012) and blood pressures (2014/2016) within each stratum of gender, race/ethnicity, and age cohort. The analyses adjusted for pre-volunteering covariates (2006/2008) as well as selection into volunteering using Inverse Probability Treatment Weighting (IPTW). The joint exposure of volunteering and effect modifiers was estimated by the relative excess risk due to interaction (RERI). Results show that individuals volunteering 201+ hours annually had a lower likelihood of clinically high diastolic (PR=0.84, p=0.04) and systolic (PR=0.98, p=0.04) blood pressure. Moderate level of volunteering was predictive of low systolic blood pressure. The combination of volunteering 201+ hours and being female was protective against high diastolic blood pressure (RERI=-0.09, p=0.02). Given the modest effect modification, frequent volunteering has an anti-hypertensive effect for the general population of adults 50 and older.

Abstract citation ID: igae098.2889

### LONELINESS AND COGNITIVE FUNCTION IN OLDER ADULTS: A LONGITUDINAL ANALYSIS

Elnaz Abaei, and Peter Martin, *Iowa State University, Ames, Iowa, United States*

Only a few studies have confirmed cognition as a predictor of loneliness. This study investigated the dynamic relationship between loneliness and cognitive performance in older adults through a random intercept cross-lagged panel model. Data from 8,443 individuals aged 65 years and older, drawn from Waves 9–14 of the Health and Retirement Study (HRS), were analyzed. Loneliness was assessed using the UCLA Loneliness Scale, and cognitive function was measured using delayed memory recall. Age, gender, and education were included as covariates. We combined waves 9-10, waves 11-12, and waves 13-14 to include all participants. Bivariate correlations revealed that older age was negatively correlated with cognitive function, loneliness was positively correlated with older age, and male gender and higher education were positively correlated with cognitive function, and negatively correlated with loneliness. Cognition and loneliness were negatively associated. Using Mplus, we computed an unconditional random intercept cross-lagged panel model (RI-CLPM) with no exogenous predictors (X2=.186, df=1, p=0.67, RMSEA=0.000 [.000,.018], CFI=1.0, TLI=1.0) and a conditional model (X2=13.431, df=7, p<.06, RMSEA=0.009 [.000,.015], CFI=.999, TLI=.997) including age, gender, and education as predictors. Results indicated individuals reporting higher loneliness exhibited lower cognitive functioning scores and older age. Male gender and higher education were negatively associated with loneliness and positively with cognitive function. This study underscores the link between loneliness and cognitive decline in older adults, emphasizing the need to address loneliness to potentially reduce cognitive decline. Insights into demographic predictors of loneliness and cognitive function could inform targeted interventions for promoting successful aging.

Abstract citation ID: igae098.2890

### POWER EXPANDS FUTURE TIME PERSPECTIVE AMONG OLDER ADULTS

Liru Wang, Mengxuan Su, and Minjie Lu, *Beijing Normal University at Zhuhai, Zhuhai, Guangdong, China (People's Republic)*

Social power has been conceptualized as the ability to control or influence others in meaningful ways (Fiske,

1993). According to Socioemotional Selectivity Theory (Carstensen, 2006), when aging, individuals tend to perceive their future as more limited. This project aims to examine whether older adults with power may perceive their future as more expanded. In Study 1, 241 participants aged from 18 to 80 (154 females, Mage=50.27, SD=16.76) reported their sense of their own power and perception of the future. Results showed that the sense of power was positively associated with an expanded future time perspective, measured by a future time perspective scale and a life-axis index. In Study 2, 55 older adults (24 females, Mage=64.25,SD=3.09) who are currently in authority at work were recruited for the high-power group, and 52 older adults (25 females, Mage=65.38,SD=3.68) without authority at word were recruited for the low-power group. Consistent with Study 1, the high-power group of older adults reported a more expanded future than did the low-power group. In addition, the high-power group of older adults displayed a stronger inclination to pursue knowledge-related goals (e.g., obtaining new skills and experiences, knowing new friends). In both studies, the effect of power on expanding future time perspective was mediated by the participants' sense of control. These findings highlight that role of power in counteracting the age-related changes in future time perspective.

Abstract citation ID: igae098.2891

### PREDICTORS OF CONTINUED ENGAGEMENT IN ONE'S FAVORITE ACTIVITY DURING THE COVID-19 PANDEMIC

Jeehoon Kim[1], and Ji Hyun Lee[2], *1. Idaho State University, Pocatello, Idaho, United States*, *2. Montana State University, Bozeman, Montana, United States*

Active engagement is one of the important pillars in successful aging (Rowe & Kahn, 1997), however less is known about one's favorite activity, a key source of enjoyment and meaning, in later life. In the context of higher constraints imposed during the pandemic, continuing one's favorite activity can play a pivotal role in maintaining well-being for older adults. The current study aimed to examine which factors are associated with sustaining engagement in one's favorite activity in 2020. Data comes from Round 10 of National Health and Aging Trends Study. Participants (n= 4,389, Age M(SD)=78(6), 55% female, 83 % White) answered, during last year, whether they were able to participate in their favorite activity previously reported at Round 5 (84% yes). Survey weighted logistic regression models were performed stratified by gender. We found that women who reported health better (OR= 1.28, p=.018) and engaged in vigorous physical activity (OR=1.96, p =.007) were more likely to continue their favorite activity. For men, younger age (OR=.97, p=.017), social engagement (OR=1.22, p =.011) and physical activity participation (walking OR=1.91, p=.014; vigorous OR=2.07, p=.001) were positively, but depressive symptoms (mild OR=.57, p =.006; major OR=.50, p=.028) were negatively linked to continuing in favorite activities. The findings show that engagement in physical activity supports continued enjoyment of one's favorite activity; social engagement and mental health are also protective factors for men. Implications for how to support older women and men's continued activity engagement will be discussed.

Abstract citation ID: igae098.2892

### SUPPORT FROM FRIENDS, BUT NOT CHILDREN, BUFFERS AGAINST DEPRESSION AMONG LEFT-BEHIND OLDER ADULTS

Suyi Wang[1], Minjie Lu[1], and Helene H. Fung[2], *1. Beijing Normal University at Zhuhai, Zhuhai, Guangdong, China (People's Republic)*, *2. The Chinese University of Hong Kong, Hong Kong, China (People's Republic)*

As adult children migrate, old parents are facing the choice of migrating with their children or remaining in their hometowns (being left-behind). Being left-behind is associated with deteriorating psychological well-being among older adults (Adhikari et al., 2011; Song, 2016; Song, 2017; Zhang, 2016). This study aims to examine whether support from children and friends may benefit the well-being of left-behind older adults. The study recruited 52 left-behind older adults (30 females, Mage = 64.96, SD = 5.291), 69 older adults migrated with their children (54 females, Mage = 64.78, SD = 4.465), and 64 older adults who were currently living with their children in the city they grew up (34 females, Mage = 66.28, SD = 5.072, local group). Consistent with previous findings, the results showed that left-behind older adults reported a higher depression level compared to the local group, and the migration group was in-between. In addition, perceived support from children was negatively associated with depression level among the migration group and the local group, but not among the left-behind group. However, only among the left-behind older adults was perceived support from friends living in the same city negatively associated with depression level. The difference in depression level between the left-behind group and the local group was mitigated among participants who perceived strong support from friends. These results highlight the salutary effect of support, especially from friends, on buffering against depression among left-behind older adults.

Abstract citation ID: igae098.2893

### THE MEDIATING EFFECT OF SELF-TRANSCENDENCE IN THE RELATIONSHIP BETWEEN SOCIAL SUPPORT AND SUCCESSFUL AGING

Do Thi Thu Huyen, Moonkyoung Park, and Xianqi Gao, *Chungnam National University, Daejeon, Republic of Korea*

**Background:** The migration of old adults Chinese often involves accompanying their children to assist with caregiving for the younger generation, a strategy that addresses the shortfall in familial support.However, it remains unclear whether these old adults migrants can effectively adapt to their new environments, maintain their daily lives, and age successfully. Access to psychological and social resources is crucial for successful aging. Among these, transcendence is highlighted as a key psychological component. Purpose To verify the mediating effect of transcendence in the relationship between social support and successful aging among Chinese migrant old adults.

**Methods:** Participants consisted of 306 Chinese migrant old adults aged 60 and above, and data on demographic information, social support, successful aging, and transcendence were collected using a structured questionnaire from August to December 2023. Analyzed using the SPSS 26.0

and AMOS 26.0 software programs. Results Social support was significantly correlated with successful aging and transcendence. Social support had a significant direct effect on both successful aging (β=.480, p<.001) and transcendence (β=.54, p<.001). Transcendence also had a significant direct effect on successful aging (β=.660, p<.001). The mediating effect of transcendence between social support and successful aging was significant (β=.306,p=.008,95%CI [.220-.392]).

**Conclusion:** This study indicate that transcendence has a mediating effect on achieving successful aging among Chinese migrant old adults. Since social support can enhance the capacity for transcendence, it is necessary to develop interventions that support transcendence, enhanced by social support, to promote successful aging among Chinese migrant old adults in new environments.

### SESSION 7360 (POSTER)

### TECHNOLOGY: OLDER ADULT INTERFACE AND USE II

Abstract citation ID: igae098.2894

### ATTITUDES TOWARD MOTIVATIONAL MESSAGING IN A DIGITAL HEALTH ACTIVITY PROGRAM FOR OLDER FAMILY CARE PARTNERS

Dawon Baik, Paul Cook, and Catherine Jankowski, *University of Colorado, Aurora, Colorado, United States*

Older family care partners of patients with heart failure (HF-FCPs) often experience physical, psychological, and social health issues due to age-related health needs, less emotional support, and social isolation. We developed a tailored digital health physical activity (PA) program, TPA4You that includes videoconferencing with a health coach, wearable sensor, and motivational messaging. To refine TPA4You, a 1-week, two-phase, iterative field test of TPA4You was conducted. Herein, we report on older HF-FCPs' perceptions and attitudes toward the motivational messaging. We used an existing motivational messaging system. Prior to generating messages, the system queried PA barriers. The system automatedly generated a message to overcome the barrier rated highest by the participant at the time of the survey. The seven participants in Phase 1 were women aged 68.3 ±7.1 yrs. (M±SD), predominantly non-Hispanic White (86%), and caring for a spouse (71%). Some attitudes toward the motivational messages were favorable, such as encouraged PA, provided logical and applicable advice, and reinforced a committed to exercise, while others were unfavorable, such as the messages were easily ignored, ineffective for increasing PA, and not sufficiently tailored to the perceived barrier. The messaging system was perceived as easy to use and quick to complete by some, while others felt that answering questions within the system was confusing, questions were repetitive, and response options too limited. Many reported twice weekly messaging was sufficient. These data will be used to modify the frequency, content, and system (automated vs. manual) of motivational messaging in Phase 2 of TPA4You modifications.

Abstract citation ID: igae098.2895

### BRIDGING THE DIGITAL DIVIDE: EXPLORING DETERMINANTS OF TELEHEALTH UTILIZATION AMONG US OLDER ADULTS

Misun Hwang, Philip Veliz, and Yun Jiang, *University of Michigan, Ann Arbor, Michigan, United States*

The growing older population emphasizes the need for effective integration into the digital health ecosystem. Multiple factors can affect technology adoption among older adults; however, few studies have comprehensively examined their use of telehealth. This study aimed to explore determinants of telehealth utilization among older adults based on the Senior Technology Acceptance Model. Logistic regression was conducted to analyze a nationally representative survey of 6,327 community-dwelling older adults (≥65 years) from the 2022 National Health and Aging Trend Study (NHATS). Telehealth utilization was defined as communicating with health professionals online to manage health. Findings indicated that 46.1% of respondents reported telehealth utilization last year. Respondents who were older, non-white, living in non-metropolitan areas, had lower levels of education and income, or lacked access to technologies were less likely to utilize telehealth. Additionally, poor cognitive function and a lack of social networks or social participation were associated with the non-use of telehealth. However, poorer health status and multiple comorbidities, including cancer (OR=1.47, 95% CI=1.12-1.92) and anxiety (OR=1.56, 95% CI=1.10-2.26), were associated with greater odds of telehealth utilization. Gender, marital status, sensory impairment, limitation of strength or movement, instrumental activities of daily living, and depression were not associated with telehealth utilization. This study confirms the digital divide among vulnerable older adults in the US. Although they have an increased need for telehealth services with their health conditions, they might not fully benefit from telehealth. There is an urgent need to improve digital inclusion and advance health equity in this population.

Abstract citation ID: igae098.2896

### EFFECTIVENESS OF USING TECHNOLOGIES FOR COMMUNITY DWELLING OLDER ADULTS: A PILOT STUDY

Pei-Fen Chang[1], Lisa Thomas[2], Jeff Feng[3], and Kai-Li Tsai[4], *1. Texas Woman's University Houston Campus, Houston, Texas, United States*, *2. The University of Texas Health Houston, Houston, Texas, United States*, *3. University of Houston, Houston, Texas, United States*, *4. Texas Woman's University, Houston, Texas, United States*

Many challenges arise with an aging population, particularly the increase of isolation and lack of social relationships. A strong sense of social connections is considered as a protective factor that is associated with general well-being. One novel approach that has been shown to increase social connection in older adults is the utilization of technology. During this post-pandemic period, we lack an understanding of whether or not older adults use technologies for daily living, especially for social connections. The aim of the study was to examine the effectiveness of an information and communication technology (ICT) program for community dwelling older adults for social connection by decreasing loneliness and increasing quality of life. A one-group pre and post-tests design was used including the inclusion criteria: older adults aged 70 and older, living alone in the community, and able to speak and understand English. Outcome measures included the SF-12 for physical and mental health, UCLA 3-item loneliness Scale for loneliness, and Technology Acceptance Model questionnaire for the self-perceived acceptance and ease of use of the technology. After receiving a 6-week intervention by using iPads, all participants demonstrated significant increase in quality of life and decreased loneliness scores. However, results of the self-perceived acceptance and ease of use of the technology were mixed. Results from this pilot study demonstrated that community dwelling older adults who live alone could benefit from using technology for communication to decrease loneliness and increase quality of life. Future study with larger sample size is needed.

Abstract citation ID: igae098.2897

### EMBRACING THE DIGITAL ROUTE: EMPOWERMENT THROUGH EHEALTH LITERACY AMONG OLDER ADULTS

Gul Seckin, *University of North Texas, Denton, Texas, United States*

Browsing the internet for health information is one of the most common online activities, with people aged 50 and above leading the technology used for health purposes. This study was conducted to examine how much older adults trust electronic sources of health information (e-health trust) and whether e-health trust had significant associations with electronic health literacy (e-health literacy), consumption of electronic health information (e-health consumerism), and sense of empowerment. The study used selective optimization with compensation theory and corrective and proactive models of successful aging as conceptual frameworks. The respondents (N= 499) were drawn from the national probability-based research panel for online surveys. The study conducted subsample analyses of middle-aged (45≤age≤59, n = 233) and older respondents (age≥60, n= 194). T-tests, multivariate regression analyses, and structural equation modeling were performed. Middle-aged respondents reported greater empowerment than older adults (M=3.31 vs. M=3.05; $\chi^2$=5.709, p<.001). No significant association was found between e-health consumerism and perceived empowerment (β=.134, p=.115). However, perceived empowerment has positive associations with e-trust (β=.252, p<.001) and e-health literacy (β=.326, p <.0010). E-trust is positively associated with e-health consumerism (β=.551, p <=.001), suggesting an indirect mediational association between e-health consumerism and perceived empowerment. By empowering older patients to become informed users of digital information and developing educational interventions to improve their health literacy, they may be more likely to take action to improve their health. This is also consistent with the literature that older adults with a higher sense of empowerment have significantly slower rates of health decline over time.

Abstract citation ID: igae098.2898

### EXPLORING THE RELATIONSHIP BETWEEN INTERNET USE AND BELIEF IN CONSPIRACY THEORIES AMONG OLDER ADULTS IN JAPAN

Masumi Takeuchi[1], Ai Fukuzawa[2], and Tetsuro Kobayashi[3], *1. Tokyo Metropolitan Institute for Geriatrics and Gerontology, Tokyo, Japan*, *2. Musashino University, Nishitokyo, Tokyo, Japan*, *3. Waseda University, Tokyo, Japan*

The use of the internet among older adults in Japan has become increasingly prevalent, leading to both positive and negative effects, such as the association between internet use and belief in conspiracy theories (Toriumi, 2023). In Japan, it has been observed that older adults are more susceptible to conspiracy theories when exposed to extreme content, such as online videos. However, there is a lack of empirical studies examining this phenomenon. This study aims to investigate the relationship between internet use and belief in conspiracy theories among older adults. An online survey was conducted in March 2024, involving 1,200 participants aged 20-79 residing in Japan. The survey assessed internet usage, belief in conspiracy theories, the UCLA Loneliness Scale, and basic demographic information. Participants were categorized into three age groups: young (20-39), middle-aged (40-59), and older adults (60-79). The results revealed no significant difference in belief in conspiracy theories among the age groups. However, multiple regression analysis indicated that among older adults, those who spent more time watching online videos were more likely to believe in conspiracy theories, particularly those who experienced higher levels of loneliness. No similar pattern was observed among the young and middle-aged participants. The results indicate that older adults, who have fewer social relationships due to retirement and other factors, may come to believe in conspiracy theories through viewing online videos. Further extensive research is required to establish protective measures that mitigate the potential risks associated with internet usage for the older population.

Abstract citation ID: igae098.2899

### EXPLORING USE OF IMMERSIVE VIRTUAL REALITY IN OLDER ADULTS WITH COGNITIVE IMPAIRMENT

A'mie Preston[1], Kalpana Padala[1], Scott Mooney[1], Christopher Parkes[1], C. Heath Gauss[2], and Prasad Padala[1], *1. Central Arkansas Veterans Healthcare System, North Little Rock, Arkansas, United States*, *2. University of Arkansas for Medical Sciences, Little Rock, Arkansas, United States*

**Background:** The innovative use of immersive virtual reality (iVR) has the potential to offer individualized, low-cost, accessible care to the older adult population. Virtual reality has gained tremendous interest for its potential uses with older adults who have cognitive impairment, though significant gaps still remain in the literature for this population. This research study aims to explore the feasibility, acceptability, and perception of technology for using iVR in an older adult, racially underrepresented, Veteran population with cognitive impairment.

**Methods:** Assessments for participants and their caregivers explored mood, quality of life, cognitive functioning, neuropsychiatric symptoms (NPS) related to cognitive impairment, and perception of the iVR device. Veterans used a head-mounted iVR device during the study visit.

**Results:** Participant demographics included race: Black (36.7%), White (63.3%). Participants reported enjoyment of iVR device [mean=111.7(± 14.05)]. Perception of iVR significantly changed after use (p=0.0001), with apparent improvement. Participants tolerated the experience well with an average score of 1.1 (± 1.34) depicted on the Simulation Sickness Questionnaire. No significant effect of the presence of NPS on the participants' ability to immerse themselves in the iVR experience was found.
**Conclusion:** Older adults can experience minimal symptoms during iVR use and may report high enjoyability. Reported NPS may not have a significant impact on the immersive experience of iVR. Perception of iVR can be positively improved following use of the device. Future research is needed in this area to identify use of iVR as a potential intervention for those with NPS associated with cognitive impairment.

Abstract citation ID: igae098.2900
### FRAMEWORK-BASED EVALUATION OF MOBILE HEALTH APPS FOR OLDER ADULTS: IMPLICATIONS FOR TECHNOLOGY-ENABLED SELF-CARE

Priya Nambisan[1], Akshat Kapoor[2], Gary Kreps[3], and Reshma Sahithi Dangeti[1], *1. University of Wisconsin Milwaukee, Milwaukee, Wisconsin, United States*, *2. East Carolina University, Greenville, North Carolina, United States*, *3. George Mason University, Fairfax, Virginia, United States*

The rapidly growing older adult population which is burdened with multiple chronic conditions (MCC) and declining physical and mental functionality, portends opportunities for developing better technologies to cater to their self-care support needs. However, it is not clear whether the current set of health apps targeted at older adults are suitable, or whether they fulfill their health and self-care support needs of older adults with MCC. We conducted a systematic review using the PRISMA method and also used a theoretical framework to evaluate currently available health apps for older adults. Many studies that review apps merely review their features, however, to get a more in-depth and nuanced understanding of whether the apps are meeting the needs of older adults, we utilize a needs-based theoretical framework. This framework was specifically developed to assess the needs of this population group, and the technological elements that could be leveraged for meeting their health needs. The framework delineates seven needs of older adults in the context of technology applications: 1) prevention focus; 2) comprehensiveness; 3) education and training for self-care; 4) community and home-based care; 5) social and emotional support; 6) privacy and security; and 7) positive experiences. Our results indicate that many apps in the market do not fulfill the needs of older adults with MCC. Our systematic review did not yield even a single comprehensive app for older adults with MCC which covered all the needs comprising the framework, thus highlighting the digital exclusion of this population and their needs for support.

Abstract citation ID: igae098.2901
### GENDERED PATHWAY TO LIFE SATISFACTION VIA HELPING SPOUSES WITH DIGITAL TECHNOLOGY USE

Hayoung Park[1], Susanna Joo[1], and Sun Ah Lee[2], *1. Yonsei University, Seoul, Republic of Korea*, *2. The Pennsylvania State University, University Park, Pennsylvania, United States*

Digital capabilities are closely related to the quality of life, and due to rapid digitalization, the exchange of help regarding digital technology use frequently occurs within close relationships in later life. As digital technology penetrates deeply into daily life, exploring the role of exchange of help regarding digital technology use within couple relationships could help specify daily interaction strategies improving the quality of life of individuals. This study explored how the husband and wife's digital capabilities affect life satisfaction via helping their spouses with digital technology use within dyadic associations. For this, we used the Digital Aging in Dyads (DAID) data collected through an online survey in May 2023, targeting 257 older Korean straight couples aged 45 to 74. According to the modified Actor-Partner Interdependence Mediation Model analysis with bootstrapping, two mediating paths were found to be significant; the higher the digital capabilities of husbands, the more help with digital technology use provided by husbands to wives, which increases the life satisfaction of both husbands and wives. However, for wives, only the direct association between helping their husbands with digital technology use and wives' life satisfaction was significant. These findings indicate gender asymmetric associations as the husband's digital capabilities serve as greater power within later-life couple relationships whereas the wife's digital capabilities nor helping their husbands with digital technology use do not play significant roles. This study is meaningful in presenting a new model for improving life satisfaction in the context of couple relationships in the digital era.

Abstract citation ID: igae098.2902
### GOING OUTDOORS AND COMPUTER USE PREDICT CHANGES IN SELF-RATED CHEERFULNESS OF OLDER ADULTS OVER 10 YEARS

Yufang Tu, Melissa O'Connor, and Rachel Grace, *North Dakota State University, Fargo, North Dakota, United States*

Positive affect decreases mortality risks and enhances wellness and longevity among older adults. Research indicates that adopting digital technology increases social inclusion among older individuals while going outdoors may benefit physical and psychological health. The current study explored whether the frequency of going outside and computer ownership/use relate to changes in older adults' cheerfulness over ten years. Data from the first 11 waves (2011-2021) from the National Health and Aging Trends (NHATS) Study were used. The study included 35,687 participants at baseline. Self-rated cheerfulness on a 5-point scale was measured. Longitudinal multilevel modeling was conducted with the frequency of going outside of home as a time-variant predictor (5-point scale) and computer ownership as a time-invariant predictor (yes/no). Covariates included sex, age groups, education level, and self-rated health. Interactions between time and go-out frequency, and time and computer ownership/use were also examined. The effect of go-out frequency on cheerfulness depends on different time points within (Estimate within=-0.01, p=0.002) and across individuals over time (Estimate between= -0.01, p=0.008). The trajectory of cheerfulness for individuals without computer use did not change over time (p=0.810). However, the cheerfulness of those with computer use did change over time (p=0.023). This study found that the go-out frequency and computer ownership/use predict changes in and improve older adults' cheerfulness over ten years, beyond demographic and health factors. This suggests the potential to include outdoor activities and computer adoption in efforts to promote healthy aging.

Abstract citation ID: igae098.2903

### MOBILE APP-BASED SURVEY DEPLOYMENT PATTERNS AND LONGITUDINAL ENGAGEMENT: A RANDOMIZED CONTROLLED TRIAL

Yuankai Zhang[1], Jian Rong[2], Xuzhi Wang[1], Eric Schramm[3], Chathurangi Pathiravasan[1], Ludovic Trinquart[4], Chunyu Liu[1], and Joanne Murabito[5], *1. Boston University School of Public Health, Boston, Massachusetts, United States*, *2. Boston University Chobanian & Avedisian School of Medicine, Boston, Massachusetts, United States*, *3. Care Evolution, Ann Arbor, Michigan, United States*, *4. Tufts University, Medford, Massachusetts, United States*, *5. Chobanian and Avedisian School of Medicine, Boston University, Boston, Massachusetts, United States*

Smartphone apps are increasingly used to collect digital survey data. Limited evidence is available on effective strategies to improve response rates especially among older adults. We conducted a randomized controlled trial (RCT, NCT04752657) embedded in the electronic Framingham Heart Study (eFHS) to test the effect of administering half of smartphone surveys every 2 weeks (experimental group) compared to administering all surveys every 4 weeks (control group) on participant response rate over 32 weeks. In all, 492 participants (mean age 73 years, 58% women, 16% non-White) were randomized. The primary outcome was the proportion of surveys returned per participant assessed longitudinally across four periods (baseline to week 8, week 8 to 16, week 16 to 24, week 24 to 32). We used mixed-effect regression models with random intercept to compare the mean proportions between groups across the 4 periods, adjusting for age (≤75 versus >75 years) and phone type (Android versus iPhone) that were used to stratify randomization. We observed significant effect modification by time (P-value=0.003), indicating that the difference in response rates between the two groups increased over time. The response rates decreased over time in both groups, but less so in the experimental group with a 7% point difference in the last period. The mean outcome decreased from 85% to 66% in the experimental group, and from 85% to 59% in the control group across the four time periods. This RCT demonstrated that the survey deployment patterns impacted the response rates over 32 weeks among older adults.

Abstract citation ID: igae098.2904

### OLDER DRIVER REPORTS OF EXPERIENCE WITH SENSORS IN VEHICLES TO RECORD DRIVING BEHAVIOR

Kelley Jackson, Ruth Tappen, David Newman, Jinwoo Jang, Borko Furht, KwangSoo Yang, Mónica Rosselli, and Michelle Villar, *Florida Atlantic University, Boca Raton, Florida, United States*

In a 5-year study of the degree to which changes in driving behavior herald a change in cognition, participants were asked to evaluate the telematic and video sensors (both driver facing and forward facing) installed in their vehicles in terms of any distraction or other concerns the sensors may have created. Both an 8-item survey and open-ended questions were asked of study participants every three months for the duration of their enrollment (three years). The questions concerned acceptability of sensors in one's vehicle including the aesthetics, source of distraction and potential for invasion of privacy. Mean overall rating of acceptability of the sensors was 3.5 out of a possible high score of 5. This rating was 3.5 at baseline, increased to 3.7 at the end of 6 months [t(110)=1.09, p=.004]. and was maintained over time. Of some interest is that there were no statistically significant differences in acceptability ratings by gender, race, ethnicity, age or education. A general inductive approach was employed to code the qualitative responses. The majority were concerned with the wires connecting sensors to power sources, both their visibility and tendency to loosen over time, creating both aesthetic and operational concerns as well as a tripping hazard for some. These were addressed early in the study to the satisfaction of most participants. The size of both the sensors and wires was mentioned a number of times and several participants suggested using smaller, wireless sensors if possible. Overall, most participants quickly acclimated to having sensors in their vehicles.

Abstract citation ID: igae098.2905

### PRELIMINARY FEASIBILITY OF AN MHEALTH INTERVENTION FOR OLDER ADULTS WITH SERIOUS MEDICAL ILLNESS AND CAREGIVERS

Danielle Llaneza[1], and Elissa Kozlov[2], *1. University of Houston, Houston, Texas, United States*, *2. Rutgers, The State University of New Jersey, Piscataway, New Jersey, United States*

Managing serious illness is often distressing and time-consuming for older adults and their caregivers. Up to 70% of adults with serious illness have symptoms of anxiety. Additionally, patients with high symptom burden and anxiety heavily impact family caregivers, which nearly 8 million older adults in the U.S. rely on for assistance. Mindfulness Therapy (MT) is a promising, non-pharmacological technique with proven efficacy and effectiveness in managing stress and anxiety in diverse populations. Mindfulness Coach is an mhealth delivered mindfulness therapy intervention developed by the Veterans Affairs National Center. The objective of this study is to report the preliminary feasibility of an eight-week trial of mHealth-delivered mindfulness therapy to alleviate anxiety and stress symptoms in this population. Thirty-one older adults with serious medical illness or their caregivers were recruited to participate in this study. After receiving an orientation to using the app, participants were instructed to complete two levels per week to learn about and practice mindfulness skills. At the end of the eight weeks, the Mindfulness Coach participant users ranged from zero minutes to 1154 minutes with an average use of 173.4 minutes. Additionally, changes in anxiety levels were also observed. This study offers preliminary evidence that an mHealth mindfulness app is being utilized by older adults with a serious medical illness or their caregivers to learn mindfulness skills to help manage the stress and anxiety associated with living with a serious medical condition.

Abstract citation ID: igae098.2906

### TECHNOLOGY ATTITUDES, PROFICIENCY, AND USAGE PATTERNS AMONG OLDER ADULTS WITH A COGNITIVE IMPAIRMENT

Xin Yao Lin[1], Walter Boot[1], Wendy Rogers[2], Neil Charness[3], and Sara Czaja[4], *1. Weill Cornell Medicine, New York, New York, United States*, *2. University of Illinois Urbana-Champaign, Champaign, Illinois, United States*, *3. Florida State University, Tallahassee, Florida, United States*, *4. Weill Cornell Medicine/Center on Aging and Behavioral Research, New York, New York, United States*

Older adults with cognitive impairment (OAwCI) may be challenged by increasing demands to engage with technology to accomplish daily activities. We investigated technology attitudes, proficiency, and usage across health, social, transportation, leisure, and domestic domains among OAwCI, including those with mild cognitive impairment (MCI), traumatic brain injury (TBI), and post-stroke cognitive impairment (PSCI). We examined if age, gender, health, cognition, and attitude towards technology (comfort, interest, efficacy) predicted technology proficiency and use in each domain and technology usage patterns. Data were gathered from 159 OAwCI, age range 60-93 years who participated in the Everyday Needs Assessment for Cognitive Tasks (ENACT) study. We found that OAwCI were frequent users of technology, were largely proficient in the use of mobile devices, and had generally positive attitudes toward technology. With respect to use of technology, the data indicated that the highest degree of engagement was with social technologies (e.g., telephone, email, text) and the least engagement was in the use of technologies in the transportation domain (e.g., using apps to purchase tickets or schedule rides). Data from the regression analyses showed that younger age, more comfort and interest towards technology predicted more technology proficiency. Perceptions of memory problems predicted more proficiency. These findings underscore that OAwCi are able to and are receptive towards using technology. The findings also provide valuable insights for tailoring interventions to meet the needs and preferences of OAwCI.

Abstract citation ID: igae098.2907

### TECHNOLOGY-RELATED COVID-19 EXPERIENCES OF OLDER VETERANS

Saanvi Lamba[1], Pranjal Tyagi[2], Diana Ruiz[3], and Stuti Dang[4], *1. Flint Hill School, Oakton, Virginia, United States*, *2. South Florida Veteran Affairs Foundation for Research and Education (SFVAFRE), Miami, Florida, United States*, *3. Miami Veterans Affairs Healthcare System, Miami, Florida, United States*, *4. Miami VA Healthcare System, Miami, Florida, United States*

We fielded the HERO CARE survey to 20,000 community-dwelling Veterans from five Veterans Affairs' (VA) Medical sites. Veterans were asked an open-ended question to "share anything else that you would like us to know about your experience with the COVID-19". We received responses from 1,992 (95.8%) males and 88 (4.2%) female veterans; 23 responses were technology-related. Technology-related comments were made by 18 male respondents [79.2 + 10.2y on average, 66.6% non-Hispanic White (NHW)], and 5 female respondents [75.8±14.0y on average, 42.9% NHW, 42.9% non-Hispanic Black]. Three coders classified these through inductive coding into emergent themes. The most common themes were trouble with technology access, technology-related usability challenges, using technology for communication during isolation, using technology for healthcare/appointments, and an inability/preference to see providers in person. A positive impact was the technology use for staying connected during isolation, endorsed by the statements, "It has reduced my ability to attend church in person I now attend via Facebook Live.", and "Have made more frequent use of the Internet and have communicated with family and friends worldwide." Older Veterans used technology to connect to VA care and with friends and family, despite challenges with technology access and usability, and sometimes a preference for in-person visits. The COVID-19 pandemic accelerated the adoption and innovative uses of technology by older veterans to enhance communication, combat social isolation, and for healthcare visits. Assisting older adults with tools and training to address access and usability challenges may allow more equitable use of technology use for enhancing access and communication.

Abstract citation ID: igae098.2908

### THE IMPACT OF COMPANION ROBOTIC PETS ON WELL-BEING AMONG COMMUNITY-DWELLING WOMEN AGED 65 AND OLDER

Suk-Young Kang[1], Jeungkun Kim[2], and Melanie Kuhl[1], *1. Binghamton University, Binghamton, New York, United States*, *2. Kangnam University, Yongin-Si, Kyonggi-do, Republic of Korea*

This study investigates the impact of companion robotic pets on depression, anxiety, loneliness, and self-rated physical health in community-dwelling women aged 65 and older. Employing a time series design for baseline stability and treatment effects (Knapp, 2018, p.237), 120 respondents from the local Office for Aging were screened, and 45 with depressive symptoms (GDS-15) were selected. Selected participants received a dog or cat robotic pet during a home visit, with follow-up visits scheduled one month later, constituting three data collection points. Analyzing depression data, baseline mean was 9.16 (SD = 2.70). Pre-test mean increased slightly to 9.40 (SD = 2.75), but significantly decreased to 5.29 (SD = 3.18) post-test. For anxiety, baseline mean was 13.18 (SD = 5.27). Pre-test mean decreased to 12.24 (SD = 4.99), and significantly decreased to 7.33 (SD = 4.35) post-test. Loneliness revealed a baseline mean of 4.31 (SD = 1.58), a pre-test mean of 4.62 (SD = 1.43), and a significant decrease to 3.13 (SD = 2.04) post-test. All reductions were statistically significant. Conversely, self-rated physical health exhibited a baseline mean of 1.44 (SD =.81), a pre-test mean of 1.42 (SD =.84), and a significant increase to 1.73 (SD =.84) post-test. These results affirm that companion robotic pets may enhance mental well-being in older women dealing with depression and anxiety. Furthermore, companion robotic pets may alleviate feelings of loneliness and contribute to improved self-rated physical health, substantiating their overall beneficial impact.

Abstract citation ID: igae098.2909

### USABILITY OF AN AFFECT-AWARE MULTIMODAL PERSONALIZED INTERACTIVE MODEL AMONG FAMILY CAREGIVERS

Yu-Ping Chang, Aditya Kumar Dwibedi, Bhushan Mahajan, Skylar Bard, and Sreyasee Das Bhattacharjee, *University at Buffalo, SUNY, Buffalo, New York, United States*

Long-term family caregiving for people with dementia is often exhausting and poses risks to caregivers' overall health. Most informal caregivers are not well-equipped to perform their caregiving responsibilities at home, facing numerous challenges, feeling stressed and overwhelmed, and receiving limited support. Artificial Intelligence, specifically following the release of 'generative pre-trained transformer' (GPT), has demonstrated promise in delivering assistive technology solutions in various domains in healthcare. Our project aims to evaluate the usability of an affect-aware multimodal interactive prototype that can facilitate human-like sensemaking, providing emotional support, and generating personalized recommendations to family caregivers. The prototype can receive multimodal inputs (speech, text) from family caregivers and evaluate their personalized emotional and task-related contexts to deliver short, machine-driven, affect-aware messages augmented with multimodal (text, video) recommendations on a pre-defined set of topics. Six healthcare providers and family caregivers were invited to evaluate the prototype. They were instructed to test the prototype on a computer for 1.5 to 2 hours and provide feedback regarding their experiences (e.g., content, accuracy, format, and technology). Participants commented on the usefulness and accuracy of information generated by the model for supporting family caregivers, the level of personalization, the level of detail for various caregiving tasks, and the response time. Overall, participants expressed enthusiasm and satisfaction regarding the model. Further refinement is needed to improve the model's accuracy and diversity of content related to caregiving tasks, level of engagement, recommended videos, and the response time. Our prototype has potential to provide greatly needed personalized support for family caregivers.

Abstract citation ID: igae098.2910

### USEFULNESS OF PATIENT-GENERATED HEALTH DATA BY DEPRESSION AND FRAILTY IN OLDER ADULTS WITH LUNG CANCER SURGERY

Mona Choi, Yesol Kim, Hyeonmi Cho, and Yeonju Kim, *Yonsei University, Seoul, Republic of Korea*

This study aimed to identify the perceived usefulness of patient-generated health data (PGHD) according to depression and frailty in older adults with lung cancer surgery. This cross-sectional study recruited patients aged 65 and over who were diagnosed with lung cancer and underwent lung resection at a tertiary hospital in the Republic of Korea. Depression was measured using the Center for Epidemiological Studies Depression Scale-10; frailty was assessed using the Korean FRAIL version and categorized into robust, prefrail, and frail. The perceived usefulness of PGHD was measured through 37 items from seven domains. Data were analyzed through one-way ANOVA, correlation, and linear regression analyses using the SPSS program. Among 132 participants, the average age was 71.79±5.20 years, with 53% men. The average depression score was 4.18±4.15 out of 30, and more than half were classified as robust (60.6%), 34.8% as prefrail, and 4.5% as frail. The higher the level of depression, the greater the perceived usefulness of Medication-related Factors (MF) among PGHD was identified (p=.028). Participants who were prefrail perceived the usefulness of Physical and Mental Well-being Factors (PMWF) (p=.015) and MF (p=.001) among PGHD to be higher compared to those who were robust. A higher perceived usefulness of PGHD was significantly associated with being prefrail (p=.032), older age (p=.017), and higher life satisfaction (p=.039). Our findings indicated that understanding the levels of depression and frailty in older adults after lung cancer surgery may help healthcare providers identify the perceived usefulness of PGHD and facilitate its application in research.

### SESSION 7400 (POSTER)

### ACUTE CARE (POSTERS)

Abstract citation ID: igae098.2911

### ADAPTING EDUCATION ON ELDERSPEAK COMMUNICATION FOR FORMAL CAREGIVERS IN THE HOSPITAL SETTING

Clarissa Shaw, and Katie Buchheit, *University of Iowa, Iowa City, Iowa, United States*

Elderspeak is communication to older adults that sounds like babytalk and perpetuates rejection of care. The Changing Talk (CHAT) intervention educates nursing home staff on elderspeak communication and effectively reduces both elderspeak by nursing home staff and rejection of care by residents with dementia. The purpose of this study was to adapt CHAT to CHAT-Acute and pilot-test the new educational program in the hospital setting. To create CHAT-Acute, we piloted CHAT with hospital nursing staff. Qualitative feedback was largely positive indicating that elderspeak was a new and important concept, virtual discussions were helpful, and elderspeak identification activities were valuable. Suggested changes included more challenging content, greater focus on the impact of elderspeak on behavior, and condensed modules with less repetition. Within the modules nearly all participants skipped activities with only one participant completing all in-module activities. We adapted CHAT to CHAT-Acute by adding hospital specific content, adjusting the language to reflect acute care, filming new exemplar videos of care encounters, updating the overall format and activities, and shortening the content for increased acceptability. CHAT-Acute was then piloted with N=57 hospital nursing staff. Confidence in dementia care measured by the CODE scale increased significantly from before to after the modules (p=.002). Greater than 90% of participants agreed that they would use the training in their job and were satisfied overall. Comments from nursing staff included: "eye opening discussion on how we speak to patients" and "it was beneficial because if gave examples and scenarios that can be applied to my work."

Abstract citation ID: igae098.2912

### CLINICAL PRESENTATION OF INTERSTITIAL LUNG DISEASE AS AN INITIAL MANIFESTATION OF ANTISYNTHETASE SYNDROME

Pamela Salcido De Pablo[1], Carolina Escorcia Encarnación[2], and Alfredo Olvera[3], *1. University Anahuac Queretaro, Queretaro, Queretaro de Arteaga, Mexico*, *2. Universidad Anahuac, Querétaro, Queretaro de Arteaga, Mexico*, *3. Universidad Anáhuac Querétaro, Educación, Distrito Federal, Mexico*

Antisynthetase syndrome (ASS) is an autoimmune disease, categorized as a type of idiopathic inflammatory myopathy, where interstitial lung disease is the most common manifestation, presenting in up to 80% of cases. It is related to antibodies and aminoacyl-transfer RNA (tRNA) and the most common one, anti-Jo. It has an incidence of 0.56 per 100,000 inhabitants, commonly in adults with an average age of 45 to 70 years old. A 72-year-old female, with a medical history of dyslipidemia, depression, and gonarthrosis, presents with dyspnea, hypoxemia, wheezings, asthenia, arthralgias, and myalgias. A chest radiograph showed abnormal parenchyma, with bilateral opacities in peripheral and subpleural zones, diagnosis was compatible with pneumonitis, treated with quinolones, steroids, and short-acting bronchodilators. With partial recovery, TC scan showed patchy ground-glass-like pattern, bronchi ecstasies, and honeycomb zones, spirometry showed restrictive pattern and rheumatology tests reports were negative for antibody anti-Jo-1 and positive in antinuclear antibodies (ANAs). After several differential diagnoses and studies; a diagnosis of antisynthetase syndrome was reached. Although its low prevalence in the general population, the importance of knowing about antisynthetase syndrome lies in understanding the pathology and encouraging research on it, thus achieving a good diagnosis and adequate interdisciplinary management that these patients should require in a syndrome that still exists. For this reason, this case report intends to mention some of the most used tools currently in use, which helped to diagnose this interesting and uncommon case.

Abstract citation ID: igae098.2913

### CULTURAL DIFFERENCES IN MEDICATION BURDEN OF OLDER HOSPITALIZED PATIENTS IN MEDICAL UNITS

Juliana Smichenko[1], Ksenya Shulyaev[1], Efrat Shadmi[1], Nosaiba Rayan-Gharra[1], Nurit Gur-Yaish[2], and Anna Zisberg[1], *1. University of Haifa, Haifa, Hefa, Israel, 2. Oranim Academic College, Kiryat Tivon, HaZafon, Israel*

Cultural differences are significantly related to the management of illness and symptoms, particularly in hospitalized older adults where medications are often employed for health stabilization. This study aimed to examine cultural variations in medication burden, specifically focusing on sedative and anticholinergic medications, among older patients admitted to medical units. A secondary analysis was conducted on data from 672 cognitively intact older adults (age 65 and above) who were functionally independent. The cohort comprised 104 Israeli Arabs, 165 former-USSR immigrants, and 403 veteran Israeli Jews. Upon admission, socio-demographic, functional, and cognitive assessments were performed, alongside daily evaluations of symptom severity. Medical records provided data on comorbidities, illness severity, and medication prescriptions, allowing for the calculation of the Drug Burden Index (DBI) to assess medication burden. Multiple regression analysis was employed to predict medication burden, considering cultural group, sex, age, functional and cognitive status, symptom severity, illness severity, comorbidities, and length of hospital stay. The model demonstrated significance ($F(10, 662)=11.647$, $p<.001$, $R2=.151$), revealing that being Israeli Arab ($t=-3.174$, $p=.002$) or former-USSR immigrant ($t=-2.157$, $p=.031$) acted as protective factors against medication burden. Symptom severity, illness severity ($t=-5.613$, $p<.001$; $t=4.102$, $p<.001$), and comorbidities ($t=3.775$, $p<.001$) emerged as significant predictors of higher medication burden. These findings underscore the presence of cultural disparities in medication prescribing during hospitalization. Despite similar presentations of symptoms and medical statuses across cultural groups, certain cultural backgrounds are associated with lower medication burdens. Understanding and exploring patient-physician dynamics within cultural contexts are essential for optimizing treatment approaches in diverse patient populations.

Abstract citation ID: igae098.2914

### DEVELOPING OF AN SDM NURSING PRACTICE MODEL FOR OLDER PATIENTS WITH DEMENTIA IN ACUTE CARE HOSPITALS

Taeko Saito[1], and Harue Masaki[2], *1. Nippon Medical School, Inzai, Chiba, Japan, 2. Chiba University, Chiba, Chiba, Japan*

This study aims to implement a Shared Decision-Making (SDM)nursing practice model for older patients with dementia in acute care hospitals among acute care nurses and evaluate its feasibility. As a preliminary step, a hybrid concept analysis was conducted, and a SDM nursing practice model was developed and refined based on the results of semi-structured interviews with nursing practitioners and researchers. In this study, five acute care nurses were recruited as research participants to confirm the feasibility of this model. Data analysis involved conducting content analysis from verbatim transcripts of individual interviews with the five participants after practice to assess feasibility. Additionally, both before and after the practice, surveys based on SDM-Q9 and Group's SDM, validated tools for assessing the SDM process, were administered to the five research participant nurses and five health care professionals involved in support (MD, MSW), generating data from the perspectives of users and stakeholders. The results indicated the feasibility of implementing the SDM process within the constraints of the acute care setting. Moreover, the study demonstrated that the SDM nursing practice model effectively adheres to the SDM process during acute phases, thereby clarifying the role of clinical nurses in facilitating the Sharad decision-making for older patients with dementia.

Abstract citation ID: igae098.2915

### EXPLORING THE FREQUENT VISITS AND ASSOCIATED FACTORS OF OLDER ADULTS VISITING EMERGENCY ROOM

Ying-Chun Li[1], Po-Yen Chang[1], Wang-Chuan Juang[2], and Yu-Qi Cheng[1], *1. National Sun Yat-sen University, Kaohsiung, Kaohsiung, Taiwan (Republic of China), 2. Kaohsiung Veterans General Hospital, Kaohsiung Veterans General Hospital, Kaohsiung, Taiwan (Republic of China)*

Older adults may frequently visit the emergency department (ED) due to numerous comorbidities. Studies pointed out that frequent ED visits are one of the main reasons for emergency overcrowding which will affect the service capability of medical institutions. Therefore, this study mainly explored the risk factors of frequent ED visits by older adults. A retrospective study with an ED database from a medical center in southern Taiwan collected older than 65 years of adults who visited the ED at least once in 2019 and 2020. They were classified into frequent users with more than four ED visits per year. The propensity-score-matching was used in a 1:1 ratio to compare frequent ED users to the control group. Research results indicated that male, veterans, triage 3 status, summer, and day shifts visited the ED more frequently (p< 0.01). Multivariate logistic regression results showed that those who entered the ED on their own and were admitted for digestive, genitourinary systems, and factors influencing health status had a higher odds ratio of visiting the ED frequently. Negative binomial regression results presented that frequent ED users will have longer hospital stays than those non-ED frequent users. In conclusion, frequent ED users consume more medical resources thus may lead to ED overcrowding. We suggest that hospitals and physicians should pay more attention to this group. Through efficient policy advocacy and disease prevention to improve the quality of care for older adults.

Abstract citation ID: igae098.2916

### IMPROVING RECOVERY FROM HOSPITALIZATION IN GERIATRIC PATIENTS THROUGH WHEY SUPPLEMENTATION AND TESTOSTERONE

Michelle Yang[1], Pratik Gongloor[1], Rachel Deer[2], Elena Volpi[3], Shuang Li[1], and Erin Hommel[1], *1. University of Texas Medical Branch, Galveston, Texas, United States*, *2. The University of Texas Medical Branch, Galveston, Texas, United States*, *3. University of Texas Health San Antonio, San Antonio, Texas, United States*

**Background:** Sarcopenia, the age-related loss of muscle mass and strength, remains a significant concern among hospitalized geriatric populations due to functional decline and increased mortality. A previous pilot study demonstrated potential benefits for testosterone or whey supplementation (WS) during and post-hospitalization towards improved physical function and reduced re-admissions. Our follow-up study assesses the feasibility and preliminary efficacy of these interventions.

**Methods:** This Phase I, single center, randomized clinical trial enrolled 80 participants, 65 years+, hospitalized for acute conditions. Participants were randomly assigned to one of four interventions: placebo oral and injectable (PO/PI) supplement, PI-WS, PO-testosterone injection (TI), or WS-TI. WS was provided twice daily during and post-hospitalization. TI was provided once pre-discharge. Testing was conducted at baseline and 4-weeks post-hospitalization. Primary outcome included Short Physical Performance Battery (SPPB) score change. Secondary outcomes included readmission rate and additional measures of physical function and body composition.

**Results:** Mean age was 71.5 years with 70% female. Age-adjusted Charlson Comorbidity Index was 4. Fifty-two participants (65%) completed the study; 15 were re-hospitalized and 13 withdrew or were lost to follow-up. Among those completing the 4-week intervention, we found no statistically significant differences in SPPB score or readmission rate.

**Conclusion:** We failed to demonstrate significant improvements in recovery among geriatric patients receiving testosterone, WS, or combination therapy. Compared to the previous study, participants were younger, healthier, and less likely to complete follow-up. We need further studies with larger samples of frail older adults to elucidate the relationship between these interventions and post-hospital functional recovery.

Abstract citation ID: igae098.2917

### RISK FACTORS FOR NINE-YEAR MORTALITY OF OLDER PATIENTS WITH COGNITIVE IMPAIRMENT AT ADMISSION

Hiroshi Yamamoto[1], Kenichi Ogawa[1], Yasushi Hisamatstu[1], and Tatsuya Ishitake[2], *1. Yamamoto Memorial Hospital, Imari, Saga, Japan*, *2. Kurume University School of Medicine, Kurume University, School of Medicine, Fukuoka, Japan*

**Background:** Dementia can be a major cause of mortality and morbidity in geriatric patients. So, it would be essential to assess their mental state in the early. Aims: We aim to appraise the impact on mortality and hospital outcomes by revised simplified short-term memory recall test (STMT-R) and collecting clinical data simultaneous.

**Methods:** The subjects were 612 acute inpatients ≥65 from December 2014 to September 2015. Following the collection of clinical data, survival was subsequently measured for 8-9years until December 2023. An STMT-R score of ≤4 were considered to indicate cognitive dysfunction. To explore the association between the risk factors and mortality in cognitive impairment subjects, Kaplan-Meier method and Cox's proportional hazards regression models were used to examine mortality and survival rates.

**Results:** The mean age of the subjects was 82.1 (±7.94), and 55.9% were female. Cognitive function was classified into three categories according to severity respectively; Incomplete Testing Group (Incomplete), Cognitive dysfunction (Abnormal) and Non-Cognitive dysfunction Group (Normal). 349 patients (57.0%) died during follow-up. Kaplan-Meier and the log-rank tests showed the negative prognostic effect of cognitive impairment, malnutrition, pneumonia and cancer-bearing state (p< 0.01). After adjustment for potential covariates the Cox regression showed that mortality hazard is increased with "Incomplete" (HR 3.526 CI 95% 2.388-5.206 p< 0.0001) and "Abnormal" (HR 1.676 1.210-2.321 p=0.0019).

**Conclusion:** Malnutrition, pneumonia and cancer-bearing state can significantly decrease the survival rate in patients with cognitive impairment at admission. Cognitive impairment also has independently an impact on survival rate in acutely ill geriatric patients.

### SESSION 7405 (POSTER)

### ADULT PROTECTION AND ELDER ABUSE

Abstract citation ID: igae098.2918

### A QUALITATIVE STUDY OF ADULT PROTECTIVE SERVICES PRACTITIONERS RESPONDING TO CASES OF ELDER ABUSE AND SELF-NEGLECT

Andie MacNeil[1], Erin Salvo[2], and David Burnes[1],
*1. University of Toronto, Toronto, Ontario, Canada*,
*2. Public Consulting Group, Portland, Maine, United States*

Adult Protective Services (APS) practitioners play a critical role in supporting older adults experiencing elder abuse and self-neglect (EASN), however, very little research has examined their experiences, from their perspectives. The purpose of this study was to examine the experiences of APS practitioners responding to allegations of EASN. Using a descriptive phenomenological qualitative methodology, semi-structured interviews were conducted with APS practitioners (n = 14) from the state of Maine. To enhance trustworthiness, two independent assessors analyzed transcript data to code transcripts into themes. Two domains, each with various subthemes, were identified: (1) rewarding elements of role and (2) challenging aspects of role. The findings of this study emphasize how APS practitioners are motivated by their capacity to help elicit positive change in the lives of their clients and support the well-being of older adults experiencing EASN. However, APS practitioners must navigate numerous challenges and barriers in their role, including time constraints, high and complex caseloads, limited resources, and broader misconceptions on APS. These findings highlight the importance of addressing these stressors to support the well-being of APS practitioners, which, in turn, can help support the vulnerable older adults they serve.

Abstract citation ID: igae098.2919

### "WE'RE THE TARGET": THE IMPACT OF CYBERCRIME AMONG OLDER ADULTS IN GHANA

Akwasi Adjei Gyimah[1], Eric Frimpong[2], and Mathias Adjei[3],
*1. Miami University, Oxford, Ohio, United States*,
*2. University of Massachusetts Boston, Boston, Massachusetts, United States*, *3. Miami University, Oxford, Ohio, United States*

Among Older adults, the impact of experiencing cybercrime affects greatly on their quality of lives. Whilst maximum attention is directed towards issues relating to aging and experiences of cybercrime in the Western world, there is insufficient attention in the academic literature in Africa. This study explores the emotional and personal constraints endured by older adults in Ghana who have been victims of cybercrime. This study will serve to fill an important gap in knowledge and aid in the development of effective strategies to address the issue. Using a purposive criterion sampling and a semi-structured interview, 16 older adults aged 60 years and older who had reported cases of cybercrimes to the Asokwa Police Service Division, Kumasi-Ghana were recruited. An interpretative phenomenological analysis (IPA) was used. Older adults who are cyber-crime victims experience feelings of anger, frustration, violation and sometimes lead to them being depressed. Additionally, financial losses resulting from cybercrime significantly causes distress and financial difficulties to continue to age successfully. Especially, older adults who worked in the informal sector, have no social security returns to benefit from but only depends on their savings. Generally, respondents held divergent views on the effectiveness of civil and criminal justice systems in combating this phenomenon in Ghana. Interventions to curb cybercrimes are fundamentally significant. Especially, when most older people are at increased risk. There is the need for education and effective training on cyber security for older people and their caregivers.

Abstract citation ID: igae098.2920

### ADULT PROTECTIVE SERVICES PROFESSIONALS WITH CLIENTS AND FAMILIES AT THE END OF LIFE: CHALLENGES AND SUPPORT NEEDS

Wei- Lin Xue[1], and Joy Ernst[2], *1. Purdue University, West Lafayette, Indiana, United States*, *2. Wayne State University, Detroit, Michigan, United States*

This study examines the experiences of Adult Protective Services (APS) workers confronting elder mistreatment at the end of life, a period of heightened vulnerability and increased abuse risk. While previous research highlights the necessity of multifaceted strategies to tackle elder abuse, the specific challenges faced by APS professionals in these critical circumstances remain underexplored. The research team conducted seven focus groups and one individual interview with APS workers and those closely associated with them. The 36 participants, including social workers, nurses, and other allied professionals, provided information on the types of elder abuse encountered, challenges or barriers in serving these clients, and the personal or professional effects these cases have on them. The qualitative analysis revealed that caregiver neglect and financial exploitation are the most frequent forms of abuse, along with self-neglect, emotional, physical, and sexual abuse. APS professionals face significant challenges such as balancing client self-determination, navigating family issues, accessing adequate care levels, addressing service refusal, and coping with a lack of services. The personal and professional impacts on APS workers include heightened empathy, trauma from client deaths, and a noted lack of preparation for such events. The workers also identified the need for increased interagency collaboration, organizational support, mental, and emotional support, and peer support to assist APS workers in dealing with clients at the end of life. These insights aim to guide the enhancement of strategies and policies to better support APS workers as they work with clients and families at the end of life.

Abstract citation ID: igae098.2921

### CAREGIVER NEGLECT IN OLDER ADULTS WITH COGNITIVE IMPAIRMENT: THEMES FROM EXAMINATION OF CASES

Amy Shaw, Alyssa Elman, and Tony Rosen, *Weill Cornell Medicine, New York, New York, United States*

Cognitively impaired older adults are at higher risk for caregiver neglect than other older adults. Characteristics of these neglected older adults and the common themes and surrounding circumstances have not been well described. We used case records from a hospital-based elder mistreatment response team to describe themes in caregiver neglect among older adults with cognitive impairment. We quantitatively and qualitatively examined records from 146 older adults with cognitive impairment for whom the response team found moderate or high suspicion for caregiver neglect. The median age was 82 (IQR 75–90) years; 53.4% were white, 19.2% were Black/African American, and 8.2% were Asian/Asian American; 21.9% had Hispanic ethnicity. Most patients (113, 77.4%) were women. Suspicion of at least one other form of elder mistreatment (verbal/emotional, physical, financial, or sexual abuse) was present for 48.67% of patients. The neglecting caregiver was most often the patient's child (42.5%), partner/spouse (19.9%), or aide (15.1%). We identified seven key themes about neglect: 1) delays in seeking medical care, 2) inadequate supervision, 3) attempts to honor patient preferences, 4) conflict between caregivers, 5) caregiver impairment (various types), 6) interference with medical or social care, and 7) "mystery friend" or relative moving into patient's home. Our work suggests that neglected older adults with cognitive impairment are commonly also victims of other forms of elder mistreatment. Themes in elder neglect may be identified that offer insight into the phenomenon. Multiple types of intervention may be needed to address the wide range of situations that may lead to neglect.

Abstract citation ID: igae098.2922

### COLLABORATION FACTORS IN ADDRESSING ELDER IPV: COMMUNITY CENTERS AND DV SUPPORT AGENCIES IN JAPAN

Asako Katsumata[1], Kiyoko Fukushima[1], and Noriko Tsukada[2], *1. Japan Lutheran College, Tokyo, Japan*, *2. Nihon University, Tokyo, Japan*

This study explores the determinants influencing collaboration between Community Comprehensive Support Centers (CCSCs) and Domestic Violence (DV) support agencies in addressing cases of older Intimate Partner Violence (IPV) in Japan. Utilizing data from a national survey conducted in November 2022, responses from 1,472 Community Social Workers (CSWs) out of 3,563 randomly selected CCSCs were analyzed. The study found that 5.8% of respondents frequently consulted with DV support organizations, 25.9% occasionally, 24.3% almost never, and 44.0% never at all. Logistic regression analysis, using gender, years of support experience, existence of a DV manual, number of husband-to-wife violence cases, and participation in training as independent variables, revealed that the presence of a domestic violence manual was the most significant factor influencing collaboration. CCSCs with a domestic violence manual were significantly more likely to consult DV support organizations (odds ratio = 2.158, 95% confidence interval: 1.425-3.266). These findings emphasize the importance of developing manuals on responding to domestic violence within CCSCs to facilitate collaboration with DV support agencies. This collaborative approach presents a viable option to enhance support mechanisms for older victims of intimate partner violence, indicating the potential impact of strategic interventions in addressing this critical societal issue.

Abstract citation ID: igae098.2923

### ELDER MISTREATMENT RISK FACTORS AMONG HOME HEALTH CARE SERVICE RECIPIENTS

Karen Schlag[1], Monique Pappadis[1], Jordan Westra[1], Yong-Fang Kuo[2], Leila Wood[3], Rebecca Czyz[1], Mukaila Raji[4], and Charles Mouton[1], *1. The University of Texas Medical Branch, Galveston, Texas, United States*, *2. University of Texas Medical Branch, Galveston, Texas, United States*, *3. The University of Texas Health Science Center, Houston, Texas, United States*, *4. University of Texas Medical Branch, Galveston, Galveston, Texas, United States*

Elder mistreatment (EM) research has often focused on EM risk factors among older adults in nursing homes and less on those homebound in Medicare-based home health care (HHC). We aimed to apply and improve a Medicare claims-based EM risk assessment model previously developed for primary care settings using the Outcomes and Assessments Information Set (OASIS) data in addition to Medicare claims. Drawing from the 20% national Medicare data, beneficiaries aged 66 and older receiving HHC in 2016, without an EM diagnosis in 2015 or 2016, and Medicare Part A/B/D eligible with no HMO through 2018 were included (N=159,278). Logistic regression models predicting 2-year EM diagnosis were evaluated. The cohort was split into training (50%), testing (25%), and validation (25%) samples. Risk factors from claims (e.g., demographics, health conditions, frailty scores (0-1), medical screening/procedure codes, and social determinants of health) and the OASIS (e.g., cognition, self-care, mobility, behavioral problems, living situation, and readmission risk score) were included. EM diagnosis prevalence was 0.4%. Our models' discriminatory power, c-statistic, of the claims-based model and the claims-based model with HHC assessments was 71.2 and 76.2, respectively. Medicare dual eligibility, disability entitlement, certain medical conditions (e.g., hepatitis, learning disabilities, post-traumatic stress disorder, COPD), and housing and income problems were associated with increased risk of EM in both models. OASIS data showed that patients living alone and with greater disruptive behaviors were at increased EM risk. Including assessment data can improve the claims-based algorithm to predict EM among HHC recipients.

Abstract citation ID: igae098.2924

### EMPOWERING OLDER ADULTS AND REDUCING HARM THROUGH PERSON-CENTERED ENDEAR PROGRAM

Julie Kim[1], Julie Rousseau[1], Jorge Sole[2], Sharnette Raye-Brown[2], Elvira Dodd[3], David Sanchez[3], and Lisa Gibbs[1], *1. University of California Irvine, Irvine, California, United States*, *2. University of California Irvine Health, Irvine, California, United States*, *3. OC Health Care Agency, Santa Ana, California, United States*

In Orange County, California, the Office of Public Guardian (OPG) investigates whether persons referred to OPG have resources and support to manage their financial and personal affairs. In the 2021-2022 fiscal year, OPG investigated 613 referrals. The UC Irvine Center for Excellence on Elder Abuse and Neglect and OPG partnered in a quality improvement project providing a person-centered case management program, ENding and Disrupting Elder Abuse Recidivism (ENDEAR), supported by the Administration for Community Living. A

case manager and referred participants collaborated to identify health and safety improvement areas that could obviate a court petition to establish conservatorship where a conservator is appointed to manage the older adult's financial affairs, personal affairs, or both. Participants (n=20, 70% female, mean age=78) receiving case management had a mean ADL score of 1.8 (0 independence to 6 total dependence), IADL score of 6.5 (0 independence to 8 total dependence), and financial capacity score of 4.9 (0 independence to 7 total dependence). All were at risk of experiencing self-neglect. Most had access to social support (n=15), including family, friends, and neighbors but lived alone (n=16). The case manager coordinated services based on home visits, needs assessments, and participants' communicated goals. On average, the case manager made 4.6 home visits over a mean of 123.9 days (range 18-386). Paired t-tests showed improvement in clients' safety ($t(19)=4.88$, $p< 0.001$) and lowered risk of self-neglect ($t(19)=-4.41$, p< 001). Findings suggest case management services support OPG goals by protecting older adults through improved safety and reduction in self-neglect.

Abstract citation ID: igae098.2925

### EXPLORING GENDER DISCORDANCE AND RISK OF CAREGIVING NEGLECT IN DEMENTIA CAREGIVING: A DYADIC PERSPECTIVE

David Hancock[1], E-Shien Chang[2], and Sara Czaja[3], *1. Weill Cornell Medicine, New York, New York, United States*, *2. Weill Cornell Medical College, New York, New York, United States*, *3. Weill Cornell Medicine/Center on Aging and Behavioral Research, New York, New York, United States*

Neglect in caregiving relationships, defined as unmet Activities of Daily Living (ADL) or Instrumental Activities of Daily Living (IADL) needs, remains a critical impediment undermining the safety of persons living with dementia (PLWD). This study explores the occurrence of neglect in gender-concordant and discordant caregiving dyads, where the caregiver and care recipient are of same or different genders. Data were drawn from 244 dyads of racially/ethnically diverse caregivers (CR) and PLWD. We operationalized neglect as CG's failure to meet any of the 6 ADL or 8 IADL for PLWD. Chi-squared tests were employed to compare neglect occurrences across three groups: female-male, male- female, and same-gender dyads. Overall, female-male caregiving dyads were most common in our study sample (n=103), followed by same gender dyads (n=102), and male-female dyads (n=39). The chi-square test indicated significant differences in neglect occurrences across the three groups ($p<.05$). Specifically, male caregivers caring for female care recipients exhibited more instances of one-week prevalence of neglect. ADL/IADL item-level analyses did not reach statistical significance due to small sample sizes; they generally supported the overall pattern. The findings suggest that gender concordance and discordance between CG and PLWD may be an important area for dementia caregiving intervention research. Future studies should focus on vulnerabilities and strengths of male CG caring for female PLWD to better support and mitigate risks of neglect in this population. These results have implications for policy and caregiving research, emphasizing the need to consider gender dynamics in caregiver support and training programs.

Abstract citation ID: igae098.2926

### HETEROSEXUAL AND SEXUALLY DIVERSE OLDER ADULTS' LIFETIME EXPERIENCE OF IPV: FINDINGS FROM THE CLSA

Gloria Gutman, Mojgan Karbakhsh, and Heather Stewart, *Simon Fraser University, Vancouver, British Columbia, Canada*

This study addresses knowledge gaps concerning prevalence and risk factors for intimate partner violence (IPV) among lesbian, gay and bisexual (LGB) compared to heterosexual Canadians aged 50+. Data derive from the Canadian Longitudinal Study on Aging (CLSA), a national cohort study with follow-up every 3 years for 20 years or until death. Participants who reported having ever been in an intimate partner relationship (n=11,727) who answered 'yes' to at least one item on the Composite Abuse Scale (Revised) – Short Form (CASR-SF) which was used to assess the prevalence of lifetime and past-year exposure to IPV and IPV types were included in this analysis. Main explanatory variables are sexual orientation and gender identity. Overall prevalence (exposure to any type) of IPV was 23.1% (16.6% among males; 29.1% among females) ($p< 0.001$). The lifetime prevalence rate was highest among LB women (35.8%), followed by GB men (29.3%), heterosexual women (29.0%) and heterosexual men (16.2%) ($P< 0.001$). The most common type of IPV was psychological (18%), with highest prevalence among LB women (27.5%) followed by heterosexual women (23.2%), GB men (20.4%) and heterosexual men (12.8%) ($P < 0.001$). The pattern of higher rates among sexually diverse woman and men then among their heterosexual age mates was also seen in lifetime experience of physical and sexual IPV. Recognition and prevention of IPV among sexually diverse older adults has been largely overlooked despite the fact that the IPV rates among same sex couples are reported to be similar or higher in comparison to opposite sex couples.

Abstract citation ID: igae098.2927

### IMPACT OF A POLICY CHANGE ON ADULT PROTECTIVE SERVICES CASE VOLUME AND SERVICES IN CALIFORNIA

Zachary Gassoumis[1], Erin Thayer[2], and Jeanine Yonashiro-Cho[3], *1. University of Southern California, Los Angeles, California, United States*, *2. Keck School of Medicine of USC, University of Southern California, Department of Family Medicine, Keck School of Medicine of USC, University of Southern California, California, United States*, *3. University of Southern California Keck School of Medicine, Los Angeles, California, United States*

The adult protective services (APS) system in the U.S. investigates and provides services to older adults and younger adults with a disability or vulnerability who are experiencing abuse, neglect, self-neglect, or financial exploitation. APS systems are undergoing shifts in their scope and nature of services, due to national movements toward program standardization, population aging, and changing population dynamics. California recently implemented a policy change, effective January 2022, that expanded APS services to all adults age 60 and older (reduced from 65) and expanded self-neglect to include individuals experiencing homelessness. We analyzed publicly available APS records from October

2019 through October 2023, using segmented linear regression on month-level data to assess the impact of this policy on overall APS case volume, number of APS clients aged 60-64, and clients referred to or receiving housing services. Following the January 2022 implementation, there was an increase in the growth of overall APS cases over time (b=103.9, p=.015) but no significant increase in the level. Among clients aged 60-64, there was a significant increase in both level and growth of cases over time, jumping from roughly 375 to 550 monthly cases immediately following the policy implementation and growing to 800 cases by October 2023. Similar results were seen for housing services, with significant increases in the growth of cases over time (b=10.2, p=.005). This growth in volume and service type has implications for programmatic refinements and for better understanding resource needs in the light of national discussions on APS standardization and scope of services.

Abstract citation ID: igae098.2928

### OPENING PANDORA'S BOX: VIRTUAL UNCOVERING OF GERIATRIC SYNDROMES

Ronan Factora, *Cleveland Clinic, Cleveland, Ohio, United States*

Virtual Capacity Evaluations (VCE) were implemented during the COVID-19 pandemic at the Cleveland Clinic Center for Geriatric Medicine to facilitate Adult Protective Services' investigations of elder abuse and neglect where a client's decision making capacity was in question. During the natural process of the evaluation, the unaddressed medical issues were uncovered, specifically identification of cognitive impairment or dementia, gait impairment and falls risk, unexplained weight loss, and non-adherence to medications that increased risk of exacerbation of chronic illness. As many of these persons were also not connected to medical care, these problems were not recognized or managed. In the initial pilot of this program involving 52 individuals, 40 were eventually diagnosed with dementia, 24 had vision impairment, 14 had hearing impairment, 28 were at risk for falls, 24 had unintentional weight loss, and 29 were not taking their medications consistently. Each of these factors potentially limits the person's ability to function independently, threatens their physical/mental health, and increases their risk of hospitalization and institutionalization. Using the Clinical Frailty Scale (CFS) as an aggregate marker of overall health status, 28 were mildly frail (CFS 5), 14 were moderately frail (CFS 6), and 1 was severely frail (CFS 7). Despite the limited focus and purpose of the VCE, communicating the presence and status of these uncovered medical issues to that person's medical provider could facilitate evaluation of management of these problems to mitigate their potential impact on that person's health, functional status, and independence.

Abstract citation ID: igae098.2929

### PROVIDING ALLEGED VICTIMS AND PERPETRATORS WITH CASE MANAGEMENT TO PREVENT FUTURE CARETAKER NEGLECT

Samantha Tuft[1], Farida Ejaz[2], Courtney Reynolds[1], and Jessica Bibbo[1], *1. Benjamin Rose, Cleveland, Ohio, United States*, *2. Benjamin Rose Institute on Aging, Cleveland, Ohio, United States*

Caretaker neglect is the second most common type of adult abuse nationwide, including Utah, where 74% of cases resulted in an 'inconclusive' disposition in FY 23. Practitioners from Utah Adult Protective Services (APS) are collaborating with researchers in an ongoing study to offer four months of telephone-based case management to alleged victims and perpetrators when APS closes their case as 'inconclusive'. The goal is to prevent future reports of caretaker neglect. The study uses a four-month, pre-post design. Care coordinators interview study participants, develop and modify individualized care plans, and make referrals to services throughout the study. Preliminary data from 18 alleged victims shows their average age is 64 (range 18-59), 50% are female, and 28% live in rural areas. They experienced a significant reduction in depressive symptoms using the Patient Health Questionnaire – 2 during the study (p =.038). Data from 9 alleged perpetrators shows that their average age is 60 years (range 43-70), 78% are female, and 11% live in rural areas; their depression scores did not decline significantly, but trended downwards over time. Discussion will focus on the value of providing case management services to improve psychosocial outcomes, such as depression. Implications for practice and policy include the value of multidisciplinary collaborations to develop interventions to potentially prevent future reports of caretaker neglect, and, perhaps, other forms of abuse, neglect or exploitation.

Abstract citation ID: igae098.2930

### UNDERSTANDING THE ROLE OF TASK BURDEN IN CAREGIVER NEGLECT OF PERSONS LIVING WITH DEMENTIA

David Hancock[1], E-Shien Chang[2], and Sara Czaja[3], *1. Weill Cornell Medicine, New York, New York, United States*, *2. Weill Cornell Medical College, New York, New York, United States*, *3. Weill Cornell Medicine/Center on Aging and Behavioral Research, New York, New York, United States*

Understanding factors influencing caregivers' perceptions of how bothersome a caregiving task is, hereafter called task burden, is vital for improving support, reducing strain, and potentially decreasing risk of caregiving neglect in persons living with dementia (PLWD). This study examines the cross-sectional associations between caregiver characteristics and perceived task burden. Data were drawn from a 6-month multicomponent, randomized controlled trial of psychosocial intervention including 244 caregivers (CG) and PLWD. Baseline data included CGs' self-perceived task burden of 6 activities of daily living and 8 activities of instrumental activities of daily living (i.e, how much does helping PLWD with this task bother or upset you), and psycho-social assessments including depression, caregiving burden, compassion, mutualism, caregiver preparedness, spirituality, and social support. We used correlation and multivariable regression analyses to identify risk factors for task burden. Significant correlations were observed between task burden and all variables of interest. When entered simultaneously into a multivariable regression analysis, only higher depression and greater caregiving burden were independently associated with task burden (p<.05). Taken together, addressing depression and caregiving burden is crucial for reducing perceived task burden. Policy and caregiving research should prioritize interventions targeting psychological and emotional aspects of caregiving, such as mental health counseling, stress management, and caregiver support groups. Additional research should explore downstream consequences of task burden with respect to other adverse caregiving outcomes.

Abstract citation ID: igae098.2931

### UNVEILING PSYCHOLOGICAL INTIMATE PARTNER VIOLENCE RISK FACTORS AMONG OLDER WOMEN

Rujeko Machinga-Asaolu, and Kathryn Showalter,
*University of Kentucky, Lexington, Kentucky, United States*

This conference poster presentation delves into the critical yet often overlooked issue of intimate partner violence (IPV) among older women. Despite its historical presence, IPV against older women aged 50 and above remains understudied, with less than 10% of IPV global data addressing this target population. Psychological IPV (IPVPA) emerges as the predominant form of IPV, significantly impacting the physical and mental well-being of older women. Through an integrated literature review, synthesizing recent IPV literature, this study aimed to illuminate the unique risk factors associated with IPVPA among older women worldwide. Utilizing the Preferred Reporting Items for Systematic Reviews and Meta-Analyses (PRISMA) framework, our research identified seven pertinent articles out of an initial pool of 355 from PubMed, CINAHL, PsycINFO, and Ageline databases. These articles met strict inclusion criteria focusing on women aged 50 and older, employing quantitative, qualitative, or mixed methods to explore IPVA. After synthesizing the included articles, we organized the descriptive risk factors into three distinct categories: vulnerabilities, stressors, and maladaptive outcomes, providing a comprehensive understanding of the multifaceted unique nature of IPVPA risk factors among older women. By meticulously evaluating existing recent research, we aim to shed light on the complexities of IPVPA and identify gaps in the literature, ultimately contributing to a more nuanced understanding of this pervasive issue. Through this presentation, we seek to raise awareness of the urgent need to address IPVPA among older women and advocate for targeted interventions and further research initiatives to better prevent IPVPA among older women.

### SESSION 7410 (POSTER)

### AGE-FRIENDLY

Abstract citation ID: igae098.2932

### CARE NEEDS AND LIFE SATISFACTION IN FRAIL OLDER ADULTS: THE MODERATING EFFECT OF AGE-FRIENDLY ENVIRONMENTS

Inhye Jung, Miseon Kang, Seolah Lee, and Hyo Jung Lee,
*Yonsei University, Seoul, Republic of Korea*

Frail older adults often face challenges in performing Activities of Daily Living (ADL) and Instrumental Activities of Daily Living (IADL), leading to diminished quality of life. Age-friendly environments, designed to facilitate aging in place, aim to enhance the well-being of older adults and their family members of all ages by modifying their surroundings and promoting adaptation. This study investigates how age-friendly environments moderate the association between care needs and life satisfaction among frail older adults, elaborating on their role in the process. Using data from the 2020 National Survey of Older Koreans, we identified 1,435 frail respondents. Being frail was defined according to the scores summed with three questionaries asking respondents' physical abilities, which were based on the Study of Osteoporotic Fracture criteria. Age-friendly environments were categorized into Physical, Service, and Sociocultural domains following WHO principles. Multi-regression analysis, employing the Process Macro Model 4, revealed that age-friendly service and sociocultural environments mitigate the negative impact of care needs on life satisfaction among frail older adults. These findings underscore the potential of age-friendly environments to improve the quality of life and support aging in place for frail older adults. Creating supportive environments for older adults with frailty is essential for promoting the overall well-being of these older adults and their care providers.

Abstract citation ID: igae098.2933

### CHARACTERISTICS AND INFLUENTIAL FACTORS OF ANXIETY AND BURNOUT AMONG CAREGIVERS AND OLDER ADULTS

Wenyu Zhang, and Meihua Ji, *Capital Medical University, Beijing, Beijing, China (People's Republic)*

With rapid aging of the society worldwide, emerging research has suggested that mental disorders and high turnover among caregivers and older adults are substantial, however evidence in this area is limited. This study aimed to assess the characteristics and influential factors of burnout and anxiety among caregivers and older adults. A total of 184 caregivers, and 200 older adults from nursing homes as well as family settings were enrolled in this cross-sectional study. Participants' job burnout and anxiety were assessed using the Maslach Burnout Inventory, and the Self-Rating Depression Scale, respectively, along with their sociodemographic information. Multiple linear regression was used to analyze the influential factors of job burnout in caregivers, and anxiety in both caregivers and older adults. As a result, there were 33.7%, 47.3% and 22.3% of participating caregivers reported high emotional exhaustion, depersonalization and low personal accomplishment, respectively. In terms of anxiety, 61.4% of caregivers reported their anxiety as moderate, 95% and 57% of older adults from nursing homes and family settings, respectively, were with moderate anxiety. Among caregivers, age, working hours, occupational stress and professional training significantly affected their level of burnout, while education and working experience prominently affected the level of anxiety. Meanwhile, the satisfaction to caregivers and self-care ability markedly affected the anxiety of older adults. In conclusion, there were high prevalence of job burnout and anxiety existed in both caregivers and older adults, more effective strategies should be taken into account to minimize this issue in the future.

Abstract citation ID: igae098.2934

### INTERGENERATIONAL COMMUNITY DEVELOPMENT: LESSONS LEARNED FROM THE SENIOR TO SENIOR DINNER DANCE PROGRAM

Youjung Lee, and Jacqueline McGinley, *Binghamton University, Binghamton, New York, United States*

Intergenerational engagement is a universal human development process that helps older adults' successful aging (Krzeczkowska et al., 2021) and children's optimal development (McAlister et al., 2019). As part of an age-friendly initiative in a small city in New York State, an intergenerational engagement program (Senior to Senior Dinner Dance) involved a multi-year partnership between community agencies, including a local Office for Aging, university, high school, senior center, and church. As part of this partnership, a community dance was held in 2022 and 2023, where high school students and older adults participated in a variety of intergenerational activities. Findings from a process evaluation of this interorganizational initiative (e.g., focus groups with six community partners; surveys with 56 older adults), using a thematic analysis (Braun & Clarke, 2006), revealed: (a) social cohesion included mutual trust amongst community stakeholders; (b) social capital was achieved by leveraging existing resources with collective goals; and (c) intergenerational engagement was promoted through the equal status, common goals, and interorganizational cooperation of participants, in addition to sanctions and support from organizers. Findings from this study highlight important lessons for those, particularly those in communities with limited resources, who wish to develop and sustain intergenerational programs. Lessons learned include the importance of mutual trust among community partners, the utilization of existing resources, and the necessity of buy-in from upper administration in each agency. Lastly, intergenerational activities (e.g., dining, dancing) can promote initial positive contact between distinct age cohorts, which may help to advance larger age-friendly initiatives.

Abstract citation ID: igae098.2935

### OPTIMIZING CARE OF OLDER ADULTS BY USING 4MS AT A COMMUNITY HEALTH CENTER

Karla Tejada Arias[1], Elizabeth Hernandez[2], Jane Borrero De Jesus[1], Andrea Rink[3], Richard Marottoli[4], Jennifer Ouellet[3], Noelle Fry[3], and Barry Wu[3], *1. Fair Haven Community Health Care, New Haven, Connecticut, United States*, *2. Yale New Haven Hospital, New Haven, Connecticut, United States*, *3. Yale University, New Haven, Connecticut, United States*, *4. Yale University School of Medicine, New Haven, Connecticut, United States*

Given the increasing needs of the aging population and limited availability of geriatricians, it is crucial for community healthcare centers to develop effective approaches for geriatric care. Fair Haven Community Health Care, implemented the 4Ms Framework of Age-Friendly Care, from the Institute for Healthcare Improvement for patients 65 and older starting July 2023 at two different clinical sites. We employed screening tools and interdisciplinary team including patient-access, medical assistants, nurses, clinicians and resident physicians, to evaluate cognitive health, mobility, and medication regimens, prioritizing patient-centered care. The Electronic Medical Record was enhanced to include fall risk and cognitive assessments. Feasibility was assessed through surveys. This intervention has been applied to 196 patients, 54% of whom identified as Hispanic, 29% white and 13% African American. A staff survey (N=23) revealed a 100% satisfaction rate, with no adverse effects on workflow. Overall visit times remained consistent, with rooming times for patients under the 4Ms framework increasing by only 11%, indicating no substantial impact on workflow efficiency. Additionally, patient satisfaction increased considerably, with patients reporting a greater sense of involvement in their healthcare decision-making process and felt their care was aligned with their priorities, demonstrating a successful transition to patient-centered care. Thus, we have demonstrated that the 4Ms framework can be integrated into standard care for older adults in a busy community health center. This successful integration highlights our ability to deliver evidence-based care and boost patient involvement, signaling a positive transition towards personalized, patient-centered healthcare delivery for this vulnerable demographic.

Abstract citation ID: igae098.2936

### PARTICIPANT EVALUATION OF 4MS EDUCATION DELIVERED BY AGE-FRIENDLY CARE, PA USING PROJECT ECHO: 2020–2023

Diane Berish[1], Jennifer Knecht-Fredo[1], Erica Husser[1], Donna Fick[1], Marie Boltz[1], Jacqueline Sabol[1], George Garrow[2], and Angela Houge[2], *1. The Pennsylvania State University, University Park, Pennsylvania, United States*, *2. The Primary Health Network, Sharon, Pennsylvania, United States*

Older adults, the largest segment of the US rural population, face significant disparities in health and healthcare compared to their non-rural peers. They have limited access to healthcare and social services for prevention, management, and treatment of chronic conditions. Age-Friendly Care, PA, a collaboration of Primary Health Network, the largest Federally Qualified Health Center in Pennsylvania, and Penn State Nese College of Nursing, aims to bring reliable, high-quality, age-friendly care to all older adults living in rural PA. Sponsored by HRSA through its Geriatric Workforce Enhancement Program, Age-Friendly Care, PA utilizes the Project ECHO (Extension for Community Health Outcomes), all-teach-all-learn, platform to engage isolated rural providers in incorporating the Institute for Healthcare Improvement's 4Ms framework (What Matters, Mentation, Medication, Mobility) into their practice. Annual participant evaluation data from the last four years (2020 n=10, 2021 n=14, 2022 n=22, and 2023 n=6) shows respondents largely agreed or strongly agreed that attending the education session helped: Improve their ability to provide appropriate care (77%-100%), develop clinical knowledge (83%-100%), and 100% recommended attendance to colleagues. Participants were asked to rate their knowledge related to the 4Ms after each session. After the 2020 session, mobility screening, depression screening, and dementia training (66.6%) had the highest ratings. For 2021, depression screening (66.6%) was highest. In 2022, dementia screening and treatment (55%) were highest and for 2023, the top-rated knowledge area was treating mobility limitations (33.3%). Project ECHO has been shown to be an effective tool to reach rural healthcare providers and promote age-friendly education.

Abstract citation ID: igae098.2937
**PHYSICIANS' PERCEPTIONS OF AGE-FRIENDLY CARE IMPLEMENTATION FOR OLDER ADULTS LIVING WITH CANCER**
Kylie Sloan, *University of California San Francisco, San Francisco, California, United States*
This qualitative pilot study investigates physicians' perceptions about the implementation of age-friendly care (e.g., 4Ms framework) within an Age-Friendly Health System (AFHS), with a specific focus on care for older adults living with cancer. The pilot uses a critical grounded theory approach to conduct and analyze virtual, semi-structured, one-on-one interviews with 6 physicians, recruited using purposive snowball sampling between October 2023-March 2024. Participants currently provide care to older adults with diagnosed cancer and work in an AFHS. Half the participants are geriatricians and the remaining have no geriatric training. All are between ages 30 and 60, 4 identify as White, 1 as Asian, 1 as biracial (Asian and White), 5 as female, 1 as male, and all worked at their organization for between 6 and 15 years. Three preliminary findings emerged: 1) Participants offer differing definitions for the "age" in "age-friendly" and look beyond a number for who to consider an "older" adult; 2) Participants (without geriatric training) express limited knowledge about AFHS and suggest making the health system's physical and digital spaces more accessible for older patients with cancer; 3) Participants indicate implementation of some "M's" (e.g., What Matters, Medication) more than other "M's" (e.g., Mentation, Mobility) within cancer care. Money, time, and staffing are key barriers to age-friendly care implementation in cancer care according to participants. Findings inform potential gaps in age-friendly care implementation (e.g., depression screening/referral process) to address within cancer care and future research exploring perceptions of older patients with cancer about age-friendly care.

Abstract citation ID: igae098.2938
**PRESERVING CELL HOMEOSTASIS AS AN AGING MODULTATION STRATEGY**
Dennis Lox, *Sports and Regenerative Medicine, Clearwater, Florida, United States*
The cell desires to maintain homeostasis. The most efficient and productive method to achieve cell homeostasis, is utilization of the Longevity Pathway AMPK-SIRT1. This allows processes to repair, induce positive patterns that lead to systemic efficiency. The Longevity Pathway may be activated by exercise, drugs, MOTS-c (a mitochondrial derived peptide), Irisin (a adipomyokine Sestrin), and circadian clock genes. Some activators display synergistic effects with others and exercise. Upon activation of AMPK-SIRT1, molecular and cellular functions occur, allowing nuclear transfer of P53 and FOXO's, this prevents cytoplasmi binding of P53-FOXO4, avoiding the deleterious effects of overactive mTOR, cell senescence and the SASP, upregulation of NF-kB, production of inflammatory cytokines, DAMP, and HMGB. This sets up the continuous feedback loop of continuing the cycle, and interactions with the of Hallmarks of Aging, compounding the process of driving further aging and disease development. Homeostasis occurs when P53 and the FOXO's are translocated to the nucleus, allowing DNA repair, mitochondrial biogenesis, NRF1,2, and PPAR transformation, production of more NAD+ occurs, which is needed in the pathway to function, and lastly increased exercise capacity. As the GI Biome is now a Hallmark of Aging, Starting with a proper diet and exercise to initiate the Longevity Pathway to allow cell homeostasis, increasing cell efficiency, and decreasing deleterious pathways. Further targets for personalized treatment may better address human diversity.

Abstract citation ID: igae098.2939
**TELLING A STORY THROUGH SPATIAL ANALYSES OF AGE-FRIENDLY PHYSICAL, SOCIAL, AND SERVICE ENVIRONMENTS IN PORTLAND, OR**
Alan DeLaTorre[1], and Eiji Toda[2], *1. Portland State University, Portland, Oregon, United States*, *2. Portland State Uniervsity, Portland, Oregon, United States*
Age-friendly communities continue to expand in number and evolve with respect to research, planning, policy, and practice. Both researchers and practitioners should understand how to collect, organize, interpret, and visualize geospatial data that elucidate geographic patterns of age-friendly features and barriers to age friendliness to guide a data-driven, evidence-based approach to community planning and policy making. This presentation will highlight the university-local government partnership that resulted in data collection, analyses, and release of an ESRI-based age-friendly story map of Portland, Oregon. Portland State University – an age-friendly university – and the City of Portland – a member of the U.S. and global age-friendly networks – collaborated to produce a series of raster maps visualizing the level of age-friendliness physical, social, and service environments. Data collection was supported by various city, county, and state agencies, as well as age-friendly partners operating in the age-friendly community, university, and health care spaces. This effort calls attention to challenges and opportunities related to research, planning, and future implementation. The analyses revealed differing spatial distributions of age-friendly physical, social, and service features across Portland, Oregon that offer insights into identifying higher-need neighborhoods and types of desired interventions. Challenges include indicator selection, data availability and collection, measurement and weighting of each indicator, and translation to policy decisions.

### SESSION 7415 (POSTER)

### AGE-INCLUSIVITY IN HIGHER EDUCATION

Abstract citation ID: igae098.2940
**CO-CREATING: THE VALUE OF INTERGENERATIONAL ART-BASED EXPERIENCES**
Joann Montepare[1], and Deborah Baldizar[2], *1. Lasell University, Newton, Massachusetts, United States*, *2. Lasell University, Seekonk, Massachusetts, United States*
Intergenerational classroom activities are common in the traditional aging curriculum. However, contemporary higher education initiatives (Age-Friendly University/AFU, Age Inclusivity Domains of Higher Education/AIDHE) advocate for the value of reciprocal intergenerational exchange across the curriculum to enhance the teaching and learning experience. Moreover, as ageism persists there is a need to extend intergenerational contact (a tool for disrupting ageism) for the benefit of all students. This poster will present an art-based classroom case study to show how incorporating intergenerational exchange in classes without a specific aging focus is not only possible, but also an effective educational strategy. In this case, younger students and older adults (residing in a local university-based retirement community) co-enrolled in a Ceramics class over the course of several semesters. Following the classes, a group of younger and older learners were invited to complete a survey about their experience. The survey included open-ended response questions (e.g., about benefits, challenges, how to enhance future classes, etc.) and scaled-response questions (e.g., about how the experience impact age attitudes, the value of intergenerational classes, interest in other intergenerational classes). Younger students' responses were very positive, reinforcing the positive educational and social values of intergenerational interaction and indicating they would welcome more such class opportunities. Older adults' responses were likewise positive, noting how the experience nurtured mutual respect and the sharing of different perspectives. Both groups offered useful recommendations for future classes, to be described.

Abstract citation ID: igae098.2941

### LEVERAGING HIGHER EDUCATION IN OUR AGE-FRIENDLY WORLD

Kathy Black[1], Patricia Oh[2], Joann Montepare[3], and Lenard Kaye[4], *1. University of South Florida Sarasota-Manatee Campus, Sarasota, Florida, United States*, *2. University of Maine, Bangor, Maine, United States*, *3. Lasell University, Newton, Massachusetts, United States*, *4. University of Maine Center on Aging, Bangor, Maine, United States*

There is increasing interest in better understanding the connection between higher education and age-friendly community efforts. The global age-friendly community (AFC) movement calls for multi-sectoral engagement in a multi-year model encompassing four core phases (engage, plan, act, measure) to improve livability in domains of community life pertaining to the built, social, and service environment. However, there is limited empirical knowledge regarding the involvement of higher education and how it supports AFC efforts. We used qualitative inquiry to assess the engagement of U.S. institutions as reported by 80 AFCs that completed a five-year cycle of participation. We conducted directed content analysis using paired AFC action plans and progress reports (n=56) and classified engagement using a priori indicators by higher educational core activities (teaching, research, and service), core phases (e.g., measure) and clustered domain areas (e.g., built environment). Results reveal engagement across all core activities of higher education with most efforts in research, in all areas of the AFC model with most reported in the action phase, and across all clustered domains of practice with the greatest amount identified in the social environment. We identify opportunities for greater engagement and leadership by higher education in our age-friendly world.

Abstract citation ID: igae098.2942

### OLDER AND YOUNGER AS OTHER: OBSTACLES TO AGE-FRIENDLINESS ON CAMPUS

Summer Roberts[1], and Cindy Lahar[2], *1. University of South Caroline Beaufort, Bluffton, South Carolina, United States*, *2. University of South Carolina Beaufort, Bluffton, South Carolina, United States*

The University of South Carolina Beaufort (USCB) drafted an endorsement of the Age-Friendly University Principles in 2020; however, this effort has yet to advance beyond the working group. USCB, whose main campus is situated intentionally between two retirement communities, and with a large and active Osher Lifelong Learning Institute (OLLI) offering courses across three campuses, clearly reflected the principles on paper. Over the next three years, students in a sociology research seminar studied, at the request of the county, the needs of the region's growing and diverse older adult population. Yet, these data collection efforts reflected limited exposure to people of older ages, resulted in primarily white and high SES samples, and separated USCB from community efforts to support older adults. Additionally, anecdotal accounts of staff and students commonly note that visitors to campus disparage traditional college-aged students. As a result, focus has shifted to more fully integrating students in shaping age-inclusive campus culture beyond coexisting age-segregated environments. This presentation explores efforts through three courses to develop an action plan. Qualitative analysis of papers from gerontology classes identifies the attitudes of students surrounding their relationship with older adults in the community and on campus, particularly highlighting the consistent use of othering and ageism in talking about those of different chronological ages. Students' on-going research of fellow students' and community members' perceptions of age-friendliness provides the basis for promoting intergenerational solidarity at USCB.

Abstract citation ID: igae098.2943

### TRACING THE FOOTSTEPS OF THE SCHOLARS FROM DE TORNYAY CENTER FOR HEALTHY AGING AT UNIVERSITY OF WASHINGTON

Yanjing Liang[1], Priscilla Carmiol-Rodriguez[2], Sarah McKiddy[1], Paige Bartlett[1], Heather Wicklein Sanchez[1], and Basia Belza[1], *1. University of Washington, Seattle, Washington, United States*, *2. University of Washington, Washington, United States*

The de Tornyay Center for Healthy Aging has been pivotal in advancing research, education, and practice since 1998. The center faculty has mentored over 165 students, connected them to community partners, awarded them over $757,000 in research funding, and supported the dissemination of their work through travel scholarships. This paper presents the evolution of the scholar community by showcasing scholars' contributions over time. An online survey was distributed to 87 former de Tornyay Center scholars, resulting in 32 responses. Results indicate that after graduating scholars pursued careers in practice (n=21, 65.6%), teaching (n=18, 56.2%), and research (n=13, 40.6%). Contributions include publishing in high-impact journals, securing research funding from institutions like the National Institutes of Health, and pioneering innovative practice models. Scholars demonstrated leadership in the

COVID-19 response and made impactful changes in clinical settings. Scholars have engaged in shaping the next generation of nursing professionals through teaching, developing educational models, and contributing to professional service globally. Scholars credited the de Tornyay Center with boosting their research skills (n=24, 75%) and providing crucial career guidance and mentorship (n=24, 75%). Early involvement in scientific research at the center influenced students' academic paths, fostering a commitment to gerontological nursing. These findings contribute to understanding scholars' journeys and form a foundation for shaping the center's strategic plan. The results underscore the center's role in nurturing scholars who significantly contribute to healthcare, cultivating a talented workforce, and influencing scholars' career paths. The de Tornyay scholar program is instrumental in fostering professionalism in scholars' careers.

Abstract citation ID: igae098.2944

### UNDERSTANDING AND ENDING AGEISM: AN INNOVATIVE COLLEGE COURSE

Jennifer Sasser, *Portland Community College, PORTLAND, Oregon, United States*

Understanding and Ending Ageism is an online community college course developed as part of a grant-funded project that aimed to raise awareness about aging and ageism, as well as to make the case for integrating these dimensions of human experience into college-wide diversity, equity, and inclusion initiatives. Designed and first offered in 2016, the course continues to be offered two terms per year and has been periodically refreshed to incorporate new perspectives and resources (including the Reframing Aging Initiative and updated AGHE Competencies). Course content includes a comprehensive overview of the characteristics, mechanisms and effects of ageism (and how ageism is experienced across the life course and at different ages and stages); examination of how ageism intersects with other "isms" in forming a system of oppression and yet is not often enough considered in discussions about diversity, equity, and inclusion; identification and planning of interventions and projects connected to increasing age-appreciation and challenging ageism; themes of aging-awareness, social justice and intergenerational solidarity; and an emphasis on reflection, critical thinking, and praxis. Unique features of the course include its collaborative learning design and its focus on "practices" that we can engage in at every level at which ageism operates and at which we can intervene: the individual; interpersonal; social/organizational; societal/political; and cultural. In addition to weekly assigned readings and roundtable discussions, students create an Ending Ageism Synthesis & Action Project as their capstone assignment.

### SESSION 7420 (POSTER)

### AGING IN PLACE (POSTERS)

Abstract citation ID: igae098.2945

### "THIS IS NOT MY WAR": MORAL CHALLENGES FACED BY MIGRANT HOME CARE WORKERS IN ISRAEL AT TIMES OF WAR

Liat Ayalon[1], and Natalie Ulitsa[2], *1. Bar Ilan University, Bar Ilan University, HaMerkaz, Israel, 2. Bar Ilan University, Ramat Gan, HaMerkaz, Israel*

The October 7 massacre had a tremendous impact on all persons residing in Israel, including the migrant workers community, which was specifically targeted by the Hamas terrorists. Subsequently, migrant workers have left the country in unprecedented numbers. This paper is based on interviews with 25 migrant home care workers who stayed in Israel during the Israel-Hamas war. Thematic analysis of in-depth interviews was conducted. Analysis revealed three main themes concerning moral dilemmas in the context of the war. The first moral dilemma concerned views of the war. Whereas migrant home care workers of Muslim background, attempted to distance themselves from the war, others expressed a strong identification with Israel. A second moral issue concerned the need to seek shelter within a short period of time, which often prevented older persons from reaching a safe room on time. This resulted in some workers leaving their care recipient during a missile attack to ensure their own safety. Workers attributed this behavior to specific instructions provided by the children of the care recipient. A third dilemma concerned the decision not to leave the country. Although many workers reported a wish to leave Israel following the war, this dilemma was largely resolved through the understanding that they have a financial debt that prevents them from returning to their home country. The findings highlight the challenges faced by migrant home care workers, who transitioned for financial reasons, but find themselves in a war situation with financial, emotional, and social commitments that challenge their loyalties.

Abstract citation ID: igae098.2946

### BENEFITS OF SERVICE LEARNING FOR STUDENT PARTICIPANTS AND OLDER ADULT RECIPIENTS

Krista Cline[1], and Catherine Bain[2], *1. Butler University, Indianapolis, Indiana, United States, 2. University of Oklahoma, Norman, Oklahoma, United States*

It is clear that service learning benefits college students, while also benefiting the community. While research on intergenerational service learning has focused on the benefits for the students, very few studies have focused on the older adults who are the recipients of the service learning. For the current study, we were interested in the benefits of service learning for both the college students and the older adults who participated in a service learning course. Data for the current study is qualitative and were collected from both the students in a sociology of aging and the life course service learning class (n=22), and the older adults who participated as recipients of the service learning (n=22). Data from the students was collected via student journals and open-ended questionnaire responses written by the students. Data from the older adults were collected via interviews by the students as well as open-ended questionnaire responses written by the older adults. The following themes emerged as benefits to students; (1) a better understanding and less fear of aging; (2) a desire to learn more about older adults; (3) a desire to engage more with older adults. The themes for the benefits to the older adults included; (1) improved social connections and companionship, and; (2) becoming family. We found that engaging in intergenerational service learning courses is beneficial to all those who are involved.

Abstract citation ID: igae098.2947

### COMMUNICATION OF LONG-TERM-CARE PLANS AMONG OLDER ADULTS

Charlie Olvera[1], Amber Miller-Winder[2], Alaine Murawski[2], Vanessa Ramirez-Zohfeld[2], and Lee Lindquist[2], *1. Northwestern University Feinberg School of Medicine, Division of Geriatrics, Evanston, Illinois, United States*, *2. Northwestern University, Evanston, Illinois, United States*

Older Adults (OAs) making long-term care (LTC) plans for post-hospitalization or Alzheimer's Disease (AD) care must communicate their plans to ensure their wishes are honored. What factors increase the likelihood of this communication? The PlanYourLifespan (PYL) study examines OA LTC decision-making, employing a tool proven effective in helping OAs make LTC decisions. Subjects were administered PYL after baseline assessment, with follow-up assessments every 6 months thereafter. Subjects indicating they made LTC plans were asked if they communicated these plans. We employed GLMM to identify significant ($p< 0.05$) factors increasing the likelihood of plan communication. 293 subjects enrolled, mean age 73.5, 72.7% female, 40.4% URM. Subjects were more likely to communicate AD LTC plans if they were married (OR2.131), had advanced directives (OR2.004) or living wills (OR1.562), or had higher health literacy (OR2.174). Subjects were more likely to communicate AD in-home care plans if they were married (OR1.871), displayed higher health activation (OR2.425) or health literacy (OR1.883), had advanced directives (OR1.651) or living wills (OR1.596). Subjects who perceived adequate social support (OR2.163), or had advanced directives (OR1.828), living wills (OR1.565), or power of attorney (OR1.529) were more likely to communicate post-hospitalization LTC plans. Married subjects (OR2.034) and those with high health activation (OR1.96) or health literacy (OR1.929) were more likely to communicate post-hospitalization in-home care plans. OAs with higher health literacy and activation, stronger social support, and pre-existing legal directives in place are more likely to communicate LTC plans. These results can help future interventions optimize communication of LTC plans among OAs.

Abstract citation ID: igae098.2948

### HOME AND COMMUNITY-BASED SERVICE USE AMONG VETERANS WITH DEMENTIA VARIES BY COMORBIDITY LEVEL AND HEALTH CARE TEAM

Emma Quach[1], Lauren Moo[2], Emily Franzosa[3], Pengsheng Ni[4], Shibei Zhao[5], and Christine Hartmann[6], *1. VA Bedford Healthcare System, Bedford, Massachusetts, United States*, *2. Department of Veterans Affairs, Bedford, Massachusetts, United States*, *3. James J Peters VA Medical Center, Bronx, New York, United States*, *4. Boston University, Boston, Massachusetts, United States*, *5. Center for Healthcare Organization and Implementation Research, Bedford, Massachusetts, United States*, *6. VA Bedford Healthcare System & University of Massachusetts Lowell, Bedford, Massachusetts, United States*

Home and community-based services (HCBS) such as home care and adult day centers (adult day) are vital to supporting adults with dementia in community settings. Using 2019 data of 143,281 patients with dementia from the Veterans Health Administration, we investigated HCBS use (use of both home care and adult day, use of only one service, and use of neither service) in older adults with dementia. These adults may receive care from one of three types of case-managed health care teams (Home Based Primary Care, geriatrics-based outpatient primary care, and dementia-focused outpatient specialty care). They had various comorbidity levels, measured as the number of (adapted) Elixhauser comorbidities index and dichotomized as $< 4$ or $\geq 4$ comorbidities. We compared HCBS use (use of neither service as referent) by patients' type of case-managed team (patients in no team as referent), stratified by patients' non-dementia comorbidity levels ($< 4$ or $\geq 4$). Use of both home care and adult day was associated with each type of health care team at each comorbidity level. Home Based Primary Care and dementia-focused specialty care were more consistently associated with HCBS use--only adult day and of only home care--than geriatrics-based primary care for patients with $\geq 4$ non-dementia comorbidities. Our findings suggest that, in patients who have both dementia and other comorbidities, case-managed health care teams in primary and specialty care settings contribute to use of critical community supports. Among these, implementation of case management faced more challenges within outpatient primary care teams.

Abstract citation ID: igae098.2949

### HOW MEDICAL OBJECTS, MEAL PREP ASSISTANCE, AND RELATIONAL CULTURE IMPACT THE MEANING OF "HOME" FOR HOME CARE CLIENTS

Lauren Spring[1], Stephanie Mason[2], Kaitlyn Kuryk[3], Janice Keefe[1], and Kelly O'Neil[1], *1. Mount Saint Vincent University, Halifax, Nova Scotia, Canada*, *2. Brock University, St. Catharines, Ontario, Canada*, *3. University of Manitoba, Winnipeg, Manitoba, Canada*

Home care policies, programming, and services in Canada are designed to support older adult clients to age well in their homes and communities. Because the medical model dominates programming and policy decisions about home care services, the delivery of home care services is rarely viewed as relational and intra-active, in which interdependent relationships and interactions occur between clients and care workers. This paper applies a relational lens that incorporates a symbolic interactionist perspective to analyze qualitative data from a series of three interviews over an 18-month period (2019-2021) with 12 older adult home care clients and 10 of their paid caregivers. The overall study explored care pathways for older adult home care clients who received publicly-funded home care services in Manitoba and Nova Scotia. Our analysis revealed that this exchange of affect notably materialized through meal preparation and medical devices in the client's home. The inter-/intra-actions between clients and care workers inscribed food-related practices and medical objects with symbolic values/meanings of home that were often unique and surprising. Better awareness of the relational cultures of meal preparation and medical devices to meanings of home and how these meanings for clients are sometimes in tension with those held by caregivers, can benefit clients and enrich home care policies and service delivery

Abstract citation ID: igae098.2950

### IMPACT OF ALTERED ROUTINES ON SOCIAL AND MENTAL HEALTH AMONG OLDER ADULTS DURING THE COVID-19 PANDEMIC

Jessica Finlay[1], Lindsay Kobayashi[2], Rebecca Milan[2], Mallory Sagehorn[1], Grace Bowman[3], and Rohini Perera[2], *1. University of Colorado Boulder, Boulder, Colorado, United States*, *2. University of Michigan, Ann Arbor, Michigan, United States*, *3. University of Colorado BioFrontiers Institute, Boulder, Colorado, United States*

The COVID-19 pandemic has altered the daily lives of older adults, necessitating adaptations in social, creative, and exercise routines while highlighting the importance of shared 'third places' (sites outside of home and work/school) as vital community support. This mixed methods study delves into the nuanced relationship between lifestyle shifts and mental health outcomes among older Americans. The COVID-19 Coping Study's national three-year follow-up survey was conducted April-May 2023 among 1,915 participants aged 59 years and above. The Beck Anxiety Inventory, Center for Epidemiologic Studies Depression Scale (CES-D), and UCLA Loneliness Scale assessed anxiety, depression, and loneliness. Reflexive thematic analysis of open-ended participant responses investigated underlying motivations and experiences driving lifestyle changes. Participants, predominantly Non-Hispanic White (93%), female (71%), and college-educated (86%), reported significant modifications to their routines influenced by viral exposure concerns, personal health considerations, and broader societal challenges since the pandemic onset. Changes in social routines were most prevalent (52%) and significantly correlated with increased experiences of anxiety ($\beta$=0.30, p=0.02), depression ($\beta$=0.33, p< 0.01), and loneliness ($\beta$=0.30, p< 0.01). Once vital hubs for social interaction and civic engagement, our qualitative findings identified that fear of infection and societal tensions led many to withdraw from shared community and civic 'third places'. As older Americans navigate the ongoing challenges of COVID-19 and other infectious diseases, understanding the complex interplay between altered lifestyle routines and community support is paramount. Results may inform interventions to develop more inclusive and accessible interventions that foster resilience and wellbeing in later life.

Abstract citation ID: igae098.2951

### IS AGING IN THEIR CURRENT DWELLING THE BEST OPTION FOR OLDER ADULTS?

Joonyoung Cho[1], and Ruth Dunkle[2], *1. University of Hawaii, Hawaii, United States*, *2. University of Michigan, Ann Arbor, Michigan, United States*

Although the majority of older adults in the United States stay in their current residence, there has been an increase in the number of housing transitions among older adults since 2000. While aging in their current dwelling is the preferred option for most older adults, according to the Ecological Theory of Aging, the Person-Environment fit changes over time, and relocation is one strategy older adults may use to promote better psychological well-being. The association between housing transition and psychological well-being has yet to be fully explored and the effects of housing transition on psychological well-being may vary across different settings and individuals. Using data from the National Health and Aging Trend Study (2011-2015 n=3,549), we applied multilevel growth modeling to examine the association between housing transition and psychological well-being across different settings including within the community, non-nursing residential facilities, and nursing homes over 5 waves. We found that within-community movers tended to report higher well-being over time compared to non-movers (adj. $\beta$= 0.32, p=0.021). In contrast, compared to non-movers, non-nursing facility movers and nursing home movers were no different in terms of changes in psychological well-being over time. (adj. $\beta$= 0.23, p=0.348; adj. $\beta$= 0.41, p=0.394). Our findings suggest that within-community housing transition can be viewed as one way to achieve aging in the right place.

Abstract citation ID: igae098.2952

### MOBILITY-RELATED PHYSICAL FUNCTION AND AGING IN PLACE FOR OLDER ADULTS WITH CHRONIC CONDITIONS

Marielle Jensen-Battaglia[1], Ying Wang[1], Robert Block[1], Kah Poh Loh[2], Supriya Mohile[2], Emily Agree[3], and Christopher Seplaki[1], *1. University of Rochester School of Medicine and Dentistry, Rochester, New York, United States*, *2. University of Rochester Medical Center, Rochester, New York, United States*, *3. Johns Hopkins University, Baltimore, Maryland, United States*

Chronic conditions may influence difficulty mobilizing inside the home (mobility), which presents a barrier to the goal of aging in place for many older adults. Understanding this relationship is critical as the prevalence of chronic conditions increases. In prior work (currently under review) we used data from community-dwelling adults aged ≥65 participating in ≥2 consecutive rounds (2011-2019) of the NHATS to show self-reported mobility difficulty was positively associated with moves to homes with fewer environmental barriers or more household members, but not to other destinations (e.g., homes with more barriers, nursing home/residential care). In the current analysis we use this dataset of 25,490 person-years of follow up from 5,894 unique participants who moved 1,456 times to investigate modification of this association by chronic conditions [non-skin cancer (5,472/25,490 person-years), myocardial infarction/stroke (6,621/25,490 person-years), dementia (8,011/25,490 person-years)]. All chronic conditions were positively associated with relocation to nursing home/residential care in bivariate multinomial logistic regression models (cancer: OR 1.43; 95% CI 1.02, 2.02; myocardial infarction/stroke OR 1.43; 95% CI 1.04, 1.96; dementia OR 2.87; 95% CI 2.08, 3.96). After adjusting for confounders, only dementia remained associated with moves to nursing home/residential care (OR 1.40; 95% CI 1.06, 1.85) for those without any mobility difficulty. However, the association of mobility with relocation did not vary significantly by chronic condition (p for interaction >0.05 for all conditions and relocation destinations). These results highlight mobility difficulty and barriers in the home environment as potentially modifiable factors to promote aging in place, regardless of chronic conditions.

Abstract citation ID: igae098.2953

### MODERATING ROLE OF NEIGHBORHOOD ENVIRONMENT IN ASSOCIATIONS BETWEEN HEARING LOSS AND COGNITION AMONG OLDER ADULTS

Ethan Siu Leung Cheung[1], and Zhe Zhang[2], *1. University of Utah, Salt Lake City, Utah, United States*, *2. Columbia University, New York, New York, United States*

The relationships between hearing loss and late-life cognitive challenges have been documented. However, little is known about how the relationships may be moderated by the built and social neighborhood environments. This study examined the associations between hearing loss and cognitive challenges among community-dwelling older adults and whether neighborhood characteristics (physical disorder and low social cohesion) moderated the associations. Cross-sectional national data from Round 11 of the National Health and Aging Trends Study were adopted (N = 2,515). Multinomial logistic regressions were used to examine associations among variables and interactive analyses were conducted to examine moderating effects. Results indicated significant relationships between the experience of hearing loss and possible dementia and between severe or profound hearing loss and probable dementia. Interactive models suggested that residing in neighborhoods with physical disorder and low social cohesion were negatively associated with possible dementia among older adults with moderate and severe or profound hearing loss, respectively, compared to those without hearing loss. Findings underscore the necessity of environmental and social interventions to enhance cognitive health among older adults with varying degrees of hearing challenges.

Abstract citation ID: igae098.2954

### NEIGHBORHOODS MATTER: ASSESSING THE IMPACT OF NEIGHBORHOOD FACILITIES ON SOCIAL ENGAGEMENT AND MENTAL WELL-BEING

Chengming Han[1], and Nan Zhou[2], *1. The University of Texas Medical Branch, Galveston, Texas, United States*, *2. Case Western Reserve University, Cleveland, Ohio, United States*

Objective. This study investigated the effects of social engagement and neighborhood facilities on depressive symptoms (CESD) levels among older adults. Methods. Data were draw from the China Health and Retirement Longitudinal Study (CHARLS) 2011-2020. The analytical sample included 3,930 respondents who are 65 years older. An instrumental regression via the Generalized Method of Moments (GMM) was employed to estimate the social engagement and CESD scores at baseline, with the number of neighborhood facilities and function limitations (poverid=1.000, pendogen=0.000) serving as instrumental variables. A crossed random multilevel regression clustered within individuals and neighborhoods was used to examine the effects of neighborhood facilities, functional limitations, and social engagement on CESD scores. Results. The instrumental regression indicated a significant negative relationship between social engagement and CESD scores ($b=-16.90$, $p < 0.000$). Neighborhood facilities and functional limitations displayed significant effects on social engagement. The crossed random mixed model confirmed the negative association between social engagement ($b= -0.24$, $p = 0.008$) and CESD scores and highlighted the significant role of neighborhood facilities ($b= -0.11$, $p < 0.000$). The effects clustered at the neighborhood level (ICC=0.37) exhibit a greater impact compared to those clustered by individual ID (ICC=0.19) over survey periods. Conclusion. The findings of this study suggested that neighborhood-level factors have a more pronounced impact on social engagement and mental health than individual-level variations. These results highlighted the need for policies and interventions that enhance community facilities and promote social engagement to support the mental well-being of aging populations.

Abstract citation ID: igae098.2955

### NURSE-LED INTERVENTIONS FOR ADULTS WITH POSTSTROKE COGNITIVE IMPAIRMENT: A SCOPING REVIEW

Azza Alharrasi, and Marie Boltz, *The Pennsylvania State University, University Park, Pennsylvania, United States*

**Background:** Stroke is one of the leading causes of death and disability worldwide. Post-stroke cognitive impairment (PSCI) is a common complication of stroke, associated with increased cost of care, hospitalizations, and care dependency. Nurses play a central role in rehabilitation of adult's post-stroke; however, is unknown about nursing-led interventions in promoting global cognition. The purpose of this review to explore Nurse-led interventions (NLI) and explore their impact up on global cognition and discrete cognitive domains.

**Methods:** The search strategy identified studies within PubMed, CINHAL, and Web of Science. The search was conducted on October 3rd, 2023, and November 7th, 2023, and 12 articles were identified. Inclusion criteria were the studies that delivered nursing interventions among adults with post-stroke cognitive impairment with the outcome of improving cognitions or discrete cognitive domains. Data extraction of illegible studies that met the inclusion criteria guided by the interventions, duration and follow-up, global cognition, discrete cognitive domains.

**Results:** The results demonstrated the significant improvement in the global cognition, discrete cognitive domains, activity of daily livings (ADLs), motor function, and quality of life. Results underscore the need for early cognitive screening and rehabilitation. The results found that there is a gap in knowledge regarding the long-term benefits or effectiveness of NLI upon PSCI.

**Conclusion:** The review suggests that more rigorous multicenter research studies need to be conducted on the NLI for adults with PSCI that should be with adequate sample size.

Abstract citation ID: igae098.2956

### PERCEPTION OF NEIGHBORHOOD WALKABILITY INFLUENCES PHYSICAL THERAPISTS' WALKING RECOMMENDATIONS FOR OLDER ADULTS

Jennifer Blackwood[1], and Hannah Karczewski[2], *1. University of Michigan, Ann Arbor, Michigan, United States*, *2. University of Michigan Flint, Flint, Michigan, United States*

**Background/objectives:** Neighborhood walkability is the extent to which built and social environments support walking. Walkability influences older adults' participation in outdoor walking. Physical therapists (PTs) may identify factors that influence decisions about outdoor participation in walking. Current understanding of PTs' knowledge and perceptions of walkability, and utilization of walkability assessments is not described and serves as the purpose of this study.

**Methods:** A cross-sectional survey was sent via email to 5000 PTs nationwide with IRB approval. The 40-item survey covered the following: walking and walkability perceptions and assessments, perception of responsibilities, and demographics. Categorical variables were compared using Chi-square analyses.

**Results:** After inclusion and exclusion criteria, a total of 122 outpatient PTs who worked in geriatrics were used for analysis. A significant difference was found between perceptions of whether PTs should assess walkability and whether they assess walkability ($x2=78.7$, $p< 0.001$). Components that influenced decisions to prescribe outdoor walking were prioritized in this order: availability (n=79, 64.8%) and maintenance (n=11, 9.0%) of sidewalks, crime (n=9, 7.4%), hills (n=7, 5.7%), aesthetics (n=6, 4.9%), and other (n=10, 8.2%). Objective measures were not used to assess walkability.

**Conclusion:** Respondents had a significant difference between perceptions of their responsibility to assess walkability and whether they do assess walkability in older adults before prescribing walking. Built environment constructs were prioritized over the social environment. Significance/Implications: Assessment of walkability may allow PTs to identify barriers and make more informed recommendations concerning outdoor walking for older adults. Objective measures are available for PTs when prescribing outdoor walking.

Abstract citation ID: igae098.2957

### PROBLEMS AND ISSUES FOR HOSTS OF SOCIAL PARTICIPATION ACTIVITIES: A CASE STUDY FROM JAPAN

Mai Takase[1], and Jun Goto[2], *1. University of Tokyo, Tokyo, Japan*, *2. Tokai University, Hiratsuka, Kanagawa, Japan*

Social participation is important for maintaining the health of older adults. In Japan, many recreational activities and hobbies are available for community-dwelling older adults for social participation. Meanwhile, little is known about the problems and issues that hosts face when conducting these activities. In this study, we aimed to clarify the problems related to hosting activities and possible measures that could be taken to alleviate these problems. Activity hosts providing activities for older adults in a community space in Chiba Prefecture (Japan) were approached. The activity content included musical events, movie, crafts, exercise, and cafés. This community space is operated by city's Social Welfare Council and all of the activities are hosted voluntarily by citizens. Semi-structured interviews were conducted with ten hosts. All hosts were ≥ 65 years old and the average years of operation was 9 ± 2.7 (mean ± SD) years. As a result, nine out of ten hosts reported that the decline of members who supported the activity owing to aging and death was the major problem. Directly asking friends for help in the operation, receiving help from attendees, and decreasing the frequency of activities were measures that was taken to continue with the activities. The hosts also found it difficult to recruit younger generations who could become the next leaders of the activity group because of their lack of personal connections with the younger generation. Social Welfare Council mediating the connection between activity hosts and the younger generation who are interested in voluntary activities were anticipated.

Abstract citation ID: igae098.2958

### PROSPECTIVE ASSOCIATIONS BETWEEN MULTIDIMENSIONAL NEIGHBORHOOD CONTEXTS AND PSYCHOSOCIAL OUTCOMES IN LATER LIFE

Ethan Siu Leung Cheung[1], Ming Wen[2], David Curtis[1], Sara Grineski[1], and Yehua Dennis Wei[1], *1. University of Utah, Salt Lake City, Utah, United States*, *2. University of Hong Kong, Pok Fu Lam, Hong Kong*

Despite extensive literature having reported on contextual place effects on older adults' individual health and well-being outcomes, the longitudinal relationships between multidimensional neighborhood environments and diverse psychosocial outcomes remain less known. This study addresses two questions: 1) What are the prospective associations between multidimensional neighborhood environments and psychosocial outcomes (i.e., purpose in life, depression, generalized anxiety disorder, life satisfaction) among older adults? 2) Do these relationships differ based on age groups (Youngest-old 65-74, Middle-old 75-84, Oldest-old 85 or over) and biological sex? This investigation adopts individual data from waves 2 (2004-2006) and 3 (2013-2014) of the core sample in Midlife Development in the United States, supplemented with an oversample of African Americans in Milwaukee. Tract-level neighborhood data come from multiple public sources and are measured in 2010. The analytic sample includes 1,249 individuals aged 65 and above at wave 3. Time-random-effect linear and logistic regressions are used to examine the main place effects, and interaction analyses by age and sex are conducted for each psychosocial outcome. Results suggested that area-level economic disadvantage is associated with a lower level of purpose in life. Social disorganization is a significant risk factor for generalized anxiety disorder. Some of these effects significantly vary by female. The findings highlight the importance of neighborhood social, built, and natural environments for various psychosocial outcomes and underscore the need for age-sensitive environmental interventions and programs to enhance psychosocial well-being in later life.

Abstract citation ID: igae098.2959

### SUPPORTING AGING IN PLACE THROUGH IWISH: LESSONS FROM THE SUPPORTIVE SERVICES DEMONSTRATION

Melissa Vandawalker[1], Elizabeth Giardino[1], Meghan Hendricksen[1], and Ian Breunig[2], *1. Abt Global, Cambridge, Massachusetts, United States*, *2. Abt Global, Nashville, Tennessee, United States*

Most Americans prefer to live independently in their homes as long as possible. The U.S. Congress sponsored the Supportive Services Demonstration to learn whether the Integrated Wellness in Supportive Housing (IWISH)

model can help adults aged 62 and older remain in HUD-assisted housing developments longer and delay transitions to long-term care. After three years of the demonstration, we found little evidence that IWISH affected residents' tenure or healthcare utilization as intended, but there are reasons for optimism about IWISH's long-term impact and lessons learned from 40 properties that implemented IWISH between 2017–2020. Based on enrollment and services data for 2,927 IWISH participants, 21 focus groups with 180 residents, and interviews with property management and onsite wellness staff, we conducted an implementation analysis of IWISH properties' fidelity to the model and identified IWISH's strengths and weaknesses. We observed substantial variation in resident participation and how properties implemented the model. We identified challenges with hiring and retaining onsite wellness staff for a diverse multilingual resident population, developing partnerships with local healthcare organizations, and addressing residents' privacy concerns over their health information. Residents and staff reported numerous benefits from IWISH suggesting a promising strategy for helping residents age in place, including examples of preventative actions by IWISH staff that helped residents avoid unnecessary emergency care, transitional care after returning from the hospital, and feeling safe and secure with a dedicated, onsite medical professional.

Abstract citation ID: igae098.2960

### THE RELATIONSHIP BETWEEN ACTIVE AGING INDEX AND PHYSICAL FUNCTION OF COMMUNITY-DWELLING OLDER ADULTS

Ji Yeong Park, and Kyung Hee Lee, *Yonsei University, Seoul, Republic of Korea*

Physical function is a key factor for older adults to live longer in their own place maintaining independence. Active Aging Index (AAI) is a multidimensional composite index, that can capture human capacity and enabling environment for active aging. Nevertheless, research rarely addresses the individual-level AAI related to the physical function of older adults living in the community. The aim of this study was to investigate the relationship between AAI and physical function of older adults living in community. We utilized the 2020 Survey of Living Conditions and Welfare Needs of Korean Older Persons, employing complex sampling. The physical function of older adults in the community refer to their ability to perform mobility and self-care duties in their daily life. It was measured using the Physical Functioning Assessment of Community-Dwelling Older Persons, a validated and reliable tool. The AAI, which consisted of 4 Domains (i.e., employment, participation in society, independent, healthy and secure living, and capacity and enabling environment for active aging) and 17 Indicators was entered as explanatory variables. The multiple regressions were performed by complex sampling design. The total active aging index was significantly related to the physical function of older adults living in the community. Three out of four domains (employment, Independent, healthy and secure living, and capacity and enabling environment for active aging) were associated with physical function. The social environment must be considered when establishing policies promoting physical function of the older adults living in the community.

Abstract citation ID: igae098.2961

### THE SOCIAL ROLES AND ITS RELATED FACTORS OF COMMUNITY-DWELLING OLDER ADULTS DURING THE COVID-19 PANDEMIC

Dai Segawa[1], Hayato Uchida[2], Ippei Suganuma[3], Keisuke Itotani[1], Atsushi Kitayama[4], and Yuki Watanabe[1], *1. Yamato University, Suita, Osaka, Japan*, *2. University of Hyogo, Himeji, Hyogo, Japan*, *3. Kyoto Tachibana University, Kyoto, Japan*, *4. Iryo Sosei University, Iwaki, Fukushima, Japan*

This study focused on the social roles of community-dwelling older adults during the COVID-19 pandemic in Japan and aimed to clarify their actual conditions and related factors. We compared the two groups of the values of four social roles of the Tokyo Metropolitan Institute of Gerontology Index of Competence and various survey items in 127 elderly subjects aged 65 years or older. The group who talked to young people tended to eat alone more (p = 0.00), to be more depressed (p = 0.00), and had lower food intake diversity scores (p = 0.04) than the group who didn't talk to young people. No significant associations were found between the three other social roles and the values of the various survey items. The results of the Logistic regression analysis showed that the no-involvement group was significantly associated with eating alone (OR=4.28, 95%CI=1.69-10.83, p=0.00) and depression-prone individuals (OR=3.73, 95%CI=1.02-6.96, p=0.01). Tanaka et al. (2016) reported that even at the level of intergenerational interaction of "talking to younger people" in daily life, the "with opportunity" group had a 1.2 to 1.5 times lower risk of future anxiety than the "without opportunity" group. In the present study, the results support the previous studies, although the interaction was at the level of "talking to younger people. It suggested that ensuring opportunities for intergenerational exchange is important to maintain the physical and mental health of the elderly during the pandemic.

Abstract citation ID: igae098.2962

### UNDERSTANDING THE HOME-BASED SERVICE NEEDS FOR OLDER ADULTS: A SCOPING REVIEW FROM CHINA

Hengyuan Zhang, and Sifeng Zhang, *Xi'an Jiaotong University, Xi'an, Shaanxi, China (People's Republic)*

The range of home-based services for older adults in China haven't been clearly defined, which adversely affects their effectiveness in enhancing well-being. It is crucial to develop a better understanding of the home-based service needs of older adults. This paper employs a scoping review methodology to explore the service needs of older adults aging in place. We searched empirical research articles from four Chinese databases, including CNKI, CQUVIP, CSDP and ChaoXing. A framework based on pecking order preference theory and demand spillover theory was established to categorize and analyze the literature findings. The final analysis includes 134 studies selected from a pool of 6,565 articles. By encoding the results of the articles, home-based services for older adults were categorized into four domains: medical and health services, family replacement services, disability care services, and individual auxiliary services. Among these, medical and health services emerge as the most urgently needed category for older adults. Family replacement services follow closely, as their benefits extend to family members. While the need for disability care services remains relatively low, they exhibit high rigidity. Individual auxiliary services, although least needed, consume significant public resources. We advocate for targeted public policies that allocate resources effectively, enhance older adults' health, and provide care services for those with disabilities in daily living activities.

### SESSION 7425 (POSTER)

### ALZHEIMER'S DISEASE AND RELATED DEMENTIAS III (POSTERS)

Abstract citation ID: igae098.2963

### A COMPREHENSIVE DIGITAL SELF-CARE SUPPORT SYSTEM FOR CAREGIVERS AND PERSONS WITH DEMENTIA/MCI AND MCC

Priya Nambisan[1], Yura Lee[2], Julie Ellis[1], and Murad Taani[1], *1. University of Wisconsin Milwaukee, Milwaukee, Wisconsin, United States*, *2. University of Wisconsin-Milwaukee, Milwaukee, Wisconsin, United States*

Research indicates that 95% of people with Alzheimer's and other dementias also suffer from one or more other chronic conditions, increasing the risk of hospitalizations and complications such as contraindicated health care and drug interactions. Effective self-management practices are vital for maintaining cognitive function and preventing the onset of new chronic conditions among those with Multiple Chronic Conditions (MCC). However, managing MCC alongside cognitive impairment (CI) can make it very challenging for both patients and caregivers. Studies suggest that computer usage can enhance cognitive functioning and potentially delay further deterioration and digital apps are beneficial for self-care support. Despite this, there is a severe lack of technological applications that support those with CI and MCC and are often digitally excluded due to lack of understanding of their needs. We present the findings of a pilot test of a Comprehensive Digital Self-care Support System (CDSSS) known as myHESTIA. This system offers caregivers and persons with Alzheimer's/Dementia a range of personalized tools including: 1) Health management tools: daily trackers for monitoring health conditions, reminders, to- do lists and educational resources; 2) Healing technologies: e.g. breathing exercises for relaxation, music therapy, learning activities like dancing or Yoga, and memory games and 3) Online social support forum: for connecting with others. This qualitative study aims to shed light on the perceived benefits of the system for both caregivers and persons with dementia/MCI, its utility in addressing unmet needs among those with CI, and its potential to improve the quality of life of caregivers.

Abstract citation ID: igae098.2964

### ADAPTED CONVERSATION ANALYSIS TO EVALUATE DYADIC DEMENTIA-RELATED ADVANCED CARE PLANNING DISCUSSIONS

Sara Bybee[1], Rebecca Utz[2], Jordana Clayton[2], Nancy Aruscavage[2], Katherine Supiano[2], Eli Iacob[2], and Kara Dassel[3], *1. University of Utah College of Nursing, Salt Lake City, Utah, United States*, *2. University of Utah, Salt Lake City, Utah, United States*, *3. University of Utah, Salt Lake Cty, Utah, United States*

Persons living with dementia (PLWD) and their care partners should engage in advance care planning (ACP) prior to serious cognitive decline. This pilot study assessed the effectiveness of the Life-Planning in Early Alzheimer's and Other Dementias (LEAD) Guide in promoting ACP concordance among PLWD and their care partners. Discussions of dyads' (N=7) responses to the LEAD Guide were audio/video-recorded and analyzed by two coders for the following elements of Conversation Analysis (CA) (related to ACP concordance and relationship quality, respectively): turn-taking (listening leading to understanding, respect/mutual regard); Repair (giving and taking to gain shared understanding, apology); Action formation (assumed topic of discussion and norms, agreement on discussion subject and scope); Action ascription (trusting, understanding of previous turn); Action sequencing (acceptance of discussion norms towards goal, unconditional love). Coders demonstrated moderate agreement across all recordings (M=73.1%, SD=4.4), with unanimous agreement on: the presence of listening leading to understanding, assumed topic of discussion and norms, agreement on the subject and scope of discussion, and understanding of previous turn in all recordings, and the absence of apology and blind trust in 3 (42.8%) recordings. CA may be unable to capture ACP concordance as some elements may be inherent to the LEAD Guide discussion itself while others are unobservable in ACP conversations. To objectively examine how interactions foster ACP concordance, CA methodology should account for 1) multiple ACP discussions, 2) factors related to either the LEAD Guide or dementia diagnosis that impact turn-taking, and 3) the dynamics between the PLWD and their care partner.

Abstract citation ID: igae098.2965

### ADVANCING CULTURALLY INCLUSIVE RESEARCH AND TOOLS IN ALZHEIMER'S DISEASE AND RELATED DEMENTIAS (ADRD)

Maryam Abdel Magid, Jean Taylor, and Anne Turner, *University of Washington, Seattle, Washington, United States*

**Background and Significance:** Existing research underscores cultural norms' influence on healthcare decisions but lacks depth in exploring their impact on dementia care transitions, critical for improving ADRD care outcomes. Research Aims include (1) Analyze interviews with dementia care providers and staff to better understand how cultural norms influence the decision-making processes for care transitions. (2) Explore methods used to facilitate inclusive and engaging discussions regarding care transitions. **Methods:** Nested in the NIA-funded Decision Making in Alzheimer's Research (DMAR) study, we examined interviews with 18 providers and staff on dementia care transitions. Prioritizing inclusivity, we leveraged the Alzheimer's Disease Research Center (ADRC) registry and community partnerships coupled with tailored methods including diversifying the team and targeted outreach efforts. We're employing a novel framework to analyze dementia care transitions, integrating societal and cultural aspects for nuanced understanding. This approach accounts for intergenerational culture, cognition perception, and care expectations, evolving to capture cultural nuances. Qualitative findings will be enriched with quantitative sentiment analysis, validated manually for accuracy.

**Key findings:** Novel framework will allow us to identify and analyze cultural norms affecting care transitions, as perceived by ADRD providers. Implications: The study's findings advocate for inclusive decision-making tools in ADRD care to accommodate diverse patient preferences. Future steps involve applying this framework to patients and caregivers and integrating findings into diverse settings involved in care decision-making. Funding statement: Funded in part by NIA grant #R01 AG066957 and the NLM T15 training grant #5T155LM007442-22. Content responsibility lies with authors, not representing NIH views.

Abstract citation ID: igae098.2966

### ASSOCIATION BETWEEN DIET AND RETINAL IMAGING MARKER IN THE PREVENT DEMENTIA STUDY

Sarah Gregory[1], Samuel Gibbon[1], Thomas MacGillivray[1], and Graciela Muniz Terrera[2], *1. University of Edinburgh, Edinburgh, Scotland, United Kingdom*, *2. Ohio University, Athens, Ohio, United States*

Eating a healthy diet is associated with a lower risk of neurodegenerative diseases, such as Alzheimer's disease (AD). Identifying associations between diet and AD biomarkers is key to understanding and identifying critical periods for interventions. The retina provides a unique, non-invasive, window into brain health. This study explored cross-sectional and longitudinal associations between diet and retinal imaging markers in the PREVENT Dementia cohort (UK/Ireland). We calculated a Mediterranean diet (MedDiet) adherence score (Pyramid) for each participant and tested for associations with measures of retinal vessel morphology (CRAE Hubbard Zone C (HZC) and artery tortuosity) using generalised additive models due to abnormally distributed residuals. Additionally, we tested for associations with dietary intake of manganese. All analyses were completed cross-sectionally and then longitudinally in the participants with follow-up data (data collection ongoing). In a preliminary study, we included 86 participants, mean age 50.87 (5.63) years, majority female (57%), with 31 APOEe4 carriers (36%). Participants had a moderate mean Pyramid score (8.16 (SD = 1.40)), which was comparable to scores in other UK cohorts, and a dietary intake of 4.77 mg (SD = 3.05) manganese. There was evidence of an association between higher dietary manganese intake and lower CRAE HZC density in the right eye. There was no evidence of associations between the Pyramid score in either eye or dietary manganese in the left eye, and no signs of longitudinal associations (n=47). Further research is needed to explore any these analyses in a larger sample, and expand with other variables.

Abstract citation ID: igae098.2967

### ASSOCIATION BETWEEN GUT MICROBIOTA, DIET, AND COGNITION IN ELDERLY PATIENTS WITH MILD COGNITIVE IMPAIRMENT

Lili Chen[1], Zhaoyi Hou[2], Jinxiu Liu[2], Xiuli Li[2], Yuping Zhang[2], Shuhui Yang[2], and Peiling Zhang[2], *1. Fujian Provincial Hospital, Fuzhou, Fujian, China (People's Republic)*, *2. Fujian Medical University, Fuzhou, Fujian, China (People's Republic)*

**Objectives:** To compare the gut microbiota in individuals with mild cognitive impairment (MCI) and normal controls (NCs) and explore the association between dietary intake and various cognitive domains across identified enterotypes, providing a basis for dietary interventions based on the Microbiota-Gut-Brain Axis.

**Methods:** We analyzed fecal samples and 16S ribosomal RNA sequences for microbiota among 100 participants (NCs: 50, MCI: 50). Cognitive function was evaluated using the Montreal Cognitive Assessment (MoCA), Mini-Mental State Examination (MMSE), and Auditory Verbal Learning Test (AVLT). A semi-quantitative food frequency questionnaire was used to assess participants' dietary intake, and differences in dietary habits and cognitive function among distinct enterotypes were analyzed.

**Results:** Participants with MCI exhibited lower bacterial richness. Linear Discriminant Analysis of Effect Sizes (LEfSe-LDA) revealed different major microbial genera between the groups, and a random forest model based on dominant bacterial genera could distinguish between MCI and NCs (AUC=0.743, 95%CI:0.646-0.840). Cluster analysis identified two enterotypes: Escherichia-Shigella (enterotype E) and Bacteroides (enterotype B). When grouped by enterotypes, similar dietary intake was associated with different cognitive domains: notably, egg intake positively correlated with MOCA and AVLT scores for enterotype E, but not for enterotype B. Conclusions: Significant differences exist in the gut microbiota of individuals with MCI compared to those with normal cognition, with dominant genera showing good predictive performance. The impact of different enterotypes on the relationship between dietary intake and cognitive function should be considered when making dietary recommendations in clinical practice.

Abstract citation ID: igae098.2968

### COGNITIVE IMPAIRMENT AND SOCIAL DETERMINANTS OF HEALTH AMONG INDIGENOUS WOMEN

Soonhee Roh[1], Yeon-Shim Lee[2], Heehyul Moon[3], Donald K. Warne[4], Serene Thin-Elk[5], and Sasheen T. Stone[6], *1. University of South Dakota, Sioux Falls, South Dakota, United States*, *2. San Francisco State University, San Francisco, California, United States*, *3. University of Louisville, Louisville, Kentucky, United States*, *4. Johns Hopkins University, Baltimore, Maryland, United States*, *5. South Dakota Urban Indian Health, Sioux Falls, South Dakota, United States*, *6. Yankton Sioux Tribe, Wagner, South Dakota, United States*

The current study aimed to examine 1) differences between cognitively impaired and cognitively intact groups regarding demographics, education, health literacy, social and community involvement (i.e., social engagement and religious activity/participation), and health (i.e., chronic conditions),

2) the amount to Alzheimer's Disease and Related Dementias (ADRD) knowledge among the cognitively impaired group, and 3) predictors of cognitive impairment. We considered three dimensions of social determinants of health (SDOH) to predict cognitive impairment: education and health literacy, social and community involvement, and health (i.e., depression and chronic conditions). Collaborating with a Northern Plains tribe, participants were recruited 123 self-identified Indigenous women aged 40 to 70 through a comprehensive recruitment strategy. Employing the SDOH framework, the research assessed cognitive impairment and Alzheimer's disease knowledge, utilizing the Ascertain Dementia 8 and AD knowledge scales (ADK-30). The investigation examined the relationships between selected SDOH variables and cognitive impairment status. More than half of the participants showed signs of cognitive impairment, which correlated with lower income and education levels. Increased knowledge about Alzheimer's disease, particularly in terms of treatment management and its life impact subscales, was associated with lower odds of cognitive impairment. Conversely, higher levels of depressive symptoms and participation in religious activities were linked to increased odds of cognitive impairment. Findings underscore the importance of culturally grounded tools and SDOH frameworks tailored to Indigenous contexts in addressing ADRD disparities. Future research should integrate historical and cultural factors to advance health equity within Indigenous communities, ultimately mitigating the impact of ADRD and promoting overall well-being.

Abstract citation ID: igae098.2969

### COPING STRATEGIES AMONG HISPANIC AND LATINX DEMENTIA CAREGIVERS

Nury Rodriguez Colmenares[1], Danny Wang[2], Loreli Alvarez[1], Katie Estes[1], Natashia Bibriescas[3], and Frank Puga[1], *1. University of Alabama at Birmingham, Birmingham, Alabama, United States*, *2. The Pennsylvania State University, University Park, Pennsylvania, United States*, *3. The University of Texas Austin, Austin, Texas, United States*

The objective of this study was to examine coping strategies employed by Hispanic and Latinx (H&L) family caregivers (FCGs) of people living with dementia (PLWD) and associations with sociodemographic characteristics. A nonparametric analysis was used to analyze data from an ongoing study on the daily experiences of H&L dementia caregivers, entitled Nuestros Días. A total of 52 H&L FCGs were assessed using Brief-COPE. Most H&L FCGs identified as female (92.2%), with a mean age of 56 years (SD = 8.6), were adult children caring for biological parent (59.6%), and provided care for 3 or more years (61.5%). Most PLWD were female (68%) with a mean age of 78 years (SD= 8.6). Overall coping strategies most frequently utilized by H&L FCGs included acceptance (M= 3.14, SD= 1), religion (M= 2.98, SD= 1), positive reframing (M=2.91, SD=0.9), and planning (M=2.8, SD=1). Conversely, substance use (M=1.13, SD=0.3), behavioral disengagement (M=1.39, SD=0.72), denial (M=1.44, SD=0.8), and humor (M=1.47, SD=0.8) were the least utilized by H&L FCGs. The use of acceptance as a coping strategy was found to have a significant association with employment status with working caregivers more frequently endorsing acceptance as a coping strategy (p=.04; Cramer's V=.409). These findings suggest that H&L caregivers utilize specific coping strategies and that sociodemographic factors, such as employment, may influence coping strategies use d. Additional studies are needed to examine the relationship between additional contextual factors (e.g., caregiving-related stressors and burden) and types of coping strategies used by H&L FCGs to inform ecologically valid and cultural interventions.

Abstract citation ID: igae098.2970

### DEPRESSION AND COGNITIVE FUNCTION IN OLDER MEXICAN DESCENT LATINOS: THE MEDIATING ROLE OF SOCIAL ENGAGEMENT

Liza Talavera-Garza, *The University of Texas Rio Grande Valley, Edinburg, Texas, United States*

Mexican descent Latinos have significantly higher rates of Alzheimer's disease and related dementias (ADRD) compared to the general population. Latinos also report higher rates of depressive symptoms in older adulthood relative to non-Latino Whites. Depression in older age is associated with poor cognitive health; however, the mechanisms that underlie this relationship are not well-understood. The current study examined social engagement, which has been linked with better cognitive health, as a mediator of the association between depressive symptoms and cognitive function among older Mexican descent Latinos. Method: A convenience sample of Mexican descent Latinos (N=159), aged 55 to 88 years (M=68.2, SD=7.05) and without diagnosed cognitive problems, were recruited from a community in South Texas. They were administered in-person interviews, in their choice of English or Spanish in Summer 2023 that included demographic questions and measures of cognitive function, depressive symptoms, and social engagement.

**Results:** Pearson correlations found that depressive symptoms were significantly negatively associated with social engagement (r = -.25, p <.002) and cognitive function (r = -.39, p <.001). Hierarchical Bayesian simulations indicated strong indirect effects of depressive symptoms on cognitive function through social engagement (unstandardized 95% CI = -.2408, -.0119, p<.0001).

**Conclusion:** The current findings suggest that cognitive decline in individuals with depressive symptoms may occur because of reduced participation in social activities. Community-based approaches, with a focus on increasing social engagement in individuals who exhibit symptoms of depression, may be viable interventions that could be leveraged to reduce the burden of ADRD in Latinos.

Abstract citation ID: igae098.2971

### DIFFERENCES OF RANKINGS IN DEATH CERTIFICATES AMONG PERSONS WITH ALZHEIMER'S DISEASE

Candace Brown[1], Yinghao Pan[1], Maggi Miller[2], and Matthew Lohman[2], *1. University of North Carolina Charlotte, Charlotte, North Carolina, United States*, *2. University of South Carolina, Columbia, South Carolina, United States*

A 2021 report indicates that 19.1% of people ages 76-84 and 34.6% of people aged 85 and older have Alzheimer's disease (AD). Data provided on death certificates using the International Statistical Classification of Diseases is guided by the WHO for coding cause and contributing comorbidities of death. Death rankings assist in monitoring which diseases need attention for better informed decision making in healthcare. However, classifying immediate cause of death presents challenges when the likelihood of polymorbidity occurs with advancing age in patients with presenting AD but inconclusive diagnoses of the disease. We used the Alzheimer's Disease and Related Dementia South Carolina (SCADR) registry and linked it with state death certificates to examine sociodemographic variables and survival probabilities of decedents between the years 2014-2019. Cox proportional hazards models were fitted and hazard ratios (HRs) computed, adjusting age at diagnosis and race. Turnbull's method estimated the survival probabilities or curves for subgroups as the time from AD diagnosis to death. Of the 78,534 patients in the study 94% were diagnosed with AD at age 61 or older. AD was listed as the immediate cause of death among all racial groups, except for Native American/American Indian, contrasting from previous research of underreporting of the disease by factor of 2.7 with underestimations more likely for people identifying as Black and Latino. However, only 1.4% had a conclusive diagnosis of the disease by autopsy. Survival probabilities drop precipitously in the first few years after diagnosis with only 25% of the cohort surviving beyond 5 years.

Abstract citation ID: igae098.2972

### EARLY CLINICAL UTILITY DATA OF A MULTI-ANALYTE BLOOD BIOMARKER TEST FOR EVALUATING COGNITIVE IMPAIRMENT

Mark Monane[1], David Merrill[2], Kim Johnson[3], Darren Gitelman[4], Demetrius Maraganore[5], Leslie Jacobs[1], Yan Jiang[6], and Joel Braunstein[1], *1. C2N Diagnostics, St. Louis, Missouri, United States*, *2. Pacific Brain Health Center, Santa Monica, California, United States*, *3. Duke University, Durham, North Carolina, United States*, *4. Advocate Lutheran General Hospital, Park Ridge, Illinois, United States*, *5. Tulane University School of Medicine, New Orleans, Louisiana, United States*, *6. Stat4ward, Pittsburgh, Pennsylvania, United States*

There is an important unmet need for timely, noninvasive, low-burden diagnostic tools to aid in Alzheimer's disease evaluation. The AAA-BBM2 (PrecivityAD2™) blood test combines plasma amyloid beta (AB)42/40 Ratio and phosphorylated-tau217/non-phosphorylated-tau217 Ratio (%p-tau217) into a clinically-validated algorithm. The test result, the Amyloid Probability Score 2 (APS2), determines the likelihood of brain amyloid, the pathological hallmark of Alzheimer's disease, on amyloid PET scan: the test result has previously demonstrated 88% sensitivity and 89% specificity. The objective of the QUIP II Study (NCT06025877) is to evaluate the clinical utility of this blood biomarker test with APS2 result among cognitively symptomatic patients presenting to memory specialists. To date, 52 symptomatic patients (median age 75, 65% female, 77% white) from 7 memory centers received the AAA-BBM2 test. Concordance with intended use of the test was 100% (52/52). Positive APS2 results were noted in 26 patients (50%). From pre- to post-test, clinician-reported probability of AD decreased from 48% to 16% among negative APS2 patients ($p< 0.0001$), and increased from 71% to 93% among positive APS2 patients ($p< 0.0001$). The composite endpoint of change in AD diagnostic certainty, drug therapy, or brain amyloid evaluation pre- and post-AAA-BBM2 testing was 79% ($p < 0.0001$ versus a pre-specified threshold of 20% clinically meaningful change). We believe that the AAA-BBM2 test showed clinical utility in its association with changes in clinicians' diagnostic certainty and clinical management among patients presenting with cognitive impairment. This blood biomarker test may allow memory specialists to guide patients to anti-AD therapies and facilitate other diagnostic considerations.

Abstract citation ID: igae098.2973

### ENABLING PRIORITIZATION FOR AMYLOID POSITIVITY TESTING WITH DIGITAL COGNITIVE ASSESSMENT

Ali Jannati[1], Karl Thompson[1], Claudio Toro-Serey[1], Russell Banks[1], John Showalter[1], David Bates[1], Sean Tobyne[1], and Alvaro Pascual-Leone[2], *1. Linus Health, Boston, Massachusetts, United States*, *2. Hebrew SeniorLife, Boston, Massachusetts, United States*

Primary care providers currently wait for a complaint to initiate a 'for-cause' cognitive evaluation and often refer the patient to a specialist. Specialist evaluation then leads to the diagnosis, but treatment occurs late and is unlikely to meaningfully prevent or reduce disability. We hypothesized a brief digital cognitive assessment (DCA), the Digital Clock and Recall (DCR), could concurrently identify CI and predict PET Aβ status, thereby providing an efficient means for timely identification and prioritization of patients for disease-modifying treatments. 930 participants (age 72.0±6.7; 56.8% female; 23% minorities) were classified as cognitively unimpaired, mild cognitive impairment, or probable Alzheimer's dementia, and 35.1% were Aβ+ on 18F-florbetapir PET scan. DCR-based models were compared with blood-based biomarkers (BBMs) Aβ42/40, pTau-181, and pTau-217, which poorly classified CI (AUCs=0.63, 0.66, 0.72) but accurately classified Aβ status (AUCs=0.81, 0.78, 0.89). DCR accurately classified CI and predicted Aβ status (AUCs=0.85, 0.83). Moreover, combining the DCR with Aβ42/40, pTau-181, and pTau-217 improved their Aβ-status prediction performance to AUCs of 0.882, 0.835, and 0.905, respectively. DCR was superior to BBMs in detecting cognitive impairment and boosted their Aβ-PET prediction ability to levels comparable to CSF biomarkers. We propose a workflow in which implementation of DCAs such as the DCR facilitates early identification of individuals with MCI or early dementia likely due to AD, thanks to the concurrent classification of CI and accurate prediction of Aβ PET status.

Abstract citation ID: igae098.2974

### EXAMINING KNOWLEDGE ABOUT FIVE ALZHEIMER'S DISEASE–RELATED SERVICE AREAS AND ITS CORRELATES IN KOREAN AMERICANS

Michin Hong[1], and Sang Lee[2], *1. Indiana University Indianapolis, Indianapolis, Indiana, United States*, *2. San Jose State University, San Jose, California, United States*

This study examined knowledge about Alzheimer's disease (AD) related services and its predictors among Korean Americans (KAs). A total of 268 KAs in the Greater

Washington metropolitan area participated and completed a cross-sectional survey. Knowledge about AD related services was assessed by asking how well they knew about each of the following areas: Diagnostic services, Alzheimer's specialists, community-based services, home-based services, and institutional care, with four categories of responses from don't know at all to know well. Multiple regression analyses were conducted for each service area with predictors including education, English proficiency, exposure to AD, sources and frequency of health-related information, and AD knowledge about treatment and management. KAs were more knowledgeable about community- and home-based services and institutional care than diagnostic services and Alzheimer's specialist. All regression models except for diagnostic services were significant: Having more exposure to AD is related to being more knowledgeable in Alzheimer's specialist, community- and home-based services, and institutional care; having more sources and frequency of health information is related to being more knowledgeable about Alzheimer's specialist; and being more knowledgeable about AD management and treatment is related to having more knowledge about community- and home-based services, respectively. Our findings revealed specific areas of services more and less known to KAs, which needs to be addressed in educational outreach. Multivariate analyses identified variations in the predictors of different service areas while confirming the robust role of exposure to AD across the service areas, suggesting ways to increase knowledge about certain types of AD services.

Abstract citation ID: igae098.2975

### EXAMINING THE RELATIONSHIP BETWEEN PERSONALITY FACTORS AND NEUROCOGNITIVE FUNCTIONING IN DEMENTIA CAREGIVERS

Alayna Jump[1], Lauren Elliott[1], Sydnie Schneider[1], Elisabeth McLean[1], Lauren Chrzanowski[1], Amir Abu-Samaha[1], Volker Neugebauer[2], and Jonathan Singer[1], *1. Texas Tech University, Lubbock, Texas, United States*, *2. Texas Tech University Health Sciences Center, Lubbock, Texas, United States*

More than 5.8 million Americans currently live with a neurodegenerative disorder such as Alzheimer's Disease or Alzheimer's Disease Related Dementia (AD/ADRD). Family caregivers of persons with AD/ADRD may be at increased risk for accelerated age-related cognitive decline compared to their peers. However, the risk and protective factors associated with neurocognitive functioning within this population are poorly understood. One factor yet to be explored is the relationship between personality traits and neurocognitive functioning. Certain personality traits, such as high neuroticism and lower openness have been linked to worse neurocognitive functioning in older adult populations, but these findings have not been expanded to family caregivers of persons with AD/ADRD. This study enrolled 52 family caregivers of persons with AD/ADRD who completed the NEO Five-Factor Inventory-3 (NEO-FFI-3), a measure of personality traits, and the Repeatable Battery for the Assessment of Neuropsychological Status (RBANS), a measure of overall neurocognitive functioning. A Pearson correlation revealed a positive trend ($r(50) = .27$, $p = .054$), such that individuals scoring lower on openness had worse neurocognitive functioning. However, the other four personality traits (i.e., neuroticism, agreeableness, extraversion, conscientiousness) were not significantly related to neurocognitive functioning (p's >.27). Future studies should aim to investigate the relationship between personality factors and neurocognitive functioning within other samples as personality traits, which remain relatively stable over time, may provide medical providers the ability to easily identify individuals who might be more susceptible to neurocognitive decline, thus streamlining preventative measures.

Abstract citation ID: igae098.2976

### EXPLORING THE LANDSCAPE OF GENERATIVE AI AND LARGE LANGUAGE MODELS IN ALZHEIMER AND DEMENTIAS RESEARCH

Duo Wei[1], Jeannine Elmasri[1], Bianca Hernandez[2], Riya Goyal[1], and David Burdick[1], *1. Stockton University, Galloway, New Jersey, United States*, *2. New Jersey Institute of Technology, Newark, New Jersey, United States*

With the introduction of ChatGPT in November 2022 and Google Bard (now Gemini) in March 2023, the artificial intelligence (AI) landscape has undergone a significant and rapid evolution. An in-depth scoping review was utilized to investigate the application of generative AI and Large Language Models (LLM) in research on Alzheimer's Disease and Related Dementias (ADRD) among older adults. The objectives were twofold: first, to investigate specific applications of generative AI and LLM in ADRD research, and second, to delineate the diverse usage of these technologies within this domain. Leveraging PubMed, search terms such as "AI," "Dementia/Alzheimer," and "Older Adults" were employed, resulting in the retrieval of 196 articles published in January 2022-December 2023. Of 196 articles found, 145 meeting eligibility criteria were meticulously categorized into two topics: ADRD domain and AI-related tasks. Statistical analysis revealed compelling insights, with significant differences in the application of generative AI ($\chi 2 = 21.91$, $p < .005$) and LLM ($\chi 2 = 21.42$, $p < .005$) in ADRD research. Notably, generative AI predominantly focused on "Research/Drug Discovery" (34.38%) and "Improved Treatment & Care" (37.50%), while LLM emphasized "Challenges/Opportunities" and "Early Detection/Diagnosis." Within the ADRD domain, articles were categorized into distinct areas such as "Care," "Cognitive Decline," "Diagnosis," "Genetics," "Imaging," and "Other." Caregiving and support emerged as predominant themes, comprising 55% of the reviewed articles, followed by diagnosis (34%). This comprehensive analysis sheds light on the multifaceted applications of AI in ADRD research, offering valuable insights into potential areas for further investigation.

Abstract citation ID: igae098.2977

### FUNCTIONAL OUTCOME IN HOME HEALTH AMONG TRADITIONAL MEDICARE AND MEDICARE ADVANTAGE BENEFICIARIES WITH DEMENTIA

Rashmita Basu, *East Carolina University, Greenville, North Carolina, United States*

This study examines the difference in functional outcomes among Traditional Medicare (TM) and Medicare Advantage (MA) beneficiaries with Alzheimer's disease and related dementias (ADRD) during a home health admission, which is unknown. This cohort study used data from the

2019 Outcome and Assessment Information Set (OASIS) and Medicare claims (Home Health (HH) and inpatient), enrollment, and Chronic Condition Warehouse (CCW) files from the CMS. Functional outcome was measured as a change in the composite ADL score (grooming, bathing, toileting, dressing, walking, transferring, ambulation, and hygiene) from HH admission to discharge. A binary logistic model with an outcome variable (1/0: if the composite score was ≤0 (improvement or static) at discharge (1), and 0 if score >0 (decline) from HH admission to discharge) was estimated. The independent predictor was a binary indicator of MA enrollment (if enrolled ≥1 month in 2019). The ADRD cohort was identified based on CCW criteria and ICD-10 codes from inpatient and HH claims. The analysis included 81,735 Medicare beneficiaries; 30.5% had MA, and 69.5% had TM. There was no difference in the number of HH visits between TM and MA enrollees (2.14 vs. 2.13; P=0.39). Adjusting for patients' demographics, total number of diagnoses, and availability of caregiving support, MA enrollees were 26% less likely to report improvement or maintenance of ADL composite score (95% CI: 0.71-0.77) than TM. A less favorable outcome for MA enrollees highlights the importance of addressing the inefficient use of home health services among people with ADRD in MA.

Abstract citation ID: igae098.2978

### HEALTH CARE PROVIDER ADOPTION OF COGNITIVE ASSESSMENT AND CARE PLAN SERVICES FOR PATIENTS LIVING WITH DEMENTIA

John Mulcahy, *University of Minnesota, Minneapolis, Minnesota, United States*

In 2017 the Centers for Medicare and Medicaid Services introduced a new billable code for Cognitive Assessment and Care Plan (CACP) Services to encourage healthcare providers to take a more active role in identifying their patients who are living with Alzheimer's Disease and Related Dementias (ADRD), and to work with these patients and their care partners to develop a comprehensive long-term care plan. This CACP service has seen very low adoption with 0.5% of Medicare beneficiaries with an ADRD diagnosis having received CACP in 2017 and only 2.9% of beneficiaries with ADRD having received it by the end of 2020. Receipt of CACP was even more dismal among beneficiaries living with ADRD who are Hispanic/Latino, dual-eligible, or living in a rural county with 1.8%, 1.9%, and 1.8% of these beneficiaries receiving CACP by 2020 respectively. It is unknown if there are specific barriers to adoption driving this low uptake. This analysis uses a 100% samples of Medicare fee-for-service claims and Medicare Advantage encounters to identify provider and practice level predictors correlated with CACP adoption. The model predicts adoption of CACP using provider specialty, peer adoption of CACP, a previously denied CACP claim, number of patients living with ADRD treated by the provider, percent of patients in Medicare Advantage, overall patient volume, and practice size. Understanding the relative contribution of these potential barriers/facilitators to provider adoption will help inform dissemination efforts and changes that can be made to billing requirements to encourage widespread CACP uptake.

Abstract citation ID: igae098.2979

### INNOVATIVE COLLABORATION: DEVELOPING DEMENTIA TRAINING WITH STUDENTS, SCHOLARS, AND PRACTITIONERS

John Shean[1], Eva Jackson[1], Tara Redd[2], Madiha Shakir[3], and Shelby Roberts[1], *1. Alzheimer's Association, Chicago, Illinois, United States*, *2. Emory University, Atlanta, Georgia, United States*, *3. Full Tilt Ahead, Arlington, Virginia, United States*

The growing prevalence of Alzheimer's and other dementias and the urgent need for public health awareness necessitates innovative, interactive, and accessible dementia training for students entering the workforce. The curriculum, A Public Health Approach to Dementia, is a new tool to meet this need. Through partnership between the Alzheimer's Association, CDC, and Emory Centers, four new modules were launched in 2023 and 2024. These free, asynchronous, online learning modules support the training needs of the current and future public health workforce. Throughout the development, the team relied on students, researchers, professors and practitioners to ensure the modules provide accessible, comprehensive knowledge on the public health approach to dementia. The curriculum includes courses on the public health impact of dementia, health equity and dementia, and public health and dementia caregiving. Data from the first two modules released in 2023, show that they are impactful. Health Equity in Dementia — Using a Public Health Lens to Advance Health Equity in Alzheimer's and Other Dementia was released in August and has over 520 people enrolled. Public Health and Dementia Caregiving was released in December and has over 200 people enrolled. Both modules had over 900 registrants for each launch webinar. Across both modules, most learners reported an increase in knowledge after taking the course (89% and 92%) and reported that it will be useful in their work or studies (87% and 90%). This curriculum demonstrates the strengths of partnering across backgrounds and expertise to support innovative learning opportunities for students entering the workforce.

Abstract citation ID: igae098.2980

### INTENTIONAL SUPPORT REDUCES TECHNICAL BARRIERS TO BEHAVIORAL INTERVENTIONS FOR DEMENTIA CAREGIVERS

Emily Tupper[1], Stephanie Irish[1], Sarah Gothard[1], Christina Zonker[1], Aimee Mooney[2], and Allison Lindauer[1], *1. Oregon Alzheimer's Disease Research Center, Oregon Health & Science University, Portland, Oregon, United States*, *2. Oregon Health & Science University, Portland, Oregon, United States*

Utilizing technology to provide research interventions can reduce historic barriers to participation, such as travel for study visits, but also presents new challenges for participant retention. Technological issues, including malfunctioning equipment or operator error, are routinely identified as a source of attrition in telehealth studies, and are often associated with a lack of technical support. The Tele-STELLA study, a telehealth intervention aimed at supporting caregivers of persons with dementia, found employment of a tech-support staff eliminated technological issues as a source of attrition. In addition to an interventionist leading each session, a support person was assigned to monitor and address any technical issues. Technical difficulties and subsequent support were recorded for each session. Of 374 recorded sessions, interventionists reported 64(17%) technical difficulties, of which 62(16.5%) were resolved in less than 15 minutes, allowing the session to continue as scheduled; 2(0.5%) sessions required rescheduling. Participant feedback about overall technical experience was gathered at 8 and 16 weeks, and at the time of withdraw. Of 150 enrolled participants, 49 withdrew before completing the study(33%), and experience data was available for 30(61%). Of those, none reported technical issues as a reason for withdrawing, and 28 reported to “strongly agree” or “agree” that they had good technical support(93%). Although attrition is higher than those reported in comparable studies, Tele-STELLA circumvented the barrier of technological issues with targeted technical support during sessions. These findings suggest that purposeful technical support could eliminate a known cause for similar interventions.

Abstract citation ID: igae098.2981

### INVESTIGATING DRUG-INDUCED DEMENTIA IN A CNS MPS ASSESSING DEFICITS IN LTP FROM ANTICHOLINERGIC COGNITIVE BURDEN

Kaveena Autar[1], Maria Grisales[2], Chelsea Honore[2], Xiufang Guo[2], David Morgan[3], Veronique Michaud[4], Jacques Turgeon[4], and James Hickman[2], *1. Hesperos, Clermont, Florida, United States*, *2. Hesperos, Orlando, Florida, United States*, *3. Michigan State University Grand Rapids, Grand Rapids, Michigan, United States*, *4. GalenusRx Inc, Orlando, Florida, United States*

Microphysiological systems (MPS) provide an avenue for small-scale modelling of organ constructs. In conjunction with human induced-pluripotent stem cells (hiPSCs) cultured on microelectrode arrays (MEAs), MPS enable the exploration of functional deficits in concurrence with drug efficacy. Central nervous system (CNS) neural network MPS can evaluate compound-induced cognitive impairment via synapse-dependent, long-term potentiation (LTP). The present work employed a previously validated hiPSC-cortical neuron LTP model to investigate drug-induced dementia onset by Anticholinergic Cognitive Burden (ACB). Utilizing an established CNS model of LTP, we have successfully been able to evaluate a dose-response relationship between ACB drug concentration and cognitive deficits. After discovering that many ACB drugs cause clinical drowsiness, caffeine was employed to re-awaken neuronal activity, akin to increased vigilance. Donepezil, an acetylcholinesterase inhibitor commonly used in the treatment of Alzheimer’s disease (AD), confirmed that LTP deficits resulted from cholinergic neurotransmission, demonstrating the ability of our CNS MPS to assess mechanism-specific synaptic decline. Synergistic effects of ACB drugs and amyloid-beta42 oligomers, which agglomerate to produce neurotoxic plaques in AD, depicted an exacerbation of LTP deficits, suggesting a higher risk of cognitive decline in patients with early-onset AD using anticholinergics. This study illustrated the ability of our human, CNS-based MPS to recapitulate cognitive dysfunction preceded by neurotransmitter homeostatic imbalance, further validating our LTP model for investigating mechanism-dependent disease phenotypes. This model can be expanded to assess synergistic effects of drugs effecting neural network integrity on preexisting conditions causing cognitive impairment, which were observed to be translatable to clinical symptoms.

Abstract citation ID: igae098.2982

### INVESTIGATING MEDIATORS OF SYSTEMIC MITOCHONDRIAL BIOENERGETICS IN COGNITIVE IMPAIRMENT AND ALZHEIMER’S DISEASE

Gargi Mahapatra[1], Jaclyn Bergstrom[2], Antonio Michel Pinto[3], Jolene Diedrich[3], Suzanne Craft[4], and Anthony Molina[1], *1. University of California San Diego, La Jolla, California, United States*, *2. UC San Diego, La Jolla, California, United States*, *3. Salk Institute for Biological Studies, San Diego, California, United States*, *4. Wake Forest University School of Medicine, Winston-Salem, North Carolina, United States*

We previously showed peripheral blood mononuclear cell (PBMC) and platelet mitochondrial bioenergetic capacities are lower in older adults with Alzheimer’s disease (AD), with notable changes in fatty acid oxidation (FAO)-mediated respiration. The metabolic pathways underlying mitochondrial functional differences remain un-identified. This study investigated lipid metabolites that are differently expressed across different stages of cognitive impairment (CI). Targeted lipidomics analyzed 51 fatty acids (FAs) in PBMCs and platelets of older adults with normal cognition (NC), mild cognitive impairment (MCI), and AD dementia (DEM). 10 FAs were selected in each cell type based on significant concentration differences between different cognitive groups. In PBMCs, 9 FAs exhibited higher concentrations in MCIs than NC and DEM, with significantly lower concentration in DEM than MCI, while 1 FA exhibited progressively declining concentrations from NC to MCI to DEM. Significant positive correlations were observed between FA concentrations and FAO and maximal mitochondrial respiration (Max). In platelets, 7 FAs showed progressively declining concentrations from NC to MCI to DEM, and significant positive correlations with FAO and Max, while 3 FAs showed progressively increasing concentrations with cognitive impairment, and significant negative correlations with FAO and Max. Significant negative correlations were also observed with mPACC5 cognitive assessment scores and hippocampal volumes. Pathway analyses identified FA biosynthesis, alpha-linolenic acid, and alpha-linoleic acid metabolism as involved pathways differently impacted across cognitive groups. These findings emphasize the importance of FAs and related metabolic pathways in systemic bioenergetic decline in CI, and may facilitate development of preventative, diagnostic, and therapeutic approaches in AD.

Abstract citation ID: igae098.2983

### THE IDA (INTERFERENCE WITH DAILY ACTIVITIES) SCALE: A NEW MEASURE OF FUNCTION IN MILD COGNITIVE IMPAIRMENT

Ruth Tappen[1], Robin Jarvis-Powers[2], David Newman[1], Mónica Rosselli[1], Joshua Conniff[1], KwangSoo Yang[1], Jinwoo Jang[1], and Ashlee Li[1], *1. Florida Atlantic University, Boca Raton, Florida, United States*, *2. St. Mary’s Medical Center, West Palm Beach, Florida, United States*

The functional decrements associated with mild cognitive impairment (MCI) are subtle, difficult to detect and often not reported by patients, yet may interfere with daily activities and be associated with patient concern, sometimes distress. Development of the IDA (Interference with Daily Activities) Scale began with a review of Memory Center patient records and interviews of 53 individuals who attended cognitive screenings sponsored by a second state-supported Memory Disorder Center. It was noted that participants reported few decrements when asked directly but endorsed far more when presented with a list of possible changes. Of 125 original items developed, 85 were retained for further testing based upon the interview results, participant endorsement of the item or supporting literature. They were then tested in two community samples, first reducing them to 65 items and then to 28 on the basis of significant ($p \leq .05$) differences between those with MCI and those who were unimpaired and frequency of endorsement by those with MCI. The final version contains 22 items related directly to cognitive function and 6 items related to the psychosocial effects of the functional difficulties encountered. Internal consistency (Cronbach's alpha) was.87, test-retest reliability over three months was.81, and criterion validity in comparison with the ECog scale was excellent ($r=.72$). IRT analysis indicated good fit and item discrimination. Use of the IDA Scale can contribute to a more comprehensive picture of the individual patient's condition and current situation in clinical and research contexts.

Abstract citation ID: igae098.2984

### THE IMPACTS OF VIRTUAL REALITY (VR) INTERVENTION ON DEMENTIA CARE EDUCATION: A SYSTEMATIC REVIEW

Xiaoli Li[1], Sandra Collins[1], Xiaoyu Liu[2], Ramesh Mutthina[3], and Chung-Yi Chiu[4], *1. Southern Illinois University Carbondale, Cabondale, Illinois, United States*, *2. Saint Louis University, St. Louis, Missouri, United States*, *3. Southern Illinois University Carbondale, Carbondale, Illinois, United States*, *4. University of Illinois Urbana-Champaign, Champaign, Illinois, United States*

VR technology has been explored for its potential to deepen the understanding of individuals living with dementia, allowing students and caregivers to immerse themselves in the lives of those affected by this condition. To date, research evidence regarding the implementation and effectiveness of VR interventions in this domain have not been evaluated and synthesized. We aimed to address this gap in the evidence. The study protocol was registered with PROSPERO, and literature published from 2000 onwards was searched from six databases: Academic Search Complete, APA PsycINFO, CINAHL, MEDLINE, Web of Science, and PubMed. Additionally, hand searches of keywords and citation tracking were conducted in Google Scholar. Both qualitative and quantitative literature were considered, and study reporting quality was assessed using the JBI Critical Appraisal Checklists. Among the 318 articles initially identified, 22 were selected for the review. The review encompassed 17 experimental studies and five qualitative studies, involving formal / informal caregivers and university students. Sample sizes ranged from 20 to 278 participants, originating from seven countries and regions, utilizing 11 distinct VR prevention programs. Overall, the incorporation of VR in dementia care education emerges as a novel and underexplored research avenue. VR interventions demonstrated enhancements in caregivers' and students' empathy, knowledge, attitude, and understanding of people with dementia, although not all experimental studies found statistically significant changes. VR-based education emerged as a valuable complement to traditional teaching and training methodologies, offering a more engaging and realistic learning experience. However, further research in larger and more diverse samples is needed.

### SESSION 7430 (POSTER)

### ARTIFICIAL INTELLIGENCE (AI)

Abstract citation ID: igae098.2985

### A QUALITATIVE STUDY ON THE EXPERIENCE OF SMART CARE TECHNOLOGY UTILIZATION AMONG OLDER ADULTS IN KOREA

Sungwan Kang, Boram Hwang, Haklyoung Kim, Seongsu Choi, Kyoungmi Mun, and Yeong Ju Lee, *Pusan National University, Busan, Republic of Korea*

To address the increasing desire among older adults who live alone to remain in the community, the Korean government and Korean companies have implemented pilot projects aimed at expanding smart technology integrated care services. Evaluations of these efforts have primarily focused on cost savings and the number of emergency rescue cases through the replacement of care personnel. However, there is a lack of research on users' experiences with various smart technologies. In particular, research on the experience of using services based on artificial intelligence (AI) and Internet of Things (IoT) among low-income, low-educated older adults remains limited. The purpose of this study is to explore the experiences of low-income, low-educated older adults on using AI-based emotional support services and IoT-based abnormal symptom monitoring services. We conducted focus group interviews with 13 participants who used the smart care services for three months in Korea. Using thematic analysis, three main themes emerged: (1) emotions and values, which represent ambivalence toward artificial intelligence (gratitude, connection, indifference, disconnection). (2) social support, which describes the alleviation of negative emotions; government workers including social workers provided detailed explanations about the services, including purposes, usage procedure, and addressing concerns related to the system. (3) new experiences, which discuss the usefulness and value that were newly discovered over the period of use; study participants emphasized that they could lessen their burden on family members. These findings serve as foundational insights for the development and dissemination of smart care services tailored to the needs of low-income, low-educated older adults.

Abstract citation ID: igae098.2986

### ART, AGING, AND ARTIFICIAL INTELLIGENCE: EXPLORING AGE SCRIPTS IN THE UNIVERSE OF AI-GENERATED ART

Laura Allen[1], Wenqian Xu[2], Mariko Nishikitani[3], Vaishnavi Atul Patil[3], Sunil Hule[3], and Dana Bradley[3], *1. University of Maryland Baltimore County, Baltimore,*

*Kentucky, United States*, *2. Lund University, Lund, Skane Lan, Sweden*,*3. University of Maryland Baltimore County, Baltimore, Maryland, United States*

This study investigates age-related biases in AI-generated art through a mixed method investigation of images of older and younger individuals. Employing 19 text prompts associated with social exclusion for older adults, we collected 465 images from an AI-art generator, MidJourney, and conducted quantitative and qualitative analyses to understand differences between age group depictions. We used Amazon Rekognition software to detect the numerical image properties. Results that were compared statistically (STATA and Python) revealed significant differences in attributes: images with older people exhibited lower overall brightness (M = 46.54) and sharpness (M = 58.58) compared to younger individuals (brightness: M = 60.50; sharpness: M = 72.55). Moreover, older people were more likely to be smiling (16%) and with eyeglasses (56%) compared to younger individuals (6% and 12%), all with $p < 0.001$. Next, we conducted a qualitative semiotics analysis with a randomly selected sample of 76 images. Older people were often depicted with a strong emphasis on nostalgia, vulnerability, and aloneness, reflected in vintage-style clothing, worn objects, and worried expressions. In contrast, younger individuals were often portrayed with a focus on modernity, energy, and independence, characterized by vibrant clothing, digital technology, and confident expressions. Together, these findings demonstrate the perpetuation of negative and reductionist age scripts in AI-generated art, such as older adults' incompetence, loneliness, fear, and outdatedness. Implications of this research extend to societal representations of aging, emphasizing the importance of challenging age-related biases in AI-generated content. Future research should explore refinement of generative models which foster more equitable outcomes.

Abstract citation ID: igae098.2987

### CAN CHATGPT IDENTIFY AND MANAGE FALL RISKS IN OLDER ADULTS?

Huai Cheng, *Minneapolis VA Hospital, Playmouth, Minnesota, United States*

Fall is one of the most common geriatric syndromes in older adults. Identifying and managing fall risks in older adults is very important but can be time-consuming. ChatGPT can answer complex questions such as passing USMLE and clinical questions. In this study, the author prompted ChatGPT to identify and manage fall risks in one old adult via a clinical vignette from a published curriculum and test whether ChatGPT can be used as a screening and management tool in daily practice. ChatGPT was prompted to respond to this clinical vignette with three approaches. First, ChatGPT was prompted to answer one general question on fall risks in old adults; Second, ChatGPT was prompted to answer 5 pre-test questions on fall prevention in older adults; Last, ChatGPT was prompted to answer 5 questions based on this clinical vignette. The author was the only person to determine the correctness of output from ChatGPT. ChatGPT can provide a comprehensive summary of 10 common fall risks in older adults and correctly answer 5 out of 5 pre-test questions with only a few answers missing. For a clinical vignette, ChatGPT provided correct answers on all five questions with only a few answers missing. ChatGPT can be used as a screening and management tool in daily practice.

Abstract citation ID: igae098.2988

### CAN CHATGPT IDENTIFY POTENTIALLY INAPPROPRIATE MEDICATIONS (PIM) IN OLDER ADULTS?

Huai Cheng, *Minneapolis VA Hospital, Playmouth, Minnesota, United States*

Reducing prevalent polypharmacy in older adults is critically important in daily practice, but it takes time to do it. Pharmacy care and other practice models can be helpful but costly. ChatGPT passed the USMLE exam and could be used to identify PIM in older adults in daily practice. Based on a case from a published curriculum by the author, ChatGPT was prompted to answer multiple clinical questions with two approaches. First, ChatGPT was prompted to answer four general questions on appropriate use of medications 1) Which of the commonly used 8 drug (s) is (are) PIM and should be avoided in older adults? 2) What is PIM in older adults? 3) How to identify PIM in older adults? 4) What to do with PIM? Second, ChatGPT was prompted to answer additional four specific questions related to the case 1) any drug-drug interactions for this patient? 2) any drug is ineffective for this patient? 3) this patient was taking so many medications. Any way to reduce the number of her medications? 4) Any drug-disease interaction for this patient? The author was the only person to determine the correctness of output from ChatGPT. Overall, ChatGPT can provide the principles of prescribing and de-prescribing for older adults. ChatGPT identified harmful medications and drug-drug interactions, but not drug-disease interactions. This indicated ChatGPT can be helpful in identifying certain PIM, but not drug-disease interactions in older adults. ChatGPT needs to be trained before providers use it to identify all PIM at daily practice.

Abstract citation ID: igae098.2989

### CAN CHATGPT PERFORM GERIATRIC KNOWLEDGE TESTS AS WELL AS MEDICAL STUDENTS, RESIDENTS, AND GERIATRICS FELLOWS?

Huai Cheng, *Minneapolis VA Hospital, Playmouth, Minnesota, United States*

ChatGP was released to the public in November of 2022 and immediately emerged as a quick information resource and application to medical education. ChatGPT passed USMLE step 1, 2 and 3. This study was to examine whether ChatGPT can perform well on 18 published validated geriatric knowledge questions compared to medical students, residents, and geriatrics medicine fellows. These 18 geriatric knowledge questions with 8 True or False questions and 10 clinical vignettes were developed to assess geriatric knowledge for medical students, residents, and geriatrics medicine fellows. In this study, ChatGPT was treated as a human or study participant and prompted to answer these 18 questions four times from April to November of 2023. Outputs from ChatGPT were collected for analysis. The mean scores of 18 questions were compared between ChatGPT and the trainees including medical students, residents, and geriatrics medicine fellows. It was found that the mean score (14.25, n=4 prompts) from ChatGPT was higher than the scores from medical students and similar to residents, however lower than geriatrics medicine fellows. Additionally, half of output from ChatGPT gave good rational for their response to the questions. This indicated ChatGPT performed reasonably well on 18 validated geriatric knowledge questions. ChatGPT could be used as a supplemental resource in geriatrics for trainees at their early stage of training and perhaps for lay persons as well.

Abstract citation ID: igae098.2990

### CONTENT AND QUALITY OF CHATGPT AS A SOURCE OF DEMENTIA INFORMATION: COMPARATIVE ANALYSES

Jill Dosso, Jaya Kailley, Payton Angus, and Julie Robillard, *University of British Columbia, Vancouver, British Columbia, Canada*

There is significant demand for easily accessible, reliable online health information from older adults and their families (Soong et al., 2020). Online information about Alzheimer's Disease and dementia ranges in quality from misleading and predatory to supportive and evidence-based (Robillard & Feng, 2016). ChatGPT was released to the public in November 2022 and is a well-known example of a new class of online tools, Large Language Models, which are generative artificial intelligence systems that can produce fluent, coherent answers to users' questions based on extensive training data. In this work, we use a validated evaluation tool (Robillard et al., 2018) to evaluate the quality and content of information about dementia from ChatGPT-3.5 at two time points (2023, 2024) and from two versions of the model (free GPT-3.5, paid GPT-4). Prompts were developed using questions extracted from the FAQ pages of national dementia organization websites in Canada, USA, and Mexico. We found that all versions of ChatGPT were unbiased, remind the user to speak to a physician, and took an appropriately balanced tone. GPT-4, unlike GPT-3.5, links to specific, identifiable, dated sources for its claims including scientific research. All forms of ChatGPT had a high Flesch-Kincaid Grade Level, indicating moderate readability. Results indicate that ChatGPT, particularly GPT-4, can be a relatively high-quality source of dementia information for non-expert users. However, it is limited in its ability to link users to local resources. Findings can inform older adults, care partners, and healthcare professionals in their decision-making around use of this new tool.

Abstract citation ID: igae098.2991

### DEVELOPMENT OF THE SWALLOWING ACTIVITY CLASSIFICATION MODEL FOR OLD ADULTS BASED ON ACOUSTIC ANALYSIS

Dan Li[1], Yaqing Zhang[1], Junhui Wu[2], Wei Luo[2], Tao Liu[2], Benshuai Lyu[1], and Shaomei Shang[2], *1. Peking University, Beijing, Beijing, China (People's Republic)*, *2. Peking University Health Science Center, Beijing, Beijing, China (People's Republic)*

Dysphagia poses a significant health threat for old people worldwide, often resulting in severe complications like aspiration, choking, and aspiration pneumonia, thereby increasing hospital readmission rates and mortality. Swallowing function assessment serves as an effective means to reduce the occurrence of complications and improve patients' survival rate. However, current assessment tools have pronounced limitations. Acoustic diagnosis, as a non-invasive method, has shown promising effectiveness in dysphagia screening and aspiration detecting, rendering it a key technology for addressing this issue. This study aims to develop a swallowing activity classifier based on acoustic recording and analysis using machine learning algorithms, which constitutes an efficient tool for non-invasive, remote, and continuous assessment of swallowing activities in old adults. The study recorded and annotated swallowing audio data from 61 old individuals, resulting in 120 acoustic samples. Signal features in time, frequency, time-frequency, and statistical domains were analyzed and extracted as inputs. Multiple models, including the Multi-Layer Perceptron (MLP), Convolutional Neural Network (CNN), and Convolutional Recurrent Neural Network (CRNN), were used for classification. The MLP, CNN, and CRNN models achieved accuracy values of 74%, 68%, and 54%, respectively, on the validation set. Results indicate that swallowing, coughing, throat clearing, choking, wet breathing, and speech are characterized by distinct acoustic patterns, which can be effectively classified and identified using machine learning methods. These findings hold promise for the research and practical application of acoustic and machine learning-based swallowing activity classification models, facilitating continuous monitoring in old people, thereby providing guidance for clinical treatment and care.

Abstract citation ID: igae098.2992

### THE EXPERIENCE OF SOCIAL WORKERS IN BIG DATA–BASED CARE SERVICES ON PREVENTING SOCIAL ISOLATION FOR OLDER ADULTS

Haklyoung Kim, Boram Hwang, Sungwan Kang, Seongsu Choi, Kyoungmi Mun, and Yeong Ju Lee, *Pusan National University, Busan, Republic of Korea*

In response to the increasing occurrence of social isolation among older adults in South Korea, local governments and ICT companies are collaborating to introduce various intelligent care initiatives based on big data, including AI care calls, AI speakers, and activity detection sensors. This study investigates the experiences of social workers providing on-site intelligent care services to older individuals living alone to prevent social isolation. Five focus group interviews involving 15 welfare officials and social workers were conducted. The findings are as follows. Firstly, they recognized biases regarding intelligent technology, such as concerns about privacy infringement, as barriers to identifying high-risk groups and accepting services. Secondly, challenging living environments with limited internet access and low digital literacy were acknowledged as obstacles hindering the sustained use of intelligent care services. Thirdly, the presence of personnel capable of providing education and support for smart device usage was deemed essential for the continued utilization of services. Fourthly, it was acknowledged that additional personnel and support beyond smart devices are necessary for groups with impaired vision, hearing, or mobility. Lastly, the involvement of social workers with a deep understanding of high-risk groups, from planning to evaluation of service models, was recognized as essential for enhancing the effectiveness and efficiency of services. Based on these findings, this study suggests practical and policy implications regarding intelligent living environments, digital literacy, ethics of artificial intelligence, caregiver training, and the involvement of social workers.

### SESSION 7435 (POSTER)

### ASSISTED LIVING

Abstract citation ID: igae098.2993

### A COMPARISON OF HEARING AID USERS AND NONUSERS AMONG PERSONS LIVING WITH COGNITIVE IMPAIRMENT

Jayeong Kim, and Yeji Hwang, *Seoul National University, Seoul, Republic of Korea*

Hearing impairment is common in older adults, including those with cognitive impairment. While hearing aids can alleviate the challenges posed by hearing impairment, less is known about the usage of hearing aids among individuals living with cognitive impairment. Therefore, this study was conducted with two main

**objectives:** To identify the prevalence of hearing aid usage among individuals with both cognitive and hearing impairment, and to examine the characteristics of those utilizing hearing aids. Secondary data analyses were conducted using 2020 National Survey of Older Koreans, which includes a nationally representative sample of community-residing older adults. This study included older adults aged 65 years and older who had both cognitive and hearing impairment (n=755). Chi-square tests and independent t-tests were employed to compare the characteristics of hearing aid users to non-users. Among those experiencing both cognitive and hearing impairment, only 32.8% used hearing aids. Individuals using hearing aids were older (t=-4.69, p<.001) and had a higher number of chronic diseases (t=-2.10, p=.036) and medications (t=-2.57, p=.010) compared to non-users. Furthermore, hearing aid users exhibited poorer cognition (t=4.17, p<.001), reported greater depressive symptoms (t=-3.7247, p<.001), and participated less in leisure activities (Chi2=11.73, p<.001). Given that only a small portion of individuals facing hearing difficulties utilized hearing aids, there is a need for greater access to these devices to enhance overall quality of life. Additionally, this study revealed that individuals using hearing aids had poorer health conditions compared to non-users. Future research should consider objectively measuring hearing impairment to better examine this relationship.

Abstract citation ID: igae098.2994

### ASSISTED LIVING STAFF VIEWS OF FAMILY INVOLVEMENT IN PALLIATIVE CARE FOR RESIDENTS WITH DEMENTIA

Jessica Yauk, Debra Dobbs, and Lindsay Peterson, *University of South Florida, Tampa, Florida, United States*

Of the close to 300,000 residents in assisted living (AL) living with dementia, an estimated 40% will die there and be in need of end-of-life care. Family of residents living with dementia are an important part of the care convoy in AL. This qualitative study aimed to understand staff perspectives about family involvement in end-of-life care. The Palliative Care Education in Assisted Living for Dementia Care Providers intervention study (2019-21) included a sample of 17 AL staff from six treatment sites in Florida who completed four homework assignments. Two of the assignments were writing assignments that 1) asked about a good death for residents and 2) asked staff specifically to identify one resident with dementia who they could do one thing to make them comfortable. A priori codes were used based on the physical, emotional, social and spiritual care domains of palliative care asked about in the homework assignments. The data was coded using Atlas.ti version 23. Family was involved in all four domains of palliative care. Social and emotional aspects of care were the most prominent (e.g., playing music, visits, care coordination, delivery of food) followed by spiritual care (e.g., pray together, attend religious services). Physical care tasks by family were reported less often. Less physically demanding care tasks were performed by the family (e.g., grooming, dressing) with family reliance on direct care staff and hospice providers for more physically demanding care (bathing, transfers), and for pain management. We will discuss the role family play in AL care provision.

Abstract citation ID: igae098.2995

### COMPARISON OF QUALITY INDICATORS IN RURAL AND URBAN ASSISTED LIVING FACILITIES

Alexa Stuifbergen, Stephanie Morgan, Katie Trainum, and Heather Becker, *The University of Texas Austin, Austin, Texas, United States*

As the population of older adults grows, the demand for assisted living facilities (ALFs) is expected to increase. This growth will disproportionately affect rural ALFs, as the proportion of older adults living in rural areas is growing at a faster rate than urban areas, and older adults living in rural areas are in poorer health compared to their urban counterparts. This exploratory descriptive study examined differences between facility characteristics and resident perspectives of care between rural and urban ALFs using data collected as part of a major statewide assessment of quality of care in ALFs. The research team conducted site visits to collect facility level data from staff and resident interviews regarding perspectives of quality of care. The sample included 517 facilities from 8 geographic regions of a large Southwestern state with 143 of the facilities located in rural settings. Overall, licensed bed size ranged from 3 to 177 with the number of occupied beds significantly greater in urban ALFs (Mean=33.71, SD 28.51) than in rural ALFS (Mean=22.54, SD 15.93) (t=5.37, p<.001). Overall, residents rated their health as good, very good, or excellent (78%) although a greater percentage of rural residents (23%) rated their health as fair or poor compared to urban residents (16%). Resident satisfaction with quality of health care and quality of experience in the facility was higher among residents of rural vs. urban ALFs. Additional research is needed to more fully explore factors influencing quality of life and quality of care in rural and urban ALFs.

Abstract citation ID: igae098.2996

### FAMILY PERCEPTIONS OF THE EFFECTS OF COVID-19 ON OHIO LONG-TERM CARE FACILITIES

Oksana Dikhtyar, and Jennifer Heston-Mullins, *Miami University, Oxford, Ohio, United States*

Family and friends are often the main source of emotional support and social contact for residents in long-term care facilities. The visitation restrictions imposed during the COVID-19 pandemic negatively impacted staff, family members, and residents, particularly those with cognitive impairment. However, there is limited literature on the impact that COVID-19 had on communication between residents and their family and friends, and communication between long-term care facilities and residents' family and friends. To understand more about the effects of COVID-19, an online, state-wide survey was conducted which included questions regarding communication. A total of 1,686 involved family members or friends responded to the survey between September 2021 to May 2022. Phone calls were the most common method of communication between residents and their family and friends during visitation restrictions, followed by window visits and dropping off food and snacks. The frequency of communication also increased as a way for families to compensate for the lack of regular visits. Staff in most facilities used emails or postal mail, phone calls or text messages, and social media to communicate with family members. The amount of information respondents received from the facility affected their perceptions about the facility. These study findings can help improve facility-family communication and help facilities explore new ways of working with and engaging residents and families beyond the pandemic.

Abstract citation ID: igae098.2997

### NURSING HOME ADMISSION FROM RESIDENTIAL CARE FACILITY: NATIONAL HEALTH AGING TRENDS STUDY (NHATS) 2011–2019

Jung Yoen Son, Deanna J Marriott,
Laura Struble, Chen Weiyun, and Janet Larson, *University of Michigan, Ann Arbor, Michigan, United States*

Limited studies have examined risk factors for nursing home admission among residents in residential care facilities (RCF), and prior research is limited by short follow-ups, which are insufficient to observe changes in health and nursing home admission. This study aimed to (1) identify baseline factors associated with transfers to a nursing home and (2) identify time-varying factors associated with transfer to a nursing home over 8 years, using a national dataset from the National Health Aging Trends Study (NHATS) collected between 2011 and 2019. We conducted Cox proportional hazards regression to examine candidate predictors (chronic conditions, hospitalization, sleep disturbances, mental health, physical performance, difficulties in activities of daily living (ADL), self-reported health, social and physical activity, and sociodemographic) associated with nursing home admission. Employing backward elimination, we built parsimonious final models for analysis. The analytic sample included 970 participants (mean age at baseline=85.7 years, 67.2% women, 83.1% White, mean length of stay at baseline=2.3 years) and 143 had transferred to nursing homes over 8 years. Those who had better physical performance at baseline (HR=0.83,CI=0.79-0.88) and were college educated (HR=0.58,CI=0.37-0.86) demonstrated a significantly lower risk for nursing home admission over 8 years. Residents who maintained physical activity (HR=0.56,CI=0.37-0.86), better physical performance (HR=0.87,CI=0.80-0.94), and fewer ADL difficulties (HR=1.13,CI=1.02-1.26) were at lower risk for nursing home admission over 8 years. Our findings can assist in identifying older adults in RCFs at risk of nursing home admission. Strategies to promote physical function and physical activity could be helpful to avoid/delay such admission.

Abstract citation ID: igae098.2998

### OLDER ADULTS' EXPERIENCES OF LONELINESS AND ITS UNDERLYING MECHANISMS IN RESIDENTIAL CARE FACILITIES

Amarjot Gill[1], and Suzanne Sullivan[2], *1. University at Buffalo, SUNY, Buffalo, New York, United States*, *2. State University of New York Upstate Medical University, Syracuse, New York, United States*

Older Adults' (OAs') transition into residential care facilities (RCFs) detaches them from their meaningful social relationships, often precipitating feelings of loneliness. This prevalent issue in RCFs can lead to poor health outcomes, significantly disrupting OAs' well-being and quality of life. Consequently, loneliness in RCFs emerges as a serious public health issue, necessitating a comprehensive understanding of its mechanism. This systematic review aims to explore OAs' experiences and perceptions of loneliness and characterize mechanisms underlying OAs' loneliness experiences through a comprehensive loneliness model. This review adhered to PRISMA guidelines. Articles from CINAHL, PubMed, PsycINFO, and Web of Science, published in English between 2018 and 2023, were examined. Of the 7,285 articles identified, 10 met the inclusion criteria. Quality appraisal for the selected studies was conducted using the Critical Appraisal Skills Programme (CASP) checklist. Three core themes of loneliness were identified: relational and individualized loneliness experiences, perception and emotional distress, and the influence of context and cognitive processes in modulating loneliness. A conceptual model delineating the mechanisms of OAs' loneliness in RCFs was developed. This review highlights the influence of OAs' residential context and cognitive processes, particularly their perceptions, in triggering loneliness and associated behavioral responses. OAs' perceptions of reduced social control and insufficient social connections in RCFs exacerbate their loneliness, precipitating distressing emotional responses and diminished quality of life. Future research must explore strategic transformations of OAs' perceptions through mechanistic targets and tailored care plans to reshape their social expectations within the context of RCF, potentially mitigating loneliness.

Abstract citation ID: igae098.2999

### PHYSICAL ACTIVITY AND SEDENTARY BEHAVIOR IN RESIDENTS OF ASSISTED LIVING: A PILOT LONGITUDINAL STUDY

Jung Yoen Son, She'Lon Tucker, Carol Vos, and
Janet Larson, *University of Michigan, Ann Arbor, Michigan, United States*

Low levels of physical activity (PA) and high sedentary behavior (SB) place older assisted living (AL) residents at risk for functional decline and premature transfer to a higher level of care. This study aimed to examine the feasibility and acceptability of conducting a longitudinal study using two objective measures of PA and SB. We recruited participants from four AL facilities in Michigan.

Participants wore two devices (ActiGraph at the wrist and ActivPal on the anterior thigh) for seven consecutive days twice, once at baseline and again six months later, and completed questionnaires. We evaluated recruitment, retention, and compliance with and acceptability of wearing devices, and conducted a longitudinal multilevel model to estimate preliminary changes in PA and SB over 6 months. Forty-two people volunteered, 19% were excluded with a low Mini-Cog score, 62% completed baseline (77% female; mean age=80.1), and 60% completed 6 months data collection. Most participants were willing to perform two measures of PA and SB and generally found the procedures to be acceptable. Over 6 months, moderate to vigorous PA significantly declined by 8 min/day (p=0.02), light PA declined by 18 min/day (p=0.13), and SB increased by 15 min/day (p=0.48). In conclusion, the results support the feasibility and acceptability of this research protocol. Participants had a small decline in PA over a six-month period of time, which was not clinically meaningful but could become significant if the trend continued.

Abstract citation ID: igae098.3000

### RECRUITMENT STRATEGIES IN LONG-TERM CARE: NEW AND NOVEL APPROACHES

Cathy Maxwell[1], Judith Tate[2], Lisa Juckett[3], Kelley Colopietro[4], Abbey Kilpatrick[2], Lorraine Mion[2], and Nilanjan Sarkar[4], *1. University of Utah College of Nursing, Salt Lake City, Utah, United States*, *2. The Ohio State University, Columbus, Ohio, United States*, *3. The Ohio State University Medical Center, Columbus, Ohio, United States*, *4. Vanderbilt University, Nashville, Tennessee, United States*

Older adults residing in long term care (LTC) can benefit from social and behavioral research to improve health outcomes and promote evidence-based practice; however, recruitment is often difficult and time/cost-intensive. Systematic reviews of literature report recruitment barriers, as well as specific recruitment strategies. This presentation extends this work by reporting on new outreach endeavors within two state-based regions with over 300 LTCs. Our work is based on three longitudinal studies in LTC facilities to promote engineering and technology-based interventions that address apathy and loneliness in LTC residents. Relationship-building is the core of successful recruitment with establishment of trust and altruistic outreach. Our strategies include: 1) utilization of personnel with longstanding presence in the community and associated organizations (e.g., Alzheimer's Association), 2) outreach opportunities to regional, state-based health care professional organizations, 3) repeated phone contacts to gauge interest, 4) provision of health screenings for residents (e.g., blood pressure, fall risk assessment), and 5) educational and enjoyable activities for residents (i.e., games, health-related topics [medications, nutrition, mind/body health, safety]). Research opportunities include studies using socially-assistive robots to address apathy among older adults with mild to moderate cognitive impairment, and augmented reality/avatar-based interventions to address loneliness among LTC residents. Our strategies established trust and promoted relationship-building between local and regional LTC facilities and the specific universities, resulting in successful recruitment of 17 (45%) out of 37 LTCs who responded to initial contact.

**Conclusion:** Successful recruitment in LTC requires multiple strategies. Trust and relationship-building promote reciprocity that can benefit both LTC facilities and universities.

Abstract citation ID: igae098.3001

### SENSE OF AESTHETICS AMONG OLDER ADULTS NEW TO LONG-TERM CARE SERVICES AND SUPPORTS

Charlotte Weiss, Karen Hirschman, and Mary Naylor, *University of Pennsylvania, Philadelphia, Pennsylvania, United States*

Engagement in aesthetic experiences and activities can contribute to older adults' quality of life and social connection. Few studies explore older adults' sense of aesthetics (the extent to which one obtains pleasure from sensory awareness and appreciation of beauty) and how this information may be useful in supporting older adults during transitions to long-term services and supports (LTSS; in home, assisted living, or nursing home). We conducted a secondary analysis of interviews with older adults new to LTSS (R01AG025524) to examine the factors associated with Sense of Aesthetics (SOA) [a Dementia Quality of Life subscale]. A total of 376 LTSS recipients had complete data. The average score of the SOA was 3.44 (SD: 0.98, range= 1-5, median=4; higher scores indicate a greater sense of aesthetics. Using multivariable linear regression, we controlled for the influence of important health-related quality of life covariates (e.g., LTSS setting, sex, age, education, number of comorbidities, sensory impairment [vision/hearing], cognitive status). Older adults with greater positive interaction with social support (p>0.001) and a greater spiritual/religious disposition (p=0.004) had higher SOA. Older adults receiving LTSS in the nursing home had lower SOA compared to those in the home (p=0.029) and no difference between those receiving LTSS in home vs assisted living (p=0.49). The findings highlight the opportunity for LTSS intradisciplinary teams to consider the value that older adults assign to aesthetics and increase access to aesthetic experiences, especially within the nursing home setting. Implications for innovative ways to increase older adults' sense of aesthetics will be discussed.

Abstract citation ID: igae098.3002

### THE COGNITIVE DECLINE TOWARD DEMENTIA AMONG NURSING HOME RESIDENTS

Audai Hayajneh, *Jordan University of Science and Technology, Irbid, Irbid, Jordan*

A scarcity of studies have examined the factors associated with mild cognitive impairment (MCI) as well as moderate cognitive impairment-as considered as dementia- among nursing home residents (NHRs). The aim of this study was to recognize the nature of relationship between the health risk variables, such as comorbidities, length of stay at hospital, the quality of life, the occurrence of depression, disability, and frailty, and dementia in NHRs. The study deployed cross-sectional design, using a convenient sample of 182 nursing home residents. The settings of the study were in the middle region of Jordan. Both stages of the MCI and dementia were determined through Montreal cognitive assessment (MoCA). Bivariate and multivariate analyses were utilized to explore the relationship between the health risk variables, and each of the MCI and dementia. There were significant differences between the stages of cognitive impairment (The MCI and dementia) in terms of age, the quality of life, comorbidities, depression, frailty. The variation in mental ability scores was dependent on the marital status of the participants, the monthly income, recently hospitalized, the level of depression, and frailty among older adults residing at nursing homes. The results of this study contribute significantly to building a healthcare management protocol to deal with nursing home residents with depressive symptoms as well as the deferral of the trajectory of development of dementia among those risky residents.

### SESSION 7440 (POSTER)

### CHRONIC DISEASE MANAGEMENT (POSTERS)

Abstract citation ID: igae098.3003

### "WE ARE SYMBIOTIC": THE IMPACT OF MULTIPLE CHRONIC CONDITIONS ON RELATIONSHIP DYNAMICS AMONG OLDER COUPLES

Angelica Eagle, and Courtney Polenick, *University of Michigan, Ann Arbor, Michigan, United States*

Despite the growing recognition of the impact of multiple chronic conditions (MCC) on individuals' lives and relationships, there is limited understanding about the effects on spousal relationships when both spouses live with MCC. This qualitative study evaluated how older couples living with MCC perceive the impact of their conditions on their relationship. A total of 56 wives (M = 66.84 years, SD = 9.73) and husbands (M = 68.16 years, SD = 10.69), who both reported having two or more chronic conditions, completed separate semi-structured interviews that included open-ended questions about the effect of their conditions on their relationship. We separately evaluated couples that included a partner living with early-stage dementia (n = 15) and couples not living with dementia (n = 41) to assess whether the presence of dementia affects couples with MCC differently due to distinct care needs and communication challenges. The data were coded using qualitative content analysis to develop major themes. Themes for the relationship impacts of MCC among couples not living with dementia included: 1) adapting daily routines around functional limitations; 2) changes in emotional and physical intimacy; 3) changes in shared activities; and 4) stronger relationship impact on one partner. Themes for couples living with dementia included: 1) changes in emotional and physical intimacy; 2) stronger relationship impact on spouses of people living with dementia; and 3) increased time spent together. These findings identify potential targets for dyadic interventions to improve relationship functioning and quality among older couples living with MCC.

Abstract citation ID: igae098.3004

### A MODIFIED MULTIFACTORIAL INTERVENTION FOR OLDER ADULTS WITH DIFFERENT KNEE OSTEOARTHRITIS SEVERITY

Suparb Aree-Ue[1], Inthira Roopsawang[1], and Natthayawadee Saraboon[2], *1. Mahidol University, Bangkok, Krung Thep, Thailand*, *2. Boromarajonani College of Nursing, Nakhon Phanom, Nakhon Phanom, Nakhon Phanom, Thailand*

Knee osteoarthritis (OA) is a common health condition affecting older adults' health worldwide. Several modalities to mitigate disease severity have been implemented; however, whether those interventions benefit all stages of disease severity has not been explored. This quasi-experimental study aimed to compare the effect of a modified multifactorial intervention program on health outcomes between older adults with mild and moderate knee OA severity. Ninety prospective participants who met the inclusion criteria were assigned into Group 1 (mild symptoms) or Group 2 (moderate symptoms). The modified multifactorial intervention program—health education about knee OA, weight control, quadriceps exercise, and home visits—was delivered to both groups lasting 12 weeks. The health outcomes (weight, psychological well-being, muscle strength, and knee OA severity) were assessed at baseline, 8-, and 12-week after enrollment using standard self-reported questionnaires and a 30-second sit-to-stand test. Data were analyzed using descriptive, independent-t-test, MANCOVA, and repeated measure MANOVA. Results revealed that the remaining 85 participants' age ranged from 60 to 85 years, with a mean age of 68.8 years (SD = 8.6 years); almost were females (95.2%; n = 79). Although knee OA severity and psychological well-being significantly differed between the groups at baseline, there was no interaction effect on other dependent variables. Weight and muscle strength evaluated by a 30-second sit-to-stand test has no significant difference between groups. Results can be concluded that the modified multifactorial intervention program is beneficial to promote better knee joint function and health among older people with mild and moderate knee OA.

Abstract citation ID: igae098.3005

### AN INVESTIGATION OF CDSMP OUTCOMES FOR PARTICIPANTS ENROLLING WITH DIFFERENT LEVELS OF NEED

Abby Teply, Christine McKibbin, Joshua Clapp, Stacy Carling, Laurance Goodwin, Catherine Carrico, Barbara Dabrowski, and Elizabeth Punke, *University of Wyoming, Laramie, Wyoming, United States*

The Self-Management Resource Center's evidence-based Chronic Disease Self-Management Program (CDSMP) empowers people to actively engage in their own care through a variety of evidence-based solutions. CDSMP strategies and foci align with the Age-Friendly framework - an evidence-based approach to guide health care with older adults that emphasizes 4Ms: "What Matters," "Medication," "Mentation," and "Mobility." In prior analysis of CDSMP baseline data, participants were represented by two distinct latent profiles based on age-friendly measures: individuals needing support ("NS") and those who were coping well ("CW"). The purpose of this study was to examine differences in CDSMP outcomes among the participant profiles. Those completing baseline and follow-up assessments (n = 147) were included. Participants were predominantly White (n = 139; 94.6%) females (n = 18; 80.3%) with a mean age of 75.21 (SD = 18.83). Repeated measures ANOVAs revealed significant interactions between CW (n = 42) and NS (n = 105) groups from pre- to post-intervention. Relative to those in the CW group, those in the NS group had greater improvement on measures of interference with daily activities, $F(1,144) = 5.15$, $p = .03$, and self-efficacy, $F(1,136)$

= 7.39, p =.01. Despite greater improvement, those in the NS profile still had greater interference with daily activities (M = 1.94, SD = 1.00) than those in the CW group (M = 1.19, SD = 1.08) at post-intervention, t(144) = 1.99, p =.05. Additional strategies may be needed to further improve outcomes for those belonging to the Age-Friendly, "needs support" profile.

Abstract citation ID: igae098.3006

### APPLYING THE AGE-FRIENDLY 4MS FRAMEWORK TO IDENTIFY NEEDS OF INDIVIDUALS ENROLLED IN CDSMP

Abby Teply, Christine McKibbin, Joshua Clapp, Stacy Carling, Sabine Schenck, Catherine Carrico, Barbara Dabrowski, and Elizabeth Punke, *University of Wyoming, Laramie, Wyoming, United States*

The Age-Friendly framework is an evidence-based approach to guide health care with older adults that emphasizes 4Ms: "What Matters," "Medication," "Mentation," and "Mobility." The Self-Management Resource Center's evidence-based Chronic Disease Self-Management Program (CDSMP) aligns with this framework. Therefore, this study applied the 4Ms to identify needs of participants from CDSMP. Participants (n = 147) were predominantly White (n = 139; 94.6%) females (n = 18; 80.3%) with a mean age of 75.21 (SD = 18.83). The sample included caregivers/supporters (n = 68; 45.3%) and individuals with chronic illnesses (n = 79; 53.7%). Participants completed pre-post measures consistent with the 4Ms (e.g., provider communication, medication adherence, depressive symptoms, and exercise). A latent profile analysis was conducted to extract unobserved subgroups identified by common profiles of response at baseline. Indices of model fit suggested a two-profile model was appropriate. Profile 1 (labeled "Needs Support") was characterized by poor medication adherence and mild depressive symptoms. High medication adherence and minimal depressive symptoms, in contrast, distinguished profile 2 (labeled "Coping Well"). T-tests revealed a significant difference between profiles on frontier and remote area (FAR) ratings, t(91.999) = -2.107, p =.038. On average, those in profile 1 lived in areas with higher FAR ratings (M = 1.10, SD = 1.24), suggesting greater remoteness, compared to those in profile 2 (M =.68, SD =.99). No differences were found on age, gender, education, etc. Rural living may complicate self-management. Additional research is needed to understand the implication of profile membership for program outcomes.

Abstract citation ID: igae098.3007

### ASSOCIATIONS OF PROTEIN INTAKE WITH THE RISK OF ALL-CAUSE, CARDIOVASCULAR, AND CANCER MORTALITY: A META-ANALYSIS

Huili Yang[1], Meng Cao[2], Chongmei Huang[1], Yaping Jin[2], and Minhui Liu[2], *1. Ningxia Medical University, Yinchuan, Ningxia, China (People's Republic)*, *2. Ningxia Medical University, Yinchuan, Ningxia, China*

The association of the amount and sources of protein consumption with mortality for cardiovascular diseases (CVD) and cancer has been extensively studied. However, the outcomes remains controversial. The present meta-analysis was conducted to synthesize findings on the associations between total, animal, and plant protein intake and the risk of mortality from all causes, and cancer. Pubmed and Web of Science were searched to identify prospective cohort studies published up to November 2022. Random effects models were used to calculate pooled effect sizes and 95% confidence intervals for the highest versus lowest categories of protein intake. Heterogeneity were evaluated with I2 statistic, and publication bias was evaluated with funnel plots and Egger's test. A total of 28 cohort studies were identified. The results showed that total protein (HR=0.99, 95%CI: 0.93, 1.04; HR=0.95, 95%CI: 0.87, 1.02; HR=0.97, 95%CI: 0.84, 1.11), animal protein (HR=1.02, 95% CI: 0.98, 1.07; HR=1.05, 95% CI: 0.99, 1.12; HR=1.00, 95% CI: 0.98, 1.02) were not associated with the all-cause, CVD and cancer mortality. However, the intake of plant protein was inversely associated with the risk of all-cause (HR=0.92, 95% CI: 0.87, 0.96) and CVD mortality (HR=0.87, 95% CI:0.81, 0.93), but not cancer mortality (HR=0.98, 95% CI: 0.94, 1.01). This study demonstrates that higher plant protein intake is associated with a reduced risk of all-cause and CVD mortality. Increasing plant protein intake is a potential way to decrease the risk of death.

Abstract citation ID: igae098.3008

### CHRONIC OBSTRUCTIVE PULMONARY DISEASE SELF-MANAGEMENT USING TECHNOLOGY IN DAILY LIFE: A PILOT STUDY

Seoyoon Woo, Anastasiya Ferrell, and Jamy Chulak, *University of North Carolina Wilmington, Wilmington, North Carolina, United States*

**Rationale:** Self-management is crucial to preventing lung function decline and improving the quality of life but is rarely performed daily in people with chronic obstructive pulmonary disease (COPD). During the COVID-19, they were forced to use telemedicine and telerehabilitation for support. However, the extent to which such technology is used to manage COPD remains unknown. This study aimed to examine the use of technology for COPD self-management and its barriers and facilitators.

**Methods:** A cross-sectional study using an online survey method was conducted. People with COPD(N=102; mean age=44years) completed a set of questionnaires, including the Self-Management Assessment Scale and the Media and Technology Usage and Attitudes Scale, and open-ended questions. Descriptive statistics, multiple regressions, and the interpretative thematic approach were used to analyze the data.

**Results:** Compared to mobile device usage(94.2%), only 3.9% used it to self-manage COPD. However, over 60% expressed a willingness to use mobile apps for self-management. eHealth literacy significantly predicted both Media and Technology Usage(β=0.70,p=0.001) and Attitudes(β=0.20,p< 0.001). COPD self-efficacy significantly predicted all five domains of self-management needs: knowledge(β=0.33,p=0.003), goals for future(β=0.36,p=0.02), daily routines(β=03,p=0.004), emotional adjustment(β=0.53,p< 0.001), and social support(β=0.51,p=0.02). Barriers to using mobile apps included their reliability, forgetfulness, and usability, just to name a few. Mobile apps for COPD self-management can be more attractive if they feature monitoring and recording of symptoms and provide relevant resources.

**Conclusion:** This study identified factors influencing the use of technology for COPD self-management. The findings can be considered in designing self-management intervention tools to help better manage COPD.

Abstract citation ID: igae098.3009

### DIETARY BEHAVIORS OF AFRICAN IMMIGRANTS LIVING WITH DIABETES AND/OR HYPERTENSION: A QUALITATIVE STUDY

Onome Osokpo[1], Lisa Lewis[2], Danny Bracy[3], Dolapo Adeniji[4], Ayomide Bankole[5], and Barbara Riegel[3], *1. University of Illinois Chicago, Chicago, Illinois, United States*, *2. Rutgers, The State University of New Jersey, Camden, New Jersey, United States*, *3. University of Pennsylvania, Philadelphia, Pennsylvania, United States*, *4. Indiana State University, Avon, Indiana, United States*, *5. Duke University, Durham, North Carolina, United States*

Healthy dietary behaviors (e.g., low-salt diet) are a critical element of self-care and the cornerstone in managing diabetes and/or hypertension. Social determinants (e.g., cultural preferences and social support) influence dietary behaviors. However, little is known about the influence of social determinants on dietary behaviors of African immigrants living with diabetes and/or hypertension accounting for their level of acculturation. We conducted a descriptive qualitative study of a purposive sample of 24 self-identified sub-Saharan African immigrant adults in the Northeastern U.S. with symptomatic hypertension and/or diabetes who spoke and understood English. Based on scores from the Short Acculturation Scale, African immigrants were classified as more or less acculturated. The sample was mainly Nigerian (75%) and males (67%), with a mean age of 59 years [40-72]. The sample was highly educated, acculturated, employed, and reported having enough income to meet their needs. Compared to more-acculturated immigrants, less-acculturated immigrants regularly ate more African or "traditional food," vegetables and rice, and avoided salt and fatty/fast/processed American foods. Less-acculturated immigrants cooked their meals and learned their diets from previous education or their workplace. Compared to the less acculturated, more acculturated participants ate fruits regularly and avoided meat or red meat. Interestingly, the more acculturated participants reported learning about their diet from family and friends and noted that family members cook the meals in their household. Interventions to promote healthy diets among immigrants should take into consideration cultural dietary preferences, influence of family, friends, and non-familial social connections, and level of acculturation.

Abstract citation ID: igae098.3010

### EXPLORING AND CHARACTERIZING MULTIMORBIDITY PROFILES AMONG ADULTS WITH HEART FAILURE

Shirin Hiatt[1], Paula Meek[2], Christopher Lee[3], and Quin Denfeld[1], *1. Oregon Health & Science University, Portland, Oregon, United States*, *2. University of Utah, Salt Lake City, Utah, United States*, *3. Boston College, Chestnut Hill, Massachusetts, United States*

Multimorbidity (MM) or coexistence of ≥2 chronic conditions is prevalent among older adults and results in poor outcomes. Among adults with heart failure (HF), MM poses exponentially more challenges, but our understanding of MM in HF is limited. The purpose of this study was to explore and characterize MM profiles in a cohort of adults with HF. We performed a secondary analysis of combined data from 6 HF studies. We used latent class analysis to identify MM profiles based on comorbidities identified with the Charlson Comorbidity Index. After identifying MM profiles, we examined sociodemographic, clinical and symptom characteristics across each profile. The sample (n=523) was 37% women, mean age 58.3±14.0 years, and mean of 2.1±1.1 comorbidities. Most patients (n=480; 92%) had MM, including HF. Among patients with MM, we identified four distinct MM profiles: Class 1-high complexity (4.6%), Class 2-moderate complexity (31.7%), Class 3-low complexity (31.9%), and Class 4-no complexity (31.9%). Class 1 was older, living alone, significantly comorbid, reported poor/fair health and depressive symptoms, and frail. Classes 2 & 3 were the next oldest but majority lived with someone. Class 4 was the youngest with the shortest duration of HF diagnosis. In conclusion, we found four distinct profiles of MM among patients with HF with marked differences in characteristics. As the prevalence of MM increases among older adults and those with chronic conditions such as HF, it is imperative to understand profiles of MM and how they contribute to function and wellbeing in order to develop effective interventions.

Abstract citation ID: igae098.3011

### EXPLORING STRESSORS AND SELF-MANAGEMENT BEHAVIORS IN RURAL AGING AFRICAN AMERICANS WITH DIABETES: A PILOT STUDY

Idethia Shevon Harvey[1], Sophia Nittinger[1], Maude Harris[1], Arika Wiggins[2], and Rahma Mkuu[3], *1. University of Missouri, Columbia, Missouri, United States*, *2. Southeast Missouri State University, Cape Girardeau, Missouri, United States*, *3. University of Florida, Gainesville, Florida, United States*

**Background:** Rural African Americans with diabetes encounter unique challenges related to managing their condition, including diabetes-specific distress, financial stress, and interpersonal stressors. This study investigated the associations between these stressors and self-management behaviors in a small population sample.

**Methods:** Fifteen rural African American adults (mean age = 66.08 yrs.; SD = + 6.16) with diabetes participated in this cross-sectional study. Participants completed validated measures, including the Summary of Diabetes Self-Care Activity (SDSCA), Center for Epidemiologic Studies Depression Scale (CES-D), Perceived Stress Scale (PSS), Partner Strain, Parental Stress Scale, Diabetes Distress, Everyday Discrimination, and Cost-FACIT. Descriptive statistics and correlational analyses examined the relationships between stressors and self-management behaviors.

**Results:** Participants reported moderate levels of financial, relational, and perceived stress, and diabetes distress. Higher levels of financial stress were associated with poorer diabetes self-care activities ($r = -0.628$, $p < 0.05$). Emotional burden ($r = 0.804$, $p < 0.01$) and interpersonal distress ($r = 0.600$, $p < 0.05$) are significantly correlated with perceived stress. Financial stress is significantly associated with everyday discrimination ($r = 0.508$, $p < 0.05$).

**Conclusion:** Despite the small sample size, our findings highlight the intricate interplay between diabetes distress, financial stress, relationship stress, and self-management behaviors among rural African Americans with diabetes. Tailored interventions addressing these stressors and promoting effective coping strategies may be crucial in improving diabetes management outcomes in this population. Further research with larger samples is warranted to confirm these findings and inform targeted interventions for better diabetes care in rural African American communities.

Abstract citation ID: igae098.3012

### FACTORS ASSOCIATED WITH COMMUNITY-DWELLING OLDER ADULTS' VIEWS OF DEPRESCRIBING: A PILOT STUDY

Hyunjin Noh, Lewis Lee, Haelim Jeong, Lauryn Emrick, Madison Hasnani, and Wendy Waddell, *University of Alabama, Tuscaloosa, Alabama, United States*

Ample evidence exists on the risks of adverse drug interactions, calling for reducing or stopping ineffective or harmful medications (i.e., describing). However, limited research exists on the views of deprescribing and associated factors among older adults. To fill this gap, this study aimed to examine factors associated with the views of deprescribing among older adults living with multimorbidity and chronic pain. Using purposeful sampling, 132 participants were recruited for a phone survey, who were 65+, living outside of nursing homes, cognitively intact, have two or more chronic illnesses and chronic pain, and take medications for the illnesses, including pain medicine. The revised patient attitudes towards deprescribing (rPATD) questionnaire encompassed five dependent variables: Burden, appropriateness, concerns about stopping, involvement, and willingness to discontinue regular medications. Independent variables included sociodemographic/pain-related variables. A series of regression analyses showed a positive association between the number of medications and the burden factor. Pain scores were positively associated with the belief of medication-induced side effects. The number of chronic health conditions was inversely associated with concerns about cessation, while pain scores were positively related to the same outcome. Age and race were negatively related to the involvement factor. Lastly, gender and marital status were significantly related to the inclination to discontinue regular medications. Findings suggest targeted education efforts for those with a higher number of illnesses, pain, and medications to demystify their concerns about deprescribing and reduce their medication-related burden. Furthermore, advocacy efforts are needed to support older and Black older adults' involvement in medication decision-making.

Abstract citation ID: igae098.3013

### FILLING THE CARE GAP FOR OLDER ADULTS LIVING IN A RURAL COMMUNITY: A NURSING CARE MODEL

Walter Dawson, Dana Womack, Lace Velk, Alexis Fifield, and Seiko Izumi, *Oregon Health & Science University, Portland, Oregon, United States*

Ensuring older adults living with multiple chronic conditions are able to remain in their own homes and communities for as long as possible is a health policy priority. Registered nurses (RNs) play a key role in assisting older adults manage their health while living in the community. Yet, the substantial contributions of nursing care such as care management and care coordination in achieving better health outcomes and reductions in healthcare costs remains largely invisible to healthcare systems and policymakers alike. To demonstrate value of nursing care and build a model of nursing services that is effective and sustainable, we conducted a study describing nursing services needed by the patients living in community settings with complex care needs and evaluated the impact of these services on outcomes. Nurses delivering community-based nursing service (CBNS) to patients living with multiple chronic conditions in rural coastal Oregon were interviewed between July and December, 2022. Outcome data about health service utilizations and expenditure of patients who received CBNS are collected between November 2023-March 2024. Interview data describe care nurses deliver to keep older adults in the community while managing their health by filling care gaps in the rural community. Preliminary outcome data analysis suggests that CBNS is a potentially effective model helping older adults remain in their own homes and communities. These findings may inform policy development regarding reimbursement to make CBNS delivery sustainable as well as increase the options for providing optimal care for older adults living with chronic conditions across multiple geographic settings.

Abstract citation ID: igae098.3014

### HEALTH CARE PROVIDER PERSPECTIVES ON MENTAL HEALTH CARE WITHIN CHRONIC CARE MANAGEMENT: A RURAL STUDY

Elizabeth Punke[1], Lucas Wall[2], Barbara Dabrowski[1], Abby Teply[1], Katy Richardson[2], Catherine Carrico[1], and Christine McKibbin[1], *1. University of Wyoming, Laramie, Wyoming, United States*, *2. UWYO, Laramie, Wyoming, United States*

Approximately 59% of older adult Medicare beneficiaries experience two or more chronic conditions. This rate is even higher among those dually-enrolled in Medicare and Medicaid (i.e., 77%; Boersma et al. 2021). In 2015, the Centers for Medicare and Medicaid Services introduced Chronic Care Management (CCM), a value-based care program, to provide reimbursement to practices for care coordination services that support the management of patients with multimorbidity. Importantly, physical and mental health conditions are often interrelated; however, providers often struggle to adequately treat mental health needs in the context of physical illness (Lattie et al., 2020). The purpose of this study was to examine healthcare providers' perspectives on serving individuals with mental health diagnoses enrolled in CCM within primary care practices. Investigators developed an interview for this qualitative study. Employees of six practices that have implemented CCM participated. Participants completed sociodemographic, professional, and practice history measures, and a qualitative interview. Participants (n = 13) were predominantly White, cisgender females (n = 12; 92%) and represented care coordinators, primary care providers, allied health professionals, and administrators. All participants completed a 60-minute interview, which was audio recorded, transcribed verbatim, and subjected to thematic analysis. Four themes were identified: Types of Chronic Health Conditions Treated, Access to Mental Health Services, Provider Experience and Comfort, and Educational Needs. Participants expressed a willingness to complete mental health training but faced challenges identifying particular training needs. Future training efforts should address foundational knowledge in mental health disorders and strategies to serve people with common mental health comorbidities.

Abstract citation ID: igae098.3015

### HEART FAILURE AND SIT-TO-STAND SCORE ON LOWER BODY MOBILITY DISABILITY: NATIVITY DIFFERENCES

Melanie Navos[1], and Soham Al Snih[2], *1. The University of Texas Medical Branch, Baytown, Texas, United States, 2. University of Texas Medical Branch in Galveston, Galveston, Texas, United States*

We examined nativity differences in the relationship of heart failure and five-times sit-to-stand scores with lower body mobility disability over 12-years of follow-up among Mexican-American aged ≥ 75 years who were non-disabled at baseline. Participants (N=987) were from the Hispanic Established Population for the Epidemiologic Study of the Elderly (2004/05-2016). Measures included socio-demographics, heart failure (HF), multimorbidity, falls, depressive symptoms, cognitive function, handgrip strength, sit-to-stand (STS) test, and lower body disability (limitations in ≥ 1 of transferring, bathing, toileting, and walking across the room). Participants were grouped into four groups: No HF-high STS (n=402), no HF-low STS (n=381), HF-low STS (99), HF-high STS (n=105). We used the generalized estimating equation models to estimate the odds ratio (OR) and 95% confidence interval (CI) of lower body disability as a function of HF and STS groups. Compared to those with No HF-high STS group, foreign-born in the HF and high STS group had lower odds (OR=0.48, 95% CI=0.24-.95) of reporting mobility disability over time after controlling for all covariates. Foreign-born and US-born in the groups of HF and low STS had greater odds (OR=2.31, 95% CI=1.14-4.67 and 7.03, 95% CI=3.57-13.81) of reporting mobility disability over time after controlling for all covariates. In conclusion, Mexican-American older adults with HF who performed high in five-times STS were at lower risk for lower body disability and the risk differed by nativity. Interventions to improve strength in lower extremities in those with HF may prevent or reduce disability in this population

Abstract citation ID: igae098.3016

### KNEE OSTEOARTHRITIS: PERCEPTIONS OF PSYCHOSOCIAL CONSEQUENCES, PAIN, AND MANAGEMENT COMPLEXITIES

Mark Luborsky[1], Marc Hochberg[2], Alice Ryan[2], Yu Dong[2], Brock Beamer[2], Michelle Shardell[2], and Alan Rathbun[2], *1. Wayne State University, Detroit, Michigan, United States, 2. University of Maryland School of Medicine, Baltimore, Maryland, United States*

Knee Osteoarthritis (KOA) afflicts 27 million U.S. adults and has adverse health and psychosocial outcomes. Depression is twice as common among persons with KOA compared to the general population and is a barrier to pain management and physical activity. While guidelines advise depression treatment, directions on co-managing depression and pain are seldom offered; a viable adjunctive treatment, duloxetine, treats both depression and pain, yet is not routinely prescribed and could facilitate physical activity. Progress in reducing KOA distress is hampered by inadequate knowledge of perceptions and experiences of persons' daily living with KOA especially when pain and depression co-occur. Qualitative interviews were conducted at baseline in a program evaluating aerobic exercise plus duloxetine to learn participants' perceptions, challenges, and how depression and pain interact in managing KOA. The sample included: n=12 (5 African American, 1 Asian, 6 White) participants, aged 41-71 years (10 female, 2 male) with self-report pain severity, median 3 (scale 0-4) and moderate to severe difficulty with Activities of Daily Living. Analytic strategies featured topic, theme, and critical case comparative methods. Results identified four key constructs: (a) unpredictability and temporal variability of impacts of KOA; (b) management requiring flexible strategies encompassing a wide range of self-image and life domains beyond pain and depression; (c) barriers including concerns with medications; depression eroding energy; a sense of isolation socially and from meaningful activities; and (d) challenges to program participation. Pathways to address the constellation of intersecting domains perceived in living with KOA and treatment program participation are needed.

Abstract citation ID: igae098.3017

### LONELINESS AND SELF-EFFICACY AMONG INDIVIDUALS WITH CHRONIC CONDITIONS: THE MEDIATING ROLE OF DEPRESSION

Barbara Dabrowski, Christine McKibbin, Catherine Carrico, Laurance Goodwin, Stacy Carling, Elizabeth Punke, Abby Teply, and Sabine Schenck, *University of Wyoming, Laramie, Wyoming, United States*

Approximately 60% of Americans live with one or more chronic conditions (CDC, 2023). Chronic disease self-management programs empower individuals to take an active role in their health management. Self-efficacy is an important construct determining self-management behaviors. Recently, low self-reported ratings of self-efficacy for managing chronic disease have been associated with high loneliness ratings (Lee et al., 2023); however, other variables that may be important regarding the relationship between loneliness and self-efficacy are less known. Depressive symptoms are associated with both loneliness and management of chronic disease. This study examined the mediating role of depressive symptoms in the relationship between loneliness and self-efficacy among individuals with chronic disease participating in the Self-Management Resource Centers, Chronic Disease Self-Management Program (CDSMP). The sample ($n = 48$, $M = 63.0$ years old, $SD = 18.0$) comprised primarily White females ($n = 37$; 77%) who presented with chronic conditions and completed a CDSMP workshop. Regression analyses revealed that baseline loneliness predicted baseline depressive symptoms ($\beta = .49$, $p < .001$). Both baseline loneliness ($\beta = -.46$, $p < .006$) and depressive symptoms ($\beta = -.75$, $p < .001$) predicted baseline self-efficacy. The relationship between loneliness and self-efficacy became non-significant ($\beta = -.12$, $p = .301$) after controlling for depressive symptoms. Examination of loneliness and depression to changes in self-efficacy pre- to post-intervention showed similar results. Depressive symptoms are important in the relationship between loneliness and self-efficacy among participants in the CDSMP. Addressing depressive symptoms may support improvement in self-efficacy among CDSMP participants experiencing loneliness.

Abstract citation ID: igae098.3018
### MULTIMORBIDITY RESEARCH: CHALLENGES IN RECRUITMENT AND RETENTION OF OLDER ADULTS
Binu Koirala[1], Chitchanok Benjasirisan[1], Arum Lim[1], Jordan Tebay[1], Cheryl Dennison Himmelfarb[1], and Patricia Davidson[2], *1. Johns Hopkins University, Baltimore, Maryland, United States*, *2. University of Wollongong, Wollongong, New South Wales, Australia*

With an aging population, medical advancement, and longer life expectancies, the prevalence of multimorbidity is increasing. This demands high-quality health research to tailor and develop effective multimorbidity prevention and management programs. Recruitment and retention are always a concern in research, and it could be more challenging and time-consuming for older adults. This session aims to identify and discuss challenges for the recruitment and retention of older adults with multimorbidity in health research from the learnings of our projects. We conducted four studies among older adults living with multimorbidity: two experience-based co-design studies to understand disease trajectory and symptom burden (n=9 patients; n=2 family caregivers; n=11 healthcare providers) and gender differences on care preferences and outcome (n=24 patients; n=7 family caregivers;15 healthcare providers); a quasi-experimental study implementing a care-coordination and symptom management (COORDINATE) program (n=25); and a cohort study in gender differences in health outcomes (n=353). Recruiting and retaining older adults in research could be affected by multi-level factors including social and structural determinants of health. These challenges can be discussed under 1) individual factors (participants' biological, physical, psychological, social status, work commitments, financial strain/availability of research instruments such as phones and capacity to use digital devices and electronic portals), research-related factors (availability of the research team, effective communication techniques, and survey design) and system-level factors (limited universal definition of multimorbidity, health and technology access). Our study demonstrated the feasibility of research among older adults living with multimorbidity, yet challenges exist in recruitment and retention and require significant resources and planning.

Abstract citation ID: igae098.3019
### NETWORK ANALYSIS OF INTEROCEPTIVE SENSIBILITY IN YOUNGER AND OLDER LIVER TRANSPLANT RECIPIENTS
JiYeon Choi, Seongmi Choi, Hanna Lee, and Sang Hui Chu, *Yonsei University College of Nursing, Mo-Im Kim Nursing Research Institute, Seoul, Republic of Korea*

Due to surgical advancements and post-transplant therapeutics, liver transplantation (LT) is increasingly viable for older adults with end stage liver conditions, such as hepatocellular carcinoma and non-alcoholic steatohepatitis. Effective post-LT symptom management is crucial for optimizing benefits of LT for older liver transplant recipients (LTRs). Interoceptive sensibility, which involves perceiving and being aware of internal body sensations, plays a vital role in symptom perception and response. Understanding interoceptive sensibility patterns across age groups may help tailor symptom management strategies for older LTRs. This study explored interoceptive sensibility in LTRs using network analysis on a sample of 200 Korean LTRs divided into two age groups: younger LTRs (< 60 years; n=122), and (2) older LTRs (≥ 60 years; n=78). Interoceptive sensibility was assessed using the Multidimensional Assessment of Interoceptive Awareness (version 2), comprising eight domains: noticing, not distracting, not worrying, attention regulation, emotional awareness, self-regulation, body listening and trusting. Network analysis examined centrality of nodes in the network for each age group. Bootstrapping (1,000 iterations) assessed network stability and accuracy. In younger LTRs, attention regulation reported the largest values for betweenness (rs=1.882), closeness (rs=1.585) and strength (rs=1.140). In older LTR, attention regulation domain reported the largest values for betweenness (re=1.666) and closeness (rs=1.907), while emotional awareness domain (rs=1.180) had the largest values for strength. Networks exhibited stability (correlation stability coefficient ≥ 0.5) and accuracy (small confidence interval). Addressing central interoceptive sensibility domains, particularly emotional awareness, may improve the tailored symptom management strategies for older LTRs, potentially enhancing post-LT outcomes.

Abstract citation ID: igae098.3020
### OLDER AGE AND DEPRESSION AS THE RISK FACTORS FOR HYPERTENSION AMONG NORTH KOREAN DEFECTORS
Sang Hui Chu[1], Oksim Kim[2], and Hokon Kim[2], *1. Yonsei University College of Nursing, Mo-Im Kim Nursing Research Institute, Seoul, Republic of Korea*, *2. Yonsei University College of Nursing, Seoul, Republic of Korea*

Observations indicate a low obesity prevalence among North Korean Defectors (NKRs), who fled to South Korea, attributed to undernourishment prior to their arrival. With an aging NKR demographic, there's growing concern over chronic disease management, including hypertension. Previous studies reported a high or comparable prevalence of elevated blood pressure among NKRs, yet the influence of mental health on hypertension has been underexplored. Given the high rates of trauma-related conditions and depression within this group, these factors could significantly elevate hypertension risk. Understanding hypertension risk factors across different age groups could inform tailored management strategies for older NKRs. This study assessed the impact of mental health on hypertension among 449 NKRs categorized into three age groups: younger (< 30 years; n=141), middle-aged (40-59 years; n=243), and older (≥ 60 years; n=65). The assessment of Post-Traumatic Stress Disorder (PTSD)/complex PTSD (CPTSD) employed the International Trauma Questionnaire (ITQ), covering six domains: re-experiencing, avoidance, sense of threat, affective dysregulation, negative self-concept, and disturbance in relationships. Hypertension and depression were identified through self-reported diagnoses. Logistic regression analysis revealed that, in addition to older age, depression was significantly associated with hypertension, but not PTSD/CPTSD, after controlling for gender, living alone, and residency period in South Korea. These findings suggest the necessity of prioritizing mental health improvement, especially depression, in hypertension management among NKRs. This work was supported by the Basic Science Research Program through the National Research Foundation (NRF) of Korea funded by the Ministry of Education (2019R1I1A2A01058746).

Abstract citation ID: igae098.3021

**OUTCOMES OF AN INTEGRATED HEALTH CARE AND COMMUNITY PROVIDER INTERPROFESSIONAL TEAM**

Jennifer Drost[1], Michelle Hughes[2], Sue Fosnight[1], Joseph Marchiano[1], Susan Hazelett[1], Michele Gareri[1], and Brandi Chrzanowski[2], *1. Summa Health, Akron, Ohio, United States*, *2. Direction Home, Akron Canton Area Agency on Aging and Disability, Uniontown, Ohio, United States*

Traditional medical care accounts for 10% of health outcomes, while social determinants of health account for over 60%. To address complex interactions between medical illness, social and behavioral health issues, and medications, effective care requires collaboration between medical and community-based providers. We have developed a collaborative interprofessional model of care between a health system and the local Area Agency on Aging (AAoA) called the Care Management Interdisciplinary Team (CMIT). CMIT care managers (social workers and nurses) complete evaluations of consumers focused on concerns around recent health care utilization (ED visits/hospitalizations), falls, medications, home safety, and behavioral health concerns. Care managers (CM) then present these complex older adults to the team, including geriatric medicine and pharmacy specialists, which develops care plans that are implemented by the CM and the consumer's primary care physician. CM presented cases between January-December 2023 and worked to follow up at 3 months. Eighty-one consumers were presented to the CMIT team over a 1-year period. A 3-month follow up is available for 44 consumers (54%). Of those with 3-month follow ups, at baseline, 10 (23%) consumers had recent health care utilization episodes, 17 (39%) had concerns about falls, 17 (39%) had concerns about medications, 21 (48%) had home safety concerns, and 10 (23%) had behavioral health concerns. At the 3-month follow up, 5 consumers had improved health care utilization, 6 fewer had falls, 5 had medication changes, 13 had improved home safety concerns, and 7 had improved behavioral health concerns.

Abstract citation ID: igae098.3022

**REDUCING OR STOPPING MEDICATIONS FOR CHRONIC PAIN: VIEWS AMONG COMMUNITY-DWELLING OLDER ADULTS**

Hyunjin Noh, Haelim Jeong, Denise Kan, and Lewis Lee, *University of Alabama, Tuscaloosa, Alabama, United States*

Effective pain control is vital in enhancing quality of life. As the dangers of adverse drug interactions are recognized, there is a shift towards reducing reliance on medications that could be ineffective or harmful (known as deprescribing) and a push for embracing non-drug methods in managing pain. However, limited research exists on how older adults perceive deprescribing. Therefore, this study aimed to understand the views of deprescribing among community-dwelling older adults living with multiple health conditions and chronic pain. Participants were recruited from the Alabama Area Agency on Aging offices. Eligible participants were 65+, Alabama residents living outside of nursing homes, cognitively intact, have more than one chronic illness and chronic pain, and take medications for the illnesses, including pain medicine. Individual, open-ended interviews were conducted by phone, asking about their experiences with taking multiple medications and thoughts about deprescribing. Thematic analysis of interviews with 30 participants revealed facilitators, barriers, and needs for deprescribing. Facilitators for deprescribing included interest in more holistic methods for pain management, concerns for long-term side effects, and desire for better quality of life, while barriers included fear of worsening pain, reluctance to change in medical regimen, and lack of support from healthcare providers. Needs for deprescribing included credible information from their healthcare providers about deprescribing and social support in deprescribing decision-making. These findings suggest that older adults may benefit from education and advocacy efforts designed to improve their knowledge and self-efficacy and healthcare providers' willingness to openly communicate with older adults about deprescribing.

Abstract citation ID: igae098.3023

**SYMPTOM BURDEN AND HEALTH CARE UTILIZATION AMONG OLDER ADULTS LIVING WITH MULTIMORBIDITY: THE COORDINATE PROGRAM**

Chitchanok Benjasirisan[1], Arum Lim[1], Jordan Tebay[1], Cheryl Dennison Himmelfarb[1], Patricia Davidson[2], and Binu Koirala[1], *1. Johns Hopkins University, Baltimore, Maryland, United States*, *2. University of Wollongong, Wollongong, New South Wales, Australia*

The increasing challenges of multimorbidity in older adults underscore the need for sustainable approaches to address care coordination and symptom management. The Care cOORDInatioN And sympTom managEment (COORDINATE) program was developed using experience-based co-design and human-centered principles by considering the needs of patients, family caregivers, and healthcare providers. The program included a need assessment, question prompt list, goals discussion, and symptom assessment in 5 visits over 6-week periods employed among adults aged 55 years and older who were living with at least 2 chronic conditions and were discharged from the intermediate care unit. Twenty-five adults agreed to participate and completed the baseline survey. The change in symptom burden and healthcare utilization at 3 months follow-up compared to baseline was examined using the Wilcoxon Signed-Rank test. The participants' mean (± SD) age was 67 ± 7 years; 52% were women, 44% had some college/associated degree; 92% were retired/unemployed, and 72% were Black. For the goals discussion component of the program, 83% of the measurable goals were fulfilled. A preliminary analysis of the symptom burden found that the level of energy significantly improved at the 3-month follow-up ($p = .032$). Significant decreases in hospitalization ($p = .01$) and critical care admission ($p < .01$) were found at 3 months post-discharge compared to 3 months before program initiation. In summary, the COORDINATE study could be a pivotal initiative in addressing the holistic care needs of older adults living with multimorbidity. This intervention demonstrates promising outcomes, signaling the need for a larger effectiveness trial.

Abstract citation ID: igae098.3024
### THE CORRELATION BETWEEN SHBG AND BLOOD LIPID AND GLUCOSE AMONG CHINESE MIDDLE-AGED AND OLDER ADULTS
Meng Cao[1], Yaping Jin[1], Huili Yang[2], Yuanbin Li[1], Minhui Liu[1], and Chongmei Huang[1], *1. Ningxia Medical University, Yinchuan, Ningxia, China*, *2. Ningxia Medical University, Yinchuan, Ningxia, China (People's Republic)*

To investigate the correlation between sex hormone binding globulin (SHBG) of serum lipid and blood glucose in middle-aged and older adults (≥ 45 years old) We recruit participants in five urban communities in Ningxia province, using a cluster stratified sampling method. Data from 1681 participants were analysed, with an average age of 63.53 ± 9.5 years old. The results showed the average level of TC, HDL, LDL, and blood glucose were lower in men than in women (P=0.000,P=0.000,P=0.000,P=0.004),LDL-c and FBG levels increased with age in men, but SHBG level decreased with age in women (P< 0.05). The results of spearman correlation analysis showed a negative correlation between SHBG and FBG (r=-0.73 P< 0.01). There was a positive correlation between age (r= 0.054 P< 0.05) and HDL-C (r=0.049 P< 0.05) and a positive correlation between SHBG level and TC (r=0.218 P< 0.05) and TG (r=0.254 P< 0.05) in Chinese women aged 75 years and more. Multiple linear regression analysis with SHBG as the dependent variable and gender, age, TC, TG, HDL-C, LDL-C and BMI as independent variables did not find statistically significant associations. Logistic regression analysis showed that sex, age, weight, SHBG,FBG and SH were risk factors for dyslipidemia. However, there was no significant correlation between SHBG level and dyslipidemia.

Abstract citation ID: igae098.3025
### THE IMPACT OF EHEALTH ON SELF-MANAGEMENT AND HEALTH OUTCOMES OF PATIENTS WITH TYPE 2 DIABETES: A SYSTEMATIC REVIEW
Yu-Shan Cheng, and Heather Cuevas, *The University of Texas Austin, Austin, Texas, United States*

Self-management is crucial to maintain glycemic control in type 2 diabetes (T2DM). Prior studies have shown that various electronic health (eHealth) technologies promote diabetes self-management (DMSM) and continuity of care. However, previous reviews focused mainly on telehealth, a single aspect, and were published more than ten years ago. Therefore, the purpose of this study was to summarize the effect of eHealth interventions on improving aspects of DMSM and health outcomes, such as depression and A1C, for adults over 60 years of age with T2DM. A systematic review was conducted on papers published from October 2018 to October 2023. Three electronic databases were used. 2020 Preferred Reporting Items for Systematic Reviews and Meta-Analyses (PRISMA) served as guides. Twenty randomized control trials in diverse ethnic communities (n=3978) across 20 countries were included in the narrative synthesis. Seven health technologies were employed to provide DMSM across studies, including mobile health applications, text messaging, phone calls, online conferences, video, wearable devices, and virtual worlds. Overall, improvement in DMSM and health outcomes was mixed. In terms of depression, only three studies showed significant improvement, while four studies did not exhibit significant improvement in A1C. Additionally, positive outcomes were not maintained after seven months (the furthest post-intervention measurement). These findings differed according to eHealth literacy, economic burden, willingness, and social support. Further work is needed to consider other variables to address both short- and long-term effects for the patients while implementing healthcare technology.

### SESSION 7445 (POSTER)

### CIVIC ENGAGEMENT

Abstract citation ID: igae098.3026
### BLACK STRUCTURAL RESILIENCE ACROSS THE LIFESPAN
Boeun Kim[1], Alicia Cooke[2], Tiffany Riser[2], and Sarah Szanton[2], *1. University of Iowa, Iowa, United States*, *2. Johns Hopkins University, Baltimore, Maryland, United States*

Resilience, which is the ability to resist, recover, or adapt in response to adverse events, can be cultivated through interactions with societal, community, family, and individual factors across the life course. However, structural resilience, signifying the extent of recruiting resources at societal and community levels, has been overlooked. Furthermore, no study has examined structural resilience using a life course approach, although structural resilience is likely to change over time. This study employed a qualitative descriptive design to understand better the structural resilience and their supportive roles in maintaining or promoting health over the life course through older African Americans' lived experiences. This study focused on African Americans, who were known to have higher levels of individual resilience than White Americans, to learn what resources helped them to thrive despite adverse circumstances. We conducted semi-structured interviews with 15 self-identified African American adults aged 50 and older living in Baltimore City. Two study team members coded the transcripts using an inductive approach, and themes were generated from the constant comparative approach. The five themes emerged: 1) underwent extraordinary adversities over the life course, 2) accessible resources during childhood, adulthood, and late adulthood, 3) emergence of grassroots organizations to provide support, 4) roles of structural resilience in response to adversities, and 5) dynamics of structural resilience across the life course. This study contributes to a better understanding of the mechanisms that preserve health under stressors. The findings may help to resolve the public health tragedy of health equity faced by older African Americans.

Abstract citation ID: igae098.3027
### DEVELOPING A MODEL FOR COLLEGE STUDENT VOLUNTEERING IN RESPITE CARE
Marta Rodriguez-Galan, *St. John Fisher University, Rochester, New York, United States*

The purpose of our study is to evaluate college student volunteer's learning on aging and caregiving through EXHALE, a grant-funded pilot respite program for family caregivers developed by Lifespan or Greater Rochester and St. John Fisher University. We are sharing the results from the first year of student participation (the program will be funded for 3 years). In addition to promoting student service learning in the field of aging, this evaluation will help in the development of a toolkit that can be used by other higher education institutions. The specific aims of the evaluation research are: 1) To evaluate students' changes regarding attitudes towards aging, specifically towards frail older adults 2) To evaluate specific areas of learning regarding aging that were more salient in their volunteer experience (e.g. working with older adults with dementia, working with low-income minority elders, etc.) 3) To identify content areas for the development of the "toolkit" that will be used as a model for training future volunteers in higher education institutions. To assess student learning in the program we used the following three
**methods:** 1) The Kogan's attitudes towards older people scale; 2) Student Reflections after each volunteer session at the respite care; 3) Bi-weekly debrief sessions with the professor. Furthermore, one of the student volunteers also served as a research assistant during the summer and received more personalized mentoring. Our findings show that students challenge some of their initial attitudes on aging, and gain knowledge about caregiving and cultural competency through this volunteering program.

Abstract citation ID: igae098.3028

### EXPLORING GENERATIVITY AMONG CULTURALLY DIVERSE OLDER ADULT VOLUNTEERS

Eireann O'Dea[1], Andrew Wister[1], Sarah Canham[2], Lun Li[3], and Barbara Mitchell[1], *1. Simon Fraser University, Vancouver, British Columbia, Canada*, *2. University of Utah, Salt Lake City, Utah, United States*, *3. MacEwan University, Edmonton, Alberta, Canada*

Generativity, the desire to look beyond the self, and teach and guide the next generation, has been described by researchers as an important component of successful aging and a key part of why older adults choose to engage in activities such as volunteering. Recent research on generativity, including Rubinstein et al's generativity framework (2015), describes how the expression of generativity can be influenced by cultural context, including traditions, sense of heritage, and family relationships. Despite this, so far little research has focused on generative expression among older adults with diverse cultural backgrounds, such as those belonging to ethnocultural minorities. Using Rubinstein's framework and life history interview as a guide, this study explores generativity among older adult volunteers who belong to ethnocultural minority communities in Vancouver, British Columbia, Canada. 14 participants aged 65 and over participated in in-depth interviews about various life course experiences, in order to bring contextual understanding to their generativity and volunteer activities in later life. Preliminary findings suggest the unique ways in which generative action can develop over the life course, and how it can potentially be influenced by a sense of belonging to an ethnocultural group. Results also speak to the contributions that are made by ethnocultural minority older adults as volunteers in their communities.

Abstract citation ID: igae098.3029

### RACIAL DIFFERENCES IN THE RELATIONSHIPS AMONG VOLUNTEERING, DISCRIMINATION, AND LIFE SATISFACTION

Huei-Wern Shen[1], and Yi Wang[2], *1. University of North Texas, Denton, Texas, United States*, *2. University of Iowa, Iowa City, Iowa, United States*

Discrimination, whether overt or subtle, has been consistently linked to lower life satisfaction across racial groups. While volunteering has been proven to bring physical and mental health benefits, its potential role in mediating the relationship between discrimination and life satisfaction across different racial groups is unknown. The present study included a subsample of 3115 older adults (65-98) who were non-Hispanic Whites (n=2374), non-Hispanic Blacks (n=449), and Hispanics (n=292) from the 2016 Health and Retirement Study. Discrimination was measured by the six-item Everyday Discrimination Scale (e.g. In your day-to-day life, how often are you treated with less courtesy or respect than other people) and was dummy-coded due to skewness (never experienced vs. ever experienced). Controlling for health-related variables (e.g., self-rated health), SES (e.g., income), contextual features (e.g., urban/rural), and sociodemographics (e.g., gender), findings from stratified multivariate linear regression models showed that non-Hispanic Whites who experienced discrimination reported lower life satisfaction; although older volunteers had higher life satisfaction, volunteering did not mediate the relationship between discrimination and life satisfaction. Among non-Hispanic Black older adults, neither experiencing discrimination nor volunteering was related to life satisfaction. In the Hispanic group, however, experiencing discrimination was related to lower life satisfaction, and volunteering partially mediates the negative impacts of discrimination on life satisfaction. These findings shed light on the possible extended benefits of volunteering in old age. Further research is warranted to better understand the underlying mechanisms through which volunteering may mitigate the negative impact of discrimination on life satisfaction, particularly among Hispanic older adults.

Abstract citation ID: igae098.3030

### WHAT FACILITATES INFORMAL SUPPORT AMONG COMMUNITY-DWELLING OLDER PEOPLE? THE ROLE OF CARING INITIATIVES

Wenran Xia, Jeroen van Wijngaarden, Martina Buljac-Samardzić, and Robbert Huijsman, *Erasmus University Rotterdam, Rotterdam, Zuid-Holland, Netherlands*

**Background:** In the context of accelerated global ageing and cutbacks in healthcare budgets, older people are encouraged to provide informal support to each other within their communities. However, the mechanism facilitating such informal support among older people is still not clear. This study aims to address this gap by investigating the perspectives and experiences of various stakeholders involved in initiatives aimed at stimulating informal support in the community setting. Method: A multiple case study was conducted in five initiatives in the Netherlands focused on facilitating informal support for older people. Semi-structured interviews were conducted with different stakeholders, including older residents who are informal support givers and receivers as well as coordinators and managers of the initiatives. A total of twenty-four participants were interviewed in twenty-one interviews. An inductive-deductive thematic analysis approach was conducted for the qualitative data.
**Results:** Analysis revealed that older people are facilitated to participate in the community-based informal support when they have abilities, are being motivated, and have opportunities to participate in informal support. Social cohesion in the community strengthens this mechanism. Additionally, community-based formal organizations contribute to stimulate on both the individual and community levels. Conclusions: This study explores the mechanisms facilitating informal support among community-dwelling older people. It emphasizes the combination of factors at the individual, community, and organizational levels. Relevant policies and practice should be formulated with consideration of these multi-level factors.

### SESSION 7450 (POSTER)

### CLINICAL TRIALS

Abstract citation ID: igae098.3031
### EFFECTIVENESS OF TARGETED WEB-BASED ADVERTISING WHEN RECRUITING OLDER ADULTS FOR A CLINICAL TRIAL
Kathryn Baldyga[1], Courtney Millar[1], Ike Iloputaife[1], George Taffet[2], and Lewis Lipsitz[3], *1. Hebrew SeniorLife, Roslindale, Massachusetts, United States*, *2. Houston Methodist Hospital, Houston, Texas, United States*, *3. Hebrew SeniorLife, Boston, Massachusetts, United States*

Recruiting older adults for clinical trials can be particularly challenging. Our objective was to determine if targeted web-based advertising is an effective recruitment strategy. The STAMINA Study (Senolytics To Alleviate Mobility Issues and Neurological Impairments in Aging) is a pilot 12-week study of the effects of intermittent Quercetin and Dasatinib on cognition and mobility in older adults with mild cognitive impairment and slow gait. Individuals were initially recruited using traditional strategies such as presentations and advertisements in clinical practices, subject registries, and community sites. After 10 months of slow recruitment rates, a web-based advertising company was used to deliver targeted online study advertisements based on an individual's browsing history. Recruitment rates were calculated based on the total number of individuals engaged in each stage of the recruitment process, divided by the number of months the strategy was utilized. Web-based advertising reached more individuals (~40 individuals/month) compared to traditional methods (~5 individuals/month). Compared to traditional methods, web-based methods had at least twice the rate of expressed interest, telephone and in-person screening completion, eligibility, and enrollment. All individuals recruited from traditional methods completed the intervention, while 3 individuals recruited from web-based advertising dropped out. Those recruited using web-based advertisements tended to be younger compared to traditional methods, but were similar in racial distribution and education. Targeted web-based advertisements may be more effective in recruiting older adults for clinical trials than traditional recruitment methods, but need further evaluation of compatible study designs, potential population bias, and cost effectiveness.

Abstract citation ID: igae098.3032
### IMPACT OF ART-BASED INTERVENTION ON BRAIN FUNCTIONAL CONNECTIVITY IN OLDER ADULTS ALONG THE ALZHEIMER'S CONTINUUM
Chenshan Huang[1], Yuanjiao Yan[2], Yanhong Shi[1], Rong Lin[1], and Hong Li[1], *1. Fujian Medical University, Fuzhou, Fujian, China (People's Republic)*, *2. Fujian Provincial Hospital, Fuzhou, Fujian, China (People's Republic)*
**Background:** Combined art-based interventions (CAIs) are considered effective treatment options for older adults along the Alzheimer's disease (AD) continuum; however, the neural mechanisms underlying associated changes in neurocognitive performance remain unclear. Thus, we aimed to investigate the impact of a CAI programme in older adults along the AD continuum and to understand its mechanism.
**Methods:** This parallel-arm randomised controlled trial was conducted between April 2021 and January 2023. Participants were randomised in a 1:1 ratio to either intervention group (IG) or waitlist control group (WG). The IG underwent a 16-week CAI programme. Neuropsychological assessments and magnetic resonance imaging were conducted before and after the intervention.
**Results:** After the intervention, the IG showed greater improvement in general cognitive function, language, and memory than the WG. Significant differences were observed in the functional connectivity (FC) values in the temporal and cerebellar anterior lobes, fusiform, inferior occipital, and lingual gyri, and perirhinal and visual cortices between the groups. Further analyses showed that FC values reduced in these regions in the IG. In addition, changes in FC values were positively correlated with those in neuropsychological test scores in the IG.
**Conclusions:** Our study suggests that the CAI programme can effectively improve general cognitive function, language, and memory in older adults along the AD continuum. These improvements may be changed due to decreases in FC in key brain regions, deepening the understanding of the neurocentral mechanisms that act as a tool for improving cognitive function.

Abstract citation ID: igae098.3033
### RECOMMENDATIONS TO IMPROVE AGING PAIN INTERVENTION RESEARCH ON MINORITIES: A SCOPING REVIEW
Avani Shah, and McKenzie Brown, *University of Alabama, Tuscaloosa, Alabama, United States*

Limited information is available for evidence supported psychological treatment options for pain in minority populations. The purposes of this scoping review were to determine if psychological treatments were 1) evaluated for minority populations; 2) if minorities were sufficiently included in clinical trials 3) if there were trials that were specific to minority populations. A search was conducted in PsycINFO, MEDLINE, and CINAHL databases for psychological interventions for pain in older adults over the age of 50. We also searched review articles. Search terms were chronic pain OR pain; older adults OR elderly OR geriatric OR aged OR human; pain management OR nonpharmacological pain management OR intervention OR psychotherapy; randomized control trial. Papers had to be in English, a randomized clinical trial, with pain as the primary outcome, and the treatment had to be a psychological intervention. We coded studies that had at least a 90% minority sample as a trial specific to a minority population or if one of the groups was minority. From 14,000 studies identified by the search, we reviewed 485 full texts. Of those,129 psychological interventions studies on older adults with chronic pain met inclusion and exclusion criteria. There were 6 African-American, 7 Asian,1 Turkish, and 2 Iranian samples. Studies represented 24 countries. However, 72 studies did not provide enough information about ethnicity or race. The lack of reporting of race and ethnicity took place across many disciplines and journal types. All journals should require reporting ethnic and racial demographics of participants to increase generalizability of findings.

Abstract citation ID: igae098.3034

### TESTING THE IMPLEMENTATION FRAMEWORK FOR BEHAVIORAL AND LIFESTYLE INTERVENTIONS IN ALZHEIMER'S DISEASE (MOBILIZE)

Dereck Salisbury[1], Vankee Lin[2], and Fang Yu[3], *1. University of Minnesota, University of Minnesota, Minnesota, United States*, *2. Stanford University, Stanford, California, United States*, *3. Arizona State University, Phoenix, Arizona, United States*

**Introduction:** Implementing multi-site behavioral intervention trials in Alzheimer's disease (AD) has many unique challenges, leading to substantial variations in delivered intervention doses and cognitive findings. These issues can be addressed by the IMplementation Framework fOr Behavioral and LIfestyLe Interventions In AlZheimer's DiseasE (MOBILIZE) that was developed to guide the design and implementation of behavioral interventions in AD. **Methods:** This study systematically evaluated the implementation outcomes of the multi-site aerobic exercise and cognitive training (ACT) trial that employed MOBILIZE. Building on the person-centered care principle, MOBILIZE includes three implementation outcomes (screening, intervention adherence, and safety) with corresponding team processes to guide trial implementation. Screening was operationalized as the duration (days) between each screening visit, last screening visit to enrollment, and enrollment to first intervention session. Intervention adherence outcomes included: adherence (attendance), session dose adherence (calculated by dividing the number of sessions that achieved >=70% of the prescribed session dose / total number of sessions completed), and days required to complete the prescribed 72 sessions. Safety was operationalized as the type, number, and severity of study-related adverse events (AEs). RESULTS. The sample (n=146) was 73.8±5.7 years in age and 23.4±2.1 on Montreal Cognitive Assessment score, with 48.0% female, and 91.8% white. Screening-to-enrollment averaged 117.4±71.7 days. Intervention adherence was 77.4%±28.3%. There was 9-study related adverse events. Screening-to-enrollment and intervention adherence differed across sites, mainly due to COVID-19 influence. CONCLUSIONS. MOBILIZE helped the ACT Trial achieve high intervention adherence and safety and may be particularly important for early-stage and multi-site trials in AD.

### SESSION 7455 (POSTER)

### COGNITION II (POSTERS)

Abstract citation ID: igae098.3035

### ANALYZING THE INFLUENCE OF MIDLIFE EMPLOYMENT TRAJECTORIES ON COGNITIVE AGING IN CHILE

Magdalena Delaporte, and Jere Behrman, *University of Pennsylvania, Philadelphia, Pennsylvania, United States*

As the global population ages, understanding the factors contributing to healthy aging becomes increasingly crucial. This paper delves into the relationship between mid-life employment trajectories and later-life cognition, utilizing a Chilean longitudinal dataset. Employing the Group-Based Trajectory Model (GBTM), distinct employment trajectories for males and females between 1980 and 2016 are estimated. Subsequently, we link these trajectories to cognitive function scores to investigate the associations between individual trajectories and cognitive function in later life. Preliminary findings reveal significant associations between certain cognitive domains and labor force participation trajectories. We find four distinct labor force participation trajectories for men and women. Results show that men that have a high probability of continued employment demonstrate better cognitive outcomes, including orientation, memory, attention, and executive function. For women, a higher likelihood of working during between ages 30 to 60 correlates with improved performance in delayed memory, attention, and executive function. These results provide valuable insights for policymakers and practitioners seeking to enhance cognitive well-being among older adults.

Abstract citation ID: igae098.3036

### ASSOCIATION OF COGNITIVE LEVEL AND QUALITY OF LIFE INDICATORS POSTSTROKE IN OLDER ADULTS: A CROSS-SECTIONAL STUDY

Azza Alharrasi, and Diane Berish, *The Pennsylvania State University, University Park, Pennsylvania, United States*

Stroke is one of the leading causes of death and disability worldwide. Post-stroke cognitive impairment (PSCI) is a common complication of stroke; however, mild cognitive impairment is highly prevalent within three months post-stroke and is associated with increased cost of care, hospitalizations, and care dependency.

This study examined the association between cognition level (TICS total score) and quality of life indicators (Depression yes=1 no=0, physical limitations yes=1 no=0, socialization yes=1 no=0) among adults aged 65+. Therefore, a cross-sectional study using the 2020 wave of Health and Retirement Study (HRS) was used to conduct logistic regression Analysis using R.4.2.2. Total sample was 1259 adults 65+ who had reported experiencing a stroke, 58% were female and average TICS score of 6.5 (SD=3.5). Covariates of having high blood pressure, diabetes, or heart disease, sex, working outside the home, education level and age were included in all models. For every one-point increase in TICS score the odds of experiencing functional impairment were 21% lower (OR =0.89, p <.0001). The association for TICS scores and odds of feeling depressed (OR=0.97, p=0.27) and odds of socialization (OR 1.02, p=0.68) were not statistically significant. These results indicate that older adults who had lower cognition scores post stroke were more likely to experience physical limitations. More rigorous research studies need to be conducted to evaluate the quality of life of older adults during post-stroke based on their cognition status. This will inform medical management to individualize the rehabilitation and treatment according to the older adults' needs.

Abstract citation ID: igae098.3037

### ASSOCIATIONS BETWEEN SELF-RATED HEALTH, COGNITIVE FUNCTION, RISK OF COGNITIVE IMPAIRMENT, AND DEMENTIA

Kayla Garner[1], Eileen Graham[1], Kathryn Jackson[1], William Chopik[2], and Emorie Beck[3], *1. Northwestern University, Evanston, Illinois, United States*, *2. Michigan State University East Lansing, East Lansing, Michigan, United States*, *3. University of California Davis, Davis, California, United States*

Psychosocial researchers have long sought to identify individual differences in early and middle adulthood that robustly predict health in later life. Our current pre-registered study builds on these efforts by assessing whether an individual's subjective perception of their health is related to their cognitive health, both concurrently and later in life. Using the coordinated data analysis (CDA) methodological framework, we have identified 17 longitudinal studies (including 7 from the Gateway to Global Aging network) that had the requisite data to test our models. We conceptually harmonized all study variables and tested identical linear and logistic regression models across all study datasets. Our preliminary results suggest that individuals who have better subjective beliefs about their health have better cognitive function both concurrently and at longitudinal follow-up (ranging from 4 to 27 years after baseline). These associations were moderated by participant gender and age at baseline, and the results were somewhat robust to the inclusion of objective health indicators. We will discuss our results within the context of Lifespan Developmental Theory and the Health Behavior Model. Overall, our study serves to elucidate how subjective perceptions of health may manifest into later cognitive health outcomes, and if subjective perceptions of health could be used as an early risk factor for identifying individuals who may be vulnerable to cognitive senescence in later life.

Abstract citation ID: igae098.3038

### CARDIOMETABOLIC DISEASE RISK PARTIALLY MEDIATES RELATIONSHIP BETWEEN FINE PARTICULATE MATTER AND COGNITION

Ethan Siu Leung Cheung, Sara Grineski, and David Curtis, *University of Utah, Salt Lake City, Utah, United States*

PM2.5 pollution is expected to worsen in many places due to climate change. This is because of hotter temperatures, less precipitation, and increases in windspeed. PM2.5 exposure is associated with many adverse health effects. Less is known about how physical and mental health challenges associated with aging, like the onset of cardiometabolic disease (CMB) and changes in psychological wellbeing, might mediate linkages between PM2.5 exposure and aging-related cognitive and physical limitations. Longitudinal data from the Midlife in the United States study (MIDUS 1: 2004-05; MIDUS 2: 2013-14) were adopted. A sample of 4,272 individuals aged 32 to 84 was included. Based on individuals' residential addresses at each wave, we linked census tract identifiers to PM2.5 exposure, which was defined by five-year annual averages. Using structural equation models, we examined cross-sectional and longitudinal associations between PM2.5 exposure and executive functioning and instrumental activities of daily living (IADL) limitations, and tested CMB risk and psychological well-being as mediators using the product of coefficients method. Results suggested that higher PM2.5 exposure was associated with lower executive functioning cross-sectionally and with larger decreases over time. No significant associations were found between PM2.5 and IADL limitations. The cross-sectional association between PM2.5and executive functioning was partially mediated by CMB risk, accounting for 14% of the estimate, and the longitudinal association showed a similar, albeit nonsignificant mediation pattern. Psychological wellbeing was not a significant mediator. Findings suggest the importance of considering the complex and indirect ways in which climate change may impact health of older adults.

Abstract citation ID: igae098.3039

### CAREGIVING AFFECTS WORKING MEMORY, HOPELESSNESS, AND PERCEIVED CONTROL: A MEDIATION MODEL

Valerie Luskey[1], William Zimbinski[1], Michael Buxton[1], Hannah Apostolou[2], and M. Lindsey Jacobs[3], *1. University of Alabama, Tuscaloosa, Alabama, United States*, *2. University of Alabama, Northport, Alabama, United States*, *3. The University of Alabama, Tuscaloosa, Alabama, United States*

Caregiver burden can have negative impacts, both on the caregiver and the person receiving care. One of these effects could include more depressive symptoms in the care recipient, which could impact everyday functioning and quality of life. To explore these relationships, data was used from the Health and Retirement Study in the 2014 Core. Participants included people who answered the questions regarding caregiving, perceived control, hopelessness, and completed a working memory task (n=772; female=59.5%, male=40.5%; age=71.14 (SD=11.88); years of education=11.55 (SD=3.53); race/ethnicity were masked due to confidentiality). Participants with Alzheimer's or Dementia were excluded, and age was used as a covariate. In a mediation analysis, there was an indirect effect of increased hours of caregiving on hopelessness in the care recipient, b=0.10, SE=0.36, p=0.004, which, in turn, lowered working memory in the person being cared for, b=-0.09, SE=0.04, p=0.03. Additionally, increased caregiving led to decreased perceived control, b=-0.08, SE=0.04, p=0.02; however, this did not translate to decreased working memory, b=-0.051, SE=0.04, p=0.21. Apart from these outcomes, increased caregiving had a direct effect on cognition, b=-0.098, t(767)=-2.73, p=0.007, suggesting it influences other mechanisms that decrease working memory not included in the model. A diagnosis of depression could be an explanatory factor that we were not able to include in the model; however, the model indicates a possible area for intervention. If caregivers receive more support and learn skills to promote hope, that could offer positive contributions to increased working memory—thus, translating to overall increased functioning.

Abstract citation ID: igae098.3040

### COGNITION PROFILES AMONG OLDER BLACK ADULTS, BY SCHOOL DESEGREGATION STATUS, EDUCATIONAL, OCCUPATION, INCOME

Collin Perryman[1], Roland Thorpe[1], and Keith E. Whitfield[2], *1. Johns Hopkins University, Baltimore, Maryland, United States*, *2. University of Nevada Las Vegas, Las Vegas, Nevada, United States*

Objective: We sought to identify the subgroups regarding the school environment (desegregated or segregated), educational attainment, occupation type, income, and cognition among older Black adults.

**Methods:** Data from older Black adults (n=602; age range: 48 to 95 years) from the Baltimore Study of Black Aging—Patterns of Cognitive Aging (BSBA-PCA) was used to explore the various cognitive function subgroups. We conducted a latent class analysis (LCA) to understand the cognitive domain subgroups. The continuous indicators were several cognitive domains (global cognition, reasoning, memory, immediate recall, working memory, language, and perceptual speed), and the categorical indicators were school type, educational attainment, occupation type, and income.

**Results:** We identified subgroup for each cognitive domain. For global cognition (1) highest educational attainment, (2) untrained occupation, and (3) most segregated; working memory (1) high educational attainment, (2) segregated, and (3) low monthly income; reasoning (1) high educational attainment; (2) mostly desegregated, and (3) segregated; memory (1) highly educated, (2) lowest income, and (3) segregated; perceptual speed (1) highly educated, (2) lowest income, and (3) segregated. Given all the indicators, each subgroup exhibited an incremental increase in mean cognition scores.

**Conclusion:** Our study provides insights to how cognition varies by school setting, educational attainment, occupation type, and income among older Black adults who attended desegregated or segregated schools in their early-life.

Abstract citation ID: igae098.3041

### COUPLING OF WITHIN-PERSON CHANGES IN SLEEP QUALITY AND SUBJECTIVE COGNITION IN COMMUNITY-DWELLING ADULTS

Jose Diaz[1], Soomi Lee[1], Lynn Martire[1], and Martin Sliwinski[2], *1. The Pennsylvania State University, University Park, Pennsylvania, United States*, *2. Pennsylvania State University, University Park, Pennsylvania, United States*

Subjective cognitive decline is not a mere complaint by older adults; it is predictive of later development of objective cognitive decline and dementia. Sleep plays a pivotal role in maintaining cognitive, physical, and mental health, especially in older adults. While studies document age-related declines in sleep quality, limited research explores whether within-person changes in sleep quality are associated with within-person changes in subjective cognition ("coupling effect"). In this study, we examined the dynamic coupling relationship between sleep quality and subjective cognition in older adults over multiple years, adjusting for potential influence of self-report bias (gauged by self-rated health). We used nine waves' longitudinal data from the Transitions in Health and Relationships study (n=131, Mage=77.09). Multilevel models indicated that changes in sleep quality were significantly associated with changes in subjective cognitive function (β=0.044, 95% CI [0.004, 0.084], p=.031), signifying a coupling effect independent of baseline sleep quality, sociodemographic covariates, and self-rated health. Furthermore, changes in self-rated health moderated the association between changes in sleep quality and subjective cognition (β=-0.047, 95% CI [-0.088, -0.005], p=.028), such that increases in self-rated health weakened the effect of increases in sleep quality on subjective cognition. These findings underscore the significance of within-person changes in sleep quality in predicting subjective cognitive decline in older adults. Notably, while the association was independent of self-report bias, it was weaker for those who reported their overall health was improved. Future work should focus on maintaining or improving sleep quality in order to prevent or delay subjective cognitive decline.

Abstract citation ID: igae098.3042

### DIET AND COGNITION IN AGING: EFFECTS OF HIGH-FAT-SUGAR DIETS ON MEMORY AND EXECUTIVE FUNCTIONING

Selen Atak[1], Alyssa Boye[2], Anda Botoseneanu[3], Susana Pecina[2], and Zhong-Xu Liu[2], *1. University of Michigan Dearborn, Michigan, United States*, *2. University of Michigan Dearborn, Dearborn, Michigan, United States*, *3. University of Michigan-Dearborn, Dexter, Michigan, United States*

High-fat and high-sugar (HFS) diets may affect the hippocampus, and consequently the hippocampus-dependent memory. Previously, we found that higher HFS diet consumption in young adults was associated with lower performance on hippocampus-dependent memory tasks, but not with their subjective memory report. However, whether similar associations exist in older adults has not been yet investigated. In this online study, 472 participants (age: 50 ~ 85 years; average = 62.6) reported their HFS consumption, measured by the Dietary Fat and Sugar Short Questionnaire, and their subjective memory issues. They also completed pattern-separation recognition and face-name associative memory tasks, which have been found to robustly engage the hippocampus. Participants also completed executive functioning tasks, i.e., Trail Making (TMT) and Stroop tasks. Results showed that different from young adults, higher HFS consumption was strongly associated with more subjective memory complaints in older adults after controlling for potential covariates. Moreover, age modulated associations between HFS consumption and memory/executive functioning. Specifically, higher HFS consumption was associated with worse hippocampus-dependent recognition memory and executive function, only in participants with age below 65, not in those with age above 65. Notably, HFS diet effects on recognition memory were also associated with participants' health measured by multi-morbidity status, in that higher HFS predicted lower memory performance only in participants with no multi-morbidity. These results suggest that HFS diet effects on cognition are more complex in older than younger adults and call for further research to explore age-/health-related factors that uniquely modulate the effects of HFS diet in older adults.

Abstract citation ID: igae098.3043

### DIMENSIONALITY, PROXIES, AND PREDICTORS OF COGNITIVE RESERVE IN THE MANCHESTER LONGITUDINAL STUDY

Stephen Aichele, *Colorado State University, Fort Collins, Colorado, United States*

In a sample of N=113 older adults (ages 62-86 years at MRI scan; 58.4% women) from the United Kingdom, we compared the dimensionality of general cognition before (COG) vs. after (COGr; cognitive reserve) residualization on global brain status (as indicated by brain volume loss, cerebral blood flow, and white matter lesion prevalence). Cognitive performance was assessed by 15 tasks spanning five domains: processing speed, fluid intelligence (e.g., spatial reasoning), crystallized intelligence (vocabulary), visuospatial memory, and verbal memory. Statistical tests were conducted in a series of structural factor analyses with progressive constraints on measurement invariance. Results showed that although COG and COGr were highly correlated (r =.84), item-factor loadings differed such that COGr more strongly emphasized fluid intelligence. We further examined differential associations of COG and COGr with 23 socio-demographic, health, and lifestyle variables in a series of regression models. Results from these latter tests showed consistent associations of education level, occupational class, and hobbies/interests as proxies both for COG and COGr. Difficulty with personal mobility was a salient risk factor for both COG and COGr, but only in models unadjusted for chronological age. In sum, results supported the role of intelligence as a potential compensatory factor for brain-related reductions in processing speed and memory. Efforts to preserve personal mobility and a diversity of hobbies and interests may serve as protective factors against brain-related reductions in cognitive performance.

Abstract citation ID: igae098.3044

### DOES SOCIAL ACTIVITY PARTICIPATION MODERATE THE RELATIONSHIP BETWEEN AGE AND COGNITION? EVIDENCE FROM THE HRS

Layla Katharine Santana, Hongdao Meng, Lindsay Peterson, Minh Quan Le, Mingyang Li, and Debra Dobbs, *University of South Florida, Tampa, Florida, United States*

Active participation in social activities contributes to increased social engagement and may have a positive impact on cognitive health in later life. Despite the well-documented association between social activity and cognitive function, the potential moderating role of social activity in age-related cognitive decline has not been well understood. In this cross-sectional study, we used data from the Health and Retirement Study (HRS), a nationally representative dataset of U.S. adults aged 50 and above (n=9,919), to examine the hypothesis that social activity participation moderates the relationship between age and cognition. Cognition was assessed by using a 27-point cognitive functioning. Social activity participation was measured by a validated 10-point scale, which measures self-reported activity participation. We estimated multivariable linear regression models in which cognitive functioning was regressed on age, and social activity participation while controlling for individual characteristics and potential confounders. We used the Hayes conditional process analysis and Johnson-Neyman plot to test the hypothesized relationships. The average age of the study sample was 67 years (SD=10.27), 59.5% were female, and 66.1% were non-Hispanic White. As expected, age was negatively associated with cognition ($\beta$ = -0.924; p <.001), and social activity participation was positively associated with cognition ($\beta$ = 0.122; p <.001). The interaction between age and social activity participation was statistically significant ($\beta$ = 0.0409; p = 0.005), indicating a significant moderating relationship. These findings suggest that social engagement may be a protective factor. Future studies should examine whether the findings can be replicated in a longitudinal study.

Abstract citation ID: igae098.3045

### DOES THE IMPACT OF COGNITIVE INTERVENTION ON MOBILITY VARY BY CHRONIC DISEASE?

Deborah Dadson, and Karlene Ball, *University of Alabama at Birmingham, Birmingham, Alabama, United States*

Mobility loss has been associated with chronic diseases, sedentary behavior, and fear of falls among older adults with approximately one-third of 70-year-olds and 80-year-olds reporting restrictions on mobility. Even though physical activity and exercise have been shown to enhance mobility and improve activities of daily living, only a few older adults are able to exercise due to several limiting factors. Cognitive training has been shown to enhance cognition, gait and motor functions, and consequently mobility among older adults. As such, this study aimed to examine how the impact of cognitive interventions on life space mobility varies by chronic disease. Using data from the Advanced Cognitive Training for Independent and Vital Elderly (ACTIVE) Clinical Trial, a sample of 2802 community dwelling older adults who were 65 years and older were examined using Multiple Linear Regression with interaction. Analyses included predicting life space mobility from the cognitive intervention trainings (reasoning, memory, and speed of processing), with gender, age, race, and mobility at baseline as covariates. Diabetes, cancer, and heart disease were entered into the model as moderators. Results indicated that speed of processing training, years of education and heart disease significantly predict Life Space Mobility (all p <.05). Reasoning training, memory training, gender, age, race, diabetes, and cancer were not uniquely associated with Life Space Mobility. These findings highlight the need to consider cognitive interventions, specifically speed of processing to ensure functional performance in everyday activities of older adults. Keywords: Mobility, cognitive intervention, chronic diseases, ACTIVE Clinical Trial

Abstract citation ID: igae098.3046

### EFFECTIVENESS OF DUAL-LANGUAGE COGNITIVE INTERVENTION IN COGNITIVE HEALTHY AND DEMENTIA BILINGUAL OLDER ADULTS

W. Quin Yow, Chi Shuen Ang, and Xiaoqian Li, *Singapore University of Technology & Design, Singapore, Singapore*

There is an increasing interest in using touch-screen devices to conduct cognitive training and collect measurements of cognitive performance. Although research with technology-based intervention has shown benefits in improving cognitive performance with older adults, overall results are mixed (Ge et al, 2018). This study aims to investigate whether our elder-friendly, multi-modal touch-screen, computerized dual-language intervention program would help to improve cognitive skills in cognitive healthy and dementia bilingual older adults in a multilingual society. Fifty-eight older adults, 26 with dementia (Mage 82.77 ± 7.45) and 32 healthy older adults (Mage 72.44 ± 8.42), completed 24 sessions of gameplay over 8-12 weeks. Each session took about 30-45 minutes to complete, and consisted of two cognitive tasks: Object Categorization (OC), and Utility of things (UoT). Participants' performance was assessed by reaction time (RT) and number of prompts used to reduce task difficulty. Using a two-way ANOVA, we compared the performance of the first 4 and last 4 sessions of the two groups for both tasks. By the last 4 sessions, there were significant improvements in performance of the dementia group (i.e., faster RT and fewer prompts for OC, and fewer prompts for UoT; post-hoc ps<.002). Performance in the cognitively-healthy group remained stable (ps>.11), and they consistenly outperformed the dementia group across the intervention (ps<.001). These improvements suggest that our cognitive intervention program could be effective for older adults with dementia. In sum, bilingual elder-friendly touch-screen platforms can be a novel yet effective method to study cognitive interventions in populations of older multilinguals.

Abstract citation ID: igae098.3047

### INFLAMMATION AND DOMAIN-SPECIFIC COGNITIVE PERFORMANCE: RESULTS FROM THE EINSTEIN AGING STUDY

Molly Wright[1], Erin Harrington[2], Jennifer Graham-Engeland[3], Martin Sliwinski[3], Richard Lipton[4], Mindy Katz[4], and Christopher Engeland[1], *1. The Pennsylvania State University, University Park, Pennsylvania, United States*, *2. University of Wyoming, Laramie, Wyoming, United States*, *3. Pennsylvania State University, University Park, Pennsylvania, United States*, *4. Albert Einstein College of Medicine, Bronx, New York, United States*

Inflammation may serve as a biological precursor to the development of mild cognitive impairment (MCI) and dementia. Although interleukin (IL)-6 may be more strongly associated with executive functioning (EF) or processing speed (PS) measures versus other cognitive domains, this has not been assessed simultaneously in a structural equation modeling (SEM) framework among older adults. The present study sought to address this gap. As part of the ongoing Einstein Aging Study, older adults (n=159, 65.4% female, 49.7% non-Hispanic White) without MCI at baseline completed neurocognitive testing and had two blood draws spaced two weeks apart at their baseline wave of data collection. Inflammation was assessed by averaging IL-6 levels obtained from these blood draws. Based on prior factor-analytic studies, a confirmatory factor analysis (CFA) was conducted that included an attention factor (Number Span Forward and Back, Verbal Fluency Test), memory factor (Free Selective Reminding Test, Logical Memory Test, Verbal Fluency Test), and EF/PS factor (Trail Making Test Parts A and B, Digit Symbol Test). A SEM model with pathways from IL-6 predicting cognitive factors was then specified, adjusting for relevant covariates based on fit statistics (age, years of education, sex, race/ethnicity). Both the CFA and SEM models fit the data well (RMSEA< 0.05, CFI>0.95). As expected, IL-6 was negatively associated with the EF/PS factor (β=-0.251, S.E.=0.073, p< 0.01). IL-6 was not associated with either the attention or memory factors (p>0.5). Based on these results, we predict that higher levels of IL-6 may predict nonamnestic MCI but not amnestic MCI.

Abstract citation ID: igae098.3048

### IS VARIABILITY IN COGNITIVE PERFORMANCE ALWAYS MALADAPTIVE? THE MODERATING ROLE OF SOCIODEMOGRAPHIC FACTORS

Laura Klepacz[1], Eric Cerino[2], Matthew Pierce[1], and Jeremy Hamm[1], *1. North Dakota State University, Fargo, North Dakota, United States*, *2. Northern Arizona University, Flagstaff, Arizona, United States*

Cognitive dispersion refers to an individual's fluctuations in performance across a range of neuropsychological domains. Although past research has linked greater dispersion to cognitive decline in clinical populations, less is known about the characterization of dispersion in unimpaired, younger samples. The current study addresses this gap by identifying sociodemographic populations for whom dispersion may not be maladaptive and potentially linked to better cognitive performance over time. We pursued this aim by examining how age, sex, income, and education moderated the association between longitudinal changes in dispersion and corresponding shifts in cognitive performance using MIDUS data (n=2,518; age 55±11.2, Range: 33 – 83 years). Respondents completed a telephone-based battery of cognitive tests measuring several neuropsychological domains. Dispersion was calculated by generating an intraindividual standard deviation across 6 unique cognitive tasks. Results of OLS regression models showed that age (b=.01, SE=.00, p<.001), sex (b=.26, SE=.07, p<.001), income (b=-.01, SE=.01, p=.056), and education (b=-.02,SE=.01, p=.066) moderated the positive association between regressed change in dispersion and regressed change in mean performance. Specifically, increases in dispersion were most strongly associated with shallower 9-year declines in cognitive performance among comparatively older adults (b=.41, SE=.05, p<.001), women (b=.40, SE=.04, p<.001), individuals with less education (b=.35, SE=.05 p<.001), and those with lower income (b=.39, SE=.04, p<.001). Our study extends the literature by contributing to a more nuanced understanding of the populations for whom dispersion may not be maladaptive. These findings suggest that dispersion may reflect healthier cognitive aging in populations that are at greater risk for cognitive impairment.

Abstract citation ID: igae098.3049

### LINKS BETWEEN SELF-REPORTED HEARING WITH LEARNING AND MEMORY IN THE HEALTH AND RETIREMENT STUDY

Ariana Stickel[1], Carlos Araujo-Menendez[2], Carmen Chek[3], Laura Coco[1], Hector González[4], and Wassim Tarraf[3], *1. San Diego State University, San Diego, California, United States*, *2. SDSU/UC San Diego, San Diego, California, United States*, *3. Wayne State University, Detroit, Michigan, United States*, *4. University of California San Diego, La Jolla, California, United States*

Growing evidence links hearing loss to increased risk for dementia, but the influence of sociodemographic characteristics (e.g., race/ethnicity, insurance status) on this relationship is understudied. Therefore, we examined the relationships between self-rated hearing with learning and memory in a large, population-based sample. Participants included 3,417 individuals (mean age = 74.5 years, SD = 7.9; 9.4% Black, 8.1% Latino, 82.5% White) from the Health and Retirement Study who also completed the Harmonized Cognitive Assessment Protocol (HCAP) in 2016. Individuals rated their hearing as “excellent”, “very good”, “good”, “fair”, or “poor”. Outcomes of interest included scores from each of the learning (1-3) and the delayed memory trials from the Consortium to Establish a Registry for Alzheimer’s Disease (CERAD) Word List Memory Task. Probability weighted linear regression models were fit to examine the associations between self-reported hearing with learning and memory. We fit a series of models adjusting for hearing aid use (M1), M1 + education (M2), and M2 + race/ethnicity, age, sex, and insurance (M3). Fair and poor self-reported hearing were associated with a steeper drop from trial 3 recall to delayed recall compared to those who reported excellent hearing in fully-adjusted models (M3: $\Delta$=-0.27, se=0.12, $p < 0.05$; $\Delta$=-0.47, se=0.16, $p < 0.01$, respectively). Self-reported poor hearing was associated with worse delayed recall after taking into account hearing aid use and several sociodemographic factors. Hearing loss may be a ubiquitous risk factor for worse delayed recall, suggesting an increased risk for Alzheimer’s disease.

Abstract citation ID: igae098.3050

### ROLE OF GAIT SPEED, OLFACTION, AND PSYCHOLOGICAL FACTORS IN COGNITIVE FUNCTION AMONG OLDER ADULTS

Leah Hanson[1], Kyle Palmberg[1], Clarissa Howe[1], Sally Gustafson[1], Aleta Svitak[1], and Terry Barclay[2], *1. HealthPartners Institute, St. Paul, Minnesota, United States*, *2. HealthPartners, St. Paul, Minnesota, United States*

Slowing of gait speed, loss of smell and increased depression, anxiety and stress have been associated with lower cognitive performance among older adults. The Minnesota Memory Project is a longitudinal study of community-dwelling adults 55 and older that investigates lifestyle factors associated with cognitive aging. Annual assessments include the Montreal Cognitive Assessment (MoCA), Alberta Smell Test (AST), gait speed test (GST) and self-report inventories, including the Cognitive Change Checklist (3CL) and measures of social support, resilience, stress, anxiety, and depression. Significant associations were identified between AST and gait scores versus MoCA. Both scores differed significantly between cognitively impaired (MoCA < 26) versus cognitively intact (MoCA ≥26) participants. On average, impaired participant AST scores were significantly lower than intact participants (6.5 ± 3.9 SD, 8.7 ± 3.6 SD) and gait scores were significantly slower (4.0 ± 1.0 SD, 3.65 ± 0.8). A greater number of self-reported cognitive complaints was associated with lower MoCA scores at baseline ($p < 0.0001$). Participants reporting higher resilience and social support reported significantly fewer cognitive complaints ($p < 0.0001$), while higher stress, anxiety and depression were associated with more cognitive complaints (all $p < 0.0001$). Significant effects of olfaction, gait and cognitive complaints on future cognitive decline were also identified. A one-unit increase in AST score was associated with an 11% decrease in the hazard of cognitive impairment within 5 years. A one-unit increase in the GST score was associated with a 60% increase. Screening that includes gait speed, olfaction and subjective cognitive complaints could identify those at risk for future cognitive decline.

Abstract citation ID: igae098.3051

### ROLE OF INFLAMMATION AS SEX-SPECIFIC MEDIATOR BETWEEN CHRONIC STRESS AND COGNITIVE FUNCTION

Laura Laiton Jimenez[1], Idan Shalev[1], Martin Sliwinski[2], and Yogasudha Veturi[1], *1. The Pennsylvania State University, University Park, Pennsylvania, United States*, *2. Pennsylvania State University, University Park, Pennsylvania, United States*

Long-term stress has been associated with poorer cognitive outcomes and increased risk of dementia, especially among women. Chronic stress is also known to lead to inflammation, whose role in neurodegeneration and associated comorbidities affecting immune function is well-established. Our investigation probes the mediating role of circulating inflammatory biomarkers (IB) in the social isolation stress (SIS) to cognitive function (CF) pathway, incorporating sex and age as moderating factors. Leveraging the MIDUS Refresher 1 cohort (N=3,800), we analyzed the role of SIS (frequency of social interactions with friends) on five CF tasks (word list recall, category verbal fluency, backward digit span, 30-second counting, and task-switching) and two IBs (C-reactive protein and interleukin-6). We employed a three-step approach to model these relationships: (1) CF~SIS; (2) IB~SIS; (3) CF ~SIS+IB. Poisson and linear regressions were employed as appropriate. After Bonferroni correction, we identified SIS as a significant predictor for the last three CF measures (p-value~0.01), with a pronounced sex effect in the last two (p-value~0.001). SIS was a significant predictor for interleukin-6 (p-value=0.0238), with a significant sex effect for C-reactive protein (p-value=0. 0.0003). Importantly, in step (3), the SIS-CF association diminished with the inclusion of IL6, highlighting its role as a putative mediator in this pathway. The sex effect remained unchanged. Next, we aim to analyze additional stressors (e.g. financial stress, perceived discrimination), examine the role of immune-related gene expression as a mediator, as well as investigate the role of inflammation-related comorbidities in the stress-CF pathway from electronic health records.

Abstract citation ID: igae098.3052

### STATE OF THE EVIDENCE FOR MULTIVITAMIN/MULTIMINERAL SUPPLEMENTATION AND COGNITIVE AGING

Rachael Patusco[1], and Ankita Srivastava[2], *1. Haleon, Warren, New Jersey, United States*, *2. WNS Global Services, Gurugram, Haryana, India*

In 2017, nearly 40% of Americans aged 60 years and over consumed a multivitamin/multimineral (MVM) product. Nutrient inadequacies, for which older adults are at greater risk, can negatively influence cognitive aging. Bringing together the converging themes of MVM popularity and cognitive aging, this review synthesized evidence on the effects a broad spectrum MVM has on cognitive aging. A literature search was conducted using ProQuest (Embase, Medline) and PubMed. Seven studies met inclusion criteria. All studies were double-blind, placebo-controlled, randomized clinical trials utilizing an MVM product (range = 10 - 26 essential nutrients). Duration of interventions ranged from 6 months to 12 years across 63 to 5,974 participants aged 60 years and older from the U.S. and European countries. There was heterogeneity in cognitive assessments utilized with some overlap of tested domains, such as episodic memory & executive function. Shorter and smaller trials (≤ 1 year, n=3), found no overall effect of MVM supplementation on cognitive performance, however some domains were sensitive to MVM intervention. Among longer duration trials (≥ 3 years, n=4), 75% reported significant improvements in episodic memory & global cognition. When measured across studies, MVM supplementation significantly improved serum vitamin levels, which is consistent with broader MVM supplementation research. Despite heterogeneity in treatment effects in shorter duration trials, in longer duration trials (>1 year), MVMs can elucidate beneficial effects, suggesting dose & duration may contribute to the treatment effect. Evidence gaps exist in exposing mechanism of action.

Abstract citation ID: igae098.3053

### THE LINK BETWEEN INTRAINDIVIDUAL VARIABILITY IN COGNITIVE PERFORMANCE AND MOBILITY IN CHRONIC STROKE

Vrinda Dimri[1], Nárlon Cássio Boa Sorte Silva[1], Guilherme Balbim[1], and Teresa Liu-Ambrose[2], *1. University of British Columbia, Vancouver, British Columbia, Canada*, *2. University of British Columbia, University of British Columbia, British Columbia, Canada*

Intraindividual variability (IIV) is the within-person trial-to-trial variation in reaction time during cognitive tasks. Higher IIV represents reduced consistency in responses, which may be due to lapses in attention, and is associated with reduced mobility in older adults. IIV may also be a more sensitive measure of cognitive performance versus traditional summary scores. A stroke can significantly impact one's cognitive function and mobility. Whether IIV in cognitive performance is associated with mobility among individuals who have experienced a stroke is unknown. We aimed to examine this relationship by using baseline data from a six-month single-blinded, 3-group parallel randomised controlled trial. Participants included community-dwelling adults (N= 119, 38.7% female) with a history of stroke, aged 55 years and older (mean= 70.71 years, SD= 8.59), able to walk 6 meters, and without dementia. Mobility was assessed based on timed up and go (TUG) performance. Residualised intra-individual standard deviation (rISD) was used as measure of IIV and computed using trial latencies of a computerised Stroop Task. TUG scores significantly predicted rISD of congruent (beta= 0.03, SE= 0.01, p-value = 0.001) and neutral trials (beta= 0.02, SE= 0.01, p-value = 0.04), but not incongruent trials (beta= 0.01, SE= 0.01, p-value = 0.18). However, these effects were not found when age was included as a covariate. Overall, these findings suggest that in adults with stroke, higher IIV measured from a simple reaction time task is associated with worse mobility. Further longitudinal studies are needed to determine if higher IIV is predictive of mobility decline post-stroke.

Abstract citation ID: igae098.3054

### USE OF A BRAIN CARE SCORE TO IDENTIFY RISK AND PROTECTIVE FACTORS FOR MAINTAINING COGNITION AND HEALTH IN LATER LIFE

Kylie Schiloski, Morgan Taylor, and Margie Lachman, *Brandeis University, Waltham, Massachusetts, United States*

The Massachusetts General Hospital McCance Center for Brain Health recently developed a Brain Care Score (BCS) to assess the risk of dementia and stroke and to promote healthier lifestyles. The BCS includes a combination of modifiable physical (e.g., blood pressure, blood glucose, cholesterol, Body Mass Index), environmental (e.g., nutrition, exercise, smoking status, sleep, alcohol consumption), and psychosocial factors (e.g., perceived stress, social support, purpose in life) that have been shown to predict dementia and stroke. The goal of the present study was to develop and test a comparable measure using data from the Midlife in the United States study (MIDUS). We examined whether the MIDUS version of the BCS predicted changes in cognition and health from the second (M2; 2004-2005) to the third wave (M3; 2013-2014) of the Biomarker sub-study sample (M2: N=1,167; Mage=54.46; SD=11.60; Age Range=34-83). Multiple regressions showed that higher BCSs predicted less decline in episodic memory (immediate and delayed recall), fluid intelligence (number series), processing speed, (backwards counting), and verbal fluency (category fluency) over time as well as less decline in functional health and fewer chronic conditions after 8 to 10 years. These findings indicate that the MIDUS version of the BCS is a promising tool to help identify those at risk of declines in cognition and health. Moreover, these findings highlight the importance of adopting a healthy lifestyle in order to reduce one's risk of experiencing declines in cognitive and physical health in middle and later adulthood.

Abstract citation ID: igae098.3055

### USING THE MONTREAL COGNITIVE ASSESSMENT IN MILD AGE-RELATED HEARING LOSS

Shraddha Shende[1], and Raksha Mudar[2], *1. Illinois State University, Normal, Illinois, United States*, *2. University of Illinois, Champaign, Illinois, United States*

Age-related hearing loss (ARHL) is a modifiable risk factor for dementia. There is a need for early detection of cognitive concerns in older adults with ARHL. The Montreal Cognitive Assessment (MoCA) is a well-validated screening tool that has been used to screen cognition in the ARHL population. However, few studies have examined the value of both MoCA total score (MoCA-TS) and MoCA-Memory Index Score (MoCA-MIS) score in ARHL and its relation to complex hearing functions, especially in those with mild hearing loss. We administered MoCA on 15 older adults with mild-to-moderate ARHL and 15 matched normal hearing controls. MoCA-TS and MoCA-MIS were calculated. Participants also completed the Quick Speech-in-Noise (QuickSiN) test and the Hearing Handicap Inventory for Adults (HHIA). Relative to controls, worse scores were obtained in the mild ARHL group on MoCA-TS (p =.031) and MoCA-MIS (p =.039). Bivariate correlations revealed a significant negative association between QuickSiN and MoCA-TS scores (p <.001), with similar trends on MoCA-MIS (p =.051). When the peripheral hearing measure was controlled using partial correlations, the negative correlation between QuickSiN and MoCA-TS remained (p =.004). The correlations of MoCA-TS and MoCA-MIS with HHIA were not significant. Our findings reveal changes in global cognition beyond encoding memory in mild ARHL. Additionally, our findings suggest that complex listening functions assessed using QuickSiN in related to overall performance on MoCA.

### SESSION 7460 (POSTER)

### DEATH, DYING, AND BEREAVEMENT

Abstract citation ID: igae098.3056

### A CONTENT ANALYSIS OF CHILDREN'S LITERATURE ON DEATH AND DYING

Celine Manternach, and Hallory Domnick, *University of Northern Iowa, Cedar Falls, Iowa, United States*

The purpose of this project was to analyze children's literature on death and dying published between 2014-today. This project builds on previous research published prior to 2014. N = 40 books were reviewed for the following content: Biological facts including type of loss, emotional responses, socio-cultural practices and beliefs, and the number of euphemisms used. Books available from local libraries and Amazon were used for this project. Examples from each book were included in Excel and frequencies and percentages were summed after analysis. A total of 10 (25%) books included biological facts, such as "heartbeat stops," 18 (45%) included emotional responses such as "Dad's eyes were red," and 8 (20%) included socio-cultural practices and beliefs such as "we all have beliefs and we don't know what happens when we die." A total of 5(8%) books included euphemistic terms such as "gone to a better place" for death. Overall, findings demonstrated that authors tended to write about emotional responses related to grief most often, whereas fewer wrote about socio-cultural practices and beliefs. Given the diversity and complexity of practices and beliefs on death and dying, additional children's books published in this area are needed to capture the diversity of experiences. While explaining death to children can be challenging, literature is a tool that parents can use to guide the conversation. However, not all children's books are created with the same intent, and it is important for adults to pre-screen books to determine how appropriate the book is for their child.

Abstract citation ID: igae098.3057

### A CROSS-CULTURAL ANALYSIS OF PREFERENCES AND ATTITUDES TOWARD ADVANCED DIRECTIVES IN TAIWAN AND THAILAND

Hui-Ching Weng[1], Duan-Rung Chen[2], Krungkraipetch Kiiti[3], and Inchai Puangtong[3], *1. National Cheng Kung University, Tainan City, Tainan, Taiwan (Republic of China)*, *2. National Taiwan University, Taipei, Taipei, Taiwan (Republic of China)*, *3. Burapha University, Mueng, Chon Buri, Thailand*

**Introduction:** Advance directives (ADs) are pivotal in healthcare planning, indicating preferences for medical treatment in critical conditions. This study examines ADs attitudes and experiences between Taiwan and Thailand, highlighting cultural influences on end-of-life care.

**Method:** Employing pairing matching, the study selected 259 matched pairs from an initial Taiwanese pool of 1488 and a Thai sample of 268 based on gender, age, education, and medical work background. Demographics included 72.2% females, with an average age of 32.91 (range 18-72), 92.3% holding a bachelor's degree or higher, and 95% working in health-related fields.

**Results:** The comparison revealed no significant differences in the perceived necessity of ADs at the current life stage or in the preference for familial decision-making during hospitalization. However, significant disparities emerged in several areas, including comfort in discussing death (Taiwan 95.8%, Thailand 84.6%) and willingness to discuss end-of-life care (Taiwan 96.1%, Thailand 91.5%). A substantial gap was found in the belief regarding peers' consideration of ADs, with 84.6% of Taiwanese versus 49% of Thais believing their age group would consider such directives. Furthermore, all eight aspects related to the desired qualities of healthcare agents showed significant differences, with Taiwanese respondents valuing trust, communication, understanding, and support more highly than their Thai counterparts.

**Conclusions:** The study underscores cultural differences in attitudes towards advance directives between Taiwan and Thailand, particularly in discussions about end-of-life care and qualities desired in healthcare proxies. These findings highlight the need for culturally tailored approaches to enhance awareness and acceptance of advance directives in both regions.

Abstract citation ID: igae098.3058

### CHOOSING TO FORGO: LIFE-SUSTAINING TREATMENT DECISIONS FOR SELVES AND RELATIVES AMONG TAIWANESE ADULTS

Duan-Rung Chen[1], Su-I Hou[2], and Yuchi Young[3], *1. National Taiwan University, Taipei, Taipei, Taiwan (Republic of China)*, *2. The University of Central Florida,*

*Orlando, Florida, United States*, *3. State University of New York at Albany, Albany, New York, United States*

**Purpose:** Since its implementation in 2019, less than 2% of adults in Taiwan have signed Advance Directives (ADs) under the Patient Right to Autonomy Act (PRAA). This research explores how the public views the PRAA and its effect on the decision to decline life-sustaining treatments (LSTs) in hypothetical end-of-life scenarios. Method: In August-November 2022, an online survey was administered to 3,922 adults in Taiwan, of which approximately 1,488 (37%) were familiar with the PRAA. These 1488 respondents completed questions regarding their perceptions of the PRAA. They responded to questions about three hypothetical end-of-life situations: terminal cancer, irreversible severe brain damage, and terminal dementia.

**Results:** Over half of the participants declined to receive LSTs for themselves and chose similarly but less for their family members. A specific difference was noted in choices concerning terminal dementia, showing a greater reluctance to withhold LSTs, especially for parents, than for themselves. There were gender-based differences, with women more likely to choose against LSTs for themselves in various situations. In contrast, gender did not affect decisions made for family members. The perception of the PRAA and the degree of ACP engagement significantly influenced the refusal of LSTs for both the participants and family members. A positive view of the PRAA was significantly linked to decisions against LSTs for spouses.

**Conclusion:** These findings underscore the complexity of decisions around LSTs, influenced by personal beliefs, gender, legal perceptions, and engagement for advance care planning.

Abstract citation ID: igae098.3059

### EXAMINING COGNITIVE FUNCTION IN BEREAVED OLDER PARENTS: THE MEDIATING ROLE OF DEPRESSIVE SYMPTOMS

Stephanie Rubinstein, and Elizabeth Zelinski, *University of Southern California, Los Angeles, California, United States*

Losing a child is a unique and exceptionally traumatic experience, often resulting in chronic and severe depressive symptoms. This is of increasing concern as the loss of adult children has become increasingly more common since 2010. Bereaved parents represent a psychologically vulnerable group about whom we know relatively little. In this study, we use data from a nationally representative sample of 5,265 community-dwelling older adults over age 50 from the Health and Retirement Study (HRS) to examine the relationship between child loss, depressive symptoms, and cognitive function. We hypothesize that being bereaved is significantly associated with worse cognitive scores and that this relationship will be mediated by the number of reported depressive symptoms. We conducted a cluster-adjusted path analysis on repeated cross-sectional data from 1995-2020. Controlling for sociodemographic and health factors, our findings confirmed these expectations. Both bereaved individuals and those with depressive symptoms exhibited significantly worse cognitive scores, respectively ($\beta$=-0.20, p<.001; $\beta$=-0.15, p<.001). Bereaved individuals were also significantly more likely to report depressive symptoms ($\beta$=0.09, p=.001). Depressive symptomology was found to partially mediate the relationship between bereavement and cognitive function, explaining approximately 7% of the variance. These results suggest that losing a child is associated with poorer cognitive function in older adults. This relationship is partially explained by the presence of depressive symptoms, likely associated with said loss. Our findings highlight the importance of addressing mental health concerns, such as depressive symptoms, within the context of bereavement to mitigate potential adverse effects of cognitive well-being in later life.

Abstract citation ID: igae098.3060

### EXAMINING THE INTERPERSONAL-PSYCHOLOGICAL THEORY OF SUICIDE AMONG OLDER KOREANS: DOES AGE MATTER?

Jiyoung Lyu[1], Hye-Mi Kim[1], Keung-Ja Hong[2], and Byoung-Jun Park[2], *1. Hallym University, Chuncheon, Kangwon-do, Republic of Korea*, *2. Sogang University, Seoul, Republic of Korea*

The interpersonal–psychological theory of suicide proposes that perceived burdensomeness, thwarted belongingness, and the acquired capability for suicide leads to suicide attempts. However, whether this applies to all age groups is not clear. Therefore, this study was aimed to apply the interpersonal-psychological theory of suicide to older Koreans of different age groups. Participants of this study were 1,200 older adults aged 65 years and above (347 Young-old, 853 Old-old) who completed 2020 surveys on Elderly Life Conditions in Chuncheon, Korea. The Interpersonal Needs Questionnaire, the Suicidal Ideation Scale, and the Acquired Capability for Suicide Scale were used to assess participants' perceived burdensomeness, thwarted belongingness, suicide attempts, and the capability for suicide. A multivariate regression results showed that thwarted belongingness was significantly associated with suicide attempts among young-old group, while perceived burdensomeness was significantly associated with suicide attempts among old-old group. These results suggest that the mechanisms around suicide attempts among older Koreans may be different by different age groups.

Abstract citation ID: igae098.3061

### JOINT ASSOCIATION OF VISIBLE AND SELF-PERCEIVED AGING WITH PREMATURE MORTALITY

Minghao Kou, Hao Ma, Xuan Wang, and Lu Qi, *Tulane University, New Orleans, Louisiana, United States*

This study quantifies the association of a combination of visible and self-perceived aging indicators with the risk of all-cause and cause-specific premature mortality. Utilizing data from 369,741 UK Biobank participants initially free of cardiovascular disease and cancer, with follow-up until December 31, 2022, we analyzed four aging indicators: hearing loss, teeth loss, self-perceived age, and falls. Using Cox proportional hazards models, we assessed their joint association with mortality outcomes over a median follow-up of 13.74 years, during which 22,934 cases of premature mortality were documented. Compared to participants without aging indicators, those exhibiting all indicators experienced a 81% (95% confidence interval, 59%–107%), 96% (47%–160%), 55% (26%–91%), and 114% (73%-165%) higher risk of all-cause, cardiovascular disease, cancer, and other causes of premature mortality, respectively. Notably, these associations were more pronounced in younger participants, those with unhealthy lifestyles, and lower socioeconomic status (P < 0.05 for interactions). Additionally, we identified a significant additive interaction between aging indicators and frailty index, contributing an additional 16.08% (95%CI, 7.91-24.25%) risk of premature mortality. In summary, the combination of visible and self-perceived aging indicators was associated with a higher risk of all-cause and cause-specific premature mortality. These findings highlight the importance of considering both visible and self-perceived aging indicators in health risk assessments and intervention planning.

Abstract citation ID: igae098.3062

### PROLONGED GRIEF DISORDER IN ADULTS OVER 65: A REVIEW IN LIGHT OF POST-COVID-19 LOSSES

Hannah Friedland, Ashley Stripling, and Jillian Crocker, *Nova Southeastern University, Fort Lauderdale, Florida, United States*

Prolonged Grief Disorder (PGD) entered the DSM-5-TR in 2022, standardizing criteria and research. As COVID-19 continues to disproportionately affect adults over 65, both directly and by disrupting traditional grieving rituals, understanding PGD in this group is essential. The review seeks to synthesize existing research on PGD in adults over 65 to guide research and practice. A PRISMA review utilizing “older adults” and “prolonged grief disorder” was conducted within APA PsychInfo and Google Scholar from 2013 to 2023. All English-language journal articles relating to the area of study were read, and relevant exclusion criteria were applied (i.e., empirical, mean age >65, prolonged grief symptoms for >1 year). Among the 15 articles meeting inclusion, PGD prevalence rates ranged between 0.5% and 21%. Participants were predominantly White women recruited from community and hospice samples. PGD risk factors included preexisting depression and type of relationship with the deceased, with the highest incidence rates following partner or child deaths. Co-occurring symptoms included sleep disturbances, lower quality of life, loneliness, and dysregulated cortisol. Given PGD’s addition to the DSM and inconsistent prevalence rates, accurate diagnosis and effective treatment are imperative, particularly for more impacted groups. Notably, death rates from COVID-19 are disproportionate among age, race, and ethnicity. As a true understanding of how PGD manifests in adults over 65, future research is warranted following the COVID-19 pandemic. Implications of the current presentation include translating findings into recommendations focused on the provision of adapted PGD interventions and assessments for adults over 65.

Abstract citation ID: igae098.3063

### PUBLIC ATTITUDES TOWARD PHYSICIAN-ASSISTED DYING: A CROSS-SECTIONAL SURVEY IN TAIWAN

Duan-Rung Chen, Kevin Chien-Chang Wu, and Chun-Tung Kuo, *National Taiwan University, Taipei, Taipei, Taiwan (Republic of China)*

**Purpose:** This study aims to assess public attitudes towards Physician-Assisted Dying (PAD) in Taiwan, where legal support for PAD is notably absent despite its public endorsement in broader Asia, including Japan, South Korea, and China. Method: Utilizing an online survey, we gathered data from 3,922 Taiwanese adults from August to November 2022 to evaluate their support for PAD across different contexts: terminal illnesses, unbearable non-terminal pain, and severe cognitive impairments.

**Results:** The findings revealed substantial support for PAD, with 86.2% in favor of terminal illnesses, 79.2% for unbearable non-terminal pain, and 72.6% for severe cognitive impairments. Support for PAD correlated with younger age, male gender, lower levels of religiosity, non-medical professional background, and a positive attitude towards advance directives.

**Conclusion:** The public attitudes towards Physician-Assisted Dying (PAD) in Taiwan reveal a significant cultural transition towards valuing individual autonomy in end-of-life decisions. The study indicates a cultural shift towards valuing individual autonomy in end-of-life decisions among the Taiwanese public. It underscores the need for policymakers to align legal frameworks with public opinion on PAD, emphasizing the prioritization of individual rights in end-of-life care.

Abstract citation ID: igae098.3064

### TAILORED CARE FOR TERMINALLY-ILL OLDER ADULTS: INTEGRATED COMMUNITY-BASED END-OF-LIFE SUPPORT TEAM MODEL (ICEST)

Anna Yan Zhang, Estelle Xun Fang, and Gloria Ka Ming Chun, *The University of Hong Kong, Hong Kong, China (People’s Republic)*

**Background:** The integrated community-based end-of-life support team (ICEST) tailored the physical-psychosocial-practical care for community-dwelling terminally ill patients and enhanced the capacity of family caregiving through ongoing assessments and seamless interdisciplinary cooperation. The study evaluated the deceased patients’ first three-month care profiles and outcomes.

**Methods:** An intervention study with three-time-point repeated measures. Public hospitals identified and referred eligible older adults (age ≥ 60, life expectancy ≤12 months) to receive ICEST services. Patients’ clinical symptoms were measured at baseline, one month, and three months after, and the care they received was recorded. Deceased cases (n=756) were extracted for analysis. Repeated ANOVA was used to analyze the changes in care profiles and clinical outcomes using SPSS 27.0.

**Results:** The ICEST could significantly reduce patients’ physical and psycho-social-spiritual symptoms. Except for patients with a short survival period (≤3 months), they experienced a sharp worsening of pain (F (3,188)=2.82,p<.05) and weakness (F (3,188)=5.32,p<.01) and drowsiness remained consistently high (F (3,190)=6.29,p<.001). Depression levels of patients with a longer survival period (>12 months) decreased more after 1st month (F(3,168)=2.68,p<.05). The highest input was in psychosocial care. No significant change was found in its input or physical care, and practical support increased after the 1st month. Psychosocial care for patients with shorter survival periods (≤3 months) was higher initially and decreased later, whereas others showed the opposite trend.
**Conclusion:** Various care profiles indicated the moment-by-moment changes in care demands among community-dwelling terminally ill patients. Timely assessments and corresponding adjustments of interventions to address real-time needs are essential.

Abstract citation ID: igae098.3065

### THE ROLE OF FAMILY AND SOCIAL INTERACTIONS IN THE ASSOCIATION OF FAMILY BEREAVEMENT AND PSYCHOLOGICAL WELL-BEING

Nahyun Kim, Haeun Shin, and Kyungmin Kim, *Seoul National University, Seoul, Republic of Korea*

Older adults may experience the death of family members more frequently than ones in earlier stages of life, and such family losses can affect psychological well-being in late life. After experiencing family losses, social interactions with remaining family members and acquaintances can be important resources for psychological adjustment. Bereaved individuals may interact more frequently with remaining family members following family deaths. Some people may be more socially engaged to cope with the negative effects of family losses, while others may reduce their social activities. This study examined the mediating role of intergenerational contact and social participation in the association between the total number of family deaths (occurred in the past 10 years) and psychological well-being (i.e., life satisfaction and depressive symptoms). We analyzed a sample of 2,279 respondents (aged 65–80) from the 2020 Korean Longitudinal Study of Ageing. The results indicated that there was no direct association of the number of family deaths with life satisfaction and depressive symptoms. However, we found one significant indirect path; experiencing more family deaths was linked to more frequent physical contact with non-coresident children, which in turn, led to higher levels of life satisfaction. Yet, the frequency of participation in social meetings was not significant as a mediator in the link between the number of family deaths and psychological well-being. Our findings suggest the importance of considering multiple family losses in late life and investigating underlying mechanisms linking accumulative life stressors to psychological adjustment among older adults.

### SESSION 7465 (POSTER)

### DEMENTIA II (POSTERS)

Abstract citation ID: igae098.3066

### A REFLECTION OF A STUDENT-LED VIRTUAL REALITY ADOPTION PROGRAM FOR PEOPLE LIVING WITH DEMENTIA IN LONG-TERM CARE

Joey Wong, Karen Lok Yi Wong, Mary Van, Yumna Hayat, Christine Wallsworth, Jim Mann, Lily Wong, and Lillian Hung, *University of British Columbia, Vancouver, British Columbia, Canada*

Previous research suggests that virtual reality (VR) holds promise in improving residents' well-being in long-term care (LTC). With ongoing staff shortages, there is a critical need to explore, evaluate and share learnings on innovative approaches for adopting technologies, including VR, in LTC. In our research study conducted at two LTC homes in Vancouver, Canada, undergraduate students supported staff members in VR adoption. This study reflects on the opportunities and challenges of VR adoption from the students' perspectives. Utilizing a critical reflection framework, our interdisciplinary team engaged in extensive discussions and reflections via Zoom meetings, resulting in the following five themes: (1) Challenging the stigma towards dementia in LTC, (2) Addressing ageism in older adults' technology usage, (3) Building relationships through consistent and repeated visits to facilitate technology adoption, (4) Recognizing students' value in technology implementation and their potential impact on LTC's future workforce, and (5) Recognizing the importance of emotional and organizational support. Based on these reflections, our team devised the "ENRICH" framework, offering six practical tips to support student involvement in enhancing technology adoption in LTC. The team's insights highlight the potential of engaging university students in promoting technology adoption in LTC settings. The "ENRICH" framework provides a practical approach to fostering technology uptake while offering valuable learning experiences for students. This study encourages further exploration of student participation in LTC research to advance future initiatives in this field.

Abstract citation ID: igae098.3067

### "UKE CAN DO IT": A REFLECTION ON A UKULELE CLASS INITIATIVE FOR OLDER ADULTS LIVING WITH DEMENTIA AND CARE PARTNERS

Joey Wong, Jason Fu, Mario Gregorio, and Lillian Hung, *University of British Columbia, Vancouver, British Columbia, Canada*

Individuals with different stages of neurocognitive disorder or dementia respond to music even at later stages of the diagnosis. Music can offer collective experiences for people from diverse cultural backgrounds who have dementia. However, limited studies explore the journey of learning musical instruments by people living with dementia. Our team, co-led by a person living with dementia, initiated a 12-week basic ukulele program to explore the potentials of music and learning to foster social connections, alleviate loneliness and bring joy to older adults living with dementia and their care partners in Vancouver, British Columbia. Eighteen older adults from diverse backgrounds (15 individuals living with different stages of dementia and three care partners) participated in the classes facilitated by a ukulele instructor. Our team critically reflected on the ukulele program based on the in-class observations and summaries from the instructor through multiple in-person and Zoom meetings. The reflection resulted in five themes: 1) Social connections through music, 2) Respite for care partners, 3) Peer support and empowerment to contribute to collective learning experiences, 4) An emphasis on enjoyment over skills, and 5) Inclusiveness in creating programs for people living with dementia. This innovative ukulele program shows the positive impact of music and collective learning. The team reflections highlight the potential of offering musical instrument programs for older adults living with dementia and their care partners to alleviate loneliness and foster social connections. Future research can investigate the long-term impact of group musical learning on individuals living with dementia and care partners.

Abstract citation ID: igae098.3068

### ACCEPTABILITY AND FEASIBILITY OF IMPLEMENTING THE DEMENTIA CARE PRACTICE RECOMMENDATIONS IN CARE COMMUNITIES

Boeun Kim[1], Basia Belza[2], Lorna Prophater[3], Emily Waddington[3], Shawn Johnson[4], and Sam Fazio[3], *1. University of Iowa, Iowa, United States*, *2. University of Washington, Seattle, Washington, United States*, *3. Alzheimer's Association, Chicago, Illinois, United States*, *4. The Alzheimer's Association, Chicago, Illinois, United States*

With growing numbers of residents living with dementia in care communities, providing quality care is challenging. Alzheimer's Association developed an intervention to increase the adoption of person-centered care in care communities using a coaching approach based on the Dementia Care Practice Recommendations (DCPR). A cluster randomized-controlled study has been implemented to evaluate the intervention since December 2022. This study aims to report interim findings. Care communities in an intervention group met with a coach monthly over 6 months to assess the level of adoption of DCPR and increase and maintain person-centered care practices. Care communities in a control group received monthly emails not relevant to person-centered care. Employee satisfaction (job satisfaction, team building and communication, scheduling and staffing, training, management and leadership), person-centered practices (workplace practices, individualized care and services, caregiver-resident relationships), and confidence in dementia scale were assessed pre-intervention (time1), post-intervention (time2), and 3-month follow-up after completion of the intervention (time3). As of February 2024, 292 care providers from 53 care communities enrolled, 164 care providers from 35 care communities completed the 6-month intervention, and 113 care providers from 25 care communities filled out 3-month follow-up assessments. We found that scheduling and staffing satisfaction (time3), management and leadership satisfaction (time3), overall employee satisfaction (time3), and person-centered practices in individualized care and services (time2 and 3) were significantly higher in intervention groups than control groups according to generalized estimating equation estimates. This study provides preliminary evidence of the efficacy of the DCPR intervention in promoting employee satisfaction and person-centered practices.

Abstract citation ID: igae098.3069

### CARING FOR CANCER PATIENTS LIVING WITH DEMENTIA: CHALLENGES AND WAYS FORWARD

Shelley Canning[1], Michael McKenzie[2], Genevieve St-Martin[3], Jagbir Kaur[2], Nicole Percival[4], Lillian Hung[5], Lynn Jackson[5], and Rachel Wan[6], *1. University of the Fraser Valley, Abbotsford, British Columbia, Canada*, *2. BC Cancer, Vancouver, British Columbia, Canada*, *3. BC Cancer, Abbotsford, British Columbia, Canada*, *4. Maplewood Care Society, Abbotsford, British Columbia, Canada*, *5. University of British Columbia, Vancouver, British Columbia, Canada*, *6. Vancouver Coastal Health, Vancouver, British Columbia, Canada*

The purpose of this study is to address the inequity of care outcomes for patients living with both cancer and dementia through exploring current challenges and barriers, with the goals of creating dementia-friendly cancer care practice recommendations and education. Cancer patients also living with dementia have been found to experience poor outcomes, receiving less cancer screening, staging, and curative treatment than patients without dementia, thus they are typically diagnosed at later stage cancers with lower survival rates. We are conducting a qualitative applied health study drawing on Interpretive Description methodology underpinned by a person-centred philosophy. Phase one of the study has involved conducting interviews with patients, their care-givers, and cancer care providers. In this presentation we are focusing on the findings from our interviews with cancer care providers. Our sample includes 15 RNs, and 15 participants including physicians, therapists, and counsellors. Although they are experts in cancer, care providers often lack confidence and knowledge regarding dementia-care. Findings from their interviews led to themes reflecting both challenges and barriers to providing care. Specific themes related to care challenges include: Difficulty Following Instructions; Balancing Treatment Risks with QOL; Including Care-givers; and Ethical Dilemmas. Additionally, specific themes related to barriers include: Lack of Communication Processes; Lack of Continuity of Care; Lack of Resources; and Lack of Dementia Education. These themes will inform Phase two of the study which will involve creating dementia aware education and recommendations for dementia-friendly policies and processes to better tailor cancer care for patients living with dementia ensuring equity.

Abstract citation ID: igae098.3070

### CHALLENGES IN SUPPORTIVE CARE DECISION-MAKING IN THE CONTEXT OF ALZHEIMER'S DISEASE AND RELATED DEMENTIAS

Jean Taylor, and Anne Turner, *University of Washington, Seattle, Washington, United States*

In the United States, persons with ADRD face multiple decisions regarding supportive care transitions; however, their involvement in decision-making often declines as their condition progresses. The aim of the Decision Making in Alzheimer's Research (DMAR) study is to center the voice of persons with dementia by understanding preferences and key factors influencing supportive care decisions. We conducted in-depth online interviews (n=79) with 24 older adults with dementia (aged 65 years and older), 37 informal caregivers, and 18 professionals who work with individuals with dementia. Interviews focused on decision making and identifying factors that related to supportive care decisions. Qualitative analysis of interview transcripts revealed that persons with dementia and caregivers were often not ready to think about or plan for supportive care changes. Challenges participants described in moving forward in planning supportive care included lack of understanding or acceptance of the diagnosis, inability to imagine future scenarios, and lack of openness to planning for the future. Tailored information about the course of dementia and potential future care options are needed. Suggestions by professionals for the best timing for provision of a decision tool ranged from "as soon as possible" to "soon after diagnosis". Some professionals emphasized the need for ongoing conversations with trusted medical professionals as the disease progresses. Our study highlights the importance of early, tailored, and ongoing support in transition planning. We will share specific suggestions for how and when to implement a decision-making tool to assist with decisions about possible future needs.

Abstract citation ID: igae098.3071

### COMMUNITY-BASED DEMENTIA SCREENING AND SUPPORTIVE SERVICES

Traci Wilson, and Mary Ek, *USAging, Washington, District of Columbia, United States*

Early detection of dementia is important to help consumers and caregivers access information, treatment and supportive services. The majority of people living with dementia (PLWD) live at home and in the community. Community-based aging services providers, such as Area Agencies on Aging (AAAs), are positioned to play a critical role in screening for cognitive impairment or dementia and referring individuals for medical cognitive assessments. Past surveys have shown that AAAs provide specialized services for PLWD and caregivers, but little is known about the prevalence of screenings and the process for referrals. This poster shares findings from a 2024 survey of AAAs about their dementia screening and supports (n=180, response rate 28%) describing the formal and informal cognitive screening, assessment, and referral services that AAAs provide, the feedback available to consumers and the agency after referrals are made, and the types of services and supports available that are tailored specifically for PLWD. The most common screening or assessment service is referrals to external sources of information (84%), followed by agency and provider staff observing for signs and symptoms of dementia (55%), referring individuals to medical cognitive assessments (43%), and conducting screenings (31%). The results demonstrate that AAAs are involved in a variety of dementia-related screenings, services, and interventions.

Abstract citation ID: igae098.3072

### DEMENTIA SEVERITY AND QUALITY OF LIFE OF CARE PARTNERS OF OLDER ADULTS WITH DIABETES AND DEMENTIA

Oluwaseun Adeyemi[1], Caroline Blaum[2], Thaddeus Tarpey[1], Victoria Dickson[3], and Joshua Chodosh[1], *1. New York University Grossman School of Medicine, New York, New York, United States*, *2. NCQA, Ann Arbor, Michigan, United States*, *3. University of Connecticut, Storrs, Connecticut, United States*

Caring for persons living with dementia (PLWD) is known to adversely affect care partner health. Health impacts specific to care partners of PLWD who also have diabetes is unknown. To examine this issue further, we assessed the association between dementia severity and care partner quality of life when caring for PLWD with diabetes. We extracted cross-sectional care partner survey responses enrolled in the Enhanced Quality in Primary Care for Elders with Diabetes and Dementia study (N=311). We defined dementia severity as a three-level categorical variable (mild, moderate, severe) using the Dementia Severity Rating Scale. Quality-of-life outcome measures were treatment burden score (scored 0 to 15; higher score = higher burden) and physical and mental health state (scored 0 to 20; higher score = better health). We controlled for both care partner and PLWD's relationship, age, sex, race/ethnicity, and educational level. We performed multivariable quantile regression analyses and reported adjusted median difference (aMD) in quality-of-life measures (plus 95% confidence interval (CI)). Mean (standard deviation) ages of PLWD and their care partners were 81.5 (7.4) and 55.2 (12.4) years, respectively. Care partners were predominantly women (77.5%) and non-Hispanic Whites (38.9%). Compared to care partners of those with mild dementia, care partners of persons with severe dementia had more treatment burden (aMD: 1.41; 95% CI: 0.49, 2.32), and poorer mental health (aMD: -2.34; 95% CI: -3.60, -1.08). Caring for PLWD with diabetes exerts substantial burden on care partners especially when dementia is more severe and may benefit from more diabetes-focused education and support.

Abstract citation ID: igae098.3073

### DEVELOPING AN ONLINE TRAINING COURSE FOR OLDER-ADULT VOLUNTEERS TO LEAD DEMENTIA ACTIVITY PROGRAMMING

Lacey DiFranco[1], Silvia Orsulic-Jeras[1], Gregg Gorzelle[2], Sara Powers[1], and Michael Skrajner[2], *1. Benjamin Rose, Cleveland, Ohio, United States*, *2. Hopeful Aging, Winchester, Massachusetts, United States*

Research over the last decade has strongly supported the use of music intervention to treat symptoms of dementia. Making Connections Through Music (MCTM), a group music intervention designed for persons living with dementia (PLWD), utilizes older adult volunteers as session leaders, addressing both staff shortages in residential care and the high need for engaging socialization activities. This poster describes the development of MCTM's online volunteer training course and presents findings from a focus group study (N=7 focus groups) aimed to test course prototypes. Two prototypes of the training were developed and tested with older adult volunteers (n=25; Mage = 76.5; 76% female) and long-term care staff (n=25; Mage = 47.2; 88% female). A thematic analysis yielded a list of recommended changes and identified critical content areas for preparing volunteers to be effective session leaders. Volunteer and staff focus group findings determined a large number of themes including the following: 1) Specific training modules on responsive behaviors, communication challenges, and dementia education; 2) Format preferences such as a self-paced structure and interactive features; 3) Hesitancy with technology-based training; 4) Desire for having supplementary materials available; and 5) Importance of leveraging technology to support long-term program sustainability. Results are currently being utilized in the development of the complete MCTM volunteer online training course. Development of such a training is critical not only for MCTM but also in providing non-professionals the tools to adequately facilitate engaging activities, including group programming, for PLWD across a variety of care settings.

Abstract citation ID: igae098.3074

### EATING EXPERIENCES IN PEOPLE LIVING WITH DEMENTIA: A CONCEPT ANALYSIS USING RODGERS'S METHODOLOGY

Zih-Ling Wang, Jenna McHale, Basia Belza, and Jennifer Sonney, *University of Washington, Seattle, Washington, United States*

In people living with dementia, eating has often been portrayed in the literature as a time-consuming process or task-centered activity. However, it is important to distinguish the experience of eating from the process and task-centered approach of feeding. This study clarifies the concept of eating experience by using Rodgers' evolutionary approach to analyzing factors associated with eating experiences in people living with dementia present in the literature. Twenty-two articles met the inclusion criteria, identifying three key attributes of eating experiences (self-connection, the special journey of life, and self-interpretation). Antecedents, as framed by the socio-ecological model, were categorized to represent Intrapersonal (personal preferences, individual culture, mealtime routines), Interpersonal (social interaction), and Environmental (dining room environment, policies) factors. Consequences were divided into external (quality of life, nutritional health, and physical health) and internal (personhood, autonomy and independence, dignity and feeling valued, and mental well-being) domains. The identified attributes, antecedents, and consequences can be utilized in healthcare education, research, and intervention approaches. This presentation allows nurses and other health care professionals to understand better people living with dementia through the relationship between eating and interpersonal, intrapersonal, and environmental aspects to develop personalized interventions and care strategies to achieve an optimal quality of life.

Abstract citation ID: igae098.3075

### EFFECTS OF DEMENTIA DIAGNOSIS ON HOSPITAL READMISSION AFTER DISCHARGE FROM A SKILLED NURSING HOME

Jennifer Carnahan[1], James Slaven[2], Emily Fox Ludden[3], Corinne Bowditch[4], Healey Ryan[2], Li Jiangqiong[2], Wanzhu Tu[2], and Alexia Torke[5], *1. Indiana University/ Regenstrief Institute, Indianapolis, Indiana, United States*, 2. *Indiana University Indianapolis, Indianapolis, Indiana, United States*, 3. *Regenstrief Institute, Inc., Indianapolis, Indiana, United States*, 4. *Oakland University William Beaumont School of Medicine, Rochester, Michigan, United States*, 5. *Indiana University School of Medicine, Indianapolis, Indiana, United States*

High acute care utilization has been associated with dementia, but the risk of repeat acute care use following a skilled nursing home (SNH) to home transition is not well understood. We examined the association of dementia with 30-day hospital readmission after leaving a SNH. Data from the Health and Retirement Study from 2000-2018 was linked to Medicare and SNH claims. We analyzed the events of hospital readmission following SNH discharge using logistic regression while controlling for repeated within-subject readmissions. There were 5,912 discharges to the community from an SNH and 941 hospital readmissions. ICD codes for dementia were present with 1,754 of the SNH-to-home transitions and 314 of the hospital readmissions. In bivariate analysis, people with dementia were more likely to be readmitted (p=0.0074). Post-SNH readmissions were also more likely among people who were Black, male, dual eligible beneficiaries, longer pre-SNH hospital stays, higher Charlson Comorbidity Index, and greater activities of daily living deficits. In the multivariable logistic regression model, the odds of readmissions were still greater among persons with dementia, but significance was attenuated (OR 1.09; 0.90, 1.32; p=0.4065). Significant associations with readmissions include the Charlson Comorbidity Index (OR 1.07; 1.04, 1.09; p< 0.0001) and activities of daily living deficits (OR 1.11 (1.04, 1.17; p=0.0005). People with dementia may be at greater risk of readmission after SNH discharge but not when controlling for factors such as socioeconomic status, activities of daily living, and other comorbidities.

Abstract citation ID: igae098.3076

### EXPLORING THE DIAGNOSTIC AND THERAPEUTIC JOURNEY OF PRIMARY PROGRESSIVE APHASIA: A CASE STUDY FROM A RURAL SETTING

Abigail Avendaño Villaseñor[1], and Ladan Ghazi Saidi[2], *1. Universidad Autónoma de Occidente, Culiacan, Sinaloa, Mexico*, 2. *University of Nebraska Kearney, Kearney, Nebraska, United States*

Primary Progressive Aphasia (PPA) is a young-onset neurological disorder that affects a person's language, behavior, and executive function abilities, caused by a focal degeneration of the frontal and temporal lobes in the brain. It is considered a frontotemporal dementia (FTD) due to the neuroanatomical areas it affects and the similar etiology it has with other neurodegenerative disorders. Differential diagnosis is difficult for health professionals due to the recent description of clinical variants, and the limited options for clinical treatment. In this cross-sectional and descriptive study, we reviewed the case of a 76-year-old male patient diagnosed with agrammatic primary progressive aphasia (nfvPPA) who lives in a rural area in Nebraska, living with his caregiver. The objective of this case study was to analyze the difficulties experienced by the patient before and after he was diagnosed with nfvPPA, as well as his current progress with treatment. We identified the main challenges they encountered during the diagnostic and therapeutic processes were: a delayed differential diagnosis, inadequate communication from practitioners, inefficient interprofessional communication, and limited treatment/management options. In addition, they had limited access to educational and support group resources, a lack of intervention implementation, failed efforts in the follow-up of treatment by each care provider, and difficult access to health care services. Our findings highlight the necessary changes needed in the management of patients through a collaborative model, to provide sufficient and accessible resources to navigate this diagnosis, and the importance of following up on the therapeutic progress of patients.

Abstract citation ID: igae098.3077

### HEALTH CARE UTILIZATION AND COSTS FOR HOSPITALIZED DEMENTIA PATIENTS IN CHINA

Zhuoer Lin[1], Fang Ba[2], Heather Allore[2], and Xi Chen[2], *1. University of Illinois Chicago, Chicago, Illinois, United*

*States, 2. Yale University, New Haven, Connecticut, United States*

**Background:** Dementia poses challenges to patients and healthcare systems; yet little is known about the healthcare utilization and costs for dementia patients in China and their major contributors.

**Methods:** We identified 51,530 inpatient records of Chinese older adults who were admitted to 1,917 hospitals during 2017-2019 with a primary diagnosis of dementia. Multivariable logistic regressions estimated associations for emergency room (ER) admission and tertiary hospital admission; and linear regressions were performed to examine the determinants of length of stay (LOS), daily and total costs in Chinese Yuan (CNY). The potential contributors included age, sex, insurance type, working status, comorbidity, admission type and hospital level.

**Results:** The sample had a median age 78 (IQR: 72-84), of whom 46.2% were females, 20.2% were admitted through an ER, and 69.0% were admitted to tertiary hospitals. LOS, daily and total costs per admission were 21 days, 948 CNY and 12,602 CNY, respectively. Increased adjusted odds of ER admission were associated with having lung diseases or lower insurance coverage. LOS was longer for older patients, females, retirees, or those with more insurance coverage or admitted to lower-level hospitals. The total costs were higher among older patients, males, patients with lung diseases, more insurance coverage, and patients admitted to ER or tertiary hospitals.

**Conclusion:** There are large variations in inpatient care utilization and costs across dementia patients of different demographics, insurance types, and comorbidities. Our findings highlighted the importance of promoting chronic disease management and insurance coverage, as well as providing affordable and equitable dementia care.

Abstract citation ID: igae098.3078

### HOME-BASED REMINISCENCE INTERVENTION IN DEMENTIA: QUALITATIVE THEMATIC ANALYSIS

Xianglan Jin, Kuan-Ching Wu, Shao-Yun Chien, and Oleg Zaslavsky, *University of Washington, Seattle, Washington, United States*

Reminiscence intervention has shown promising potential in alleviating dementia-related morbidities, thanks to the relatively intact remote memories of persons living with dementia (PLWDs). However, reminiscence interventions have been primarily delivered in group settings by healthcare professionals, which is costly and hard to scale up. One innovative solution is training family caregivers to carry out reminiscence conversations with their loved ones in their homes at their convenience. This thematic analysis aims to examine the participants' perspectives of a home-based reminiscence intervention to inform future research on dyadic reminiscence interventions. A pilot study on a home-based reminiscence intervention was conducted between November 2022 and May 2023. Qualitative data were collected through semi-structured individual interviews with nine dyad participants at the end of the intervention. The interviews were audio-recorded and transcribed verbatim. Initial codes and categories were developed inductively, and queries on Atlas.ti were used for theme identification and recontextualization. Peer debriefing and external review were utilized to ensure rigor. Participants reported positive experiences of the home-based intervention. Centering on and engaging the PLWD in conversations was perceived as positive brain stimulation. Participants highly appreciated that it was conducted in home settings and acknowledged that the intervention content, duration (1-1.5 hours for 24 weeks), frequency (biweekly), and logistics were acceptable. PLWDs expressed willingness to share stories with their families. Most caregivers showed great interest in training on reminiscence conversations. Future studies should empower family caregivers to initiate and lead reminiscence conversations at home along with the provision of adequate caregiving support.

Abstract citation ID: igae098.3079

### IMPLEMENTATION OF DEMENTIA FRIENDS IN WASHINGTON STATE

Katie Zeitler, *University of Washington, Seattle, Washington, United States*

Dementia Friends (DF) is a global movement whose mission is to raise awareness about dementia and the challenges associated with its stigma. The purpose of this presentation is to trace the journey including the evidence supporting the implementation of DF in Washington State and describe next steps. The movement has 13.5 million participants worldwide and utilizes a train-the-trainer model to deliver information sessions to members of the community that discuss what dementia is, five key messages, and communication tips. From 2018-2019, DF was piloted in Washington with the support of the Dementia Action Collaborative. Data showed that Dementia Attitudes Scale scores improved after a DF session and that this improvement was maintained through the one-month follow up. The results from the pilot program led to the approval of funding from Washington state for the implementation of the program in 2022. The state lead for DF Washington, the UW Memory and Brain Wellness Center, implemented the program by partnering with Regional Lead Organizations to assist in deploying the train-the-trainer model to expand DF in their regions. Each Regional Lead Organization nominated a Regional Coordinator to oversee the DF program in their region and train new volunteer "DF Champions" to lead information sessions. Funding from the state and partnerships with Regional Lead Organizations have facilitated the expansion of DF program from 214 participants in 3 counties in 2019 to 2,170 participants in 24 counties in 2024. A key challenge for DF will be cultivating community partnerships in rural areas and underserved populations.

Abstract citation ID: igae098.3080

### IN-HOSPITAL MORTALITY AMONG DEMENTIA PATIENTS IN CHINA: EVIDENCE FROM HOSPITAL ADMINISTRATIVE DATA

Zhuoer Lin[1], Fang Ba[2], Heather Allore[2], and Xi Chen[2], *1. University of Illinois Chicago, Chicago, Illinois, United States, 2. Yale University, New Haven, Connecticut, United States*

Despite the fast-growing dementia patient population, dementia care in China is still inadequate and inequitable that may lead to undesirable disease outcomes. We assessed the in-hospital mortality for dementia patients in China and its variations across patient and hospital characteristics. Inpatient medical records of Chinese older adults admitted to 1,917 hospitals during 2017-2019 with a primary diagnosis of dementia were analyzed; and multivariate logistic regression was used to assess the association of in-hospital mortality with age bands, sex, working status, insurance type, major comorbidity, admission type, and hospital level. Out of 51,525 included admissions, 363 dementia patients died in a hospital (0.70%, 95% CI, 0.63% - 0.78%). The odds of in-hospital mortality were significantly higher for patients aged 80 and over, having lung diseases, and for those admitted through emergency room or hospital transfers, or those admitted to lower-level hospitals compared to their higher-level counterparts. These findings suggest large variations in dementia in-hospital mortality that can be associated with both patient-level and hospital-level factors. Targeted and coordinated dementia care should be promoted to reduce in-hospital mortality and provide more equitable and quality dementia care.

Abstract citation ID: igae098.3081

### INTERGENERATIONAL BENEFITS OF DISASTER PREPAREDNESS CAREGIVER INTERVENTION

Maria Donohoe[1], Morolake Adeagbo[1], Brittany Anderson[1], Aaron Seaman[2], and Sato Ashida[1], *1. University of Iowa, Iowa City, Iowa, United States*, 2. *University of Iowa, Iowa CIty, Iowa, United States*

The evaluation of interventions focuses on understanding how intervention contents and components impact participant outcomes through specified mechanisms of influence. Social network literature highlights the importance of quality interactions; however, little attention has been paid to how the characteristics of interpersonal interactions may influence intervention processes and impacts. Implementation of a disaster preparedness intervention with caregivers of persons living with dementia (PLWD) began as part of a randomized controlled trial. The Disaster PrepWise-Caregiving (DPW-CG) program helps caregivers develop a household disaster and emergency management plan considering the unique family caregiving needs. Currently, DPW-CG is being delivered by trained AmeriCorps members who are college students. As part of a larger trial, a team of implementation scientists are concurrently conducting observational evaluations of the intervention delivery. The preliminary data reveals key characteristics of intergenerational interactions between the caregivers and the AmeriCorps members that influence the quality of intervention delivery. Our initial findings show that caregivers of PLWD, especially spousal caregivers who are older, looked forward to the company of AmeriCorps members and shared their life experiences and values throughout the visit. Before the commencement of intervention delivery, older caregivers strategically welcomed and eased AmeriCorps members into the process through small talk, offering food/drinks, and confidence-building compliments. This often resulted in better engagement during the intervention and positive learning experiences for the AmeriCorps members. Future intervention trials may seek to understand how characteristics of interpersonal interactions benefit program deliverers and recipients and impacts intervention effectiveness and outcomes.

Abstract citation ID: igae098.3082

### P. GINGIVALIS-LPS PROMOTES AD/ADRD VIA CASPASE-4-MEDIATED NONCANONICAL INFLAMMASOME PATHWAY

Ambika Verma, Gohar Azhar, Pankaj Patyal, Xiaomin Zhang, and Jeanne Y Wei, *University of Arkansas for Medical Sciences, Little Rock, Arkansas, United States*

Alzheimer's disease (AD) and related dementias (ADRD) are significant contributors to illness and death in the older adult population worldwide. Chronic periodontitis and its keystone pathogen, Porphyromonas gingivalis, have increasingly been linked with AD/ADRD. P. gingivalis releases various virulence factors, including lipopolysaccharide (LPS), which have been shown to trigger systemic inflammatory events responsible for neuroinflammation and AD/ADRD. Caspase-4 directly senses and binds cytosolic LPS, promoting its oligomerization and activation, leading to the formation of the non-canonical inflammasome. However, the role of caspase-4 in P. gingivalis- LPS mediated AD-related neuropathology remains poorly defined. We hypothesized that P. gingivalis- LPS may be responsible for neuroinflammation via activation of caspase-4 mediated non-canonical inflammasome pathway through activation of its downstream NOD-like receptor family pyrin domain containing 3 (NLRP3) and caspase-1 inflammasome dependent cell signaling. Our overall hypothesis is exploring the cell signaling pathway by which P. gingivalis- LPS are able to affect the microglial/neuronal cells via activating caspase-4, that might be responsible for neuroinflammation and lead to AD/ADRD. Our results suggest that P. gingivalis-LPS activated caspase-4 and cleaved gasdermin D (GSDMD) to form the GSDMD pores to trigger pyroptosis, a form of programmed lytic cell death. The GSDMD pores in the plasma membrane also promoted $K^+$ influx, sensed by the NLRP3 inflammasome to activate caspase-1, resulting in IL-1β maturation and secretion, as well as pyroptosis. This P. gingivalis- LPS induced caspase-4-mediated NLRP3/caspase-1 activation and associated IL-1β secretion, promotes neuroinflammatory environment that drives AD/ADRD pathogenesis.

Abstract citation ID: igae098.3083

### REDUCING CARE REFUSAL IN OLDER ADULTS WITH DEMENTIA THROUGH HUMANITUDE CAREGIVER TRAINING

João Pärtel Araújo[1], Catherine Van Son[2], Helena Luz[3], and Rosa Melo[4], *1. Coimbra University, Aljubarrota, Coimbra, Portugal*, *2. Washington State University, MARYSVILLE, Washington, United States*, *3. University of Coimbra, Coimbra, Coimbra, Portugal*, *4. Escola Superior de Enfermagem de Coimbra, Coimbra, Coimbra, Portugal*

**Background:** Care refusal by older adults with cognitive impairment creates significant challenges for professional caregivers. Humanitude is a multimodal care method that promotes well-being during care for both care recipients and caregivers. This study evaluated the impact of Humanitude training for professional caregivers on refusal of care behaviors in older adults with cognitive impairment in an acute care setting.

**Methods:** Two 4-day Humanitude care method (HCM) trainings were conducted for 23 healthcare professionals from six geriatric wards of an acute hospital. Training included theory, simulation, and clinical practice of Humanitude techniques with identified older adults. The participants identified 17 older adults with cognitive impairment who routinely refuse care. Refusal of care was measured at baseline and after Humanitude training using the Refusal of Care Informant Scale (RoCIS).

**Results:** Most of the 23 healthcare professionals participating in the training were nurses, 78%, with an average of seven years of experience. The older adults identified were women (65%), with an average age of 83. All the older adults identified had a diagnosis of a major neurocognitive disorder, and the average Functional Assessment Staging Tool score was 4.9. The average RoCIS scores decreased by 71.4% after the participants used Humanitude techniques during the training's clinical practice.

**Conclusion:** A brief 4-day HCM training reduced care refusal by older adults with cognitive impairment. Implementing Humanitude care methods could improve caregiver capacity to address care refusal challenges and mitigate its effects on caregiving. Studies on Humanitude's sustainability and outcomes are needed for caregiver and organizational adoption.

Abstract citation ID: igae098.3084

### THE CONTINUOUS LEARNING NEEDS OF PERSONAL SUPPORT WORKERS WHO CARE FOR PEOPLE LIVING WITH DEMENTIA IN LONG-TERM CARE

Grace Norris, Marie Savundranayagam, Afshin Vafaei, and Gail Teachman, *Western University, London, Ontario, Canada*

Personal support workers (PSWs) in long-term care (LTC) homes comprise over half of the workforce responsible for providing care to people living with dementia. Compared to other healthcare professionals, PSWs receive the least education, which fails to equip them with the necessary competencies for quality dementia care. To provide optimal care to people living with dementia, PSWs need to be offered opportunities for continuous education that addresses their specific learning needs. Therefore, this study identified and examined the dementia-specific learning needs of PSWs in LTC. Interpretive description guided the secondary qualitative analysis of 22 focus groups with 'mid-career' PSWs (n = 39) in LTC. The analysis identified specific learning needs along with ways in which those needs are best met. The learning needs include: 1) addressing responsive behaviours, 2) person-centered communication and attitudes, 3) delirium, and 4) dementia as a chronic condition. Learning needs were most commonly attributed to limited preparation during formal PSW education and a lack of continuous training opportunities throughout their career. Learning needs are best met through experiential methods involving peer learning, feedback, and evaluation within a supportive environment. For the learning needs to mediate practical outcomes, there needs to be an openness to dementia education and a good teamwork culture within LTC. The data generated from this research study will be essential to developing future continuing education for PSWs, contributing to knowledge about dementia care within LTC settings, and improving the quality of care provided to people living with dementia.

Abstract citation ID: igae098.3085

### TRAINING, SUPPORTING, AND ENGAGING VOLUNTEERS 55+ IN VIRTUAL COMPANIONSHIP FOR PEOPLE WITH DEMENTIA

Jessica Herpst[1], Quincy Samus[2], Katherine Marx[2], and Kathleen Kirchner[2], *1. Alzheimer's Association - Greater Maryland Chapter, Baltimore, Maryland, United States*, *2. Johns Hopkins University, Baltimore, Maryland, United States*

Volunteers play an essential role in our communities and societal health. Engaging, training, and mobilizing a critical mass of volunteers to address social isolation and other dementia care needs may be a potent approach to address the dementia care crisis, and one that is attractive to older volunteers seeking to remain in impactful post-retirement roles. A collaboration between Johns Hopkins University and the Alzheimer's Association evaluated the feasibility and acceptability of a virtual companionship program for community-residing people with dementia (PWD) delivered by trained Volunteers 55 years and older in the MEMORI Corps trial. We aim to describe the feasibility and lessons learned for recruitment, training, and support of the program volunteers. 52 eligible volunteers were recruited and 35 completed initial volunteer onboarding and training modules and were matched to provide regular virtual visits to a person living with dementia for a 12-week intervention. An approachable and responsive training program and learning environment was created that included didactic learning modules as well as initial and experiential learning opportunities supported by an interdisciplinary clinical team and weekly group-based training support. Common implementation challenges included volunteer long term commitment, difficulty using technology or low confidence using Zoom, scheduling of sessions, maintaining professional boundaries, and addressing dementia symptoms during sessions. Perceived benefits included camaraderie among volunteers; building confidence in using technology, in working with PWD and interpersonal skills; learning about healthy aging; and feeling more productive and connected to the community. Overall, volunteer engagement was feasible for volunteers and had perceived benefits.

Abstract citation ID: igae098.3086

### VISUALIZING EATING EXPERIENCES IN DEMENTIA: USING MASLOW'S HIERARCHY OF NEEDS AND THE MUNSELL COLOR SYSTEM

Zih-Ling Wang, and Jenna McHale, *University of Washington, Seattle, Washington, United States*

Eating experiences among individuals with dementia represent a multifaceted, subjective, and unique life journey. Despite their complexity, studies analyzing these experiences are limited. This study aims to explore the various domains that shape the trajectory of eating experiences in individuals living with dementia, adapting the Maslow's Hierarchy of Needs five-stage model and Munsell Color System to map these experiences visually. This presentation introduces an innovative model for visualizing the antecedents of eating experiences. It proposes strategies to enhance positive outcomes and nutritional health in individuals with dementia throughout their experience trajectory. Eating experiences are described across five domains: Aesthetic, Esteem, Sociocultural and Belonging, Safety, and Physiological and Biological. As dementia progresses, increased dependency, the domains measured by degree inward towards the center, can significantly affect these eating experiences. These domains are situated by their degree, with each domain independently fluctuating in size and shape based on the degree of maintained personhood as dependency needs shift. Minimal variation within these domains is essential for optimizing the eating experience. Each domain-related experience is subject to fluctuation over time, influenced by both external and internal life events. The trajectory's shape is a visual representation of changes in experience over time. By identifying positive and negative cases within the eating experiences, this presentation illustrates how deficiencies in specific domains can lead to negative eating experiences. Additionally, the presentation offers health professionals a method to tailor care strategies for enhancing eating experiences by interpreting current and trend domain mappings.

Abstract citation ID: igae098.3087

### WELL-BEING AND FUNCTIONING PROFILE AMONG OLDER ADULTS BETWEEN DIFFERENT LIVING SETTINGS

Wan-Ling Hsu, and SangNam Ahn, *Saint Louis University, St. Louis, Missouri, United States*

Addressing the care needs of older adults in the United States is complex and challenging, especially considering that more than half of Americans aged 65 years and older have three or more chronic diseases, often accompanied by cognitive impairment. Thus, it is imperative to identify the types of care that may be more suitable for older adults who are potentially cognitively impaired. This study examined well-being and functioning profile (WFP) among older adults with probable or possible dementia between nursing home and comprehensive senior living settings, using the living settings as a proxy for types of care. The WFP includes assessments of emotional well-being, bothersome pain, general health, and functional limitations. The analysis is based on data collected from the National Health and Aging Trends Study spanning from 2018 to 2022. Linear regression with survey weights revealed a significant association between living settings and WFP among the 338 participants with probable or possible dementia. Specifically, older adults residing in comprehensive senior living settings exhibited notably enhanced emotional well-being (Coef= -5.276, $p < 0.001$), general health (Coef= -1.117, $p = 0.006$), and limitations with mobility (Coef=-3.180, $p = 0.003$) compared to those in nursing home settings. Findings emphasize the potential strength of comprehensive senior living settings in caring for older adults with probable or possible dementia, enhancing improved outcomes. This prompts further research into specific care components and strategies to integrate successful practices from comprehensive senior living settings into nursing home settings, fostering well-being for this growing demographic facing cognitive challenges.

### SESSION 7470 (POSTER)

### ECONOMICS OF AGING

Abstract citation ID: igae098.3088

### ASSISTANCE, PLANNING, AND UNMET NEEDS: FACTORS SHAPING OLDER ADULTS' PET CARE BEHAVIORS IN THE UNITED STATES

Jessica Bibbo, Sarah Nicolay, and Samantha Tuft, *Benjamin Rose, Cleveland, Ohio, United States*

Pets continue to be an important relationship for many people throughout life, while the abilities and resources available to provide for a pet change over the life course. This study surveyed people ages 50 and older on the assistance they had with pet care, whether they had gone without necessities to provide for their pet, and whether they had plans in place for their pet in case of emergencies or major life events. Participants (N=1,039) were enrolled in Foresight 50+, an omnibus survey representative of the US household population aged 50 or older. Descriptive statistics determined the proportion of adults who had experienced pet care behaviors along with specific types of assistance, necessity, and planning. A series of multivariate logistic regressions were conducted to identify associations between demographics and having assistance, going without, and planning. Most pet owners (72.4%) had some type of help with pet care, 18.8% had gone without at least one necessity to provide for their pet, and 23.7% had at least one plan in place for their pet. Larger household size was associated with having assistance (OR=1.96, p=.026). Lower income (OR=1.20, p=.024) and younger age (OR=1.06, p=.038) were associated with going without a necessity. Being female (OR=4.54, p=.003), smaller household size (OR=1.76, p=.036), and older age (OR=1.06, p=.010) were associated with having at least one plan in place. Available resources and personal characteristics significantly impact pet care behaviors. These results are a step toward better understanding how to support people and pets age in place.

Abstract citation ID: igae098.3089

### COST EFFECTIVENESS ANALYSIS OF IMPLEMENTING A SCREENING PROGRAM FOR EARLY-STAGE ALZHEIMER'S DISEASE DETECTION

Yeongin Jung, Kaili Wu, Mashael Alnufeay, Xiaotong Jin, Jeromie Ballreich, and Emmanuel Drabo, *Johns Hopkins University, Baltimore, Maryland, United States*

Recent advancements in Alzheimer's Disease(AD) diagnosis have been propelled by the advent of disease-modifying therapies and the emergence of plasma-based biomarkers, notably P-tau217, which offers diagnostic accuracy comparable to cerebrospinal fluid assays and enables early disease detection. This study evaluates the cost-effectiveness of two AD early detection strategies for individuals aged 65 and above showing early-stage neuropathological abnormalities: solely using blood biomarker testing(P-tau217) and a sequential approach starting with plasma biomarker followed by confirmation via Aβ-PET scan. The cost-effectiveness of two early screening methods in a representative cohort of older adults in the US aged 65 and above with early-stage brain abnormalities(n=1000) is evaluated: 1) P-tau217 blood biomarker testing alone(Strategy 1); and 2) P-tau217 blood biomarker testing followed by confirmatory Aβ-PET scan(Strategy 2). Employing a hybrid model combining a decision tree and a Markov model, we simulated screening decisions, outcomes, costs, and quality-adjusted-life-years(QALYs) across AD progression stages(preclinical, mild, moderate, severe) and death over 35 years. Results suggest that Strategy 1(blood biomarker testing alone) dominates Strategy 2, producing 891 incremental QALYs at the additional cost of $49.2million translating to an incremental cost-effectiveness ratio(ICER) of $55,194/QALY gained and an incremental net monetary benefit of $39.9million at the decision threshold of $100,000/QALY gained. These results were attributable to the direct medical costs from delayed AD identification by false-negative misclassifications, and the avoided disutility from Aβ-PET. The findings were robust to uncertainties in model input parameters and highlight the utility of blood biomarkers as a cost-effective preclinical AD screening option in US older adults.

Abstract citation ID: igae098.3090

### DISCHARGE PREPARATION FOR OLDER ADULT IN PROLONGED EMERGENCY: A CASE-CONTROL STUDY

Jinming Liu[1], Fengli Gao[1], and Zijing Chen[2], *1. Beijing Tsinghua Changgeng Hospital, Beijing, Beijing, China (People's Republic)*, *2. The First Hospital Of Jilin University, Changchun, Jilin, China (People's Republic)*

This study aims to explore the effectiveness of discharge preparation services in older adult with prolonged emergency observation and functional impairment. From August 2020 to June 2023, older adult (age ≥60 years) with Barthel Index < 100 and a hospital stay of >7 days in the emergency observation ward at a tertiary hospital in Beijing, China were included. A retrospective case-control study was conducted. The intervention group, guided by dedicated nurses, underwent personalized assessments, comprehensive care planning, and follow-up, while the control group received standard care. Primary outcomes included average hospitalization costs, length of stay, and 30-day readmission rates. A total of 628 patients were included after one-to-one nearest-neighbor matching without replacement. Among them, 314 received routine discharge preparation, and 314 received specialized nurse-led discharge preparation. Both groups demonstrated no statistically significant differences in baseline characteristics. The intervention group showed no significant difference in average hospitalization costs (72030 ± 63533RMB vs. 72446±68282RMB, t = 1.99, P = 0.906) or average length of stay (36 ±32days vs. 36 ±27days, t = 1.159, P=0.803). However, the intervention group exhibited a significantly lower 30-day readmission rate compared to the control group (6.7% vs. 15%, t = 3.36, P = 0.008). The findings suggest that while discharge preparation services may not impact immediate costs or length of stay for older adult with prolonged emergency observation and functional impairment, they significantly contribute to reducing the 30-day readmission rate. This emphasizes the importance of specialized nurse-led interventions in enhancing post-discharge outcomes and reduce healthcare system burden.

Abstract citation ID: igae098.3091

### FAMILY TIES AND HEALTH CARE DECISION-MAKING IN LATE LIFE: EXAMINING FINANCIAL DEPENDENCY IN HEALTH FOR INDIA

Atul Pandey, and Isha Sharma, *Indian Institute of Technology Jodhpur, Jodhpur, Rajasthan, India*

With the increasing number of age 60 years and above population, it is becoming important to improve healthcare and economic assurance for grandparents/parents. Family ties and culture is a way of consideration of healthcare choices and providing financial support. Family member's financial stability assure the finance for healthcare for aged population. The dissolution of traditional extended families in India is closely related to the above-described issues, which also lead to rising economic inequality, urbanization, modernization, growing individualism, and a greater emphasis on healthcare choices. The rise in nuclear families and longer life expectancies has led to an increase in the cost of caring for the late ageing population. This study is examining the role of family ties and healthcare decision making through the lens of financial dependency. This study uses Longitudinal Ageing Study in India (LASI) i.e. secondary data source capturing 31464 respondents with the age of 60 years and above. It again classified into the male (16366) and female (15098) cohort. The data provide significant information related to the coverage of financial security. One out of every five individuals with age 60 years and above is enrolled in an organized sector's pension plan. A sizable share (20.65%) of the population is covered by central and state pension programs. In contrast, only a small percentage of India's retired population (2.1%) is covered by private pension, or employer-funded pension schemes. Just 7% of individuals believe their family's financial situation is adequate, whereas 53% comes from middle class family.

Abstract citation ID: igae098.3092

### FINANCIAL TRANSFERS BETWEEN OLDER FILIPINOS AND THEIR ADULT CHILDREN: THE ROLE OF EDUCATION AND PUBLIC PENSIONS

Kent Jason Cheng, *The Pennsylvania State University, University Park, Pennsylvania, United States*

**Objectives:** The Philippines is expected to transition to an aging society in less than two decades. Provision of resources and safety nets largely befalls family members since government support is limited. This study examines how education and public pensions shape older Filipinos' financial transfers to and from their adult children.
**Methods:** Data on Filipinos aged 60+ with at least one living child came from the 2018 Longitudinal Study of Ageing and Health in the Philippines (n=5,645). Logistic regressions interacting educational attainment and public pension receipt (no pensions, have contribution-based pensions, have social pensions, have both) were ran, controlling for relevant sociodemographic factors, average characteristics of children, and living arrangements. Predicted probabilities were generated and pairwise comparisons were conducted.
**Results:** Higher educational attainment and having contribution-based public pensions were associated with higher (decreased) odds of giving (receiving) transfers. Interactive models suggest a modest moderating effect of education and public pension types on giving and receiving financial transfers. Within education level, public pension receivers, especially those with contribution-based pensions, tend to have the highest probability of transferring to children and lowest probability of being exclusively on the receiving end of money transfers.

**Discussion:** Public pension receivers being more likely to give downward financial transfer across all educational levels suggests that public transfers for older adults benefit both the intended beneficiaries and their descendants. Additionally, older parents having contribution-based pensions as an income source spares their adult children from providing upward transfers. Results support crowding in – that public transfers facilitate private transfers.

Abstract citation ID: igae098.3093

### GROWTH OF THE PROGRAM OF ALL-INCLUSIVE CARE FOR THE ELDERLY AND THE ROLE OF PRIVATE EQUITY

Katherine Miller[1], Ravi Gupta[2], and Daniel Polsky[1], *1. Johns Hopkins University, Baltimore, Maryland, United States*, *2. Johns Hopkins University School of Medicine, Baltimore, Maryland, United States*

The Program of All-Inclusive Care for the Elderly (PACE) is a managed care program for adults 55+ who require nursing home level of care and are mostly dual-eligible for Medicare and Medicaid. PACE programs receive capitated payments per person for comprehensive medical and social services to enrollees, primarily in adult day health centers. In 2016, the Centers for Medicare & Medicaid began allowing for-profit ownership of PACE. Our objective is to describe the growth of PACE from 2010 to 2022 in the context of the 2016 policy change allowing for for-profit investment. We also describe the share of for-profit programs receiving private equity investments. We use Refinitiv and Medicare Advantage contract and enrollment data. Enrollment in for-profit programs from 2016 to 2022 increased by an annual average of 13% compared to 5.6% in non-profit programs. This is the context of an annual rate of increase in enrollment of 11% in the 6 years pre-policy change and 7% in the 6 years post-policy change. Among enrollees at for-profit programs in 2022, 47% received care at programs with private equity investments. Despite the growth of for-profit ownership and private equity investments, most enrollees received care from non-profit programs (78%) in 2022. Since allowing for-profit ownership, expansion has been driven by for-profit entities, ~50% of which received private equity investments. While expanding access to PACE may be important for certain adults, examining the effects of growth in for-profit and private equity ownership of PACE programs for patient outcomes is an important next step.

Abstract citation ID: igae098.3094

### HEALTH CARE USE IN DIVERSE OLDER ADULT POPULATIONS WITH ALZHEIMER'S DISEASE AND RELATED DEMENTIAS

Gee Kim, *FAIR Health, New York, New York, United States*

Average medical costs for people aged 65 and older with a diagnosis of Alzheimer's disease and related dementias (ADRD) are far higher than for people in the same age range without an ADRD diagnosis. Prior research shows the incidence of ADRD is significantly higher for Black and Hispanic populations than for non-Hispanic white and Asian populations. It is unclear, however, whether there are significant racial and ethnic disparities in the overall utilization and cost of care among patients with ADRD. An independent nonprofit organization, with the nation's largest collection of private healthcare claims, studied the relationship between imputed race and ethnicity and overall healthcare utilization and spending in over one million patients aged 65 and older with a diagnosis of ADRD. Among the preliminary findings: In comparison with patients in other racial/ethnic groups, Black patients with ADRD had (1) on average poorer health, as measured by a higher average number of chronic conditions and a higher Hierarchical Conditions Category (HCC) score; (2) more intensive healthcare utilization, i.e., on average more admissions, longer admissions, more total days spent in the hospital, more claims filed, more distinct procedures received and more distinct providers seen; and (3) higher spending even after controlling for race/ethnicity-related HCC scores. These findings suggest race/ethnicity influences healthcare utilization and spending directly and indirectly for patients with ADRD. Results can be used to inform healthcare research, clinical guidelines, care delivery and benefits for older people with ADRD, as well as inform health equity approaches among minority and diverse populations.

Abstract citation ID: igae098.3095

### LABOR MARKET PROSPECTS AND FINANCIAL AND HEALTH OUTCOMES OF ADULT CHILDREN CAREGIVERS AFTER THEIR PARENTS' DEATH

Stipica Mudrazija[1], Katherine Ornstein[2], and Fernando Hernandez[3], *1. University of Washington, Seattle, Washington, United States*, *2. Johns Hopkins University, Baltimore, Maryland, United States*, *3. Urban Institute, Washington, District of Columbia, United States*

As the population ages, an increasing number of adult children provide unpaid care to their parents who require help with health or function. While research and policy attention has focused on improving the caregiving experience, including workplace leave practices, little is known about the outcomes and challenges of family members once their caregiving role ends post-death. The context of caregiving, including factors such as the age of deceased parent, duration of care, level of (family) support, and caregiver gender, among others, may all impact the labor force trajectory and other adult children caregivers' outcomes following their parents' death. For this analysis, we use data from the 1998-2020 waves of the Health and Retirement Study, focusing on labor market trajectories, financial wellbeing and health outcomes of former caregivers ages 53-61 at the time of their parents' death. Our preliminary results suggest that within two years of their parents' death, adult children caregivers remain 6 percentage points less likely to work than those whose parents have not passed away. This suggests that former caregivers continue to be less likely to work than non-caregivers following the death of their parents. Additional analyses will focus on examining longer term labor force trajectory of former family caregivers as well as other outcomes of interest, including their financial wellbeing and physical and mental health status.

Abstract citation ID: igae098.3096

### LIVING WITH MULTIMORBIDITY AND FINANCIAL TOXICITY: A PATIENT- AND CLINICIAN-INFORMED FRAMEWORK

Cara McDermott, Michael Lourie, Hudson Hazlewood, and Caroline Sloan, *Duke University, Durham, North Carolina, United States*

Most older adults in the US have multimorbidity (2+ serious illnesses). Living with multimorbidity impacts patient quality of life, specifically managing the symptoms of multiple diseases and accompanying medications, emotional strain, and financial toxicity, or the financial stress of having unaffordable medical expenses. We developed an initial framework describing financial toxicity in multimorbidity to inform a financial toxicity screening tool and associated interventions for the clinical setting. We then iteratively refined the framework with patient and clinician feedback. We recruited 8 patients with multimorbidity taking 5+ medications from Duke Primary Care, along with 8 clinicians. We conducted semi-structured interviews with patients to describe their experiences living with multimorbidity and financing/prioritizing healthcare needs. In focus groups with clinicians, we explored ways to adapt existing financial toxicity screening questionnaires plus barriers/facilitators to future implementation of financial toxicity interventions in primary care clinics. Our initial framework included the dynamic nature between acute stressors (e.g., heart failure exacerbation), financial coping (e.g., borrowing money), financial reserve (e.g., insurance coverage), and patient outcomes (e.g., quality of life). We added/clarified the following domains following patient interviews and clinician focus groups: caregiver support/involvement (e.g. help with bills), health system constraints (e.g. cannot waive copays), health system-based support (e.g. charity care), community-level supports/programs (e.g. food banks), clinician moral distress (e.g. anger at not being able to provide optimal care), clinician financial coping (e.g. changing prescription based on cost). Next steps include using the framework to adapt screening questionnaires and interventions to detect and address financial toxicity in multimorbidity.

### SESSION 7475 (POSTER)

### EDUCATION AND TRAINING: WORKFORCE DEVELOPMENT

Abstract citation ID: igae098.3097

### ADDRESSING THE NURSING WORKFORCE GAP IN SKILLED NURSING FACILITIES WITH AN AGE-FRIENDLY CURRICULUM

Diane Brown[1], Sue Fosnight[2], Jennifer Drost[2], Mary Gergis[3], Cynthia Hovland[3], Margaret Sanders[4], Denise Kropp[4], and Michele Gareri[2], *1. University of Akron, Akron, Ohio, United States*, *2. Summa Health, Akron, Ohio, United States*, *3. Cleveland State University, Cleveland, Ohio, United States*, *4. Northeast Ohio College of Medicine, Rootstown, Ohio, United States*

Skilled nursing home quality and staffing are positively correlated. Staffing shortages have been a long-standing problem however, COVID-19 exacerbated the issue. Currently, more than one in four US nursing homes report staff shortages, especially those in direct care to residents, nurse aides and nurses. To encourage workforce growth, a 12-module virtual curriculum was developed including Age-Friendly Health Systems care, interprofessional teamwork, and other relevant topics. Each module included a recorded didactic, pre-post module quizzes, discussion boards, and supplemental videos and articles. Trainees also spent 10 hours shadowing the interprofessional team in a skilled nursing facility. Two cohorts of undergraduate senior nursing students (n=18, and n=35), nurse assistants in training (n=9, cohort 1), and social work students (n=9, cohort 2) have been trained. Outcome measure were pre and post-education using surveys: Nursing Home Attitude, PES-NWI work environment, and ISVS-9 for teamwork attitude. Results of the first nursing cohort rated the highest nursing home attitude items: ability to provide comfort, flexible scheduling and spending time with clients with the lowest rating for working with persons with dementia. The PES-NWI survey showed highest concern for teamwork, adequate staffing and supportive administration with the lowest concern related to use of nursing diagnoses. The ISVS showed similarities pre and post with highest scores for client involvement in care and importance to work as a team, and lowest for comfort leading a team. Administrators of skilled nursing and educators can use this information to close the gap on improving interest in this setting for new graduates.

Abstract citation ID: igae098.3098

### AGEIST ATTITUDES AND PREPARATION AS INDIRECT PREDICTORS OF TRAINEES' INTEREST IN CLINICAL WORK WITH OLDER ADULTS

Grace Caskie[1], Mackenzie Kirby[1], and Eve Root[2], *1. Lehigh University, Bethlehem, Pennsylvania, United States*, *2. Lehigh University, Clayton, Missouri, United States*

The striking discrepancy between the number of older adults in need of mental healthcare and the small number of psychologists with specialized training in providing this care (Moye et al., 2019) underscores the importance of identifying modifiable factors that could increase psychology trainees' interest in future clinical work with older adults. We tested a structural equation model specifying ageist attitudes and preparation regarding aging as having indirect effects on interest in working with older adults through three measures of perceived work with older adults. Doctoral trainees (N=187; age=22-43 years) in clinical/counseling psychology completed the Fraboni Scale of Ageism, a 10-point item assessing trainees' perceptions of how well their program prepared them to work with older adults, the Working with Older Adults Scale (assessing perceptions of ease/difficulty of work, social approval for work, favorable evaluation of work, and interest in work), the Social Desirability Scale, and demographic items. Controlling for trainees' age and social desirability tendencies, the model demonstrated good fit (CFI=.95, TLI=.94, RMSEA=.07). Significant indirect effects were found for ageist attitudes ($p<.001$) and preparation ($p=.01$) on interest. More ageist attitudes related significantly to greater perceived difficulty ($p<.001$), less favorable evaluations (p<.001), and less social approval (p=.02) regarding working with older adults, while greater preparation related only to less difficulty of working with older adults. Difficulty, social approval, and evaluation of work with older adults each significantly predicted interest (p<.001). Implementing training opportunities for trainees that aim to reduce ageist attitudes could increase interest in working with the older adult population.

Abstract citation ID: igae098.3099

### AGING SCIENCE EDUCATION: OLLI COLLABORATION BENEFITS UNDERREPRESENTED STUDENTS

Maeva Laflamme, Melanie Horn Mallers,
Laura Zettel-Watson, and Jennifer Piazza, *California State University Fullerton, Fullerton, California, United States*

The aging population is growing and diversifying, generating a need for workforce specialists (e.g., scientists, physicians) prepared to integrate knowledge about aging into their respective fields. Unfortunately, the fields that bring innovative change to improve the lives of older adults are the fields that continue to lack diversity due to a "leaky pipeline," whereby underrepresented students are often lost or pushed out academically from MSTEM fields. To increase diversity in the aging-science workforce, a critical goal is to implement training programs that advance the study of aging within MSTEM. With the support of NIH funding, we designed an empirically derived, multi-pronged program to purposefully intersect the study of aging with science and research. To increase student involvement with older adults, we partnered with our campus-based Osher Lifelong Learning Institute (OLLI) members, many of whom are scientists themselves, regularly serve as panelists, interview participants, proofread students' works, and act as audience members for our students. They also informally interact with students through community-based service learning opportunities, outreach, and volunteer activities. After collaborating with OLLI for one year, students report greater empathy and joy when interacting with older adults, increased interest and motivation to work in aging sciences, and decreased ageism. The MSTEM-OLLI collaboration has served as a critical component of advancing our goal of recruiting, retaining, and preparing underrepresented undergraduates for a future in MSTEM aging research. Our findings indicate that strengthening and expanding meaningful partnerships between older adults and students is a key priority to enhancing aging science programs.

Abstract citation ID: igae098.3100

### BUILDING CAPACITY TO ADDRESS HOARDING AND CLUTTER AMONG OLDER ADULTS: DEVELOPING A NETWORK OF TRAINED PROVIDERS

Bronwyn Keefe, Megan Nizza, and Jordana Muroff, *Boston University, Boston, Massachusetts, United States*

Hoarding disorder (HD) is a challenging mental health and public health problem characterized by difficulty discarding possessions, and excessive acquisition that contribute to substantial clutter and impaired functioning. HD occurs in 2-5% of adults and worsens with age. Clutter limits the ability to utilize space and function in a home, poses significant health risks, and creates housing instability. The lack of people trained in HD limits access to evidence-based assessments and interventions and home-based support that may mitigate risks. To address this, the Center for Aging and Disability Education and Research developed an online course on Hoarding Disorder in Older Adults to increase the knowledge and skills of those working in community-based agencies, including housing and local health departments. A total of 110 people completed the training, with 96% strongly agreeing it expanded their knowledge and understanding and 93% saying this training will help them to apply new practice skills. The learners showed significant improvements (p<.001) in all learning competencies from the pre-post-course self-assessments. The largest change was in the area of being able to identify intervention options and current treatment approaches with hoarding. This program can serve as a model to improve the attitudes, knowledge, and practice behaviors of a wide range of people who work with older adults. The goal is that this will lead to successfully engaging those with hoarding, which may increase openness to services, improve their mental health, and reduce public health risks so older adults can continue to live independently.

Abstract citation ID: igae098.3101

### DEVELOPMENT OF A TRAINING MODEL WITHIN HOSPICE AND PALLIATIVE CARE FOR NURSE PRACTITIONERS

Alex Laffer[1], Chandrika Kumar[2], Tracy Shamas[2],
Sarah Gillespie-Heyman[2], Alexandra Podosek[2], and
Andrea Ruskin[2], *1. VA Connecticut Healthcare System, New Haven, Connecticut, United States*, *2. VA Connecticut Healthcare System, West Haven, Connecticut, United States*

Workforce shortages within palliative care restricts availability of this service. Although clinicians may develop an interest in this specialty later in their careers, acquiring the necessary education and training is challenging. In academic medical centers like the Veteran's Administration (VA), interprofessional palliative care teams are well-positioned to deliver high-quality, accessible palliative care services when patient needs surpass what can be addressed by primary care and surgical subspecialities. Given workforce shortages, this quality improvement project aims to increase competency for nurse practitioners (NPs) in hospice and palliative care. A 12-month, minimum 500 clinical hour, training program designed to provide clinical and skill-based education to NPs currently working on the VA Community Living Center was developed. The predominantly practical curriculum focuses on interprofessional teamwork, communication skills, and application of medical, psychosocial, cultural, spiritual, and ethical content. The curriculum is designed in a longitudinal manner that supports the NP's ability to maintain their routine clinical care responsibilities. The program includes two hours of protected time each day consisting of hospice and palliative care clinical experiences alongside interprofessional learning. The training cohort, beginning March 2024, consists of two NPs. The team developed a mixed methods approach to assess competencies and progress across the training period. This project has resulted in an innovative approach to enhance hospice and palliative care training that will advance mid-career clinicians' skills and help address workforce shortages without requiring clinicians to leave their jobs for extended training. Implementation will have implications for the feasibility, sustainability, and replication of this model.

Abstract citation ID: igae098.3102

**NUMERACY SKILL USE AND SKILL PROFICIENCY AMONG OLDER WORKERS IN THE UNITED STATES**

Takashi Yamashita[1], Donnette Narine[1], Runcie Chidebe[2], Phyllis Cummins[2], Jenna Kramer[3], and Rita Karam[4], *1. University of Maryland Baltimore County, Baltimore, Maryland, United States*, *2. Miami University, Oxford, Ohio, United States*, *3. RAND Corporation, Arlington, Virginia, United States*, *4. RAND Corporation, Santa Monica, California, United States*

Recent studies linked numeracy—the ability to understand numeric information -- and a variety of economic well-being outcomes (e.g., employment, income). However, aging is generally associated with lower numeracy skills, and in turn, older workers may face disadvantages in the labor market. The key to maintaining and promoting numeracy skills over the life course is continuous use in everyday life as well as at work. Yet relatively little is known about how older workers use their numeracy skills. Nationally representative data of older workers aged 50 years and older (n = 1,760) were obtained from the 2012/2014/2017 U.S. Program for the International Assessment of Adult Competencies (PIAAC) restricted-use-file pooled data. Latent class analysis was conducted to identify underlying numeracy skill use patterns. Results showed that three classes or subgroups with distinctive numeracy skill use patterns were identified. Subgroup 1 (infrequent users) only used specific and basic numeracy skills, such as calculating prices and costs at home. Subgroup 2 (occupational users) used a variety of numeracy skills, such as preparing graphs and tables, and using formulas, mostly at work. Subgroup 3 (ubiquitous users) used a variety of basic to advanced numeracy skills both at work and at home. Subgroups 1, 2, and 3 had significantly different numeracy skill proficiency scores, 247, 265, and 280 out of 500 points, respectively. Detailed demographic and socioeconomic characteristics were also examined by the identified latent classes to inform education interventions and policies to promote numeracy skills and in turn, economic well-being of older workers.

Abstract citation ID: igae098.3103

**OLDER WORKERS' DEMOGRAPHIC AND SOCIOECONOMIC CHARACTERISTICS BY DIGITAL SKILL USE PATTERNS IN THE UNITED STATES**

Takashi Yamashita[1], Donnette Narine[1], Runcie Chidebe[2], Phyllis Cummins[2], Jenna Kramer[3], and Rita Karam[4], *1. University of Maryland Baltimore County, Baltimore, Maryland, United States*, *2. Miami University, Oxford, Ohio, United States*, *3. RAND Corporation, Arlington, Virginia, United States*, *4. RAND Corporation, Santa Monica, California, United States*

In the current technology-rich society, having sufficient skills to use information and communication technology (ICT) for problem-solving is advantageous in the labor market from a human capital perspective. However, older workers tend to have lower digital problem-solving skills than their younger counterparts. Practice engagement theory suggests that continuous use of skills can promote and maintain skill proficiency over the life course. The current study examined the digital problem-solving skills use patterns among older workers in the United States. Nationally representative samples of workers aged 50 years and older (n = 1,700) were obtained from the 2012/2014/2017 U.S. Program for the International Assessment of Adult Competencies (PIAAC) restricted-use-file pooled data. Latent class analysis with seven ICT use indicators, such as email, spreadsheet, and virtual discussions, at home and work (14 in total) identified two distinctive underlying subgroups, including ubiquitous ICT users and infrequent ICT users. The ubiquitous ICT users had a greater average digital problem-solving skills score (280 out of 500 points) than infrequent ICT users (244 points). About 25% of the ubiquitous users used virtual discussions at work, while only 3% of the infrequent users did. Subsequent binary logistic regression showed that the ubiquitous users were more likely to be younger, women, college-educated, and higher income earners than the infrequent users. Findings are useful to identify older workers who may be at risk of being digitally left behind in the labor market, as well as to inform digital problem-solving skills promotion interventions based on the ubiquitous users' characteristics.

### SESSION 7480 (POSTER)

### EDUCATION: GERONTOLOGY/GERIATRIC EDUCATION (POSTERS)

Abstract citation ID: igae098.3104

**ADAPTING TO HEALTH CHANGE: STUDENT RESPONSES TO DIFFERENT DELIVERY MODALITIES**

Keith Kleszynski, Lee Jennings, and Shirley James, *University of Oklahoma Health Sciences Center, Oklahoma City, Oklahoma, United States*

**Background:** Adapting to Health Change (AHC) is a two-hour aging sensitivity simulation training designed to improve student empathy and understanding of common age- and chronic illness-related functional changes. AHC includes role-play with props and 4 activity stations: hearing loss, neuropathy and dexterity, vision loss and medication management, and mobility and transfer. Training is facilitated by interdisciplinary faculty and staff.

**Methods:** AHC was delivered in-person in 2 sessions to 38 speech pathology students and delivered virtually to 136 social work students in 4 sessions. The simulation was modified for online education by using common household items for props in the activity stations. Post-training evaluation results were analyzed between delivery modalities and student groups.

**Results:** Students strongly agreed that the simulations helped them better understand how it feels to age with health changes (79%) and potential stereotypes and biases associated with aging (80%). Students strongly agreed the simulations provided useful insight into the aging experience (82%) and that the experience will help them develop into better health care providers of patients adapting to changes associated with aging (83%). The majority of students felt that their knowledge of age-related challenges had improved (94%). There were no significant differences between student groups or between those who received virtual versus in-person delivery.

**Conclusion:** Aging empathy simulations improve student understanding of age-related health changes and can be effectively delivered in-person or online, making wider delivery to health professions students using virtual training platforms more feasible.

Abstract citation ID: igae098.3105

### AGE-FRIENDLY ESCAPE ROOM: A POLYPHARMACY QUANDARY

Betsy Yang[1], Caroline Park[2], Carter Davis[2], Joanne Weith[3], Simeon Kang[4], Silvia Tee[5], Christine Gould[2], and Vinita Shastri[2], *1. Palo Alto VA Medical Center, Palo Alto, California, United States*, *2. VA Palo Alto Healthcare System, Palo Alto, California, United States*, *3. Department of Veterans Affairs Palo Alto, Palo Alto, California, United States*, *4. Geriatrics Research and Educational Clinic Center (GRECC), Palo Alto, California, United States*, *5. Stanford University School of Medicine, Stanford, California, United States*

Gamification, or adding elements like interactivity and puzzle-solving to teaching, has the potential to promote engagement in geriatric medical education. We organized a 60-minute escape room at a regional internal medicine conference to teach the 4Ms (What Matters, Medications, Mobility, and Mentation) Age-Friendly framework to interprofessional providers through gamification. 15 interprofessional participants received a short patient case and formed small teams to "Escape the Room" by rotating through each of the respective stations of 4Ms, moderated by interdisciplinary preceptors, to learn about salient points of geriatric care with the specific "M" in mind for the patient case. At the end of their 4 station rotations, participants received a clue to solve the final puzzle using the 4Ms framework which answers the question "Which medication will we deprescribe first to treat our patient experiencing cognitive impairment, polypharmacy, and falls?" From the retrospective post-then-pre questionnaire, we evaluated participants' confidence and knowledge of 4Ms and workshop effectiveness. Results showed significant improvement in participants' confidence (2.73) and knowledge (2.24) of 4Ms (on a 10-point Likert Scale, $p< 0.01$, n=15, Wilcoxon Signed-Rank Test). Participants strongly agreed with recommending the workshop to others ($\mu=4.9$ out of 5, SD=0.4) and applying 4Ms to their practice ($\mu=4.9$ out of 5, SD=0.3). Qualitative feedback indicated the escape room was both "useful" and "engaging", though some participants recommended allotting more time to the activity. With this workshop, we demonstrated that an interprofessional escape room can teach important geriatric concepts in an effective and engaging manner.

Abstract citation ID: igae098.3106

### BUILDING BRIDGES ACROSS GENERATIONS: THE INTERGENERATIONAL CLASSROOM

Jessica Hsieh[1], Raza Mirza[1], Christopher Klinger[1], Julianna Hill[1], Heather Janes[2], and Claudia Osmond[2], *1. University of Toronto, Toronto, Ontario, Canada*, *2. Christie Gardens, Christie Gardens, Ontario, Canada*

The issue of aging demands greater attention in education systems, and intergenerational approaches help to combat ageism and improve the lives of older adults, now and in the future. Taking an intergenerational approach to contextualizing the experiences of older adults within post-secondary classrooms settings is a novel approach towards building interest in the field of aging. The University of Toronto (UofT) partnered with Christie Gardens, a retirement community and long-term care home, to launch an innovative experiential learning initiative: The Intergenerational Classroom. Half the learners were UofT undergraduate students (n=24); the other half were older adults residing at Christie Gardens (n=25). Through interactive seminar-style discussions, collaborative projects and mentorship, the course, which was held at Christie Gardens, provided a semester-long exploration on aging. To evaluate the program, pre/post-surveys were administered to all learners, and following the semester, students (n=6) and older adults (n=6) participated in focus group discussions. Outcomes of program success were identified across domains, including meaningful friendships and bonds created, increased awareness of aging issues, reduced ageist attitudes, and greater sense of community and civic engagement. Evaluations revealed that 92% had an excellent learning experience, 95% found the course intellectually stimulating, and that 100% would recommend the course to others. The Intergenerational Classroom explored aging from a viewpoint that considered the perspectives of both older and younger generations. Intergenerational approaches can help to create a brighter and more inclusive future for all generations, ensuring that individuals can grow up and grow older with dignity, rights, and opportunity.

Abstract citation ID: igae098.3107

### CAN NOVICE LEARNERS BE CLINICAL CHANGE AGENTS FOR DEMENTIA CARE PRACTICE?

Edmund Duthie[1], Kathryn Denson[1], Deborah Simpson[2], Stacy Barnes[3], Wendy Betley[4], Amanda Szymkowski[1], and Steven Denson[1], *1. Medical College of Wisconsin, Milwaukee, Wisconsin, United States*, *2. University of Wisconsin School of Medicine and Public HealthH, Milwaukee, Wisconsin, United States*, *3. Marquette University College of Nursing, Milwaukee, Wisconsin, United States*, *4. Alzheimer's Association, Chicago, Illinois, United States*

**Intro:** Caregiver support is an essential component in dementia care and yet clinicians' referrals to community-based support services are lacking. Direct Connect (DC) is a clinician-initiated referral process for dementia patients and caregivers, offered by the Alzheimer Association to make the process simple and efficient.

**Methods:** We created a brief educational intervention targeting medical, nursing, and pharmacy students to develop them into DC champions at clinical rotation sites in Wisconsin. Content focused on dementia detection, caregiver burden, available resources, and DC referrals. Delivery included online, in-person, and hybrid formats. Data was collected with a post-intervention survey and student interviews. At baseline (2021), 277 DC referrals were made by clinicians in Wisconsin.

**Results:** Following a 2-year rollout to students (N=375), the annual number of DC referrals received from clinicians increased (300 in 2022; 351 in 2023). However, review of referral data revealed the increase was primarily from licensed clinicians not at student champion sites. Follow-up interviews with students indicated they find it daunting to suggest changes in busy clinical settings where they lack experience and autonomy. Despite incorporating scripting and role playing into our educational session, students continued to lack confidence.

**Discussion:** Although student champions have proven effective in other clinical conditions, our results suggest different strategies are needed for dementia care. Based on these findings, we propose to refocus training efforts toward more senior learners such as medical residents who have more experience and clinical autonomy, and to embed the DC referral within the electronic health record to facilitate usage.

Abstract citation ID: igae098.3108

### EFFECTS OF ADVANCE CARE PLANNING TRAINING ON STUDENTS' KNOWLEDGE, COMFORT, AND CONFIDENCE

Stacy Barnes[1], and Susan Breakwell[2], *1. Marquette University College of Nursing, Milwaukee, Wisconsin, United States*, *2. Marquette University, Milwaukee, Wisconsin, United States*

Students report feeling unprepared to engage in advance care planning (ACP).

**Methods:** A 2-hour, interprofessional workshop was held in-person in April 2018 and 2019, and virtually in 2021 and 2022 for students from medicine, nursing, physician assistant, speech pathology, counseling psychology, biomedical sciences, communication, and sociology (N=223). The workshop aimed to educate students about ACP, foster exploration of personal preferences for end-of-life care, and empower them to initiate ACP conversations with others. It incorporated highly engaging activities including personal reflection, drawing, writing, video clips, case presentations, as well as small and large group discussions. A Qualtrics survey, using a retrospective post- then pre- questionnaire design, elicited feedback 1-day post-workshop.

**Results:** Regardless of delivery format, respondents rated the workshop highly (97% excellent or good) and liked interacting with peers from other disciplines in small group discussions best. Paired-samples t-tests revealed statistically significant differences following the workshop in four of five areas ($p< 0.001$) i.e., increased understanding of advance directives, comfort completing their own advance directive, comfort talking with their family and/or personal doctor, and confidence in starting an ACP conversation with others. No significant change was detected in students' comfort exchanging ACP ideas in a team discussion ($p=0.19$).

**Discussion:** A workshop can increase students' self-reported knowledge, comfort, and confidence with ACP, as applied to themselves and/or family. However, results suggest working in an interprofessional healthcare team requires additional training and experience. These findings can help guide ACP curricula in health professions education.

Abstract citation ID: igae098.3109

### EFFORTS TO INCREASE GERONTOLOGY DEGREE-SEEKERS AND DEVELOP THE LOCAL WORKFORCE

Lisa Hall, *Missouri State University, Springfield, Missouri, United States*

Since 1990, Gerontology majors at Missouri State University (MSU) have served their practicums at a variety of sites, which we consider to be "community partners". These community partners consist of public and private entities that serve older adults, ranging from the local Area Agency on Aging, which is authorized by the Older Americans Act, to caregiving individuals who own Limited Liability Companies (LLC). The services offered range from high-activity recreation to end-of-life care. The MSU Gerontology Program has routinely surveyed our community partners to understand their expectations for and opinions about our program and students. In 2023, we added two additional areas of inquiry: (1) their current networking practices with each other and (2) the degree to which they and their employees were interested in taking aging-related courses at our university. Results from our online survey and follow-up interviews will be shared. Next steps in our continued efforts to increase Gerontology degree-seekers and to develop the local age-related workforce will be discussed.

Abstract citation ID: igae098.3110

### ENGAGING THE PAST FOR THE FUTURE: APPLYING ANTIRACIST PEDAGOGY TO HEALTH DISPARITIES IN LATER LIFE

Rona Karasik[1], Kyoko Kishimoto[1], Isaac Asirifi Boateng[1], Camryn Hafner[2], and Claudia Burgos Zuniga[1], *1. Saint Cloud State University, Saint Cloud, Minnesota, United States*, *2. Bournewood Health Systems, Somerville, Massachusetts, United States*

Pervasive health disparities among racial groups are well documented throughout the life course and across generations. The continued persistence of these disparities, however, suggests that simply recognizing their presence is insufficient for preparing future gerontologists, care-providers, and policymakers to address them. How can educators help gerontology students not only acknowledge the existence and impact of such disparities, but also to consider their root causes - an essential step toward effecting social change? Moreover, how can we prepare gerontology educators to guide students through this exploration? Building on previous content compilations regarding housing (Kishimoto & Karasik, 2018) and retirement (Karasik & Kishimoto, 2022), and integrating emerging research and educational approaches from fields such as Public Health (e.g., Braveman et al., 2022; Jones et al., 2018; Yearby et al., 2022), this presentation showcases newly developed/adapted educational materials and activities geared toward facilitating gerontology students' exploration of racial health disparities from an antiracist perspective. Central to the provided lesson plans is an annotated timeline pairing key historical events with health care related policies and programs in the U.S., as well as multi-media resources on related topic areas. Given the current political climate (e.g., the dual influences of Black Lives Matter and anti-DEI legislation) and the varying extent of students' (both domestic and international) prior understanding of US history, strategies for supporting productive classroom discussions on racial health disparities in later life are also shared from both faculty and student perspectives.

Abstract citation ID: igae098.3111

### IMPROVING ELDER HEALTH BEHAVIORS AND STUDENT LEARNING OUTCOMES WITH HOPE-BASED INTERPROFESSIONAL EDUCATION PROGRAM

Britteny Howell[1], Leslie Redmond[2], Amber Worthington[1], Najma Musa[1], and Allexis Mahanna[3], *1. University of Alaska, Anchorage, Alaska, United States*, *2. University of Manitoba, Winnipeg, Manitoba, Canada*, *3. University of Alaska Anchorage, Anchorage, Alaska, United States*

Although Interprofessional Education (IPE) and research enhances learning, communication, and care of patients, it remains underutilized in higher education, demonstrating the need for extracurricular IPE opportunities. This presentation describes an interprofessional research project that brought together faculty, undergraduate, and graduate students from several health and social science disciplines to design and deliver a 15-week healthy aging program for older adults living in the urban Circumpolar North. Five faculty and one graduate research assistant led the project while eight students team-taught weekly, 1-hour sessions in the community focusing on healthy lifestyles within a framework of Persuasive Hope Theory. We will present the results of the student satisfaction survey regarding their involvement with the research as well as the participant satisfaction with the program. Students reported gains in "thinking like a scientist," increased confidence conducting research tasks, benefits from teamwork, and greater consideration of the needs of older adults in their field of study. Older adult participants (N=39) also indicated favorable opinions of the program, responding that they were satisfied with the student-instructors, learning, application, impact, and value of the program. Despite small sample sizes, results from student and participant surveys both demonstrate that the IPE and intergenerational learning opportunities were meaningful. Additionally, results suggest that students may be more likely to consider a career working with older adults if given hands-on research experiences.

Abstract citation ID: igae098.3112

### OCCUPATIONAL THERAPY AND NEUROPSYCHOLOGY: INTERDISCIPLINARY COLLABORATION IN CURRICULUM DEVELOPMENT

Theresa Christopher[1], and Jennifer Christopher[2], *1. North Central College, Naperville, Illinois, United States*, *2. Jesse Brown VA Medical Center, Chicago, Illinois, United States*

Interdisciplinary care models have a positive impact on older adult care (Harris, 2006). This is especially true for people diagnosed with dementia (Galvin et al., 2014). Neuropsychologists and occupational therapists are vital team members in providing care to people diagnosed with dementia. However, there is a lack of research highlighting the collaboration between these two professions. The current authors believe that graduate education is an opportune time to initiate these collaborations. The current poster aims to showcase a partnership between a neuropsychologist and occupational therapy professor. This collaboration resulted in a one-hour lecture provided by a neuropsychologist to masters of occupational therapy students in a geriatric theory course. The lecture outlined the role of the neuropsychologist in differential diagnosis of dementia and the opportunities for interdisciplinary collaboration between occupational therapists and neuropsychologists. The authors believe that this lecture is beneficial to routinely include in standard occupational therapy geriatric curriculum. In the future, the authors hope to conduct a quantitative study to examine the effectiveness of this lecture in promoting students' understanding of dementia and the partnership between neuropsychology and occupational therapy. References Galvin, J. E., Valois, L., & Zweig, Y. (2014). Collaborative transdisciplinary team approach for dementia care. Neurodegenerative disease management, 4(6), 455-469. doi: 10.2217/nmt.14.47. Harris, B. A. (2006). Interdisciplinary education: What, why, and when? Journal of Physical Therapy Education, 20(2), 3-8.

Abstract citation ID: igae098.3113

### PRIORITIZING GERIATRIC EDUCATION FOR HEALTH CARE PROVIDERS

Lana Brown, Wanda Morrison, Julie Smith, and Dennis Sullivan, *Central Arkansas Veterans Healthcare System, North Little Rock, Arkansas, United States*

Within the United States, there is a deficit of Geriatricians providing care for older adults, and this deficit will only grow as the population continues to age, meaning all health care providers will be responsible for the care of older adults. A learner needs assessment is a systematic approach to examining what individuals or groups need to learn in relation to health care practice. A learner needs assessment is a core element of designing effective, targeted geriatric educational interventions. In 2022, a brief six question online survey was distributed to all health care providers within the South Central Veterans Affairs (VA) Health Care Network to determine the top geriatric education needs. Within the survey, provider demographics requested were primary discipline and primary work setting (inpatient/outpatient). Respondents were requested to choose at least 5 out of 50 topics provided that would be the most useful for future geriatric education. Responses were received from 143 health care providers with the majority of the responses being from social workers 59(41.3%) and nurses 45(31.5%). There were 103 health care providers from outpatient areas and 40 from inpatient areas. The top geriatric education topics requested were mental health (50.4%), Alzheimer's disease/dementia (48.3%), legal issues (38.5%), chronic pain (35.0%), depression (32.2%), and management of chronic conditions (30.1%). Experts were recruited to provide geriatric education on the highest priority requested topics. Providing geriatric education improves care for older adults ensuring health care providers are prepared to care and advocate for this diverse and often vulnerable population.

Abstract citation ID: igae098.3114

### PROMOTING GERONTOLOGICAL TRAINING AND POSITIVE ATTITUDES OF AGING THROUGH HIGH-IMPACT TEACHING PRACTICES

Aimee Fox, and Bethany Riding, *Utah Valley University, Orem, Utah, United States*

Despite being the youngest state, Utah, along with the rest of the United States, has a growing aging population. There is demand from state leaders and employers for individuals, families, and a workforce that are knowledgeable about the experiences and needs of older adults. Utah Valley University (UVU) is Utah's largest public university, with a dual-mission of a first-rate teaching university and open-admissions promoting easy access to higher education. Yet, efforts at UVU to promote gerontological training in the social sciences have been minimal. Students at UVU are often unaware of what gerontology is and the importance of gerontological training, and report negative attitudes and fears about aging and older adults. This descriptive case study is the first stage of a larger project aimed at promoting enrollment in UVU's gerontology certificate and shifting student attitudes about aging to promote positive perceptions of and interactions with older adults and aging experiences. During this first stage, the gerontology certificate was adjusted to align with AGHE gerontology competencies, and an Adult Development and Aging course was re-designed with high-impact practices, including a common intellectual experience focused on service- and experiential-learning activities. This study reports on re-design efforts, the engagement and feedback of 80 students across two sections of the course, and the impact of learning activities on students' attitudes about aging and older adults. Also addressed are theoretical and practical suggestions for promoting gerontological training, potential pitfalls to avoid, and recommendations for implementing these changes across a variety of higher education settings.

Abstract citation ID: igae098.3115

### TEACHING STRATEGIES FOR INTERDISCIPLINARY COLLABORATION THROUGH A PUBLIC HEALTH LENS

Elaine Jurkowski, *Southern Illinois University Carbondale, Carbondale, Illinois, United States*

The Social Determinants of Health (SDOH) have become increasingly important in workforce training for health/medical professionals working with older adults to improve health outcomes. Inter-professional collaboration across disciplines such as primary care medicine, physician assistants, social workers etc. does not occur naturally since educational programs are siloed. However, these disciplines are required to work collaboratively with each other, targeting SDOH's and patient outcomes. The objective was to promote communication across disciplines and help each discipline understand the roles played in practice with older adults. Three specific strategies to address Interprofessional Education were devised and implemented within a rural context to address the goal of improving collaboration and communication. An educational seminar was conducted using cases and guide questions. A second strategy was through "Student hot-spotting" where students worked with a high-need complex case over the course of a school year weekly, as a team-based approach. A third strategy made use of a weekly medical clinic for team-based patient care with students from medicine, social work, PA, nursing and nutrition. Findings suggest professionals were surprised at what they learned from the other disciplines they were collaborating with. They also learned about community-based resources and improved health outcomes when the lens of public health was in the forefront and an intentional part of the curricula. All participants felt that the chance to collaborate outside of their disciplines strengthened their skills and impact. In conclusion, curricula intentionally devised using a public health lens, in collaboration with other disciplines improves overall collaboration among professionals.

Abstract citation ID: igae098.3116

### THE RAFT DEBATE: GETTING UNDERGRADUATE STUDENTS TO THINK CRITICALLY ABOUT AGING

Andrew Fiscella, *University of South Florida, Tampa, Florida, United States*

A student-centered approach has been advocated to help students develop as learners, as it allows students to think critically, beyond what might be required in an essay. However, it can be difficult to achieve in larger classes, where a teacher-centered approach is more often utilized. The current study explores adapting a traditional debate format for students in a Physical Changes & Aging course as a tool to engage them in critical analysis of various topics related to biological aging, allowing the incorporation of student-centered techniques. In the Raft Debate, students represent gerontologists who become stranded on a desert island. There is a raft which can take them off the island, but it is only big enough for one group. To determine who gets off the island, students are split into teams, with pairs of teams assigned a topic which had been briefly introduced over the course of the semester. Students will be asked to prepare not only arguments in favor of their position but must also effectively counter-argue their opponents. After arguments by both teams have been presented, the audience will vote for the team which made the most convincing argument. Additionally, students will complete a peer review of their teammates. The goal of this assignment is that as students collaborate with each other and ultimately, compete against their classmates, they will gain a deeper understanding of the biological aging process and how it may be applied to both their professional and personal lives.

Abstract citation ID: igae098.3117

### THE RELATIONSHIP BETWEEN BIOGRAPHY, EDUCATION, AND ICT USE IN AGE: A LIFE COURSE PERSPECTIVE

Christina Klank[1], Tjard de Vries[1], Ines Himmelsbach[1], and Michael Doh[2], *1. Catholic University Freiburg, Freiburg, Baden-Wurttemberg, Germany*, *2. Catholic University Freiburg, Freiburg im Breisgau, Baden-Wurttemberg, Germany*

The digital transformation of society is challenging for many older adults, especially those living in institutionalized settings. Support systems are needed, but often lacking. This study first presents a learner-centered approach that focuses on individual interests and abilities of the target group: older adults in institutionalized settings. In order to sustain ICT use (information and communication technology), professionals need to develop programs based on the needs of the target group. Therefore, this study secondly uses a narrative approach to analyze biographies of older adults in order to exploit their potential for conclusions about ICT use. An interdisciplinary German research project established a participatory design for the target group. This design pairs advanced peers with beginners to introduce ICT (e.g. tablets, Internet) based on beginners' interests and needs. Advanced peers were recruited as volunteers and participated in a train-the-trainer program beforehand. In this study, biographical-narrative and problem-centered interviews were conducted with 42 older German adults (White; age=62-95; 57% female; beginners=24, advanced=18). The interviews were analyzed within a narrative approach to compare subjective narratives about biography, education, and ICT use. Different biographical patterns were identified and are illustrated by two contrasting cases from each group. The results provide evidence that older adults' biographies influence and partially predict older adults' motivation and educational interests, which in turn influence ICT use. Additionally, ICT is perceived as either a lifestyle (e.g. meeting communication needs) or a tool (e.g. maintaining independence). When developing educational activities for older adults, we recommend including biographical components in curricula.

### SESSION 7485 (POSTER)

### ENVIRONMENT AND AGING (POSTERS)

Abstract citation ID: igae098.3118

### BEYOND BORDERS: LONG-DISTANCE CAREGIVING AND SLEEP HABITS IN SOUTH ASIAN COMMUNITIES

Aziza Siddiqui, Srujana Chekuri,
Akankshya Chataut, Dinesh Karki, and
Julie Blaskewicz Boron, *University of Nebraska Omaha, Omaha, Nebraska, United States*

Caregiving is generally altruistic in South Asian (SA) communities; however, caregiving activities can still create significant strain for caregivers depending on situational factors, including sleep and built environments (Hossain et al., 2020). Particularly, long-distance caregivers may grapple with the added complexities of not being physically present to provide care. This study utilized an online survey to investigate sleep habits and routines, caregiving activities, neighborhood satisfaction, and access to community resources for the SA community located in Omaha, Nebraska (N=74, MAge=25.8, SD=4.58). Caregivers reported on their perceived burdens, highlighting the unique challenges faced as they logistically navigate from afar, which remains underrepresented in research for the SA community (Hossein et al., 2020). Results revealed that SA caregivers' sleep satisfaction was significantly related to caregiver responsibilities, including familial and professional expectations ($\chi2(6, N=74)=20.74,p<.05$). Caregivers concerned about their sleep patterns were significantly more likely to have impaired quality of life ($r(72)=0.62$, $p<.001$). Self-rated home cleanliness was also considerable in caregivers' controlling their bedtime ($r(72)=0.36$, $p<.001$). Thus, control over sleep length and quality are essential factors to reduce caregiver burden. This highlights that how better sleep habits and a clean surrounding environment can potentially reduce strain experienced by many long-distance caregivers. The impact of home environments for the long-distance SA caregiving community is yet to be fully understood. It would be valuable to investigate this community's caregiver norms and cultural expectations further to promote environments conducive to sleep quality and overall quality of life.

Abstract citation ID: igae098.3119

### CLEAN AIR ACT 1990 AND ITS LONG-TERM IMPACT ON MORTALITY RISK AMONG OLDER ADULTS IN THE UNITED STATES

Siyao Lu, and Emma Zang, *Yale University, New Haven, Connecticut, United States*

**Background:** Air pollution significantly influences public health, being a predominant cause of illness and death in the United States. The 1990 Clean Air Act (CAA) marks a pivotal regulatory initiative aimed at confronting this issue by enhancing air quality and public health outcomes. This study investigates the long-term effects of the CAA 1990 on mortality rates among the elderly, paying special attention to racial differences in health outcomes.

**Methods:** This study adopts a difference-in-differences approach, analyzing county-level administrative data from 1982 to 2000. This approach evaluates the long-term impact of the CAA 1990's implementation on the mortality rates of older adults across counties classified as "attainment" or "nonattainment" regarding the National Ambient Air Quality Standards (NAAQS) in 1990.

**Results:** The analysis reveals that the CAA 1990 significantly lowered mortality risks among the older population. Black men experienced the most substantial health benefits, although the impacts on Black older adults manifested more slowly compared to their White counterparts. The next phase of this research will explore potential mechanisms by examining the CAA 1990's influence on air pollution reduction.

**Discussion:** This study underscores the CAA 1990's role in mitigating public health burdens among older adults and highlights the essential need for policies that address racial disparities in the effects of environmental health. The insights are crucial for guiding future environmental policy, promoting environmental justice, and narrowing health disparities.

Abstract citation ID: igae098.3120

### DEVELOPING A TYPOLOGY OF SENIOR CENTERS: CONCEPTUAL AND METHODOLOGICAL CONSIDERATIONS

Ceara Somerville, and Jan Mutchler, *University of Massachusetts Boston, Boston, Massachusetts, United States*

Senior centers are important resources for older adults and their families, intended to serve their communities by tailoring programs and services to meet the needs and interests of those in their local environment. The network of senior centers across the nation and within states is heterogenous, but there is limited research focused on senior centers' organizational capacity. This project uses a novel dataset from a comprehensive database of all senior centers in Massachusetts (n=342) to explore the commonalities and differences in models of senior center operations. A factor-cluster approach was applied to a subsample of 278 senior centers. Nearly 40 indicators of organizational goals, structure, people, resources, and policies were included in a nonlinear principal components analysis, resulting in 11 standardized scales representing components of organizational capacity (e.g., leadership, governance, core programs and services). These scores were then entered into a two-step cluster algorithm to determine the optimal grouping of senior centers into distinct types of operational models. Three clusters emerged; the most notable differences between them occurred on measures of wellness programs, core programs and services, staff capacity, space capacity, and social and financial resources. Differences between clusters on several demographic characteristics of the community (e.g., population size and density, racial and ethnic composition) were observed, providing empirical support of senior centers operating in response to their local populations. Senior centers are locally focused, but identifying common operational types and their relation to the wider community can inform research, policy, and practice to better support the network.

Abstract citation ID: igae098.3121

### IMPACT OF ENVIRONMENTAL SATISFACTION ON SLEEP QUALITY AMONG SOUTH ASIAN CAREGIVERS FOR OLDER ADULTS

Srujana Chekuri, Aziza Siddiqui, Dinesh Karki, and Julie Blaskewicz Boron, *University of Nebraska Omaha, Omaha, Nebraska, United States*

Caregivers for older adults rely heavily on good sleep quality for their general health and ability to provide care. This study investigated the impact of environmental satisfaction, both at home and in the surrounding neighborhood, on sleep quality among South Asian caregivers for older adults, specifically those not living with the care recipients. Participants completed a survey focused on South Asian caregivers' sleep quality and satisfaction with their surroundings, such as the cleanliness of living spaces, noise levels, and the condition of neighborhood walkways and cycling lanes. A series of regression analyses were used to examine relationships. Results demonstrated a statistically significant relationship between environmental satisfaction and sleep quality. Specifically, high satisfaction with home cleanliness, and low noise levels were positively associated ($p< 0.05$) with increased sleep duration. Similarly, the state of outdoor infrastructure, such as well-kept sidewalks, and cycling routes were also positively associated ($p< 0.05$) with more hours of sleep among caregivers. This highlights the significant role of favorable indoor and outdoor environments in enhancing sleep quality among South Asian caregivers for older adults, pointing to the potential benefits of targeted environmental interventions. These findings further emphasize the need of a supportive environment for the well-being of South Asian caregivers, even when they are not living with their care recipient. The focus on a specific cultural group may limit generalizability. Thus, future studies should incorporate diverse caregiver samples to explore the generalizability of results across different cultural contexts and living situations (e.g., residing with or without the care recipient).

Abstract citation ID: igae098.3122

### NEIGHBORHOOD ENVIRONMENT AND COGNITIVE FUNCTION AMONG OLDER ADULTS IN BRAZIL

Lourdes Romañach Álvarez[1], Mateo Farina[2], and Elizabeth Muñoz[1], *1. The University of Texas Austin, Austin, Texas, United States*, *2. University of Texas at Austin, Austin, Texas, United States*

Neighborhood characteristics, such as social cohesion and community resources, are linked to better cognitive functioning in older adulthood. However, little is known about the significance of neighborhoods for cognitive function in lower- and middle-income countries where this association may be augmented by limited resources and higher economic inequality. The present study used data from the 2016 Brazilian Longitudinal Study of Aging (ELSI-Brazil) to examine the association between self-reported neighborhood characteristics and cognitive functioning. ELSI-Brazil is a nationally representative sample of community-residing adults in Brazil aged 50 years and older ($n = 9,412$). We fit a series of linear regression models to test the hypothesis that more favorable neighborhood characteristics are associated with better cognitive functioning. Participants completed self-reported questionnaires of their neighborhood's social cohesion and availability of healthy foods. A global cognitive score was calculated with a latent variable (g) that was created with confirmatory factor analysis. Adjusted models showed that social cohesion, specifically appraising their neighborhood as a good place to live and trusting most people in their neighborhood, was associated with higher cognitive functioning. Likewise, those who reported greater availability of fresh fruits and vegetables within their neighborhood displayed better cognitive function. Findings provide support for the protective role of neighborhood social cohesion and availability of healthy foods on cognitive function among adults in Brazil. Further research is needed to elucidate what mechanisms may explain this association in the Brazilian context.

### SESSION 7490 (POSTER)

### FAMILY CAREGIVING III (POSTERS)

Abstract citation ID: igae098.3123

### AN OBJECTIVE MEASURE OF STRESS REACTIVITY/ RESILIENCE IN BLACK WOMEN DEMENTIA FAMILY CAREGIVERS

Kathy Wright, Karen Moss, Nathan Helsabeck, Ingrid Richards Adams, Karen Rose, and Maryanna D. Klatt, *The Ohio State University, Columbus, Ohio, United States*

Real-time objective measures of stress are crucial for identifying mechanisms applicable to health behavior change research for dementia caregivers. We explore the Pittsburgh Stress Battery (PSB) in a non-laboratory setting (R21AG077069) as an objective measure of stress reactivity/ resilience. The PSB was administered to 16 Black American women caregivers with hypertension (40-65 years old). PSB includes three challenging tasks (Stroop, Mental Math, and Mirror Tracing). An automatic cuff measured systolic blood pressure (SBP), diastolic blood pressure (DBP) and heart rate (HR) at three intervals during the 20-minute battery followed by a 30-minute recovery. Average participant age was

62.4 (SD= 6.1). Ten assessments were completed in an office and six at home. One Stroop and three Mental Math were not completed due to computer/internet issues or refusal. Mean SBP, DPB, and HR at baseline were 128.9 mmHg, 81.6 mmHg, and 71.3 (beats per minute). The difference from baseline in SBP was 10.5 mmHg (SD= 8.3) for Stroop, Mental Math -.94 mm Hg (SD= -0.6), and Mirror Tracing 1.6 mmHg (SD= 9.4). For DBP, Stroop was 5.0 mm Hg (SD=4.6), Mental Math 0.2 mm Hg (SD= 3.2), and Mirror Tracing 0.3 mm Hg (SD= 7.0). HR difference from baseline was 3.8 (SD= 0.5), Mental Math -4.24 (SD= 8.2), and Mirror test -0.75 (SD= -3.4). Findings indicate that PSB elicits SBP/DBP/HR stress reactivity/resilience. PSB was portable for use in community settings. Study results will help guide interventions for behavior change, aiming to improve health outcomes in Black Americans.

Abstract citation ID: igae098.3124

### ASSESSING THE IMPACT OF THE WYOMING DEMENTIA TOGETHER CAREGIVER NETWORK ON RURAL CAREGIVERS

Barbara Dabrowski[1], Christine McKibbin[1], Catherine Carrico[1], Sabine Schenck[1], Stacy Carling[1], Abby Teply[1], Elizabeth Punke[1], and Katherine Kitchen Andren[2], *1. University of Wyoming, Laramie, Wyoming, United States*, *2. Craig H. Neilsen Rehabilitation Hospital, University of Utah Health, Salt Lake City, Utah, United States*

Approximately 15.7 million people care for a family member or friend living with dementia (CDC, 2023). Many programs have been developed to support and educate dementia caregivers, but fewer are designed to reach rural areas. The Wyoming Dementia Together (WDT) Caregiver Network is an adaptation of the Extension for Community Health Outcomes (ECHO) model, created to enhance capacity for care in rural areas. The specific adaptation of the model for caregivers includes a Hub team of dementia care experts and rural Spoke Sites (individual caregivers). This study investigated outcomes associated with caregiver participation in the WDT network. The sample (n = 13, M = 61.4 years old, SD = 10.2) comprised White female dementia caregivers (n = 12; 92.3%) who completed at least six months of the WDT program. Caregivers completed pre-post measures regarding self-efficacy, caregiver burden, depressive symptoms, and unmet needs. Chi-square analyses revealed significant reductions in unmet needs related to family (chi-square = 6.77, df = 1, p =.009) and daily living needs (chi-square = 8.55, df = 1, p =.003). Changes in self-efficacy, burden, or depressive symptoms were non-significant; however, means did change in the expected direction. Effect sizes for self-efficacy (p =.23, d =.25), burden (p =.38, d =.39), and depression (p =.12, d =.48) were small to moderate. These findings suggest promise for improved outcomes associated with participation in WDT. Future research is needed with larger samples to further examine the impacts of the WDT program.

Abstract citation ID: igae098.3125

### ASSESSMENT OF CAREGIVER REPORTED VISIT-ASSOCIATED LOGISTICAL CHALLENGES FOR CLINICAL VISIT COORDINATION

Victoria Ngo[1], Elizabeth Marfeo[2], Steven Shirk[1], Elizabeth Chamberlin[1], and Lauren Moo[1], *1. Department of Veterans Affairs, Bedford, Massachusetts, United States*, *2. Tufts University, Medford, Massachusetts, United States*

Family caregivers (FCG) of persons living with dementia face high care burden as the care recipient ages, especially with care coordination of in-person healthcare visits. As FCG burden increases, there is an increased likelihood of missed appointments and negative health outcomes for both the FCG and the care recipient. Limited evidence exists describing the impact of managing complex visit-associated logistical challenges FCGs face when coordinating appointments. To address this gap, this study uses a care coordination framework to develop a new assessment—the Visit-Associated Logistics Assessment (VALA). We adopted a three-phased approach: (1) multidisciplinary expert feedback to establish content coverage; (2) cognitive interviews to refine item wording; and (3) field testing to establish the feasibility and usability of the VALA among a clinical sample of FCG of persons living with dementia (n = 20). Results from the first two development phases yielded six functional domains: (1) scheduling, (2) visit preparation, (3) time/travel, (4) occupational impact, (5) routine disruption, and (6) psychosocial well-being. Initial end-user feasibility was established. The VALA had a low respondent burden, shown by minimal missing data, and appropriate content coverage, as indicated by an acceptable distribution of responses across items and response categories. The VALA represents the first FCG-focused assessment characterizing logistical factors for helping older persons attend medical visits. Initial phases of content validation and usability testing support the use the VALA within clinical and community-based settings to better support management and care coordination of in-person clinical visits for FCGs of persons living with dementia.

Abstract citation ID: igae098.3126

### BURDEN AND BENEFITS OF INFORMAL CAREGIVING AND OLDER CAREGIVING RECIPIENTS' SUBJECTIVE WELL-BEING

Weidi Qin[1], Linh Dang[2], and Briana Mezuk[2], *1. University of Wisconsin Madison, Madison, Wisconsin, United States*, *2. University of Michigan, Ann Arbor, Michigan, United States*

Informal caregiving plays an important role in and has implications for the subjective well-being (SWB) of older caregiving recipients. Using data from the 2021 National Study of Caregiving (N = 991) linked to the National Health and Aging Trend Study (N = 3,321), this study addressed three questions: 1) Is having an informal caregiver associated with better SWB among older adults? 2) Are informal caregivers' perceptions of caregiving burden and benefits associated with older caregiving recipients' SWB? 3) Does the relationship between perceived caregiving burden/benefits and caregiving recipients' SWB differ by gender and relationship types (close family members vs. non-close family)? SWB was measured with a 7-item scale. Caregiving burden was measured with 9 items regarding difficulties in providing caregiving (i.e., emotional difficulty). Caregiving benefit was measured with 4 items indexing beneficial experiences (i.e., closeness to caregiving recipients). Weighted multivariable OLS regressions were used to examine the relationships between caregiving burden, benefit, and SWB.

Older adults with a caregiver reported lower levels of SWB compared to those without (B = –0.73, p = 0.001). Expectedly, caregiving burden was associated with lower levels of subjective well-being in caregiving recipients (B = –1.22, p < 0.001). The relationship between caregivers' perceived benefits and caregiving recipients' SWB differ by caregiving recipients' gender (B = 2.01, p = 0.044) and relationship types (B = -2.25, p = 0.032). The findings highlight the importance of considering caregiving recipients' gender and family relationships in developing interventions to improve informal caregiving experiences.

Abstract citation ID: igae098.3127

### CAREGIVER ROLE TRANSITIONS IN HOME-BASED ALZHEIMER'S CARE: A SHANGHAI CASE STUDY

Qingwei Wang[1], Fengzhi Zhao[2], and Zixuan Wu[3], *1. Xi'an Jiaotong-Liverpool University, Suzhou, Jiangsu, China*, *2. Xi'an Jiaotong-Liverpool University, Suzhou, Jiangsu, China (People's Republic)*, *3. Johns Hopkins University, Baltimore, Maryland, United States*

Alzheimer's disease poses a growing health crisis in China, exacerbated by the country's rapidly aging population and cultural emphasis on family-based care. Caregivers shoulder immense physical and psychological burdens, often struggling to reconcile personal expectations with the demands of caregiving. This study, based on ethnographic data collected through prolonged engagements with a family in Shanghai caring for an older adult diagnosed with Alzheimer's disease, explores the application of Role Theory. This framework explains the sequential stages of role assumption, enactment, ambivalence, and disengagement, offering insights into the complex dynamics of caregiving within China's cultural context. While existing studies have identified factors influencing caregivers' "willingness" and "psychological stress" primarily through assessments of psychiatric distress, the practical applications of theoretical models in caregiving practices for this population in China, especially those documented through first-hand experiences, remains underexplored. Drawing from diverse sources, including academic research and firsthand accounts, this study discloses the dynamic process of role transitions in practice, highlighting in particular the transition from a career-focused individual to a full-time caregiver and the subsequent role reversal between parents and children as the illness progresses. By clarifying the dynamic interplay of roles and responsibilities within caregiving relationships, insights gained through this in-depth case study applying Role Theory in practice can offer a roadmap for enhancing caregivers' well-being and optimizing patient care outcomes.

Abstract citation ID: igae098.3128

### EFFECT OF AN AI-BASED NEGOTIATION TRAINING FOR FAMILY CAREGIVERS OF OLDER ADULTS WITH ALZHEIMER'S DISEASE

Alaine Murawski[1], Celie Joblin[2], Marianne Tschoe[1], Vanessa Ramirez-Zohfeld[1], Charlie Olvera[3], Johnathan Mell[4], Jeanne Brett[1], and Lee Lindquist[1], *1. Northwestern University, Evanston, Illinois, United States*, *2. Northwestern University, Chicago, Illinois, United States*, *3. Northwestern University Feinberg School of Medicine, Division of Geriatrics, Evanston, Illinois, United States*, *4. Custom Technologies LLC, Orlando, Florida, United States*

Family caregivers (CGs) of older adults with ADRD (OADRD) may experience conflicts when providing care, yet are often ill-equipped to handle these challenges. We evaluated NegotiAge (NA), a web-based intervention leveraging artificial intelligence (AI) to teach CGs negotiation skills, and assessed the impact that NA had on how CGs resolve conflicts and retain negotiation knowledge. CGs of OADRD were recruited nationally and enrolled in a randomized controlled trial. CGs accessed negotiation training resources through NA and were randomized to negotiate conflict scenarios in real-time, with AI-powered avatars exhibiting emotions and behaviors simulating real-life interactions. CGs were assessed at baseline (T1) and 1-week post-intervention (T2) with the Dutch Test for Conflict Handling (which examines yielding, compromising, forcing, problem-solving, and avoiding conflict domains) and with a 15-item panel consisting of negotiation terminology and conflict statement (interests, rights, power) identification. Matched pair t-tests were performed between T1-T2. 119 CGs (mean age 53.5, 75.6% female, 32.8% Black) completed both surveys. CGs receiving more negotiation exercises scored lower in avoiding (Δ=-0.97, t=-2.2, p=0.03) and compromising (Δ=-0.53, t=-3.5, p=0.0006) at T2. Mean total negotiation knowledge score increased between T1-T2 (Δ= 2.07, t=1.18, p = 0.07), as did identification of interests-based conflict statements (Δ=4.49, t= 4.48, p = 0.01). NA positively impacted CG conflict resolution skills: CGs improved both in avoiding and compromising during conflict, and in identifying common interests, essential to determining root causes of conflicts. Future research will examine NA's impact on long-term CG outcomes (e.g. well-being, stress, burden) and care of the OADRD.

Abstract citation ID: igae098.3129

### EXAMINING THE FEASIBILITY AND BENEFITS OF SELF-QUANTIFICATION FOR FAMILY CAREGIVERS: A MIXED-METHOD APPROACH

Tomoko Wakui[1], Suguru Okubo[2], Satoko Fujihara[1], Yoko Moriyama[3], Takeshi Nakagawa[4], Shuichi Obuchi[1], Shuichi Awata[1], and Ichiro Kai[5], *1. Tokyo Metropolitan Institute for Geriatrics and Gerontology, Tokyo, Japan*, *2. BMS Yokohama Inc., Yokohama, Kanagawa, Japan*, *3. National Institute of Public Health, Wako, Saitama, Japan*, *4. National Center for Geriatrics and Gerontology, Obu, Aichi, Japan*, *5. University of Tokyo, Tokyo, Japan*

Supporting family caregivers poses significant challenges because of their inability to seek assistance amid limited time and burden. This study aimed to explore the feasibility of implementing a self-quantification program for family caregivers of community-dwelling older adults. We developed the Caregiving Visualization Program (Care-VIP) allowing family caregivers to document their daily caregiving and life activities and emotional well-being. Initially, an online survey was conducted with 3,256 family caregivers, who were proportionately distributed based on the percentage of the nation's older population requiring care, to investigate their caregiving situations. Subsequently, 201 participants, who requested the program participated, engaged in the self-quantification program for 60 days and provided feedback in a follow-up survey. Feasibility was evaluated based on participant characteristics, completion rates, and the qualitative benefits of self-quantification. Logistic regression analysis revealed that participants were more likely to be fully employed (OR:1.79; p<.01) and experience higher levels of burden (OR:1.01; p<.01) and daily caregiving demands (OR:1.50; p<.01). The completion rate after 60 days was 87.9%, with a 90% completion rate at 98.5%. The content analysis of open-ended responses identified several benefits, including "purposeful and attentive care", "identifying improvements", "establishing daily routines", and "enhancing emotional and self-awareness" derived from "systematic care analysis" facilitated by "reflection". Furthermore, self-quantification led to "instrumental record-keeping", encouraging "articulated insight," fostering "information sharing". This study discusses the significance and limitations of reflective practices and documentation activities with Care-VIP in supporting more frequent and heavily burdened family caregivers.

Abstract citation ID: igae098.3130

### EXPLORING THE HEALTH CARE UTILIZATIONS AND OUTCOMES OF FAMILY CAREGIVERS FOR OLDER ADULTS WITH DEMENTIA

Yu-Qi Cheng, and Ying-Chun Li, *National Sun Yat-sen University, Kaohsiung, Kaohsiung, Taiwan (Republic of China)*

Family caregivers of older adults with dementia usually face more negative issues, such as family relationships, personal career, financial status, and mental and physical problems. Therefore, family caregivers could increase healthcare utilizations and worsen healthcare outcomes. Nevertheless, previous studies results were inconsistent, which could be due to different health policies or healthcare systems in the studied countries. Whether family caregivers receive better care and reduce their burdens under Taiwan's universal healthcare system with free access to healthcare. This is a very important policy research question. We linked outpatient data, inpatient records, medical care facility factors, and registration files from the National Health Insurance Program Data to form the analyzed datasets. Family caregivers for people over 65 with dementia were identified in 2008 and followed for five years. As a result, Family caregivers of older persons with dementia faced long time pressure which led to an increased probability of diseases, such as depression, anxiety, and chronic diseases (all were p< 0.01). These caregivers with disease disorders also significantly consumed more total, outpatient, inpatient, and emergency expenditures (all were p< 0.01). In addition, caregivers for those with dementia who had low and middle-income status used more medical resources (OR=2.16, p< 0.01) and had worse mental health outcome (OR=1.11, p< 0.01). In summary, the empirical results indicated that family caregivers who stay in high stress for a longer time are more likely to cause physical and mental health problems. These family caregivers will use more healthcare resources and experience poorer healthcare outcomes.

Abstract citation ID: igae098.3131

### GRANDCHILDREN WITH DISABILITIES: THE ROLE OF GRANDPARENTS' RELATIONSHIPS

Kingsley Mbam[1], Janelle Fassi[2], Caitlin Coyle[1], Gary Siperstein[1], Jeff Ramdass[1], and Jason Rodriquez[1], *1. University of Massachusetts Boston, Boston, Massachusetts, United States*, *2. University of Massachusetts Boston, Goffstown, New Hampshire, United States*

A body of research, largely in the disability and rehabilitation fields, has shown that grandparents support their grandchildren with disabilities (e.g., autism, down syndrome, and physical disability). However, how grandparents' experiences of helping raise their grandchildren with disabilities influence their identity development in later life is largely unaddressed. This study examined grandparents' relationships with their grandchildren under 21 with disabilities to learn how their experiences contribute to their identity development and perceptions of aging. We recruited 8 grandparents through senior centers across Massachusetts and conducted in-depth qualitative interviews with them by Zoom or phone. Interview questions asked about grandparents' aging experiences as they relate to work, family and social relationships and self-perception, detailed questions about the nature of their relationships with grandchildren, identity development, and demographics. After transcribing the interviews, we used the NVivo 14 software program for qualitative data analysis to analyze our data, identify themes, and draw conclusions. We approached analysis using a constant comparative method that sorts data into various groups, according to specified attributes, and then organizes them in a way to formulate new ideas or theory. Themes that emerged were grandparents' resilience, attitude of gratitude, role conflict of grandparental with parental influence, and the desire to live longer to remain a support for the grandchild. These findings highlight the paradox of grandparents' emotions in supporting their grandchildren with disabilities for improved quality of life and functioning. Furthermore, these findings have implications for practitioners providing support services to grandparents raising their grandchildren with disabilities.

Abstract citation ID: igae098.3132

### GRANDPARENT CAREGIVING AND EPIGENETIC AGING AMONG MIDLIFE AND OLDER ADULTS IN THE UNITED STATES

Aarti Bhat[1], Kaitlin Trexberg[1], Abner Apsley[1], David Almeida[2], and Idan Shalev[1], *1. The Pennsylvania State University, University Park, Pennsylvania, United States*, *2. Pennsylvania State University, University Park, Pennsylvania, United States*

In recent decades, grandparents have taken more active caregiving roles, with over 10% of U.S. grandparents raising a grandchild for 6+ months. Role theory postulates grandparent caregivers face unique strain due to complex roles as second-time parents, and physical demandingness of caring for children. Given grandparents are already vulnerable to aging-associated health deterioration, it is important to understand how grandparent caregiving may affect aging processes. Epigenetic aging is an indicator of healthspan and mortality risk that has not been studied in context of grandparent caregiving. DNA methylation patterns at sites associated with markers of aging have led to development of epigenetic "clocks", which estimate biological age. We examined associations between grandparent caregiving and several epigenetic clocks among a sample of grandparents participating in the Midlife in the United States (MIDUS) study (n=488; mean age=59.41; Black=30.94%; other non-white race=6.56%; female=60.04%). 18.65% of grandparents reported taking on major responsibility for raising grandchild(ren) for 6+ months; among these individuals, mean number of years caring for grandchild(ren) was 5.27 (median=3, range=0-21). Preliminary analyses found, after controlling for sociodemographic characteristics (chronological age was compared against biological age estimates for each clock; race, gender, socioeconomic and marital status were covariates) and BMI (covariate), significant associations between grandparent caregiving and some epigenetic clocks (i.e., DunedinPACE), but not all. Among grandparents who reported providing instrumental care, number of years of care (using median split) was also significantly associated with DunedinPACE. These results indicate prolonged grandparent caregiving may contribute to increased stress and health deterioration at a molecular level.

Abstract citation ID: igae098.3133

### HEALTH CARE USE AMONG SPOUSE CAREGIVERS OF OLDER ADULTS LIVING WITH DEMENTIA

Lianlian Lei, Kenneth Langa, Edward Norton, Helen Levy, Hyungjin Myra Kim, Frederic Blow, and Donovan Maust, *University of Michigan, Ann Arbor, Michigan, United States*

Little is known about how caregiving is associated with caregivers' health care use, particularly where the care recipient is a person living with dementia [PLWD]. This study aims to characterize health care use of spouses of PLWD. Using the 2000–2018 Health and Retirement Study linked with Medicare claims data, we identified community-dwelling dyads where one member had dementia, both members were aged ≥65 years, and enrolled in fee-for-service Medicare; for dyads included in multiple waves, we randomly selected one wave (N=924 unique dyads). We examined the association of spouse care intensity with spousal health care use, adjusted for spousal socio-demographic, health, and functional characteristics. Forty-three percent of spouses did not provide care; and among those providing care, the average monthly hours were 27, 150, and 430 for the first, second, and third tertiles, respectively. Adjusted for spousal characteristics, spouses not providing help had higher total annual Medicare costs ($5,728 and $5,776 higher than those in the second and third tertiles, respectively, p<.05 for both), acute hospitalization (8.2% and 10.4% higher than those in the second and third tertiles, respectively, p<.05 for both), and sub-acute care (7.2% higher than those in the second tertile, p<.01). The spouses who did not provide caregiving hours had the highest health care costs and use. It is unclear whether PLWD spouses do not need care or whether these spouses, by virtue of their own health problems, do not have the capacity to provide caregiving.

Abstract citation ID: igae098.3134

### MEASURING CAREGIVING INTENSITY

Pamela Nadash, Eileen Tell, and Sung Park*, University of Massachusetts Boston, Boston, Massachusetts, United States*

This paper discusses measurement of caregiving intensity, using empirical data to illustrate the need to develop a consensus around measurement of this construct. "Caregiving intensity" differs from the related construct, "caregiver burden", which measures the subjective experience of caregiving; caregiving intensity focuses instead on objective measures of the caregiving experience. Caregiving intensity is operationalized in various ways, employing hours of care provided, the perceived difficulty of care, care recipient characteristics, co-residence, or combinations thereof. We use a unique dataset comprising a representative panel of 385 family caregivers, which was compiled to help understand the decision-making process behind hiring professional home care. Consequently, the survey collected rich data about the caregiving experience of participants, including caregiving-related variables and respondents' employment situations, enabling the construction of variables representing caregiver intensity, including the frequency of care provided for activities of daily living (ADLs) and instrumental activities of daily living (IADLs) and the level of perceived difficulty in providing those activities, as well as co-residence, long-distance caregiving, and having a child under 21. Using multivariate techniques employing these measures of intensity as predictor variables, the study found that while the frequency of helping with ADLs and IADLs mattered in predicting use of paid home care, the likelihood of being employed was not predicted by frequency of ADL and IADL support, but rather by the perceived difficulty of these caregiving tasks (as well as other variables). These findings suggest the need for further work in conceptualizing caregiving intensity.

Abstract citation ID: igae098.3135

### PREVALENCE OF FIREARM OWNERSHIP AND MENTAL HEALTH CHALLENGES AMONG CAREGIVERS: INSIGHTS FROM A NATIONAL STUDY

Jessika Dayrit, Cesz Islaya, Robyn Medcalf, Lauren Charitou, and Renuka Alladi*, U.S. Department of Veterans Affairs/Palo Alto Healthcare System, Palo Alto, California, United States*

Despite studies on the association between firearm accessibility and suicide, there is a gap in understanding the prevalence of firearm ownership among caregivers. This study aims to analyze the prevalence of firearm ownership and mental health challenges among civilian and U.S. military veteran caregivers using data from CDC Behavioral Risk Factor Surveillance System (BRFSS) 2021. Employing a cross-sectional study design, with a total sample size of N=17,625, data analysis was conducted using Stata 17, incorporating appropriate weighting and 95% confidence intervals for demographic variables. Binary logistic regression was employed to analyze the data. Findings suggest that both civilian caregivers (OR=1.17, 95% CI=1.07-1.29) and U.S. military veteran caregivers (OR=1.98, 95% CI=1.55-2.54) were more likely to own a firearm at home compared to non-caregivers. Furthermore, civilian caregivers (OR=1.26, 95% CI=1.14-1.40) and U.S. military veteran caregivers (OR=2.14, 95% CI=1.64-2.78) exhibited higher odds of experiencing depression rates, along with an increased likelihood of serious difficulties in concentrating, remembering, or making decisions due to depression (civilian caregivers: OR=1.40, 95% CI=1.23-1.59; U.S. military veteran caregivers: OR=1.65, 95% CI=1.18-2.30). This nationally representative survey highlights critical insights into firearm accessibility, mental health challenges, and safety among caregivers, emphasizing the importance of interventions to prevent unintentional death and suicide. Firearm safety discussions with patients and caregivers help reduce the risk of accidental death or suicidal behavior. Assessment of firearm ownership and safety, as part of the practitioner-patient-caregiver relationship, is as crucial as evaluating chronic illnesses. Promoting comprehensive approaches to addressing firearm safety in clinical practice ensures patient-caregiver well-being.

Abstract citation ID: igae098.3136

### PRIORITIES TO IMPROVE DEMENTIA CAREGIVING IN CHINESE IMMIGRANT FAMILIES: REPORT OF A CAREGIVER WORKSHOP

Kris (Pui Kwan) Ma[1], Anne Saw[2], and Joyce Cheng[3], *1. University of Washington, Seattle, Washington, United States*, *2. DePaul University, Chicago, Illinois, United States*, *3. Chinese Community Health Resource Center, San Francisco, California, United States*

Linguistically diverse caregivers of people with dementia represent a growing but under-recognized segment of the dementia care workforce in the U.S. There is a continued shortage of culturally and linguistically responsive dementia caregiving interventions for older U.S. Chinese immigrants with limited English proficiency. To address the unmet needs of Chinese-speaking caregivers, integrating caregivers' input is vital to studies to ensure that research aligns with their experiences. This was a focus group study of 10 Chinese-speaking caregivers (mean age 73.7, eight women) conducted during a dementia caregiving workshop, in partnership with a community health resource center in California. During the in-language workshop, caregivers identified their needs in surveys, then discussed with one another in the focus group. Consensus was built to establish priority areas of research and clinical practice to address caregivers' experiences and needs. We used descriptive and thematic analyses to summarize caregivers' priorities. Caregiver-identified priorities included information and practical support in a) management of behavioral and psychological symptoms of dementia (BPSD), b) communication with care recipients and other family members, c) care for caregivers' physical and mental health, and d) assistance to connect to in-language community resources. We identified ways in which these priorities can inform dementia caregiving research and practice for Chinese-speaking caregivers and immigrant families in the U.S. It is recommended that future research and interventions for Chinese-speaking immigrant caregivers in the U.S. prioritize BPSD management skills training, adopt a family-centered approach and include family outcomes, and address service barriers via community-clinic partnerships and outreach.

Abstract citation ID: igae098.3137

### PUTTING NEGOTIAGE IN ACTION: APPLYING SKILLS FROM AN AI-BASED NEGOTIATION TRAINING FOR CAREGIVERS OF OLDER ADULTS

Celie Joblin[1], Alaine Murawski[2], Marianne Tschoe[2], Vanessa Ramirez-Zohfeld[2], Johnathan Mell[3], Jeanne Brett[2], and Lee Lindquist[2], *1. Northwestern University, Chicago, Illinois, United States*, *2. Northwestern University, Evanston, Illinois, United States*, *3. Custom Technologies LLC, Orlando, Florida, United States*

Family caregivers provide crucial support for older adults, yet this can sometimes lead to conflict. We created NegotiAge, an online AI-based negotiation training to help caregivers resolve conflicts. Through instructional resources and practice with AI-based avatars, caregivers learn to navigate conflicts. We characterized how family caregivers utilized NegotiAge in addressing real-life conflicts with their older adults. Family caregivers participated in a randomized controlled trial of the NegotiAge intervention. A month later, they were surveyed with open-ended questions about their use of knowledge and skills gained with NegotiAge with the older adult they support. Two coders analyzed the data using constant comparative thematic analysis. 119 caregivers (mean age 53.5 yrs, 75.6% female, 32.8% Black) completed the training and the one-month survey. 73% (n=119) of participants managed conflicts related to caregiving in the month following NegotiAge. Conflict themes involved: 1) health-related concerns, 2) daily living tasks, 3) finances, 4) acceptance of assistance, and 5) familial duties. 86% (n=119) of participants reported handling conflicts differently because of the NegotiAge training. Themes that surfaced included: 1) changing behaviors away from power dynamics to shared interests, 2) uncovering the causes of conflict, and 3) enhancing communication. Participants utilized their negotiation skills in various contexts, including professional environments, retail, real estate, and other relationships. NegotiAge positively influenced how family caregivers manage conflicts. They applied skills acquired from NegotiAge to manage conflicts with older adults, family, and others. Subsequent research will explore NegotiAge's impact on caregiver burden, stress, and the care of older adults.

Abstract citation ID: igae098.3138

### SANDWICH GENERATION CAREGIVING: NEGATIVE RELATIONSHIP QUALITY AND BURNOUT

Sabine Lohmar, Erika Fenstermacher, Montgomery Owsiany, Catherine Ju, and Barry Edelstein, *West Virginia University, Morgantown, West Virginia, United States*

The sandwich generation provides caregiving for both their children and their aging parents. These caregivers often experience low satisfaction, depression, and burnout, yet little is known about their contributing factors. For example, researchers have not investigated the role of relationship quality among sandwich generation caregivers, who face unique challenges. This study is the first to investigate relationship quality as a moderator between informal caregiver burnout and depression. Regression analyses included measures of positive and negative relationship quality and informal caregiver burnout as predictors and depressive symptoms as the criterion. Correlational analyses indicated that positive relationship quality ($r = -.414$, $p < .001$; $r = -.274$, $p = .005$) and negative relationship quality ($r = .721$, $p < .001$; $r = .467$, $p < .001$) were significantly associated with informal caregiver burnout and depression, respectively. Positive relationship quality significantly moderated the association between caregiver burnout and depression for sandwich generation caregivers only ($R^2 = .323$, $F[3, 99] = 15.741$, $p < .001$, $\beta = .019$, $p = .006$), as did negative relationship quality ($R^2 = .307$, $F[3, 96] = 14.184$, $p < .001$, $\beta = -.016$, $p = .046$). Among sandwich caregivers, positive relationship quality buffered the relation between caregiver burnout and depression, while negative relationship quality enhanced this relation. Positive and negative relationship quality were not significant moderators among individuals who care for parents only or children only. These results suggest that, for sandwich generation caregivers, relationship quality is an important contributing factor for caregiver burnout and depression.

Abstract citation ID: igae098.3139

### SOCIAL MEDIA–BASED BIBLIOTHERAPY FOR INFORMAL CAREGIVERS OF PEOPLE WITH DEMENTIA: A PILOT RCT

Shanshan Wang[1], Leung Sze Him Isaac[2], and Fan Avis[2], *1. The Hong Kong Polytechnic University, Hong Kong, Hong Kong, China (People's Republic)*, *2. The Chinese University of Hong Kong, Hong Kong, China (People's Republic)*

**Background:** Informal caregivers of individuals with dementia are often overlooked as they themselves experience mental health issues. This randomized controlled trial aimed to assess the efficacy of social media-based bibliotherapy in improving the mental health of informal caregivers.
**Methods:** A total of 60 informal caregivers were enrolled. Participants were randomly assigned to either the social media-based bibliotherapy group, which received eight weekly sessions of electronic bibliotherapy, or the usual care group, which only received routine services from community centers. Outcomes were assessed at baseline and immediately post-intervention. Descriptive statistics, t-tests, Mann-Whitney U tests, $\chi 2$ tests, and generalized estimating equations were used for quantitative data analysis.
**Results:** The average age of caregivers was 57.41 (SD, 13.63), with a majority being female (79.3%). Baseline characteristics were similar between groups. The findings demonstrated that social media-based bibliotherapy was well received for informal caregivers, with WhatsApp being the preferred medium in this study. The efficacy on mental health (Wald $\chi 2$=8.918, p=.003) and all the subscales of stress (Wald $\chi 2$=4.198, p=.040), anxiety (Wald $\chi 2$=7.667, p=.006), depression (Wald $\chi 2$=9.127, p=.003) was statistically significant. The efficacy on caregiving appraisal was only significant on the perceived caregiving burden subscale (Wald $\chi 2$=4.954, p=.026). The efficacy on the mental component scale of health-related quality of life approached significance (Wald $\chi 2$=3.634, p=.057). Conclusions: The utilization of social media-based electronic bibliotherapy was well received among informal caregivers of individuals with dementia, demonstrating potential positive effects on their mental health within this specific cohort.

Abstract citation ID: igae098.3140

### THE FEASIBILITY OF A PEER-DELIVERED PSYCHOSOCIAL INTERVENTION FOR FAMILY CARE PARTNERS OF HOSPICE CANCER PATIENTS

Jacquelyn Benson, Kyle Pitzer, Ryan Lindsay, Karla Washington, and Debra Parker Oliver, *Washington University in St. Louis, St Louis, Missouri, United States*

Providing care and support to a hospice patient with cancer can exact an enormous psychological toll on families. Approximately 50% of advanced cancer family care partners report moderate to severe depression and anxiety. Many studies indicate that the psychological suffering of family care partners can be alleviated by enhancing their coping skills. However, mechanistically informed psychosocial interventions that are better tailored to the unique needs of cancer caregivers are needed. Study objectives were to: (1) evaluate the feasibility and acceptability of a 1:1 peer-delivered psychosocial support intervention for hospice family care partners of cancer patients, and (2) determine a range of potential effects of the intervention on mechanisms of change and care partners' psychological distress symptoms to guide variable inclusion for a future trial. The study was carried out using a convergent parallel mixed methods design. Qualitative data were analyzed using deductive thematic analysis. Descriptive statistics were performed for demographic characteristics, and rates of recruitment, attrition, and survey completion. Parametric and non-parametric t-tests were conducted to examine mean differences of study outcomes between baseline and midpoint, and endpoint. Mean differences above 0, in the expected direction, and confidence intervals with a 10% or greater change in the total outcome were used to guide decision-making about future trial inclusion. Results demonstrated that the intervention is acceptable, appropriate, and feasible to deliver to hospice family caregivers of cancer patients. Depressive symptoms, anxiety symptoms, benefit finding, and self-efficacy were also determined to be appropriate measures for future use in a larger trial.

Abstract citation ID: igae098.3141

### THE IMPACT OF PAID FAMILY LEAVE ON INFORMAL AND FORMAL CARE RECEIPT FOR MIDDLE-AGED AND OLDER ADULTS WITH DISABILITIES

Yuting Qian, and Xi Chen, *Yale University, New Haven, Connecticut, United States*

About one-sixth of middle-aged and older adults in the U.S. have difficulty with at least one activity of daily living (ADL), and this prevalence increases with age. Working caregivers face a trade-off between spending time earning income and providing care for their disabled family members. Recognizing this challenge, thirteen states and Washington, D.C. have implemented or recently passed mandatory paid family leave (PFL) programs, which provide partially paid leave for workers caring for ill family members. This paper analyzes the impact of PFL policies on both informal and formal care receipt among middle-aged and older adults with disabilities in the U.S., and the heterogeneous effects by family availability. We use data from the 1998-2018 public and restricted-use version of the Health and Retirement Study (HRS) and leverage the PFL programs implemented in California (2004), New Jersey (2009), and New York (2018) in a difference-in-differences (DiD) design. We find that, among individuals with disabilities who have both a spouse and children, PFL access is associated with an 8.0 percentage point increase in the likelihood that disabled individuals report receiving informal care from their children. We also show that PFL access significantly increases the receipt of formal care and increases the use of home care services and nursing home care for disabled individuals who have a spouse. Our findings demonstrate that PFL policies support healthy aging for middle-aged and older adults with disabilities, but its impact is limited for some vulnerable groups, such as disabled individuals with children only.

Abstract citation ID: igae098.3142

### THE IMPACT OF TRAUMA AND POSTTRAUMATIC STRESS SYMPTOMS ON CAREGIVER WELL-BEING

Christina Hugenschmidt[1], Natasha Collier[2], and Gretchen Brenes[1], *1. Wake Forest University School of Medicine, Winston-Salem, North Carolina, United States*,

*2. Atrium Health Wake Forest Baptist, Winston-Salem, North Carolina, United States*

There is a high level of variability in caregiver experiences and outcomes, and up to 50% of variance in caregiver well-being remains unexplained. We propose that exposure to traumatic events and experience of post-traumatic stress symptoms (PTSS) may be a previously unrecognized dimension of caregiver burden. We surveyed 25 caregivers of persons with dementia (PWD) to assess 1) lifetime exposure to traumatic events, 2) post-traumatic stress symptoms (PTSS) related to the caregiving experience, and 3) associations between trauma exposures and PTSS and caregiver burden, depression, and grief. Caregivers averaged 66.4 years old (range: 36-81) and had been caring for their loved one an average of 5.5 years (range: 1-23 years). Caregivers had different relationships with the care recipients (daughter: n=9, spouse/partner: n=9, son: n=4, sister: n=2, friend: n=1) and n=11 (44%) lived with the care recipient. Forty percent of the sample had experienced at least one adverse childhood experience (ACE) and 96% reported at least one trauma as an adult. Four participants (16%) stated that caregiving for their PWD or a caregiving-related event was the most stressful event of their life, and 35% had elevated PTSS related to caregiving. ACEs and PTSS in response to caregiving were highly predictive of important outcomes for caregiver well-being, including depression (ps ≤ 0.03), prolonged grief (ps ≤ 0.03), and caregiver strain (ps ≤ 0.02). These findings suggest that trauma experienced before and during caregiving and PTSS are significantly associated with caregiver well-being.

### SESSION 7495 (POSTER)

### FINANCIAL WELLNESS

Abstract citation ID: igae098.3143

### ENTORHINAL CORTEX THICKNESS IS ASSOCIATED WITH FINANCIAL EXPLOITATION VULNERABILITY IN OLDER ADULTS

Laura Fenton[1], Lauren Salminen[2], Aaron Lim[2], Gali Weissberger[3], Annie Nguyen[4], Jenna Axelrod[2], Daisy Noriega-Makarskyy[2], and Duke Han[2], *1. University of Southern California, California, United States*, *2. University of Southern California, Los Angeles, California, United States*, *3. Bar-Ilan University, Ramat Gan, HaMerkaz, Israel*, *4. University of California San Diego, La Jolla, California, United States*

Research suggests that increased financial exploitation vulnerability (FEV) due to declining decision making may be an early behavioral manifestation of brain changes occurring in preclinical Alzheimer's disease (AD). One of the earliest documented brain changes during this preclinical phase is neurodegeneration of the entorhinal cortex. The objective of the current study was to examine the association between a measure of FEV and thickness of the entorhinal cortex in 97 older adults without cognitive impairment (mean age (SD) = 68.72 (7.45); mean education (SD) = 16.52 (2.14); 76% female; 76% White). We also investigated FEV associations with frontal regions typically associated with decision making (e.g., dorsolateral and ventromedial prefrontal cortices). Exploratory analyses investigated the interactive effect of age and cortical thickness on FEV. Results showed that greater FEV was associated with significantly lower entorhinal cortex thickness (B = -0.05, SE = 0.02, p = 0.04), but not with thickness in frontal regions. There a significant interaction between age and entorhinal cortex thickness on FEV, whereby lower entorhinal cortex thickness was associated with greater FEV in older participants (B = -0.01, SE = 0.003, p = 0.04). When the group was split according to age using a median split (70+; < 70 years old), lower entorhinal cortex thickness was associated with greater FEV only in the older group (B = -0.10, SE = 0.04, p = 0.01). Collectively these findings support the notion that FEV may serve as an early behavioral manifestation of preclinical AD before noticeable cognitive impairment.

Abstract citation ID: igae098.3144

### MEDICAL FINANCIAL HARDSHIP AND EMOTIONAL WELL-BEING AMONG US OLDER ADULTS WITH AND WITHOUT ALZHEIMER'S DISEASE

Shinae Choi[1], and Maria Pisu[2], *1. University of Alabama, Tuscaloosa, Alabama, United States*, *2. University of Alabama at Birmingham, Birmingham, Alabama, United States*

This study investigated the impact of medical financial hardship (MFH), specifically the inability to make medical payments, on various dimensions of emotional well-being (EWB), including positive and negative affect, life satisfaction, and sense of meaning and purpose in life, among U.S. older adults with and without Alzheimer's disease and related dementias (ADRD). Data were from the 2016-2020 waves of the Health and Retirement Study (N=12,904). Multivariate regression analyses were computed adjusting for health and sociodemographic factors. Overall, 884 respondents faced MFH, and 352 self-reported a physician diagnosis of ADRD. In people without ADRD, 6.7% faced MFH, while among those with ADRD, the percentage rose to 12.2% ($\chi 2(1)=16.324$, p< 0.001). In people without ADRD, MFH was significantly associated with lower life satisfaction (b=-1.195, p< 0.001), positive affect (b=-0.301, p< 0.001), sense of meaning and purpose in life (b=-0.417, p< 0.001), and with higher negative affect (b=0.454, p< 0.001). In those with ADRD, MFH was significantly associated with lower life satisfaction (b=-1.193, p< 0.01) and higher negative affect (b=0.661, p< 0.01); associations with positive affect (b=-0.459) and sense of meaning and purpose in life (b=-0.315) were nonsignificant at the p< 0.05 level. Findings suggest that MFH is twice as likely in people with ADRD, potentially impacting several aspects of their EWB, including life satisfaction and negative affect. The impact is broader for older adults without ADRD, where MFH may also affect their evaluation of meaning and purpose in life. We discuss implications for further research and interventions to improve EWB of older adults facing MFH, both with and without ADRD.

Abstract citation ID: igae098.3145

### SOCIAL NETWORK SIZE, SOCIAL FULFILLMENT, AND FINANCIAL EXPLOITATION VULNERABILITY IN OLDER ADULTS

Daisy Noriega-Makarskyy[1], Shaneen Upal[1], Elizabeth Erdman[1], Laura Fenton[2], Aaron Lim[1], Gali Weissberger[3], Laura Mosqueda[4], and Duke Han[1], *1. University of Southern California, Los Angeles, California, United States*, *2. University of Southern*

*California, California, United States*, 3. *Bar-Ilan University, Ramat Gan, HaMerkaz, Israel*, 4. *University of Southern California Keck School of Medicine, Pasadena, California, United States*

This study tested whether aspects of social functioning, including breadth of current social roles (e.g., being a family member, co-worker, neighbor, etc.), and fulfillment in various social supports (i.e., relationship depth), are associated with perceived financial exploitation vulnerability (FEV) among older adults. Adults aged 50 or older (N=115; 69% of sample >65 years) completed a laboratory visit consisting of questionnaires assessing relationship breadth, depth, and perceived FEV. The relationship depth measure included a total score as well as subscales for: 1) Appraisal, or availability of interpersonal advice or guidance; 2) Belonging, or availability of interpersonal empathy and acceptance; and 3) Tangible, or material/financial support resources. Hierarchical linear regressions separately tested associations between relationship depth or breadth and FEV. After covarying for demographics, depression/anxiety symptoms, and general cognition, FEV was not significantly associated with relationship breadth, or the Appraisal and Tangible subscales (p's=.13-.93). FEV was negatively associated with Relationship Depth Total Score (B(SE) = -.08(.03), p<.01) and Belonging subscale (B(SE) = -.28(.06), p<.001). In summary, among a sample of older adults, perceived financial exploitation vulnerability was not associated with the number of different regular social roles a person had, or the availability of social resources for material/financial assistance, or interpersonal advice/guidance. Rather, financial exploitation vulnerability was greatest among individuals with a weaker sense of social belonging, marked by instances such as not having someone to go with on a spontaneous day trip. Such results corroborate the benefits of close interpersonal relationships that may reduce vulnerability to financial exploitation in older age.

Abstract citation ID: igae098.3146

### THE IMPLEMENTATION OF A STATEWIDE INTERDISCIPLINARY OLDER ADULT FINANCIAL JUSTICE COALITION

Briana Kohlbrenner, Jodi Catlow, Eric Chess, and Carson De Fries, *University of Denver, Denver, Colorado, United States*

Financial fraud and exploitation have devastating impacts that are felt acutely among older adults. To address this issue in Colorado, a coalition was formed in March of 2023 to bring together the state's strong but historically disconnected contingent of experts across disciplines. This coalition includes 60 individuals representing 30 agencies including law enforcement, financial institutions, adult protective services, government, and non-profit agencies. The goals of the coalition are to 1) convene and grow this network to include all key stakeholders and leaders in financial fraud and exploitation across the state, 2) provide support, training, actionable tools, and resources for front-line workers and 3) streamline resources and create a collaborative state-wide hub for resources around financial abuse. Through this initiative we have identified barriers to collaborative efforts in this area including sharing data across agencies, building trust between industries, and an overall lack of shared language. Lessons learned from the implementation of this coalition include how to communicate across disciplines, elevating the expertise of those in the field without replicating work or operating in silos, and utilizing an academic institution to facilitate these collaborative efforts. To provide an overview of the innovative and interdisciplinary coalition, we will showcase the process of developing, maintaining, and expanding this coalition with the purpose of providing a framework for other states to replicate.

Abstract citation ID: igae098.3147

### UNDERSTANDING OLDER AMERICANS' DEBT: INSIGHTS FROM COGNITIVE AND IN-DEPTH INTERVIEWS

Hector Ortiz, *Consumer Financial Protection Bureau, Washington, District of Columbia, United States*

Debt is a common and important factor in older adults' financial lives and balance sheets. According to the Survey of Consumer Finances, in 2022, 63 percent (31.3 million) of households headed by adults ages 60 and older held debt. In total, they held over $4.1 trillion in debt. Existing research and data suggest that there is also a strong relationship between debt and aging. This relationship is growing and changing. Despite the importance of debt in older adults' financial lives, there are foundational gaps in qualitative data that limit researchers, policymakers, and educators' ability to understand and monitor the level of debt held by older adults and the risk that debt poses to older adults. To address this gap, and provide insights into the growing and changing relationship between aging and debt, the Consumer Financial Protection Bureau (CFPB), an agency of the United States government, conducted cognitive and in-depth interviews with 54 participants, a majority (81 percent) of whom were age 62 or older, in June and July 2023. The findings revealed important gaps in the completeness of existing data sources that serve as a tool for monitoring trends and risks related to older adults' debt. Moreover, the study findings also have important implications for areas where greater focus on consumer protection may be warranted.

### SESSION 7500 (POSTER)

### GENDER

Abstract citation ID: igae098.3148

### ALZHEIMER'S DISEASE: GENDER AND GENERATION DISPARITIES IN ATTITUDES AND HELP-SEEKING AMONG AMERICAN INDIAN ADULTS

Yeon-Shim Lee[1], Soonhee Roh[2], Heehyul Moon[3], Donald K. Warne[4], Serene Thin-Elk[5], and Sasheen T. Stone[6], 1. *San Francisco State University, San Francisco, California, United States*, 2. *University of South Dakota, Sioux Falls, South Dakota, United States*, 3. *University of Louisville, Louisville, Kentucky, United States*, 4. *Johns Hopkins University, Baltimore, Maryland, United States*, 5. *South Dakota Urban Indian Health, Sioux Falls, South Dakota, United States*, 6. *Yankton Sioux Tribe, Wagner, South Dakota, United States*

**Purpose:** While the benefits of knowledge about health and preventive measures are widely acknowledged, little attention has been paid to the knowledge and attitudes of American Indian adults towards Alzheimer's Disease and other dementias

(ADRD). Therefore, this study examines the attitudes towards Alzheimer's Disease and help-seeking intentions among American Indian adults, with a specific focus on gender differences. Method: Convenience sampling was used to recruit 227 American Indian adults in the Northern Plains. We employed t-tests and chi-square tests to examine the differences in attitudes toward ADRD (including stigma, knowledge, and attitude) and help-seeking intentions by gender and generation (Millennial or Younger vs. Gen X or older).

**Results:** More than two-thirds of the participants showed signs of cognitive impairment. Among the respondents, 60% were female, and 54% had completed education beyond high school. Approximately 50% were from the Millennial or younger generation. Female American Indian adults were more likely to report higher levels of ADRD knowledge, positive attitudes toward ADRD, and help-seeking intentions compared to their male counterparts. No significant differences were found in attitudes and help-seeking intentions by generation.

**Conclusion:** The findings underscore the gender disparities in ADRD-related attitudes and help-seeking intentions among American Indian adults. Tailored educational support programs should account for these variations, focusing on strategies to encourage American Indian adults to seek an ADRD diagnosis.

Abstract citation ID: igae098.3149

### ASSOCIATIONS OF PHYSICAL ACTIVITY ENGAGEMENT WITH CEREBRAL AB AND TAU PET FROM MIDLIFE

Mitzi Gonzales[1], Daniel Kojis[2], Nicole Spartano[3], Emma Thibault[4], Charles DeCarli[5], Keith Johnson[4], Alexa Beiser[2], and Sudha Seshadri[6], *1. Cedars-Sinai Medical Center, Los Angeles, California, United States*, *2. Boston University, Boston, Massachusetts, United States*, *3. Boston University School of Medicine, Boston, Massachusetts, United States*, *4. Massachusetts General Hospital, Boston, Massachusetts, United States*, *5. University of California Davis, Davis, California, United States*, *6. The University of Texas Health San Antonio, San Antonio, Texas, United States*

Higher midlife physical activity engagement has been associated with lower dementia risk in late life. However, the underlying mechanisms contributing to the protective effect remain unclear. The goal of the current study was to evaluate the associations of physical activity with cerebral amyloid beta (Aβ) and tau in a predominately middle-aged community-based cohort, as well as to explore whether the associations differ by age or sex. Participants from the Framingham Heart Study underwent 11C-Pittsburgh Compound B amyloid and 18F-Flortaucipir tau positron emission tomography (PET) imaging. Total physical activity levels were evaluated by self-report using the Physical Activity Index (PAI). Cross-sectional associations between total PAI with regional Aβ and tau PET retention were evaluated using linear regression models adjusted for demographic and cardiovascular risk factors. Interactions with sex and age group were examined and stratified analyses were performed when significant. The sample included 354 participants (mean age 53±8 years, 51% female). Higher total PAI scores were associated with lower entorhinal cortex (β(SE)=-0.021(0.008), p=0.013), inferior temporal lobe (β(SE)=-0.017(0.008), p=0.040), and rhinal cortex (β(SE)=-0.018(0.008), p=0.030) tau PET binding. There were significant interactions with sex with only males displaying inverse associations between total PAI with entorhinal cortex, inferior temporal lobe. and rhinal cortex tau. The results suggest that higher midlife physical activity may confer resistance against tau pathology in regions susceptible to early neuropathological changes. However, the effects may vary based on sex, highlighting the importance of better understanding and tailoring lifestyle interventions to address sex disparities.

Abstract citation ID: igae098.3150

### GENDER DIFFERENCES IN DISEASE BURDEN AND QUALITY-OF-LIFE TRAJECTORIES AMONG OLDER ADULTS LIVING WITH MULTIMORBIDITY

Arum Lim[1], Chitchanok Benjasirisan[1], Jordan Tebay[1], Cheryl Dennison Himmelfarb[1], Patricia Davidson[2], and Binu Koirala[1], *1. Johns Hopkins University, Baltimore, Maryland, United States*, *2. University of Wollongong, Wollongong, New South Wales, Australia*

Heart failure (HF) is a leading cause of hospitalization and is associated with higher multimorbidity. Despite gender disparities and differences in HF research participation and health outcomes, differences in the trajectories of their lives have not been studied enough. This study aimed to examine the 1-year trajectories of disease burden and quality of life (QoL) for older adults with multimorbidity. Multimorbidity was defined as the presence of HF and one or more conditions among common chronic conditions in older adults. The study prospectively collected socio-demographic, clinical, and outcome data at baseline, 30-day, and 1-year follow-up using an electronic patient portal among adults aged 50 years and older living with multimorbidity. Disease burden and QoL data were collected using a modified disease burden impact scale and the EuroQoL (EQ)-5D-5L. Linear mixed-effect models were used for statistical analyses. A total of 200 patients (102 women and 98 men) completed surveys at the three time points. Participants' mean±SD age was 71±9.5 years; men were older than women. The disease burden decreased at the 30-day follow-up (23.7±14.9) and the 1-year follow-up (24.6±15.7) compared to baseline (26.3±16.3). In an unadjusted analysis, disease burden was higher in women than men (beta=2.516; p=0.017). QoL was not significantly different by gender and time. After adjusting for age and social support, there were no significant gender differences in disease burden and QoL trajectories and no time and gender interaction. There is a need for gender-based research on multimorbidity to promote health equity.

Abstract citation ID: igae098.3151

### IDENTIFYING GENDER-BASED DIFFERENCES IN CHARACTERISTICS OF OLDER ADULT CANNABIS USERS

Juliamaria Coromac-Medrano, Elizabeth Anquillare, Adrianna Neiderman, and Rachel Thayer, *University of Colorado Colorado Springs, Colorado Springs, Colorado, United States*

As cannabis use continues to rise among older adults, understanding different patterns of cannabis use is essential for interpreting health outcomes and treatment expectations for this population. This study explored gender differences in age of onset, frequency of use, primary methods of cannabis ingestion, and scores on substance use disorder and cognitive questionnaires between male and female older adult cannabis users (OACU). This sample included 52 OACU ages 60+ (Mage=67.98, SD=5.77; 50%=female; 90%=White). Chi-square analyses and independent samples t-tests were performed to identify gender differences in patterns of cannabis use. The percentage of men who endorsed primarily inhalation methods (76%) was significantly greater than women (46%), and the percentage of women who endorsed primarily consuming edibles (54%) was significantly greater than men (24%), $\chi 2(1, N=49)=4.69$, $p<.05$. Men were more likely to use cannabis on more days of the previous week (M=5.42, SD=2.42) compared to women (M=3.88, SD=2.83), $t(50)=2.11$, $p<.05$. Men also generated significantly greater scores on the Cannabis Use Disorder Identification Test-Revised (CUDIT-R) compared to women (Mscore=12.85, SD=6.75 and Mscore=8.96, SD=5.85, respectively), $t(50)=2.22$, $p<.05$. Women (Mscore=195.48, SD=46.88) reported significantly greater cognitive self-efficacy compared to men (Mscore=168.35, SD=44.54), $t(49)=-2.18$, $p<.05$. There was no difference regarding the age of first ever cannabis use between men (Mage=22) and women (Mage=26). These findings elucidate several differences in use patterns between male and female OACU. Future research can explore additional gender differences to better inform clinical teams about the individual characteristics of an older adult cannabis user.

Abstract citation ID: igae098.3152

### MALE SPOUSAL CAREGIVERS ARE ON THE RISE BUT THEY PROVIDE MOSTLY SECONDARY, INSTRUMENTAL, AND LOW-INTENSITY CARE

Hyungmin Cha, and Jennifer Ailshire, *University of Southern California, Los Angeles, California, United States*

Despite demographic projections that more men are adopting caregiving roles than ever before, there is a lack of population-level estimates regarding the prevalence of male caregivers over time. It is also unclear which types of caregiving tasks men typically provide. Using data from the Health and Retirement Study (2002-2018), we investigate trends in the prevalence of spousal caregiving among both men and women. We also analyze subcategories of caregiving, including primary versus secondary caregiving, activities of daily living versus instrumental activities of daily living, and high-intensity versus low-intensity care. Our results indicate that the prevalence of male caregivers has been increasing from 2002 to 2018, but these caregivers primarily focus on secondary, instrumental, and low-intensity caregiving. Further descriptive statistics comparing male caregivers in 2002 to those in 2018 reveal that this trend may be attributed to a growing number of Hispanic individuals, younger cohorts, highly educated, retired, and wealthy men, as well as those with older spouses. Our findings reveal the increase in male caregivers over time, which reshapes the gendered profile of caregivers. Nevertheless, male caregivers still primarily provide secondary, instrumental, and low-intensity care.

Abstract citation ID: igae098.3153

### SLEEP AND STRESS IN MIDDLE-AGED AND OLDER CAREGIVERS OF CHILDREN, PARENTS, AND SPOUSES: EXAMINING SEX DIFFERENCES

Melanie Stearns[1], Kevin McGovney[2], Ashley Curtis[1], Amy Costa[1], Mary Beth Miller[2], Uma Nair[1], and Christina McCrae[1], *1. University of South Florida, Tampa, Florida, United States*, *2. University of Missouri, Columbia, Missouri, United States*

Informal caregiving is associated with sleep and stress, particularly among women, but no studies have examined the impact of caregiving for different individuals (child, parent, spouse) on sleep and stress. This study fills that gap. Caregivers of a child (N=151, Mage=40.50, 52%-female), parent (N=99, Mage=44.45, 52%-female), and spouse (N=71, Mage=55.26, 45%-female) completed the Perceived Stress Scale and reported bed/waketime consistency and night awakenings. Three-way ANOVAs evaluated interactions (caregiver-type x sleep/stress x sex) and main effects, controlling for age. Main effects also were examined separately for men and women. Pairwise comparisons clarified significant differences. Interactions not significant. A significant main effect (p=.049) occurred for night awakenings, consistent bedtime (p=.025), consistent waketime (p=.049), and stress (p=.045). Caregiver-child reported less consistent bedtimes (M=3.66+\-1.73) than caregiver-parent (M=2.93+\-1.63; p<.001) or caregiver-spouse (M=3.20+\-1.78; p=.009). Caregiver-child reported less consistent waketimes (M=3.23+\-1.62) than caregiver-parent (M=2.61+\-1.52; p=.003). Caregiver-child (M=4.39+\-2.87; p=.009) and caregiver-parent (M=4.28+\-2.91; p=.031) reported more night awakenings than caregiver-spouse (M=3.35+\-2.46). Caregiver-child reported more stress (M=19.91+\-5.95) than caregiver-spouse (M=17.56+\-7.46; p=.013). For women, a significant main effect (p=.008) occurred for stress and consistent waketimes (p=.047). Among women, caregiver-child reported more stress (M=21.25+\-6.02) than caregiver-parent (M=20.75+\-6.0; p=.014) or caregiver-spouse (M=16.63+\-7.4; p<.001). Caregiver-child reported less consistent waketimes for women (M=3.25+\-1.71) than caregiver-parent (M=2.83+\-1.30; p=.010). Thus, child and parent caregivers, particularly women, may experience increased sleep problems/stress. This may be in part due to caregiving burdens placed on women. Thus, women caregivers may need additional monitoring and support. We encourage longitudinal research examining what contributes to increased sleep and stress among caregivers.

### SESSION 7505 (POSTER)

### IMMIGRATION

Abstract citation ID: igae098.3154

### AGING AMONG JAPANESE OVERSEAS/EXPATRIATES: A COMPREHENSIVE MIXED METHODS STUDY

Itsuko Toyama, *St. Andrew's University/Momoyamagakuin University, Kyoto, Kyoto, Japan*

The challenges of aging for Japanese individuals living abroad have received limited attention in both Japan and their countries of residence, and they are underrepresented in academic research. This marginalization derives from the small population of Japanese expatriates and the ambiguity surrounding the term "Japanese," as a nationality and cultural identity. As there are currently no available data on this topic, original research is necessary. Additionally, a holistic perspective is essential to approach aging as a life course. This study employs a convergent parallel mixed methods design to examine four Japanese communities in multicultural societies across the US, the UK, the Netherlands, and Brazil. Quantitative data were collected through nearly identical questionnaires on aging conditions and analyzed in Brazil (2002), the UK (2013), the Netherlands (2014), and New York (2018). Qualitative data, including interviews and oral histories, were collected in the respective communities since 1993. This presentation demonstrates the validity of using mixed methods in minority aging research. The comparison of quantitative research indicates ethnic strategies for supporting aging individuals and preserving dignity, including culturally oriented facilities or residences that use the Japanese language and offer food, activities, and care for older adult. Qualitative research interviews and oral histories reveal individual resilience in terms of aging and escaping isolation. For instance, some participants use their internment experiences to adapt to group living in a retirement or nursing home, while others maintain the hope of returning to Japan. Thus, the mixed methods approach provides a detailed and comprehensive conceptualization of aging.

Abstract citation ID: igae098.3155

### BARRIERS TO SOCIAL ENGAGEMENT OF OLDER IMMIGRANTS IN THE UNITED STATES: A SYSTEMATIC REVIEW OF LITERATURE

Dolapo Adeniji[1], Vivian Miller[2], Betty Tonui[3], and Emma Barnhart[2], *1. Indiana State University, Avon, Indiana, United States*, *2. Bowling Green State University, Bowling Green, Ohio, United States*, *3. Oakland University, Rochester, Michigan, United States*

Social engagement is crucial in older adulthood. Social engagement strengthens a sense of belonging, fosters meaningful participation in activities, enhances physical and mental health, alleviates stress and anxiety, and promotes a healthy lifestyle. However, older immigrants often face challenges in engaging socially due to acculturation stress and unfamiliar cultural norms. Using a systematic review of existing literature, this study seeks to identify existing literature that discusses older immigrants' involvement and participation in community programming, synthesize findings on the accessibility of such programs, and present recommendations for modifications in programs and policies to serve this population best. Guided by the PICO framework and PRISMA guidelines, a systematic review of 11 university databases yielded (N=20) relevant peer-reviewed journal articles after completing a three-stage review process. These articles document social engagement practices identified among various ethnic backgrounds, including older Korean Americans, Chinese Americans, Mexican Americans, and Vietnamese, as well as immigrants and refugees originating from Africa and Asia. Findings suggest that ageism, social disintegration, and language barriers hinder the effectiveness of existing community programs, thus leading to social isolation and loneliness. While our study encompassed various ethnic groups, it is imperative to recognize the potential unique social engagement practices within each of these groups. This acknowledgment is essential in planning community programs to accommodate languages and cultural preferences that can help alleviate social isolation among aging immigrants. Future studies may benefit from a more targeted examination of social engagement practices within distinct ethnic groups to inform targeted interventions.

Abstract citation ID: igae098.3156

### EFFECTIVENESS OF AN AGING-RELATED PREPARATION EDUCATION PROGRAM FOR OLDER CHINESE MIGRANTS IN JAPAN

Li Yao, and Harue Masaki, *Chiba University, Chiba, Chiba, Japan*

**Introduction and objectives:** To resolve the long-term care issues of older Chinese migrants in Japan, we developed an aging-related education program focused on preparation for long-term care consisting of six modules with lectures and discussions through real-time videoconferences. This study aimed to evaluate the effectiveness of this developed education program among older Chinese in Japan.
**Methods:** Older Chinese people and their family members living at home independently in the Tokyo and Kansai areas were recruited. The before-after study was conducted to collect the data from the Preparation for Future Care Needs Scale-14 (PFCN-14) and Attitudes to Aging Questionnaire (AAQ). Data was analyzed by descriptive statistics and the Friedman non-parametric repeated measures ANOVA test.
**Results:** Twenty-two participants with an average age of 73.0 (SD= ±7.18) years completed all questionnaires, of which 14 (63.6%) were female. The median (IQR) PFCN-14 total scores before intervention, after intervention, and one month after intervention were 40.00 (7)%, 46.50 (13)%, and 48.50 (8)% ($\chi^2$=18.644, P< 0.001). There were significant differences between before and after intervention (P=0.016) and between before and one month after intervention (P< 0.001). The median (IQR) AAQ total scores were 82.00 (23)%, 80.50 (22)%, and 79.00 (21)%, respectively, with no significant differences ($\chi^2$=2.533, P=0.279).
**Conclusion:** This education program improved participants' status of preparing for long-term care by promoting awareness, decision-making, and concrete planning actions. It can be used to improve health literacy and the autonomy of later life for older Chinese migrants in Japan.

Abstract citation ID: igae098.3157

### NATIVITY STATUS AND SUCCESSFUL AGING IN LATINX OLDER ADULTS: FINDINGS FROM A CROSS-SECTIONAL SURVEY

Ajla Basic, and Mushira Khan, *Mather Institute, Evanston, Illinois, United States*

Previous research has underscored the dynamic relationship between health and aging, highlighting the significant influence of social, cultural, and environmental factors on aging outcomes in racial and/or ethnic minority older adults. Building on the extant literature, this cross-sectional study explores the relationship between nativity status and successful aging in community-dwelling Latinx adults 50+, examining whether being born in the U.S. or having immigrated (first-generation immigrant) influences the process of aging well. A total of 141 Latinx older adults (mean age = 62.83; 65.9% female) residing in Chicagoland completed paper surveys. Measures included the successful aging scale, short acculturation scale, well-being indicators including affiliation (social connectedness), autonomy (personal control), and achievement (personal growth), and sociodemographic variables. Findings from multiple regressions revealed that nativity status (U.S. born) moderated the relationship between successful aging and age ($F(3, 130)=3.56$, $p < 0.05$), gender($F(3, 133)=5.08$, $p< 0.05$), autonomy ($F(3, 134)=2.56$, $p < 0.05$), and self-reported health ($F(3,136)=8.83$, $p < 0.05$). These findings suggest that experiences of aging in Latinx older adults can differ depending on whether a person was born in the US or immigrated, indicating the importance of considering factors such as cultural attitudes toward aging, migration and settlement processes, and differential access to resources. Furthermore, cultural norms and expectations surrounding gender roles may be experienced differently by U.S. born Latinx older adults and first-generation immigrants, impacting caregiving, health maintenance, and social support networks. Future research should explore in-depth the mechanisms through which nativity status influences perceptions of aging well in Latinx older adults.

Abstract citation ID: igae098.3158

### STATE OF HEALTH AND FUNCTIONAL AGING IN THE IMMIGRANTS AND MIGRANTS IN UKRAINE

Olena Tomarevska, and Oleksandr Poliakov, *D. F. Chebotarev Institute of Gerontology of the National Academy of Medical Sciences of Ukraine, Kyiv, Kyyiv, Ukraine*

The study of migration factors and the likelihood an impact on aging and health of respondents is important for the healthcare. This study is aimed to evaluate differences in the health of immigrants, migrants, and non-migrants among the 31-105 years. Online study will be perspectives to assess the health state and effectiveness of rehabilitation in migrant's groups. Analysis of functional aging and health, sensory, cognition and physical capabilities of migrants and non-migrants in 31-105 years (1995-2023) offline and online study in Ukraine. For participants after 90, the impact of immigration processes of interstate and internal migration on health was determined. In persons 60-89 years old was relationship between functional aging and interstate immigration to Ukraine. Primary health assessment was analyzed the impact of outward migration and comparison with people who remained inside the country in Ukraine. In the results the trend are accelerator characters of functional aging. The leading indicators in monitoring health status and functional aging processes were the ability in centenarians to sit-to-stand up test ($r=0.176$, $p< 0.05$), in the older adults ($r=-0.210$, $p< 0.05$), in the online study ($P=0.006$) as well as the ability to remember three words after over time ($r=0.174$, $p< 0.05$) in ninety-year-olds. We found changes in these parameters, characterized by better indicators for migrants who emigrated and immigrated to the territory of Ukraine. This fact shows that people migrate with better health indicators, and these relationships health status at older persons and centenarian require monitoring and for rehabilitation programs.

Abstract citation ID: igae098.3159

### UNVEILING THE AGING EXPERIENCES OF CHINESE IMMIGRANTS WITH SOCIAL MEDIA POSTS

Xiayu Chen, and Wan Wen, *University of Illinois Urbana-Champaign, Champaign, Illinois, United States*

Chinese immigrants are among the largest immigrant groups worldwide. As they age, they may encounter a complex array of cultural and social challenges, striving to find a balance between maintaining their cultural values and adapting to the social norms of the migrated countries. With the aim of understanding the community integration and well-being of the aging Chinese immigrants, this study analyzed social media data to uncover topics that reflect their daily living experience. We collected 3,756 posts from January 1, 2013, to January 1, 2024, from the "Evergreen Life" subforum on Wenxuecity.com, a leading Chinese-language BBS forum catering to the overseas Chinese community. Using Latent Dirichlet Allocation topic modeling approach, this study identified five key topics reflecting their aging experiences, including (1) society and life, focusing on navigating societal norms and healthcare systems in migrated countries and life comparisons between China and abroad; (2) overseas cultural experiences, emphasizing personal adaptation stories; (3) family daily life, highlighting intergenerational dynamics and the importance of family support in aging life; (4) physical activities and personal growth, showing efforts towards well-being improvement through exercise and hobbies; (5) religion and spiritual life, demonstrating the role of spirituality in community connection. These findings offer a comprehensive view of the social, cultural, familial, physical, and spiritual aspects of aging among Chinese immigrants, emphasizing the need for culturally sensitive support services to foster a more inclusive environment for this population.

### SESSION 7510 (POSTER)

### LONG-TERM CARE II (POSTERS)

Abstract citation ID: igae098.3160

### A COMPARATIVE STUDY OF INTEGRATED LONG-TERM CARE SERVICES IN TAIPEI CITY AND NINGBO CITY

Huan Liu[1], and Hong Mi[2], *1. NingBo University of Technology, NingBo, Zhejiang, China (People's Republic), 2. Zhejiang University, Hangzhou, Zhejiang, China (People's Republic)*

This study aims to compare the integrated long-term care service models in Taipei and Ningbo, exploring their similarities, differences, and experiences for mutual learning. Through literature review and field research, it was found that Taiwan's long-term care system has advantages such as comprehensive coverage, diversified services, professional teams, smart technology applications, community participation, policy support, and a supportive social and cultural environment. On the other hand, Ningbo's integrated long-term care services also have unique features in terms of service content and format, emphasizing family care, promoting the integration of medical care and older people care, and developing community rehabilitation services. The comparative study reveals that Taiwan's long-term care system is more mature and comprehensive, with higher service quality and coverage, while Ningbo has certain advantages in the integration of medical care and older people care, as well as in community rehabilitation services. Based on this, the study proposes recommendations for the complementary development of long-term care service models in Taipei and Ningbo, including strengthening policy coordination, promoting inter-departmental cooperation, and enhancing service quality and efficiency, aiming to provide reference for the improvement and development of long-term care services in both regions.

Abstract citation ID: igae098.3161

### COMPARING HEALTH CARE WORKFORCE IN NURSING HOMES BETWEEN TAIWAN AND THE UNITED STATES: A PILOT STUDY

Bianca Shieu[1], Ya-Wen Lee[2], and John Harris[3], *1. UT Health San Antonio, San Antonio, Texas, United States*, *2. Changhua Christian Hospital, Changhua, Changhua, Taiwan (Republic of China)*, *3. Magee-Womens Research Institute, Pittsburgh, Pennsylvania, United States*

Nursing homes (NHs) play a pivotal role in providing long-term care for elderly and chronically ill individuals. However, limited research has focused on understanding resident composition, staffing levels, and quality of care across different cultures. Therefore, this study aims to comprehensively compare the NH workforce between Taiwan and the United States, identifying improvement opportunities, enhancing staffing practices, and ultimately improving the lives of NH residents. This comparative analysis specifically examines two hospital-affiliated NHs in the US and three Christian-affiliated hospital-affiliated General-NHs in Taiwan. The first author conducted observations and interviews at two Hospital-affiliated NHs located in the Northeastern region of the United States, while the second author conducted observations and interviews at three Christian-based hospital-affiliated General NHs situated in Central Taiwan. Both authors spent several hours each day walking through the NHs, documenting their observations through field notes after each observation setting, conducting interviews with NH staff, and engaging with administrators to gather additional demographic information. We used Atlas.ti software for data analysis of our interviews. This study identified five distinct aspects warranting discussion due to their differences: ownership structure, NH resident demographics, medication administration protocols, staff training practices, and staffing shortages. In conclusion, the analysis of the healthcare workforce in NHs between Taiwan and the United States reveals critical insights into the challenges and opportunities faced by both nations. Additionally, this study contributes to a deeper understanding of NHs' workforce dynamics, with implications for policy, practice, and resident well-being.

Abstract citation ID: igae098.3162

### CONTINUOUS USERS OF LONG-TERM CARE 2.0 IN TAIWAN: PATTERNS AND HEALTH COSTS OVER TIME

Ya-Mei Chen[1], Shih-Cyuan Wu[2], Shiau-Fang Chao[2], Ming-Jen Lin[2], and Chien-Fang Chu[3], *1. National Taiwan University, Taipei City, Taipei, Taiwan (Republic of China)*, *2. National Taiwan University, Taipei, Taipei, Taiwan (Republic of China)*, *3. Ministry of Health and Welfare, Taipei City, Taipei, Taiwan (Republic of China)*

Taiwan has implemented the Long-Term Care 2.0 (LTC 2.0) Plan since 2017, but only about one third of users continuous to remain in the system after 6 months. The current study examined the patterns of the continuous user and their associations to older adults' health costs overtime. This study utilized Taiwan's LTC Plan 2.0 database and the National Health Insurance Plan claim dataset, and included 155,220 clients who continuously utilized any LTC Plan 2.0 services for six months between 2017 and 2019. These users' health costs were examined in the 12 months after 6 months of HCBS continuous use. Latent class analysis and generalized estimating equations were used to investigate the associations between different service use patterns and the changes in health costs. Five subgroups of LTC use patterns were identified, including home-based personal care (PC) group (n= 99,163, 63.9%), home-based medical care (MC) group (n= 13,304, 8.5%), home-based personal care and medical care (PC/MC) group (n = 9,269, 6.0%), Reablement (RA) group (n= 20,132, 13.0%), and community care (CC) group (n= 13,352, 8.6%). When compared to care recipients in PC/MC group, those in the PC group ($5.84/month, p< 0.001), MC group ($8.21/month, p< 0.001), and CC group ($6.79/month, p< 0.001) had increased more health costs overtime. Such trends are more prominent among those with moderate and severe disability, except those in RA group. The integrated services (PC/MC group) and reablement (RA group) demonstrate slower growth in health costs, particularly among those with moderate and severe disabilities.

Abstract citation ID: igae098.3163

### CURRENT TRENDS IN THE DIRECT CARE WORKFORCE

Christopher Kelly, and Jerome Deichert, *University of Nebraska Omaha, Omaha, Nebraska, United States*

We used data from the 2020-2022 American Community Survey to examine trends among direct care workers (DCWs) in both occupation and industry. We found the number of nursing assistants, psychiatric aides, and home health aides decreased 5.8%, to nearly 1.9 million, while the number of personal and home care aides decreased 0.8%, to more than 1.3 million. Nursing assistants, psychiatric aides, and home health aides were more likely than personal and home care aides to be female, under age 25, African American, recently divorced, and to receive health insurance from their employer. They were less likely to be age 65 and over, to receive health insurance from Medicare or Medicaid, and to have a disability. Nearly 1.9 million DCWs were employed in home and community-based services, as compared to under 900,000 in residential care facilities and under 500,000 in hospitals. There were increases in the number of DCWs employed by outpatient care centers (10.7%), hospitals (7.2%) and individual and family services (3.7%). The largest decreases were in the number of DCWs employed by skilled nursing facilities (17.4%), assisted living facilities (12.3%), and private households (6.1%). DCWs employed by residential care facilities were more likely to be nursing assistants, psychiatric aides, or home health aides and they were more likely to work year-round and full-time. These findings suggest that changes already underway in the direct care workforce accelerated during/after the COVID-19 pandemic. DCWs are shifting towards jobs requiring less training and/or licensure and away from jobs in residential care facilities.

Abstract citation ID: igae098.3164

### DEVELOPMENT OF A MEASURE OF ALF RESIDENTS' ACTIVITY PREFERENCES AND REINFORCERS

Nicole Praska[1], Daniel Houlihan[2], Jeff Buchanan[2], and Rachel Weiskittle[1], *1. University of Colorado, Colorado*

*Springs, Colorado Springs, Colorado, United States, 2. Minnesota State University Mankato, Mankato, Minnesota, United States*

The population of individuals aged 65 and older has grown steadily since the 1990s, consequating a corresponding rise in the resident population of Assisted Living Facilities (ALFs). This significant increase has highlighted the importance of monitoring residents' quality of life and reinforcers of enjoyable activity engagement. However, the resident preference assessments currently used for this purpose are outdated and lack specificity. To address this limitation, this study aimed to create an updated measure of ALF residents' activity engagement reinforcers. The study involved a two-step process. First, 10 subject matter experts in the geriatric field compiled a list of 107 potential reinforcers. This list was then refined into a likert-scale survey of 60 selected items and administered to 612 older adults. Exploratory factor analysis identified five factors (social, traditions, food and activities, family and friends, and outings), and resulted in a 33-item survey. Results of this study provide an effective tool for ALF activity directors and staff to accurately assess ALF residents' interests and motivators for activity engagement. Clinical implications include connecting at risk ALF residents with individualized program scheduling that may improve their quality of life. This poster reviews the methodology and further results of this survey development.

Abstract citation ID: igae098.3165

### EXPLORING THE ASSOCIATION BETWEEN GOOGLE RATINGS AND NURSING HOME QUALITY

Neeraj Dayama[1], Shivani Gupta[2], and Rohit Pradhan[3], *1. Texas Tech University Health Sciences Center, Lubbock, Texas, United States, 2. University of Houston - Clear Lake, Houston, Texas, United States, 3. Texas State University, San Marcos, Texas, United States*

Online review platforms such as Google ratings have become a resource for consumers seeking nursing home (NH) care for their loved ones. As these ratings reflect subjective experiences, it is important to explore how they may correlate with the more rigorous and systematic measures of NH quality. The primary purpose of this study was to explore the association of Google ratings with NH deficiencies. This study is structured as a cross-sectional analysis (2018) utilizing the following datasets: Brown University's LTC Focus, Ohio nursing home Google rating data, and Area Health Resource File. The dependent variable was deficiencies which reflect a facility's failure to meet specified resident care standards, known as federal participation requirements (FPRs). Examples of deficiencies include a facility's failure to adhere to infection control protocols or improper medication management. The independent variable was consumer reported Google ratings for Ohio NHs. We used negative binomial linear regression, adjusting for NH organizational (size, occupancy, ownership, chain affiliation, payer mix) and market factors at the county level (per capita income, urban/rural location) to assess the relationship. Mean Google rating was 3.55 out of 5 and mean total deficiency count was 11.98. We found that Google ratings were negatively associated with deficiencies (Incidence Rate Ratio=0.93, $p< 0.05$) (as the number of deficiencies increased, Google ratings declined). From a policy perspective, our findings highlight the need for regulatory frameworks to incorporate consumer feedback mechanisms in evaluating and improving nursing home care quality.

Abstract citation ID: igae098.3166

### GRIEF AND BEREAVEMENT SUPPORT FOR STAFF IN LONG-TERM CARE HOMES: IDENTIFYING BARRIERS AND FACILITATORS

Cari Randa-Beaulieu[1], Habib Chaudhury[2], Gloria Puurveen[3], and Theresa Pauly[2], *1. Alzheimer Society of British Columbia, Vancouver, British Columbia, Canada, 2. Simon Fraser University, Vancouver, British Columbia, Canada, 3. University of British Columbia Okanagan, Kelowna, British Columbia, Canada*

Admissions of older adults to long-term care are occurring at more advanced ages with more complex care needs, resulting in shorter lengths of stay before death and an increased demand for a palliative approach to care. However, long-term care staff often lack access to sustainable educational, organizational, and interpersonal support in end-of-life care, and must learn on the job while facing death in the workplace. To address this gap, we conducted a scoping review of literature on grief and bereavement support for long-term care staff. Following PRISMA-Scoping Review guidelines and the six-step framework by Levac and colleagues, social sciences databases were searched and data were analyzed through narrative synthesis and the creation of infographics. Fifty-eight studies met inclusion criteria. Strategies, interventions, and rituals were organized into five domains:1) Cultural Attitudes Toward Death and Unmet Need for Support; 2) Organizational Policy and Practice; 3) Formal Peer-support; 4) Informal Peer-support; and 5) Individual Beliefs and Self-Care Practices influenced by temporal aspects. Identifying barriers and facilitators of grief support for long-term care staff, and hospice and palliative care specialists' practices, can offer more holistic and effective approaches to long-term care staff wellness to combat burnout and staff turnover. Grief rituals present scalable, holistic approaches fostering long-term care staff wellness that could be explored in future research to collaboratively build long-term care staff capacity for person-centered end-of-life care.

Abstract citation ID: igae098.3167

### IDENTIFYING OPPORTUNITIES TO OPTIMIZE PERSON-CENTERED CARE IN LONG-TERM CARE

Tonya Roberts[1], Jingxi Li[2], Justin Boutilier[2], and Yonatan Mintz[2], *1. University of Wisconsin - Madison, Madison, Wisconsin, United States, 2. University of Wisconsin Madison, Madison, Wisconsin, United States*

Person-centered care (PCC), or care consistent with preferences, improves long-term care (LTC) resident quality of life, family satisfaction, and staff job satisfaction. Despite benefits, PCC has not been fully adopted across homes. One barrier is a lack of knowledge regarding patterns in resident preferences that can meaningfully inform operational decision-making to improve PCC. This study aimed to take the first step toward filling that gap by: 1) describing LTC residents' preferences; 2) describing the impact of aligning care with preferences, and 3) identifying gaps in care that could be improved with operational interventions. Preference surveys were administered, and audio recorded with 27 LTC residents, or family proxies, over the course of >70 sessions. Descriptive analysis of survey and audio data was conducted. Residents/families rated the majority (84%) of preferences on daily routines, social relationships, caregiving, and leisure as important; however, specific preferences endorsed were highly unique. Residents/families reported nearly half (42%) of preferences were rarely or never met and nearly all (94%) of their current preferences were not the same as before admission. Residents/families described negative impacts on mental well-being, quality of life, or physical health when preferences were not met and engaging in a process of adapting preferences to what was available at the facility. Findings highlight that both idiosyncratic and common preferences and care gaps exist across residents, and different interventions may be needed to address PCC at individual and organizational levels. Possible targets for operational interventions to address these different needs and improve PCC will be discussed.

Abstract citation ID: igae098.3168

### INDIVIDUAL AND ORGANIZATIONAL BENEFITS OF CO-LOCATING NURSING HOME AND CHILD CARE SERVICES

Helen Wang[1], Shannon Jarrott[2], and Mengyao Ma[3], *1. University of Michigan, Ann Arbor, Michigan, United States*, *2. The Ohio State University, Columbus, Ohio, United States*, *3. Hong Kong University of Science and Technology, Hong Kong, Hong Kong*

Co-location nursing homes with childcare services has gained recognition for fostering positive intergenerational relationships. Researchers have identified benefits to individual participants, but little attention has been paid to organizational outcomes. Using econometrics methods, we analyzed historical data of all 19,641 unique US nursing homes from 2015Q1 to 2020Q1 to assess the association between co-location and nursing home quality measures, including deficiency reports and resident performance measures. Leveraging Centers for Medicare & Medicaid Services (CMS) and Data Axle data, our empirical model tested the association between on-site childcare and nursing home quality. We controlled for potential confounds, including nursing home characteristics (e.g., capacity and ownership), regional socioeconomic status, and year and zip code fixed effects. Combining business location data and public information sources, we identified 139 nursing homes with on-site childcare services operating in our sample period. Regression analyses indicated that nursing homes with on-site childcare had 9.94% lower yearly deficiency case reports than homes without childcare ($p < 0.01$) due to lower health deficiency rates but not fire safety deficiencies. Considering quarterly resident and institution measures, nursing homes with on-site childcare centers demonstrated 1.1% to 5.8% higher resident vaccination rates ($p < 0.01$) and significantly lower psychotropic medication use ($p < 0.001$) than those without childcare. Our study highlights that co-locating childcare and nursing homes is associated with better nursing home performance, benefiting residents and organizations. Further research should address mechanisms by which outcomes are achieved. Intergenerational program co-location may support benefits beyond those experienced by younger and older participants.

Abstract citation ID: igae098.3169

### LONG-TERM CARE TRAJECTORIES AFTER STROKE IN SWEDEN AND THE UNITED STATES

Karin Modig[1], and Greg Arling[2], *1. Karolinska Institutet, Stockholm, Stockholms Lan, Sweden*, *2. Purdue University, West Lafayette, Indiana, United States*

Stroke is a major cause of mortality and disability in old age worldwide, often requiring ongoing formal long-term care (LTC). Only a handful of studies examining longitudinal patterns of institutional and community based LTC beyond the immediate post-stroke period. In order to better understand the impact of LTC use by stroke survivors, we compared findings from studies of LTC trajectories among people discharged from the hospital after a stroke in Sweden and the United States. The Swedish study modeled LTC trajectories over a three-year period for a nationally representative cohort of 31,560 stroke survivors age 70 and older during 2015 to 2017, while the US study modeled LTC trajectories over 12 months among a sample of 3,811 Veterans discharged from Veterans Administration (VA) hospitals after stroke during 2007. Approximately half of the sample in each study was alive and without a history of LTC use at one year. Use of LTC in both studies was associated with LTC use prior to stroke, advanced age, number of comorbid conditions including dementia, and frailty/dependency. In the Swedish study, transitions into formal LTC tended to be unidirectional - few people entering formal LTC ever returned to no care, and few people entering care homes ever transitioned back to home care. In the VA study, frequent transitions occurred between nursing homes and home-based LTC, as well as out of the LTC system entirely. We discuss how differences in LTC policies, particularly the role of the nursing home, affect LTC trajectories in the two countries.

Abstract citation ID: igae098.3170

### STAFF PERSPECTIVE ON VIRTUAL TEAM-BASED CARE IN THE PROCESS OF TRANSITION TO LONG-TERM CARE

Lillian Hung[1], Chin Pang Ian Chan[1], Marie-Lee Yous[2], Denise Connelly[3], Karen Wong[1], Mariko Sakamoto[4], Paulina Santaella-Tafolla[1], and Huini Ge[1], *1. University of British Columbia, Vancouver, British Columbia, Canada*, *2. McMaster University, Hamilton, Ontario, Canada*, *3. Western University, London, Ontario, Canada*, *4. University of Victoria, Victoria, British Columbia, Canada*

Transitioning from hospital to long-term care (LTC) is a vulnerable time for older patients and families. Successful care transitions require interdisciplinary team and cross-sectoral coordination, as well as engagement of patients and families in care planning. This study aimed to identify enablers and barriers to delivering virtual team-based care to support older adult care transitions from hospital to LTC. Patton's utilization-focused approach informed the study design. Our research methods included semi-structured interviews, focus groups, and organizational policy reviews. The study involved 52 multidisciplinary team members, including nurses, physicians, rehabilitation practitioners, healthcare leaders, and older patients and family members. Thematic analysis identified key enablers (team engagement, training and support, and access to digital equipment) and barriers (technology infrastructure, resources, privacy and security). Our findings suggest that virtual team-based care is perceived by healthcare staff as acceptable, efficient, and cost-saving. Older persons and their families have complex care needs, and prioritizing their involvement in virtual team-based care planning is key to addressing barriers in the process of care transition success. Clear policies and guidelines are needed to support the integration of virtual team-based care into practice to optimize support to families and patient health outcomes. Future research should investigate effective strategies to include older persons and families through virtual team-based care planning technology.

Abstract citation ID: igae098.3171

**TAKING CHARGE AND FLEXING PROTOCOLS: SNF NURSING STAFF'S UTI IDENTIFICATION**

Kimberly Delgado[1], and Donna Roberson[2], *1. East Carolina University College of Nursing, Greenville, North Carolina, United States*, *2. East Carolina University, Greenville, North Carolina, United States*

Skilled Nursing Facility (SNF) residents are at increased risk of adverse reactions, including death, due to inappropriate antibiotic use, which accounts for up to 75% of all SNF antibiotic prescriptions; the most common indication being urinary tract infection (UTI). The structure of SNFs create barriers to appropriate diagnosis and treatment of UTIs, with limited registered nurse availability and health care providers (HCP) being off-site. The purpose of this study was to describe SNF nursing staff experiences with residents whom they suspected were presenting with a UTI. A better understanding of what takes place prior to HCP contact can assist SNF leadership with improving nursing-led interventions. This qualitative descriptive study used a semi-stratified purposive sample, snowball sampling, and social media recruitment. Single, semi-structured interviews were completed using open-ended questions. Data analysis resulted in three themes: way out of the norm, taking ownership, and flexing protocols. Participants identified SNF residents' behaviors that were 'way out of the norm,' as a key indicator of a UTI. Approaches to care included nursing staff taking ownership, or responsibility, and flexing protocols to prioritize the care and well-being of their residents. SNF nursing staff spend more time with their residents than the HCP, which results in them being heavily involved in the process of UTI identification. Inclusion of SNF nursing staff in future research is critical to the exploration of practices and improving outcomes. Research study recruitment barriers illustrate the tremendous need for education among SNF facility leadership to garner support and permission to access facilities.

Abstract citation ID: igae098.3172

**THE EFFECT OF MEDICAID MANAGED LONG-TERM SERVICES AND SUPPORT ON PARTICIPANT QUALITY OF LIFE AND UNMET NEED**

Howard Degenholtz[1], Anny Treat[2], Keri Kastner[1], and John Yauch[1], *1. University of Pittsburgh, Pittsburgh, Pennsylvania, United States*, *2. Sebastian Mann Associates, Pittsburgh, Pennsylvania, United States*

In 2018 the Commonwealth of Pennsylvania began a three year transition of Medicaid Aging and Disabled Waiver programs to Managed Long-Term Services and Supports (MLTSS). The new program, Community HealthChoices, serves adults with disabilities who receive home and community based services (HCBS), live in a nursing facility, and people dually eligible for both Medicaid and Medicare who do not use long-term services and supports ("duals"). Three managed care organizations operate the program through state-wide contracts. The program was implemented statewide over a three year period in three regional phases, creating a unique natural experiment. In other analyses, we have demonstrated significant changes to care management and slowing in the rate of growth of HCBS spending. This is the first study of HCBS participants' subjective experience. A total of 14,706 prospective telephone interviews were conducted with random samples of participants before the transition to CHC and 18 and 30 months post-transition. We found that psychological well-being was unchanged, but depressive symptoms decreased over time. There was no change in the consequences of unmet need (e.g., having a hot meal or a bath). In general, when compared duals, HCBS users report lower access to primary care and lower self-reported health status. After the implementation, duals users reported higher health status and HCBS users were more likely to report having a medical care coordinator. The transition to MLTSS brought substantial changes to the way Medicaid LTSS is financed and delivered in Pennsylvania. However, the experience of the average participant has been relatively stable.

Abstract citation ID: igae098.3173

**THE HEALTH OF DIVERSE CALIFORNIANS WITH NEEDS FOR LONG-TERM SERVICES AND SUPPORTS**

Kathryn Kietzman[1], Lei Chen[2], and Preeti Juturu[1], *1. University of California Los Angeles, Los Angeles, California, United States*, *2. University of California San Francisco, San Francisco, California, United States*

This study used data from the 2019-2020 California Long-Term Services and Supports (CA-LTSS) survey to identify differences in health outcomes within a population of older adults and adults with disabilities. Our study sample included 2030 adults who lived in home and community settings in California, and experienced at least one type of LTSS difficulty, including cognitive impairment and difficulties performing activities of daily living and/or instrumental activities of daily living. We conducted chi-square tests using weighted representative California population-level data. Among people with LTSS difficulties, we found significant racial/ethnic differences in health insurance coverage, usual source of care, and psychological distress. For example, a larger proportion of Asian adults with LTSS difficulties reported having no health insurance (9.0%), having no usual source of care (21.2%), and experiencing serious psychological distress (37.3%) compared to other racial/ethnic groups. We also found significant differences by employment status. Specifically, adults with LTSS difficulties who were in the labor market were more likely to experience serious psychological distress (28.8%), have no health insurance (11.3%), experience delays in receiving care or prescriptions (26.1%), have unmet needs (41.1%), and report financial worries (68.7%), compared to adults with LTSS difficulties who were not in the labor market. Income level was also significantly associated with health and well-being, health insurance coverage, usual source of care, and unmet needs. These results can be used to inform the development of programs and policies for diverse people with LTSS needs, and further suggest the need for innovative strategies to advance health equity.

Abstract citation ID: igae098.3174

**THE USE OF MINDFULNESS-BASED INTERVENTION FOR LONG-TERM CARE RESIDENTS: A REVIEW OF THE LITERATURE**

Audrieanna Raciti[1], and Yu-Ping Chang[2], *1. Buffalo, New York Mills, New York, United States*, *2. University at Buffalo, SUNY, Buffalo, New York, United States*

Physiological and psychological health is a significant concern among people residing in long term care. Researchers have begun to design and test mindfulness-based interventions (MBIs) as a strategy to lessen the burden of these impacts on long-term care residents as well as our already strained healthcare system. The purpose of this review is to synthesize the use of MBIs and their impact on health-related outcomes and quality of life among long term care residents. Six databases: CINAHL, PUBMED, Cochrane, Web of Science, Psychinfo, and Embase were searched to identify original studies leveraging the use of mindfulness to improve health-related outcomes for long term care residents. Multiple keywords were used including mindfulness-based stress reduction, meditation, mindfulness intervention, long-term care, nursing home, residential care, older adult, elderly, and aged. Nine studies meeting the selection criteria were included in this systematic review. Most of the studies have a small to medium sample size. The types of MBIs include mindfulness-based cognitive therapy, silent meditations, mountain meditations, loving-kindness meditations, awareness exercises, breathing exercises, visualization, and guided imagery. The majority of those MBIs demonstrate a significant improvement in alleviating symptoms of stress, depression, anxiety, relocation anxiety, worsening dementia, and other mental health concerns, while one study also showed significant improvement in A1C levels among residents with type 2 diabetes. However, some studies have a high attrition rate. Future research is needed to utilize a more rigorous design with a large sample size and longitudinal studies. Participant engagement should be considered as part of the intervention activities.

Abstract citation ID: igae098.3175

### TRAJECTORY OF DECLINING PHYSICAL FUNCTION AMONG NURSING HOME RESIDENTS IN JAPAN

Kasumi Ikuta, Maiko Noguchi-Watanabe, Miya Aishima, and Sakiko Fukui, *Tokyo Medical and Dental University, Bunkyo-ku, Tokyo, Japan*

The timing and speed of physical function decline among nursing home residents vary, and the decline trajectory remains unclear. Therefore, this multicenter retrospective study was aimed at identifying the trajectory of declining physical function among Japanese nursing home residents. We included new nursing home residents who were admitted between July 2021 and March 2023, who had a high physical function score (Barthel Index [BI]: >60), and whose physical function declined during the period from admission to 6 months after. We evaluated the BI monthly as an indicator of the residents' physical function status. We conducted a group-based trajectory model analysis to find the physical functional decline. Four types of models were identified. Furthermore, we compared the trajectory model to other trajectory models using the Akaike Information Criterion. Based on the results, we included 163 new residents, including 106 women (65.0%), with a mean age of 86.16 years (SD = 5.84). We selected the five groups trajectory model: slight decline, slightly larger decline, maintenance after decline, decline after maintenance, and large decline. The characteristics of the large-decline group were a diagnosis of dementia and low motivation and those of the maintenance-after-decline group were female sex and low body mass index and oral function. We identified the trajectory group based on the timing and speed of physical function decline. We believe that nursing home staff assess the trajectories of physical function decline and individualize residents' interventions accordingly.

Abstract citation ID: igae098.3176

### TRENDS IN US NURSING HOMES RESIDENTS WITH LIMITED ENGLISH PROFICIENCY (LEP)

Maureen Mickus[1], and Jade Nguyen[2], *1. Western Michigan University, Kalamazoo, Michigan, United States, 2. Western MIchigan University, Kalamazoo, Michigan, United States*

Ethnic elders residing in U.S. nursing homes experience significantly lower quality of life compared to their white counterparts. Among the most vulnerable are those with Limited English Proficiency (LEP) who may be unable to communicate even basic needs to staff. Based on the Minimum Data Set (MDS), this study examined the prevalence of LEP residents in the U.S. between 2020 and 2023 as defined by those needing language interpreter services. From 2020 to 2021, the number of LEP residents decreased by 25.3% from 46,877 to 35,021, in part due to the disproportionate rate of death from COVID-10 among minorities. After 2021 an increase was reported by an estimated 2,000 residents per year with an estimated 39,584 LEP residents recorded in 2023. The need for interpreter services in nursing homes varied significantly by state with 14.4% of California and 7.71% of New York residents as compared with 14 states reporting less than 1%. Not surprisingly, U.S. Census statewide data of individuals age 65+ in the general population reporting speaking English less than "very well" was positively correlated with the percentage of LEP residents in nursing facilities, r (47) =.91, p <.001. Based on the unmet needs that are likely associated with LEP residents and the unavailability of interpreters 24/7 in facilities, increased cultural education and sensitivity is warranted for staff in long-term care facilities. Future research determining which specific languages are spoken in facilities in addition to ways to improve communication is needed.

### SESSION 7515 (POSTER)

### POLICY

Abstract citation ID: igae098.3177

### FROM PAST TO PRESENT: REFINING LONG-TERM CARE POLICIES IN RESPONSE TO CHINA'S AGING POPULATION

Qingwei Wang[1], Yu Ma[2], and Jiayu Chen[3], *1. Xi'an Jiaotong-Liverpool University, Suzhou, Jiangsu, China, 2. University of Bristol, Bristol, England, United Kingdom, 3. Cambridge University, Cambridge, England, United Kingdom*

The evolution of long-term care policy in China is a complex response to the nation's demographic shift and economic changes. Despite extensive policy development, discrepancies between policy intentions and real-world demands of older adults persist. This study employs a longitudinal analysis to dissect the dynamics that have shaped China's aging policies from the 1940s to 2023. By critically examining legislative documents and top-level directives, this research identifies gaps between policy formulations and market response and the challenges of navigating a fragmented regulatory landscape. A misalignment has emerged between the supply of Home and Community-Based Services and institutional care demand, resulting in resource underutilization and diverging from intended policy outcomes. The research further explores the regulatory fragmentation within China's long-term care sector, indicating that decentralized supervision is a significant barrier to achieving cohesive governance and effective policy implementation. The findings illustrate the critical need for adaptive policy mechanisms that better align with demographic realities and consumer expectations. This paper contributes to the discourses on long-term care policy by offering insights into the effectiveness of China's strategic initiatives in recent years and suggesting directions for future policy refinement. It aims to ensure sustainable care by addressing both the discrepancies in policy and market response and the challenges of fragmented authority.

Abstract citation ID: igae098.3178

### INTERGENERATIONAL EQUITY: AN URGENT CALL FOR AGING POLICIES IN SUB-SAHARAN AFRICA

Abraham Teshome[1], Dolapo Adeniji[2], Gifty Ashirifi[1], and Margaret Adamek[1], *1. Indiana University Indianapolis, Indianapolis, Indiana, United States*, 2. *Indiana State University, Avon, Indiana, United States*

Contrary to popular belief, family-based care and support for older people in Sub-Saharan Africa is gradually eroding due to various factors, including urbanization, migration, and modernization. As the issues of older people and aging are not mainstreamed into national economic and development agenda and policies, most older people in the region live in abject poverty, which has undermined their healthy aging and quality of life. Although many Sub-Saharan African countries are signatories to the 2002 Madrid International Plan of Action on Ageing (MIPAA), only a few have developed national aging policies to meet the needs of their older population. Against this backdrop, we conducted cross-sectional qualitative study to learn why public policy in Sub-Saharan Africa is sluggish in responding to the needs of the region's growing older population. We conducted an online survey (n=78) and three FGDs (n=13) with African scholars and practitioners from 12 nations. Based on descriptive and thematic analyses, our study revealed five themes: a) lack of political will, b) disconnect between researchers and policymakers, c) lack of research and comprehensive data related to older people, d) inappropriate aging policy, and e) budget constraints. Our study's findings call for more research and comprehensive data regarding the needs and challenges of the region's older population, policy advocacy, and awareness-raising about older adults' issues among policymakers, involvement of all stakeholders in aging policy development, and regular dialogues between policymakers, researchers, older adults, and other stakeholders.

Abstract citation ID: igae098.3179

### POLICIES FOR SUPPORTING CAREGIVERS OF OLDER ADULTS WITH LONG-TERM CARE NEEDS: A MIXED-METHODS SYSTEMATIC REVIEW

Marco Albertini, Eva Bei, and Federico Toth, *University of Bologna, Bologna, Emilia-Romagna, Italy*

**Background:** In an era marked by the imperative to restrain costs within health and social care services throughout Europe, informal caregivers of older adults assume a pivotal role as primary providers of long-term care. Nevertheless, there remains a shortage in mapping the various social policies pertaining to both direct and indirect support for informal carers across the various EU member states. Objective: This mixed-method systematic review synthesizes data on care policies related to diverse support forms implemented across EU countries to assist caregivers.

**Methods:** A comprehensive search strategy was conducted in three electronic databases and grey literature to minimise publication bias.

**Results:** Preliminary findings of studies published between 2010 and 2023 indicate that various forms and levels of support have been introduced or reformed over the past 13 years across EU countries to support caregivers. Despite the challenges posed by population ageing, there is significant policy development disparity among EU members, with some countries to have established mechanisms aside financial assistance to support carers, while others to only now starting to express interest in developing such services. Financial support, primarily through cash-for-care schemes, emerges as the predominant form of assistance, followed by respite care, while other policies in terms of training and mental health services exhibit a notably lower level of development within the EU social policy landscape.

**Conclusion:** The review is expected to provide significant implications for policymaking, thoroughly mapping the implementation of diverse social care policies across Europe to ensure the sustainability of informal care.

Abstract citation ID: igae098.3180

### RURAL DISPARITIES IN ELDERLY AMBULATORY CARE READMISSIONS: EVIDENCE FROM NATIONAL AMBULATORY MEDICAL CARE SURVEY

Sunny Kang[1], Nancy Miller[2], and Ting Zhang[1], *1. University of Baltimore, Baltimore, Maryland, United States*, *2. University of Maryland Baltimore County, Baltimore, Maryland, United States*

**Objective:** We examined factors associated with older patients' readmission to ambulatory care within one week to two months' discharge, and how this readmission pattern primarily varied by locality.

**Methodology:** We analyzed 7,619 older patients (age 50 and older) from the 2016 National Ambulatory Medical Care Survey. Logistic regression analyses were utilized to examine the risk factors for ambulatory readmission. Findings: Compared to the residence in Metropolitan Statistical Areas, the residence in rural areas (or micropolitan areas) will increase the odds of readmissions (OR=1.61, 95%CI[1.30, 1.99]), after controlling for other variables. Being Black was not statistically significant in predicting the likelihood of readmission, while black patients using Medicaid as the primary payment experienced higher risks of readmission (OR=2.09, 95%CI[1.02, 4.32]). The higher facility percentage (>50%) of Medicare beneficiaries was associated with smaller odds of readmission (OR=0.65, 95%CI[0.58, 0.73]). Nursing home visiting within the prior week or seeing a physicians' assistant each increased the odds of readmission, whereas seeing a mental healthcare provider decreased the risks for readmissions (OR=0.40, 95%CI[0.19, 0.83]). Patients diagnosed with cardiovascular disease and Type II diabetes were associated with smaller odds of readmission. Policy Implications: Residence in rural areas (or micropolitan statistical areas) or being black Medicaid beneficiaries predicted greater odds of readmission to ambulatory care among older patients. Rural patients experienced lower likelihood of staff responsiveness after adjusting for other factors. Further research should explore the basis of these disparities. Mental health diagnoses in depressive and psychosis disorders also called upon additional studies regarding special care needs.

### SESSION 7520 (POSTER)

### RESEARCH METHODS AND ISSUES: QUALITATIVE

Abstract citation ID: igae098.3181

### CO-DESIGNING RESEARCH IN COLLABORATION WITH PEOPLE WHO ARE RESIDENTS AND STAFF IN LONG-TERM CARE FOR OLDER ADULTS

Kay Shannon[1], David Jones[2], and Katie Featherstone[3], *1. Auckland University Of Technology, Auckland, Auckland, New Zealand*, 2. *Manukau Institute of Technology, Ōtara, Auckland, New Zealand*, 3. *University of West London, London, England, United Kingdom*

One New Zealand provider of long-term care for older adults has shown fortitude in overcoming obstacles to developing a homelike facility where 81 residents live in small houses and continue with activities that connect them with lifelong identities. The CARE Village, Te Manaaki a Tura is the host organization for the sequential mixed methods study, which aims to compare resident experiences and outcomes in homelike and traditional facilities. Phase one determined the impact of the homelike model of care on International Resident Assessment Instrument (interRAI) quality indicators. The phase two qualitative study to understand the operationalization of nursing care at both types of facilities was co-designed with residents and staff of The CARE Village, Te Manaaki a Tura, including the staff-led Māori Cultural Awareness Team. The presentation reports on the co-design process. This presentation will discuss the co-design process and the meaningful contributions and insights to designing the qualitative research including developing questions for research participants and developing the strategy for analysing the data. Co-designers who are residents demonstrated their fortitude in continuing to provide service to the community. Co-design is becoming increasingly important as researchers and practitioners must include the perspectives of clients and other stakeholders in their work. Therefore, this presentation offers advice for those wanting to learn about the practical elements of the process.

Abstract citation ID: igae098.3182

### DECODING DEMENTIA STIGMA THROUGH NETNOGRAPHY: LESSONS FROM THE DEMENTIA DIARIES

Kelly Marnfeldt, *University of Southern California, Los Angeles, California, United States*

Worldwide, 10 million people are diagnosed with dementia each year. The experience of receiving a dementia diagnosis is commonly described in terms of lost identity and self-worth, with feelings of grief, fear of decline, and worries of becoming a burden. Such feelings are exacerbated by pervasive stigmatization and narratives that perpetuate the misconception that a diagnosis equals the end of a meaningful life. Moreover, people with dementia (PWD) are not only absent from stigma-reduction campaigns but they are underrepresented and overlooked across the research-practice-public health continuum. The present study aims to (1) explore perspectives and experiences of dementia-related stigma among PWD and (2) to examine how active participation in awareness and education campaigns by PWD influences their feelings/experiences of stigma. Netnographic methods were employed to analyze archived reflections (~3k) gathered from the UK-based Dementia Diaries—an online platform dedicated to fostering dialogue and combating stigma surrounding dementia. Between 2015 and 2023, 109 individuals contributed 2,946 diary entries to the platform, with 1 to 332 (M=64.04; SD=96.3) entries per person. This analysis identified themes of avoidance, abandonment, and invisibility in PWD's encounters with stigma, and references to the transformative impact of self-advocacy efforts. PWD attest that involvement in awareness activities fosters humanization and shifts attitudes, promoting inclusion, empathy, and solidarity. Future research should examine the mechanisms underpinning these effects, with an emphasis on personal narratives, advocacy, and stigma-countering strategies. Policy interventions could have broader societal impact by prioritizing meaningful social interactions with PWD and harnessing positive representations in stigma-reduction campaigns.

Abstract citation ID: igae098.3183

### DESIGN ELEMENTS OF EDUCATIONAL TOYS FOR PATIENTS WITH MILD ALZHEIMER'S DISEASE: A QUALITATIVE STUDY

HongFei Jia, YaPing Ding, and Ling Zhu, *Nanjing Medical University, Nanjing City, Jiangsu, China (People's Republic)*

**Background:** Alzheimer's disease (AD) is a fatal chronic neurodegenerative disease, and its incidence and mortality rate are closely related to age. Once diagnosed, patients need to take long-term medication, which seriously affects the quality of life of those who are ill. Research has demonstrated that the educational toy is an effective intervention to improve cognitive function among patients with mild Alzheimer's disease. Despite the rapid growth in developing educational toys, incorporation of stakeholders' views has been limited. Objective: This project aimed to identify the design elements that should be considered during development of educational toys for patients, from the perspectives of patients with mild Alzheimer's disease and their caregivers. Materials and

**methods:** A total of 10 patients with mild Alzheimer's disease and 12 caregivers were selected. Participants were recruited using purposive sampling. Semi-structured interviews were conducted between August and November 2023. The content analysis method was used to summarize the interview data and refine the themes.

**Results:** Main themes that emerged from the data analysis included the following: (1) materials; (2) structure; (3) function; (4) color; (5) shape.
**Conclusion:** Collecting patients' and caregivers' perspectives through qualitative interviews, the developed puzzle toy can serve as a user-friendly and engaging tool to potentially complement traditional interventions in slowing cognitive deterioration in patients with mild Alzheimer's disease. These design elements can also be considered by developers and researchers when developing and evaluating educational toys for people with mild Alzheimer's disease.

Abstract citation ID: igae098.3184

### PRELIMINARY RESEARCH ON TOUCHPOINT METHOD TO ENGAGE CHINESE OLDER ADULTS IN PARTICIPATORY DESIGN ACTIVITIES

Jiabei Jiang[1], Tongxin Sun[2], Jianing Yin[2], and Yuanyuan Liu[3], *1. Nanjing university of the arts, Nanjing, Jiangsu, China (People's Republic)*, *2. Tsinghua University, Beijing, Beijing, China (People's Republic)*, *3. School of Mechanical Engineering & Automation, Beihang University, Beijing, Beijing, China (People's Republic)*

Collaborative engagement with older adults in design research activities is an effective and beneficial method for researchers to understand the needs of this demographic. Participatory design, originally proposed within Western value systems, faces challenges in application across different regions and cultural contexts, particularly in participatory design activities targeting older adults within the Chinese societal context. However, limited literature focuses on the methodologies of participatory design practices with older adults in non-Western countries. This study views the organizational process of participatory design activities as a form of service design research involving older adults, where design researchers serve as providers and older adults as the recipients of the service. Drawing upon service design thinking, this paper introduces the concept of 'participation touchpoints'—moments within activities that encourage active involvement of older adults—to explore the interactions between participants and the organizers, procedures, and environments of activities. This approach aims to optimize the experience of older participants and enhance their engagement. Furthermore, based on the Theory of Planned Behavior, we proposed a framework of participation touchpoints including attitude guidance, incentive motivation, and capability compensation. We evaluated this framework through a participatory design workshop with older adults and multiple stakeholders, focusing on the dynamic interactions among organizers, participants, and the activities themselves. This research endeavors to foster more effective collaboration with older adults in design research, aiming to develop a participatory design framework that ensures mutual empowerment for older adults and the research team.

Abstract citation ID: igae098.3185

### SECONDARY EMOTIONAL DISTRESS: SUPPORTING YOUR RESEARCH TEAM THROUGH THE QUALITATIVE RESEARCH PROCESS

Kathryn Coccia[1], Verna Hendricks-Ferguson[1], Stephanie Wladkowski[2], and Cara Wallace[1], *1. Saint Louis University, St. Louis, Missouri, United States*, *2. Bowling Green State University, College of Health & Human Services, Bowling Green, Ohio, United States*

Conducting qualitative interviews with older adults experiencing serious illness as a patient or caregiver may include distressing subject matter for researchers. A growing body of evidence shows researchers are at risk of developing secondary emotional distress from eliciting or processing participants' distressing experiences. Novice researchers or graduate research assistants may be particularly vulnerable to developing secondary emotional distress. Many qualitative research plans include protocols for managing participant distress, but very few include formal considerations for mitigating psychological risk for members of the research team. Using a case study from an ongoing NIH-funded research study on live discharge from hospice, this presentation will focus a wider discussion of how difficult emotional experiences can occur both during the data collection process and again in analysis. This phenomenon will be discussed from the lens of a graduate research assistant, the primary investigator, and senior co-investigators. Presenters will share how these situations were processed, insights gleaned from these experiences, and considerations for developing protocols and cultivating resources to support team members in future studies.

Abstract citation ID: igae098.3186

### THE OPPORTUNITIES AND CHALLENGES OF CONDUCTING OBSERVATIONAL RESEARCH IN LONG-TERM RESIDENTIAL CARE ENVIRONMENTS

Kirsty Haunch, and Karen Spilsbury, *University of Leeds, Leeds, England, United Kingdom*

Observational research has potential to offer new insights when addressing questions that matter for older people living in long term residential care settings (or care homes); moving beyond what people "say they do" to offer data of what is seen in every-day activities and interactions. However, there are challenges associated with why, how, where and in what context observations should be used in this setting. Care homes are dynamic care environments, and approximately 70% of older people living in care homes will live with dementia or cognitive impairment. These contextual and 'people' factors require careful consideration when choosing this method. Drawing on the experiences of two research teams, we share real world examples of the ethical, methodological, and practical concerns when using the observation method in the UK context. We offer examples from our work to guide researchers on: i) 'making a case' for observation; ii) the observational 'gaze' (or focus); iii) including people who lack capacity; iv) managing concerns about care or practices; v) the role of researcher; and vi) addressing observational sensitivities. As researchers, what we bring, what we do, and what we leave behind when using this method can have a significant impact on the lives (and work) of people in care homes. Our paper addresses a significant gap in the existing literature. It will be of interest to researchers worldwide and has the potential to benefit those living (and working) in care homes by enhancing the quality of research endeavours.

Abstract citation ID: igae098.3187

### WEARABLES IN LONG-TERM DEMENTIA RESEARCH: A MIXED METHOD STUDY OF USER EXPERIENCES AND SUPPORT NEEDS

Colleen Peterson, *University of Michigan Transportation Research Institute, Ann Arbor, Michigan, United States*

Passive wearables data collection may be specifically beneficial to aging research featuring dementia populations, who have caregiving and cognitive burdens that can make study participation and reliable data collection more difficult, especially as dementia progresses. This three-phase project aims to inform best practice recommendations to enhance recruitment and adherence in long-term wearables research featuring this population. Based on our systematic review and preliminary in-house data testing, we selected three wearables offering different capabilities and form (from Garmin, Pulse HR, and AngelSense) to test real world usability, data quality, and support needs. This is the first study to recruit persons living with dementia and their caregivers to evaluate multiple devices outside of a laboratory or focus group setting (N=12 dyads). The person living with dementia assented to wearing each wearable for two weeks. Their caregiver rated many facets of each device following its use with the Quebec User Evaluation of Satisfaction with Assistive Technology measure. Open-ended questions and a cumulative semi-structured interview provided context and in-depth comparative perspectives of their experiences in the study. Wearable durability, simplicity, and data availability/monitoring capacity were important to participant favorability and adherence. Technical help and check-ins were also deemed highly valuable. Data indicate how the devices suited the dyads' needs or caused issues, as well as how study staff could better support ongoing use. Collectively, our findings suggest ideal criteria to guide wearable selection and key protocol factors that can enhance participant recruitment and adherence in long-term dementia research.

### SESSION 7525 (POSTER)

### RESEARCH METHODS AND ISSUES: QUANTITATIVE

Abstract citation ID: igae098.3188

### ACTIVITY RECORDING CAPILLARY FEEDER (ARC): A HIGH-THROUGHPUT BEHAVIORAL PHENOMIC ASSESSMENT IN DROSOPHILA

Serena Wu, Vivian Pham, Tiffany Nguyen, and Mahtab Jafari, *University of California Irvine, Irvine, California, United States*

Drosophila melanogaster is not only a well-established model organism in aging research but it is also considered an emerging animal model to screen and evaluate potential anti-aging compounds. Our lab developed an algorithm to screen and identify natural products and botanical extracts with lifespan extension and healthspan-improving properties. Once a compound or extract was shown to increase lifespan, we performed various phenotypic assays to determine its impact on biomarkers of health in Drosophila. This work established Drosophila as a model system in pre-clinical aging research. However, traditional behavioral phenotypic assays such as geotaxis are laborious and time-consuming, hampering screening efficiency. To address this challenge in the emerging field of phenomics, we have utilized an automated system, originally developed by Ja Lab, University of Florida, called the Activity Recording Capillary feeder assay (ARC). This recording system leverages high-resolution machine vision to monitor Drosophila behaviors such as movement, sleep, and feeding dynamics. This innovative approach enables rapid and simultaneous assessment of multiple behavioral parameters, offering greater reproducibility and scalability than conventional methods. In this work, we present preliminary data on the feasibility of using ARC as a high-throughput phenomics screening modality to evaluate natural product induced changes in aging-related phenotypes such as locomotion. This work was supported by UCI Calit2.

Abstract citation ID: igae098.3189

### DEVELOPING THE CHINESE VERSION OF THE ASSESSMENT OF INTERPROFESSIONAL TEAM COLLABORATION SCALE IN TAIWAN

Hsiao-Wei Yu[1], Carole Orchard[2], and Yu-Ching Hu[3], *1. National Taiwan University, Taiwan (Republic of China)*, *2. University of Western Ontario, London, Ontario, Canada*, *3. Chang Gung University of Science and Technology, Taoyuan City, Taoyuan, Taiwan (Republic of China)*

The delivery of long-term care reablement services emphasizes an interprofessional collaboration (IPC) approach. This study aims to use the existing Assessment of Interprofessional Team Collaboration Scale (AITCS) as an IPC measurement tool, assessing its alignment with Taiwan's unique caring culture compared to its original use in Canada. Brislin's Back-Translation Method was employed to create Chinese versions of the AITCS questionnaires, which were then administered to 201 professionals and paraprofessionals involved in reablement services in Taiwan to evaluate reliability and validity. Consistent with the original AITCS, the Chinese Version scale comprises three primary domains—partnership, cooperation, and coordination—with 23 question items. It demonstrates satisfactory reliability (Cronbach's alpha=0.97 for total scale, ICC=0.91 for test-retest reliability) and acceptable convergent validity (AVE=0.68-0.80, CR>0.90), along with discriminant validity (coefficients < 0.80 between domains). Analysis of IPC performance among participants shows that collaboration (3.82 ± 0.75) and partnership (3.78 ± 0.77) exhibit more favorable scales compared to cooperation (3.56 ± 0.63) (p<.001). This study has preliminarily developed a Chinese Version of the IPC measurement tool, suggesting the need for further investigation into strategies for enhancing cooperation in IPC performance within the context of reablement services in Taiwan.

Abstract citation ID: igae098.3190

### EXPLORING CIND STABILITY ACROSS TIME: INSIGHTS FOR IMPROVING COGNITIVE IMPAIRMENT CLASSIFICATIONS

Cynthia McDowell, Nicholas Tamburri, Jodie Gawryluk, and Stuart MacDonald, *University of Victoria, Victoria, British Columbia, Canada*

Objective: Cognitive impairment no dementia (CIND) classifications are susceptible to false positives and may not be indicative of progressive cognitive decline or dementia risk. The current project sought to investigate the dynamic nature of CIND across six years and to elucidate whether baseline impairment severity (single vs. multidomain impairment) predicts CIND stability patterns such as reversion and fluctuation between cognitive statuses. Method: Utilizing data from Project MIND, participants (N=259; 65-90 years)

were classified as either Normal Cognition (NC) or CIND across five annual assessments using a battery of five cognitive tasks. Participants were classified as either Stable NC, Stable CIND, Progressors (NC—CIND), Reverters (CIND—NC) or Fluctuaters (CIND—NC—CIND) according to their across-time stability patterns. Participants initially classified as CIND were further subdivided based on impairment severity: single-task (CIND-S) or multiple-task (CIND-M) impairment. Multinomial logistic regression was utilized to explore whether baseline CIND severity differentiated Reverters and Fluctuaters from those who remained Stable CIND.
**Results:** Most individuals were unstable in their CIND status for several years following baseline assessment. Compared to those classified as Stable CIND, Fluctuaters (-1.52, p=.01) and Reverters (-1.68, p=.01) were more likely to be classified as CIND-S (72% and 74%, respectively). Conclusions: Reverting back to NC or fluctuating between cognitive statuses is partly a function of instability in CIND-S classifications. Moving forward, CIND classifications could be improved through careful consideration of CIND severity at initial assessment. In particular, those classified as CIND-M may represent an at-risk group most likely to exhibit progressive cognitive impairment.

Abstract citation ID: igae098.3191

### FACTORS LEADING TO DISCONTINUOUS USE OF GOVERNMENT-SPONSORED LONG-TERM CARE 2.0 SERVICE PLAN IN TAIWAN

Ya-Mei Chen[1], and Shih-Cyuan Wu[2], *1. National Taiwan University, Taipei City, Taipei, Taiwan (Republic of China), 2. National Taiwan University, Taipei, Taipei, Taiwan (Republic of China)*

The Long-Term Care 2.0 (LTC 2.0) Plan, initiated in Taiwan in 2017, respectively, aimed to provide home- and community-based services (HCBS) for individuals in need. However, a significant proportion discontinued usage within six months, prompting an investigation into influencing factors. This study, analyzing LTC 2.0 data from 2018 to 2021 for older adults in two cities, tracked 59,151 service users for up to a year after their initial assessments through three application stages (assessment to service prescription, service utilization, and continuous service utilization for more than six months). Key factors affecting discontinuation included age, middle-low income family status, behavioral problems, informal caregivers, and foreign caregivers. Individuals with informal caregivers had a higher likelihood of obtaining service prescriptions (OR=1.82***) and utilizing LTC services (OR=1.33***), but were prone to discontinuing LTC 2.0 during continuous service utilization (OR=0.78***). Those with foreign caregivers were less likely to progress beyond the service utilization stage (OR=0.77***), but were inclined to maintain usage once started (OR=1.16***). Similarly, those aged over 85 and individuals with behavioral problems were less likely to progress beyond utilization but inclined to maintain usage (Age OR=1.22***; Behavioral problems OR= 1.14***). In contrast, individuals from middle-low income families were more likely to be prescribed and initiate usage but less likely to maintain it (OR= 0.77***). The presence of informal/foreign caregivers significantly influenced utilization status across these three stages, emphasizing the need for services development that address not only users' needs but also the needs of both formal and informal caregivers.

Abstract citation ID: igae098.3192

### INVESTIGATING WITHIN-PERSON MEDIATION OF GAIT-COGNITION ASSOCIATIONS IN A WALKING INTERVENTION FOR OLDER ADULTS

Nicholas Tamburri, Cynthia McDowell, Sandra Hundza, and Stuart MacDonald, *University of Victoria, Victoria, British Columbia, Canada*

Objective: Between-person associations between gait and cognitive function are well-documented, with select interventions (e.g., exercise) known to benefit both physiological function and cognitive health. Comparatively few studies, however, have investigated links between within-person changes in gait and cognition in the context of exercise interventions, or the mechanisms that might underlie this relationship. Method: The current study employed data from the Healthy Bodies Healthy Minds study -- a longitudinal walking intervention for initially-sedentary older adults (N = 118, 65-87 years). The study employs an intensive repeated-measures design, with physiological and cognitive function indexed at 5 assessments: baseline, 6, 9, 12, and 16 weeks. Gait velocity was assessed using a GAITRite Computerized Walkway, executive functioning was measured via the Groton Maze Learning (GML) Test, with aerobic capacity indexed by converting performance on the Rockport 1-Mile Walk Test to a V02 max proxy.
**Results:** Employing longitudinal multilevel mediation (1-1-1) models, we explored the within-person relationship between gait velocity and executive functioning, with particular consideration of aerobic capacity as a mediator. The indirect effect (ab+σajbj=-0.25) was significant, with aerobic capacity mediating 14.5% of the within-person gait-cognition time-varying association. Consistent with hypotheses, increases in gait velocity were associated with increases in aerobic capacity, which, in turn, conferred downstream benefits on GML test performance. Conclusions: These results further our understanding of the gait-cognition relationship, identifying aerobic health as an important within-person mediator in the context of a walking intervention. Key implications include the potential benefits of simple lifestyle interventions for promoting cognitive health in older adults.

Abstract citation ID: igae098.3193

### MEMORY ISSUES IN RETROSPECTIVE LIFE HISTORY SURVEYS: DO QUESTION TYPE AND TOPIC MATTER?

Wenqing Qian[1], Wenshan Yu[2], Mary Beth Ofstedal[1], Jacqui Smith[1], Marina Larkina[1], and Amanda Sonnega[3], *1. University of Michigan, Ann Arbor, Michigan, United States, 2. Duke University, Ann Arbor, Michigan, United States, 3. University of Michigan, University of Michigan, Michigan, United States*

Collecting retrospective information from the older population is critical yet challenging, and especially in self-administered surveys. Older adults may have difficulty retrieving autobiographical memories, navigating skip patterns in mail surveys, or using the complex technology often required for web surveys. These factors may lead to potential data quality concerns. Our study investigates data quality in the Health and Retirement Study (HRS) Life History Mail Survey (LHMS: N = 14934), that asks people over age 50 about events and experiences during childhood and midlife. We investigate characteristics of survey items that are related to item-missingness. Items were coded on 4 dimensions: (1) format: whether it was part of a grid or not, (2) life period: childhood vs. midlife, (3) detail: semantic vs. episodic, and (4) topic: residence, education, work, partnership, health. Overall, the item missing rate (IMR) was low (7.8%). Grids had higher IMRs than non-grid items (14.2% vs 6.4%). Controlling for this, we found that the average IMR for midlife questions was higher than for those pertaining to childhood in non-grid formats (8.9% vs. 4.9%), but similar in grid formats (11.6% vs. 12.2%). Additionally, episodic questions had a higher IMR compared to semantic questions in grid formats (13.7% vs. 10.3%), but lower in non-grid formats (3.1% vs. 6.1%). Among the five sections, items related to work and health history exhibited higher IMRs for both grid and non-grid formats. We discuss the implications of these results for the design of self-administered surveys and for analyses based on data from the LHMS.

Abstract citation ID: igae098.3194

### USING DRIED BLOOD SPOT ANALYSIS IN OLDER ADULTS WITH MILD COGNITIVE IMPAIRMENT: A FEASIBILITY STUDY

Miranda McPhillips[1], Junxin Li[2], Nalaka Gooneratne[3], and Nancy Hodgson[3], *1. Villanova University, Villanova, Pennsylvania, United States*, *2. Johns Hopkins School of Nursing, Baltimore, Maryland, United States*, *3. University of Pennsylvania, Philadelphia, Pennsylvania, United States*

Dried blood spots (DBS) are whole blood collected on filter paper and dried. We sought to determine the feasibility of using DBS for a virtually delivered intervention, 4 week, Multicomponent Behavioral Sleep Intervention (MBSI) for insomnia in older adults with mild cognitive impairment, during Covid-19. We explored the mechanisms by which MBSI affected sleep via inflammatory biomarkers (IL-6, IL-1ß, TNF-α, and C-reactive protein). Twenty-seven participants were enrolled; 3 dropped out. Participants were mailed supplies and instructions for collecting DBS at 3 study time points (baseline, immediately post-intervention, and 3-month follow-up). Participants were instructed to keep the filter paper lying flat at room temperature for 24 hours to dry before putting in a biohazard bag with a desiccant and mailing back in a pre-stamped envelope. All samples were labeled, transported, and stored in a -70°C freezer until ready to be analyzed. We used published methods to extract the blood spots in conjunction with Meso Scale Discovery (MSD) multiplex immunoassay for analysis. 20/27 (74%) of participants mailed the DBS cards back at baseline; 14 of those 20 (70%) mailed back DBS cards at timepoint 2 and timepoint 3. Only 10 participants completed all 3 timepoints (10/27; 37%). For all (n=48) samples, CRP and IL-1β values were all within normal limits. For IL-6, 22/48 (46%) values were below the lower limit of detection (LLOD) and 12/48 (25%) were too low to calculate. For TNF-α, 26/48 (54%) values were below LLOD. We will discuss barriers and facilitators to this type of collection and analysis.

Abstract citation ID: igae098.3195

### USING SIMULATION IN DEMENTIA CAREGIVER RESEARCH: LESSONS LEARNED

Cheryl Lee, and Rita Jablonski, *University of Alabama at Birmingham, Birmingham, Alabama, United States*

The purpose of this methods presentation is to describe how simulation-based training was used to impart critical skills for two members of a dementia caregiver research team: the interventionist and the person monitoring treatment fidelity. To have sufficient rigor and reproducibility in a randomized clinical trial, highly detailed protocols and training procedures must be developed and consistently followed. Inadequate training of interventionists may result in delivery variations of the same intervention, negatively affecting outcomes and the study's generalizability. Simulations followed the Healthcare Simulation Standards of Best Practice's general criteria for prebriefing and debriefing. Four pre-licensure nursing students role-played as simulated family caregivers who faced care-resistant behaviors by persons living with dementia. They co-created their simulated identities with the principal investigator. A treatment fidelity checklist was created to ensure the interventionist adhered to the key components of the intervention protocol. Interrater reliability for the treatment fidelity checklist was 100%. Treatment fidelity scores for the interventionist ranged from 23.1% to 52.9%, indicating poor adherence to the intervention protocol. Debriefings with the interventionist revealed that the simulated caregivers were passive during the simulations and that she had difficulty suspending disbelief during the simulations, which contributed to poor adherence. Students shared during their debriefing that their simulated identities should have had more details. Simulation training was continued using two family caregivers with better treatment fidelity results (90%-95%). Simulation is a viable method for training research personnel, but care is needed to select actors with experiences that mirror the research conditions.

### SESSION 7530 (POSTER)

### SOCIAL AND HEALTH EQUITY, DIVERSITY, AND INCLUSION

Abstract citation ID: igae098.3196

### A CAGED DEATH: EFFORTS TO IMPROVE QUALITY OF LIFE AND DEATH FOR AGING INCARCERATED WOMEN

Tosha Big Eagle[1], L. Shatswell[2], Kelly O'Sullivan[3], Raven Weaver[3], and Cory Bolkan[3], *1. Washington State University, Vancouver, Washington, United States*, *2. University of Puget Sound, Tacoma, Washington, United States*, *3. Washington State University, Pullman, Washington, United States*

Older incarcerated women are overlooked in research despite facing significant challenges; many remain incarcerated until death, even though prisons are not designed to provide sufficient geriatric or end-of-life care. This scoping review investigated the intersectionality between aging, incarceration, and health outcomes, focusing on older women, particularly those from historically marginalized social identities. With limited research on the effects of incarceration, especially for older, minoritized populations, we adopted a comprehensive approach, spanning between 1990 - 2023 and utilizing Academic Search Complete, APA PsycINFO, CINAHL, and PubMed databases. The search employed tiered keywords related to incarceration, jail/prison; aging, older adults; support, services, health; and women, race/ethnicity, culture. Two independent reviewers screened and identified 85 relevant articles for detailed analyses. Findings suggest that health outcomes, including disruption to physical, cognitive, and psychosocial development, are exacerbated among older women. Further, women 's prisons lack peer caregiving programs available in some men's prisons, highlighting gender inequities. Results underscore the critical role of adequate healthcare access to improve quality of life and death during incarceration, and successful re-entry into society, when applicable. There is also a dire need for gender-specific care, preventative healthcare, and early intervention strategies to address the unique health needs of incarcerated women as they age and die in prison. Few examples of programs/policies providing equitable access to comprehensive healthcare services are available. Future research contextualizing the intersectionality of aging, incarceration, and health outcomes, can contribute to this underexplored field, urging an inclusive and holistic approach to the well-being of incarcerated older women.

Abstract citation ID: igae098.3197

### ASSOCIATION BETWEEN SOCIAL POLICY, STIGMA, SOCIAL ACCEPTANCE, AND MENTAL HEALTH AMONG OLDER MEN WHO HAVE SEX WITH MEN

Alex Siu Wing Chan[1], Mark Lachmann[2], Hok Bun Ku[1], and Elsie Yan[1], *1. The Hong Kong Polytechnic University, Hong Kong, Hong Kong, China (People's Republic)*, *2. Mount Sinai Hospital, Toronto, Ontario, Canada*

**Background:** Older MSM (Men who have Sex with Men) often face discrimination and marginalization due to ageism and homophobia, impacting their psychological well-being. This study investigates the influence of social factors on the psychological well-being of older MSM, considering variations in social policy and cultural contexts across China, Hong Kong, and Taiwan.

**Methods:** A comparative approach was utilized, gathering data (N=453) from older MSM in the aforementioned regions. Measures assessing social policy, stigma, social acceptance, and psychological well-being were employed. Control variables, such as age, education level, and income, were included to mitigate potential influences. The data were analyzed using descriptive statistics, ANOVA, and regression analysis.

**Results:** The findings demonstrate significant negative effects of stigmatization across all three regions. F-statistics indicate the regression models' overall significance: China ($F = 69.583$, $p < 0.001$), Hong Kong ($F = 51.971$, $p < 0.001$), and Taiwan ($F = 57.725$, $p < 0.001$). This highlights stigmatization's strong influence on dependent variables in each region. Additionally, high adjusted R-squared values suggest substantial explained variance: China (Adj R2 = 0.632), Hong Kong (Adj R2 = 0.514), and Taiwan (Adj R2 = 0.565), emphasizing the model's ability to account for variability in dependent variables.

**Conclusion:** The study underscores the need for targeted interventions and policy changes to combat stigma and promote social acceptance, thereby enhancing the psychological well-being of this vulnerable population.

Abstract citation ID: igae098.3198

### ASSOCIATION OF NEIGHBORHOOD CHARACTERISTICS AND RACE ON HEALTH STATUS AND HEALTH CARE USE FOR OLDER ADULTS

Mohammad Usama Toseef, *Oakland University/Corewell Health, Farmington Hills, Michigan, United States*

Research suggests that neighborhood characteristics are important social determinants of health. However, there is limited evidence that explores the interaction of race/ethnicity and neighborhood characteristics on healthcare use. We study this issue and specifically focus our analysis on how older Arab Americans use healthcare relative to their White counterparts. We conducted a retrospective study among patients 65 years and older (N = 112,056) who were tested for SARS-CoV-2 at the emergency departments of a large integrated midwestern healthcare system between March 1, 2020, and July 31, 2022. We used area deprivation index (ADI) quintiles that allows ranking of neighborhoods based on socioeconomic disadvantage. The outcomes for our analysis were infection with SARS-CoV-2, hospitalization, ED revisit 30 days and readmission 30 days. We estimated logistic regression models controlling for race/ethnicity, ADI and interaction of these two. We found that older Arab Americans in the 3rd and 4th quintiles were more likely than Whites to test positive for SARS-CoV-2. Arab Americans in the 4th quintile were more likely to be admitted as compared to Whites while no significant differences were observed in the other quintiles. Arab Americans in the 3rd quintile of ADI were more likely to have an ED revisit within 30 days and re-hospitalized within 30 days as compared to Whites. This shows how culture and perception of healthcare provision can play a role in the use of healthcare resource and policymakers and stakeholders may find it useful to focus on neighborhood characteristics as captured by ADI or similar measures.

Abstract citation ID: igae098.3199

### EXPLORING EARLY BARRIERS TO KIDNEY TRANSPLANT: THE MORAL ECONOMIES OF DIALYSIS CLINICS

Jenny McDonnell, Ellen Idler, and Adam Wilk, *Emory University, Atlanta, Georgia, United States*

Kidney transplant conveys steep survival and quality of life benefits, yet only one-third of patients with kidney failure are referred for transplant evaluation. We explore the dialysis clinic as a site of inequities in transplant, wherein dialysis care professionals' (DCPs') early resource allocation decisions shape patients' downstream access to transplant. Using a modified grounded theory approach, we conducted 39 in-depth interviews during summer 2022 with DCPs (e.g., nephrologists, social workers, nurses) about their processes leading to transplant referral or non-referral. Phone interviews were recorded and transcribed verbatim. Textual data were coded using MAXQDA software. Combining within- and across-case strategies for analysis, we uncovered tension between DCP's reported referral philosophies and their tacit strategies for selectively allocating resources. While most DCPs reported that they "refer all patients", in practice, they selectively direct limited resources (e.g., time, education, advocacy, empowerment) toward "ideal" patients. DCPs described "ideal" patients as compliant, activated, and motivated, compared to "risky" patients who "don't do what they're supposed to do", struggle with self-management, and may "get less use out of a transplanted kidney" due to advanced age. We extend previous research by framing the dialysis clinic as a moral economy in which resources flow through a web of specific norms, meanings, and practices related to DCPs' perceptions of patients' deservingness and worth. Findings indicate that within this moral economy, DCPs direct limited resources toward "ideal" patients and away from "risky" patients most in need of DCPs' time and advocacy. Calls for future research and policy implications are discussed.

Abstract citation ID: igae098.3200

### HEALTH CARE WORKERS' PERSPECTIVES ON PROVIDING PALLIATIVE CARE TO MULTILINGUAL POPULATIONS WITH DEMENTIA

Katyayani Strohl[1], Neeta Ghosh[2], Syeda A Fatima Ahmed[2], Danae Dotolo[3], and Rashmi Sharma[2], *1. University of Houston, Houston, Texas, United States*, *2. University of Washington, Seattle, Washington, United States*, *3. University of Washington Medicine, Seattle, Washington, United States*

Importance: Persons living with dementia (PLWD) who prefer a language other than English (PLOE) are at high risk for sub-optimal palliative care. Although data suggest that multi-level (individual, clinical, healthcare system, community/policy-level) interventions may help address inequities, few studies have identified specific targets. Objective: To identify targets for multi-level interventions to improve palliative care for PLWD and PLOE.

**Methods:** We conducted qualitative interviews with social workers and medical interpreters who work with Chinese, Spanish, or Vietnamese speaking PLWD. Interviews were conducted from October 2022-October 2023, audiotaped, transcribed, and coded by an interdisciplinary investigator team using an inductive iterative approach.

**Results:** Interviews were conducted with 5 Spanish, 2 Chinese, and 2 Vietnamese interpreters and 5 social workers. Four themes emerged: 1) interpretation when the patient 'wasn't making sense'; 2) complexity with patient/family dynamics in decision-making; 3) barriers to anticipatory communication about dementia trajectory and quality of life; and 4) lack of culturally- and language appropriate resources. These data suggest multi-level intervention targets to improve palliative care in the context of dementia and linguistic diversity: 1) training interpreters about palliative care and how to navigate interpretation with PLWD, and providing clinicians communication skills training; 2) facilitating clinician-interpreter pre-meetings; 3) improving staffing and resources for social workers to shift from a crisis-focus to facilitating goals-of-care communication; and 4) increasing culturally and linguistically-appropriate and accessible resources. Impact: Our findings contribute to a gap in the literature about how to address the unique barriers PLWD with PLOE face, informing future intervention development.

Abstract citation ID: igae098.3201

### HEALTH EQUITY APPROACH TO EXAMINE COGNITIVE DIFFICULTY WITH AND WITHOUT A MENA CHECKBOX IN THE US

Tiffany Kindratt, and Basma Tnesh, *The University of Texas Arlington, Arlington, Texas, United States*

Federal guidelines for collecting race/ethnicity in the US include White, Black, Hispanic/Latino, Asian, American Indian/Alaskan Native (AI/AN), and Native Hawaiian/Other Pacific Islander (NH/OPI) as minimum reporting categories. Middle Eastern and North African (MENA) Americans are included in the definition for the White race group, which includes other individuals of European ancestry. In 2023, the Office of Management and Budget proposed adding a separate checkbox for MENA individuals. Several studies have identified that MENA Americans have a higher burden of cognitive health concerns compared to Whites. Having a separate checkbox for MENA Americans may provide a truer assessment of health disparities for other racial/ethnic groups. We examined whether removing MENA Americans from the White race group altered comparisons of cognitive difficulty between racial/ethnic minoritized populations and Whites. Using national data from the American Community Survey (ages >=45 years; N=7,284,988), we identified 67,213 adults (ages 45+ years) with MENA ancestry/birthplace. Most self-reported as White (n=60,993) followed by some other race (n=3,699), Black (n=1,957), Asian (n=468), AI/AN (n=72), and NH/OPI (n=24). Cognitive difficulty was higher among Black, Asian, AI/AN, and NH/OPI compared to White adults before and after removing adults with a MENA ancestry or birthplace from the sample. Marginal changes were observed. For example, for Black adults, odds increased from 1.23 to 1.24. While we did not find a difference in cognitive difficulty when we removed MENA adults from the national sample, further research is needed to determine whether this finding differs in smaller samples (e.g., states) and for other health outcomes.

Abstract citation ID: igae098.3202

### INCREASING DIGITAL EQUITY IN RURAL EASTERN NORTH CAROLINA

Abby Schwartz, Alice Richman, Leslie Cofie, Nate Mizell, and Leslie Osorio, *East Carolina University, Greenville, North Carolina, United States*

Rural eastern North Carolina is largely an economically distressed region with limited access to broadband internet. In most places in the US, access to internet and technology is integral to daily life and is critical to accessing healthcare and social services, however in the 18 counties included in the present study, fewer than 25% of the households have broadband access. Residents in these counties experience a dire digital divide. We aim to bridge this divide by delivering an educational intervention and through the development of a technology (iPads, hotspots) library lending program in each of the counties. This poster presents results from the formative evaluation that was completed to identify the internet connectivity, technology, and resource needs in the study counties. Twenty-five interviews were completed with key informants including trusted leaders, heads of libraries/staff, and residents. The mean age of resident respondents was 51 years old, and most were female (70%) with all identifying as African American. Interviews were completed by telephone or virtually and lasted ~45 minutes each. Thematic analysis was used to analyze the interview transcripts, and key themes that emerged across the interviews highlighted the critical need and interest in a digital lending program, desired topics for educational trainings (e.g., online job searches, support in completing social service forms online), and ways to engage residents in the programs. The poster concludes with a discussion of how the themes informed the development of lending library and educational programming, and considerations when implementing these programs in rural settings.

Abstract citation ID: igae098.3203

### RACIAL DIFFERENCES IN ADVANCE CARE PLANNING IN SAN ANTONIO, TEXAS

Jonathan Dao, Cory Kittleman, Pratima Gangupantula, Ninette Siby, Avinash Kakulavar, Rhea Gogia, and Jason Morrow, *The University of Texas Health Science Center San Antonio, San Antonio, Texas, United States*

Advance directives (AD) convey patient's preferences about end-of-life care to physicians and loved ones when they lose capacity. Only 45.6% of older adults (OA) have filled out an advance directive due to lack of time, knowledge, or comfort discussing the topic. Our organization prepared and delivered presentations on ADs at seven community centers in San Antonio, Texas to primarily caregivers, community health workers, and OA. Pre- and post-surveys were developed to assess demographics and evaluate attitudes (Likert scale) regarding ADs. Of 119 surveys, 50 respondents were OAs over 65 years old with a mean age of 74.8; 48% (n=24) identified as Hispanic/Latino, 30% (n=15) as African American (AA), and 25% (n=13) as White. 37.5% of Hispanic, 40% of AA, and 61.5% of White respondents reported previously completing an AD. 29.17% of Hispanic, 46.7% of AA, and 69.2% of White respondents reported previously completing an MPOA. 37.5% of Hispanic, 29.7% of AA, and 46.2% of White respondents reported being offered help from a physician to complete an AD. A higher proportion of White OAs had completed an MPOA, AD, and were offered help by a physician than AA or Hispanic/Latino OAs. This data remains consistent with previous studies on racial disparities in AD completion. A majority of respondents (68%) were never offered help from a doctor to complete an AD indicating a need to continue engaging patients, especially those of lower socioeconomic status.

Abstract citation ID: igae098.3204

### SOCIAL CLASS AND SOCIAL TRUST: THE CASE OF A NORDIC WELFARE COUNTRY

Marja Jylha, *Tampere University, Tampere, Pirkanmaa, Finland*

Trust in societal institutions and other people is an important foundation of democratic systems and contributes to the quality-olf-life. Finland has been considered as a high-trust society, with a reasonably egalitarian society and educational system, and the welfare state, including universal public services. In recent years the availability of health care and home and long-term care for older people has decreased, and"care poverty" has increase. In the project"Aging and social well-being" we investigated the trust of older individuals in societal instutions and health and old people´s services, and the differences in trust between social classes, measured as education, income, and occupational social class. The nation-wide survey included 3085 invdividuals aged from 65 to 84 years. Of the five societal institutions incluced, schools and educational system was the most trusted, and business life the least trusted. Trust in each five societal institutions and for the summary score showed a linear association with each measure of social class. For health and care services, a clear majority in each social class trusted acute health care and care services for chronic conditions, but a clear minority trusted home care, and only one in fourth trusted long-term care. Trust in health care was associated with social class, but trust in older people´s services was low in all social classes. The findings show that even in a Nordic welfare state, trust in societal institutions is highly dependent on social position, but low trust in old people´s care is shared by all social classes.

Abstract citation ID: igae098.3205

### THE IMPACT OF CHILDHOOD ADVERSITIES ON INTERPERSONAL AND INSTITUTIONAL DISCRIMINATION IN MIDDLE AND LATE ADULTHOOD

Wenxing Wei, *Case Western Reserve University, Cleveland, Ohio, United States*

Introduction: Research on the impact of childhood adversities on later-life experiences of interpersonal and institutional discrimination is scarce. Drawing on the life course perspective, this study examines how childhood adversities influence interpersonal and institutional discrimination experienced in middle and late adulthood.

**Methods:** Utilizing the Health and Retirement Study (HRS), this study included a nationally representative sample of 14,268 adults aged 45 and older. Ordinary Least Squares (OLS) regressions were conducted to examine whether childhood adversities were associated with interpersonal and institutional discrimination, controlling for sociodemographic characteristics.

**Results:** Findings indicate a significant relationship between childhood adversities and both interpersonal and institutional discrimination in later life. Specifically, increased experiences of childhood adversities were associated with higher levels of reported discrimination, underscoring the persistent effects of early adversities on individuals' later life experiences.

**Discussion:** This study contributes to the existing literature by focusing on the impact of childhood adversities on discrimination in an age group frequently neglected in studies. It underscores the importance of developing comprehensive prevention and intervention to mitigate the long-term effects of childhood adversities in middle and late adulthood. Additionally, the research identifies directions for future studies to delve into the mechanisms of these associations, to consider a wider range of childhood adversities (e.g., bullying), and to examine discrimination on broader levels (e.g., systemic). These explorations are crucial for the development of more effective support strategies and policy frameworks for individuals affected by childhood adversities and experiencing discrimination.

### SESSION 7535 (POSTER)

### SOCIAL DETERMINANTS OF HEALTH AND AGING II

Abstract citation ID: igae098.3206

### A SCOPING REVIEW OF HOME AND COMMUNITY-BASED SERVICES AND OLDER ADULTS' HEALTH OUTCOMES

Seon Kim, *Drexel University, Philadelphia, Pennsylvania, United States*

**Background and Objectives:** Most older adults aspire to age in their homes and communities rather than in institutions. However, as functional limitations grow with aging, older adults often find independent living challenging. The demand for home- and community-based services (HCBS) has steadily increased to alleviate the challenges faced by older adults. This scoping review explores the relationship between HCBS and the health outcomes of older adults. **Methods:** A search of the literature was conducted across major electronic databases between January 1, 2005, and August 1, 2023. 11,317 relevant peer-reviewed articles were identified, and 12 articles were selected for the final sample. **Results:** HCBS is consistently found to be related to better health outcomes, including physical, mental, and hospitalization. However, black and Asian older adults reported lower accessibility to HCBS and SRH compared to their White counterparts. Also, the low density and lack of HCBS in the neighborhoods are also related to the lower health of older adults. **Discussion and Implications:** HCBS is helpful in enhancing older adults' health outcomes; however, the better accessibility of HCBS in neighborhoods and older individual demographic characteristics should be considered. Also, policymakers should consider addressing the diverse needs of older adults and tailor HCBS.

Abstract citation ID: igae098.3207

### CHILDHOOD DIFFICULTIES AND LATE-LIFE DEPRESSIVE SYMPTOMS: THE ROLE OF EDUCATION AND FAMILY COPING

Seolah Lee, Minju Seong, Miseon Kang, Inhye Jung, and Hyo Jung Lee, *Yonsei University, Seoul, Republic of Korea*

This study aims to examine how childhood difficulties are associated with late-life psychological well-being. Both adverse experience in origin family, such as family dysfunction and economic hardship, and adulthood economic hardship are known to impede healthy coping and family satisfaction. Thus, using the Cumulative Advantage/Disadvantage Theory, current study further explores how individual's socio-economic status and family relational aspects in late life mediates the associations between childhood difficulties and depressive symptoms among older Koreans.We use the nationally representative Korean Welfare Panel Study (KoWePS), and select respondents aged 60-79 years old (n = 3,234). A serial mediation model was performed using the Hayes's PROCESS Macro, bootstrapped 5,000 resamples. The indirect and total effects of childhood difficulties on depressive symptoms in late life were significant. Level of education and coping strategies of family conflict in late life had mediating effects on the relationship between childhood difficulties and older adults' depressive symptoms. Income in late life, however, hadn't a mediating effect on the relationship. Family satisfaction in late life had a mediating effect only thorough level of education and coping strategies of family conflict in late life sequentially. Findings suggest that experience of childhood difficulties has an adverse impact on older adults' psychological well-being through deprivation of education opportunities in childhood and non-constructive coping strategies of family conflict in late life. Tackling inequality in education over one's life course, as well as helping individuals develop healthy coping strategies, may be considered to mitigate the negative impacts of childhood difficulties on late-life psychological well-being.

Abstract citation ID: igae098.3208

### CLINICIANS' PERSPECTIVES REGARDING THE INFLUENCE OF ADVERSE SOCIAL DETERMINANTS ON ACUTE CARE RESOURCE USE

Onome Osokpo[1], Karen Hirschman[2], Elizabeth Shaid[3], Kathleen McCauley[4], and Mary Naylor[2], *1. University of Illinois Chicago, Chicago, Illinois, United States, 2. University of Pennsylvania, Philadelphia, Pennsylvania, United States*, *3. Penn Nursing, Merion Station, Pennsylvania, United States*, *4. University of PA School of Nursing, Ardmore, Pennsylvania, United States*

Adverse Social Determinants of Health (SDoH) factors place older adults living with multiple chronic conditions at risk for poor outcomes during transitions from hospital to home. The Transitional Care Model (TCM) intervention, an advanced practice registered nurse (APRN)–led, team-based care-management strategy, improves outcomes throughout these transitions. However, little is known about the SDoH factors identified by APRNs delivering the TCM that may influence acute-care resource use. Guided by the Healthy People 2030 SDoH framework, we present the results of a descriptive qualitative analysis of 36 quality improvement and support meetings with clinical team members (i.e., APRNs, clinical coordinators) at three healthcare systems (7 hospitals/4 states) responsible for the delivery of the TCM to older adults hospitalized with heart failure, COPD, or pneumonia as part of the MIRROR-TCM trial. A review of clinical team meetings from January-June 2022, where teams met and reviewed and completed root cause analyses related to TCM care delivery challenges and acute-care resource use, revealed that most of the SDoH factors occurred within the Healthcare Access and Quality and Social and Community Context domains of Healthy People 2030 SDoH framework. These challenges include barriers to scheduling appointments, communication lapses amongst providers, reluctance or refusal by patients and/or families for home visits, and language barriers with non-English speakers. Strategies to address these challenges include, advocating for and coordinating follow-up appointments, use of telehealth, and healthcare-community partnerships to address patients' concerns and unmet health-related social needs. Engaging these transitional care strategies may result in improved posthospitalization outcomes.

Abstract citation ID: igae098.3209

### CONCEPTUALIZING THE HEALTH CARE WORKFORCE AS A SOCIAL DETERMINANT OF HEALTH IN LONG-TERM CARE SETTINGS

Roy Thompson[1], Sherif Olanrewaju[2], and Allison Squires[3], *1. Hunter College, City University of New York, Philadelphia, Pennsylvania, United States*, *2. The Pennsylvania State University, University Park, Pennsylvania, United States*, *3. New York University, New York, New York, United States*

The social determinants of health include environmental conditions where: individuals reside and age that affect their health, functioning, quality of life, and risks. We propose a conceptual model of how the health workforce becomes a social determinant of health resulting from the sustained person-clinician relationship. To illustrate the application of this proposed model, we examine the case of long-stay residents in long-term care settings. These residents spend ≥101 days, oftentimes until death in these facilities and have prolonged healthcare staff interactions which impact their health outcomes. Evidence suggests that sustained contact between residents and the healthcare workforce does impact their quality of life, health outcomes, and risks. We posit that the health workforce can be a contributing factor in inequitable outcomes in long-term care settings, thus becomes a social determinant of health. Their interactions and practices with residents may explain disparities in Black and Hispanic residents who typically have significantly higher rates of pain, falls, antipsychotic use, and pressure injuries relative to their Non-Hispanic White counterparts. Additionally, Black and Hispanic residents receive care in geographically segregated nursing homes. Through multi-level exemplars, we will illustrate how as a social determinant the workforce characteristics like racial and ethnic concordance, skill mix, and hours per resident impact health outcomes. Therefore, studies that conceptualize and operationalize the healthcare workforce as a social determinant of health at the individual, interpersonal and organizational levels are needed to improve staffing interventions to mitigate health disparities across in long-term care settings.

Abstract citation ID: igae098.3210

### HETEROGENEOUS EFFECTS OF CHILDHOOD ADVERSITY ON LATE-LIFE BRAIN HEALTH: A CAUSAL FOREST APPLICATION

Eleanor Hayes-Larson[1], Ryo Ikesu[2], Kosuke Inoue[3], Paola Gilsanz[4], Rachel Whitmer[5], M Maria Glymour[6], Dan Mungas[5], and Elizabeth Rose Mayeda[2], *1. USC Leonard Davis School of Gerontology, Los Angeles, California, United States*, *2. University of California Los Angeles, Los Angeles, California, United States*, *3. Kyoto University, Kyoto, Kyoto, Japan*, *4. Kaiser Permanente Northern California, Oakland, California, United States*, *5. University of California Davis, Davis, California, United States*, *6. Boston University, Boston, Massachusetts, United States*

Effect heterogeneity across individuals may help explain inconsistent evidence regarding effects of childhood adversity on brain health. We used harmonized Kaiser Healthy Aging and Diverse Life Experiences and Study of Healthy Aging in African Americans (n=617) data to evaluate heterogeneity in effect estimates of childhood adversity (z-score of 7 adverse childhood events factor score, dichotomized at median) on brain white matter hyperintensity volume (WMH, log transformed). We used an honest causal forest with augmented inverse probability weighting and 10-fold cross-validation to estimate conditional average treatment effects (CATEs); this approach captures complex heterogeneity better than traditional regression models. Candidate sources of heterogeneity included age at MRI, demographics, US southern birth, childhood SES measures and interactions between variables. Overall, exposure to at least median childhood adversity was associated with 0.14 higher log units of WMH (95% CI -0.06, 0.35) after covariate adjustment. The best linear fit model for the observed treatment effect had an out-of-bag predicted treatment effect coefficient of 0.81 (P-value=0.02), indicating the heterogeneity in the association. Individuals with estimated CATEs below the median estimated CATE were older (mean age 76.4y vs. 72.5y), more likely to be male (56% vs 63%), and more likely to report low childhood SES (55% vs. 74% average/well-off, 16% vs 7% ever went hungry). This is preliminary work in a relatively small sample; more work is needed to understand the impact of childhood adversity on late-life brain health, including differences across individual characteristics.

Abstract citation ID: igae098.3211

### INCOME INEQUALITY, INCOME, AND HEALTH OVER THE LATER LIFE COURSE IN EUROPE: A LONGITUDINAL AND CROSS-NATIONAL STUDY

Mengling Cheng[1], Nicolas Sommet[2], and Dario Spini[2], *1. University of Lausanne / East China University of Science and Technology, Switzerland*, *2. University of Lausanne, Lausanne, Vaud, Switzerland*

Evidence from the U.S., UK, and Europe documented that income inequality has modest effects on the social gradient of health. However, few studies investigate how the effect of income inequality on the social gradient of health evolves over the life course. This study aims to examine to what extent, and in what ways, does the effect of income on later-life health trajectory vary across more unequal and less unequal societies. This study used data from the Standardized World Income Inequality Database (SWIID, 2004-2020) and the Survey of Health, Ageing and Retirement in Europe (SHARE, 2004-2010). We used national-level Gini index to measure income inequality. We used life satisfaction as the health outcome. We performed multilevel modelling to analyze the data in which observations (level 1) are nested in participants (level 2), country-years (level 3), and countries (level 4). Our analysis revealed three findings. First, the effect of income on life satisfaction among older adults is more pronounced in unequal countries. Second, the effect of income inequality on life satisfaction weakens as individuals age. Third, the importance of income on life satisfaction across societies of varying levels of income inequality decreases over the life course of an individual. Findings from this study show that contextual-level income inequality can modify the effects of individual income on health as individuals age. This can help the policy makers and practitioners to target the vulnerable population and offer more effective intervention.

Abstract citation ID: igae098.3212

### LEISURE ENGAGEMENT AND HAPPINESS IN LATER LIFE BASED ON A LEVEL OF FLOW EXPERIENCE

Jungsu Ryu, *Marshall University, Huntington, West Virginia, United States*

There has been a growing body of literature that examines various environmental predictors (e.g., time spent outdoors, and social interaction) of happiness. Furthermore, scholars have reported on the contributing role of leisure activities to happiness. However, research has rarely examined the associations between different types of leisure activity and happiness based on a different extent of flow experiences at an advanced age. Therefore, the purpose of this study was to explore what kinds of leisure activity contribute to happiness in later life based on the level of experiencing flow. A convenience sample of 188 participants was recruited from senior centers in South Korea, which consisted of 67 males and 121 females with ages ranging from 60 to 90 years (M = 74.99, SD = 5.49). The results showed that the participants in the high flow state significantly feel happier than those in the low flow state. Additionally, home-centered and social activity was the only significant contributor to happiness for the high flow state. For the low flow state, there was no significant relationship between each leisure engagement and happiness. Our findings elucidate that the importance of social engagement in happiness should be emphasized than any other activities in later life, and experiencing flow during social engagement considered meaningful also needs to be accentuated. It implies that older adults are more likely to be happy when social engagement is a meaningful and motivating activity compared to when simply participating in socializing without any motivation.

Abstract citation ID: igae098.3213

### LIFETIME TRAUMA AND ALLOSTATIC LOAD: PSYCHOSOCIAL RESOURCES AS BUFFERS?

Mallory Bell, Wencheng Zhang, and Kenneth Ferraro, *Purdue University, West Lafayette, Indiana, United States*

Although past studies report a relationship between stress and allostatic load, less research examines whether psychosocial resources buffer this relationship. Further, most studies examine allostatic load cross-sectionally. The current study uses sixes waves (2006-2016) of the Health and Retirement Study to examine the relationship between lifetime traumas and allostatic load over time. We also examine whether a variety of psychosocial resources, such as neighborhood cohesion, social support, and volunteering, help protect against the deleterious consequences of trauma on physiological dysregulation. Results indicate that higher lifetime trauma is associated with higher allostatic load. Allostatic load did not change over time Further, we found little evidence that psychosocial resources moderate the relationship between lifetime traumas and allostatic load.

Abstract citation ID: igae098.3214

### MODERATING THE EFFECTS OF COGNITIVE DECLINE ON DAILY FUNCTIONING IN OLDER ADULTS: THE ROLE OF SOCIAL SUPPORT

Youngmin Cho[1], Bei Wu[2], Baiming Zou[3], Lixin Song[4], Ruth Anderson[5], and Anna Beeber[1], *1. Johns Hopkins University, Baltimore, Maryland, United States*, *2. New York University, New York, New York, United States*, *3. University of North Carolina, Chapel Hill, North Carolina, United States*, *4. University of Texas Health Science Center at San Antonio, San Antonio, Texas, United States*, *5. Duke University, Durham, North Carolina, United States*

As cognitive impairments increasingly affect community-dwelling older adults (CDOA), it becomes significant to understand the longitudinal impact of social support factors—such as living arrangements, social network size, and participation in social activities—on the relationship between cognitive status and daily functioning. Daily functioning includes activities of daily living (ADL), instrumental activities of daily living (IADL), physical capacity, and mobility. This study aims to determine whether social support factors moderate the relationship between cognitive status and daily functioning. We analyzed data from the National Health and Aging Trends Study (NHATS), a longitudinal survey of a nationally representative cohort of Medicare beneficiaries aged 65 and over in the US. Our analysis included eight years of data from 6,238 community-dwelling participants. Using linear mixed-effects models, we explored the interaction effect between cognitive status and social support factors on trajectories of daily functioning. Overall, all areas of daily functioning significantly declined over time, with faster decreases observed as cognitive status worsened. Living arrangements moderated the relationship between cognitive status and IADL, specifically mitigating the negative impact of cognitive decline on IADL. Increased participation in social activities eased the decline in ADL for individuals with probable dementia and in physical capacity for those with mild cognitive impairment (MCI); however, the size of the social network had no significant impact. Our findings highlight the importance of incorporating social support factors into tailored interventions to mitigate declines in daily functioning among CDOA, tailored to their specific cognitive status.

Abstract citation ID: igae098.3215

### NEIGHBORHOOD POLLUTING INFRASTRUCTURE AND COGNITIVE HEALTH IN OLDER ADULTS

Vivian Ku, Ketlyne Sol, and Laura Zahodne, *University of Michigan, Ann Arbor, Michigan, United States*

Air pollution is known to be associated with poorer brain and cognitive health. Modifiable sources of pollution in neighborhoods such as industrial and hazardous waste facilities may serve as intervention targets. However, less is known about how polluting infrastructure relates to different cognitive domains. This study examines the relationship between polluting infrastructure and cognition within older adults. 475 adults aged 55+ from the Michigan Cognitive Aging Project (MCAP) completed a comprehensive neuropsychological battery measuring cognition across five domains (episodic memory, executive function, processing speed, language, visuospatial function). Participant geocoded addresses were linked to data on polluting infrastructure in the National Neighborhood Data Archive. Polluting infrastructure was operationalized as the presence of Toxic Release Inventory sites and the proportion of highway length to the total length of streets in a census tract. Generalized estimating equations accounting for geographic clustering modeled relationships between polluting infrastructure and cognitive health, controlling for age, sex, education, income, population density, and neighborhood disadvantage. Higher proportions of highways were related to poorer episodic memory ($\beta$= -0.660, p= 0.040). A trend was also found between the presence of at least one toxic site and poorer language ($\beta$= -0.121, p= 0.056). Both highways and toxic sites represent polluting infrastructure that could reduce cognitive health among older adults. Differential associations between pollution sources and cognitive domains may indicate that different types of pollutants (e.g., airborne fine particulates versus others) influence unique brain pathways.

Abstract citation ID: igae098.3216

### PARTNER CORRESPONDENCE IN SOCIAL INTERACTION TIME AND WELL-BEING: EVERYDAY EVIDENCE FROM OLDER DYADS

Carlotta Grünjes[1], Elizabeth Zambrano Garza[2], Rachel Murphy[2], Kenneth Madden[2], Denis Gerstorf[3], Gizem Hueluer[1], and Christiane Hoppmann[4], *1. University of Bonn, Bonn, Nordrhein-Westfalen, Germany*, *2. University of British Columbia, Vancouver, British Columbia, Canada*, *3. Humboldt University Berlin, Berlin, Berlin, Germany*, *4. The University of British Columbia, Vancouver, British Columbia, Canada*

Social interactions play an important role in older adults' daily well-being. Longer duration of interaction has been associated with higher well-being at the individual level. However, less is known about the extent to which partners differ in their convergence in interaction reports and whether such differences might be associated with daily well-being. Positive Illusion theory states that individuals are more content in relationships if they idealize their partner leading to the assumption that overreporting interaction time may benefit well-being. Based on the literature, we expect that longer social interaction time is associated with higher well-being at the individual level. At the partner level, we look at discrepancies and similarities. We assess whether individuals report higher well-being on days when they overreport the interaction time with their partner. We further test whether partners who are more similar in evaluating the time spent together are also more similar in their well-being reports. This study uses dyadic end-of-day diary data from 136 Canadian older adults (M = 66.68 years, SD = 13.11 range: 18-87 years, 60% women) and a close other of their choice. Preliminary analyses indicate that longer duration of interaction was associated with higher well-being. Correlations show differences in interaction time reports between partners. Multilevel models will examine whether overreporting interaction time may be associated with well-being. Further analyses will investigate how synchrony in interaction time reports shape joint well-being. As data collection is completed, these results will be ready to be presented by the time of GSA conference in November.

Abstract citation ID: igae098.3217

### SELF-REPORTED WELL-BEING AMONG OLDER ADULTS WITH A PERSONAL OR FAMILY HISTORY OF INCARCERATION

Louisa Holaday[1], Kimberly Stone[1], Brita Roy[2], Brie Williams[3], Albert Siu[4], and Emily Wang[5], *1. Icahn School of Medicine at Mount Sinai, New York, New York, United States*, *2. NYU Langone Health, New York, New York, United States*, *3. University of California San Francisco, San Francisco, California, United States*, *4. Icahn School of Medicine at Mount Sinai, New York, New York, United States*, *5. Yale University, New Haven, Connecticut, United States*

The United States has one of the highest incarceration rates in the world. Prior work has demonstrated adverse health effects associated with personal or family history of incarceration, but none has focused on older adults. We conducted a cross-sectional study using data from the nationally representative Family History of Incarceration survey. Among the 1,114 adults aged 55 or older, we tested the association between personal incarceration and/or family member incarceration and overall; financial; social; physical; and mental wellbeing controlling for age; gender; race/ethnicity; income; education; employment; and marital status. We accounted for time since last incarceration, and duration of incarceration. Overall, 21% of respondents had been incarcerated (of whom 78% returned to the community over 10 years ago), and 64% had had an immediate family member incarcerated (of whom 30% were incarcerated 1 year or longer). "Thriving" overall wellbeing, which is associated with life expectancy, was less common among those with personal or family incarceration exposure (63.3% of those without incarceration exposure vs. 47.7-55.7% of those with incarceration exposure). In fully adjusted models, personal or family incarceration exposure was associated with lower odds of reporting "excellent" or "very good" physical health (OR: 0.50-0.55; all P< 0.05); and "thriving" social wellbeing (OR: 0.44-0.60; all P< 0.008). There existed a dose response relationship between longer durations of family member incarceration and lower wellbeing. Exposure to incarceration is independently associated with self-reported physical health and social wellbeing among older adults. Policy makers should consider methods to mitigate the harms associated with incarceration.

Abstract citation ID: igae098.3218

### SOCIAL CAPITAL PROFILES AMONG CHINESE OLDER ADULTS: A LATENT CLASS ANALYSIS

Yuekang Li[1], and Nancy Morrow-Howell[2], *1. University of Chinese Academy of Social Sciences, Beijing, Beijing, China (People's Republic)*, *2. Washington University in St. Louis, St. Louis, Missouri, United States*

Existing literature often overlooks the multifaceted social engagements of older adults, neglecting how well-being is intricately influenced by diverse combinations of social capital. This study investigates social capital profiles among Chinese older adults, examining how these profiles vary in relation to demographic characteristics and health outcomes. The China Family Panel Study (CFPS) 2016 wave provided data for 7,295 adults aged 60 and above. Based on three levels of social capital (family, community, and societal), Latent Class Analysis (LCA) was employed to identify the social capital profiles of older adults. Multinomial logistic and linear regression models analyze the influence of personal and neighborhood factors on social capital profiles and their correlation with depressive symptoms. Through LCA, three distinct social capital profiles emerged: "Family-centered," "Family Plus Society," and "Diverse." These profiles, each with their unique configurations of social capital, are linked to specific demographic and community features as well as mental health statuses. Notably, this research underscores the particular vulnerability of the "Family-centered" group, characterized by their restricted access to various forms of social capital. Meanwhile, older adults' social capital is highly connected with the neighborhood they live in. The findings from this study deepen our understanding of social capital by adopting an ecological perspective. The methodology employed to discern social capital compositions and profiles more accurately reflects the diverse social engagements of older adults, offering important insights into the intricate interplay between different levels of social capital and varied older populations.

Abstract citation ID: igae098.3219

### SOCIAL DETERMINANTS OF HEALTH AMONG CUSTODIAL GRANDMOTHERS: ADVANCING AN INTERSECTIONAL APPROACH

Tenesha Littleton, *University of Alabama, Tuscaloosa, Alabama, United States*

Racial disparities in custodial grandparenting, as well as disparities in the physical and mental health outcomes of custodial grandparents, are well-documented. As a result of long-standing socio-structural inequalities across the life course, custodial grandparents are disproportionately single, Black women with low socio-economic status. These categories of difference and the process of racialization contribute both to the need for grandparent caregiving in the Black community and the negative health consequences of caregiving for Black custodial grandmothers. Macro level structures and systems of oppression impact access to the social determinants of health shaping the life trajectories of racialized groups and contributing to unequal aging which suggests the importance of an intersectional lens when understanding the lived experiences of Black custodial grandmothers. A scoping review was conducted to determine the extent to which an intersectional framework is utilized in research examining the experiences of Black custodial grandmothers. A search of relevant databases (PsychINFO, SociINDEX, PubMed, and Web of Science) was performed using the following search terms and Boolean logic: ("grandparent" OR "grandmother" OR "grandfather" OR "custodial grandparent") AND ("intersectionality"). Quantitative, qualitative, and mixed-methods peer-reviewed journal articles published in English through January 2024 were included. Findings suggest that research integrating intersectionality with large representative samples is limited. This presentation will propose a conceptual framework that integrates the social determinants of health with an intersectional life course perspective as proposed by Ferrer and colleagues (2017). Recommendations for future research using intersectional approaches will be offered.

Abstract citation ID: igae098.3220

### SOCIOECONOMIC AND GENETIC DETERMINANTS OF COGNITIVE FUNCTION IN THE AGING POPULATION IN KAZAKHSTAN

Adil Supiyev[1], Angela Gutierrez[2], and Graciela Muniz Terrera[3], *1. Ohio University Heritage College of Osteopathic Medicine, Athens, Ohio, United States*,*2. Western University of Health Sciences, Pomona, California, United States*, *3. Ohio University, Athens, Ohio, United States*

Objective There is limited evidence about cognitive performance of older adults in Kazakhstan, a Central Asian nation that was formerly part of the Soviet Union. Our study aims to fill the gaps by assessing cognitive function and its determinants in the population of Astana city and its surrounding rural areas. Methods Between 2012 and 2015, 954 older adults aged 50–74 from urban and rural areas in Central Kazakhstan participated in this study (response rate: 59%). Trained practitioners administered four neuropsychological tests to assess cognitive function, including verbal memory, verbal fluency, attention, and concentration. Additionally, a standardised protocol was used, consisting of a structured questionnaire, objective physical examination, and blood sample collection. Results After controlling for age, sex, and cardiometabolic risk factors, our analysis revealed significant associations between cognitive function and residence, ethnicity, education levels, and selected socioeconomic factors. Notably, APOE4 status showed a strong association with cognitive decline, consistent with prior research. Furthermore, individuals with higher deprivation levels, men, rural residents, and those with lower education levels demonstrated significantly poorer cognitive function outcomes. Conclusion Understanding the association between cognitive function and selected socio-demographic factors within the Central Asian region contributes significantly to global health insights. The contrasts observed in residential environments and levels of deprivation emphasize the urgency of targeted interventions aimed at mitigating cognitive decline risks among vulnerable populations worldwide.

Abstract citation ID: igae098.3221

### THE INTERPLAY OF SOCIAL DETERMINANTS, DIGITAL TECHNOLOGY, AND DEPRESSIVE SYMPTOMS IN KOREAN OLDER ADULTS

Donghee Shin[1], and Giyeon Kim[2], *1. Chung-Ang University; Korea Disabled People's Development Institute, Seoul, Republic of Korea*, *2. Chung-Ang University, Seoul, Republic of Korea*

The purpose of this study was to address the mechanisms between digital inequality and mental health disparities among Korean older adults, recognizing that social determinants of health (SDH) also contribute to the digital divide. We investigate whether older adults' digital capacity exacerbates or mitigates health inequalities in later life. Drawn from the 2020 National Survey of Older Koreans, a nationally representative sample of 9,885 adults aged 65 and older was analyzed. We employed hierarchical multiple regression, moderation, and mediation analyses. SDH factors including sex, income, education and geographic location (rural-urban residence) were examined. Results showed that capacity to use digital technology significantly moderates and mediates the relationship between income, education, geographic location and depressive symptoms. Notably, digital technology use buffers against the negative effects of low income ($B=.021$, $p<.05$) and living in metropolitan areas ($B=.095$, $p<.001$, $B=.067$, $p<.01$) on depressive symptoms, while it also amplifies the negative effect of low educational attainment on depressive symptoms ($B=-.030$, $p<.001$). Specifically, heightened digital technology use among older adults augments the detrimental effects of education on depressive symptoms. Findings suggest that the importance of developing diverse support programs to improve digital literacy among Korean older adults, offering possible ways for reducing the digital divide and addressing mental health disparities.

Abstract citation ID: igae098.3222

**USING LATENT CLASS GROWTH ANALYSIS TO IDENTIFY SUBGROUPS OF OLDER ADULTS WITH SHARED TRAJECTORIES OF FRAILTY STATUS**

Hillary Spangler[1], Wenyi Xie[2], David Lynch[1], Feng-Chang Lin[2], and John Batsis[2], *1. University of North Carolina School of Medicine, Chapel Hill, North Carolina, United States*, *2. University of North Carolina, Chapel Hill, North Carolina, United States*

**Background:** Frailty is a geriatric syndrome with significant clinical heterogeneity among older adults. Identifying groups experiencing different frailty trajectories is critical for identifying those at high risk of frailty progression and prevention.

**Methods:** We used data from the National Health and Aging Trend Study (2011-2020), a cohort of Medicare beneficiaries >65 years. Individuals were categorized using Fried's frailty phenotype. We included older adults (n=6,904) with ≥2 interviews with frailty status (improving, stable, worsening). We used latent class growth analysis (LCGA) to identify patterns of change in frailty status and the impact of social determinants of health (SDOH: transportation, family support, income, education) on frailty trajectories. We defined rural by The Office of Management and Budget's standards.

**Results:** Participants were 56.9% female, median age of 70-74. At baseline, 24.6% were robust, 50.4% were pre-frail, and 25.0% were frail. LCGA identified three trajectories over 10 years. Most participants (54.1%) had a stable trajectory, with almost no change in frailty status. Fewer participants (29.1%) had an improving trajectory, initially experiencing a decrease in the number of frailty items at ~6 years, followed by a slow increase over time. The smallest group (16.9%) had a worsening trajectory, with an increasing number of frailty components over time. The worsening trajectory group was older at baseline, majority female. Marital status, lower education and income levels, and Medicaid enrollment were associated with higher odds of worsening frailty trajectory.

**Conclusion:** SDOH and rurality were associated with progressive frailty trajectories, highlighting the need for policy-level interventions for frailty mitigation.

### SESSION 7540 (POSTER)

### SOCIAL ISOLATION AND LONELINESS II

Abstract citation ID: igae098.3223

**A SYSTEMATIC REVIEW OF MEASURES USED IN STUDIES LINKING SOCIAL ISOLATION, LONELINESS, AND PHYSICAL HEALTH**

Priya Nambisan[1], Sushma Gangula[1], Julie Ellis[1], Murad Taani[1], and Yura Lee[2], *1. University of Wisconsin Milwaukee, Milwaukee, Wisconsin, United States*, *2. University of Wisconsin-Milwaukee, Milwaukee, Wisconsin, United States*

Social isolation (SI) and loneliness significantly impact public health, particularly among older adults, leading to adverse physical health outcomes. Despite extensive research in this field, there is considerable variability in how these factors are measured. Our systematic review, following the PRISMA method, identified 22 relevant studies. Among these, 18 primarily utilized secondary data and assessed social isolation through questions covering marital status, contact frequency with family, friends, and participation in social activities. Composite indices and scales were often constructed from these measures, with scores indicating low/high social isolation. However, it's essential to note that loneliness, which can be felt despite social interaction, requires validated scales for accurate measurement. Out of the 22 studies, 4 didn't employ validated scales for either SI or loneliness, while 6 lacked validation for SI, and 5 out of 20 for loneliness. Measurement scales varied for loneliness, from simple classifications to Likert scales, with higher scores indicating heightened loneliness. Some studies adopted established scales like the Bude and Lantermann scale, while others employed modified versions or analogues. Our analysis highlights the importance of using validated measures to assess these variables accurately. Markedly, different measurement approaches yielded distinct impacts on health outcomes. Moving forward, standardization and validation of measurement tools are critical for enhancing the reliability and comparability of research findings in this area.

Abstract citation ID: igae098.3224

**A SYSTEMATIC REVIEW OF QUANTITATIVE STUDIES OF LONELINESS AND SOCIAL ISOLATION IN LONG-TERM CARE**

LaTonya Smith[1], and Laurie Theeke[2], *1. George Washington University, Washington, District of Columbia, United States*, *2. The George Washington University, Ashburn, Virginia, United States*

Loneliness and social isolation, though conceptually different, are both prevalent conditions for adults in long-term care facilities. These individuals, often separated from their previous lives, communities, and families, can experience a significant disconnect, leading to a pervasive sense of isolation and abandonment. Both conditions elicit a stress response, exacerbate physical health issues, lead to anxiety and depression, and significantly decrease quality of life and longevity; making it critical to understand these conditions in this population. The purpose of this paper is to present a systematic review of quantitative studies that operationalized loneliness or social isolation in samples of older adults in long-term care. The literature search used EbscoHost and included MEDLINE Complete, CINAHL complete, APA PsycArticles, and APA PsycINFO and resulted in 20 articles for inclusion. The synthesis of study findings makes it clear that loneliness and social isolation are associated with harmful health outcomes with up to 56% of residents reporting loneliness. Older adults with more functional ability and higher resilience scores were less lonely. Those with pain and those with rumination, deterministic thinking and confabulation reported increased loneliness. Intervention programs that included gardening, music, videoconferencing with families, laughter yoga, and peer mentor programs were effective for diminishing loneliness. Future studies are needed for comparative effectiveness of current interventions and for the testing of new interventions designed for this setting that are more precise to each condition. Clinical protocols for assessment and treatment of loneliness and social isolation in long-term care could enhance resident experience and potentially, longevity.

Abstract citation ID: igae098.3225

### ASSOCIATION BETWEEN PATTERNS OF SOCIAL ACTIVITIES AND FRAILTY STATUS AMONG OLDER ADULTS IN CHINA

Sok Leng Che, Ka Kei Chao, and Ka Kit Wong, *Kiang Wu Nursing College of Macau, Macao, Macao, Macau*

Loneliness has been identified as a public health priority by WHO. Studies have indicated that participating in social activities could reduce loneliness and the probability of being frail. This study examines social activity among Chinese older adults and its impact on frailty. This investigation utilizes data from 10,762 participants aged 60 years and over in the 2018 wave of the China Health and Retirement Longitudinal Survey (CHARLS). Frailty status was assessed by constructing a Frailty Index using health deficit accumulation model. Latent class analysis was employed to explore patterns of social activities, and logistic regression was used to identify factors associated with frailty status. The study identified five distinct types of social activity pattern, e.g. face-to-face interaction, socially active and diverse, socially inactive, socializing through the role of caregiver, and socializing through the Internet. Older adults in China are characterized by being socially inactive, accounting for 82% (n=8,827) of the total participants. Compared to older adults who practice face-to-face interaction, older adults who are identified as socially inactive (aOR=2.05, $p< 0.001$, 95% C.I.=1.68 to 2.51), and socialize in the role of caregiver (aOR=1.43, $p=0.01$, 95% C.I.=1.08 to 1.88) showed significantly higher odds of being frail. Older adults who hold the role of caregiver are particularly susceptible to frailty. The caregiving role may restrict their social network, while social inactivity may increase the risk of social isolation. Therefore, older individuals exhibiting these patterns of social activity may benefit from enhanced community and family support.

Abstract citation ID: igae098.3226

### ASSOCIATION OF LIVING ALONE AND SOCIAL ISOLATION WITH ALL-CAUSE AND CARDIOVASCULAR MORTALITY: THE OTASSHA STUDY

Keigo Imamura[1], Hisashi Kawai[1], Manami Ejiri[1], Hiroyuki Sasai[1], Hirohiko Hirano[1], Yoshinori Fujiwara[1], Kazushige Ihara[2], and Shuichi Obuchi[1], *1. Tokyo Metropolitan Institute for Geriatrics and Gerontology, Tokyo, Japan*, *2. Hirosaki University, Hirosaki, Aomori, Japan*

Living alone is considered a risk factor for all-cause and cardiovascular mortality, but this association may be confounded by social isolation status. This study examined how living alone while also being socially isolated affects older adults' risk of all-cause and cardiovascular mortality. A total of 4,075 community-dwelling older adults (mean age: 72 years, male: 46%) who had participated in the 2012 or 2013 mail survey of the health checkup of the Otassha Study were included; the study period was October 2012 to November 2020. Social isolation was assessed by frequency of interaction with others, and less than once a week was defined as social isolation. Participants were classified into four groups: no social isolation – not living alone, no social isolation – living alone, social isolation – not living alone, and social isolation – living alone. A Cox proportional hazards model was used to analyze the association of combined living alone and social isolation with all-cause and cardiovascular mortality. A total of 203 (5.0%) of the participants were found to be living alone while being socially isolated. Social isolation was significantly associated with all-cause and cardiovascular mortality, regardless of whether the participants lived alone or not ($p< 0.05$). On the other hand, there was no association with mortality among those who lived alone but were not socially isolated. These results indicate that living alone is not necessarily a risk factor for all-cause or cardiovascular mortality. In conclusion, engaging in regular social interactions could help older adults reduce mortality.

Abstract citation ID: igae098.3227

### CONNECTING THREADS: SOCIAL PARTICIPATION'S ROLE IN REDUCING LONELINESS AMONG OLDER ADULTS

Nahyun Kim, and Keiko Katagiri, *Kobe University, Kobe, Hyogo, Japan*

Social participation among older adults occurs within the context of local community networks, suggesting that engaging in such activities can expand neighborhood networks, enhancing social connections and fostering a sense of belonging. Active social participation serves to both bridge and bond social networks by expanding them to diverse communities and reinforcing close-knit within groups. Despite the acknowledged advantages of social participation in alleviating loneliness, research gaps persist regarding its specific function and effects on loneliness. This study explores the effects of social participation on loneliness, particularly through the lens of bridging new communities and reinforcing internal group bonds, focusing on relationships with weak neighborhood ties and their impact on reducing loneliness. An online survey was conducted with Japanese individuals aged 60–79 years (n=1000), focusing on 570 respondents who were actively involved in social participation. The survey collected data on variables such as bridging to new communities, internal group bonds, the size of weak neighborhood ties (greetings, casual conversations), and loneliness. A path analysis revealed that strong internal group bonds were negatively related to loneliness, highlighting the importance of deep, supportive relationships within groups. Although direct links between the extent of bridging to new communities and immediate loneliness reduction were not found, an indirect positive effect through the expansion of neighborhood networks was identified, associated with decreased loneliness. This highlights the dual benefits of social participation: fostering close-knit group connections and expanding community engagement through weak ties; both are crucial for reducing loneliness and enhancing well-being in older adults.

Abstract citation ID: igae098.3228

### DAILY EMOTION EXPRESSION, MODES OF SOCIAL CONTACT, AND WELL-BEING IN LATE LIFE

Shiyang Zhang[1], Nicole Richards[2], Sibo Gao[2], and Karen Fingerman[2], *1. University of Texas at Austin, Austin, Texas, United States*, *2. The University of Texas Austin, Austin, Texas, United States*

Older adults engage in in-person and remote forms of contact (phone, text). In-person contact may provide opportunities for sharing emotions and promoting well-being in ways that other modes of contact do not. This study examined expressing positive and negative emotions during different modes of social contact (in-person, phone), and the association between emotion expression and well-being throughout the day. Community-dwelling older adults aged 65 - 90 (N = 286, Mage = 73.95) from the Daily Experiences and Well-being Study completed ecological momentary assessments (EMA) during which they indicated their social contact as well as positive and negative mood every 3 hours for 5 to 6 days. Participants also wore Electronically Activated Recorders (EAR) to unobtrusively capture ambient sounds. Sound snippets that contained participants' speaking (N = 30,311) were transcribed verbatim and processed using the Linguistic Inquiry and Word Count (LIWC) to obtain linguistic features pertaining to emotion expression. Multilevel models revealed that in-person and phone contact were both associated with an increased use of positive words (e.g., love, sweet), yet only in-person contact was associated with more words expressing negative feelings (e.g., worried, sad). Using both positive and negative words was associated with a better mood in the same 3 hours. No associations were found between either positive or negative words and negative mood. Findings suggest that older adults may benefit emotionally from expressing both positive and negative feelings during in-person contact. Interventions for older adults' emotional well-being may focus on fostering emotionally meaningful social ties and providing in-person contact.

Abstract citation ID: igae098.3229

### EFFECTS OF SOCIAL ISOLATION ON THE WELL-BEING AND COPING MECHANISMS OF OLDER ADULTS IN BISHOFTU TOWN, ETHIOPIA

Messay Kotecho[1], Abraham Kassay[1], and Margaret Adamek[2], *1. Addis Ababa University, Addis Ababa, Adis Abeba, Ethiopia*, *2. Indiana University Indianapolis, Indianapolis, Indiana, United States*

Research concerning older adults is gaining attention in Ethiopia. Nevertheless, previous research has disregarded the issue of social isolation among elders in Ethiopia. The prevalence of social isolation among older adults is now a significant concern worldwide due to its impact on both individuals and society at large. This study examined how social isolation affects the well-being of older adults and their coping mechanisms, focusing specifically on the experiences of older adults in Bishoftu Town, Ethiopia. A phenomenological approach was used to investigate the experiences of 10 older adults (5 male and 5 female) aged 60 and above, purposefully selected for in-depth interviews and observations. The results indicate that participants are increasingly susceptible to social isolation due to factors such as negative perceptions of aging, loss of meaningful relationships, economic hardships, and age-related health issues. Consequently, elders experience a reduction in social connections, health issues, psychological distress, and challenges in meeting basic needs. Despite these obstacles, older adults have developed various coping strategies, including engaging in prayer and spiritual practices, depending on intergenerational support, participating in traditional savings associations (Idir), and sleeping excessively. Nonetheless, many participants express feelings of pessimism, weakness, lack of affection, and a sense of worthlessness about their future. Additionally, participants report facing disengagement and a loss of social ties alongside grappling with severe poverty and inadequate access to healthcare services. The study underscores the urgent need to bolster intergenerational solidarity and social capital within the community to tackle the multifaceted challenges confronting older adults in Ethiopia.

Abstract citation ID: igae098.3230

### FAMILISM MODERATES RELATIONS BETWEEN SOCIAL NETWORK AND COGNITION IN MIDLIFE LATINE ADULTS

Adrienne Grudzien, Samantha Gonzales, Shanna Burke, Celine Cambara, Alexandra Briceno, Daniel Jimenez, Stephanie Scott, and Sabrina Sales Martinez, *Florida International University, Miami, Florida, United States*

Larger social network size, a measure of social isolation, has been linked to better cognitive function in older adults. Research indicates that stress-related immune and endocrine pathways may activate in socially isolated adults and negatively impact brain tissue. This study examined whether Latine adults reporting poorer social network quality but holding familial cultural values that prioritize closeness and loyalty in the family group experienced worse cognitive function. Cross-sectional multiple regression and moderation analyses were used to evaluate the influence of familism (via MACVS Obligations and Support scale) on relations between Lubben Social Network Scale score and National Alzheimer's Coordinating Center cognitive domains in midlife Latine adults (n=51). Participants were predominantly female (92.16%), white (80.39%), and married or partnered (62.75%). The mean age of this sample was 52.84 (SD=4.89) years. Differential interaction effects were identified after adjusting for age, sex, race, income, and education level: familism strengthened relations between social network and episodic memory ($\beta$ =.112, p = 0.019), and semantic memory ($\beta$ =.059, p = 0.022) and weakened those with visuospatial ($\beta$ =-.017, p = 0.044), executive function ($\beta$ =-.04, p = 0.05), and verbal fluency ($\beta$ = -.075, p = 0.046). These findings suggest that familism may be protective of visuospatial, executive function, and fluency domains, while semantic and episodic memory are more vulnerable in familism-aligned but socially isolated, aging Latine adults. Further evidence is necessary, however, to support the development of targeted interventions and support systems addressing cognitive health disparities in Latine adults.

Abstract citation ID: igae098.3231

### GENDER DIFFERENCES IN COVID-19'S EFFECTS ON LONELINESS, ISOLATION, AND COGNITIVE HEALTH AMONG TAIWANESE RETIREES

Wan-Chen Hsu, Nuan-Ching Huang, and Susan C. Hu, *National Cheng Kung University, Tainan, Tainan, Taiwan (Republic of China)*

**Objectives:** This study investigates the impacts of lock-down policies during the COVID-19 pandemic on the loneliness, social isolation, and cognitive function of retirees in Taiwan, focusing on the differences between men and women. Method: We conducted a two-stage stratified sampling survey to collect 1,115 valid retirees aged 50-74 in Taiwan in early 2023. A mixed-methods approach, combining focus groups and quantitative analysis, was employed. Loneliness was evaluated using the UCLA Loneliness Scale, and social isolation was measured through interaction with family members and social participation. Cognitive function was assessed using the Saint Louis University Mental Status. The statistical analysis included network analysis and multinomial logistic regression.

**Results:** This study revealed that approximately 30% of participants experienced loneliness during the COVID-19 pandemic, and about 20% of men and women reported less time allocated to volunteering and group activities. Although regression analysis showed no gender differences in loneliness scores and cognitive functions, network analysis found gender-specific relationships among related factors and sub-item of cognitive tests. For example, women's feelings of loneliness negatively correlated with geometric judgment performance, while men showed a positive correlation between orientation test scores and volunteer involvement. Qualitative research further underscored the crucial role of community support in facilitating recovery from the pandemic, particularly for individuals who were less engaged in community activities.

**Discussion:** This study highlights the need to explore gender-specific correlates of loneliness and isolation across different cognitive items and emphasizes strengthening community support for post-pandemic recovery.

Abstract citation ID: igae098.3232

### HOW DOES DIGITAL USE AFFECT THE DAILY ACTIVITIES AND LONELINESS AMONG CHINESE OLDER ADULTS?

Xiayu Chen[1], Zhan Yu[2], and Qingwen Xu[3], *1. University of Illinois Urbana-Champaign, Champaign, Illinois, United States*, *2. East China Normal University, Shanghai, Shanghai, China (People's Republic)*, *3. New York University, New York, New York, United States*

The increasing integration of digital technology into everyday life has significant implications for the aging population. This study explores the relationship between Internet use and the psychosocial well-being of older adults, particularly examines whether digital engagement would have replaced (or enhanced) traditional activities and social interactions, and to what extent digital engagement has contributed to alleviating loneliness among older adults within the Chinese community-dwelling context. Utilizing data from the Chinese General Social Survey, which included 4,283 respondents aged 60 and over, we performed various regression models to examine the relationship among Internet use and daily activities, as well as Internet use and loneliness. Our research reveals the digital divide within the older population in China, where Internet users were the majority those with access to resources and learning and physical capacity, suggesting a deeper concern for technology equity. Our findings also indicate that Internet use among older adults is associated with increased participation in traditional activities, such as shopping, information seeking, and socializing with friends and relatives. Additionally, a higher intensity of Internet use correlates with lower levels of reported loneliness, indicating that Internet use could enhance social connections and overall well-being among older adults. These results highlight the potential of digital technologies to support active aging by fostering enhanced social interactions and engagement in daily life. It calls for developing holistic digital integration strategies and creating an age-friendly digital environment. The findings also provide valuable insights for policymakers and stakeholders in China and potentially in other contexts.

Abstract citation ID: igae098.3233

### LONELY AND SOCIALLY DISCONNECTED OLDER ADULTS ARE AT HIGHER RISK FOR PREVALENT AND INCIDENT DEMENTIA

Carmen Chek[1], Katherine Knauft[1], Ariana Stickel[2], Carlos Araujo-Menendez[3], Hector Gonzalez[4], and Wassim Tarraf[1], *1. Wayne State University, Detroit, Michigan, United States*, *2. San Diego State University, San Diego, California, United States*, *3. SDSU/UC San Diego, San Diego, California, United States*, *4. University of California San Diego, La Jolla, California, United States*

A 2020 report from the National Academies indicates that nearly one in four adults over age 65 are lonely. Loneliness has important health implications, including for cognitive aging. However, loneliness might not manifest uniformly in the population. Herein, we address a gap in the existing literature by examining the link between heterogeneities in the risk of loneliness and dementia outcomes. We used data from n = 8,805 adults aged 65+ from the Health and Retirement Study (average age 74.7; 56.3% women) to estimate latent classifications of loneliness (11 items from the UCLA loneliness index) and tested, using generalized linear regression models, whether different manifestations of loneliness were differentially associated with dementia (Langa-Weir criteria) prevalence and incidence (over 3-waves of data). A four-class solution best fits the data: "No/Low loneliness" (NL; 51.6%); "Socially disconnected" (SD; 18.0%); "Lonely but not socially disconnected" (LNSD; 12.6%), and "Lonely and socially disconnected" (LSD; 17.8%). Notably, these classifications and their prevalence rates were largely consistent across waves. Adjusting for sociodemographic covariates, compared to individuals in the NL group, the LNSD (OR = 1.68; 95%CI = 1.22;2.32) and LSD (OR = 1.71; 95%CI = 1.33;2.20) groups were more likely to meet criteria for prevalent dementia at baseline. The LSD group was also more likely to meet criteria for incident dementia at follow-ups (within 2 and 4 years from baseline). Loneliness is not uniformly distributed in the population and its subtypes confer differential risk for dementia outcomes. Focusing on the subtypes can potentially pinpoint targetable groups for interventions.

Abstract citation ID: igae098.3234

### NEIGHBORHOOD AND PSYCHOSOCIAL PREDICTORS OF ALLOSTATIC LOAD AMONG LATINX ADULTS IN THE UNITED STATES

Angela Gutierrez[1], Adil Supiyev[2], Courtney Thomas Tobin[3], Barış Sevi[4], and Graciela Muniz Terrera[5], *1. Western University of Health Sciences, Pomona, California, United States*, *2. Ohio University Heritage College of Osteopathic Medicine, Athens, Ohio, United States*, *3. University of California Los Angeles, Los Angeles, California, United States*, *4. MEF University, İstanbul, Istanbul, Turkey*, *5. Ohio University, Athens, Ohio, United States*

Prior research highlights the independent roles of neighborhood and psychosocial risk and protective factors for accelerated physiological aging. However, the combined role of risk and protective neighborhood and psychosocial factors on allostatic load (AL) among middle-aged and older Latinx adults in the U.S. remains unclear. Utilizing data from Latinx adults ages 50 and older (n = 319) in the Health and Retirement Study (Time 1 = 2006/2008; Time 2 = 2010/2012; Time 3 = 2014/2016), this study aimed to examine the role of neighborhood (perceived physical disorder, neighborhood social cohesion) and psychosocial (loneliness, social support) factors in shaping baseline AL and change in allostatic load. Linear mixed models were used to estimate baseline AL and rate of change in AL, adjusting for sociodemographic factors. Higher loneliness was associated with higher baseline AL, p = 0.028. This association persisted when considering neighborhood factors, p = 0.025. When social support was added to the model, the association between loneliness and AL was attenuated and no longer statistically significant, p = 0.174. Findings provide evidence of a positive association between loneliness and baseline allostatic load. Finding also highlight social support as a protective factor for baseline AL among Latinx adults. Health promotion efforts should consider implementing culturally relevant approaches to foster social support and to promote healthy aging among Latinx adults.

Abstract citation ID: igae098.3235

### THE EQUITABLE AGING IN HEALTH CONCEPTUAL FRAMEWORK: CASE STUDIES OF SOCIAL ISOLATION AND LONELINESS

Angela Perone[1], Leyi Zhou[1], Mo'e Yaisikana[1], and Leixuri Urrutia-Pujana[2], *1. University of California Berkeley, Berkeley, California, United States*, *2. University of the Basque Country, Bilbao, California, Spain*

Research has well-documented health disparities associated with social isolation and loneliness among older adults. However, much of this research focuses on the United States, and conceptual and theoretical frameworks incorporating power are underdeveloped. This poster presents a new conceptual framework that bridges theories of power and social and political determinants of health to provide a new vision for developing interventions that incorporate power and justice from a global perspective. The Equitable Aging in Health Conceptual Framework foregrounds power as a core driver of social and political determinants of health and the interventions addressing health disparities that flow from power inequities. We argue that interventions that infuse justice must inherently address power, and this conceptual framework aims to help researchers, policymakers, and practitioners accomplish that objective. To illustrate the utility of this conceptual framework, this poster presents three case studies. The first case study applies this conceptual framework to loneliness among rural indigenous older adults in Taiwan. The second case study applies this framework to social isolation among urban community-dwelling older adults in mainland China. The final case study applies this framework to social isolation and loneliness among nursing home residents in Spain. These case studies illustrate how this framework can serve as a tool to visually identify and map how five domains of power can shape social and political determinants of health and a blueprint for conceptualizing interventions that infuse justice.

Abstract citation ID: igae098.3236

### THE ROLE OF LATE-LIFE SOCIAL PARTICIPATION IN THE RISK OF DEMENTIA: A LONGITUDINAL STUDY

Xi Wang[1], Sita Lujintanon[1], Pablo Martinez Amezcua[1], and Jennifer Schrack[2], *1. Johns Hopkins University, Baltimore, Maryland, United States*, *2. Johns Hopkins Bloomberg School of Public Health, Baltimore, Maryland, United States*

Social connectedness may prevent dementia in community-dwelling older adults. However, few studies have long-term follow-up and repeated assessments of social participation. We analyzed the data of adults aged 65 years and over in the United States from the National Health and Aging Trends Study (n = 5,893) from 2012 to 2019. We assessed social connectedness annually with questions about engagement in various activities ("Yes"/"No"), including visiting family/friends, attending religious services, organized activities, and going out for enjoyment. We created a binary composite variable indicating participation in at least one of the activities. Incident dementia was defined using the NHATS dementia algorithm (reported dementia diagnosis, AD8, and neurocognitive test results). We estimated the longitudinal association between social participation and dementia using generalized estimating equation models adjusted for characteristics measured in 2011 (one year before the first exposure assessment), including age, sex, race, marital status, educational attainment, household income, depressive symptoms, and self-rated health. Composite social participation was associated with lower odds of dementia (odds ratio [OR]: 0.41; 95% confidence interval [CI]: 0.31, 0.54). Individually, visiting family/friends (0.53; [0.45, 0.63]), attending religious services (0.64 [0.55; 0.75]), participating in organized activities (0.74; [0.61, 0.89]), and going out for enjoyment (0.38; [0.32, 0.45]) were all associated with lower odds of dementia. Among community-dwelling older adults in the United States, multiple forms of social participation may be targets for dementia prevention interventions or serve as early markers of dementia risk.

### SESSION 7545 (POSTER)

### WORKFORCE

Abstract citation ID: igae098.3237

### ASSOCIATIONS BETWEEN NURSING HOME WORKPLACE SAFETY CULTURE AND PATIENT SAFETY CULTURE

Brandon Hesgrove, Emily Tyler, Naomi Yount, Joann Sorra, and Susan Hassell, *Westat, Rockville, Maryland, United States*

An influential report from the Institute for Healthcare Improvement purports that a strong workplace safety culture is a precondition of a strong culture of safety in healthcare. However, existing empirical evidence is limited. We used data from a 2023 pilot test of the Workplace Safety Supplemental Item Set administered with the Surveys on Patient Safety Culture (SOPS) Nursing Home Survey to examine associations between all 9 workplace safety culture and all 14 resident safety culture measures. Data were obtained from 2,515 staff (40% response rate) in 48 nursing homes across 20 states. Associations were estimated using multiple linear regressions, where, because of strong multicollinearity, the dependent variable for each regression was one patient safety culture measure and the independent variables were one workplace safety culture measure, nursing home bed size, and ownership. The analysis was at the nursing home level and nursing home percent positive scores were calculated as the percentage of staff that answered with the two most positive response options (out of five). 123 out of 126 associations were statistically significant ($p < 0.05$). The two workplace safety culture measures most highly associated with resident safety culture were Management Support for Workplace Safety (mean $\beta = 0.78$) and the Overall Rating on Workplace Safety for Staff (mean $\beta = 0.73$). The very strong associations between resident and workplace safety culture measures imply that the two are interconnected and improving a culture of safety for nursing home staff will also improve safety for residents.

Abstract citation ID: igae098.3238

### ECONOMIC INSECURITY DURING THE COVID-19 PANDEMIC AMONG HEALTH CARE WORKERS BY EDUCATIONAL ATTAINMENT

Tracy Mroz, Ben Dunlap, and Bianca Frogner, *University of Washington, Seattle, Washington, United States*

The COVID-19 pandemic had unprecedented impacts on healthcare workers. Prior to the pandemic, healthcare workers in lower skilled jobs (e.g., nursing assistants, home care aides) versus higher skilled jobs (e.g., physicians, nurses) were more likely to experience economic insecurity. We examined economic insecurity among healthcare workers between July 2021 and August 2023 via secondary analysis of Household Pulse Survey data. We used multivariable logistic regression to investigate the relationship between economic insecurity and educational attainment as a proxy for higher versus lower skilled workers, controlling for other sociodemographic characteristics. Of the 108,206 respondents who reported working in healthcare, representing a weighted sample of 10,656,917 healthcare workers, 49.5% had less than an associate degree, 10.0% had an associate degree, 21.7% had a bachelor's degree, and 18.9% had a master's degree or greater. Among workers with less than an associate degree, associate degree, bachelor's degree, and master's degree or higher: an estimated 78.7%, 69.9%, 56.1%, and 40.0% reported difficulty with usual expenses; 53.6%, 42.7%, 27.6%, and 17.4% reported food insufficiency; and 11.1%, 8.1%, 4.6%, and 2.8% reported being behind on their rent or mortgage payments, respectively. Lower educational attainment was significantly associated with greater economic insecurity across all measures. This relationship was attenuated, but still significant, after controlling for other sociodemographic characteristics. Lower skilled workers are critical to the healthcare workforce, especially in long-term care. Policies to promote economic security among these workers are needed to ensure a robust workforce is available to care for older adults.

Abstract citation ID: igae098.3239

### NURSING HOME WORKER MENTAL HEALTH AND TURNOVER INTENTION AMONG PARENTS

Katherine Kennedy[1], David C. Mohr[2], and Whitney Mills[3], *1. VA Providence Healthcare System, Center of Innovation in Long-Term Services and Supports, Providence, Rhode Island, United States*, *2. Veterans Health Administration, Mason, Ohio, United States*, *3. VA Providence Health Care System, VA Providence Health Care System, Rhode Island, United States*

Nursing home (NH) staff job dissatisfaction and turnover are associated with lower care quality. However, little is known about the impact of staff mental health, parenthood, and marital status. Guided by the Job Demands-and-Resources Model (assumes positive effects of job resources and negative effects of job demands on individual and work outcomes), we compared psychological distress and job satisfaction for single parents and married parents. NH employee and manager data from Wave 4 (2011-12) of the Work Family Health Network study were combined (N=1,144) to define parents (N=682). Strata were compared on demographics, job characteristics, turnover intention, job satisfaction, and psychosocial health using bivariate tests and logistic regression models. Single parents comprised 51.8% (n=353) of the sample. In bivariate tests, single parents reported higher turnover intention and psychological distress as well as lower job satisfaction and social support than married parents ($p<.001$). Turnover intention was more likely endorsed when staff reported greater psychological distress (OR=1.07, 95% CI: 1.03, 1.12, $p<.01$). Distress was more likely with increasing work-to-family conflict (OR=1.68, 95% CI: 1.22, 2.31, $p<.01$) and increasing family-to-work conflict (OR= 1.78, 95% CI: 1.21, 2.63, $p<.01$). Distress was less likely with higher household income (OR= 0.92, 95% CI=0.87, 0.94, $p<.05$). Additional research is needed to understand and develop strategies to mitigate psychological distress and increase job resources among single parents to improve NH worker outcomes.

Abstract citation ID: igae098.3240

### OUTCOMES ASSOCIATED WITH PARTICIPATION IN THE ILLINOIS WORKPLACE WELLNESS STUDY

Laura Payne[1], Do Yun Kim[2], Jaesung An[3], Megan Janke[4], Elizabeth Orsega-Smith[5], Julie Bobitt[6], and Neha Gothe[7], *1. University of Illinois Urbana-Champaign, Urbana, Illinois, United States*, *2. University of Illinois Urbana-Champaign, Champaign, Illinois, United States*, *3. California State University East Bay, Hayward, California, United States*, *4. Berry College, Rome, Georgia, United States*, *5. University of Delaware, Newark, Delaware, United States*, *6. University of Illinois Chicago, Chicago, Illinois, United States*, *7. Northeastern University, Boston, Massachusetts, United States*

Workplace wellness (WW) programs assert health benefits; however, most available evidence is based on observational studies comparing participants to non-participants

(Reif et al., 2020). Experiences and outcomes associated with WW program participation may vary by the age. This study examined factors that shaped participation among younger (under 50) and older employees (age 50+) in a workplace wellness program. Using data from the Illinois WW Study (N=4,834), we examined several health behaviors (physical activity, smoking, alcohol use), participation rates, and presenteeism. Participants were randomly assigned to treatment and control groups; they completed a baseline survey and annual follow-up surveys for three years. Physical activity (PA) was measured with three questions (compared to other people your age are you more active, about the same, less active? Are you trying to increase your PA? In the last 12 months, has your doctor told you to increase your PA?). Smoking and alcohol use were assessed with the number of days smoked/drank in a week and the number of smokes/drinks/day. Presenteeism was measured with the 6-item Stanford presenteeism scale. The 50 and over age group scored significantly higher (M = 3.31, SD = 0.63) than the under 50 group (M = 3.07, SD = 0.70) on the composite health behavior score. For all four study periods (i.e., fall 2016, spring 2017, fall 2017, and spring 2018), there was no significant association between age and workplace wellness program participation. Results show no significant difference in absenteeism between the control and treatment group over the study period.

Abstract citation ID: igae098.3241

### THE EXPERIENCES OF DISCLOSING ALZHEIMER'S DISEASE IN THE US WORKPLACE

Allison Gibson[1], Kathryn Coccia[2], and Lauren Hasten[3], *1. Saint Louis University, St Louis, Missouri, United States, 2. Saint Louis University, Chicago, Illinois, United States, 3. Saint Louis University, St. Louis, Missouri, United States*

With the shift of aging adults delaying retirement, more individuals are being diagnosed with Alzheimer's disease and related dementia (ADRD) while still in the workforce. Using a modified grounded theory approach, 16 people living with ADRD were interviewed about their experience receiving a diagnosis while still in full-time employment (mean age 66). Thematic analysis was employed to examine the study's aims with member checking and an audit trail to enhance study credibility and trustworthiness. Many study participants shared their diagnosis with employers, resulting in positive experiences. Study findings indicated that participants were more likely to disclose their diagnosis to their employer when they felt supported by their supervisor, perceived their employer would offer them reasonable accommodations to continue working, or felt they were well positioned to retire. Those who did not disclose their diagnosis did so because they feared reasonable accommodations were either not available or accessible, and they reported significant financial stress. Study implications suggest those newly diagnosed need support regarding ADRD and employment, and human resources personnel need more training on ADRD workers. While not all participants were able to continue in the workforce following diagnosis, many could through flexible work roles and supportive environments. Many study participants were able to continue in their full-time positions until they retired or elected to shift employment to a new role or employer. However, some experiences highlight the continued challenges of accessing disability resources. This presentation will conclude with recommendations for specialized policies, training, and future research.

Abstract citation ID: igae098.3242

### THE IMPACT OF AGE-SPECIFIC PSYCHOLOGICAL SELF-SUFFICIENCY ON ECONOMIC WELL-BEING AMONG OLDER WORKER JOB SEEKERS

Mina Lee[1], Hyesu Yeo[2], and Philip Hong[2], *1. Binghamton University, Binghamton, New York, United States, 2. University of Georgia, Athens, Georgia, United States*

**Aim:** Federal and state workforce policies prioritize Economic Self-Sufficiency (ESS) but often overlook crucial psychological dimensions. Psychological Self-Sufficiency (PSS) provides an empowerment framework, especially for low-income jobseekers, integrating psychological and economic well-being. While PSS represents a transformative path from Employment Barrier (EB) to Employment Hope (EH) and positively impacts ESS, the age-specific impact of PSS measures on economic well-being remains unexplored. This study validates PSS measures and investigates their relationship with ESS among older worker job seekers.
**Methods:** Data were collected from 1006 participants aged 50+, engaged with community-based agencies offering employment training services (2012-2016. Exploratory Factor Analysis (EFA) and Confirmatory Factor Analysis (CFA) identified older-worker PSS using 27 EB and 24 EH items. ESS was assessed using the 15-item WEN ESS scale. Structural Equation Modeling (SEM) examined the relationship between older-worker PSS and ESS pre- and post-program participation.
**Results:** EFA and CFA revealed a 16-item 4-factor EB and a 15-item 3-factor EH structure. EB factors included human capital, personal balance, physical and mental health, and labor market exclusion barriers, while EH factors encompassed self-assurance, skill and resource utilization, and goal orientation. SEM indicated significant PSS, highlighting the transformative path from EB to EH, significantly affecting ESS with EH fully mediating EB.
**Conclusion:** This study highlights the distinct age-specific composition of PSS factors among low-income older workers, emphasizing the need for age-friendly intervention strategies by workforce development practitioners. By enhancing psychological self-sufficiency, these interventions can contribute to the economic well-being of older workers in the context of employment transition.

Abstract citation ID: igae098.3243

### THE IMPACT OF THE TEACHING NURSING HOME ON THE NURSING HOME WORKFORCE

Howard Degenholtz[1], Kenna Campbell[1], and Anny Treat[2], *1. University of Pittsburgh, Pittsburgh, Pennsylvania, United States, 2. Sebastian Mann Associates, Pittsburgh, Pennsylvania, United States*

The Teaching Nursing Home (TNH) is designed to improve the long-term care workforce and improve quality of care in nursing homes. The initiative has multiple components: enhanced clinical rotations for nursing students with partner schools of nursing, implementation of the Institute for Healthcare Improvement Age-Friendly Health System “4M” quality improvement model, and an online learning network. CMS Payroll Based Journal (PBJ) data were analyzed using a propensity weighted difference in difference model to determine if there was an effect on attrition or staffing levels. Staff satisfaction and burnout surveys were conducted at participating nursing homes in the winter of 2022 using a combination of web-based and in-person paper administration. Follow-up surveys were collected in 2023. Response rates ranged from 14% to 54%. A total of 190 surveys were available at baseline, and 183 at follow-up. All surveys were anonymous; cross-sectional analyses were conducted to identify trends. The findings for burnout were mixed. Emotional exhaustion decreased, but depersonalization increased and personal accomplishment was unchanged. Overall job satisfaction was unchanged but the management and leadership component of fob satisfaction declined. Attrition among registered nurses, licensed practical nurses and certified nurse assistants was lower than among matched facilities in the same market areas. After the first year of implementation, the TNH program is showing mixed benefits in terms of burnout and job satisfaction, but some benefit in terms of reducing turnover. As the Age-Friendly Nursing Home program is disseminated across the US, it is important to track the impact on the workforce.

Abstract citation ID: igae098.3244

### THE ROLE OF PSYCHOLOGICAL CAPITAL IN MODULATING CAREER ADAPTABILITY AND TURNOVER AMONG NURSING ASSISTANTS

Changxian Sun[1], and Yurong Xing[2], *1. Jiangsu Vocational Insititue of Commerce, Nanjing, Jiangsu, China (People's Republic)*, *2. Nanjing Medical University, Nanjing, Jiangsu, China (People's Republic)*

**Background:** High turnover intention of nursing assistants was detrimental to the sustainability of long-term care. Career adaptability is an important determinant in reducing turnover intention, but little research has explored the mechanism from the perspective of psychological capital. The aim of this study was to analyze the association between career adaptability and turnover intention and to examine the mediating role of psychological capital between career adaptability and turnover intention among nursing assistants in mainland China.

**Methods:** A cross-sectional online study was conducted among 276 nursing assistants from eight nursing homes in Nanjing, China. The participants' career adaptability, psychological capital, and turnover intention were obtained. SPSS 26.0 and Amos 24.0 software were employed for statistical analysis.

**Results:** Career adaptability was positively related to psychological capital and negatively linked to turnover intention ($P < 0.01$). Psychological capital played a fully mediating role ($\beta = -0.085$, $P < 0.05$) in the relationship between career adaptability and turnover intention, and the largest indirect effect was generated through the curiosity dimension. Conclusions: The management of long-term care facilities should focus on assessing the level of career adaptability of nursing assistants. The overall improvement of career adaptability and psychological capital is conducive in reducing turnover intention. Targeted interventions are recommended to improve career adaptability and reduce turnover intentions by increasing career curiosity. Online career adaptability programs can be developed for nursing assistant students to improve their psychological capital and facilitate career transitions.

### SESSION 7600 (POSTER)

### ADVOCACY

Abstract citation ID: igae098.3245

### COMPONENTS OF SUPPORTED DECISION-MAKING BY ADULT GUARDIANS FOR ADULTS UNDER GUARDIANSHIP IN JAPAN

Sachiko Kasahara, *Shitennoji University, Habinko, Osaka, Japan*

The adult guardianship system in Japan is designed to protect the rights and property of elderly individuals or those with diminished decision-making capacity. The system also allows for property management and personal care considerations. Adult guardians include relatives and non-relative guardians, but in Japan non-relative guardians account for over 80% of the total. For non-relative adult guardians who often meet the individual after being appointed as their guardian, respecting the individual's wishes is an extremely important task in guardianship activities. The purpose of this research is to visualize the components of supported decision-making by non-relative guardians. The previous studies indicated that it was important to know what the specifics of supported decision-making for people under wards. The research was conducted from September to October of 2022 by self-administered questionnaires. The survey was mailed to 1,180 non-relative adult guardians in Japan. The data was collected Multistage sampling. The response rate was 25.2%. The statistical analysis was an exploratory factor analysis. Three factors in the specifics of supported decision-making were extracted by the factor analysis: Supporting for forming, expressing, and realizing of their wishes of the person under wards; Assessment and relationship with them; and Supporting by a team. The Cronbach's alpha for internal consistency was greater than 0.8 for each factor. In addition, the three factors were associated each other. In conclusions, the present study found that the specifics of supported decision-making were Supporting their wishes as process, Preconditions for supported decision-making, and Supporting as a team.

Abstract citation ID: igae098.3246

### DISCRIMINATION AND RESILIENCE ON HEALTH ACROSS THE LIFESPAN

Yeonjung Lee[1], Cindy Vang[2], and Youhung Her-Xiong[3], *1. University of Hawai'i at Mānoa, Honolulu, Hawaii, United States*, *2. California State University Chico, Chico, California, United States*, *3. Q&C Research Consulting, LLC, Eau Claire, Wisconsin, United States*

Asian and Asian American (AAA) older adults have encountered varying forms of discrimination in the United

States. However, discrimination across the life course shaping the lives of AAA is an emerging area in understanding health disparities. This study aimed to shed light on this area by using the Life Course Perspective and the Asian Critical Theory to characterize the levels of discrimination, resilience, and health of AAA older adults before and after the COVID-19 pandemic. The preliminary data were collected through convenience and snowball sampling methods. Participants (N=39) completed a survey using discrimination, resilience, and health measures before and during the pandemic. Descriptive results indicated increased perceived mild discrimination post-pandemic compared to the pre-COVID-19 pandemic. The findings, however, indicate that participants showed increased resilience during the COVID-19 pandemic. Based on the preliminary data focusing on hard-to-reach older adults, the present study findings can help understand the unique association between discrimination and resilience experiences among AAA older adults.

Abstract citation ID: igae098.3247

### EXAMINING GUARDIANSHIP RISK OF THE UNBEFRIENDED UTILIZING A SOCIOECOLOGICAL MODEL TO IMPROVE ADVOCACY AND POLICY

Solange Marcel, *Hunter College, CUNY, New York, New York, United States*

**Background:** Adults without decisional capacity, having no surrogate, are known as unbefriended persons and are often referred for guardianship. Guardianship is meant to serve as a means of protection; however, a guardian's decision-making authority is conversely a threat to autonomy. The aim of this literature review is to examine the prevalence of unbefriended persons at risk for guardianship while exploring the gaps, trends, and inconsistencies related to healthcare provider and/or institutional knowledge deficits.

**Methodology:** A literature review was performed using electronic databases CINAHL, PubMed, Medline, and PsychInfo. Publication parameters were set between 2010 and 2024. Additionally, relevant articles with a special medicolegal focus were hand-selected without publication date restrictions.

**Results:** Search terms resulted in 258 articles for title and abstract screening. Utilizing the socioecological model, 17 studies were synthesized. Neurocognitive disorders and psychotic disorders were intrapersonal antecedents. Social isolation increased vulnerability at the interpersonal level. Organizational factors included inadequate healthcare knowledge. Lack of guardian access was a notable environmental factor. At the policy level, health facilities lacked effective policies to promote ethical and timely decision-making.

Conclusions: At each socio-ecological level, there are vastly different experiences for the unbefriended compared to decisional-incapacitated persons with identified surrogates. These differences should be the impetus for future education and serve as a springboard for advocacy initiatives. A multidisciplinary approach consisting of clinicians, healthcare ethics committees, and the guardianship system is compulsory to address the evolving needs of the unbefriended.

Keywords: unbefriended, unrepresented, persons without proxies (PWP), decisional capacity, incapacitation, guardianship, and surrogates.

Abstract citation ID: igae098.3248

### RECONSIDERING MANDATORY RETIREMENT FOR POLITICIANS: A MULTIDISCIPLINARY APPROACH

Roy Tzemah-Shahar[1], and Aviad Menashe[2], *1. University of Haifa, Haifa, HaZafon, Israel, 2. The Open University, Israel, Ra'anana, HaMerkaz, Israel*

The upcoming 2024 U.S. presidential election has ignited a widespread discourse on the age of candidates; globally, the political arena has witnessed seasoned leaders surpassing the age of 80 and continuing to hold office. The advanced age of politicians raises multifaceted questions across cultural, social, legal, political, and gerontological research fields. Is there a case for considering mandatory retirement age for politicians, and under what circumstances? We review available literature, federal and state laws, media reports (e.g., social networks), and health research focusing on the impact of age on work-related performance. Moreover, we explore theoretical and practical aspects of age and ageism in the workforce. The debate surrounding the advanced age of politicians has intensified in recent decades: older politicians may hinder the rise of new generations and raise concerns about their physical or cognitive ability in old age; however, these leaders are often perceived as experienced and well-connected, providing an irreplaceable wealth of knowledge. While retirement age varies globally, only a few countries have set mandatory retirement age for politicians. Recent health studies suggest that chronological age alone is insufficient to determine an individual's ability to participate in the workforce; however, there are no objective criteria other than chronological age for mandatory retirement of most professions, including politicians. There is no consensus on ability or disability of older politicians to perform adequately, but their experience remains unparalleled. Physical and cognitive function, as reflected in biological age metrics, may serve as an alternative to chronological age concerning mandatory retirement.

Abstract citation ID: igae098.3249

### USING CANCER SURVIVORSHIP EXPERIENCES TO INFORM CANCER ADVOCACY IN NIGERIA

Chizobam Nweke[1], Chinelo Nduka[2], Runcie Chidebe[3], and Candi Nwakasi[1], *1. University of Connecticut, Storrs, Connecticut, United States*, *2. Nnamdi Azikiwe University Teaching Hospital, Nnewi, Anambra, Nigeria*, *3. Miami University, Oxford, Ohio, United States*

**Background:** Despite having a high cancer burden that is still rising, Nigeria, like many rapidly aging countries in sub-Saharan Africa, due to a very high burden of infectious diseases, is not paying adequate attention to non-communicable diseases, especially cancer. This study explored the experiences of cancer survivors for informing tailored cancer advocacy programs in Nigeria.

**Methods:** A qualitative descriptive approach was used on a sample of 19 women with age ranging from 29 – 55 years, with a history of breast cancer. We had four focus group discussions during data collection and used reflexive thematic analysis for data analysis.

**Results:** The following themes were identified from the analysis: 1) strengthening care quality, 2) addressing delayed presentation, 3) expanding access to informal support, and 4) alleviating the cost burden of cancer care.
**Discussion:** A better understanding of cancer survivorship experiences among Nigerian women can help strengthen cancer advocacy in Nigeria. In turn, strong cancer advocacy will help to prioritize cancer control, and mitigate a looming cancer crisis in Nigeria.

### SESSION 7605 (POSTER)

### AGEISM

Abstract citation ID: igae098.3250

### "ACT YOUR AGE!" YOUNG ADULTS' CONCERN REGARDING PRESCRIPTIVE AGE STEREOTYPE VIOLATION

Amy Gourley, and Alison Chasteen, *University of Toronto, Toronto, Ontario, Canada*

A younger subjective or 'felt' age is often associated with positive health outcomes among adults over 60. However, a dearth of research examines young adults' attitudes towards older people who report feeling 'young at heart'. The degree to which a young adult feels concerned about violations of prescriptive age stereotypes (i.e., that older people should act their age) may predict their reactions to older adults who feel young at heart. The present work examines young adults' (N = 296) attitudes toward older vignette characters described as feeling either their age (65), 45, or 25. Participants evaluated one of six possible characters and completed a measure of their concern that the character would violate prescriptive age stereotypes (e.g., "I am concerned that older people like the person described in the vignette will come to places where I like to hang out."). Participants were more concerned that characters who felt 25 or 45 would violate prescriptive age stereotypes compared to characters who felt 65. Moreover, concern about characters' potential stereotype violation predicted participants' attitudes toward the character. Specifically, the more concerned participants felt, the lower they rated characters' competence, particularly when the character felt 25 or 65. These findings suggest that older adults' felt ages and younger adults' concerns about preserving strict age-group boundaries may be key predictors of evaluations of older people. To develop interventions to reduce ageism, more work must consider younger people's reactions to older adults' felt ages as well as their concern regarding prescriptive age stereotypes.

Abstract citation ID: igae098.3251

### AGE-RELATED VALUE ORIENTATIONS IN LARGE LANGUAGE MODELS (LLMS)

Xin Zhang, Yuanyi Ren, and Guojie Song, *Peking University, Beijing, Beijing, China (People's Republic)*

Large Language Models (LLMs) are transforming diverse fields and gaining increasing influence as human proxies. This development underscores a critical scientific need to evaluate their value orientations and understanding, which are major determinants of individual and collective human behavior. We used a newly developed evaluation pipeline grounded in realistic human-AI interactions to probe age-related value orientations (e.g., hostile and benevolent ageism) of six different LLMs (including GPT-3.5, GPT-4, Llama2-7B, Llama2-70B, Mistral-7B and Mixtral-8x7B). The results indicated that compared with human participants, these Large Language Models exhibited significantly less hostile ageism in terms of both descriptive and prescriptive ageism, while these Large Language Models exhibited a similar level of benevolent ageism to the degree of human participants. Moreover, we also observed a reversed aging stereotype pattern such that in contrast to typical High-Warmth-Low-Competence stereotype according to stereotype content model, Large Language Models exhibited a High-Warmth-High-Competence stereotype on older adults. Further experiment was conducted to examine how such age-related value orientation of LLMs (especially the benevolent ageism and the high-competence stereotype) could influence individuals' attitudes as well as behavioral tendencies toward older adults. 80 younger adults were randomly assigned to discuss age-related topics with either LLMs or humans, who will present previously generated contents expressing benevolent ageism and high competence stereotype toward older adults. Results indicated that participant's attitudes (as measured by SCM and AAS) as well as helping behaviors (in terms of SVO and third-party punishment game) toward older adults were most influenced by LLMs compared with humans.

Abstract citation ID: igae098.3252

### DO MINDFULNESS ACTIVITIES IMPROVE HANDGRIP STRENGTH AMONG OLDER ADULTS: A PROPENSITY SCORE MATCHING APPROACH

Muhammad Thalil[1], Waad Ali[2], Boadi Agyekum[3], and Shobhit Srivastava[4], *1. The Pennsylvania State University, University Park, Pennsylvania, United States*, *2. Sultan Qaboos University, Muscat, Masqat, Oman*, *3. University of Ghana, Acra, Greater Accra, Ghana*, *4. International Institute for Population Sciences, Mumbai, Maharashtra, India*

The integration of mindfulness practices into the daily routine of the elderly population has been proven to enhance their overall well-being and vitality. There is limited evidence regarding the association between mindfulness activity and physical strength among community-dwelling older adults. Since hand grip strength is an important measure for evaluating the health and well-being of older adults, we explored the association between yoga/mindfulness practices and handgrip strength among older Indians using nationally representative data (N=27,707; mean age: 68.6 [SD:7.3]). Handgrip strength was assessed using a handheld Smedley Hand Dynamometer and mindfulness activities were self-reported. Key findings showed that for the matched sample (ATT), handgrip strength increased by 0.55 points for those who were involved in mindfulness activities compared to older adults who were not involved in mindfulness activities. The results of the Average Treatment Effect on the Untreated (ATU) showed that among individuals who were not involved in mindfulness activities, if they were involved in mindfulness activities, their handgrip strength was likely to increase by 1.16 points. The findings of the current study highlight the importance of integrating mindfulness techniques, including yoga, into public health programs aimed at ageing populations. By tailoring personalized programs that improve both mental and physical health, older adults can maintain an active lifestyle, thereby reducing healthcare costs and enhancing societal well-being.

Abstract citation ID: igae098.3253

### DOES CAREGIVING HASTEN AGING? EXPLORING THE LINK BETWEEN TRIPLE-DUTY CAREGIVERS' TRANSITIONS AND SUBJECTIVE AGE

Xin Sun[1], and Zhan Hu[2], *1. Fudan University, Shanghai, Shanghai, China (People's Republic)*, *2. Fudan Institute on Ageing, Shanghai, Shanghai, China (People's Republic)*

Perceptions of aging serve as crucial indicators for evaluating positive aging. This study examines the relationship between various caregiving transition types and subjective aging over time. Utilizing data from the 2018 and 2020 waves of the China Longitudinal Aging Study, we examine six transition types, incorporating both intra- and extra-familial caregiving behaviors, by constructing three subjective aging change patterns: "Consistent over time", "Accelerated growth", and "Relative decline". The findings reveal that sustained caregiving is associated with a relative decline in subjective age, with a more pronounced effect observed in the reduction of ongoing extramural caregiving. Caregiver role transitions, such as exiting caregiving and transitioning between intra- and extra-familial caregiving, lead to an accelerated increase in subjective age, while entering caregiving aligns subjective age with the passage of time. In the context of caregiving role transitions, a positive self-perception of aging enhances the subjective age of female caregivers, whereas a positive general aging attitude reduces the subjective age of male caregivers. The study underscores the significance of caregiving experiences as crucial predictive factors for subjective aging. Establishing comprehensive family support policies throughout the caregiving lifecycle, promoting voluntary caregiving participation, can contribute to enhancing adaptive aging

Abstract citation ID: igae098.3254

### GENDER DIFFERENCES IN THE EXPERIENCE OF AGE-RELATED MICROAGGRESSIONS

Hannah Lewis[1], Crystal Hecht[2], and Jeff Buchanan[2], *1. University of North Dakota, Grand Forks, North Dakota, United States*, *2. Minnesota State University Mankato, Mankato, Minnesota, United States*

Microaggressions are subtle forms of discrimination which occur in interpersonal interactions. This construct has been examined individually in the context of sexism, and more recently, ageism. Despite society's gendered view on aging, the intersection of how gender impacts the experience of age-related microaggressions is understudied. The current study utilized existing survey data from Lewis et al. (2023) to examine how intersectional identities of age and gender influence the experience of age-related microaggressions. The original study utilized an online survey to ask older adults (n=200, 57% females) about their experiences with 20 age-related microaggressions. Frequency analyses were used to identify gender differences by item, with a difference of >20% serving as a threshold for defining meaningful gender differences. Gender differences existed on items in all six variables measured (frequency, cause, relationship, location, emotional response, and behavioral reaction). Chi-squares tests of independence were also run to examine these differences on a larger scale. The results suggested gender had a significant relationship with frequency $\chi2(5)=6.64$,p =.005, cause $\chi2(7)=157.61$,p <.001, and emotional reaction $\chi2(2)=7.37$, p =.03. Namely, women more often reported experiencing microaggressions, believed these interactions occurred because of their gender, and had negative emotional responses. Overall, the results suggest a difference in the experience and perception of these interactions. These results highlight a need for educational programming on ageism to incorporate a discussion on intersectional identities. Future studies should utilize focus groups to gain a better understanding of the role gender identity plays in the context of age-related microaggressions.

Abstract citation ID: igae098.3255

### LONG-TERM EFFECT OF HEALTH CARE DISCRIMINATION ON LIFE SATISFACTION OF FUNCTIONALLY LIMITED OLDER ADULTS IN ENGLAND

Felipe Sandoval Garrido, *University of Tsukuba, Tsukuba, Ibaraki, Japan*

Healthcare discrimination, e.g. receiving poorer service compared to others, detrimentally affects older adults, particularly those with greater care needs. In the UK, the long-term interplay of healthcare discrimination and functional limitations is under-researched, leading to potential care gaps and poorer health outcomes. This study fills this gap by examining the effects of healthcare discrimination and functional limitations on the life satisfaction of 12,690 individuals aged 60+ from the 2010-2019 English Longitudinal Study of Ageing. The 2010-11 survey assessed healthcare discrimination and functional limitations using ADL and IADL scales. Life satisfaction was measured using the Satisfaction with Life Scale, with scores from 2010-11, 2014-15, and 2018-19 analyzed via a linear mixed-effect model to account for the longitudinal design. Findings show that 2.9% (368 respondents) experienced discrimination. A preliminary bivariate logistic regression showed that those with more significant functional limitations were twice as likely to face discrimination (odds ratio: 2.010). The mixed-effect model showed a 1.580-point decrease in life satisfaction due to discrimination and a 1.134-point decrease from ADL/IADL difficulties. An interaction effect led to a larger 2.399-point drop when combining both factors, adjusted for demographic and health variables. These results highlight the intersection of healthcare discrimination and functional limitations, emphasizing the need for targeted policy and educational reforms. Incorporating these insights into training programs for healthcare professionals and establishing more inclusive, sensitive healthcare policies could significantly enhance care quality and outcomes for the elderly. This study underscores the imperative for such initiatives to better cater to the aging population's unique needs.

Abstract citation ID: igae098.3256

### PREVALENCE OF AMBIVALENT AGEISM AMONG AGING SERVICES PROVIDERS AND ASSOCIATION WITH STRESS

Emily Kinkade, and Heather Fuller, *North Dakota State University, Fargo, North Dakota, United States*

Ageism is an ambivalent construct consisting of positive and negative attitudes towards older adults. It is well documented that ageism is harmful to the physical and mental well-being of both the holder and target of ageist beliefs and behaviors. However, most ageism research focuses on hostile ageism within specific contexts (e.g., the workplace and medical settings). Little to no research investigates the prevalence and implications of ambivalent ageism among other populations that provide important resources to older adults. This study investigates how common ambivalent ageism is among aging service providers, and whether ambivalent ageist beliefs are associated with their feelings of stress and burnout. A small sample of Midwestern aging service providers (n=40) was recruited. Ambivalent ageism was measured using the Ambivalent Ageism Scale, while service provider stress and burnout were measured with the Perceived Occupational Stress Scale and the Copenhagen Burnout Inventory, respectively. A paired-samples t-test found that benevolent ageism was significantly higher than hostile ageism among aging service providers. Multiple regression analyses indicated that among aging services providers, benevolent and hostile ageism were associated only with personal burnout, and not with perceived stress, perceived occupational stress, nor work-related burnout. Such findings suggest that benevolent ageism may be more common among service providers because it is less likely to be viewed as problematic, and thus is more normalized and unquestioned. Further, ambivalent ageist beliefs may be associated with personal burnout due to exacerbating stress-related conditions such as compassion fatigue.

Abstract citation ID: igae098.3257

### PREVALENCE OF AMBIVALENT AGEISM AMONG FAMILY CAREGIVERS AND RELATIONSHIP TO STRESS

Emily Kinkade, and Heather Fuller, *North Dakota State University, Fargo, North Dakota, United States*

Informal family caregivers play an important role in the healthy aging of many older adults. However, little to no research focuses on how family caregivers' beliefs and attitudes about older adults impact their experience as a caregiver. This study investigates the prevalence of ambivalent ageism - both benevolent and hostile - among informal family caregivers, whether ambivalent ageist beliefs are associated with feelings of stress and burnout, and whether factors such as the relationship quality between the caregiver and care recipient and perceived burden mediate the relationship between ambivalent ageist attitudes and caregiver stress. A sample of informal family caregivers (n=137) was collected from the Midwest. Ambivalent ageist attitudes were measured using the Ambivalent Ageism Scale, family caregiver stress and burden were measured with the Kingston Caregiver Stress Scale and the Zarit Burden Interview respectively, and relationship quality was measured using the Dyadic Relationship Scale. A paired-samples t-test indicated that benevolent ageism was significantly higher than hostile ageism. Multiple regression analyses indicated benevolent ageism was not associated with caregiver stress or burden; however, hostile ageism was significantly associated with caregiver stress and burden. Further, it was found that relationship quality did not mediate the relationship between hostile ageism and caregiver stress, but perceived caregiver burden did mediate this relationship. These findings are discussed and interpreted within the framework of the Informal Caregiving Integrative Model.

Abstract citation ID: igae098.3258

### RELATIONSHIP OF AGING KNOWLEDGE TO AGE BIAS VARIES ACROSS KNOWLEDGE DOMAIN FOR DOCTORAL PSYCHOLOGY TRAINEES

Benjamin Johnson, and Grace Caskie, *Lehigh University, Bethlehem, Pennsylvania, United States*

Doctoral psychology trainees often express low interest in clinical work with older adults, which may be attributed to ageist stereotypes they hold. Levy's (2018) PEACE model states that one of the two most effective ways to reduce age bias is to increase accuracy of knowledge about aging. Few studies have examined psychology trainees' aging knowledge or whether it relates to their age bias. We assessed the aging knowledge of 192 doctoral psychology trainees (aged 21-30 years), using Palmore's Facts on Aging Quiz (FAQ), which includes three domains (biological, psychological, social). We also investigated the relationship of trainees' aging knowledge to age bias (measured by FSA, ASD, and AmbAS). On average, trainees answered 13 FAQ items correctly (52%), indicating low aging knowledge. Trainees had more accurate aging knowledge in biological (M=70%) than psychological (M=60%) or social (M=40%) domains. They were significantly more likely (p<.001) to respond incorrectly to 10 facts and correctly to 11 facts; the social domain showed the most incorrect knowledge. Trainees' total aging knowledge was not significantly correlated with any age bias measure; however, greater biological aging knowledge was positively correlated with more age bias (FSA and AmbAS), while greater psychological and social aging knowledge were negatively correlated with all age bias measures. Thus, to reduce age bias by increasing aging knowledge, interventions may need to be domain-specific. Additionally, psychology training may overemphasize biological aging and physical decline (i.e., medical model), suggesting interventions address other aspects of aging and focus on age-related gains in addition to losses.

Abstract citation ID: igae098.3259

### THE RELATIONSHIPS BETWEEN INFORMATION SOURCES, PERSPECTIVES ON PUBLIC HEALTH POLICIES, AND AGEISM

Cherrie Park, *The Ohio State University, Columbus, Ohio, United States*

Global concerns over heightening ageism have been raised throughout and after the pandemic. While it was argued that age-based public health policies during the pandemic contributed to intergenerational tension, little evidence was available about these concerns. In response, this study aimed to understand younger adults' ageism in the context of public health crisis by examining the relationships between younger adults' perspectives on health policies, sources of health information, and the intensity of their ageism. For a quantitative inquiry, survey data were collected in the United States from 317 individuals ages 18 to 44. Their ageism levels were measured with the Ambivalent Ageism Scale, which had been developed to capture multifaceted attitudes (i.e., hostile and benevolent) toward older adults. Multiple regression analysis revealed that benevolent ageism was more intense than hostile ageism among the survey respondents. The intensity of ageism was significantly associated with their beliefs about safety measures and the prioritization of older adults in distributing medical resources, as well as with their choice of information sources (e.g., social media) to receive health information. The findings inform people doing advocacy work for older adults, including those in the helping profession, about future directions for combating ageism in times of public health crisis. For example, they should guide people to utilize various information sources and accept health information critically. This study can be applied to developing effective education models, policies, and practices for alleviating ageism.

Abstract citation ID: igae098.3260

### THE ROLE OF SOCIAL NETWORKS ON THE RELATION BETWEEN AGE DISCRIMINATION EXPERIENCE AND DEPRESSION: A BUFFER OR A STRESS?

Junmin Park[1], and Yeonjung Lee[2], *1. Chung-Ang University, Seoul, Republic of Korea*, *2. Chung-Ang University, Seoul, Republic of Korea*

A Confucian culture in Korea, which holds the older people in high esteem has been eroded due to the disruption of the traditional family structure since industrialization and modernization, resulting in the diminished respect for older adults. However, given these cultural background, experiences of age discrimination might be devastating especially for older adults. While social capital theory suggests that individuals obtain material, cultural, and human capital through their social networks, it is unclear whether social networks play a role as a buffer or a stress for the association between age discrimination experience and depression. Therefore, the objective of this study is to examine the moderating role of social networks (formal & informal) in the relationship between age discrimination experience and depression. Specifically, formal social networks are the frequency of participation in civic society, and informal social networks are the frequency of contact and interaction with family and non-family members. Andrew Hayes' PROCESS macro analytical approach was applied using a selected sample who are aged 65 and older from the 2020 National Survey of Older Koreans (n=10,097). The results show that older adults who experienced age discrimination are likely to have more depressive symptoms. While formal social networks had no significant moderating effect, informal social networks had a significant moderating effect. Specifically, the association between age discrimination experience and depression is stronger at a high frequency of informal social network. Findings have implication that social networks might worsen the consequences of age discrimination for depression among Korean older adults.

Abstract citation ID: igae098.3261

### UNDERMINING OLDER ADULTS' AUTONOMY IN THE NAME OF HELP: NEGATIVE AGING ATTITUDES AND PHYSICAL HEALTH OUTCOMES

Zizhuo Chen[1], and Xin Zhang[2], *1. Peking University, beijing, Beijing, China (People's Republic)*, *2. Peking University, Beijing, Beijing, China (People's Republic)*

Older adults often face challenges in maintaining autonomy, such as having others make decisions for them and receiving paternalistic help. This research aimed to investigate whether such phenomena, although usually presented in the guise of benevolent help, reflect negative aging attitudes and threaten older adults' physical health. Study 1 surveyed 89 younger adults on their preference for providing paternalistic (e.g., Developing strict meal plans) versus agentic help (e.g., Offering dietary recommendations with explanations) for different age groups. Results indicated that participants believed older adults (compared with younger adults) were more acceptable to paternalistic help and deemed it more effective than agentic help. Such inclination was positively associated with participants' demeaning attitudes (i.e., disregarding ones' advanced psychological needs) towards older adults and fixed essentialist beliefs about aging. Study 2 analyzed a 4-wave, 10-year, nationally representative dataset with 4288 cases to explore the impact of compromised autonomy (having others make decisions for them) on older adults' physical health. Although cross-lagged model revealed a mutual positive prediction between older adults' autonomy and their health status, further analysis using instrumental variables confirmed the causal relationship of autonomy on health, rather than the reverse. Moreover, older adults who were less able to make decision for themselves exhibited lower survival probabilities, even after controlling for other variables, including age, health status, ADL, etc. These findings underscored negative attitudes underlying some benevolent helping behaviors that undermined older adults' autonomy and their detrimental physical outcomes, and shed light on the significance of protecting and promoting older adults' autonomy.

### SESSION 7610 (POSTER)

### ATTITUDES ABOUT AGING

Abstract citation ID: igae098.3262

### "I'M GOING TO LIVE HOW LONG?" EXPLORING GAPS AND INFLUENTIAL FACTORS IN PERCEPTIONS OF LIFE EXPECTANCY

Lauren Cerino[1], Adam Felts[1], Lisa D'Ambrosio[2], Taylor Patskanick[1], and Joseph Coughlin[2], *1. Massachusetts Institute of Technology, Cambridge, Massachusetts, United States*, *2. MIT AgeLab, Cambridge, Massachusetts, United States*

As the population ages and life expectancy increases, it becomes increasingly important to understand the public's perceptions and expectations of their own aging, longevity, and life expectancy. Research shows that most people tend to underestimate their own life expectancy (Bago d'Uva, O'Donnell, and van Doorslaer, 2017; Society of Actuaries, 2020). Having an accurate sense for how long one may live plays an important role in informing decision-making and planning around finances, health, and general aspirations. The MIT AgeLab conducted focus groups (N=69, ages 27-76) and a survey (N=1184, ages 20-79) examining participants' perceived life expectancy and the factors influencing their expectations. The survey sample's overall perceived life expectancy was 79.84 years, with differences emerging among demographics; for example, older participants reported significantly higher life expectancy compared to younger participants. Health status, lifestyle, and familial health history emerged as top factors influencing participants' expectations. Further analysis will explore differences in perceived life expectancy among participants with varying life experiences (e.g., caregiving status, employment, family history), self-reported health, hopes and goals (e.g., desired life expectancy), and personality traits (e.g., optimism), as well as demographic characteristics (e.g., age, gender, race/ethnicity, residential environment). We will suggest both psychological (via construal theory) and sociological (via analyzing sociocultural knowledge about old age as a life stage) explanations for these findings. This session will emphasize practical implications for researchers and practitioners to inform how they might guide individuals toward more accurate perceptions of their life expectancy, enabling people to engage in improved decision-making and planning.

Abstract citation ID: igae098.3263

### ACCEPTABILITY AND FEASIBILITY OF WEARABLE SENSORS IN AN AGING INDIVIDUAL LIVING COMMUNITY

Rebecca Koszalinski[1], Ruth Tappen[2], Behnaz Ghoraani[2], Edgar Vieira[3], Borko Furht[2], David Newman[2], and Muhammad Tanveer Jan[2], *1. University of Central Florida, Greenacres, Florida, United States*, *2. Florida Atlantic University, Boca Raton, Florida, United States*, *3. Florida International University, Miami, Florida, United States*

The use of health technology to detect and measure outcomes in aging individuals offers safe, efficient, and cost-effective detection of changes. There are numerous barriers to acceptability and engagement of technology including participant lack of knowledge, insufficient support, and challenging functionality. It is unclear if barriers of the use of smartwatches stem from participants' unfamiliarity, inability to use technology, disinterest, or if bias contributes to inadequate engagement and testing with older adults. This team of researchers designed a pilot study to investigate the acceptability of smartwatches among aging individuals residing in adult living community in the Southeastern United States. Out of 27 participants who consented to the study, 7 (25.9%) were requested repeats and all (100%) wore the smartwatches for a period of 1 to 5 days. Engagement was high throughout the data collection period (May – December, 2023), with participants expressing interest in the smartwatches' data output. Verbatim comments were predominantly positive ("like the look"; "like the technology"; "comfortable"). However, concerns were raised about smartwatch's size (n = 2), weight (n = 1), and the necessity of maintaining the correct time on the device to facilitate independent time management (n = 5). This study contributes to the literature by highlighting the successful engagement and deliberate inclusion of aging individuals in health technology research, and the acceptability of technology (smartwatches) in this population. It suggests the importance of consulting aging individuals in the planning stages of deploying specific sensor technologies.

Abstract citation ID: igae098.3264

### AGE BIAS IN PEOPLE'S DAILY ACROSS 72 YEARS

Chunyan Mai[1], Amber X. Chen[2], Ji Cao[1], and Helene H. Fung[1], *1. The Chinese University of Hong Kong, Hong Kong, China (People's Republic)*, *2. University of California Santa Barbara, Santa Barbara, California, United States*

**Background:** Older adults were traditionally portrayed to be respected in China, a collectivistic and culturally tight society. However, findings are mixed on attitudes towards the older adults in contemporary China, amidst socio-economic transformation and cultural shifts. Addressing this gap, our study investigates the evolution of societal attitudes towards the older group, as reflected in narratives from the People's Daily Newspaper (Renmin Ribao) from 1950 to 2021.

**Methods:** Societal attitudes were measured with three metrics: moral judgment (virtue versus vice), ageist evaluations (positive versus negative), and stereotypes (warmth versus competence), utilizing natural language processing. Age biases (Virtue Bias, etc.) refer to the association between attitude-related words (virtue, etc.) and the older group relative to the younger group. To explain the overtime change in the Age Biases, time series models and Granger tests were applied to examine the correlation and precedence between cultural values and age biases.

**Results:** The results of time series models revealed that after accounting for GDP per capita, collectivistic values were positively correlated with Warmth Bias. Additionally, cultural looseness exhibited positive correlations with Virtue Bias, Positive Ageist Bias, and Warmth Bias. Granger tests revealed that declines in cultural looseness preceded declining Virtue Bias and Positive Ageist Bias.

**Discussion:** This is the first known study longitudinally exploring societal attitudes towards the older group in China. Our findings highlight the significance of the temporal dynamics of societal attitudes along with cultural changes.

Abstract citation ID: igae098.3265

### AGING ATTITUDES: THE EFFECTS OF INTERGENERATIONAL RELATIONSHIPS AND VILLAGE ECONOMIC CONTEXT IN RURAL CHINA

Xiaoyu Fu, Ying Xu, and Merril Silverstein, *Syracuse University, Syracuse, New York, United States*

Understanding the dynamics between intergenerational relationships and attitudes toward aging is crucial for developing supportive policies for older adults, particularly in rapidly changing rural contexts (Wu & Yuan, 2023). This study investigated how parent-adult child relationships (level 1 predictors) and village economic development (level 2 predictor) influence older adults' attitudes toward aging in rural China. From the 2021 wave of the Longitudinal Study of Older Adults in Anhui Province, we sampled 1394 older adults aged 60 and over across 64 villages in Anhui. Using mixed-effects multilevel regression models, we found that the quality of parent-adult child relationships, support from adult children, and the level of economic development in villages positively affect older adults' attitudes toward aging. Importantly, the effect of parent-adult child relationship on aging attitude is shaped by the village's economic development level, indicating a stronger positive association in more economically developed contexts. These findings highlight the significant contextual effect of economic conditions on the impact of intergenerational relationships on aging attitudes, underscoring the importance of considering both individual and village-level factors in aging research in rural China.

Abstract citation ID: igae098.3266

### AS YOUNG AS YOU FEEL? TRACKING SUBJECTIVE AGE AND PHYSICAL WELL-BEING BEFORE A LUNG CANCER SCAN

Victoria Dunsmore[1], and Shevaun Neupert[2], *1. University of North Carolina, Chapel Hill, North Carolina, United States*, *2. North Carolina State University, Raleigh, North Carolina, United States*

Research has not yet considered how cancer-related events trigger poor quality of life. Here, researchers examined whether the discrepancy between felt and chronological age was related to physical well-being, and if these fluctuated as patients with lung cancer approached a triggering event – repeated CT scans. 25 patients treated with curative intent treatment for lung cancer were recruited within 6 months of their upcoming CT scan. Patients were predominantly white (80%; Black: 12%), women (96%), between 43 and 78 years old (M = 62.33, SD = 8.10). Baseline surveys collected age, and repeated monthly surveys gathered physical well-being and felt age every 30 days until one's scan. Following a monthly response rate of 100% (59 of 59 received), researchers calculated proportional discrepancy felt age scores by subtracting chronological age from felt age and dividing by chronological age. Using this score, researchers investigated the monthly relationship between age discrepancy and physical well-being. Results showed that on months when patients felt closer to, or older than, their chronological age they reported increases in physical well-being ($\gamma10 = 1.75$, $t = 2.13$, $p = .04$). The present model accounted for 33% of the within-person, and 39% of the between-person variance of physical well-being. In contrast to studies in non-clinical populations, results showed that feeling older was associated with better physical well-being in the months prior to a CT scan. Qualitative interviews could identify themes in coping with repeated scans that may mitigate the effects cancer treatment can have on physical well-being.

Abstract citation ID: igae098.3267

### ASSOCIATIONS BETWEEN OBJECTIVE HEARING FUNCTION AND SUBJECTIVE VIEWS OF AGING

Jana Koch, Brooke Brady, and Kaarin Anstey, *University of New South Wales, Sydney, New South Wales, Australia*

Subjective views of aging have been associated with positive health-promoting behaviours and well-being. While age-related changes are considered fundamental to views of aging, the role of declining hearing function in shaping these age views remains a largely unexplored area. Within the scope of this study, we hypothesized that poor hearing function would contribute to less favorable views of aging, as measured on multiple related constructs. Data were obtained from the app-based, longitudinal 'Labs without Walls' study, including 184 participants with a mean age of 55.6 years (17.1 SD). Subjective views of aging were measured by the Self-perceptions of aging scale, Expectations regarding aging scale (with subscales on physical health, mental health, and cognitive function), subjective age, and ideal age. Hearing function was measured on a validated app-based hearing task, and a pure-tone average was calculated in the better-hearing ear on frequencies 500, 1000, 2000, and 4000 Hz. Structural equation modelling was employed to examine the unique contributions of hearing function on subjective views of aging while controlling for chronological age, sex-at-birth, loneliness, cognition and socioeconomic status. As hypothesized, poor hearing function was associated with negative expectations regarding aging, especially regarding maintaining cognitive function and physical health. Hearing function was also associated with an older ideal age. However, poor hearing function was not predictive of any other constructs of subjective views of aging. These findings highlight that hearing loss affects expectations of the aging process. Future interventions should examine whether addressing these negative expectations could lead to improved hearing health behaviors.

Abstract citation ID: igae098.3268

### CAREGIVER STATUS MODERATION OF THE RELATION BETWEEN PERCEPTIONS OF COGNITION AND COGNITIVE PERFORMANCE

Maia McLin, and Danielle Nadorff, *Mississippi State University, Mississippi State, Mississippi, United States*

Custodial grandparents have additional stressors associated with becoming a caregiver later in life. Caregiving grandparents use cognitive strategies that non-caregiving older adults may not regularly utilize. Concerns regarding aging are common to older adults, however these concerns may be elevated amongst older adults who have the additional concern of responsibility over grandchildren. In general, those who report more control over cognitive aging are more likely to have better cognitive performance. These perceptions may affect behaviors such as participating in cognitively-taxing tasks. Those who have additional stressors, such as caregiving to grandchildren, may have additional burden that lessens perceptions of cognitive control. This study assesses whether caregiving grandparent status affects the relation between perceptions of control over aging and cognitive performance. Data included 234 caregiving and 1591 non-caregiving participants from the third wave of a longitudinal data collection survey Midlife in the United States study (MIDUS III). MIDUS III included self-administered surveys assessing caregiver status, social factors, and phone interview assessment of cognitive performance. Moderation of caregiver status on the relation between perceptions of cognition control and cognitive performance was tested using SPSS Process macro. A significant interaction was found ($t=2.08$, $p<.05$), indicating that caregiver status moderates this relation. Perceptions of cognitive control were not predictive of cognitive performance for grandparent caregivers ($p=.06$), but were predictive for non-caregivers ($p<.001$). These results suggest that grandparent caregiver status affects the impact of perceptions of cognitive control on cognitive performance.

Abstract citation ID: igae098.3269

### DAILY HEALTH PROBLEMS, SUBJECTIVE AGING, AND THE MODERATING ROLE OF AWARENESS OF AGE-RELATED CHANGES

Maiken Tingvold, and Anna Kornadt, *1. University of Luxembourg, Esch-sur-Alzette, Grevenmacher, Luxembourg*

Indicators of subjective aging, such as a persons' subjective age or their perception of accelerated aging, are related to health problems, in that experiencing health issues can lead to more negative subjective aging. This has been shown for longitudinal relationships as well as in daily assessments. In the current study, we were interested in moderators of this relationship. We assumed that on days in which participants experienced more age-related gains, the coupling of daily subjective aging and health problems should be weaker, whereas it should be amplified on days in which participants experienced more age-related losses. A sample of N = 70 participants aged 52 – 75 years (Mage = 62.74, SD = 5.53) reported on their daily subjective age (uni- and multidimensional), accelerated aging, health problems and awareness of age-related gains and losses on up to 14 days of daily diary assessments. Age, gender, education, and baseline health were included as covariates. Multilevel models showed that as expected, the perception of age-related losses increased the impact of daily health problems on multidimensional subjective age and accelerated aging. Contrary to expectations, however, more daily gains also increased the impact of daily health problems on subjective age and accelerated aging. Our findings attest to the importance of both the perception of age-related gains and losses in daily life, and show that irrespective of valence, perceiving changes as age-related might be influential for the interpretation of daily experiences and their impact on developmental outcomes.

Abstract citation ID: igae098.3270

**DOES CHATGPT HAVE AGEISM?**

Huai Cheng, *Minneapolis VA Hospital, Playmouth, Minnesota, United States*

Ageism is prevalent in the media and clinical practice. AI is defined as thinking and acting like humans. ChatGPT as one type of AI was released to public in 11/2022 and immediately emerged as a quick information resource and interaction with humans. It is very important to know whether ChatGPT has discrimination against older adults and offers non-age biased health information in aging and geriatrics. To explore these, ChatGPT's responses to geriatric attitudes inquiry were assessed by using a validated instrument. This geriatrics attitude instrument is composed of 6 positive attitude questions and 10 items negative attitude questions. They have been tested among medical students, residents, and geriatric fellows. In the present study, ChatGPT was prompted to answer these 16 geriatric attitude questions on a scale of 1-5 (1-strongly disagree and 5=strongly agree). Prompt is based on the directions and questions from the originally published paper. The outputs from ChatGPT were collected to compare the results from medical students, residents and geriatric fellows published in the literature. The total geriatrics attitude score from ChatGPT was significantly lower than humans (2.68 vs 3.77). However, the mean positive geriatric attitude score was 4.1 (the higher the better). The mean negative geriatric score was 1.8 (the lower the better). About half outputs from ChatGPT also provide positive rational for their responses. Our results demonstrate that ChatGPT is less likely to produce an age-biased output. Therefore, we should be comfortable to trust non-age biased output on aging and geriatrics from ChatGPT.

Abstract citation ID: igae098.3271

**EXPLORING RACIAL DIFFERENCES IN WILLINGNESS FOR COGNITIVE TESTING IN OLDER ADULTS**

Amy Costa, Whitney Kasenetz, Alex Crisci, Ashley Curtis, and Jennifer O'Brien, *University of South Florida, Tampa, Florida, United States*

Knowledge and attitudes about dementia contribute to diagnosis and treatment outcomes. People of color (POC) are less likely to receive dementia screening and an official dementia diagnosis compared to white adults, leaving them vulnerable to missed diagnosis and early intervention. This study tested whether racial group (0=white, N=255; 1=POC, N=56) moderates associations between knowledge about Alzheimer's disease (AD) and willingness to undergo cognitive testing. Older adults (N=364, Mage=69.5±6.3 years, 171 women) completed the Perceptions Regarding Investigational Screening for Memory (PRISM; acceptance of AD screening sub-score [PRISM-accept]) and the Alzheimer's Disease Knowledge Scale (ADKS). After completing the questionnaires, participants were invited to the lab to complete the Montreal Cognitive Assessment (MoCA; 0=not completed, 1=completed). A linear regression determined whether race moderated associations between ADKS and PRISM-accept sub-score, controlling for education and gender. A logistic regression determined whether race moderated associations between ADKS and MoCA completion. Race did not moderate associations between ADKS and PRISM-accept ($p>.05$). Race moderated associations between ADKS and whether they completed the MoCA ($\chi2=12.2$, $p<.001$). Specifically, higher knowledge about AD had higher odds of MoCA completion for white older adults ($B=.10$, $p<.001$), but lower odds of MoCA completion for POC ($B=-.21$, $p=.03$). Results reveal increased knowledge about AD may increase willingness to be screened for white older adults but decrease willingness for POC. Results may be due to factors influencing screening acceptance in POC (e.g., lack of trust). The present study highlights the need to tailor recommendations to increase willingness to be screened for dementia based on race.

Abstract citation ID: igae098.3272

**FUTURE ORIENTATION MEDIATES ASSOCIATIONS BETWEEN ATTITUDE TOWARD OLDER ADULTS AND IMPULSIVITY IN YOUNG ADULTS**

Xin Wang[1], and Tianyuan Li[2], *1. The Chinese University of Hong Kong (Shenzhen), ShenZhen, Guangdong, China (People's Republic)*, *2. The Chinese University of Hong Kong (Shenzhen), Shenzhen, Guangdong, China (People's Republic)*

Attitude towards older adults (ATOA) is related to young adults' future expectation, which could also influence their current behaviors. Impulsivity is a risk factor related to a number of health concerns in young adulthood. The current study investigated how ATOA was related to young adults' impulsive behaviors and explored whether future orientation was a mediator. A total of 371 Chinese young adults were recruited and completed a survey on their demographic characteristic, ATOA, consideration of future consequences (CFC) and impulsiveness. The results showed that ATOA had a significant negative curvilinear relationship with impulsiveness ($\beta = -.04$, $p =.01$). Notably, participants with very positive or very negative ATOA both reported lower impulsiveness level compared with those who were in the middle. Furthermore, CFC mediated this curvilinear relationship ($\beta = -.02$, $p =.02$). There was a significant curvilinear relationship between ATOA and CFC

(β =.08, p < 0.001), with both very positive and very negative ATOA related to higher levels of CFC. Higher CFC was further related to lower impulsiveness (β = -.20, p < 0.001). The results suggest that young adults holding very positive or very negative ATOA are both concerned about their future and are more capable of restraining from impulsive behaviors. The inverted U-shaped pattern between ATOA and young adults' impulsiveness provides a new perspective to understand the role of ATOA in early adulthood. Future studies could further investigate the different motives driving the future orientation for young adults with either positive or negative ATOA.

Abstract citation ID: igae098.3273

### HEALTH AND ENERGY OUTLOOK PREDICT FUNCTIONAL HEALTH 20 YEARS LATER

Morgan Taylor, and Margie Lachman, *Brandeis University, Waltham, Massachusetts, United States*

Research has shown that older adults who hold positive self-perceptions of aging have better cognitive and health outcomes. However, few studies have investigated this in a long-term longitudinal and full adult lifespan sample. We used data from the Midlife in the United States (MIDUS) study to investigate whether outlooks of one's health and energy assessed at MIDUS 1 (M1; 1995-1996) would be associated with health limitations (e.g., trouble carrying groceries, difficulty climbing stairs) decades later at MIDUS 3 (M3; 2013-2014). At M1, participants were asked to rate their health and energy on a scale from 0 to 10 at three different time points: the past (10 years prior), the present, and the future (10 years hence). To determine their outlook, we computed a difference score by subtracting participants' past rating from their future rating. Scores ranged from -10 to 10 and represented a 20 year change that modeled the actual gap between M1 and M3. Using multiple linear regression, we found that health and energy outlook was negatively associated with residual change in health limitations at M3, such that those with more positive outlooks reported fewer health limitations, controlling for age, sex, education, and baseline health limitations. Participant age did not interact with this effect, demonstrating that positive outlooks can benefit the health of individuals across the adult lifespan.

Abstract citation ID: igae098.3274

### HOW DO OLDER ADULTS PRIORITIZE DIFFERENT ASPECTS OF THEIR PHYSICAL HEALTH?

Rachel Logue Cook, and Susan Brown, *University of Michigan, Ann Arbor, Michigan, United States*

Older adults often manage multiple health concerns and must decide which aspects of health to prioritize. While gerontologists and geriatricians are typically concerned about the development of cancer, cognitive and mobility impairments, and mental health disorders (Kotsani et al., 2021), little is known about which aspects of health are important to older adults. We developed an online survey that asked respondents to rank 10 health areas (arm and shoulder function, balance, bladder and bowel function, hand function, memory and cognition, mental health and mood, sleep, speech and communication, vision, walking) from most (#1) to least (#10) important in their daily life. Participants were also asked to explain why these rankings were selected in open-ended questions. Five hundred and ninety-five older adults (mean age 71.5 ± 5.5y) completed the survey. Health areas most often ranked as #1 were memory and cognition (36%), vision (19%), and mental health and mood (18%). Areas ranked as #10 were most commonly bladder and bowel function (22%), arm and shoulder function (21%), and sleep (21%). Open-ended responses indicated that health areas were given higher priority if respondents felt they impacted their ability to maintain independence, participate in valued activities, or they had experienced an impairment. Results from this study indicate that many older adults consider memory, vision, and mental health as important components of healthy aging. Further, these findings underscore the importance of understanding how and why older adults prioritize their health in order for healthcare providers to better serve the aging population.

Abstract citation ID: igae098.3275

### HOW GROWING OLDER WITH THEIR "BEST FRIEND" CHANGES OLDER ADULT MEN'S PERSPECTIVES ON AGING

Ranell Mueller, *Flathead Valley Community College, Kalispell, Montana, United States*

Knowledge of the deeper meaning of how growing older alongside companion animal dogs affects men's perspectives of aging is limited. This study employed qualitative research methods utilizing individual interviews and panel discussions with older adult men to investigate the dynamic phenomenon of their personal experience of growing older alongside their aging companion animal dogs. Individual audio-recorded and in-depth interviews and repeated panel discussions with a subgroup of participants, convened as a panel over a three-month period, explored behavioral and emotional manifestations of aging along with a companion animal. Analysis involved open, axial, and selective coding of transcripts to reveal underlying patterns within the data. Outcomes included insight into the role of dogs in the men's perspectives of attitudes toward aging, toward not only themselves as older adult men, but also toward their aging dogs. Findings reveal that the older adult men felt empathy for their aging dogs, which translated to feeling empathy for themselves as well as other older adults. The men revealed attitudes of being sympathetic and understanding of the aging process simply because of aging alongside their aging dogs. A prominent idea that emerged was a feeling of grace toward oneself, others, and their aging dogs. This study offers insight into the deeper understanding of one effect caring for older companion animals has on older adult men's attitudes toward aging. This study provides movement toward a theory of the role of dogs in the development of older adult men's attitudes toward aging.

Abstract citation ID: igae098.3276

### IS A UNIVERSAL TERM FOR OLDER ADULTS CULTURALLY APPROPRIATE? CONSIDERING PREFERRED TERMS IN AFRICA

Margaret Adamek[1], Gifty Ashirifi[1], Dolapo Adeniji[2], and Abraham Teshome[1], *1. Indiana University Indianapolis, Indianapolis, Indiana, United States*, *2. Indiana State University, Avon, Indiana, United States*

Terminology matters. For consistency's sake, one may argue that aging scholars should use one universal term for older adults regardless of what part of the world they are studying. Others may argue that the terms used by scholars in the Global

South to refer to older adults should not be dictated by scholars in the Global North. If the term “elder” is a term of respect for older adults in Sub-Saharan Africa, should African scholars writing about aging be required to use the preferred term of the Global North? Can a universal term for older adults be culturally appropriate globally? With such questions in mind, we conducted a mixed methods study of preferred terms for older adults. Respondents included 78 African scholars and practitioners who completed an online survey. For this study we analyzed responses of 13 scholars from 6 African nations who participated in online focus groups. Using descriptive narrative analysis, our results indicate a wide variety of preferred terms for older adults including elder (Ethiopia), elderly, senior citizen, seniors, pensioners, and golden agers (Malawi). Many terms for older adults exist in local dialects—some connoting respect for elders, others that are derogatory. Respondents indicated older Africans prefer terms that convey respect, honor, and endearment. In scholarly dissemination efforts, we recommend a balanced approach with a universal term such as older adults for international audiences and yet leeway for native scholars to use the term for older people (e.g., “elder”) that is a best fit for their culture.

Abstract citation ID: igae098.3277

### PERCEPTIONS OF AGING: INVESTIGATING GROUP DIFFERENCES AND PREDICTORS OF SUBJECTIVE AGE

Grace da Rosa[1], and Peter Martin[2], *1. Georgia State University, Atlanta, Georgia, United States*, *2. Iowa State University, Ames, Iowa, United States*

While aging is a universal process, individuals perceive and experience aging in markedly different ways. Subjective age reflects how people feel (younger or older) compared to their actual age. The purpose of this study was to assess the association of subjective age (younger vs. older) with education, actual age, self-report health, personality, loneliness, chronic stress, and psychological well-being. This study used data from 2000 wave of the Health and Retirement Study. A total of 3,113 adults 65-years and older were included in this study. The results revealed that older adults who feel younger compared to their chronological age reported lower depression scores, lower Neuroticism, lower levels of loneliness, and lower chronic stress, compared to individuals who perceived themselves as older than their chronological age. In addition, older adults who felt younger compared to their chronological age reported higher levels of Extraversion, Agreeableness, Conscientiousness, Openness to Experience, Life Satisfaction, Control over their health, Control over their social life, and Control over their financial situation. Significant linear regression results confirmed that high levels of Neuroticism, low Agreeableness, and low levels of hopefulness were associated with older perceived age. Higher levels of Extraversion and Openness to Experience and reported higher Control over health were associated with younger perceived age. In summary, this study offers valuable insights on the importance of subjective age. Interventions that target perceptions of aging may have positive effects on health behaviors, psychological functioning, and overall quality of life of older adults.

Abstract citation ID: igae098.3278

### READINESS FOR AGING: A CONCEPT ANALYSIS

Yanjing Liang, and Basia Belza, *University of Washington, Seattle, Washington, United States*

As awareness of aging grows alongside healthcare advancements, advocacy for older adults’ quality of life intensifies. However, people focus more on the physiological, psychological, and social challenges associated with the aging process, limited attention has been paid to entering the pre-aging stage. Understanding of the phenomenon and concept of readiness for aging will enable us to appreciate the opportunities and challenges individuals face as they approach aging. This concept analysis conceptualizes readiness for aging. PubMed, Web of Science, PsycINFO, and CINAHL were systematically searched by using combinations of the terms “ready”, “readiness”, “prepared”, “prepare”, “preparedness”, “preparation”, “aging” and “ageing” in the abstract or title (from the earliest date available to February 2024). Rodgers’ approach was employed to clarify the antecedents, attributes, and consequences of readiness for aging. Fifteen eligible articles were reviewed. The findings were categorized into antecedents, attributes, and consequences. Antecedents of readiness for aging were classified into five categories: self-efficacy, social support, aging in place, financial preparedness, and health awareness. Attributes were characterized by psychological resilience, social connectedness, future orientation, financial plan, and health maintenance. The consequences of readiness for aging include quality of life, social well-being, financial security, and healthy aging. Given the limited attention readiness for aging has received, this concept analysis offered a refined and clarified conceptual definition of readiness for aging. Through literature reviews, qualitative and quantitative data collection, and collaboration with healthcare professionals, and legislators, future research can prioritize and enhance conceptual development, fostering holistic approaches to aging.

Abstract citation ID: igae098.3279

### THE LIFESTYLE AND UNMET NEEDS AMONG UNMARRIED MIDDLE-AGED AND OLDER ADULTS IN TAIWAN

Ching-Ju Chiu, *National Cheng Kung University, Tainan, Tainan, Taiwan (Republic of China)*

This study used in-depth interviews and questionnaires to explore the lifestyle and psychological state of Generation X singles in urban areas of Taiwan, using a mixed methodology that combines qualitative and quantitative research methods. The interview focuses on the individual’s understanding and planning of single life, as well as the acceptance of emerging technologies, while the questionnaire includes personality traits and loneliness tests to supplement the qualitative information in a quantitative way. This mixed research strategy maximizes the understanding of the target group, focusing on the target group’s preparation for future changes in the life stage of legal old age (65 years old) and their attitudes towards emerging technologies, and also provides a scientific basis for formulating more effective policies. After conducting in-depth conversations with multiple participants and understanding their life choices, expectations and challenges, the results of this study revealed the participants’ diverse attitudes towards single life and their flexible preparations for the future, as well as their acceptance of new technologies in daily life. It also provides many valuable insights to financial planners and government policy makers.

Abstract citation ID: igae098.3280
### THE MEDIATING ROLE OF PERCEIVED MASTERY ON THE RELATIONSHIP BETWEEN SELF-PERCEPTIONS OF AGING AND COGNITION

Hannah Apostolou[1], Michael Buxton[2], Valerie Luskey[2], and M. Lindsey Jacobs[3], *1. University of Alabama, Northport, Alabama, United States*, *2. University of Alabama, Tuscaloosa, Alabama, United States*, *3. The University of Alabama, Tuscaloosa, Alabama, United States*

Internalized ageism can be understood as a negative perception of one's own aging experience. Negative perceptions of aging may lead to disengagement in daily tasks and exacerbate decreases in cognition. Positive self-perceptions of aging, however, may serve as a protective mechanism for cognition. Additionally, the relationship between self-perceptions of aging and cognition may be impacted by one's sense of control in areas such as perceived mastery. Researchers used data from the 2020 Health and Retirement Study to examine the relationship between self-perceptions of aging on total cognition and the mediating influence of perceived mastery in a sample of older Americans (n=4369). Specific measures from the Psychosocial and Lifestyle Questionnaire and cognitive battery were selected to conduct the analyses in PROCESS for SPSS Version 4.2. The results showed that there was a significant total effect between self-perceptions of aging and total cognition (B=0.018, p< 0.001), and path a (i.e., self-perceptions of aging on perceived mastery) (B=0.004, p< 0.001) and path b (i.e., perceived mastery on total cognition) (B=0.795, p>0.001) were both significant. The direct effect (B=0.014, p< 0.001) was significant when adding perceived mastery to the relationship between self-perceptions of aging and total cognition. This suggests that more positively rated self-perceptions of aging are associated with higher total cognition scores independently, and this relationship may also operate via perceived mastery. Interventions aimed at increasing positive attitudes towards one's own aging experience could lead to increased cognitive functioning. Further, skills training aimed at increasing one's sense of control may further benefit cognitive outcomes.

Abstract citation ID: igae098.3281
### THE RELATIONSHIP BETWEEN CAREGIVING TRANSITION AND SUBJECTIVE AGE: THE ROLE OF SENSE OF CONTROL

Hyojin Choi[1], and Kristin Litzelman[2], *1. University of Vermont, Vermont, United States*, *2. University of Wisconsin Madison, Madison, Wisconsin, United States*

Subjective age (SA) – how old one feels in relation to their chronological age – varies considerably in challenging situations. The strain and demands of family caregiving might therefore influence patterns of SA. Furthermore, psychological resources that enable family caregivers to deal with stressors, such as sense of control, may buffer these adverse effects. The purpose of this study was to examine the relationship between transitions in caregiving status and SA, and the moderation role of sense of control (perceived constraint and mastery) in this relationship. Using longitudinal data from the Health and Retirement Study (2006–2020), multilevel analyses were conducted to assess both within- and between-person relationships among caregiving transition, sense of control and subjective age. The sample consisted of married/partnered respondents (n=14,944 observations), and we categorized them into five caregiving transition groups: new caregivers, previous caregivers, long-term caregivers, potential caregivers, and non-caregivers based on caregiving status for their spouse/partner with limitations on daily living activities in consecutive waves. The findings show that caregiving transition interacted with perceived constraints and mastery to correlate with SA. Namely, among new caregivers, higher average levels of perceived constraints were associated with older SA (Beta=1.36, p=0.017). Among previous caregivers, higher within-person levels of perceived mastery were associated with younger SA (Beta=-2.66, p=0.001). These findings highlight the importance of developing and implementing interventions tailored to support spouses across the caregiving trajectory in having healthy levels of psychological resources, such as skills training programs and psychoeducation.

Abstract citation ID: igae098.3282
### WHO'S AFRAID OF LIVING TO 100? ATTITUDES TOWARD A CENTENARIAN LIFE ACROSS GENERATIONS

Adam Felts[1], Lauren Cerino[1], Taylor Patskanick[1], Lisa D'Ambrosio[2], and Joseph Coughlin[2], *1. Massachusetts Institute of Technology, Cambridge, Massachusetts, United States*, *2. MIT AgeLab, Cambridge, Massachusetts, United States*

The U.S. centenarian population is expected to increase fourfold over the next three decades, according to Pew Research Center (2024). Amid this increasing likelihood of living to 100, and given the general mystique that a century-long life possesses, how do people think and feel about the possibility of a 100-year life? We explore results from a survey that asked respondents a battery of questions related to their attitudes toward a 100-year lifespan, including their interest in living a 100-year life, their emotions, their concerns, and their perceived level of preparedness. Further, we examine the sources of advice that respondents trusted to guide them through a 100-year life. Finally, we review archetypes respondents chose that reflect the kind of person they would like to embody at age 100, to begin to explore questions (and how to ask questions) about how people conceptualize the "the good life" in later old age. Reporting and discussion of survey results (N=1,184) will be paired with qualitative findings from focus groups (N=69) on the same topic. In the discussion of these results, we will focus on whether people hope for or fear a 100-year life, what their aspirations would be in old age given a lifespan of such great length, and how they would prepare for it. Particular attention will be paid to generational or life course differences in responses, especially in the sources of advice that people prefer, which suggest profound generational shifts in people's preferred sources of expertise.

### SESSION 7615 (POSTER)

### BIOBEHAVIORAL HEALTH

Abstract citation ID: igae098.3283
### ADHD SYMPTOMOLOGY AND COGNITIVE PERFORMANCE IN LATER LIFE

Marrium Mansoor, and Ben Katz, *Virginia Tech, Blacksburg, Virginia, United States*

Attention Deficit Hyperactivity Disorder (ADHD) is a neurodevelopmental disorder affecting up to 6.7% of adults worldwide. Validated questionnaires to diagnose ADHD often include scales for both the inattention and hyperactivity symptoms which characterize this disorder. Although inattention symptoms have been previously linked to cognitive performance in younger samples, very few studies have examined links between these ADHD symptoms and cognitive performance in adult samples, and very few have done so with middle aged and older adults. In this study, we drew from a nationally representative sample from the Health and Retirement Study (HRS) of ~ 1400 middle to older adults (M = 69 years, SD = 10.3; Range = 55-107 years) who completed a set of cognitive measures and an ADHD symptomology questionnaire. We found statistically significant associations between self-reported symptoms of inattention and cognitive measures including Number Series (B=-0.056, p=0.036), Serial 7s (B=-0.088, p< 0.001), Delayed Recall (B=-0.112, p< 0.001), Immediate Recall (B=-0.116, p< 0.001), and global cognition (B=-0.148, p< 0.001) while controlling for age, gender, years of education, and self-rated health. Hyperactivity symptoms were only significantly predictive of global cognition (B=-0.077, p=0.01) and no other measures. These results are consistent with previous work on the links between ADHD symptomology and cognitive performance in younger populations and add to the literature on ADHD in later life. This may have implications for clinicians and practitioners as well as future research on older adults with ADHD.

Abstract citation ID: igae098.3284

### DEPRESSIVE SYMPTOMS, SLEEP, AND QUALITY OF LIFE IN ADULTS WITH CHRONIC CONDITIONS

Idiatou Diallo[1], Judy Bass[1], Rashelle Musci[1], Leslie Adams[1], Joseph Gallo[2], and Adam Spira[1], *1. Johns Hopkins University, Baltimore, Maryland, United States*, *2. Johns Hopkins Bloomberg School of Public Health, Baltimore, Maryland, United States*

Physical, mental, and sleep problems threaten well-being; however, there is limited research on the temporal connections of these issues. We investigated the longitudinal associations among depressive symptoms, sleep disturbances, and quality of life (QoL) among middle-aged and older adults (aged 50+) with diabetes, hypertension, and/or cardiovascular disease in the United States. Participants (N=17,283) drawn from the Health and Retirement Study (HRS) reported at least one of these medical conditions, and data from the 12th (T1), 13th (T2), and 14th (T3) waves (2014-2018) was used. Depressive symptoms were assessed via a modified version of the 8-item Center for Epidemiological Studies-Depression (CES-D) scale (scores: 0-7). Sleep disturbance was measured using the 4-item Jenkins Sleep Scale (JSS) (scores: 2-6). QoL was evaluated through the 5-item Satisfaction with Life Scale (SWLS) (scores: 5-35). Within-person associations showed short-term reciprocal links: higher depressive symptoms predicted higher sleep disturbance (T2–T3: $\beta$=0.036, p< 0.05), and higher sleep disturbance predicted higher depressive symptoms (T2–T3: $\beta$=0.035, p< 0.01). Higher QoL predicted lower sleep disturbance (T2–T3: $\beta$=-0.076, p< 0.001), and higher sleep disturbance predicted lower QoL (T2–T3: $\beta$=-0.064, p< 0.001). Within-person associations also indicated a one-way association over time: higher depressive symptoms predicted lower QoL (T1–T2: $\beta$=-0.104, p< 0.001; T2–T3: $\beta$=-0.141, p< 0.001). Future studies should evaluate the dynamic nature of the associations over longer study periods while assessing the influence of mediators/moderators at different time points. Policies should prioritize integrating sleep, depression, and QoL programs/services into primary care due to their temporal associations among individuals with physical illnesses.

Abstract citation ID: igae098.3285

### GAME ENGAGEMENT AMONG MIDDLE-AGED AND OLDER ADULTS PREDICTS LATER-LIFE COGNITIVE PERFORMANCE

Marrium Mansoor, and Ben Katz, *Virginia Tech, Blacksburg, Virginia, United States*

Although previous literature has linked intellectually stimulating activities to better cognitive outcomes for older adults through cross-sectional and experimental paradigms, fewer studies have looked at the relationship between gameplay at earlier ages and cognitive performance many years later in life. Here, a nationally representative sample of ~1200 middle and older adults drawn from the Health and Retirement Study (M=64.91 years, SD=7.68; Range = 50-91) was used to examine links between global cognition 14 years following self-reported gameplay frequency of two different types of gameplay - word games (WG) and card games (CG) - while accounting for baseline global cognition, age, gender, and years of education. Results indicated that only WG predicts global cognition (B= 0.05, p=0.039) such that more frequent gameplay earlier on was linked to better global cognition scores 14 years later. Although this association is modest and requires further exploration through cross-lagged models and more granular longitudinal studies, these results indicate that word games may play a role in cognitive engagement in later life that is similar to the protective effects of other intellectually stimulating activities. As computer and videogame play become more pervasive in older adult populations, future research should also focus on different types of games (including video games, mobile games, and/or board and puzzle games) to explore the nuances of game engagement throughout the life course and cognitive performance in later life.

Abstract citation ID: igae098.3286

### HISPANIC DEMENTIA CAREGIVERS INVOLVEMENT IN EVERYDAY DECISION-MAKING

Abigail Poe[1], Loreli Alvarez[1], Danny Wang[2], Natashia Bibriescas[3], and Frank Puga[1], *1. University of Alabama at Birmingham, Birmingham, Alabama, United States*, *2. The Pennsylvania State University, University Park, Pennsylvania, United States*, *3. The University of Texas Austin, Austin, Texas, United States*

This analysis aimed to examine the degree of decision-making involvement (DMI) of Hispanic and Latinx (H&L) dementia caregivers of people living with dementia (PLWD). Data was used from an ongoing study on the daily mental health experiences of H&L caregivers of PLWD, entitled “Nuestros Días” (Our Days). Analysis of variance was used to examine differences in caregiver involvement in everyday decisions, such as determining meals (Decision-Making Involvement Scale), based on H&L dementia caregiver age (e.g., 40-49, 50-59, etc.). A subset of 47 H&L dementia caregivers from the main study who completed the DMI scale were included in the analysis. The majority of the H&L caregivers identified as female (91.3%), with a mean age of 47 (SD = 17.4). Around half (48.6%) of the H&L caregivers were working full-time (30+ hours a week). H&L dementia caregivers reported moderate involvement (M = 22.9, SD = 7.1, Range 0-36) in DMI for the PLWD. Caregiver age was significantly associated with DMI (F(3, 40) = 3.08, p =.03). Post hoc comparisons using the Tukey HSD test indicated that the mean DMI for caregivers in the 60-69 age group was significantly higher than the mean DMI for caregivers in the 70+ age group (MD = 11.05, SD = 2.93, p =.027). Age may influence H&L caregiver DMI for the PLWD. Further studies are needed to identify additional factors (e.g., contextual, cultural, contextual) associated with H&L dementia caregiver's DMI for the PLWD and potential associations with caregiver stress and burden.

Abstract citation ID: igae098.3287

### PHYSICAL ACTIVITY AND SERUM BIOMARKERS IN OLDER WOMEN WITH CARDIOVASCULAR DISEASE EXPERIENCING FATIGUE

Shamatree Shakya[1], Nathan Tintle[1], Lisa Sharp[2], and Shannon Halloway[1], *1. University of Illinois Chicago, Chicago, Illinois, United States*, *2. University of Illinois Urbana-Champaign, Champaign, Illinois, United States*

Fatigue is a common symptom of cardiovascular disease (CVD) and can be an early indicator of frailty. Physical activity (PA) can improve fatigue and frailty-related serum biomarkers: brain-derived neurotrophic factor (BDNF), vascular endothelial growth factor-A (VEGF A), and insulin-like growth factor-1 (IGF-1). However, variation in PA and serum biomarkers among patients with CVD who are experiencing fatigue is understudied. We aimed to investigate (a) differences in PA and serum biomarkers in women with CVD experiencing fatigue and (b) the influence of PA on fatigue. We used cross-sectional, baseline data from older women (≥ 65 years, N=253) with CVD participating in the MindMoves trial (NCT04556305). PA included ActiGraph accelerometer measures of sedentary behavior, light PA, moderate-to-vigorous PA, and daily step count, and cardiorespiratory fitness. Serum biomarkers included BDNF, VEGF-A, and IGF-1. Fatigue was assessed using two items from the Center for Epidemiologic Studies-Depression Scale. Two-sample t-tests examined differences in PA and biomarkers across subgroups with and without fatigue. Logistic regression was used to examine associations between significant PA variables and fatigue. Fatigue was prevalent in 17% of participants. There were significantly fewer daily minutes of MVPA (p=0.0005) and lower daily step count (p=0.0142) in those experiencing fatigue. No significant differences were observed in serum biomarkers. After adjusting for sociodemographic characteristics, no PA variable was associated with fatigue. Women with CVD who experience fatigue had lower engagement in PA. Future research is needed to investigate fitness measures (e.g., strength) and other biomarkers to understand underlying physiological processes influencing PA in fatigue.

Abstract citation ID: igae098.3288

### SOCIAL DETERMINANTS OF CHINESE OLDER ADULTS' SOCIAL PARTICIPATION: A MULTIPLE-GROUP ANALYSIS

Jianling Liang, Jia Zhuang, and Jie Zhuang, *The Hong Kong Polytechnic University, Hong Kong, Hong Kong*

In China, as the nation's aging population grows, its family structure diversifies, with more older adults living apart from their children. While social participation is widely acknowledged as a crucial channel for older adults to maintain their social and psychological well-being, its social determinants among older adults from different family structures remain obscure. This study addresses this knowledge gap with data from the Telephone Support Service in Jiangmen City, China (N = 9,871). First, one-way ANOVA results reveal that older adults living alone demonstrate a higher level of social participation than those living with spouses or those with their children (F = 23.441, p <.001). Further, multiple-group analysis (MGA) was performed to determine the group difference across these three elderly groups regarding the relationship between their social participation and health status (i.e., vision, hearing, and mobility) and demographic features (i.e., age and gender). Three group differences were identified. First, compared with those living alone, the relationship between vision and social participation is significantly stronger among older adults living with spouses (CMIN = 4.317, p =.038). Second, the relationship between mobility and social participation among older adults living alone is significantly stronger than that demonstrated among those living with children (CMIN = 5.772, p =.016). Third, the relationship between hearing and social participation is significantly stronger among older adults living alone than those living with children (CMIN = 5.741, p =.017). These findings offer fresh insight to understand the social participation of older adults with different features.

### SESSION 7620 (POSTER)

### BIOSTATISTICS

Abstract citation ID: igae098.3289

### ANALYTIC METHODS FOR HEALTH DISPARITIES IN ALZHEIMER'S DISEASE AND RELATED DEMENTIA

Igor Akushevich[1], Arseniy Yashkin[1], and Julia Kravchenko[2], *1. Duke University, Durham, North Carolina, United States*, *2. Duke University School of Medicine, Durham, North Carolina, United States*

Analysis of Medicare data reveals persistent health disparities related to Alzheimer's disease and related dementias (AD/ADRD) in the United States. These disparities were measured using empirically estimated age-adjusted rates or hazard ratios (HRs) obtained from proportional hazard (PH) models. However, methods for explaining disparities based on comparisons of HR derived from different PH models have strong limitations and observed differences in HRs cannot be causally interpreted. To overcome these limitations, we developed and applied a series of approaches utilizing both traditional workflows as well as novel methodologies such as the Powers-Yun approach, approaches for population attributable fractions, rank-and-replace methods, and parametric g-formula modeling. We sequentially applied these approaches to two multi-racial cohorts of Medicare beneficiaries with baseline ages 67 and 75 and 10 and 15 years of follow-up, respectively. The estimates of the effects of factors contributing to racial disparities in AD risk showed that social determinants of health were an important predictor of disparity with strong competing effects from risk-related diseases, such arterial hypertension and diabetes mellitus. Traditional methods proved to be useful for univariable and bivariate comparisons but did not allow for clear conclusions on multiple inter-related factors. Estimates obtained through alternative methods while generally consistent were not identical. The Powers-Yun approach and approaches for population attributable fractions demonstrated the most promising results. The parametric g-formula showed great potential for establishing causality. However, further analytic work has to be completed to understand how specific assumptions associated with specific methods impact the estimates.

Abstract citation ID: igae098.3290

### DESIGN AND MODELING FOR SPARSELY SAMPLED PHYSIOLOGICAL SYSTEMS DATA

Charlotte Clapham[1], Ravi Varadhan[2], and Karen Bandeen-Roche[2], *1. Johns Hopkins University Bloomberg School of Public Health, Baltimore, Maryland, United States*, *2. Johns Hopkins University, Baltimore, Maryland, United States*

Physical resilience is a pressing research area in aging. The Study of Physical Resilience and Aging (SPRING) posits that resilience is rooted in the fitness of specific physiological systems: It is studying resilience by introducing stressors on selected systems and monitoring their return to homeostasis as time series. We aim to use data from these dynamical stimulation tests to model the fitness of underlying dynamical systems; However, the extreme sparsity and measurement error in SPRING presents a methodological challenge. This presentation reports studies to evaluate alternative methods for recovering system dynamics. We first explored the limits of Local Linear Approximation (LLA) for this purpose using data simulated from curves sampled with varying levels of sparsity and measurement error. We found that LLA could not accurately or precisely recover the curves' dynamics when data was extremely sparsely sampled or had moderate measurement error, making it unviable for SPRING. Our presentation also reports work to develop more nuanced methodologies that implement integro-differential equation modeling, smoothing techniques, and leveraging of ancillary data from other studies that performed dynamic stimulation tests on older adults. These strategies aim to enhance signal, reduce measurement error and borrow strength incorporating existing knowledge. This work has implications on our ability to better understand and predict resilience.

Abstract citation ID: igae098.3291

### PAN-CANADIAN ESTIMATES OF THE PREVALENCE ASSOCIATED WITH CRITICAL WANDERING AMONG HOME CARE CLIENTS

Antonio Miguel-Cruz[1], Hector Perez[2], Micaela Jantzi[2], Lili Liu[2], and John P. Hirdes[2], *1. University of Alberta, Edmonton, Alberta, Canada*, *2. University of Waterloo, Waterloo, Ontario, Canada*

Wandering is a behaviour common in people living with dementia. Persons living with dementia are at increased risk of getting lost and going missing due to critical wandering. However, the evidence is inconclusive. We used 17 years of cross-sectional clinical assessment records from home care services based on interRAI Home Care assessments to provide Pan-Canadian estimates of the prevalence and clinical risks associated with recent critical wandering. (n=1,598,191). The main clinical variable of interest was recent occurrence of critical wandering. We tested associations between wandering and dementia and cognitive impairment. In all the study regions combined, the overall rate of recent wandering is 3.0%, representing 48,641 unique individuals. The sample was comprised of mainly older adults, with 84.3% over the age of 65. The sample was also predominately female (60.9%). A dementia diagnosis including Alzheimer's disease or other dementias was present in 23.6% of the sample. Dementia was strongly associated with the prevalence of recent wandering Chi-squared(1, N=1,598,191) = 101,298.866, $p < 0.0001$). Cognitive impairment was also strongly associated with the prevalence of recent wandering Chi-squared(1, N=1,598,191) = 155,414.738, $p < 0.0001$). Our analyses also show that persons with wandering behaviour experience many different additional clinical problems that can pose important risks to their health and would require attention of first responders and health professionals. interRAI HC offers insights into wandering risk and health profiles of home care client. This information should be used to manage risk in the community and should be shared with first responders where appropriate.

Abstract citation ID: igae098.3292

### TIME-TO-EVENT ANALYSIS IN EVALUATING THE ASSOCIATION OF FALL RISK APPRAISALS AND PHYSICAL ACTIVITY IN OLDER ADULTS

Tho Nguyen[1], Ladda Thiamwong[1], Chang Liu[1], Jethro Raphael Suarez[2], Joon-Hyuk Park[1], and Rui Xie[1], *1. University of Central Florida, Orlando, Florida, United States*, *2. University of Central Florida*

Older adults are encouraged to participate in moderate-to-vigorous physical activity (MVPA) for 75 to 300 minutes per week for health benefits. However, the association of fall risk appraisal (FRA) with meeting the recommended activity levels remains uncertain. We assessed FRA in 134 participants aged 61 to 89 (mean age=74.26±6.85 years, 88.8% female, and 70.15% no history of falls) using the Short Fall Efficacy Scale-International and a static balance assessment, and categorized them into four FRA groups: rational (10.5%, low fear of falling (FOF) and normal balance), irrational (42.9%, high FOF but normal balance), incongruent (25.6%, low FOF and poor balance), and congruent (21.1%, high FOF but poor balance). Using time-to-event analysis based on daily accelerometer activity count data, we examined the impact of FRA on the duration of MVPA, light physical activity (LPA), and sedentary behavior (SB) across seven consecutive days. Kaplan-Meier curves demonstrate that older adults in the rational FRA group had the highest probability of sustaining MVPA throughout the day, whereas those in the congruent group had the lowest probability (log-rank $p < 0.001$). Conversely, participants in the congruent group exhibited the highest likelihood of extending SB duration, while those in the rational group had the lowest (log-rank $p < 0.001$). No significant differences were observed among FRA groups regarding LPA duration. The findings underscore the significance of mitigating a sedentary lifestyle and emphasize the importance of implementing targeted interventions to enhance the duration of MVPA as a pivotal determinant in diminishing the fall risks among older adults.

Abstract citation ID: igae098.3293

### XGBOOST MACHINE LEARNING TO IDENTIFY PREDICTIVE VALUES OF CARDIOMETABOLIC RISK FOR COGNITIVE DECLINE AND MORTALITY

Longjian Liu[1], Saishi Cui[1], Ruifeng Wang[1], Kaidi Xu[1], and Howard Eisen[2], *1. Drexel University, Philadelphia, Pennsylvania, United States*, *2. Thomas Jefferson University Hospital, Philadelphia, Pennsylvania, United States*

**Aim:** To investigate the crucial biomarkers of cardiometabolic disorders that contribute to the risk of cognitive decline and mortality among adults with cognitive decline and dementia.

**Methods:** We analyzed a cohort of 6814 participants aged 45 to 84 years at the baseline of 2000-2002 in the Multi-Ethnic Study of Atherosclerosis. Cognitive function was assessed in 4,591 during 2010-2012. Dementia included Alzheimer's Disease (AD) and AD-related dementia. All participants were followed through to December 2015. XGBoost based machine learning (ML) approach, a novel technique, was used to identify the important features of various biomarkers for assessing the risk of cognitive decline and mortality in those with cognitive decline and dementia.

**Results:** Among 5723 with valid follow-up information, 227 died in those with cognitive decline or dementia. The mortality rate (95%CI) was 3.8 (3.2-4.5) per 1000 person-years in males, and 2.1 (1.7-2.5) per 1000 person-years in females. XGBoost models identified the top five critical biomarkers: carotid intimal-medial thickness, elevated glucose, urinary albumin to creatine ratio (UACR), interleukin 6, and factor VIII. The XGBoost models demonstrated an accuracy, sensitivity, specificity, and area under the receiver operating characteristic curve (AUC) of 74%, 73%, 75%, and 85%, respectively, in classifying cognitive decline. XGBoost survival models identified top five biomarkers: CIMT, UACR, homocysteine, cystatin-C, and D-Dimer for mortality risk, with a C-index of 0.86% and a mean time-dependent AUC of 90%. Conclusions: XGBoost based ML approaches effectively identified the pivotal features among multiple biomarkers of cardiometabolic disorders linked to the risk of cognitive decline and mortality.

### SESSION 7625 (POSTER)

### CARDIOVASCULAR DISEASE

Abstract citation ID: igae098.3294

### ASSOCIATION OF SYSTEMIC IMMUNE-INFLAMMATION INDEX ON THE RISK OF STROKE IN AMERICAN ADULTS

Xichenhui Qiu[1], Yiqing Huang[1], Changning Wei[2], and Qun Wang[1], *1. Shenzhen University, Shenzhen, Guangdong, China (People's Republic)*, *2. Shenzhen Polytechnic University, Foshan, Guangdong, China (People's Republic)*

**Background:** Chronic inflammation is associated with various health consequences, including cardiovascular diseases and cancer. The systemic immune-inflammation index (SII) and system inflammation response index (SIRI) have emerged as novel inflammatory and prognostic markers. However, the relationship between SII, SIRI, and stroke risk in representative populations remains understudied. This study aimed to investigate the association between SII, SIRI, and stroke risk in US adults.

**Methods:** A cross-sectional investigation utilized datasets from the National Health and Nutrition Examination Survey (NHANES) conducted between 2005 and 2018. Multivariate regression models were employed to assess the relationship between SII, SIRI, and stroke risk. Nonlinear relationships were described using smoothing curves and a two-tailed linear regression model. Subgroup analysis and interaction tests were performed to assess relationship stability.

**Results:** The study included 36,129 adults aged 20 to 85 years. The analysis revealed a significant positive association between stroke risk and both SII (OR = 1.27, 95% CI = 0.98 to 1.65, p for trend = 0.0438) and SIRI (OR = 1.49, 95% CI = 1.13 to 1.97, p for trend= 0.0016) after adjusting for confounding factors.

**Conclusion:** Elevated levels of SII and SIRI are associated with an increased risk of stroke in US adults. These findings underscore the importance of managing inflammatory indices as potential interventions to mitigate stroke risk.

Abstract citation ID: igae098.3295

### COMPARE FRAILTY IN OLDER ADULTS AND NONOLDER ADULTS WITH HEART FAILURE

Jenny Hsieh[1], and Pi-Ling Chou[2], *1. University of Washington, Seattle, Washington, United States*, *2. Kaohsiung Medical University, Kaohsiung, Kaohsiung, Taiwan (Republic of China)*

Frailty is commonly presented among patients with chronic diseases, including heart failure(HF), leading to poor prognosis. However, the association between age and frailty in patients with HF remains unclear. Therefore, our study aims to compare the difference in frailty status in older and non-older adults with chronic HF symptoms. A cross-sectional study was conducted, where data was collected between January 1, 2023, and January 31, 2024. Participants were recruited from cardiology departments of two hospitals in southern Taiwan (N=110). Instruments measuring frailty included the Frailty Phenotype Criteria (FPC) and Clinical Frailty Scale(CFS). Data analysis was conducted using R version 4.3.2. In our study, we observed pre-frailty and frailty in older adults with proportions of 49.1% (n=26) and 22.6% (n=12) according to FPC, respectively. For non-older adults, the proportions were 36.4% (n=16) and 18.1% (n=5), respectively. The CFS revealed that 50.8% (n=31) of older adults exhibited frailty, while 38.8% (n=19) of non-older adults did. No significant difference was observed in frailty status between older and non-older adults, as indicated by both the FPC (p = 0.21) and the CFS (p = 0.29). The findings suggested a high prevalence of frailty in patients with heart failure, irrespective of age group. Our results indicated that frailty was a functional decline not limited to older adults. This study highlighted the importance of frailty assessments in patients with heart failure symptoms across all age groups, providing an evidence base to innovate potential strategies to promote improvement in prognosis.

Abstract citation ID: igae098.3296

### EXAMINING THE RELATIONSHIPS BETWEEN RESILIENCE, CARDIOVASCULAR HEALTH, AND RACE AMONG US ADULTS

Briana Sprague[1], and Kelly Mosesso[2], *1. Indiana University Indianapolis, Indianapolis, Indiana, United States, 2. Indiana University School of Medicine, Indianapolis, Indiana, United States*

Modifiable risk and resilience factors explain up to 90% of cardiovascular disease risk. These may contribute to racial cardiovascular health (CVH) disparities, but there has been limited examination of the role of resilience on CVH and disparities. Our goal was to examine (1) which risk and resilience measures were associated with CVH and (2) whether there were differences in these measures by race among a sample of US adults. Aim 1's sample included adults aged 34-84 from the MIDUS biomarker substudy (N = 1255). Aim 2's sample comprised of adults aged 28-84 from the MIDUS parent study (N = 4702). The primary outcome of interest was CVH, operationalized as the AHA's Life's Essential 8 total score, behavior, and health subscores. The hypothesized resilience measures were psychological well-being ("PWB"; assessed with Ryff and Multidimensional Personality Scale), purpose in life, mindfulness, gratitude, and optimism (MPS subscales). Of those, greater PWB (Ryff), purpose in life, and optimism correlated with better Essential 8 total score and behavioral subscore (ps <.01). Higher PWB (MPS), as well as higher gratitude, were associated with better Essential 8 behavioral scores (p <.05). Of these, PWB (MPS), gratitude, and optimism were significantly differences by race, where Black adults had significantly greater values on all measures (p <.001). Implications for future research and practice will be discussed.

Abstract citation ID: igae098.3297

### PLANT-BASED DIETS AND CARDIOVASCULAR DISEASE RISK FACTORS IN OLDER ADULTS: AN INTEGRATIVE LITERATURE REVIEW

Tricia VanCleef, *Fran and Earl Ziegler College of Nursing, University of Oklahoma Health Sciences, Oklahoma City, Oklahoma, United States*

Cardiovascular disease (CVD) is the leading cause of mortality globally, disproportionately impacting older adults. Plant-based diets (PBDs) have emerged as a dietary strategy to mitigate CVD risk, characterized by foods derived from plants and minimal to no animal-derived foods. This integrative review assesses the effectiveness of PBDs for improving CVD risk factors in older adults. A search was conducted in CINAHL, PubMed, and Medline databases for studies of PBDs and CVD risk factors in individuals aged 65 years and older. Quality assessment was performed using the Mixed Methods Appraisal Tool (MMAT). 17 publications were analyzed, 7 experimental and 10 non-experimental. All studies showed at least one positive association between PBDs and cardiovascular health in older adults. Findings highlighted significant associations between PBD adherence and reductions in key CVD risk factors such as low-density lipoprotein cholesterol (LDL-C), blood pressure, and body mass index (BMI), alongside potential benefits in reducing coronary artery calcium and enhancing endothelial function and serum isoflavone concentrations. There is evidence for the potential of PBDs as an effective dietary strategy to prevent the onset and progression of CVD in older adults and reduce the risk of CVD mortality. This review indicates the need for further research, specifically larger randomized control trials focused on older adults to strengthen the evidence base and to guide healthcare recommendations. PBDs should be considered as a part of holistic CVD prevention and management strategies for older adults, encouraging dietary patterns that are rich in fruits, vegetables, legumes, beans, nuts, seeds, and whole grains.

Abstract citation ID: igae098.3298

### PREDICTORS OF CHANGE IN CAROTID ARTERY ULTRASOUND MEASURES IN THE LONG LIFE FAMILY STUDY

Allison Kuipers, Caitlyn Kline, Ryan Cvejkus, Emma Barinas-Mitchell, and Joseph Zmuda, *University of Pittsburgh, Pittsburgh, Pennsylvania, United States*

Early vascular changes of cardiovascular disease can be assessed using carotid ultrasound. The Long Life Family Study (LLFS) may help elucidate factors associated with decelerated vascular aging. Therefore, we identified predictors of change in carotid intima-media thickness (IMT) and interadventitial diameter (IAD) over ~6.0 years of follow-up (range 3.2-8.4) in 674 participants from the LLFS. Common carotid artery B-mode ultrasound was conducted using a GE LOGIQ 3 ultrasound system. We calculated change and annualized percent change (APCT) in mean carotid IMT and IAD. Age, sex, and study site were included in all models, and baseline measures of lifestyle, cardiometabolic conditions, and medication were tested in linear regression models ($\alpha=0.05$). Final models were adjusted for relatedness using GEE. Participants were 67.5 years old (range 46-90), 43% male, 35% hypertensive, 6% diabetic, and 30% were on lipid-lowering medication, 96% didn't smoke and 80% regularly walked >3hours/week. IMT increased 0.046mm or 1.08% annually, while IAD increased 0.166mm or 0.37% annually (all P< 0.0001). IMT APCT change was greater in those aged < 65 (1.37%) than over 65 (0.95%, P=0.004) but did not differ by sex (P=0.20). Whereas IAD APCT change did not differ by age group (P=0.10) but was greater in females (0.44%) than males (0.29%, P=0.006). Outside of age, sex, and site, independent predictors of IMT and IAD APCT included fasting glucose and smoking, plus triglycerides for IMT and systolic blood pressure for IAD. These findings highlight the need for continued research into the role of glucose homeostasis in vasculature aging.

Abstract citation ID: igae098.3299

### RACIAL/ETHNIC DISPARITIES IN TRAJECTORIES OF CARDIOVASCULAR RISK FACTORS IN US ADULTS

Radha Dhingra, Hanzhang Xu, Scott Lynch, Qing Yang, Michael Green, Jessica West, and Matthew Dupre, *Duke University, Durham, North Carolina, United States*

Racial/ethnic disparities in major cardiovascular disease (CVD) risk factors are well documented. However, studies have not considered racial/ethnic differences in the interrelationships among these CVD risk factors and how they vary across age. We used six waves of longitudinal data from the Health and Retirement Study ([HRS] 2006-2016; n=10,292) to examine co-occurring changes in systolic blood pressure ([BP] mmHg), non-HDL cholesterol (mg/dl), diabetes (y/n), and smoking (y/n) at ages 50-80. Group-based multi-trajectory models were used to identify age-related trajectories of CVD risk factors and multinomial logistic regression models were used to identify participant characteristics associated with the trajectories of CVD risk factors. In our cohort (mean (SD) age: 62 (7.2) years; 41% male; 66% non-Hispanic White, 17% non-Hispanic Black, 13% Hispanic, and 3% other race/ethnicity), we found eight distinct trajectories of age-related changes in CVD risk factors (37% of adults had optimal CVD levels). We also found that non-Hispanic Black and Hispanic adults were significantly more likely to exhibit elevated age-related risks related to BP, cholesterol, diabetes, and/or smoking compared with non-Hispanic White adults. The associations were differentially attenuated with the inclusion of multiple covariates and showed that education, household income, and nativity status (among Hispanics) contributed most to the excess CVD risks observed in non-Hispanic Black and Hispanic adults. Overall, our findings demonstrate the impact of social determinants on racial/ethnic disparities in age-related changes in CVD risk factors in middle-aged and older adults in the United States.

Abstract citation ID: igae098.3300

### RACIAL/ETHNIC DISPARITIES IN TRAJECTORIES OF HOSPITALIZATIONS AMONG PATIENTS WITH HEART FAILURE

Radha Dhingra, Hanzhang Xu, Bradley Hammill, Scott Lynch, Jessica West, Michael Green, and Matthew Dupre, *Duke University, Durham, North Carolina, United States*

Little is known about racial/ethnic differences in the long-term patterns of hospitalization following the diagnosis of heart failure (HF). We examined racial/ethnic differences in trajectories of hospital admissions following a diagnosis of HF and assessed the factors contributing to the long-term risks of hospitalization. We included 5,606 patients with newly-diagnosed HF between January 2015 and July 2018 in the Duke University Health System and followed them for up to five years. Group-based trajectory models were used to identify trajectories of all-cause admissions following the diagnosis of HF. Multinomial logistic regression models were used to identify patient characteristics associated with the trajectories of admissions. Approximately 45.6% (N=2,556) of HF patients had low risks of hospitalization, 36.6% (N=2,052) had elevated risks of admission shortly after diagnosis (early risk), 9.9% (N=553) had elevated risks at later stages of illness (late risk), and 7.9% (N=445) had consistently high risks of hospitalization. Non-Hispanic Black patients were more likely to exhibit early risks of hospitalization (odds ratio, [95% CI]: OR=1.33, [1.16-1.52]; P<.001), late risks of hospitalization (OR=1.92, [1.58-2.34]; P<.001), or consistently high risks of hospitalization (OR=1.89, [1.52-2.35]; P<.001) compared with non-Hispanic White patients. Diabetes, chronic kidney disease, and residence in a disadvantaged neighborhood significantly contributed to the excess risks of admissions among non-Hispanic Black patients. Overall, these findings have important implications for identifying the social and clinical factors associated with racial disparities in the timing and frequency of hospital admissions in HF patients.

Abstract citation ID: igae098.3301

### SOCIOECONOMIC DISADVANTAGE AND EARLY AND RECURRENT ADMISSIONS IN PATIENTS WITH HEART FAILURE

Radha Dhingra, Hanzhang Xu, Bradley Hammill, Scott Lynch, Jessica West, Michael Green, and Matthew Dupre, *Duke University, Durham, North Carolina, United States*

The association between socioeconomic status and 30-day readmissions is well-documented in patients with heart failure (HF). However, it is less clear whether socioeconomic disadvantage has an immediate and/or lasting impact on risk of hospital admissions following the diagnosis of HF. We examined whether area-level disadvantage was associated with early and recurrent risks of hospitalizations in patients diagnosed with HF. We included 5,889 patients (ages 65-85 years) with newly diagnosed HF between January 2015 and July 2018 in the Duke University Health System and followed them for up to eight years. We assessed the association of area-level socioeconomic disadvantage (low, moderate, or high) with hospital admissions within 30-days after HF diagnosis using multivariable logistic regression models. We also assessed the risks of recurrent admissions over the entire duration of follow-up using Prentice, Williams, and Peterson models with total time. In our cohort (mean (SD) age: 75 (6) years; 51% female; 67% non-Hispanic White), 71% patients had at least one admission and 40% patients died over a median follow-up of 5.6 years. Area-level disadvantage was not associated with risks of admission within 30-days after HF diagnosis (hazard ratio [95% CI]: HR=1.09 [0.90-1.31]; P=.371). However, patients living in high-disadvantaged areas had significantly greater risks of recurrent admissions over follow-up (HR=1.11 [1.05-1.16]; P<.001). We observed similar patterns for 30-day mortality and mortality over the entire follow-up period. Overall, results from this study suggest that area-level socioeconomic disadvantage should be considered in clinical decision-making and guidelines to improve long-term outcomes in patients managing HF.

Abstract citation ID: igae098.3302

### STAYING ACTIVE IN DAILY LATER LIFE: BENEFITS ON HYPERTENSION AND DEPRESSION DURING THE COVID-19 PANDEMIC

Jeehoon Kim[1], and Mi-Jeong Lee[2], *1. Idaho State University, Pocatello, Idaho, United States*, *2. University of Hawai'i at Mānoa, Honolulu, Hawaii, United States*

Maintaining active engagement is important to reduce disease risks in later life. Given greater restrictions during the COVID-19 pandemic, adults ages of 71-100 might have limited opportunities to engage in daily exercise. However, less is known whether physical activity is related to cardiometabolic diseases and mental health in 70-plus adults during the pandemic. Using the 2021 National Health and Aging Trends Study (NHATS) accelerometry data (n=740), this study conducted a latent profile analysis based on four activity-related variables including total activity count per minute (TACM). Multinomial logistic regression analysis was performed (base outcome=high level group). Predictors included BMI, incidence of hypertension, type 2 diabetes, cancer, heart diseases, depression and dementia, NHATS summary score of Short Physical Performance Battery, and demographic variables. Three classes were profiled based on the activity level: low

(26%, 683 TACM), middle (56%, 1,227 TACM), and high level (18%, 1,829 TACM). Low- and middle-level older adults were more likely than high-level to be overweight and had higher incidence of hypertension and mild depression. Low-level older adults tended to have worse summary scores of physical performance than high-level group. Self-reported walking or vigorous physical activity participation was not associated with physical activity level measured with accelerometer. P-values are less than.05 for all findings. Staying active in daily life was correlated with better health outcomes, lower prevalence of hypertension and mild depression, in 70-plus adults during the COVID-19 pandemic. Intervention strategies to promote physical activity in later life, thus helping reduce risks of cardiometabolic diseases, will be discussed.

Abstract citation ID: igae098.3303

### TESTING THE ROLE OF MITOCHONDRIAL DYSFUNCTION IN AGE-RELATED HYPERTENSIVE AORTIC DISEASE

Arjune Dhanekula, Scott DeRoo, Christopher Burke, Billie Hwang, Michael Mulligan, Jay Pal, and David Marcinek, *University of Washington, Seattle, Washington, United States*

Aging is a risk factor for hypertension, aortic aneurysm, and dissection. However, the age-related changes in aortic biology underlying this risk are poorly understood. Mitochondrial dysfunction is an important contributor of both aging and cardiovascular pathology. However, most models of aortic disease are in young animals, lacking the context of age-related changes. To test whether mitochondrial changes drive aortic aneurysm, we developed a unique mouse model replicating age-related hypertensive aortic disease. A pilot study used multiple doses of angiotensin II (AngII) for 4 weeks to test the development of aortic disease in 20 month C57Bl/6 mice (n=8). These data revealed a statistically significant increase in ascending aortic size for AngII doses ⩾1000 ng/kg/min (1.603 +/- 0.054 mm versus 1.433 +/- 0.064 mm, p< 0.05). A cohort of older C57Bl/6 mice (23-24 months, n=22) were then treated with AngII (1500 ng/kg/min) or saline, with no change in aortic size over the 4 week study period. The older cohort also revealed no difference in maximal oxygen consumption rate (OCR) between the AngII and saline groups (30 +/- 3.6 versus 29.4 +/- 0.9 pmol/(sec*mg), p=0.99). However, the 20 month mice in the pilot data had a significantly higher maximal OCR (58 +/- 13.4 pmol/(sec*mg), p< 0.0001). These results demonstrate that angiotensin II administration in older mice results in the development of aortic aneurysm in an age-dependent manner, with an unexpected increase in maximal mitochondrial respiratory capacity associated with disease development. These results highlight the importance of testing age-related disease in the context of aging biology.

### SESSION 7630 (POSTER)

### COGNITIVE IMPAIRMENT (POSTERS)

Abstract citation ID: igae098.3304

### A NEW MEDICATION-RELATED CUMULATIVE ANTICHOLINERGIC EXPOSURE INDEX EVALUATING RISK OF COGNITIVE IMPAIRMENT

Veronique Michaud[1], Kaveena Autar[2], Gerald Condon[1], Chelsea Honore[3], Maria Grisales[3], James Hickman[3], and Jacques Turgeon[1], *1. GalenusRx Inc, Orlando, Florida, United States*, *2. Hesperos, Clermont, Florida, United States*, *3. Hesperos, Orlando, Florida, United States*

Given the associations between anticholinergic exposure and poor clinical outcomes in older adults, precise quantification of the cumulative anticholinergic burden appears advisable to evaluate the risk–benefit ratio of prescribing or deprescribing some medications. Numerous examples of anticholinergic quantification scales have been used for the past two decades. These were all developed using varying methods of measuring the anticholinergic activity of drugs and varying methods of classifying drugs into distinct potency categories. Recently, we have performed a systematic review of scales and models used to quantify anticholinergic burden in older adults. We have compiled the most comprehensive list of drugs with anticholinergic activities (N=642 drugs); the AntiCholinergic and Sedative Burden Catalog (ACSBC). Using human-on-a-chip technology, we have computed a quantitative Potency AntiCholinergic Score (PACS) by performing a dose-response LTP- suppression for each drug. Furthermore, to better inform our proprietary Aggregated PolyPharmacy Regimen Assessment Index for Safety and Equity (APPRAISE™), we have developed a new unique Medication-Related Cumulative Anticholinergic Exposure (MeReCAE) index. Using drug regimen data from 12,383 patients extracted from the Medical Expenditure Panel Survey (MEPS), we have sequentially proceeded to the virtual addition of amitriptyline, amantadine, asenapine and caffeine to the drug regimen of these patients. A more important shift toward higher MeReCAE values was observed when amitriptyline and asenapine were added to the drug regimen of patients. The quantitative analysis performed allowed us to determine exactly how many patients were in the Low, Moderate and High risk of the MeReCAE index following the addition of the tested drugs.

Abstract citation ID: igae098.3305

### A SCOPING REVIEW OF MACHINE LEARNING STUDIES ON DEMENTIA RISK FOR ETHNORACIAL MINORITIES

Michin Hong[1], Soo-Yeon Ji[2], Seon Kim[3], and Kyeongmo Kim[4], *1. Indiana University Indianapolis, Indianapolis, Indiana, United States*, *2. Bowie State University, Bowie, Maryland, United States*, *3. Drexel University, Philadelphia, Pennsylvania, United States*, *4. Virginia Commonwealth University, Richmond, Virginia, United States*

This study aims to provide an integrated review about dementia risk for ethnoracial minorities from machine learning (ML) based-studies. While ML methods are widely used in dementia research, their benefit for ethnoracial minorities remains unclear. We located prior research using combinations of the key words related to ML (e.g., neural network, deep learning, artificial intelligence), dementia and ethnoracial minorities from major electronic databases. Out of 599 initially searched articles, 389 remained after removing duplicates, and finally, 14 met the inclusion criteria. The interdisciplinary research team employed reiterative data analysis to identify key themes. All included studies, published since 2020, aimed to identify essential risk factors and generate predictive models for cognitive impairments. Most studies and treated race as a predictor after aggregating it into two or three categories, finding a lower risk of dementia among non-Hispanic Whites compared to Hispanics or Non-Hispanic Blacks. One study aimed to develop a predictive model of cognitive impairment applicable across non-Hispanic White, no-Hispanic Black, and Hispanic groups. Various ML techniques were used, such as neural network, random forest, support vector machine, and gradient boosting with some adopting Synthetic Minority Over-sampling Technique to oversample minority groups. Our review indicated that ML-based studies have made limited progress in understanding dementia risk among ethnoracial minorities. National data sets used in most reviewed studies, have lacked minority samples, and this seems to persist with the ML approaches, possibly contributing to deteriorating the existing racial gap. Proactive efforts should focus on ethnoracial variations in dementia risk in ML-based studies.

Abstract citation ID: igae098.3306

### ACTING WITH AWARENESS AND VISUOSPATIAL LEARNING IN MILD COGNITIVE IMPAIRMENT

Melissa Zammitti[1], Emily Bower[2], and Geoffrey Tremont[3],
*1. Pacific University, Portland, Oregon, United States,*
*2. Pacific University, Hillsboro, Oregon, United States,*
*3. Brown University, Providence, Rhode Island, United States*

Mindfulness practice is a potentially low-risk intervention for mild cognitive impairment (MCI), with numerous studies indicating its robustness in addressing modifiable risk factors associated with the progression of MCI. Evidence suggests a positive association between mindfulness and cognitive ability, including within the visuospatial processing domain; however, which facets of mindfulness are associated with aspects of visuospatial processing are not well understood. We aimed to assess the relationship between the five facets of mindfulness and visuospatial learning and memory (VSLM). We hypothesized that stronger endorsement of the acting with awareness facet would correlate with improved VSLM, as present-mindedness could enhance the processing of one's physical surroundings. In this secondary analysis, we conducted a hierarchical linear regression using baseline data from a pilot intervention study involving older adults with MCI. 46 participants with amnestic MCI (65% female, mean age 71.6 years) completed the Five Facet Mindfulness Questionnaire (FFMQ) and the Brief Visuospatial Memory Test-Revised (BVMT-R). We controlled for age, race, gender, education, and depression. The analyses revealed a negative relationship between BVMT-R learning scores and FFMQ Acting with Awareness subscale scores (FFMQ-A) such that higher learning was related to lower awareness ($\beta$= -.540, p=.021. Associations between BVMT-R scores and other mindfulness facets were not significant (p's>.05). Findings suggest that the specific domain of mindfulness, acting with awareness, may be associated with poorer visuospatial learning over repeated trials in individuals with MCI. The findings encourage further investigation of the acting with awareness facet and its role in interventions for older adults with MCI.

Abstract citation ID: igae098.3307

### BARRIERS AND FACILITATORS TO CARE PEOPLE WITH COGNITIVE IMPAIRMENT IN HOSPITALS OF CHINA: A QUALITATIVE STUDY

Yalin Lv[1], Yinuo Ou[2], Zhijiang Li[1], Jie Jia[1], and Juan Li[1],
*1. Huashan Hospital Fudan University, Shanghai, Shanghai, China (People's Republic), 2. Huashan Hospital Fudan University, National Center for Neurological Disorders of China, Shanghai, Shanghai, China (People's Republic)*

Cognitive impairment refers to various degrees of cognitive decline in memory, attention, language, execution, and orientation, significantly impacting people's daily activities and quality of life. With the aggravation of aging population, China has a huge number of people with cognitive impairment and high demand for care. Although there are several international guidelines of care individuals with cognitive impairment, class A tertiary hospitals in China have not well applied them to the clinical care. This study aimed to understand the context of care people with cognitive impairment in hospitals and to explore potential barriers and facilitators to evidence-informed practices. Semi-structured interviews were conducted with nurses from various departments in a Class A tertiary hospital in China. We developed an interview guide based on the Theoretical Domain Framework and guidelines. Data were analyzed using a framework analysis and domains were identified as salient based on their frequency. Finally, 11 domains related to the clinical care of people with cognitive impairment as well as 6 "barrier" and 11" facilitator " domains were identified. The "barrier" domains were social influences, environmental context and resources, social/ professional role and identity, beliefs about consequences, beliefs about capabilities and intentions. The "facilitator" domains were knowledge, physical skills, cognitive, interpersonal skills, social influences, environmental context and resources, social/ professional role and identity, behavioral regulation, memory attention and decision processes, beliefs about consequences, beliefs about capabilities and intentions. These will inform collaboration of multiple disciplines and evidence-based intervention to improve the practice of caring cognitive impairment individuals in hospitals.

Abstract citation ID: igae098.3308

### CO-CREATING RESOURCES TO RAISE AWARENESS OF RISKS OF GOING MISSING AMONG PERSONS WITH DEMENTIA

Christine Daum[1], Vanessa Vahedi[1], Isabella Chawrun[1], Noelannah Neubauer[1], Emily Rutledge[1], Adebusola Adekoya[1], Antonio Miguel-Cruz[2], and Lili Liu[1],
*1. University of Waterloo, Waterloo, Ontario, Canada,*
*2. University of Alberta, Edmonton, Alberta, Canada*

Persons living with dementia are at risk of getting lost and going missing due to wayfinding challenges. Yet, there are few accessible resources available that raise awareness of the risks of going missing and strategies to manage these risks. The purpose of this project was to develop engaging and informative learning resources for first responders, search and rescue personnel, persons living with dementia, care partners, and health and social care providers. Co-design and co-production approaches guided the development of these resources and were informed by reviews of the academic and grey literature, dialogue with persons with lived experiences, and content analysis of missing person cases. We produced three sets of resources: 1) nine dementia-friendly first responder videos for police and search and rescue personnel to enhance their awareness of dementia and risks of going missing; 2) a toolkit of strategies and resources for persons living with dementia and care partners to reduce the risks associated with getting lost and going missing; 3) eight personas and scenarios that depict contextual factors within missing events that can be utilized by service providers and the public in their interactions with persons living with dementia and care partners. All resources were reviewed by participants through interviews, focus groups, and written feedback to identify essential content and ensure accuracy, relevance, and comprehensibility. These sets of learning resources can elevate awareness and offer practical strategies to support persons living with dementia, care partners, first responders, and service providers.

Abstract citation ID: igae098.3309
### COLLEGE EDUCATION AND LEISURE ACTIVITIES PREDICT LATE-LIFE COGNITIVE STATUS: DO BIRTH COHORTS DIFFER?
Jacqui Smith, Kimson Johnson, and Marina Larkina, *University of Michigan, Ann Arbor, Michigan, United States*

Efforts to determine potentially modifiable factors associated with heterogeneity in cognitive status in old age point to the importance of participation in cognitive, physical, and social leisure activities and the relevance of historical changes in completion of post-secondary education. We use data from two birth cohorts in the US Health and Retirement [HRS: Baby Boomers (BB) born 1948-1959; Older cohorts (OC) born before 1948] to examine this proposal. Eligible participants (N=8503: Mage=71) provided post-secondary education information in the HRS Life History Mail Survey (LHMS, 2015-2017) and cognitive status and participation in leisure activities in the 2016 or 2018 HRS biennial waves. Cohorts differed in rates of college education (BB 53% vs OC 42%) and overall leisure activity participation (BB>OC, p<.001). A multiple regression analysis predicting cognitive status (normal vs impaired: Langa-Weir algorithm) revealed significant effects for college education (OR=O.47), leisure activities (OR=0.87), and cohort (ps <.001), after controls for age, gender, wealth, race, marital status, chronic illnesses, and depression. In follow-up cohort-stratified analyses, we grouped people by level of participation in specific activity types (top quartile vs rest). Interestingly, within the older cohort, the risk of cognitive impairment was significantly reduced by high participation in cognitive (OR=0.42) and physical (OR=0.71) leisure activities in addition to having college education (OR=0.47). Within the BB cohort, only people with high participation in cognitive leisure activities (OR=0.65) in addition to college education (OR=0.38) had a reduced risk. Future research should investigate why some leisure activities seem to confer benefits for cognitive resilience whereas others don't.

Abstract citation ID: igae098.3310
### CONFLICT MONITORING IN AMNESTIC MILD COGNITIVE IMPAIRMENT: FINDINGS FROM A PICTURE–WORD INTERFERENCE PARADIGM
Elizabeth Lydon[1], and Raksha Mudar[2], *1. University of Illinois Urbana-Champaign, Champaign, Illinois, United States*, *2. University of Illinois, Champaign, Illinois, United States*

Older adults with amnestic mild cognitive impairment (MCI) experience a decline in episodic memory but do not meet diagnostic criteria for dementia. Mounting evidence suggests other aspects of cognition may be impacted, including behavioral and neural alterations in conflict monitoring. Neural underpinnings of these changes have been captured through the use of electroencephalography (EEG) by most frequently examining event-related potentials (ERPs). Fewer studies have examined event-related spectral perturbations (ERSPs) that underlie conflict processes in MCI. We examined differences between 27 individuals diagnosed with amnestic MCI (age: 75.3 ± 5.6) and 27 age- and education-matched cognitively healthy controls (HC; age: 72.6 ± 7.9) using a novel picture-word interference paradigm. Stimuli for this task included line drawings of animals and objects with matched or mismatched word labels. Participants judged if the picture-word pairs were matched or mismatched using a response box while continuous EEG was recorded. We examined differences between HC and MCI on behavioral performance (accuracy and reaction time) and ERSP measures (mean spectral power in theta, alpha, and beta frequency bands). Behavioral results reveled that the MCI group took longer to respond and was less accurate than the HC group on mismatched (conflict) trials across both tasks. Additionally, both groups were less accurate when responding to mismatched versus matched trials across tasks. Preliminary analysis of ERSP measures showed different patterns of neural oscillatory activity within and across the two groups. Findings support the presence of behavioral and neural alterations in conflict processing in amnestic MCI.

Abstract citation ID: igae098.3311
### CORRELATIONS AMONG SLEEP BIOMETRICS, DAILY STEPS, AND SELF-REPORTED FUNCTION IN OLDER COUPLES WITH AND WITHOUT MCI
Kourosh Alimohammadbeik, and Cynthia Jacelon, *University of Massachusetts Amherst, Amherst, Massachusetts, United States*

Altered sleep patterns are common among older individuals and those with Mild Cognitive Impairment (MCI). Sleep changes can lead to fatigue, and alterations in physical and social function. In couples, disrupted sleep of one partner can affect the other's sleep patterns. We compared measures of sleep and steps, captured by actigraph, with self-reported fatigue, physical function, and social roles among cognitively intact couples, and those with one partner who had MCI. The WHO International Classification of Functioning, Disability, and Health was the framework. Using a correlational design, we compared biometric measures with scales on the PROMIS 29 at three points in time in 13 couples >70 years old. Eight couples had one partner who scored < 25 on the MoCA. Significance was set at P =0.05. In cognitively intact couples, total and deep sleep time were correlated with fatigue (negative) and social roles. Deep sleep time was associated with physical function. Bedtime and time awake were correlated with fatigue (negative) and social roles. In couples with MCI, total and deep sleep time were associated with fatigue (negative). Steps correlated with physical activity, fatigue (negative), and social roles. Time awake was positively associated with fatigue. Bedtime and number of awakenings were associated with social role (negative). Sleep quality and steps have significant effects on self-reported function of couples with and without MCI. Total and deep sleep time were most important for cognitively intact couples, and number of steps was most important for couples with MCI.

Abstract citation ID: igae098.3312

### DIFFERENCES IN ADVANCED CARE PLANNING BY LEVEL OF COGNITIVE IMPAIRMENT

Isabella Arellanes[1], and Mireille Jacobson[2], *1. University of Southern California Leonard Davis School of Gerontology, Los Angeles, California, United States*, *2. University of Southern California, Los Angeles, California, United States*

Advance Care Planning (ACP) allows patients to make decisions regarding their health care while they still have the ability to do so. ACP may be particularly important for individuals experiencing cognitive decline who are likely to become unable to make decisions for themselves. This study identifies differences in advance care planning based on cognitive impairment status. This sample includes 6,650 older adults between the age of 65-90 years from the 2018 wave of the Health and Retirement Study. Cognition was categorized based on the Langa-Weir cognition classifications, with normal cognition classified by scores of 12-27 (n= 5,313), “cognitive impairment, not dementia” by scores of 7-11 (n= 1,085), and dementia by scores between 0-6 (n= 252). Among respondents with a normal cognition score, 65% reported having either a living will or power of attorney. Among those categorized as having “cognitive impairment, not dementia” and dementia, the percentages of ACP completion were lower, 59% and 54%, respectively. In a regression model controlling for race/ethnicity, gender, years of education, marital status, AD or dementia diagnosis, income, and healthcare utilization, all but diagnosis were significant predictors of ACP. There was a significant difference in ACP between those with normal cognition as those classified as having “cognitive impairment, not dementia” (p< 0.05). Compared to those in the normal cognition group, those in the “cognitive impairment, not dementia” group are 1.3 times more likely to engage in ACP (p < 0.05). These findings suggest differences in significant predictors of ACP based on level of cognitive impairment.

Abstract citation ID: igae098.3313

### DIGITAL INTERVENTIONS FOR PEOPLE WITH MILD COGNITIVE IMPAIRMENT: A SYSTEMATIC REVIEW OF RCTS

Chanchan He[1], Nan Jiang[2], Ke Chen[2], Yue Yu[3], and Yexuan Xiao[2], *1. Tsinghua University, Shenzhen, Beijing, China (People's Republic)*, *2. Tsinghua University, Beijing, Beijing, China (People's Republic)*, *3. Tsinghua Shenzhen International Graduate School, Shenzhen, Guangdong, China (People's Republic)*

**Background:** Digital interventions aim to improve outcomes in irreversible Mild Cognitive Impairment (MCI) with limited evidence on effective strategies. This review evaluates the effectiveness of such interventions through randomized controlled trials (RCTs), identifying key characteristics of success.
**Methods:** The search was conducted on CINAHL, Embase, MEDLINE, PubMed, PsycINFO, and grey literature from January 1, 2010, to November 20, 2023, to identify randomized controlled trials that examined the effects of digital interventions on global cognitive function outcomes in individuals with MCI. Mean differences and standard deviations of neuropsychological assessment scores were extracted to derive standardized mean differences.
**Results:** Out of 2,859 studies, 19 were analyzed. Digital interventions improved global cognitive function in MCI patients (SMD (95% CI) = 0.40 (0.22-0.59)), executive function (-0.48 (-0.76 to -0.21)) and attention (0.39 (0.16-0.61)). Four critical factors contribute to the success of these interventions: (1) Ease of use; (2) Opportunities for social interactions; (3) Human support; (4) Engagement in physical exercises; and (5) interventions tailored to the participants' needs. Additionally, methodological considerations for future randomized controlled trials on digital MCI interventions include: (1) Inclusion of both a healthy control group and an intervention group with clinically diagnosed MCI; (2) Collecting support-related data throughout the intervention period; (3) Assessing intervention success qualitatively and quantitatively; (4) Conducting follow-up interviews and surveys up to 1-year post-intervention to evaluate long-term outcomes.
**Conclusion:** The insights from this systematic review empower future digital MCI intervention researchers, health professionals, clinicians, and patients to design, develop, and implement successful interventions for older users.

Abstract citation ID: igae098.3314

### EFFECTS OF FRAILTY AND FUNCTIONAL IMPAIRMENT ON THE ASSOCIATIONS OF COGNITIVE IMPAIRMENT AND DEMENTIA WITH HEALTHCARE COSTS

Howard Fink[1], Kerry Sheets[2], Lisa Langsetmo[1], John Schousboe[3], Allyson Kats[4], and Kristine Ensrud[4], *1. Minneapolis Veterans Affairs Health Care System, Minneapolis, Minnesota, United States*, *2. Hennepin Healthcare, Minneapolis, Minnesota, United States*, *3. HealthPartners, St. Paul, Minnesota, United States*, *4. University of Minnesota, Minneapolis, Minnesota, United States*

Dementia and cognitive impairment (DCI) are associated with higher total healthcare costs (THC). We sought to determine the fraction of incremental THC of DCI accounted for by frailty and/or functional impairment. We utilized a multi-cohort dataset of 8165 community-dwelling, fee-for-service Medicare beneficiaries (53% women, 79% non-Hispanic White, mean age 79 years) who had index exams in 1 of 4 prospective cohort studies linked with Medicare claims. Cohort-based DCI was defined by self-or-proxy report of a dementia diagnosis or abnormal cognitive test results. Multimorbidity was count of chronic conditions. Frailty was a claims-based deficit accumulation index (CFI) or the Cardiovascular Health Study phenotype (3+ components of shrinking, slowness, weakness, poor energy, low physical activity). Functional impairment was self-reported difficulty performing 4 ADLs (with none, 1, 2, 3 or 4 of walking, climbing steps, transferring, bathing/showering).

Annualized THC were ascertained for 36 months following index exams. After adjustment for age, race, geographic region, and multimorbidity, those with cohort-based DCI vs. no DCI had higher incremental THC ($6883 in women and $7276 in men). Respectively, 40%, 31%, 33% and 63% of incremental THC in women and 28%, 29%, 40% and 58% of incremental THC in men were attributable to CFI, phenotypic frailty, functional impairments or a combination of all 3 measures. In conclusion, in both men and women, most of the incremental THC of DCI were attributable to frailty and functional impairment. Further attention should be given to assessment of these geriatric syndromes and potential mitigation of their impact among those with DCI.

Abstract citation ID: igae098.3315

### EXAMINING THE IMPACT OF POSTSTROKE COGNITIVE IMPAIRMENT ON FUNCTIONAL MOBILITY

Anjali Tiwari, and Neha Lodha, *Colorado State University, Fort Collins, Colorado, United States*

Approximately, 70% of individuals experience some degree of cognitive impairment following stroke. The study aims to determine the impact of post-stroke cognitive impairment on functional mobility.

**Methods:** Twenty-nine stroke survivors (cognitively impaired; N = 15 and cognitively normal; N = 14) participated in this study. Participants performed (1) Overground walking while simultaneously performing a secondary cognitive task. We measured gait speed (m/s) and stride time variability (%). 2) Simulator driving task: Participants performed a car-following task and responded to the brake lights of the lead car. We measured the mean (pounds), and variability in brake force (%). To examine whether post-stroke cognitive impairment influences performance on walking and driving tasks, we conducted a one-way ANCOVA test while adjusting for the stroke motor severity assessed by the Modified Rankin Scale. Results – The cognitively impaired stroke group showed slower gait speed ($F(1, 26) = 9.594$, $p=0.005$), increased stride length variability ($F(1,26) = 5.505$, $p=0.033$), and brake force variability ($F(1, 26) = 3.316$, $p= 0.035$) compared with the cognitively normal stroke group. The two groups showed no significant difference in mean brake force ($F(1, 26) = 3.345$ $p= 0.087$). Conclusion- Post-stroke cognitive impairment significantly impacts functional mobility and contributes to impaired walking and braking function, even after controlling for the severity of motor impairment post-stroke. Significance- The findings underscore the critical role of cognitive function in post-stroke recovery of functional mobility, above and beyond motor function. Intervention targeting cognitive function alongside motor function would be important for optimal functional recovery following stroke.

Abstract citation ID: igae098.3316

### IMPACT OF RTMS ON CAREGIVER BURDEN

Prasad Padala[1], Christopher Parkes[1], C. Heath Gauss[2], and Kalpana Padala[1], *1. Central Arkansas Veterans Healthcare System, North Little Rock, Arkansas, United States, 2. University of Arkansas for Medical Sciences, Little Rock, Arkansas, United States*

**Background:** Caregivers of persons with cognitive impairment experience a high level of burden. While the deleterious psychosocial and mental health effects of dementia caregiving are firmly established, very little is known about the burdens or psychiatric outcomes of providing care to a spouse with mild cognitive impairment (MCI). These individuals may be ideal targets for selective preventive interventions to maximize their psychological well-being as caregiving burdens related to their spouses' cognitive impairment increase. The objective is to assess the changes in caregiver burden after the single session repetitive transcranial magnetic stimulation (rTMS) in participants with MCI.

**Methods:** Participants with MCI (N=32) along with their caregivers were recruited as part of an interventional study for apathy using rTMS. The ongoing study is a double-blind randomized sham-controlled study of 20 treatments with rTMS/sham coil. In order to test the use of single session as a prognosticator of the full treatment, all participants received a single active treatment 2-weeks prior to the full treatment. Baseline assessments included measures of memory, behavioral problems, functioning and caregiver burden.

**Results:** Mean age was 76.7(±7.3) years, 90% were Caucasian and 10% were female. Mean Mini Mental State Examination (MMSE) score was 25.7(±2.9), Apathy Evaluation Scale-Clinician version (AES-C) was 47.1(±7.7), and the Zarit Burden Score was 43(±6.1) at baseline. There was a significant improvement in the caregiver burden and apathy after the single session rTMS although there was no statistically significant change in MMSE. Conclusions: Noninterventional neuromodulation such as rTMS may play a role in alleviating caregiver burden.

Abstract citation ID: igae098.3317

### MODERATING EFFECT OF DEPRESSION ON THE ASSOCIATION OF COGNITIVE IMPAIRMENT WITH EVERYDAY FUNCTION IN OLDER ADULTS

Thomas McIntyre[1], Jacqueline Becker[1], Katherine Hackett[2], Lili Chan[1], Fernando Carnavali[1], Juan Wisnivesky[1], Laura Curtis[3], and Alex Federman[2], *1. Ichan School of Medicine at Mount Sinai, New York, New York, United States, 2. Icahn School of Medicine at Mount Sinai, New York, New York, United States, 3. Northwestern University Feinberg School of Medicine, Chicago, Illinois, United States*

Cognitive impairment (CI) and depression often co-occur, and each is known to impair older adults' ability to perform activities of daily living. We sought to determine whether co-morbid depression moderates the association of CI with impairments in instrumental activities of daily living (IADL). We recruited English speaking adults ages ≥55 years without preexisting dementia or CI from primary care practices in New York City and Chicago (n=1,045). IADLs were assessed using Lawton's 8-item scale and impairment was defined as ≥1 activity requiring assistance. Cognitive function was measured using Montreal Cognitive Assessments (MoCA); moderate-severe CI was defined as MoCA score ≥1.5 standard deviations below the age and education adjusted z-score. Depression was defined as a score ≥10 using Patient Health Questionnaires (PHQ)-8. We fit linear regression models estimating the association of mild CI, moderate-severe CI, and depression with counts of IADL impairments, adjusting for age and sex. We then added an interaction term for CI and depression. The mean age was 67 (8.0) years, 54% were female, 35% Black and 25% Hispanic. Median IADL impairments were 0 (IQR 0-7); 16% had mild CI, 5% moderate-severe CI, and 15% had depression. Moderate-severe CI (β=0.84, p< 0.01) and severe depression (β=1.31, p< 0.01) were each independently associated with IADL impairments in the adjusted model. The interaction term of moderate-severe CI with depression was also significant (β -0.85, p < 0.01). Depression significantly worsens the association of CI with IADL impairment, highlighting the importance of identifying and treating this common condition in older adults.

Abstract citation ID: igae098.3318

### MULTIDIMENSIONAL SOCIAL RELATIONSHIP AND LONELINESS AMONG OLDER ADULTS WITH COGNITIVE IMPAIRMENT

Soobin Park[1], Sojung Park[1], BoRin Kim[2], Takashi Amano[3], and Jihye Baek[1], *1. Washington University in St. Louis, St. Louis, Missouri, United States*, *2. University of New Hampshire, Durham, New Hampshire, United States*, *3. Rutgers, The State University of New Jersey, Newark, New Jersey, United States*

As a modifiable risk factor of cognitive impairment, loneliness has received growing attention in research. By focusing on older adults with cognitive impairment, this study aims to add to the knowledge about the complex association among cognitive impairment, social relationships, and loneliness We explored distinct patterns of multidimensional social relationships and examined how these patterns influence social and emotional loneliness among older adults with cognitive impairments. Data came from the 2018 Health and Retirement Study and aged over 65 years. To focus on older adults with cognitive impairments, we excluded respondents with high cognitive function measured by the Telephone Interview for Cognitive Status (TICS) score (N=640). Latent class analysis was used to identify subgroups of social relations using multidimensional indicators of social relations including quantity (e.g., frequency of social contact with varying modes, social network size) and quality (e.g., positive and negative social support). Five patterns of social relationships emerged: Lack Friends/Weak (16%), Lack Children/Positive Friends (10%), Lack Family (11%), Virtual Diverse (52%), and Diverse/Negative (11%). Regression analyses showed Diverse/Negative group (p< 0.01) was significantly associated with a higher level of emotional loneliness compared to the Virtual Diverse group. All three groups (p< 0.05) except Lack Children/Positive Friends (p>.1) had significantly a higher level of social loneliness. Interestingly, more than half had a quantitatively diverse social relationship, maintaining rich social networks despite their cognitive impairment. Our findings highlight the importance of efforts that help decrease negative and secure positive or meaningful support as ways to reduce loneliness among cognitively impaired older adults.

Abstract citation ID: igae098.3319

### NEIGHBORHOOD DISORDER AND COGNITIVE HEATH: THE CAUSAL MEDIATING EFFECTS OF ALLOSTATIC LOAD AND PHYSICAL ACTIVITIES

Jiao Yu, Yi Wang, and Xi Chen, *Yale University, New Haven, Connecticut, United States*

Numerous studies have attempted to establish a link between neighborhood disorder and later life cognitive decline, with stress accumulation and reduced physical activity hypothesized as potential mediators. However, there remains a significant gap in our understanding of the causal mechanisms underlying this link. This research utilizes data from the Health and Retirement Study (2006-2008 and 2010-2012) to investigate whether stress accumulation, as measured by allostatic load, and physical activity mediate the association between perceived neighborhood disorder and cognitive health. Older Americans aged 65 years and older without dementia at baseline were selected (n=4,346). Cognitive function was measured using the Telephone Interview for Cognitive Status (TICS). Employing propensity score matching followed by causal mediation analyses, we find that higher levels of perceived neighborhood disorder at baseline are associated with poorer cognitive health at a subsequent wave. Under a counterfactual framework, if every respondent had experienced one standard deviation above the mean level of neighborhood disorder, the cognitive score would be 0.68 lower than the score if everyone had experienced one standard deviation below the mean (total effect: -0.68, 95% CI: -0.94, -0.42). Approximately three percent (natural indirect effect = -0.02, 95% CI: -0.04, -0.01) of this effect is attributable to physical activity. Notably, we find no significant mediating effect of allostatic load (natural indirect effect = -0.005, 95% CI: -0.02, 0.004) after adjusting for individual-level characteristics. Our findings underscore the significant role of the behavioral factor in mediating the relationship between disadvantaged neighborhoods and cognitive health among older adults.

Abstract citation ID: igae098.3320

### NEUROCOGNITIVE CORRELATES OF COMORBIDITIES IN OLDER ADULTS WITH CHRONIC PAIN AND NEGATIVE EMOTIONS

Irina Mindlis[1], Lisa Ravdin[2], M. Carrington Reid[3], and Dimitris Kiosses[2], *1. Weill Cornell Medicine, New York, New York, United States*, *2. Weill Cornell Medicine, New York, New York, United States*, *3. Weill Cornell Medicine, Weill Cornell Medicine, New York, United States*

Chronic pain and depression are associated with poor cognition in older adults, yet little is known regarding the additional contribution of comorbidities to performance on specific neurocognitive domains in this population. We examined specific neurocognitive domain performance using baseline data from the Problem Adaptation Therapy for Pain Collaborative Care Program (PATH-Pain) trial, which tested a psychosocial intervention designed to improve emotion regulation in 100 adults ≥ 60 years with comorbid chronic pain and negative emotions (vs. usual care). Participants completed questionnaires on comorbidities (Charlson Comorbidity Index), depressive symptoms (Montgomery-Asberg Depression Rating Scale), pain intensity, and were remotely administered the Montreal Cognitive Assessment with no visual elements (MoCA-blind) and the Repeatable

Battery for Neuropsychological Status. Regression assessed the relationship between specific neurocognitive domains and comorbidities, adjusting for depressive symptoms and pain intensity. In adjusted models, greater comorbidities remained associated with poorer immediate memory (b = -.352, p <.001) adjusting for pain intensity (b =.217, p =.040) and depressive symptoms (ns). After excluding participants with neurologic conditions, immediate memory remained associated with comorbidities (b = -.304, p =.019), but not pain intensity. Similar patterns emerged for attention, with greater comorbidities associated with poorer performance among all participants (b = -.266, p =.015), and for participants without neurological diagnoses (b = -.301, p =.020). No associations were found with language, delayed memory, abstraction, or orientation. Findings suggest difficulties with initial encoding and verbal learning; further research is needed to understand the potential impact on health behaviors and outcomes.

Abstract citation ID: igae098.3321

### OLFACTION AND COGNITIVE FUNCTION IN NONDEMENTED OLDER ADULTS: FINDINGS FROM HEALTH ABC

Yaqun Yuan[1], Keran Chamberlin[2], Chenxi Li[1], Zhehui Luo[1], Anna Kucharska-Newton[3], Srishti Shrestha[4], Eleanor Simonsick[5], and Honglei Chen[6], *1. Michigan State University East Lansing, East Lansing, Michigan, United States*, *2. Michigan State University, United States*, *3. University of North Carolina at Chapel Hill, Chapel Hill, North Carolina, United States*, *4. University of Mississippi Medical Center, Jackson, Mississippi, United States*, *5. National Institute on Aging, Baltimore City 0400 Baltimore City, Maryland, United States*, *6. Michigan State University, Michigan State University, Michigan, United States*

We examined olfaction in relation to longitudinal change in cognitive function among 1987 community-dwelling participants of the Health, Aging and Body Composition study (aged 71-82 years, 52.8% women, and 33.1% Black race), free of dementia and Parkinson's disease. Participants completed the Brief Smell Identification Test at the year 3 clinic visit in 1999-2000. Olfaction was defined as good (test score 11-12), moderate (9-10), hyposmia (7-8), or anosmia (0-6). Cognitive function was assessed using the Modified Mini-Mental State examination (3MS) up to 5 times at clinic visits in year 3, 5, 8, 10 and 11. We conducted multivariable joint model analyses to account for potential bias due to death and loss to follow-up, in addition to potential confounders. Olfaction status was inversely associated with 3MS test score in both cross-sectional and longitudinal analyses over 8 years of follow-up. At baseline, compared with participants with good olfaction, the 3MS test score was 0.80 (95%CI, 0.21-1.38) points lower for those with moderate olfaction, 1.82 (95%CI, 1.08-2.57) points lower for those with hyposmia, and 1.87 (95%CI, 1.00-2.75) points lower for those with anosmia. Longitudinally, the annual decline was 0.09 (95%CI, 0.01-0.17) faster for those with moderate olfaction, 0.22 (95%CI, 0.11-0.32) faster for those with hyposmia, and 0.30 (95%CI, 0.17-0.43) for those with anosmia. Similar associations were observed across subgroups defined by sex, race, self-reported general health status, and APOE-ε4 genotype. Our study provides important evidence that poor olfaction is associated with a faster decline in cognitive function in non-demented older adults.

Abstract citation ID: igae098.3322

### POTENTIAL MENTAL BENEFITS OF REMOTE EXPRESSIVE ARTS PROGRAM IN OLDER ADULTS WITH MILD COGNITIVE IMPAIRMENT

Yuting Luo, and Hong Li, *Fujian Medical University, Fuzhou, Fujian, China (People's Republic)*

The use of expressive arts program (EAP) in mild cognitive impairment may positively impact the psychological well-being. A future direction for EAP may be to explore a new channel for MCI patients to participate via the mobile internet, as this could improve accessibility. The study explores the impact of a remote expressive arts program (rEAP) on the mental health of older adults with mild cognitive impairment (MCI). Conducted through a two-arm, single-blinded, pilot, randomized controlled trial, participants were assigned to either a 12-week rEAP or health education (HE) group. Pre- and post-treatment psychological evaluations were conducted. Results from 73 MCI patients showed significant improvements in anxiety mood (Self-Rating Anxiety Scale, P=0.032) and potential benefits in subjective well-being, depression, self-esteem, and self-efficacy in the rEAP group compared to the HE group. Qualitative interviews with rEAP participants highlighted enthusiasm, feasibility, usability, and content satisfaction. Positive cognitive and mental benefits were reported, with themes including enhanced cognitive function perception, relaxation, creativity, self-confidence, and social engagement. The findings suggest that a 12-week rEAP can enhance the psychological well-being of older adults with MCI, indicating its feasibility and applicability for this population. The study underscores the significance of rEAP as a feasible intervention leveraging mobile internet to improve mental health in older adults with MCI.

Abstract citation ID: igae098.3323

### PREDICTING MILD COGNITIVE IMPAIRMENT AND DEMENTIA: MACHINE LEARNING VERSUS TRADITIONAL METHODOLOGIES

Bada Kang[1], Dahye Hong[1], Jennifer Kim[1], and Sarah Oh[2], *1. Yonsei University, Seoul, Republic of Korea*, *2. Harvard T.H. Chan School of Public Health, Boston, Massachusetts, United States*

Although mild cognitive impairment (MCI) faces significant challenges in terms of early diagnosis and intervention, machine learning (ML) shows promise in managing complex data for MCI and dementia prediction. This study assessed and compared the predictive accuracy of ML models with traditional logistic regression in identifying MCI and dementia onset using the Korean Longitudinal Study of Aging (KLoSA) dataset. The sample comprised 4,975 older adults aged 60 years or older. Identification of MCI and dementia relied on self-reported diagnoses with sociodemographic, lifestyle, and health-related variables as key covariates. The dataset was categorized into training and test sets to predict MCI and dementia, employing models such as logistic regression, light gradient-boosting machine, XGBoost, CatBoost, and Random Forest. Shapley additive explanation values were used to determine the contribution of each feature to the prediction rate. Logistic regression excelled in predicting dementia onset, whereas XGBoost performed better in predicting MCI (XGBoost AUC: 0.7271; Random

Forest AUC: 0.6741). Increased assets reduced MCI risk, however, lower education, reduced activities of daily living functional ability, and pain in daily life increased MCI risk. Predictors of developing dementia were being female, older age, reduced activities of daily living functional ability, and lower education. The ML algorithms, particularly XGBoost, demonstrated potential for predicting MCI onset using KLoSA data. This study underscores the crucial role of sociodemographic factors in the early prediction and prevention of cognitive impairment. By leveraging readily available sociodemographic and health-related characteristics, more cost-effective and targeted interventions can be provided to at-risk community-dwelling older adults.

Abstract citation ID: igae098.3324

### RACIAL/ETHNIC DIFFERENCES IN SUBJECTIVE COGNITIVE DECLINE SYNDROMES

Moyosoreoluwa Jacobs[1], Peter Lichtenberg[2], and Wassim Tarraf[2], *1. Wayne State University, Melvindale, Michigan, United States*, *2. Wayne State University, Detroit, Michigan, United States*

Subjective cognitive decline (SCD) is a precursor to Alzheimer's Disease and Related Dementias (ADRD). SCD rates vary by race/ethnicity. SCD is also heterogenous in the population and can yield different syndromes. We used Behavioral Risk Factor Surveillance System data (2015/2016 and 2019/2020) from non-Hispanic White (NHW), Black (NHB), and Hispanic individuals aged 50+ years (unweighted-n=44,152) who endorsed SCD, to fit latent class analysis (LCA) and derive SCD syndromes based on measures of activities limitations, need for assistance, and consulting with healthcare providers. We used multinomial logistic regression to model differences in syndrome prevalence by R/E and assess the contributions of predisposing (e.g., age), enabling (e.g., insurance), and health need (E.g., cardiovascular) factors to explaining these racial/ethnic (R/E) differences. Three groupings (social limitations [13.3%], mixed functional limitations [26.2%] and uncomplicated [60.5%]) fit the data well. Compared to NHWs (21.9%), Hispanics and NHBs were significantly more likely to meet criteria for mixed functional limitations [38.2% and 41.2% respectively] and less likely to be in the uncomplicated group. Predisposing, enabling, and health need factors only partially attenuated (10-22%) the Relative Risk Ratios for classification in the mixed functional limitations for NHBs and Hispanics. Hispanics and NHBs presented with more mixed SCD-related functional limitations compared to NHWs, suggesting higher risk for developing ADRDs. Since nearly 2/3 individuals in the mixed functional limitations group reported discussing SCD with healthcare providers, there are opportunities to provide interventions for this high risk group at their place of care, and reduce R/E disparities in SCD and ADRD.

Abstract citation ID: igae098.3325

### SUBJECTIVE COGNITIVE DECLINE AND DAILY AFFECTIVE WELL-BEING

Mijin Jeong[1], Jacqueline Mogle[2], Heejung Jang[2], Nikki Hill[3], and Mindy Katz[4], *1. Keimyung University, Daegu, Republic of Korea*, *2. Clemson University, Clemson, South Carolina, United States*, *3. The Pennsylvania State University, University Park, Pennsylvania, United States*, *4. Albert Einstein College of Medicine, Bronx, New York, United States*

Subjective cognitive decline (SCD) is self-reported cognitive decline in the absence of objective cognitive deficits. Although SCD (especially SCD accompanied by worry about the decline) is considered as the earliest progress of dementia, less is known about daily experiences of affect among older adults with SCD. Considering depression is deeply associated with progress of dementia, it is important to understand how individuals with SCD have experienced daily affect. We examined differences in levels of daily affective well-being for older adults with and without SCD. In the Einstein Aging Study (EAS), a total of 210 participants reported whether they perceived SCD and if so, indicated whether were worried by this decline. Daily positive affect (PA) and daily negative affect (NA) were measured using 8 items (happy; relaxed; energetic, enjoy; anxiety; loneliness; depression; and frustration) each day for 14 continuous days (between May 2017 and March 2020). We tested hypotheses by using multilevel models (MLM). For daily PA, SCD (either with or without worry) was associated with lower average daily reports of total PA specifically driven by differences on the items "happy" and "relaxed" compared to individuals without SCD. For daily NA, endorsement of SCD with worry was associated with higher total NA, specifically for the items "anxiety," "depression," and "frustration" compared to individuals without SCD and those with SCD but without worry. This study helps to better understand the impact of perceiving early cognitive changes on different aspects of daily affect to identify targets for promoting psychological well-being among older adults.

Abstract citation ID: igae098.3326

### SUBJECTIVE COGNITIVE DECLINE AND ITS IMPACTS AMONG MIDDLE-AGED AND OLDER WOMEN VETERANS AND NONVETERANS

Amy Henion[1], Amanda Cheney[2], Kim Sundstrom[1], Mohammed Alshanbari[1], and Erin Bouldin[1], *1. University of Utah, Salt Lake City, Utah, United States*, *2. University of Utah, Orem, Utah, United States*

Veterans more frequently experience traumatic brain injuries than their non-veteran peers and therefore may be at higher risk of cognitive decline. Little is known about subjective cognitive decline (SCD) among women veterans. We aimed to describe the prevalence and impacts of SCD among women veterans and compare them with non-veteran women using 2021-2022 Behavioral Risk Factor Surveillance System data from 29 jurisdictions that included the optional Cognitive Decline module. Respondents aged ≥45 years were classified as having SCD if they experienced increased memory problems or confusion over the past year. We calculated weighted estimates among 1,905 women veterans (n=268 with SCD) and 66,880 women non-veterans (n=7,609 with SCD). Women veterans were slightly younger than non-veterans (mean 61.8 versus 64.0 years, p=0.045) and more often had depression history (65.8% versus 47.9%, p=0.003). Other demographic and health characteristics – including risk factors for dementia – were similar. SCD prevalence was 13.3% among veterans and 11.7% among non-veterans (p=0.24). Among women with

SCD, there were no significant differences in SCD-related social or household limitations by veteran status. Similarly, the prevalence of needing help because of SCD was similar for women veterans and non-veterans (34.6% and 35.4%, p=0.89). Veterans had more frequently discussed their SCD with a healthcare provider than non-veterans (65.7% versus 47.4%, p< 0.001). SCD prevalence and SCD-related impacts were similar among veteran and non-veteran women. The question of why veterans are more likely to discuss their SCD with providers warrants exploration and may inform strategies to facilitate such discussions in other groups.

Abstract citation ID: igae098.3327

### TRENDS IN THE PREVALENCE OF CARDIOMETABOLIC COMORBIDITIES AMONG INDIVIDUALS WITH COGNITIVE IMPAIRMENTS

Yaguang Zheng, Xiang Qi, and Bei Wu, *New York University, New York, New York, United States*

Individuals with cognitive impairment have common cardiometabolic comorbidities; however, their prevalence trends remain unknown. We aimed to address this knowledge gap by selecting participants with cognitive impairment from the Health and Retirement Study. Cognitive status was evaluated using the validated Telephone Interview for Cognitive Status (TICS), categorizing participants into normal cognition (TICS≥12), mild cognitive impairment (MCI) (TICS 7-12), and dementia (TICS < 7). The study analyzed data from 40,170 participants with cognitive impairment, with an average age of 73.4±11.2 years. Between 1996 and 2018, there was an observed increase in the prevalence of hypertension, diabetes, and stroke among adults with dementia and MCI. Specifically, hypertension prevalence rose from 60.5% to 77.0% in the dementia group and from 55.7% to 72.9% in the MCI group, diabetes prevalence increased from 21.8% to 37.9% among those with dementia and from 19.1% to 34.5% among those with MCI, and stroke prevalence escalated from 13.5% to 18.0% in the dementia group and from 8.7% to 12.3% in the MCI group. Conversely, heart disease prevalence decreased in individuals with dementia (from 35.3% to 32.2%) but slightly increased in those with MCI (from 28.4% to 29.6%). Our study found that adults with dementia or MCI had an increased prevalence trend in hypertension, diabetes, and stroke over the past two decades, although those with dementia had a decreased prevalence trend in heart disease. Further action is needed to reduce the cardiometabolic burden in this vulnerable population.

Abstract citation ID: igae098.3328

### WHAT TYPES OF SUPPORT BURDENED DEMENTIA CAREGIVERS LOOK FOR?

Yeji Hwang[1], and Hannah Cho[2], *1. Seoul National University, Seoul, Republic of Korea*, *2. University of Pennsylvania, Philadelphia, Pennsylvania, United States*

As dementia caregivers provide excessive amount of care daily to persons living with dementia, many caregivers feel physical or emotional burden. However, lack of information is available on what kind of support that caregivers seek for when they feel physical or emotional burden. Therefore, this study aimed to investigate support that burdened dementia caregivers look for. We conducted a secondary data analysis of the National Study of Caregiving (NSOC) IV, focusing on caregivers of persons living with possible or probable dementia (n=518). Descriptive data analyses, chi square tests, and logistic regression analyses were performed for data analysis. In this study, when caregivers felt emotional burden (42%), they were more likely to use service that take care of the care recipient so that they could take some time away from caregiving (OR= 3.160, p=0.001) or find paid helper to do household chores or personal care (OR= 2.259, p=0.004). Similarly, when caregivers felt physical burden (23.98%), they were more likely to use service that take care of the care recipient so that they could take some time away from caregiving (OR= 3.050, p=0.004) or find paid helper to do household chores or personal care (OR= 2.651, p=0.006). Other support such as support group or training for caregivers did not show any significance. This study found dementia caregivers seek support services when they feel emotional or physical burden. Policy makers should consider these services readily accessible to dementia caregivers.

### SESSION 7635 (POSTER)

### COMMUNICATION AND LANGUAGE

Abstract citation ID: igae098.3329

### A QUICK NOVEL MOBILE HEARING IMPAIRMENT ASSESSMENT FOR DIGITAL SPEECH HEARING SCREENING

Russell Banks[1], Barry Greene[2], Isaiah Morrow[1], Marissa Ciesla[1], David Woolever[3], Sean Tobyne[1], Ali Jannati[1], and Alvaro Pascual-Leone[4], *1. Linus Health, Boston, Massachusetts, United States*, *2. Fire1, Glasnevin, Dublin, Ireland*, *3. Lucid Hearing, Wake Forest, North Carolina, United States*, *4. Hebrew SeniorLife, Boston, Massachusetts, United States*

It is estimated that 1 in 4 people worldwide will be living with hearing impairment by 2050. We propose a digital Speech Hearing Screener (dSHS) using short nonsense word recognition to measure speech-hearing ability. We compare dSHS outcomes with standardized pure-tone averages (PTA) and speech-recognition thresholds (SRT). 50 participants aged 55 or older underwent PTA and SRT measurement. One-way ANOVA was used to compare differences between hearing impaired and hearing not-impaired groups, by the dSHS, with a clinical threshold of moderately impaired hearing at 35dB and severe hearing impairment at 50 dB. dSHS results significantly correlated with PTAs/SRTs. ANOVA results revealed the dSHS was significantly different ($F(1,47)=38.1$, $p< 0.001$) between hearing impaired and unimpaired groups. Classification analysis using a 35dB threshold, yielded accuracy of 85.7% for PTA-based impairment and 81.6% for SR-based impairment. At a 50dB threshold, dSHS classification accuracy was 79.6% for PTA-based impairment (NPV-93%) and 83.7% (NPV-100%) for SRT-based impairment. The dSHS successfully differentiates between hearing-impaired and unimpaired individuals in under 3 minutes. This hearing screener offers a time-saving, in-clinic hearing screening to streamline the triage of those with likely hearing impairment to the appropriate follow-up assessment, thereby improving the quality of services. Additionally, this tool can help to rule out hearing impairment as a cause or confounder of cognitive impairment.

Abstract citation ID: igae098.3330
### CAREGIVER CONVERSATIONS WITH PERSONS WITH MILD COGNITIVE IMPAIRMENT, ALZHEIMER'S DISEASE, AND PARKINSON'S DISEASE

Marie Savundranayagam[1], Annette Schumann[1], Joseph Orange[1], Sharon Pritchard[1], M. Kathleen Pichora-Fuller[2], Walter Wittich[3], Paul Mick[4], and Jennifer Campos[5], *1. Western University, London, Ontario, Canada*, *2. University of Toronto, Toronto, Ontario, Canada*, *3. University of Montreal, Montreal, Quebec, Canada*, *4. University of Saskatchewan, Saskatoon, Saskatchewan, Canada*, *5. The KITE Research Institute, University Health Network, Toronto, Ontario, Canada*

This study describes conversations between persons living with mild cognitive impairment (MCI), Alzheimer's Disease (AD), Parkinson's Disease (PD) and their family caregivers, and the possible contributions of sensory loss. Conversations (N=130) between a participant (60% male) and a family caregiver (75% female; spouses) in the COMPASS-ND study were analyzed. Conversations were transcribed verbatim, segmented into communication units (c-units), and analyzed using the trouble-source repair (TSR) paradigm that includes analyses of communication breakdowns, repair initiators, and repair strategies. A one-way analysis of variance was conducted to evaluate between-group differences among sensory and communication variables. The AD group had poorer hearing than the MCI and PD groups. Both AD and MCI groups had poorer vision than the PD group. The MCI group used significantly more c-units than the AD and PD groups. The mean proportion of the TSR sequences was 13% across all groups, but with significantly more sequences in the AD and PD groups than the MCI group. Notably, the proportion of trouble sources created by caregivers was significantly greater for conversations in the AD group than the MCI or PD groups. The proportion of repair initiators used was significantly greater for the AD group than the MCI or PD groups. The intersection of cognition and sensory variables will be illustrated using three case studies. The findings highlight the active participation of persons living with AD and sensory loss in signalling misunderstandings created by caregivers. However, the interface between cognitive and sensory abilities influences the communication among both conversational partners.

Abstract citation ID: igae098.3331
### COMMUNITY PREVALENCE OF HEARING LOSS AMONG OLDER ADULTA IN MA, RI, CT, AND NH: COMPARING MEASURES

Eric Frimpong[1], Alexa Fleet[2], Constanza Tamara Matta Gallero[1], Taylor Jansen[1], and Elizabeth Dugan[1], *1. University of Massachusetts Boston, Boston, Massachusetts, United States*, *2. University of Massachusetts Boston-- Gerontology, Boston, Massachusetts, United States*

**Background:** Hearing impairment and hearing loss may negatively impact social engagement and social relationships. Understanding community rates of hearing loss would identify disparities and may inform service delivery and potentially improve quality of life for adults with hearing loss. The aim of this study is to describe community prevalence of hearing loss and to compare two measurement approaches: self-report and clinically derived from Medicare claims.

**Methods:** Data were from the Healthy Aging Data Report (healthyagingdatareports.org) for the New England states (MA, NH, CT, and RI) with indicators calculated from the American Community Surveys (MA: 2012-2016; RI:2016-2018; CT: 2014-2018 and NH: 2012-2016), and the Medicare Master Beneficiary Summary Files (MA: 2014-2015; RI: 2016-2017; CT: 2016-2017 and NH: 2014-2015).

**Results:** rates derived from CMS clinical diagnosis were higher (MA: 16.1%; RI: 16.4%; CT: 15.7%, and NH: 14.4%) compared to the self-reported ACS rates (MA: 14.2%; RI: 13.9%: CT: 12.4% and NH: 15.0%). The highest community prevalence rate of self-reported hearing loss (36.36%) was in Monroe MA. For clinically diagnosed hearing loss, Hanover NH (home to Dartmouth College and health system) reported the highest rate (26.12%). Having excellent access to health care is associated with higher community rates of clinically diagnosed hearing loss. Results showed that the community prevalence of hearing impairment was variable by community. The potential impact of hearing loss on social, emotional, and physical well-being indicates a need for interventions to diagnose and treat it.

Abstract citation ID: igae098.3332
### FEASIBILITY AND ACCURACY OF A POINT-OF-CARE COMMUNICATION FEEDBACK APP FOR NURSING HOME STAFF

Kristine Williams, Carissa Coleman, Jinxiang Hu, and Xi Chen, *University of Kansas Medical Center, Kansas City, Kansas, United States*

Educating the long-term-care workforce in dementia care skills is critical. Research has established that reinforcing learned skills in practice is important for successful adoption. However, on-site, expert feedback is costly and not always feasible. The 3-hour Changing Talk (CHAT) educational intervention decreases staff elderspeak (communication that sounds like babytalk) which in turn, reduces negative behavioral responses of care recipients with dementia. We developed an automated feedback IOS application that records staff and uses natural language processing to analyze their speech, providing just-in-time feedback on their use of elderspeak diminutives (terms of endearment). This study evaluated feasibility and accuracy of the SPEEKO for Elderspeak app for point-of-care feedback on staff elderspeak communication. Long-term-care staff (n=17) self-recorded their communication during resident care. Amazon Translate language processing and a customized elderspeak algorithm were used to identify staff use of diminutives, a common part of elderspeak, in the recordings. Trained coders also completed manual transcription and psycholinguistic coding of diminutives in the recordings that were compared to the app results with chi-squared tests. The relationship of total diminutives comparing human and app identification was significant $\chi 2=12.21$, $p<.001$. Associations were significant for elderspeak diminutive word roots ("dear" $\chi 2 =0.01$, $p=.004$); "sweet" ($\chi 2=20.20$, $p<.001$); and "girl" ($\chi 2=5.08$, $p=.02$). Other terms were not significantly correlated ("honey" $\chi 2=2.70$, $p=.10$) and "baby" ($\chi 2=0.85$, $p=.36$). With improvements to overcome issues in accuracy of recording, transcription, and analyses, the SPEEKO for Elderspeak app has potential to provide self-monitoring feedback promoting implementation of communication skills in dementia care.

Abstract citation ID: igae098.3333

**RECOLLECTING AND REFLECTING: STORY ASIDES IN YOUNG AND OLDER ADULTS' AUTOBIOGRAPHICAL STORIES**

Teagan McCune, and Nicole Alea, *University of California Santa Barbara, Santa Barbara, California, United States*

The ways people tell autobiographical stories changes with age. One change is the amount of tangentially-related information included in these stories. These pieces of information–story asides–add context (e.g. knowledge about the world or storyteller). Story asides are expressed more frequently in the autobiographical stories of older adults compared to young adults, but the underlying mechanism is unclear. One potential mechanism is how people remember the story. The current study tested two potential mediators of this expression in young and older adults' autobiographical stories. As part of a larger study, 61 young (M = 19.9 years) and 35 older (M = 73.8 years) adults shared an autobiographical story about the same event, a positive vacation. Participants then completed a modified version of the Autobiographical Memory Questionnaire, including 5 items about recollection (e.g., feeling the emotions they felt then) and 3 items about reflection (e.g., how significant this event is) on their autobiographical story. Mediation regression analyses indicated that story aside expression, recollecting about events, and reflecting on events all increased with increasing age. However, recollection and reflection did not predict story asides, nor were there any mediating effects of these variables between age and story aside expression in autobiographical stories. These findings suggest the way people remember and reflect on their stories is perhaps not an underlying mechanism for age group differences in story aside expression. If not the subjective indicators of memory quality and experience (recollection, reflection), then other cognitive and goal-oriented variables might explain these changes.

Abstract citation ID: igae098.3334

**RELATIONAL COLLABORATION BEYOND PROFESSIONAL TEAMS: NEGOTIATING HYBRID EXPERTISE WITH INFORMAL CAREGIVERS**

Laura Ginoux, and Kirstie McAllum, *Université de Montréal, Montréal, Quebec, Canada*

When conceptualizing collaboration in the context of health and social care, most definitions privilege interactions among members of the interprofessional team. To achieve joint goals and solve problems more effectively, these professionals must combine and integrate their disciplinary knowledge with that of others, including those outside the immediate healthcare team. Over the last three decades, the health communication literature has insisted on the need for patient-centered healthcare practices. Patient-centeredness includes appreciation for and inclusion of patients' experiential knowledge (based on their daily experiences with health conditions). We note that the literature neglects the idea of experiential expertise of informal caregivers (ICG). Yet, ICGs are precious aids and mediators between the older adults they care for and healthcare workers (HW). Unfortunately, ICGs are not always included in decision-making processes, and hierarchies of knowledge remain within relationships with HWs. Following a literature review, our research highlights the need to develop a relational vision of collaboration that would enable interprofessional teams to consider ICGs as part of the team, enhance the quality of interactions, and, therefore, improve the quality of care provided to older adults. The objectives of this paper are threefold. First, we define our theoretical model of relational collaboration. Second, we explain why developing relational collaboration between ICGs and HWs benefits teams as well as older adults. Third, we contend that nurturing the relational dimensions of collaboration (respect, cultural humility, empathy, compassion, and trust) enables ICGs and HWs to negotiate a hybrid form of expertise that integrates medical and experiential knowledge.

### SESSION 7640 (POSTER)

### COMMUNITY-BASED RESEARCH

Abstract citation ID: igae098.3335

**A PARTICIPATORY STUDY TO CO-DESIGN SOCIAL NORM NUDGES FOR IMPROVING MENTAL HEALTH HELP-SEEKING IN OLDER PEOPLE**

Tianyin Liu, and Jia Wang, *The Hong Kong Polytechnic University, Hong Kong, Hong Kong*

Mental health help-seeking is often delayed in older people, and different theories have hypothesized diverse mechanisms to improve help-seeking. The theory of planned behavior (TPB) and nudge theory (NT) converge on social norms as promising targets for intervention. Despite accumulating evidence of social norm nudges in promoting health behaviors, no intervention has utilized social norms to promote mental health help-seeking in older adults. This study aims to (a) co-design social norm-nudges with different stakeholders and (b) pilot their feasibility and effects in improving mental health help-seeking intention in older people. The participatory study recruited ten older people, two social workers, and two experienced researchers to co-design the social norm nudges. All participants received three-hour training on TPB and NT principles, followed by ten interactive co-design workshops over three months. A set of 14 social norm nudges was designed and piloted among 20 older people for feasibility and effects. The participants completed a self-administered baseline questionnaire, received 14 social norm nudges over smartphones in two weeks (one message per day), completed the post-intervention questionnaire and provided feedback to the nudges afterwards. The primary outcomes were changes in subjective norms and help-seeking intention assessed using a revised Chinese version of the TPB questionnaire (C-TPB); secondary outcomes included perceived behavioral control, help-seeking attitude, perceived barriers to help-seeking (C-TPB subscales), and mental health assessed by PHQ-9, GAD-7 and UCLA-3. Preliminary results suggested the feasibility of the social norms to be delivered digitally and the effects in increasing help-seeking intention and attitude.

Abstract citation ID: igae098.3336

### A QUALITATIVE ANALYSIS OF PERCEIVED BEREAVEMENT NEEDS OF COMMUNITY-DWELLING OLDER ADULTS

Nicole Praska[1], Marcus Chur[2], Caroline Collins-Pisano[1], and Rachel Weiskittle[1], *1. University of Colorado, Colorado Springs, Colorado Springs, Colorado, United States, 2. University of Colorado Colorado Springs, Colorado Springs, Colorado, United States*

This paper presentation will discuss the qualitative findings of a city-wide community needs assessment on available grief support with particular emphasis on service needs for older adults. The purposive sampling of this survey targeted local professionals who frequently interact with bereaved individuals in their work, such as mental health providers, funeral directors, faith leaders, and healthcare providers. Survey items asked participants to identify local grief support demand, engagement, barriers, and interests. Of the 141 participants who completed the survey, 80 (57%) provided free response answers eligible for qualitative analysis. Themes were extracted using manifest coding across three raters. Major themes on older adult bereavement include: perceived gaps in available services, service format suggestions (e.g., virtual vs. in-person, faith-based vs. secular), and suggestions for the creation of older adult grief support based on type of loss (e.g., pet loss, kinshiplessness), and perceived educational interest, such as substance use and emotions in grief. In this presentation, qualitative methodology of the study, as well as its resulting themes and subthemes, will be further described to offer insight into participant perspectives on service needs for older adult bereavement.

Abstract citation ID: igae098.3337

### ACADEMIC-NGO PARTNERSHIP IN CONSTRUCTING A LOGIC MODEL FOR EVALUATION OF A CARER-CENTRIC COMMUNITY-BASED PROJECT

Vera Mun Yu Tang, Vivian Weiqun Lou, Tarani Chandola, Clio Yuen Man Cheng, Iris Xiaoyu Ruan, and Vincy Wing Sze Pang, *The University of Hong Kong, Hong Kong, China (People's Republic)*

Evaluation is the cornerstone of demonstrating a particular project's success and paving the way for continuous improvement. However, most of the existing evaluation in social service sector focused on outcome, whereas some evaluated process and a very few included both process and outcome evaluation. Logic models have been found useful in programme evaluation but little is known about how to construct and apply logic models for guiding the process and outcome evaluation of community-based projects, and even less for projects with academic-NGO partnership. A pilot project adopting a carer-centric approach has been launched in 2023, which is anticipated to serve carers and older adults with caring needs in the district-based caregiver support units operated by five NGO partners in Hong Kong. This paper reports how the university as a project partner constructs a comprehensive logic model for evaluation through engaging different stakeholders (including NGO partners, funder, and potential beneficiaries) in two rounds of small group semi-structured interview. Deductive approach is applied for thematic analysis using MAXQDA to identify and prioritize inputs, activities, outputs, and outcomes of the project at initiate phase. Findings provide academics, NGOs and funding bodies with fresh insights into new elements of logic model, including volunteers as important inputs, co-creation workshops as important activities for project positioning and service alignment, number of referral cases as important outputs, and experience consolidation for service adjustment as important intermediate outcomes. This study highlights that process and outcome evaluation can be advanced by promoting stakeholder engagement to co-construct logic model.

Abstract citation ID: igae098.3338

### COMPARISON OF ENERGY AND NUTRIENT INTAKE WITH DIETARY GUIDELINE OF RURAL ELDERS AND ITS CONTACT WITH OXIDATIVE STRESS

Jing Shi, Yanhua Ning, and Weijuan Kong, *Ningxia Medical University, Yinchuan, Ningxia, China (People's Republic)*

The aging in rural areas has surpassed that in urban areas, becoming increasingly severe, and the health status of the older should be of concern. Diet and nutrient intake are important influencing factors for the occurrence and development of chronic diseases, and are currently relatively easy to control. Previous studies have shown that oxidative stress is a common pathogenic mechanism of chronic diseases. This cross sectional study invited 376 older residents from 3 rural communities to complete a dietary survey and collect blood samples for calculating dietary intake and laboratory testing of oxidative stress levels, respectively. Compared the energy and nutrient intake of the participants with recommendation in the Dietary Guidelines, and employed latent analysis based on whether the intake meets the recommended. The results revealed three classes, "imbalanced nutrient-high energy" (37.50%, imbalanced in intake of energy and nutrients with high energy and protein intake), "sufficient nutrient-low energy and protein" (18.35%, sufficient and balanced intake of other nutrients except for energy and protein), and "low nutrient" (44.15%, low intake of energy and various nutrients). Among the oxidative stress biomarkers, imbalanced nutrient-high energy had higher value than did the other classes for 8-iso-PGF2α; sufficient nutrient-low energy and protein valued higher than imbalanced nutrient-high energy and low nutrient classes for SOD. Oxidative stress can be measured based on the different classes of energy and nutrient intake classes and their predictors.

Abstract citation ID: igae098.3339

### CULTURAL ADAPTATION: THE POWER OF COMMUNITY VOICE IN DESIRING A DEMENTIA SUPPORT INTERVENTION FOR LATINE CAREGIVERS

Quinton Cotton[1], Paul Kissell[2], Elma Johnson[1], Manka Nkimbeng[1], and Joseph Gaugler[1], *1. University of Minnesota, Minneapolis, Minnesota, United States, 2. University of Minnesota School of Public Health, Minneapolis, Minnesota, United States*

Intro: Latine family caregivers of persons living with Alzheimer's disease and other related dementias (ADRD) need access to culturally congruent support services. Interventions incorporating community engagement have shown effectiveness in providing ADRD-specific education, supporting caregivers, and mitigating accessibility concerns (transportation and meeting occupational demands).

**Methods:** Data was collected via structured conversations (World Cafe style discussions) with youth, adult caregivers, and older adults (n=52) on their perspectives, needs, and preferences about aging and dementia needs in the community. Data were coded deductively and analyzed via directed content analysis.

**Results:** Latine community members shared their interest in developing a community-engaged program that addressed training on health conditions, emphasized the importance of patience and compassion in dementia caregiving, and language concerns. Content analysis revealed community youth emphasizing interpersonal connection and integration of older adults with the broader community; adult caregivers suggesting a need for caregiver self-care and the mitigation of daily scheduling conflicts; and older adults emphasizing need for emotional support, respectful communication, and language barriers in medical, occupational, and service environments. Implications: Community-engaged feedback is vital in developing interventions reflecting Latine community needs and cultural values. Culturally responsive interventions are necessary because they integrate community member experiences and needs while concurrently promoting community ownership over the research process.

Abstract citation ID: igae098.3340

### DEVELOPING A HEALTH PROMOTION PROGRAM WITHIN THE CARE LIVING LAB FOR COMMUNITY-DWELLING OLDER ADULTS

Ji Yeon Lee[1], Eunhee Cho[2], Jiyoung Shin[2], Hyeonmi Cho[2], and Bada Kang[2], *1. Inha University, Incheon, Republic of Korea*, *2. Yonsei University, Seoul, Republic of Korea*

Health promotion for community-dwelling older adults is necessary to facilitate aging in place as the world moves towards an aging society. Substantial efforts have been made to increase the participation of older adults in health promotion programs; however, they were often fragmented due to a lack of active participation of older adults and stakeholders in community settings. This project aimed to co-create a health promotion program for older adults in the community within a care living lab focusing on the five principles: co-creation, engaging end-users and stakeholders, multiple methods, and real-life settings. Older adults as end-users and stakeholders were included in the co-creation of the health promotion program. Multiple methods, such as workshops, meetings, and interviews, were conducted within the existing community program. In total, 37 older adults, 19 frontline staff members, and stakeholders engaged in the co-creation process. The health promotion program ultimately included education for dementia, insomnia, and falls, cognitive worksheets, and exercise, which improved the level of frailty in older adults. The developed program was applied to another group of older adults, effectively improving their physical and cognitive functions, as well as their quality of life. Satisfaction with the program was high, and frontline staff reported positive experiences with the co-creation process. The health promotion program, developed within the care living lab, improved older adults' health status and brought positive experiences among both older adults and staff. The living lab can be an effective method to improve older adults' health status by facilitating co-creation and engagement.

Abstract citation ID: igae098.3341

### EFFECT OF ACTIVITY-BASED INTERVENTION ON QUALITY OF LIFE AMONG OLDER ADULTS LIVING IN COMMUNITY

Jisu Seo, Rhayun Song, Yuelin Li, and Eunna Oh, *Chungnam National University, Daejeon, Republic of Korea*

With age, older people experience reduced participation in activities. According to the Activity theory of aging, older adults can age successfully by engaged in their favorite activities. This study was conducted to systematically review RCTs that applied activity-based interventions (ABIs) to older adults living in community, and was to classify the ABIs into interaction-based social activities, exercise-based physical activities and self-directed individual activities, as well as compare and analyze the size of the effect on quality of life based on three types of activities. The analysis included 76 randomized studies from 6 Korean databases as well as 5 English databases. Cochrane's Risk of Bias tool was used to assess the quality of the risk of bias. A CMA version 4 was used to calculate the effect size and a 95% confidence interval using a random effect model and to assess heterogeneity and publication bias. A network meta-analysis was conducted using R version 4.3.1 software's "netmeta" package. Using GRADE, we assessed the quality of the evidence. ABIs applied to older adults living in community significantly improved their quality of life (SMD=0.56), and all three types of activity-based interventions significantly improved their quality of life. According to the result of ranking of network meta-analysis, self-directed individual activities was found to have the highest probability of being selected at 91.7%. As older people spend more time alone as aging, it is important to enhance their quality of life by encourage them to choose and participate in activities according to their own interests.

Abstract citation ID: igae098.3342

### ENHANCING DEMENTIA-FRIENDLY COMMUNITIES IN TOKYO, JAPAN THROUGH COMMUNITY-BASED PARTICIPATORY RESEARCH

Mika Sugiyama, Hiroki Inagaki, Kae Ito, and Shuichi Awata, *Tokyo Metropolitan Institute for Geriatrics and Gerontology, Tokyo, Japan*

In Japan, grappling with the challenges of an increasingly aging population, the Basic Law on Dementia was enacted in June 2023 to foster the development of dementia-friendly communities (DFCs) nationwide. Over the past decade, starting from 2013, a community-based participatory research (CBPR) initiative has been underway in Chiyoda Ward, Tokyo, aimed at establishing a comprehensive dementia support system. Monthly team meetings have served as forums for discussing institutional progress and addressing challenges, with efforts toward DFCs guided by the PDCA cycle. In partnership with Chiyoda Ward, a longitudinal mail survey was conducted to assess the needs of approximately 10,000 older adults. For non-respondents, visiting nurses conducted home visits to provide early-stage dementia support. Additionally, a novel system was implemented to extend "early support" and "watchful waiting support by visiting nurses" to individuals at high risk of dementia who did not engage with public consultation services. Chiyoda Ward has actively organized "dementia cafes" and support meetings for affected individuals, along with training "dementia supporters" for community volunteering. Additionally, the introduction of the "Chiyoda Dementia Support Company/University Certification System" in 2022 has acknowledged outstanding efforts, resulting in the certification of six entities. Notably, there has been a positive trend in the proportion of elders feeling confident about living safely within their community despite dementia, as indicated by the mail survey increasing from 28% in 2016 to 35% in 2021. As a result of CBPR efforts, significant progress has been made in the social implementation of DFC in Chiyoda Ward.

Abstract citation ID: igae098.3343

**FORTY YEARS OF CARING: THE STATE OF THE VOLUNTEER CAREGIVING MOVEMENT**

Ronald Berkowsky, *California State University Channel Islands, Camarillo, California, United States*

With the expansion of the older adult population and its impacts on demands and costs of healthcare in the US, there has been a shift from relying on paid care work towards supporting informal unpaid caregiving. While there is an abundance of research that examines the impacts and value of informal caregiving provided by close family and friends, there is little that examines the impacts and value of volunteer caregiving (i.e., unpaid caregiving services provided by community members who do not have a close relationship with the care recipient). Services provided by community volunteers can provide needed assistance to community-dwelling older adults who encounter significant barriers to other care sources (e.g., cannot afford paid caregiving, lack access to family/friends, etc.). This community-based model of caregiving was first piloted in 1984 through support from the Robert Wood Johnson Foundation's Faith in Action program and, in addition to promoting services that support older adults aging in place, is seen as an avenue to promote intergenerational collaboration, civic engagement, and well-being among community members. In this presentation, I present data collected in collaboration with the National Volunteer Caregiving Network (NVCN) from member sites to assess the current state of volunteer caregiving in the US. More specifically, I present data which elucidates the reach of volunteer caregiving, the characteristics of care recipients and volunteers, the services utilized, and the economic value of these services. I conclude with a discussion on the impacts of the volunteer caregiving movement as it enters its 40th year.

Abstract citation ID: igae098.3344

**GRIEF LITERACY: COMMUNITY-BASED STRATEGIES TO REFRAME GRIEF IN OLDER ADULTS**

Katherine Supiano, and Bradbury Laura, *University of Utah, Salt Lake City, Utah, United States*

Grief Literacy is the ability to be compassionately present with a person who is suffering. Older adults realize more grief experiences than the population in general, and have the highest likelihood of poor bereavement outcomes, including risk for Prolonged Grief Disorder. Recognizing that our society is fragmented, and the varying norms and traditions to support one another in times of suffering are unclear, increasing grief knowledge would equip both the public and professionals to identify grief and suffering more readily and to activate appropriate supports to be proactive in avoiding complications from the grief and suffering such as isolation and suicide-risk. In addition to formal care offered by palliative and hospice care professionals, grief counselors, and mental health clinicians, the Grief Literacy Movement equips all citizens to support one another in times of loss, suffering and bereavement. We developed and implemented a year-long State-funded program to promote grief literacy across the State of Utah, targeting persons most impacted by the pandemic; older adults, indigenous persons, Veterans, rural communities—and their overlapping constituencies. Rather than confining this effort to provision of support, we developed grassroots strategies for mutual empowerment of community members to reframe and address individual and collective loss. We present applications including education of mental health providers/health providers, first responders, religious communities, and the engagement of citizen groups. We will provide outcomes of our framework applied across multiple social and community settings that enhance supportive care and contribute to mitigating risk of interpersonal harms that can affect bereaved older persons.

Abstract citation ID: igae098.3345

**HEALTH CARE UTILIZATION BEFORE AND AFTER ENROLLMENT IN HOME-BASED PRIMARY CARE**

Samantha Rizzo[1], Anne Wahl[2], Thomas Chameli[3], Lindsay Ayers[3], Parsa Mahmoudi[3], Yijung Kim[4], Lauren Bangerter[5], and Karl De Jonge[6], *1. Beth Israel Deaconess Medical Center, Brookline, Massachusetts, United States*, 2. *Washington Hospital Center, Washington, District of Columbia, United States*, 3. *Georgetown University School of Medicine, Washington, District of Columbia, United States*, 4. *Medstar Health Research Institute, Hyattsville, Maryland, United States*, 5. *Medstar Health, Hyattsville, Maryland, United States*, 6. *MedStar Washington Hospital Center, Washington, District of Columbia, United States*

Homebound older adults typically exhibit higher rates of emergency department (ED) visits and hospital admissions compared to non-homebound individuals. This study evaluated health care utilization rates before and after patients enrolled in the MedStar Washington Hospital Center House Call Program, a home-based primary care (HBPC) model. Data were drawn from the electronic medical record for 12-months before and 12-months after enrollment in the HBPC program. A total of 530 patients enrolled in HBPC between June 30, 2018, and July 1, 2023. Most patients were female (76.4%) and African American (80.8%), with an average age of 84.3 at enrollment. Over 40% of the patients were living with dementia, 23% had experienced a stroke, and 81% had hypertension. Using paired t-tests, we found a decrease in the average annual rate of ED visits (1.58 vs. 0.92, $p < 0.001$) and hospital admissions (0.91 vs. 0.53, $p < 0.001$) from the pre-12-month enrollment period to the post-12-month period. Linear regression revealed important predictors of post-enrollment ED visits, including greater pre-enrollment ED use, history of chronic obstructive pulmonary lung disease (COPD), and coronary artery disease (CAD). Post-enrollment hospital admissions were predicted by the frequency of pre-enrollment admissions, presence of chronic kidney disease (CKD), and CAD. Patients with CAD, CKD, or COPD are more likely to present to the ED or hospital during enrollment in HBPC than those without these comorbidities The overall utilization results suggest that enrollment in a HBPC model lessens the rate of ED visits and hospital admissions in a homebound population.

Abstract citation ID: igae098.3346

### IMPROVING AWARENESS OF FOOD RESOURCES FOR OLDER ADULTS THROUGH REGIONAL COLLABORATIVE EFFORT

Joan Ilardo, and Angela Zell, *Michigan State University East Lansing, East Lansing, Michigan, United States*

The Successful Nutrition Programs Across the Lifespan (SNP-AL) created a roadmap with recommendations for selecting sustainable, successful, evidence-informed nutrition programs that meet the community's needs and foster cross-sector collaboration; implementing interventions; and gathering data to measure program outcomes. The Capital Area Coalition (CAC) addressed lack of communication around its regional food resources. The pandemic changed many community resources, so residents weren't aware of current food resources or how to access emergency food. 2-1-1 coordinates with regional resources and provides information about local health and human services resources. CAC's strategy included a media campaign, implemented in Spring and Summer 2023, directing residents with food insecurity to contact 2-1-1 for available resources, using local TV and radio spots and including 1400 leaflets containing information about food resources in produce boxes delivered to income-eligible residents. They provided 2-1-1 information to appropriate agencies. We used the Collaborative Effectiveness Assessment from the Prevention Institute to measure cohesiveness. Results demonstrated CAC was highly motivated with trust, strong communication, and conflict resolution skills. They perceived their mission was simultaneously overarching and comprehensive, aligning with their overall objective. CAC's sustainability plan for 2-1-1 is helping local agencies recognize the importance of providing current information to 2-1-1. Calls to 2-1-1 was the indicator of impact of their media campaign. We compared data from 2-1-1 for 4/2022-6/2022 and 4/2023-6/2023. Food Lines referrals increased from 32 to 37. Food Stamps/SNAP decreased to 49 from 55. Food Pantries referrals increased the most, increasing 29% from 342 in 2022 to 479 in 2023.

Abstract citation ID: igae098.3347

### PEER-LED EXERCISE RESULTS IN IMPROVED SLEEP EFFICIENCY WITHOUT CONCURRENT CHANGES IN MOOD

Chitra Banarjee[1], Kworweinski Lafontant[1], Jethro Raphael Suarez[2], Joon-Hyuk Park[1], David Fukuda[1], Jeffrey Stout[1], Nichole Lighthall[1], and Ladda Thiamwong[1], *1. University of Central Florida, Orlando, Florida, United States*, *2. University of Central Florida*

Sleep is an important physiological process for recovery and regulation of homeostatic processes in the human body. Changes in sleep have additionally been shown to affect mood, including anxiety and depression. The present study implements multimodal assessment of sleep and mood using the wearable FitBit® Charge 5, as well as subjective surveys, the PHQ-9 and GAI, evaluating anxiety and depression, respectively. The study sample included 108 community-dwelling older adults (mean age = 74.19 years ± 6.96; 89.8% female). Of these adults, 58 participated in weekly peer-led exercise and exercise, (strength-training and walking) at least 30 mins twice a week for eight weeks. Participants wore a FitBit® for a mean of 76.5 days ± 59.24, including at night. Using the FitBit®, the study evaluated time asleep and sleep efficiency, calculated based on the time asleep, deep and REM sleep, and restoration. Participants undergoing intervention exhibited a significant increase in both sleep duration ($p < 0.001$; intervention=297.2 minutes, control=274.8 minutes) and efficiency ($p < 0.001$; intervention=93.6, control=92.2), as evaluated by Wilcoxon tests. However, subjective assessments of anxiety ($p=0.288$) and depression ($p=0.986$) did not reveal significant differences. These results indicate that peer-led exercise results in a significant improvement in sleep, which is consistent with previous research. The lack of concordant results in mood suggests that more intensive exercise may be necessary to improve mood. Additionally, the study was limited by individual variability in FitBit® wear time. Future research should evaluate the long-term effects of increasing the time and intensity of peer-led exercise on sleep and mood.

Abstract citation ID: igae098.3348

### RECIPROCALITY OF COGNITIVE IMPAIRMENT, PAIN, AND DEPRESSION: MODELING WITHIN-PERSON EFFECTS

Miharu Nakanishi[1], Marieke Perry[2], Rachele Bejjani[3], Satoshi Yamaguchi[4], Satoshi Usami[5], and Jenny van der Steen[1], *1. Leiden University Medical Center, Leiden, Zuid-Holland, Netherlands*, *2. Radboud University Medical Center, Nijmegen, Gelderland, Netherlands*, *3. Impatients N.V., Amsterdam, Noord-Holland, Netherlands*, *4. Tokyo Metropolitan Institute for Geriatrics and Gerontology, Tokyo, Japan*, *5. University of Tokyo, Tokyo, Japan*

Cognitive impairment, pain, and depressive symptoms are common and interrelated factors in older adults. However, the directionality and specificity of their association remains unclarified. This study explored whether these factors prospectively increase reciprocal risk and examined the longitudinal association between these factors and quality of life (QoL). We used longitudinal data from The Older Persons and Informal Caregivers Survey Minimal Data Set (TOPICS-MDS; the Netherlands). The data of 11,582 home-dwelling older adults with or without cognitive impairment were analyzed. Older adults self-reported cognitive impairment, pain, depressive symptoms, and QoL at baseline and after 6 and 12 months of follow-up. A Random Intercept Cross-Lagged Panel Model was used to assess the prospective association between the three factors, while a multilevel linear regression analysis in a two-level random intercept model was used to examine the longitudinal associations between the three factors and QoL at the within-person level. At the within-person level, pain at 6 months was associated with subsequent depressive symptoms ($\beta=.04$, $P=.024$). The reverse association from depression to pain, and longitudinal associations between pain and cognitive impairment and between depressive symptoms and cognitive impairment were non-significant. Pain, depressive symptoms, and cognitive impairment showed a significant association with poor QoL 6 months later. A directional relationship was observed from pain to depressive symptoms. Pain reduction may be important in the prevention of depressive symptoms, ultimately optimizing the QoL of older adults.

Abstract citation ID: igae098.3349

**THE CONTRIBUTION OF NIGROSTRIATAL DOPAMINERGIC TRANSMISSION ON APATHY IN OLDER ADULTS**

Yixiao Song[1], Peggy Cawthon[2], Meryl Butters[1], Lana Chahine[1], and Caterina Rosano[1], *1. University of Pittsburgh, Pittsburgh, Pennsylvania, United States*, *2. California Pacific Medical Center, San Francisco, California, United States*

Apathy is a common and impairing symptom in neurological disorders and its connection with striatal dopamine levels has been examined in Parkinson's and Alzheimer's disease. Little is known about the nature of this relationship in older adults free from neurological diseases. This study explores the link between apathy and nigrostriatal dopaminergic neurotransmission in community-dwelling older adults without overt neurological symptoms. This was a cross-sectional study of community-dwelling older adults (N=230, mean age 75.3yrs; 60% female) from Monongahela-Youghiogheny Healthy Aging Team (MYHAT) and Study of Muscle, Mobility and Aging (SOMMA) studies. The nigrostriatal dopamine binding site density of VMAT2 was quantified via (+)-[11C]DTBZ PET imaging. Apathy was assessed with Apathy Evaluation Scale, an 18-item questionnaire using a 4-point Likert scale (range 18-72; higher score indicates greater apathy). Latent class analysis was used to identify latent subtypes of apathy. Multinomial logistic regression was used to investigate the relationship between dopaminergic transmission and latent classes. Three latent classes were identified, namely, low apathy (mean 20.7; SD 2.36; N=110), moderate apathy (mean 25.5; SD 2.83; N=89), and high apathy (mean 32; SD 4.82; N=31). Each SD decrement in nigrostriatal dopaminergic transmission corresponded to 39% and 42% lower odds of having high apathy compared to low apathy (OR=0.61; 95%CI: 0.39-0.95; p-value=0.028) and moderate apathy (OR=0.58; 95%CI: 0.36-0.90; p-value=0.015), respectively, after adjusting for age at the time of MRPET scan, gender, and race. Our study highlights apathy's inverse relationship with dopaminergic neurotransmission, which underscores the importance of considering dopaminergic pathways in managing apathy in older adults.

Abstract citation ID: igae098.3350

**URINARY INCONTINENCE AS A RISK TO PHYSICAL, MENTAL, AND SOCIAL HEALTH AMONG OLDER KOREAN AMERICANS IN SENIOR HOUSING**

Juyoung Park[1], and Yuri Jang[2], *1. USC Suzanne Dworak-Peck School of Social Work, Los Angeles, California, United States*, *2. University of Southern California, Los Angeles, California, United States*

Urinary incontinence (UI) or involuntary leakage of urine, is a common geriatric condition. Despite its harmful effect on health and well-being, UI has often been neglected in geriatric care. In the present study, we examined the risks posed by UI to the physical, mental, and social health of older Korean Americans living in subsidized senior housing. Survey data were collected from 313 older Korean-American residents in subsidized senior housing in the Los Angeles area. UI was assessed with a question on the frequency of experiencing involuntary leakage of urine. Physical, mental, and social health risks were indicated by a single item self-rated health (fair/poor rating), the Patient Health Questionnaire–9 (probable depression), and the Lubben Social Network Scale–6, (isolation from family and isolation from friends). More than half of the sample reported UI, with the experience being infrequent (i.e., seldom) for 46.3% and frequent (i.e., sometimes or often) for 10.3%. Regardless of the frequency, UI posed a significant risk to physical and mental health. The odds of reporting fair/poor health and having probable depression were 1.82 to 6.81 times higher among those with infrequent/frequent UI than among those without. Family isolation was not associated with UI; however, the odds of being isolated from friends were 2.95 times higher among those with frequent UI compared with the reference group. Our findings underscore the importance of addressing UI in the health promotion effort in senior housing, calling for interdisciplinary approaches for resident care.

### SESSION 7645 (POSTER)

### DELIRIUM

Abstract citation ID: igae098.3351

**CO-DESIGNING AN MHEALTH-BASED CAREGIVING MASTERY PROGRAM FOR DELIRIUM PREVENTION IN PERSONS LIVING WITH DEMENTIA**

Yong Kyung Choi[1], Shih-Yin Lin[2], Kate Santoso[3], Jennifer Pruskowski[1], Donna Fick[4], and Abraham Brody[5], *1. University of Pittsburgh, Pittsburgh, Pennsylvania, United States*, *2. New York University, New York, New York, United States*, *3. NYU Langone Health, New York, New York, United States*, *4. The Pennsylvania State University, University Park, Pennsylvania, United States*, *5. HIGN, New York University Rory Meyers College of Nursing, New York, New York, United States*

Caregivers play an essential role in the prevention and early detection of Delirium Superimposed on Dementia (DSD) in community settings. To support caregivers, our study utilized a co-design methodology to adapt an existing evidence-based comprehensive dementia symptom management program originally designed for clinicians (Aliviado Dementia Care) for use by caregivers with a specific focus on DSD (Aliviado DSD Caregiving Mastery). We enrolled 10 caregivers of persons living with dementia (70% female; mean age=57.2; 5 caregivers having previously cared for their care recipient when experiencing delirium) to participate in a 5-week co-design workshop via Zoom. Over the 5 weeks, participants co-designed a series of family education videos, care plans, and communication guides; and refined the existing caregiver education articles and Aliviado mobile app. Session transcripts were analyzed immediately post-session using Instant Data Analysis to generate key themes for improvements. The main themes included caregiver-friendly language across program elements (e.g. changing "non-pharmacological" to "non-medication-based"), clear next steps (e.g. whether to go to the ER, timing to start a new care plan, etc.), and fine-tuning the app's user interface (e.g. improved navigation between caregiver-administered delirium assessment questions). The co-design approach not only increased person- and family-centeredness of this program but also produced more actionable program components that empower caregivers to deliver DSD care and be better advocates for their loved ones. This collaborative process underscores the importance of engaging caregivers as co-designers, yielding a more accessible and user-friendly set of tools to support caregivers and their care recipients.

Abstract citation ID: igae098.3352

### FACTORS RELATED TO DELIRIUM AMONG OLDER ADULTS IN NEUROLOGICAL INTENSIVE CARE UNITS

Aeyoung Cho[1], Kyung Hee Lee[1], JiYeon Choi[2], and Jung Yeon Kim[3], *1. Yonsei University, Seoul, Republic of Korea*, *2. Yonsei University College of Nursing, Mo-Im Kim Nursing Research Institute, Seoul, Republic of Korea*, *3. Severance Hospital, Seoul, Republic of Korea*

Delirium, an acute confusion states, significantly increases in intensive care unit (ICU) patients and older adults due to COVID-19 pandemic. However, patients with neurological disorders face a challenge due to the difficulty in distinguishing symptoms caused by encephalopathy; there is particularly a lack of research about older adults in ICU. We investigated the incidence of delirium and related factors among older adults in a neurological ICU during the COVID-19 pandemic. A retrospective study was conducted using electronic medical record data from 945 older adults aged 65 to 93 years admitted to the neurological ICU at a tertiary hospital in Seoul, South Korea from January 2020 to July 2023. Delirium was evaluated using the Intensive Care Delirium Screening Checklist score. Logistic regression was performed using the SPSS/WIN 26.0 program. The incidence rate of delirium was 16.9%, and 49.8% of patients were categorized as subsyndromal delirium during ICU stay. Logistic regression revealed significant factors affecting delirium among older adults in the neurological ICU, including dexmedetomidine use (OR=8.13), length of ICU stay (OR=2.89, OR=3.61 and OR=6.05 for 3-5days, 6-10days and >10days, respectively), blood urea nitrogen (BUN) levels (OR=4.55), visual impairment (OR=2.13), APACHE II score (OR=1.07) and age (OR=1.05). Accurate awareness of delirium symptoms by health professionals and identification of delirium-related factors are crucial for effective patient management. Pre-classification of delirium high-risk groups among older adults admitted to neurological ICUs is essential for providing high-quality delirium care.

Abstract citation ID: igae098.3353

### IS DELIRIUM EDUCATION IMPORTANT: MEDICAL STUDENTS' ATTITUDES FOLLOWING A DELIRIUM WORKSHOP

Benjamin Rosenstein, *University of Minnesota, Department of Family Medicine and Community Health, Minneapolis, Minnesota, United States*

Delirium is an all too frequent syndrome for which older adults, particularly those with dementia, are at an increased risk. As the population ages, medical students will encounter adults affected by delirium increasingly frequently during their rotations. Unfortunately, education related to delirium is inconsistent within US medical schools. Prior studies have shown students gain knowledge and skills following specific delirium education courses. However, medical students' attitudes related to delirium as an educational topic have not previously been explored. This study used a thematic analysis to review 4th-year medical students' reflections of a workshop focused on delirium evaluation, management, treatment, and outcomes. The review revealed 8 primary themes including aspects such as: students frequently encountered older adults experiencing delirium but were unaware of associated poor outcomes, they have limited to any education on delirium during their rotations, they feel this is an often overlooked yet important topic, and they found the workshop provided valuable skills applicable to their current and future practices. Most surprising was medical students' recognition and reflection of ageist practices they had encountered, though it was not a specific topic within the workshop. This study demonstrates that delirium should be a standard topic in medical education. It provides significant clinical value and may improve medical students' knowledge, practices, and attitudes, including counteracting ageism, thus promoting age-friendly care of older adults.

Abstract citation ID: igae098.3354

### NURSING PRACTICE AND OPTIMAL DELIRIUM CARE AMONG OLDER ADULTS IN ACUTE CARE SETTINGS

Tanya Mailhot[1], Zineb Bouaouina[1], Tania Valentine Joannette[1], Imene Khetir[2], and Patrick Lavoie[1], *1. Université de Montréal, Montreal, Quebec, Canada*, *2. Montreal Heart Institute, Montréal, Quebec, Canada*

Studies have emphasized the limited availability of data regarding the current practices of nurses in delirium care for patients in acute care settings. Similarly, there is a lack of information regarding the obstacles and facilitators in providing optimal care for the prevention, detection, and management of delirium.

**Aim:** To describe nurses' practices about delirium care in acute care patients and their perceptions about barriers and facilitators regarding optimal care. Method: A two phase, multi method design was used. The quantitative phase utilized a self-reported survey to assess nurses' knowledge, practice, confidence, and collaboration regarding delirium care. The qualitative phase employed focus groups to complement and explore survey data in-depth. Recruitment took place on nine acute surgical and medical units across two university-affiliated hospitals in Canada, involving nurses in direct patient care.

**Results:** 231 nurses reveal diverse insights into delirium care in acute settings and survey participants showed solid knowledge of delirium symptoms and outcomes, yet 23% did not identify the hypoactive form's prevalence. While 81% reported receiving information on delirium screening, challenges included time constraints and misuse of detection tools. Qualitative themes highlighted communication's vital role, challenges posed by delirium presentations, the significance of staff support, time constraints, the impact of experienced staff, the role of families, and the importance of additional resources for optimal delirium care.

**Conclusion:** Findings align with existing literature, emphasizing the multifaceted nature of delirium care and the need for tailored approaches, education, and collaborative strategies to improve overall care quality.

### SESSION 7650 (POSTER)

### EDUCATION AND TRAINING: PROGRAM EVALUATION

Abstract citation ID: igae098.3355

### CHATO NATIONAL TRIAL: USING OPEN-ENDED RESPONSE TO DEVELOP VIDEO TUTORIALS TO IMPROVE NURSING HOME IMPLEMENTATION

Maria Jaros[1], Carissa Coleman[1], Frances Yang[1], Clarissa Shaw[2], Maria Hein[2], Yelena Perkhounkova[2], and Kristine Williams[1], *1. University of Kansas Medical Center, Kansas City, Kansas, United States*, *2. University of Iowa, Iowa City, Iowa, United States*

The Changing Talk: Online (CHATO) intervention was developed to increase staff access to person-centered dementia care communication education in a national nursing home sample. A qualitative review of the post-evaluation survey responses (N=1605) from participants in the first year of the study was performed to determine issues associated with the modules to improve implementation across nursing homes. Three open-ended questions addressed technical difficulties and what participants liked most and least about the program. Technical difficulties were reported by 21.1% of participants, including internet connection (5%), video difficulties (3.8%), non-specific technical difficulties (3.7%), website interface (3.1%), registration (1.8%), and navigating the modules (1.1%). Sixty-nine percent of participants reported what they liked the best about the course. Participants indicated real-world application (22.4%) was liked best, followed by the videos in the modules (18.8%), and the presentation of the material (5.9%). When asked what participants liked least about the course, 54.3% of participants provided input. The length of the three-hour course (21.4%) was liked least, followed by the presentation of the material (7.7%), and the interactive components of the modules (6.5%). In response to these findings, video tutorials were created addressing components most reported in the post-evaluation which included outlining navigation features within the CHATO training, registering for the course, and navigating to the modules. Tracking new users accessing the video tutorials via the CHATO website as well as annual coding will provide ongoing monitoring of implementation success and will provide input for re-design of the modules in future research.

Abstract citation ID: igae098.3356

### EMPOWERING AN AGE-FRIENDLY HEALTH CARE WORKFORCE: AN INTERPROFESSIONAL PROGRAM APPROACH

Leah Tobey-Moore, Melanie Beehler, Kathryn Neill, Karen Dickinson, Laura Spradley, and Robin McAtee, *University of Arkansas for Medical Sciences, Little Rock, Arkansas, United States*

Growing interest in caring for the geriatric population is a challenge in healthcare education. In Arkansas (AR), 18% of the population is over 65. The Arkansas Geriatric Education Collaborative (AGEC) is a HRSA-funded Geriatric Workforce Enhancement grant recipient with a main goal to encourage health professions students toward careers in geriatrics. AGEC's Geriatric Student Scholar Program exists to expand exposure and foster interest regarding older adults (OA) through experiential learning and interprofessional training. Each year, 4-5 students are chosen to further their knowledge and interest in the field of geriatrics. This year's team of 5 students (pharmacy, physician assistant, medical doctor, audiology, and physical therapy) completed two geriatric-focused didactic events, two experiential community events, and an interprofessional education (IPE) team project. Their 2024 IPE project focused on Mobility from the 4Ms Framework of Age-Friendly Healthcare and was delivered via an active learning "escape room." Reflection essays were submitted following each attended activity. AGEC activities supported students' understanding and application of interprofessional, collaborative skills needed to care for OAs and allowed students to take on the role of educator by creating, designing, and evaluating a learning activity called "Falls and Fall Prevention in the OA." Programmatic surveys at the conclusion of the 7-month experience demonstrated 1) positive overall experience, 2) enhanced interprofessional collaboration skills, 3) strong teamwork dynamics and 4) deepened interest in geriatrics.

Abstract citation ID: igae098.3357

### EVALUATION OF A SOCIAL WORK AND NURSING INTERPROFESSIONAL TRAINING ON DEMENTIA-FRIENDLY CARE

Leslie Hasche, Pilar Ingle, and Michael Talamantes, *University of Denver, Denver, Colorado, United States*

While social work and nursing education offer generalist professional degrees, several initiatives over the last 20 years have infused geriatric education and specialty training to counter the long-standing geriatric workforce shortages. However, these training programs are frequently separated within distinct disciplinary silos with limited opportunities for interprofessional training. Interprofessional education involves a critical pedagogy that increases knowledge about principles, ethics, and values of other professions, while also developing critical thinking skills to promote collaborative working relationships that improve care. The purpose of this study is to evaluate an annual interprofessional cohort training specific to Alzheimer's Disease and Related Dementias for social work and advanced practice nursing students. From 2019 to 2024, five cohorts (total n= 43, with 24 social work students and 19 advanced practice nursing students) have completed this training. Approximately 30 hours of learning activities supplemented their existing professional degree program education over a 6-month period. These activities included: (1) an immersive training with experiential Second Wind Dreams' Virtual Dementia Tour, (2) webinar didactic content on dementia-friendly and interprofessional care and interprofessional dyad case consultations, and (3) individual, personalized coaching. Using a pre/post survey adapted from measures of interprofessional collaborative practice and geriatric social work competencies, results demonstrated consistent improvement across each of the domains: values and ethics, roles/responsibilities and communication, assessment and treatment, and teamwork. The positive evaluation findings for increasing interprofessional competencies in dementia-friendly care offer support for expansion to other disciplines, geographical regions, and over sustained time and for strengthening the geriatric workforce.

Abstract citation ID: igae098.3358
### FROM KNOWLEDGE TO PRACTICE: TRAINING FAITH LEADERS IN AGING AND MENTAL HEALTH
Annalee Wilson[1], Bronwyn Keefe[1], and Erin Emery-Tiburcio[2], *1. Boston University, Boston, Massachusetts, United States*, *2. Rush University, Chicago, Illinois, United States*

Faith leaders can play a leading role in recognizing and supporting the mental health of their older congregants. Older adults attend faith services more frequently than members of other age groups and often turn to faith leaders for mental health support. To increase faith leaders' knowledge and skills pertaining to mental health and aging, the Center for Aging and Disability Education and Research (CADER) developed an online course, Mental Health and Aging for Faith Leaders. In this course, participants learn to identify mental health conditions, to describe barriers to treatment, to identify services and resources available for treating mental health conditions in older adults, and to explain the role that faith leaders can play in supporting older adult mental health. CADER piloted the course with 44 faith leaders from Massachusetts (n=7) and Chicago, Illinois (n=37). Of the Chicago-based faith leaders, all except one indicated that they serve a congregation that is majority Black. Faith leaders showed significant improvements ($p< 0.05$) in all learning competencies from the pre-course competency self-assessment to the post-course competency self-assessment. When asked what changes they anticipate making after taking the course, faith leaders reported that they intended to make changes on the individual level (e.g., by continuing to learn more about aging and mental health); the interpersonal level (e.g., by identifying the signs of mental health concerns in their older congregants); the organizational level (e.g., by implementing mental health programming at their faith organizations); and at the societal level (e.g., by promoting awareness about mental health).

### SESSION 7655 (POSTER)

### FALLS II (POSTERS)

Abstract citation ID: igae098.3359
### A GRAPHICAL MODEL APPROACH TO UNVEILING FALL TRIGGERS IN COMMUNITY-DWELLING OLDER ADULTS
Tho Nguyen, Chang Liu, Yi Liu, Ladda Thiamwong, Qian Lou, and Rui Xie, *University of Central Florida, Orlando, Florida, United States*

We aim to enhance understanding of fall risk factors and examine their interrelationships in community-dwelling older adults from various domains: sociodemographics, mental well-being, body composition, self-assessed and performance-based fall risk assessments, and physical activity patterns. Our study included 120 subjects aged 60 to 96 years old from Central Florida (average age of 74.8, SD 7.38), 77.5% female, and 29.2% with a history of falling. We used a novel machine learning technique known as the Graphical Model (GM) to analyze the association among 37 fall risk factors. GM reveals the higher-order dependence structure and graphical relationships among variables, including continuous and categorical types. This approach goes beyond traditional correlation analysis, providing innovative insights into sophisticated interactions within a high-dimensional dataset. Among the identified features, 34 exhibited pairwise relationships, while COVID-19-related factors and housing composition remained conditionally independent. Prominent relationships include sedentary behavior having strong negative links to both light physical activity and moderate-to-vigorous physical activity. Participant age was positively correlated with balance score but negatively correlated with body composition factors and Senior Technology Acceptance. The CDC's Stopping Elderly Accidents, Deaths, and Injuries score had moderate positive relationships with the Rapid Assessment of Physical Activity (strength and flexibility) and Mindful Attention Awareness Scale, while it had moderate negative relationships with the Short Falls Efficacy Scale-International score and history of falls. The findings of this study serve as an initial exploration, laying the foundation for future research, including the longitudinal dynamics of these features that contribute to fall prevention in older adults.

Abstract citation ID: igae098.3360
### ADAPTING STEADI TOOLKIT FOR LONG-TERM CARE: A DELPHI STUDY
Hilaire Thompson[1], Elizabeth Phelan[1], Ellen McGough[2], and Kushang Patel[1], *1. University of Washington, Seattle, Washington, United States*, *2. University of Rhode Island, Kingston, Rhode Island, United States*

The prevalence of falls for older adults in long-term care facilities (LTCF) is estimated to be 50-60%. However, fall prevention programs, such as the CDC's STEADI, have not been validated for use in LTCF. Lacking are screening, assessment, and decision-support toolkits to be used with older adults residing in LTCF. This project seeks to address this gap by developing a refined toolkit for use in LTCF. As a first step, an initial list of adaptations to the STEADI toolkit were identified following four separate scoping reviews. Interventions were categorized in six areas: Assessment, Education/Training, Facility, Medication Management, Resident Lifestyle, and Referral. Subject matter experts from medicine, nursing, physical therapy, and long-term care administration from across the US were invited to provide feedback on the initial draft of toolkit interventions. Interviews (n=9) were transcribed and recommendations for changes to assessment and management strategies were identified. Following these revisions, we recruited individuals working in LTCFs to participate in two rounds of surveys using a modified Delphi technique. Respondents (n=51) were asked to rate 69 interventions as either not at all helpful, possibly helpful, helpful or extremely helpful. We established consensus as =/>60% of individuals agreeing that the intervention was "extremely helpful." In round one, 37 interventions reached consensus for inclusion (Assess: 16; Education/Training: 4; Facility: 8; Medication Management: 2; Resident Lifestyle: 4; Referral: 3). In round two, three additional items reached consensus for inclusion. The toolkit is now being tested for feasibility, acceptability and impact on falls and fall-related injuries.

Abstract citation ID: igae098.3361
### ASSESSING MOBILITY AND FALL RISKS IN TAIWAN'S OLDER ADULT POPULATION: A LONGITUDINAL STUDY
Fang-Lin Kuo[1], and Zih-Yong Liao[2], *1. National Health Research Institutes, Yunlin, Yunlin, Taiwan (Republic of*

*China)*, 2. *National Yang Ming Chiao Tung University, Taipei, Taiwan (Republic of China)*

Mobility difficulties impact older adults' health significantly, leading to adverse outcomes and loss of independence. In Taiwan, 21% of community-dwelling older adults report falls, and over half have a fear of falling, highlighting the importance of identifying individuals at risk. This study aimed to explore mobility changes and repeated falls in Taiwan's rural areas, where the older population exceeds 24%. A longitudinal study was conducted annually from 2022 to 2023, focusing on physical ability and gait assessments in community-dwelling older adults (65+) who were able to walk (n=250). We used linear mixed-effects regression models and logit models to examine whether personal characteristics are associated with changes in mobility and falls. The findings showed no significant differences in falls and walking performance at the 1-year follow-up after confounder adjustments. However, individuals living in buildings without elevators, using walking devices with cognitive impairment and exhibiting poorer gait performance such as slower gait speed, peak angle velocity at swing phase and toe clearance were more likely to experience repeated falls. Furthermore, about 14.78% of participants who were independently walking at baseline began using a walking device by 2023, indicating a transition to new walking dependence (NWD). The NWD group tended to have a higher number of chronic illnesses, repeated falls and slower walking speed. This study highlights the need to consider environmental and gait characteristics in addressing the mobility dependence and fall risks. Interventions to prevent falls and facilitate the adaptation to walking assistive devices could help manage mobility decline among older adults.

Abstract citation ID: igae098.3362

### CHARACTERIZING THE CIRCUMSTANCES OF FALLS AMONG COMMU ITY-DWELLING OLDER ADULTS

Abigail Kehrer-Dunlap[1], Rebecca Bollinger[1], Audrey Keleman[2], Beau Ances[1], and Susan Stark[1], *1. Washington University in St. Louis, St. Louis, Missouri, United States*, *2. Department of Veterans Affairs, St. Missouri, United States*

Falls are the leading cause of injury, institutionalization, and injury-related mortality among older adults. As the older adult population is expected to grow exponentially in the next decade, the cost and consequences of falls will continue to rise. While promising fall prevention interventions exist, the death rate due to falls continues to climb. Limited population health effectiveness of these interventions may be attributable to a poor understanding of contributing factors to falls, such as the environmental and contextual circumstances in which falls occur. The objective of this study was to characterize the circumstances of falls among community-dwelling older adults. We completed monthly, prospective fall monitoring with 350 cognitively normal (Clinical Dementia Rating=0) older adults for up to four years. Standardized interviews were conducted to gather the circumstances of all reported falls, including the location, time of day, and activity at the time of the fall. We qualitatively analyzed fall interviews to determine the reason for the fall and factors of limited or divided attention preceding the fall. There were a total of 1,133 falls reported by 212 (61%) participants. The majority of falls occurred outdoors (53%), in the afternoon (45%), while walking or running (50%), and due to tripping over or catching feet on an object (37%). Additionally, 37% of falls occurred during instances of limited or divided attention. Taken together, these findings provide a greater understanding of the circumstances of older adult falls that may inform future classifications of types of falls and lead to tailored fall prevention approaches.

Abstract citation ID: igae098.3363

### CLINIMETRIC PROPERTIES OF THE ABC-6, ABC-16, AND MODIFIED FALLS EFFICACY SCALE WHEN DELIVERED ELECTRONICALLY

Jennifer Blackwood[1], and Matthew Carpenter[2], *1. University of Michigan, Ann Arbor, Michigan, United States*, *2. University of Michigan Flint, Flint, Michigan, United States*

The Activities Specific Balance Confidence Scales (ABC, ABC-6) and the Modified Falls Efficacy Scale (M-FES) are outcome measures used to assess self-efficacy in maintaining balance in older adults. All three measures have established reliability and validity when delivered in a clinician-driven paper assessment. However, it is unknown what the clinimetric properties of these measures are when delivered electronically without verbal direction. Therefore, the purpose of this study is to establish the reliability, validity, standard error of measurement, and the minimal detectable change (95% confidence interval) of these measures when delivered electronically in community-dwelling older adults. 67 participants were recruited electronically through the University of Michigan's Health Research platform. Older adult participants were English-speaking; aged 65+ years; had access to a computer or electronic device with reliable internet; and were diagnosed with various health conditions. A Qualtrics survey was distributed to participants that consisted of the ABC-16 and M-FES with written directions. Results from the 67 participants demonstrated excellent test-retest reliability (ICC2,1 = ≥0.94) and excellent internal consistency ($\alpha$ = ≥0.94) for all measures. The ABC-16 had a smaller SEM (3.85) and MDC95 (10.67) compared to the ABC-6 (SEM = 4.99, MDC95 = 13.83). For the MFES, the SEM was 0.27 and MDC95 was 0.75. All measures had significant Pearson correlations ($p < 0.01$) with the falls history indicating construct validity. These results indicate the ABC-16/6 and M-FES can be utilized in an electronic format when assessing older adults' self-efficacy in balance.

Abstract citation ID: igae098.3364

### ENHANCING DEMENTIA CARE PARTNER ENGAGEMENT IN FALL PREVENTION PROGRAMS: A SYSTEMATIC REVIEW

Xiaoyi Zeng, Yuanjin Zhou, and Namkee Choi, *The University of Texas Austin, Austin, Texas, United States*

Community-dwelling older people with cognitive impairment/dementia (CI/D) experience a higher risk of falls than age-matched peers without CI/D. Fall prevention programs for this population often engage their family/friend care partners. Care partner engagement (CPE) has five components: care partners' recruitment, retention, attendance, active participation, and maintenance of changes. Little is known about the facilitators and barriers that impact CPE in those programs and strategies used to enhance CPE. We conducted a mixed-methods systematic review following PRISMA guidelines (PROSPERO: CRD42023422200). Twenty-nine unique fall prevention programs from 32 studies were included. Most studies described retention (n=19, 60%), attendance (n=12, 38%), and active participation (n=32, 100%) aspects of CPE. Fewer studies illustrated factors related to care partner recruitment (n=6, 19%) and maintenance of change (n=6, 19%). Commonly reported CPE facilitators were older people with CI/D and care partners' perceived benefits and suitableness of the fall prevention programs and positive relationships between the dyads. The main barriers to CPE are the dyads' poor health and the burden related to the study logistics of participating in the programs. Program characteristics (e.g., structured/group format, social engagement opportunities, delivery mechanisms, timing) and providers' characteristics are commonly reported organizational-level factors impacting CPE. Twenty-seven studies mentioned the use of educational (e.g., training, consultation, booklet), relational (e.g., strengthening care relationships, engaging paid caregivers), and behavioral strategies (e.g., action plans, visual reminders, cognitive approach, tracking logs) to promote CPE. Findings proposed a theoretical framework and identified potential strategies to enhance CPE in fall prevention programs for older people with CI/D.

Abstract citation ID: igae098.3365

### FALL RISK FACTORS IN COMMUNITY-DWELLING OLDER ADULTS: AN UMBRELLA REVIEW OF SYSTEMATIC REVIEWS

Stephanie Saunders[1], Cassandra D'Amore[2], Qiukui Hao[1], Ayse Kuspinar[1], Julie Richardson[1], and Marla Beauchamp[1], *1. McMaster University, Hamilton, Ontario, Canada*, 2. *McMaster Univeristy, Hamilton, Ontario, Canada*

Falls are an imperative public health concern, resulting in disability, mental health challenges, and increased mortality risk. An extensive body of literature has examined falls risk factors; however, it has become challenging to navigate. We summarized systematic reviews examining risk factors for falls among community dwelling older adults. We searched seven databases (Medline, Embase, CINAHL, Cochrane Library, PsychINFO, Ageline) for systematic or scoping reviews including community-dwelling older adults (≥60 years) and reporting risk factors for falls in prospective studies. Three independent reviewers screened 8,173 records and included 57 reviews. Thirty-three reviews conducted meta-analyses and 24 reported relationships from primary studies. Reviews were published between 2004-2023 and examined patients with stroke (n=2), diabetes (n=2), frailty (n=3), and cognitive impairments (n=3). Types of falls examined included recurrent falls (n=45) and injurious falls (n=18). Reviews reported risk factors across every construct of the WHO's ICF model; health conditions (n=20 reviews), body structure and function (n=26), activity (n=23), participation (n=11), environmental factors (n=10), and personal factors (n=11). Most commonly conducted meta-analyses included: mobility impairments (demonstrating increased risk across gait, strength, and clinical performance tests, n=12/20), balance impairments (increased risk n=15/16), BMI (null risk n=12/12), frailty (increased risk n=9/10). Heterogeneity of reported effect sizes prohibited the calculation of pooled magnitudes for risk factors. Narrative synthesis results are summarized separately. Physical risk factors for falls are well established and can be addressed through interventions. Findings highlight narrow examination of psychosocial and environmental risk factors for falls in community dwelling older adults, indicating areas for future research.

Abstract citation ID: igae098.3366

### FALLS IN ADULTS: STEADI AND DURABLE MEDICAL EQUIPMENT PREDICTIVE ANALYSIS

Karen Probst, and Kathryn Unger, *Gannon University, Erie, Pennsylvania, United States*

Falls are the leading cause of death with fall rates continuing to rise. The Center for Disease Control, (CDC, 2020) predicted that by the year 2030 there will be 53 million falls among older adults. This alarming rate of falls resulted in a comparative study of the Stopping Elderly Accidents, Deaths & Injuries (STEADI) assessments as well as the sensitivity and specificity of the tools. Research was conducted (n=116) to determine if a condensed 3-question questionnaire could provide a brief comprehensive evaluation of fall risk. The data was then analyzed to determine if the provision of specific durable medical equipment (DME) impacted the results of either STEADI assessment. The study involved distributing both versions of the STEADI electronically to participants at Chosen Mission Incorporated, a non-profit that distributes DME at no cost to their local community. The condensed STEADI was found to have a strong correlation to the comprehensive STEADI as well as the acquisition of DME. The assessment demonstrated a high sensitivity and low specificity indicating a connection between fall risk and actual falls within six months. The findings from this research may benefit healthcare professionals to better and more quickly identify individuals with increased fall risk and potential for falls within six months.

Abstract citation ID: igae098.3367

### INFLUENCE OF SATISFACTION WITH INTERVENTIONS ON THE OCCURRENCE OF FALLS IN OLDER PEOPLE: RANDOMIZED CLINICAL TRIAL

Juliana Ansai, Leticia Teodoro Maciel, Mariana Ignácio Sossai, Mariana Luiz de Melo, Graziele Norberto Pereira, Mel Silva de sá, Ana Beatriz Simões Pereira, and Karina Gramani-Say, *Federal University of São Carlos, São Carlos, Sao Paulo, Brazil*

Falls are recurrent among older people, and there is a need of more personalized interventions to be adopted to prevent falls. The purpose of this study was to analyze the influence of satisfaction with the interventions on occurrence of falls in older people with a history of falls. This is a randomized clinical trial, with older people with at least 2 falls in the past year. Participants were allocated into Control Group (CG) (with monthly monitoring about falls and general health) and Intervention Group (IG). The IG was submitted to a case management intervention over 16 weeks, including a multidimensional assessment, intervention proposal and individualized care plan. The assessment consisted of falls data (falls calendar and monthly phone calls) over 16 weeks and satisfaction with interventions (Short Assessment of Patient Satisfaction SAPS 0-28, and overall satisfaction with care 0-10) after 16 weeks. The final sample consisted of 56 older people. The median SAPS score in the GC was 22.00 and in the IG 25.00 (p=0.046). The median overall satisfaction score was 10.00 in both groups (p=0.782). After 16 weeks, 34.5% of the IG and 48.1% of the CG fell (p>0.05), and 6.9% of the IG and 14.8% presented harmful falls (p>0.05). There was no influence of satisfaction with intervention on falls data over the short follow-up. Future studies that identify other factors related to improvements in falls data among brazilian faller older people are needed.

Abstract citation ID: igae098.3368

### MACHINE LEARNING APPROACHES FOR FALL PREDICTION IN KOREAN COMMUNITY-DWELLING OLDER ADULTS

Jungwon Cho, Minhee Yang, and Eunhee Cho, *Yonsei University, Seoul, Republic of Korea*

Falls cause significant physical, psychological, and financial burdens for older adults. They represent the second leading cause of death worldwide. On average, approximately 27% of older adults fall annually. To prevent fall-related injuries, identifying older adults at risk for falls and engaging them in fall-prevention interventions are important measures. This study aimed to develop and evaluate the fall prediction model for older adults in the community with machine learning approach. A total of 9,884 participants were included in the analysis from the 2020 Korean National Survey of Older Adults. The dataset included 48 predictor variables, including demographics, health status, health behavior, and environmental factors. We employed several prediction models, including logistic regression, decision tree, random forest, and support vector machine. Synthetic minority oversampling technique (SMOTE) approaches were employed to address class imbalance. The area under the receiver operating characteristic curve (AUC) ranged from 0.84 to 0.98 across four algorithms. The random forest model had the highest AUC value of 0.98, followed by logistic regression (0.91), support vector machine (0.91), and decision tree (0.84). Marital status, the presence of visual impairment, economic activity status, sex, and diagnosis of hypertension were the five most important features for predicting falls. Our findings suggest that machine learning methods can accurately identify older adults at a high risk for falls, potentially enabling early interventions and targeted preventive measures. This approach could ultimately lead to reductions in fall-related injuries and healthcare costs and improvements in the overall quality of life of older adults.

Abstract citation ID: igae098.3369

### SYMPTOM BURDEN AND RECURRENT FALLS IN OLDER CANCER SURVIVORS: A LONGITUDINAL POPULATION-BASED STUDY

Myeongjin Bae[1], Kushang Patel[2], and Nancy Gell[1], *1. University of Vermont, Burlington, Vermont, United States*, *2. University of Washington, Seattle, Washington, United States*

Older adults with a cancer history may be at greater fall risk as activity-limiting symptoms from cancer and cancer treatment often persist, even after treatment has ended. Therefore, we aimed to prospectively determine the effects of pain, fatigue, and sleep difficulty on fall risk in older adults with a cancer history. Data from the National Health and Aging Trends Study Rounds 1-5 (2011-2015) were analyzed. The study sample (n=1443) was limited to community-dwelling Medicare beneficiaries who self-reported any history of cancer, other than skin cancer only. Symptoms were assessed through questions regarding pain, fatigue, and sleep difficulty. The main outcome measure was the cumulative incidence of recurrent (≥2) falls in at least one year from 2011. All analyses were weighted and accounted for complex sampling design. Incidence of recurrent falls was significantly higher among those with pain, fatigue, and sleep difficulty. In adjusted models, compared with those with no symptoms, the adjusted risk ratios for recurrent falls were 1.80 (95% confidence interval [CI] = 1.31–2.48), 2.10 (95% CI = 1.61–2.75), and 1.66 (95% CI = 1.25–2.20) for older cancer survivors with pain, fatigue, and sleep difficulty, respectively. For those with all three symptoms, the adjusted risk ratio for recurrent falls was 4.58 (95% CI = 2.82–7.42), compared to those with no symptoms. This study revealed the significant impact of aging and cancer-related symptoms on fall risk in older adults with a cancer history. Reducing symptom burden is needed to mitigate fall risk in this population.

Abstract citation ID: igae098.3370

### THE ASSOCIATION BETWEEN PATIENT-LEVEL PROACTIVITY AND RISK OF CANCER-RELATED FALLS

Chuan Lu[1], and Qianyan Zheng[2], *1. Dana Farber Cancer Institute, Boston, Massachusetts, United States*, *2. Harvard T. H. Chan School of Public Health, Boston, Massachusetts, United States*

Cancer-related falls are prevalent among elder patients given physiological and functional compromise undergoing formidable antineoplastic treatment process. However, effective fall prevention can be challenging without a clear risk-navigating system and sufficient patient self-awareness. The purpose of this cross-sectional study was to evaluate fall-related burden and identify potential risk factors among elders undergoing active cancer treatment, based on the public Medicare Health Outcome Survey (MHOS) collecting patient-reported outcomes provided by Medicare health programs in the United States. The study compared the distributions of clinically implemented fall prevention and patient proactive consultation regarding fall management using beneficiaries from 2018 (14937; 6.55%) to 2022 (21683; 7.74%) undergoing active cancer treatment. Multivariable-adjusted logistic regressions were performed using 2022 cohort to explore potential risk factors of falls. Fall occurrence rates sustained around 30%. Patient's consultation rate revealed a significant growth over the 5-year period (2018: 29.39% vs. 2022: 34.27%; p< 0.001), while prevention implementation rates were without significant change. Lung cancer (OR=1.995, 95%CI=1.803-2.208) and colon cancer (OR=1.648, 95%CI=1.478-1.837) were associated with greater risk of falls compared to non-cancers. Patients who were advised to increase physical activeness (OR=0.719, 95%CI=0.664-0.779) and with active fall consultants (OR=0.171, 95%CI=0.157-0.186) exhibited significantly lower fall occurrences. This study substantiated the potential role of proactive measures, namely promoting patient's physical activeness and risk awareness, in reducing fall risk among elders under antineoplastic treatment. Colon or lung cancer may be priority risk identifiers for fall prevention. Future prospective trials are warranted to establish clearer clinical guidance to minimize fall risks targeting elder cancer populations.

### SESSION 7660 (POSTER)

### FAMILY CAREGIVING IV (POSTERS)

Abstract citation ID: igae098.3371

### A BALANCING ACT: QUALITATIVE DESCRIPTIONS OF DAILY STRESS AMONG BLACK AND WHITE DEMENTIA CAREGIVERS

Angelica Eagle[1], Emma Weber[1], Emily Noyer[2], Angela Turkelson[1], and Kira Birditt[1], *1. University of Michigan, Ann Arbor, Michigan, United States*, 2. *Survey Research Center, Ann Arbor, Michigan, United States*

ADRD caregiving is often stressful and time consuming, having potentially negative implications on the lives of caregivers. This qualitative study described negative daily experiences among ADRD caregivers, considering variations by relationship type and race. Participants from the SWELCare study included 159 ADRD caregivers (97 White, 62 Black) comprising 52 child caregivers, 91 spouse/partner caregivers, and 16 other family or friend caregivers, aged 21 to 86 (M = 62.72, SD = 12.34). Caregivers completed up to 5 interactive voice response interviews for 5 consecutive evenings, providing open-ended responses regarding their most negative event that occurred that day. The data revealed 7 major themes with the most frequently reported regarding: 1) caregiving responsibilities, followed by 2) other daily problems, 3) interpersonal tension with their care recipient, 4) study participation, 5) interpersonal tension with others, 6) physical health problems, and 7) emotional health problems. Over half of the responses included 2 or more themes, indicating negativity across multiple domains. The majority of the descriptions involved issues outside of caregiving tasks. Additionally, White caregivers were significantly more likely to report tension with care recipients than Black caregivers (30% vs 19%). Further, spousal/partner caregivers were significantly more likely to report tension with care recipients than adult child caregivers (31% vs 19%), which suggests interpersonal tension with care recipients vary by race and relationship type. These data identify areas outside of caregiving responsibilities that caregivers may need assistance with, which provides potential targets for improving caregivers' daily lives.

Abstract citation ID: igae098.3372

### ADVANCING RESPITE RESEARCH TO SUPPORT FAMILY CAREGIVERS OF OLDER ADULTS

Kim Whitmore[1], and Sarah Swanson[2], *1. Marquette University, Sun Prairie, Wisconsin, United States*, 2. *University of Nebraska Medical Center/Munroe-Meyer Institute, Omaha, Nebraska, United States*

One of the most frequently expressed needs of family caregivers of older adults is respite – a temporary break from the responsibilities of caregiving. ARCH National Respite Network and Resource Center (ARCH) has advanced efforts to enhance the respite evidence base to assist with sustainability, promote continuous quality improvement, and help translate research into best practices. The Committee for Advancement of Respite Research (CARR) advises ARCH on the execution of its respite research initiative and is comprised of former members of the ARCH Expert Panel on Respite Research, research scholars and evaluators, and foundation representatives. This session will highlight the efforts of the CARR to address the following identified priority areas: • Define and measure the value (cost-effectiveness) of respite; • Recommend common data elements (CDE) for respite-related research; and • Expand culturally appropriate research with historically underrepresented populations This session will also provide an overview and discuss the application of the following products that have developed from the work of the CARR: • Measuring the Value of Respite which includes a proposed framework for research; • Recommended Common Data Elements (CDEs) for Respite-related Research; • Resources for Culturally and Linguistically Competent Respite Research; and • Ensuring Cultural and Linguistic Competence: A Guide for Respite Researchers. Finally, we will highlight how this work aligns with the research recommendations in the 2022 National Strategy to Support Family Caregivers.

Abstract citation ID: igae098.3373

### CAREGIVERS' POOR SLEEP AND PLASMA BIOMARKERS FOR NEURODEGENERATION

Yeonsu Song[1], Sarah Choi[1], Raeanne Moore[2], Alexander Erickson[3], Karen Camacho[4], Monica Cappelletti[1], Cathy Alessi[3], and Jennifer Martin[3], *1. University of California Los Angeles, Los Angeles, California, United States*, 2. *University of California San Diego, La Jolla, California, United States*, 3. *VA Greater Los Angeles Healthcare System, Los Angeles, California, United States*, 4. *Greater Los Angeles VA Health Care System, North Hills, California, United States*

Family caregivers of people living with dementia (PLWD) have an increased risk of dementia. Poor sleep is common among caregivers, but the relationship between sleep and biomarkers of neurodegeneration in this population is unknown. In a secondary analysis of data from a behavioral sleep intervention trial among caregivers of PLWD, we tested the relationship between subjective and objective sleep and plasma biomarkers of neurodegeneration and explored the preliminary effects of the intervention on these biomarkers. Participants included 18 caregivers of PLWD who received a sleep education program (active treatment versus control). Sleep measures included the Pittsburgh Sleep Quality Index, total nights of good sleep (sleep diary), and wrist- actigraphy-measured sleep duration, sleep efficiency (SE), and total wake time (TWT). Plasma biomarkers included amyloid beta-40 (Aβ40), amyloid beta-42 (Aβ42), Glial Fibrillary Acidic Protein (GFAP), Neurofilament light (NfL), and Aβ42/40 ratio. All measures were collected at baseline (n=18), post-intervention (n=11), and three months (n=7). Pearson correlations and t-tests were performed. At baseline, greater (worse) TWT was associated with higher Aβ40 (r=0.60, p=0.009) and Aβ42 (r=0.62, p=0.006) levels. Lower (worse) SE was associated with higher Aβ40 (r= -0.52, p=0.026), Aβ42 (r= -0.61, p=0.008), and NfL (r= -0.48, p=0.043). Less nights of good sleep were associated with a lower Aβ42/40

ratio (r=0.51, p=0.032). Biomarker levels were not significantly different between treatment and control at follow-ups. In conclusion, poor sleep is associated with increased biomarkers of neurodegeneration among caregivers of PLWD. Larger studies are needed to test whether sleep interventions reduce this population's neurodegeneration risk.

Abstract citation ID: igae098.3374

### DAILY AFFECT AND SLEEP AMONG FAMILY CARE PARTNERS OF PEOPLE LIVING WITH DEMENTIA

Yeonsu Song[1], Kevin Kuehn[2], Nicole Lewis[1], Alexander Erickson[3], Brent Mausbach[2], Jennifer Martin[3], and Raeanne Moore[2], *1. University of California Los Angeles, Los Angeles, California, United States*, *2.University of California San Diego, La Jolla, California, United States*, *3. VA Greater Los Angeles Healthcare System, Los Angeles, California, United States*

Caregiving can be a positive and negative experience simultaneously. However, many of the family care partners of people living with dementia (PLWD) often experience poor sleep, leading to heightened levels of depression and caregiving strain. This study examined daily variations in positive and negative affect and the bidirectional role of sleep and daily affective states. A total of 35 care partners (mean age 63 years, 39% non-White, 81% women) completed ecological momentary assessment surveys of their positive and negative affect and subjective sleep three times a day for seven days. Variables included positive (currently feeling energetic, happy, motivated, rested, relaxed) and negative affect (currently feeling anxious, depressed, helpless, hopeless, nervous, tense, worried, worthless; range 1-5), and 'good sleep' (i.e., no trouble with falling asleep and staying asleep) on previous nights. Descriptive and mixed-effect logistic regressions were performed. Mean levels of positive affect were significantly lower in the evening compared to the morning/afternoon (F (df= 1, 491) = 54.24, p< 0.001); no time-of-day differences were found for negative affect (F (df= 1,492) = 1.33, p=0.25). Higher levels of mean positive affect were associated with a higher probability of good sleep (OR=2.23, 95%CI=1.26-3.94, p< 0.01). No significant relationships were found between negative affect and good sleep. This study demonstrates a within-day variation in positive affect among family care partners, which is related to the quality of sleep the night before. Understanding these dynamics is crucial for developing targeted interventions to improve the sleep and emotional well-being of care partners of PLWD.

Abstract citation ID: igae098.3375

### DEMENTIA CAREGIVING STYLES: USING EXPERT FEEDBACK TO ADAPT THE PARENTING STYLES MODEL

Darby Simon[1], Emilee Ertle[2], and Benjamin Mast[2], *1. Massachusetts General Hospital, Boston, Massachusetts, United States*, *2. University of Louisville, Louisville, Kentucky, United States*

Identifying how family caregivers provide care to people living with dementia has important implications for the quality of life for both parties. This project adapted the well-researched parenting styles framework to create a new measure reflecting Dementia Caregiving Styles. Although there are important differences between people with dementia and children, there are similarities between parents and family caregivers in how they approach these caring relationships. This creates comparable experiences such as balancing autonomy versus safety, making decisions on behalf of their care-recipient, and changing family roles. The current framework measures caregivers' levels of responsiveness, demandingness, and autonomy-granting during interactions with their care-recipients. This poster reviews the process of adapting the Parenting Styles Inventory-II into the proposed Dementia Caregiving Styles Inventory by collecting feedback from dementia caregiving experts on this topic. For each item, experts rated the relevance to dementia caregiving and provided qualitative feedback. Experts could also provide their overall thoughts about adapting the parenting styles framework for dementia caregivers. Lynn's (1986) standards were used to establish each item's content validity via expert agreement. Several themes emerged from the experts' feedback. Most notably, the demandingness subscale's items required major revisions to apply to dementia caregiving. Conversely, the subscales regarding responsiveness to the care-recipient's needs and balancing autonomy versus safety were applicable to dementia caregivers. At the end of the revision process, the experts were overwhelmingly in support of the items suggesting that adapting parenting styles to dementia caregiving could provide a framework that helps family caregivers provide better dementia care.

Abstract citation ID: igae098.3376

### DEVELOPING A TAILORED STRAIN INDEX FOR LONG-DISTANCE FAMILY CAREGIVERS OF PERSONS WITH DEMENTIA

Francesca Falzarano[1], Verena Cimarolli[2], Kathrin Boerner[3], and Richard Chunga[4], *1. University of Southern California, Los Angeles, California, United States*, *2. LeadingAge, Washington, District of Columbia, United States*, *3. Carl von Ossietzky University of Oldenburg, Oldenburg, Niedersachsen, Germany*, *4. University of Massachusetts Boston, Boston, Massachusetts, United States*

Most research has examined the experiences and consequences of dementia family caregiving among proximate caregivers, while the unique needs of the growing number of long-distance caregivers (LDCs) remain understudied. Since the vast majority of research and evidence-based intervention efforts have specifically focused on caregivers who reside in close proximity to their care-recipients, existing measures of caregiver strain do not adequately capture the unique distance-related challenges experienced by LDCs. Thus, the current study aims to evaluate the suitability of a nine-item strain index tailored for LDCs. The scale is comprised of: 1) a three-item measure assessing level of difficulty experienced with the physical, financial, and emotional aspects of care from the National Study on Caregiving (NSOC); and 2) six novel items assessing difficulty with LDC (e.g., monitoring care, traveling to the care-recipient). Baseline data (n=40) derived from a study examining the feasibility of LDCare - a virtual, multicomponent intervention for LDCs - were used to conduct an exploratory factor analysis (EFA) with the NSOC and LDC-specific items. Initial findings suggested the exclusion of two factors that did not significantly contribute to the scale's underlying covariance structure. Further analyses suggested that a seven-item scale comprised of two components yielded the best model fit, accounting for 57.1% of the total variance. The final scale included three items capturing distance challenges and four items assessing general caregiving challenges. Findings provide evidence for the scale's suitability among LDCs and underscore the importance of tailored assessments to adequately measure the unique challenges associated with LDC.

Abstract citation ID: igae098.3377

### GRANDMOTHER INITIATIVES IN FAMILY TRANSFORMATION: OUTCOMES OF AN INTERVENTION STUDY

Carol Musil, Alexandra Jeanblanc, Christopher Burant, and Jaclene Zauszniewski, *Case Western Reserve University, Cleveland, Ohio, United States*

To address the well-documented stress and health challenges faced by grandmothers raising grandchildren, a sample of 342 grandmothers living with grandchildren participated in a national online, NIH funded randomized clinical trial testing two methods to reduce stress. We evaluated whether the 4-week Grandmother Initiatives in Family Transformation (GIFT) intervention (Resourcefulness Training © via video plus structured journaling) compared to a 4-week unstructured journal-only condition (with video instruction) improved grandmothers' individual and family well-being: a) mental health (general mental health and depressive symptoms), b) physical health (self-rated and general health), c) family well-being (family functioning) and d) resources (resourcefulness, support, and reward) at 2, 12, and 24 weeks post-intervention. We ran RM-ANOVA for each outcome, looking at changes over time, time by arm, time by days journaling, and time by number of video views for their arm. Grandmothers in both arms showed significant improvements on general mental health and family functioning and reduced depressive symptoms and stress across time, establishing that both arms received helpful interventions; there were no changes in physical health indicators. Participants in both arms reported improved resourcefulness, support, and caregiving reward. Significant improvements over time in mental health and support were reported by participants journaling more frequently. The number of video views was significant over time only for resourcefulness, although there were between-subjects effects for video views for general mental health and depressive symptoms. Implications for online interventions to reduce stress, and the impact of active participation (journaling, practice and video support) are discussed.

Abstract citation ID: igae098.3378

### LIFE COURSE APPROACH TO CAREGIVING FOR AGING PARENTS: THE ROLE OF ADVERSE CHILDHOOD EXPERIENCES

Jooyoung Kong, Stephanie Robert, and Jason Fletcher, *University of Wisconsin Madison, Madison, Wisconsin, United States*

The high strain and burden of caring for aging parents have been well-documented. Few but growing studies have found the effects of earlier distal factors, such as childhood maltreatment, on negative mental health for caregivers of aging parents. Taking a life course approach to intergenerational caregiving, this study examined the long-term impact of adverse childhood experiences (ACEs) on adult caregivers of aging parents. Using data from the Midlife in the United States (MIDUS), we analyzed a sample of 412 caregivers of aging mothers. We conducted a series of latent class analyses (LCA) for a holistic assessment of the patterns of ACEs among the caregivers. Respondents' exposure to ACEs available in MIDUS included parental neglect, emotional abuse, physical abuse, sexual assault (perpetrator not specified), living with a household member with a substance use issue, and parental divorce/separation/death during childhood. Among the caregivers of mothers, we identified a five-latent-class structure: No/low ACEs (60%), Neglect and Household Challenges (12%), Paternal Abuse (5%), Parental Physical Abuse (16%), and Substantial ACEs (7%). The respondent's education was significantly associated with latent class membership. Caregivers in the Substantial ACEs class showed consistently worse physical and mental health than those in the No/low ACEs class. The current study contributes to raising awareness of adult-child caregivers who experienced childhood trauma, who may deal with accumulated disadvantages from childhood adversities, their potential correlates, and current caregiving. Further investigations will help find ways to reduce ACEs' impact in order to promote the health of these caregivers and strengthen their well-being.

Abstract citation ID: igae098.3379

### PARTICIPANT EVALUATION OF A SOCIAL ENGAGEMENT PROGRAM FOR FAMILY CARE PARTNERS OF PERSONS WITH DEMENTIA

Eliza Baby[1], Elizabeth Lydon[2], Sarah Jones[2], Xiayu Chen[2], Vincent F. Mathias[2], Wendy Rogers[2], Minakshi Raj[2], and Raksha Mudar[2], *1. University of Illinois Urbana-Champaign, Urbana, Illinois, United States*, *2. University of Illinois Urbana-Champaign, Champaign, Illinois, United States*

Caregivers of persons with dementia (PwD) often experience increased social isolation, loneliness, and negative health outcomes. While support groups provide critical education and connection for care partners, there is less opportunity for engagement outside of the caregiving role. We developed and assessed the benefits of a technology-based social engagement program, CareEngage, designed to be convenient and accessible to older adult caregivers (60+ years) of a family member with dementia. Sixty-four older caregivers completed the intervention. Participants attended eight social engagement sessions over 4-6 weeks. During each session, participants met remotely via Zoom for casual, unmoderated conversations on various topics of interest. The topics were intentionally unrelated to caregiving and covered five categories: Arts and Culture; Life Experiences; Nature, Health, and Wellness; Recreation and Sports; and Science and Technology. Immediately following each session, participants completed a survey to assess self-reported enjoyment, engagement, and connection on a five-point scale in addition to providing an open-ended comment. Participants also completed a post-program survey to capture overall program satisfaction. A mixed methods approach was employed to assess post-session and post-program surveys. High scores were reported on enjoyment (mean = 4.41 ± 0.89), engagement (mean = 4.52 ± 0.78), and connection (mean = 4.44 ± 0.84). On the post-program survey, the majority reported they enjoyed the program (75%), and they would recommend it to others (72%). Findings suggest virtual social engagement opportunities may be beneficial for enhancing the social well-being of older care partners of PwD.

Abstract citation ID: igae098.3380

**PERCEIVED EXPERIENCES OF MINORITIZED FAMILY DEMENTIA CAREGIVERS IN MENTALIZING IMAGERY THERAPY**

Miranda Zea[1], Paulina Gutierrez-Ramirez[1], Liliana Ramirez Gomez[1], Kimberly Dueck[1], Maria Melero-Dominguez[1], and Felipe Jain[2], *1. Massachusetts General Hospital, Boston, Massachusetts, United States*, *2. Depression Clinical and Research Program / Massachusetts General Hospital, Boston, Massachusetts, United States*

Family dementia caregivers often experience stress and caregiver burden as a result of their responsibilities. Specifically, family dementia caregivers from racial and same sex minoritized groups face challenges including a lack of access to culturally concordant resources. Mentalizing Imagery Therapy (MIT) is a new mindfulness and guided imagery therapy that is designed to enhance self and other understanding, and alleviate psychological symptoms. We aimed to describe the perceived impact of MIT on three minoritized caregivers: a Black female, an Asian male, and a White gay female partnered to a Black woman. Across virtual and in-person trials of MIT (n=99), the proportion of racial and ethnic minority individuals was ~70%, and at least 4% sexual minorities. MIT comprised four, weekly, two-hour sessions, incorporating mindfulness and imagery exercises. Semi-structured exit interviews were conducted for perceived experiences by two independent raters, and consensus achieved. We purposefully selected three participants who could clearly express their thoughts and who described cultural factors for in-depth examination. Individuals from minoritized groups participating in MIT reported experiencing positive changes in well-being and an increased capacity for mentalization in relation to themselves and others (particularly the person living with dementia). They also reported gaining insights into unique cultural factors that impacted caregiving. These results suggest potential promise of MIT for aiding the distinct challenges confronted by minoritized family dementia caregivers. Participants demonstrated heightened well-being, and an increased capacity for mentalization. Future research should validate these findings with minority populations in larger samples.

Abstract citation ID: igae098.3381

**SEEKING SUPPORT FOR MENTAL HEALTH IN EAST ASIAN AMERICAN DEMENTIA CAREGIVERS: A GROUNDED THEORY STUDY**

Eunjung Ko[1], Judith Tate[2], Kathy Wright[2], and Karen Rose[2], *1. New York University, New York, New York, United States*, *2. The Ohio State University, Columbus, Ohio, United States*

Asian Americans in general are reluctant to seek mental health support. This can be detrimental to those who are also dementia caregivers. Unfortunately, Asian Americans and each subgroup in this population are underrepresented in dementia caregiving research. We explored help-seeking behaviors for mental health in East Asian American dementia caregivers using a qualitative analysis based on constructivist grounded theory. We conducted 60–90 minute interviews with each participant. Interviews were recorded and transcribed verbatim. To date, 16 participants have completed the interviews using purposive sampling. Thirteen were females, including 12 daughters and one daughter-in-law. Twelve were first-generation immigrants. The average caregiving duration was 40 months. We developed seven themes—"providing care," "individual values," "a need for support," "seeking support," "barriers to using support," "benefits from support," "living now and the future"—comprising 18 categories. While providing care, caregivers observed care recipient's symptoms, experienced caregiving challenges, and encountered personal life events, leading them to need and seek help. Individual values of caregivers—cultural acceptance, faith, caregiving competence, dyadic connectedness—could influence how they perceive their situations. Caregivers then sought formal, informal, and self-support separately or in combination, albeit with several barriers to accessing support. Through the support, caregivers could achieve benefits (connecting, learning, relieving, reassuring). The benefits, along with individual values while providing care, could affect caregivers' viewpoints on the present and future. These initial findings can provide researchers with valuable insights into the imperative need for focused research on Asian American communities and the development of strategies to bolster mental health support for this population.

Abstract citation ID: igae098.3382

**SELF-CARE AND WELL-BEING INTERVENTIONS FOR FAMILY CAREGIVERS OF FRAIL OLDER ADULTS IN A RURAL CONTEXT**

Siqi Yang[1], Nicolas Cuevas[2], Shatakshi Shelar[1], Yu Lu[1], Bhagyashree Katare[3], and Nasreen Lalani[3], *1. Purdue University, West Lafayette, Indiana, United States*, *2. Universidad Nacional de Colombia, Bogota, Bogota, Colombia*, *3. Purdue University, Purdue University, Indiana, United States*

The rapid aging of the population and limited access to healthcare in rural communities pose significant challenges for caregivers caring for older family members suffering from serious chronic illnesses. The heavy burden of care and multiple responsibilities cause poor coping, self-care and wellbeing outcomes and therefore need interventions to promote their resilience and overall quality of life. Our systematic review aims to explore interventions available in the existing literature to promote the self-care and well-being of family caregivers of frail older adults residing in rural underserved areas. Arksey & O'Malley's methodological framework of scoping studies was employed for the analysis. Databases such as PubMed and Scopus were used to search the articles. PRISMA and Covidence tools were followed to review and analyze the study. The CASP tool was used to examine the quality of the study and consensus was established among three research assistants and the advisor in the team. Our findings include 24 selected articles, with the majority of studies conducted in the U.S. (63%). Interventions were categorized into four groups as digital-web-based interventions (7), telehealth interventions (6), psychoeducational interventions (9), and other interventions (2). The most common outcomes observed across these interventions were increasing caregiver knowledge, self efficacy and reducing caregiver burden. Our review emphasizes the need for more research using community-engaged and culturally based approaches to promote the wellbeing and resilience of rural caregivers.

Abstract citation ID: igae098.3383

### STRUCTURAL INFLUENCES ON WHY OLDER ASIAN IMMIGRANTS PROVIDE CARE FOR SPOUSES WITH DEMENTIA

Julie Kim, and Jung-Ah Lee, *University of California Irvine, Irvine, California, United States*

Elder care and dementia caregiving studies on Asian immigrant families commonly cite cultural expectations, filial piety, and Confucianism as motivations for substantial reliance on informal caregivers such as adult children and children-in-law. Yet in Asian immigrant families of older adults with dementia, spouses assume primary caregiving roles as much as daughters and daughters-in-law do. This study moves beyond cultural arguments by exploring the influence of structural forces that leave caregiving responsibilities to spouses rather than adult children, or other caregivers, in Asian immigrant families of older adults with dementia. Data come from a larger dementia caregiver home-visit-based intervention study with bilingual and bicultural community health workers (CHW) visiting caregivers. The present study employs content analysis of CHW's home visit logs with 14 Korean immigrant (10 wives) spousal caregivers and 18 Vietnamese immigrant (10 wives) spousal caregivers, both ethnic groups associated with strong cultural norms of filial piety. Detailed home visit logs contain observations of living arrangements and environments and recount conversations and events from each home visit, contextualizing the social and physical environments of caregivers and spouses with dementia. Findings show Asian immigrant spousal caregivers do not expect high levels of care support from their adult children, as assumed in cultural arguments. Instead, structural influences, such as ethnic enclaves and resources therein, the couples' relative socioeconomic positions, access to institutional resources, and changing structures in family relationships, shape spousal caregiving arrangements and modify older immigrants' expectations of adult children.

Abstract citation ID: igae098.3384

### SUPPORTING SOCIAL INTERACTION OF OLDER FAMILY CARE PARTNERS THROUGH A DIGITAL HEALTH PHYSICAL ACTIVITY PROGRAM

Dawon Baik, *University of Colorado, Aurora, Colorado, United States*

Older Family care partners (FCPs) experience more social isolation, mental and physical health issues than younger FCPs. Loneliness and social disconnectedness in older FCPs can lead to an increased risk of poor health and low well-being. To support social interaction and connectedness in older FCPs of persons with heart failure (HF-FCPs), we identified their needs and preferences on a technology-supported physical activity (PA) program using scenario-based design methods. The program included one-on-one PA coaching, using a fitness tracker and motivational text messaging. Fifteen HF-FCPs were recruited at a University Hospital between September and December 2022. We conducted semi-structured qualitative interviews. Participants were aged 65.9±4.4 years (M±SD), female (73%), and spouses (87%). HF-FCPs perceived that the program could support their social connectedness. Participants reported that interaction with a health coach via videoconferencing would be valuable for their social health in the context of caregiving (e, g., time constraints, caregiving duties limiting FCPs to the home). A group-based program was suggested to increase motivation for support and feedback from other FCPs. They indicated that texting would be useful to share their PA and caregiving experience and provide support and encouragement to other FCPs. Participants highlighted the need for their own social groups, because as one described: "There is a support group for people with the same condition and their caregivers, but it's really talking about the condition itself, not about how we take care of ourselves." Future studies should develop and test technology-based, caregiver-centered interventions for sedentary and socially isolated older FCPs.

Abstract citation ID: igae098.3385

### THE HEALING POWER OF COMPASSIONATE LOVE: LATER-LIFE CAREGIVING STATUS AND MENTAL HEALTH IN THE UNITED STATES

Nirmala Lekhak[1], Tirth Bhatta[1], Foster Kamanga[1], Cecilia Fernandes[2], and Eva Kahana[3], *1. University of Nevada Las Vegas, Las Vegas, Nevada, United States, 2. PrismaHealth Children'sHospital, Richland, South Carolina, United States, 3. Case Western Reserve University, Cleveland, Ohio, United States*

The detrimental effects of family caregiving on mental health have been extensively established, with research focusing on emotional support as a mechanism to explain these relationships. However, previous research has not investigated such mechanisms across caregiving statuses especially among older caregivers. Furthermore, the significance of compassionate love in understanding the negative impacts of caregiving on mental health lacks attention. Using nationwide web-based data (n=1,861), we examined the unique role of compassionate love in explaining the differences in depressive symptoms, anxiety, and loneliness across caregiving status. Our ordinary least squares regression (OLS) estimates indicate that not caregivers, former caregivers, and caregivers who were not living with care-recipients (caregivers not in-residence) had significantly lower levels of anxiety, loneliness, and depressive symptoms than caregivers who were living with care-recipients (caregivers in-residence), with the most notable difference observed in loneliness. After controlling for socioeconomic status, health, and emotional support in our models, compassionate love entirely accounted for the remaining differences in depressive symptoms and anxiety between caregivers in-residence and not caregivers. Additionally, it fully explained the remaining differences in loneliness between former caregivers, caregivers not in-residence, and those living with them. Although considerably weaker (changed from $b=-0.48$, $p< 0.001$ to $b=-0.38$, $p< 0.01$), the loneliness gap between not caregivers and caregivers in-residence remained statistically significant even after accounting for love. Our findings highlight the importance of recognizing differences in caregiving status when designing future interventions to improve family caregivers' feelings of love and mental health in later life.

Abstract citation ID: igae098.3386

### THE PARADOX OF PAID CARE: BENEFITS AND CHALLENGES FOR SPOUSAL CAREGIVERS' EMOTIONAL WELL-BEING

Leslie Fontaine, Jyoti Savla, and Karen Roberto, *Virginia Tech, Blacksburg, Virginia, United States*

Recent research suggests that social factors may buffer negative outcomes of caregiving, including social isolation. In particular, spouse caregivers of partners living with moderate to severe dementia (PLwD) often find themselves confined to their homes, which reduces their opportunities for social interactions and intensifies feelings of loneliness and caregiver stress and burden. For many spouse caregivers, their primary in-person interactions are with paid caregivers. The aim of this study was to investigate if the use of paid helpers mitigated feelings of loneliness and overload among caregivers. Fifty-one rural spouse caregivers (61% wives, 98% White, 87% residing only with PLwD), answered structured interview questions that assessed their levels of loneliness, role overload, and degree of assistance from paid helpers. Low levels of overload and loneliness was experienced by 16% spouses, with 88% of them not utilizing paid services. In contrast, 65% of spouses reported significant levels of both overload and loneliness. Within this group, 62% of wives used paid helpers, whereas 75% of husbands did not. Multinomial regressions indicated that husbands without paid helpers experienced more pronounced feelings of loneliness and overload, whereas wives with paid helpers reported increased feelings of loneliness and overload, suggesting that paid support does not uniformly alleviate the emotional strain and loneliness of caregiving. Findings underscore the complex gender differences associated with paid support on the emotional well-being of spouse caregivers. Discussion highlights the benefits of paid assistance in addressing multifaceted caregiving outcomes and the need for more tailored and nuanced support strategies for spouse caregivers.

### SESSION 7665 (POSTER)

### FORMAL CAREGIVING

Abstract citation ID: igae098.3387

### BLURRING BOUNDARIES: QUALITATIVE RESEARCH ON DIRECT CARE WORKERS' EXPERIENCES

Brittney Pond, *University of California San Francisco, San Francisco, California, United States*

In the United States, there are 4.8 million direct care workers who provide care and services to older adults and people living with disabilities (PHI, 2023). Research suggests that direct care workers in home and community-based services occupy a unique role between family and non-family. In this poster, I discuss how direct care workers' close relationships with their clients often led to blurring personal and professional boundaries that created challenges through semi-structured interviews with 43 (n=43) direct care workers providing care and services to older adults in the U.S. This research addresses two primary research questions: 1) what is the nature of work and relationships with clients for paid direct care workers? 2) how do direct care workers for non-family older adults navigate personal and professional boundaries? Through these interviews, I found that direct care workers unique roles within home care settings often caused challenges such as doing or feeling pressure to do additional labor on and off their shifts and hesitancy to be away from their clients. These findings support and expand upon existing literature that direct care workers often form close, reciprocal relationships with non-family clients and often participate in additional labor beyond their work requirements.

Abstract citation ID: igae098.3388

### EXPLORING THE MEDIATION EFFECT OF SOCIAL SUPPORT ON CAREGIVERS' PHYSICAL HEALTH FOR PERSONS WITH DEMENTIA

Hannah Cho[1], Liming Huang[1], Karla Washington[2], Debra Parker Oliver[2], and George Demiris[1], *1. University of Pennsylvania, Philadelphia, Pennsylvania, United States, 2. Washington University in St. Louis, St. Louis, Missouri, United States*

In the U.S., over 11 million families provide 18 trillion hours of unpaid care yearly to Persons with Dementia (PwD). Families, often primary caregivers, face overwhelming stress and health decline due to caregiving demands and employment. This study examines how social support affects caregivers' stress and physical health in hospice settings for caregivers of PwD. From September 2022 to February 2024 the parent study (n= 167) was assessed using the Medical Outcomes Survey and the Short-Form 12 Questionnaire. The correlation analysis revealed that social support is positively correlated with caregiving duration and negatively correlated with physical health in hospice care. Consequently, a path analysis was conducted to examine the impact of social support on the relationship between caregiving duration and physical health of caregivers of PwD during hospice care. We controlled for age and gender in regression. Preliminary results showed the mediation effect of social support is 0.12 ($p = .12$). The direct and total effects of caregiving duration on physical health are 0.04 ($p = 0.9$) and 0.16 ($p = .58$), respectively. The model is just identified with an RMSEA of 0 and a TLI and CFI of 1. Non-significant results imply that the mediation effect may not be a substantial component of the total effect or that with the sample size statistical power is not high enough to detect it. Given these findings, further investigation is needed, and the data should be re-run as the intervention is ongoing with the full dataset for more definitive insights.

Abstract citation ID: igae098.3389

### EXPLORING USE OF COMMUNITY SUPPORT SERVICES BY BLACK FAMILY DEMENTIA CAREGIVERS: FACILITATORS AND BARRIERS

Florence Johnson, and Sheria Robinson-Lane, *University of Michigan, Ann Arbor, Michigan, United States*

In this qualitative study, we delve into the factors influencing community support services (CSS) utilization, including respite care and support groups. We conducted focus group discussions to uncover the barriers and facilitators that lead individuals to engage with or abstain from these services. Analyzing these factors will provide insights to enhance the effectiveness of CSS and its impact on the community. Our focus group involved six current or former

Black family caregivers of persons with dementia (PWD). The virtual meeting, lasting approximately 1.5 hours, collected information on community support use, dementia care knowledge, and self-care practices. Transcriptions were thematically analyzed using the Rigorous and Accelerated Data Reduction (RaDAR) method—a team-based strategy to extract relevant data for specific project deliverables. Four key themes emerged to aid caregivers in managing their responsibilities: Education on Dementia Stages: Understanding different stages of dementia helps caregivers effectively manage challenging behaviors. Empowerment through Dementia Subtypes: Knowledge of various dementia subtypes enables caregivers to seek support groups for guidance. Respite Care Access: Learning about and accessing respite care prevents burnout and promotes well-being. Emotional Support: Addressing guilt provides emotional well-being for caregivers. As the older adult population grows, dementia caregiving becomes a critical public health issue. Family members caring for PWD in the community face significant challenges, emphasizing the importance of effective support services.

Abstract citation ID: igae098.3390

### IMPACT OF COMMUNICATION ON INTERACTIONS BETWEEN FAMILY CAREGIVERS AND HEALTH CARE PROFESSIONALS

Nadia Khan[1], Donna Benton[2], and Yujun Zhu[2], *1. University of Southern California Leonard Davis School of Gerontology, Los Angeles, California, United States*, *2. University of Southern California, Los Angeles, California, United States*

Family members providing care to older adults with progressive conditions are at an increased risk for caregiver burnout and poorer health. Few studies have examined how healthcare professionals interact with and support the family caregivers of their patients. This study investigated the perceived attitudes of healthcare professionals toward family caregivers and the experiences of family caregivers in healthcare settings where their loved ones receive medical treatment. We used a voluntary, anonymous survey through the software REDCap composed of Likert-scale questions to obtain the opinions of family caregivers to older adults diagnosed with a memory impairment (N=199). We conducted linear regression to examine the relationship between demographic characteristics and the quality of communication between caregivers and healthcare providers. Results showed that 57% of participants strongly disagreed or disagreed that their healthcare team gave them information on how to care for themselves while caring for loved ones. Preliminary linear regression analysis showed that there is a statistically significant positive relationship ($p < 0.05$) between the discussion of caregiver self-care and the demographic variables of income and education level. Additionally, 48% of participants strongly disagreed or disagreed that their healthcare team asked them about their well-being and quality of life. Our initial findings have implications for how healthcare professionals communicate with family caregivers about their well-being and the emotional burden of family caregiving. Additionally, our results demonstrate the need for new communication strategies between healthcare professionals and family caregivers that will effectively address their diverse needs. Word Count: 244

Abstract citation ID: igae098.3391

### LONGITUDINAL CARE ARRANGEMENT PATTERNS AND SUBJECTIVE WELL-BEING AMONG COMMUNITY-DWELLING OLDER ADULTS

Esther Shin[1], BoRin Kim[2], and Sojung Park[3], *1. Illinois State University, Normal, Illinois, United States*, *2. University of New Hampshire, Durham, New Hampshire, United States*, *3. Washington University in St. Louis, St. Louis, Missouri, United States*

Many older adults with health problems receive assistance from caregivers in their communities. The availability and nature of caregiving (e.g., formal or informal) is contingent on varying individual and contextual factors with important consequences in the care recipients' life quality. Existing research tends to examine single sources of caregiving. Even when examined together, the majority of current investigations focus on older adults with dementia. Using the National Health and Aging Trends Study (2011-2022), this study identified distinct longitudinal trajectories of informal and formal caregiving and their association with subjective well-being among community-dwelling older adults aged over 70. We restricted our sample to those with caregiver support throughout the study period (n=1,200). Using group-based multiple trajectory modeling, we identified four longitudinal care patterns: (1) Consistently limited care (9.3%), (2) Predominantly sole informal caregiver (57.5%), (3) Multiple informal caregivers (17.2%), and (4) Dual caregivers (16%). In the consistently limited care group, older adults were more likely unmarried, educated, non-white, living alone, and with mobility issues. Those with multiple informal caregivers tended to be older, female, in poor health, and living in independent/assisted living. Conversely, older adults in dual caregiving were likely educated, covered by Medicaid, and residing in retirement communities. Findings highlight the role of sociodemographic factors in shaping patterns of caregiving arrangements, and having dual caregiving was associated with increased well-being among care recipients. Creating an affordable care scheme would be essential in supporting healthy aging among older adults who wish to remain at home with adequate caregiving support.

Abstract citation ID: igae098.3392

### WHAT COMES AFTER BEING NAMED EMERGENCY CONTACT: EXPERIENCES OF CURRENT AND POTENTIAL SURROGATE DECISION-MAKERS

Maresi Berry-Stoelzle[1], Sarah Murray[1], Daniel Karr[2], Tammy Walkner[3], and Bruce Alexander[1], *1. Iowa City VA Medical Center, Iowa City, Iowa, United States*, *2. Department of Veterans Affairs, Iowa City, Iowa, United States*, *3. Iowa City VA Health Care System, Iowa City, Iowa, United States*

Veterans have high rates of chronic health conditions than non-veterans. As their comorbidity progresses, recommended therapies are often more complex and may not contribute to the Veteran's quality of life. During hospital admissions, a combination of medication and illness can result in the Veteran being unable to make decisions for themselves. Their designated Durable Power of Attorney (DPOA) or, if none is designated, the Emergency Contact/Next of Kin (EC/NoK) then makes these decisions. This mixed-methods study identified Veterans and their EC/NoK who had been hospitalized at a VAMC during the last year. Semi-structured interviews with EC/NoKs focused on identifying support needs, challenges of the health care system, perceptions of "goals of care" conversations, and the responsibilities of being a medical decision-maker. Questions were determined from prior literature on 1) experience of caregivers and 2) medical decision making for others. Four themes were identified via Consolidated Framework for Implementation Research: 1) when EC/NoK have higher social connections with the Veteran's healthcare team, they report higher confidence and preparedness for their role; 2) most preparation consisted of conversations with the Veteran about terminal decisions, 3) EC/NoK with the highest self-report of preparedness reported previous experience with medical decision-making; 4) those whose self-reported lower confidence also reported higher levels of isolation and a lack of support in their caregiver role. These interviews with EC/NoK demonstrate opportunities to increase knowledge of medical decision-making and the opportunities to support EC/NoK's in their role as DPOA's within a healthcare system.

### SESSION 7670 (POSTER)

### HEALTH AND SOCIAL SERVICES INTERVENTIONS

Abstract citation ID: igae098.3393

### ASSOCIATIONS BETWEEN UNMET NEED AND SELF-REPORTED HEALTH STATUS AND QUALITY OF LIFE IN CHINESE OLDER ADULTS

Siou-Tang Huang[1], Weiguo Chong[2], and Herng-Chia Chiu[2], *1. China Medical University, Taichung, Taichung, Taiwan (Republic of China)*, *2. Tsinghua University, Beijing, Beijing, China (People's Republic)*

**Background:** The demand for home-based community health services has become more critical as the population ages. To what extent are associations between unmet need and health status crucial for health planning for older adults. The study aimed to examine the degree of unmet need and self-reported health status and quality of life for an older adult population.

**Methods:** Population-based data from the China Health and Longevity Longitudinal Survey (CLHLS) was used. Older persons who participated in both the 2014 and 2018 surveys were included. After match and exclusion, 2,271 subjects were analyzed. Community health services include services of home visits, mental and health prevention.

**Results:** The baseline age is 80.3 years old, 84.17 years old on average four years after. More than 10% of older persons lost their spouses during this period. There are 46.40% and 46.63% reported unmet need in 2014 and 2018, respectively. LMM analysis reveals that compared with the unmet group, those reported needs were fully met and partially met older appeared significantly better in health status (ß = 0.12, ß =0.01) and quality of life (ß =015, ß =0.11).

**Implication:** Our findings conclude that unmet services are highly negatively associated with health status as well as quality of life. To improve the older population's health and quality of life, a community services survey is needed to design programs to meet the needs of various older populations.

Abstract citation ID: igae098.3394

### EFFECTS OF A THEORY-ORIENTED SOCIAL FACILITATION INTERVENTION ON SOCIAL ISOLATION: A RANDOMIZED CONTROLLED TRIAL

Huimin Xiao, Huangqin Liu, and Qiyuan Huang, *Fujian Medical University, Fuzhou, Fujian, China (People's Republic)*

Transitioning to a nursing home represents significant life changes, which often result in a significant reduction and potential dissolution of pre-existing emotional bonds with family, friends, and social networks. Social facilitation is essential to help nursing home residents adapt a new life in nursing home. Thus, this study aimed to develop and examine the effects of the social facilitation program based on the cognitive-behavioral model of social isolation (SFCBSI), on social isolation, subjective well-being, cognitive tendencies, psychological adjustment, self-efficacy, and social support of nursing home residents. A randomized, controlled, assessor-blinded, parallel-arm trial was conducted in two nursing homes Fujian, Southeast of China from September 2023 to February 2024. 180 participants were randomly assigned to the intervention group (n = 90) and the control group (n = 90). The control group received routine institutional care. The intervention group received the SFCBSI program involving cognitive reconstruction, emotional management, and behavioral change, which was implemented by group for 4 weeks. Participants were assessed using the Cognitive Appraisal Orientation Test, Nursing Home Adjustment Scale, General Selfefficacy Scale, Perceived Social Support Scale, Social Isolation Scale and the Memorial University of Newfoundland Scale of Happiness Survey. Outcomes were evaluated at baseline and immediately after the intervention. The intervention participants showed significant differences in social isolation, subjective well-being, cognitive tendencies, psychological adjustment, self-efficacy, and social support, compared to the control group ($P < 0.001$). It indicates that the SFCBSI could reduce social isolation and improve psychological well-being among nursing home residents.

Abstract citation ID: igae098.3395

### INSIDE THE BLACK BOX OF MANAGED CARE: SERVING AT-RISK OLDER ADULTS IN MASSACHUSETTS' SENIOR CARE OPTIONS PROGRAM

Pamela Nadash[1], Marc Cohen[1], Edward Miller[1], Janelle Fassi[2], Maryssa Pallis[1], Megan Siebecker[1], and Elizabeth Simpson[1], *1. University of Massachusetts Boston, Boston, Massachusetts, United States*, *2. University of Massachusetts Boston, Goffstown, New Hampshire, United States*

People dually eligible for Medicare and Medicaid are at risk for poorly coordinated care, high-cost health service utilization, and poor outcomes. Commonwealth Care Alliance (CCA), a not-for-profit managed care organization, serves approximately 35,000 people dually eligible for Medicare and Medicaid in the Commonwealth of Massachusetts. CCA has two primary products: Senior Care Options, which has served people 65 years or older since 2003, and One Care, which has served people 18-64 years of age since 2013. Drawing from key informant interviews and other data collection, this study sought to understand the organizational experience of the CCA model. It identifies the challenges CCA faces in ensuring that its vulnerable membership has access to both appropriate clinical care and the broad range of health-related services that are critical to the health of members. It also identifies the plan's mechanisms for addressing these challenges. The evolution of CCA's model of care and the various mechanisms it adopted over time to adapt to the needs of its membership and comply with state and federal requirements are discussed. These changes are then placed in the context of ongoing conversations about the most effective models of integrated care. Findings highlight CCA's focus on health-related social needs; the impact of member advocacy on program design; and the implications of non-profit status for workplace culture and resources.

Abstract citation ID: igae098.3396

### THE RELATIONSHIP BETWEEN DYADIC COPING AND INTIMACY OF PATIENTS WITH STROKE AND SPOUSES: A DYADIC APPROACH

Xiaohui Liu[1], Nana Liang[2], Xuan Du[3], Haihua Gao[1], Xiaoping Yang[1], Huijuan Wang[1], and Jialin Yuan[1], *1. Ningxia Medical University, Yinchuan, Ningxia, China (People's Republic)*, *2. Gansu Medical College, Pingliang, Gansu, China (People's Republic)*, *3. HeXi University, Zhangye, Gansu, China (People's Republic)*

Most studies have focused on coping in middle-aged and elderly stroke patients and spouses in isolation. However, marital intimacy is more prominent when middle-aged and elderly stroke patients and spouses are coping with the disease together. This study conducted from December 2020 to July 2021,included 203 stroke patients and their spousal caregivers. They completed the General Data Questionnaire, Dyadic Coping Inventory, the Quality of Relationship Index. Data was analyzed using SPSS 26.0 and Amos 22.0, including descriptive statistics, paired t test, Pearson correlation analysis. And this research used actor-partner interdependence mediation model to explore the relationship of intimacy, among stroke patients and their spousal caregivers.The actor-partner interdependence model revealed actor effects of stress communication, supportive coping, delegated coping.and common coping on intimacy for both patients and their spouses($\beta$ =0.572; $\beta$ = 0.500; $\beta$=1.283; $\beta$ = 0.665, $p < 0.05$). There were no similar partner effects ($p > 0.05$). Although patients' stress communication had a partner effect on spouses intimacy ($\beta$ = 0.221, $p < 0.05$), spouses had no similar partner intimacy effect ($p >0.05$). There were actor and partner effects of negative dyadic coping on intimacy for both patients and their spouses($\beta$ = -0.587; $\beta$ = -0.206, $p < 0.05$). Our results describe the middle-aged and elderly patients and their spouses as an interactional unit in which positive dyadic coping supports a satisfactory relationship. This study highlights the need for couple-based interventions, including strategies combined with individual and dyadic therapy for both partners.

### SESSION 7675 (POSTER)

### HEALTH BEHAVIOR CHANGES (POSTERS)

Abstract citation ID: igae098.3397

### FACTORS ASSOCIATED WITH LIFE SATISFACTION IN VETERANS IN THE HEALTHY AGING PROJECT: BRAIN PSYCHOEDUCATIONAL CLASS

Maulika Kohli[1], Alice Verstaen[2], and Emily Trittschuh[1], *1. VA Puget Sound Healthcare System, Seattle, Washington, United States*, *2. Lyra Health, Burlingame, California, United States*

The Healthy Aging Project-Brain (HAP-B) was developed to encourage engagement in activities associated with successful aging. Across 6 sessions, HAP-B targets sleep, socialization, physical, and cognitive activity through myth-busting, developing SMART goals, and tracking behavioral change. This study aims to examine associations between physical and mental health factors with life satisfaction. Veteran participants (n=50, mean age = 70.6 years) were predominantly male-identity (88%) and White (76%). Linear regressions examined associations between physical and emotional functioning factors and life satisfaction at baseline, covarying for age. Results indicate that higher life satisfaction is significantly associated with lower depressive symptoms, higher emotional well-being, and higher self-efficacy in our sample. HAP-B is effective in facilitating behavioral change that has downstream positive effects on emotional functioning. It is feasible, accessible, and scalable, supporting a model of care among older adult Veterans that combines psychoeducational and skill-based approaches. Future groups may emphasize emotional well-being as a direct intervention target. There is a significant need to prioritize engagement with, and accessibility of, mental healthcare services to improve both emotional well-being and overall satisfaction with life. Healthcare providers are well-positioned to encourage engagement with activities that are protective against adverse cognitive aging.

Abstract citation ID: igae098.3398

### LONGITUDINAL CHANGES IN ALCOHOL USE ACROSS 20 YEARS: A DAILY DIARY STUDY

Sara Miller[1], and David Almeida[2], *1. The Pennsylvania State University, Pennsylvania, United States*, *2. Pennsylvania State University, University Park, Pennsylvania, United States*

Longitudinal studies often identify declines in alcohol consumption with aging. However, drinking trajectories may vary across alcohol use indicators and person-level characteristics. The current study utilized three bursts of daily diary surveys from the National Study of Daily Experiences to examine changes in alcohol consumption across 20 years among a sample of adults aged 24 to 81. Participants (N=1,429; Mage=49.9) completed 2+ bursts of 8-day telephone diary interviews. Outcome measures included burst-level abstaining, drinking frequency, frequency of exceeding guidelines of the National Institute on Alcohol

Abuse and Alcoholism (1+/2+ drinks for females/males), and average drinking quantity (i.e., number of drinks on drinking days). Moderating variables included baseline age, biological sex (1=Male), education (1=Less than high school to 5=Graduate school), and perceived physical health (1=Poor to 5=Excellent). Multilevel linear and logistic regression analyses revealed that alcohol use quantity declined over time, while frequency of drinking, frequency of exceeding guidelines, and likelihood of alcohol abstention were stable, adjusting for baseline sociodemographic, health behavior, psychosocial, and health characteristics. There were also individual differences in drinking trajectories. Older age was associated with drinking more frequently and consuming fewer drinks per occasion at baseline as well as stronger declines in most alcohol use indicators across time. Furthermore, graduate education was linked with increasing drinking frequency, while male sex and poorer physical health were associated with stronger declines in drinking quantity. Future studies should evaluate mechanisms of age-related declines in alcohol use and examine contributing factors of individual differences in alcohol use trajectories across adulthood.

Abstract citation ID: igae098.3399

### PATIENT PORTALS PROMOTE SHARED DECISION-MAKING AND ADOPTION OF HEALTHY HABITS RELATED TO MENOPAUSE

Roshni Singh[1], Fei Tang[2], Diana Ruiz[2], and Stuti Dang[3], *1. University of Miami, Miami, Florida, United States*, *2. Miami Veterans Affairs Healthcare System, Miami, Florida, United States*, *3. Miami VA Healthcare System, Miami, Florida, United States*

Perimenopausal women would benefit from a better understanding of menopause, and associated conditions and therapies. We implemented a menopause educational intervention called “MyHealtheVet to Enable And Negotiate for Shared decision-making” (MEANS) using the Veterans Affairs (VA) patient portal MyHealtheVet (MHV), in the Miami VA Healthcare System (MVAHS). Intervention participants received weekly secure messages with information on menopause. Here, we examine the effect of the MEANS intervention on SDM and adoption of healthy behaviors. We enrolled 269 women at MVAHS in MEANS. Participants’ average age was 53.2 years; 42.4% white, 43.1% black, and 24.2% Hispanic; 63.2% had a college education, and 95.9% already used MHV; 160 completed the six-month intervention and evaluation. Post-intervention, 57.5% reported that MEANS allowed them to get information that would otherwise be difficult to obtain, 79% said it increased their understanding of menopause, 80% felt more confident to discuss menopause treatments with their provider. Many reported discussing menopause-related conditions and treatments with their providers: 39% reported having discussed hormone therapy, 46% discussed osteoporosis, 33% discussed urinary incontinence. Many had adopted positive health behaviors: 8.1% had started taking hormones, 48% started calcium, 78% started Vitamin D; 38% started doing Kegel’s exercise; 69% began exercising, and 70% improved their diet. This pilot patient portal intervention shows that patient portals may offer a scalable tool to enhance SDM between patients and providers regarding menopause-related topics and to promote healthy behaviors among racially and ethnically diverse menopausal women. This strategy may be extrapolated to other non-urgent conditions.

Abstract citation ID: igae098.3400

### PERCEIVED AND ACTUAL BMI IN AGING ADULTS: A SCOPING REVIEW

Sarah Hubner[1], and Julie Blaskewicz Boron[2], *1. Oregon Health Authority, Public Health Division, Health Promotion and Chronic Disease Prevention, Camas, Washington, United States*, *2. University of Nebraska Omaha, Omaha, Nebraska, United States*

Sustaining a healthy weight across the lifespan is an important aspect of disease prevention and quality of life in aging. Still, approximately 77% of US adults deviate from ideal weight recommendations, contributing to disease and disability. Improving lifestyles is critical to reducing this chronic disease burden. Self-perception of weight status (perceived-BMI) and its accuracy (versus actual-BMI) may influence behavior through goal setting and motivation; however, this relationship remains understudied in aging adults. Examining BMI dissonance and its broader context within the aging population may contribute to better understanding how self-perception shapes behavior and wellness. The current study contributes to this goal by assessing the field via scoping review. Electronic database searches were carried out in: PubMed, APA PsycInfo, Sports Medicine & Education Index. Peer reviewed white literature published in English from database inception to May 2024 were included in the search. Eligible research was related to aging adults’ body form and included primary measures for perceived- and actual-BMI. Populations were community-dwelling adults, aged 45+. The search revealed 4160 titles. After title, abstract, and full text screen, 13 articles were selected for inclusion. Evaluation of the selected studies showed that diverse aspects of perceived-BMI were described, including diagnostic accuracy and alignment with actual-BMI, association with health literacy, behavioral impacts, and correlations to lifestyle change strategies. These synthesized results may contribute to understanding how self-perception shapes behavior and wellness; discussion will include how this review can inform interventions to promote healthier lifestyles and reduce chronic disease burden across the adult lifespan.

Abstract citation ID: igae098.3401

### PREVALENCE OF LIFESTYLE-BASED MODIFIABLE DEMENTIA RISK FACTORS AMONG CANADIAN ADULTS AGES 50+

Danielle D’Amico[1], Brian Tan[2], Malcolm Binns[2], Howard Chertkow[2], and Nicole Anderson[2], *1. Baycrest Academy for Research and Education, Toronto, Ontario, Canada*, *2. Baycrest Academy for Research & Education, Toronto, Ontario, Canada*

Modifiable lifestyle behaviours are key determinants of cognitive health and dementia risk. The prevalence of lifestyle-based dementia risk factors is currently unknown among the Canadian adult population. The purpose of this study was to establish age- and sex-adjusted prevalence of risk in five domains using validated scales: 1) physical activity (CHAMPS); 2) brain-healthy eating (Eating Pattern Self-Assessment); 3) cognitive engagement (Florida Cognitive Activities Scale); 4) social connections (UCLA Loneliness

Scale); and 5) mental well-being including depression, anxiety, distress, and perceived stress (DASS-21 and PSS-10). Males ages 70-79 (n=120) and females ages 60-69 (n=145), 70-79 (n=217), and 80+ (n=115) completed each scale online. Depending on the age decade x sex group, the percentage of individuals who exhibited mild depression, anxiety, distress, and perceived stress were 20.0-28.7%, 14.7-22.6%, 13.0-20.0%, and 43.3-54.7%, respectively. 33.1-54.5% did not meet the physical activity recommendations of 150 minutes/week of moderate to vigorous exercise. No participants met the brain-healthy eating recommendations. High-risk cut-off scores (below the 10th percentile) ranged from 8.0-11.6 for cognitive engagement and 23.4-24.4 for social engagement. The number of domains upon which individuals were at high risk ranged from 1 to 5 (median = 4). Data are currently being collected for females ages 50-59 and males ages 50-69 and 80+. This study is part of the first-of-its-kind research-based community centre at Baycrest delivering personalized dementia risk reduction programming. These data will be used to identify domains in which participants are at-risk in order to guide personalized dementia risk reduction program strategy.

### SESSION 7680 (POSTER)

### HEALTH PROMOTION

Abstract citation ID: igae098.3402

### A PILOT TEST OF A NOVEL MEDICAL EDUCATION DEVICE FOR LVAD PATIENTS

Jennifer Crittenden[1], Hannah Longley[2], Asheesh Lanba[3], Amarpreet Kohli[3], Nihar Kumthekar[3], Jacob Inger[3], and Killian Hadsell[3], *1. University of Maine, Orono, Maine, United States*, *2. University of Maine, Bangor, Maine, United States*, *3. University of Southern Maine, Portland, Maine, United States*

Medical assist devices, like the Left Ventricle Assist Device (LVAD), are increasingly used by individuals 65 and older. This project entails a pre-clinical trial feasibility study of a novel, non-invasive, mock LVAD device (the XVAD). Phase I testing consisted of adults under the age of 65 (N = 8, M = 28.5 years) and Phase II expanded study enrollment into the 65-90 age group (N = 4, M = 71 years). All participants wore the device for three to five days and experienced random device alarms similar to those provided by an LVAD device. Each alarm and participant response time was recorded by the XVAD device. Real time response data was supplemented with survey data, qualitative diary data, and feedback gathered via a debrief interview. Qualitative findings underscore the following: Importance of accessible device design for older adults, the influence of participant activity on alarm response times, the need for clarity related to alarm responses, and the impact of XVAD usage on daily activities. Findings indicate that feedback across phases, though they engaged different age groups, yielded convergence around accessibility concerns. Examination of response time device data indicates that while older and younger participants demonstrated different reaction times at the start of the trial, the groups demonstrated equivalent reaction times to device alarms at the end of each trial indicating the potential for effective patient training over time. Findings will be used to inform device development and can inform similar mock device trials that use a multi-generational phased testing approach.

Abstract citation ID: igae098.3403

### ANALYSIS OF DIFFERENCE OF FOOT PLANTAR PRESSURE IN OLDER WOMEN WITH DIFFERENT BONE MINERAL DENSITY LEVELS

Min Liu[1], Gong Chen[2], Dongmin Wang[1], and NING KANG[1], *1. Peking University, Beijing, Beijing, China (People's Republic)*, *2. Peking University, Beijing, Beijing, China*

**Objectives:** To explore the characteristics of foot plantar pressures in the older women with different bone mineral density levels.

**Methods:** 50 older women (68.84 ± 3.73 yr) were recruited from one community in Beijing, China. T value of the bone mineral density and average force of 10 plantar partitions (toe 1, toe 2-5, metatarsal 1, metatarsal 2 and metatarsal 3, metatarsal 4, metatarsal 5, midfoot, medial heel, and lateral heel) in two feet were tested. Taking the median of T value, the participants were divided into low and high bone mineral density group (L group: N = 27, T = -3.43 ± 0.80, 68.62 ± 3.42 yr; H group: N = 23, T = -1.37 ± 0.68, 69.09 ± 4.12 yr). Independent T test was used to analyze the difference of foot plantar pressures between two groups.

**Results:** (1) The average force of toe 1 on the dominant side in the L group was significantly lower than that in the H group (t = -2.161, p = 0.036). (2) The average force of metatarsal 5 on the dominant side and that of metatarsal 1 on the non-dominant side were significantly higher than that in the H group (t = 2.413, p = 0.020; t = 2.182, p = 0.034). Conclusions: The difference of average force in the older women with different bone mineral density levels was greater on the dominant side than that on the non-dominant side, and greater on the forefoot than that on the arch and heel.

Abstract citation ID: igae098.3404

### APPLYING A SALUTOGENIC MODEL TO EXPLORE RESOURCES UTILIZATION IN OLDER ADULTS WITH PREDIABETES: A QUALITATIVE STUDY

Yaqian Liu, Angela Leung, and Jed Montayre, *The Hong Kong Polytechnic University, Hong Kong, Hong Kong*

**Background:** Older adults with pre-diabetes face unique health challenges, including limited awareness and resources for disease management. The Salutogenic model of Health focuses on identifying and utilising available resistance resources surrounding people to cope with tension created by stressors. This approach is crucial for this vulnerable population addressing both prevention and effective management of pre-diabetes.

**Objectives:** To explore pre-diabetes population's experience towards receiving diabetes risk information, and what available resources employed to cope with stressful pre-diabetes situation.

**Methods:** This descriptive phenomenological qualitative study using six-step inductive thematic analysis was conducted in Hong Kong. Individual in-depth semi-structured interview guided by Salutogenic model were conducted to explore the experience of coping with prediabetes through the lens of sense of coherence (comprehensibility, manageability, meaningfulness), available resources and their utilization. Purposive sampling was used to select adults above 65 years diagnosed with pre-diabetes.
**Results:** The study revealed that older adults with prediabetes primarily leverage personal resilience, community support, and healthcare guidance as coping resources. Comprehensibility was enhanced through tailored health education, enabling participants to grasp their condition's implications. Manageability was facilitated by accessible healthcare services and social support networks, aiding in lifestyle adjustments. Meaningfulness emerged as participants perceived their proactive health behaviors as future investments. Despite varying levels of resource accessibility, a common thread was the pursuit of a balanced, informed approach to pre-diabetes management.
**Conclusion:** This study provided resource map and coping mechanisms of pre-diabetic population in Hong Kong. This informs the development of a Resources-Oriented Salutogenic intervention tailored to prediabetes population.

Abstract citation ID: igae098.3405

### DEVELOPMENT AND ASSESSMENT OF AN INTERVENTION PROGRAM USING PAIR GO: RESULTS OF AN EXPLORATORY STUDY

Ai Iizuka[1], Kristine Mulhorn[2], Koki Ito[1], Moe Kitago[1], Chiaki Ura[1], Tsuyoshi Okamura[1], Kenji Toba[1], and Hiroyuki Suzuki[1], *1. Tokyo Metropolitan Institute for Geriatrics and Gerontology, Tokyo, Japan*, *2. Drexel University, Philadelphia, Pennsylvania, United States*

The board game Go, a traditional board game that originated in Asia, is associated with positive effects on cognitive function in older adults. However, as a one-on-one game, Go usually requires a single winner and a single loser. Pair Go creates a team-based game where winning and losing is a shared experience. Pair Go enhances social interaction because it is played by pairs of players. Social interaction has a positive impact on the mental health of older adults. This study aimed to develop a new intervention program using Pair Go and to explore the impact on mental health and social interaction. This single-arm intervention study was conducted from October 2023 to January 2024 in Tokyo, Japan. Twenty-eight community-dwelling older adults (71-86 years) attended Pair Go classes once a week for 90 minutes, for 12 sessions. Each session involved attending lectures, solving exercises, and playing Pair Go. Mental health, and social interaction were assessed before and after the intervention. Comparison of pre- and post-test scores using paired t-tests revealed that scores on the University of California, Los Angeles Loneliness Scale were statistically significant lower in the post-test (mean [SD] score, 38.5 [6.6]) than the pre-test (36.6 [5.9]) ($p<.05$). In addition, 50% of the participants made new social connections in the Pair Go class, such as having tea or making phone calls after the program. These results indicate that the Pair Go program may affect mental health and enhance social interaction not seen with the original Go.

Abstract citation ID: igae098.3406

### ECOLOGICAL MOMENTARY ASSESSMENT TO MEASURE SOCIAL CONNECTEDNESS IN OLDER ADULTS: AN INTEGRATIVE REVIEW

JiYeon Choi[1], Seongmi Choi[1], Hun Kang[2], and Jiyoung Shin[3], *1. Yonsei University College of Nursing, Mo-Im Kim Nursing Research Institute, Seoul, Republic of Korea*, *2. Yale University School of Public Health, New Haven, Connecticut, United States*, *3. Yonsei University, Seoul, Republic of Korea*

While the importance of social connectedness in the lives of older adults is well-established, more comprehensive evidence is needed regarding its real-world operation. This study utilizes Ecological Momentary Assessment (EMA), increasingly employed in social and health sciences, to capture real-time social interactions in everyday environments, aiming to conduct an integrative review to understand multifactorial components of social connectedness and identify implementation strategies for older adults. Five databases (PubMed, Embase, Web of Science, CINAHL, and PsycINFO) were searched to retrieve studies published up to the year 2023. Of the 2,208 studies identified, 21 were selected for final analysis. Our analysis focused on identifying the multifactorial components of social connectedness (structural, functional, and quality) and assessment of EMA methods and participant adherence to the EMA. The components of social connectedness using EMA included the following: structural components such as social contacts or interactions (n=18), loneliness (n=9) as a functional component, and quality components, such as quality of social interactions (n=3). Additionally, as a context for understanding social connectedness, location at the time of assessment, and physical (e.g., pain, fatigue) and psychological states (e.g., anxiety, depression) were evaluated. Data were mostly collected using an app on digital devices (e.g., smartphone), with assessments conducted 5-6 times per day for 5 to 21 days, achieving an adherence rate of over 70%. Findings of this review highlight the current state of science in measuring social connectedness in older adults through EMA, focusing on its structural, functional, and quality components, and indicating its feasibility in real-world contexts.

Abstract citation ID: igae098.3407

### ECONOMIC IMPACT OF FRAILTY OR PREFRAILTY IN THE ELDERLY AGED 75 OR OVER IN JAPAN

Hiroto Yoshida[1], and Yuriko Matsuzaki[2], *1. Tohoku Bunka Gakuen University, Sendai, Miyagi, Japan*, *2. Japan Health Care College, Sapporo, Hokkaido, Japan*

This study examined the impact of frailty or pre-frailty on medical and long-term care expenditures in an older Japanese population. The subjects were those aged 75 years and over who responded to the survey (March 2018) in Bibai, Hokkaido, Japan (n=1,203) and have never received certification of long-term care insurance at the survey. We followed up 758 individuals (63.0%) who used medical services at least once during the follow-up period until the end of August 2019 (17 month-period). We defined frailty as a state in performing 4 items or more and pre-frailty 2 or 3 items of 15 items which were composed of un-intentional weight loss, history of falls, etc. Among 758 subjects, 202 subjects (26.6%) were judged to be frailty group, 210 subjects (27.7%) pre-frailty group, and 346 subjects (45.6%) non-frailty group. We compared period to the new certification of long-term care insurance (LTCI), accumulated medical and long-term care expenditures between the three groups during the follow-up period. Cox proportional hazard models were used to examine the association between baseline frailty and the new certification of LTCI. The relative hazard ratio (HR) was higher in pre-frailty group than non-frailty group (HR=3.93, 95% CI:1.37-11.22, P=.011). The average cumulative medical and long-term care expenditures per capita during the follow-up period were significantly (P=.025) larger for those in the frailty group (1,190,000 yen). We confirmed the accessibility of pre-frailty to long-term care service use and the strong economic impact of frailty in the elderly aged 75 or over in Japan.

Abstract citation ID: igae098.3408

### EHEALTH LITERACY AMONG OLDER ADULTS: THE ROLE OF DIGITAL LITERACY AND SELF-EFFICACY

Linda Göbl[1], Mario R. Jokisch[2], Hans-Werner Wahl[3], David Leopold[1], and Michael Doh[1], *1. Catholic University Freiburg, Freiburg im Breisgau, Baden-Wurttemberg, Germany*, *2. Kempten University of Applied Sciences, Freiburg im Breisgau, Baden-Wurttemberg, Germany*, *3. Heidelberg University, Heidelberg, Baden-Wurttemberg, Germany*

Particularly for older adults with an increased risk of multimorbidity, digital health technologies can provide added value for health management. Successful use of these technologies requires appropriate skills. Key constructs are eHealth literacy and internet self-efficacy, which become particularly relevant in older age. This study investigated the relationship between these two multifactorial constructs in older adults, with eHealth literacy as a dependent variable, focusing on the mediating effect of digital literacy, and task-specific eHealth self-efficacy (i.e., contacting doctors via video communication and online health information seeking). A cross-sectional dataset with 327 internet-savvy German adults (mean age=71.75±6.06; 41% female) was analyzed using a structural equation model. After controlling for age, sex and subjective health, general internet self-efficacy showed a positive direct association on eHealth self-efficacy (β=.45, p< 0.01). General internet self-efficacy showed no indirect association on eHealth literacy, mediated by digital literacy, but a positive direct association on digital literacy (β=.72, p< 0.001). Digital literacy (β=.30, p< 0.05) was the strongest predictor of eHealth literacy. The model fit the data good (CFI=0.980; RMSEA=0.044) and explained 22.9% of the variance of eHealth literacy and 69.7% of the variance of digital literacy. These results point to the need for programs improving eHealth literacy to also focus on the use of digital devices and the promotion of general and task-specific internet self-efficacy. Implications for public health strategies will be discussed in order to improve eHealth literacy among older adults in a targeted manner and enable the successful use of digital health technologies.

Abstract citation ID: igae098.3409

### HEALTH PROMOTION BEHAVIORS AMONG SPOUSAL CAREGIVERS WITH HIGH DAILY MANAGEMENT CHRONIC CONDITIONS

Jinmyoung Cho[1], Molly Horstman[2], Alan Stevens[3], Laura Sands[4], and Heather Allore[5], *1. Saint Louis University School of Medicine, Ballwin, Missouri, United States*, *2. Baylor College of Medicine, Houston, Texas, United States*, *3. Baylor Scott & White Health, Belton, Texas, United States*, *4. Virginia Tech, Blacksburg, Virginia, United States*, *5. Yale University, New Haven, Connecticut, United States*

As the disease trajectory of dementia increases caregiving intensity, family caregivers are less likely to take care of their own health. Although the potential health threats from chronic caregiving distress are known, far less is known about health behaviors among caregivers with chronic conditions while caring for a person with dementia (PWD). This study aims to identify caregivers diagnosed with chronic conditions requiring high daily self-management and examine their health promotion behaviors associated with the intensity of caregiving among spousal caregivers for PWD. Using ICD-10 codes from medical records of 152 spousal caregivers aged 65 and older, six chronic conditions requiring high daily self-management (CCHDSM) were asthma, chronic kidney disease, chronic obstructive pulmonary disease, diabetes, heart failure, and ischemic heart disease. The intensity of caregiving was identified by cluster analysis (high vs. low). Self-rated health promotion behaviors were assessed as: health responsibility, physical activity, nutrition, interpersonal relations, and stress management. Ninety-nine caregivers (61.5%) were diagnosed with at least one CCHDSM. No significant difference in the intensity of caregiving was observed between CCHDSM and non-CCHDSM caregivers. A significant interaction between the intensity of caregiving and CCHDSM in performing physical activities (F=5.03, p<.01) indicates that caregivers with CCHDSM and providing high intensity of caregiving were the least engaged in physical activities compared to others. Findings highlight caregiving role hinders physical activities for spousal caregivers in the most needs of physical activities. Future studies, interventions, and support systems are urgently needed to reduce burden of caregiving for spousal caregivers struggling with chronic conditions.

Abstract citation ID: igae098.3410

### INTERGENERATIONAL DIFFERENCES: HPV KNOWLEDGE AND PERCEIVED HEALTH STATUS AT A MIDWESTERN UNIVERSITY, 2016–2022

Whitney Nesser[1], and Scott Snyder[2], *1. Indiana State University, Terre Haute, Indiana, United States*, *2. University of Alabama at Birmingham, Birmingham, Alabama, United States*

Studies of Human Papillomavirus (HPV) knowledge and health status have been conducted with university students, but typically exclude faculty and staff. The study aim was to assess HPV knowledge and perceived health status among students, staff, and faculty at a Midwestern university. Three cross-sectional surveys were conducted at a Midwestern university in 2016, 2019, and 2022 assessing HPV knowledge and perceived health status. A total of 2678 student, staff, and faculty responses were received. Perceived health status was measured using a standard health status question. Knowledge of HPV was measured as total score on a 15-item true/false test. There were 145 respondents 61 years or older. A factorial ANOVA revealed that HPV knowledge was significant for age group (p <.001) but not cohort or interaction. Respondents 61 and older performed better than those aged 18-21 but lower than groups between ages 27-45. Main effects for minority status and age group (p <.001) were found but no interaction (p =.655). No cohort effect was found for perceived health status. Chi-square analyses showed perceived health status relates to age group (p <.01) with significantly more individuals 61 and older rating their health status as excellent than expected (SD = 2.6). Perceived health status for this age group was independent of cohort (p =.22). These findings highlight the need to increase HPV education to individuals under age 27, further investigate health status perceptions in individuals 61 and older, and explore perceived health status in minority respondents.

Abstract citation ID: igae098.3411

### PREVALENCE AND ASSOCIATED FACTORS FOR POSSIBLE SARCOPENIA IN KOREAN OLDER ADULT FALLERS

Jae Jun Lee[1], Gwang Suk Kim[1], Min Kyung Park[2], Layoung Kim[1], Namhee Kim[1], and Ji Yeon Lee[3], *1. Yonsei University, Seoul, Republic of Korea*, *2. Yonsei University College of Nursing, Seoul, Republic of Korea*, *3. Inha University, Incheon, Republic of Korea*

Sarcopenia causes adverse health outcomes in older adults, especially in those who have fall experience. Asian Working Group for Sarcopenia (AWGS) introduced possible saropenia for screening sarcopenia in 2019. Identifying the prevalence and associated factors of possible sarcopenia is essential to prevent and manage sarcopenia, however research on possible sarcopenia is still lacking. Therefore, the purpose of this study is to identify the prevalence and associated factors for possible sarcopenia in Korean older adult fallers. This is a cross–sectional study with 184 community-dwelling older adults who aged 65 or over and have at least one fall experience within the last two years. Possible sarcopenia was evaluated based on the AWGS 2019 using calf circumference, five times sit-to-stand test, and handgrip strength. Age, gender, number of fall, depression, cognitive impairment, nutritional status, physical inactivity, and fear of falling were collected and assessed as covariates, and logistic regression analysis was conducted. The mean age of the participants was 74.3±6.5 and 79.3% was female. Among the 184 participants, 47 (25.5%) were possible sarcopenia. Using logistic regression analysis, physical inactivity (odds ratio = 2.55, 95% confidence level = 1.15–5.67) and fear of falling (odds ratio = 1.71, 95% confidence level = 1.01–2.92) were significantly associated with possible sarcopenia, whereas other covariates were not significant. Possible sarcopenia is prevalent in Korean older adult fallers. Our results indicated that encouraging physical activity and making efforts to reduce the fear of falling can be effective strategies for preventing possible sarcopenia in older adult fallers.

Abstract citation ID: igae098.3412

### RESIDENTS' PERSPECTIVES ON PROGRAM IMPACT OF A GROUP-BASED MIND–BODY INTERVENTION IN CCRCS

Calli Mitchell, Riley Psenka, Reid Anctil, Madeleine Scully, Barrett Laird, Elyse Park, and Lara Traeger, *Massachusetts General Hospital, Boston, Massachusetts, United States*

Older adults in continuing care retirement communities (CCRCs) may experience critical stressors related to biological and social aging. Few interventions have been established to assist residents with managing life stressors, daily functioning, and sense of community. We conducted an analysis of exit feedback from intervention participants in a randomized trial of group-based stress management skills training (SMART-Relaxation Response Resiliency Program [SMART-3RP]; 9 weekly sessions delivered by a community clinician) versus waitlist control for independent living residents at nine CCRCs across the United States (N=288; 8/2022-1/2023). SMART-3RP is an evidence-based mind-body resiliency program that integrates relaxation response training with cognitive-behavioral and positive psychology principles. Intervention participants (n=148) completed a post-intervention questionnaire including 3 categorical items and open-text fields to evaluate program helpfulness with managing life stressors, daily functioning, and sense of community. Responses were evaluated using descriptive statistics and qualitative content analysis. 131/148 (88.5%) intervention participants provided feedback (77% female, M age=81 years [range=66-93 years], 91.5% White non-Hispanic). Most endorsed that the program helped manage life stressors (82.4%) and daily functioning (77.9%); participants further identified benefits of learning or strengthening skills for practicing gratitude, relaxation, emotion regulation, mindful awareness, self-compassion, and cognitive restructuring. Over half (56.9%) felt the program enhanced sense of community; residents reflected the value of connecting with group leaders and/or other group members and increasing motivation to engage in other community social and physical activities. Findings support that community residents valued participation in a group-based intervention to enhance skills for managing life stressors, daily functioning, and social connection.

### SESSION 7685 (POSTER)

### IMPLEMENTATION SCIENCE

Abstract citation ID: igae098.3413

### FACTORS AFFECTING THE IMPLEMENTATION OF THE PRITAH INTERVENTION: LESSONS LEARNED

Klarissa Ponstein, Michel Bleijlevens, Petra Erkens, and Jan Hamers, *Maastricht University, Maastricht, Limburg, Netherlands*

The implementation of effective healthcare interventions into routine practice is challenging and influenced by various factors, including the context of implementation, individuals involved, and intervention characteristics. This study aims to explore challenges by implementing the Prevention and Reduction of Involuntary Treatment At Home (PRITAH) intervention. After a careful preparation phase, we faced serious challenges that temporarily delayed implementation. To gain insights into the reasons and factors that led to implementation delay of PRITAH, two separate focus groups were conducted with 1) two policy staff members, a district nurse, two case managers dementia, and a middle manager, and 2) two senior managers and three directors. We examined both barriers and facilitators using the Consolidated Framework for Implementation Research (CFIR). Results show that relevant stakeholders, including members from focus group one, were not involved in a timely manner. Moreover, communication gaps between senior management and middle management, policy staff, district nurses and dementia case managers led to inconsistencies in knowledge of the implementation steps being taken and hindered the decision-making processes. Nevertheless, the members of both focus groups acknowledged the shared goal of preventing involuntary treatment at home as facilitator, emphasizing the importance of goal alignment. Furthermore, a clear implementation plan from the intervention developers ensured that implementation efforts were guided by organizational expectations. The delays in implementing PRITAH emphasize timely stakeholder engagement, effective communication, joint alignment of goals and a clear implementation plan. Based on the results an adapted implementation plan has been developed.

Abstract citation ID: igae098.3414

### IMPLEMENTATION OF ENGAGE IN A GERIATRIC MENTAL HEALTH SERVICE LINE WITH NONSPECIALIST PROVIDERS

Matthew Schurr, Emily Post, Teresa Walker, and Brenna Renn, *University of Nevada Las Vegas, Las Vegas, Nevada, United States*

Despite development of efficacious psychosocial treatments for depression in older adults, implementation in real-world settings is limited, particularly in the context of patient comorbidities and across interprofessional teams. Engage is an empirically-supported psychotherapy for late-life depression but has yet to translate from randomized controlled trials to widespread implementation. Engage's utility as a brief, structured behavioral protocol holds potential for feasible dissemination across multiple provider disciplines. This study evaluated a real-world training and pilot implementation of Engage psychotherapy across a geriatric mental health care hospital team. Care managers (N = 22) across interprofessional healthcare backgrounds (occupational therapy, social work, nursing) participated in training, consultation, and implementation of Engage with older adult patients with depression. Upon completion of the implementation trial, providers participated in a 1-hour focus group to provide feedback about their experience. Focus group interviews were double-coded using thematic analysis to extract implementation themes informed by the Consolidated Framework for Implementation Research (CFIR). Thirteen themes emerged related to CFIR constructs of innovation adaptability, innovation design, critical incidents, compatibility, access to knowledge and information, need, and capability. Overall findings suggest that Engage is a feasible treatment method that fits the needs of providers, patients, and larger health care systems. Barriers to implementation included depressive symptom burden, patient complexity, and provider concerns related to self-efficacy and previous related experiences. Implementation trials and elicitation of provider feedback to inform commensurate modifications should be a staple procedure to ensure optimal implementation of newly adopted mental health treatment services.

Abstract citation ID: igae098.3415

### OPTIMIZING FUNCTION AND INDEPENDENCE: NATIONAL IMPLEMENTATION OF THREE CLINICAL PROGRAMS WITHIN VA

Jaime Hughes[1], Caitlin Kappler[2], Connor Drake[2], Leah Zullig[2], Kasey Decosimo[3], Sara Webb[3], and Susan Hastings[4], *1. Wake Forest University School of Medicine, Winston-Salem, North Carolina, United States, 2. Durham VA Health Care System, Durham, North Carolina, United States, 3. Durham VA Health Care, Durham, North Carolina, United States, 4. Durham VA, Durham, North Carolina, United States*

Older Veterans aged 65+ account for roughly one-half of Veterans Affairs (VA) Healthcare users. Comprised of more than 170 medical facilities, VA promotes programs to support function and independence for both Veterans and their caregivers. The VA Optimizing Function and Independence Quality Enhancement Research Initiative (Function QUERI) focuses on the national scale-up of three evidence-based programs (EBPs) for older Veterans and their caregivers: STRIDE (hospital-based mobility), Caregivers FIRST (caregiver education and training), and Group PT (group-based rehabilitation for knee osteoarthritis). As part of the Function QUERI program, three individual Type III hybrid effectiveness-implementation trials were conducted to, first, identify baseline organizational characteristics (organizational readiness, implementation climate, and implementation experience) associated with successful program adoption of each EBP; and, second, to evaluate strategies to adopt and sustain each EBP. To date, 142 VA facilities have participated in the implementation of one or more EBPs (STRIDE=67, Caregivers FIRST=142, Group PT N=19). The trials tested a similar implementation approach where all sites received foundational support tools (e.g. program toolkits, documentation templates, technical assistance); and, sites that did not adopt their respective EBP by pre-specified benchmarks received enhanced implementation support via virtual external facilitation (STRIDE= 13, Caregivers FIRST=13, Group PT =9). Barriers to program adoption were similar across EBPs (e.g., competing demands, staffing challenges, coordination between disciplines, poor leadership support). Offered as part of both foundational tools and enhanced support activities, implementation strategies (e.g., adaptation, engage and train stakeholders, monitor and evaluate feedback) were used to address adoption barriers across all EBPs.

Abstract citation ID: igae098.3416

**QUALITATIVE DATA INTEGRATION IN DISASTER PREPAREDNESS CAREGIVER INTERVENTION**

Brittany Anderson[1], Morolake Adeagbo[1], Maria Donohoe[1], Tiffany Wunderlich[1], Sato Ashida[1], and Aaron Seaman[2], *1. University of Iowa, Iowa City, Iowa, United States*, *2. University of Iowa, Iowa CIty, Iowa, United States*

Disaster PrepWise Caregiving (DPW-C) is an online disaster preparedness program for caregivers of persons with dementia (PWD). DPW-C works with collegiate AmeriCorps volunteers to develop and build caregivers' household disaster plan in a location of their choosing, whether at home, in public, or through online platforms. The program utilizes an all-hazards approach, which highlights all-encompassing disaster preparedness, while accommodating the specific needs of caregivers and the importance of maintaining their caregiving tasks during and after a disaster. Within the hybrid type one trial, an evaluation of the program's potential determinants of implementation is being conducted. Guided by the PRISM (Practical, Robust Implementation, and Sustainability Model) framework, the team is utilizing the triangulation of qualitative methods to assess the implementation process and fidelity through observational evaluation of the social interactions between the AmeriCorps program interventionists and the caregiver participants. This entails examining interventionists' communication and engagement with caregivers regarding their disaster plans. Observation data elucidate traits and themes in approach and engagement that were more successful in leading to positive social interactions that facilitated in-depth discussion and careful development of the household disaster plan. In response to the project's needs, the research team is using observation data to adapt the program and intervention processes to improve the overall success of interventions. As the project progresses, the qualitative team will expand the project evaluation by including examinations of the program's effectiveness and long-term sustainability. This poster will discuss how the qualitative team helped integrate gained knowledge to improve the program implementation.

Abstract citation ID: igae098.3417

**STRENGTHENING FAMILY CAREGIVER SUPPORTS: QUALITATIVE FINDINGS FROM THE ADULT DAY SERVICES PLUS PROGRAM TRIAL**

Dionne Bailey[1], Quinton Cotton[1], Katherine Marx[2], Laura Gitlin[3], and Joseph Gaugler[1], *1. University of Minnesota, Minneapolis, Minnesota, United States*, *2. Johns Hopkins University, Baltimore, Maryland, United States*, *3. Drexel University, Philadelphia, Pennsylvania, United States*

Home and community-based services, such as adult day services (ADS), that support people with dementia (PLWD) and their family caregivers frequently fail to meet the full range of dementia care and management needs. There is insufficient data on implementation mechanisms that achieve desirable results among existing interventions targeting dementia family caregivers. In a pragmatic trial, we evaluated the feasibility and acceptability of integrating a caregiver-specific intervention [ADS Plus] delivered through adult day services. Post-intervention, we conducted a directed content analysis using the Consolidated Framework for Implementation Research, drawing from semi-structured interviews with dementia family caregivers (n=15) and ADS site staff (n=14). Our findings demonstrate that key aspects of ADS Plus supported delivery and uptake: intervention adaptability, personalization, and structure (innovation); responsive to external changes and intervention marketability (outer domain); aligned goals and familiarity (inner setting); involvement of research staff, connections among practitioners, and meeting caregiver needs (individual domain), and; understanding caregivers needs and addressing staff capacity to take action (implementation process). ADS Plus offers a viable community-based solution for supporting dementia family caregivers. Future studies can assess fidelity across diverse implementation contexts to ensure greater transferability of findings.

Abstract citation ID: igae098.3418

**TOWARD SUCCESSFUL IMPLEMENTATION OF A QUALITY IMPROVEMENT INTERVENTION IN NURSING HOMES: EXTERNAL FACILITATION**

Regina George[1], Jacob Miranda[2], A. Lynn Snow[1], Michelle Hilgeman[3], Megan McCullough[4], and Christine Hartmann[5], *1. University of Alabama, Tuscaloosa, Alabama, United States*, *2. California State University East Bay, Hayward, California, United States*, *3. Tuscaloosa VA Medical Center, Tuscaloosa, Alabama, United States*, *4. University of Massachusetts Lowell, Lowell, Massachusetts, United States*, *5. VA Bedford Healthcare System & University of Massachusetts Lowell, Bedford, Massachusetts, United States*

Nursing home interventions are challenging to implement and sustain. Implementation scientists have identified facilitation as a strategy that impacts the success of an intervention. Facilitation is a complex and dynamic process that requires facilitators to develop expertise in a wide range of activities necessary to effectively support the implementation. There is limited understanding of how these activities are executed in a dynamic facilitation process. This understanding gap impacts training of facilitators and consequently, efficient delivery and sustainment of interventions. A qualitative analysis of the real-time interactions between implementation facilitators and recipient sites would contribute to our understanding of the practice of facilitation in nursing homes. In this study an expert external facilitator's interactions with nursing home teams were recorded and the activities of the external facilitator (EF) were analyzed. Data comes from a pilot three-site pragmatic clinical trial of a complex quality improvement and sleep intervention for residents living with dementia in nursing homes. The intervention is based in relational coordination, person-centered care, and adult learning principles. EF largely took place in leadership team 30-minute coaching sessions via videoconference. Reflexive thematic analysis of 24 EF session transcripts is ongoing: preliminary code clusters include metacognition (connecting actions and impact), strategic compliments (praise that encourages specific action) and problem solving (connecting causes and developing individualized solutions). Transcripts provide real-world examples of EF activities. This project provides insight into the practice of facilitation and may inform training of implementation facilitators, thus increasing incorporation and sustainment of interventions in nursing homes.

### SESSION 7690 (POSTER)

### LIFE COURSE AND DEVELOPMENTAL CHANGE

Abstract citation ID: igae098.3419

### AGE DIFFERENCES IN THE TEMPORAL PATTERNS OF SELF-CONTINUITY AND TEMPORAL DISCOUNTING

Yi Lu[1], and Corinna Loeckenhoff[2], *1. Cornell University, ITHACA, New York, United States*, *2. Cornell University, Cornell University, New York, United States*

Self-continuity, the perceived connectedness with past and future selves, varies across the life span. Previous research found that both past and future self-continuity are positively associated with age and that such effects are more pronounced for more distant time intervals (Rutt & Löckenhoff, 2016). Questions remain about the appropriate mathematical modeling of such patterns and potential associations between age differences in self-continuity and temporal discounting, the tendency to devalue future outcomes. In response, the present study assessed both past and future self-continuity as well as temporal discounting across four temporal distances (0.5, 1, 5, 10 years) in a U.S. adult life-span sample (N=461, aged 18–96, M = 51.38, SD =19.54, 52% female, 65% Non-Hispanic White). When compared to linear and exponential models, hyperbolic models provided the best fit for both self-continuity and temporal discounting. Subsequent analyses therefore focused on hyperbolic slope parameters (k) derived for each participant. Chronological age was negatively associated with k for both self-continuity and temporal discounting, indicating that, with age, the perceived connectedness with past/future selves and the perceived value of delayed rewards show fewer decrements with increasing temporal distance. However, although k parameters for past/future self-continuity were significantly associated with each other, they were not significantly associated with temporal discounting. Moreover, age differences in temporal discounting remained significant after controlling for age differences in past/future self-continuity. The present study extends our understanding of previously reported age differences in self-continuity and temporal discounting but suggests that they cannot be traced to the same underlying mechanism.

Abstract citation ID: igae098.3420

### DOMINANT BUT NOT NONDOMINANT HANDGRIP ASYMMETRY INFLUENCES ACTIVITY AND COGNITIVE FUNCTION CHANGE

Liangchi Chen[1], Sae Hwang Han[2], and Jeffrey Lockman[1], *1. The University of Texas Austin, Austin, Texas, United States*, *2. University of Texas at Austin, Austin, Texas, United States*

Handgrip strength (HGS) is commonly used in clinical and research settings for examining muscle function. Additionally, HGS asymmetry is often used as a screening tool for identifying functional and cognitive limitations among older adults. This longitudinal study examined changes in activity limitations and cognitive function associated with baseline handgrip asymmetry. We analyzed 8 waves of biennial data collected from adults 51 and older in the nationally representative Health and Retirement Study (2006–2020, N=7,053). HGS asymmetry ratio was measured at baseline and was calculated as nondominant HGS/dominant HGS; following earlier works, individuals with HGS asymmetry ratio smaller than 0.80 and higher than 1.20 were considered to have dominant and nondominant handgrip asymmetry, respectively. We used multilevel models to investigate whether HGS asymmetry was linked to a faster rate of change in activity limitations (evaluated with activities and instrumental activities of daily living items; range: 0–11) and cognitive function (evaluated with Telephone Interview for Cognitive Status; range: 0–27) during the observation period. Results indicated that individuals with dominant HGS asymmetry, but not non-dominant HGS asymmetry, developed activity limitations at a faster rate (b=.015, p=.030) and also showed a faster rate of cognitive decline (b=-.053, p=.005) compared to individuals with HGS symmetry. While the present findings are based on a U.S. sample, future research endeavors are aimed at investigating similar issues beyond the confines of the U.S. population.

Abstract citation ID: igae098.3421

### INTERDISCIPLINARY COLLABORATION TO SUPPORT AGE FRIENDLINESS AND PREPARATION FOR INTERGENERATIONAL PROGRAMMING

Kelly Munly, Jared Frederick, and Harriett Gaston, *Penn State Altoona, Altoona, Pennsylvania, United States*

Human Development and Family Studies (HDFS) and History Programs at a small campus collaborated to support Adult Development and Aging students (N=72) to understand the life course context of older adults they prepared to work with in a long-term care practicum experience. This effort to prepare students for intergenerational programming is part of a larger effort toward Age Friendliness at this campus and in the surrounding community. Life course theory is the foundation of project aims and design. After HDFS faculty established a plan for collaboration with the History Program, they were able to provide two classes of Adult Development and Aging students (N=72) with historical insights on the life course experiences of the older adults that students would be working with. Context and life course experiences discussed included dynamics related to diversity—often considered invisible—resulting from the Great Migration, as well as socioeconomic moments in time that would also influence the life course for multiple generations. After each class of discussion facilitated by the History Program, students were asked to provide reflections on their Canvas course online platform on what they had heard in light of their imminent practicum experiences. Initial thematic analysis of student reflections suggest that discussions facilitated between the History and Human Development and Family Studies Programs across two classes enhanced student understanding and supported a stronger foundation toward Age Friendliness and readiness to engage in intergenerational programming.

Abstract citation ID: igae098.3422

### LATE-LIFE OUTCOMES OF FEELINGS OF BELONGING THROUGHOUT THE LIFE COURSE

Emily Briggs, and Jacqui Smith, *University of Michigan, Ann Arbor, Michigan, United States*

Belonging is a fundamental human need that encourages fortitude throughout the life course. In the literature, belonging has been studied primarily from two perspectives: place attachment (Chawla, 1992, Scannell, 2021) and belonging within a social group (Allen, 2020). Less research, however, has been conducted about how belonging is experienced by the individual – self-perceptions of belonging. Using data from the Health and Retirement Study Life History Mail Survey (N=3,091, M Age =70.48 [50-98]) we investigated retrospective self-reports of belonging from three life stages, childhood, adolescence and midlife, as predictors of belonging and activity engagement in late life. First, we conducted linear regressions controlling for sociodemographic variables (gender, age, race, marital status). This revealed that self-reported feelings of belonging in childhood (β=.11, p<.001), adolescence (β=.13, p<.001), and midlife (β=.23, p<.001) were significantly predictive of heightened feelings of belonging in late life (Adj R2=.043,.051,.068). Second, we assessed self-reported belonging in childhood, adolescence, midlife and late life as predictors of late life engagement. These analysis revealed that belonging in childhood (β=.06, p<.001), midlife (β=.15, p<.001), and late life (β=.13, p<.001) were significantly predictive of heightened activity engagement in late life (Adj R2=.026,.037,.040). Overall this study demonstrates the importance of assessing how individuals think about their belonging retrospectively throughout their lifespan as a predictor of subsequent late-life belonging and activity engagement– both protective factors as a person ages.

Abstract citation ID: igae098.3423

### SUPER AGE: THE RISE OF BIOSOCIAL SUPER AGE ESTIMATES AS ALTERNATIVES TO CHRONOLOGICAL AGE

Adam Perzynski[1], Douglas Gunzler[2], Jarrod Dalton[3], and Kristen Berg[4], *1. MetroHealth and Case Western Reserve University, Cleveland, Ohio, United States*, *2. CWRU, Cleveland, Ohio, United States*, *3. Cleveland Clinic, Cleveland, Ohio, United States*, *4. CWRU and MetroHealth, Cleveland, Ohio, United States*

Recent discoveries have established conceptual and empirical linkages between the social characteristics of communities, biological and physiological variation and disease onset and outcomes. Cellular and molecular markers appear to be at least partially indicative of an accelerated aging process. We are approaching the widespread availability of "Super Ages", highly accurate biosocial estimates of the aging of a human person. Super Ages have more predictive validity for disease and disability than chronological age. As compared with chronological age, Super Age estimates can be described as having an accelerated (hyper-aging) or decelerated (hypo-aging) character. We ask: What types of social changes– beneficial or detrimental– might be postulated based upon a shift toward incorporating biosocial measures of aging in age-graded policy and care delivery? We present case-based scenarios illustrative of the coming biosocial dilemma. Consider two matched neighborhoods each containing 400 adults with a chronological age between 40 and 50 years who have aged in place (Neighborhood A and Neighborhood B). All persons in Neighborhood A experience uniform social and environmental hazards while hazards are minimal in Neighborhood B. Molecular analyses find Neighborhood A adults experience hyper-aging of chronological age plus 4 to 7 years. Neighborhood B are as much as 3 to 5 years "younger" creating between community biosocial age gap of 9.5 years. Both neighborhoods experience equal access to health services. Guideline-based screening (i.e. breast, lung and colon cancer screening) is age-graded but Neighborhood A is more vulnerable. Super Age estimates are fraught with new policy and care delivery challenges.

Abstract citation ID: igae098.3424

### THE GENDERED LONG ARM OF CHILDHOOD: EARLY PARENT–CHILD RELATIONSHIP AND LATER-LIFE MENTAL HEALTH IN RURAL CHINA

Jia Wang, and Mingyue Ma, *The Hong Kong Polytechnic University, Hong Kong, Hong Kong*

The large body of literature examining adverse childhood experiences (ACE) on late-life health has paid relatively little attention to gender variation. We contend that a gender perspective is crucial for understanding if women's disadvantages in health have an early origin from childhood. In particular, we suggest that non-material aspects of ACE, such as poor parent-child relationships, are more likely to exhibit gendered patterns in their influences on health, particularly in developing countries with traditional gender norms and higher gender inequality. Using five waves of data from the China Health and Retirement Longitudinal Study (CHARLES), we examine the associations between parent-child relationships in childhood and later-life mental health among Chinese men and women aged 45 and above in rural areas. Our analyses yield three important findings: 1) Compared to rural men, rural women tend to exhibit worse mental health in terms of both the baseline level and the developmental trajectory of mental health over time; 2) From a snapshot perspective, poorer parent-child relationship during early life is related to worse baseline level of mental health among both rural men and women, and this association is more pronounced among rural women; 3) From a temporal perspective, poorer parent-child relationship in early life is associated with higher likelihoods of experiencing depression throughout the whole survey period, particularly among rural women. Our study sheds new light on the significance of a gender lens when examining the early-life origins of health inequality.

### SESSION 7695 (POSTER)

### MENTAL HEALTH (POSTERS)

Abstract citation ID: igae098.3425

### ACT FOR OLDER VETERANS: TELEHEALTH-BASED ACCEPTANCE AND COMMITMENT GROUP THERAPY FOR OLDER VETERANS

Johnna Kelley, Deanna Dragan, and Katherine Luci, *Salem VA Healthcare System, Salem, Virginia, United States*

Acceptance and Commitment Therapy (ACT) is a transdiagnostic intervention with promise for use with older adults due to its relevance to a variety of mental health conditions, overall well-being, and adjustment to life changes. This pilot study (n = 10) sought to demonstrate that a novel group therapy intervention for older adults, delivered via telehealth, is efficacious and acceptable to Veterans aged 60 years of age or older. Retention rates (10/10) and attendance (M = 12.7/16 sessions) of study completers suggest feasibility and acceptability of the group. Six participants demonstrated high engagement (attended $^{3}$ 12 sessions) and four demonstrated average engagement (attended 5-11 sessions). Participants completed a structured, post-intervention interview; feedback was analyzed for themes and generally supported acceptability of the intervention. Additional themes referred to participants' experience of the virtual format, connections made with other group members, and areas for improvement. Finally, paired sample t-tests were conducted to analyze pre-post measures of anxiety, depression, loneliness, well-being, and engagement in meaningful activities. Outcome data suggests no statistically significant changes on these measures; however, the decrease in scores on a measure of depression reflects a small to moderate effect size ($t(9) = 1.467$, $p = .18$, $d = .46$). Future studies with larger sample sizes are needed to determine the impact of treatment engagement on these outcome measures. Overall, preliminary outcome data combined with qualitative feedback from participants, retention rates, and attendance rates, suggest that ACT delivered in a virtual group format is acceptable, feasible, and likely beneficial to older Veterans.

Abstract citation ID: igae098.3426

### ADVERSE CHILDHOOD EXPERIENCES AND MENTAL HEALTH IN MIDDLE AGE AND OLDER ADULTS: THE MEDIATIONAL EFFECT OF RESILIENCE

Christopher Griffith, Tiana Broen, Julia Scott, and Leilani Feliciano, *University of Colorado Colorado Springs, Colorado Springs, Colorado, United States*

Adverse childhood experiences (ACEs), physical health problems, and poor health perception are known predictors of subsequent poor mental health later in life. To support aging adults in their mental wellbeing, it is crucial to understand how they may recruit internal resources, such as resilience, to cope with past adverse experiences and current health difficulties. The current study used structural equation modeling (SEM) to examine the mediational effect of resilience on the pathways between adverse childhood experiences, sociodemographic variables, and health-related variables to mental wellbeing (a latent factor represented by depression, anxiety, and stress scores, higher score = lower mental wellbeing). Survey data were analyzed from a sample of adults aged 40 to 94 years (N = 334; Mage = 60.46; 48.8% women; 86.2% white). Number of ACEs were significantly correlated with age ($\beta = -8.86$, $p < .001$), number of physical health conditions ($\beta = 0.44$, $p < .05$) and mental health conditions ($\beta = 1.11$, $p < .001$), and poorer subjective health perception ($\beta = 0.47$, $p < .001$). Resilience mediated the pathways from subjective health perception to poor mental wellbeing ($\beta = .05$, $p < .01$), number of mental health conditions to poor mental wellbeing ($\beta = .11$, $p < .01$), and number of ACEs to poor mental wellbeing ($\beta = 0.03$, $p < .05$). Overall, these results highlight the significance of ACEs on mental wellbeing outcomes, and the importance of enhancing resilience in older clients with a history of ACEs.

Abstract citation ID: igae098.3427

### ASSOCIATION BETWEEN FUNCTIONAL LIMITATIONS AND SOCIAL ISOLATION AMONG PEOPLE WITH AND WITHOUT DEPRESSIVE SYMPTOMS

Chuwen Zhong, Briana Mezuk, and Linh Dang, *University of Michigan, Ann Arbor, Michigan, United States*

Functional limitations are prevalent among older adults with depression, and prior research suggests that social isolation can be an underlying mechanism. This study examines whether the structure of linkages between functional limitations and social isolation differs by the presence of cardinal depressive symptoms (dysphoria/anhedonia) using network analysis. Data came from the 2014/2016 Health and Retirement Study (n=7,270). Cardinal depressive symptoms were assessed using the Composite International Diagnostic Interview. Isolation (n=14 nodes) and functioning (n=16 nodes) were measured using multiple indicators (e.g., having a partner, balance test, feeling isolated, having difficulty dressing). We compared the centrality, network density, network structure, global strength, and correlation of edges between the networks of respondents who did vs. did not have cardinal symptoms of depression. Respondents who had depressive symptoms (n=628, 8.64%) were younger and more likely to be female. Compared to those without depressive symptoms, the network of those with depressive symptoms had a lower density of edges (ratio = 0.14 vs 0.20) and had different central nodes (lack of common peers vs difficulty taking medications, respectively). However, the network comparison tests showed that the two networks were similar in terms of correlation of edges (Pearson $r = 0.81, p < 0.01$), network structure (Network Invariance Test statistic = 1.82, $p = 0.48$), and strength (Global Strength Invariance Test statistics = 35.28, $p = 0.79$). Findings suggest that the network linkages between functional limitations and social isolation do not substantially differ as a function of cardinal depressive symptoms.

Abstract citation ID: igae098.3428

### CHINESE–WHITE DIFFERENCES IN SOCIAL ACTIVITY, SOCIAL SUPPORT, AND MENTAL HEALTH AMONG OLDER ADULTS

Lingming Chen, Elizabeth Procter-Gray, Qun Le, Danielle LoPilato, Marianella Ferretto, Meng Zhang, Kevin Kane, and Wenjun Li, *University of Massachusetts Lowell, Lowell, Massachusetts, United States*

Studies on healthy aging of older Asian Americans, especially Chinese Americans, are rare. This study evaluated Chinese – Non-Hispanic White differences in social activity, social support and mental health. The study enrolled 554 community-dwelling older adults (434 Non-Hispanic White and 120 Chinese) aged 65-95 years old in central and northeastern Massachusetts (2018-2023). Social activity and social support were measured by the Lifestyle Questionnaire. Mental health includes depressive symptoms assessed by the Center for Epidemiologic Studies Depression Scale, anxiety symptoms assessed by the Beck Anxiety Inventory, perceived stress assessed by the Perceived Stress Scale, and resilience assessed by the modified Brief Resilience Scale. Chinese - White differences in these factors were analyzed using linear regression models adjusting for age, gender, self-rated health, physical activity, body pain, and living alone. Results showed that in both unadjusted and adjusted models, older Chinese had less social support, lower frequency of social activities, and lower level of resilience compared to Non-Hispanic Whites. In the unadjusted models, older Chinese had higher levels of perceived stress, anxiety, and depressive symptoms compared to Non-Hispanic Whites, but there were not differences in these three factors after adjusting for covariables. In conclusion, social activity, social support, and mental health were different between Chinese and Non-Hispanic Whites among older adults in the study population. The findings from this study can inform the design and implementation of health programs tailored to specific groups, thereby reducing health disparities and improving overall well-being for older adults.

Abstract citation ID: igae098.3429

### CHRONIC DISEASE, DEPRESSION, AND SUICIDAL IDEATION IN OLDER ADULTHOOD: THE ROLE OF INTERPERSONAL FACTORS

Kallisse Dent, Linh Dang, Viktoryia Kalesnikava, Briana Mezuk, and Leah Richmond-Rakerd, *University of Michigan, Ann Arbor, Michigan, United States*

Suicide risk increases in older adulthood, and new chronic-disease diagnoses can be a precipitating factor. Motivated by the interpersonal theory of suicide, we tested interpersonal constructs --thwarted-belongingness, perceived-burdensomeness, and hopelessness -- as mediators of associations of chronic-disease with depression and passive suicidal ideation (PSI). Data were from the Health and Retirement Study, a nationally-representative, longitudinal cohort of older adults. We identified incident chronic-disease diagnoses and changes in interpersonal factors over a four-year period (2010-2014 or 2012-2016). Depression and PSI were assessed two years later (2016 or 2018) using the Composite International Diagnostic Interview- Short Form. We measured thwarted-belongingness by social contact, positive and negative social support, loneliness, and community engagement; perceived-burdensomeness by activities of daily living (ADLs), instrumental ADLs, financial strain, number of helpers, and employment status; and hopelessness using a four-item scale. Statistical analyses accounted for complex survey design and adjusted for demographics, baseline health characteristics, and the outcome at baseline. Older adults with a newly diagnosed chronic-disease, relative to those without a new diagnosis, were at elevated risk for depression (odds ratio (OR)=1.34 [1.03-1.75]) and PSI (OR=1.63 [1.26-2.11]). They also experienced greater increases in thwarted-belongingness (β=0.11 [0.01-0.20]), perceived-burdensomeness (β=0.12 [0.06-0.15]), and hopelessness (β=0.07 [0.01-0.13]) following diagnosis. Accounting for these changes in interpersonal factors modestly attenuated associations of new chronic-disease diagnoses with depression and PSI. New chronic-disease diagnoses antedate depression and PSI, even within a population with pre-existing chronic-disease burden. Targeting interpersonal factors, including thwarted-belongingness, perceived-burdensomeness, and hopelessness, following incident chronic-disease diagnoses may modestly reduce depression and PSI risk.

Abstract citation ID: igae098.3430

### COMMUNITY PREVALENCE OF POSTTRAUMATIC STRESS DISORDER, VETERAN STATUS, AND FALLS IN MA, CT, AND RI

Josephine Boateng[1], Alexa Fleet[2], Megan Siebecker[3], Taylor Jansen[3], and Elizabeth Dugan[3], *1. University of Massachusetts Boston, Dorchester, Massachusetts, United States*, *2. University of Massachusetts Boston-- Gerontology, Boston, Massachusetts, United States*, *3. University of Massachusetts Boston, Boston, Massachusetts, United States*

Post-traumatic stress disorder (PTSD) is a debilitating condition that is not well-understood in older people and often undetected. PTSD may arise from military service, falls, or other traumatic events. Understanding the prevalence of PTSD is important to identify service gaps. This study uses data from the Healthy Aging Data Report (healthyagingdatareports.org). Indicator data sources included: Medicare Beneficiary Summary Files (2014-2015 MA, 2016-2017 CT and RI), the American Community Survey (2012-2016 MA, CT, RI) and BRFSS (2012 and 2014 MA, 2012-2018 CT, 2012-2017 RI). Small area estimation techniques were used to calculate rates using Stata and ArcGIS was used for mapping and geographic analyses. Results showed that community rates for PTSD were: CT 1.2% (0.41-3.10%), RI 1.7% (0.91-4.59%), MA 1.8% (0.58%-5.71%). Massachusetts had both the widest range in PTSD community rates and the highest state rate. Military service: MA 18.8% (3.24-42.58%), CT 17.4%, (6.70-33.7%), and RI 19.0% (9.51-31.70%). Falls: MA 10.6% (6.47-18.18%), CT 26.3% (19.31-32.39%), and RI 26.3% (21.51-31.17%). Disparities in access to care or clinicians who could diagnose PTSD may contribute to some of the differences observed. Understanding these regional disparities would inform collaborative efforts from researchers, policymakers and stakeholders. Further research is warranted on this unexplored topic.

Abstract citation ID: igae098.3431

### EXPLORING CENTRAL SYMPTOMS OF SUBTHRESHOLD DEPRESSION IN OLDER ADULTS: A NETWORK ANALYSIS

Rendong He[1], Li Chen[2], Yiming Qiu[2], and Junxin Li[3], *1. Jilin University School of Nursing, Changchun, Jilin, China*, *2. Jilin University, Changchun, Jilin, China (People's Republic)*, *3. Johns Hopkins School of Nursing, Baltimore, Maryland, United States*

Subthreshold depression (StD), a precursor to major depressive disorder, has not been well studied in older adults. Identifying central symptoms among various symptoms within the mental disorders enhances understanding of mental disorder structures and informs prevention strategies. We investigated the central symptoms of StD in community-dwelling older adults in China in a cross-sectional study. Depressive symptoms were assessed by prime care physicians during older adults' well visits using the Hamilton Depression Scale-17 (HAMD-17). Those with StD (7≤ HAMD-17< 17) were included in the study (n = 424). We also collected data on demographics, depressive symptoms, anxiety symptoms, and cognitive function. The network model was constructed using the "least absolute shrinkage and selection operator" technique with the tuning parameters minimizing the extended Bayesian information criterion. Node centrality was assessed using Expected Influence (EI). Network stability was examined with a case-dropping subset bootstrap featuring a correlation stability coefficient (CS). The sample consisted of 53.5% females who, on average, were 69.69 ± 6.80 years and with a HAMD-17 score of 10.46 ± 3.21. Among the 17 symptoms, the two most central symptoms were "Loss of interest in work or activities" (EI = 0.51) and "Depressed Mood" (EI = 0.41). The correlation between these two symptoms is the strongest (Edge weight = 0.26) among all symptoms. The estimated network has a good stability (CS = 0.44). Our findings suggest that "Loss of interest in work or activities" and "Depressed Mood" are potential intervention targets for StD in older adults.

Abstract citation ID: igae098.3432

### GERIATRIC PSYCHIATRY OUTPATIENT REACTIONS TO A POST-COVID TRANSITION FROM VIRTUAL TO IN-PERSON CARE

Kathryn Mertz, Samantha Baldinger, Jennifer RIvas, Amir Nikzad, Michael Scarpelli, and Blaine Greenwald, *Zucker Hillside Hospital, Glen Oaks, New York, United States*

Because of social distancing/quarantining, the COVID-19 pandemic catalyzed necessary shifts to telepsychiatry in outpatient mental health settings. To enable access and reimbursement for elders, the Centers for Medicaid and Medicare Services (CMS) liberalized restrictive telemedicine regulations. In our Geriatric Psychiatry Clinic, in-person visits quickly shifted to virtual. Tele-video and audio-only services became universally operative between Spring 2020 through May 2023, when – with the public health emergency (PHE) ending - CMS determined hospital-based clinics were ineligible for the 'facility fee' if patients were seen virtually, so return to in-person care was strongly encouraged. Shortly thereafter, to determine impact, consecutive geriatric psychiatry clinic patients over a several days period were surveyed re: satisfaction with virtual versus in-person care, and about challenges experienced returning in-person. Of 134 patients surveyed, 43% had engaged in tele-video, 43% in audio-only, and 17% utilized both. Utilizing a 5 -point Likert scale (very unsatisfied = 1 to very satisfied = 5), virtual (video, audio or both) (mean = 4.1) and in-person (mean = 4.2) care did not differ (p-value = 0.82 [Wilcoxon signed-rank test]). Amongst challenges associated with returning to in-person, 75% identified medical issues as very difficult/difficult, 46% financial issues, 25% distance to clinic, 23% transportation concerns, and 16% caregiving responsibilities. Taken together, findings suggest that geriatric psychiatry outpatients are comparably satisfied with virtual and in-person visits, however notable proportions experienced challenges reverting to in-person, especially because of medical and financial issues. As such, continuing advocacy supporting virtual options in psychiatric care of elders is warranted.

Abstract citation ID: igae098.3433

### HEARING LOSS AND SPOUSE'S MENTAL HEALTH WITH GENDER DIFFERENCES

Jaemin Jeon, and Kyuho Lee, *Daegu University, Gyeongsan, Kyongsang-bukto, Republic of Korea*

This research investigated the influence of individuals' auditory acuity on the occurrence of depressive symptoms in their spouses, mediated by the provision of support within marital relationships among elderly couples. Data from a sample of 1,069 older adult couples participating in the Health and Retirement Study (HRS) were analyzed. Utilizing three-wave dyadic data collected in 2010, 2014, and 2018, we conducted path analyses employing Mplus 7.0 to estimate the mediating effects of spousal support while controlling for autoregressive effects. Our findings indicate that husbands' superior auditory acuity at the initial assessment (T1) was associated with a reduction in depressive symptoms among their wives at the third assessment point (T3). Spousal support served as a mediator in this relationship. However, wives' auditory acuity did not exhibit a longitudinal impact on their husbands' depressive symptoms. This study provides empirical evidence supporting the significant role of older adults' auditory abilities within the context of marital relationships and the mental well-being of spouses. Keywords: hearing, spousal support, depressive symptoms

Abstract citation ID: igae098.3434

### INTEGRATED BEHAVIORAL AND PSYCHOLOGICAL INTERVENTIONS IN VHA COMMUNITY LIVING CENTERS

Kyle Page[1], HyeRim Ryu[1], Elizabeth MacDonald[2], Benjamin Szymanski[3], Kimberly Curyto[4], and Michele Karel[5], *1. Edward Hines, Jr. VA Hospital, Oak Park, Illinois, United States*, *2. Office of Mental Health and Suicide Prevention, Department of Veteran Affairs, Ann Arbor, Michigan, United States*, *3. Office of Mental Health and Suicide Prevention, Department of Veterans Affairs, Ann Arbor, Michigan, United States*, *4. Western New York VA Healthcare System, Batavia, New York, United States*, *5. VA Central Office, Washington, District of Columbia, United States*

Veterans Health Administration (VHA) Community Living Centers (CLC), or nursing home care settings, care for Veterans with high rates of mental illness and behavioral concerns. VHA requires the integration of psychologists on CLC teams and access to psychiatric services. An anonymous national survey of CLC mental health professionals aimed to characterize the status of CLC mental health practice, including intervention services. One hundred seven of 221 (48.4%) psychologists and psychiatric providers responded to the survey. Half or more of respondents reported these psychological interventions to be most helpful in the CLC: supportive therapy (76%), behavior therapy (75%), behavior management (66%), reminiscence/narrative therapy (65%), relaxation training (59%), cognitive behavioral therapy (54%), problem-solving therapy (52%), and motivational interviewing (50%). Respondents also identified barriers to utilizing Evidence Based Psychotherapies (EBPs) in the CLC, including Veteran cognitive impairment (57%), illness (48%), and preference for another form of intervention (32%). About a third (32%) reported offering group interventions. Respondents reported frequent collaboration with CLC team members in mental health interventions, most commonly by asking about their observations (76%), co-signing others on progress notes (71%), having them monitor signs and symptoms (68%), and many other strategies for team-based care. One third reported having regular, interdisciplinary behavior rounds and 67% reported that their teams used a team-based behavioral intervention ("STAR-VA") for managing dementia-related behaviors. Findings suggest a wide range of mental health intervention practice patterns in the CLC, the importance of team engagement, and opportunities for additional clinical and training resources.

Abstract citation ID: igae098.3435

### INTEGRATION OF PSYCHOLOGICAL ASSESSMENT SERVICES IN VHA COMMUNITY LIVING CENTERS

Kyle Page[1], HyeRim Ryu[1], Elizabeth MacDonald[2], Benjamin Szymanski[3], Kimberly Curyto[4], and Michele Karel[5], *1. Edward Hines, Jr. VA Hospital, Oak Park, Illinois, United States, 2. Office of Mental Health and Suicide Prevention, Department of Veteran Affairs, Ann Arbor, Michigan, United States, 3. Office of Mental Health and Suicide Prevention, Department of Veterans Affairs, Ann Arbor, Michigan, United States, 4. Western New York VA Healthcare System, Batavia, New York, United States, 5. VA Central Office, Washington, District of Columbia, United States*

Psychologists embedded in Veterans Health Administration (VHA) Community Living Centers (CLC), or nursing home care settings, serve a crucial role in providing neurocognitive, clinical capacity, suicide risk and other evaluation services for residents with medical, psychiatric, and cognitive comorbidities. An anonymous national survey of CLC mental health professionals aimed to characterize the status of CLC mental health practice, including psychological assessment services. Eighty-two percent of respondents were psychologists (n=88). The majority (92%) do not complete portions of the Minimum Data Set (MDS) assessment. Reported practice varied regarding use of brief cognitive tests, with 57% using these tests when a resident they are following exhibits dementia warning signs. Focused or expanded cognitive testing batteries were conducted by the majority (75%), with 26% of respondents conducting them at least monthly. Eighty-seven percent of respondents reported that their cognitive evaluations had some to great impact on informing treatment plans. The large majority (94%) reported conducting medical decision-making evaluations at least sometimes, and at least half reported evaluating other decision-making or functional capacities. At the same time, a significant minority of psychologists reported that additional training regarding neurocognitive (27%) and capacity (46%) evaluations would be helpful. The majority (85%) indicated that, if the suicide risk screening upon admission to the CLC was positive, the psychologist typically completed a comprehensive suicide risk evaluation. Findings highlight the extent and importance of psychological assessment services to inform person-centered treatment planning for Veterans with complex care needs, pointing to areas of resource development to support these evaluation practices.

Abstract citation ID: igae098.3436

### MENTAL HEALTH AS A FORTITUDE FACTOR

Tobi Abramson[1], Jackie Berman[1], Karen DeBell[2], and Christina Hatten[2], *1. NYC Department for the Aging, New York, New York, United States, 2. New York State Office of Mental Health, Albany, New York, United States*

NYS' Geriatric Mental Health Act has led to the establishment of a series of Geriatric Demonstration grants. To build fortitude, this round of grants focuses on the development of a Partnership to Support Aging in Place (PSAP) as a model to provide in-community services and supports to address unmet behavioral health, physical health, aging, and social support needs of older adults. The triple partnership of providers includes three core service areas: (1) OMH licensed mental health services (2) OASAS licensed SUD service providers (3) NYS Area Agency on Aging (AAA). The grant components also includes providing certified peer specialists, utilizing technology, and wrap around funding. These grants address disparities in access to services experienced by diverse, vulnerable, marginalized populations, those disproportionately affected by the COVID-19 pandemic. For the 6 grants, there have been over 280 admissions of older adults who need either mental health, aging services, or substance use disorder services. Of enrollees, 72% suffered from depression, 73% suffered from anxiety, 27% suffered from substance abuse, and 50% had an aging need. Innovative technologies includes the implementation of a created curriculum surrounding Fitbit use, and Elli-Q. Both substance use disorder and mental health peers have been central in these grants and many of these peers have been instrumental in conducting the peer-led program known as 'Do More Feel Better'. Each component of this initiative and the outcome data will be discussed in this session. All of these factors strengthen older adults mental health fortitude.

Abstract citation ID: igae098.3437

### ROLES OF GENDER AND SOCIAL MEDIA USE: MODERATING THE RELATIONSHIP BETWEEN FRUSTRATION AND DEPRESSIVE SYMPTOMS

Vivian Weiqun Lou, and Clio Yuen Man Cheng, *The University of Hong Kong, Hong Kong, China (People's Republic)*

Depressive symptoms among older adults pose a significant public health challenge, requiring thorough investigation of contributing factors. This study aimed to examine the role of gender and social media use as moderators in the relationship between frustration of basic psychological needs and depressive symptoms among young-olds ($N = 686$; Mage = 61.9 years; $SD = 5.82$), with implications for the development of targeted and tailored interventions. The regression model yielded statistical significance ($F(6, 679) = 46.74$, $p < 0.001$), accounting for 28.61% of the variance in depressive symptoms among young-olds. Notably, a significant interaction effect emerged between frustration and social media use ($\beta = -0.042$, $p = 0.019$). Young-olds who reported higher levels of frustration and increased social media use displayed elevated depressive symptoms, suggesting the potential detrimental impact of excessive social media consumption on mental well-being. Furthermore, the interaction effect between gender and social media use was also significant ($\beta = 2.161$, $p < 0.001$), indicating distinct group differences. Among males, a significant negative interaction effect was observed between frustration and social media use on depressive symptoms ($\beta = -.099$, $p =.006$). Conversely, no significant interaction effect between frustration and social media use was found among females ($\beta = -.017$, $p =.392$). Overall, these findings highlight the complex interplay of frustration, social media use, gender, and depressive symptoms among young-olds. Tailored interventions addressing the unique needs and vulnerabilities of different gender groups and promoting healthy social media use is crucial in reducing depressive symptoms in this population.

Abstract citation ID: igae098.3438

### SELF-ACCEPTANCE: FORTITUDE FOR MIDDLE-AGED AND OLDER ADULTS, ENHANCING MENTAL WELL-BEING AMID RECESSION

Ilan Kwon, *Our Lady of the Lake University, San Antonio, Texas, United States*

Self-acceptance serves middle-aged and older adults to manage adversity and maintain mental well-being during Recession. Embracing both positive and negative aspects of oneself can cultivate a sense of self-worth, reduces vulnerability to external stressors, and bolster resilience. This positive attitude enables individuals to navigate adversity with greater adaptability and emotional stability and reinforce their coping mechanisms that enhance their capacity to manage adversity and maintain mental well-being despite economic hardship. Using the national data, Midlife in the United States (MIDUS 3, 2013-2014), this study aims to identify different patterns of socio-economic challenges experienced by middle-aged and older Americans (aged 50-64) during the 2008 recession and examine the interaction effect of the different types of challenging experiences and self-acceptance levels on later-life mental well-being. Latent class analysis of twenty-six socio-economic challenge items revealed five patterns of the Recession experiences: People encountering various difficulties, job transitions, financial hardships, homebuyers burdened with reduced debts, and those experiencing no significant challenges. Multiple regression results showed that people facing financial hardship and job mobility during the Recession reported heightened mental health issues compared to those with fewer challenges, even after controlling for other variables. Higher levels of self-acceptance correlated with better mental health outcomes. Notably, individuals with elevated self-acceptance demonstrated resilience in maintaining mental well-being despite economic adversity. By highlighting the protective role of self-acceptance, this study offers valuable insights into effective coping strategies and informs interventions aimed at bolstering the resilience and psychological well-being of middle-aged and older adults facing socio-economic challenges and adversity.

Abstract citation ID: igae098.3439

### THE RELATIONSHIP BETWEEN VOLUNTEERING AND SUBJECTIVE WELL-BEING AMONG OLDER ADULTS IN JAPAN

Koji Fujita, Yuri Yokoyama, Mariko Nishi, Hiroko Matsunaga, and Yoshinori Fujiwara, *Tokyo Metropolitan Institute for Geriatrics and Gerontology, Tokyo, Japan*

The aim of this study is to examine the relationship between the frequency of volunteer activities among older adults and their subjective well-being. We mailed a questionnaire survey to 354 people aged 60 and over who belonged to volunteer organizations registered with a social welfare council within Saitama Prefecture. We received 235 responses for a response rate of 66.4%. Out of 215 participants, we excluded non-consenting and invalid responses, and subsequently analyzed the responses of 204 participants. We categorized these participants into three groups by frequency of volunteer activities: high activity, low activity, and non-activity. We performed a Kruskal-Wallis test with a multiple comparison test on the WHO–Five well-being index (range: 0-25 points). The high activity group accounted for 45.6% (n = 93), the low activity group for 35.8% (n = 73), and the non-activity group for 18.6% (n = 38). A significant difference was observed among the three groups in the WHO–5 (p=.013); the non-active group had the lowest mean score, while the high-activity group had the highest mean score. According to the multiple comparison test, this difference between the high and low activity groups was significant (p=.013). More frequent participation in volunteering among older adults is associated with higher subjective well-being. Volunteering may help maintain the physical and mental health of older adults and can reduce social isolation and loneliness. Therefore, participating in volunteer activities may help older adults to maintain and improve their mental health and subjective well-being.

Abstract citation ID: igae098.3440

### THE ROLE OF GENDER IN THE RELATIONSHIP BETWEEN VIOLENCE EXPOSURE AND PSYCHOLOGICAL DISTRESS IN MIDDLE AGE

Khalil Iktilat[1], Michal Isacson[2], Roy Tzemah-Shahar[3], and Maayan Agmon[4], *1. Haifa University, Dabburiya, HaZafon, Israel*, *2. Massachusetts Institute of Technology, Cambridge, Massachusetts, United States*, *3. University of Haifa, Haifa, HaZafon, Israel*, *4. University of Haifa, University of Haifa, HaZafon, Israel*

Middle age is a critical period in the lifecycle, with unique dynamics in men and women respectively. It represents a sensitive window for health prevention that can improve the aging process. To date, it remains unclear how environmental factors, like exposure to violence, influence psychological distress in this age group. In other age groups, women were observed to develop heighter distress than men following violence exposure. Here we aim to examine this gender-specific association between levels of exposure to violence and psychological distress in a population of middle-aged Muslims in Israel, who have been exposed to increasing violence in the past decade. A cohort of 363 middle-aged adults (223 women) from three Muslim villages in northern Israel was recruited. Psychological distress was assessed by the Kessler 6 Psychological Distress questionnaire, and violence exposure was assessed by the Screen for Adolescent Violence Exposure (SAVE) questionnaire. We controlled for age, sex, education level, and socioeconomic status using multivariate linear regression models with post-hoc models for each gender separately. Middle-aged males reported significantly greater exposure to violence whereas women reported significantly heightened psychological distress levels. A positive link between exposure to violence and psychological distress ($\beta=0.145$, $p=0.017$) was found using a multivariate model, and sex was a significant covariate ($\beta=0.132$, $p=0.045$). Post-hoc modelling revealed that this relationship was only significant among women ($\beta=0.160$ $p=0.041$). Our findings demonstrate that middle-aged women are at risk for violence-associated psychological distress. This research provides evidence for gender-based prevention and intervention programs.

Abstract citation ID: igae098.3441

### THE SALUTOGENIC EFFICACY OF THE VOICE-ACTIVATED INTELLIGENT PERSONAL ASSISTANT INTERVENTION: A CASE STUDY

Terence Lau, and Angela Leung, *The Hong Kong Polytechnic University, Hong Kong, Hong Kong*

Existing Parkinson's disease (PD) interventions are primarily focused on physical rehabilitation and pathogenic-based. The lack of positive, health-orientated psychosocial interventions prompted the research team to develop a salutogenic VIPA user protocol and examine its preliminary efficacy through an 8-week pilot RCT. This case report selected the participants with the largest sense of coherence-13 item scale (SOC-13) improvement to explore their user experience through the salutogenic perspective. A 32-minute face-to-face, post-intervention semi-structured interview adopting the interpretive descriptive approach was conducted on 31st Jan, 2024. The interviewee was a 63-year-old male, level 3 Hoehn and Yahr Scale with 2 years of PD history, documenting a 100% compliance rate and a 16-point improvement in SOC-13 across all comprehensibility, manageability, and meaningfulness subdomains. The transcripts were coded deductively through thematic analysis referencing the salutogenesis and psychosocial well-being framework. Written consent was signed before the research, and the participant agreed to be audiotaped during the interview. The encyclopedia-like features within VIPA facilitated participants' comprehensibility by retrieving desired online information. On manageability and psychological well-being, the participant metaphorically described the VIPA intervention as an electric wheelchair for physically disabled people to that of people with Parkinson's Disease (PWP). Thus, the intervention could benefit the autonomy and environmental mastery domain within psychological well-being alongside the manageability domain. For meaningfulness, the participant further elaborated the VIPA intervention could minimize the difference between PWP and other citizens. The salutogenic-based VIPA intervention can contribute a salutogenic force, lessening the effect of constantly deteriorating PD progression experienced by PWP.

Abstract citation ID: igae098.3442

### TRANSFORMING TELEHEALTH ACCESS: INTEGRATING BEHAVIORAL HEALTH AND TECHNOLOGY COACHING

Pedro Carbajal-Madrid, and Rachel Holben, *SCAN Health Plan, Long Beach, California, United States*

Research indicates only 21% of older adults in the U.S. utilize telehealth services, with economic, linguistic or literacy challenges exacerbating barriers to accessing quality health care. The Insights Program at SCAN / Independence at Home is a behavioral health program that provides individual and group therapy for people aged 55 and over and their caregivers. Serving over 2,400 individuals in 2023, the Insights program offers no cost services to those who otherwise may not be able to access mental health treatment, with a reduction in symtoms of depression and anxiety over 50%. During the Covid-19 pandemic, transitioning from in-person to telehealth sessions highlighted clients' struggles with technology as they were expected to engage with online platforms without prior training. This led to older adults avoiding scheduling appointments or missing scheduled visits due to a lack of understanding of technology. To meet this unmet need, Independence at Home launched the T.E.C (Technology, Education and Coaching) program in 2021. This program pairs trained Digital Coaches with Insights' clients, providing individualized support to help them use virtual platforms for online therapy sessions and access other health and social services. Early outcomes show the T.E.C program significantly increased digital literacy (1.13 vs 2.94, p<.0001 [eHealth Literacy Scale]) and technology engagement (.44 vs.75, p=.0014 [Program's Technology Engagement Survey]) based on pre (n=22) and post (n=22) testing. These findings demonstrate the program's positive impact on the clients' understanding and proficiency in utilizing technology and navigating electronic health platforms.

Abstract citation ID: igae098.3443

### USING AN MBSR MOBILE APP FOR MENTAL HEALTH: EXPERIENCES OF OLDER ADULTS IN UNDERSERVED COMMUNITIES

Cristina de Rosa, and Yu-Ping Chang, *University at Buffalo, SUNY, Buffalo, New York, United States*

Older adults living in underserved and racial/ethnic minority communities during the COVID-19 pandemic and beyond faced challenges regarding access to mental health care, compounded by additional vulnerability to disease. Technological solutions such as mobile applications provide great opportunities to address mental health concerns. This qualitative, descriptive analysis explores experiences of participating in an interactive and culturally-tailored mindfulness-based stress reduction (MBSR) mobile application intervention for mental health among older adults from an underserved, primarily African American community. Individual semi-structured interviews were conducted with participants who completed an 8-week MBSR mobile app intervention. Interviews were transcribed verbatim and analyzed using Braun and Clarke's reflexive thematic analysis. A total of 17 participants were included in the analysis. Participants were ages 60-84 (M = 65.76, SD = 6.02); 94.1% were female; 82.4% reported their race/ethnicity as Black or African American. Four themes were identified in our preliminary analysis: app technology facilitated an openness to mindfulness and deconstructed barriers to access, enabled participants to come into a greater awareness of self and surroundings, allowed on-demand practices particularly during prime opportunities and stressful situations, and encouraged personal habits for maintaining and revisiting calm and relaxing states. Findings suggest feasibility and acceptability of a mobile app-delivered MBSR intervention addressing mental health needs for older adults, as well as satisfaction with app content, delivery, and support. Innovative technologies such as mobile apps have immense potential to bridge gaps in care and should be further evaluated for effectiveness and possible implementation to ameliorate mental health symptoms in underserved communities.

### SESSION 7700 (POSTER)

### MINORITY AND DIVERSE POPULATIONS

Abstract citation ID: igae098.3444

### "I WON'T GO THROUGH THAT OR TAKE MY FAMILY THROUGH THAT": FAMILY RELATIONSHIPS AND KIDNEY DISEASE DECISION-MAKING

Tyrone Hamler[1], Asia Cutforth[1], Emily Miller[2], and Kari O'Donnell[3], *1. University of Denver, Denver, Colorado, United States*, *2. Case Western Reserve University, Cleveland, Ohio, United States*, *3. MetroHealth, Cleveland, Ohio, United States*

Chronic kidney disease (CKD) uniquely impacts older Black Americans, who are nearly four times more likely to develop kidney failure than older White Americans and more likely to have family members also diagnosed with CKD. Utilizing a phenomenological approach, this study explored participants' perceptions of how family members' prior experiences with CKD influenced treatment-related decision-making. This study aimed to (1) describe how older Black adults viewed the experiences of family and friends previously diagnosed with CKD and (2) to understand how these social complexities informed their own choices for future CKD care. A reflective, thematic content analysis was conducted to identify patterns across participant responses (N=52). Participants were pre-dialysis, diagnosed with stage four or five chronic kidney disease, and were receiving outpatient nephrology care at a large, urban midwestern hospital. Three primary themes emerged: (1) Decision-Making Factors, (2) Health Inequities, and (3) Continuity of Care. These three interconnected themes centered on factors that influenced why and how decisions related to chronic kidney disease were made and how racial identity and prior family experiences with CKD influenced these choices. Over the next 40 years, the population of Black Americans aged ≥ 65 years will nearly triple, and CKD will continue to be a major public health concern. Addressing the complex support requirements and burdens of CKD care is critical for reshaping the negative and disorienting experiences of older Black Americans and reducing future decision-making conflicts.

Abstract citation ID: igae098.3445

### A PRELIMINARY EXAMINATION OF SOCIAL MEDIA USE AMONG HISPANIC/LATINX DEMENTIA CAREGIVERS

Sofia Mildrum Chana, Loreli Alvarez, Andres Azuero, Michael Crowe, and Frank Puga, *University of Alabama at Birmingham, Birmingham, Alabama, United States*

Hispanic/Latinx (H&L) family caregivers of people living with dementia (PLWD) experience elevated stress levels that can negatively affect their mental health. Caregivers are also prone to loneliness and social isolation and may rely on technology and social media to enhance social ties, seek support or cope with stressors. However, no studies to date have examined social media use patterns in this population. Thus, this analysis presents a preliminary description of social media use habits among H&L caregivers of PLWD (n=45) employing data from the ongoing national-level, NIA-funded study entitled, "Nuestros Días (Our Days)." We examined bivariate associations between social media use, distress from behavioral symptoms of dementia, perceived general stress, and depressive symptoms. Participants (93.3% female, Mage=55.69, SDage=8.75) reported using social media an average of 158.18 minutes (SD=131.44) per day, with Facebook (31.55%) and Instagram (27.8%) being the most used platforms. Average time spent using social media was positively associated with behavioral symptom distress (r=.37, p=.04) and depressive symptoms (r=.41, p=.02). Passive social media use (i.e., consumption of social media content without active engagement, such as 'doomscrolling') was positively correlated with behavioral symptom distress (r=.49, p<.001) as well as perceived stress (r=.33, p=.03) and depressive symptoms (r=.36, p=.02). But active social media use (i.e., engagement with content and deliberate interaction with other users) was not significantly correlated with any mental health outcomes. Findings highlight the different contributions of active versus passive social media use to mental health outcomes, with passive use being more closely linked to worsened mental health.

Abstract citation ID: igae098.3446

### "OUR ELDERS LOVE WHEN WE MAKE THE BUFFALO DINNERS AND SOUP": TRADITIONAL FOODS AND TITLE VI NUTRITION PROGRAMS

Traci Wilson, *USAging, Washington, District of Columbia, United States*

Title VI Native American Aging Programs (Title VI programs), funded by Title VI of the Older Americans Act, support the health, wellness and social engagement needs of Native elders by providing nutrition, supportive services and caregiver supports to American Indian, Alaska Native and Native Hawaiian people. There are over 290 Title VI grantees, representing a diverse group of more than 400 tribal nations. Title VI programs provide culturally-relevant services to address the health-related social needs of elders, such as meals, transportation, in-home services, social engagement opportunities, and family caregiver supports, helping to ensure that elders can continue to live in their homes and communities as long as possible. A large portion of Title VI funding is allocated to nutrition programs, including congregate and home-delivered meals. Every three years, USAging, with funding from the Administration for Community Living, surveys Title VI directors about their program funding, staffing, services, and unmet needs. The most recent survey, conducted in late 2023 with an 80% response rate, included a new module on if and how Title VI nutrition programs incorporate traditional foods. This poster will describe the prevalence and types of traditional foods in OAA Title VI nutrition programs, involvement of elders in providing recipes and passing on knowledge, and the challenges and successes Title VI programs have when sourcing and incorporating traditional foods into their nutrition programs.

Abstract citation ID: igae098.3447

### CONCEPTUALIZING BLACK FEMINIST-WOMANIST GERONTOLOGY

Brandy Wallace[1], Cassandra Ford[2], and Tamara Baker[3], *1. University of Maryland Baltimore County, Baltimore, Maryland, United States*, *2. University of Alabama, Tuscaloosa, Alabama, United States*, *3. University of North Carolina, Chapel Hill, Chapel Hill, North Carolina, United States*

There is a long history documenting Black women being particularly vulnerable to negative implicit and/or explicit behaviors, which marginalizes their social positioning and puts them at an increased risk of feeling powerlessness, and physically and emotionally stressed. This has created a chasm, particularly between this group and the healthcare industry - resulting in poorer health outcomes across generations of Black women. Recent scholarship has questioned the lack of culturally responsive, theory-guided research addressing the connections between aging, Black womanhood, and what is needed to advance health equity. Models that utilize traditional theories of aging often do not account for cultural context that undergirds the aging experience specifically among Black women. To understand the ways in which Black women thrive, we must consider various approaches that define their well-being. Dichotomizing aging into concrete categories as healthy/unhealthy may unintentionally isolate this group where aging successfully presents as a contradiction, thus perpetuating further marginalization. It is important that scholarship and intervention projects reflect cultural humility in dissemination. Applying Black-Feminist-Womanist methods in gerontology provides a curation of thought that creates a foundation by which Black women survive, live, and age, despite the 'gold standard' of aging being dominated by white ethnocentric context that pathologizes older Black women's lived experiences. In this presentation, we summarize the principles and methodological approaches of Black Feminist and Womanist perspectives and use them to conceptualize "Black Feminist-Womanist Gerontology," a culturally relevant model for older Black women. Factors of the model and recommendations of its use will be discussed.

Abstract citation ID: igae098.3448

### DISCRIMINATION AND COGNITIVE HEALTH DISPARITIES AMONG OLDER AMERICANS: A FOCUS ON NATIVITY AND RACE/ETHNICITY

Yi Wang[1], Man Guo[1], Huei-Wern Shen[2], and Morgan Stangl[1], *1. University of Iowa, Iowa City, Iowa, United States*, *2. University of North Texas, Denton, Texas, United States*

Discrimination, characterized by unfair or prejudicial treatment based on perceived differences, has been identified as a significant stressor with potential detrimental effects on cognitive health in the aging population. Disproportionately experienced by immigrants and racial/ethnic minorities, discrimination can exacerbate existing health disparities. Using data from the 2016 and 2018 Leave-Behind Questionnaire of the Health and Retirement Study (N=9,440), this study aims to examine 1) What is the associations between discrimination and cognitive health (global cognitive functioning score), and whether such relationships vary by nativity (US born: yes/no)? 2) Does race/ethnicity (White, Black, Hispanic) moderate the above relationships? and 3) Do contextual resources (neighborhood social cohesion, social support from family and friends) and activity engagement (volunteering, cognitively stimulating activities) serve as protective factors for cognitive health in the face of discrimination? Results from linear regression models revealed that discrimination is negatively linked to cognitive health and this relationship is stronger for immigrants than non-immigrants. Across nativity and race/ethnicity groups, only Hispanic immigrants reported a significantly lower cognitive score (in comparison with U.S. born Whites) when facing higher levels of perceived discrimination. As for protective factors, both activity engagement indicators were associated with higher cognitive scores among immigrants. These findings emphasize the heightened vulnerability of immigrant populations, particularly Hispanic immigrants, to the negative effects of discrimination on cognition. The protective factors, such as volunteering and engaging in cognitively stimulating activities, highlights avenues for intervention and support to mitigate the adverse consequences of discrimination on cognitive health among older immigrants in the U.S.

Abstract citation ID: igae098.3449

### DISPARITIES IN TYPE 2 DIABETES ONSET AMONG MIDLIFE AND OLDER ADULTS IN THE US: A FOCUS ON NATIVITY AND RACE/ETHNICITY

Charlotte Asiedu, and Adam Davey, *University of Delaware, Newark, Delaware, United States*

Type 2 Diabetes Mellitus (T2DM) disproportionately affects midlife and older adults in the US and globally and is marked by significant disparities across certain nativity groups (foreign-born, native-born, and second-generation immigrants (SGI)). While previous research has predominantly focused on diabetes prevalence across racial groups, there exists a significant gap in understanding how this disease prevalence and onset may differ based on immigration status and nativity. This study utilized data from a nationally representative sample of the California Health Interview Survey (CHIS, 2003–2022) to understand disparities in the onset of diabetes among midlife (45–64) and older adults (65+) in the US. Survey-weighted analyses suggested an association between nativity and age of diabetes onset (early, mid-life, and late). The study sample, N = 200,730, comprised 42.35% males and 57.65% females within ages 40-85+. The overall survey-weighted chi-square tests revealed a statistically significant association between nativity and diabetes onset ($p < 0.001$). Foreign-born adults had the highest prevalence of diabetes (15.1%), followed by SGI (13.3%). Foreign-born adults were also more likely to have received their diabetes diagnosis before age 40 (23.1%, $P < 0.001$) followed by SGI (20.9%, $p < 0.001$). Native-born adults were more likely to receive their diagnosis after age 60 (30.5%) compared to SGI (29.7%) and foreign-born adults (21.8%). Findings highlight disparities in T2DM prevalence and onset by nativity. The study underscores the necessity for further research on the socioeconomic, biological, psychological, cultural, and behavioral determinants of these disparities to inform targeted policies and interventions.

Abstract citation ID: igae098.3450

### EXAMINING CHANGES IN CAREGIVING NETWORKS BY RACE AND ETHNICITY BETWEEN 2015 AND 2022

Alexa Bragg, and Chanee Fabius, *Johns Hopkins University, Baltimore, Maryland, United States*

The growing heterogeneity of the aging population in the United States exists alongside an increasing demand for family and unpaid caregivers, particularly for those living with disability in the community. Caregivers often "role-share," whereby multiple caregivers assist with the same tasks. Given well-documented differences in caregiving networks by race and ethnicity, it is important to assess the evolution of these arrangements over time. Leveraging the National Health and Aging Study (NHATS), a nationally representative study of Medicare beneficiaries, we compare sociodemographic and caregiving network characteristics of community-dwelling Non-Hispanic Black, Hispanic, and Non-Hispanic White groups living with disability in 2015 (n=1851) and 2022 (n=1475). Role-sharing was examined across four domains: medical, household, mobility, and self-care. While few older adult characteristics changed, Black and Hispanic older adults consistently experienced disparities in income, Medicaid-enrollment, and probable dementia. Fewer children were identified as caregivers in 2022 than in

2015 among older Black and White adults; this proportion remained stable among Hispanic older adults. More "other family" members were identified as helpers across all groups. The greatest amount of role-sharing was observed in household tasks; roughly 30% of Black, White, and Hispanic older adults experienced role-sharing in these activities in 2015 and 2022. Race differences in role-sharing patterns were evident across both time points. For instance, Hispanic older adults experienced greater role-sharing with self-care tasks than Black and White older adults in both years. Findings demonstrate reliance on alternative support structures and better inform caregiver policies for those caring for diverse groups of older adults.

Abstract citation ID: igae098.3451

### LIFE EXPECTANCY AND COVID-19 MORTALITY DISPARITIES AMONG NATIVE AMERICAN OLDER ADULTS IN WYOMING

Megan Siebecker[1], Josephine Boateng[2], Alexa Fleet[3], Taylor Jansen[1], and Elizabeth Dugan[1], *1. University of Massachusetts Boston, Boston, Massachusetts, United States*, *2. University of Massachusetts Boston, Dorchester, Massachusetts, United States*, *3. University of Massachusetts Boston-- Gerontology, Boston, Massachusetts, United States*

Accumulated inequities contribute to health disparities. Striking evidence of this is found in Native older adults in Wyoming. The history of violence and racism against Native peoples in the US is a national shame. Documenting the health challenges experienced by Native older adults brings awareness and potential remedy to this racist history. American Indian and Alaska Native communities are a historically under-researched population and are at greater risk than non-Hispanic white older adults for many chronic conditions. The current study reports on results from the Wyoming Healthy Aging Data Reports (healthyagingdatareports.org). We compared community rates of life expectancy and COVID-19 mortality in Wyoming and in Fremont County (home to the largest proportion of older Native adults in WY). Data sources include US Census Bureau American Community Survey 2016-2020 (population characteristics), Robert Wood Johnson Foundation (community life expectancy), and the Wyoming Department of Health (COVID-19 infection and mortality rates). Small area estimation techniques were used to calculate rates, and ArcGIS software was used for mapping and geographic analyses. Results show an estimated 2,430 Native American older adults who are 55+ are living in Wyoming, and 56% of them reside in Fremont County. Life expectancy rates in Fremont County (73) are 5 years lower than the state average (78.1). There is also a 1.5x higher COVID mortality rate in Fremont County (513.7) as opposed to the state average (340.4). Communities that have experienced systematic discrimination and inadequate health care services have poor outcomes. Policy interventions to address such inequities are warranted.

Abstract citation ID: igae098.3452

### LONELINESS, SOCIAL ENGAGEMENT, AND COGNITIVE STATUS AMONG OLDER BLACK AND WHITE ADULTS

Bettina Beech[1], Marino Bruce[1], Charles Udo[1], Ana Velazquez[1], and Roland Thorpe[2], *1. University of Houston, Houston, Texas, United States*, *2. Johns Hopkins University, Baltimore, Maryland, United States*

Social connection has been consistently shown to influence health status and recent research findings suggest that an absence of relationships with significant others can increase risks for cognitive impairment. Few studies have examined how the presence or absence of social connections have implications for cognitive impairment and even fewer have considered how these associations can vary by race and gender. The purpose of this study was to examine race and gender differences in the associations between loneliness, social engagement, and cognitive impairment among Black and White adults in the Health and Retirement Study (HRS). Data were drawn from participants who completed the Core and Leave Behind Questionnaire items in the 2016 HRS. Any cognitive impairment, a dichotomous variable derived from the Telephone Interview for Cognitive Status, was the outcome of interest. Loneliness and social engagement were the primary independent variables. Black men (35.5%) and Black women (27.0%) were disproportionately represented among respondents with any cognitive impairment relative to White men (14.6%) and White women (16.2%). Results from race- and gender-stratified Modified Poisson Regression models indicated that loneliness was associated with a higher prevalence of any cognitive impairment (PR=1.29, CI:1.07-1.57) among White women. Social engagement was inversely related with any cognitive impairment among White men (PR=0.97, CI:0.95-0.99). Neither of the main independent variables were significant for Black men or women in the study. Studies with robust and inclusive social connection measures are needed to further examine the association between loneliness, social engagement, and cognitive functioning among diverse populations.

Abstract citation ID: igae098.3453

### MARKETING ISN'T MAGIC: HOW ALTER LEVERAGED THESE BEST PRACTICES TO INCREASE RECRUITMENT AND RETENTION

Fayron Epps[1], and Andy Suggs[2], *1. The University of Texas Health San Antonio, San Antonio, Texas, United States*, *2. Reckon Branding, Norcross, Georgia, United States*

Dementia-Friendly Faith Villages was launched in 2018 to address the lack of resources and awareness of dementia in African American and faith communities in the State of Georgia. The program struggled to meet recruiting, retention, and program goals in its inaugural year. In response to less-than-desirable progress, the principal investigator partnered with Reckon Branding to define a messaging focus for the program that better connected with each of its key audiences: Church leaders, caregivers, and partner programs within their space. Considering 71% of customers recommend a brand based on their emotional connection to it, Reckon knew they had to tell a story that extended beyond facts and figures. The program was provided with a new verbal and visual focus through internal ideation sessions, audits of competitive programs, and external interviews of Church leadership and caregivers of those with cognitive impairments. Armed with the more memorable name ALTER, a new logo, and more consumer-friendly marketing materials, the team was able to secure 83 partnerships over the next 4 years. This strategic pivot has helped ALTER exceed its partner goals by over 300% and empowered the program to develop consistently impactful marketing materials, which provided improved recruitment reach, increasing the participants from 7,500 to more than 100,000. Thanks to this success, ALTER has extended its reach from Georgia, to many more African American communities of faith across 14 states.

Abstract citation ID: igae098.3454

### MIDDLE AGED AND OLDER BLACK AMERICAN PERCEPTIONS OF DISCRIMINATION

Heather Hunter[1], Corey L. Nagel[2], and Tracie Harrison[2], *1. University of Arkansas for Medical Sciences, Fayetteville, Arkansas, United States, 2. University of Arkansas for Medical Sciences, Little Rock, Arkansas, United States*

The purpose of this study was to examine discrimination reported by middle aged and older non-Hispanic Black Americans by sex, region, type, and frequency throughout the United States. Understanding the contextual and regional influences on recent discriminatory outcomes perceived by older Black adults provides context for future intervention. Hence, we conducted an analysis of 3,761 Non-Hispanic Black adults aged 50+ who participated in the Health and Retirement Study between the years of 2008-2018, examining participants responses to the Perceived Everyday Discrimination Scale. This 6-item scale assesses the frequency of specific types of discrimination with follow up questions that query respondent attributions for experienced discrimination. A binary measure indicating discrimination several times per month or greater was created and used to calculate descriptive statistics stratified by sex and census region of residence. The majority (67%) of respondents reported an experience with one of the listed types of discrimination, with a slightly higher portion of the men (69%) reporting discrimination than the women. Of the sample 28% reported having experienced one of the types of discrimination a few times per month or greater. Highest ranked reasons for discrimination ranged from ancestry (N=200, 29%), gender (N=614, 25%), race (N=1.540, 63%), age (N=998. 41%), financial (N=725, 30%), and disability 465 (19%). Never experiencing discrimination of any type was reported by 34.1% (South), 32.5% (Northeast), 31.9% (Midwest), and 29.8% (West) of respondents by region. Middle aged and older Black Americans perceived discrimination resulting in feelings of less respect, greater fear, heightened threat, and/or poor healthcare.

Abstract citation ID: igae098.3455

### MULTIMORBIDITY AND ADL DISABILITY AMONG MEXICAN AMERICAN OLDER ADULTS: SEX DIFFERENCES

Eduardo Silveyra[1], and Soham Al Snih[2], *1. The University of Texas Medical Branch, Galveston, Texas, United States, 2. University of Texas Medical Branch in Galveston, Galveston, Texas, United States*

Medical advancements in the 20th century allowed the development of treatments for many diseases, increasing people's average life expectancy. We examined sex differences in the relationship between multimorbidity and disability among Mexican American older adults ages ≥75 years who were non-disabled at baseline over 12-years of follow up. Participants (N=999) were from the Hispanic Established Population for the Epidemiologic Study of the Elderly (2004/2005-2016). Measures included socio-demographics, body mass index, pain, falls, depressive symptoms, cognitive and physical function, handgrip strength, multimorbidity and limitations in activities of daily living (≥ 1 ADL's). Participants were divided into four groups according to the number of chronic conditions (CC): 0-1, 2, 3, and ≥ 4). Generalized Estimating Equation model were used to estimate the odds ratio and 95% Confidence Interval of ADL disability over time as a function of multimorbidity and number of chronic conditions controlling for all covariates. We found that female participants reporting 3 CC had greater odds of ADL disability (OR=1.60, 95% CI=1.08-2.38) over time than those with 0-1 CC after controlling for all covariates. Female and male participants reporting ≥4 CC had greater odds of ADL disability (OR=2.13, 95% CI=1.44-3.16 and OR=1.46, 95% CI=0.94-2.27, respectively) over time than those with 0-1 CC after controlling for all covariates. In conclusion, female participants were at increased risk for ADL disability when reporting ≥3 CC while the association was non-significant among males. Our findings indicate the need for evidence-based prevention and interventions targeting multimorbidity to prevent or delay ADL disability in this population.

Abstract citation ID: igae098.3456

### MULTIMORBIDITY, MUSCLE STRENGTH, AND FALLS AMONG OLDER MEXICAN AMERICANS

Alondra Uribe[1], and Soham Al Snih[2], *1. The University of Texas Medical Branch, Galveston, Texas, United States, 2. University of Texas Medical Branch in Galveston, Galveston, Texas, United States*

**Abstract objectives:** To determine the relationship of multimorbidity and muscle strength with falls among older Mexican Americans without a history of falls at baseline. Design: Longitudinal study. Setting and Participants: This 12-year prospective cohort study included 899 non-institutionalized Mexican Americans aged ≥75 residing in Arizona, California, Colorado, New Mexico, and Texas from the Hispanic Established Population for the Epidemiologic Study of the Elderly.

**Methods:** Measures include socio-demographics, medical conditions, Body Mass Index, disability, handgrip strength (HGS), depressive symptoms, pain, cognitive function, and multimorbidity (≥2 self-reported medical conditions). Participants at baseline were divided into four groups: high HGS and multimorbidity (N=349), low HGS and multimorbidity (N=263), high HGS and without multimorbidity (N=181), and low HGS and without multimorbidity (N=104). Generalized estimating equation models estimated the odds ratio (OR) and 95% Confidence Interval (CI) of falls as a function of multimorbidity and HGS groups controlling for all covariates.

**Results:** The OR of falls was 0.72 (CI=0.54-0.95) for those with multimorbidity and high HGS, 0.52 (95% CI=0.33-0.82) for those without multimorbidity and high HGS, and 0.46 (95% CI=0.29-0.74) for those without multimorbidity and low HGS, versus those with multimorbidity and low HGS, after controlling for all covariates. Conclusions and Implications: Mexican American older adults with multimorbidity and high HGS had a 28% decreased risk of falls over time. Increasing muscle strength through exercise may help prevent falls among those with multimorbidity.

Abstract citation ID: igae098.3457

**NATIVITY DIFFERENCES IN OLDER MEXICAN AMERICAN HOSPITALIZATIONS: STRENGTH AND FUNCTION AS MARKERS**

Juan Ventura[1], and Soham Al Snih[2], *1. The University of Texas Medical Branch, Galveston, Texas, United States*, *2. University of Texas Medical Branch in Galveston, Galveston, Texas, United States*

Hospitalizations are a difficult occurrence for many older adults. Additionally, health differences have been noted in adults according to their country of origin. This study aims to compare nativity differences in hospitalizations using handgrip strength (HGS) and short physical performance battery (SPPB) among Mexican American older adults. This prospective cohort study included 1,324 Mexican Americans aged ≥75 years from the Hispanic Established Population for the Epidemiological Study of the Elderly. Measures included socio-demographics, body mass index, multi-morbidity (≥2 medical conditions), depressive symptoms, disability, cognitive function, and HGS–SPPB performance groups. Generalized estimating equation models were utilized to estimate the odds ratio (OR) and 95% Confidence Interval (CI) of hospitalization as a function of HGS and SPPB performance groups. Foreign-born participants in the high HGS–low SPPB (OR=0.62, 95% CI=0.40-0.97) and high HGS–high SPPB (OR=0.42, 95% CI=0.18-0.97) groups experienced significantly lower odds of hospitalizations compared to those in the lowest performing group (low HGS–low SPPB). No association was found between HGS-low SPPB (OR=0.71, 95% CI=0.50-1.00) and HGS-high SPPB (OR=0.63, 95% CI=0.36-1.11) with hospitalizations among US-born. Those with Multimorbidity and falls had greater odds of hospitalizations among both US and foreign-born. High levels of education were associated with lower odds of hospitalization among foreign-born and high depressive symptoms were associated with greater odds of hospitalizations among US-born. Overall, older Mexican Americans who performed high in HGS and high in both HHS and SPPB experienced relatively decreased odds of hospitalizations, with the effect being significant in those who were foreign-born.

Abstract citation ID: igae098.3458

**PREDICTORS OF LIVER FUNCTION AMONG MEXICAN AMERICAN WITH DISABLING ARTHRITIS**

Tracie Harrison[1], Shelley Blozis Villareal[2], and Heather Hunter[3], *1. University of Arkansas for Medical Sciences, Little Rock, Arkansas, United States*, *2. University of California Davis, Davis, California, United States*, *3. University of Arkansas for Medical Sciences, Fayetteville, Arkansas, United States*

In this secondary analysis of data from our recent biobehavioral study of Mexican American men and women with disabling arthritic conditions, we analyzed the relationships between mental health predictors, inflammation and liver enzymes, after controlling for age and sex. Consistent with the theoretical paradigm evaluating the prevalence and impact of diseases of desperation, the relationships between grief, anxiety, depression, inflammation (c-reactive protein, CRP) and liver function (Serum glutamic oxaloacetic transaminase (SGPT)) were analyzed. The 67 men (N=18) and women (N=46) 46-83 years of age (age M=62.4 years) were primarily unemployed (55%), married (43%) and high school graduates (36%). Participants were community residing and recruited through flyers, word of mouth, and known key informants. After IRB approval, surveys and blood samples were collected in homes and then analyzed. Results using Pearson correlations with reliable measures indicated significant relationships between SGPT and CRP (r=.46, P< 0.01), SGPT and anxiety (r=.365, p<.01), SGPT and depression (r=.34, p< 0.02), and SGPT and grief (r=.30, p<.04). Using a regression analysis, after controlling for age and sex, mental health (anxiety, depression, and grief) explained an additional 16% (R2 change =.16, F = 2.4, p =.08) of the variance in liver enzymes. This study was limited to a small sample size. In conclusion, psychological variables may influence physiological outcomes in Mexican Americans aging with severe arthritis, and further research is needed on mental health needs among Mexican Americans aging with disability in the community setting.

Abstract citation ID: igae098.3459

**UNRAVELING THE THREADS OF DEMENTIA PERCEPTION AMONG OLDER ASIAN INDIAN IMMIGRANTS IN THE UNITED STATES**

Malinee Neelamegam, Shilpa Patil, RoiSan Nhpang, and Stacey Griner, *University of North Texas Health Science Center, Fort Worth, Texas, United States*

The Asian Indian (AI) population, the second-largest immigrant group in the US, includes a substantial proportion aged 50 and older. Despite facing cognitive risks, they're underrepresented in aging research. Our study delves into dementia perceptions among older AI immigrants in the US. We conducted in-depth interviews with 12 Asian Indian immigrants aged 50 and above in the Dallas-Fort Worth Metroplex. Guided by a semi-structured interview guide, we explored their perceptions of dementia and aging in the US. Thematic analysis, utilizing both predetermined and emerging codes, was employed to analyze the data. Of the participants, the majority were women (n=21), with an average age of 68.9 years (SD=5.3) and an average length of stay in the US of 35.1 years (SD=13.1). Alarmingly, there was a notable lack of awareness regarding cognitive aging and dementia. Emerging themes included concerns about the financial burden of screening and treatment, societal acceptance, and access to support services. Moreover, participants' immigration journeys significantly influenced their social networks and caregiving choices as they aged. Addressing the expense of care, enhancing caregiving resources, and addressing the low awareness of brain health among older AI immigrants in the US are crucial focal points for future research. These findings can inform culturally tailored initiatives and interventions aimed at dementia and aging within South Asian communities.

### SESSION 7705 (POSTER)

### NURSING SCIENCE

Abstract citation ID: igae098.3460

**EXPLORING CAREGIVING PATHS AMONG RACIALLY DIVERSE FAMILY CAREGIVERS OF OLDER ADULTS WITH DEMENTIA**

Jung-Ah Lee[1], Hyun Jung Kim[2], and Young-Shin Lee[3], *1. University of California Irvine, Irvine, California, United States*, *2. Seattle University, Seattle, California, United*

*States*, *3. San Diego State University, San Diego, California, United States*

Ethnic minority family caregivers who directly care for community-dwelling older adults experiencing dementia, navigate distinct paths in their caregiving journey. Our aim was to discern the unique aspects of caregiving experienced by ethnic minorities in comparison to White caregivers. The study employed a descriptive qualitative design utilizing thematic analysis. Participants were recruited from diverse California ethnic communities. Bilingual nursing students offered emotional support to home-bound caregivers through weekly calls for two months during the pandemic crisis. Twenty-three participants included 7 White, 8 Latino, 5 Korean, and 3 Vietnamese caregivers. The mean age was 60, ranging from 27 to 79 years old. Eighty-one percent of minority persons with dementia (PWD) were Medicaid beneficiaries, while none of the White PWD were low-income. Significant differences in caregiving experiences were identified across three key areas: problem-solving skills, social boundaries, and dynamics between PWD and their caregivers. White caregivers reported more active problem-solving skills, establishing social boundaries that extended beyond their immediate family members, and dedicating more time to engaging PWD in shared activities. Conversely, minority caregivers with limited English proficiency exhibited a tendency to endure problems, carefully constructed social boundaries with selective membership, and seemed to prefer solitary activities (e.g., watching TV) over actively engaging with PWD (e.g., gardening). The findings showed that low-income minority caregivers had lower problem-solving skills, limited social boundaries, and less PWD engagement at home compared to counterparts. We underscore the importance of culturally sensitive support and tailored interventions in addressing the diverse needs of caregivers across different ethnic backgrounds.

Abstract citation ID: igae098.3461

### EXPLORING MEDICATION CONVERSATIONS IN NURSING HOME CARE CONFERENCES: A QUALITATIVE STUDY

Te-Lien Ku[1], Jingxi Li[1], James Ford[1], and Tonya Roberts[2], *1. University of Wisconsin Madison, Madison, Wisconsin, United States*, *2. University of Wisconsin - Madison, Madison, Wisconsin, United States*

Care conferences (CCs) represent a structured opportunity for nursing home (NH) staff, residents, and families in collaborative discussions about medication care planning, a fundamental component ensuring resident disease management and safety. Yet, how medication issues are addressed within CCs remains unexplored. This study aimed to describe how medications were discussed and who discussed them through a convenience sample of naturally observed CCs (n=15) in a 40-bed, nonprofit, short-stay, Midwestern NH. Observations were audio-recorded, transcribed verbatim, and analyzed using thematic analysis. CCs lasted 20 to 55 minutes each (mean=36 minutes). The number of attendees in CCs ranged from 5 to 15, with 6 being the most frequent (33.3%). Of these, 14 CCs (93%) involved medication conversations. A variety of staff attended CCs; however pharmacists were absent. Social workers and registered nurses were primarily engaged in medication conversations. Residents and families typically participated passively. Medication conversations centered around three main themes: (1) Information Seeking and Sharing—unifying participants' understanding regarding current medication and offering opportunities for consultation; (2) Decision Making—negotiating and making decisions about adding, changing, or stopping the resident's medication during NH stay; and (3) Discharge Planning—confirming the continuity of the resident's medication post-discharge and navigating access to managing medication resources. The findings of this study could enhance our understanding of how medications-related discussions are conducted during CCs in NHs. Future studies can further identify the different professional roles of NH staff in medication care planning according to residents' and families' needs, and explore the inclusion of pharmacists in CC.

Abstract citation ID: igae098.3462

### PERSON-CENTERED PRACTICE OF NURSES IN LONG-TERM CARE HOSPITALS

Su Hyun Kim, and Yoon Saeng Choi, *Kyungpook National University, Daegu, Republic of Korea*

**Objective:** This study investigates how professional competence, professional commitment, and nursing organizational culture influence the implementation of person-centered care by nurses in long-term care facilities. Method: A sample of 131 nurses from seven long-term care hospitals in South Korea participated in this research. Standardized measures were utilized to assess professional competence, professional commitment, nursing organizational culture, and person-centered care. Data analysis was conducted using hierarchical multiple regression analysis.

**Results:** Nurses in long-term care hospitals displayed average scores of 4.77±0.84 for professional competence and 4.27±0.89 for professional commitment on a scale of 1 to 7, and 3.45±0.44 for person-centered care on a scale of 1 to 5. Notably, nurses scored highest in relation-oriented culture and lowest in task-oriented culture. Professional competence (β=.59, p<.001) and innovation-oriented culture (β=.36, p<.001) significantly impacted person-centered care, explaining 52.5% of the variance.

**Conclusion:** Improving person-centered care in long-term care settings requires interventions aimed at enhancing nurses' professional competence and cultivating an innovation-oriented nursing organizational culture.

### SESSION 7710 (POSTER)

### OBESITY/OVERWEIGHT

Abstract citation ID: igae098.3463

### BODY COMPOSITION CHANGE AND PHYSICAL FUNCTION AFTER INTENSIVE BEHAVIORAL WEIGHT LOSS INTERVENTION

Kacey Chae[1], Amie Bettencourt[1], Denise Houston[2], Eleanor Simonsick[3], Luigi Ferrucci[4], Rita Kalyani[1], Jeanne Clark[1], and Kimberly Gudzune[1], *1. Johns Hopkins University School of Medicine, Baltimore, Maryland, United*

*States*, 2. *Wake Forest University School of Medicine, Winston Salem, North Carolina, United States*, 3. *National Institute on Aging, Baltimore City 0400 Baltimore City, Maryland, United States*, 4. *National Institute on Aging, Baltimore, Maryland, United States*

The LookAHEAD trial, an RCT of adults with obesity and diabetes, has demonstrated weight loss (WL) efficacy through intensive lifestyle intervention (ILI), but also a decline in lean mass (LM) after ILI. One potential concern with LM reduction is worsening physical function (PF), particularly among older adults. Our study objective was to evaluate the change in PF across different categories of body composition changes during ILI. We conducted a secondary analysis within the LookAHEAD trial, among ILI participants with dual-energy X-ray absorptiometry (DXA). The independent variables were 1). percent WL, 2). percent FM change, and 3). percent appendicular lean mass (ALM) change at 12 months (12M). The dependent variable was the change in self-reported PF at 12M, measured by the SF-36 (higher scores indicating better PF). At baseline, 628 participants (63% women) had mean age 58.3 years (SD 6.7), weight 96.1 kg (16.8), FM 39.8 kg (10.7), ALM 22.9 kg (5.1), and PF score 81.5 (17.6). Mean WL was 9.2% (6.3); PF scores increased in the WL ≥10% and 5-9.9% groups. Mean change in FM was -14.8% (11.7); PF scores increased in the high FM-loss, moderate FM-loss, and low FM-loss groups [high-loss vs. low-loss (p=0.02); high-loss vs. gain (p=0.04)]. Mean change in ALM was -4.8% (4.9); PF score increased in the high ALM-loss, moderate ALM-loss, and low ALM-loss groups, but the between-group differences were not significant (p=0.06). Results suggest that LM loss does not necessarily contribute to PF declines; FM loss may be a greater contributor to PF improvement.

Abstract citation ID: igae098.3464

### CHARACTERIZING PERFORMANCE VARIABILITY DURING A SIX-MINUTE WALK TEST

Tina Brinkley[1], Fang-Chi Hsu[1], Dalane Kitzman[1], Denise Houston[2], and Jason Fanning[3], *1. Wake Forest University School of Medicine, Winston-Salem, North Carolina, United States*, 2. *Wake Forest University School of Medicine, Winston Salem, North Carolina, United States*, 3. *Wake Forest University, Winston-Salem, North Carolina, United States*

The six minute walk (6MW) is a test of sub-maximal exercise capacity that reflects the level at which most activities of daily living are performed. While 6MW outcomes traditionally focus on distance covered or average gait speed, recent efforts explore methods to capture performance variability. We used functional principal component analysis (fPCA) to characterize patterns of lap-to-lap performance in 33 adults with overweight/obesity (mean age: 64.2±7.7 yrs, mean BMI: 35.5±6.1 kg/m2, 79% male, 82% White). The 6MW was performed on a 20-m course using standardized procedures, and the time to complete each half lap was recorded. Spearman correlation coefficients were used to examine associations between fPCA components and physical function measures including the expanded Short Physical Performance Battery (eSPPB) and self-reported mobility. Two fPCA components were extracted, explaining 72% (PC1) and 15% (PC2) of the variance in lap-to-lap performance. PC1 captured both speed and consistency of performance, where higher scores were correlated with greater 6MW distance (r=0.93, p< 0.0001), faster initial 6MW gait speed (r=0.80, p< 0.0001), 4-meter usual gait speed (r=0.64, p< 0.0001), and repeated chair stands time (r=-0.40, p=0.02), and higher eSPPB (r=0.50, p-0.027) and mobility (r=0.54, r=0.001) scores. PC2 captured consistency in performance, with higher scores correlating with less gait change speed during the 6MW (r=-0.63, p< 0.0001) and better balance (r=0.43, p=0.0122). These results suggest faster gait speed overall as well as during the initial stages of the 6MW are associated with better physical function. Future research should examine whether intervening on gait speed and gait consistency benefits long-term independence.

Abstract citation ID: igae098.3465

### DIETARY DIVERSITY AND OVERWEIGHT ARE ASSOCIATED WITH HIGH INTRINSIC CAPACITY AMONG CHINESE URBAN OLDER ADULTS

Siyang Lin[1], Jingyi Chen[1], Xinye jiang[2], Xiaoming Huang[1], Yin Yuan[3], Na Li[3], Feng Huang[3], and Pengli Zhu[4], *1. Shengli Clinical Medical College of Fujian Medical University, Fuzhou, Fujian, China (People's Republic)*, 2. *Fujian University of Traditional Chinese Medicine, Fuzhou, Fujian, China (People's Republic)*, 3. *Fujian Provincial Hospital, Fuzhou, Fujian, China (People's Republic)*, 4. *The Shengli Clinical Medical College of Fujian Medical University, Fuzhou, Fujian, China (People's Republic)*

**Background and objectives:** The associations of intrinsic capacity (IC) with dietary diversity and body mass index (BMI) remain unclear in older adults. This study aimed to examine the associations of dietary diversity and BMI on high IC.

**Methods:** The cross-sectional study used data from the Fujian Prospective Aging Cohort, which included 1972 individuals aged 60-98 from 2020 to 2021. The dietary diversity score (DDS) was constructed with eight food varieties, and consuming ≥five varieties of food daily was considered a high DDS. BMI was grouped into underweight, normal weight, overweight, and obesity according to the Chinese guidelines. High IC was defined as ≥three unimpaired domains of cognition, locomotion, sensory, vitality, and psychology.

**Results:** Compared with low DDS, high DDS had a positive association with high IC (OR = 1.42, 95 % CI = 1.16-1.74). Compared with normal weight, underweight was inversely related to high IC (OR = 0.18, 95 % CI = 0.09-0.36), overweight was positively related to high IC (OR = 1.65, 95 % CI = 1.33-2.06), while no significant association was observed between obesity and high IC. The restricted cubic spline model exhibited an inverted U-shaped nonlinear curve of BMI and high IC and identified an optimal BMI of 25.7 kg/m2 for high IC.

**Conclusions:** High DDS is a protective factor of high IC in older adults. Overweight had the most protective association with high IC among the four BMI subgroups. Individuals with overweight and higher dietary diversity had higher IC.

Abstract citation ID: igae098.3466

### METABOLOMIC CHARACTERIZATION OF UNINTENTIONAL WEIGHT LOSS AMONG COMMUNITY-DWELLING OLDER ADULTS

Shanshan Yao[1], Megan Marron[2], Samaneh Farsijani[1], Ravi Shah[3], Venkatesh Murthy[4], and Anne Newman[5], *1. University of Pittsburgh, Pittsburgh, Pennsylvania, United States*, *2. University of Pissburgh, Pittsburgh, Pennsylvania, United States*, *3. Vanderbilt University Medical Center, Nashville, Tennessee, United States*, *4. University of Michgan, Ann Arbor, Michigan, United States*, *5. University of Pittsburgh, University of Pittsburgh, Pennsylvania, United States*

Unintentional weight loss is associated with higher mortality and impaired mobility in older adults. Metabolomics can provide insight into yet unknown pathways of unintentional weight loss and inform its early prediction and targets for intervention. Here we investigated plasma metabolite associations of subsequent unintentional weight loss over 2 years (mean annual change [SD]=-1.5 [2.0] kg) in 2330 participants (mean age 74.6 years, 51% women, 37% Black) from the Health, Aging and Body Composition study. Metabolites were measured by liquid chromatography-mass spectrometry. Multinomial logistic regression models were used to identify the associations of each of the 478 metabolites with weight loss with/without an intention and weight gain (>3% annual weight gain/loss), relative to weight stability. The metabolite associations of unintentional weight loss differed from those of intentional weight loss and weight gain. Forty-seven metabolites were only significantly associated with unintentional weight loss after age, sex, race, current obesity, and multiple comparison adjustments compared with weight stable (FDR < 0.05). Lower levels of branched-chain amino acids, aromatic amino acids, phospholipids, long-chain poly-unsaturated triglycerides, and higher levels of amino acid derivatives, poly-unsaturated fatty acids, and carbohydrates were associated with higher odds of unintentional weight loss relative to weight stable. The identified metabolite associations were independent of smoking, energy expenditure, diet, inflammation, chronic conditions, and medication (< 20% attenuation). Altered amino acid metabolism, impaired mitochondrial fatty acid oxidation, and inflammaging implicated by the identified metabolites, which have been linked to insulin resistance and sarcopenia in literature, may precede unintentional weight loss in older adults.

Abstract citation ID: igae098.3467

### THE ASSOCIATION BETWEEN WEIGHT-ADJUSTED WAIST INDEX AND COGNITIVE FUNCTION IN OLDER ADULTS

Xichenhui Qiu[1], Jiahao Kuang[1], Changning Wei[2], and Xujuan Zheng[1], *1. Shenzhen University, Shenzhen, Guangdong, China (People's Republic)*, *2. Shenzhen Polytechnic University, Foshan, Guangdong, China (People's Republic)*

**Background:** The impact of obesity on cognitive function has generated interest, and weight-adjusted waist index (WWI) has emerged as a novel marker of obesity. However, the association between WWI and cognitive function in older adults remains unclear. This study aims to explore the relationship between WWI and cognitive performance in older adults using data from the National Health and Nutrition Examination Survey (NHANES) 2011–2014.

**Methods:** A cross-sectional investigation was conducted using NHANES data. Multivariate regression and smoothing curve fitting were used to analyze the linear and nonlinear relationship between WWI and cognitive function, assessed by neuropsychological tests. Subgroup analysis and machine learning models were employed to evaluate the stability and associations between WWI and cognitive metrics.

**Results:** The analysis included 3472 participants. Results showed that higher WWI was significantly associated with lower scores on cognitive tests, including CERAD-WL (learning), AFT (memory), and DSST (processing speed). This association remained stable after considering WWI as a categorical variable. The negative association was more pronounced in men and decreased with advancing age. Non-linear threshold effects were observed, with stronger correlations between WWI and cognitive function above specific thresholds. Conclusions: Increased abdominal obesity, as indicated by higher WWI, was linked to deficits in learning, memory, verbal fluency, and processing speed among older adults. These findings suggest that abdominal obesity may play a crucial role in cognitive decline in this population. Interventions targeting abdominal obesity could be important for preserving cognitive performance in older adults.

### SESSION 7715 (POSTER)

### PERSONALIZED/PRECISION AGING

Abstract citation ID: igae098.3468

### APP PROMOTING NONPHARMACOLOGICAL INTERVENTIONS FOR PEOPLE LIVING WITH DEMENTIA IN NURSING HOMES

Minhee Yang, Sinwoo Hwang, EunKyo Kim, Jungwon Cho, and Eunhee Cho, *Yonsei University, Seoul, Republic of Korea*

Mobile app-based interventions have emerged as a promising approach for providing individualized non-pharmacological interventions to people living with dementia (PLWD) to manage behavioral and psychological symptoms of dementia (BPSD). Offering such interventions can be challenging in nursing homes because of the lack of support from family members and heavy workload when composing and implementing individualized non-pharmacological interventions. This study aimed to develop and evaluate a decision support application for nurses to facilitate non-pharmacological interventions. The research team developed an app that provides symptom-specific non-pharmacological interventions, and experts provided feedback on the app's usability. Finally, the app was tested with four dyads of PLWD and nurses for four weeks (two weeks of the intervention period only) and was assessed for its overall quality and usability using questionnaires and interviews. Nurses recorded BPSD diaries ranging from 17 to 78 for four weeks. The implementation of symptom-specific, individualized non-pharmacological interventions using the application led to a reduction in BPSD. The nurses most frequently provided individualized music programs, followed by reminiscence programs. The engagement level of each dyad ranged from 3.1 to 3.6 out of 5. Overall, the app was perceived as beneficial in facilitating symptom-specific, individualized non-pharmacological care in nursing homes. Collectively, this study highlights the usefulness of the app in nursing homes by assisting nurses in their decisions regarding non-pharmacological interventions. However, further enhancements are warranted to improve usability and efficacy within the context of nursing homes.

Abstract citation ID: igae098.3469

### EPIGENETIC AGING AND XENOBIOTIC METABOLISM IN THE MOUSE LIVER

Sara Abudahab[1], Dalia Al Saeedy[2], Ola AlAzzeh[2], and Joseph McClay[2], *1. UST Global, Richmond, Virginia, United States*, *2. Virginia Commonwealth University, Richmond, Virginia, United States*

Hepatic xenobiotic metabolism declines with age, and while intact metabolism is associated with longevity, few studies have explored the genome-wide impact of epigenetic aging on these processes. In this study, we used liver tissues from male C57BL/6 mice (4, 24, and 32 months), obtained from the National Institute on Aging. We mapped DNA methylation changes using Reduced Representation Bisulfite Sequencing (RRBS) to identify age-associated differentially methylated sites (a-DMS). Our findings demonstrate significant age-associated methylation changes, with younger mice (4 months) displaying different patterns compared to older counterparts. First, we found that genes encoding Phase I and II drug-metabolizing enzymes, in addition to ABC and SLC transporters, showed differential methylation with age. Targeted analysis of important pharmacogenes (Cyp1a2, Cyp2d9, and Abcc2) showed that aging led to Cyp2d9 hypermethylation and reduced expression, while Abcc2 expression remained unchanged. Cyp1a2 was hypomethylated with age, yet its expression decreased. Bioinformatic analysis revealed that a-DMS hypomethylated with age were enriched for HNF4α binding sites. HNF4α is downregulated with age, potentially explaining the reduction in Cyp1a2 expression despite hypomethylation. Pathway analysis of a-DMS revealed disruptions in xenobiotic metabolism pathways, including small molecule catabolism, are evident in both normally aged (24 months) and super-aged (32 months) mice when compared to the younger group (4 months). This study underscores the impact of epigenetic modifications in aging on liver function and xenobiotic metabolism.

Abstract citation ID: igae098.3470

### PROTEOMIC BRAIN AGE GAP, DEMENTIA RISK, AND BRAIN VOLUME MEASUREMENTS

Minghao Kou, Hao Ma, Xuan Wang, and Lu Qi, *Tulane University, New Orleans, Louisiana, United States*

Aging is the most profound risk factor for dementia, yet the molecular basis for brain aging and dementia remains largely unknown. We utilized levels of proteins primarily originating from the brain to measure brain-specific aging signature (age gap) and assessed its associations with all-cause dementia, Alzheimer's disease (AD), and vascular dementia. We analyzed Olink Explore 1536 proteomics data from 45,113 UK Biobank participants, followed until December 31, 2022. Machine learning models estimated the brain age gap based on 53 brain-enriched proteins, demonstrating strong correlation with chronological age (Spearman r = 0.84). Per-unit increment in the brain age gap z-score was associated with significantly elevated risks of all-cause dementia (hazard ratio [95% confidence interval], 1.82 [1.69-1.96]), AD (2.12 [1.88-2.39]), and vascular dementia (1.91 [1.58-2.31]), respectively. Notably, 2.1% of the study population exhibited extreme brain aging defined as brain age gap z-score <-2 or >2, correlating with over 4-fold increased risks of all-cause dementia and vascular dementia (4.39 [2.94–6.55] and 4.16 [1.27–13.64], respectively), and up to 6-fold increased risk of AD (HR 6.15 [3.35–11.28]). These findings were corroborated by Magnetic resonance imaging (MRI) assessments showing that a higher brain age gap aligns with cortex atrophy and increased white matter hyperintensity. Conclusively, the proteomic brain age gap serves as a meaningful indicator of brain structural degeneration and dementia risk, independent of chronological age.

Abstract citation ID: igae098.3471

### THE INTERPLAY BETWEEN GENETIC VARIANTS IN MIR146A AND ABCG2 GENES IS ASSOCIATED WITH HUMAN SURVIVAL

Anatoliy Yashin, Deqing Wu, Konstantin Arbeev, Hongzhe Duan, Rachel Holmes, Igor Akushevich, and Svetlana Ukraintseva, *Duke University, Durham, North Carolina, United States*

The ABCG2 gene is well-known for its role in developing drug resistance and for its impact on the survival of cancer patients. To modulate the effects of ABCG2 on health and survival different intervention strategies were suggested. Among them, the use of non-coding RNAs that are involved in posttranscriptional modification of gene expression was suggested. MiR146a is known as a posttranscriptional regulator of expressions of many genes involved in aging, health, and survival traits. In this paper, we evaluate the role of interplay between variants in miR146a and ABCG2 genes in human survival. We apply the logistic regression model with the interaction term to the analysis of Framingham Heart Study data to test the null hypothesis that the interplay between variants in miR146a and ABCG2 genes is not associated with human survival. As an outcome we use the survival trait, Sur1, defined by dichotomizing a person's lifespan, LS, as follows: Sur1=1, if LS >=85 years; Sur1=0 if LS < 85. We used the clumping procedure to reduce the number of tests and increase the Bonferroni correction threshold (BCT). The analysis detected a statistically significant association of interaction between rs13120400 in the ABCG2 gene and rs2910163 in the miR146 gene, β=-0.46. p=3.74E-03, BCT = 1.00E-02. Interactions of rs13120400 also showed nominally statistically significant associations with two other SNPs from the miR146a gene. rs2910164: β=-0.38, p=1.96E-02; and rs2961920: β=-0.36, p=2.45E-02. It turns out that all four SNPs are well known in scientific literature and play important roles in many health and longevity-related traits.

Abstract citation ID: igae098.3472

### USE OF OCULOMETRICS AS AN AT HOME BRAIN HEALTH MONITORING TOOL: A CASE STUDY

Quinn Kennedy[1], Beatriz Hernandez[2], and Dorion Liston[3], *1. neuroFit, Carmel, California, United States*, *2. NA,*

*Cupertino, California, United States*, 3. *neuroFit, Mountain View, California, United States*

Quick, objective brain health assessments are needed to monitor long term cognitive function. Oculometrics, the measurement of eye movements, may be one solution as they can quantify the health of cortical circuitry associated with perception and motor tracking. Our previous work demonstrated that an oculometric task is sensitive to a wide range of neurocognitive function. The purpose of this case study was to examine the feasibility of using an oculometric task as an at home brain health monitoring tool. The five-minute task is based on a classic step-ramp visual tracking paradigm that was administered on a PC computer with a camera and eye tracking capability. It yields 10 z-scored eye tracking metrics that are computed into a summary score. A 51-year-old woman completed the oculometric task at home on 138 days over the course of six months and recorded notes regarding anything that may have impacted performance. Data was successfully captured on 111 days (mean score = 0.02 (s =.48)). Distractions in the environment (n = 15 instances) led to worse performance (mean score = -0.37 (s =.51)). Poor sleep the night before (n = 7 instances) also may impair performance (mean score = -.051 (s =.52). Anecdotally, worse scores after poor sleep motivated the participant to improve her sleep habits. Longitudinal plots indicated improvement in the task over the first few weeks, then scores stabilized. Results provide preliminary support for oculometrics as an at-home brain health monitoring tool but point to challenges in controlling environmental factors.

### SESSION 7720 (POSTER)

### PHYSICAL ACTIVITY AND EXERCISE (POSTERS)

Abstract citation ID: igae098.3473

### A REINFORCEMENT LEARNING APPROACH TO ENHANCE PHYSICAL ACTIVITY AND REDUCE FALL RISK IN OLDER ADULTS

Chang Liu[1], Ladda Thiamwong[1], Tho Nguyen[1], Jethro Raphael Suarez[2], Joon-Hyuk Park[1], Qian Lou[1], and Rui Xie[1], 1. *University of Central Florida, Orlando, Florida, United States*, 2. *University of Central Florida*

The proliferation of sensing technology enables us to accurately track physical activity (PA) using wearable devices. Consequently, based on the PA patterns provided, it offers us an opportunity to design personalized interventions delivered via notifications, aiming to promote healthy behaviors and eventually reduce fall risk among older adults. We assessed minute-level accelerometer-based PA data, in total 1.24 million observations, from 134 participants (average age 74.26, SD 6.85, female 88.8% and 70.15% have no history of falls) in our Physio-Feedback Exercise Program (PEER) and control groups. The study targets the reduction of fall risk and the improvement of PA levels in economically disadvantaged older adults recruited in 2023. We employed a reinforcement learning (RL) approach with a policy network that continuously learns steps and vector magnitude from participants, aiming to find an optimal policy to encourage participants to take the action of 'standing'. We assigned a positive reward when participants achieved Moderate to Vigorous Physical Activity status, a neutral reward for Light Physical Activity, and a negative reward for sedentary behavior. A three-layer fully connected neural network with sigmoid activation functions identified a significant difference in action probabilities, with the control group averaging 0.6456 and the PEER group at 0.7192. Significantly, from 9 AM to 6 PM, probabilities surged for both groups, peaking at 0.7438 (control) and 0.8188 (PEER). After 6 PM, probabilities declined across all groups. The probability of an event is directly proportional to its likelihood; therefore, a higher probability corresponds to the greater likelihood of the event occurs.

Abstract citation ID: igae098.3474

### BIOPSYCHOSOCIAL DETERMINANTS OF PHYSICAL ACTIVITY IN BLACK WOMEN WITH CARDIOVASCULAR DISEASE

Hyun Jung Kim, and Shweta Gore, *MGH Institute of Health Professions, Boston, Massachusetts, United States*

Cardiovascular disease (CMD) remains the primary cause of death among Black women in the United States. Given the well-recognized benefits of physical activity (PA) in reducing CVD mortality, PA levels among Black women remain notably low. This study aims to explore factors associated with low PA in Black women with CMD via secondary analysis of subjective Global Physical Activity Questionnaire data in the 2013-2028 National Health and Nutrition Examination Survey (NHANES) sample. The prevalence of CVD among Black women (26,100,000 ± 2,171,143) in the estimated total population of United States (US) was significantly greater than in Black men (21,300,000 ± 1,592,102) ($t = -5.02$, $p < 0.001$). Significantly greater proportion of Black women in the US did not meet the recommended PA guidelines (79.9% versus 89.3% in Black men and women; $t = 3.51$, $p = 0.001$). Among Black men and women, age, self-perceived health, and difficulty walking emerged as significant predictors of meeting PA guidelines ($F5, 40 = 10.07$, $p < 0.001$, whereas age and high glycosylated hemoglobin (HbA1c) were significant predictors among Black women ($F8, 36 = 2.62$, $p = 0.023$). Every unit increase in age and HbA1c decreased the odds of Black women meeting the PA guidelines by 5% and 27.6% (OR = 0.96, $p = 0.014$ and OR = 0.96, $p = 0.014$).

Abstract citation ID: igae098.3475

### BLOOD-BASED CARDIAC BIOMARKERS AND PHYSICAL ACTIVITY: PRELIMINARY RESULTS

Jennifer Shearon, Baylie Rushing, Madeleine Love, and Denise Head, *Washington University in St. Louis, St. Louis, Missouri, United States*

Mixed findings regarding effects of physical activity on the brain in later adulthood motivate further research into mechanisms and moderators of potential effects. Cardiovascular health could be part of a mechanistic pathway and/or a moderator of physical activity effects. While there are myriad indicators of cardiovascular health, blood-based cardiac markers may prove particularly sensitive. One goal of this study was to investigate associations of two blood-based markers of cardiovascular health with physical activity/cardiorespiratory fitness. The second aim was to examine the moderating effect of cardiac biomarkers in associations of physical activity/fitness with neurotrophins, considered molecular mechanisms of physical activity effects, as well as with hippocampal volumes.

Cognitively normal participants in the Knight ADRC Adult Children Study (n=56, ages 43-85) completed a blood draw for assessment of cardiac biomarkers (high sensitivity cardiac troponin T, N-terminal probrain natriuretic peptide) and neurotrophins (brain-derived neurotrophic factor, insulin-like growth factor, vascular endothelial growth factor). Composites were created for cardiac biomarkers and for neurotrophins. Participants completed a submaximal exercise test to estimate cardiorespiratory fitness and wore an actigraphy watch for seven days, from which a physical activity composite score was created. Hippocampal volumes were from the most recent MRI scan. Higher physical activity was associated with lower levels of cardiac biomarkers (p=0.03). There was no moderating effect of cardiac biomarkers on associations between physical activity and neurotrophin levels (p=0.74) or physical activity and hippocampal volumes (p=0.24). Blood-based cardiac biomarkers may be useful for considering mechanistic associations between physical activity and cardiovascular health in larger samples.

Abstract citation ID: igae098.3476

### CHARACTERIZATION OF NOVEL ACCELEROMETER-MEASURED PHYSICAL ACTIVITY METRICS BY COGNITIVE STATUS IN OLDER ADULTS

Yurun Cai, Beth Snitz, and Ann Cohen, *University of Pittsburgh, Pittsburgh, Pennsylvania, United States*

Although levels of physical activity (PA) are associated with risk of mild cognitive impairment (MCI) and Alzheimer's disease (AD), most studies have used self-report PA and/or focused solely on intensities (e.g., MVPA) which may lead to recall bias or measurement error. We aim to characterize novel PA metrics among adults aged □50y with various cognitive status in the Connectomics in Brain Aging and Dementia study. A total of 166 (mean age 65.3□8.9y, 65.9% women) participants with neuropsychological and valid accelerometer data in 2016-2021 were included in the analysis. PA metrics included total activity counts (TAC), logged TAC (LTAC), activity fragmentation, active minutes/day, number of active bouts, and PA complexity calculated using multiscale entropy. Participants were classified into normal control (NC)(n=84), impaired without complaints (IWOC) or subjective cognitive complaints (SCC)(n=36), and MCI/AD(n=44). Analysis of variance analysis found that IWOC/SCC had fewer number of active bouts than NC (85.34 vs. 91.83, p=0.043), lower PA complexity than NC (52.57% vs. 64.33%, p=0.001) and MCI/AD (52.57% vs. 60.68%, p=0.045). Participants with MCI/AD had lower LTAC than NC (14.38 vs. 14.50, p=0.042) and higher activity fragmentation than the other two groups (25.89% vs. 23.70%, p=0.035). Logistic regression models showed that each 1% higher in PA complexity was associated with 4% lower odds of IWOC/SCC compared to NC (OR=0.96, 95%CI:0.93-0.99, p=0.004), adjusted for sociodemographics. Our study highlights the importance of assessing novel PA metrics particularly PA complexity which may be more sensitive than conventional metrics to identify adults at early stages of pathological progression to dementia.

Abstract citation ID: igae098.3477

### DECLINE IN EXERCISE IN OLD AGE: LONGITUDINAL EVIDENCE OF DISTANCE TO DEATH AND THE ROLE OF SELF-PERCEPTIONS OF AGING

Zheyuan Zhang[1], Wan Wen[2], and Shannon Mejia[3], *1. Beijing Normal University, Beijing, Beijing, China (People's Republic)*, *2. University of Illinois Urbana-Champaign, Champaign, Illinois, United States*, *3. University of Illinois Urbana-Champaign, Urbana, Illinois, United States*

As individuals age, they are more likely to face health issues and physical mobility constraints associated with mortality, resulting in a decrease in engagement in physical activities during their later life stages. This decrease in activity can adversely impact their longevity and well-being. Self-perceptions of aging (SPA) constitute a crucial psychological resource for older adults. More positive SPAs have a protective effect against health decline and encourage the adoption of healthier behaviors. However, the impact of SPA on health behaviors towards the end of life, especially in moderating the relationship between the proximity to death and exercise engagement, remains insufficiently explored. This study used 10-year longitudinal data from deceased participants of the Chinese Longitudinal Healthy Longevity Survey (CLHLS) (N=9,778, age range:62~117, 59% female). After excluding observations with missing data, a total of 12,027 observations were analyzed. Multilevel models showed that as older adults approached the end of life, their likelihood of engaging in physical activity decreased. However, positive SPA can mitigate this decline. These insights underscore the importance of SPA in health promotion interventions aimed at enhancing physical activity among older adults, offering strategies to increase exercise participation in their later years.

Abstract citation ID: igae098.3478

### EFFECT OF EXERCISE OR MENTAL AND SOCIAL ACTIVITIES ON INTRAINDIVIDUAL VARIABILITY OF COGNITIVE PERFORMANCE IN STROKE

Vrinda Dimri[1], Nárlon Cássio Boa Sorte Silva[1], Guilherme Balbim[1], Janice Eng[1], and Teresa Liu-Ambrose[2], *1. University of British Columbia, Vancouver, British Columbia, Canada*, *2. University of British Columbia, University of British Columbia, British Columbia, Canada*

Stroke is associated with increased risk of cognitive impairment. Evidence has shown exercise to be a useful strategy for improving cognitive function in this population. Intraindividual variability (IIV), a sensitive measure of cognitive performance, is the within-person trial-to-trial variation in reaction time during cognitive tasks. Whether exercise training also improves IIV of cognitive performance in stroke is unknown. The study was a six-month single-blinded, 3-group parallel randomised controlled trial. Participants included community dwelling adults (N= 119, 38.7% female) with a history of stroke, aged above 55 years (mean= 70.71, SD= 8.59), able to walk 6 meters, and without dementia. Participants were randomly allocated to twice-weekly supervised classes of 1) exercise training (EX); (2) cognitive and social activities (ENRICH); or (3) a balance and tone (BAT; control). Residualised intraindividual standard deviation (rISD) was used as measure of IIV and computed using trial latencies of a computerised Stroop Task. IIV was assessed at baseline, 6-and-12 months. At 6-and-12 months, there was no significant reduction in IIV for all three groups. Furthermore, there were no significant differences in the IIV scores of the EX and BAT (estimated mean difference= 0.30, SE= 0.53, p-value = 0.89) or ENRICH and BAT (estimated mean difference= 0.64, SE= 0.55, p-value = 0.40) groups. The findings suggest that a 6-month program of exercise or mental and social activities does not significantly impact IIV of cognitive performance in stroke. Further studies are required to determine if IIV can be improved using other types of exercise or cognitive training programs.

Abstract citation ID: igae098.3479

### EFFECTS OF PHYSICAL ACTIVITY AND CVD RISK ON MEMORY CONCERNS: RESULTS FROM THE NATIONAL HEALTH INTERVIEW SURVEY

Joel Anderson[1], Robin Harris[2], and Jennifer Smith[3], *1. University of Tennessee-Knoxville, Knoxville, Tennessee, United States*, *2. University of Tennessee, Knoxville, Tennessee, United States*, *3. University of Tennessee, Knoxville, Knoxville, Tennessee, United States*

Common pathways exist between cardiovascular and cerebrovascular diseases in aging adults; therefore, this study examined the associations between the frequency of physical activity (PA) and memory issues in older adults with or at risk for cardiovascular disease (CVD). Data from the 2022 National Health Interview Survey were used to examine the influence of CVD risk and physical activity on self-reported experiences of memory challenges using logistic regression. The sample (N = 13,432) was limited to adults ≥50 years of age. Most of the sample was female, White, married/partnered, and reported a good to excellent health status. Roughly a quarter (24.9%) reported some difficulty remembering or concentrating while the majority (89.1%) had some level of increased CVD risk based on Framingham criteria. Half of the sample (51%) did not meet the current PA guidelines for older adults. After controlling for age, sex, race, ethnicity, and region, those with a poor or fair health status (OR = 3.24, CI = 2.94–3.56, p < 0.001), increased CVD risk (OR = 1.26, CI = 1.09–1.47, p = 0.002), or who did not meet current PA guidelines (OR = 1.67, CI = 1.46–1.90, p < 0.001) more frequently reported challenges with remembering or concentrating. Given these findings, further research is warranted into the role of PA in memory issues, particularly for those at risk for CVD. Following guidelines for PA for these older adults may be helpful.

Abstract citation ID: igae098.3480

### EFFECTS OF PHYSIO-FEEDBACK EXERCISE PROGRAM (PEER) ON PHYSICAL ACTIVITY LEVELS IN OLDER ADULTS

Jethro Raphael Suarez[1], Joon-Hyuk Park[2], Rui Xie[2], Kworweinski Lafontant[2], and Ladda Thiamwong[2], *1. University of Central Florida*, *2. University of Central Florida, Orlando, Florida, United States*

Adults aged 60 years and older are the most sedentary group in the United States. Low levels of physical activity (PA) and high levels of sedentary behavior (SB) increase the risk for developing diseases. Therefore, it is crucial to investigate intervention strategies that help increase PA and decrease SB levels in older adults. This study was an 8-week, Physio-fEedback Exercise pRogram (PEER), aimed at improving PA levels and balance while reducing the fall risk of community-dwelling older adults by providing visual physio-feedback on balance and body composition, cognitive reframing to lower the fear of falling, and a peer-led group exercise. We conducted a preliminary analysis of 55 community-dwelling older adults aged 61 to 89 years (49 women, 6 men; mean age = 73.75 ± 7.28 years) in low-income settings to determine if the intervention influenced PA levels. Total PA levels were measured across 7 consecutive days using the ActiGraph GT9X Link wireless activity monitor at baseline and post-intervention for a control (n = 31) and intervention group (n = 24), and categorized as sedentary behavior (SB), light PA (LPA), and moderate-to-vigorous PA (MVPA). PA was compared between groups using Student's t-test and Mann-Whitney U test, but significant differences were not found. However, the PEER group showed a decrease in total SB mean (208.64 min), increase in total LPA mean (89.99 min), and increase in total MVPA median (8.75 min) between baseline and post-intervention. The results show that PEER has the potential to impact PA levels, but more research is warranted.

Abstract citation ID: igae098.3481

### EXPLORING BARRIERS AND FACILITATORS TO PHYSICAL ACTIVITY IN EUROPEAN MIDLIFE AND OLDER ADULT SAMPLES

Tiana Broen[1], Stephen Aichele[2], and Leilani Feliciano[1], *1. University of Colorado Colorado Springs, Colorado Springs, Colorado, United States*, *2. Colorado State University, Fort Collins, Colorado, United States*

Physical activity (PA) is an adaptable and accessible health-promoting behavior with wide-ranging benefits (e.g., reduced risk for diabetes, heart disease, and all-cause mortality). However, only 24.3% of U.S. adults meet current recommendations for PA (150 minutes of moderate-intensity PA and 2 days of muscle strengthening activity per week). It is therefore crucial to understand factors that may support increased engagement in PA. The current study used a random forest machine learning analysis (RFA) alongside a conventional parametric methodology (i.e., linear multiple regression) to evaluate the relative importance of 58 risk/protective factors for PA (moderate and strenuous activity) in a population-representative sample of middle-aged and older adults (N = 67,603; 56.1% women; ages 45-105y; 18 countries) from the Survey of Health, Ageing and Retirement in Europe (SHARE Wave 6). For both women and men, top predictors identified through RFA included variables related to socio-demographics (age, sex, education, country), physical and functional health (mobility, self-rated health, grip strength), as well as variables related to mental health (cognitive performance, depressive symptoms) and socio-relational engagement (caring for others outside of the home, social connectedness). Follow-up hierarchical regressions showed that key predictors accounted for 35% of variability in PA, with contributions as follows: socio-demographic (20%), health/mobility (12%), and cognitive and socio-relational (3%). Together, these results indicate that PA is influenced by multiple bio-psycho-social factors and suggest that future interventions to promote PA may benefit by considering such constellations.

Abstract citation ID: igae098.3482

### FEASIBILITY AND EFFECTS OF HIGH-INTENSITY INTERVAL TRAINING ON COGNITIVE FUNCTION AND BRAIN HEALTH IN PERIMENOPAUSE

Victoria Cirone[1], Nárlon Cássio Boa Sorte Silva[1], Cindy Barha[2], Todd Handy[1], Ryan Stein[1], Sarah Heath[1], and Teresa Liu-Ambrose[3], *1. University of British Columbia,*

*Vancouver, British Columbia, Canada, 2. University of Calgary, Calgary, Alberta, Canada, 3. University of British Columbia, University of British Columbia, British Columbia, Canada*

Neuroendocrine changes during perimenopause negatively impact cognitive function and brain health (e.g., reduced verbal episodic memory and myelin catabolism in the brain). Exercise promotes cognitive performance and mitigates vascular risk factors for cognitive impairment. High-intensity interval training (HIIT) involves short bursts of high-intensity exercise interspersed with active recovery; it improves cardiovascular fitness and vascular risk profile. HIIT addresses the “lack of time” barrier to exercise that perimenopausal females report. This pre-post pilot study assessed the feasibility and potential effects of 12-weeks of HIIT on cardiovascular fitness, verbal episodic memory, hippocampal volume, and myelin content. Feasibility metrics included recruitment rate (i.e., 1/month), withdrawal rate (i.e., < 15%), adherence (i.e., >60%). Training fidelity was assessed by cardiovascular fitness (VO2max.) Verbal episodic memory was assessed by Rey Auditory Verbal Learning Test delayed recall. Hippocampal volume was measured by T1-weighted magnetic resonance imaging. Myelin content was measured by myelin water fraction. Cohen’s d and one-tailed paired t-tests assessed effect sizes and potential changes. Recruitment rate was 0.35 participants/month (six participants in 17 months). Withdrawal rate was 16.67% and adherence was 88.2%. The intervention had a negligible effect on VO2max (Cohen’s d=0.06, p=0.35), large and significant effect on delayed recall (Cohen’s d=0.94, p=0.04), negligible effect on hippocampal volume (Cohen’s d=0.06, p=0.18), and medium to large effect on sagittal stratum myelin content (Cohen’s d=0.68, p=0.06). Future studies should consider home-based exercise and tailored recruitment methods (e.g. social media) to meet recruitment targets. HIIT may be beneficial for cognitive function and myelin content.

Abstract citation ID: igae098.3483

### FEASIBILITY OF A REMOTE SOCIAL NETWORK-BASED OCCUPATIONAL THERAPY PHYSICAL ACTIVITY INTERVENTION

Noah Webster[1], Margaret Santioni[2], Lisa Petroff[1], Maham Adnan[1], Katrina McGuire[1], and Neil Alexander[1], *1. University of Michigan, Ann Arbor, Michigan, United States, 2. University of Michigan School of Medicine, Ann Arbor, Michigan, United States*

Despite physical activity being associated with personal and societal benefits, only 16 percent of people age 65 and older engage in recommended levels. To assist in increasing this percentage, we developed a six-week social network-based occupational therapy intervention to be administered remotely with older adults living in subsidized housing. The intervention was designed to improve physical activity through incorporation of socially engaged residents as advisory committee members and addressing activity barriers such as pain through group and individual meetings with an occupational therapist. In summer/fall 2023 a pilot feasibility study was conducted with a cohort of older adults (n=5) living in a Southeast, Michigan housing community. Process outcomes were examined and secondary health and activity outcomes were assessed via pre- and post-intervention interviews and accelerometry. Participants attended on average 9.2 (SD=1.3) intervention activities out of 10. None of the participants knew each other prior to intervention enrollment. However, post-intervention, four of the five participants reported knowing three or more other participants. In terms of secondary outcomes, three out of five participants reported a decline in the extent to which pain interfered with activities and perceived barriers to physical activity decreased for two participants, increased for two, and stayed the same for one. Step counts increased for two participants, but decreased for three. Secondary outcomes were mixed and suggest needed modifications. Additionally, while participation rates exceeded expectations, feasibility was not supported for the social network approach. However, newly formed social ties resulting from the intervention highlight potential for future behavior change.

Abstract citation ID: igae098.3484

### GENETIC RISK, MODIFIABLE FACTORS, AND COGNITIVE PERFORMANCE IN MIDDLE- AND OLDER-AGED CANADIANS

Shawna Hopper[1], Cindy Barha[2], Mari DeMarco[3], Theodore Cosco[1], Andrew Wister[1], and John Best[1], *1. Simon Fraser University, Vancouver, British Columbia, Canada, 2. University of Calgary, Calgary, Alberta, Canada, 3. University of British Columbia, Vancouver, British Columbia, Canada*

Whether the apolipoprotein E (APOE) e4 allele interacts with modifiable factors to predict cognitive performance is unclear. This study examined 26,448 participants (45 to 86 years of age at baseline) in the Canadian Longitudinal Study on Aging who had their blood genotyped for the APOE e4 allele, of whom 6,305 (24%) were e4 carriers. Cognitive outcomes were Rey Auditory Verbal Learning Test 5-minute delayed recall, Mental Alterations Test, and Animal Fluency Test, which were collected at baseline (December 2011 to July 2015), follow-up 1 (median 2.9 years after baseline), and follow-up 2 (median 5.8 years after baseline). We examined interactions between time from baseline, APOE e4 status and the following exposures: self-reported physical activity and a composite risk score comprised of sedentary behavior, smoking, depressive symptoms, social isolation, whole blood hemoglobin A1c fraction, and percent body fat. Models were adjusted for age, sex, ethnicity, study language (French/English), residential setting (urban/rural), and educational attainment. Across cognitive outcomes, an age by APOE e4 interaction was observed (p values ranging from <.001 to.01), such that the difference in cognitive performance between carriers and non-carriers increased as participant age increased. Although physical activity and composite risk were associated with cognitive performance (all p values <.01), there was not convincing evidence that these associations varied by e4 carrier status after correcting for multiple comparisons. Together, these findings confirm that APOE e4 carriers show greater reductions in cognitive performance but do not suggest that e4 status alters associations between modifiable risk and cognitive decline.

Abstract citation ID: igae098.3485

### LEISURE ACTIVITY AND POSITIVE MOOD ACROSS ADULTHOOD: RESULTS FROM THE KOREAN TIME USE SURVEY

Seung-Eun Cha[1], Kyungmin Kim[2], and Hyejoong Kim[3], *1. University of Suwon, Hwasung si, Kyonggi-do, Republic*

*of Korea*, *2. Seoul National University, Seoul, Republic of Korea*, *3. Gachon University, Seongnam-si, Kyonggi-do, Republic of Korea*

Adults in middle and later life may look forward to dedicating more time to outdoor leisure activities, which can contribute to their health and well-being. This study utilized 2-day time diaries from the 2019 Korean Time Use Survey (respondent N = 22,821; age 20–84) to examine how Korean adults engage in various outdoor activities (e.g., social visits, attending cultural events, and exercising) and whether their activity engagement (indicated by outdoor activity diversity and total hours of outdoor activity) is associated with positive mood. Further, we investigated whether these associations varied by age groups (age 20–39, 40–64, and 65–84), considering differences in family and work demands. We accounted for respondents' total TV viewing hours, total "doing-nothing" hours, and sociodemographic characteristics as control variables in the models for positive mood. The findings showed that total hours of outdoor activities and watching TV were positively associated with a positive mood in younger adults, supporting the psychological benefits of detachment from work. Yet, for older individuals, diversity in outdoor activities was positively associated with positive mood, while total hours of outdoor activities were not significant—which indicates that engaging in various activities throughout the day can be beneficial for psychological well-being in later life. Notably, we found that total TV viewing hours were negatively associated with the positive mood of older adults. This study sheds light on the differential impacts of leisure activities based on life stages, underscoring the importance of diverse leisure activities for the psychological health of older individuals.

Abstract citation ID: igae098.3486

### MAPPING THE LITERATURE FOR THE PHYSICAL ACTIVITY SCALE FOR THE ELDERLY (PASE): A SCOPING REVIEW

Cassandra D'Amore[1], Lexie Lajambe[1], Noah Bush[2], Sydney Hiltz[3], Justin Laforest[4], Isabella Viel[3], Qiukui Hao[3], and Marla Beauchamp[3], *1. McMaster Univeristy, Hamilton, Ontario, Canada*, *2. Fairway Physiotherapy, Thunder Bay, Ontario, Canada*, *3. McMaster University, Hamilton, Ontario, Canada*, *4. Choice Health Centre, Halifax, Nova Scotia, Canada*

The Physical Activity Scale for the Elderly (PASE) is a commonly used and recommended self-report measure of physical activity in older adults. This scoping review aimed to map the nature and extent to which the PASE has been used in community-dwelling older adults, including the evidence for its psychometric properties. Review eligibility criteria included: i)community-dwelling older adults (60+ years), ii)physical activity integrated into study aims and measured with the PASE, and iii)published in English. Pairs of independent reviewers screened abstracts, full-texts, and extracted data. Where possible, weighted mean PASE scores were calculated for subgroups based on age, sex, and clinical population. Seven electronic databases were searched in January 2023; 4,124 studies were screened, and 232 articles from 35 countries were included. Most studies were completed in high-income countries (86.4%) and North America (49.57%). A variety of clinical conditions were included (n=21), the most common populations being osteoarthritis (n=13), Parkinson's disease (n=11), and cognitive impairment (n=7). Psychometric properties for ten versions of the PASE were reported. All versions demonstrated acceptable test-retest reliability. Evidence for construct validity reported moderate correlations with self-reported physical activity and fair to moderate correlations with accelerometry-derived activity. Pooled means were reported in graphs and forest plots by age, sex, and clinical populations. The PASE was widely used across geographical locations and culturally adapted to several populations. Some psychometric properties have been evaluated; however, further research is required to examine responsiveness and predictive validity. The weighted mean PASE scores presented can improve interpretability of scores in similar populations.

Abstract citation ID: igae098.3487

### MEASUREMENT OF EXERCISE SELF-REGULATORY EFFICACY IN OLDER US VETERANS WITH COPD

Patricia Bamonti[1], Mariana Wingood[2], Stephanie Robinson[3], Grace Rose[1], and Marilyn Moy[1], *1. VA Boston Healthcare System, Boston, Massachusetts, United States*, *2. Wake Forest University School of Medicine, Winston-Salem, North Carolina, United States*, *3. VA Bedford Healthcare System, Bedford, Massachusetts, United States*

Physical activity promotion and exercise are essential in the management of chronic obstructive pulmonary disease (COPD), a life-limiting disease that causes dyspnea and fatigue. Exercise self-regulatory efficacy is an individual's confidence to exercise despite barriers and is an essential component to increasing physical activity/exercise. Exercise Self-Regulatory Efficacy scale (Ex-SRES) was developed and initially validated to measure one underlying factor in a small sample of adults with COPD. Research is needed examining the psychometric properties of the scale with larger samples, utilizing objective measures of physical functioning, to further establish its psychometric properties. Secondary analyses were performed on baseline assessment of two cohorts of U.S. Veterans with COPD who participated in a physical activity intervention (n=191; age M =68±8; 97% male;93.2% White). The Ex-SRES asked how confident participants felt they could exercise given 16 unique barriers. Daily steps were assessed for 7-10 days, and exercise endurance was assessed via the 6-minute walk test (6MWT). The St. George Respiratory Questionnaire (SGRQ) assessed health-related quality of life and participants self-reported frequency of days walking for exercise/week. The Ex-SRES demonstrated reliability (Cronbach's $\alpha$ =.94) and convergent validity: average steps/day (r=.22), 6-MWT distance (r=.25), SGRQ (r=-.20), and walking (r=.30) (p's<.01). Confirmatory factor analysis demonstrated acceptable fit of a unidimensional factor structure, $\chi 2$(458.07, p <.001; SRMR = 0.06; $\chi 2$/df = 4.4). Findings are consistent with prior research demonstrating a unidimensional factor structure. However, room for improved model fit is noted. Future research is needed exploring whether a multi-dimensional factor structure may improve fit.

Abstract citation ID: igae098.3488

### MONTH-LONG FITNESS CHALLENGES IMPROVE ENDURANCE AND STRENGTH FOR VETERANS PARTICIPATING IN GEROFIT

Julie Rekant[1], Jamie Giffuni[2], and Odessa Addison[3], *1. Baltimore VA Medical Center, Baltimore, Maryland, United States*, *2. Baltimore and Loch Raven VAMC, Baltimore, Maryland, United States*, *3. University of Maryland School of Medicine, Baltimore, Maryland, United States*

Older adults are vulnerable to loneliness and low physical activity engagement. Peer accountability can help encourage participation in exercise and foster a sense of community. Two, month-long fitness challenges were offered to older Veterans (65+ years) in Gerofit: (1) "The Long March" was a walking challenge with daily step goal targets increasing each week, (2) "Squatober" was a sit to stand challenge with daily bodyweight squat repetition goals increasing each week. Veterans were provided with cardio and strength workouts to complete in addition to the daily challenges. Thirty-one unique Veterans participated in these challenges (9 Veterans participated in both). Veterans in The Long March challenge (N=18, Age: 72.8±4.5 years, BMI: 30.2±5.9 kg/m2, 83% Male, 78% Black) participated in 88.3±28.0% of challenge days and met their step goals 50.2±36.1% of the days they participated. Veterans in The Long March challenge increased their Six Minute Walk Test distances by 28.0±48.7 meters after four weeks. Twenty-two Veterans participated in the Squatober challenge (Age: 74.3±6.3 years, BMI: 30.7±5.2 kg/m2, 81% Male, 86% Black). Sixty-three percent (N=14/22) completed every workout. Veterans participating in the Squatober challenge reported an overall rating of perceived exertion of 5.0±2.5 out of 10, decreased their Five Time Sit to Stand times by 1.3±2.3 seconds, and were able to complete an average of 2.6±4.1 more sit to stands during the 30 Second Chair Stand Test after four weeks. Older Veterans found fitness challenges engaging and showed functional improvements in just four weeks with daily movement goals.

Abstract citation ID: igae098.3489

### PEER-LED DIET AND EXERCISE INTERVENTION FOR OLDER VETERANS WITH DYSMOBILITY

Ben Friedman[1], Monica Serra[2], Rhianna Patel[3], Elizabeth Dennis[4], Lisa Kilpela[2], and Odessa Addison[4], *1. University of Maryland Baltimore, Baltimore, Maryland, United States*, *2. The University of Texas Health San Antonio, San Antonio, Texas, United States*, *3. VA Medical Center, Baltimore, Maryland, United States*, *4. University of Maryland School of Medicine, Baltimore, Maryland, United States*

**Purpose:** Older adults with mobility impairments have increased falls, higher hospitalization rates, and elevated healthcare costs. Veterans face higher obesity rates and poorer health outcomes. This study evaluated the adherence and preliminary efficacy of a peer-led diet and exercise intervention targeting older Veteran with dysmobility in two urban areas with a high proportion of racial and ethnic minorities.

**Methods:** Twelve older Veterans (78.9 ±6.4 years) from San Antonio, TX, and Baltimore, MD, participated in a 12-week peer-led intervention. The groups met twice a week for one hour and focused on behavioral modification, nutrition, and whole-body aerobic and band-resisted exercises. Baseline and post-intervention assessments included the 6 Minute Walk Test (6MWT), Timed Up and Go (TUG), grip strength,10-Meter Walk Test (10mWT), Four Square Step Test (4SST), and Short Physical Performance Battery (SPPB). Descriptive statistics were computed for each outcome and paired sample t-tests assessed within-subject changes. Adherence was measured by session attendance and completion rate.

**Results:** Significant improvements were observed in the 10mWT ($p < 0.05$). While not statistically significant, most participants demonstrated improvements in the TUG (5/9), 6MWT (6/9), and 4SST (7/9). For those that improved on the SPPB (4/9), half were no longer below the cut-off score of ≤ 10. Session attendance was 60.96% (TX) and 76.47% (MD). Study completion rate was 83.33% (1 drop out per site). Implications: This pilot study supports the need for further investigation to explore the long-term effects of a peer-led diet and exercise intervention addressing multiple health domains among older Veterans.

Abstract citation ID: igae098.3490

### PHYSICAL ACTIVITY AND EXERCISE-INDUCED NEUROTROPHIC AND CORTISOL RESPONSES: PRELIMINARY RESULTS

Thinh Huynh, Baylie Rushing, Madeleine Love, Denise Head, and Marta Stojanovic, *Washington University in St. Louis, St. Louis, Missouri, United States*

Neurotrophic and cortisol responses to a bout of exercise are a pathway for understanding the potential mechanisms underlying the beneficial effects of regular physical activity on cognitive and brain health. However, there is limited understanding of the factors that moderate such responses. This study examined whether long-term exercise history and current moderate-to-vigorous physical activity (MVPA) levels predicted neurotrophic and cortisol responses to acute exercise. In addition, we investigated whether these associations were moderated by age and sex. Clinically normal middle-aged and older adults from the Knight ADRC Adult Children Study (n=54; age=43-85) completed a questionnaire about exercise engagement over a 10-year period and underwent a submaximal VO2 assessment, with both representing a combined estimate of their long-term physical activity. Participants also completed seven days of actigraphy, which was used to estimate current MVPA. Participants engaged in a bout of exercise, with neurotrophic factors and cortisol measured at baseline, 10 minutes post-exercise, and 30 minutes post-exercise. Long-term physical activity did not influence neurotrophic (p=0.538) or cortisol responses (p=0.276), regardless of age and sex (p's >0.143). Higher current MVPA was associated with a greater neurotrophic response (p=0.007) across sex (p=0.165). Additionally, there was a moderation by age (p=0.014), with less of an association as age increased. Greater current MVPA was also associated with a smaller cortisol response (p=0.044), across age and sex (p's>0.144). These results suggest that current MVPA may be particularly important when considering pathways between a single bout of exercise and neurocognitive health.

Abstract citation ID: igae098.3491

### PHYSICAL ACTIVITY FRAGMENTATION AND FALLS IN OLDER ADULTS

Braden Popelsky[1], Kelley Pettee Gabriel[2], Erin Dooley[2], and Kelly Ylitalo[3], *1. Baylor University, Bryan, Texas, United States*, *2. University of Alabama at Birmingham,*

*Birmingham, Alabama, United States*, *3. Baylor University, Waco, Texas, United States*

Physical activity (PA) may be an important fall prevention strategy. Current PA guidelines emphasize total PA dose, but daily patterning of PA is underappreciated. With aging, PA bouts become less frequent and shorter in duration (i.e., more fragmented). PA fragmentation may be an indicator of fall risk, but the relationship is not well understood. This study examined daily PA patterns and fall risk in a sample of older adults from the National Health and Aging Trends Study (NHATS; (n=685, 54.3% female, 61.5% aged 70–79 years). Wrist-worn accelerometry from Round 11 (2021) measured PA and surveys from Round 12 (2022) measured falls. PA variables were categorized into tertiles and incident falls were defined as ≥1 self-reported fall in the year following the PA assessment. A modified Poisson approach was used to estimate the relative risk of total PA and PA fragmentation with falls. Overall, 40.0% reported ≥1 incident fall. After adjustment for sociodemographic and health characteristics, those in the highest tertile of total PA accumulation had lower fall risk (aRR=0.74; 95% CI: 0.57, 0.95) and those in the highest tertile of PA fragmentation had increased fall risk (aRR=1.33; 95% CI: 1.02, 1.74) compared to those in the lowest tertiles, respectively. Models were attenuated after the adjustment for physical functioning. PA fragmentation may identify fall risk in older adults. More work is needed to understand the complex relationship between PA and physical functioning across the life-course.

Abstract citation ID: igae098.3492

### RUNNING, HEALTHY AGING, AND SOCIOCULTURAL CHANGE OVER 50 YEARS: PERSPECTIVES FROM OLDER ADULTS

Barbekka Hurtt, Seth Hudes, Shana Deters, Josie Allen, Asia Cutforth, and Leslie Hasche, *University of Denver, Denver, Colorado, United States*

Running is a relatively easily accessible and perceived low barrier to entry sport for any age. This project goal was to understand how running is conceptualized as part of healthy aging over the past 50 years, given sociocultural and demographic changes that have occurred. Through recruitment on social media, with running groups, and at marathon events, participants completed survey and interview data on their life course experiences with running. Out of 37 survey participants, 5 were aged 61 or older with an average age 71 years old; of 21 interview participants, 5 were aged 61 or older. Survey results indicated that running was perceived as contributing to: a) positive increases in physical health attributes such as bone, cardiovascular, and immune health, muscle strength, and metabolism; and b) slightly or significantly more positive mood; conversely, all participants indicated their mood worsened when taking time off from running. Interview data supported the survey data and revealed positive perspectives of older athletes through phrases such as "motivating, strong, badass, happy, healthy, cool, active;" conversely, the majority of respondents felt "angry, upset, or frustrated" at the phrase "You play/run like an old person." Slightly more than 50% of participants desired improvement for older adult community athletic opportunities, particularly those related to running. Yet, most older adult participants agreed that they "feel comfortable in my community to achieve my full athletic potential." Community leaders, health, and aging services should consider opportunities to promote running groups for older adults that support both new and experienced runners.

Abstract citation ID: igae098.3493

### SKELETAL MUSCLE OXIDATIVE CAPACITY AND LIFE COURSE PHYSICAL ACTIVITY IN PARTICIPANTS OF THE BLSA

Ann Zenobia Moore[1], Teresa Tian[1], Eleanor Simonsick[2], and Luigi Ferrucci[1], *1. National Institute on Aging, Baltimore, Maryland, United States*, *2. National Institute on Aging, Baltimore City 0400 Baltimore City, Maryland, United States*

Physical activity has been associated with measures of mitochondrial quality and mitochondrial dysfunction and is connected to many aging processes. However, most studies relating physical activity to mitochondrial measures only capture physical activity over a limited period. The impact of engaging in physical activity across the life course on mitochondrial function has not been fully characterized. Here, in participants of the Baltimore Longitudinal Study of Aging we evaluate the association between a summary measure of life course physical activity (LCPA) and mitochondrial oxidative capacity, represented by postexercise phosphocreatine recovery rate (kPCr) in the quadriceps measured through 31P magnetic resonance spectroscopy. The study sample included 416 participants (43.5% male) with complete data. LCPA, derived from a physical activity history questionnaire by ranking participants' sequences of reported activity intensity, starting with the current decade and ending with the response for physical activity during adolescence, was statistically significantly associated with kPCr ($p < .01$) in multivariable linear regression models adjusted for demographic characteristics and indicators of current physical activity. The positive association between LCPA and kPCr independent of current physical activity suggests that mitochondrial oxidative capacity in later life may have a cumulative dimension. Our observations demonstrate that even limited information on prior activity may be informative for understanding mitochondrial variability and highlight the importance of exercise across the life course.

Abstract citation ID: igae098.3494

### THE ASSOCIATION OF PHYSICAL ACTIVITY AND MORTALITY AMONG RECENTLY HOSPITALIZED OLDER ADULTS WITH DEMENTIA

Brittany Burch, and Barbara Resnick, *University of Maryland Baltimore, Baltimore, Maryland, United States*

Even though the period following hospitalization is a critical time for physical activity, recently hospitalized older adults with dementia spend 83.3% of their days sedentary. Filling a research gap, this study aims to test the hypothesis that greater amounts of physical activity at one month post discharge is associated with lower mortality over the first year post discharge. We obtained the variable of "physical activity one month post discharge" through application of the MotionWatch8 accelerometer for 24 hours between 30-60 days after hospital discharge. We obtained the variable of "mortality status over one year post discharge" through care partners' report of participant death or alive status at 6 months and 1 year post hospital discharge. We performed simple logistic regression and multiple logistic regression, controlling for comorbidities, age, and cognitive status. Among 42 recently discharged older adults with dementia, we found that participants' amount of time spent in physical activity one month post discharge was not statistically significantly associated with mortality within one year post discharge (Simple Logistic Regression: OR=.996, CI=.992,1.000; p=.053; Multiple Logistic Regression: OR=.996, CI=.992,1.001; p=.095). However, we did observe a strong trend. In particular, the data suggests that as a recently discharged person with dementia increases their time spent in physical activity by 10 daily minutes, the odds that they will die over the next year decreases by 4%. This demonstrates the critical importance of physical activity following hospital discharge among older adults with dementia and the need to test this association in a larger sample.

Abstract citation ID: igae098.3495
**THE EFFECTS OF EXERCISE ON BRAIN FUNCTIONAL CONNECTIVITY IN ADULTS AGED 55–65 EXPOSED TO EXPERIMENTAL BED REST**
Guilherme Balbim[1], Chun-Liang Hsu[2], Daria Tai[1], Simon Duchesne[3], Kenneth Madden[1], and Teresa Liu-Ambrose[1], *1. University of British Columbia, Vancouver, British Columbia, Canada*, *2. The Hong Kong Polytechnic University, Hong Kong, Hong Kong*, *3. Université Laval, Quebec, Quebec, Canada*

Bed rest can occur because of chronic conditions, injury, or hospitalization and initiate a downward spiral of deleterious changes in several body systems. Exercise is a countermeasure to many of these changes. However, its impact on middle-aged and older adults' brain functional connectivity (FC) during bed rest remains unexplored. We examined the effects of exercise on brain resting-state FC in adults exposed to 14 days of experimental 6° head-down bed rest (HDBR). We conducted a non-blinded, parallel-group, randomized controlled trial with 23 healthy adults aged 55-65. Participants were randomized to either 14 days of HDBR (CON) or 14 days of HDBR with daily aerobic and strength exercise training (EX). At baseline and HDBR completion, participants underwent brain structural and resting-state functional magnetic resonance (MRI). Resting-state MRI preprocessing was performed using the FMRIPREP version 20.2.3. We extracted preprocessed resting-state time-series from a priori networks of interest based on Yeo's 7-network parcellation. We conducted complete-case data analysis (NEX=11; NCON=8). Analysis of covariance explored EX effects on resting-state FC after controlling for baseline resting-state FC and baseline physical activity assessed by the Physical Activity Scale for the Elderly. Changes in EX resting-state FC within- and between- the Visual, Sensorimotor, Dorsal Attention, Ventral Attention, Fronto-Executive, Frontoparietal, and Default Mode Networks were not statistically significant compared with CON (all ps > 0.05). Exposure to 14 days of experimental HDBR or 14 days of daily aerobic and strength exercise training in HDBR did not elicit changes in brain resting-state FC of healthy middle-aged and older adults.

Abstract citation ID: igae098.3496
**THE ROLE OF AGE AND EXERCISE HISTORY IN AFFECTIVE RESPONSE TO ACUTE EXERCISE: PRELIMINARY RESULTS**
Marta Stojanovic, Baylie Rushing, Madeleine Love, John Morris, and Denise Head, *Washington University in St. Louis, St. Louis, Missouri, United States*

A more positive affective response to acute exercise has been linked to greater physical activity levels. Older adults might experience increased physiological symptoms (e.g., shallow breathing) during acute exercise and subsequently less positive affect post-exercise. Additionally, sedentary individuals were shown to have a more negative affective response. The goals of the study were to examine whether age and exercise history impact the affective response to exercise and its association with overall physical activity levels. Cognitively normal individuals from the Knight ADRC Adult Children Study (n=65; age range 44-85 years) completed an acute bout of moderate-intensity exercise. Affect was measured at baseline, immediately after, 15 minutes, and 30 minutes post-exercise. Participants also completed one week of actigraphy, which provided an objective estimate of their physical activity levels, and a self-report questionnaire about their exercise history over the past 10 years. While positive affect increased immediately post-exercise similarly across age (p=0.103), older individuals showed a quicker return to baseline (p=0.012). Age did not moderate the association between affect and physical activity (p>0.119). Exercise history did not predict affective response (p's>0.847), however, individuals with greater exercise history showed a stronger association between a more positive affective response and greater physical activity levels (p< 0.001). These results suggest that older individuals might experience a less positive affective response to moderate-intensity exercise. Furthermore, exercise history might be a relevant moderator of the link between affective response and physical activity. Future studies should use a larger sample size and consider additional moderators, including cardiovascular health and sleep.

Abstract citation ID: igae098.3497
**USING QUALITATIVE METHODS TO CULTURALLY ADAPT A COMMUNITY-ENGAGED PHYSICAL ACTIVITY INTERVENTION FOR LATINOS**
Zvinka Zlatar[1], Lazaro Martinez Lujan[2], Mikael Anne Greenwood-Hickman[2], Julie Cooper[3], Stefani Florez Acevedo[4], Andrea-Paula Vargas[1], and Dori Rosenberg[2], *1. University of California San Diego, La Jolla, California, United States*, *2. Kaiser Permanente Washington Health Research Institute, Seattle, Washington, United States*, *3. Kaiser Permanente Washington Health Research Institute, Kaiser Permanente Washington Health Research Institute, Washington, United States*, *4. University of Washington, Seattle, Washington, United States*

Hispanics/Latinos are at increased risk of developing Alzheimer's Disease and related dementias (ADRD), but culturally adapted interventions to prevent ADRD in this high-risk population are scarce. We aimed to develop a community-engaged physical activity (PA) intervention to lower ADRD risk among middle-aged and older Hispanic/Latino adults through qualitative feedback from a community advisory board (CAB) and pilot study participants. Ten Hispanic/Latino community members (average age = 67.2, 90% female, 90% Spanish-speaking) participated in a 3-week pre-post remote PA study. Participants received a Fitbit and two health coaching calls from a bilingual/bicultural health coach.

Qualitative data were collected through two focus groups with pilot participants and two CAB meetings (seven members) to inform the culturally adapted intervention. Thematic analysis of transcribed and translated focus groups was conducted. Themes from notes taken at CAB meetings were also compiled. Focus group data themes highlighted that the intervention was well-received, with participants recognizing the cognitive benefits of PA engagement, the significance of prioritizing time to oneself, an increased sense of commitment, and identifying the Fitbit as an effective tool as facilitators. Barriers to engagement included life obligations, ingrained habits, adverse weather, and challenges related to physical and mental health factors. The CAB helped to design the study logo and recruitment materials while pilot participants identified a desire for group activities and face-to-face interaction with study staff. Participant and CAB feedback was instrumental in the cultural adaptation process to refine the intervention. Community-engaged approaches are crucial for ADRD prevention in high-risk Hispanic/Latino older adults.

### SESSION 7725 (POSTER)

### PSYCHOSOCIAL WELL-BEING

Abstract citation ID: igae098.3498

### AGE-RELATED DIFFERENCES IN EVERYDAY SOLITUDE SEEKING WHEN TIME WITH OTHERS FEELS LESS DESIRABLE

Yoonseok Choi[1], Hansol Kim[2], and Christiane Hoppmann[3], *1. University of British Columbia, Vancouver, British Columbia, Canada*, *2. Sogang University, Seoul, Republic of Korea*, *3. The University of British Columbia, Vancouver, British Columbia, Canada*

People spend a significant amount of time by themselves. This project seeks to investigate one potential motive underlying solitude seeking in daily life, namely, to disengage from undesirable social interactions. Social relationships and interactions often involve obligations to others, particularly so in collectivistic cultures; meeting obligations can be taxing when social demands exceed one's capacity. Seeking solitude may be a way to reduce obligation-related social strains. Additionally, there may be age-related differences in motive strength considering age-related differences in social relationship structures, with higher obligatory demands placed on younger adults (e.g., work, childcare). To examine these ideas, we used 610 repeated daily life assessments (2 times per day for 10 days) collected from an adult lifespan sample of 51 Koreans (Age range: 22-71 years; 65% female, 98% some postsecondary education). Everyday assessments of preference for solitude were predicted by the extent to which previous-day social interactions were deemed voluntary and by their interaction with age. Results based on multilevel modeling showed that while previous-day reductions in voluntary social interaction ratings were not significantly associated with next-day increases in preference for solitude, those who reported generally lower levels of voluntary social interactions showed generally higher preferences for solitude (OR = 0.70, p =.001). Age moderation was significant in the predicted direction with younger age being associated with higher preference for solitude from lower voluntary social interactions (OR = 1.02, p =.002). These results highlight a possibly restorative function of solitude that plays out differently across the adult lifespan.

Abstract citation ID: igae098.3499

### ARE LIFE ACCOMPLISHMENTS RELATED TO WELL-BEING: FINDINGS FROM THE HEALTH AND RETIREMENT STUDY (HRS)?

Marina Larkina, and Jacqui Smith, *University of Michigan, Ann Arbor, Michigan, United States*

Butler (1963) proposed that engaging in a whole life review is especially beneficial for older adults because it promotes a sense of self-worth. Previous research highlights links between reminiscing and psychological well-being, however, life review narratives are rarely collected in the context of large, population-based aging studies. This study uses data from the HRS Life History Mail Survey (2017, 2019) to 1) examine factors that predict older adults (N=12,815; Mage= 68 years; range 50-101) willingness to share their life's most important accomplishments (up to three); and 2) establish relations between content and complexity (content variety) of reported accomplishments and participants' well-being. Participants who reported their accomplishments (59%), compared to those who didn't, were more likely to be better educated, currently married, wealthier, have better memory and fewer depressive symptoms. Importantly, they also were more likely to perceive their life-as-a-whole as either completely or very satisfied (OR=1.315, CI=1.214-1.425, p<.001). Multinomial analysis of content and complexity of reported accomplishments revealed that people with higher life satisfaction were more likely to report relationship-related accomplishments (47%; family, friendship, or caring for others) compared to those who were most proud of their societal achievement (35%; education, work, military services), adjusted RRR=1.207, CI=1.061-1.372, p<.005. People, who wrote about personal qualities (e.g., their faith, health) did not differ from other two groups. The complexity of reported accomplishments was not associated with life satisfaction but was related to respondents' education and memory. Our findings support the proposal that reviewing one's life accomplishments enhances well-being in later life.

Abstract citation ID: igae098.3500

### DOES THE SENSE OF MATTERING BUFFER DEPRESSIVE SYMPTOMS? EVIDENCE FROM THE CHAT PROGRAM SURVEY

Mengzhao Yan, Sheila Salinas Navarro, Sindy Lomeli, and Kathleen Wilber, *University of Southern California, Los Angeles, California, United States*

Sense of mattering refers to a feeling of being valued and important to others. Recognized as a unique construct and a form of psychological capital, mattering has profound implications on health and well-being. Previous research has demonstrated the protective effect of high level of mattering against mental health issues. However, this construct remains understudied in older adults. Employing the baseline survey (N = 2,385, average age = 71) of the "Connecting, Health, Aging, & Technology (CHAT) Program," an initiative to bridge the digital divide for underserved community-dwelling older adults launched by the California Department of Aging, we examined the association between sense of mattering and depressive symptoms. Older adult participants identified by local area agencies on aging, were assessed and screened into the program. The relationship between participants' scores on mattering (range 4 to 20) and having depressive symptoms was examined using multivariate logistic regression. After controlling for sociodemographic factors (i.e., age, gender, race/ethnicity, education, marital status), self-rated health, and anxiety, each unit higher level of mattering was associated with 12% lower odds of having depressive symptoms (OR = 0.88, 95% CI: 0.85 – 0.92). The five-item general mattering scale (Marcus, 1991) showed adequate internal consistency in older adults (Cronbach's $\alpha$ = 0.80). Our findings highlight the importance of mattering to mental health. We recommend that future policies and programs aimed at improving older adults' health and well-being (e.g., age-friendly movements, combating ageism, interventions) take boosting sense of mattering into account.

Abstract citation ID: igae098.3501

### FUNCTIONAL LIMITATIONS AND WELL-BEING THROUGHOUT THE ADULT LIFESPAN: THE ROLE OF PERCEIVED BURDENSOMENESS

Claire Williams, and Natalie Dautovich, *Virginia Commonwealth University, Richmond, Virginia, United States*

Functional limitations represent individuals' difficulty with completing essential activities of daily living. Though functional limitations have been linked to poorer well-being outcomes, less is known about potential protective factors for well-being in the lived experience of functional limitations. This study aimed to examine the potential moderating effect of perceived burdensomeness on the association between functional limitations and life satisfaction, a common indicator of well-being, across the adult lifespan. The present study used archival data from 696 individuals who participated in the Midlife in the United States Refresher study. Participants' perceived burdensomeness significantly moderated the association between functional limitation status and life satisfaction (B = 0.18, p =.0486) after controlling for gender identity (B = 0.003, p = 0.9461) and racial identity (B = 0.03, p = 0.0724). The negative association between functional limitations and life satisfaction was stronger for those with a higher degree of perceived burden. Interestingly, age did not emerge as a significant secondary moderator in this model. Therefore, it seems that the buffering effect of a low level of perceived burden may be salient across age groups. The current study provides support for the importance of perceived burdensomeness in the lived experience of functional limitations across the adult lifespan. In the future, clinicians should consider integrating mental health screeners in healthcare settings in order to identify at-risk individuals who are experiencing functional limitations, and potentially consider establishing preventative, education-based interventions concerning sleep in the experience of functional limitations.

Abstract citation ID: igae098.3502

### INTERGENERATIONAL QUALITY OF LIFE: SIMILARITIES AND DIFFERENCES AMONG MUSICIANS

Whitney Nesser[1], and Scott Snyder[2], *1. Indiana State University, Terre Haute, Indiana, United States*, *2. University of Alabama at Birmingham, Birmingham, Alabama, United States*

Music has been shown to improve Quality of Life (QoL) for various populations. However, few studies have studied older musicians' QoL or QoL in an intergenerational sample of musicians. To address this gap this study assessed four QoL domains (physical, psychological, social, and environmental) in a nationally representative intergenerational sample of musicians using the WHOQOL-BREF scale. Data were obtained via a survey of professional and amateur musicians (N=407) across the United States (US). Results demonstrated that while the oldest subgroup of musicians had the highest mean scores for environmental, psychological and social QoL, there were no significant differences between age groups for any of the QoL domains. Rating scale responses for all QoL items were independent of age group except for "How often do you have negative feelings" (p=.01). Examination of standardized residual showed that significant effect was due to the older musicians responding "never" more frequently than expected. The Spearman correlations of environmental QoL with psychological, and social QoL were significantly lower (p <.05) in the older musicians (r=.442, r=.494) than in the aggregate of other age groups (r=.715, r=.733). Furthermore, there was a significantly lower Spearman correlation between "enjoyment of being a musician" and "impact of being a musician on QoL" for older respondents (r=.384) compared to younger respondents (r=.678). Future research is needed to establish normative QoL data for the US on all age groups as measured by the WHOQOL-BREF and to examine the role of music as a mediator of QoL across the lifespan.

Abstract citation ID: igae098.3503

### LONGITUDINAL ASSOCIATIONS BETWEEN LONELINESS AND DEPRESSIVE SYMPTOMATOLOGY IN THE OLDEST-OLD

Morgan Lynch, and Christopher Beam, *University of Southern California, Los Angeles, California, United States*

Prior research suggests that loneliness is a precursor for depressive symptomatology (hereafter "depression") in older adults (Cacioppo et al. 2010). Yet, whether their findings extend to the oldest-old is an open question. Moreover, recent work suggests that loneliness does not predict depression after accounting for trait-level components of each variable (Griffin et al., 2022). In the current study, we model five waves of loneliness and depression scores, measured two years apart, from 702 pairs of Swedish twins 80+ years of age from the Origin of Variances in the Oldest-Old study (OCTO). We use a Trait-State-Error (TSE) model to estimate trait, correlated time-specific, and uncorrelated time-specific components of loneliness and depression in the oldest-old. Lag-1 phenotypic correlations between loneliness and depression were small to moderate (rs:.23-.42). Univariate models were first compared; a State Error (SE) model best fit both variables. Model comparison tests indicated between-person differences (i.e., trait-like components) were not significant for either loneliness or depression. In a bivariate SE model, we found bidirectional cross-trait effects; depression positively predicted loneliness (b = 0.19, 95% CI [0.03,

0.46]), and loneliness negatively predicted depression after accounting for within-timepoint correlations (b = -0.33, 95% CI [-0.61, -0.09]). Within-variable autoregressive components were also significant; higher scores at the previous timepoint predicted an increase in loneliness (b = 0.91, 95% CI [0.84, 0.96]) and depression (b = 1.19, 95% CI [1.11, 1.27]). Findings suggest that in the oldest-old, loneliness does not presage depression as it does in young-old, alternatively, depression may precede loneliness.

Abstract citation ID: igae098.3504

### MOMENTARY SOLITUDE AND WELL-BEING ACROSS ADULTHOOD: THE MODERATING ROLE OF TRAIT MINDFULNESS

Xinyuan Peng[1], Da Jiang[1], Dwight Tse[2], Yuen Wan Ho[3], and Jennifer Lay[4], *1. The Education University of Hong Kong, Hong Kong, China (People's Republic)*, *2. University of Strathclyde, Glasgow, Scotland, United Kingdom*, *3. Lingnan University, Hong Kong, Guangdong, China*, *4. University of Exeter, Exeter, England, United Kingdom*

Solitude (an objective state of being alone without social contact) evokes positive and negative experiences. People spend more time alone as they age. Little is known about factors influencing affective experiences in solitude. Trait mindfulness facilitates emotion regulation in various social contexts. The person-environment fit theory posits that individuals enjoy better well-being in environments aligning with their traits. Mindfulness emphasizes self-awareness and stress reduction, which seems consistent with the nature of solitude. Thus, we aimed to investigate whether trait mindfulness would moderate the relationship between solitude and affective well-being in a lifespan sample. An experience sampling study (N = 191; Mage = 51.49, SDage = 17.70, range = 19-93, 66% women) was conducted. Participants completed smartphone-based assessments measuring momentary social situations (in solitude vs. not) and affective experiences five times per day for seven consecutive days. High- and low-arousal positive (PA) and negative affect (NA) were assessed with nine items. Five facets of trait mindfulness were examined as moderators. Results revealed that mindfulness facets played protective roles in maintaining well-being in solitude. Higher levels of "describing" and "acting with awareness" weakened the negative association between solitude and high-arousal PA. A higher level of "nonreactivity to inner experiences" amplified the positive association between solitude and low-arousal PA. A higher level of "observing" weakened the positive association between solitude and low-arousal NA. Age did not moderate these relationships. These findings enhance understanding of affective well-being in solitude across adulthood by highlighting the roles of different facets of mindfulness.

Abstract citation ID: igae098.3505

### OLDER ADULTS' INTERACTIONS ON SHORT-FORM VIDEO PLATFORMS ENHANCE THEIR SOCIAL ADAPTATION: A CHAIN MEDIATION MODEL

Jingxuan Wu, and Huamao Peng, *Beijing Normal University, Beijing, Beijing, China (People's Republic)*

**Background:** An increasing number of older adults are embracing short-form video platforms like TikTok. Beyond passive watching, how active engagement by older adults on short-form video platforms influences their social adaptation deserves significant attention. Objective: Explore the effect of interactions on short-form video platforms (e.g., liking, commenting, sharing, and rewarding, etc.) on social adaptation among older adults and examine the chain mediating roles of basic psychological needs satisfaction (competence, autonomy, and relatedness) and attitudes towards self ageing to reveal the underlying psychological mechanism.

**Method:** Two thousand older adults (aged 55–85 years old, Mage = 60.20±3.88) completed the Social Adaptation Self-evaluation Scale, the Short-form Video Interactive Behavior Questionnaire, the Basic Psychological Needs Scale, and the Attitudes to Ageing Questionnaire. PROCESS Model 6 was used to test a chain multiple mediation model.

**Results:** Frequent short-form video interactions of older adults not only directly influence their social adaptation level positively but also indirectly contribute to its improvement through more satisfaction of basic psychological needs and greater positive attitudes towards self ageing.

**Discussion:** More interactions on short-form videos platforms contribute to the satisfaction of older adults' basic psychological needs. This, in turn, encourages a comparatively positive attitude towards ageing, and ultimately bolstering adaptability across various aspects of later life. This insight motivates older adults to immerse themselves more deeply and actively participate in short-form videos, utilizing the new media application as an emerging space for social engagement, thereby accumulating psychosocial well-being to achieve active and successful aging.

Abstract citation ID: igae098.3506

### PROMOTING PURPOSEFUL RETIREMENT IN NORTH DAKOTA AND SOUTH DAKOTA

Leacey Brown[1], and Jane Strommen[2], *1. South Dakota State University Extension, Rapid City, South Dakota, United States*, *2. North Dakota State University Extension, Fargo, North Dakota, United States*

A sense of purpose is correlated with a variety of outcomes in older age. Outcomes associated with purpose include mortality, longevity, psychological health, dementia/memory, physical health, and health promotion. Yet, many adults may not view older age and retirement as a time to live with purpose. As a result, educational programs are needed to help adults learn how they might incorporate purpose into their retirement planning. As the land-grant universities in their respective states, North Dakota State University and South Dakota State University are well positioned to fill this void. Both organizations are tasked with providing education and outreach on a variety of topics for individuals and families. For this reason, they came together to develop the Purposeful Retirement Book Club. This program was first piloted in 2020 and has been offered five times, reaching 81 adults. This poster will describe the value of including purpose in retirement planning, discuss evaluation results and lessons learn piloting the program, as well as strategies other organizations might use to implement this program in their community. Participants will also learn about the facilitator guide that was developed following the pilot of the program. This guide is available for anyone interested in offering the program.

Abstract citation ID: igae098.3507

**PSYCHOLOGICAL RESILIENCE AMONG OLDER ADULTS WITH TRAUMATIC BRAIN INJURY: A CONCEPT ANALYSIS**

Premgamon Kuntajak, *University of Washington, Seattle, Washington, United States*

**Aim:** To examine the concept of psychological resilience among older adults with Traumatic Brain Injury (TBI).

**Background:** Psychological resilience fosters adaptation after TBI, serving as a crucial indicator of recovery and success in rehabilitation. Despite being recognised for over decades, resilience remains a poorly defined concept within the realm of TBI. Thus, there is an urgent need for further exploration of psychological resilience within TBI to enhance the understanding and clarify the meaning of psychological resilience and its significance to older adults with TBI.

**Method:** Walker and Avant's modified method of concept analysis was used to guide this analysis. Literature from multiple scholarly databases was identified keywords: "Resilience," "Psychological Adaptation," "Hardiness," and "Psychological Well-Being," which were imputed using established inclusion criteria: full text, peer-reviewed, and printed in English from 2013-2023. Eligible articles were examined to determine the context of resilience and to comprehend and analyse its attributes, antecedents, and consequences. Outcomes: Antecedents of psychological resilience include exposure to adverse events and a social support system. Two principal attributes consisted of personal traits and the adaptation process. Psychological resilience facilitates improved physiological and psychological health outcomes, enhances recovery, and decreases complications among older adults with TBI.

**Conclusion:** Research in TBI is transitioning from a deficit-oriented perspective to a strengths-based approach. A firm knowledge of psychological resilience from this analysis offers valuable insights that can guide nursing interventions, inform the application of theory, influence policymaking, and drive research possibilities aimed at improving health outcomes for older adults with TBI and their families.

Abstract citation ID: igae098.3508

**THE PSYCHOSOCIAL EFFECTS OF EXPRESSIVE ARTS THERAPY ON PATIENTS WITH AGE-RELATED MACULAR DEGENERATION**

Rainbow Tin Hung Ho[1], Temmy L. T. Lo[1], Wai Chi Chan[1], Allen M. Y. Cheong[2], Qing Li[3], and Adrian H. Y. Wan[1], *1. The University of Hong Kong, Hong Kong, China (People's Republic)*, *2. The Hong Kong Polytechnic University, Hong Kong, Hong Kong*, *3. Grantham Hospital, Grantham Hospital, Hong Kong*

Age-related macular degeneration (AMD) is the leading cause of central vision loss in older adults. With irreversible vision loss, patients with AMD also experience psychosocial distress, which is given less attention. This study aims to evaluate the psychosocial effects of Expressive arts therapy (EXAT) on patients with AMD. 33 participants aged 51–74 were randomized into the EXAT (N=20) and control (N=13) group. Participants in the EXAT group received eight weekly 1.5-hour sessions, whereas participants in the control group maintained their usual routine. The psychosocial condition of participants was assessed at baseline (T0) and post-intervention for the EXAT group or 8-week follow-up for the control group (T1). Mann-Whitney U Tests revealed that participants in the EXAT group had significantly higher social support and quality of life in mental health than those in the control group at T0 (Z = -3.231 - -2.093). It also showed that the EXAT group had significantly higher resilience, depressive symptoms, psychosocial adaptation, social support, and quality of life in the ocular pain aspect compared to the control group at T1 (Z = -2.838 - -2.157). Wilcoxon Signed-Ranks Tests found significant improvement in the EXAT group in resilience, psychosocial adaptation in anxiety/depression aspect, and quality of life in role difficulties aspect (Z = -2.255 - -2.017), with no significant changes in the control group across the two times. The results demonstrated that EXAT can potentially enhance patients' psychosocial well-being with AMD. A larger sample size and follow-up assessments are needed for further investigation.

Abstract citation ID: igae098.3509

**USING THE INTERPRETIVE FRAMEWORK TO CREATE AND DELIVER MEANINGFUL GROUP ACTIVITIES FOR OLDER ADULTS**

Anna Tulloh[1], Megan Westmore[2], Keith Anderson[1], and Rebecca Mauldin[3], *1. University of Mississippi, Oxford, Mississippi, United States*, *2. The University of Texas Arlington, Arlington, Texas, United States*, *3. University of Texas at Arlington, Arlington, Texas, United States*

Activity programming is recognized as a vital component in supporting engagement and improving quality of life for older adults. Despite the ubiquity of such programs and a plethora of research exploring both why meaningful activities are important for older adults and what makes an activity meaningful, current guidelines for activity development fail to offer a standardized framework. Without a clear framework for developing meaningful group activities, a potential for inconsistencies in activity appeal, quality, and effectiveness exists. This paper suggests an innovative solution by drawing on the field of interpretation—a discipline traditionally associated with environmental, historical, and cultural education. Interpretation provides principles and practical guidance for creating engaging, relevant, enjoyable, and personalized experiences for a wide range of audiences. In this paper, we present a brief history of interpretation and describe its principles, applications, and the "interpretative equation." Using data from field notes collected during a study of an online nature-based group activity in an assisted living community, we describe how the program was developed, implemented and adapted in alignment with the elements of the interpretive equation. This equation combines activity-related knowledge (e.g. gaining a deeper understanding of the environment presented online), an understanding of participants (e.g. increasing knowledge of individual's dementia-related support needs), and appropriate techniques

(e.g., incorporating reminiscence) to achieve an interpretive "opportunity" (i.e., a meaningful activity in our formulation). Our results demonstrate how interpretation can be a powerful tool in bridging the gap between the theory and practice of meaningful activity development.

### SESSION 7730 (POSTER)

### QUALITY MEASUREMENT/IMPROVEMENT

Abstract citation ID: igae098.3510

### FLOURISHCARE MODEL OF INTEGRATED CARE: THE VALIDATION OF THE FLOURISH INDEX-REVISED (FI-R)

Anna Faul[1], Samantha Cotton[1], Pamela Yankeelov[2], and Barbara Gordon[2], *1. University of Louisville, Louisville, Kentucky, United States*, *2. University of Louisvlle, Louisville, Kentucky, United States*

The FlourishCare (FC) Model for geriatric primary care brings professional disciplines across the continuum of care together to provide quality integrated age-friendly healthcare to older adults with multiple chronic conditions (MCCs). This integrated care model is backed by research demonstrating the efficacy of a comprehensive approach to healthcare by ensuring that services are well-coordinated across the entire spectrum of a person's health needs. This study validates the Flourish Index–Revised (FI-R), a tool evaluating integrated care models. The FI-R uses 25 quality-of-care indicators and 7 contextual community indicators. The FI-R was validated with Categorial Principal Components Analysis (CATPCA) using a sample of 949 patients 50+ who were mostly female (73%), Non-Hispanic White (70%), living in urban areas (90%), and married (29%), single (22%) or divorced (19%). The mean age was 73.46 (SD=10.86) and mean years of education was 14.30 (SD=2.14). CATPCA showed a four-dimensional structure of biological, psychological, and two social determinants of health (SDOH) subdomains: health behaviors and community. Final selection of indicators was based on total variance accounted for >0.30, absolute values of item loadings >0.45, and not having cross loadings >0.45 on two factors. Internal consistency (Cronbach Alpha) for the determinants were: biological=0.75, psychological=0.76, SDOH: community=0.70, SDOH: Health Behaviors=0.50 and total FI-R=0.95. Sensitivity to change was shown for the total FI-R, psychological determinants, and SDOH:health behaviors but not for biological determinants. The validation of the FI-R shows promise for its usability to evaluate integrated health care models using existing measures in electronic health systems.

Abstract citation ID: igae098.3511

### GENDER DIFFERENCES IN YEARS OF GOOD LIFE AMONG OLDER ADULTS IN INDIAN SUBNATIONAL LEVEL POPULATION

Ruchira Chakraborty, *International Institute for Population Sciences, Mumbai, Maharashtra, India*

The study investigates the heterogeneities in healthy life among older-adults in India, using a novel concept of counting 'expected years of good life'. Grounded in Amartya Sen's 'capable longevity' approach, the index of YoGL (Years of Good Life) is an amalgamation of objective and subjective dimensions of health. A year is counted as 'good' if a person is simultaneously out of poverty, free from severe activity limitations and cognitive impairments, and experiences positive life satisfaction. After attaining a certain age, remaining good life years within life expectancy are computed by employing Sullivan's method using Longitudinal Ageing study in India,2017-18 (LASI wave-I) dataset. The results show, at age 50, YoGL for male is 13.9 years (55.6% of remaining life expectancy) and for females, 11.3 years (41.7% of remaining life expectancy). Supporting the 'gender-health-mortality paradox', results reveal that women's advantage in life expectancy doesn't translate into equal advantage in 'good-years' for all older age-groups. Inter-state variability is higher in terms of YoGL, than in life expectancy; from 64% years counts as 'good life' in Punjab to 34% of remaining years as 'good life' in Odisha. Regression decomposition indicates functional limitations contributes 32%, cognitive impairment 20% and birth-cohorts contributes around 18% in explaining the regional heterogeneity.

Abstract citation ID: igae098.3512

### HOW FRONTLINE STAFF PERCEIVE AND REDUCE GAPS IN KNOWLEDGE AND PRACTICE IN LOCALLY INITIATED QUALITY IMPROVEMENT

B Josea Kramer, and Joseph Douglas, *VA Greater Los Angeles Healthcare System, Los Angeles, California, United States*

We evaluated local quality improvement projects that were conducted as part of continuing professional development (CPD) for disciplines that support primary care in Veterans Health Affairs (VA) clinics and medical centers with the goal of understanding how the workforce perceives areas for improvement. The VA Geriatric Scholars Program (GSP) provides core training in its longitudinal CPD through intensive didactic courses in geriatrics, interactive workshop in quality improvement (QI) and coaching to implement a local QI project. Unlike most CPD that includes a QI component in focused training on a specific new clinical intervention, participants in GSP had wide latitude to determine the change to be implemented to enhance healthcare for older Veterans. Using a qualitative approach and applying thematic analysis, we analyzed 740 QI projects at 468 VA facilities undertaken by 809 GSP participants. Clinician-initiated projects indicated the workforce perception of gaps in primary care knowledge and prior training of its interdisciplinary workforce to provide care for older adults in the choice of clinically oriented projects, such as reducing potentially inappropriate medications or instituting fall prevention activities. Other projects focused on enhancing processes within the healthcare system, such as timely lab reports or referral protocols. Our results indicate that educational needs assessments can be conducted at the interprofessional team level, identifying the needs for CPD that are perceived as relevant by the workforce.

Abstract citation ID: igae098.3513

### TWO-LEVEL CONFIRMATORY FACTOR ANALYSIS OF THE POSITIVE AND NEGATIVE AFFECT SCHEDULE (PANAS) IN MID- AND LATE LIFE

Madeline Nichols, and John Geldhof, *Oregon State University, Corvallis, Oregon, United States*

The Positive and Negative Affect Schedule (PANAS; Watson et al., 1988) is widely used in the study of well-being; however, its factor structure continues to be a source of debate (Wedderhoff et al., 2021). The present study examined the factor structure of the PANAS using data from the third wave of the National Study of Daily Experiences (NSDE; N=1176, Mage=62.48, SD=10.20, Range=43-91, %Female=57.38) in which participants indicated how much of the time they felt positive and negative emotions each day for eight days. We conducted two-level confirmatory factor analyses in MPlus informed by the 45-degree rotation hypothesis (Yik et al., 1999) that classifies emotion words by both valence (negative or positive) and activation (high or low). Results revealed that a five-factor structure at both the within and between levels exhibited good fit ($\chi2(188) = 989.76$; $p < .001$; RMSEA = 0.02; CFI = 0.960; TLI = 0.949). These factors were: high activation negative (upset, angry, frustrated); negative (worthless, sad, hopeless, lonely); high activation positive (enthusiastic, attentive, proud, active, confident); positive (happy, cheerful); and low activation positive (calm, satisfied). Latent correlations indicated factor separation at both the within- and between-person levels (median absolute correlations of.44 and.60, respectively). These results emphasize the importance of considering both valence and activation in the study of daily affective well-being. The authors recommend that researchers using the PANAS consider the implications of valence and activation when examining associations between affect and health-related outcomes.

Abstract citation ID: igae098.3514

### VETERAN- AND CAREGIVER-REPORTED FACTORS IMPACTING VETERAN-DIRECTED CARE (VDC) PROGRAM ENROLLMENT AND EXPANSION

Wendy Hathaway[1], Pranjal Tyagi[2], Erin Bouldin[3], Samer Nasr[4], and Stuti Dang[5], *1. VA Providence Healthcare System, Tampa, Florida, United States*, *2. South Florida Veteran Affairs Foundation for Research and Education (SFVAFRE), Miami, Florida, United States*, *3. University of Utah, Salt Lake City, Utah, United States*, *4. Veterans Integrated Service Network (VISN) 8. , St. Petersburg, Florida, United States*, *5. Miami VA Healthcare System, Miami, Florida, United States*

The Department of Veterans Affairs (VA) is increasing access to Home- and Community-Based Services (HCBS) as an alternative to long-term institutional care (LTIC). Veteran Directed Care (VDC) is a self-directed HCBS program that empowers veterans at risk of LTIC to hire their own care providers. The VA is increasing VDC access by expanding the program to all VA medical centers (VAMCs) nationally. As part of a multi-aim and mixed-method evaluation project examining barriers and facilitators to expanding VDC, qualitative semi-structured interviews were conducted across seven VAMCs in Florida, South Georgia, and Puerto Rico with two groups of veterans who were referred to VDC: those that enrolled in the program (n=2) and those that declined (n=2). We also interviewed family caregivers of veterans in both groups (n=2 each). Interviews were conducted via Microsoft Teams from January-March 2024 and recorded with auto-transcription. Data were analyzed using rapid qualitative analysis techniques. Enrolled veterans and their caregivers pointed to several facilitators including the ease of enrollment; user-friendly management tools; compensation for care; role recognition; the ability to choose their caregiver and schedule; the reliability and trustworthiness of self-selected care providers; and relationships formed. Those who declined to enroll identified several barriers such as the program complexity; burdensome employee management tasks; fear of losing eligibility for other programs and services; and feeling the compensation was inadequate. Interviews are still ongoing. Understanding the veteran and caregiver perspectives on enrollment will inform strategies and mechanisms to support VA's goal of increasing VDC access and veteran participation.

### SESSION 7735 (POSTER)

### SLEEP

Abstract citation ID: igae098.3515

### ASSOCIATION OF SLEEP MEASURES WITH IMMUNE CELL PHENOTYPES IN THE FRAMINGHAM HEART STUDY

Ahmed Ragab[1], Jiachen Chen[2], Yumeng Cao[1], Margaret Doyle[3], Kathryn Lunetta[1], and Joanne Murabito[4], *1. Boston University, Boston, Massachusetts, United States*, *2. Boston University School of Public Health, Boston, Massachusetts, United States*, *3. University of Vermont Larner College of Medicine, Colchester, Vermont, United States*, *4. Chobanian and Avedisian School of Medicine, Boston University, Boston, Massachusetts, United States*

Sleep plays a significant role in regulating immune functions. Disordered sleep is linked to higher risk of infection and chronic inflammation. The relationship between sleep and the immune system is reciprocal, with each influencing the other. Adequate sleep is crucial for maintaining a balanced immune response and overall health. The associations between sleep measures and comprehensive immune cell phenotypes have not been well studied. Among Framingham Heart Study (FHS) Offspring participants who participated in the Sleep Study (1996-1998) and had immune cell data, we investigated five different sleep measures, average weighted sleeping hours per week, apnea hypopnea index with 3% oxygen desaturation, self-reported snoring >3 times a week, percentage of sleep time with oxygen saturation below 90% (TST90%) and the Epworth Sleepiness Scale (ESS) in relation to fourteen immune cell phenotypes. At exam 7 (1998-2001) using flow cytometry immune cells were profiled including T cells, B cells, NK cells, CD4+, CD8+, CD4+CD28-CD27-, CD8+CD28-CD27-, CD4+ TEMRA, CD8+ TEMRA, CD4+/CD8+ ratio, CD4+(TN/TM), CD8+ (TN/TM), CD8+ Tc17/Treg ratio and CD4+ Th17/Treg ratio. Linear Mixed Effects models were used for analysis with adjustment for covariates. Among 199 participants (mean age 62 years, range 42 to 83 years, 51% female), CD8+ TEMRA and CD8+CD28-CD27- were significantly associated with ESS and TST90% after adjusting for age, sex, CMV status, time difference between exams, family relationships, sleep medications and cardiovascular risk factors (Beta Coeffs:0.05, -0.02; FDRs=0.03, 0.03) respectively. We conclude that CD8+ TEMRA and CD8+CD28-CD27- are associated with daytime sleepiness and sleep-related hypoxemia. Further investigation is justified.

Abstract citation ID: igae098.3516

### ASSOCIATIONS BETWEEN SLEEP HYGIENE INDEX SCORE AND WHITE MATTER MICROSTRUCTURE

Lisa Weimar[1], Meina Zhang[1], Vincent Magnotta[1], Eric Axelson[2], Josh Cochran[1], Lauren Hopkins[1], and Chooza Moon[1], *1. University of Iowa, Iowa City, Iowa, United States*, *2. University of Iowa College of Medicine, Iowa City, Iowa, United States*

Sleep complaints are common among older adults. Sleep hygiene refers to practices that promote adequate sleep and may represent an interventional target for improving sleep quality. Individuals with Alzheimer's disease often exhibit white matter (WM) microstructure degenerations observable through diffusion tensor imaging (DTI). As people age, poor sleep quality can increase the risk of developing Alzheimer's disease and damage WM microstructures. However, few studies have investigated the relationship between sleep hygiene and the structural integrity of WM among older adults. Data was collected from 49 older adults (51% female) with mean age 70.4 years (SD = 6.50). Diffusion-weighted MRI sequences were acquired using a 3.0T scanner. Fractional anisotropy (FA) scalar maps were generated through FSL-DTIFIT, and the Johns Hopkins University-ICBM white matter atlas was utilized to assess FA values within WM tracts. Participants completed the Sleep Hygiene Index (SHI), which is a self-reported instrument. In this preliminary analysis, poorer sleep hygiene was associated with lower FA values in the bilateral cerebral peduncles and posterior limb internal capsules, right anterior limb internal capsule, and left uncinate fasciculus compared to those who have better sleep hygiene. This preliminary data suggests that among this small, homogenous sample poor sleep hygiene is associated with reduced WM integrity. Future studies should include larger samples, longitudinal designs, comprehensive sleep measures, and multimodal imaging methods to confirm these findings and identify the mechanisms underlying these associations.

Abstract citation ID: igae098.3517

### CONCEPT ANALYSIS: SLEEP QUALITY IN OLDER ADULTS

Suah Park[1], and Eeeseung Byun[2], *1. University of Washington, Seattle, Washington, United States*, *2. University of Washington School of Nursing, Seattle, Washington, United States*

Sleep quality is crucial in determining one's overall quality of life, particularly among older adults disproportionately affected by poor sleep. Despite its significance, there exists a gap in the analysis of the concept of sleep quality in older adults. This concept analysis was guided by Walker and Avant's method to conceptualize sleep quality in older adults comprehensively. A systematic search of studies published between 2018 and 2023 was conducted across databases, including CINAHL, EMBASE, PubMed, and Web of Science. Inclusion criteria encompassed quantitative and qualitative studies exploring sleep quality in older adults. The analysis identified key elements, such as defining attributes, antecedents, and consequences of sleep quality in older adults, based on the content of the articles. Fifty articles provided data for sleep quality in older adult conceptualization. Six defining attributes were identified: (a) number of awakenings, (b) sleep duration, (c) sleep disturbance, (d) sleep efficiency, (e) sleep latency, and (f) sleep satisfaction. Antecedents encompassed factors such as Diet, Physical activity, Circadian rhythm, Perceived health, Chronic disease, Psychosocial factors, Sociodemographic factors, and Environmental factors. Consequences included Fall, Daytime dysfunction, Physical health, Mental health, Mortality, and Quality of life. The bidirectional relationships between consequences and sleep quality indicated that some antecedents could be part of consequences and vice versa. This concept analysis presents a conceptual model of sleep quality in older adults based on defining attributes, antecedents, and consequences. The model provides a foundation for further testing and developing measures and interventions for addressing poor sleep quality in older adults.

Abstract citation ID: igae098.3518

### DAILY SLEEP AND EMOTION INERTIA IN LATE LIFE

Zexi Zhou, and Karen Fingerman, *The University of Texas Austin, Austin, Texas, United States*

Emotion inertia, conceptualized as the extent to which specific emotional states persist over time, is a crucial aspect of emotion dynamics associated with psychological well-being. This study examines the associations between sleep and emotion inertia in older adults' daily life. We used the ecological momentary assessment data over 5–6 days from the Daily Experiences and Well-being Study (N = 251, Mage = 73.7). Each morning, older adults indicated their sleep (i.e., duration, quality, disturbances) the prior night. At each three-hour assessment, they rated feelings of positive (e.g., loved, content) and negative (e.g., worried, lonely) emotion. They also reported depressive symptoms in the baseline interview. Multilevel models showed that higher sleep quality was associated with lower emotion inertia on days when older adults had lower averaged positive emotion or higher averaged negative emotion. In contrast, on days featuring higher averaged positive emotion or lower averaged negative emotion, high sleep quality was associated with increased emotion inertia. Furthermore, more overall hours of sleep predicted less emotion inertia across the study period only among older adults who had more depressive symptoms but not among those with fewer depressive symptoms. Findings suggest that good sleep may benefit older adults' everyday emotional well-being by maintaining good mood on days characterized by favorable emotional states, and facilitating susceptibility to change in mood on days with less favorable emotional states. Results also underscore the heightened protective role of good sleep in older adults experiencing more depressive symptoms.

Abstract citation ID: igae098.3519

### EDUCATION AS A MODERATOR IN THE ASSOCIATION OF SLEEP DURATION AND DEPRESSIVE SYMPTOMS

Wuyi Dong, Yan-Jhu Su, ChangPu Liang, and Dongfang Hong, *University of Massachusetts Boston, Boston, Massachusetts, United States*

Optimal sleep duration is critical for older adults' health and either too long or too short sleep duration potentially leads to mental disorders such as depression, anxiety, and distress. This study explores the association between sleep duration and depression among older adults, with a focus on whether education achievement buffers the association.

To create a pooled cross-sectional dataset, we combined data from the National Health and Nutrition Examination Survey (NHANES) of two consecutive waves (2015-16 and 2017-18). Our analysis includes a weighted sample of individuals aged 60 and older without missing responses (n=9,254). We measured sleep duration for three categories based on weekday reports: short sleep (< 7 hours), optimal sleep (7 to 9 hours), and long sleep (>=9 hours). Depression was evaluated using total scores from a 9-item Patient Health Questionnaire (PHQ; range: 0-27). Through multiple linear regression models—adjusting for demographics characteristics, self-reported health, health behaviors, and living arrangements, we found either people with short sleep duration or long sleep duration is associated with higher depression symptoms (β=1.28, p<.001 and β=1.24, p<.001, respectively), compared to optimal sleep duration individuals. The interaction effect showed that higher education levels (college or above) significantly decrease depression associated with longer sleep (β=-1.66, p<.05). These findings suggest that both insufficient sleep and excessive sleep increase the depression risks among older adults, while higher education may have protective effects against mental health challenges related to non-optimal sleep durations.

Abstract citation ID: igae098.3520

### EXAMINING SELF-REPORTED SLEEP QUALITY WITH COGNITIVE PERFORMANCE IN OLDER HISPANIC ADULTS: THE COMPADRE STUDY

Vanessa Young[1], Carlos Gaona[1], Luis Serrano-Rubio[1], Chen-Pin Wang[1], Tiffany Kautz[1], Jeffrey Kaye[2], Zachary Beattie[2], and Mitzi Gonzales[3], *1. The University of Texas Health Science Center San Antonio, San Antonio, Texas, United States*, *2. Oregon Health & Science University, Portland, Oregon, United States*, *3. Cedars-Sinai Medical Center, Los Angeles, California, United States*

Poor sleep quality has been associated with worse cognition performance. As sleep quality is multidimensional, examining separate factors captured by the PROMIS™ Sleep Disturbance 8b scale, insomnia, and sleep dissatisfaction, may provide greater insight into aspects most pertinent to cognitive health. Herein, we evaluated the associations between self-reported sleep quality, dissatisfaction, and insomnia with cognition in older Hispanic adults and examined whether self-reported physical activity modifies these associations. Participants completed self-report measures of sleep quality (PROMIS-Sleep Disturbance 8b) and physical activity (Rapid Assessment of Physical Activity). The National Alzheimer's Coordinating Center UDSv3 measured cognitive abilities. Associations between self-reported sleep with cognition were examined using linear regression models adjusted for age, sex, and education. Interactions with physical activity were evaluated. A total of 85 participants were included (mean age 69.9[SD 4.8] years; 72% Female; 100% Hispanic). Self-reported sleep quality, dissatisfaction and insomnia were not significantly associated with any cognitive outcomes (p>0.05) and there were no significant interactions with physical activity (p>0.05). Although self-reported sleep quality measures were not associated with cognitive performance in this exclusively Hispanic cohort, previous research using objective sleep measures has shown a connection. This highlights the need for objective assessments that capture various aspects of sleep, such as architecture, microarousals, and sleep-disordered breathing. Digital biomarkers of sleep and physical activity, currently underway in the COMPADRE study, may increase precision into their impact on cognitive health in this at-risk, underrepresented, population; reduce cultural bias in measurement and outcomes; and facilitate targeted interventions to promote healthy aging.

Abstract citation ID: igae098.3521

### EXPLORING THE ASSOCIATIONS BETWEEN DAILY SLEEP AND THE PITTSBURGH SLEEP QUALITY INDEX (PSQI) AMONG OLDER ADULTS

Hye Won Chai[1], Alyssa Gamaldo[1], Charlene Gamaldo[2], Abigail Stephan[1], Christine Phillips[3], and Lesley Ross[1], *1. Clemson University, Clemson, South Carolina, United States*, *2. Johns Hopkins Howard County Medical Center, Columbia, Maryland, United States*, *3. Clemson University Institute for Engaged Aging, Seneca, South Carolina, United States*

While the Pittsburgh Sleep Quality Index (PSQI) is widely used in sleep research, it is based on adults' retrospective memory which may be subject to recall bias. Daily measures of sleep using smartphones may supplement PSQI by providing a more ecologically valid snapshot of respondents' sleep patterns. However, there is a limited understanding about whether and how daily reports of sleep (over 14 days) map onto PSQI scores among older adults. This study aims to explore the associations between diverse measures of daily sleep and PSQI in later life. We used baseline survey and diary data from the Everyday Function Intervention Trial on 95 older adults (age range: 60-88). Daily measures of sleep included overall means and day-to-day variations in sleep quality, difficulty falling asleep, difficulty staying asleep, and feeling refreshed after wake across two weeks. Results revealed that higher overall mean levels of difficulty falling asleep was associated with higher total PSQI score (i.e., worse sleep). Higher overall mean levels of sleep quality were associated with several PSQI subscales (less sleep disturbance, better sleep efficiency, and higher subjective quality of sleep). Higher day-to-day variations in sleep quality was associated with better PSQI sleep efficiency and higher variations in feeling refreshed after wake was associated with longer PSQI sleep latency. These results suggest that daily measures of sleep may be tapping onto different dimensions of PSQI and further highlight the need to incorporate diverse measures of sleep to have a more holistic understanding of sleep patterns in later life.

Abstract citation ID: igae098.3522

### EXPLORING THE EFFECTS OF AN EIGHT-WEEK LIFESTYLE INTERVENTION ON REST-ACTIVITY RHYTHM IN ADULTS

Meina Zhang, Michelle Voss, Lucas Carr, Kara Whitaker, Jenna Springer, and Chooza Moon, *University of Iowa, Iowa City, Iowa, United States*

Rest activity rhythm (RAR) describes the pattern of physical activity and sleep in 24 hours. Disruptions in RAR have been associated with various age-related chronic conditions, including sleep disorders, depression, and increased risk for cognitive decline. Few studies examined if RAR changes from intervention that includes both physical activity and sleep components. This study investigated the effects of an 8-week lifestyle intervention targeting the full 24-hour activity profile (physical activity, sedentary behavior, and sleep) on RAR. Participants engaged in up to five sessions with a student health coach, viewed three health education videos targeting physical activity, sedentary behavior, and sleep, and received an Acttrust2 activity monitor. A cohort of 26 participants (mean age = 49.3 years, SD = 11.1; 73.1% female) was monitored using wrist-worn Acttrust2 devices for one week pre- and post-intervention to observe changes in RAR. RAR parameters were analyzed using cosinor approach, such as amplitude (maximum activity level), mesor (mean activity level), pseudo-F-statistic (the robustness of RAR), and acrophase (peak activity time). The intervention showed mixed effects on RAR parameters. While not statistically significant, positive changes were observed in amplitude (Cohen's d=0.24) and mesor (Cohen's d=0.12). Conversely, the pseudo-F-statistic (Cohen's d = -0.002) and acrophase (Cohen's d=-0.02) remained stable post-intervention. Although statistically significant changes in RAR parameters were not observed, encouraging trends suggest the potential for the intervention to influence maximum and mean physical activity level. These findings contribute to our understanding of lifestyle interventions in adults and highlight areas for further research and intervention refinement.

Abstract citation ID: igae098.3523

### PARENTHOOD AND SLEEP IN A POPULATION-BASED SAMPLE OF OLDER ADULTS

Jacquelyn Duckett, and Rodlescia Sneed, *Wayne State University, Detroit, Michigan, United States*

Numerous studies have examined the association between parenthood and health-related outcomes in young and middle-aged adults; however, there has been less inquiry into how parenthood is associated with health-related outcomes among adults aged >50. Further, few studies have explored how various aspects of the parenthood experience (e.g., number of children, geographic proximity, frequency/nature of contact, relationship quality) are differentially associated with health-related outcomes. The current study aimed to evaluate associations between parenthood and sleep, a critical factor in maintaining health among adults aged >50. We addressed our research questions via secondary analysis of data from the 2016 and recent waves of the Health and Retirement Study, a nationally representative population-based study of community-dwelling U.S. adults aged >50 (n= 4,201). Both parenthood measures and sleep outcomes were measured via self-report. We used multiple linear regression to investigate how various aspects of parenthood were differentially related to sleep outcomes, controlling for demographic characteristics, chronic health conditions, and baseline sleep scores. We observed that more positive interactions and fewer negative interactions with children were associated with better sleep. None of our other parenthood measures were associated with sleep outcomes. Our findings suggest that interaction quality, but not other aspects of the parent-child relationship, is important for the sleep hygiene of older adults.

Abstract citation ID: igae098.3524

### SLEEP AND CORTISOL IMPACT COGNITIVE OUTCOMES IN MIDLIFE HISPANIC/LATINE ADULTS AT RISK FOR ALZHEIMER'S DISEASE

Shanna Burke, Samantha Gonzales, Adrienne Grudzien, Celine Cambara, Alexandra Briceno, Daniel Jimenez, Stephanie Scott, and Sabrina Sales Martinez, *Florida International University, Miami, Florida, United States*

Sleep disturbance has been associated with an increased risk of Alzheimer's disease (AD). Repeated stressors, including ongoing sleep disturbance, trigger persistent increases in glucocorticoid levels, which affect brain function. Various cognitive domains appear to be significantly impacted by sleep. This study examined the effect of sleep and cortisol levels on cognitive test outcomes. Hispanic/Latine adults (n=39; mean age 53 yrs (SD: 5.12)) with first-degree relatives with AD were recruited from Miami-Dade and Broward counties, FL. Cortisol was measured from saliva obtained three times in one day (waking, 30 minutes post-waking, and bedtime). Cognition was measured using the NACC Neuropsychological Battery (UDS3). Sleep was measured using wrist-worn actigraphy (ActiGraph wGT3X-BT). Multiple linear regression was performed to examine correlations between sleep, cognition, and cortisol. Average sleep duration was 6.40 (SD = 1.37) hours. Average efficiency was 92% (SD = 3%). Average sleep fragmentation was 27.79 (SD = 9.59) minutes post-sleep onset. Sleep efficiency was significantly associated with executive attention after adjusting for education and income ($\beta$ = -18.73, p =.046). Sleep fragmentation was significantly associated with immediate memory ($\beta$ = -.045, p =.0181). The effect of cortisol at waking on episodic memory delayed was dependent on sleep efficiency ($\beta$ = 211.004, p =.031). The interaction between sleep duration and cortisol at bedtime was significantly associated with nonverbal memory ($\beta$ =10.37, p =.023). The identification of modifiable risk factors for AD, including poor sleep and increased stress, will inform the development of intervention efforts to prevent or delay AD onset.

Abstract citation ID: igae098.3525

### SLEEP ARCHITECTURE IN OLDER ADULT INTENSIVE CARE UNIT SURVIVORS

Priscilla Carmiol-Rodriguez[1], and Maya Elias[2], *1. University of Washington, Washington, United States*, *2. University of Washington, Seattle, Washington, United States*

Sleep is highly fragmented in older adults recovering from critical illness, despite discharge out of an intensive care unit (ICU). The aim of this study was to measure nighttime sleep architecture in older ICU survivors after ICU discharge to a post-ICU inpatient unit. The sample consisted of 13 older ICU survivors who were functionally independent prior to hospitalization, mechanically ventilated in ICU, and enrolled within 24-48 hours after ICU discharge at an academic medical center. Sleep architecture was measured via a portable polysomnography sleep monitoring device (Sleep Profiler) between 10:00 PM-6:00 AM over two consecutive nights. Data were analyzed using descriptive statistics, and the one-sample Wilcoxon signed rank test evaluated the differences between the time spent in each sleep stage in our sample of older ICU survivors versus the established norms for healthy older adults. Among our sample, participants were awake approximately 53.4% (interquartile range [IQR]: 45.0) of nighttime hours. The median percentages of the sleep stages were: stage N1 = 2.1% (IQR: 6.65); stage N2 = 26.35% (IQR: 41.63); stage N3 = 0.8% (IQR: 6.33); and stage REM

= 0.15% (IQR: 7.6). Results indicated that the median percentages of the sleep stages N1, N2, N3, and REM were significantly lower than the expected reference values for each sleep parameter ($p < 0.05$). Therefore, older ICU survivors may experience insufficient slow wave sleep and REM sleep, which are vital for recovery from critical illness. Further interdisciplinary research is needed to optimize sleep in the post-ICU inpatient setting.

Abstract citation ID: igae098.3526

### SLEEPLESS SUPPORT: CAREGIVER SLEEP QUALITY AND ITS CORRELATES

Alexander Erickson[1], Susan McCurry[2], Monica Kelly[3], Anna Papazyan[3], Karen Camacho[3], Michael Mitchell[3], Jennifer Martin[1], and Yeonsu Song[4], *1. VA Greater Los Angeles Healthcare System, Los Angeles, California, United States*, *2. University of Washington, Seattle, Washington, United States*, *3. Greater Los Angeles VA Health Care System, North Hills, California, United States*, *4. University of California Los Angeles, Los Angeles, California, United States*

Adult caregivers of people living with dementia (PLWD) experience poor sleep, negatively impacting their ability to take a caregiving role. This study explored associations between caregiver sleep quality and factors at caregiver and PLWD levels. Participants were 45 caregiver-PLWD dyads, with a majority identifying as female (n=36; 75%), non-Hispanic white (n=19;39%), and having some college education (n=39; 87%). Caregiver sleep quality was measured with the Pittsburgh Sleep Quality Index (PSQI global score and three subscales: Sleep Efficiency, Perceived Sleep Quality, and Daily Disturbances). Other measures included the Zarit Burden Interview (ZBI), the Revised Memory and Behavior Problems Checklist (RMBPC) frequency and caregiver distress subscales, PLWD activities of daily living (ADLs), and PLWD Sleep Disorders Inventory (SDI) total score. Analyses included descriptive statistics, Pearson correlations, and t-tests. On average, caregivers endorsed poor global sleep quality (PSQI; mean of overall score). Higher caregiver Daily Disturbances scores were associated with higher ZBI scores (r=.33, p=.03), lower PLWD's ADL functioning (r=-.35, p<.001), higher RMBPC frequency (r=.31, p=.04), and higher SDI (r=.39, p=.008). Higher caregiver age was associated with better PSQI global sleep quality (r=-.35, p=.02) and lower Daily Disturbance subscale scores (r=-.49, p<.001). Poor Sleep Efficiency subscale scores were associated with more sleep problems of PLWD (r=.38, p=.01), and caregivers of non-White race had significantly worse sleep efficiency (t(43)=3.17, p=.003). Caregivers of PLWD generally reported poor sleep, which was associated with caregiver burden, care recipient function and behavioral impairments. Addressing PLWD's sleep and dementia-related behavioral challenges may help caregivers improve their sleep quality.

Abstract citation ID: igae098.3527

### SUBJECTIVELY AND OBJECTIVELY ASSESSED SLEEP IN OLDER ADULTS WITH AND WITHOUT MILD COGNITIVE IMPAIRMENT

Kworweinski Lafontant[1], James Brightman[1], Jethro Raphael Suarez[2], Amber Blount[1], David Fukuda[1], Jeffrey Stout[1], and Ladda Thiamwong[1], *1. University of Central Florida, Orlando, Florida, United States*, *2. University of Central Florida*

Mild cognitive impairment (MCI) has been proposed as a potential confounding factor when subjectively assessing sleep in older adults. However, it is unclear whether older adults with MCI are less accurate in self-reporting sleep than those without MCI. This cross-sectional investigation compared the mean absolute difference (MAD) between subjectively and objectively assessed sleep duration, efficiency, and latency among community-dwelling low-income older adults with MCI (n = 13, females = 11, age = 77.5 ± 7.83 years, BMI = 29.0 ± 4.29 kg/m2) and those without MCI (n = 77, females = 69, age = 73.8 ± 7.07 years, BMI = 30.6 ± 7.38 kg/m2). The Pittsburgh Sleep Quality Index (PSQI), wrist-worn actigraphy (GT9X Link, ActiGraph LLC, Pensacola, FL, USA) and the Rowland Universal Dementia Assessment Scale were used to assess subjective sleep, objective sleep, and MCI, respectively. A Kolmogorov-Smirnov test revealed that data were non-normally distributed, so Mann-Whitney U tests were used for between-group comparisons. There was a significant difference in sleep duration MAD between older adults with MCI (6.04 ± 2.22 hrs) and those without MCI (6.42 ± 1.47 hrs; p = 0.03). However, sleep efficiency and latency MAD were not significantly different between the groups. While older adults with MCI may be less accurate at recalling sleep duration, their ability to distinguish sleep duration from time spent in bed is comparable to that of older adults without MCI. Future research should include older adults with MCI when assessing specific subdomains of sleep quality subjectively.

Abstract citation ID: igae098.3528

### THE LONGITUDINAL EFFECTS OF PERSISTENT PAIN AND SLEEP HEALTH ON FRAILTY AMONG COMMUNITY-DWELLING OLDER ADULTS

Tuo Yu Chen[1], Philile Mgabhi[1], Feng-Jen Tsai[1], Chyi-Huey Bai[1], Hui-chuan Hsu[1], Yi-Hua Chen[1], and Yi-Chun Kuan[2], *1. Taipei Medical University, Taipei, Taipei, Taiwan (Republic of China)*, *2. Shuang Ho Hospital, New Taipei, New Taipei, Taiwan (Republic of China)*

Research has shown that persistent pain can affect frailty of community-dwelling older adults. Additionally, evidence supports the significant association of sleep health with persistent pain and frailty. However, the relationship between persistent pain, sleep health, and frailty is not yet fully understood, and it remains unclear whether better sleep health could alleviate the impact of persistent pain on frailty. We investigated whether baseline sleep health and persistent pain predicted frailty at 4-year follow-up among community-dwelling older adults. Besides, we examined whether the relationship between persistent pain and frailty differed by sleep health. Data were from the Taiwan Longitudinal Study on Aging (N=2,562;2011-2015). A frailty index was built using 37 items on functioning and physical and mental health. Persistent pain was measured with a 5-level categorical variable (no pain [reference], mild pain, mild persistent pain, moderate persistent pain, severe persistent pain). Sleep health was assessed using the SATED model (satisfaction-alertness-timing-efficiency-duration). Covariates included baseline sociodemographic status (age,sex,education), errors in cognitive tests, frailty, and poor health behaviors (drinking,lack of exercise). Results showed that baseline moderate persistent pain (OR=1.44,95%CI=1.03-2.01), severe persistent pain (OR=1.65, 95%CI=1.06-2.57), and poor sleep health (OR=1.14,95%CI=1.04-1.26) increased the risks of frailty at 4-year follow-up. A significant synergistic effect between persistent pain and sleep health was observed, such that the risks of frailty were reduced among individuals with better sleep health, even with more severe persistent pain. Overall, persistent pain and sleep health predicted frailty. Improving sleep health among older adults with persistent pain may help mitigate the risks of frailty.

Abstract citation ID: igae098.3529

### THE ROLE OF SOCIAL SUPPORT IN SLEEP HEALTH FOLLOWING SPOUSAL LOSS: A LONGITUDINAL STUDY

Kristin Calfee, and Soomi Lee*, The Pennsylvania State University, University Park, Pennsylvania, United States*

The loss of a spouse is consistently rated as one of the most stressful life events. Both spousal bereavement and divorce have been associated with poorer physical and mental health, including worsened sleep. However, the impact of spousal loss on sleep health may be mitigated by robust social support networks. This study utilizes a multidimensional measure of sleep health to examine its association with spousal loss, considering potential moderation by social support. 2,462 adults (Mage=66yrs) from the Midlife in the United States study provided longitudinal data at 2004-2005 and 2013-2016, 277 of whom lost a spouse to death or divorce between the two timepoints. A composite score of sleep regularity, satisfaction, alertness, efficiency, and duration was used to measure sleep health (higher=better). Social support was measured using Affectual Solidarity (AS), capturing both support and strain. General linear regression adjusted for sociodemographic covariates and depression was used to examine the association between spousal loss and sleep health, and the interaction between spousal loss and AS. Spousal loss was not a predictor of sleep health at follow-up. However, there was significant moderation by AS, such that the loss of a spouse was associated with worse sleep health for those who experienced a decrease in AS since the spousal loss (Slope=0.19, SE=.09, p=.03), but not for those who experienced an increase or no change in AS. Findings suggest that spousal loss may not necessarily degrade sleep health when social support is stronger. Future analyses will examine these associations separately by bereavement vs. divorce.

### SESSION 7740 (POSTER)

### SOCIAL SERVICES: POLICY, FINANCING, AND DELIVERY SYSTEMS

Abstract citation ID: igae098.3530

### BARRIERS TO COMMUNITY FOOD RESOURCES AMONG OLDER ADULTS: PERCEPTIONS OF PROVIDERS AND OLDER ADULT STAKEHOLDERS

Sheryl Groden, *University of MIchigan Flint, Flint, Michigan, United States*

Finding proper nutrition for individuals living in or near a food desert is of particular concern for older adults, many who experience chronic health problems. This qualitative study explores barriers and challenges in food accessibility for older adults in Flint, Michigan. Qualitative data was collected at four focus group sessions in early 2022. Two focus groups included 15 older adults, 65 years of age or older. They shared their experiences and challenges regarding food accessibility. The other focus groups included six professionals employed at agencies connected to food distribution. Based on responses from both groups, we revealed challenges to food access/distribution among older adults. Data analysis highlights gaps in communication and knowledge of community food provision as barriers to older adults accessing food. Community providers noted while Flint doesn't have adequate grocery stores, the community was not short of community-provided meals. Despite numerous food assistance programs in the community, older adults were unaware of all food distribution options. They were also unaware of digital apps to find food distribution or the best pricing for goods at grocery stores. As non-profit agencies and businesses rely more on digital communication, written communication is often forgotten, leaving out many older adults. Other identified communication barriers included literacy issues, language barriers (growing Arabic and Spanish-speaking populations), and lack of overall knowledge on where to go for community food. Implications for addressing gaps in communication will help to address food access and nutrition security for older adults in marginalized communities such as Flint, MI.

Abstract citation ID: igae098.3531

### FACILITATORS AND BARRIERS IN NAVIGATING COMMUNITY AND HEALTH CARE SYSTEM AMONG INFORMAL CAREGIVERS OF OLDER ADULTS

Boah Kim[1], Andrew Wister[1], Barbara Mitchell[1], Lun Li[2], and Laura Kadowaki[1], *1. Simon Fraser University, Vancouver, British Columbia, Canada*, *2. MacEwan University, Edmonton, Alberta, Canada*

Informal caregivers are fulfilling important support roles for older adults who tend to require a greater range of services while navigating these community and healthcare systems to fulfill their growing complex needs. Yet, there is sparse knowledge on the most significant facilitators and barriers to effective system navigation (SN) among caregivers. This paper aims to fill these gaps through an investigation of the key factors affecting SN difficulties among informal caregivers of older adults. The Behavioural-Ecological Framework of Healthcare Access and Navigation (BEAN) model is used to frame the study. Using the General Social Survey (GSS) on Caregiving and Care Receiving 2018, a total of 2,733 informal caregivers whose primary care receivers were aged 65 or older were analyzed. Hierarchical logistic regression showed that the probability of reporting SN difficulties was lower for caregivers with social capital/cohesion compared to those without social capital/cohesion. In comparison, the probability of reporting SN difficulties was higher among caregivers with caregiving supports and among caregivers whose care receivers use a higher amount of formal health services. Several sociodemographic covariates (e.g., sex, education level, self-rated stress) were also identified. Given the significance of the social capital and utilization factors, as well as several key covariates as facilitators/barriers in SN among informal caregivers, our findings support certain aspects of the BEAN model. To address the challenges and to fill the care gaps on SN among informal caregivers, there is a need to implement coordinated schemes and health policy for older adults and their caregivers.

Abstract citation ID: igae098.3532

### GOALS OF CARE IN A MEDICAID HCBS PROGRAM: VARIATIONS BY RACE, ETHNICITY, AND DEMENTIA

Julie Robison[1], Victor Elisha[1], Kathy Kellett[2], Kristin Baker[2], and Richard Fortinsky[1], *1. University of Connecticut, Farmington, Connecticut, United States*, *2. UConn Health, Farmington, Connecticut, United States*

Medicaid-funded home and community-based services (HCBS) for older adults aim to prolong independent living despite physical and cognitive disabilities. HCBS person-centered care plans ideally align services provided with recipients' goals of care. Medicaid-eligible HCBS populations are racially and ethnically heterogenous, with multiple chronic conditions. This study aims to determine how living with dementia, and racial and ethnic group membership, are associated with meeting self-identified goals of care and person-centered outcomes based on their HCBS-related experiences. 148 ethnically diverse adults ages 65+ with and without dementia were interviewed 9-10 months after enrolling in Connecticut's Medicaid HCBS waiver program about the goals they set at enrollment. Structured interviews were conducted in person or virtually, based on respondent preference. Assistant or proxy unpaid caregiver respondents participated when warranted. 90% of respondents said they had as much input as they wanted in setting their goals. 76% felt they had met their goal, including 49% doing better than they had expected, and 16% reporting changing their goal. 83% said that thinking about goals was helpful in planning future care and many had discussed their goals with another health care provider (45%) or friends and family (76%). Goal achievement was equally likely for people with dementia compared to people without dementia as well as between race and ethnicity groups. However other aspects differed both by dementia status and race/ethnicity. Examples of specific goals for various subgroups will be presented.

Abstract citation ID: igae098.3533

### POTENTIALLY PREVENTABLE HOSPITALIZATIONS IN A CONNECTICUT MEDICAID HOME AND COMMUNITY-BASED SERVICES POPULATION

Ellis Dillon[1], Richard Fortinsky[1], Lisa Barry[2], Chae Man Lee[1], Martha Porter[1], Deborah Migneault[3], and Julie Robison[1], *1. University of Connecticut, Farmington, Connecticut, United States*, *2. UConn Center on Aging, Farmington, Connecticut, United States*, *3. University of Connecticut Health, Farmington, Connecticut, United States*

State Medicaid programs address the needs of older adults, and people with disabilities or chronic illnesses through a range of home and community-based services (HCBS) and aim to prevent adverse health outcomes. This study determines rates of potentially preventable hospitalizations (PPH) for Connecticut residents receiving Medicaid-funded HCBS and compares rates across HCBS programs using a CMS Home Health quality measure. This retrospective cohort analysis linked Medicaid and Medicare fee-for-service claims, Medicaid administrative data, and uniform clinical and functional assessment data for 33,688 Connecticut residents. PPH rates between 4/1/2022-9/30/2022 were compared across six HCBS programs via logistic regressions adjusted for the 8-point InterRAI Risk of Hospital Use Scale (Hosp-RiskHC) score. Rates of PPHs ranged from 0.8% (Autism Waiver) to 15.7% (Personal Care Attendant Waiver, PCA). Individuals with PPHs represented over half (55.6%) of all hospitalized individuals. PCA participants were more likely (OR=1.3, 95% CI:1.13-1.58) and Acquired Brain Injury Waiver participants were less likely (OR=0.7, 95% CI:0.48-0.93) to have a PPH compared to Elder Waiver recipients. Likelihood of experiencing a PPH increased with each successive Hosp-RiskHC score (OR=1.2, 95% CI:1.16-1.22). Controlling for risk factors, rates of PPH varied across HCBS programs, potentially due to the nature of their disabilities, differing services provided by each program, or a combination of individual and structural factors. Identifying variations in PPH rates is a critical step toward building a Value-Based Payment (VBP) program for HCBS providers, who have historically been excluded from VBP opportunities, and identifying systemic gaps and opportunities for coordinated interventions across healthcare providers.

Abstract citation ID: igae098.3534

### RESEARCH ON THE IMPACT OF INTERGENERATIONAL DEMOGRAPHIC EVOLUTION ON COMMUNITY SERVICES IN CHINA (2024–2050)

Yuxia Wu[1], Wanwan Si[2], and Hong Mi[2], *1. Ningbo University of Technology, Ningbo, Zhejiang, China (People's Republic)*, *2. Zhejiang University, Hangzhou, Zhejiang, China (People's Republic)*

Facing the dual challenges of population aging and a declining birthrate, China is actively promoting mutual assistance and support among multiple generations through a combination of policies and coordinated services. Utilizing datasets from multiple Chinese censuses and the China Family Panel Studies (CFPS), this study employs microsimulation models to establish predictive frameworks for both population intergenerational shifts and service demands. It delves into the trajectories of generational transitions and their subsequent impact on community-based services over the forthcoming 25 years in China. Building upon the long-term care practices and experiences in generational integration within OECD nations, the study proposes phased service policy alignments and synergistic strategies. The research underscores that defining parameters such as demographic structures, health statuses, care paradigms, and multi-generational dynamics, along with segmenting service recipients and fine-tuning policies and service collaborations, enables precision targeting and flexible adjustments in community services. This, in turn, fosters "upward intergenerational" connections — where individuals in their early senior years provide informal caregiving to those who are significantly older and may have physical limitations — and "downward intergenerational" bonds — in which grandparents contribute to the upbringing of their grandchildren. Such arrangements not only reinforce societal bonds but also alleviate the caregiving responsibilities of families, particularly those with members in their mid-30s to mid-40s, who often juggle between child-rearing and older adults care responsibilities.

Abstract citation ID: igae098.3535

### UNMET SERVICE NEEDS AND HOME AND COMMUNITY-BASED SERVICE USE IN RURAL AND URBAN DEMENTIA CAREGIVERS

Christina Miyawaki[1], Angela McClellan[1], David Russell[2], and Erin Bouldin[3], *1. University of Houston, Houston, Texas, United States*, *2. Appalachian State University, Boone, North Carolina, United States*, *3. University of Utah, Salt Lake City, Utah, United States*

The lack of resources and services for caregivers of people with dementia (PWD) in rural areas in the U.S. has been well-documented. However, less research has explored the conditions of caregivers in urban areas. The purpose of this study was to identify the unmet caregiver service needs of urban caregivers of PWD, explore factors associated with their service use, and compare the outcomes with those of rural counterparts. Guided by Andersen's theory of health service use, we interviewed 20 caregivers in rural Western North Carolina and 18 caregivers in urban Houston, Texas. Using the same interview guide, we inquired about their service needs and use, and contextual factors that facilitate their service use, and conducted a thematic analysis of the interviews. Interviews revealed that rural caregivers wished for more information and caregiver support whereas urban caregivers searched for information until they found satisfactory solutions. Rural caregivers expressed guilt about using services because they were limited and zero-sum while urban caregivers sought to assist their fellow caregivers through sharing available resources. Urban caregivers needed more culturally tailored services while rural caregivers' cultural needs were more place-specific. Increasing options for online and virtual support groups and expanding the reach across the U.S. might address the unique concerns of rural caregivers. Developing ethnic-specific resources for their diverse population and PWD represents a priority among urban caregivers. Racially/ethnically diverse health professionals, including nurses and social workers with multi-linguistic skills, are needed to meet the needs of racially/ethnically diverse patients and caregivers in urban areas.

### SESSION 7745 (POSTER)

### SUCCESSFUL AGING/PRODUCTIVE AGING: THEORIES AND CONCEPTS

Abstract citation ID: igae098.3536

### BEYOND PHYSICAL ABILITY: PROMOTING INCLUSION IN DEFINITIONS OF SUCCESSFUL AGING

Payton Rule, and Emily Willroth, *Washington University in St. Louis, St. Louis, Missouri, United States*

Physical function and the absence of disability have long been conceptualized as key components of successful aging in most frameworks. However, prior work in disability studies and psychology have suggested that physical functioning is not necessary for successful aging and that this conceptualization may be ableist in nature. Specifically, this definition implies that people with congenital disabilities are not able to age successfully and reinforces society's view that able-bodied lives are better, happier, or more worthy than disabled people's lives. This stands contrary to prior research demonstrating high engagement and wellbeing among individuals with disabilities. Moreover, this definition fails to acknowledge the role of inclusive environments (e.g., no stairs, wide doors/walkways) in promoting participation and successful aging. All these factors may lead to increased disability shame, stigma, and discrimination, which can decrease wellbeing, health, and longevity for people with disabilities. In this conceptual presentation, we draw from research in psychology, gerontology, and disability studies to suggest a broader and more inclusive definition of successful aging as continued engagement in activities one needs and wants to participate in. We describe how this definition promotes inclusion by acknowledging that participation can take many forms (e.g., through adaptive devices) and recognizing the power of the environment for promoting successful aging. In this way, we argue that this definition of successful aging takes the onus of functioning off the individual and places it on society to create inclusive environments, allowing all individuals the potential to age successfully, regardless of physical ability.

Abstract citation ID: igae098.3537

### LAYPERSON'S UNDERSTANDING OF GEROTRANSCENDENCE

Oscar Ribeiro[1], Taiane Abreu[1], Laetitia Teixeira[2], and Lia Araujo[3], *1. University of Aveiro, Aveiro, Aveiro, Portugal*, *2. University of Porto, Porto, Porto, Portugal*, *3. Polytechnic Institute of Viseu, Viseu, Viseu, Portugal*

Gerotranscendence is an adaptive theory of aging that posits a mindset shift across three dimensions: cosmic, coherence, and solitude. Despite its consolidation in recent years, the theory's depth continues to evolve, stimulating a need for broader perspectives to enhance its practical comprehension. This study aimed to explore laypersons' perceptions regarding gerotranscendence's aspects. Three focus groups were conducted with a total of 18 participants, ranging in age from 58 to 98 years old (mean=79.5). Open-ended questions were used to collect information regarding what gerotranscendence and its dimensions meant to them; thoughts on the components of the theory after a brief explanation were also obtained. Transcripts from recorded sessions underwent content analysis and organized under two overarching themes: general assumptions and personal perceptions. The latter theme encompassed two sub-themes highlighting participants' identification of personal experiences, as well as their diverse and complementary interpretations of the theory. Initially, participants encountered challenges grasping the theory's terminology. Subsequent clarification facilitated their recognition and resonance with gerotranscendence's components. Further discussions exposed associations between concepts of gerotranscendence and other theoretical frameworks of successful aging, notably the life cycle theory and socio-emotional selectivity theory. This interconnection highlights the theory's relevance within broader aging frameworks. Engaging lay individuals in gerotranscendence discussions yields profound insights into both its fundamental principles and culturally embedded nuances. By associating theoretical concepts with lived experiences, such dialogues enrich the understanding of aging paradigms, paving the way for more holistic approaches to aging research and practice.

Abstract citation ID: igae098.3538

LIFE SATISFACTION AMONG BLACK AND WHITE AMERICANS: FINDINGS FROM THE 2020 HEALTH AND RETIREMENT STUDY

Chesney Ward, *Utah State University, Logan, Utah, United States*

The U.S. population is living longer, yet data are limited regarding life satisfaction among older adults. Understanding this construct for older adults is essential given the greater longevity and notable risks of reduced satisfaction as one ages. The purpose of this study was to examine racial differences between middle-aged and older White and Black Americans on life satisfaction and explore the correlation of religious beliefs and perceived control over one's life and health with self-reported life satisfaction. The study presents results utilizing data from the 2020 Health and Retirement Study, a nationally representative sample. The sample (N=1163) was a comparative analysis of Black and White respondents. Findings indicated factors that contribute to overall life satisfaction, differences in race, and gender differences. Black respondents when compared to White respondents reported lower overall life satisfaction but higher religious beliefs. The current study helps to fill this gap by examining the relationship between psychosocial and demographic factors and life satisfaction among the sample. By drawing on the literature and the study data, implications on the impact of religious belief on overall life satisfaction are discussed. This study proposes ways in which researchers, practitioners, and policy advocates can improve life satisfaction for Black and African American populations in the United States by employing a religious aspect.

Abstract citation ID: igae098.3539

POSTING SHORT-FORM VIDEOS PROMOTES OLDER ADULTS' PSYCHOLOGICAL HEALTH: A MODEL BASED ON SELF-DETERMINATION THEORY

Jingxuan Wu, and Huamao Peng, *Beijing Normal University, Beijing, Beijing, China (People's Republic)*

**Objectives:** This study focuses on how posting behaviors of older adults on short-form video platforms (e.g., TikTok) influences depression and reveals the psychological mechanism (satisfying basic psychological needs of autonomy, competence, and relatedness) from the perspective of the Self-Determination Theory and uncover whether browsing behavior moderates this effect. Method: A total of 1705 older adults aged 55 to 83 years were instructed to complete the Center for Epidemiologic Studies Depression Scale, the Short-form Video Usage Behavior Questionnaire, and the Basic Psychological Needs Scale. Data analysis was conducted using SPSS 25.0.

**Results:** 1) Older adults posting short-form videos exhibit lower levels of depression compared to those who have never posted short-form videos through the mediations of greater competence (indirect effect = -.15) and autonomy (indirect effect = -.05), and the indirect effect of autonomy becomes insignificant when the diversity of browsing behaviors is high. 2) Among older adults posting short-form videos, posting short-form videos frequently decreases depression through competence (indirect effect = -.16), autonomy (indirect effect = -.04), and relatedness (indirect effect = -.06), regardless of the diversity of browsing behaviors.

**Discussion:** This study highlights the positive effect of posting short-form videos on older adults' psychological health by satisfying basic psychological needs. For those unable or unwilling to post videos, diversifying browsing behaviors can improve autonomy and safeguard psychological health. These findings emphasize older adults' initiative and creativity, along with the importance of lifelong competence development, and present new strategies to support successful aging in the era of Internet-based media.

Abstract citation ID: igae098.3540

SYNTHESIZING LIFESTYLE MEDICINE AND SUCCESSFUL AGING: A PERFECT PAIRING FOR THEORIZING OPTIMAL AGING?

Teri Undem, and Heather Fuller, *North Dakota State University, Fargo, North Dakota, United States*

Within gerontology, various theoretical perspectives on what it means to age successfully have emerged over recent decades (e.g. Rowe & Kahn, etc.) and garnered empirical support. Within medicine and related healthcare fields, research is prolific regarding engaging in healthy living in terms of physical health, mental health, and well-being. In recent years, a theoretical perspective of Lifestyle Medicine has emerged to describe the role of such lifestyle factors on health. The present work includes a scoping review of both literatures with the ultimate goal of determining overlaps and distinctions between theoretical concepts of successful aging and lifestyle medicine. We take a holistic approach to develop a model that identifies linkages between the principles of successful aging and lifestyle medicine. The resultant 'Lifestyle Model of Optimal Aging' merges the core pillars of Lifestyle Medicine (nutrition, physical activity, stress management, restorative sleep, social connection, and avoidance of risky substances) with the core principles of successful aging from the models put forth by Rowe and Kahn (1997) and expanded upon by Urtamo et.al., (2019). This new, holistic approach to exploring the linkages between the principles of LM and the essential components of successful aging highlights how proactive behavioral health strategies related to nutrition, exercise, sleep, stress, and substances can foster optimal aging in cognitive and physical functioning and disease prevention, thereby potentially yielding more positive outcomes for aging well. Discussion explores how concepts underlying successful aging may be enhanced by incorporating a lifestyle medicine approach.

Abstract citation ID: igae098.3541

THE MULTIDIMENSIONAL SUCCESSFUL AGING SCALE: A LONGITUDINAL VALIDATION STUDY

Edwin Ka Hung Chung, and Dannii Y. Yeung, *City University of Hong Kong, Hong Kong, China (People's Republic)*

The diversified measurement tools of successful aging have restricted comparison of findings between studies and hindering development of this field advancement (Cosco et al., 2014). This study thereby aimed to validate the 25-item Multidimensional Successful Aging Scale (MSAS) through examining its overall structure, measurement invariance, and predictive validity. Four hundred and fourteen community-dwelling older Chinese adults (Mage=68.92 years, SD=6.64, range=60–91; 59.9% females) were assessed on the MSAS in conjunction with demographic, physical and psychological measures at baseline (Time 1) and at 6 months (Time 2). The results of confirmatory factor analysis first revealed a good model fit of the 9-factor model of the MSAS in the two assessments, and indicated that the 9-factor model fitted the data better than other higher-order factor models. In addition to the metric invariance across age and gender groups as well as strict invariance across groups of individuals with and without chronic illness, a strict form of longitudinal invariance was shown at the 6-month interval. Furthermore, the overall results of hierarchical linear regression analyses indicated that, among all dimensions of MSAS in Time 1, positive attitudes and perceived constraints were more predictive of depressive symptoms, functional ability, and pain in Time 2. Overall, the present study advances the previously established psychometric properties of MSAS (Chung & Yeung, 2021; 2022) by demonstrating its temporal stability and predictive value. Findings also support the multidimensional nature of successful aging, and provide health care practitioners and policymakers a more holistic understanding of what is important to older adults.

Abstract citation ID: igae098.3542

### THEORETICAL ANALYSIS OF THE DISABLEMENT PROCESS MODEL IN THE CONTEXT OF VISION LOSS IN LATE LIFE

Haruno Suzuki, and Margaret Wallhagen, *University of California San Francisco, San Francisco, California, United States*

Vision loss experienced by older adults is associated with poor biopsychosocial health, yet the mechanisms underpinning adjustment to this disability are poorly understood. One framework that may facilitate the exploration of the adjustment is the disablement process model (DPM) (Verbrugge & Jette, 1994), which describes how a chronic condition leads to disability. However, limited studies have evaluated the model's applicability to older adults living with vision loss. This study critically appraises the theoretical assumptions of DPM for its applicability to studying vision loss acquired in late life. We utilized the criteria adapted from Chinn and Kramer (2011), which include 6-items of description of theory and 5-items of critical reflection. The central pathway of the DPM describes moving from pathology to impairments, functional limitations, and disability with intra- and extra-individual factors influencing the experience of disability. Definitions of each concept and subconcept are explicit yet general enough to use in various populations with disabilities, increasing the model's generalizability. However, the magnitude of the effects of each subconcept on disability and health outcomes is unclear. Furthermore, it is based on a medical model of disability, focusing on preventing disability. It lacks the nuance of social models that include strengths and capacities, thus limiting its use in developing inclusive communities that reinforce intrinsic capability and extrinsic environmental supports, such as age-friendly community. The findings imply the necessity of modifying the model to improve its utility in exploring the lived experience of disability among older adults living with vision loss in late life.

Abstract citation ID: igae098.3543

### TOPIC CLUSTERS OF SUCCESSFUL AGING STUDIES: RESULTS OF A TOPIC MODELING APPROACH

Ha Neul Kim, and Paul Freddolino, *Michigan State University, East Lansing, Michigan, United States*

Literature regarding successful aging reflects a wide variety of fields and perspectives. Given the range of definitions and approaches found in the published literature, it is important to investigate clusters of topics studied over time. This study aimed to show the change of topic clusters within successful aging studies. The study used topic modeling methodology to analyze vast amounts of abstract data. Among publications collected from Scopus (4,458) and Web of Science (5,187), 5,610 publications were analyzed. Topic clusters were analyzed in two ways: by a) division of time (1960s – 1990s, 2000s, 2010s, 2020s) and b) all years combined. In the 1960s – 1990s, 11 topic clusters ranging from health to emotional well-being emerged without any dominant domain. In the 2000s, two clusters related to social support and health appeared as major clusters. In the 2010s, one topic cluster that included words related to health and social participation was the biggest. In the 2020s, emotional health and social participation appeared again as one of the major clusters and health-related topics started to diverge into subgroups like physical health and mental health. In all years of publications combined, the major cluster involved words that are related to either health or social domains. Results revealed that successful aging has been studied in many fields using multidimensional perspectives. The dominant categories were health and social domains. These findings suggest interprofessional practice, an interdisciplinary approach in research, and multi-sector involvement in policy.

### SESSION 7750 (POSTER)

### TECHNOLOGY: RESEARCH APPLICATION/ MEASUREMENT/DEVICES

Abstract citation ID: igae098.3544

### A KNOWLEDGE GRAPH–BASED DEMENTIA CARE INTELLIGENT RECOMMENDER SYSTEM FOR MANAGING DEMENTIA CARE

Yue Sun[1], and Zhi-wen Wang[2], *1. Peking University, Beijing, Beijing, China (People's Republic)*, *2. Peking University Health Science Center, Beijing, Beijing, China (People's Republic)*

**Background:** Caring for people with dementia is perceived as one of the most challenging caring roles, so effective and practical support is essential. One such innovative approach to internet-based tailored health intervention is the use of recommender system. Objective: This study develops a mobile-based dementia care intelligent recommender system (DCIRS) that can provide personalized and timely care interventions recommendations for caregivers when they encounter difficult various care problems.

**Methods:** A knowledge graph was constructed using the 1023 personalized care plan set for dementia patients as the source material. The graph embedding method was used to introduce the constructed domain knowledge graph of dementia care into the recommender model. The graph embedding module learned the features of the knowledge graph through the TransR translation model, and the recommender module interacts with the learned features of the graph and the recommended items to realize personalized recommendation of care plans with the help of the above recommender algorithm.

**Results:** The core function of the DCIRS was to recommend personalized care plans according to the personalized characteristics of people with dementia, such as gender, disease severity, hobbies, walking ability, and care problems. Conclusions: This study was a pioneering research to develop a comprehensive recommender system for caregivers of people with dementia. According to the evaluation results, our DCIRS showing high specificity and accuracy. This system can provide a novel perspective for patient-centered and needs-based support of caregivers of people with dementia.

Abstract citation ID: igae098.3545

### ASSOCIATIONS OF PREVENTION FOCUS TRAITS WITH SEDENTARY BEHAVIOR IN COMMUNITY-DWELLING OLDER ADULTS

Jethro Raphael Suarez[1], Amber Blount[2], Joon-Hyuk Park[2], and Ladda Thiamwong[2], *1. University of Central Florida, 2. University of Central Florida, Orlando, Florida, United States*

Sedentary behavior is prevalent among older adults and can increase the risk of developing health conditions, such as muscle atrophy and heart disease. Beyond physical factors, it is important to determine mental factors that could be associated with excessive sedentary behavior. The Regulatory Focus Questionnaire (RFQ) is an 11-item instrument that allows an individual to self-report the frequency of occurrences of specific events in their life. The RFQ yields 2 separate scores: a promotion focus score and prevention focus score. A higher promotion focus score describes an individual who is goal-motivated, open to change, and willing to take risks. A higher prevention focus score describes an individual who is motivated by avoiding losses, more cautious, prefers constant routine, detail-oriented, and avoids risks. A cross-sectional study was conducted with 94 community-dwelling older adults aged 61 to 89 years (84 women, 10 men; mean age = 74.87 ± 6.76 years) to determine the relationship between total measured sedentary behavior and promotion and prevention focus, respectively. Sedentary behavior was objectively measured for each participant using the ActiGraph GT9X Link wireless activity monitor over 7 consecutive days. Kendall's Tau test revealed that sedentary behavior had a significant positive correlation with prevention focus scores ($p = 0.00819$, $\tau = 0.194$), but not with the promotion focus scores ($p = 0.647$, $\tau = -0.0335$). These findings suggest that certain personality traits, such as being loss-avoidant, detail-oriented and less accepting of change, can be associated with older adults that engage in increased sedentary behavior.

Abstract citation ID: igae098.3546

### DEVELOPMENT OF THE SELF-EFFICACY IN SMARTPHONE USE SCALE FOR OLDER ADULTS

Yutian Cheng, and Yasuyuki Gondo, *Osaka University, Suita, Osaka, Japan*

Currently, a large proportion of older adults are still unable to use smartphone functions, except for calling or dialing. Considering the strategy to accelerate smartphone use, developing a tool to assess users' confidence is important for future intervention studies. Thus, this study aimed to develop the scale for measuring the self-efficacy of smartphone use for older adults. We came up with 38 items first, derived former researches and some of developed own experience from smartphone lecture held for older adults. We conducted an internet survey for Japanese older adults aged 60 to 89, totally 400 participants (50% are female). Exploratory factor analysis and correlation analysis with general self-efficacy were conducted. By deleting those items which factor loading was too low or correlation with general analysis was not significant, the final questionnaire turned out to be 26 items. Further factor analysis revealed a three-factorial solution with good reliability (Cronbach alpha is 0.926). Three factors are named as Confidence (Cronbach alpha is 0.928), Problem Solving (Cronbach alpha is 0.904), and Realization of Benefit (Cronbach alpha is 0.855). Scores of self-efficacy of smartphone use showed significant positive correlations between general self-efficacy, which means high level of construct validity. With the development of this scale, it found out to be more than one dimension of self-efficacy in smartphone use, provided more precise assessment for further study. In further application research, we hope to develop a shorter version so it can reduce older adults' burden.

Abstract citation ID: igae098.3547

### DEVELOPMENT OF THE TECHNOLOGY INTEGRATION BELIEF INDEX FOR FAMILY CAREGIVERS

Tomoko Wakui[1], Kentaro Watanabe[2], Hiroyasu Miwa[2], and Sakiko Itoh[3], *1. Tokyo Metropolitan Institute for Geriatrics and Gerontology, Tokyo, Japan*, *2. National Institute of Advanced Industrial Science and Technology, Kashiwa, Chiba, Japan*, *3. Tokyo Medical and Dental University, Tokyo, Japan*

In highly aged societies, the technology integration into community care settings is promoted. Despite this, real-world adoption faces hurdles because of the common belief that care provided by humans is inherently superior. This gap highlights the absence of specific measures to assess this belief. This study aims to develop the Technology Integration Belief Index for Family Caregivers (TIBI-FC), which evaluates family caregivers' attitudes towards technology use in long-term care. We conducted a survey with 2,002 family caregivers of older adults living in the community, selected in proportion to the national percentage of older population in need of care. Using SPSS 29.0 and Amos 29, we carried out exploratory and confirmatory factor analyses. Model fit was evaluated using the comparative fit index (CFI) and the root-mean-square error of approximation (RMSEA). The participant group was 42.2% female, with the average age of 60.0 (SD = 10.7). The finalized TIBI-FC, comprising 14 items, was categorized into two dimensions: efficiency and stereotype. The first factor, efficiency, included six items, while the second, stereotype, comprised eight items, demonstrating a robust two-factor structure (CFI =.972, RMSEA =.060). This structure suggests that the TIBI-FC effectively captures the nuanced beliefs about technology integration in care. Importantly, the development of the TIBI-FC not only sheds light on existing challenges but also sets the stage for future strategies to enhance technology acceptance among family caregivers, potentially transforming the landscape of long-term care.

Abstract citation ID: igae098.3548

**EFFECTS OF A GAIT TRAINING PROGRAM USING A WALKING-ASSIST WEARABLE ROBOT ON COMMUNITY-DWELLING OLDER ADULTS**

Sinwoo Hwang, and Eunhee Cho, *Yonsei University, Seoul, Republic of Korea*

Two-thirds of people aged 65 and older require help with daily activities such as eating, bathing, and getting in and out of bed or a chair. Walking-assist wearable robots have shown significant improvements in physical function in controlled settings for patients. In this study, we aimed to determine the feasibility and evaluate the effectiveness of a gait assistance and gait resistance training program using a walking-assist wearable robot for community-dwelling older adults. A total of 23 community-dwelling older adults aged 65 and older (30 participants recruited, 7 dropped out) enrolled in a 12-session, 6-week gait assistance and gait resistance training program using a walking-assist wearable robot. A single-group, pre- and post-test design was employed to evaluate effectiveness. Paired t-tests and linear mixed model analysis reveal that the functional performance (assessed using the 10-Meter Walk Test, Timed Up-and-Go, and Four Square Step Test) and leg muscle strength of the participants improved significantly at the end of the program compared to the baseline values ($P < 0.01$). This study stands out for applying gait assistance and resistance training across various terrains, unlike previous studies that only tested gait assistance in controlled environments. The results demonstrated significant improvements in functional performance and muscle strength in older adults, suggesting the effectiveness of preventive healthcare services utilizing a walking-assist wearable robot as an intervention that can contribute to improving independent functioning and frailty among community-dwelling older adults.

Abstract citation ID: igae098.3549

**EXPLORING BED SENSOR SLEEP TECH: INSIGHTS FROM INTERDISCIPLINARY TEAM IN A GERIATRIC ASSESSMENT INPATIENT SETTING**

Cromwell Acosta[1], Yong Zhao[2], Joanna Lawrence[3], Michelle Towell[3], Marion MacKay-Dunn[3], Heather D'Oyley[3], Bryan Chow[3], and Lillian Hung[2], *1. Vancouver General Hospital, Vancouver, British Columbia, Canada*, *2. University of British Columbia, Vancouver, British Columbia, Canada*, *3. University of British Columbia Hospital, Vancouver, British Columbia, Canada*

Poor sleep can affect cognitive function and decrease overall quality of life. Evaluating sleep is critical in the care of hospitalized older adults. This study compared the use of traditional paper-based logs charted by nurses to record sleep patterns with smart sensors offering continuous monitoring and analysis of sleep patterns. The goal was to investigate the perspectives of staff members in an interdisciplinary team on sleep monitoring methods. We also asked participants about barriers and facilitators in implementing bed sensor technology in an Inpatient setting. Employing Interpretive Descriptive methodology, we conducted interviews and focus groups involving 29 staff members with diverse roles in one inpatient setting. The Consolidated Framework for Implementation Research (CFIR) guided our data analysis. Our findings indicate that while the traditional paper-based method, somnolog, is prone to inaccuracies from subjective estimates and sleep disturbances, bed sensor sleep technology is perceived as efficient, data-driven, and evidence-supported. Barriers to technology adoption include resistance to change, consent issues, patient comfort and safety concerns, and familiarity with the technology. On the other hand, facilitating factors comprise orientation and training, trial integration, effective communication, and evidence-based incentives. Utilizing the CFIR framework provides valuable insights into the challenges and aids in incorporating technology into care settings. Future research should focus on practical strategies involving interdisciplinary teams to facilitate innovative practices.

Abstract citation ID: igae098.3550

**FACTORS ASSOCIATED WITH PERCEPTION OF PATIENT-GENERATED HEALTH DATA AMONG OLDER PATIENTS POST LUNG CANCER SURGERY**

Mona Choi, Hyeonmi Cho, Yesol Kim, and Yeonju Kim, *Yonsei University, Seoul, Republic of Korea*

This study examines factors related to the perceived usefulness of patient-generated health data (PGHD) among older patients who underwent lung cancer surgery. This descriptive cross-sectional study involved individuals aged ≥65 years who had lung cancer surgery at least 4 weeks prior. Participants were recruited from an outpatient clinic, and survey data from 102 patients were analyzed. Thirty-seven PGHD items were categorized into 7 groups: Environmental Factors (EF), Lifestyle Factors (LF), Body Composition and Activity Level (BCAL), Medication-related Factors (MF), Physiological Parameters (PP), Physical and Mental Well-being Factors (PMWF), and Digital Engagement (DE). Stepwise linear regression models were used to identify factors associated with patients' perceptions of PGHD. Higher digital literacy was associated with greater perceived usefulness in EF and DE categories. Older individuals perceived PGHD in LF and BDAL categories as less useful. Unemployed individuals found PGHD less useful in LF compared to employed individuals. Higher household income was associated with greater perceived usefulness in PMWF category. Non-smartphone users perceived PGHD as less useful in BCAL and PP categories. Patients who underwent surgery more than 5 years ago found PGHD less useful in MF and PP categories than those with more recent surgeries. Individuals with lower education levels and those living alone showed lower perceived usefulness in PP compared to their counterparts. Understanding patients' personal characteristics, digital literacy, and technology access is crucial for utilizing PGHD effectively for health promotion among older patients with lung cancer surgery. This understanding can assist healthcare providers in identifying and addressing potential challenges.

Abstract citation ID: igae098.3551

**FAMILY EXPERIENCE IN USING TELEPRESENCE ROBOTS WITH RESIDENTS IN LONG-TERM CARE**

Lillian Hung[1], Grace Hu[2], Joey Wong[1], Haopu (Lily) Ren[1], Jim Mann[1], and Lily Wong[1], *1. University of British*

*Columbia, Vancouver, British Columbia, Canada*, 2. *Columbia University, New York, New York, United States*

Informal care, most often provided by family or friends, remains a hidden and underrecognized undertaking within long-term care (LTC) settings. As care recipients transition into LTC, informal care partners experience a parallel shift in their caregiving roles: transitioning from primary care partner to visitor. Many care partners report this transition to be difficult, as they must surrender an unpredictable level of control and involvement over their loved one's care. The COVID-19 pandemic intensified this experience by introducing new challenges: individual health concerns, fluctuating care home protocols, and isolating government policies. Telepresence robots are emerging as a tool that can ease the care partner transition. The main aim of this study is to examine the experiences of care partners who used telepresence robots with loved ones living in LTC settings, during the COVID-19 pandemic. This study took place between May 2021 and August 2023 in five urban LTC homes located in British Columbia, Canada. A total of 20 care partners were recruited through purposive sampling. The care partners participated in semi-structured interviews, in-person or virtually, and thematic analysis was employed to identify four key themes characterizing their experiences using the robot: 1) Decreases care partner burden, 2) Facilitates care partner-staff relationship, 3) Creates relational autonomy, and 4) Expands the scope of what is possible. Findings capture the ability of the robot to enhance the caregiving experience for informal care partners. Further research on the sustainability of robot implementation amongst diverse geographic regions and care home compositions is needed.

Abstract citation ID: igae098.3552

### INFORMATIVE, DULL, INCOMPREHENSIBLE: SEEKING A COMMON VOCABULARY AROUND DATA IN LATER LIFE

Nicole Dalmer, and Cal Biruk, *McMaster University, Hamilton, Ontario, Canada*

With the domestication of digital devices into the everyday, this paper offers critical reflections from two, related studies that both sought to explore how older Canadians communicate their experiences and understandings about the digital technologies and related data they encounter and use. The first study, a virtual, qualitative survey with 70 older Canadians highlighted their understandings of data, and the second, a pilot interview study with 7 older adults in their homes explored how and where data circulates in their everyday lives. Not only do our daily activities and routines rely on data, our routines are often, in turn, converted into data through our many devices (Burgess et al., 2022). Living with data, however, is an experience that differs from person to person and from group to group. Given these differences, how might we communicate about data with different groups? In both studies, we sought to explore and document participants' own diction, images, metaphors, and concepts they articulate when thinking with and about their own data; giving them a space to speak and share "their own data stories louder than those that are being told about them by others" or by other devices (Thorp, 2021, p. 19). In this paper, we offer our observations on the challenges and difficulties (and potential ways forward) in finding a common vocabulary in research relationships between participants and researchers to speak about data and to the many ways that data circulate in and around older adults' everyday lives.

Abstract citation ID: igae098.3553

### LEVELING UP ADHERENCE PREDICTION: MOTIVATION'S INFLUENCE ON ENGAGEMENT WITH TECH-BASED COGNITIVE INTERVENTIONS

Rinanda Shaleha[1], Nelson Roque[1], and Erin Harrell[2], 1. *The Pennsylvania State University, University Park, Pennsylvania, United States*, 2. *National Institute on Aging, Baltimore, Maryland, United States*

In this study, comprising a sample size of N=118 older adults with an age range of 65 years and above, we investigated the impact of individual motivation on adherence behavior within a technology-based cognitive training intervention. Despite previous research indicating limited effects of framing conditions (negative, positive, and disabled) on adherence (Harrell et al., 2021), we hypothesized that individual motivation levels would significantly influence adherence behavior. Motivation is operationally defined based on participants' performance in cognitive training games, where high motivation is indicated by a pattern of low failure rates and successful game performance. Utilizing advanced multilevel modeling techniques, we compared adherence models with and without motivation as a predictor. Our findings supported our hypothesis. The linear mixed model analysis revealed a significant effect of motivation on adherence (Estimate = 1.35939, SE = 0.24490, t (1422.78) = 5.551, p <.001), indicating that for each unit increase in motivation, there is a significant increase in adherence. Likelihood ratio tests (LRTs) comparing the model fit showed significantly divergent log-likelihoods between the two models ($\Delta\chi^2$ (1) = 27.559, p <.001). These findings suggest that high levels of motivation play a crucial role in driving adherence behavior within the context of technology-based cognitive training interventions. By leveraging motivational factors and harnessing the power of gamification, researchers and practitioners can design more effective interventions that promote adherence, facilitate behavior change, and ultimately improve outcomes for participants.

Abstract citation ID: igae098.3554

### NAMING AND STORYTELLING IN SOCIAL ROBOT INTERACTIONS: A NARRATIVE ANALYSIS OF ENGAGEMENT AND PERSONAL CONNECTIONS

Clio Yuen Man Cheng, and Vivian Weiqun Lou, *The University of Hong Kong, Hong Kong, China (People's Republic)*

Social robots have emerged as promising tools with gerontology, offering the potential to enhance human-computer interaction and improve the well-being of older adults. This study employs narrative analysis approach to examine the impact of naming and storytelling in social robot interactions among older singletons. A total of 17 participants, aged between 60 to 73, engaged in 15-minute interaction sessions with a social robot (LOVOT), during which they provided a name for the robot and crafted a story around it. Through a comprehensive narrative analysis, this study uncovers the intricate dynamics of naming and storytelling in shaping older adults' engagement and personal connections within social robot interactions. The analysis reveals that naming and storytelling serve as powerful vehicles for expressing expectations, personal experiences, and emotional connections. Participants drew upon their individual backgrounds, memories, and cultural references to create narratives that reflected their unique perspectives and desires. The narratives showcased the participants' desires for intelligent and emotionally responsive robots while evoking nostalgia through connections to past experiences. Additionally, the narratives revealed a longing for companionship and emotional support, exemplified by associations of robot naming with deceased pets. Understanding the role of naming and storytelling in social robot interactions is crucial for designing robots that effectively cater to the needs and desires of older adults, ultimately enhancing their engagement and overall well-being. This study contributes to the field of human-robot interaction in gerontology by shedding light on the significance of personal narratives in fostering meaningful connections between older adults and social robots.

Abstract citation ID: igae098.3555

### PREDICTING ACCIDENTS OF OLDER ADULTS LIVING WITH DEMENTIA USING ASSISTIVE TECHNOLOGIES AND MACHINE LEARNING

Kyung Hee Lee[1], Eunjin Yang[2], Ji Yeon Lee[3], and Aeyoung Cho[1], *1. Yonsei University, Seoul, Republic of Korea*, *2. Gachon University, Incheon, Republic of Korea*, *3. Inha University, Incheon, Republic of Korea*

Although older adults living with dementia prefer to age in their own homes, they face many challenges such as falls, injuries, and night-time disturbances. Predicting and preventing those challenges could help both older adults and their caregivers to maintain aging in place. This study aimed to develop and validate machine learning models in predicting accident subgroups among individuals with dementia using assistive technologies (i.e., in-home motion sensors and actigraphy). Data were collected from 75 older adults living with dementia and their family caregivers in South Korea. Utilizing 966 days of data on sensors, sleep, and survey from dyads, various machine learning models were developed to predict three accident subgroups—physical impact, night-time behaviors/wandering, and risky behaviors. The performances of five machine learning classification models were compared and important features were extracted. Python 3.9 was used for all analyses. The Gradient Boosting Model excelled in predicting physical impact and night-time behaviors, while CatBoost performed best for risky behaviors, achieving F1-scores of 0.50, 0.71, and 0.87, respectively. Important features were different by type of accidents. For physical impact, caregiver's age, comorbidity severity, and total sleep time were the three top features; for night-time behaviors/wandering, cognitive function and number of medications were the most important features; neuropsychiatric and depressive symptoms were the most important features for risk behaviors. In addition, activity patterns recorded by in-home motion sensors emerged as important features. The findings highlight the potential of these assistive technologies to identify high-risk individuals, provide a safer environment, and support family caregivers.

Abstract citation ID: igae098.3556

### PREFERENCE FOR DIGITAL DEVICES AND PATIENT-GENERATED HEALTH DATA UTILITY IN OLDER PATIENTS AFTER LUNG CANCER SURGERY

Mona Choi, Yeonju Kim, Hyeonmi Cho, and Yesol Kim, *Yonsei University, Seoul, Republic of Korea*

This study aimed to investigate the preference for digital devices and the utility of patient-generated health data (PGHD) among older patients who have undergone lung cancer surgery. A cross-sectional descriptive study included patients at least four weeks after lung cancer surgery and excluded those with cognitive impairments. Participants in this study were recruited from a tertiary hospital outpatient clinic and conducted using a structured questionnaire: 1) patient's general characteristics; 2) experience and duration of use of digital devices; 3) preference for wearable or attachable digital devices for PGHD collection; and 4) PGHD usability. Descriptive and bivariate analyses were performed. 135 patients were analyzed (age = 70.19 ± 11.80 years; 47.0% female). Smartphones (94.1%) were the most experienced digital devices, and the period of use varied from 4 months to 15 years. The wristwatch type was the most preferred wearable digital device (61.5%), and men preferred the belt type. Depending on the level of education, there was a difference in preference between the watch type and the skin patch type, and there was a difference in preference for the glasses type depending on the quality of sleep. The most informative PGHDs were lung function (91.1%), blood pressure (83.7%), and step count (83.7%). The usefulness of PGHD, such as drug side effects and social relationships, differed depending on the number of drugs taken. This study may help healthcare providers select appropriate digital health devices and utilize useful PGHD to improve health outcomes among older patients after lung cancer surgery.

Abstract citation ID: igae098.3557

### THE FEASIBILITY OF PERFORMING A VIRTUAL REALITY MEAL-PREPARING TASK IN YOUNG AND OLDER ADULTS

Oshadi Jayakody[1], Judy Pa[2], Joe Verghese[1], Hussain Doriwala[3], Robert Carrera[3], Sunil Agrawal[3], and Helena Blumen[1], *1. Albert Einstein College of Medicine, Bronx, New York, United States*, *2. University of California, San Diego, La Jolla, California, United States*, *3. Columbia University, New York, New York, United States*

Virtual reality (VR) is an innovative technology to use in Alzheimer's clinical trials to improve cognitive and behavioral skills in older adults. However, the application of VR in older adults raises questions regarding their ability to use VR platforms and concerns regarding simulator sickness (i.e., dizziness). We compared the task completion time, accuracy, simulator sickness and acceptability of a virtual meal-preparing task between 8 young (M age 25, SD 2.6, M years of education 17.1 SD 1.6, 50% women) and 8 older adults at risk for dementia (M age 76.3, SD 7.9, M years of education 12.9 SD 8.3, 62.5% women, 62.5% Black or Hispanic, screened with AD8 dementia screening). Following a practice task, all participants completed the meal-preparing task developed using Unity 3D (V2022 LTS). Participants were seated, wore a headset, and used wireless hand controllers to navigate and perform the task. Results from two-sample t-test showed that i) older adults took a longer time to complete the task (75 Vs 235 seconds; $p < 0.001$) and ii) made more errors ($p=0.04$) compared to young adults but no participants reported simulator sickness (i.e., dizziness, headache, eyestrain, etc.). All participants reported high self-efficacy in performing similar virtual tasks. This pilot study shows that, even though older adults required more guidance during the practice task, they were still able to complete a simple virtual task within an average of < 5 minutes. Additionally, they reported high acceptability (i.e., self-efficacy, level of enjoyment) and willingness to engage in future VR interventions.

Abstract citation ID: igae098.3558

### THE IMPACT OF IMMERSIVE VIRTUAL REALITY MEDITATION ON MENTAL HEALTH OF PEOPLE LIVING WITH DEMENTIA

Yongseop Kim[1], Jungjoo Lee[2], Dylan Kim[3], and Junhyoung Kim[4], *1. University of Utah, Salt Lake City, Utah, United States*, *2. University of Southern Mississippi, Hattiesburg, Mississippi, United States*, *3. Choate Rosemary Hall, Wallingford, Connecticut, United States*, *4. Texas A&M University, College Station, Texas, United States*

Non-immersive, technology-based mindfulness meditation programs have been shown to effectively reduce depressive symptoms and improve the emotional health of older adults. Still, little research has been conducted to assess the mental health benefits of an immersive virtual reality meditation (IVRM) program among persons living with dementia (PLWD). The present study investigates the impact of the IVRM on depression, emotional health, and quality of life among PLWD. This study is based on a single-arm design. PLWD (n=8) received six sessions (three times a week for 2 weeks) of an IVRM program lasting 30-40 minutes each. The IVRM program is used as an individualized, customized meditation tool that provides more than 300 audio tracks, meditation process monitoring, and a selection of meditation environments. The findings indicated significant changes between pre and post interventions, showing a decrease in depression ($M = 2.25$, $SD = 2.12$; $t(7) = 3.0$, $p < .05$, $d = 1.06$) and an increase in emotional health ($M = -3.87$, $SD = 4.05$; $t(7) = -2.71$, $p < .05$, $d = -0.95$). However, a significant difference was not found in quality of life. This study provides suggestive evidence that the IVRM program led to the improvement in emotional health and a reduction in depression among PLWD. Healthcare professionals need to become more aware of the impact of the use of individualized VR-based meditation programs and learn how they might be integrated into their services to improve the emotional health and well-being of their clients.

Abstract citation ID: igae098.3559

### VESTA: A GROUNDBREAKING TOOL FOR ENHANCING FINANCIAL LITERACY AND SCAM RESILIENCE AMONG OLDER ADULTS

Michael Barnett[1], Samuel Van Vleet[2], Rebekah Griffin[1], Matthew Fontanese[1], William Mallender[1], Harrison Boynton[1], and Allyson Coldiron[1], *1. The University of Texas Tyler, Tyler, Texas, United States*, *2. Miami University, Oxford, Ohio, United States*

VESTA (Virtual Economic Simulation and Trust Assessment) is a groundbreaking assessment tool designed to address the escalating challenges faced by older adults in the realm of modern financial capacity and digital literacy. VESTA replicates real-world scenarios in a smartphone setting to assess older adults' ability to safely navigate virtual financial transactions, detect financial scams, and share information securely. This preliminary study explores older adults' ($N = 20$) initial reactions and 5-point ratings of VESTA. Results indicate that older adults positively rated VESTA as applicable to real life ($M = 3.70$, $SD = 0.92$) with realistic representations of messaging, mobile banking, and a smartphone layout – demonstrating the effectiveness of VESTA in simulating real-world scenarios. Older adults agreed that the application provides a good assessment of users' risk for financial scams ($M = 4.05$, $SD = 0.76$), as well as their money management, ability with banking technology, trust judgment, and ability to share information safely. Additionally, older adults viewed VESTA as easy to use, easy to interact with, easy to understand, and an enjoyable gameplay experience, reaffirming its applicability across diverse older adult populations. Older adults positively rated VESTA as useful for everyday life by gathering useful information, testing abilities with financial technology, and educating people on their risk for financial scams ($M = 4.70$, $SD = 0.57$). In the modern financial landscape dominated by new technologies, VESTA enhances financial literacy and detects scam susceptibility among older adults, offering tangible benefits for their financial well-being in the modern financial technology landscape.

### SESSION 7900 (POSTER)

### FLASH POSTER SESSION: ATTITUDES ABOUT AGING AND AGE-FRIENDLY EDUCATION

Abstract citation ID: igae098.3560

### ADDRESSING THE NURSING WORKFORCE GAP IN SKILLED NURSING FACILITIES WITH AN AGE-FRIENDLY CURRICULUM

Diane Brown[1], Sue Fosnight[2], Jennifer Drost[2], Mary Gergis[3], Cynthia Hovland[3], Margaret Sanders[4], Denise Kropp[4], and Michele Gareri[2], *1. University of Akron, Akron, Ohio, United States*, *2. Summa Health, Akron, Ohio, United States*, *3. Cleveland State University, Cleveland, Ohio, United States*, *4. Northeast Ohio College of Medicine, Rootstown, Ohio, United States*

Skilled nursing home quality and staffing are positively correlated. Staffing shortages have been a long-standing problem however, COVID-19 exacerbated the issue. Currently, more than one in four US nursing homes report staff shortages, especially those in direct care to residents, nurse aides and nurses. To encourage workforce growth, a 12-module virtual curriculum was developed including Age-Friendly Health Systems care, interprofessional teamwork, and other relevant topics. Each module included a recorded didactic, pre-post module quizzes, discussion boards, and supplemental videos and articles. Trainees also spent 10 hours shadowing the interprofessional team in a skilled nursing facility. Two cohorts of undergraduate senior nursing students (n=18, and n=35), nurse assistants in training (n=9, cohort 1), and social work students (n=9, cohort 2) have been trained. Outcome measure were pre and post-education using surveys: Nursing Home Attitude, PES-NWI work environment, and ISVS-9 for teamwork attitude. Results of the first nursing cohort rated the highest nursing home attitude items: ability to provide comfort, flexible scheduling and spending time with clients with the lowest rating for working with persons with dementia. The PES-NWI survey showed highest concern for teamwork, adequate staffing and supportive administration with the lowest concern related to use of nursing diagnoses. The ISVS showed similarities pre and post with highest scores for client involvement in care and importance to work as a team, and lowest for comfort leading a team. Administrators of skilled nursing and educators can use this information to close the gap on improving interest in this setting for new graduates.

Abstract citation ID: igae098.3561

### AGE-FRIENDLY ESCAPE ROOM: A POLYPHARMACY QUANDARY

Betsy Yang[1], Caroline Park[2], Carter Davis[2], Joanne Weith[3], Simeon Kang[4], Silvia Tee[5], Christine Gould[2], and Vinita Shastri[2], *1. Palo Alto VA Medical Center, Palo Alto, California, United States*, *2. VA Palo Alto Healthcare System, Palo Alto, California, United States*, *3. Department of Veterans Affairs Palo Alto, Palo Alto, California, United States*, *4. Geriatrics Research and Educational Clinic Center (GRECC), Palo Alto, California, United States*, *5. Stanford University School of Medicine, Stanford, California, United States*

Gamification, or adding elements like interactivity and puzzle-solving to teaching, has the potential to promote engagement in geriatric medical education. We organized a 60-minute escape room at a regional internal medicine conference to teach the 4Ms (What Matters, Medications, Mobility, and Mentation) Age-Friendly framework to interprofessional providers through gamification. 15 interprofessional participants received a short patient case and formed small teams to "Escape the Room" by rotating through each of the respective stations of 4Ms, moderated by interdisciplinary preceptors, to learn about salient points of geriatric care with the specific "M" in mind for the patient case. At the end of their 4 station rotations, participants received a clue to solve the final puzzle using the 4Ms framework which answers the question "Which medication will we deprescribe first to treat our patient experiencing cognitive impairment, polypharmacy, and falls?" From the retrospective post-then-pre questionnaire, we evaluated participants' confidence and knowledge of 4Ms and workshop effectiveness. Results showed significant improvement in participants' confidence (2.73) and knowledge (2.24) of 4Ms (on a 10-point Likert Scale, $p< 0.01$, $n=15$, Wilcoxon Signed-Rank Test). Participants strongly agreed with recommending the workshop to others ($\mu=4.9$ out of 5, $SD=0.4$) and applying 4Ms to their practice ($\mu=4.9$ out of 5, $SD=0.3$). Qualitative feedback indicated the escape room was both "useful" and "engaging", though some participants recommended allotting more time to the activity. With this workshop, we demonstrated that an interprofessional escape room can teach important geriatric concepts in an effective and engaging manner.

Abstract citation ID: igae098.3562

### BUILDING BRIDGES ACROSS GENERATIONS: THE INTERGENERATIONAL CLASSROOM

Jessica Hsieh[1], Raza Mirza[1], Christopher Klinger[1], Julianna Hill[1], Heather Janes[2], and Claudia Osmond[2], *1. University of Toronto, Toronto, Ontario, Canada*, *2. Christie Gardens, Christie Gardens, Ontario, Canada*

The issue of aging demands greater attention in education systems, and intergenerational approaches help to combat ageism and improve the lives of older adults, now and in the future. Taking an intergenerational approach to contextualizing the experiences of older adults within post-secondary classrooms settings is a novel approach towards building interest in the field of aging. The University of Toronto (UofT) partnered with Christie Gardens, a retirement community and long-term care home, to launch an innovative experiential learning initiative: The Intergenerational Classroom. Half the learners were UofT undergraduate students (n=24); the other half were older adults residing at Christie Gardens (n=25). Through interactive seminar-style discussions, collaborative projects and mentorship, the course, which was held at Christie Gardens, provided a semester-long exploration on aging. To evaluate the program, pre/post-surveys were administered to all learners, and following the semester, students (n=6) and older adults (n=6) participated in focus group discussions. Outcomes of program success were identified across domains, including meaningful friendships and bonds created, increased awareness of aging issues, reduced ageist attitudes, and greater sense of community and civic engagement. Evaluations revealed that 92% had an excellent learning experience, 95% found the course intellectually stimulating, and that 100% would recommend the course to others. The Intergenerational Classroom explored aging from a viewpoint that considered the perspectives of both older and younger generations. Intergenerational approaches can help to create a brighter and more inclusive future for all generations, ensuring that individuals can grow up and grow older with dignity, rights, and opportunity.

Abstract citation ID: igae098.3563

### OPTIMIZING CARE OF OLDER ADULTS BY USING 4MS AT A COMMUNITY HEALTH CENTER

Karla Tejada Arias[1], Elizabeth Hernandez[2], Jane Borrero De Jesus[1], Andrea Rink[3], Richard Marottoli[4], Jennifer Ouellet[3], Noelle Fry[3], and Barry Wu[3], *1. Fair Haven Community Health Care, New Haven, Connecticut, United States*, *2. Yale New Haven Hospital, New Haven, Connecticut, United States*, *3. Yale University, New Haven, Connecticut, United States*, *4. Yale University School of Medicine, New Haven, Connecticut, United States*

Given the increasing needs of the aging population and limited availability of geriatricians, it is crucial for community healthcare centers to develop effective approaches for geriatric care. Fair Haven Community Health Care, implemented the 4Ms Framework of Age-Friendly Care, from the Institute for Healthcare Improvement for patients 65 and older starting July 2023 at two different clinical sites. We employed screening tools and interdisciplinary team including patient-access, medical assistants, nurses, clinicians and resident physicians, to evaluate cognitive health, mobility, and medication regimens, prioritizing patient-centered care. The Electronic Medical Record was enhanced to include fall risk and cognitive assessments. Feasibility was assessed through surveys. This intervention has been applied to 196 patients, 54% of whom identified as Hispanic, 29% white and 13% African American. A staff survey (N=23) revealed a 100% satisfaction rate, with no adverse effects on workflow. Overall visit times remained consistent, with rooming times for patients under the 4Ms framework increasing by only 11%, indicating no substantial impact on workflow efficiency. Additionally, patient satisfaction increased considerably, with patients reporting a greater sense of involvement in their healthcare decision-making process and felt their care was aligned with their priorities, demonstrating a successful transition to patient-centered care. Thus, we have demonstrated that the 4Ms framework can be integrated into standard care for older adults in a busy community health center. This successful integration highlights our ability to deliver evidence-based care and boost patient involvement, signaling a positive transition towards personalized, patient-centered healthcare delivery for this vulnerable demographic.

Abstract citation ID: igae098.3564

### PROMOTING GERONTOLOGICAL TRAINING AND POSITIVE ATTITUDES OF AGING THROUGH HIGH-IMPACT TEACHING PRACTICES

Aimee Fox, and Bethany Riding, *Utah Valley University, Orem, Utah, United States*

Despite being the youngest state, Utah, along with the rest of the United States, has a growing aging population. There is demand from state leaders and employers for individuals, families, and a workforce that are knowledgeable about the experiences and needs of older adults. Utah Valley University (UVU) is Utah's largest public university, with a dual-mission of a first-rate teaching university and open-admissions promoting easy access to higher education. Yet, efforts at UVU to promote gerontological training in the social sciences have been minimal. Students at UVU are often unaware of what gerontology is and the importance of gerontological training, and report negative attitudes and fears about aging and older adults. This descriptive case study is the first stage of a larger project aimed at promoting enrollment in UVU's gerontology certificate and shifting student attitudes about aging to promote positive perceptions of and interactions with older adults and aging experiences. During this first stage, the gerontology certificate was adjusted to align with AGHE gerontology competencies, and an Adult Development and Aging course was re-designed with high-impact practices, including a common intellectual experience focused on service- and experiential-learning activities. This study reports on re-design efforts, the engagement and feedback of 80 students across two sections of the course, and the impact of learning activities on students' attitudes about aging and older adults. Also addressed are theoretical and practical suggestions for promoting gerontological training, potential pitfalls to avoid, and recommendations for implementing these changes across a variety of higher education settings.

Abstract citation ID: igae098.3565

### READINESS FOR AGING: A CONCEPT ANALYSIS

Yanjing Liang, and Basia Belza, *University of Washington, Seattle, Washington, United States*

As awareness of aging grows alongside healthcare advancements, advocacy for older adults' quality of life intensifies. However, people focus more on the physiological, psychological, and social challenges associated with the aging process, limited attention has been paid to entering the pre-aging stage. Understanding of the phenomenon and concept of readiness for aging will enable us to appreciate the opportunities and challenges individuals face as they approach aging. This concept analysis conceptualizes readiness for aging. PubMed, Web of Science, PsycINFO, and CINAHL were systematically searched by using combinations of the terms "ready", "readiness", "prepared", "prepare", "preparedness", "preparation", "aging" and "ageing" in the abstract or title (from the earliest date available to February 2024). Rodgers' approach was employed to clarify the antecedents, attributes, and consequences of readiness for aging. Fifteen eligible articles were reviewed. The findings were categorized into antecedents, attributes, and consequences. Antecedents of readiness for aging were classified into five categories: self-efficacy, social support, aging in place, financial preparedness, and health awareness. Attributes were characterized by psychological resilience, social connectedness, future orientation, financial plan, and health maintenance. The consequences of readiness for aging include quality of life, social well-being, financial security, and healthy aging. Given the limited attention readiness for aging has received, this concept analysis offered a refined and clarified conceptual definition of readiness for aging. Through literature reviews, qualitative and quantitative data collection, and collaboration with healthcare professionals, and legislators, future research can prioritize and enhance conceptual development, fostering holistic approaches to aging.

Abstract citation ID: igae098.3566

### TRACING THE FOOTSTEPS OF THE SCHOLARS FROM DE TORNYAY CENTER FOR HEALTHY AGING AT UNIVERSITY OF WASHINGTON

Yanjing Liang[1], Priscilla Carmiol-Rodriguez[2], Sarah McKiddy[1], Paige Bartlett[1], Heather Wicklein Sanchez[1], and Basia Belza[1], *1. University of Washington, Seattle, Washington, United States*, *2. University of Washington, Washington, United States*

The de Tornyay Center for Healthy Aging has been pivotal in advancing research, education, and practice since 1998. The center faculty has mentored over 165 students, connected them to community partners, awarded them over $757,000 in research funding, and supported the dissemination of their work through travel scholarships. This paper presents the evolution of the scholar community by showcasing scholars' contributions over time. An online survey was distributed to 87 former de Tornyay Center scholars, resulting in 32 responses. Results indicate that after graduating scholars pursued careers in practice (n=21, 65.6%), teaching (n=18, 56.2%), and research (n=13, 40.6%). Contributions include publishing in high-impact journals, securing research funding from institutions like the National Institutes of Health, and pioneering innovative practice models. Scholars demonstrated leadership in the COVID-19 response and made impactful changes in clinical settings. Scholars have engaged in shaping the next generation of nursing professionals through teaching, developing educational models, and contributing to professional service globally. Scholars credited the de Tornyay Center with boosting their research skills (n=24, 75%) and providing crucial career guidance and mentorship (n=24, 75%). Early involvement in scientific research at the center influenced students' academic paths, fostering a commitment to gerontological nursing. These findings contribute to understanding scholars' journeys and form a foundation for shaping the center's strategic plan. The results underscore the center's role in nurturing scholars who significantly contribute to healthcare, cultivating a talented workforce, and influencing scholars' career paths. The de Tornyay scholar program is instrumental in fostering professionalism in scholars' careers.

### SESSION 7905 (POSTER)

### FLASH POSTER SESSION: LIVING WITH DEMENTIA

Abstract citation ID: igae098.3567

### ADAPTED CONVERSATION ANALYSIS TO EVALUATE DYADIC DEMENTIA-RELATED ADVANCED CARE PLANNING DISCUSSIONS

Sara Bybee[1], Rebecca Utz[2], Jordana Clayton[2], Nancy Aruscavage[2], Katherine Supiano[2], Eli Iacob[2], and Kara Dassel[3], *1. University of Utah College of Nursing, Salt Lake City, Utah, United States*, *2. University of Utah, Salt Lake City, Utah, United States*, *3. University of Utah, Salt Lake Cty, Utah, United States*

Persons living with dementia (PLWD) and their care partners should engage in advance care planning (ACP) prior to serious cognitive decline. This pilot study assessed the effectiveness of the Life-Planning in Early Alzheimer's and Other Dementias (LEAD) Guide in promoting ACP concordance among PLWD and their care partners. Discussions of dyads' (N=7) responses to the LEAD Guide were audio/video-recorded and analyzed by two coders for the following elements of Conversation Analysis (CA) (related to ACP concordance and relationship quality, respectively): turn-taking (listening leading to understanding, respect/mutual regard); Repair (giving and taking to gain shared understanding, apology); Action formation (assumed topic of discussion and norms, agreement on discussion subject and scope); Action ascription (trusting, understanding of previous turn); Action sequencing (acceptance of discussion norms towards goal, unconditional love). Coders demonstrated moderate agreement across all recordings (M=73.1%, SD=4.4), with unanimous agreement on: the presence of listening leading to understanding, assumed topic of discussion and norms, agreement on the subject and scope of discussion, and understanding of previous turn in all recordings, and the absence of apology and blind trust in 3 (42.8%) recordings. CA may be unable to capture ACP concordance as some elements may be inherent to the LEAD Guide discussion itself while others are unobservable in ACP conversations. To objectively examine how interactions foster ACP concordance, CA methodology should account for 1) multiple ACP discussions, 2) factors related to either the LEAD Guide or dementia diagnosis that impact turn-taking, and 3) the dynamics between the PLWD and their care partner.

Abstract citation ID: igae098.3568

### ADVANCING CULTURALLY INCLUSIVE RESEARCH AND TOOLS IN ALZHEIMER'S DISEASE AND RELATED DEMENTIAS (ADRD)

Maryam Abdel Magid, Jean Taylor, and Anne Turner, *University of Washington, Seattle, Washington, United States*

**Background and Significance:** Existing research underscores cultural norms' influence on healthcare decisions but lacks depth in exploring their impact on dementia care transitions, critical for improving ADRD care outcomes. Research Aims include (1) Analyze interviews with dementia care providers and staff to better understand how cultural norms influence the decision-making processes for care transitions. (2) Explore methods used to facilitate inclusive and engaging discussions regarding care transitions.
**Methods:** Nested in the NIA-funded Decision Making in Alzheimer's Research (DMAR) study, we examined interviews with 18 providers and staff on dementia care transitions. Prioritizing inclusivity, we leveraged the Alzheimer's Disease Research Center (ADRC) registry and community partnerships coupled with tailored methods including diversifying the team and targeted outreach efforts. We're employing a novel framework to analyze dementia care transitions, integrating societal and cultural aspects for nuanced understanding. This approach accounts for intergenerational culture, cognition perception, and care expectations, evolving to capture cultural nuances. Qualitative findings will be enriched with quantitative sentiment analysis, validated manually for accuracy.
**Key findings:** Novel framework will allow us to identify and analyze cultural norms affecting care transitions, as perceived by ADRD providers.
**Implications:** The study's findings advocate for inclusive decision-making tools in ADRD care to accommodate diverse patient preferences. Future steps involve applying this framework to patients and caregivers and integrating findings into diverse settings involved in care decision-making. Funding statement: Funded in part by NIA grant #R01 AG066957 and the NLM T15 training grant #5T155LM007442-22. Content responsibility lies with authors, not representing NIH views.

Abstract citation ID: igae098.3569

### CO-CREATING RESOURCES TO RAISE AWARENESS OF RISKS OF GOING MISSING AMONG PERSONS WITH DEMENTIA

Christine Daum[1], Vanessa Vahedi[1], Isabella Chawrun[1], Noelannah Neubauer[1], Emily Rutledge[1], Adebusola Adekoya[1], Antonio Miguel-Cruz[2], and Lili Liu[1], *1. University of Waterloo, Waterloo, Ontario, Canada*, *2. University of Alberta, Edmonton, Alberta, Canada*

Persons living with dementia are at risk of getting lost and going missing due to wayfinding challenges. Yet, there are few accessible resources available that raise awareness of the risks of going missing and strategies to manage these risks. The purpose of this project was to develop engaging and informative learning resources for first responders, search and rescue personnel, persons living with dementia, care partners, and health and social care providers. Co-design and co-production approaches guided the development of these resources and were informed by reviews of the academic and grey literature, dialogue with persons with lived experiences, and content analysis of missing person cases. We produced three sets of resources: 1) nine dementia-friendly first responder videos for police and search and rescue personnel to enhance their awareness of dementia and risks of going missing; 2) a toolkit of strategies and resources for persons living with dementia and care partners to reduce the risks associated with getting lost and going missing; 3) eight personas and scenarios that depict contextual factors within missing events that can be utilized by service providers and the public in their interactions with persons living with dementia and care partners. All resources were reviewed by participants through interviews, focus groups, and written feedback to identify essential content and ensure accuracy, relevance, and comprehensibility. These sets of learning resources can elevate awareness and offer practical strategies to support persons living with dementia, care partners, first responders, and service providers.

Abstract citation ID: igae098.3570

### COST EFFECTIVENESS ANALYSIS OF IMPLEMENTING A SCREENING PROGRAM FOR EARLY-STAGE ALZHEIMER'S DISEASE DETECTION

Yeongin Jung, Kaili Wu, Mashael Alnufeay, Xiaotong Jin, Jeromie Ballreich, and Emmanuel Drabo, *Johns Hopkins University, Baltimore, Maryland, United States*

Recent advancements in Alzheimer's Disease(AD) diagnosis have been propelled by the advent of disease-modifying therapies and the emergence of plasma-based biomarkers, notably P-tau217, which offers diagnostic accuracy comparable to cerebrospinal fluid assays and enables early disease detection. This study evaluates the cost-effectiveness of two AD early detection strategies for individuals aged 65 and above showing early-stage neuropathological abnormalities: solely using blood biomarker testing(P-tau217) and a sequential approach starting with plasma biomarker followed by confirmation via Aβ-PET scan. The cost-effectiveness of two early screening methods in a representative cohort of older adults in the US aged 65 and above with early-stage brain abnormalities(n=1000) is evaluated: 1) P-tau217 blood biomarker testing alone(Strategy 1); and 2) P-tau217 blood biomarker testing followed by confirmatory Aβ-PET scan(Strategy 2). Employing a hybrid model combining a decision tree and a Markov model, we simulated screening decisions, outcomes, costs, and quality-adjusted-life-years(QALYs) across AD progression stages(preclinical, mild, moderate, severe) and death over 35 years. Results suggest that Strategy 1(blood biomarker testing alone) dominates Strategy 2, producing 891 incremental QALYs at the additional cost of $49.2million translating to an incremental cost-effectiveness ratio(ICER) of $55,194/QALY gained and an incremental net monetary benefit of $39.9million at the decision threshold of $100,000/QALY gained. These results were attributable to the direct medical costs from delayed AD identification by false-negative misclassifications, and the avoided disutility from Aβ-PET. The findings were robust to uncertainties in model input parameters and highlight the utility of blood biomarkers as a cost-effective preclinical AD screening option in US older adults.

Abstract citation ID: igae098.3571

### EXPLORING THE LANDSCAPE OF GENERATIVE AI AND LARGE LANGUAGE MODELS IN ALZHEIMER AND DEMENTIAS RESEARCH

Duo Wei[1], Jeannine Elmasri[1], Bianca Hernandez[2], Riya Goyal[1], and David Burdick[1], *1. Stockton University, Galloway, New Jersey, United States*, *2. New Jersey Institute of Technology, Newark, New Jersey, United States*

With the introduction of ChatGPT in November 2022 and Google Bard (now Gemini) in March 2023, the artificial intelligence (AI) landscape has undergone a significant and rapid evolution. An in-depth scoping review was utilized to investigate the application of generative AI and Large Language Models (LLM) in research on Alzheimer's Disease and Related Dementias (ADRD) among older adults. The objectives were twofold: first, to investigate specific applications of generative AI and LLM in ADRD research, and second, to delineate the diverse usage of these technologies within this domain. Leveraging PubMed, search terms such as "AI," "Dementia/Alzheimer," and "Older Adults" were employed, resulting in the retrieval of 196 articles published in January 2022-December 2023. Of 196 articles found, 145 meeting eligibility criteria were meticulously categorized into two topics: ADRD domain and AI-related tasks. Statistical analysis revealed compelling insights, with significant differences in the application of generative AI ($\chi 2 = 21.91$, $p < .005$) and LLM ($\chi 2 = 21.42$, $p < .005$) in ADRD research. Notably, generative AI predominantly focused on "Research/Drug Discovery" (34.38%) and "Improved Treatment & Care" (37.50%), while LLM emphasized "Challenges/Opportunities" and "Early Detection/Diagnosis." Within the ADRD domain, articles were categorized into distinct areas such as "Care," "Cognitive Decline," "Diagnosis," "Genetics," "Imaging," and "Other." Caregiving and support emerged as predominant themes, comprising 55% of the reviewed articles, followed by diagnosis (34%). This comprehensive analysis sheds light on the multifaceted applications of AI in ADRD research, offering valuable insights into potential areas for further investigation.

Abstract citation ID: igae098.3572

### FUNCTIONAL OUTCOME IN HOME HEALTH AMONG TRADITIONAL MEDICARE AND MEDICARE ADVANTAGE BENEFICIARIES WITH DEMENTIA

Rashmita Basu, *East Carolina University, Greenville, North Carolina, United States*

This study examines the difference in functional outcomes among Traditional Medicare (TM) and Medicare Advantage (MA) beneficiaries with Alzheimer's disease and related dementias (ADRD) during a home health admission, which is unknown. This cohort study used data from the 2019 Outcome and Assessment Information Set (OASIS) and Medicare claims (Home Health (HH) and inpatient), enrollment, and Chronic Condition Warehouse (CCW) files from the CMS. Functional outcome was measured as a change in the composite ADL score (grooming, bathing, toileting, dressing, walking, transferring, ambulation, and hygiene) from HH admission to discharge. A binary logistic model with an outcome variable (1/0: if the composite score was ≤0 (improvement or static) at discharge (1), and 0 if score >0 (decline) from HH admission to discharge) was estimated. The independent predictor was a binary indicator of MA enrollment (if enrolled ≥1 month in 2019). The ADRD cohort was identified based on CCW criteria and ICD-10 codes from inpatient and HH claims. The analysis included 81,735 Medicare beneficiaries; 30.5% had MA, and 69.5% had TM. There was no difference in the number of HH visits between TM and MA enrollees (2.14 vs. 2.13; P=0.39). Adjusting for patients' demographics, total number of diagnoses, and availability of caregiving support, MA enrollees were 26% less likely to report improvement or maintenance of ADL composite score (95% CI: 0.71-0.77) than TM. A less favorable outcome for MA enrollees highlights the importance of addressing the inefficient use of home health services among people with ADRD in MA.

Abstract citation ID: igae098.3573

### HEALTH CARE WORKERS' PERSPECTIVES ON PROVIDING PALLIATIVE CARE TO MULTILINGUAL POPULATIONS WITH DEMENTIA

Katyayani Strohl[1], Neeta Ghosh[2], Syeda A Fatima Ahmed[2], Danae Dotolo[3], and Rashmi Sharma[2], *1. University of Houston, Houston, Texas, United States*, *2. University of Washington, Seattle, Washington, United States*, *3. University of Washington Medicine, Seattle, Washington, United States*

**Importance:** Persons living with dementia (PLWD) who prefer a language other than English (PLOE) are at high risk for sub-optimal palliative care. Although data suggest that multi-level (individual, clinical, healthcare system, community/policy-level) interventions may help address inequities, few studies have identified specific targets. Objective: To identify targets for multi-level interventions to improve palliative care for PLWD and PLOE.

**Methods:** We conducted qualitative interviews with social workers and medical interpreters who work with Chinese, Spanish, or Vietnamese speaking PLWD. Interviews were conducted from October 2022-October 2023, audiotaped, transcribed, and coded by an interdisciplinary investigator team using an inductive iterative approach.

**Results:** Interviews were conducted with 5 Spanish, 2 Chinese, and 2 Vietnamese interpreters and 5 social workers. Four themes emerged: 1) interpretation when the patient 'wasn't making sense'; 2) complexity with patient/family dynamics in decision-making; 3) barriers to anticipatory communication about dementia trajectory and quality of life; and 4) lack of culturally- and language appropriate resources. These data suggest multi-level intervention targets to improve palliative care in the context of dementia and linguistic diversity: 1) training interpreters about palliative care and how to navigate interpretation with PLWD, and providing clinicians communication skills training; 2) facilitating clinician-interpreter pre-meetings; 3) improving staffing and resources for social workers to shift from a crisis-focus to facilitating goals-of-care communication; and 4) increasing culturally and linguistically-appropriate and accessible resources. Impact: Our findings contribute to a gap in the literature about how to address the unique barriers PLWD with PLOE face, informing future intervention development.

Abstract citation ID: igae098.3574

### IMPLEMENTATION OF DEMENTIA FRIENDS IN WASHINGTON STATE

Katie Zeitler, *University of Washington, Seattle, Washington, United States*

Dementia Friends (DF) is a global movement whose mission is to raise awareness about dementia and the challenges associated with its stigma. The purpose of this presentation is to trace the journey including the evidence supporting the implementation of DF in Washington State and describe next steps. The movement has 13.5 million participants worldwide and utilizes a train-the-trainer model to deliver information sessions to members of the community that discuss what dementia is, five key messages, and communication tips. From 2018-2019, DF was piloted in Washington with the support of the Dementia Action Collaborative. Data showed that Dementia Attitudes Scale scores improved after a DF session and that this improvement was maintained through the one-month follow up. The results from the pilot program led to the approval of funding from Washington state for the implementation of the program in 2022. The state lead for DF Washington, the UW Memory and Brain Wellness Center, implemented the program by partnering with Regional Lead Organizations to assist in deploying the train-the-trainer model to expand DF in their regions. Each Regional Lead Organization nominated a Regional Coordinator to oversee the DF program in their region and train new volunteer "DF Champions" to lead information sessions. Funding from the state and partnerships with Regional Lead Organizations have facilitated the expansion of DF program from 214 participants in 3 counties in 2019 to 2,170 participants in 24 counties in 2024. A key challenge for DF will be cultivating community partnerships in rural areas and underserved populations.

Abstract citation ID: igae098.3575

### THE EXPERIENCES OF DISCLOSING ALZHEIMER'S DISEASE IN THE US WORKPLACE

Allison Gibson[1], Kathryn Coccia[2], and Lauren Hasten[3], *1. Saint Louis University, St Louis, Missouri, United States*, *2. Saint Louis University, Chicago, Illinois, United States*, *3. Saint Louis University, St. Louis, Missouri, United States*

With the shift of aging adults delaying retirement, more individuals are being diagnosed with Alzheimer's disease and related dementia (ADRD) while still in the workforce. Using a modified grounded theory approach, 16 people living with ADRD were interviewed about their experience receiving a diagnosis while still in full-time employment (mean age 66). Thematic analysis was employed to examine the study's aims with member checking and an audit trail to enhance study credibility and trustworthiness. Many study participants shared their diagnosis with employers, resulting in positive experiences. Study findings indicated that participants were more likely to disclose their diagnosis to their employer when they felt supported by their supervisor, perceived their employer would offer them reasonable accommodations to continue working, or felt they were well positioned to retire. Those who did not disclose their diagnosis did so because they feared reasonable accommodations were either not available or accessible, and they reported significant financial stress. Study implications suggest those newly diagnosed need support regarding ADRD and employment, and human resources personnel need more training on ADRD workers. While not all participants were able to continue in the workforce following diagnosis, many could through flexible work roles and supportive environments. Many study participants were able to continue in their full-time positions until they retired or elected to shift employment to a new role or employer. However, some experiences highlight the continued challenges of accessing disability resources. This presentation will conclude with recommendations for specialized policies, training, and future research.

### SESSION 7910 (POSTER)

### FLASH POSTER SESSION: ADRD AND LONG-TERM CARE

Abstract citation ID: igae098.3576

### A REFLECTION OF A STUDENT-LED VIRTUAL REALITY ADOPTION PROGRAM FOR PEOPLE LIVING WITH DEMENTIA IN LONG-TERM CARE

Joey Wong, Karen Lok Yi Wong, Mary Van, Yumna Hayat, Christine Wallsworth, Jim Mann, Lily Wong, and Lillian Hung, *University of British Columbia, Vancouver, British Columbia, Canada*

Previous research suggests that virtual reality (VR) holds promise in improving residents' well-being in long-term care (LTC). With ongoing staff shortages, there is a critical need to explore, evaluate and share learnings on innovative approaches for adopting technologies, including VR, in LTC. In our research study conducted at two LTC homes in Vancouver, Canada, undergraduate students supported staff members in VR adoption. This study reflects on the opportunities and challenges of VR adoption from the students' perspectives. Utilizing a critical reflection framework, our interdisciplinary team engaged in extensive discussions and reflections via Zoom meetings, resulting in the following five themes: (1) Challenging the stigma towards dementia in LTC, (2) Addressing ageism in older adults' technology usage, (3) Building relationships through consistent and repeated visits to facilitate technology adoption, (4) Recognizing students' value in technology implementation and their potential impact on LTC's future workforce, and (5) Recognizing the importance of emotional and organizational support. Based on these reflections, our team devised the "ENRICH" framework, offering six practical tips to support student involvement in enhancing technology adoption in LTC. The team's insights highlight the potential of engaging university students in promoting technology adoption in LTC settings. The "ENRICH" framework provides a practical approach to fostering technology uptake while offering valuable learning experiences for students. This study encourages further exploration of student participation in LTC research to advance future initiatives in this field.

Abstract citation ID: igae098.3577

### A SYSTEMATIC REVIEW OF QUANTITATIVE STUDIES OF LONELINESS AND SOCIAL ISOLATION IN LONG-TERM CARE

LaTonya Smith[1], and Laurie Theeke[2], *1. George Washington University, Washington, District of Columbia, United States, 2. The George Washington University, Ashburn, Virginia, United States*

Loneliness and social isolation, though conceptually different, are both prevalent conditions for adults in long-term care facilities. These individuals, often separated from their previous lives, communities, and families, can experience a significant disconnect, leading to a pervasive sense of isolation and abandonment. Both conditions elicit a stress response, exacerbate physical health issues, lead to anxiety and depression, and significantly decrease quality of life and longevity; making it critical to understand these conditions in this population. The purpose of this paper is to present a systematic review of quantitative studies that operationalized loneliness or social isolation in samples of older adults in long-term care. The literature search used EbscoHost and included MEDLINE Complete, CINAHL complete, APA PsycArticles, and APA PsycINFO and resulted in 20 articles for inclusion. The synthesis of study findings makes it clear that loneliness and social isolation are associated with harmful health outcomes with up to 56% of residents reporting loneliness. Older adults with more functional ability and higher resilience scores were less lonely. Those with pain and those with rumination, deterministic thinking and confabulation reported increased loneliness. Intervention programs that included gardening, music, videoconferencing with families, laughter yoga, and peer mentor programs were effective for diminishing loneliness. Future studies are needed for comparative effectiveness of current interventions and for the testing of new interventions designed for this setting that are more precise to each condition. Clinical protocols for assessment and treatment of loneliness and social isolation in long-term care could enhance resident experience and potentially, longevity.

Abstract citation ID: igae098.3578

### DESIGN ELEMENTS OF EDUCATIONAL TOYS FOR PATIENTS WITH MILD ALZHEIMER'S DISEASE: A QUALITATIVE STUDY

HongFei Jia, YaPing Ding, and Ling Zhu, *Nanjing Medical University School of Nursing, Nanjing City, Jiangsu, China (People's Republic)*

**Background:** Alzheimer's disease (AD) is a fatal chronic neurodegenerative disease, and its incidence and mortality rate are closely related to age. Once diagnosed, patients need to take long-term medication, which seriously affects the quality of life of those who are ill. Research has demonstrated that the educational toy is an effective intervention to improve cognitive function among patients with mild Alzheimer's disease. Despite the rapid growth in developing educational toys, incorporation of stakeholders' views has been limited. Objective: This project aimed to identify the design elements that should be considered during development of educational toys for patients, from the perspectives of patients with mild Alzheimer's disease and their caregivers.

**Materials and Methods:** A total of 10 patients with mild Alzheimer's disease and 12 caregivers were selected. Participants were recruited using purposive sampling. Semi-structured interviews were conducted between August and November 2023. The content analysis method was used to summarize the interview data and refine the themes.

**Results:** Main themes that emerged from the data analysis included the following: (1) materials; (2) structure; (3) function; (4) color; (5) shape.

**Conclusion:** Collecting patients' and caregivers' perspectives through qualitative interviews, the developed puzzle toy can serve as a user-friendly and engaging tool to potentially complement traditional interventions in slowing cognitive deterioration in patients with mild Alzheimer's disease. These design elements can also be considered by developers and researchers when developing and evaluating educational toys for people with mild Alzheimer's disease.

Abstract citation ID: igae098.3579

### EFFECTIVENESS OF AN AGING-RELATED PREPARATION EDUCATION PROGRAM FOR OLDER CHINESE MIGRANTS IN JAPAN

Li Yao, and Harue Masaki, *Chiba University, Chiba, Chiba, Japan*

Introduction and

**objectives:** To resolve the long-term care issues of older Chinese migrants in Japan, we developed an aging-related education program focused on preparation for long-term care consisting of six modules with lectures and discussions through real-time videoconferences. This study aimed to evaluate the effectiveness of this developed education program among older Chinese in Japan.
**Methods:** Older Chinese people and their family members living at home independently in the Tokyo and Kansai areas were recruited. The before-after study was conducted to collect the data from the Preparation for Future Care Needs Scale-14 (PFCN-14) and Attitudes to Aging Questionnaire (AAQ). Data was analyzed by descriptive statistics and the Friedman non-parametric repeated measures ANOVA test.
**Results:** Twenty-two participants with an average age of 73.0 (SD= ±7.18) years completed all questionnaires, of which 14 (63.6%) were female. The median (IQR) PFCN-14 total scores before intervention, after intervention, and one month after intervention were 40.00 (7)%, 46.50 (13)%, and 48.50 (8)% ($\chi^2$=18.644, P< 0.001). There were significant differences between before and after intervention (P=0.016) and between before and one month after intervention (P< 0.001). The median (IQR) AAQ total scores were 82.00 (23)%, 80.50 (22)%, and 79.00 (21)%, respectively, with no significant differences ($\chi^2$=2.533, P=0.279).
**Conclusion:** This education program improved participants' status of preparing for long-term care by promoting awareness, decision-making, and concrete planning actions. It can be used to improve health literacy and the autonomy of later life for older Chinese migrants in Japan.

Abstract citation ID: igae098.3580

### INNOVATIVE COLLABORATION: DEVELOPING DEMENTIA TRAINING WITH STUDENTS, SCHOLARS, AND PRACTITIONERS

John Shean[1], Eva Jackson[1], Tara Redd[2], Madiha Shakir[3], and Shelby Roberts[1], *1. Alzheimer's Association, Chicago, Illinois, United States*, *2. Emory University, Atlanta, Georgia, United States*, *3. Full Tilt Ahead, Arlington, Virginia, United States*

The growing prevalence of Alzheimer's and other dementias and the urgent need for public health awareness necessitates innovative, interactive, and accessible dementia training for students entering the workforce. The curriculum, A Public Health Approach to Dementia, is a new tool to meet this need. Through partnership between the Alzheimer's Association, CDC, and Emory Centers, four new modules were launched in 2023 and 2024. These free, asynchronous, online learning modules support the training needs of the current and future public health workforce. Throughout the development, the team relied on students, researchers, professors and practitioners to ensure the modules provide accessible, comprehensive knowledge on the public health approach to dementia. The curriculum includes courses on the public health impact of dementia, health equity and dementia, and public health and dementia caregiving. Data from the first two modules released in 2023, show that they are impactful. Health Equity in Dementia — Using a Public Health Lens to Advance Health Equity in Alzheimer's and Other Dementia was released in August and has over 520 people enrolled. Public Health and Dementia Caregiving was released in December and has over 200 people enrolled. Both modules had over 900 registrants for each launch webinar. Across both modules, most learners reported an increase in knowledge after taking the course (89% and 92%) and reported that it will be useful in their work or studies (87% and 90%). This curriculum demonstrates the strengths of partnering across backgrounds and expertise to support innovative learning opportunities for students entering the workforce.

Abstract citation ID: igae098.3581

### THE CONTINUOUS LEARNING NEEDS OF PERSONAL SUPPORT WORKERS WHO CARE FOR PEOPLE LIVING WITH DEMENTIA IN LONG-TERM CARE

Grace Norris, Marie Savundranayagam, Afshin Vafaei, and Gail Teachman, *Western University, London, Ontario, Canada*

Personal support workers (PSWs) in long-term care (LTC) homes comprise over half of the workforce responsible for providing care to people living with dementia. Compared to other healthcare professionals, PSWs receive the least education, which fails to equip them with the necessary competencies for quality dementia care. To provide optimal care to people living with dementia, PSWs need to be offered opportunities for continuous education that addresses their specific learning needs. Therefore, this study identified and examined the dementia-specific learning needs of PSWs in LTC. Interpretive description guided the secondary qualitative analysis of 22 focus groups with 'mid-career' PSWs (n = 39) in LTC. The analysis identified specific learning needs along with ways in which those needs are best met. The learning needs include: 1) addressing responsive behaviours, 2) person-centered communication and attitudes, 3) delirium, and 4) dementia as a chronic condition. Learning needs were most commonly attributed to limited preparation during formal PSW education and a lack of continuous training opportunities throughout their career. Learning needs are best met through experiential methods involving peer learning, feedback, and evaluation within a supportive environment. For the learning needs to mediate practical outcomes, there needs to be an openness to dementia education and a good teamwork culture within LTC. The data generated from this research study will be essential to developing future continuing education for PSWs, contributing to knowledge about dementia care within LTC settings, and improving the quality of care provided to people living with dementia.

Abstract citation ID: igae098.3582

### THE IMPACTS OF VIRTUAL REALITY (VR) INTERVENTION ON DEMENTIA CARE EDUCATION: A SYSTEMATIC REVIEW

Xiaoli Li[1], Sandra Collins[1], Xiaoyu Liu[2], Ramesh Mutthina[3], and Chung-Yi Chiu[4], *1. Southern Illinois University Carbondale, Cabondale, Illinois, United States*, *2. Saint Louis University, St. Louis, Missouri, United States*, *3. Southern Illinois University Carbondale, Carbondale, Illinois, United States*, *4. University of Illinois Urbana-Champaign, Champaign, Illinois, United States*

VR technology has been explored for its potential to deepen the understanding of individuals living with dementia, allowing students and caregivers to immerse themselves in the lives of those affected by this condition. To date, research evidence regarding the implementation and effectiveness of VR interventions in this domain have not been evaluated and synthesized. We aimed to address this gap in the evidence. The study protocol was registered with PROSPERO, and literature published from 2000 onwards was searched from six databases: Academic Search Complete, APA PsycINFO, CINAHL, MEDLINE, Web of Science, and PubMed. Additionally, hand searches of keywords and citation tracking were conducted in Google Scholar. Both qualitative and quantitative literature were considered, and study reporting quality was assessed using the JBI Critical Appraisal Checklists. Among the 318 articles initially identified, 22 were selected for the review. The review encompassed 17 experimental studies and five qualitative studies, involving formal / informal caregivers and university students. Sample sizes ranged from 20 to 278 participants, originating from seven countries and regions, utilizing 11 distinct VR prevention programs. Overall, the incorporation of VR in dementia care education emerges as a novel and underexplored research avenue. VR interventions demonstrated enhancements in caregivers' and students' empathy, knowledge, attitude, and understanding of people with dementia, although not all experimental studies found statistically significant changes. VR-based education emerged as a valuable complement to traditional teaching and training methodologies, offering a more engaging and realistic learning experience. However, further research in larger and more diverse samples is needed.

Abstract citation ID: igae098.3583

### THE USE OF MINDFULNESS-BASED INTERVENTION FOR LONG-TERM CARE RESIDENTS: A REVIEW OF THE LITERATURE

Audrieanna Raciti[1], and Yu-Ping Chang[2], *1. Buffalo, New York Mills, New York, United States*, *2. University at Buffalo, SUNY, Buffalo, New York, United States*

Physiological and psychological health is a significant concern among people residing in long term care. Researchers have begun to design and test mindfulness-based interventions (MBIs) as a strategy to lessen the burden of these impacts on long-term care residents as well as our already strained healthcare system. The purpose of this review is to synthesize the use of MBIs and their impact on health-related outcomes and quality of life among long term care residents. Six databases: CINAHL, PUBMED, Cochrane, Web of Science, Psychinfo, and Embase were searched to identify original studies leveraging the use of mindfulness to improve health-related outcomes for long term care residents. Multiple keywords were used including mindfulness-based stress reduction, meditation, mindfulness intervention, long-term care, nursing home, residential care, older adult, elderly, and aged. Nine studies meeting the selection criteria were included in this systematic review. Most of the studies have a small to medium sample size. The types of MBIs include mindfulness-based cognitive therapy, silent meditations, mountain meditations, loving-kindness meditations, awareness exercises, breathing exercises, visualization, and guided imagery. The majority of those MBIs demonstrate a significant improvement in alleviating symptoms of stress, depression, anxiety, relocation anxiety, worsening dementia, and other mental health concerns, while one study also showed significant improvement in A1C levels among residents with type 2 diabetes. However, some studies have a high attrition rate. Future research is needed to utilize a more rigorous design with a large sample size and longitudinal studies. Participant engagement should be considered as part of the intervention activities.

### SESSION 7915 (POSTER)

### FLASH POSTER SESSION: LONG-TERM CARE

Abstract citation ID: igae098.3584

### A COMPARATIVE STUDY OF INTEGRATED LONG-TERM CARE SERVICES IN TAIPEI CITY AND NINGBO CITY

Huan Liu[1], and Hong Mi[2], *1. NingBo University of Technology, NingBo, Zhejiang, China (People's Republic)*, *2. Zhejiang University, Hangzhou, Zhejiang, China (People's Republic)*

This study aims to compare the integrated long-term care service models in Taipei and Ningbo, exploring their similarities, differences, and experiences for mutual learning. Through literature review and field research, it was found that Taiwan's long-term care system has advantages such as comprehensive coverage, diversified services, professional teams, smart technology applications, community participation, policy support, and a supportive social and cultural environment. On the other hand, Ningbo's integrated long-term care services also have unique features in terms of service content and format, emphasizing family care, promoting the integration of medical care and older people care, and developing community rehabilitation services. The comparative study reveals that Taiwan's long-term care system is more mature and comprehensive, with higher service quality and coverage, while Ningbo has certain advantages in the integration of medical care and older people care, as well as in community rehabilitation services. Based on this, the study proposes recommendations for the complementary development of long-term care service models in Taipei and Ningbo, including strengthening policy coordination, promoting inter-departmental cooperation, and enhancing service quality and efficiency, aiming to provide reference for the improvement and development of long-term care services in both regions.

Abstract citation ID: igae098.3585

### FACTORS LEADING TO DISCONTINUOUS USE OF GOVERNMENT-SPONSORED LONG-TERM CARE 2.0 SERVICE PLAN IN TAIWAN

Ya-Mei Chen, and Shih-Cyuan Wu, *National Taiwan University, Taipei City, Taipei, Taiwan (Republic of China)*

The Long-Term Care 2.0 (LTC 2.0) Plan, initiated in Taiwan in 2017, respectively, aimed to provide home- and community-based services (HCBS) for individuals in need. However, a significant proportion discontinued usage within six months, prompting an investigation into influencing factors. This study, analyzing LTC 2.0 data from 2018 to 2021 for older adults in two cities, tracked 59,151 service users for up to a year after their initial assessments through three application stages (assessment to service prescription, service utilization, and continuous service utilization for more than six months). Key factors affecting discontinuation included age, middle-low income family status, behavioral problems, informal caregivers, and foreign caregivers. Individuals with informal caregivers had a higher likelihood of obtaining service prescriptions (OR=1.82***) and utilizing LTC services (OR=1.33***), but were prone to discontinuing LTC 2.0 during continuous service utilization (OR=0.78***). Those with foreign caregivers were less likely to progress beyond the service utilization stage (OR=0.77***), but were inclined to maintain usage once started (OR=1.16***). Similarly, those aged over 85 and individuals with behavioral problems were less likely to progress beyond utilization but inclined to maintain usage (Age OR=1.22***; Behavioral problems OR= 1.14***). In contrast, individuals from middle-low income families were more likely to be prescribed and initiate usage but less likely to maintain it (OR= 0.77***). The presence of informal/foreign caregivers significantly influenced utilization status across these three stages, emphasizing the need for services development that address not only users' needs but also the needs of both formal and informal caregivers.

Abstract citation ID: igae098.3586

### GRIEF AND BEREAVEMENT SUPPORT FOR STAFF IN LONG-TERM CARE HOMES: IDENTIFYING BARRIERS AND FACILITATORS

Cari Randa-Beaulieu[1], Habib Chaudhury[2], Gloria Puurveen[3], and Theresa Pauly[2], *1. Alzheimer Society of British Columbia, Vancouver, British Columbia, Canada*, *2. Simon Fraser University, Vancouver, British Columbia, Canada*, *3. University of British Columbia Okanagan, Kelowna, British Columbia, Canada*

Admissions of older adults to long-term care are occurring at more advanced ages with more complex care needs, resulting in shorter lengths of stay before death and an increased demand for a palliative approach to care. However, long-term care staff often lack access to sustainable educational, organizational, and interpersonal support in end-of-life care, and must learn on the job while facing death in the workplace. To address this gap, we conducted a scoping review of literature on grief and bereavement support for long-term care staff. Following PRISMA-Scoping Review guidelines and the six-step framework by Levac and colleagues, social sciences databases were searched and data were analyzed through narrative synthesis and the creation of infographics. Fifty-eight studies met inclusion criteria. Strategies, interventions, and rituals were organized into five domains:1) Cultural Attitudes Toward Death and Unmet Need for Support; 2) Organizational Policy and Practice; 3) Formal Peer-support; 4) Informal Peer-support; and 5) Individual Beliefs and Self-Care Practices influenced by temporal aspects. Identifying barriers and facilitators of grief support for long-term care staff, and hospice and palliative care specialists' practices, can offer more holistic and effective approaches to long-term care staff wellness to combat burnout and staff turnover. Grief rituals present scalable, holistic approaches fostering long-term care staff wellness that could be explored in future research to collaboratively build long-term care staff capacity for person-centered end-of-life care.

Abstract citation ID: igae098.3587

### LONG-TERM CARE TRAJECTORIES AFTER STROKE IN SWEDEN AND THE UNITED STATES

Karin Modig[1], and Greg Arling[2], *1. Karolinska Institutet, Stockholm, Stockholms Lan, Sweden*, *2. Purdue University, West Lafayette, Indiana, United States*

Stroke is a major cause of mortality and disability in old age worldwide, often requiring ongoing formal long-term care (LTC). Only a handful of studies examining longitudinal patterns of institutional and community based LTC beyond the immediate post-stroke period. In order to better understand the impact of LTC use by stroke survivors, we compared findings from studies of LTC trajectories among people discharged from the hospital after a stroke in Sweden and the United States. The Swedish study modeled LTC trajectories over a three-year period for a nationally representative cohort of 31,560 stroke survivors age 70 and older during 2015 to 2017, while the US study modeled LTC trajectories over 12 months among a sample of 3,811 Veterans discharged from Veterans Administration (VA) hospitals after stroke during 2007. Approximately half of the sample in each study was alive and without a history of LTC use at one year. Use of LTC in both studies was associated with LTC use prior to stroke, advanced age, number of comorbid conditions including dementia, and frailty/dependency. In the Swedish study, transitions into formal LTC tended to be unidirectional - few people entering formal LTC ever returned to no care, and few people entering care homes ever transitioned back to home care. In the VA study, frequent transitions occurred between nursing homes and home-based LTC, as well as out of the LTC system entirely. We discuss how differences in LTC policies, particularly the role of the nursing home, affect LTC trajectories in the two countries.

Abstract citation ID: igae098.3588

### OLDER ADULTS' EXPERIENCES OF LONELINESS AND ITS UNDERLYING MECHANISMS IN RESIDENTIAL CARE FACILITIES

Amarjot Gill[1], and Suzanne Sullivan[2], *1. University at Buffalo, SUNY, Buffalo, New York, United States*, *2. State University of New York Upstate Medical University, Syracuse, New York, United States*

Older Adults' (OAs') transition into residential care facilities (RCFs) detaches them from their meaningful social relationships, often precipitating feelings of loneliness. This prevalent issue in RCFs can lead to poor health outcomes, significantly disrupting OAs' well-being and quality of life.

Consequently, loneliness in RCFs emerges as a serious public health issue, necessitating a comprehensive understanding of its mechanism. This systematic review aims to explore OAs' experiences and perceptions of loneliness and characterize mechanisms underlying OAs' loneliness experiences through a comprehensive loneliness model. This review adhered to PRISMA guidelines. Articles from CINAHL, PubMed, PsycINFO, and Web of Science, published in English between 2018 and 2023, were examined. Of the 7,285 articles identified, 10 met the inclusion criteria. Quality appraisal for the selected studies was conducted using the Critical Appraisal Skills Programme (CASP) checklist. Three core themes of loneliness were identified: relational and individualized loneliness experiences, perception and emotional distress, and the influence of context and cognitive processes in modulating loneliness. A conceptual model delineating the mechanisms of OAs' loneliness in RCFs was developed. This review highlights the influence of OAs' residential context and cognitive processes, particularly their perceptions, in triggering loneliness and associated behavioral responses. OAs' perceptions of reduced social control and insufficient social connections in RCFs exacerbate their loneliness, precipitating distressing emotional responses and diminished quality of life. Future research must explore strategic transformations of OAs' perceptions through mechanistic targets and tailored care plans to reshape their social expectations within the context of RCF, potentially mitigating loneliness.

Abstract citation ID: igae098.3589

### POLICIES FOR SUPPORTING CAREGIVERS OF OLDER ADULTS WITH LONG-TERM CARE NEEDS: A MIXED-METHODS SYSTEMATIC REVIEW

Marco Albertini, Eva Bei, and Federico Toth, *University of Bologna, Bologna, Emilia-Romagna, Italy*

**Background:** In an era marked by the imperative to restrain costs within health and social care services throughout Europe, informal caregivers of older adults assume a pivotal role as primary providers of long-term care. Nevertheless, there remains a shortage in mapping the various social policies pertaining to both direct and indirect support for informal carers across the various EU member states. Objective: This mixed-method systematic review synthesizes data on care policies related to diverse support forms implemented across EU countries to assist caregivers.
**Methods:** A comprehensive search strategy was conducted in three electronic databases and grey literature to minimise publication bias.
**Results:** Preliminary findings of studies published between 2010 and 2023 indicate that various forms and levels of support have been introduced or reformed over the past 13 years across EU countries to support caregivers. Despite the challenges posed by population ageing, there is significant policy development disparity among EU members, with some countries to have established mechanisms aside financial assistance to support carers, while others to only now starting to express interest in developing such services. Financial support, primarily through cash-for-care schemes, emerges as the predominant form of assistance, followed by respite care, while other policies in terms of training and mental health services exhibit a notably lower level of development within the EU social policy landscape.
**Conclusion:** The review is expected to provide significant implications for policymaking, thoroughly mapping the implementation of diverse social care policies across Europe to ensure the sustainability of informal care.

Abstract citation ID: igae098.3590

### THE EFFECT OF MEDICAID MANAGED LONG-TERM SERVICES AND SUPPORT ON PARTICIPANT QUALITY OF LIFE AND UNMET NEED

Howard Degenholtz[1], Anny Treat[2], Keri Kastner[1], and John Yauch[1], *1. University of Pittsburgh, Pittsburgh, Pennsylvania, United States*, *2. Sebastian Mann Associates, Pittsburgh, Pennsylvania, United States*

In 2018 the Commonwealth of Pennsylvania began a three year transition of Medicaid Aging and Disabled Waiver programs to Managed Long-Term Services and Supports (MLTSS). The new program, Community HealthChoices, serves adults with disabilities who receive home and community based services (HCBS), live in a nursing facility, and people dually eligible for both Medicaid and Medicare who do not use long-term services and supports ("duals"). Three managed care organizations operate the program through state-wide contracts. The program was implemented state-wide over a three year period in three regional phases, creating a unique natural experiment. In other analyses, we have demonstrated significant changes to care management and slowing in the rate of growth of HCBS spending. This is the first study of HCBS participants' subjective experience. A total of 14,706 prospective telephone interviews were conducted with random samples of participants before the transition to CHC and 18 and 30 months post-transition. We found that psychological well-being was unchanged, but depressive symptoms decreased over time. There was no change in the consequences of unmet need (e.g., having a hot meal or a bath). In general, when compared duals, HCBS users report lower access to primary care and lower self-reported health status. After the implementation, duals users reported higher health status and HCBS users were more likely to report having a medical care coordinator. The transition to MLTSS brought substantial changes to the way Medicaid LTSS is financed and delivered in Pennsylvania. However, the experience of the average participant has been relatively stable.

Abstract citation ID: igae098.3591

### THE ROLE OF PSYCHOLOGICAL CAPITAL IN MODULATING CAREER ADAPTABILITY AND TURNOVER AMONG NURSING ASSISTANTS

Changxian Sun[1], and Yurong Xing[2], *1. Jiangsu Vocational Insititue of Commerce, Nanjing, Jiangsu, China (People's Republic)*, *2. Nanjing Medical University, Nanjing, Jiangsu, China (People's Republic)*

**Background:** High turnover intention of nursing assistants was detrimental to the sustainability of long-term care. Career adaptability is an important determinant in reducing turnover intention, but little research has explored the mechanism from the perspective of psychological capital. The aim of this study was to analyze the association between career adaptability and turnover intention and to examine the mediating role of psychological capital between career adaptability and turnover intention among nursing assistants in mainland China.

**Methods:** A cross-sectional online study was conducted among 276 nursing assistants from eight nursing homes in Nanjing, China. The participants' career adaptability, psychological capital, and turnover intention were obtained. SPSS 26.0 and Amos 24.0 software were employed for statistical analysis.

**Results:** Career adaptability was positively related to psychological capital and negatively linked to turnover intention ($P < 0.01$). Psychological capital played a fully mediating role ($\beta = -0.085$, $P < 0.05$) in the relationship between career adaptability and turnover intention, and the largest indirect effect was generated through the curiosity dimension. Conclusions: The management of long-term care facilities should focus on assessing the level of career adaptability of nursing assistants. The overall improvement of career adaptability and psychological capital is conducive in reducing turnover intention. Targeted interventions are recommended to improve career adaptability and reduce turnover intentions by increasing career curiosity. Online career adaptability programs can be developed for nursing assistant students to improve their psychological capital and facilitate career transitions.

### SESSION 7920 (POSTER)

### FLASH POSTER SESSION: ACUTE AND PRIMARY CARE

Abstract citation ID: igae098.3592

### CLINICAL PRESENTATION OF INTERSTITIAL LUNG DISEASE AS AN INITIAL MANIFESTATION OF ANTISYNTHETASE SYNDROME

Pamela Salcido De Pablo[1], Carolina Escorcia Encarnación[2], and Alfredo Olvera[3], *1. University Anahuac Queretaro, Queretaro, Queretaro de Arteaga, Mexico*, *2. Universidad Anahuac, Querétaro, Queretaro de Arteaga, Mexico*, *3. Universidad Anáhuac Querétaro, Educación, Distrito Federal, Mexico*

Antisynthetase syndrome (ASS) is an autoimmune disease, categorized as a type of idiopathic inflammatory myopathy, where interstitial lung disease is the most common manifestation, presenting in up to 80% of cases. It is related to antibodies and aminoacyl-transfer RNA (tRNA) and the most common one, anti-Jo. It has an incidence of 0.56 per 100,000 inhabitants, commonly in adults with an average age of 45 to 70 years old. A 72-year-old female, with a medical history of dyslipidemia, depression, and gonarthrosis, presents with dyspnea, hypoxemia, wheezings, asthenia, arthralgias, and myalgias. A chest radiograph showed abnormal parenchyma, with bilateral opacities in peripheral and subpleural zones, diagnosis was compatible with pneumonitis, treated with quinolones, steroids, and short-acting bronchodilators. With partial recovery, TC scan showed patchy ground-glass-like pattern, bronchi ecstasies, and honeycomb zones, spirometry showed restrictive pattern and rheumatology tests reports were negative for antibody anti-Jo-1 and positive in antinuclear antibodies (ANAs). After several differential diagnoses and studies; a diagnosis of antisynthetase syndrome was reached. Although its low prevalence in the general population, the importance of knowing about antisynthetase syndrome lies in understanding the pathology and encouraging research on it, thus achieving a good diagnosis and adequate interdisciplinary management that these patients should require in a syndrome that still exists. For this reason, this case report intends to mention some of the most used tools currently in use, which helped to diagnose this interesting and uncommon case.

Abstract citation ID: igae098.3593

### CULTURAL DIFFERENCES IN MEDICATION BURDEN OF OLDER HOSPITALIZED PATIENTS IN MEDICAL UNITS

Juliana Smichenko[1], Ksenya Shulyaev[1], Efrat Shadmi[1], Nosaiba Rayan-Gharra[1], Nurit Gur-Yaish[2], and Anna Zisberg[1], *1. University of Haifa, Haifa, Hefa, Israel*, *2. Oranim Academic College, Kiryat Tivon, HaZafon, Israel*

Cultural differences are significantly related to the management of illness and symptoms, particularly in hospitalized older adults where medications are often employed for health stabilization. This study aimed to examine cultural variations in medication burden, specifically focusing on sedative and anticholinergic medications, among older patients admitted to medical units. A secondary analysis was conducted on data from 672 cognitively intact older adults (age 65 and above) who were functionally independent. The cohort comprised 104 Israeli Arabs, 165 former-USSR immigrants, and 403 veteran Israeli Jews. Upon admission, socio-demographic, functional, and cognitive assessments were performed, alongside daily evaluations of symptom severity. Medical records provided data on comorbidities, illness severity, and medication prescriptions, allowing for the calculation of the Drug Burden Index (DBI) to assess medication burden. Multiple regression analysis was employed to predict medication burden, considering cultural group, sex, age, functional and cognitive status, symptom severity, illness severity, comorbidities, and length of hospital stay. The model demonstrated significance ($F(10, 662)=11.647$, $p<.001$, $R2=.151$), revealing that being Israeli Arab ($t=-3.174$, $p=.002$) or former-USSR immigrant ($t=-2.157$, $p=.031$) acted as protective factors against medication burden. Symptom severity, illness severity ($t=-5.613$, $p<.001$; $t=4.102$, $p<.001$), and comorbidities ($t=3.775$, $p<.001$) emerged as significant predictors of higher medication burden. These findings underscore the presence of cultural disparities in medication prescribing during hospitalization. Despite similar presentations of symptoms and medical statuses across cultural groups, certain cultural backgrounds are associated with lower medication burdens. Understanding and exploring patient-physician dynamics within cultural contexts are essential for optimizing treatment approaches in diverse patient populations.

Abstract citation ID: igae098.3594

### DEVELOPING OF AN SDM NURSING PRACTICE MODEL FOR OLDER PATIENTS WITH DEMENTIA IN ACUTE CARE HOSPITALS

Taeko Saito[1], and Harue Masaki[2], *1. Nippon Medical School, Inzai, Chiba, Japan*, *2. Chiba University, Chiba, Chiba, Japan*

This study aims to implement a Shared Decision-Making (SDM)nursing practice model for older patients with dementia in acute care hospitals among acute care nurses and evaluate its feasibility. As a preliminary step, a hybrid concept analysis was conducted, and a SDM nursing practice model was developed and refined based on the results of semi-structured interviews with nursing practitioners and researchers. In this study, five acute care nurses were recruited as research participants to confirm the feasibility of this model. Data analysis involved conducting content analysis from verbatim transcripts of individual interviews with the five participants after practice to assess feasibility. Additionally, both before and after the practice, surveys based on SDM-Q9 and Group's SDM, validated tools for assessing the SDM process, were administered to the five research participant nurses and five health care professionals involved in support (MD, MSW), generating data from the perspectives of users and stakeholders. The results indicated the feasibility of implementing the SDM process within the constraints of the acute care setting. Moreover, the study demonstrated that the SDM nursing practice model effectively adheres to the SDM process during acute phases, thereby clarifying the role of clinical nurses in facilitating the Sharad decision-making for older patients with dementia.

Abstract citation ID: igae098.3595

### IMPROVING RECOVERY FROM HOSPITALIZATION IN GERIATRIC PATIENTS THROUGH WHEY SUPPLEMENTATION AND TESTOSTERONE

Michelle Yang[1], Pratik Gongloor[1], Rachel Deer[2], Elena Volpi[3], Shuang Li[1], and Erin Hommel[1], *1. University of Texas Medical Branch, Galveston, Texas, United States*, *2. The University of Texas Medical Branch, Galveston, Texas, United States*, *3. University of Texas Health San Antonio, San Antonio, Texas, United States*

**Background:** Sarcopenia, the age-related loss of muscle mass and strength, remains a significant concern among hospitalized geriatric populations due to functional decline and increased mortality. A previous pilot study demonstrated potential benefits for testosterone or whey supplementation (WS) during and post-hospitalization towards improved physical function and reduced re-admissions. Our follow-up study assesses the feasibility and preliminary efficacy of these interventions.

**Methods:** This Phase I, single center, randomized clinical trial enrolled 80 participants, 65 years+, hospitalized for acute conditions. Participants were randomly assigned to one of four interventions: placebo oral and injectable (PO/PI) supplement, PI-WS, PO-testosterone injection (TI), or WS-TI. WS was provided twice daily during and post-hospitalization. TI was provided once pre-discharge. Testing was conducted at baseline and 4-weeks post-hospitalization. Primary outcome included Short Physical Performance Battery (SPPB) score change. Secondary outcomes included readmission rate and additional measures of physical function and body composition.

**Results:** Mean age was 71.5 years with 70% female. Age-adjusted Charlson Comorbidity Index was 4. Fifty-two participants (65%) completed the study; 15 were re-hospitalized and 13 withdrew or were lost to follow-up. Among those completing the 4-week intervention, we found no statistically significant differences in SPPB score or readmission rate.

**Conclusion:** We failed to demonstrate significant improvements in recovery among geriatric patients receiving testosterone, WS, or combination therapy. Compared to the previous study, participants were younger, healthier, and less likely to complete follow-up. We need further studies with larger samples of frail older adults to elucidate the relationship between these interventions and post-hospital functional recovery.

Abstract citation ID: igae098.3596

### MOBILITY-RELATED PHYSICAL FUNCTION AND AGING IN PLACE FOR OLDER ADULTS WITH CHRONIC CONDITIONS

Marielle Jensen-Battaglia[1], Ying Wang[1], Robert Block[1], Kah Poh Loh[2], Supriya Mohile[2], Emily Agree[3], and Christopher Seplaki[1], *1. University of Rochester School of Medicine and Dentistry, Rochester, New York, United States*, *2. University of Rochester Medical Center, Rochester, New York, United States*, *3. Johns Hopkins University, Baltimore, Maryland, United States*

Chronic conditions may influence difficulty mobilizing inside the home (mobility), which presents a barrier to the goal of aging in place for many older adults. Understanding this relationship is critical as the prevalence of chronic conditions increases. In prior work (currently under review) we used data from community-dwelling adults aged ≥65 participating in ≥2 consecutive rounds (2011-2019) of the NHATS to show self-reported mobility difficulty was positively associated with moves to homes with fewer environmental barriers or more household members, but not to other destinations (e.g., homes with more barriers, nursing home/residential care). In the current analysis we use this dataset of 25,490 person-years of follow up from 5,894 unique participants who moved 1,456 times to investigate modification of this association by chronic conditions [non-skin cancer (5,472/25,490 person-years), myocardial infarction/stroke (6,621/25,490 person-years), dementia (8,011/25,490 person-years)]. All chronic conditions were positively associated with relocation to nursing home/residential care in bivariate multinomial logistic regression models (cancer: OR 1.43; 95% CI 1.02, 2.02; myocardial infarction/stroke OR 1.43; 95% CI 1.04, 1.96; dementia OR 2.87; 95% CI 2.08, 3.96). After adjusting for confounders, only dementia remained associated with moves to nursing home/residential care (OR 1.40; 95% CI 1.06, 1.85) for those without any mobility difficulty. However, the association of mobility with relocation did not vary significantly by chronic condition (p for interaction >0.05 for all conditions and relocation destinations). These results highlight mobility difficulty and barriers in the home environment as potentially modifiable factors to promote aging in place, regardless of chronic conditions.

Abstract citation ID: igae098.3597

### OPENING PANDORA'S BOX: VIRTUAL UNCOVERING OF GERIATRIC SYNDROMES

Ronan Factora, *Cleveland Clinic, Cleveland, Ohio, United States*

Virtual Capacity Evaluations (VCE) were implemented during the COVID-19 pandemic at the Cleveland Clinic Center for Geriatric Medicine to facilitate Adult Protective Services' investigations of elder abuse and neglect where a client's decision making capacity was in question. During the natural process of the evaluation, the unaddressed medical issues were uncovered, specifically identification of cognitive impairment or dementia, gait impairment and falls risk, unexplained weight loss, and non-adherence to medications that increased risk of exacerbation of chronic illness. As many of these persons were also not connected to medical care, these problems were not recognized or managed. In the initial pilot of this program involving 52 individuals, 40 were eventually diagnosed with dementia, 24 had vision impairment, 14 had hearing impairment, 28 were at risk for falls, 24 had unintentional weight loss, and 29 were not taking their medications consistently. Each of these factors potentially limits the person's ability to function independently, threatens their physical/mental health, and increases their risk of hospitalization and institutionalization. Using the Clinical Frailty Scale (CFS) as an aggregate marker of overall health status, 28 were mildly frail (CFS 5), 14 were moderately frail (CFS 6), and 1 was severely frail (CFS 7). Despite the limited focus and purpose of the VCE, communicating the presence and status of these uncovered medical issues to that person's medical provider could facilitate evaluation of management of these problems to mitigate their potential impact on that person's health, functional status, and independence.

Abstract citation ID: igae098.3598

### PERCEPTION OF NEIGHBORHOOD WALKABILITY INFLUENCES PHYSICAL THERAPISTS' WALKING RECOMMENDATIONS FOR OLDER ADULTS

Jennifer Blackwood[1], and Hannah Karczewski[2], *1. University of Michigan, Ann Arbor, Michigan, United States*, *2. University of Michigan Flint, Flint, Michigan, United States*

**Background/objectives:** Neighborhood walkability is the extent to which built and social environments support walking. Walkability influences older adults' participation in outdoor walking. Physical therapists (PTs) may identify factors that influence decisions about outdoor participation in walking. Current understanding of PTs' knowledge and perceptions of walkability, and utilization of walkability assessments is not described and serves as the purpose of this study.

**Methods:** A cross-sectional survey was sent via email to 5000 PTs nationwide with IRB approval. The 40-item survey covered the following: walking and walkability perceptions and assessments, perception of responsibilities, and demographics. Categorical variables were compared using Chi-square analyses.

**Results:** After inclusion and exclusion criteria, a total of 122 outpatient PTs who worked in geriatrics were used for analysis. A significant difference was found between perceptions of whether PTs should assess walkability and whether they assess walkability ($x2=78.7$, $p< 0.001$). Components that influenced decisions to prescribe outdoor walking were prioritized in this order: availability (n=79, 64.8%) and maintenance (n=11, 9.0%) of sidewalks, crime (n=9, 7.4%), hills (n=7, 5.7%), aesthetics (n=6, 4.9%), and other (n=10, 8.2%). Objective measures were not used to assess walkability.

**Conclusion:** Respondents had a significant difference between perceptions of their responsibility to assess walkability and whether they do assess walkability in older adults before prescribing walking. Built environment constructs were prioritized over the social environment. Significance/Implications: Assessment of walkability may allow PTs to identify barriers and make more informed recommendations concerning outdoor walking for older adults. Objective measures are available for PTs when prescribing outdoor walking.

Abstract citation ID: igae098.3599

### RISK FACTORS FOR NINE-YEAR MORTALITY OF OLDER PATIENTS WITH COGNITIVE IMPAIRMENT AT ADMISSION

Hiroshi Yamamoto[1], Kenichi Ogawa[1], Yasushi Hisamatstu[1], and Tatsuya Ishitake[2], *1. Yamamoto Memorial Hospital, Imari, Saga, Japan*, *2. Kurume University School of Medicine, Kurume University, School of Medicine, Fukuoka, Japan*

**Background:** Dementia can be a major cause of mortality and morbidity in geriatric patients. So, it would be essential to assess their mental state in the early. Aims: We aim to appraise the impact on mortality and hospital outcomes by revised simplified short-term memory recall test (STMT-R) and collecting clinical data simultaneous.

**Methods:** The subjects were 612 acute inpatients ≥65 from December 2014 to September 2015. Following the collection of clinical data, survival was subsequently measured for 8-9years until December 2023. An STMT-R score of ≤4 were considered to indicate cognitive dysfunction. To explore the association between the risk factors and mortality in cognitive impairment subjects, Kaplan-Meier method and Cox's proportional hazards regression models were used to examine mortality and survival rates.

**Results:** The mean age of the subjects was 82.1 (±7.94), and 55.9% were female. Cognitive function was classified into three categories according to severity respectively; Incomplete Testing Group (Incomplete), Cognitive dysfunction (Abnormal) and Non-Cognitive dysfunction Group (Normal). 349 patients (57.0%) died during follow-up. Kaplan-Meier and the log-rank tests showed the negative prognostic effect of cognitive impairment, malnutrition, pneumonia and cancer-bearing state ($p < 0.01$). After adjustment for potential covariates the Cox regression showed that mortality hazard is increased with "Incomplete" (HR 3.526 CI 95% 2.388-5.206 $p< 0.0001$) and "Abnormal" (HR 1.676 1.210-2.321 $p=0.0019$).

**Conclusion:** Malnutrition, pneumonia and cancer-bearing state can significantly decrease the survival rate in patients with cognitive impairment at admission. Cognitive impairment also has independently an impact on survival rate in acutely ill geriatric patients.

### SESSION 7925 (POSTER)

### FLASH POSTER SESSION: GLOBAL CONTEXTS OF AGING

Abstract citation ID: igae098.3600

### ANALYZING THE INFLUENCE OF MIDLIFE EMPLOYMENT TRAJECTORIES ON COGNITIVE AGING IN CHILE

Magdalena Delaporte, and Jere Behrman, *University of Pennsylvania, Philadelphia, Pennsylvania, United States*

As the global population ages, understanding the factors contributing to healthy aging becomes increasingly crucial. This paper delves into the relationship between mid-life employment trajectories and later-life cognition, utilizing a Chilean longitudinal dataset. Employing the Group-Based Trajectory Model (GBTM), distinct employment trajectories for males and females between 1980 and 2016 are estimated. Subsequently, we link these trajectories to cognitive function scores to investigate the associations between individual trajectories and cognitive function in later life. Preliminary findings reveal significant associations between certain cognitive domains and labor force participation trajectories. We find four distinct labor force participation trajectories for men and women. Results show that men that have a high probability of continued employment demonstrate better cognitive outcomes, including orientation, memory, attention, and executive function. For women, a higher likelihood of working during between ages 30 to 60 correlates with improved performance in delayed memory, attention, and executive function. These results provide valuable insights for policymakers and practitioners seeking to enhance cognitive well-being among older adults.

Abstract citation ID: igae098.3601

### CAREGIVER ROLE TRANSITIONS IN HOME-BASED ALZHEIMER'S CARE: A SHANGHAI CASE STUDY

Qingwei Wang[1], Fengzhi Zhao[2], and Zixuan Wu[3], *1. Xi'an Jiaotong-Liverpool University, Suzhou, Jiangsu, China, 2. Xi'an Jiaotong-Liverpool University, Suzhou, Jiangsu, China (People's Republic), 3. Johns Hopkins University, Baltimore, Maryland, United States*

Alzheimer's disease poses a growing health crisis in China, exacerbated by the country's rapidly aging population and cultural emphasis on family-based care. Caregivers shoulder immense physical and psychological burdens, often struggling to reconcile personal expectations with the demands of caregiving. This study, based on ethnographic data collected through prolonged engagements with a family in Shanghai caring for an older adult diagnosed with Alzheimer's disease, explores the application of Role Theory. This framework explains the sequential stages of role assumption, enactment, ambivalence, and disengagement, offering insights into the complex dynamics of caregiving within China's cultural context. While existing studies have identified factors influencing caregivers' "willingness" and "psychological stress" primarily through assessments of psychiatric distress, the practical applications of theoretical models in caregiving practices for this population in China, especially those documented through first-hand experiences, remains underexplored. Drawing from diverse sources, including academic research and firsthand accounts, this study discloses the dynamic process of role transitions in practice, highlighting in particular the transition from a career-focused individual to a full-time caregiver and the subsequent role reversal between parents and children as the illness progresses. By clarifying the dynamic interplay of roles and responsibilities within caregiving relationships, insights gained through this in-depth case study applying Role Theory in practice can offer a roadmap for enhancing caregivers' well-being and optimizing patient care outcomes.

Abstract citation ID: igae098.3602

### CHOOSING TO FORGO: LIFE-SUSTAINING TREATMENT DECISIONS FOR SELVES AND RELATIVES AMONG TAIWANESE ADULTS

Duan-Rung Chen[1], Su-I Hou[2], and Yuchi Young[3], *1. National Taiwan University, Taipei, Taipei, Taiwan (Republic of China), 2. The University of Central Florida, Orlando, Florida, United States, 3. State University of New York at Albany, Albany, New York, United States*

**Purpose:** Since its implementation in 2019, less than 2% of adults in Taiwan have signed Advance Directives (ADs) under the Patient Right to Autonomy Act (PRAA). This research explores how the public views the PRAA and its effect on the decision to decline life-sustaining treatments (LSTs) in hypothetical end-of-life scenarios. Method: In August-November 2022, an online survey was administered to 3,922 adults in Taiwan, of which approximately 1,488 (37%) were familiar with the PRAA. These 1488 respondents completed questions regarding their perceptions of the PRAA. They responded to questions about three hypothetical end-of-life situations: terminal cancer, irreversible severe brain damage, and terminal dementia.

**Results:** Over half of the participants declined to receive LSTs for themselves and chose similarly but less for their family members. A specific difference was noted in choices concerning terminal dementia, showing a greater reluctance to withhold LSTs, especially for parents, than for themselves. There were gender-based differences, with women more likely to choose against LSTs for themselves in various situations. In contrast, gender did not affect decisions made for family members. The perception of the PRAA and the degree of ACP engagement significantly influenced the refusal of LSTs for both the participants and family members. A positive view of the PRAA was significantly linked to decisions against LSTs for spouses.

**Conclusion:** These findings underscore the complexity of decisions around LSTs, influenced by personal beliefs, gender, legal perceptions, and engagement for advance care planning.

Abstract citation ID: igae098.3603

### DIETARY BEHAVIORS OF AFRICAN IMMIGRANTS LIVING WITH DIABETES AND/OR HYPERTENSION: A QUALITATIVE STUDY

Onome Osokpo[1], Lisa Lewis[2], Danny Bracy[3], Dolapo Adeniji[4], Ayomide Bankole[5], and Barbara Riegel[3],

*1. University of Illinois Chicago, Chicago, Illinois, United States, 2. Rutgers, The State University of New Jersey, Camden, New Jersey, United States, 3. University of Pennsylvania, Philadelphia, Pennsylvania, United States, 4. Indiana State University, Avon, Indiana, United States, 5. Duke University, Durham, North Carolina, United States*

Healthy dietary behaviors (e.g., low-salt diet) are a critical element of self-care and the cornerstone in managing diabetes and/or hypertension. Social determinants (e.g., cultural preferences and social support) influence dietary behaviors. However, little is known about the influence of social determinants on dietary behaviors of African immigrants living with diabetes and/or hypertension accounting for their level of acculturation. We conducted a descriptive qualitative study of a purposive sample of 24 self-identified sub-Saharan African immigrant adults in the Northeastern U.S. with symptomatic hypertension and/or diabetes who spoke and understood English. Based on scores from the Short Acculturation Scale, African immigrants were classified as more or less acculturated. The sample was mainly Nigerian (75%) and males (67%), with a mean age of 59 years [40-72]. The sample was highly educated, acculturated, employed, and reported having enough income to meet their needs. Compared to more-acculturated immigrants, less-acculturated immigrants regularly ate more African or "traditional food," vegetables and rice, and avoided salt and fatty/fast/processed American foods. Less-acculturated immigrants cooked their meals and learned their diets from previous education or their workplace. Compared to the less acculturated, more acculturated participants ate fruits regularly and avoided meat or red meat. Interestingly, the more acculturated participants reported learning about their diet from family and friends and noted that family members cook the meals in their household. Interventions to promote healthy diets among immigrants should take into consideration cultural dietary preferences, influence of family, friends, and non-familial social connections, and level of acculturation.

Abstract citation ID: igae098.3604

### DIMENSIONALITY, PROXIES, AND PREDICTORS OF COGNITIVE RESERVE IN THE MANCHESTER LONGITUDINAL STUDY

Stephen Aichele, *Colorado State University, Fort Collins, Colorado, United States*

In a sample of N=113 older adults (ages 62-86 years at MRI scan; 58.4% women) from the United Kingdom, we compared the dimensionality of general cognition before (COG) vs. after (COGr; cognitive reserve) residualization on global brain status (as indicated by brain volume loss, cerebral blood flow, and white matter lesion prevalence). Cognitive performance was assessed by 15 tasks spanning five domains: processing speed, fluid intelligence (e.g., spatial reasoning), crystallized intelligence (vocabulary), visuospatial memory, and verbal memory. Statistical tests were conducted in a series of structural factor analyses with progressive constraints on measurement invariance. Results showed that although COG and COGr were highly correlated (r =.84), item-factor loadings differed such that COGr more strongly emphasized fluid intelligence. We further examined differential associations of COG and COGr with 23 socio-demographic, health, and lifestyle variables in a series of regression models. Results from these latter tests showed consistent associations of education level, occupational class, and hobbies/interests as proxies both for COG and COGr. Difficulty with personal mobility was a salient risk factor for both COG and COGr, but only in models unadjusted for chronological age. In sum, results supported the role of intelligence as a potential compensatory factor for brain-related reductions in processing speed and memory. Efforts to preserve personal mobility and a diversity of hobbies and interests may serve as protective factors against brain-related reductions in cognitive performance.

Abstract citation ID: igae098.3605

### EFFECTIVENESS OF DUAL-LANGUAGE COGNITIVE INTERVENTION IN COGNITIVE HEALTHY AND DEMENTIA BILINGUAL OLDER ADULTS

W. Quin Yow, Chi Shuen Ang, and Xiaoqian Li, *Singapore University of Technology & Design, Singapore, Singapore*

There is an increasing interest in using touch-screen devices to conduct cognitive training and collect measurements of cognitive performance. Although research with technology-based intervention has shown benefits in improving cognitive performance with older adults, overall results are mixed (Ge et al, 2018). This study aims to investigate whether our elder-friendly, multi-modal touch-screen, computerized dual-language intervention program would help to improve cognitive skills in cognitive healthy and dementia bilingual older adults in a multilingual society. Fifty-eight older adults, 26 with dementia (Mage 82.77 ± 7.45) and 32 healthy older adults (Mage 72.44 ± 8.42), completed 24 sessions of game-play over 8-12 weeks. Each session took about 30-45 minutes to complete, and consisted of two cognitive tasks: Object Categorization (OC), and Utility of things (UoT). Participants' performance was assessed by reaction time (RT) and number of prompts used to reduce task difficulty. Using a two-way ANOVA, we compared the performance of the first 4 and last 4 sessions of the two groups for both tasks. By the last 4 sessions, there were significant improvements in performance of the dementia group (i.e., faster RT and fewer prompts for OC, and fewer prompts for UoT; post-hoc ps<.002). Performance in the cognitively-healthy group remained stable (ps>.11), and they consistenly outperformed the dementia group across the intervention (ps<.001). These improvements suggest that our cognitive intervention program could be effective for older adults with dementia. In sum, bilingual elder-friendly touch-screen platforms can be a novel yet effective method to study cognitive interventions in populations of older multilinguals.

Abstract citation ID: igae098.3606

### FINANCIAL TRANSFERS BETWEEN OLDER FILIPINOS AND THEIR ADULT CHILDREN: THE ROLE OF EDUCATION AND PUBLIC PENSIONS

Kent Jason Cheng, *The Pennsylvania State University, University Park, Pennsylvania, United States*

**Objectives:** The Philippines is expected to transition to an aging society in less than two decades. Provision of resources and safety nets largely befalls family members since government support is limited. This study examines how education and public pensions shape older Filipinos' financial transfers to and from their adult children.

**Methods:** Data on Filipinos aged 60+ with at least one living child came from the 2018 Longitudinal Study of Ageing and Health in the Philippines (n=5,645). Logistic regressions interacting educational attainment and public pension receipt (no pensions, have contribution-based pensions, have social pensions, have both) were ran, controlling for relevant sociodemographic factors, average characteristics of children, and living arrangements. Predicted probabilities were generated and pairwise comparisons were conducted.
**Results:** Higher educational attainment and having contribution-based public pensions were associated with higher (decreased) odds of giving (receiving) transfers. Interactive models suggest a modest moderating effect of education and public pension types on giving and receiving financial transfers. Within education level, public pension receivers, especially those with contribution-based pensions, tend to have the highest probability of transferring to children and lowest probability of being exclusively on the receiving end of money transfers.
**Discussion:** Public pension receivers being more likely to give downward financial transfer across all educational levels suggests that public transfers for older adults benefit both the intended beneficiaries and their descendants. Additionally, older parents having contribution-based pensions as an income source spares their adult children from providing upward transfers. Results support crowding in – that public transfers facilitate private transfers.

Abstract citation ID: igae098.3607

**HEALTH CARE UTILIZATION AND COSTS FOR HOSPITALIZED DEMENTIA PATIENTS IN CHINA**

Zhuoer Lin[1], Fang Ba[2], Heather Allore[2], and Xi Chen[2], *1. University of Illinois Chicago, Chicago, Illinois, United States*, *2. Yale University, New Haven, Connecticut, United States*

**Background:** Dementia poses challenges to patients and healthcare systems; yet little is known about the healthcare utilization and costs for dementia patients in China and their major contributors.
**Methods:** We identified 51,530 inpatient records of Chinese older adults who were admitted to 1,917 hospitals during 2017-2019 with a primary diagnosis of dementia. Multivariable logistic regressions estimated associations for emergency room (ER) admission and tertiary hospital admission; and linear regressions were performed to examine the determinants of length of stay (LOS), daily and total costs in Chinese Yuan (CNY). The potential contributors included age, sex, insurance type, working status, comorbidity, admission type and hospital level.
**Results:** The sample had a median age 78 (IQR: 72-84), of whom 46.2% were females, 20.2% were admitted through an ER, and 69.0% were admitted to tertiary hospitals. LOS, daily and total costs per admission were 21 days, 948 CNY and 12,602 CNY, respectively. Increased adjusted odds of ER admission were associated with having lung diseases or lower insurance coverage. LOS was longer for older patients, females, retirees, or those with more insurance coverage or admitted to lower-level hospitals. The total costs were higher among older patients, males, patients with lung diseases, more insurance coverage, and patients admitted to ER or tertiary hospitals.
**Conclusion:** There are large variations in inpatient care utilization and costs across dementia patients of different demographics, insurance types, and comorbidities. Our findings highlighted the importance of promoting chronic disease management and insurance coverage, as well as providing affordable and equitable dementia care.

Abstract citation ID: igae098.3608

**NEIGHBORHOOD ENVIRONMENT AND COGNITIVE FUNCTION AMONG OLDER ADULTS IN BRAZIL**

Lourdes Romañach Álvarez[1], Mateo Farina[2], and Elizabeth Muñoz[1], *1. The University of Texas Austin, Austin, Texas, United States*, *2. University of Texas at Austin, Austin, Texas, United States*

Neighborhood characteristics, such as social cohesion and community resources, are linked to better cognitive functioning in older adulthood. However, little is known about the significance of neighborhoods for cognitive function in lower- and middle-income countries where this association may be augmented by limited resources and higher economic inequality. The present study used data from the 2016 Brazilian Longitudinal Study of Aging (ELSI-Brazil) to examine the association between self-reported neighborhood characteristics and cognitive functioning. ELSI-Brazil is a nationally representative sample of community-residing adults in Brazil aged 50 years and older (n = 9,412). We fit a series of linear regression models to test the hypothesis that more favorable neighborhood characteristics are associated with better cognitive functioning. Participants completed self-reported questionnaires of their neighborhood's social cohesion and availability of healthy foods. A global cognitive score was calculated with a latent variable (g) that was created with confirmatory factor analysis. Adjusted models showed that social cohesion, specifically appraising their neighborhood as a good place to live and trusting most people in their neighborhood, was associated with higher cognitive functioning. Likewise, those who reported greater availability of fresh fruits and vegetables within their neighborhood displayed better cognitive function. Findings provide support for the protective role of neighborhood social cohesion and availability of healthy foods on cognitive function among adults in Brazil. Further research is needed to elucidate what mechanisms may explain this association in the Brazilian context.

### SESSION 7930 (POSTER)

### FLASH POSTER SESSION: ADRDS

Abstract citation ID: igae098.3609

**A COMPREHENSIVE DIGITAL SELF-CARE SUPPORT SYSTEM FOR CAREGIVERS AND PERSONS WITH DEMENTIA/MCI AND MCC**

Priya Nambisan[1], Yura Lee[2], Julie Ellis[1], and Murad Taani[1], *1. University of Wisconsin Milwaukee, Milwaukee, Wisconsin, United States*, *2. University of Wisconsin-Milwaukee, Milwaukee, Wisconsin, United States*

Research indicates that 95% of people with Alzheimer's and other dementias also suffer from one or more other chronic conditions, increasing the risk of hospitalizations and complications such as contraindicated health care and drug interactions. Effective self-management practices are vital for maintaining cognitive function and preventing the onset of new chronic conditions among those with Multiple Chronic Conditions (MCC). However, managing MCC alongside cognitive impairment (CI) can make it very challenging for both patients and caregivers. Studies suggest that computer usage can enhance cognitive functioning and potentially delay further deterioration and digital apps are beneficial for self-care support. Despite this, there is a severe lack of technological applications that support those with CI and MCC and are often digitally excluded due to lack of understanding of their needs. We present the findings of a pilot test of a Comprehensive Digital Self-care Support System (CDSSS) known as myHESTIA. This system offers caregivers and persons with Alzheimer's/Dementia a range of personalized tools including: 1) Health management tools: daily trackers for monitoring health conditions, reminders, to- do lists and educational resources; 2) Healing technologies: e.g. breathing exercises for relaxation, music therapy, learning activities like dancing or Yoga, and memory games and 3) Online social support forum: for connecting with others. This qualitative study aims to shed light on the perceived benefits of the system for both caregivers and persons with dementia/MCI, its utility in addressing unmet needs among those with CI, and its potential to improve the quality of life of caregivers.

Abstract citation ID: igae098.3610

### ASSOCIATION BETWEEN DIET AND RETINAL IMAGING MARKER IN THE PREVENT DEMENTIA STUDY

Sarah Gregory[1], Samuel Gibbon[1], Thomas MacGillivray[1], and Graciela Muniz Terrera[2], *1. University of Edinburgh, Edinburgh, Scotland, United Kingdom*, *2. Ohio University, Athens, Ohio, United States*

Eating a healthy diet is associated with a lower risk of neurodegenerative diseases, such as Alzheimer's disease (AD). Identifying associations between diet and AD biomarkers is key to understanding and identifying critical periods for interventions. The retina provides a unique, non-invasive, window into brain health. This study explored cross-sectional and longitudinal associations between diet and retinal imaging markers in the PREVENT Dementia cohort (UK/Ireland). We calculated a Mediterranean diet (MedDiet) adherence score (Pyramid) for each participant and tested for associations with measures of retinal vessel morphology (CRAE Hubbard Zone C (HZC) and artery tortuosity) using generalised additive models due to abnormally distributed residuals. Additionally, we tested for associations with dietary intake of manganese. All analyses were completed cross-sectionally and then longitudinally in the participants with follow-up data (data collection ongoing). In a preliminary study, we included 86 participants, mean age 50.87 (5.63) years, majority female (57%), with 31 APOEe4 carriers (36%). Participants had a moderate mean Pyramid score (8.16 (SD = 1.40)), which was comparable to scores in other UK cohorts, and a dietary intake of 4.77 mg (SD = 3.05) manganese. There was evidence of an association between higher dietary manganese intake and lower CRAE HZC density in the right eye. There was no evidence of associations between the Pyramid score in either eye or dietary manganese in the left eye, and no signs of longitudinal associations (n=47). Further research is needed to explore any these analyses in a larger sample, and expand with other variables.

Abstract citation ID: igae098.3611

### ASSOCIATION BETWEEN GUT MICROBIOTA, DIET, AND COGNITION IN ELDERLY PATIENTS WITH MILD COGNITIVE IMPAIRMENT

Lili Chen[1], Zhaoyi Hou[2], Jinxiu Liu[2], Xiuli Li[2], Yuping Zhang[2], Shuhui Yang[2], and Peiling Zhang[2], *1. Fujian Provincial Hospital, Fuzhou, Fujian, China (People's Republic)*, *2. Fujian Medical University, Fuzhou, Fujian, China (People's Republic)*

**Objectives:** To compare the gut microbiota in individuals with mild cognitive impairment (MCI) and normal controls (NCs) and explore the association between dietary intake and various cognitive domains across identified enterotypes, providing a basis for dietary interventions based on the Microbiota-Gut-Brain Axis.

**Methods:** We analyzed fecal samples and 16S ribosomal RNA sequences for microbiota among 100 participants (NCs: 50, MCI: 50). Cognitive function was evaluated using the Montreal Cognitive Assessment (MoCA), Mini-Mental State Examination (MMSE), and Auditory Verbal Learning Test (AVLT). A semi-quantitative food frequency questionnaire was used to assess participants' dietary intake, and differences in dietary habits and cognitive function among distinct enterotypes were analyzed.

**Results:** Participants with MCI exhibited lower bacterial richness. Linear Discriminant Analysis of Effect Sizes (LEfSe-LDA) revealed different major microbial genera between the groups, and a random forest model based on dominant bacterial genera could distinguish between MCI and NCs (AUC=0.743, 95%CI:0.646-0.840). Cluster analysis identified two enterotypes: Escherichia-Shigella (enterotype E) and Bacteroides (enterotype B). When grouped by enterotypes, similar dietary intake was associated with different cognitive domains: notably, egg intake positively correlated with MOCA and AVLT scores for enterotype E, but not for enterotype B. Conclusions: Significant differences exist in the gut microbiota of individuals with MCI compared to those with normal cognition, with dominant genera showing good predictive performance. The impact of different enterotypes on the relationship between dietary intake and cognitive function should be considered when making dietary recommendations in clinical practice.

Abstract citation ID: igae098.3612

### ASSOCIATIONS OF PHYSICAL ACTIVITY ENGAGEMENT WITH CEREBRAL AB AND TAU PET FROM MIDLIFE

Mitzi Gonzales[1], Daniel Kojis[2], Nicole Spartano[3], Emma Thibault[4], Charles DeCarli[5], Keith Johnson[4], Alexa Beiser[2], and Sudha Seshadri[6], *1. Cedars-Sinai Medical*

*Center, Los Angeles, California, United States*, 2. *Boston University, Boston, Massachusetts, United States*, 3. *Boston University School of Medicine, Boston, Massachusetts, United States*, 4. *Massachusetts General Hospital, Boston, Massachusetts, United States*, 5. *University of California Davis, Davis, California, United States*, 6. *The University of Texas Health San Antonio, San Antonio, Texas, United States*

Higher midlife physical activity engagement has been associated with lower dementia risk in late life. However, the underlying mechanisms contributing to the protective effect remain unclear. The goal of the current study was to evaluate the associations of physical activity with cerebral amyloid beta (Aβ) and tau in a predominately middle-aged community-based cohort, as well as to explore whether the associations differ by age or sex. Participants from the Framingham Heart Study underwent 11C-Pittsburgh Compound B amyloid and 18F-Flortaucipir tau positron emission tomography (PET) imaging. Total physical activity levels were evaluated by self-report using the Physical Activity Index (PAI). Cross-sectional associations between total PAI with regional Aβ and tau PET retention were evaluated using linear regression models adjusted for demographic and cardiovascular risk factors. Interactions with sex and age group were examined and stratified analyses were performed when significant. The sample included 354 participants (mean age 53±8 years, 51% female). Higher total PAI scores were associated with lower entorhinal cortex (β(SE)=-0.021(0.008), p=0.013), inferior temporal lobe (β(SE)=-0.017(0.008), p=0.040), and rhinal cortex (β(SE)=-0.018(0.008), p=0.030) tau PET binding. There were significant interactions with sex with only males displaying inverse associations between total PAI with entorhinal cortex, inferior temporal lobe. and rhinal cortex tau. The results suggest that higher midlife physical activity may confer resistance against tau pathology in regions susceptible to early neuropathological changes. However, the effects may vary based on sex, highlighting the importance of better understanding and tailoring lifestyle interventions to address sex disparities.

Abstract citation ID: igae098.3613

### COPING STRATEGIES AMONG HISPANIC AND LATINX DEMENTIA CAREGIVERS

Nury Rodriguez Colmenares[1], Danny Wang[2], Loreli Alvarez[1], Katie Estes[1], Natashia Bibriescas[3], and Frank Puga[1], *1. University of Alabama at Birmingham, Birmingham, Alabama, United States*, 2. *The Pennsylvania State University, University Park, Pennsylvania, United States*, 3. *The University of Texas Austin, Austin, Texas, United States*

The objective of this study was to examine coping strategies employed by Hispanic and Latinx (H&L) family caregivers (FCGs) of people living with dementia (PLWD) and associations with sociodemographic characteristics. A nonparametric analysis was used to analyze data from an ongoing study on the daily experiences of H&L dementia caregivers, entitled Nuestros Días. A total of 52 H&L FCGs were assessed using Brief-COPE. Most H&L FCGs identified as female (92.2%), with a mean age of 56 years (SD = 8.6), were adult children caring for biological parent (59.6%), and provided care for 3 or more years (61.5%). Most PLWD were female (68%) with a mean age of 78 years (SD= 8.6). Overall coping strategies most frequently utilized by H&L FCGs included acceptance (M= 3.14, SD= 1), religion (M= 2.98, SD= 1), positive reframing (M=2.91, SD=0.9), and planning (M=2.8, SD=1). Conversely, substance use (M=1.13, SD=0.3), behavioral disengagement (M=1.39, SD=0.72), denial (M=1.44, SD=0.8), and humor (M=1.47, SD=0.8) were the least utilized by H&L FCGs. The use of acceptance as a coping strategy was found to have a significant association with employment status with working caregivers more frequently endorsing acceptance as a coping strategy (p=.04; Cramer's V=.409). These findings suggest that H&L caregivers utilize specific coping strategies and that sociodemographic factors, such as employment, may influence coping strategies use d. Additional studies are needed to examine the relationship between additional contextual factors (e.g., caregiving-related stressors and burden) and types of coping strategies used by H&L FCGs to inform ecologically valid and cultural interventions.

Abstract citation ID: igae098.3614

### DIFFERENCES OF RANKINGS IN DEATH CERTIFICATES AMONG PERSONS WITH ALZHEIMER'S DISEASE

Candace Brown[1], Yinghao Pan[1], Maggi Miller[2], and Matthew Lohman[2], *1. University of North Carolina Charlotte, Charlotte, North Carolina, United States*, 2. *University of South Carolina, Columbia, South Carolina, United States*

A 2021 report indicates that 19.1% of people ages 76-84 and 34.6% of people aged 85 and older have Alzheimer's disease (AD). Data provided on death certificates using the International Statistical Classification of Diseases is guided by the WHO for coding cause and contributing comorbidities of death. Death rankings assist in monitoring which diseases need attention for better informed decision making in healthcare. However, classifying immediate cause of death presents challenges when the likelihood of polymorbidity occurs with advancing age in patients with presenting AD but inconclusive diagnoses of the disease. We used the Alzheimer's Disease and Related Dementia South Carolina (SCADR) registry and linked it with state death certificates to examine sociodemographic variables and survival probabilities of decedents between the years 2014-2019. Cox proportional hazards models were fitted and hazard ratios (HRs) computed, adjusting age at diagnosis and race. Turnbull's method estimated the survival probabilities or curves for subgroups as the time from AD diagnosis to death. Of the 78,534 patients in the study 94% were diagnosed with AD at age 61 or older. AD was listed as the immediate cause of death among all racial groups, except for Native American/American Indian, contrasting from previous research of underreporting of the disease by factor of 2.7 with underestimations more likely for people identifying as Black and Latino. However, only 1.4% had a conclusive diagnosis of the disease by autopsy. Survival probabilities drop precipitously in the first few years after diagnosis with only 25% of the cohort surviving beyond 5 years.

Abstract citation ID: igae098.3615

### EARLY CLINICAL UTILITY DATA OF A MULTI-ANALYTE BLOOD BIOMARKER TEST FOR EVALUATING COGNITIVE IMPAIRMENT

Mark Monane[1], David Merrill[2], Kim Johnson[3], Darren Gitelman[4], Demetrius Maraganore[5], Leslie Jacobs[1], Yan Jiang[6], and Joel Braunstein[1], *1. C2N Diagnostics, St. Louis, Missouri, United States*, *2. Pacific Brain Health Center, Santa Monica, California, United States*, *3. Duke University, Durham, North Carolina, United States*, *4. Advocate Lutheran General Hospital, Park Ridge, Illinois, United States*, *5. Tulane University School of Medicine, New Orleans, Louisiana, United States*, *6. Stat4ward, Pittsburgh, Pennsylvania, United States*

There is an important unmet need for timely, noninvasive, low-burden diagnostic tools to aid in Alzheimer's disease evaluation. The AAA-BBM2 (PrecivityAD2™) blood test combines plasma amyloid beta (AB)42/40 Ratio and phosphorylated-tau217/non-phosphorylated-tau217 Ratio (%p-tau217) into a clinically-validated algorithm. The test result, the Amyloid Probability Score 2 (APS2), determines the likelihood of brain amyloid, the pathological hallmark of Alzheimer's disease, on amyloid PET scan: the test result has previously demonstrated 88% sensitivity and 89% specificity. The objective of the QUIP II Study (NCT06025877) is to evaluate the clinical utility of this blood biomarker test with APS2 result among cognitively symptomatic patients presenting to memory specialists. To date, 52 symptomatic patients (median age 75, 65% female, 77% white) from 7 memory centers received the AAA-BBM2 test. Concordance with intended use of the test was 100% (52/52). Positive APS2 results were noted in 26 patients (50%). From pre- to post-test, clinician-reported probability of AD decreased from 48% to 16% among negative APS2 patients ($p< 0.0001$), and increased from 71% to 93% among positive APS2 patients ($p< 0.0001$). The composite endpoint of change in AD diagnostic certainty, drug therapy, or brain amyloid evaluation pre- and post-AAA-BBM2 testing was 79% ($p < 0.0001$ versus a pre-specified threshold of 20% clinically meaningful change). We believe that the AAA-BBM2 test showed clinical utility in its association with changes in clinicians' diagnostic certainty and clinical management among patients presenting with cognitive impairment. This blood biomarker test may allow memory specialists to guide patients to anti-AD therapies and facilitate other diagnostic considerations.

Abstract citation ID: igae098.3616

### EFFECTS OF A STAGED INTEGRAL ART-BASED COGNITIVE INTERVENTION FOR OLDER ADULTS ON THE ALZHEIMER'S DISEASE SPECTRUM

Yuanjiao Yan[1], Chenshan Huang[2], Yanhong Shi[2], Rong Lin[2], and Hong Li[2], *1. Fujian Provincial Hospital, Fuzhou, Fujian, China (People's Republic)*, *2. Fujian Medical University, Fuzhou, Fujian, China (People's Republic)*

**Background:** The combined art activities can prevent or reverse the cognitive decline. However, few studies have systematically examined its effects. To evaluate the effects of a staged integral art-based cognitive intervention (SIACI) in older adults on the Alzheimer's disease (AD) spectrum.

**Methods:** Participants were randomised in a 1:1 ratio into an intervention group (n=72) and a wait-list control group (n=72). The intervention group underwent a 16-week, 24-session SIACI program, while the control group underwent the program after the follow-up assessment. General and specific domains of cognitive function, and other health-related outcomes were measured at baseline (T0), immediately after the intervention (T1), and 6 months follow-up (T2).

**Results:** Of the 144 participants (mean [SD] age, 71.6 [5.8] years; 50 [34.7%] males and 94 [65.3%] females), 130 and 115 completed the questionnaires at T1 and T2, respectively. The average attendance rate in the intervention group was 86.0%. At T1, the intervention group showed greater improvement than the control group in general cognitive functions (MoCA, between-group mean difference, 95% CIs, 0.4 to 2.5, p=0.009). Additionally, the intervention group demonstrated statistically significant improvement compared to the control group in language, memory, quality of life, sleep quality and physical activity level at T1. Statistically significant group differences remained in sleep quality (PSQI, between-group mean difference, -1.3 [95% CIs, -2.5 to -0.1], p=0.035) at the 6-month follow-up. Conclusions: In this randomised controlled trial of the SIACI program, older adults on the AD spectrum who participated in the program experienced improved cognition and psychological health.

Abstract citation ID: igae098.3617

### ENABLING PRIORITIZATION FOR AMYLOID POSITIVITY TESTING WITH A DIGITAL COGNITIVE ASSESSMENT

Ali Jannati[1], Karl Thompson[1], Claudio Toro-Serey[1], Russell Banks[1], John Showalter[1], David Bates[1], Sean Tobyne[1], and Alvaro Pascual-Leone[2], *1. Linus Health, Boston, Massachusetts, United States*, *2. Hebrew SeniorLife, Boston, Massachusetts, United States*

Primary care providers currently wait for a complaint to initiate a 'for-cause' cognitive evaluation and often refer the patient to a specialist. Specialist evaluation then leads to the diagnosis, but treatment occurs late and is unlikely to meaningfully prevent or reduce disability. We hypothesized a brief digital cognitive assessment (DCA), the Digital Clock and Recall (DCR), could concurrently identify CI and predict PET Aβ status, thereby providing an efficient means for timely identification and prioritization of patients for disease-modifying treatments. 930 participants (age 72.0±6.7; 56.8% female; 23% minorities) were classified as cognitively unimpaired, mild cognitive impairment, or probable Alzheimer's dementia, and 35.1% were Aβ+ on 18F-florbetapir PET scan. DCR-based models were compared with blood-based biomarkers (BBMs) Aβ42/40, pTau-181, and pTau-217, which poorly classified CI (AUCs=0.63, 0.66, 0.72) but accurately classified Aβ status (AUCs=0.81, 0.78, 0.89). DCR accurately classified CI and predicted Aβ status (AUCs=0.85, 0.83). Moreover, combining the DCR with Aβ42/40, pTau-181, and pTau-217 improved their Aβ-status prediction performance to AUCs of 0.882, 0.835, and 0.905, respectively.

DCR was superior to BBMs in detecting cognitive impairment and boosted their Aβ-PET prediction ability to levels comparable to CSF biomarkers. We propose a workflow in which implementation of DCAs such as the DCR facilitates early identification of individuals with MCI or early dementia likely due to AD, thanks to the concurrent classification of CI and accurate prediction of Aβ PET status.

Abstract citation ID: igae098.3618

### ENTORHINAL CORTEX THICKNESS IS ASSOCIATED WITH FINANCIAL EXPLOITATION VULNERABILITY IN OLDER ADULTS

Laura Fenton[1], Lauren Salminen[2], Aaron Lim[2], Gali Weissberger[3], Annie Nguyen[4], Jenna Axelrod[2], Daisy Noriega-Makarskyy[2], and Duke Han[2], *1. University of Southern California, California, United States*, *2. University of Southern California, Los Angeles, California, United States*, *3. Bar-Ilan University, Ramat Gan, HaMerkaz, Israel*, *4. University of California San Diego, La Jolla, California, United States*

Research suggests that increased financial exploitation vulnerability (FEV) due to declining decision making may be an early behavioral manifestation of brain changes occurring in preclinical Alzheimer's disease (AD). One of the earliest documented brain changes during this preclinical phase is neurodegeneration of the entorhinal cortex. The objective of the current study was to examine the association between a measure of FEV and thickness of the entorhinal cortex in 97 older adults without cognitive impairment (mean age (SD) = 68.72 (7.45); mean education (SD) = 16.52 (2.14); 76% female; 76% White). We also investigated FEV associations with frontal regions typically associated with decision making (e.g., dorsolateral and ventromedial prefrontal cortices). Exploratory analyses investigated the interactive effect of age and cortical thickness on FEV. Results showed that greater FEV was associated with significantly lower entorhinal cortex thickness (B = -0.05, SE = 0.02, p = 0.04), but not with thickness in frontal regions. There was a significant interaction between age and entorhinal cortex thickness on FEV, whereby lower entorhinal cortex thickness was associated with greater FEV in older participants (B = -0.01, SE = 0.003, p = 0.04). When the group was split according to age using a median split (70+; < 70 years old), lower entorhinal cortex thickness was associated with greater FEV only in the older group (B = -0.10, SE = 0.04, p = 0.01). Collectively these findings support the notion that FEV may serve as an early behavioral manifestation of preclinical AD before noticeable cognitive impairment.

### SESSION 7935 (POSTER)

### FLASH POSTER SESSION: MINORITY ISSUES IN AGING

Abstract citation ID: igae098.3619

### "I WON'T GO THROUGH THAT OR TAKE MY FAMILY THROUGH THAT": FAMILY RELATIONSHIPS AND KIDNEY DISEASE DECISION-MAKING

Tyrone Hamler[1], Asia Cutforth[1], Emily Miller[2], and Kari O'Donnell[3], *1. University of Denver, Denver, Colorado, United States*, *2. Case Western Reserve University, Cleveland, Ohio, United States*, *3. MetroHealth, Cleveland, Ohio, United States*

Chronic kidney disease (CKD) uniquely impacts older Black Americans, who are nearly four times more likely to develop kidney failure than older White Americans and more likely to have family members also diagnosed with CKD. Utilizing a phenomenological approach, this study explored participants' perceptions of how family members' prior experiences with CKD influenced treatment-related decision-making. This study aimed to (1) describe how older Black adults viewed the experiences of family and friends previously diagnosed with CKD and (2) to understand how these social complexities informed their own choices for future CKD care. A reflective, thematic content analysis was conducted to identify patterns across participant responses (N=52). Participants were pre-dialysis, diagnosed with stage four or five chronic kidney disease, and were receiving outpatient nephrology care at a large, urban midwestern hospital. Three primary themes emerged: (1) Decision-Making Factors, (2) Health Inequities, and (3) Continuity of Care. These three interconnected themes centered on factors that influenced why and how decisions related to chronic kidney disease were made and how racial identity and prior family experiences with CKD influenced these choices. Over the next 40 years, the population of Black Americans aged ≥ 65 years will nearly triple, and CKD will continue to be a major public health concern. Addressing the complex support requirements and burdens of CKD care is critical for reshaping the negative and disorienting experiences of older Black Americans and reducing future decision-making conflicts.

Abstract citation ID: igae098.3620

### A SCOPING REVIEW OF MACHINE LEARNING STUDIES ON DEMENTIA RISK FOR ETHNORACIAL MINORITIES

Michin Hong[1], Soo-Yeon Ji[2], Seon Kim[3], and Kyeongmo Kim[4], *1. Indiana University Indianapolis, Indianapolis, Indiana, United States*, *2. Bowie State University, Bowie, Maryland, United States*, *3. Drexel University, Philadelphia, Pennsylvania, United States*, *4. Virginia Commonwealth University, Richmond, Virginia, United States*

This study aims to provide an integrated review about dementia risk for ethnoracial minorities from machine learning (ML) based-studies. While ML methods are widely used in dementia research, their benefit for ethnoracial minorities remains unclear. We located prior research using combinations of the key words related to ML (e.g., neural network, deep learning, artificial intelligence), dementia and ethnoracial minorities from major electronic databases. Out of 599 initially searched articles, 389 remained after removing duplicates, and finally, 14 met the inclusion criteria. The interdisciplinary research team employed reiterative data analysis to identify key themes. All included studies, published since 2020, aimed to identify essential risk factors and generate predictive models for cognitive impairments. Most studies and treated race as a predictor after aggregating it into two or three categories, finding a lower risk of dementia among non-Hispanic Whites compared to Hispanics or Non-Hispanic Blacks. One study aimed to develop a predictive model of cognitive impairment applicable across non-Hispanic White, no-Hispanic Black, and Hispanic groups. Various ML techniques were used, such as neural network, random forest, support vector machine, and gradient boosting with some adopting Synthetic Minority Oversampling Technique to oversample minority groups. Our review indicated that ML-based studies have made limited progress in understanding dementia risk among ethnoracial minorities. National data sets used in most reviewed studies, have lacked minority samples, and this seems to persist with the ML approaches, possibly contributing to deteriorating the existing racial gap. Proactive efforts should focus on ethnoracial variations in dementia risk in ML-based studies.

Abstract citation ID: igae098.3621

### AN OBJECTIVE MEASURE OF STRESS REACTIVITY/RESILIENCE IN BLACK WOMEN DEMENTIA FAMILY CAREGIVERS

Kathy Wright, Karen Moss, Nathan Helsabeck,
Ingrid Richards Adams, Karen Rose, and Maryanna D. Klatt,
*The Ohio State University, Columbus, Ohio, United States*

Real-time objective measures of stress are crucial for identifying mechanisms applicable to health behavior change research for dementia caregivers. We explore the Pittsburgh Stress Battery (PSB) in a non-laboratory setting (R21AG077069) as an objective measure of stress reactivity/resilience. The PSB was administered to 16 Black American women caregivers with hypertension (40-65 years old). PSB includes three challenging tasks (Stroop, Mental Math, and Mirror Tracing). An automatic cuff measured systolic blood pressure (SBP), diastolic blood pressure (DBP) and heart rate (HR) at three intervals during the 20-minute battery followed by a 30-minute recovery. Average participant age was 62.4 (SD= 6.1). Ten assessments were completed in an office and six at home. One Stroop and three Mental Math were not completed due to computer/internet issues or refusal. Mean SBP, DPB, and HR at baseline were 128.9 mmHg, 81.6 mmHg, and 71.3 (beats per minute). The difference from baseline in SBP was 10.5 mmHg (SD= 8.3) for Stroop, Mental Math -.94 mm Hg (SD= -0.6), and Mirror Tracing 1.6 mmHg (SD= 9.4). For DBP, Stroop was 5.0 mm Hg (SD=4.6), Mental Math 0.2 mm Hg (SD= 3.2), and Mirror Tracing 0.3 mm Hg (SD= 7.0). HR difference from baseline was 3.8 (SD= 0.5), Mental Math -4.24 (SD= 8.2), and Mirror test -0.75 (SD= -3.4). Findings indicate that PSB elicits SBP/DBP/HR stress reactivity/resilience. PSB was portable for use in community settings. Study results will help guide interventions for behavior change, aiming to improve health outcomes in Black Americans.

Abstract citation ID: igae098.3622

### BLACK STRUCTURAL RESILIENCE ACROSS THE LIFESPAN

Boeun Kim[1], Alicia Cooke[2], Tiffany Riser[2], and
Sarah Szanton[2], *1. University of Iowa, Iowa, United States, 2. Johns Hopkins University, Baltimore, Maryland, United States*

Resilience, which is the ability to resist, recover, or adapt in response to adverse events, can be cultivated through interactions with societal, community, family, and individual factors across the life course. However, structural resilience, signifying the extent of recruiting resources at societal and community levels, has been overlooked. Furthermore, no study has examined structural resilience using a life course approach, although structural resilience is likely to change over time. This study employed a qualitative descriptive design to understand better the structural resilience and their supportive roles in maintaining or promoting health over the life course through older African Americans' lived experiences. This study focused on African Americans, who were known to have higher levels of individual resilience than White Americans, to learn what resources helped them to thrive despite adverse circumstances. We conducted semi-structured interviews with 15 self-identified African American adults aged 50 and older living in Baltimore City. Two study team members coded the transcripts using an inductive approach, and themes were generated from the constant comparative approach. The five themes emerged: 1) underwent extraordinary adversities over the life course, 2) accessible resources during childhood, adulthood, and late adulthood, 3) emergence of grassroots organizations to provide support, 4) roles of structural resilience in response to adversities, and 5) dynamics of structural resilience across the life course. This study contributes to a better understanding of the mechanisms that preserve health under stressors. The findings may help to resolve the public health tragedy of health equity faced by older African Americans.

Abstract citation ID: igae098.3623

### CHINESE–WHITE DIFFERENCES IN SOCIAL ACTIVITY, SOCIAL SUPPORT, AND MENTAL HEALTH AMONG OLDER ADULTS

Lingming Chen, Elizabeth Procter-Gray,
Qun Le, Danielle LoPilato, Marianella Ferretto,
Meng Zhang, , Kevin Kane, and Wenjun Li, *University of Massachusetts Lowell, Lowell, Massachusetts, United States*

Studies on healthy aging of older Asian Americans, especially Chinese Americans, are rare. This study evaluated Chinese – Non-Hispanic White differences in social activity, social support and mental health. The study enrolled 554 community-dwelling older adults (434 Non-Hispanic White and 120 Chinese) aged 65-95 years old in central and northeastern Massachusetts (2018-2023). Social activity and social support were measured by the Lifestyle Questionnaire. Mental health includes depressive symptoms assessed by the Center for Epidemiologic Studies Depression Scale, anxiety symptoms assessed by the Beck Anxiety Inventory, perceived stress assessed by the Perceived Stress Scale, and resilience assessed by the modified Brief Resilience Scale. Chinese - White differences in these factors were analyzed using linear regression models adjusting for age, gender, self-rated health, physical activity, body pain, and living alone. Results showed that in both unadjusted and adjusted models, older Chinese had less social support, lower frequency of social activities, and lower level of resilience compared to Non-Hispanic Whites. In the unadjusted models, older Chinese had higher levels of perceived stress, anxiety, and depressive symptoms compared to Non-Hispanic Whites, but there were not differences in these three factors after adjusting for covariables. In conclusion, social activity, social support, and mental health were different between Chinese and Non-Hispanic Whites among older adults in the study population. The findings from this study can inform the design and implementation of health programs tailored to specific groups, thereby reducing health disparities and improving overall well-being for older adults.

Abstract citation ID: igae098.3624

### DEPRESSION AND COGNITIVE FUNCTION IN OLDER MEXICAN DESCENT LATINOS: THE MEDIATING ROLE OF SOCIAL ENGAGEMENT

Liza Talavera-Garza, *The University of Texas Rio Grande Valley, Edinburg, Texas, United States*

Mexican descent Latinos have significantly higher rates of Alzheimer's disease and related dementias (ADRD) compared to the general population. Latinos also report higher rates of depressive symptoms in older adulthood relative to non-Latino Whites. Depression in older age is associated with poor cognitive health; however, the mechanisms that underlie this relationship are not well-understood. The current study examined social engagement, which has been linked with better cognitive health, as a mediator of the association between depressive symptoms and cognitive function among older Mexican descent Latinos. Method: A convenience sample of Mexican descent Latinos (N=159), aged 55 to 88 years (M=68.2, SD=7.05) and without diagnosed cognitive problems, were recruited from a community in South Texas. They were administered in-person interviews, in their choice of English or Spanish in Summer 2023 that included demographic questions and measures of cognitive function, depressive symptoms, and social engagement.

**Results:** Pearson correlations found that depressive symptoms were significantly negatively associated with social engagement ($r = -.25$, $p <.002$) and cognitive function ($r = -.39$, $p <.001$). Hierarchical Bayesian simulations indicated strong indirect effects of depressive symptoms on cognitive function through social engagement (unstandardized 95% CI = -.2408, -.0119, $p<.0001$).

**Conclusion:** The current findings suggest that cognitive decline in individuals with depressive symptoms may occur because of reduced participation in social activities. Community-based approaches, with a focus on increasing social engagement in individuals who exhibit symptoms of depression, may be viable interventions that could be leveraged to reduce the burden of ADRD in Latinos.

Abstract citation ID: igae098.3625

### EXPLORING STRESSORS AND SELF-MANAGEMENT BEHAVIORS IN RURAL AGING AFRICAN AMERICANS WITH DIABETES: A PILOT STUDY

Idethia Shevon Harvey[1], Sophia Nittinger[1], Maude Harris[1], Arika Wiggins[2], and Rahma Mkuu[3], *1. University of Missouri, Columbia, Missouri, United States*, *2. Southeast Missouri State University, Cape Girardeau, Missouri, United States*, *3. University of Florida, Gainesville, Florida, United States*

**Background:** Rural African Americans with diabetes encounter unique challenges related to managing their condition, including diabetes-specific distress, financial stress, and interpersonal stressors. This study investigated the associations between these stressors and self-management behaviors in a small population sample.

**Methods:** Fifteen rural African American adults (mean age = 66.08 yrs.; SD = + 6.16) with diabetes participated in this cross-sectional study. Participants completed validated measures, including the Summary of Diabetes Self-Care Activity (SDSCA), Center for Epidemiologic Studies Depression Scale (CES-D), Perceived Stress Scale (PSS), Partner Strain, Parental Stress Scale, Diabetes Distress, Everyday Discrimination, and Cost-FACIT. Descriptive statistics and correlational analyses examined the relationships between stressors and self-management behaviors.

**Results:** Participants reported moderate levels of financial, relational, and perceived stress, and diabetes distress. Higher levels of financial stress were associated with poorer diabetes self-care activities ($r = -0.628$, $p < 0.05$). Emotional burden ($r = 0.804$, $p < 0.01$) and interpersonal distress ($r = 0.600$, $p < 0.05$) are significantly correlated with perceived stress. Financial stress is significantly associated with everyday discrimination ($r = 0.508$, $p < 0.05$).

**Conclusion:** Despite the small sample size, our findings highlight the intricate interplay between diabetes distress, financial stress, relationship stress, and self-management behaviors among rural African Americans with diabetes. Tailored interventions addressing these stressors and promoting effective coping strategies may be crucial in improving diabetes management outcomes in this population. Further research with larger samples is warranted to confirm these findings and inform targeted interventions for better diabetes care in rural African American communities.

Abstract citation ID: igae098.3626

### NATIVITY STATUS AND SUCCESSFUL AGING IN LATINX OLDER ADULTS: FINDINGS FROM A CROSS-SECTIONAL SURVEY

Ajla Basic, and Mushira Khan, *Mather Institute, Evanston, Illinois, United States*

Previous research has underscored the dynamic relationship between health and aging, highlighting the significant influence of social, cultural, and environmental factors on aging outcomes in racial and/or ethnic minority older adults. Building on the extant literature, this cross-sectional study explores the relationship between nativity status and successful aging in community-dwelling Latinx adults 50+, examining whether being born in the U.S. or having immigrated (first-generation immigrant) influences the process of aging well. A total of 141 Latinx older adults (mean age = 62.83; 65.9% female) residing in Chicagoland completed paper surveys. Measures included the successful aging scale, short acculturation scale, well-being indicators including affiliation (social connectedness), autonomy (personal control), and achievement (personal growth), and sociodemographic variables. Findings from multiple regressions revealed that nativity status (U.S. born) moderated the relationship between successful aging and age ($F(3, 130)=3.56$, $p < 0.05$), gender($F(3, 133)=5.08$, $p< 0.05$), autonomy ($F(3, 134)=2.56$, $p < 0.05$), and self-reported health ($F(3,136)=8.83$, $p < 0.05$). These findings suggest that experiences of aging in Latinx older adults can differ depending on whether a person was born in the US or immigrated, indicating the importance of considering factors such as cultural attitudes toward aging, migration and settlement processes, and differential access to resources. Furthermore, cultural norms and expectations surrounding gender roles may be experienced differently by U.S. born Latinx older adults and first-generation immigrants, impacting caregiving, health maintenance, and social support networks. Future research should explore in-depth the mechanisms through which nativity status influences perceptions of aging well in Latinx older adults.

Abstract citation ID: igae098.3627

### PRIORITIES TO IMPROVE DEMENTIA CAREGIVING IN CHINESE IMMIGRANT FAMILIES: REPORT OF A CAREGIVER WORKSHOP

Kris (Pui Kwan) Ma[1], Anne Saw[2], and Joyce Cheng[3], *1. University of Washington, Seattle, Washington, United States*, *2. DePaul University, Chicago, Illinois, United States*, *3. Chinese Community Health Resource Center, San Francisco, California, United States*

Linguistically diverse caregivers of people with dementia represent a growing but under-recognized segment of the dementia care workforce in the U.S. There is a continued shortage of culturally and linguistically responsive dementia caregiving interventions for older U.S. Chinese immigrants with limited English proficiency. To address the unmet needs of Chinese-speaking caregivers, integrating caregivers' input is vital to studies to ensure that research aligns with their experiences. This was a focus group study of 10 Chinese-speaking caregivers (mean age 73.7, eight women) conducted during a dementia caregiving workshop, in partnership with a community health resource center in California. During the in-language workshop, caregivers identified their needs in surveys, then discussed with one another in the focus group. Consensus was built to establish priority areas of research and clinical practice to address caregivers' experiences and needs. We used descriptive and thematic analyses to summarize caregivers' priorities. Caregiver-identified priorities included information and practical support in a) management of behavioral and psychological symptoms of dementia (BPSD), b) communication with care recipients and other family members, c) care for caregivers' physical and mental health, and d) assistance to connect to in-language community resources. We identified ways in which these priorities can inform dementia caregiving research and practice for Chinese-speaking caregivers and immigrant families in the U.S. It is recommended that future research and interventions for Chinese-speaking immigrant caregivers in the U.S. prioritize BPSD management skills training, adopt a family-centered approach and include family outcomes, and address service barriers via community-clinic partnerships and outreach.

Abstract citation ID: igae098.3628

### A SURVEY OF MICROGLIA ASSOCIATION WITH AMYLOID PLAQUE IN 3XTG ALZHEIMER'S DISEASE MODELS

Anthony Asante-Danso[1], Mofida Abdelmageed[2], and Anirban Paul[2], *1. Howard University, Washington, District of Columbia, United States*, *2. Penn State College of Medicine, Hershey, Pennsylvania, United States*

Brain aging is characterized by a progressive functional decline of cellular and molecular mechanisms that affect most living organisms. These mechanisms' decline is the primary risk factor for many neurodegenerative diseases, including Alzheimer's Disease (AD). In neurodegenerative diseases like AD, extracellular deposition of amyloid-beta (Aβ) and microglial activation are critical facets that influence disease progression. Microglia, the resident immune cells of the central nervous system, play a key role in maintaining brain homeostasis and eliminating these Aβ forms through phagocytosis. However, increased microglia association with amyloid plaque can lead to neuroinflammation, thus creating a more neurotoxic environment in the brain, leading to further AD progression and neuronal loss. Little research examines how this association of microglia and amyloid plaque differs with sex or age. To address research gaps, we are using wild-type and triple transgenic human-induced AD models (3xTg-AD) of female and male mice to gain a strong understanding of the changes in microglia activation in brain aging and AD.

### SESSION 8000 (POSTER)

### LATE BREAKING POSTER SESSION I

Abstract citation ID: igae098.3629

### ABSENCE OF ANGIOTENSIN TYPE 2 RECEPTOR IS LINKED TO HIGHER BODY WEIGHT AND POOR AEROBIC PERFORMANCE IN AGED MICE

Michael Abadir[1], Michael Bene[2], Adam Said[3], Ruth Marx[4], Laura Powell[4], and Jeremy Walston[4], *1. University of Maryland, Woodstock, Maryland, United States*, *2. Johns Hopkins School of Medicine, Baltimore, Maryland, United States*, *3. Emory College of Arts and Sciences, Atlanta, Georgia, United States*, *4. Johns Hopkins University, Baltimore, Maryland, United States*

Age-related physical decline significantly impacts the quality of life and increases dependence in older adults. The role of angiotensin receptors in the decline of strength and endurance in older organisms has not yet been fully explored. The two main angiotensin receptors are: Angiotensin 1 (AT1) receptor, and Angiotensin 2 (AT2) receptor. This study aimed to investigate the effects of each angiotensin receptor on physical performance in older mice. We compared aged (24 months old) Losartan (an AT1 receptor antagonist) treated wild type (WT) mice to age- and sex-matched mice lacking the AT1 or AT2 receptor (AT1KO or AT2KO). Mice underwent treadmill running until exhaustion or a maximum duration of one hour, with number of falls off belt (NOF), number of gentle stimuli (NGS), and total time on the treadmill belt (TOB) recorded. NGS indicates how often mild stimuli were used to encourage mice to return to the treadmill belt. Body weights showed significant differences between groups by ANOVA ($p=0.03$), with Tukey HSD indicating AT2KO mice were significantly heavier than WT ($p=0.04$). TOB analysis indicated significant differences (Kruskal-Wallis, $p=0.03$), with post-hoc analyses revealing the AT2KO group spent less TOB than WT ($p=0.008$) and WT los ($p=0.01$). Differences in NGS were detected, with the AT2KO requiring significantly more stimuli compared to WT ($p=0.003$), WT los ($p=0.001$), and AT1KO ($p=0.01$). These findings highlight the impact of angiotensin receptor modifications on physical performance and suggest that loss of AT2 may result in decreased aerobic performance, potentially due to increased body weight.

Abstract citation ID: igae098.3630

### ACTIVATED SKN-1 ALTERS THE LONGEVITY TRAJECTORY OF LONG-LIVED C. ELEGANS MUTANTS

Chris Turner, and Sean Curran, *University of Southern California, Los Angeles, California, United States*

In the presence of stressful environments, the SKN-1 cytoprotective transcription factor is activated to induce the expression of gene targets that can restore homeostasis. However, constitutive activation of SKN-1 results in diminished health and a reduction of lifespan. Here we demonstrate the necessity to regulate the activity of SKN-1 for maintaining the longevity promoting responses associated with impaired daf-2/insulin receptor signaling, the eat-2 model of caloric restriction, and glp-1-dependent loss of germ cell proliferation. A hallmark of animals with constitutive SKN-1 activation is the age-dependent loss of somatic lipids and this phenotype is linked to the general reduction in survival in animals harboring the skn-1gf allele, but surprisingly, daf-2lf; skn-1gf double mutant animals do not redistribute somatic lipids which suggests the insulin signaling pathway functions downstream of SKN-1 in the maintenance of lipid distribution. As expected, eat-2lf; skn-1gf double mutant animals, which independently activate SKN-1, continue to display somatic lipid depletion in older ages with and without the skn-1gf activating mutation but animals lacking a proliferating germline do not redistribute somatic lipids, which supports a genetic model where SKN-1 activity is an important regulator of lipid mobilization in response to food availability to fuel the developing germline by engaging the daf-2/insulin receptor pathway.

Abstract citation ID: igae098.3631

### ACTIVE IMMUNIZATION AGAINST MYOSTATIN AND ACTIVIN A IMPROVES SKELETAL MUSCLE STRENGTH DESPITE GH/IGF-1 DEFICIENCY

Mishfak Abdullah Mohamed Mansoor[1], Sarah Ashiqueali[2], Md Tanjim Alam[1], Amanda Lasseter[3], Brett Thibodeaux[3], and Michal Masternak[4], *1. University of Central Florida, Orlando, Florida, United States*, *2. Mayo Clinic, Rochester, Minnesota, United States*, *3. Vaxxinity, Merritt Island, Florida, United States*, *4. University of Central Florida College of Medicine, Orlando, Florida, United States*

Muscle wasting is a significant hurdle in patients due to immobilization, prolonged bed rest, and injury. In particular, the older population develops sarcopenia, characterized by progressive loss of muscle strength due to natural aging. However, there are no FDA-approved drugs for sarcopenia, and it is critical to explore novel therapeutics. Myostatin inhibition is one such strategy that has had varying degrees of success previously, yet the desired levels of muscle strength have not been achieved. Here, we employed a novel vaccine (V13) that generates anti-myostatin antibodies and another (V07) that generates anti-activin A; myostatin and activin A regulate muscle growth negatively. We administered seven doses of V13, V07, and V35 (generates both anti-myostatin and anti-activin A) to long-living, GH-deficient Ames dwarf mice (df/df) and their normal littermate controls (N/df). By week 24 post-first dose, we saw increased forelimb grip strength in V13, V07, and V35 vaccinated df/df mice and V07 and V35 vaccinated N/df mice. Additionally, almost 40% of the vaccinated df/df mice completed the accelerating rotarod trials, compared to the 20% of placebo-treated counterparts. We observed increased lean body mass and reduced fat mass in vaccinated N/df mice, yet surprisingly there were no changes in lean and fat mass in df/df mice. In summary, our results indicate that while myostatin and activin A inhibition play an intimate role in fat metabolism in the presence of GH/IGF-1, this immunization strategy improves muscle motor performance in GH/IGF-1 deficient mice providing promising treatments in older animals regardless age-associated suppression of GH/IGF-1 axis.

Abstract citation ID: igae098.3632

### ACUTE NAD+ SUPPLEMENTATION MODULATES SYSTEMIC INFLAMMATION IN A LPS-INDUCED DELIRIUM MOUSE MODEL

M. K. Kirsten Chui, Prasanna Vadhana Ashok Kumaar, Eric Verdin, and John Newman, *Buck Institute for Research on Aging, Novato, California, United States*

Delirium is a serious neuropsychiatric condition that lacks an effective treatment intervention. A confusional state often brought on by acute illness, delirium is associated with acute inflammation and metabolic dysfunction. NAD+ is a metabolite involved in both cellular energy generation and immunomodulation, that has previously been found to promote brain health, metabolic functions, and reduce inflammation. Whether NAD+ supplementation may be beneficial for delirium has not been explored yet. In the present study, we investigate the effect of acute supplementation of NMN, a direct precursor of NAD+, in a LPS-induced acute inflammation mouse model for delirium. While NAD+ did not rescue the LPS-induced sickness behavior and metabolic dysfunction in mice, a comprehensive cytokine profile analysis did reveal rescue of plasma IFNγ levels by NMN supplementation. We also observed partial improvement on the levels of IL-12p40, LIX, and IL-17 which were sex dependent. Overall, we demonstrated that acute NAD+ boosting can lessen the LPS-induced cytokine storm in aged mice.

Abstract citation ID: igae098.3633

### AGE AND LOSARTAN EFFECTS ON MITOCHONDRIAL PROTEOME IN MICE

Adam Said[1], Michael Bene[2], Michael Abadir[3], Simion Kreimer[4], Ruth Marx[5], Laura Powell[5], and Jeremy Walston[5], *1. Emory College of Arts and Sciences, Atlanta, Georgia, United States*, *2. Johns Hopkins School of Medicine, Baltimore, Maryland, United States*, *3. University of Maryland, Woodstock, Maryland, United States*, *4. The Mass Spectrometry and Proteomics Facility Johns Hopkins University School of Medicine, Baltimore, Maryland, United States*, *5. Johns Hopkins University, Baltimore, Maryland, United States*

Loss of mitochondrial function with age is associated with reduced organ functional performance and predispose individuals to age-related diseases. Current studies suggest the renin-angiotensin system significantly interacts with mitochondrial function. This study investigated the effects of aging and Losartan, an angiotensin II type 1 receptor blocker, on the mitochondrial proteome in mice. Mitochondrial samples from young mice (3 months), aged mice (24 months), and aged mice treated with Losartan (24 and 33 months) were analyzed using Cytoscape. Aged (24 months) mice had a marked downregulation of beta-oxidation and amino acid degradation proteins compared to young (3 months) mice.

In contrast, chaperone and muscle contraction proteins were upregulated in older mice. Additionally, the TCA cycle proteins showed significant changes with age, with some proteins becoming increased in abundance while others became significantly less abundant. Overall, there was not a clear regulation pattern, suggesting a further complexity in mitochondrial energy production with age. Losartan treatment in 24-month-old mice upregulated the TCA cycle and beta-oxidation proteins. In contrast, chaperone and muscle contraction proteins were downregulated. This trend opposes the aging effect and suggests the regulatory effects of Losartan. At 33 months, there were no significant changes in any protein groups of losartan-treated mice. This difference in expression suggests that more advanced age may prevent the regulatory effects of losartan. In line with previous studies, these results suggest that Losartan has the potential to compensate for age-related functional decline. Losartan is a potential pharmacological intervention that may reverse aging-related mitochondrial proteome decline.

Abstract citation ID: igae098.3634

### AGE-RELATED DIFFERENCES IN KETONE METABOLISM: A STUDY OF KETONE ESTER RESPONSES ACROSS LIFESPAN

Ester Hernandez[1], Aydan Jordan[2], Chatura Senadheera[3], Elizabeth Stephens[4], John Newman[4], Madison Kackley[2], Jeff Volek[2], and Brianna Stubbs[3], *1. Buck Institute for research on aging, Cotati, California, United States*, *2. Ohio State University, Columbus, Ohio, United States*, *3. Buck Institute, Novato, California, United States*, *4. Buck Institute for Research on Aging, Novato, California, United States*

Ketone supplements, such as ketone esters, induce ketosis without other dietary changes and have demonstrated many potential applications in younger adults. However, metabolism by older adults is unknown. This study investigated the metabolic responses to ketone ester consumption across the lifespan, to understand how aging influences blood ketone responses. This study was an open-label, single-arm, observational study of n=82 adults (37=Male, age=20-70y). After an overnight fast, subjects consumed 360 mg/kg/lean body mass of ketone ester (bis-octanoyl-(R)-1,3-butanediol) in a beverage. Then, capillary blood samples were collected at regular intervals for 4h and beta-hydroxybutyrate (BHB) was assayed using a handheld device. Endpoints were peak BHB concentration (Cmax), area under the curve (AUC). The analysis of BHB Cmax across five decades showed no overall effect (p=0.5), with the highest Cmax in the 41-50y group (1.68 mM) and the lowest in the 51-60y group (1.32 mM). In contrast, although there was no overall difference in BHB AUC between age groups (p=0.24, mean mm.min-1, age 20-30y =184, 31-40y =194, 41-50y =221, 51-60y =203, 61-70y =223) there was a notable difference between the 21-30y and 61-70y groups (p=0.03). Linear regression analysis revealed a positive relationship between BHB AUC and age ($R^2$=0.08, p=0.01), but not between BHB Cmax and age ($R^2$=0.01, p=0.37). We cautiously interpret our data to suggest a relationship between increasing age and changes to ketone metabolism that increases AUC but not Cmax. We plan to expand the sample size to 400 subjects to improve our ability to detect-age related differences in exogenous ketone metabolism.

Abstract citation ID: igae098.3635

### ARABINOSE MITIGATE METABOLIC STRESS IN INFLAMMAGING

Brenda Landvoigt Schmitt, Patricia da Silva Pantoja, and Brandt D Pence, *University of Memphis, Memphis, Tennessee, United States*

Arabinose (Ab) is a monosaccharide found in vegetables, fruits, and grains. It has been showed that Ab can manage blood sugar levels, improve gut health, potential anti-aging, antidepressant, antioxidants. Inflammaging is a chronic low graded inflammation commonly seen in aging, contribute significantly to age related disease. We aimed to investigate the impact of Ab on the gene expression of pro-inflammatory cytokines (IL-1β, IL-6, and TNF-α) which are implicated in inflammaging. Additionally, we investigate if the Ab impacted the bioenergetic cell metabolism using Seahorse methodology to assess mitochondrial oxidative phosphorylation (OxPhos) activity, measured as the oxygen consumption rate (OCR) and extracellular acidification rate (ECAR) mitigating inflammation induced by lipopolysaccharide (LPS). U937 cell line was cultured in RPMI media with Pen-Strep 1% and FBS 10%. RTq-PCR and mito-stress-test using Seahorse XF technology were performed. The data analysis was performed in Prism 9. Cells were pre-treated with Ab 30 min following the addition of LPS 3h incubation time at 37°C, 5% CO2 A dose response test was performed were the pre-treatment showed significative response with Ab 50uM in pro-inflammatory cytokines genes downregulating IL-1β, IL-6, and TNF-α. The OCR metabolic phenotype and maximal respiration, under stress conditions, was improved by pre-treatment with Ab in presence of LPS. Ab protected cell to increase ECAR even in presence of LPS. Overall, these results suggest an improvement on mitochondrial OxPhos capacity maintaining the glycolysis performance of monocytic cells under inflammatory stress offering potential benefits for managing inflammaging in older populations.

Abstract citation ID: igae098.3636

### AWE-INDUCTION IMPROVES RECOVERY FROM STRESS AND IMPROVES COGNITIVE PERFORMANCE

Kristina Hash[1], S. Alison Bolling[2], Leslie Tower[2], and Julie Hicks Patrick[1], *1. West Virginia University, Morgantown, West Virginia, United States*, *2. WVU, Morgantown, West Virginia, United States*

Portable, affordable and nonpharmacological interventions are needed to support physical and cognitive wellbeing across the lifespan. A promising psychosocial intervention to accomplish such goals includes awe inductions. Using our previously-validated video inductions, data are provided by 61 adults (M age = 28, range 18-81 yrs). Continuous measures of heart rate variability (HRV), including respiration, Baevsky's stress index and other indices of sympathetic and parasympathetic functioning were collected via Polar H10 heart rate monitors. Participants completed flanker and choice reaction time (RT) tasks. Manipulation checks verified adherence to the protocol. As expected, performance was slower in the cognitively challenging incongruent flanker conditions relative to the easier congruent condition. A series of moderated regressions examined whether age altered the relations between HRV and cognitive performance. Differences in Baevsky's stress index between the Neutral and Awe conditions interacted with age to explain 27% of the variance in post-awe incongruent flanker reaction time, F (3, 51)= 6.30, p<.001. Both age and the interaction term uniquely contributed to the equation. Probing the interaction revealed that although younger adults increased their speed of response under the awe condition, older adults increased their accuracy and slowed their response times accordingly. HRV indices suggested that across age, the awe induction resulted in decreased parasympathetic functioning, suggesting that awe might improve health and recovery from stress.

Abstract citation ID: igae098.3637

### BLOOD MULTIOMIC PROFILES REFLECT SYSTEM STATES OF ORGANS IN MICE

Kengo Watanabe[1], Lance Pflieger[2], Max Robinson[3], Jodi Lapidus[4], Richard Miller[5], Oliver Fiehn[6], Robert Moritz[7], and Noa Rappaport[1], *1. Institute for Systems Biology, Seattle, Washington, United States*, *2. Phenome Health, Seattle, Washington, United States*, *3. ISB, Seattle, Washington, United States*, *4. OHSU-PSU School of Public Health, Portland, Oregon, United States*, *5. University of Michigan, Ann Arbor, Michigan, United States*, *6. UC Davis, Davis, California, United States*, *7. Institute For Systems Biology, Seattle, Washington, United States*

The rate of aging can vary among organs, as evidenced by organ-specific biological age models. Such models are typically constructed based on the blood biomarkers or phenotypes that are known to be specific to a particular organ. However, this approach potentially limits the models to reflecting only a narrow scope of organ system states. Herein, we report the potential of blood omics data to reflect alterations in organ systems, using the NIA Longevity Consortium mouse prolongenvity proteomics and metabolomics data which were derived from kidney, liver, gastrocnemius muscle, and plasma samples from each mouse. While correlation analysis mainly identified the correlations of matched analytes between organs and blood, machine learning models to predict the organ analyte abundance or system state from the plasma analytes revealed the differences between sexes or prolongevity interventions. Our findings suggest the power of blood omics for identifying and characterizing diverse system states of organs involved in aging and longevity.

Abstract citation ID: igae098.3638

### BUILDING AN ELEPHANT BRAIN DATABASE: ADVANCING INSIGHTS INTO COGNITIVE AGING IN ELEPHANTS

Daniella Chusyd[1], Patrick Hof[2], Shannon Risacher[3], Hu Cheng[4], and Chet Sherwood[5], *1. Indiana University, Bloomington, Indiana, United States*, *2. Mount Sinai, New York, New York, United States*, *3. Indiana University, Indianapolis, Indiana, United States*, *4. Indiana University, Indiana University, Indiana, United States*, *5. The George Washington University, Washington DC, District of Columbia, United States*

Cognitive aging is a complex phenomenon, and a comparative approach leverages the strengths of various species to identify conserved and divergent mechanisms associated with cognitive health. Elephants are an informative species for cognitive aging research because they are analogous to humans in that they live to comparable ages, display complex cognition, have strong long-term memory, and rely on social bonds. They differ, however, in their evolutionary path to arrive at these same outcomes. Studying elephants can potentially reveal different trajectories for cognitive performance maintenance and impairment compared to humans, and provide information on brain aging in elephants, contributing missing knowledge on aspects of well-being in a long-lived species. We initiated the Elephant Brain Database to enhance the use of elephants as a comparative species to further understand the structure, function, and evolution of the brain, and to increase the availability of elephant postmortem brain tissue, MRI scans, and related datasets for investigators. This repository is for postmortem fixed and frozen brain specimens and when possible, postmortem structural MRI scans and diffusion tensor images. In collaboration with North American zoos and partners in Zambia, we obtained fixed or frozen brain specimens from 23 individuals (age: stillborn to 75 years). Of those, 12 are from Asian elephants (Elephas maximus; 8 females, 4 males) and 11 are from African savanna elephants (Loxodonta africana; 4 females, 7 males). A structural MRI scan was conducted on one left hemisphere from a 52-year-old female Asian elephant. This collection represents the largest consolidation of elephant brain resources.

Abstract citation ID: igae098.3639

### CANINE COGNITION ASSESSMENTS IN THE TEST OF RAPAMYCIN IN AGING DOGS (TRIAD)

Sydney Holland[1], Anna Ferguson[2], Kate Creevy[1], Rozalyn Anderson[3], and May Reed[4], *1. Texas A&M University College of Veterinary Medicine and Biomedical Sciences, College Station, Texas, United States*, *2. University of Washington School of Medicine, Seattle, Washington, United States*, *3. University of Wisconsin-Madison, Madison, Wisconsin, United States*, *4. University of Washington School of Medicine, University of Washington, Washington, United States*

The primary objective of the Dog Aging Project's Test of Rapamycin in Aging Dogs (TRIAD), is to assess whether rapamycin treatment extends lifespan in healthy, middle-aged, medium-to-large companion dogs. A secondary objective is to determine whether rapamycin impacts cognitive function in aging dogs. This placebo-controlled, double-blinded, randomized clinical trial is underway; however, baseline data allows us to investigate relationships between demographic factors and cognitive function. Dogs who met medical records eligibility criteria for TRIAD were assessed during an in-person examination by veterinary neurologists. Baseline cognitive functional assessment involved two validated clinical assessments: the Sustained Gaze test (possible scores 1 to 60 seconds; higher scores indicate greater attention span), and the CAnine DEmentia Scale (CADES) (possible scores of 0-85; higher scores indicate greater impairment). Thirty-five dogs were examined for inclusion in TRIAD, including baseline assessment of cognitive function. The median age of evaluated dogs was 9.2 years (8.1-10.2) and the median weight was 27.7 kg (24.5-30.2). The median CADES score was 1.0 (0.0-4.0) and median Sustained Gaze score was 9.0 (5.3-14.0). Post hoc analysis did not identify significant correlations between age, weight, sex, or breed and scores for either cognitive test. CADES and Sustained Gaze tests do not reveal an influence of demographic factors on cognition at middle-age in dogs being assessed for enrollment in TRIAD. Accordingly, the TRIAD clinical trial will enable robust assessment of rapamycin's impact on age-related cognitive decline.

Abstract citation ID: igae098.3640

### CARDIAC SPECIFIC OVEREXPRESSION OF SERUM RESPONSE FACTOR ALTERS MITOCHONDRIAL STRUCTURE AND FUNCTION

Pankaj Patyal, Gohar Azhar, Ambika Verma, Xiaomin Zhang, and Jeanne Y Wei, *University of Arkansas for Medical Sciences, Little Rock, Arkansas, United States*

Serum response factor (SRF) is a transcription factor required for the regulation of genes important for cardiac structure and function. Previously, we have shown that increased levels of SRF in transgenic mouse carrying human SRF under Myh6 promoter resulted in cardiomyopathy and early mortality. Although, there is a rapidly growing body of literature investigating the effects of aging on the heart and how age-related alterations affect cardiac and mitochondrial function, and underlying mechanisms remain unclear. Herein, we tested the hypothesis whether cardiac specific overexpression of SRF alters mitochondrial function. We utilized SRF transgenic mice at different postnatal ages to demonstrate temporal increase in the SRF expression in the heart. Western blot analysis demonstrate the significant increase in the BNP, marker for left ventricular cardiac hypertrophy ($p < 0.01$). Electron microscopy revealed mitochondrial abnormalities. Further, we used high-resolution respirometry to determine mitochondrial function in SRF overexpressed mouse heart. Our data indicate the significant decrease in the mitochondrial respiratory chain complexes (I-IV) respiratory rates and maximal electron transport system capacity ($p < 0.01$). The markers for mitochondria biogenesis PGC1α and PGC1β expression levels were reduced analyzed by western blot analysis ($p < 0.05$). Further, protein expression levels of Opa1 and DRP1, markers for mitochondrial fusion and fission were significantly reduced ($p < 0.01$). Our findings implicate that the increase in the SRF expression in cardiac aging induce mitochondrial alterations and functional decline. Thus, SRF is likely to be one of the downstream effectors of the signaling pathways involved in mediating mitochondrial function.

Abstract citation ID: igae098.3641

### CHARACTERIZING PROTEIN STRUCTURES IN BETA-AMYLOID TREATED NEURONS WITH LIMITED PROTEOLYSIS-MASS SPECTROMETRY

Kuren Anderson-Steward[1], Ying Hao[2], Deyaan Guha[3], Ziyi Li[2], Benjamin Jin[2], Andrew Singleton[2], and Andy Qi[2], *1. Howard University, Bowie, Maryland, United States, 2. National Institutes of Aging, National Institute of Health, Bethesda, Maryland, United States, 3. Brandeis University, Waltham, Massachusetts, United States*

Limited Proteolysis by Mass Spectrometry (LiP-MS) is a technique used to observe the structural changes of proteins on a proteome-wide scale. Limited proteolysis consists of a specific, or limited, number of proteases performing hydrolyzation on a large portion of proteins. This causes the protease to detect the weak portion of the hydrolyzed protein and cut it out. The technique of limited proteolysis coupled mass spectrometry aims to alleviate the limitations of classic proteomic analysis, such as being unable to detect protein structural alterations on a wide scale and at high throughput. In this experiment, we used iPSC-derived neurons treated with beta-amyloid and performed classic proteomics and LiP-MS in parallel to observe the proteome scale of structural changes caused by beta-amyloid. Through experimentation, it was found that the control group consisted of 180 protein profiles that the beta-amyloid treated group did not possess using the limited proteolysis-mass spectrometry technique. Additionally, various cleavage sites by proteinase K in the beta-amyloid treated neurons compared to the control neurons, as well as multiple molecular pathways, such as neuron projection cytoplasm, were identified.

Abstract citation ID: igae098.3642

### CIRCULATING MIRNA PROFILING FOR THE DETECTION OF SARCOPENIA COMPONENTS IN OLDER ADULTS

Hyung Eun Shin, Jae Young Jang, Daehyun Lee, Heeeun Jung, Hyunjin Cho, Nahyun Lim, Chang Won Won, and Miji Kim, *Kyung Hee University, Seoul, Republic of Korea*

MicroRNAs (miRNAs), short, non-coding RNAs that regulate gene expression at translation, are recently emerging as potential biomarkers for sarcopenia. The miRNAs have been extensively studied in etiology of sarcopenia; however, the role of miRNAs on sarcopenia components has not yet been explored. We aimed to examine the differentially expressed miRNAs in plasma of older adults in relation to muscle mass, strength, and performance status. A total of 96 older adults (women: 50.0%; mean age: 76.6 ± 3.6 years) were randomly selected and matched by age and sex from the Korean Frailty and Aging Cohort Study (KFACS). Participants were categorized into four groups based on criteria of Asian Working Group for Sarcopenia 2019: "Normal" ($n = 25$), "Low muscle mass (LMM) only" ($n = 23$), "Low muscle strength (LMS) only" ($n = 25$), "Low physical performance (LPP) only" ($n = 23$). MiRNA profiles, derived from miRNA sequencing, were established via screening for differentially expressed miRNAs in four groups, and candidate differentially expressed miRNAs were validated using RT-qPCR. The miRNA profiling and validation analysis showed that three differentially expressed miRNAs, including miR-144-3p, miR-122-3p, and miR-210-3p, were significantly increased in plasma of "LMS only" group, compared to "LMM only" and "LPP only" groups ($p < 0.05$). The Kyoto Encyclopedia of Genes and Genomes revealed that three miRNAs were mainly related to neurotrophin signaling pathway ($p < 0.05$). These results suggest that increased levels of miR-144-3p, miR-142-3p, and miR-122-3p could serve as candidate plasma biomarkers for the decline of muscle strength in older adults.

Abstract citation ID: igae098.3643

### COMMUNITY CENTERED PERSPECTIVES ON ALZHEIMER'S DISEASE: A SYNTHESIS OF DATA FROM FOUR COMMUNITY ADVISORY BOARDS

Ivy Ahmed[1], Diane DeVincentis[1], Teresa Cronin[1], and Jennifer Pettis[2], *1. Eisai, Nutley, New Jersey, United States, 2. Gerontological Society of America, Clifton Park, New York, United States*

As the number of people with Alzheimer's disease in the United States continues to grow, they and their caregivers face increasingly complex challenges in the health care ecosystem. To understand the impact of Alzheimer's disease on communities, Eisai with participation of the Gerontological Society of America convened four Community Advisory Boards (CABs) inviting a variety of communities of interest across multiple disciplines that serve the community. These informative day-long meetings shed light on the local impact of this potentially devastating disease and allowed for meeting participants to share many creative solutions in place through grassroots dedication. The team identified four different communities based on their unique population and location: Dade County, Florida; Summit County, Ohio; Greater Atlanta, Georgia; Los Angeles, California. During the meetings, participants reflected on the myriad needs within their community; highlighting their most pressing unmet needs; and spotlighted successful programs, initiatives, and interactions that occur within the community to support their community members with AD and their caregivers. While each community's needs were unique, several common themes emerged uniting these four communities illustrating shared issues and creative solutioning. Shared themes included the need for materials that address brain health to raise awareness of AD prevention and the importance of early detection, culturally tailored resources, and information delivered by trusted community champions in settings that leverage existing social outlets/meeting places. This poster will illustrate key findings from each individual meeting and highlight commonalities identified among them.

Abstract citation ID: igae098.3644

### CONFORMAL PREDICTION OF RESIDUAL COGNITION IN AGING AND ALZHEIMER'S DISEASE

Ime Essien[1], Laura McDaniel[1], Jeremy Walston[1], David Bennett[2], Peter Abadir[1], and Rama Chellappa[1], *1. Johns Hopkins University, Baltimore, Maryland, United States*, *2. Rush University Medical Center, Chicago, Illinois, United States*

Cognitive reserve (CR) and resilience in aging and Alzheimer's disease (AD) have gained significant attention in recent research. We explore cognitive resilience by examining how individuals maintain cognitive function despite neuropathological burden, particularly in the context of AD in Religious Orders Study and Rush Memory and Aging Project (ROSMAP) cohort. Our approach involves modeling residual cognition, defined as the difference between observed cognitive function and expected function based on neuropathological burden. Conformal prediction is applied to generate prediction intervals for global cognitive scores based on beta-amyloid and tau protein levels and demographic data. This provides a valid measure of uncertainty to derive our residual cognition. This method allows us to identify cases where beta-amyloid and tau protein levels are misaligned with final cognitive diagnoses, providing valuable feature interpretability. The study stratifies participants using the Clinical consensus diagnosis of cognitive status at time of death into control, asymptomatic AD (AsymAD), and AD groups to investigate cognitive maintenance in the presence of AD-associated neuropathological changes. By comparing residuals across different diagnostic groups, we gain insights into factors contributing to cognitive resilience. We use the clinical diagnosis of cognitive status as a validation measure to examine how well our residual cognition measure predicts clinical outcomes. Our research builds upon previous work on CR measurement, including decomposition approaches, the Alzheimer's Disease Cognitive Resilience Score, and the residual reserve index. The findings will help inform interventions to promote cognitive health in aging populations.

Abstract citation ID: igae098.3645

### CROSSTALK BETWEEN HYALURONAN AND ITS REGULATORS AFFECTS AGING

Kaustuv Basu, and Paraskevi Heldin, *Uppsala University, Uppsala, Uppsala Lan, Sweden*

The regulation of extracellular matrix (ECM) turnover is crucial in aging. Hyaluronan (HA) is a large, viscoelastic polysaccharide and a major component of ECM. It lubricates and organizes tissue structure in different body parts (e.g. skin) and orchestrates multiple cellular functions through its receptors CD44 and RHAMM and binding proteins. HA is synthesized by HA synthase (HAS). The synthesis of HA in the longest-lived rodent naked mole rat (NMR) and the extended lifespan of a transgenic mouse overexpressing the NMR-HAS2 gene illustrates the importance of HA in longevity. HA level is significantly modulated with increasing age. Thus, understanding the complex crosstalk between HA and its regulators is crucial to regulating aging. Here, we revealed a vital connection between the function of p53 and its paralogue p63 and HA biosynthesis and CD44 signaling. Previously, it was reported that p53 suppressed the expressions of CD44 and RHAMM. We identified that CD44 interacted with the apoptosis-stimulating protein of p53 (iASPP) and affected p53-mediated apoptosis. We also revealed that the mutant p53 suppressed HAS2 and -3 mRNA expression. The mutation status of endogenous p53 and the balance between p53 and p63 levels affected HAS2 and HAS3 expression differently after TGF-β1 stimulation. We noticed that the activation of p63 and Ras was essential for regulating HAS and CD44 without TGF-β1 stimulation. We observed the importance of the HA receptor in regulating aging and aging-related disease. Our study unveiled the significance of the interplay between HA and its regulators, vital in understanding the complex aging process.

Abstract citation ID: igae098.3646

### DAILY CONSUMPTION OF KETONE ESTER, BIS-OCTANOYL (R)-1,3-BUTANEDIOL, IS SAFE AND TOLERABLE IN HEALTHY OLDER ADULTS

Chatura Senadheera[1], Traci Blonquist[2], Brianna Stubbs[1], Stephanie Roa Diaz[1], Sawyer Peralta[3], Elizabeth Stephens[4], and John Newman[4], *1. Buck Institute, Novato, California, United States*, *2. Biofortis Mérieux NutriSciences, Addison, Illinois, United States*, *3. Albert Einstein College of Medicine, New York, New York, United States*, *4. Buck Institute for Research on Aging, Novato, California, United States*

Ketone bodies are endogenous metabolites produced during fasting or a ketogenic diet that have pleiotropic effects on aging pathways. Ketone esters (KEs) are compounds that induce ketosis without dietary changes, but KEs have not been studied in an older adult population. The primary objective of this pilot study was to assess tolerability and safety of KE ingestion in older adults. We carried out a randomized, placebo-controlled, double-blinded, parallel-arm trial (NCT05585762) with community-dwelling, independent older adults in stable health. N=30 (M=15, F=15; age=76y, range 65 –90y) were randomized and n=23 completed. Participants were randomly allocated to consume either KE (25g bis-octanoyl (R)-1,3-butanediol) or a taste, appearance, and calorie-matched canola oil placebo (PLA) daily for 12 weeks. Tolerability was assessed using a daily log for 2-weeks, and then via a bi-weekly phone interview. Safety was assessed by vital signs and lab tests at screening and weeks 0, 4 and 12, along with tabulation of adverse events. There was no difference in the prespecified primary outcome of proportion of participants reporting moderate or severe nausea, headache, or dizziness on more than one day in a two-week reporting period (KE n=2 (14.3% [90% CI=2.6–38.5]);PLA n=1 (7.1% [90% CI=0.4–29.7]). Dropouts numbered four in the PLA group and two in the KE group. More symptoms were reported in both groups during the first two weeks; symptoms were reported less frequently between 2–12 weeks. There were no clinically relevant changes in safety labs or vital signs in either group.

Abstract citation ID: igae098.3647

### DEFINING A NOVEL HOMEOSTAT THAT SENSES THE BARRIERS OF PARENTAL REPRODUCTIVE-SPAN AND SETS PROGENY STRESS CAPACITY.

Bennett Van Camp[1], and Sean Curran[2], *1. University of Southern California, Chandler, Arizona, United States*, *2. University of Southern California, Los Angeles, California, United States*

The ability to respond to stressors quickly and adequately is a core component to survival, and dysregulation of these resistance pathways can be debilitating. The cytoprotective transcription factor SKN-1 plays a critical role in response to multiple types of stress that can impact Caenorhabditis elegans fitness (e.g., developmental timing, reproductive output). However, activation of SKN-1 is antagonistically pleiotropic, driving multiple age-dependent pathologies. For example, as compared to wildtype animals, SKN-1 activation in early adulthood provides enhanced resistance to oxidative stress, but greater sensitivity in midlife and an early loss of motility, imbalanced lipid homeostasis and diminished lifespan. Because the negative phenotypes associated with constitutive SKN-1 activation manifest at the end of reproduction, we use long-term parental age selection of late progeny (last quartile of total progeny) as a tool to investigate the differential health of early and late progeny across the reproductive span. Selection for >50 generations resulted in significant decreases of multiple SKN-1 age-related measures of health; most prominently, acute oxidative stress resistance, lipid homeostasis, movement, developmental timing, starvation resistance, and lifespan. Perhaps most surprising, acutely releasing the age selection resulted in instantaneous consequences on some, but not all, health parameters, of the immediate brood. This observation suggests the existence of a novel homeostatic set point that can bookmark the temporal boundaries of the parental reproductive span. These findings reshape the way we think about SKN-1 and the parental role in homeostatic control in offspring and provide additional details of its complex role in the regulation of healthspan.

Abstract citation ID: igae098.3648

### DETERMINANTS OF AGE AT NATURAL MENOPAUSE AMONG POSTMENOPAUSAL GHANAIAN WOMEN IN OKAIKWEI NORTH DISTRICT, GHANA.

Okyere Kontor[1], Veneranda Nyarko[2], and Paul Botwe[3], *1. University of Ghana School of Public Health, ACCRA, Greater Accra, Ghana*, *2. KORLE-BU TEACHING HOSPITAL, ACCRA, Greater Accra, Ghana*, *3. University of Ghana, Accra, Greater Accra, Ghana*

The age at natural menopause, influenced by genetic and modifiable factors is linked to the risk of certain chronic conditions. While menopause has been studied extensively in other countries, there is limited data on its determinants among women in developing countries like Ghana, creating a theory gap for investigations. This study aims to assess post-menopausal women's knowledge of menopause, determine the average age at natural menopause, and identify factors influencing this age among postmenopausal Ghanaian women in the Okaikwei North Municipality of Ghana. A cross-sectional study design with a quantitative approach was used. Consecutive sampling selected 296 post-menopausal women attending Achimota Hospital's (municipal referral hospital) outpatient clinic. Data were collected via a structured questionnaire and analyzed using Stata version 16. Descriptive statistics, bivariate analysis (Chi-square and Fisher's exact test) and multiple logistic regression (crude and adjusted odds ratios) were used, with statistical significance set at $p < 0.05$. The study found that 48.3% of participants had adequate knowledge about menopause. Most women experienced the "expected" natural menopause, 46-55 years (93.2%), with early menopause (40-45 years) at 3.7% and late menopause (>55 years) at 3.1%. The average age at natural menopause was 49.5 years (±0.17 SD). Significant factors associated with natural menopausal age included age at menarche, age at first pregnancy and contraceptive use. The study highlighting low knowledge about menopause among the participants therefore recommends an enhanced practical education, cost-effective health care, and women's empowerment to better prepare women for menopause, particularly those approaching this transition.

Abstract citation ID: igae098.3649

### DEVELOPMENT OF SCAFFOLD FOR A ISCHEMIC STROKE MICROFLUIDIC CHIP PLATFORM

Lidadi Agbomi[1], Thomas Nathaniel[2], Richard Goodwin[3], and Bruce Gao[4], *1. Clemson University, Clemson, South Carolina, United States*, *2. University of South Carolina School of Medicine Greenville, Greenville, South Carolina, United States*, *3. University of South Carolina, Columbia, South Carolina, United States*, *4. Clemson University, Clemson, South Carolina, United States*

Age-related brain diseases are rising, with strokes, especially ischemic strokes (IS), being a leading cause of death. Accurate modeling of IS is crucial for diagnosis, but animal models have limitations. When creating a new, human, cell-based microfluidic chip platform for modeling IS, a suitable 3D scaffold for cell growth is essential. For this, we chose to generate a 3D scaffold using a hydrogel platform that contains microchannels that will model the cerebral vasculature. 3D Agarose hydrogels were fabricated for testing. Hydrogels of 0.5%, 1%, and 2% agarose were polymerized according to the manufacturer's protocol. Hydrogels were placed in a 60mm Petri dish. Testing was done on each hydrogel to determine the best agarose concentration after frozen for 10, 15, and 20 hours, then lyophilized for 8 hours. This method helped make the hydrogel more porous. The carbohydrate glass was fabricated by combining 53g of sucrose, 25g of glucose, 10g of Dextran, and 50 ml of Reverse Osmosis water and heated to 165°C. This solution was then poured into a heated 50mL glass syringe to be extruded into the hydrogel later. The 2% agarose hydrogel yielded the best results after lyophilization with the agarose maintaining some of its gel-like characteristics, while being more porous to help with fluid flow later. Preliminary tests were successful. Further tests will determine the best method for extruding carbohydrate glass into the hydrogel. Once it is extruded, the scaffold will be integrated into a microfluidic chip platform, and flow experiments will be initiated.

Abstract citation ID: igae098.3650

### DIETARY METHIONINE RESTRICTION STARTED LATE IN LIFE IMPROVES NEUROMUSCULAR FUNCTION AND PROMOTES HEALTHY AGING

Andrey Parkhitko[1], Ulalume Hernandez Arciga[1], Ceda Stamenkovic[2], Shweta Yadav[1], Gene Ables[3], Alessandra Sacco[2], Michael Jurczak[1], and Stacey J Sukoff Rizzo[1], *1. University of Pittsburgh, Pittsburgh, Pennsylvania, United States*, *2. Sanford Burnham Prebys Medical Discovery Institute, San Diego, California, United States*, *3. Orentreich Foundation for the Advancement of Science, Inc, Cold Spring, New York, United States*

Methionine metabolism is negatively affected by aging and restricting methionine consumption (MetR) extends lifespan across diverse species. Similarly, levels of enzymes in the tyrosine degradation pathway (TDP) increase with age, the targeting of which extends Drosophila lifespan. However, we have limited information on the effects of either metabolic manipulation when started late in life in mice, and we lack information on the effects of dietary MetR on the aging process in humans. To investigate the efficacy of targeting either methionine metabolism or the TDP for healthspan improvement in advanced age, we initiated long-term dietary MetR or treatment with the TDP inhibitor, nitisinone, in 18-mo male and female C57BL/6J mice. Our data demonstrate multiple healthspan benefits of dietary MetR started late in life with the strongest improvements observed in neuromuscular function, metabolic health, lung function, and frailty. By contrast, we observed no such signal of benefit with nitisinone treatment. Using single-nucleus RNA and ATAC sequencing of muscle tissues, we identified subtype-specific processes and transcription factors activated by dietary MetR. Epigenetic clocks can be used to evaluate longevity interventions across different species including humans. Surprisingly, dietary MetR did not significantly affect the mouse epigenetic clock. An analysis of human samples obtained from a randomized clinical trial (NCT04701346) examining an 8-week dietary MetR intervention also did not reveal a significant MetR-dependent effect on the epigenetic clocks. These findings establish beneficial effects of MetR started late in life and provide translational rationale to develop MetR mimetics as an anti-aging intervention in humans.

Abstract citation ID: igae098.3651

### DIETARY PROTEIN RESTRICTION IN AGED MALE MICE REDUCES MARKERS OF SASP IN ADIPOSE TISSUE

Molly Easter[1], Khristina Young[1], Hanna Sothers[2], Jolaiya Aldridge[3], Yvann Batamack[1], Prerana Vaddi[4], Christopher D. Morrison[5], and Cristal Hill[1], *1. University of Southern California, Los Angeles, California, United States*, *2. University of Alabama at Birmingham, Birmingham, Alabama, United States*, *3. University of Southern California, University of Southern California, California, United States*, *4. University of Southern California, Leonard Davis School of Gerontology, Los Angeles, California, United States*, *5. Pennington Biomedical Research Center, Baton Rouge, Louisiana, United States*

Aging is associated with cellular senescence and metabolic impairment of adipose tissue. Moreover, the detriments of aging are further exacerbated by obesity on metabolic function in adipose tissue. Our lab and others have shown that dietary protein restriction (DPR), without reducing calorie intake, protects against age-related metabolic decline and improves lifespan. To determine if DPR reduces cellular senescence in adipose tissue during aging and aging with obesity, 16 month old male C57BL/6 mice were fed either a normal-protein (20%), low-protein (5%), high-fat (60% fat) or a low-protein/high fat (5% protein; 60%fat) diet for 24-weeks, then adipose depots were analyzed via real time quantitative PCR (RT-qPCR) for cellular senescence markers. We found significant decreases in p21, senescence associated secretory phenotype markers(SASP) and reduced SA-Bgal staining in adipose tissue of mice that were fed a low protein diet compared to normal protein diet fed mice. Additionally, mice on a low protein diet and high fat diet demonstrated significant decreases in cellular senescence markers compared to mice on a normal protein and high fat diet, suggesting that a low protein diet decreases the burden of cellular senescence on adipose tissue in aged mice and aged obese mice. Overall, these data suggests that DPR reduces the burden of cellular senescence on adipose tissue function during aging and aging with obesity. Given the negative impact of cellular senescence on adipose tissue, DPR offers a potential dietary intervention to prevent the detriments of cellular senescence on adipose tissue function during aging and aging with obesity.

Abstract citation ID: igae098.3652

### DIFFERENTIAL EFFECT OF SIRT1 ON MITOCHONDRIAL FUNCTION: INSIGHTS INTO MITOCHONDRIAL RESPIRATORY COMPLEXES

Xiaomin Zhang[1], Jyotsna Shrivastava[2], Pankaj Patyal[1], Ambika Verma[1], Shakshi Sharma[3], Gohar Azhar[1], and Jeanne Y Wei[1], *1. University of Arkansas for Medical Sciences, Little Rock, Arkansas, United States*, *2. University of Arkansas for Medical Sciences, little rock, Arkansas, United States*, *3. University of Arkansas for Medical Sciences, LITTLE ROCK, Arkansas, United States*

Sirtuin-1 gene (SIRT1) regulates various cellular functions that influence metabolic homeostasis and aging. SIRT1 dysfunction has been implicated in cardiovascular diseases and mitochondrial dysfunction. However, mechanism of SIRT1 in the regulation of cellular respiration, metabolic homeostasis, and mitochondrial respiratory complexes is not well-known.

We tested whether SIRT1 gene knockout (KO) in HEK293T affects mitochondrial respiratory complex function and expression profiles of associated genes compared with wildtype (WT) HEK293T cells. Oxygen consumption rate (OCR, assayed by XFe96 Analyzer and Oxygraph-O2K) was reduced ($p < 0.001$ and $p < 0.05$, respectively) in HEK293T SIRT1-KO cells compared to WT. Basal extracellular acidification rate (ECAR) was also reduced in SIRT1-KO cells relative to WT ($p < 0.001$), with higher levels of ATP in SIRT-1 KO ($p < 0.01$). SIRT1-KO significantly reduced the expression of glycolytic genes, including Phosphofructokinase P ($p < 0.01$), Hexokinase 2 ($p < 0.05$), and Phosphofructokinase Mv4 relative to WT ($p < 0.05$). Mitochondrial respiratory complexes are vital for ATP production through oxidative phosphorylation. Complex IV is crucial for the final step of electron transport chain. Compared to WT, significantly reduced OCR for complex IV in SIRT1-KO cells indicated mitochondrial dysfunction and impaired energy production ($p < 0.05$). SIRT1-KO also repressed the expression of mitochondrial respiratory complex I genes (NDUFAB1, NDUFV1, and NDUFV2), essential for efficient cellular respiration. This repression suggests compromised mitochondrial bioenergetics and metabolic homeostasis. Interestingly, SIRT1-KO also downregulated mitofusin-1, a key protein in maintaining mitochondrial morphology and size. Our data highlights that SIRT1 is crucial for regulating mitochondrial respiration, metabolism, and bioenergetics.

Abstract citation ID: igae098.3653

### DIFFERENTIAL MICRORNAS PROFILING OF MARSHALLESE IN ARKANSAS IS ASSOCIATED WITH CHRONIC DISEASES OF AGING

Gohar Azhar[1], Ambika Verma[1], Pankaj Patyal[1], Sheldon Riklon[2], Philmar M Kabua[3], Pearl McElfish[3], and Jeanne Y Wei[1], *1. University of Arkansas for Medical Sciences, Little Rock, Arkansas, United States*, *2. UAMS Northwest Regional Campus, UAMS Northwest Campus, Arkansas, United States*, *3. UAMS Northwest Regional Campus, Fayetteville, Arkansas, United States*

Arkansas is home to one of the largest populations from the Republic of the Marshall Islands (RMI), who have settled in the United States. Marshallese communities have a disproportionately high prevalence of many chronic diseases including diabetes, obesity, cardiovascular disease and inflammatory conditions. In the era of RNomics, miRNAs are critical in determining the cellular fate in many pathological conditions. Here we report for the first time, miRNAs expression profiling and differential expression in the Marshallese population. Blood samples were collected from 47 Marshallese subjects after obtaining informed consent and subjected to Illumina based next generation RNA sequencing. According to the sequencing reads annotation in the miRBase database, the most significantly expressed miRNAs were screened using log2 fold-change values selected with Bonferroni corrected p-value< 0.01. A total of 63 human miRNAs were differentially expressed in Marshallese subjects, and among them, a total of 52 human miRNAs were significantly upregulated and 11 were significantly downregulated in males versus female subjects. Two most abundant human miRNAs families were significantly upregulated in Marshallese population including hsa-miR-548 and hsa-let-7, which play important role in regulating several inflammatory responses. Interestingly, the differentially expressed miRNAs have known associations with diabetes mellitus (hsa-miR-21, hsa-miR-26, hsa-miR-93, hsa-miR-96, hsa-miR-126), hypertension (hsa-miR-17, hsa-miR-21), Alzheimer's disease and related dementias (AD/ADRD) (hsa-miR-144-3p, hsa-miR-144-5p) and also cardiovascular diseases (hsa-miR-548j-5p, hsa-miR-584-3p, has-let-7a-5p). Diabetes mellitus remains a major health concern in Marshallese and might accelerate co-morbidities with cardiovascular conditions and ADRD. Hence, the role of specific miRNAs associated with these conditions merits further investigation.

Abstract citation ID: igae098.3654

### E3 UBIQUITIN LIGASE2 SIAH2 MAY REGULATE AGE-ASSOCIATED LIPID TURNOVER IN ADIPOSE TISSUE MACROPHAGES

Bhaswati Ghosh, *Pennington Biomedical Research Center, BATON ROUGE, Louisiana, United States*

The global adult population with obesity is rapidly increasing. One of the aging hallmarks is accumulation of pro-inflammatory cells, which disrupts the balance between lipid storage and lipolysis leading to adipose tissue (AT) expansion and obesity-related insulin resistance. The primary inflammatory contributors in AT are adipose tissue macrophages (ATMs). As in obesity, ATM accumulation increases with aging. Notably, the ubiquitin-proteasome system, the non-lysosomal degradative machinery that maintains protein homeostasis, tightly regulates inflammation and declines with age. We hypothesized that aging downregulates proteasomal activity in ATMs resulting in dysfunctional lipid metabolism. We previously showed the E3-ubiquitin ligase Seven-in-Absentia mammalian homolog2 (Siah2) is crucial for promoting adipogenesis in pre-adipocytes and regulates obesity-mediated AT inflammation. Recently we found Siah2 is present in immune cells including macrophages. Here, we generated a macrophage-specific Siah2 knockout (SKM) mouse model to investigate the role of Siah2 in ATM lipid metabolism. We co-cultured primary SKM-bone marrow-derived macrophages (SKM-BMDMs) or wild-type-bone marrow-derived macrophages (WT-BMDMs) in presence of gonadal white adipose tissue from lean mice as a model of bone marrow-derived ATMs. Interestingly, SKM-ATMs accumulate more, and larger lipid droplets than WT-ATMs via increased lipid uptake. Furthermore, Siah2KO in ATMs suppresses lysosomal delivery of lipid droplets, and the SKM-ATM lysosomes are enlarged compared to WT-ATMs. Consistently, SKM-BMDMs are pro-inflammatory and show decreased oxygen consumption rate indicative of a switch to glycolytic metabolism. Altogether, our findings indicate Siah2 functions as a critical lipid sensor in ATMs, raising the possibly that aging downregulates Siah2 activity in ATMs leading to declined lipid turnover.

Abstract citation ID: igae098.3655

### EARLY DIAGNOSIS OF ALZHEIMER'S DISEASE USING A SIMPLE AND SENSITIVE NANOBIOSENSOR

Xiaohu Xia, *University of Central Florida, Orlando, Florida, United States*

Existing diagnostic methods for Alzheimer's disease (AD), such as PET/MRI imaging and cerebrospinal fluid tests, are invasive and/or costly. Recent studies have demonstrated that exosomes (nanoscale extracellular vesicles carrying specific biomarkers that can traverse blood-brain barrier) are ideal sources of AD biomarkers, which can be conveniently collected from peripheral body fluids like blood and saliva. Detection of AD exosomal biomarkers opens a new avenue for non-invasive diagnosis of AD. However, the concentrations of AD exosomal biomarkers in body fluids are extremely low. Therefore, highly sensitive analytical techniques such as mass spectrometry are required to measure the exosomal biomarkers accurately. Unfortunately, these sensitive techniques come with high costs and can be cumbersome, limiting their widespread use in clinical settings. In this research, we report a simple and low-cost nano-biosensor for ultrasensitive detection of AD exosomal biomarkers. The nano-biosensor relies on metal nanoparticles as labels or signal transducers. After conjugation of the metal nanoparticles with bioreceptors specific to AD exosomal biomarkers, the nanoparticle-bioreceptor conjugates can specifically catalyze the formation of colorimetric signal that can be conveniently quantified by an inexpensive spectrophotometer. The nano-biosensor is expected to be able to detect AD exosomal biomarkers from blood and/or saliva samples, representing a minimally invasive and accessible technology for early diagnosis of AD.

Abstract citation ID: igae098.3656

### EFFECTS OF SPINELESS ON SURVIVAL AND REPRODUCTION IN DROSOPHILA MELANOGASTER

Shelton Swint, and Jessica Hoffman, *Augusta University, Augusta, Georgia, United States*

The aryl hydrocarbon receptor (AhR) is a widely conserved, ligand-activated mammalian transcription factor involved in numerous physiological processes, including maintaining stem cell pluripotency during development and regulating xenobiotic detoxification. One group of commonly studied activators of AhR are tryptophan and its downstream metabolites. Recent studies have shown that AhR-deficient mice display similar survival trends to control animals during early life (≤12 months) and then display a premature aging phenotype. As mice live for two to three years, we can advance our knowledge of AhR and aging using the fruit fly (D. melanogaster) as an alternative. Flies possess an ortholog of the mammalian AhR called spineless (ss), but the effects of ss on survival have yet to be shown. Additionally, many tryptophan-derived metabolites, like tryptamine, have been shown to decrease lifespan in control flies, potentially due to activation of AhR. We found that ss-deficient flies are shorter-lived than wildtype flies, and similar to trajectories in mice, ss flies have normal survival until around 40 days of age. The lifespan-shortening effects of tryptophan metabolites were reduced in ss-deficient flies, suggesting that the negative effects of tryptophan metabolites may be dependent on ss. Furthermore, oxidative stress assays showed that ss flies are more stress resistant, significantly outliving the wildtype flies at an early age. The effects of ss are also being investigated using a potential AhR-antagonist, cis-Jasmone. Overall, our work demonstrates that ss-deficient flies have reduced lifespan and altered responses to tryptophan metabolites, suggesting a conserved role of AhR in the aging process.

Abstract citation ID: igae098.3657

### EVALUATING BIOLOGICAL AGE METRICS: ROBUST PREDICTORS OF AGING PROCESSES IN A CLINICAL COHORT

Elizabeth Stephens[1], Brianna Stubbs[2], Chatura Senadheera[2], Laura Alexander[2], Wendie Silverman-Martin[2], Sawyer Peralta[3], Stephanie Roa Diaz[3], and John Newman[3], *1. Buck Institute for Research on Aging, Rohnert Park, California, United States*, *2. Buck Institute, Novato, California, United States*, *3. Buck Institute for Research on Aging, Novato, California, United States*

Biological age (BA) metrics, constructed by physical and biochemical markers, are increasingly recognized as powerful tools to quantify the biological processes associated with aging. Health and function in a population of the same chronological age (CA) varies between individuals. BA metrics, such as Klemera-Doubal Method (KDM), PhenoAge, and Homeostatic Dysregulation (HD) provide an age independent assessment of health status. Here, we aimed to examine the robustness and utility of BA metrics using data from a 12-week, randomized, parallel group, double-blind, placebo-controlled pilot study evaluating the safety and tolerability of a ketone ester (n=23; M=14, F=9; age=76y, range 65-90y). KDM and PhenoAge, and their residuals, showed strong correlations with CA (r=0.80 and r=0.89, respectively). In contrast, HD and its residual exhibited minimal correlation with CA (r=0.04). Using LASSO regression models, we identified several significant predictors of BA, including red cell distribution width, monocyte percentage, blood urea nitrogen, pulse and blood pressure, C-reactive protein, and total cholesterol and high-density lipoprotein. The models' reliability was confirmed by minimizing prediction errors through cross-validated mean squared error paths, validating their robustness and generalizability. Our results validate KDM and PhenoAge as reliable BA metrics, emphasizing their potential use in clinical settings to assess aging and predict health outcomes. The biomarkers we found to predict BA reflect key biologic processes, such as inflammation, immune, kidney, and cardiac function, and metabolism, underscoring the relevance of BA as a complement to CA in assessing aging and age-related health risks.

Abstract citation ID: igae098.3658

### FEASIBILITY OF RECRUITING PEOPLE WITH MILD COGNITIVE IMPAIRMENT IN THE CONTEXT OF HEART FAILURE

Miyeon Jung[1], Susan Pressler[2], Dustin Hammers[3], and Liana Apostolova[3], *1. Indiana University School of Nursing, Indianapolis, Indiana, United States*, *2. Indiana University, Indianapolis, Indiana, United States*, *3. Indiana University School of Medicine, Indianapolis, Indiana, United States*

Recruiting people with mild cognitive impairment (MCI) with another chronic condition such as heart failure (HF) can be arduous. Our investigative group will discuss the challenges encountered while recruiting older adults with both MCI and HF using data from a pilot study testing the efficacy of cognitive interventions to improve cognitive function and the strategies to overcome them. Initially, eligibility criteria included age ≥65 years, HF confirmed by echocardiography, and MCI defined using a 2-step process: (1) Montreal Cognitive Assessment (MoCA) ≤23; and (2) diagnostic consensus of MCI based on the presence of cognitive impairment in the absence of functional decline. Enrollment began on 4/3/2023 by screening Cardiology and Neurology clinics patients. Only 12 participants were enrolled over the next 7 months (rate=1.5 participants/month) due to high screen failure rates (59%) owing to MoCA performances above the eligibility threshold and low recruitment rate (5%). To meet recruitment goals (8 participants/month), eligibility criteria were modified by lowering the age cutoff from 65 to 55 years and removing the MoCA screen and the MCI requirements, while adding the requirement of subjective cognitive concern allowing both those with normal cognition and MCI but not dementia. Phone recruitment was added by screening electronic health records of people who diagnosed with HF. 7 months after implementing the modifications, additional 58 participants were consented exceeding our recruitment goals (69% of those consented=MCI, 26%=normal cognition, 5%=dementia/excluded from the study). In conclusion, feasibility of our original strategies recruiting older adults with both MCI and HF was not supported.

Abstract citation ID: igae098.3659

### FECAL TRANSPLANT FROM LONG LIVING AMES DWARF MICE ALTERS THE MICROBIOME AND BIOMARKERS OF LIVER HEALTH IN CONTROL MICE

Sarah Ashiqueali[1], Natalie Hayslip[2], Diptaraj Chaudhari[3], Augusto Schneider[4], Xiang Zhu[2], Sarah Noureddine[5], Hariom Yadav[6], and Michal Masternak[7], *1. Mayo Clinic, Rochester, Minnesota, United States*, *2. University of Central Florida, Orlando, Florida, United States*, *3. Wayne state university, Wayne State University, Michigan, United States*, *4. Universidade Federal de Pelotas, Pelotas, Rio Grande do Sul, Brazil, 5. Hesperos Inc., Orlando, Florida, United States*, *6. University of South Florida, Tampa, Florida, United States*, *7. University of Central Florida College of Medicine, Orlando, Florida, United States*

Aging is associated with intestinal dysbiosis, a condition characterized by diminished microbial biodiversity and inflammation. This leads to increased vulnerability to extraintestinal manifestations such as autoimmune, metabolic, and neurodegenerative conditions and accelerates mortality. As such, modulation of the gut microbiome is a promising way to extend healthspan. In this study, we explore the effects of fecal microbiota transplant (FMT) from long-living Ames dwarf donors to their normal littermates, and vice versa, on the recipient gut microbiota and liver transcriptome. Importantly, our previous studies highlight differences between the microbiomes of Ames dwarf relative to their normal siblings, potentially contributing to their extended lifespan and healthspan. Our findings demonstrate that FMT from Ames dwarf mice to normal mice significantly alters the recipient's gut microbiota, reprograms bacterial metabolic functions related to healthy aging, and changes the liver transcriptome, indicating improved metabolic health. Our liver mRNA sequencing and RT-PCR validation reveals that FMT in normal recipient animals administered dwarf donor stool may contribute to the significant downregulation of p21, Elovl3, and Insig2, genes involved with cellular senescence and liver metabolic pathways. Our data suggest a regulatory axis exists between the gut and liver, highlighting the potential of microbiome-targeted therapies in promoting healthy aging. Future research should validate these findings and study the underlying mechanisms that confer geroprotection.

Abstract citation ID: igae098.3660

### FROM GEROSCIENCE TO GEROTHERAPEUTICS: KUAKINI CENTER OF RESEARCH EXCELLENCE FOR TRANSLATIONAL RESEARCH ON AGING

Bradley Willcox[1], Kazuma Nakagawa[2], Yeonjung Lee[3], Kamal Masaki[1], and Richard Allsopp[4], *1. Kuakini Medical Center, Honolulu/Kuakini Medical Center, Hawaii, United States*, *2. Kuakini Medical Center, Honolulu, Hawaii, United States*, *3. University of Hawai'i at Mānoa/ Kuakini Medical Center, Honolulu, Hawaii, United States*, *4. University of Hawaii, Honolulu, Hawaii, United States*

The primary aim of the Kuakini HHP Center of Excellence for Translational Research on Aging is to utilize geroscientific principles to improve human healthspan, including pharmacology-based gerotherapeutics. This research leverages an exceptional cohort of 8,006 American men of Japanese and Okinawan ancestry, aged 45-68 years at the baseline exam in 1965. Fourteen comprehensive clinical exams have been conducted. Over 500,000 biological samples and over 900 brains have been collected over six decades. The primary aim of our Center is to translate these largely NIH-funded resources into therapeutic innovations that modify aging's biological pathways, potentially preventing or alleviating multiple age-related diseases and optimizing human healthspan. Our early-stage investigators have novel, recent data on risk factors for premature cognitive aging. Our research approach, with our biotech collaborators, has also contributed to a data science ecosystem with AI and ML-enabled insights on healthy aging from novel multi-omic studies and aging-related health outcomes from our human aging cohort. Key findings are consistent with the geroscience hypothesis and have identified specific pathways for targeted gerotherapeutic interventions, now in Phase 2 human trials, that may contribute to healthier human aging. This presentation will present the latest findings regarding the specific pathways, the molecular targets, and the gerotherapeutic agents that hold promise to mitigate major aging-related diseases, including sarcopenia and cognitive impairment.

Abstract citation ID: igae098.3661

### FUNCTIONAL CHARACTERIZATION OF ENDOCANNABINOID RELATED 2-AG SYNTHASES IN DOPAMINERGIC NEURONS

Quentin Price[1], Disa Basu[2], and Huaibin Cai[2], *1. Howard University, Los Angeles, California, United States*, *2. National Institute of Aging, Bethesda, Maryland, United States*

We previously linked several loss of function mutations of the enzyme diglycerol lipase beta (DAGLB) to early onset Parkinsonism. Parkinson's disease is defined as the loss of dopaminergic neruons (DANs) in the substantia nigra compacta (SNC). DAGLB is the main synthase of 2-arachidonyl glycerol (2-AG) in nigral DANs. 2-AG from the nigral DANs disinhibits presynaptic inputs such as the GABAergic inputs from the direct spiny projection neurons (dSPNs) of the striatum. However, DAGLA is expressed in nigral DANs as well. We asked ourselves if decreasing the expression fo both DAGLA and DAGLB in the nigral DANs of mice induce motor phenotypes of Parkinson's disease.

Abstract citation ID: igae098.3662

### GREATER DOPAMINERGIC SIGNALING IN PUTAMEN IS RELATED TO BETTER GAIT QUALITY IN ADULTS WITHOUT PARKINSON'S DISEASE

Mauricio Gil-Silva[1], Caterina Rosano[1], Nicolaas Bohnen[2], Stephen Kritchevsky[3], Eni Halilaj[4], Kayla Bohlke[5], and Andrea Rosso[1], *1. University of Pittsburgh, Pittsburgh, Pennsylvania, United States*, *2. University of Michigan, Ann Arbor, Michigan, United States*, *3. Wake Forest University School of Medicine, Wake Forest University School of Medicine, North Carolina, United States*, *4. Carnegie Mellon University, Pittsburgh, Pennsylvania, United States*, *5. University of Pittsburgh Swanson School of Engineering, Pittsburgh, Pennsylvania, United States*

Gait quality aspects beyond pace like walking smoothness (harmonic ratio), regularity (entropy rate), and complexity (Lempel-Ziv complexity) may be useful in capturing the motor control strategies important in successful everyday walking. Dopaminergic signaling in the central nervous system plays a crucial role in motor function. Age-related declines in dopamine within the putamen may contribute to concurrent declines in gait quality in older adults without Parkinson's. We investigated the relationship between putamen DA signaling and gait quality and hypothesized that lower DA signaling was associated with poorer gait quality. In 199 participants (Age: 75.00 (4.63), Sex: 123 F (61.81%) BMI: 28.22 (5.41)), (+)-[11C] (DTBZ) PET imaging estimated the striatal binding site density of the type 2 Vesicular Monoamine Transporter (VMAT2). Accelerometers on the L3 segment of the lumbar spine during walking over 15 meters measured entropy rate, Lempel-Ziv complexity, wavelet entropy, and harmonic ratio of the signal in the medial/lateral, anterior/posterior, and vertical anatomical directions. Multiple linear regression estimated the cross-sectional association between the posterior putamen and each gait measure, adjusted for age, sex, BMI, race, education, and hours walking per week. Lower posterior putamen DA binding site density was associated with both greater vertical wavelet entropy (-0.243 [-0.425, -0.060]) and greater medial/lateral Lempel-Ziv complexity (-0.045 [-0.083, -0.007]). Positive trending associations (p=< 0.10) for posterior putamen DA binding site density were found in medial/lateral entropy rate (0.072 [-0.010, 0.155]) and medial/lateral wavelet entropy (0.256 [-0.059, 0.571]). These results suggest that dynamic complex gait functions are more stable with higher putaminal DTBZ binding.

Abstract citation ID: igae098.3663

### HOW CAREGIVING SHAPES THE RELATIONSHIP BETWEEN ACTIVITY DIVERSITY AND COGNITION

Cassidy Doyle[1], and Brent Small[2], *1. University of South Florida, Tampa, Florida, United States*, *2. University of North Carolina at Chapel Hill, Chapel Hill, North Carolina, United States*

Research indicates frequent participation in leisure activities may buffer age-related cognitive declines but activity variety (activity diversity) may have a stronger relationship with cognitive health. The activity diversity-cognition relationship is understudied in caregivers, who may be susceptible to cognitive declines and therefore benefit from leisure activity participation. This study examines cognitive differences between caregivers and non-caregivers and explores the roles of activity diversity and frequency in this relationship. The Health and Retirement Study was used to investigate the relationship between activity diversity, activity frequency, and cognition among caregivers (N=6,472) and non-caregivers (N=4,343). From the Psychosocial and Lifestyle Questionnaire, 17 leisure activities were assed to create activity diversity and frequency measures. Activity diversity was quantified using Shannon's entropy, scores ranging from 0 to 1, with higher scores indicating greater diversity. Activity frequency was measured by summing the frequencies of 17 activities (0=not at all to 5=daily). Moderation analysis assessed the relationships between activity diversity-cognition and activity frequency-cognition by caregiving status. The sample was predominantly female, White, educated, and engaged in diverse activities (M=0.49). Caregivers had lower cognition than non-caregivers (p <.001). Moderation analyses showed that higher activity diversity and frequency were associated with better cognition for both groups, with stronger effects for non-caregivers (p <.001). Greater leisure frequency and diversity were related to better cognition. Unexpectedly, caregivers engaged in more frequent and diverse leisure compared to caregivers, but associations between activity engagement-cognition were stronger in non-caregivers. Investigating the potential for activity engagement to benefit multiple domains (wellbeing, stress, cognition) among caregivers is warranted.

Abstract citation ID: igae098.3664

### HYPERTENSION, AGE AND SEX INFLUENCE REGIONAL CEREBRAL BLOOD FLOW IN OLDER ADULTS

Jasroop Miglani[1], Nathanial Ashford[1], Kristen Farris[1], Swati Rane Levendovszky[1], and Angela Hanson[2], *1. University of Washington School of Medicine, Seattle, Washington, United States*, *2. University of Washington, Kenmore, Washington, United States*

**Background:** Reduced cerebral blood flow (CBF) is a potential early biomarker for dementia and older adults with hypertension (HTN) are at increased risk of accelerated cognitive decline. This study explored the influence of HTN, age, and sex on CBF following lipid ingestion.

**Methods:** 36 older adults (age 66.9 ± 7.2, 44% hypertensive, 67% women) underwent arterial spin labeling 3D pCASL MRI to measure CBF at baseline (t0) and 2 hours after drinking heavy cream (t2). Regions examined: total cerebrum (tCBF), angular gyrus (AGBF), hippocampus (HBF), posterior cingulate (PCBF), and temporal lobe (TBF). Statistics included Pearson correlation in SAS and generalized linear modeling in R.

**Results:** Age negatively correlated with tCBF at t0 (CC -0.33, p=0.05) which was magnified after lipid ingestion (CC -0.41, p=0.01); similar findings noted for other regions. Men had lower tCBF than women at t0 (Coefficient -8.918, p=0.003) and t2 (Coefficient -11.3, p< 0.001); pattern held for all regions examined. When covariates were added to the model, men were more likely to be hypertensive which partially explained this finding; however, the sex effect remained significant after controlling for factors known to affect CBF (age, heart rate, hematocrit, SBP).

**Conclusion:** Age correlated with lower CBF both fasting and after lipid feeding. Men had lower CBF than women which was only partially explained by HTN; a finding that has been reported in the literature. Further investigation into how various demographic variables influence CBF is crucial in order to utilize CBF as an early biomarker in clinical trials.

Abstract citation ID: igae098.3665

### IGF1R VARIANT R407H PREVENTS COGNITIVE IMPAIRMENT AND NEUROINFLAMMATION IN CRISPR MICE WITH AAV AB42 AND PTAU

Joo Young Park[1], Jackson Wezeman[1], Ruby Sue Mangalindan[1], and Warren Ladiges[2], *1. University of Washington, Seattle, Washington, United States*, *2. University of Washington, University of Washington, Washington, United States*

Late-onset Alzheimer's disease (AD) is an age-related disease. The geroscience concept dictates that interventions that slow aging should also slow age-related diseases. This concept was tested in C57BL/6 mice expressing a CRISPr engineered IGF1R variant R407H shown to be enriched in centenarians. A total of 14 females, 25 to 28 months of age, carrying the R407H variant or wild type, were given intravenous injections of AAV9-PHP.eb serotype concoctions at 10^12 vg per dose, containing either a mCherry-sham construct or Aβ42 and pTau constructs associated with neuropathology of AD. Mice were followed for 11 weeks and then tested for cognition. Following euthanasia, brains were collected, and hippocampal tissues processed for RNA sequencing (Novogene, Sacramento). The mice with the IGF1r variant, and infected with AAV Aβ42 and pTau, showed significantly better performance in a spatial navigation learning task compared to wild type cohorts. RNA sequencing analysis in the same cohorts using DES Seq2 showed a significant downregulation of inflammatory cytokines and chemokines including Ccl21b (log2 fold change = -2.3, adjusted p-value < 0.05). These preliminary observations suggest the potential of further studying naturally occurring rare variants such as IGF1r R407H as a way to help slow or prevent AD, in line with the geroscience concept.

Abstract citation ID: igae098.3666

### IMPACT OF LOW PROTEIN DIETS ON GUT HEALTH

Prerana Vaddi[1], Khristina Young[2], Molly Easter[2], and Cristal Hill[2], *1. University of Southern California, Leonard Davis School of Gerontology, Los Angeles, California, United States*, *2. University of Southern California, Los Angeles, California, United States*

Introduction: Studies on the gut-brain axis report that changes in the microbiome contribute to age-related diseases. One of the most significant factors affecting microbiome composition is diet. Prior work from our lab has demonstrated that low protein (LP) diets induce fibroblast growth factor 21, improve metabolic health, and extend lifespan in male mice. However, the molecular adaptations of a low-protein diet on gut health and their systemic effects on protecting against age-related diseases remains unclear. Here, we investigated the impact of an LP diet on the microbiome and the associated effects on metabolic and immune health.

**Methods:** At 15 months of age, female mice (n=12 mice/diet) were fed either a normal protein (NP) or LP diet for 20-weeks. Feces were collected to assess microbiota, species richness, and species diversity. Metabolic profiling on food intake, fasted glucose levels, and glucose tolerance tests were conducted to assess the relationship between microbiome profiles and metabolic health.

**Results:** Relative abundances of Bacteroides and Defferibacterota were reduced in LP diets and increased in Verrucomicrobiota. Gene set enrichment analyses (GSEA) reveal that mice on the LP diets regulate T-Cell dependent immune and inflammatory pathways. Consistent with our previous studies in male mice, we show that aging female have improved glucose clearance during LP-diet.

**Conclusion:** Notably, previous studies show Bacteroides species promote T-Cell dependent immune pathways, and Verrucomicrobiota species regulate glucose homeostasis. Collectively, GSEA data, the improvements in glucose homeostasis, and changes in microbiota significantly benefit metabolic and immune health.

Abstract citation ID: igae098.3667

### INTER-RELATIONSHIPS OF CIRCULATING PLASMA METABOLITES, INTERMUSCULAR FAT, AND PROCESSING SPEED: HEALTH ABC STUDY

Richard Xu[1], Megan Marron[2], Shanshan Yao[1], Seyoung Kim[3], Ravi Shah[4], Venkatesh Murthy[5], Anne Newman[3], and Caterina Rosano[1], *1. University of Pittsburgh, Pittsburgh, Pennsylvania, United States*, *2. University of Pitssburgh, Pittsburgh, Pennsylvania, United States*, *3. University of Pittsburgh, University of Pittsburgh, Pennsylvania, United States*, *4. Vanderbilt University Medical Center, Nashville, Tennessee, United States*, *5. University of Michgan, Ann Arbor, Michigan, United States*

Higher intermuscular fat (IMF) is associated with worse processing speed, measured by the digit symbol substitution test (DSST) in older adults. However, the underlying biological mechanisms are not well understood. Since muscles and the brain are both highly metabolically active, we sought to identify metabolites that would explain the IMF-DSST association. We assessed 613 known plasma metabolites in 2388 participants from the Health, Aging and Body Composition Study (average age ± standard deviation: 74.7 ± 2.9 years, 50% men, 63% white), using liquid chromatography-mass spectrometry. Using linear regression, we first identified metabolites associated with both IMF and DSST, and then used mediation analyses to assess to what extent these metabolites explained the IMF-DSST association. Higher IMF was associated with worse DSST scores (standardized beta (95% CI): -0.08 (-0.12, -0.03), p< 0.001) adjusting for age, sex, and race. Adjusting for covariates and multiple comparisons, 66 metabolites were associated with both IMF and DSST and 24 of these significantly attenuated the IMF-DSST association. Four attenuated the association by ≥10-14%: higher levels of adrenic acid (omega-6 polyunsaturated fatty acid), and lower levels of maslinic acid, 1-methylnicotinamide (myokine derived from vitamin B3), and C20:5 lysophosphatidylcholine (lysophospholipid containing omega-3 fatty acid) were associated with higher IMF

and worse DSST. Prior studies suggested adrenic acid may have pro-inflammatory effects, while the other three metabolites have neuroprotective, anti-inflammatory, and antioxidant effects. This study provides hypothesis-generating evidence that metabolites related to myokines, fatty acids, and phospholipids may partially explain the negative association of intermuscular fat with processing speed.

Abstract citation ID: igae098.3668

### LOSARTAN REJUVENATES SERUM AND HEART METABOLOME OF AGED MICE BY ANGIOTENSIN DEPENDENT AND INDEPENDENT MECHANISMS

Michael Bene[1], Ruth Marx[2], Laura Powell[2], Luigi Ferrucci[3], Mohammed Khadeer[3], Ruin Moaddel[3], Michelle Rudek[2], and Jeremy Walston[2], *1. Johns Hopkins School of Medicine, Baltimore, Maryland, United States*, *2. Johns Hopkins University, Baltimore, Maryland, United States*, *3. National Institute on Aging, Baltimore, Maryland, United States*

Emerging evidence suggests losartan, a selective angiotensin II receptor type 1 (AT1) antagonist, may reduce frailty risk and improve physical function in older adults. Losartan is metabolized to EXP3174 (a potent AT1 antagonist) and EXP3179 (a PPARγ agonist). To elucidate mechanisms through which losartan may reduce frailty and improve physical function we performed targeted metabolomics in serum and heart samples from young (4 months) and aged (24-28 months) mice. Aged mice treated with losartan displayed a global shift towards a more "youthful" serum metabolome ($\rho$=-0.22, p=0.01) and a stronger "youthful" shift in the heart metabolome ($\rho$= -0.49, p =3.6e-8), with 71/120 and 74/115 measured metabolites shifting in abundance opposite to age respectively. Serum of AT1KO mice showed no significant metabolomic change but displayed a similar "youthful" shift when treated with losartan, indicating losartan's effect is AT1 independent. Supporting this, a mediation analysis of serum levels of losartan and EXP3174 revealed serum losartan had a significant correlation to the metabolomic aging signature that was not mediated by EXP3174. In contrast to serum, AT1KO alone shifted the heart metabolome to a more 'youthful" state suggesting losartan's effect on the heart may be partially AT1 dependent. Interestingly, AT2KO mice also displayed more "youthful" serum and heart metabolomes, but when treated with losartan produced an interference effect blocking the "youthful" shift seen with either AT2KO or losartan alone. Losartan's "rejuvenating" effect on serum and heart metabolome was associated with improved survival in WT mice (log-rank, p =0.015) further supporting improvement in aging trajectory.

Abstract citation ID: igae098.3669

### MARKERS OF INFLAMMATION ARE MODIFIED BY SEX, FRAILTY AND EXERCISE IN A HEAD-DOWN TILT BEDREST STUDY

Susan Howlett[1], Maddison L Hodgins[1], Judith Godin[2], Kenneth Rockwood[1], and Scott Grandy[1], *1. Dalhousie University, Halifax, Nova Scotia, Canada*, *2. Nova Scotia Health Authority, Halifax, Nova Scotia, Canada*

Prolonged bedrest can induce physiological stress thereby exacerbating frailty and inflammation. We explored whether Head-Down Tilt (HDT) bedrest with or without exercise would alter inflammatory markers implicated in frailty. Twenty 55-65-year-old males (baseline frailty index (FI)=0.02±0.02) and females (FI=0.03±0.03) were subjected to HDT bedrest (2-weeks). Half were randomly assigned to a high-intensity interval/aerobic/strength training exercise intervention for 60 minutes/day (Control: n=4 males, 5 females; Exercise n=5 males, 6 females). FI and serum inflammatory cytokines were measured (multiplex assay) during bedrest (days 0,1,3,7,14), and recovery (days 0,4,32). Data were analyzed with linear mixed models. Proinflammatory marker levels (interleukin (IL)-6, IL-8) increased during bedrest in all groups but were higher in males than females (IL-6: ß=8.87, IL-8: ß=12.93; p< 0.01). IL-8 declined during recovery in all groups, although IL-8 and IL-6 remained higher in males (IL-6: ß=9.32, IL-8: ß=2.85, p< 0.01). The anti-inflammatory cytokine IL-10 was higher in males than females during bedrest, declined in recovery and remained highest in males. During bedrest proinflammatory cytokines (IL-1ß, IL-12) increased as frailty increased in all participants (IL- ß: ß=0.01, IL-12: ß=0.02, p< 0.05) whereas monocyte chemoattractant protein-1 levels declined (ß =-20.54, p< 0.05). The anti-inflammatory protein IL-1ra increased with frailty throughout bedrest in all, especially those who exercised (ß=3.15, p< 0.01). In older adults, inflammatory markers increased during bedrest, declined during recovery and were higher in males than females. Some inflammatory markers increased in parallel with frailty during bedrest. Exercise also increased IL-1ra levels, which may help reduce inflammation in older adults during prolonged bedrest.

Abstract citation ID: igae098.3670

### MECHANISMS OF GUT MICROBIOTA IN FRAILTY: FINDINGS FROM MENDELIAN RANDOMIZATION AND MOUSE MODEL STUDIES

Ting Xu[1], Shulin Song[2], Dan Zhao[3], Yaguang Zheng[4], Xiang Qi[4], Minghui Ji[2], Qin Xu[2], and Bei Wu[4], *1. nanjing medical university, harrison, New Jersey, United States*, *2. nanjing medical university, nanjing, Jiangsu, China (People's Republic)*, *3. Shandong University, Jinan, Shandong, China (People's Republic)*, *4. New York University, New York, New York, United States*

Frailty is a prevalent syndrome among older adults, and recent evidence suggests a connection between gut microbiota and frailty. However, the underlying mechanisms remain unclear. This study aimed to investigate the relationship between gut microbiota and frailty, with a focus on how serum metabolites and inflammatory markers might mediate the association. We used a combination of Mendelian randomization and animal models in our approach. Mice frailty was induced via chronic inflammation and assessed by the frailty index based on mice model. We used high-throughput 16S rDNA sequencing to identify alterations in gut microbiota. Differences between groups were examined through Wilcoxon rank-sum tests. We then used spearman correlation analysis to examine the association between gut microbiota changes and frailty-related indicators and metabolites. MR methods, including inverse variance weighted, MR-Egger, and weighted median approaches were used to estimate causal effects. Our animal experiments revealed gut microbiota dysbiosis in frail mice, notably a reduction in Ruminococcus. We found a positive correlation between Ruminococcus abundance and frailty scores ($\rho$ = 0.716, p

< 0.001) and inflammatory markers. Through metabolomic profiling, we identified several key metabolites potentially involved in frailty, including S-Methyl-5'-thioadenosine, L-anserine, and indoleacetic acid. Further analysis using MR methods confirmed a causal relationship between Ruminococcus obeum abundance and frailty index (p = 0.004, OR = 1.035, 95% CI: [1.011, 1.059]), underscoring the role of inflammatory proteins and metabolites as incomplete mediation. This study provides insights into how gut microbiota contribute to frailty and highlights potential biomarkers and therapeutic targets to support healthy aging.

Abstract citation ID: igae098.3671

### MEETING THE CHALLENGE: NEURAL RESPONSE ASSOCIATED WITH MINIMAL DUAL-TASK COST IN AGING

Claudia Schneider[1], Sophie Molholm[1], John Foxe[2], Johanna Wagner[3], and Pierfilippo De Sanctis[1], *1. Albert Einstein College of Medicine, Bronx, New York, United States*, *2. University of Rochester Medical Center, Rochester, New York, United States*, *3. Zander Labs, Cottbus, Brandenburg, Germany*

Walking while engaged in a cognitively challenging task is a common daily activity that becomes harder as we grow older. The cost associated with performing two tasks simultaneously, called dual-task cost (DTC), can predict fall risk and cognitive decline. However, findings are mixed and predictive validity is modest. This study aims to define a neural marker of DTC that, when combined with a behavioral measure, could provide a more robust predictor. To this end, we acquired event-related potentials (ERP) in 36 healthy individuals aged 65 years and older who performed a cognitive Go/NoGo response inhibition task while sitting (i.e., single task) or walking (i.e., dual-task). To determine a neural marker of DTC, we considered differences in DTC between participants. Guided by findings in young adults showing a link between low behavioral DTC and reduction in NoGo-ERP amplitude during walking, we tested this prediction in individuals aged 65 years and older. Participants experienced dual-task cost when performing the Go/NoGo task while walking. They also exhibited a reduced ERP amplitude performing the Go/NoGo task during walking compared to sitting. When participants were split into two equal groups defined by their Go/NoGo performance, however, only the group with minimal DTC revealed a NoGo-ERP modulation during walking. Our data suggest that low DTC is linked to walking-related change in neurocognitive processing (i.e., NoGo-ERP amplitude reduction). The NoGo-ERP may serve as neural DTC marker to help improve early prediction of fall risk and cognitive decline in aging.

Abstract citation ID: igae098.3672

### MF-300 (15-PGDH ENZYME INHIBITOR) REVERSES AGE-RELATED MUSCLE WEAKNESS IN MICE BY RESTORING MUSCLE QUALITY

Micah Webster[1], Jennifer Martin[2], Bruce Fahr[1], and Ramzi Khairallah[2], *1. Epirium Bio, San Diego, California, United States*, *2. Myologica LLC, Baltimore, Maryland, United States*

Sarcopenia is an age-related disease that causes muscle weakness and reduced muscle quality with disproportionate impact on fast-twitch muscle. Gene expression of 15-hydroxyprostaglandin dehydrogenase (15-PGDH), an enzyme that metabolizes prostaglandins including PGE2, is elevated in older adult muscle and may contribute to sarcopenia. The study tested whether MF-300, a 15-PGDH inhibitor, could improve muscle quality (force per unit of mass, defined as specific force) in an ageing mouse model. C57BL/6J mice, aged 90-92 weeks, were stratified on body weight and muscle force into groups that were administered oral MF-300 (10, 30 or 60mg/kg every other day) or vehicle (control). A group of 39-54 week-old mice administered vehicle served as a younger adult comparator. Nerve evoked force production of the plantar flexors was measured and normalized to muscle mass to assess muscle specific force. Aged-vehicle mice had lower absolute and specific force relative to the adult-vehicle group. After 12 weeks of treatment, MF-300 10mg/kg significantly increased maximal muscle force (p< 0.0001) and specific force (p=0.02) in aged animals compared to vehicle. MF-300 10 mg/kg showed a significant increase in absolute (p=0.0003) and specific muscle force (p=0.02), as well as contraction rate (p=0.0068), of the extensor digitorum longus (a fast-twitch muscle) compared to the aged-vehicle group. This experiment demonstrates that MF-300 enhances the intrinsic properties of aged fast twitch muscle fibers independently of muscle mass. These findings suggest that MF-300 holds potential as therapeutic strategy for restoring muscle quality and strength in sarcopenia.

Abstract citation ID: igae098.3673

### MICRORNA BIOMARKERS AND POTENTIAL MECHANISMS ASSOCIATED WITH VO2PEAK VARIATION IN OLDER ADULTS

Genesio Karere[1], Fang-Chi Hsu[1], Peggy Cawthon[2], Xiping Zhang[3], Karyn Esser[4], Laura Cox[5], and Stephen Kritchevsky[6], *1. Wake Forest University School of Medicine, Winston-Salem, North Carolina, United States*, *2. California Pacific Medical Center, San Francisco, California, United States*, *3. University of Florida, University of Florida, Florida, United States*, *4. University of Florida, Gainesville, Florida, United States*, *5. Wake Forest University School of Medicine, WINSTON-SALEM, North Carolina, United States*, *6. Wake Forest University School of Medicine, Wake Forest University School of Medicine, North Carolina, United States*

Peak oxygen consumption during exercise (VO2peak) is an indicator of cardiorespiratory and overall fitness. However, some older adults are intolerant to exercise, impeding VO2peak measurement and necessitating development of alternative methods to screen patients for VO2peak and define pathways that accompany VO2peak variation. We used RNA sequencing to identify expressed microRNAs (miRNAs) and mRNAs in serum and skeletal muscle from 72 participants (70-91 years old; females=53%) in the Study of Muscle, Mobility and Aging, with low (11-14.7 ml/kg/min) or high (28.3-39.4) VO2peak (N=18/group) as well as 36 random samples spanning the entire spectrum of VO2peak. We analyzed samples discordant for VO2peak using the Linear Models for Microarray/RNAseq Data analysis package and identified five serum miRNAs (miR-1301-3p, -431-5p, -501-5p, -519a-3p, and -18a-3p) that discriminate individuals with low or high VO2peak (adjusted p< 0.05). In muscle, 14 miRNAs also displayed differential expression in low and high VO2peak samples. We identified sex differences in expression of 8 miRNAs in muscle (adjusted p< 0.05). Ten differentially expressed (DE) miRNAs, predicted to regulate 50 DE miRNAs, showed an inverse expression pattern in muscle, consistent with direct participation in gene regulation and VO2peak variation. We used Weighted Gene Co-expression Network Analysis and Ingenuity Pathway Analysis tools and identified ME Cyan as the top-most gene module correlated with VO2peak (r=0.7, p=4e-12). Pathways enriched with the module genes included nitric oxide synthesis and signaling. In conclusion, we identified a panel of potential circulating and tissue miRNA biomarkers of VO2peak in older adults and potential pathways that associate with VO2peak variation.

Abstract citation ID: igae098.3674

### MITOCHONDRIAL DYSFUNCTION IN DYSREGULATED MONOCYTE IMMUNE RESPONSE

Rafael Moura Maurmann, and Brandt D Pence, *University of Memphis, Memphis, Tennessee, United States*

Monocytes are key innate immune cells involved in inflammatory signaling. This function is dysregulated in older adults, which thus correlates with increased basal inflammation and disease severity. This phenotype coincides with the buildup of mitochondrial dysfunction markers and defects on mitochondrial dynamics. However, a link between those phenomena remains to be explored. We thus aimed to identify a connection between impaired monocyte immune function and the accumulation of defective mitochondria. We used U937 human monocytic cell line to establish a mitochondrial dysfunction model in vitro comparable to aged monocytes. Cells were treated for 3h with 1 uM FCCP, a mitochondrial depolarized inducer, in the presence or absence of 100 nM bafilomycin A1, an autophagy inhibitor, followed by a 3h recovery in the absence of FCCP. Bafilomycin A1 was kept in cell culture media during recovery. A mitochondrial stress test was then performed on a Seahorse XFp to evaluate extracellular acidification rate (ECAR) and oxygen consumption rate (OCR) responses. FCCP solely treatment led to compromised mitochondrial maximal respiration, which was restored after 3h recovery. However, bafilomycin A1 addition prevented restoration and further aggravated mitochondrial function. The combined treatment induced a decrease in mitochondrial oxygen consumption rate both at basal and stressed conditions, negatively impacting ATP production. Besides, a reduction mitochondrial proton leak may indicate a deficiency on membrane potential maintenance, which further impacts ATP production. While we have not assessed immune function markers to the present, these results validate our model of mitochondrial dysfunction in vitro similar to aged cells.

Abstract citation ID: igae098.3675

### MODULATING CELLULAR SENESCENCE AND MTOR PATHWAY: THE THERAPEUTIC POTENTIAL OF FINGOLIMOD IN NPC DISEASE

Morteza Abyadeh, and Alaattin Kaya, *Virginia Commonwealth University, Richmond, Virginia, United States*

Niemann-Pick type C (NPC) disease is a genetically-driven neurodegenerative disorder with no effective treatment. Neurodegeneration, particularly in the cerebellum, is a key pathological feature. Fingolimod phosphate (FTY720), initially approved for multiple sclerosis, functions mainly as an immunomodulator but shows potential in treating neurodegenerative conditions. This study investigates FTY720's therapeutic potential in mitigating cellular senescence and neurodegeneration in NPC using both in vitro and in vivo models. Our findings reveal that FTY720 pretreatment reduces cellular senescence in fibroblasts, delays muscle mass decline, and improves motor neuron function in NPC mouse models, as evidenced by performance in balance beam and vertical screen tests. Omics analyses indicate that FTY720 exerts anti-inflammatory and anti-aging effects by modulating mTOR signaling and complement coagulation pathways. Importantly, FTY720's effects were sex-specific, with greater efficacy observed in male mice. Immunohistopathological studies further confirmed FTY720's protective effects, showing more pronounced benefits in males. In conclusion, FTY720 demonstrates significant promise as a treatment for NPC, particularly in males, by delaying disease symptoms through its protective effects against cellular senescence and inflammation.

Abstract citation ID: igae098.3676

### MOUSE SKELETAL MUSCLE OVEREXPRESSION OF TFEB: AN EXERCISE MIMETIC PROMOTING BRAIN HEALTH INDEPENDENT OF AHN

Mia Hakian[1], Ian Matthews[1], Catherine Cai[1], and Constanza Cortes[2], *1. University of Southern California, Los Angeles, California, United States*, *2. University of Southern California, Cortes Lab, Los Angeles, California, United States*

Adult hippocampal neurogenesis (AHN), which forms new neurons in the dentate gyrus of the hippocampus, is important for cognition and decreases with age. This reduction in AHN can be reversed with voluntary wheel running in mice, increasing the expression of Transcription Factor E-B (TFEB) within skeletal muscle. TFEB regulates lysosomal and metabolic function through the expression of genes controlling mitochondrial oxidation, fatty acid oxidation, and oxidative phosphorylation. Our lab has recently shown that a transgenic mouse model that overexpresses TFEB only in skeletal muscle mimics many of the neuroprotective benefits of exercise during aging compared to the control, but the effect of TFEB on AHN was unknown. Therefore, we hypothesized that overexpression of TFEB in skeletal muscle has the potential to increase AHN in our exercise mimicking TFEB model. Levels of AHN were measured by using immunofluorescence approaches in hemibrain cryosections to quantify the number of doublecortin (DCX) positive cells, a marker for immature neurons, in the dentate gyrus of the hippocampus of 3-month-old control and muscle-TFEB overexpressing mice. If skeletal muscle-TFEB overexpression is an exercise mimetic, then we expect to see increased levels of AHN in the DG of the hippocampus in TFEB mice compared to the control. We found no significant difference of AHN in TFEB mice compared to the control. These results contradict our hypothesis, but are interesting because we show the maintenance of memory function in our old muscle TFEB mice. This suggests our muscle TFEB mice mimic exercise's improvement of hippocampal function independent of AHN.

Abstract citation ID: igae098.3677

### NO PHONE, MORE STRESS: EXPLORING ANXIETY AS THE MEDIATOR BETWEEN NOMOPHOBIA AND STRESS

Rinanda Shaleha, and Nelson Roque, *Pennsylvania State University, University Park, Pennsylvania, United States*

This study (N=567) examines how nomophobia, the fear of being without one's mobile phone, affects mental health by influencing anxiety and stress. Through regression and mediation analyses with nonparametric bootstrap, we found that anxiety mediates 69.24% of the impact of nomophobia on stress (ACME =.0724, 95% CI [.0568,.09], p <.00001). This substantial mediation underscores the central role of anxiety in the relationship between nomophobia and stress. Analysis by age and gender revealed variable mediation strengths: Young adults of both genders exhibited robust effects, with males showing a mediation proportion of 73.3% (ACME = 0.07424, p <.0001) and females 66.9% (ACME = 0.06940, p <.0001). Middle-aged females demonstrated a particularly high proportion at 88% (ACME = 0.05532, p =.002), contrasted by less pronounced effects in middle-aged males (ACME = 0.02218, p =.196). Older females also displayed significant effects, with a mediation proportion of 82.8% (ACME = 0.09425, p =.004). The observed demographic variations highlight the role of contextual factors such as age and gender in the psychological impacts of nomophobia. These insights are crucial for developing tailored interventions that more effectively counteract the adverse effects of mobile device dependency. Notably, the consistent mediation of stress by anxiety across most groups suggests that strategies focused on reducing anxiety could be particularly effective in managing nomophobia-related stress.

Abstract citation ID: igae098.3678

### NOVEL PLASMA PROTEIN BIOMARKERS: A TIME-DEPENDENT PREDICTIVE MODEL FOR ALZHEIMER'S DISEASE

Tianchi Zhuang[1], Ting Xu[2], Xichenhui Qiu[3], Yingqi Yang[1], Xiang Qi[4], Qin Xu[1], and Minghui Ji[1], *1. nanjing medical university, nanjing, Jiangsu, China (People's Republic), 2. nanjing medical university, harrison, New Jersey, United States, 3. Shenzhen University, nanjing, Jiangsu, China (People's Republic), 4. New York University, New York, New York, United States*

The prediction of Alzheimer's disease (AD) onset is crucial for the efficient disease and the management of its progression. While current diagnostic methods often rely on neuroimaging and cerebrospinal fluid (CSF) biomarkers, there is a growing need for less invasive and more accessible predictive tools. The study aimed to construct a risk predictive model for AD onset using novel plasma protein biomarkers. We analyzed data from 440 participants aged 60 and above from the Alzheimer's Disease Neuroimaging Initiative cohort. Using a combination of Cox regression, LASSO regression, and cross-validation, we identified seven protein signatures (APOE, CGA, CRP, CCL26, CCL20, NRCAM, PYY) predictive of AD risk. Cox model demonstrated robust predictive performance in AD onset, with area under the curve (AUC) values of 0.77, 0.76, and 0.77 at 4, 6, and 8 years, respectively, and stable up to 12 years (AUC≥0.75). The model's effectiveness was further validated using time-dependent ROC curves and Kaplan-Meier curves, which showed that low-risk individuals had significantly delayed AD onset compared to the high-risk group (P<.001). All identified protein signatures were significantly associated with AD risk (P<.001) and time of onset (P<.01). However, we observed weak correlations between plasma and CSF protein levels, suggesting potential differences in peripheral and central nervous system biomarker profiles. Additionally, three proteins (APOE, CGA, CRP) showed lower expression in APOE ε4 carriers (P<.05). These findings highlight the complex interplay between genetics and protein expression in AD pathogenesis and concludes that plasma protein signatures have potential as valuable predictors of AD risk.

Abstract citation ID: igae098.3679

### NOVEL SERUM BIOMARKERS FOR SARCOPENIA: INSIGHTS FROM CLINICAL DATA AND STUDIES USING C2C12 MYOBLASTS

Hsien-Hao Huang, *Taipei Veterans General Hospital, Taipei, Taipei, Taiwan (Republic of China)*

**Introduction:** Sarcopenia, the progressive loss of skeletal muscle mass and function, is a critical public health issue among older adults with underlying functional decline and increased risk of adverse health outcomes. While roles have been established for gut hormones in age-associated anorexia, their direct influence on sarcopenia remains unknown. We evaluated the associations between elevated glucagon-like peptide-1 (GLP-1) and cholecystokinin (CCK) serum levels and sarcopenia and investigated their direct effects on myogenesis and muscle fusion.

**Methods:** Total 179 patients with sarcopenia were enrolled in this study. This study utilized clinical biomarkers, Bioelectrical Impedance Analysis (BIA), and comprehensive geriatric assessment. Concurrently, C2C12 myoblasts were treated with varying concentrations of GLP-1 and CCK (0, 1, 10, and 100 ng/mL) to examine their influence on myoblast differentiation and myocyte fusion, as assessed by myosin heavy chain (MYH1/2) expression using western blot, and fusion index using immunostaining. Results Patients with sarcopenia exhibit significantly elevated levels of GLP-1 and CCK. GLP-1 impedes the differentiation of myoblasts into mature myocytes in vitro, thereby potentially restricting myogenesis, and GLP-1 treatment negatively impacts myocyte fusion with myotubes. In contrast, CCK inhibits myocyte fusion, a crucial step in muscle formation, without altering the initial differentiation of myoblasts. These findings underscore the critical role of these hormones in muscle development and provide novel insights into their contribution to sarcopenia.

**Conclusion:** This study supports the use of GLP-1 and CCK as key biomarkers for sarcopenia, suggesting that their impact extends beyond metabolism and directly affects muscle integrity.

Abstract citation ID: igae098.3680

### PANDEMIC HARDSHIP AMONG SENIOR LIVING RESIDENTS: WHO WAS MOST VULNERABLE?

Mollie George[1], Lindsay Wilkinson[2], Julie Masters[2], and Julie Blaskewicz Boron[2], *1. University of Nebraska Omaha,*

*Lincoln, Nebraska, United States*, 2. *University of Nebraska Omaha, Omaha, Nebraska, United States*

The COVID-19 pandemic presented unprecedented challenges for older adults. While there has been an abundance of research on the pandemic-related experiences of community-dwelling older adults, little research has examined those in senior living communities (SLCs). This is significant given the restrictions enforced in SLCs to reduce the risk of infection and potential hospitalization or death. The current research aimed to investigate the pandemic-related experiences and correlates of pandemic hardship among those residing in SLCs. Survey data were collected during the pandemic from adults age 60 or older living in a group of SLCs in Nebraska. Most respondents in the analytic sample (n=726; Mage=84, SD=7, range: 60-100; 73% female) resided in independent living (86%). Drawing on descriptive statistics and linear regression analysis, the results revealed a moderate to high amount of pandemic hardship (M=4, scale: 1-6). The most prevalent negative pandemic-related experiences included difficulty visiting family and friends (91% of respondents agreed), missing out on important events (83%), and perceiving little control over their lives (68%). In models accounting for sociodemographic and health characteristics, residents most vulnerable to the hardship of the pandemic were less likely to be never married (ps< 0.05), had lower perceived health (p< 0.05), and scored lower on resilience (p< 0.001). This research reveals the pandemic-related experiences of an understudied population of independent and assisted living residents, including areas of their lives they perceived to be most impacted by social distancing. This can be helpful in identifying vulnerable groups in need of support during times of crisis.

Abstract citation ID: igae098.3681

### PARTICIPATION IN VALUED ACTIVITIES, RESTRICTION, AND SOCIAL ISOLATION IN DEMENTIA CAREGIVING

Sarah Won[1], Valerie Cotter[2], Chakra Budhathoki[3], and Natalie Regier[1], *1. Johns Hopkins University School of Nursing, Baltimore, Maryland, United States*, 2. *Johns Hopkins University School of Nursing, Johns Hopkins School ofNursing, Maryland, United States*, 3. *Johns Hopkins University, Baltimore, Maryland, United States*

This study investigated the associations between five indicators of social determinants of health (SDOH) and participation in valued activities, participation restriction, and social isolation among family and other unpaid caregivers of older adults with dementia. Data from the 2022 National Health and Aging Trends Study and National Study of Caregiving IV (Round 12) were analyzed. The sample comprised 757 caregivers of community-dwelling care recipients aged 65 and older with possible/probable dementia. SDOH indicators included household income, education level, health insurance coverage, and neighborhood characteristics (neighborhood physical disorder and social cohesion of care recipients). Multivariable logistic regression models examined associations between SDOH indicators and caregiver outcomes, adjusting for demographics (race/ethnicity, age, gender, and marital status), health conditions, and caregiving characteristics (relationship with the care recipient, duration of caregiving year, hours helped last month, and informal/formal social support). Higher household annual income was significantly associated with increased odds of participation in valued activities (OR=3.46 for $30,000-$70,000, OR=3.40 for $70,000+, p< 0.01) and decreased odds of social isolation (OR=0.19 for $30,000-$70,000, OR=0.26 for $70,000+, p< 0.05) compared to income <$30,000. Higher education level (associate's degree or above) was associated with significantly higher odds of participation in valued activities (OR=6.17, p< 0.001) compared to high school or less. No significant associations were found between SDOH indicators and participation restriction. Household income and education level were crucial SDOH factors associated with participation and social isolation. These findings highlight the importance of considering SDOH factors in developing interventions and policies to support dementia caregiving.

Abstract citation ID: igae098.3682

### PATHWAYS LINKING ICT UTILIZATION TO CHRONIC DISEASES SELF-MANAGEMENT AMONG OLDER ADULTS IN CHINA

Qingru Chen[1], Yan-Yan Chen[2], Ke Gong[3], Xuefeng Zhang[4], and Lirong Zhao[4], *1. Huadong Hospital Affiliated to Fudan University, Shanghai, Shanghai, China (People's Republic)*, 2. *Fudan University, Shanghai, Shanghai, China (People's Republic)*, 3. *Huadong Hospital affiliated to Fudan University, Huadong Hospital Affiliated to Fudan University, Shanghai, China (People's Republic)*, 4. *Huadong Hospital affiliated to Fudan University, Shanghai, Shanghai, China (People's Republic)*

The underlying mechanisms linking information and communication technology (ICT) utilization to self-management are currently unclear. This cross-sectional study aims to examine the mediating role of health literacy, self-efficacy, and social support in the relationship between ICT utilization and chronic disease self-management among older adults in China. A total of 590 older adults (≥60 years) with at least one chronic disease were recruited from a tertiary hospital in Shanghai between July 2023 and June 2024. Questionnaire data were recorded on self-reported socio-demographics, self-management, social support, health literacy, self-efficacy, ICT utilization. Pearson's correlation analysis identified the relationship between these factors and self-management. Serial multiple mediation model was used to test the hypothesized relationships. The results show ICT utilization, health literacy, self-efficacy, social support, and chronic disease self-management were found significantly correlated (p< 0.001). ICT utilization not only has a direct positive impact (effect=0.2377; 95%CI=0.1826-0.2927) on chronic disease self-management, but also has an indirect impact on chronic disease self-management through the independent mediating role of health literacy (effect=0.0912; 95%CI=0.0608-0.1240) and social support (effect=0.0186; 95%CI=0.0048-0.0367) and the chain mediating role of health literacy and self-efficacy (effect=0.0223; 95%CI=0.0128-0.0342), the chain mediating role of health literacy and social support (effect=0.0104; 95%CI=0.0044-0.0187), the chain mediating role of health literacy, self-efficacy, and social support (effect=0.0020; 95%CI=0.0008-0.0036).These findings suggest that ICT utilization can enhance chronic disease self-management among older adults and indirectly affects it through increasing health literacy, self-efficacy while providing more social support.

Abstract citation ID: igae098.3683

### PERCEIVED BENEFITS OF TREATMENTS OF ALZHEIMER'S DISEASE AND RELATED DEMENTIAS FOR COST-EFFECTIVENESS ESTIMATES

Faika Zanjani[1], Madeline McIntyre[2], Tracey Gendron[1], and Eline van den Broek-Altenburg[3], *1. Virginia Commonwealth University, Richmond, Virginia, United States*, *2. VCU, Richmond, Virginia, United States*, *3. University of Vermont, Burlington, Vermont, United States*

Objective: This study aims to identify important non-Quality-adjusted life years (QALYs) benefits of novel Alzheimer's Disease and Related Dementia (AD/ADRD) treatments. Cost-effectiveness analysis of AD/ADRD is mostly based on QALYs gained. However, other benefits in the International Society for Pharmacoeconomics and Outcomes Research value flower, such as severity of disease, hope, and option value are not included. Which of these benefits are most important for AD/ADRD is unknown.

**Methods:** Twelve semi-structured focus groups were conducted with at-risk individuals (60%) and caregivers (40%). Study protocol/interview guide was designed to target AD/ADRD knowledge and attitudes. Focus groups were recorded. transcribed, and analyzed thematically using ATLAS.ti and two coders.

**Results:** There was a lack of knowledge about both the condition and treatments. When considering treatment, cost and side effects were important, but impact on family dynamics was also critical. Participants lack trust in advertising, confused by the healthcare system, feel primary care providers are undertrained and lack time, and specialists are unavailable; the whole system lacks coordination, fails to communicate effectively, and provides conflicting information. Participants indicated AD/ADRD is associated with grief, sadness, fear, frustration, and hopelessness. They also expressed feeling alone and confused. Caregiving at a distance presents a particular challenge. Conclusions: Omitted elements of hope and option value from the value flower are important for AD/ADRD, suggesting that current cost-effectiveness estimates may systematically undervalue important components of treatments. Yet even with new medications, access challenges may undermine potential value by creating systematic challenges in receiving diagnoses and identifying treatment alternatives.

Abstract citation ID: igae098.3684

### PERCEIVED DISCRIMINATION ON DEPRESSION IN OLDER ADULTS: MODERATING EFFECT OF GEOGRAPHIC ACCESS TO SOCIAL SERVICES

Seo-Yun Choi[1], and Soondool Chung[2], *1. University of Southern California, Los Angeles, California, United States*, *2. Ewha Womans University, Seoul, Republic of Korea*

Age discrimination against older adults represents a significant issue in South Korea, with notable implications for their mental health and well-being. Addressing ageism is critical as it limits resources for detecting and treating depression in older adults, affecting their health behaviors and willingness to seek help. On the other hand, despite a high prevalence of depression among this population, only 2.2% utilize mental health services. This study examined how geographic accessibility to social services for diagnosing and treating depression can improve mental health service utilization among older adults experiencing age discrimination. About 9,920 individuals 65 and above from the National Survey of Older Persons were included. It explored the moderating effect of geographic accessibility on the relationship between perceived age discrimination and depression. Correlation and moderating effect analyses using SPSS PROCESS Macro Model 1 were computed, and the results demonstrated that perceived ageism is positively associated with depression ($r=.220$, $p<.01$). More importantly, geographic access to social services significantly moderated this relationship ($B=-.180$, $p<.05$), reducing the impact of perceived ageism on depression. These findings underscore the importance of enhancing geographic access to social services to mitigate the effects of ageism on depression among older adults. The study recommends direct measures, such as expanding social service infrastructure in rural areas, and indirect measures, like improving public transportation connectivity and introducing age-friendly technology. Further research will be necessary to understand regional characteristics better and enhance access to adequate social services.

Abstract citation ID: igae098.3685

### PERCEPTIONS OF DIABETES SELF-MANAGEMENT AMONG MIDDLE AGED ADULTS

Willencia Louis-Charles, Hye Soo Lee, and Wendy Rogers, *University of Illinois Urbana-Champaign, Champaign, Illinois, United States*

Older adults with diabetes are at higher risk of heart and kidney failure that arise due to mismanagement of the condition. Understanding the factors that influence self-management among middle-aged adults may enable healthcare providers to intervene before complications arise. We investigated whether knowledge, access to healthcare, illness identity, and diabetes distress emerged as factors that influence self-management for 11 middle-aged adults (aged 52-63, $M=59.77$, $SD=5.17$). All participants had type 2 diabetes. These participants completed questionnaires and a semi-structured interview either in-person or through Zoom during a one-time 2-hour session. Questionnaires included the Diabetes Knowledge Questionnaire (DKQ), Barriers to Access to Care Evaluation (BACE), Illness Identity Questionnaire (IIQ), and Diabetes Distress Scale (DDS). Following the questionnaires, they then were asked to elaborate on their experiences related to those themes and topics. Participants felt that they lacked knowledge, even though their scores on the DKQ were above 12, which is considered as satisfactory in the literature. Similarly, their BACE scores reflected enough access to healthcare, but many felt that they were not getting enough assistance. The IIQ data revealed that many participants felt more enrichment and rejection than engulfment and acceptance in reference to their diabetes. Many participants acknowledged feelings of distress that were not captured in their DDS. The mixed methods approach revealed that the standard measures are not diagnostic of the experiences, evidenced by the interview data. More in-depth evaluations of factors that affect health self-management for this population will be valuable to guide interventions before health complications arise.

Abstract citation ID: igae098.3686

### PERSONALITY TRAITS, SCREEN TIME, AND MOBILE PHONE DEPENDENCE ACROSS THE LIFESPAN

Stephanie Washburn[1], Hailey Andrews[2], and Nelson Roque[2], *1. University of Florida, Gainesville, Florida, United States*, *2. Pennsylvania State University, University Park, Pennsylvania, United States*

Digital technologies have become integral to daily life worldwide, with many individuals increasingly dependent on mobile devices like smartphones and tablets. The widespread growth of technology use worldwide highlights the need to understand how individual traits contribute to mobile device dependence and its potential impacts on mental health, paving the way for interventions to mitigate these effects. This study examined the association between self-reported screen time, mobile phone dependence (Nomophobia Questionnaire), and personality traits (Ten Item Personality Inventory), across the lifespan. Data were collected from 550 adults (Mage=39yrs, Rangeage=19-77; 54% female; 59% non-Hispanic White) through an online survey, delivered on Prolific. Linear regression models, adjusted for age and gender, assessed the relationship between personality traits and two outcomes: self-reported screen time and mobile phone dependence. For screen time (N = 299), significant predictors were age ($\beta$ = -0.06, p =.001), conscientiousness ($\beta$ = -0.44, p =.013), and agreeableness ($\beta$ = -0.47, p =.014). For mobile phone dependence (N = 550), significant predictors were age ($\beta$ = -0.33, p =.001), emotional stability ($\beta$ = -5.07, p <.001), and agreeableness ($\beta$ = 2.59, p =.021). These results suggest that three of the Big Five personality traits influence screen time and mobile phone dependence. Understanding these factors may guide interventions to reduce dependency and its mental health effects. Future longitudinal studies are needed to explore these relationships over time and establish causality.

Abstract citation ID: igae098.3687

### PHYSICAL ACTIVITY, PURPOSE, AND COGNITION: TESTING POSSIBLE MEDIATION PATHWAYS WITH CROSS-LAGGED PANEL ANALYSIS

Farima Sadeqi, and Ryan Best, *West Virginia University, Morgantown, West Virginia, United States*

Numerous studies have emphasized the cognitive benefits older adults experience from engaging in regular physical activity. Previous research has largely highlighted the physiological mechanisms of this relationship, like improvements in cardiovascular health and brain structure, but recent work has begun investigating possible psychological pathways. Prior work from the authors supported the hypothesis that purpose in life acts as a mediator between physical activity and cognitive function, though research suggests a bidirectional relationship between physical activity and purpose in life. Given the bidirectional nature of the relationship between these two variables, the current work tests the possibility that physical activity could also act as the mediator in a single model using a structural equation modeling framework. In a sample of 2960 adults aged 33 to 92 collected through two waves of the Midlife in the United States (MIDUS) study, we examined two potential mediation pathways in a cross-lagged panel model where purpose in life mediates the effect of physical activity on cognition and vice versa. Two separate models test mediation pathways on executive functioning and episodic memory. Results show that purpose in life acted as a significant mediator between physical activity and both cognitive abilities (p <.021), while the indirect effects of physical activity between purpose in life and both cognitive abilities were not significant (p >.67). Findings from this study suggest that among physical activities with similar cardiovascular benefits, the one that also nurtures individuals' purpose in life and boosts psychological well-being should be prioritized.

Abstract citation ID: igae098.3688

### PHYSICAL FUNCTION AND MITOCHONDRIAL BIOENERGETICS IN PEOPLE LIVING WITH HIV AND CANCER

Alice Ryan[1], Kathleen Griffith[2], and Sausan Jaber[3], *1. University of Maryland School of Medicine, Baltimore, Maryland, United States*, *2. GWU and Baltimore VAMC, Baltimore, Maryland, United States*, *3. Baltimore VAMC, Baltimore, Maryland, United States*

**Purpose:** To determine physical fitness and function, and potential mechanisms of frailty including bioenergetic health that may differ between older adults living with HIV (PLW) without and with a history of cancer (PLW+C).
**Methods:** Participants (n=34, mean±SD, 64±6.6 years) underwent a cancer-specific geriatric assessment, physical function and fitness testing. In a subset (n=21, PLW n=10 and PLW+C n=11 lung, anus, colon, prostate), platelets were assayed for parameters of bioenergetic functions (Agilent Seahorse XFe96) in the presence of substrates for either glycolysis, the citric acid cycle, or fatty acid oxidation (FAO). Compounds were injected to determine basal, maximal, ATP-linked, and spare respiratory capacity.
**Results:** PLW (100% male, 82% African American, BMI 28.7±6.3 kg/m2) enrolled. There were no differences between PLW (n=16) and PLW+C (n=18) in VO2peak (19.6±5.7 vs. 20.3±5.4 ml/kg/min), 6-minute walk distance (1400±385 vs.1273±415 ft), Short Physical Performance Battery score (10.8±1.5 vs.10.0±1.9), Berg Balance Scale score (52.2±3.1 vs 51.9±5.0), and Timed Up and Go (8.26±2.32 vs.10.2±5.3 s). Mito stress maximal (243.1±79.2 vs. 251.9±93.6 pmolO2/min/1x107cells) and spare respiratory capacity (113.8±61.5 vs. 121.3±51.4 pmolO2/min/1x107cells) are not significantly different between PLW and PLW+C; whereas FAO maximal (42.4±28.7 vs. 61.6±30.9 pmolO2/min/1x107cells) and spare respiratory capacity (30.6±25.0 vs. 56.9±29.5 pmolO2/min/1x107cells) tended to be higher in PLW+C. Conclusions: Although Mito Stress bioenergetic parameters of platelets from PLW+C or PLW are not different, the trend toward higher maximal and spare respiratory capacity in the presence of the FAO substrate in PLW+C may imply that their platelets have a higher reliance on FAO for fuel.

Abstract citation ID: igae098.3689

### PLASMA PROTEOMIC MEDIATORS LINKING INTERMUSCULAR FAT WITH PSYCHOMOTOR SPEED

TANAKA TOSHIKO[1], Xianging Huang[2], Sadiya Khan[2], Iva Miljkovic[3], James Greg Terry[4], Teresa Tian[5], Caterina Rosano[3], and Luigi Ferrucci[5], *1. NIA/IRP/TGB/*

*LSS, Baltimore, Maryland, United States*, 2. *Northwestern University, Northwestern University, Illinois, United States*, 3. *University of Pittsburgh, Pittsburgh, Pennsylvania, United States*, 4. *Vanderbilt University Health Sciences, Nashville, Tennessee, United States*, 5. *National Institute on Aging, Baltimore, Maryland, United States*

Intermuscular fat (IMF), or muscular fat infiltration, has been inversely associated with measures of cognitive function. However, the mechanisms linking higher IMF and poor cognitive function are not fully understood. To explore this, we characterized plasma proteomic profile of IMF in Baltimore Longitudinal Study on Aging (BLSA, n=942), and validated the findings in the Coronary Artery Risk Development in Young Adults Study (CARDIA; n=2453) study. Plasma proteomics was measured using 7K SOMAscan, IMF was measured on CT scans of the thigh (BLSA) or abdomen (CARDIA), and processing speed was assessed with digit symbol substitution test (DSST). Associations between measures of IMF and proteins were assessed using linear regression adjusting for age, sex, education, self-reported race, and total muscle area. Of the 7268 plasma proteins tested, 718 proteins were significantly associated with IMF in both discovery and validation studies (FDR p<=0.05). Mediation analysis of the IMF -associated proteins, 28 proteins significantly mediated the inverse association between IMF and DSST. Pathway analyses show the biological process and molecular functions most enriched by these proteins were the synaptic structure and function (STLITRK3, CBLN2, NTRK3, EPHA4, C1QL3) pathway, and growth factor binding (HTRA1, IGFBP2, NTRK2, EGFR, IGFBP4, IGAFLS) pathways. These results suggest a unique proteomic signature of IMF that is consistent across two independent cohorts. Further, the association of higher IMF with lower cognitive processing speed may be at least partially mediated through dysregulation of synaptic functions and growth factors binding.

Abstract citation ID: igae098.3690

### PREDICTING EXCESSIVE SPENDING IN OLDER ADULTS: THE IMPACT OF COGNITIVE STATUS AND MENTAL HEALTH

Emily Flores, and Peter Lichtenberg, *Wayne State University, Detroit, Michigan, United States*

Excessive spending among older adults poses a significant risk to financial stability, particularly in the context of cognitive and psychological factors. This study utilized data from the WALLET study to examine predictors of excessive spending, focusing on sociodemographic variables, cognitive status, and mental health as measured by the Geriatric Depression Scale (GDS). The sample included 150 older adults, predominantly African American (65%) and female (80%), with an average age of 72.9 years (SD = 7.9). Correlation analyses found significant associations between excessive spending and cognitive status (r =.18, p <.05) and GDS scores (r =.21, p <.01). Regression analyses showed that in the first model, cognitive status (β =.18, p <.05) was a significant predictor of excessive spending when measured as a percentage, while education was also significant (β =.19, p <.05). However, the second model revealed that higher levels of depression (β =.21, p <.05) significantly predicted excessive spending, even after controlling for sociodemographic and cognitive status. These findings highlight the role of depression in financial risk behaviors, and it underscores the need for targeted interventions focusing on cognitive and psychological assessments to address excessive spending in older adults.

Abstract citation ID: igae098.3691

### PREDICTIVE MODELING OF HEALTH STATUS OVER LIFESPAN BASED ON SENESCENCE BIOMARKERS

Lingzi Tang[1], Siarhei Hladyshau[2], Allison Ross[3], Kirsten Nyrop[4], Amy Entwistle[5], Hyman Muss[6], and Denis Tsygankov[7], 1. *Georgia Institute of Technology, Atlanta, Georgia, United States*, 2. *St. Jude Children's Research Hospital, St. Jude Children's Research Hospital, Tennessee, United States*, 3. *University of North Carolina, Chapel Hill, North Carolina, United States*, 4. *UNC Medicine, Chapel Hill, North Carolina, United States*, 5. *Sapere Bio, Durham, North Carolina, United States*, 6. *University of North Carolina - Chapel Hill, Pittsboro, North Carolina, United States*, 7. *Georgia Institute of Technology and Emory University, Atlanta, Georgia, United States*

Cellular senescence is known to play a significant role in aging and the development of age-related diseases. However, the complex interplay between the accumulation and clearance of senescent cells and the relationship of these processes to the physiological decline during natural aging is not understood. Here, we report a study that explores the potential of cellular and immune senescence biomarkers (p16, p14, LAG3, CD28, and CD244) in predicting health metrics as determined by standard clinical assessments. Data were collected from a cohort of 250 healthy participants aged 25 to 85 years and included gene expression in T-cells from peripheral blood, comprehensive blood panels, quality of life (QOL) surveys, body composition analysis, and physical performance evaluations. A combination of computational techniques, including correlation analysis, unsupervised hierarchical clustering, and machine learning techniques (such as an ensemble of bagged decision trees), was employed to assess the predictive capabilities of the biomarkers relative to the health assessments and vice versa. Our findings indicate that blood and QOL assessments generate distinct participant clusters, both strongly associated with p16 expression. The highest accuracy in predicting p16 expression was achieved by integrating measurements across various assessments, suggesting a systemic role for this biomarker. Furthermore, our analysis led to a scoring system that produces integrative metrics strongly and specifically correlating with different senescence biomarkers, suggesting a simple, practical method to differentiate between varying levels of cellular and immune senescence in naturally aging individuals.

Abstract citation ID: igae098.3692

### PRELIMINARY FINDINGS OF A MINDFUL WALKING PROGRAM TO PROMOTE HEALTH IN AFRICAN AMERICAN OLDER ADULTS

Jenna Dzwierzynski, Meda Zabielaite, Jongwon Lee, Halle Prine, and Chih-Hsiang Yang, *University of South Carolina, Columbia, South Carolina, United States*

African American adults aged 60 and older are nearly twice as likely to develop Alzheimer's disease and other dementias compared to non-Hispanic Whites. Regular physical activity, like walking, benefits brain health and may delay cognitive decline. Mindfulness training is cost-effective and can be easily integrated with a walking program. This study explored whether 24 sessions of 30-minute mindful walking could improve cognitive function, sleep, depressive symptoms, and physical functioning in older African Americans compared to a control group by using pre-post assessment comparisons. After randomization, the intervention group incorporated mindfulness techniques during each walking session over 12 weeks. The control group did not receive any intervention. At Week 13, both groups were reassessed. The study included 66 participants (34 intervention, 32 control, 91% female). Descriptive statistics at Week 13 indicated that the intervention group had slightly better sleep (4.74 ±.44 vs 5.09 ±.51), perceived cognition (1.21 ±.29 vs 1.81 ±.31), depression (6.32 ±.53 vs 6.66 ±.59), and timed-up-and-go (TUG) (11.09 ±.38 vs 11.95 ±.50) scores compared to the control group. The intervention group also had a higher mean MoCA score compared to the control group (25.18 ±.46 vs 24.31 ±.53), with a p-value of.080. The sample size was insufficient for formal statistical testing, and further research with a larger sample is needed to evaluate the efficacy of mindful walking interventions. The preliminary results suggest that mindful walking may support healthy aging and reduce health disparities among older African Americans.

Abstract citation ID: igae098.3693

### PREVALENCE AND PREDICTORS OF LONELINESS AMONG CAREGIVERS BY CARE RECIPIENT'S LEVEL OF COGNITIVE IMPAIRMENT

Elyse Couch[1], Rebecca Howe[2], and Jodi L. Southerland[3], *1. Brown University, Providence, Rhode Island, United States*, *2. VA Providence Healthcare System, Providence, Rhode Island, United States*, *3. East Tennessee State University, Johnson City, Tennessee, United States*

Loneliness is increasingly being recognized as a public health issue. However, little is known about the prevalence or predictors of loneliness among caregivers in the United States (US). This study aimed to assess the prevalence and predictors of loneliness among US caregivers by the care recipient's level of cognitive impairment. We conducted a cross-sectional study of caregivers for Medicare beneficiaries in the US using data from the National Study of Caregiving (NSOC). Caregivers were divided into three groups by the care recipient's level of cognitive impairment: non-dementia, MCI, and ADRD caregivers. Logistic regression models were used to identify predictors of severe loneliness (defined as feeling lonely every day or most days) among caregivers. Sample weights were applied to all analyses. Overall, 8.5% of caregivers reported feelings of severe loneliness, equating to over 750,000 caregivers when sample weights were applied. The prevalence of severe loneliness was higher in dementia caregivers (13%) compared to MCI (7.4%) and non-dementia (7.1%) caregivers. Adjusted logistic regression models found dementia caregiving, being without a current partner, and reporting emotional difficulties with the caregiving role were significantly associated with severe loneliness. Increasing age and caring for a parent/other relative (vs spouse) were associated with reduced odds of loneliness. There was no difference in odds of severe loneliness by gender or race. Dementia caregiving is associated with an increased risk of loneliness. Future research is needed to understand how loneliness affects caregiver health and wellbeing outcomes and to develop interventions to address caregiver loneliness, particularly for dementia caregivers.

Abstract citation ID: igae098.3694

### PRO-INFLAMMATORY CYTOKINES IN OLDER AFRICAN AMERICAN WOMEN IN A PAIN AND DEPRESSION INTERVENTION

Rebekah Bhansali[1], Melissa Hladek[2], Catherine Clair[3], Sarah Szanton[4], Jessica Gill[4], and Janiece Taylor[2], *1. Johns Hopkins University, Severn, Maryland, United States*, *2. Johns Hopkins University, Johns Hopkins University, Maryland, United States*, *3. Johns Hopkins Bloomberg School of Public Health, Baltimore, Maryland, United States*, *4. Johns Hopkins University, Baltimore, Maryland, United States*

Aging, pain, and depression are interconnected health concerns that significantly impact older women. African American women face additional risks of comorbid pain and depression, severe depressive symptoms, and less frequent pain treatment. Pro-inflammatory cytokines and inflammatory mediators are associated with both conditions. The DAPPER (Depression and Pain Perseverance through Empowerment and Recovery) pilot study tailored nurse-led interventions to meet the needs of older African American women with chronic pain and depression. This pilot study assessed non-pharmacological interventions among African American women aged 50+ with chronic pain and depressive symptoms. The intervention consisted of eight nurse-led sessions. Participants were randomized into waitlist control and immediate intervention groups. Ten participants in the immediate intervention group completed the program and provided pre- and post-intervention saliva samples. Outcomes, including goal attainment, pain, depression, and stress, were measured using validated tools such as PROMIS-8 and PSS-14. Saliva samples were collected for cytokine analysis, including TNFα, IL-8, IL-6, and IL-1β. Although under-powered for direct conclusions, data shows a downward trend in psychosocial and pro-inflammatory biomarkers. Pre- to post-intervention mean levels of psychosocial and cytokine markers (TNFα, IL-8, IL-6, IL-1β) decreased in a subset of the immediate intervention group, supported by median sensitivity analyses. The DAPPER pilot study acknowledged the intersectionality of race, age, and gender to provide personalized care and considered the biochemical mechanisms involved in pain and depression. Further research is needed to explore the potential biochemical impact of nurse-led, home-based interventions, particularly for older African American women.

Abstract citation ID: igae098.3695

### PROMOTING EXECUTIVE FUNCTIONS IN OLDER ADULTS: THE ROLE OF GAMIFICATION AND INDIVIDUAL DIFFERENCES

Morgan Gomez[1], Anja Pahor[2], Audrey Carrillo[1], Aaron Seitz[1], and Susanne Jaeggi[1], *1. Northeastern University, Boston, Massachusetts, United States*, *2. University of Maribor, Maribor, Maribor, Slovenia*

Evidence suggests that cognitive training can be beneficial for older adults to maintain and improve executive functions (EFs). EFs are important for many everyday functions, and they are impacted by aging and dementia. Integrating game-like features can boost motivation and engagement during cognitive training, promoting learning outcomes. Yet, incorporating game design elements may also increase cognitive load and distraction for some individuals. Using EF training, we investigated individual differences and the impact of gamification in older adults, including those at risk for Alzheimer's disease and related dementias (AD/ADRD). We conducted two crossover randomized controlled trials (143 older adults; M=69 years, SD=7.5) comparing the effects of gamified and non-gamified EF interventions. We tested whether and how individual differences in inhibitory control (IC) or general cognitive ability (GCA) at baseline predict the effectiveness of these interventions. We hypothesized that participants with greater difficulty inhibiting distractions would benefit more from non-gamified training, whereas those with higher distractor tolerance would perform better with gamified tasks. Results showed that IC significantly predicted transfer. Furthermore, high IC individuals benefitted more from gamified training, whereas low IC individuals benefitted more from non-gamified training. Conversely, GCA did not predict transfer, with no significant interaction between GCA and training condition. These findings support the IC model over the GCA model on predicting transfer effects. Our findings will inform the development of personalized cognitive interventions to mitigate EF impairments, particularly in populations at risk for AD/ADRD.

Abstract citation ID: igae098.3696

### PURPOSE IN LIFE, PHYSICAL ACTIVITY AND FUTURE THOUGHTS: RESULTS FROM DAILY LIFE ASSESSMENT IN YOUNG AND OLDER ADULTS

Nathan Lewis[1], Yoonseok Choi[1], Jennifer Lay[2], Peter Graf[1], Da Jiang[3], Atiya Mahmood[4], Helene H. Fung[5], and Christiane Hoppmann[5], *1. University of British Columbia, Vancouver, British Columbia, Canada*, *2. University of Exeter, Exeter, England, United Kingdom*, *3. Education University of Hong Kong, Hong Kong, China (People's Republic)*, *4. Simon Fraser University, Vancouver, British Columbia, Canada*, *5. The Chinese University of Hong Kong, Hong Kong, China (People's Republic)*

Theoretical work suggests a sense of purpose in life may encourage health-promoting behaviors by facilitating a more future-oriented time perspective about one's everyday goals and actions. However, little research has investigated whether people with a strong sense of purpose are thinking more about the future in their everyday lives and whether this changes across adulthood. Using ecological momentary assessment (EMA), the present study aimed to investigate associations between a sense of purpose in life, age, thoughts about the future, and physical activity. A total of 256 younger and older adults living in Canada and Hong Kong (Mage=48.0, SD=24.6, range=18-85, 68% women) completed electronic assessments 3x/day for 10 days, recording their current thoughts and how much these were directed towards the future. A subset of participants also completed a 6-month follow-up where they reported their physical activity over the past week. Multilevel models indicated those with a higher in sense of purpose reported more future-oriented thoughts across the EMA period, adjusting for self-rated health and demographics (b=3.12, 95% CI=0.33–5.90, p=.028). Associations between sense of purpose and thoughts of the future were consistent across younger and older adults. While a sense of purpose was associated with more future-oriented thoughts, these thoughts did not predict subsequent physical activity 6 months later (b=-0.00, p=.998). Findings support the notion that adults higher in purpose direct their thoughts more towards the future in daily life, yet more work is needed to uncover if and how this impacts health behavior decisions.

Abstract citation ID: igae098.3697

### QUANTIFYING DIFFERENCES IN MITOCHONDRIAL RETICULUM OF HEALTHY OLD AND PERTURBED HUMAN MUSCLE USING AI SEGMENTATION

Lisa Hartnell, Alexander Maltsev, and Luigi Ferrucci, *National Institutes on Aging, Baltimore, Maryland, United States*

Several studies have shown that mitochondrial health and function decline with aging and chronic disease having deleterious consequences on mobility and cognition. To better understand these changes, we examined the structure of skeletal muscle mitochondria in participants in the Genetic and Epigenetic Signatures of Translational Aging Laboratory Testing (GESTALT) study and in older individuals with peripheral arterial disease (PAD). Image analyses was performed on volumes of muscle generated by Focused Ion Beam (FIB)-Scanning Electron Microscopy (SEM). FIB-SEM yields isotropic volumes that allow analysis with deep learning aided segmentation at near membrane resolution. As previously reported using volumetric imaging, we found that mitochondrial reticulum is a highly organized continuum of individual mitochondria around myofibers found close to the location of energy utilization. To perform a quantitative analysis, we developed a deep learning segmentation workflow and applied the process to image volumes of gastrocnemius muscle ranging from 10-20 □m3 collected from healthy old (75-89y) participants in GESTALT, in frail individuals and in patients diagnosed with peripheral artery disease (PAD). We found substantial differences in the reticulum size, with fragmentation in frailty and PAD patients compared to healthy controls. We present our progress toward developing a measure of the breakdown of continuity of reticulum we termed "Fragmentation Index". Future work aims to increase the number of individuals sampled and to apply the Fragmentation Index along with other measures of mitochondrial activity to evaluate the association of continuous mitochondria with healthy aged muscle vs muscle from frail individuals or individuals with PAD.

Abstract citation ID: igae098.3698

### RACE AND ETHNIC DIFFERENCES IN ATTITUDES TOWARD FAMILY-BASED SUPPORT TO OLDER ADULTS IN THE U.S.

Nekehia Quashie[1], and Neil Mehta[2], *1. University of Rhode Island, University of Rhode Island, Rhode Island, United States*, *2. The University of Texas Medical Branch, Galveston, Texas, United States*

Prior research suggests cultural differences in norms of family responsibility to older adults and familial norms being more salient among minority families such as Black Americans relative to non-Hispanic Whites. However, there is little understanding of ethnic variations in attitudes toward familial responsibility for older adults among the

Black American population. Using data drawn from the National Survey of American Life- Reinterview conducted between 2001 and 2003 and an analytic sample of African Americans (N=1,235), Caribbean Blacks (N=356), and non-Hispanic Whites (N=411) aged 40 and older, I examine race and ethnic differences in attitudes toward supporting older adults in need with finances, household chores, and personal care. Results show that for financial support to older adults, Caribbean Blacks were more likely than African Americans to support mixed family and government responsibility, relative to mainly familial responsibility. In contrast, non-Hispanic Whites were less likely than African Americans to support mainly government relative to mainly family responsibility to older adults. Regarding attitudes toward household support, Caribbean Blacks were more likely than African Americans to support mainly government responsibility, relative to family. There were no statistically significant race and ethnic differences in mixed or mainly government versus mainly family responsibility in supporting older adults with personal care needs. The findings underscore ethnic heterogeneity among Black Americans in attitudes toward the family being mainly responsible for some forms of support to older adults, which is increasingly important to inform culturally relevant policy initiatives to support vulnerable older adults and their families.

Abstract citation ID: igae098.3699

### RACE, PRODUCTIVE ACTIVITIES, AND COGNITIVE FUNCTIONING IN THE HEALTH ABC STUDY

Ronica Rooks[1], Mirza Ishrat Noor[2], and Elizabeth Vasquez[3], *1. University of Colorado Denver, Denver, Colorado, United States*, *2. University at Albany, Albany, New York, United States*, *3. University at Albany, SUNY, Rensselaer, New York, United States*

Productive activities (working and volunteering) promote older adults' purpose in life, physical activity, and social support and may improve cognitive functioning. We hypothesized older adults working or volunteering will have higher cognition, and each productive activity will attenuate the relationship between race and cognition. We used the Health ABC study, a cohort of community-dwelling, well-functioning Black (48.4%) and White (51.6%) older adults aged 70-79 in year 1 (n=2,996). Our linear mixed-effects models included race, working or volunteering, gender, age, education, smoking, the 400-meter walk, diabetes, and racial interactions with each productive activity in year 1 and the Teng 3MS in year 11. In our working model, working and its racial interaction were not significant. Older Black vs. White adults scored -4.4 points lower; women, a high school degree or some post-secondary education, and former vs. never smokers related to higher; and each additional year of age and diabetes related to lower 3MS scores. In our volunteering model, volunteering was not significant. Older Black vs. White adults scored -5.2 points lower; women, a high school degree or some post-secondary education, and former vs. never smokers related to higher; each additional year of age and diabetes related to lower; and older Black vs. White volunteers scored 2.0 points higher 3MS scores. While racial disparities in 3MS scores existed in both models, older Black volunteers were cognitively protected. Our policy implication is to invest in older adults' volunteering as social determinant interventions to maintain cognitive functioning and reduce racial disparities.

Abstract citation ID: igae098.3700

### RACIAL CLIMATE AND WILLINGNESS TO PARTICIPATE IN CLINICAL RESEARCH AMONG AFRICAN AMERICAN ADULTS

Sanaz Dabiri[1], Rema Raman[2], and Jevay Grooms[3], *1. University of Southern California, Arlington, Virginia, United States*, *2. University of Southern California, Los Angeles, California, United States*, *3. Howard University, Washington DC, District of Columbia, United States*

In the US, there is a striking inequity in morbidity and mortality rates among racial and ethnic minoritized populations, particularly African American (AA) individuals, compared to White individuals. Despite these disparities, recruiting AA communities for clinical research remains challenging. Using the social-ecological model as a framework, this project aimed to examine whether the racial climate of the geographical area-including police shootings, neighborhood vulnerabilities, and individual trust in institutions- is significantly associated with willingness to participate in clinical studies. We analyzed data from the University of Florida HealthStreet registry, which assesses community perceptions toward research participation. This data was aligned with police shootings from the Fatal Encounters dataset and the Social Vulnerability Index by county and zip code. Participants had an average age of 42.6 years (SD=16.49) with an average education of 13.12 years (SD=2.5). Most were female (64%) and identified as AA (53.7%). Results showed that higher rates of police shootings were significantly associated with lower willingness to participate in studies involving blood samples (b=-1.39,se=.004, p<.01), taking medication (b=-.01,se=.006, p<.05), genetic studies (b=.001,se=.00, p<.05), and using medical equipment (b=-.02,se=.00,p<.001) among AA adults. Higher social vulnerability was also linked to lower research participation (b=-3.53,se=1.05, p<.001). Trust in research (ACME b=-.0004, p<.01) and researchers (ACME b=-.0003, p<.01) mediated the relationship between police shootings and willingness to take medication. Racial climate's contribution to research participation among AA communities is poorly understood. Our findings underscore the need to address these factors to improve community engagement and reduce the diversity gap in research.

Abstract citation ID: igae098.3701

### RACIAL DIFFERENCES IN PREDICTIVE CAPACITY OF EDUCATIONAL POLYGENIC SCORES ON PHYSICAL LIMITATIONS FOR OLDER ADULTS

Wade Catt[1], Micah Williams[1], and Kenzie Latham-Mintus[2], *1. Indiana University School of Medicine, Indianapolis, Indiana, United States*, *2. Indiana University Indianapolis, Indiana University Indianapolis, Indiana, United States*

This research examined whether educational polygenic scores were associated with physical limitations among older adults with European or African ancestry. In the European ancestry sample, we found that education polygenic scores were significantly associated with physical limitations in the age- and sex-adjusted models; however, education polygenic scores were no longer associated with physical limitations after adjusting for current socioeconomic status and health risk factors. In the African ancestry sample, education polygenic scores were not associated with physical limitations. Observed educational attainment was a robust predictor of physical limitations in both samples. This research demonstrates the racial inequalities in the predictive capacity of educational polygenic scores. We hypothesize that this disparity is a result of structural barriers to educational attainment by race and/or racial inequities in data collection. Both explanations stem from structural racism and highlight the limited usefulness of polygenic scores for clinical decision-making. We use these findings to explore competing theoretical explanatory frameworks of the utility and limitations of the use of polygenic scores in social and health sciences.

Abstract citation ID: igae098.3702

### RACIAL//ETHNIC DIFFERENCES IN WILLINGNESS TO PARTICIPATE IN ALZHEIMER'S DISEASE RESEARCH

Eleanor Skemp, Tanish Sathish, Yichen Zhang, and Brian Carpenter, *Washington University in St. Louis, St. Louis, Missouri, United States*

The underrepresentation of minority populations in Alzheimer disease (AD) research has implications for both science and practice, as diversity and inclusion are crucial to ensure generalizable findings and equitable healthcare. However, various factors influence the reluctance of minoritized individuals to participate in AD research, making it important to understand the attitudes that hinder their participation. This study investigated racial/ethnic differences in attitudes about participating in AD research. Results are based on data from a 2022 national survey of U.S. adults (N = 1,274), with a deliberate oversampling of Black respondents. Participants were surveyed about their willingness to join an AD research registry, participate in an AD cohort study, and about reasons underlying their willingness. Participants self-identified as White (47.3%), Black (43.9%), and Hispanic (8.8%). A higher percentage of Blacks were unwilling to participate in a research registry or a cohort study than Whites and Hispanics (p's <.006), and Blacks were less likely to perceive participation as a contribution to society (p <.001). Hispanics expressed greater concern in cohort studies about the time commitment, the potential to receive negative health news, and fear that research results could be used against them (p's <.002). As a note of caution, statistically significant differences were relatively small. Despite the limitations a relatively crude racial/ethnic categorization, results could inform AD research recruitment strategies by identifying factors that may cause hesitance among potential participants, with the goal of enhancing the presence of underrepresented groups in AD research.

Abstract citation ID: igae098.3703

### RECALLING YOUTH IN VIRTUAL REALITY IN OLDER ADULTS WITH MILD COGNITIVE IMPAIRMENT

DaEun Kim, Hsiao-Wen Liao, Leila Aflatoony, and Jeff Wilson, *Georgia Institute of Technology, Atlanta, Georgia, United States*

As people age, their autobiographical memory becomes less vivid and detailed. We developed a virtual reality (VR) environment that recreates a nostalgic lounging area from the 1970s. Aside from examining feasibility, we tested whether cognitively healthy older adults (n = 16; Mage = 66.7) and individuals with mild cognitive impairment (MCI; n = 12; Mage = 75.3) differ in VR experience, memory phenomenology, and memory details, and whether VR experience correlates with memory variables. The virtual lounge comprised standard cues and was customized with personal photos. After the VR session, participants rated VR experience (i.e., presence, time travel, and bringing back memories), described a positive memory from their youth, and rated memory phenomenology (i.e., vividness, reliving, and significance). Memories were content-coded for semantic and episodic details. Regarding feasibility, most participants were able to follow the instructions to navigate the environment, use the controller to turn on a virtual lamp and record player (healthy controls: 100%, 91.7%, 86.7%; MCIs: 75%, 90.9%, 90%), and reported low discomfort in VR. Mann-Whitney's U tests indicated no group differences in VR experience, memory phenomenology, and semantic details. Healthy controls recalled more episodic details than the MCI group. Overall, sensing presence and experiencing time travel in VR correlated with stronger memory reliving. The extent to which VR brought back memories correlated with greater memory significance. Similar levels of VR experience and memory phenomenology between the two groups, along with the correlations between the two variables, suggest the potential utility of VR for facilitating memory recollection.

Abstract citation ID: igae098.3704

### REDEFINING SUCCESSFUL AGING: INTEGRATING DISABILITY AND THE ROLE OF MODIFIABLE FACTORS

Madina Khamzina[1], Shannon Mejia[2], and Wendy Rogers[3], *1. University of Maryland College Park, Silver Spring, Maryland, United States*, *2. University of Illinois Urbana-Champaign, Urbana, Illinois, United States*, *3. University of Illinois Urbana-Champaign, Champaign, Illinois, United States*

Disability is a common experience in older adulthood, yet traditional models often neglect its role in successful aging. This study integrates disability into the successful aging framework, utilizing subjective well-being and self-rated health as key indicators. We aim to link disability and successful aging and examine modifiable factors that could foster successful aging among individuals with disabilities over time. We conducted cross-sectional analyses using data from two year periods, the 2011 (N=6,440) and 2020 (N=3,220) waves of the National Health and Aging Trends Study. Disabilities related to mobility, hearing, and vision were self-reported. Indicators of successful aging included subjective well-being and self-rated health. Physical activity and social engagement were examined as modifiable protective factors. Moderation analyses tested whether these factors had stronger effects among individuals with disabilities. The prevalence of successful aging among those with disabilities was 40% in 2011 and 38% in 2020. Regression analyses showed that each type of disability was negatively associated with successful aging indicators. Social engagement and physical activity consistently supported successful aging across both time periods. Social engagement was especially beneficial in 2011 for those with mobility, hearing, and multiple disabilities. Findings underscore the importance of recognizing successful aging with disability and highlight the role of modifiable factors like physical activity and social engagement in promoting successful aging. This study supports the inclusion of disability in aging paradigms, emphasizing adaptive strategies for older adults with disabilities.

Abstract citation ID: igae098.3705

### RESULTS OF A DOUBLE-BLIND RCT OF THE FUNCTIONAL EFFECT OF KETONE ESTERS IN OLDER ADULTS

Brianna Stubbs[1], Elizabeth Stephens[2], Chatura Senadheera[1], Sawyer Peralta[1], Stephanie Roa Diaz[1], Wendie Silverman-Martin[1], Laura Alexander[1], and John Newman[2], *1. Buck Institute, Novato, California, United States*, *2. Buck Institute for Research on Aging, Novato, California, United States*

**Background:** Ketone bodies are metabolites produced during fasting or on a ketogenic diet that have pleiotropic effects on the inflammatory and metabolic aging pathways underpinning frailty in in vivo models. Ketone esters (KEs) are compounds that induce hyperketonemia without dietary changes and may impact physical and cognitive function in young adults. The functional effects of KEs have not been studied in older adults.

**Objectives:** Our long-term goal is to examine if KEs modulate aging biology mechanisms and clinical outcomes relevant to frailty in older adults. Here, we report the exploratory functional and quality-of-life outcome measures collected during a 12-week safety and tolerability study of KE (NCT05585762). Design: Randomized, placebo-controlled, double-blinded, parallel-group, pilot trial of 12-weeks of daily KE ingestion. Setting: The Buck Institute for Research on Aging, California. Participants: Community-dwelling older adults (≥ 65 years), independent in activities of daily living, with no unstable medical conditions (n = 30). Intervention: Subjects were randomly allocated (1:1) to consume 25 g daily of either KE (bis-octanoyl (R)-1,3-butanediol) or a taste, appearance, and calorie-matched canola oil placebo (PLA). Measurements: Longitudinal change in physical function, cognitive function and quality of life were analyzed as exploratory outcomes in completers (n = 11 PLA, n = 12 KE). A composite vigor-frailty functional outcome was calculated. Heart rate and activity were followed using digital wearables.

**Results:** There were no statistically significant longitudinal differences between groups in exploratory functional, activity-based or quality of life outcomes in this pilot study.

Abstract citation ID: igae098.3706

### RISKY DECISION-MAKING AND ALZHEIMER'S DISEASE FAMILY MEDICAL HISTORY

Heaven Cauble, and Sheila Black, *University of Alabama, Tuscaloosa, Alabama, United States*

Alzheimer's Disease is associated with a decline in multiple cognitive domains including executive function (EF). This decline in EF often results in a decline in decision-making capacity (DMC). It can affect daily functioning in every aspect of life and oftentimes has extreme consequences related to healthcare and financial decisions. However, unlike other impairments of AD (e.g., memory and executive function), DMC has not been extensively researched as an early symptom. By studying people who are genetically at-risk of developing AD via family, we may be able to detect early differences in DMC as a possible result of genetic predisposition of AD. 60 middle-aged adults were recruited into groups based on family medical history of AD and completed the Game of Dice Task, a gambling task that measures risky decision-making. We found that family medical history of AD contributes to variances between scores, indicating that having an increased genetic predisposition to AD is associated with risky decision-making in mid-adulthood for those individuals. This research could support looking at decision-making capacity as a possible early symptom of AD, thus allowing for earlier intervention.

Abstract citation ID: igae098.3707

### ROLE ENGAGEMENT IN VOLUNTEERING: IMPACTS ON SUBJECTIVE WELL-BEING AND COGNITION IN MIDDLE AND LATER LIFE

Shiyu Lu, *The University of Hong Kong, Hong Kong, China (People's Republic)*

The existing literature predominantly emphasizes the quantitative aspect of volunteering, such as volunteer hours, overlooking the depth of role engagement and psychological dimensions of volunteer involvement and their impact on health. The study focused on the impacts of volunteer hours, role engagement, and engagement profiles on the well-being and health of midlife and older adults. The research encompassed 665 participants aged 55 and above in Hong Kong. Initially, participants were classified into three categories based on volunteer hours (non-volunteers, 1-49 hours annually, 50 hours or more annually). Subsequently, they were segmented into four levels of role engagement (non-volunteers, low, medium, high) and six volunteering profiles (non-volunteers, occasional, regular, engaged, active, dedicated volunteers) considering volunteer hours and engagement levels. Results from multivariate regression analyses indicated that individuals volunteering over 50 hours annually exhibited enhanced subjective well-being ($\beta = 0.18$, $p < 0.001$) and self-rated health ($\beta = 0.10$, $p < 0.01$) compared to non-volunteers. Those with medium and high engagement levels also reported improved subjective well-being (Medium: $\beta = 0.13$, $p < 0.001$; High: $\beta = 0.18$, $p < 0.001$) and self-rated health (Medium: $\beta = 0.08$, $p < 0.05$; High: $\beta = 0.07$, $p < 0.05$) compared to non-volunteers. Cognitive benefits were consistent across volunteer hours and engagement levels, with active and dedicated volunteers experiencing the most significant advantages in well-being, health, and cognition. The study underscores the importance of role engagement in volunteering for late-life well-being and health, suggesting implications for integrating volunteering into social prescribing for healthy aging.

Abstract citation ID: igae098.3708

### SELF-CARE AS A FORTITUDE FACTOR AGAINST COMPASSION FATIGUE: LESSONS LEARNED FROM COVID-19

Cathy Scott[1], Junrong Shi[2], and Linda Samuel[3], *1. University of Tennessee at Chattanooga, Chattanooga, Tennessee,*

*United States*, 2. *University of Tennessee Chattanooga, Chattanooga, Tennessee, United States*, 3. *North Carolina A & T State University, North Carolina A & T State University, North Carolina, United States*

Gerontology practitioners did not receive the national attention as others, but they were on the frontlines during the COVID-19 pandemic caring for older adults, one of our nation's most vulnerable populations. They helped older adults manage health complications, isolation and navigate limited services. This mixed-methods study examined self-care among gerontology practitioners. Qualitative data were collected through semi-structured interviews with 13 gerontological practitioners. The thematic content analysis demonstrated the overall well-being of gerontology practitioners during the COVID-19 pandemic including fear, physical exhaustion, and anxiety coupled with an unsupportive workplace are associated with compassion fatigue. Gerontology practitioners described spiritual/faith practices, leisure activities, and the support of family as self-care activities that sustained them and contributed to their constancy while serving older adults. Quantitative data were collected using an online survey. The statistical analysis with a small sample (N= 37) showed that gerontological practitioners engaged in self-care activities (M=31.9, SD=4.83, range=22-41), measured by a 10-item Likert-scale (Cronbach alpha=0.60). They showed a medium level of compassion fatigue (M=29.7, SD=7.86, range:16-45), measured by a 10-item Likert-scale (Cronbach alpha=0.88). The linear regression showed a strong negative correlation between self-care and compassionate fatigue (b=-0.8, p< 0.01) controlling for age, race, professional field, and years of working with older people (gender is not included with only two males). Findings suggest self-care as a protective factor against compassion fatigue and the need for increased administrative support/resources, communication, and recognition of the psychological impact on clients and practitioners, especially for agencies working with chronic and terminally ill clients during crisis situations.

Abstract citation ID: igae098.3709

### SENOLYTIC EFFECTS ON COGNITION, MOBILITY, AND BIOMARKERS IN OLDER ADULTS WITH MCI AND SLOW GAIT: A PILOT STUDY

Courtney Millar[1], Ike Iloputaife[1], Kathryn Baldyga[1], Amani Norling[2], Afroditi Boulougoura[3], Theodoros Vichos[3], Tamara Tchkonia[4], and Jim Kirkland[4], 1. *Hebrew SeniorLife, Roslindale, Massachusetts, United States*, 2. *Hebrew SeniorLife, Marcus Institute of Aging, Harvard Medical Schools, Roslindale, Massachusetts, United States*, 3. *BIDMC, Boston, Massachusetts, United States*, 4. *Cedars-Sinai Medical Center, Los Angeles, California, United States*

One aim of this single-arm pilot study, called the STAMINA Study, evaluates the preliminary effects of two senolytic drugs, Dasatinib and Quercetin (DQ), on phenotypic and biological outcomes in older adults with slow gait speed and mild cognitive impairment. Twelve participants took six cycles of 100 mg of Dasatinib and 1250 mg of Quercetin for two days every two weeks over 12 weeks. Absolute changes in cognition and mobility, and percent changes in biomarkers were calculated. Spearman correlations between changes in phenotypic and biomarker outcomes were performed. Montreal Cognitive Assessment (MoCA) scores (mean change = 1.0 point, 95%CI: -0.7, 2.7) and stride length (mean change = 0.031 meters, 95% CI: -0.003, 0.066) tended to improve following DQ, without reaching statistical significance. However, MoCA scores did improve significantly by 2 points (95%CI: 0.1, 4.0) in those with the lowest baseline MoCA scores (18-25 points). Mean percent change in tumor necrosis factor-alpha (TNF-α), a key product of the senescence associated secretory phenotype (SASP), decreased following DQ (mean change = -3.0%, 95%CI: -13.0, 7.1), which was significant when compared to biomarker controls (p=0.04). Reduction in TNF-α was significantly correlated with increases in MoCA score (r=-0.65, p=0.02).This study suggests that intermittent DQ treatment may have functional benefits in older adults with slow gait speed and mild cognitive impairment. The observed reduction in TNF-α and its correlation with increases in MoCA scores suggests that DQ may improve cognition by modulating the SASP. However, these data are preliminary and must be interpreted with caution.

Abstract citation ID: igae098.3710

### SENSE OF PURPOSE IN LIFE PREDICTS HIGHER ENGAGEMENT IN COGNITIVELY STIMULATING ACTIVITIES

Nathan Lewis[1], Kyrsten Hill[2], Gabrielle Pfund[3], Payton Rule[2], Mathias Allemand[4], and Patrick Hill[2], 1. *University of British Columbia, Vancouver, British Columbia, Canada*, 2. *Washington University in St. Louis, St. Louis, Missouri, United States*, 3. *Auburn University, Auburn, Alabama, United States*, 4. *University of Zurich, Zurich, Switzerland*

Several studies have noted associations between having a strong sense of purpose or direction in life and better cognitive health outcomes including better memory, executive functioning, and reduced dementia risk. However, limited research has considered why these associations occur. Engaging in cognitively stimulating activities such as reading is believed to promote cognitive reserve—the brain's ability to compensate for damage and maintain function even in the face of aging or neurological disease. The present study aimed to test this potential mechanism by examining whether a stronger sense of purpose predicts higher engagement in cognitively stimulating activities, and whether these associations differed based on age and retirement status. Data were collected from an online survey of 787 American adults on Prolific, with over-recruitment of older adults (Mage=50.31, SD=16.34, Range=19-85, 49.30% women, 71.08% White). Multiple regression tested whether those with higher in sense of purpose reported more frequent engagement in 15 activities from the Cognitive Leisure Activity Scale (e.g., reading a book/newspaper, doing puzzles, attending a club or group activity). Having a stronger sense of purpose in life was associated with more frequent participation in cognitive leisure activities (β=0.25, 95% CI [0.19, 0.32], p<.001) adjusting for age, gender, race, income, and retirement status. Additional models found no significant interactions with age (β= 0.00, 95% CI [-0.00, 0.01], p=.156) and retirement status (β=0.09, 95% CI [-0.09, 0.24], p=.236). These findings suggest that a strong sense of purpose may encourage cognitive activity, regardless of age or retirement, potentially supporting cognitive health.

Abstract citation ID: igae098.3711

### SEROTONIN DEFICIENCY FROM CONSTITUTIVE SKN-1 ACTIVATION DRIVES PATHOGEN APATHY

Brandy Weathers, Tripti Nair, Sean Curran, James Nhan, and Nicole Sthur, *University of Southern California, Los Angeles, California, United States*

When an organism encounters a pathogen, the host innate immune system activates to defend against pathogen colonization and toxic xenobiotics produced. In humans, nuclear factor erythroid 2-related factor (Nrf2) is a critical regulator in innate immune responses under stress; it regulates gene expression in response to the oxidative stress induced by pathogens and is activated within innate immunity cells in response to pathogenic stress. Caenorhabditis elegans activate defense responses to ensure survival upon exposure to pathogens, including activation of the cytoprotective transcription factor SKN-1, a homolog of human Nrf2. SKN-1 directs homeostasis against cellular stress, including pathogenic stress. Exposure to Pseudomonas aeruginosa (PA14) revealed that animals with constitutive activation of SKN-1 develop an apathy-like behavior towards the pathogenic bacteria. Though wild-type C. elegans adapt to avoid pathogens, animals with constitutive SKN-1 activation choose not to leave while continuing to feed on the pathogen. This behavior is mediated by tissue-specific actions of SKN-1 that initiate cell non-autonomous outcomes for pathogen avoidance, survival, and metabolic adaptation. Remarkably, although animals with SKN-1 activation are initially apathetic to leaving behaviors associated with PA14 exposure, treatment with exogenous serotonin significantly reduces this phenotype. SKN-1 activation in neurons and intestinal tissues triggers a distinct transcriptional defect in serotonin signaling from both neurons and non-neuronal tissues, and a significant reduction of serotonin production. This altered signaling is the driving force behind the pathogen apathy behavior observed. Taken together, this work defines the role(s) of the SKN-1-serotonin-immunomodulatory pathway in organismal health and aging and in pathogen defenses.

Abstract citation ID: igae098.3712

### SERUM PTAU181/NEUROFILAMENT LIGHT ELEVATIONS CORRELATE WITH OPIOID USE AND FECUNDITY IN A PSYCHIATRIC CLINIC

Emily Hanson[1], Karl Berner[2], and Jon Berner[3], *1. Woodinville Psychiatric Associates, Federal Way, Washington, United States*, *2. CONQUER Clinics LLC, Monroe, Washington, United States*, *3. Woodinville Psychiatric Associates, Woodinville, Washington, United States*

Chart review of initial N=73 cohort screening for dementia risk revealed expected positive control with age across standard ATN measures. All ATN measures were independent of insulin sensitivity (insulin/BMI) Amyloid ratio was uncorrelated with ptau181/Nfl. Broad chart review revealed a divergence between amyloid and tau serum markers: tau associates with opioid use (r. 259, p. 01) and increased fecundity (r. 215, p. 079) while adverse amyloid ratio associates with decreased fecundity (r. -0.223, p.067) and absent statistical association with opioid use. This data is consistent with previous CSF studies documenting elevated ptau181 and TNFa in patients with pain. These results have implications for patient stratification and treatment selection. Granular psychosexual histories may provide an assay for the biochemical tradeoffs associated with harem vs. monogamous mating strategies, obviously implicated the current size disparities in human males vs. females. Ptau 181 elevations associated with a pain behavioral phenotype and CSF elevations of TNFa direct attention to prophylactic medications with known efficacy in human neurogenic pain likely associated with equilibrium distributions of the "M1-like" and "M2-like" microglia: ketamine, rapamycin, and dimethylfumarate. Acute cortisol challenge may also provide useful clinical information regarding the potentially post- translational equilibrium distribution, opaque to RNAseek in autopsy specimens, of the "M2c- like" microglia vs. the IL-4 dependent "M2a-like" microglia.

Abstract citation ID: igae098.3713

### SEX- AND AGE-SPECIFIC METABOLIC EFFECTS OF EARLY-LIFE RAPAMYCIN TREATMENT IN UM-HET3 MICE

Yun Zhu[1], Robert Stockwell[2], David Medina[1], Rong Yuan[2], and Andrzej Bartke[1], *1. Southern Illinois University School of Medicine, Springfield, Illinois, United States*, *2. Southern Illinois university Shool of Medicine, Springfield, Illinois, United States*

UM-HET3 mice were treated with rapamycin (4 mg/kg, i.p., 3 times/week) from 15 to 56 days of age. At day 15, no significant differences in body weight (BW) between the treatment and control groups within each sex. During the treatment, males in the treated group consistently had lower BW than controls (pairwise-t test, $P < 0.01$), while no differences were seen in females. Interestingly, the treatment significantly reduced the relative weights of fat pads (visceral, subcutaneous, and brown fat) in males, with a suggestive reduction in the relative pancreas weight and significant increases in heart, liver, and kidney relative weights. No significant organ weight changes were observed in females. At 2 months, rapamycin significantly raised fasting glucose in males (t test, $P = 0.0002$) but not in females. By 6 months, fasting glucose levels were significantly reduced in treated mice compared to controls, with a 26.7% reduction in males and 15.7% in females (t test, $P = 0.0001$ and 0.041, respectively). Glucose tolerance, assessed by the area under the curve (AUC) of relative glucose levels, was significantly increased by rapamycin in both sexes at 2 and 6 months (t test, $P < 0.05$), except in 6-month-old males. No significant effects or interactions were found in the insulin tolerance test. Indirect calorimetry showed no significant changes in heat production, but a suggestive increase in RQ was observed in treated males. These findings suggest that early-life rapamycin treatment may have long-term effects on metabolic parameters, and the effects are sex- and age-specific.

Abstract citation ID: igae098.3714

### SEX AS A BIOLOGICAL VARIABLE IN CARDIAC AGING: CORRELATIONS AMONG ECHOCARDIOGRAM, FRAILTY, AND ANESTHESIA RECOVERY

Grecia Garcia Marquez[1], Thuy Pham[2], Katarzyna Cieslik[3], and George Taffet[4], *1. Houston Methodist Hospital, houston, Texas, United States*, *2. Houston Methodist*

*Hosptital, Houston, Texas, United States*, *3. Methodist Hospital Research Institute, Houston, Texas, United States*, *4. Houston Methodist Hospital, Houston, Texas, United States*

Cardiovascular dysfunctions are associated with advanced age. By 2050, the number of people older than 65 will triple. With this increased number of older individuals, it becomes crucial to understand how different organs respond to aging and if the processes are different between men and women. If so, then we need sex-specific approaches to extend patients' healthspan and lifespan through effective management and treatment strategies. We and others have found sex-specific distinct cellular responses in cardiac aging in mice. In this study, we examined the correlation between anesthesia recovery time, frailty index, and cardiac parameters obtained by echocardiography and Doppler imaging performed under 1.5% isoflurane anesthesia in 25-month-old mice. Post-anesthesia recovery time and the frailty index (involving recordings of 24 different parameters) are markers of "whole body resilience". Using matrix analysis with non-parametric Spearman correlation with two-tailed p values, we found notable differences between sexes. In males, a strong correlation was observed between diastolic parameters, anesthesia recovery time, and frailty index. In females, the correlation was strongest for systolic parameters. These findings suggest that anesthesia recovery time and frailty index are associated with different cardiac parameters in older male and female mice, underscoring the implications of cardiovascular aging differing between sexes and highlighting the importance of consideration of sex as a biological variable (SABV) in geroscience research.

Abstract citation ID: igae098.3715

### SEX DIFFERENCES IN FACTORS ASSOCIATED WITH FALLS AMONG COMMUNITY-DWELLING OLDER ADULTS WITH OSTEOARTHRITIS

Aya Yoshikawa[1], and Horng-Shiuann Wu[2], *1. Texas Woman's University, Dentin, Texas, United States*, *2. Michigan State University, East Lansing, Michigan, United States*

Osteoarthritis elevates the risk of falling among older adults due to joint pain and stiffness. Research suggests that sleep problems, depression, and fatigue may also relate to falls among those with osteoarthritis, yet sex influences in these associations remain understudied. This study investigated factors associated with falls by sex (male vs. female). A sample of 3,895 community-dwelling older adults with osteoarthritis in the 2016 Health and Retirement Study (2,624 females; 1,271 males) were analyzed using survey-weighted logistic regression. All analyses were controlled for sociodemographic characteristics, including geographic residence, and health-related issues (e.g., balance problems, chronic diseases, and medication use). Most participants were female (64.5%), in their 70s (M = 73.74, SD = 6.4), White (89.0%), had a high school or higher education (89.3%), and non-rural residents (70.9%). For both men and women, balance problems, functional limitations, and cancer increased the risk of falling, while pain, sleep problems, depression, or fatigue were not associated with falls. Among women, being White (OR = 3.87, 95% CI; 1.86, 8.05 compared to Black), rural residence (OR = 1.59, 95% CI: 1.01–2.50 compared to urban residence), and opioid use (OR = 1.60, 95% CI: 1.00, 2.54) increased the risk of falling. Among men, having heart problems (OR = 1.84, 95% CI: 1.24, 2.72) increased the risk of falling. Sex-tailored fall prevention strategies, such as providing rural programs and opioid education for women and increasing awareness and fall education for men with heart problems, are encouraged to support active living among older adults with osteoarthritis.

Abstract citation ID: igae098.3716

### SEX DIFFERENCES IN LINKS BETWEEN AGING EXPECTATIONS, PURPOSE IN LIFE, AND HEALTHY BEHAVIORS IN MIDLIFE KOREANS

Sun-ui Shin, Hyun-E Yeom, Eunyoung Park, and Mi Sook Jung, *Chungnam National University, Daejeon, Republic of Korea*

Establishing a purpose in life and fostering healthy behaviors are critical for enhancing the quality of life in middle-aged populations. Negative perceptions of aging are closely linked to active self-care and overall well-being in aging individuals. This study aimed to investigate the mediating role of purpose in life for the relationship between aging expectations and healthy behaviors and to explore sex-related differences in these associations among midlife Koreans. A descriptive cross-sectional study was conducted with 298 community-dwelling adults aged 50 to 64 (mean age: 56.1 years; 154 males, 144 females). Data were collected through self-administered surveys and analyzed using Pearson's correlation and PROCESS macro in SPSS version 26.0. Results indicated that aging expectations were significantly associated with purpose in life (r = -0.335, p < 0.001), and purpose in life was positively correlated with healthy behaviors (r = 0.276, p < 0.001). The influence of aging expectations on purpose in life differed by sex (β = -0.047, p = 0.020), with a more pronounced impact observed in males compared to females. Aging expectations indirectly affected healthy behaviors through purpose in life across both sexes, confirming the mediating role of life purpose. These findings suggest that aging expectations significantly influence the purpose in life, which subsequently leads to healthy behaviors in midlife adults, especially in men. The study underscores the importance of fostering positive perceptions of aging and a sense of purpose in life to support healthy aging, emphasizing the need to consider sex-related differences in these associations.

Abstract citation ID: igae098.3717

### SHARING THE CARE: SOCIAL MEDIA USE FOR SELF-DISCLOSURE IN FAMILY CAREGIVERS OF PEOPLE WITH DEMENTIA

Gianna Kohl[1], Katrina Scior[2], and Georgina Charlesworth[2], *1. University of Illinois Urbana-Champaign, Champaign, Illinois, United States*, *2. University College London, London, England, United Kingdom*

Family caregivers of people with dementia often face unique challenges in their caregiving role, including the complex task of sharing their caregiving experiences and identity with their social networks. Social media platforms provide a valuable avenue for self-disclosure of stigmatized identities such as dementia, enabling access to support. This study explores the patterns and purposes of self-disclosure by family caregivers of people with dementia on social media. A cross-sectional online survey was conducted with 323 respondents, aged 20 to 86 (M = 59.2, SD = 12.34), of whom 78% were female. Among the whole sample, 255 (79%)

reported using social media, with 66% of them using it daily. Facebook was the most frequently used platform (81%), followed by Twitter (42%) and Instagram (33%). Of the social media users, 121 (47%) also posted dementia-related content. Content analysis of open-ended questions revealed that these caregivers use social media to share their dementia caregiving journey, including providing insights into the lives and experiences of the person they support, while also engaging in awareness raising and mutual support activities with other caregivers. The findings show that social media is an important resource for family caregivers of people with dementia, facilitating the sharing of personal experiences, building support networks, and engaging in advocacy. The high rate of regular use underscores its value in navigating caregiving experiences and challenges. Healthcare professionals and organizations should leverage these platforms to offer targeted support, while future research should explore how social media can be integrated into support strategies.

Abstract citation ID: igae098.3718
### SKN-1 ISOFORM-C IS ESSENTIAL FOR C. ELEGANS DEVELOPMENT
Marisa Gaglio[1], Vandita Gorla[2], Tripti Nair[2], Carmen Ramos[2], Chris Turner[2], and Sean Curran[2], *1. University of Southern California, Los angeles, California, United States*, *2. University of Southern California, Los Angeles, California, United States*

The transcription factor SKN-1 in Caenorhabditis elegans is a critical regulator of various biological processes, impacting development, diet and immune responses, cellular detoxification, and lipid metabolism; thereby playing a pivotal role in regulating the health and lifespan of the organism. The primary isoforms of SKN-1 (SKN-1a, SKN-1b, and SKN-1c) exhibit distinct functions resembling mammalian Nrf transcription factors. This study investigates the specific role of the SKN-1c isoform in development by generating mutants with targeted missense mutations in the skn-1c and skn-1a isoforms. The skn-1c Met1Ala mutants, which replaces a start methionine with alanine, renders SKN-1c non-functional while preserving other isoforms, produced inviable embryos, requiring a balancer chromosome for proper embryonic development. In contrast, skn-1a Met1Ala mutants, which replaces the start methionine with alanine for this isoform, displayed normal embryonic development and hatching. Moreover, the data suggest that SKN-1c plays a crucial role in embryonic development, as strains without maternally deposited SKN-1c lead to embryos that are developmentally arrested. Together, these findings contribute to our understanding of SKN-1c's specific role in influencing embryogenesis and development in C. elegans.

Abstract citation ID: igae098.3719
### SLEEP DISTURBANCES AND PAIN ON DEPRESSION IN OLDER ADULTS WITH AND WITHOUT ALZHEIMER'S DISEASE
YingHua Wu[1], Benjamin Hougaard[2], Bárbara Gómez Cáceres[3], Jamie Kiefer[3], and Rowena Gomez[3], *1. Palo Alto University, Watsonville, California, United States*, *2. Palo Alto University, Florence, Wisconsin, United States*, *3. Palo Alto University, Palo Alto, California, United States*

Pain is associated with depression and sleep disturbance in older adults (OAs), including those with Alzheimer's disease (AD; Cao et al., 2019; Tsai et al., 2021). However, limited research examines how various sleep disturbances moderate the relationship between pain and depression in OAs and those with and without AD. From the Health and Retirement Study, the sample included 15,512 OAs without AD (59% female; Mage = 69.28; SDage = 10.81) and 211 OAs with Alzheimer's disease (64.9% female; Mage = 81.15; SDage = 9.82). Depression was measured based on 14 questions about depressive symptoms corresponding with criteria A of major depressive episode. Pain was assessed by two survey questions about present pain and severity. The severity of different sleep disturbances was also assessed. Custom ANOVAs indicated that trouble waking up too early ($p < .001$), trouble falling asleep ($p < .001$), trouble waking up during the night ($p < .001$), and feeling rested in the morning ($p < .001$) significantly moderated the effect of pain on depression severity in OAs without AD. However, the moderation was not significant in OAs with AD, though there was a main effect of trouble falling asleep, feeling rested in the morning, and pain on depression. Interventions aimed at improving specific aspects of sleep may help mitigate the effects of pain on depression severity in OAs without AD. The absence of this moderation in OAs with AD still suggests the impact of sleep disturbance and pain on depression.

Abstract citation ID: igae098.3720
### SLEEP QUALITY, COGNITIVE AGING, AND MORTALITY AMONG MIDDLE TO OLDER AGED US ADULTS
Rio Tate[1], and May Beydoun[2], *1. University of South Florida, Tampa, Florida, United States*, *2. National Institute on Aging, Baltimore, Maryland, United States*

**Background:** Sleep and dementia may interact with each other to determine mortality risk, perhaps differentially by sex and by race.

**Methods:** The study examined bi-directional associations of dementia, sleep and mortality, while testing both interactions and mediating effects in a large sample of older US adults using the nationally representative Health and Retirement Study (HRS) dataset, and middle-aged US adults using the Healthy Aging in Neighborhoods of Diversity across the Life Span (HANDLS) dataset. 6,911 older participants and 1,364 middle-aged participants were included for analysis.

**Results:** Regarding HRS, poor sleep quality was associated with higher mortality risk with a dose response relationship, and a strong association between dementia status and mortality risk was found. Dementia status' positive association with mortality risk was stronger in the "better sleep" group (lowest tertile of "poor sleep" variable), with significant interaction between dementia status and poor sleep tertile. Regarding HANDLS, poorer sleep was associated with lower survival probability over time compared to better sleep, particularly among individuals with above median global mental status.

**Conclusions:** Poor cognition was positively associated with mortality risk, particularly among males and among White older adults, and individuals with better sleep quality in both HANDLS and HRS studies. Future studies should uncover some of the underlying mechanisms behind the dementia-sleep antagonistic interactions.

Abstract citation ID: igae098.3721

### SOCIAL DETERMINANTS OF HEALTH ASSOCIATED WITH 30-DAY REHOSPITALIZATION BY USING DISCHARGE SUMMARIES.

Youjeong Kang, and Jiaying Lu, *Emory University, Atlanta, Georgia, United States*

**Background:** There has been increasing interest in the use of Social Determinants of Health (SDOH) in cardiovascular disease outcomes by reducing health disparities. For example, Centers for Medicare and Medicaid Services will require the hospitals reporting five SDOH components (housing, intimate partner violence, utilities, food insecurity and transportation insecurity) in 2025. There is limited evidence which SDOH components are associated with 30-day rehospitalization in HF patients who were in the ICU. Objective: To assess associations between the SDOH and 30-day rehospitalization in HF patients who were in the ICU by using the Medical Information Mart for Intensive Care III (MIMIC-III) data.

**Methods:** We identified HF patients by using ICD-9 codes related to HF from the MIMIC-III data that is de-identified and is publicly available. We used python to extract SDOH features from discharge summaries and used Chi-square statistical test (whether or not keyword is contained in discharge summaries vs whether or not HF patients have 30-day hospital rehospitalization).

**Results:** Mean age was 71 (± 14.2), 53% were male, and 73% were white. In particular, keyword "daughter" contributes to the 30-day rehospsitalization. It is unclear how daughter contributes 30-day rehospitalization but "daughter can be one of the SDOH components as social support.

**Conclusion:** It is difficult to identify how "daughter" is related to 30-day rehospitalization. We will utilize natural language processing models to analyzing the real-world social meaning of SDOH keywords to understand the correlation (e.g. the "daughter" reflecting patients have daughter accompany or other status).

Abstract citation ID: igae098.3722

### SOCIOEMOTIONAL FACTORS DRIVING SEXUAL INTEREST AND SATISFACTION IN OLDER ADULTHOOD

Annie Nguyen[1], Malinee Neelamegam[2], and Matthew Smith[3], *1. University of California San Diego, La Jolla, California, United States*, *2. University of North Texas Health Science Campus, Fort Worth, Texas, United States*, *3. Texas A&M University, College Station, Texas, United States*

Research shows that sexual interest declines with age, while sexual satisfaction remains relatively stable in later life. Declines in sexual interest can result from health deficits that pose barriers to sex; however, for older adults who are healthy and able to have sex, socioemotional factors may influence sexual satisfaction. This study examined the associations between socioemotional variables, sexual interest, and sexual satisfaction among older adults. Data were drawn from the subset of adults ages 60+ in UCSD SAGE study (n=359). A series of linear regression models were fitted to examine sexual interest and sexual satisfaction as separate outcomes while accounting for demographics (age, gender, marital status, race/ethnicity), socioemotional variables (morale, self-compassion, satisfaction with life), and health indicators (physical function, general health). Older adults who were younger ages (B=-.172, p=.001), men compared to women (B=.283, p<.001), married/partnered compared to single (B=.213, p<.001), and had better physical functioning (B=.214, p<.001) reported higher sexual interest. In the model for sexual satisfaction among sexually active older adults (n=148), those with greater morale (B=-.027, p=.03) and better physical function (B=.271, p=.003) reported higher sexual satisfaction. Findings reinforce the importance of physical function for sexual interest and satisfaction among older adults. Socioemotional factors, such as morale, are more strongly associated with sexual satisfaction. However, these relationships may be bidirectional or recursive in nature (i.e., those with higher morale are satisfied with sexual encounters, and that sexual satisfaction may lead to higher morale), requiring additional longitudinal studies.

Abstract citation ID: igae098.3723

### SPATIAL NAVIGATION IN ALZHEIMER'S DISEASE - A META-ANALYSIS

Dorota Kossowska-Kuhn[1], Gillian Gouveia[1], Neil Charness[2], and Richard Wagner[1], *1. Florida State University, Tallahassee, Florida, United States*, *2. Weill Cornell Medicine, New York, New York, United States*

The global population of individuals aged 65 and over is projected to nearly double by 2050, reaching 1.6 billion. This demographic shift is expected to drive a significant increase in dementia cases, with Alzheimer's disease (AD) accounting for 60-80% of these cases. While episodic memory loss is a hallmark of AD, spatial disorientation is often one of the earliest symptoms (Coughlan et al. 2018).This meta-analysis investigates the differences in spatial navigation performance between cognitively healthy older adults and those with AD. The effect size is measured using Hedge's g. The analysis considers various moderators, including the mode of test administration (real-world, virtual reality, computer screen), the type of measure (time, accuracy), and the nature of the task (maze navigation, environmental navigation), alongside basic study characteristics such as publication year, study location, participant age, gender, and education.The analysis includes 124 effect sizes from 42 studies, involving 2409 participants, of which 941 were diagnosed with AD and 1468 were cognitively healthy older adults. The results show that cognitively healthy older adults have significantly better navigation abilities ($p < 0.05$) compared to those with AD, as indicated by a standardized mean difference (SMD) of 1.97.This study shows a significant decline in spatial navigation abilities in individuals with AD compared to healthy older adults, consistent across various test methods and tasks. Addressing these deficits is vital to reduce the growing impact of dementia.

Abstract citation ID: igae098.3724

### STAFF FOCUS GROUP TO INFORM A DYADIC PSYCHOLOGICAL INTERVENTION FOR SHORT-STAYS IN NURSING HOME

Evan Plys, Makenna Law, Morgan Seward, and Ana-Maria Vranceanu, *Massachusetts General Hospital, Boston, Massachusetts, United States*

**Background:** Subacute/post-acute nursing home (NH) stays represent a missed opportunity for psychological intervention with patient-caregiver dyads at a highly stressful time. Existing interventions are not designed specifically for this setting and dyadic population. This study aimed to identify: (1) psychological needs to inform intervention content; and (2) context-specific considerations for implementation.
**Methods:** We conducted 5 focus groups with 28 NH staff (e.g., leadership, clinical, administrative). Interview guides were iteratively developed and aligned with the Designing for Accelerated Translation of Emerging Innovations (DART) framework. Data were analyzed using hybrid indicative-deductive reflexive thematic analysis to map emergent themes onto the DART framework for intervention development (i.e., demand, user-centered design, and context-sensitive).
**Results:** A dyadic psychological intervention was identified as filling a gap in care, demonstrating value added. Two themes related to demand: (1) staff need help addressing psychological concerns, and (2) psychological services are lacking for short-stay dyads. Four themes related to user needs: (1) fear and worry related to declining health; (2) emotional responses to changes in independence; (3) impact of psychological distress on rehabilitation progress; and (4) impact of psychological distress on discharge. For context-sensitive approaches, staff provided feedback for intervention delivery (e.g., session length), implementation (e.g., referrals), and the research protocol (e.g., randomization).
**Conclusion:** Qualitative data was matched with theory and evidence-based skills to develop a dyadic psychological intervention for the NH setting: Building Resilience In SKilled nursing care (BRISK). This presentation will present intervention content, implementation, and protocol considerations to be tested in an upcoming open pilot.

Abstract citation ID: igae098.3725

### STEREOTYPE EMBODIMENT AFFECTS FEAR OF FALLING: COMPARISON OF EXPLICIT AND IMPLICIT MEASURES

Amber Blount, Eman Ali, Carla Leinbach, Dahee Kim, Kworweinski Lafontant, Nichole Lighthall, and Ladda Thiamwong, *University of Central Florida, Orlando, Florida, United States*

Stereotype Embodiment Theory posits that societal stereotypes about aging affect older adults' self-perceptions of aging (SPA). The implicit belief of negative stereotype associations may be less influential than explicit stereotype beliefs. Stereotype affirming beliefs can be implicit in which there's no self-identification, while stereotype embodiment is explicit self-identification with negative stereotypes. Little is known about the role of implicit associations of aging in older adults. Negative SPA is associated with fear of falling (FOF) in older adults. This study explored the association between stereotype embodiment and implicit associations, and FOF among low-income community-dwelling older adults (N=162, MeanAge = 74.6, SD = 7.15). Stereotype embodiment was assessed using the Consequences-control-negative subdomain of the Brief Ageing Perceptions Questionnaire, while implicit associations of aging was measured using the Age Implicit Association Test. FOF was assessed using the Short Fall Efficacy Scale-International. Most participants (86.9%) exhibited implicit aging stereotype beliefs, with 41.4% also displaying stereotype embodiment. Among those with both negative implicit associations and stereotype embodiment, 48.3% had high FOF. No significant correlation was found between stereotype embodiment and implicit associations. However, a significant association between stereotype embodiment and FOF was found ($p < 0.05$), whereas the association between implicit associations and FOF was non-significant. These findings support stereotype embodiment theory, highlighting that self-identification with negative aging stereotypes can contribute to self-fulfilling prophecies more than mere stereotype beliefs do. This holds implications for clinical gerontologists by indicating a need for interventions targeting maladaptive self-stereotyping to mitigate overestimated FOF in older adults.

Abstract citation ID: igae098.3726

### STRESS AND IDENTITY FOR LGBTQ+ HEALTH: MODELING RISK AND RESILIENCE

Erin Clancy[1], M. Aaron Guest[2], Aanika Dhawan[1], and Rachel Koffer[2], *1. Arizona State University, Tempe, Arizona, United States*, *2. Arizona State University, Phoenix, Arizona, United States*

Marginalized communities, including lesbian and gay populations, may experience unique experiences and expectations associated with growing older. The present study aimed to provide a preliminary model of how lesbian and gay adults view their own aging and identify psychosocial risk and resilience factors specific to this community. Data are from a pilot study of social perceptions of aging in lesbian and gay middle-aged and older adults, in which 45 gay and 46 lesbian adults participants (ages = 55-87, 70% white) completed questionnaires on their health, perceptions of aging, community engagement and identity congruence, and stressful experiences. Self-perceptions of aging were measured with an 8 item scale ranging from 1 to 6, where higher scores indicate greater agreement with positive perceptions of aging (e.g., "So far, I am satisfied with the way that I am aging."). In a regression model accounting for self-rated health, subjective socioeconomic status, and race/ethnicity, lesbian women reported better self-perceptions of aging than gay men ($b = 0.50$, $SE = 0.16$, $p <.01$). Further, greater outness was associated with better self-perceptions of aging ($b = 0.04$, $SE = 0.01$, $p <.01$), LGBTQ+ community engagement did not relate to self-perceptions of aging ($b = -0.10$, $SE = 0.12$, $p =.40$), and more stressful experiences in daily life were associated with worse self-perceptions of aging ($b = -0.15$, $SE = 0.05$, $p <.01$). Results will be discussed in the context of a model of risk and resilience for "accelerated social aging" among LGBTQ+ populations.

Abstract citation ID: igae098.3727

### SUBJECTIVE COGNITIVE DECLINE AND RISK OF MORTALITY AMONG BEREAVED PARENTS IN OLD AGE

Jieun Song, Seung Eun Cha, and Carol Ryff, *University of Wisconsin-Madison, Madison, Wisconsin, United States*

The death of a child is a highly traumatic event that may impact the subsequent health of parents. Prior research shows greater risk of early deaths in bereaved parents relative to non-bereaved parents. This study examined whether the associations between the death of a child and parents' mortality risk are mediated by subjective cognitive decline (SCD), known to be predictive of mortality and Alzheimer's disease, contingent on parents' age. The analytic sample consists of participants in Midlife in the U.S. (MIDUS) waves II and III (2004-2005, 2013-2014, respectively); parents who experienced the death of a child prior to MIDUS II (n=341) and non-bereaved parents who did not experience the death of a child (n=2,219). SCD was assessed at MIDUS III and mortality data were obtained via National Death Index 2022 files. Results of moderated mediation analysis revealed significant mediation effects of SCD between death of a child and parental mortality risks over time among parents who were aged 65 and older at SCD assessment, but not among parents who were younger than 65. Bereaved parents who reported a decline in memory at age 65 or older had greater risks of mortality over 8-9 years relative to their counterparts. Findings suggest that increased risks of mortality in bereaved parents during old age can be detected by a relatively simple assessment of SCD, which can be utilized to screen vulnerable bereaved parent groups who may benefit from interventions to mitigate detrimental impacts of bereavement on mortality risks.

Abstract citation ID: igae098.3728

### SYMPTOM OVERLAP BETWEEN SLEEP DISORDER AND DEPRESSIVE SYMPTOMS

Abolade Oladimeji[1], Kari O'Donnell[2], Douglas Gunzler[3], Kristen Berg[4], Douglas Einstadter[2], Elizabeth Pfoh[5], Jarrod Dalton[5], and Adam Perzynski[2], *1. Case Western Reserve University, cleveland, Ohio, United States, 2. MetroHealth, Cleveland, Ohio, United States, 3. CWRU, Cleveland, Ohio, United States, 4. CWRU and MetroHealth, Cleveland, Ohio, United States, 5. Cleveland Clinic, Cleveland, Ohio, United States*

Introduction: The PHQ-9, a common screening tool for depression, asks about sleep and fatigue. An under-recognized concern is the overlap between sleep and fatigue depressive symptoms and sleep disturbances. We investigated whether elevated depression screening scores were due to sleep disorders rather than depression itself in older adults.

**Methods:** The study cohort included 2,257 adults (65+) with at least two outpatient primary care visits and at least one Medicare Annual Wellness Visit, who initially screened positive on the PHQ-2 (≥2) and completed the PHQ-9. A multiple indicator multiple cause (MIMIC) analysis was used to examine differential item functioning of the sleep and fatigue items of the PHQ-9, attributable to sleep disorders, based on diagnosis codes.

**Results:** The MIMIC analysis revealed a positive association between sleep disorders and depression ($\beta = 0.19$, $p < 0.001$), and a small positive effect on the "feeling tired" fatigue item of the PHQ-9 ($\beta = 0.06$, $p < 0.001$). No significant relationship was observed between sleep disorders and the PHQ-9 "sleep" item. The model exhibited a good fit, with RMSEA=0.040 (90% CI: 0.034-0.047), CFI=0.99, TLI=0.99, and SRMR=0.04.

**Discussion:** Sleep disorders and depressive symptoms are associated but no meaningful differential item functioning was detected in this sample of older adults. The PHQ-9 is an adequate screening tool for depression in older adults with sleep disorders.

Abstract citation ID: igae098.3729

### SYNCHRONIZED ACTIVITIES PROMOTE POSITIVE SENTIMENTS OF YOUNGER ADULTS TOWARDS OLDER ADULTS

Sarah Kim, and Hsiao-Wen Liao, *Georgia Institute of Technology, Atlanta, Georgia, United States*

Studies have shown that younger adults often hold negative perceptions toward older adults, which can compromise intergenerational relationships and social harmony. The present study centers on interpersonal synchrony as a potential intervening venue. It adopted a pre- and post-test experimental design to test whether synchronous activities, specifically synchronous walking, promote positive sentiments of younger adults toward older adults. Components essential for forming positive interpersonal relationships regarding impression, perceived self-other closeness, connectedness, prosociality, and conversation engagement were examined. Participants (N = 51; 64.7% female) first completed a battery of questionnaires online assessing background information. After a few days, they were paired up with an older confederate and engaged in tasks. Participants were randomly assigned to one of the three conditions (i.e., synchronous walking, asynchronous walking, and no walking) and completed standard questionnaires assessing their sentiments toward the older confederate throughout experiments. A series of one-way repeated measures ANOVA, one-way ANOVA, and chi-square test of independence were conducted. Results revealed that synchronous walking significantly enhanced participants' impressions, perceived self-other closeness, connectedness, and prosocial attitudes and behavior toward their older partner compared to asynchronous and no walking. The results remained significant when a set of individual difference factors was considered. This study extends past research on interpersonal synchrony to the context of aging. It sheds light on intervention practices for improving young people's sentiment and behavior toward older adults, promoting intergenerational relationships.

Abstract citation ID: igae098.3730

### TARGETING SOCIALLY ISOLATED OLDER ADULTS: THE INTERNET-BASED CONVERSATIONAL ENGAGEMENT TRIAL (I-CONECT)

Hiroko Dodge[1], Chao-Yi Wu[2], Liu Chen[3], and Kexin Yu[4], *1. Harvard Medical School, Massachusetts General Hospidal, Framingham, Massachusetts, United States, 2. Massachusetts General Hospital, Harvard Medical School, Massachusetts General Hospital, Harvard Medical School, Massachusetts, United States, 3. Massachusetts general hospital, Boston, Massachusetts, United States, 4. Oregon Health and Science University, Portland, Oregon, United States*

Social isolation is a risk factor for dementia. In the recently completed randomized controlled trial named I-CONECT (www.i-conect.org; ClinicalTrials.gov: NCT02871921), we investigated the impact of frequent social interactions via webcam/internet on the cognitive function and emotional well-being, recruiting socially isolated older adults aged 75 and above. Participants in the experimental group engaged in semi-structured conversations with interviewers, prompted by daily themes and associated pictures, 4 times per week (30 minutes/session) for 6 months. The control group received only brief weekly phone check-ins. A total of 186 subjects (86 with normal cognition, 100 with mild cognitive impairment [MCI]) were randomized. We earlier reported that global cognitive function (the primary outcome), as measured by the Montreal Cognitive Assessment (MoCA), improved by nearly 2 points in the MCI experimental group compared to the control group at 6 months (effect size: Cohen's d = 0.73) (Dodge et al., 2024, doi: 10.1093/geront/gnad147). Functional MRI data suggested a trend toward increased connectivity in the dorsal attention network favoring the experimental group. Social satisfaction improved in both groups. Our new results not reported in the topline results paper include: (1) Participants in the experimental group increased the frequency of their social interactions over time compared to the control group, and (2) Individual Treatment Response analysis showed that participants in the top 30% of responders could delay cognitive decline by 6 months or more. We will summarize all findings from this trial thus far and discuss future directions.

Abstract citation ID: igae098.3731

### TECHNOLOGY AND PROSPECTIVE MEMORY: ATTITUDES AMONG OLDER ADULTS WITH SUBJECTIVE COGNITIVE IMPAIRMENT

Edie Sanders, and Walter Boot, *Weill Cornell Medicine, New York, New York, United States*,

Prospective memory (PM), the ability to remember to execute an intention in the future, is critical for the performance of everyday tasks important for independence and quality of life. PM failures are more frequent with increased age and can be even greater for older adults with a cognitive impairment such as subjective cognitive decline (SCD). Technology holds promise for providing everyday PM support for older adults with SCD, but little is known regarding their attitudes and preferences toward technology-based PM support. The present work utilized an online survey and focus groups to examine these attitudes. 188 older adults with SCD and 190 older adults without SCD completed an online survey on a variety of technologies to support PM in different areas of activities. A separate sample of 24 older adults with SCD participated in online focus groups and were probed about their perception of the use of technology devices for supporting PM. Results show that the attitudes of older adults with SCD 1) differed based on the type of device and the type of activity the technology is meant to support, 2) were more positive than attitudes of older adults without SCD, and 3) were associated with a variety of individual difference factors. Results indicate that older adults with SCD may be a promising population to target using technology-based solutions, and the type of device and type of activity being supported are important to consider in the development of technology solutions to enhance independence and well-being.

Abstract citation ID: igae098.3732

### TECHNOLOGY PREFERENCES AMONG MILD COGNITIVE IMPAIRMENT (MCI) DYADS LIVING IN A MEMORY CARE DESERT

Michael McCarthy[1], Eric Cerino[1], Megan McCoy[2], Margarita Martinez[2], Thomasina Seaton[1], Amanda Goldtooth[1], Raechel Livingston[1], and Rasheera Dopson[3], *1. Northern Arizona University, Flagstaff, Arizona, United States*, *2. Northern Arizona University, Northern Arizona University, Arizona, United States*, *3. Northern Arizona University, Flagtsaff, Arizona, United States*

Digital health technologies hold promise for early detection and support of individuals and family members contending with Mild Cognitive Impairment (MCI), which may be an early step in the progression toward dementia. These technologies may be especially relevant for care dyads living in "memory care deserts" who are often isolated, under-resourced, experience geographic barriers to care, and lack access to health information and services. The aim of this study is to explore technology preferences of MCI care dyads participating in the Northern Arizona Memory Study in order to better understand the feasibility and acceptability of these technologies for intervention development and delivery. We used a mixed methods approach with semi-structured interviews conducted with 11 dyads comprised of an older adult with MCI and their caregiver (n=22). These data were combined with survey data about participant knowledge and use of technologies, as well as their preferences for mode of delivery for interventions (e.g., interactive website with professional coaching, text messages with peers dealing with memory loss). Qualitative data suggest that there is general interest in technology among MCI care dyads. However, comfort levels with specific technologies vary due to demographic, social, economic, personal, and structural factors. Survey data corroborate these findings with most participants indicating agreement about the usefulness of technologies for MCI detection and support, as well as access to and knowledge of these technologies. Researchers may benefit from these insights as they develop technology-based supportive interventions and resources for underserved MCI care dyads.

Abstract citation ID: igae098.3733

### TEST-RETEST RELIABILITY OF THE NIH TOOLBOX - COGNITION BATTERY OVER SHORT RETEST INTERVALS.

Aaron Bowes[1], Bruno Giordani[2], and Voyko Kavcic[1], *1. Wayne State University, Detroit, Michigan, United States*, *2. University of Michigan, Ann Arbor, Michigan, United States*

The NIH Toolbox-Cognition Battery (NIHTB-CB) is a commonly used computerized cognitive assessment tool in clinical research, but its reliability over short retest intervals has not been thoroughly assessed. This study evaluates the reliability of NIHTB-CB subtests over a 3-week retest interval in participants from an ongoing longitudinal study with biannual NIHTB-CB assessment. Thirteen community-dwelling African American/Black adults aged 65 or older (M = 70.61/SD = 3.36), recruited from the Institute of Gerontology at Wayne State University (R01AG054484; Kavcic, PI) and the Michigan Alzheimer's Disease Research

Center (MADRC, P30AG072931; Paulson, PI), participated. Participants had previously completed NIHTB-CBv2 on iPad tablets every six months for approximately 18 months (M = 17.46/SD = 7.52). The distribution of diagnoses was: 9 normal cognition, 2 non-amnestic MCI and 3 Cognitively Impaired, not MCI. For this study, they now completed the NIHTB-CB twice over a 3-week interval. Reliability was assessed using intraclass correlations (ICCs). Paired t-tests were used to assess overall change in scores over time. All NIHTB-CB ICCs ranged from 0.75 to 0.90, indicating good reliability, except List Sorting Working Memory that had an ICC in the moderate range (0.55). No significant differences were found for any paired t-tests. As clinical trials increasingly use computer-based testing measures, understanding their reliability over short intervals is crucial. Our findings indicate that the NIHTB-CB, when participants are familiar with the test, still shows acceptable retest reliability for both summary indices and subtests over even short retest intervals.

Abstract citation ID: igae098.3734

### THE ASSOCIATION BETWEEN RACE, SEX, AGE, AND PERSONAL FINANCES DURING THE COVID-19 PANDEMIC

Lena Simon[1], Alexa Allan[2], Junyan Tian[3], Orfeu Buxton[4], Alan Zonderman[5], Michele Evans[5], and Alyssa Gamaldo[6], *1. Institute of Engaged Aging, Snellville, Georgia, United States*, *2. The Pennsylvania State University, University Park, Pennsylvania, United States*, *3. Penn State University, State College, Pennsylvania, United States*, *4. Pennsylvania State University, University Park, Pennsylvania, United States*, *5. National Institute on Aging, NIA, Maryland, United States*, *6. Clemson University, Clemson, South Carolina, United States*

The current study explored (a) the association between personal finances during the COVID-19 pandemic and depressive symptoms and (b) whether this association varies by sociodemographic characteristics. The sample included 158 aging adults (60% African Americans; 72% Female; age 46-83) from the Healthy Aging in Neighborhood of Diversity across the Life Span Sleep Study (HANDLSleep). Participants were asked to self-report changes in their finances during the COVID-19 pandemic on a scale from 0 (unstable or consistent decline) to 2 (consistent increase). The Patient Health Questionnaire-9 (PHQ-9) measured depressive symptomology and severity. Linear regression models indicated reports of unstable/consistent decline in finances during the COVID-19 pandemic were significantly associated with greater depressive symptomology even after adjusting for age, sex, and race ($\beta$ = -.24, p =.034). Additionally, a significant interaction between race and COVID-19 finances item was observed ($\beta$ =.91, p =.033). Nonsignificant interactions were observed between the COVID-10 finances item and other demographic variables (age, sex). Subsequent race-stratified analyses suggested that the significant association between unstable/consistent decline in finances during COVID-19 and greater depressive symptoms were observed only among White adults ($\beta$ -.52, p =.008) but not within Black/African American adults ($\beta$ = -.10, p >.05). These findings highlight the financial consequences of sociohistorical events (e.g., structural factors in a COVID-19 pandemic context) for mental health. These differential outcomes in groups of community dwelling adults suggest different public health measures and policies may be warranted under these circumstances.

Abstract citation ID: igae098.3735

### THE ASSOCIATION BETWEEN SOCIAL DETERMINANTS OF HEALTH AND SOCIAL FUNCTIONING AMONG OLDER ADULTS WITH STROKE

Lalipat Phianhasin[1], Chitchanok Benjasirisan[2], Suebsarn Ruksakulpiwat[3], and Eeeseung Byun[4], *1. University of Washington, Seattle, Washington, United States*, *2. Johns Hopkins University, Baltimore, Maryland, United States*, *3. Faculty of Nursing, Mahidol University, Nakhon Pathom, Nakhon Pathom, Thailand*, *4. University of Washington School of Nursing, Seattle, Washington, United States*

Older adults with stroke often experience physical and mental disabilities, resulting in limitations on social functioning, such as participating in social roles and activities. Social determinants of health (SDOH), such as education and income, are shown to have an impact on morbidity and mortality in stroke survivors. However, little is known about the impact of SDOH on social functioning in older adults with stroke. We aimed to examine the associations between SDOH (i.e., marital status, race/ethnicity, education, poverty income ratio [PIR], work status, and health insurance status) and social functioning (i.e., difficulty doing errands alone, participating in social activities, and work limitations due to health problems) in older adults with stroke. We used the National Health Interview Survey 2019-2022 data, which had 61,957 participants aged 60 years and older, including 4,163 stroke survivors. We used logistic regression to examine the associations between SDOH and social functioning after adjusting for covariates, including age, sex, and perceived health status. Being a racial/ethnic minority and not working over the previous week were associated with difficulty doing errands alone. Not being married, being Hispanic or non-Hispanic Asian, and not working over the previous week were associated with difficulty participating in social activities. Not being married, having a PIR < 1 (income below the federal poverty threshold), and not working over the previous week were associated with work limitations due to health problems. Addressing SDOH in care plans for older adults with stroke may improve social functioning and quality of life in this at-risk population.

Abstract citation ID: igae098.3736

### THE ASSOCIATIONS BETWEEN LONELINESS, SOCIAL ISOLATION AND FIVE DOMAINS OF COGNITION

Dokyung Yoon[1], Elizabeth Zelinski[2], and Teal Eich[3], *1. University of Southern California, Leonard Davis School of Gerontology, Los Angeles, California, United States*, *2. University of Southern California, Los Angeles, California, United States*, *3. USC, Los Angeles, California, United States*

This study aimed to investigate the associations between loneliness, social isolation, and five cognitive domains by using a comprehensive neuropsychological battery. Participants included 2,884 older adults (mean age = 72.8) in the 2016 Health and Retirement Study Harmonized Cognitive Assessment

Protocol. The 11-item UCLA Loneliness Scale was used for loneliness. For social isolation, several indicators were scaled (e.g., marital status, contact frequency with children, other family members, and friends, and participation in social organizations) using the Steptoe's Social Isolation Index. Based on the confirmatory factor analysis findings from Zahodne et al. (2020)'s article, the average z-scores of the test results corresponding to each cognitive domain (memory, executive function, visuoconstruction, language, and processing speed) were calculated. Multiple regression for each cognitive domain was conducted. The results revealed that there were significant associations between loneliness and the cognitive scores across all five domains, indicating that the lonelier older adults were, the lower their cognitive scores tend to be. Even after controlling for demographics and depression, these associations remained significant for memory, executive function, and language. Social isolation showed a significant association only with processing speed. However, the interaction term between loneliness and social isolation was not significant across all five cognitive domains. This study provides that links between social isolation, loneliness, and cognition differ across domains. Furthermore, loneliness might have an association with memory, executive function, and language independently of depression.

Abstract citation ID: igae098.3737

### THE BENEFITS OF GENERATION IDENTITY OVER AGE IDENTITY IN OLDER ADULTS: THE MEDIATING ROLE OF NOSTALGIA

Yuanqing Chang[1], Jiehua Lu[1], and Xin Zhang[2], *1. Peking university, Peking University, Beijing, China (People's Republic)*, *2. Peking University, Beijing, Beijing, China (People's Republic)*

**Background and Objectives:** As individuals age, they develop two types of self-identification: age identity and generation identity. Previous research suggests generation identity is more beneficial in promoting well-being of older adults. However, the underlying mechanisms and the generalizability in the Chinese cultural context remain underexplored.

**Research Design and Methods:** This study employed a randomized single-factor between-subjects design with 160 community-dwelling Chinese older adults (M = 67.61 years, SD = 5.63 years; 31.88% men). Participants were randomly assigned to focus on similarities or differences with peers (age identity salience condition) or contemporaries (generation identity salience condition), followed by assessments of nostalgia and subjective well-being including life satisfaction, loneliness, meaning in life, and awareness of age-related changes.

**Results:** Word frequency based on lexicon text analysis revealed that participants in generation identity salience condition mentioned more era-specific events. The Biterm Topic Model (BTM) identified two themes: "Hardship of the Past vs. Present Happiness" and "Present Happy Life" in both groups. However, the generation group had a higher proportion of the first theme (56.25% vs. 42.5%). Independent T-tests indicated significant group differences in nostalgia and subjective well-being ($p <.05$). Structural equation modeling revealed nostalgia could function as a significant mediator between identity salience condition and subjective well-being ($\beta$ =.092, 95% CI = [0.000, 0.184]).

**Discussion and Implications:** The findings suggest that emphasizing generation identity can significantly enhance older individuals' life satisfaction, meaning in life, and awareness of age-related gains, offering empirical support for strategies aimed at promoting active and successful aging.

Abstract citation ID: igae098.3738

### THE CLOSER THE BETTER? AGE DIFFERENCES IN EXPRESSING EMOTIONS TO OTHERS

Jane Stephenson[1], Enna Chen[2], Kathrine Whitman[3], and Laura Carstensen[4], *1. Stanford University, Stanford, California, United States*, *2. Stanford University, Menlo Park, California, United States*, *3. Stanford University, Palo Alto, California, United States*, *4. Stanford University, Stanford University, California, United States*

Socioemotional Selectivity Theory posits that as people age and future time horizons shorten, they prioritize emotional meaning over exploration. As a result, they selectively narrow their social networks to focus on close and emotionally meaningful relationships. Thus, older adults may be more likely to express their feelings to close social partners and benefit more emotionally from sharing their feelings in close relationships as compared to more distant ones. In an experience sampling study collected at five randomly selected times per day for seven days, 180 participants (aged 18 to 93, M = 57.3) reported whether they talked to someone about their emotions, their relationship to that person (spouse/partner, family, friend, acquaintance, coworker, stranger), and whether they felt better after talking to that person. On average, participants felt better after talking to someone about their emotions regardless of their relationship with that person, although they felt significantly better when they shared with close social partners (spouse/partner, family, friend) as compared to peripheral social partners (acquaintances, coworkers, strangers). While age was not significantly associated with the likelihood of expressing feelings to another person, sharing feelings with a family member was associated with feeling significantly better among older participants as compared to younger ones. These findings indicate that sharing feelings with others, no matter their relationship, can benefit emotional well-being at all ages. However, close social relationships provide the most emotional benefit and familial ones appear to play an especially important role at relatively advanced ages.

Abstract citation ID: igae098.3739

### THE CONNECTION BETWEEN SEROTONIN SIGNALING, AGING, AND IMMUNITY WHEN OBSERVED FROM C. ELEGANS AND HUMAN STUDIES

Liyat Kebbede[1], Brandy Weathers[1], and Tripti Nair[2], *1. University of Southern California, Leonard Davis School of Gerontology, Los Angeles, California, United States*, *2. University of Southern California, Los Angeles, California, United States*

As humans age, various environmental threats impact healthspan. Therefore maintaining an intact immune system is vital. Efficiency of human immune response can decline over time leading to complications with age-related diseases. Homeostatic mechanisms are in place to combat threats to the immune system. Serotonin, a neurotransmitter that critically regulates functions in inflammation and immune response, begins to lack functionality with aging. Signaling dysfunction in neurotransmitters can lead to complications in immune response and negatively impact healthspan. Using C. elegans, the connection between immune response and serotonin signaling can be investigated, as this model organism shares homology with the innate human immune system. The Curran lab designed a mutation in the homeostatic pathway resulting in low serotonin signaling. Consequently, impairment to pathogen sensing occurs. C. elegans rely upon the innate immune system to avoid and survive exposure to harmful bacteria. Their transparent nature and 30 day lifespan, allows for visualization of protein signaling and genetic tracing across the life. With a mutation in SKN-1, it has been observed that they become unaware of the dangers of a lethal pathogen, Pseudomonas aeruginosa (PA14), and are even apathetic towards its threat. The serotonin mutant animals remain on PA14 significantly longer than wild type (WT) animals. This study covers therapeutic interventions with serotonin administration and depletion to observe the neurotransmitters necessity in human clinical trials and C.elegans laboratory studies conducted in the Curran lab. Understanding how serotonin signaling impairs the behaviors of C. elegans underscores its importance in maintaining efficient immune response in humans.

Abstract citation ID: igae098.3740

### THE DIGITAL DIVIDE AMONG CHINESE OLDER ADULTS: INTERNET USAGE AND EHEALTH LITERACY

Jiaan Zhang, *Fudan University, Fudan University, Shanghai, China (People's Republic)*

In recent years, particularly due to the COVID-19 pandemic, digital dependency has grown significantly in China, exacerbating the digital divide as a pressing social issue. This study examines Internet usage and eHealth literacy among Chinese older adults aged 60 and above compared to their younger counterparts, using data from the newly available 2021 China General Social Survey. Internet usage was assessed by asking respondents whether they currently use the Internet. eHealth literacy was measured using the 8-item eHealth Literacy Scale, with each item scored on a 5-point Likert scale. Age groups were compared using chi-square tests and t-tests. Correlates of Internet usage and eHealth literacy were analyzed with logistic regression and OLS regression models, respectively. The results indicate that older adults significantly lag in Internet usage, with only 35.25% of those aged 60 and above using the Internet, compared to 89.09% of the under-60 group. Additionally, the younger group demonstrated significantly higher eHealth literacy scores. Regression analyses further revealed that the negative association between older age and Internet usage is more pronounced among rural residents, and that older age is more strongly associated with lower eHealth literacy scores in both women and rural residents, controlling for sociodemographic and socioeconomic status, social support, and health status. This study provides the most recent evidence of significant disparities between older adults and other age groups in China regarding Internet usage and eHealth literacy. It underscores the need for policies and programs focused on bridging the digital divide among older adults.

Abstract citation ID: igae098.3741

### THE EFFECTS OF HEALTH COACHING ON PATIENT ACTIVATION AND WELL-BEING OF COMMUNITY-DWELLING OLDER ADULTS

Edwin Ka Hung Chung, Dannii Y. Yeung, and Frank Youhua Chen, *City University of Hong Kong, Hong Kong, China (People's Republic)*

Health coaching appears as a promising intervention to initiate behavioural change and improve health outcomes for outpatients (Krok-Schoen et al., 2017; Sharma et al., 2016). However, its effectiveness in improving self-management of chronic diseases were mainly examined in young and middle-aged adults with specific chronic diseases (Lin et al., 2021; Tülüce & Kutlutürkan, 2018). Whether health coaching is still effective in community-dwelling older adults, particular those with lower education and multiple chronic diseases, remains uncertain. This study thereby aims to examine the effects of a health coaching intervention in a sample of 194 older Hong Kong Chinese adults (Mage = 79.1, SD = 6.90, range = 62–95). Depending on their health needs, participants received 12 – 18-week home-based health coaching intervention during July 2022 – July 2024. Their patient activation, physical activity, nutrition behaviours, loneliness, and depression were measured before and immediately after the intervention. Repeated measures ANCOVAs showed that participation in the health coaching intervention significantly increased patient activation and nutrition-related health behaviours, and reduced loneliness and depression. Theses results remained significant even after controlling for age, sex, education, living arrangement, receipt of community centre and home care services, numbers of chronic diseases, polypharmacy, and frequency of intervention sessions. The findings of this study thus revealed beneficial effects of health coaching in enhancing older adults' competence in managing their health, engagement in health behaviours, and psychological well-being. Policymakers and health-related professionals should implement health coaching in the primary healthcare system to maintain older adults' well-being and promote ageing-in-place.

Abstract citation ID: igae098.3742

### THE EVOLUTION OF FILIAL PIETY TO INTERGENERATIONAL SUPPORT IN TAIWAN

Yunann Lin[1], Hui-Ching Weng[2], and Wang Chih-Liang[3], *1. National Chung Cheng University, TAINAN, Tainan, Taiwan (Republic of China)*, *2. National Cheng Kung University, Tainan City, Tainan, Taiwan (Republic of China)*, *3. Spring Sun, Spring Sun clinic, Tainan, Taiwan (Republic of China)*

**Objective:** The practice of filial piety concept may vary with sociocultural transformation under aging impact. It's crucial to interpret how filial behaviors affect parent–child interaction. Thereby, this study aimed to explore associations between filial piety and intergenerational support in Taiwan.

**Methods:** This longitudinal study analyzed data collected from the 2016 Taiwan Social Change Survey, consisting of demographics, self-rated health, sibling number, children number, coresident with parents or children, reciprocal filial piety (RFP), authoritarian filial piety (AFP), financial, household, and emotional support. Participants were categorized into three cohorts: the traditional generation (born before 1945), the baby boomer generation (1946-1964), and Generation X (1965-1980). ANOVA and multilevel regression were adopted to analysis intergenerational associations. Findings: AFP present significant decrease in the baby boomer (2.55 vs 2.9, p< 0.001) and generation X (2.4 vs 2.9, p< 0.001) when comparing with the traditional generation; while RFP show no difference among three cohorts. After adjusting gender, education, marriage, self-rated health, income, sibling number, and coresident with parents, RFP of generation X was positively associated with household (p=0.05) and emotional support (p< 0.001) to parents. Moreover, AFP of baby boomer generation was marginal associated with financial (p=0.053) and household (p=0.058) support to parents.
**Conclusion:** The diminish of AFP embodied the trend of mutual affection and equality in the parent–child relationship. The mediation effect of RFP in generation X was practiced in household and emotional support; while the mediation effect of AFP in baby boomer generation was practiced in financial and household support.

Abstract citation ID: igae098.3743
### THE HEALTH DISPARITIES AMONG OLDER ADULTS AGING IN PLACE: A RURAL-URBAN COMPARISON IN KOREA

Mi Sun Choi[1], and Hyunmin Lee[2], *1. Silla University, Busan, Republic of Korea*, *2. University of Seoul, Seoul, Republic of Korea*

Aging in place (AIP) has emerged as a pivotal focus of welfare policies for the aging population in Korea, with health being a crucial determinant of AIP success. Regional variations can significantly impact the health of older adults who choose to remain in their communities. This study aims to elucidate the differences in factors affecting the health of older adults who continue to reside in their communities between urban and rural areas. Utilizing the ecological model of aging and the push and pull framework, we performed a multiple regression analysis on data from 647 older adults (aged 65 and above) from the Korean Welfare Panel Study, all of whom have lived in the same residence since the first wave in 2006. The analysis identified that, for both urban (n=342) and rural (n=305) older adults, work ability, levels of depression, and housing satisfaction significantly affect subjective health status. Specifically, for urban residents, male gender, higher income, compliance with minimum housing standards, and fewer outpatient visits were positively associated with health perception. The results show differences in health characteristics between urban and rural AIP older adults. In urban settings, both subjective perceptions and the physical housing environment play substantial roles in health, whereas, in rural areas, subjective perceptions are more pronounced. To promote health equity among AIP older adults, urban areas may benefit from targeted housing improvement initiatives, whereas enhancing the living environment in rural communities is essential for supporting the well-being of the aging population in these areas.

Abstract citation ID: igae098.3744
### THE IMPACT OF DIETARY PROTEIN RESTRICTION ON WHITE ADIPOSE TISSUE FUNCTION DURING AGING

Khristina Young[1], Molly Easter[1], Prerana Vaddi[2], Michael Donkor[3], Meghan Wong[3], and Cristal Hill[1], *1. University of Southern California, Los Angeles, California, United States*, *2. University of Southern California, Leonard Davis School of Gerontology, Los Angeles, California, United States*, *3. Univeristy of Southern California, Los Angeles, California, United States*

White adipose tissue (WAT) regulates metabolic health, lipid storage and energy mobilization, yet these functions decline during aging and aging with obesity. Studies show that interventions such as dietary protein restriction (DPR) can improve metabolism, benefit overall health, and extend lifespan. Hence, the unique impact of DPR on the physiological function of adipose tissue represents a plausible target to support general health. Data from our lab and others show that DPR induces browning in WAT through thermoregulatory genes. This modification within the WAT produces beige adipocytes by marked increases in UCP1 (Uncoupling protein 1) gene expression and other related genes, such as the coregulator PR-domain containing 16 (PRDM16). Specifically, PRDM16 regulates adipogenic differentiation, thus playing a key role in regulating the function of adipose tissue. Our goal is to investigate if browning of WAT via Prdm16 is essential to improve metabolic health during DPR, and if DPR-induced improvements in metabolic health are sex dependent. Here, 3-months of age, male and female normal-control littermates and Prdm16 KO mice were fed either a normal protein (20%) or a low-protein (5%) diet for 7 months. Collectively, these data suggest that Prdm16 contributes to dependent and independent metabolic endpoints on body weight, glucose and lipid homeostasis, and adipokine signaling in WAT in middle aged mice. Furthermore, transcriptomic profiling highlights that DPR induces biological processes that are sex-dependent on energy storage and usage. The work is supported by the National Institute on Aging (NIA) of the National Institutes of Health (NIH) - MOSAIC– NIA R00AG070273.

Abstract citation ID: igae098.3745
### THE INTERACTION BETWEEN SHORT SLEEP AND EXERCISE FREQUENCY WITH HEART RATE VARIABILITY: CROSS-SECTIONAL STUDY

Taylor Fein[1], Muhammad Thalil[2], and Soomi Lee[3], *1. Pennsylvania State University, Rock Hill, New York, United States*, *2. Pennsylvania State University, State College, Pennsylvania, United States*, *3. The Pennsylvania State University, University Park, Pennsylvania, United States*

Exercise has been shown to be a powerful tool that can improve cardiovascular health. Both exercise and adequate sleep have been associated with increased heart rate variability (HRV), while insufficient amounts of both are associated with decreased HRV. Yet, the combined influence of exercise and sleep on HRV is less clear. This study explores if exercise levels and sleep duration interact to be associated with HRV. 391 adults (Mage=57yrs) from the Midlife in the United States Biomarker study 2004-2009 provided sleep actigraphy, electrocardiogram (ECG) HRV measurements and answered questions on exercise habits. Participants were grouped as short (< 6 hours) or adequate sleepers (≥ 6 hours) and inadequate (< 150 minutes per week) or adequate exercise frequency (≥150 minutes per week) based on CDC guidelines. ANCOVA analysis adjusted for demographic and health-related covariates was used to examine the interaction between sleep duration and exercise frequency on HRV. There were no significant individual associations of sleep duration or exercise frequency with HRV, however combined associations were found. For individuals with less than 6 hours of sleep, those with inadequate exercise frequency had significantly lower HF-HRV (B=-0.59, SE = 0.21, p=0.005) and RMSSD (B=0.29, SE = 0.10, p=0.003) compared to those with adequate exercise. Among adequate sleeper's, no significant differences in HRV were observed between each exercise group. Findings suggest that for those with short sleep, lack of exercise may exacerbate the negative consequences of short sleep on HRV. Future studies should explore whether and how exercise intensity may change this relationship.

Abstract citation ID: igae098.3746

### THE MEDIATING EFFECT OF BRAIN CONNECTIVITY ON DUAL-TASK PERFORMANCE: A PILOT STUDY

John Manning[1], and Jennifer Yentes[2], *1. Texas A&M University, Bryan, Texas, United States*, *2. Texas A&M University, Texas A&M University, Texas, United States*

Neural connections between the prefrontal cortex and the striatum, known as cortico-striatal (CSTR) connections, are important in both cognitive tasks and gait. The purpose of this study was to investigate the mediating role of CSTR resting-state functional connectivity in the relationship between dual-tasking and gait in older adults. We made two primary hypotheses: 1) set shifting dual-task would exert a significant direct effect on gait measures and 2) task would exert a significant indirect effect on gait through CSTR functional connectivity. Five older adults completed a set shifting task where a letter-number combination (e.g. B7) was presented and subjects verbalized the number if the background was orange, or the letter if the background was blue, while standing and walking (dual-task). Later, fMRI was collected and CONN toolbox was used to calculate CSTR. A mediation analysis was performed with task as the exposure variable, CSTR as the mediator variable, and dual-task performance as the outcome variable. Results of the simple linear regression indicate no relationship between dual-task and gait (β=-0.03, p=0.62). Results of the mediation analysis indicate there is not a significant indirect effect of dual-task on gait through CSTR connectivity (i.e.=-0.024, p=0.77). This provides foundational knowledge on how these neural connections may or may not be directly related to dual-task performance during a set-shifting dual-task, which is an important step in understanding how these methods could be used for identifying early warning signs of neurodegeneration. Data collection is ongoing and updated results will be presented at the conference.

Abstract citation ID: igae098.3747

### THE MODERATING RELATIONSHIP BETWEEN FUTURE SELF-CONTINUITY AND NUMERACY ON RETIREMENT SAVINGS

Laken Mooney, and Ryan Best, *West Virginia University, Morgantown, West Virginia, United States*

Worldwide, lifespan is increasing while birth rates are declining resulting in an increasing proportion of older adults in the population. This demographic shift will manifest in an increased proportion of adults in retirement who will rely on non-employment sources of income. Though Social Security provides a key source of retirement income, it is often insufficient and requires supplementation by other sources. Since the late 1970s, the most common retirement savings programs have largely shifted from employers (e.g., pensions) to individuals (e.g., 401k and IRA programs). As such, a greater responsibility for retirement savings now falls on individuals and is more susceptible to variance in individual decision making. Individual difference factors which predict retirement savings include numeracy, the ability to understand and work with numbers, and future self-continuity (FSC), the sense of connectedness we have to our future selves. Using a zero-inflated Poisson model to analyze data from the RAND American Life Panel (n=798), we found a significant interaction between numeracy and FSC when predicting retirement savings (p<.001) after controlling for age and income. Among those that had retirement savings, increased numeracy predicted greater savings for individuals with high, but not low, FSC. This demonstrates that highly numerate individuals appear to have the skills necessary to maximize retirement savings but may not utilize these skills unless they have a strong connection to their future self. Results are discussed in light of the current landscape of retirement preparation and how to best promote individual saving.

Abstract citation ID: igae098.3748

### THE PARADOX OF DIGITAL CONNECTIVITY: COMPARING THEORIES ON LONELINESS AND MOBILE PHONE DEPENDENCE

Hailey Andrews, and Nelson Roque, *Pennsylvania State University, University Park, Pennsylvania, United States*

In the Digital Age, smartphones and tablets are widespread, but their impact on well-being, particularly the relationship between loneliness and mobile phone dependence (nomophobia), remains debated. The direction of this relationship is unclear, with limited research across the lifespan. This study examines two theories: the Displacement Hypothesis, which suggests that nomophobia leads to loneliness, and the Theory of Compensatory Internet Use (CIUT), which proposes that loneliness drives nomophobia. Data were collected from 550 adults (Mage = 40.15, SD = 12.23, 54% female; 59% non-Hispanic White) via an online survey, delivered on Prolific. Linear regression models, adjusting for sociodemographics (age, gender), evaluated the relationship between measures of mobile phone dependence (Nomophobia Questionnaire) and loneliness (UCLA Loneliness Scale). For the model with loneliness as an outcome, (N = 550), age (β = -0.10, p =.028) and nomophobia (β = 0.06, p =.003) were significant predictors. For the model with nomophobia as an outcome, (N = 550), age (β = -0.35, p <.001), gender (β = -6.40, p =.009), and loneliness (β = 0.28, p =.003) were significant predictors. Both directions showed a significant relationship, however, the model with loneliness as a predictor of nomophobia explained slightly more variance (4.6%) in the outcome (vs 2.5% for nomophobia predicting loneliness). Results suggest that reducing loneliness could help mitigate mobile phone dependence. Future longitudinal research could further clarify the relationship between loneliness and nomophobia, providing more evidence for either the CIUT or the Displacement Hypothesis.

Abstract citation ID: igae098.3749

### THE POWER OF SHARED EXPERIENCE: VALIDATION AS A CATALYST FOR MEANING MAKING AMONG ADRD CAREGIVERS

Jacquelyn Benson[1], Abigail Haber[2], Karla Washington[3], and Abigail Rolbiecki[4], *1. Washington University in St. Louis, St Louis, Missouri, United States*, *2. Albert Einstein College, New York, New York, United States*, *3. Washington University in St. Louis, St. Louis, Missouri, United States*, *4. University of Colorado Anschutz, Aurora, Colorado, United States*

Caregiver Speaks is a professionally facilitated online group intervention designed to support hospice family caregivers of patients with dementia by using photos as a tool for expressing and processing their caregiving experiences. The structured intervention enables ADRD caregivers to articulate their feelings, challenges, and experiences, with the primary goals of reducing caregiver distress, alleviating grief, and enhancing overall well-being. The intervention emphasizes helping ADRD caregivers find personal significance in their roles through photo-elicited communication, fostering meaningful emotional expression and reflection. The primary objective of the current study was to evaluate ADRD caregivers' descriptions of their experiences in the Caregiver Speaks intervention to determine how their interactions in the group facilitated meaning making. Forty-one (41) exit interviews were analyzed using a constant comparative method involving an iterative process of deductive and inductive coding. Results indicated that caregivers' interactions with others in the group primarily evoked a sense of solidarity, although some outliers—particularly those with strained relationships with their care recipients—reported feelings of alienation or disconnection. While direct dialogue between group members was infrequent, caregivers found reconciliation in their caregiving experiences and their care recipient's illness by seeing their own experiences reflected in the photos and stories shared by others. The indirect validation provided by fellow participants was particularly impactful for many caregivers, who often lacked connections with other dementia caregivers. These findings underscore the critical roles of shared lived experience and validation in the meaning-making process.

Abstract citation ID: igae098.3750

### THE RELATIONSHIP BETWEEN AGING PERCEPTIONS AND COGNITION AMONG OLDER ADULTS

Ava Mcvey[1], Christine Phillips[2], and Lesley Ross[1], *1. Clemson University, Clemson, South Carolina, United States*, *2. Clemson University Institute for Engaged Aging, Seneca, South Carolina, United States*

Maintaining cognition is critical to overall health and independence. While behavioral predictors (e.g., cognitive engagement, social engagement, physical activity) of cognition well-established in research, the relationship of one's perceptions of aging and cognition have been less thoroughly investigated. The current study cross-sectionally explored the relationships between self-perceptions of aging (SPA; including awareness of age-related change and expectations regarding aging) and cognition, including performance-based measures of executive function (Matrix Reasoning, Letter/Number Switching, Digit Symbol, Trails A & B, Visual Paired Associates) and self-reported cognition (Cognitive Self Report Questionnaire). Participants (n=334) included community dwelling older adults from the: Elucidating the Necessary Active Components of Training (ENACT) and Everyday Function Intervention Trials (E-FIT) studies. A series of four multivariate linear regressions were conducted, one for each facet of SPA and both executive function and self-reported cognition. Results found that neither awareness of age-related change nor expectations regarding aging predicted performance-based executive functioning. However, both awareness of age-related change (β =.65, p <.001, 95% CI [.29,1.03]) and expectations regarding aging (β =.17, p <.001, 95% CI [.11,.23]) predicted self-reported cognition. The current study addresses a gap in understanding how different facets of SPA may associate with different aspects of cognition (executive functioning and self-reported cognition). Results suggest that SPA do not correlate with objective executive functioning performance in this sample. However, one's perception of the aging process may give insight into how they perceive their own current cognitive abilities, which can be an important indicator of well-being.

Abstract citation ID: igae098.3751

### THE RELATIONSHIP BETWEEN CELLULAR GLYCOLYSIS AND AGE IN BLOOD CELLS OF ADULTS

Ben Keri[1], Howard Phang[2], Jaclyn Bergstrom[3], Stephen Dozier[4], Lina Scandalis[1], and Anthony Molina[1], *1. University of California San Diego, San Diego, California, United States*, *2. University of California, San Diego, La Jolla, California, United States*, *3. UC San Diego, La Jolla, California, United States*, *4. University of California San Diego, la jolla, California, United States*

Mitochondrial dysfunction is a hallmark of aging that leads to inefficient cellular energy production, causing a shift towards aerobic glycolysis termed the Warburg Effect. While most studies examined the effect of aging on oxidative phosphorylation (OXPHOS), the relationships between glycolysis and age are less understood. Blood cells are a promising avenue for establishing biomarkers for their accessibility and versatility. We hypothesized that blood cells from older participants would have higher glycolytic function compared to blood cells from younger donors. We used blood collected from adults across the human lifespan through the San Diego Nathan Shock Center. We isolated platelets, peripheral blood mononuclear cells (PBMCs), monocytes, and lymphocytes, and measured glycolytic proton efflux rate (glycoPER) using the Agilent SeahorseXFe96 which is comparable to lactate efflux rate. Pearson correlation analysis showed moderate positive associations between age and glycoPER for platelets (r=0.31, p=0.48) and PBMCs (r=0.44, p=0.004) in women. There were no correlations between age and glycoPER for monocytes or lymphocytes in women, or any cell type in men.

Our findings support our hypothesis that the glycolytic function of cells from older donors is higher than that of younger donors, but only for certain cell populations in women and not for men. Interestingly, gPER was positively correlated with OXPHOS in most cell types, in contrast to the Warburg Effect premise. In conclusion, our study suggests a relationship between glycolysis and age that should be further investigated in the context of age-related bioenergetic decline.

Abstract citation ID: igae098.3752

### THE RELATIONSHIP BETWEEN LIVING ALONE AND STRESS FROM A LIFE COURSE PERSPECTIVE

Junyub Lim[1], Ross Andel[2], Katerina Sheardova[3], Jan Pavlik[4], Jana Amlerova[5], and Jakub Hort[6], *1. Arizona State University, Phoenix, Arizona, United States*, *2. Arizona State University, Phoenix, Arizona, United States*, *3. International Clinical Research Center, St. Anne's University Hospital, Brno, Jihomoravsky kraj, Czech Republic*, *4. AlzheimerChain Foundation, Prague, Hlavni mesto Praha, Czech Republic*, *5. Second Faculty of Medicine, Motol Epilepsy Center, Charles University, Prague, Czechia, Prague, Hlavni mesto Praha, Czech Republic*, *6. Second Faculty of Medicine, Charles University, Motol University Hospital, Prague, Czechia, Prague, Hlavni mesto Praha, Czech Republic*

**Background/objectives:** Social isolation may affect psychological well-being, but understanding this link across adulthood is limited. We examined living alone in relation to dealing with daily stress among younger, middle-aged, and older adults.

**Methods:** Data from Terrapino, a new mobile application designed to support cognitive health, were utilized. Psychological well-being was assessed with a 10-item index of dealing with daily stress over the past month (get angry, feel like things are piling up, feel like being on top of things, etc.) reported as never/sometimes/often/very often. Demographic, health, and lifestyle information was also collected.

**Results:** Complete data were available for 6,154 users who gave consent to allow reports of aggregate data (mean=57.2±14.4, range 18-103 years, 73% were female). Users were categorized as young (18-30 years, n=331), middle-aged (31-60 years, n=3,058), and older (61+ years, n=2,765) adults, of whom 45% (52% male/42% female), 20% (17% male/21% female), and 32% (15% male/39% female) young, middle-aged, and older adults, respectively, lived alone. Controlling for sex, education, and comorbidity, young adults who lived alone had poorer ability to deal with daily stress (Estimate=0.22, $p<.05$), no differences existed among middle-aged adults ($p>.05$) and better ability to deal with daily stress emerged among older adults living alone (Estimate=-0.11, $p<.01$).

**Discussion:** Using data from a freely available mobile application, young users who lived alone dealt with daily stress relatively poorly, whereas their older counterparts showed opposite trends regardless of comorbidity. This study sets the stage for research into mobile application use to improve well-being of older adults living alone.

Abstract citation ID: igae098.3753

### THE ROLE OF APOEE4 IN THE ASSOCIATION BETWEEN PRODUCTIVE AND LEISURE ACTIVITIES AND COGNITIVE HEALTH

Yeonjung Lee[1], Ernest Gonzales[2], Yanyan Wu[3], Kathryn Braun[4], Peter Martin[5], Bradley Willcox[6], and Ross Andel[7], *1. University of Hawaii at Mānoa, UH Mānoa, Hawaii, United States*, *2. New York University, New York University, New York, United States*, *3. University of Hawaiʻi at Mānoa, Honolulu, Hawaii, United States*, *4. University of Hawaii, Honolulu, Hawaii, United States*, *5. Iowa State University, Ames, Iowa, United States*, *6. Kuakini Medical Center, Honolulu/Kuakini Medical Center, Hawaii, United States*, *7. Arizona State University, Phoenix, Arizona, United States*

Cognitive health is influenced by genetic and environmental factors. This study fills a gap of knowledge by examining the role of the apolipoprotein E gene, APOEε4, within the context of how employment, civic engagement, and leisure activities relate to cognitive health. Using pooled data from the Health and Retirement Study (HRS) Psychosocial and Lifestyle Questionnaires (2010/2012: Time 1 and 2014/2016: Time 2) and the HRS data on APOEε4 alleles, linear regression models with a lagged dependent variable were performed to examine associations between productive and leisure activities with cognitive functioning at the follow-up time point, as well as the role of APOEε4 in these associations. Among all participants (n=7,605), employment, low or high-intensity volunteering, cognitive leisure activities, and social leisure activities were associated with higher levels of cognitive functioning. The presence of at least one ε4 allele was related to poorer cognitive functioning at Time 2. Among people without the APOEε4 allele (n=5,630), employment, high-intensity volunteering, cognitive/social leisure activities were significantly associated with cognitive functioning. Among people with at least one APOEε4 allele (n=1,975), low-intensity volunteering, and cognitive/physical leisure activities were significantly associated with better cognitive functioning. We found that employment, civic engagement, and leisure activities all contribute to cognitive health, although the benefits may be restricted to low-intensity volunteering and cognitive/physical leisure activities among individuals with at least one APOEε4 allele who are known to be inherently at a greater risk of cognitive problems, highlighting an avenue to a relatively easily-implementable strategy for cognitive health promotion.

Abstract citation ID: igae098.3754

### THE ROLE OF FILIAL PIETY IN AGING AND CAREGIVING AMONG CHINESE AND CHINESE AMERICANS

Chunhong Xiao[1], Rita Jablonski[2], Patricia Patrician[1], Aoyjai Montgomery[2], Youhua Wang[3], and Adelais Markaki[4], *1. University of Alabama at Birmingham/School of Nursing, Birmingham, Alabama, United States*, *2. University of Alabama at Birmingham, Birmingham, Alabama, United States*, *3. Southwest University, Chongqing, Chongqing, China (People's Republic)*, *4. University of Alabama at Birmingham, Birimingham, Alabama, United States*

Understanding filial piety as it pertains to aging and caregiving among Chinese and Chinese Americans (CCAs) is expected to bridge the cultural chasm in health care and improve the quality of rendered services. This concept analysis aims to understand the role of filial piety relevant to caring for aging CCAs and to propose an operational definition. We used four databases and followed Walker and Avant's method for data extraction and analysis. A total of 26 studies met inclusion criteria and were chosen for synthesis. Synthesis of evidence identified four antecedents: (a) filial obligation as a 'cultural gene', (b) sense of altruism, (c) familial solidarity, and (d) societal expectation of 'birth right'. Attributes included: familial material and emotional support, obedience, pious reverence, and societal norm. Consequences were related to caregiver burden, psychological and physical well-being, quality of life, and health equity. Operational definition of filial piety is: an intrinsic desire of adult children to support parents/elders materially and emotionally as well as an extrinsic desire to adhere to the Chinese societal moral tenet to honor family and be honorable children. Future study should examine dual roles of filial piety as a moderator of role appraisal to caregiver burden and as a mediator of resources. The permeating cultural value of filial piety affects help-seeking behavior, caregiving, family relationships, and caregiver health for CCAs. Understanding the meaning, importance, and effect of filial piety for Asians and CCAs is essential for healthcare providers to provide culturally sensitive social and psychological support to caregivers.

Abstract citation ID: igae098.3755

### TIME SPENT FOR OTHERS: VARIED ASSOCIATIONS WITH WELL-BEING ACROSS TWO TIME SCALES AND SOCIODEMOGRAPHIC GROUPS

Kyoungmin Cho[1], David Almeida[2], and Soomi Lee[3], *1. PSU, state College, Pennsylvania, United States*, *2. Pennsylvania State University, University Park, Pennsylvania, United States*, *3. The Pennsylvania State University, University Park, Pennsylvania, United States*

Existing research on spending time for others yields inconsistent findings regarding its effects on well-being. Some studies report positive effects, while others find negative effects. To address this inconsistency, our study explored whether spending time for others differentially impacts overall eudaimonic well-being and day-to-day hedonic well-being, considering potential differences across sociodemographic groups. 665 participants (Mage=48.27) in the Midlife in the United States Study completed both monthly survey and eight-day diary survey. Monthly and daily time spent for others, overall eudaimonic well-being (psychological and social domains), and daily hedonic well-being (positive affect, negative affect, daily stressors) were measured. OLS regression and multilevel models evaluated the associations of time spent for others with eudaimonic and hedonic well-being at the two time scales, adjusting for sociodemographic and health covariates (e.g., age, gender, education, self-reported physical health, marital status, day of week, and number of missed diary). Results indicated that, at the monthly level, participants who spent more time for others reported higher overall psychological and social well-being. However, at the daily level, they experienced more negative affect and stressors, with these effects more pronounced in men and adults under 56-year old. This study clarifies inconsistencies in prior research, highlighting differential associations of spending time for others with individual well-being across different time scales and sociodemographic groups. While short-term responses tend to be negative, particularly among men and younger individuals, long-term outcomes appear to be positive.

Abstract citation ID: igae098.3756

### TIME TO ENHANCE THE MESSAGE? KNOWLEDGE OF DEMENTIA RISK AND SYMPTOMS IN CENTRAL NORTH CAROLINA

Cassandra Germain[1], and Travonia Brown-Huges[2], *1. North Carolina A&T State Univeristy, Greensboro, North Carolina, United States*, *2. North Carolina A&T State University, Greensboro, North Carolina, United States*

It is well known that Black-American and Latino adults are at higher risk for Alzheimer's disease compared to their White counterparts. Yet, misconceptions about AD persist in ethnic-minority communities. For example, a recent Alzheimer's Association Report (2021) suggests that concern about developing Alzheimer's is lower among Native Americans (25%), Blacks (35%) and Hispanics (41%), compared with Whites (48%) and more than half of non-White Americans believe significant loss of memory or cognitive abilities is a normal part of aging. The current data is part of an ongoing study designed to evaluate existing knowledge about dementia risk factors and the early warning signs of dementia in low-moderate income communities in Central North Carolina. A 17-item true/false questionnaire framed as a "Brain Health Trivia" quiz was used to assess knowledge. Participants had the choice of completing an electronic (cell phone or tablet) or paper version. The electronic version provided respondents with an immediate completion score as well as the correct answers to incorrect questions. To date (N=307) adults aged 18+ have completed the survey. Results show that community members are knowledgeable about many preventive factors such as the importance of diet and exercise, but are less knowledgeable about the impact of chronic disease on brain health. Nearly 45% of respondents surveyed were not aware that diabetes was a risk factor for dementia and 34.4% were not aware that hypertension was a risk factor. These results demonstrate the need for ongoing education and outreach efforts on brain health particularly in low-moderately resourced communities.

Abstract citation ID: igae098.3757

### TRADITIONAL COMPLEMENTARY AND ALTERNATIVE MEDICINE USE OF KOREAN AMERICAN OLDER ADULTS: CULTURAL AND HEALTH NEEDS

Grace Yi[1], Yuri Jang[2], and Leah Martinez[3], *1. California State University Fullerton, Fullerton, California, United States*, *2. University of Southern California, Los Angeles, California, United States*, *3. California State University, Fullerton, California State University, Fullerton, California, United States*

The Korean American population, primarily comprised of first-generation immigrants, is rapidly growing and aging. Literature underscores the significant challenges faced by KAs, largely due to limited English proficiency and cultural differences, which impact their accessibility to the healthcare system and, as a result, health disparity. This study examines the use of Traditional Complementary and Alternative Medicine (TCAM), such as acupuncture and traditional herbal medicines, as a culturally appropriate means to address health inequality. Using data from the Study of Older Korean Americans, a multisite survey conducted in 2017-2018 across five states (California, New York, Texas, Hawaii, and Florida), this study analyzed factors influencing TCAM use among Korean American older adults (KAOA) aged 60 and above (N=2,176) through logistic regression analysis using StataBE18. Over one-quarter (27.2%) of participants reported TCAM use. After controlling demographic factors (age, gender, marital status, education, financial status, and spirituality), significant predictors of TCAM use included acculturation factors, such as ethnic culture closeness (Odds Ratio [OR]=1.04, p<.05), the need for a translator in healthcare settings (OR=.69, p<.01), and residing in areas with a high density of Korean Americans (OR=1.40, p<.05). Also, health and mental health-related factors included cancer (OR=.53, p<.05), arthritis (OR=1.69, p<.001), needing help with daily activities (OR=1.10, p<.05), and experiencing a lack of interest in life (OR=1.72, p<.05). These findings highlight the significance of acculturation and health/mental health factors in determining TCAM use among KAOA and call for health policymakers and providers to adopt culturally competent approaches when working with ethnic minority older adults.

Abstract citation ID: igae098.3758

### TRANSITION INTO RECEIVING CARE AND DEPRESSIVE SYMPTOMS AMONG KOREAN OLDER ADULTS

Haeun Yoo, and Kyungmin Kim, *Seoul National University, Seoul, Republic of Korea*

Although transitioning into receiving care in late life is common, its psychological impact remains underexplored. Adaptation theory suggests that depressive symptoms may peak when individuals start receiving care but diminish over time as they adapt. On the other hand, they may become more depressed over time as their health conditions decline. Furthermore, the availability of a larger care resource network, including formal and informal caregivers, may buffer the negative impact of receiving care on depressive symptoms. This study aims to examine how transitioning into receiving care is associated with changes in depressive symptoms, and how the association varies by perceived care resources. Utilizing panel data from the Korean Longitudinal Study of Ageing (biennial surveys; 2008–2022), we compared two groups of older adults (aged 65–105; observation N = 3,739), based on care status at two consecutive waves: (a) received care at both waves and (b) newly received care at later wave. We found that older adults who transitioned into receiving care showed greater increases in depressive symptoms than those who continued receiving care, adjusting for participants' sociodemographic/health characteristics as well as depressive symptoms at prior wave. Moreover, the association between transitioning into receiving care and changes in depressive symptoms differed depending on perceived care resources; thus, the negative psychological impact of transitioning into receiving care was significant only for participants with fewer perceived care resources at prior wave. The findings highlight the importance of a well-established support network in the context of disability.

Abstract citation ID: igae098.3759

### TRANSNATIONAL PARENT-CARE AND FILIAL PIETY: THE PARADOX OF IDEALIZED TRADITIONAL IMMIGRANT VALUES

Joonsik Yoon, *Syracuse University, Syracuse, New York, United States*

For families from highly filial societies, such as those from East Asian countries, providing care to parents in later life has long been considered an important value and duty. However, providing parent-care to older adults in modern societies can present many practical challenges for families. When adult children emigrate abroad while the parents stay, the physical distance separating the transnational families presents additional difficulties in meeting filial expectations and providing practical care. In this qualitative ethnographic study, ten first-generation Korean Americans were interviewed in depth using an open-ended, semi-structured protocol. These interviews focused on respondents' experiences and concerns surrounding transnational parent-caregiving for parents living back in South Korea. A grounded theory approach was used to adapt the research focus to accommodate emerging thematic elements as the interviews progressed. Most respondents expressed a strong preference for idealized and traditional models of filial piety. They not only felt a strong obligation to support and care for their parents as they aged, but many even stated that they wanted to co-reside with their parents. This is notable since the practice is not only outdated in the West, but it has rapidly fallen out of favor and use in Korea. This preference for seemingly outdated traditional filial values stood in stark contrast to the respondents' acknowledgement that there were significant obstacles in achieving their preferred care goals. I conclude with a discussion of the respondents' preferences for seemingly outdated and unachievable filial ideals.

Abstract citation ID: igae098.3760

### UNDERSTANDING BARRIERS TO PARTICIPATE IN DEMENTIA RESEARCH AMONG OLDER ADULTS IN THE BRONX, NEW YORK

Oshadi Jayakody, Mirnova Ceide, Morgan Grabanski, and Claudene George, *Albert Einstein College of Medicine, Bronx, New York, United States*

The Bronx County in New York (NY) has the highest prevalence of dementia in the state and is considered the least healthy of NY counties. This study aims to identify the barriers to participation in dementia research among older adults in the Bronx and explore strategies for improving their research readiness. We are distributing a brief survey to 100 older adults (aged ≥60) visiting ambulatory practice and memory clinics at Montefiore Medical Center and community-based older adult centers in the Bronx. This survey, which will be completed in September 2024, assesses participants' knowledge of dementia research, awareness of local dementia research studies, willingness to participate in different types of research and barriers for participation. Currently, we have collected survey data from 15 older adults (n=46% White,15% Black, 30% Hispanic). Our preliminary results show that 50% of older adults were aware of available local dementia research studies. Only 17% reported prior participation while 80% reported willingness to participate in future research. Most respondents (81%) were interested in studies investigating cognitive function and healthy aging, whereas 36% were interested in research involving brain imaging, drug trials or lifestyle interventions and genetic studies. The main barrier to participation was limited access to our study centers (55%) followed by lack of time (27%). Interestingly, 82% indicated that free transportation or parking would incentivize their participation. These findings highlight the knowledge gaps and what barriers to address (i.e. enhance accessibility) to improve readiness to participate in future dementia research among older adults in the Bronx.

Abstract citation ID: igae098.3761

**UNDERSTANDING THE ROLE OF CULTURE IN SHAPING ATTITUDES AND BELIEFS ON URINARY INCONTINENCE: A SCOPING REVIEW.**

Simran Panesar, Saima Rajabali, and Adrian Wagg
*University of Alberta, Edmonton, Alberta, Canada*

Urinary incontinence (UI) is a common condition among older adults with diverse consequences for health and well-being. Shame, stigma, and cultural perspectives can prevent treatment-seeking behaviour. There is a scarcity of literature that explores the role that culture, beyond stigma, plays in shaping attitudes to, beliefs about, and management practices of UI. This review investigates and describes what is known about the role of culture in shaping the attitudes and beliefs on UI to identify gaps in the literature and direct future research. Following the Joanna Briggs Institute (JBI) method for scoping reviews, the MEDLINE, EMBASE, PsychINFO, CINAHL, EBSCOhost, and SCOPUS databases were searched, without any restriction on language and publication date. A full extraction was conducted of seventeen articles that met the inclusion criteria. Key themes revealed were the stress of managing UI and devout cleanliness for religious activities among Muslim women, a norm of secrecy/silence around UI for Latina women, the potent role of caste discrimination on pelvic health-seeking behaviours in parts of India, and the intersection of UI with cultural belief systems like Confucianism, which prioritize maintaining respectability, challenged by the taboo condition of UI. Although several studies emphasized the surface-level stigma of UI, there is a dearth of literature exploring culture and UI. Further research is needed to inform health promotion efforts regarding UI, which consider the severe emotional toll of UI stemming from the isolation it can create, segregating those that experience it from full participation in society.

Abstract citation ID: igae098.3762

**UNVEILING THE ASSOCIATION BETWEEN SOCIAL ISOLATION AND SLEEP DISTURBANCES AMONG LATINX OLDER WOMEN AND MEN**

Luciana Giorgio[1], Erick da Luz Scherf[2], and Nicole Ruggiano[2], *1. University of Alabama School of Social Work, Tuscaloosa, Alabama, United States*, *2. University of Alabama, Tuscaloosa, Alabama, United States*

**Background:** Sleep disturbances are associated with an increased risk of cardiovascular, cardiometabolic, and neurodegenerative conditions. Aging involves physiologic changes associated with increased sleep disturbances, however, sociocultural stressors, such as social isolation, can exacerbate these disturbances. Although Latinx older adults are more likely to experience social isolation and poorer sleep than their non-Latinx White counterparts, they remain underrepresented in sleep health research. We examined the association between social isolation and sleep disturbances and explored gender differences in this relationship.

**Methods:** We used data from Latinx older adults participating in the Hispanic Community Health Study/Study of Latinos Sociocultural Ancillary Study (n=323). We conducted weighted linear regression analyses adjusted for sociodemographics and selected health conditions to examine the relationship between social isolation and sleep disturbances. Sleep disturbances were measured using the Women's Health Initiative Insomnia Rating Scale(WHIIRS). Social isolation was assessed using validated loneliness and social support scales. Gender differences were examined using cross-products.

**Results:** Most participants were women (54.30%), immigrants (97.56%), Cuban (40.33%), and Spanish-speaking (86.00%). Participants reported mean loneliness of 1.44(SD=1.66) and social support of 26.40(SD=6.68). Average WHIIRS scores were 7.53(SD=5.68). Increasing loneliness was associated with a 0.48-point increase in WHIIRS (β=0.48, SE=0.24, p=0.04) in adjusted models. Gender was not a significant moderator in this relationship. Social support was not significantly associated with WHIIRS.

**Conclusions:** Loneliness was a robust predictor of sleep disturbances among Latinx older adults. Psychological and community-level interventions aimed at increasing social connection may improve sleep, thus reducing the burden of health conditions in this population.

Abstract citation ID: igae098.3763

**USER-CENTRED DIGITAL TRANSFORMATION: JAPANESE OLDER ADULTS' PERSPECTIVES ON DIGITIZATION**

Mineko Wada[1], and Kazuya Takeda[2], *1. Hiroshima University, Hiroshima, Hiroshima, Japan*, *2. Kaneda Hospital, Maniwa, Okayama, Japan*

This community-based study aimed to explore how Japanese community-dwelling older adults perceived the use of digital technologies in daily life. Although the importance of including older adults in aging and technology research and development has been increasingly emphasized in recent years and the Japanese government has been using the slogan "No One Left Behind" to promote user-centred digital transformation, older adults' perspectives on a digitized society have not been explored in depth to date. In this qualitative study, 18 Japanese older adults participated in either semi-structured individual interviews (10 participants) or focus groups (8), depending on their preferences. Both the interviews and focus groups elicited participants' experiences of adapting to digitization in their daily lives and their related perceptions of this shift (i.e., challenges, benefits, and concerns). All the interviews and focus group discussions were digitally recorded, transcribed verbatim, and analyzed thematically. Our analysis generated five themes that illustrate Japanese older adults' negative and positive perspectives on digital transformation: 1) disapproval of dehumanization, 2) concerns about being monitored and controlled through the digitizing of personal information, 3) increasing risks of scams and fraud, 4) lack of access to immediate support for issues relating to digital adaptation, and 5) potential for expanding social connection and entertainment. We discuss strategies for addressing negative perspectives among older adults that are related to low digital literacy, including, for example, increasing and improving the accessibility of immediate support to help with challenges relating to digitization.

Abstract citation ID: igae098.3764

### USING COMPANION CARE TO ADDRESS THE DUAL BURDEN OF LONELINESS AND CHRONIC ILLNESS

Kelsey McNamara[1], Jill Bongiorni Meadows[2], and Brian Smith[2], *1. Papa Inc., Brooklyn, New York, United States*, *2. Papa Inc., Miami, Florida, United States*

Chronic illness is a risk factor for loneliness. Concurrently, loneliness can increase the risk of and exacerbate chronic conditions, especially among older adults. Special Supplemental Benefits for the Chronically Ill (SSBCI) are benefits that can be offered to qualifying Medicare Advantage members to improve health and function. This study's objective was to evaluate the impact of a companion care program, designed to address loneliness and lack of social support, on SSBCI recipients' quality of life. Members received companion care through their health plan and were paired with "Papa Pals" for companionship and assistance with instrumental activities of daily living. Members were assessed at enrollment and ≥8 weeks later for loneliness (UCLA Three-Item Loneliness Scale, Range: 3-9, =3 "not lonely", 4-6 "lonely", 7-9 "severely lonely") and health-related quality of life (CDC's Healthy Days Measure, Range: 0-30 days). Change in outcomes were analyzed using the one-sample t-test. Analysis included 531 SSBCI recipients who were lonely at baseline, used companion care any time between 2021-2023, and completed a reassessment. Average age was 74, 63% were female. Average baseline values: 5.7 loneliness score, 9.6 mentally unhealthy days, 14.1 physically unhealthy days. On average, outcomes improved: -0.99 loneliness score, -1.4 mentally unhealthy days, -1.6 physically unhealthy days. In regards to loneliness score, 66% of members improved, 13% maintained, 22% regressed. Our results indicate companion care can significantly improve loneliness among a population experiencing a high burden of physical and emotional stress; research should continue to evaluate scalable interventions to address loneliness among this population.

Abstract citation ID: igae098.3765

### VALIDATION OF DEMENTIA CARE-RELATED SCALES AMONG INFORMAL CAREGIVERS OF LATINOS WITH DEMENTIA OR MCI

Jaime Perales-Puchalt[1], Monica Fracachan Cabrera[2], Christina Baker[2], Mariana Ramirez Mantilla[2], and Jeffrey Burns[1], *1. University of Kansas Medical Center, Fairway, Kansas, United States*, *2. University of Kansas Medical Center, Kansas City, Kansas, United States*

We aimed to test the psychometric properties of several dementia care-related scales among Latinos. We leveraged secondary baseline data from a one-arm mHealth trial on dementia caregiver support. We included 100 responses for caregiver-focused scales and 88 responses for care recipient-focused scales. Scales included the Neuropsychiatric Inventory Questionnaire Severity and Distress scales, six-item Zarit Burden Inventory, Ten-item Center for Epidemiologic Studies Depression Scale, Geriatric Depression Inventory, Quality of Life in Alzheimer's Disease, and Single-item Satisfaction With Life Scale. We calculated concurrent validity using Pearson and Spearman correlations and expected correlations amongst all variables in line with the Stress Process Framework. We calculated internal consistency reliability using Cronbach's alpha. All concurrent validity correlations followed the expected directionality, with 19/21 inter-scale correlations in the total sample reaching statistical significance ($p< 0.05$), and 17/21 reaching at least a low correlation (0.3). Cronbach's alpha ranged from 0.832 and 0.879 in all scales in the total sample. Findings suggest that these English and Spanish caregiver-administered scales have good psychometric properties.

Abstract citation ID: igae098.3766

### VIEWS ON AGING AND PERCEIVED DISCRIMINATION: THE MODERATING ROLE OF GENDER TYPICALITY

Kelly Smith, and JoNell Strough, *West Virginia University, Morgantown, West Virginia, United States*

Age and gender are outwardly visible personal characteristics upon which people make social judgments (Cuddy & Fiske, 2002). As such, these different characteristics may be associated with perceived discrimination. Here, we examined whether perceived discrimination varied depending on individuals' views of their own aging (VoA) and their gender typicality when each was measured as a multidimensional construct. VoA was comprised of aging-related cognitions, self-perceptions of aging, and subjective age. Gender typicality was comprised of gender roles and expectations, gender self-concept, and gender social presentation. Participants ($N = 616$; 40-93 yrs., $M = 53.43$ yrs.; 56% women, 85% White) completed self-report measures collected online via Prolific. Interactions between VoA and gender typicality were assessed using an SEM framework in Mplus, and there were two significant interactions among components of VoA and gender typicality. First, perceptions of discrimination were greater for participants who reported less positive aging-related cognitions and highly-gender-typical gender roles and expectations. Second, perceptions of discrimination were greater for those with less positive self-perceptions of aging, and this was amplified for those with lower gender typicality (roles and expectations). Together, these findings suggest that high and low gender typicality in the form of conformity to gender roles and expectations are differentially associated with perceptions of discrimination depending on VoA. The importance of considering multiple personal characteristics to better understand perceptions of discrimination is discussed along with recommendations for future research that considers the intersection of VoA and other individual difference characteristics.

Abstract citation ID: igae098.3767

### WEALTH, DEBT, AND COGNITIVE AND SELF-CARE DIFFICULTY AMONG AMERICANS AGED 65+

Connor Sheehan[1], Jiwon Jang[2], Joseph Saenz[3], and Dylan Connor[4], *1. Arizona State University, Scottsdale, Arizona, United States*, *2. Arizona State University, Tempe, Arizona, United States*, *3. Arizona State University, Phoenix, Arizona, United States*, *4. Arizona State University, Arizona State University, Arizona, United States*

As the American population ages, the number of individuals with cognitive and self-care challenges is projected to increase substantially, stressing the importance of understanding their social determinants. Previous research has connected socioeconomic status, specifically educational attainment and income, to cognitive and self-care problems. However, considerably less research has examined the role of wealth in older adulthood. This is an oversight, as wealth reflects cumulative life course exposure and accumulation of socioeconomic resources, and debt may be a unique stressor. We use novel geographic machine learning-based estimates of wealth for every American household to predict cognitive and self-care difficulties among Americans aged 65+. We linked GEOWEALTH data, a machine learning estimate of household wealth, to the 2019-2021 American Community Survey (n=742,348) to create one of the largest data sources of wealth and health heretofore analyzed. Regression models predict cognitive and self-care difficulties adjusting for demographic and socioeconomic characteristics, including income and education. Cognitive and self-care difficulties decreased with increasing wealth, while being in debt nearly doubled the probability of these difficulties at every age. This pattern is amplified among White Americans and those in areas with concentrated debt. Wealth consistently shows stronger associations with cognitive and self-care difficulties than educational attainment or income. Our findings emphasize the importance of wealth and debt for well-being in older adulthood. Future research should explore the directionality of this association and the impact of different sources of wealth and debt.

Abstract citation ID: igae098.3768

### WHAT MAKES YOU FEEL AFRAID OF DYING: A SYSTEMATIC REVIEW OF DEATH ANXIETY AMONG KOREAN OLDER ADULTS

Haeun Kim, and Yeonjung Lee, *Chung-Ang University, Seoul, Republic of Korea*

Death anxiety (DA) refers to negative emotional responses such as anxiety, fear, and denial toward death that result from the inevitability of death. Studies have suggested that older adults experience higher levels of DA than other age groups in Korea, and DA is related negatively with suicidality. Given that the suicide rate among Korean older adults is extremely high as about 39.9 suicides per 100,000 people aged 65 and older as of 2022, DA and related studies need to be appraised and synthesized. This study aims to examine recent research and provide a theoretical basis for various intervention programs by conducting a systematic literature review on factors influencing DA among Korean older people. The three key electronic databases in Korea (e-Article, KCI, and RISS) were extensively searched using the selected keywords. Among the peer-reviewed articles written in Korean since 2015, a total of 21 eligible articles were included for analysis after excluding qualitative studies, dissertations, overlapping studies, and gray literature. Findings show that the factors influencing DA can be classified as followings: sociodemographic, psychological, and medical factors, intervention programs, attitudes toward death, and preparation for death. Among the psychological factors, depression was found to relate significantly and positively with DA. Regarding the DA interventions, some programs implemented at the community such as positive psychology improvement program, dance sports, music and physical activities have a significant effect on reducing DA. Findings provide a theoretical basis for developing the intervention programs and practical strategies and thus, alleviating DA among older adults.

Abstract citation ID: igae098.3769

### WHEN I'M 64 – LONGITUDINAL OBSERVATIONS FROM AN INTERDISCIPLINARY COURSE ON AGING FOR FIRST-YEAR STUDENTS

Karuna Thomas, Matthew Picchiello, Nancy Morrow-Howell, Susan Stark, and Brian Carpenter, *Washington University in St. Louis, St. Louis, Missouri, United States*

Research has highlighted the value of education for college students on the aging process. Curricula have the potential to address misconceptions about aging and draw students toward further coursework and experiences with older adults. This study explored the impact of participation in an interdisciplinary course on aging across one year for the Fall 2023 cohort (N = 50) with a control group that did not participate in the course (N = 33), and after five years for the Fall 2017 cohort (N = 59) and control group (N = 57). The course was offered to first-year undergraduate students. Weekly classes included older adults from the community. Surveys assessing attitudes towards aging and participation in aging-related activities were administered prior to and at the end of the academic year, and an annual follow-up. Group-by-time analyses showed that participants from the class had significantly more positive attitudes towards aging [$F(1, 82) = 13.67$, $p < .05$, $\eta p2 = .03$], and significantly fewer ageist beliefs [$F(1, 82) = 9.21$, $p < .05$, $\eta p2 = .02$] at the end of the academic year compared to the control group. In the 5-year follow-up, students in the course also reported more engagement in aging-related fields compared to the control group [$t(114) = 4.65$, $p < .05$]. The course content was reported to be relevant to 45.8% of former students in their professional life, and 57.6% in their personal lives. These results substantiate the short and long-term positive impact of engaging undergraduate students in curriculum on aging.

Abstract citation ID: igae098.3770

### YOU MADE IT TO A BIG NUMBER": PERCEPTIONS OF OLDER ADULTHOOD AMONG ADULTS WITH SICKLE CELL DISEASE

Elizabeth Linton[1], Joseph Gallo[1], Jennifer Schrack[1], Lydia Pecker[2], and Sophie Lanzkron[3], *1. Johns Hopkins*

*Bloomberg School of Public Health, Baltimore, Maryland, United States*, *2. Johns Hopkins School of Medicine, Baltimore, Maryland, United States*, *3. Sidney Kimmel Medical College, Philadelphia, Pennsylvania, United States*

This study categorizes how adults with sickle cell disease (SCD) define older adulthood and identifies topics important for future gerontology research. Individuals aged 18 and older were recruited from a pilot mixed-methods study in an outpatient SCD clinic, selected for representation across age, sex, genotype, and frailty (physically robust, prefrail, frail) according to the 5-item FRAIL scale. Interviews were analyzed using Lieblich et al's interpretive categorical-content approach. Five thematic categories defining the onset of older adulthood emerged: (1) SCD's cumulative impact, (2) Observed lifespan of others with SCD, (3) Social determinants of health and lifestyle, (4) Statistical life expectancy, and (5) Social roles and transitions. Proposed research to promote healthier aging were (1) studying SCD sequelae and age-related comorbidities, (2) developing new SCD treatments and cure, and (3) identifying non-opioid pain management. Population aging for people living with SCD presents novel opportunities to promote dignity across the lifespan. This study's findings may inform the content of health communications to facilitate goal-concordant SCD care.

### SESSION 8010 (POSTER)

### LATE BREAKING POSTER SESSION II

Abstract citation ID: igae098.3771

### 1. ENGAGEMENT: A PRIORITY NEED IN CARE TRANSITIONS EXPERIENCED BY OLDER ADULTS AND CAREGIVERS

Claire Regan[1], Karen Hirschman[1], Rachel Harding[1], Molly McHugh[1], Onome Osokpo[2], Kathleen McCauley[1], Elizabeth Shaid[3], and Mary Naylor[1], *1. University of Pennsylvania, Philadelphia, Pennsylvania, United States*, *2. University of Illinois Chicago, Chicago, Illinois, United States*, *3. Penn Nursing, Merion Station, Pennsylvania, United States*

Care transitions are a vulnerable time for older adults, particularly transitions from hospital to home. The Transitional Care Model (TCM) is designed to support older adults and their informal caregivers during this period, consistently demonstrating improvements in health outcomes while lowering care costs. This multi-component, advanced practice registered nurse (APRN)-led, team-based care intervention was recently implemented in a large-scale RCT in three U.S. healthcare systems (9/2020-3/2023). Content analysis of APRN intervention documentation for 480 patients revealed key barriers to delivering the TCM as designed. The lack of patient and/or informal caregiver engagement theme was the most common documented challenge (231/480, 48.1%), higher than issues of complexity of care (166/480, 34.6%) and social determinants of health (SDOH, 138/480, 22.8%). The lack of engagement theme was most often identified as a patient issue (197/231, 85.3%) or patient and caregiver issue (23/231, 10.0%). Subcategories included limited participation in care, patient refusal of services, and lack of adherence to plan of care (e.g. lifestyle changes, medication management and motivation). Lack of engagement was complicated by the complexity of care and/or SDOH (101/231, 43.7%). These findings demonstrate the challenges of patient engagement in the presence of complexity of care and SDOH within transitional care and how difficult engagement of patients and caregivers can be to achieve. Future research is needed to understand the factors contributing to and mediating lack of engagement as well as this challenge's impact on health outcomes. Addressing this knowledge gap will inform the design of more effective transitional care interventions.

Abstract citation ID: igae098.3772

### 48 HOUR HOSPICE HOME IMMERSION RESEARCH KEY MEDICAL STUDENT LEARNING OUTCOMES: UNE COM 2019-20 STUDENT COHORT

Amanda Okpoebo, Jennifer Berkowitz, and Marilyn Gugliucci, *University of New England College of Osteopathic Medicine, Biddeford, Maine, United States*

Traditional palliative and end-of-life care education in US medical schools is often inadequate in preparing students to care for patients. To address this, the University of New England College of Osteopathic Medicine (UNE COM) introduced the Learning by Living 48-Hour Hospice Home Immersion (HHI) Project. This innovative program immerses students into an 18-bed acute care (ICU) hospice home for 48 hours, where they provide care to patients, support families, and engage in postmortem care while working with an interprofessional staff team and independently. Utilizing qualitative ethnographic/autobiographic research designs, two key research questions included: (1) What significant lessons were learned from the 48-hour home hospice immersion?; and (2) What skills will they apply in their future practice? Students documented their experiences in journals. Eleven second-year medical student journals from the 2019-20 cohort were selected for analysis; 300+ pages of data. During each of three stages: pre-fieldwork, fieldwork, and post-fieldwork, qualitative analysis included content review, creating a codebook, and conducting a final analysis with NVIVO 14 software. Three key themes emerged for question one: (a) Holistic comfort care/medicine, (b) Subjectivity of suffering, and (c) Religion/spirituality. For question two, students reported: (a) Increased confidence in caring for dying patients and their families, (b) Improved ability to discuss death, and (c) Enhanced understanding of the physician's role in curing versus caring. Each student's experience at Gosnell Hospice Home was unique, yet all gained critical insights that will shape their development into compassionate and skilled physicians in palliative and end-of-life care.

Abstract citation ID: igae098.3773

### A CHALLENGE HONORING SELF-DETERMINATION OF NURSING HOME RESIDENTS WITH DEMENTIA; WHICH SELF -- THEN SELF OR NOW SELF?

Mercedes Bern-Klug[1], and Meredith Levine[2], *1. University of Iowa, Iowa City, Iowa, United States*, *2. The Harry and Jeanette Weinberg Center for Elder Justice at The Hebrew Home at Riverdale, Bronx, New York, United States*

Medicare and/or Medicaid certified nursing homes (NHs) are required to inform residents they have a right to an advance directive. The government's Resident Rights requires staff to honor resident self-determination. What happens when instructions in an advance directive are in opposition to the current expressed requests of a person with advanced dementia? Using qualitative descriptive methodology, in this pilot study, 12 NH staff members in various roles engaged in individual interviews and responded to questions that speculated they had a resident with dementia who, when they had decisional capacity, indicated in a written advance directive they did not want to be fed, once they lost the ability to feed themselves. Staff were unanimous in declaring the need to honor the resident's self-determination, and yet could foresee instances in which a person with said advance directive, and unable to self-feed, would indicate through words or gestures, the desire to eat. Many staff indicated they would go ahead and feed the person; others worried that would dishonor the advance directive. Staff also expressed other challenges including how "self-feed" is operationalized, the fluctuating nature of functional capacity in dementia, and the potential conflict that could result with families and NH surveyors. Some staff indicated it would be against their religion or sense of morality to not feed a person who expressed an interest in eating. We conclude that although such advance directive templates are readily available online, the potential for logistical and moral challenges in the NH context are real and serious.

Abstract citation ID: igae098.3774

### A CLIENT-CENTERED HOME TRAINING APP OF MUSIC-BASED THERAPY FOR UPPER EXTREMITY FUNCTIONS IN OLDER ADULTS WITH STROKE

Yi-An Chen[1], Sara Lynn Johnstone[1], Annie Solomon[1], Martin Norgaard[1], Gil Weinberg[2], Dhruv Pargai[2], Evan Szypulski[1], and Jon Sanford[1], *1. Georgia State University, Atlanta, Georgia, United States*, *2. Georgia Institute of Technology, Atlanta, Georgia, United States*

Music-based therapy (MBT) has been shown to be effective in improving upper extremity (UE) functions for older adults with stroke in clinical settings. To expand the MBT benefits to home, we designed a client-centered, stroke-specific MBT app and examined the app feasibility with stroke survivors in this study. During the 2-week pilot home testing, the participants (n=5, mild to moderate UE motor impairments) actively used the app for rehab exercises around 71% of the time, which equals 26.6 ± 16.7 min per day, 5 days per week, over 2 weeks. Motor outcomes portrayed a positive trend from pre- to post-intervention. Participants' average Fugl-Meyer Assessment scores increased from 53 to 57, and Nine-Hole Pegboard Test time decreased from 38 seconds to 32 seconds. Additionally, participants provided generally positive feedback, commenting on the app user-friendliness (e.g., easy to navigate, clear exercise instructions, convenience) and motivational music feature (e.g., engaging and a new way to experience therapy). They also enjoyed the flexibility of training scheduling – participants could do rehab exercises at any time they wanted. Based on the positive findings, we anticipate that the app will serve as a feasible tool for older adults with stroke to engage in a personalized program to improve UE functions at home. This will minimize the burden of travel and wait times to healthcare clinics and increase rehab access for older adults. We are currently continuing the data collection and app refinement to optimize the app to support stroke recovery in older adults.

Abstract citation ID: igae098.3775

### A FRAMEWORK FOR UNDERSTANDING PALLIATIVE CARE PREFERENCES AMONG AFRICAN AMERICAN PATIENTS WITH CANCER: THE 6S MODEL

Elisha Oduro[1], and Joan Carpenter[2], *1. University of Maryland, Baltimore, Baltimore city, Maryland, United States*, *2. University of Maryland, Baltimore, Baltimore, Maryland, United States*

African American (AA) patients with cancer have the highest mortality rates and poorest outcomes among all racial groups in the US, yet they are almost one-third less likely to receive palliative care (PC) than their White counterparts. AAs are less likely to receive PC due to systemic racism, discrimination, provider bias, and culturally insensitive healthcare services. Additionally, research has primarily focused on highlighting the disparities in PC access among AA patients with cancer rather than understanding the experiences and preferences of those who do receive PC services. Grounded in 6S-Model for Person-Centered Palliative Care, this presentation aims to illustrate a theoretically derived approach for clinicians and researchers to develop culturally sensitive PC for AA patients with cancer. The model comprises six concepts elucidating different aspects of patient care to ensure sensitive care and overcome disparities: self-image, symptom relief, social relations, self-determination, synthesis, and strategies. Self-image is the model's central concept, reflecting the patient's personal experiences and identity. To preserve self-image, it is crucial to alleviate the patient's symptoms and physical suffering while maintaining their social connections. Self-determination ensures patient involvement in their care, and synthesis and strategies address their care preferences, spiritual and existential needs. Utilizing the 6S Model for Person-Centered Palliative Care in studies and clinical care, will allow AA patients with cancer to share their PC experiences and collaborate in creating culturally sensitive PC, thereby integrating AA patients with cancer preferences and needs into PC, improving quality of life, care outcomes, and promoting equity in PC services.

Abstract citation ID: igae098.3776

### A LONGITUDINAL ANALYSIS OF DOMAIN-SPECIFIC CHANGES IN PROMIS® SELF-EFFICACY AND PREDICTORS IN PARKINSON'S DISEASE

Haesung Kim[1], Lisa Shulman[2], Laurence Magder[1], John Schumacher[3], George Unick[1], Sunita Shakya[1], and Ann Gruber-Baldini[2], *1. University of Maryland Baltimore, Baltimore, Maryland, United States*, *2. University of Maryland School of Medicine, Baltimore, Maryland, United States*, *3. University of Maryland Baltimore County, Baltimore, Maryland, United States*

**Background:** There is limited research on the magnitude and predictors of longitudinal changes in Self-Efficacy (SE) among Parkinson's disease (PD) patients. OBJECTIVE: To describe longitudinal changes in Patient-Related Outcomes Measurement Information System -SE (PROMIS-SE®): managing daily activities (SEM-DA), emotions (SEM-E), medications and treatments (SEM-MT), social interactions

(SEM-SO), and symptoms (SEM-S) and examine predictors of changes in PD.

**Methods:** A longitudinal analysis was conducted on 239 PD patients using two-time points (3years±180days). Baseline predictors included age, sex, race, education, PD duration, PD severity, medical comorbidities, and cognitive ability. Student T-tests and Pearson's correlation coefficient examined the relationship between PROMIS-SE domains and predictors of changes.

**Results:** SE decreased by more than 5 T-score units (0.5SD) across all domains [Mean±SD: SEM-DA (-6.13±8.66), SEM-E (-5.32±10.49), SEM-MT (-7.2±10.11), SEM-SO (-5.69±9.96), and SEM-S (-5.18±10.25)]. Significant larger mean declines ($p<.05$) were associated among older age (age< 65 vs. 65+) in SEM-E (-3.84 vs. -6.53) and SEM-MT (-5.63 vs. -8.47), higher cognition (MoCA< 26 vs.26+) in SEM-E (-1.54 vs. -7.30), SEM-SO (-3.22 vs. -7.03), and SEM-S (-3.17 vs. -6.58), and higher education (no college vs. college) in SEM-SO (-2.10 vs. -6.00). Change in UPDRS total score correlated with change in SEM-S ($r=-0.26$, $p<.05$).

**Conclusions:** Over three years, PD patients experienced significant SE changes across all domains, with the greatest decline in SE for managing medical treatments. Greater declines in SE were associated with older age, higher cognition, and higher education. This may indicate a higher sensitivity of the SE measure to change among those with higher cognition and education.

Abstract citation ID: igae098.3777

### A QUALITATIVE STUDY EXPLORING TECHNOLOGICAL SUPPORT IN THE DISTANCE CAREGIVING CONTEXT

Andrea Budnick, and Farina Bünning, *Charité - Universitätsmedizin Berlin, Berlin, Berlin, Germany*

As demographic change progresses, fewer and fewer relatives are available to provide care locally. Consequently, distance caregiving has increasingly attracted the attention of the gerontological research community. Technological support plays a role in enabling the distance caregiving arrangement. However, there is a paucity of research on this type of support in the distance caregiving context. Hence, this qualitative study explores actors' experiences, perceptions, and attitudes in performing their roles within the distance caregiving arrangement by utilizing technological support. One-on-one guided interviews were conducted face-to-face with distance caregivers (N=20; mean age=51.8 years), care recipients (N=20; mean age=82.4 years), and representatives of the supportive local network (N=18; mean age=55.4 years). Interviews were transcribed verbatim. Data were analyzed using phenomenological analysis. Our findings show that distance caregivers successfully implement various forms of communication technology to keep the care arrangement running by using devices like landline phone, smartphone, and/or e-mail. In everyday life, aids such as dementia clocks, smart home applications for automated door and/or window control, or smart speakers are used. Around-the-clock video surveillance of care recipients is used in only two distance caregiving arrangements. Potentials and risks of technological support are viewed by all actors and include maintaining independence or social participation, but concerns are also expressed regarding the triggering of false alarms, possible misuse, or data protection. Fear of technology or lack of tech literacy prevent actors from using technological support. Triangulated findings imply that improved tech literacy could create more reliability and simplify distance caregiving for all actors involved.

Abstract citation ID: igae098.3778

### A SCOPING REVIEW OF OBESITY INTERVENTIONS AND EPIDEMIOLOGICAL STUDIES AMONG OLDER RURAL ADULTS

Maki Karakida, and Jaqueline Avila, *University of Massachusetts Boston, Boston, Massachusetts, United States*

Rural communities are known for their limited access to care, transportation, and healthy foods, which can increase obesity risks. Obesity among older adults is associated with mobility impairment, falls, diabetes, hypertension, and can further deteriorate the health of older rural residents. This scoping review assessed studies targeting obesity/overweight among rural adults aged 50+ to: 1) analyze the prevalence of obesity and the highest risk groups in epidemiological studies, and 2) assess the efficacy of clinical/community interventions to reduce obesity/overweight in this population. CINAHL Ultimate, PubMed, Google Scholar, and MEDLINE databases were searched from January 1, 2000 to June 27, 2024. Fifteen articles met the eligibility criteria. Selected studies were then divided into: community interventions (n= 3), remote interventions (n= 3), hybrid intervention (n= 1), and cross-sectional epidemiological studies in the US (n= 3) and abroad (n= 5). Epidemiological studies consistently showed that the prevalence of obesity/overweight was higher among older women than men. While one US study found significant associations between lower educational attainment and higher obesity rates, one non-US study reported no associations between obesity and educational levels. Community interventions included nutrition and exercise sessions. One randomized-controlled intervention significantly reduced participant's weight compared to those who did not receive face-to-face counselling. Remote/hybrid interventions, including telehealth and video conferencing, were cost- and time-effective, but only three of them successfully lowered participants' weight and/or improved physical functions. Further interventions need to focus on the prevention and control of obesity among high-risk groups, such as women and people with lower educational attainment.

Abstract citation ID: igae098.3779

### A SYSTEMATIC REVIEW OF RESILIENCE IN FAMILY CAREGIVERS OF AUTISTIC ADULTS: IMPLICATIONS FOR RESEARCH AND PRACTICE

Daphne Chakurian, and Lori Popejoy, *University of Missouri - Columbia, Columbia, Missouri, United States*

Family caregivers (FCGs) of autistic adults provide lifelong care and experience chronic stress, stigma, and social isolation, placing them at risk of poor health, quality of life, and well-being. Resilience, stress, and coping research have primarily been limited to FCGs of autistic children with inconsistent use of theory, definition, and measurement. This systematic review aimed to examine resilience in FCGs of autistic adults. Guided by the Transactional Model of Stress and Coping, this review followed PRISMA guidelines, and the protocol was registered with PROSPERO. Comprehensive searches of 10 databases identified 1,935 records. Risk of bias was appraised using the Johns Hopkins Evidence-Based Practice Model and Guide. Nineteen studies meeting inclusion criteria were included in the narrative analysis and were mapped to the conceptual model. Designs were cross-sectional and phenomenological. We found that higher FCG quality of life and well-being was associated with increased problem and meaning-focused coping, social support, future planning, and less perceived burden and stigma. A variety of FCG and autistic adult person and environment factors provided context for the stress and coping process. Social support and coping strategies were key to FCG resilience outcomes and may inform clinical guidelines and policy development. Research is warranted to examine social support, coping strategy, future planning, and perceived burden and stigma as modifiable factors to enhance FCG resilience. The use of well-defined theoretical constructs and measures could facilitate future systematic reviews, as well as contribute to clinical guidelines and policy development.

Abstract citation ID: igae098.3780

### "A NEVER-ENDING CYCLE": ANXIETY AND SLEEP AS PSYCHOSOCIAL CORRELATES OF FREEZING OF GAIT

Sarah Ghose[1], Natalie Dautovich[2], Claire Williams[2], Samantha Luehrsen[2], John Karlsen[2], GinaMari Blackwell[2], Leslie Cloud[2], and Ingrid Pretzer-Aboff[2], *1. Cleveland Clinic, Cleveland, Ohio, United States*, *2. Virginia Commonwealth University, Richmond, Virginia, United States*

More than 50% of people diagnosed with Parkinson's disease (PD) suffer from Freezing of gait (FoG). This debilitating symptom is characterized by an intermittent inability to initiate voluntary forward movement. Much research has focused on biological mechanisms underlying FoG, but less on psychosocial correlates. This study used a mixed-method design to elucidate associations between sleep, anxiety, and FoG. Participants were engaged in a more extensive NIH-funded parent study protocol. Thirteen people with PD (76.9% male; M age = 69 years old, SD = 6.73 years) participated. Sleep measures using actigraphy were collected one week before the parent study started. Measures of anxiety and FoG were collected pre- and post-walking through a FoG-triggering protocol. On average, participants self-reported higher State Anxiety scores at baseline and lower anxiety scores post-task. Contrarily, salivary alpha-amylase levels measuring anxiety increased steadily from baseline to post-task. Additionally, on average, participants' self-reported (sleep diaries) better sleep across indices when compared with actigraphic measurement. Qualitatively, participants reported noticing strong associations between anxiety, sleep, and worsened FoG outcomes. Higher levels of anxiety were associated with worsened FoG. Poor sleep was associated with worsened next-day functioning and FoG. A few participants reported noticing no association between their sleep and FoG. Findings highlight the importance of anxiety and sleep for FoG outcomes, supporting prior research suggesting that higher anxiety and worse sleep may exacerbate FoG. These results suggest a need and utility for (1) further investigation and (2) the assessment and treatment of anxiety and sleep concerns beginning at PD diagnosis.

Abstract citation ID: igae098.3781

### ACCURACY OF MINIMUM DATA SET FALL ASSESSMENTS IN LONG-TERM CARE RESIDENTS

Laura Graham[1], Yongmei Li[2], Chintan Dave[3], and Michelle Odden[4], *1. VA HERC, VA Palo Alto Health Care System, VA Palo Alto Health Care System, California, United States*, *2. Stanford, Palo Alto, California, United States*, *3. Rutgers University, New Brunswick, New Jersey, United States*, *4. Stanford University, Palo Alto, California, United States*

The Minimum Data Set (MDS) is commonly used to ascertain falls among long-term care residents for research and quality improvement. However, studies have shown that MDS may underreport falls compared to chart abstraction. Natural Language Processing (NLP) algorithms to detect falls are limited but have demonstrated effectiveness in identifying falls in medical records. Thus, we aimed to develop and validate a rule-based NLP algorithm to identify falls in Veterans Health Administration (VA) long-term care residents and compare it to MDS. To do so, we identified a national cohort of patients receiving care at 114 VA long-term care facilities (10/1/2006-9/30/2019). Using text mining in VA clinic notes, we developed a rule-based NLP algorithm to identify falls. The algorithm was validated using manual chart abstraction on 200 patients (positive predictive value [PPV] 0.99). We then compared the results of the NLP algorithm to the MDS assessment, which identifies a fall within the past 30 days at the time of the assessment, using sensitivity, specificity, and PPV. In our cohort of 45,183 patients, we identified 154,165 falls (7.8 per 1,000 days) among 22,331 residents using NLP compared to 16,356 falls (0.8 per 1,000 days) among 8,859 residents using MDS. Compared to NLP, the MDS had a sensitivity of 0.30, a specificity of 0.91, and a PPV of 0.77. Our findings suggest that MDS may dramatically undercount falls among long-term care residents, potentially leading to misclassification in research studies. Future studies should focus on alternative methods of fall detection in retrospective cohorts.

Abstract citation ID: igae098.3782

### ADVANCES IN GERONTOLOGY CURRICULUM DESIGN: CENTRING LIVED EXPERIENCES OF LGBTQ+ OLDER ADULTS

Kimberley Wilson[1], Noah Pevie[2], and Emma Hofstra[1], *1. University of Guelph, Guelph, Ontario, Canada*, *2. Memorial University, St. John's, Newfoundland and Labrador, Canada*

While research on LGBTQ+ aging in Canada has increased, post-secondary educators have identified gaps in pedagogical aids to facilitate their teaching in this area. Older LGBTQ+ individuals have unique social/historical contexts compared to their majority peers, often involving minority stress experiences (e.g., stigma, discrimination, physical violence) that contribute to inequalities during late life. To create a future workforce that understands and appreciates the unique needs and social/historical contexts of aging LGBTQ+ individuals, our team prioritized developing curriculum resources that center the voices of LGBTQ+ older adults. We interviewed 26 LGBTQ+ older adults (M = 65.9, Range = 51-89). Participants shared stories about their own life courses and how they reflected the landscape of current curricula. They offered strategies for enhancing curriculum to ensure that post-secondary education include diverse LGBTQ+ narratives. Through conventional content analysis we identified topics that LGBTQ+ older adults named as requirements for students to understand the experiences of LGBTQ+ aging within hetero- and cis-normative health/social contexts. Through our analysis we articulated the importance of co-construction of educational materials with LGBTQ+ communities. Participants also identified the development of tools to be used within classrooms to challenge instructors to unpack their own assumptions about LGBTQ+ aging. They shared the importance of highlighting unique experiences of aging within these communities including the histories of queer/trans discrimination and the experiences of survivors of the HIV/AIDs crisis. Findings from this project will inform curriculum design and resources that move beyond normativity and include narratives of the lived/living experiences of LGBTQ+ older adults.

Abstract citation ID: igae098.3783

### AGE-FRIENDLY HEALTHCARE: AN EVOLUTIONARY CONCEPT ANALYSIS

Anna Zisberg, Paule Sarah Fraiman, Nosaiba Rayan-Gharra, Alexandra Danial-Saad, Dikla Segel-Karpas, Sharon Ost-Mor, and Shoshi Keisari, *University of Haifa, Haifa, Hefa, Israel*

Current global aging trends emphasize the need to adapt healthcare systems to address older adults' specific needs. Numerous age-friendly healthcare initiatives and approaches have emerged, but the field lacks a unified framework. The current study used Rodger's evolutionary concept analysis model to explore 'age-friendly healthcare', examining the concept's characteristics, components and structure. An initial literature search in multiple databases to identify articles relevant to age-friendly healthcare retrieved 1,407 articles. After screening for duplicates and relevance, 140 articles were examined for eligibility based on inclusion criteria related to age-friendly care, language, and full-text availability. Following full-text screening, 65 articles were included for data extraction by multiple researchers to synthesize theoretical, methodological and design elements. We identified similar terms, related cases, antecedents and consequences of age-friendly healthcare, as well as distinguishing attributes: Respect for older adults' autonomy and needs; Leadership and organizational knowledge and support; Proactive policies and processes of care; Holistic care environments; and Communication and follow-up with awareness of challenges and barriers as well as prioritization of continuity-of-care. An overarching definition was crafted based on these findings. The concept of age-friendly healthcare is still in development, with much research focused on development and implementation but not yet on real-world patient and health-system outcomes. However, our proposed definition and analysis of the concept may help unify the field, clarify future research directions, and identify areas requiring further study. It may also enable development of improved practices and policies for the implementation of age-friendly healthcare in a variety of settings.

Abstract citation ID: igae098.3784

### AGE-SPECIFIC DETERMINANTS OF PHYSICAL ACTIVITY ENGAGEMENT IN BREAST CANCER SURVIVORS: A MULTIGROUP ANALYSIS

Mi Sook Jung, Xirong Cui, Kyeongin Cha, Ahrim Lee, Eunyoung Park, and Hyun-E Yeom, *Chungnam National University, Daejeon, Republic of Korea*

Physical activity is highly beneficial for breast cancer survivors. However, many survivors often have insufficient levels of physical activity due to fatigue, psycho-social barriers, limited knowledge, and low self-efficacy. These barriers vary in type and severity due to age-related factors, potentially leading to differences in physical activity levels and health-related quality of life (HRQOL). This study drew upon the Individual and Family Self-Management Theory to explore the interrelationships between contextual and process factors and proximal outcomes (physical activity, HRQOL). Community-dwelling 450 breast cancer survivors were recruited to assess cancer stigma, health literacy, social support, exercise self-efficacy, behavioral regulation, physical activity engagement, and HRQOL. Structural equation modeling and multigroup analysis with the moderating effect of age were performed. Greater stigmatization of cancer, lower social support, and worse behavioral regulation were directly associated with lower physical activity engagement while health literacy, behavioral regulation, and physical activity engagement had direct effects on HRQOL. Behavioral regulation mediated the association between contextual factors (stigma, health literacy) and physical activity. After confirming full measurement invariance between the groups, significant differences in factor loadings were found in specific pathways from contextual (stigma) and process factors (behavioral regulation, social support) to physical activity, highlighting age-related differences in how these factors interact. The findings underscore how complex patterns of contextual and process correlates influence physical activity in older and middle-aged women with breast cancer, respectively. The age-specific uniqueness should be considered when developing targeted strategies to promote active living and achieve optimal health in later life.

Abstract citation ID: igae098.3785

### AGING IN AMERICA: CARE AND COMMUNICATION ACROSS THE FIELD

Brianna Margulis[1], Alexandra Garcia[2], Richelle Matarazzo[3], Lynn Tepper[4], Carol Kunzel[1], and Stacey Whalen[4], *1. Columbia University Medical Center, New York, New York, United States*, *2. Columbia University, New York, New York, United States*, *3. Columbia School of Social Work, New York, New York, United States*, *4. Columbia University College of Dental Medicine, New York, New York, United States*

Northern Manhattan has the largest population of individuals over 65. Students within the graduate programs of Columbia University electively participated in the workshop "Aging in America: Care and Communication Across the Field." The workshop aimed to provide future professionals with communication techniques and best practices when caring for the aging population. Before and after participating in the workshop, participants completed a survey asking them to self-assess their readiness to communicate with the aging population. The workshop also taught participants how to recognize at-risk-for lower health literacy individuals and diminish barriers whenever possible. The workshop randomized participants into small groups composed of different participating programs (College of Dental Medicine, Programs in Physical Therapy, Programs in Occupational Therapy, School of Nursing, MD Program, Clinical Pastoral Education Program, Program in Genetic Counseling, Institute of Human Nutrition, Mailman School of Public Health, and School of Social Work). Participants facilitated discussions about how they would utilize the tools provided to navigate scenarios concerning older adults. The pre and post-surveys indicated that our efforts impacted participants by making them feel more prepared to communicate with an older adult who has experienced sensory, cognitive, physical, and/or behavioral changes. Participants reported that they learned more about their future colleagues' scope of practice, which promotes better communication. The limitations of our workshop were within the smaller sample size of responses we received. Graduate programs should implement this workshop among health professional students in the future.

Abstract citation ID: igae098.3786

### AGING IN PLACE WITH A WARMING CLIMATE: HOUSING POLICY AND DESIGN FOR AGING WITH EXTREME HEAT

Nichole Kain, *Antioch University New England, Saint Paul, Minnesota, United States*

Aging populations—particularly in areas prone to extreme heat—are facing unique residential challenges. In the past 10 years, extreme heat has killed more people in the United States (U.S.) than all other weather hazards combined, and it is expected to get hotter, with extreme heat events predicted to happen more often in the future. People over the age of 65 are disproportionately represented as the majority of heat victims, and are likely to perish in their own homes during these events. This study reveals that age-adaptive and climate-adaptive housing designs contribute to our quality of life and ability to survive as we age in a warming world. In addition to advancing our understanding of aging in places with extreme heat, this research offers two pieces of applied materials: A residential guide/handout for the general public that combines mitigation for both extreme heat and age-friendly design, as well as a policy research brief promoting the future development of energy efficient and age-friendly housing.

Abstract citation ID: igae098.3787

### AGING WITH DISABILITY AND AGING INTO DISABILITY: HOW INCOME AND MISTREATMENT EXPERIENCE ALTER SELF-PERCEIVED AGE?

Yalu Zhang[1], and Ziyan Chen[2], *1. Stony Brook University, Stony Brook, New York, United States*, *2. Peking University, Beijing, Beijing, China*

Individuals who experience early-onset disability (aging with disability) might have a lifetime of adaptation and resilience that shapes their views on aging differently from those who encounter disability in later life (aging into disability). Conversely, older adults with late-onset disability might maintain higher financial stabilities which could significantly impact their aging experience. It remains understudied if these contrasting effects tend to offset each other or if one has a more significant impact. Using 2018 China Longitudinal Aging Social Survey data, this study examined whether the onset timing of disability shapes attitudes towards aging differently, and how the experience of elder abuse could alter this association. Attitudes toward aging were measured both in subjective age and an index of self-perceived capabilities and values. Consistent with previous findings, this study found that the majority of older adults with impairments in (instrumental) activities of daily living had the onset of disabilities after the age of 65 (n=660, 91%). This study uniquely identified that, compared to older adults with early-onset disabilities, those who had later-onset disabilities perceived their aging, capabilities, and social values more negatively (O.R.=2.56, p=0.003). The odds drastically increased to 9.03 times if limiting the analytical sample to those who experienced elder abuse in the last 12 months (p=0.013). This study provides valuable insights into how the timing of disability onset influences older adults' attitudes toward aging. The marked impact of elder abuse on these perceptions highlights an urgent need for targeted prevention and interventions among those who have disabilities with aging.

Abstract citation ID: igae098.3788

### ALZHEIMER'S DIAGNOSIS AND CARE BY RACIAL AND ETHNIC GROUP IN THE MEDICARE POPULATION

Margaret Hoyt, Melissa Bailey-Taylor, Julie Chandler, and Joseph Johnston, *Eli Lilly and Company, Indianapolis, Indiana, United States*

Little is known about differences in healthcare resource utilization (HCRU) among racial/ethnic groups with Alzheimer's disease (AD). Our study aims to address this gap with an incident AD cohort in Medicare claims data. We identified Medicare patients ≥65 years old with a first (index) AD code (ICD-10 G30*) or medication (galantamine, rivastigmine, donepezil, or memantine) in 2018 or 2019 who were continuously enrolled (gap < 45 days) one year pre- and post-index. Descriptive analyses of HCRU and cost data were stratified by race/ethnicity. Our analysis included 887,984 patients, the majority female (64.2%), with mean (sd) age 80.6 (7.5) years. 72.0% identified as non-Hispanic White, 9.9% non-Hispanic Black, 12.8% Hispanic, and 3.8% Asian. In the 2018-2019 Medicare population, the incidence of Alzheimer's disease per 100,000 person-years was 1,349 for White, 1,649 Black, 2,003 Hispanic, and 1,500 for Asian patients. Fewer White patients (18.8%) were dual-Medicare/Medicaid eligible than non-White patients (46.0-64.4%), and non-White patients had higher rates of many chronic comorbid illnesses. More White patients had at least one neurologist visit in the year pre-index (39.0%) compared to Black (35.1%), Hispanic (37.5%), and Asian (33.1%) patients. More Black patients had an inpatient admission with AD as the primary diagnosis post-index (11.0%) than

White (9.5%), Hispanic (7.6%), and Asian (5.8%) groups, and costs for long-term care and hospice were highest for Black and Hispanic patients, respectively, which could indicate more severe disease. These statistically significant results suggest discrepancies between racial/ethnic groups in the Medicare AD population; further research is needed to understand the differences.

Abstract citation ID: igae098.3789

### ALZHEIMER'S DISEASE AND DISPARITY: A RURAL UNDERCOUNTING

Noah Brenman, Mengyuan Cheng, and Nasim Ferdows, *Northeastern University, Boston, Massachusetts, United States*

Rising life expectancy has led to an increasing prevalence of age-related diseases, particularly Alzheimer's disease and related dementia. The more rapid increase of the older population in rural areas, combined with a higher prevalence of dementia risk factors, and limited access to healthcare services, may lead to higher rates of dementia in these communities. This project examines the differences in dementia mortality rates between rural and urban areas over a three-year period. We used county-level data on age-adjusted mortality rates from the CDC Wonder Database, merging it with County Health Rankings from 2018-2020. Using Rural-Urban Commuting Area (RUCA) we defined the level of rurality as: Urban, rural-adjacent to an urban area, and rural non-adjacent to an urban area. We found that rural areas had higher dementia age-adjusted death rates compared to urban areas, even after accounting for select county characteristics. The percent difference between rural and urban dementia mortality ranged between 7.1 to 9.8%, showing a persistent widening gap in rural-urban dementia mortality rates.

Abstract citation ID: igae098.3790

### AN INTERNATIONAL COMPARISON AND CHINA'S REGIONAL DEVELOPMENT: EVALUATION OF QINGDAO'S LONG-TERM CARE POLICY

Huiru Song[1], and Hong Mi[2], *1. King's College London, Shandong, China (People's Republic)*, *2. Zhejiang University, Hangzhou, Zhejiang, China (People's Republic)*

Qingdao is the first city in China to fully implement long-term care insurance, and its system has important reference significance for the healthy and equity development of the elderly population in Shandong Province. The policy divides long-term care insurance benefits into medical services and nursing services, with the medical service fee being 3,500 yuan per year for employees and 3,000 yuan per year for residents, and the nursing service fee being 18,000 yuan per year for employees and 5,000 yuan per year for residents. According to forecasts, the GDP of Shandong Province is expected to reach 14,145.63 billion yuan in 2035. Therefore, according to the ratio of one employee to two permanent residents, under the fixed disability rate program, the province's disabled elderly population is 1,554,800, including 513,100 employees and 1,041,700 permanent residents, and under the variable disability rate program, the province's disabled elderly population is 1,728,700, including 570,500 employees and 1,158,200 permanent residents. At the same time, under the fixed disability rate plan, the long-term care insurance expenditure in Shandong Province in 2035 will be 8826,153,360 yuan, accounting for 6.23/10,000 of GDP. Under the variable disability rate scenario, long-term care insurance expenditure is 981,3526,560 yuan, accounting for 6.937 per 10,000 of GDP, which is much lower than that of OECD countries. In the future, Shandong Province should implement precise positioning based on Qingdao's policies and combine the regional characteristics of each city to promote long-term care insurance from wide coverage to full coverage.

Abstract citation ID: igae098.3791

### ARE STATINS THAT GOOD? ASSESSING THE PROTECTIVE EFFECT OF STATINS AMONG OLDER ADULTS WITH SERIOUS ILLNESS OR INJURY

Sandra Sanchez-Reilly[1], Siva Naga Srinivas Yarrarapu[2], Erin Sidle[3], Marla Khalikov[4], Jennifer LaCoss[5], Elizabeth Reilly[6], Shannon Crenshaw[3], and Ashley McGinity[7], *1. South Texas Veterans Health Care System and the University of Texas Health Science Center at San Antonio, San Antonio, Texas, United States*, *2. UTHSCSA, San Antonio, Texas, United States*, *3. University Health, San Antonio, Texas, United States*, *4. University Health System, University Health System, Texas, United States*, *5. UT Health San Antonio, UT Health San Antonio, Texas, United States*, *6. Health Careers High School, Health Careers High School, Texas, United States*, *7. University of Texas Health San Antonio, San Antonio, Texas, United States*

**Background:** Older Adults (OA) constitute the majority of Emergency Department (ED) visits, largely due to fall-related injuries and acute serious illnesses (ASI). OA's ED visits lead to potentially extended hospitalizations and increased healthcare costs. Statins, commonly prescribed for cholesterol-lowering effects/cardiovascular benefits, are widely used among OA. Few inconclusive studies have explored the relationship between statins and OA falls/ASI. Objective: To investigate the relationship between statins and its protective effects among OA who visit ED due to injury/ASI.

**Methods:** OA ED visits (age>65) were collected for seven weeks (summer 2024) at level-1 trauma center. Variables included acuity score (1-4, 1 being worse), diagnosis, ICU admission need, death and statins usage prior to event.

**Results:** 1771 OA-ED. Median age 73 (68-80), 52% female (923), 34% White, 55% Hispanic. Acuity: 1(3%), 2(33%), 3(59%), 4(5%). OA statins usage was 50%. 25% visits were falls, followed by infections (13%), GI complaints (11%) among others. 50% OA went home, 43% admitted. 12% became ICU admissions, 2% died. Among statin-takers, 13.2% were admitted to ICU vs.11.4% not-on-statins (p=0.27,NS). Interestingly, 1.3% statin-takers died because of the injury/ASI that brought them to ED, vs. 2.6% not-on-statins (p=0.04). Falling was the most common reason for seeking ED: 21.6% statin-takers vs. 27.9% not-on-statins (p=0.002), suggesting a protective effect.

**Conclusions:** Statins appear to have a significant protective effect on falls/ASI among OA, specifically pertaining to decreasing their fall risk and in-hospital mortality rate. Further studies should explore prophylactic ways to use statins in improving hospital-related outcomes and decreasing risk of geriatric syndromes.

Abstract citation ID: igae098.3792

### ARE WE WAREHOUSING PEOPLE WITH SERIOUS MENTAL ILLNESS? EXPLORING US NURSING HOMES IN 2000 AND 2021

Kim Hadson[1], Marissa Bergh[2], and Jasmine Travers[3], *1. NYU Rory Meyers College of Nursing, New York, New York, United States*, *2. New York University, Long Island City, New York, United States*, *3. New York University, New York, New York, United States*

The 1999 Supreme Court Olmstead decision reaffirmed people with serious mental illness (SMI) have the right to care integrated into community settings. The growth of SMI in nursing homes (NHs) suggests care has instead shifted to NHs. Insufficient attention has been given to the facility characteristics and state environments to understand if individuals with SMI in NHs are receiving high quality, equitable care. We used cross-sectional LTCFocus data from 2000 and 2021 to identify NHs with high volumes of SMI (more than 50% of residents with a diagnosis of schizophrenia or bipolar) and compared them to those below this cutoff. High volume NHs grew from 1.00% (161) of all NHs in 2000 to 3.10% (461) in 2021. While most states gained one or fewer high volume NHs, California gained 54, Illinois 44, Missouri 42, and Ohio 35, suggesting state policies play a role. High volume facilities were fundamentally different from other facilities. Wilcoxon rank sum tests and Pearson's Chi-squared test of the 2021 data showed significantly ($p< 0.001$) lower acuity and higher restraint use, 13 year lower average resident age, 0.21, 0.13, and 0.29 fewer registered nurse, licensed practical nurse, and certified nursing assistant hours per resident day respectively, 20% fewer female residents, 16% fewer White residents, 7% fewer residents with dementia, 22% more residents on Medicaid and 22% more facilities being for-profit. More investigation is needed to determine if high volume NHs offer appropriate care for people with SMI, or if they are warehouses for inappropriate segregation from community living.

Abstract citation ID: igae098.3793

### ASSESSING RACIAL DISPARITIES IN UTILIZATION OF HEALTHCARE SERVICES, 2010-2020

Yanjun Dong[1], Kun Wang[2], and Victoria Rizzo[3], *1. SUNY Albany, Albany, New York, United States*, *2. College of Arts & Sciences News, The University of Alabama at Birmingham, Alabama, United States*, *3. University at Albany, State University of New York, University at Albany, State University of New York, New York, United States*

**Objectives:** Little is known about the intersectional effects of race, gender, income, and education on healthcare service utilization among BIPOC (Black, Indigenous, and People of Color) older adults. This study fills this gap by analyzing longitudinal data of older adults in the U.S. from 2010 to 2020.

**Methods:** Using six waves of longitudinal data from the Rand Health and Retirement Study (n= 26,304), this study employed random-effect Poisson and logistic models to examine disparities across seven healthcare services, focusing on the interactions of BIPOC with gender, education, and income.

**Results:** The findings show disparities in healthcare utilization, with BIPOC older adults consistently reporting lower utilization across different types of services, including hospital stays, prescription drug use, outpatient surgery, home healthcare, physician visits, and dental care. For the interactions, the results indicate that, compared with others, low-income BIPOC older adults are less likely to access prescription drugs ($\beta = -1.10$, $p < 0.01$) but more likely to use home healthcare ($\beta = 0.52$, $p < 0.05$) and physician services ($\beta = 0.75$, $p < 0.05$). BIPOC women were less likely to utilize nursing home services ($\beta = -0.84$, $p < 0.05$).

**Discussion:** This study underscores the effects of socioeconomic factors and systemic inequities on healthcare utilization. Policy implications include the need for targeted interventions to reduce financial barriers, culturally competent care models, and enhanced health literacy programs. The findings highlight the urgent need for systemic changes to create an equitable healthcare system that addresses the needs of marginalized populations.

Abstract citation ID: igae098.3794

### ASSESSING READINESS OF BLACK OLDER ADULTS TO DISCUSS ADVANCE CARE PLANNING WITH THEIR SURROGATES

Ekenechukwu Akabike[1], Rebecca Howe[2], Alicia Agnoli[1], Julie Bidwell[1], and Grace Amadi[1], *1. University of California, Davis, Sacramento, California, United States*, *2. VA Providence Healthcare System, Providence, Rhode Island, United States*

Advance care planning (ACP) is a tool to identify health care goals in serious situations or if anticipating end-of-life circumstances. ACP is not always utilized by those who might most benefit from it and appears underutilized by black older adults for unclear reasons. This study explores 3 aims to better understand the readiness of black older adults to complete ACP including examining patient readiness to participate in ACP using the stages of change model, types of barriers and promoting factors to patient participation in ACP, and patients and surrogates' concordance on ACP readiness. A sub-analysis explores how readiness for ACP participation varies based on the patient-surrogate relationship. A mixed methods design used surveys and qualitative interviews to assess black older adults aged 60 – 91 and their associated surrogate selected from outpatient clinics within an academic hospital and a local community church in Sacramento, CA. Participants included 30 older adult respondents with 12 surrogates consisting of 7 adult children and 5 spouses/partners. Results suggest variation of end-of-life care discussion readiness where 63% of surrogates report already completing this discussion but only 45% of patients with surrogates report similar findings. Variation also occurs with patient readiness to discuss end-of-life care with adult children (17% of patients with children surrogates report not thinking about having these discussions yet) compared to those with a spouse/partner surrogate (100% of patients with partners/spouses have already had discussions). Additional research may help increase understanding of themes of patient and surrogate readiness and engagement in ACP.

Abstract citation ID: igae098.3795

### ASSESSMENT OF A U.S. NURSING STUDENT EXCHANGE PROGRAM FOCUSED ON JAPANESE OLDER ADULTS

Keiko Ono, *Aomori University of Health and Welfare, Aomori-City, Aomori, Japan*

From May 13 to 15, 2024, undergraduate nursing students from the U.S. participated in an international exchange at a university in Japan. A one-day program explicitly focused on learning about services for older adults in Japan: this study aimed to evaluate that program. On May 14, 2024, an intervention program was conducted in Japan, featuring lectures on Long-Term Care Insurance and other issues concerning older adults, including visits to nursing care facilities for older adults. The study involved seven undergraduate American nursing students. A qualitative descriptive research design was employed to assess the program's impact. Before the intervention, participants individually responded to several questions via QR Codes using Microsoft Forms. These responses served as the basis for a group discussion, during which students summarized their thoughts on a sheet. After the intervention, the group discussed and revised their initial thoughts on the same questions. Finally, participants answered several questions using QR codes. The survey results indicated that none of the students were familiar with services for older Japanese adults before the program. However, after the program, the percentage of students interested in nursing care services for older adults in Japan increased from 57.1% to 71.4%. Additionally, the program positively influenced the student's perception of older adults. They described older adults with terms such as "sociable," "active," "happy," "willing to live," and "well cared for." This program may have made students perceive older adults in Japan as positive and content.

Abstract citation ID: igae098.3796

### ASSOCIATION BETWEEN NEIGHBORHOOD PHYSICAL ENVIRONMENT SATISFACTION AND AGING ATTITUDES IN OLDER CHINESE ADULTS

Sophie Zhijing Xu[1], Dan Zhao[2], Weiyu Mao[3], Jing Wang[4], Zhongfang Yang[5], Yaguang Zheng[1], and Bei Wu[1], *1. New York University, New York, New York, United States, 2. Shandong University, Jinan, Shandong, China (People's Republic), 3. University of Nevada, Reno, Reno, Nevada, United States, 4. University of New Hampshire, Durham, New Hampshire, United States, 5. Fudan University, School of Nursing, Fudan University, Shanghai, China (People's Republic)*

With the aging population and increasing prevalence of chronic diseases in China, understanding the factors influencing aging attitudes among elderly individuals is crucial. This study examines the role of neighborhood environment satisfaction in shaping aging attitudes among elderly Chinese individuals with chronic diseases. Using data from the latest China Longitudinal Aging Social Survey (CLASS, 2020), demographic characteristics including gender, marital status, education level, residence type, employment status, and age were analyzed. Regression analysis explored associations between aging attitudes and satisfaction with various neighborhood environment factors, controlling for demographic variables. The sample included 7,111 participants aged 60 years and older. Among them, 5,574 (78.38%) had chronic diseases. Higher satisfaction with road conditions ($\beta = 0.815$, $P < 0.001$) and accessibility facilities ($\beta = 0.339$, $P = 0.003$) showed significant positive associations with attitudes toward aging in this group. Conversely, none of the neighborhood environment satisfaction factors were significantly associated with aging attitudes among older adults without chronic diseases. This study underscores the importance of neighborhood environment satisfaction in shaping aging attitudes among elderly Chinese individuals with chronic diseases. Creating age-friendly environments and tailored interventions can foster positive aging experiences and well-being in this demographic. These findings have implications for policymakers, urban planners, and healthcare professionals striving to create supportive environments conducive to healthy aging in rapidly aging societies like China.

Abstract citation ID: igae098.3797

### ASSOCIATION BETWEEN SARCOPENIA AND NEUROPSYCHIATRIC SYMPTOMS IN OLDER ADULTS WITH ALZHEIMER'S DISEASE

Derong Zeng[1], Nan Zhang[2], Bingbing Qi[3], Teruaki Kawasaki[4], Ichiro Akiguchi[4], and Ayae Kinoshita[5], *1. Kyoto University, Kyoto City, Kyoto, Japan, 2. The University of Manchester, Manchester, England, United Kingdom, 3. Villanova University, Villanova University, Pennsylvania, United States, 4. Kyoto Clinical and Translational Research Center for Neurocognitive Disorders, Kyoto Clinical and Translational Research Center for Neurocognitive Disorders, Kyoto, Japan, 5. Kyoto University, Kyoto University, Kyoto, Japan*

Sarcopenia is a vulnerability state that increases the risk of many adverse health outcomes, but little is known about the effects of sarcopenia on neuropsychiatric health. To explore the association between sarcopenia and neuropsychiatric symptoms in older adults with Alzheimer's Disease, data from 110 participants were assessed using the Asian Working Group for Sarcopenia criteria. The participants had a mean age of 83.15 years (SD = 5.49), a mean MMSE score of 20.69 (SD = 5.03). The study focused on the relationship between sarcopenia and 12 neuropsychiatric inventory items, including delusions, hallucinations, aggression, depression, anxiety, elation, indifference, disinhibition, lability, aberrant motor behaviors, nighttime disturbances, and eating disturbances. The relationships between sarcopenia and these neuropsychiatric symptoms were explored using logistic regression models. Logistic regression analyses revealed significant associations between sarcopenia and both aberrant motor behaviors (Estimate=-1.5612, p=0.0141) and nighttime disturbances (Estimate= -1.2096, p=0.0194). Further analysis incorporating age, sex, MMSE, and grip strength showed that while the association between sarcopenia and eating disturbances was not significant (Estimate=1.51272, p=0.1248), age emerged as a significant predictor (Estimate = 0.18272, p = 0.00869), suggesting that age may be a more critical factor in the development of eating disturbances in this population. These findings suggest that sarcopenia may play a role in the development or exacerbation of specific neuropsychiatric symptoms in AD patients. Further research is needed to understand the underlying mechanisms and to develop targeted interventions to improve the quality of life for this population.

Abstract citation ID: igae098.3798

### ASSOCIATION BETWEEN WAIST-BASED ANTHROPOMETRIC INDICES AND MILD COGNITIVE IMPAIRMENT FROM THE KFACS

Daehyun Lee, Hyung Eun Shin, Heeeun Jung, Jae Young Jang, Hyunjin Cho, Nahyun Lim, Chang Won Won, and Miji Kim, *Kyung Hee University, Seoul, Republic of Korea*

While several studies have explored the association between obesity and cognitive function in older adults, definitive evidence linking waist-based anthropometric indices to impaired cognitive function remains lacking. Our study aimed to investigate the association between the waist-based anthropometric indices and mild cognitive impairment (MCI) in older adults according to sex. We conducted a retrospective study on 1,192 community-dwelling older adults (51.8% women; mean age 75.1±3.6 years) from the Korean Frailty and Aging Cohort Study during a 6-year follow-up. The waist-based anthropometric indices at baseline include waist circumference (WC), waist-to-height ratio (WHtR), waist-to-weight ratio (WWR), and weight-adjusted-waist index (WWI). These indices were calculated as follows: WHtR= WC (cm) / height (cm), WWR= WC (cm) / body weight (kg), and WWI= WC (cm) /√(body weight (kg)). MCI incidence was measured from Wave 2 to 4, with MCI defined as Mini-Mental State Examination< 24. Multivariate logistic regression analysis was utilized to examine the association between these waist-based anthropometric indices and MCI, and restricted cubic spline curve was used to assess the potential nonlinear relationship. The incidence of MCI was 21.1% in men and 34.1% in women during 6-year follow-up (p< 0.01). The highest WWI quintile was significantly associated with MCI only in men (odds ratio: 4.49, 95% confidence interval: 1.39–14.47) in the fully adjusted models. However, no significant associations were found in WC, WHtR, and WWR. Our findings suggest that WWI is more strongly associated with MCI compared to other waist-based anthropometric indices in men, proposing its potential as a clinical indicator for MCI.

Abstract citation ID: igae098.3799

### ASSOCIATION OF ALCOHOL CONSUMPTION WITH IMMUNE CELL PHENOTYPES IN THE FRAMINGHAM HEART STUDY

Ahmed Ragab[1], Jiachen Chen[2], Yumeng Cao[1], Margaret Doyle[3], Kathryn Lunetta[1], and Joanne Murabito[4], *1. Boston University, Boston, Massachusetts, United States, 2. Boston University School of Public Health, Boston, Massachusetts, United States, 3. University of Vermont Larner College of Medicine, Colchester, Vermont, United States, 4. Chobanian and Avedisian School of Medicine, Boston University, Boston, Massachusetts, United States*

Alcohol consumption, prevalent among 2.3 billion people worldwide, accounts for 4.7% of global mortality. While excessive drinking is harmful, moderate intake may have nuanced effects on immune function, though this relationship is not well-studied. We analyzed the relationship between average daily alcohol consumption and immune cell phenotypes among Framingham Heart Study Offspring participants. Consumption was examined in multiple ways: categorized and ordinalized (nondrinkers, moderate, at-risk, heavy), using average grams per day, and dichotomized as light vs heavy drinkers, with and without non-drinkers omitted. We profiled immune cell phenotypes using flow cytometry, including T, B, and NK cells, and T subtypes: CD4+, CD8+, CD4+CD28-CD27-, CD8+CD28-CD27-, CD4+ TEMRA, CD8+ TEMRA, CD4+/CD8+ ratio, CD4+(TN/TM), CD8+ (TN/TM), CD8+ Tc17/Treg ratio, CD4+ Th17/Treg ratio and GranzymeB CD8+/CD4+ratio. We used linear mixed effects models adjusting for age, sex, and cytomegalovirus status to test for associations, and FDR≤0.05 within an analysis set (defined by consumption definition) to declare significance. Among 928 participants (mean age 62 years, range 40-88, 52% female), 64% consumed alcohol. Compared to abstainers, CD4+(TN/TM) ratio was significantly increased in the moderate and at-risk daily consumption categories (β=0.26 for both, FDR=0.03). There were no significant linear associations between consumption and any of the immune cell phenotypes. However, among drinkers, we observed a significant negative association between average daily alcohol consumption and CD8+(TN/ TM) ratio (β= -0.14 for each higher category of consumption, FDR=0.03). The effects of consumption appeared stronger in males than females. Our findings suggest alcohol consumption may impact immune cell phenotypes.

Abstract citation ID: igae098.3800

### ASSOCIATION OF CARDIOMETABOLIC RISK FACTORS AND TRANSITION OF DISABILITY STATUS AMONG CHINESE ADULTS

Heming Pei[1], Gong Chen[2], Jiajia Li[3], and Bo Liang[3], *1. Columbia University, New York, New York, United States, 2. Peking University, Beijing, Beijing, China, 3. Peking University, Beijing, Beijing, China (People's Republic)*

**Background:** Chronic diseases significantly increase the risk of functional disability. Understanding the relationship between cardiometabolic risk factor (CMRF) comorbidities and transitions in disability status is crucial for devising early intervention strategies at the population level.

**Method:** This study utilized a prospective cohort design, drawing data from the China Health and Retirement Longitudinal Study (CHARLS) spanning from 2011 to

2018. A total of 6,397 Chinese middle-aged and older adults were followed for a total of 44,779 person-years. A Multi-state Markov model was employed to simulate longitudinal disability status transitions and to estimate the effect sizes of CMRFs, along with demographic, socioeconomic, and behavioral factors.

**Results:** Over the 7-year follow-up, CHARLS participants experienced an increased likelihood of transitioning from normal physical function to IADL (Instrumental Activities of Daily Living) impairment, rising from 7.6% to 11.0%. Moreover, the transition intensity from intact ADL (Activities of Daily Living) to IADL impairment was 2.5 times higher than that from intact ADL to ADL impairment. Unlike other chronic conditions, CMRFs particularly influenced ADL by significantly reducing the probability of recovery from ADL impairment to a better physical function state. Specifically, the probability of shifting from IADL impairment to intact ADL decreased by 30% (HR=0.70, 95% CI [0.53-0.92]) among individuals with two CMRF components.

**Conclusion:** The findings suggest that CMRFs act as risk factors by impeding the recovery process of ADL impairment. Early intervention targeting obesity, hypertension, hyperlipidemia, and diabetes could help reduce or delay the incidence of functional disability.

Abstract citation ID: igae098.3801

### ASSOCIATION OF CREATININE- AND CYSTATIN-C–BASED INDICES WITH INCIDENT SARCOPENIA: A LONGITUDINAL STUDY

Jae Young Jang, Hyung Eun Shin, Daehyun Lee, Heeeun Jung, Hyunjin Cho, Nahyun Lim, Chang Won Won, and Miji Kim, *Kyung Hee University, Seoul, Republic of Korea*

Creatinine and cystatin-C–based indices have been reported to be associated with sarcopenia; however, the long-term relationship between these indices and sarcopenia remains unclear. This study examined the longitudinal relationship between baseline serum creatinine and cystatin-C–based indices and incidence of sarcopenia during a 6-year follow-up to compare which indicator well predicts sarcopenia in community-dwelling older adults. This study included 698 participants (mean age=75.1±3.7years; women=54.4%) from the Korean Frailty and Aging Cohort Study (KFACS). Baseline Creatinine-to-cystatin-C ratio estimated glomerular filtration rate (eGFR) ratio (eGFRcystatin-C[mL/min/1.73m2]/eGFRcreatinine[mL/min/1.73m2]), sarcopenia-index (serum creatinine[mg/dL]×eGFRcystatin-C[mL/min/1.73m2]), predicted skeletal muscle mass index (pSMI), and total-body muscle mass index (TBMM) were measured. Sarcopenia was defined by following the guidance of the Asian Working Group for Sarcopenia 2019. The incidence of sarcopenia during 6-year follow-up was 22.5% in men and 16.7% in women. The pSMI and TBMM showed higher area under the receiver operating characteristic curves for predicting the incidence of sarcopenia in men (pSMI==0.634; TBMM=0.635) and women (pSMI=0.727; TBMM=0.724) than other indices. Higher pSMI and TBMM were associated with the incidence of sarcopenia in both men (pSMI, odds ratio [OR]=0.370, 95% confidence interval [CI]=0.223–0.614; TBMM, OR=0.928, CI=0.896–0.962) and women (pSMI, OR=0.149, CI=0.072–0.307; TBMM, OR=0.896, CI=0.857–0.936), after adjusting confounders. pSMI and TBMM showed higher predictive capabilities for the incidence of sarcopenia compared to other indices. Also, higher pSMI was strongly associated with a lower risk of incident sarcopenia. These findings suggest that monitoring pSMI could help predict the onset of sarcopenia in community-dwelling older adults.

Abstract citation ID: igae098.3802

### ASSOCIATION OF HEALTHCARE ACCESS AND DUAL ELIGIBILITY STATUS BASED ON ENROLLMENT IN MANAGED CARE

Aastha Dharia[1], Wassim Tarraf[2], Ramin Homayouni[1], and Mohammad Usama Toseef[3], *1. Oakland University William Beaumont School of Medicine, Rochester, Michigan, United States*, *2. Wayne State University, Detroit, Michigan, United States*, *3. Corewell Health, Royal Oak, Michigan, United States*

Dual-eligible beneficiaries (enrolled in both Medicare and Medicaid) remain a growing population with complex care needs. However, there are limited studies evaluating self-reported access to healthcare among these individuals. Using six key outcomes that describe the characteristics of beneficiaries' usual source of care from the 2021 Medical Expenditure Panel Survey, we retrospectively analyzed self-assessment questionnaire data among individuals age 18 and older (N = 12,924) to evaluate the association between healthcare access and dual-eligibility status based on enrollment in managed care. A series of survey-weighted multivariable logistic regression models were estimated. After adjusting the models for demographic factors, socioeconomic factors, and interaction term between dual-eligible and managed care status, we calculated marginal effects of managed care and dual-eligibility on healthcare access outcomes and used analysis of variance contrast tests to estimate the difference in the probability of outcomes based on managed care and dual-eligibility status. The results demonstrate non-dual managed care beneficiaries were significantly less likely to seek care [4.1% versus 2.5%, p-value = 0.0338] and experienced greater delays in care [6.6% versus 4.9%, p-value = 0.0398] due to cost. We also noted no significant impact on established and regular care access independently based on dual-eligibility status or managed care status. These findings suggest managed care may not significantly influence dual-eligibles' access to healthcare but should be analyzed further for associations with healthcare disparities before making policy recommendations. Future research should also examine associations between quality of care measures and dual-eligibility status to continue holistically evaluating healthcare equity.

Abstract citation ID: igae098.3803

### ASSOCIATION OF IMMUNE CELLS AND INFLAMMATORY BIOMARKERS WITH DEPRESSIVE SYMPTOMS IN THE FRAMINGHAM HEART STUDY

Sandhya Iyer[1], Yumeng Cao[2], Kathryn Lunetta[2], Ryan Liebegott[3], Margaret Doyle[4], Jiachen Chen[3], Ahmed Ragab[2], and Joanne Murabito[5], *1. Boston University School of Medicine, Boston, Massachusetts, United States*, *2. Boston University, Boston, Massachusetts, United States*, *3. Boston University School of Public Health, Boston, Massachusetts, United States*, *4. University of Vermont Larner College of Medicine, Colchester, Vermont, United States*, *5. Chobanian and Avedisian School of Medicine, Boston University, Boston, Massachusetts, United States*

Depression is a common mental health disorder with substantial impact on older adults. Immune dysfunction has been studied as a mechanism for depressive symptoms, providing the opportunity for new targeted treatment approaches. This study investigated the associations between immune cell phenotypes, determined by flow cytometry, and peripheral inflammatory biomarkers, measured using the OLINK Inflammation panel, with Center for Epidemiologic Studies Depression Scale (CES-D) scores, indicative of depressive symptoms, in the Framingham Heart Study Offspring Cohort. Participants (n=996) had a mean age 62, were 52% female, and had a mean CES-D score 5 (SD=7) with 8% having a CES-D score >/=16. A linear mixed-effects model was used (generalized estimating equation model for the dichotomous CES-D) with the proteins and immune cells as predictors and CES-D scores as the outcome, adjusting for age, sex, BMI, smoking status, CVD, depression medication use, NSAID use, and cytomegalovirus (CMV) status (immune cells only). We used Bonferroni adjusted p< 0.005 to declare significance. Effect estimates (beta) are reported in standard deviation units. Our results suggested a positive association between FGF-19 and CES-D score (beta=0.11, p=0.0004), as well as a suggestive negative trend between the CD8+IL17+:CD8+CD25+FoxP3+ ratio (Tc17/CD8+Tregs) and CES-D score (beta=-0.1, p=0.03). In addition, we observed a positive suggestive trend between IL-17C and depressive symptoms (CES-D >/= 16) (beta=0.35 p=0.009), that strengthened with higher levels of symptoms (p< 0.005). This study helps to expand our understanding of the role of inflammation in the pathophysiology of depression.

Abstract citation ID: igae098.3804

### ASSOCIATIONS BETWEEN 10-YEAR PHYSICAL FUNCTION TRAJECTORIES AND 24-HOUR ACTIVITY CYCLE BEHAVIORS IN OLDER ADULTS

Mikael Anne Greenwood-Hickman[1], Weiwei Zhu[1], Abisola Idu[1], Laura Harrington[1], Susan McCurry[2], Andrea LaCroix[3], Pamela Shaw[1], and Dori Rosenb erg[1], *1. Kaiser Permanente Washington Health Research Institute, Seattle, Washington, United States*, *2. University of Washington, Seattle, Washington, United States*, *3. University of California, San Diego, San Diego, California, United States*

Physical activity (PA), sedentary behavior (SB), and sleep comprise an individual's 24-hour activity cycle, which is likely bidirectionally associated with physical function over the life course. To explore how function predicts activity, we examined whether self-reported (activities of daily living [ADLs]) and objective (short Performance-based Physical Function [sPPF]) measures of physical function predicted PA, SB, and sleep in the Adult Changes in Thought (ACT) cohort. We fit linear mixed effects models to define trajectory slopes for each functional measure over the prior 10 years. We used multivariable linear regression to investigate the relationship between trajectories and device-measured PA and SB and self-reported sleep. Participants (N=905) were 77.6 (SD=6.9) years old, 55% female, 91% White, and reported impairment on a median of 1 ADL (IQR=[0,2]) with a median score of 9 (IQR=[8,11], higher=better functioning) on the sPPF at activity measurement. A steeper increasing slope of ADL impairment was associated with more sitting time (35.0 mins/day [4.3,65.0] per 0.4-unit increase in slope), longer mean sitting bout duration (3.5 min/bout [0.8,6.2]), fewer steps (-1372 [-2223,-638]), less MVPA (-13 min/day [-22.6,-5.0]), and more time in bed (25.5 min/day [6.5,43.5]). More steeply decreasing sPPF slope was associated only with steps and MVPA (-1180 steps [-2853,-185] and -15.7 min/day [-35.6,-2.3] per 0.3-unit decrease in slope). Standing time, Light-intensity PA, and self-reported sleep quality were not associated with either measure. Combined with prior literature suggesting associations with future function, these findings support the bidirectional nature of the relationship between the late-life 24-hour activity cycle and physical function.

Abstract citation ID: igae098.3805

### ASSOCIATIONS BETWEEN PULMONARY FUNCTION AND PHYSICAL REHABILITATION OUTCOMES AMONG OLDER ADULTS

Lindsey Mathis[1], Na Sun[1], Simon Ho[1], Lane White[1], Odessa Addison[2], Douglas Savin[1], and Jason Falvey[2], *1. University of Maryland Baltimore, Baltimore, Maryland, United States*, *2. University of Maryland School of Medicine, Baltimore, Maryland, United States*

Physical rehabilitation is crucial for older adults with functional impairments, as persistent impairments are linked to increased risks of hospitalization, mobility disability, and mortality. Impaired pulmonary function and associated symptoms of dyspnea may limit participation in rehabilitation programs, but it is unclear whether these impairments negatively impact outcomes. To answer this question, our analysis included 819 community-dwelling rehabilitation users (63% female; weighted n=3,882,191) from Round 5 (2015) of the National Health and Aging Trends Study (NHATS). Peak expiratory flow (PEF) categorized participants into "normal," "moderately impaired," or "severely impaired" lung function. Participants reported whether they met rehabilitation goals during their latest episode of care (yes/no). Multivariable survey-weighted logistic regression, adjusting for demographics and medical complexity, was used to estimate these relationships. In our sample, 71% had normal PEF, 22% had moderate PEF impairment, and 7% had severe PEF impairment. Severe impairment had 57% lower odds (aOR=0.4, 95% CI 0.2-0.9) and moderate impairment had 35% lower odds (aOR=0.6, 95% CI 0.4-1.0) of meeting rehabilitation goals compared to normal PEF. Similar trends were observed when limiting rehab users to only musculoskeletal condition (e.g., joint replacement). This study found that nearly 4 million older adults in the U.S. use rehabilitation annually, with 29% having impaired lung function. Our data suggest impairments in PEF may limit progress with rehabilitation, even for those with musculoskeletal indications, and thus suggests care pathways to more optimally manage older adults with limited lung function in rehabilitation settings may be needed to help them achieve their goals.

Abstract citation ID: igae098.3806

### ASSOCIATIONS OF MEAN COGNITIVE PERFORMANCE AND INTRAINDIVIDUAL VARIABILITY WITH WHITE MATTER LESIONS

Uriel Urias[1], Kelsey Thomas[2], Mary Ellen Garcia[3], Amanda Gonzalez[3], Britney Luu[1], Victoria Gutierrez[1], and Katherine Bangen[4], *1. San Diego State University, San Diego, California, United States*, *2. University of California San Diego, La Jolla, California, United States*, *3. University of California, San Diego, San Diego, California, United States*, *4. University of California, San Diego, La Jolla, California, United States*

White matter lesions (WMLs) are magnetic resonance imaging markers of small vessel cerebrovascular disease and have been associated with Alzheimer's disease and related dementias. Intraindividual variability (IIV), often measured as variability in performance across neuropsychological tests at a single time point, has emerged as a sensitive marker of early cognitive difficulties that may have utility beyond measures of mean neuropsychological performance (MP). However, the associations of IIV with neuroimaging variables remains understudied. This study included 70 older adults without dementia (mean age=68.57) who completed a comprehensive neuropsychological battery and underwent MRI brain scans. WMLs were adjusted for estimated total intracranial volume and transformed to reduce skew. Linear regression models examined the associations of IIV, MP, and WMLs both across and within the cognitive domains of memory, attention/processing speed, and executive function. Covariates included age, sex, and education. IIV showed no significant association with WMLs, neither within nor across the domains examined (all p's>.05). In the domains of memory (B=-.035, p=.025), executive function (B=-.039, p=.018), and in global cognition (B=-.065, p=.003) lower MP was associated with more WMLs. In the attention/processing speed domain, MP was not significantly associated with WMLs (all p's >.05). The present study only examined cross-sectional data, so future studies should re-evaluate the associations with IIV, MP, and WMLs longitudinally. While the small sample size may limit the generalizability of this study, these findings suggest that measures of MP may better reflect WMLs relative to measures of IIV.

Abstract citation ID: igae098.3807

### ASSOCIATIONS OF SOCIAL RELATIONSHIPS WITH INFLAMMATORY, CARDIOVASCULAR AND IMMUNE CELL BIOMARKERS

Ahmed Ragab[1], Jiachen Chen[2], Yumeng Cao[1], Margaret Doyle[3], Joanne Murabito[4], and Kathryn Lunetta[1], *1. Boston University, Boston, Massachusetts, United States*, *2. Boston University School of Public Health, Boston, Massachusetts, United States*, *3. University of Vermont Larner College of Medicine, Colchester, Vermont, United States*, *4. Chobanian and Avedisian School of Medicine, Boston University, Boston, Massachusetts, United States*

Social relationships are essential for health, as social isolation and loneliness are linked to higher risks of morbidity and mortality. These associations may result from behavioral changes, physiological alterations, increased inflammation, and a weakened immune system. Community-based cohorts investigating the association between social isolation and inflammation have not included immune cell phenotypes and have been limited to a small number of inflammatory and cardiovascular biomarkers. We investigated the relationship between the Social Network Index (SNI), and 3 biomarker datasets: 1) 68 inflammatory biomarkers (N=875), 2) 71 cardiovascular biomarkers (N=3295), and 3) 28 immune cell phenotypes(N=996), among Framingham Heart Study Offspring participants (mean age 61). SNI was calculated as the sum of four components: marital status, the presence of close friends or relatives, attendance at religious services, and participation in social groups and treated as an ordinal predictor (1=lowest level of connections, 4= highest). We used Linear Mixed Effects models with SNI as the predictor and biomarkers/immune phenotypes as the outcomes, adjusting for age and sex and CMV (immune phenotypes). We used FDR≤0.05 to declare significance within each biomarker dataset. SNI was positively associated with inflammatory biomarkers HGF, IL-6, OSM, and IL-10RB. SNI was also positively associated with cardiovascular proteins CNTN1, IGFBP3, FGF23, CD56, sRAGE, and IGF1, and negatively associated with GDF15, MMP9, FBN, MCP1, CD14, A1M, IGFBP2, CRP, EFEMP1, and PPBP. SNI was not significantly associated with any of the immune cell phenotypes in the full sample. We conclude that social isolation is associated with multiple inflammatory and cardiovascular biomarkers.

Abstract citation ID: igae098.3808

### BARRIERS AND FACILITATORS TO PHYSICAL ACTIVITY OF OLDER ADULTS IN KOREAN NURSING HOMES: A QUALITATIVE STUDY

Young Ko[1], Seon Kyung Yun[2], Geun Sil Yook[1], and Seo Young Kim[1], *1. Gachon University, Incheon, Republic of Korea*, *2. Saekyung University, Yeongwol, Kangwon-do, Republic of Korea*

Various factors can either reduce or improve the physical activity level of older people living in nursing homes. Although it is critical to manage the risk of inactivity, only a few studies have investigated the multifaceted factors associated with physical activity among nursing home residents. The purpose of this study was to investigate directors' and care workers' perceptions of barriers and facilitators to physical activity for older adults in Korean nursing homes. This was a qualitative descriptive study. We collected data from January 13 to February 14, 2023. We used thematic analyses to analyze data from 4 directors and 22 long-term care workers in nursing homes in South Korea. We extracted 4 themes and 11 sub-themes reflecting the factors related to older adults in nursing homes: (a) "safety is more important than activity," (b) "encourage activities tailored to their preference," (c) "activities in harmony," and (d) "meaning of living at a nursing home." Individual, interpersonal, environmental, and policy factors influence older adults' physical activity in nursing homes. These factors guide policymakers, healthcare providers, and care workers in developing and implementing activity planning to help improve recipients' physical activity level in nursing homes. This research was supported by the Basic Science Research Program through the National Research Foundation of Korea (NRF) funded by the Korean government (MSIT) (No. 2022R1F1A1074082).

Abstract citation ID: igae098.3809

### BEHIND THE WALLS: THE IMPLICATIONS OF ASSISTED LIVING BUILDING CODES ON RESIDENT SAFETY

Adam Pennavaria[1], Lindsey Smith[2], Cassandra Hua[3], Paula Carder[4], Eric Jutkowitz[5], and Kali Thomas[6], *1. Oregon Health and Science University, Portland, Oregon, United States*, *2. Oregon Health & Science University, Portland, Oregon, United States*, *3. University of Massachusetts Lowell, Lowell, Massachusetts, United States*, *4. OHSU-PSU School of Public Health, Portland, Oregon, United States*, *5. Brown University, Brown University, Rhode Island, United States*, *6. Johns Hopkins University, Baltimore, Maryland, United States*

As the US population collectively ages, issues related to assisted living (AL) resident safety have become an increasingly pressing issue for the general public, reflected by major media reporting in the last year. The building codes that govern ALs play an important role in mitigating the risks associated with falls, wandering, and other hazards, especially for residents living with Alzheimer's disease and related dementias. We sought to understand how states vary in their approaches to building code regulations that might impact resident safety. We used search term-based text retrieval to identify areas of text relevant to locks, alarms, and egress restriction. A preliminary analysis of 2023 AL policy text by state suggests that a majority of states' AL policy regulations contain some kind of reference to requirements for locks, alarms, or other type of egress restriction, with a few outliers – regulations in Nebraska, Minnesota, South Carolina, and Tennessee appear to make no mention of any of these subjects, while New Mexico, Texas, Washington, and others include multiple references to each. For instance in NM, residents must have the ability to safely leave the building, so memory care units rely on alarms and provide access to secure outdoor environments. 38 states' regulations (75%) refer to locks, 40 states (78%) refer to alarms, and 27 states (53%) refer to limiting/restricting egress. This research has implications for understanding the relationships between building code requirements and resident safety. It provides insight for those who seek to improve safety standards in AL communities.

Abstract citation ID: igae098.3810

### BEREAVED DEMENTIA CAREGIVERS MAKE MEANING THROUGH RELIGION IN A PHOTO-ELICITATION INTERVENTION

Annabelle Yang[1], Taryn Bogdewiecz[2], Jacquelyn Benson[1], Karla Washington[1], and Abigail Rolbiecki[3], *1. Washington University in St. Louis, St. Louis, Missouri, United States*, *2. University of Colorado Anschutz Medical Campus, Aurora, Colorado, United States*, *3. University of Colorado Anschutz, Aurora, Colorado, United States*

Many persons living with dementia (PLWD) rely on informal caregivers, who undergo extensive personal upheaval approaching and after end-of-life for their PLWD. To promote successful adaptation in bereavement, caregivers can engage in a process of meaning making as they make sense of their loss, find "silver linings" in their experiences, or undergo shifts in identity. Religious and spiritual coping can facilitate this meaning making. Prior evidence has found that successful meaning making improves bereavement outcomes, reducing risk for grief complications. Caregiver Speaks is an intervention designed to facilitate such meaning making in caregivers of PLWD through photo-elicitation, whereby hospice family caregivers share stories and images narrating their experiences within a private Facebook group during active caregiving and bereavement. The purpose of the current study was to explore how caregivers from the bereavement arm of the Caregiver Speaks project made meaning of their experiences. Participants were recruited from hospices across the Midwest and Northeast US. A deductive and inductive qualitative analysis of Facebook posts was performed by two independent coders. They engaged in consensus coding to refine and categorize codes into themes. Religious and spiritual coping was a primary theme, as caregivers expressed feelings of peace knowing their loved ones were in Heaven, hope for the future because God has a plan, and comfort in scripture. Others described inner peace found in nature or meditative practices that provided comfort. These findings hold promise for meaning making interventions designed to facilitate religious and spiritual coping to improve bereavement outcomes.

Abstract citation ID: igae098.3811

### BLACK-WHITE RACIAL DISPARITIES IN MORTALITY: FINDINGS FROM THE SAINT LOUIS PERSONALITY AND AGING NETWORK (SPAN)

Isaiah Spears[1], Megan Wolk[1], Aaron Gorelik[1], Jayne Siudzinski[1], Sara Norton[1], Michael Esposito[2], Thomas Oltmanns[1], and Ryan Bogdan[1], *1. Washington University in St. Louis, St. Louis, Missouri, United States*, *2. University of Minnesota, Minneapolis, Minnesota, United States*

Black-White racial health disparities in America are striking. Black Americans have worse physical health and lower life expectancy than White Americans. However, the potential factors contributing to increased mortality in later life among Black Americans are rarely investigated. Here, we examined Black-White racial mortality disparities in the longitudinal St. Louis Personality and Aging Network (SPAN) Study of older adults (n=1,630; aged 55-65 at baseline; 67.4% White; 32.6% Black), with mortality data collected from the National Death Index. Black SPAN participants had greater mortality over the course of the study than White participants($\chi^2$ = 47.667, p = <.0001). Specifically, 26.9% of Black participants (n = 142) passed away relative to 12.7% of White participants (n = 139). Indices of socioeconomic status (i.e., annual household income; educational attainment) partially accounted for this difference. Additional planned analyses will examine whether environmental (e.g., discrimination, stress exposure), biological (e.g., inflammation), and psychological/behavioral (e.g., health behaviors, personality) factors may also contribute to these Black-White mortality disparities. These findings replicate prior studies documenting racial health disparities, the potential contributions of socioeconomic status, and position the SPAN study to evaluate plausible environmental, behavioral, and biological mechanisms. Ultimately, better understanding the environmental, psychological/behavioral, and biological mechanisms that may contribute to Black-White racial health disparities may help facilitate interventions and policy to improve racial health equity.

Abstract citation ID: igae098.3812

### BLOOD BIOMARKERS ASSOCIATED WITH POSTOPERATIVE DELIRIUM INCIDENCE, SEVERITY, DURATION AND SUBTYPE

Hyangkyu Lee, Hyeonmi Cho, Jeongeun Choi, and Hyunki Park, *Yonsei University, Seoul, Republic of Korea*

To evaluate the associations of neurological biomarkers —specifically tau, phosphorylated tau, amyloid β40, amyloid β42, amyloid β42/40 ratio, glial fibrillary acid protein (GFAP), neurofilament light (NfL), and brain-derived neurotrophic factor (BDNF) —with the incidence, severity, duration, and subtype of postoperative delirium. In a group of patients over 70 years old who had spine surgery, delirium patients (n=95) and non-delirium patients (n=95) were matched 1:1 with propensity score matching using demographic, lifestyle, and clinical characteristics. Neurological biomarkers were measured after collecting peripheral blood samples immediately after the surgical procedure in the recovery unit. Delirium was observed twice a day from the first to the third postoperative day, and once a day thereafter. Although there were no significant associations between biomarkers and the incidence of postoperative delirium, patients who developed postoperative delirium, each 1 standard deviation increase in NfL was associated with a 1.51 increase in the peak DRS-R-98 severity score (95% confidence interval: 0.07 to 2.95). Each 1 standard deviation rise in GFAP and NfL was associated with an odds ratio of 2.08 (95% confidence interval: 1.15 to 3.74) and 2.35 (95% confidence interval: 1.13 to 4.92), respectively, for delirium duration of 4 days or more. The odds ratio for the hypoactive subtype of delirium increased by 1 standard deviation in A□42 (95% CI: 1.28 to 6.66). Our study demonstrated that blood biomarkers could be useful in predicting the severity, duration, and subtype of postoperative delirium among older adults.

Abstract citation ID: igae098.3813

### BRICKS FOR BETTER BRAINS©: STRATEGIES TO ENHANCE ENGAGEMENT IN PERSONS LIVING WITH DEMENTIA

Natalia Kasperovich[1], and Jennifer Sasser[2], *1. Bricks for Better Brains©, Beaverton, Oregon, United States*, *2. Portland Community College, PORTLAND, Oregon, United States*

The Bricks for Better Brains© activity is an intervention to slow down the progression of cognitive decline for persons living with dementia. The activity utilizes various carefully selected, interlocking bricks (LEGO®) in bright colors as an innovative medium for stimulating participants' creative capacities, using visual perception and motor sensory skills, as well as strengthening social engagement. Cognitive impairments can present unique challenges for effectively engaging this population in meaningful activities. An in-person observational study was conducted as a preliminary exploration of strategies to increase engagement of persons living with dementia in activities with viable therapeutic potential using The Bricks for Better Brains© protocol. The study consisted of two groups (five males and fifteen females total) of people 75 years of age and older living with moderate-to-severe dementia and residing in an institutional care setting. Four activity modification strategies were tested – modification to objects and property; modification to space demands; modification to social demands; and modification to sequence and timing. All modifications in space demands increased engagement. Modification in objects and property, sequence and timing, and social demands were positive for the group but showed mixed efficacy for individual. These results support the person-centered care model and highlighted the importance of creating stimulating environments by understanding how people living with dementia interact with their surroundings. By identifying efficient activity modification strategies we can increase activity engagement and improve the quality of life of the people living with dementia. Future studies will need to include the reliability of observational measures.

Abstract citation ID: igae098.3814

### BRIDGING GAPS IN GERIATRIC EXPERTISE: OREGON ECHO NETWORK'S IMPACT ON INTERPROFESSIONAL EDUCATION AND CARE

Leah Brandis[1], Anders Herreid-O'Neill[2], Maryan Carbuccia Abbott[2], Laura Byerly[2], and Kerri Smith Slingerland[2], *1. OHSU, Portland, Oregon, United States*, *2. Oregon Health & Science University, Portland, Oregon, United States*

The United States faces a critical shortage of healthcare professionals trained in geriatric care, exacerbated by a rapidly aging population. In response to this pressing need, the Oregon ECHO Network (OEN) has forged partnerships with local and statewide organizations to pioneer a series of ECHO programs aimed at disseminating geriatric care principles across diverse care settings. Over six years, the network's reach has expanded from primary care to encompass post-acute and long-term care communities, culminating in a portfolio of geriatric care ECHO courses. This presentation will share results from an Older Adult ECHO Impact Report covering 28 cohorts of 11 different older adult-related ECHO programs since 2018. OEN has engaged more than 50 faculty and guest speakers and 881 registrants for older adult health care education alone. Faculty panels are interdisciplinary and have included prescribing clinicians, such as physicians and nurse practitioners, along with occupational therapists, pharmacists, registered nurses, behavioral health professionals, physical therapists, peer support specialists and others. Emergent themes, ongoing gaps, lessons learned and program impacts will be shared from participant surveys and interviews. Highlighted are new collaborations with the Oregon Center of Excellence for Behavioral Health & Aging and the Geriatric Workforce Enhancement Program, underscoring efforts to address gaps in geriatric expertise through innovative educational models.

Abstract citation ID: igae098.3815

### BUILDING AN EVALUATION FRAMEWORK FOR THE OREGON CENTER OF EXCELLENCE FOR BEHAVIORAL HEALTH AND AGING

Lindsey Smith[1], Julia Unsworth[1], Allyson Stodola[2], Dani Himes[2], Leah Brandis[3], Karen Cellarius[2], Paula Carder[4], and Walter Dawson[3], *1. Oregon Health & Science University, Portland, Oregon, United States*, *2. Portland State University, Portland, Oregon, United States*, *3. OHSU, Portland, Oregon, United States*, *4. OHSU-PSU School of Public Health, Portland, Oregon, United States*

The Oregon Center of Excellence for Behavioral Health and Aging (OCEBHA) was established in 2024 with the mission to serve as a backbone organization, coordinating efforts to improve behavioral health services for older adults in Oregon. Housed at Portland State University and Oregon Health & Science University, the Center is focused on enhancing workforce training, supporting community partner organizations, advancing research, and promoting policies that improve access to care. We created an evaluation plan aligned with the Collective Impact model. The evaluation framework was developed to assess OCEBHA's effectiveness in growing workforce capabilities, coordinating partnerships, promoting health equity, and driving systemic change. By constructing the Center's role as a backbone organization, the evaluation plan was designed to measure both the process and impact of the Center's efforts across multiple sectors. Key elements of the plan include the integration of community feedback, collaboration metrics, and health equity outcomes. This approach ensures that the evaluation reflects the complex, collaborative nature of the Center's work and provides actionable insights for continuous improvement.

Abstract citation ID: igae098.3816

### CANDIDACY 2.0-UNRAVELING HEALTH INEQUITIES IN CHRONIC CONDITION CARE ACCESS

Sharon Koehn[1], C. Allyson Jones[1], Claire Barber[2], Lisa Jasper[1], Anh Pham[1], Cliff Lindeman[3], and Neil Drummond[1], *1. University of Alberta, Edmonton, Alberta, Canada*, *2. University of Calgary, Calgary, Alberta, Canada*, *3. College of Physicians and Surgeons of Alberta, Edmonton, Alberta, Canada*

The Inverse Care Law, positing that those most in need of healthcare are least likely to receive it, continues to describe persistent health inequities among older adults with chronic conditions. This study introduces Candidacy 2.0 (Chronic Condition (CC)), an innovative framework designed to unravel the complex mechanisms of these inequities in healthcare access for diverse older adult populations. Using Critical Interpretive Synthesis, we analyzed qualitative and mixed methods literature on rheumatoid arthritis experiences across various populations, including racial and ethnic minorities, LGBTQI+ individuals, and those with disabilities. Our key finding was the identification of a crucial eighth dimension: the "embodied relational self," which transforms the framework into a powerful tool for understanding intersectional experiences of health inequity. Candidacy 2.0 (CC) offers a comprehensive understanding of how patients' and care providers' experiences are shaped by systemic injustices and social determinants of health. By integrating approaches like intersectionality, concordance, and recursivity, this model provides a new lens to conceptualize and address health disparities in chronic condition management for older adults. The framework's significance lies in its potential to guide the development of culturally relevant health promotion and intervention efforts, offering concrete explanations for access challenges rooted in socially patterned influences. Our findings have substantial implications for enhancing health equity in geriatric healthcare delivery, informing policy, and driving further research. By implementing Candidacy 2.0 (CC), stakeholders can develop targeted interventions that address the unique healthcare needs of diverse older adults, working towards eliminating disparities across the life course.

Abstract citation ID: igae098.3817

### CARDIOVASCULAR DISEASE RISK AND FALLS IN MEN AND WOMEN LIVING WITH AND WITHOUT HIV: MACS/WIHS COMBINED COHORT STUDY

Elizabeth Vasquez[1], Mark Kuniholm[2], Allison Appleton[3], Anjali Sharma[4], Michael Yin[5], Todd Brown[6], Phyllis Tien[7], and Deborah Gustafson[8], *1. University at Albany, SUNY, Rensselaer, New York, United States*, *2. University at Albany (SUNY), Rensselaer, New York, United States*, *3. University at Albany, Albany, New York, United States*, *4. Albert Einstein College of Medicine, Bronx, New York, United States*, *5. Columbia University, New York, New York, United States*, *6. Johns Hopkins, Baltimore, Maryland, United States*, *7. UCSF, San Francisco, California, United States*, *8. State University of New York Downstate Health Sciences University, BROOKLYN, New York, United States*

Falls risk assessment tools underestimate the risk among People Living with HIV (PLWH). The aim of this cross-sectional analysis was to measure associations between American College of Cardiology/American Heart Association (ACC/AHA) 10-year cardiovascular disease (CVD) risk scores and history of falls in a multi-center United States cohort of PLWH and similar people living without HIV (PLWOH). CVD risk was categorized as low, borderline, intermediate and high (< 5%, 5-7.4%, 7.5-19.9% and ≥20%, respectively) and history of falls was ascertained using a standardized protocol across study sites. Among n=2728 participants (Men, n=939, 578 MLWH/361 MLWOH; (Women, n= 1789, 1274 WLWH/515 WLWOH), median ages were 58 (IQR:51-65) and 55 (IQR:49-60) years for men and women, respectively. Median CVD risk within 10 years was 11% (IQR:6%-18%) and 5% (2%-11%) for men and women, respectively. All analyses were adjusted for age and stratified by sex and HIV status. Higher CVD risk was associated with increased history of falls (high vs low CVD risk: OR=2.31; 95%CI:1.16,4.62; intermediate vs low CVD risk OR=2.13; 95%CI:1.11,4.07) among MLWOH. Findings were similar in MLWH after adjustment for CD4+ T cell count and HIV-1 RNA detectability (<=200). In WLWH, only high CVD risk was associated with falls (OR:1.76; 95%CI:1.13,2.7), while among WLWOH, no association of CVD risk with falls was observed when compared with a low-risk score (< 5%). Results suggest CVD risk scores may be beneficial to guide providers' lifestyle or clinical management recommendations to decrease risk of falls and preserve and/or improve quality of life among MLWOH, MLWH, and WLWH.

Abstract citation ID: igae098.3818

### CARDIOVASCULAR RISK FACTORS AND MENTAL HEALTH OUTCOMES AMONG LGB+ OLDER ADULTS

Jennifer Smith[1], Robert Rodriguez[2], and Joel Anderson[3], *1. University of Tennessee, Knoxville, Knoxville, Tennessee, United States*, *2. University of Texas Medical Branch: School of Public and Population Health, Galveston, Texas, United States*, *3. University of Tennessee-Knoxville, Knoxville, Tennessee, United States*

Emerging evidence highlights that sexual and gender minority (SGM) adults exhibit a higher prevalence of cardiovascular disease (CVD) and its risk factors compared with their non-SGM peers. This increased susceptibility underscores a critical intersection with cognitive health given the link between CVD and cognitive decline and early-onset dementia. Additionally, mental health challenges such as depression and anxiety not only heighten CVD risk but also predict severe cardiovascular complications. Utilizing cross-sectional data from the National Health Interview Survey (NHIS), this study examined these interconnected health issues among older adult LGB+ populations to inform comprehensive interventions aimed at addressing both cardiovascular and cognitive health. This cross-sectional study analyzed data from the 2022 NHIS data to describe cardiovascular and cognitive health characteristics among LGB+ and non-LGB+ older adults using chi-squared tests. The sample was limited to adults who self-reported being age 50 years or older. A CVD risk variable was created based on Framingham criteria. Data from 13,426 participants, including 341 LGB+ individuals, revealed significant differences in demographic characteristics, health, and lifestyle. LGB+ older adults were younger and more frequently formally educated. They exhibited lower rates of CVD risk factors and increased physical activity and preventative health behaviors, yet higher incidence of depression and anxiety and higher rates of smoking and alcohol use. These preliminary findings highlight the unique characteristics of LGB+ older adults in terms of cardiovascular health, offering potential targets for culturally tailored interventions.

Abstract citation ID: igae098.3819

### CARE MANAGER PERCEPTIONS OF A CARE COORDINATION INTERVENTION IN THE ED FOR RESIDENTS IN ASSISTED LIVING COMMUNITIES

Grace Wittenberg[1], Peter Serina[2], Nichole Stetten[3], Ann Reddy[2], and Ellen McCreedy[4], *1. Brown University School of Public Health, Eau Claire, Wisconsin, United States*, *2. Brown University School of Public Health, Providence, Rhode Island, United States*, *3. Brown University, Brown University, Rhode Island, United States*, *4. Veterans' Administration, Providence, Rhode Island, United States*

Of the 800,000 residents in US assisted living communities, nearly half are transferred to the Emergency Department (ED) each year, often without a support person or up-to-date medical information. This lack of information can lead to more testing in the ED, increased ED length of stay, and unnecessary hospitalizations. To address this problem, Bluestone Physician Services, providing care to ALC residents in Florida, Minnesota, and Wisconsin, developed the ED Early Response Program for patients in its accountable care organization. In the program, a complex care manager (CCM) is notified when one of Bluestone's ALC patients registers at an ED. Bluestone staff then provide standardized, clinical information to the ED via phone and fax. To analyze the feasibility and acceptability of the program, we conducted semi-structured interviews with 12 CCMs involved in the program. Using directed content analysis, we identified five overarching themes: populations most likely to benefit from the program (e.g. people living with dementia, hospice), key components of the program, program strengths, areas of opportunity, and impact on patient care. CCMs described that the program positively impacted care transitions to the ED by providing added clinical information and fostering interprofessional communication. However, they also noted areas for improvement, including enhancing ED provider receptivity by increasing awareness of the program in the ED. Overall, by using CCM insights gained during implementation, this program can be used as a model to address key communication gaps for ALC residents during transitions of care, including ED visits.

Abstract citation ID: igae098.3820

### CARE TRANSITION EXPERIENCES OF HISPANIC PATIENTS WITH DEMENTIA FOLLOWING EMERGENCY DEPARTMENT DISCHARGE

Jacqueline Sandoval[1], Juliette Brook[2], Zoe Lien[1], Joan Monin[3], Cameron Gettel[4], Ashley Hagaman[1], and Ula Hwang[5], *1. Yale University, New Haven, Connecticut, United States*, *2. University of Pennsylvania, Perelman School of Medicine, Philadelphia, Pennsylvania, United States*, *3. Yale School of Public Health, New Haven, Connecticut, United States*, *4. Yale University School of Medicine, New Haven, Connecticut, United States*, *5. New York University, New York, New York, United States*

Older patients report fragmented emergency department (ED) care, including insufficient communication with clinicians and inadequate referrals to outpatient care and social services. These challenges are likely even greater for those with cognitive impairment (CI) and their care partners (CPs) and especially for non-English speaking populations, where language differences bring additional challenges. This study seeks to fill a gap in the literature by exploring ED discharge care transition experiences for Hispanic patients with dementia. Ten dyads - consisting of a patient with CI or dementia diagnosis and their CP - were enrolled in the ED and completed semi-structured interviews (via video or telephone) 1 week later addressing their ED and post-discharge experiences. Interviews investigated participants' experiences in the ED and subsequent care transitions back to the community. Interviews were recorded, transcribed and coded by three research team members for thematic content, adopting a combined deductive-inductive approach until thematic saturation was reach across all dyads. Prespecified codes were adapted using the National Quality Forum's Quality Measurement Framework on ED Transitions of Care and included eight outcomes for ED transitions of care: accessibility of services, shared decision-making, connection and alignment, care coordination, information sharing/communication, safety, healthcare utilization/costs, and experience of care. Key study findings emphasize patient and care partner priorities of shared decision-making with the ED treating team, how patients and care partners manage language barriers and cognitive impairment challenges and identifying factors that either support or hinder the quality and safety of ED discharge care and transitions back home.

Abstract citation ID: igae098.3821

### CARE TRANSITIONS FROM HOSPITAL TO HOME: PERSPECTIVES FROM OLDER ADULTS, CAREGIVERS, AND HEALTHCARE PROFESSIONALS

Taghreed Alotaibi[1], Judith Sixsmith[2], roseanne cetnarskyj[1], and mei Fang[3], *1. Dundee University, Dundee, Scotland,*

*United Kingdom*, 2. *University of Dundee, Dundee, Scotland, United Kingdom*, 3. *SIMON FRASER UNIVERSITY, Simon Fraser University, British Columbia, Canada*

**Background:** Saudi Arabia switched from hospital-focused to home-based care for older persons with long-term care needs due to a 25% increase in acute hospital bed occupancy for non-acute older adults. Healthcare professionals, older adults, and families are affected by the change in the care transition. Improvement in health outcomes depends on understanding all three groups.

**Aim:** This study aims to investigate the hospital-to-home transition of older adults with chronic conditions. The objective is to identify factors that facilitate or hinder the transition, with the aim of improving care during this critical phase.

**Methods:** The research employed a descriptive case study analysed with thematic analysis and an institutional ethnography. Pre- and post-discharge data were obtained from older adults, carers, and healthcare workers at King Saud Medical City using semi-structured interviews, focus groups, participatory observation, and documentary analysis.

**Findings:** The findings indicate that culture, family, and national healthcare policies play a crucial role. Older adults and their caregivers encounter obstacles during the transition process, including gender segregation causing difficulties with care coordination. The lack of standardised post-discharge follow-up and fragmented care provision further contribute to these hurdles.

**Conclusion:** The research recommends creating a unified administration system as a necessary foundation for solving the largest problems in the transition. This may help Saudi Arabia's care policymakers and planners create a family-centred healthcare system. Future research should focus on targeted strategies to improve the care transition experiences of older adults and their caregivers.

Abstract citation ID: igae098.3822

### CAREGIVING IMPACT ON MEDICARE OLDER ADULTS: RESULTS OF THE US HEALTH AND WELLNESS SURVEY

Lycia Tramujas Vasconcellos Neumann[1], Kathy Annunziata[2], and Lauren Stratton[1], *1. Alzheimer's Association, Chicago, Illinois, United States*, 2. *Oracle Life Sciences, Austin, Texas, United States*

As the number of older adults caring for someone with a serious illness increases, there is a growing need to understand better the impact of caregiving on older adults' health and finances. Based on responses from older Medicare recipients (65+) to the US 2022 National Health and Wellness Survey, an internet-based, cross-sectional survey, this study investigated differences in self-reported health status, healthcare utilization, and out-of-pocket costs between (1) older adults who are caregivers (n = 1,159) and those who are not (n = 11,443), and (2) older caregivers of individuals living with Alzheimer's disease or other dementias - ADRD (n = 255) and those caring for an adult with other conditions (n = 904). Results were weighted to the population by sex, age, and race/ethnicity. Caregiver and non-caregiver cohorts had comparable median and mean ages. Compared with older non-caregivers, older caregivers had significantly higher comorbidities and significantly lower global health, physical health, and mental health T scores ($p< 0.05$). There were no significant differences in reported use of ER, hospitalizations, and median number of medical visits in the six months before the survey. However, older caregivers reported higher mean out-of-pocket Rx expenses than non-caregivers ($44.51 vs. $34.02). Comparative analyses of older dementia caregivers with non-dementia caregivers did not indicate significant differences in health and financial impact. In conclusion, caregiving impacts older adults' health in various ways, and health plans and providers should acknowledge and consider the impact of caregiving when assessing older adults' health conditions, managing and coordinating their care.

Abstract citation ID: igae098.3823

### CAREGIVING STRESS IN ADRD CAREGIVERS: THE INFLUENCE OF SOCIAL DETERMINANTS OF HEALTH AND SOCIAL SUPPORT

Kyungmi Lee[1], and Chunhong Xiao[2], *1. Case Western Reserve University Frances Payne Bolt, Cleveland, Ohio, United States*, 2. *University of Alabama at Birmingham/School of Nursing, Birmingham, Alabama, United States*

Alzheimer's disease and related dementias (ADRD) caregivers face significant physical, emotional, and financial challenges. These stressors are influenced by social determinants of health (SDOH), such as income and service accessibility, as well as caregiver-specific factors like health status and caregiving burden. Social support can help mitigate caregiving stress. This study aimed to examine the relationships among SDOH, caregiver-specific factors, social support, and caregiving stress in ADRD caregivers. Utilizing data from the Caregiving in the U.S. 2020 study (n = 357 ADRD caregivers), we conducted a multiple regression analysis ($F = 15.236$, $p < 0.001$). Results show that social support significantly reduces caregiving stress ($\beta = -0.261$, $p = 0.004$). Caregiver health status ($\beta = -0.592$, $p < 0.001$), difficulty accessing services ($\beta = 0.770$, $p < 0.001$), and caregiving burden ($\beta = 0.511$, $p < 0.001$) are also significant predictors of stress. Although age did not directly impact caregiving stress, it significantly influenced service access ($\beta = -0.010$, $p = 0.036$), which in turn affected stress levels. Financial strain ($\beta = 0.270$, $p < 0.001$) and physical strain ($\beta = 0.171$, $p = 0.005$) were also associated with service accessibility issues. These findings suggest that enhancing social support and improving service access could effectively reduce caregiving stress. Future research should further investigate these areas to develop targeted interventions for ADRD caregivers.

Abstract citation ID: igae098.3824

### CARING FOR NURSING HOME RESIDENTS WITH DEMENTIA AND SERIOUS MENTAL ILLNESS: A QUALITATIVE STUDY

Laura Block[1], Andrea Gilmore-Bykovskyi[2], Donovan Maust[3], and Tonya Roberts[4], *1. University of Wisconsin-Madison, Madison, Wisconsin, United States*, 2. *University of Wisconsin-Madison School of Medicine and Public Health, Madison, Wisconsin, United States*,

*3. University of Michigan, Ann Arbor, Michigan, United States, 4. University of Wisconsin - Madison, Madison, Wisconsin, United States*

Over half of nursing home (NH) residents are living with dementia, and a growing number also experience serious mental illness (SMI) and/or comorbid dementia and SMI. Despite the heightened vulnerability of this population, studies indicate NHs struggle to meet their needs, yet little research has documented specific challenges or strategies for their care. By employing a qualitative descriptive design, this study aims to describe NH staff perspectives on caring for residents with dementia and SMI. Partnerships were developed with NH and provider networks servicing rural and urban areas of a Midwest state, with purposive sampling of not-for-profit, county-run and for-profit facilities. Staff completed semi-structured interviews, which were analyzed applying thematic analysis. Ten individuals from three facilities have participated. While all participants identified dementia as common among residents, recognition of SMI as prevalent varied by staff and facility type. Participants described an interaction whereby a resident's past (e.g., trauma) interacts with their mental health, functional, behavioral, and cognitive needs, which then influences selection of care and symptom management strategies. Staff described person-centered care as a universal best practice, while prescribing clinicians were attuned to appropriate pharmacology that reflected the resident diagnosis. Staff in some facilities described developing tailored behavioral and crisis plans. Findings suggest potential best practices to support residents with dementia and SMI, yet ongoing challenges caring for individuals with more complex behaviors and/or comorbid SMI. Future research should integrate further frontline nurse and nursing assistant perspectives, as additional strategies and challenges translating care plans to practice may exist.

Abstract citation ID: igae098.3825

### CELEBRATING THE 100TH DAY OF SCHOOL: PILOTING AN AGEISM-REDUCING TOOLKIT FOR PREK TO SECOND GRADERS

Tina Newsham[1], Cynthia Hancock[2], Daniel Alston[2], Lisa Borrero[3], Katherina Nikzad-Terhune[4], Sarah Tesar[2], and Elizabeth Fugate-Whitlock[1], *1. University of North Carolina Wilmington, Wilmington, North Carolina, United States, 2. University of North Carolina Charlotte, Charlotte, North Carolina, United States, 3. University of Indianapolis, Indianapolis, Indiana, United States, 4. Northern Kentucky University, Lexington, Kentucky, United States*

Upon learning that some celebrations of the 100th day of school involve young children dressing up "like a 100-year-old," our team questioned the ageist ideas this activity could reinforce. Children adopt beliefs about various social groups at an early age. They are inundated with ageist messages through books, TV, and other sources and internalize those ideas without questioning them. Those who hold negative views of aging live, on average, 7.5 fewer years than those who hold positive views. To prevent negative outcomes, children must be taught accurate and positive information about aging and older adulthood from an early age. The purpose of our research project is to provide teachers with evidence-based educational materials related to aging (centenarians in particular). We created a toolkit about aging and ageism to give PreK and elementary teachers options for celebrating the 100th day of school in a way that reinforces academic content while supporting age-inclusivity. The toolkit includes lesson plans celebrating centenarians. We used a mixed-method approach to pilot test the toolkit with 40 teachers and obtained a complete data set from 25 of them. Quantitative data were collected pre- and post-pilot using the 12-item Expectations Regarding Aging Survey. Qualitative data were collected through online pre-and post-pilot survey questions and post-pilot semi-structured interviews. We provide details on the toolkit and findings from the pilot study, which revealed a statistically significant improvement in expectations about aging ($p< 0.05$) and strong qualitative support for the value of the toolkit.

Abstract citation ID: igae098.3826

### CHALLENGES FACING PRIMARY CARE NURSE PRACTITIONERS CARING FOR PERSONS LIVING WITH DEMENTIA

Kyle Featherston, Maura Dougherty, Siqing Wang, and Lusine Poghosyan, *Columbia University School of Nursing, New York, New York, United States*

The nurse practitioner (NP) workforce plays a vital role in caring for the growing population of persons living with dementia (PLWD). However, organizational and structural barriers hinder their contribution to patient care. This study presents results from a national sample of 968 primary care NPs who care for PLWD. NPs were asked to report on the organizational support and resources available in their practices to care for PLWD. NPs also reported on the relationships between NPs and administration, as well as NP job satisfaction, burnout, and intentions of leaving their jobs. NPs reported their organizations lacked the resources to care for PLWD that they had for patients with other chronic diseases. Specifically, only 14.67% reported a registry of PLWD overdue for services and fewer than 60% had tools, templates, and reminders for PLWD that were largely available for chronic conditions like hypertension and dementia. Crucially, these deficits in the organizational context of care resulted in NPs rating the quality of the care provided to PLWD at their organization lower than the overall quality of care the organization delivered. Although NPs were largely satisfied with their jobs, over one-third reported being burned out, and over one-fifth reported they intended to leave their jobs within the next year. Addressing these barriers to care and creating work environments conducive to NP practice are necessary to fully utilize the capacity of the NP workforce to improve care of the older adult population living with dementia.

Abstract citation ID: igae098.3827

### CHARTING NEW PATHS: LGBTQIA+ GERIATRICS FELLOWSHIPS

Roy Noy[1], and Noelle Marie Coloma Javier[2], *1. Tel Aviv Sourasky Medical Center, Tel Aviv, Israel, 2. Icahn School of Medicine at Mount Sinai, NY, New York, United States*

**Background:** As the global population aged 65 and older continues to grow, so does the number of individuals identifying as LGBTQIA+. Despite this increase LGBTQIA+ health content are often overlooked in medical education, leading to significant disparities in care. Recognizing this gap, the American Geriatrics Society emphasized addressing LGBTQIA+ health disparities as early as 2015.

**Methods:** Our pilot program, a collaboration between the Brookdale Department of Geriatrics and Palliative Medicine at Mount Sinai in New York and the Tel Aviv Medical Center (TAMC), is designed to address disparities in LGBTQIA+ healthcare. As part of this initiative, a geriatric resident from TAMC completed a four-month observership at Mount Sinai, receiving specialized training tailored to LGBTQIA+ healthcare for older adults. The hands-on curriculum allowed the resident to shadow various services, including geriatric and palliative care, rehabilitation, and specialized clinics such as the Institute for Advanced Medicine and the Center for Transgender Medicine. In addition, the resident engaged in one-on-one educational sessions focused on LGBTQIA+ care.

**Results:** The outcomes of this pilot program include the creation of a tailored and replicable curriculum for geriatric residency that can be applied to other programs, scholarly peer-reviewed publications, sustainable collaborations, partnerships with advocacy organizations, and the establishment of Tel Aviv's first LGBTQIA+ geriatric clinic.

**Conclusions:** Pilot programs like ours hold significant promise for advancing LGBTQIA+ health education and improve the quality of care for older LGBTQIA+ adults, as evidenced by the establishment of Tel Aviv's first LGBTQIA+ geriatric clinic.

Abstract citation ID: igae098.3828

### CHATGPT ANALYSIS OF AGEISM IN THE 2024 PRESIDENTIAL ELECTION: RESEARCH, POLITICS, AND POLICY IMPLICATIONS

Faith Hopp, *Wayne State University, Detroit, Michigan, United States*

Gerontologist Robert Butler introduced the concept of Ageism, described as "the great sleeper in American life" in a 1968 interview with journalist Carl Bernstein. Ageism refers to discrimination based on age and has been particularly evident during the 2024 election season. To further examine this relevant phenomenon, we used ChatGPT to analyze instances of ageism related to President Biden's campaign. We instructed ChatGPT to collect a sample of articles from mainstream media mentioning President Biden (N=23), asking the program to score the frequency and type of ageist language during the month before and after the June 27 2024 Biden-Trump debate. Ageist themes included statements about Biden being a bad leader, not trustworthy, not mentally and/or physically fit, and not competent and/or relevant as a candidate. The number of ageist statements about Biden increased in the month following the debate compared with the pre-debate month. We found fewer ageist-related statements about Biden in the African American press both before and following the debate, but access to these articles was limited due to paywall restrictions. These findings suggest that ageism continues to be relevant and will continue to drive age-related discourse, impacting policy, practice, and older adult life experiences. Future researchers should consider the role of internet paywalls in limiting public access to news sources and the breadth of content available for ChatGPT analysis. We recommend that researchers obtain full-text articles using newspaper databases rather than relying on ChatGPT internet scans to ensure comprehensiveness and analytical depth.

Abstract citation ID: igae098.3829

### COMBATING AGEISM: EVALUATING THE IMPACT OF AN AGE-POSITIVE TRAINING IN HEALTHCARE

Barbara Lester, *Intermountain Health, Salt Lake City, Utah, United States*

Ageism among health and social service providers can have a negative impact on the services and outcomes for older adults navigating these systems. The U.S. population aged 65 and older is expected to nearly double by 2060, underscoring the need to address ageism in healthcare. To address this issue, this study explored the impact of "Building Momentum with Age Positive Teams," an evidence-informed one-hour employee assistance training program designed to reduce ageism and foster an age-positive culture among healthcare employees. The program, adapted from the "Ending Ageism Together" workshop, was implemented from December 2023 to June 2024 at Intermountain Health, a health system with almost 70,000 employees. A total of 76 participants participated in the training and completed both the pre-test and immediate post-test, showing a significant improvement in the total score on the Expectations Regarding Ageing Scale (ERA-12) ($p < 0.0001$). Furthermore, 38 participants also completed a third follow-up post-test administered 30 days after completing the program. Findings indicated that there were sustained significant improvements from pre-test to both immediate post-test and 30-day post-test ($p < 0.0001$). The study's findings highlight the potential effectiveness of targeted educational and workforce development interventions in reducing ageism, promoting more inclusive healthcare practices, and aligning with broader goals of reframing aging narratives. Additionally, findings demonstrated that although knowledge about aging improved, retention of this knowledge varied over time across participants. These results support the ongoing need for educational strategies that combat ageism and promote positive attitudes towards aging in healthcare.

Abstract citation ID: igae098.3830

### COMMUNITY ENGAGEMENT, SOCIAL SUPPORT, AND RELATIONSHIP DYNAMICS AMONG OLDER ADULTS POST-COVID-19

Limei Chen[1], Mengzhao Yan[2], and Philip Rozario[3], *1. George Mason University, Fairfax, Virginia, United States*, *2. University of Southern California, Los Angeles, California, United States*, *3. Adelphi University School of Social Work, Adelphi University School of Social Work, New York, United States*

Social integration theory and social capital theory suggest the positive effect of community participation and the benefits of social connections gained in the community on health and wellbeing in later life. However, it is unclear whether these positive effects persist under the social restrictions imposed by the COVID-19 pandemic. To understand if the benefits of community participation can withstand such unprecedented disruptions, this study explores how community participation influences spousal/partner relationships, physical health, and mental health among older adults before and after COVID-19. Using linked data of the National Social Life, Health, and Aging Project (NSHAP) Round 3 and Covid-19 Study, we assess the impacts of volunteering, attending meetings, and religious activities using multinomial regrenssion models. Findings reveal that community participation, particularly volunteering and attending meetings, is strongly associated with improved relationship outcomes, even under stress from events related to the COVID-19 pandemic. Religious activities also support better relationships, especially when stressful events are present. While social support further enhances these positive effects, it can diminish the independent benefits of volunteering. For physical and mental health, community engagement serves as a protective factor, mitigating the adverse effects of stressful events. These results underscore the crucial role of active community involvement in promoting well-being and maintaining positive spousal/ partner relationship dynamics among older adults, highlighting its importance as a buffer against stressful events.

Abstract citation ID: igae098.3831

### COPING WITH PERSISTENT NURSING HOME CHALLENGES: TEAM FUNCTIONING STRATEGIES TO EMPOWER DIRECT CARE WORKERS

Alfred Boakye[1], and Jennifer Morgan[2], *1. University of Maryland, Baltimore, Baltimore, Maryland, United States*, *2. Georgia State University, Atlanta, Georgia, United States*

Nursing homes continue to experience staffing shortages, heavy workloads, poor quality indicators, and infection control challenges. Increasing levels of acuity and numbers of residents with challenging behaviors requires high-functioning teams in order for nursing homes to deliver high-quality person-centered care. Extant literature has described the benefits of developing and building teams to reduce staff conflict, reduce staff turnover and absenteeism, improve financial outcomes, and increase quality of care, motivation, and job satisfaction. Team functioning and improved communication can play a crucial role in delivering positive resident care outcomes. The aim of this study was to identify strategies that nursing homes can use to improve team functioning and communication in service of improved resident outcomes. Using a phenomenological qualitative approach with semi-structured interviews, twenty-five (25) nursing assistants were recruited from two high-quality nursing centers to understand team-related coping strategies adopted in order to combat persistent stressors. Interviews lasted 30-45 minutes. Interviewees were predominately Black (23), women (23), and had modal age range of 41-50 years old. Results of the analysis suggest that strategies such as formalizing team structures, dismantling cliques, encouraging the use of collaboration strategies, and tailoring check-in options for individual workers within teams (e.g., emails, phone calls and daily huddles) are critical to maximizing team functioning. These themes support our recommendation that nursing home leaders could profitably implement trauma-informed approaches to build more resilient teams. Trauma-informed principles such as safety, choice, collaboration, trustworthiness and empowerment offer values that can be infused into positive work cultures and support high-functioning teams.

Abstract citation ID: igae098.3832

### CREATING DIGITAL STORIES ON AGING EXPERIENCES: A RAPID METHODOLOGICAL REVIEW OF DEVELOPMENT PROCEDURES

Nisha Gopalakrishnan[1], Katlyn Mellett[1], Noeman Mirza[2], Wendy Hulko[1], Daniel Gan[3], Karen Lok Yi Wong[4], Anthony, L. Kupferschmidt[5], and Heather Lee[6], *1. Thompson Rivers University, Kamloops, British Columbia, Canada*, *2. University of Winsdor, Toronto, Ontario, Canada*, *3. University of Toronto, Toronto, Ontario, Canada*, *4. University of British Columbia, Vancouver, British Columbia, Canada*, *5. City of Vancouver, Vancouver, British Columbia, Canada*, *6. City of Kamloops, Kamloops, British Columbia, Canada*

Digital storytelling is increasingly used to showcase the life histories of older adults and their communities. The Mobilizing Aging in Place (MAP) project team was tasked to engage older adults in the creation of five digital stories as a means of sharing the findings of two studies on aging in place with municipal and regional planners and health care policy/decisionmakers. While there were numerous examples of digital stories on aging experiences, the best way to develop digital stories with older adults was unclear. To create clarity in effective methods, we conducted a rapid methodological review of existing literature on digital storytelling involving older adults and experiences of aging. After searching in the Discovery search engine, we reviewed 61 abstracts and articles based on specific inclusion and exclusion criteria. This yielded 18 articles that we included in the review. The included literature discussed digital stories on various health and intergenerational knowledge transfer topics. Researchers used a variety of software, editing tools, and recording methods to combine images, videos, and sounds. Digital stories were developed both in collaboration with older adults and by the older adult participants themselves. The review showed that there are no set criteria or best practices for creating digital stories, and that workshops and interviews are commonly used to engage older adults in digital storytelling. We recommend a more comprehensive and broader review be undertaken with a focus on all forms of digital storytelling regardless of type of population and context.

Abstract citation ID: igae098.3833

### CULTURAL COMPETENCE TRAINING AND EVALUATION AMONG NURSING HOME STAFF

Tochukwu Okolie[1], Tina Sandri[2], Staja Booker[3], Prince Ekoh[4], and Kingsley Udeh[5], *1. Miami University, Oxford, Ohio, United States*, *2. Forest HIlls of DC, Washington DC, District of Columbia, United States*, *3. University of Florida, University of Florida, Florida, United States*, *4. University of Calgary, Calgary, Alberta, Canada*, *5. MIAMI UNIVERSITY OHIO, oxford, Ohio, United States*

The dynamics of cultural and ethnic identity in healthcare has become more salient in the United States due to the growing population diversity. In particular, significant number of nursing home staff are from diverse sociocultural groups, which necessitates cultural competency in nursing homes in order to provide quality care to older adults. Cultural competency in nursing homes is usually analyzed in terms of the interplay between staff members and the older adults, but this study investigated the relational cultural competency between staff members in the workplace. First, cultural competency training on promoting inclusion and respecting diversity, and communication/teamwork was provided to staff members working in a nursing home in Washington, D.C. To evaluate the level of cultural competency post-training, a semi-structured in-depth interview was conducted with 16 nursing home staff. Participants were purposefully selected to capture variation in cultural identity and ethnicity, national origin, work experience and position at the organization. The interviews were conducted individually and audio-recorded after consent was obtained. The audio files were transcribed verbatim and analyzed with the aid of Nvivo12. Findings show that the training was positively perceived as having impacted their outlook of culture and identity in the workplace. Most of the participants were comfortable with working with individuals from different cultures and identified techniques used in communicating when there are language barriers, including utilizing interpreters and translation tools. Participants recommended that organization's leadership should create more opportunities for intercultural exchange among staff members through potlucks, and recognizing national holidays of staff members.

Abstract citation ID: igae098.3834

### DEVELOPMENT AND ASSESSMENT OF THE VIRTUAL INTERGENERATIONAL COMPARATIVE GLOBAL AGING CURRICULUM

Grzegorz Zurek[1], Alina Zurek[2], and Lyn Holley[3], *1. Wroclaw University of Health and Sport Sciences, Wroclaw, Dolnoslaskie, Poland*, *2. University of Wroclaw, Wroclaw, Dolnoslaskie, Poland*, *3. University of Nebraska at Omaha, Omaha, Nebraska, United States*

This presentation describes didactic process in the international, experimental, hybrid course, Comparative Global Aging. Created by the University of Nebraska at Omaha and two Polish universities – the University of Wroclaw and the University School of Physical Education in Wroclaw, it was structured to nurture intra- and intergenerational relationships and understanding between students and older adults on different continents. Classes are conducted in a "real time" audiovisual format designed to accommodate geographical and temporal distance and cultural diversity of professors and students. Methods to track independent learning and conscious self-improvement of students were custom-fitted to the course and to the requirements of information/communication technology. The course has been conducted each year from 2018 to 2023. Qualitative data from students' courses in each edition of the Post-Class Reflections and Learning Journals were used to evaluate and improve a series of planned and systematic teaching methods inspired by both teachers and students. Data are from 44 UNO students, 23 students from Poland and 35 students from European universities in 9 European countries participating in the ERASMUS academic student exchange program.. Although courses were conducted in English, student teams were intentionally multinational and multicultural. Qualitative evaluation of data from students informed continuous improvement of methods and resources in subsequent editions of the course, and indicate it meets accepted standards for international, intergenerational, comparative, gerontological, service-learning university and virtually synchronous pedagogies, and could provide a template for courses that give learners the opportunity to work collaboratively international teams and communicate effectively.

Abstract citation ID: igae098.3835

### DIFFERENCES IN HOME HEALTH SERVICES AND OUTCOMES FOR MEDICARE ADVANTAGE PLANS WITH EPISODIC VS. PER-VISIT PAYMENTS

Rachel Prusynski[1], Anthony D'Alonzo[2], and Michael Johnson[2], *1. University of Washington, Seattle, Washington, United States*, *2. BAYADA Home Health Care, Pennsauken, New Jersey, United States*

For millions of older adults receiving home health (HH) services annually, those with Medicare Advantage (MA) plans receive fewer visits and have worse functional outcomes compared to similar patients with Traditional Medicare (MA) plans. However, the thousands of MA plans available to beneficiaries are not created equal. MA plans with episodic payments have fewer administrative restrictions for HH providers than MA plans that pay per visit. This study of 285,297 HH episodes from 2019-2022 used robust inverse probability of treatment weighting to detect differences in HH service provision and patient outcomes between TM, episodic MA, and per-visit MA plans. Patients with both MA plans had shorter HH episodes and fewer visits from nurses, physical, occupational, and speech therapists, and home health aides than TM. Compared to TM, patients with episodic MA were less likely to improve in mobility and self-care function (Odds ratio [OR] 0.92 and 0.94, respectively) but had lower rates of inpatient admissions during HH (OR=0.95). Patients with per-visit MA had higher rates of community discharge (OR=1.06) and inpatient admissions (OR=1.06) than TM. Per-visit MA plans had higher rates (OR=1.12) of inpatient admissions than episodic MA. While both MA plans provide fewer HH services than TM, outcomes for MA plans versus TM are mixed depending on the MA plan payment structure. However, when comparing MA plans, per-visit plans have higher rates of inpatient admissions than episodic plans, a negative patient outcome. HH providers negotiating with MA plans should prioritize episodic payments to maximize outcomes and reduce administrative burden.

Abstract citation ID: igae098.3836

### DIGITAL HEALTH AND OLDER ADULT WELL-BEING: THE MEDIATING ROLE OF SOCIAL TECHNOLOGY USE

Shannon Power, *University of Georgia, Athens, Georgia, United States*

Older adults engage in digital health use (DHU) and social technology use (STU) to connect with health care providers and loved ones. DHU has expanded as an accessible health care option, though its effects on well-being are underexplored. Meanwhile, STU is known to improve quality of life for older adults. This study investigates the association between DHU and well-being and determines whether STU mediates this association. Data from the 2022 National Health and Aging Trends Study were analyzed, with a sample of 5,773 U.S. Medicare beneficiaries, ages 65 and older. The sample was primarily White (64%), female (58%), 65-79-years-old (52%), and urban-living (84%). Structural equation modeling captured the complex relationships of the latent constructs of DHU (e.g., telehealth visit, sending portal messages), STU (e.g., video chat, texting, social media), and well-being (e.g., belief in one's life purpose, self-determination, confidence). Confirmatory factor analyses with sufficient fit indices established validity of the measurement models. In the full model, DHU was negatively associated with well-being (β=-0.22, p<.001); however, a mediation analysis revealed a strong partial mediation (β=0.32 (CI 0.24-0.46), SE=0.055, z=6.163, p<.001) with acceptable model fit. Adding STU as a mediator reversed the direction and increased the magnitude of the association between DHU and well-being. The significant negative path between DHU and well-being may indicate that DHU is not a preferred option for older adults, but the partial mediation suggests that STU offers familiarity with communication technology that strengthens the association towards well-being. Future research should clarify the reasons underlying these significant associations.

Abstract citation ID: igae098.3837

### EARLY CHILDHOOD EXPERIENCES AND LATE LIFE COGNITIVE FUNCTION AMONG OLDER CHINESE ADULTS

Duy Nguyen[1], Rui Liu[2], Limei Chen[3], and Yookyong Lee[4], *1. Sacred Heart University, Teaneck, New Jersey, United States*, *2. Sacred Heart University, Fairfield, Connecticut, United States*, *3. George Mason University, Fairfax, Virginia, United States*, *4. University of Alabama - Birmingham, Birmingham, Alabama, United States*

Extensive research has documented the negative impacts of adverse childhood experiences on individual well-being, yet Asian older adults have been overlooking. For the current study, we used the China Health and Retirement Longitudinal Study (CHARLS), a national, longitudinal study of representative residents and applied the Life Course Perspective to test how childhood conditions and social relationship indicators affect cognitive health at later life. This study combined data from three datasets: Wave 1 (2011), The Life History Survey Data (2014), and Wave 5 (2020). The study sample (N=9,941) included respondents 50 and over at baseline, without cognitive impairment in the baseline, and still living at Wave 5. Women constituted 52.4% of the sample, and the mean age was 60.2 (SD=6.77). Two outcome variables (self-rated memory and cognitive health status) were used in data analyses. The multivariate analyses results showed that weak neighborhood quality and social cohesion, poor social relationships, poor childhood health status were associated with worse cognitive function. The study findings demonstrate the effects of early childhood conditions on late-life cognitive function. As aging in China accounts for most global aging, public health approaches that stimulate positive childhood memories can have a meaningful protective effect for older adult well-being. Our findings underscore the global importance of childhood health, childhood experiences, and social relationships, which are not only those older adults in China but globally. Further cross-national research is needed to understand the diversity in sociohistorical experiences across global aging populations.

Abstract citation ID: igae098.3838

### EFFECTS OF DISCRIMINATION ACROSS THE LIFE COURSE: A CASE STUDY OF NARRATIVE METHOD ON TAIWANESE MOVIE BANANA PARADISE

Sheng-Chin HSU, *Ming Chuan University, Taipei, Taipei, Taiwan (Republic of China)*

The effects of discrimination across the life course are one of the products from the cumulations of diverse individuals and groups. This paper aims to present the results of effects of discrimination across the life course on Taiwan movie "Banana Paradise" through a narrative theory. "Banana Paradise" expressed the changes of main character in his life course, and showed diversity of his identity and discrimination via these changes during 1949 to 1989. The main objective is to offer an alternative to understand what effects of discrimination are constructed by the shifting across the life course and at the same time to show how effects of discrimination were embodied by interactions with others across one's life course.

Abstract citation ID: igae098.3839

### ENHANCING SOCIAL CAPITAL IN OLDER ADULTS: STRATEGIES IN THE ASIAN CONTEXT

On Fung Chan, Terry Lum, Shiyu Lu, and Hiu-kwan Chui, *The University of Hong Kong, Hong Kong, China (People's Republic)*

Research on enhancing social capital among older adults in East Asia often neglects the aspects of trust, norms, and reciprocity, focusing primarily on social participation. Moreover, it frequently overlooks the capacity of older adults for collective action within society. This study addresses these gaps by exploring strategies that enable older adults in Hong Kong to shift from being passive service recipients to active participants in community and collective action. Using a qualitative research design. We conducted focus groups with 43 participants over 60, who were committee members of various socially beneficial groups, including self-initiated and NGO-led groups. These groups engaged in activities such as promoting age-friendliness, ageing in place and ecological conservation. Data were collected through semi-structured interviews and analyzed using deductive and inductive thematic analysis based on Nahapiet and Ghoshal's framework. The findings identified five key strategies to enhance social capital among older adults: establishing connections with trusted intermediaries, transforming individuals to share collective goals, providing stable platforms for trust-building, facilitating informal interactions to enhance cohesion, and leveraging influential figures to reinforce group norms. These strategies empower older adults and integrate them into the broader social fabric, thereby contributing to collective action. The study provides actionable insights for policymakers and social workers to develop interventions that enhance social capital among older adults, thereby leveraging their potential as active societal contributors.

Abstract citation ID: igae098.3840

### ETHNIC AND RACIAL DIFFERENCES IN END-OF-LIFE CARE IN TEXAN COLORECTAL CANCER DECEDENTS

Bethany Leach[1], Efstathia Polychronopoulou[2], Yong-Fang Kuo[3], Mukaila Raji[4], and Myrna Serna[2], *1. University of Texas Medical Branch at Galveston, Galveston, Texas, United States*, *2. The University of Texas Medical Branch, Galveston, Texas, United States*, *3. University of Texas Medical Branch, Galveston, Texas, United States*, *4. University of Texas Medical Branch, Galveston, Galveston, Texas, United States*

There are known racial and ethnic disparities in advance care planning (ACP) and downstream outcomes. We hypothesized that among Texan Medicare decedents with colorectal cancer (CRC), there would be racial and ethnic disparities in ACP, palliative care (PC) consultation and hospice enrollment. Data from the Texas Cancer Registry linked with Medicare claims records for patients age ≥66 years who died 6 months to 5 years after a primary CRC diagnosis between 2010 and 2018 was analyzed. Number (proportion) of Non-Hispanic Black (NHB), Non-Hispanic White (NHW) and Hispanic decedents with ACP, PC consultation and hospice enrollment in the last 30 days of life are reported and chi-square tests used to assess statistically significant differences. A multivariable logistic regression model was used to assess racial/ethnic differences in odds of hospice enrollment, adjusting for CRC stage and other demographic variables. Among 5,757 Medicare decedents, ≤2.2% had ACP with no significant racial/ethnic differences noted. While NHBs were most likely to receive PC consultation (106 (18%), p=0.017), they were least likely to be hospice enrolled (369 (62%), p< 0.001). The multivariable logistic regression model showed NHBs had decreased odds of hospice enrollment when compared to NHWs (OR 0.67, 95% CI 0.55-0.81). Among Medicare decedents with CRC, there are low rates of ACP and racial/ethnic disparities in PC consultation and hospice care. More and earlier ACP is needed to provide patient-centered care. While more NHBs received PC consultation in the last 30 days of life, it is unclear if PC consultations were provided earlier in life course.

Abstract citation ID: igae098.3841

### EXPLORING RELUCTANCE: HEALTH PROFESSIONS STUDENTS AND GERIATRIC CARE

Mingyang Zheng[1], Pamela Frasier[2], and H. George Philippi Jr.[3], *1. Radford University, Blacksburg, Virginia, United States*, *2. Radford University, Radford, Virginia, United States*, *3. Radford University, Radford University, Virginia, United States*

The aging population in the United States is growing rapidly, yet a shortage of healthcare workers persists, with health profession students showing limited interest in geriatric care. It is urgent to understand the reluctance of working with older adults among health profession students to develop interventions to address the workforce shortage issue. This study aims to understand the factors influencing these students' intentions to work with older adults using the Theory of Planned Behavior. Using a cross-sectional survey design, the study surveyed 244 health profession students at a mid-sized public comprehensive university in the Southeast. Structural equation modeling demonstrated a good fit ($\chi^2$ = 625.981, df = 340, p < 0.001, CFI = 0.918, RMSEA = 0.059, SRMR = 0.064, TLI = 0.908). The results indicated that attitudes (β = 0.930, p < 0.001) and subjective norms (β = 0.864, p < 0.001) play critical roles in shaping health profession students' intention toward geriatric care, while perceived behavioral control (β = 0.169, p = 0.118) and self-efficacy (β = 0.008, p = 0.976) were not significant predictors. These findings suggest that educational interventions should focus on improving attitudes toward older adults and leveraging social influences to enhance the desirability of geriatric careers. Future research should explore longitudinal changes in health profession students' attitudes and evaluate targeted interventions on students' career intentions. Addressing biases and enhancing the educational curriculum are crucial steps toward mitigating the workforce shortage in geriatric care.

Abstract citation ID: igae098.3842

### FACILITATORS OF AND BARRIERS TO ADULT DAY SERVICES PROVISION: PROVIDERS' PERSPECTIVES.

Kingsley Udeh[1], Molly Noble[2], Heather Menne[3], Randi Hamill[4], and Sara McLaughlin[5], *1. MIAMI UNIVERSITY OHIO, oxford, Ohio, United States*, *2. Miami University, Oxford, Ohio, United States*, *3. Miami University, Miami University, Ohio, United States*, *4. LeadingAge Ohio, Columbus, Ohio, United States*, *5. Miami university, Oxford, Ohio, United States*

Adult day services (ADS) can help people living with various chronic conditions remain in their community, and may improve well-being among participants and their family caregivers. Despite the potential benefits, knowledge of the ADS landscape is underdeveloped, especially as it pertains to the experiences of ADS providers since the COVID-19 pandemic. This study aimed to understand the facilitators and barriers experienced by ADS providers. We conducted two focus groups with seventeen ADS providers in Ohio. Thematic analysis was used to identify themes in the data. Several facilitators for providing ADS were identified, including dedicated, consistent, and committed staff; partnerships with local organizations; sufficient reimbursement; and diverse funding sources. Barriers included: staffing issues (e.g., recruitment and retention); referral and recruitment of ADS participants; limited knowledge, awareness, and misconceptions about ADS among families and case managers; transportation services (e.g., inadequate and safe escort of participants to and from ADS centers); and insufficient or delayed reimbursement. Providers identified the following solutions with the potential to mitigate perceived barriers: educational initiatives to foster understanding of ADS among key stakeholders (e.g., regulators, case managers, community members) and additional funding and media publicity support from local, state, and federal authorities. As one of the only studies that seeks to understand the landscape of ADS in Ohio, this research is uniquely situated to provide crucial insights for tailoring interventions that consider ADS providers' unique challenges. It also informs future research focusing on improving the quality of ADS.

Abstract citation ID: igae098.3843

### FACTORS ASSOCIATED WITH HOME HEALTH CARE VERSUS SKILLED NURSING FACILITY REFERRAL FOLLOWING HOSPITAL DISCHARGE

Natalie Turner, and Amber Sabbatini, *University of Washington, Seattle, Washington, United States*

Among post-acute services, home health care has markedly increased following hospitalization. Home health care is less expensive compared to other post-acute services, namely skilled nursing facilities, and is more aligned with older adults' desire to remain at home. The objective of this study was to identify individual, hospital, and community factors associated with home health care referral among Medicare beneficiaries. Multivariate logistic regressions with hospital-level fixed effects were used to estimate the patient, hospital and community-level factors associated with home health care referral among Medicare beneficiaries at hospital discharge. Older age, being female, and dual enrolled beneficiary status were associated with decreased odds of home health care referral. Beneficiaries living in rural compared to urban settings had higher odds of home health care referral. Communities with a higher proportion of people of color had a lower odds of home health referral, while having a greater percentage of residents living in poverty was associated with higher odds. The decision to refer to home health care was largely driven by patient-level factors. However, the racial and ethnic composition of a community as well its economic status and rurality may affect the odds of a home health care referral beyond both the patient characteristics and the specific hospital in which they were treated. Ensuring equitable access to home health care necessitates increased investment in care infrastructure and policies that dismantle structural barriers to preferred post-acute care settings.

Abstract citation ID: igae098.3844

### FINANCIAL FORTITUDE: IMPACT OF CAREGIVING ACROSS GENERATIONS

Quynh Vo[1], Shikha Bhurtel[2], Janice Bell[2], Pauline DeLange Martinez[3], Robin Whitney[4], and Heather Young[5], *1. UC Davis, Elk Grove, California, United States*, *2. UC Davis, Davis, California, United States*, *3. UC Davis, Sacramento, California, United States*, *4. San Jose State, San Jose, California, United States*, *5. UC Davis, Ashland, Oregon, United States*

There is growing evidence of the economic impact of unpaid family caregiving on physical, mental, social and financial wellbeing. The financial burden of caregiving has not however been fully examined nationally by generation. To address this gap, we examined relationships between generation and selected measures of financial fortitude among caregivers. This cross-sectional study used the 2020 Caregiving in the U.S. Survey to examine any reported impact of caregiving on savings, debt, and bills among adult caregivers who cared for an older adult. The primary independent variable was generation categorized as millennial, Gen X, and Baby Boomer. Survey-weighted logistic regression was used to model outcomes as functions of generation, controlling for socio-demographic and caregiving variables. Compared to Baby Boomers, millennials had higher odds of reporting impact on savings (OR=1.8; p=.006; 95%CI [1.17, 2.70]), debt (OR=2.2; p< 0.01; 95%CI [1.45, 3.40]), and bills (OR=2.6; < 0.01; 95%CI [1.69, 4.24]). Generation X also had higher odds of reporting impact on savings (OR=1.6; p=.006; 95%CI [1.15, 2.3]) and debt (OR=1.4; p=.03; 95%CI [1.02, 2.17]). Caregivers with higher incomes reported lower odds of impacts on all outcomes while those supporting more activities of daily living and caregiving for longer hours had higher odds. Additionally, caregivers with college degrees reported lower odds of impacts on bills. These findings highlight the role of generational status, socio-demographic and caregiving factors on financial outcomes, emphasizing the need for targeted interventions and policies that provide financial support to younger caregivers.

Abstract citation ID: igae098.3845

### FROM POLICY TO PRACTICE: EVALUATING THE EFFECTIVENESS AND EXECUTION GAPS OF TLTCP 2.0 IN TAIWAN

Yu-Ting Chou[1], Kaichen Yao[1], Hsiu-Jung Wang[2], and Ya-Mei Chen[1], *1. National Taiwan University, Taipei City, Taipei, Taiwan (Republic of China)*, *2. Ministry of Health and Welfare Shuang-Ho Hospital, NEW TAIPEI CITY, Yunlin, Taiwan (Republic of China)*

Taiwan has implemented The Ten-Year Long-term Care Plan version 2.0 (TLTCP 2.0) for 7 years, focusing on integrated long-term care (LTC) services. Case managers at Community Integrated Service Center is the key and responsible for proposing managing service plan. However, gaps persist between policy and execution. To understand these gaps, related challenges, and outcomes, we conducted mixed-method study, including qualitative interviews with care managers and analysis of case managements outcomes using data from one a major center in northern Taiwan. Paired T-tests assessed differences between the Case Management Level (CML) scores at discharge and the first re-evaluation. Interviews revealed that the biggest gap and challenge is to become “one-stop shop.” TLTCP 2.0 users still face confusion due to multiple contacts during their service period. Case managers coordinated at least 140 cases monthly, yet the governments’ Care Services Management Information Platform is poorly designed to facilitate this coordination. Additionally, policy design flaws have led to service misuse, such as unclear requirement for range of motion services. Quantitative findings showed that while case mangers handle a higher caseload than in advanced countries, users demonstrate significant improvements in activities of daily living, primary caregivers’ burden, and CML scores after receiving integrated services. However, instrumental activities of daily living scores declined, likely due to reliance on service like house chores, leading to disuse these skills. Overall, the TLTCP 2.0 seems to be effective, but addressing policies and platform design and the issue of skill disuse is crucial for future improvement.

Abstract citation ID: igae098.3846

### GERIATRICS EDUCATION MENTOR (GEM) PROGRAM: OUTCOMES FROM PAIRING STUDENTS AND OLDER ADULTS DURING MEDICAL SCHOOL.

Hannah Griffin[1], David Wihry[2], Marilyn Gugliucci[3], and Susan Wehry[4], *1. UNECOM, Biddeford, Maine, United States*, *2. University of Maine, South Portland, Maine, United States*, *3. University of New England College of Osteopathic Medicine, Biddeford, Maine, United States*, *4. University of New England, Portland, Maine, United States*

Currently, only 15% of medical schools have mentor programs that educate students to work with older adults and their health. The University of New England College of Osteopathic Medicine Geriatrics Education Mentor (GEM) program is an experiential education component within the 1st and 2nd year curriculum whereby students and older adults are paired to conduct five “home-visit” GEM assignments. A pre/post survey design assessed students’ competence and confidence with older people (assigned GEMs). The survey was emailed to first-year medical students before they began GEMs and was sent again upon completion of the program at the end of their second year. This process was carried out through academic years 2020-2021 through 2023-2024 with an average of 174 first-year students per class, and this data was analyzed in RStudio. Results had a 26% response rate that included overall data (N= 133/

Pre & N=138/ post), 24 responses were matched assessments. No significant change in students' interest (p > 0.4), perceived readiness and competence (p > 0.07), or confidence in working with older adults (p > 0.5) was revealed. However, perceived readiness and confidence increased from pre- to post-test, indicating that a larger sample size might reveal significance. Self-reported knowledge of older adult healthcare needs increased, pre- to post-test, with the paired subset showing a significant increase (p< 0.015). Although a small sample, these findings provide a foundation for medical schools to consider implementing a mentor type program to augment medical student skill/knowledge in older adult health care to advance age-friendly health care.

Abstract citation ID: igae098.3847

### HEALTH AND WELL-BEING OUTCOMES OF MEALS ON WHEELS: A SYSTEMATIC REVIEW FOR A COMPLEX INTERVENTION

Bernadette Wright, Victoria Owens, and Sanjaya Wilson, *Meals on Wheels America, Arlington, Virginia, United States*

Meals on Wheels programs deliver nutritious meals to older adults' homes, providing the opportunity to address social and safety needs that affect health. To make informed decisions on programs and policies, decision-makers need to understand which Meals on Wheels services lead to specific impacts, identifying the affected populations, locations, and mechanisms involved. We investigated these questions through a systematic review of studies demonstrating Meals on Wheels' effects on older adults' health and health-related outcomes. Forty-eight publications met inclusion criteria, representing 47 studies and 105 outcome analyses. Outcomes frequently examined included diet quality (20), nursing home use/living independently (17), food insecurity (12), healthcare utilization (11), social connection (10), falls/home safety (6), nutrition (5), and other outcomes (24). Study designs included single group quasi-experiments (13), analyses of perceived outcomes (12), comparison group quasi-experiments (9), single-group pre-post studies (8), and randomized experiments (5). Majorities of studies were from the U.S. (38), in peer-reviewed publications (35), and published in the past 10 years (27). Risks of bias included unaccounted-for variables (11) and underpowered samples (9). Eighty-eight analyses (45 studies) found evidence of beneficial effect. Studies attributed success in part to meeting participant preferences and nutritional needs, serving those with greatest need, offering more meals/food, and ensuring opportunity for social connection. Seventeen analyses (9 studies) did not find positive effects, most frequently explained by methodological issues or participants not receiving enough services. These findings suggest that Meals on Wheels services that meet participants' preferences and needs can reduce barriers to health and aging in place.

Abstract citation ID: igae098.3848

### HEALTH OUTCOMES AND RISK FOR MORTALITY IN OLDER ADULTS WITH ATTENTION DEFICIT/ HYPERACTIVITY DISORDER SYMPTOMS

Tara Cooper, and Andrea Piccinin, *University of Victoria, Victoria, British Columbia, Canada*

Attention deficit/hyperactivity disorder (ADHD) is a neurodevelopmental disorder that persists into older adulthood with important implications for healthy ageing. Previous research documents significant disparities for children and adolescents with ADHD compared to their neurotypical peers on various social determinants of health. Critically, previous research reports a significantly higher risk for mortality in adults with ADHD followed since childhood. The literature on health outcomes of older adults with ADHD is sparse. Therefore, the purpose of this research was to examine social determinants of health and risk for mortality associated with ADHD symptoms in a sample of older adults. Participants from the Longitudinal Aging Study Amsterdam (n= 986) were screened for childhood and current ADHD symptoms in 2008/2009 and followed until death or most recent assessment (August, 2021). Based on symptoms reported currently and evidence of childhood onset, participants were assigned to either an ADHD symptom (ADHD-sx) group (n= 147) or a non-ADHD group (n= 839). Survival analysis revealed no significant risk for mortality associated with ADHD-sx (HR:.90, 95% CI [.63, 1.29], p=.58). Furthermore, although older adults with ADHD-sx reported more chronic health conditions (p <.001), group differences were not observed in terms of income (p=.49), education (p=.95), social participation (p=.63) or occupational attainment (p=.94). These results suggest that selection and survival bias are important considerations when studying older adults with neurodevelopmental disorders. Future directions for research on ADHD in healthy ageing are presented.

Abstract citation ID: igae098.3849

### HEALTH, SOCIAL ISOLATION, AND 12-YEAR MORTALITY ACROSS DIFFERENT COMMUNITY-BASED SENIOR HOUSING TYPES

Seoyeon Ahn[1], Sojung Park[2], Eunsun Kwon[2], and BoRin Kim[3], *1. Duke-NUS Medical School, Singapore, Singapore*, *2. Washington University in St. Louis, St. Louis, Missouri, United States*, *3. University of New Hampshire, Durham, New Hampshire, United States*

The literature on subsidized senior housing for low-income older adults primarily focuses on affordability and health outcomes like mortality. In contrast, research on other senior housing models often emphasizes the social environment, creating a disconnect that may lead to misunderstandings about factors influencing health and well-being across different housing types. This study examines the influence of socio-economic status, health conditions, and social isolation on 12-year mortality across various community-based senior housing types. Data were drawn from twelve waves of the National Health and Aging Trend Study (2011-2022), involving 6,534 community-dwelling adults (obs=29,929) aged 70 or older who died during the study period. Housing types were categorized into five groups: 1) subsidized senior housing (SSH), 2) senior housing with low-income status and housing cost burden (HCB), 3) senior housing without low-income status and non-HCB, 4) traditional homes with low-income status or HCB, and 5) traditional homes without low-income status and non-HCB. A hierarchical discrete-time event history model using logistic regression was employed to assess the influence of health conditions, social isolation, and housing type on mortality risk. We found that (1) low income or HCB significantly increased mortality risk across all housing types; (2) chronic health conditions and severe social isolation were strong predictors of higher mortality; and (3) socially isolated individuals had a lower mortality risk when living in senior housing compared to traditional homes. These findings highlight the importance of housing affordability policies and suggest that congregate senior housing offers protective benefits for socially vulnerable older adults.

Abstract citation ID: igae098.3850

### HEALTHY DIETARY PRACTICE AND SELF-RATED HEALTH AMONG KOREAN OLDER ADULTS: THE MEDIATING ROLE OF PHYSICAL ACTIVITY

Joosuk Chae[1], Hyunmi Kim[2], and In-Taek Lim[3], *1. SCELA (Comprehensive Support Center for the Elderly Living Alone), Seoul, Republic of Korea*, *2. SCELA (Comprehensive Support Center for the Elderly Living Alone), Seoul, Republic of Korea*, *3. Catholic University, Seoul, Republic of Korea*

This study explored the relationship between healthy eating habits, physical activity, and self-rated health perception among older Korean adults, with a specific focus on whether physical activity level, as measured by the Global Physical Activity Questionnaire (GPAQ), serves as a mediating factor. We examined whether healthy eating habits influenced self-rated health perceptions through increased physical activity using Process Macro 4.3. The analysis included 1,468 older adults aged ≥65 years from the first year (2022) of the 9th Korea National Health and Nutrition Examination Survey (KNHANES), a nationally representative panel survey conducted in Korea from 2022 to 2024. The findings confirmed that healthy eating habits affected self-rated health perceptions via increased physical activity. This suggests that healthy eating habits positively help increase physical activity, which in turn enhances self-rated health status. These results highlight the importance of an integrated approach that incorporates healthy eating education and physical activity promotion when developing future care service plans for older adults in Korea. Based on these findings, more comprehensive guidelines can be established for social workers to design individualized programs within the older adults' care service delivery system.

Abstract citation ID: igae098.3851

### HEARING LOSS AND COGNITIVE DECLINE: POLICY RECOMMENDATIONS

Girish Hemrajani, and Debra Dobbs, *University of South Florida, Tampa, Florida, United States*

Hearing loss is the third most common health condition in older age after arthritis and heart disease. With a rapidly increasing older adult population, hearing loss cases are expected to grow from 38 million to 73 million by 2060. Hearing loss in midlife has been estimated to account for 9% of cases of dementia, a huge – but potentially reversible – disease burden given that dementia affects 47 million people worldwide.

**Methods:** The systematic literature review was included from the Ageline database, containing articles published in English from January 2013 to June 2024. The search terms 'hearing loss,' 'cognitive decline' and 'older adults' produced 161 results and out of these 18 relevant articles were chosen for the review.

**Results:** In eight articles, hearing interventions were found to reduce cognitive decline in cognitively healthy older adults with hearing loss and slow down the progression of cognitive decline. One article found the provision of hearing aids would have a 48% protective effect on cognitive changes compared to the control over three years. The other articles were equally divided as "not sure" and "need further investigations". Implications: The review highlights the importance of addressing hearing loss as a preventative measure and suggests policy measures and prevention strategies to maximize cognitive well-being in older adults. Age related sensorineural hearing loss is widely treatable with hearing aids and targeted intervention for hearing loss may play a fundamental role in dementia prevention. Key words: hearing loss, cognitive decline, hearing aid.

Abstract citation ID: igae098.3852

### HIGH CONFIDENCE IN MANAGING COMMON GERIATRIC PROBLEMS AMONG RURAL FRONTLINE CLINICIANS

Alison Stone[1], Eileen Dryden[2], Lauren Moo[3], William Hung[4], Meaghan Kennedy[5], Camilla Pimentel[6], Jessica Riley[7], and Thomas Caprio[8], *1. Veterans Affairs, Dracut, Massachusetts, United States*, *2. Veterans Health Administration, Bedford, Massachusetts, United States*, *3. Department of Veterans Affairs, Bedford, Massachusetts, United States*, *4. James J Peters VA Medical Center, James J Peters VA Medical Center, New York, United States*, *5. Veterans Affairs, Bedford, Massachusetts, United States*, *6. VA Bedford Healthcare System, Bedford, Massachusetts, United States*, *7. Veteran's Affairs, Bedford, Massachusetts, United States*, *8. University of Rochester Medical Center, Rochester, New York, United States*

**Background:** Older rural Veterans often have less access to specialty care services, including geriatrics. The Veterans Health Administration's (VHA) GRECC Connect is a national program that provides virtual geriatric services for patients and geriatric education for rural clinicians through a case conference series focused on the 5Ms (Mind, Mobility, Medications, Multicomplexity, and Matters Most), a hallmark of high-quality geriatric care. We aimed to measure rural frontline clinicians' confidence in managing common geriatric problems to inform future education efforts.

**Methods:** We conducted an electronic survey-based needs assessment during three case conferences in Spring, 2024. The survey included a brief demographic section and 16 questions on respondents' confidence addressing clinical issues within the 5Ms (e.g., polypharmacy, falls prevention, goals of care).

**Results:** Forty nine percent (211/430) of conference participants completed the survey, including social workers, pharmacists, nurses, and physicians. On average, 69% of respondents reported being 'confident' or 'very confident' (range 53%-84%) across the 16 questions. Highest confidence levels were in the 'Matters Most' domain. Topics rated lower included managing depression, deprescribing, and managing behaviors in patients with dementia. Confidence varied according to health professions and the responsibilities associated with each role. Providers who serve a higher percentage of rural Veterans reported feeling more confident than their urban colleagues across all questions.
**Conclusion:** The high level of confidence aligns with findings in the literature that report high self-efficacy ratings in the 5Ms domains among other VA providers and may be related to the VHA's focus on Age Friendly Health Systems.

Abstract citation ID: igae098.3853

### HOSPITAL AT HOME PROGRAMS: CAREGIVER INSIGHTS ON SUCCESSES AND ENHANCEMENTS

Julia Hart[1], Brittni Howard[2], Jeannys Nnemnbeng[1], David Levine[3], Ksenia Gorbenko[1], Bruce Leff[4], Albert Siu[1], and Abigail Baim-Lance[5], *1. Icahn School of Medicine at Mount Sinai, New York, New York, United States*, *2. University of Massachusetts at Amherst, Amherst, Massachusetts, United States*, *3. Brigham and Women's Hospital, Boston, Massachusetts, United States*, *4. Johns Hopkins Medicine, Baltimore, Maryland, United States*, *5. Veterans Health Administration, New York, New York, United States*

Hospital at Home (HaH), an alternative to hospital-based care, continues to expand across the US. While the caregiver role is of great interest, little is known about their experiences. This is the first qualitative study, to our knowledge, examining caregiver experiences with HaH in a national sample. Beginning October 2023 (and ongoing), we conducted semi-structured interviews with caregivers via Zoom lasting 30-60 minutes, recruited through the HaH User's Group, a national collaborative focused on implementation. This analysis uses rapid strategies including: systematic development of analysis templates, their synthesis to identify preliminary themes, and group consensus to ascertain meaning. To date, we completed 13 interviews with spouses, adult children, and grandchildren representing 5 programs (5 NE, 7 MW, 1 SE). 92% were female and 85% white. The mean age is 68 (range: 57-83). Program duration ranged from 3 days to 1 month. Overall, participants felt positive about HaH, reporting they would repeat the experience. Main benefits include: convenience, patient goal alignment, high quality care, and improved patient outcomes compared to brick and mortar experiences. Caregivers reported that providers were responsive, reliable and communicative. Yet, participants described lacking communication about caregiver roles and expectations, and desired additional information. Caregivers also reported needing more clarity about the timing of visits, tasks and procedures. They suggested that HaH may be more challenging to implement in contexts of greater caregiver strain or higher patient acuity. While caregivers generally reported positive HaH experiences, clearer and more explicit communication about the caregiver role could improve their experiences.

Abstract citation ID: igae098.3854

### HOW DO KOREANS PERCEIVE THE EXPERIENCE OF CAREGIVING? A SYSTEMATIC REVIEW OF THE OUTCOMES OF INFORMAL CAREGIVING

YoungBin Koh, Sunghyun Ko, and Yeonjung Lee, *Chung-Ang University, Seoul, Republic of Korea*

Globally, several research have been conducted to show the evidence of caregiving impact. In general, caregiving responsibilities have negative impacts on caregivers' well-being, and further research has investigated the differences in the outcomes in terms of gender, race, and socioeconomic status. However, there exists not enough efforts to explain these differences at a macro level. For example, how countries differ in the extent to which they rely on informal care services and whether there are differences in caregiving impact among countries. These differences might be related to differences in cultural and policy background. Specifically, South Korea promotes formal long-term care through its national insurance. However, the support for informal caregivers is somewhat weaker compared to other countries, and there is limited research. The purpose of this study is to evaluate the research evidence in Korean contexts and summarize current findings on the outcomes of informal caregiving. A systematic review was performed with a structured literature search following PRISMA guidelines in five Korean databases. Our systematic search yielded 1,143 articles, of which 31 were included for synthesis. Findings show evidence of a negative impact of caregiving including caregivers' deteriorating physical and mental health as well as reduced employability, especially for female caregivers. However, there are also some positive aspects such as having more satisfied relationship with caregiver recipient. The findings underscore the need for interventions to reduce the negative impact, particularly among female caregivers. We also suggest further research to investigate the cultural contexts to make caregiving experience positive.

Abstract citation ID: igae098.3855

### IMPACT OF ORGANIZATIONAL FACTORS ON HEALTH ACROSS FRAILTY LEVELS IN TAIWAN'S DISABILITY PROGRAM

Wan-Yu Chiu[1], Hui-Fen Mao[1], Ling-Hui Chang[2], and Ya-Mei Chen[3], *1. National Taiwan University, Taipei, Taipei, Taiwan (Republic of China)*, *2. National Cheng-Kung University, Tainan, Tainan, Taiwan (Republic of China)*, *3. National Taiwan University, Taipei City, Taipei, Taiwan (Republic of China)*

Taiwan's nationwide Prevention and Delay of Disability (PDD) program aims to maintain the physical and mental health of older adults through community-engaged partnerships. This study explored key organizational factors within the PDD program and their impact on health outcomes across different frailty levels. A cross-sectional study (2019-2020) involved 392 administrators from PDD service centers and 13,213 participants, categorized into frail (563), pre-frail (2,397), and healthy (10,253) groups. Over a 12-week period, multiple centers provided a consistent, multi-domain intervention across service centers, universities, and communities. This intervention included physical exercise, nutrition guidance, cognitive training, and social participation, all uniformly applied to the participants. Data were analyzed using multilevel and regression models, employing a tool with twenty items and six dimensions: internal efficiency, responsiveness, professionalism, community cooperation, environmental factors, and sustainability. Results showed that organizational professionalism significantly benefited pre-frail participants by reducing depression ($\beta = 0.347$, $p < 0.001$), fall risk ($\beta = 0.010$, $p = 0.007$), and improving overall health ($\beta = 0.625$, $p = 0.005$). Universities enhanced independent function for both frail ($\beta = 1.215$, $p = 0.024$) and pre-frail (β = 1.287, p = 0.006) participants. Additionally, organizations with favorable environmental factors and strong professionalism had the most positive impact on pre-frail participants, while providing limited benefits to frail participants and minimal impact on healthy participants. It is recommended that healthy participants engage in community activities better aligned with their health status, while frail participants may require more intensive and personalized interventions to maintain functionality and support health improvements.

Abstract citation ID: igae098.3856

### IMPACT OF PROVIDING IPADS TO OLDER ADULTS: LESSONS CALIFORNIA FUNDED PROGRAM

Sheila Salinas Navarro[1], Mengzhao Yan[1], Sindy Lomeli[1], Carly Roman[2], and Kathleen Wilber[1], *1. University of Southern California, Los Angeles, California, United States*, *2. Archstone Foundation, Long Beach, California, United States*

The California Department of Aging innovative Connections, Health, Aging, & Technology (CHAT) program has bridged the digital divide by providing free iPads with internet access to older adults aged 60 and over. This initiative sought to enhance mental and physical well-being through increased community engagement, information, and access to services. Our research employed a mixed-method approach to understand participants' usage and motivations. We used Stata 18 to analyze data from 980 participants who completed a pre and post-test. Most used their iPads more than once a day (n=454; 46%) or once a day (n=211; 22%); 62 participants (6%) did not use the iPad. In a qualitative analysis conducted with Dedoose using grounded theory, we identified key themes for using the iPad, including the role of pre-test expectations and motivators identified in the post-test. Initially, participants anticipated using their iPads for communication, learning, well-being, technology, and simplifying life. However, post-test results revealed a fascinating shift in motivations, now centered on technology, education, entertainment, communication, and health. This shift underscores the profound impact of the CHAT program on the participants' attitudes and behaviors. Understanding what motivates users is crucial for tailoring programs that support older adults in mastering advanced features and enhancing their connection to loved ones, information, the community, and healthcare providers. Our findings suggest that older adults are not only interested in but also actively engaged with technology for a variety of activities.

Abstract citation ID: igae098.3857

### IMPROVING STUDENT CONFIDENCE IN APPLYING GERIATRICS BEST PRACTICES USING THE 4MS FRAMEWORK

Jennifer DeGennaro[1], Leonard Powell[2], Christian White[2], and Kevin Overbeck[2], *1. Rowan-Virtua School of Osteopathic Medicine, Stratford, New Jersey, United States*, *2. Rowan University, Stratford, New Jersey, United States*

Recognized by the Institute for Healthcare Improvement as a Level II Age-Friendly Health System, the Rowan-Virtua School of Osteopathic Medicine (RVSOM) Department of Geriatrics and Gerontology is committed to infusing the 4Ms framework (What Matters, Medication, Mentation, Mobility) into all education initiatives as well as clinical practice. RVSOM medical students are exposed to the 4Ms in the second year of undergraduate courses and the framework is further emphasized during the required 4-week clerkship. To standardize content delivered to students rotating at a host of rotation sites and to augment the assigned self-study modules, a new initiative was introduced at the beginning of academic year (AY) 2024-2025. Students now participate in a full day of in-person didactics designed to teach them to use evidence-based screening tools and employ best practices when caring for older patients. A post-clerkship survey on the 4Ms approach was administered in AY 2023-2024 which allows an examination of differences between cohorts of students who received the in-person didactics and those who did not. Analysis of the data indicates that students benefit from the focused didactics as significant differences emerged on nearly half of the survey items [e.g., sufficient knowledge to perform a Geriatric Assessment [t(109) = 2.45, p =.016, d =.47] and confidence in initiating a conversation about Advance Care Planning [t(109) = -2.69, p =.008, d = -.51]). Findings indicate that enhanced training and exposure to Geriatrics best practices bolsters students' confidence in understanding geriatrics issues and assessing older patients.

Abstract citation ID: igae098.3858

### INFECTIOUS DISEASE CONTROL REGULATIONS IN ASSISTED LIVING COMMUNITIES: BEFORE AND AFTER COVID-19

Danielle Gerken[1], Lindsey Smith[1], Paula Carder[2], Cassandra Hua[3], Eric Jutkowitz[4], and Kali Thomas[5], *1. Oregon Health & Science University, Portland, Oregon, United States*, *2. OHSU-PSU School of Public Health, Portland, Oregon, United States*, *3. University of Massachusetts Lowell, Lowell, Massachusetts, United States*, *4. Brown University, Brown University, Rhode Island, United States*, *5. Johns Hopkins University, Baltimore, Maryland, United States*

Assisted living (AL) residents are at high risk for adverse health outcomes due to infection, particularly those living in memory care. Despite this, at the outset of the COVID-19 pandemic, most states had minimal or no regulations regarding infectious disease control within AL communities. In this study, we sought to determine if state agencies responded to the COVID-19 pandemic by increasing infectious disease control regulations for AL. We systematically sourced AL regulations for all 50 states and Washington D.C. from the legal policy database Westlaw. We then used search-term driven text retrieval to identify sections of policy relevant to infection control to determine the applicability and scope of each policy. In 2019, there were 13 regulations from 9 states regarding how AL providers should respond to pandemics and epidemics. In 2020, 13 regulations from 10 different states were found. In 2023, this number increased to 18 regulations from 11 different states. The majority of regulations required AL providers to inform a local health department or official about any disease outbreak occurring within the facility. In 2019, 7 of the 9 states (77.8%) had regulations requiring reporting. In 2020, that number decreased to 6 out of the 10 states (60%), and in 2023 the number of states requiring reporting was 8 out of the 11 (72.7%). Our analysis revealed that while a few states have made improvements, there is still a gap in regulatory protection that could result in poor health outcomes for AL residents.

Abstract citation ID: igae098.3859

### INNOVATIVE PERSON-CENTERED SERVICES THROUGH THE KŪPUNA SUPPORT NAVIGATORS

Jenny Jinyoung Lee[1], and Christy Nishita[2], *1. University of Hawaii at Manoa, Honolulu, Hawaii, United States*, *2. University of Hawaii, Honolulu, Hawaii, United States*

The Kūpuna Support Navigator Program in Hawai'i employs Community Health Workers, known as Kūpuna Support Navigators, to connect kūpuna (older adults in Hawaiian) with essential community resources tailored to their specific needs. This innovative, person-centered approach significantly enhances the aging support network in Hawai'i through comprehensive education, seamless integration of care services with advanced tracking software, and rigorous impact evaluation. Its strategic design offers scalable, replicable, and sustainable solutions that enhance the ability of older adults across diverse communities to age-in-place. This program increases the availability of trained Community Health Workers embedded in the aging support network, fostering a supportive environment that enhances the well-being and independence of older adults, ensuring they receive better support and care. The program encompasses a holistic framework that addresses immediate health and social needs while focusing on long-term sustainability and community empowerment. In this presentation, we will detail the setup, structure, and processes of the Kūpuna Support Navigator Program. We will leverage data visualization to present specific outcomes that illustrate the program's effectiveness in meeting the complex needs of its target population. Additionally, we will discuss the lessons learned and best practices developed, emphasizing the program's potential as a model for similar initiatives. This overview aims to highlight the significant role of the Kūpuna Support Navigator Program in promoting and enhancing the well-being and independence of older adults.

Abstract citation ID: igae098.3860

### INTERGENERATIONAL CARE PROVISION AND THE CARE PLANNING OF SANDWICHED AGEING CARERS

Xue Bai, and Mengyu Liu, *The Hong Kong Polytechnic University, Hong Kong, Hong Kong*

An increasing number of people in their 50s and 60s are sandwiched between surviving parents and children. However, there is a research gap on the relationship between upward and downward care provision of sandwiched ageing carers and the impacts of intergenerational care provision on their care planning. Integrating theories on intergenerational transfer, resource depletion, and proactive coming, this study aims to answer two questions in Hong Kong's context: (a) How do sandwiched ageing carers provide care across multiple generations? (b) Does intergenerational care provision influence sandwiched ageing carers' care planning? Using data from the Survey on Sandwiched Ageing Carers in Hong Kong (N = 1500), we find that overall levels of upward and downward care provision of sandwiched ageing carers are positively correlated. However, the high care intensity attenuates such a positive relationship. When the percentage of household income spent for upward and downward care exceeds 12.2%, the association turns to negative. Moreover, the sandwiched ageing carers who provide more upward and downward care are less prepared for their future care. These findings deepen the understanding of intergenerational care provision and care planning experiences among ageing families in Hong Kong and enhance professionals' and service providers' awareness of care planning in multi-generational family contexts.

Abstract citation ID: igae098.3861

### INTERGENERATIONAL TUTORING: HEALTH BENEFITS FOR OLDER ADULTS IN A TWO-YEAR QUASI-EXPERIMENTAL STUDY

Peter Sun[1], Nancy Morrow-Howell[2], Mary Click[3], and Kendra Minch[3], *1. Bar-Ilan University, Ramat Gan, Tel Aviv, Israel*, *2. Washington University in St. Louis, St. Louis, Missouri, United States*, *3. The Oasis Institute, St. Louis, Missouri, United States*

The COVID-19 pandemic has significantly impacted older adult volunteering. It is important to understand if prior relationships between volunteering and health remain consistent in a post-pandemic context. We surveyed Oasis tutors aged 51 and over before and after two years of volunteering and used three matching algorithms—nearest-neighbor matching, optimal full matching, and subclassification—to match them with a comparison group of non-volunteers in the 2016 and 2018 waves of the Health and Retirement Study. G-computation was then used on the merged sample (N = 518) to estimate the effect of volunteering on depression, functional limitations, and self-rated health. With all three matching algorithms, Oasis tutors experienced lower levels of depression and fewer functional limitations, compared to the HRS group of non-volunteers. When using nearest-neighbor matching, Oasis tutors had better self-rated health than the HRS comparison group. Volunteering in the Oasis Intergenerational Tutoring Program positively impacts older adults' health, supporting the social model of health promotion.

Abstract citation ID: igae098.3862

### INVESTIGATING THE BUILT ENVIRONMENT SURROUNDING NATURALLY OCCURRING RETIREMENT COMMUNITIES (NORCS) IN TORONTO

Laura Fusca[1], Paula Rochon[2], Tai Huynh[3], Shoshana Hahn-Goldberg[3], Lavina Matai[4], Longdi Fu[4], and Rachel Savage[2], *1. University of Toronto, Mississauga, Ontario, Canada*, *2. Women's College Hospital, Toronto, Ontario, Canada*, *3. University Health Network, Toronto, Ontario, Canada*, *4. ICES, Toronto, Ontario, Canada*

A positive built environment can improve the health of older adults and support aging in place by increasing access to amenities, physical activity, and social interaction. We investigated the walkability and amenity density surrounding Naturally Occurring Retirement Communities (NORCs) in Toronto, areas where many older adults live. This population-based descriptive study linked two built environment datasets with a provincial registry of high-rise NORC buildings in Ontario, Canada by postal code (PC). Mean (SD) walkability index scores and quartiles, as well as amenity density categories were compared for NORC and non-NORC PCs. Building and resident characteristics were also compared for NORC PCs in the least and most favourable categories for each outcome. Our analysis of walkability and amenity density included 49,295 (488 [9.9%] NORC) and 49,945 (489 [9.8%] NORC) Toronto PCs respectively. Walkability was significantly higher for NORC (mean 7.1 (SD 9.1)) versus non-NORC (4.9 (8.3)) PCs; although 57 (11.7%) NORC PCs were in low walkability neighbourhoods. NORC PCs were also more amenity dense, with 63.0% being classified as medium/high density compared to 50.5% of non-NORC PCs. Low walkability and amenity-poor NORCs tended to house older, immigrant, ethnically diverse and lower-income populations. Low walkability NORCs were also more likely to be apartments with supports and have residents without access to basic needs. Overall, our findings suggest that many NORCs are well-positioned to support aging in place given their walkability and amenity access, however action should be taken to support NORCs with sub-optimal environment conditions.

Abstract citation ID: igae098.3863

### LEADERSHIP EXCELLENCE AND ADVANCEMENT OF DIRECTORS (LEAD) PROGRAM: FIRST INSIGHTS FROM A NEW MENTORSHIP MODEL

Juanita Bacsu[1], and Harleah Buck[2], *1. Thompson Rivers University, Kamloops, BC Canada, British Columbia, Canada*, *2. University of Iowa, Iowa city, Iowa, United States*

Mentorship is critical to advancing leadership skills and building confidence in academia. However, there is a paucity of programs focused on supporting new center directors in the field of aging. Working with the Gerontological Society of America, we created the Leadership Excellence and Advancement of Directors (LEAD) Program as a mentorship model to provide peer support, networking opportunities, professional growth, and skill development for new center directors. This presentation aims to: 1) identify critical planning steps and strategies to develop high quality mentorship programs to support new center directors in the field of aging; and 2) recognize the LEAD Program as an emerging prototype to elevate leadership skills, build confidence, and advance research excellence to support new directors of gerontological research centers. The Lead Program is a groundbreaking GSA model that is geared towards new directors by providing peer support, leadership development, mentorship, and a dynamic network of fellow directors. The need for the LEAD Program was identified during a recent GSA Center Directors meeting where several directors shared that they were retiring and new directors were looking for mentorship support. The development of the LEAD Program was cultivated by: i) identifying priority topic areas and needs; ii) establishing meeting forma and frequency; iii) addressing potential program barriers and obstacles; and iv) exploring opportunities for peer support and collaboration to advance new directors in the field of aging. The next steps are to implement and evaluate the LEAD Program to strengthen the mentorship model for future cohorts of center directors.

Abstract citation ID: igae098.3864

### LONELINESS AND DEPRESSION AMONG SEXUAL AND GENDER MINORITY OLDER ADULTS WITH COGNITIVE IMPAIRMENT

Hyun-Jun Kim[1], Kilohana Haitsuka[1], Hailey Jung[1], and Karen Fredriksen-Goldsen[2], *1. University of Washington, Seattle, Washington, United States*, *2. University of Washington, University of WA, Goldsen institute, Washington, United States*

Sexual and gender minority (SGM) older adults are at elevated risks of cognitive impairment (CI). High rates of loneliness and depression are particularly concerning for SGM older adults living with CI as they are linked to decreased physical health, lower life satisfaction, and accelerated cognitive decline. Yet little is known about the risk and protective factors of loneliness and depression for SGM older adults with CI. This study, utilizing the Health Equity Promotion Model, analyzes the Aging with Pride: National Health, Aging, and Sexuality/Gender Study to examine socioeconomic status, adverse life course experiences, and psychological and social factors that predict depressive symptomatology and the mediating role of loneliness. Data were a sub-sample of 160 participants with CI assessed by the Montreal Cognitive Assessment. Approximately 50% reported feeling isolated from others, 33% were experiencing depression, 40% reported physical or sexual child abuse, 70% were single, and 25% had a network size of 3 or less. A bootstrapped mediation analysis using Mplus revealed that child abuse, day-to-day discrimination, perceived stress, not being partnered, reduced network size, and lack of social support and community engagement were indirectly associated with depressive symptomatology via loneliness. Those with adverse childhood experiences, ongoing distressing stigma, and social isolation are of major concern. Improving support networks may reduce the risk of loneliness and depression. These findings can be utilized to develop culturally tailored interventions to enhance the mental health and overall quality of life of this understudied and underserved population.

Abstract citation ID: igae098.3865

### LONGITUDINAL ASSOCIATIONS OF HEARING PROBLEM AND DEMENTIA IN COMMUNITY-DWELLING OLDER ADULTS IN THE UNITED STATES

Jun Chu[1], Christine Mair[1], and Michel Boudreaux[2], *1. University of Maryland, Baltimore County Baltimore, Maryland, United States*, *2. University of Maryland College Park, College Park, Maryland, United States*

There is evidence linking hearing problem to greater risk of dementia among older adults. However, no study has assessed such association longitudinally using nationally representative survey data. In addition, studies examining racial/ethnic differences in the association are limited. Using data from 2011-2022 National Health and Aging Trends Study and cox proportional hazards model, we examined the longitudinal association between having hearing trouble and dementia among older adults, and whether this association varied by race the ethnicity. Adjusting for demographics characteristics, number of chronic conditions, hearing aid status and social isolation status, we found that having hearing trouble was significant associated with a 2.29 (Hazard ratio=2.29, $p < 0.001$) higher risk of developing dementia over 11 years. After interacting race/ethnicity and hearing problem in a separate model, we did not find any statistically significant difference in the association by race and ethnicity. The findings suggest that older adults with hearing problem may be at a greater risk of developing dementia. Future studies should assess the effects of policies that promotes hearing tests and hearing aid usage among older adults on dementia delay and prevention.

Abstract citation ID: igae098.3866

### LONG-TERM PATTERNS OF FAMILY AND PAID CAREGIVING BY CARE TYPES AND SOURCES

Esther Shin[1], Sojung Park[2], Soobin Park[2], BoRin Kim[3], Eunsun Kwon[4], and Seoyeon Ahn[5], *1. Illinois State University, Normal, Illinois, United States*, *2. Washington University in St. Louis, St. Louis, Missouri, United States*, *3. University of New Hampshire, Durham, New Hampshire, United States*, *4. Fairleigh Dickinson University, Hackensack, New Jersey, United States*, *5. Duke-NUS Medical School, Singapore, Singapore*

To support aging in place for community-dwelling older adults, sustainable long-term care and support are crucial. The existing literature often focuses on family caregiving, formal caregiving, or their relationship, such as whether they substitute for each other. This study aims to broaden the understanding of collaborative care by exploring the intersection of different care forms and caregiver sources, an area that is less studied. Using data from the National Health and Aging Trends Study (2011-2022), this research examines longitudinal patterns of caregiving, focusing on the types of services (ADL/IADL support) and their sources (unpaid informal, paid formal, and paid family caregivers). The study sample included individuals who received consistent caregiver support throughout the study period. Group-based multiple trajectory (GBMT) modeling identified four distinct care mix patterns: (1) consistently low care (72.2%), (2) increasing ADL informal care (18.3%), (3) increasing formal care (7.4%), and (4) decreasing formal care with increasing paid informal care (2.1%). Compared to the relatively healthy and less vulnerable "consistently low care" group, those receiving "increasing ADL informal care" were more likely to be married women, while those with "increasing formal care" tended to live alone, be male, and have dementia. Notably, individuals with "decreasing formal care but increasing paid family care" were often racial minorities, in poor health, and older, possibly utilizing consumer-directed care programs. These findings provide an initial empirical foundation for understanding care mix, highlighting the need for further research on gender-specific caregiving patterns and the impacts of these care trajectories on health outcomes.

Abstract citation ID: igae098.3867

### MONTESSORI-BASED APPROACH FOR ALZHEIMER'S AND DEMENTIA: HANDBOOK FOR IMPLEMENTATION IN AN ADULT DAY CARE PROGRAM

Annette Rodrigues, *California State University, Long Beach, Long Beach, California, United States*

Adult day care (ADC) programs serve as an intervention that offers a solution to the challenges faced by an increasing population of individuals living with dementia. Including activities for mental and physical stimulation such as games, exercises, and art, ADC offers dual benefits by providing community-based care for older adults and respite for caregivers. Studies indicate, however, that a lack of quality and standardization negatively impacts the quality of ADC programs, thereby reducing utilization. As program directors expand their services to address the needs of the current demographic, a Montessori approach to dementia care can serve as an effective method of delivering deliberate, evidence-based care. However, most existing applications focus on in-home and long-term care settings rather than ADCs. To address this gap, this project aimed to develop a handbook for implementation in an ADC setting. Montessori-Based Approach for Alzheimer's and Dementia: Handbook for Implementation in an Adult Day Care Program was developed based on research, observations, and feedback from expert panelists. In the handbook, Montessori principles are presented, barriers and challenges are addressed, organizational strategies are offered, as well as recommendations for developing meaningful person-centered programs, preparing the environment, and addressing participants, staff, and family. By providing a comprehensive guide for an evidence-based standard of care, this handbook aims to advance the field of aging by combining existing research that supports Montessori-based dementia care and facilitating its implementation into an ADC program, with a goal of increasing utilization and improving quality of life.

Abstract citation ID: igae098.3868

### MOVING BEYOND DISCRIMINATION: BUILDING A COALITION FOR LGBTQ+ OLDER PERSONS LIVING IN EAST TENNESSEE

Joseph Winberry[1], Sharon Bowland[2], Jennifer Smith[3], and Namrata Mukherjee[4], *1. University of North Carolina at Chapel Hill, Durham, North Carolina, United States*, *2. University of Tennessee, Knoxville, Tennessee, United States*, *3. University of Tennessee, Knoxville, Knoxville, Tennessee, United States*, *4. University of Tennessee Knoxville, Knoxville, Tennessee, United States*

**Aims:** Research with 50+ older members of the LGBTQIA+ community, conducted by Winberry (Forthcoming), aimed to identify important needs and healthcare gaps in services in East Tennessee. This poster describes establishing a coalition to address their complex needs and gaps.

**Methods:** As part of a larger community-based participatory research project, a strategic plan created through interviews and focus groups with LGBTQIA+ older persons identified the type of partners needed to accomplish what this population wanted around aging services.

**Results:** The coalition was formed in a three-step process. First, the earliest members identified organizations and individuals who could address participants' needs: Culturally competent healthcare, services specifically for LGBTQIA+ people, social support, life planning, and caregiver services. Second, potential members who could address those needs were engaged. Those with personal ties or compelled by a social justice ethos around the LGBTQIA+ community were particularly interested in joining. Last, the role of the coalition members had to be negotiated, given limitations around staff, money, and time. Understanding exactly how members will contribute and when this needs to be renegotiated is essential to coalition success.

**Conclusion:** The Aging Rainbow Coalition (ARC)—consisting of older members from the LGBTQIA+

community, the local Pride agency, the state flagship land grant university, and other aging services organizations—was established at the county Office on Aging. This poster on the coalition creation process provides details across the three steps of the creation process and highlights insights useful for researchers and practitioners.

Abstract citation ID: igae098.3869

### MULTIGENERATIONAL DYNAMICS AND FUTURE CARE PLANNING: A QUALITATIVE STUDY OF THREE-GENERATION FAMILIES IN HONG KONG

Xue Bai, Rui Kang, Ke Ma, Kun Tian, and Jing Hu, *The Hong Kong Polytechnic University, Hong Kong, Hong Kong*

Numerous ageing adults need to provide care for older and/or younger generations while also facing life challenges associated with the transition to old age. However, no systematic investigation has examined aging sandwich carers' future care planning, nor the impacts of intergenerational care provision on their care planning. A purposive sample of 120 individuals from 40 multigenerational families in Hong Kong was recruited through various channels. The families all filled the following inclusion criteria: (a) had at least one adult aged 50–69 years with both surviving parents and children, (b) all three generations were willing to participate, and (c) the participants spoke Chinese and lived in Hong Kong. For each eligible family, an older parent from the oldest generation, an adult from the sandwiched, and a child from the youngest generation were interviewed separately. Both inductive and deductive coding approaches were used for data analysis. The findings reveal that lifelong multigenerational care transfer and intergenerational relationships shaped the future care planning and preparation processes and experiences. The upward care provision and substantial investment in child-rearing often lead to delayed and indirect reciprocity expectations from the sandwich generation. Moreover, the experience of caring for aging parents prompts sandwich generation to make financial, health, and social life-related care plans. However, traditional family communication patterns often obstruct open discussions about care plans between sandwich generation and their children. Anticipated future circumstances of both aging parents and children further shape the intended care arrangements of the sandwich generation.

Abstract citation ID: igae098.3870

### NARRATIVES OF WARTIME VOLUNTEERING AMONG OLDER ADULTS IN ISRAEL

Peter Sun[1], and Liat Ayalon[2], *1. Bar-Ilan University, Ramat Gan, Tel Aviv, Israel*, *2. Bar Ilan University, Bar Ilan University, HaMerkaz, Israel*

Volunteering is an important vehicle for the health promotion of older adults, yet research on its role during times of war remain limited. This research aimed to explore the representation and rhetoric of older adult volunteers in the Israeli media during Operation Swords of Iron. Using the LexisNexis database, four Israeli newspapers were searched for English-language articles published between October 7, 2023 and August 7, 2024: Haaretz, The Times of Israel, Yediot Achronot, and The Jerusalem Post. After removing duplicates and articles that were not relevant to older adult volunteering, a total of 23 articles were retained for thematic analysis. To probe the sentiments of these narratives, sentences containing keywords about older adult volunteers were compared against a preexisting lexicon of semantic orientations. Thematic analysis revealed three themes: (1) Older adult volunteers as heroic and self-sacrificial amid the risks of war, (2) the virtue of being productive versus unproductive, and (3) older adults as agile first responders in situations of distress. Sentiment analysis further uncovered both positive and negative feelings that were collocated with the rhetoric on volunteering, such as the juxtaposition of older volunteers with the lack of supervision or training. The rhetoric of heroism, sacrifice, and productivity suggest the need to attend to age-related stereotypes of older adults. This research not only has implications for the health of older adults, but also for supporting older adult volunteers contributing towards large-scale public health challenges.

Abstract citation ID: igae098.3871

### NEIGHBORHOOD ENVIRONMENTAL CORRELATES OF FALLS AMONG OLDER ADULTS – IMPLICATIONS FOR FALLS PREVENTION PROGRAMS

Leland Ackerson, Elizabeth Procter-Gray, Qun Le, Kevin Kane, and Wenjun Li, *University of Massachusetts Lowell, Lowell, Massachusetts, United States*

Neighborhood sociodemographic environmental factors may influence the risk of falling in older adults, which has important policy implications. We investigated the associations of neighborhood physical and social characteristics with rates of indoor and outdoor falls in a multi-racial cohort of 729 community-dwelling adults aged 65 to 99 residing in Massachusetts (2018-2024). Associations of Census tract-level sociodemographic and built environmental characteristics with fall rates were analyzed using negative binomial models. The overall rates of any, indoor and outdoor falls were 67.9 (95% CI: 63.9 – 72.2), 32.1 (95% CI: 29.4-35.1) and 35.6 (95% CI: 32.7-38.7) falls per 100 person-years, respectively. In crude analyses rates of any, indoor and outdoor falls were significantly associated with 12, 3 and 20 of the 80 neighborhood characteristics under analysis, respectively. After adjusting for individual age, sex, and race, 4, 7 and 6 remained statistically significant for any, indoor and outdoor falls, respectively (e.g., for outdoor falls % male, Hispanic population, % commuting 25-34 minutes, % of Black-occupied homes that are rented, % of Hispanic-occupied homes that are rented, and total number of Hispanic-occupied homes). After further adjustments for health-related risk factors, only a few neighborhood factors remained significant for either indoor or outdoor falls. In conclusion, neighborhood characteristics are more likely associated with outdoor falls than indoor falls. The observed associations likely result from compositional effects rather than contextual effects. When designing community-based falls prevention programs, the population compositional effects and geographic distribution of older adults with higher fall risks should be carefully considered.

Abstract citation ID: igae098.3872

### NURSES' PERSPECTIVES ON MEDICATION ADMINISTRATION SAFETY FOR RESIDENTS IN NURSING HOMES: A QUALITATIVE STUDY

Bianca Shieu[1], Mei-Chen Wang[2], John Harris[3], and Ya-Wen Lee[4], *1. UT Health San Antonio, San Antonio, Texas, United States*, *2. Zoe Liu & Eric Zhou Medical*

*Office PC, New York, New York, United States*, *3. Magee-Womens Research Institute, Pittsburgh, Pennsylvania, United States*, *4. Changhua Christian Hospital, Changhua, Changhua, Taiwan (Republic of China)*

Medication administration (MA) in nursing homes (NH) offers several key benefits to residents. However, the complexity of MA often overwhelms existing systems and individual capacities, leading to dissatisfaction among NH residents and staff. Enhancing safety and efficacy requires a robust, user-centered evaluation of the NH MA system, focusing on safety, efficiency, and staff satisfaction issues and opportunities. This qualitative study examined NH staff nurse perspectives regarding MA safety concerns and potential solutions for NH residents, including those with Alzheimer's Disease and Related Dementias (ADRD). We used purposeful sampling to recruit 12 nurses employed at two NHs in the northeastern United States to participate in phone interviews. The Systems Engineering Initiative for Patient Safety (SEIPS) constructs informed the question guide, coding, and qualitative theme identification. We audio-recorded and transcribed the interviews verbatim. We organized the themes based on SEIPS components, which include (1) Technology-related concerns (non-user-friendly charting systems). (2) Organization-related concerns (teamwork, communication, and leadership). (3) Person-related concerns (variability in staff training and backgrounds). (4) Task-related concerns (different care approaches for ADRD residents, extreme workload variation throughout the day, task prioritization). (5) Environment-related concerns (staff shortages). Overall, the key themes identified by participants included the significance of user-friendly charting systems, customizing MA to accommodate diverse nursing backgrounds, and appropriate nurse-to-patient staffing ratios.

Abstract citation ID: igae098.3873

### NURSING HOME BARRIERS AND ENABLERS TO USING THE GOALS OF CARE VIDEO INTERVENTION

Latarsha Chisholm[1], Susan Hickman[2], and Kathleen Unroe[3], *1. University of Central Florida, Orlando, Florida, United States*, *2. Regenstrief Institute, Inc., Indianapolis, Indiana, United States*, *3. Indiana University, Indianapolis, Indiana, United States*

Advance care planning (ACP) is a process that prepares individuals for communicating and making decisions about personal goals regarding medical care. Despite the benefits, ACP is limited across nursing homes (NHs) due to implementation challenges. It is essential to understand how to implement ACP, like Goals of Care (GOC) evidence-based intervention. The GOC video is intended to supplement ACP discussions to educate patients living with dementia and families. This study was performed to identify enablers and barriers to using the GOC video. We interviewed 12 staff from six Florida NHs. A pre-implementation guide was developed using constructs from the Consolidated Framework for Implementation Research 2.0. Enablers included: mission alignment (6 out of 6 NHs); physical space, use of information technology, and learning-centeredness (5 out of 6 NHs); and work infrastructure, communication, readiness, recipient-centeredness, and implementation facilitators (4 out of 6 NHs). Barriers included: design of the GOC video (6 out of 6 NHs; access to health education (5 out of 6 NHs); and patient readiness and culture as barriers (4 out of 6 NHs). NH staff reported various barriers and enablers to use the GOC video; however, some barriers and enablers were similar across NHs. Developing a standardized protocol that allows flexibility maybe helpful to nursing homes implementing the GOC video intervention.

Abstract citation ID: igae098.3874

### OLDER ADULT ACCESS TO SELF-DIRECTED SERVICES THROUGH MEDICAID HOME AND COMMUNITY BASED SERVICE WAIVERS

Natalie Turner, *University of Washington, Seattle, Washington, United States*

Self-Direction (SD) is an underused service delivery model shown to improve health outcomes and increase care satisfaction for older adults. Rooted in choice, empowerment, and control over long-term services and supports (LTSS), SD epitomizes person-centeredness. And, because of its ability to provide LTSS in a culturally responsive manner, has the potential to enhance racial equity in LTSS provision. Since each state determines what services can be self-directed and how many beneficiaries can self-direct, state policy impacts access to and use of SD. This mixed-method study identified state differences in access to SD among 1915(c) waivers that serve older adults using Framework Analysis approach and descriptive statistics. Findings indicate potential access barriers to SD provided through 1915(c) waivers. Of the 60 waivers serving older adults in the U.S., 35 allowed SD (58%), representing 26 states and DC. While states described the benefits of SD within waiver applications and highlighted its alignment with person-centeredness, most set very low goals for the number of beneficiaries expected to use SD. Seventy-five percent of waivers set goals for participation in SD at less than 25% of beneficiaries. Furthermore, states acknowledged SD's flexibility and ability to tailor services to participant needs, while limiting the types of services individuals could self-direct. In light of the established benefits of SD, these findings raise questions about potential underutilization of SD among older adults. Further research is needed to better understand apparent discrepancies between state descriptions of SD and perceived and actual access to these services.

Abstract citation ID: igae098.3875

### OLDER PEDESTRIANS' PERSPECTIVES ON WALKING SAFETY IN KAMPALA: A QUALITATIVE INQUIRY INTO THE BUILT ENVIRONMENT.

Juliet Namukasa[1], and Heather Mulkey[2], *1. Makerere University Business School, Kampala, Kampala, Uganda*, *2. University of Southampton, University of Southampton, England, United Kingdom*

Safe, accessible, and inclusive urban environments are important elements in walking as a means of transport. Limited studies are focusing on the walking safety of older people especially in African cities. This study explored the older pedestrians' perceptions and experiences about walking safety in the built environment of Kampala. A qualitative design was used where purposive and convenience sampling methods were used to recruit fifteen participants from the streets of Kampala and its neighbourhood. Findings showed that older pedestrians had negative perceptions and experiences of walkability and safety in Kampala and its neighbourhoods citing inadequate pedestrian facilities e.g. the lack of sidewalks on most roads, insufficient lighting, uncovered drainage systems, and uneven surfaces. Additionally, the general lack of respect for pedestrians and traffic rules, including speed driving affects the older pedestrians' sense of safety. Despite these challenges, older pedestrians valued safe mobility within their neighbourhoods for accessing services and sustaining social connections similar to other community members. The study concludes that enhancing the safety of older pedestrians is essential for promoting equitable road access for all members of the community, it urges city policymakers to prioritize improvements in pedestrian infrastructure. Keywords: older pedestrians, walking safety, Kampala City, built environment, perceptions, experiences, qualitative study, equitable road access.

Abstract citation ID: igae098.3876

### ORGANIZATIONAL CULTURE AND SOCIAL ISOLATION IN NURSING HOMES

Ebenezer Martey[1], Jennifer Morgan[2], Antonius Skipper[2], and Candace Kemp[2], *1. University of Alabama at Birmingham, Birmingham, Alabama, United States*, *2. Georgia State University, Atlanta, Georgia, United States*

Social isolation is a prevalent issue among nursing home residents, which can lead to various chronic diseases, such as depression, dementia, and chronic pain. Social integration refers to the active engagement in social ties, institutional connections, and participation in community-based activities. Organizational culture is a critical factor in providing person-centered care and fostering communication, teamwork, and quality improvement processes. Using a case study methodology, this study surveyed 49 nursing home staff and interviewed eight residents in one nursing center to understand the context of organizational culture and social integration among nursing home residents. Analysis of resident interview data indicated that communication challenges between staff and residents exist, as some residents report being uninformed, while others report great appreciation for staff responsiveness and personalized care. Social isolation was found to be a challenge for some residents due to a lack of meaningful engagement often exacerbated by health or mobility issues. Relationships between roommates often challenged resident well-being while supportive relationships among residents were essential to well-being. Residents noted that staff were very involved with encouraging residents to participate in activities and even take on specific responsibilities to foster a sense of community. The new availability of private rooms and bathrooms in this case study home was a big source of satisfaction for both residents and families. Private rooms appear to enhance satisfaction by enabling residents to exercise autonomy around daily schedules and promote dignity. Resident feedback on communication challenges revealed the need for organizations to practice greater transparency and consistent messaging.

Abstract citation ID: igae098.3877

### OUTCOMES OF A PILOT WELLNESS PROGRAM FOR LOW-INCOME OLDER ADULTS IN INDEPENDENT LIVING: A MIXED METHODS STUDY

Khalid Yusuf, Isabelle Erb, Joan Tunningley, and Victor Ronis-Tobin, *Xavier University, Cincinnati, Ohio, United States*

By 2030, one-fifth of U.S. residents will be of retirement age, leading to increased demand for residential services for older adults (OAs). Effective wellness programming is crucial to support the physical and psychological well-being of low-income residents, yet there is limited evidence to guide such program development and evaluation. This pilot study details the first phase of a program development initiative in two independent living communities in Southwest Ohio managed by Episcopal Retirement Services (ERS). A mixed-methods approach was used to inform program design. Participants were 57 residents, with an average age of 75.71 years (SD = 7.75); 89.5% were female, and 59.6% were Black or African American. Most participants (86%) reported incomes below $25,000, and 52.6% were divorced. Baseline engagement levels were low (M = 19.82, SD = 6.92), as measured by the Social Engagement and Activities Questionnaire (SEAQ). SEAQ scores did not correlate with program attendance; however, significant positive correlations were observed between going outside the home and both the number of friends ($r(55) = .380$, $p = .004$) and having friends within the residence ($r(55) = .314$, $p = .018$). Focus group analysis (n = 13) identified eight themes: Holistic Engagement, Activities for Social Connection, Participation Challenges, Personalization Desires, Impact of Isolation, Male Engagement Issues, Strategies for Boosting Engagement, and Self-Improvement Aspirations. These findings emphasize the need for wellness programs that address physical health, foster social connections, and promote personal growth. Active resident-staff collaboration in the development and maintenance of such programming is crucial for success.

Abstract citation ID: igae098.3878

### PARTNERING WITH COMMUNITY ON A SUCCESSFUL FEDERAL FUNDING APPLICATION FOR MESOTHELIOMA CAREGIVERS

Amanda Woodward[1], Maria Towe[2], Jen Hirsch[1], and Julie White[3], *1. Michigan State University, East Lansing, Michigan, United States*, *2. Mesothelioma Applied Research Foundation, Washington, DC, District of Columbia, United States*, *3. Mesothelioma Applied Research Foundation, Washington, District of Columbia, United States*

The Michigan State University School of Social Work (MSUSSW) and the Mesothelioma Applied Research Foundation (Meso Foundation) collaborated on a successful application for federal funding to develop and conduct feasibility testing of an online caregiver support intervention. The process of developing the proposal and the design of the four-year project follow key principles of the Community-Engaged Research Framework described by NORC at the University of Chicago. The research questions emerged from a survey the Meso Foundation administered to its community, thus aligned with a foundational component of community engaged research where the community drives the research focus. The team includes members with lived experiences whose time and expertise are incorporated through processes that promote shared decision making (e.g., co-principal investigators) and budgeted compensation for their work. Information about research processes, resources, and timelines are openly shared with all team members through password-protected cloud-based storage. Meeting schedules, modes of communication, workplans, and decision-making protocols are established collaboratively and revisited regularly throughout the work. The project itself engages patients, caregivers, those who have lost a loved one, and health professionals through in-depth interviews and focus groups to better understand the caregiver experience from diagnosis through end of life. Participant experiences and feedback will be used to develop and refine an online caregiver support intervention. The feasibility of the intervention will then be tested in an intervention-only design. This poster will describe the six key principles of community-engaged research and the ways we are implementing them in more detail.

Abstract citation ID: igae098.3879

### PERSPECTIVES ON POLICIES TO IMPROVE EQUITY IN HOSPITAL AT HOME

Jeannys Nnemnbeng[1], Abigail Baim-Lance[1], Brittni Howard[2], Julia Hart[1], Bruce Leff[3], David Levine[4], Albert Siu[1], and Ksenia Gorbenko[1], *1. Icahn School of Medicine at Mount Sinai, New York, New York, United States*, *2. University of Massachusetts at Amherst, Amherst, Massachusetts, United States*, *3. Johns Hopkins Medicine, Baltimore, Maryland, United States*, *4. Brigham and Women's Hospital, Boston, Massachusetts, United States*

Hospital at Home (HaH) delivers acute hospital-level care at home as a substitute for traditional hospital care. Compared to traditional hospital care, HaH care demonstrates better patient satisfaction and care experience, lower readmission rates, and lower costs of care. There has been limited research on equity issues in HaH care delivery. We explored HaH program leaders' views on the policy landscape and opportunities for equitable delivery of HaH across the US. We conducted a qualitative study using semi-structured interviews with healthcare leaders (1-4 leaders per program) at 18 HaH programs diverse by geography (region, rurality), size, and safety-net status. We conducted interviews via Zoom and used rapid template analysis and focused coding to identify key themes. We identified four areas supporting equitable HaH care delivery: (1) Technology access to promote more complete broadband coverage, particularly in rural areas; (2) Medicaid expansion to allow the enrollment of Medicaid beneficiaries, a frequent recommendation to increase lower-income individuals' access to the program, including younger age groups that did not typically participate and would benefit; (3) Workforce role diversity to align with the needs of communities and therefore to include services like paramedicine familiar with delivering care at home; and (4) Sustainable and broadening of HaH funding including making the waiver permanent and expanding support for a package to connect patients with community-based care at discharge. HaH success in advancing equitable care delivery requires greater policy support to ensure equal access and solid funding mechanisms to support its ongoing growth.

Abstract citation ID: igae098.3880

### POPULATION AGING AND INCREASING CHRONIC DISEASE BURDEN AMONG UNAUTHORIZED AGRICULTURAL WORKERS

Nicholas Bishop[1], Baltazar Campos[1], Daniel Martinez[1], and Jenjira Yahirun[2], *1. University of Arizona, Tucson, Arizona, United States*, *2. Bowling Green State University, Bowling Green, Ohio, United States*

The US and Mexico are experiencing rapid population aging and increased chronic disease burden. We hypothesize these dynamics are magnified among undocumented farmworkers who work in challenging conditions and have limited access to healthcare. We used data from the National Agricultural Workers Survey (2001–2018) to examine whether 1) the age of undocumented farmworkers has increased over time (n=19,434), and 2) chronic disease burden has increased among undocumented workers aged 50+ (n=1,463). Multiple regression was used to examine the association between respondent age and survey year, and Poisson regression was used to estimate the association between number of doctor-diagnosed conditions (asthma, diabetes, hypertension, and heart disease) and survey year among respondents aged 50+. Models were adjusted for covariates (sex, migrant type, health service use, region, and age (in the chronic disease model)), and person weights. The mean age of the sample in survey years 2001–2002 was 28 (SD=9.7), which increased to 39 (SD=11.0) by 2017–2018. After adjusting for covariates and weights, there was a monotonic positive increase in age across survey years (e.g., respondents surveyed in 2017–2018 were on average 8.36 years older (95% CI:7.61–9.12, p<.001) than those surveyed in 2001–2002). Among undocumented agricultural workers aged 50+, respondents in the 2017–2018 survey waves had an expected condition count 202% greater than respondents in the 2001–2002 waves (IRR=3.02, 95% CI:1.45–6.29, p=0.003). An aging population of undocumented farmworkers with increasing chronic disease burden represents a significant health equity issue and strain on US agricultural output.

Abstract citation ID: igae098.3881

### PREPARING FUTURE HEALTH PROFESSIONALS FOR AGE, CULTURAL, AND TECHNOLOGY (ACT) FRIENDLY CARE IN HEALTH SCIENCES

Nosaiba Rayan-Gharra[1], Shoshi Keisari[1], Lia Ring[2], Shelly Engdau[1], Dikla Segel-Karpas[1], Anna Zisberg[1], and Alexandra Danial-Saad[1], *1. University of Haifa, Haifa, Hefa, Israel*, *2. Ashkelon Academic College, Ashkilon, HaDarom, Israel*

The aging population emphasizes the need for tailored training in welfare and health sciences, particularly for students who will work with culturally diverse older populations using advanced technological tools. This study examines how well the curriculum at the Faculty of Welfare and Health Sciences prepares students for Age, Cultural, and Technology (ACT) Friendly care. An online survey was conducted among 208 faculty lecturers, teaching mandatory undergraduate and/or graduate courses (BA only=65, MA only=82, both BA and MA=61). Respondents rated how extensively they address aging, multiculturalism, and technology in their courses. Only 15.4% had received training in aging studies, while 16.4% were involved in aging research. Aging-related topics were included in 31% of undergraduate and 27% of graduate courses. Multiculturalism was similarly integrated (28% for both programs), while technology was less addressed (13% for BA and 8% for MA courses). Only 17.3% of respondents extensively incorporated ACT topics in their teaching, and 65.7% did not address them at all. Multivariate analysis controlling for gender, seniority, and research area showed that involvement in aging, multiculturalism, or technology research predicted teaching these topics (p< 0.05). The findings highlight a significant gap in preparing students for ACT-friendly care, particularly at the graduate level, and a lack of integration of the three domains. This study underscores the need for ongoing assessment and curriculum development to equip future health professionals for age-appropriate, culturally sensitive, and technologically integrated care. Faculty development initiatives are also recommended to enhance educators' capacity to incorporate these critical areas into their teaching.

Abstract citation ID: igae098.3882

### PREPARING THE NEXT GENERATION OF THE DEMENTIA CARE WORKFORCE VIA SERVICE LEARNING: A SCOPING REVIEW

Makenna Green[1], Jamie Mayer[2], Laura White[3], and Gail Kouame[4], *1. Northern Illinois University, Saint Charles, Illinois, United States*, *2. Northern Illinois University, DeKalb, Illinois, United States*, *3. University of Alabama at Birmingham, Birmingham, Alabama, United States*, *4. University of South Alabama*

The prevalence of Alzheimer's Disease and Related Dementia's (ADRD) is rising rapidly with the aging of the baby boomer generation. A large workforce gap in dementia providers is widening with education as a frequently cited barrier to entry into the ADRD workforce (Mayer et al, 2023). This scoping review aimed to explore the extant literature on the use of service learning (SL) pedagogy related to ADRD in higher education settings for students in health-related majors. A systematic search was conducted using seven databases. Sources were included if participants were undergraduate/graduate students in any health-related discipline and if SL was utilized in conjunction with specific instructional paradigms (e.g., coursework, on-site workshops). SL, though individually defined by authors of included studies, was required to include community engagement with adults with ADRD; studies specific to clinical rotations were excluded. Included sources (n=32) were systematically extracted for characteristics including student demographics, SL components (learning instruction methods/training, experiences, reflection), and outcomes. Analysis of training revealed most ADRD SL paradigms relied on setting-specific training (n=15) over didactic academic instruction (n=7) or a combination (n=7). SL experiences ranged in type with informal activities (n=13) and creative arts (n=9) most commonly utilized to engage participants. The reflection component most commonly included open-ended reflective journals (n=15). Analysis revealed variability in how SL is represented in the literature. Despite this variability, sources frequently report SL as a viable option for educating the next generation of dementia service providers, with observed improvements in student attitudes and knowledge related to ADRD.

Abstract citation ID: igae098.3883

### PROFESSIONAL CAREGIVER SATISFACTION AND BURNOUT IN ASSISTED LIVING

Elizabeth Morton[1], Sheryl Zimmerman[2], Lea Efird-Green[1], and Scott Davis[3], *1. University of North Carolina at Chapel Hill, Chapel Hill, North Carolina, United States*, *2. University of North Carolina at Chapel Hill (UNC), Chapel Hill, North Carolina, United States*, *3. University of North Carolina Eshelman School of Pharmacy, Asheville, North Carolina, United States*

Professional caregivers (direct care workers) perform most of the care for residents in assisted living (AL) and have consistently high turnover rates. Because high turnover is associated with low job satisfaction and decreased quality of care, understanding professional caregiver satisfaction can inform approaches to improve the well-being of both staff and residents. This study obtained information from 559 professional caregivers from 69 AL communities to explore job satisfaction, burnout, and how satisfaction varies based on professional caregiver and AL characteristics (e.g., resident-case mix, staffing ratio). It also established the validity of the Direct Care Worker Job Satisfaction Scale (scored 1-4) as applied to this population through a principal components analysis. Principal components analysis produced a single-factor solution with an eigenvalue of 10.8, the only eigenvalue above 1. All scale items loaded at least 0.70 on the single factor. Overall, professional caregivers had an average satisfaction of 3.05 ("satisfied"), and 18.5% were burned out; satisfaction and burnout were negatively correlated (-0.46, p< 0.0001; adjusted -0.43, p< 0.0001). Satisfaction was positively correlated with the percent of residents with dementia (0.19, p< 0.0001) and provision of special/memory care at their AL community (0.17, p< 0.0001); it was negatively correlated with the professional caregiver-to-resident staffing ratio (-0.18, p< 0.0001) and presence of licensed nursing staff (-0.27, p< 0.0001). Results suggest that caring for individuals with dementia is particularly rewarding for professional caregivers, while being responsible for more residents and working alongside licensed nursing staff may lessen satisfaction.

Abstract citation ID: igae098.3884

### PROXIMITY, CARE VALUES, AND CAREGIVING STRAIN: EVIDENCE FROM THE 2020 NAC/AARP NATIONAL SURVEY

Tingan Jin, *The London School of Economics and Political Science, London, England, United Kingdom*

The concept of distant caregiving emerged as family caregivers can care for older adults in settings other than living with their parents. Previous studies have illustrated that differences in geographical distance could lead to different caregivers' experiences in terms of physical, emotional, and financial strain. This study uses the most recent nationally representative data on caregivers in the US from the National Alliance for Caregiving and AARP to explore the characteristics and values of caregivers with varying distances to care recipients and test the effect of geographical distance on various aspects of caregiving strain. The preliminary analysis conducted in June 2024 suggests that caregivers providing care at a distance reported lower physical and financial strain levels regardless of proximity intervals. Long-distance carers, namely those who live more than one hour away from their parents, show more physical strain (b=-0.236, p< 0.05) and financial strain (b =-0.228, p< 0.05) than those who live with or close to their parents. Findings regarding emotional strain were against previous studies, and we found that while those who live close to their parents reported lower levels of emotional strain (b=-0.171, p< 0.05), the pattern does not apply to long-distance caregivers with a positive, statistically insignificant relationship. Adding components regarding caregivers' willingness and perceptions towards care provision, it is found that whether the respondents feel they had a choice to take care of their parents is a statistically significant predictor of physical (b=0.508, p< 0.05), financial (b=0.446, p< 0.05), and emotional strain (b=0.810, p< 0.05).

Abstract citation ID: igae098.3885

### REALITIES OF DIVERSITY, INCLUSION, AND BELONGING IN ASSISTED LIVING: SAFETY, CONNECTION, AND COMMUNITY

Jacklyn Kohon, Dani Himes, and Sarah Dys, *Portland State University, Portland, Oregon, United States*

This study centers the voices and experiences of those working and living in assisted living, residential care, and memory care (AL/RC) settings in Oregon to understand how workplace diversity, equity, inclusion, accessibility, and belonging (DEIAB) can promote well-being. From January to April 2024, we conducted individual and focus group interviews with a total of 68 people, including 25 direct care staff, 9 former direct care staff, 9 administrators, 7 management representatives and 18 current residents from 23 AL/RC communities. We highlight six primary themes: 1) Care staff face discrimination, physical and verbal abuse from residents and colleagues; 2) those experiencing discrimination or not belonging were more likely to be from a minoritized group; 3) there is inconsistency in the existence, awareness, and currentness of diversity, equity, and inclusion policies, making implementation challenging; 4) company culture can support feelings of connection and belonging among staff; 5) care staff rely on peers and coworkers for support; and 6) workplaces need infrastructure that reflects language diversity of residents and care staff. Participants noted discrimination can happen overtly and subtly. In many instances, overt discrimination can be easier to address and handle, while subtle forms of discrimination happen daily and many times are invisible or look like unspoken norms. Recommendations include comprehensive DEIAB policies with clear implementation plans, DEIAB training programs for residents, investing in linguistic accessibility resources, and training administrators and managers in inclusive leadership practices.

Abstract citation ID: igae098.3886

### RECIPROCAL RELATIONSHIP BETWEEN LONELINESS AND LIFE SATISFACTION IN HONG KONG OLDER ADULTS

Foong Yee Vivien Tang, and Kee Lee Chou, *The Education University of Hong Kong, Hong Kong, China (People's Republic)*

Given the limited research on the reciprocal relationship between loneliness and life satisfaction in the Chinese community, this study examines this dynamic and explores the mediating effect of neighbourhood collective efficacy. Loneliness, a prevalent issue among the aging population, significantly impacts psychological well-being and overall quality of life. Conversely, life satisfaction reflects an individual's overall contentment with life and is a crucial component of successful aging. Utilizing a two-wave secondary data from 1,696 Chinese older adults aged 60 and above (53.9% female), this study analyzed how changes in loneliness predict subsequent changes in life satisfaction, and vice versa. Participants were assessed on their levels of loneliness, life satisfaction, sociodemographic factors, health, social resources, and social support. The findings indicate a reciprocal relationship: loneliness was associated with lower life satisfaction two years later (B = -1.44, p = 0.014), and reduced life satisfaction led to increased loneliness (B = -1.106, p < 0.001). Furthermore, neighbourhood collective efficacy was found to fully mediate the relationship between loneliness and life satisfaction (β = 0.18, p < 0.001). These results underscore the importance of addressing loneliness and life satisfaction simultaneously. Maintaining strong neighbourhood collective efficacy may help mitigate the negative impact of loneliness on life satisfaction. This highlights the potential for targeted, community-based intervention programs designed to enhance life satisfaction and alleviate loneliness among older adults.

Abstract citation ID: igae098.3887

### REFINING CARE NETWORK MEASURES FOR DIVERSE DEMENTIA CAREGIVERS USING CROSS-CULTURAL COGNITIVE INTERVIEWING

Melissa Howe[1], Alan Rodriguez Tiburcio[2], Kelly Pudelek[3], Selena Zhong[3], Nell Compernolle[3], and Lissette Piedra[4], *1. NORC at the University of Chicago, Penn Valley, California, United States*, *2. NORC at the University of Chicago, NORC at the University of Chicago, Illinois, United States*, *3. NORC at the University of Chicago, Chicago, Illinois, United States*, *4. University of Illinois Urbana-Champaign, University of Illinois at Urbana-Champaign, Illinois, United States*

It is often assumed that American English speakers universally understand validated instruments and survey items used in previous research. However, comprehension can vary widely due to differences in dialect, education background, and other factors, impacting both survey data quality and respondent burden. To address this, we used cross-cultural cognitive interviewing methods to: (1) facilitate the inclusion of caregivers from diverse cultural and linguistic groups and (2) ensure that survey items elicit comparable data across these groups for accurate analysis. We conducted ~60-minute interviews with 20 informal caregivers to test new and existing survey questions designed to characterize the care networks and caregiving experiences of adults who regularly assist a family member or friend living with dementia. We assessed their ease or difficulty when answering survey questions with close-coded response options and probed into their understandings of key terms. Initially, we interviewed ten caregivers purposefully selected to represent diverse ethnoracial and gender identities, education backgrounds, and geographic locations (urban/rural). After analyzing the transcripts to identify and remedy problematic wording, we conducted ten additional cognitive interviews with new respondents, focusing on Latinx caregivers who primarily speak American English. This iterative process informed specific changes, such as refining the description of "caregiver" in a question designed to be maximally inclusive and replacing misunderstood words such as "dominant" and "primary" with more familiar words. Variations in participant comprehension underscore the importance of rigorously pre-testing research materials. Post-survey development, our data and findings will provide valuable context for interpreting survey results.

Abstract citation ID: igae098.3888

### REFRAMING AGING: THROUGH THE LENS OF ETHICAL ARTIFICIAL INTELLIGENCE

Cynthia McNellis, *American Public University System, Egg Harbor Twp, New Jersey, United States*

Artificial Intelligence (AI) technology is advancing at a rapid pace. In addition, our older adult cohort is growing at an unprecedented rate. The purpose of my research is to address the ethical concerns when AI intersects with this population, offering policy recommendations to bridge this ethical digital divide. To address this AI ethical dilemma, my research did not reach back prior to 2019 in order to provide a concise policy analysis that includes existing AI ethical policies/recommendations from the European Union, United States, and U.S. States, key differences, and how ethical AI policies are central to Area Agencies on Aging's development of aging services. My findings indicated gaps in ethical AI design with developed policy recommendations. These gaps include inclusive data collection, user-centric design, algorithmic biases and understanding, digital literacy, New Jersey AI Task Force membership, and a need for a New Jersey AI Ethical Framework. With my five policy recommendations, New Jersey will be better positioned in assuring AI systems will be equitable, ethical, and effective in addressing the present and future needs of older adults. With this ethical AI framework, New Jersey will help to better older adults' quality of life, as well as save valuable time and resources through the new industrial revolution that is Artificial Intelligence. Keywords: Artificial Intelligence, ethics, older adults, algorithmic bias, AI ageism

Abstract citation ID: igae098.3889

### REHOSPITALIZATION RISK AMONG VETERANS RECEIVING POST-ACUTE HOME HEALTH SERVICES: DIFFERENCES BY PAYOR SOURCE

Ellen McCreedy[1], Frank Devone[2], Pedro Gozalo[1], Stefan Gravenstein[2], Marguerite Daus[3], Christine Jones[4], Kate Magid[5], and Kali Thomas[6], *1. Veterans' Administration, Providence, Rhode Island, United States*, *2. United States Department of Veterans Affairs, Providence, Rhode Island, United States*, *3. Department of Veterans Affairs, Aurora, Colorado, United States*, *4. VA Eastern Colorado Healthcare System, Aurora, Colorado, United States*, *5. Rocky Mountain Regional VA, Aurora, Colorado, United States*, *6. Johns Hopkins University, Baltimore, Maryland, United States*

Medicare-eligible Veterans who require post-acute skilled home health (HH) after a hospitalization can have HH paid for by the Veterans' Administration (VA) or Medicare. Our prior work showed Veterans enrolled in Medicare Advantage (MA) are more likely to have the VA pay for HH than MA. In this analysis, we look at differences in 90-day rehospitalization among MA-enrolled Veterans receiving VA-paid post-acute HH versus MA-paid post-acute HH. All Veterans enrolled in MA and receiving HH after an index hospitalization at a VA medical center between October 1, 2016 and September 30, 2019 were included. Data were extracted from the VA electronic medical record. We used inverse probability weighting to address baseline differences in age, patient complexity, and past utilization. We used cox proportional hazards models to estimate the effect of VA- versus MA-paid HH on the risk of 90-day rehospitalization. There were 14,501 eligible hospitalizations requiring HH; 8,753 (60.4%) received VA-paid HH, and 5,748 (39.6%) received MA-paid HH. On average, Veterans receiving VA-paid HH waited 3.7 days (SD: 3.2) to start HH, compared to 4.8 days (SD: 3.8) for Veterans receiving MA-paid HH. After preliminary weighting, Veterans who received VA-paid HH had a lower risk of 90-day rehospitalization, compared to Veterans who received MA-paid HH (adjusted hazard ratio=0.88; 95% CI: 0.83,0.93). Veterans whose HH was paid for by MA received services one day later, on average, and had higher risk of 90-day rehospitalization compared to Veterans receiving VA-paid HH. Potential implications on quality of post-acute care for MA-enrolled Veterans discussed.

Abstract citation ID: igae098.3890

### ROLE OF MACHINE LEARNING (ML) IN AGING IN PLACE RESEARCH: A SCOPING REVIEW

Sojung Park[1], Eunhye Ahn[1], Tae-Hyuk Ahn[1], SangNam Ahn[2], Soobin Park[1], Eunsun Kwon[3], Seoyeon Ahn[4], and Yuanyuan Yang[1], *1. Washington University in St. Louis, St. Louis, Missouri, United States*, *2. Saint Louis University, St. Louis, Missouri, United States*, *3. Fairleigh Dickinson University, Hackensack, New Jersey, United States*, *4. Duke-NUS Medical School, Singapore, Singapore*

As global aging accelerates, Aging in Place (AIP) is increasingly central to improving older adults' quality of life. Machine Learning (ML) is widely used in aging research, particularly in health monitoring and personalized care. However, most studies focus on clinical settings, leaving a gap in understanding ML's application in non-clinical AIP contexts. This review addresses this gap by exploring the themes, policy implications, and ethical concerns of AIP-ML studies, including AI bias and fairness. The review examined 32 peer-reviewed studies sourced from databases like PsycINFO, MEDLINE, and PubMed. Through thematic analysis, three main themes emerged: successful aging, managing depressive symptoms, and fostering social connectedness. The studies employed various ML techniques, including classification algorithms, random forest, SVM, and LASSO, to predict health outcomes such as depression, cognitive decline, and functional disabilities in older adults. They also identified key risk factors and examined disparities in healthcare access and digital inclusion. However, the studies often lacked validation across diverse populations, which limits their policy impact. Most research focused on healthy, community-dwelling older adults, excluding those with dementia or disabilities, thereby introducing biases that could disproportionately affect marginalized groups The review highlights that while ML models are useful for prediction, they may overlook the needs of less healthy older adults, reducing their accuracy and fairness. These findings emphasize the need for transparency and participant involvement in data usage, suggesting that despite its limitations, the current literature offers valuable insights for advancing AIP research and guiding the development of more inclusive AIP policies.

Abstract citation ID: igae098.3891

### SERVICE AND PROGRAM NEEDS TO SUPPORT OLDER, LOW-INCOME HOMEOWNERS AGING IN PLACE

Sung-Jin Lee[1], Renee Robinson[1], Elizabeth Hopfer[1], Minyong Lee[1], and Kathleen Parrott[2], *1. North Carolina*

*A&T State University, Greensboro, North Carolina, United States*, 2. *Virginia Tech, Blacksburg, Virginia, United States*

An interdisciplinary team of the Housing, Kinesiology, and Textile programs at North Carolina A&T State University is collaborating on a multi-year research project to examine the person-environment fit perspective for older, low-income homeowners in North Carolina. Using a qualitative phenomenological study approach, researchers conducted 19 in-home and personal interviews with urban low-income, older homeowners in community-dwelling units. A local nonprofit housing agency providing home modification services to low-income older adults assisted with sample recruitment. Researchers conducted a content analysis using interview transcripts from recorded voices, home assessment data, photos taken during home visits, and interviewers' notes. Data analysis revealed personal elements (e.g., living alone, informal support, mortgage payments, technology capability levels) and environmental elements (e.g., steps, dust/unclean home conditions, clutter, continuous upkeep needs) that can be associated with respondents' aging-in-place practices. The qualitative data also highlighted the need for additional services and programs to improve their home environment or housing conditions beyond home modifications. Examples include cleaning services (e.g., dust on floors, carpets, ceilings, or vents), laundry services for home textiles (e.g., fabric drapes on windows), lawn care programs, and services for placing trash bins at the curb for pickup and returning them near the home. This study provides an opportunity to understand older, low-income homeowners aging in place and their home environments, which have been less explored using a qualitative approach. This research presentation will be supported by qualitative comments from study participants, along with photos of home environments.

Abstract citation ID: igae098.3892

### SHEMANCIPATION: A GROUNDED THEORY OF AFRICAN AMERICAN WOMEN'S PROCESS OF TRANSITIONING TO AGE 50

Lisa Bradley, *Henry Ford College, Southfield, Michigan, United States*

The study of adult development has produced numerous theories to describe participants' experiences, processes, and related developmental milestones. Despite the number of studies found in the literature, few have explored adult development for African American women, and fewer still have explored African American women's midlife experience. The present study, rooted in theories of intersectionality and critical gerontology, used constructivist grounded theory to investigate a discrete aspect of midlife aging for African American women, the age-50 transition. Guided by the research question, "How do African American women describe their experience of turning 50?", eighteen semi-formal interviews were conducted with 11 African American women, age 50 to 55, which coincides with Levinson's (1986/1996) Middle Adulthood. The women confirmed that they were born, raised, and currently lived in the United States. During data collection, constant comparison and memoing were used to evaluate the data for gaps and overlapping instances. Initial coding generated the assignment of 161 unique codes. After four rounds of coding, the three processes of Reflection, Realization, and Resolution, and the theoretical concept of "Shemancipation" emerged. Findings of the present study were partially aligned with Cohen's (2005/2006) stages of Midlife Reevaluation and Liberation, and Howell & Beth's (2002) midlife development stages of External Awareness and Internal Awareness stages. The findings report includes several direct quotes from the participants to intentionally amplify voices long omitted from the literature. Further study with additional cohorts was recommended, as well as an investigation of the role played by the global pandemic on introspective processes.

Abstract citation ID: igae098.3893

### SOCIAL SUPPORT NETWORKS AND LONELINESS: DIFFERENCES ACROSS RACIAL/ETHNIC GROUPS

Orah Burack, and Wingyun Mak, *The New Jewish Home, New York, New York, United States*

Higher levels of social support (SS) has been associated with a number of positive health and well-being outcomes including lower levels of loneliness. Additionally, SS networks have been found to vary across race/ethnicity. Researchers have therefore begun to examine how specific aspects of SS differentially impact outcomes among racial/ethnic groups. In this study we examined whether visits with different types of SS providers (children, siblings, friends), impact loneliness differentially across race/ethnicity. Data were drawn from the AARP Loneliness and Social Connections Survey with a national sample of community dwelling adults age 45+ years (M=61.84, SD=10.12; N=4040). Participants self-identified as White non-Hispanic (N=3149), Black non-Hispanic (N=420), or Hispanic (N=472). A series of hierarchical linear regressions (one for each racial/ethnic group) examined the relationship between loneliness and frequency of in-person visits from: children, siblings, or friends, after controlling for age, gender, marital status, and perceived health. While older, married, healthier adults reported less loneliness across all groups, differences were found by SS provider type. Less loneliness was significantly related to more visits from: siblings ($\beta$=.05, p<.001) and friends ($\beta$=.11, p<.001) for Whites ($\Delta$R2=.03, p<.001); friends ($\beta$=.09, p=.008) for Hispanics ($\Delta$R2=.02., p=.01); while for Black subjects there were similar size trends for children ($\beta$=.08, p=.08), siblings ($\beta$=.07, p=.03., p=.001), and friends ($\beta$=.07, p=.09). These results highlight that SS providers are not interchangeable, with differing impacts on loneliness across ethnicity/race. Implications and reasons for these findings will be discussed.

Abstract citation ID: igae098.3894

### STATE-LEVEL OPPORTUNITIES TO ADDRESS BEHAVIORAL HEALTH NEEDS OF OLDER ADULTS: A NEW CENTER OF EXCELLENCE FOR OREGON

Walter Dawson[1], Allyson Stodola[2], Karen Cellarius[2], Dani Himes[2], Lindsey Smith[1], Leah Brandis[3], Nirmala Dhar[4], and Paula Carder[5], *1. Oregon Health & Science University, Portland, Oregon, United States*, *2. Portland State University, Portland, Oregon, United States*, *3. OHSU, Portland, Oregon, United States*, *4. Oregon Health Authority, Portland, Oregon, United States*, *5. OHSU-PSU School of Public Health, Portland, Oregon, United States*

Serious mental illness and substance use disorders among older adults frequently intersect with neurocognitive changes and complex physical health issues. At the same time, access to behavioral health services is often poor for many older adults. Multiple underserved populations such as older adults from communities of color and individuals living in rural/remote communities experience substantial barriers to access and use of behavioral health services. There is a need for enhanced program and policy actions to ensure access to behavioral health for these populations of older adults. This session describes the state of behavioral health and aging services for older adults in Oregon and efforts to address local and statewide needs. This includes the impetus for establishing the Oregon Center of Excellence for Behavioral Health and Aging (OCEBHA), the goals of OCEBHA, and the long-term vision of the Center to address behavioral health disparities.

Abstract citation ID: igae098.3895

### SUBJECTIVE FINANCIAL STRAIN AMONG CAREGIVERS OF INDIVIDUALS WITH ALZHEIMER'S DISEASE AND DEMENTIA

Yujun Zhu, Susan Enguidanos, Kathleen Wilber, Donna Benton, and Francesca Falzarano, *University of Southern California, Los Angeles, California, United States*

Caregivers of individuals with Alzheimer's disease and dementia often face significant caregiving burdens. While the cost of care is admittedly high for dementia patients, research on the financial strain experienced by family caregivers remains limited. This cross-sectional study used publicly available data from the "Caregiving in the U.S. 2020" survey of 1,241 caregivers who identified as caring for an adult age 50 and older. Comparative analyses were conducted to assess subjective financial strain and caregiving intensity among caregivers of individuals with and without Alzheimer's disease/dementia. Logistic regression models were used to explore the association between caring for individuals with Alzheimer's disease/dementia and family caregivers' subjective financial strain after controlling for caregivers' demographic characteristics, care recipients' conditions and care intensity. The average age of caregivers was 56.1 (SD=16.5) years and 59% were female. About 61% of caregivers for individuals with Alzheimer's disease/dementia reported financial strain, significantly higher than the 51% reported by caregivers of individuals without these conditions (P<.001). Caregivers assisted in more activities of daily living (2.1 vs. 1.4) and instrumental activities of daily living (4.9 vs. 4.3) in the Alzheimer's group compared to the non-Alzheimer's caregivers. Caring for recipients with Alzheimer's disease/dementia was associated with a higher (OR=1.5, 95% CI: 1.1-2.0) likelihood of experiencing subjective financial strain. This study highlights the amplified financial challenges faced by caregivers of individuals with Alzheimer's disease and dementia. The results underscore the necessity for comprehensive support systems, including financial assistance programs to alleviate the burdens on this population.

Abstract citation ID: igae098.3896

### TECHNOLOGY THAT SUPPORTS EXTENDING DEMENTIA-FRIENDLY COMMUNITY BASED CARE: A SCOPING REVIEW

Nina Buckley, Elizabeth Hewitt, Limei Chen, and Sang Choi, *George Mason University, Fairfax, Virginia, United States*

With global aging on the rise, there is increasing interest in technological interventions that support persons living with dementia (PLwD) to age in place. This scoping review identifies and examines technologies specifically designed for dementia care, explores their usage contexts, and assesses their potential for implementation in community settings. A systematic search was conducted across nine electronic databases—APA PsycInfo, AgeLine, MEDLINE, SocINDEX, Global Health, Web of Science, Google Scholar, and CINAHL—following the WHO (2023) framework for the classification of digital health interventions. The review included peer-reviewed articles published between 2014 and 2024, using search terms related to "dementia," "digital technology," "quality of life," and "community." Articles were screened for eligibility based on predefined criteria, such as being peer-reviewed and written in English. Out of 1,009 articles originally identified, 54 met the inclusion criteria and were included in the review. The findings reveal a range of technologies aimed at keeping PLwD safe and supported at home. However, the majority of studies highlighted a significant reluctance among PLwD to adopt these technologies, often due to a lack of familiarity and comfort with digital tools. This hesitancy, particularly among family caregivers, emerged as a major barrier to the successful implementation of technological interventions in community settings. To enhance the effectiveness of technology in dementia care, there is a need for strategies that address caregiver concerns and improve digital literacy. Future research should focus on developing user-centered interventions that are adaptable to the diverse needs and preferences of both PLwD and their caregivers.

Abstract citation ID: igae098.3897

### THAT'S ANTHROPOLOGY: CONFRONTING AGEISM AND STUDENT DISCOMFORT IN SERVICE LEARNING

S.M. Cho[1], Cara Williams[1], Andrea Freidus[2], and Dena Shenk[1], *1. University of North Carolina at Charlotte, Charlotte, North Carolina, United States*, *2. Michigan State University, Michigan State University, Michigan, United States*

Undergraduate anthropology majors participated in a 15-hour, semester-long service-learning project at an adult day and health care center during Spring 2024. While a research-based model of intergenerational service-learning has been incorporated in the undergraduate gerontology program curriculum at UNC Charlotte for 25 years, this was the first time anthropology undergraduates engaged in intergenerational research as part of their senior seminar. With no prior coursework in gerontology, and limited intergenerational experiences, anthropology senior seminar students began the semester with uncertainty and ageist biases. The founding director of the gerontology program, a professor emerita of anthropology, guided students' practical development by providing lectures, leading exercises, and facilitating discussions throughout the semester. During the course, the students learned about and confronted complex presentations of ageism. Their time at the day center involved socializing with participants, assisting staff, creating and tending a therapeutic garden, working with participants to create memory boxes, and building rapport. These steps were essential for building a reciprocal, collaborative intergenerational research relationship that integrates anthropological pedagogy. We suggest their initial resistance to this work is the result of age segregation and internalized sociocultural narratives of ageism, as well as misguided expectations of the frustrating, messy, challenging reality of undertaking anthropological research using participant observation. Consistent narrative themes of unprecedented gratitude, personal growth, and learning about ageism emerged from student field notes, class discussions, and written reflections. This program evaluation presents novel insights for future integration of intergenerational programming and gerontological knowledge into anthropological pedagogy.

Abstract citation ID: igae098.3898

### THE ASSOCIATION BETWEEN DIGITAL TECHNOLOGY ACTIVITIES AND COGNITIVE DOMAINS OF COMMUNITY DWELLING OLDER ADULTS

Erh-Chi Hsu[1], Eric Jutkowitz[2], and Kali Thomas[1], *1. Johns Hopkins University, Baltimore, Maryland, United States*, *2. Brown University, Brown University, Rhode Island, United States*

While prior research has established a relationship between digital technology use and improved cognitive function among older adults, the impacts of different technology activities on specific cognitive domains remain underexplored. This study examines associations between various technology activities and cognitive trajectories in community-dwelling older adults without dementia. We utilized repeated cross-sectional data from 5,596 participants in the National Health and Aging Trends Study (2015-2022). Technology activities included online shopping, banking, medication refills, social network site visits, and checking health conditions online. Cognitive domains assessed were episodic memory, executive function, and orientation. Covariates included sex, age, race, education, living arrangement, income, daily and instrumental activity difficulties, self-rated health, rurality, and device ownership. Survey-weighted mixed-effect models with random intercepts evaluated the associations between technology activities and cognitive trajectories, with descriptive stratification by sex. In 2015, online banking (28%) and visiting social network sites (25%) were the most popular activities, with females generally showing lower usage except for social network visits (F: 27%; M: 23%). Social engagement online had the strongest positive association with episodic memory ($\beta$=0.50, 95% CI: 0.33-0.67, p< 0.001) and orientation ($\beta$=0.15, 95% CI: 0.10-0.21, p< 0.001) independent of time. Online banking showed the strongest positive association with executive function ($\beta$=0.12, 95% CI: 0.06-0.18, p< 0.001) independent of time. Notably, all five activities were more beneficial for females regarding episodic memory (p< 0.001). These findings suggest that interventions promoting online instrumental activities (shopping, banking, medication refills) and social engagement online could enhance cognitive function in older adults, particularly females.

Abstract citation ID: igae098.3899

### THE EFFECT OF SOCIAL PARTICIPATION OF OLDER ADULTS ON DEPRESSION: FOCUSED ON THE MODERATING EFFECT OF HOUSEHOLD TYPE

Sehyun Baek[1], and Sungkyu Lee[2], *1. New York University, New York, New York, United States*, *2. Soongsil University, Seoul, Republic of Korea*

Previous studies have found that the social participation of older adults is positively associated with perceived health, happiness, and life satisfaction (Kim et al., 2018). However, as they age, their social activities tend to decrease, leading to increased depression (Choi & Kim, 2023; Kim & Jung, 2017). Given that older adults living alone show a gradual decline in social activities as they age (Shin, 2022), the household type may intervene in the relationship between social participation and depression among older adults. However, little is known about the structural associations of these variables. Therefore, we examined the extent to which household type (i.e., living alone vs. not living alone) moderates the relationship between social participation and depression among older adults. We utilized data from the 2022 Korean Longitudinal Study of Aging (n=6,055) and conducted moderated regression analysis using Process Macro. The study found a negative relationship between social participation and depression of older adults (b=-.136, p<.01), and a moderating effect of household types on this relationship was statistically significant (p <.05). More specifically, the effect of social participation on depression was greater for older adults living alone than for those who do not live alone. These results highlight the need for policy efforts aimed at increasing the social participation of older adults to reduce their depression. Also, considering that the negative association between social participation and depression is more pronounced among older adults living alone, the interventions targeting the growing number of older adults living alone should be prioritized in policy planning.

Abstract citation ID: igae098.3900

### THE FORTITUDE FACTOR FOR CAREGIVERS: UNDERSTANDING THE EFFECT OF EMPLOYMENT AND CAREGIVING ON CAREGIVER WELLBEING

Sarah Clem[1], Joan Davitt[1], Stephanie McCorvey[1], Todd Becker[2], Jennifer Eastman[3], and Greg Sesek[4], *1. University of Maryland Baltimore, Baltimore, Maryland, United States*, *2. Washington University School of Medicine in St. Louis, St. Louis, Missouri, United States*, *3. Maryland Department of Disabilities, Maryland Department of Disabilities, Maryland, United States*, *4. Department of Human Services, Baltimore, Maryland, United States*

Shifting support services from community-based facilities to home-based settings has highlighted the dual roles of family/friend caregivers. This study investigated challenges experienced by caregivers of vulnerable adults in one east coast state. A convenience sample (N= 2,129) of caregivers, recruited through state and local agencies, completed an anonymous online survey to enhance understanding of caregiving experiences, challenges, and service utilization patterns. This paper focused on employed caregivers who reported greater service utilization than caregivers who were not employed. Within employed caregivers, we found they made workplace compromises to balance caregiving responsibilities including: going in late, taking a less demanding job, taking a leave of absence, and going from full to part-time work. There were positive associations between the number of compromises made in the workplace with caregiver-reported stress levels, financial strain, and service utilization. To address these challenges, employed caregivers reported using various coping strategies including exercise, mental health support, spiritual practice, hobbies, and support groups. Employer support to caregivers included: flexible hours, remote work options, paid sick days, paid family leave, referral/counseling supports, and unpaid family leave. Mixed results were found for relationships between employer supports and caregiver compromises, stress levels and financial strain. Research shows that multiple demands on employees can reduce worker productivity and efficiency. Responding to trends indicating an increase of informal caregivers in the workforce, this study highlights the needed support to promote caregiver resilience and their ability to remain employed. Implications for employers, community agencies, and government systems will be discussed.

Abstract citation ID: igae098.3901

### THE INTERSECTION OF OLDER ADULTS, DISABILITIES, AND MENTAL HEALTH

Onyinye Mbanefo[1], and Pamela Teaster[2], *1. Virginia Tech, Blacksburg, Virginia, United States*, *2. Virginia Tech., BLACKSBURG, Virginia, United States*

The number of individuals aged 65 and older is projected to reach 82 million by 2050. This demographic shift is accompanied by an increase in the prevalence of disabilities and mental health issues among older adults. Nearly 46% of adults aged 75 and older report having a disability, and 25% of those aged 65 and older are living with a mental health condition such as anxiety or depression. These challenges are exacerbated by cumulative disadvantage, ageism, and a shortage of mental health providers trained to work with this population. To address these issues, we apply a strengths-based approach to explore the intersection of aging, disability, and mental health. Our approach emphasizes the resilience and potential of older adults, challenging the dominant narratives of decline and loss. Utilizing the Socio-Ecological Model, we examine how individual, relational, community, and societal factors influence the mental health of older adults, highlighting the need for integrated and holistic care strategies. We explore key concepts such as cumulative advantage and disadvantage, ageism, and intersectionality, which are critical in understanding the complexities faced by older adults. Our work advocates for comprehensive mental health care that addresses both the physical and psychological well-being of older adults, promoting dignity, equity, and quality of life. Our findings suggest that a strengths-based approach can effectively enhance mental health outcomes for older adults, providing a framework for mental health practitioners and policymakers alike.

Abstract citation ID: igae098.3902

### THE LIFE STAGE AT MIGRATION MATTERS: A STUDY OF OLDER IMMIGRANTS' SOCIAL WELLBEING BASED ON THE CLSA

Yeonjung Lee[1], Lun Li[2], and Boah Kim[3], *1. Chung-Ang University, 2. MacEwan University, Edmonton, Alberta, Canada*, *3. Simon Fraser, Vancouver, British Columbia, Canada*

Based on life course theory, this study examines the different social wellbeing trajectories among older immigrants in Canada based on their life stage at migration. The study applies Linear Mixed Models (LMM) to three waves of data from the Canadian Longitudinal Study on Aging (2011 to 2021). Among the sample of 4,248 older immigrants, most of them (two-thirds) migrated before the age of 30 (young adults), one-fifth migrated between 30 and 45 years old (adulthood), and about five percent of them migrated after 45 years and older (middle and older age). Results from LMM reveal that older immigrants who migrated during middle and older age reported the lowest level of social wellbeing, measured by social participation, social support, and social network size. This group of older immigrants also experienced the greatest decline in social participation, social support, and network size over ten years compared to those who migrated before 45 years old. Older adults who migrated to Canada before the age of 30 reported the best situation with higher levels of social wellbeing, and well-maintained social connection over time. In addition, employment, income level, education, and family responsibilities are significant factors related to the changes in social wellbeing over time among older immigrants. The findings from this study highlight the need to support older immigrants who come to Canada in a later life stage (i.e., after a middle-aged period), and who lack the social resources and capabilities to accumulate social capital and enhance social wellbeing.

Abstract citation ID: igae098.3903

### THE ROLE OF HEALTH IN RETIREMENT PLANNING TOWARDS EXTENDED WORKING LIVES

Rachel Crossdale[1], and Nehle Penning[2], *1. The University of Sheffield, UK, University of Sheffield, England, United Kingdom*, *2. TU Dortmund University, Dortmund, Nordrhein-Westfalen, Germany*

Decades of research have shown that health is a key determinant of labour force participation in later life. As the ageing global population necessitates extended working lives (EWLs) for broader economic sustainability, the importance of the older population for the labour market is emphasised. Understanding the role of health and health inequality across the life course is crucial for impactful policy development to protect the health of the older workforce and encourage participation in EWLs. Using 100 interviews on risks and turning points in working life with older workers in Germany, Poland, Sweden, and the UK, this research explores the role of health as a barrier and facilitator of EWLs across the working life course. Drawing on policy and literature to contextualise our findings, this research reveals the role of health in retirement planning, including assumptions about health decline, impact on work, and the importance of partner's health. Health as a 'push and pull' factor is also explored, with good health both fostering labour market engagement and encouraging early retirement, while physical and mental health can be both positively and negatively impacted by work. Inequalities by sector of employment, gender, and education level are also explored, with all four countries facing a 'double bind' whereby the most vulnerable must work longer for financial security but are limited by poor health. For future older populations to be healthier and more productive the current treatment-based model of health care needs to be challenged with preventative policy interventions throughout the life course.

Abstract citation ID: igae098.3904
### THE ROLE OF HOUSING IN LONG-TERM NURSING HOME UTILIZATION IN THE UNITED STATES: AN INTEGRATIVE REVIEW
Marissa Bergh[1], and Jasmine Travers[2], *1. New York University, Long Island City, New York, United States*, *2. New York University, New York, New York, United States*

Given the growing housing crisis, researchers increasingly point to housing as a possible risk factor for nursing home (NH) utilization, yet the exact pathways from housing to NH utilization are unclear. The purpose of this integrative review was to develop an understanding of how housing acts as a mechanism that impacts NH utilization in the United States. Five databases were searched in April 2024 and 16 total articles were included. This review highlights four pathways for this relationship: 1) Financial Asset vs. Burden, represents how some leverage home equity to help finance necessary NH placements, while for others the cost-burden of housing may lead to early nursing home placements; 2) Access to Affordable Housing, represents how the supply of affordable housing impacts the ability of NH residents to return to community living; 3) (Mis)match Between Needs and Housing, represents how functional needs rapidly evolve, leading to housing transitions and NH placements; and 4) Compounding Systemic Inequities, represents how systemic inequities in access to affordable, quality housing lead to disparities in NH placement for socio-economically marginalized older adults. The results from this review provide evidence of the relationship between housing and NH utilization across these four pathways; however, due to weak quality of evidence, and limited methodological diversity, more research is needed to strengthen this evidence. Clinicians, researchers, and policymakers should still recognize the importance of housing in NH utilization and seek to target policies and interventions towards improving housing conditions for those at risk for NH placement.

Abstract citation ID: igae098.3905
### THE SENIOR COMPANION JOURNEYS PROJECT: A TRAINING TO ENHANCE VOLUNTEERS' KNOWLEDGE OF AD/ADRD
Noelle Fields[1], Ling Xu[1], Pingnan Li[2], Mary Stringfellow[3], and Melissa Gomez[4], *1. The University of Texas at Arlington, Arlington, Texas, United States*, *2. University of Texas at Arlington, Arlington, Texas, United States*, *3. UT Southwestern Medical Center, Dallas, Texas, United States*, *4. The Senior Source, Inc., Dallas, Texas, United States*

Research suggests that trained volunteers may be equipped to serve as interventionists for family care partners (CPs) of persons living with dementia (PLWD). The Senior Companion Journeys Project (SCP-J) was developed to help bridge gaps in knowledge of AD/ADRD and CP support. The purpose of this study was to evaluate the SCP-J training (n = 49) as part of a volunteer-led intervention for CPs of PLWD. The Alzheimer's Disease Knowledge Scale (ADKS) and open-ended questions were used to examine the SCs' knowledge before and after the SCP-J training. Non-parametric McNemar tests were conducted for each item and the overall correct response rate of the ADKS scale, respectively. Qualitative interview data were analyzed using RaDAR analysis. The results of the quantitative data indicated overall significant improvements in the overall correct response rate of ADKS after the SCP-J training (p <.001). Some specific ADKS items showed a significant increase after the training including items related to early responses to AD/ADRD behaviors (p <.001), high cholesterol and risk for developing AD/ADRD (p <.01), and high blood pressure and risk for developing AD/ADRD (p <.01). Qualitative findings revealed three themes: improved knowledge or reinforced knowledge of AD/ADRD, improved coping skills for problematic behaviors associated with AD/ADRD, and improved confidence in providing CP support. Overall findings suggest that the SCP-J Training is a promising intervention for enhancing knowledge about AD/ADRD for SC volunteers. Recommendations for future research and strategies for other volunteer-led AD/ADRD interventions in community-based settings are offered.

Abstract citation ID: igae098.3906
### THE WHO INTEGRATED CARE FOR OLDER PEOPLE (ICOPE) FRAMEWORK AND THE ASSOCIATION WITH FRAILTY IN OLDER ADULTS.
Wan Yun Chou[1], and Su-I Hou[2], *1. University of Central Florida, Orlando, Florida, United States*, *2. The University of Central Florida, Orlando, Florida, United States*

The World Health Organization (WHO) introduced the Integrated Care for Older People (ICOPE) framework, designed to enhance the intrinsic capacity (IC) of older adults, focusing on their physical and mental health. This shift in care approach, from disease-centered to person-centered, reflects the rapid increase in the global aging population. The objective of the current study was to explore how IC relates to frailty in older adults. In a medical center in Taiwan in 2021, this cross-sectional study involved patients aged 65 and older admitted to the geriatric ward. Participants completed a questionnaire that included an IC assessment based on the six domains of ICOPE (cognition, mobility, nutrition, visual, hearing, and depressive symptoms), Fried's frailty scale, and demographic information. Fried's frailty scale categorized participants into robust (score=0), prefrail (score=1-2), or frail (score ≥3) groups. Logistic regression analysis was employed to examine the association between individual ICOPE domains and frailty, adjusting for age, gender, and number of comorbidities. Of the 210 participants, 39.0% were categorized as prefrail and 28.6% as frail. Notably, mobility loss and depressive symptoms were strongly linked to prefrail and frail conditions, with significant adjusted odds ratios (aOR) indicating higher risks. Additionally, malnutrition and hearing loss were significantly associated with frailty. These findings underscore the critical need for attention to IC and frailty in older adults and advocate for regular implementation of the WHO ICOPE screening pathway. While the convenience sampling method may limit generalizability, further research is necessary better to understand the utility of ICOPE in frailty identification.

Abstract citation ID: igae098.3907
### TOO SICK TO BE TRUE? EVALUATING PROBLEMATIC CODING PRACTICES IN MEDICARE'S PATIENT DRIVEN PAYMENT MODEL
Harsha Amaravadi[1], Rachel Prusynski[1], Paul Fishman[2], and Tracy Mroz[1], *1. University of Washington, Seattle, Washington, United States*, *2. University of Washington, University of Washington, Washington, United States*

The 2019 Patient Driven Payment Model (PDPM) aimed to promote budget neutral, patient-centered care by adequately reimbursing skilled nursing facilities (SNFs) for patient clinical complexity. Though PDPM mitigated financial incentives to provide excessive care, it may have introduced new incentives to falsely inflate or "upcode" clinical complexity. We hypothesize that upcoding may occur for common, chronic conditions associated with high reimbursement under PDPM. High reimbursement encompasses both diagnosis codes and services for complex conditions (e.g., diabetic wound care or IV use for severe anemia). Example conditions include complicated diabetes, blood loss anemia, and obesity. We sought to quantify the degree of documentation changes for conditions likely to be upcoded. Using a difference-in-difference design, we analyzed Traditional Medicare claims from 2018-2021 for all patients with a hospital-SNF care episode (n = 5.43 million), comparing diagnoses on the index hospital claim to the corresponding SNF claim for each patient. By using hospital claims, which are not subject to PDPM, as a control, we mitigate all patient-level confounding. We detected increased documentation for the selected conditions, representing an additional 32.18% [95%CI: 31.55, 32.82] in SNF coding for complicated diabetes, 54.59% [95%CI: 52.33, 56.95] for severe anemia, and 83.08% [95%CI: 82.00, 84.15] for obesity. PDPM sought to improve payment adequacy for caring for clinically complex patients, but Medicare's financial sustainability also depends on accurate documentation of clinical factors that influence resource utilization and care provision. Our investigation of potential upcoding may inform future policy refinement to support sustainable, patient-centered care delivery for older adults.

Abstract citation ID: igae098.3908

### TOWARDS AN AGE-FRIENDLY UNIVERSITY: INSIGHTS FROM CULTURALLY DIVERSE COMMUNITY-DWELLING OLDER ADULTS

Joey Wong, Mario Gregorio, Paulina Santaella, Albin Soni, Jason Fu, Ilan Meghelli, and Lillian Hung, *University of British Columbia, Vancouver, British Columbia, Canada*

Lifelong learning is essential for enhancing older adults' self-esteem and social connections and expanding their capabilities. Including older adults on university campuses fosters intergenerational learning, where younger students benefit from their experiences and mentorship. This project addresses the growing emphasis on creating age-friendly universities by improving inclusiveness, diversity and equity at the University of British Columbia (UBC) Vancouver campus. The study, conducted in June and July 2024, engaged 25 community-dwelling older adults from diverse backgrounds—including Indigenous groups, multi-ethnic groups and people of color, LGBTQIA2+, and individuals with various physical and mental capacities—in audit walks with younger students across the campus. These walks assessed features related to "age-friendliness." Group conversations before and after the walks provided valuable insights into participants' perceptions of age-friendliness and feedback on current age-friendly university principles. Findings revealed a strong interest among older adults in continued learning, research collaboration, and mentorship opportunities. However, significant barriers were also identified regarding campus navigation, course registration, affordability, and communication. Additional concerns included safety issues, more accessible features, and improved transportation infrastructure. Participants also highlighted the importance of cultural inclusivity, such as multilingual resources and diverse art representations. This study not only reaffirms established age-friendly principles but also underscores the need to address environmental safety and accessibility from the perspectives of older adults. It advocates for a collaborative approach to developing an age-friendly university campus that effectively meets the needs of all its members, ensuring an inclusive environment for learners of all ages.

Abstract citation ID: igae098.3909

### TRAJECTORIES OF CHRONIC PAIN AMONG OLDER VETERANS: IDENTIFYING PAIN-WORSENING PREDICTORS VIA MACHINE LEARNING

Chiyoung Lee[1], Yeri Kim[2], Seoyoung Kim[2], Beth Cohen[3], kent Kwoh[1], Hyochol Ahn[1], Juyoung Park[1], and Heewon Kim[2], *1. University of Arizona, Tucson, Arizona, United States*, *2. Soongsil University, Seoul, Republic of Korea*, *3. University of California, San Francisco, San Francisco, California, United States*

Chronic pain is a major public health problem affecting approximately 100 million Americans and United States military Veterans, who constitute a particularly vulnerable group. While pain research in Veterans is actively underway, information on the longitudinal course of pain in this population is limited. This study aimed to 1) identify the various longitudinal pain status trajectories among older Veterans over a 10-year period and 2) detect factors predicting membership in the worsening trajectory of chronic pain. We analyzed data from 619 Veterans (mean age: 58.5 years) participating in the Mind Your Heart Study, an ongoing prospective cohort study examining diverse health outcomes among Veterans. Initially, we employed a generalized mixture model to identify pain trajectory classes using Brief Pain Inventory (BPI) pain intensity subscale score collected at 2-, 5-, and 10-year intervals. Two distinct trajectories were identified—low and high—both of which remained relatively stable. Subsequently, several feature selection methods extracted the predominant features from participants' baseline characteristics that predicted membership in the high vs. low pain trajectory. These included: prior arthritis diagnosis; prior post-traumatic stress disorder (PTSD) diagnosis; depression symptoms; PTSD symptoms of avoidance, hyperarousal, and negative mood alterations; physical functioning; sleep quality; and overall health. The scikit-learn RandomForestClassifier, utilizing the refined feature set, achieved a classification accuracy of 0.79, yielding results nearly identical to those obtained using all 261 features. These findings are clinically informative and pertinent, highlighting potential intervention targets warranting intensive pain care plans based on probable long-term prognosis and discussing early treatment strategies among older Veterans.

Abstract citation ID: igae098.3910

### TRANSCENDING 'INTERDISCIPLINARY PARADOX': TOWARDS AN AUTONOMOUS BODY OF KNOWLEDGE THROUGH TRANSDISCIPLINARITY

Shiyu Chen[1], and Shandong Zhou[2], *1. Hunan Women's University, Changsha, Hunan, China (People's Republic)*, *2. Xiangtan University, Changsha, Hunan, China (People's Republic)*

Autonomous body of knowledge (BoK) in gerontology is not only an extrinsic requirement to address the challenges of an aging society, but also an intrinsic need for the discipline to achieve its independency. The establishment of such a BoK requires an introspective reflection. This poster presents a review of the progress on the establishment of gerontology's autonomous BoK, and explores the evolvement of the discipline following the "multidisciplinary-interdisciplinary-transdisciplinary" continuum. The study finds a 'interdisciplinary paradox' in current gerontological BoK, which manifests as: programmatic discourse on interdisciplinarity in gerontology continues to proliferate and disseminate, yet disciplinary and sub-disciplinary differentiation and specialization are ever-increasing. Moreover, there remains structural barriers in academia supporting interdisciplinary research in gerontology. To resolve this paradox and reinforce the establishment of gerontological autonomous BoK, a transdisciplinary research model (ISOE model) consisting of 3 phases could be adopted. The 3 phases in the model are as following: a. Breaking down barriers between organizations, interacting with diverse stakeholders and framing the real-world problem collectedly b. Integrating research methodologies and perspectives, and collaborating on producing solution-orientated knowledge and transferable results; c. Translating and applying research results to real world practice. In conclusion, transdisciplinary method offers a promising pathway to establish an autonomous BoK in gerontology.

Abstract citation ID: igae098.3911

### TREATMENT BURDEN AMONG DIVERSE MIDDLE-AGED AND OLDER ADULTS WITH MULTIMORBIDITY

Ana Quiñones[1], Sheila Markwardt[1], Anda Botoseneanu[2], Viet-Thi Tran[3], and Victor Montori[4], *1. Oregon Health & Science University, Portland, Oregon, United States*, *2. University of Michigan-Dearborn, Dexter, Michigan, United States*, *3. Université Paris Cité, Université Paris Cité, Ile-de-France, France*, *4. Mayo Clinic, Rochester, Minnesota, United States*

The demands associated with ongoing disease management may overwhelm middle aged and older adults living with two or more chronic conditions. Treatment burden refers to both (a) the numerous demands—such as the time and costs of travel to clinical appointment, administrative burden, and coordinating fragmented care—healthcare makes on patients and (b) the negative impact on the patient's wellbeing. We evaluated data from respondents of the treatment burden experimental module included in the 2022 Health and Retirement Study data collection wave (N=1,888). We analyzed responses to the 15-item Treatment Burden Questionnaire (TBQ; 15 questions scored 0-10; total sum 0-150; >59 considered unsustainable burden). We compared mean total TBQ score by sociodemographic and multimorbidity combination groups using either a t-test (among 2 groups) or ANOVA (among >2 groups). We compared sustainability across groups using a chi-square test. Respondents (mean 69 years; 62% female, 71% living with 2+ chronic conditions) had a mean TBQ score of 20.3/150 (SD 22.7) with 125 (6.6%) reporting unsustainable burden. Administrative and financial tasks and lifestyle changes were the most burdensome activities. Unsustainable burden was significantly more likely among younger (51-64 vs. older), lower educated (< high school vs. ≥high school), and Black and Latino (vs. non-Latino White) respondents, and in those living with 2+ chronic conditions. In this nationally-representative sample of US adults, participants reported less treatment burden than previously reported in international studies. Certain sociodemographic groups reported high and unsustainable burden of treatment, informing future interventions to reduce treatment burden in mid and late life.

Abstract citation ID: igae098.3912

### TRENDS AND CHARACTERISTICS OF WORKING CAREGIVERS USING PAID FAMILY LEAVE PROGRAMS IN RHODE ISLAND AND WASHINGTON

Jennifer Im[1], Edwin Wong[2], Paul Fishman[2], Heather Hill[1], and Bryan Weiner[1], *1. University of Washington, Seattle, Washington, United States*, *2. University of Washington, University of Washington, Washington, United States*

Many family caregivers are part of the workforce and integral to older adults' care. State Paid Family Leave (PFL) programs offer partial wage replacement to working caregivers. However, data on caregiving leave use is limited. This study examines trends and characteristics of workers who sought caregiving leave in Rhode Island (RI) and Washington (WA) using PFL program administrative data. Outcomes were application and approval for caregiving leave. Covariates included age, sex, ethnicity (WA only), income, primary language, and industry. Logit models with average marginal effects were used. In RI, 14,323 claims were recorded between 2015-22; 82.3% were approved. In WA, 75,739 claims were recorded between 2020-23; 76.7% were approved. Overall, caregiving applications increased annually. Applicants' mean ages were mid-to-late 40s (RI=48.3; WA=46.9). Most applicants were female (RI=68.3%; WA=61.5%) and English-speaking (RI=95.1%; WA=89.2%). WA applicants were predominantly White (57.5%), Hispanic (13.8%), and mixed race (8.3%). Applications for spousal caregiving was most common in RI (36.3%); caregiving for children was most common in WA (39.1%). The lowest-earning workers had the highest percentage of denied claims (RI=35.5%; WA=29.8%). In both states, older age and higher income were significantly associated with claim approval. In RI, worker caring for spouse, parent, or grandparent had significantly lower probabilities of claim approval, compared to children; In WA, the opposite was observed. The results suggest caregiving PFL is rising among workers, with claim approval more likely for older workers. Higher income was associated with claim approval suggesting programs may not benefit workers that need it the most.

Abstract citation ID: igae098.3913

### TRENDS IN CHRONIC DISEASE SPENDING AND HEALTH OUTCOMES AMONG MEDICARE FFS BENEFICIARIES IN PUERTO RICO

Matthew Campos, and Maricruz Rivera-Hernandez, *Brown University School of Public Health, Providence, Rhode Island, United States*

There is growing recognition of disparities in chronic disease outcomes between Puerto Ricans on the island and those on the US mainland, yet comparative data are limited. We assessed the prevalence of key chronic conditions—Alzheimer's disease and related dementias (ADRD), diabetes, and chronic kidney disease (CKD)—and total care costs for Medicare fee-for-service (FFS) beneficiaries in Puerto Rico (PR) compared to states with high older adult (65+) Puerto Rican populations [Florida (FL), New York (NY), Georgia (GA), New Jersey (NJ), California (CA)] from 2015 – 2020. We used data from the Mapping Medicare Disparities Tool from the Center for Medicare and Medicaid Services. Outcomes of interest included age-adjusted prevalence and risk-adjusted total costs per beneficiary. From 2015 – 2020, age-adjusted prevalence trends in PR showed decreases in ADRD by three percentage points (13% to 10%) and diabetes by six percentage points (46% to 40%). Conversely, CKD prevalence increased by 12 percentage points from 21% to 33%. Despite ADRD trends aligning with national averages, diabetes (PR: 40%, Nation: 26%, FL: 28%, NY: 27%, GA: 29%, NJ: 29%, CA: 24%) and CKD (PR: 33%, Nation: 25%, FL: 29%, NY: 24%, GA: 30%, NJ: 25%, CA: 23%) rates in Puerto Rico remain notably higher. Additionally, PR risk-adjusted average total spending per beneficiary in 2020 was below national averages for all assessed chronic conditions (ADRD: $13,037 vs. $20,678; diabetes: $11,363 vs. $15,525; CKD: $13,181 vs. $18,376). These disparities suggest potential impacts on access and quality of care and highlight the need for policy interventions.

Abstract citation ID: igae098.3914

### UNCERTAINTY IN HOME HEALTH CARE FOR OLDER VETERANS: VA PERSPECTIVES ON AGENCY SELECTION AND CONTRACTING

Chelsea Leonard[1], Kate Magid[2], Lexus Ujano-De Motta[3], Marguerite Daus[4], Cari Levy[5], and Christine Jones[1], *1. VA Eastern Colorado Healthcare System, Aurora, Colorado, United States*, *2. Rocky Mountain Regional VA, Aurora, Colorado, United States*, *3. VA Eastern Colorado Healthcare System, aurora, Colorado, United States*, *4. Department of Veterans Affairs, Aurora, Colorado, United States*, *5. CU Anschutz, Aurora, Colorado, United States*

Older Veterans discharged from the hospital with skilled home health care (HHC) have their HHC delivered by community home health agencies (HHA) and financed primarily through the Department of Veteran's Affairs (VA) or Medicare. We sought to understand perspectives of VA personnel on the process of HHC agency selection and financing for Veterans referred for HHC. We used a sequential mixed methods study design and conducted in-person and virtual site visits at three VA Medical Centers (VAMCs) in the highest decile of VA-paid HHC services (3 in-person) and three VAMCs in the highest decile of Medicare-paid HHC services (1 in-person, 2 virtual). We observed processes related to HHC referrals and conducted interviews with key informants. Interviews were recorded, transcribed, and analyzed using inductive-deductive content analysis to identify themes related to referral processes, agency selection, and financing. We conducted 29 interviews with 39 personnel at 6 VAMCs. We identified 4 themes related to uncertainty around agency and payor selection: 1) VA roles are siloed and next steps are a "black box;" 2) VA personnel are uncertain how HHAs are selected for local networks; 3) Local VAMCs develop strategies to determine HHA quality and reputation; and 4) Veterans' role in selecting HHA or financing is unclear. Understanding points of uncertainty has implications for VA's ability to provide high-quality HHC for older Veterans. Our findings suggest that providing accessible information on HHA quality and clear guidance around Veterans' role in HHA and payor selection may streamline the process of providing HHC to Veterans.

Abstract citation ID: igae098.3915

### UNDERSTANDING AND EXPERIENCING AGEISM: PERSPECTIVES FROM OLDER ADULTS

Aaron Li, Nancy Morrow-Howell, Natalie Galucia, Khrystal Johnson, and Brian Carpenter, *Washington University in St. Louis, St. Louis, Missouri, United States*

Interventions to reduce ageism usually target younger people and service providers, with little research on interventions directed explicitly at older people to identify and resist interpersonal and internalized ageism. This study increases knowledge to guide the development of such interventions by elucidating how older people think about ageism and clarifying motivations to confront it. We also need to know the language older people use to describe ageism and the experiences they report in employment, health care, and family setting. This knowledge is important for designing acceptable and effective anti-ageism programs. We employed a focus group methodology, gathering data on the lived experience of ageism among a diverse group of 50 people over the age of 60. Questions elicited participants' thoughts about interpersonal and internalized ageism, examples of how they experienced it, and how they reacted. Findings suggest that older people are not clear about what constitutes interpersonal ageism, and internalized ageism is difficult to identify. Instead, participants resorted to talking about their experiences of growing older and frequently mentioned a lack of respect as the hallmark of ageism. Examples of ageism within their families and among younger acquaintances were more common than examples in other domains of daily life. Older age did not rise to the level of importance of other marginalized identities; instead, racism, ableism and homophobia were more central to their experiences of discrimination. Findings suggest that interventions with older people must include consciousness-raising about ageism and its consequences well as common examples and skills to combat it.

Abstract citation ID: igae098.3916

### UNDERSTANDING COMMUNITY RESOURCES FOR OLDER ADULTS WITH DISABILITIES

Sunghei Han[1], Sojung Park[1], Soobin Park[1], Esther Shin[3], Sarah Kang[4], Eunsun Kwon[5], Seoyeon Ahn[6], and BoRin Kim[7], *1. Washington University in St. Louis, St. Louis, Missouri, United States*, *3. Illinois State University, Normal, Illinois, United States*, *4. University of California Los Angeles, Los Angeles, California, United States*, *5. Fairleigh Dickinson University, Hackensack, New Jersey, United States*, *6. Duke-NUS Medical School, Singapore, Singapore*, *7. University of New Hampshire, Durham, New Hampshire, United States*

For older adults with various disabilities—physical, mental, cognitive, and others—diverse community resources are essential for daily functioning and their Aging in Place (AIP). This scoping review analyzed 21 peer-reviewed studies from databases like PsycINFO, CINAHL, MEDLINE, Scopus, and PubMed to identify gaps in research on community resources for this population. Thematic analysis revealed four main themes: 1) the development of partnerships among local governments, service providers, and stakeholders to coordinate resources; 2) evaluation of community-level interventions such as telerehabilitation and mental health programs; 3) factors affecting access to and use of community resources; and 4) the impact of these resources on health and well-being. The studies primarily focused on home and community-based services (HCBS), but while some addressed specific care aspects like fall prevention and dementia care, broader research often overlooked the comprehensive needs of older adults with disabilities. Only two studies focused on resources for mental health and substance use disorders, highlighting significant gaps, particularly in primary care. Additionally, many studies lacked clear definitions of disabilities, leading to a fragmented understanding of community resources. This lack of clarity hinders efforts to address the unmet needs of older adults with disabilities, including diverse subgroups like LGBTQ individuals and ethnic minorities. The study highlights the need for more systematic research into the availability and accessibility of community resources to support older adults with varying disabilities better, ensuring no critical needs are left unmet in this vulnerable population.

Abstract citation ID: igae098.3917

### UNDERSTANDING OLDER ADULTS' INTEREST IN USING VIRTUAL REALITY TO SUPPORT SOCIAL ENGAGEMENT ACTIVITIES

George Mois[1], Emre Eraslan[2], Qiyuan Cheng[2], Tongxin Sun[2], Willencia Louis-Charles[2], Avinash Gupta[2], and Wendy Rogers[2], *1. University of Georgia, Athens, Georgia, United States*, *2. University of Illinois Urbana-Champaign, Champaign, Illinois, United States*

The experience of loneliness affects one in four adults over age 65. These experiences can have significant impacts on wellbeing and quality of life. Changes associated with aging such as retirement, relocation, loss of friends and family can impact access to social engagement opportunities. Moreover, communities often lack resources required to meet individuals' social needs. Leveraging immersive information communication technologies such as virtual reality (VR) presents a unique opportunity in bridging the resource gap, enabling individuals to connect with others, meet new people, and engage in diverse virtual activities. However, due to a limited body of research, there is little understanding of the types of VR activities older adults are interested in partaking in with in-network (e.g., family, friends) and out-of-network (e.g., strangers, new acquaintance) individuals. We explored older adults' interests in using VR to engage with others and the types of activities they might be interested in engaging in. Participants were 21 older adults, aged 61-80 (M=69, SD=5.45). Our mixed methods approached included questionnaires, passive virtual reality demonstrations, and a semi structured interview. The older adults had positive attitudes about the VR and were interested in using it with family and friends. They could also see the potential of using VR to interact with other people with shared interests (e.g., hobbies, traveling). Participants reported interest in a diverse set of VR based activities to engage with others. These findings illustrate the potential of VR for social engagement activities for older adults, accessible from their homes, to reduce loneliness.

Abstract citation ID: igae098.3918

### UNIVERSITY-CITY PARTNERSHIP TO RECRUIT DIVERSE, LOW-INCOME SENIORS TO ALLEVIATE HEALTH DISPARITIES

Carla Leinbach, Abigail Tice, and Janet Lopez, *University of Central Florida, Orlando, Florida, United States*

Recruitment challenges are the top cited reason that research studies miss their goals. Recruiting under-represented groups including people of color, those with cognitive or physical challenges, socio-economically marginalized persons, and older persons exacerbates recruitment challenges. We aimed to explore strategies to recruit diverse, low-income, older participants for our NIH-funded clinical trial study and compared recruitment numbers between pre-post recruitment implementations. We collected data from May to August 2024 from Orlando, Florida. Pre-implementation, locations had fewer than 10 participants per site and required 8 to 12 weeks to organize. Our findings indicated four recruitment strategies 1) creating university-city partnerships resulted in 17 new senior programs to recruit from; 2) recruiting untapped places resulted in an uptick in new participants; 3) Multi-lingual marketing increased enrollment inquiries, and 4) Efficiently captured inquiries increased enrollment. Pre-implementation, the study struggled to identify locations with sufficient senior population density, identified limited physical spaces to conduct assessments, struggled to obtain permission to recruit on-site, and faced market rental rates for use of space. Enrollment rates were low due to the facilities' leadership and potential participants viewed researchers as "vendors", not trusted partners. Post-implementation, cohorts ranged from 25 – 40+ seniors and required approximately 2 weeks to organize. This partnership expanded the reach of recruitment into historically difficult to access populations and contributed to a net regain of committed completion timelines. Strategic utilization of local city and county government for participant recruitment and facility access can aid research projects in reaching their goals and diversifying the participant pool.

Abstract citation ID: igae098.3919

### UNMET NEEDS AND HEALTHCARE SERVICE UTILIZATION AMONG OLDER ADULTS: A SYSTEMATIC REVIEW

Maham Khan[1], Laura Sands[2], Cozette Comer[3], and Jackson Hoch[3], *1. Virginia Tech., Blacksburg, Virginia, United States*, *2. Virginia Tech, Blacksburg, Virginia, United States*, *3. Virginia Tech, Virginia Tech, Virginia, United States*

Unmet needs refer to the absence of necessary assistance for completing Activities of Daily Living (ADLs), and they are associated with severe health consequences, such as hospitalizations, emergency room visits, nursing home placement, and mortality among older adults. The high cost of unmet needs pose challenges at both individual and systemic levels. While the prior literature has focused on immediate consequences of unmet needs (such as going without bathing or toileting), this systematic review consolidates evidence to assess the impact of unmet needs on healthcare utilization and emphasizes the importance of improving community-based care to mitigate (costly) consequences. A comprehensive search of multiple databases, including Scopus, Web of Science, PubMed, and CINAHL, using appropriate keywords (unmet needs, acute healthcare utilization) identified 6,475 studies. After a rigorous screening process, eight studies met the inclusion criteria for analysis, focusing on non-institutionalized older adults in the U.S. with at least one ADL difficulty. Results show that individuals with unmet needs experience double the hospitalization rates compared to those with met needs. Emergency room visits are also 31% higher, especially among those with unmet self-care needs. From the hospital deaths, 74 % report to have experienced unmet needs at end-of-life. Sociodemographic factors, such as older age, being minority race, living alone, and cognitive impairment further exacerbate the risks acute healthcare utilization. Addressing unmet needs is crucial not only for improving quality of life among older adults but also for alleviating the strain on healthcare resources through targeted policy interventions.

Abstract citation ID: igae098.3920

### UNTOLD STORIES OF AGING – FOSTERING INTERGENERATIONAL DIALOGUE THROUGH AN ART-BASED COMMUNITY ACTION PROJECT

Sarah Jen, *University of Kansas, Lawrence, Kansas, United States*

Individuals who can imagine their aging futures are better prepared for the aging process. However, stories of later life often go untold, limiting public understanding of this complex phase of life. The Untold Stories of Aging Project was created to combat societal ageism through encouraging the creation of aging-focused art and intergenerational dialogue. Between 2021 - 2024, we have amassed a digital art exhibition including over 50 pieces of artwork from over 40 artists. Seven individual artists or groups have received awards for their submissions, honoring the evocative impact of their pieces, their connections to aging-related insights, and their creativity and originality. We present findings from interviews with awardees who discussed their artistic process, lessons learned about their own aging futures, and the intergenerational dialogue surrounding their pieces. Artists often cited intergenerational dialogue or the importance of family relationships as the motivation for their pieces. For example, one 26-year-old chose to challenge ageist stereotypes through her art following a conversation with her mother who described being perceived as “incapable” as she aged. Older artists shared how meaningful it was to be recognized as artists, with one 90-year-old stating, “It’s thrilling to know that something we are doing in such a common and ordinary way is impressive to anybody.” Through this project, artists have cultivated space for older adults to share their stories and legacies, illustrating how art and surrounding dialogue can combat societal ageism, foster intergenerational healing, and inspire audiences to consider and plan for their own aging futures.

Abstract citation ID: igae098.3921

### USE OF REHABILITATION SERVICES AMONG OLDER CANCER SURVIVORS IN THE NATIONAL HEALTH AND AGING TRENDS STUDY

Min-Hui Huang, and Thomas Chung, *University of Michigan-Flint, Flint, Michigan, United States*

Older cancer survivors (OC) have higher risks of physical impairments than non-cancer older adults (NC). Cancer rehabilitation utilization remains a challenge. This study examined rehabilitation usage in OC using the National Health and Aging Trends Study (NHATS) round 12 (2022) dataset. Objectives were: 1) estimate rehabilitation prevalence in OC and NC; 2) identify factors associated with rehabilitation in each group; 3) compare rehabilitation duration, areas, and outcome between OC and NC. Community-dwelling adults ≥65 years with rehabilitation and cancer diagnosis responses were included (OC: N=653, age=78.7±7.33 years; NC: N=5168, age=79.9±7.63 years). General linear models (Objectives 1 and 2), Chi-square tests and t-tests (Objective 3) were conducted in IBM-SPSS version 28. Rehabilitation prevalence in the past year was similar between groups (OC: 26%; NC: 23%). Pain (OC: p=0.037; NC: p=0.004), higher education (OC: p< 0.001; NC: p< 0.001), and lower physical function (OC: p< 0.001; NC: p< 0.01) were linked to rehabilitation use in both groups. Unique factors linked to rehabilitation included low energy (p=0.030) for OC, and impaired lower-body strength (p=0.037) and balance (p=0.050) for NC. OC had longer rehabilitation durations (2.2±0.94 vs. 2.1±0.80 months, p< 0.001) and were more likely to rehabilitate for low energy (p=0.004), falls (p=0.040), walking inside (p=0.035) and outside the home (p=0.027). More OC (81.1%) reported meeting rehabilitation goals than NC (70.1%)(p=0.010). Despite similar rehabilitation prevalence between groups, distinct factors linked to rehabilitation usage in OC highlight the need for tailored programs. Positive rehabilitation outcomes in OC support more rehabilitation referrals to improve physical function.

Abstract citation ID: igae098.3922

### USING AN OLDER ADULT MENTORING PROGRAM TO INCREASE INTEREST IN GERIATRICS AMONG MEDICAL STUDENTS

Shannon Arnette[1], and Madeline McIntyre[2], *1. Virginia Commonwealth University, Richmond, Virginia, United States*, *2. VCU, Richmond, Virginia, United States*

Older adults are the largest consumers of healthcare, yet there is a projected shortage of geriatricians in the future. The Older Adult Mentoring Program at a large, urban-serving, mid-Atlantic university aims to educate first-year medical students by increasing their awareness of internal and external ageism within healthcare through three semi-structured interviews conducted by the students with their mentors over two semesters, allowing them to interact with community-dwelling older adults and gain a person-centered perspective of aging. We conducted a quantitative study to determine the program’s impact on the students’ openness to working in geriatrics as a specialty. In April 2024, at the end of the program, we asked the students several questions, such as how they think about their healthcare goals as they age, their comfort level working with older adults, and their openness to pursuing geriatrics. Our findings suggest that the program positively impacted students’ attitudes to working with older adults. Over 75% of students agreed that the program changed how they think about their healthcare needs and/or goals as they age. Approximately 77% of students agreed that they feel more confident working with older adults. Over 60% of students either somewhat agreed or agreed that they are more open to pursuing geriatrics as a specialty, and over 90% were more open to working with older adults regardless of their future specialty. This shows how meaningful a program like this can be in changing students' attitudes towards older adults and geriatrics.

Abstract citation ID: igae098.3923

### USING DIGITAL HEALTH TO REDUCE BURDEN IN OLDER ADULTS WITH CANCER UNDERGOING CHEMOTHERAPY: A SYSTEMATIC REVIEW

Hyebeen Sim[1], Jinhee Shin[2], and Kennedy Diema Konlan[3], *1. Kangbuk Samsung Hospital, Seoul, Republic of Korea*, *2. Woosuk university, Jeollabuk-do, Ch'ungch'ong-bukto, Republic of Korea*, *3. University of Health and Allied Sciences, Ho, Volta, Ghana*

Digital health interventions have the potential to enhance health outcomes and support older adults with cancer in managing their conditions. However, the specific benefits and components of these interventions for this population remain inadequately defined. This systematic review aims to evaluate the effectiveness of digital health interventions in alleviating the burden of chemotherapy for older adults with cancer. This systematic review used PRISMA guidelines and the PICO framework. The Searches were conducted across five databases (CINAHL, Embase, PubMed, Cochrane Library, and Web of Science). Of the 5,683 articles screened, 13 were selected based on the inclusion criteria. Data extraction was performed by two authors using a predetermined matrix. The analysis was done using the interpretative thematic synthesis design. The digital health technologies examined included mobile apps, remote monitoring, and games, utilizing devices such as tablets, smartphones, and smartwatches. Significant benefits were observed for older adults with cancer undergoing chemotherapy, including improvements in symptom management, physical activity, nutritional support, and quality of life. Additionally, patients in the intervention groups experienced reductions in hospitalizations and treatment-related toxicities, along with improved efficacy in symptom monitoring. Digital health interventions show considerable promise in supporting older adults undergoing chemotherapy. However, further research is essential to clearly define the components of these digital health interventions for older adults with cancer and their specific impacts. Developing and adopting accessible digital health intervention applications that can be used at any time and place will be crucial in effectively preventing or alleviating the adverse effects of cancer treatment.

Abstract citation ID: igae098.3924

### USING GEOGRAPHIC INFORMATION SYSTEMS ANALYSIS TO UNDERSTAND THE BURDEN OF PROVIDING RIDES FOR OLDER ADULTS

Rebecca Mauldin[1], Swasati Handique[1], Soeun Jang[1], and Rupal Parekh[2], *1. University of Texas at Arlington, Arlington, Texas, United States*, *2. University of Connecticut, New York, Connecticut, United States*

Transportation is a significant factor for older adults' mobility and quality of life. However, some older adults need rides from others for transportation and ride providers may experience burdens when providing rides regularly. This study explores the use of geographic information systems to develop geospatial models (or "activity spaces") of routine travel patterns, both for regular activities and for providing transportation to older adults. This exploratory study was a cross-sectional survey among people who provided rides to older Vietnamese immigrants in the Dallas-Fort Worth metroplex area (N=20). Geospatial analyses using ArcGIS were employed to examine activity spaces (AS) and the geospatial burden of providing rides. Results indicated that nearly half of the ride providers experienced transportation costs, missed work, and increased personal stress from giving rides. Significant portions of ride-provision activity spaces were found outside the provider's regular activity spaces. We present and compare three potential indicators for assessing ride providing burden based on the ride providers' regular and ride-provision activity spaces. The percentage of the ride-provision AS outside the AS was associated with giving fewer rides per month. Findings suggest potential ways to measure ride provider burden for research and practice and have the potential to inform transportation subsidy policies. This study has implications a policy subsidizing private ride providers which could reduce costs and improve accessibility without additional infrastructure. Geospatial tools can assess ride-provision burdens, guide subsidy eligibility, and provide valuable data for city planning and service location optimization.

Abstract citation ID: igae098.3925

### USING QUALITATIVE ANALYSIS TO DEVELOP HIRING GUIDELINES TO IMPROVE CAREGIVING FOR PEOPLE WITH DEMENTIA AT HOME

Deborah Watman[1], and Jennifer Reckrey[2], *1. Icahn School of Medicine at Mount Sinai, New York, New York, United States*, *2. Icahn School of Medicine at Mount Sinai, Icahn School of Medicine at Mount Sinai, New York, United States*

Many people living with dementia are being cared for at home by hired paid caregivers. While paid caregivers frequently become an integral part of the care team, little is known about how they are selected by families of people living with dementia. We conducted a secondary analysis of qualitative data collected during two studies that examined family caregiver perspectives on the role of paid caregivers in home-based dementia care (study 1 n=15, study 2 n=9). We developed a codebook focused on emergent themes related to the selection and hiring of paid caregivers, specifically noting practical advice from family caregivers. We analyzed results using thematic analysis. Family caregivers frequently described difficulty setting up care and hiring paid caregivers. This was true for both those who hired paid caregivers privately and those who worked with agencies who provided Medicaid-funded paid care. Many had to cycle through several caregivers, increasing stress for the family, and reducing constancy for the person with dementia. Our findings point to the need for improved hiring guidelines for paid caregivers. Informational materials, including sample interview questions for both paid caregivers as well as family members, can improve the efficiency and quality of the hiring process. It behooves family members, home health agencies, and privately paid caregivers to approach the hiring process deliberately and thoughtfully. Creating and distributing formalized guidelines and protocols can serve as a helpful resource at a stressful time, and improve the overall quality of care for the person with dementia at home.

Abstract citation ID: igae098.3926
VALIDITY AND RELIABILITY OF THE MULTIDIMENSIONAL FUNCTIONAL ASSESSMENT QUESTIONNAIRE (OARS) IN ANGOLAN ELDERLY
Rita Bárbara[1], Rui Gonçalves[2], Carlos Sousa[3], Luciano Chingui[4], Antonio Armando[3], and Mario Lopes[1], *1. Universidade de Aveiro, Aveiro, Portugal*, *2. Escola Superior de Tecnologia da Saúde de Coimbra do Instituto Politécnico de Coimbra., Coimbra, Portugal*, *3. Universidade Privada de Angola, Luanda, Angola*, *4. Universidade Metodista Angola, Luanda, Angola*
**Introduction:** The Older American Resources and Services (OARS) questionnaire assesses functionality and cognition in the elderly. Validating and culturally adapting instruments like the OARS is crucial to ensure their accuracy across different contexts. Objective: This study aimed to translate, culturally adapt, and validate the OARS for elderly individuals in Angola, focusing on cognitive and functional domains.
**Methods:** The adaptation of the OARS involved a rigorous process, including translation, cultural adaptation, and clinimetric analysis. A multidisciplinary panel of experts, including professionals from internal medicine, public health, epidemiology, physiotherapy, and nursing, reviewed and validated the adapted version. The sample comprised 102 elderly residents of Luanda, randomly selected to ensure representativeness. Reliability was assessed using Cronbach's alpha coefficient, while validity was examined through exploratory factor analysis, with particular attention to item factor loadings.
**Results:** The physical activity domain demonstrated high internal consistency (Cronbach's alpha = 0.80) and robust factor loadings, indicating reliability and validity in the Angolan context. The mental health domain showed moderate internal consistency (Cronbach's alpha = 0.59) and variable factor loadings, suggesting the need for further refinement for better cultural and linguistic adaptation.
**Conclusion:** The adapted OARS version proved reliable for assessing physical activity among Angolan elderly, but the mental health domain requires adjustments. These findings highlight the importance of clinimetrics and factor analysis in the cultural adaptation of instruments, ensuring their effectiveness across diverse sociocultural contexts. Keywords: Cognition, clinimetrics, validation

Abstract citation ID: igae098.3927
VIRTUAL COMPARATIVE GLOBAL AGING CURRICULUM/COURSE – STUDENT GLOBAL COMPETENCES QUALITATIVE THEMATIC ANALYSIS
Alina Zurek[1], Grzegorz Zurek[2], and Lyn Holley[3], *1. University of Wroclaw, Wroclaw, Dolnoslaskie, Poland*, *2. Wroclaw University of Health and Sport Sciences, Wroclaw, Dolnoslaskie, Poland*, *3. University of Nebraska at Omaha, Omaha, Nebraska, United States*
Presents global competences acquired by students of the international virtual course Comparative Global Aging implemented in the years 2018-2023. The course is the result of American-Polish cooperation between the University of Nebraska in Omaha and two Polish universities - the University of Wrocaw and the University School of Physical Education in Wrocław. The content of thirty-seven Learning Journals of students from 10 European countries studying in Poland in the ERASMUS student exchange program and UNO students was subjected to qualitative analysis using MAXQDA software. Discuss the results in the three distinguished dimensions of global competences - cognitive, socio-emotional and ethical, and five thematic clusters: Critical and Constructive Thinking, Self-Awareness and Self-Direction in Education, Effective Cross-Cultural Cooperation, Open Intergenerational Communication, Innovation in Action and Problem Solving. Learning outcomes achieved by students, i.e. the ability to examine local, global and cross-cultural issues in the topic of ageing, understand and appreciate the perspectives of people living in other countries and cultures, engage in open and effective interactions, and work to solve the problems of older people using a broad global perspective of ageing countries and societies

Abstract citation ID: igae098.3928
VISUAL FUNCTIONS AND PERFORMANCE ON COGNITIVE CHALLENGE TEST IN OLDER ADULTS
Diane Zheng, Rosie Curiel, Alexandra Ortega, Kirsten Crenshaw, Denise Carballea, Brooke Bosworth, Emory Neer, and David Loewenstein, *University of Miami, Miami, Florida, United States*
The Loewenstein-Acevedo Scales for Semantic Interferences and Learning (LASSI-L) is a culturally sensitive Cognitive Challenge Test (CCT) that has been shown to be predictive of preclinical Alzheimer's disease (AD). Visual impairment is a risk factor for AD and cognitive decline. Associations between LASSI-L and visual functions in older adults have never been evaluated. We assessed 133 adults aged 61 to 88 diagnosed with normal cognition, amnestic mild cognitive impairment (MCI), or pre-MCI using a comprehensive clinical evaluation and cognitive battery. The mean age was 73.5 years, 68% female, the average education was 15 years, and mean MMSE was 28.3. About 60% of the sample identified as Hispanic/Latino. Participants were administered objective vision tests measuring distance visual acuity and contrast sensitivity, and LASSI-L. Multiple linear regressions were performed to examine associations between visual function and LASSI-L measures. After adjusting for age, sex, and education, contrast sensitivity was statistically significantly associated with the LASSI-L maximum storage capacity (A2 cued recall, $\beta$=2.80, se=1.1, p=0.01), proactive semantic interference (PSI) (B1cued intrusions, $\beta$=-2.99, se=1.25, p=0.02), and failure to recover from proactive semantic interference (frPSI) (B2 cued recall, $\beta$=3.64, se=0.24, p=0.004). Distance visual acuity was not associated with LASSI-L measures. Contrast sensitivity, or the ability to discern characters on a similar color background or brightness, is an important visual function that facilitates daily activities. The association between contrast sensitivity and subtle cognitive decline such as PSI and frPSI on CCT suggests that visual changes co-occur during pre-dementia stages and may be considered alongside other diagnostic tools.

Abstract citation ID: igae098.3929
WHAT ARE FAMILY CAREGIVERS NOT PREPARED FOR?
Roshni Singh[1], Saanvi Lamba[2], Richard Munoz[3], Pranjal Tyagi[4], Sandra Garcia-Davis[1], Stuti Dang[5],

Diana Ruiz[6], and VA Elizabeth Dole Center of Excellence[7], *1. University of Miami, Miami, Florida, United States*, *2. Flint Hill School, Oakton, Virginia, United States*, *3. South Florida Veteran Affairs Foundation for Research and Education, Miami, Florida, United States*, *4. South Florida Veteran Affairs Foundation for Research and Education (SFVAFRE), Miami, Florida, United States*, *5. Miami VA Healthcare System, Miami, Florida, United States*, *6. Miami Veterans Affairs Healthcare System, Miami, Florida, United States*, *7. VA, VA HSR, Texas, United States*

Informal family caregivers of patients have a myriad of non-medical needs that are burdensome to caregivers and critical for disease management. Yet, caregivers are often not adequately prepared to address the needs of their Veterans. This study aimed to identify what caregivers feel unprepared for. We used round one data from the HERO CARE Survey. Here, we describe the preparedness of caregivers of Veterans, who responded to the following open-ended question: Out of all the tasks that you help the Veteran with, Is there anything specific you would like to be better prepared for? Qualitative responses to the open-ended question were coded and analyzed thematically. We received responses from 690 caregivers, 68.2 (SD: 12.2) years-old on average, 601 (87.1%) female, 70.7% non-Hispanic White, 62.0% spousal caregivers, 83.8% primary caregivers and 46.8% had provided care for >5 years. Twenty-one (3.0%) felt not prepared, 345 (50.0%) somewhat prepared, and 342 (49.6%) very prepared. Caregivers most important concerns reported were dealing with changes related to declining health (20.4%), equipment/home modifications (10.6%), dealing with memory loss (9.6%), advocating for services (8.8%), preparing for the future (7.8%), managing finances/legal matters (7.1%), mobility/falls/transfers (7.0%), respite/HHA (6.8%), handle emergencies (5.2%), and financial assistance (4.3%). Caregivers will be able to better meet the needs of patients if they are more prepared with the knowledge and skills to do their caregiving tasks. Developing ways to provide support, education, training, and resources will be pivotal in improving outcomes for veterans and their caregivers.

Abstract citation ID: igae098.3930

### WHAT KIND OF HEALTH SCREENING IS USEFUL FOR PERSONS AGING WITH HIV?

Manuel Ramos[1], Shelby Brage[2], Christian Nouryan[3], Bruce Hirsch[3], Joseph McGowan[3], Ashwin Mattam[3], and Edith Burns[4], *1. The Donald and Barbara Zucker School of Medicine at Hofstra/Northwell, Floral Park, New York, United States*, *2. The Feinstein Institutes for Medical Research, Manhasset, New York, United States*, *3. Zucker School of Medicine at Hofstra/Northwell, Manhasset, New York, United States*, *4. Northwell Health, Manhasset, New York, United States*

**Background:** In the United States, 54% of people aging with HIV (PAWH) are age >/= 50 and 89.7% are virally suppressed. HIV is linked to increased risk of developing comorbidities and may accelerate aging. This population may not receive adequate screening for changes related to advancing age. We describe initial findings of a “Maturity Screen” for PAWH.

**Methods:** Setting and population. Convenience sample of PAWH aged 50 years or older receiving care at an HIV specialty clinic in a major metropolitan area. Patients completed a series of measures administered by trained care team members. Measures. sociodemographic (e.g., age, sex), comorbid conditions. Maturity Screen: MoCA, PHQ2, ADL, IADL, GAD2, gait speed and Edmonton Frail Scale.

**Results:** 82 participants were screened to-date; 41 males, 36 females, 4 unknown. Mean age 63 (SD: 7.6 yrs.); mean # comorbid conditions 1.8 (SD: 1.3, range 0-5); top four comorbidities were hyperlipidemia (56%), hypertension (43%), diabetes (11%), and CKD (11%). Mean MoCA 24.9 (SD: 3.7, range 15-30); mean PHQ2 0.6 (SD: 1.2); ADL 5.9 (SD: 0.5); IADL 7.8 (SD: 0.8, max score 9).

**Conclusion:** In this sample of PAWH maturity screening suggests healthier group than anticipated and low levels of depression. Borderline mean cognitive screen suggests need to monitor for emerging issues likely to impact quality of life. Examining social support in the context of PAWH and potential cognitive decline may identify those with increased future healthcare needs and poor outcomes. Gait speed and frailty measures are currently being analyzed and may identify additional functional needs.

Abstract citation ID: igae098.3931

### WHY NOT LONG-TERM CARE? EXPLORING STUDENT NURSES’ PERSPECTIVES ON CAREERS CARING FOR OLDER ADULTS

Kristen Childress[1], and Angela Kokinakos[2], *1. University of Washington, Seattle, Washington, United States*, *2. Phoenix Psychiatric Services, PLLC, Seattle, Washington, United States*

The aging population and growing numbers of people living with Alzheimer’s Disease and related dementias (ADRD) are increasing the demand for licensed nurses, especially in the long-term care (LTC) sector. More than 89% of long-term care facilities in the United States (US) report ongoing staffing shortages that persist despite recruitment and retention efforts, directly impacting quality of care and outcomes for people with ADRD. Existing evidence describes factors influencing nursing staff retention in LTC but there is a paucity of information related to factors that impact nursing students’ decision-making related to entering careers in LTC. The purpose of this qualitative study was to understand student nurses’ perceptions of LTC as a career path. The first of four focus groups was conducted with five individuals. Interviews revealed that most participants plan to work in the hospital following graduation. Themes related to lack of plans to work in LTC included limitations in career advancement, past experiences with LTC culture, and perceptions of the care provided in LTC. Pay was a mixed factor, with quality of the work environment being more important to participants. Participants felt that faculty representation of LTC careers was positive, but participants described limited exposure to gerontologic nursing and LTC as part of their training, indicating that modification of these factors might have changed their perspective of careers in LTC. These early results identify key areas where crafting and delivering positive messaging and implementing educational interventions could boost nursing students’ interest in LTC careers.

Abstract citation ID: igae098.3932

**WORKPLACE RETENTION FRAMEWORKS FOR NURSING IN LONG-TERM CARE: AN INTEGRATIVE REVIEW**

Winnie Sun[1], Lucas Martignetti[2], and Jen Calver[3],
*1. Ontario Tech University, Lindsay, Ontario, Canada,*
*2. Ontario Tech University, OSHAWA, Ontario, Canada,*
*3. Ontario Tech University, Oshawa, Ontario, Canada*

**Background:** Nurses and Personal Support Workers are essential for care and services provided for Long term Care (LTC) homes. Concerns regarding unsafe living and working condition resulting in high turnover rates. Despite high turnover rate, very little guidance to help improve staffing stability and workforce retention planning. The purpose of this integrative review is to synthesise the existing literature regarding LTC staff retention to guide the development of a comprehensive framework addressing LTC staffing issues.

**Methods:** This review was conducted using Whittemore and Knafl's integrative review methodology to assess both empirical, theoretical and grey literature. Databases were searched such as CIHNL, PubMed, PyschINFO. Included articles are published in English; use empirical and theoretical methodologies; and focuses on workplace retention frameworks, models, and strategies for the LTC workforce. Articles involving non-facility based LTC and those focused on recruitment rather than retention was excluded. Thematic analysis using Braun & Clark was used to identify themes related to retention frameworks and models influencing turnover.

**Results:** A total of 1196 articles were retrieved, with 1086 articles screened, while 17 articles were included in the final sample. Examples of identified models including Huber's Structural Model and the Dual-Driver Model. Retention framework focuses on five group factors: Organizational and personal characteristics, leadership/management, social systems and labour market conditions.

**Conclusion:** The results of this study will be useful to guide subsequent stages of this research to gain consensus of LTC staff using Delphi methodology to support the co-creation of a workforce retention framework in LTC sector.

### SESSION 8015 (POSTER)

### LATE BREAKING POSTER SESSION III

Abstract citation ID: igae098.3933

**BRIDGING THE GAP IN OBESITY TREATMENT FOR OLDER PEOPLE: THE ROLE OF THE TREAT AND REDUCE OBESITY ACT (TROA)**

Samuel C. Van Vleet[1], Jeein Jang[2], and Maizonne Fields[3],
*1. Miami University, Oxford, Ohio, United States,*
*2. University of Massachusetts, Boston, Boston, Massachusetts, United States, 3. The University of Alabama at Birmingham, Birmingham, Alabama, United States*

Obesity is a chronic disease with significant impacts on health outcomes and economic burdens throughout the life course. Obesity is prevalent and often undertreated among older people, especially those with lower socioeconomic status and those living in rural areas. Current coverage for obesity treatment is sparse and inconsistent across states, creating barriers to care for many. The Treat and Reduce Obesity Act (TROA), a public policy initiative that has been in development for over a decade, seeks to address these gaps in care among Medicare beneficiaries. TROA, which has garnered bipartisan support and is bicameral, aims to improve access to comprehensive treatment options for older people. This presentation explores the benefits of treating obesity on various comorbidities, such as heart disease, sleep apnea, and diabetes, and will emphasize the need for expanded Medicare coverage for obesity treatments. Utilizing data from the 2024 Obesity Coverage Nexus Platform, we highlight the high prevalence and associated risks of obesity among older people and examine how state-level variations in insurance coverage for obesity treatments impact access. The passage of TROA would provide a more equitable and effective approach to obesity management, helping to alleviate the economic burden of out-of-pocket costs for obesity treatments and improving health outcomes for lower-income and underserved populations.

Abstract citation ID: igae098.3934

**CHINESE FAMILY CAREGIVERS' EXPERIENCE SUPPORTING FAMILY MEMBERS WITH DEMENTIA TRANSITIONING INTO US NURSING HOMES**

Yiyang Yuan[1], Catherine Dubé[1], Emily Lim[2], Shu Xu[3], Shan Qu[2], Emily McPhillips[1], and Kate Lapane[1],
*1. University of Massachusetts Chan Medical School, Worcester, Massachusetts, United States, 2. University of Massachusetts Boston, Boston, Massachusetts, United States, 3. University of Michigan, Ann Arbor, Michigan, United States*

Asian American family caregivers of individuals with dementia experience a greater burden compared to other racial/ethnic groups, underscoring the need for formal long-term care options including nursing homes. Transitioning a family member with dementia into a nursing home can be stressful due to cultural expectations and the lack of linguistically and culturally appropriate care. This qualitative study focused on caregivers of Chinese descent, the largest Asian community in the U.S., and explored their experiences as they support their family members with dementia through the nursing home transition. We conducted fourteen in-depth, semi-structured interviews with Mandarin- or English-speaking family caregivers. Through thematic analysis, we identified six major themes: the role of filial piety, allocation of caregiving duties, reasons for and against nursing homes, decisions about nursing home care, the impact of the COVID-19 pandemic, and advice and suggestions. Filial piety remained a significant influence across all aspects of the caregiving experience. Caregivers reported physical, emotional, and financial struggles, limited availability of culturally and linguistically appropriate nursing home care, concerns about nursing home quality, and challenges in family discussions regarding the transition to a nursing home. Caregivers noted that COVID-19-related social isolation might have worsened their family members' dementia. Many expressed the need for the Chinese community to become more familiar with healthcare options for the aging population and called for additional nursing home staff training to enhance the quality of culturally and linguistically appropriate nursing home care. Understanding caregivers' experiences can also help identify areas for improvement in dementia care services.

Abstract citation ID: igae098.3935

### CLINICAL CORRELATION OF MMSE AND TAU RESULTS FROM BLOOD OR IMAGING REPORTS

Surya Sunil, Malini Nair, Rui Huang, Viviana Obando, Kyra Ferreira, Jennifer Glynn, and Anil Nair, *Boston Neuropsychiatry, Boston, Massachusetts, United States*

**Background:** A novel blood test for Alzheimer pathology is now available. Abnormal tau measurements from blood or brain PET may indicate brain tau. We analyzed the correlation of Mini Mental Status Examination (MMSE) to tau results disclosed to patients in a memory clinic.

**Objectives:** To assess the correlation of blood or PET tau positivity to MMSE test scores. To evaluate an MMSE score cut-off that translates optimally to tau positivity.

**Method:** Retrospective observational chart review of patients attending a memory clinic in Boston, MA, with a MMSE test score and blood tau or tau-results were included for analysis. Baseline MMSE and a binary blood or PET scan result were analyzed with covariates age, sex and race. Spearman correlation of MMSE to blood/PET tau. Kruskal Wallis/Chi square tests for relationship of age, sex, gender and education with MMSE score and tau-PET status. ROC analyses and sensitivity/specificity were obtained.

**Results:** We disclosed 45 positive and 10 negative tau results. 58.2% were female and 82.7% were white. Mean age was 70.12 ± 14.17, mean MMSE was 21.35±7.36. Tau abnormality trended negatively with MMSE score (r= -0.26, p=0.07). A cut point of 28 for MMSE estimated tau deposition in the brain by PET scan or blood tau, with an AUC of 0.694 (95%CI 0.48 to 0.90).

**Conclusion:** In a small pilot study, tau blood or PET results trended negatively with MMSE scores, possibly indicating a ceiling effect as we only analyzed very mildly impaired subjects.

Abstract citation ID: igae098.3936

### CLINICAL EFFICACY OF A DIGITAL COGNITIVE ASSESSMENTS IN DETECTING COGNITIVE IMPAIRMENTS: A POOLED ANALYSIS

Duong Huynh, Mary Patterson, and Bin Huang, *BrainCheck, Austin, Texas, United States*

Dementia has become a public health concern worldwide with significant social and economic impacts. Early detection of dementia, which involves diagnosis of mild cognitive impairment (MCI), a possible prestage of dementia, is crucial for timely interventions, care planning, and reducing healthcare costs. To promote proactive screening, it is important to equip primary care providers with assessment tools that are not only validated and reliable but also easy-to-administer and seamlessly integrated into their routine workflows. BrainCheck's cognitive assessment tool (BC-Assess), with its demonstrated high diagnostic efficacy, usability, and feasibility of implementation, offers a promising solution. This study aimed to re-evaluate the efficacy of BC-Assess in identifying MCI and dementia based on a pooled dataset. The dataset included BC-Assess data of 193 individuals (age mean=73.7; SD=11.2), comprising those with normal cognition (NC; N=48) and those diagnosed with MCI (N=53) or dementia (N=92), acquired from two previous studies and from BrainCheck's clinical partners. Each individual's cognitive status was determined based on results from provider clinical diagnoses (N=158; 82%) or from the Montreal Cognitive Assessment (N=35; 18%). Significant differences in BC-Assess overall scores were found between the three groups (p<.001). ROC analysis shows that BC-Assess can achieve high sensitivity (>=85%) and specificity (>=81%) in distinguishing MCI and dementia patients from those with normal cognition, comparable with performance level obtained previously from a single data source. These results demonstrate the ability of BC-Assess to hold its diagnostic efficacy over combined data collected from different settings.

Abstract citation ID: igae098.3937

### CLINICAL TRIALS USING MUSIC IN DIVERSE COMMUNITIES OF OLDER ADULTS

Tara Rose, *University of Southern California, Los Angeles, California, United States*

Music is being integrated into health care services and nonpharmacological treatment options as part of physical health and well-being programs for older adults. When health care professionals and older adult providers try to locate successful programs to initiate, they look to clinicaltrials.gov as a resource to find the most rigorous and robust programs implemented. In large US cities, such as Los Angeles, where there are multiple ethnic populations with specific cultural music interests, it is important to have knowledge of culturally specific interventions in order to create evidence-based programs for multiple community members. The purpose of this review is to analyze music programs and interventions for African American, Asian American, and Latino/Hispanic older adults to better understand emerging best practices. A search of all trials registered in the clinicaltrials.gov was conducted using key words describing these ethnic populations and also the word "music." Of the 146 studies generated, 15 met the eligibility criteria, with all studies enrolling older adults along with other age groups. Studies were conducted across four regions of the US, 53% in Northeast, 20% South, 13% Midwest, and 13% West. Music interventions for specific medical conditions (20% dementia and 27% other diseases) and mental health/well-being (53%) were the primary foci in studies. Data on intervention types, demographics, and methodology will be presented. Results to date suggest that more research is needed on best practices in music interventions for specific cultural groups and that new approaches would benefit cities and communities, especially in areas with diverse multicultural populations.

Abstract citation ID: igae098.3938

### CLINICIAN AWARENESS, ATTITUDES, AND INTEREST IN BLOOD TESTS FOR ALZHEIMER'S DISEASE

Karen Tracy[1], Julie Wood[2], Ben Tiede[3], and Emily Scholler[4], *1. Gerontological Society of America, Gerontological Society of America, District of Columbia, United States*, *2. American*

*Academy of Family Physicians, Leawood, Kansas, United States*, *3. CEOi, Wayne, Pennsylvania, United States*, *4. High Lantern Group, Wayne, Pennsylvania, United States*

Clinical research demonstrates the value of early detection of dementia, including dementia caused by Alzheimer's disease (AD) and related disorders. Yet, timely and accurate diagnosis of AD in clinical practice remains challenging. The most widely used biomarkers to aid diagnosis of AD are positron emission tomography (PET) scans and cerebrospinal fluid (CSF) biomarkers. However, the clinical community recognizes that these tests have some limitations. Blood tests, also known as blood-based biomarkers (BBMs), are emerging as an aid for detecting and diagnosing AD. These include tests that measure circulating levels of amyloid or phosphorylated tau protein. The Global CEO Initiative on Alzheimer's Disease (CEOi) is leading a global effort to prepare for the widespread adoption of BBMs for diagnosis of AD into clinical practice. As part of this effort, CEOi commissioned the American Academy of Family Physicians and GSA to issue surveys to their members in May and June 2024 to explore current perceptions and experience with BBM testing. Members were also asked to share their thoughts on how BBMs might be used in clinical practice and what is needed to strengthen their knowledge about BBMs. Insights from compatible surveys issued by AAFP and GSA are shared along with what clinicians would need to employ BBM tests in their practices.

Abstract citation ID: igae098.3939

### COGNITIVE AND PRACTICE EFFECTS OF IMMERSIVE VIRTUAL REALITY USE IN OLDER ADULTS: PRELIMINARY RESULTS

Ferdinand Delgado[1], and Jeremiah Greenberg[2], *1. University of New Hampshire, University of New Hampshire, New Hampshire, United States*, *2. University of New Hampshire, Durham, New Hampshire, United States*

Virtual reality through head-mounted displays (VR-HMD) is emerging as a tool for cognitive training in older adults. However, little is known about the impact of commercially available VR-HMD games on cognitive function. The purpose of this study was to examine preliminary results on the practice effects of VR-HMD games and their influence on cognition. Six participants (aged 71-81 years; 5 males) played VR-HMD games twice a week for eight weeks, for 60 minutes each session. Using the Meta Quest 3, participants played First Steps, featuring three laser shooting tasks (single-laser, dual-laser, and automatic laser) on weeks 1 and 5, and Fruit Ninja (Classic and Zen modes) on weeks 2 and 6. A Tai Chi and table tennis game were also played, but game scores were not recorded. Their average game score after each week was used for analysis. NeuroTrax BrainCare Tests were used to measure executive function (EF), information processing speed (IPS), attention, and visual-spatial ability before and after the intervention. Paired sample t-tests revealed significant practice effects in the single-laser (4251.24±1114.80 to 5066.36±1553.75, p=0.02) and automatic laser (4930.24±1311.34 to 6059.54±1219.53, p=0.018) tasks in First Steps, and Fruit Ninja's classic mode (30.46±5.80 to 43.48±12.07, p=0.017). Visual-spatial ability showed a significant improvement (124.22±11.08 to 129.5±9.23, p=0.014), but not the other cognitive domains (p≥0.05). Improvement in Fruit Ninja's classic mode showed a correlation trend with the change in visual-spatial ability (p=0.052). In conclusion, older adults can improve game performance in VR-HMD. Further research is needed to clarify VR-HMD effects on cognitive function.

Abstract citation ID: igae098.3940

### COGNITIVE ASSESSMENT OF MARSHALLESE PEOPLE USING QDRS (QUICK DEMENTIA RATING SCALE)

Gohar Azhar[1], Shakshi Sharma[2], Sheldon Riklon[3], Philmar M Kabua[4], Pearl McElfish[4], and Jeanne Y Wei[1], *1. University of Arkansas for Medical Sciences, Little Rock, Arkansas, United States*, *2. University of Arkansas for Medical Sciences, LITTLE ROCK, Arkansas, United States*, *3. UAMS Northwest Regional Campus, UAMS Northwest Campus, Arkansas, United States*, *4. UAMS Northwest Regional Campus, Fayetteville, Arkansas, United States*

Health disparities in Marshallese living in the US are common due to language barriers, cultural differences, lack of insurance, and inability to pay. Marshallese face strikingly high rates of heart attacks, diabetes, depression, and anxiety. As cognition in this population has been understudied, we used the Quick Dementia Rating Scale (QDRS), to determine aspects of their cognition. QDRS is a dementia staging tool of 10 items (30 points). Forty-nine subjects consented to the study, which included 34 younger adults (YA) and 15 older adults (OA). Self-reported co-morbidities of diabetes were 67% in OA vs 38% in YA; heart diseases were 67% in OA vs 9% in YA; hypertension was 33% in OA vs 18% in YA; anxiety and depression were 60% in OA vs 26% in YA. Cognitive assessment showed that 13% of OA had moderate to severe difficulty in memory and recall compared to only 2% of YA. Additionally, 7% OA had moderate to severe problems in orientation, attention, concentration, language, and decision-making abilities. Moderately severe problems were also found in behavior with personality changes and reduced personal hygiene in OA. Only 3 % of YA had problems performing tasks or activities outside the home compared to 7% in OA. Furthermore, 13 % of OA had moderate to severe problems with anxiety or depression with no issues reported by YA. Our results demonstrated that higher risks associated with cardiovascular diseases and diabetes mellitus corelated with greater cognitive, behavioral, and functional impairment in older adult Marshallese in Arkansas.

Abstract citation ID: igae098.3941

### COMPARATIVE ANALYSIS OF FALL RATES AMONG COMMUNITY-OLDER ADULTS IN SIX COUNTRIES

Hadi Kooshiar, *Texas A&M University-Corpus Christi, Mission Viejo, California, United States*

The number of older people who desire to live in their homes is rising. So, this study aimed to evaluate fall rates in community-dwelling older adults across six English-speaking countries. This cross-sectional survey was conducted with 114 older adults residing in Canada, the USA, Australia, Ireland, New Zealand, the UK, and Northern Ireland. Participants recruited online through social media and passive snowball sampling completed self-rated fall risk (FRQ) and Activities-specific Balance Confidence (ABC-6) surveys, along with sociodemographic and health-related questions. Canadian and Irish respondents comprised the highest and lowest proportions of the sample, at 37% and 9%, respectively. The mean age was 67 (SD = 7.8), with a balanced gender distribution. Chronic diseases, notably joint pain and abnormal blood pressure, were reported by 25.4% of respondents. 44.8% of respondents reported falls in the past year, with 35.1% reporting falls in the past six months. Multiple falls were reported by 25.2% of respondents, with 45.8% reporting fractures primarily in the hands, forearms, or arms. Fall rates varied significantly among the countries: Canada (54.1%), USA (44.4%), Australia (53.8%), Ireland (40%), New Zealand (41.7%), and the UK and Northern Ireland (23.5%). Geographic, demographic, and methodological factors influence these rates. Higher fall rates in Canada may be due to an aging population and adverse weather conditions. The study's findings have direct implications for research, clinical practice, and education. Recommendations for a prospective design with a large sample size are essential for a more comprehensive understanding of falls rate and effective prevention of falls.

Abstract citation ID: igae098.3942

### COMPARING STROKE MORTALITY TRENDS IN RURAL AND URBAN AREAS DURING AND BEFORE THE COVID-19 PANDEMIC

Vivian Lin, Sasha DiVall, Mengyuan Cheng, and Nasim Ferdows, *Northeastern University, Boston, Massachusetts, United States*

COVID-19 has undeniably disrupted healthcare services, with many individuals advised to avoid hospital visits unless necessary. However, timely treatment following a stroke is critical to minimize damage or fatality, raising concerns about how these disruptions may have affected stroke outcomes. This study explores the association between COVID-19 and stroke mortality rates across different ruralities across a three-year period (2018-2020). Using data from the CDC WONDER database and County Health Rankings, we examined stroke mortality trends, adjusting for county-level characteristics. The study classified rurality using Rural-Urban Commuting Area codes from the Economic Research Service into three categories: urban, rural-adjacent to an urban area, and rural non-adjacent to an urban area, given the generally lower access to medical care in rural settings. Our analysis, which utilized data up to 2020—covering less than a full year of the COVID-19 pandemic—found no significant differences in stroke mortality rates across the different rurality levels. However, it is important to note that the limited data from the early stages of the pandemic may not fully capture the potential impact of COVID-19 on stroke mortality. As more recent data from 2021-2022 become available, we anticipate that a more comprehensive analysis could reveal different trends, particularly as the effects of prolonged lockdowns and healthcare disruptions become more evident. This research aims to provide a clearer understanding of stroke mortality trends during the pandemic, offering insights that may guide healthcare practices and policy decisions to ensure that all populations, especially those in rural areas, have access to necessary care.

Abstract citation ID: igae098.3943

### COMPREHENSIVENESS AND READABILITY OF CHATGPT'S ANSWERS TO DEMENTIA FAMILY CAREGIVERS' QUESTIONS

Soojeong Han[1], Bayu Aryoyudanta[2], and Yong Kyung Choi[2], *1. Columbia University School of Nursing, New York, New York, United States*, *2. University of Pittsburgh, Pittsburgh, Pennsylvania, United States*

Family caregivers experience challenges in deciding on care transitions. As ChatGPT is a powerful tool for obtaining information, this study aims to evaluate the comprehensiveness and readability of ChatGPT's answers to dementia family caregivers' questions regarding transitioning to hospice and palliative care. We extracted data from a public online forum, ALZConnected.org, dedicated to caregivers and individuals affected by dementia. Our dataset comprised 35,482 original posts collected from 2011 to 2023. By filtering with the search terms "palliative", "hospice", or "end of life", 123 questions regarding transitioning were discovered. Of 123 questions, 24 were selected with convenience sampling and entered into ChatGPT. The comprehensiveness of ChatGPT's answers was evaluated using single-turn and then multi-turn conversations. The readability of all final answers was assessed using the Flesch Reading Ease (FRE) (Rouhi et al., 2024). Results revealed that 41.67 % (n = 10) was scored as comprehensive, 33.33 % (n = 8) as adequate, and 25.00 % (n = 6) as incomplete. The median comprehensiveness score was 2.00 (mean 2.08, SD 0.88). The mean (SD) FRE score was 25.37 (10.39), indicating 'very difficult to read'. We further investigated whether ChatGPT can provide answers that are easier to read on a different literacy level. The mean (SD) FRE score of revised answers was 46.42 (12.99), indicating 'difficult to read'. The t-test revealed significant differences between the two answers on the FRE scale ($t = -6.20$; $p < 0.0001$). Further studies can explore how to maximize the benefits of using ChatGPT for family caregivers.

Abstract citation ID: igae098.3944

### CULTURAL AND PSYCHOMETRIC VALIDATION FOR COGNITIVE ASSESSMENT OF OLDER ADULTS

Rita Bárbara[1], RUI GONÇALVES[2], and MARIO LOPES[1], *1. Universidade de Aveiro, AVEIRO, Aveiro, Portugal*, *2. Escola Superior de Tecnologia da Saúde de Coimbra do Instituto Politécnico de Coimbra., Coimbra, Coimbra, Portugal*

**Introduction:** Cognitive assessment is vital for the early detection and management of cognitive disorders in older adults. Instruments developed in one cultural context may not be applicable to other populations without proper adaptation. This study aims to culturally adapt the cognitive domain of the Older Americans Resources and Services (OARS) instrument for use with Angolan elderly, ensuring it accurately captures the cultural and specific needs of this group. Objective: The primary goal is to validate the cognitive domain of the OARS for its cultural relevance and clinical reliability in Angola. This involves modifying the instrument to better suit the Angolan cultural context.

**Methods:** The study included 127 Angolan older adults. An initial factor analysis identified items misaligned with the local cultural context. These items were revised and adapted. A follow-up factor analysis evaluated the coherence of the revised instrument. Reliability was measured using Cronbach's alpha.

**Results:** The initial analysis revealed several problematic items, which were modified for improved cultural fit. The revised factor analysis demonstrated a more coherent structure, with Cronbach's alpha rising to 0.82, indicating good internal consistency among the updated items.

**Discussion:** Culturally adapting the OARS was crucial for improving its sensitivity to Angolan older adults' needs, leading to a more accurate and equitable cognitive assessment.

**Conclusion:** The adaptation and psychometric validation of the OARS have produced a more suitable tool for cognitive assessment in Angola, highlighting the importance of considering cultural factors in instrument development. Keywords: Cognitive assessment, cultural adaptation, elderly.

Abstract citation ID: igae098.3945

### DELIRIUM AND CARDIOVASCULAR DISEASE IN OLDER ADULTS WITH COVID-19

Esther Park, Milenko Petrovic, and Gohar Azhar, *University of Arkansas for Medical Sciences, Little Rock, Arkansas, United States*

SARS-COV-2 is often associated with cardiovascular diseases such as arrhythmias and congestive cardiac failure (CHF), and cardiovascular disease is itself linked with the development of delirium. In this retrospective study we explored the relationship between heart disease and acute delirium in 319 COVID-19 patients 65 and older, seen at the University of Arkansas for Medical Sciences from January 7, 2018 to January 5, 2021. We reveiwed the frequency of cardiac conditions (atrial fibrillation, atrial flutter, tachycardia, bradycardia, CHF, CAD, other) in this cohort with emphasis on timing before and concurrent with COVID-19 delirium. We found a strong association of acute delirium during COVID-19 infection in patients with cardiac disease as compared to patients without cardiac disease (p< 0.00001). 128/319 (40.1%) COVID-19 patients suffered from delirium during or after COVID-19, and 88 of these patients had cardiac disease. 121/319 (37.9%) COVID-19 patients had no other cardiac conditions excluding essential hypertension. The majority (83/121, 68.6%) of those with no cardiac conditions listed in EMR also had no delirium documented during or after infection. Patients with cardiac conditions had a risk ratio of 1.38 of developing delirium during or after COVID-19 infection compared to patients without COVID-19. In conclusion, having a preexisting cardiac condition increases the likelihood of delirium in older adults with COVID-19 infection. Improved treatment strategies that optimize cardiovascular care during COVID-19 will also benefit cognition and enhance outcomes in older adults. A better understanding of these complex interactions should improve treatment of delirium in older adults with SARS-COV-2.

Abstract citation ID: igae098.3946

### DEPRESSIVE SYMPTOMS AND HANDGRIP STRENGTH IN MEXICAN AMERICAN OLDER ADULTS: SEX DIFFERENCES

Jamil Okada[1], Joshua Patino[2], Ariza Martinez[2], and Soham Al Snih[3], *1. UTMB, Bedford, Texas, United States*, *2. The University of Arizona, Tucson, Arizona, United States*, *3. University of Texas Medical Branch in Galveston, Galveston, Texas, United States*

The aim of this study was to examine the role of sex in the relationship between depressive symptoms and handgrip strength decline among Mexican American older adults aged ≥65 who scored normal or high in handgrip strength (≥17 Kg in females and ≥27 Kg in males) at baseline over 23 years of follow up. Participants (N=1226) were from the Hispanic Established Population for the Epidemiologic Study of the Elderly (1993/1994-2016). Measures included high depressive symptoms as the independent variable assessed with the Center for Epidemiological Study Depression Scale (CES-D ≥16), handgrip strength in kilograms as outcome variable, and socio-demographics, comorbidities, body mass index, cognitive function, physical function, and disability as covariates. General linear mixed models were used to estimate the population average change in handgrip strength as a function of high depressive symptoms over time. Female participants with high depressive symptoms experienced a greater [β= -1.34, Standard Error (SE)= 0.26, p-value= < 0.0001] decline in handgrip strength over time than females without high depressive symptoms after controlling for all covariates. No significant decline (β= - 0.52, SE= 0.39, p-value= 0.1812) in handgrip strength as a function of high depressive symptoms was found among male participants over time after controlling for all covariates. In conclusion, we found that the effect of high depressive symptoms was greater among female than male participants. Screening and treatment of depressive symptoms may delay decline in muscle strength to further delay disability, maintain independence, prevent institutionalization, and reduce the risk of mortality in this underserved population.

Abstract citation ID: igae098.3947

### DETERMINATION OF PHYSICAL ACTIVITY AND SLEEP LEVELS FROM ACTIGRAPH LEAP USING OPEN-SOURCE R PACKAGE

Jethro Raphael Suarez[1], Chitra Banarjee[2], Joon-Hyuk Park[2], and Ladda Thiamwong[2], *1. University of Central Florida, 2. University of Central Florida, Orlando, Florida, United States*

Accelerometers are the most common devices used for objectively assessing physical activity (PA) and sleep levels, but cleaning and analysis of the data takes time and requires expertise. Raw Accelerometer Data Analysis (also called GGIR) is an open-source R package that helps researchers process and analyze raw multi-day data from accelerometers and calculates PA and sleep levels. GGIR can process various formats of raw data (e.g.,.csv,.gt3x) from several types of accelerometers but not the raw.csv data from the ActiGraph LEAP device. We aimed to determine PA and sleep levels from LEAP devices by investigating how the raw.csv data file could be manipulated to be compatible with GGIR. We used the pilot data from our NIH-funded study with 24 community-dwelling older adults aged 62 to 91 (mean age = 74.63 ± 7.73 years) who wore the LEAP device for 7 consecutive days. The raw.csv data files were analyzed using a code created in R language that read each raw.csv data file in chunks using the "read.csv" function. We found that manipulation of the column and row structure of the raw.csv file in R resulted in compatibility with GGIR only after removal of the Time Stamps column and matching the header formatting of raw accelerometer.csv files exported from the

ActiLife program. Our findings indicated that GGIR can be used to analyze data from LEAP devices and allows for creating a detailed report of PA and sleep levels that benefits both researchers and older adults.

Abstract citation ID: igae098.3948

**DEVELOPMENT AND ASSESSMENT OF THE PERFORMANCE OF A LARGE LANGUAGE MODEL FOR ADMINISTERING THE SHORT BLESSED TEST**

Rafeeul Jaman[1], Sarah Nessen[2], Michael Adjei-Poku[2], Joella Byerley[2], Olivia Sailors[2], Jason Karlawish[2], Kyra O'Brien[2], and Ari Friedman[2], *1. University of Pennsylvania, Lansdoqne, Pennsylvania, United States, 2. University of Pennsylvania, Philadelphia, Pennsylvania, United States*

Universal health system-based screening for mild cognitive impairment (MCI) or impairment consistent with dementia can modify care and increase access to disease-modifying therapies and memory centers, potentially reducing disparities. Constraints on nurse and clinician time have hindered existing efforts. Large language models (LLMs) present a potential solution. However, they are subject to confabulation and poor arithmetic accuracy, and were not trained on data containing definitive assessments of cognitive impairment. We hypothesized that using an LLM to administer the Short Blessed Test (SBT) and calculating test scores from item scores would mitigate these errors. We conducted prompt engineering on OpenAI GPT4o to construct an interactive tool. A team member then simulated being patients with different levels of cognitive impairment across an orthogonal matrix spanning the 6 SBT item scores, answering each question to achieve the intended item score. We summed the LLM-reported item scores to the total score that should have been obtained based on the input answers. We evaluated 57 sets of item scores with total scores of 2-26. Of these, 54.7% had an LLM score equal to the true score. All errors except 2 were within 3 points of the true score. Using a cutoff of >=5 points for mild cognitive impairment, specificity was 100.0%, and negative predictive value was 50%. LLMs may play a role in reducing nurse and clinician screening burden in hospital settings, where the benefit of being able to conduct screening at all outweighs the possibility of missed diagnoses, but requires human confirmation.

Abstract citation ID: igae098.3949

**DEVICE MEASURED SLEEP QUALITY AND PHYSICAL ACTIVITY IS ASSOCIATED WITH COGNITION IN CHRONIC STROKE**

Ryan Stein[1], Ryan Falck[1], Daria Tai[1], Jennifer Davis[2], Ging-Yuek Hsiung[1], Janice Eng[1], Laura Middleton[3], and Teresa Liu-Ambrose[1], *1. University of British Columbia, Vancouver, British Columbia, Canada, 2. The University of British Columbia - Okanagan, Kelowna, British Columbia, Canada, 3. University of Waterloo, Waterloo, Ontario, Canada*

Having a stroke increases risk for dementia two-fold. Both moderate to vigorous physical activity (MVPA) and sleep are positively associated with cognitive function. These associations may vary by biological sex. Notably, evidence shows physical activity mitigates the negative impact of poor sleep on cognitive function. Poor sleep quality is a common consequence of stroke and may contribute to cognitive impairment and dementia poststroke. We examined if MVPA attenuates the relationship of device-measured sleep quality and cognitive function in older males and females living with chronic stroke (i.e., > 12 months since stroke). We used baseline data acquired from 97-community-dwelling older adults with chronic stroke (71±9 years; n=38 female) enrolled in a 6-month randomized controlled trial. We measured MVPA and sleep duration using wrist-worn actigraphy. Global cognitive function was measured with the 13-item Alzheimer's Disease Assessment Scale (ADAS-Cog). We assessed whether MVPA moderated the association between sleep duration and the 13-item ADAS-Cog score in: 1) the full sample; and 2) among males and females separately. Fugl-Meyer score, Montreal Cognitive Assessment score, age, and biological sex were included as covariates. In the full sample, MVPA significantly moderated the association between sleep duration and the 13-item ADAS-Cog score (ß=0.0006±0.0003; p=0.041). Specifically, among individuals with shorter sleep duration, those with greater MVPA had better global cognitive function. This interaction was significant in males (ß=0.0013±0.0005; p=0.016) but not females (ß=0.0004±0.0004; p=0.259). We suggest that MVPA is beneficial for preserving global cognitive function among stroke survivors with shorter sleep duration, particularly among males.

Abstract citation ID: igae098.3950

**DIGITAL INTERVENTIONS FOR URINARY TRACT INFECTIONS PREVENTION IN PERSONS LIVING WITH DEMENTIA: A SCOPING REVIEW**

Kuan-Ching Wu, Basia Belza, Donna Berry, Frances Lewis, and Oleg Zaslavsky, *University of Washington, Seattle, Washington, United States*

People with dementia are significantly more prone to hospitalizations for urinary tract infections (UTIs) and experience more severe health outcomes compared to those without dementia. Digital interventions, such as smartphone applications, wearable devices, and telehealth platforms, show potential for transforming UTI detection, monitoring, and adherence to preventive measures. However, there is a lack of studies examining how existing digital interventions support UTI prevention in persons living with dementia. This paper reviewed various digital interventions tested in current literature for managing or preventing UTIs in this population and identified key outcomes. Utilizing the PRISMA-ScR framework, this scoping review searched five databases for studies from January 1998 to January 2024 that included digital interventions using technological devices. After screening 1800 citations, we included seven studies and evaluated three digital interventions: Technology Integrated Health Management (TIHM), a real-time locating system (RTLS), and a smart diaper system. These studies reported various outcome variables and AI algorithms for health monitoring and UTI prediction, with sensitivities up to 91%. TIHM and RTLS were highly effective in UTI detection, while the smart diaper system had lower sensitivity for urine voiding detection. Despite the prevalence of sensor-based technology and AI algorithms for early UTI detection, most interventions lack theoretical foundations and preventive strategies, highlighting the need for comprehensive approaches involving dementia caregivers. Future research should diversify its scope and integrate preventive measures guided by clinical guidelines and behavioral theories to enhance UTI management, improve quality of life, and reduce healthcare costs for persons with dementia and their caregivers.

Abstract citation ID: igae098.3951

### DOCUMENTING REPORTS OF SUSPECTED ABUSE AND NEGLECT: THE EVALUATION OF A STANDARDIZED PATIENT NOTE

Amy Webster[1], Sara Webb[1], Nina Sperber[2], Kasey Decosimo[1], Jaime Halaszynski[3], Jennifer Koget[4], Jennifer Silva[5], and Lena Makaroun[6], *1. Durham VA Health Care, Durham, North Carolina, United States*, *2. Durham VA, Durham, North Carolina, United States*, *3. Butler VA Health Care System, Butler, Pennsylvania, United States*, *4. Veterans Health Administration, Pittsburgh, Pennsylvania, United States*, *5. Department of Veterans Affairs, Washington, District of Columbia, United States*, *6. VA Pittsburgh Healthcare System, Pittsburgh, Pennsylvania, United States*

Elder abuse (EA) impacts 10% of older adults in the U.S. annually with devastating health consequences, yet few healthcare systems systematically document reports of EA in the electronic health record (EHR). We present acceptability and usability findings from the first of a two-phase Veterans Health Administration (VHA) quality improvement pilot of a standardized note template developed by the National Social Work Program for documenting reports of suspected abuse/neglect in the EHR. In spring 2024, we conducted a usability evaluation through semi-structured interviews with 22 staff from 3 sites including the following roles: 19 social workers, 1 nurse, 1 physical therapist, and 1 mental health case manager. Overall, participants found the note easy to access and use describing it as "really straightforward", "simple", and "self-explanatory." Participants reported the standardized note made it easier to identify that a report was made and facilitated follow-up care for patients who needed it. Half of participants completed the note in ≤5 minutes. Staff in one location said that the template streamlined the reporting process, making it "quicker and easier." Several staff suggested improving procedures to ensure patient privacy and safety when documenting. Modifications based on findings were incorporated to improve usability. Following the second phase of the pilot with 5 additional sites (ongoing), the refined note template will be disseminated for national use in VHA, creating opportunities for real-time monitoring of EA at a population level. Lessons learned from this initiative could inform other healthcare systems interested in systematizing clinical documentation for abuse/neglect reports.

Abstract citation ID: igae098.3952

### DOES THE PERCEIVED DOSAGE OF BZRAS DURING TAPERING AFFECT SLEEP QUALITY IN OLDER ADULTS?

Sarah Wong[1], Michael Mitchell[2], Karen Camacho[2], Thien Nguyen[3], and Constance Fung[4], *1. Touro University California, California, United States*, *2. Greater Los Angeles VA Health Care System, North Hills, California, United States*, *3. University of California, Los Angeles, TORRANCE, California, United States*, *4. VA Greater Los Angeles, North Hills, California, United States*

Reducing adverse events (e.g., falls) is a primary motivator to deprescribe benzodiazepine receptor agonists (BZRAs) in older adults, but success rates vary despite intensive approaches (e.g., cognitive behavioral therapy for insomnia [CBTI]). Mechanisms such as placebo effects may be unexplored targets for improving deprescribing success. A masked taper is one approach to disrupting these mechanisms. Using data from the masked tapering arm of a 5-year randomized clinical trial that augmented CBTI with a masked taper (clinicaltrials.gov NCT03687086; n=89, 67% male, mean age = 69.2 years, 77.5% White, mean Insomnia Severity Index =13.6), we investigated relationships among BZRA pharmacokinetics (duration of action, receptor localization, lipophilicity), perceived BZRA dosage, and sleep quality. Daily, participants guessed their BZRA dosage taken nightly relative to their baseline dosage (guesses classified as over/concordant/underestimation) and completed diaries assessing sleep quality (sleep efficiency, sleep latency, wake after sleep onset). Mixed models examined the effects of pharmacokinetics on underestimation and perceived dosage (over/concordant/underestimation) on sleep quality. Pharmacokinetic measures were not significantly associated with dosage underestimation (all p-values >.05). Dosage overestimation was associated with improved sleep efficiency, reduced sleep latency and reduced wake after sleep onset) (all p-values <.001). Dosage underestimation was associated with worse sleep quality (all p-values <.001). Pharmacokinetics were not associated with estimation of BZRA dosage in a masked taper, but participants who perceived taking a higher dosage of BZRA reported better sleep quality. Future studies may explore the effects of perceived dosage of other drugs (such as analgesics) to aid in masked tapering efforts.

Abstract citation ID: igae098.3953

### DUAL SENSORY IMPAIRMENT AND PHYSICAL FUNCTION AMONG MEXICAN AMERICAN OLDER ADULTS

Arati Bendapudi[1], and Soham Al Snih[2], *1. University of Texas Medical Branch, Galveston, Texas, United States*, *2. University of Texas Medical Branch in Galveston, Galveston, Texas, United States*

The study objective was to examine the relationship between hearing impairment (HI) and vision impairment (VI) on physical function over 12-years of follow-up among Mexican Americans aged 75 years and older. Participants (N=662) were from the Hispanic Established Population for the Epidemiological Study of the Elderly who scored 7 or more on the Short Physical Performance Battery (SPPB) at baseline. Measures included socio-demographics, medical conditions, pain, depressive symptoms, falls, cognitive function, disability, and handgrip strength. Dual Sensory Impairment (DSI) was defined as the presence of sensory and visual impairment. Physical function impairment (PFI) was defined as having a score of less than 7 on the SPPB. Participants were grouped into four groups: No HI - No VI (n=503), HI only (n=70), VI only (n=32), and VI-HI (n=17). Generalized Estimating Equation models estimated the odds ratio (OR) and 95% Confidence Interval (CI) of PFI as a function of DSI over time after controlling for all covariates. Participants with HI only, and those with DSI had greater odds (OR=1.64, 95% CI=1.17-2.31, p-value=0.0043 and OR=2.35, 95% CI=1.38-4.83, p-value=0.0209, respectively) of developing PFI over time compared to those without DSI

after controlling for all covariates. No significant association was found between those with VI only and PFI. In conclusion, Mexican American older adults with DSI were at higher risk of developing PFI over time. These findings highlight the importance of culturally informed clinical practices that address DSI in an underserved population with language barriers and limited access to health care.

Abstract citation ID: igae098.3954

### DYNAMIC STANDING BALANCE AND MOBILITY IMPROVES WITHIN SIX MONTHS OF EXERCISE PARTICIPATION IN OLDER VETERANS

Kathleen Dondero[1], Jamie Giffuni[2], Megan Kelly[3], and Odessa Addison[4], *1. Towson University, Towson, Maryland, United States*, *2. Baltimore and Loch Raven VAMC, Baltimore, Maryland, United States*, *3. Baltimore Veterans Affairs Medical Center, Baltimore, Maryland, United States*, *4. University of Maryland School of Medicine, Baltimore, Maryland, United States*

Older veterans are particularly vulnerable to declines in balance and mobility. Gerofit is a nationwide clinical exercise program aimed at improving physical function. However, it is unclear if Gerofit has an impact on balance, particularly in those that are already at high risk for falls. The Four-Square Step Test (FSST) is a valid and reliable measure of dynamic balance where completion times >12 seconds indicates a high risk for falls. The FSST was assessed at baseline, 6-months, and 1-year in 140 older Veterans who participated in the Gerofit exercise program. This sample included 88 "low fall risk" veterans (FSST < 12 seconds; age 71 ± 5.8 yrs; SPPB Score 11.08 ± 1.4; gait speed 1.2 ± 0.22 m/s) and 52 "high fall risk" veterans (FSST > 12 seconds; age 74 ± 8.1 yrs; SPPB Score 8.65 ± 2.2; gait speed 0.95 ±0.24 m/s). On average, FSST times improved ~13% from baseline (13.31 ± 7.6 seconds) to 6-months (11.55 ± 7.1seconds; P< 0.001) and was retained through 1 year of exercise participation (11.54 ± 7.1 seconds; P=1.0). Importantly, high fall risk veterans showed clinically meaningful improvements in FSST times from baseline (19.1 ± 1.4) to 6 months (15.2 ± 1.4; P=.001) in the program and maintained these improvements through 1-year (15.8 ± 1.4; P=1.0). Veterans, regardless of fall risk, can experience improvements in balance and mobility within 6 months of regular exercise participation and retain these improvements with continued exercise participation.

Abstract citation ID: igae098.3955

### EARLY DETECTION: A COMPREHENSIVE COGNITIVE SCREENING TOOL FOR ADRD THROUGH INNOVATIVE MOBILE APP TECHNOLOGY

Lenora Smith, and Charles O'Brien, *University of Alabama in Huntsville, Huntsville, Alabama, United States*

Currently, there are no curative treatments for Alzheimer's Disease; it is imperative to detect cognitive decline early to provide lifestyle factors that may play a protective role in older individuals, delaying the progression of mild cognitive impairment (MCI). Neurospective is a mobile application platform being designed to facilitate the early detection of cognitive decline, including MCI and pre-clinical Alzheimer's disease and related dementias (ADRD). It will utilize a unique combination of engaging cognitive assessment games, diverse user data collection, and machine learning to establish a robust and accessible screening tool. The app is being built around cognitive assessments from a suite of mini-games. The activities are inspired by established screening tests, like 3MS, MoCA, and SLUMS, to assess language, problem-solving, reasoning, memory, and motor function. This innovative app will be easily accessible for screening MCI and assess outcomes based on an evolving machine learning algorithm. The continuously evolving models, based on user data, will lead to increasingly accurate predictions of cognitive health and potential ADRD risk. This app will initially establish a relative measure of cognitive health within the population, eventually progressing into a reliable screening tool for early detection of MCI. Neurospective presents a unique and promising approach to early detection of MCI through its accessible mobile app format, comprehensive data collection, and evolving machine learning models. This project has the potential to significantly improve our understanding of cognitive health and contribute to earlier intervention for individuals at risk of ADRD.

Abstract citation ID: igae098.3956

### EFFECT OF TIME-RESTRICTED EATING ON NEUROVASCULAR COUPLING AND METABOLIC PROFILE IN OLDER ADULTS

Ana Clara da Costa Pinaffi Langley[1], Camila Bonin Pinto[1], Keith Kleszynski[2], Stefano Tarantini[1], and Andriy Yablu chanskiy[1], *1. University of Oklahoma Health Sciences, Oklahoma City, Oklahoma, United States*, *2. University of Oklahoma Health Sciences Center, Oklahoma City, Oklahoma, United States*

Vascular cognitive impairment and dementia (VCID) account for up to 30% of dementia cases worldwide, representing an important public health challenge. Normal cognitive function requires adequate cerebral blood flow (CBF) regulation. Therapeutical strategies aimed at rescuing age-related CBF dysregulation can delay or mitigate the incidence of VCID. Time-restricted eating (TRE) is an accessible alternative to caloric restriction that has demonstrated benefits on cardiometabolic outcomes. However, the effect of TRE on cerebrovascular function in older adults remains unexplored. Therefore, this pilot study aimed to investigate whether TRE improved cerebrovascular function and metabolic profile in heathy older adults. Participants were instructed to maintain a 10:14 intermittent fasting regimen daily for 6 months. Outcomes of interest were changes from baseline to endpoint in neurovascular coupling (NVC) response assessed with near-infrared spectroscopy and metabolic profile assessed using metabolomics analysis. Six participants (100% female, mean age: 59±5 years) completed the pilot study. Six months of TRE were associated with a significant increase in NVC responses (n=5) in the dorso-lateral prefrontal cortex, the region relevant to the cognitive stimulation paradigm. TRE was also associated with an increase in the levels of tetrahydrofolate, L-arginine and nicotinamide, metabolites involved in vascular health (n=5). TRE resulted in an increase in several metabolites directly implicated in antioxidant, anti-inflammatory, or anti-senescence pathways in the vasculature. Further, principal component analysis revealed a distinct clustering of the before and after TRE data. Our limited dataset indicates that TRE may improve NVC and the availability of metabolites relevant to vascular health in older adults.

Abstract citation ID: igae098.3957

### EFFECTIVENESS OF A NOVEL MEDITERRANEAN DIET APP IN ENHANCING ADHERENCE: RCTS BASED ON SCT

Yan Su[1], and Oleg Zaslavsky[2], *1. University of Massachusetts Darmouth, Dartmouth, Massachusetts, United States*, *2. University of Washington, Seattle, Washington, United States*

Adherence to the Mediterranean diet is a proven nutritional intervention that supports healthy aging and reduces chronic disease risk among community-dwelling older adults. Two pilot RCTs were conducted to evaluate the feasibility and efficacy of a novel mobile health intervention aimed at enhancing adherence to this diet among older users. A total of 34 participants, aged 56-88, were randomized into intervention and control groups. The intervention group used a mobile app for 3 months, receiving feedback on food choices, personalized recipe recommendations, and in-app messaging. Outcomes measured included adherence to the Mediterranean diet, five Social Cognitive Theory (SCT) constructs, and Mediterranean diet knowledge. Linear mixed models (LMMs) showed the intervention group had a significantly greater improvement of 1.52 points in Mediterranean diet adherence compared to the control group after the intervention ($P<.05$). The intervention group also increased their whole grain intake by 1.53-ounce equivalents more than the control group ($P<.01$) and their legume intake by 0.14-ounce equivalents more than the control group ($P<.01$). Additionally, the intervention group reduced their red meat consumption by 0.67 ounces more than the control group ($P<.05$). However, when each SCT construct or Mediterranean diet knowledge was included in the models individually, none were significant predictors, nor did they affect the group-time interaction, suggesting these factors did not mediate the intervention's effectiveness. The novel mobile intervention effectively improved dietary adherence among community-dwelling older adults, likely due to the combined influence of multiple behavior change techniques or other factors not captured by SCT.

Abstract citation ID: igae098.3958

### EFFECTIVENESS OF A WEB-BASED INTERVENTION FOR LEWY BODY DEMENTIA CAREGIVERS: IMPACT BY DEPRESSION STATUS

Yan Su[1], Annie T. Chen[2], and Oleg Zaslavsky[2], *1. University of Massachusetts Darmouth, Dartmouth, Massachusetts, United States*, *2. University of Washington, Seattle, Washington, United States*

Lewy Body Dementia (LBD) affects 1.4 million Americans, with 80% of care provided by family members who often face significant mental health challenges. Few digital interventions are tailored specifically for LBD or offer a fully remote, asynchronous format. This pilot study evaluated the effectiveness of an 8-week web-based intervention among 39 LBD caregivers, comparing its impact on those with and without depression. The intervention included online discussions on caregiving and problem-solving skills, moderated by a trained facilitator, with participants required to post at least twice weekly. Measurements were taken at baseline, post-intervention, and one month later. Of the 39 caregivers, 21 were classified as having depression, and 18 as without depression. Among those with depression, the intervention significantly reduced depressive scores from baseline to post-intervention ($d$ = –1.35, 95% CI: –3.36, –0.61) and to follow-up ($d$ = –2.01, 95% CI: –3.36, –1.25). Reductions in burden ($d$ = –0.47, 95% CI: –1.04, –0.02) and perceived stress ($d$ = –1.12, 95% CI: –2.05, –0.54) were also observed, with sustained stress reduction at follow-up ($d$ = –1.33, 95% CI: –3.17, –0.36). Improvements in self-efficacy ($d$ = 0.48, 95% CI: 0.00, 1.32) and social support ($d$ = 0.71, 95% CI: 0.08, 1.38) were noted from baseline to follow-up. In contrast, caregivers without depression showed no significant improvements in these areas, except for a modest increase in social support at follow-up ($d$ = 0.33, 95% CI: 0.10, 0.57). This study highlights the potential of the intervention to effectively support LBD caregivers, particularly those with depression.

Abstract citation ID: igae098.3959

### EFFECTIVENESS OF INTERVENTIONS FOR CHRONIC DISEASE MANAGEMENT AMONG OLDER ADULTS IN THAILAND: A META-ANALYSIS

Noppawan Piaseu, and Phatcharaphon Whaikid, *Mahidol University, Bangkok, Krung Thep, Thailand*

This study aims to assess effectiveness of interventions in managing chronic diseases in older Thais. We searched PubMed, Embase, Scopus, and Thai-Journal Citation Index Centre databases. This systematic review included articles published between 2018 and 2023, focusing on randomized controlled trials or quasi-experimental designs. Research quality was assessed using JBI critical appraisal tools. Meta-analysis employed JBI-SUMARI software. The protocol was registered on PROSPERO under identification code CRD42023475031. Thirty-seven articles met inclusion criteria, showing a significant effect favoring interventions. Systolic blood pressure (SBP) decreased by -12.27 mmHg (MD), 95% CI: -16.06, -8.47), and diastolic blood pressure (DBP) by -7.53 mmHg (95% CI: -9.72, -5.34). Subgroup analyses indicated a significant decrease in SBP due to self-management (MD -16.79 mmHg, 95% CI: -25.21, -8.38), lifestyle modification (MD -16.40 mmHg, 95% CI: -22.24, -10.56), physical and exercise (MD -8.77 mmHg, 95% CI: -13.02, -4.51), and application use (MD -3.49 mmHg, 95% CI: -5.73, -1.25). DBP decreased with self-management (MD -9.34 mmHg, 95% CI: -10.95, -7.72), lifestyle modification (MD -9.88 mmHg, 95% CI: -15.29, -4.47), physical and exercise (MD -4.65 mmHg, 95% CI: -6.51, -2.78), and application use (MD -4.09 mmHg, 95% CI: -5.92, -2.25). T2DM management decreased fasting blood sugar (SMD -0.78, 95% CI: -1.27, -0.311) and HbA1c (SMD -0.56, 95% CI: -0.81, -0.311). Lifestyle modification (SMD -0.70, 95% CI: -1.17, -0.24) and physical and exercise (SMD -0.46, 95% CI: -0.80, -0.13) decreased HbA1c. Self-management is effective in reducing BP in older Thais with hypertension, while lifestyle modification proves optimal for those with T2DM.

Abstract citation ID: igae098.3960

### EFFECTS OF ACUTE EXERCISE AND A DUAL COGNITIVE TASK ON A NOVEL CIRCULATING BIOMARKER

Madison Croucher, Jennifer R. McCall, Kathryn Sausman, Lidio L. de Albuquerque, Tamatha Arms, and Matthew Peterson, *University of North Carolina Wilmington, Wilmington, North Carolina, United States*

This study reports on the effect of aerobic exercise, with or without a concurrent cognitive task on circulating levels of beta hydroxybutyric acid (b-HBA) and glucose in older adults with normal cognition.

**Methods:** Two bouts of exercise one week apart were performed to examine pre-post b-HBA and glucose in, 1) 10-minutes of stationary bicycling, and 2), 10-minutes of stationary bicycling with a cognitive challenge. Variables included cognitive screening (MoCA), fasting blood samples, health status, physical performance (SPPB), hand function, and depressive symptoms.

**Results:** N=10 female participants were 90% Caucasian, 71.6 +/-4.3 years old with 3.2 +/-2.1 comorbidities, normal cognitive functioning (MoCA=27 +/-1.5) and good physical performance (SPPB 11.1 +/-1.1). There was a clinically meaningful positive association between change in circulating concentrations of b-HBA and MoCA scores with the dual task condition (r=0.32). Glucose was utilized more in the exercise only task compared to the dual condition task (Visit 2 – Visit 1 difference: mean increase of 9.5% +/- 16.4% in pre-post % change), whereas b-HBA was depleted in the dual task condition (Visit 2-Visit 1 difference: mean decrease of 17.0% +/- 33.4% in pre-post % change). Both b-HBA and glucose concentrations changes had moderate effect sizes (Cohen's D=0.51 and 0.57, respectively). Conclusions: This study provides proof of concept for differing energy substrate utilization, when the brain is tasked with a cognitive challenge added to an already moderately intense whole-body cycling exercise. Future studies will objectively quantify energy substrate utilization in the brain using this experimental design and imaging studies.

Abstract citation ID: igae098.3961

### EFFECTS OF AN IMMERSIVE VIRTUAL REALITY TAI CHI INTERVENTION ON THE MENTAL HEALTH OF OLDER ADULTS

Jaehyun Kim[1], Marcos Ardon Lobos[1], Serim Park[2], Richard Kim[3], Jinsil Seo[2], and Junhyoung Kim[2], *1. East Carolina University, Greenville, North Carolina, United States*, *2. Texas A&M University, College Station, Texas, United States*, *3. St. Mark's School, Southborough, Massachusetts, United States*

A significant proportion of the older population (approximately 1 in 5) suffers from diagnosed and sub-clinical mental health conditions. However, evidence indicates that older adults are less likely to seek mental health services than younger adults, primarily due to stigma and limited mental health literacy. As researchers have highlighted the urgent need for innovative solutions in geriatric mental health care, we explored the effectiveness of the use of an immersive virtual reality Tai Chi intervention (IVTC) as a potential solution. Eighteen older adults from senior centers in North Carolina, United States participated in eight 15-minute IVTC sessions over a month and completed surveys at the start (T1) and end (T2) of the program. Analysis using two-tailed paired t-tests revealed significant reductions in depressive symptoms following participation in the IVTC ($t(17) = 4.594$; $p < .001$; SE =.12), with a large effect size ($d = 1.10$). Additionally, perceived stress levels were significantly decreased after participation ($t(17) = 2.541$; $p < .001$; SE =.09), showing a moderate effect size ($d = .60$). Participants also reported high usability scores, indicating a favorable response to use of the IVTC program. These findings suggest that the IVTC program effectively reduces the stress and depressive symptoms of older adults, highlighting its potential as an innovative, technology-based intervention for those at higher risk of developing mental disorders. Study findings also provide important implications for future research and emphasize the need for further exploration of IVTC as a promising approach to geriatric mental health care.

Abstract citation ID: igae098.3962

### EFFECTS OF IZUMIOTSU CITY'S COGNITIVE DECLINE PREVENTION DANCE AS A STRUCTURED ACTIVITY IN ADULT DAY SERVICES

Atsuko Miyazaki[1], Kaori Ueki[2], and Atsushi Hiyama[3], *1. The University of Tokyo, Tokyo, Japan*, *2. TOPPAN Inc., Tokyo, Japan*, *3. Hitotsubashi University, Kunitachi, Tokyo, Japan*

This study examined the effects of an innovative cognitive decline prevention dance program in adult day services. Developed through a community co-creation living lab in Izumiotsu City, Japan, this Hip-Hop-inspired dance is specifically designed for individuals with mild cognitive impairment (MCI), who often struggle with imitation tasks. The program replaced 40 minutes of a 2-hour high-intensity circuit training session in day services. Twenty-six day service users (3 males, 23 females; mean age 82.62 years, SD=4.09) participated in a 4-month intervention study, divided into a dance group (n=14) and a fitness group (n=12, continuing full circuit training). Results showed significant improvements in the dance group compared to the fitness group in cognitive function tasks, including arithmetic operations ($F(1,21) = 5.65$, $p = 0.018$, $\eta 2 = 0.205$) and spatial memory accuracy ($F(1,21) = 4.23$, $p = 0.036$, $\eta 2 = 0.168$). Postural assessments revealed significant improvements in the dance group, including increased shoulder height symmetry ($F(1,21) = 3.53$, $p = 0.046$, $\eta 2 = 0.143$) and reduced left knee hyperflexion in standing position ($F(1,21) = 19.19$, $p = 0.001$, $\eta 2 = 0.477$), indicating better postural alignment. This dance program is a product of industry-academia-government-civil collaboration, combining university research, municipal policy-making, corporate development, and citizen participation. As a result of this collaborative approach, an effective program tailored to the needs of older adults was created. The significant improvements observed suggest that innovating structured activities in adult day services, through such collaborative efforts, offers a promising approach to addressing health challenges in super-aged societies.

Abstract citation ID: igae098.3963

### EFRAILTY.ORG: ANALYSIS OF INITIAL USERS

Megan Cheslock[1], Stephanie Sison[2], Lily Zhong[3], Natalie Newmeyer[4], Kuan-Yuan (Ken) Wang[4], Andrea Schwartz[5], Ariela Orkaby[6], and Dae Hyun Kim[4], *1. Veterans Affairs-Bedford (Edith Nourse Rogers Memorial Veterans Hospital), Bedford, Massachusetts, United States*, *2. University of Massachusetts Chan Medical School, Worcester, Massachusetts, United States*, *3. University of Connecticut School of Medicine, Farmington, Connecticut, United States*, *4. Hebrew SeniorLife, Boston, Massachusetts, United States*, *5. Harvard Medical School, Boston,*

*Massachusetts, United States, 6. Brigham and Women's Hospital, Boston, Massachusetts, United States*

In March 2024, eFrailty.org was launched to the public with the goal of making frailty assessment tools accessible to clinicians and researchers. We report the uptake and initial user experiences of eFrailty between January 1, 2024 and July 31, 2024, as measured by Google Analytics. eFrailty was viewed over 23,000 times by more than 5,400 users. More than 1000 users (18%) returned after their initial visit. There were 4 distinct spikes in usage coinciding with the airing of a GeriPal Podcast episode, two international geriatrics conferences, and publication of a NEJM review article—all of which mentioned eFrailty. Analytics data indicated the site was accessed from over 60 different countries. The highest number of users were from the United States (2,400 users), followed by China (802), and Canada (522). The most frequently visited site feature was the 'Help Me Choose a Frailty Tool' page, which guides the user through a series of questions to identify an assessment tool that is best aligned to their purpose and available information. Of the 15 validated frailty assessment tools featured, the three most frequently viewed were the CGA-FI (2,105 views), Clinical Frailty Scale (709), and FRAIL Scale (680). User feedback was gathered informally from local and international colleagues using the site, and via e-mails sent through the site's 'Contact Us' link. In response to feedback about the CGA-FI, the authors created a dedicated instruction manual. These initial data suggest the utility of eFrailty website for clinical care, research, and education.

Abstract citation ID: igae098.3964

### ELEMENTS OF DIGNITY FROM THE VIEWPOINT OF STAFF IN LONG TERM CARE FACILITIES AND THE INTERACTION DIGNITY MODEL

Nanako Hasegawa[1], Yukari Niimi[2], Chikako Sone[3], Eriko Otake[4], and Cynthia Jacelon[1], *1. University of Massachusetts Amherst, Amherst, Massachusetts, United States, 2. Nagoya Women's University, Nagoya, Aichi, Japan, 3. Nagano College of Nursing, Komagane, Nagano, Japan, 4. National College of Nursing, Japan, Kiyose, Tokyo, Japan*

Japan has the highest proportion of older people of any country in the world. Culturally, it is important that older adults who enter long term care (LTC) facilities maintain their dignity as they did in their previous lives. However, providing dignified is challenging. Lack of dignity in care threatens not only the dignity and health of residents, but also that of staff. To contextualize and visualize elements of dignity from the perspective of staff, and using a hermeneutic phenomenological prospective approach, we conducted individual and follow-up interviews (18 interviews total) with 9 staff members (4 nurses and 5 certified care workers; age 25–43) working in LTC. Interviews were recorded and transcribed verbatim. Codes were extracted and organized into subthemes and themes. Member checking, blind checking, and a panel of seven judges were used to reduce bias and improve the validity of the findings. Two themes emerged as the core of individual dignity: "Individual dignity not affected by others" and "The individual dignity of the staff." Three themes were identified as representing the interactions between staff and society: "Dignified care in the narrow sense," "Dignified care by LTC facilities and the LTC system," and "Dignity in relation to family members, friends, society, and other residents". Identifying the "individual dignity of the staff" was identified as a significant finding. Dignity in LTC facilities includes not only dignity of residents but also of the staff, and "Dignified care in the narrow sense" was identified in the interaction between these two.

Abstract citation ID: igae098.3965

### ENHANCING VISUOMOTOR INTEGRATION IN OLDER ADULTS: INSIGHTS FROM A MULTI-FACETED TRAINING PROTOCOL

Edward Ofori[1], and Justin Foster[2], *1. Arizona State, Phoenix, Arizona, United States, 2. Excelling Edge, Phoenix, Arizona, United States*

This investigation rigorously assessed the impact of an 8-week training regimen on visual-motor integration and balance in older adults, particularly focusing on individuals diagnosed with Parkinson's Disease (PD). Seven participants over the age of 50 years (mean age: 62.4 ± 8.6 years for Control, 73.0 ± 5.7 years for PD) underwent pre- and post-intervention evaluations using the Senaptec Sensory Station and Sway Medical systems. The assessments targeted Eye-Hand Coordination (EHC), Multiple Object Tracking (MOT), and balance metrics. Participants engaged in weekly in-person training sessions and bi-weekly at-home exercises designed to enhance dynamic balance, visual-motor integration, and cognitive function. Each at-home session included activation (saccades, brisk walk, dynamic balance), dynamic vision (customized Senaptec app exercises), and a final phase tailored to individual needs, focusing on either eye-hand coordination or parasympathetic nervous system (PNS) activation. Results indicated no statistically significant improvements in balance assessments ($p>0.10$); however, significant improvements were observed in Central EHC within the PD group ($t = 14.35$, $p = 0.04$), with combined analysis across groups also showing significance ($t = 2.73$, $p = 0.03$). For peripheral EHC, the Control group exhibited significant task time improvements ($t = 3.61$, $p = 0.02$), with combined group analysis confirming significance ($t = 3.14$, $p = 0.02$). MOT scores showed the Control group outperforming the PD group ($p < 0.02$, 1712±605°/s vs 893±220°/s). The findings suggest that targeted visual-motor training can mitigate cognitive and motor declines in aging and neurodegenerative conditions, warranting further large-scale studies.

Abstract citation ID: igae098.3966

### EVALUATING FRAILTY INDEX INTEGRITY: INSIGHTS FROM AN INTERNATIONAL NETWORK STUDY

Robert Cavanaugh[1], Chen Yanover[2], Chelsea Wong[3], Tal El-Hay[2], Ariela Orkaby[4], Maytal Bivas-Benita[2], Pinchas Akiva[2], and Brianne Olivieri-Mui[5], *1. Northeastern University, South Portland, Maine, United States, 2. KI Institute, Tel Aviv, Israel, 3. Beth Israel Deaconess Medical Center, Brookline, Massachusetts, United States, 4. Brigham and Women's Hospital, Boston, Massachusetts, United States, 5. Northeastern University, Boston, Massachusetts, United States*

Deficit-accumulation Frailty indices (FIs) are a key tool in population-level translational geroscience approaches to understanding function, health, and aging. International applications of Fis have been enabled by the uptake of common data models (CDMs) but the validity of such applications is not guaranteed. We compared frailty prevalence using two validated electronic health record based FIs - the United States (US)-based Veterans Affairs FI (VAFI) and United Kingdom (UK)-based electronic FI (eFI) across 5 Observational Medical Outcomes Partnership CDM databases. Frailty was calculated by age for patient-records 40+ years old with 1+ years of data in the 1) US All of Us database (n=211,568), 2) US IQVIA Pharmetrics+ (n=5,292,854), 3) UK IMRD-THIN (n=3,080,557), 4) UK IMRD-EMIS (n=843,928), 5) UK BioBank (n=209,566). Code and results are available in an open-source R package and interactive website. The VAFI identified higher prevalence of frailty in US databases (All of US: VAFI 10.3% v eFI 2.5%, Pharmetrics+: 9.6% v 2%) while the eFI identified higher prevalence in UK databases (IMRD-THIN: < 0.003% v 0.08%, IMRD-EMIS: 0.09% v 0.4%, UK BioBank: 0.03% v 0.1%). eFI prevalence was lower than previously reported (2.2% v 15-20%, >65 years old; Clegg et al., 2016). FIs appear to be dependent on their development context, limiting external validity for international network studies. FIs must be vetted for consistency and validity when used across CDM sources if they are to contribute to the study of health and aging at scale. Future work will extend this comparison to additional international data sources.

Abstract citation ID: igae098.3967

### EVALUATION OF PRECLINICAL ALZHEIMER'S DEMENTIA USING PLANTAR PRESSURE REGULATION ABILITY

Jieun Yoon[1], Kazuki Ota[1], Tetsuaki Arai[2], Miyuki Nemoto[3], Kiyotaka Nemoto[2], Miho Ota[2], Mariko Miyashita[3], and Tomohiro Okura[1], *1. Uiversity of Tsukuba, Tsukuba, Ibaraki, Japan*, 2. *Institute of Medicine/University of Tsukuba, Tsukuba, Ibaraki, Japan*, 3. *Tanita Corporation, Itabashi, Tokyo, Japan*,

Strong connectivity between peripheral sensors and the central nervous system assists in cognitive load distribution. Aging and neurological disorders, including various peripheral neuropathies cause decreased plantar skin sensitivity. The early detection and prevention of Alzheimer's disease, particularly during the preclinical period, is highly important. The preclinical Alzheimer's cognitive composite 5 (PACC5) test formula is an effective test for assessing the preclinical AD period. However, completing the PACC5 is time consuming and psychologically and physically challenging for 60- to 87-year-old patients. We developed a plantar pressure performance test to evaluate the correlation between PACC5 scores and graded plantar distributions in the preclinical period. Participants (n = 210) aged 60–87 years (mean: 71.0 ± 7.0) provided informed consent. PACC5, gap_total, gap_downslope, gap_upslope, and error (deviation from the target value) values for each relaxing- and exerting-force movement of the legs were evaluated. We used the BM-220 (prototype, Tanita Co. Ltd) for analyzing plantar pressure. The participants were divided into three groups (upper, middle, and lower) based on plantar pressure. Group differences were assessed using Spearman's correlation and the Kruskal-Wallis tests, with $P < 0.05$ considered statistically significant. All items showed a weak negative correlation value trend ($R = -0.370$ to $-0.234$). PACC5 scores among the upper, middle, and lower groups (differences ordered from lowest to high) showed significant differences, and the PACC5 scores of the lower group were less than those of the upper group. Thus, plantar pressure movement may serve as a preliminary performance index for preclinical period selection.

Abstract citation ID: igae098.3968

### EVERYDAY CHALLENGES IN OLDER ADULTS WITH TRAUMATIC BRAIN INJURY: A CASE STUDY OF TWO DYADS

Tooba Umar, Cassie Payonk, Olivia Kuhl, Raksha Mudar, and Wendy Rogers, *1. University of Illinois Urbana Champaign, Champaign, Illinois, United States*

Traumatic Brain Injury (TBI) results in various cognitive, somatic, and psychological challenges that affect daily life. We explored self-perceptions of these challenges in older persons with TBI (PwTBI) and their care partners (CareP), using data from two dyads (N=4). We interviewed participants about daily activities and related cognitive challenges in five domains: health, social engagement, transportation, domestic life, and leisure activities. Participants rated the difficulty of activities for the PwTBI on a scale from 1 (very easy) to 5 (very difficult). Thematic and content analyses revealed discordances in perceptions between PwTBI and CareP on several domains in both dyads, with concordance only in diet and nutrition activities in Dyad 1, which are crucial aspects of the health domain. Other activities showed discordances across both participants. In Dyad 1, PwTBI reported cognitive challenges more frequently than their CareP. However, in Dyad 2, the PwTBI reported cognitive difficulties much less frequently during activity ratings. Conversely, their care partner reported cognitive difficulties related to episodic memory and social cognition affecting the domains of health, social engagement, and transportation. These findings underscore the complexity of cognitive challenges faced by PwTBI and the differing perspectives between participants and care partners. The study highlights the need for tailored interventions that address both the challenges expressed by PwTBI and those noticed by their CareP to better support daily functioning and quality of life in older adults with TBI. This dyadic approach provides valuable insights for developing personalized care strategies.

Abstract citation ID: igae098.3969

### EXPERIENCES OF FAMILY CAREGIVERS OF INDIVIDUALS WITH HYPERTENSION AND DIABETES: A META-ETHNOGRAPHY

Ashwag Alhabodal[1], Confidence Francis- Edoziuno[1], Marsha Williamson[2], Wendy Henderson[3], and Ruth Lucas[1], *1. University of Connecticut, Storrs, Connecticut, United States*, 2. *Pace University, New York, New York, United States*, 3. *University of Pennsylvania, Philadelphia, Pennsylvania, United States*

The number of older adults diagnosed with chronic diseases such as hypertension or diabetes mellitus is growing, and their home care is often managed by family caregivers. The purpose of this meta-ethnography is to synthesize qualitative evidence, published between 2014 and 2024, and identify common challenges, motivations, and coping strategies of caregivers. Ten articles were included, conducted in diverse settings across high-, middle-, and low-income countries with 261 caregivers, mostly females. Through Noblit and Hare's approach, five overarching themes were identified: caregiving expertise and knowledge, psychosocial impact, support and coping mechanisms, financial and physical burdens, and intrinsic motivations and values. Many caregivers receive inadequate formal training and resources, which often lead to intense emotional, financial, and physical burdens. These personal burdens were balanced by their strong personal values and motivation to provide care. The voices of caregivers reveal not only the depth of their commitment but also the significant cost caregiving takes on their mental and emotional health. Caregivers speak of isolation and anxiety, as well as the resilience they draw from family bonds and religious beliefs. Their narratives highlight the universal challenges experienced by caregivers globally. However, the availability and quality of support systems may differ widely. Caregivers have a critical role in the management of chronic diseases for their older adult family members, and they require interventions and policy changes to ensure their well-being and the quality of care they provide.

Abstract citation ID: igae098.3970

### EXPLORATION OF VARIABILITY IN STRESS AND SLEEP QUALITY IN OLDER PEOPLE WITH MILD COGNITIVE IMPAIRMENT

Sonoko Kabaya, Chieko Greiner, Yuko Yamaguchi, and Masahide Nakamura, *Kobe University, Kobe, Japan*

Mild cognitive impairment (MCI) is common, affecting 10%-35% of people over 65, since medical technology has advanced. Previous studies have reported that stress in older people with MCI exacerbates cognitive decline and can lead to daily depressive affect contagion between family caregivers (FCs) caring for older people with MCI. They have also indicated that stress adversely affects sleep quality and duration among older people with MCI. However, it is not clear how stress and sleep quality in older people with MCI fluctuate within a day especially throughout night. The aim of this study was to explore the characteristics of variability in stress and sleep quality in older people with MCI. We recruited dyads of older adults with MCI over the age of 65 and FCs. Data collection was performed continuously for 7 consecutive days. We adopted smart watches (GARMIN vívosmart 4) to measure Heart Rate Variability (HRV)-based stress and sleep quality. The Mann-Whitney U test was performed using Python 3.12. Results showed that stress levels were significantly higher during wakefulness compared to nighttime sleep (p=0.05e-89); however, no significant differences were observed based on the depth of sleep (p >.05). This finding is beneficial for considering how to reduce their stress especially during deep sleep and how to improve sleep quality.

Abstract citation ID: igae098.3971

### EXPLORING DIET'S IMPACT ON CARDIOMETABOLIC AND COGNITIVE HEALTH IN AMERICAN INDIANS

Marianne Chanti-Ketterl[1], Jennifer Jones-Locklear[2], Jada Brooks[3], Cierra Williams[1], Jessica Locklear[2], Leslie Strickland-Yanick[2], and Heather Whitson[1], *1. Duke University, Durham, North Carolina, United States*, *2. University of North Carolina Pembroke, North Carolina, United States*, *3. university of North Carolina, Chapel Hill, North Carolina, United States*

**Objective:** This study explores the link between cardiometabolic biomarkers and neurocognition in older American Indians, focusing on diet quality as measured by the Healthy Eating Index (HEI).

**Methods:** Participants provided biological specimens and completed health, diet, and exposome questionnaires in a mobile clinic. Cardiometabolic biomarkers (lipids, glucose) were measured with a Cholestech LDX Analyzer and log-transformed. Neurocognition was assessed using the Montreal Cognitive Assessment (MoCA). OLS regressions, adjusted for age and education, examined the association between biomarkers and neurocognition across HEI tertiles to assess effect modification.

**Results:** Across three months, we enrolled 41 participants, predominantly women (76%), with a mean age of 72 years (SD 7.8) and an average of 12.6 years of education (SD 3.2). HDL-Cholesterol levels were higher in women than in men (n=39, p=0.008). The mean HEI score was 64.3(SD 11.4), with varied diet quality among participants; lowest HEI tertile (44.2-58.1), highest HEI tertile (71.4-84). In the lowest HEI-tertile, higher MoCA scores were associated with higher LDL-C, lower triglycerides, and higher glucose. In contrast, the mid-HEI-tertile showed higher MoCA scores associated with lower TC and lower glucose, but higher LDL-C and triglycerides. For the highest HEI-tertile, higher MoCA scores were associated only with lower triglycerides.

**Discussion:** These preliminary findings suggest the relationship between cardiometabolic markers and cognitive function may depend on diet quality. Understanding the role of nutrition on brain and cardiometabolic health among older American Indians may lead to culturally tailored nutritional interventions to promote brain health in aging. Further analysis of specific nutrients, the microbiome, and the metabolome are forthcoming.

Abstract citation ID: igae098.3972

### EXPLORING FALL RISK MANAGEMENT OF OLDER ADULTS IN A SAMPLE OF RURAL HEALTH CLINICS

Dawn Venema, Yao Yao, and Victoria Kennel, *College of Allied Health Professions, University of Nebraska Medical Center, Omaha, Nebraska, United States*

Fall risk management (FRM) is an important component of primary care for older adults. Rural populations are aging more rapidly than urban ones, increasing the importance of FRM in rural communities. The Centers for Disease Control's (CDC) Stopping Elderly Accidents, Deaths, and Injuries (STEADI) toolkit supports FRM implementation, including fall risk screening, assessing, and intervening. However, factors such as gaps in knowledge, time and financial constraints, and lack of patient engagement can limit FRM implementation. Using questions based on the STEADI toolkit, we surveyed Nebraska Rural Health Clinics (RHCs) to explore current FRM practices, facilitators, and barriers; 27% responded. Eighty-two percent screened older adult patients for fall risk. The percent of responding RHCs that conducted the following assessments for over half of their patients identified at-risk were: home environment (70%), comorbidities (45%), gait/strength/balance (40%), vitamin D (34%), orthostatic hypotension (21%), medication (17%), and footwear (17%). External reporting of quality measures focused on screening, assessment, and care plan documentation. Long-term FRM impacts (fall-related emergency department visits, hospitalizations) were rarely tracked, nor required for external reporting. Facilitators of FRM were availability of rehabilitation therapists in the community, time to conduct screening and assessments, and the Medicare Wellness Visit. Barriers were time to provide interventions, reimbursement, and patient receptivity to FRM activities. We are developing a quality improvement program called Coordinated Action Toward Community Health: RedUce Risk And Limit (CATCH RURAL) Falls to facilitate implementation of the CDC's STEADI toolkit in RHCs for older adults including possible adaptations for FRM in rural communities.

Abstract citation ID: igae098.3973

### EXPLORING TRANSITIONAL CARE UNITS IN ONTARIO CANADA: A QUALITATIVE STUDY

Katherine McGilton[1], Alexandra Krassikova[2], Lydia Yeung[2], Sandra McKay[3], Martine Puts[4], Sara Guilcher[4], Kerry Kuluski[4], and Jennifer Bethell[1], *1. Toronto Rehabilitation Institute – University Health Network, Toronto, Ontario, Canada*, *2. KITE Research Institute, Toronto, Ontario, Canada*, *3. VHA Home HealthCare, Toronto, Ontario, Canada*, *4. University of Toronto, Toronto, Ontario, Canada*

Transitional care units (TCUs) provide short-term, low-intensity, restorative care to older adults who are medically fit to leave the hospital but cannot do so due to various factors, such as the availability of home care support or long-term care beds. TCUs are similar to skilled nursing facilities in the US, also referred to as intermediate care models, post-acute care or alternate care units depending on the country of origin. In Ontario, Canada, TCUs were introduced in the past decade, and little is known about them. The objectives of this study were to examine the processes of care of TCUs in Ontario employing an exploratory qualitative design. Semi-structured interviews were conducted with seven TCU managers. A five-step inductive thematic analysis adapted from Braun and Clarke was applied to the data to identify themes. Interviewees were on average 43 years of age; four identified as men and three as women. Years of experience as a TCU manager at the time of the interview ranged from 2 months to 5 years. Four themes were identified: a) going above and beyond; 2) managing patients' expectations; 3) creating a team that works together; 4) navigating a constantly changing environment. Qualitative findings demonstrate the important role of TCUs in helping patients transition from acute care to other settings. TCUs are filling in a gap in the healthcare system of Ontario and need to be recognized as a core service. Qualitative findings may be used by healthcare organizations and unit managers to guide the operation of TCUs.

Abstract citation ID: igae098.3974

### FACTORS ASSOCIATED WITH LONGITUDINAL DIGITAL SURVEY ENGAGEMENT IN THE ELECTRONIC FRAMINGHAM HEART STUDY

Yuankai Zhang[1], Chathurangi Pathiravasan[1], Jian Rong[2], Belinda Borrelli[3], Jamie Faro[4], Xuzhi Wang[1], Chunyu Liu[1], and Joanne Murabito[2], *1. Boston University School of Public Health, Boston, Massachusetts, United States*, *2. Boston University Chobanian & Avedisian School of Medicine, Boston, Massachusetts, United States*, *3. Boston University Henry M. Goldman School of Dental Medicine, Boston, Massachusetts, United States*, *4. University of Massachusetts Chan Medical School, Worcester, Massachusetts, United States*

Smartphone surveys are increasingly used in health research, but factors affecting long-term survey response among older adults, are not well understood. We conducted a study of 611 participants (mean age 74 years, 57% women, 86% White) from the electronic Framingham Heart Study to identify sociodemographic, health-related, and technological factors associated with survey response over 32 weeks assessed longitudinally across four 8-week periods. The same number of surveys were sent to the participants with one group receiving half of surveys every 2 weeks and the other group receiving all surveys every 4 weeks. The outcome was the proportion of surveys each participant returned during each period (up to 22 surveys of 20 unique types were sent during each period). Multivariable mixed-effects regressions with random intercepts examined associations between multiple factors and response rates, adjusting for age group (≤75 versus >75 years), sex, and time periods. Lower depressive symptom score (20-item CES-D; $\beta$=-0.04, p-value=0.028), younger age group (≤75 years; $\beta$=0.08, p-value=0.009), and use of iPhone (vs. Android; $\beta$=0.24, p-value< 0.001) were significantly associated with higher response rates. Significant effect modification by time periods (p-value< 0.05) indicated that over time, non-drinkers, iPhone users, and the 2-week survey group had less decline in response rates compared to their counterparts. Findings suggest that sociodemographic, health-related, and technological factors significantly influence digital survey engagement among older adults, with interactions between these factors and time. This highlights the need for targeted strategies to improve long-term engagement, particularly among older participants and those with higher depressive symptom scores.

Abstract citation ID: igae098.3975

### FACTORS ASSOCIATED WITH OBJECTIVELY MEASURED LIFE-SPACE AND GREEN SPACE IN AGING ADULTS

Michelle Odden[1], Annabel Tan[1], Sylvie Dobrota Lai[1], Sheila Mwanda[1], David Rhodes[2], Suzanne Judd[2], and Vishnu Ravi[1], *1. Stanford University, Palo Alto, California, United States*, *2. University of Alabama Birmingham, Birmingham, Alabama, United States*

Life-space is an important aging health outcome, and measurement of the life space allows for a better characterization of participants' environments than their home address. We developed a mobile phone application to passively capture life-space in older adults and sampled Google street view images from participants' life-space to characterize the environments where they spent their time. We recruited 82 participants aged 65 and older from the REasons for Geographic And Racial Differences in Stroke (REGARDS) study. They were followed for 2 weeks and invited to complete nightly mobile questionnaires. We measured features of the built environment that capture walkability (sidewalks, benches, streetlights), neighborhoods (houses), and green space. Among 82 participants, 76 completed all 14 days of data collection. Median daily maximum distance from home was 6.6 km (IQR: 1.3 to 15.5, range: 0 to 5074.9 km). Median distance traveled over two weeks was 477.1 km (IQR: 234.4, 817.13; range: 39.1 to 31,743.1 km). Adults younger than 70 years, women, Black participants, and those with income >$75,000 per year had larger life-space and traveled further from home. Engagement in exercise, number of chronic conditions, and depressive symptoms were not associated with life-space metrics. Participants with 2 or more chronic conditions spent less time in green space, although the effect sizes did not reach statistical significance after multivariable adjustment (standardized effect sizes -0.61 (95% CI: -1.35, 0.14) and -0.05 (95% CI:-0.11, 0.01), respectively). In summary, there was wide variation in objectively-measured life-space and we found sociodemographic patterning of life-space movement.

Abstract citation ID: igae098.3976

### FACTORS IN ABDOMINAL OBESITY, SARCOPENIA, AND SARCOPENIC OBESITY AMONG KOREAN ELDERLY: KNHANES 2022

Do Thi Thu Huyen, Xianqi Gao, and Moonkyoung Park, *Chungnam National University, Daejeon, Republic of Korea*

**Background:** Abdominal obesity, sarcopenia, and sarcopenic obesity are prevalent among the elderly, contributing to significant health risks. Understanding the nutritional, lifestyle, and biomarker profiles associated with these conditions is crucial for developing targeted interventions.

**Purpose:** This study explores the associations between nutritional intake, lifestyle factors, and biomarkers with abdominal obesity, sarcopenia, and sarcopenic obesity among Korean elderly individuals, using data from the 2022 Korea National Health and Nutrition Examination Survey (KNHANES).

**Methods:** The analysis included 845 participants (293 normal, 360 with abdominal obesity, 160 with sarcopenia, and 32 with sarcopenic obesity). Variables analyzed included hand grip strength, body composition, dietary habits (e.g., caloric intake, protein intake, supplement use), lifestyle factors (e.g., physical activity, sedentary behavior, smoking), psychological factors (e.g., depression, anxiety, stress), and biomarkers (e.g., hsCRP, cholesterol, triglycerides, blood pressure). Statistical analyses were conducted using SPSS 29.0, applying descriptive statistics and logistic regression within a complex sampling design.

**Results:** The prevalence of abdominal obesity, sarcopenia, and sarcopenic obesity was 34.7%, 43.6%, 18.0%, and 3.8%, respectively. Sarcopenia was more prevalent among males, those aged 75 and older, and those living alone. Abdominal obesity was more common among those with hypertension or diabetes. Sarcopenic obesity was associated with living alone, high hsCRP, stress, and low physical activity.

**Conclusion:** Comprehensive nutritional and lifestyle assessments are essential in managing abdominal obesity, sarcopenia, and sarcopenic obesity among the elderly. Public health strategies that improve diet quality and promote physical activity may mitigate these conditions.

Abstract citation ID: igae098.3977

### FALLS PREDICT FASTER PROGRESSION OF ALZHEIMER DEMENTIA

Audrey Keleman[1], Melody Li[2], Rebecca Bollinger[2], Melissa Krauss[2], Elizabeth Grant[2], John Morris[2], and Susan Stark[2], *1. Department of Veterans Affairs, St. Louis, Missouri, United States*, *2. Washington University in St. Louis, St. Louis, Missouri, United States*

Preclinical Alzheimer disease (AD) is a decades-long period in which pathological changes accumulate in the brain (e.g., amyloid plaques) but individuals remain cognitively unimpaired (CU; Clinical Dementia Rating®[CDR®]=0). Motor impairments such as falls have been associated with preclinical AD and may offer early intervention opportunities. However, as aging affects cognition, motor function, and falls, differentiating between trajectories of healthy aging and preclinical AD is complex. This longitudinal cohort study investigated the impact of falls and preclinical AD on dementia symptom onset. We prospectively monitored falls for 1 year among 124 CU older adults and assessed preclinical AD status using amyloid positron emission tomography. We followed this cohort for 12 years and evaluated CDR annually. The cohort was grouped by baseline preclinical AD and faller status: 34 Preclinical AD-/Fall- ("healthy agers"), 53 Preclinical AD-/Fall+, 15 Preclinical AD+/Fall-, and 22 Preclinical AD+/Fall+. Kaplan-Meier survival analysis examined time to progression to CDR 1 (mild dementia) by group. Participants were on average 74 years old at baseline, 78% female, and 96% White with no significant demographic differences between groups. Preclinical AD+/Fall+ individuals progressed to CDR 1 most rapidly, while Preclinical AD-/Fall- ("healthy agers") progressed least quickly. Preclinical AD+/Fall- and Preclinical AD-/Fall+ groups had similar progression rates. Each group's proportional hazard of dementia progression differed significantly from healthy agers, unadjusted and controlling for age and gender (ps< 0.5). These findings suggest falls associate with faster progression of AD dementia, warranting further research to disentangle motor function and falls in healthy agers and those with preclinical AD.

Abstract citation ID: igae098.3978

### FEASIBILITY AND ACCEPTABILITY OF A TELEHEALTH-BASED GROUP INTERVENTION FOR VETERANS WITH MCI

Katherine Luci[1], Patricia Pilkinton[2], Deanna Dragan[3], M. Lindsey Jacobs[4], Hannah Apostolou[5], Allyshia Hinton[6], Jamie Tedder[7], and Teresa Granger[2], *1. Salem VA Healthcare System, Salem, Virginia, United States*, *2. Tuscaloosa VA Medical Center, Tuscaloosa, Alabama, United States*, *3. Salem VAMC, Salem, Virginia, United States*, *4. The University of Alabama, Tuscaloosa, Alabama, United States*, *5. University of Alabama, Northport, Alabama, United States*, *6. Tuscaloosa Research Education and Advancement Corporation, Tuscaloosa, Alabama, United States*, *7. James H. Quillen VA Medical Center, Johnson City, Tennessee, United States*

MInds Navigating the Diagnosis of Mild Cognitive Impairment (MiND-MCI) is a nine-session telehealth intervention developed for older Veterans experiencing stress related to MCI. MIND-MCI targets memory and thinking abilities, stress/coping, and lifestyle risk factors through peer support, education, and skills training. This DoD-funded study aims to evaluate the feasibility, acceptability, and preliminary effectiveness of MIND-MCI to improve QoL among Veterans with

MCI. At baseline, participants (n = 16) in this pilot study were ages 61-79 years old and scored in the mild cognitive impairment range (M = 22.8) on the Montreal Cognitive Assessment. Retention rates (88%) and attendance (M = 7/9 sessions) of study completers suggest feasibility and acceptability of the intervention. Eleven participants demonstrated high engagement (attended ≥ 75% of sessions). Participants reported a high level of acceptability (120/147 total score possible) on the Telehealth Usability Questionnaire, and a majority of participants indicated that they agree or completely agree with the acceptability of the intervention on two additional measures (Acceptability of Intervention Measure and the Intervention Appropriateness Measure). Preliminary feedback was obtained from study interventionists to identify barriers and facilitators for delivering this treatment over telehealth. Themes included: range of severity of MCI across participants, session interruptions and delays related to troubleshooting technological issues, strategies used to support older adults' use of a telehealth platform, and the importance of peer support. Preliminary results support strong engagement with and acceptability of telehealth modalities in this older adult population, a population often presumed to lack telehealth capability and proficiency.

Abstract citation ID: igae098.3979

### FEASIBILITY AND ACCEPTABILITY OF GAMIFIED CYCLING EXERCISE IN A LONG-TERM CARE HOME: A QUALITATIVE STUDY

Lillian Hung, Yong Zhao, Michelle Lam, Jamie lam, Arwen Fong, Tiffany Wu, Michelle Xiao, and Haopu (Lily) Ren, *University of British Columbia, Vancouver, British Columbia, Canada*

Gamification can motivate older adults to exercise by transforming physical activities into enjoyable experiences. Incorporating gaming elements in cycling exercises can foster a sense of interest and achievement, potentially improving health outcomes. This study investigated the acceptability and feasibility of motivating residents living in long-term care homes with a gamified cycling exercise. Fourteen residents (13 living with dementia) completed a 4-week gamified cycling exercise twice a week. Safety during exercise was addressed by monitoring heart rate and symptom observation. With an interpretive description approach, we conducted interviews with residents and family members and focus groups with staff and leadership. The thematic analysis identifies three themes representing the feasibility and acceptability of gamified cycling exercise among LTC residents: ease of use and accessibility, physical and mental health benefits, fun engagement and community building. Future research should explore dementia-friendly design, culture-related game content, family orientation and engagement, group exercise and organization support. This study showed promising acceptability and feasibility of gamified cycling exercise in a long-term care home. Successful implementation relies on tailoring interventions to meet residents' specific needs and preferences while acquiring rapport with interdisciplinary staff in the care home.

Abstract citation ID: igae098.3980

### FEASIBILITY OF SMART AMBIENT BRIGHT LIGHT (SABL) FOR NURSING HOMES RESIDENTS WITH DEMENTIA

Yo-Jen Liao, Ying-Ling Jao, and Marie Boltz, *The Pennsylvania State University, University Park, Pennsylvania, United States*

Ambient bright lighting interventions have shown some positive benefits on nursing home (NH) residents with dementia. A feasible implementation approach and stakeholder buy-in have not been well evaluated. This study examined NH staff's perspectives on the feasibility of implementing the Smart Ambient Bright Light (SABL). The SABL was installed in participants' bedrooms and common areas in two NHs to automatically deliver bright light during the day and dim light during the night. This convergent mixed methods study enrolled 22 participants, including nursing, activity, maintenance, and administrative staff. Quantitative data were collected using the Acceptability of Intervention Measure, Feasibility of Intervention Measure, and Intervention Appropriateness Measure. Qualitative data were collected via semi-structured interviews and analyzed using content analysis. Quantitative results showed good feasibility (mean=4.4, SD=0.51), acceptability (mean=4.1, SD=0.9), and appropriateness (mean=4, SD=0.7) on a 1-5 scale. Qualitative results were categorized in installation, light brightness, automatic system, and resident responses categories. Maintenance staff reported that installing the SABL was relatively easy. Brightness wise, some reported that the lights were too bright while others thought it made people more awake during the day. Most staff reported that the lighting system was easy to use while others preferred manual control to better accommodate residents' various routine. Some reported that residents showed improved neurobehavioral symptoms, sleep, mood, and engagement. Overall, staff perspectives on SABL were positive, but some improvements to the light brightness and automatic system are needed. Limitations on the facility's building structure and lighting fixtures should be considered in future studies.

Abstract citation ID: igae098.3981

### FEASIBILITY OF USING AN E-INTERVENTION PLATFORM FOR LONGITUDINAL RESEARCH ON DEMENTIA CARE DYADS

Constanca Paul, and Soraia Teles, *University of Porto, Porto, Portugal*

Remote Measurement Technologies (RMT) are increasingly used in research, offering cost-effective and unobtrusive data collection. This study examines the feasibility of using the e-intervention "iSupport-Portugal," adapted from WHO's "iSupport" for longitudinal research on dementia care dyads. A prospective cohort study was designed to predict the institutionalization of persons with dementia (PwD) at 9 months using baseline data from informal caregivers (IC) registering on isupport-portugal.pt. Participation rates, loss to follow-up, and event rates were analyzed. Completers and non-completers of baseline and follow-up assessments were compared on sociodemographic and psychosocial variables. In 16 months, 253 IC registered on isupport-portugal.pt, with 82% agreeing to research. Most are women (82.6%), middle-aged (M=51.6), and children of the PwD (70.0%). The baseline protocol was completed by 52.9% of IC. Baseline participation rate was higher among spouses/children of PwD compared to other IC (46.5% vs. 27.5%; $\chi^2(1, N=253)=4.194$, $p=.041$). Compared to caregivers lost to follow-up, completers

(65.3%) were significantly older (M 54.5 vs. M 46.7, t(174) =–4.220, p< 0.001), more often male (89.7% vs. 60.3%; $\chi^2$(1, N=175)=7.950, p=.005), children/spouses of PwD (69.9% vs. 34.8% others, $\chi^2$(1, N=176)=9.413, p=.002), cohabitating with PwD (82.4% vs. 60.0%, $\chi^2$(1, N=118)=6.197, p=.013), and had cared longer (Mdn 43 vs. Mdn 18; U=847, p=.008). The institutionalization rate was 7.8% at 9 months. iSupport-Portugal is a pioneering e-program exploited as an RMT that enabled cost-effective data collection from IC across national territories. High loss to follow-up calls for innovative retention strategies, especially for younger IC, which may intersect with platform user experience. Funding:FCT (2022.07587.PTDC)

Abstract citation ID: igae098.3982

### FEASIBILITY OF WHEY PROTEIN SUPPLEMENTATION TO IMPROVE OLDER ADULT FUNCTION POST-HOSPITALIZATION

Michelle Yang, Pratik Gongloor, Rachel Deer, Elena Volpi, Shuang Li, and Erin Hommel, *University of Texas Medical Branch, Galveston, Texas, United States*

Sarcopenia, the age-related involuntary loss of muscle mass and function, is frequently accelerated following hospitalization. Whey protein supplementation may reduce sarcopenia and improve functional recovery. We performed a pilot randomized-controlled trial to assess feasibility of providing whey protein to older adults during and following an acute hospitalization with the goal to improve function. Subjects ages 65+ were recruited from a single academic hospital's medical-surgical units. Subjects were randomized into three arms: maltodextrin, whey protein, or collagen supplementation, provided twice daily during hospitalization and for 30-days post-hospitalization. Primary outcomes were recruitment, retention, adherence, and data collection feasibility rates. Secondary outcomes were preliminary efficacy on physical function and body composition. From June 2019 to February 2024, 116 subjects were enrolled, a rate of ~2 per month. Retention was 76.8%. Adherence rates were 58.5% for maltodextrin, 58.6% for whey protein, and 60.6% for collagen. Adherence was significantly higher for subjects completing the study compared to those that withdrew or were re-hospitalized. Data collection feasibility rates were moderate to high with SPPB completed on 100% of participants and DEXA collection on 61% of participants. Preliminarily, there were no statistically significant differences in physical function or body composition between groups. Overall, we demonstrated slow recruitment, low adherence, and moderate retention rates in this study of adults 65+ following an acute hospitalization. A prior trial suggested potential efficacy of whey protein on improving function of frail hospitalized older adults. However, strategies to optimize recruitment, retention, and supplement adherence are necessary before performing further assessment of efficacy.

Abstract citation ID: igae098.3983

### FINANCIAL HEALTH AND WELL-BEING OF RURAL CAREGIVERS OF OLDER ADULTS WITH CHRONIC ILLNESSES

Evans Osei, Nasreen Lalani, Siqi Yang, Sampada Wagle, and Bhagyashree Katare, *Purdue University, West Lafayette, Indiana, United States*

Rural caregivers experience various financial stressors and challenges in providing care to their ill family members. Rural health disparities such as limited access to care, economic opportunities, resources, and support pose increased financial threats and insecurity resulting in poor patient and caregiver outcomes. Our study aimed to explore perceived financial stressors/challenges and their impacts on the self-care and well-being of rural caregivers of older family members with serious chronic illness. Using a purposive sampling approach, we did qualitative interviews among N=20 rural female caregivers caring for their older family member with any serious chronic illness. Interviews were done in-person and online, interview duration was about 30-45 minutes. Data was transcribed and analyzed deductively and inductively using thematic content analysis. Major financial impacts of caregiving included: leaving job or reducing work hours, time constraints on caregivers' self-care, inability to balance family relations and caregiving roles, and difficulty navigating financial processes in the existing healthcare system. These challenges led to cutting down on groceries, reliance on retirement savings, and lack of personal time. Participants faced sleep and diet disruptions, shouldered financial decisions alone with limited or no support, and felt guilty over unfulfilled care recipient wishes due to financial constraints. Adequate self-care, coping, and well-being are associated with improved financial well-being and reduced financial threat. Caregiver support programs should focus on strategies and interventions to improve the financial well-being and overall quality of life of caregivers in the rural communities.

Abstract citation ID: igae098.3984

### FINE TUNING OF A PRETRAINED GAIT MODEL FOR FRAILTY CLASSIFICATION PREDICTION

Laura McDaniel, Ime Essien, Yuxiang Guo, Ayush Gupta, Vineet Shenoy, Peter Abadir, and Rama Chellappa, *Johns Hopkins University, Baltimore, Maryland, United States*

This study presents a multimodal gait model designed to identify the frailty status of individuals based on their gait observed in video footage. Frailty is defined as a clinically recognizable state of increased vulnerability resulting from age-related declines in reserve and function across multiple interrelated physiological systems. These risks manifest as an increased likelihood of falls, hospitalization, need for long-term care, disability, and mortality. Although deep learning has advanced in medical applications, its potential in early frailty detection through gait analysis is limited by the need for extensive data, which is currently limited. To address this issue, we leveraged the OpenGait framework, GaitBase, a pretrained gait model typically used for gait surveillance and recognition applications, by fine-tuning it with medical data for frailty classification. Data were collected using the marker-less Qualisys Motion Capture system, resulting in a dataset of 64 individuals classified by frailty status—non-frail, frail, and pre-frail—according to the Fried Frailty Phenotype. Detectron2 models were used for panoptic image segmentation and detection, generating silhouette and box predictions from video frames. By integrating both silhouette and RGB data, our model enhances robustness and generalization across the three frailty classes. To address the challenges of a small dataset, we employed data augmentation, hyperparameter tuning, and cross-validation to improve classification accuracy. This model aims to advance frailty classification using deep learning, offering a time-efficient method for clinicians to assess frailty. Future steps include incorporating metadata such as height and weight to further enhance prediction accuracy.

Abstract citation ID: igae098.3985

### FRAILTY TRAJECTORIES IN BRAZILIAN COMMUNITY-DWELLING OLDER ADULTS: A 9-YEAR FOLLOW-UP STUDY.

Ruth Melo[1], Daniela Assumpção[2], Meire Cachioni[3], Deusivania Falcão[4], Samila Sathler Tavares Batistoni[5], Flávia Silva Arbex Borim[6], Anita Liberalesso Neri[7], and Mônica Sanches Yassuda[5], *1. University of São Paulo, São Paulo, Sao Paulo, Brazil*, *2. Universidade Estadual de Campinas (UNICAMP), Campinas, Sao Paulo, Brazil*, *3. University São Paulo, São Paulo, Sao Paulo, Brazil*, *4. USP - University of Sao Paulo, USP - University of Sao Paulo, Sao Paulo, Brazil*, *5. University of São Paulo, University of São Paulo, Sao Paulo, Brazil*, *6. Universidade Estadual de Campinas, Universidade Estadual de Campinas, Sao Paulo, Brazil*, *7. State University of Campinas, State University of Campinas, Sao Paulo, Brazil*

This prospective observational study analyzed the factors influencing frailty trajectories among Brazilian community-dwelling older adults over a nine-year follow-up period. Sociodemographic data, self-assessed health, multimorbidities, social support, advanced daily living activities (AADL), cognition, and frailty phenotype were selected for analysis from the Fibra (Frailty in Brazilian Elderly) study database. Incidence was calculated based on new cases (pre-frailty/frailty) at follow-up versus phenotype (robust/pre-frailty) at baseline. Pearson's chi-square test was used for intergroup comparison (i.e., progression status). The relative risk (RR) for frailty progression and reversal was calculated using logistic regression, both in crude (c) and adjusted (a) forms. After nine years, the proportion of older adults classified as frail increased (11.9% to 18.1%), while the proportion of those classified as robust decreased (30.1% to 13.8%). The incidence of frailty and pre-frailty was 22.9% and 49.4%, respectively. Advanced age (RRa:3.41; p=0.001), cognitive impairment (RRa:2.06, p=0.002), illiteracy (RRa:1.70; p=0.012), male sex (RRa:1.68; p=0.044) and non-performance/abandonment of AADL (RRa:1.48; p=0.007) were predictors of worsening/death in follow-up. Only living alone was protective against frailty. After nine years, there was an increase in older adults living with frailty and a reduction in the ones considered robust, with an incidence of frailty of 22.9%. Different factors predicted worsening/death after nine years. Future longitudinal studies should improve the understanding of risk factors for frailty progression.

Abstract citation ID: igae098.3986

### FRAILTY, FALL RISK, AND FEAR OF FALLING POSITIVELY ASSOCIATED IN COMMUNITY-DWELLING OLDER ADULTS

Abigail Tice, Norma Conner, Wei Zhang, Qun Huo, Yingru Li, Christopher Emrich, Rui Xie, and Ladda Thiamwong, *University of Central Florida, Orlando, Florida, United States*

More than one in four older adults fall annually, resulting in 3 million emergency department visits. Frailty, a biological syndrome resulting from declining physiological systems, increases vulnerability to adverse outcomes in older adults. Frailty has been identified as a risk factor for fall occurrence in older adults. Many community-dwelling older adults report heightened fear of falling (FOF), and FOF is recognized as a risk factor for fall occurrence. While frailty is significantly, positively correlated with fall risk and FOF, the relationship between all three measures is less understood. The current objective was to examine relationships between frailty, fall risk, and FOF using the Fatigue, Resistance, Ambulation, Illness, and Loss of weight (FRAIL), Stopping Elderly Accidents, Death, and Injuries (STEADI), and short Fall-Efficacy International (short FES-I) questionnaires, respectively, in 178 older adults 65 years or older in Orlando, Florida. Spearman correlation analysis revealed significant associations between FRAIL and STEADI scores (p< 0.0001, r=0.6291), FRAIL and short FES-I scores (p< 0.0001, r=0.5294), and STEADI and short FES-I scores (p< 0.0001, r=0.7230). Linear regression analysis supports the significant associations between FRAIL and STEADI scores (p< 0.0001, r2=0.4407), FRAIL and short FES-I scores (p< 0.0001, r2=0.3293), and STEADI and short FES-I scores (p< 0.0001, r2=0.4636). The significant positive relationships suggest that as frailty increases, fall risk and FOF increase and that as FOF increases, fall risk increases. This work implicates the importance of screening in older adults to prevent the progression of frailty to reduce fall risk and FOF. Addressing FOF is important to reduce fall risk in older adults.

Abstract citation ID: igae098.3987

### FUNCTIONAL IMPROVEMENT AMONG NURSING HOME RESIDENTS WITH DEMENTIA AFTER SKILLED NURSING FACILITY STAY

Ana Montoya, Pil Park, Megan Jensen, and Julie Bynum, *University of Michigan, Ann Arbor, Michigan, United States*

Dementia is associated with decline in physical function that may be exacerbated during a hospitalization. Physical function is potentially modifiable through physical therapy during a short-term skilled nursing facility (SNF) stay after hospitalization. While many nursing home (NH) residents experience functional improvement after a SNF stay, some experience no improvement or even decline. It remains unclear whether long-stay NH residents with dementia benefit from a SNF stay prior to transitioning back to long-term care. This study aimed to determine if NH residents with dementia are as likely to experience functional improvement in activities of daily living (ADL) as those without dementia after a SNF admission. Using Michigan state Minimum Data Set (MDS) data from 2017-2019, we identified long-stay NH residents with Medicare-covered SNF stay in 2018 and qualifying ADL variables (n=2,127 residents with dementia; n=1,595 residents without dementia). We analyzed NH residents' assessments within 120 days after SNF admission and calculated the Long-Form ADL Self-Performance score. We defined functional improvement as one point decrease in ADL score from the first SNF assessment. Almost two thirds of residents experienced functional improvement (61.2% with dementia vs. 63.3% without dementia; p=0.189). The median time to achieve improvement did not differ between the two groups (16 vs. 14 days, respectively; p=0.063). Cox proportional-hazards models revealed no effect of dementia on functional improvement after incorporating demographic characteristics and clinical indicators (HR=0.94, 95%CI [0.85-1.04]). These results support prior findings that NH residents with dementia achieve similar benefit from a SNF stay compared to those without dementia.

Abstract citation ID: igae098.3988

### GENDER DIFFERENCES IN DEPRESSIVE SYMPTOMS' IMPACT ON CHRONIC DISEASES, FUNCTIONAL LIMITATIONS, AND MORTALITY

Seoyeon Ahn, Abhijit Visaria, Rahul Malhotra, and Angelique Chan, *Duke-NUS Medical School, Singapore, Singapore*

Despite extensive research on chronic diseases, functional limitations, and depressive symptoms at older ages, the patterns of change and interconnectedness among them remain unclear. We examined the trajectories of chronic diseases (cardiometabolic conditions and chronic pain) and functional limitations in individuals aged 60-69 years at baseline and explored how depressive symptoms influenced the onset of chronic diseases, functional limitations, and mortality using data from three waves of the Panel on Health and Aging of Singaporean Elderly (2009-2015; n=386 men and 471 women). We used multichannel sequence analysis to investigate gender-specific health trajectories and random-effects logistic regression to examine depressive symptoms' role in the onset of chronic diseases, functional limitations, and mortality. Inverse probability of attrition weights were used to account for selection bias due to loss to follow-up. Our findings reveal distinct gender-specific health trajectories. Men had higher mortality rates but experienced slower progression in chronic pain and physical functional decline, despite an earlier onset and higher prevalence of cardiometabolic diseases. Women lived longer but experienced earlier onset and faster progression of chronic pain and functional decline. Depressive symptoms were linked with faster onset of cardiometabolic diseases and functional limitations in both genders, and with mortality only in men. Individuals, families, providers, and policymakers should be mindful of the gender differences in the onset and progression of health deterioration and mortality in old age. Our findings also highlight the need for gender-specific research on the complex interactions between chronic diseases, functional limitations, mortality, and depressive symptoms.

Abstract citation ID: igae098.3989

### GENETIC AND SEROLOGICAL BIOMARKERS AS PREDICTORS OF PARKINSON'S DISEASE COGNITIVE IMPAIRMENT: A SYSTEMATIC REVIEW

Hajir Mohammed[1], Mohamed Atif Mohamed Yousif[1], Mohamed Elhassan[2], Yassein Elhussein[3], Dina Badawi[4], Sarra Gabralla[1], and Nadir Abdelrahman[5], *1. Tele-Geriatric Research Fellowship, East Lansing, Michigan, United States*, *2. Royal College of Surgeons Ireland, Waterford, Dublin, Ireland*, *3. Dubai Medical College for Girls (DMCG)), Dubai, United Arab Emirates*, *4. University of Texas Health (UTHealth) Houston, Texas, United States*, *5. Michigan State University, East Lansing, Michigan, United States*

Parkinson's disease (PD) is the second most common neurodegenerative disorder in the elderly population. Cognitive impairment (CI) is one of the most common and important non-motor symptoms in Parkinson's disease and the identification of predictive biomarkers for it would help in its management. Databases were searched systematically down to Jan 2019. Selection criteria focused on studies with serological and genetic biomarkers. Other radiographically or clinically related papers were excluded. Total of 9 studies with 11 different biomarkers were assessed and found to be significantly associated with cognitive decline in PD. These include APOE4 allele mentioned in 2 studies (RR= 2.98), (RR= 7.4). miR-203a-3p/miR-16-5p ratio (AUC= 0.7593), Serum high mobility group box-1 levels (RR= 1.398), ganglion cell inner plexiform layer complex in the macula thickness (RR= 0.553), B12 (RR= 0.793), CSF NfL (HR= 2.82). MIA, CRP and albumin group (HR= 4.3), Neuroexosomal EAAT-2 (P= 0.003), and P16 (P= 0.003). Biomarkers can serve as potential predictive tools. Their integration into routine assessments could enhance the stratification of patients at risk for cognitive impairment, allowing for tailored therapeutic approaches and preventive strategies aimed at mitigating cognitive decline. However, they warrant further investigation to elucidate their roles in the pathophysiology of cognitive impairment in PD. Longitudinal studies are needed to validate these biomarkers and determine their utility in predicting cognitive decline over time. Additionally, exploring the interplay between these biomarkers and other neurobiological factors may uncover novel therapeutic targets.

Abstract citation ID: igae098.3990

### GEOGRAPHIC DISTRIBUTION OF THE CERTIFIED NURSING ASSISTANT WORKFORCE IN U.S. COUNTIES

Mallory Johnson, *University of California Davis, Davis, California, United States*

Although geographic disparities in LTSS access are well documented, no studies exam the spatial variation in the workforce primarily responsible for delivering direct care. This study assesses the distribution of the certified nursing assistants (CNA) and the community characteristics associated with CNA employment. A cross-sectional design is used to explore the relationship between CNA employment and health care delivery and training infrastructure, local labor market structure, and population characteristics. Data from CMS's Payroll Based Journal, the American Community Survey, and the National Conference of State Legislatures are fitted to a negative binomial hurdle model. 2,871 counties employ 1,616,798 CNAs ("count locations"). Only 271 counties employ no CNAs ("zero locations"). Zero locations are more likely to be rural and have lower population, less hospitals, and fewer CNA training programs even though their aging population is higher. Regression results demonstrate that the number of hospitals, nursing home beds, and total population are positively associated with CNA employment in both count and zeros locations. Medicaid expansion, the foreign-born population, and the proportion of the workforce employed in hospitality or public sector jobs, and land area are negatively associated with CNA employment in both count and zero locations. Differential effects are seen for the number of CNA training programs, population with a disability, and insurance status. CNA employment is highly variable. These findings confirm the "inverse care law" by demonstrating that CNA employment is not associated primarily with population need but with other health care, labor market, and population factors.

Abstract citation ID: igae098.3991

### GIS ANALYSIS OF PHYSICAL ACTIVITY AND FALL RISK AMID GLOBAL WARMING: ROLES OF COMMUNITY ENVIRONMENTS

Yingru Li, Ladda Thiamwong, Dahee Kim, Christopher Emrich, Rui Xie, Jennifer Crook, and YunYing Zhong, *University of Central Florida, Orlando, Florida, United States*

Regularly engaging in physical activity (PA) may reduce fall risk among older adults. However, hot weather amid global warming and/or less walkable community environments may negatively impact their participation in PA, especially those who live in low-income or otherwise socially vulnerable areas. This study aims to visualize spatial variations of PA and fall risk among low-income older adults (LOAs) across four communities in Orlando, Florida, during the summer months and to examine the roles of community environments on fall risk and PA. This study used data from 63 LOAs collected from May to September 2023. Self-reported fall risk was measured using the CDC STEADI fall risk checklist. PA was assessed through average active and sedentary minutes recorded by FitBit over five months. Community environments were assessed using walk scores and 31 different social vulnerability index characteristics. This study employed ArcGIS pro to visualize spatial variations and SPSS Amos to examine the roles of community environments. LOAs' PA and fall risk varied across the four communities. Higher PA and lower fall risk were observed among LOAs in communities with higher walk scores and lower social vulnerability indexes. The results of path analysis revealed recursive relationships: higher community social vulnerability was associated with lower community walkability, which led to less active and more sedentary time among LOAs, contributing to their higher fall risk. These findings highlight the impact of community environments on LOAs' health and well-being and suggest that actions should be taken to improve community walkability, particularly in socially vulnerable communities.

Abstract citation ID: igae098.3992

### GROUND REACTION FORCE IN SIT-TO-STAND MOVEMENT PREDICT SARCOPENIA

Kazuki Ota[1], Jieun Yoon[1], Kyohei Shibuya[1], Mariko Miyashita[2], Tomoko Miyama[2], and Tomohiro Okura[1], *1. University of Tsukuba, Tsukuba, Ibaraki, Japan*, *2. Tanita Corporation, Itabashi, Tokyo, Japan*

New method for evaluating lower-limb muscle strength has been attempted using ground reaction force during sit-to-stand movement. The specific variables used are the vertical peak force, the rate of force development (RFD), and the stable time. These variables have also been shown to be associated with physical function and experience of falling. However, their relationship to sarcopenia, the loss of skeletal muscle mass and muscle strength, and predictors of sarcopenia have not been investigated. In this study, we aimed to investigate the relationship between ground reaction force during sit-to-stand movement and sarcopenia diagnostic factors, as well as identify predictors of sarcopenia. The subjects were 1462 people aged 46-89 years (71.0±8.9 years). The sarcopenia was determined by grip strength, waking speed, and skeletal muscle mass (SMI) index. Force variables were calculated using the Zaritz (BM-220, Tanita Corporation). The statistical methods used was the Pearson correlation coefficient test, with $P < 0.05$. In addition, ROC curve analysis was performed, the area under the curve (AUC) and accuracy were calculated. The results showed that there were 1411 people without sarcopenia and 51 people with sarcopenia. Significant positive correlations were found between grip strength, walking speed, and SMI index and vertical peak force (r = 0.446, 0.306, 0.338), RFD (r = 0.264, 0.389, 0.159). Furthermore, the RFD had high discriminatory accuracy (AUC: 0.841, accuracy: 83.3 %). These results suggest that ground reaction force in sit-to-stand movement is a useful predictor of sarcopenia.

Abstract citation ID: igae098.3993

### GUIDING FUTURE AUTONOMY-SUPPORTIVE BEHAVIOR BASED ON HISTORICAL KNOWLEDGE OF NURSING THEORIES

Michel Bleijlevens[1], Melissa Botana Gronek[1], Stan Vluggen[2], Esmee Moonen[1], Aileen van Dijk[1], Jan Hamers[1], and Judith Meijers[1], *1. Maastricht University, Maastricht, Limburg, Netherlands*, *2. Zuyd University of Applied Sciences, Limburg, Netherlands*

Autonomy in care provision is central to providing person-centered, ethically sound care that meets the needs and preferences of older people. By stimulating autonomy, nurses can help maintain dignity, enhance quality of life, and promote overall well-being. Nevertheless, there remains a lack of knowledge on what autonomy is and how nurses could show autonomy-supportive behavior. This review examines the concept of autonomy and how nurses can effectively foster it in their ADL practice. In this scoping review, leading nursing theories that shaped the nursing profession and mentioned autonomy or related terms were selected from nursing textbooks, internet searches, and expert consultations. Two researchers independently conducted the screening and analysis using inductive coding. Ultimately, nine theories were included that mentioned related concepts to autonomy such as self-governance. None of the theories explicitly described autonomy or autonomy-supportive behaviors. However, we did find themes related to autonomy such as: being oneself, having a sense of freedom and control, expressing and making choices, and engaging deliberately in actions. Furthermore, six actions were identified to guide nurses in enhancing autonomy: being aware of their own behavior; respecting individual uniqueness; fostering interpersonal connections; facilitating open communication; allowing patients to choose the best course of action; and providing collaborative guidance and assistance. While nursing theories may lack explicit guidelines on autonomy-supportive behaviors, the actions identified in this review offer a practical framework for enhancing autonomy in long-term care. However, further knowledge development in this area is essential in order to stimulate autonomy-supportive behavior.

Abstract citation ID: igae098.3994

### HEALTH SEEKING BEHAVIOR FOR YOUNG ONSET DEMENTIA IN WESTERN COUNTRIES AND EAST ASIA: A SCOPING REVIEW

Ko-Xin Chen[1], Han-Yun Chang[2], and Valerie Cotter[2], *1. National Cheng Kung University, Tainan, Taiwan (Republic of China)*, *2. Johns Hopkins University School of Nursing, Baltimore, Maryland, United States*

**Aim:** We aimed to investigate the impact of culture in Western countries and East Asia on the health-seeking behavior in young onset dementia.

**Background:** While previous research has largely focused on the impact of culture on health-seeking behaviors in late onset dementia, a gap exists in understanding these influences in young onset dementia since they present unique challenges and needs.
**Design and Methods:** A scoping review was conducted using Arksey and O'Malley's framework. Databases searched included Embase, Ovid MEDLINE, Cochrane Trials, CINAHL, PsycINFO, Scopus, Airiti Library, and Taiwan's National Digital Library of Theses and Dissertations, with coverage up to July 22, 2024. The PRISMA-ScR checklist was employed to ensure the rigor of this scoping review.
**Results:** From 1,611 retrieved articles 27 studies were included. The synthesized results were analyzed using the Health Belief Model. Two main themes emerged from the analysis: (i) perceived susceptibility, severity, and barriers, with subthemes including stigma, financial impact of working, psychological and emotional effects, diagnostic challenges, and access to healthcare; and (ii) perceived benefits, self-efficacy, and cues to action, with subthemes including welfare, individualistic or collective values, meaningful activities, and environmental support.
**Conclusion:** Health-seeking behavior for young onset dementia is influenced by cultural differences between Western countries and East Asia. Specifically, Western cultures emphasize individualistic values, while East Asian cultures prioritize collective decision-making. Clinical implication: It is crucial to develop informed and culturally appropriate care for persons with young onset dementia that is tailored to the distinct needs of Western and East Asian populations.

Abstract citation ID: igae098.3995
### HELPING AGING VETERANS PLAN AHEAD (HAVE)
Dennis Sullivan[1], Bonnie Dawson[2], Angela McFadden[2], Ramona Rhodes[2], Linda Sawyer[2], and Christina Crawford[1], *1. Central Arkansas Veterans Healthcare System, North Little Rock, Arkansas, United States*, *2. Department of Veterans Affairs, North Little Rock, Arkansas, United States*

It is estimated that up to 60% of older Veterans (OVs) do not have an Advance Directive (AD). The HAVE clinical program initiative was developed to address this problem by helping OVs understand the importance of having an ACP. Initially, OVs at high risk for institutionalization (per Office of GEC PLI List) were invited (by letter and phone call) to participate in HAVE. At the first visit (conducted in-person or by VVC), the OV (and Caregiver) met with a HAVE lay health advisor (HLHA) who conducted a baseline assessment including administration of Brief Health Literacy, Knowledge of Care options, and Quality of Life rating questionnaires. Education focused on incorrect responses was provided and AD materials were provided for home review. The HLHA then used motivational interviewing techniques to help the OV articulate his/her desires for ACP and to indicate what he/she thought was important that he/she could do to ensure these wishes were honored and to then create SMART goals for doing this. Up to three follow-up phone visits were conducted. In the first four months of the program, 218 eligible OVs were identified. Of these, 17 refused or could not be contacted, 40 already had an AD, and 55 were scheduled for a first visit. To date, 16 of the 17 OVs completing the HAVE program created a new AD and 6 OVs updated their older AD. Given the early success of HAVE, the program is being expanded to Veterans over age 60.

Abstract citation ID: igae098.3996
### HETEROGENEOUS METABOLOMIC AGING ACROSS THE SAME AGE AND PEDICTION OF HEALTH OUTCOME
Xueqing Jia[1], Jiayao Fan[1], Xucheng Wu[1], Joris Deelen[2], Dan Zhou[1], and Zuyun Liu[1], *1. Zhejiang University School of Medicine, Hangzhou, Zhejiang, China (People's Republic)*, *2. University of Cologne, Cologne, Nordrhein-Westfalen, Germany*

Existing metabolomic clocks exhibit deficiencies in capturing the heterogeneous aging rates among individuals with the same chronological age. Yet, the modifiable and non-modifiable factors in metabolomic aging have not been systematically studied. Here, a new aging measure—MetaboAgeMort—was generated and validated using metabolomic profiles from 239,291 UK Biobank participants for 10-year all-cause mortality prediction. The MetaboAgeMort showed significant associations with all-cause mortality, cause-specific mortality, and diverse incident diseases. Adding MetaboAgeMort to a conventional risk factors model improved the predictive ability of 10-year mortality. A total of 99 modifiable factors across seven categories were identified for MetaboAgeMort. Among these, 16 factors representing pulmonary function, body composition, socioeconomic status, dietary quality, smoking status, alcohol intake, and disease status showed quantitatively stronger associations. The genetic analyses revealed 99 genomic risk loci and 271 genes associated with MetaboAgeMort. The tissue-enrichment analysis showed significant enrichment in liver. This study illuminates heterogeneous metabolomic aging across the same age, providing avenues for identifying high-risk individuals, developing anti-aging therapies, and personalizing interventions, thus promoting healthy aging and longevity.

Abstract citation ID: igae098.3997
### HOW CHILDHOOD NEGLECT AFFECTS LATER LIFE LONELINESS
Karina Tavares, Gina Barbera, and Cindy Tsotsoros, *University of Rhode Island, Kingston, Rhode Island, United States*

Individuals who experience neglect within the first eighteen years of life are at increased risk for loneliness later in life. This study explores whether the type of neglect experienced (emotional, physical, or both), and the age at which it occurs, affects later-life loneliness. This was accomplished by using a subsample of 169 women aged 18–86 who reported experiencing at least one form of neglect. Demographic indicators, the 17-item ACEs questionnaire (Felitti et al., 1998), and the UCLA Loneliness Scale (Russell, 1996) were examined. Neglect was categorized into three groups: emotional neglect, physical neglect, and both emotional and physical neglect. Age of first experienced neglect was categorized into three groups: ages 0–5, 6–11, and 12–18 years. When comparing the three categories of neglect, an ANOVA revealed that those who experience both emotional and physical neglect experience the highest levels of later-life loneliness. A multiple linear regression model showed that age alone predicted 2.3% of the variance in loneliness. When including both age and neglect, the model accounted for 9.8% of the variance, and adding their interaction improved the prediction to 12.3%. This study underscores that experiencing both emotional and physical neglect, particularly during early childhood, significantly increases the risk of loneliness in later life.

Abstract citation ID: igae098.3998

### IDENTIFYING UNKNOWN STAGED CANCER IN THE UNITED STATES BY SELECTED VARIABLES DEPENDS ON THE TYPE OF REPORTING SOURCE

Ray Merrill, *Brigham Young University, Provo, Utah, United States*

Appropriate site-specific cancer treatment depends on the stage of disease at diagnosis. However, some cancer is unstaged, especially in older age. This study examines the level of unstaged cancer incidence for 24 cancer sites in the US according to type of reporting source and, for each source, the rate of unstaged cancer by sex, age, race, ethnicity, and household income. The rate of unstaged cancer according to cancer lethality is also investigated. Analyses were based on 2,719,800 male and 2,727,223 female malignant cancer cases diagnosed during 2015–2021. Data was collected by 22 population-based cancer registries in the National Cancer Institute's SEER Program. Poisson regression estimated rate ratios adjusted for sex, age, race, ethnicity, and household income. Approximately 9.4% of males and 7.2% of females had unstaged cancer. The adjusted rate of unstaged cancer was 60% (95% CI 59%-61%) higher in males than females, significantly increased with age, decreased with household income, and was 11% (95% CI 10%-12%) higher in Blacks (vs. Whites) and 11 (95% CI 10%-12%) higher in Hispanics (vs. non-Hispanics). Several adjusted rate differences were identified according to the type of reporting source. Finally, there was a significant negative association between site-specific percentages of unstaged cancer and 5-year relative survival rates for males and females. Thus, the level of unstaged cancer is negatively associated with cancer lethality and depends on the type of reporting source. The type of reporting source should be considered when identifying the level of unstaged cancer according to selected variables.

Abstract citation ID: igae098.3999

### IMMEDIATE AND SUSTAINABLE EFFECTS OF TDCS ON PAIN IN OLDER ADULTS WITH ALZHEIMER'S DISEASE AND RELATED DEMENTIAS

Chiyoung Lee[1], Juyoung Park[1], Mindy Fain[1], James Galvin[2], and Hyochol Ahn[1], *1. University of Arizona, Tucson, Arizona, United States*, *2. University of Miami, Miami, Florida, United States*

Chronic pain management is a complex clinical process requiring thorough assessment. Especially for older adults with Alzheimer's disease and related dementias (ADRD), behavioral pain indicators are crucial in complementing self-reports. We simultaneously assessed the impact of transcranial direct current stimulation (tDCS) on mitigating both self-reported pain and pain behaviors to more objectively and comprehensively explore its effects in older adults with ADRD. The analysis investigated 40 participants randomly (1:1) subjected to active and sham tDCS for 20 min on 5 consecutive days. The numeric rating scale (NRS) and Mobilization–Observation–Behavior–Intensity–Dementia (MOBID-2) scale were used to assess self-reported pain and pain behaviors, respectively. Multi-group latent transition analysis enabled the simultaneous evaluation of both pain domains in a single model and analysis of their changes as a function of intervention exposure by modeling the transition probabilities of latent classes and comparing these changes between groups. Two latent pain categories ("high pain" and "low pain") were identified based on NRS and MOBID-2 scores. Active group participants with "high pain" exhibited a 47.1% probability of transitioning to "low pain" immediately after intervention completion compared with no transition in the sham group. From immediate to 1-month follow-up, both groups exhibited positive transitions (44.3% vs. 39.2%). Active group participants with "high pain" at 1-month follow-up had a 58.4% likelihood of transitioning to "low pain" by 3 months post-intervention, whereas sham group participants exhibited no change. Our findings support the immediate and enduring effects of tDCS on pain reduction in older adults with ADRD.

Abstract citation ID: igae098.4000

### IMPACT OF 4MS ON OUTCOMES FOR HOSPITALIZED OLDER ADULTS IN AN AGE-FRIENDLY SYSTEM

Justin Lam[1], Susan Kwiatek[2], Maria Carney[3], Philip Solomon[3], Mark Tursi[4], Christian Nouryan[5], and Edith Burns[6], *1. Zucker School of Medicine at Hofstra/Northwell, Brooklyn, New York, United States*, *2. Northwell Health, New Hyde Park, New York, United States*, *3. Zucker School of Medicine at Hofstra/Northwell, New Hyde Park, New York, United States*, *4. Krasnoff Quality Management Institute/Northwell Health, New Hyde Park, New York, United States*, *5. Zucker School of Medicine at Hofstra/Northwell, Manhasset, New York, United States*, *6. Northwell Health, Manhasset, New York, United States*

**Introduction:** The Institute for Healthcare Improvement identifies four essential components (the 4Ms) of high-quality care for older adults within an Age-Friendly Health System (AFHS): What Matters, Medication, Mentation, and Mobility. This framework allows healthcare providers to address and act on key issues, such as goals-of-care discussions, the in-patient use of potentially harmful medications (e.g. benzodiazepines, opioids, hypnotics), delirium prevention, and mobility maintenance.

**Methods:** EMR data pulled manually and through aggregated dashboards. Population/Setting: patients ≥65 admitted June-July 2024 to eight hospitals (5 community, 2 tertiary, 1 quaternary) within a large integrated health system, all committed to care excellence. Patients grouped into categories based on the number of assessed M's. Positive screens were reviewed for "act-ons." Patient outcomes: LOS, disposition, 30-day readmissions and ED visits.

**Results:** Of 2,337 adults aged ≥65, 0.3% had no assessments, 10.6% had one assessment, 39.6% two assessments, 40.3% three assessments, and 9.2% had all four assessments. Of the 118 patients who had all 4Ms assessed, chart review showed 42% had an 'act-on' for What Matters, 43% act-on for Medication, 22% act-on for Mentation, 83% act-on for Mobility. Mean LOS 11.8 days (range: 3-72); June 30-day readmissions: 10.2%. Conclusions: A minority of patients had all 4Ms assessed. Act-ons were documented most frequently for Mobility, less for other M's. Data on outcomes for ≤3M assessments is being analyzed and compared to those with 4Ms. This data will contribute to the growing evidence base for all 4Ms in AFHS transformation.

Abstract citation ID: igae098.4001

### IMPACT OF IMMIGRATION TIMING ON MEDICATION NON-ADHERENCE AND COGNITIVE FUNCTION IN OLDER ADULTS

Yuan Fang[1], and Jayoung Han[2], *1. Binghamton University, State University of New York, Binghamton, New York, United States*, *2. Fairleigh Dickinson University School of Pharmacy and Health Sciences, Florham Park, New Jersey, United States*

The aging U.S. immigrant population exhibits higher rates of both diagnosed and undiagnosed Alzheimer's Disease and related dementia compared to U.S.-born residents. Traditional diagnostic methods often fail to detect cognitive decline in immigrants due to cultural and linguistic barriers. Immigration is a multifaceted risk factor affecting cognitive health through acculturation, language proficiency, and healthcare access. This study examines the moderating role of immigration timing in the relationship between medication non-adherence and cognitive function. We analyzed data from the Health and Retirement Study (HRS), focusing on adults >65 years who participated in interviews between 2012-2016 (n=10,869). Cognitive function was assessed using recently developed standardized latent cognitive scores. The relationship between medication non-adherence and cognitive scores was modeled using a moderated moderation model, examining interactions between age at immigration, education, and medication non-adherence, controlling other confounders. Non-immigrants were assigned a high placeholder value for age at immigration, and this variable was rank-normalized for analysis. A sensitivity analysis using the raw age at immigration variable was also conducted. Medication non-adherence, immigrant, and older age at immigration were associated with lower cognitive scores, while higher education was positively associated. Significant negative two-way interactions indicated that immigration status (β=-0.38±0.14, p=0.0006), timing (β=-0.13±0.03, p< 0.0001), and education (β=-0.91±0.03, p=0.0003) moderated the impact of medication non-adherence on cognitive decline. In conclusion, our study showed that medication non-adherence is a significant predictor of cognitive decline, with later immigration worsening cognitive vulnerability, highlighting the moderating effect of immigration age on the relationship between medication non-adherence and cognitive decline.

Abstract citation ID: igae098.4002

### IMPACT OF NUMBER OF FUNCTIONAL TEETH ON INCIDENT MOBILITY DISABILITY IN OLDER ADULTS: FINDINGS FROM THE KFACS

Nahyun Lim, Hyung Eun Shin, Daehyun Lee, Heeeun Jung, Jae Young Jang, Hyunjin Cho, Chang Won Won, and Miji Kim, *Kyung Hee University, Seoul, Republic of Korea*

The prevalence of mobility disability among older adults has a detrimental impact on their ability to live independently. It has been reported that losing functional teeth (FT) is linked to mobility disability. Nevertheless, there is insufficient longitudinal research investigating an association between the number of FT and incident mobility disability. We aimed to evaluate the association between the number of FT at baseline (2016–2017) and incident mobility disability after 6-year (2022-2023) in community-dwelling Korean older adults. A total of 1,090 older adults without mobility disability at baseline (50.2% women; mean age 75.3 ± 3.4 years) were analyzed from the Korean Frailty and Aging Cohort Study. FT were defined as total teeth, including remaining natural teeth and prosthetically restored teeth on panoramic radiography. Mobility disability was defined after 6-year as gait speed less than 1.0 m/s. The multivariate logistic regression analysis evaluated the association between FT and incident mobility disability. Participants having FT less than 20 were 26.3% (n=287), and the incident mobility disability was 30.2% (n=329). FT less than 20 were significantly associated with a higher risk of incident mobility disability in un-adjusted model (odds ratio [OR]: 2.27, 95% confidence interval [CI]: 1.71–3.00). After adjusting for confounders, FT less than 20 had a higher risk of incident mobility disability (OR: 1.95, 95% CI: 1.38–2.75). In conclusion, the number of FT was independently associated with incident mobility disability. Our findings suggest that maintaining FT at least 20 is essential to prevent mobility disability in older adults.

Abstract citation ID: igae098.4003

### IMPACTS OF HANDGRIP ON REVERSING FRAILTY PROGRESSION IN OLDER ADULTS: A TWO-YEAR FOLLOW-UP STUDY

Wan Yun Chou[1], and Su-I Hou[2], *1. University of Central Florida, Orlando, Florida, United States*, *2. The University of Central Florida, Orlando, Florida, United States*

Frailty is characterized by a significant decline in the functional reserves of multiple organ systems, which increases vulnerability to stressors and elevates the risk of adverse health outcomes. Addressing frailty is becoming crucial for both intervention and policymaking. This prospective cohort study, conducted between 2018 and 2020 at a medical center in Taiwan, aimed to assess the needs of individuals at risk of frailty within the community. The study involved 318 participants aged 65 and older who attended annual health examinations. Frailty was measured using Fried's frailty scale, cumulative deficits, and physical risk variables, assessed through self-reported questionnaires and objective measures at admission, 6, 12, and 24 months. Based on frailty scores, participants were categorized into low-risk and high-risk groups, with 0 indicating low risk and 1 or higher indicating high risk. The study analyzed differences between these groups concerning the five criteria of Fried's frailty scale: weight loss, low handgrip strength, exhaustion, slow walking speed, and physical inactivity. Wilcoxon signed-rank tests and Generalized Estimating Equation (GEE) models were employed for analysis. 60% (n=193) of participants were considered high-risk. Notably, the decline in grip strength was significantly greater in the high-to-low-risk group compared to the low-to-high-risk group at 12 and 24 months (p=0.036 and 0.016). GEE models revealed a borderline significant reduction in frailty risk associated with improvements in cognitive function (aOR=0.69, 95% CI=0.46-1.04). These findings suggest that enhancing grip strength and cognitive function can contribute to reversing frailty, highlighting the benefits of targeted interventions for older adults.

Abstract citation ID: igae098.4004
### IMPLEMENTING THE LOOK AHEAD LIFESTYLE INTERVENTION IN PRACTICE AND COMMUNITIES: A SCOPING REVIEW

Yan Du[1], Lina Paola Buitrago Ubaque[2], Valeria Medrano[3], Erin Finley[3], Helen Hazuda[3], Sara Espinoza[4], Lixin Song[5], and Elena Volpi[6], *1. UT Health San Antonio, San Antonio, Texas, United States*, *2. University of Miami, Miami, Florida, United States*, *3. UT Health SA, San Antonio, Texas, United States*, *4. Cedars-Sinai Medical Center, Los Angeles, California, United States*, *5. University of Texas Health Science Center at San Antonio, San Antonio, Texas, United States*, *6. University of Texas Health San Antonio, San Antonio, Texas, United States*

Diabetes is a public health concern as the population aging rapidly. The Look AHEAD study demonstrated that a lifestyle intervention could improve glucose and lipid control, mental health, physical function, quality of life, and the need for diabetes medications. We conducted a scoping review to explore the implementation of the Look AHEAD intervention in other populations and settings. We searched PubMed, Scopus and CINAHL in December 2023. Two researchers screened each article independently. The data extracted from the included studies were systematically tabulated and analyzed to identify study characteristics, key themes, trends, and gaps. The review included 21 articles from 8 study projects. Twelve articles reported on study methods or qualitative findings. Seven projects focused on adults (RCT), and one in older adults aged 65 and over (one arm, pre-and post- test). Interventions were delivered in clinical (4 projects), community (3 projects), and academic (1 project) settings, with sample sizes ranging from 20 to 331 participants. The number of educational sessions ranged from 10 to 60. Most studies incorporated self-monitoring, goal setting, psychological strategies, and social support for physical activity and diet. Three projects used meal replacements. Weight improved significantly in all intervention groups except one pilot trial. HbA1c improved across all groups, with only one study reporting significant results. Two studies assessed reduced diabetes medication use. The studies varied in sample size, intervention sessions, and settings. Overall, adapting Look AHEAD to other settings is feasible, but the effectiveness, adoption, and sustainability of implementing the adapted interventions warrant further investigation.

Abstract citation ID: igae098.4005
### IMPROVE TECHNOLOGY AND EHEALTH LITERACY IN A RETIREMENT COMMUNITY VIA THE USE OF LLM-POWERED CHATBOT

Ray-Yuan Chung, Feng Chen, Yein Jeon, and Oleg Zaslavsky, *University of Washington, Seattle, Washington, United States*

The low levels of technology and eHealth literacy of retirement community residents are a barrier to technology use and information retrieval. In response, we proposed the development of an LLM-powered chatbot to improve the technology and eHealth literacy of the residents. We adopted a human-centered design approach and aimed to complement the existing technological infrastructure of the community app by providing straightforward, conversational assistance. To enhance user accessibility, we emphasize the interface's features including large text, white and black interface for vision support, voice interaction capabilities, and simplified navigation, catering to the varied technological proficiency levels within the community. The chatbot's functionalities are divided into two primary goals: facilitating information navigation within the retirement community and enhancing technology and eHealth literacy among its residents. For information navigation, the chatbot allows users to easily access essential information such as dining menus and contact details. To improve technology literacy, the chatbot delivers simple, up-to-date guides and information on specific questions posed by the users, providing them with easy-to-follow instructions. To evaluate our chatbot, residents were asked to complete tasks during the trial session and complete a survey. The survey collected demographics, digital literacy scores, user satisfaction, and feasibility. Residents successfully utilized our chatbot to complete the tasks. Furthermore, they reported high levels of satisfaction and a strong likelihood of recommending it to peers. Therefore, we suggest that our chatbot may serve as a valuable tool for older adults with low technology and eHealth literacy.

Abstract citation ID: igae098.4006
### INADEQUATE SLEEP ASSOCIATED WITH FRAILTY DEVELOPMENT AND FALL RISK IN LOW-INCOME OLDER ADULTS

Abigail Tice, Chen Chen, Varadraj Gurupur, Boon Peng Ng, David Fukuda, Janet Lopez, Hwan Choi, and Ladda Thiamwong, *University of Central Florida, Orlando, Florida, United States*

Sleep, which is important for improving brain performance and overall health, declines with aging, and older adults from disadvantaged socioeconomic groups are more likely to have poorer sleep. Frailty, the decline of physiological systems, is associated with poor sleep parameters (e.g., less deep sleep and sleep duration) in older adults. As frailty progresses, fall risk and fear of falling (FOF) increase, increasing the likelihood of a fall that can result in serious injury, hospitalization, and even death. The relationship between sleep duration and frailty, fall risk, and FOF is not well understood, therefore, the objective of this study was to investigate the relationships between them. The Fatigue, Resistance, Ambulation, Illness, and Loss of weight (FRAIL), Stopping Elderly Accidents, Death, and Injuries (STEADI), short Fall-Efficacy International (FES-I), and Pittsburgh Sleep Quality Index (PSQI) questionnaires were administered to 124 community-dwelling, low-income older adults aged 60 years or more in Orlando, Florida. Spearman correlation analysis revealed associations between sleep duration and FRAIL ($p=0.0258$, $r=-0.1741$), STEADI ($p=0.0010$, $r=-0.2537$), and short FES-I ($p=0.0083$, $r=-0.2056$). While unpaired t-test analysis revealed no difference in short FES-I between low sleep (< 7 hours; n=83) and healthy sleep (7-8 hours; n=72), FRAIL ($p=0.0248$) and STEADI scores ($p=0.0338$) significantly increased with low sleep duration. In conclusion, inadequate sleep duration appears to increase the development of frailty and fall risk in community-dwelling older adults. Screening older adults for sleep problems and interventions to improve sleep duration have the potential to reduce frailty and fall risk in low-income older adults.

Abstract citation ID: igae098.4007

### INDOOR TEMPERATURES AND MENTAL HEALTH IN COMMUNITY-DWELLING OLDER ADULTS: A SYSTEMATIC REVIEW

Min Kyung Park[1], Dahye Hong[2], Seolah Yoon[2], Yubeen Kim[1], Minsub Kim[1], and Bada Kang[1], *1. Yonsei University College of Nursing, Seoul, Republic of Korea*, *2. Yonsei University, Seoul, Republic of Korea*

The relationship between indoor temperature and mental health is becoming increasingly important as climate change leads to extreme temperature fluctuations. Older adults are particularly vulnerable to indoor temperature changes because of their diminished ability to regulate body temperature and because they spend a significant amount of time indoors. This systematic review examined the relationship between indoor temperature and mental health outcomes among community-dwelling older adults. A comprehensive search of seven electronic databases (MEDLINE, Embase, Cochrane Library, CINAHL, PsycINFO, Google Scholar, and PROQUEST) was conducted on April 4, 2024. Of the 2,328 identified studies, 15 met the inclusion criteria. Mental health outcomes associated with indoor temperature were categorized into four groups: sleep problems such as insomnia and sleep disturbance; emotional problems such as emotional distress and negative mood; social interaction problems such as social exclusion and low social participation; and other mental health issues such as anxiety, agitation, and annoyance. Sleep problems were the most frequently reported mental health outcome related to indoor temperature (n=9). Older adults living in substandard housing conditions, those with economic difficulties, and those residing in urban areas were vulnerable to exposure to hot or cold indoor temperatures because of housing-related risks such as low energy efficiency, inadequate heating or cooling, and limited access to green spaces. These findings highlight the need for evidence-based guidelines to improve mental health by ensuring appropriate indoor temperatures in the community. Improving housing conditions through policy support could be an effective strategy for enhancing the mental health of community-dwelling older adults.

Abstract citation ID: igae098.4008

### INSIGHTS FOR IMPROVING OLDER ADULT NUTRITION WITH MULTIVITAMIN/MINERAL SUPPLEMENTS

Roger Fielding[1], Robert Blancato[2], Kathryn Porter Starr[3], Shelley McDonald[4], Rachel Zimmer[5], Jennifer Pettis[6], and Karen Tracy[7], *1. Jean Mayer USDA Human Nutrition Research Center on Aging at Tufts University, Boston, Massachusetts, United States*, *2. National Association of Nutrition and Aging Services Programs (NANASP), National Association of Nutrition and Aging Services Programs (NANASP), District of Columbia, United States*, *3. Duke University School of Medicine, Hillsborough, North Carolina, United States*, *4. DUKE UNIVERSITY MEDICAL CENTER, Durham, North Carolina, United States*, *5. Wake Forest Baptist Health, Wake Forest Baptist Health, North Carolina, United States*, *6. Gerontological Society of America, Clifton Park, New York, United States*, *7. Gerontological Society of America, Gerontological Society of America, District of Columbia, United States*

In June 2024, GSA convened an expert roundtable to address the role of multivitamin/mineral supplements (MVMs) as part of complete nutrition for older adults. Older adults often do not obtain adequate amounts of micronutrients from their regular dietary patterns and are at risk for nutritional deficiencies. Reasons cited by experts included physiologic changes; comorbid conditions; conditions associated with aging (e.g., sarcopenia, frailty); socioeconomic factors; declines in physical function; and interrelated changes in cognition, functional capacity, mental health, and mobility. Nutrition has wide-ranging effects on health and well-being, and nutrition screening would aid in identifying deficiencies. However, nutritional status is not consistently assessed and documented in clinical practice. Experts proposed expanding interdisciplinary care and expanded payment opportunities as strategies to improve nutritional assessments and identified emerging care models that successfully address the nutritional needs of older adults. Expanded education and training opportunities related to nutrition for clinicians and implementing strategies for effectively educating older adults about nutrition were also cited as important needs. Experts noted that MVMs may be recommended to help meet nutritional needs in older adults. Providers should consider polypharmacy, potential medication/MVM interactions, and chronic conditions when making recommendations, and educational supports are needed to enhance acceptance of and adherence to recommendations. Experts also identified strategies for enhancing nutritional research and incorporating nutritional information into clinical practice guidelines to increase the clinician confidence around MVM recommendations.

Abstract citation ID: igae098.4009

### INTRINSIC CAPACITY TRANSITION AND ITS ASSOCIATION WITH INCIDENT DISABILITY: FINDINGS FROM THE KFACS

Hyunjin Cho, Hyung Eun Shin, Heeeun Jung, Daehyun Lee, Jae Young Jang, Nahyun Lim, Chang Won Won, and Miji Kim, *Kyung Hee University, Seoul, Republic of Korea*

Intrinsic capacity (IC) progressively decreases with age, accelerating the risk of disability. However, it is less known whether IC transition is associated with disability. We aimed to investigate the association between IC transition and incident disability among community-dwelling older adults. A retrospective analysis was conducted over a 6-year follow-up with biennial measurements, including 2,104 participants (52.7% women; mean age 76.1 ± 3.7 years) from the Korean Frailty and Aging Cohort Study. Participants were categorized based on the IC transition from the baseline to Wave 2: “Remained well and improved”, “Worsened”, and “Remained poor”. Disability was defined as a need for assistance in any items in activity of daily living. Incident disability was measured at Wave 3 and 4. Multivariate logistic regression was used to assess the relationship between IC transition and incident disability. The IC transition probability for “Remained well and improved” was the highest at 72.0%, followed by 15.8% for “Remained poor” and 12.2% for “Worsened”. Furthermore, over a 4-year follow-up, the incidence of disability among participants was 6.2% (n = 131). Compared with the “Remained well and improved”, the “Worsened” (odds ratio [OR]: 1.73, 95% confidence interval [CI]:1.01–2.98) and the “Remained poor" (OR: 2.34, 95% CI:1.45–3.79) had an increased risk of incident disability after adjusting covariates. Worsened IC and Remained poor groups were significantly associated with an increased risk of incident disability, compared to Remained well and improved group. Our findings suggest that early monitoring of IC is meaningful to prevent disability.

Abstract citation ID: igae098.4010

### JOURNEY TO GUIDE: ONE PRACTICE'S ROAD TO IMPLEMENTATION

Carolyn Clevenger, and Laura Medders, *Emory University, Atlanta, Georgia, United States*

In July 2023, Guiding and Improved Dementia Experience (GUIDE) was announced by the Center for Medicare and Medicaid Innovation Center. GUIDE was proposed to test the efficacy of an alternate payment methodology for participating dementia care models committed to providing an enhanced quality of care. The program is hoping to improve quality of life, reduce total cost of care, reduce inappropriate prescribing and healthcare utilization, and delay nursing home placement. GUIDE will pay for comprehensive clinical services as well as caregiver support services. Emory's Integrated Memory Care has been providing comprehensive dementia care services in our unique model since 2015. However, the IMC has had to adapt our current practice to meet the new requirements of GUIDE. The IMC will discuss implementing the necessary requirements for the GUIDE program and how we adapted our current clinic model to meet the GUIDE requirements. The IMC has had to establish additional processes for intake—including a home visit, utilize different required assessments and measures, continue providing caregiver support, and respite care.

Abstract citation ID: igae098.4011

### KEY INSTRUMENTAL ACTIVITIES OF DAILY LIVING AND COGNITIVE DOMAINS IN NON-DEMENTED OLDER ADULTS: A NETWORK ANALYSIS

Jiaying Li[1], Rendong He[2], Erh-Chi Hsu[1], and Junxin Li[3], *1. Johns Hopkins University, Baltimore, Maryland, United States*, *2. Jilin University School of Nursing, Changchun, Jilin, China*, *3. Johns Hopkins School of Nursing, Baltimore, Maryland, United States*

**Background:** Interconnected relationships within and between instrumental activities of daily living (IADL) and cognitive domains indicate central IADLs, cognitive domains, and bridge IADLs, which we aim to identify as modifying them can effectively enhance holistic cognitive and IADL functions.

**Methods:** A cross-sectional analysis of adults aged 65 and older in United States on five IADL and six cognitive domains. Network analysis identified central and bridge variables. Nonparametric and case-dropping bootstrap methods checked network stability. Network comparison tests assessed sex differences with Benjamini-Hochberg adjustments.

**Results:** Of the 2,239 participants, 56.4% were female. We computed and tested three networks: IADL, cognition, and bridge. The Correlation Stability Coefficients for these were 0.67, 0.75, and 0.44 respectively, all exceeding the threshold of 0.25. Meal preparation (centrality: 3.87), visual attention (0.86), and shopping (0.41) were identified as central IADL, central cognition, and bridge IADL respectively, as they had the highest centrality indices and were significantly higher than others in their networks ($p < 0.05$). Notably, gender differences emerged in the IADL network, with stronger associations between laundry and meal preparation in females (1.69 vs. males: 0.74, $p = 0.001$) and higher centrality in meal preparation among females (difference = 1.99, $p = 0.007$).

**Conclusion:** While broad enhancements in all IADL and cognitive domains are beneficial, targeting meal preparation, visual attention, and shopping optimizes cost-effectiveness in improving holistic IADL, holistic cognition, and holistic cognition function via IADL interventions among older adults. Notably, meal preparation interventions may be less effective in males, requiring tailored approaches.

Abstract citation ID: igae098.4012

### KNOWLEDGE GAPS REGARDING NALOXONE USE AMONG OLDER ADULTS IN HIGH OPIOID PRESCRIBING REGIONS IN SOUTHERN USA

Gohar Azhar[1], Naomi Armstrong[1], Lauren Camp[1], Karen Coker[1], Patricia E Savary[1], Shakshi Sharma[2], Regina Gibson[1], and Jeanne Y Wei[1], *1. University of Arkansas for Medical Sciences, Little Rock, Arkansas, United States*, *2. University of Arkansas for Medical Sciences, LITTLE ROCK, Arkansas, United States*

Arkansas has one of the highest opioid prescribing rates in the country. Nevertheless, there is a dearth of knowledge regarding opioid use and naloxone among older adults. We surveyed community dwelling older adults about opioid risks and naloxone use in rural Arkansas. Total number of respondents was n=461, and the age ranged from 50-59 (22%), 60-69 (34%), 70-79 (30%), or 80+ (14%) with 77% females and 52% black or African Americans. The percentage correct answers received from survey respondents decreased as age increased. Older adults underestimated their risk for opioid overdose (older vs younger; 35% vs 16%, P=0.020). In addition, more older adults believed that only heroin or fentanyl users were likely to overdose on opioids (older vs younger; 85%,vs 68% P=0.029). The use of naloxone as an overdose antidote was much less recognized by older adults (older vs younger; 66% vs 87%, P=0.016), as well as the requirement to administer as second dose of naloxone if the initial dose was unsuccessful (older vs younger; 40% vs 63%, P=0.033). Most older adults were unaware that naloxone could be obtained from the pharmacy without a prescription (older vs younger; 47% vs 71%, P=0.018). In summary, knowledge gaps regarding opioids and naloxone use were significantly higher in older adults. Since older adults are frequently prescribed opioids for pain, it is imperative that greater educational efforts are directed towards ameliorating the knowledge deficits about opioids and correct use of naloxone to prevent adverse effects and deaths due to opioid overdose.

Abstract citation ID: igae098.4013

### LIGHT EXPOSURE IN NURSING HOME RESIDENTS WITH DEMENTIA: IMPACT ON AFFECT AND NEUROBEHAVIORAL SYMPTOMS

Ying-Ling Jao[1], Yo-Jen Liao[2], Diane Berish[2], Julian Wang[1], Margaret Calkins[3], Ching-Ju Chiu[4], Yun-Han Kuan[5], and Marie Boltz[2], *1. Pennsylvania State University, University Park, Pennsylvania, United States*, *2. The Pennsylvania State University, University Park, Pennsylvania, United States*, *3. IDEAS Institute, Cleveland Heights, Ohio, United States*, *4. National Cheng Kung University, Tainan, Tainan, Taiwan (Republic of China)*, *5. Yang-ming Branch of Taipei City Hospital, Taipei, Taipei, Taiwan (Republic of China)*

Persons with dementia are at a higher risk for circadian disturbances and need more bright light to regulate circadian rhythm due to age-related vision deficiencies and reduced activity in suprachiasmatic nuclei. However, the impact of light exposure on nursing home (NH) residents with dementia has not been well evaluated. This secondary analysis examined the associations of daytime light exposure with affect and neurobehavioral symptoms in this population. Circadian stimulus (CS) was included as a lighting measure. Data of 13-week repeated measures were from a clinical trial with 27 residents with dementia. Participants wore light sensors to measure individual light exposure (lux, CCT, and CS) and Philadelphia Affect Rating Scale and Neuropsychiatric Inventory were used to assess affect and neurobehavioral symptoms every other week. Correlation and multilevel modeling (MLM) analyses were performed. Participants' average age was 87 and 74% were female. Results showed that higher lux levels were significantly correlated with more contentment (r=0.162, p=0.0399) and less sadness (r=-0.185, p=0.0183), less depression (r=-0.157, p=0.0459), and less anxiety (r=-0.207, p=0.0081). Higher CCT levels were significantly correlated with less delusion (r=-0.182, p=0.0205) and appetite changes (r=-0.159, p=0.0434). Higher CS levels were significantly correlated with aberrant motor behavior (r=-0.171, p=0.0293) and anxiety (r=-0.210, p=0.0072). MLM showed that CS significantly predicted decreased aberrant motor behavior (β=-5.04, p< 0.05), nighttime behavior (β=-4.85, p< 0.05), and anxiety (β=-6.08, p< 0.05). The results revealed the importance of light exposure in this population. The findings can guide environmental design to improve affect and neurobehavioral symptoms for NH residents with dementia.

Abstract citation ID: igae098.4014

### LIVING WITH YOUNG ONSET DEMENTIA IN RESIDENTIAL CARE: A SCOPING REVIEW

Linda Johansson[1], and Therése Bielsten[2], *1. School of Health and Welfare, Jönköping University, Jönköping, Jonkopings Lan, Sweden*, *2. Jönköping University, Jönköping, Jonkopings Lan*, *Sweden*

About 3.9 million people live with Young Onset Dementia (YOD) worldwide. As dementia progresses, there may be a need for transition to residential care. It can be challenging for persons with YOD to access age-appropriate services, even though they might have different needs compared to persons with late-onset dementia. This scoping review aimed to present the experiences of people with YOD living in residential care facilities. Peer-reviewed research articles focusing on either the perspective of people with dementia onset before the age of 65 living in residential care or their family members were included. A literature search in MEDLINE, CINAHL, PsycINFO, Scopus, ProQuest, and Web of Science conducted in December 2023 yielded 13 qualitative studies from Europe, North America, and Australasia. Four studies included experiences of persons with YOD, three included experiences of family members, and six had a mix of participant experiences. In total, 90 (Md = 9) persons with dementia and 292 (Md = 17) family members participated. A synthesis of findings indicates that a home-like environment and age-adjusted activities based on the interests of the individual were highly valued. This review provides a first step towards understanding what person-centered care means for people with YOD in residential care. However, even though people with YOD can communicate their experiences and needs, there were few studies focusing on their perspectives. To improve care, further research needs to focus on the voices of individuals with dementia.

Abstract citation ID: igae098.4015

### LIVING WITH YOUNG ONSET DEMENTIA IN RESIDENTIAL CARE: ANALYSIS OF DATA FROM THE SWEDISH DEMENTIA REGISTER

Linda Johansson[1], Therése Bielsten[2], Jimmy Lindberg[2], and Deborah Finkel[3], *1. School of Health and Welfare, Jönköping University, Jönköping, Jonkopings Lan, Sweden*, *2. Jönköping University, Jönköping, Jonkopings Lan, Sweden*, *3. University of Southern California, Los Angeles, California, United States*

Approximately 10,000 people in Sweden have Young Onset Dementia (YOD), i.e., dementia onset before the age of 65. As a result of disease progression, relocation to a residential care facility might be necessary. In Sweden, age-adapted residential facilities are only available in larger cities, and the specific needs of persons living with YOD might not be adequately addressed. The study aimed to describe characteristics of residents with YOD living in residential care and the quality of care. National quality registry data from the Swedish Dementia Register (SveDem) between 2012 and 2022 were used. In total, 124 people with YOD living in residential care units designed for persons with dementia (n=109) or in residential care facilities for older persons (n=15) were identified. The average age of admittance was 61.3±3.9 years, with no significant differences found between women and men. On average, six medications were used, and a medication review had been conducted within the past 12 months for 84% of the participants. Care received in accordance with one's life story (71%) and individual environmental adaptations (64%) were common. However, few participants (22%) had advanced care plans, and restraints were present in 44% of the persons with YOD. While it is not possible to draw general conclusions from this small study, it indicates that person-centered care is central in residential care for persons with YOD in Sweden. Future research should focus on increasing the understanding of why staff use restraints to such a high extent and how this practice can be prevented.

Abstract citation ID: igae098.4016

### LONGITUDINAL ASSOCIATIONS BETWEEN HAND GRIP STRENGTH AND DEPRESSIVE SYMPTOMS AMONG OLDER ADULTS USING KLOSA

SOOYOUNG KWON[1], and Gwang Suk Kim[2], *1. YONSEI UNIVERSITY, Seoul, Republic of Korea*, *2. Yonsei University, Seoul, Republic of Korea*

Many studies have reported an inverse association of hand grip strength (HGS) and depressive symptoms.

However, the longitudinal directionality between these two factors remains unclear. This study was performed using a random intercept cross-lagged panel model to investigate the longitudinal reciprocal associations and autoregressive relationships between HGS and depressive symptoms in older Korean adults. We conducted secondary data analysis using the data of 3,353 older adults (mean age=74.38±6.484 [range:65~101] years; 1,480 [44.1%] were men at baseline) from the Korean Longitudinal Study of Aging (KLoSA) across 2016, 2018, 2020, and 2022 (four waves). Measures included general characteristics, mean values of HGS, and the Center for Epidemiologic Studies Depression scale-10. Result showed that no significant cross-lagged path was observed between HGS and depressive symptoms, however, across individuals, HGS was inversely correlated with depressive symptoms (β=-9.041, p<.001), and the concurrent negative relationship between these two conditions was significant only at baseline (β=-1.578, p=.004), indicating that individuals with lower HGS had higher depressive symptoms. All autoregressive paths were significant except from 2020 to 2022, suggesting the severities in one's level of HGS and depressive symptoms carried over to the next time point. When considering gender differences, the same pattern of HGS and depressive symptoms was observed in men, while women showed a partially inverse association between them. Our findings suggest that HGS and depressive symptoms have a negative cross-sectional relationship, and gender has a different effect on the longitudinal correlation between HGS and depressive symptoms.

Abstract citation ID: igae098.4017

### LONGITUDINAL ASSOCIATIONS OF HEARING IMPAIRMENT WITH SOCIAL ISOLATION AMONG BLACK AND WHITE OLDER ADULTS

Mary Thoma[1], Erin Ferguson[2], Jacqueline Torres[1], Nicole Armstrong[3], Kristine Yaffe[1], and Willa Brenowitz[4], *1. University of California San Francisco, San Francisco, California, United States*, *2. University of California, San Francisco, San Francisco, California, United States*, *3. Warren Alpert Medical School of Brown University, Providence, Rhode Island, United States*, *4. Kaiser Permanente, Portland, Oregon, United States*

Social isolation has been declared an epidemic by the U.S. Surgeon General. Hearing impairment (HI) may be an important risk factor for isolation among older adults. Studies of HI and isolation have been inconclusive and use primarily cross-sectional and self-reported data. We used data from the HealthABC study (N=2227 Black and White adults aged 70-79 at recruitment) to examine associations of baseline objective HI (average pure-tone audiometry >40 dB, with and without use of hearing aids) with frequency of contact with family and friends (< weekly vs. at least weekly), depressive symptoms (CESD-10), and loneliness (frequency in past week; 1-4) across 6 visits (6-7 years). We used mixed effects models adjusted for demographic and clinical variables. Untreated objective HI was associated with increased odds of < weekly contact with friends (OR=1.47; 95% CI: 1.08,2.00) and more depressive symptoms (B=0.64; 95% CI: 0.26,1.01) across follow-up, but was not with frequency of family contact or loneliness. Objective HI treated with hearing aids was not associated with any outcome. We additionally found that self-reported HI ("hearing limits personal/social life") was associated with more depressive symptoms (B=1.49; 95% CI: 1.03,1.95) and loneliness (B=0.09; 95% CI: 0.01,0.16) across follow-up. Our results provide novel evidence that untreated objective hearing loss is associated with social isolation and subjective HI is associated with psychosocial wellbeing. Treatment for HI may reduce the likelihood of social isolation.

Abstract citation ID: igae098.4018

### LONGITUDINAL AUTOREGRESSIVE AND CROSS-LAGGED EFFECTS BETWEEN FRAILTY AND COGNITIVE FUNCTION OVER 14 YEARS

Min Jung Kim[1], and Chang Gi Park[2], *1. Gachon University, Incheon, Republic of Korea*, *2. University of Illinois Chicago, Chicago, Illinois, United States*

Frailty and cognitive impairment are common geriatric conditions with a strong association, though it remains unclear whether frailty leads to cognitive impairment or vice versa. Both conditions are typically irreversible and follow worsening trajectories that vary among individuals. This study examined the longitudinal autoregressive and reciprocal causal relationships between frailty and cognitive impairment, accounting for within-person processes, using a nationally representative sample, Korean Longitudinal Study of Aging 2006 to 2020. Frailty Index (FI) scores were derived from 33 items, while cognitive impairment was assessed using the Korean Mini-Mental Status Examination (K-MMSE). The sample included 3,853 adults aged 45 years or older at baseline with complete FI and K-MMSE data. A random-intercept cross-lagged panel model with robust maximum likelihood estimation was employed to address non-normality in the outcomes. The analysis revealed significant reciprocal causal relationships between FI and K-MMSE scores, with the strength of these relationships gradually increasing over time. The magnitudes of these relationships were comparable at each time point. Both FI and K-MMSE exhibited significant autoregressive effects, with FI showing consistently higher autoregressive coefficients than K-MMSE over the 14-year period. These findings suggest that frailty and cognitive function may serve as therapeutic targets for each other, with frailty being more persistent over time and cognitive impairment potentially influenced by other factors, including changes in frailty. Early interventions targeting either condition may yield synergistic preventive effects on both frailty and cognitive impairment, offering a valuable approach to managing these debilitating conditions.

Abstract citation ID: igae098.4019

### LONGITUDINAL STUDIES OF COGNITIVE AGING: STUDY DESIGNS TO OPTIMIZE STATISTICAL POWER TO DETECT COGNITIVE DECLINE.

Jasmin Duehring[1], Lawrence Ong[2], and Steven Edland[1], *1. University of California San Diego, La Jolla, California, United States*, *2. University of Callifornia San Diego, La Jolla, California, United States*

Practice effects in longitudinal studies of cognitive function in aging and Alzheimer's disease refer to the observation that performance on cognitive measures may improve on repeated assessments, because of increased familiarity with testing procedures and content of the tests. We have previously shown that a clinical trial run-in period intended to wash out practice effects can dramatically improve the efficiency of clinical trials. We now show that a run-in period can dramatically improve the efficiency and statistical power of longitudinal cohort studies of cognitive aging as well. Data are 1,094 participants with a baseline diagnosis of amnestic mild cognitive impairment (aMCI) enrolled in the National Alzheimer Coordinating Center (NACC) cohort (mean age 74.8, range 60 – 97, 44.1% female). In these data, the signal-to-noise in annual change after a run-period was approximately twice (2.4-fold) that observed without a run-in period. This increased signal translates to an 80% reduction in sample size required to detect a given effect of exposure, or, conversely, to an increase in statistical power given available sample size. For example, in the NACC cohort, p-values for detection of a one year change were p=0.086 without run-in, and p=8e-5 with a run-in period. Incorporating an early run-in period to longitudinal cohort studies of aging and Alzheimer's disease can dramatically improve the statistical power of these studies to detect predictors of cognitive decline.

Abstract citation ID: igae098.4020

### LONGITUDINAL TRANSITIONS IN FRAILTY STATES OVER SIX-YEAR FOLLOW-UP: RESULTS FROM THE KFACS

Heeeun Jung, Hyung Eun Shin, Daehyun Lee, Jae Young Jang, Hyunjin Cho, Nahyun Lim, Chang Won Won, and Miji Kim, *Kyung Hee University, Seoul, Republic of Korea*

The longitudinal nature of frailty reveals its dynamic process through transitions of frailty states over time. However, the transitions between frailty states, including their progression and reversibility, remain unclear in Korea. We investigated these longitudinal transitions in frailty states among Korean community-dwelling older adults. We conducted a 6-year follow-up analysis of 2,633 older adults (52.0% women; mean age 76.5 ± 3.9 years) from the Korean Frailty and Aging Cohort Study (KFACS). Participants who had frailty status measured at baseline and experienced at least one frailty state transition during follow-up were included. Frailty states are categorized as follows: robust, pre-frail, and frail according to the Fried frailty phenotype and death. Multi-state Markov models estimated 1-year transition probabilities and explored associations between sociodemographic factors (age, sex, education level) and frailty transitions. Over a mean follow-up period of 4.6 years, a total of 5,942 frailty state transitions were observed. Of these, 3,568 (61.5%) were maintained, 1,358 (22.9%) worsened, and 926 (15.6%) improved. The 1-year progression probability to pre-frail was higher than the probability of moving to frail (20.2% vs. 0.9%) in the robust state. Pre-frail individuals were more likely to revert to robust than frail individuals (17.7% vs. 2.6%). Furthermore, the 1-year transition to death was highest for those in the frail state compared to other states. Frailty progression was associated with older age, being female, and lower education level. In conclusion, understanding the dynamic changes between frailty states and the sociodemographic factors influencing these changes may offer opportunities to reverse frailty progression.

Abstract citation ID: igae098.4021

### MACHINE LEARNING-BASED PREDICTION MODEL FOR IN-HOSPITAL MACCE IN OLDER PATIENTS WITH CHD

Wen Tang[1], Xuedong Wang[2], Xuebing Yang[3], and Ying Sun[4], *1. Beijing Friendship Hospital, Capital Medical University, Beijing, Beijing, China (People's Republic)*, *2. Hainan University, Haikou, Hainan, China (People's Republic)*, *3. Chinese Academy of Sciences, BEIJING, Beijing, China (People's Republic)*, *4. Beijing Friendship Hospital, Capital Medical University, BEIJING, Beijing, China (People's Republic)*

**Objectives:** Older patients with coronary heart disease (CHD) are at a higher risk of major adverse cardiac and cerebrovascular events (MACCE) during hospitalization. This study aims to develop a risk prediction model for in-hospital MACCE events in older patients with CHD using emerging machine learning methods.

**Methods:** The study included 885 older inpatients with CHD (74.68±6.89 years, 58.3% male). We created prediction models via XGBoost, Support Vector Machine(SVM), random forests, Histogram-Based Gradient Boosting Classifier(HGBC) and Gradient Boosting Decision Trees(GBDT) using 38 features, and evaluated its performance using five-fold cross-validation..

**Results:** MACCE was identified in 106 patients. The XGBoost model demonstrated the best performance, achieving an accuracy (ACC) of 93.56% and an area under the curve (AUC) of 0.8736 (95% confidence interval: 0.756–0.967). The SVM model achieved an ACC of 90.17% and an AUC of 0.8028 (95% CI: 0.684–0.906); the Random Forest model achieved an ACC of 91.53% and an AUC of 0.8675 (95% CI: 0.764–0.953); the HGBC model achieved an ACC of 93.45% and an AUC of 0.8550 (95% CI: 0.741–0.953); and the GBDT model achieved an ACC of 93.11% and an AUC of 0.8674 (95% CI: 0.756–0.957). Finally, SHAP analysis revealed that the top three most important features were troponin T, the FRAIL score, and the NYHA functional classification.

**Conclusion:** Machine learning models can effectively predict in-hospital MACCE events in older patients with CHD. Troponin levels, frailty status, and cardiac function are strong predictive indicators.

Abstract citation ID: igae098.4022

### MALNUTRITION RISK AMONG OLDER AMERICANS ACT (OAA) NUTRITION PROGRAM PARTICIPANTS IN 2022 AND 2023

Jaime Gahche[1], Lydia McGrath[2], Laura Borth[3], Shirley Chao[4], Judy Simon[3], MaryBeth Arensberg[5], and Johanna Dwyer[6], *1. NIH Office of Dietary Supplements, Bethesda, Maryland, United States*, *2. Tufts University, Boston, Massachusetts, United States*, *3. National Association of Nutrition and Aging Services Programs, Washington DC, District of Columbia, United States*, *4. FoodPolicy Insights, Boston, Massachusetts, United States*, *5. Abbott Nutrition Division of Abbott, Abbott Nutrition Division of Abbott, Ohio, United States*, *6. National Institutes of Health, Bethesda, Maryland, United States*

Identifying older adults at risk for malnutrition is essential to combat poor outcomes associated with malnutrition. In 2022, the National Survey of Older Americans Act Participants (NSOAAP) included the Malnutrition Screening Tool (MST), a validated, self-report of unintentional weight loss and poor appetite. This provided the first opportunity to evaluate malnutrition risk (MR, defined as a score ≥2) consistently in a representative sample of Older Americans Act (OAA) program participants. In this cross-sectional analysis of 2022 (n=3531) and 2023 (n=4159) NSOAAP data, MR prevalence was estimated for total sample and within OAA programs included in NSOAAP (home-delivered meals (HDM), congregate meals (CM), homemaker (HM), case management (CS), transportation services (TR)). 2022 overall MR prevalence was 19.4%, with no significant differences between programs. 2023 overall MR prevalence remained fairly stable (17.2%) with no significant difference vs. 2022 (p< 0.0984). 2023 MR prevalence was 20.8% (HDM), 9.9% (CM), 26.6% (HM), 21.1% (CS), 17.7% (TR). 2023 MR prevalence among CM participants was significantly lower compared to MR risk in other OAA programs and also significantly lower compared to 2022 (9.9% vs. 16.7% p< 0.0195). Further research is needed to understand these trends, which may be partially affected by pandemic-related program changes. These data indicate a substantial proportion of OAA program participants were at MR in 2022—with very little change in 2023, except decreased prevalence in CM program participants. The findings highlight the continued need for including MR measures in the NSOAPP to evaluate program effectiveness and help reduce malnutrition in older adults.

Abstract citation ID: igae098.4023

### MARITAL TRAJECTORIES, RACE/ETHNICITY, NATIVITY, AND CARDIOVASCULAR COMORBIDITIES AMONG US ADULTS

Maki Karakida[1], Jeffrey Stokes[2], and Qian Song[2], *1. University of Massachusetts Boston, UMass Boston, Massachusetts, United States*, *2. University of Massachusetts Boston, Boston, Massachusetts, United States*

Marital loss – one of the most stressful and emotional life events – often ties to negative cardiovascular health outcomes, which can be comorbid with other chronic conditions, like diabetes. Cardiovascular complications, spousal bereavement, and grey divorce are increasingly common in older adulthood, yet their effects may vary among US-born and foreign-born racial-ethnic minorities. The present study examines the longitudinal relationships between marital quality, race/ethnicity, nativity, and cardiovascular comorbid conditions among 6,953 Americans aged 58 and over. Leveraging the RAND HRS Longitudinal File 2020 (2010–2020) and binomial logistic regressions, we analyzed how married or partnered adults in 2010 undergone marital loss and developed heart disease, high blood pressure and hypertension, stroke, and/or diabetes by 2020, during the COVID-19 crisis. Across all the models, the main effects highlighted that the widowed group had a significantly higher likelihood of cardiovascular comorbidities (all p < 0.026) unlike the married and divorced groups. The model, examining the intersection of race/ethnicity and nativity – interacted with marital status – revealed that compared with US-born White married adults, US-born Hispanic divorcees (OR = 4.39; p < 0.022) and foreign-born non-Hispanic Other divorcees (OR = 10.49; p < 0.038) indicated 4-10 times greater cardiovascular comorbidity risks. Findings suggest that among older nonnative and/or racial-ethnic minorities, spousal bereavement and martial dissolution/separation developed worse cardiovascular comorbid conditions, especially during the first year of the pandemic outbreaks. Cardiovascular comorbidities, interacted with race/ethnicity, nativity, and marital status, are warranted to be further investigated.

Abstract citation ID: igae098.4024

### MEDIAL-LATERAL POSTURAL SWAY INCREASE AMONG COMMUNITY-DWELLING OLDER ADULTS WITH SELF-REPORTED ANXIETY

Jethro Raphael Suarez[1], Amber Blount[2], Kworweinski Lafontant[2], Nichole Lighthall[2], Helen Huang[2], Joon-Hyuk Park[2], and Ladda Thiamwong[2], *1. University of Central Florida, 2. University of Central Florida, Orlando, Florida, United States*

Anxiety is a prevalent and concerning issue for older adults. Previous research has shown that anxiety negatively affects overall balance, but the effect of self-reported anxiety on postural sway direction for older adults is unclear. This study aimed to compare sway direction between community-dwelling older adults that were categorized as having moderate-to-high anxiety and those that had low-to-no anxiety. The BTrackS Balance System (BBS), a stationary and simple balance assessment instrument, was used for measuring ranges of sway in the medial-lateral and anterior-posterior directions during a non-visual static balance test. The Geriatric Anxiety Inventory Short Form (GAI-SF) is a self-reported assessment of anxiety and was used for categorization. We cross-sectionally compared sway direction from 170 community-dwelling older adults aged 61 to 91 (152 women, mean age = 74.3 ± 7.1 years) who completed a BBS assessment and completed the GAI-SF using Mann-Whitney U tests. A total of 133 older adults were categorized as having low-to-no anxiety (GAI-SF ≤ 2), while 37 were categorized as having moderate-to-high anxiety (GAI-SF ≥ 3). The groups significantly differed in the medial-lateral direction (p = 0.03), but not the anterior-posterior direction (p = 0.14). These results show that older adults with increased self-reported anxiety may move in the medial-lateral direction more than those with less self-reported anxiety. However, these results may be limited by other factors (e.g., past injuries, depression). Future research should consider such factors among older adults that show symptoms of anxiety.

Abstract citation ID: igae098.4025

### MONITORING CHRONIC PAIN AND QUALITY OF LIFE USING TABLET SURVEYS IN OLDER ADULTS LIVING IN RURAL ARKANSAS

Regina Gibson[1], Gohar Azhar[1], Shakshi Sharma[1], Riley Swafford[2], Karen Coker[1], and Jeanne Y Wei[1], *1. University of Arkansas for Medical Sciences, Little Rock, Arkansas, United States*, *2. University of Arkansas for Medical Sciences, Litttle Rock, Arkansas, United States*

Arkansas has the second highest legal opioid prescribing rate in the U.S. We did a pilot study to assess the feasibility of using tablets to measure chronic pain and its impact of cognition, sleep, depression, and quality of life in older adults.

Seventy-one older adults were trained in the use of tablets to use at home for 90 days that were pre-loaded with surveys on opioids, daily pain diary, sleep Pittsburgh Sleep Quality Index (PSQI), and Geriatric Depression scale (GDS-15). 1,944 responses were recorded over three months by 71 participants with mean age ± SD 70 ± 8 years (range 55-91). Males 23%, females 50%. Of females, 51% were African American and 49% white. All but one male was white. 80% of participants reported some pain with most ratings between 5 or 6 out of 10 (10 being worst). None of the participants were on opioids during the period of the study. Memory symptoms were reported most frequently in 28% of respondents, followed by daytime sleepiness in 33%. PSQI scores indicated poor sleep quality in 28%. Only one participant's GDS score suggested depression. The feasibility of using tablet-based assays in older adults living in rural regions was successfully demonstrated, showing that older adults are receptive to learning new information and recording their health parameters on a tablet. The data obtained can provide clinicians a broader picture of the older adults pain-associated symptomatology and thereby assist them in designing better personalized pain management programs.

Abstract citation ID: igae098.4026

### MULTILEVEL ANALYSIS OF AARC GAINS AND LOSSES IN OLDER ADULTS

Natsue Shiraishi[1], Yasuhiro Yamamoto[2], Takeshi Nakagawa[3], and Takashi Horiuchi[1], *1. Okayama University, Okayama, Okayama, Japan*, *2. Hyogo University of Teacher Education, Kato, Hyogo, Japan*, *3. Osaka University, Suita, Osaka, Japan*

This study investigates the factors associated with Awareness of Age-Related Change (AARC) gains and losses in older adults. Numerous educational institutions offer programs tailored to older adults, fostering continuous learning. Therefore, in the case of older people, lifelong learning, and not just educational history, should be investigated. Thus, this study examined whether lifelong learning as social participation is associated with AARC. We conducted a comprehensive analysis using an 12-month 3-wave survey collected between 2023 and 2024 (N=878, ages 70 and above at baseline). The independent variables were those measured at Time 1. AARC was analyzed in association with initial values. As a result, initial AARC-gain values were higher for those with more lifetime learning at Time 1. Our multilevel model results indicate that age ($b = 0.02$, $p < 0.001$) and lifelong learning ($b = 0.11$, $p < 0.001$) significantly are associated with levels of AARC-gains, while physical activity ($b = -0.0002$, $p < 0.001$) and IADL ($b = -1.49$, $p < 0.001$) are associated with levels of AARC-losses. No individual differences in slope were observed. These findings underscore the importance of continuous education and physical activity in mitigating age-related losses and enhancing gains, providing valuable insights for interventions aimed at improving the well-being of older adults.

Abstract citation ID: igae098.4027

### MULTISENSORY LOSS AND DEPRESSION IN THE ATHEROSCLEROSIS RISK IN COMMUNITIES NEUROCOGNITIVE STUDY (ARIC-NCS)

Joseph Shen[1], Alison Huang[1], Jennifer Deal[2], Honglei Chen[3], and Anna Kucharska-Newton[4], *1. Johns Hopkins Bloomberg School of Public Health, Baltimore, Maryland, United States*, *2. Johns Hopkins University, Baltimore, Maryland, United States*, *3. Michigan State University, Michigan State University, Michigan, United States*, *4. University of North Carolina at Chapel Hill, Chapel Hill, North Carolina, United States*

Multisensory loss is a potentially modifiable risk factor for depression, but population-level evidence is lacking. This study quantified the association between multisensory loss across four senses (hearing, vision, smell, touch) and depressive symptoms in older US adults. Data (N=812) were from the ARIC-NCS and the Eye Determinants of Cognition study joint cohort (2016-2017), an observational study of older adults from Washington County, MD and Jackson, MS. 812 participants with complete data on hearing, vision, olfaction, peripheral neuropathy, depression, and demographic and health covariates were included. Sensory loss was measured using objective tests (pure tone audiometry [hearing], Early Treatment of Diabetic Retinopathy Study chart [vision], Sniffin' Sticks tests [olfaction], and monofilament tests [peripheral neuropathy]). Multisensory loss was analyzed as a count (0-4). Depressive symptoms were measured by the 11-item Center for Epidemiologic Studies Depression (CES-D-11) scale. Scores were modeled continuously. The incidence rate ratio of depressive symptoms associated with number of sensory losses was assessed using covariate adjusted negative binomial regression. Of 812 participants (aged 71-93 years, 62.7% female, 58.6% White, 43.3% with higher than a high school education) 30.3% had one sensory loss, 31.4% had two, 17.6% had three, and 5.3% had four. Each additional sensory loss was associated with a 10% (Incidence rate ratio of CES-D-11 Score: 1.10; 95% CI: 1.02-1.20) increase in CES-D-11 score in the fully adjusted model. Multisensory loss is associated with depression in older adults. Clinical awareness of this association is valuable for the promotion of mental well-being among patients with sensory loss.

Abstract citation ID: igae098.4028

### NAVIGATING POST-ACUTE CARE: LIVED EXPERIENCES OF MINORITIZED OLDER ADULTS

Dominique Como, *University of Pittsburgh, Pittsburgh, Pennsylvania, United States*

Annually, over 40% of Medicare beneficiaries use post-acute care (PAC) after hospitalization to aid functional and medical recovery. Disparities in PAC access, care quality, and patient outcomes are evident across social identities (e.g., gender, ethnicity) and social determinants of health indicators (e.g., housing, food access). However, limited research has delved into the lived experiences of older adults using PAC services. Understanding barriers and facilitators from the patient perspective is crucial for addressing health disparities. This study explored the lived experiences of older adults in PAC settings. As part of a mixed-methods study, older adults (aged 55 or older) who self-identified as Black/African American or Hispanic/Latino/Spanish origin and were actively receiving care in a PAC facility in Southern California were invited to participate in interviews. A semi-structured interview guide was developed based on information obtained from a literature review and revised following content review from community member expert. Eight participants were interviewed between May and July 2024. Thematic analysis revealed four key themes, highlighting salient areas, influencing post-acute experiences: Patient Involvement in Healthcare Experiences (e.g., active participation, patient autonomy, and self-advocacy in healthcare decisions); Provider Competence & Attitude (e.g., provider communication, knowledge, and disposition); Care Delivery Considerations (e.g., staffing levels, time constraints, physical handling practices); and Support Networks & Resources (e.g., familial support, facility resources). These findings offer valuable insights for enhancing PAC patient health and satisfaction, by providing comprehensive high-quality care, improving the effectiveness of care delivery, and strengthening patient-provider interactions to better serve minoritized older adults.

Abstract citation ID: igae098.4029

### NAVIGATING THE HOME CARE LANDSCAPE FOR MEDICARE BENEFICIARIES: FINDINGS FROM A SECRET SHOPPER STUDY

Abigail Shilvock[1], Peggy Leung[2], and Madeline Sterling[2], *1. Cornell University Medical College, New York, New York, United States, 2. Weill Cornell Medicine, New York, New York, United States*

An estimated 2.9 million patients access home health care annually through Medicare, primarily provided by certified home health care agencies (CHHAs). Despite Medicare covering 60-day episodes of home health care, patients often experience difficulty obtaining adequate and timely care. We investigated the real-world experience of Medicare beneficiaries and their caregivers seeking care through CHHAs by conducting a cross-sectional Secret Shopper study of CHHAs in New York State between February and March 2024. A trained investigator, posing as a family caregiver seeking home care for an older adult, contacted CHHAs using a standardized script informed by the Levesque conceptual framework of access to healthcare. Out of 110 identified CHHAs, 86 were eligible for analysis. 72% of CHHAs reported availability to provide skilled home health nursing services, while 66% reported availability to provide unskilled services (i.e., home health aide). Only 5% offered private pay skilled services with nursing visits costing \$115-\$300 and physical therapy \$145-\$230 per visit. One in five CHHAs provided an hourly rate for aide services, with a mean rate of \$35.60 per hour. Nearly 80% of CHHAs could obtain insurance approval and over 75% could perform an in-home nursing evaluation within a week of referral placement. However, only 50% reported the patient would start receiving services within a week of evaluation. Qualitative analysis of field notes collected by the caller revealed three main themes: 1) numerous barriers to accessing home health care, 2) variability in access and timeliness of services across agencies, and 3) inconsistent customer service.

Abstract citation ID: igae098.4030

### NEIGHBORHOOD SOCIOECONOMIC STATUS AND SARCOPENIA IN COMMUNITY-DWELLING OLDER ADULTS

Xiangyu Zhai, Moyao Wang, Shiyin Zhan, and Doris Sau Fung Yu, *The University of Hong Kong, Hong Kong, China (People's Republic)*

Individual-level socioeconomic characteristics are known to predict sarcopenia, but less is known about how the neighborhood context shapes this debilitating problem among older adults. This study aims to examine the association between nSES and sarcopenia. We included 943 older adults aged over 60 years (56.0% female, mean age 72 ± 8.1). Sarcopenia was diagnosed according to the Asian Working Group for Sarcopenia 2019 (AWGS2019) algorithm covering skeletal muscle mass, muscle strength, and physical performance. nSES, the sum of the standardized values of the proportion of single-person households, never-married status, low education attainment, households with low income, unemployment, non-professional vocation, and non-owner occupiers, were obtained from the Census and Statistics Department and divided into distribution-based tertiles of all 292 Tertiary Planning Units in Hong Kong (T1 = highest nSES). There were 89 participants (8.4%) diagnosing sarcopenia. The sample was distributed across three tertiles of nSES (T1: 157 participants [16.6%]; T2: 193 participants [20.5%]; T3: 593 participants [62.9%]). Compared with T1, the lower nSES tertiles showed higher odds of sarcopenia, after adjusting for age, sex, body mass index, and individual-level socioeconomic characteristics (T2: OR = 1.38 [95% CI, 0.49 – 3.87], T3: OR = 3.53 [95% CI, 1.51 – 8.28], T2: aOR = 1.53 [0.51 – 4.54], T3: aOR = 3.12 [95% CI, 1.21 – 8.03]). This is the first study to identify the negative relationship between comprehensive nSES and the prevalence of sarcopenia. The findings urge the need to develop socio-environmental sensitive risk stratification and preventive care to manage later-age sarcopenia.

Abstract citation ID: igae098.4031

### NOVEL NUTRITION EQUITY METHOD: OLDER ADULT NUTRITION EQUITY INDEX (NEI) DEVELOPED IN HEALTH ABC & EXPANDING TO PHRESH

Aarohee Fulay[1], Tamara Dubowitz[1], Samaneh Farsijani[2], Kerri Freeland[2], Jimmie Roberts[3], Andrea Rosso[2], Denise Houston[4], and Elsa Strotmeyer[2], *1. University of Pittsburgh School of Public Health, Pittsburgh, Pennsylvania, United States, 2. University of Pittsburgh, Pittsburgh, Pennsylvania, United States, 3. Hebrew SeniorLife, Boston, Massachusetts, United States, 4. Wake Forest University School of Medicine, Winston Salem, North Carolina, United States*

Nutrition equity – no barriers to obtaining healthy foods – is critical for older adults. Current nutrition equity research lacks sufficient attention to multiple and concurrent processes that may contribute to acquiring healthy food. For example, improving food security, i.e. fiscal ability to obtain healthy foods, alone may not improve diet without accounting for geospatial food access or individual physical limitations. We designed a novel nutrition equity index (NEI) for older adults which combines food security, food access, and physical limitations data from older Black and White adults in Pittsburgh, PA and Memphis, TN from the Health, Aging, and Body Composition Study (Health ABC; 51.7% women; 37.9% Black; 74.7±2.9 years). In multivariable linear regression, low (12.1%) and moderate nutrition equity (32.5%) vs. high were associated with lower dietary quality (measured via Healthy Eating Index), though after adjusting for race (which was included as a marker for social/structural factors), this NEI association attenuated to non-significance, suggesting effect modification. We will extend our analysis to examine nutrition equity with a historically marginalized population living in two predominantly Black neighborhoods by using recent data from Pittsburgh Hill/Homewood Research on Neighborhood Change and Health (PHRESH; 80.7% women; 94.1% Black; 64.8±9.3 years). We will expand the NEI to include additional food access (e.g. distance to major food shopping venue) and functional ability measures in an expanded population of middle-aged to older adults. Expansion of the NEI and its application in this new population/setting will highlight the structural nutrition equity components which are critical to address.

Abstract citation ID: igae098.4032

### NURSE PERCEPTIONS OF A SYSTEMS-BASED OLDER ADULT MOBILITY INTERVENTION IN ACUTE CARE UNITS

Olivia Erickson[1], David Tucker[1], Barbara King[2], and Linsey Steege[1], *1. University of Wisconsin-Madison, Madison, Wisconsin, United States*, *2. UW-Madison, Madison, Wisconsin, United States*

Lack of ambulation during a hospital stay is the leading cause of hospital-acquired disability for older adult patients. Acute care nursing staff are identified as responsible for maintaining patient functional ability. However, nurses infrequently walk patients due to system barriers. Multiple interventions to improve patient ambulation have been tested with mixed results. Few researchers have designed interventions that target nurse barriers or addressed factors that may impact nurse acceptance of a mobility intervention or sustainability. This study aimed to understand how nurses experienced implementing a systems-based mobility intervention and identify which components of the intervention need further adaptation to create the right "fit" for inpatient units. A qualitative study using directed content analysis was conducted. Four adult medical inpatient units from 2 academic medical centers in the Midwest served as the study sites. 53 staff members participated in 8 focus groups that were audio recorded and transcribed. Four categories that describe the nursing staff experience were identified: Needing the Right Dose, Fostering Communication, Being Valued, and Making Ambulation Visible. Nurses perceived the intervention as adaptable to fit their unit needs, improved communication between staff members, patients, and healthcare providers, and aligned with their value of achieving good patient outcomes. It is critical to understand nurses' experiences with implementing a systems-based intervention when promoting behavior change to improve older adult patient ambulation. Failed implementation efforts can have serious consequences on nurses' perceptions of patient ambulation, the sustainability of programs, and the quality of care for older adults.

Abstract citation ID: igae098.4033

### NURSE STAFFING LEVELS AND OTHER FACTORS ASSOCIATED WITH NURSING HOME QUALITY

Juh Hyun shin[1], Charlene Harrington[2], Soo-Kyoung Lee[3], Jinhyun Ahn[4], Yeonwoo Lee[4], and Sanna Ali[5], *1. George Washington University, Ashburn, Virginia, United States*, *2. University of California, San Francisco, San Francisco, California, United States*, *3. Seoul National University, Seoul, Republic of Korea*, *4. Jeju National University, Jeju-si, Cheju-do, Republic of Korea*, *5. George Washington University, WASHINGTON DC, District of Columbia, United States*

This study evaluated the quality of nursing homes (NHs) by analyzing deficiency citations reported by the Centers for Medicare and Medicaid Services (CMS). Key factors examined included CMS nurse staffing standards, ownership types, demographic characteristics, facility size, occupancy rates, geographic location, and Medicaid funding. Data from all U.S. NHs for Fiscal Year 2019 were sourced from CMS and LTCFocus, encompassing 13,086 facilities. Using ordinary least squares (OLS) regression, the study found that NHs with a total hours per resident day (HPRD) of 4.1 and registered nurse (RN) HPRD of 0.75 experienced fewer deficiency citations (1.18 and 0.8, respectively). In contrast, the CMS 2024 standards predict 3.48 total HPRD and RN HPRD of 0.5, with deficiencies of 0.82 and 0.6. The analysis showed that higher RN, licensed nurse, and certified nursing assistant HPRD correlated with reduced deficiency scores, indicating better quality. Conversely, higher bed occupancy rates were associated with fewer deficiencies, while increased proportions of non-White residents and urban locations correlated with more deficiencies. Additionally, larger facilities and those with higher Medicaid coverage had more deficiencies. Not-for-profit NHs generally had lower deficiency scores. These results emphasize the crucial role of adequate nurse staffing in maintaining NH quality and illustrate how various provider characteristics influence deficiency levels.

Abstract citation ID: igae098.4034

### NURSING HOME CONTEXTUAL FACTORS IMPACTING PALLIATIVE CARE CONSULTATION IMPLEMENTATION

Joan Carpenter[1], Elisha Oduro[1], Ruth Lopez[2], and Kathleen Unroe[3], *1. University of Maryland Baltimore, Baltimore, Maryland, United States*, *2. MGH Institute of Health Professions, Boston, Massachusetts, United States*, *3. Indiana University, Indianapolis, Indiana, United States*

Palliative care (PC) consultation has been shown to improve the quality of life of nursing home (NH) residents. However, its implementation in NHs is uneven and poorly characterized. Grounded in the Integrated Promoting Action on Research Implementation in Health Services framework, we used a qualitative descriptive approach to explore how the inner context (organization leadership, staff, culture) and the outer context (policy, finances) impact the implementation of PC consultation in NHs. We conducted semi-structured interviews with key partners in fourteen NHs in the Eastern US. We used directed content analysis to analyze transcripts and identify key themes. Participants (N=33) were NH leaders, nurses, social workers, and palliative care agency leaders and nurse practitioners, predominantly female (93.9%), White (93.9%), and one-third had at least 20 years of experience working in NHs. Our findings reveal factors within the inner and outer contexts that either pull consultations toward or push consultations away from residents. In the inner context, factors that pull consultations toward residents were supportive organizational leadership, staff referrals with detailed residents' information and assistance during consultations, and a welcoming NH environment. However, the absence of a reliable screening tool for staff to identify residents for consultation was seen as pushing away from residents. In the outer context, policies that levy monetary penalties for hospital readmission were seen to pull consultations toward residents while financial "burdens" and "drains" were seen as pushing consultations away from residents. These fndings will be used to develop a sustainable and scalable PC consultation model in NHs.

Abstract citation ID: igae098.4035

### OLDER ADULTS' PERSPECTIVES ON USING AN AI-AIDED SMARTPHONE

Yi-Ling Hu[1], and I-Chen Chen[2], *1. Chag Gung University, Toayuang, Taoyuan, Taiwan (Republic of China)*, *2. Da-Yeh University, Changhua, Changhua, Taiwan (Republic of China)*

Falls and injurious falls are expected to increase with the aging population, causing significant distress. Developing an artificial intelligence (AI)-aided smartphone system to minimize home visits may reduce home visits and costs. Older adults' perspectives on AI and related implementation tasks are crucial. Therefore, focus groups were conducted to explore older adults' acceptance and concerns about using an AI-aided smartphone system. Community-dwelling older adults (over 65 years old) without cognitive impairment were recruited from local senior centers, hospitals, and the Department of Occupational Therapy at Chang Gung University (CGU), Taiwan. Two coders independently analyzed transcripts using a framework analysis approach. Data saturation was reached when no new ideas emerged in the final focus groups. The research team has conducted three focus groups with older adults (n=15). The main finding showed that older adults viewed AI-aided Smartphones as helpful but preferred traditional home programs with human contact; they were highly aware of falls but were concerned that new AI technology would steal their smartphone information. Other themes, such as fear of scams, lack of knowledge, and lack of age-friendly smartphone platforms, also emerged. The result of this study indicates implementing the AI-aided smartphone program for fall prevention is possible, but multiple barriers need to be removed for older adults. The finding may help health professionals aid traditional fall prevention programs with new technology and may advance program effectiveness.

Abstract citation ID: igae098.4036

### ORAL FRAILTY ASSESSMENT TOOL: INSTRUMENT DEVELOPMENT AND CROSS-SECTIONAL VALIDATION STUDY

Qiaoqin Wan[1], Yue Yang[2], and Chengfengyi Yang[3], *1. Peking University Health Science Center, Beijing, Beijing, China (People's Republic)*, *2. Peking University School and Hospital of Stomatology, Beijing, Beijing, China (People's Republic)*, *3. Peking University, Beijing, Beijing, China (People's Republic)*

Oral frailty is widespread in older adults, which will gradually contribute to multiple adverse health outcomes. The current assessment tools may not comprehensively assess oral frailty, thus a practical and feasible oral frailty scale is needed. The study aimed to develop an oral frailty assessment tool and evaluate its psychometric properties. The scale was developed in two stages. First, based on the Gilbert Multidimensional Conceptual Model of Oral Health, the items were generated through a literature review and Delphi expert correspondence. For psychometric analysis, content validity, structure validity, concurrent validity, convergent and discriminant validity, and internal consistency reliability of the scale were assessed. Data were collected from a convenient sample of 372 Chinese older adults, and exploratory and confirmatory factor analyses were used. The diagnostic accuracy was further conducted through receiver operating characteristic curve analysis. The newly developed scale consisted of five dimensions and 16 items. The content validity of the scale was 0.974, with 0.649 for concurrent validity, and the aggregation and differentiation validity were ideal as well. Besides, the samples were cross-validated by exploratory factor and confirmatory factor analyses. The internal consistency reached 0.865, and the retest reliability was 0.940. Meanwhile, by comparing the diagnostic accuracy with Oral Frailty Instrument-6 (OFI-6), it was found that the cutoff value was 9.5. Findings demonstrate that the Oral Frailty Assessment Tool has acceptable content validity, structure validity and reliability in measuring oral frailty. A wider range of cultures and samples are needed to refine the scale.

Abstract citation ID: igae098.4037

### ORAL FRAILTY INDEX-8 QUESTIONNAIRE'S VALIDITY & ASSOCIATION WITH SARCOPENIA & PHYSICAL PERFORMANCE IN OLDER ADULTS

Herb Howard Hernandez, Celestine Lim, Jun Pei Lim, Jia Qian Chia, Audrey Yeo, Cai Ning Tan, Wee Shiong Lim, and Justin Chew, *Tan Tock Seng Hospital, Singapore, Singapore*

Oral frailty (OF) is the age-related decline in oral function, and is associated with negative outcomes, including aspiration, malnutrition, and physical frailty. Despite its importance, validated tools for OF assessment are limited, preventing early detection and intervention. The Oral Frailty Index-8 (OFI-8) is a questionnaire assessing tooth loss, chewing, swallowing and oral health behaviours. We aim to evaluate OFI-8's construct validity and determine its associations with muscle strength, physical performance, sarcopenia, and functional outcomes. We conducted a cross-sectional analysis of 300 community-dwelling older adults (mean age 67.4+/-7.1years), using exploratory factor analysis (EFA) to determine OFI-8's factor structure. We assessed OFI-8's correlations with dual-energy X-ray absorptiometry (DXA)-derived appendicular lean mass corrected for height (ALM/ht2), maximum handgrip strength and usual gait speed. We then examined the associations of OFI-8 with outcomes of sarcopenia (self-reported questionnaire,SARC-F), physical performance (Short Physical Performance Battery,SPPB) and IADL function (Lawton scale) using multiple linear regression adjusted for age and sex. EFA revealed a 3-factor structure for OFI-8 (Factor-1:Oral and Swallowing Problems; Factor-2:Dental Care; Factor-3:Dietary and Social Habits). Higher OFI-8 scores, indicating OF, demonstrated weak but significant correlations with low lean mass(r=-0.119,P=0.038), impaired handgrip strength(r=-0.218,P=0.001), and slow gait speed(r=-0.203,P=0.004), and was associated with sarcopenia (Beta=0.044,95%CI 0.0012 to 0.089,P=0.044), impaired physical performance (Beta=-0.088,95%CI -0.156 to -0.021,P=0.011), and IADL impairment (Beta=-0.080,95%CI -0.15 to -0.010,P=0.026). Our results provide preliminary evidence for the factor structure and validity for the OFI-8, as well as its association with sarcopenia and functional impairment. Further research is required to evaluate its predictive validity for longitudinal outcomes.

Abstract citation ID: igae098.4038

### OUTCOMES OF A PALLIATIVE CARE INTERVENTION FOR ADULTS WITH DEMENTIA IN SKILLED NURSING FACILITIES

Elisha Oduro[1], Joan Carpenter[1], Nancy Hodgson[2], Laura Hanson[3], Shijun Zhu[4], Merve Gurlu[1], and Mary Ersek[5], *1. University of Maryland Baltimore, Baltimore, Maryland, United States*, *2. University of Pennsylvania, Philadelphia, Pennsylvania, United States*, *3. University of North Carolina School of Medicine, Chapel Hill, North Carolina, United States*, *4. University of Maryland School of Nursing, Baltimore, Maryland, United States*, *5. Department of Veterans Affairs, Philadelphia, Pennsylvania, United States*

Older adults living with Alzheimer's disease and related dementias (ADRD) often receive disease-focused rehabilitative care in post-acute skilled nursing facilities (SNF) without access to palliative care (PC). To address this gap, our team developed a primary PC intervention and tested it among SNF patients with ADRD. We conducted a pilot pragmatic clinical trial comparing the primary PC intervention to usual care in 9 SNFs in the MidAtlantic US. Using a nonequivalent group design, SNF resident participants (n=18) were clustered by site and assigned to intervention or control group in a 2:1 (respectively) ratio. We measured patient quality of life with the Palliative Outcome Scale Version 2 (POSv2, range 0-40; higher scores reflect poorer quality of life) and analyzed data using linear mixed effects model. Patients (n=13 intervention, n=5 control) had a mean age 84 years, were mostly White (94%) and female (72%). Our results showed that quality of life had a trend of improvement for the intervention group (POSv2 baseline=12, follow up=10.5) but not for the control group (POSv2 baseline=6.6, follow up=9.3); the intervention demonstrated a clinically meaningful effect yet was not statistically significant (effect size=-.51, p=.20). Our findings suggest that a primary PC intervention is promising in its effect to improve quality of life among patients with ADRD during SNF care. Future work will focus on testing the intervention in a larger more diverse sample and exploring the most valuable intervention components as assessed by patients with ADRD and their care partners.

Abstract citation ID: igae098.4039

### PAIN AND MOBILITY OF OLDER ADULTS IN MEDICAL AND SURGICAL UNITS: AN EHR-BASED DATA ANALYSIS

Urszula Snigurska[1], Sarah Ser[2], Mattia Prosperi[3], Ragnhildur Bjarnadottir[4], Robert Lucero[5], and Todd Manini[6], *1. University of Florida Clinical and Translational Science Institute, Gainesville, Florida, United States*, *2. University of Florida College of Public Health and Health Professions & College of Medicine, Gainesville, Florida, United States*, *3. College of Public Health and Health Professions & College of Medicine, Gainesville, Florida, United States*, *4. University of Florida College of Nursing, Gainesville, Florida, United States*, *5. University of California Los Angeles, Los Angeles, California, United States*, *6. University of Florida College of Medicine, Gainesville, Florida, United States*

Pain accelerates age-related decline in mobility. One in five hospitalized older adults have pain. Pain experience varies by the type of hospitalization. The purpose of this study was to describe pain and mobility in hospitalized older adults. We hypothesized that pain would be higher in patients with lower mobility. We analyzed electronic health record data from an academic health center in Florida between 2012 and 2021. We used the mean pain score and mean score on the mobility subscale of the Braden Scale for each encounter. We used the Wilcoxon Rank-Sum Test and Pearson's correlation coefficient to measure the differences and relationship between pain and mobility. The cohort included 19,854 older adults (13,878 medical and 5,976 surgical patients). The mean mobility score was 3.05 ± 0.57 in medical and 2.88 ± 0.51 in surgical patients ($W = 48697407$, $p < 0.001$). The mean pain score was 1.93 ± 2.72 in medical and 2.25 ± 2.61 in surgical patients ($W = 36893487$, $p < 0.001$). There was no correlation between pain and mobility in either medical ($r = 0.01$, $p = 0.427$) or surgical patients ($r = 0.02$, $p = 0.085$). Mean pain intensity may not be associated with mobility in hospitalized older adults. Instead, the highest level of pain or uncontrolled pain may be associated with mobility decline. Our findings provide motivation for a future study to account for confounders of the relationship between pain and mobility, such as analgesics, and temporality of pain and mobility assessments in the hospital.

Abstract citation ID: igae098.4040

### PAIN ASSESSMENT AND MANAGEMENT IN NURSING HOME RESIDENTS WITH ADRD: INSIGHTS FROM INTERDISCIPLINARY TEXT MESSAGES

Ashley Woods[1], Matthew Farmer[2], Mihail Popescu[3], and Kimberly Powell[4], *1. University of Missouri, Santa Fe, New Mexico, United States*, *2. University of Misosuri, Colombia, Missouri, United States*, *3. University of MIssouri, University of Missouri, Missouri, United States*, *4. University of Missouri, Columbia, Columbia, Missouri, United States*

The study aims to evaluate the relationship between the representation of pain assessment and management in interdisciplinary text messages about older adults residing in Nursing Homes (NH) with and without Alzheimer's Disease and Related Dementias (ADRD) before NH to hospital transfer. A retrospective cohort study and secondary data analysis of (n=21,000) text messages exchanged among healthcare team members caring for NH residents who transferred to the hospital from 2016 to 2020 was performed. Natural language processing was used to identify mentions of pain assessment, management, and pain medications in text messages. A two-sample independent T-test was used to test our hypothesis. A negative binomial generalized linear model was used to assess the association between pain mentions and various resident characteristics, including ADRD status. The analysis included 694 transfer events involving 414 residents, 39% of whom had ADRD. Significant differences were found in the t-test with pain assessment and management mentions, with ADRD residents having fewer mentions ($t = 2.38$, $p=0.02$) of pain assessment and management than those without ADRD. The generalized linear model identified statistically significant predictors (age, race, and specific comorbidities) of the number of text messages exchanged among NH residents, but interestingly, dementia stage and avoidable transfers were not significant. Our findings indicate that pain is underrepresented in communications regarding ADRD residents, as well as Black patients, potentially contributing to disparities in pain assessment and management in these populations.

Abstract citation ID: igae098.4041

### PARTNERS IN CARE MODEL FOR PERSONS WITH DEMENTIA AND MULTIMORBIDITY (PCM)

Ingelin Testad[1], Martha Gjestsen[2], and Lise Birgitte Holteng[2], *1. Centre for Age-Related Medicine - SESAM, Stavanger University Hospital, Stavanger, Rogaland, Norway*, *2. Stavanger University Hospital, Stavanger, Rogaland, Norway*

The increasing prevalence of old age, multimorbidity and dementia over time suggest a pressing need for interventions which can help mitigate the health challenges associated with an aging population. One of the challenges adhere to the complexity and frequency of interactions with- and transitions between multiple health services, which further increases the vulnerability to coordination breakdowns, thus representing organisational challenges for the health system as well as to the care of this vulnerable group of people. This project represents a development of a Partner in Care Model (PCM) to meet the challenges persons with multimorbidity and dementia are met with. The Partner in Care Model will organize the care within the existing health care system, to increase the health care professionals expertise in involving carers to give high quality and cost effective care to persons with multimorbidity and dementia, to meet the major challenges this vulnerable group of people. Furthermore, PCM will have a holistic approach to meet all the challenges in this vulnerable group of people, on the management of medical-, lifestyle and psychosocial-health care needs. More specifically we aim to: • Identify the most common care models used in managing persons with multimorbidity with dementia • Describe the effectiveness of existing care models to develop a Partner in Care Model • Test the feasibility of a Partner in Care Model

Abstract citation ID: igae098.4042

### PATIENT AND CARE PARTNER VALUES WHEN CONSIDERING DEMENTIA TREATMENT OUTCOMES

Maayra Butt[1], Josiah Drakes[2], Paola Rosa[2], Melanie Bahti[2], Kristin Harkins[2], Jason Karlawish[2], Scott Halpern[2], and Catherine Auriemma[2], *1. University of Nevada Las Vegas, Mechanicsburg, Pennsylvania, United States*, *2. University of Pennsylvania, Philadelphia, Pennsylvania, United States*

We sought to understand how persons living with dementia (PWD) and their family care partners value hypothetical treatment outcomes relative to one other using a point allocation task. Participants distributed 100 points across six potential outcomes based on perceived relative importance. Outcomes included slowed decline in brain function, reduced agitation and combativeness, reduced family caregiver stress, prolonged ability to perform activities of daily living, increased time living at home, and increased socialization and engagement. Equality of distributions between outcome scores and between subgroups were compared using Wilcoxon signed-rank and rank-sum tests, respectively. 201 individuals participated (n=154 (77%) care partners and n=47 (23%) PWD). Across all participants, slowing declines in brain function scored highest (20, IQR 10, 30, $p < 0.05$ for all pair-wise comparisons). Care partners compared to patients more highly valued reduced agitation and combativeness (15.5, (IQR 10, 25) vs 10 (IQR 3, 16), $p < 0.001$). Patients compared to care partners more highly valued reducing family caregiver stress (16 (IQR 10, 30) vs 10 (IQR 5,20), p=0.003). Among care partners, those whose loved one had mild dementia more highly valued slowing declines in brain function (20 (IQR 10, 37.5) vs 15 (IQR 10, 25), p=0.02). Conversely, care partners of those with moderate or severe dementia more highly valued reducing agitation and combativeness (20 (IQR 10, 25) vs 12.5 (7.5, 21) p=0.007). PWD and dementia care partners expressed differing values when considering treatment outcomes. The development of future treatments should consider the most valued outcomes and disease severity to enhance potential treatment satisfaction.

Abstract citation ID: igae098.4043

### PERFORMANCE ON THE MOCA IS NOT ASSOCIATED WITH COGNITIVE RESERVE IN COMMUNITY-DWELLING OLDER ADULTS

Marcella Barneclo[1], A. Lisette Isenberg[2], Joy Stradford[3], Kimberly Espejo[4], and Judy Pa[2], *1. University of California, San Diego, San Diego, California, United States*, *2. University of California, San Diego, La Jolla, California, United States*, *3. University of California San Diego, La Jolla, California, United States*, *4. UC San Diego, San Diego, California, United States*

The Cognitive Reserve (CR) hypothesis suggests that more experiential resources (e.g. education, occupation, recreational activities) can help individuals combat age-related cognitive decline and maintain higher cognitive function with age. As such, higher CR has been implicated in greater cognitive resilience potentially correlated to delaying the onset and progression of Alzheimer's Disease (AD). Previous research in cognitively normal populations found that the Montreal Cognitive Assessment (MoCA), a well-established neuropsychological assessment of global cognition used to identify Mild Cognitive Impairment (MCI) or mild AD in older adults, may reflect CR. However, limited information exists on the association between MoCA and CR for MCI. The present study investigates the relationship between MoCA and CR in older adults with early MCI using the Cognitive Reserve Index questionnaire (CRIq), a proxy measure of CR determined by education, occupational activity, and recreational activity. The study includes 138 older adults aged 50-85 with early MCI, comprising 50 males and 88 females, with an average age of 66.1 ± 7.40. No correlation between MoCA and total CRIq scores ($r = 0.12$, $p = 0.17$), or the subcategories of working activity ($r = 0.08$, $p = 0.35$), education ($r = 0.05$, $p = 0.54$), and leisure time ($r = 0.11$, $p = 0.19$) were observed. Age was included as a covariate in the models. There were no differences between sexes for CRIq scores, MoCA scores, or years of education, respectively. These findings suggest that performance on the MoCA is not associated with CR in older adults with early MCI.

Abstract citation ID: igae098.4044

### PHYSICAL ATTRIBUTES LINKED TO FRAILTY IN NURSING HOME RESIDENTS: INSIGHTS FROM A CROSS-SECTIONAL STUDY

Francis Kin Tung Chan[1], Chi Tat Fung[1], Ka Po Chan[2], and Yijian Yang[2], *1. Hong Kong Sheng Kung Hui Welfare Council Limited, Hong Kong, Hong Kong, China (People's Republic)*, *2. The Chinese University of Hong Kong, Hong Kong, China (People's Republic)*

Frailty is a complex geriatric condition characterized by reduced physiological reserve and increased risk of adverse outcomes. Older adults in nursing homes are generally frail and have poor mobility. However, research on frailty in nursing homes is scarce compared to community settings, particularly on its association with mobility and postural stability. A cross-sectional analysis of baseline data from a two-arm cluster randomized controlled trial was conducted, involving 133 participants aged 60 and above from 20 Hong Kong residential care homes. The FRAIL-NH scale was used to assess frailty through factors such as fatigue, mobility, and nutrition for nursing home residents. Walking speed was assessed by the GAITRite computerized walkway system, postural stability with the Swaymeter. Multiple regression statistical analysis was conducted to identify associations between FRAIL-NH and physical attributes. Walking speed (Standardized Coefficients = -0.38, p< 0.001), anterior-posterior body sway (Standardized Coefficients = -0.288, p=0.02), and BMI (Standardized Coefficients = -1.98, p=0.018) were significantly associated with FRAIL-NH, with walking speed as the strongest predictor account for 19.6% of total variance. However, medial-lateral body sway did not show significant association with FRAIL-NH. Additionally, no significant correlation was observed between specific muscle strength measures and FRAIL-NH. Our study highlights walking speed and anterior-posterior sway as key indicators of frailty in nursing home residents. These results identify the need for focused interventions that improve balance and mobility, which may potentially decrease frailty and risk of falling for this vulnerable population. Further research is crucial for developing effective frailty interventions in nursing homes.

Abstract citation ID: igae098.4045

### PHYSICAL, COGNITIVE AND MENTAL CHARACTERISTICS AND DIGITAL HEALTH TECHNOLOGY USE AMONG PREFRAIL OLDER ADULTS

Takahisa Ohta[1], Sho Hatanaka[1], Takashi Shida[1], Narumi Kojima[1], Yosuke Osuka[2], Tsuyoshi Okamura[1], Hirohiko Hirano[3], and Shuichi Awata[1], *1. Tokyo Metropolitan Institute for Geriatrics and Gerontology, Itabashi, Tokyo, Japan*, *2. National Center for Geriatrics and Gerontology, Obu, Aichi, Japan*, *3. Tokyo Metropolitan Institute for Geriatrics and Gerontology, Tokyo Metropolitan Institute for Geriatrics and Gerontology, Tokyo, Japan*

Remote interventions for prefrail older people are more feasible than for frail older people, but the benefits of using digital health technologies (DHT) have not been comprehensively investigated. This cross-sectional study examined whether DHT use in prefrail older people is associated with better physical/cognitive/mental function. Participants aged ≥70 years with prefrailty, as defined by the Japanese Cardiovascular Health Study criteria, were recruited from the Itabashi Longitudinal Study on Aging between 2022–2024. Exclusion criteria comprised impaired activities of daily living, Mini-Mental State Examination (MMSE) scores < 23, or physician-diagnosed dementia. DHT ownership and usage, such as messaging, searching, video calls, and video viewing, were self-reported. Physical characteristics included muscle strength, body mass index, and skeletal muscle mass index. Cognitive function was assessed by MMSE, and mental health was evaluated using WHO-5. Binomial logistic regression, adjusted for demographics, lifestyle, and socioeconomic status, calculated adjusted odds ratios (aORs) and 95% confidence intervals (CIs), with non-DHT ownership as the reference. Among 2,681 participants, eligible 862 (mean age 77.0±4.5 years, 44.0% women) were analyzed. DHT ownership was inversely associated with lower handgrip strength (< 28kg for men, < 18kg for women) (aOR: 0.68; 95% CI: 0.47–1.00) and lower MMSE (< 27) scores (aOR: 0.65; 95% CI: 0.44–0.98). The use of searching (aOR: 0.49; 95% CI: 0.28–0.83) and video calls (aOR: 0.58; 95% CI: 0.38–0.88) were inversely associated with lower WHO-5 scores. These findings suggest DHT may support physical/cognitive/mental health in prefrail older adults.

Abstract citation ID: igae098.4046

### PHYSIO-FEEDBACK EXERCISE PROGRAM (PEER) ON ACCELEROMETER-BASED DATA: A SMOOTHING SPLINE ANOVA MODEL

Chang Liu, Yi Liu, Ladda Thiamwong, Tho Nguyen, Wei Zhang, and Rui Xie, *University of Central Florida, Orlando, Florida, United States*

Exercise is crucial for reducing falls in older adults. Physio-fEedback Exercise pRogram (PEER) consists of physio-feedback, cognitive reframing, and peer-led exercises that can reduce fall risk. However, there is a lack of data on the outcome of PEER on physical activity (PA) that may provide valuable insights for immediate and long-term improvements. We examined the effectiveness of PEER intervention on the daily PA level from 64 low-income older adults (mean age = 73.1 ± 6.26 years, female 87.5%), randomized into either PEER or control groups. The PEER group participated in weekly exercise sessions led by a trained peer for 8 weeks, followed by 13 weeks of follow-up, while the control group data was collected over a similar 21-week period without intervention. Physical activity was measured using a commercial accelerometer (Fitbit device). Data were categorized into sedentary, lightly active, moderately active, and very active minutes. We evaluated the peer-led group exercise days on four daily activity levels using a Smoothing Spline ANOVA (SSANOVA) model with partial splines and incorporated parameter covariates into the classic SSANVOA model. The results indicated a significant increase in moderately active and very active minutes in the PEER group during the 8-week intervention period, highlighting the short-term effectiveness of the program. We also observed the attenuation of the intervention's effects over the 13-week follow-up across all four activity levels. PEER intervention significantly boosts daily activity levels among older adults in the short term, and sustained efforts may be necessary to maintain these benefits over the long term.

Abstract citation ID: igae098.4047

### PILOT RESULTS OF A GAMIFIED TRAINING FOR CERTIFIED NURSING ASSISTANTS ON AGE-FRIENDLY HEALTHCARE

David Wihry[1], Susan Wehry[2], Angela Hunt[3], Judith Metcalf[4], and Victoria Spofford[4], *1. University of Maine, South Portland, Maine, United States*, *2. University of New England, Portland, Maine, United States*, *3. The Cedars, Portland, Maine, United States*, *4. University of New England, Biddeford, Maine, United States*

Certified Nursing Assistants (CNAs) are an integral part of supporting the health and wellbeing of older adults within long-term care (LTC) settings. As the Age-Friendly Healthcare movement expands to include LTC, it will be important to ensure effective and accessible training for CNAs on Age-Friendly Healthcare competencies. This poster will present the results of a pilot study examining the usability and satisfaction with an app-based training on the 4Ms of Age-Friendly Healthcare (What Matters, Medication, Mentation, Mobility). The modules are tailored to the practice needs of CNAs and use principles of gaming to increase uptake of the training. A post training survey was distributed to 127 CNAs from eight LTC communities who completed the curriculum, with a 79% response rate. 78% agreed they would prefer to learn this way versus alternative learning methods such as online learning, classroom learning, etc. (12% neither agreed nor disagreed, 10% disagreed). 85% agreed that they believe gamification is a successful delivery method for learning (8% neither agreed nor disagreed, 7% disagreed). 80% agreed that they would recommend the training program to their co-workers (13% neither agreed nor disagreed, 7% disagreed). The number of participants stating that they had a “very good” level of knowledge of Age-Friendly Healthcare increased from 25% to 39% using a retrospective pre-post rating. Qualitative feedback highlighted interactivity, brevity, and ability to engage the individual as key themes about the value of the training modules. Implications for use of gamification in Age-Friendly Healthcare training will be presented.

Abstract citation ID: igae098.4048

### PREDICTING ALL-CAUSE MORTALITY USING THE SARCOPENIA PHENOTYPE: EVIDENCE FROM THE KFACS

Miji Kim, Hyung Eun Shin, Daehyun Lee, Heeeun Jung, Jae Young Jang, Hyunjin Cho, Nahyun Lim, and Chang Won Won, *Kyung Hee University, Seoul, Republic of Korea*

The Global Leadership Initiative on Sarcopenia (GLIS) has sought to establish a consensus on sarcopenia diagnosis, which involves reduced muscle mass and strength. We investigated the association between sarcopenia phenotypes (low muscle mass, muscle strength, and physical performance) and all-cause mortality. We conducted a prospective 6-year follow-up analysis of 2,400 older adults (52.8% women; mean age 76.0±3.9 years) enrolled in the Korean Frailty and Aging Cohort Study. Participants were included if they had undergone dual-energy X-ray absorptiometry, handgrip strength testing, and physical performance assessments at baseline (2016–2017). Discrete-time Cox proportional hazards models evaluated the association between sarcopenia and all-cause mortality. According to the GLIS criteria, the prevalence of sarcopenia was 15.4% in men and 7.5% in women. Totally, 203 deaths occurred, with 6-year mortality rates of 2.1 (95% confidence interval [CI]: 1.9–2.5) per 100 person-years in individuals without sarcopenia, and 4.8 per 100 person-years (95% CI: 3.6–6.3) in those with sarcopenia. After adjusting for potential confounders, reduced muscle mass and muscle strength were significantly associated with an increased risk of all-cause mortality (hazard ratio [HR]: 1.56, 95% CI: 1.09–2.24). Moreover, the presence of all three criteria of sarcopenia was associated with an increased risk of all-cause mortality (HR: 1.87, 95% CI: 1.27–2.67), while reduced muscle mass and physical performance were not significantly associated with mortality (p=0.06). In conclusion, the conceptual definition of sarcopenia outlined by the GLIS, which includes both reduced muscle mass and muscle strength, is associated with premature mortality among community-dwelling older adults.

Abstract citation ID: igae098.4049

### PREDICTING SOCIAL ISOLATION IN OLDER ADULTS WITH COGNITIVE DECLINE

Bada Kang[1], Min Kyung Park[2], Jennifer Kim[1], Seolah Yoon[1], Seok-Jae Heo[3], Chaeeun Kang[1], and Dahye Hong[1], *1. Yonsei University, Seoul, Republic of Korea*, *2. Yonsei University College of Nursing, Seoul, Republic of Korea*, *3. Yonsei University College of Medicine, Seoul, Republic of Korea*

Social isolation in older adults, which encompasses limited social interaction and loneliness, is a significant risk factor for dementia. Subjective cognitive decline (SCD) and mild cognitive impairment (MCI) stages are considered focal points of preventive interventions, at which cognitive and physical functions can be restored. Thus, preventing social isolation via prompt identification of high-risk individuals is crucial. This study aimed to develop and validate machine learning models to predict social interaction and loneliness levels among older adults with SCD and MCI. This study included 99 community-dwelling older adults (n=67 SCD and 32 MCI). While demographic and health-related data were collected via survey, we applied a mobile Ecological Momentary Assessment approach for real-time measurement of social interaction and loneliness levels and wrist-worn actigraphy for sleep and activity data. While the Random Forest model was the most suitable for predicting social interaction level (area under the receiver operating characteristic curve [AUC]: 0.935), the Gradient Boosting Machine model was the most suitable for predicting high levels of loneliness (AUC: 0.887). Time-specific analyses demonstrated the association between a low frequency of physical movement in the morning and a low level of social interaction, and decreased sleep quality during night time and high levels of loneliness. This study showed the potential of machine learning models to predict the social interaction and loneliness of older adults at risk for dementia. The algorithms can be applied to develop mobile preventive interventions that utilize real-time sleep and behavioral data to prevent social isolation in these at-risk individuals.

Abstract citation ID: igae098.4050

### PRELIMINARY EFFICACY OF AN OT INTERVENTION TO PREVENT FUNCTIONAL DECLINE AMONG OLDER ADULTS WITH CHRONIC DISEASES

Dalmina Arias[1], Mansha Mirza[2], and Heidi Fischer[2], *1. University of Illinois at Chicago, Chicago, Illinois, United States*, *2. University of Illinois Chicago, Chicago, Illinois, United States*

Chronic disease affects 95% of older adults in the US, with 80% having two or more conditions. Poor management can lead to a decline in health, mobility, and ADL/IADL limitations, contributing to lower self-efficacy in disease management. Early identification and intervention of ADL/IADL limitations could help break the cycle of chronic disease and functional decline. This 2-arm parallel RCT explored the preliminary efficacy of an occupational therapy (OT) intervention for adults aged 55 and over with chronic conditions. The 12-week program included a comprehensive ADL assessment and ten intervention sessions focused on health management, physical deconditioning, and ADL/IADL limitations. Forty community-dwelling adults (mean age = 67 years) with diabetes or heart disease were recruited. Outcomes measured included physical functioning (Patient-Specific Functional Scale, Physical Performance Test, PROMIS Physical Functioning), self-efficacy for chronic disease management, health-related quality of life (PROMIS Global Health Measure), and ADL/IADL performance (Late-Life Functioning and Disability Index). ANCOVA was used for data analysis with baseline scores as covariates and Sidak correction for multiple comparisons. A significant effect favoring the intervention group was observed for the main outcome, the Patient-Specific Functional Scale (p =.04, d = 0.82, 95% CI [-1.5, -0.17]). Other outcomes showed promising trends with smaller effect sizes. These preliminary findings support the potential of OT interventions in managing chronic diseases among aging adults. Larger trials are needed to confirm these results.

Abstract citation ID: igae098.4051

### PRESENTING VISION IMPAIRMENT AND RESIDENTIAL CARE USE AMONG OLDER ADULTS

Matthew Brault, *NORC, Columbia, Maryland, United States*

Nursing home and assisted-living facility use is common among older adults but is financially burdensome. Identifying risk factors for residential care use may focus attention on interventions that reduce or delay need for these services. Using data from the National Health and Aging Trends Study (NHATS) from 2021 (Round 11) and 2022 (Round 12), we measured the association of presenting visual acuity and contrast sensitivity on residential care use. Survey participants were assessed for presenting distance and near visual acuity and contrast sensitivity using a tablet-base examination tool. Residential care status, determined by the location of the Round 12 interview, was modeled as a function of vision impairment and relevant confounders using logistic regression. Of 2,727 participants, 2.1% were in residential care in 2022. Presenting near (19%) and distance acuity impairment (9%) were not associated with residential care, with adjusted odds ratios (aOR) of 1.48 (95% CI: 0.68 – 3.22) and 0.55 (0.17 – 1.81) respectively. Presenting contrast sensitivity impairment (24%) was associated with higher odds of residential care (aOR: 2.16 [95% CI 1.20 – 3.88]). This association was mediated by covariates for gait speed and recurrent falls. Results suggest that contrast sensitivity impairment may represent a separate risk factor for residential care use, apart from physical frailty or cognitive decline. Interventions to address or prevent eye diseases associated with impaired contrast sensitivity could reduce or delay residential care placement, a possibility that requires further investigation.

Abstract citation ID: igae098.4052

### PREVALENCE OF POSSIBLE SARCOPENIA AND ASSOCIATED FACTORS AMONG OLDER HOMELESS ADULTS IN HOME FOR DESTITUTE

Phatcharaphon Whaikid[1], Noppawan Piaseu[2], and Wasana Srisuk[2], *1. Mahidol University, Bangkok, Krung Thep, Thailand*, *2. Mahidol University, Bangkok, Nakhon Pathom, Thailand*

Sarcopenia is a significant problem leading to increased morbidity and mortality risk, particularly in older homeless adults in home for destitute as they are vulnerable and are experiencing premature degeneration. The Asian Working Group for Sarcopenia (AWGS) 2019 introduces possible sarcopenia for early prevention of sarcopenia. Therefore, this study aimed to describe the prevalence and risk factors of possible sarcopenia among older homeless adults. This cross-sectional study included 163 older homeless adults aged 50 and above in homes for destitute in Thailand. The participants completed a questionnaire on personal and health-related factors, and assessments according to AWGS 2019 criteria. Data were analyzed using descriptive statistics and multinomial logistic regression. Results revealed that the prevalence of case finding, and possible sarcopenia was 74.9% and 71.2%, respectively. Among participants aged 50-59, the prevalence rates were 60.8% and 55.1%, while those aged 60 years and over showed a significantly higher prevalence of 90.8% and 89.5%, respectively. Multinomial logistic regression analysis revealed four risk factors significantly associated with possible sarcopenia, including age, gender, BMI, and number of comorbidities (odds ratio (OR) = 1.253, 95% confidence interval (CI) = 1.117-1.405, OR =.222, 95% CI =.056-.874, OR =.584, 95% CI =.458-.745, and OR =.308, 95% CI =.109-.868, respectively). This study confirmed that possible sarcopenia can begin as early as age 50, aligning with indications of premature degeneration in this population. Therefore, it is recommended to start screening earlier than usual to ensure proper management and delay the progression of sarcopenia.

Abstract citation ID: igae098.4053

### PREVENTION OF SEVERE EVENTS IN OLDER PEOPLE IN SWEDEN – POSSIBILITIES AND CHALLENGES

Marie Ernsth Bravell[1], Linda Johansson[2], and Deborah Finkel[3], *1. Jonkoping University, Jönköping, Jonkopings Lan, Sweden*, *2. School of Health and Welfare, Jönköping University, Jönköping, Jonkopings Lan, Sweden*, *3. Jönköping University, Jönköping University, Jonkopings Lan, Sweden*

Older adults are at high risk of malnutrition, falls, pressure ulcers and poor oral health. In Sweden the quality register Senior Alert is trying to improve the preventive work among older people. This study used data from Senior Alert to understand how the preventive work process functions in different care settings. Data included 2957 persons that were assessed in Senior Alert: 864 in nursing homes, 226 in home care and 1829 in hospital. Descriptive and comparative analyses were performed to understand how many were assessed as having risk, and then the actions planned and follow-ups. The results demonstrated that people who were assessed in hospital had risks of some kind in almost half of the cases (46%), in comparison to nursing homes (29%) and home care (8%). Planned actions differed between the settings: in nursing homes, actions were more often planned to prevent poor oral health (29%) whereas hospital staff more often planned actions to prevent falls (73%), malnutrition (71%) and pressure ulcers (57%). Planned actions were significantly less in home care. Follow-ups follow the same patterns, where follow-up in home care were significantly smaller than in nursing homes and hospitals in all areas. Home care provides good opportunities to provide preventive care, as it is more continuous than, for example, hospital care. Additional analyses identified particular actions planned and performed, for a fine-grained investigation of preventive care in Sweden.

Abstract citation ID: igae098.4054

### PRIMARY LANGUAGE'S ROLE IN SOCIAL INTEGRATION AND FUNCTIONAL INDEPENDENCE AFTER TRAUMATIC BRAIN INJURY

Wonkyung Jung[1], and Janiece Taylor[2], *1. Boston College, Chestnut Hill, Massachusetts, United States*, *2. Johns Hopkins University, Johns Hopkins University, Maryland, United States*

Social integration is an ultimate goal of neurorehabilitation and linked to better functional outcomes. However, older immigrants whose primary language is not English may have difficulties reintegrating into society after their injuries. This study investigated the moderating role of primary language in the relationship between social integration and functional independence following traumatic brain injury (TBI) among older adults at the first-year post-injury. Data were obtained from the Traumatic Brain Injury Model System National Database (TBIMS-NDB). Using Hayes's PROCESS macro-Model 1, data were analyzed to assess the impact of social integration and primary language on functional independence. We finally had 1442 older adults aged between 65 and 88 years at the time of TBI. The results revealed that social integration significantly influenced functional independence ($\beta = 11.399$, $p < 0.001$), while primary language had an insignificant impact ($\beta = -3.735$, $p = 0.051$). Additionally, a significant interaction effect was found between social integration and language ($\beta = 6.912$, $p = 0.006$), indicating that the relationship between social integration and functional independence was moderated by primary language. The model explained a substantial proportion of the variance in functional independence (R-Square = 0.328). Further analysis showed that the effect of social integration on functional independence varied based on language proficiency, with a stronger association observed among non-native English speakers ($\beta = 25.224$) compared to native English speakers ($\beta = 18.311$). These findings highlight the need to consider primary language in interventions to improve social integration and functional outcomes for older adults with TBI.

Abstract citation ID: igae098.4055

### PROBIOTICS AND NUTRIENT SUPPLEMENTS IN MILD COGNITIVE IMPAIRMENT: A SYSTEMATIC REVIEW AND META-ANALYSIS.

Mohamed Elhassan[1], Linda Ahmed[2], Rawaa Adil[2], Leena Mohamed[2], Ahmed Mussaad[2], and Nadir Abdelrahman[3], *1. Royal College of Surgeons Ireland, Waterford, Dublin, Ireland*, *2. Tele Geriatric Research Fellowship, Tele Geriatric Research Fellowship, Michigan, United States*, *3. Michigan State University, East Lansing, Michigan, United States*

Mild cognitive impairment (MCI) is a syndrome marked by cognitive decline beyond normal expectations for age and education, without significantly disrupting daily activities. MCI is a recognized risk factor for dementia, and early detection may allow for secondary prevention. This study evaluates the impact of nutrient supplements and probiotics on MCI patients. Databases including PubMed, Cochrane, Google Scholar, and Science Direct were searched for studies published between 2019 and 2024. Randomized controlled trials (RCTs) involving MCI patients treated with nutrient supplements or probiotics, with cognitive function assessed via the MMSE, were included. Studies with dementia patients were excluded. Data extraction was done independently by two reviewers. A random-effects meta-analysis was conducted to pool standardized mean differences (SMDs). Seven RCTs, totaling 523 participants, were included. Both probiotics and nutrient supplements significantly improved cognitive function, as measured by MMSE, compared to controls (SMD = 0.77, 95% CI: 0.33 to 1.21). Subgroup analysis revealed that nutrient supplements significantly enhanced MMSE scores compared to controls (SMD = 0.8, 95% CI: 0.46 to 1.14). MCI remains a complex issue in geriatric medicine due to ongoing debates over diagnosis and treatment. Currently, no FDA-approved medication exists for MCI. Most studies and guidelines advise against using Dementia drugs like Memantine and Donepezil for MCI due to limited efficacy and potential side effects. However, promising results from recent trials on nutrient supplements and probiotics suggest these could be integrated into future treatment protocols.

Abstract citation ID: igae098.4056

### PROFILING THE ORAL MICROBIOME IN OLDER ADULTS WITH DEMENTIA

Sol-ah Jeong[1], Bock-Young Jung[2], Insuk Lee[1], Jun Hyung Cha[1], and Yejun Choi[3], *1. Yonsei University, Seoul, Republic of Korea*, *2. Yonsei University College of Dentistry, Seoul, Republic of Korea*, *3. Yonsei University*

Early diagnosis and monitoring of clinical progression are crucial for managing dementia. While the relationship between oral health, brain health, and dementia has recently received increased attention, the oral microbiome in dementia patients remains uncharacterized. Therefore, this study aimed to identify the diversity and composition of the oral microbiome in older adults with dementia using next-generation sequencing, and compare it to healthy individuals without dementia to determine whether the oral microbiome could serve as a potential biomarker for the diagnosis and prevention of dementia. A total of 58 older adults aged 65 years and older participated in the study, 30 with dementia and 28 cognitively intact healthy individuals. All participants were screened and clinically diagnosed by a neurologist using the Korean version of the Mini-Mental State Examination (K-MMSE), the Clinical Dementia Rating scale (CDR), and brain magnetic resonance imaging (MRI). 1ml of unstimulated saliva was collected from each subject, the tongue was swabbed with a sterile swab for 20 seconds to collect samples, and full-length 16S rRNA amplicon sequencing was performed on all the samples. Statistical analysis was performed using MaAsLin2, and it was found that Fusobacteriota was more abundant in both the tongue and saliva of the dementia group compared to the healthy group, while Pseudomonadota is more dominant in the healthy group. Fusobacterium periodonticum was found to be more abundant as the K-MMSE scores decreased. Prevotella jejuni, Streptococcus anginosus, and Streptococcus gordonii are more enriched in the Dementia group compared to the healthy group.

Abstract citation ID: igae098.4057

### PROGRESSION TO FRAILTY – THE ROLE OF MUSCLE RESILIENCE

LaDora Thompson[1], and Lais Perazza[2], *1. Boston University, Boston, Massachusetts, United States*, *2. Boston University, Boston University, Massachusetts, United States*

The impact of a stressful event within the progression to frailty is unknown. In this study we determined the response to an acute stress in Robust, Prefrail, and Frail mice. We hypothesized the Robust mice would show greater resilience compared to Prefrail and Frail mice following a stressful event; whereas the Prefrail mice would show an intermediary resilience status. 17-27-month-old C57BL/6 female mice were classified as Robust, Prefrail or Frail using the Vitality Phenotype (strength, endurance, activity, walking speed, health deficits.) Stress was induced by downhill running (DR). Resilience was determined by muscle contractility and bone mineral density (BMD). 38% of cohort was classified as Prefrail, 33% as Frail and 29% as Robust. Performance parameters were different between the 3 categories of frailty. Tetanic force (soleus) in Frail mice was significantly reduced compared to Prefrail and Robust mice. When tetanic force was normalized to muscle mass, the frailty-driven reduction in force was still observed, suggesting impairment in protein quality. BMD was similar between Prefrail and Frail, while both differed from Robust, suggesting that BMD loss occurred early in the progression to frailty. Following DR, the Robust and Prefrail mice showed a significant reduction in tetanic force compared to non-damaged control Robust and Prefrail mice. In contrast, soleus tetanic force in Frail mice following DR was not different between non-damaged control Frail mice, likely due to the already preexisting muscle dysfunction. In sum, our data suggests that resilience after acute stress is dependent on the frailty status.

Abstract citation ID: igae098.4058

### PROLONGED EFFECTS OF THE COVID-19 PANDEMIC ON FRAILTY TRENDS IN A VETERAN SUBPOPULATION

Nalini Bhalla[1], and Janet Fawcett[2], *1. Phoenix VA Medical Center, Phoenix, Arizona, United States*, *2. Phoenix VA Health Care System, Phoenix, Arizona, United States*

The first year of the Covid-19 pandemic resulted in an increase in frailty when compared to a recovering trend the prior year in our previously studied subpopulation of 3532 older veterans. We used an automatically generated Veterans Affairs (VA) Metrics computed assessment score to monitor frailty trends for an additional two years to see if this increase persists. We studied the one year difference in mortality Care Assessment Need (CAN) scores in 2652 veterans (mean(SD) age 71.7(1.3) years, 98.8% Male) from 2019 to 2023. Inclusion criteria were age 70-75 and CAN score >=75 as of 2/8/19. Veterans who died during the study period or did not have a 1-year mortality CAN score for any of the years were excluded. The year to year difference between the mortality CAN scores was compared with a one-way ANOVA with repeated measures with a Greenhouse-Geisser correction and found to be significant for increasing frailty ($p < 0.001$). We also compared the last mean (SD) CAN score from the 2652 patients alive on 2/10/23 with the 735 patients deceased between 2/12/21 and 2/10/23 and found a significantly higher CAN score in the patients who died ($p < 0.001$). The sequelae of repeated Covid infections involve a broad array of organ systems with an unclear duration of excess risk. Additionally, an uneven post pandemic recovery in the diagnosis and management of chronic conditions may explain the persistent increase in frailty in our studied veterans up to 3 years after the pandemic was declared.

Abstract citation ID: igae098.4059

### QUANTIFYING NURSING HOME CARE QUALITY FOR OLDER HIV AND SEXUAL AND GENDER MINORITY POPULATIONS: A SCOPING REVIEW

Haley Moriarty[1], Chelsea Wong[2], and Brianne Olivieri-Mui[3], *1. Northeastern University, Northeastern University, Massachusetts, United States*, *2. Beth Israel Deaconess Medical Center, Brookline, Massachusetts, United States*, *3. Northeastern University, Boston, Massachusetts, United States*

Standardized quantitative measures of nursing home (NH) care quality for older people with human immunodeficiency virus (HIV) or sexual and gender minority (SGM) populations are sparce. This scoping review identified the current literature empirically measuring quality of care in NHs for people with HIV (PWH) and SGM populations including, but not limited to, validated measures endorsed by national organizations. The initial librarian-assisted search strategy across four databases resulted in 764 articles. Literature that quantified care quality for HIV (8 studies) or SGM (1 study, 1 report) populations in the NH setting were included. No studies examined the overlap of HIV and SGM populations nor were there studies of general clinical care for either population. The lowest quality was seen with measures of ART prescriptions (n=4/8 studies) and CD4/viral load monitoring (n=2/8 studies). NHs tended to have lower overall care quality (e.g. antipsychotic prescribing, star rating, rehospitalizations) for PWH v people without HIV unless PWH were the majority patient population (n=4/8). One study and one report measured staff/clinicians' levels of discrimination toward SGM populations finding low willingness to provide care and high rates of refusal to admit SGM patients, respectively. There is insufficient empirical data measuring clinical care quality for SGM using NHs. In conclusion, HIV care quality in NHs has been measured but general clinical care quality for PWH using NHs has not. The growth of the aging HIV and SGM populations demands more research empirically evaluating NH care quality beyond HIV care and including intersecting HIV and SGM identities.

Abstract citation ID: igae098.4060

### RACIAL AND RURAL-URBAN DISPARITIES IN DISCHARGE AMONG MEDICARE PATIENTS HOSPITALIZED FOR ISCHEMIC STROKE

Mengyuan Cheng, Winston Kennedy, and Nasim Ferdows, *Northeastern University, Boston, Massachusetts, United States*

Disparities in post-acute stroke care are a critical public health issue, as they can profoundly affect patient outcomes and long-term recovery. Understanding how race and rurality shape discharge destination patterns is essential for addressing these inequities. This study explores these disparities among Medicare beneficiaries hospitalized for ischemic stroke in 2021, using data from the 5% Medicare Fee-For-Service sample. We conducted multinomial logistic regression, stratifying by survival status and adjusting for age, sex, stroke severity, comorbidity, dual-eligibility status, depression, hospital length of stay, and state Medicaid expansion status. Rurality was categorized using Rural-Urban Commuting Area codes into urban, rural-adjacent, and rural non-adjacent areas. Significant disparities emerged among patients who died during or shortly after hospitalization. Hispanic and Black patients had 62% lower odds of being discharged to hospice, another hospital, or other settings compared to White patients (OR=0.38, 95% CI 0.20-0.73). Patients classified as 'Others' (Asian, Native American, etc.) had 74% lower odds of being discharged to such settings (OR=0.26, 95% CI 0.11-0.62). Rural-adjacent patients had 44% lower odds of being discharged to hospice or other settings compared to urban patients (OR=0.56, 95% CI 0.31-1.01), while rural non-adjacent patients showed a 36% reduction in odds, though this was not statistically significant. Our findings highlight the critical need for tailored interventions to address racial and rural-urban disparities in post-stroke care, particularly for patients who die during or shortly after hospitalization, to promote equitable care.

Abstract citation ID: igae098.4061

### REACTANCE/HEIGHT AS A POTENTIAL BIOIMPEDANCE INDICATOR OF FRAILTY AMONG COMMUNITY-DWELLING OLDER ADULTS

Kworweinski Lafontant[1], Estefania Zamarripa[1], Abigail Tice[1], Amber Blount[1], Jethro Raphael Suarez[2], Dahee Kim[1], Jeffrey Stout[1], and Ladda Thiamwong[1], *1. University of Central Florida, Orlando, Florida, United States*, *2. University of Central Florida*

Frailty is prevalent among older adults and is characterized by reductions in physical function and muscle quality. Despite the emerging clinical utility of bioelectrical impedance analysis (BIA), little is known about how bioimpedance indices, such as phase angle (PhA), Levi's Muscle Index (LMI), reactance/height (Xc/Height), and resistance/height (R/Height), relate to physical function (i.e., handgrip strength, postural sway, Timed-Up-and-Go, Sit-to-Stand, Short Physical Performance Battery) and frailty. This study aimed to investigate these relationships in community-dwelling older adults. We cross-sectionally compared physical function measures and bioimpedance indices between frail, pre-frail, and robust categories as determined by the FRAIL questionnaire in 150 community-dwelling older adults (female, n = 137; age = 73.9 ± 6.9 years; BMI = 30.5 ± 6.4 kg/m2) using Kruskal-Wallis H and one-way ANOVA tests. Spearman rho (ρ) and Pearson r correlation coefficients were used to assess relationships between variables. PhA ($r = -0.15$, $p = 0.07$) and LMI ($\rho = 0.05$, $p = 0.57$) were not significantly associated with FRAIL scores, unlike Xc/Height ($r = -0.29$, $p < 0.001$) and R/Height ($r = -0.20$, $p = 0.02$). Among the bioimpedance indices, only Xc/Height differed significantly between frailty categories ($F(2,147) = 6.55$, $p = 0.002$) while all physical function assessments except postural sway differed between the categories ($p < 0.05$). These results suggest that only Xc/Height may be indicative of frailty among older adults. However, these conclusions may be limited by the use of the FRAIL questionnaire; future research should compare LMI and PhA using multiple frailty assessments.

Abstract citation ID: igae098.4062

### RED BLOOD CELL DISTRIBUTION WIDTH: FURTHER INSIGHTS

Matt Oberdier[1], Marta Zampino[2], Michelle Shardell[3], Majd AlGhatrif[4], Edward Lakatta[1], Eleanor Simonsick[1], and Luigi Ferrucci[1], *1. National Institute on Aging, Baitmore, Maryland, United States*, *2. University of Maryland, Baltimore, Maryland, United States*, *3. University of Maryland School of Medicine, Baltimore, Maryland, United States*, *4. Johns Hopkins University, Baltimore, Maryland, United States*

Red blood cell distribution width (RDW) measures erythrocyte size variability and strongly predicts cardiovascular disease (CVD) morbidity and mortality. Our prior cross-sectional analysis showed that inflammation, metabolic rate, and body habitus are independently associated with RDW. To better understand determinants of RDW, we performed a longitudinal analysis associating changes in RDW within the normal range (11.5 - 15.0%) with age, sex, race, and changes in other erythrocyte properties, major CVD risk factors, and other physiologic measures. Data were from 452 men and 482 women, aged 50 to 94 years enrolled in the Baltimore Longitudinal Study of Aging. For each participant and each predictor, change was the regression coefficient of time from a linear model. Coefficients of candidate correlates were then used to predict change in RDW in multiple linear regression models that were reduced by backward selection. In the final model, increasing erythrocyte number (STβ=1.4980, P< 0.001), mean platelet volume (STβ=0.2019, P< 0.001), anion gap (STβ=0.1754, P< 0.001), blood urea nitrogen (STβ=0.0684, P=0.0395), urine albumin-creatinine ratio (STβ=0.1007, P=0.0015), alanine transaminase (STβ=0.1580, P< 0.001), interleukin-6 (STβ=0.2338, P< 0.001), and diastolic blood pressure (STβ=0.0769, P=0.0107) and decreasing glucose (STβ=-0.2425, P< 0.001), vitamin B12 (STβ=-0.1310, P< 0.001), and C-reactive protein (STβ=-0.1801, P< 0.001) were associated with increasing RDW. The association of age with increasing RDW was independently explained by decreasing glucose (STβ=-0.2310, P< 0.001). Taken together, these findings suggest that rising RDW may reflect accelerated aging. Studies in larger, more diverse populations are necessary to confirm these findings and determine their role in mechanisms leading to age-associated increases in RDW.

Abstract citation ID: igae098.4063
**REDUCING AVOIDABLE ED VISITS IN OLDER ADULTS—A COLLABORATIVE APPROACH BETWEEN ED AND NURSING HOMES**
Hanzhang Xu[1], Chong Yau Ong[2], Angus Jun Jie Ng[2], Gayathri Devi D/O Nadarajan[3], Marcus Eng Hock Ong[4], and Jean Mui Hua Lee[2], *1. Duke University, Durham, North Carolina, United States*, *2. Sengkang General Hospital, Singapore, Singapore*, *3. Singapore General Hospital, Singapore, Singapore*, *4. Health Services and Systems Research, Singapore, Singapore*
Singapore has one of the fastest aging populations in the world. As the number of older adults increases, so does the number of older adults living in nursing homes (NH). Prior research has found that NH residents have a high rate of emergency department (ED) visits and close to half of these visits might be avoidable. To promote high-quality care for NH residents and to address the overcrowding in the ED, we developed a telehealth-based care coordination program named EAGLEcare ACT (Enhancing Advance care planning, Geriatric and end-of-Life care - Acute Care Team). In brief, a public health institution - Sengkang General Hospital has partnered with NHs in their catchment area and delivered telehealth consultations to help NH staff manage acutely-ill NH residents on-site instead of transporting them to the ED. Between Aug 2020 – Dec 2023, we engaged 11 out of the 35 NHs in the catchment area, 7 of which have come on board, and 1166 telehealth consultations were delivered. After the implementation of EAGLEcare ACT, there was a significant decrease (pre: 51/9%, post: 40.5%, P<.001) in the proportion of ED visits from partnering NHs. The impact largely remained throughout the program period. We also found that the effect of EAGLEcare ACT varies by NHs and might be related to differences in the characteristics of NH residents and experiences of NH staff. Findings from this project provided much-needed evidence to enhance care transitions, improve high-quality tele-ED care, and promote health and well-being for older adults residing in NHs.

Abstract citation ID: igae098.4064
**REFINING BP MONITORING: IMPACT OF HYPERTENSION AND VARIABILITY ON COGNITIVE TEST PERFORMANCE IN OLDER ADULTS**
Mitchell Roberts, Erica Sappington, Pruthvinath Jeripity Venkata, Roma Tremblay, and Carla VandeWeerd, *UF Health Precision Health Research Center- The Villages, The Villages, Florida, United States*
Blood pressure (BP) management is crucial in mitigating cognitive decline, yet the relationship between hypertension and cognitive function is complex. To further explore this, data were collected on cognitive function (via MoCA), demographics, hypertension, and BP (measured 3 times concurrently) from older adults 50-89 between 5/27-7/19/24 at the UFHealth PHRC in The Villages (UFIRB202400472) as part of a broader study to test and refine Casana's Heart Seat (THS) algorithm for passive in-home BP monitoring. MoCA scores as the dependent variable, were examined using multiple regression. Predictors included age, gender, hypertension (self-reported diagnosis, systolic >160mmHg), BP variability (standard deviation of 3 BP's), and pulse pressure, adjusted for interaction effects and confounding variables. A cross-sectional analysis of 502 older adults (M=71.98, SD=6.77) revealed that 54.2% (n=272) self-reported hypertension, with 95% of this subgroup reporting antihypertensive medication use. The mean MoCA score for all participants was 25.7 (SD=2.4). Regression analyses identified age and hypertension as significant predictors of lower MoCA scores (β = -0.621, p =.004). Higher mean systolic pressure was linked to lower MoCA scores (β = -0.193, p <.001). Older age (80+) correlated with greater BP variability (β = 0.067, p <.001) however, variability in systolic (p=.569) and diastolic (p=.280) BP was not significantly associated with MoCA scores. These findings highlight the importance of managing hypertension to preserve cognitive health in older adults. Implications will be discussed in terms of policy and practice.

Abstract citation ID: igae098.4065
**RELATING SPECIFIC MEDICATIONS TO RECORD OF FALLS IN A LARGE COHORT OF OLDER SWEDISH ADULTS**
Daniel Peterson[1], Linda Johansson[2], Bjorn Westerlind[3], Thais Lopes De Oliveira[4], and Deborah Finkel[5], *1. Arizona State University, Arizona State University, Arizona, United States*, *2. School of Health and Welfare, Jönköping University, Jönköping, Jonkopings Lan, Sweden*, *3. Jonkoping University, Jönköping, Jonkopings Lan, Sweden*, *4. Karolinska Institutet, Stockholm, Stockholms Lan, Sweden*, *5. University of Southern California, Los Angeles, California, United States*
Falls are common and devastating for older adults and can be impacted by medication. However, analyses relating medication and falls often focus on a subset of medications. We assessed relationships between medication prescription and prospectively captured falls within a large, Swedish National Quality Register (Senior Alert) sample. Participants (N=441) were tracked for approximately 2.5 years. The observation period was separated into 30-day windows, within which the presence of medication prescription and number of falls were noted. To relate medication prescription and falls, we used Logistic Generalized Estimating Equation (GEE) self-comparison models, considering the dependence of longitudinal data as a cluster to correct confidence intervals and individuals as the cluster. Total number of medications for each 30-day window was included in the model. 286 unique medications were identified and initially considered. However, for model convergence, only medications that were prescribed 100 or more times across all participants and timeframes were included in the analyses, leaving 86 medications. After correction for multiple comparisons, 3 classes of medications were related to falls (annotated with 5-digit ATC codes): B01AB (antithrombotics), B03BB (folic Acid), and A03AX (functional gastro-intestinal [GI] disorder medications). Findings are partially consistent with previous work, as antithrombotic agents were related to falls. Folic acid and GI medications were not previously related to increased fall-risk. Results extend previous work in several ways. 1) data were analyzed via a robust, within subject design, 2) data were collected in older adults living in a novel, Swedish cohort, and 3) we interrogated many common medications.

Abstract citation ID: igae098.4066

### RELIABILITY OF REMOTE SELF-ADMINISTRATED DIGITAL COGNITIVE ASSESSMENTS

Duong Huynh, Mary Patterson, and Bin Huang,
*BrainCheck, Austin, Texas, United States*

Early diagnosis of cognitive impairment and dementia relies on comprehensive, evidence-based cognitive assessment, which currently requires a clinic visit and access to skilled healthcare providers. This poses a challenge for people who live in areas with inadequate primary care services and those who have economic, insurance, or other transient hardships (transportation, time, etc.) that limit their access to healthcare services. Digital cognitive assessments (DCAs) with remote testing capabilities have emerged as an efficient and cost-effective solution. The aim of this study was to validate the reliability of BrainCheck, a platform for DCAs, when self-administered remotely. A total of 46 participants (60.9% female; age range 52-76) remotely completed a battery of eight BrainCheck cognitive assessments twice on the same device (iPad=8, iPhone=5, laptop=33): the participants self-administered in one session and were administered by a research coordinator (examiner) in the other session. The inter-session interval (ISI) varied across participants, from within the same day to 21 days apart. Testing outcomes, including the duration of time needed to complete the battery, the raw score from each assessment, and the raw overall score, were compared between the two sessions. We found moderate or good agreement between self- and examiner-administered performance, with intraclass correlation ranging from 0.59 to 0.83. Results from mixed-effects modeling further confirm the non-significant difference between self- vs. examiner-administered testing performance, which is independent of other factors including testing order, ISI, device, and participants' demographic characteristics. These results demonstrate the feasibility of remote self-administration using BrainCheck.

Abstract citation ID: igae098.4067

### RESILIENCE COMPONENTS, POSTOPERATIVE COMPLICATIONS, AND QUALITY OF LIFE DETERIORATION AFTER PANCREATECTOMY

Jung-Yeon Choi[1], and Kwang-il Kim[2], *1. Seoul National University Bundang Hospital, Seongnam, Kyonggi-do, Republic of Korea*, *2. Seoul National University College of Medicine, Seoul National University, Seoul, Republic of Korea*

**Background:** Resilience, the ability to adapt positively to adversity, remains a multisystemic process with no standardized objective measurement methods. The aim of this study was to identify the association between resilience components and frailty, postoperative complications, and quality of life changes after pancreatectomy in older patients.

**Methods:** This study evaluated older patients (aged ≥ 65) scheduled for pancreatectomy between August 2020 and December 2023. Patients who underwent a Comprehensive Geriatric Assessment and signed informed consent were included. Frailty was determined by multidimensional frailty score more than 5. Neurohumoral resilience was measured using the ACTH stimulation test, cardiovascular autonomic function using orthostatic blood pressure measurement, and cognitive-motor function using dual-task gait tests. The primary outcome was postoperative complications, and the secondary outcome was the deterioration in quality of life one year after pancreatectomy.

**Results:** A total of 57 patients were included in the analysis. Among them, 17 (29.8%) were classified as frail, 10 patients (17.5%) experienced postoperative complications, and 12 patients (24.5%) had worsened quality of life after one year. Low blood pressure and slow usual gait speed was associated with frailty. Diminished cortisol responsiveness correlated with frailty and postoperative complications. Quality of life deterioration was associated with differences between dual-task (serial 7) gait speed and fast gait speed.

**Conclusion:** This study highlights the potential association between multidomain resilience components, frailty, and clinical outcomes in older patients undergoing pancreatectomy. Future research should focus on developing robust, objective, and reliable resilience metrics for clinical use.

Abstract citation ID: igae098.4068

### RESULTS OF A STEPPED EXERCISE PROGRAM AMONG VETERANS WITH KNEE OSTEOARTHRITIS AND CO-OCCURRING BACK PAIN

Brett Burrows[1], Sandra Woolson[2], Cynthia Coffman[2], and Kelli Allen[2], *1. Duke University School of Medicine, Durham, North Carolina, United States*, *2. Durham VA Health Care System, Durham, North Carolina, United States*

Co-occurring back pain can exacerbate an already high risk of disability among patients with knee osteoarthritis (OA). We described the effect of a stepped exercise program for patients with knee OA (STEP-KOA) versus arthritis education (AE) on Western Ontario and McMaster Universities Osteoarthritis Index (WOMAC) scores for participants with and without co-occurring back pain. Veterans with knee OA (n= 345) were randomized 2:1 to STEP-KOA or AE control group that received mailed materials. STEP-KOA began with 3 months of internet-supported home exercise (Step 1), followed by 3 months of bi-weekly physical activity coaching (Step 2), then 3 months of physical therapy (Step 3) if participants did not make clinically relevant improvement in pain or function with the prior step. WOMAC, which assesses lower-extremity pain, stiffness and function was the primary outcome at 9 months. Self-reported back pain was reported at baseline. Differences in mean WOMAC scores at 9-months were compared between arms in those with and without back pain using t-tests. Overall, 72.4% (n=250) reported having co-occurring back pain. Among participants with co-occurring back pain (n= 176), 9-month mean WOMAC score was 10.1 points lower (95% CI -15.5, -4.7; p< 0.001) in STEP-KOA vs. AE. Among participants without co-occurring back pain (n= 79), 9-month mean WOMAC score did not differ between arms (Mean Difference= 4.3; 95% CI -5.3, 13.9; p=0.37). In conclusion, among participants with co-occurring back pain, the STEP-KOA intervention resulted in a significant reduction in mean WOMAC score at 9-months compared to AE alone.

Abstract citation ID: igae098.4069

### SAFETY AND EFFECTS OF HEAT-INACTIVATED LACTOBACILLUS PARACASEI D3.5 (LPD3.5) ON MENTAL WELLBEING IN OLDER ADULTS

Amanda Lloyd[1], Alina Warren-Walker[1], Nigel Holt[1], Doug Lynch[2], Denis Kay[3], Kevin Stephens[4], Shalini Jain[5], and Hariom Yadav[5], *1. Aberystwyth University, Ceredigion, Wales, United Kingdom*, *2. Postbiotics Inc, Winston Salem, North Carolina, United States*, *3. Neurodyn Life Sciences Inc, Charlottetown, Prince Edward Island, Canada*, *4. Agroceutical Products Ltd, Glasbury, England, United Kingdom*, *5. University of South Florida, Tampa, Florida, United States*

A healthy gastrointestinal (GI) tract and its microbiome are crucial for maintaining overall health, while microbiome abnormalities can lead to various health issues including brain health and impact quality of life (QOL). These issues are often linked to dysregulation of the gut-brain axis. Although probiotics and prebiotics are common strategies to improve gut and brain health, they have limitations. Postbiotics, offering stable and predictable exposure, present an attractive alternative. In this open-label pilot study, we tested PoZibio®, a postbiotic containing heat-inactivated Lactobacillus paracasei D3.5 (LpD3.5), in 13 healthy older adults (≥50 years) over 30 days to assess its safety, tolerability, and impact on GI function and QOL. Participants consumed four LpD3.5/PoZibio capsules daily and completed the SF-36 and GSRS surveys before and after the study. PoZibio was found to be safe and well-tolerated, with notable improvements in emotional well-being and gastrointestinal symptoms. However, due to the study's open-label design, small sample size, and short duration, these findings should be interpreted cautiously. Larger, placebo-controlled trials are needed to confirm PoZibio's potential benefits for the gut-brain axis and overall health.

Abstract citation ID: igae098.4070

### SEABIRD: RECRUITING A DIVERSE COMMUNITY-BASED POPULATION FOR AN ALZHEIMER'S BLOOD TEST STUDY

Matthew Larsen[1], Melody Li[1], Melanie Burton[2], Lisa Soke[2], Rickey George[1], and Randall Bateman[1], *1. Washington University in St. Louis, St. Louis, Missouri, United States*, *2. Washington University School of Medicine, St. Louis, Missouri, United States*

Older adults, often isolated and facing health challenges, are less integrated into society and research, making it crucial for Alzheimer's disease (AD) studies to effectively recruit them. The Study to Evaluate Amyloid in Blood and Imaging Related to Dementia (SEABIRD) enrolled 1,122 participants to determine the participant acceptability and accuracy (relative to amyloid PET) of a plasma amyloid-β 42/40 assay in a diverse, community-based sample of older adults. Participants completed a blood collection, cognitive screening, and a survey about their study experience and perceptions of the blood test. A subset of participants completed additional blood collection for reproducibility, amyloid PET and MRI for validation of the blood test, and the Clinical Dementia Rating® for validation of the cognitive screening measures. To ensure a diverse sample of participants, demographic factors such as race and education were prioritized during the recruitment process. Participants were recruited through existing study registries, electronic medical record chart reviews, newspaper postings, flyers, and community events. Of the participants enrolled, 23.5% self-identified as Black or African American, exceeding the study's goal of 14% for the US population and 5.2% for a large AD research cohort (the Alzheimer's Disease Neuroimaging Initiative [ADNI] study in 2022). Additionally, SEABIRD participants were more likely to have a high school education or less (25.3%) compared to those in ADNI (15.5%). SEABIRD achieved broader diversity than typical AD cohorts and may help inform practices to recruit underrepresented populations. These results demonstrate the feasibility of recruiting a diverse, community-based population for AD blood collection studies.

Abstract citation ID: igae098.4071

### SELF-RATED SUCCESSFUL AGING AMONG OLDER TRANSGENDER WOMEN AND TRANSFEMININE PERSONS

Anna Rubtsova[1], Don Operario[1], Stefan Baral[2], Sarah Murray[2], Iaah Lucas[1], Mariah Valentine-Graves[1], and Travis Sanchez[1], *1. Emory University, Atlanta, Georgia, United States*, *2. Johns Hopkins University, Baltimore, Maryland, United States*

Successful aging (SA) refers to older adults' own holistic appraisals of how well they are aging. Research suggests that SA is achievable in historically marginalized communities. However, relatively little is known about SA among transgender older adults who may experience challenges related to gender affirming therapy, barriers to health care, intersectional stigmas, and discrimination. This study aimed to examine prevalence and correlates of self-rated SA (SRSA) among older transgender women and transfeminine persons, using data from the 2023/2024 cycle of Transgender Women's Internet Survey and Testing (TWIST) study. Our sample (n=46) included TWIST participants aged 50+ who completed an SRSA assessment: average age 57.8, 89.1% white, 69.6% with ≥college education, 60.9% employed, and 54.55% with annual income ≥$75,000. SRSA was assessed using a single-item scale, asking participants to rate themselves in terms of SA from 1 to 10 (SRSA≥7=high SA). We used Fisher's exact and t-tests to examine group differences by SRSA≥7 status. Pearson correlations were computed to determine psychosocial correlates of SRSA. We found that 63% of our sample reported SRSA≥7. While there were no group differences in sociodemographic variables, higher proportion of participants with SRSA≥7 reported no comorbidities (18% v. 4%, p=.016) and were comfortable with how others perceived their gender identity (65.5% v. 29.4%, p=.019). Higher SRSA was associated with higher levels of social capital (r=.57, p<.001) and social participation (r=.33, p=.029), and lower levels of depression (r=-.42, p=.005). These results suggest the potential for interventions to enhance positive psychosocial factors to promote SA in transgender people.

Abstract citation ID: igae098.4072

### SELF-REPORTED FALL RISK AND FEAR OF FALLING OF OLDER ADULTS: THE MEDIATING AND MODERATING IMPACT OF HEALTH LITERACY

Dahee Kim, Ladda Thiamwong, Christopher Emrich, Yingru Li, and Rui Xie, *University of Central Florida, Orlando, Florida, United States*

**Objectives:** Older adults who perceive more fall risks in their daily lives may have a higher fear of falling (FOF). Despite, as a human agency, health literacy of older adults may improve their health and well-being outcomes, but the role of health literacy in fall risk-FOF association has been less highlighted. This study investigated mediating and moderating impacts of their health literacy on the fall risk-FOF association. Methods. This study used data from 176 community-dwelling older adults (MeanAge = 74.49, SDAge = 7.16). Self-reported fall risk was measured using the CDC STEADI fall risk checklist. FOF was assessed using the Short Falls Efficacy Scale International. Health literacy was reported as the level of confidence in filling out a medical form by oneself. This study employed the SPSS PROCESS Macro to test the mediation and moderation models. Results. Older adults' health literacy moderated their self-reported fall risk-FOF association. The more fall risks older adults perceive, the higher FOF the Low health literacy group showed, whereas the lower FOF the High health literacy group reported. This indicates that health literacy would buffer the impact of fall risk in daily life on FOF in late adulthood. However, we found no indirect impact of self-reported fall risk on FOF via health literacy. Conclusion. These findings highlight the role of health literacy in mitigating the impact of self-reported fall risk on FOF of older adults. This study suggests health literacy as an agency that enhances health and well-being in older adults' remaining lives.

Abstract citation ID: igae098.4073

### SENSOR-BASED MONITORING OF PHYSICAL ACTIVITY IN OLDER ADULTS SIX MONTHS POST-FALL: MODERATING FACTORS

Laura Schmidt[1], Tim Stuckenschneider[1], Robert Kwiecien[2], and Tania Zieschang[1], *1. Carl von Ossietzky University Oldenburg, Oldenburg, Niedersachsen, Germany*, *2. University of Muenster, Muenster, Nordrhein-Westfalen, Germany*,

Older individuals who have experienced a fall often experience concerns about falling, impaired activities of daily living, and reduced physical activity (PA). Changes in PA following a fall are often influenced by moderators such as age or fall history; however, these moderators remain understudied. Therefore, this project investigates the association between moderators and changes in PA in older adults after a fall. Participants aged ≥60 years were recruited if they presented to the emergency department following a fall without hospital admission. Moderators were collected through a home-based geriatric assessment conducted within four weeks after the fall. PA was measured within four weeks and six months after the fall using an accelerometer (activPAL4, PAL Technologies Ltd., UK). Associations between moderators and changes in PA were calculated fitting Linear Mixed Model (LMM) in R (R Core Team, 2023 Version 4.3.2 (2023-10-31). A total of 178 valid datasets were analyzed. LMM analysis revealed a significant average increase in step count over time (p=0.006). Notably, female participants showed a significant larger increase in steps per day (+1,777.9, 95% CI: 292.2 – 3263.7, p=0.019) than male participants. In women, fall history (95% CI: -1009.5 – (-207.4), p=0.003) and age (95% CI: -315.8 – (-82.5), p< 0.01) were negatively associated with step counts. Given the healthcare challenges associated with falls in older adults, our findings underscore the importance of early identification of moderators in emergency department settings. Identifying these factors may enable targeted interventions for individuals at risk of functional decline post-fall, potentially preventing further functional decline.

Abstract citation ID: igae098.4074

### SEX DIFFERENCES IN THE ASSOCIATION BETWEEN COMORBIDITIES AND COGNITIVE STATUS IN OLDER BLACK AMERICANS

Albert Junior Nyarko[1], and Darlingtina Esiaka[2], *1. University of Kentucky, Lexington, Kentucky, United States*, *2. University of Kentucky College of Medicine, University of Kentucky, Kentucky, United States*

The decline in cognitive status among aging populations is a growing concern, particularly for Black Americans who experience a higher burden of chronic conditions that are known precursors of cognitive impairment. Despite the known disparities in brain health outcomes among aging Black Americans, limited research has explored how sex interacts with comorbid conditions to impact cognitive status in this population. We investigated sex differences in the association between comorbidities (stroke, diabetes, hypertension, sleep apnea, PTSD, active depression, cancer, asthma, and brain tumors) and cognitive status (presence of abnormal cognitive status) among older Black Americans. We analyzed data from participants (N= 448, 330 females; Mage = 75.79, SDage = 7.40; mean years of education = 15.89) who are a part of the University of Kentucky ADRC cohort. Our analysis showed significant sex differences in the association between comorbidities and cognitive status. Having a higher number of comorbidities was associated with greater presence of abnormal cognitive status in Black American males (b =.016, p =.131) than females (b =.002, p =.580). Further analysis showed that hypertension was significantly associated with a greater presence of abnormal cognitive status in Black American males (b =.295, p =.007). The presence of comorbidities, especially hypertension, may impact cognition more in older Black American males than females. Our findings highlight the complexity of comorbidities' impact on cognition and underscore the importance of considering sex differences in research on aging and brain health in Black Americans.

Abstract citation ID: igae098.4075

### SINGLE AND DUAL-TASK SPATIOTEMPORAL GAIT ANALYSIS OF OLDER ATHLETES PARTICIPATING IN THE NATIONAL SENIOR GAMES

Susan Leach[1], Deborah Doerfler[1], Jodi Schilz[1], Rose Vallejo[1], Becca Jordre[2], and Beth Moody Jones[1], *1. University of*

*New Mexico, Albuquerque, New Mexico, United States*, 2. *University of South Dakota, Vermillion, South Dakota, United States*

One purpose of this study was to gather normative spatiotemporal gait parameters in older athletes and determine differences under three walking conditions: Forward Usual Single Task, Forward Usual Dual Task, and Forward Fast Single Task. Post hoc analyses were explored to determine a) the impact of a cognitive dual task during usual speed on gait parameters b) to determine the impact of a change in gait speed from usual to fast on gait parameters. A secondary purpose was to determine if Open, Closed or Continuous sport category influenced gait parameters. It was hypothesized that gait parameters would change with the addition of a dual task, with increased speed, and sport category. Regarding methodology, participants included 335 athletes 50 years or older in this single center cross-sectional, quasi-experimental study. Spatiotemporal gait parameters were quantified using ProtoKinetics' Zeno Walkway system. A multivariate analysis of variance compared gait parameters under three walking conditions. Post-hoc analyses determined the impact of a cognitive dual task during usual walking speed, and a change in gait speed from usual to fast. Results indicated a significant effect of walking condition on all spatiotemporal gait parameters. From single to dual task, all gait parameters were significant with the exception of stride width coefficient of variation. From usual to fast speed, select gait parameters were significant. Sport category also demonstrated changes in select gait parameters. To conclude, normative spatiotemporal gait parameters were established for older athletes in three walking conditions and differences identified.

Abstract citation ID: igae098.4076

### SLEEP IN FOCUS: EVALUATING WEARABLE BIOMETRIC DEVICES FOR DEMENTIA SLEEP MONITORING- A SCOPING REVIEW OF EFFICACY

Leroy Griffiths[1], Ellen McCreedy[2], and Ann Reddy[3], *1. Brown University, Providence, Rhode Island, United States*, *2. Veterans' Administration, Providence, Rhode Island, United States*, *3. Brown University School of Public Health, Providence, Rhode Island, United States*

Sleep disturbances are prevalent among individuals with Alzheimer's disease and related dementias (ADRD) and adversely affect their physical and cognitive functioning. Monitoring sleep patterns, therefore, is imperative for assessing cognitive deterioration. Wearable devices may provide a cost-effective, non-invasive approach for conducting sleep studies in people with ADRD, provided they are tolerable and accurate. We conducted a scoping review to evaluate the efficacy of wearable devices in measuring sleep among people with ADRD, compared to gold standard measures: polysomnography (PSG) or sleep diaries. Utilizing Pubmed, we identified 140 potentially relevant articles. Of these, 104 were excluded during abstract review, 33 during full-text review, and 3 met the inclusion criteria for analysis. The primary reasons for full-text review exclusion were: lack of a gold standard comparator or direct measure comparison (76%), incorrect study population (12%), and studies involving randomized controlled trials without baseline data (6%). The included studies indicated that actigraphy consistently overestimated total sleep time and sleep disturbances compared to sleep diaries and questionnaires. Results are consistent with previous research that suggests actigraphy may overestimate sleep in low-mobility populations. It may also indicate that sleep diaries are ineffective for this population. Since none of the studies utilized PSG, more research is necessary to establish efficacy. Similarly, if sleep diaries are imprecise, the effectiveness of wearables may be insufficiently substantiated. Furthermore, none of the included studies report sample characteristics regarding race, despite the optical technology utilized in wearables being linked to racial bias in individuals with darker skin tones.

Abstract citation ID: igae098.4077

### SMARTPHONE OVERUSE AMONG OLDER ADULTS: A SCOPING REVIEW

Jeongeun Choi, Hyangkyu Lee, Seolah Yoon, and Hyeonmi Cho, *Yonsei University, Seoul, Republic of Korea*

The rapid advancement of technology and widespread adoption of smartphones have led to increased smartphone usage among older adults. While smartphones offer many benefits, excessive use can lead to health issues, especially for older adults who may experience reduced social interactions and higher levels of anxiety or depressive symptoms. However, most existing research has focused primarily on adolescents and younger adults. This scoping review aimed to investigate the terms and definitions, factors associated with smartphone overuse, and its outcomes in older adults. We searched seven databases, including PubMed, PsycINFO, Embase, Cochrane Library, Web of Science, Scopus, and CINAHL, covering studies published from 2007 to 2024. We followed the Joanna Briggs Institute scoping review methodology, and sixteen studies were selected. Smartphone overuse was characterized by various terms and definitions, including 'smartphone overdependence', 'smartphone addiction', and 'problematic smartphone usage'. According to the biopsychosocial model, factors related to smartphone overuse were classified into biological, behavioral, psychological, and social categories. Most commonly reported factors included biological aspects such as age, sex, and BMI; behavioral factors like types of smartphone activities; psychological factors including loneliness, anxiety, and depressive symptoms; and social factors such as income and educational level. Smartphone overuse can negatively affect physical, psychological, and social health, resulting in pain in the musculoskeletal system, sleep disturbances, anxiety, depressive symptoms, and loneliness. Future research should focus on developing standardized definitions and exploring effective interventions to mitigate the negative effects of smartphone overuse in older adults.

Abstract citation ID: igae098.4078

### SOCIAL CONNECTIONS AND FALLS AMONG OLDER WOMEN WITH AND WITHOUT HIV IN THE UNITED STATE

Lien Quach[1], Patricia Lloyd[1], Kristine M. Erlandson[2], Tracey Wilson[3], Anna Rubtsova[4], Anjali Sharma[5], Jeffrey Burr[1], and Mark Siedner[6], *1. University of Massachusetts Boston, Boston, Massachusetts, United States*, *2. University of Colorado - Anschutz Medical Center, University of Colorado Anschutz Medical Campus, Colorado, United States*, *3. Downstate Health*

*Sciences University, Brooklyn, New York, United States*, *4. Rollins School of Public Health, Emory University, Atlanta, Georgia, United States*, *5. Albert Einstein College of Medicine, Bronx, New York, United States*, *6. Massachusetts General Hospital, Boston, Massachusetts, United States*

Falls affect one in four older adults annually and are linked to biological and environmental risk factors. Women with HIV (WWH) may be particularly vulnerable due to lower social support and higher loneliness but their relationship with falls is not well understood. We analyzed data from 1,068 women aged 50 and older in the 2017 Women's Interagency HIV Study (WIHS). Social connections were evaluated using the Multidimensional Social Support Scale, which queries respondents about issues such as support for sharing private worries and help with medical appointments. Loneliness was defined as a score of 6-9 on the UCLA Loneliness Scale (range 0-9). Logistic regression models estimated associations between social connections and loneliness with falls, adjusting for age, education, alcohol use, smoking, and total of comorbidities. Falls were reported by 31.5% of participants, with similar rates between WWH and women without HIV (WWoH) (adjusted odds ratio [aOR]=1.08; 95%CI 0.79,1.49). Loneliness was associated with a higher likelihood of falls (aOR=1.28; 95%CI 1.04,1.82). Some social connection aspects, such as having someone with whom to provide information or share worries (aOR=0.90; 95%CI 0.81, 0.996), or to receive assistance with medical appointments (aOR=0.89; 95%CI 0.80, 0.989), was associated with a lower odd of falls. WWH experience falls at rates similar to WWoH. However, loneliness and lack of social connections are key contributors to falls in older women in the US. Future research should explore whether interventions to mitigate loneliness and enhance social connections impact falls in older women in the US.

Abstract citation ID: igae098.4079

### SOCIAL SUPPORT USING SOCIAL NETWORKING SERVICES FOR OLDER ADULTS WITH MCI

Seolah Yoon[1], Jeongeun Choi[1], Min Kyung Park[2], Dahye Hong[1], and Bada Kang[2], *1. Yonsei University, Seoul, Republic of Korea*, *2. Yonsei University College of Nursing, Seoul, Republic of Korea*

Older adults with mild cognitive impairment (MCI) are more likely to experience negative health-related outcomes. Social support is an important strategy for preventing adverse outcomes. While social support interventions using social networking services (SNSs) have potential benefits for social relationships and health, their impact on older adults with MCI, along with usability challenges and strategies, has not been comprehensively synthesized. The aim of this scoping review was to map the use of social support interventions using SNSs for health and social care of older adults with MCI and examine the usability challenges and strategies of SNS platforms. Following the Joanna Briggs Institute methodology, eight studies published between January 2005 and June 2024 were selected from such databases as PubMed, CINAHL, Web of Science, EMBASE, PsycINFO, SCOPUS, and Google Scholar. Social support interventions were categorized into five types: social networking, informational, esteem, emotional, and tangible support. Positive outcomes of the interventions included enhanced social interaction, increased physical activity, and improved memory and alertness. Usability challenges, such as technical skill deficits and privacy concerns, were identified, and strategies, such as weekly email checklists, training sessions, and simplified design features, have been proposed to address them. This review highlights the potential of social support interventions using SNSs to positively impact the physical, cognitive, and social outcomes of older adults with MCI. We discuss gaps in social support interventions attributed to SNSs and implications for future interventions aimed at improving the health and social well-being of older adults with MCI.

Abstract citation ID: igae098.4080

### SOCIOECONOMIC INEQUALITIES IN HEALTHY AGING OF OLDER KOREAN ADULTS: AN AGE-PERIOD-COHORT ANALYSIS

Yunhwan Lee, Eunsaem Kim, Jinhee Kim, Sukyung Kim, YooGyung Shin, and Jimin Han, *Ajou University School of Medicine, Suwon, Kyonggi-do, Republic of Korea*

As South Korea's healthy life expectancy continues to increase, reaching 73 years in 2021 and ranking third in the world, there is growing interest in assessing functional ability as well as its disparity across various older population segments. The study aimed to investigate healthy aging trends by socioeconomic status in the recent decade among Koreans aged 65 years or older and to analyze the age, period, and cohort effects. Data were from the four waves, about 10,000 people in each wave, of the National Survey of Older Koreans conducted every three years from 2011 to 2020. The physiological, physical, cognitive, psychological, and social dimensions were combined to create the healthy aging index (HAI), with higher scores indicating a healthier state. Socioeconomic status included education, household income, and wealth. A multilevel analysis with a cross-classified random effects model was performed. An overall trend of healthy aging in the past decade was evident, but the gap between the highest and lowest levels of education has widened. The 1935-1944 birth cohort showed a significant decrease in HAI, suggesting a long-lasting negative effect of early life adversity in the latter years of colonial rule. There was a strong age effect, demonstrating a quadratic decline in HAI over the life course. Even though the last ten years have shown a general trend toward healthy aging, socioeconomic disparity persists. The study's findings underscore the need for policies on healthy aging to consider the life-long vulnerability of certain aging population segments characterized by adverse early life experiences.

Abstract citation ID: igae098.4081

### STANDARDIZED NURSING LANGUAGE—KNOWLEDGE-BASED CLINICAL DECISION SUPPORT SYSTEM MODULE FOR LONG TERM CARE NURSES

Juh Hyun shin[1], Chung Hyuk Park[1], Myungeun Lee[1], Soo-Kyoung Lee[2], Crystel Farina[1], Suhyun Park[3], Burton Korer[1], and Melissa Batchelor[1], *1. George Washington University, Washington, District of Columbia, United States*, *2. Seoul National University, Seoul, Republic of Korea*, *3. University of Minnesota, Minneapolis, Minnesota, United States*

Clinical decision support systems (CDSS) integrate patient health information with clinical expertise to guide referrals, reduce prescription medication errors, and ensure adherence to guidelines. Standardized nursing languages, such as NANDA-I (North American Nursing Diagnosis Association International), NIC (Nursing Interventions Classification), and NOC (Nursing Outcomes Classification), enhance evidence-based practice by providing essential diagnostic and outcome data. However, the adoption of CDSS in long term care settings, incorporating standardized nursing languages and culturally specific scenarios to improve care for diverse racial and ethnic groups. We employed machine learning techniques, including support vector machines, random forests, and large language modeling, to train and test the CDSS using 130 scenarios of simulated nursing home residents generated by Generative Pre-Trained Transformer 4.0. Usability was assessed using the System Usability Scale (Brooke, 1996) and a questionnaire by Lund (2001) evaluating usefulness, satisfaction, and ease of use. The System Usability Scale (SUS) yielded a mean score of 60 (SD = 3.65), while the USE score was 6.62 (SD = 0.55), with high ratings for 'ease of learning.' Usability issues were also identified through heuristic evaluation with nursing faculty experts. All ratings fell within a range of 5 to 7, indicating overall positive feedback. The CDSS has the potential to significantly enhance care quality, marking a pivotal advancement in applying healthcare technology to support nursing professionals. Future research should reflect on a more nursing care-specific application in developing and applying new technology in long term care settings

Abstract citation ID: igae098.4082

### STUDY DESIGN, PROTOCOL, AND HUMAN HEALTH EFFECTS OF PFAS IN THE TSUKUBA CITY COHORT

Heesoo Eun[1], Jieun Yoon[2], Mayumi Kameyama[1], Hiroshi Hata[1], Tetsuaki Arai[3], Tomohiro Okura[3], and Hiroyuki Nishiyama[3], *1. National Agriculture and Food Research Organization, Tsukuba, Ibaraki, Japan, 2. Uiversity of Tsukuba, Tsukuba, Ibaraki, Japan, 3. Institute of Medicine/University of Tsukuba, Tsukuba, Ibaraki, Japan*

PFAS (perfluoroalkyl and polyfluoroalkyl substances) are toxic chemicals that are urgently regulated worldwide. While several adverse effects on the human body are known, including impacts on liver, kidney, and reproductive functions, the effects of 30 different multicomponent PFAS on the human body remain largely unexplored. Recently, it has been suggested that PFAS may affect Alzheimer's disease, necessitating more rapid investigation. Given that PFAS exposure in humans primarily occurs through food intake, we initiated a cohort study in collaboration with various medical specialists to understand and address the actual situation of PFAS exposure in Japanese individuals who consume seafood with relatively high PFAS concentrations. The ongoing Tsukuba Happiness Life Study involves a large group of citizens aged 45 to 89 years from Tsukuba City, Ibaraki Prefecture. The study will investigate the molecular species and concentrations of more than 30 PFAS in blood and urine samples collected from 1,000 healthy, consenting participants. Additionally, cognitive function and motor performance will be assessed. Specialists from various medical departments at the University of Tsukuba Hospital will evaluate the results, relating the molecular species and concentrations of PFAS to their health effects. This research will, for the first time, reveal the effects of PFAS derived from dietary habits on the human body, providing comprehensive knowledge on PFAS, including their impact on cognitive and motor functions.

Abstract citation ID: igae098.4083

### SUBJECTIVE COGNITIVE CONCERNS AND NEUROPSYCHOLOGICAL PERFORMANCE IN PRODROMAL PARKINSON'S DISEASE

Britney Luu[1], Katherine Bangen[2], Mary Ellen Garcia[2], Uriel Urias[1], Douglas Galasko[2], and Kelsey Thomas[2], *1. San Diego State University, San Diego, California, United States, 2. University of California, San Diego, La Jolla, California, United States*

While subjective cognitive concerns (SCC) are commonly observed in preclinical Alzheimer's disease, SCC in cognitively unimpaired individuals at risk for Parkinson's disease (PD), as defined by reduced sense of smell (hyposmia) or REM behavior sleep disorder (RBD), is less understood. Therefore, this study examined associations between SCC and individual neuropsychological measures in the prodromal PD cohort (n=715, mean age=67.75) from the Parkinson's Progression Markers Initiative (PPMI) study. SCC (n=199) or no SCC (n=516) was based on participant or caregiver report for item 1 on the Unified Parkinson's Disease Rating Scale, part 1. Analyses of covariance, adjusting for age, education, sex/gender, race/ethnicity, psychiatric factors, motor symptoms, and prodromal classification (hyposmia or RBD), examined the association between SCC and measures of memory, language, visuoperception, processing speed, and executive functioning. Relative to participants without SCC, those with SCC had lower performance on Animal Fluency (B=-1.02, p=.009, np2=.011) and Symbol Digit Modalities (B=-1.68, p=.036, np2=.007). When examining the SCC x prodromal classification interaction, hyposmia or RBD status did not moderate the associations for Animal Fluency or Symbol Digit Modalities. Overall, SCC in prodromal PD participants was only associated with two measures requiring speeded retrieval and processing. Unlike in preclinical Alzheimer's disease, where SCC is often linked to memory, no such association was found in prodromal PD. Instead, SCC was associated with speeded measures that are likely more sensitive to PD related changes. Future work will investigate the utility of SCC to predict conversion to PD and longitudinal rates of cognitive decline.

Abstract citation ID: igae098.4084

### THE CONTRIBUTION OF COMMUNITY/NEIGHBORHOOD TO COMMON PREDICTORS OF NON-COMMUNICABLE AGING RELATED DISEASE

Alicia DeLouize[1], Paul Kowal[2], Nirmala Naidoo[3], Srean Chhim[4], Heng Sopheab[4], Josh Snodgrass[1], and Savuth Chin[4], *1. University of Oregon, Eugene, Oregon, United States, 2. The Australian National University, Canberra, Australian Capital Territory, Australia, 3. World Health Organization, Geneva, Geneve, Switzerland, 4. National Institute of Public Health, Phnom Penh, Phnom Penh, Cambodia*

Non-communicable diseases (NCDs) are now the leading causes of death globally. Their increasing rates have been associated with market integration and they tend to cluster in certain neighborhoods and communities, yet research has mostly focused on individual level causes. The aim of this research was to identify neighborhood/community level associations with NCDs that are often attributed to individual lifestyle factors. The World Health Survey Plus (WHS+) was conducted in Cambodia in 2023 and is among the first studies to collect nationally representative point-of-care biomarker data along with anthropometric, performance test, and survey data (N = 5,275). Multi-level models were run, with 98 regions and 276 communities as random intercepts, predicting rates of diabetes, hypercholesterolemia, hyperlipidemia, hypertension, arthritis, angina, stroke, and frailty level. Between 22% to 77% of the variance usually attributed to fixed effect independent variables (education, log of household income, salt, alcohol, tobacco, waist-to-stature-ratio (WSR), anxiety, stress, and sedentary behavior) was attributed to group-level fixed effects. Alcohol, sedentary behavior, stress, anxiety, WSR, and education were group-level variables that were conflated at the individual level for at least one NCD. Prevention and treatment of non-communicable diseases have mostly centered around individual lifestyle changes, yet there are likely ecological causes that can be targeted.

Abstract citation ID: igae098.4085

### THE EFFECT OF PICEATANNOL FROM PASSION FRUIT SEED EXTRACT ON SIRTUIN EXPRESSION

Motoki Tsukiashi, Shinpei Kawakami, Hiroko Maruki-Uchida, and Naoki Iemoto, *Morinaga & Co., Ltd., Yokohama, Kanagawa, Japan*

Piceatannol, a polyphenol abundantly found in passion fruit seeds, is structurally similar to resveratrol. It has four hydroxyl groups, is highly water-soluble, and is expected to have various effects, including antioxidant properties. Sirtuins are known to promote longevity and prevent various chronic diseases associated with aging. Given the growing interest in longevity research, this study aimed to explore the potential of piceatannol to induce sirtuins. We hypothesized that piceatannol might function as a more potent activator of sirtuins than resveratrol. Using THP-1 cells, we performed RT-PCR and Western blotting to determine sirtuin mRNA expression and protein levels after the addition of piceatannol. Our in vitro results showed that piceatannol and its metabolites significantly induced sirtuin mRNA expression and increased sirtuin protein levels, surpassing the effects of resveratrol. To validate these findings in humans, we examined changes in sirtuin mRNA expression in blood after the ingestion of a test food containing 100 mg of piceatannol or a placebo food for two weeks in a double-blind, placebo-controlled, parallel-group study. The results of the analysis of 281 participants confirmed the upregulation of sirtuin mRNA expression due to piceatannol intake. Moreover, stratification by menopausal status revealed that sirtuin mRNA expression was significantly higher as a result of piceatannol intake among postmenopausal women. These findings suggest that piceatannol intake may promote longevity through sirtuin activation, offering potential for longevity therapies and dietary supplements. This study emphasizes the need for further research on piceatannol's mechanisms of action in the growing field of longevity research.

Abstract citation ID: igae098.4086

### THE FREQUENCY OF RAW VEGETABLE AND FRUIT CONSUMPTION AND ASSOCIATES WITH DEPRESSIVE MOOD IN JAPANESE OLDER ADULTS

Mai Takase[1], Yuri Yokoyama[1], Yu Nofuji[1], Takumi Abe[2], Kumiko Nonaka[1], and Hiroshi Murayama[1], *1. Tokyo Metropolitan Institute for Geriatrics and Gerontology, Tokyo, Japan*, *2. Meiji University, Tokyo, Japan*

The consumption of vegetables and fruits are known to associate with better health. It is also proven that the intake of raw and unprocessed vegetables and fruits relate to better mental state in younger adults, due to retainment of micronutrients that could be lost from cooking. However, little is known about the relationship between intake of raw vegetables and fruits and mental state in older adults. In this research, we aimed to clarify the association between the frequency of raw vegetable and fruit consumption and depressive mood in Japanese older adults. A questionnaire survey was conducted at the Wako Cohort Study, which targets community dwelling older adults (≥65 years old) living in Saitama prefecture, Japan (September 2023, N=1,004). The frequency of raw vegetable and fruit consumption (every day vs. less than every day) was used as independent variable, and Geriatrics Depression Scale 5 (score of 2 and above as depressive mood vs. 1 and below as none) was used as dependent variable. The percentage of those who consumed raw vegetable and fruit everyday was 27.2% (n=273) and the percentage of those with depressive mood was 27.4% (n=275). Adjusted with age, sex, marital status, education, self-rated health, number of teeth, comorbidity, BMI, cohabitant, income status, and dietary variation score, a binary regression analysis (n=896) revealed that frequency of raw vegetable and fruit consumption negatively associated with depressive mood (OR=0.62, 95%CI=0.40-0.97). This result suggests the importance of frequent raw vegetable and fruit consumption for better mental state in older adults.

Abstract citation ID: igae098.4087

### THE IMPACT OF DEPRESSION ON OBJECTIVE AND SUBJECTIVE ORAL HEALTH INDICATORS IN OLDER INDIVIDUALS

Boyun Choi[1], and Bock-Young Jung[2], *1. Yonsei University, Seoul, Republic of Korea*, *2. Yonsei University College of Dentistry, Seoul, Republic of Korea*

Depression significantly affects overall health and quality of life, including oral health and subjective discomfort. This study examines how objective and subjective oral health factors differ based on depression diagnosis in individuals aged 65 and older. Using data from the 2018-2020 National Health and Nutrition Survey (n=3324) by the Korea Centers for Disease Control and Prevention, this study compared oral health in patients with and without depression. The research assessed subjective factors like chewing difficulties, self-perceived oral health, and recent tooth pain, alongside objective factors such as the number of natural teeth and the need for prosthetics. Analyses included Rao-Scott chi-square tests, survey logistic regression, and linear regression. Significant differences were observed in subjective oral health factors between the depressed and non-depressed groups. Depression was associated with recent tooth pain ($p = 0.0022$), chewing discomfort ($p < 0.0001$), and self-perceived poor oral health (p = 0.0136). Regardless of gender, age, BMI, depression group reported the same pattern. However, no significant differences were observed in objective factors such as the number of natural teeth or the need for prosthetics. These findings suggest that depression could influence oral subjective and functional ability factors more sensitively and how older individuals perceive their oral health.

Abstract citation ID: igae098.4088

### THE IMPACT OF INDIVIDUAL AND NEIGHBORHOOD SES ON A SMOKING CESSATION INTERVENTION IN A LUNG CANCER SCREENING SETTING

Jaqueline Avila[1], Efren Flores[2], Yan-Jhu Su[1], Jennifer Haas[2], Elyse Park[2], and Nancy Rigotti[2], *1. University of Massachusetts Boston, Boston, Massachusetts, United States*, *2. Massachusetts General Hospital, Boston, Massachusetts, United States*

Lung cancer screening (LCS) offers a teachable moment for smoking cessation, but socioeconomic status (SES) may affect treatment success. This study assesses whether educational level or neighborhood SES are associated with smoking abstinence and completion of a smoking cessation intervention offered in the LCS context. This is a secondary analysis of a clinical trial (NCT03611881) testing the effectiveness of smoking cessation treatment for older smokers scheduled for LCS (N=615). Outcomes were self-reported 7-day smoking abstinence and study follow-up completion at 6-months. Independent variables were educational level (high school graduation or less [low] vs. post-high school education [high]); neighborhood SES (Area Deprivation Index [ADI], ranging 1 [low SES] to 100 [high SES] categorized as: highest 15% vs. remaining 85% scores), and a combination of both measures. Logistic regression models tested the association between outcomes and SES measures, adjusted for covariates. 32.7% of the sample had low educational level, and the mean sample ADI was 19.9 (SD: 12.8). Smoking cessation was higher among those with higher vs. lower neighborhood SES (15.7% vs. 7.4%, p-value=0.03). Study completion was lower among those with lower vs. higher educational level (78.1% vs. 84.5%, p=0.05). In multivariable models, these associations were not significant but individuals with high educational level and low neighborhood SES were more likely to complete the study than those with both low educational level and low neighborhood SES (OR: 6.04, 95%CI: 1.47-24.7). Smoking cessation in the LCS context might be improved by targeting community factors that affect neighborhood SES and educational level.

Abstract citation ID: igae098.4089

### THE IMPACT OF PARTICIPATION IN AN IMMERSIVE VIRTUAL REALITY TAI CHI PROGRAM ON THE BALANCE OF OLDER ADULTS

Jaehyun Kim[1], Marcos Ardon Lobos[1], Serim Park[2], Seungjun Oh[3], Jay Kim[2], and Junhyoung Kim[2], *1. East Carolina University, Greenville, North Carolina, United States*, *2. Texas A&M University, College Station, Texas, United States*, *3. Chadwick International School, Incheon, Republic of Korea*

Falls are the leading cause of injury-related mortality in older adults aged over 65 years, with ~36 million older adults suffering from falls each year that result in more than 34,000 deaths. Tai Chi, an ancient Chinese martial art, is shown to reduce the risk of falls by improving physical functioning (e.g., balance, flexibility, muscle strength). Despite the well-recognized health benefits of Tai Chi instruction, older adults often encounter barriers including limited access to programs, a lack of instructors, and environmental distractions. To address these barriers, this study examined the impact of an immersive virtual reality Tai Chi intervention (IVTC) on the balance of older adults. Eighteen older adults from senior centers in North Carolina participated in eight 15-minute IVTC sessions over a month and completed the Berg Balance (BB) test before and after the IVTC intervention program. The results showed significant improvement in BB scale scores after the IVTC sessions (p <.001) with a large effect size (d = -2.75). Holden's System Usability Scale indicated that participants were very favorable towards using the program. Our findings suggest that the IVTC program is a very usable intervention that could effectively improve the balance of older adults, highlighting its potential as an innovative intervention to reduce fall risks for older adults.

Abstract citation ID: igae098.4090

### THE PREDICTORS OF SEDENTARY BEHAVIOR AMONG OLDER ADULTS WITH POOR MOBILITY

Albert Okrah[1], Elizabeth Nelson-Twakor[1], Sadanand Fulzele[1], Samantha Landwehr[2], Karrie Zhao[2], Katherine Hsieh[2], and Deborah Jehu[1], *1. Augusta University, Augusta, Georgia, United States*, *2. Georgia State University, Atlanta, Georgia, United States*

Older adults with poor mobility are typically more sedentary, which poses increased risk for disability. Identifying the predictors of objectively measured sedentary behavior may affect targeted treatment strategies. This study aimed to identify the predictors of sedentary behavior in older adults with poor mobility. We recruited 18 older adults with self-reported poor mobility (78.3±8.6 years, 44.4% female) from the community and residential care facilities. Participants reported demographic information (years of education, age, sex, fall history) and completed a battery of assessments, including cognition (Rey Auditory Verbal Learning Test), depression (Geriatric Depression Scale), and gait (gait speed). Participants wore an activity monitor on their non-dominant ankle for 7 days (ActiGraph, Pensacola, FL). We used a minimum wear time of ≥4 days, including 2 weekend days in the analysis. We conducted a linear regression with sedentary behavior percentage as the dependent variable, with moderate-to-vigorous physical activity (MVPA), fall history, depression, memory, and gait speed as variables of interest, and we controlled for age, sex, and education,. All variables were correlated at r≤±0.3. The overall model was significant (R2=0.93, p=0.004). The predictors of high sedentary behavior were: greater depression [β=0.59, CI=0.04,1.14], lower MVPA [β=-0.16, CI=-0.2,-0.13], male sex [β=2.71, CI=0.03,5.39], and better memory [β=1.02, 0.29,1.74]. Our results suggest that older adults with poor mobility who are male, more depressed, less physically active, and have better memory are more at risk for being sedentary. Targeted interventions to reduce sedentary behavior and increase exercise, especially those most at-risk for a sedentary lifestyle, may improve health outcomes.

Abstract citation ID: igae098.4091

### THE RELATIONSHIP BETWEEN CREATININE-TO-CYSTATIN C RATIO AND FRAILTY: THE MEDIATING ROLE OF MUSCLE STRENGTH

Shuli Jia[1], Mei-Ling Ge[1], Qian-Li Xue[2], and Bigrong Dong[1], *1. West China Hospital, Sichuan University, Chengdu, Sichuan, China (People's Republic)*, *2. Johns Hopkins University, Baltimore, Maryland, United States*

**Background:** The creatinine to cystatin C ratio (CCR) has recently been suggested as a predictor for many adverse clinical outcomes. This study aimed to investigate the association between CCR and frailty, as well as the mediating role of muscle strength.

**Methods:** Cross-sectional and longitudinal analyses with 4-year follow-up of community-dwelling adults aged ≥60 years were conducted. Frailty was assessed using the physical frailty phenotype, which includes five binary criteria (weakness, slowness, exhaustion, low activity level and weight loss). Participants with three or more criteria were defined as frail. Muscle strength was measured by the maximum handgrip strength (HGS) of two hands. Logistic regression was used to analyze the associations between baseline CCR and prevalent and incident frailty. Mediation analyses were performed to examine the mediating role of HGS in the relationship between baseline CCR and incident frailty.

**Results:** Higher CCR was associated with lower risk of frailty (OR 0.41(0.18,0.92), p=0.030) in cross-sectional analysis, but this relationship was not significant when adding HGS in the regression model. After excluding participants who were frail at baseline and during the mid-follow-up, and those met the criteria for weakness at baseline, a total of 2428 participants were included in the longitudinal analysis. The association between baseline CCR and incident frailty was partially mediated by the mid-follow-up HGS (mediation proportion: 14%, p < 0.001). Conclusions: HGS partially mediated the associations between CCR and frailty in Chinese populations. The findings reveal a novel biomarker for frailty, which may also facilitate the identification of frailty subtypes.

Abstract citation ID: igae098.4092

### THE ROLE OF CANCER-RELATED FATIGUE IN PERCEIVED AND ACTUAL ATTENTION AMONG OLDER COLORECTAL CANCER SURVIVORS

Mi Sook Jung, Kyeongin Cha, Ahrim Lee, Xirong Cui, Dahyeon Lee, Jeongeun Hwang, Eunyoung Park, and Hyun-E Yeom, *Chungnam National University, Daejeon, Republic of Korea*

Older people treated for cancer often experience greater fatigue compared to younger survivors, partly due to age-related physiological changes and reduced physical resilience. Our previous study found that both perceived cognition and actual test performance were significantly associated with cancer-related fatigue (CRF) in middle-aged cancer survivors. However, it remains unclear whether the impact of fatigue on cognition is more pronounced in older cancer survivors compared to their younger counterparts. This study aims to compare attention function between different age groups and to examine whether increased fatigue contributes to poor attention. We assessed middle-aged (n=131) and older (n=133) colorectal cancer survivors using self-rated questionnaires (Attentional Function Index, Functional Assessment of Cancer Therapy-Fatigue, Patient Health Questionnaire) and a computerized test (Attention Network Test, ANT). Data were analyzed using multivariable linear regression and linear mixed models. Older participants exhibited poorer executive attention performance than middle-aged participants, while perceived attention and fatigue levels were similar between age groups. Additionally, older participants did not show improvement across trials unlike middle-aged participants showing faster performance with repeated trials. Fatigue was significantly associated with performance on ANT. This association remained significant even when controlling for educational level, comorbid condition, depressed mood, and restorative activity. The findings indicate that older cancer survivors had reduced executive attention function and may be more vulnerable to the effect of cancer-related fatigue, which is intrinsically linked to attention dysfunction. This enduring challenge suggests a need for targeted intervention addressing both cancer-related fatigue and cognitive impairment to improve outcomes for older cancer survivors.

Abstract citation ID: igae098.4093

### THE VALUE OF IN-PERSON VA MEDICAL FOSTER HOME NATIONAL CONFERENCE FOR RURAL PROGRAM COORDINATORS

Chelsea Manheim, Rachel Fix, and Leah Haverhals, *Rocky Mountain Regional VA Medical Center, Aurora, Colorado, United States*

The Department of Veterans Affairs (VA) Medical Foster Home (MFH) program provides long-term care for nursing home eligible Veterans who desire to reside in home-like environments. In MFHs, Veterans are matched with non-VA caregivers to live in caregivers' homes as part of their family, with caregivers providing around-the-clock care for up to three patients. MFH coordinators are responsible for full program implementation including recruiting, screening, and monitoring caregivers; recruiting Veterans; and partnering with VA's Home Based Primary Care teams who provide Veterans' healthcare in MFHs. Balancing these many duties, the MFH coordinator position can prove isolating. In 2024, the VA Office of Geriatrics and Extended Care (GEC) sponsored two, two-day, conferences for MFH staff to encourage goal setting, cross-site collaboration, and foster support. The first conference hosted 83 attendees from east coast programs in Orlando, Florida in March, and the second hosted 98 attendees from Midwest and western programs in Chicago, Illinois in May. Conferences coincided with VA efforts to expand MFH programs to all VA Medical Centers (VAMCs) by 2026. Additionally, the VA Office of Rural Health (ORH) currently funds expansion of four rural MFH programs. To assess experiences of MFH coordinators from the four rural expansion programs, we conducted interviews with them in June 2024. Coordinators reported making strong connections with more seasoned coordinators in nearby areas, developed innovative marketing ideas and created action plans, and improved motivation to serve in rural areas. Interviews showed that in-person conferences provide needed boosts to coordinators working in solitary positions.

Abstract citation ID: igae098.4094

### TIME-OF-DAY MOVEMENT AMOUNT AND VARIABILITY BY COGNITIVE STATUS IN A SAMPLE OF BLACK OLDER ADULTS

Amal Wanigatunga[1], Francesca Marino[2], Fangyu Liu[1], Nicola Shen[3], Marta Karas[4], Lauren Parker[1], Marilyn Albert[5], and Jennifer Schrack[1], *1. Johns Hopkins Bloomberg School of Public Health, Baltimore, Maryland, United States*, *2. Boston University School of Medicine, Boston, Massachusetts, United States*, *3. Boston Public Health Commission, Boston, Massachusetts, United States*, *4. Takeda Pharmaceuticals, Boston, Massachusetts, United States*, *5. Johns Hopkins University School of Medicine, Baltimore, Maryland, United States*

Reduced daily movement, particularly in the mornings, is linked with elevated Alzheimer's disease risk, yet prior studies have consisted of primarily non-Hispanic White participants. Whether this association exists in Black older adults, who often are underrepresented in research and experience delays in cognitive impairment diagnosis, remains unexplored. Wrist-worn accelerometry was analyzed from 21 Black participants (median age: 73 years, 83% women) referred from the Johns Hopkins Alzheimer's Disease Research Center who evaluate each participant for mild cognitive impairment (MCI). Separate age and sex adjusted linear regression models estimated movement differences by MCI status in mean: 1) total physical activity amount or variability (counts/d) over 24-hrs and 2) amount or variability of activity (counts/d) from 12am-6am, 6am-12pm, 12pm-6pm, and 6pm-12am. Variability measures were calculated as the SD of minute-level activity during each time period. Thirteen participants (62%) were identified with MCI. Participants with MCI averaged 1,700,927 (SD=721,021.9) counts/d compared to cognitively unimpaired participants who averaged 1,727,588 (SD=841,627.9) counts/d. Amount of activity counts/d did not differ between those living with and without MCI overall or by time-of-day (p for all>0.56). However, there was a significant difference in physical activity variability between 6am-12pm, where variability was higher among those with MCI by 66,451 (95% CI: 883, 132,020) counts/d. Our findings warrant further investigation into whether variability, rather than amount, in everyday movement occurring during early morning hours could serve as a digital biomarker to enhance cognitive impairment screening for Black older adults living independently.

Abstract citation ID: igae098.4095

### TOGETHER, WE DO SAVE LIVES: EVALUATING THE IMPACT OF A GERIATRIC TRAUMA PROGRAM

Sandra Sanchez-Reilly[1], Erin Sidle[2], Shannon Crenshaw[2], Elizabeth Reilly[3], Marla Khalikov[3], Jennifer LaCoss[3], Nicolina Martinez[3], and Ashley McGinity[4], *1. South Texas Veterans Health Care System and the University of Texas Health Science Center at San Antonio, San Antonio, Texas, United States*, *2. University Health, San Antonio, Texas, United States*, *3. Health Careers High School, Health Careers High School, Texas, United States*, *4. UT Health San Antonio, UT Health San Antonio, Texas, United States*

**Background:** Trauma, the fifth cause of death among Older Adults (OA), accounts for up to 25% of all trauma admissions. Multi-comorbidities, polypharmacy, decreased function, and increased mortality are some challenges requiring geriatric expertise. Objective: To evaluate the clinical effectiveness of a geriatric trauma service consultation (GTSC) program in a level-1-trauma hospital.

**Methods:** 1321 GTSC were collected December/22-June/24. Variables included length-of-stay, morbidity/mortality and place-of-discharge.

**Results:** Median age 78 (73-85). 58% female (765); 73% White, 20% Hispanic. 62% GTSC occurred within the first day of hospital admission, but 2% GTSC occurred >6 days after admission (delayed consults-DC). Within 30-days-of-admission, 3% and 2% hospital-readmission-rate. Falls constituted 74% GTSC, 17% were motor vehicle accidents. 87% GTSC came from home but only 35% returned home with 56% discharged to rehabilitation facilities. When comparing GTSC performed within 2 days of admission vs. DC, DC showed higher mortality (3.1% vs. 6.9%, p=0.24,NS); higher ICU stays (38.8% vs. 75%,p< 0.0001); higher 30-day-hospital-readmission (2.1% vs. 3.4%,p=0.47,NS); higher total length-of-stay (mean 6.9 days vs. 24.5,p< 0.0001); higher length-of-stay from day-of-GTSC (mean 6 days vs. 10.5,p=0.0004). OA with delirium had the longest hospital length-of-stay (mean 21.3 days,p< 0.0001) and higher ICU length-of-stay (15.9 days,p< 0.0001). Conclusions: When consulted early, GTSC showed to decrease mortality, hospital readmissions; and being significantly effective in decreasing ICU time and lowering length-of-stay among OA. Delirium poses a serious dilemma showing significantly worse length-of-stay and ICU need. Further studies need to focus on GTSC as an earlier intervention and to target specific geriatric syndromes such as delirium.

Abstract citation ID: igae098.4096

### TOWARDS GAIT-BASED SCREENING FOR COGNITIVE IMPAIRMENT

Haley Hentnik, Christine Phillips, Abigail Stephan, Lesley Ross, and Reed Gurchiek, *Clemson University, Clemson, South Carolina, United States*

While cognitive impairment is a hallmark feature of Alzheimer's disease, it can be difficult to detect in preclinical stages. Gait metrics, like speed and variability, have been suggested to augment early detection efforts and could be used in combination with existing cognitive-based screening tools such as the modified Telephone Interview for Cognitive Status (TICS-m). However, the specificity of these gait metrics and their relationship with cognition remains unclear. This study aimed to quantify the relationship between gait and a cognitive screening assessment (TICS-m) and cognitive attention (Useful Field of View Test, UFOV) in a sample of 106 older adults (71.9 +/- 5.9 years old). Three gait metrics were significantly (p < 0.05) correlated with TICS-m including usual gait speed (r = 0.27), gait symmetry (r = 0.30), and gait smoothness (r = -0.23). These metrics were also correlated with a composite UFOV score; the strongest relationship indicated that individuals with worse attention and slower processing speed presented with a less smooth gait (r = -0.42). Interestingly, gait smoothness was more strongly related to UFOV than was TICS-m (r = -0.30). These results corroborate previous research reporting similar relationships between visual-spatial processing and gait variability in individuals with cognitive impairment. However, gait smoothness was correlated with gait speed, and thus it remains unclear if our findings are independent of this relationship. Future research should explore the specificity of gait-based digital biomarkers of cognitive impairment and their utility for early detection and screening.

Abstract citation ID: igae098.4097

### VOICES FROM THE FIELD: EXPLORING THE VALUE OF GERONTOLOGISTS IN INTERPROFESSIONAL GERIATRIC CARE TEAMS

Alfred Boakye, and Diane Martin, *University of Maryland Baltimore, Baltimore, Maryland, United States*

Interprofessional healthcare teams effectively deliver safe and high-quality care to patients with complex healthcare needs. Through collaborative team-based efforts, doctors, nurses, social workers, pharmacists, and other professionals address multiple aspects of health, including physical, mental, and social well-being. Team-based care is now recognized as a national priority to optimize the health and social well-being of older adults and their families; however, geriatric care teams often do not include professionals with expertise in aging, such as gerontologists. The aim of this study was to explore what gerontologists can contribute to a geriatric interprofessional care team. Sixteen (16) gerontology students, faculty, and healthcare professionals completed semi-structured surveys, and the results were analyzed using a modified grounded theory. Respondents highlighted that the unique interdisciplinary perspective on the aging process held by gerontologists can promote person-centered care and bridge the gap between older adults and community resources. These findings illustrate the valuable role that gerontologists can have as members of interprofessional geriatric healthcare teams. Additional research is needed to evaluate whether integrating gerontologists can lead to enhanced care, improved care coordination, and advance evidence-based practices, research, and policy.

### SESSION 8020 (POSTER)

### LATE BREAKING POSTER SESSION IV

Abstract citation ID: igae098.4098

### "IT'S NOT THE AGE, IT'S THE APTITUDE: FACTORS INFLUENCING ADOPTION OF DIGITAL HEALTH APP AMONG OLDER ADULTS"

Yosefa Birati, Hod Orkibi, Lola Kolpina, and Anna Zisberg, *University of Haifa, Haifa, Hefa, Israel*

The rapid growth of digital health services has established mobile health (mHealth) apps as a key communication channel between healthcare providers and patients in primary care. These apps offer features such as appointment scheduling, access to medical records, and administrative task management. This study examines how digital health literacy, perceived ease of use, and perceived usefulness of mHealth apps explain mHealth apps and online services usage among older adults. Our study involved 179 community-dwelling older adults (mean age 73.5±5.05). The sample was relatively well-educated, with 33.2% holding a bachelor's degree and 31.5% having a master's or higher degree. Notably, 80% of participants reported high to very high confidence in using mHealth apps. Multiple regression analysis showed that age and health status alone explained only 4% of the variance in mHealth app use. However, when accounting for digital health literacy ($\beta$ =.228, p <.001), ease of use ($\beta$ =.369, p <.001), and usefulness ($\beta$ =.283, p <.001) the model accounted for 58.7% of the variance ($\Delta R^2$ =.493, p <.001). These findings underscore the critical importance of addressing digital health literacy and usability challenges among older adults. By focusing on these factors over age alone, we can enhance the design and personalization of mHealth apps and facilitate independent engagement with digital health services among the older adult population.

Abstract citation ID: igae098.4099

### A LATENT CLASS ANALYSIS OF INSTRUMENTAL ACTIVITIES OF DAILY LIVING (IADL) CAREGIVING NEEDS

Suyoung Kim[1], Sunshine Rote[2], Phillip Cantu[3], Flávia Andrade[4], and Jacqueline Angel[1], *1. University of Texas at Austin, Austin, Texas, United States*, *2. University of Louisville, Louisville, Kentucky, United States*, *3. University of Texas Medical Branch, Galveston, Texas, United States*, *4. University of Illinois at Urbana Champaign, Urbana, Illinois, United States*

**Objectives:** Mexican American caregivers experience time-intensive caregiving situations yet studies on heterogeneity in caregiving needs are limited. This study (1) utilizes latent class analysis (LCA) to classify caregivers into groups based on care recipient IADL intensity and type of support need and (2) examines caregiver psychological outcomes by care recipient support need.

**Methods:** Data from the Hispanic Established Populations for the Epidemiologic Study of the Elderly (Wave 7, N = 885) was analyzed using LCA. Characteristics of both caregivers and care recipients were included as predictors for a dyadic analysis, and caregiver perceived stress, depression, overall life satisfaction were investigated as tertiary outcomes.

**Results:** Four distinct groups of Mexican American caregivers were identified by care recipient IADL assistance need: comprehensive (39%), social task only (29%), minimal (23%), and physical mobility only (9%). Care recipient cognitive functioning and nativity status as well as caregiver homeownership status were associated with IADL assistance need. Caregiver perceived stress was highest for comprehensive IADL assistance need and lowest for social task only need. Caregiver life satisfaction peaked for those providing care for care recipients with minimal need, and caregiver depressive symptoms were more prevalent for comprehensive and physical mobility only IADL assistance need.

**Discussion:** Caregiving demands, especially for mobility and comprehensive social, household, mobility, and financial care needs, are associated with greater caregiver psychological burden. Targeted, culturally competent interventions aimed at improving the mental health of Mexican American caregivers should address the unique challenges with mobility and comprehensive care needs.

Abstract citation ID: igae098.4100

**A LIFE COURSE PERSPECTIVE ON FACTORS UNDERLYING NURSING HOME STAFF RETENTION**

Waqar Ahmad, Jennifer Morgan, Elisabeth Burgess, and Eric Wright, *Georgia State University, Atlanta, Georgia, United States*

Nursing homes continue to experience persistent challenges in retaining nursing home staff. Understanding the life course context of nursing home staff has the potential to shed needed light on this persistent issue. The life course perspective emphasizes understanding the dynamic interplay of personal, social, organizational, and historical factors and how this dynamism influence lives. Using cross-sectional survey data from 678 nursing home staff working in 23 nursing homes, we use ordinal logit regression to estimate a predictive model consisting of demographic, job quality and life course factors predicting employment intentions. Life course-relevant findings include: intergroup differences in employment intentions by life course markers and identities with career stage, benefits status, and breadwinner status demonstrating significant impact on intent to stay. Further, we find evidence that cumulative disadvantage, emotional burnout and other care roles have additional significant impacts on intent to stay. This study has practical implications for nursing home leadership and policy makers. Nursing home leaders need to amend their conventional strategies of retaining the LTC workforce by creating tailored supports for workers while simultaneously addressing job quality deficits. Access to mental health services, increased leave for caregiving and respite, improved access to high-quality health insurance and benefits that assist this workforce to deal with contingency factors and career mobility (e.g. small grants, tuition remission) have the potential to alleviate some of the instability in this often-vulnerable workforce.

Abstract citation ID: igae098.4101

**A MOBILE CLINIC STUDY ON BRAIN HEALTH IN OLDER ADULTS IN A RURAL, SOCIOECONOMICALLY DISADVANTAGED COUNTY.**

Marianne Chanti-Ketterl[1], Jennifer Jones-Locklear[2], Jada Brooks[3], Cierra Williams[1], Misty Bullard[2], Keenya Locklear[2], Scott Davis[1], and Heather Whitson[1], *1. Duke University, Durham, North Carolina, United States*, *2. UNIVERSITY OF NORTH CAROLINA AT PEMBROKE, University of North Carolina Pembroke, North Carolina, United States*, *3. UNIVERSITY OF NORTH CAROLINA, Chapel Hill, North Carolina, United States*

**Objective:** This collaborative pilot project assessed the feasibility of enrolling 50 older adults from underrepresented populations in Robeson County, North Carolina (NC), considered a racially diverse and socioeconomically disadvantaged area.

**Methods:** The study protocol, adapted from the Alzheimer's Gut Microbiome Project, was modified to fit local cultural contexts with input from community and local academic partners. The Lumbee Tribe of NC, headquartered in Robeson County, and its Tribal IRB endorsed the project and community-informed recruitment materials. Participants were recruited through the NC Registry for Brain Health, community-based referrals, and local meetings. A mobile clinic facilitated study visits. After consenting, participants underwent immediate fasting glucose and cholesterol testing via finger prick and biomarker collection through venipuncture. They completed a Montreal cognitive assessment and answered demographics, lifestyle, medical conditions, environmental exposures, and diet questionnaires. Instructions for home stool sample collection and shipment were provided. A nurse then reviewed participants' physical measures, offering health counseling and resources. Participants received a printed summary of most study results except those from venipuncture, which were sent to the lab. Compensation with merchant cards was provided after shipment of stool samples.

**Results:** Fifty-seven individuals expressed interest; nine declined participation, two were ineligible, and five are pending the visit. To date, we have 41 people enrolled, nearly all recruited through community outreach.

**Discussion:** This study demonstrated the feasibility of enrolling older adults in research from a rural, underserved area. The strong community-academic engagement, including endorsement from the Lumbee Tribe and using a local mobile clinic, was key to the recruitment process.

Abstract citation ID: igae098.4102

**A NETWORK ANALYSIS OF HOW COMPONENTS OF WELL-BEING DIFFER ACCORDING TO EDUCATION ATTAINMENT**

Shuna S. Khoo[1], Zhihao Ma[2], and Lile Jia[1], *1. National University of Singapore, Singapore, Singapore*, *2. Nanjing University, Nanjing, Jiangsu, China (People's Republic)*

Singapore is on the cusp of ushering in a new wave of older adults. As one of the Four Asian Tigers, the country underwent rapid industrialization in the 1950s - 90s and buttressed its development upon a foundation of high education attainment for its citizens. This has created a disparity in the educational profile of our current (low educational attainment) and future (high educational attainment) older adults. To future-proof our aging interventions and programs, there is an urgent need to understand how different factors define and affect individuals' well-being based on their educational attainment. We surveyed a representative community sample (N = 5,224, Mage = 52.8 years) and, employing a network analysis, mapped out the relationships among the key elements of well-being. Machine learning techniques revealed distinct network patterns between low- and high-education adults ($p < .001$). Low-education residents' well-being revolves around factors relating to their present life conditions (e.g., chronic illnesses, eating habits, quality of life). By contrast, high-education residents' well-being is centered around factors that relate to an overall sense of balance (e.g., feelings of harmony) and a future orientation (e.g., anticipated quality of life in ten years). Our findings can be used to assess well-being interventions based on how they impact the central factors for low- and high-education residents, respectively. The present methods and findings would be relevant to any fast-developing country; understanding the residents' well-being profiles is critical to finetuning support for current older adults while anticipating future older adults' needs regarding longevity.

Abstract citation ID: igae098.4103

### A PILOT RCT TO EXAMINE THE IMPACT OF A THERAPY DOG INTERVENTION ON LONELINESS IN HOSPITALIZED OLDER ADULTS

Nancy Gee[1], Lisa Townsend[2], Erika Friedmann[3], Sandra Barker[2], and Megan Mueller[4], *1. Virginia Commonwealth University School of Medicine, Richmond, Virginia, United States*, *2. Virginia Commonwealth University, Richmond, Virginia, United States*, *3. University of Maryland Baltimore, Baltimore, Maryland, United States*, *4. Tufts University, North Grafton, Massachusetts, United States*

Loneliness is linked to significant health threats and is potentially more dangerous than obesity; it affects as many as 29% of noninstitutionalized older adults. Loneliness is exacerbated for those who require inpatient rehabilitation, are displaced from their social networks, spend little time receiving therapy, and are physically inactive and socially isolated. Evidence suggests that companion animals provide a number of health and wellbeing benefits and that interacting with a trained therapy dog may reduce loneliness. Older adult (59+ years) medical inpatients (N=42) were randomly assigned to receive one of three conditions: dog and handler interaction (AAI), handler-only Conversational Control (CC), or Usual Care (UC), for 20 minutes per day over three days. The UCLA Loneliness Scale and Short Form as well as an analog rating scale were used to assess loneliness. Linear Mixed Models with random intercepts were applied to examine differences in the changes from before to after the conditions. Changes in the UCLA-SF [t (200.356) = 1.851, p = 0.033] and the analog loneliness scale [t (194.407) = 2.651, p = 0.004], differed significantly between the AAI condition and the UC conditions but not between the CC and UC conditions (p =.175). Trajectories of changes in loneliness showed more improvement in the AAI than in the UC condition. These results indicate that AAI was effective for reducing loneliness in hospitalized older adults. Human handler only visits did not result in similar findings, indicating that there is something unique and beneficial about the presence of the dog.

Abstract citation ID: igae098.4104

### A QUALITATIVE EXPLORATION OF A PSYCHOSOCIAL INTERVENTION FOR LONELINESS AND SOCIAL ISOLATION IN OLDER ADULTS

Max Zubatsky, Marla Berg-Weger, Dimitra Tziarli, Rachel Livingston, and Jen Lauck, *Saint Louis University, St. Louis, Missouri, United States*

Loneliness and social isolation is a public health epidemic in older adults. Although providers are exploring more psychosocial interventions to help curb this trend in the community and healthcare settings, very few evidence-based approaches have been able to deliver effective interventions and strategies for this population. Circle of Friends (CoF) is an emerging global intervention that has been implemented in a variety of settings for older adult socialization and improving overall mood. The CoF group consists of weekly one hour sessions over 12 weeks, targeting different themes around health and lifestyle, arts and hobbies, and theraeutic writting and activities. The purpose of this study is to explore the experiences of participants in their involvement of Circle of Friends from three different group settings (university, virtual, and primare care). A focus group format helped collect qualitative themes from 11 participants across three completed groups between 2022-2024. Overall, a consensus of main themes were found across all three groups regarding access to a social group, motivation to improve other health and lifestyle activities, and better coordination with other providers and resources in their area. Over 50% of the reports from these focus groups mentioned the lack of any social group options or resources given by their healthcare team (Geriatrics or Primary Care). This study highlights not only the impact that a psychosocial group has for older adult patients, but the need for more older adult group options in integrated care and community settings in the older adult population.

Abstract citation ID: igae098.4105

### A SPECTRUM OF AGING: INTRODUCTION OF A REFLECTED EXPERIENCE MODEL OF AGING WELL

Patricia Davis Chilton, Priscilla Magrath, Matthew Grilli, Valeria Pfeifer, Daniel Sullivan, and Matthias Mehl, *University of Arizona, Tucson, Arizona, United States*

Current methods to study aging lack a personalized approach in investigating the question of what it means to age well - the narrated, lived experience of those doing the aging. The current study collected narratives from 92 individuals between the ages of 62 and 95. Participants were interviewed via Zoom Health and provided four minutes of spontaneous (i.e. unprepared) answers to the question, "In your own words, what does aging well mean to you?". The responses were recorded, transcribed using speech-to-text, deidentified, and checked for accuracy by trained research assistants. The responses were analyzed qualitatively using an interpretivist approach, inductively identifying recurring ideas from the narratives, and extracting themes that represent a broad spectrum of aging well experiences and meaning. The main themes that emerged were: Lifestyles/Health/Wellness, Compensation/Adaptation, Social Connection, Social Contribution, Existential, and Time Awareness. A thematic map was created representing the main themes and their 24 subthemes. Using MAXQDA software, a descriptive analysis of the coded themes indicated that out of 1,618 coded segments, 28% represented Lifestyle/Health/Wellness themes (e.g. physical/cognitive health, travel, finances), 25% reflected Existential themes (e.g. purpose/meaning, emotional wellbeing, spirituality, openness/growth), 15% represented Social Connection, 10% were Compensation/Adaptation (e.g. interdependence, technology, adjusting environment/mindset), while 6% represented both social contribution (e.g. volunteerism) and time awareness. These analyses constitute a first step towards a reflected experience model of aging and can facilitate a richer understanding of the diversity and lived experience of aging well.

Abstract citation ID: igae098.4106

### ACCEPTABILITY AMONG DEMENTIA CAREGIVERS OF THE ONLINE SELF-GUIDED ACT FOR CAREGIVERS PROGRAM

Gavin Green, Josey Batura, Heather Kelley, Jacob Gossner, Ty Aller, Michael Levin, and Elizabeth Fauth, *Utah State University, Logan, Utah, United States*

Acceptance and Commitment Therapy (ACT) is an effective therapeutic intervention for stress and mental health concerns across various populations. Our study evaluates the acceptability of an online self-guided ACT program tailored specifically for dementia caregivers via a longitudinal mixed-method design. The study was advertised to community programs (e.g. Area Agencies on Aging, Alzheimer's Association), and N = 83 caregivers completed the post-intervention survey (Mean age = 62.6; 82% female; 89% White, 57% spouse caregivers). The system usability scale averaged 86.1/100, indicating high acceptability, with the majority of participants reporting they would like to use the program again (76%), that the information was helpful (86%), and that they would refer the program to other caregivers (85%). We used a team-based approach to thematic analysis of in-depth interviews from 28 participants at both post-intervention and follow-up. Qualitative data supported the quantitative findings as participants expressed a high level of satisfaction with the program's online asynchronous structure and content. Participants emphasized the practical skill-building exercises and emotional education as particularly helpful. Further, many participants reported continued engagement with the program by revisiting notes, reviewing session summaries, and reflecting on specific analogies or information. Those who maintained engagement away from the program reported increased motivation to apply skills taught in the program in their daily lives. The online self-guided ACT for Caregivers program demonstrates the potential for broader implementation of ACT in community and healthcare settings through its mobile and cost-effective nature.

Abstract citation ID: igae098.4107

### ACCEPTABILITY OF A CHATBOT FOR INFORMATION AND ADVICE ON DEMENTIA CAREGIVING

Sheung-Tak Cheng[1], Peter Ng[2], and Tomoko Wakui[3], *1. The Education University of Hong Kong, Tai Po, Hong Kong, 2. Hong Kong Polytechnic University, Hong Kong, Hong Kong, 3. Tokyo Metropolitan Institute for Geriatrics and Gerontology, Tokyo, Japan*

Providing ongoing support to caregivers as their needs change in the long-term course of dementia is a serious challenge to any healthcare system. Conversational artificial intelligence (AI) operating 24/7 may help to tackle this problem. This presentation describes the development of a generative AI chatbot (called Chatbot-A) using the GPT-4o large language model, with a personality agent to constrain its behavior to providing advice on dementia caregiving based on a revised version of the Benefit-Finding Intervention manual (an intervention reported in JG:PS and other journals). The chatbot's responses to 21 common questions were compared with those of another chatbot (called Chatbot-B) using a collection of authoritative sources (World Health Organization iSupport, By Us For Us Guides, and 185 webpages by Alzheimer's Association, National Institute on Aging, and UK Alzheimer's Society) as its knowledge base. Results showed highly context-sensitive and rather sophisticated answers by both chatbots, with Chatbot-A performing slightly better on mental health-related questions. Subsequently, 10 caregivers used Chatbot-A for two weeks and provided ratings and comments on its acceptability. They found Chatbot-A highly user-friendly, and its responses quite helpful and easy to understand. They were rather satisfied with it and would strongly recommend it to other caregivers. During the 2-week trial period, the majority used Chatbot-A more than once per day. Results supported conversational AI as a viable approach to improve support to caregivers. Due to space limitation, this poster will present the two chatbot's answers to selected questions and the caregivers' feedback on Chatbot-A in detail.

Abstract citation ID: igae098.4108

### ACTIVITY ENGAGEMENT FOR OLDER ADULTS WITH COGNITIVE IMPAIRMENT: UNDERSTANDING NEEDS AND SUPPORTS

Elizabeth Lydon[1], Carlyn Ellison Keeler[2], Raksha Mudar[2], Sara Czaja[3], Walter Boot[4], Neil Charness[5], and Wendy Rogers[2], *1. Miami University, Cincinnati, Ohio, United States*, *2. University of Illinois, Champaign, Illinois, United States*, *3. Weill Cornell Medicine/Center on Aging and Behavioral Research, New York, New York, United States*, *4. Weill Cornell Medicine, New York, New York, United States*, *5. Florida State University, Tallahassee, Florida, United States*

Understanding the daily challenges experienced by older adults living with cognitive impairment is essential for developing strategies to support their health and quality of life. These impairments involve cognitive changes that can pose barriers to daily activities, especially complex tasks such as managing medications. However, the specific difficulties faced by this population are not well understood. The Everyday Needs Assessment for Cognitive Tasks (ENACT) Study examines these challenges in community-dwelling older adults with probable cognitive impairment determined by reporting recent changes in their cognition and scoring as mildly impaired on global cognitive screening. Participants were aged 60 and older (N = 91). We used a mixed methods needs assessment approach to identify difficulties across five activity domains: Health, Social Engagement, Transportation, Domestic Life, and Leisure and Recreation. Participants rated the level of difficulty they experienced with activities within these domains using a Likert scale and then participated in in-depth interviews to explore the underlying causes, focusing on memory, planning, organizing, and communication challenges. We examined participants' response strategies to challenges, such as using visual reminders, technology, personal habits, and seeking assistance from others. Difficulties related to memory and executive functioning were key contributors to the participants' challenges. Details from the interview data provide insights about the context of the challenges. Their most common response strategy was utilizing support from others. Interventions and technology solutions designed to support this population should prioritize features that aid memory recall and task organization and facilitate functional independence.

Abstract citation ID: igae098.4109

### ADDRESSING CAREGIVER TRANSPORTATION BARRIERS TO ENHANCE HEALTH CARE ACCESS FOR OLDER ADULTS

Renée St. Louis, Nicole Zanier, Jennifer Zakrajsek, and Colleen Peterson, *University of Michigan Transportation Research Institute, Ann Arbor, Michigan, United States*

Providing transportation assistance to an aging family member who no longer drives remains a pivotal caregiving task. Caregiver challenges related to coordinating and providing this transportation can have an impact on the frequency and level of health care an older adult can access. Applying the social ecological model, this qualitative study aimed to gain a contemporary, in-depth understanding of caregiver experiences facilitating health care access for those who no longer drive to inform health care solutions across various levels of the system. Transcribed audio recordings of four focus groups divided between urban and rural areas (N=21 individuals providing transportation assistance to aging adults) were iteratively reviewed to draw out themes to meet this aim. Participants described their health care transportation burden and barriers to health care access for their care recipients as well as their visions for more viable options and services. Participants expressed the need for streamlined care services including, for example, organization-based policy changes to improve safe transference from a personal or service vehicle and into the hospital/clinic. Perceived inadequacy of community transportation options led to participants filling the gaps by providing rides, asking friends for help, or posting on social networking sites to ensure health care access for their care recipients. On an interpersonal level, participants highlighted the benefits of intangible yet critical support, such as compassionate employees. Findings underscore multifaceted challenges caregivers face in providing transportation assistance, emphasizing the need for systemic improvements and policy changes to enhance health care access and support services.

Abstract citation ID: igae098.4110

### ADVERSE CHILDHOOD EXPERIENCES AND DEPRESSION: AGING PERCEPTIONS' MEDIATING ROLE IN OLDER KOREAN IMMIGRANTS

Hajin Lee[1], Juyoung Park[2], and Yuri Jang[2], *1. Ewha Womans University, Seoul, Republic of Korea*, *2. University of Southern California, Los Angeles, California, United States*

In response to the sustained mental health impact of Adverse Childhood Experiences (ACEs) in later life and the heightened vulnerability of older adults in socially disadvantaged positions, we examined how early life adversities shape self-perceptions of aging and mental health among low-income older Korean immigrants. We conceptualized self-perceptions of aging as a mediator and hypothesized that the impact of ACEs on depressive symptoms would be mitigated by negatively internalized perceptions of aging. Using data from 315 older Korean residents in subsidized housing in the Los Angeles area (Mean age = 79.4), we found supportive evidence for our hypotheses. In addition to the direct effects of ACEs (B [SE] =.80 [.18], p <.001) and self-perceptions of aging (B [SE] = –.68 [.16], p <.001) on depressive symptoms, the indirect effect of ACEs on depressive symptoms through self-perceptions of aging was found to be significant (B [SE] =.12 [.05], bias-corrected 95% bootstrap confidence interval = 0.02, 0.22). Those with greater ACE exposures were more likely to hold negative perceptions of aging, which in turn led to greater symptoms of depression. Our findings elucidate a potential mechanism through which early life adversities manifest the impact on the current mental health state of low-income older Korean immigrants and highlight the importance of culturally sensitive community-based interventions to foster resilience and positive views on aging for the protection and promotion of community mental health.

Abstract citation ID: igae098.4111

### AGE AND EMOTION: AGE MODERATES THE RELATIONSHIP BETWEEN MIXED EMOTIONAL EXPERIENCES AND PSYCHOLOGICAL WELL-BEING

Kathrine Whitman, Enna Chen, Jane Stephenson, and Laura Carstensen, *Stanford University, Palo Alto, California, United States*

Aging is associated with higher levels of emotional well-being, with older adults experiencing more positive emotions, fewer negative emotions, and more mixed emotions in daily life. Mixed emotional experiences (reporting more than one type of emotion during the same period) may indicate more emotional complexity and adaptive functioning. Alternatively, it may indicate degraded emotional experience. Here, we examined the relationship between everyday positive, negative, and mixed emotional experiences with life satisfaction, trait-level positive emotion, psychological well-being, and depression. Participants in an experience sampling study (n =180, aged 18 to 93, M = 56.9) reported their emotions at five randomly selected times each day for seven days. As expected, reports of positive emotions were associated with life satisfaction and trait-level positive emotion, while negative emotions were associated with depression and inversely correlated with psychological well-being. Interestingly, the association of negative emotions with depression was weaker in older participants, suggesting that older people may be more resistant to clinical depression even in the face of negative emotions. Most interestingly, we found that mixed emotional states were associated with increased psychological well-being among older adults but decreased psychological well-being in younger adults. Older adults' increased psychological well-being in relation to mixed emotions and their resilience to the effects of negative emotion are consistent with adaptive, age-related changes in emotional experience and well-being. These findings highlight the differential effects of experiential positive, negative, and mixed emotions on various measures of well-being, with younger and older adults experiencing key differences in emotional drivers of well-being.

Abstract citation ID: igae098.4112

### AGE AND RACE PATTERNS IN TWO TYPES OF OBJECTIFICATION OF WOMEN

Aurora Sherman, *Oregon State University, Corvallis, Oregon, United States*

Objectification of women can take several forms, including denial of autonomy, sexualization, and self-objectification. Objectification is a dehumanizing experience with significant negative psychological and physical health consequences which has largely been studied in majority White and younger samples of women. This study surveyed 349 women aged 50 and older, who are Latina (n= 107), African American (n= 125), or White (n= 117). Objectification was measured by the Self-Objectification Beliefs and Behaviors Scale (SOBBS, Lindner & Tanleff-Dunn, 2017) and by the Invisibility subscale from the Self-Objectification Scale (SOS-I, Talmon & Ginsburg, 2016). The web-based survey was administered by Qualtrics Panel Services. Results indicate that the scales are reliable for all three groups and across mid-life and older women. Further, ANCOVAs with self-rated health as a covariate revealed no significant differences in either objectification measure based on race or ethnic group, suggesting that objectification is a shared experience for women from these three backgrounds. Finally, the ANCOVA also showed a significant age-group difference in the SOBBS scores ($F(1, 324) = 5.18$, $p < .03$) but not in Invisibility scores ($F(1, 325) = 2.06$, ns). Mid-life women reported higher self-objectification experiences (EMM = 42.62, SE = 1.33) compared to women older than 65 (EMM = 38.32, SE = 1.33). There were no significant interactions of race group with age group for either measure. Results are discussed in the context of Objectification and Life Course Theories.

Abstract citation ID: igae098.4113

### AGE DIFFERENCES IN DECISION SUPPORT NETWORK SIZE: A STUDY OF TOTAL JOINT REPLACEMENT CANDIDATES

Tess Wild[1], Catherine Riffin[2], and Corinna Löckenhoff[1], *1. Cornell University, Ithaca, New York, United States, 2. Weill Cornell Medicine, New York, New York, United States*

Arthritis, which affects tens of millions of middle-aged and older adults, is one of the most common causes of activity limitation, disability, and chronic pain. Despite evidence that total joint replacement (TJR) can improve quality of life and physical functioning in arthritis patients, many eligible TJR candidates hesitate to pursue surgical intervention, and this reluctance is higher among older patients. Research shows that willingness to undergo TJR is influenced by a variety of psychosocial factors. Social relationships in particular can be an important source of support and information for patients when considering surgery. It is well documented that people's social networks tend to be smaller and more close-knit as they age, and recent research suggests that decision support networks for hypothetical healthcare decisions show the same trend. The present study examined whether this pattern extends to realistic decisions about TJR. Using a name-elicitation approach, we collected information on the size of patients' decision support networks ($M_{size}$ = 3.30; $range_{size}$ = 0-8) in a sample of potential TJR candidates (N = 98; $M_{age}$ = 67.88; $range_{age}$ = 40-93 years). As predicted, linear regression revealed that, compared to younger potential TJR candidates, older potential TJR candidates identified fewer members in their decision support networks (B= -0.033; p =.048). Additional analyses examined the role of potential covariates including demographics (e.g., gender, marital status), health (e.g., pain, functional status), and psychosocial variables (e.g., personality, health literacy). Theoretical implications and ramifications for clinical practice are discussed.

Abstract citation ID: igae098.4114

### AGE DIFFERENCES IN OPEN-ENDED DECISION EVALUATIONS AND THEIR IMPLICATIONS FOR DECISION AVOIDANCE

Julia Nolte[1], and Corinna Löckenhoff[2], *1. Tilburg University, Tilburg, Noord-Brabant, Netherlands, 2. Cornell University, Ithaca, New York, United States*

Decision avoidance is more frequent with age, but associated mechanisms remain underexplored. To address this gap, a mixed-methods study captured decision makers' subjective evaluation of choice options prior to making or avoiding choices. An adult lifespan sample (N = 227, 18-88 years, M = 50.71, SD = 19.22, 55% women) evaluated four health and consumer decision scenarios. Specifically, participants were asked to write down what they liked and disliked about the available choice options before making or avoiding a choice. Decision avoidance was positively associated with age. Open-ended responses were on average 33.08 words long (SD = 22.38) and contained a sum of 21.18 statements (SD = 16.42). The number of words or statements was unrelated to age. Responses were coded for remarks about positive ($\kappa$ =.79-.88), negative ($\kappa$ =.60-.74), and fixable/acceptable ($\kappa$ =.32-.85) choice attributes, as well as choice preferences ($\kappa$ =.34-.67). No significant findings emerged with respect to the latter two categories; among the former, the number of negative remarks dominated the positive ones. Decision makers who focused relatively more on negative attributes were older and reported poorer mental well-being. Among middle-aged and older adults, but not younger adults, a stronger focus on negative compared to positive attributes was associated with more pronounced decision avoidance tendencies. All findings remained significant after accounting for the number of words or statements. In combination, this may suggest that older age groups hold stricter decision standards or are less willing to trade-off between favorable and unfavorable choice attributes.

Abstract citation ID: igae098.4115

### AGE DIFFERENCES IN SOCIOCULTURAL DETERMINANTS OF INDIGENOUS HEALTH

Amanda Goldtooth, Raechel Livingston, and Eric Cerino, *Northern Arizona University, Flagstaff, Arizona, United States*

Healthy aging frameworks often lack a culturally-informed theoretical approach to investigate how sociocultural resources advance health equity, particularly for Indigenous adults. Our work addressed this gap by empirically testing Yellow Bird and colleagues' (2023) Cultural Determinants of Healthy Indigenous Aging model. Specifically, we examined age differences in associations between sociocultural resources and health. In a sample of 85 Native American or Alaska Native adults from the Midlife in the United States study (average age=54.25 years, SD=12.86, 52% female), respondents completed surveys on sociocultural resources (spirituality, neighborhood quality, purpose) and self-reported health outcomes (physical health, negative affect, positive affect). We regressed each health outcome on the sociocultural resources and adjusted for age, sex, and education. We also examined whether age moderated these associations. People with higher purpose reported better self-rated physical health (Est.=0.06, SE=0.01, p<.001), lower negative affect (Est.=-0.05, SE=0.01, p<.001), and higher positive affect (Est.=0.04, SE=0.01, p<.001). Age moderated the relationship between spirituality and positive affect (Est.=0.01, SE=0.002, p<.01) such that higher spirituality related to higher positive affect among comparatively older adults (Est.=0.14, SE=0.04, p<.01). People with better neighborhood quality reported better self-rated physical health (Est.=0.61, SE=0.20, p<.01). Age moderated the relationship between neighborhood quality and positive affect (Est.=0.02, SE=0.01, p<.05) such that better neighborhood quality related to higher positive affect among comparatively older adults (Est.=0.48, SE=0.19, p<.05). Consistent with Yellow Bird and colleague's (2023) culturally-informed framework, results suggest that purpose, neighborhood quality, and spirituality may serve as sociocultural pathways to promote healthy Indigenous aging and advance health equity.

Abstract citation ID: igae098.4116

### AGEISM AND PERSONALITY AS UNIQUE PREDICTORS OF OLDER ADULT WELLBEING

Claire Wininger, Megan Wolk, Jennifer Beatty, Judy Kwak, and Patrick Hill, *Washington University in St. Louis, St. Louis, Missouri, United States*

Ageism is a primary risk factor for poorer psychological wellbeing in older adulthood. However, the pernicious effects of ageism prove complicated to alleviate given they move beyond whether one experiences discrimination due to age, as older adults often internalize the negative stereotypes associated with these discriminatory acts. The current study evaluated whether certain personality traits may exacerbate or mitigate the wellbeing concerns associated with ageism. Participants (n = 250, ages: 60-80) completed measures of ageist discrimination, internalized ageism, the Big Five personality traits, and psychological wellbeing. Results indicated that older adults who reported greater ageist discrimination and internalized ageism reported worse psychological wellbeing, as did adults who scored lower on extraversion but higher on neuroticism. Multiple regression tests of moderation though found that the Big Five personality traits failed to moderate ageism-wellbeing associations. Put differently, personality traits and ageism were independent predictors of wellbeing. Moreover, the current study found that experiences of ageist discrimination and internalized ageism were positively correlated, and both held unique negative associations with psychological wellbeing. These findings clearly demonstrate that both forms of ageism hold independent pernicious effects for older adults largely regardless of their personality.

Abstract citation ID: igae098.4117

### AGING AND RADICAL ENVIRONMENTAL CHANGE: "WHY DON'T THEY JUST LEAVE?"

Marlene Friis[1], Jeanette Gustat[2], Katie Cherry[3], James Cronin[4], and William Bertrand[1], *1. Tulane University, New Orleans, Louisiana, United States*, *2. Tulane University School of Public Health and Tropical Medicine, New Orleans, Louisiana, United States*, *3. Louisiana State University, Baton Rouge, Louisiana, United States*, *4. XXX* AQ5

Coastal Louisiana has experienced extensive land loss due to oil and gas extraction, flood protection measures, and repeated exposure to hurricanes, flooding, and other severe weather events. Radical socioeconomic changes further negatively impact older residents. Projected relative sea-level rise, increased intensity of weather events, and large infrastructure projects may further alter the ecosystem, leaving residents with the option to either adapt or retreat. Governmental agencies are working towards reducing population vulnerability through risk reduction measures, disincentivizing further population growth, or promoting outmigration to higher ground. Critically, older adults may be uniquely vulnerable to the hazards associated with coastal living as they are both overrepresented in population statistics yet may also be less sensitive to policies designed to move people out of harm's way. Regarding migration decisions, older adults are often deeply embedded in the socioecological systems of historic communities and typically value the benefits of continuity over disruption. Using the Environmental Distress Scale on a sample of geographically vulnerable adult residents in lower Plaquemines Parish, Louisiana (n=161) and in-depth interviews with older adults (n=45), results indicate that Place Attachment, Solastalgia, Threat Perception, Felt Impact, and Action may undergird decisions to stay put in the face of radical environmental change. Results show that most adults ages 55 and over (75%) intend to stay in the area. Although some older adult may qualify as trapped (involuntarily immobile), this research supports older adults' agency in choosing to be voluntarily immobile in the face of slow-onset environmental changes. This distinction has policy implications.

Abstract citation ID: igae098.4118

### AN OPEN PILOT STUDY OF A COMMUNITY-ENGAGED BRAIN HEALTH EDUCATION PROGRAM FOR CHINESE AMERICANS AGED 50 OR ABOVE

Kaipeng Wang[1], Carson De Fries[1], Xiaoyouxiang Li[1], Fei Sun[2], and Jie Zhu[3], *1. University of Denver, Denver, Colorado, United States*, *2. Michigan State University, East Lansing, Michigan, United States*, *3. Texas State University, San Marcos, Texas, United States*

Chinese Americans, the largest Asian American subgroup in the U.S., face linguistic, cultural, and socio-economic barriers to dementia prevention. To promote brain health in this population, a community-engaged approach is essential. This study evaluates a community-engaged education program for Chinese Americans aged 50 or older, designed to enhance brain health knowledge and motivate lifestyle changes to prevent dementia. The program is structured into four phases. First, we assessed participants' interests in brain health topics, availability, and preferred modes of delivery. Next, experts in the identified topics developed educational content and outcome assessments. The third phase involved implementing a six-session program over three months, offered in Mandarin both virtually and in-person. The sessions covered general knowledge about Alzheimer's disease and related dementias, diet, sleep, physical exercise, health checks, and mindfulness. Finally, we evaluated the program's feasibility and effectiveness using mixed methods, including pre-post surveys, session feedback questionnaires, and focus groups. Seventy-seven participants completed the pretest survey, and 47 (61%) attended at least four sessions and completed the post-test survey. The results of paired t-tests showed statistically significant improvements in knowledge of brain health, diet, exercise, and health checks, as well as increased motivation for lifestyle changes. While supporting these results, qualitative findings further revealed the applicability, understandability, comprehensiveness, cultural relevance, elder friendliness of the sessions, and participants' motivation for attending and self-improvement. Findings highlight feasibility and effectiveness of a community-engaged education approach in brain health promotion and lifestyle change and underscore the need for continued community outreach among Chinese Americans.

Abstract citation ID: igae098.4119

### ASSESSING HEALTH BEHAVIORS AND WELLBEING OF NON-SPOUSAL LGBTQIA+ CAREGIVERS OF PEOPLE WITH AD/ADRD

Clover Caldwell, Heather Schwartz, AJ Slowey, Nicole Werner, and Andrew Pickett, *Indiana University Bloomington, Bloomington, Indiana, United States*

Informal caregivers of people living with Alzheimer's Disease (AD) or AD-Related Dementias (AD/ADRD) face high levels of burden and stress, while LGBTQIA+ identifying individuals often experience stigmatization and discrimination. Young adults are increasingly involved in caregiving for older adult relatives. To date, there is limited research related to the experiences LGBTQIA+ dementia caregivers, particularly those who are not spousal partners of care recipients. We conducted an online survey of LGBTQIA+, non-spousal caregivers, recruited through the Prolific platform. Participants (n=116) responded to items about health-behaviors (e.g., physical activity, sleep hygiene, alcohol use) and caregiving experiences (e.g., burden). Participants most commonly identified as white (n=63, 53.4%), women (n=64, 54.2%), and bisexual (n=71, 60.2%). The average age was 37.64 (SD=12.44) years and participants reported 25.54 (SD=25.17) average weekly hours caring for parents (n=56, 48.3%), grandparents (n=40, 34.5%), or others (n=24, 20.7%). Participants most commonly were 'moderately active' (n=70, 60.3%), had poor sleep quality (M=2.32 / 4, SD=.87), moderate alcohol use (n=52, 50.5% reported 3-6 drinks per session) and caregiver burden (M=13.54, SD=7.19). Participants reported feelings of loneliness (M=5.39/ 9, SD=2.04) and depressive symptoms (M=9.32, SD= 7.22), both of which were predicted by caregiver burden (Loneliness: F=2.73, p<.001, Adj. R2=.30; Depressive Symptoms: F=2.92, p<.001, Adj. R2=.32). Non-spousal LGBTQIA+ dementia caregivers reported suboptimal health behaviors, partially associated with time and other constraints related to caregiving. Further, caregiver burden was associated with both loneliness and depressive symptoms. Additional research and interventions are needed to promote health and reduce burden among this population.

Abstract citation ID: igae098.4120

### ASSESSING THE PREDICTIVE POWER OF VOLUNTEER FREQUENCY CATEGORIES ON HEALTH: AN OUTCOME-WIDE APPROACH

Samuel Nemeth[1], Seoyoun Kim[2], Cal Halvorsen[3], and Isha Patel[2], *1. Rutgers School of Public Health, Piscataway, New Jersey, United States*, *2. Texas State University, San Marcos, Texas, United States*, *3. Washington University in St. Louis, St. Louis, Missouri, United States*

Later life volunteering contributes not only to community betterment, but also significant health benefits for volunteers themselves. However, researchers have used various methods for measuring volunteering frequency—often with data from the Health and Retirement Study (HRS)—ranging from binary to eight-level categories. These diverse categorizations can obscure an accurate understanding of the relationship between volunteering and health while causing confusion among authors and reviewers on the ideal way to specify volunteering frequency. In response, we conducted a longitudinal outcome-wide analysis using nationally representative panel data from the HRS from 2006-2016 (N=17,062). We examined five different categorizations (2, 3, 4, 5, and 8 categories) of volunteering frequency at "baseline" (i.e., 2012, 2014) to examine how they differentially predicted 12 health outcomes (e.g., self-rated health, depressive symptoms, blood pressure, cholesterol) in the following wave (i.e., 2014, 2016). We found that as the number of levels of volunteering increased, the estimated effects on health become more attenuated (i.e., the effect sizes reduced). However, the confidence intervals become increasingly tighter, indicating that multiple categories of volunteering explain a larger proportion of variance in health outcomes more effectively. Importantly, and regardless of the measurement strategy used, the substantive conclusions for the health benefits of volunteering did not change. Rather than recommending a "best" practice, we advocate for a carefully reasoned selection of volunteering categorizations when designing studies and interpreting results that most optimally apply to researchers' objectives.

Abstract citation ID: igae098.4121

### ASSOCIATION OF COGNITION WITH FALLS RISK MODERATED BY RACE AND SOCIAL DETERMINANTS OF HEALTH

Sheila Black[1], Olivio Clay[2], Tyler Bell[3], Jason Blake[2], Kyle Kraemer[4], Barbara Jackson[5], Heaven Cauble[1], and Teairra Evans[6], *1. The University of Alabama, Tuscaloosa, Alabama, United States*, *2. The University of Alabama at Birmingham, Birmingham, Alabama, United States*, *3. University of California San Diego, San Diego, California, United States*, *4. Texas Education Agency, Austin, Texas, United States*, *5. NORC, Chicago, Illinois, United States*, *6. Louisiana State University, Baton Rouge, Louisiana, United States*

This study examined the extent to which social determinants of health and race moderated the effects of cognitive decline on fall risk. Our sample included 681 participants from the control arm of the Advanced Cognitive Training for

Independent and Vital Elderly (ACTIVE) study who received five assessments across ten years. Participants in this study received measures to assess their cognitive status and history of falls. The cognitive evaluation included measures to assess processing speed, reasoning ability, and episodic memory. The active database included measures that allowed us to assess social determinants of Health (SDOH). SDOH included economic stability, education, social and community context, access to healthcare, neighborhood and built environment. We found that changes in cognition (e.g., processing speed) were associated with greater fall risk. The effects of cognitive decline were moderated by race and social determinants of health. That is, slower processing speed over time was related to increased fall risk for people with above average educational/ occupational status, access to health care, and social community contexts. Regarding race, cognitive decline over time was related to a lower fall risk in White older adults but higher fall risk in Black/African American older adults. These findings were discussed in terms of racial disparities in SDOH resources and in terms of the relation between life space and SDOH resources.

Abstract citation ID: igae098.4122

**ASSOCIATIONS BETWEEN LIFE COURSE ADVERSITIES AND SUBJECTIVE VIEWS ON AGING**

Takeshi Nakagawa[1], Daisuke Ito[2], and Saori Yasumoto[1], *1. Osaka University, Suita, Osaka, Japan*, *2. Tokyo Metropolitan University, Tokyo, Japan*

Recent research indicates that how individuals perceive their own aging may be influenced by negative life events or adversities in early life, such as war (Korniek et al., 2024). However, little is known about whether and when the impacts of adversities become salient across the lifespan. This study examined the associations between life course adversities and subjective views on aging. Data was derived from a nationally representative survey of Japanese older adults aged 60 years and above. Life course adversities (including occurrence and age at first occurrence) were measured retrospectively in 2006. Subjective views on aging and covariates (including sociodemographic, social, and health-related factors) were measured in 1996. A total of 782 participants (60-84 years at baseline; female 54.2%) completed the two surveys. Of these participants, 88.9% experienced at least one adversity in their lifetime. We conducted multiple linear regression analysis to examine the associations of life course adversities, classified as childhood (ages 0-12), adolescence (ages 13-19), young adulthood (ages 20-34), midlife (ages 35-54), and older adulthood (ages 55+), with subjective views on aging. We found that, while individuals who experienced more adversities in older adulthood perceived their own aging as more negative (Beta = -.076, p = 0.035), those who experienced more adversities in childhood perceived their own aging as more positive (Beta =.095, p = 0.009). Results suggest that adversities at different ages may have differential effects on subjective experiences in late life. Further examinations are required to better understand the resilience and vulnerability of older adults.

Abstract citation ID: igae098.4123

**ATTACHMENT SECURITY AND COPING SKILLS IN INTERACTIONS BETWEEN DAUGHTERS AND THEIR MOTHERS WITH COGNITIVE CHANGE**

Dustin Gad[1], Joan Monin[2], and Jenna Wells[1], *1. Cornell University, Ithaca, New York, United States*, *2. Yale School of Public Health, New Haven, Connecticut, United States*

The caregiving literature shows that daughters with secure attachment to their mothers with cognitive change report decreased stress and caregiver burden. However, the behavioral patterns that underlie this link remain undiscovered. In the present study, we examined the associations between daughters' apparent security, an observational attachment code developed by Brooke Feeney and colleagues indicating comfortability and openness, in interactions with their mothers and their tendency to use various coping strategies. Daughter-mother dyads (N = 69) engaged in video-recorded discussions about their concerns regarding their mothers' memory loss. Apparent security was rated by trained coders. Daughters completed questionnaires about their use of coping styles. Results showed that daughters' greater apparent security was associated with less use of self-distraction (B = -.470, p =.047), denial (B = -.471, p =.005), and behavioral disengagement (B = -.422, p =.010). Daughter's greater apparent security was also associated with greater displays of positive affect (r =.487, p <.001), emotional disclosure (r =.352, p =.003), descriptive disclosure (r =.293, p =.015), open discussion (r =.496, p <.001), confidence (r =.262, p =.030), and receptiveness to mothers' support attempts (r =.476, p <.001). These results suggest that interventions and targeted efforts to make daughters feel more open and accepting with their mothers during discussions about memory loss may increase their ability to cope in the context of their mother's dementia.

Abstract citation ID: igae098.4124

**ATTRIBUTED DIGNITY ACROSS THE LIFESPAN: COMPARING DIGNITY IN OLDER ADULTS AND COLLEGE-AGE INDIVIDUALS.**

Emefa Adawudu[1], Chunfang Chen[2], Jeungok Choi[1], and Cynthia Jacelon[1], *1. University of Massachusetts, Amherst, Amherst, Massachusetts, United States*, *2. University of Mount Saint Vincent, USA, University of Mount Saint Vincent, USA, New York, United States*

Attributed Dignity is a core value in human interaction. Researchers have shown that the perception of dignity changes across the lifespan. To understand attributed dignity across the lifespan, we compared the factor structure of the Jacelon Attributed Dignity Scale (JADS) between samples of older adults and college-aged individuals. The theory of attributed dignity guided this study. Data was collected from 289 older adults from senior centers in New England and 380 college students at a large public university in New England using JADS, social desirability scale (SDS), and Rosenberg Self Esteem Scale (SES). SDS and SES were used to determine construct validity. Exploratory Factor Analysis (EFA), construct validity, and reliability of the scale were analyzed. EFA resulted in an 18-item scale with four factors including 'self-value', 'perceived value from others', 'behavior with respect towards others', and 'self in relation to others' for both older adults and college-age individuals. Older adults rated 'behavior with respect towards others' as the most important factor, while 'self-value' was considered the most important attribute of dignity in college-age individuals. Correlations between the JADS, SDS, and SES indicate attributed dignity is a unique concept. Findings indicate that the perception of attributed dignity varies across the lifespan. Consistent with a developmental perspective, older adults value their behavior most highly, while college-aged individuals are more focused on their own self-value. Understanding how the emphasis among factors of attributed dignity changes across the lifespan can provide direction for interventions to effectively enhance attributed dignity.

Abstract citation ID: igae098.4125

### AWARENESS OF PERSONAL GAINS, MINDFULNESS, AND PSYCHOLOGICAL WELLBEING AMONG DEMENTIA CAREGIVERS

Darby Simon, Evan Plys, Elizabeth Allen, Morgan Seward, and Ana-Maria Vranceanu, *Massachusetts General Hospital, Boston, Massachusetts, United States*

The current study tested if increased awareness of personal gains from caregiving and mindfulness skills each independently related to psychological well-being among caregivers of people living with dementia, beyond caregiver role-related factors. We conducted a secondary analysis of baseline data from 92 stressed caregivers, aged 22 to 83 years old (M=53.90, SD= 14.53), who participated in a clinical trial. Three separate multiple regression models tested the relationship between awareness of personal gains (Caregiver Reaction Scale- Personal Gain subscale), mindfulness (Applied Mindfulness Process Scale), and outcomes of depression (Hospital Anxiety and Depression Scale [HADS]-Depression), stress (Perceived Stress Scale-10), and anxiety (HADS-Anxiety), while controlling for role overload, caregiving competence, and hours of providing direct care per week. Results revealed that, in the full model, higher awareness of personal gains ($\beta$ = -.31, $p$ <.001) was significantly related to lower levels of depression. Higher awareness of personal gains ($\beta$ = -.23, $p$ <.05) and higher levels of mindfulness ($\beta$ = -.18, $p$ <.05) were significantly related to lower levels of stress. Neither awareness of personal gains nor mindfulness predicted caregivers' anxiety. These findings provide evidence that awareness of personal gains and mindfulness uniquely predict aspects of dementia caregivers' psychological well-being, even after controlling for well-established predictors. Psychological interventions may target caregivers' awareness of their personal gains and mindfulness to improve their caregiving experience and psychological health. Anxiety may require different treatment approaches.

Abstract citation ID: igae098.4126

### BARRIERS AND FACILITATORS TO SOCIAL PARTICIPATION WHEN AGING WITH A LONG-TERM DISABILITY: A SCOPING STUDY

Samuel Turcotte[1], Sirine Kheroua[1], Angéline Labbé[1], Mia Lapointe[1], Manh Hung Nguyen[1], Megan Veilleux[1], Pascale Simard[1], and Melanie Levasseur[2], *1. Université Laval, Québec, Quebec, Canada*, *2. Université de Sherbrooke, Sherbrooke, Quebec, Canada*

**Introduction:** Supporting the social participation of individuals aging with a long-term neurological disability contributes to active and healthy aging. However, knowledge about factors influencing the social participation of this population remains scarce.

**Objectives:** Integrate knowledge regarding the barriers and facilitators to social participation for people aging with a long-term neurological disability.

**Methods:** A scoping review following the Joanna Briggs Institute guidelines was conducted across four databases (MEDLINE, CINAHL, PsycInfo, and EMBASE). Analysis relied on the Human Development Model - Disability Creation Process (HDM-DCP). This study is reported according to the PRISMA extension for scoping review (PRISMA-ScR).

**Results:** Eighteen articles (8 quantitatives and 10 qualitatives) representing 1,319 participants and eight neurological conditions (e.g., stroke, multiple sclerosis, traumatic brain injury, spinal cord injury, aphasia, post-polio, spina bifida, and cerebral palsy) were included. Participants' average age ranged from 53.4 to 72 years and the average time since their disability ranged from 5 to 46.26 years. A total of 26 barriers and 42 facilitators to social participation were identified. The most recurrent barriers were associated with the organic system (e.g., presence of comorbidities) and the societal environment (e.g., transportation accessibility). The most frequent facilitators included identity factors (e.g., acceptance of one's condition) and the community environment (e.g., available social participation opportunities).

**Conclusion:** This study lays the groundwork for developing interventions aimed at promoting active and fulfilling aging among older adults with long-term disabilities. Supporting social participation for this population should involve harnessing individual strengths and environmental resources as well as eliminating environmental systematic barriers.

Abstract citation ID: igae098.4127

### BARRIERS AND SOLUTIONS TO VOLUNTEERING FOR OLDER ADULTS WITH DISABILITIES

Megan Nickrent[1], Haseeb Rehman[1], Bethanie Sharp[2], Lyndsie Koon[2], and Wendy Rogers[1], *1. University of Illinois Urbana-Champaign, Champaign, Illinois, United States*, *2. University of Kansas, Lawrence, Kansas, United States*

Volunteering is associated with improved health, decreased symptoms of depression, and increased companionship for older adults. Volunteering can empower people with disabilities, and both the volunteer and the recipient of services experience benefits. Unfortunately, older adults with disabilities often experience barriers to participating in volunteering. Understanding those barriers can highlight areas wherein we can facilitate volunteerism for older adults with disabilities and enable their participation in community activities. We performed an archival analysis of data from Aging Concerns, Challenges, and Everyday Solution Strategies study. Older adults aged 60-79 with vision, hearing, or mobility disabilities were asked about challenges they faced and their responses to those challenges. They provided detailed information in an interview across a range of daily activities, discussing in-depth those that were most difficult. Across the three disability groups, 17 participants discussed difficulty volunteering. Common challenges included organizers of the event not providing accommodations, supervisors not knowing what tasks to assign to persons with disabilities, and other people doubting the abilities of persons with disabilities. Participants expressed frustration with these barriers to volunteering. We explored their responses to these barriers as well. Many participants reportedly used tools or sought assistance from others, but some responded by simply not participating in the activity. Understanding the range of challenges experienced by older adults with disabilities when engaging in the community and exploring their current solutions can help determine ways to increase volunteer engagement for this population. In turn, this can help improve quality of life for older adults with disabilities.

Abstract citation ID: igae098.4128

### BATTLING HEAT: COMMUNITY ENGAGEMENT AND HEALTH STRATEGIES FOR LOW-INCOME OLDER ADULTS LIVING ALONE

YunYing Zhong[1], Jianwen Li[2], Ajani Jackson[3], Dahee Kim[1], Ladda Thiamwong[1], Jennifer Crook[1], Christopher Emrich[1], and Rui Xie[1], *1. University of Central Florida, Orlando, Florida, United States*, *2. City University of Macau, Macau, Macao, Macau*, *3. UCF Intrepid Scholar, Orlando, Florida, United States*

Low-income older adults living alone are at higher risk for falls, social isolation, and worsening functions. The impacts of extreme heat on their health are worthy of further investigation. This study investigates the data from 45 low-income older adults living alone (M=76.6 years old, SD =7.42) in Florida to understand their responses and adaptation strategies to extreme heat or hot days. Most respondents (66.7%) reported discomfort, ranging from slightly uncomfortable (24.4%), uncomfortable (13.3%), very uncomfortable (24.4%), to extremely uncomfortable (4.4%). Less than half of participants (42.2%) reported feeling hot or very hot, and 71.1% reported feeling moderately thirsty to very very thirsty. Overall, participants rated the threat of hot weather on health and day-to-day activities as 5.44 out of 10 (SD=3.25). Despite participants recognizing the potential health risks, there is a disconnect between the perceived threat and actions taken to decrease it. Majority of them did not stay inside (64.4%), nor reduce physical activity (75.6%), nor visit a cool place (93.3%), nor stay hydrated (60.0%), nor take cold bath/shower (95.6%). They appeared lack of knowledge of managing heat in multiple ways, with most respondents reporting only one way to manage heat (57.8%). Most respondents are participating in programs in local senior centers (53.3%) and they have future interested in community programs on heat prevention (53.3%). These findings emphasize the need for specific community-based interventions that provide health strategies to adapt to the extreme heat to address the immediate comfort and health concerns of low-income older adults living alone.

Abstract citation ID: igae098.4129

### BEDROOM ENVIRONMENTAL QUALITY PARAMETERS ASSOCIATED WITH OLDER ADULTS' BEHAVIORAL AND PSYCHOLOGICAL SYMPTOMS

Wan-Tai Au-Yeung[1], Zachary Beattie[2], and Jeffrey Kaye[2], *1. Oregon Health and Science University, Portland, Oregon, United States*, *2. Oregon Health & Science University, Portland, Oregon, United States*

Behavioral and psychological symptoms are common among older adults with apathy, depression and anxiety being the most prevalent. Identifying factors associated with such symptoms may help mitigate them. The study, Monitoring Apathy, Depression and Anxiety Using Technology Evaluations, aimed to identify indoor environmental quality factors that may trigger these symptoms. The study was a 9-month observational study in 12 participants' personal residences. Participant mean age was 79.0 (6.4) years, 7 were males (58.3%), 7 had normal cognition, 4 had MCI and 1 had early-stage AD. The participants self-reported the severity of their apathy, depression and anxiety weekly online by answering standard surveys sent via email, which consisted of the 6-item Withdrawal-Apathy-Vigor subscale from the Geriatric Depression Scale for assessing apathy, and depression, and anxiety subscales from Patient-Reported Outcomes Measurement Information System for assessing depression and anxiety respectively. Bedroom environments were assessed with environmental sensors (Awair Omni, San Francisco) which measured light level, noise level, temperature, humidity and air quality every 5 minutes. Individual mixed-effect models were built with each symptom severity being the outcome, and each weekly mean bedroom environmental quality factor being the predictor, while controlling for age, sex and the week the data was collected. Lower light level in the bedroom was significantly associated with more severe depression scores ($p< 0.01$) and higher temperature was associated with more severe apathy scores ($p< 0.05$). Optimizing specific aspects of indoor environmental quality in older adults' personal homes can potentially assist in improving management of behavioral and psychological symptoms.

Abstract citation ID: igae098.4130

### BELIEFS ABOUT AGING AND TRAUMA-INFORMED CARE WITHIN SKILLED NURSING FACILITIES

Melissa Meynadasy[1], Hannah Bashian[2], Jennifer Moye[2], Jennifer Sullivan[3], Whitney Mills[4], Jane Driver[5], Marcus Ruopp[5], and Kelly O'Malley[2], *1. VA Boston Healthcare System, West Roxbury, Massachusetts, United States*, *2. VA Boston Healthcare System, Boston, Massachusetts, United States*, *3. VA MC Providence, Providence, Rhode Island, United States*, *4. VA Providence Health Care System, VA Providence Health Care System, Rhode Island, United States*, *5. VA Boston Healthcare System, Brockton, Massachusetts, United States*

Pessimism about aging is ubiquitous and impacts emotional well-being and willingness to implement healthcare strategies with older adults. Individuals experiencing increased stress may be motivated to differentiate themselves from an older, more vulnerable group, which may further intensify pessimistic beliefs. This is particularly important to consider in skilled nursing facilities (SNFs) due to vulnerabilities residents experience. Healthcare environments/approaches can contribute to re-engagement with trauma symptoms, and pessimism about aging are related to trauma symptom endorsement. To explore how expectations about aging may impact staff perceptions of trauma-informed care (TIC), we assessed staff across disciplines (n = 78; nursing, rehabilitation, psychosocial, medical) in a sample of VA SNFs. Staff completed

Expectations Regarding Aging (e.g., cognition, physical) and Attitudes Related to Trauma-Informed Care (e.g., beliefs about behavior) questionnaires. Higher total and subscale scores indicate positive expectations of aging and greater TIC endorsement. Expectations of aging differed significantly by discipline ($F(3, 65) = 6.79$, $p <.001$, $\eta 2 =.24$); nurses had lower expectations compared to other disciplines – and a significant difference compared to rehabilitation staff ($p =.001$; Bonferroni correction). Expectations about aging were not significantly related to overall perceptions of TIC ($p =.09$). However, staff expectations about cognitive functioning, were positively correlated with TIC attitudes about underlying causes of behavior ($r =.25$, $p =.037$), suggesting accurate knowledge about cognitive aging relates to more accurate understanding of resident behavior. This preliminary work suggests beliefs about aging may be related to some but not all aspects of TIC attitudes.

Abstract citation ID: igae098.4131

### BETTER TOGETHER? PERCEIVED PROSPECTIVE MEMORY FAILURES RELATE TO COGNITIVE COLLABORATION IN MIDDLE AND LATE LIFE

Anisha Sharma[1], Celinda Reese-Melancon[1], Jennifer Margrett[2], Riley Wyatt[1], Erin Harrington[3], Jennifer Byrd-Craven[1], Rachael Turner[4], and Peter Rendell[5], *1. Oklahoma State University, Stillwater, Oklahoma, United States*, *2. Iowa State University, Ames, Iowa, United States*, *3. University of Wyoming, Laramie, Wyoming, United States*, *4. University of Nebraska Kearney, Kearney, Nebraska, United States*, *5. Australian Catholic University, Australian Catholic University, Victoria, Australia*

Research examining everyday memory among older couples indicates that partners rely on one another for cognitive support (e.g., Harris et al., 2022). We built upon this body of work to determine whether perceptions of a partner's everyday prospective memory (PM) forgetfulness are related to the perceived cognitive support (e.g., planning, reminders) one offers the partner. Participants were 196 middle-aged and older married individuals ($M = 64.51$ years, Range = 40–88 years) who completed self and proxy versions of the Prospective and Retrospective Memory Questionnaire (Smith, 2000) as well as a measure of self-reported collaborative prospective memory (adapted from Reese-Melancon et al., 2018 and Harrington & Reese-Melancon, 2022). Regression analyses indicated that age and perceptions of one's partner's forgetfulness predicted significant variance in the amount of PM support one reports offering one's partner. Participants who perceived greater PM forgetting in their partner reported providing more help to them, with older adults reporting providing more assistance to their partners. Additional analyses indicated biological sex and one's own forgetfulness were significant predictors of respondents' perceived support from their partners. Participants who reported more PM forgetting reported receiving more partner assistance with males reporting receiving more assistance than females. These findings contribute to our understanding of collaborative cognition in adulthood, especially prospective memory, an aspect of memory that is instrumental to the maintenance of independence in late life.

Abstract citation ID: igae098.4132

### BEYOND THE STARTING LINE: AN INTERSECTIONAL LIFE COURSE ANALYSIS OF OLDER WOMEN RECREATIONAL RUNNERS IN RURAL MAINE

Jennifer Jain, *University of Maine, Orono, Maine, United States*

This study offers a close examination of the experiences of women runners, age 65 and older, who have witnessed the vast changes to the landscape of recreation and sports across their lifespans. The enactment of Title IX in 1972 made it more acceptable for women to pursue an active lifestyle. This qualitative study uses a grounded theory approach to examine the experiences of 13 women runners, ages 65-78, who live in rural Maine. The study aimed to understand the turning points along their life course that inspired them to run, the personal and social challenges, and the strategies that enabled them to continue running. The women participated in focus groups, journaled, and participated in follow-up interviews. Preliminary findings show that many older women were introduced to unorganized sports through family members at a young age. As adults, they turned to recreational running to maintain and improve their mental and physical health. Older women runners face challenges such as health issues, concerns related to public perception of aging bodies, slowed running pace that impedes participation in club running, and a lack of running companions due to living in rural locations. Despite their challenges, a majority of the women continued to run by drawing upon their commitment, planning, and preparations, self-awareness of their body's abilities, and acceptance and adaptation to challenges. The results of this study can help to improve future community programming and lower the systemic and geographical barriers to maintaining physical activity for older women.

Abstract citation ID: igae098.4133

### BIOMARKERS OF HEALTH AND AGING IN A COHORT OF VIETNAMESE ADULTS AGED 60 AND OLDER

Robert Tennyson[1], Melanie Martin[2], Tiffany Pan[2], Kim Korinek[3], Zachary Zimmer[4], Alan A. Cohen[5], and Toan Tran[6], *1. Vanderbilt University, Nashville, Tennessee, United States*, *2. University of Washington, Seattle, Washington, United States*, *3. University of Utah, Salt Lake City, Utah, United States*, *4. Mount Saint Vincent University, Halifax, Nova Scotia, Canada*, *5. Columbia university, New-York, New York, United States*, *6. Hanoi Medical University, Hanoi, Ha Noi, Vietnam*

War and traumatic events have negative health impacts across the life course, and most wars are situated in lower- and middle-income countries, where many survivors are now or will soon be entering old age. To date, however, most aging research has focused on health effects in high-income countries, neglecting the burdens of war exposure for the broader global population. The Vietnam Health and Aging Study (VHAS), a longitudinal study of 2,447 individuals aged 60+, investigates how stress and trauma experienced during the American War impacts health and aging of Vietnamese survivors. Using biomarker and anthropometric data collected in 2021-2022, we examined associations of recruitment characteristics (age, sex, district, military participation) with select cardiometabolic and inflammatory biomarkers (e.g., C-reactive protein [CRP], Interleukin-6 [IL-6]). Preliminary analyses revealed variation in associations of key demographic and outcome measures. Hypertensive risks were significantly higher for males, and overweight/obesity (OW/OB) risk significantly higher for females, whereas age was positively associated with hypertensive risk and inversely associated with OW/OB risk. Hypertensive risk was lower for those with formal military experience, while proinflammatory markers (i.e., CRP, IL-6) increased with age but did not vary by sex or military participation. District residence, which reflects differences in bombing intensity during the war, showed varied associations with inflammatory and cardiometabolic biomarkers. These findings suggest inflammatory and cardiometabolic risks are not uniformly coupled across participants in this study, underscoring the complexities of how prior war trauma interacts with other factors to influence health and well-being during aging.

Abstract citation ID: igae098.4134

### BODY MASS INDEX AND OLDER WOMEN AND FEMMES EXPERIENCES WITH DISCRIMINATION IN THE HEALTHCARE SETTING

Tavia Roache, Lauren Brown, Amor Mathershed, Heaven Stewart, Leilani Benton, and Mekiayla Singleton, *University of Southern California, Los Angeles, California, United States*

BMI is used widely both within and outside of healthcare settings to understand health despite the contradictory evidence around the impact of obesity on mortality in middle age and older adulthood. We examine how BMI impacts reports of older white, Black and Latine women and femmes' self-reports of ever experiencing discrimination in the health care setting over their lifetime using the Health and Retirement Study (HRS) leave behind questionnaire (n=7,614). We also examine if experiencing this type of discrimination in the healthcare setting impacts these women's mental health. Having ever been unfairly denied health care or treatment was more commonly reported by older Black (n=1,059) and Latine (n=588) women/femmes relative to older white women. Regardless of race/ethnicity, women and femmes with a higher BMI have an increased likelihood of reporting being unfairly denied health care (OR=1.03, p=0.01). Women who report being unfairly denied treatment in the doctor's office also report more depressive symptoms (B=0.93; p< 0.001). Findings demonstrate that women with higher BMI are at increased risk of experiencing discrimination in a health care setting over their lifetime. This is especially true for Black and Latine women/femmes who are more likely to report discrimination. Anti-racist efforts in healthcare must also address unfair treatment of women and femmes based on their BMI.

Abstract citation ID: igae098.4135

### BRIDGING THE DIGITAL DIVIDE: A USER-CENTERED DESIGN APPROACH TO ENHANCING SOCIAL CONNECTIVITY AMONG OLDER ADULTS

Edward Chanquin, Sheila Salinas Navarro, and Francesca Falzarano, *University of Southern California, Los Angeles, California, United States*

Social isolation is highly prevalent among older adults, leading to negative health outcomes and increased healthcare costs. With the U.S. Census Bureau projecting a near doubling of adults aged 65 and older by 2060, addressing this issue is crucial. Innovative, user-centered solutions are imperative because older adults often face health, cognitive, and mobility limitations that diminish opportunities for engagement. Technologies can help overcome these barriers. This study aimed to develop a potential intervention to enhance social connectivity among older adults through user-centered design, understanding their needs in using digital technologies. This qualitative study employed an ethnographic approach, engaging 50 older adults in Los Angeles. Semi-structured interviews and participant observations were conducted. Thematic analysis informed the iterative design of a prototype named TwinFlame. The study revealed three key insights: 1. Older adults prioritized user-friendly interfaces facilitating communication with loved ones. 2. Participants expressed a need for educational support in using digital devices, particularly for advanced features. 3. Cultural competence was critical in ensuring inclusivity and acceptance of the proposed solution. TwinFlame, a device resembling a candle with integrated digital communication features, was developed to address these needs. Its design incorporates simplified interfaces, built-in tutorials, and culturally sensitive elements. While not yet tested, TwinFlame's potential for enhancing social connectivity lies in its user-centered approach, directly addressing the identified needs of older adults. This research contributes to understanding how tailored digital tools can potentially reduce the digital divide and social isolation among older adults, highlighting the importance of user-centered design in gerontechnology.

Abstract citation ID: igae098.4136

### BUILDING THE OLDER ADULT BEHAVIORAL HEALTH WORKFORCE: OREGON'S CENTER OF EXCELLENCE FOR BEHAVIORAL HEALTH & AGING

Leah Brandis[1], Walter Dawson[1], Allyson Stodola[2], Dani Himes[2], Nirmala Dhar[3], Lindsey Smith[4], and Paula Carder[4], *1. OHSU, Portland, Oregon, United States*, *2. Portland State University, Portland, Oregon, United States*, *3. Oregon Health Authority, Portland, Oregon, United States*, *4. OHSU-PSU School of Public Health, Portland, Oregon, United States*

The need for behavioral health workforce support is prevalent nationwide. Older adult Oregonians have a 26% prevalence of anxiety disorders, 12% prevalence of serious mental illness and Oregon has the 4th highest rate in the nation for older adult deaths due to intentional self-harm. The newly established Oregon Center of Excellence for Behavioral & Aging (OCEBHA) is designed to support the behavioral health needs of older adults. A primary objective is to boost the capacity of the workforce to support older adults living with behavioral health needs. The workforce is defined broadly and includes behavioral health and aging subject matter experts, clinical and administrative health care workers, direct care workers in long-term care settings, first responders, care managers, and older adults themselves. There are already workforce training efforts happening across Oregon, therefore, in order to collaborate and align efforts, OCEBHA has launched a coalition of organizations

(n= 15) who already train this workforce. This coalition will meet quarterly to share upcoming training opportunities in order to support the sectors' efforts, identify gaps, as well as brainstorm future collaborations. In addition, Oregon has recently received the state's first Geriatric Workforce Enhancement Program (GWEP) award and OCEBHA is already working closely with the Oregon GWEP to collaborate on future training endeavors. Based on identified gaps, a comprehensive workforce training plan for the next four years is in development. The intention is to collaborate with partners across the state to ensure training is available to meet basic older adult behavioral health competencies.

Abstract citation ID: igae098.4137

### CAN NUDGES REDUCE THE SHARING OF MISINFORMATION ONLINE? EXPERIMENTAL EVIDENCE ACROSS THE LIFESPAN

Ryan Moore[1], Li Chu[1], Jeffrey Hancock[1], and Laura Carstensen[2], *1. Stanford University, Stanford, California, United States*, *2. Stanford University, Stanford University, California, United States*

Observational data on online behavior indicates that older adults are more likely to share misinformation than younger adults. Thus, identifying scalable strategies to reduce older adults' sharing of misinformation is imperative. This study investigates the potential for 1-sentence text-based nudges appearing alongside online news to reduce intentions to share misinformation. A lifespan sample of American adults (aged 18-99, M=49.1, SD=20.8) was shown online news headlines fact-checked as true or false by professional fact-checkers and asked about their intentions to share each headline. Alongside each headline, participants saw nudges that varied in two ways: (1) whether they were emotionally meaningful or not and (2) whether they were presented in a novel manner or not. Drawing on socioemotional selectivity theory, which posits that as people's time horizons shrink they prioritize emotional meaning, we expected that emotionally meaningful nudges would be more likely to reduce older adults' sharing of misinformation. Interestingly, in contrast to studies based on online behavioral data, we found that in a survey context older adults were less likely to report intentions to share misinformation online than younger adults, even after controlling for baseline sharing tendencies and other covariates. We found that novel nudges were more effective than non-novel nudges at reducing intentions to share misinformation across all ages, and that novel nudges were more effective than accuracy nudges, another popular intervention (Pennycook et al. 2021). Emotionally meaningful nudges were not more effective for older adults. This study draws implications for designing effective interventions against misinformation sharing across the lifespan.

Abstract citation ID: igae098.4138

### CARING FOR ONE, TWO, OR THREE GENERATIONS? PREVALENCE OF VARIOUS CAREGIVING ROLES AND CAREGIVER CHARACTERISTICS

Hua Zan[1], Loriena Yancura[1], and Melissa Barnett[2], *1. University of Hawaii at Manoa, Honolulu, Hawaii, United States*, *2. University of Arizona, Tucson, Arizona, United States*

With increasing diversification in household composition and living arrangements over recent decades, the roles of family caregivers have become more complex, sometimes requiring individuals to assume multiple caregiving responsibilities such as caring for both grandchildren and older parents. While caregiving can be a rewarding experience, it can negatively affect caregiver well-being, especially for those providing high-intensity care. Given that family caregivers are the backbone of our care system, it is crucial to understand who these caregivers are and their health and wellbeing. This study provides a profile of U.S. intergenerational caregivers using data from 2018 RAND Health and Retirement Study (HRS) family files and HRS core public files. We estimate the prevalence of four different caregiver types (i.e., caring for older parents only, spouses only, grandchildren only, and those filling more than one caregiving role) overall and by socio-demographic characteristics. We also compare the socio-demographic characteristics and health outcomes (e.g., self-reported health and depression) of different caregiver types to non-caregivers. Our results showed that one in three adults age 50 or older were caregivers, the majority of which only cared for one generation: grandchildren, spouses, or older adults. Approximately 14% of caregivers cared for multiple types of family members. Caregiver profiles and health outcomes vary by caregiver type. For example, spousal caregivers had the highest depression score compared to individuals only caring for grandchildren or older parents and those filling more than one caregiving role. This knowledge is essential for establishing a foundation for targeted interventions to support caregivers.

Abstract citation ID: igae098.4139

### CHALLENGES AND INSIGHTS: EVALUATING LIFE STORY WORK'S ROLE IN DEMENTIA CARE POST-PANDEMIC

Lacey DiFranco[1], Silvia Orsulic-Jeras[1], Sara Powers[1], and Lisbeth Sanders[2], *1. Benjamin Rose, Cleveland, Ohio, United States*, *2. LifeBio, Marysville, Ohio, United States*

Decades of research have demonstrated that life story work can be an invaluable tool for residential care staff and individuals living with dementia. This study examines outcomes from 53 residential care staff (Mage=46.2 years, 92.5% female) implementing LifeBio Memory™ – an application that utilizes voice recorded interviews and artificial intelligence to automate production of resident story books and resources. In a pre-and post-intervention analysis utilizing resident proxy and staff evaluations, we observed an interesting distinction between the importance of person-centered care at the individual versus organizational level. We found a significant increase from baseline to follow-up among staff reporting they knew the resident's life story [t(105) = -10.24, p <.001] and care preferences [t(106) = -8.91, p <.001] better after implementing the intervention. However, when asked about their feelings about and ability to provide person-centered care at their organization, staff reported a significant decrease from baseline (M = 39.70, SD = 5.54) to follow-up (M = 37.96, SD = 6.99) [t(52) = -1.42, p = 0.033]. More importantly, staff that reported higher agreeance to providing personalized care, also reported stronger organizational support at baseline (r = 0.427, p =.001) and follow-up (r = 0.606, p <.001). Additional feedback revealed organizational culture and direction for utilizing materials varied, and care facilities prioritized personal care over socialization and engagement when short-staffed. Discussion will focus on the role of organizational support for sustaining the use of life story materials in the provision of person-centered care within residential care settings.

Abstract citation ID: igae098.4140

### CHILDHOOD SPEECH IMPAIRMENT AND DEMENTIA RISKS AMONG US OLDER ADULTS

Haowei Wang[1], Shu Xu[2], and Yalian Pei[1], *1. Syracuse University, Syracuse, New York, United States*, *2. University of Michigan, Ann Arbor, Michigan, United States*

Speech problems in childhood have profound implications for learning, communication, as well as the development of social and cognitive skills in adulthood. However, research has yet examined how early life speech problems may impact subsequent dementia risks in later life. Using a nationally representative data from the longitudinal Health and Retirement Study, this study investigated how the experience of speech problems before age 16 was associated with the risk of dementia among older adults aged 50 and older. We pooled the full life history information about childhood speech problems for N = 19,082 participants who had 1-11 observations for cognitive and sociodemographic information from 2000 to 2020. We constructed person-interval data and estimated discrete-time event history models to examine risks for developing dementia, which was measured using a validated Langa-Weir classification of cognitive function. We found that about 3.45% of US older adults had speech problems in childhood, with a higher prevalence for younger cohorts, male, and non-Hispanic White, compared to their counterparts. Results showed that older adults with childhood speech problems had higher risks of developing dementia in later life (RRR = 1.49, $p < 0.01$), compared to those who never had speech problems in childhood. The effects of childhood speech problem on dementia risks were partially explained by BMI, activities of daily living (ADL) limitations, and cardiometabolic and depressive symptoms. Findings highlight the long-arm impact of early life health adversity and suggest the needs for screening and early intervention programs for childhood speech impairment.

Abstract citation ID: igae098.4141

### CLOSED-INCISION NEGATIVE PRESSURE WOUND THERAPY (NPWT) IN OLDER PATIENTS FOLLOWING PRESSURE SORE RECONSTRUCTION

Young Min Jee[1], Seungkeun Lee[2], and Jun Ho Park[3], *1. Kangdong Sacred Heart Hospital, Seoul, Republic of Korea*, *2. Soonchunhyang University Bucheon Hospital, Bucheon, Kyonggi-do, Republic of Korea*, *3. SMG-SNU Boramae Medical Center, Seoul, Republic of Korea*

Pressure sores significantly affect older patients, with the sacrum being especially vulnerable due to its proximity to the anus and potential for fecal contamination. Despite preventive measures, some sores still require surgical intervention. Postoperative care focuses on monitoring, hygiene, and pressure alleviation. This study assessed the efficacy of Closed-Incision Negative Pressure Wound Therapy (CI-NPWT) for postoperative wound management in patients with sacral pressure sores treated with local flaps. A retrospective analysis was performed on sacral sore patients who underwent reconstructive surgery from March 2019 through April 2023. Surgical procedures involved debridement and wound coverage using gluteal artery perforator-based fasciocutaneous flaps, followed by postoperative monitoring. Patients were grouped into either conventional monitoring or NPWT management using the INFOV.A.C. Data on patient demographics, flap metrics, fluid drainage amounts, and six-month postoperative outcomes were collected, and then analyzed with SPSS Statistics. In this study of 52 patients, 25 received NPWT and 27 received conventional dressing after surgery. While flap survival rates were 100% in both groups, the NPWT group demonstrated a significantly lower incidence of flap complication and reduced drainage amounts on the seventh postoperative day (17.2 vs. 27.8 cc, $P< 0.05$). The conventional dressing group exhibited one case of wound infection and three cases of dehiscence, while the NPWT group had no complications. Closed-incision NPWT may outperform conventional monitoring in treating immobilized sacral pressure sore patients following local flaps. This technique holds promise as a potential gold standard in sacral sore flap care in older patients.

Abstract citation ID: igae098.4142

### COMMUNICATING DEMENTIA ACROSS THREE GENERATIONS WITHIN CHINESE FAMILIES

Shicheng Xu[1], Vivian Weiqun Lou[2], and Ernest Gonzales[1], *1. New York University, New York, New York, United States*, *2. The University of Hong Kong, Hong Kong, China (People's Republic)*

Despite the benefit of early detection and diagnosis of dementia, globally, nearly 75% of people living with dementia are not diagnosed, indicating the barriers to help-seeking behaviors in the early stages. While in individualistic culture, help-seeking is subject to self-determination, in collectivist culture like China, help-seeking is subject to family determination that requires communication among multigenerational family members, including adult grandchildren. Research focusing on pre-diagnostic family communication is scarce. We aimed to understand how the family communicated to get the initial screening, disclose the diagnostic result, address the uncertainties, and manage emotions across three generations. Guided by constructivist grounded theory, we conducted semi-structured interviews with 28 Chinese participants (6 adult grandchildren, 5 adult children, 9 people with early-stage dementia, and 7 spouses). The results revealed "disconnected" and "connected" family communication patterns between the message sender and receiver. Barriers to connected communication include hierarchical family relationships, limited emotional expression, preference for implicit and crisis-induced communication. Family members tend to put an invisibility clock on dementia symptoms, the diagnostic result, and even the self-consciousness of people living with dementia. Nonetheless, older adults who received an early-stage dementia diagnosis expressed a strong desire to engage in family communication. Adult grandchildren also exerted a key role in family communication and decision-making processes. Social workers should empower intergenerational family members with communication and persuasion strategies to enhance diagnosis, prognosis, and emotional communication. This study underscores the fundamental role of family communication in early-stage dementia to increase screening and facilitate the subsequent care arrangement.

Abstract citation ID: igae098.4143

### COMPARING GAINS AND LOSSES: EXAMINING THE EFFECTS OF INCENTIVES ON WORKING MEMORY CAPACITY IN OLDER ADULTS

Julie Pham[1], and Holly Bowen[2], *1. Southern Methodist University, Dallas, Texas, United States*, *2. Southern Methodist Univeristy, Dallas, Texas, United States*

Aging is associated with declines in cognition, particularly in areas of working memory responsible for the temporary storage, processing, and manipulation of information. Prior studies have shown that the use of incentives can be successful at modulating cognition in older age. However, much of the previous work has largely focused on reward incentives. Limited research on loss avoidance incentives has been done to understand whether its use can differentially improve cognition, especially in working memory capacity. Arguably, this is an important area of focus as older adults can experience greater losses with age, such as in health, independence, and social networks, that may shift decision-making priorities towards conserving resources and minimizing decline. The current study aimed to investigate whether older adults prioritize gains or losses differently while completing a spatial working memory task. An online sample of 75 older adults (ages 60+) were recruited using the Cloud Research Connect Platform. Each participant completed both a reward and loss avoidance block using a single-item change detection task discriminating between different colored squares. A paired samples t-test was conducted to evaluate the effect of reward and loss avoidance conditions. Findings did not indicate a significant difference in working memory performance between conditions. This suggests that use of either rewards or loss incentives may not differentially improve working memory capacity and can both have potential benefits in modulating cognitive function in an older adult sample.

Abstract citation ID: igae098.4144

### COMPARISON OF HEALTH OUTCOMES AMONG TWO SEPTUAGENARIAN COHORTS A DECADE APART IN JAPAN

Can Wang[1], Yasuyuki Gondo[1], Takeshi Nakagawa[1], Yukie Masui[2], Kei Kamide[1], Ayaka Kasuga[3], Saori Yasumoto[1], and Madoka Ogawa[1], *1. Osaka University, Suita, Osaka, Japan*, *2. Tokyo Metropolitan Institute of Gerontology, Tokyo, Japan*, *3. Ritsumeikan University, Osaka, Japan*

**Background:** Previous studies have suggested that the health status of older adults living in the community has improved significantly over the past two decades. To reassess whether this trend continues, this study examined the health profiles of a current cohort of 70-year-olds and compared them with a cohort born a decade earlier.

**Method:** This cohort comparison study was part of the observational Septuagenarians, Octogenarians, Nonagenarians Investigation with Centenarians (SONIC) study. Data were collected from two groups of 69 to 71-year-old community-dwelling older adults in 2010 (born between 1938 and 1941 (n=483)) and in 2023(born between 1951 and 1954 (n=283)), using identical methodologies. All participants underwent comprehensive physical, mental, social, and cognitive assessments.

**Result:** Overall, the later cohort was healthier than the earlier cohort. In physical performance, the later cohort showed 21.08% higher grip strength, 13.65% higher gait speed, 7.02% higher chair-seated stepping, and in cognitive function, the later cohort showed 7.93% higher MOCA-J score. The later cohort also reported higher levels of social support($\eta 2=0.013$) and showed higher perceived health($\eta 2=0.011$). However, mental health assessed by WHO-5 was lower in later cohort($\eta 2=0.008$).

**Conclusion:** Our findings regarding functional capacity were in-keeping with previous studies, which showed later cohort exhibited superior health outcomes across multiple domains compared to earlier cohort. However, there is an inconsistency with previous studies, which reported that later cohorts had better mental health than earlier cohorts at the same age. Future research should explore the reasons for this discrepancy.

Abstract citation ID: igae098.4145

### CONSCIENTIOUSNESS MODERATES THE RELATIONSHIP BETWEEN INTERLEUKIN-6 AND MEMORY DECLINE

Suzanne Segerstrom, *Oregon State University, Corvallis, Oregon, United States*

Evidence supports the role of the pro-inflammatory cytokine interleukin-6 in accelerating cognitive decline and increasing ADRD risk. Personality may influence this relationship in a pathoplastic way, by protecting against or exacerbating the cognitive consequences of inflammation. Conscientious (i.e., controlled, planful, organized) people are characterized by healthier behaviors, which may play a protective role. Among 182 older adults (Mage=74 years, 41% female), Rey Auditory Verbal Learning Test (RAVLT) performance was measured annually for up to 16.6 years (M=6.5 years). Total learning and delayed recall were regressed on expected performance (norms based on age, gender, and IQ), time, IL-6, conscientiousness, and their interactions with each other and with gender. There was a significant interaction among time, IL-6, and conscientiousness ($F(1,167.51)=5.36$, $p=.021$) such that people with high conscientiousness and low IL-6 maintained total learning and thus increased their performance relative to norms over time; people with low conscientiousness and/or high IL-6 maintained performance relative to norms. The same interaction was observed for delayed recall ($F(1,165.22)=5.20$, $p=.024$). The recall effect decreased after adjusting for learning, but in a gender-specific way (interaction with gender, $F(1,164.74)=4.97$, $p=.027$). Adjusting for total learning eliminated the interaction for men but not for women. Rather than a pathoplastic relationship, IL-6 and conscientiousness had a synergistic effect on memory decline over time. Neither low IL-6 nor high conscientiousness alone were sufficient to preserve memory. The healthy habits of conscientious older people may need an advantageous physical environment in order to affect memory and possibly other cognitive functions.

Abstract citation ID: igae098.4146

### CONTROL AT EVERY AGE: HOW EMOTIONAL RESPONSES VARY ACROSS LIFESPAN

Ryan Muskin, David Popivker, and Eric Allard, *Cleveland State University, Cleveland, Ohio, United States*

Control may play a crucial role in regulating emotional reactivity, with this regulation potentially differing between older and younger adults across various emotional subtypes. Research suggests that perceptions of control tend to decrease with age, which may result in older adults being more sensitive to low-control situations. To explore this, we recruited 60 younger adults (18-35) and 50 older adults (60+) who were asked to relive strong emotional memories involving either loss or failure. Emotional reactivity was assessed through self-reported affect, heart rate variability, and electrodermal activity. Our findings support the hypothesis that as perceived control increases, emotional reactivity decreases for older adults but increases for younger adults (p <.05). Further analysis of discrete emotions revealed a positive correlation between perceived control and positive affect for older adults (Pearson's r =.34), but not for younger adults. These results suggest that lower perceived control in older adults may lead to greater emotional reactivity and reduced positive affect. Future research should investigate how the interplay between age, control, and emotions influences emotion regulation strategies, particularly those that involve accepting diminished control.

Abstract citation ID: igae098.4147

### CULTURALLY ADAPTING A CREATIVITY AND WELL-BEING ARTS CURRICULUM FOR MANDARIN SPEAKING OLDER ADULTS

Doria Qu[1], Jeffrey Graupner[1], Renee Kroplewski[1], Ashley Mensik[2], and Katherine Thompson[1], *1. University of Chicago, Chicago, Illinois, United States*, *2. Loyola University Chicago, Chicago, Illinois, United States*

Creativity Circle program is an English-language arts curriculum previously shown to reduce social isolation in older adults. This curriculum was translated, culturally adapted, implemented, and evaluated for a group of 16 Mandarin-speaking older adults over eight weeks. Modifications included incorporating Cantonese songs, Chinese poetry, and culturally significant art activities such as clay modeling of traditional items and illustrating group values using meaningful symbols. Of the 16 initial participants, 15 completed the program, with one dropout due to illness. Participants found the adapted curriculum helpful and culturally appropriate. Key qualitative findings reveal that participants prioritized intrinsic values such as happiness, health, and family over materialistic pursuits, and approaching aging and death with acceptance. Participants reported feeling less lonely after the series (Median=1, n=12; Likert: 5=Completely Agree, 1=Completely Disagree: "I feel lonely"),) when compared to the pre-series survey (Median=2, n=10though the difference did not reach significance (Mann Whitney U Test, U=85, p =.077). This study highlights the effectiveness of cultural adaptation in enhancing program relevance and participant well-being, underscoring the need for gerontological practices that respect diverse cultural perspectives. It demonstrates the feasibility of culturally adapted creative activities to engage older adults, reinforce cultural identity, and promote overall well-being in later life. Future research will focus on larger-scale implementation and quantitative assessment of impact on social isolation.

Abstract citation ID: igae098.4148

### DAILY CAREGIVING TIME AND SPOUSAL CAREGIVERS' WELL-BEING: THE MODERATING ROLES OF DAILY MARITAL INTERACTIONS

Iris Yang[1], and Stephanie Wilson[2], *1. SMU, DALLAS, Texas, United States*, *2. UNIVERSITY OF ALABAMA AT BIRMINGHAM, Birmingham, Alabama, United States*

With more individuals providing informal care (i.e., providing unpaid care to an adult with functional limitations), understanding the impacts of this role is crucial. The literature on the well-being of informal caregivers is divided, with some studies reporting primarily harmful effects and others concluding that caregiving is fundamentally beneficial. This study examined how positive and negative marital interactions moderate the association between caregiving time and caregiver affect in both within- and between-person processes, aiming to clarify the mechanisms behind the varied outcomes observed in caregiver well-being. In the Midlife in the United States Survey (MIDUS), 212 spousal caregivers participated in an 8-day daily diary study, resulting in 1689 days of data. A daily, within-person approach captured the day-to-day fluctuations of caregivers' experiences and emotional well-being. Multi-level modeling was employed, with covariates including caregiver health, gender, employment status, minority status, education, and time spent caring for people besides a spouse. Results indicated that daily marital strain did not significantly moderate the relationship between daily caregiving time and negative affect, while marital uplifts did, reducing the association between increased caregiving time and negative affect. Conversely, daily marital strain and uplifts did not moderate the association between caregiving time and positive affect. At the between-person level, the proportion of days a caregiver experienced marital strain moderated the association between providing more care and having less positive affect. The results generally support the view that caregiving is predominantly linked with psychological burden while highlighting the potential role of marital uplifts in buffering this association.

Abstract citation ID: igae098.4149

### DAILY DYADIC COPING AND SALIVARY CORTISOL RHYTHMS IN COUPLES LIVING WITH EARLY-STAGE DEMENTIA

Courtney Polenick[1], Angela Turkelson[1], Charity Garner[1], Yin Liu[2], and Kira Birditt[1], *1. University of Michigan, Ann Arbor, Michigan, United States*, *2. Utah State University, Logan, Utah, United States*

Dyadic coping (i.e., how couples cope with stress) is associated with subjective well-being in dementia care dyads. However, we know little about dyadic coping and physiological stress reactivity in the context of daily dementia care. We evaluated how daily perceptions of individual and partner supportive (when one partner provides problem-focused and/or emotion-focused coping to help the other) and delegated (when one partner takes over responsibilities to help the other) dyadic coping are associated with salivary cortisol among couples living with early-stage dementia. Participants included 34 people living with dementia (PLWD; M = 72.2 years, SD = 7.5) and their spouse caregivers (M = 69.5 years, SD = 8.7) who completed 7 days of morning and evening phone interviews and collected saliva samples (four times per day) during four of those days. We estimated multi-level models with outcomes including the cortisol awakening response (AR; awakening to 30-min post-awakening) and wake-evening slope (WES; 30-min post-awakening to bed-time). PLWD had a higher next-day AR when they reported higher individual supportive and delegated dyadic coping. PLWD also had a higher same-day and next-day AR and a steeper decline in WES when they reported higher partner delegated dyadic coping. On average across days, PLWD had a flatter decline in WES when caregivers reported higher partner delegated dyadic coping. Finally, on average across days, caregivers had a higher AR when they reported higher individual supportive and delegated dyadic coping. These findings identify targeted strategies to improve health and well-being in couples navigating early-stage dementia.

Abstract citation ID: igae098.4150

### DAILY VARIABILITY OF MULTI-DIMENSIONAL SUBJECTIVE AGE ACROSS THE ADULT LIFE-COURSE: IMPACT OF SCORING METHODS

Shally Zhou, Brooke Brady, Lidan Zheng, and Kaarin Anstey, *University of New South Wales, Sydney, New South Wales, Australia*

Subjective age (SA), measured as how old you feel, is associated with positive health and developmental outcomes. Despite the increased use of multi-dimensional items, there is great heterogeneity in how it is implemented. SA measures often use different scoring methods which may impact subsequent analyses and interpretations. To understand the daily fluctuations of multi-dimensional SA and evaluate the impact of scoring differences, 210 participants aged 18 to 84 (M = 54.50, SD = 18.03, 60.95% female) reported six SAs over two week-long bursts of daily surveys. Common SA scoring methods including raw, discrepancy, proportional and composite average scores were compared. Multi-level models demonstrate chronological age is a significant predictor for all scoring methods across all SA dimensions. Examining raw and discrepancy scores, younger participants felt older than their chronological age, but this difference decreased and reversed with increasing age leading to older participants feeling younger. Proportional scores reveal the percentage difference between felt SA and chronological age reduced 0.17% every year, indicating felt discrepancies were largest at both extremes of the sample. Furthermore, intercepts and significant associations varied across scoring methods and SA dimensions for sex, expectations regarding aging, self-perceptions of aging, daily stress, and daily positive and negative affect. Exploring multi-dimensional SA provides deeper insights into life-course variability, and a more nuanced understanding of ageing. Scoring approaches led to somewhat different interpretations in a life-course sample. Future studies are encouraged to consider the study's design and main research question when determining the most appropriate questions and scoring method.

Abstract citation ID: igae098.4151

### DAYTIME ACTIVITY ATTENUATES ASSOCIATIONS BETWEEN SLEEP EFFICIENCY AND MORTALITY AMONG OLDER ADULTS

Collin Sakal, and Xinyue Li, *City University of Hong Kong, Hong Kong, China (People's Republic)*

Adequate sleep is crucial for promoting longevity, and yet epidemiological understanding of the relationship between sleep and mortality is largely based on a single metric: sleep duration. This study examined associations between sleep efficiency and mortality in a cohort of 33,428 older adults in the UK Biobank over approximately 9-years of follow-up. Sleep efficiency was derived from wrist-worn accelerometer data using a Hidden Markov Model-based sleep-wake detection algorithm and was summarized based on sufficiency (average) and irregularity (standard deviation) across the wear periods. Associations were examined using Cox Proportional Hazard models adjusting for sociodemographic factors, smoking, drinking, depression, social support, medications, cancer, and sleep duration. In fully adjusted models, average accelerometer-assessed total daytime activity was further included using restricted cubic splines to examine its potential to attenuate observed associations. In adjusted Cox models without daytime activity, low average (< 0.85) sleep efficiency was associated with greater risk of mortality (Ref: ≥0.85, HR: 1.31, 95% CI: 1.18, 1.46) as was high irregularity (top quartile) in sleep efficiency (Ref: bottom quartile, HR: 1.35, 95% CI: 1.20, 1.53). However, neither measure of sleep efficiency was significant after adjusting for daytime activity. L-shaped relationships were observed between daytime activity and mortality where low activity levels were associated with an increased risk and high levels of activity were associated with a decreased risk. Our findings suggest that daytime activity attenuates associations between both sufficiency and irregularity in sleep efficiency with mortality among older adults in the United Kingdom.

Abstract citation ID: igae098.4152

### DAY-TO-DAY CHANGES IN A SIMPLIFIED MEDITERRANEAN DIET: IMPLICATIONS FOR NEXT DAY COGNITIVE FUNCTION

Kelly Parker, and Jeremy Hamm, *North Dakota State University, Fargo, North Dakota, United States*

Although individual differences in Mediterranean diet (MD) have been linked to better cognitive functioning over extended periods of time, little is known about day-to-day fluctuations in MD adherence and how it may influence daily cognitive functioning. This daily diary study examined a simplified Mediterranean diet (sMD) and its impact on cognitive functioning the following day over the course of one week. Participants (N=217, Mage = 54±15, 65% female) completed daily surveys and cognitive tasks over one week to measure their daily adherence to the sMD and their daily cognitive function. Lagged multilevel models assessed the impact of (within-person centered) daily sMD on next day cognitive functions that included episodic memory, executive functioning, and processing speed. Age, sex, income, education, and race were controlled in all models. Results showed that on days following higher than usual adherence to the sMD, participants had faster processing speed (b = -45.10, p < 0.01) and greater letter fluency (b = 0.49, p = 0.04) than on days after lower than usual sMD adherence. These findings show that day-to-day shifts in sMD adherence have implications for lagged changes in cognition for middle-aged and older adults, suggesting even small changes in sMD add up and could contribute to improved cognitive aging over time. Small, but simple changes such as increasing fruit, vegetable, and fish intake and reducing sugar-sweetened beverage consumption may thus result in improved cognitive functioning even over very brief time periods.

Abstract citation ID: igae098.4153

### DECISION TO SCREEN: CAREGIVERS VOICES IN MAMMOGRAPHY DECISION-MAKING WITH DEMENTIA

Adele Crouch, and Nicole Fowler, *Indiana University, Indianapolis, Indiana, United States*

The USPSTF recommends mammograms through 74 years old and data suggest that individuals should have a life expectancy of 10 years for a mortality benefit. Mammography beyond 75 may increase burden and risk of overdiagnosis and overtreatment; dementia may exacerbate this burden. Despite guidelines and changing risks and benefits, many over 75 who have life limiting illnesses receive mammograms; it's unclear what motivates these decisions. Therefore, this descriptive qualitative analysis explored dementia caregivers descriptions about the decision to undergo or not undergo mammography screening for their relatives with dementia. Sixty dyads from an IRB-approved study on mammography decision-making completed interviews. Regarding the decision to screen, a main theme emerged: unconscious choice. Sub-themes included: 1) automatic reoccurring event; 2) healthcare provider recommended; 3) hospital/healthcare organization recommended, 4) family history. Some identified it as an automatic reoccurring event ("It was needed for general health, and it's something that we do in our household religiously."). In consultation with a healthcare provider some decided to proceed with mammograms ("Her doctor said it was time for her annual mammogram and he would schedule it"). Numerous were prompted to receive a mammogram by a hospital/healthcare organization ("We received a reminder in the mail that it was time for me, and it was time for her, and I just made the appointment. There was no discussion."). Lastly, participants identified that their family history of cancer prompted them. Overall, many participants may not have recognized mammography as a choice.

Abstract citation ID: igae098.4154

### DECODING EHR DATA: THE ROLE OF SOCIAL SUPPORT IN PRESSURE INJURIES AMONG HOMEBOUND OLDER ADULTS

Christine Chow[1], Yijung Kim[2], Lauren Bangerter[3], and Karl De Jonge[4], *1. Georgetown University Medical Center, Arlington, Virginia, United States*, *2. Medstar Health Research Institute, Hyattsville, Maryland, United States*, *3. Medstar Health, Hyattsville, Maryland, United States*, *4. MedStar Washington Hospital Center, Washington, District of Columbia, United States*

Homebound older adults are increasingly susceptible to acquiring pressure injuries (PIs) due to a variety of pathological and physiological changes associated with aging. However, the impact of social risk factors of PIs among homebound older adults remains largely understudied. This research was conducted to explore how unstructured data via House Call clinical notes can provide insights of psychosocial factors that influence pressure ulcer conditions compared to formal diagnoses via ICD-10 codes. Using electronic health records and clinical notes (N= 1,770) from patients enrolled in a home-based primary care program (04/01/2021-03/31/2024), we evaluate the impact of a novel natural language process (NLP) algorithm to identify PIs among older adults (N= 664 patients, Mean age= 86.55, 78% female, 84% Black or African American). These clinical notes were analyzed using bigram analysis to identify patterns of psychosocial and other factors that influence documentation of pressure ulcers. Themes included known common risk factors for PIs (functional impairment, malnutrition, co-morbidities, frailty, and poor tissue perfusion). However, social support was a unique category identified with multiple associated bigram phrases, suggesting a strong relationship with PIs and underscores a need for further investigation. This study highlights the emerging potential of NLP methodology in clinical research for the systematic analysis of large volumes of clinical narratives, offering valuable insights from these interpersonal relationships. Furthermore, understanding social risk factors can contribute to the early identification of PIs, inform clinical practices, and help reduce the incidence of PIs in homebound older adults.

Abstract citation ID: igae098.4155

### DEMENTIA CARE SHARED BY MULTIPLE CHINESE FAMILY CAREGIVERS: EXPLORING FAMILY-LEVEL PREPAREDNESS FOR CAREGIVING

Jacky C. P. Choy, and Dipsy H. S. Wong, *The University of Hong Kong, Hong Kong, China (People's Republic)*

Involvement of multiple family members as caregivers is commonly observed and often essential in families living with dementia. While preparedness for caregiving was found to be associated with better caregiver outcomes, previous studies centred on one primary caregiver and rarely enquired their preparedness at a family level. This qualitative study aims to explore the preparedness of family as a unit for dementia care. Participants were 30 caregivers (12 spousal caregivers; 31 to 86 years old) who took care of a person living with dementia (PLwD) in the community and reported having more than one family members involved (3.8 caregivers involved in average). We conducted semi-structured in-depth interviews to understand the care needs of PLwD, their care arrangements, family dynamics, and perceived family preparedness. Thematic analysis with an inductive approach was adopted. Seven themes suggesting 7 components of family preparedness emerged: 1) Common understanding of PLwD care needs, 2) Aligned caregiving roles and expectations, 3) Identification of primary caregiver, 4) Flexibility for care task sharing and substitution, 5) Conflict resolution in care arrangement decision making, 6) Exchange of caregiving resources and information, and 7) Sharing of feelings and emotional support. Proximity, composition, family values, and prior communication patterns among members substantially shape their preparedness for caregiving. These components largely resonate with the dimensions of communication, roles, problem solving and affective exchange in McMaster model of family functioning and resemble concepts in models of team effectiveness. Family-centered interventions targeting family preparedness for caregiving can potentially improve caregiver as well as family outcomes.

Abstract citation ID: igae098.4156

### DEMENTIA DIAGNOSIS, HEALTHCARE USE, AND OUTMIGRATION OF OLDER ADULTS IN PUERTO RICO AFTER HURRICANE MARIA

Jeung Hyun Kim[1], Yoojin Lee[1], Yanru Liao[2], and Maricruz Rivera-Hernandez[3], *1. Brown University, Providence, Rhode Island, United States*, *2. Center for Gerontology and Healthcare Research, Brown University, Rhode Island, United States*, *3. Brown University School of Public Health, Providence, Rhode Island, United States*

Puerto Rico's historic net migration loss spiked after Hurricane Maria, which devastated its economy and health system (e.g., from 1.4% to 3.4% outmigration among all Puerto Rican Medicare beneficiaries and about 60% increase in the net-migration loss before/after September 2017). This research examined whether the diagnosis of Alzheimer's Disease and Related Dementias (ADRD) among the older adults in Puerto Rico associated with their decision to migrate to the U.S. mainland, particularly after the year of the Hurricane. The data was derived from the Master Beneficiary Summary File (MBSF), and focused on fee-for-service enrollees in Puerto Rico (n=72,117 from the MBSF 2017/67,985 from the MBSF 2018). Logistic regression showed that older adults who were diagnosed with ADRD were significantly more likely to move to the mainland compared to the non-ADRD individuals in 2017 and 2018. In addition, higher levels of healthcare utilization were strongly related to the outmigration decision. When stratified by age (i.e., 65+ or 80+), these associations remained consistent. These results may suggest that older adults with ADRD in Puerto Rico may have moved to the mainland to seek better healthcare (i.e., long-term care) in the years after the Hurricane, and higher levels of their healthcare use may have contributed to their outmigration decisions. While many studies focus on the young generation to capture the Hispanic migration flow to the U.S., our study shows that the complex health needs of older adults, including the very old ones, propel the emerging outmigration trend in Puerto Rico.

Abstract citation ID: igae098.4157

### DEMENTIA FRIENDS IN INCARCERATED SETTINGS: RESULTS OF A STAFF E-LEARNING SESSION

Jessica Bibbo[1], Sarah Nicolay[1], Bonnie Burman[2], Marty Williman[2], Elizabeth Kinzig[2], and Salli Bollin[2], *1. Benjamin Rose, Cleveland, Ohio, United States*, *2. Ohio Council for Cognitive Health, New Albany, Ohio, United States*

Individuals aging in incarcerated settings such as prison develop dementia at a higher rate than those outside of such settings. The percentage of inmates aged 60 or older is rapidly increasing, yet there is little training for staff about dementia. The Ohio Council for Cognitive Health worked with the Ohio Department of Rehabilitation and Correction (ODRC) to develop Dementia Friends for Incarcerated Settings, an e-learning course made available to all ODRC staff. These results are from the optional and anonymous pre- and post-surveys embedded in the session; 125 of the 364 respondents completed both (Mage=53.2, SDage=9.1, Female=53.6%; Full time=95.2%). A series of McNemar Tests indicated significant improvement in one of the four true/false items measuring knowledge about dementia (Changing the environment will make no difference to a person who has dementia [False]), p <.001. A series of Wilcoxon Signed Rank Tests indicated significant improvement in five of the sliding-scale items (range: 0 indicating no agreement to 100 indicating total agreement) measuring attitudes about dementia (e.g., I am confident interacting with people living with dementia; It is possible to improve the lives of people living with dementia) (p ≤.010). Most respondents agreed or strongly agreed that the material covered in the session would be useful in their work (90.2%), that they learned new information (88.6%), and that they would recommend the session to coworkers (89.5%). These results indicate the potential impact of and underscore the need for dementia education within incarcerated settings.

Abstract citation ID: igae098.4158

### DETECTION OF AI-GENERATED IMAGES: A MIXED METHODS STUDY ON AGE-RELATED DIFFERENCES

Enilda Velazquez[1], Gabriela Flores-Cruz[1], and Nelson Roque[2], *1. University of Central Florida, Orlando, Florida, United States*, *2. Pennsylvania State University, University Park, Pennsylvania, United States*

Generative artificial intelligence (AI) content has become widely accessible but continues to face misuse for spreading misinformation that often targets older adults. This is concerning considering that AI generated content is projected to occupy 10% of the internet by 2025 (Gartner, 2024). Previous research has implicated age and analytical thinking as potential predictors in how well individuals identify misinformation (Pehlivanoglu et al., 2022; see CISDA model, Frazier et al., 2019). This study uses a mixed methods approach to examine the vulnerability of individuals across age groups (young adulthood: 18-39, middle adulthood: 40-59, old adulthood: 60-89; within-subjects) to discern AI manipulated images (AI-generated, non-AI generated; between-subjects) by using (1) signal detection analyses (i.e., hit, miss, false alarm, correct rejection) and (2) response time measures while controlling for analytical thinking (Cognitive Reflection Test score, Frederick, 2005) as an external variable (N = 190). A mixed methods ANOVA suggests that older adults (M = 0.426, SD = 0.150) exhibited significantly higher false alarms than younger adults (M = 0.306, SD = 0.180, p = 0.007, n2p = 0.053). However, there were no differences of age cohort on the rate of hits, misses, or correct rejections, nor on image discernment reaction time while controlling for analytical thinking. The increased false alarm rate among older adults may reflect AI sensitivity toward images, potentially indicating that AI sensitivity acts as a protective factor in the AI interactions of older adult's AI interactions. Future research should explore this sensitivity as a safeguard against misinformation exploitation.

Abstract citation ID: igae098.4159
### DETERMINANTS OF SLEEP HEALTH IN THE CONTEXT OF THE COVID-19 PANDEMIC AMONG OLDER CHINESE IMMIGRANTS IN THE U.S.

Stephanie Lyn Marquez[1], Wendi Da[2], Fengyan Tang[3], and Yanping Jiang[1], *1. Rutgers, The State University of New Jersey, New Brunswick, New Jersey, United States*, *2. Rutgers, the State University of New Jersey, New Brunswick, New Jersey, United States*, *3. University of Pittsburgh, Wexford, Pennsylvania, United States*

Good sleep is crucial for healthy aging. While recent studies have examined psychosocial factors related to sleep health, their impact on older Asian immigrants is not well understood. Notably, few studies have examined how psychosocial factors and immigration factors may intensify the COVID-19 pandemic's effect on sleep among this population. This study investigated how these factors, assessed prior to the pandemic, affected sleep health during COVID-19 among older Chinese immigrants. Data were drawn from the Population Study of Chinese Elderly (PINE), focusing on participants who completed the Pittsburgh Sleep Quality Index and Women's Health Initiative Insomnia Rating Scale in wave 6 (2021-2023) and at least one prior wave between wave 4 (2017-2019) and wave 5 (2019-2021). A mixed-effects linear spline model analyzed predictors across five domains: individual characteristics (age, sex, marital status, education, income, health literacy, chronic conditions), psychological distress (depressive symptoms, anxiety, perceived loneliness), immigration factors (years living in the U.S., acculturation), interpersonal factors (social support, social strain, filial piety receipt and expectations), and perceived neighborhood environment (disorder and cohesion), all adjusted in the context of COVID-19. Results were stratified by residential environment (living in Chinatown versus living outside of Chinatown). The findings revealed that the pandemic worsened sleep health, with depressive symptoms being significantly related to a faster decline in sleep quality and faster increase in insomnia, regardless of residential environment. These findings suggest that the pandemic intensified the influence of psychosocial and immigration factors on sleep health among older Chinese immigrants, highlighting the need for targeted interventions.

Abstract citation ID: igae098.4160
### DIFFERENCES IN PATIENTS WHO FAIL A COGNITIVE SCREEN IN PRIMARY CARE VS. THOSE WHO "ALMOST FAIL"

Anna Higbie[1], Christina Baucco[2], Anthony Perkins[2], and Nicole Fowler[2], *1. Regenstrief Institute, Indianapolis, Indiana, United States*, *2. Indiana University, Indianapolis, Indiana, United States*

In primary care, short cognitive screening tools are used to measure potential cognitive impairment. These tools have been validated in patients aged 65+ and have published cut points to determine if further assessment is needed. We conducted a secondary analysis of data from the Caregivers Outcomes in Alzheimer's Disease (COADS) trial to compare characteristics of patients who screened positive using established cut points versus those who scored one point away. Screening tests included the Mini-Cog, MIS-T, and MIS-T & Virtual Clock Draw. We also compared mild cognitive impairment (MCI) and Alzheimer's Disease and Related Dementias (ADRD) diagnoses between the two groups at 24 months. Among the sample, n=44 failed based on standard cut points, and n=94 scored one point from fail. There were no significant differences in the age, gender, or Charlson Comorbidity Index (CCI) between the groups. Patients one point from failing were significantly more likely to have more education and report daily in-person interactions (p=0.013 and p=0.020). At 24 months, no significant differences in the rate of MCI or ADRD diagnoses were found in electronic medical records. Although the group who had a positive screen at baseline had statistically significant differences in cognitive impairment at 24 months, as measured by the IQCODE (4.4 times more likely to score above 3.6, p=0.009, with higher cognitive decline indicated by their IQCODE scores, p=0.014). These findings suggest better performance on cognitive screeners is associated with reduced cognitive decline over time, underscoring the importance of early detection for at-risk populations.

Abstract citation ID: igae098.4161
### DIFFERENTIAL DIGITAL DIVIDES: AGE GAPS ACROSS EXISTING AND EMERGING TECHNOLOGIES

Edie Sanders[1], George Mois[2], Wendy Rogers[3], and Walter Boot[1], *1. Weill Cornell Medicine, New York, New York, United States*, *2. University of Georgia, Athens, Georgia, United States*, *3. University of Illinois Urbana-Champaign, Champaign, Illinois, United States*

As the necessity of digital technology and the number of devices needed to participate in everyday life increases, understanding age differences in the use of familiar and emerging technologies is crucial for implementing technology-based solutions to enhance independence and wellbeing. We conducted an online survey to understand the types of technology categories used across age groups to support everyday life. Forty younger adults (aged 18-35 years), 40 middle-aged adults (35-64 years), and 40 older adults (65+ years) reported technology use across 13 categories (e.g., smart phones, robots) within the past year. Age-related differences in technology use varied across technology categories. For example, nearly every participant in each age group reported using a computer, a navigation device, a smartphone, and a TV, devices commonly used for everyday activities. Several technologies, including virtual reality and wearable devices, were reported most frequently by middle-aged adults. The use of tablets was reported least frequently by younger adults, voice-activated assistants were reported most frequently by older adults, and gaming consoles were reported less frequently with each increasing age group. Robots were less commonly used, and this pattern was similar for all age groups. These data challenge the idea that older adults are overall less likely to adopt and use technology compared to younger age groups. Understanding technology use at the level of an individual may be more important than at the level of age group for tailoring technology interventions that support everyday activities.

Abstract citation ID: igae098.4162
### DIGITAL AGEISM PERSISTS IN ARTIFICIAL INTELLIGENCE-GENERATED IMAGES

Nicole Derenne, Lindsey Martens, Nicole Virgin, Phillip Hoffarth, Richard Van Eck, Marilyn Klug, Gunjan Manocha, and Donald Jurivich, *University of North Dakota, Grand Forks, North Dakota, United States*

Artificial intelligence systems (AI), including text-to-image models, often reflect and amplify societal age-related biases, a phenomenon known as digital ageism. This study examines if instances of digital ageism in AI-generated representations of older adults lessen over time. Examining how digital ageism evolves in AI-image generation can provide insight into the interplay between technology advancements, societal attitudes toward aging, and the well-being of older adults interacting with the dynamic digital landscape. This mixed methods project compared 164 images generated by Open AI's DALL-E 2 image generator at two points, one year apart. Text prompts, identical at both points of inquiry, included terms from the geriatric lexicon (e.g., frail older adult, dementia, successful aging). Images were qualitatively evaluated to identify perceived gender, race, socioeconomic status, and emotional expression. Quantitative analysis of the frequency with which each category was represented found significant differences in gender and socioeconomic representation at both points of inquiry but no difference between the two points. The number of white racialized representations of older adults was 5-fold higher than other racialized representations at both points, with an increase of Asian racialized representations at the second point of inquiry. There was no significant difference between emotional expressions between the inquiry points. Despite the increased social emphasis on positive views on aging, AI text-to-image generators persistently generated predominantly white racialized middle-class male figures. A limitation of this study is that it focuses only on AI text-to-image generation and no other AI-generated content.

Abstract citation ID: igae098.4163

### DIGITAL SOCIAL PRESCRIBING LITE: ADDRESSING BARRIERS OF PREVENTIVE INTEGRATED CARE THROUGH COMMUNITY ENGAGEMENT

Daniel Gan[1], Adam Hoverman[2], and Kate Mulligan[3], *1. University of Toronto, Toronto, Ontario, Canada*, *2. University of Washington, University of Washington, Washington, United States*, *3. University of Toronto, University of Toronto, Ontario, Canada*

Digital social prescribing solutions attempt to integrate health and social care by targeting both preventive and complex health challenges. However, a lack of community engagement impedes referral uptake. This paper uses "customer discovery" to explore and address current barriers of digital social prescribing from the perspectives of service users and staff of health and community sectors. Phase 1 prioritized community perspectives and led to a community-generated place-based wellness model to align interests across sectors. Phase 2 conducted market research with over 70 health and community stakeholders in Metro Vancouver through snowball sampling. Insights were gathered on current referral processes, barriers to social prescribing, and stakeholder perspectives. From the insights shared, it was evident that achieving integrated care faces twin challenges of resource allocation and staff burnout. Funding-related barriers include inadequate partnership, intermittent funds, lack of billable codes, variations in cost, and lack of monitoring and evaluation. Staffing-related barriers include time-intensive processes, outdated information, inappropriate referrals and lack of guidance for quality improvement. Based on insights from stakeholders, initial cross-sectoral implementation may fruitfully focus on preventing the effects of social isolation. This "lite" version of digital social prescribing requires an automated referral and monitoring platform which allows prevention for the masses while flagging complex cases for intervention. This reduces clinician burden and empowers community sector to support wellbeing. The place-based model "Connectedness, feeling At home, and joyful Play" was incorporated into a web app. Digital social prescribing lite may pave the way for data-driven, organic scaling up of preventive integrated care.

Abstract citation ID: igae098.4164

### DIRECTION MATTERS: THE ASYMMETRICAL EFFECT OF SOCIAL ISOLATION ON COGNITION

Jay Kayser, and Kimson Johnson, *University of Michigan, Ann Arbor, Michigan, United States*

Social isolation poses significant risks to cognitive health in older adults, with isolated individuals facing higher risks of cognitive impairment. However, existing research assumes symmetrical effects of increases and decreases in social isolation. This study examines the impact of changes in social isolation on cognition using data from the National Health and Aging Trends Study (2011-2018). Social isolation was measured using a 4-item scale based on Berkman and Syme's Social Network Index, and cognition was assessed using a dichotomous classification of dementia (no dementia vs possible or probable dementia) and domain-specific cognitive testing. Fixed-effects models with first differencing and robust standard errors were used to estimate the asymmetrical effects of variations in social isolation on cognition. Increases in social isolation increased the odds of possible or probable dementia (OR=1.13, p =.02), while decreases in isolation did not decrease odds of dementia classification. For specific cognitive domains, increased social isolation predicted decreased recall ($\beta$ = -0.07, p =.02) but decreased social isolation did not predict increased recall. Both increases and decreases in social isolation were associated with changes in orientation such that decreased social isolation predicted improved orientation ($\beta$ = 0.06, p <.001), and increased social isolation predicted lower orientation ($\beta$ = - 0.06, p <.001). Executive function was not predicted by changes in social isolation. These findings highlight the asymmetrical nature of changes in social isolation on cognition. Social isolation primary prevention efforts may yield a greater impact in reducing adverse cognitive outcomes than secondary and tertiary interventions.

Abstract citation ID: igae098.4165

### DISPARITIES IN LOW DOSE COMPUTED TOMOGRAPHY LUNG CANCER SCREENING IN THE UNITED STATES: A MULTI-LEVEL ANALYSIS

Tung-Sung Tseng[1], Chien-Ching Li[2], Hui-Yi Lin[3], Kelsey N. Witmeier[4], Yu-Wen Chiu[5], Michael Celestin[6], and Edward J. Trapido[7], *1. LSUHSC, New Orleans, Louisiana, United States*, *2. Rush University, Chicago, Illinois, United States*, *3. Louisiana State University Health Sciences Center,*

*New Orleans, Louisiana, United States, 4. LSU Health Science Center New Orleans, New Orleans, Louisiana, United States, 5. LSU Health Sciences Center, School of Public Health, Mew Orleans, Louisiana, United States, 6. LSU Health New Orleans, New Orleans, Louisiana, United States, 7. LSU Health Sciences Center, New Orleans, Louisiana, United States*

Low-dose computed tomography (LDCT) screening is recommended for high-risk smokers to decrease lung cancer-related mortality and increase prognosis. In the U.S., the uptake of LDCT screening among eligible smokers is suboptimal. The impacts of social-environmental and individual factors on LDCT screening uptake using nationally representative datasets are understudied. The current study investigated multi-level factors associated with LDCT screening using national data.

**Methods:** The 2017-2021 Behavioral Risk Factor Surveillance System (BRFSS) data, as well as other state-level variables (Medicaid expansion status and the number of screening facilities), were applied. The final study sample consisted of 15640 respondents from 29 states. All analyses were weighted to account for the complex sampling design applied in BRFSS.

**Results:** The overall utilization rate of LDCT screening is only 18.4%. The LDCT screening rate varied by state (6.2 -31.1%). LDCT screen rates were positively associated with the number of lung cancer screening facilities per 10,000 smokers ($r=0.67$, $p< 0.001$). Among the respondents, individuals who were employed, never married, reported good health status, did not have primary care physicians, economic concerns like low income, did not have routine checkups, and did not have certain chronic conditions (i.e., cancer, asthma, COPD) had a lower utilization rate of LDCT screening compared with their counterpart.

**Conclusion:** The use of LDCT screening among eligible smokers remains low. Enhancing access to care for high-risk individuals, promoting services to diverse racial and socioeconomic groups, and expanding Medicaid coverage to incorporate annual LDCT screening can be used to guide future lung cancer screening programs.

Abstract citation ID: igae098.4166

### DOES LONELINESS MEDIATE THE RELATIONSHIP BETWEEN EARLY-LIFE ADVERSITY AND COGNITIVE FUNCTION IN LATER LIFE?

Jinnan Ren[1], and Kenneth Ferraro[2], *1. Purdue University - West Lafayette, West Lafayette, Indiana, United States, 2. Purdue University, West Lafayette, Indiana, United States*

Research has consistently linked negative childhood experiences to cognitive decline in later life, but the mechanisms that may mediate this relationship remain unclear. This study explores the influence of early-life adversity (ELA) and loneliness on cognitive function in later life. We analyzed three waves of data (2006-2010) from the Health and Retirement Study (N=10,787) to compare the potential influence of an ELA composite and six distinct ELA domains (SES disadvantage, disruptive home environment, risky parental behavior, chronic disease, impairment, and risky adolescent behavior) on cognitive function in later life. In addition, we examined whether loneliness in later life mediates this relationship using structural equation modeling. We found that the ELA composite was not associated with a decline in cognition two years later. However, in terms of ELA domains, SES disadvantage, disruptive home environment, risky parental behavior, and impairment in childhood are each associated with cognitive function in later life. Further, results indicated that loneliness partially mediated the relationship between two specific ELA domains — (a) disruptive home environment and (b) risky parental behavior — and cognitive function. However, the direct effects of ELA composite and risky adolescent behavior on cognitive function were insignificant, indicating complete mediation by loneliness in these cases. This study demonstrates how loneliness mediates the relationship between certain early-life risk factors and later-life cognitive decline. It highlights the importance of policy interventions aimed at reducing harmful family environments and addressing adolescent behavior issues. Strategies to reduce loneliness in later life may protect cognitive function among older adults.

Abstract citation ID: igae098.4167

### DOMAINS OF PERSON-CENTERED CARE IN LONG-TERM CARE COMMUNITIES

Elizabeth Seidel[1], Shih-Yin Lin[2], and Tara Cortes[2], *1. HIGN at NYU Rory Meyers College of Nursing, New York, New York, United States, 2. New York University, Rory Meyers College of Nursing, New York, New York, United States*

Person-centered care (PCC) in long-term care (LTC) has been integrated in a patchwork fashion. No standard nomenclature or quality indicators exist for PCC culture, policy, training, or practice. As part of the 2nd phase of a large study to establish the standards of care for PCC, we conducted 8 resident (n=56, age 65-102) and 9 staff (n=66) in-person focus groups, at 5 LTC communities in NY, PA, IL, AZ, and CA to validate essential PCC domains and concepts. Deductive content analysis was applied to validate person-centered domains previously established by the University of Maine during phase 1 of the study: personhood; resident care, choice, and empowerment; food and dining; enrichment and socialization; staff empowerment; physical environment, and family engagement. Phase 2 focused on populations that are more inclusive of residents from historically marginalized groups. We successfully validated all domains previously proposed with minor revisions to the domain definitions and a clearer understanding of the elements within them. For instance, consistent quality across all levels of care was added to the resident, care, choice, and empowerment domain, and the personhood domain was expanded to include staff advocating for the residents when their care preferences differ from their family. The top three domains discussed across the focus groups were resident care, choice and empowerment; personhood; and enrichment and socialization. By identifying elements that drive PCC, measures can be developed to ensure standards are in place to guide excellence in PCC in LTC communities.

Abstract citation ID: igae098.4168

### DYADIC DISCORDANCE ABOUT DAILY LIVING CHALLENGES AMONG PERSONS WITH COGNITIVE IMPAIRMENT

Ana Sophia Garces Torres[1], E-Shien Chang[2], Jerad Moxley[3], Walter Boot[3], Wendy Rogers[4], Sara Czaja[5], and Neil Charness[6], *1. Weill Cornell Medicine/Center on Aging and Behavioral Research, New York, New York, United States*, *2. Weill Cornell Medical College, New York, New York, United States*, *3. Weill Cornell Medicine, New York, New York, United States*, *4. University of Illinois Urbana-Champaign, Champaign, Illinois, United States*, *5. Weill Cornell Medicine/Center on Aging and Behavioral Research, New York, New York, United States*, *6. Florida State University, Tallahassee, Florida, United States*

As the prevalence of older adults with a cognitive impairment (PwCI) continues to increase, it is critical to identify cognitive tasks which are challenging for them, as well as areas of needed support for both the PwCI and their care partner (CP). Identifying discordant reports about daily challenges among dyads may also flag risks for potentially adverse caregiving experiences. This presentation will report on sources of dyadic discordance about daily cognitive tasks. Data were drawn from baseline quantitative and qualitative data from an ongoing, longitudinal mixed methods needs-assessment study called Everyday Needs Assessment for Cognitive Tasks (ENACT). PwCI and their CPs (n=22 dyads) rated the difficulty level that the PwCI experienced across five daily activity categories. The average ages of PwCI and CPs were 74.2 and 64.7 years respectively. Dyads were predominantly spouses/partners (64.7%). Both PwCIs and CPs identified health-related activities (e.g., health information activities, appointment activities, health management activities) as the most challenging. Additionally, the findings indicate that dyadic congruence regarding activity challenges varies according to activity. There was agreement between the PwCI and CPs regarding transportation, $r(20)=0.53$, $p< 0.05$ and domestic life, $r(20)=0.72$, $p< 0.01$ activities. Despite significant correlation in leisure and recreation activities, $r(20)=0.71$, $p< 0.01$, CPs underestimated PwCI's experienced difficulty, $t(21) = 2.70$, $p< 0.05$. Additional qualitative thematic analyses revealed the dynamic role of technology in navigating cognitive challenges. Improved understanding of the areas of dyadic discordance can assist in guiding support interventions for individuals experiencing cognitive challenges and their CP.

Abstract citation ID: igae098.4169

### EARLY-LIFE EDUCATION CONTEXTS AND LATE-LIFE COGNITIVE STATUS: TOWARD IDENTIFYING MODIFIABLE RISKS

Kimson Johnson, *University of Michigan, Ann Arbor, Michigan, United States*

The complexity of K-12 school environments arises from their diverse student compositions, historical and geographical contexts, all of which may influence late-life cognition. Despite this, the long-term impacts of these factors on cognitive status in later life are not well understood. This study utilized data from the Health and Retirement Study (HRS) 2015-2017 Life History Mail Survey (N = 6,980; Mage = 72.5) to investigate how K-12 educational contexts—specifically school racial composition (White, Black, Hispanic, Other, and Mixed) and school setting (Public, Private, Mixed)—affect cognitive status in late life. Cognitive status was assessed in the 2020 HRS biennial wave using the Langa-Weir algorithm, classifying participants into Normal, Cognitive Impairment but No Dementia (CIND), and Dementia categories. After adjusting for sociodemographic and childhood factors, multinomial logistic regression analysis revealed that participants who attended predominantly Black K-12 schools had a higher risk of CIND and Dementia (RRR=2.28, 95% CI: 1.36, 3.80 and RRR=2.09, 95% CI: 1.08, 4.07, respectively). In contrast, those who attended private and mixed schools had a lower relative risk for CIND compared to public school attendees (RRR=0.43, 95% CI: 0.27, 0.68 and RRR=0.54, 95% CI: 0.37, 0.79). Additionally, participants from mixed school settings exhibited the lowest relative risk for dementia (RRR=0.51, 95% CI: 0.30, 0.88). Rural education was associated with a higher relative risk for CIND (RRR=1.26, 95% CI: 1.02, 1.58) compared to urban education. These findings highlight the need for targeted interventions and policies to create equitable educational environments that support cognitive health in later life.

Abstract citation ID: igae098.4170

### EFFECTS OF PSYCHOEDUCATION ON BURDEN, DEPRESSION, AND ANXIETY IN DEMENTIA FAMILY CAREGIVERS: A SYSTEMATIC REVIEW

Hyeyeon Shin[1], Chanchanok Wandee[2], and Donal O'Mathuna[2], *1. Ohio State University, Columbus, Ohio, United States*, *2. Ohio State University, Ohio State University, Ohio, United States*

As dementia cases rise globally, caregiving becomes increasingly prolonged and demanding compared to other chronic illnesses, leading to heightened risks of caregiver burden, depression, and anxiety. Evaluating psychoeducation is crucial as it provides caregivers with knowledge, coping strategies, and support to manage their mental health and caregiving responsibilities. This review assesses the effectiveness of psychoeducation interventions on caregiver burden, depression, and anxiety among family caregivers of dementia patients. Following PRISMA guidelines, we searched PubMed, CINAHL, MEDLINE, and PsycINFO using relevant terms and assessed study quality with the Joanna Briggs Institute (JBI) tool. We included only randomized controlled trials (RCTs) where the control group received usual care or other interventions. We reviewed 23 articles involving 2,651 informal caregivers. All articles evaluated caregiver burden, with 11 studies showing statistically significant reductions. Depression was assessed in 16 articles, with 8 demonstrating statistically significant positive effects, while anxiety was measured in 9 articles, with 4 reporting significant improvements. Among these, one study targeting a specific population showed significant effects on both burden and depression. In another study, although overall depression scores in the intervention group were not statistically significant, a subgroup with scores above the cutoff line showed positive effects, whereas the control group's outcomes worsened. Overall, 50% of the studies reported significant positive effects of psychoeducation. Despite some studies lacking statistical significance, reductions in scores indicate that psychoeducation can alleviate caregiver burden, depression, and anxiety. Further research and development of targeted interventions for vulnerable populations are needed.

Abstract citation ID: igae098.4171

### EFFECTS OF SMARTHEALTH INTERVENTION ON DEMENTIA CAREGIVERS' REACTIONS AND EMOTIONS: A PILOT STUDY

Eunjung Ko[1], Kathy Wright[2], Kristina Gordon[3], John Stankovic[4], Hongning Wang[5], Ashley Gao[6], Peng Wang[4], and Karen Rose[2], *1. New York University, New York, New York, United States*, *2. The Ohio State University, Columbus, Ohio, United States*, *3. The University of Tennessee Knoxville, Knoxville, Tennessee, United States*, *4. University of Virginia, Charlottesville, Virginia, United States*, *5. University of Virginia, University of Virginia, Virginia, United States*, *6. William & Mary, Williamsburg, Virginia, United States*

Dementia care can cause emotional distress among caregivers. As technology advances, innovative interventions to alleviate stress in this group have emerged, highlighting the need for more investigation. We aimed to develop a deep learning-based smarthealth intervention using acoustic monitoring and ecological momentary assessment to detect stress and deliver stress management messages. We also intended to identify the intervention effects on the reactions and emotions of caregivers of people living with dementia. During the four-month intervention period, we collected data about participants' emotional status and reactions to their care recipients' symptoms through pre- and post-questionnaires, periodic phone surveys, and semi-structured interviews. Descriptive analysis, Wilcoxon signed-rank test, and abductive thematic analysis were used. Ten of 11 participants completed the study. The average age was 60 years, and participants reported providing care for approximately three years. Participants were predominently female (72.3%), White (81.8%), and spouses (63.6%). Pre-and post-questionnaires showed significant changes in caregivers' perceived frequency of care recipients' disruptive symptoms (p=.026) and their reactions to these symptoms (p=.006), but not in other emotional-related variables. Although answers to periodic phone surveys varied, participants tended to consider self-care when they reported better physical health and less stress and depressive mood. Interviews revealed seven themes—affective attitudes, burden, ethicality, intervention adherence, intervention coherence, perceived effectiveness, and suggestions—containing 27 categories. While participants' feedback on our smarthealth intervention was mixed, the intervention improved participants' awareness of emotions and caregiving situations. The findings suggest the potential benefits of the smarthealth intervention on caregivers' awareness of emotions and self-care.

Abstract citation ID: igae098.4172

### ENGAGING RURAL OLDER ADULT COMMUNITY CENTERS TO SUPPORT COGNITIVE HEALTH: CBPR LESSONS AND BARRIERS

Megan McCoy[1], Eric Cerino[2], Michael McCarthy[2], Margarita Martinez[1], Louis Lucero[3], Thomasina Seaton[2], Amanda Goldtooth[2], and Raechel Livingston[2], *1. Northern Arizona University, Northern Arizona University, Arizona, United States*, *2. Northern Arizona University, Flagstaff, Arizona, United States*, *3. Joe C. Montoya Community and Senior Center, Flagstaff, Arizona, United States*

Academic-community partnerships grounded in community-based participatory research (CBPR) methods can further health equity and improve cognitive health for older adults and caregivers in rural communities. Older adult community centers, specifically, can serve as partners to advance preventative memory screening and early interventions to address cognitive health disparities and provide support for caregivers. The aim of this study is to describe a model for researchers in the early stages of partnership development, aspiring to use CBPR, to better align activities with community needs. Informed by the Quality Improvement and Innovation Partnership (QIIP) model, we describe the process of engaging older adult community centers as research partners to advance the Northern Arizona Memory Study. Descriptive findings from surveys with 6 center leaders, combined with thematic analysis of partnership development process notes, illuminate lessons and barriers to engagement, as well as conceptual advances for CBPR methods. Lessons include the importance of honoring mutual expertise, informal engagement with center staff and participants to establish trust, and "word of mouth" as affirmation of shared vision. Contextual barriers specific to rurality also impact partnership development capacity (limited staffing/resources) and logistics (travel distances). Findings suggest community centers serving older adults in rural communities are interested in collaborating with researchers to support cognitive health. However, logistical and capacity barriers limit the extent to which centers can commit to CBPR partnerships. Consideration of unique community contextual factors is necessary, requiring researchers to tailor partnership development to each center rather than adopting a "one size fits all" approach.

Abstract citation ID: igae098.4173

### EVALUATING CPLA2 ACTIVITY IN APOE4 CSF AFTER PREVENTE4 DHA SUPPLEMENTATION

Temi Ogunade[1], Hussein Yassine[2], and Marlon Vincent Duro[2], *1. USC Leonard Davis School of Gerontology, Los Angeles, California, United States*, *2. USC Keck School of Medicine, Los Angeles, California, United States*

Alzheimer's disease (AD) is a neurodegenerative disorder characterized by cognitive decline, neuroinflammation, amyloid plaques, and neurofibrillary tangles. The apolipoprotein E ε4 (APOE4) allele is the greatest genetic risk factor for late-onset AD. Most intervention studies involving omega-3 supplements share inconsistent results in APOE4 carriers, calling for a deeper examination of the mechanisms behind its anti-inflammatory properties. Phospholipase A2 (PLA2) enzymes are involved in the hydrolysis of membrane phospholipids, releasing fatty acids like docosahexaenoic acid (DHA) and arachidonic acid (AA) which modulate intracellular signaling pathways that influence differences in inflammation and lipid metabolism similar to AD pathology. The PreventE4 trial is a randomized, double-blind study that investigates the relationship between the APOE4 genotype and brain DHA delivery. 368 cognitively unimpaired adults with cardiovascular risk factors for dementia are given high DHA supplementation or a placebo. Among the group, 184 participants are APOE4 carriers and 184 are non-APOE4 carriers. The study questions if APOE4 limits DHA delivery to the brain. Our lab has demonstrated that calcium dependent PLA2 activity (cPLA2) is increased in APOE4 carriers with AD dementia. We hypothesize that APOE4 is associated with greater cPLA2 activity in cerebrospinal fluid (CSF), resulting in a lower increase in CSF DHA levels after supplementation. I propose to measure cPLA2 activity in CSF samples from PreventE4 participants using a liposomal-based fluorescent assay. The results will provide better insight on how APOE4 complicates responses to DHA supplementation. Knowing this, we will be able to target DHA's metabolism and identify who benefits most from omega-3 supplementation.

Abstract citation ID: igae098.4174

### EXAMINING SITUATION SELECTION ACROSS THE LIFESPAN USING A NOVEL DAY RECOLLECTION METHOD

Judy Kwak, and Patrick Hill, *Washington University in St. Louis, St. Louis, Missouri, United States*

Situation selection is the process by which people approach or avoid certain people, places, or objects to regulate their emotions. Prior research suggests that older adults engage in situation selection more frequently than younger adults because they become more adept at avoiding situations that provoke negative emotions. However, research on these behaviors is limited, in part due to the lack of a reliable scale. The current study addressed this gap by surveying 304 adults ranging 18-79 years old (M=46, SD=16) from Prolific to test a novel measure of day recollection situation selection. First, participants reflected upon the previous day's events and wrote about how they felt, as if writing in a diary. Next, their diary entry was presented in a read-only format at the top of the following page, where they answered questions about how they chose to approach or avoid situations. Exploratory factor analyses revealed a 3-factor structure reflecting distinct forms of situation selection: (1) approaching positive, (2) approaching negative, and (3) avoiding negative. Internal consistency of these subscales was high (alphas = 0.81–0.89), and intercorrelations were small-to-moderate (rs = -0.04–0.35). No age differences were found in approaching positive or avoiding negative situations (rs = -0.02– -0.07). However, older adults reported approaching negative situations less frequently than younger adults (r = -0.14). These findings suggest that older adults strategically select environments to enhance their well-being. With age, adults may prioritize emotional goals by selecting situations conducive to minimizing stressors while increasing positive experiences.

Abstract citation ID: igae098.4175

### EXAMINING SPIRITUALITY, COGNITIVE, AND FUNCTIONAL HEALTH IN OLDER U.S. ADULTS

Katherine Britt[1], Fanghong Dong[2], Lauren Massimo[1], Jill Hamilton[3], Nancy Hodgson[1], Shana Stites[1], and Dawn Mechanic-Hamilton[1], *1. The University of Pennsylvania, Philadelphia, Pennsylvania, United States*, *2. Washington University at St. Louis, St. Louis, Missouri, United States*, *3. Emory University, Atlanta, Georgia, United States*

Used as a salient resource among Black communities to cope with stress, spirituality and religion are associated with better overall cognitive function. However, their impact on individual cognitive domains and functional health is limited, and understanding these relationships could inform targeted interventions for optimizing cognitive and functional health. Using data from cognitively unimpaired older Black and White U.S. adults in 2021-2022 from the Aging Brain Cohort (ABC) at The University of Pennsylvania Alzheimer's Disease Research Center, we examined cross-sectional associations between spirituality (measured with one item, "To what extent do you consider yourself a spiritual person?"), religiousness (measured with one item, "To what extent do you consider yourself a religious person?"), cognition (i.e., memory measured with Benson Delayed Recall and executive function measured with Trail Making B) and functional health (measured with the Functional Rating Scale (FRS) and Functional Activities Questionnaire (FAQ) (N=158) using multivariable regression analysis. Participants were 27.8% Black, 64.6% women, with mean age (74.4 years) and education (16.7 years). Controlling for age, education, sex, and social interaction, spirituality was positively associated with memory ($\beta$ = 0.19, 95%CI [0.05, 0.33], p<.007) but not executive function or functional health. In comparison, religiousness was positively associated with functional health (FAQ only)($\beta$ = 0.31, 95%CI [0.01, 0.61], p<.046)) but not memory or executive function. Older Black and White adults with greater spirituality had higher memory scores, and those with greater religiousness had a higher functional health score. Longitudinal studies are needed to examine associations further.

Abstract citation ID: igae098.4176

### EXAMINING THE CHARACTERISTICS OF UNIVERSITY EMPLOYEES AND STUDENTS PROVIDING CARE TO ADULTS WITH ADRD

Jodi L. Southerland[1], Erin Mauck[1], Shimin Zheng[1], and Matthew Smith[2], *1. East Tennessee State University, Johnson City, Tennessee, United States*, *2. Texas A&M University, College Station, Texas, United States*

University employee and student caregivers face challenges balancing caregiving with employment and educational responsibilities. With over 4,000 degree-granting institutions in the United States, this represents a growing need for intervention. Data were collected via a Qualtrics survey from university employees and students (n=350) who provided unpaid care to a relative or friend 18 years or older. Participants completed assessments including the Zarit Burden Index, Center for Epidemiologic Studies 4-item Depression Scale, Generalized Anxiety Disorder 2-item scale, and Connor-Davidson Resilience Scale. Chi-square and independent t-tests were employed to compare personal, socio-emotional, and caregiving characteristics between caregivers to people with Alzheimer's Disease and related dementias (ADRD) and non-ADRD caregivers. Compared to non-ADRD caregivers, the care recipients of ADRD caregivers were older and had more health conditions. ADRD caregivers also had been caregivers for fewer years than non-ADRD caregivers. Although ADRD caregivers were more likely to provide personal care, they also had more caregiving support (e.g., unpaid and paid) than non-ADRD

caregivers. Compared to non-ADRD caregivers, ADRD caregivers reported significantly higher caregiver burden (t=-2.39, P=0.02). ADRD caregivers reported a greater need for caregiving resources and were more likely to disclose their caregiving status to university personnel compared to non-ADRD caregivers. Findings highlight the need for university-based resources to support the unique challenges of employee and student ADRD and non-ADRD caregivers. Opportunities exist to introduce programs and services (e.g., respite care, flexible scheduling, problem-solving interventions) to help employees and students succeed in their work/studies, manage caregiver burden, and provide high-quality care to care recipients.

Abstract citation ID: igae098.4177

### EXAMINING THE EXPERIENCES AND UNIQUE CARE NEEDS OF SPEC POPS IN AL: FACILITATORS AND BARRIERS TO MAINTAINING THE SELF

Jeffrey Lentz[1], Alexis Bender[1], Candace Kemp[2], and Molly Perkins[1], *1. Emory University, Atlanta, Georgia, United States*, *2. Georgia State University, Atlanta, Georgia, United States*

The assisted living (AL) population is growing more diverse. This diversity is reflected in the increasing use of AL by African Americans, Latinos, and other cultural groups who have traditionally "cared for their own," as well as a rising number of members of other special populations (e.g., sexual minorities and persons aging with HIV) requiring and using residential care. As part of a 5-year study of care at the end of life in AL funded by the National Institute on Aging (R01AG047408), we use the Sort and Sift, Think and Shift method to investigate the experiences and unique care needs of AL residents who represent various special populations within our sample. Data include in-depth and informal interviews with residents. These cases (three females and two males) reflect intersecting social statuses (e.g., race, class, ethnicity, sexual orientation, illness, and disability). Our analysis identified facilitators and barriers to maintaining the self. These statuses make them particularly vulnerable to stigma and other challenges that adversely affect their quality of life, including trauma earlier in life, language barriers, cultural marginalization, discrimination, and inadequate social support. Strengths that contribute to their ability to negotiate these risks and maintain a positive sense of self include religiosity and spirituality, as well as having a sense of community or group pride (e.g., as an HIV survivor or member of a racial/ethnic group). Findings have implications for interventions to improve the quality of life at the end of life for residents who represent members of special, often hidden populations.

Abstract citation ID: igae098.4178

### EXAMINING THE IMPACT OF TRAUMA-INFORMED CARE ON NEUROPSYCHIATRIC SYMPTOMS IN THE LONG-TERM CARE SETTING

Sahebi Saiyed, and Alexis Bender, *Emory University, Atlanta, Georgia, United States*

Major life changes, such as transitioning into skilled nursing care, can trigger a traumatic stress response in older adults. In 2019, CMS released the requirement for trauma-informed care (TIC) in Skilled Nursing Facilities (SNF). This exploratory project aimed to assess the effectiveness of TIC on reducing neuropsychiatric symptoms, assessed using the NPI-NH, among patients with a history of trauma in one SNF. We conducted a retrospective chart review and trauma screening of 59 patients; primarily female (n=51, 86%), white (n=35, 59%) and ranged in age from 67-102 (Mean=82.97). 43 (73%) patients reported trauma and 27 (46%) patients were defined as "high risk" (i.e., history of trauma and either an NPI-NH score (≥ 20) or ≥2 psychotropic medications). 12 (44%) "high-risk" patients received a targeted care plan intervention (i.e., consistent staffing, music therapy, spiritual support, and specialized nursing care) based on the trauma reported and input from patients or their care partners. NPI-NH scores we reassessed one month after the intervention initiation. The NPI-NH score for the intervention group was 36.00 (SD=19.34) at baseline and 24.56 (SD=19.68) at follow-up. We conducted a paired samples t-test (pre- to post-intervention) with a more lenient p-value of 0.10 due to the exploratory nature of this intervention. We found there was a significant difference with a moderate effect between the mean NPI-NH scores from pre- to post-intervention, t(11) = 1.95, p=.08, Cohen's d = 0.59, indicating promise for TIC interventions to improve the quality of life for patients admitted into SNF with a trauma history.

Abstract citation ID: igae098.4179

### EXERCISE IN RURAL COMMUNITIES: A QUALITATIVE STUDY OF HOW PAST EXPERIENCES IMPACT CURRENT EXERCISE PRACTICES

Bryant O'Leary, Tasha Shaffer, Brianna Routh, Justin Whitten, and Dawn Tarabochia, *Montana State University, Bozeman, Montana, United States*

As the aging population continually grows, understanding how to keep older adults engaged in exercise is of increasing importance. This is critical as exercise trends show that engagement dramatically decreases with age. Moreover, older adults are more likely to live in rural communities as compared to urban communities (Cohen & Greaney, 2023). Previous studies explored barriers related to the overall decrease in exercise engagement, yet few have focused on older adults' motivation to maintain exercise practices. The purpose of this project was to explore how past experiences with exercise influence current exercise participation among rural, older adults. Residents engaging in exercise and aged 55+ (n=42) from a western state were recruited using convenience sampling (partnership with local Extension agents and social media outlets). As part of a larger online survey, participants completed an open-ended questionnaire specific to past experiences with exercise. Participant results were analyzed using Van Manen's (1990) hermeneutical approach with three members of the research team developing themes from participant responses. Trustworthiness was established by a peer reviewer to assess the themes for accuracy. Researchers identified five themes associated with how past experiences influenced current exercise practices. These included health and wellness (18), accessible instruction/resources (17), friends and family (13), community programming (10), and youth activity (4). By understanding past experiences, outreach professionals may find more impactful early or later life supports to promote exercise across the lifespan.

Abstract citation ID: igae098.4180

### EXPLORING DELIBERATE PRACTICE TO REDUCE AGE-RELATED PERFORMANCE DIFFERENCES IN A COMPLEX TASK

Michael Prevratil[1], Neil Charness[1], and Walter Boot[2], *1. Florida State University, Tallahassee, Florida, United States*, *2. Weill Cornell Medicine, New York, New York, United States*

Can age-related differences in complex task performance be overcome with simple behavioral interventions? We monitored differences between older and younger adults' performance in a complex psychomotor task with the intent of developing a behavioral intervention grounded in principles of deliberate practice. An older (N = 24, M age = 71) and younger (N = 28, M age = 19.1) sample were taught to play a complex videogame called Space Fortress Participants also completed several cognitive tests and a semi-structured interview. The older adults performed significantly worse on Total score (Cohen's d = 1.45) and three of four subscore metrics (Control, Points, and Speed). Like the differences between men and women (Harwell, Boot, and Ericsson, 2018), score differences between younger and older adults appeared to be driven by strategic and potentially modifiable behaviors. For example, older adults held control inputs for twice as long compared to the younger sample (Cohen's d = 0.98), a common strategic error. Older adults primarily implemented variants of the "drifter" strategy, letting their ship float uncontrolled through the game environment, while most younger adults implemented the optimal "orbiter" strategy, piloting their ship in a clockwise path around the central fortress. These differences demonstrate likely modifiable aspects of performance that can be improved with targeted behavioral interventions. Findings will be used to develop practice environments and instructions anticipated to greatly reduce or eliminate age-related performance differences.

Abstract citation ID: igae098.4181

### EXPLORING EARLY LIFE EXPOSURES AND THEIR IMPACT ON PHYSICAL ACTIVITY AMONG NATIVE HAWAIIAN ELDERS

Phoebe Hwang[1], Samantha Keaulana-Scott[2], Ilima Ho-Lastimosa[2], Jane Chung-Do[2], and Kathryn Braun[2], *1. University of Hawaii at Manoa, Honolulu, Hawaii, United States*, *2. University of Hawaii, Honolulu, Hawaii, United States*

In the United States, Native Hawaiians (NH) tend to face more barriers to meeting physical activity (PA) recommendations than other ethnic groups. Previous studies have shown that national evidence-based PA interventions do not resonate with Native Hawaiians and are not effective in increasing physical activity levels. Adverse childhood experiences (ACEs) and early life exposures significantly impact PA behaviors and health outcomes in adulthood, particularly among NH populations. The purpose of this study is to explore how early life exposures experienced by NH may affect their perceptions and behaviors towards PA. In-depth interviews were conducted with 20 NH community-dwelling elders ages 55 years and older recruited from an urban area and a rural area in O'ahu, Hawai'i. Interviews were audio-recorded, transcribed, and independently coded by two researchers. Three major themes were identified in this study. First, most elders were engaged in PA post-retirement, but PA type varied by the residential type (urban versus rural). Second, barriers to childhood PA were contextualized by the residential type that participants grew up in. Third, PA perspectives and behaviors were influenced by gender roles and expectations. Separately, stories of racial discrimination and ACEs naturally arose during the interviews among NH participants who were raised in urban areas. Findings suggest that PA behaviors are shaped by early life social norms, which are influenced by the residential type. Future studies should focus on exploring the connection between early life perceived discrimination and later life health behaviors.

Abstract citation ID: igae098.4182

### EXPLORING EPIGENETIC CLOCKS AND COGNITIVE CHANGE IN MIDDLE ADULTHOOD

Daisy Zavala[1], Zita Oravecz[2], Jennifer Graham-Engeland[2], Christopher Engeland[2], Martin Sliwinski[2], Krishna Veeramah[1], and Stacey Scott[1], *1. Stony Brook University, Stony Brook, New York, United States*, *2. The Pennsylvania State University, University Park, Pennsylvania, United States*

Positive age acceleration, in which a person's epigenetic age is older than their chronological age, was associated with worse mean-level cognitive performance and higher moment-to-moment intraindividual variability in our prior work utilizing only a single burst of data. The present study extends this work by utilizing a Bayesian Double Exponential Model (BDEM) to examine longitudinal changes in cognition while accounting for practice effects. In a diverse sample of 142 midlife adults, baseline age acceleration was derived from five DNA methylation epigenetic clocks (Horvath 1, Horvath 2, Hannum, PhenoAge, GrimAge). Across four annual bursts of data, ambulatory processing speed and working memory was determined through the symbol search and n-back task, respectively. Using the BDEM framework, for both tasks we found that peak performance (at burst 1) was slower for individuals with greater age acceleration and those who were chronologically older. However, we found no longitudinal changes in peak performance on average, and neither age acceleration nor chronological age were predictive of differences in longitudinal change. Interestingly, greater age acceleration (but not chronological age) was associated with more fluctuations in scores across the study period, but greater chronological age (not age acceleration) was linked with more forgetting between bursts in the symbol search task. Although it is unclear what is driving opposing findings, these results reveal that even in an age heterogeneous sample spanning midlife (prior to expected cognitive declines, or onset of impairment) differences in age acceleration predict differences in peak performance and variability.

Abstract citation ID: igae098.4183

### EXPLORING SELF-COMPASSION AND EXPANDED OUTCOMES: A THEORETICAL MODEL OF CAREGIVING

Claire Grant, and Katherine Judge, *Cleveland State University, Cleveland, Ohio, United States*

Expanding the theoretical understanding of the caregiver stress process is integral to the development and tailoring of evidence-based interventions for caregivers of people living with dementia (PLWD). Recent calls within the literature have suggested testing new theoretical models to expand the investigation of positive predictors and mechanisms of action to a broader range of outcomes including positive aspects of caregiving. The proposed poster will address both gaps within the literature by testing a model of self-compassion developed by Cha and colleagues (2023) adapted for caregivers of PLWDs. Self-compassion is a construct relating to positive self-concept including key tenets of self-kindness, common humanity, and mindfulness. It has been identified as a strong predictor of various caregiver wellbeing outcomes and is increasingly used in intervention research. The theoretical model investigates self-compassion as a main predictor and proposes two parallel lines of mediation (general resources allowing for self-control behavior and specific psychological resources including coping and emotion regulation strategies) to impact a range of outcomes including depression, anxiety, and perceived stress, along with more novel positive outcomes including social support, and positive aspects of caregiving. Testing the model in a sample of caregivers of PLWDs will provide important insights into predictors of both positive and negative outcomes with important implications in development of evidence-based interventions as potential mechanisms of change are investigated. The poster will present a review of the literature supporting the model and discuss ongoing data collection with analysis plans. Further implications across caregiver theory and intervention development will be discussed.

Abstract citation ID: igae098.4184

### EXPLORING SOCIAL CONNECTEDNESS, PERSONALITY, HEALTH, AND LONELINESS AMONG OLDER ADULTS

Willencia Louis-Charles[1], Pallabi Bhowmick[1], Avinash Gupta[1], Wendy Rogers[1], and George Mois[2], *1. University of Illinois Urbana-Champaign, Champaign, Illinois, United States*, *2. University of Georgia, Athens, Georgia, United States*

As people age, changes in their social network may occur due to retirement, the loss of family and friends, and reduced social participation stemming from health issues, potentially leading to social isolation and feelings of loneliness. Approximately one in four older adults in the US alone are considered socially isolated. Being socially isolated is related to loneliness, but they are distinct constructions. Personality traits and perceptions of health may relate to loneliness perceptions. Personality traits are the enduring characteristics of a person and can predict how they will connect with their network. Older adults are also more likely to experience chronic conditions that impact their ability to socialize. As part of a larger study, we explored the relationships between social connectedness, personality, health, and loneliness for 21 older adults (61-80, M=69.0, SD=5.5) Participants completed the Ten-Item Personality Inventory and the Social Connectedness Scale, which integrates the Social Disconnectedness Scale and the Social Isolation Scale. A descriptive analysis was conducted to identify the participants' personality types and self-reported social connectedness. Emotional stability was negatively correlated (r=-0.55) with perceived loneliness with participants having higher emotional stability also scoring high on the Social Connectedness Scale. No significant association was found between the other personality traits and loneliness. However, perceived health was positively linked to better social connection (r=0.51). These results provide insights into the potential role of personality and health for social connectedness that might help tailor potential interventions that could reduce loneliness among older adults, leading to a better quality of life.

Abstract citation ID: igae098.4185

### EXPLORING THE EFFECTIVENESS OF COGNITIVE STIMULATION THERAPY FOR DEMENTIA IN UNITED STATES, BRAZIL, AND HONG KONG

Ebow Nketsiah, *Saint Louis University, St. Louis, Missouri, United States*

Cognitive Stimulation Therapy (CST) is a cost-effective non-pharmacological intervention for dementia developed in the United Kingdom. Though CST has been adopted and implemented all over the world, there has not been a study to evaluate its effectiveness across multiple counties. This study analyzes existing data from CST groups in USA, Hong Kong, and Brazil to evaluate the primary outcomes CST for persons with dementia. Paired sample t-tests were conducted to evaluate the effect of CST on cognition, quality of life, physical function, and depression across the countries. Among the USA CST groups (N=288), significant t-test were calculated for cognition, t(261) = -7.412, p< 0.01, quality of life, t(199) = -8.707, p< 0.01, and depression, t(254) = 6.347, p< 0.01. A non-significant t-test was calculated for physical function, t(103) = 1.262, p=0.21. Among the Hong Kong CST groups (N=408), significant t-tests were calculated for cognition, t(204) = 3.125, p< 0.01, depression, t(236) = 3.084, p< 0.01. However, non-significant t-tests were calculated for quality of life, t(376) = -0.614, p=0.539, physical function, t(84) = -0.888, p=0.377. On the other hand, among Brazil CST groups (N=47), significant t-test was calculated for cognition, t(46) = -2.961, p< 0.01. However, non-significant t-tests were calculated for quality of life, t(46) = -1.760, p=0.085, physical function, t(46) = -0.250, p=0.804, and depression, t(46) = 0.345, p=0.690. There are cultural and demographic factors that influence the effectiveness of interventions like CST. As CST is being implemented as a cost-effective non-pharmacological intervention for dementia and Alzheimer's worldwide, its cultural applicability is worth exploring.

Abstract citation ID: igae098.4186

### EXPLORING THE LINK BETWEEN PLACE ATTACHMENT AND SOCIAL WELLBEING IN OLDER ADULTS

Mary Cox, Jennifer Beatty, and Patrick Hill, *Washington University in St. Louis, St. Louis, Missouri, United States*

Many residents of St. Louis feel strong emotional bonds with the city, a phenomenon known as 'place attachment.' The relationship between place attachment and social well-being in older adults is a crucial area of study, particularly in an urban center like St. Louis, which has been the stage for important sociocultural events in Missouri. Research indicates that place attachment is positively correlated with social well-being and belonging. This connection is significant for older adults, as environments that promote social connection, autonomy, and security can support cognitive health and overall well-being in older adults. The current study tested our prediction that older adults with increased attachment to St. Louis will report higher levels of social well-being. Using survey data from 492 participants ages 65-85, we conducted correlational analyses to examine the relationship between intergroup anxiety and social well-being, exploring whether these associations differ based on levels of attachment to St. Louis. There were significant correlations between attachment to St. Louis (M=4.39, SD=1.31) and the well-being facets of social acceptance, social integration, social contribution, and social actualization (p< 0.001); however, social coherence was not significantly associated with place attachment. Moreover, place attachment was not associated with other wellbeing indicators, such as self-reported intergroup anxiety. While place attachment has beneficial social outcomes, it does not improve all wellbeing indicators. This suggests that a strong bond with St. Louis may not address all social challenges, highlighting the need for further exploration of how urban environments can better support older adults' psychological and social needs.

Abstract citation ID: igae098.4187

### FAMILIAL VISITATION OF LONG TERM CARE FACILITY RESIDENTS: DIFFERENTIATING SOCIAL BEHAVIORS FROM CARE COORDINATION

Caroline Collins-Pisano, and Rachel Weiskittle, *University of Colorado, Colorado Springs, Colorado Springs, Colorado, United States*

High rates of loneliness persist among residents of long-term care (LTC) facilities despite frequent familial visitation. Most studies on LTC visitation do not differentiate social behavior (e.g., talking to the resident about their interests) from care coordination (e.g., attending the resident's care conferences). It is possible but unknown if these distinct forms of visitation impact resident loneliness differently. Past research has identified barriers to overall LTC facility visitation, such as family member demographics (e.g., gender, income) and relational factors (e.g., closeness, history of care provision). The aim of the present study was to determine whether these demographic and relational factors related to social behavior and care coordination differently. Community-dwelling adults aged 18 and older who endorsed having a close friend or relative currently residing in a LTC facility (N = 175) recruited via ResearchMatch completed an online survey regarding their demographics, LTC visiting behaviors, and relationship with the resident. Overall, results indicated demographic variables were not significantly associated with either social behavior or care coordination, but relational factors were significant predictors of both outcomes. Individuals who reported higher closeness with the resident and who had been their primary caregiver demonstrated significantly greater care coordination involvement and social behavior involvement. Immediate family members were more likely to be involved in care coordination than friends or extended family, but they were not more likely to socially interact with the resident. These findings imply there are distinct facilitators and barriers for care coordination and social involvement that warrant further investigation.

Abstract citation ID: igae098.4188

### FAMILY HEALTH STRESS AND INSOMNIA SYMPTOMS AMONG OLDER ADULTS: THE ROLE OF RELATIONSHIPS WITH THEIR CHILDREN

Jeein Jang, *University of Massachusetts Boston, Quincy, Massachusetts, United States*

Insomnia symptoms that arise from stress pose a significant physical and psychological health concern for older adults. Yet, little empirical research has explicitly examined the association between stress caused by family health concerns and insomnia symptoms, along with the role that relationships with children play in moderating this relationship. This study uses Pearlin's stress process model and data from the 2018 and 2020 Health and Retirement Study (N=7,755) to investigate these associations in adults aged 50 and above. Ordered logistic regressions assess the link between family health stress and insomnia severity using four items (i.e., trouble falling asleep, nighttime awakening, early morning awakenings, and feelings of nonrestorative sleep). Additionally, the moderating role of two relationships with children, positive and negative, is included in the analysis. All statistical analyses are adjusted for sociodemographic, health-related factors, caregiver status, and their children's socioeconomic status. Results show older adults experiencing 'somewhat upsetting' and 'very upsetting' family health stress had 24% and 50% higher odds, respectively, of experiencing more severe insomnia symptoms compared to those with no family health stress. Interestingly, both positive and negative relationships only moderated the association between 'somewhat upsetting' stress and insomnia symptoms. These findings underscore the need for targeted clinical and public health interventions to help older adults with family health stress reduce their insomnia symptoms by fostering positive relationships with their children.

Abstract citation ID: igae098.4189

### FAMILY HISTORY OF ALZHEIMER'S DISEASE AND LOCUS COERULEUS ACTIVATION IN HEALTHY OLDER AND YOUNGER ADULTS

Elayna Seago, Ya-Yun Chen, Tae-Ho Lee, and Ben Katz, *Virginia Tech, Blacksburg, Virginia, United States*

The locus coeruleus (LC) is a small nucleus in the brainstem that produces the majority of norepinephrine in the brain. It engages in reciprocal communication with the frontoparietal network and the salience network, both networks that are critical for attention and undergo significant structural and functional changes during aging. Additionally, the LC is suggested as the primary region of early Alzheimer's Disease (AD) pathology and its dysfunction may contribute to AD symptomology. It is largely unknown whether family history of AD is linked to LC activity or moderates the association between age and LC functional activity. In this study, functional MRI scans were collected while participants completed the Attentional Network Task. Neural activity in the LC was compared in congruent and incongruent conditions. Twenty-three out of 77 participants had a family history of AD. Participants age ranged from 18 to 74. Multiple regression analysis, controlling for gender, showed no main effect of age on left LC activation (p = 0.289); critically, there was a marginal main effect of family history of AD on left LC activation (p =.088) such that the activation in incongruent compared to congruent condition was higher in participants with a family history of AD than in participants without a family history of AD. We did not find an interaction between family history of AD and age (p =.180) or any right LC effects. These results demonstrate that AD family history is related to left LC activity in both younger and older adults.

Abstract citation ID: igae098.4190

### FAMILY MATTERS: EFFECTS OF UNFAIRNESS AND DISAGREEMENT ON DEMENTIA CAREGIVERS' GAINS AND BURDEN

Suyoung Nah, and Jyoti Savla, *Virginia Tech, Blacksburg, Virginia, United States*

Although caregiving occurs within a family, the influence of family contexts on caregiver outcomes remains understudied. Building on the distributive justice framework and stress process theory, we examined whether an unequal division of care among adult siblings, perceived unfairness in the division, and family disagreement over parental care are associated with caregivers' gains and role overload. We further explored whether perceived unfairness and family disagreement interact in predicting caregiver outcomes. Using data from the 2015 and 2017 National Health and Aging Trends Study and National Study of Caregiving, we examined adult child caregivers of persons living with dementia (N = 364 for cross-sectional analysis and N = 173 for longitudinal analysis; Mage = 54.0; White = 51%). Multivariate multilevel analysis was conducted to investigate the effects of these family contexts on caregivers' gains and role overload. Caregivers who felt they were providing more care than their fair share experienced higher overload at initial assessment ($\beta = 0.20$, p =.001) and at follow-up ($\beta = 0.23$, p =.04). Greater family disagreement was associated with higher role overload at initial assessment ($\beta = 0.33$, p <.001). Family disagreement moderated the effect of perceived unfairness, such that the perception of providing less care was linked to lower gains at follow-up only when family disagreement was high ($\beta = -0.67$, p <.001). Findings highlight the importance of perceived unfairness and family disagreement in caregiver outcomes. Interventions should promote shared caregiving responsibilities and facilitate open communication within families about care dynamics.

Abstract citation ID: igae098.4191

### FAMILY STRENGTH AND LIFE SATISFACTION IN OLDER ADULTS WITH VISUAL IMPAIRMENTS: A STRUCTURAL EQUATION MODELING

Eunjin Yang[1], and Seohyeon Kim[2], *1. Gachon University, Incheon, Republic of Korea*, *2. Seoul National University, Seoul, Republic of Korea*

Although family strength has been recognized as one of the crucial factors in the well-being and life satisfaction of people with disabilities, studies regarding its specific role in the life satisfaction of older adults with visual impairment are still limited. Therefore, this study aimed to explore how family strength affects life satisfaction, examining the mediating roles of self-esteem and disability acceptance in this relationship. Data were obtained from the 4th Disability and Life Dynamics Panel in South Korea, analyzing 386 participants aged 50 and above. Structural equation modeling was employed to analyze the direct and indirect effects among family strength, self-esteem, disability acceptance, and life satisfaction. The overall model fit indices met the recommended criteria, indicating the hypothesized model appropriately represented the relationships between family strength and life satisfaction among older adults with visual impairments (Normed $\chi2=1.876$, TLI=.985, CFI=.993, RMSEA=.048, SRMR=.017). The results revealed that family strength enhances life satisfaction, both directly and indirectly through promoting self-esteem and disability acceptance. These findings suggest that an integrated approach focusing on strengthening family support, as well as enhancing self-esteem and disability acceptance, is recommended to improve life satisfaction for older adults with visual impairment. This study contributes to a better understanding of mechanisms underlying life satisfaction in older adults with visual impairments. In addition, it provides valuable insights for developing targeted interventions to enhance family strengths and to create supportive strategies for this vulnerable population.

Abstract citation ID: igae098.4192

### FEASIBILITY OF A PHYSICAL ACTIVITY GOAL SETTING INTERVENTION AMONG INSUFFICIENTLY ACTIVE MIDLIFE ADULTS

Molly Maxfield[1], Keenan Pituch[1], Dereck Salisbury[2], Fang Yu[1], and Rodney Joseph[1], *1. Arizona State University, Phoenix, Arizona, United States*, *2. University of Minnesota, University of Minnesota, Minnesota, United States*

Physical activity (PA) during midlife can reduce risk for Alzheimer's disease and related dementias. However, adherence to national PA guidelines (≥150 min/week of moderate-to-vigorous intensity aerobic PA [MVPA]) among midlife adults is rarely achieved through behavioral PA interventions, which may be due to inconsistent or unsuitable approaches to goal setting. The purpose of this study was to assess the feasibility of a 6-month virtual coaching intervention designed to test the effectiveness of three different goal setting techniques to enhance mechanisms of self-regulation and self-efficacy and increase PA among insufficiently active midlife adults with obesity. Study participants (n=24) were provided a Fitbit to self-monitor their MVPA, received weekly virtual PA coaching conducted via Zoom, and were assigned to 1 of 4 groups: i) static weekly goal of 150 min/week of MVPA, ii) weekly self-selected MVPA goals, iii) modest incremental increase in weekly MVPA goal (20% min/week greater than previous week's MVPA); or iv) non-goal setting comparison group. Results showed favorable outcomes for retention (91.3% [n=21] retained at 6-months), coaching sessions attendance (85.9% of sessions completed), and Fitbit wear adherence (Fitbit worn for > 10 hour/day on 87.1% of intervention days). Although the small sample precludes use of inferential statistics or examination of group differences, patterns of change suggested that participants experienced improvements in MVPA and psychosocial mediators of self-efficacy and self-regulation. Building upon these results, a forthcoming Stage 2 proof-of-concept trial (n=120)

will be conducted to examine the effectiveness of these goal-setting techniques to increase MVPA among midlife adults.

Abstract citation ID: igae098.4193

### FEASIBILITY, ACCEPTABILITY, AND UTILITY OF THE HOME ALONE INTERVENTION: A PRELIMINARY MIXED METHODS EVALUATION

Grace Savard[1], Robyn Birkeland[2], Stephanie Ingvalson[2], Renèe Pepin[3], Jill Cigliana[4], Laura Gitlin[5], and Joseph Gaugler[2], *1. University of Minnesota, 2. University of Minnesota, Minneapolis, Minnesota, United States, 3. Geisel School of Medicine at Dartmouth, Lebanon, New Hampshire, United States, 4. Memory Care Home Solutions, Memory Care Home Solutions, Missouri, United States, 5. Drexel University, Philadelphia, Pennsylvania, United States*

Research has established that living alone with cognitive impairment in later life presents an increased risk for social isolation, decline in mobility, and other adverse health outcomes. While the number of older adults living independently with cognitive impairment is expected to increase, few programs are available to address the specific needs of this growing population. Home Alone—a novel intervention that includes seven coaching sessions focused on home safety, lifestyle planning, and meaningful engagement through behavioral activation—was designed to support older adults living alone at home with memory concerns. A feasibility pilot phase of Home Alone was conducted between June 2023 and February 2024. Fifteen participants (average age: 73; range: 55-88) living across the United States completed the intervention via telehealth or local in-home sessions. Data and feedback were provided through surveys (baseline, 1, and 3 months) and semi-structured interviews. Mixed methods results showed that the intervention was feasible, acceptable, and useful to participants. Interviews, open-ended survey responses, and coaching sessions yielded insights into program effectiveness and benefits. While in the program, participants restructured home environments to enhance safety, connected with previously unfamiliar resources, and incorporated technology to assist with tasks of daily living. Nearly all participants noted appreciation for tailored activities, educational information, and personal companionship as they navigated transitions and changes. Incorporating participant and staff perspectives from the feasibility phase, this poster will expand on the Home Alone program's feasibility, acceptability, and utility and highlight modifications made to the intervention for the ongoing larger pilot study (April 2024-Present; n=20).

Abstract citation ID: igae098.4194

### FOUR TYPES OF FINANCIAL SUPPORT AMONG "SANDWICHED" MIDDLE-AGED ADULTS: LIFE AND FAMILY RELATIONSHIP SATISFACTION

Hyeonji Cho[1], Meejung Chin[2], and Catherine García[1], *1. Syracuse University, Syracuse, New York, United States, 2. Seoul National University, Seoul, Republic of Korea*

Middle-aged adults who provide supports to both their parents and children – often referred to as the "sandwiched generation" – are at higher risk for poor mental health. However, within the Korean cultural context, where strong expectations for intergenerational financial support exists, may influence the well-being of individual's who either fulfill or fail to meet these expectations. This study classified middle-aged adults into four groups based on their financial support patterns: (a) Supporting both parents and children, (b) supporting only parents, (c) supporting only children, and (d) Supporting neither. Using data from the 25th wave (2022) of the Korean Labor and Income Panel Study (KLIPS), we analyzed 1,975 middle-aged householders aged 40-64, all of whom have at least one living parent and co-reside with at least one child ([a]=72.6%, [b]=14.2%, [c]=9.8%, [d]=3.4%). Controlling for sociodemographic and economic characteristics, we conducted linear regression analyses to examine the association between financial support groups (group [a] as the base level) and life satisfaction indicators (i.e. overall life satisfaction and family relationship satisfaction). Regarding overall life satisfaction, group (a) was more satisfied than group (b) (B=-0.09, p=.031) and (c) (B=-0.16, p<.001). Additionally, group (a) was more satisfied in their family relations than group (c) (B=-0.14, p=.002) and (d) (B=-0.14, p=.046). These results suggest that, in Korea, the societal expectation to fulfill financial responsibilities towards parents may contribute to a greater sense of life satisfaction among those who successfully meet these obligations, whereas those unable to do so might experience diminished well-being.

Abstract citation ID: igae098.4195

### FREQUENCY OF SOCIAL MEDIA USE IS ASSOCIATED WITH DELAY DISCAOUNTING IN OLDER ADULTS

Allison Walton[1], Daniel Moss[2], Abigail Stephan[3], Lesley Ross[3], and Christine Phillips[2], *1. Clemson Institute for Engaged Aging, Seneca, South Carolina, United States, 2. Clemson University Institute for Engaged Aging, Seneca, South Carolina, United States, 3. Clemson University, Clemson, South Carolina, United States*

Delay discounting is the decline in the perceived value of a reward as the time to reward receipt increases. Individuals who discount more steeply favor smaller more immediate rewards (i.e., impulsive choices) over larger delayed rewards (i.e., self-controlled choices). Social media and smartphone engagement, whereby users have instant access to information or social networks, are linked with higher delay discount rates in younger adults. However, there is a dearth of research surrounding this relationship in older adults. The present study examined associations between delay discounting, use of health and financial tracking apps, and social media use, as well as age as a potential moderator, in community-dwelling adults ages 55 years and older (M=68.64, SD=8.2, n=120) enrolled in the Tracking Real-world Activities in Life Study. Delay discount rate was assessed using the Monetary Choice Questionnaire. Three self-report items assessed frequency of app and social media use. Multiple linear regression results indicated delay discount rate was positively associated only with social media use, statistically controlling for age ($\beta$ =.17, t(118) = 2.14, p =.035). Age did not moderate this relationship. Results suggest that regardless of age, more frequent social media use is linked with greater preference for immediate rewards. Future research should explore whether frequent engagement with social media increases delay discount rate or if those who discount more steeply are more inclined to engage with social media. Additionally, potential interventions targeting impulsivity and delay discounting are needed in this population who are at risk of online scams

Abstract citation ID: igae098.4196

### FROM THE PERSPECTIVES OF PROVIDERS TO OLDER ADULTS: CONVERSATIONS ABOUT BEHAVIORAL HEALTH SERVICES IN OREGON

Ellis Hews[1], Dani Himes[1], Allyson Stodola[1], Sarah Dys[1], and Walter Dawson[2], *1. Portland State University, Portland, Oregon, United States*, *2. Oregon Health & Science University, Portland, Oregon, United States*

The Older Adult Behavioral Health Initiative (OABHI) is an innovative program funded by the Oregon Health Authority to address gaps in services for older adults and people with physical disabilities with behavioral health (BH) issues. The Initiative consists of 24 Behavioral Health Specialists who work with community partners across Oregon to improve BH services through workforce development, health and wellness promotion, complex care consultations, and promoting coordination and collaboration between aging and BH agencies. The Institute on Aging (IOA) at Portland State University (PSU) has evaluated the effectiveness of the Initiative since its establishment in 2015. Prior evaluation efforts included interviews and surveys from key community partners, staff from Area Agencies on Aging (AAA), Aging and Disability Resource Centers (ADRC), Aging and People with Disabilities (APD), and Community Mental Health Programs (CMHPs). As the Initiative comes upon nearly ten years of existence, the evaluation team conducted a new evaluation approach by facilitating focus groups with these key community partners in addition to primary care providers, older adults (65+) with lived experience, and senior centers representing 25 rural and urban Oregon counties (n=54). Participants were asked about the top challenges in their communities and how the Initiative can better address their biggest needs when supporting older adults and people with physical disabilities living with behavioral health issues. Findings from these data identify recommendations to address systems-level barriers and opportunities to promote intra-agency collaboration and outreach to community partners and consumers.

Abstract citation ID: igae098.4197

### FROM TWINS TO DIGITAL TWINS: LLM-DRIVEN COGNITIVE PERFORMANCE PREDICTION

Anqing Zheng, Daniel Gustavson, Robin Corley, and Chandra Reynolds, *University of Colorado Boulder, Boulder, Colorado, United States*

Human digital twins, virtual representations of individuals that simulate one or more aspects of human behavior and characteristics, have gained increasing attention for their potential applications in training optimization and personalized interventions. This study explores the use of Large Language Models (LLMs) with in-context learning to simulate cognitive performance, leveraging the power of real human sibling pairs. Our study aims to assess the accuracy of these simulated representations by comparing their predictive power against actual performance and their siblings' performance across various cognitive domains in those approaching midlife. We utilize the CATSLife dataset of 1,040 individuals from 505 sibships (including twins and non-twin pairs, ages 28-49). Our dataset encompasses over 60 characteristics with empirical associations with cognitive maintenance, spanning across lifestyle, psychosocial, environmental, and demographic domains, serving as the foundation for simulating cognitive performance. For evaluation, we employ intraclass correlation coefficients to quantify similarities between sibling-sibling, individual-simulation, and sibling-simulation pairs. Preliminary analyses reveal strong cognitive performance correlations among monozygotic pairs (MZ; rs =.37~.85), establishing a benchmark for our simulations. We expect our LLM-based simulation will outperform traditional statistical approaches in predicting individual cognitive performance, while remaining within the bounds of observed MZ similarities. By comparing sibling-sibling and sibling-simulation similarities, we aim to demonstrate a novel evaluation metric for human digital twins and provides insights into the capabilities of advanced computational methods in predicting individual cognitive performance. This approach could open avenues for developing more precise, personalized strategies in cognitive health management and targeted cognitive enhancement interventions.

Abstract citation ID: igae098.4198

### GENDER DIFFERENCES IN THE PATHWAYS FROM DAILY STRESS TO CARDIOVASCULAR HEALTH IN CAREGIVERS

Kelsey Biernot[1], Erin Clancy[1], and Rachel Koffer[2], *1. Arizona State University, Tempe, Arizona, United States*, *2. Arizona State University, Phoenix, Arizona, United States*

Caregivers' risk for cardiovascular disease may be increased because of stress. Furthermore, less is known about gender differences in the effects of caregiving on stress and cardiovascular health. This study examined the associations between gender and daily stressors on cardiovascular health in caregivers, first cross-sectionally at two time points 10 years apart and then longitudinally. Data from two waves of the Midlife in the United States Study (Wave 2: n = 2,087, 13.56% caregivers; age = 33-84 years; Wave 3: n= 3,437, 13.73% caregivers; age= 39-93 years) included caregiving status in the past 12 months, daily diary stressor reports, measures for the American Heart Association's Life's Simple 7. Cross-sectional regressions found caregiving was only related to increased stressors in women, not in men, at Wave 2 (BWave2 = -0.08, p= 0.02; BWave3 = -0.01, p= 0.84). Both waves showed significant interactions between stressor exposure and caregiving status on cardiovascular health (BWave2 = 0.46, p = 0.047; BWave3 = -0.95, p< 0.001). In Wave 2, caregiving was related to lower cardiovascular health for those with more stressors, while in Wave 3 caregiving was related to worse cardiovascular health for those with fewer stressors. The interactive effects of caregiving and stressor exposure did not differ by gender. Follow-up analyses revealed the effects of wave 2 caregiving and stress did not relate to wave 3 cardiovascular health. Findings indicate caregiving status is most important for concurrent cardiovascular health, and differs by the context of daily stressor experiences.

Abstract citation ID: igae098.4199

### GENTLE MOVES: AN INNOVATIVE IN-HOME PHYSICAL ACTIVITY PROGRAM INCLUSIVE OF OLDER ADULTS WITH ADRD

Elise Hu[1], Lijuan Yin[2], Woojin Song[2], Jordan Skowronski[2], Jane Stocks[3], Hanaan Bing-Canar[4], Maria Caceres[2], and Naoko Muramatsu[2], *1. University of Illinois at Chicago, Chicago, Illinois, United States*, *2. University of Illinois Chicago, Chicago, Illinois, United States*, *3. Northwestern University Feinberg School of Medicine, Chicago, Illinois, United States*, *4. Icahn School of Medicine at Mount Sinai, New York, New York, United States*

While regular physical activity (PA) is vital for older adults' health, older adults with Alzheimer's disease and related dementias (ADRD) often find regular PA unattainable due to prolonged inactivity, functional limitations, and/or lack of motivation. Pro-Home(TM) Gentle Moves for Healthy Aging (GM) is an in-home PA program inclusive of inactive/frail older adults with ADRD, adapted from an evidence-based PA program. GM is innovative because (1) GM applies behavioral learning theory to guide learning, managing, and reinforcing regular PA; (2) GM features PA goalsetting to enhance motivation and builds PA into participants' daily routine; (3) the in-home PA moves are appropriate for inactive/frail older adults with ADRD; and (4) participants receive a personalized tablet to remind and support daily completion of GM. We remotely pilot-tested GM with 8 participants and their caregivers. Participants had a mean age of 79.3 (SD=9.67), 5 were female, and caregivers were participants' children (n=4), spouses (n=3), or grandchildren (n=1). Despite varying degrees of ADRD, all dyads actively engaged with our interventionist to set PA goals, learn GM, and incorporate GM into their daily routines. Additionally, all dyads completed the 3-month program (100% retention). Participants felt safe doing GM at home and found GM's intensity appropriate for their abilities. While most participants with mild ADRD independently completed the 3-month intervention with daily tablet use after initial instruction, participants with moderate ADRD required greater caregiver support using the tablet and reminding and motivating participants daily. Future directions include implementing GM into healthcare settings and investigating further motivational strategies.

Abstract citation ID: igae098.4200

### GENTRIFICATION AND COGNITIVE DECLINE IN OLDER ADULTS: MULTI-ETHNIC STUDY OF ATHEROSCLEROSIS

Jana Hirsch[1], Kari Moore[1], Reyhaneh Akbar Nejad Yazdi[1], Lilah Besser[2], Timothy Hughes[3], and Yvonne Michael[1], *1. Drexel University Dornsife School of Public Health, Philadelphia, Pennsylvania, United States*, *2. University of Miami, Boca Raton, Florida, United States*, *3. Wake Forest University School of Medicine, Winston-Salem, North Carolina, United States*

Gentrification, transition of neighborhoods from a state of disinvestment to reinvestment, introduces new amenities potentially beneficial for health of residents who are able to remain. However, the displacement of existing residents may result in stress and diminished social connection. These disparate influences of gentrification may positively or negatively influence older adults' brain health. We used a sample of 1,292 participants from the Multi-Ethnic Study of Atherosclerosis (MESA) to examine associations between gentrification from 2000 to 2010 and cognitive function change between Exams 5 (2010-2012) and 6 (2016-2018). Neighborhoods were categorized as continually advantaged (ineligible to gentrify), continually disadvantaged (eligible to gentrify but not gentrified), and gentrified. We categorized three continuous cognitive measures as maintained/improved score versus decline in score from Exam 5 to 6: CASI (version 2), global cognitive function; Digit Symbol Coding (DSC) task, processing speed; and Digit Span (DS), short term memory. Compared to those living in continually disadvantaged neighborhoods, participants residing in gentrified neighborhoods had 5% lower risk of CASI decline and 2% lower risk of DSC decline. More pronounced, those residing in continually advantaged neighborhoods had 10% lower risk of CASI decline, 6% lower risk of DS decline, and 8% lower risk of DSC decline. After adjustment for age, gender, race/ethnicity, language of administration, education, income, working status, and APOE4, only DSC declines remained statistically significant. Among a multi-ethnic and geographically diverse population, we observed a linear relationship through which risk of cognitive decline decreased with higher level and improvement of neighborhood socioeconomic conditions.

Abstract citation ID: igae098.4201

### GLOBAL DEMOGRAPHIC-CULTURAL SHIFTS AND 'KINLESSNESS': RECONSIDERING INTERGENERATIONAL THEORIES

Christine Mair[1], and Rose Anderson[2], *1. University of Maryland Baltimore County, Baltimore, Maryland, United States*, *2. University of Maryland Baltimore, Baltimore, Maryland, United States*

Demographic and cultural shifts such as declining fertility, expanding longevity, and shifts toward individualism (e.g., living alone) have contributed to changes in intergenerational relationships among aging populations cross-nationally. Specifically, the proportion of older adults who do not have children or partners ("kinless") is expected to increase substantially in nearly all regions of the globe over the next 50 years. Yet, these shifts do not occur evenly across low-, middle-, and high-income countries. Such substantial demographic-cultural changes and cross-national inequalities will require that scholars reconsider existing concepts and theories of intergenerational relationships. In this paper, we analyze publicly available aggregate, country-level data from Our World in Data and the World Values Survey from multiple time periods to explore trends in fertility, longevity, population age composition, rates of living alone and one-person households, rates of "kinlessness", and cultural endorsement of values associated with individualism and friendship in low-, middle-, and high-income countries. The results illustrate differences by countries and regions, which highlight areas for future conceptual development. Based on these results, we propose new directions for gerontological theories, measurement, and policy regarding intergenerational relationships, parenthood and partnership, childlessness and "kinlessness," and siblings, extended family, and friendships that address the shifting characteristics of intergenerational relationships globally.

Abstract citation ID: igae098.4202

### HEARING LOSS AND LONELINESS AMONG OLDER ADULTS: LIVING ALONE AS A RISK FACTOR

Kira Birditt[1], Angela Turkelson[1], Emily Noyer[2], Emma Weber[1], and Crystal Ng[1], *1. University of Michigan, Ann Arbor, Michigan, United States*, *2. Survey Research Center, Ann Arbor, Michigan, United States*

Hearing loss is a leading source of disability within the United States, affecting up to 65% of adults aged 71 and older in the United States. Hearing loss is associated with greater loneliness which is also common among older adults and predicts a range of negative health outcomes. However, little is known about whether hearing loss predicts changes in loneliness over time. Further, adults who live alone with hearing loss may be particularly at risk for loneliness. Using newly released longitudinal data from the Health and Retirement Study (2018 and 2022), this study seeks to examine the implications of hearing loss for loneliness over time. Participants included respondents (N = 2,753) who participated in 2018 and 2022 waves (ages 50 to 97; 60% women). Participants completed an in person hearing test and questionnaires in which they reported loneliness with the three item UCLA loneliness scale. A total of 72% of the respondents had mild to severe hearing loss and 20% lived alone. Multilevel models controlling for age, gender, race and education showed that having worse hearing predicted greater loneliness. Worse hearing predicted increased loneliness over time among older adults living alone but not those living with others. Hearing loss is an important risk factor for loneliness among older adults with those who live alone at greatest risk. Further research should examine whether using hearing aids reduces the negative implications of hearing loss for loneliness and examine factors that predict hearing aid use among older adults who live alone.

Abstract citation ID: igae098.4203

### HEIGHTENED STRESS IN FOOD CHOICES: HOW ACUTE EXERCISE IMPACTS OLDER ADULTS' DECISION-MAKING

Hyeon Jung (Judith) Heselton[1], Annelie Persson[2], and Julie Blaskewicz Boron[3], *1. University of Nebraska Omaha, Lincoln, Nebraska, United States*, *2. University of Nebraska, Omaha, Omaha, Nebraska, United States*, *3. University of Nebraska Omaha, Omaha, Nebraska, United States*

Both habitual and acute exercise have a subjective stress-reducing effect in young and older adults. Exercise is associated with improved cognitive performance related to decision-making, while stress correlates with poorer decision-making regarding food intake in some individuals. Previous research with young adults found that acute exercise reduced perceived stress, as well as increased the amount selected for consumption both immediately after and 30 minutes post exercise. The current study assessed the impact of acute exercise on perceived stress in older adults while making hypothetical choices related to the amount and timing of food intake. Thirty-one healthy older (Mage=68.67; SD=3.08; Range=64-75) participants (54.8% female; Mean BMI=26.16kg/m2; SD=2.82kg/m2) completed 45-minutes of aerobic exercise and a resting control condition in randomized order. Perceived stress was surveyed (scale 0-10) before the exercise bout, immediately after, and 30-minutes post-condition. Food amount and intertemporal food preferences (e.g., immediate vs. delayed consumption) were assessed. Repeated-measures of analysis of variance (SPSS version 29) indicated no main effect on food amount preference, preferred timing of consumption, or stress; however, an interaction of condition and time on stress (p=0.013) showed when compared to rest, exercise resulted in increased stress from pre-to-post+30 min (F=7.58; p=0.02). Results suggest that acute exercise may transiently increase perceived stress in the context of decision-making around food choices in older adults highlighting the importance of available food options for this population. After replication, future studies should seek to elucidate the etiology and duration of increased stress, and the source of stress, in older adults.

Abstract citation ID: igae098.4204

### HELP THAT HURTS: HOW BENEVOLENT AGEISM AND ABLEISM INTERSECT

Devin Thompson[1], and Carla Strickland-Hughes[2], *1. University of the Pacific, Modesto, California, United States*, *2. University of the Pacific, Stockton, California, United States*

Older adults and people with disabilities are common victims of overaccommodative and unwanted help, partially due to their shared stereotypes of incompetence. This research replicated past work on perceptions of benevolent ageism (Chasteen et al., 2021), extending with examination of ableism and ratings of need for help. We aimed to test how younger and older persons' impressions of an older woman (e.g., warmth) were affected by her reaction to benevolent ageism, depending on her physical disability. Younger (n=205; 18-35 years old) and older (n=216; 55+ years old) participants were recruited via MTurk, CloudResearch, and participant pools. Participants were randomly assigned to read a benevolent ageism scenario where an older woman had a cane or not and reacted by politely confronting or reluctantly accepting unnecessary help. Participants rated the older woman before and after her reaction. Consistent with preregistered hypotheses and past research, ratings of warmth, competence, and overall impression increased whereas ratings of perceived need for help decreased when the older women moderately confronted the offer of help. These effects were more pronounced for older participants than younger participants. However, the presence of the cane influenced impressions by younger, but not older, participants, who rated the older women as more warm after confronting ageism only when she had a cane. This age difference in perception based on the cane might be explained by experience with mobility aids. Implications of results for understanding how ableism and ageism interact for perceivers of different ages and abilities will be discussed.

Abstract citation ID: igae098.4205

### HELPING BEHAVIORS AND COGNITIVE FUNCTIONING: IMPLICATIONS FOR INDIVIDUALS WITH COGNITIVE IMPAIRMENT AND DEMENTIA

Shiyang Zhang, HaeJin Jang, and Sae Hwang Han, *University of Texas at Austin, Austin, Texas, United States*

Formal volunteering is widely recognized for its health benefits, including improved cognitive functioning and delayed onset of cognitive impairment and dementia. However, the extent to which these cognitive benefits vary across individuals with different levels of cognitive functioning remains unclear, and the impact of informal helping (e.g., helping friends, neighbors) on cognition is even less well understood. To address these gaps, we analyze data from the U.S. Health and Retirement Study (1998–2020; 199,126 observations from 35,004 individuals) using unconditional quantile regression models. This allowed for investigating the heterogeneous effects of two forms of helping behaviors–formal volunteering and informal helping–on cognitive functioning, measured with the Telephone Interview for Cognitive Status (TICS; range 0–27), across the cognitive functioning distribution. Our findings reveal that the cognitive benefits of both helping behaviors become progressively more pronounced for individuals experiencing worse cognitive functioning, including those with cognitive impairment (TICS score: 7–11) and dementia (score: 0–6). These unique results challenge the prevailing assumption that healthier individuals are more likely to engage in helping behaviors and thus accrue more benefits. Instead, this study highlights that helping behaviors may be particularly beneficial for individuals at the lower end of the cognitive functioning distribution, and shed a positive light on many older adults who continue to help others despite having health challenges. Our findings support recommendations for healthcare practitioners to devise and implement innovative strategies that integrate helping behaviors as part of healthcare to promote cognitive health and well-being among older adults, especially those at risk of cognitive impairment.

Abstract citation ID: igae098.4206

### HIV STIGMA AND COGNITION AMONG OLDER WOMEN LIVING WITH HIV

Thi Vu[1], Jenni Wise[2], Deborah Jones Weiss[3], Monica Diaz[4], Aruna Chandran[5], Sheri Weiser[6], Leah Rubin[5], and Joan Monin[7], *1. Yale University School of Public Health, Charlotte, North Carolina, United States*, *2. University of Alabama at Birmingham, Birmingham, Alabama, United States*, *3. University of Miami Miller School of Medicine, Miami, Florida, United States*, *4. School of Medicine, University of North Carolina at Chapel Hill, Chapel Hill, North Carolina, United States*, *5. The Bloomberg School of Public Health, Johns Hopkins University, Baltimore, Maryland, United States*, *6. University of California San Francisco, San Francisco, California, United States*, *7. Yale School of Public Health, New Haven, Connecticut, United States*

Internalized HIV stigma refers to the negative beliefs, feelings, and attitudes that people living with HIV (PLHIV) adopt about themselves due to societal HIV stigma. Internalized HIV stigma negatively impacts mental health. Up to 55% of PLWH experience HIV-associated neurocognitive disorders. This study examines longitudinal associations between internalized HIV stigma and cognition among women aged 50+ living with HIV in the historic Women's Interagency HIV Study. Internalized HIV stigma was measured between 2013-2015 using the negative self-image sub-scale of the HIV stigma scale. Seven cognitive domains (executive function, processing speed, attention/working memory, verbal learning, verbal memory, verbal fluency, and fine motor function) were assessed using a validated cognitive battery at baseline (2013-2017) and 2-3 years later. Demographically adjusted T-scores were calculated for each domain with higher scores indicating better performance. A global cognition score was computed by averaging the domain-specific scores. Linear regression models adjusted for age, race, ethnicity, cognition at baseline, average annual income, undetectable viral load, smoking history, recent non-prescription drug use, menopausal status, depression, and alcohol use. Participants' (N=760) mean age was 54 years; 61% identified as Black/African American; 13% were Hispanic; and 54% had an annual income below $12,000. The mean HIV stigma score was 1.78 (SD=0.64). Higher internalized HIV stigma was associated with poorer global cognitive function (B= -0.68, p< 0.05), verbal learning (B= -1.42, p< 0.05), and verbal memory (B= -1.40 p< 0.05) at time 2. Findings suggest assessing and monitoring HIV stigma could have beneficial cognitive effects for older women aging with HIV.

Abstract citation ID: igae098.4207

### HOME MAINTENANCE STRESS AND BURDEN IN A SAMPLE OF INFORMAL CAREGIVERS OF PERSONS WITH DEMENTIA

Patricia Griffiths[1], Louis Tennenbaum[2], Seymour Turner[3], Susan Kimmel[2], and Jon Sanford[4], *1. PDGRIFF, LLC, Blairsville, Georgia, United States*, *2. Homes Renewed Ventures, Washington, DC, District of Columbia, United States*, *3. HomesRenewed Ventures, Washington, District of Columbia, United States*, *4. Georgia State University, atlanta, Georgia, United States*

Home maintenance (HM) as it relates to stress, burden and aging in place (AiP) of informal caregivers (ICGs) of persons with dementia (PwD) is undervalued and understudied, yet a crucial component of a safe and reliable home environment. Homes Renewed Ventures surveyed a convenience sample of 14 ICGs caring for PwDs to assess stress and burden associated to HM and the relationship between HM, disruptions to, and AIP. HM Stress and burden were rated on a 5-point scale (none to a great deal) and were highly correlated (r=.92). ICGs reported high stress (mean = 3.9 + 1.4) and burden (3.6 + 1.5) in relation to HM (median and mode stress = 5, burden 4 and 5). Almost three-quarters (73%) had HM problems that disrupted daily routines with five instances resulting in relocations. ICGs rated the likelihood of doing more HM if provided with information (a) customized to their home or (b) on who to call and financing. The means, modes and medians were four or more indicating a high likelihood to do more HM if provided with helpful, personalized information. A Fisher's exact test indicated a significant association (p=.027) between HM Stress and the likelihood of doing more if provided with option a) customized information about what needs to be done. Although a small convenience sample, these preliminary findings provide strong evidence that HMI are disruptive and stressful, suggesting that enhancing caregiver mastery of these issues is a potential mechanism for reducing ICG stress and burden.

Abstract citation ID: igae098.4208

### HOW DOES AWARENESS OF SOCIAL SERVICES PLAY A ROLE IN THE RELATIONSHIP BETWEEN RELATIONAL RESOURCES AND THE AIP?

Junmin Park[1], and Yeonjung Lee[2], *1. Chung-Ang University, Seoul, Republic of Korea*, *2. Chung-Ang University, Seoul, Republic of Korea*

The process of 'Aging in Place (AIP)' encompasses a variety of declines in physical capabilities, adaptability to environmental changes, and the breadth of interpersonal relationships. The AIP is not just about staying in the community; it also involves the availability of requisite resources to address the needs that emerge throughout the AIP journey. In this context, relational resources such as an individual's social capital, may serve as a significant asset. Nonetheless, the availability of relational resources is not uniform among older adults, and it might be risky to solely rely on relational resources, potentially resulting in an excessive burden on the enhancement of AIP sustainability. Instead, social services can act as supportive resources. The purpose of this study is to examine the moderating role of perceived awareness of social services in the relationship between relational resources and the intention to AIP. Logistic regression was conducted using data from the 2020 National Survey of Older Koreans (n=10,097). The results show that older adults who have more relational resources are likely to have more intention to AIP and specifically, the association is stronger at a higher awareness of services. The findings show that there is a need for a range of social engagement programs to provide more opportunities to build relational resources in later life, thus increasing the likelihood of AIP. Policy efforts are also needed to increase awareness of social services among older adults and so, they can plan their AIP with the assurance of various available supports.

Abstract citation ID: igae098.4209

### HOW DOES SOCIAL INTEGRATION SHAPE HEALTHCARE USE AMONG OLDER ADULTS IMMIGRANTS?

Aditi Patel[1], Sungjae Hong[2], and Shannon Mejia[3], *1. University of Illinois at Urbana Champaign, Urbana, Illinois, United States*, *2. University of Illinois at Urbana-Champaign, Champaign, Illinois, United States*, *3. University of Illinois Urbana-Champaign, Urbana, Illinois, United States*

Immigrant populations often face significant health challenges due to language or financial barriers, creating unique vulnerabilities that impact community health. This study focuses on social integration among immigrant older adults, which indicates how a relationship between an individual and their community is built and maintained. The aim is to understand how social integration, which has shown varying support for healthcare access, affects immigrant older adults and their use of novel healthcare measures like telehealth. We interviewed 22 Korean immigrant older adults living in the Chicago Metropolitan Area. The interviews covered respondents' life experiences, focusing on affiliated communities and healthcare experiences. We conducted a content analysis to explore approaches to developing social integration and its implications for the healthcare experience. Our analysis compared the healthcare and telehealth experiences by the types of communities they belonged to and to what extent they felt affiliated with it. The analysis revealed three findings. First, strong affiliations in Korean communities, such as the Korean church, led to access to healthcare information, like available healthcare resources. Second, telehealth services were not associated with the social integration of immigrant older adults because they adopted telehealth during the COVID-19 pandemic based on healthcare providers' recommendations. Third, strong affiliations with adult children supported the continued utilization of telehealth services in the years that followed the COVID-19 pandemic. These findings highlight the critical role of social networks and social integration in shaping healthcare decisions, offering valuable insights for developing strategies to improve telehealth accessibility and support services for immigrant populations.

Abstract citation ID: igae098.4210

### HOW FORMAL AND INFORMAL HEALTHCARE KNOWLEDGE IMPACT DEMENTIA CAREGIVER PREPAREDNESS

Zoe Thomas[1], and Francesca Falzarano[2], *1. University of Washington, Seattle, Washington, United States*, *2. University of Southern California, Los Angeles, California, United States*

Dementia family caregivers frequently experience significant burden and depressive symptoms, which can harm their health and impair their ability to fulfill caregiving responsibilities. Previous research has shown that greater dementia knowledge or health literacy improves caregivers' psychological symptoms and helps them feel more prepared for their role. Socioeconomic status and education are also strong predictors of health literacy. However, much of this research has been conducted in Asia, indicating there is a need to investigate dementia literacy further in the United States. This qualitative study aims to explore how formal versus informal healthcare system knowledge affects dementia family caregivers' feelings of preparedness. Formal knowledge is defined as being obtained from a job or school education before becoming a caregiver. Informal knowledge includes doing independent research online, learning from support groups, and any past caregiving experience or managing personal healthcare. Knowledge as a theme emerged in 8 out of 30 caregiver semi-structured interviews, with excerpts subsequently analyzed and coded using reflexive thematic analysis. Results show that caregivers with formal knowledge of the healthcare system had a better grasp of dementia progression, felt more confident in discussions with physicians, and were better prepared to mitigate safety risks in the person with dementia. In contrast, those with informal knowledge displayed greater uncertainty and relied more heavily on the physician to validate their decisions. The findings highlight the advantages of formal healthcare knowledge in facilitating preparedness and indicate the need for educational interventions to improve health literacy among dementia family caregivers.

Abstract citation ID: igae098.4211

### I FOLLOW THROUGH: CONSCIENTIOUSNESS IS ASSOCIATED WITH ADHERENCE

Sarada Lakshmanan[1], Sheryl Flynn[2], Joel Reyes[2], Stephanie Rubinstein[1], and Elizabeth Zelinski[1], *1. University of Southern California, Los Angeles, California, United States*, *2. Blue Marble Health Inc., Altadena, California, United States*

Conscientiousness has been linked to positive health benefits, as conscientious individuals have high self-control and are determined to follow a plan (Roberts et al., 2009). Therefore, we tested a sample of 44 older adults aged 57-95, to see whether conscientious individuals were more likely to adhere to a time-intensive, self-guided, digital, home-based fall prevention program. The fall efficacy program contained 11 lessons with 60 5-10-minute-long classes and an avatar-guided program based on the Otago Exercise Program consisting of lower extremity strengthening and balance exercises. Participants completed up to three lessons and exercised up to three days per week. Conscientiousness was assessed using the Big 5 Personality Test. Lesson adherence reflects the number of lessons completed and exercise adherence was measured from baseline to 3 months and from 3 months to 6 months, creating a 3-month and 6-month adherence statistic. We then used Pearson's r-correlation coefficients to assess the relationships between personality traits and exercise and lesson adherence. None of the traits were significantly related to lesson adherence. Intellect/imagination and extraversion were significant for 3-month exercise adherence ($p<.05$) while emotional stability was significant for 6-month exercise adherence ($p<.05$). Conscientiousness was significant for both 3-month ($p<.05$) and 6-month exercise adherence ($p<.05$). Conscientious individuals appear to have a stronger tendency to adhere to exercise programs over both 3 and 6 months, suggesting a potential link between conscientiousness and sustained habits. Further analysis is needed to make more definitive and directional conclusions regarding this relationship.

Abstract citation ID: igae098.4212

### I THINK I KNOW HOW YOU FEEL: NEUROPSYCHOLOGICAL CORRELATES OF EMOTION PERCEPTION IN FRONTOTEMPORAL DEMENTIA

Anna Toledo[1], Nishita Paruchuri[1], Sae Yokoyama[2], Enna Chen[3], Alice Hua[4], Joel Kramer[5], Robert Levenson[6], and Casey Brown[7], *1. Georgetown University, Washington, District of Columbia, United States*, *2. Princeton University, Princeton, New Jersey, United States*, *3. Stanford University, Stanford, California, United States*, *4. UC Berkeley, UC Berkeley, California, United States*, *5. University of California, San Francisco, University of California, San Francisco, California, United States*, *6. University of California, Berkeley, Berkeley, California, United States*, *7. Georgetown University, Georgetown University, District of Columbia, United States*

Neurodegenerative diseases lead to deficits in cognitive functioning that could disrupt the ability to perceive others' emotions. We examined whether cognitive deficits in semantic knowledge and executive function relate to two aspects of emotion perception. Individuals with frontotemporal dementia and healthy controls (N = 110; 33 behavioral variant, 23 non-fluent variant, 30 semantic variant, and 24 healthy controls) completed cognitive tests of semantic word knowledge (Peabody Picture Vocabulary Test and Boston Naming Test) and executive function (Digit Span Backwards, Stroop, Design Fluency, and Trail-Making). To assess different facets of emotion perception, participants completed an emotion labeling task that measured the ability to identify specific emotions (sad, happy) experienced by characters in films and an emotion tracking task that measured the ability to continuously track the emotional valence of a character in a film using a rating dial. Regression analyses revealed that semantic word knowledge was associated with emotion labeling but not emotion tracking, even after accounting for relevant covariates (diagnosis, age, sex, global cognition, and dementia severity). Executive function was associated with both emotion labeling and emotion tracking, even when accounting for covariates. Steiger's Z test revealed that executive function was more strongly associated with emotion valence tracking than with emotion category labeling. Findings reveal how distinct neurocognitive abilities are linked with unique facets of emotion perception. Semantic word knowledge may subserve the ability to distinguish between specific emotion categories and executive function may be more important when continuously updating one's unfolding emotions than making a single emotional judgment.

Abstract citation ID: igae098.4213

### I? AI? EXPLORING ARTIFICIAL INTELLIGENCE USAGE, PERSONALITY AND OTHER CHARACTERISTICS IN OLDER ADULTS

Sharon Ost-Mor[1], Nir Wittenberg[2], and Yossefa Birati[3], *1. University of Haifa, University of Haifa, Hefa, Israel*, *2. Ariel University, Ariel University, HaMerkaz, Israel*, *3. University of Haifa, Haifa, HaZafon, Israel*

As Artificial intelligence (AI) becomes increasingly prevalent in modern society, understanding its adoption among older adults is crucial. Therefore, this study examines the relationship between AI usage, Big 5 personality traits, and positive solitude, among adults aged 60 and over in Israel. Grounded in the Technology Acceptance Model (TAM), the study explore how personality traits and positive solitude influences perceived AI usability and adoption. A sample of 260 older adults participated in the study. Data were collected through online validated questionnaires and open-ended questions, using Qualtrics. Results revealed that AI users (mean age 69.91, SD=6.7) were younger than non-users (M=74.91, SD=7.6). Of the AI users, 68% were women, 94% were home-dwelling, 65% married, and 98% independent. Additionally, 76% reported using AI for 30-120 minutes daily. Non-users felt subjectively older both physically (M=62.82, SD=11.7) and mentally (M=62.82, SD=10.7) compared to users (physical: M=54.84, SD=13.5; mental: M=58.65, SD=12.7). Significant correlations were found between attitude to life and perceived ability to use AI ($r=0.315$, $p< 0.01$), as well as between attitude to life and aging perception ($r=0.495$, $p< 0.01$). The qualitative analysis revealed varied responses to AI use. Participants noted significant benefits, including new opportunities and skill enhancement. However, they also encountered technical challenges, concerns about data reliability, and difficulties in formulating effective queries. These findings offer valuable insights into promoting AI adoption among older adults, potentially enhancing their quality of life and independence in the digital age. Future research should explore interventions to increase AI acceptance and usage among this population.

Abstract citation ID: igae098.4214

### IDENTIFYING MULTIMORBIDITY RISKS IN OLDER ADULTS: TEN YEAR REVIEW USING THE RGA TOOL

Jinmyoung Cho[1], Wenjin Wang[1], Max Zubatsky[2], Allison Gibson[2], and Marla Berg-Weger[2], *1. Saint Louis University School of Medicine, Ballwin, Missouri, United States*, *2. Saint Louis University, St Louis, Missouri, United States*

Approximately one in two older adults in the U.S. experiences multimorbidity, defined as the coexistence of two or more chronic diseases. The consequences of multimorbidity are significant, including increased vulnerability to acute illness, exacerbation of existing conditions, frequent hospitalizations, and elevated medical costs. Identifying the risk factors for multimorbidity in advance can guide healthcare service decision-making and help prevent adverse outcomes. This project utilized geriatric syndromes from the Rapid Geriatric Assessment (RGA) tool to assess the risk of multimorbidity. The RGA includes four geriatric syndromes: frailty, sarcopenia, geriatric anorexia, and cognitive decline. It was administered to a total of 16,615 individuals aged 65 years and over across Missouri from 2015 to 2024. Multimorbidity was defined as having five or more illnesses, which is one of frailty screening measures. Nearly 40% of the participants reported having five or more illnesses (37.3%). After controlling for age group, sex, race, and ethnicity, logistic regression analysis showed that the prevalence of sarcopenia (OR=3.807 [3.488 – 4.156], p <.001), geriatric anorexia (OR = 1.329 [1.224 – 1.443], p <.001), and dementia (OR=1.273 [1.158 – 1.401], p <.001) were significantly associated with having multimorbidity. This project provides evidence that the RGA is a valid screening tool for identifying multimorbidity across different practice settings and geographic regions of Missouri. The findings underscore the importance of early detection of geriatric syndromes to prevent further morbidity and disability among older adult populations.

Abstract citation ID: igae098.4215

### ILLNESS INTRUSIVENESS AND PERCEIVED CONTROL ON QUALITY OF LIFE IN OLDER ADULTS WITH ARTHRITIS AND MULTIMORBIDITY

Sama Joshi[1], M. Carrington Reid[2], and Irina Mindlis[2], *1. University of Maryland School of Medicine, Rockville, Maryland, United States*, *2. Weill Cornell Medicine, New York, New York, United States*

Arthritis is independently associated with diminished quality of life (QOL) among older adults; and QOL is even worse among those with arthritis and multimorbidity (MM). Illness intrusiveness—i.e., the perception that diseases and their treatments interfere with valued roles and activities—leads to poor QOL among those with chronic illnesses. Further, the perceived control individuals have over their illnesses has been proposed as a mechanism underlying the relationship between illness intrusiveness and QOL. While illness intrusiveness and perceived control are amenable to change, they are understudied among older adults with arthritis and MM. We investigated the role of perceived control as a potential mediator or moderator in the relationship between illness intrusiveness and QOL among older adults with arthritis and MM. We conducted a secondary analysis on a cross-sectional sample of older adults with MM (N=228) using PROCESS macro for SPSS. Participants were on average 72.0 years (SD=5.5), largely women (66.1%), with 2.8 chronic illnesses (SD=0.4), and high levels of pain intensity (M =7.92, SD=2.29) and illness intrusiveness (M=38.86, SD=16.55). Perceived control was a significant mediator in the relationship between illness intrusiveness and QOL, even after adjustment for pain intensity [β = -0.16, 95% CI (-0.13, -0.06)]. Perceived control did not moderate the relationship. In this sample of older adults with arthritis and MM, with high levels of illness intrusiveness and pain intensity, perceived control mediated the relationship between illness intrusiveness and QOL. Perceived control may be a target for future behavioral interventions to improve QOL in this population.

Abstract citation ID: igae098.4216

### IMPACT OF COGNITION AND DEPRESSION ON PHYSICAL ACTIVITY IN OLDER ADULTS WITH CVD: NHANES 2011-2014

Hyun Jung Kim[1], and Shweta Gore[2], *1. MGH Institute of Health Professions, CAMBRIDGE, Massachusetts, United States*, *2. MGH Institute of Health Professions, Boston, Massachusetts, United States*

Cardiovascular disease (CVD) is a major cause of morbidity and mortality. Physical activity (PA) is crucial for its prevention and management. Cognitive impairment and depression, common in older adults with CVD, impact PA and sedentary behaviors (SB). This study explores the associations between cognitive function, depression, PA, and SB in 632 older adults with CVD, using data from the NHANES 2011-2014. PA was assessed using the Global Physical Activity Questionnaire – meeting PA guideline as ≥ 500 MET-min/wk and SB as spending ≥ 7 hr/day in sedentary time. Cognitive function was measured as a composite z-score from four standardized tests (CERAD Word List Learning and Recall, Animal Fluency Test, and Digit Symbol Substitution Test). Depression was measured using the Patient Health Questionnaire (PHQ-9) and categorized into none (score: 0), minimal (1-9), moderate (10-14), and severe depression (15-27). Survey design methods were used to adjust for the complex sample design. Significant predictors from preliminary bivariate analyses were included in logistic regression models. When adjusting for covariates, higher cognitive function increased the odds of meeting PA guidelines by 79% (OR = 1.79, 95% CI = 1.01-3.17, p =.047) and moderate depression by 79% (OR = 0.21, 95% CI = 0.08-0.56, p =.003). Self-perceived general health was also a significant predictor of PA. On the contrary, cognitive function and depression were not significantly associated with SB. These results highlight the importance of considering cognitive and mental health to promote adherence of PA in this population.

Abstract citation ID: igae098.4217

### IMPACT OF PROJECT ECHO ON SELF-ASSESSED KNOWLEDGE IN LONG-TERM CARE AND PRIMARY CARE WORKFORCES

Jacqueline Telonidis[1], Cherie Brunker[2], Christopher Hernandez[1], Kara Dassel[3], and Linda S. Edelman[4], *1. University of Utah, Salt Lake City, Utah, United States*, *2. Internal Medicine Division of Geriatrics, Salt Lake City, Utah, United States*, *3. University of Utah, Salt Lake Cty, Utah, United States*, *4. College of Nursing, Salt Lake City, Utah, United States*

Project ECHO is a widely accepted method for transforming care through education. The Utah Geriatric Education Consortium (UGEC), a Health Resources and Services Administration funded Geriatric Workforce Enhancement Program, developed a monthly Age-Friendly

ECHO series to educated clinicians about the Institute for Healthcare Improvement Age-Friendly Health Systems Initiative 4M's Framework. The purpose of this program evaluation was to measure knowledge gained over the 12-month series ending in June 2024.

**Methods:** We used retrospective pre-post testing via Zoom polling to measure knowledge gained for each ECHO topic. This approach measures participants' learning after the session by asking them to assess their knowledge from two viewpoints – BEFORE and AFTER participating.

**Results:** The UGEC hosted 11 ECHOs with a total of 461 participants: Allied Health (3.04%), Behavioral Health (20.61%), Medicine (10.63%), Nursing (20.17%), Paraprofessionals (6.07%), and other professions including facility administrators, informal caregivers, etc. (39.48%); students made up 3.08% across all disciplines. Overall, results revealed significant improvements in knowledge gained (t = -12.5273, df = 208, p < 0.001). The greatest changes were observed for presentations about Advanced Treatment Options for Parkinson's Disease (t = -5.9847, df = 18, p < 0.001), Helping Patients Age Well (t = -5.4349, df = 20, p < 0.001), and Keys to Addressing Ageism (t = -4.8190, df = 18, p < 0.001). Conclusions: Retrospective pre-post is a valid method to measure learning compared to a traditional pre-post approach. Project ECHO is an effective learning model for best-practice dissemination to increase knowledge about the 4Ms Framework.

Abstract citation ID: igae098.4218

### IMPACT OF WALKING GROUP INTENSITY & TIMING ON EXERCISE ATTITUDES IN OLDER ADULTS: MIXED-EFFECTS ANALYSIS

Jingchuan Wu[1], David Conroy[2], and Margaret Danilovich[3], *1. Penn State University, State College, Pennsylvania, United States*, *2. University of Michigan, Ann Arbor, Michigan, United States*, *3. Northwestern, Chicago, Illinois, United States*

How does physical activity intensity influence pre-frail and frail older adults' attitudes toward physical activity? Casual-speed walking is enjoyable but more intense walking programs can stimulate greater physiologic adaptations and benefits. However, intensity might negatively impact motivation if it leads older adults to evaluate physical activity less favorably. These attitudes can be affective (involving emotional responses, such as enjoyment) or instrumental (involving perceived practical benefits, such as health improvements) and influence future physical activity through their effects on intentions. Whether high-intensity exercise alters the dynamics of attitude change is unclear. This study was a secondary analysis of the effects of 16-week casual-speed versus high-intensity walking intervention on attitudes toward walking in pre-frail and frail older adults. Participants (n = 156; M age = 79.9 years, SD = 7.0) were randomized into casual-speed or high-intensity walking groups and reported attitudes toward the intervention at four time points (baseline, 6, 12, and 16 weeks). Generalized linear mixed models (GLMM) with a gamma distribution assessed attitude changes over time. Results showed instrumental attitudes initially declined (Estimate = -0.006, p <.001) but followed a quadratic pattern, stabilizing or slightly improving later (Estimate = 0.0002, p =.008). Affective attitudes remained stable, with no significant changes over time. Intensity did not affect the rate of change for either attitude type, suggesting similar changes regardless of intensity. We concluded that high intensity walking did not influence attitudinal changes toward walking, and concerns about adverse motivational impacts of high-intensity activity in older adults may be overstated.

Abstract citation ID: igae098.4219

### IMPROVING FUTURE PLANNING AMONG OLDER ADULTS WITH VISION-THREATENING EYE DISEASE

Silvia Sörensen[1], and Yunxiang Chen[2], *1. University of Rochester, Warner School of Education and Human Development, Rochester, New York, United States*, *2. University of Rochester,, Warner School for Education and Human Development, Rochester, New York, United States*

Preparation for Future Care Needs (PFCN) can protect older adults' well-being as they face age-related challenges like Age-related Macular Degeneration (AMD), which progressively impacts the loss of their activities, functioning, and quality of life. However, rates of PFCN are lower among AMD patients than non-diagnosed community-dwelling older adults. We examined whether PFCN can be increased in a sample of 180 AMD patients aged 60-96 (63% women). Participants were randomly assigned to an 8-week in-home Preventive Problem-Solving Intervention (PREPSI), designed to enhance PFCN, or to an in-home Life Review intervention (attention control). Both conditions received 4 weeks of vision loss education before the intervention. We used Latent Growth Modeling to test whether the intervention would lead to change in the trajectories of the five dimensions of PFCN compared to the control condition over 5 measurement points (two post-intervention). Trajectories were significantly different for PREPSI versus control participants on Decision Making (DM). DM improved significantly for the intervention group and continued to improve at 6 month follow-up (p<.05). Although the trajectories for Concrete Planning (CP) diverged, they were not significantly different from one another (Wald $\chi 2$ =1.511, p=.219). Gathering Information (GI) improved significantly for both PREPSI and control group. Awareness showed significant and surprising decrease only in the intervention group, whereas Avoidance showed non-significant changes. Within the intervention group, covariate analyses showed that older participants had slower rates of GI increase and men had a faster increase in CP than women. PREPSI effectively increases engagement with future care needs for this population.

Abstract citation ID: igae098.4220

### IMPROVING LITERACY OF ALZHEIMER'S DISEASE AND RELATED DISORDERS (ADRD) AMONG OLDER ASIAN AMERICANS IN MICHIGAN

Fei Sun[1], Kangyi Zhou[2], Arienne Patano[3], Daniel Velez-Ortiz[3], and Zhiqi Yi[4], *1. Michigan State University, Haslett, Michigan, United States*, *2. Walt Whitman High School, Potomac, Maryland, United States*, *3. Michigan State University, East Lansing, Michigan, United States*, *4. University of Kansas, Lawrence, Kansas, United States*

This study evaluates a community-based participatory intervention designed to enhance ADRD knowledge and reduce ADRD stigma and worry among older Asian Americans in Michigan. Participants were recruited from senior centers and housing, religious congregations, and community Asian service agencies. They engaged in a 2.5-hour program comprising an educational session and a selected culturally tailored activity (e.g., group meals, mindfulness yoga. Pre- and post-intervention surveys focused on ADRD knowledge, worry, and stigma. The intervention was administered to 15 Korean participants (76.4±10.0 years old, 80% female), 82 Chinese participants (82.2±6.6 years old, 60% female), and 32 Indian participants (74.4±7.27 years old, 56% female) delivered in the participants' native language; the images and examples were also culturally relevant to participants. For the Korean group, ADRD knowledge significantly increased (Hedges' g =.60, p <.05), and stigma significantly decreased (Hedges' g =-1.0, p<.01), though changes in worry were not statistically significant. The Chinese participants showed a similar pattern, with an increase in knowledge (Hedges' g=.32, p<.01) and a decrease in stigma (Hedges' g=-0.45, p<.01). The Indian community experienced an improvement in ADRD knowledge (Hedges' g=.50, p<.01) but no significant changes in stigma or worry. Findings underscore the importance of culturally sensitive community-based interventions to address ADRD literacy gaps among older Asian Americans. While knowledge levels increased across all Asian groups, reducing stigma was only effective in the Korean and Chinese communities. Addressing dementia-related worry remains challenging, suggesting the need for more innovative strategies.

Abstract citation ID: igae098.4221

### INCOME-BASED POVERTY AND MATERIAL HARDSHIP PREDICT REDUCED COGNITIVE PERFORMANCE IN OLDER AMERICAN ADULTS

George Daniel[1], Cedric Williams[2], Aneesah Lawrence[1], Kiera Buckley[2], Delaney Leonard[1], Darren Bernal[1], Olivia Bailey[1], and Denee Mwendwa[1], *1. Howard University, Washington, District of Columbia, United States*, *2. Howard University, Howard University, District of Columbia, United States*

**Background:** Research has demonstrated a deleterious impact of lower socioeconomic status (SES) on cognitive functioning. SES has primarily been conceptualized and defined in research as education, income, and/or wealth. This study extends our understanding of the association between SES on cognitive functioning by examining income-based poverty and material hardship as proxies for SES. An additional aim investigated the moderating effects of race and sex on these relationships.

**Methods:** Participants (n=10,974) in the Health and Retirement Study (HRS) completed sociodemographic and neuropsychological assessments. Analyses determined demographic and health differences by poverty-based income. Subgroup analyses investigated the influence of race and sex. Linear regression determined the impact of income-based poverty and material hardship on cognitive functioning, while controlling for demographic and other covariates.

**Results:** Mean age was 74 years old. Sample characteristics were 15% Black, 10% Hispanic, and 58% female. Those below the income-based poverty threshold (n=960) were younger, Black, less wealthy, less educated, and unhealthier. Income-based poverty was the strongest predictor of poorer cognitive functioning (B = -1.65, p < 0.001), and this relationship was stronger in Blacks and women. Indicators of material hardship (e.g., food scarcity, rationing medication, and difficulty paying bills) also predicted poorer cognitive functioning.

**Discussion:** Income-based poverty and material hardship predicted poorer cognitive functioning. Novel measures of SES may help to provide further insight into the negative impact SES has on cognitive functioning, particularly in Blacks and women. Future studies should focus on multidimensional poverty and its implications on long-term cognitive functioning and in older adults.

Abstract citation ID: igae098.4222

### INDIVIDUAL AND RELATION-INFERRED SELF-EFFICACY FOR PHYSICAL ACTIVITY INCREASE IN OLDER ADULT COUPLES

Elizabeth Zambrano Garza[1], Tiana Broen[2], Maureen Ashe[1], Kenneth Madden[1], Denis Gerstorf[3], and Christiane Hoppmann[1], *1. University of British Columbia, Vancouver, British Columbia, Canada*, *2. University of Colorado Colorado Springs, Colorado Springs, Colorado, United States*, *3. Humboldt University Berlin, Berlin, Berlin, Germany*

Older adults encounter daily problems which may hinder them to engage in physical activity. Individual self-efficacy has been positively associated with physical activity, but it does not consider the social world we live in; relation-inferred self-efficacy does, as it captures one's perception about whether a close other believes in their abilities to engage in these behaviors. With a sample of 110 community-dwelling older adult couples who wore accelerometers and reported daily ratings of problems for up to 7 days, this project analyzes associations of both types of self-efficacy and daily problems with physical activity (MVPA and steps). Assuming that both modalities of self-efficacy may be particularly useful to motivate individuals, interactions of self-efficacy and daily problems were examined. We controlled for well-established associations with physical activity (age, gender, and physical limitations). Daily problems were not associated with physical activity. When modelled separately, both types of self-efficacy were associated with more physical activity; when modelled together, only relation-inferred self-efficacy was significant. Unfortunately, there were no significant interaction effects. Findings replicate previous evidence and shed light on the importance of social context for physical activity engagement in older adulthood.

Abstract citation ID: igae098.4223

### INDIVIDUAL AND SOCIAL FACILITATORS OF PHYSICAL ACTIVITY IN STROKE SURVIVORS: EVIDENCE FROM DAILY LIFE ASSESSMENTS

Elizabeth Zambrano Garza[1], Theresa Pauly[2], Rachel Murphy[1], Maureen Ashe[1], Kenneth Madden[1], Wolfgang Linden[1], Denis Gerstorf[3], and Christiane Hoppmann[1], *1. University of British Columbia,*

*Vancouver, British Columbia, Canada*, 2. *Simon Fraser University, Vancouver, British Columbia, Canada*, 3. *Humboldt University Berlin, Berlin, Berlin, Germany*

Physical activity is a means through which stroke survivors can reduce their future stroke risk. Individual intentions (purposeful aims to engage in a behavior) and self-efficacy (beliefs about their capacity to successfully perform the behavior) have been associated with more physical activity. Spouses are underrecognized resources that may facilitate physical activity engagement. We specifically examined dyadic intentions (shared aims to engage in a behavior) and dyadic self-efficacy (beliefs about the capacity to engage in a behavior with a partner) to explore the important role of spouses in facilitating physical activity. 88 Canadian stroke survivors (Mage = 67.84, SD = 10.79; 73% male; 35% with college degrees; 82% White) wore physical activity monitors for up to 14 consecutive days and completed morning and evening questionnaires including daily individual and dyadic intentions and self-efficacy. Results reveal positive associations between individual morning intentions and physical activity (self-report and accelerometer based) but not for individual self-efficacy. Dyadic variables (intentions and self-efficacy) were positively associated with self-reported physical activity but not with accelerometer assessed physical activity. Notably, associations differed by analytical level: within-person effects were significant for individual intentions and dyadic self-efficacy, whereas between-person effects were significant for both individual and dyadic intentions. This project highlights and discusses the importance of considering within and between person effect to provide more granular insights on the complexity of the targeted phenomenon as well as issues of physical activity measurement (self-report, device-derived) to light on avenues for increasing physical activity engagement in stroke survivors.

Abstract citation ID: igae098.4224

### INSIGHTS FROM A NATIONAL COMPARISON STUDY ON MENTAL HEALTH CONDITIONS OF AUSTRALIAN AGED CARE USERS

Lok Man Leung, and Elsie Yan, *The Hong Kong Polytechnic University, Hong Kong, Hong Kong*

**Background:** The mental health status of aged care service users has important implications for service planning and care outcomes. Objective: This study examines the prevalence of mental health conditions among Aboriginal and Torres Strait Islanders aged 50 and above who accessed aged care services in Australia between 2017 and 2022. Comparison was made between Home Care(HC) users and Residential Aged Care(RAC). Method: Data were obtained from the Australian Institute of Health and Welfare(AIHW) database on Mental Health in Aged Care(n=10,535 for HC and n = 6,757 for RAC users). Mental health conditions were assessed by Aged Care Eligibility Assessments and, where applicable, the Geriatric Depression Scale(GDS), Kessler-10(K10), and Cornell Scale for Depression in Dementia(CSDD).

**Results:** Mental health conditions were more prevalent in RAC than HC users (58.6% vs. 31.1%, $X^2$=1276.41, p<.01), with mood disorders being the most common, affecting 43.1% of RAC and 20.9% of HC users ($X^2$=970.89,p <.01). The same pattern was observed for anxiety/stress disorders (26.5% vs. 11.2%, $X^2$ = 682.97, p<.01) and psychotic disorders(6.2% vs. 4.1%, $X^2$ = 34.99, p<.01). Over the five years (2017–2022), a steady increase in recorded mental health conditions was observed in both settings (from 21.0% to 34.5% for HC and 56.0% to 58.4% for RAC). The increase is particularly sharp for mood disorders (from 12.3% to 24.1% for HC and 40.2% to 42.7% for RAC).

**Conclusion:** These findings highlight the need for targeted mental health interventions in residential care. Further research should develop interventions tailored to the specific needs of this group.

Abstract citation ID: igae098.4225

### INSIGHTS GAINED FROM A VIRTUAL INTERGENERATIONAL YOGA INTERVENTION DURING COVID-19

Nancy Martens[1], and Debra Schutte[2], *1. University of Michigan, Flint, Michigan, United States*, 2. *Wayne State University, Detroit, Michigan, United States*

Studies focused on social isolation, intergenerational, and virtual interventions revealed lack of rigor (Cohen-Mansfield et al., 2018, Conn & Grove, 2019; Chipps et al., 2017). The Shine Through2 pilot study aimed to determine feasibility and acceptability of an intergenerational yoga intervention delivered virtually, twice weekly, for six weeks. The Shine Through2 project was tailored for populations who may be experiencing social isolation. The project was developed as a novel community and university collaboration pilot and is a starting point for establishing partnerships aimed to improve older adult and college student health, and subsequently community health. The project included 23 older adults and 12 college students. The data suggest older adults and college students found this virtually - delivered socialization and yoga intervention feasible and acceptable. Insights gained from the project include: 1) Pre-screening for social isolation and depression are impactful assessment modalities, 2) Utilizing a Senior Certified Yoga Instructor added (necessary) expense and scalability challenges, 3) Sampling bias can be overcome with intentional recruitment strategies 4) Specialized yoga programming may be cost-prohibitive, 5) Intergenerational component challenging to deliver virtually, 6) Study design did not allow direct comparison of efficacy between older adults and college students, and 7) Provision of community yoga resources was limited by COVID-19. Based on the results explored in ShineThrough2, we conclude that socialization and yoga interventions can be used to improve health outcomes including measures of social isolation, depressive symptoms and meaning in life.

Abstract citation ID: igae098.4226

### INTERGENERATIONAL SILENT DISCO PARTIES FOR OLDER ADULTS LIVING IN LONG-TERM CARE

Ilan Meghelli[1], Rynnie Cin Ee Son[1], Julia Banco[1], Oliver Hang Kin Yu[1], Shambhavi Arora[2], Swastika Mondol[1], and Lillian Hung[1], *1. University of British Columbia, Vancouver, British Columbia, Canada*, 2. *UBC, Vancouver, British Columbia, Canada*

Silent disco headphone parties in long-term care (LTC) settings offer a unique way to bring residents together to dance and share musical experiences through wireless, multichannel headphones. Unlike traditional group activities, these parties allow residents to choose their music, creating a personalized yet communal experience. Older adults living in LTC often experience loneliness and social isolation. Silent disco parties can offer a unique opportunity for socialization and enjoyment. This study explored the experiences of 22 older adults in silent disco parties with an intergenerational group of volunteers on the dance floor. Over six weeks, data were collected through video ethnography, including video recordings, conversational interviews, observations, and focus groups with 40 staff members. Thematic analysis identified three themes: intergenerational togetherness, social capacity, and inclusivity. Findings suggest that intergenerational silent disco parties hold promising potential to address loneliness and social isolation in LTC settings. Future research should investigate how these parties can influence residents' social health and quality of life.

Abstract citation ID: igae098.4227

### INTERGENERATIONAL VERSUS PEER-LEARNING IN OLDER VOLUNTEER TRAINING: A MIXED-METHODS EVALUATION

Xinxin Cai, Nianzi Sun, and Xue Bai, *The Hong Kong Polytechnic University, Hung Hom, Kowloon, Hong Kong*

Intergenerational learning activities enhance older adults' well-being and intergenerational relationships, but their impact on learning outcomes is less understood. This explanatory sequential mixed-methods study evaluates a volunteer training program conducted between March and December 2023. Older adults were assigned to either a peer-learning group (n = 73) or an intergenerational learning group (n = 49), the latter including undergraduate students participating alongside them in a two-stage program: 1) thematic training workshops on family communication, later-life planning, or healthy living, and 2) designing and organizing a service project. Quantitative assessments were conducted before and after the workshops (T0, T1) and after the volunteer activities (T2). Outcomes included self-rated volunteering abilities, thematic knowledge acquisition, well-being (loneliness, happiness, life control), and intergenerational and peer relationships. After the program, 12 participants attended two focus group interviews. Repeated measures ANOVA revealed significant improvements in volunteer abilities and thematic knowledge only in the peer-learning group. A significant between-group effect was observed for thematic knowledge. MANOVA results indicated a significant time*group interaction, with the intergenerational group showing reduced loneliness. Qualitative analysis suggested older adults were highly motivated to learn and contribute to the community. While they valued interaction with younger generations, they did not perceive it as influencing their learning. However, they believed student commitment influenced their volunteer experiences. This study highlights the learning capabilities of older adults in delivering skill-based volunteer activities. While intergenerational components may not directly enhance learning outcomes, they can improve well-being. Their inclusion should align with program goals for maximum impact.

Abstract citation ID: igae098.4228

### INTERIM ANALYSIS: VALIDATION OF THE AGITATION IN ALZHEIMER'S SCREENER FOR CAREGIVERS (AASC®)

W. Clay Jackson[1], Jared Stroud[2], Malaak Brubaker[3], Mehul Patel[3], Sheri Fehnel[4], Sue Peschin[5], George Grossberg[6], and Carolyn Clevenger[7], *1. University of Tennessee College of Medicine, Memphis, Tennessee, United States*, *2. OhioHealth Physician Group, Columbus, Ohio, United States*, *3. Otsuka Pharmaceutical, Princeton, New Jersey, United States*, *4. RTI Health Solutions, Research Triangle Park, North Carolina, United States*, *5. Alliance for Aging Research, Washington, District of Columbia, United States*, *6. St. Louis University, St. Louis, Missouri, United States*, *7. Emory University, Atlanta, Georgia, United States*

Agitation in Alzheimer's dementia often goes unrecognized or underreported, contributing to delayed diagnosis and treatment. The International Psychogeriatric Association (IPA) set a standard for defining agitation in cognitive disorders. The Agitation in Alzheimer's Screener for Caregivers (AASC®), the first clinical tool based on IPA criteria, was developed to support caregiver-healthcare professional (HCP) discussion and timely recognition of agitation in Alzheimer's dementia. It has 2 questions assessing presence and impact of agitation behaviors. This ongoing, prospective, multisite, single-visit observational study will calculate predictive metrics for recognizing agitation by the AASC® versus IPA criteria. Interim (n=50 caregiver-HCP dyads) and final (n=150 dyads) analyses will determine agreement rates (yes/no) and sensitivity/specificity. This ongoing interim analysis included 44 dyads (caregivers completed AASC®; HCPs completed IPA criteria). Most patients/caregivers were female (70%/50%) and 74 (±7.3)/58 (±18.9) years old. Most caregivers were White (57%), a spouse/partner (43%) or child (23%), and spent a mean (SD) of 40.8 (44.3) hours/week providing care. Percentage agreement between AASC® and IPA was 70.45%; Cohen's kappa coefficient (level of agreement) was 0.35 (95% CI: 0.08, 0.62). The number of false positives/negatives using AASC® was 3/10; positive/negative predictive values (95% CI) were 0.727 (0.464, 0.991)/0.697 (0.540, 0.854). The strongest contributor to false negatives on the AASC® was caregivers' minimization of the impact of agitation behaviors on patient functioning, underscoring a key educational gap. These results suggest that the AASC® can improve caregiver-HCP communication and awareness of agitation behaviors and their impact, thereby advancing early recognition of agitation in Alzheimer's dementia.

Abstract citation ID: igae098.4229

### INTERNET USE, PERCEIVED CONTROL, AND INFORMATION ANXIETY AMONG CHINESE OLDER ADULTS

Jiaan Zhang, *Fudan University, Fudan University, Shanghai, China (People's Republic)*

Despite increasing evidence that Internet use may contribute to Internet-related anxiety in the digital age, studies specifically focusing on older adults remain limited. This study investigates the association between Internet use and information anxiety among older adults, and examines whether this relationship is mediated by perceived control.

We analyzed newly collected data from a random sample of adults aged 55 and older living in Shanghai, China in 2024. The total sample comprised 428 older Internet users. Internet use was assessed by measuring the duration of Internet use, frequency of Internet use, and types of Internet use. Information anxiety was measured using a 23-item scale based on Wurman's theory of Information Anxiety, with each item rated on a 5-point scale ranging from strongly disagree to strongly agree. Perceived control was evaluated by asking respondents to indicate their agreement with statements including four items of personal mastery and eight items of perceived constraints, using a 7-point scale. The results indicate that the duration of Internet use is significantly associated with lower levels of information anxiety, and that Internet use for communication and social use reduces information anxiety. Additionally, perceived control is negatively associated with information anxiety. Further analysis reveals that perceived control partially mediates the relationship between Internet use duration and information anxiety. These findings have important implications for developing effective interventions to alleviate Internet-related anxiety among older adults.

Abstract citation ID: igae098.4230

### LGBTQIA+ AD/ADRD CAREGIVERS' PERCEPTIONS OF THE ACCEPTABILITY OF A LEGAL AND FINANCIAL PLANNING TRAINING TOOL

Clover Caldwell[1], Richard Chunga[2], Sydney Hoel[3], Heather Schwartz[1], AJ Slowey[1], Nicole Werner[3], and Andrew Pickett[3], *1. Indiana University Bloomington, Bloomington, Indiana, United States*, *2. University of Massachusetts Boston, Boston, Massachusetts, United States*, *3. Indiana University, Bloomington, Bloomington, Indiana, United States*

LGBTQIA+ identifying informal caregivers for people living with Alzheimer's Disease (AD) and AD-related dementias (AD/ADRD) face multiple, overlapping stressors related to identity stigmatization and role-based burden. This includes legal/financial planning, as the legal landscape surrounding LGBTQIA+ identity is inconsistent and ever-changing. Part of a larger project, this study examined LGBTQIA+ identifying caregivers' perceptions of the acceptability of CareVirtue Planner, a web-based legal and financial planning support tool for AD/ADRD caregivers. Semi-structured interviews with LGBTQIA+ caregivers (n=9) were conducted after three months of use. We conducted an inductive content analysis using the following process: open coding, collaborative generation of the initial codebook, dual coding with the codebook, consensus discussion on refinements to finalize codebook. Our analysis resulted in four overarching themes among users: sense of commonality and personal validation related to legal and financial challenges of caregiving, Planner features promoting caregiver preparedness, inclusive language and need for LGBTQIA+ specific tailoring, and individual characteristics and personal histories impacting relative usefulness of the Planner. Participants broadly found Planner material to be well organized and its features useful (e.g., document storage 'vault'), with content best suited for those early in the caregiving journey. Users provided specific recommendations to increase LGBTQIA+ appropriateness, including creation of additional content to specifically address unique legal challenges facing LGBTQIA+ caregivers, inclusion of diverse families in materials (e.g., vignettes), and vetting of externally linked resources for LGBTQIA+ inclusiveness. Further research is needed to support LGBTQIA+ AD/ADRD caregivers navigating complex legal and financial situations.

Abstract citation ID: igae098.4231

### LIFELONG LEARNING: HOW HEALTH LITERACY SHIELDS OLDER ADULTS FROM THE MORTALITY RISKS OF LOWER EDUCATION

Hee Lee[1], Jieun Song[2], and Jonghwa Do[2], *1. The University of Alabama, Tuscaloosa, Alabama, United States*, *2. University of Wisconsin-Madison, Madison, Wisconsin, United States*

Research has demonstrated the significant impacts of education and health literacy (HL) on mortality risks. However, the combined effects of education and HL on mortality in older age, particularly using population samples, remain underexplored. This study investigates whether the association between education and mortality is moderated by varying levels of HL among older adults, using data from the Wisconsin Longitudinal Study (1957-2020). The analytic sample includes participants aged 60 and older at the 2010-2012 data collection (n=3,381, mean age=70.8). Mortality data were obtained from the National Death Index 2021 files, and HL was measured using the Newest Vital Sign (NVS) Health Literacy Score. Within this cohort, 531 respondents had limited HL, 893 had possibly limited HL, and 1,957 had adequate HL. Survival analysis using Cox Proportional Hazard models revealed that older adults with limited HL who completed only high school or some college education faced 22% and 49% higher mortality risks, respectively, compared to their peers with a college degree. Notably, high school graduates with adequate HL exhibited a 20% lower risk of mortality compared to those with a college degree but limited HL. These findings suggest that the adverse effects of lower education on mortality can be significantly mitigated by adequate health literacy, even in older age. This underscores the importance of interventions focused on enhancing health literacy, particularly among older adults with relatively lower educational attainment.

Abstract citation ID: igae098.4232

### LIVED EXPERIENCE OF DEMENTIA FAMILY CAREGIVERS IN RURAL CHINA: FINDINGS FROM A QUALITATIVE STUDY

Dexia Kong, *The Chinese University of Hong Kong, Hong Kong, China (People's Republic)*

**Background:** Currently, China has the highest prevalence of dementia worldwide. The prevalence of dementia is greater in rural areas than in urban ones, mostly because over two-thirds of Chinese older adults reside in rural regions. Moreover, the cultural preference for family-based eldercare, in conjunction with the absence of dementia-related services in rural areas of China, has resulted in the responsibility of caring for older adults with dementia falling solely on family caregivers. Nevertheless, there has been no prior research that has examined the lived experiences of caregivers of individuals with dementia in rural China. This study investigates the lived experiences of dementia family caregivers in rural China, thereby addressing the knowledge gap.

**Methods:** In-depth qualitative data were collected from 25 family caregivers residing in a rural county in southwestern China. All interviews were recorded and transcribed verbatim. The interview narratives were analyzed using the conventional content analysis approach. All transcripts were coded by two experienced researchers with qualitative training. Discrepancies in coding were resolved by consensus.

**Results:** Main themes that emerged from the data include caregivers' perceptions of dementia, motivation for caregiving, challenges in caregiving, dynamics within family relationships, coping strategies, and unmet needs.

**Conclusion:** In rural China, families are crucial to the care of older adults living with dementia. The findings underscore the pressing need to develop psychoeducational intervention strategies for supporting family caregivers of individuals with dementia in rural or low-resource regions in China. Policy and service implications will also be discussed.

Abstract citation ID: igae098.4233

### LONGITUDINAL ASSOCIATION OF FOOD INSECURITY AND MOBILITY LIMITATIONS AMONG US OLDER ADULTS

Usha Dhakal[1], Khalil El Asmar[2], and Carlos Mendes de Leon[1], *1. Georgetown University, Washington, District of Columbia, United States*, *2. American University of Beirut, Beirut, Beyrouth, Lebanon*

Food insecurity among older adults is associated with a range of adverse health outcomes, including cognitive impairment, poor mental health, and mortality. The role of food insecurity in impairments in functional status, such as mobility limitations, remains unclear. Therefore, we used weighted generalized linear models to investigate the association between food insecurity and mobility limitations. Considering the 2014 Health and Retirement Study (HRS) wave as the baseline, we extracted data from an HRS subsample who completed the 2013 Health Care and Nutrition Study (n=6476) and three additional HRS waves through 2020. Mobility limitations scores were based on five items (range:0-5); higher scores indicated more limitations. Food insecurity scores were derived from a validated scale of food access and availability. On average, older adults had difficulty performing at least one mobility task at baseline, and their mobility limitations score increased by 0.5 points per year. After adjustment for demographic and socio-economic factors, greater food insecurity was significantly associated with greater mobility limitations at baseline ($\beta$=0.19, 95%CI[.15,.23], p<.0001), but it was not significantly associated with greater increase in mobility limitations over time ($\beta$=0.004, 95%CI[.002,.009], p=.22). Associations remained mostly unchanged after controlling for chronic conditions and depressive symptoms. Our findings suggest that food insecurity is associated with mobility limitations, although its role in continued mobility decline over time was small and not statistically significant. Early identification of at-risk older adults for food insecurity may still offer benefits for improved mobility and thereby contribute towards better overall physical functioning and prevent disability onset.

Abstract citation ID: igae098.4234

### LONGITUDINAL ASSOCIATIONS OF HEALTH BEHAVIOR ADHERENCE WITH PSYCHOLOGICAL WELL-BEING AND NUMBER OF ILLNESSES

Fumiko Hamada[1], and Soomi Lee[2], *1. USF HPCC/ USF School of Aging, Tampa, Florida, United States*, *2. The Pennsylvania State University, University Park, Pennsylvania, United States*

Lifelong healthy habits are vital for healthy aging. This study examined the longitudinal associations of adherence to three key health behaviors (i.e., sleep, physical activity, healthy eating) on psychological well-being (PWB) and illnesses 10 years later. Data were from Midlife in the United States II and III (2005-2006, 2013-2017) participants who provided daily diary data at both timepoints (n=858; Mage=54 at T1). Over eight consecutive days, participants reported nightly sleep duration and the daily amount of vigorous physical activity. A subsample (n=428) reported weekly fast-food consumption. Adherence rates for each health behavior were calculated at each timepoint by summing the number of days with optimal sleep (6< sleep hours≤9), vigorous physical activity (≥30 minutes), and not eating fast food. Participants also completed a survey for PWB and the number of illnesses. A series of general linear or Poisson models examined the associations between the adherence variables and PWB and the number of illnesses, adjusting for sociodemographic and health covariates, baseline PWB or illnesses, and 10-year change in each adherence behavior. Adherence to optimal sleep and not eating fast food were stable across 10 years, while adherence to physical activity decreased. Greater adherence to optimal sleep (B=1.31; SE=0.45; p=0.003) and physical activity (B=1.16; SE=0.43; p=0.007) were each associated with better PWB 10 years later but not with the number of illnesses. Greater adherence to not eating fast food was associated with fewer illnesses (B=-0.12; SE=0.03; p< 0.001) but not PWB. Findings suggest that adherence to healthy lifestyles may be crucial for healthy aging.

Abstract citation ID: igae098.4235

### LONG-TERM EFFECTS OF LOCAL AREA NEW DEAL WORK RELIEF IN CHILDHOOD ON LATE LIFE DEPRESSION

Sepideh Modrek[1], and David Rehkopf[2], *1. San Francisco State Univeresity, San Francisco, California, United States*, *2. Stanford University, Palo Alto, California, United States*

Objective To investigate whether childhood exposure to local area New Deal emergency employment work relief activity was associated with lower depressive symptoms in late life. Methods This study utilized individual-level data from the Wisconsin Longitudinal Study (WLS) linked to the full count 1940 census. New Deal emergency employment programs were the largest non-wartime expansion in government led infrastructure, services, and employment policy in U.S. history. We used within county variation in WLS participants' exposure to emergency employment work relief activity during childhood (ages 0-3) to examine its association with depressive symptoms in late life. We examined depressive symptoms at three ages, 53-55, 65-67, and 72-74 but with a focus on depressive symptoms at ages 72-74 as a marker for late-life depression. Results Children who lived in neighborhoods with moderate or high levels of emergency employment work relief activity exhibited 14-18% lower depressive symptom scores at ages 72-74 compared to those from neighborhoods with low activity. These findings were consistent across various measures of late-life depressive symptoms, different model specifications, and after accounting for prior depressive symptoms. Discussion The study highlights the long-term mental health benefits of economic policies aimed at mitigating concentrated economic downturns among the most impacted individuals. Childhood exposure to New Deal work relief reduced depressive symptoms in older age, particularly new onsets of depressive symptoms at ages 72-74. These results suggest social policies aimed at maintaining economic activity in downturns can have long-term positive impacts on population mental health.

Abstract citation ID: igae098.4236

### LOW-INTENSITY PEER-LED EXERCISE DOES NOT CHANGE PHYSICAL ACTIVITY OR BALANCE IN OLDER ADULTS

Chitra Banarjee[1], Jethro Raphael Suarez[2], Renoa Choudhury[3], Joon-Hyuk Park[1], Rui Xie[1], Jeffrey Stout[1], and Ladda Thiamwong[1], *1. University of Central Florida, Orlando, Florida, United States*, *2. University of Central Florida*, *3. Virginia Polytechnic Institute and State University, Blacksburg, Virginia, United States*

Decreased physical activity in aging adults is associated with increased fear of falling (FOF) and decreased static balance. These results varied by the level of engaged physical activity (i.e., light, moderate, vigorous). This study examined the effect of a low-intensity peer-led intervention on step count, FOF, and static balance. We recruited 65 community-dwelling older adults (mean age = 73.52 years ± 5.66; 87.7% female) at two-time points (mean interval = 177.66 days ± 80.33) to evaluate longitudinal changes in step count, perceived fear of falling (Short Falls Efficacy Scale-International (FES-I)), and objective static balance (BTrackS Balance System (BBS)). Step count was recorded over a 7-day interval at each time point. For FOF, participants completed the short FES-I, a 7-item self-report questionnaire. For static balance, participants stood on a balance plate for 3 20-second trials that were averaged to determine the center of pressure path length. Forty-one participants attended a once-weekly peer-led exercise for one hour. Wilcoxon tests revealed no significant effect of this intervention in step count (p=0.984), FOF (p=0.1972), or static balance (p=0.8136). This suggests once-weekly low-intensity exercise did not significantly affect physical activity and balance measures. Limitations include a predominantly female sample and the low-intensity nature of the intervention. Further research should investigate the effects of a longer duration, higher-intensity peer-led exercises in a larger, more diverse sample.

Abstract citation ID: igae098.4237

### MAKING BUILT ENVIRONMENT MORE AGE-FRIENDLY -- FIRST TASK OF UNIVERSITY OF COLORADO AGE-FRIENDLY TASKFORCE

Kathryn Nearing[1], and Jodi Waterhouse[2], *1. University of Colorado Anschutz Medical Campus, Denver, Colorado, United States*, *2. CU Anschutz Multidisciplinary Center on Aging, Aurora, Colorado, United States*

When the Age-Friendly University (AFU) Taskforce at the University of Colorado Anschutz Medical Campus initiated strategic planning in 2023, the built environment – an antecedent condition for achieving AFU priorities – emerged as priority #1. The taskforce tailored an existing tool available from University of Massachusetts-Boston to align the built-environment assessment with the broader mission-focused areas of CU Anschutz – one of the first four academic medical campuses in the world to receive the AFU designation. During April 2023, each taskforce representative paired with an older adult community member; each dyad was assigned a campus zone to assess using the adapted Age-Friendly Inventory and Campus Climate Survey. The community member provided first-hand accounts of how they experienced aspects of the built environment, while the taskforce representative recorded observations. Once compiled, the taskforce reviewed data and identified the following areas of concern: wayfinding supports (e.g., signs and campus maps -- printed, online); parking kiosk accessibility; distances (e.g., from parking to campus buildings); campus building and restroom accessibility. Taskforce members presented results and recommendations to the Chancellor, campus architect, Vice Chancellor for Facilities, and Communications team. Through campuswide collaborations that expanded to include parking managers and the campus shuttle service, issues with campus maps, parking kiosks, crosswalks, walking distances, and building/restroom accessibility are being addressed, with a progress report provided to the Chancellor in July 2024. This work is essential. The built environment fundamentally influences campus accessibility across the lifespan and the age-inclusivity of events, classes, research studies and healthcare services.

Abstract citation ID: igae098.4238

### MAPPING RELIGIOUS AND SPIRITUAL CARE IN WISCONSIN LONG-TERM CARE: A DESCRIPTIVE STUDY

Kathryn Lyndes[1], Sarah McEvoy[2], and Louis Arata[3], *1. Saint Mary's College, Notre Dame, IN, Notre Dame, Indiana, United States*, *2. Rush University Medical Center, Chicago, Illinois, United States*, *3. Saint Mary's College, Notre Dame, Indiana, United States*

The importance of religion and spirituality (R/S) for older adults is well-recognized (Pew Research Center, 2015); however, little is known about specific types of R/S care and who provides it across a range of long-term centers. The aim of this study was to expand on a pilot study that explored the feasibility of gathering details about R/S care of residents in long-term care centers in northern Indiana (Lyndes & Fitchett, 2021), and on a single Tennessee county study that enabled the researchers to hone the study design (Lyndes, McEvoy, & Arata, 2023). The current study in this abstract extended the sample to Wisconsin, employing a systematic selection of diverse residential care centers (including skilled nursing homes, assisted living, independent living, and senior housing), and developing a preliminary description of the types of services as well as the training and criteria of the staff and volunteers who provide them. Results indicate that the majority of multi-disciplinary staff who participated in this study identified R/S care as beneficial to the health and well-being of residents. Many centers rely on staff chaplains and/or volunteer community members, with a range of credentials and training such as Clinical Pastoral Education, to provide an array of R/S services, for example one-on-one visits and weekly worship. The data has important implications for allied healthcare teams and long-term care providers who deliver services to older adults, including the need for culturally competent training across faith traditions to meet the needs of diverse residents.

Abstract citation ID: igae098.4239

### MEANINGFUL ACTIVITY IN ASSISTED LIVING RESIDENTS WITH DEMENTIA: RESULTS FROM THE MAC-4-BSD STUDY

Sarah Holmes[1], Elizabeth Galik[1], Barbara Resnick[1], Susan Scherr[2], Erin O'Brien[3], Sorah Levy[1], Merve Gurlu[1], and Shijun Zhu[1], *1. University of Maryland School of Nursing, Baltimore, Maryland, United States*, *2. Stevenson University, Owings Mills, Maryland, United States*, *3. Post-Acute and Long-Term Care Medical Association, Columbia, Maryland, United States*

Engagement in meaningful activity is beneficial for assisted living (AL) residents with dementia and may help to reduce behavioral symptoms and improve quality of life. The purpose of this study was to test the feasibility and preliminary efficacy of a theory-based approach, Meaningful Activity for Managing Behavioral Symptoms of Distress (MAC-4-BSD), for decreasing behavioral symptoms and increasing engagement in meaningful activity. It was hypothesized that residents exposed to MAC-4-BSD will demonstrate reduced behavioral symptoms, increased meaningful activity engagement and quality of life at 4 months compared to those exposed to education only. Using a cluster randomized controlled trial with repeated measures design, this study included 71 residents with dementia from 5 communities. Residents were evaluated at baseline and 4 months. Descriptive statistics were used to describe the sample and evaluate feasibility. Linear mixed models were used to determine effectiveness. Most participants were female (n=52, 73%), and White (n=62, 87%) with a mean age of 85 years (SD=8.2). The MAC-4-BSD intervention was feasible to implement with regard to delivery, receipt, and enactment. Preliminary effectiveness was demonstrated based on increased engagement in meaningful activity ($b=7.69$, $p=.005$), improved quality of life ($b=3.74$, $p=.03$), and decreased staff distress related to residents' behavioral symptoms ($b=-1.83$, $p=.037$) among those exposed to MAC-4-BSD versus the control group. There was no change in the severity or number of behavioral symptoms. These findings demonstrate that engagement in meaningful activity is a promising approach that can be used to help improve the quality of life for AL residents with dementia.

Abstract citation ID: igae098.4240

### MINDFULNESS PRACTICE IN OLDER ADULTS: MIXED-METHOD FINDINGS FROM AN OUTPATIENT GROUP INTERVENTION

Christopher Griffith, Jonathan Sundby, Tiana Broen, and Leilani Feliciano, *University of Colorado Colorado Springs, Colorado Springs, Colorado, United States*

Mindfulness can be thought of as a skill that centers on directing attention, in the present moment, with non-judgment. Mindfulness skills are effective strategies for addressing psychological symptoms, including improving mood, stress, chronic pain, sleep, and well-being in older adults. To further examine the effects of mindfulness practice in a group setting, two 8-week mindfulness skills building groups were conducted at a community outpatient clinic for older adults in Southern Colorado. Participants ($n = 11$) completed pre-post measures on depression, anxiety, stress, well-being, pain, sleep, and trait-mindfulness. The final session was recorded and transcribed to allow for thematic analysis. During this session, participants were asked how mindfulness practice influenced them and what changes they may have noticed. Some responses included themes related to outcome measures (e.g., stress, well-being), with additional themes emerging related to: 1) overall effectiveness, 2) greater insight, 3) interpersonal functioning, 4) group cohesion, 5) context, and 6) filler. Paired-samples t-tests were conducted demonstrating significant improvements in depressive symptoms [$t(5) = 4.84$, $p <.01$], anxiety [$t(8) = 2.79$, $p <.05$], perceived stress [$t(9) = 3.03$, $p <.05$], well-being [$t(8) = -3.90$, $p <.01$], and trait mindfulness [$t(9) = -6.01$, $p <.001$]. Study findings support mindfulness for symptom reduction and improved mental health and well-being in older adults, and provide a better understanding of subjective benefits to participating in mindfulness groups. Thematic results indicated that older adults value mindfulness practice and appreciate the shared experience of learning and practicing with their peers.

Abstract citation ID: igae098.4241

### MISREMEMBERING SOLITUDE IN OLDER AGE: AGE AND CULTURE SHAPE RECALL ACCURACY FOR LONELINESS AND HAPPINESS IN SOLITUDE

Jennifer Lay[1], Yuen Wan HO[2], Dwight Tse[3], and Da Jiang[4], *1. University of Exeter, Exeter, England, United Kingdom*, *2. Lingnan University, Hong Kong, Guangdong, China*, *3. University of Strathclyde, Glasgow, Scotland, United Kingdom*, *4. Education University of Hong Kong, Hong Kong, China (People's Republic)*

Recalling past affective experiences is key to guiding behaviour – including social and solitary activities – in ways that maximise wellbeing. However, affect recall is not always accurate. Previous research suggests that older (vs. younger) adults show a positivity effect in memory, depending on whether the stimuli are culturally meaningful. It is unclear, however, whether this age-related positivity effect applies to recalling experiences of solitude (time without social contact), which become increasingly prevalent in older adulthood. An age-stratified sample of adults aged 18-81 (M age = 40.8 years, 60.8% female) in the United Kingdom ($N = 214$) and Hong Kong ($N = 220$) reported their current affective experiences and social situations 5 times per day over 7 days using a smartphone app. Participants then recalled how they felt on average over the 7-day period at times when they were in solitude. Models controlling for peak and recent affect, overall time in solitude, and independent/interdependent self-construal showed that whereas younger adults tended to retrospectively overestimate how lonely they felt in solitude, this effect reversed with age such that older adults tended to underestimate their loneliness in solitude. Moreover, whereas UK older adults tended to retrospectively overestimate their happiness in solitude, HK older adults tended to underestimate their happiness in solitude. Findings suggest individuals misremember their solitude experiences in ways that align with age-normative and cultural attitudes to solitude, and that there is an age-related positivity effect which is shaped by culture. We discuss implications for loneliness and wellbeing in older adulthood.

Abstract citation ID: igae098.4242

### MOBILE HEALTH AND RESOURCE FAIRS TO ADDRESS SOCIAL DETERMINANTS OF HEALTH OF LOW-INCOME OLDER ADULTS

Angie Sardina, Jeffrey Meadows, Lena Simon, Marsha Hampton, Christine Phillips, Lesley Ross, and Alyssa Gamaldo, *Clemson University, Clemson, South Carolina, United States*

Mobile Health and Resource Fairs (MHRFs) were designed to provide resources, education, and social engagement to low-income communities through reducing traditional barriers and constraints for older adults. We (1) explored effective marketing approaches and rationale for resident attendance; (2) examined satisfaction and expectations of the MHRFs, and (3) examined the likelihood for attending future events. MHRF events comprised 2 sites in Greenville, SC. Ninety-four participants (ages 50-90+) registered (site 1=77%; site 2=23%). Participants were asked to complete survey items on how they learned of the event and their reason for attending (aim 1). At the end of the MHRF, participants reported on event expectations and satisfaction (aim 2) using a Likert scale ranging from 0 (not all) to 10 (very much), and the likelihood of attending future events (aim 3) using a single Likert-Scale question ranging from 0 (very unlikely) to 4 (very likely). Attendees were predominately female (64%), Black/African American (73%), with a high school education (55%). Effective marketing approaches (aim 1) were event flyers (62%), other (14%; e.g., MHRF-specific staff/activities or housing activities/advertisements), walking by the event (11%), family/friends recommendations (9%), and housing staff recommendations (5.3%). Attendance rationale included obtaining healthy-aging information (49%), social engagement (28%), access to necessities (19.1%); and volunteerism (16%). Participants reported high expectations (M=9.72) and satisfaction (M=9.58) with the MHRF. Participants reported they were "somewhat-very likely" to attend future events (83%, aim 3). These findings affirm MHRFs can successfully provide critical health/social service resources, while simultaneously engaging older adults from underserved lower-income communities.

Abstract citation ID: igae098.4243

### MODIFIABLE DEMENTIA RISK FACTORS: THE EFFECTS OF BEHAVIOR MODIFICATION ON PERCEPTIONS OF AN OLDER ADULT AT RISK

Karli Poettcker, and Molly Maxfield, *Arizona State University, Phoenix, Arizona, United States*

Alzheimer's disease and related dementias are progressive and have significant effects on those diagnosed and their caregivers. Increasing emphasis on modifiable lifestyle factors to reduce dementia risk (e.g., physical activity, managing blood pressure, blood sugar, and weight) offers a sense of personal control and self-efficacy. Potential disadvantages include negative perceptions of individuals who do not modify their behaviors and labeling of dementia as a "lifestyle disease." We examined whether behavior modification to reduce dementia risk affects attributions of responsibility and capability. In an online study, 242 participants (18-64 years, Mage=41.28, SDage=12.60) read a vignette describing 'Patrick's' concerns about cognitive decline as reported to his physician. Although determined to be normal age-related change, the physician recommended exercise and diet changes to reduce Patrick's dementia risk by improving cardiovascular health. Participants were randomly assigned to read about 'Patrick,' who either did or did not follow recommended lifestyle changes. Participants then rated Patrick's responsibility for his current condition, capability for basic tasks, their willingness to socialize with him, and emotional reactions he provoked. Multivariate analysis of variance revealed that not modifying behavior led to rating Patrick as more responsible for his condition and less capable of decision-making compared to Patrick with behavior change; additionally, reading about Patrick without behavior modification resulted in lower willingness to socialize and greater levels of negative emotional reactions compared to Patrick with behavior modification (ps <.05). Findings highlight how behavior modification affects perceptions, suggesting the need for careful public health messaging about dementia risk and lifestyle modifications.

Abstract citation ID: igae098.4244

### NAVIGATING HOSPICE CARE: PERSPECTIVES FROM OLDER BLACK AMERICANS

Channing Tate[1], Monica Perez-Jolles[1], Laura Schere[1], Tsion Shiferaw[1], Gwendolyn Mami[2], Daniel Matlock[1], and Amy Huebschmann[1], *1. University of Colorado AMC, Aurora, Colorado, United States*, *2. Global Collaborations, LLC, Aurora, Colorado, United States*

Misperceptions about hospice care are universally prevalent and disproportionately impact communities of color. Hospice remains an underutilized healthcare service, especially among older Black Americans. Potential cultural barriers to enrollment among Black Americans are important to identify. The study goal was to assess older Black adults' perspectives of hospice care across four dimensions of hospice eligibility. A total of 144 participants responded to the written prompt, "In your own words, describe hospice care." Written responses were evaluated to assess any misconceptions in their perspectives of hospice care. Participants were eligible if they were ≥65 years of age and self-identified as Black or African American. Participant ages ranged from 65 to 97 years (M =74.62, SD = 6.94), were predominately female (81%) and widowed (33%). Most participants accurately described hospice care/eligibility (80%), goals of hospice (89%), what services hospice provides (83%). However, only 39% of participants correctly identified locations of hospice services highlighting a potential barrier to hospice enrollment among older Black Americans. Additionally, some participants (8%) reported certain myths and conspiracies pertaining to hospice care, such as "hospice was created by the KKK". These results highlight potential misunderstandings about hospice among older Black Americans that may serve as barriers to hospice enrollment and perpetuate inequities. These barriers go beyond hospice knowledge and include misconceptions and conspiracies rooted in medical mistrust and structural racism. The misconception that hospice care cannot be provided in the patients' home is modifiable with interventions that use strategies tailored to support enhanced communication between patients and clinicians.

Abstract citation ID: igae098.4245

### NON-PHARMACOLOGICAL DEMENTIA CARE APPROACHES: IMPLEMENTING STAR-VA IN A SHORT-STAY, SUBACUTE MEDICAL SETTING

Jennifer Christopher[1], Theresa Christopher[2], and Thomas Yuelling[1], *1. Jesse Brown VA Medical Center, Chicago, Illinois, United States*, *2. North Central College, Naperville, Illinois, United States*

Staff Training in Assisted Living Residences for Veterans Affairs Medical Centers (STAR-VA) is an evidenced-based, interdisciplinary, non-pharmacological approach to managing dementia-related behavioral and psychological symptoms in long-term care settings (Karlin et al., 2017). The program involves a psychologist and nurse who provide dementia education, facilitate behavioral analysis, and implement person-centered behavior plans. This study highlights the piloting of STAR-VA in a short-stay, subacute, medical rehabilitation setting. The case study involves a 74-year-old African American, male, Veteran diagnosed with Dementia due to Multiple Etiologies (Frontotemporal Degeneration and Vascular Disease). The Veteran exhibited elopement attempts, refusal to follow directions, falls, verbal aggression, and emotional distress. Behavioral analysis suggested these were related to lack of independence, boredom, psychosocial stressors, and medication side effects. Behavior plan interventions included individual psychotherapy, scheduled recreation therapy sessions focused on preferred pleasant activities, and chaplain services. The clinical pharmacist reviewed the medication list and psychiatry and general medicine providers adjusted medications. Care providers were encouraged to use effective nonverbal and verbal communication strategies, including the "listen with respect, comfort, and redirect" approach. After two months, assessments (i.e., Neuropsychiatric Inventory Questionnaire, Cohen-Mansfield Agitation Inventory–Short Form, Geriatric Depression Scale–Short Form) revealed discontinued elopement attempts, compliance with care, improved mood, reduced falls, and decreased nursing staff distress. These interventions facilitated the Veteran's transition to long-term community placement. Six-month follow-up assessments revealed the Veteran remained behaviorally stable. This case demonstrated that STAR-VA can be effectively adapted for short-stay, subacute medical rehabilitation settings. It further supports interdisciplinary, non-pharmacological approaches for dementia.

Abstract citation ID: igae098.4246

### OLDER ADULTS WHO PLAY VIDEO GAMES: WHO ARE THEY AND HOW DO THEY FARE?

Amanda Sonnega[1], and John Sonnega[2], *1. University of Michigan, Ann Arboar, Michigan, United States*, *2. Eastern Michigan University, Plymouth, Michigan, United States*

Video games for leisure are often considered the purview of younger adults. When older adults are the focus of video game use it is often for rehabilitation or prevention. Growing literature explores the potential of video games to improve cognition and health in older adults but very little that is population-based. Available literature shows that fewer older adults engage in gaming when compared to younger adults but that the older adults play with a similar frequency to younger populations. Internet use provides psychosocial benefits to older adults, and we propose that gaming for leisure does so as well. We use data from the Health and Retirement Study (HRS), a biennial survey of a nationally representative sample of adults over age 50 in the United States begun in 1992. HRS, in an add-on module, asked respondents if they played video games such as X-box or PlayStation (n=1,734). We describe the socio-demographics and other individual differences of respondents who report playing video games. We find that those who report using video games are more likely to be male (64%) and younger than those who do not report using these games (mean age of gamers compared to nongamers is 62 year v. 74 years; p<.01). The gender gap shrinks as adults age. We subsequently examine the relationships between gaming and psychosocial outcomes, and find no difference between gamers and nongamers in depressive symptoms (p=.12). Interestingly, gamers have slightly higher cognitive scores (p<.02) longitudinally. We discuss the potential role of gaming in retirement.

Abstract citation ID: igae098.4247

### OPTIMIZING VOLUNTARY WORK DESIGN FOR HEALTHY AGING: RETHINKING VOLUNTEER ENGAGEMENT

Shiyu Lu, *The University of Hong Kong, Hong Kong, China (People's Republic)*

While existing research primarily focuses on the health benefits of volunteering in terms of quantity (e.g., participation or volunteer hours), the psychosocial aspects of role engagement in volunteering and its impact on healthy aging remain underexplored. Additionally, insights on establishing a supportive volunteering environment at the organizational level to facilitate healthy aging are lacking. This innovative study connected voluntary work design, role engagement in volunteering, and subjective well-being (SWB) among older adults. In an ongoing cross-sectional study involving 258 volunteers aged 60 and above in Hong Kong, the research revealed that the role engagement of volunteers ($\beta = 0.08$, p = 0.014) significantly correlated with SWB, while no significant association was observed between volunteer hours in the past 12 months and SWB ($\beta = 0.001$, p = 0.297), after adjusting for factors like age, gender, and cognitive function. Introducing elements of voluntary work design, such as task significance, organizational support, and autonomy, into the model unveiled a direct link between organizational support and improved SWB ($\beta = 0.15$, p = 0.003). Task significance (0.32, p =0.028) and organizational support ($\beta = 0.04$, p = 0.016) were further found to influence SWB through the role engagement of volunteering. The study underscores both theoretical and practical implications concerning the creation of a supportive volunteering environment conducive to healthy aging.

Abstract citation ID: igae098.4248

### ORAL HEALTH, PERCEIVED STRESS, AND SUBJECTIVE MEMORY COMPLAINTS ACCORDING TO DIABETES STATUS

Min Jung Kim, *Gachon University, Incheon, Republic of Korea*

Oral health status has been linked to cognitive impairment. People with diabetes often experience more oral health issues and higher emotional stress than those without, that may increase the risk of cognitive impairment. This study explored the relationship between oral health, perceived stress, and subjective memory complaints (SMC), and whether these relationships differed by diabetes status, using data from the 2021 Korean National Health and Nutrition Examination Survey. Perceived oral health, stress levels, and SMC were assessed via questionnaires, and the number of natural teeth was counted during exams. Tobit regression models were used, with subgroup analyses by diabetes status. Among 1,444 adults, after controlling for demographic factors, perceived oral health ($r = 0.16$, $p < .05$) and stress levels ($r = 0.16$, $p < .05$) significantly increased SMC in healthy adults ($n = 504$). However, only stress levels increased SMC in those with prediabetes ($n = 602$, $r = 0.29$, $p < .001$) and diabetes ($n = 260$, $r = 0.19$, $p < .05$). The number of natural teeth did not affect SMC in any group. These findings suggest perceived oral health may indicate early cognitive decline in healthy adults, while psychological status is a more critical factor in those with prediabetes or diabetes. These results suggest that perceived oral health may be an early indicator of cognitive decline in healthy adults, while psychological health plays a more dominant role in cognitive decline in adults with prediabetes or diabetes. Tailored interventions to prevent cognitive decline should consider diabetes status.

Abstract citation ID: igae098.4249

### PICOLINIC ACID, A CATABOLITE OF TRYPTOPHAN, HAS AN ANABOLIC EFFECT ON MYOBLASTS AND INCREASES MOBILITY IN C. ELEGANS

Gustavo Duque[1], Daniel Rivas[1], Abdelrahman AlOkda[1], Ivan Baltasar-Fernandez[2], and Jeremy Van Raamsdonk[1], *1. Research Institute of the McGill University Health Centre, Montreal, Quebec, Canada*, 2. *University of Castilla-La Mancha, Toledo, Castilla-La Mancha, Spain*

Osteoporosis, sarcopenia, and osteosarcopenia (simultaneous occurrence of these diseases) are highly prevalent in older persons and associated with falls and fractures. Compounds with dual anabolic effects on muscle and bone could become the treatment of choice for these conditions. We have previously reported the anabolic effect of picolinic acid (PIC) on bone in vitro and in vivo. However, its effect on muscles remains to be elucidated. Therefore, the aim of the study was to examine the effect of PIC on muscle. C2C12 myoblasts were treated with PIC (1, 50 and 100 µM) or vehicle for 7 days. Transcription factors involved in myogenesis were evaluated by western blot, and the fusion index and myotubules' length were calculated in confocal microscopy images at timed intervals (Day 1, 3, 5 and 7). In addition, C. elegans were treated with 1mM PIC, and the frequency of thrashing (mobility) was evaluated at timed intervals (Day 1, 8 and 16). PIC-treated myoblasts showed a higher and earlier expression of myosin II and MyoD transcription factors ($p < 0.001$). On day 5, PIC-treated myotubes were significantly longer when treated with 50 µM (+80.7 µm, $p=0.001$) and 100 µM (+73.0 µm, $p=0.002$) than vehicle-treated controls. The frequency of thrashing was also significantly increased in the PIC-treated C. elegans at all timed intervals ($p < 0.001$). In conclusion, this study demonstrates that, in addition to its anabolic effect on bone, PIC also has an anabolic effect on muscle, which opens up exciting possibilities for further exploration in animal models and future geroscience research.

Abstract citation ID: igae098.4250

### PROTEASOME DYSFUNCTION A KEY PATHOLOGIC FEATURE OF ALZHEIMER'S DISEASE MITIGATED BY PROTEASOME ACTIVATORS

Andrew Pickering, *University of Texas Health Science Center at Houston, Houston, Texas, United States*

Alzheimer's Disease (AD) is the leading cause of dementia worldwide, affecting nearly 50 million people and characterized by cognitive decline, β-amyloid plaques, and hyperphosphorylated tau. The failure of numerous clinical trials targeting β-amyloid (Aβ) underscores the need for novel therapeutic strategies. A consistent feature of AD, particularly in the hippocampus, is reduced proteasome activity. This impairment is thought to result from direct inhibition by Aβ or hyperphosphorylated tau, disrupting essential neuronal processes like memory and synaptic plasticity. This study explores the hypothesis that AD-related deficits are driven, in part, by Aβ-induced proteasome dysfunction. We demonstrate that oligomeric Aβ inhibits 20S proteasome activity while destabilizing the 26S proteasome into free 20S units. Treatment with proteasome activators enhances 20S and 26S function, reducing Aβ42-induced toxicity in SK-N-SH cells. In Drosophila overexpressing Aβ42, oral administration of proteasome agonists delayed mortality and restored cognitive function. Similarly, in mouse models, chronic treatment with proteasome activators protected against Aβ42-induced working memory deficits, and acute treatment significantly improved spatial learning deficits in hAPP(J20) mice. Our findings reveal that Aβ exerts dual inhibitory effects on 20S and 26S proteasomes, contributing to AD pathology. Proteasome activation mitigates these deficits, suggesting that enhancing proteasome activity could be a promising therapeutic approach for AD.

Abstract citation ID: igae098.4251

### PSYCHOTHERAPY AND CARE QUALITY OF LONG-STAY NURSING HOME RESIDENTS WITH MENTAL ILLNESS OR DEMENTIA

Tianwen Huan[1], Orna Intrator[2], Adam Simning[1], Kenneth Boockvar[3], David C. Grabowski[4], and Shubing Cai[1], *1. University of Rochester, Rochester, New York, United States*, 2. *Icahn School of Medicine at Mount Sinai, New York, New York, United States*, 3. *University of Alabama, Birmingham, Alabama, United States*, 4. *Harvard Medical School, Boston, Massachusetts, United States*

This study aims to fill the knowledge gap on the effect of psychotherapy on the quality of care of long-stay nursing home (NH) residents with mental illness or Alzheimer's Disease and Related Dementias (ADRD). The 2017-2018 MDS and Medicare claims (e.g., Carrier file) and Residential History File were used to identify mental illness and ADRD diagnoses, patient characteristics, psychotherapy and quality of care. Fee-for-service Medicare beneficiaries aged 65 and older enrolled in Medicare Parts B who had six quarterly MDS assessments with/ without psychotherapy in the last four quarters were compared on two subgroups of residents: those with mental illness but no ADRD and those with ADRD. Five quarterly outcomes were examined: any 1) physical behavior; 2) verbal behavior; 3) other behavior; 4) hospitalization; 5) emergency-room visits. Covariates included predisposing, enabling characteristics and need factors. Propensity score matching was used to balance covariates between treatment and control groups. Difference-in-difference models of quarterly data, clustered within patients, were estimated for each outcome among each cohort. Analyses included 525,114/180,000 resident-quarter observations from 87,519/30,000 unique NH long-stay residents with ADRD/mental-illness-only. Among the ADRD/mental-illness-only group, 579/469 unique residents received psychotherapy. Psychotherapy was associated with lower likelihood of hospitalization (Odds ratio: 0.73, 95%CI: 0.53, 0.99) in residents with mental-illness-only. No significant results were found for the other outcomes nor the ADRD group. Findings suggest that psychotherapy may have limited effects on the quality of care for residents with ADRD but may contribute to lower hospitalization risk among residents with mental-illness-only.

Abstract citation ID: igae098.4252

### RACE DIFFERENCES IN WALK SPEED, CARDIORESPIRATORY FITNESS, AND MUSCLE MITOCHONDRIAL RESPIRATION

Paul Coen[1], Giovanna Distefano[1], Sofhia Ramos[2], Peggy Cawthon[3], Steven R Cummings[3], Anne B Newman[4], and James DeLany[1], *1. AdventHealth, Orlando, Florida, United States*, *2. AdventHealth, AdventHealth, Florida, United States*, *3. California Pacific Medical Center, San Francisco, California, United States*, *4. School of Public Health, University of Pittsburgh, Pittsburgh, Pennsylvania, United States*

Older adults who self-identify as Black have a disproportionately higher incidence of mobility disability. Whether older adults who are Black also have lower fitness and mitochondrial energetics compared to those who are White has not been adequately investigated. The Study of Muscle, Mobility and Aging (SOMMA) examined 879 older adults (≥70 years old), including 116 who self-identified as Black, who could complete a 400m walk within 15 minutes. Mitochondrial respiration (MaxOXPHOS) was measured in permeabilized fibers from biopsies of the vastus lateralis. A cardiopulmonary exercise test assessed maximum oxygen consumption (VO2peak). Education, income, financial resources, race, sex and age were determined by self-report. Propensity score matching was used to match Blacks with Whites with a 1:1 ratio. Black (n=90) and White (n=90) groups were well matched for age, sex, SOMMA multimorbidity index, BMI, muscle mass, physical activity, marital status, educational achievement, and whether financial needs were met (all p>0.05). Despite being well matched, those who identified as Black had a slower 400m walking speed (0.97 vs. 1.03 m/s, p=0.014), lower MaxOXPHOS (50.8 vs. 60.9 (pmol/(s*mg)), p< 0.001), and lower cardiorespiratory fitness (1391 vs 1566 ml/min, p=0.007), when compared to those who identified as White. Multivariate regression showed that VO2peak and MaxOXPHOS, but not socioeconomic factors, attenuated the race difference in 400m walking speed. In conclusion, while the etiology of race differences in mobility is multifactorial, our data indicate that muscle mitochondrial respiration and cardiorespiratory fitness may contribute to slower walking speed of individuals who identify as Black compared to White.

Abstract citation ID: igae098.4253

### RURAL OLDER ADULT CARE TRANSITIONS: A SCOPING REVIEW

Amiritha Kumar, and Daniel Liebzeit, *The University of Iowa, Iowa City, Iowa, United States*

Older adult (age 65 and older) care transitions are frequent and complex, particularly in rural areas lacking equitable access to health and other services. This scoping review: 1) evaluates research on older adult rural care transitions and 2) synthesizes types of transitions studied, challenges and opportunities in rural health care, and gaps in the literature. Of a total of 6,976 articles retrieved in July 2024 from PubMed, CINAHL, PsycINFO, Dissertations and Theses, AgeLine, and Embas, 91 articles were included. Eligible articles focused on older adults, rural setting, a care transition, and provided empirical data. Findings revealed a primary focus on acute care transitions; care transitions involving long-term care and assisted living were vastly underrepresented. Most were observational, with few interventions to address the specific challenges faced by rural older adults. The evidence base on acute care transitions reveals disparities in readmission rates between rural and other populations, with limited focus on patient-centered outcomes. Limited literature on long-term care and assisted living transitions primarily focused on losses experienced, such as loss of independence. Although improved outcomes are often associated with collaborative, individualized planning, significant gaps remain in application of effective models of transitional care. Follow-up or post-acute care appears key for rural older adults, offering benefits comparable to urban settings; however, rural access barriers underscore the need for intervention development to reduce health disparities. Developing patient-centered interventions for rural older adults during care transitions is crucial, and addressing research gaps will create opportunities to further reduce disparities and improve outcomes.

### SESSION 8025 (PAPER)

### LATE BREAKING: ALZHEIMER'S DISEASE, INCLUDING ALZHEIMER'S DISEASE—RELATED DEMENTIAS I

Abstract citation ID: igae098.4254

### ENHANCING DEMENTIA CLASSIFICATION FOR DIVERSE GROUPS: TRANSFORMER-BASED CONTINUOUS SCORING OF CLOCK DRAWING TEST

Mengyao Hu[1], Yi Murphey[2], Tian Qin[2], Laura Zahodne[3], Richard Gonzalez[3], and Vicki Freedman[3], *1. UTHealth at Houston, Houston, Texas, United States*, *2. University of Michigan-Dearborn, Dearborn, Michigan, United States*, *3. University of Michigan, Ann Arbor, Michigan, United States*

Alzheimer's disease and related dementias (ADRD) significantly impact older adults' quality of life and pose challenges to public health systems. The clock-drawing test (CDT) is a widely used dementia screening tool due to its ease of administration and effectiveness. However, manual CDT-coding in large-scale studies can be time-intensive and prone to coding errors. In this study, we developed a deep learning neural network (DLNN) system based on Vision Transformer (ViT) to automate CDT-coding using continuous scoring and investigate its value for dementia classification, compared to ordinal CDT scores. Using a nationally representative sample of older adults from the National Health and Aging Trends Study (NHATS), we trained ViT models on CDT images to generate both ordinal and continuous scores. We compared the predictive power of these scores for dementia classification and identified demographic-specific thresholds. Continuous CDT scores provided more precise thresholds for dementia classification than ordinal scores, varying by demographic characteristics. Lower thresholds were identified for Black individuals, those with lower education, and those aged 90 or older. Compared to ordinal scores, continuous scores also allowed for a more balanced sensitivity and specificity. This study demonstrates the potential of continuous CDT scores generated by DLNN models in enhancing dementia classification. By identifying demographic-specific thresholds, it offers a more inclusive and adaptive approach for dementia classification. Results of this study could lead to improved guidelines for using CDT in dementia screening, allowing more accurate dementia classification across diverse demographic groups.

Abstract citation ID: igae098.4255

### IMPLEMENTING AND EVALUATING MULTILINGUAL, COMPETENCY-BASED DEMENTIA TRAINING FOR IHSS CAREGIVERS IN CALIFORNIA

Jarmin Yeh[1], Matthew Beld[1], Brittney Pond[1], Mel Neri[1], Suzanna Martinez[1], Andrea Garcia[2], Moraima Castaneda[2], and Corinne Eldridge[2], *1. University of California, San Francisco, San Francisco, California, United States*, *2. Center for Caregiver Advancement, Los Angeles, California, United States*

The rising prevalence of Alzheimer's dementia in California's aging population underscores the urgent need for a well-trained direct care workforce to help older adults age in place. This study implemented and evaluated competency-based dementia training for caregivers in California's Medicaid-Funded In-Home Supportive Services (IHSS) program from 2019 to 2024. The program, offered in English, Spanish, Cantonese, and Mandarin, transitioned from in-person to online delivery due to COVID-19, providing an opportunity to assess this novel online approach. Using a quasi-experimental, mixed-methods design guided by the RE-AIM framework (Reach, Effectiveness, Adoption, Implementation, and Maintenance), we evaluated training outcomes, curriculum, staff roles, and delivery logistics. Of 722 caregivers who completed the training, 488 were included in the analytic sample, and 50 participated in interviews. Additionally, 11 key informant stakeholders were interviewed. Mean scores from validated instruments evaluating caregivers' dementia knowledge (Toye et al., 2014) and self-efficacy (Fortinsky et al., 2002) showed statistically significant increases between pre-post-training and pre-3-month follow-up ($p< 0.001$). Thematic analysis of caregiver interviews revealed diverse pathways into caregiving, emotional rewards, enhanced advocacy skills, and improved interactions with medical providers. Stakeholder interviews uncovered implementation barriers and facilitators to intervention fidelity, and strategies for addressing the critical need for a skilled dementia direct care workforce. The training's adaptability to online formats and multiple languages demonstrates its real-world efficacy and potential readiness for trial design. This study highlights the importance of evidence-informed, culturally and linguistically appropriate training for IHSS caregivers in managing the health and well-being of older adults living with Alzheimer's dementia.

Abstract citation ID: igae098.4256

### PSYCHIATRIC DISORDERS IN EARLY-ONSET VS. LATE-ONSET DEMENTIA: AN ANALYSIS OF THE ALL OF US RESEARCH PROGRAM

Xiang Qi[1], Ana-Maria Vranceanu[2], and Bei Wu[1], *1. New York University, New York, New York, United States*, *2. Massachusetts General Hospital, Boston, Massachusetts, United States*

Early-onset dementia (EOD), defined as being diagnosed with dementia before age 65, affects approximately 200,000 Americans. While EOD is characterized by an earlier and more severe onset, late-onset dementia (LOD) patients have longer exposure to potential risk factors for psychiatric disorders. This study investigates the relationship between EOD and psychiatric disorders, comparing it to LOD and individuals without dementia. We conducted a retrospective cohort study using electronic health records of 286,826 participants from the NIH All of Us Research Program. This diverse cohort included 59.7% women (mean age 52.3). Using multivariable logistic regression models, we found that compared to LOD, individuals with EOD showed increased risks of depression (OR 1.47, 95% CI 1.23-1.76), anxiety (OR 1.47, 95% CI 1.23-1.75), bipolar disorder (OR 2.63, 95% CI 2.04-3.38), schizophrenia (OR 2.76, 95% CI 1.96-3.88), and substance use and addiction (OR 1.86, 95% CI 1.96-3.88). The differences were more pronounced when comparing EOD to the no dementia group, with notably higher odds for depression (OR 4.82, 95% CI 4.37-5.33), anxiety (OR 3.79, 95% CI 3.30-4.36), bipolar disorder (OR 3.42, 95% CI 2.90-4.03), schizophrenia (OR 3.48, 95% CI 3.03-4.00), and substance use and addiction (OR 2.92, 95% CI 2.64-3.22). These findings persisted after controlling for sociodemographic characteristics, lifestyle factors, functional ability, and chronic conditions. Our results highlight the need for comprehensive mental health screening and targeted interventions for individuals with EOD. This study emphasizes the importance of early detection and specialized care for this vulnerable population, potentially informing healthcare policies and improving patient outcomes.

Abstract citation ID: igae098.4257

### SEE SOMETHING, SAY SOMETHING: A QUALITATIVE STUDY OF NEIGHBORHOOD PERCEPTIONS AND BRAIN HEALTH

Sierra Heuer[1], Lilah Besser[2], Briana Dominguez[1], Stephanie Huynh[1], Bao Ngoc Le[1], Boi-san Nguyen[1], Jason Pham[1], and Oanh Meyer[1], *1. University of California, Davis, Sacramento, California, United States*, *2. University of Miami, Boca Raton, Florida, United States*

Studies have shown the importance of neighborhood factors, such as residential segregation and green spaces, to older adult brain health. In this qualitative study, we examine older adults' perceptions of their neighborhood, with a particular focus on segregation. We recruited participants from the University of California Davis Alzheimer's Disease Research Center and the local Northern California community to participate in semi-structured interviews over Zoom or telephone. They answered questions such as, "What kinds of activities do you do in your neighborhood?" and "Are neighbors segregated or separated based on age, race/ethnicity, SES, or language?" On average, interviews lasted 22.5 minutes, and were audiotaped, transcribed verbatim, and analyzed for recurring themes. Among the 32 participants, 62.5% were female, 59.4% non-Hispanic White, and 40.6% Black/African American. Mean age was 79.0 years (SD=8.3). The most prominent themes were (1) Accessibility to public stores and similar third spaces (e.g., churches, libraries, coffee shops); (2) Accessibility to green space, including parks, trees, etc.; (3) Staying active in the community, both physically and socially; (4) Neighborhood awareness/cohesion; and (5) Stereotypes, discrimination, and segregation, a sample quote being, "I'm happy with the diversity of the neighborhood…a lot of people would say it's not very diverse, it's…segregated, mostly economically," (Male, 73 years-old). These findings highlight the social and built environment factors that are salient for older adults and may potentially affect brain health. Future research will examine how these themes might differ depending on older adults' cognition and with objective measures of the neighborhood.

Abstract citation ID: igae098.4258

### SOCIAL NETWORK BRIDGING AND COGNITIVE RESERVE IN SPECIFIC DOMAINS: BENCHMARKING TO EDUCATIONAL ATTAINMENT

Haosen Sun[1], and Brea Perry[2], *1. University of Nevada, Reno, Reno, Nevada, United States*, *2. Indiana University, Bloomington, Indiana, United States*

Cognitive reserve helps delay and slow cognitive decline despite neuropathology, with diverse social networks that have rich bridging potential enhancing this reserve. However, the benefits to different cognitive domains from such networks and the relative impact compared to other known resiliency factors are unclear. This study aims to identify which domain-specific cognitive reserve benefits most from social networks with higher bridging capital, with a comparison with education. Data from 217 participants in the Social Networks in Alzheimer's Disease (SNAD) project were analyzed. Cognitive reserve was assessed using the residual approach, comparing cognitive domain performance against brain structure metrics from MRI. Social network bridging capital was measured through network characteristics such as structural holes, diversity, weak ties, and network size. Linear regressions examined the association between bridging capital and cognitive reserve across cognitive domains, comparing the effect size to educational attainment. Network bridging capital was significantly associated with general cognitive reserve and cognitive domains such as executive function, visuospatial skills, language, and episodic memory. The impact of bridging capital was comparable to or greater than that of educational attainment in these domains. However, it did not significantly enhance cognitive reserve in attention and processing speed, where education had a stronger influence. Overall, social networks with high bridging potential are particularly beneficial for enhancing cognitive reserve in certain domains. Interventions aimed at slowing cognitive decline in these areas could leverage diverse networks, especially early in disease trajectories, constituting a late-life modifiable protective factor.

Abstract citation ID: igae098.4259

### WORK TOLL: AN INTERSECTIONAL ANALYSIS ON ABSENTEEISM AND PRESENTEEISM AMONG FAMILY CAREGIVERS FOR PLWD

Mary Milnamow, *University at Buffalo, Buffalo, New York, United States*

Working family caregivers for PLwD experience higher rates of work productivity loss—absenteeism and presenteeism—compared to their non-caregiving counterparts. Accessible workplace supports remain out of reach for many caregivers making them vulnerable for compensation loss. Only recently has an intersectionality approach been used in quantitative research to address persistent inequalities across disciplines. This cross-sectional study examined 1) relationship between caregivers' intersecting social identities and access to flexible hours; and 2) extent caregivers' intersecting social identities impact experiences of absenteeism and presenteeism. Dyads (n=891) from 2017 wave of National Health and Aging Trends Study (NHATS) and National Survey of Caregivers (NSOC) were analyzed using the validated WPAI-CG measure. Mixed-effect logistic regression models and generalized linear models were conducted. Findings suggest caregivers with one full-time job were less likely to have flexible hours compared to caregivers with part-time or multiple jobs ($p<.05$). Black female caregivers were more likely to have flexible hours compared to Black male caregivers (OR=9.3, p=.024), but the opposite relationship was found for White caregivers (OR=0.19, p=.002). Caregivers who were female, post-HS education, with flexible hours, and multiple health conditions had greater presenteeism ($p<.05$). Caring for someone with dementia was associated with greater presenteeism ($p<.05$). Caregiving situations vary drastically. Flexible hours are popular but may not adequately address work productivity loss; furthermore, findings suggest disparate access to them. As the number of PLwD is expected to rise over the next 25 years, policies and workplaces need to consider inclusive solutions to meet the needs of working family caregivers.

### SESSION 8030 (PAPER)

### LATE BREAKING: QUALITY IMPROVEMENT INNOVATIONS IN HEALTH CARE I

Abstract citation ID: igae098.4260

### CMS AND ASTP: FEDERAL PARTNERSHIPS FORGED TO SUPPORT THE DEVELOPMENT OF INTEROPERABLE E-CARE PLANS.

Elizabeth Palena Hall[1], and Michael Wittie[2], *1. The Centers for Medicare and Medicaid Services, Baltimore, Maryland, United States*, *2. Assistant Secretary for Technology*

*Policy and Office of the National Coordinator for Health Information Technology (ASTP/ONC), Washington, District of Columbia, United States*

Across the United States, more than 25% of Americans have multiple chronic conditions that often impact quality of life, mortality, complexity of care and cost. The need for seamless interdisciplinary communication and data driven decision-making continues to grow, however, health care often remains fragmented, poorly coordinated and inefficient. A comprehensive, shared, interoperable e-care plan has the potential to improve patient outcomes, satisfaction, and quality of care by making comprehensive health data available across providers and settings when and where it is needed most. At the Federal level, the Centers for Medicare & Medicaid Services (CMS) and the Assistant Secretary for Technology Policy/Office of the National Coordinator for Health IT (ASTP) are engaged in efforts to improve interoperable e-care plans. This session will describe the current state of e-care plans and challenges, promising practices from the field, and opportunities for advancement. This presentation will discuss how ASTP programs interact with CMS' work to support an e-care plan environment where patients' care plan information is securely and readily available to support patient outcomes. The session will include an overview of updates and proposals to ASTP's Health IT Certification Program, a description of how the United States Core Data for Interoperability (USCDI) and USCDI+ Program can support the collection and exchange of e-care plans with the care team and patients, support quality measurement and quality reporting programs, and highlight how interested parties can engage in ongoing standards and policy development.

Abstract citation ID: igae098.4261

### ENHANCING AGE-FRIENDLY CARE IN VALUE-BASED HEALTH SYSTEMS: CMS DELIRIUM ASSESSMENT AND POLICY STRATEGY

Tara McMullen, Shari Ling, and Lenise Cummings-Vaughn, *Centers for Medicare & Medicaid Services, Baltimore, Maryland, United States*

Delirium is a clinical syndrome characterized by alteration in consciousness and cognitive function, frequently observed in older adults. This condition is associated with a risk of mortality and can lead to severe and persistent functional impairments in survivors. The incidence of delirium is notably high, affecting up to 80% of older adults in intensive care units (ICUs) and up to 35% of those with general inpatient admissions. Routine and standardized cognitive assessments are essential for optimizing patient management and implementing delirium prevention strategies aimed at mitigating risk factors. To enhance person-centered care in delirium management, it is crucial to adopt age-friendly care frameworks that emphasize evidence-based approaches to assessment and management. In this presentation, Federal officials from the Centers for Medicare & Medicaid Services (CMS) will address the ongoing clinical and policy initiatives aimed at developing a comprehensive strategy for the screening and assessment of delirium, integrated within the framework of high-quality, age-friendly healthcare. This presentation will explore the essential attributes that health systems must possess to effectively translate age-friendly principles into improved health outcomes for patients with delirium. Officials will also discuss the development of age-friendly measures that address the specific needs of older adults to enhance both individual outcomes and population-level health. Additionally, the discussion will focus on policy levers that should be considered to promote age-friendly, value-based care, thereby improving the screening and prevention of delirium.

Abstract citation ID: igae098.4262

### ENHANCING HEALTHCARE VALUE THROUGH INTEROPERABLE E-CARE PLANS: A FRAMEWORK FOR QUALITY IMPROVEMENT

Arlene Bierman[1], Shari Ling[2], and Johanthan Nebeker[3], *1. Agency for Healthcare Research and Quality, Rockville, Maryland, United States*, *2. Centers for Medicare & Medicaid Services, Baltimore, Maryland, United States*, *3. Department of Veterans Health Affairs, Washington, District of Columbia, United States*

Patient-centered data is critical for providing high-value care and assessing quality including functional and cognitive status, goals, and outcomes that matter most to people; these are often missing in Electronic Health Records (EHRs). Furthermore, lack of interoperability makes it challenging to share and aggregate data across clinicians and care settings, leading to fragmented and/or duplicative care. E-care plans (eCP) and data standards that focus on goal setting and outcome management are in development, and hold promise to support person- centered care planning across sites and settings of care. E-Care Plans support care coordination and quality improvement and inform future healthcare value-focused policies—such as those related to equitable coverage, quality improvement, measurement, payment, and innovation model planning. Interoperable eCPs could be integrated into clinical care and across the healthcare ecosystem. This integration will enhance care coordination and facilitate seamless information exchange. In this session, Federal officials from the CMS, VA, and AHRQ will explore the benefits of eCPs and discuss the work underway to support the implementation of these plans in clinical care. This presentation will also discuss efforts to develop value sets and FHIR mappings to enable bidirectional information exchange and the concurrent pilot testing of applications with patient data. E-care plans have the potential improve patient safety, reduce burden, support the efficient exchange of patient data such as cognitive function, physical function, and individual goals to support improving person-centered outcomes, advancing health equity, conducting economic analysis, and tailored program development.

### SESSION 8035 (PAPER)

### LATE BREAKING: CLINICAL PRACTICE INNOVATIONS I

Abstract citation ID: igae098.4263

### ASSESSING THE EFFECTS OF VR URBAN GREENNESS WITH PEER SUPPORT MODEL FOR MENTAL HEALTH IN AGING POPULATION

Shiyu Lu[1], Hiu-kwan Chui[1], and Linyan Li[2], *1. The University of Hong Kong, Hong Kong, China (People's*

*Republic), 2. City University of Hong Kong, Hong Kong, China (People's Republic)*

Engaging with green spaces has shown to benefit mental health in later years. However, global urbanization has limited city-dwellers' access to nature, especially older adults who may struggle to reach such spaces. Virtual Reality (VR) urban greenness offers a cost-effective solution for promoting mental well-being in this demographic. This study aimed to assess the effectiveness of the VR urban greenness with peer support model in enhancing mental health among the aging population. From October 2023 to July 2024, we collaboratively developed a 5-minute 360-degree VR video of urban green spaces with older individuals in Hong Kong, using the photovice method. Subsequently, we conducted a 3-arm randomized controlled trial involving 25 participants with mild depressive symptoms in each arm: VR urban green space with peer supporters (A), VR urban green space without peer supporters (B), and non-biophilic VR intervention without peer supporters as a control group. Both intervention groups (A and B) reported significant reductions in stress levels, anxiety, and negative emotions, coupled with an increase in positive emotions. Conversely, no significant changes were observed in the control group. Following the qualitative study, it emerged that participants in both intervention groups displayed favorable attitudes towards VR and showed strong acceptance of the 360-degree VR urban green spaces. The research further delved into the policy and practical implications of integrating VR technology into mental health promotion strategies for the elderly.

Abstract citation ID: igae098.4264

### AWARENESS AND ATTITUDES ABOUT MEDICAL AID IN DYING IN THE U.S.

Elissa Kozlov[1], Samuel Nemeth[2], and Elizabeth Luth[3], *1. Rutgers University, Piscataway, New Jersey, United States, 2. Rutgers School of Public Health, Piscataway, New Jersey, United States, 3. Rutgers University, New Brunswick, New Jersey, United States*

Medical Aid in Dying (MAID) allows terminally ill patients to seek a prescription for medication to end their lives. Despite being legal in 10 states and Washington DC, public awareness of and attitudes towards MAID remain unclear. This study aims to assess the level of awareness of MAID laws and attitudes about MAID in the United States. Using mTurk and Prime Panels, we collected a purposive national sample of n= 2,343 people living in states where MAID is legal and n=1151 living in states where MAID is not legal. The sample was normally distributed for income, education, political affiliation, and had diverse religious representation. We over sampled for adults living in MAID states and adults over 60 because this is the population most likely to use MAID. In states where MAID is legal, 14.4% of respondents knew that MAID was legal in their state. In the full sample, 30% knew that MAID was legal anywhere in the United States. In the full sample, 20% reported that MAID was not morally acceptable, and 25% reported that they would probably or definitely not pursue MAID personally if they were faced with a terminal illness. This study revealed a significant gap in public awareness, with many residents unawares that they live in states where MAID is legal. However, attitudes towards MAID are mostly favorable with a 44% expressing support for MAID personally. These findings suggest the increased need for public education on MAID laws to ensure that residents are informed about their end-of-life-options.

Abstract citation ID: igae098.4265

### FEASIBILITY AND ACCEPTABILITY OF IMPLEMENTING DEPRESCRIBING IN POST-ACUTE HOME HEALTH CARE

Jinjiao Wang[1], Jenny Shen[2], Fang Yu[3], Kobi Nathan[4], Yeates Conwell[2], Sandra Simmons[5], Amanda Mixon[5], and Thomas Caprio[2], *1. University of Rochester - School of Nursing, Rochester, New York, United States, 2. University of Rochester Medical Center, Rochester, New York, United States, 3. Arizona State University, Phoenix, Arizona, United States, 4. Saint John Fisher University, Rochester, New York, United States, 5. Vanderbilt University Medical Center, Nashville, Tennessee, United States*

Polypharmacy is common in home health care (HHC), but no deprescribing intervention has been tested in HHC. In a prior study, we identified essential components of deprescribing by interviewing patients, primary, acute and post-acute care providers, pharmacists, and HHC nurses in 12 U.S. states. This study assessed the feasibility and acceptability of deprescribing in HHC through the collaboration of an HHC nurse and a pharmacist to conduct home medication review, deprescribing assessment, primary care provider discussion, patient education, deprescribing implementation, and safety monitoring. In 11 patients who received HHC following hospital discharge, we assessed feasibility and acceptability of deprescribing through surveys and qualitative interviews with patients, providers, and study interventionists. Patients received deprescribing components over 3-4 home visits. On average, the HHC nurse spent 1 hour/home visit/patient, the pharmacist spent 1 hour on deprescribing assessment/patient, and the provider spent 5 minutes / patient to review and implement deprescribing recommendations. Over 70% of deprescribing recommendations were accepted by providers, with 30% not accepted mostly due to patient preference. Providers found deprescribing "very feasible," but noted barriers such as time constraints and patient preferences. The HHC nurse found deprescribing components "very feasible," citing the multidisciplinary approach as a facilitator but noting communication challenges with providers. Providers and patients both generally reported that deprescribing components were helpful and led to patients feeling empowered. In conclusion, deprescribing components were feasible in HHC and highly acceptable to patients and providers. Effective communication between patients, HHC nurses, and providers is crucial for deprescribing in HHC.

Abstract citation ID: igae098.4266

### INTEGRATING CERTIFIED NURSING ASSISTANTS INTO INTERDISCIPLINARY CARE TEAM AND RESIDENT CARE PLANNING

Aasha Raval[1], Mia Ermolenko[2], Andreina Jimenez[1], Anisha Balaji[3], Shivani Shenoy[1], Marissa Bergh[1], and Jasmine Travers[1], *1. New York University, New York, New York, United States, 2. SUNY Downstate College of Medicine, Brooklyn, New York, United States, 3. Albert Einstein College of Medicine, New York, New York, United States*

Integrating certified nursing assistants (CNAs) into interdisciplinary care team (IDT) and resident care planning is essential for delivering person-centered care in nursing homes. In 2016, the Centers for Medicare and Medicaid Services mandated this integration, but the lack of clear guidelines made implementation challenging. As a result, a specific road map is needed for nursing homes to optimize how best to integrate CNAs into IDT and resident care planning. This qualitative study, guided by Donabedian's Quality of Care Model, explored the barriers and facilitators in integrating CNAs into IDT and resident care planning. Seventeen participants including CNAs, directors of nursing, social workers, nursing home surveyors, ombudsmen, and physicians were recruited from across the United States using the State Survey Agency Directory, LinkedIn, and a referral program initiated by our research team. One-on-one, 30-minute virtual Zoom interviews were conducted between June-2022 and June-2023. Interview data were analyzed using directed content and thematic analyses. Five key themes emerged from the data and included: recognition of challenges for CNA Integration, nursing home leadership and structure, integration of CNAs, care coordination and communication, and care integrity. The findings led to the development of two INTEGRATE guides in August 2024: one for nursing home executive leadership which offers strategies for effectively integrating CNAs into IDT and resident care planning, and another for CNAs, which provides practical guidance on contributing to IDT meetings and care planning processes. The next steps of this work involve testing the efficacy and acceptability of the INTEGRATE guides in nursing homes.

Abstract citation ID: igae098.4267

### MEALTIME EXPERIENCES DURING AML/MDS TREATMENT: LONGITUDINAL QUALITATIVE STUDY PRELIMINARY RESULTS

Victoria Crowder[1], Youngmin Cho[2], Chiao-Hsin Teng[3], Melissa Batchelor[4], Anna Beeber[2], Lorinda Coombs[1], Daniel Richardson[1], and Ashley Bryant[1], *1. University of North Carolina at Chapel Hill, Chapel Hill, North Carolina, United States*, *2. Johns Hopkins University, Baltimore, Maryland, United States*, *3. National Health Research Institutes, Miaoli, Miaoli, Taiwan (Republic of China)*, *4. George Washington University, Washington, District of Columbia, United States*

Older adults (aged ≥60) with acute myeloid leukemia (AML) and myelodysplastic syndromes (MDS) face varied experiences related to eating and mealtimes during treatment with hypomethylating agents and Venetoclax (HMA+VEN). Family/friend carepartners may provide extensive support to patients during this time and undergo challenges of their own. A gap exists in our understanding of eating and mealtime experiences over time, and how this affects quality of life (QOL); further information is crucial to guide patients, clinicians and carepartners in their management. We aimed to understand patient's eating and mealtime experiences with AML/MDS and their carepartners during Cycles 1 and 2 of HMA+VEN treatment, the efforts made to address challenges, and the impacts on QOL. We conducted semi-structured interviews with 11 older adult patients and ten carepartners at Cycles 1 and 2 and conducted directed content analysis with the Adaptive Leadership Framework for Chronic Illness. We identified five preliminary categories: (1) adaptive challenges for patients in eating and mealtimes, (2) changes with eating and mealtimes over Cycles 1 and 2, (3) adaptive challenges for carepartners in care of patients, (4) how they collaboratively address challenges, and (5) the impact of experiences or changes in eating and mealtimes on QOL. Findings highlight and expand our knowledge of eating/mealtime experiences during chemotherapy for patients with AML/MDS and carepartners, including their experiences with appetite loss, adaptations of meals, and the extensive involvement of carepartners. These findings may guide future intervention development to support symptom management, enhance support and services, and maintain QOL.

Abstract citation ID: igae098.4268

### NAVIGATING AND ENHANCING HEALTHCARE COMMUNICATION: CHALLENGES AND STRATEGIES FOR LATE-DEAFENED OLDER ADULTS

Gianna Kohl, Erin Heniff, Maya Dye, and Wendy Rogers, *University of Illinois Urbana-Champaign, Champaign, Illinois, United States*

Effective communication in healthcare is crucial, especially for older adults with complex health issues. Late-deafened older adults, who develop severe to profound bilateral hearing loss post-lingually, face unique challenges in communicating their health needs. This issue is not well understood, and research describing strategies to manage these communication challenges in clinical settings is lacking. Understanding these challenges and responses for healthcare activities is essential to support late-deafened older adults and health professionals, ensuring quality care. Data were collected through the Aging Concerns, Challenges, and Everyday Solutions (ACCESS) study wherein questionnaires and interviews were completed by 56 late-deafened participants aged 60 to 80 ($M = 69.4$, $SD = 6.0$), living with a diagnosis of hearing loss for at least ten years (age of onset: $M = 31.8$, $SD = 19.9$). We conducted a detailed content analysis of interview data about challenges and response strategies in healthcare settings. The most difficult activity was participating in healthcare appointments ($n = 38$), followed by managing health, diet, and nutrition ($n = 13$). Communication barriers included conversational partners using verbal communication and insufficient visual cues, lack of support, and unclear speech. Response strategies included using external assistive technology, adaptive communication strategies (e.g., lip-reading), and educating healthcare providers about their needs. Our findings highlight the need for health professionals in clinical practice to understand and adapt to the specific communication challenges faced by late-deafened older adults. Implementing effective communication strategies and providing tailored support can lead to more accurate diagnosis and treatment, ultimately enhancing patient care.

Abstract citation ID: igae098.4269

### UNDERSTANDING AND ADDRESSING COMMUNITY-BASED PALLIATIVE CARE REFUSALS: A MULTI-PERSPECTIVE STUDY

Kira Sheldon[1], and Elizabeth Luth[2], *1. Rutgers University--New Brunswick, New Brunswick, New Jersey, United States*, *2. Rutgers University, New Brunswick, New Jersey, United States*

More than half of older adults have multimorbid chronic conditions, resulting in disability, functional limitations, and reduced quality of life. Community-based palliative care, increasingly part of Medicare Advantage value-based care plans, supports seriously ill individuals and their caregivers to improve symptom management and satisfaction and reduce acute care use. Yet, many potentially eligible individuals decline free services when offered. To understand why eligible individuals refuse palliative care, we conducted semi-structured interviews and focus groups with 22 Medicare Advantage plan members and caregivers who refused palliative care and five palliative care team members at a non-profit healthcare agency. Transcripts were analyzed to identify reasons for refusal. We identified three overarching reasons plan members and caregivers declined palliative care: (mis)associating it with end of life, unclear benefit of palliative care, and not wanting to change current care. Palliative care team members confirmed these findings and contributed additional insights. Medicare Advantage plan members and families who refused palliative care represent a key resource for understanding how to better explain services and increase acceptance of palliative care. Palliative care team members' perspectives provide insights from interactions with seriously ill individuals who may be disinclined or unable to engage in research. In clinical practice, clearly differentiating palliative care from hospice, providing an individualized explanation for how it will address an individual's particular needs, and emphasizing integration of palliative care with existing care teams may improve engagement in palliative care among seriously ill older adults.

### SESSION 8040 (PAPER)

### LATE BREAKING: ARTIFICIAL INTELLIGENCE AND EMERGING TECHNOLOGIES

Abstract citation ID: igae098.4270

### AGEISM SOURCES AFFECT TECHNOLOGY ACCEPTANCE ON WEARABLE ROBOTS: A RANDOMIZED BETWEEN-SUBJECT EXPERIMENT

Clio Yuen Man Cheng, and Vivian Weiqun Lou, *The University of Hong Kong, Hong Kong, China (People's Republic)*

Existing literature shows that ageism contributes to the widening of digital divide. However, it remains largely mysterious how ageism specifically impacts technology acceptance, particularly in the context of soft wearable robots. This study aims to fill this gap by conducting a randomized between-subject experiment with six animation vignettes: self-directed ageism, ageism from family and friends, ageism from technology developers, ageism from strangers, unframed, and control. A total of 237 older adults aged 50 to 74 in Hong Kong joined the experiment. The Kruskal-Wallis tests show group significant difference in the intention to use wearable robots, $\chi2(5) = 12.470$, $p = .029$. Specifically, the study found that ageism from technology developers had a significant negative impact on the intention to use wearable robots, in contrast to ageism from family and friends or strangers. Additionally, ageism from family and friends was associated with a significantly higher perceived external control of wearable robots, $\chi2(5) = 12.184$, $p = .032$, as well as a significantly higher perceived performance of wearable robots, $\chi2(5) = 14.767$, $p = .011$, when compared to ageism from technology developers and the control condition. Ageism from family and friends yielded a notably higher perception of health benefits associated with wearable robots, $\chi2(5) = 23.766$, $p < .001$, surpassing self-directed ageism, ageism from technology developers, and the control condition. These findings highlight the distinct effects of ageism from different sources on wearable robot acceptance, underscoring the importance of considering the specific origins of ageism when examining its impact on technology acceptance.

Abstract citation ID: igae098.4271

### IN-HOME ASSISTIVE TECHNOLOGY PROTECTS DEMENTIA CAREGIVERS FROM WORSENING SLEEP EFFICIENCY

Julian Scheffer[1], Darius Levan[2], Jenna Wells[3], Dolores Gallagher-Thompson[4], Kevin Grimm[5], Kuan-Hua Chen[6], Gene Wang[7], and Robert Levenson[8], *1. University of Western Ontario, London, Ontario, Canada*, *2. University of California, Berkeley, Berkeley, California, United States*, *3. Cornell University, Ithaca, New York, United States*, *4. Stanford University, Stanford, California, United States*, *5. Arizona State University, Tempe, Arizona, United States*, *6. University of Nebraska Medical Center, Elkhorn, Nebraska, United States*, *7. Care Daily Company, Kirkland, Washington, United States*, *8. University of California, Berkeley, San Francisco, California, United States*

Caregivers for people with dementia or mild cognitive impairment often report sleep problems due to heightened vigilance concerning worrisome behaviors (e.g., falls, wandering) by their care recipients (CRs). Interventions are needed to help alleviate these issues and the associated sleep troubles for caregivers. One promising approach is to utilize scalable and affordable in-home technologies that monitor CR behaviors round-the-clock and alert caregivers to potentially dangerous situations. We conducted two randomized controlled trials with independent samples to determine whether People Power Caregiver (PPCg), a newly developed in-home monitoring and alerting system, benefitted caregivers' sleep over a six-month period. Combining the two studies, a total of 162 primary caregivers of the CRs were randomly assigned to either an active condition (PPCg system fully activated) or a control condition (Study 1: water leak detection only; Study 2: waitlist control procedure). Caregivers self-reported their sleep quality using the Pittsburgh Sleep Quality Index at baseline, three-months, and six-months. Using latent growth modeling, caregivers in the control conditions reported significantly worsening sleep efficiency compared to caregivers in the active condition (Active: $B = -0.30$, $SE(B) = 0.14$; Control: $B = 0.74$, $SE(B) = 0.12$; Condition Effect on Linear Slope: $p = .038$). We observed a similar pattern with PPCg benefitting caregivers' sleep duration; however, this result was not statistically significant (Condition Effect on Linear Slope: $p = .105$). These results suggest that in-home technologies such as PPCg may offer an effective way to help caregivers achieve healthy sleep as they contend with the demands of caregiving.

Abstract citation ID: igae098.4272

### INNOVATING THE DETECTION OF CARE PRIORITIES IN HEART FAILURE USING LARGE LANGUAGE MODELS

Alaa Albashayreh, Nahid Zeinali, and Stephanie White, *University of Iowa, Iowa City, Iowa, United States*

Engaging older adults with advanced chronic conditions, such as heart failure, in discussions about their care priorities is crucial for ensuring treatments align with their preferences, especially at the end of life. Despite the abundance of data in electronic health records (EHRs), documentation of care priorities is often inconsistent and underutilized. This study utilizes natural language processing (NLP) to detect and characterize care priorities in the EHRs of older adults with heart failure, aiming to enhance patient-centered care. We retrained Bio-Clinical-BERT, a Bidirectional Encoder Representations from Transformers (BERT) large language model, using EHR data from a Midwestern U.S. hospital to create Care-BERT, a novel model for predicting care priorities in clinical narratives. We developed a gold-standard corpus of 1,068 notes, focusing on comfort measures only and life-sustaining treatments, with the dataset divided into training (80%) and testing (20%) sets. Care-BERT outperformed BERT-base, Bio-Clinical-BERT, and PubMed-BERT, achieving the highest performance in predicting care priorities (internal validation: F1-score = 0.941, AUC = 0.978; external validation with 200 GPT-based synthetic notes: F1-score = 0.876, AUC = 0.966). Applied to 2,218,251 EHR notes for 7,984 older adults with heart failure (mean age = 76.9 years), Care-BERT revealed that 2.8% had comfort measures only and 17.3% had life-sustaining treatments documented. This study highlights the potential of Transformer-based NLP models like Care-BERT to improve the documentation of care priorities in EHRs and enhance patient-centered care. Future research will explore documentation variations across patient groups using NLP-based labels, providing further insights into care preferences.

Abstract citation ID: igae098.4273

### SCAMMED BY LOVE: THE ROLE OF LONELINESS, TRUST, AND AGE IN FINANCIAL LOSSES FROM U.S. ONLINE ROMANCE SCAMS

Rebecca Cole, *University of Texas at Rio Grande Valley, Edinburg, Texas, United States*

Online romance scam (ORS) victims are popularly characterized as lonely, overly trusting older adults targeted by scammers seeking gifts and money through fake romantic relationships. However, research focuses on middle-aged women, overlooking risks to older adults using social networking, online dating and Internet technologies. The present exploratory research aims to identify these risks by examining the associations among age, feelings of loneliness and trust, and ORS money loss from data collected in a nationwide online survey about U.S. ORS victimization. Male (n = 4) and female (n = 27) participants who were victimized in an ORS and lost any amount of money were identified through community partners and social media. Most participants reported their race as White (n = 17) or Black or African American (n = 10) and had a college degree (n = 22). Their average age was 52 years. The mean reported money lost to the ORS was $133,647. Pearson's product-moment correlation was used to identify the variables' associations, and Cohen's D was used to determine the effect size of the relationships. The only statistically significant result identified a medium-strength relationship between age and ORS money loss $r(28) = .584$, $p < .01$. This finding contributes to the limited knowledge in the U.S. of ORS by giving some foundational insight into the role of age and ORS victimization. Technology continues to be a constant in most adults' lives, and more support is needed to create a safe online Internet experience for all adults.

Abstract citation ID: igae098.4274

### TECHNOLOGY FOR HEALTHCARE WORKFORCE FOR OLDER ADULTS: CHATGPT-ASSISTED QUALITATIVE ANALYSIS

Soojeong Han[1], and Gregory Alexander[2], *1. Columbia University School of Nursing, New York, New York, United States*, *2. Columbia University, New York, New York, United States*

Rigorous guidance on ChatGPT use in qualitative research is not available. Here we examine the quality of ChatGPT versus human analysis. The International Summit on Innovation and Technology in Care of Older People (IS-ITCOP) was held on June 6-7, 2024 in New York. 47 experts in care of older adults attended IS-ITCOP. Attendees participated in structured roundtable discussions about long-term care workforce readiness to use technology in care of older adults. Each roundtable discussion included a lead facilitator and support staff who recorded discussions lasting approximately 2 hours. We used 10 roundtable discussions in our analysis. We followed the methodology established by Goyanes, Lopezosa, and Jordá(2024) for ChatGPT. Our human analysis was based on Braun and Clarke's(2006) approach. Emerging themes included data privacy and security, informed consent, balancing autonomy and safety, role of the informatics workforce, technological advancements and ethical use, and policy and regulation. Compared to human analysis, 14 out of 79 sub-themes (18%) were missing in ChatGPT analysis. Findings suggest that ChatGPT has several strengths. ChatGPT completed the qualitative analysis in a few minutes, while humans took several days. ChatGPT provided clear definitions and explanations of words and concepts. It efficiently calculated convergence and divergence rates for each theme. ChatGPT also has weak points. ChatGPT cannot create a hierarchical coding scheme. It may not be able to capture all prompts from discussions. ChatGPT's answers were derived from its training data, which may not be current. Also, ChatGPT appeared to miss some nuances or keywords, such as EHR.

### SESSION 8045 (PAPER)

### LATE BREAKING: HEALTH EQUITY ACROSS THE LIFE COURSE I

Abstract citation ID: igae098.4275

### "SURVIVING AGING" - LOW-INCOME OLDER ADULTS AND THEIR PERSPECTIVES ON THE MEANING OF LIFE: A QUALITATIVE ANALYSIS

Daniel David[1], Kim Hadson[2], and Abraham Brody[3], *1. NYU College of Nursing, New York, New York, United States*,

*2. NYU Rory Meyers College of Nursing, New York, New York, United States, 3. New York University, New York, New York, United States*

**Introduction:** Low-income older adults often face challenging life circumstances, making it essential to explore how they derive meaning in life. This study examines the perspectives of residents in Medicaid-supported assisted living facilities on what gives their lives meaning, shedding light on their psychosocial needs and well-being.

**Methods:** Qualitative interviews were conducted with 60 residents across three Medicaid-supported assisted living facilities in New York City. Participants responded to the question, "What gives your life meaning?" The sample was diverse, including individuals navigating aging, illness, and limited resources. Data were analyzed using content analysis to identify common themes.

**Results:** Five key themes emerged: 1) Overcoming Challenges, 2) Seeking Meaningful Relationships with Family and Aides, 3) Focusing on and Accepting the Present, 4) Personal Growth and Community Contribution, and 5) Maintaining Autonomy. A subset expressed an existential view, stating, "There is no meaning." Despite numerous challenges, most residents sought meaning late in life by focusing on strengths rather than barriers.

**Discussion:** The findings highlight the complexity of meaning-making in later life, particularly among low-income older adults in under-resourced settings. While challenges are significant, the ability to find meaning through relationships, personal growth, and autonomy suggests critical avenues for intervention. Existential concerns raised by some participants indicate a need to address these perspectives within psychosocial support frameworks.

**Conclusion:** This study offers insights into how low-income older adults in assisted living derive meaning. Emphasizing strengths over barriers underscores the importance of targeted interventions to support their psychosocial needs and enhance their quality of life.

Abstract citation ID: igae098.4276

### DISPARITIES AMONG LONG-TERM AND SHORT-TERM USERS OF MEALS ON WHEELS SERVICES

France Weaver[1], Jennifer Chubinski[2], and Sarah Walsh[3], *1. West Virginia University, Morgantown, West Virginia, United States, 2. Xavier University, Cincinnati, Ohio, United States, 3. Eastern Michigan University, Ypsilanti, Michigan, United States*

Home-delivered meal programs for older adults, commonly known as Meals on Wheels (MOW) services, provide nutritional benefits and are valued by clients, but most studies capture use over a short period of time, typically a few months. This study is the first to focus on duration of use and identify disparities in the long-term and short-term MOW users. It uses 35,756 observations from the 2013-2022 National Health and Aging Trends Study (NHATS), an annual survey of Medicare enrollees aged 65+. A fixed effect multinomial logit model, with complex survey design, predicts the probabilities of being a short-term or long-term MOW user across lagged sociodemographic and health characteristics. Short-term users are defined as reporting MOW use once (12.4% of the sample) and the long-term users indicate use for 2+ years (8.6%). Persons who are Black are statistically more likely to be long-term MOW users (13.7%) than non-Black (8.0%), similarly for those on Medicaid (15.7%), having 2+ instrumental activity of daily living limitations (10.9%), and pre-frail (9.5%). In contrast, no racial or health differences are found for short-term MOW users, except for those on Medicaid (18.7%). These results identify the populations more likely to be long-term MOW users, but further research is needed to determine if prolonged MOW use is an indicator of sustained independence or a consequence of barriers to additional services. This study helps to better understand the role played by MOW and the federal Older Americans Act Nutrition Program, which funds those services, at serving diverse populations.

Abstract citation ID: igae098.4277

### REVIEWING DATA DISAGGREGATION IN AGING HEALTH RESEARCH: NIA GRANTS AND RECOMMENDATIONS FOR EQUITY

Moroni Fernandez Cajavilca[1], Matthew Lee[2], and Lan Doan[3], *1. New York University, New York, New York, United States, 2. New York University Grossman School of Medicine, New York, New York, United States, 3. NYU Langone Health, NYU Grossman School of Medicine, New York, United States*

Current federal minimum standards for collecting and reporting race and ethnicity data often group diverse individuals into broad, monolithic categories. Despite advances in policy and practice and calls for inclusive research, data equity remains a significant issue in aging-related health research. Racially and ethnically diverse older adults are underrepresented in health research and rarely disaggregated by ethnicity in data collection, analysis, and reporting of aging-related health outcomes. Data disaggregation offers a promising approach to advancing data equity in aging research. We reviewed the National Institutes of Health Research Portfolio Online Reporting Tools Expenditures and Results (NIH RePORTER) database to identify extramural National Institute on Aging (NIA) grants from 1985 to 2024 that proposed disaggregating race and ethnicity data for aging research. We found only 12 NIH-funded awards, demonstrating the limited focus on this issue. Most grants that explicitly mentioned data disaggregation were awarded from 2015 onwards (n=11, 92%). Proposed data disaggregation by ethnicity in NIA-funded grants included Latino and Hispanic, Asian, Pacific Islander, and Arab ethnic groups. Grant funding mechanisms included investigator-initiated grants such as R01 grants (n=5, 42%) and predoctoral training grants/awards such as the F31 and R36 (n=3, 25%). Enhancing cultural congruence and methodological rigor in data collection and reporting that is reflective of the diversifying aging population is crucial for meaningfully addressing health equity. We provide recommendations to advance aging-related health research and highlight data disaggregation considerations for racially and ethnically minoritized communities that are historically under-engaged and underrepresented in aging health research.

Abstract citation ID: igae098.4278

### THE HEALTH TRAJECTORY OF OLDER IMMIGRANTS IN CANADA: FINDINGS FROM THE CANADIAN LONGITUDINAL STUDY ON AGING

Lun Li[1], Andrew Wister[2], and Yeonjung Lee[3], *1. MacEwan University, Edmonton, Alberta, Canada*, *2. Simon Fraser University, Vancouver, British Columbia, Canada*, *3. Chung-Ang University*

In Canada, older immigrants constitute about one-third of aging Canadians, and the population size of older immigrants is projected to keep growing. The health and wellbeing of older immigrants are critical to Canada's healthcare and social services systems. This study intends to examine the health trajectory of older immigrants in Canada over ten years (2011 to 2021) using three waves of data from the Canadian Longitudinal Study on Aging based on Linear Mixed Models. The data analysis unit contains a total of 21,480 older adults (aged 65 years and older), including 4,248 immigrants and 17,232 Canada-born. The results indicate that older immigrants report better physical health (fewer chronic diseases and better physical capability) than Canada-born older adults but experience a greater decline in physical health over ten years. When it comes to mental health, older immigrants and Canada-born older adults report similar levels of depression and loneliness, and the change of mental health is parallel over time. In addition, older immigrants report significantly lower levels of social wellbeing (e.g., social support and social participation), and a greater decline in perceived social support and social participation. The findings from this study enrich the "healthy immigrant effect" with older immigrants' health situation in Canada over ten years. The findings also emphasize the need to support older immigrants in Canada to maintain social wellbeing and enhance the positive impacts of social determinants of health during the aging process.

Abstract citation ID: igae098.4279

### THE RELATIONSHIP BETWEEN RETIREMENT AND CARDIOVASCULAR DISEASE: A MEDIATION ANALYSIS IN THE U.S. AND SOUTH KOREA

Gayoung Choi[1], Ruijia Chen[2], Hee Man Park[3], and Harold Lee[4], *1. Sungkyunkwan University, Seoul, Republic of Korea*, *2. Boston University, Lexington, Massachusetts, United States*, *3. Pennsylvania State University, State College, Pennsylvania, United States*, *4. Penn State, University Park, Pennsylvania, United States*

Retirement has been linked to increased risk for cardiovascular disease (CVD), but little is known about the pathways underlying the association. Moreover, most studies have been conducted in Western countries, and their findings might be less generalizable to Eastern countries due to different social contexts and labor and employment environment. We examined 1) whether retirement status is associated with a higher risk of CVD in South Korea and the U.S., and 2) whether health (e.g., hypertension, diabetes, and obesity), behavioral (e.g., physical activity, alcohol consumption, and smoking), and psychological factors (e.g., loneliness, depressive symptoms, and quality of life) mediate these associations. Data were drawn from the Health and Retirement Study (HRS, 2002–2014; n=11,855) and the Korean Longitudinal Study of Aging (KLoSA, 2006–2018; n=4,908). We analyzed the data using cox proportional hazard model, controlling for socio-demographic factors, health conditions, health behavior, and social isolation. We also performed causal mediation analyses based on counterfactual framework. After adjusting for all covariates, retirement (vs. employment) was associated with an increased likelihood of developing CVD (Hazard Ratio (HR): 1.22, 95% CI: 1.00-1.49) in Korea but not in the U.S. Regarding potential mediators, several physical health factors accounted for a proportion of the association in KLoSA: hypertension (17.7%, p< 0.01), diabetes (12.1%, p< 0.05), and obesity (3.7%, p=0.01). The psychological pathway, specifically quality of life, also mediated the association (4.8%, p< 0.05). Our findings highlight that public health interventions targeting physical and mental health could be an important strategy for preventing CVD among retirees, especially in Korea.

### SESSION 8050 (PAPER)

### LATE BREAKING: COMMUNITY-ENGAGED RESEARCH APPROACHES I

Abstract citation ID: igae098.4280

### A SENIOR CENTER-BASED MULTILEVEL LIFESTYLE INTERVENTION IN OLDER ADULTS WITH TYPE 2 DIABETES

Yan Du[1], Lixin Song[2], Elena Volpi[3], Helen Hazuda[4], Zenong Yin[5], Erin Finley[4], Carlos Jaen[4], and Josh Mireles[6], *1. UT Health San Antonio, San Antonio, Texas, United States*, *2. University of Texas Health Science Center at San Antonio, San Antonio, Texas, United States*, *3. University of Texas Health San Antonio, San Antonio, Texas, United States*, *4. UT Health SA, San Antonio, Texas, United States*, *5. UTSA, San Antonio, Texas, United States*, *6. House of Neighbor Service, San Antonio, Texas, United States*

Approximately 30% of adults aged 65 and over in the U.S. have Type 2 diabetes (T2D), leading to impaired physical function and reduced quality of life. This ongoing pilot project aims to assess the feasibility and acceptability of a multilevel lifestyle intervention at a day-activity senior center, examine preliminary effects, and explore implementation strategies. The study employs a 6-month, single-arm, pre- and post- test design (3 and 6 months). In collaboration with key stakeholders, including senior center staff and individuals with T2D, we are adapting the Diabetes Prevention Program curriculum and the Look AHEAD lifestyle intervention into a multilevel lifestyle intervention, targeting individual, interpersonal, and organizational levels. Nineteen older adults (mean age=68.5±5.5, Latinos/Hispanics=94.7%, female=89.5%) were enrolled. Fourteen digital lessons, facilitated by trained community health workers, are being provided separately in English and Spanish in a group format. Semi-structured interviews with key stakeholders will be conducted at 6 months to explore study acceptability and intervention adoption, implementation and sustainability. Baseline data showed that 83.3% of participants were obese, with 42.1% meeting physical activity recommendations. Self-efficacy scores for physical activity and diabetes management were 63.4±11.9 and 54.6±21.0. The mean HbA1c, extended short physical performance battery, and quality of life was 6.5±0.8, 15.7±3.8, and 40.8±7.8, respectively. The baseline data reveal significant challenges, such as high obesity rates and low self-efficacy in physical activity and diabetes management. We are leveraging these insights to tailor the multilevel lifestyle intervention to improve diabetes management, physical function, and quality of life in older adults with T2D.

Abstract citation ID: igae098.4281

### IGESS: A CO-CREATIVE INTERGENERATIONAL STRATEGY FOR ENHANCING POSITIVE INTERGENERATIONAL OUTCOMES

Alice C. Wong[1], Daniel W.L. Lai[1], Alison Y.T. Ou[1], and Xue Bai[2], *1. Hong Kong Baptist University, Hong Kong, Hong Kong, China (People's Republic)*, *2. The Hong Kong Polytechnic University, Hong Kong, Hong Kong, China (People's Republic)*

Intergenerational interaction is crucial for promoting social cohesion and mutual understanding. The Inter-Generational Engagement in Secondary Schools (i-GESS) program was created to engage older adults and university students in co-creating learning activities for students in secondary schools to enhance positive intergenerational interactions, attitude, knowledge, and skills. This study examined the changes using a quasi-experimental pre-post survey design in 129 older adults (age 60+), 110 university students (age 18 to 22), and 470 secondary school students (age 13 - 16). The results demonstrated substantial improvements in attitudes towards different age groups, as measured by the 34-item Kogan's scale. Secondary school students showed a significant 13.7% improvement in attitudes towards older adults, while university students and older adults noted increases of 4.2% and 5%, respectively, in their views towards youth. Additionally, self-rated communication skills saw notable gains, with secondary school students and university students recording increases of 7.9% and 9.5%, respectively. Trust in communication also increased substantially, evidenced by a 12.1% rise among secondary school students and a 5% improvement among older adults. The most pronounced enhancements were in the attitudes of secondary school students towards older adults and the communication skills of university students, indicating major impacts in these areas. The i-GESS initiative successfully bridged generational divides, fostering meaningful relationships across age groups via the application of the co-creation strategy. The positive outcomes further reinforce the benefits of this intergenerational approach for enhancing mutual cooperation and respect.

Abstract citation ID: igae098.4282

### INTERGENERATIONAL SOLIDARITY AND MENTAL HEALTH IN CHINESE AMERICAN FAMILIES: A DYADIC APPROACH

Mengting Li[1], Qun Le[2], Man Guo[3], Changmin Peng[4], Fengyan Tang[5], Wendi Da[6], and Yanping Jiang[7], *1. Renmin University of China, Beijing, Beijing, China (People's Republic)*, *2. University of Massachusetts Lowell, Lowell, Massachusetts, United States*, *3. University of Iowa, Iowa City, Iowa, United States*, *4. University of Massachusetts Boston, Boston, Massachusetts, United States*, *5. University of Pittsburgh, Wexford, Pennsylvania, United States*, *6. Rutgers, the State University of New Jersey, New Brunswick, New Jersey, United States*, *7. Rutgers, The State University of New Jersey, New Brunswick, New Jersey, United States*

Existing family and caregiving studies have focused on mental health of either older adults or adult children, while less is known about the effect of intergenerational relations on mental outcomes for both generations using a dyadic approach. This study examined the association between intergenerational solidarity and mental health among older Chinese Americans and their middle-aged adult children with dyadic analysis, while considering the gendered nature of these relationships. This study included 216 father-child and 337 mother-child dyads. Three indicators of intergenerational solidarity (emotional closeness, contact frequency, and upward support) were assessed by both parents and children. Mental health of both generations was assessed with anxiety, depression, and loneliness. Actor–Partner Interdependence Models were used for dyadic analysis. In father-child dyads, lower contact frequency perceived by children and greater upward support perceived by older fathers were associated with older fathers' lower levels of anxiety and loneliness; and higher emotional closeness perceived by adult children was associated with adult children's lower levels of anxiety. In mother-child dyads, higher emotional closeness and upward support perceived by older mothers were associated with older mothers' lower levels of anxiety, depression, and loneliness, while higher emotional closeness and upward support perceived by adult children were associated with adult children's lower levels of loneliness. The findings suggest that dyadic intervention promoting emotional closeness and upward support may benefit mental health for both generations and achieve optimal caregiving outcomes. The gendered pattern in intergenerational relations should also be considered in dyadic interventions.

Abstract citation ID: igae098.4283

### SOCIAL NETWORK TYPES TRANSITIONS, HEALTH AND WELL-BEING-A PERSON-CENTERED APPROACH AMONG CHINESE OLDER ADULTS

SHUTING DAI, *City University of Hong Kong, Hong Kong, China (People's Republic)*

The social networks of older individuals reflect personal life history and cultural factors. However, little is known about stability and change in social network types over time in China. The purposes of this study were to (1) investigate transitions in social network types of Chinese older adults; and (2) examine the transitions of the relationship of the social network types to the measures of health and well-being. Data were drawn from five waves of the China Health and Retirement Longitudinal Study to examine the changes in social network heterogeneity in old Chinese cohort. Data from 19395 observations of 7321 older adults (65 years and older) were considered. Latent transition analysis (LTA) was conducted using seven social network-related variables. The identified typologies were then regressed on the indicators of health and well-being. We found 5 social network types at both waves, representing the most to the least diverse types–diverse, unmarried and diverse, extended family, immediate family, and restricted. About 68% of respondents between waves retained their social network type, whereas 19% transitioned into more diverse types and 13% into less diverse types. Compared with the restricted network, older adults in all other networks were more likely to report higher self-rated health, higher life satisfaction, and lower depressive symptoms at both waves. The findings capture the dynamics in social network composition among older adults in China. A person-centered approach is recommended for policymakers to understand social network transitions, and promoting social network diversity in later life may contribute to healthy aging.

Abstract citation ID: igae098.4284

**TRANSRURAL LIVES: LESSONS FROM AN INTERDISCIPLINARY COMMUNITY-BASED DIGITAL STORYTELLING PROJECT**

Griff Tester, and M. Eliatamby-O'Brien, *Central Washington University, Ellensburg, Washington, United States*

TransRural Lives is an interdisciplinary, community-based digital storytelling project focused on transgender older adults (50+) living in non-metropolitan areas of the Pacific Northwest. Through unstructured interviews, we create audio vignettes that capture their unique experiences, which are featured on our website. While social science research often groups lesbian, gay, and transgender older adults together, older transgender individuals face distinct health and community-building challenges. Additionally, much of the scholarship overlooks the urban-rural divide, often portraying transgender people as primarily urban subjects. However, rural areas offer alternative sites for transgender identity formation that are equally significant. TransRural Lives provides a platform for non-linear and metaphorical storytelling, allowing participants to express joy, repression, and gender knowledge on their terms, without the constraints of linear, medicalized narratives. Our project highlights the socio-cultural, geographic, and historical factors that have shaped transgender experiences in rural areas, revealing the complexity of their lives. This paper reflects on the first two years of our work, including the relationships we've developed with community partners and the challenges of navigating university policies that often do not accommodate community engagement – such as debates over the use of incentives and disputes about "ownership" of stories. This issue is particularly relevant for partners like the Northwest Portland Area Indian Health Board's Paths (Re)Membered Project, which empowers the Two Spirit and Indigenous LGBTQ+ community by emphasizing their strengths, resilience, and histories in the pursuit of health equity, and actively challenges research procedures rooted in colonial practices.

### SESSION 8055 (PAPER)

### LATE BREAKING: ALZHEIMER'S DISEASE, INCLUDING ALZHEIMER'S DISEASE—RELATED DEMENTIAS II

Abstract citation ID: igae098.4285

**AGREEMENT OF MEDICARE PART D VS. MINIMUM DATA SET REPORTED MENTAL HEALTH DRUGS USE IN NURSING HOMES**

Tianwen Huan[1], Orna Intrator[2], Adam Simning[1], Kenneth Boockvar[3], David C. Grabowski[4], and Shubing Cai[1], *1. University of Rochester, Rochester, New York, United States, 2. Icahn School of Medicine at Mount Sinai, New York, New York, United States, 3. University of Alabama, Birmingham, Alabama, United States, 4. Harvard Medical School, Boston, Massachusetts, United States*

Researchers have often used the nursing home Minimum Data Set (MDS) data to measure the rate of medication use, but little evidence exists on the accuracy of the MDS-based medication items. We compared quarterly rates of antipsychotic, antidepressant, and hypnotic use between the MDS and Part D Event file (PDE) in 2018 among long-stay nursing home residents enrolled in Medicare Part D who were ≥ 65 years old with psychiatric disorders or dementia identified in the MDS data. We used Cohen kappa to assess agreement in the share of residents using mental health medications during the quarter and used PDE data as a reference to calculate validity parameters. A total of 34.0%/73.4% of study participants were identified using antipsychotic/antidepressant medications in MDS assessments vs 34.3%/71.3% identified in PDE records (kappa value: 0.90/0.83). The sensitivity, specificity, positive predictive value (PPV) and negative predictive value (NPV) of MDS data compared to PDE were 96.7%/84.5%, 92.8% /96.7%, 96.3%/91.1%, and 93.6%/94.0%. Only 3.7% of study participants identified in the MDS data, vs 32.6% PDE-based users (kappa value: 0.1), used hypnotics. By combining anxiety medications with hypnotics, the rates of MDS hypnotic or anxiety users increased to 35.0% (kappa value: 0.74). The sensitivity, specificity, PPV, and NPV were 89.5%, 85.6%, 92.8%, and 79.8%, respectively. Agreement between the MDS and PDE in antipsychotic and antidepressant use was high, suggesting that the MDS is a valid tool to measure antipsychotic and antidepressant use. Additional work is needed to understand the disagreements between MDS and PDE in hypnotic use.

Abstract citation ID: igae098.4286

**CLINICIAN TYPE AND SETTING OF CARE DELIVERED TO MEDICARE BENEFICIARIES WITH DEMENTIA IN THE U.S.**

Donovan Maust[1], Rachel Davis[2], Ulrike Muench[3], Steven Marcus[4], and Joanne Spetz[5], *1. University of Michigan, Michigan, United States, 2. University of Michigan, Ann Arbor, Michigan, United States, 3. UCSF, San Francisco, California, United States, 4. University of Pennsylvania, Philadelphia, Pennsylvania, United States, 5. University of California, San Francisco, San Francisco, California, United States*

Improving care coordination for persons living with dementia (PLWD) requires understanding of the types of clinicians delivering care and the settings in which they practice. We identified all beneficiaries with dementia in traditional Medicare in 2019. We used the Medicare Carrier file—i.e., where bills for care provided by individual clinicians are recorded—to identify clinicians providing care to PLWD, which we then linked to the National Plan & Provider Enumeration System (NPPES). Using the Carrier and NPPES files, we characterized the workforce providing care to PLWD, including specialty and licensure (e.g., primary care physician, neurologist, nurse practitioner), setting of care delivery, and whether the clinician included a dementia diagnosis in the billing information. Overall, 1,934,318 PLWD received care from 783,225 unique clinicians. The most common settings of care were office (74.8% of PLWD), emergency room (63.9%), inpatient hospital (52.1%), and skilled nursing facility (37.1%). 87.0% of PLWD received care from a primary care physician, 62.9% from a nurse practitioner, and 33.1% from a physician assistant. Among all the clinicians that care for PLWD, 20.3% are primary care physicians, 15.3% nurse practitioners, and 9.0% physician assistants. 2.4% are psychiatrists, 1.7% neurologist, and just 0.5% are geriatric subspecialist physicians. For clinicians providing care to PLWD, the majority provide care in the outpatient setting, followed by hospital-based care. Programs to improve care coordination for PLWD, such as GUIDE Model programs, must account for delivery across multiple health care settings, recognizing that geriatric subspecialists comprise an extremely small proportion of the PLWD-serving workforce.

Abstract citation ID: igae098.4287

### DEMENTIA RISK FACTORS PREDICT ALTERED WHITE MATTER MICROSTRUCTURE IN A RURAL AGING COHORT

Andrew Bender[1], Pavithran Pattiam Giriprakash[1], Dietmar Cordes[2], Jessica Caldwell[3], and Justin Miller[4], *1. Cleveland Clinic, Las Vegas, Nevada, United States, 2. Cleveland Clinic, LAs Vegas, Nevada, United States, 3. Cleveland Clinic, Cleveland Clinic, Nevada, United States, 4. University of Washington, Seattle, Washington, United States*

Diffusion-weighted magnetic resonance imaging (dMRI) studies of older adults reveal associations between dementia risk factors and altered cerebral white matter microstructure. However, these findings are based almost exclusively on residents of metropolitan areas. Older residents of rural areas face increased risk for Alzheimer's disease and related dementias, but there are few neuroimaging studies of dementia risk in this population. The present study investigated associations between dementia risk factors and white matter alterations in rural-dwelling older adults (n=76) without dementia, recruited from the Nevada Exploratory Alzheimer's Disease Research Center. We applied voxel-based analysis (VBA) to separately evaluate established risk factors (APOE e4 carriage, family history of dementia, body mass index [BMI], and diagnosed hypertension, hypercholesterolemia, and sleep apnea) as predictors of differences in dMRI measures of white matter microstructure (fiber density, crossing fiber entropy, fractional anisotropy, and mean diffusivity), while accounting for differences in age, sex, and handedness. VBA results (familywise error corrected p<.05) showed APOE e4 allele carriage, family history of dementia, higher BMI, and self-reported diagnoses of hypertension and sleep apnea are independently associated with widespread patterns of lower white matter fiber density. Moreover, we observed similar patterns of results in subsidiary analyses that also accounted for diagnosed mild cognitive impairment. These are the first findings to characterize associations between elevated dementia risk factors and altered white matter microstructure in rural-dwelling older adults. Future studies should evaluate whether the effects of elevated risk factors on cerebral microstructure differ between rural- and metropolitan-dwelling older adults.

Abstract citation ID: igae098.4288

### DNA METHYLATION AGE ACCELERATION MEDIATES THE ASSOCIATIONS BETWEEN EARLY-LIFE CIRCUMSTANCES AND DEMENTIA RISK

Fan Pu[1], Zhuoer Lin[2], Jiao Yu[1], Zuyun Liu[3], and Xi Chen[1], *1. Yale University, New Haven, Connecticut, United States, 2. University of Illinois Chicago, Chicago, Illinois, United States, 3. School of Medicine, Zhejiang University, Zhejiang, China (People's Republic)*

Dementia represents critical public health challenges and exerts an immense economic burden. While mounting evidence has linked early-life circumstances to dementia risk, prior research mainly focused on single factors, failing to capture the complex interplay of comprehensive early-life circumstances and the underlying biological pathways. This study aims to fill the gap by exploring how multidimensional early-life circumstances, spanning family education, relationships, financial status, regional context, and school experiences, contribute to dementia risk in later life. Particularly, we investigate the role of DNA methylation age acceleration (DNAmAgeAccel) as a strong mediator of the associations. Using data from 4,018 American adults aged 50 and older from the Health and Retirement Study, we assessed dementia using the Langa-Weir Classification of Cognitive Function and DNAmAgeAccel through the residuals of 11 DNAm clocks. Our multivariate regression analyses demonstrated significant associations between early-life circumstances and dementia risk. Notably, adverse family education environments, such as mother's low educational attainment and absence of time/attention from mother, along with poor family relationships, were associated with an increased risk of dementia. In contrast, positive school experiences, like engagement in creative arts in high school, were protective. Importantly, DNAmAgeAccel showed significant mediation effects, especially in education-related factors. For instance, DNAmAgeAccel mediated 15.1% to 32.8% of the association between maternal education and dementia risk. These findings highlight the enduring impact of multidimensional early-life circumstances on late-life cognitive health and suggest DNAmAgeAccel as a key biological pathway linking early adversity to dementia risk.

Abstract citation ID: igae098.4289

### EMPOWERING COLLABORATION: ENHANCING GERIATRIC CARE WITH AGE-FRIENDLY CNA TRAINING BY LTC OMBUDSMEN

Theresa Sivers-Teixeira[1], Erin Thayer[2], E-Shien Chang[3], Stella Chen[4], Debra Cherry[5], Rachel Tate[6], and Bonnie Olsen[7], *1. Keck School of Medicine of University of Southern California, Alhambra, California, United States, 2. Keck School of Medicine of USC, University of Southern California, Department of Family Medicine, Keck School of Medicine of USC, University of Southern California, California, United States, 3. Weill Cornell Medical College, New York, New York, United States, 4. Keck School of Medicine, Los Angeles, California, United States, 5. Alzheimer's Los Angeles, Los Angeles, CA 90010, California, United States, 6. Wise and Healthy Aging, Santa Monica, California, United States, 7. University of Southern California, Los Angeles, California, United States*

Long-Term Care (LTC) Certified Nursing Assistants (CNAs) who provide direct care to residents often face significant challenges in supporting daily activities and the well-being of residents with functional decline. Frequent difficult behaviors can lead to negative experiences, reducing residents' quality of life and diminishing CNAs' job satisfaction. LTC Ombudsmen (LTCO) assist residents with issues related to day-to-day care, health, safety, personal preferences, rights violations, poor quality of care, and elder abuse. The USC Geriatric Workforce Enhancement Program (GWEP) developed an innovative program to train LTCO to deliver the Age-Friendly (AF) Nursing Home curriculum to improve CNAs' geriatric competencies. A four-lesson curriculum, developed collaboratively by the Los Angeles LTCO Program administered by WISE & Healthy Aging, Alzheimer's Los Angeles, Dr. Shien Chang, and the USC GWEP team, addresses: 1) AF principles of nursing home care, 2) evidence-based strategies for managing behavior issues related to dementia, and 3) strategies for increasing culturally appropriate communication with patients and caregivers. Using a train-the-trainer model, 5 LTCOmbudsmen were trained and delivered the curriculum to 133 CNAs in eight local nursing homes. A pre-post training survey administered to CNA participants showed a statistically significant increase in confidence in applying the training topics. Focus groups with LTCO trainers revealed a positive bidirectional impact on ombudsmen and CNAs. This training model introduced AF care strategies and strengthened the shared purpose between LTCO and CNAs. The study provides a foundation for ongoing collaboration and dissemination of this scalable and sustainable model to improve care for older adult LTC residents.

Abstract citation ID: igae098.4290

### EXPLORING AI FOR DEMENTIA CARE SUPPORT IN NORTHERN ONTARIO: A LITERATURE REVIEW

Hom Shrestha, and Lucy Shrestha, *Laurentian University, Sudbury, Ontario, Canada*

Dementia is the seventh leading cause of death globally, significantly impairs cognitive abilities and memory loss. Artificial intelligence (AI) has the potential to revolutionize dementia care by assisting healthcare professionals in diagnosing, managing, and treating dementia worldwide. We conducted literature review using Google Scholar, PubMed, and Laurentian libraries (OMNI), focusing on key terms such as "dementia," "Alzheimer's disease," "artificial intelligence," and "healthcare." This review covered peer-reviewed publications from 2014 to 2023, aiming to fill gaps in this research area. The search yielded five relevant articles, identifying three core themes: AI's impact on Alzheimer's disease and related dementias (ADRD), early detection and diagnosis of ADRD, and AI's role in supporting healthcare professionals, caregivers, and patients. Zhang et al. (2023) emphasized AI's role in diagnosing Alzheimer's disease using MRI, PET scans, biomarkers, and advanced machine learning applications. Hane et al. (2020) highlighted the use of machine learning models for assessing ADRD risk by analyzing clinical notes to prevent nursing home admissions and reduce care costs. Xie et al. (2020) explored the use of assistive AI devices by caregivers to manage ADRD, reducing patient dependence. Li et al. (2020) focused on voice-activated AI tools that help caregivers manage nutrition and reduce their workload. Gustavsson, Svanberg, and Mullersdorf (2015) examined the benefits of the interactive robotic "Justo Cat" in enhancing communication in dementia care. This review suggests conducting ethnographic participatory focused-groups with thematic analysis to inform policymakers in developing specialized educational programs, integrating advanced AI, expanding digital health and telemedicine, and launching community-driven initiatives.

Abstract citation ID: igae098.4291

### TEN-YEAR ANALYSIS OF ADVERSE DRUG REACTIONS IN PEOPLE WITH DEMENTIA USING FDA ADVERSE EVENT REPORTING SYSTEM DATA

Jieyu Mao[1], Miyae Yamakawa[1], and Yasushi Takeya[2], *1. Osaka University, Suita, Osaka, Japan*, *2. Gerontological and Geriatric Nursing Research Unit, Osaka, Osaka, Japan*

Dementia often leads to polypharmacy and increased ADR risk, making ADR recognition vital since People with Dementia (PwD) struggle to communicate discomfort. This study used the FDA Adverse Event Reporting System (FAERS) database from 2013 to 2024 to extract dementia-related ADR reports. A total of 23,519 ADR reports were analyzed after data cleansing. Missing data was addressed using the MissForest algorithm and refined with KNN imputation. Signal detection calculated PRR (Proportional Reporting Ratio) for frequently reported drugs and ADRs. Over the ten years, the number of dementia-related ADR reports fluctuated significantly, while showing a sharp peak around 2023. Consumers are the most common reporters of ADRs, accounting for 33.4% of the reports, followed by medical doctors at 30.1%. The median time from treatment initiation to the occurrence of ADR was 20 days. Regarding patient outcomes, 38.2% reported hospitalization, and 12.4% reported death. The most reported ADRs were death (706 cases), drug ineffective (527 cases), off label use (502 cases) and aggression (458 cases). Approximately 48.8% of the top 100 ADR reports can be observed and reported by nurses. Exelon Patch was the drug most frequently reported to cause ADRs, strongly associated with death, while Epidiolex showed significant signals for seizure. Cholinesterase inhibitors and antipsychotics/anticonvulsants had high ADR report counts across multiple system organ classes, especially nervous system. Due to cognitive impairments, PwD often face challenges in reporting symptoms, highlighting the importance of objective observation and monitoring by healthcare professionals to ensure accurate ADR identification and improve PwD outcomes.

### SESSION 8060 (PAPER)

### LATE BREAKING: TRANSLATIONAL GEROSCIENCE

Abstract citation ID: igae098.4292

### A NOVEL DNA METHYLATION-BASED SURROGATE BIOMARKER FOR LATENT SYSTEMIC INFLAMMATION

Helen Meier[1], Eric Klopack[2], Mateo Farina[3], Belinda Hernández[4], Colter Mitchell[5], Jessica Faul[5], Cathal McCrory[4], and Eileen Crimmins[6], *1. Institute for Social Research, Institute for Social Research, Michigan, United States*, *2. University of Southern California, University of Southern California, California, United States*, *3. University of Texas at Austin, Austin, Texas, United States*, *4. Trinity College Dublin, Dublin, Dublin, Ireland*, *5. University of Michigan, Ann Arbor, Michigan, United States*, *6. University of Southern California, Los Angeles, California, United States*

Chronic low-grade systemic inflammation is a risk factor for chronic diseases and mortality and is an important biomarker in health research. DNA methylation (DNAm)

surrogate biomarkers are valuable exposure, risk factor and health outcome predictors in population studies. We generated a DNAm surrogate biomarker for chronic, systemic inflammation from a previously identified systemic inflammation latent variable derived from seven inflammatory markers and evaluated its performance relative to measured inflammatory biomarkers in predicting several age-associated outcomes of interest, including mortality, activities of daily living and multimorbidity in the Health and Retirement Study. The DNAm surrogate, Inflammation Latent Variable Methylation Surrogate (InfLaMeS), strongly correlated with individual inflammation markers and performed similarly to the latent systemic inflammation when predicting multimorbidity, disability, and 4-year mortality. These results suggest that InfLaMeS provides a robust alternative to blood-chemistry measures of inflammation that can be used for further scientific insight into understanding the role of inflammation in aging and health.

Abstract citation ID: igae098.4293

### FASTER EPIGENETIC AGING AND INCREASED RISK OF PROSPECTIVE MORBIDITY AND MORTALITY FOR U.S. VETERANS

Kyle Bourassa, *Durham VA Medical Center, Durham, North Carolina, United States*

Epigenetic measures of aging have promise as biomarkers of future morbidity and mortality, but require validation in real-world medical settings. To provide evidence of this validation, we investigated whether an epigenetic measure of aging—DunedinPACE—was associated with prospective health for 2,216 post-9/11 U.S. military veterans enrolled in the Post-Deployment Mental Health Study (472 women, 1,744 men; 1,077 non-Hispanic Black veterans, 1,139 non-Hispanic White veterans). Prospective health outcomes included chronic disease burden (Charlson comorbidity index), predicted annual VA healthcare costs (Nosos scores), chronic disease incidence, and mortality—all assessed using VA electronic health records (EHR). Veterans averaged 37.4 years old at enrollment and with an average of 13.1 years of EHR follow-up. Veterans with faster DunedinPACE aging scores developed greater chronic disease burden and greater predicted healthcare costs over the subsequent 5, 10 year, and 15 years. Faster aging scores were associated with increased risk for incident myocardial infarction, stroke, diabetes, cancer, liver disease, and renal disease, as well as greater risk of mortality due to all-causes and death due to chronic disease. Results remained when controlling for demographic and technical covariates, as well as self-reported smoking and smoking methylations scores, replicated when stratifying by sex and race/ethnicity, and replicated when using an alternative measure of epigenetic aging (PC-GrimAge). These findings suggest DunedinPACE is a biomarker of accelerated aging that associated with prospective morbidity and mortality across multiple chronic diseases and organ systems. With further refinement, epigenetic aging scores have the potential to be useful clinical biomarkers of future health.

Abstract citation ID: igae098.4294

### THE IMPACT OF HIIT ON MUSCULAR, ENDURANCE, AND QUALITY OF LIFE METRICS IN OLDER ADULTS

Rashelle Hoffman[1], Blake Murphy[2], April Krywe[2], Devon Stoffel[2], and Mitchel Magrini[2], *1. Creighton Univeristy, Creighton University, Nebraska, United States*, *2. Creighton University, Omaha, Nebraska, United States*

High-intensity interval training (HIIT) can mitigate age-related declines in muscle mass and strength that lead to decreased functional ability and loss of independence. However, previous HIIT protocols are often treadmill or recumbent bike-based with limited whole-body functional exercises and often do not include older adults (OA) beyond 70 years of age. The aim of this study was to examine the effects of a 12-week, Total Body HIIT (TB-HIIT) protocol on muscle size (cross-sectional area [CSA] of the rectus femoris [RF], vastus lateralis [VL], and vastus medialis [VM]), muscle strength (peak toque [pT] and rate of torque development [pRTD]), muscle activation (peak electromyography [pEMG] and rate of EMG rise [pRER]), cardiovascular endurance (6-minute walk test [6MWT]), and quality of life (QOL, 36-Item Short Form [SF-36]) in OA. Older adults, 65-95 years of age, were recruited and completed all outcomes at baseline and following the 12-weeks of TB-HIIT. The TB-HIIT protocol progressively increased the exercise-to-rest ratio every four weeks (20sec:40sec, 30sec:30sec, 40sec:20sec) with target intensity being 85-95% of participants' heart rate max. Twenty-four OA (74.8□6.37 years, range=65-89) completed this study. Results showed significant increases in VL-CSA (p=0.02, g=-0.77), VM-CSA (p< 0.001, g=-1.03), cardiovascular endurance (p< 0.001, g=-0.95), and health-related QOL (p=0.012, g=-0.47). No significance was revealed in RF-CSA (p=0.068, g=-0.4), pT (p=0.78, g=-0.17), pRTD: p=0.91, g=-0.07), pEMG (p=0.77, g=-0.17) or pRER (p=0.51, g=-0.39). Future research should continue to explore the benefits of TB-HIIT with consideration towards OA subpopulations with comorbidities and greater physical impairment to advance exercise prescription and function for this population.

### SESSION 8065 (PAPER)

### LATE BREAKING: HEALTH EQUITY ACROSS THE LIFE COURSE II

Abstract citation ID: igae098.4295

### "[CERVICAL CANCER], NOT AT THIS AGE...": TREATMENT EXPERIENCES OF OLDER WOMEN WITH ADVANCED CERVICAL CANCER

Runcie Chidebe[1], and Ifeoma Okoye[2], *1. Miami University, Oxford, Ohio, United States*, *2. University of Nigeria, University of Nigeria, Enugu, Nigeria*

**Background:** Cervical cancer (CC) is 90% preventable with the HPV vaccine against sub-types 16 and 18, yet an estimated 21 women die every day from CC, and 7,968 die annually in Nigeria. Most of the women diagnosed with CC are older women, yet the gerontological aspect of CC among this population is largely scarce. Older women living with CC are at risk of undertreatment and are most likely to report the worst prognosis. This phenomenological study is aimed at understanding the treatment experiences of older women living with stage IV CC and their meaning-making of this incurable disease.

**Method:** Using interpretive phenomenological analysis (IPA), 16 older women (50 – 85 years) with advanced CC

were recruited. Data was collected through semi-structured interviews, and the six steps of IPA were used for analysis. **Results:** Four identified themes: "[cervical cancer], not at this age, not at any age anyway but at this age to me is even worse", "He [the doctor] talked about surgery but because of my age, surgery was moved out", "There is a need for support and to be loved" and "When someone's [cancer] is palliative, take it as palliative, when it is critical treat it as such. Don't treat palliative and leave critical aside". **Conclusion:** This study provided a gerontological insight into the lived experiences of these older women. As the world is working to eliminate cervical cancer with the human papillomavirus vaccine, older women who already have advanced cervical cancer should not be forgotten.

Abstract citation ID: igae098.4296

### COMPARISON OF MULTIDIMENSIONAL AGING TRAJECTORIES BETWEEN PEOPLE AGING WITH AND WITHOUT DISABILITIES

Seeun Park[1], and Lisa Bratzke[2], *1. University of Washington, Seattle, Washington, United States*, *2. University of Wisconsin-Madison, University of Wisconsin-Madison, Wisconsin, United States*

**Background:** The aging trajectories of people aging with disabilities (PAwD) may involve distinct life experiences compared to those of people without disabilities (PWoD). However, the extent to which aging trajectories vary between the two groups remains unclear. This study investigates these differences, analyzing the impact of long-term disability at phenotypic and functional levels, and examining social disparities across various demographic factors using data from the 2006-2020 waves of the Health and Retirement Study.

**Methods:** Phenotypic aging was measured by the metabolic dysfunction risk score (MDRS), and functional aging was assessed through limitations in activities of daily living (ADLs) and instrumental activities of daily living (IADLs). Growth-curve modeling was used.

**Results:** A total of 7,234 participants were included. PAwD exhibited significantly higher baseline MDRS and ADLs/IADLs scores, with more rapid escalation over time, indicating premature and accelerated aging. Significant social disparities in aging were observed across cohort, gender, race/ethnicity, and educational attainment. Recent cohorts of PAwD showed more rapid increases in MDRS. Women and racial minorities, especially non-Hispanic Blacks and Hispanics, had higher MDRS. Across all race/ethnicity groups, PAwD exhibited higher MDRS and functional limitations. Lower educational attainment was associated with both higher MDRS and ADLs/IADLs.

**Conclusion:** PAwD experience an accelerated and unequal aging process compared to PWoD, with significant social disparities. These findings suggest the need for tailored health interventions and policies that address the distinct aging trajectories and vulnerabilities of PAwD, emphasizing early detection and targeted support to mitigate the compounded effects of disability and sociodemographic factors.

Abstract citation ID: igae098.4297

### EXPERIENCES WITH PARATRANSIT USE AMONG OLDER ADULTS LIVING WITH DISABILITY

Morgan Fique[1], Lindsey Mathis[2], Andrea Melgar Castilo[2], Erica Twardzik[3], Jason Falvey[1], and Jasmine Cooper-Williams[4], *1. University of Maryland School of Medicine, Baltimore, Maryland, United States*, *2. University of Maryland Baltimore, Baltimore, Maryland, United States*, *3. University of Michigan, Ann Arbor, Michigan, United States*, *4. University of Maryland School of Pharmacy, Baltimore, Maryland, United States*

Public transportation agencies are required to offer paratransit services to individuals near fixed routes, but structural barriers to use (e.g., delays in rides) may disproportionately impact older adults. This is important, as nearly 10% of urban-dwelling older adults use public transportation, many to get medical care. However, how older users interact with, benefit from, or experience barriers related to paratransit services is unclear. We completed 12 semi-structured interviews with paratransit users aged 55 and older in the Baltimore area and analyzed the transcripts using inductive thematic analysis. Two independent researchers coded each transcript. Six main themes emerged: 1) Paratransit services help older adults maintain independence and reduce social isolation by improving access to healthcare and social engagement; 2) Ableist interactions with paratransit staff and access to working equipment (e.g., wheelchair lifts) were negative determinants of paratransit use experiences; 3) Inefficiency of the service, eligibility process, and recertification process place high burdens on older riders; 4) Paratransit frequently fails to meet needs of older disabled riders who experience cognitive impairment, sensory impairment, and/or mobility device use; 5) Built and social environment factors such as sidewalk quality, weather, and crime exposure increase the burden on older riders; and 6) Formal and informal social supports are critical for older adult paratransit use. The themes identified revealed critical gaps in current paratransit systems. These findings are crucial for guiding efforts to reshape policies and practices. By addressing these barriers, we can work towards improving service inefficiencies, eliminating ableism, and better supporting older disabled riders.

Abstract citation ID: igae098.4298

### SUPPORTING AGING THROUGH HOUSING CHOICE: A DISCUSSION TOOL FOR COLLABORATION TOWARD LOCAL SOLUTIONS

Nancy Berlinger, *The Hastings Center, Garrison, New York, United States*

This abstract concerns applied research and new open-access publications that will include piloting data from late September 2024, hence its submission as late-breaking research. This paper will present a discussion tool to support local discussion and collaboration to meet the housing needs of moderate-income older Americans. It has been informed by insights from national and regional aging and policy audiences and reflects findings from a mixed-methods study presented at GSA in 2022 concerning pandemic-era innovations that held promise for policy and practice concerning housing equity for older adults. Research shows that the vast "missing middle" population of older adults who do not qualify for housing subsidies and cannot afford market solutions geared to wealthy individuals struggles to pay for both housing and care. Within the national shortage of affordable housing, there is a shortage of housing that meets the needs of this population: housing that is affordable, accessible or adaptable, and a good fit for an aging person's needs. This paper will share a slide-deck format tool and other resources that offer ways to explain housing needs, clarify realistic options, anticipate challenges, analyze and share success stories, and encourage productive collaboration among local policymakers, community members, housing developers, service providers, and other participants.

Abstract citation ID: igae098.4299

### THE GROWING DEMAND FOR AGE-FRIENDLY CARE AND UNDERSERVED OLDER ADULTS IN THE HEALTH CARE SYSTEM

Katy Terveer, *Age Wave, Orinda, California, United States*

This session will present on the challenges and opportunities to re-think our health care system based on the latest findings from "Meeting the Growing Demand for Age-Friendly Care: Health Care at a Crossroads," a study released by Age Wave and The John A. Hartford Foundation. We will explore the experiences and pain points of older adults, particularly those underserved, in today's health care system based on a recently conducted survey with over 2,500 older adults ages 65+. We will identify key findings for those who are the least satisfied with their health care, namely, those with low financial resources, women, rural older adults, and Black and Hispanic older adults. We'll summarize the key recommendations from the report for transforming health care systems to support longer healthspans and greater well-being as we age.

### SESSION 8070 (PAPER)

### LATE BREAKING: ALZHEIMER'S DISEASE, INCLUDING ALZHEIMER'S DISEASE—RELATED DEMENTIAS III

Abstract citation ID: igae098.4300

### COGNITIVE DECLINE AMONG OLDER ADULTS FOLLOWING TRAUMATIC BRAIN INJURY

Jennifer Albrecht, *University of Maryland School of Medicine, Baltimore, Maryland, United States*

Traumatic brain injury (TBI) is a risk factor for Alzheimer's disease and related dementias. However, evidence for this association among older adults is less robust, and further studies are needed to better understand cognitive recovery following TBI in this population. Retro-TBI is an ongoing prospective cohort study of adults aged 65+ diagnosed with TBI at a Level 1 trauma center. In the current study, we assessed four cognitive domains (memory, language, attention, and visuospatial/constructional) at 2 weeks, 3 and 6 months post-TBI using the Repeatable Battery for the Assessment of Neuropsychological Status (RBANS). We calculated the slope of the RBANS total age-adjusted scaled scores for each participant with ≥2 assessments (n=42) over days since TBI. Participants with a negative slope were considered to have cognitive decline. We compared participant characteristics between those with and without cognitive decline using Student's t-test or Fisher's exact test. Average age was 73.9 (standard deviation 7.8) years and 47.6% were female. Findings indicated that 31.0% (13/42) of participants had cognitive decline and were more likely to report a history of depression (61.5% vs. 13.8%, p=.003) and live alone (76.9% vs 34.5%, p=.02) than those without cognitive decline. The cognitive decline group was also more likely to experience decline in visuospatial/constructional (61.5% vs. 24.1%, p=.04) and immediate memory skills (61.5% vs. 27.6%, p=.047) compared to those without cognitive decline. Preliminary findings from this ongoing study suggest that one-third of older adults experience cognitive decline over 6 months post-TBI, with immediate memory and visuospatial skills particularly impacted.

Abstract citation ID: igae098.4301

### IMPLEMENTATION STUDY OF THE USE OF A BLOOD BIOMARKER TEST IN ALZHEIMER'S DISEASE: CLINICIAN SURVEY FROM QUIP II STUDY

Mark Monane[1], Robert Carlile[2], Kim Johnson[3], Darren Gitelman[4], Lawren VandeVrede[5], Leslie Jacobs[6], and Joel Braunstein[6], *1. C2. N DIagnostics, St. Louis, Missouri, United States*, *2. Palmetto Primary Care Physicians, Summerville, South Carolina, United States*, *3. Duke University, Durham, North Carolina, United States*, *4. Advocate Lutheran General Hospital, Park Ridge, Illinois, United States*, *5. UCSF, San Francisco, California, United States*, *6. C2. N Diagnostics, St. Louis, Missouri, United States*

Recent studies have shown that commercially available, high-performing blood biomarkers (BBMs) for Alzheimer's disease (AD) pathology represent accurate, accessible, acceptable, and equitable tools to aid health care providers (HCPs) in the evaluation of their patients with signs or symptoms of mild cognitive impairment (MCI) or dementia. However, implementation of AD BBM tests into the clinical care pathway has not been extensively evaluated. The objective of this study was to assess the incorporation of the multi-analyte blood biomarker PrecivityAD2™ test (C2N Diagnostics, LLC, St. Louis, MO) into the clinical workflow of specialty clinics. A total of 203 patients (average age 74 years) evaluated by 12 HCPs (neurologists, geriatricians, geriatric psychiatrists, and others) representing 8 outpatient sites were included as part of the real-world QUIP II Study (NCT06025877). A five-item, end-of-study survey was developed and conducted using The Technology Acceptance Model (David, 1989) and included two constructs: perceived usefulness and perceived ease of use of this BBM test. HCP-reported acceptance scores averaged 9.6 (median 10, range 7-10): contribution to clinical decision-making and ease of understanding test results received the highest ratings. The net promoter score was 75, above the benchmark of 50 associated with excellent customer satisfaction (Qualtrics, 2024). Given recent guidelines and workshop recommendations highlighting the use of AD BBM tests in clinical care, we believe these survey data provide evidence of robust usefulness and acceptance of this BBM test, supporting its incorporation into the clinical care pathway to help HCPs rule in and rule out AD in cognitively symptomatic patients.

Abstract citation ID: igae098.4302

### IMPROVING CARE THROUGH IMPROV: RESULTS OF A PILOT TEST WITH FAMILY AND FRIENDS OF PERSONS LIVING WITH DEMENTIA

Candace Kemp[1], Jennifer Morgan[1], Emerald Anglin[1], Celeste Greene[2], Crystal Williams[1], and Amanda Williams[3],

*1. Georgia State University, Atlanta, Georgia, United States, 2. Gerontology Institute at Georgia State University, Atlanta, Georgia, United States, 3. Dad's Garage Theatre Company, Decatur, Georgia, United States*

Evidence suggests that the use of improvisational (improv) theatre techniques in the dementia care context can have positive outcomes for care partners, persons living with dementia, and care relationships. We refined and pilot tested the feasibility, acceptability, and preliminary efficacy of the training program, "Improving Care through Improv," which incorporates improv fundamentals with practical dementia care knowledge, among family and friends of persons living with moderate dementia. We used multiple methods to assess the program, including a no-control pre-post design trial involving surveys and focus groups. Participants received in-person training co-designed and co-led by an improv educator and a gerontologist during weekly 2-hour group sessions held over a 4-week period. We assessed the primary outcome (caregiver mastery) and exploratory outcomes (perceived stress, anxiety, caregiver burden, perceived ability to manage behaviors, and care recipient's quality of life) at baseline; upon program completion, and 3 months post baseline. Forty care partners participated in the intervention, completed all three surveys, and participated in focus groups. Recruitment, enrollment, and completion rates alongside quantitative and qualitative findings indicate high levels of feasibility and acceptability. Dependent sample t-tests between times 1 and 3 showed significant improvement in caregiver mastery and significant reductions in perceived stress and depression. Participants characterized the program as "needed" and "very helpful." Three months post baseline, 100% reported using the skills taught, with most saying that they used them "frequently" or "nearly always." Findings demonstrate improv's promise for improving dementia care experiences, illuminate areas for refinement and application, and support further testing.

Abstract citation ID: igae098.4303

### LIVE AND DIE WITH DIGNITY: ALASKA NATIVE PERSPECTIVES AND EXPERIENCES ON DEMENTIA CAREGIVING AND FAMILY STIGMA

Steffi Kim[1], Jordan Lewis[2], Hannah Rebadulla[3], Lena Thompson[4], Murat Demir[3], Prince Iverson Alejo[1], and Zoey Hilderbrand[5], *1. University of Alaska, Anchorage, Alaska, United States, 2. University of Minnesota Medical School, Duluth, Duluth, Minnesota, United States, 3. University of Alaska Anchorage, Anchorage, Alaska, United States, 4. University of Alaska Fairbanks, Fairbanks, Alaska, United States, 5. University of Anchorage Alaska, Anchorage, Alaska, United States*

Family stigma (FS), referring to experiences of stigma by association, is the consequence of stigmatizing beliefs and attitudes. This study assessed FS among Alaska Native (AN) Alzheimer's disease and related dementias (ADRD) caregivers spanning three domains: caregiver, layperson, and structural stigma. Stigma can prevent caregivers from seeking a diagnosis, treatment, or support services. Caregivers report limited knowledge of the disease, little engagement with support and resources, and social isolation. This pilot study used a mixed methods approach to outline ADRD-related stigma and its impact AN caregivers' quality of life. Semi-structured 60-90-minute interviews guided by the adapted Family Stigma – Alzheimer's Disease scale were conducted with thirteen female AN ADRD caregivers across Alaska. The caregiver's mean age was 62, with an average of seven years of caregiving. Descriptive statistics were used to report the quantity and frequency of reported experiences of stigma. Qualitative inquiries allowed for the exploration of in-depth cultural context. Interviews were analyzed using deductive and inductive content analysis. Caregivers identified several sources of FS. Layperson and structural stigma items were endorsed most often. Caregiver stigma detailed that limited support results in high levels of distress and experiences of depression. Cultural values such as caring for the Elders were protective and led to family and community involvement in giving care. Stigma reduced AN ADRD caregiver's quality of life in the areas of family cohesion, limited participation in social events, and impacted professional engagement negatively. This study can inform future work to decrease the stigma associated with AN ADRD.

Abstract citation ID: igae098.4304

### SELF-FUNDED PERSONAL CARE IS COMMON AND COSTLY FOR PEOPLE WITH DEMENTIA ACROSS THE INCOME SPECTRUM

Jennifer Reckrey[1], Karen Shen[2], and Yang Yang[2], *1. Icahn School of Medicine at Mount Sinai, Icahn School of Medicine at Mount Sinai, New York, United States, 2. Johns Hopkins University, Baltimore, Maryland, United States*

remain in the community; those without Medicaid coverage or long-term care insurance must self-fund this care (i.e., pay for privately or out-of-pocket). Using data from the Health and Retirement Study (2002-2018), we identified the prevalence and financial burden of self-funded paid care among community-dwelling people ≥65 who received help with ≥1 activity of daily living (n=4666). Dementia status, receipt of paid care, and financial information (income, cost of self-funded paid care) were determined by survey responses. One-quarter of PLWD (versus 14% without dementia) used self-funded care. Among PLWD, the probability of self-funding care increased with income, but was significant for all income groups: 15%, 23%, 32%, and 48% of those with incomes 100%, 100-200%, 200-400%, and ≥400 of the federal poverty level respectively self-funded paid care. The amount and cost of this self-funded care was high, particularly for PLWD: 45% of PLWD (versus 22% without dementia) received ≥40 hours/week of self-funded care and nearly half spent ≥$1000/month self-funding paid care. The median PLWD that self-funded care spent 45% of their monthly income on self-funded care; in the lowest-income group, the median PLWD spent nearly 75% of their monthly income on self-funded paid care. Given self-funded paid care is common and costly throughout the income spectrum, programs and policies aimed at easing the financial burden of self-funded paid care (e.g., expanded access to Medicaid home and community-based services) are essential, particularly for low-income individuals and families.

Abstract citation ID: igae098.4305

### UNMET NEEDS & AVOIDABLE NURSING HOME PLACEMENTS AMONG BLACK, LATINO, AND WHITE PEOPLE LIVING WITH DEMENTIA

Jasmine Travers[1], Shivani Shenoy[1], Marissa Bergh[2], Aasha Raval[1], and Andreina Jimenez[1], *1. New York*

*University, New York, New York, United States*, 2. *New York University, Long Island City, New York, United States*

Many nursing home (NH) placements are avoidable considering equivalent care can be provided in the community setting. Preventing avoidable NH placements and enabling aging in the home and community has been prioritized by federal, state, and local entities. Guided by Diwan and Moriarty's conceptual frame for Identifying Unmet Healthcare Needs of Community Dwelling Older Adults, this qualitative descriptive study explored the unmet needs and related barriers that drive avoidable NH placements among Black, Hispanic/Latino, and White people living with dementia (PLWD). One-on-one semi-structured interviews were conducted March-November 2022. Participants including PLWD, family care partners (FCP), and NH staff, were recruited from two New York NHs. Policy experts were recruited from aging organizations across the country. Data were analyzed using a directed content and thematic analysis approach. Sixty-one individuals were interviewed, 19 PLWD, 17 FCP, 15 NH staff, and 10 aging policy experts. Seven distinct themes specific to unmet needs emerged from the data and included 1) support with activities of daily living and basic home maintenance; 2) resources and services; 3) treatment; 4) socialization; 5) individual preferences; 6) function of the home; and 7) equipped, available, and supported family. Related barriers that contribute to unmet needs defined within our guiding framework included awareness, knowledge, availability, affordability, and acceptability of resources and services. Participants described multifaceted factors that contribute to the unmet needs driving avoidable NH placements. Strategies to address these unmet needs and improve the understanding of the complex home and community-based services landscape may help reduce these placements.

Abstract citation ID: igae098.4306

**WHAT FAMILY CAREGIVERS FEEL UNPREPARED FOR: DIFFERENCES BETWEEN DEMENTIA AND NON-DEMENTIA CAREGIVERS**

Roshni Singh[1], Richard Munoz[2], Sandra Garcia-Davis[1], Saanvi Lamba[3], Stuti Dang[4], Diana Ruiz[5], Luci Leykum[6], and Marianne Desir[5], 1. *University of Miami, Miami, Florida, United States*, 2. *South Florida Veteran Affairs Foundation for Research and Education, Miami, Florida, United States*, 3. *Flint Hill School, Oakton, Virginia, United States*, 4. *Miami VA Healthcare System, Miami, Florida, United States*, 5. *Miami Veterans Affairs Healthcare System, Miami, Florida, United States*, 6. *South Texas Veterans Health Care System, Austin, Texas, United States*

This study identified tasks for which informal caregivers feel insufficiently prepared, stratified by Veterans' dementia status. Using the Veterans Affairs' HERO CARE Survey data, we analyzed caregivers' responses to one open-ended question: "Out of all the tasks that you help the Veteran with, is there anything specific you would like to be better prepared for?" Responses were coded thematically into ten domains and differences in reported domains between caregivers of Veterans with and without dementia were compared. 296 dementia and 426 non-dementia caregivers were included. On average, caregivers were female (87.3%), non-Hispanic white (71.4%), primary caregivers (85.9%), and had provided over five years of care (54.2%). Dementia and non-dementia caregivers were demographically similar except in age (70.9 vs 65.9 years, $p < 0.001$); spouses (69.1% vs 58.2%, $p=0.004$); working at-least part-time (18.3% vs 25.9%, $p=0.003$), and hours of care-provision per week (94.7 vs 74.5, $p < 0.001$). Preparedness concerns for both dementia and non-dementia caregivers respectively included care-coordination (24.3% vs 25.1%, $p=0.81$), nursing/medical tasks (19.3% vs 21.6%, $p=0.44$), advance planning (16.6% vs 15.7%, $p=0.77$), and assistance with Instrumental Activities of Daily Living (IADLs) (11.5% vs 9.4%, $p=0.36$) and ADLs (11.5% vs 7.3%, $p=0.05$). Dementia caregivers expressed significantly more concerns about caregiver self-care (7.4% vs 3.1%, $p=0.007$), and less about handling emergencies (1.7% vs 4.7%, $p=0.03$) than non-dementia caregivers. Informal caregivers may need additional training in several domains of care tasks ranging from direct care to service coordination to improve caregiving competency. Dementia caregivers may particularly benefit from learning self-care strategies.

### SESSION 8075 (PAPER)

### LATE BREAKING: QUALITY IMPROVEMENT INNOVATIONS IN HEALTH CARE II

Abstract citation ID: igae098.4307

**ADDRESSING THE LONG-TERM CARE POLICIES IN HAWAII: AN APPLICATION OF BARDACH'S MODEL**

Mohammad Hossain, *Grand Valley State University, Walker, Michigan, United States*

In this policy analysis paper, I utilized Bardach's (1977) model to evaluate three alternative policy options addressing the crucial needs of long-term care in Hawaii. The policy options included the Long-Term Care Insurance (LTCI), the Kupuna Caregivers Program (KCP), and the Long-Term Care Insurance Partnership Program (LTCIPP). These policies were analyzed based on Bardach's (1977) eight-step model. Further, the evaluation justified the selected policies against several criteria such as financial feasibility, effectiveness, political acceptability, policy sustainability, an overall impact to support older adults towards age in place, effects on employers, and sustainability. The findings of this policy analysis included that the LTCIPP, as a 3rd policy alternative in the list, has more potential to address the service gaps of LTC in the long run compared to the remaining policies. Though the LTCIPP has been progressively growing as a promising LTC initiative, and currently, most U.S. states are entitled to this program, it might not be affordable for potential care recipients from marginal communities such as low-income, homeless, Indigenous communities, etc. Further, this paper acknowledges that addressing the needs for long-term care necessitates comprehensive efforts. Aside from the enrollment in LTCIPP for Hawaii, this analysis recommends promoting and advocating for appropriate funding for the Kupuna Caregiver Program, which is unique even in the U.S., to support low-income families and their care-needed elderly members. The paper suggests that partnering with private entities enhances the government's chances of avoiding cost-shifting challenges.

Abstract citation ID: igae098.4308

**AN ASSESSMENT OF STATE EMPLOYEE BACKGROUND CHECK POLICIES FOR IMPROVING QUALITY IN NURSING FACILITIES**

Gul Rukh Mehboob[1], Brian Kaskie[2], and Hari Sharma[3], *1. University of Iowa - Iowa City, IA, Iowa City, Iowa, United States*, *2. University of Iowa, Iowa City, Iowa, United States*, *3. University of Iowa - Iowa City, IA, Iowa, Iowa, United States*

Employee background checks are important measures for ensuring the safety of older adults in nursing homes. Programs like the National Background Check Program (NBCP) provide federal grants to help states enhance their background check systems for long-term care receivers. While these checks require resources, their effectiveness in reducing abuse, neglect, and exploitation remains uncertain. We aim to compare NBCP grant-receiving states (N=27) with non-receiving states (N=23) to identify regulatory changes and adaptation of specific policy measures over time to improve employee background check systems. We used a policy scan approach to compare and rank states by thoroughly analyzing regulations, bills, documents and reports from 2005 to 2024. We evaluated states based on three policy domains: types of LTC providers required to conduct employee background checks, the disqualifying offenses considered by the states, and the processes used to implement and administer these checks. We also analyzed temporal changes in states' background check systems over time. We found notable differences in regulations concerning the types of LTC providers involved and disqualifying offenses between NBCP and non-NBCP states. However, we found little difference in the processes between these two groups. Overall, the NBCP states show better adaptation and temporal changes due to advanced technical systems and improved regulations. To our knowledge, this is the first study to comprehensively assess the regulatory framework of state employee background check systems. Our study has direct policy implications for state and national policymakers debating the effectiveness of NBCP in improving nursing home quality.

Abstract citation ID: igae098.4309

### CONTENT ANALYSIS - NURSING HOME (NH) STATEMENTS OF DEFICIENCIES (SODS) AS QUALITY IMPROVEMENT INNOVATION

Mary Dellefield[1], and Caroline Madrigal[2], *1. UCSF, San Francisco, California, United States*, *2. VA Boston Healthcare System, Brockton, Massachusetts, United States*

The Centers for Medicare and Medicaid (CMS) issue Statements of Deficiency (SODs) collected by trained observers detailing practices that pose harm or 'immediate jeopardy' to nursing home residents. SODs document nursing home compliance failures, such as F-tag 0678, which addresses non-compliance with federal cardiopulmonary resuscitation (CPR) requirements, a critical indicator of resident autonomy and self-determination. This study aimed to describe practice patterns and organizational characteristics linked to F-tag 0678 immediate jeopardy citations in 80 nursing homes across CMS Region 5 (Michigan [17], Minnesota [30], Ohio [22], Wisconsin [11]} from 2019-2022. An a-priori code book was developed, based on previous SOD research and American Heart Association guidelines. Content analysis was applied to SODs to identify practice patterns and organizational characteristics. Sampled nursing homes were for-profit (69%), with a mean bed size of 93 (range= 26-212) and an average star rating of 2.4 (range=1-5). Major practice pattern failures included: early detection (identifying code status/initiating CPR); logistics (communication failures; failures to follow policy/procedures); documentation (lack of clarify about code status/inaccurate documentation of CPR event); staff performance (lack of familiarity with nursing home operations); and delay in initiating CPR (13 minutes to 4 hours). Structured analysis of SODs offers a novel, efficient approach to leverage existing CMS data to drive quality improvement efforts and inform policy decisions. This research has the potential to inform the development of evidence-based interventions, enhance regulatory oversight, improve the quality of care, and protect the rights of vulnerable nursing home residents.

Abstract citation ID: igae098.4310

### EXAMINE QUALITY OF CARE AMONG OLDER ADULTS IN MEDICARE ADVANTAGE COMPARED WITH TRADITIONAL MEDICARE

Elham Mahmoudi, *University of Michigan Medical School, Ann Arbor, Michigan, United States*

About 50% of Medicare beneficiaries are enrolled in Medicare Advantage (MA), incentivized to offer more efficient and coordinated care than traditional Medicare (TM). Evidence suggests timely access to coordinated and high-quality ambulatory care is critical to preventing adverse health events (e.g., hospitalization and long-term institutionalization). In this study, we used 2017-2021 (latest available) Medicare Current Beneficiary Survey (MCBS) data to examine the effect of MA vs. TM on 10 quality metrics among older adults with and without dementia. We applied inverse propensity weighting to balance the characteristics of MA and TM enrollees and used the Average Treatment Effect method, adjusting for sociodemographic, rurality, and health-related variables. Among people without dementia, MA was associated with greater challenges receiving needed care [TM=5.06%; MA=6.50%; (diff=1.4 percentage point (PP); 95% CI:0.9%,1.9%)] and accessing specialty care [TM=5.25%; MA=6.02%; (diff=.73 PP; 95% CI:.21%,.1.26%)]. However, TM was associated with more dissatisfaction with out-of-pocket costs [TM: 13.78%; MA=12.18%; (diff=1.6PP; CI: -2.32%, -.87%)] and distance/location of different health services [TM=8.67%; MA=7.49%; (diff=1.18 pp; CI: -1.81, -.55)]. Among beneficiaries with dementia, except for dissatisfaction with ease of access to care with better results in MA (TM=7.53%; MA=5.09%; diff=2.45pp; 95%CI: -4.6%, -.26%), we did not find any significant differences in quality metrics between MA and TM. In conclusion, TM performed better in receiving the needed care and specialty care. MA was superior in lowering out-of-pocket costs and better care coordination. Dissatisfaction with care was substantially higher among enrollees with dementia than among those without, regardless of MA or TM.

Abstract citation ID: igae098.4311

### EXAMINING THE COMPLETENESS OF MEDICARE ADVANTAGE ENCOUNTER DATA FOR MEASURING POST-ACUTE CARE UTILIZATION

Hyunkyung Yun, and Cyrus Kosar, *Brown University, Providence, Rhode Island, United States*

Given the rapid growth of Medicare Advantage (MA) program, the degree to which the newly-released MA encounter data reliably capture care utilization has serious implications for the ability to evaluate MA-related policies accurately and conduct rigorous academic research. However, little is known about the quality of MA encounter data outside of the acute care setting. We assessed how well the encounter data captured post-acute skilled nursing facility (SNF) and home health (HH) utilization overall and across MA contracts. We identified 2016-2019 Minimum Data Set (MDS) and the Outcome and Assessment Information Set (OASIS) admission assessments preceded by inpatient claims and calculated the proportion of assessments matched to a SNF or HH encounter record within 7 days of the assessment's admission date. In 2019, there were 871,101 MDS assessments and 812,002 OASIS assessments following an MA inpatient stay. About 82% of MDS assessments could be matched to a SNF encounter records (up from 75% in 2016) while 63% of OASIS assessments matched to a HH encounter record (52% in 2016). There was substantial contract-level variation in match rates and a lack of correlation across services. Only 21% of contracts that had high SNF encounter completeness (match rates of 90% and above) also had high HH completeness, and 68% of contracts with high HH completeness also had high SNF completeness. Efforts to improve the quality of MA encounter data are needed as CMS prioritizes this data as a source for program oversight and payment.

Abstract citation ID: igae098.4312

### IMMERSIVE GREEN SPACE AND EMOTIONAL BENEFITS FOR OLDER PEOPLE LIVING IN RESIDENTIAL CARE HOMES FOR ELDERLY (RCHES)

Hiu Kwan Yung[1], Adedayo Ogungbile[2], Eric Ohene[1], Yujun Fan[1], Siqiang Wang[1], and Cynthia Yim[1], *1. The Hong Kong Polytechnic University, Hong Kong, Hong Kong, China (People's Republic)*, *2. Hong Kong Metropolitan University, Hong Kong, Hong Kong, China (People's Republic)*

In densely populated cities like Hong Kong where land is scare, elderly residents in care homes often do not have access to natural gardens. However, previous research has proofed that natural gardens are beneficial to people's psychological and social well-being. To address this problem, our study explores the use of a portable Cave Automatic Virtual Environments (CAVE) to simulate therapeutic effects of physical gardens, with the aim of providing elderly residents of RCHEs with the healing elements of nature, and potentially reducing stress and enhance emotional health. 18 residents living in two RCHEs were recruited to participate in the virtual garden experiments bi-weekly over two months. Questionnaire surveys were conducted to elicit their evaluation and feelings on the different features of the virtual gardens. We collected respondents' skin conductance levels by using the Shimmer wearable sensors to analyze changes in heart rates, stress levels and emotional states. The results show that the virtual garden is able to make older people feel more relax and recall the nostalgic feelings of the built environment. This study has won the award organized by the National Academy of Medicine of the United States which recognizes its contribution in catalyzing innovations to promote people's lifelong physical, mental, and social health and well-being.

### SESSION 8080 (PAPER)

### LATE BREAKING: EFFECTS OF DISCRIMINATION ACROSS THE LIFE COURSE

Abstract citation ID: igae098.4313

### COUNTY JAIL AS A SITE OF CARE AND CONTROL FOR OLDER HOMELESS SEATTLELITES

Ian Johnson, *University of Texas- San Antonio, San Antonio, Texas, United States*

An estimated 1.2% of people over age 50 living in community settings have been recently involved in the criminal-legal system. County jails detain people who have yet to face trial, have been sentenced to misdemeanors with short sentences, or who violated parole. Much of the attention to aging within the criminal-legal system has focused on long-term institutions such as prisons; this gap limits our understanding and ability to respond to the need of older adults in the homeless-jail cycle. Using electronic medical record data from a specialty street-based palliative care team and key informant interviews, this presentation will present preliminary findings from a reflexive thematic analysis. Findings include: (1) entangled health and behavioral health precedents for criminal-legal involvement; (2) jail healthcare designed for high acuity and low complexity; (3) discretionary and subjective compassion for aging issues; and (4) ageism's role in driving institutionalization of older people experiencing homelessness. Implications include guiding future abolitionist research in gerontology, workforce preparation within homeless response systems and correctional programs, and interventions to reduce jail use by older people.

Abstract citation ID: igae098.4314

### DISCRIMINATION ACROSS THE LIFE COURSE OF OLDER LONG-TERM RESIDENTS IN KOREA

Jasmyn Kim[1], and Jinmoo Heo[2], *1. Yonsei University, Seoul, Republic of Korea*, *2. Yonsei University, Seoul, Republic of Korea*

Despite being one of the world's most homogenous nations, South Korea's (hereafter Korea) international population has grown significantly over the past few decades. As of 2022, approximately 2.5 million foreign citizens reside in Korea, comprising 5% of the total population and contributing to its evolving identity as a multicultural society. This demographic shift parallels Korea's rapid socio-economic growth. However, foreign residents often encounter substantial challenges due to the persistent notion of a unified racial, ethnic, and cultural identity in Korean society, which can lead to cultural differences, social exclusion, stereotypes, and discrimination against those considered 'others.' The current qualitative case study explores the experiences of older long-term foreign residents from Western countries, focusing on discrimination across the life course. In-depth interviews were conducted with older international adults (mean age: 64.5) who had resided in Korea for over 3 years. Key findings revealed that despite experiencing alienation and exclusion from mainstream society, participants highlighted the importance of establishing social connections within both expatriate and local communities and reported a sense of belonging fostered through intercultural exchange, personal relationships and professional achievements. Although there is a growing emphasis on multiculturalism, prevailing cultural norms often hinder the complete integration of foreign residents into Korean society. This research sheds light on the complex power dynamics of ethnicity, social status, and discrimination, highlighting the need for more inclusive policies to better incorporate foreign residents in an increasingly globalized society.

Abstract citation ID: igae098.4315

**HEALTH EFFECTS OF EXTENDED WORKING LIFE AMONG OLDER ADULTS**

Rongfang Zhan, and Elias Mpofu, *University of North Texas, Denton, Texas, United States*

**Objectives:** To examine health benefits of working beyond the age of 66 in addition to the impact of socioeconomic status (SES) on potential health effects.

**Methods:** This longitudinal study utilized data from 4,492 respondents, aged 50 years and over, from 6 waves (2004 - 2014) of the Health and Retirement Study. Measures included physical activities of daily living, mental health and the prevalence of chronic diseases in older adults.

**Results:** Remaining in the workforce was associated with a reduced risk of mental illness. Socioeconomic disparities were apparent: extended working life had a beneficial effect on physical health and independence of older adults with lower SES and enhanced mental wellbeing of those with higher SES. No significant group differences in prevalence of chronic conditions was noted.

**Discussion:** The current findings provide support for public policies aimed at increasing retirement age and have implications for how best to protect older worker's health.

Keywords: Older workers, retirement, socioeconomic status, health.

Abstract citation ID: igae098.4316

**LGBTQ-RELATED NEGATIVE LIFE EVENTS AND COGNITION: THE ROLE OF SLEEP, MENTAL DISTRESS AND UNSUPPORTIVE FAMILY**

Tara McKay, and Anyah Prasad, *Vanderbilt University, Nashville, Tennessee, United States*

LGBTQ+ older adults are at increased risk of poor cognition. Using data from the LGBTQ+ Social Networks, Aging and Policy Study (QSNAPS, N=1,202), a unique panel study of 50-76 year old LGBTQ+ adults, and employing Structural Equation Modelling (SEM), we examine the association of LGBTQ-related negative life events (NLEs) with cognitive difficulty. We test whether the effect of NLEs on cognition is mediated by sleep quality or mental distress and whether it is exacerbated by the presence of unsupportive family members. About 20% of respondents report past year experiences of discrimination, harassment and/or violence and 40% report any cognitive difficulty. The association of NLEs with cognitive difficulty (e.g., trouble remembering names) is fully mediated by trouble falling asleep and mental distress (OR=1.21); NLEs increased trouble falling asleep (OR=1.96, p< 0.000), poorer sleep increased the likelihood of severe mental distress (OR=1.32, p< 0.000) and cognitive difficulty (OR=1.09, p<.001), and mental distress increased the odds of cognitive difficulty (OR=2.06, p< 0.001). The presence of unsupportive family members moderates the effect of NLEs on mental distress (main effect: OR=1.15, p=0.67; interaction effect: OR=2.86, p=0.009). The mediation effect of mental distress for the association between NLEs and cognitive difficulty for those with unsupportive family (OR=2.36) is greater than for those with no unsupportive family (OR=1.11). LGBTQ+ adults, especially those with unsupportive family, are at increased risk of cognitive impairment in later life. Interventions to support mental wellbeing, sleep hygiene, and affirmation of LGBTQ+ family members across the life course are critical to achieving health equity in aging.

Abstract citation ID: igae098.4317

**THE INFLUENCE OF DISCRIMINATION ON GAY AND LESBIAN SPOUSAL DEMENTIA CAREGIVERS**

Toni Calasanti[1], and Brian de Vries[2], *1. Virginia Tech, Blacksburg, Virginia, United States*, *2. San Francisco State University, San Francisco, California, United States*

Most research has speculated that discrimination would influence the experiences of gay and lesbian spousal caregivers (e.g., Fredriksen-Goldsen, et al, 2016), but to date little research has revealed the actual impacts of discrimination and where and how it matters. The present study of 13 gay and 16 lesbian spousal dementia caregivers finds that both past experiences and present discrimination influence how they give care. On the one hand, past experiences of discrimination have taught these caregivers to be cautious, even when they have been out for many years and even when they live in what are typically considered to be supportive areas. Further, and even though many respondents reported no accounts of discrimination presently, some reported discriminatory experiences with social service or medical providers; and one gay husband had experienced violence in a care setting. At the same time, and what is less documented, are the ways that our respondents feel that what they learned to do to respond to discrimination prepared them for the challenges of spousal dementia care. This included all that lesbians and gay men faced during the HIV/AIDs epidemic. Having been "othered," they feel they are more empathetic and sensitive to the stigma that comes with cognitive decline; they have greater self-reliance borne of the struggle with HIV/AIDs or in the face of estrangement from families or broader society; and they rely upon the resources provided by their communities. These results highlight areas both for subsequent novel research and interventions to support same-sex spousal dementia caregivers.

Abstract citation ID: igae098.4318

**THE PERCEIVED LIFE EXPERIENCES OF AFRICAN METHODIST EPISCOPAL (AME) MEMBERS**

Nia Lane-Nelson, *Hallmark University, San Antonio, Texas, United States*

The African American (AA) church has played an essential role in the AA community. Using the Life Course Theory (LCT) as a sensitizing framework, this qualitative descriptive study described how AA members of one AME church perceived their life experiences as influencing their current health status. LCT key concepts (pathways or trajectories, early life experiences, critical or sensitive periods, cumulative impacts, and risk and protective factors) concentrate on the socioeconomic and environmental contexts within the life of an individual. Individual interview questions were developed based on the LCT key concepts. Seventeen in-depth interviews were conducted. Qualitative content analysis was used to identify categories and subcategories. Within the key concept of early life experiences 3 categories (Economic hardships, Personal hardships, and Protective influences in early childhood) were identified. Within the category of Economic hardships 3 subcategories were identified; not having food, living was tight, and neglect in early childhood due to economic hardships. Within Personal hardships the subcategories were parental responsibilities in early childhood, separation in family, family trauma, and experiences with racism at an early age. Within Protective influences in early childhood the subcategories were receiving love and support, feeling safe and protected, learning from parents and others, dealing with racism, and active in childhood activities. Through using the LCT, participants were able to highlight the social, economic, and environmental issues experienced throughout their lives and the impact on their health. Participants shared narratives spanning from early childhood to the present, with a common stressor being experiences with racial discrimination.

Abstract citation ID: igae098.4319

### UNDERSTANDING RACIAL BIAS AWARENESS ACROSS ADULTHOOD: THEMES OF BIAS, JUSTIFICATIONS, AND EMOTIONS

Jennifer Beatty[1], Patrick Hill[1], Jonathan E. Doriscar[2], and Seanna Leath[1], *1. Washington University in St. Louis, St. Louis, Missouri, United States*, *2. Northwestern University, Evanston, Illinois, United States*

This project employed a mixed-methods approach to analyze how age-diverse White Americans (N = 345; age range: 19 to 85) make meaning of specific instances of personal racial bias. Across three data collections, that targeted younger, middle-aged, and older adults, we asked participants to write about a time when they were racially biased. In the samples, 98% of younger adults, 91% of middle-aged adults, and 86% of older adults indicated specific instances of racial bias, and many indicated antiblack bias. Age-diverse adults frequently assumed that racial out-group members were criminal or deviant or assumed that those individuals had an undesirable culture. The participants also often indicated numerous justifications and emotions about their bias. For example, common justifications across all ages included perceived threat or the ambiguity of the situation. Specific emotions and justifications only differed moderately by age. Our findings indicate that while age-diverse White adults may make meaning of their bias in different ways, they tend to do so with self-conscious emotions and bias justifications that do not mitigate bias. Critically, middle-aged and older adults were less likely to indicate emotions at all in their reports. These findings may reflect a positivity bias among older adults who are less willing to highlight or remember their negative emotions or it may speak to the possibility that they may feel less fear in intergroup settings. The current work offers insights into more personalized approaches to bias regulation by integrating specific bias contexts with bias justifications and emotional awareness.

### SESSION 8085 (PAPER)

### LATE BREAKING: HEALTH EQUITY ACROSS THE LIFE COURSE III

Abstract citation ID: igae098.4320

### ADVERSE CHILDHOOD EXPERIENCES AND SOCIAL PARTICIPATION ON FRAILTY STATE TRANSITIONS: A 10-YEAR PROSPECTIVE COHORT

Jiajia Li[1], Heming Pei[2], Ning Kang[1], Gong Chen[1], and Lijun Pei[1], *1. Peking University, Beijing, Beijing, China (People's Republic)*, *2. Columbia University, New York, New York, United States*

**Background:** Adverse childhood experiences (ACEs) is associated with frailty, while the association with frailty state transitions and the role of social participation remain unclear. This study aimed to investigate the association between ACEs and frailty state transition, alongside the moderation effect of social participation

**methods:** Data from 9,621 adults aged 45+ from the CHARLS (2011-2020) were analyzed. Frailty was measured with the frailty index, while ACEs and social participation were measured with a validated questionnaire. The association between ACEs and frailty state transitions was estimated with multi-state models. Interaction analysis was used to examine the moderation effects of social participation.

**Results:** Participants exposed to higher ACEs levels (≥ 4) were associated with an increased risk of forward transition (robust to pre-frail, HR=1.36, 95%CI: 1.21-1.53; prefrail to frail, HR=1.38, 95%CI: 1.17 - 1.62) and decreased risk of backward transition (pre-frail to robust, HR = 0.64, 95%CI: 0.54 – 0.76). Additionally, participants with social participation were associated with increased risk of backward transition (frail to pre-frail, HR = 1.14, 95%CI: 1.02 – 1.27). Social participation moderated the association between ACEs exposure and frailty (P for interaction < 0.05), while participants with lower ACEs exposure levels and social participation were associated with an increased risk of transition from frail to pre-frail (ACEs levels =1, HR = 1.57, 95%CI: 1.06 – 2.32; ACEs levels =2, HR = 1.49, 95%CI: 1.02 – 2.17).

**Conclusion:** High levels of ACEs exposure were associated with a greater likelihood of adverse frailty development. Older adults with ACEs exposure might benefit from intervention strategies to improve social participation.

Abstract citation ID: igae098.4321

### INVESTIGATING HEALTHCARE ACCESS DISPARITIES IN MINNESOTA THROUGH GIS ANALYSIS

Woo Jang[1], Drew Krull[2], Seonhwa Lee[3], and Merril Silverstein[4], *1. Minnesota State University, Mankato, Mankato, Minnesota, United States*, *2. ERM, Minneapolis, Minnesota, United States*, *3. Texas Women's University, Denton, Texas, United States*, *4. Syracuse University, Syracuse, New York, United States*

According to the 2020 report on population projections from the Minnesota State Demographic Center, older residents aged 85 and above are expected to more than double within the next 35 years. The report also highlights that in 2033, 32% of rural counties will experience a higher proportion of residents aged 65 years and older than 19% for urban counties. With the expected increase in the older population, the demand for healthcare services in rural areas will substantially rise. However, healthcare facilities are more dispersed in rural areas. Despite several accounts pointing to healthcare-deprived areas or the differential geographic presence of healthcare resources in recent years, GIS-based analysis in this area remains limited. To address this gap, our study utilizes data from the 2020 Census to assess various socio-demographic and economic factors shaping healthcare access inequalities for individuals aged 65 years and older in Minnesota. Employing GIS techniques, we identify unique spatial patterns and associated social factors contributing to healthcare access disparities. Our results visualize detailed maps highlighting areas facing challenges in accessing healthcare services and low-access populations across Minnesota. This study provides insights into understanding the spatial and social determinants of healthcare disparities, which assist policymakers and healthcare professionals to allocate resources more efficiently and address the unique needs of the target group.

Abstract citation ID: igae098.4322

### MARITAL STATUS AND VASCULAR EVENTS IN A DIVERSE NORTHERN MANHATTAN COHORT

Courtney Gao[1], Jackson Roberts[2], Tatjana Rundek[3], Mitchell Elkind[4], Hannah Gardener[5], and Jose Gutierrez[6], *1. Columbia Presbyterian Medical Center, New York, New York, United States*, *2. Massachusetts General Brigham, Boston, Massachusetts, United States*, *3. University of Miami, University of Miami Miller School of Medicine, Florida, United States*, *4. Columbia University Irving Medical Center, New York, New York, United States*, *5. Leonard M. Miller School of Medicine, University of Miami, Miami, Florida, United States*, *6. Columbia University, New York, New York, United States*

**Background:** Studies have shown that marital status is related to vascular outcomes but with potential variation by race/ethnicity. It remains unclear whether these associations occur in predominantly Hispanic populations.

**Methods:** In the Northern Manhattan Study prospective cohort, we assessed participants ≥40 years and stroke-free at baseline. We categorized participants as married or unmarried, a group subdivided into divorced/separated, widowed, or single. We used adjusted hazard models to relate marital status to myocardial infarction (MI), stroke, vascular death, and all-cause death.

**Results:** Of 2865 participants (53% Hispanic, mean 69 ±10 years), 32% were married, 16% were single, 28% were widowed, and 25% were divorced/separated. During a mean follow-up time of 15 years (IQR 3-28),14% had a stroke, 8% had an MI, and 68% died. Unmarried participants had a higher risk of any vascular event (aHR, 1.20 [95% CI, 1.04-1.39]) and all-cause death (aHR, 1.14 [95% CI, 1.02-1.28]) compared to married participants. The direction of the hazard ratios for stroke (aHR, 1.06 [95% CI, 0.83-1.35]) and MI (aHR, 1.11 [95% CI, 0.81-1.51]) was consistent with an increased risk. Divorced/separated participants had a higher risk of vascular events (aHR, 1.35 [95% CI, 1.14-1.61], vascular death (aHR, 1.29 [95% CI, 1.05-1.59]), and all-cause death (aHR, 1.21 [1.06-1.39]) compared to married participants.

**Conclusion:** In a predominantly Hispanic cohort, unmarried participants had a higher risk of vascular events and all-cause death. Future studies should explore the impact of risk stratification of patients based on marital status and provision of support to patients undergoing marital transition.

Abstract citation ID: igae098.4323

### SAFETY OF PUBLIC BUSES IN NATURALLY OCCURRING RETIREMENT COMMUNITIES

Tracy Chippendale[1], Ruheena Sangrar[2], Shlomit Rotenberg[2], Angela Curl[3], and Lisa Suzuki[1], *1. New York University, New York, New York, United States*, *2. University of Toronto, Toronto, Ontario, Canada*, *3. University of Otago, Christchurch, Otago, New Zealand*

Community mobility is critical to support aging-in-place. For older adults living in low-and moderate-income urban communities, driving may not be an option due to costs associated with car ownership, impracticalities of cars in urban settings, or a lack of driving history. Some older adults must retire from driving due to changes in their functional status. Safe public transportation is therefore paramount to equity in community mobility. The purpose of this Ecological theory informed study was to explore supports and limitations regarding safe public bus use in naturally occurring retirement communities (NORC). A multiple qualitative case study design was used with purposive sampling of representative cases, including four bus routes in NORC neighborhoods, two in Toronto, Canada, and two in NYC. Field observations and in-depth semi-structed interviews were conducted. Inclusion criteria for participants were: 1) Age 60 and older 2) Resident of one of the four NORC neighborhoods 3) Ride the bus on average 2 or more times per month 4) Ambulatory with or without a walking aid (e.g. cane, rollator) and 5) English speaking. A descriptive, thematic approach was used for data coding and analyses, and data was organized according to the Framework Method. Approximately 45 hours of field observations were conducted, and N=17 NORC residents participated in interviews. Results revealed four major themes: Impact of other people's behavior, Problematic passageways: obstructions, Personal impact on safety, and Space & time orientation. Several subthemes also emerged. Results suggest implications for policy and education to increase safety for older adult bus users.

Abstract citation ID: igae098.4324

### SUPPORTIVE CARE NEEDS AND INTERVENTIONS IN PANCREATIC CANCER PATIENTS AND THEIR CAREGIVERS: A SCOPING REVIEW

Liang Fu[1], Su Hyun Kim[2], Matt Hayward[2], Candice Vieira[2], Alexander Parikh[2], and Lixin Song[1], *1. University of Texas Health Science Center at San Antonio, San Antonio, Texas, United States*, *2. Kyungpook National University, Daegu, Republic of Korea*

Pancreatic cancer remains one of the most lethal cancers and predominantly affects older adults. This scoping review aims to provide a comprehensive review of the literature regarding supportive care needs and corresponding interventions in patients with pancreatic cancer and their family caregivers. We conducted this scoping review following the Joanna Briggs Institute Manual for Evidence Synthesis. We searched five English databases using 'pancreatic cancer,' 'patients/caregivers,' 'supportive care,' and 'needs' in January 2024. We summarized the data employing the Supportive Care Framework. Of the 4,342 references identified, 41 were included. Among the 31 descriptive studies, the top four highest supportive care needs were informational (n = 24), physical (n = 10), emotional (n = 9), and practical needs (n = 8). In the 10 experimental studies, most of the interventions in involved pain/symptom management (n = 8), following by adjustment/supportive counselling (n = 6), psycho-educational service or activity (n = 4), and orientation/ongoing patient and family education (n = 4). Four studies demonstrated statistically significant improvements in outcomes for intervention groups compared to control groups. Patients with pancreatic cancer and their family caregivers experienced a spectrum of supportive care needs, particularly informational needs. Several intervention strategies were applied to address these supportive care needs, but only a few demonstrated statistically significant improvements in outcomes. These findings enhance our understanding of the supportive care needs and appropriate interventions for patients with pancreatic cancer and their family caregivers, providing a foundation for further research to address these needs through a health equity lens.

### SESSION 8090 (PAPER)

### LATE BREAKING: APPLYING PRINCIPLES TO REFRAME AGING IN YOUR WORK

Abstract citation ID: igae098.4325

### "IT TAKES A SPECIAL PERSON": EXPLORING NURSING STUDENTS' END-OF-LIFE CARE EXPERIENCES AND ITS INFLUENCE ON EMOTIONS

Kalei Kowalchik, *The Pennsylvania State University, Olyphant, Pennsylvania, United States*

Navigating end-of-life care as a nursing student can results in intense emotions such as grief and despair and there is limited research on undergraduate nursing students' thoughts and experiences on the need for emotional support resources when caring for patients and families at the end of life. To explore to explore and understand what end-of- life care experiences influence undergraduate nursing students' emotions. A qualitative descriptive design study was used to interview third- and fourth-year nursing students. Purposive sampling techniques were used to recruit participants. Demographic data was collected via Qualtrics, and interviews were held via face-to-face (n=4) and Zoom (n=17). Data were transcribed verbatim, de-identified, and analyzed for themes. Results show that providing care to dying patients and their families is emotionally impactful to students and creates self-awareness about death and mortality. Students' personal, educational, occupational, and clinical experiences caring for dying patients influences how they process and regulate their emotions when professionally caring for dying patients and their families. Additionally, these experiences are influential to how they interact with death and dying topics in their personal lives. Caring for dying patients and exposure to death as a nursing student is emotionally impactful to students as an emerging professional and as a person. Nurse educators should integrate supportive resources for students caring for dying patients to help develop students' resilience, emotional intelligence, and self-awareness when providing EOL care.

Abstract citation ID: igae098.4326

### A PILOT AND FEASIBILITY STUDY OF THE AGING TOGETHER ANTI-AGEISM PEER SUPPORT PROGRAM

Andrew Steward, Connor Thomas Francisco Keane, Yura Lee, and Young Cho, *University of Wisconsin-Milwaukee, Milwaukee, Wisconsin, United States*

Ageism is internalized from an early age, with accumulating, negative health impacts across the life course. Although the theory of relational ageism describes how ageism is reinforced through interpersonal interactions, few ageism-related intervention studies have actively engaged older adults at personal and relational levels. We conducted a pilot and feasibility study of a manualized, anti-ageism peer support program facilitated by trained older adults called "Aging Together." Key components of the program include peer support, education about ageism and health, and "tell your story of aging." Participants received a 30-page workbook providing education and journaling/discussion prompts. Six small groups of five-to-eight participants (N=48 total) met for ten, weekly, in-person (60-90 minute) sessions in a medium-sized city of the U.S. Midwest. Results indicate the program was feasible and acceptable overall (attendance rate=88.91%, attrition rate=6.25%, Mean acceptability score=4.23/5). Based on 30-minute pre/post phone surveys, Mann-Whitney U tests (N=28) indicated a significant decrease in relational ageism (M=2.73/6 to 2.19/6; p<.01), decrease in internalized ageism (M=1.91/6 to 1.66/6), significant decrease in depressive symptoms (M=8.61/30 to 6.57/30; p<=.01), increase in social connectedness (M=4.19/5 to 4.26/5), increase in self-efficacy (M=3.29/4 to 3.36/4), and decrease in cognitive function (M=29.67/40 to 29.5/40). By incorporating education about ageism and storytelling within a small, peer support group setting, the Aging Together program may be particularly effective in reducing depressive symptoms and relational ageism, which is an understudied aspect of ageism. Future research should scale this program across diverse geographical settings and explore potential mediating relationships between internalized/relational ageism and psychosocial well-being.

Abstract citation ID: igae098.4327

### CONTRIBUTING FACTORS TO POST-RETIREMENT EMPLOYMENT AND LIFE SATISFACTION

Zhong Wang, *Yeshiva University, New York, New York, United States*

This study explores the factors influencing post-retirement employment and its impact on retirement satisfaction, aiming to provide insights to improve older adults' productivity and well-being, thereby enhancing their life satisfaction and contributing to the cohesion of families, communities, and society at large. Utilizing secondary data from the RAND HRS Longitudinal File 2020, this quantitative research examines a sample of 1,561 individuals born in 1948 and 1949. The longitudinal data tracks changes in retirement trajectories and self-reported well-being over time. Logistic regression analysis revealed several key findings: African American and Hispanic individuals were less likely to intend to work beyond age 65 but more likely to engage in post-retirement employment. Higher household wealth correlated with a decreased intention to work past 65 but an increased likelihood of continued labor market participation. The health and employment status of a spouse significantly impacted older adults' work intentions, retirement decisions, and satisfaction. Additionally, there were significant reciprocal relationships between respondents' work plans and their retirement decisions. The findings highlight the need to address the diverse job-related needs of older adults in gerontological social work practice. Advocating for employment as a human right, the study highlights the importance of age-friendly policies, such as flexible work arrangements and phased retirement options. Social work practice can enhance opportunities for older adults through financial literacy programs, job training, and career counseling, particularly in underserved communities. Additionally, acknowledging the correlation between family dynamics and retirement experiences, gerontological social work can better support older adults in navigating their retirement transitions.

Abstract citation ID: igae098.4328

### CREATING A SUCCESSFUL DATA PLAN: NIA BEHAVIORAL AND SOCIAL AGING RESEARCH DMS REVIEW EXPERIENCE

James McNally[1], and Paavani Jain[2], *1. University of Michigan, Ann Arbor, Michigan, United States*, *2. National Institute on Aging, Baltimore, Maryland, United States*

The revised NIH Data Management and Sharing (DMS) Policy took effect in January 2023, requiring all researchers applying for NIH funding to develop a detailed DMS plan as part of the application. In turn, NIH became responsible for reviewing and approving the submitted plan before issuing an award and monitoring compliance. Having recently completed our first year of DMS reviews, this presentation will report lessons learned by the NIA Division of Behavioral and Social Research (DBSR). One of the critical changes was the requirement for a six-element document addressing Data Type; Related Tools, Software, and Code; Standards; Data Preservation, Access, and Associated Timelines; Access, Distribution, or Reuse Considerations; and Oversight of Data Management and Sharing study. Despite efforts to educate and prepare all parties for this transition, researchers had questions about the best way to prepare a DMS plan and from NIA staff on reviewing the submitted requests. Within DBSR, the Office of Data Resources and Analytics (ODRA) established a review team to examine DMS plans, develop necessary resources, and coach grantees. The presentation will present lessons learned for best practices in DMS development. We will discuss handling primary versus secondary data, metadata, and source code, selecting an appropriate repository for socio-behavioral and interdisciplinary aging data, data restrictions, and navigating various grant mechanisms and their data prospects. A non-compliant DMS plan can delay or reverse funding decisions; NIA seeks to ensure all researchers understand this plan's importance and how to successfully create one that supports ongoing scientific discovery.

Abstract citation ID: igae098.4329

### FRAMING AGE AND IMMIGRATION: OLDER IMMIGRANT WOMEN AND WELL-BEING IN A TIME OF SOCIAL INTOLERANCE

Jasmin Tahmaseb, *West Chester University, West Chester, Pennsylvania, United States*

In today's contemporary troubling social climate, discourse against immigration is on the rise. In the U.S. older immigrants make up 13 % of the older adult population. The media often portrays both older adults and immigrants in biased and stereotypical ways, such portrayal can have a negative impact on the well-being of older immigrants and increase late life isolation and loneliness. This presentation examines the impact of how immigration and age are framed during the 2024 political campaign and the ways that those messages influence the lives of older immigrant women. Life history interviews exploring the goals, concerns, and supports of 18 older immigrant women (70+) indicate that the current political discourse on immigration has a negative influence on their lives, particularly as they navigate late life challenges. Media messages addressing the intersection of immigration and age are often negative increasing psychological distress among the women interviewed. We present examples and discuss the results of a content analysis of 10 news articles appearing over a 2-month period in several prominent newspapers, an analysis that illustrates the discriminatory nature of the ways that content is framed. We discuss the concerns, stressors, and resilience of participants as they struggle to manage late life challenges in a time of social intolerance and discrimination. The lives of older immigrants are often marked by cultural and economic challenges. Discriminatory framing of messages relating to both aging and immigration can increase life-course cumulative disadvantage negatively impact the well-being of an already vulnerable and socially marginalized group.

Abstract citation ID: igae098.4330

### IMPACTS OF INTERGENERATIONAL CARE ON FUTURE CARE EXPECTATIONS: A SURVEY-EXPERIMENT STUDY OF AGING SANDWICH CARERS

Xue Bai, and Shuai Zhou, *The Hong Kong Polytechnic University, Hong Kong, Hong Kong, China (People's Republic)*

**Abstract background:** An increasing number of people in their 50s and 60s are sandwiched between surviving parents and children. Numerous ageing adults often provide support for older and/or younger generations while also facing their own challenges and stereotypes associated with the transition to old age.

**Objectives:** This study examined the effects of multi-dimensional, multi-directional care provision on future care expectation among sandwich carers.

**Design and methods:** We implemented three factorial survey experiments within a large-scale representative survey of 1,500 sandwiched carers aged 50 and 69 (Mean age = 58.86±5.47) in Hong Kong. Care expectation was assessed in hypothetical scenarios across three key domains: financial, instrumental, and emotional care. Ordered logistic regression and multi-nominal logistic regression models were used.

**Results:** Downward support to children was positively associated with filial care expectations, especially emotional support (OR = 1.45; 95% CI = [1.21, 1.73], p< 0.001). Sandwiched carers' SES was negatively associated with expectations for financial (OR = 0.75; 95% CI = [0.62, 0.89], p< 0.01) and instrumental care (OR = 0.85; 95% CI = [0.71, 1.01], p=0.068) from children. Providing instrumental support to both parents and children reduced the expectation for future instrumental care. Having sons and greater socioeconomic resources increased the preference for self-care, while downward support reduced it.

**Discussion:** Downward support to children strengthens filial care expectations among sandwich carers, especially mothers. However, providing instrumental support to both parents and children, along with SES, promotes the pursuit for self-care. It is crucial to enhancing sandwich carers' image, caregiving capabilities and later-life preparations.

Abstract citation ID: igae098.4331

### MEDIA REPRESENTATION OF OLDER WOMEN DURING WAR CONFLICT: CASE OF UKRAINE AND ISRAEL

Ksenya Shulyaev, and Tova Band-Winterstein, *University of Haifa, Haifa, HaZafon, Israel*

Media reportage of war conflicts tends to underrepresent women, especially older women, and distort their involvement in wars through narrow role characterizations. Women are often cast less as political actors and more as helpless victims. This backdrop sets the stage for our study, which examines the media portrayal of older women during wartime, focusing on conflicts in Ukraine and Israel. By content-analyzing news from Ukraine and Israel, we identify recurring themes and highlight the roles and experiences of older women as depicted in media from the onset of the full-scale war in Ukraine (February 2022) and the "Iron Swords" in Israel (October 2022). The findings revealed five key portrayals of older women (1) Older Women as Victims: symbolizing the enemy's inhumanity and brutality (2) Older Women as Enablers of Family Continuity: guardians of family bonds with warriors, maintaining household rituals and caring for grandchildren amidst chaos (3) Older Women as Resilient Heroines: Symbols of Hope, Survival and Legacy. (4) Older Women as Bearers of Wisdom: transmitting wisdom and stability during times of conflict. (5) Older Women as Symbols of Patriotism: Volunteers, donators, symbolizing endurance and national pride. The media's portrayal of older women during wartime underscores their multifaceted roles and the profound impact of conflict on their lives and conveys symbolic messages to society to serve broader societal and political purposes. This study aims to contribute to reframing aging representation in media by enhancing knowledge, improving ageistic attitudes toward older women, and supporting addressing aging issues during wartime.

### SESSION 8095 (PAPER)

### LATE BREAKING: COMMUNITY-ENGAGED RESEARCH APPROACHES II

Abstract citation ID: igae098.4332

### ASSOCIATION OF HAVING DOMESTIC WORKERS WITH ELDERLY'S SOCIAL PARTICIPATION, VOLUNTEERING, AND LIFELONG LEARNING

Youjuan Zhang, and Xue Bai, *The Polytechnic University, Hong Kong, Hong Kong*

Due to the family caregiver shortage and life expectancy increase, there has been an emerging worldwide trend of migrant domestic workers (MDWs) providing elderly care. However, the association of having MDWs at home with older adults' psychosocial wellbeing has been under investigated. This study used data from the Panel Study of Active Ageing and Society (PAAS, conducted from June to November 2022) among 5007 Cantonese-speaking residents aged ≥50 years (64.2 ± 8.4 years, women: 53.5%) in Hong Kong. Participants self-rated their social participation frequency on a 20-item four-point scale, from "1.=never" to "4.=almost every day". Moreover, participants reported their volunteering experience by rating their monthly volunteering time (from "0.=none" to "5.≥20 hours"), and their learning activities over the past two years using a simple yes/no response. On average, participants scored 29.32 (SD: 5.01) out of 80 on social participation, 17.6% engaged in volunteering, and 4.4% had learning activities. After adjusting age, sex, living status, education level, family income, and physical status, results from linear and logistic regression models showed that having an MDW at home was associated with enhanced social participation ($\beta$=1.24, p< 0.001), and increased odds of participating in volunteering (OR=1.38, 95% CI: 1.05-1.81, p=0.02) and learning activities (OR=2.38, 95% CI: 1.60-3.53, p< 0.001) among older adults. Social participation, volunteering, and lifelong learning are crucial for fostering community engagement and quality of life. Our findings imply that integrating MDWs into elderly care not only supports older adults' psychosocial well-being but also could contribute to community development as a whole.

Abstract citation ID: igae098.4333

### CHALLENGES AND STRATEGIES FOR HOME PAID CAREGIVERS: ADVANCING CARE FOR OLDER ADULTS IN URBAN CHINA

Zixuan Wu[1], and Qingwei Wang[2], *1. Johns Hopkins University, Baltimore, Maryland, United States*, *2. Xi'an Jiaotong-Liverpool University, Suzhou, Jiangsu, China*

Older adults living in community settings often depend on family caregivers, especially those suffering from chronic illnesses. To mitigate caregiving burdens and secure professional nursing support, many families in China's urban centers opt for high-end paid caregivers who offer integrated medical-care services. This study aims to explore the challenges faced by paid caregivers in providing care to older adults in Shanghai and to develop a framework for advancing caregiving for China's aging population. Using a qualitative research design, this study draws on in-depth interviews with 10 paid caregivers from Yi Jia, a local aging care company that offers tailored nursing services. The interview data are supplemented with ethnographic observations and video recordings during home visits. Preliminary thematic analysis revealed three major themes: 1) Home-based care poses logistical challenges, including high technological demands that exceed the capabilities of caregivers from less developed regions and inadequate living conditions that compromise privacy and safety; 2) Paid caregivers face emotional burdens from the high demands of their work and must also cope with homesickness and limited social support due to their migration and separation from their families; 3) Cultural compatibility between caregivers, older adults, and families influenced by regional differences would lead to failure in accommodating local living habits and result in mismatches in health communication. Policymakers should regulate the paid caregiving market to ensure that caregivers are proficient in professional skills, emotional resilience, and cultural adaptability. Continuous collaboration between organizational supervisors, families, and caregivers is crucial to delivering comprehensive care.

Abstract citation ID: igae098.4334

### COMMUNITY-BASED GARDENING INTERVENTIONS FOR ASIAN AMERICAN IMMIGRANTS AND REFUGEES: A SCOPING REVIEW

Lan Doan[1], Casandra Chen[2], My Duyen Tran[2], Stella Yi[2], and Simona Kwon[2], *1. NYU Langone Health, NYU Grossman School of Medicine, New York, United States*, *2. NYU Grossman School of Medicine, New York, New York, United States*

Gardening and horticultural therapy have been shown to increase overall sense of a community, social engagement and quality of life, and increase physical activity. Gardening interventions are innovative means to address social isolation and loneliness by engaging adults in a meaningful way and building supportive communities that integrate informative and respectful interactions around stigmatized topics like social isolation and loneliness and culturally specific contexts and experiences way (e.g., combining gardening interests with culturally relevant foods). However, few interventions have been developed for diverse cultural communities with limited English ability and there are no known interventions targeting isolation and loneliness among older Asian American adults who face severe economic and language challenges that limit health access. This scoping review examines gardening-based interventions for Asian American populations, specifically older adults. Following PRISMA guidelines, we searched PubMed, CINAHL, PsycINFO, and Embase in June 2024 for peer-reviewed articles related to gardening, health interventions and Asian American people in the US. Of the 1419 studies identified, 25 were reviewed in full and 6 studies were included. Key themes include cultural tailoring of garden-based nutrition programs; physical and mental health benefits of community gardens for refugees and immigrants; and that role of community gardens in fostering community social support and individual sense of belonging. Studies focused on adults and older adults and Bhutanese, Karen, Nepali, and Southeast Asian communities. These findings highlight the limited studies available for Asian American communities and urgent need for developing culturally relevant healthy aging interventions for diverse refugee and immigrant populations.

Abstract citation ID: igae098.4335

### FUNCTIONAL LIMITATIONS AND MORTALITY AMONG PERSONS AGING WITH HIV INFECTION AND SUBSTANCE USE

Hema Ramamurthi[1], Ann Gruber-Baldini[2], and Denise Orwig[2], *1. Bloomberg School of Public Health, Johns Hopkins University, Baltimore, Maryland, United States*, *2. University of Maryland School of Medicine, Baltimore, Maryland, United States*

Aging individuals living with HIV experience earlier onset of adverse health outcomes, increased hospitalization rates, and mortality. However, there is limited research demonstrating the impact of HIV, substance use and functional limitations on survival. We analyzed self-reported longitudinal data collected during biannual visits from 2005 to 2020 among 2,633 participants contributing 120527 study visits in the community-engaged, AIDS Linked to the Intravenous Experience (ALIVE) cohort. Functional limitation was defined as having reported "limited a lot" in one of the 4 items of the physical activity section of Medical Outcomes Study-HIV scale. Scores from each visit were summed and categorized into: No Limitation = 0, Moderate Level of Limitation = 1-2 and Severe Limitation > 3. The sample included 751 (28.52%) People Living with HIV (PLWH), who were slightly older than HIV-negative counterparts (47.97 years vs. 44.89 years, p< 0.001). PLWH had a higher prevalence of functional limitations compared to HIV-negative individuals (44.1% vs. 38.3%, p< 0.05). In Cox Proportional Hazards models adjusted for socio-demographic, lifestyle factors and comorbidities, detectable viral load (HR=2.49, CI=2.08-2.97), and having 1-2 or >3 functional limitations (HR=1.27, CI =1.07-1.50 & HR=1.78, CI=1.51-2.10) respectively were predictive of mortality. This analysis highlights the importance of self-reported functional limitations and other potentially modifiable risk factors as important correlates of mortality among PLWH. These factors should be of key focus in the development of future interventions to effectively reduce adverse outcomes among PLWH.

### SESSION 9000 (PAPER)

### LATE BREAKING: ALZHEIMER'S DISEASE, INCLUDING ALZHEIMER'S DISEASE—RELATED DEMENTIAS IV

Abstract citation ID: igae098.4336

### A META-ANALYSIS OF EXTRACELLULAR VESICLES BIOMARKERS IN THE DIAGNOSIS OF ALZHEIMER'S DISEASE AND RELATED DEMENTIAS

Hash Brown Taha, *University of Southern California, Los Angeles, California, United States*

Accurate differential diagnosis of dementia disorders including Alzheimer's disease (AD), frontotemporal dementia (FTD), dementia with Lewy bodies (DLB), Parkinson's disease dementia (PDD), and vascular cognitive impairment and dementia (VCID), along with conditions like prodromal mild cognitive impairment (MCI) or negative controls (NCs), continues to challenge neurologists. Extracellular vesicles (EVs) have emerged as a popular tool for their capacity to encapsulate disease-specific signatures, particularly in neurodegenerative and neurological disorders. To this end, we have performed a comprehensive, PRISMA-guided systematic review and meta-analysis, utilizing sophisticated statistical modeling to determine the diagnostic accuracy, explore between-study variance and heterogeneity (I2), and investigate potential publication bias using biomarkers derived from general EVs (n = 44) or speculative CNS-enriched EVs (n = 18). For all comparative analyses, biomarkers derived from general EVs demonstrated superior diagnostic accuracy, less between-study variance, heterogeneity, and publication bias than those from speculative CNS-enriched EVs. The diagnostic accuracy was low in differentiating among different dementia disorders or from NCs. However, the analysis for diagnosing persons with AD vs. VCID achieved the highest diagnostic accuracy, suggesting that further studies may focus on this comparison. Meta-regressions revealed that studies using cerebrospinal fluid, employing citrate instead of EDTA for blood collection, using thrombin for coagulation factor depletion, isolating EVs with purer methods such as combined UC and SEC, not freezing EVs post-isolation, and quantifying miRNA biomarkers instead of proteins, achieved better diagnostic accuracy and less heterogeneity.

Abstract citation ID: igae098.4337

### ASSOCIATION OF CHILDHOOD RESIDENTIAL CHANGE WITH LATER LIFE MEMORY TRAJECTORIES IN THE HEALTH AND RETIREMENT STUDY

Abbey Hamlin[1], Dana Alhasan[2], Helen Meier[3], Mark Manning[4], Xuexin Yu[5], Sirena Gutierrez[6], and Noah Webster[5], *1. University of Texas at Austin, Austin, Texas, United States*, *2. University of North Carolina, Charlotte, Charlotte, North Carolina, United States*, *3. Institute for Social Research, Institute for Social Research, Michigan, United States*, *4. Oakland University, Rochester, Michigan, United States*, *5. University of Michigan, Ann Arbor, Michigan, United States*, *6. University of California, San Francisco, San Francisco, California, United States*

Childhood residential change may affect later life memory, although few studies have examined this relationship, and research in diverse racial/ethnic samples is sparse. Data were from the US Health and Retirement Study. Childhood residential change was measured by the number of residences before the age of 16 (0-1, 2, 3, 4 or more) (N=4020). Moving due to financial difficulties before 16 years old was categorized as yes versus no (N=4005). Memory function was measured using composite memory z-scores incorporating direct and proxy assessments from 1996-2016. We utilized mixed-effects linear regression models with subject-specific random slopes and intercepts adjusting for sociodemographic characteristics to estimate associations between residential change and memory, by race/ethnicity and parental education. The mean age was 57.5±5.9 years, 82.1% self-identified as non-Hispanic White, 12.7% as non-Hispanic Black, and 5.2% as Other/Unknown. Fewer moves during childhood were associated with a higher initial average memory level, but a faster rate of memory decline. Moving due to financial difficulties during childhood was not associated with initial memory levels, but was associated with faster memory decline. We did not observe effect modification by race/ethnicity or parental education. Results suggest that childhood residential change and the reasons for residential change may contribute to later life memory function and decline.

Abstract citation ID: igae098.4338

### CLINICAL PHENOTYPING OF THE COGNITIVELY UNIMPAIRED IN THE AD SPECTRUM USING PET IMAGING

Cynthia Felix[1], Felix Joy Kollasserry[2], George Rebok[3], Beth Snitz[1], Pamela Ferreira[1], William Klunk[1], Suzanne Baker[4], and Tharick Pascoal[1], *1. University of Pittsburgh, Pittsburgh, Pennsylvania, United States*, *2. Indian Statistical Institute, Bangalore, Karnataka, India*, *3. Johns Hopkins University, Baltimore, Maryland, United States*, *4. Lawrence Berkley National Lab, Berkley, California, United States*

Clinical phenotyping of cognitively unimpaired (CU) older adults who may progress to dementia is a pressing issue in Alzheimer's disease (AD) since early interventions are the most effective. Out-patient cognitive screening commonly uses rapid and simple tests such as the Montreal Cognitive Assessment (MoCA) but its role in preclinical AD remains unclear. We hypothesize that sub-categorizing CU based on the presence of amyloid (A) and/or tau (T) pathologies, as measured by PET, can cognitively phenotype CU using MoCA. We studied 147 CU [CDR global score 0] older adults with MK6240 and Flortaucipir tau-PET and PiB or NAV4694 amyloid PET from the ongoing HEAD study. Based on tracer uptake, individuals were divided into A-T-, A-T+, A+T- and A-T- groups. They were evaluated using the UDS-3 battery. One-way ANOVA with Tukey's correction for multiple comparisons, was used to test for significant differences between groups. With both MK6240 and Flortaucipir, MoCA total scores revealed significant differences between A+T+ and A-T- groups [mean: -1.74 (SE: 0.56), p: 0.013 and mean: -1.56 (SE: 0.6), p: 0.049] respectively. A+T- vs A+T+ was also significant with MoCA total and MoCA Delayed Recall using MK6240 [mean: -2.05 (SE: 0.77), p: 0.041 and mean: -1.15 (SE: 0.42), p: 0.033]. MK6240 shows more sensitivity than Flortaucipir. Therefore, MoCA, a simple and commonly used in-office neuropsychological test can detect cognitive dysfunction in Aβ and tau positive (A+/T+) CU older adults. This highlights the value of precisely characterizing preclinical AD subtypes that are more likely to develop AD dementia.

Abstract citation ID: igae098.4339

### HOW DO SOCIAL NETWORKS CHANGE IN THE LEAD-UP TO DEMENTIA DEATH

Zachary Baker[1], Andrew Alberth[2], M. Aaron Guest[1], Allie Peckham[1], Joahana Segundo[1], and Joseph Saenz[1], *1. Arizona State University, Phoenix, Arizona, United States*, *2. University of Massachusetts Boston, Boston, Massachusetts, United States*

Larger social networks are associated with a lower risk of dementia, but little is known about how social networks shift when someone has dementia as they approach death. We investigate how social networks among individuals with dementia change as they approach death. We hope this research will inform support systems and care practices that benefit individuals with dementia and their care partners. Participants: 2,295 deceased people with dementia from the Health and Retirement Study (2004-2018). Multilevel models estimated associations between dementia, race/ethnicity, time, and close family and friend network size while controlling for several sociodemographic variables. Social networks shrank linearly as death approached. A decrease in close friends primarily drove shrinkage. Some more unusual patterns emerged when race/ethnicity were crossed with time. Specifically, Hispanic/Latino persons with dementia showed the opposite pattern: as they approached death, the number of close extended family increased dramatically, an increase of one person every two and a half years. Dementia risk, social networks, and patterns of social network shrinking are unequal across people of different races and ethnicities. Contrasting patterns of increasing numbers of extended may be driven by familismo. Perhaps as the needs of the people with dementia increased, family members "rallied around" to provide care. Given the importance of social networks, especially during challenging times, and their tendency to shrink closer to death, if non-Hispanic/Latino individuals could adopt the effective strategies used by Hispanic/Latino older adults to maintain and grow their social networks, they might experience similar benefits of robust social networks.

Abstract citation ID: igae098.4340

### THE BURDEN OF SPOUSAL CAREGIVERS IN YOUNG-ONSET DEMENTIA: INSIGHTS FROM A MIXED-METHODS STUDY

Xiaoyan Cui[1], Junqiao Wang[1], Bei Wu[2], Ding Ding[3], Qianhua Zhao[3], Xueting Tang[1], and Jing Wang[4], *1. Fudan University, Shanghai, Shanghai, China (People's Republic)*, *2. New York University, New York, New York, United States*, *3. Huashan Hospital, Fudan University, Shanghai, Shanghai, China (People's Republic)*, *4. University of New Hampshire, Durham, New Hampshire, United States*

Becoming a spousal caregiver for a person with young-onset dementia (YOD) is an unexpected event that deviates from the normal life course trajectory, creating unique and profound challenges. Understanding their burden, the primary sources of this burden, and their coping resources is essential for developing tailored support services. We employed a mixed-methods approach to gain a comprehensive understanding of the burden experienced by eleven spousal caregivers of persons with YOD living in the community. We used the Zarit Burden Interview to quantify caregiving burden and conducted semi-structured qualitative interviews to explore the caregiving experience in depth. We utilized a joint display method to integrate and present the converging findings from quantitative and qualitative data, offering a holistic perspective on the caregiving burden. All spousal caregivers in this study experienced varying degrees of burden, driven primarily by the dynamic care needs and dependency of their loved ones. The integrated data showed that they face significant challenges in maintaining their personal identity and social life, with many sacrificing career aspirations and enduring social isolation. Societal expectations compounded the burden, leading to self-imposed guilt and dissatisfaction. While caregiving reconfigures family dynamics, it often strengthens bonds with children. The lack of support resources and overwhelming responsibilities places immense psychological stress on them. Despite these challenges, caregivers remain committed to their roles. There is an urgent need to develop family-centered community support services and respite care to provide younger spousal caregivers with the time and space to manage their personal lives and alleviate their burden.

Abstract citation ID: igae098.4341

### USING SOCIAL MEDIA POSTS TO UNDERSTAND THE HEALTH INFORMATION NEEDS FOR OLDER ADULTS WITH DEMENTIA

Ebenezer Larnyo, Karim Benyassine, and Natasha Preece, *University of California, Santa Barbara, Santa Barbara, California, United States*

Older adults with dementia and their caregivers face significant challenges, necessitating innovative approaches to support their evolving health information needs. This study leverages the power of social media to explore these needs, analyzing 664 posts from Reddit communities dedicated to dementia and Alzheimer's disease. Thematic analysis and sentiment analysis were employed to identify key themes and emotional undertones. Additionally, deep learning models (convolutional neural network (CNN), transformers, bidirectional long short-term memory (BI-LSTM), and gated recurrent unit (GRU)) were compared for sentiment prediction accuracy, with CNN demonstrating superior performance. Our findings reveal that caregivers constitute the majority of contributors, sharing experiences related to caregiving burden, dementia symptoms, and emotional challenges. Despite the inherent difficulties, a predominantly positive sentiment prevailed, highlighting the importance of social connection and support. This research underscores the potential of social media as a valuable resource for understanding the experiences of dementia caregivers. By harnessing these insights, healthcare providers and policymakers can develop more effective interventions and support systems to improve the quality of life for both patients and caregivers.

### SESSION 9010 (PAPER)

### LATE BREAKING: ALZHEIMER'S DISEASE, INCLUDING ALZHEIMER'S DISEASE—RELATED DEMENTIAS V

Abstract citation ID: igae098.4342

### BEING A SUPERWOMAN, THE EXPLORATION OF AFRICAN AMERICAN WOMEN DEMENTIA CAREGIVERS AND THEIR MENTAL HEALTH

Shanae Rhodes, *University of Texas Health Science Center San Antonio, San Antonio, Texas, United States*

Existing literature presents evidence that African American caregivers experience a health paradox where they have commonly reported having worse physical health but better mental health related to caregiver burden when compared to other racial groups. However, there are inconsistencies across the research literature regarding mental health outcomes for this population. Guided by Pearlin's Stress Process Model and the Superwoman Schema, the purpose of this study was to explore the influence of the Superwoman Schema characteristics and caregiver burden on the psychological health (anxiety and depressive symptoms) of African American female caregivers. A mixed methods design was used for this study. This presentation will focus on the quantitative segment. Cross-sectional correlations and regression analyses were conducted using data collected from 127 participants to investigate the relationship between caregiver burden, the five Superwoman characteristics, and psychological health for African American female caregivers. Results showed that caregivers who reported a higher endorsement of any of the Superwoman characteristics were more likely to report higher caregiver burden, anxiety, and depressive symptoms. Endorsement of some of the Superwoman characteristics (i.e., obligation to present an image of strength and obligation to help) partially mediated the relationship between caregiver burden and anxiety and caregiver burden and depressive symptoms. Future research should continue to examine the mediation of Superwoman characteristics on the relationship between caregiver burden and psychological health outcomes. These partial mediators can inform intervention development to mitigate the negative impact of caregiver burden on mental health.

Abstract citation ID: igae098.4343

### ENHANCING COGNITIVE AND EMOTIONAL WELL-BEING THROUGH PIANO TRAINING IN OLDER ADULTS WITH MILD COGNITIVE IMPAIRMENT

Rose Lin[1], Laura Robinson[2], Elinor Freer[1], and Kathi Heffner[1], *1. University of Rochester, Rochester, New York, United States*, *2. University of Rochester, University of Rochester, New York, United States*

Neuropsychiatric symptoms such as depressed mood are increasingly recognized alongside cognitive decline in older adults with mild cognitive impairment (MCI). In response to international guidelines advocating a holistic approach to address both emotional and cognitive symptoms, we conducted a descriptive qualitative study to assess the feasibility and acceptability of a 12-week structured piano training program for seven older adults with MCI. This program included weekly one-hour sessions alternating between individual and group formats, focusing on music theory and scales, with 15-minute daily practices. Content analysis of semi-structured interviews revealed that participants found piano training rewarding, they appreciated integrating piano practice into daily routines, which fostered a sense of purpose and enhanced their ability to manage schedules and handling everyday tasks. Notably, 71.4% of participants enrolled in community school piano class following program completion. They valued interacting with peers facing similar cognitive challenges provided comfort and shared understanding of cognitive decline. Positive reinforcement from instructors and tailored support were crucial in sustaining motivation, particularly when frustrations associated with acquiring new skills, as noted in prior and current studies, could worsen neuropsychiatric symptoms. Recognizing their abilities to learn, despite cognitive decline, boosted self-confidence that often compromised by cognitive impairment. Some adopted piano playing as a new strategy to cope with negative emotions, enhancing overall emotional resilience. Our preliminary findings suggest that piano training has potential to improve well-being in older adults with MCI, setting stage for assessing its long-term impact on cognitive and emotional well-being in larger trials.

Abstract citation ID: igae098.4344

### GENDER-SPECIFIC EFFECTS OF IL-6 AND DECLINES IN PROCESSING SPEED AND EXECUTIVE FUNCTIONING IN OLDER ADULTS

Elana Gloger[1], Patrick Smith[2], and Suzanne Segerstrom[3], *1. Pennsylvania State University, University Park, Pennsylvania, United States*, *2. University of North Carolina - Chapel Hill, Chapel Hill, North Carolina, United States*, *3. Oregon State University, Corvallis, Oregon, United States*

Elevated peripheral inflammatory markers among older adults associate with cognitive decline, particularly for women, for whom cognitive decline is more prevalent and rapid. Few prospective studies have examined gender-specific associations between inflammation and cognitive decline or examined stable differences vs. fluctuations in inflammation. Participants (N = 235) were older adults (Mage = 75.1, range = 60-92) from a longitudinal study of neuropsychological assessments and blood draws every six months across up to 12 years. Systemic inflammation was operationalized as serum IL-6. The Trail Making Test provided assessment of psychomotor processing speed (A) and executive functioning (B and B-A). Multilevel models tested associations between cognition and inflammation and moderation by gender. Higher stable differences and fluctuations over time in IL-6 were significantly associated with slower TMT-A performance for women and men (between-person: g = -.06, p =.04; within-person: g = -.02, p =.049). Higher IL-6 was more strongly associated with slower TMT-B and B-A performance for men (TMT-B: g =.006, p =.02; TMT-B-A: g =.008, p =.01). Older participants demonstrated slower TMT-A performance at higher IL-6 (g =.026, p =.01). Women experience higher rates of Alzheimer's disease diagnosis and faster age-related cognitive decline, suggesting gender-specific factors related to cognition yet to be uncovered. In the present study, executive function was sensitive to changes in systemic inflammation in a gender-specific way. Gender-specific differences in neurological aging remains a critical target for future intervention research aimed at addressing preclinical AD and accelerated aging.

Abstract citation ID: igae098.4345

### PAIN MANAGEMENT IN DEMENTIA: A NEED FOR SPECIFICITY AND SUSTAINABILITY IN EXERCISE-BASED PROGRAMS

Annalisa Na[1], Joke Bradt[2], Julie Fritz[3], Gediminas Gliebus[4], and Laura Gitlin[2], *1. Drexel University, Drexel University, Pennsylvania, United States*, *2. Drexel University, Philadelphia, Pennsylvania, United States*, *3. University of Utah, Salt Lake City, Utah, United States*, *4. Baptist Health South Florida, Boca Raton, Florida, United States*

Over 50% of people living with dementia (PLWD) experience pain associated with functional decline. Pain and dementia are heterogeneous and complex, making the development of effective nonpharmacological interventions challenging. Hence, we used Intervention Mapping (IM), an evidence-based process for designing complex behavioral interventions. This study focuses on completing Step 1 of IM (needs assessment) to define population and intervention priorities. We conducted and synthesized the findings of three studies: (1) a scoping review of 81 studies summarizing current literature, (2) an analysis of the National Health and Aging Trends Study (NHATS) database (n=9,974) to characterize pain among community-dwelling PLWD, (3) qualitative interviews with clinicians (n=19) and PLWD/caregivers (n=17) to assess perspectives on current care. The scoping review revealed that most research focuses on passive interventions in long-term care facilities and lacks specificity in reporting pain diagnosis and dementia severity. From the NHATS database, low back and knee osteoarthritis were the most prevalent pain conditions among community-dwelling PLWD. Qualitative analysis of interviews confirmed scoping review findings of an overall lack of exercise-based interventions for pain despite being standard care among cognitively intact individuals. While there was a shared interest in exercise-based interventions, clinicians expressed concerns about adapting and sustaining these programs. Triangulating data (epidemiological and qualitative sources) and following IM provides foundational support to design clinical interventions for pain in PLWD. Specifically, intervention development should prioritize community-dwelling PLWD with knee and/or low back pain using therapeutic exercises tailored to the individual's interests and abilities; however, research is needed to address sustainability.

Abstract citation ID: igae098.4346

### PREDICTORS OF INTENT TO USE DIGITAL MEMORY BOOK AMONG DEMENTIA CAREGIVERS

Yura Lee[1], Chorong Oh[2], Min Sook Park[3], and Ji Yae Bong[4], *1. University of Wisconsin-Milwaukee, Milwaukee,*

*Wisconsin, United States*, *2. Ohio University, Athens, Ohio, United States*, *3. Florida State University, Florida State University, Florida, United States*, *4. University of North Carolina at Charlotte, Charlotte, North Carolina, United States*

A memory book is a popular reminiscence approach for people with dementia (PWD) and caregivers (CGs). Recently, it has evolved into a digital form by incorporating music, videos, or other multimedia, referred to as a digital memory book (DMB). Like conventional memory books, DMBs have been found to improve quality of life, cognition, communication, and mood for PWD and CGs, while also reducing CG burden. Despite these benefits, the usage of DMBs remains low, and there is limited research identifying factors influencing intent to use DMBs in this population. Considering that family CGs are most often the facilitators of DMBs for PWD, this study aims to examine the predictors of intent to use DMB among dementia CGs. Based on the Technology Acceptance Model, we collected data from 170 dementia CGs. Findings from linear regression analyses show that dementia type, primary caregiver status, age, race, education, caregiver health, and relationship to PWD were significantly related to intent to use DMBs. We also found a significant interaction effect between dementia type (Alzheimer's disease [AD] vs. other dementias) and stage (mild vs. moderate to severe) ($b=0.493$, $SE=0.217$, $p < .05$). Specifically, differences in intent to use DMBs between mild and moderate to severe stages were significantly greater among CGs for AD compared to other dementias ($\Delta M= 0.45$ vs. 0.05). This study contributes to identifying predictors of the intent to use DMB among dementia CGs. Considering these factors will be crucial in developing and implementing DMB programs to enhance their use, thereby benefiting this population.

Abstract citation ID: igae098.4347

### USING TRANSFER LEARNING TO IMPROVE ALGORITHMIC DEMENTIA CLASSIFICATION ACROSS DIFFERENT RACIAL AND ETHNIC GROUPS

Anja Leist[1], M Maria Glymour[2], Kenneth Langa[3], and Jung Hyun Kim[1], *1. University of Luxembourg, Esch-sur-Alzette, Grevenmacher, Luxembourg*, *2. Boston University, Boston, Massachusetts, United States*, *3. University of Michigan, Ann Arbor, Michigan, United States*

**Background:** In the absence of clinical assessments, algorithmic 'probable dementia' estimation aids public health and epidemiological research on determinants and consequences of dementia. Algorithmic performances have differed by social and racial/ethnic groups due to heterogeneous sample sizes. In this study, we present improvements in the accuracy of group-specific 'probable dementia' estimation using a transfer learning approach. Transfer learning – a novel approach not previously applied in algorithmic dementia classifications – uses a large "source" dataset with imprecise (non-clinical) outcome assessments to develop an initial algorithm with a large and diverse sample, complemented by a smaller "target" dataset with high-quality (clinical) outcome assessments to develop models with refined estimation of model coefficients.

**Methods:** Data came from the Health and Retirement Study (source data: N=6,630) and the Harmonized Cognitive Assessment Protocol (target data: N=2,388). Different algorithms to estimate probable dementia were evaluated through overall accuracy (Brier score), calibration (intercept, slope), and discriminative ability (area under the receiver operating characteristic curve, AUC; area under the precision-recall curve, AUPRC).

**Results:** Among non-Hispanic Black participants, the transfer-learned algorithm had higher accuracy than the best previously reported algorithm (Brier 0.049 vs. 0.061; AUC 0.84 vs. 0.81; AUPRC 0.52 vs. 0.39). Among Hispanic participants, the transfer-learned algorithm improved model calibration (Intercept −0.07 vs. −1.19; Slope 0.88 vs. 0.08).

**Discussion:** The use of transfer learning can improve algorithms to estimate probable dementia from non-clinical assessments for groups historically underrepresented in research. More accurate classification will lead to more equitable epidemiological and public health research on dementia.

### SESSION 9015 (PAPER)

### LATE BREAKING: ALZHEIMER'S DISEASE, INCLUDING ALZHEIMER'S DISEASE—RELATED DEMENTIAS VI

Abstract citation ID: igae098.4348

### FEASIBILITY AND ACCEPTABILITY OF MBCT AND BA TO REDUCE PRE-DEATH GRIEF IN FAMILY CAREGIVERS OF PERSONS WITH DEMENTIA

Benjamin Neugebauer[1], Caroline Cummings[1], Sarah Sparks[1], Lauren Elliott[1], Volker Neugebauer[2], Lauren Chrzanowski[1], Elisabeth McLean[1], and Jonathan Singer[1], *1. Texas Tech University, Lubbock, Texas, United States*, *2. Texas Tech University Health Sciences Center, Lubbock, Texas, United States*

Family caregivers of individuals with Alzheimer's Disease and Alzheimer's Disease Related Dementia (AD/ADRD) may experience pre-death grief (PDG). Behavioral activation (BA) and mindfulness-based cognitive therapy (MBCT) improve grief outcomes for bereaved individuals but neither have been investigated in the context of PDG. Notably, there are high rates of treatment attrition for family caregivers of persons with AD/ADRD underscoring the need to improve treatment options for this population. The present study is Stage I of NIH Stage Model for Behavioral Intervention Development that examines the acceptability and feasibility of two interventions. Participants in West Texas have limited to no resources due to a lack of respite, neurologists, and caregiving interventions, as they live 5 hours from a large urban setting. Family caregivers in West Texas were randomized to receive MBCT or BA following administration of neuropsychological and psychological assessments. Participants completed a shorter battery of assessments each session, and the full battery during the final session and at all follow ups. Thirty-three participants were recruited and consented, 84.8% (n=28) completed the study, 96.0% (n=27) of participants completed the final survey, and 86% (n=24) completed the 1-month follow up.

When comparing the number of sessions attended by participants receiving MBCT vs. BA, there was no significant differences (p=0.73). Qualitatively, participants endorsed enjoying the interventions, and reported they were not burdensome. Results support the feasibility and acceptability of these evidence-based interventions for family members of persons with AD/ADRD in a unique West Texas sample.

Abstract citation ID: igae098.4349

### IMPLEMENTING SMART AMBIENT BRIGHT LIGHT FOR NURSING HOME RESIDENTS WITH DEMENTIA: ASSESSING FIDELITY

Diane Berish[1], Julian Wang[2], Shevvaa Beiglary[3], Ying-Ling Jao[2], and Yo-Jen Liao[1], *1. The Pennsylvania State University, University Park, Pennsylvania, United States*, *2. Pennsylvania State University, University Park, Pennsylvania, United States*, *3. Penn State University, University Park, Pennsylvania, United States*

Up to 90% of persons with dementia experience neurobehavioral symptoms and poor sleep which may be associated with limited daylight exposure and circadian disruptions. Lighting interventions regulate the circadian rhythm and have been shown to reduce agitation. Smart Ambient Bright Light (SABL) incorporates natural light and is an automatic lighting system that delivers bright light during the day (photopic illuminance ≥400 lux, circadian stimulus [CS]≥0.3) and dim light during the night (lux≤40, CS ≤0.1). Two methods were used to assess SABL fidelity. First, manual measurements were conducted during the daytime twice a week. Second, each participant wore a light sensor to measure individual lighting exposure for 24 hours, 7 days a week, every other week. Manual measurement results indicated that the SABL lighting conditions across different spaces generally met the targets during daytime (lux: mean=394.2, SD=49.75; CS: mean=0.302, SD=0.07). Individual sensors also showed that participants' average daytime light exposure during the intervention met the target for lux (mean=406.9, CI=369.9-443.8) and CS (mean=0.363, CI= 0.332-0.393). Individual nighttime lighting exposure during the intervention was above target for lux (mean=94.5. CI= 82.3-106.7) and CS (mean=0.111, CI=0.098-0.124). Additionally, participants received higher daytime CS (t=-8.7, p< 0.001) and daytime lux (t=-8.48, p< 0.0001) while also receiving lower nighttime CS (t=4.43, p=0.0001) and nighttime lux (t=7.4, p< 0.0001) during the intervention period than the control period. The results indicate the SABL achieved fidelity for daytime, but its fidelity for nighttime, especially lux, could be improved. Future studies should incorporate lessons learned to improve SABL implementation fidelity.

Abstract citation ID: igae098.4350

### MEDIATING PROTEINS UNDERLYING THE RELATIONSHIP BETWEEN MITOCHONDRIAL DNA COPY NUMBER AND DEMENTIA FROM TWO COHORTS

Teresa Tian[1], David Zweibaum[1], Yong Qian[2], Luke Pilling[3], Jun Ding[1], and Luigi Ferrucci[1], *1. National Institute on Aging, Baltimore, Maryland, United States*, *2. NIH, Baltimore, Maryland, United States*, *3. University of Exeter, Exeter, England, United Kingdom*

Recent reports have shown that blood mitochondrial DNA copy number (mtDNAcn) is associated with dementia risk, but the biological underpinnings remain unclear. Identifying proteins that mediate this relationship may provide mechanistic insights. In the UK Biobank, we performed mediation analyses of 2,917 proteins via Olink platform in the association between mtDNAcn estimated using fastMitoCalc and future dementia diagnosis among 33,852 participants initially free of dementia (mean age=57 years). Over an average 13.2 years of follow-up, 891 participants (2.6%) developed all-cause dementia. We found 57 mediating proteins for all-cause dementia (FDR-p< 0.05), mostly from inflammation and cardiometabolic function panels and implicated in 24 pathways with top ranked pathways being TNFs binding to physiological receptors, coagulation system, plasma lipoprotein assembly/remodeling/clearance, degradation of the extracellular matrix, and activation of matrix metalloproteinases (p< 0.05). Three protein networks with more than 10 significant proteins were related to organismal injury and abnormality, hereditary disorders, cellular function and maintenance, post-translational modification, cell death and survival, and lipid metabolism. Of 57 mediating proteins for all-cause dementia, 4 proteins (TNFRSF11B, SMOC1, Fibronectin, and SMAD1) also mediated the relationship between mtDNAcn and cognitive impairment or dementia in the validation cohort of 593 Baltimore Longitudinal Study of Aging participants with available cross-sectional data (mean age=76.8 years, SomaScan, p< 0.05). These findings may suggest that proteins important for energy metabolism, neurotransmitter synthesis, lipid and glucose homeostasis, and immunity may underlie the relationship between mitochondrial dysfunction and dementia risk. Future longitudinal studies should further validate these findings and investigate longitudinal changes in proteins.

Abstract citation ID: igae098.4351

### PREDICTING THE ONSET OF DEMENTIA WITH LONGITUDINAL OBJECTIVE MEASURES OF PHYSICAL FUNCTION

Sydney Schaefer[1], Daniel Peterson[2], Marie Ernsth Bravell[3], and Deborah Finkel[4], *1. Arizona State University, Tempe, Arizona, United States*, *2. Arizona State University, Arizona State University, Arizona, United States*, *3. Jönköping University, Jönköping, Jonkopings Lan, Sweden*, *4. University of Southern California, Los Angeles, California, United States*

Older adults with dementia have reduced physical function; however, whether physical function decline accelerates prior to dementia onset remains unclear. This study compared changes in three objective physical function measures for up to 26 years in 518 older adults who did or did not develop dementia: Time to complete a three-meter walk (Gait), grip dynamometry (Grip strength), and time to complete five functional tasks (e.g., putting coins into a slot) (Fine motor). Data from three longitudinal Swedish datasets of older adults (OCTO-Twin, GENDER, SATSA) were analyzed. During the studies, 259 participants were diagnosed with dementia (through clinical consensus); only data prior to the age of dementia onset were included. Propensity matching identified 259 participants who did not develop dementia and whose age at last observation matched the age of diagnosis in the dementia group. Linear latent growth curve models captured longitudinal changes in each measure prior to the onset of disability; thus, intercepts reflect an estimate of physical function at dementia onset or the last timepoint in the study. After controlling for sex, the fine motor test declined faster in the dementia onset group, with differences emerging 9 years prior to dementia onset. Gait and grip strength showed no differences between groups in the rate of change. Fine motor decline also occurred at the same rate as cognitive decline (measured with the Mini-mental status exam). These findings suggest that functional fine motor tasks may identify elevated risk for developing dementia, particularly in cases where memory tests may not be reliable.

Abstract citation ID: igae098.4352

### SHRINKING WORLDS: SOCIAL NETWORK OVERLAP FOR OLDER ADULTS WITH AND WITHOUT ADRD AND THEIR CLOSE TIES

Lucas Hamilton[1], and Brea Perry[2], *1. Augustana University, Sioux Falls, South Dakota, United States*, *2. Indiana University, Bloomington, Indiana, United States*

Alzheimer's disease and related dementias (ADRD) are an urgent public health issue with psychosocial consequences that reverberate through family systems, weakening key social bonds for diagnosed individuals and caregivers. Feelings of social isolation are associated with worse clinical outcomes for people with ADRD and elevated dementia risk in caregivers. Data from 124 focal-informant dyads were used to examine the characteristics and consequences of shared ties between older adults and their informants – typically a spouse or child. Focal participants were diagnosed as cognitively normal (n = 73) or experiencing ADRD-related cognitive impairment (n = 51). All participants completed separate social network interviews, naming people with whom they discuss important or health-related matters. Using multilevel logistic regression, we found that family members and individuals offering psychosocial support were significantly more likely to be shared ties (all odds ratios > 1.26, all p-values <.05). People who provided instrumental support were more likely to be shared ties only in the networks of ADRD informants (OR = 2.11, p <.01), suggesting they may mediate caregiving tasks on behalf of the person with ADRD. More network overlap was associated (p <.05) with worse general cognition and episodic memory (β = -.261; β = -.323, respectively) among ADRD focals, indicating that loss of social independence is associated with poorer cognition (or vice versa). Higher overlap was also associated with greater loneliness in ADRD informants (β =.282, p =.05), suggesting that lacking social ties outside the caregiving system may threaten caregiver wellbeing.

Abstract citation ID: igae098.4353

### THE ASSOCIATION OF EARLY LIFE ADVERSITY AND LIFE COURSE RELATIONSHIP QUALITY WITH LATE-LIFE COGNITIVE HEALTH

Monica Walters[1], Roger Wong[2], Kiana Scambray[1], Toni Antonucci[1], and Wassim Tarraf[3], *1. University of Michigan, Ann Arbor, Michigan, United States*, *2. State University of New York Upstate Medical University, Syracuse, New York, United States*, *3. Wayne State University, Detroit, Michigan, United States*

Early life adversity (ELA) relates negatively to cognition, yet how particular ELA experiences may differentially relate to cognition and whether associations may be attenuated by life course relationship quality (LCRQ) is unclear. We aimed to assess how ELA subgroups related to cognition and whether associations may be buffered by LCRQ. Adults (ages 50-64 at baseline) from the Health and Retirement Study participated (n=4,193; 2006/2008=baseline; 2018/2020=follow-up). Latent class and profile analyses identified ELA and LCRQ subgroups, respectively. We related ELA and LCRQ subgroups to global cognition (linear regression) as well as risk of cognitive impairment, not dementia (CIND) and dementia (multinomial logistic regression) at follow-up. We included sociodemographic and health-related covariates. We identified 4 ELA (High Adversity, Family Disruptions, Elevated Household Trauma, Low Adversity) and 3 LCRQ (Strong Ties, Mixed Ties, Weak Ties) classes. Of note, racially/ethnically minoritized adults were more likely to belong to Family Disruptions and Weak Ties classes than White adults. Participants with Family Disruptions (vs. Low Adversity) had worse cognition (global: b=-0.84, 95% CI[-1.25;-0.43]; CIND: RRR=1.54, 95% CI[1.16;2.03]; dementia: RRR=1.64, 95% CI[1.02;2.64]); controlling for LCRQ and sociodemographics attenuated associations. Participants with Weak Ties (vs. Strong) had worse cognition (b=-2.70, 95% CI[-3.40;-2.01]) and higher risk of CIND (RRR=3.22, 95% CI[2.11;4.89]) and dementia (RRR=4.10, 95% CI[2.16;7.81]); associations were not explained by covariates. Family Disruptions negatively impacted cognition, but associations were explained by sociodemographics, indicating potential resilience from ELA. However, negative LCRQ, particularly Weak Ties, was consistently harmful to cognition. Therefore, targeting midlife and late-life social relationships may benefit cognition.

Abstract citation ID: igae098.4354

### UNDERSTANDING THE SUBJECTIVITIES OF OLDER ADULTS WITH DEMENTIA IN A RESIDENTIAL CARE FACILITY

Lingyi Shen, *Duke Kunshan University, Kunshan, Jiangsu, China (People's Republic)*

China has the largest population of people with dementia. However, inadequate care support is available, leading to families to being overwhelmed and compelled to send their loved ones to residential care facilities. While much attention has been paid to understanding institutional dementia care from the caregivers' perspectives, seldom does research focus on the experiences of older adults with dementia, especially in China. This study aims to explore the subjectivities of older adults with dementia in a residential care facility in the Chinese context. Over a two-month period, participant observation was conducted with informal conversations in a dementia unit in eastern China. Based on the collective perception, reconstructed memory and oral histories, different individuals' realities were articulated. Through verbal and physical behaviors such as resistance to care, othering and grouping, older adults with dementia constructed the understanding towards themselves and their surroundings, as well as their own meaning of life. Constant manifestation of intersubjectivity was also observed within the group. Despite their disrupted lived experiences, older adults with dementia maintained their established relationships, with family and beyond, and connections with the broader society through their performance of embodied experiences, disclosing the complexity of family dynamics in contemporary China and the social history of the country. The study also identifies elements that constrain their subjectivities, such as the overuse of antipsychotics and therapeutic lies. A new and dialectical perspective towards the relationship between older adults with dementia and the care institution is provided, informing the development of person-centered dementia care in China.

### SESSION 9020 (PAPER)

### LATE BREAKING: MOTIVATING PATIENTS FOR HEALTH BEHAVIOR CHANGE

Abstract citation ID: igae098.4355

### ACTIVITY INTENSITY AND FALL-RELATED DEATHS AMONG OLDER ADULTS: RESULTS FROM A 12-YEAR NATIONAL SURVEY

Oluwaseun Adeyemi, and Joshua Chodosh, *New York University Grossman School of Medicine, New York, New York, United States*

Fall-related deaths among older adults are a growing public health concern, with activity intensity potentially playing a critical role in mitigating these outcomes. This study assessed the relationship between activity intensity and fall-related death among community-dwelling U.S. older adults. We pooled twelve years of survey data (2006 to 2017) from the Integrated Public Use Microdata Series of the National Health Interview Survey for this retrospective cohort study. We identified older adults (65 years and older) who sustained fall injuries (N=2,454). The outcome variable was fall-related death. We defined low activity intensity as a binary variable using the American Heart Association's weekly 500 Metabolic Equivalent of Task (MET) as a cut-off. We controlled for sociodemographic, healthcare access, and health characteristics, performed survey-weighted logistic regression analysis, and reported the adjusted mortality odds (plus 95% confidence interval (CI)). The survey comprised 2,454 older adults with fall injuries, representing 863,845 US older adults. The population was predominantly female (68%), non-Hispanic Whites (85%), and divorced/separated (54%). During the follow-up period, 45% of the study population died. Approximately 81% of the study population had low activity levels. However, between 2006 and 2017, the proportion of the study population with low physical activity decreased from 90% to 67%. After adjusting for sociodemographic, healthcare access, and health characteristics, low activity level was associated with two-fold increased odds of death (AOR: 2.11; 95% I: 1.59 – 2.81). Promoting higher physical activity levels may significantly reduce the risk of fall-related deaths among older adults.

Abstract citation ID: igae098.4356

### EXPLORING THE MODERATING ROLE OF COGNITIVE FUNCTION IN SCREEN TIME AND STRESS

Nelson Roque, and Robert McGill, *Pennsylvania State University, University Park, Pennsylvania, United States*

Understanding the interplay between brain health and digital media use is crucial in our current technological era. Prior research has shown that stress influences screen time usage and that screen time can impact cognitive function and brain structure (Neophytou, E., Manwell, L.A., & Eikelboom, R., 2021). This online survey study, conducted on Prolific, examines the relationship between screen time and stress, as moderated by momentary cognitive functioning, in a sample of 319 participants (mean age = 40.5 years, age range 19-77, 61% female). Momentary cognitive processing speed was assessed using a web-based cognitive assessment developed by M2C2 (NIA #U2CAG060408), stress via the Perceived Stress Scale, and screen time through self-reported data (yesterday's screen time reported on cell phone). The bootstrapped SEM moderation analysis found that stress significantly increases screen time (stress_effect = 0.169, p = 0.004, 95% CI [0.051, 0.287]). The interaction effect (interaction_effect = 0.153, p = 0.011, 95% CI [0.031, 0.278]) indicates that faster processing speed (better cognition) amplifies the impact of stress on screen time. For example, in moments when one has faster processing speed, stress may lead to increased screen use as a way to cope with heightened awareness of stressors. Cognition alone does not significantly affect screen time (cognition_effect = 0.022, p = 0.740, 95% CI [-0.102, 0.162]). These findings underscore the need to consider cognitive dynamics when studying the relationship between digital media use and well-being. Future research should employ real-time data collection to explore how cognitive changes (across domains), affect screen time behaviors.

Abstract citation ID: igae098.4357

### FLEXIBILITY IN PRACTICE: CASE EXAMPLES OF THE ROLE OF A BRAIN HEALTH INTERVENTIONIST IN THE AGEWISE-AP PROGRAM

Jaye McLaren, Lauren Moo, Andrew Nguyen, and Maureen O'Connor, *Department of Veterans Affairs, Bedford, Massachusetts, United States*

Healthy brain aging and reducing the risk of dementia is important to older adults. The Aging Well through Interaction and Scientific Education program (AgeWISE) provides group education (12 sessions) on lifestyle factors and techniques to manage age-related cognitive change. Participants who completed AgeWISE reported feeling that they had more control over their brain health, but many requested additional supports to help them make lifestyle changes important to them. We added an 8-session one-on-one action plan component to the AgeWISE program (AgeWISE-AP) to help participants make personalized lifestyle modifications that support healthy brain aging. AgeWISE-AP utilizes a Brain Health Interventionist (BHI) to guide participants in the creation and implementation of individualized, client-centered goals while adhering to a manualized scaffolding. Fifteen participants have engaged in AgeWISE-AP to date. We will provide case examples illustrating the flexibility required from the BHI to manage individual needs in goal setting, adapt goals as needed, and successfully guide participants in behavior change within a manualized program. The AgeWISE-AP program requires the BHI to have strong clinical skills and content-specific training to support goal setting and implementation that is client-centered while also ensuring the sessions are operating within the structure of the manualized program. Walking this fine line is imperative for the success of the program as well as for its replicability in other settings.

Abstract citation ID: igae098.4358

### OPIOID DEPRESCRIBING: QUALITATIVE INSIGHTS INTO REDUCING MOTIVATION FOR UNHEALTHY SUBSTANCE USE

Lisa LaRowe[1], Sarah Stone[1], Heily Chavez Granados[1], and Christine Ritchie[2], *1. Massachusetts General Hospital, Boston, Massachusetts, United States*, *2. Mass General Hospital and Harvard Medical School, Boston, Massachusetts, United States*

Nearly 38% of older adults in the United States have chronic pain, and opioids continue to be used frequently for chronic pain management in this population. While recommendations suggest limiting opioids in older adults, given limited effectiveness and associated harms, one potential consequence of deprescribing opioids is that patients may turn to non-prescribed opioids or nonopioid (e.g., alcohol, cannabis, nicotine) substances to self-medicate pain. We conducted 3 focus groups of older adults with chronic pain who were prescribed opioids (N = 10, 65-92 years old) to explore perspectives regarding factors that may influence motivation to use substances during opioid deprescribing. We also aimed to elicit preferences for an opioid deprescribing intervention that reduces risk for adverse substance use-related outcomes. Qualitative data were analyzed using rapid data analysis. Participants identified the following factors that may increase motivation to use substances during opioid deprescribing: intolerable pain, lack of access to alternative pain treatments, psychological distress, and history of substance use/abuse. Factors that may decrease motivation to use substances include: having access to alternative pain treatments, having access to psychosocial treatment, having adequate social support, and being aware of the risks/consequences of substance use. To mitigate risk that patients will use non-prescribed opioids and/or nonopioid substances to cope with pain during opioid deprescribing, participants highlighted the importance of having a clear plan for deprescribing, ensuring access to effective alternatives to opioids, utilizing psychosocial and other nonpharmacologic pain treatment strategies, and providing psychoeducation about the risks of substance use.

Abstract citation ID: igae098.4359

### SEX DIFFERENCES IN MULTI-DIMENSIONAL SLEEP HEALTH AND DEMENTIA RISK: A PROSPECTIVE STUDY IN THE UK BIOBANK

Tianyi Huang, May Beydoun, and Lenore Launer, *National Institute on Aging, Baltimore, Maryland, United States*

Sex differences in sleep health risk factors may contribute to differences in dementia risk between women and men. There were 409,789 dementia-free UK Biobank participants (55% women) who self-reported 7 sleep-related traits at baseline in 2006-2010. Incident dementia was identified using algorithmically defined outcomes through primary care, hospital admissions, or death records until December 31, 2022. The prevalence of insomnia symptoms and non-restorative sleep was 7-8% higher in women, while snoring, sleepiness and napping were more common in men. Over a median follow-up period of 13.8 years, there were 7,583 incident dementia cases (3,406 Alzheimer's, 1,651 vascular). After adjusting for potential confounders, the increased dementia risk associated with sleepiness, napping, and evening chronotype was higher in women, whereas the association between non-restorative sleep and dementia risk was stronger in men. For example, the multivariable-adjusted hazard ratio (95% CI) for sleepiness was 1.59 (1.35, 1.86) in women and 1.21 (1.04, 1.40) in men (p-interaction by sex=0.01), and the corresponding estimate for non-restorative sleep was 1.27 (1.17, 1.39) and 1.39 (1.26, 1.52) in women and men, respectively (p-interaction by sex=0.06). U-shaped associations between short/long sleep duration and dementia risk were similar in women and men. Similar sex differences were observed separately for Alzheimer's and vascular dementia, although the associations were generally weaker for Alzheimer's disease. In summary, most sleep health dimensions were differentially associated with dementia risk in women and men. Recognizing these sex differences may help develop sex-specific strategies to promote sleep health and moderate the risk for dementia.

Abstract citation ID: igae098.4360

### TRENDS IN DRUG-DRUG INTERACTIONS AMONG US OLDER ADULTS, 2015-2016 VS. 2021-2023

Dima Qato[1], Shyam Krishnan Ondanat Veetil[1], and Jocelyn Wilder[2], *1. University of Southern California, Los Angeles, California, United States*, *2. NORC, Chicago, Illinois, United States*

**Background:** Adverse drug events due to drug-drug interactions are a leading cause of preventable mortality in older adults yet current information on the use of interacting regimens is limited. In this study, we quantify changes in the frequency and types of potential major (contraindicated and generally avoid) drug-drug interactions among older US adults.

**Methods:** Descriptive analyses of a longitudinal, nationally representative sample of community-dwelling older adults in the U.S. In-home interviews with medication logs were conducted in 2015-2016 and 2021-2023. We used Micromedex to identify potential major drug-drug interactions. Primary outcomes were the prevalence of the concurrent use of medications with a major drug-drug interactions overall and for specific medications.

**Results:** The study comprised of 4175 participants in 2015-2016 and 2819 in 2021-2023. In 2015-2016, 12.3% of older adults were at risk for a major drug-drug interaction compared with 10.8% in 2021-2023(P =.051). While declines in the use of interacting regimens involving aspirin (3.1% to 1.7%), warfarin (1.3% to 0.4%), and hydrocodone (0.9% to 0.4%) were observed during this time period, increases in the use of citalopram (0.9% to 1.4%), tizanidine (0.2% to 0.6%) and albuterol (0.3% to 0.6%) in interacting combinations was also found (all P< 0.05). Conclusions: Although the use of interacting medications among older adults has modestly declined since 2015, 1 in 9 is still potentially at risk for a major drug-drug interaction. Improving patient awareness of commonly used drug-drug interactions has the potential to reduce preventable adverse drug events associated with their use among older adults.

Abstract citation ID: igae098.4361

### UNDERSTANDING GOAL SETTING AND BEHAVIOR CHANGE MECHANICS IN OLDER ADULT SITTING REDUCTION

Mikael Anne Greenwood-Hickman, David Arterburn, Julie Cooper, Bev Green, Erika Holden, Jennifer McClure, and Dori Rosenberg, *Kaiser Permanente Washington Health Research Institute, Seattle, Washington, United States*

While sedentary behavior (SB) is highly prevalent in older adults, scant evidence documents how participants actionably change this behavior. We explored intervention fidelity, participant satisfaction, and the types of goals participants set based on their preferences within a 6-month randomized controlled trial of a SB intervention for adults aged ≥60 years that successfully reduced participant sitting. Coaches tracked goals for all participants, and 8% of visits were randomly reviewed and coded for fidelity using a structured template. At study completion, participants completed a brief satisfaction questionnaire. Goals data were qualitatively coded and grouped by reminder strategy and topic. Fidelity and satisfaction data were summarized using frequencies and mean (standard deviation). Intervention participants (N=140, mean age 69 [6.0], 62% women, 66% Non-Hispanic White) had high satisfaction (95% Satisfied or Very Satisfied), and 83% reported perceived improvements to health and/or daily functioning. Fidelity to intervention content and techniques was also high across coded intervention sessions (SMART goals set in 98% of sessions, 93% active problem solving with coach, 95% discussed specific sitting break reminder strategies). Participants primarily set sitting reduction goals leveraging outward (e.g., fitness band prompts, using a standing desk for certain tasks) and habit (e.g., adding standing to a meal, standing during daily chores) reminder strategies tailored to individual preferences and lifestyle. Using inner cues such as awareness of pain or stiffness were less common. Specific goals to reduce SB varied widely, suggesting tailored SB reduction intervention approaches are likely important, particularly for older adults with chronic conditions.

### SESSION 9025 (PAPER)

### LATE BREAKING: QUALITY IMPROVEMENT INNOVATIONS IN HEALTH CARE III

Abstract citation ID: igae098.4362

### ADVANCING AGE-FRIENDLY CARE IN THE VA: MIXED-METHODS IMPLEMENTATION RESULTS OF 39 IMPLEMENTATION INSTANCES

Amanda Peeples[1], LauraEllen Ashcraft[1], Octavia Goodman[1], Phoebe Whiteside[1], Rebecca Brown[2], Robert Burke[4], Connor Warren[1], and Lindsay Pelcher[3], *1. Corporal Michael J. Crescenz VA Medical Center, Philadelphia, Pennsylvania, United States*, *2. University of Pennsylvania, Philadelphia, Pennsylvania, United States*, *3. Veterans Health Administration, Pittsburgh, Pennsylvania, United States*, *4. XXX*

AQ5

Age-friendly care integrates the 4Ms (what Matters, Medication, Mentation, Mobility) to promote evidence-based care for older adults. The Safer Aging through Geriatrics-Informed Evidence-Based Practices Quality Enhancement Research Initiative (SAGE QUERI) is a hybrid type-III implementation-effectiveness trial comparing an implementation strategy bundle to dissemination as usual. We implemented four evidence-based practices (EBPs) aligned with the 4Ms across 9 VA Medical Centers. We used descriptive statistics and qualitative interviews with 30 implementing clinicians to report mixed-methods outcomes for reach, adoption, implementation, and maintenance across 39 implementation instances. Reach to Veterans varied widely across EBPs: Mobility n=30; Mentation n=37; Medication n=184; what Matters n=8000+. Eligibility criteria impacted reach; clinicians reported the ease of enrolling Veterans in an EBP with wide eligibility (e.g., every Veteran who is a candidate for surgery) versus an EBP with strict eligibility (e.g., "all the moons have to be aligned" to identify/enroll Veterans). Across 39 implementation instances, 77% (n=31) initially adopted the EBP, with a total of 169 clinicians trained (what Matters n=21; Mobility n=29; Medication n=30; Mentation n=89). Clinicians described training requirements, alignment of EBPs with current practices, and organizational support as key factors influencing adoption. Implementation fidelity varied by EBP: 34% what Matters; 80% Mobility; 100% Mentation and Medication. Clinicians noted the wider impact of EBP-related practice changes, including using new skills with Veterans ineligible for the full EBPs. Across 39 implementation instances, 51% (n=20) reached full maintenance; an additional 23% (n=9) achieved implementation. Sites that had successfully integrated EBPs into practice expressed intentions to sustain EBPs.

Abstract citation ID: igae098.4363

### DESCRIBING ADVANCED PRACTICE REGISTERED NURSES' INFLUENCE ON IMPROVED NURSING HOME OUTCOMES

Alisha Johnson, Amy Vogelsmeier, and Veronica Thompson, *University of Missouri, Columbia, Missouri, United States*

Advanced practice registered nurses (APRN) embedded full-time within nursing homes (NH) improve key resident outcomes such as reducing hospitalizations, increasing advance directive use, and improving resident/family satisfaction. Despite positive evidence, this care model is not widely adopted, and there has been minimal research on how APRN practice impacts outcomes. Informed by Donabedian's Structure-Process-Outcome framework, we conducted a cross-case analysis to understand how APRNs influenced patient (direct) and facility-level (indirect) outcomes through nursing home structures and processes. We used eight years of data generated from The Missouri Quality Initiative, a CMS-funded demonstration project aimed to reduce avoidable hospitalizations through embedded APRN care in 16 midwestern nursing homes. Cases consisted of two higher-performing homes and two lower-performing NHs. Each case was analyzed using deductive coding of four databases to create a cross-database table. First and second level thematic coding was applied to the cross-database tables to create the high and low performing cases for cross-case comparison. Stark differences arose between the higher and the lower performing cases around themes including proactive/reactive processes, individual vs. group-oriented processes, and administrative supports (present/absent/lip-service). APRNs applied proactive processes at the patient (direct) and facility (indirect) level when NH structure (i.e. adequate administrative support) was present. More successful homes, even with higher turnover, demonstrated proactive mentoring processes, initiated and supported by the APRN, instead of passive, reactive processes and deference to administration. APRNs are effective caregivers if structural supports allow for proactive processes maximizing APRN expertise, supporting the inclusion of APRNs within NH care delivery models.

Abstract citation ID: igae098.4364

### LONG-TERM CARE IN-HOME: LIVED EXPERIENCES OF INDIVIDUALS IN VA MEDICAL FOSTER HOMES

Chelsea Manheim, Rachel Fix, and Leah Haverhals, *Rocky Mountain Regional VA Medical Center, Aurora, Colorado, United States*

The Department of Veterans Affairs (VA) Medical Foster Home (MFH) program provides long-term care for Veterans who are nursing home eligible but desire to reside in home-like environments. In MFHs, Veterans are matched with non-VA caregivers to live in the caregiver's home and become part of their family. As the Veteran population rapidly ages, the VA is proactively expanding the MFH program to all VA Medical Centers (VAMCs) by 2026. This work describes lived experiences of Veterans in MFHs, their caregivers, and their family members. From February-April 2024, we conducted N=23 phone interviews with these individuals from 10 sites across eight states. Sites are part of a current VA Office of Rural Health (ORH) MFH expansion program. Veterans described living fulfilling lives in MFHs and taking advantage of activities in home and in the community through assistance from their caregivers. Veterans who lived previously in nursing homes shared that their lives improved greatly once they moved into MFHs. Many caregivers found their work highly rewarding, but also shared that caregiving can be isolating and that the VA provides them with limited respite options. Therefore, those who serve as caregivers must have a great desire to do this work. Some family members described that placing their Veteran family member in a MFH allowed them to return to work and expressed deep appreciation of the program. VAMCs starting and expanding MFH programs can apply findings from this work to better design and implement MFH programs in coming years.

Abstract citation ID: igae098.4365

### RASI'S IN RECOMMENDED HIGHER TARGET DOSES MAY LOWER RISK OF DEATH AND KIDNEY FAILURE IN OCTOGENARIANS WITH HFREF

Ali Ahmed[1], Paul Heidenreich[2], Wilbert Aronow[3], Richard Allman[4], Charles Faselis[1], Mo-kyung Sin[6], Phillip Lam[4], and Gregg Fonarow[6], *1. Washington DC Veterans Medical Center, VA Medical Center, District of Columbia, United States*, *2. Stanford University, Stanford, California, United States*, *3. New York Medical College, Valhalla, New York, United States*, *4. George Washington University, Washington, District of Columbia, United States*, *5. Seattle University, Seattle, Washington, United States*, *6. UCLA Health, Los Angeles, California, United States*

Renin-angiotensin system inhibitors (RASIs) reduce the risk of death in patients with heart failure with reduced ejection fraction (HFrEF). National heart failure (HF) guidelines recommend that RASIs be used in higher target doses in patients with HFrEF. However, clinical trials of target vs. below-target dose RASIs did not include octogenarians, and target-dose group had worsening kidney function, but kidney failure (KF) was not examined. Thus, it is unknown whether RASIs in target doses may improve clinical outcomes without causing KF in octogenarians with HFrEF. Of the 311,611 Veterans with HF receiving RASIs, 32,964 were ≥80 years with ejection fraction ≤40% and without KF of whom 6655 (20.2%) received target-dose. Propensity scores for target-dose, calculated for each of 32,964 patients, were used to match 6642 (99.8% of 6655) target-dose patients with 6642 below-target-dose patients. Matched patients (N=13,284) were balanced on 66 baseline characteristics and had mean(SD) age 84.5(3.4) years, ejection fraction 31.3(8.2)%, and estimated glomerular filtration rate 58.5(18.2) mL/min/1.73m2. During 5 years of follow-up, 70.3% of the patients died, 34.1% were hospitalized for HF, and 1.7% developed KF. Target-dose RASI use was associated with a significant 5% (95% CI, 1–9%) lower risk of death (HR, 0.95; 95% CI, 0.91–0.99). There was a trend toward a lower risk of KF (HR, 0.80; 95% CI, 0.61–1.04), and consequently a lower risk of the composite endpoint of KF or death (HR, 0.94; 95% CI, 0.91–0.98). These new findings provide evidence for the use of RASIs in recommended higher target doses in octogenarians with HFrEF.

Abstract citation ID: igae098.4366

### UNDERSTANDING CHALLENGES & ADAPTIVE STRATEGIES FOR BATHING & SHOWERING AMONG ADULTS WITH MOBILITY DISABILITIES

Pallabi Bhowmick[1], Elena Remillard[2], Meghana Daliparthi[1], Kainat Shahid[1], Wendy Rogers[1], and Ian Rice[1], *1. University of Illinois Urbana-Champaign, Champaign, Illinois, United States*, *2. Georgia Institute of Technology, Atlanta, Georgia, United States*

Self-care and bathing activities are among the most challenging and dangerous activities for individuals aging with mobility disabilities, yet their technology needs remain largely unexplored. There is a need for more specificity about the nature of the challenges. Two user needs studies were conducted exploring challenges and strategies for everyday activities among individuals with long-term mobility impairments (lasting at least 10 years). We analyzed data on bathing/showering challenges from a subsample of participants from these two studies (n=28), aged 52–86 (M=64; SD=9.2), who were wheelchair users and were unable to stand due to lower body mobility impairments. Findings reveal several critical issues and adaptive strategies around bathing/showering. 82% of the participants either stopped bathing (i.e., submerging in a tub) or could not bathe without assistance, relying instead on overhead shower systems and adaptations like roll-in, no-threshold showers, grab bars, and shower chairs/benches. Secondly, the majority transitioned to handheld showerheads over fixed showerheads, and to using bathing wands/reacher sticks for greater flexibility in cleaning themselves. Lastly, several participants expressed concerns about the loss of upper body strength with age, making it difficult to use heavy showerheads or adjust water temperature with controls that are difficult to reach and press. These findings underscore the need for customizable, assistive showering devices for individuals who must shower in a seated position, a research area that has received limited attention. Designing a lightweight robotic showerhead with accessible controls could be a promising solution, which would significantly enhance independence and quality of life for these individuals.

### SESSION 9030 (PAPER)

### LATE BREAKING: ALZHEIMER'S DISEASE, INCLUDING ALZHEIMER'S DISEASE—RELATED DEMENTIAS VII

Abstract citation ID: igae098.4367

### EXPLORING SUBJECTIVE COGNITIVE DECLINE IN OLDER ADULTS: A MIXED METHODS APPROACH

Xi Wen, and Huiling Li, *Soochow University--Suzhou, Suzhou, Jiangsu, China (People's Republic)*

**Objective:** This study aims to explore the cognitive health of older adults exhibiting SCD by using a mixed methods approach to identify the factors and implications of perceived cognitive decline, thereby contributing to early intervention strategies.
**Methods:** We employed a convergent parallel mixed-method design. Quantitative data were collected using the Dementia Assessment Sheet for Community-based Integrated Care System 21 items (DASC-21) and the 9-items Subjective Cognitive Decline Questionnaire (SCD-Q9) from 1,458 older adults without clinical dementia in Soochow, China. Qualitative data were gathered through semi-structured interviews to capture detailed narratives on cognitive complaints, their perceived causes, and impacts on daily life.
**Results:** Quantitative analysis revealed that 48.7% of participants experienced SCD, with significant associations found with advanced age, low education levels, underweight, diabetes, cataract/glaucoma, osteoporosis, hyperlipidemia, and ischemic heart disease. Qualitative thematic analysis identified three primary themes: cognitive complaints, multifactorial attributions of cognitive decline, and the effects on daily life.
**Conclusion:** Our findings underscore the importance of recognizing SCD in older adults as a possible precursor to more severe cognitive impairment. The mixed methods approach provided a comprehensive understanding, suggesting that both objective and subjective assessments are critical for early detection and intervention. These insights highlight the need for tailored interventions focusing on modifiable risk factors and enhancing quality of life for older adults with SCD.

Abstract citation ID: igae098.4368

### INITIAL DEVELOPMENT OF THE WRITING TO HEAL APP: A STRUCTURED WRITING SERIES FOR CAREGIVERS OF SPOUSES LIVING WITH ADRD

Angie LeRoy[1], Samantha Weiss[2], Katherine Harris[2], Valentina Maza[3], Sierra Wickline[1], Alexandria Henderson[2], and Leona Liskovec[4], *1. University of North Florida, Jacksonville, Florida, United States*, *2. Baylor University, Waco, Texas, United States*, *3. Rice University, Waco, Texas, United States*, *4. Third i Research & Design, Tallahassee, Florida, United States*

Caregiving for a spouse living with Alzheimer's or a Related Dementia (ADRD) is incredibly stressful, and puts caregivers at risk for developing health problems, themselves. Caregivers' health issues may affect not only their own quality of life but also their ability to care for their spouse. Thus, developing interventions that support caregivers and help mitigate the stress associated with caregiving, is critical. In Part I of the study, we conducted focus groups with people caring for a spouse living with ADRD) to identify opportunities for targeted treatment and potential barriers to a cognitive-based online expressive writing intervention specifically tailored to spousal caregivers. Results from Part I indicated that caregivers had dynamic needs and requested an intervention that was efficient, mobile, and readily accessible. Collaborating with a product designer to review the qualitative results from Part I, the research team determined that an app would be the best-fitting modality for intervention delivery. In Part IIa, we conducted a second wave of focus groups, throughout which we iteratively adapted the design for an app-based writing intervention for stress and grief among spousal caregivers. To prepare for app development, we conducted preliminary usability testing (Part IIb), during which caregivers interacted with a prototype of the future app to complete a number of proposed task flows. In this article, we present methods and results from Parts I & II as well as reviewing our future plans for app development and subsequent beta testing of the intervention in a pilot RCT.

Abstract citation ID: igae098.4369

### INSIGHT FROM RECRUITING DYADS OF INDIVIDUALS WITH DEMENTIA AND CARE PARTNERS FOR A MULTI-SITE ORAL CARE INTERVENTION

Jing Wang[1], Shahrzad Siamdoust[2], Sophie Zhijing Xu[2], Ruth Anderson[3], Brenda Plassman[4], Donald Bailey[3], and Bei Wu[2], *1. The University of New Hampshire, Durham, New Hampshire, United States*, *2. New York University, New York, New York, United States*, *3. Duke University, Durham, North Carolina, United States*, *4. Duke University Medical Center, Durham, North Carolina, United States*

Maintaining oral health is essential for individuals with dementia, yet they often need support. We sought to identify lessons learned during the recruitment process for an intervention designed to teach care partners skills to guide individuals with mild dementia in proper oral hygiene techniques and provide reminders to practice oral hygiene care. Throughout the study, research team members from two research sites met regularly to discuss their experiences in recruiting participants. Notes from these meetings served as the basis for identifying key challenges and effective strategies. Recruitment involved direct patient engagement, collaboration with dementia research centers and community organizations, and the use of electronic health record systems, including MyChart. Recruitment coordinators connected study personnel with organizations and support groups, thereby broadening the recruitment base. The most effective strategies were leveraging established relationships or building new ones with clinical dementia experts to access a clinically well-characterized patient pool and presenting at support groups within dementia-focused community organizations to build connections and disseminate information about the study. Barriers to recruitment included the reduced frequency of patient visits to clinics during the COVID-19 pandemic, which limited in-person recruitment opportunities, as well as the time required to establish new relationships with non-affiliated hospitals and community organizations. Facilitating factors included building trust-based relationships with medical and community facilities, diversifying recruitment channels, maintaining flexible and clear communication, and customizing strategies for different contexts. In conclusion, successful recruitment for this intervention required a multifaceted approach that combined strong partnerships with flexibility and tailored communication strategies.

Abstract citation ID: igae098.4370

### LGBTQ+ IDENTITY SOCIAL SUPPORT AND CARE MECHANISMS AMONG LGBTQ+ MCI/ADRD CAREGIVERS

Nik Lampe[1], Ellesse-Roselee Akre[2], Harry Barbee[2], and Tara McKay[3], *1. University of South Florida, Tampa, Florida, United States*, *2. Johns Hopkins Bloomberg School of Public Health, Baltimore, Maryland, United States*, *3. Vanderbilt University, Nashville, Tennessee, United States*

LGBTQ+ caregivers for individuals living with mild cognitive impairment or Alzheimer's disease and related dementias (MCI/ADRD) experience disparities in discrimination, social isolation, healthcare, and financial strain, compared to their heterosexual and cisgender counterparts. In this study, we examine the relationship between the roles of being an LGBTQ+ MCI/ADRD caregiver has on levels of LGBTQ+-identity social support and care outcomes among LGBTQ+ older adults. Using Wave 3 data (N=1,038) of The LGBTQ+ Social Networks, Aging, and Policy Study (QSNAPS), we use descriptive analyses to estimate sample characteristics and Chi-square tests and logistic regressions to test the associations between MCI/ADRD caregiver status and outcomes concerning LGBTQ+-identity social support from family, friends, coworkers, and neighbors as well as various LGBTQ+-affirming care practices. Eighty-three respondents (8.3%) in the QSNAPS Wave 3 sample identified as caregivers for individuals with neurocognitive disorders. Compared to LGBTQ+ non-MCI/ADRD caregivers, LGBTQ+ MCI/ADRD caregivers were: 51.7% less likely to have family support (p<.01) and 49.1% less likely to have neighbor support (p<.05). Compared to LGBTQ+ non-MCI/ADRD caregivers, LGBTQ+ MCI/ADRD caregivers were more likely to receive practical help from others (p< 0.1); and less likely to report their providers' offices displaying LGBTQ-affirming materials (p< 0.1) and providers using gender-inclusive language (p< 0.1). No significant associations were found between MCI/ADRD caregiver status and other LGBTQ+-affirming care outcomes. Understanding LGBTQ+-identity social support and care mechanisms can inform targeted interventions to reduce health disparities among LGBTQ+ MCI/ADRD caregivers.

Abstract citation ID: igae098.4371

### PROSPECTIVE ASSOCIATIONS BETWEEN THE STATE MINIMUM WAGE AND COGNITIVE FUNCTION IN OLDER AMERICANS

Boram Lee[1], Lucia Petito[2], and Daniel Kim[1], *1. Northeastern University, Boston, Massachusetts, United States*, *2. Northwestern University, Chicago, Illinois, United States*

Limited research has explored how social policies influence cognitive health in older age. This study aimed to examine the associations between the state-level minimum wage and cognitive function in older US adults. We used data from the Health and Retirement Study (1998-2020) on non-self-employed workers who were age 50-75 and free of dementia at baseline, with cognitive function measured during the study period (N=7490). For cognitive function, we used data based on the Telephone Interview for Cognitive Score (TICS; 0-27, with higher values indicating better cognitive function). We explored the state-level minimum wage, adjusted to 2021 US dollars, lagged by 2, 6, and 10 years. We employed individual fixed-effects linear models and examined for differences in associations by wage, working hours, race/ethnicity, and sex. Among all workers, the state minimum wage was not associated with cognitive function. However, for low-wage workers and part-time workers, a $1 increase in the 6-year lagged state minimum wage was associated with a 0.40 (95% CI=0.17 to 0.64, p=.001) and 0.17 (95% CI=0.03 to 0.32, p=.01) point increase in the TICS, respectively, controlling for time-invariant individual factors and time-varying individual-level and state-level covariates. Among low-wage workers, stronger associations between the state minimum wage and increases in cognitive function were observed in non-Hispanic Whites (β= 0.43, p<.001) but not Black Americans (β=0.04, p=0.91) and Hispanic Americans (β=0.30, p=0.21). There were no differences by sex. Overall, our findings support that increasing the state minimum wage could benefit the cognitive health of older adults, particularly among low-wage workers.

Abstract citation ID: igae098.4372

### THE ROLE OF TELEHEALTH PAYMENT PARITY ON RECOMMENDED CARE AMONG WORKERS WITH HIGH-RISK FACTORS OF DEMENTIA

Zhang Zhang, *Johns Hopkins Bloomberg School of Public Health, Baltimore, Maryland, United States*

**Objective:** This study examines the impact of telehealth payment parity, requiring equal payment rate for telehealth and in-person services, on the use of recommended care services and emergency department (E.D.) visits among insured workers with high-risk factors of dementia.

**Methods:** We conducted a causal inference design and adopted a two-way fixed-effect difference-in-differences approach using the Merative Commercial Claims and Encounters database from 2019 to 2021. We focused on insured workers with mental health disorders or cardiometabolic risks (CMRs), which are high-risk factors for dementia.

**Results:** Telehealth payment parity significantly increased psychotherapy visits by 0.2208 visits per quarter and telepsychotherapy by 0.4107 visits per quarter. Additionally, it reduced E.D. visits among those with mental health disorders by 0.003 visits per quarter, a 25% relative decrease. However, the policy did not significantly enhance the uptake of preventive care counseling for CMRs.

**Conclusion:** Telehealth payment parity effectively boosted psychotherapy usage and reduced E.D. visits for mental health disorders. Public health Implications: Future policies and public health interventions should optimize the telehealth payment model to enhance preventive care uptake, especially for those with CMRs.

### SESSION 9035 (PAPER)

### LATE BREAKING: CLIMATE CHANGE AND AGING

Abstract citation ID: igae098.4373

### AGE DIFFERENCES IN PRO-ENVIRONMENTAL BEHAVIORS: IS IT ABOUT ME OR THE NEXT GENERATION?

Zhixuan Lin[1], and Helene Hoi-Lam Fung[2], *1. University of Zurich, Zurich, Zurich, Switzerland*, *2. The Chinese University of Hong Kong, Hong Kong, China (People's Republic)*

Aging society and climate change have emerged as two pressing concerns in modern society. However, questions remain unsolved regarding whether older and younger adults engage in pro-environmental behavior with different frequencies and/or out of different motivations. In the current study, we examined age differences in pro-environmental behaviors, as well as two underlying motivations: self-relevance and self-transcendence motivations. The former is defined as the extent to which people feel personally harmed or worried by climate change (i.e., ecological risk perceptions), and the latter is defined as the extent to which people hold concerns about the next generation. According to the positivity effect in the aging process and the socioemotional selectivity theory, older age could be correlated with lower risk perceptions and higher generativity concerns. These factors indicate opposing directions in age-related differences in pro-environmental behaviors. We conducted a survey among 126 older adults (Mean age = 70.65, SD = 5.12, 61.5% females) and 117 younger adults (Mean age = 25.43, SD = 5.93, 67.2% females). The results revealed that older adults engaged in pro-environmental behaviors more frequently and scored higher on social generativity concerns compared to younger adults. However, no significant age difference was found in ecological risk perceptions. Moreover, social generativity concerns positively correlated with pro-environmental behaviors only among younger adults, but not among older adults. Ecological risk perceptions positively correlated with pro-environmental behaviors among both younger and older adults. These results provide insights into different approaches for encouraging pro-environmental behaviors among younger and older adults.

Abstract citation ID: igae098.4374

### LOCAL USE OF POLLUTING COOKING FUELS AND ADULT COGNITIVE HEALTH

Sneha Mani[1], Aashish Gupta[2], and Irma Elo[3], *1. Johns Hopkins Bloomberg School of Public Health, Baltimore, Maryland, United States*, *2. University of Oxford, Oxford, England, United Kingdom*, *3. University of Pennsylvania, Philadelphia, Pennsylvania, United States*

Scientific understanding of the relationship between environmental hazards and cognitive health at older ages in low- and middle-income countries (LMICs) is poor. Using data from the Longitudinal Aging Study of India and the World Health Organization's Survey on Global AGEing and adult health for four LMICs, we examine the association of direct and local exposure to polluting cooking fuels with cognitive health at older ages. We document the negative influence of both: cognitive health is poorer among household members who use polluting fuels and among residents of neighborhoods where the use of polluting fuels is more common. We find that women living in households that use polluting fuels and where the local use of polluting fuels is the highest have the lowest predicted cognitive scores. Conversely, men living in households that do not use polluting fuels and in areas with no local use of polluting fuels have the highest predicted cognitive scores. These associations cannot be explained by accounting for individual or local differences in socioeconomic status. Our findings reveal the substantial direct and indirect influence of polluting fuel use in LMICs and help understand why overall cognitive health may be poor in these settings, especially for women. Additionally, the presence of negative externalities of solid fuel use for cognitive health implies a greater justification for efforts to reduce the use of such fuels.

### SESSION 9040 (PAPER)

### LATE BREAKING: CLINICAL PRACTICE INNOVATIONS II

Abstract citation ID: igae098.4375

### ADDRESSING THE HIGH RISK OF OPIOID OVERDOSE AMONG OLDER ADULTS: A RETROSPECTIVE CHART REVIEW

Bethea Kleykamp, Erin Lynch, and Hannah Smith, *University of Maryland School of Medicine, Baltimore, Maryland, United States*

**Background and Objectives:** In Maryland, fatalities due to opioid overdoses are highest among adults aged 55 and over a. This retrospective chart review evaluated 49 adults in outpatient opioid treatment in Baltimore as a function of age. Analyses: Intake characteristics and treatment outcomes were compared between 24 older (ages 55-70) and 25 younger (ages 23-35) adults using ANOVA and $\chi 2$ statistics (alpha=0.05).

**Results:** The cohort was 54% male, and most patients were prescribed methadone (84%). Older adults were more likely to be Black/African American (68% vs. 24%) and had longer histories of opioid (30 vs. 9 years) and tobacco use (45 vs. 14 years) ($ps < 0.05$). At intake, equal proportions of older and younger adults tested positive for cocaine (49%), fentanyl (61%), and benzodiazepines (27%); cannabis use was less common for older adults (17% vs. 56%; $\chi 2$ =8.15). While engaged in treatment, older adults were less likely to test positive for cocaine (26% vs. 77%) and cannabis (16% vs. 60%) ($ps < 0.05$), with no differences in fentanyl (66%) or benzodiazepine use (29%). Older adults remained in treatment longer (186 vs. 105 days, F=6.7) and had higher retention rates (21% vs. 0%) ($\chi 2$ =5.8).

**Conclusion:** Older adults showed less polysubstance use and longer retention in care. However, continued fentanyl and benzodiazepine use could put older adults at greater risk of overdose due to age-related vulnerabilities and prolonged substance use. These differences highlight the unique clinical characteristics of older adults at risk of overdose, which can inform age-friendly addiction treatment approaches.

Abstract citation ID: igae098.4376

### APP ENGAGEMENT IN TECHNOLOGY-SUPPORTED SPEECH-LANGUAGE THERAPY FOR PRIMARY PROGRESSIVE APHASIA

Ollie Fegter[1], Alfred Rademaker[1], Matthew Bona[2], Angela Roberts[3], and Emily Rogalski[2], *1. Northwestern University Feinberg School of Medicine, Chicago, Illinois, United States*, *2. University of Chicago, Chicago, Illinois, United States*, *3. Western University, London, Ontario, Canada*

As the global population ages, dementia prevalence is increasing. Primary Progressive Aphasia (PPA) is a clinical dementia syndrome characterized by progressive language decline. Access to care for individuals living with PPA is limited by a shortage of qualified clinicians and evidence-based interventions. While technology-supported interventions have the potential to overcome this barrier, there has been no systematic exploration of factors affecting web application use in this population. This study aimed to evaluate feasibility and acceptability and identify clinical and demographic factors affecting app usage in individuals with PPA. Web application data were analyzed from participants (N=95) of Communication Bridge-2, an NIH stage 2 randomized controlled trial of speech-language therapy for PPA (NCT03371706). Participants were encouraged to complete app-based practice exercises five days per week over the 12-month intervention. Data-driven user-type clustering determined engagement groups based on number of logins. Multinomial logistic regression identified demographic and clinical factors that differed between groups. On average, participants completed 13.7 (SD = 5.95) home practice exercises 3.99 days per week (SD = 1.19) and logged into the app 5.88 times per week (SD = 1.29). Older age and increased education significantly predicted increased app engagement ($p < .05$), even when accounting for work status; other clinical and demographic factors were nonsignificant. All participants logged into and completed practices exercises on the app at least weekly, demonstrating high feasibility and acceptability. These findings suggest that technology-supported interventions may be a valuable tool to improve access to evidence-based care to individuals with PPA and related conditions.

Abstract citation ID: igae098.4377

### EVALUATION OF A NURSE PRACTITIONER-LED VIRTUAL BEHAVIORAL MEDICINE PROGRAM: INNOVATIVE NEUROPSYCHIATRIC CARE.

Shirin Vellani[1], Lynn Haslam[1], Miles Luke[1], Amaya Singh[2], and Andrea Iaboni[3], *1. Toronto Rehabilitation Institute - University Health Network, Toronto, Ontario, Canada*, *2. Baycrest Health Sciences, Toronto, Ontario, Canada*, *3. University of Toronto, Toronto, Ontario, Canada*

People living with dementia in long-term care (LTC) who exhibit severe neuropsychiatric symptoms are often at risk of being transferred to the hospital from their familiar environment. The virtual Behavioral Medicine (VBM) model was designed to manage these symptoms with intensive virtual support within LTC to prevent hospitalization. We adapted this model to a nurse practitioner (NP)-led approach and conducted a program evaluation to assess its implementation and preliminary effectiveness during its first year. Between April 2023 to March 2024, 102 patients were referred for management of severe neuropsychiatric symptoms and/or consideration for admission to our psychogeriatric unit. The average wait time from referral to the first consultation was 10 days, with follow-ups occurring every 7.6 days by an NP and every 16.2 days involving a geriatric psychiatrist. The average length of stay in the program was 41 days. The average Neuropsychiatric Inventory score improved from 31 to 9.4 at discharge, and the number of medications decreased from 16 to 9. Among the 102 patients, 48 (47%) improved with VBM alone, avoiding hospital admission. In a survey of 60 LTC teams, 49 (81%) responded. Over 90% noted improvement in neuropsychiatric symptoms and expressed satisfaction with the quality of care and support provided by the VBM team. All respondents felt heard and more equipped to care for residents in-house, though some reported challenges with the virtual technology. These first-year insights into operations will inform continuous quality improvement. Further evaluation of caregiver satisfaction and the effectiveness of the NP-led model is warranted.

Abstract citation ID: igae098.4378

### NEXT STEPS: ADVANCING CLINICAL TRIALS RESEARCH IN NURSING HOMES

Kathleen Unroe[1], Susan Hickman[2], Cari Levy[3], Sheryl Zimmerman[4], Alex Floyd[2], and Debra Saliba[5], *1. Indiana University, Indianapolis, Indiana, United States*, *2. Regenstrief Institute, Inc., Indianapolis, Indiana, United States*, *3. University of Colorado School of Medicine, Aurora, Colorado, United States*, *4. University of North Carolina at Chapel Hill, Chapel Hill, North Carolina, United States*, *5. University of California Los Angeles, Los Angeles, California, United States*

The National Institute on Aging recently funded the $15.6 million NEXT STEPs (Nursing home EXplanatory clinical Trials: Supporting Transformation by Enhancing Partnerships) initiative to create the organizational structure, processes, and procedures to develop a sustainable, diverse network of nursing home (NH) partners and researchers conducting clinical trials to guide optimal care. The perspectives and participation of interdisciplinary clinical providers and researchers are critical to inform the launch and activities of this innovative network. In addition to leveraging existing infrastructure and collaborations, NEXT STEPs embraces a comprehensive perspective of NH research that addresses six key elements of successful trials: 1) Designing the intervention, including anticipation of the study's potential burden on NH staff and residents, and potential regulatory issues; 2) Ensuring equity throughout the trial's design, conduct, analyses and dissemination; 3) Recruiting both NHs and individual participants, such as assessing and communicating the value of a given intervention to residents, providers, staff or leadership; 4) Cultivating engagement throughout the project, including plans to monitor and respond to internal or external events that affect projects; 5) Assessing outcomes, including identifying metrics and reliable data sources that can be used across projects; and 6) Following up and following through on communicating results and developing sustainable paths to implement successful interventions. Activities of this project will be guided through deep involvement with partner workgroups. This presentation will describe the evidence that supported the key elements and highlight the opportunities available for researchers, providers, and advocates to participate in the NEXT STEPS initiative.

Abstract citation ID: igae098.4379

**PHYSICAL CAPACITY IS ASSOCIATED WITH ATTENTION IN MIDDLE AGED ADULTS**

Khalil Iktilat, Roy Tzemah-Shahar, and Maayan Agmon, *University of Haifa, Haifa, Hefa, Israel*

Associations between physical capacity and various aspects of executive function, including attention, have been identified in older and younger cohorts, but the relationship among middle-aged individuals at the gateway of aging is largely understudied. The primary objective of this study was to investigate this relationship in middle-aged Muslims in Israel, a group experiencing chronic health disparities. This cross-sectional study included 255 participants (159 women), aged 51.29±4.26 (45-60), assessed using the six-minute walk test (6MWT) and 30-second sit-to-stand test (30STS) for physical capacity, and the Stroop Test and Trail Making Test (TMT) for selective and alternating attention, respectively. We found a significant positive correlation between the 30STS and Stroop-C scores even when controlling for age, sex, education level, and hemoglobin A1c, indicating that physical capacity is independently and positively associated with selective attention ($\beta$=0.132, P=0.057). In contrast, no correlation was found between the 30 STS and TMT ($\beta$=-0.107, P=0.107), suggesting that alternating attention is not associated with physical capacity. Interestingly, neither attention assessment was associated with the 6MWT, suggesting that among healthy, free living, middle-aged individuals, more challenging assessments should be used when examining associations between physical capacity and executive function. Our findings analyzed this past month, in time for late-breaking submission, highlight the nuances of the relationship between physical capacity and aspects of executive function middle aged adults. Interventions that include challenging physical tasks may reduce selective attention decline in middle-aged adults.

Abstract citation ID: igae098.4380

**VETERAN AND FAMILY PERSPECTIVES OF THE VA NURSING HOME TO HOME PROGRAM**

Leah Haverhals, Chelsea Manheim, and Rachel Fix, *Rocky Mountain Regional VA Medical Center, Aurora, Colorado, United States*

Veterans meeting United States (US) Veterans Health Administration (VA) service-connected disability requirements are eligible for VA provided nursing home (NH) care, resulting in high utilization. In 2023, the VA launched the Nursing Home to Home (NHTH) pilot program at three VA Medical Centers. NHTH's objective is to identify Veterans residing in VA-contracted NHs interested in returning to live in the community. Through assessments by an interdisciplinary team, care coordination, and follow-up services, efforts are initiated to support appropriate, safe, and successful discharges. From March-July 2024, we conducted N=11 interviews with 13 Veterans and family members to describe their experiences with NHTH. Veterans felt more comfortable at home, had adequate caregiving and home health support, and said their healthcare needs were met. Several shared they were confident in bathing and dressing independently and expressed increased self-determination in improving their mobility. Families and Veterans noted the time from program referral to NH discharge ranged from three to six months, which they felt was appropriate. Families shared the NHTH team was available to answer questions, provide education, and offer help navigating the VA, including identifying services Veterans may be eligible for that families had not been aware of previously. One family member shared the NH had not provided adequate care to the Veteran and was glad the Veteran returned home, and many Veterans noted they would recommend the program to other Veterans. After one year, feedback shared about NHTH has been largely positive, highlighting several benefits and showing potential for expansion.

### SESSION 9045 (PAPER)

### LATE BREAKING: COMMUNITY-ENGAGED RESEARCH APPROACHES III

Abstract citation ID: igae098.4381

**ANALYSIS OF THE DEMAND AND INFLUENCING FACTORS FOR SMART HOME CARE AMONG COMMUNITY-DWELLING ELDERLY PEOPLE**

HongFei Jia, and YaPing Ding, *Nanjing Medical University, Nanjing Medical University, Jiangsu, China (People's Republic)*

**Objective:** To investigate the smart home care requirements for community-residing adults in their later years, analyze the factors influencing these needs, and establish a scientific basis for the development of targeted smart care solutions.
**Methods:** Between December 2023 and February 2024, a convenience sampling method was employed to select 322 community-residing adults in their later years from Jiangsu Province as participants in this study. Assessment tools included a general information questionnaire, the Mini-Mental State Examination (MMSE), the Barthel Index for activities of daily living, and a custom scale to measure the demand for smart home care within this population. Data analysis involved independent samples t-tests, one-way ANOVA, and multiple linear regression to assess the smart home care needs and influencing factors among community-residing adults with disabilities in their later years.
**Results:** The multiple linear regression analysis indicated that pre-retirement occupation, self-care ability, cognitive function, and the willingness to use smart care technologies in the future are significant factors influencing the demand for smart home care (all P-values < 0.05).
**Conclusion:** The current demand for smart home care among community-residing adults in their later years exhibits diversity. To adequately meet their multifaceted smart care needs, it is essential to consider the main influencing factors such as their occupational background, level of self-care, and cognitive abilities.

Abstract citation ID: igae098.4382

### DECOLONIZING GERONTOLOGY BY REPATRIATING EXPERTISE: THEORY, RESEARCH DESIGN, AND A RESEARCH STUDY EXAMPLE

Alexandra Crampton, *Marquette University, Milwaukee, Wisconsin, United States*

This presentation provides a rationale, a research design, and a research example of decolonizing gerontology through integration of Indigenous philosophy and practices. Colonization in gerontology is identified on three levels; reducing complexity of aging into problems for outside experts to solve; associating aging with loss that negates (and potentially threatens) modern values of youth, independence, and productivity; and limiting the research role of older adults to that of consenting research subjects. Decolonization in gerontology explores complexity of lived experience, and expands the research role of older adults to actively challenge, deepen, and redirect the research. My research design incorporates Shawn Wilson's decolonization work and lessons from the successful repatriation of Ahayu: da by Zuni negotiators. My research example is from a study conducted between July 2021 and July 2024 in a Midwestern retirement community where the average age is 86. Covid restrictions persisted through May, 2023. My research goal was to repatriate gerontological constructs, such as "successful aging" and "older adulthood" through community-engaged research. Data were collected using ethnographic methods of participant observation, in-depth interviews, archival document collection, and frequent consultation with research participants. Although "successful aging" and "older adulthood" were recognized by research participants, these constructs were rejected as non-informative by those confronting major disruption to roles, status, and/or functioning. Construct repatriation included reformulating aging as reckoning with loss of capacities, relationships, and other sources of meaning that had been integral to a sense of self and means of flourishing. Research presentations became a means of shared reckoning.

Abstract citation ID: igae098.4383

### MODERNIZING CHINA'S PRIMARY HEALTHCARE: REVISITING THE BAREFOOT DOCTOR PROGRAM TO ELEVATE SPECIALIZED NURSING

Qingwei Wang[1], and Zixuan Wu[2], *1. Xi'an Jiaotong-Liverpool University, Suzhou, Jiangsu, China*, *2. Johns Hopkins University, Baltimore, Maryland, United States*

A persistent challenge in China's primary healthcare system is the shortage of qualified healthcare professionals, a problem exacerbated by the decline of the Barefoot Doctor (BD) program. The newly innovative introduction of Specialized Nurses (SNs) in 2024 seeks to address physician shortages by equipping nurses with advanced skills, including prescription authority. This article explores how lessons from the BD program can inform the modernization of China's primary care workforce with a focus on the needs of older adults. Through a comparative analysis, the study examines how both initiatives aim to address the shortage of healthcare professionals by harnessing grassroots power. Three critical themes are explored: 1) Community integration of Specialized Nurses (SNs) may face challenges due to prevailing cultural perceptions of nursing professionalism and the confinement of SNs to tertiary hospitals, which significantly hinder their involvement in community healthcare; 2) The effectiveness of the program is constrained by insufficient financial support and unclear career progression paths; 3) Sustainable caregiving strategies are necessary that address both traditional and contemporary health needs, expanding beyond the BD's focus on environmental and infectious diseases to include non-communicable diseases. Drawing from the BD model, future efforts should prioritize community-based care, expanding opportunities for home visits, especially for older adults who may face mobility challenges.. Ensuring adequate fiscal funds and active agency involvement from the government and health administrators is crucial for sustaining the primary healthcare system. Additionally, preventive care should adopt a holistic and interdisciplinary approach that integrates diet, exercise, and psychological care.

Abstract citation ID: igae098.4384

### THE ASSOCIATION BETWEEN CLOSE RELATIONSHIPS AND HAPPINESS AMONG OLDER ADULTS

Elnaz Abaei, and Peter Martin, *Iowa State University, Ames, Iowa, United States*

Close relationships have long been recognized as crucial in shaping older adults' happiness and well-being. This study investigated the association between close relationships (including those with a spouse, children, and friends) and happiness among older adults using random intercept cross-lagged panel models (RI-CLPMs). We pooled waves 11-12, 13-14, and 15-16 from the RAND file of the Health and Retirement Study (HRS). Our analytic sample included 15,758 individuals aged 50 years and older (Mean age=67.14). We ran three RI-CLPMs to examine how close relationships with spouse, children, and friends affect the happiness of older adults. All models demonstrated a good fit with the data (spouse's model, $\chi 2$ (df=7)=6.979, p=.43, CFI=TLI=1.00; children's model, $\chi 2$ (df=8)=13.91, p=.08, CFI=TLI=1.00; friend's model, $\chi 2$ (df=8)=17.35, p=.03, CFI=.999, TLI=.996). We also conducted a sensitivity analysis by computing models without missing data imputation. The results were mostly consistent with the initial analysis. In the initial waves, we did not find carry-over and spill-over effects between close relationships and happiness. However, carry-over effects were observed for models in the later waves. We also found that married individuals tend to be happier as they age. Additionally, women were more likely to have close relationships with children and friends compared to men. Practical implications should focus on fostering positive interactions among older adults with close social contacts, like encouraging companionship and supportive communication from spouses and children. Group interventions, such as peer support groups, can enhance happiness by monitoring social interactions to promote well-being in older adults.

Abstract citation ID: igae098.4385

### TRENDS IN HEALTH CONDITIONS, HEALTH NEEDS, HEALTH SERVICES & MEDICATION USE AMONG OREGON AFH RESIDENTS (2017-2023)

Wafi Albalawi[1], Ozcan Tunalilar[2], and Paula Carder[3], *1. Oregon Health and Science University, Portland, Oregon,*

*United States*, *2. Portland State University, Portland, Oregon, United States*, *3. OHSU-PSU School of Public Health, Portland, Oregon, United States*

The aging U.S. population is increasing the demand for Long-Term Care Services. Adult Foster Homes (AFHs) offer a home-like environment for residents with complex health needs, particularly beneficial for older adults, including those with dementia. However, these settings remain understudied. This study evaluated trends in resident characteristics, chronic conditions, service utilization, medication use, activities of daily living (ADLs), and family involvement among AFH residents in Oregon from 2017 to 2023, using data from the Oregon Community-Based Care study. Data were analyzed using descriptive statistics and OLS regression. Results indicate that the proportion of female residents remained stable at 60-64%. Residents aged 75-84 increased from 18.7% to 25.7%, while those aged 85+ decreased from 41.6% to 31.5%. Hypertension (48.7-51.6%) and dementia (43.6-47.7%) are the most prevalent conditions. Arthritis declined from 37.8% to 30.0%, and multiple medication use peaked at 61.5% in 2023. Bathing (78.4-82.9%) and dressing (60.5-69.5%) are the ADLs requiring the most assistance. Family involvement saw significant changes during the pandemic, with social visits decreasing from 64.9% to 37.8% and outings from 39.8% to 17.4%. The study found both similarities and differences between AFH residents and assisted living (AL) residents in the U.S. Hypertension and dementia were the most prevalent conditions in both groups. However, AFH residents had higher rates of dementia and arthritis, as well as greater reliance on Medicaid (54%-63.2%) compared to AL residents (17%). These findings highlight the higher acuity among AFH residents and emphasize the need for further research on smaller residential settings for older adults.

### SESSION 9050 (PAPER)

### LATE BREAKING: HEALTH EQUITY ACROSS THE LIFE COURSE IV

Abstract citation ID: igae098.4386

### ADVANCE CARE PLANNING IN A SUBSIDIZED HOUSING COMMUNITY: PRELIMINARY RESEARCH RESULTS FROM A PILOT INTERVENTION

Christine Kimpel, Lorely Chavez, Christian Ketel, and Kate Clouse, *Vanderbilt University, Nashville, Tennessee, United States*

Advance care planning (ACP) can improve end-of-life outcomes, but ACP remains low among adults with low SES, who likely have had multiple adverse life experiences. To reduce the stress of ACP, we are piloting a trauma-informed advance care planning (TI-ACP) intervention in a subsidized housing community. Following IRB approval, we purposively and snowball sampled 15 adults (aged 18+) in a 150+ unit subsidized housing community in Nashville, Tennessee (May 2024-present). Using a pre-post single-arm intervention design, we conducted visits to collect mixed methods data in the onsite Resiliency Hub, a resource center. The intervention included a researcher-led conversation using a discussion prompt checklist. The median age was 60 (IQR: 57-65), seven (46.7%) identified as Black or African American, and eleven (73.3%) identified as men (n=15). At 30-day follow-up (n=9), implementation acceptability, appropriateness, and feasibility score medians and ranges were 16 (16-18), 16 (15-17), and 16 (15-18), respectively. Implementation barriers included competing priorities, limited social support, skepticism about outsiders, ACP skepticism, and medical mistrust. Potential adaptations included explaining privacy procedures and including supplementary education for challenging medical concepts. Participants rated the innovation as acceptable, appropriate, and feasible and provided insight into potential barriers and adaptations. The conversation checklist and educational materials may be utilized across clinical and community-based contexts. A team-based approach may maximize the benefit of TI-ACP by holistically addressing complex social and health barriers for older adults with low SES. Future iterations will explore using community health workers as interventionists and a community advisory council to design training.

Abstract citation ID: igae098.4387

### ASSOCIATION OF HEALTHCARE USE AND EXPENDITURE BASED ON DUAL ELIGIBILITY AND ENROLLMENT IN MANAGED CARE

Mohammad Usama Toseef[1], Aastha Dharia[2], Ramin Homayouni[2], and Wassim Tarraf[3], *1. Corewell Health, Royal Oak, Michigan, United States*, *2. Oakland University William Beaumont School of Medicine, Rochester, Michigan, United States*, *3. Wayne State University, Detroit, Michigan, United States*

Individuals enrolled in both Medicaid and Medicare, also known as duals, have complex and high healthcare needs and disproportionately account for higher state and federal spending. In recent years, most of these beneficiaries have been enrolled in some form of managed care plans. In this study, using the 2020-2021 Medical Expenditure Panel Survey, we evaluate the association of dual-eligibility status and different forms of healthcare use and expenditures: inpatient, emergency room and outpatient. We also explore if enrollment in a managed care affects this relationship. We estimate a series of Poisson regressions for use variables and generalized linear models for expenditure variables that account for the interaction between dual-eligibility status and enrollment in managed care. Postestimation, we calculate marginal effects and use analysis of variance contrast tests to estimate the differences in the predicted use events and average expenditure based on managed care and dual-eligibility status. We find that, on average, duals eligibles have a higher use of inpatient and emergency room than non-duals but similar outpatient visits. We also find that non-duals enrolled in managed care have lower outpatient and emergency room visits than their non-managed care counterparts. No significant differences are observed for dual eligibles in terms of their managed care status for both use and expenditures. While more research and years of data are needed to establish whether managed care improves utilization and lowers spending for dual eligibles, we find that managed care affects duals and non-duals differently and stakeholders should consider this to provide equitable health.

Abstract citation ID: igae098.4388

**IMPLICATIONS OF TIMING, COMMUNITY, AND DIFFUSION FOR IMMIGRANT HEALTHCARE AND TELEHEALTH EXPERIENCES**

Sungjae Hong[1], and Shannon Mejia[2], *1. University of Illinois at Urbana-Champaign, Champaign, Illinois, United States*, *2. University of Illinois Urbana-Champaign, Urbana, Illinois, United States*

Inequality in access to healthcare among immigrant older adults can evolve into health disparities, resulting in social burdens on society. This study integrates life course and diffusion of innovations perspectives to examine how the timing of immigration, global events, and interdependence intersect with social systems and communication channels to influence healthcare and telehealth adoption. We interviewed 22 Korean immigrant older adults who immigrated to the Chicago Metropolitan Area. The interviews detailed respondents' life trajectories, including immigration timing, communication channels, and social relationships. Participants also reported perceptions of and experiences with healthcare and telehealth. To discover how perceptions of and experiences with telehealth varied according to life experiences, we conducted the direct content analysis based on the diffusion of innovations and the life course theories. We coded interviews with predefined codes based on the theories but added new codes if needed. Four main themes emerged: 1) Racial/ethnic communities served as the primary communication channel for healthcare information, 2) The difference between the first and 1.5 generations in the trajectories of healthcare service experience, 3) Adult children as the primary driver of telehealth adoption, and 4) The limited scope of authority innovation diffusion, where telehealth services were adopted within the COVID-19 context, but exchanged for in-person healthcare services due to cultural norms. Our findings provide new insight into the lifelong mechanisms of inequalities in healthcare and telehealth and highlights importance of communication channels and community connections in efforts to improve access to and use of telehealth services in immigrant older adult populations.

Abstract citation ID: igae098.4389

**TRAJECTORIES OF FALLS AND ASSOCIATED FACTORS AMONG ADULTS 65 AND OLDER: INSIGHTS FROM THE HEALTH AND RETIREMENT STUDY**

Rohit Baal Balasundaram[1], Brittany Krenek-Schneider[1], Edna Patricia Mendoza[1], Anup Dharmendrakumar Patel[1], Lien Quach[2], and Uyen-Sa Nguyen[1], *1. University of North Texas Health Science Center, Fort Worth, Texas, United States*, *2. Massachusetts General Hospital, Boston, Massachusetts, United States*

Falls are the leading cause of injuries among older adults in the United States, with over 14 million older adults, or 1 in 4, reporting a fall each year. This longitudinal prospective cohort study investigated fall trajectories and associated risk factors in people with no prior history of falls. We analyzed 2006 to 2018 data from the Health and Retirement Study (HRS), restricted to participants 65+ who did not report falls prior to the baseline year of 2006. The outcome is biennial incidence of falls, and risk factors include sociodemographic and biological factors, and health behaviors. Group-based trajectory modeling identified distinct fall trajectories. Multinomial logistic regression assessed associations between trajectories and risk factors;. Three distinct trajectories of falls incidence were identified over a 12-year period. Nearly half of the population (48.9%) remained in a low-stable risk group, 22.4% experienced a moderate but steadily increasing risk, and 28.7% belonged to a high-increasing risk group. Individuals identified as Black/African American or "Other" race were associated with a lower likelihood of being in the high-risk group compared to White/Caucasian individuals (OR = 0.38, 95% CI: 0.27, 0.55; OR = 0.32, 95% CI: 0.16, 0.67, respectively). Conversely, having comorbid conditions was associated with a higher likelihood of being in the high-risk group (OR = 1.37, 95% CI: 1.08, 1.73). The study identified distinct trajectories of fall risk among older adults without a history of falls, significantly varying by race/ethnicity and comorbid conditions, highlighting the need for targeted fall prevention strategies tailored to specific groups.

Abstract citation ID: igae098.4390

**USING THE SAMHSA TRAUMA-INFORMED PRINCIPLES TO ADAPT ADVANCE CARE PLANNING FOR OLDER ADULTS WITH LOW SES**

Christine Kimpel, Lorely Chavez, Christian Ketel, and Kate Clouse, *Vanderbilt University, Nashville, Tennessee, United States*

Advance care planning (ACP) is the process of preparing for future medical care received during times of cognitive incapacity, but the process may be very stressful for low SES communities. To address lifetime traumas and the everyday stress of unmet needs, we used the Substance Abuse and Mental Health Services Administration's (SAMHSA) trauma-informed principles to adapt an ACP intervention. Using a single-arm pre-post intervention design and mixed methods approach, we continually adapted the intervention with participant feedback (n=19). The initial intervention consisted of an audio-recorded, one-time, in-person, flexible conversational approach using a discussion prompt checklist, conducted in a quiet private setting in a subsidized housing community. We thematically analyzed field notes and intervention transcripts using the SAMHSA framework. Nineteen participants completed the intervention (median age: 60, IQR: 58-63): 10 identified as Black or African American and 12 as men. Themes included trauma awareness (safety), facilitating privacy (trustworthiness), peer referral (peer support), person-centered communication (collaboration), healthcare risk-benefit analysis education (empowerment), and acknowledgment of historical trauma, stigma, and access challenges (equity). We have found the SAMHSA framework useful for enhancing trauma awareness during the ACP process. There is significant conceptual overlap between the person-centeredness of ACP and the SAMHSA principles. The universal safety precautions help to avoid re-traumatization while discussing previous death-related experiences. The equity focus provides opportunities to tailor the approach. These trauma-informed principles should be implemented in clinical provider training, organizational policy and procedures, and future research interventions with similar communities.

### Participant Index

Arabic numbers refer to session numbers.
Duplicate numbers indicate the participant is listed as an author on multiple abstracts within a session.

**A**
Østbye, Truls: 2065, 3025, 7205
Abadir, Michael: 8000
Abadir, Michael P: 8000
Abadir, Peter: 1580, 2245, 4005, 8000, 8015
Abaei, Elnaz: 7355, 9045
Abbate, Lauren M: 7155
Abbing, Jens: 1120
Abbott, Katherine M: 1160, 3380
Abdallah, Lisa: 2440
Abdel Magid, Maryam S: 7425, 7905
Abdelmageed, Mofida: 8000
Abdelrahman, Nadir: 8015
Abdulrahim, Sawsan: 1155
Abe, Takumi: 7275, 8015
Abi Nassif, Alexandra: 1155
Ables, Gene: 8000
Abraham, Alison: 7285
Abrams, Leah: 4445
Abramson, Corey: 3175, 4450
Abramson, Tobi A: 7695
Abreu, Taiane: 7745
Abu-Baker, Dania: 3115
Abudahab, Sara: 7715
Abudu-Birresborn, Diana: 4175
Abul, Yasin: 7030
Abul, Zeynep: 7070
Abu-Samaha, Amir: 7065, 7425
Abyadeh, Morteza: 8000
Acevedo-Fontanez, Adrianna I: 2220
Acharya, Jhuma: 2195
Acierno, Ron: 2520
Ackerson, Leland K: 8010
Acosta, Cromwell: 7750
Adamek, Margaret E: 7515, 7540, 7610
Adams, Leah: 2450
Adams, Leslie: 7615
Adams, Swann A: 3175, 7100
Adams, Taylor: 4170
Adams-Price, Carolyn E: 4540
Adaniya, Stephanie: 3510
Adar, Sara D: 2040, 3125, 3145
Adawudu, Emefa A: 8020
Addai-Dansoh, Stephen: 7020
Addison, Odessa: 2345, 7315, 7720, 8010, 8015
Addo-Mensah, Dorothy Ewurabena: 1465, 3300
Adeagbo, Morolake: 7465, 7685
Adekoya, Adebusola: 7630, 7905
Adelnia, Fatemeh: 7010
Adeniji, Dolapo O: 7440, 7505, 7515, 7610, 7925
Adepoju, Omolola E: 3485
Adeyemi, Oluwaseun: 7465, 9020
Adil, Rawaa: 8015
Adjei, Mathias: 7240, 7405
Adjei Gyimah, Akwasi: 7240, 7405
Adjei-Poku, Michael: 8015
Adkins, Daniel E: 1485
Adnan, Maham: 7720
Adolph, Sharada Sarah: 3430
Adorno, De'Lisia: 3235
Aflatoony, Leila: 8000
Agard, Pernilla: 3550
Agboji, Aderonke: 2360
Agbomi, Lidadi: 8000
Agerholm, Janne: 7215
Aghakhani, Elham: 4220
Agmon, Maayan: 1435, 3085, 4075, 7300, 7695, 9040
Agnoli, Alicia: 8010
Agrawal, Sunil: 7750
Agrawal, Yuri: 2080
Agree, Emily M: 7420, 7920
Aguila, Emma: 1320
Aguilar, Genevieve C: 2125
Aguilar, Vanessa: 3240
Aguirre, Alyssa: 1355, 2355, 7040
Aguirre, Annabella S: 3245, 7260
Agyekum, Boadi: 7605
Agyemang, Lillian: 2265, 7155
Ahlbrand Robinson, Amy: 4240
Ahmad, Anam: 7010
Ahmad, Waqar: 8020
Ahmadi, Matthew: 1245
Ahmed, Ali: 2160, 2380, 9025
Ahmed, Ivy: 8000
Ahmed, Linda: 8015
Ahmed, Syeda A Fatima: 7530, 7905
Ahmidouch, Mohamed: 2085
Ahn, Eunhye: 8010
Ahn, Hyochol: 2240, 8010, 8015
Ahn, Jinhyun: 8015
Ahn, SangNam: 7465, 8010
Ahn, Sangwoo: 7005
Ahn, Seon Ah: 1140
Ahn, Seoyeon: 8010, 8015
Ahn, Tae-Hyuk: 8010
Aichele, Stephen R: 7455, 7720, 7925
Ailshire, Jennifer: 1015, 1320, 2040, 2110, 2460, 2515, 3125, 3245, 3540, 7040, 7500
Aishima, Miya: 7510
Ajrouch, Kristine J: 1155, 1495, 3040, 3065
Akabike, Ekenechukwu: 8010
Akbar Nejad Yazdi, Reyhaneh: 8020
Aki, Sugiura: 7330
Akiguchi, Ichiro: 8010
Akinbami, Yewande: 3340
Akiva, Pinchas: 8015
Akre, Ellesse-Roselee: 1170, 9030
Akter, Nahida: 1080, 1160
Akushevich, Igor: 3010, 3085, 7075, 7200, 7300, 7620, 7715
Al Bcherraoui, Myriam: 3040
Al Ghazo, Ali: 7320
Al Juboori, Ruaa: 4080, 7055
Al Saeedy, Dalia: 7715
Al Snih, Soham: 2020, 7205, 7300, 7440, 7700, 8015
Alam, Md Tanjim: 8000
Alam, Rifat: 7145
Alanazi, Eman: 7200
Al-Ani, Mohammad: 1445
Alaux, Alexia: 1100
AlAzzeh, Ola: 7715
Albalawi, Wafi: 9045
Albashayreh, Alaa: 8040
Albayyaa, Mohanad: 7020
Alber, Jessica: 7205
Albers, Elizabeth A: 2015, 3130
Albert, Jeffrey: 1140
Albert, Marilyn: 2525, 3070, 3555, 7015, 7200, 8015
Albert, Steven M: 1290, 2350, 2450, 2480, 3045, 3550, 7300
Alberth, Andrew G: 2225, 3075, 4270, 9000
Albertini, Marco: 3025, 7515, 7915
Albrecht, Jennifer: 1255, 4385, 8070
Alburez-Gutierrez, Diego: 4050
Alcantar, Sandy: 3460
Alcusky, Matthew: 7320
Alderson, Jacob E: 7070, 7115
Aldossary, Heba M: 1490, 7270
Aldridge, Jolaiya: 8000
Aldridge, Melissa D: 1395
Aldwin, Carolyn M: 7030
Alea, Nicole: 7250, 7635
Alejo, Prince Iverson C: 8070
Aleksic, Sandra: 1460
Aleong, Rosanne: 7040
Alessi, Cathy: 7660
Alexander, Bruce: 7665
Alexander, Gregory L: 3090, 8040
Alexander, Karah: 2295
Alexander, Laura: 8000
Alexander, Neil B: 7720
Alexanderson, Kristina: 2365
Alfonso Lopez, Irene: 3150
Alford, Heather: 4440
AlGhatrif, Majd: 8015
Alhabodal, Ashwag Saad: 8015
Alharrasi, Azza: 7420, 7455

© The Author(s) 2024. Published by Oxford University Press on behalf of the Gerontological Society of America.
This is an Open Access article distributed under the terms of the Creative Commons Attribution License (https://creativecommons.org/licenses/by/4.0/), which permits unrestricted reuse, distribution, and reproduction in any medium, provided the original work is properly cited.

Alhasan, Dana: 1045, 9000
Ali, Akilah R: 4390, 7065, 7205
Ali, Eman: 8000
Ali, Mohab: 7325
Ali, Sanna: 8015
Ali, Talha: 2045
Ali, Waad: 7605
Alimohammadbeik, Kourosh: 7630
Alkeridy, Walid A: 1150, 3335
Alladi, Renuka: 7490
Allan, Alexa C: 1250, 2055, 2330, 8000
Allard, Eric S: 4295, 8020
Allemand, Mathias: 8000
Allemann, Alexandrea L: 3425
Allen, Elizabeth M: 1345, 2315, 8020
Allen, Josie R: 7720
Allen, Julie Ober: 1095, 2330
Allen, Kelli D: 2145, 7330, 8015
Allen, Laura D: 7430
Allen, Melissa: 7040
Allen, Nancy A: 2060, 4070
Allen, Rebecca Sue: 1210, 1325
Aller, Ty B: 4435, 7065, 7270, 8020
Allgood, Sarah: 2155
Allison, Caitlin: 4215
Allison, Matthew A: 2220
Allison, Theresa A: 4480
Allman, Richard: 9025
Allore, Heather: 3035, 3520, 7060, 7465, 7680, 7925
Allsopp, Richard: 8000
Almack, Kathryn: 1015
Almeida, David: 1255, 1350, 1525, 2385, 4150, 4180, 4405, 4410, 7255, 7350, 7490, 7675, 8000
Almenar-Queralt, Angels: 7310
Al-Naggar, Iman: 3210, 4350
Alnufeay, Mashael: 7470, 7905
AlOkda, Abdelrahman: 8020
Alotaibi, Taghreed: 8010
Al-Rousan, Tala: 3065
Alshanbari, Mohammed: 7630
Alston, Daniel: 8010
Altenmueller, Eckart: 4490
Alter, Isaac L: 7140
Altona, Janissa: 4345
Alvarez, Loreli: 7425, 7615, 7700, 7930
Alvarez-McNelis, Desiree: 3375
Alves, Gilberto: 7350
Alves da Silva do Nascimento, Mariana: 3155
Am, Linda: 7110
Amadi, Grace: 8010
Amanambu, Peace Nneka: 4295
Amano, Takashi: 4190, 4265, 7630
Amaravadi, Harsha: 7080, 8010
Amato, Francesco: 2240, 7305
Ambrosio, Fabrisia: 2165
Ameer, Fathima: 7210
Amin, Iftekhar: 1485, 7125
Amin, Vikesh: 1280
Amjad, Halima: 1255, 3215, 4100
Amlerova, Jana: 8000
Amoatey, Solomon: 4000
Amor, Corina: 3160
Amponsah, Abigail: 4360
Amy, Byers: 2190
An, Jaesung: 7545
An, Yang: 1255
Anaab, Esme: 7080
Anaya-Carmona, Israel: 7200
Ances, Beau: 7655
Anctil, Reid: 7680
Andel, Ross: 2185, 8000
Andersen, Troy C: 3175
Andersen, Zorana J: 3125
Anderson, Andrea M: 1390
Anderson, Brittany: 7465, 7685
Anderson, Joel G: 2015, 2225, 3190, 7720, 8010
Anderson, Keith A: 2260, 4345, 7725
Anderson, Kennedy: 7200
Anderson, Lindsey J: 7305
Anderson, Nicole D: 1205, 7675
Anderson, Rose: 8020
Anderson, Rozalyn: 8000
Anderson, Ruth: 7535, 9030
Anderson, Stacy: 1025
Anderson, Susan J: 1175
Anderson-Steward, Kuren A: 8000
Andrade, Flávia Cristina Drumond: 3030, 7135, 8020
Andrade Rabello, Gilciney: 4465
Andreoletti, Carrie: 2440, 3515
Andres, Lanvin: 1470
Andrews, Hailey: 8000
Andrews, Heather: 7355
Andrews, Ryan: 4380
Andrilla, C. Holly: 2500
Ang, Chi Shuen: 2100, 7455, 7925
Ang, Shannon: 1095, 2065, 3405
Angel, Cybele: 4195
Angel, Jacqueline L: 2155, 3030, 8020
Angellini, Aude: 1420
Anglin, Emerald: 8070
Angus, Payton: 7430
Anjum Ashrafi, Sadia: 7145
Annunziata, Kathy: 8010
Anquillare, Elizabeth: 7225, 7500
Ansai, Juliana Hotta: 7225, 7655
Ansie, Vera: 7335
Anstey, Kaarin J: 3185, 7610, 8020
Antenor, Angelo: 7010
Anthony, Mia: 7015
Antic, Mina: 4375
Antonakakis, Antigone: 1505
Antonucci, Toni C: 1045, 1155, 1495, 3040, 3540, 9015
Anupindi, Meenakshi: 7070
Aouad, Marion: 7080
Apostolou, Hannah: 7070, 7455, 7610, 8015
Apostolova, Liana G: 8000
Applebaum, Robert A: 1315, 4510, 7295
Appleton, Allison: 8010
Apsley, Abner T: 7010, 7490
Apuan, Victoria N: 2405
Arai, Tetsuaki: 8015
Arai, Yasumichi: 7115
Aranda, Maria P: 1320, 1335, 3030, 3280
Arata, Louis: 8020
Araujo, Lia: 7745
Araujo-Menendez, Carlos EE: 7000, 7455, 7540
Aravena, Jose M: 4235
Arbeev, Konstantin: 1025, 7075, 7210, 7715
Archambault, Dennis: 1295, 4265
Archer, Hannah: 7000
Arciniega, Adrian M: 7010
Ard, Jamy D: 2505
Ardekani, Azam: 7030
Ardon Lobos, Marcos: 8015
Aree-Ue, Suparb: 7205, 7440
Arellanes, Isabella C: 7630
Arensberg, MaryBeth: 8015
Arias, Dalmina: 8015
Arigoni, Danielle: 2460
Arivudainambi, Aishwarrya P: 4530
Arling, Greg: 7510, 7915
Armando, Antonio: 8010
Armendariz, Marina: 1495
Arms, Tamatha: 8015
Armstrong, Grace: 1490
Armstrong, Naomi: 8015
Armstrong, Nicole: 8015
Arnette, Shannon M: 1145, 3510, 8010
Arnold, Michelle: 7140
Arnold, Moriah: 7310
Aronow, Wilbert: 9025
Arora, Neeraj: 3340
Arora, Shambhavi: 8020
Arp, Patrick C: 7000
Arpawong, Thalida Em: 1455, 3220, 4375
Arreguin, Berty: 1245
Arteaga, Ignacia: 3175, 4450
Arterburn, David: 1265, 9020
Arthanat, Sajay: 3185
Arthur, Elizabeth K: 2445
Aruscavage, Nancy: 3500, 7425, 7905
Arya, Shipra: 3435
Aryoyudanta, Bayu: 8015
Asante-Danso, Anthony J: 8000
Aschenbrenner, Andrew J: 4515
Ash, Kelly: 2450
Ashcraft, Alyce S: 7080
Ashcraft, LauraEllen: 9025
Ashe, Maureen C: 8020
Ashebir, Sophia: 1095, 7065, 7090
Ashford, Nathanial K: 8000
Ashida, Sato: 2185, 3345, 3485, 4220, 7465, 7685
Ashiqueali, Sarah A: 8000
Ashirifi, Gifty D: 7515, 7610
Ashok Kumaar, Prasanna Vadhana: 8000
Ashrafi, Jaya: 7015
Asiedu, Charlotte: 7700
Askim, Torunn: 7060
Aslan, Ilker: 7160
Asraf, Kfir: 1435

Assadi Khalil, Samar: 7300
Assi, Lama: 3275
Assi, Sahar: 2525, 3275
Assumpção, Daniela de: 8015
Asthana, Sanjay: 3225
Aswani, Truphosa: 2015, 3025
Atak, Selen: 7455
Atayee, Rabia: 1435
Atchison, Chandler: 4160
Atherton, Olivia E: 4480
Atif Mohamed Yousif, Mohamed: 8015
Atkin, Andrew: 1245
Atoyebi, Oladele: 7155
Attuquayefio, Tuki: 3185
Atul Patil, Vaishnavi: 7430
Au, Daisy: 3200
Aubry, JP: 2420
Audley, John: 3340
Auriemma, Catherine L: 8015
Austad, Steven N: 4210
Autar, Kaveena: 7425, 7630
Au-Yeung, Wan-Tai M: 3285, 8020
Avendaño Villaseñor, Abigail: 7465
Avent, Elizabeth S: 2001
Avesani, Carla Maria: 3330
Avidon, Irina: 7320
Avila, Jaqueline C: 1405, 2205, 8010, 8015
Avis, Fan: 7490
Awata, Shuichi: 7350, 7490, 7640, 8015
Axelrod, Jenna: 7495, 7930
Axelson, Eric: 7735
Ayala, Alba: 4045
Ayalon, Liat: 1190, 2460, 7420, 8010
Ayers, Lindsay R: 7640
Azar, Ariel: 1110, 3410
Azhar, Gohar: 7010, 7210, 7465, 8000, 8015
Azhir, Alaleh: 1575
Azuero, Andres: 7285, 7700

### B

Ba, Fang: 7465, 7925
Bárbara, Rita Cássia: 8010, 8015
Bédard, Marie-Ève: 3320
Bünning, Farina: 8010
Baars, Jan: 1180
Babenko, Natalia: 7070
Babitzke, Jennifer: 2500
Baby, Eliza: 7660
Bachner, Yaacov: 3530
Bacon, Cindy: 7275
Bacsu, Juanita R: 3175, 8010
Badal, varsha E: 4180
Badawi, Dina T: 8015
Bae, Myeongjin: 7655
Baek, Daniel: 4355
Baek, Jihye: 1295, 2260, 4140, 4265, 7290, 7630
Baek, Jonggyu: 7320
Baek, Sehyun: 8010
Bagley, Olivia: 1025
Bahti, Melanie: 8015
Bai, Chen: 1445
Bai, Chyi-Huey: 7735
Bai, Xue: 8010, 8020, 8050, 8090, 8095
Bai, Yaru: 4055
Baier, Rosa R: 3240
Baik, Dawon: 7360, 7660
Baik, Sol: 1045
Bailey, Dionne: 3130, 4445, 7685
Bailey, Donald: 9030
Bailey, Olivia: 8020
Bailey-Taylor, Melissa: 8010
Baillargeon, Emma M: 2510, 4215
Baim-Lance, Abigail: 8010, 8010
Bain, Catherine: 7420
Baird, Lola: 2385
Baird, Sara: 7080
Bak, Katherine: 7225
Bakare, Jemimah: 7260
Baker, Christina: 8000
Baker, Hamilton: 2355
Baker, Jillian: 1365
Baker, Kristin: 7740
Baker, Natalie R: 1200
Baker, Shelby: 3450
Baker, Suzanne: 9000
Baker, Tamara A: 2110, 4000, 7700
Baker, Zachary G: 3130, 9000
Bakk, Louanne: 2265, 7155
Balaam, Kori: 2330
Balachandran, Arun: 4550
Balaji, Anisha: 8035
Balasundaram, Rohit Baal: 9050
Balbim, Guilherme M: 7455, 7720
Baldinger, Samantha: 7695
Baldizar, Deborah: 7415
Baldyga, Kathryn: 7450, 8000
Bales, Diane: 3575
Ball, Karlene: 4490, 7455
Ball, Sarah: 1490
Ballew, Shoshana: 3570
Ballreich, Jeromie: 7470, 7905
Balmuth, Alexa: 7065, 7090
Balogun, Saliu: 4000
Balogun, Zainab: 1070
Balsa, Jonah: 7010
Balser, sarah: 1060
Baltasar-Fernandez, Ivan: 8020
Balubaid, Afnan: 4095
Bamdas, Jo Ann M: 3190
Bamonti, Patricia: 7720
Banarjee, Chitra: 7205, 7640, 8015, 8020
Banaszak-Holl, Jane: 1550
Bancks, Michael P: 3180
Banco, Julia: 8020
Banday, Muneeb Ul Lateef: 3315
Bandeen-Roche, Karen J: 1130, 1205, 2230, 7620
Bandholm, Thomas Q: 4075
Bandi, Sriya: 7215
Band-Winterstein, Tova: 3015, 7265, 8090
Bandyopadhyay, Attrayee: 1485
Bangen, Katherine J: 7005, 8010, 8015
Bangerter, Lauren R: 1270, 7640, 8020
Baniassadi, Amir: 2040
Banisalman, Islam: 1465
Bankole, Ayomide Okanlawon: 3300, 7440, 7925
Banks, Jack: 2075
Banks, Russell: 7425, 7635, 7930
Banner-Lukaris, Davina: 2360
Banno, Ryoichi: 7315
Bannon, Sarah M: 3250
Banskota, Nirad: 7210, 7305
Bao, Dapeng: 4535
Bar, Rachel J: 7040
Barada, Fatmeh: 1155
Baral, Stefan: 8015
Baralt, José: 3265
Baranes, Guy: 1245, 2240
Barbakoff, Daniel: 1005
Barbee, Harry: 1095, 1170, 9030
Barber, Claire: 8010
Barber, Emma: 1130
Barbera, Gina: 2130, 8015
Barclay, Ruth: 7300
Barclay, Terry: 7455
Bard, Skylar: 7360
Bardales, Rhonda L: 3275
Bardo, Anthony R: 1480
Barha, Cindy: 3335, 7720
Barinas-Mitchell, Emma: 7625
Barker, Sandra: 8020
Barneclo, Marcella A: 8015
Barnes, Deborah E: 3010, 3175, 4230, 7005
Barnes, Lisa: 2110, 2215
Barnes, Mary E: 7225
Barnes, Stacy: 7480
Barnett, Melissa A: 8020
Barnett, Michael: 7750
Barnhart, Emma M: 7505
Baron, Aliza R: 7250
Barooah, Adrita: 3065
Barr, Ashley: 7225
Barragan, Cassandra L: 1530
Barragan, Noel: 7015, 7200
Barral Rodriguez, Sandra M: 1025
Barrett, Anne: 1010, 7160
Barrett, Tyler D: 3010
Barron-Vaya, Yolanda: 4360
Barrus, Hannah: 2505
Barry, Lisa C: 3420, 7740
Bartels, Stephen J: 2315, 3250
Bartke, Andrzej: 8000
Bartlett, Paige: 7415, 7900
Bartlett, Robin M: 3450
Bartlett, Sean: 3485
Bartley, Jenna M: 1275
Barton, Kelli N: 3565
Barton-Ort, Kelly: 7315
Basaran Sahin, Duygu: 7050
Basaria, Shezhad: 7285
Bashian, Hannah: 2385, 8020
Basic, Ajla: 7505, 7935
Basrai, Zavera: 2500
Bass, David M: 1030, 1265
Bass, Judy: 7615
Bassola, Barbara: 7245
Basu, Disa: 8000
Basu, Kaustuv: 8000
Basu, Rashmita: 7200, 7425, 7905
Batamack, Yvann: 8000
Batchelor, Melissa: 8015, 8035
Bate, Angela: 4255
Bateman, Randall: 8015
Bates, David: 7425, 7930
Batista-Malat, Eleanor: 2150, 2375
Batsis, John A: 2505, 3580, 3585, 7275, 7535
Batura, Josey M: 2065, 7065, 8020

Baucco, Christina M: 8020
Bauer, Scott R: 4350
Bauman, Alexandra: 7350
Baumann, Sara E: 7300
Baumgart, Matthew: 3000, 4235, 7005, 7200
Bayer, Thomas: 7200
Bayliss, Elizabeth: 2085
Bayly, Melanie: 7135
Bayne, Alycia: 1425
Beach, Brian: 2365
Beach, Douglas: 1265
Beagley, Lindsey: 4320
Beam, Christopher R: 1215, 3220, 7725
Beamer, Brock: 7440
Bean, Jonathan F: 3335, 4215
Beans, Jeffrey: 7315
Beare, Richard: 4100
Beatriz Simões Pereira, Ana: 7655
Beattie, Zachary: 3285, 7020, 7735, 8020
Beatty, Jennifer F: 8020, 8080
Beauchamp, Marla: 2170, 7655, 7720
Beaudreau, Sherry A: 7235
Beauregard, Lisa: 7295
Beavers, Daniel: 2505
Beavers, Kristen: 1410, 2505
Becares, Laia: 1110
Bechek, Bonnie: 3175
Beck, Emorie D: 4515, 7455
Becker, Clemens: 2170
Becker, David J: 3255
Becker, Heather: 7145, 7435
Becker, Jacqueline H: 7630
Becker, Marigrace: 3510
Becker, Todd D: 1165, 4080, 7020, 8010
Beckner, Tara: 1390
Bedard, Michel: 3185
Beeber, Anna: 7535, 8035
Beech, Bettina M: 3475, 7700
Beehler, Melanie: 7650
Beerman, Isabel: 3325
Behrend, Abigail: 4295
Behrens, LIza L: 1080, 4290, 4320
Behrman, Jere R: 1280, 7455, 7925
Bei, Eva: 7515, 7915
Beiglary, Shevvaa: 7200, 9015
Beiser, Alexa: 7500, 7930
Bejjani, Rachele: 7640
Bekelman, David: 2180
Bektas, Arsun: 7210
Belalcazar, Kimberly: 7045
Belanger, Chris: 1010
Belanger, Emmanuelle: 3535, 7295
Belazis, Laura: 1190
Beld, Matthew: 8025
Belin de Chantemele, Eric: 7285
Bell, Bethani: 2555
Bell, Janice: 2200, 7145, 8010
Bell, Mallory: 7535
Bell, Tyler: 8020
Bellelli, Giuseppe: 4315, 7010
Belleville, Sylvie: 4490
Belmonte, Juan Carlos Izpisua: 2310
Belsky, Dan: 4550
Beltran, Susanny J: 7055
Belvederi Murri, Martino: 3035
Belza, Basia: 2115, 2450, 3140, 4235, 7415, 7465, 7610, 7900, 8015
Bendapudi, Arati: 8015
Bender, Alexis: 8020
Bender, Alexis A: 8020
Bender, Andrew R: 8055
Bender, Anna E: 1490
Bene, Michael R: 7305, 8000
Benfer, Jessica: 1070
Benge, Jared: 7040
Benjamin, Cynthia: 7005
Benjasirisan, Chitchanok: 7440, 7500, 8000
Bennett, Antonia: 4080
Bennett, David: 1235, 4005, 8000
Bennett, Henrietta Armah: 7040
Bennett, Katherine A: 4470
Bennett, Stephanie: 2295
Bensenor, Isabela G: 1320
Bensimon, Moshe: 2001
Benson, Clark: 3425, 7200
Benson, Jacquelyn J: 7490, 8000, 8010
Benton, Donna: 2375, 7665, 8010
Benton, Leilani: 8020
Benyassine, Karim: 9000
Benzel, Heather: 1395
Berg, Kristen: 7690, 8000
Berg, Russell: 1400
Bergeman, Cindy S: 7255
Bergh, Marissa: 8010, 8035, 8070
Berghoff, Ashley: 4095
Bergstrom, Jaclyn: 1435, 2235, 4170, 7010, 7425, 8000
Berg-Weger, Marla: 4080, 8020
Berian, Julia R: 3335, 4530
Beridze, Giorgi: 3330, 7060
Berish, Diane: 3230, 3380, 7040, 7410, 7455, 8015, 9015
Berkman, Cathy: 2225
Berkowitz, Jennifer: 8010
Berkowsky, Ronald W: 7640
Berlinger, Nancy: 2245, 8065
Berman, Brian: 3565
Berman, Jackie: 7695
Bernacki, Gwen M: 7205
Bernal, Darren: 8020
Bernal-Alonso, Amaya: 4045
Bernard, Dominique D: 7225
Bernard, Marie A: 3400
Berner, Jon: 8000
Berner, Karl F: 8000
Bern-Klug, Mercedes: 8010
Bernstein, Isaac A: 2080
Bernstein Sideman, Alissa: 3065, 7325
Berridge, Clara: 1345, 2125, 2355, 2565, 3065, 3315
Berry, Donna L: 8015
Berry, Sarah: 1275
Berry-Stoelzle, Maresi: 2385, 7665
Berta, Whitney: 3170
Bertram, Lars: 7030, 7210
Bertrand, William: 8020
Besnier, Florent: 4490
Bessen, Sarah: 2035
Besser, Lilah M: 4430, 8020, 8025
Best, John: 7720
Best, Latrica E: 4000
Best, Ryan: 8000
Best, Tristin: 4300
Bethell, Jennifer: 8015
Bethune, Tyrone: 3430
Betley, Wendy: 7480
Bettencourt, Amie: 7710
Betz, Elizabeth: 7275
Betz, Joshua R: 1440
Beydoun, May A: 1250, 2330, 8000, 9020
Beydoun, Safa: 4005
Bhagianadh, Divya: 3115
Bhalla, Nalini S: 8015
Bhandari, Mira: 4005
Bhansali, Rebekah A: 8000
Bhasuran, Balu: 1355
Bhat, Aarti C: 7490
Bhat, Ajay: 4005
Bhatta, Tirth: 1140, 7660
Bhattacharjee, Anindita: 4170
Bhattacharyya, Kallol Kumar: 7355
Bhattarai, Pratik: 3055
Bhattarai, Preeti: 3055
Bhatti, Sumaya: 4465
Bherer, Louis: 4490
Bhowmick, Pallabi: 3530, 8020, 9025
Bhurtel, Shikha: 2200, 8010
Bibbo, Jessica: 7405, 7470, 8020
Bibriescas, Natashia: 7425, 7615, 7930
Bidwell, Julie: 8010
Bieber, Heather: 7320
Bielak, Allison A M: 4185, 7225
Bielsten, Therése: 4010, 4335, 8015
Bierman, Arlene S: 1205, 4160, 8030
Biernot, Kelsey M: 8020
Biesmans, Guido Anna Maria Carolus: 7295
Big Eagle, Tosha R: 7530
Biggerstaff, Matthew: 1065
Bilimoria, Karl: 1130
Binder, Ellen F: 1275
Bing-Canar, Hanaan: 8020
Binns, Malcolm: 7675
Birati, Yosefa: 8020
Birchander, Ellen: 3495
Birditt, Kira: 4085, 4525, 7660, 8020
Birkeland, Robyn W: 3130, 8020
Biruk, Cal: 7750
Bise, Christopher: 7330
Bishop, Alex: 1385, 7115, 7150
Bishop, Christine E: 1230, 1315
Bishop, Lauren: 4475
Bishop, Nicholas J: 3035, 3540, 8010
Bishop, Sonia K: 1480, 7200
Bishop, Teddy: 1080, 7250
Bitto, Alessandro: 7210
Bivas-Benita, Maytal: 8015
Björk, Jonas: 1285
Bjarnadottir, Ragnhildur I: 8015
Bjelka, Ava T: 1010
Black, Kathy: 7415
Black, Sheila: 8000, 8020
Blacker, Deborah: 1545
Blackshear, Chad: 1000

Blackwell, GinaMari: 8010
Blackwood, Jennifer: 7420, 7655, 7920
Blain, Hubert: 2170
Blake, Jason A: 7285
Blake, Jason: 8020
Blancato, Bob: 2390
Blancato, Robert: 8015
Blanchette, Caroll-Ann: 4490
Blank, Sabrina: 1540
Blanken, Anna E: 1325
Blaskewicz Boron, Julie: 7485, 7675, 8000, 8020
Blaum, Caroline S: 2075, 7465
Bleijlevens, Michel HC: 4255, 7295, 7685, 8015
Blind, Melissa: 2335, 2395
Blinka, Marcela D: 4300
Block, Laura: 2550, 3425, 7200, 8010
Block, Robert: 7420, 7920
Blodgett, Joanna M: 1245
Blonquist, Traci: 8000
Bloom, David: 7275
Bloom, Rachel F: 1165, 2305
Bloomberg, Mikaela: 3095
Blount, Amber: 7735, 7750, 8000, 8015
Blow, Frederic: 7490
Blozis Villareal, Shelley A: 7700
Bluck, Susan: 7020
Blue, Benjamin: 7010
Bluestein, Daniel: 4510
Blumen, Helena M: 7750
Boa Sorte Silva, Nárlon Cássio: 7455, 7720
Boafo, Jonelle: 1395
Boakye, Alfred: 8010, 8015
Boardman, Charles: 1125
Boateng, Isaac Asirifi: 2490, 7250, 7480
Boateng, Josephine: 7135, 7695, 7700
Bobitt, Julie: 3115, 7545
Bock, Jarrod: 7200
Bockting, Walter: 3075
Boddie-Forbes, Ra'Sheda: 4040
Bodner, Ehud: 1565, 2030
Boerner, Kathrin: 7040, 7065, 7660
Bogdan, Ryan: 8010
Bogdewiecz, Taryn: 8010
Boger, Jennifer: 2410, 2530
Bohlke, Kayla: 8000
Bohnen, Nicholaas: 2510, 8000
Bolkan, Cory: 1190, 2180, 2325, 7350, 7530
Bollin, Salli: 8020
Bolling, S. Alison: 8000
Bollinger, Rebecca: 7655, 8015
Boltz, Marie: 1080, 2140, 3130, 3230, 7040, 7410, 7420, 8015
Bolz, Fabio: 7230
Bona, Matthew: 9040
Bonds Johnson, Kalisha: 4165
Bong, Ji Yae: 9010
Bongiorni Meadows, Jill: 8000
Bonin Pinto, Camila: 8015
Boockvar, Kenneth: 8020, 8055
Booker, Emma: 2370
Booker, Staja: 8010
Boon, Jeffrey T: 1265, 7075
Boone, Anna: 7275
Boot, Walter: 1040, 2355, 3060, 3415, 4130, 4420, 7360, 8000, 8020
Booth, Richard: 4270
Borate, Samruddhi Nandkumar: 7200
Bordes, Piper J: 2300, 2330, 7045
Borgards, Kaila: 4525
Borgogna, Joanna-Lynn C: 7210
Borisoff, Jaimie: 7155
Boron, Julie B: 4205
Borrelli, Belinda: 8015
Borrero, Lisa: 8010
Borrero De Jesus, Jane: 7410, 7900
Borson, Soo: 1430, 7325
Bortey, Dennis: 4175
Borth, Laura: 8015
Boscardin, John: 7005
Bose Brill, Seuli: 2415
Boström, Anne-Marie: 1050, 7215
Bosworth, Brooke: 8010
Botana Gronek, Melissa P: 8015
Botoseneanu, Anda: 3035, 7455, 8010
Botwe, Paul: 8000
Bouaouina, Zineb: 7645
Bouchard, Lauren: 3195
Boucher, Nathan A: 2090, 2485, 2500
Boucher, Sarah: 7135
Boudreaux, Madison T: 3275
Boudreaux, Michel: 8010
Boulay, Marc: 4085
Bouldin, Erin: 2545, 3100, 3320, 3430, 4390, 4520, 7335, 7630, 7730, 7740
Boulougoura, Afroditi: 8000
Bouranis, Nicole: 4130, 4340
Bourassa, Kyle J: 8060
Boustani, Malaz: 4230
Boutilier, Justin J: 7510
Bouton, Lauren J: 1110
Bove, Allyn M: 3400, 7330
Bowblis, John R: 3255, 4305, 7295
Bowditch, Corinne: 7465
Bowen, Holly: 8020
Bowen, Lauren M: 3310
Bower, Emily S: 1020, 4120, 4180, 7630
Bowers, Barbara J: 4465
Bowes, Aaron: 8000
Bowland, Sharon E: 8010
Bowles, Kathryn: 3105
Bowles, Kathryn H: 4550
Bowman, Caitlin R: 7000
Bowman, Grace I: 4430, 7420
Boyd, Cynthia: 2085, 4055
Boye, Alyssa: 7455
Boyle, Julie: 2370
Boynton, Harrison: 7750
Brach, Jennifer S: 4215
Bracy, Danny: 7440, 7925
Bradley, Dana B: 3120, 7430
Bradley, Lisa D: 8010
Bradt, Joke: 9010
Brady, Brooke: 7610, 8020
Brady, Loretta: 2130
Brady, Samantha: 7050, 7065
Brage, Shelby: 8010
Bragg, Alexa: 7700
Bramajo, Octavio N: 1170, 4455, 7230, 7300
Brandas, Benedetta: 1235
Brandis, Leah M: 8010, 8020
Brandt, Anita: 4550, 7205
Brandt, Martina: 2025
Brankaert, Rens: 3425
Brannock, M. Daniel: 1370, 2150, 3270
Brashear, Jessica S: 7080
Brasher, Melanie S: 2130
Brasky, Theodore M: 2445
Brassolotto, Julia: 4445
Bratches, Reed WR: 4045
Bratsberg, Bernt: 2425
Bratzke, Lisa: 8065
Brauer, Simon G: 3040
Brault, Matthew: 8015
Braun, Kathryn L: 4015, 7055, 8000, 8020
Braunstein, Joel B: 7425, 7930, 8070
Bravo, Adrian: 7065
Brazg, Tracy: 4470
Breakwell, Susan: 7480
Breckling, Meghan: 7325
Breder, Kelseanne P: 3075
Brekke, Bailee: 1315, 3275, 4510
Brenes, Gretchen A: 7490
Brenick, Alaina: 2175
Brenman, Noah: 8010
Brennan, Daniel KM: 2010, 3150, 4420
Brennan-Ing, Mark: 3490, 4225
Brenowitz, Willa D: 8015
Brett, Jeanne: 7490
Breunig, Ian: 1015, 7420
Brewer, Judson: 2315, 3250
Brewer, Julie: 2315
Brewer, Robin N: 3315, 7030
Brewster, Glenna S: 4165, 7270
Breyfogle, Rachel: 3215
Briceno, Alexandra: 7540, 7735
Brickman, Adam: 7350
Brickner, Carlin: 4550
Briggs, Emily: 7690
Brightman, James D: 3055, 7025, 7735
Brinkley, Tina E: 7710
Brinkley-Rubinstein, Lauren: 3420
Brinton, Daniel: 1075
Bristol, Alycia: 2060
Britt, Katherine C: 2055, 2190, 8020
Britton, Karysa: 4515
Brock, Kaye: 3085
Brocklebank, Laura Ann: 3050, 4500
Brodrick, Marisa-Francesca: 3110
Brody, Abraham A: 2415, 3010, 4360, 7645
Brody, Abraham: 8045
Broen, Tiana M: 7695, 7720, 8020
Broese Van Groenou, Marjolein I: 1120
Brook, Juliette: 8010
Brooks, Holly: 7025
Brooks, Jada L: 8015, 8020
Brooks-Smith-Lowe, Sharifa: 7325

Brothers, Allyson: 3515, 3575, 4320
Brotman, Rebecca: 7060, 7210
Broughton, Susan: 3060
Broussard, Kimetha L: 2550
Brown, Cait: 7080
Brown, Candace: 7425, 7930
Brown, Casey L: 3355
Brown, Casey: 8020
Brown, Cynthia J: 4075
Brown, Diane K: 7475, 7900
Brown, Ellen L: 4060
Brown, J. Scott: 1505, 1535
Brown, Karl J: 3435, 7205
Brown, Kevin: 2505
Brown, Lana M: 7480
Brown, Lauren: 8020
Brown, Leacey E: 7725
Brown, Lynn: 7275
Brown, Maria T: 7100
Brown, McKenzie: 7450
Brown, Monique J: 2215, 4105
Brown, Nancy L: 7250
Brown, Rebecca T: 7290
Brown, Rebecca: 9025
Brown, Ryan L: 1570
Brown, Sarah E: 7060
Brown, Susan H: 1175, 3085, 7205, 7610
Brown, Susan L: 3065
Brown, Sylvia: 3150
Brown, Teneil: 4460
Brown, Todd T: 7285, 8010
Brown-Huges, Travonia: 8000
Browning, Wesley R: 1510, 2215, 7040
Broyhill, Tatum: 3115
Brubaker, Malaak: 8020
Bruce, Marino A: 3475, 7700
Bruckenthal, Patricia A: 3265
Brunet, Anne: 2310
Brunker, Cherie: 8020
Brunkert, Thekla: 4255
Brunt, Christopher: 3255
Bryan, Angela D: 3115
Bryan, Robert N: 3180
Bryant, Ashley L: 8035
Bryant, Brittni: 3150
Bryant, Elizabeth A: 1165
Bryant, Gillian: 7250
Bu, Feifei: 4440
Buchanan, Jeff: 7510, 7605
Buchanich, Jeanine M: 3375
Buchheit, Katie: 7400
Buchman, Aron: 1235
Buck, Harleah: 2370, 8010
Buckley, Kiera: 8020
Buckley, Matthew: 2310
Buckley, Nina: 8010
Buckwalter, Joseph A: 7205
Bucy, Taylor I: 4445
Buddendorff, Lauren: 7210
Budhathoki, Chakra: 8000
Budnick, Andrea: 7110, 8010
Budreau, Josyaah: 2395
Buehrer, Tabea: 1220
Buell, Susan: 1015
Buenning, Mareike: 2025
Bueno, Michael: 2340
Buffel, Tine: 1180, 3200
Bulanda, Jennifer Roebuck: 1505
Buljac-Samardzić, Martina: 7445
Bullard, Misty: 8020
Bullock, Karen: 2415, 3275, 4360
Bumbalova, Katerina: 1165
Burack, Orah R: 8010
Burant, Christopher J: 7265, 7660
Burch, Brittany: 4205, 7720
Burden, Sorrel: 2530
Burdick, David C: 2275, 3515, 7425, 7905
Burgdorf, Julia G: 3105, 4360, 4460
Burgess, Elisabeth O: 8020
Burgos Zuniga, Claudia C: 7250, 7480
Burgos-Melendez, Yesenia: 4475
Burkart, Britney J: 7240
Burke, Christopher: 7625
Burke, James: 2090
Burke, Robert E: 9025
Burke, Shanna L: 7540, 7735
Burkhardt, Summer: 7300
Burk-Leaver, Erin Y: 3080, 7285
Burman, Bonnie: 8020
Burnaevskiy, Nikolay: 7010
Burnes, David: 2150, 4365, 7405
Burnett, Jason: 2520, 4355
Burnette, Denise: 1140
Burningham, Zachary: 7135
Burns, Edith: 4100, 8010, 8015
Burns, Jeffrey: 8000
Burns, Madison: 2180
Burns, Reilly: 7000
Burns, Shane D: 4000, 4455
Buron, Bill: 2550
Burr, Jeffrey A: 1360, 1405, 2205, 3545, 7270, 7350, 8015
Burrage, Rachel: 7055
Burrows, Brett: 8015
Burton, Jordan B: 3045
Burton, Melanie: 8015
Burton, Shandra D: 3500
Bush, Noah: 7720
Buta, Brian: 1205
Butler, Rachel A: 7110
Buto, Peter T: 1545
Butt, Maayra I: 8015
Butters, Meryl: 7640
Buxbaum, Channa M: 7040
Buxton, Michael R: 1210, 7070, 7455, 7610
Buxton, Orfeu M: 1250, 2290, 7210, 8000
Buys, David R: 3575, 4040
Buysse, Daniel J: 2290
Byambaa, Altan-Ochir: 7205
Bybee, Sara G: 2225, 2480, 3400, 3500, 7425, 7905
Byerley, Joella: 8015
Byerly, Laura: 8010
Byers, Amy: 3010
Bylund, Carma: 7020
Bynum, Julie: 3000, 8015
Byrd, DeAnnah R: 2190
Byrd, Goldie S: 1315
Byrd-Craven, Jennifer: 8020
Byun, Eeeseung: 7735, 8000
Byun, Jun Y: 7285

### C

Caban-Holt, Allison: 1315
Cabral Junior, João: 7350
Cabrera Lozoya, Daniel: 1485, 7145
Cabrero Castro, Jose Eduardo: 4550, 7230
Cacchione, Pamela Z: 3555
Caceres, Maria: 8020
Cachioni, Meire: 8015
Caefer, Jacqueline: 2450
Caggiu, Giulia: 7275
Cagle, John: 1070, 4195, 7055, 7110
Cai, Catherine: 8000
Cai, Gege: 1230
Cai, Huaibin: 8000
Cai, Shubing: 8020, 8055
Cai, Wenjie: 4380, 7275
Cai, Xinxin: 8020
Cai, Xiuting: 3270
Cai, Yurun: 7720
Cain, Stephen M: 7205
Cala-Garcia, Juan: 4170
Calamia, Matthew: 7045
Calasanti, Toni: 8080
Calderón-Larrañaga, Amaia: 3035, 3330, 4045, 7010, 7060, 7215
Calderon, Russell J: 1535, 2465, 3335
Caldwell, Clover: 8020
Caldwell, Jessica ZK: 8055
Calfee, Kristin: 7130, 7735
Calhoun, Katie: 1295, 2070
Calkins, Margaret: 4325, 8015
Callaway-Lane, Carol: 2280
Calligaro, Hugo: 7015
Callow, Daniel: 3070
Calo, William: 1080
Calver, Jen: 3010, 8010
Camacho, David: 3280
Camacho, Karen: 7660, 7735, 8015
Cambara, Celine: 7540, 7735
Camell, Christina: 3160
Cameron, Erinn C: 2040
Cameron, Kathleen A: 1005
Cammer, Allison: 4195, 4440
Camp, Alyssa: 1010
Camp, Cameron: 1070
Camp, Lauren A: 8015
Campbell, Kenna: 3255, 7545
Campbell, Leah: 2265
Campbell, Matthew D: 7075
Campbell, Sarah: 2340
Campetti, Randi: 1145, 4355
Campione, Joanne R: 4450
Campos, Annalis: 2505
Campos, Baltazar: 8010
Campos, Jennifer L: 1070, 2410, 4300, 7225, 7635
Campos, Matthew: 1195, 8010
Can, Reyyan: 4020
Candrian, Carey: 3490
Canham, Sarah L: 1295, 2395, 2560, 4445, 7445
Cannell, Brad: 4355
Canning, Shelley E: 7465
Cannon, Melissa: 3560
Cannon, Rachel M: 1030
Cantave Burton, Cassandra: 3015
Cantrell, Sarah: 4530
Cantu, Phillip: 3030, 8020
Cao, Ji: 7610
Cao, Jiawei: 1215, 2030
Cao, Katy (Qiuchang): 1280
Cao, Meng: 7440
Cao, Rui: 7225
Cao, Xuemei: 3025

Cao, Yumeng: 2160, 7735, 8010
Cao, Yunqi: 3270
Cappelletti, Monica: 7660
Cappi, Carolina: 7350
Cappon, Davide: 7355
Caprio, Thomas: 8010, 8035
Caraballo, Christina: 3090
Carbajal-Madrid, Pedro: 7695
Carballea, Denise: 8010
Carballo-Casla, Adrián: 3330
Carbuccia Abbott, Maryan: 8010
Carder, Paula: 2185, 3535, 4510, 8010, 8020, 9045
Cardoso-Arias, Rafael: 2125
Carey, James: 2245
Carlile, Robert M: 8070
Carling, Stacy: 7440, 7490
Carlozzi, Noelle: 7065
Carlson, Chalise: 3110, 7135, 7235
Carlson, Michelle C: 1240, 2045, 3375
Carlston, Kelsey: 4035
Carmiol-Rodriguez, Priscilla: 2115, 7415, 7735, 7900
Carnahan, Jennifer L: 7465
Carnavali, Fernando: 7630
Carneiro Alves de Oliveira, Bruno: 7350
Carney, Maria: 8015
Carnie, Martha B: 3340
Carolan, Kelsi: 1105
Carpenter, Brian: 1325, 4000, 7055, 8000, 8010
Carpenter, Joan: 1080, 1160, 8010
Carpenter, Joan G: 8015
Carpenter, Matthew: 7655
Carpenter, Rebekah: 4140
Carpentieri, JD: 2365
Carpio, Rubi: 7000
Carr, Dawn C: 1280, 3380, 3540, 3555
Carr, Deborah S: 3410, 4030, 4330, 4505
Carr, J Jeffrey: 2220
Carr, Lucas: 7735
Carranza, Erick: 1210, 7070
Carrera, Robert: 7750
Carrero, Juan-Jesús: 3330, 7060
Carrico, Catherine: 7440, 7490
Carrillo, Audrey: 8000
Carrington-Lawrence, Stacy: 1340
Carrion, Carmen I: 1495
Carrion, Iraida V: 2445
Carroll, Isabella: 1345
Carroll, Judith: 2005
Carson, Erin: 2500
Carson, Paul: 7090
Carstarphsen, Kathy J: 3275
Carstensen, Laura: 1575, 3355, 4420, 7255, 8000, 8020
Cartledge, Anne: 2395
Carwile, Hope: 3240
Casanova, Ramon: 7015
Casati, Fabio: 1140
Case, Donna J: 7155
Case Fankhouser, Rebekah: 7035
Casey, Joan A: 4035, 4375
Caskie, Grace I L: 7055, 7475, 7605
Cassidy, Jessica: 4105, 7155, 7340
Cassidy, Terri: 7160
Cassidy-Eagle, Erin L: 1135
Castañeda-Avila, Maira A: 7060
Castaneda, Moraima: 8025
Castro, Hugo: 4235
Catlow, Jodi: 7495
Catt, Wade: 8000
Cauble, Christina: 2535
Cauble, Heaven: 8000, 8020
Cauley, Jane A: 1365, 7060
Cavalli, Stefano: 1220
Cavanagh, Lucia: 2020
Cavanaugh, Robert: 4225, 8015
Cavanaugh, Sheamus: 2335
Cawthon, Peggy M: 1090, 1240, 1435, 2080, 2220, 4005, 4260, 4310, 7060, 7640, 8000, 8020
Ceide, Mirnova: 8000
Cejtin Rosen, Francesca W: 4370
Celestin, Michael David: 8020
Cellarius, Karen: 8010
Cepukenas, Emma: 7200
Cerino, Eric S: 1525, 4165, 4180, 4405, 4410, 7225, 7255, 7455, 8000, 8020
Cerino, Lauren: 7050, 7610
Cernohous, Inessah: 3450
Cerruti, Minyoung: 7155
Cervantes, Maria: 7320
Cetnarskyj, Roseanne: 8010
Cha, Hyungmin: 1015, 3245, 3370, 7040, 7500
Cha, Jun Hyung: 8015
Cha, Kyeongin: 8010, 8015
Cha, Seung Eun: 7720, 8000
Chaaya, Monique: 1155
Chachlani, Preeti: 7030
Chadwick, Darren: 1015
Chae, Hwa Young: 1140
Chae, Joosuk: 8010
Chae, Kacey: 7710
Chahine, Lana: 2510, 7640
Chai, Hye Won: 7735
Chakraborty, Ruchira: 7730
Chakravarty, Esha: 7020
Chakurian, Daphne: 8010
Chalise, Hom Nath: 4065
Chamberlin, Elizabeth S: 3110, 7135, 7490
Chamberlin, Emma: 2175
Chamberlin, Keran W: 2525, 7060, 7090, 7630
Chambers, Kelly: 4005
Chameli, Thomas: 7640
Chamut, Steffany: 4225
Chan, Alex Siu Wing: 7530
Chan, Angelique: 3290, 3545, 7145, 8015
Chan, Athena Chung Yin: 7005, 7265
Chan, Chin Pang Ian: 7510
Chan, Ding-Cheng (Derrick): 7275
CHAN, Francis KIN TUNG: 4195, 8015
Chan, Ka Po: 4195, 8015
Chan, Lili: 7630
Chan, On Fung: 3200, 8010
Chan, Sonia Kin Lai: 4050
Chan, Wai Chi: 7725
Chandler, Chelsea: 2355
Chandler, Julie: 8010
Chandler, Kelly D: 4280
Chandola, Tarani: 7640
Chandra, Tamir: 3325
Chandran, Aruna: 8020
Chang, Arthur: 7000
Chang, E-Shien: 1060, 1270, 2045, 4355, 7065, 7405, 8020, 8055
Chang, Grace: 7300
Chang, Han-Yun: 8015
Chang, Hui: 7355
Chang, Leah: 7010
Chang, Ling-Hui: 8010
Chang, Pei-Fen J: 7360
Chang, Po-Yen: 7400
Chang, Wen-Pin: 7205
Chang, Ya-Han: 7240
Chang, Yuanqing: 8000
Chang, Yu-Ping: 7245, 7360, 7510, 7695, 7910
Chang (Gudjonsson), Milan: 2040, 2240, 7305
Chanquin, Edward: 8020
Chanti-Ketterl, Marianne: 3280, 8015, 8020
Chao, Ka Kei: 7540
Chao, Linda L: 3010
Chao, Shiau-Fang: 7510
Chao, Shirley: 8015
Chapman, Brian G: 2175
Chapman, Chelsea: 2265
Chappell, Richard J: 4300
Chapple, Hilary: 2395
Charitou, Lauren Pizzulli: 7490
Charles, Cara I: 7000
Charles, Minard G: 2235
Charles, Susan T: 1350, 4180, 7030, 7255
Charlesworth, Georgina: 8000
Charlton, Judith L: 3455
Charness, Neil: 1040, 3205, 3480, 4130, 7360, 8000, 8020, 8020
Chasteen, Alison L: 1010, 7605
Chataut, Akankshya: 7485
Chaudhari, Diptaraj: 8000
Chaudhuri, Asish: 4170
Chaudhury, Habib: 1555, 3205, 4345, 7155, 7510, 7915
Chauhan, Shekhar: 3540
Chaves, Paulo HM: 3330, 7080, 7125
Chavez, Lorely A: 9050
Chavez Granados, Heily: 9020
Chavira, Laura: 7325
Chavkin, Nick: 3325
Chawrun, Isabella: 7630, 7905
Chaytor, Naomi S: 4515
Che, Sok Leng: 7540
Che, Yan: 7205
Chee, Wonshik: 3370, 7270
Chek, Carmen: 7455, 7540
Chekuri, Srujana: 7485
Chellappa, Rama: 8000, 8015
Chen, Amber X.: 7610
Chen, Annie T.: 2530, 8015
Chen, Bangwei: 7080
Chen, Brian H: 4170
Chen, Casandra WY: 8095
Chen, Chen: 8015
Chen, Chixiang: 1535, 3035, 3330, 4385
Chen, Chun: 7010
Chen, Chunfang: 8020
Chen, Diefei: 7225
Chen, Duan-Rung: 7035, 7460, 7925

Chen, Enna: 8000, 8020, 8020
Chen, Feinian: 3360
Chen, Feng: 1560
Chen, Feng: 8015
Chen, Frank Youhua: 8000
Chen, Gong: 3050, 7680, 8010, 8085
Chen, Haiying: 1390
Chen, Hongbo: 7305
Chen, Honglei: 2525, 4440, 7060, 7090, 7630, 8015
Chen, Hongxiu: 1355
Chen, I-Chen: 8015
Chen, I-Ling: 7080
Chen, Jen-Hao: 1540
Chen, Jiachen: 2160, 7735, 8010
Chen, Jiayu: 7515
Chen, Jingyi: 7710
Chen, Jinyu: 1000
Chen, Jufu: 1235
Chen, Ke: 7235, 7630
Chen, Ko-Xin: 8015
Chen, Kuan-Hua: 3285, 8040
Chen, Lei: 7510
Chen, Li: 7695
Chen, Liangchi: 7690
Chen, Lijing: 2465
Chen, Lili: 7425, 7930
Chen, Limei: 2370, 2470, 7095, 8010
Chen, Lin: 1195, 7300
Chen, Lingming: 3245, 7260, 7695, 7935
Chen, Liu: 8000
Chen, Li-Ying: 7070
Chen, Lu: 4030
Chen, Natalie M: 7040
Chen, Qiaoxi: 3135
Chen, Qingru: 8000
Chen, Qize: 7050
Chen, Ruijia: 1545, 2045, 8045
Chen, Shiyu: 8010
Chen, Shiyun: 7005
Chen, Shuangzhou: 1560
Chen, Siting: 3540, 7300
Chen, Siyu: 4310
Chen, Stella: 8055
Chen, Tuo Yu: 7150, 7270, 7735
Chen, Will: 1455, 4045
Chen, Xi: 2020, 2120, 3165, 3370, 7055, 7465, 7490, 7630, 7925, 8055
Chen, Xi: 7040
Chen, Xi: 7635
Chen, Xiaodong: 1365, 2500
Chen, Xiaotong: 7235
Chen, Xiayu Summer: 1115, 2265, 2355, 3030, 4270, 7135, 7155, 7505, 7540, 7660
Chen, Ya-Mei: 7510, 7525, 7915, 8010
Chen, Yanjun: 4300
Chen, Yan-Yan: 8000
Chen, Ya-Yun: 8020
Chen, Yi-An: 8010
Chen, Yi-Fan: 7080
Chen, Yi-Hua: 7735
Chen, Ying: 4550
Chen, Yishu: 4535
Chen, Yu-Chih: 4190, 7120
Chen, Yu-Chun: 7280
Chen, Yuling: 7215
Chen, Yunxiang: 8020
Chen, Zhengyi: 2015
Chen, Zhirui: 7045
Chen, Zijing: 2255, 2500, 7330, 7470
Chen, Ziyan: 8010
Chen, Ziyuan: 7255
Chen, Zizhuo: 7605
Cheney, Amanda: 3430, 7630
Cheng, Catherine J: 4310
Cheng, Clio Yuen Man: 1115, 7080, 7150, 7640, 7695, 7750, 8040
Cheng, Evien: 7010
Cheng, Greta Jianjia: 3375
Cheng, Hu: 8000
Cheng, Huai Y: 7430, 7610
Cheng, Jie: 7260
Cheng, Joyce: 7490, 7935
Cheng, Kent Jason G: 7470, 7925
Cheng, Mengling: 4455, 7535
Cheng, Mengyuan: 8010, 8015
Cheng, Qiyuan: 8010
Cheng, Rong: 1025
Cheng, Sheung-Tak: 8020
Cheng, Yu-Qi: 7110, 7400, 7490
Cheng, Yu-Shan: 7440
Cheng, Yutian: 7750
Cheong, Allen M. Y.: 7725
Chern, Alexander: 7140
Cherry, Debra: 8055
Cherry, Katie E: 2300, 2330, 7045, 7225, 8020
Chertkow, Howard: 7675
Cheslock, Megan: 8015
Chesney, Margaret A: 1245, 3010
Chess, Eric: 7495
Chesser, Stephanie A: 4000
Cheung, Ethan Siu Leung: 1105, 7420, 7455
Cheung, Pui Yin: 7230
Chew, Justin: 8015
Chhim, Srean: 8015
Chi, Nai-Ching: 7040
Chi, Peggy: 3170
Chia, Jia Qian: 8015
Chidebe, Runcie CW: 2445, 4000, 7350, 7475, 7600, 8065
Chien, Shao-Yun: 7465
Chih-Liang, Wang: 8000
Childress, Kristen: 8010
Chimehi, Bahareh: 3285
Chin, Janecca: 4050
Chin, Jessie: 4420
Chin, Laurie: 1325
Chin, Meejung: 8020
Chin, Savuth: 8015
Chin, Shu Yee: 1095
Chingui, Luciano: 8010
Chintapanyakun, Teepatad: 7205
Chiow, Jobina: 7100
Chippendale, Tracy: 8085
Chiriboga, David A: 3440, 3525
Chisholm, Latarsha: 8010
Chisolm, Cindy: 7210
Chitwood, Skyla: 1425
Chiu, Ching-Ju: 7610, 8015
Chiu, Chi-Tsun: 3290
Chiu, Chung-Yi: 7425, 7910
Chiu, Herng-Chia: 7670
Chiu, Wan-Yu: 3255, 8010
Chiu, Yu-Wen: 8020
Cho, Aeyoung: 7645, 7750
Cho, Al: 3080
Cho, Emma: 1290, 7145
Cho, Eunae: 7270
Cho, Eunhee: 7295, 7640, 7655, 7715, 7750
Cho, Hannah: 7145, 7630, 7665
Cho, Hyeonji: 8020
Cho, Hyeonmi: 7360, 7640, 7750, 8010, 8015
Cho, Hyunjin: 8000, 8010
Cho, Hyunjin: 8015, 8015
Cho, Jinmyoung: 4130, 4340, 7275, 7680, 8020
Cho, Joonyoung: 3040, 7030, 7420
Cho, Jungwon: 7655, 7715
Cho, Kyoungmin: 8000
Cho, S.M.: 8010
Cho, Seungjong: 2260, 7150
Cho, Seyoung: 7005
Cho, Tsai-Chin: 2515, 3145
Cho, Young I: 8090
Cho, Youngmin: 7535, 8035
Cho, Youngtae: 4070
Chodos, Anna: 1430
Chodosh, Joshua: 3010, 7465, 9020
Choi, Ahreum: 7275
Choi, Bokyoung: 7275
Choi, Boyun: 8015
Choi, Eunhee: 7275
Choi, Eunju: 7005
Choi, Eunyoung: 1015, 2040, 2460, 3125, 3245, 3540
Choi, Gayoung: 8045
Choi, Ha Young: 1570
Choi, HwaJung: 1450, 1495, 3145
Choi, Hwan: 8015
Choi, Hyojin: 7610
Choi, Jae Woo: 3545
Choi, Jean: 3095
Choi, Jeongeun: 8010, 8015, 8015
Choi, Jeungok: 8020
Choi, JiYeon: 7205, 7440, 7645, 7680
Choi, Jung-Yeon: 8015
Choi, Mi Sun: 8000
Choi, Minhyuk: 1545
Choi, Mona: 7360, 7750
Choi, Moon: 7155
Choi, Namkee G: 2520, 7080, 7655
Choi, S. Emily: 7350
Choi, Sang D: 8010
Choi, Sarah: 7660
Choi, Seo-Eun: 2020
Choi, Seongmi: 7205, 7440, 7680
Choi, Seongsu: 7430
Choi, Seo-Yun: 8000
Choi, Shinae L: 2420, 7495
Choi, Won: 3410
Choi, Y.Joon: 7030
Choi, Yejun: 8015
Choi, Yeon Jin: 1015, 3245
Choi, Yong Kyung: 7645, 8015
Choi, Yoon Saeng: 7705
Choi, Yoonseok: 7725, 8000
Chong, Weiguo: 7670
Chopik, William: 1570, 3405, 4525, 7455
Chosed, Renee: 7000
Chou, Kee Lee: 8010, 2520
Chou, Lin-Na: 1075
Chou, Pi-Ling: 7625
Chou, Wan Yun: 8010, 8015
Chou, Yu-Ting: 8010

Choudhury, Renoa: 7205, 8020
Choun, Soyoung: 7030
Chow, Bryan: 7750
Chow, Christine: 8020
Chow, Denise: 7270
Chow, Julian Chun-Chung: 7030
Choy, Jacky C. P.: 8020
Christensen, Janelle J: 3045, 4530
Christensen, Kaare: 7010
Christensen, Keri: 1065, 3510
Christian, Thomas: 2180
Christiansen, Cory L: 7155
Christopher, Emma: 7000
Christopher, Jennifer: 7480
Christopher, Jennifer M: 8020
Christopher, Theresa R: 7480, 8020
Chrzanowski, Brandi: 7440
Chrzanowski, Lauren K: 3385, 7065, 7425, 9015
Chu, Charlene H: 4175, 4465
Chu, Chien-Fang: 7510
Chu, Jing: 2465
Chu, Jun: 8010
Chu, Li: 1575, 4420, 8020
Chu, Sang Hui: 7205, 7440
Chua, Jolin: 1095
Chubinski, Jennifer: 8045
Chui, Hiu-kwan: 3200, 8010, 8035
Chui, M. K. Kirsten: 2530, 8000
Chukwuorji, JohnBosco C: 4000, 7150
Chulak, Jamy: 2325, 7440
Chun, Gloria Ka Ming: 7460
Chung, Edwin Ka Hung: 4390, 7745, 8000
Chung, Jane: 4205, 4420
Chung, Misook: 7005
Chung, Ray-Yuan: 8015
Chung, Seohyeon S: 7275
Chung, Soondool: 1140, 3440, 8000
Chung, Thomas: 8010
Chunga, Richard E: 7040, 7065, 7660, 8020
Chung-Do, Jane: 8020
Chur, Marcus: 7640
Churchill, Linda C: 7260
Chusyd, Daniella E: 8000
Cicero, Caroline: 2440
Cicero, Ethan: 2015, 7100
Cichy, Kelly E: 4280
Cidav, Tom: 7200, 7260
Ciemins, Elizabeth L: 3215
Ciesla, Marissa: 7635
Cieslik, Katarzyna A: 1420, 8000
Cigliana, Jill: 8020
Cigolle, Christine: 7320
Cihlar, Volker: 1100
Cilluffo, Silvia: 7245
Cimarolli, Verena R: 7040, 7065, 7135, 7660
Ciolfi, Mary Lou: 3090
Cipher, Daisha: 1105, 7265
Ciro, Carrie A: 7330
Cirone, Victoria: 7720
Clair, Catherine: 2230, 8000
Clancy, Erin: 8000, 8020
Clapham, Charlotte: 7620
Clapp, Joshua: 7440
Clardy, Aisling: 1145
Clari, Marco: 7245
Clark, Alexandra L: 1495, 2045
Clark, Carolyn: 4060
Clark, Jeanne M: 7710
Clark, Kate: 3170
Clark, Melissa: 3535
Clark, Valerie: 3240
Clarke, Heather: 3215
Clarke, Philippa: 1045, 1115, 3540, 7140
Clay, Olivio: 4490, 8020
Clayton, Jordana: 3500, 7425, 7905
Clayton-Jones, Kate: 2360
Cleland, Claire L: 3125
Clem, Sarah: 7020, 8010
Clevenger, Carolyn K: 1125, 2295, 7220, 8015, 8020
Cleverdon, Amy C: 4045
Click, Mary V: 8010
Clifford, Adeline: 2130
Cline, J. Mark: 3045
Cline, Krista: 7420
Cloud, Leslie: 8010
Clouse, Kate: 9050
Cloyes, Kristin G: 2395, 4330
Clutter, Jill E: 7265
Coakley, Amanada: 4235
Coates, Martha C: 7020
Coats, Heather: 7065
Coats, Jacquelyn: 3235
Cobb, Ryon J: 3180
Coccia, Kathryn W: 4080, 7520, 7545, 7905
Cochran, Josh: 7735
Cochrane, Barbara: 4470
Coco, Laura: 7455
Coe, Norma B: 7000
Coelho, Luisa: 7310
Coen, Paul M: 1240, 1410, 2080, 2220, 4310, 8020
Coffman, Cynthia J: 2145, 8015
Cofie, Leslie: 7530
Cogliati, Tiziana: 1035, 4310
Cohen, Alan A.: 7010, 8020
Cohen, Ann D: 3375, 7720
Cohen, Beth: 8010
Cohen, Deborah: 4160
Cohen, Emma: 7350
Cohen, Joshua E: 2045, 2315, 3250
Cohen, Marc: 4305, 7670
Cohen, Steven A: 4105
Cohen, Susanna: 1565
Cohen Quay, Lisa: 3200
Cohn-Schwartz, Ella: 3530
Coker, Karen: 8015
Coldiron, Allyson M: 7750
Cole, Rebecca A: 8040
Cole, Steve W: 1060
Cole-Hunter, Thomas: 3125
Coleman, Carissa: 1530, 7635, 7650
Coleman, Rachel: 2265
Coletta, Giulia: 4260
Coley, Yates: 3010, 4230
Collette, Christopher: 7285
Colley, Alexis: 4045
Collier, Natasha: 7490
Collins, Amanda J: 3075
Collins, Amber: 1070
Collins, Sandra K: 7425, 7910
Collins-Pisano, Caroline: 7640, 8020
Colobong, Romeo: 4445, 7040, 7055
Colombowala, Fatema: 1020, 4120
Colopietro, Kelley J: 7155, 7435
Colverson, Aaron: 4285
Colvin, Meaghan E: 1185, 2130
Comer, Cozette: 8010
Comery, Alastair: 2365
Como, Dominique H: 8015
Compernolle, Nell: 3405, 8010
Condon, Gerald: 7630
Coney, Rachel: 4510
Cong, Zhen: 7045
Cong Vu, Nguen: 7265
Congedo, Benjamin: 2345
Conklin, Jamie: 1465, 3300
Connell, Cathleen M: 3145
Connelly, Denise: 4270, 7510
Conner, Laneshia: 3275
Conner, Norma: 8015
Conniff, Joshua: 7425
Connolly, Maureen: 4235
Connolly, MT: 2150
Connor, Dylan: 8000
Conroy, David E: 1545, 8020
Contreras, Cameron M: 4325
Conway, Mike: 1485, 7145
Conwell, Yeates: 8035
Cook, Diane J: 2360
Cook, Paul: 7360
Cook, Wendy: 1365
Cook-Daniels, Loree: 4495
Cooke, Alicia K: 7445, 7935
Coombs, Lorinda A: 8035
Coon, David W: 2190, 4320, 7260
Cooney, Alissa M: 2535
Cooper, Brittany: 2180
Cooper, Julie B: 7720, 9020
Cooper, Kalvry: 1185
Cooper, Lujira: 4445
Cooper, Tara R: 8010
Cooper-Williams, Jasmine: 1075
Cooper-williams, Jasmine K: 8065
Cope, Marie T: 2300
Corazzini, Kirsten: 2000, 2380
Cordes, Dietmar: 8055
Coresh, Josef: 3570
Corley, Robin P: 4095, 7225
Corley, Robin: 8020
Corley, Samantha S: 4185
Corman, Jennifer: 7030
Corneau, Emily: 4460
Cornelison, Laci: 3090
Cornelius, Talea: 1345
Cornell, Porita: 3535
Corner, Lynne: 7035
Coromac-Medrano, Juliamaria: 7225, 7500
Correa, Seneca: 2370
Cortes, Constanza: 8000
Cortes, Tara: 8020
Cortes, Tiffany M: 2505
Cosco, Theodore D: 7720
Cosentino, Stephanie: 1025
Cossett, Benoit: 7030
Costa, Amy N: 1135, 1405, 3055, 7500, 7610
Costa Grille, Alba Maria: 3150
Costantini, Helena: 4355
Costello, Kathryn: 7125
Cotten, Shelia: 2355
Cotter, Valerie T: 8000, 8015

Cotton, Quinton: 7640, 7685
Cotton, Samantha G: 2340, 3175, 4470, 7340, 7730
Couch, Elyse: 3500, 7200, 8000
Coughlin, Joseph: 1095, 3245, 7050, 7065, 7090, 7610
Coulourides Kogan, Alexis M: 2495
Couper, David: 2525
Covington, Amy: 7315
Covinsky, Kenneth e: 4480
Cowen, Nannette: 7065
Cox, Brian S: 3240
Cox, Laura: 8000
Cox, Mary A: 8020
Cox, Simon: 3325
Cox, Susan M: 4435
Coxon, Selby: 3455
Coyle, Caitlin: 7490
Cozad, Aisha B: 3015, 7125
Craft, Suzanne: 2235, 7425
Craig, Taylor: 2230
Cram, Peter: 2090
Crampton, Alexandra: 3365, 9045
Crane, Paul K: 3065, 7320
Cranley, Lisa: 4175, 4465
Crawford, Christina: 8015
Craypo, Lisa: 7200
Creevy, Kate E: 8000
Crenshaw, Kirsten: 8010
Crenshaw, Shannon M: 8010, 8015
Crespo, Eric L: 3325
Crews, Deidra C: 2010, 3150, 4420
Crimmins, Eileen: 1455, 2040, 3095, 8060
Crisafio, Mary: 7020
Crisci, Alex: 7610
Crittenden, Jennifer: 2265, 7680
Crocker, Andrew: 1065
Crocker, Jillian: 7460
Croff, Raina L: 3140
Cronin, James: 8020
Cronin, Teresa: 8000
Crook, Jennifer M: 2300, 3055, 7025, 8015, 8020
Crossdale, Rachel: 8010
Crouch, Adele: 8020
Crouch, Elizabeth: 4105
Croucher, Madison: 8015
Crouter, Scott: 7005
Crowder, Jolie: 2280
Crowder, Victoria R: 8035
Crowe, Alyssa: 7020
Crowe, Michael: 1320, 7700
Crowley, Julia: 4475
Cruz, Grace Trinidad: 7205
Cruz, Michelle: 1575
Cruz, Roberta J: 2305
Cruz Erisman, Morgan: 2560
Cruz-Almeida, Yenisel: 1445
Cruz-Carrillo, Yesenia: 4065, 4500
Cryer, Anthony Q: 3140
Crystal, Stephen: 7110
Csallner, Christoph: 7155
Csikai, Ellen L: 7055
Cudjoe, Thomas: 1225, 2010, 2230, 2520, 4190, 4220, 4265, 4520
Cuevas, Heather: 7440
Cuevas, Nicolas: 7660
Cuffaro, Taylor: 1530
Cui, Saishi: 7620
Cui, Xiaoyan: 9000
Cui, Xirong: 8010, 8015
Cummings, Caroline: 9015
Cummings, Maya Rockeymoore: 1180
Cummings, Stephen: 2220, 2510
Cummings, Steve: 1090, 1435
Cummings, Steven R: 2080, 8020
Cummings-Vaughn, Lenise: 8030
Cummins, Phyllis: 7350, 7475
Cunningham, Solveig A: 3530, 7060
Curci-Delgaro, Kathy: 3240
Curiel, Rosie: 8010
Curl, Angela L: 7160
Curl, Angela: 8085
Curran, Sean: 8000
Curriero, Frank C: 3375
Curriero, Samantha: 2010, 3150
Curtan, Shelley: 3340
Curtis, Ashley F: 1135, 1405, 3055, 3360, 7500, 7610
Curtis, David S: 7230, 7420, 7455
Curtis, Laura M: 7630
Curyto, Kimberly: 1070, 1080, 3240, 7695
Cusack, Meagan: 7290
Cutforth, Asia: 7700, 7720, 7935
Cvejkus, Ryan: 2220, 7060, 7625
Czaja, Sara J: 1040, 4130, 4340, 4355, 4370, 7040, 7065, 7080, 7360, 7405, 8020
Czyz, Rebecca: 7405

**D**

Da, Wendi: 4365, 8020, 8050
da Costa Pinaffi Langley, Ana Clara: 8015
da Luz Scherf, Erick: 8000
da Rosa, Grace: 7610
da Silva Pantoja, Patricia: 8000
Düzel, Sandra: 7030, 7210
Dabelko-Schoeny, Holly: 2070, 2195, 3080, 3485
Dabiri, Sanaz: 8000
Dabrowski, Barbara S: 7440, 7490
Daddato, Andrea: 2085
Dadgostar, Porooshat: 2430
Dadson, Deborah A: 7455
Dagne, Mahederemariam: 1300
Dahal, Arati: 7080
Dahal, Roshani: 7080
Daher-Nashif, Suhad: 1155
Dahlqvist Leinhard, Olof: 4310
Dai, Lu: 7060
Dai, Shuting: 8050
Dai, Xiaochen: 7040
Daliparthi, Meghana: 9025
Dalkin, Sonia: 4255
Dallo, Florence: 1045
Dalmer, Nicole: 7750
D'Alonzo, Anthony: 8010
Dalton, Jarrod: 7235, 7690, 8000
D'Ambrosio, Lisa: 1095, 7050, 7065, 7090, 7610
D'Amico, Danielle: 7675
D'Amore, Cassandra H: 7655, 7720
Dams-O'Connor, Kristen: 3250
Dang, Hope: 7010
Dang, Kristina: 2040
Dang, Linh: 3540, 7490, 7695
Dang, Stuti: 2545, 7360, 7675, 7730, 8010, 8070
Dangeti, Reshma Sahithi: 7360
Dangler, Kevin: 4535
Danial-Saad, Alexandra: 8010
Daniel, Gaea: 4165
Daniel, George: 8020
Daniel, Kathryn: 1105, 7265
Danielsen, Chris: 2395
Danielson, Michelle E: 1410
Danilovich, Margaret: 8020
Dannefer, Dale: 1180
Dansereau, Lisette: 4450
D'Antonio, Patricia: 2390, 3260, 3390
Dao, Elizabeth: 1535, 3335
Dao, Jonathan: 7530
Daquin, Jane C: 4060
Darby, Kevin P: 7225
Dark-Freudeman, Alissa: 2325
Darmann-Finck, Ingrid: 2140
Das, Papiya: 2050
Das Bhattacharjee, Sreyasee: 7360
Das Gupta, Debasree: 1235, 7355
Dash, Atreya: 7305
Dassel, Kara B: 3465, 3500, 4520, 7425, 7905, 8020
Dauenhauer, Jason: 2265, 7155
Daum, Christine H: 7630, 7905
Daus, Marguerite: 4460, 8010
Dautovich, Natalie: 7725, 8010
Davatzikos, Christos: 2510, 7015
Dave, Chintan: 8010
Davey, Adam: 3550, 7700
David, Daniel: 1395, 8045
Davidson, Leslie F: 3300
Davidson, Patricia: 7440, 7500
Davidson-Turner, KJ: 3235
Davie, Laura: 2000
Davies, Katharine: 1115
Davila, Heather: 1395
Davis, Andra: 7065
Davis, Carter H: 7235, 7480, 7900
Davis, Ian: 1390
Davis, Jennifer C: 1365, 8015
Davis, Kimberly D: 4470
Davis, Lewis: 1070
Davis, Rachel: 8055
Davis, Scott A: 1520, 8010
Davis, Scott: 8020
Davis, Vincent: 4340
Davis Chilton, Patricia: 8020
Davis-O'Reilly, Cynthia: 4160
Davitt, Joan K: 8010

Davlyatov, Ganisher: 1550, 3255
Davoudi, Anis: 3070
Dawson, Bonnie: 8015
Dawson, Deirdre R: 1205
Dawson, Walter D: 4235, 7440, 8010, 8010, 8020, 8020
Dayama, Neeraj: 7510
Dayley, Amber: 7085
Dayrit, Jessika G: 7490
De, Supriyo: 7210, 7305
de Albuquerque, Lidio L.: 8015
de Boer, Bram: 4345
De Filippi, Francesco A: 7275
De Fries, Carson M: 1185, 4115, 7495, 8020
De Jonge, Karl E: 7640, 8020
De Klerk-Rubin, Vicki: 7250
De Lima, Bryanna A: 3045
De Man, Ruben: 4170
de Medeiros, Kate: 7055
de Paula Couto, Clara: 1375
de Rosa, Cristina: 7245, 7695
De Sanctis, Pierfilippo: 8000
De Vito, Alyssa: 7040
de Vries, Brian: 2540, 8080
De Vries, Erica: 4255
de Vries, Tjard: 7480
de Wit, Emma J: 2255
Deal, Jennifer A: 1055, 2035, 2525, 4300, 4440, 8015
Dean, Devlon: 1530
Dean, Olivia: 1085
DeBell, Karen E: 7695
DeCarli, Charles: 4430, 7500, 7930
Deck, Monica M: 3065
Decker, Autumn E: 1190, 2180, 2325
Deckert, Amy: 7325
DeConne, Theodore M: 4005
Decosimo, Kasey: 7685, 8015
Deelen, Joris: 8015
Deer, Rachel: 7400, 7920, 8015
DeFrancesco, Erica: 1345
Degenholtz, Howard B: 2480, 3255, 7110, 7510, 7545, 7915
DeGennaro, Jennifer: 8010
DeGroot, Lyndsay: 4370
Deichert, Jerome: 7510
Deif, Reem: 1155
DeJohn, Amber: 4430
Dekel, Tal: 3015
Dekhtyar, Serhiy: 3035
Del Bene, Victor: 7285
Delaney, Erin: 7055
DeLange Martinez, Pauline: 8010
DeLany, James P: 2505, 8020
Delaporte, Magdalena: 7455, 7925
DeLaTorre, Alan: 7410
Delgado, Ferdinand: 8015
Delgado, Kimberly: 7510
Delgado, Roxana E: 2295
Delgado, Sara: 3575
Delgado-Ortiz, Laura: 2170
DeLiema, Marguerite: 1370, 2150, 3270
Delitto, Anthony: 7330
Dellefield, Mary E: 8075
Deller, Juergen: 7050
DeLongis, Anita: 4405
DeLouize, Alicia M: 8015
DeMarchis, Emilia: 4160
DeMarco, Mari L: 7720
Demir, Murat: 8070
Demircan, Kamil: 7210
Demirer, Ibrahim: 1285
Demiris, George: 1290, 2085, 4220, 7145, 7270, 7665
Demuth, Ilja: 7030, 7210
den Ouden, Mirre D: 4255
Denfeld, Quin E: 7440
Denham, Michael W: 7140
Denison, Paige: 2450
Dennis, Elizabeth A: 7315, 7720
Denno, Benjamin: 4235
Denson, Kathryn: 7480
Denson, Steven: 7480
Dent, Elsa: 2230
Dent, Kallisse R: 7695
Derboghossian, Grace: 7015
Derby, Carol A: 2160
Derenne, Nicole M: 8020
DeRoo, Scott: 7625
Dertinger, Melinda: 2395
Desai, Smit: 4420
Deshmukh, Madhyami: 2255
Desir, Marianne: 2545, 8070
Desjardins, Michael R: 2045, 3375, 4430
Desoria, Maureen G: 7080
Deters, Shana: 7720
Deturbide, Katherine: 3170
Devereaux Melillo, karen: 2440
Devi, Reena: 4255, 7295
DeVincentis, Diane: 8000
Devone, Frank: 4460, 8010
Dewansingh, Neha: 7130
Dews, Denise: 3300
Dey, Judith: 4305
Dey, Nutifafa E Y: 1450, 4065, 4500
Dey, Venance: 4175
Dhakal, Usha: 8020
Dhaliwal, Ravneet: 1020
Dhanekula, Arjune: 7625
Dhar, Nirmala: 8010, 8020
Dharia, Aastha K: 8010, 9050
Dhawan, Aanika: 8000
Dhingra, Radha: 7625
Dhoble, Abhjieet: 3135
Di Bona, Vito: 3010
Di Gessa, Giorgio: 1290, 2365, 3125
Diallo, Idiatou: 3070, 7615
Dian, Larry: 1365
Diaz, Jose A: 7455
Diaz, Joseph: 3115
Diaz, Monica: 8020
Diaz-Asper, Catherine: 2355
Diaz-Valdes Iriarte, Antonia: 3035
DiCamillo, Mark: 7015
DiCarlo, Nicholas: 4025
Dickerson, Anne E: 3185, 7160
Dickinson, Karen: 7650
Dickson, Victoria: 7465
Dieckmann, Nathan F: 7020, 7205
Diederich, Freya: 3550
Diedrich, Jolene: 7425
Diegel, Morgan: 7010
Diehl, Keegan: 4435, 7065
Diema Konlan, Kennedy: 7080
Dieterk, Kristi: 7000
DiFranco, Lacey: 7465, 8020
Dikhtyar, Oksana: 4510, 7295, 7435
Dilanchian, Andrew: 3060
Dilian, Omer: 1435
Dillon, Ellis: 4045, 7740
Dillon, Kayla: 7200
DiMaria-Ghalili, Rose Ann: 7020
Dimeo, Christina: 3460
Dimonte, Valerio: 7245
Dimri, Vrinda: 7455, 7720
Dindo, Lilian: 7205
Ding, Ding: 9000
Ding, Jun: 9015
Ding, Kedong: 7055
Ding, Kehong: 7210
Ding, YaPing: 7520, 7910, 9045
Ding, Yong: 1355
Dionne, Laura A: 1530
Dissault, Nicole: 2180
Distefano, Giovanna: 4310, 8020
DiVall, Sasha: 8015
Divecha, Ayushi: 3250
Dixon, Mandy: 3060
Dixon, Saharra: 2555
Dixon-Gordon, Katherine: 7255
Do, Jonghwa: 8020
Doan, Andrew: 7210
Doan, Lan N: 8045, 8095
Dobalian, Aram: 7045
Dobbs, Debra: 1360, 2095, 2185, 2370, 3365, 3495, 7435, 7455, 8010
Dobish, Heidi B: 4040
Dobrota Lai, Sylvie: 8015
Dodd, Elvira: 7405
Dodge, Hiroko H: 7005, 8000
Doerfler, Deborah: 8015
Doh, Michael: 7480, 7680
Dohan, Daniel: 3175, 4450
Doheny, Megan: 7215
Doherty, Aiden: 3050
Doherty, Fiona: 2195
Dokshina, Darya: 2045
Dolan, Hanne R: 7260
Dolansky, Mary A: 1490
Dolbin-MacNab, Megan L: 1190, 7005, 7265
Dole Center of Excellence, VA Elizabeth: 8010
Doll, Johannes: 3155
Domingo, Divine C: 7150
Dominguez, Briana Alejandra: 8025
Dominigue, Benjamin: 4550
Domnick, Hallory: 7265, 7460
Domoto-Reilly, Kimiko: 1480, 2530, 7200
Donahue, Patrick T: 2045, 3375
Dondero, Kathleen R: 8015
Donega, Stefano: 7210, 7305
Donelan, Karen: 1230
Donelle, Lorie: 4270
Dong, Bigrong: 8015
Dong, Fanghong: 8020
Dong, Jingyi: 2125
Dong, Sihan: 1245
Dong, Wuyi: 7070, 7235, 7350, 7735
Dong, Yanjun: 7155, 8010

Dong, Yu: 1535, 3035, 7440
Dong, Yue: 3480
Donkor, Michael: 8000
Donofry, Shannon D: 4315
Donohoe, Maria: 2185, 7465, 7685
Donorfio, Laura KM: 1185, 2175, 2325
Donovan, Heidi: 4530
Donovan, Nancy J: 4180
Dooley, Erin E: 7720
Dopson, Rasheera: 8000
Dorame, Ashley: 4375
Doran, Kelly: 1070
Dore, Peter: 1275
Doriscar, Jonathan E.: 8080
Doriwala, Hussain: 7750
Dorn, Adrianah: 7200
Dorr, David A: 4160
Dorris, Jennifer: 4540
Dosa, David M: 2185
Dose, Ann Marie: 4415
Dosso, Jill A: 2565, 7430
Dotolo, Danae: 7530, 7905
Dotson, Vonetta M: 1205, 2475
Dougherty, Maura: 8010
Dougherty, Ryan J: 3070
Douglas, Joseph: 7730
Douglas, Natalie: 1160
Dowd, James J: 1300
Dowden, Lauren: 1125
Dowling, Maritza: 3190
Dowling, N. Maritza: 2160
Downer, Brian: 1075, 1170, 1320, 4550, 7230
Downing, Kimberly: 4505
Doyle, Cassidy: 8000
Doyle, Margaret F: 2160, 7735, 8010
D'Oyley, Heather: 7750
Dozier, Mary E: 3575, 4120
Dozier, Stephen: 8000
Drabo, Emmanuel F: 7470, 7905
Draeger, Dagmar: 7110
Dragan, Deanna: 4330, 7695, 8015
Drake, Connor: 7685
Drakes, Josiah: 8015
Draughn, Melissa: 4265
Dravecz, Nikolette: 3060
Draw, Kandis: 4505
Dremann, Andrea: 3175
Drewelies, Johanna: 1350, 7030, 7210
Driscoll, Monica: 7010
Driver, Charles: 4185
Driver, Jane: 8020
Driver, Jane "Jenny": 3425
Drossel, Claudia: 3110
Drost, Jennifer: 7440, 7475, 7900
Drumheller, Jake: 7315
Drummond, Neil: 8010
Dryden, Eileen: 2430, 8010
Du, Pang: 4110
Du, Shufa: 7275
Du, Xuan: 7670
Du, Yan: 8015, 8050
Duan, Hongzhe: 7075, 7210, 7715
Duan, Liping: 7320
Duan, Siyu: 2255
Duan, Yanjun: 4465
Duan, Yinfei: 4100, 4195, 4245
Dubé, Catherine: 8015
Duberstein, Paul: 1380, 7110
Dublin, Sascha: 3010, 4230
Dubose, Kline: 4520
Dubowitz, Tamara: 8015
Dubure, Emmanuel Banbur: 7145
Duchesne, Simon: 7720
Duchow, Olivia: 7205
Duchowny, Kate: 1240, 7350
Duckett, Jacquelyn: 7265, 7735
Duckworth, Alyssa C: 7310
Dueck, Kimberly: 7660
Duehring, Jasmin: 8015
Duff, Kevin: 7200
Dufour, Alyssa B: 2080
Dugan, Elizabeth: 1305, 1405, 3265, 7065, 7135, 7240, 7635, 7695, 7700
Duggan, Katherine A: 1525, 7030
Dukkipati, Haripriya: 2180
Dunavan, John: 1265, 7075
Dunbar-Jacob, Jacqueline: 2140
Dunkle, Ruth: 7420
Dunlap, Ben: 7545
Dunlap, Janette: 7320
Dunlap, Shawn: 2345
Dunnigan, Allison: 7155
Dunsmore, Victoria J: 7610
Dupre, Matthew: 7625
Dupuis, Sherry L: 7055
Dupuy, Emma G: 4490
Duque, Gustavo: 1330, 1580, 2350, 8020
Duque Urrego, Daniel: 7205
Durkin, Maeve: 7205
Dusetzina, Stacie: 3075
Duthie, Edmund H: 2535, 7480
Dutta, Angshita: 7075
Dutta, Chhanda: 2435
Duty, Charlotte: 7215
Dwibedi, Aditya Kumar: 7360
Dwyer, Johanna: 8015
Dye, Maya E: 8035
Dyer, Jamie R: 4040
Dys, Sarah: 1230, 3535, 8010, 8020
Dzwierzynski, Jenna: 8000

**E**

Eagle, Angelica: 7440, 7660
Earles, Julie L: 7225
Easter, Molly: 8000
Eastman, Jennifer: 8010
Eaton, Charles: 4515, 7040
Eaton, Jacqueline: 3465, 4040, 7085
Eberhardt, Kenda: 1135
Ebulum, Genevieve: 7150
Eby, David W: 3455
Echevarria, Klarisse: 7000
Eckstrom, Elizabeth N: 3045
Edahiro, Ayako: 7350
Edelman, Linda S. S: 4520, 8020
Edelstein, Barry: 7115, 7490
Edison, Jos: 7275
Edland, Steven D: 8015
Edwards-Childs, Paulita L: 2480, 3100
Efird-Green, Lea: 1520, 2105, 8010
Ehlers, Diane: 3285
Ehrlich, Joshua R: 1115, 2525, 4380, 4455, 7035
Eich, Teal: 8000
Einstadter, Douglas: 8000
Eisen, Howard: 7620
Eisenberg, Chase: 1505
Eisenstein, Amy R: 2295
Eisikovits, Zvi: 3015
Ejaz, Farida K: 1145, 3340, 7405
Ejiri, Manami: 7205, 7540
Ek, Mary: 7465
Ekerdt, David J: 1375, 4330
Eklund, Nicole M: 4315
Ekoh, Prince C: 8010
El Asmar, Khalil: 8020
El Sammak, Aya: 1155
Elayoubi, Joanne: 7070
Eldadah, Basil: 1340
Eldridge, Corinne: 8025
Elfenbein, Pamela H: 3450
Elftmann, Hanley: 7110
Elhassan, Mohamed: 8015
El-Hay, Tal: 8015
Elhussein, Yassein: 8015
Elias, Maya N: 7735
Eliatamby-O'Brien, M.: 8050
Eliopoulos, Elaine M: 3015
Elisha, Victor: 7740
Elkind, Mitchell S V: 8085
Ellington, Lee: 4330, 4520, 7085
Elliot, Amy E: 3380, 7295
Elliot, Christian: 7005, 7135
Elliott, Emily: 7045
Elliott, Lauren: 1335, 3385, 4435, 7065, 7425, 9015
Elliott, Meghan R: 7030
Elliott, Susan J: 4470
Ellis, Erica J: 4510
Ellis, Jennifer L: 2455
Ellis, Julie: 7070, 7425, 7540, 7930
Ellis, Katrina R: 1060
Ellis, Sydney: 7345
Ellisman, Mark: 7015
Ellison Keeler, Carlyn: 8020
Elman, Alyssa: 4355, 7405
Elmasri, Jeannine: 7425, 7905
Elo, Irma T: 9035
Elvevåg, Brita: 2355
Emery-Tiburcio, Erin: 4470, 7650
Emezue, Chuka N: 3500
Emlet, Charles A: 4495
Emrich, Christopher: 2300, 7025, 8015, 8020
Emrick, Lauryn: 7440
Eng, Janice: 7720, 8015
Engdahl, Bo L: 2425
Engdau, Shelly: 8010
Engeland, Christopher G: 1250, 7210, 7455, 8020
Engell, Katia: 7055
Engelman, Michal: 3225
Engelmann, Corinne D: 4300
Engle, David W: 4100
Engleman, Michal: 3225
English, Tammy: 3355
Enguidanos, Susan: 4080, 8010
Enroth, Linda: 2460
Ensrud, Kristine: 4055, 4385, 7630
Enterria-Morales, Daniel: 7210
Entwistle, Amy: 8000
Eo, Yugyeong: 3545
Epps, Fayron R: 2295, 4175, 7005, 7700
Epstein, Cynthia: 1380

Epstein, Mara M: 7060
Epstein-Lubow, Gary: 1260, 1270, 2305
Eramudugolla, Ranmalee: 3185
Eraslan, Emre: 8010
Erb, Isabelle: 8010
Erdman, Elizabeth C: 7495
Erickson, Alexander J: 2345, 7660, 7735
Erickson, Kirk: 4315
Erickson, Lance: 2385
Erickson, Olivia: 8015
Erkens, Petra: 7685
Erker, Jayma: 7075
Erlandson, Kristine M. M: 7285, 8015
Ermolenko, Mia: 8035
Ernst, Joy S: 7405
Ernsth Bravell, Marie: 8015, 9015
Ernstsen, Linda: 7005
Ersek, Mary: 1160, 4195, 8015
Ertle, Emilee: 7660
Erus, Guray: 7015
Erving, Christy L: 3235, 3475
Escorcia Encarnación, Carolina: 7400, 7920
Esiaka, Darlingtina K: 1465, 1495, 3220, 3475, 4000, 8015
Espejo, Kimberly: 8015
Espejo, Luis: 7010
Espeland, Mark A: 1390
Espinoza, Sara: 1460, 8015
Espinoza, Sara E: 1410, 1460, 2505, 4210
Esposito, Michael: 3375, 4035, 7350, 8010
Esser, Karyn: 8000
Essien, Ime: 8000, 8015
Estabrooks, Carole A: 4100, 4195
Estapé, Jorge: 2445
Estapé, Tania: 2445
Estes, Katie L: 7425, 7930
Estlein, Roi: 1565
Estrada, Leah: 4480
Eun, Heesoo: 8015
Eustice-Corwin, Alexander: 4400
Evans, Michele: 1250, 2055, 2330, 8000
Evans, Teairra: 8020
Evans, Tracy L: 4340
Evans, William J: 4260
Evelyn-Gustave, Allyson: 4420
Everson-Rose, Susan A: 1265
Eymundsdottir, Hrafnhildur: 2040
Eze, John E: 7150
Ezeihuoma, Obinna: 7150
Ezeokonkwo, Francesca C: 2460

**F**

Fabbre, Vanessa: 4250
Fabius, Chanee: 3105, 3570, 7700
Factora, Ronan: 7405, 7920
Fadalla, Carol: 3385
Fadel, Meghan: 3100, 3430
Fagundes, Christopher P: 3285
Fahr, Bruce T: 8000
Fain, Mindy: 8015
Falcão, Deusivania Vieira Da Silva: 8015
Falck, Ryan S: 1365, 1535, 3335, 7030, 8015
Fallahi, Afsaneh: 7315
Falvey, Jason: 1075, 1535, 3035, 4385, 8010, 8065
Falzarano, Francesca: 1165, 2305, 7660, 8010, 8020
Famula, Jessica: 2200
Fan, Baoyu: 3330
Fan, Erica K: 3375
Fan, Jason: 2395
Fan, Jiayao: 8015
Fan, Yujun: 8075
Fang, Chao: 2365
Fang, Estelle Xun: 7460
Fang, Fang: 1485, 7145
Fang, Mei: 3060, 8010
Fang, Mei Lan: 1015
Fang, Ya: 1365, 2255, 7305
Fang, Yu: 4270
Fang, Yuan: 8015
Fanning, Jason: 2505, 7710
Farago, Fanni: 3015
Farina, Crystel L: 8015
Farina, Mateo: 1280, 2515, 3030, 3095, 3370, 4400, 7040, 7485, 7925, 8060
Farmer, Heather R: 3075, 4015
Farmer, Justin: 3035, 4440
Farmer, Matthew S: 8015
Farnham, Trish: 2500
Faro, Jamie: 8015
Farook, Jahoor: 2235
Farrants, Kristin: 2365
Farrell, Tim: 4520
Farris, Kristen M: 8000
Farsijani, Samaneh: 7060, 7710, 8015
Fasching Maphis, Christine: 2460
Faselis, Charles: 9025
Fassi, Janelle E: 1185, 2130, 7265, 7490, 7670
Fastame, Maria Chiara: 1235
Faucheux, Gavin R: 7225
Faul, Anna C: 4470, 7340, 7730
Faul, Jessica: 8060
Fauth, Elizabeth B: 4335, 4435, 7030, 7065, 7270, 7325, 8020
Fawcett, Janet: 8015
Fazio, Sam: 2105, 3000, 3000, 4125, 4200, 7465
Fears, Vanessa: 4475
Featherston, Kyle G: 8010
Featherstone, Katie: 7520
Federman, Alex: 3250, 7630
Fegter, Ollie: 9040
Fehland, Jess: 3425
Fehnel, Sheri: 8020
Fei-Fei, Li: 1575, 4420
Fein, Taylor: 8000
Feldman, Sid: 4445
Feldman, Sue: 3255
Feliciano, Leilani: 7695, 7720, 8020
Felix, Cynthia: 4490, 9000
Felt, John M: 4180
Felts, Adam: 7065, 7610
Feng, Derek: 2020
Feng, Jeff: 7360
Feng, Yali: 2355
Fennell, Charles: 3140
Fenstermacher, Erika A: 7115, 7490
Fenton, Laura: 7495, 7930
Ferdows, Nasim: 4105, 8010, 8015
Ferguson, Anna M: 8000
Ferguson, Dee: 7225
Ferguson, Dian: 3140
Ferguson, Erin: 8015
Ferguson, Giselle A: 4185
Ferguson, Lee Ann S: 1450
Ferguson, Melissa M: 2065
Fernandes, Cecilia: 7660
Fernandez, Mariana: 4050
Fernandez, Otniel: 4420
Fernandez Cajavilca, Moroni: 7085, 8045
Ferraro, Kenneth: 3405, 7535, 8020
Ferreira, Kyra: 8015
Ferreira, Pamela: 9000
Ferrell, Anastasiya: 7440
Ferreri, Jeff: 4490
Ferretto, Marianella E: 7695, 7935
Ferri, Christine: 2275, 7235
Ferrie, Joseph P: 4375
Ferrucci, Luigi: 2510, 4170, 7010, 7210, 7305, 7710, 7720, 8000, 8000, 8000, 8015, 9015
Festus, Eniola Z: 3235
Fete, Zoe F: 1030
Feyh, Lauren: 7265
Fick, Donna M: 2140, 7410, 7645
Fiehn, Oliver: 8000
Fielding, Roger: 2005, 2380, 8015
Fields, Beth: 1455, 2085, 2190, 2415, 4530, 7270
Fields, Bronwyn: 2200
Fields, Cynthia D: 4100
Fields, Eve: 1535
Fields, Maizonne: 8015
Fields, Noelle L: 1105, 2195, 7005, 7265, 8010
Fields, Torrie: 2390
Fifield, Alexis: 7440
Figueroa Gray, Marlaine S: 3065
Fike, Kayla J: 7350
Filippi, Melissa: 7325
Fillingim, Roger B: 1445
Finch, Caleb: 2040
Finchum, Tonya: 1385
Fine, Courtney: 1250
Finegan, Kerry: 4200
Fingerman, Karen: 4085, 4180, 7005, 7045, 7540, 7735
Fink, Howard: 4055, 7630
Finkel, Deborah: 4010,: 8015, 8015, 9015
Finlay, Jessica M: 3375, 4430, 7420
Finley, Erin: 8015, 8050
Finn, Laura A: 2535
Finnigan, Samuel: 4020
Finsel, Julia S.: 7050
Fiori, Katherine L: 4525, 7070
Fiorotto, Marta l: 1420
Fique, Morgan T: 8065
Fiscella, Andrew J: 7480
Fischer, Heidi: 8015
FIshbein, Kenneth W: 7210
Fisher, Gwenith: 4375
Fisher, Miranda: 3485
Fisher, Zachary: 7255
Fishman, Paul: 8010
Fisk, Calley: 1015, 3245
Fiske, Amy: 7000, 7220
Fitch, Catherine: 4035
Fitzgerald, Kathryn C: 7285
Fix, Rachel: 8015, 9025, 9040

Flanagan, Jane: 3230, 4235
Flatt, Jason: 2015, 2225, 3190, 3490, 4025, 7100
Fleet, Alexa: 7135, 7635, 7695, 7700
Fletcher, Jason: 4360
Fletcher, Jason M: 1280, 7660
Flood, Sarah: 4035
Florack, Janina: 1555
Florence, L. Carter: 1190
Flores, Carlos A: 1280
Flores, Efren: 8015
Flores, Emily V: 8000
Flores, Valerie: 4510
Flores-Cruz, Gabriela: 8020
Flores-Lagunes, Alfonso: 1280
Florez Acevedo, Stefani P: 1265, 7720
Floris, Patrizia: 7275
Floyd, Alex: 4195, 7110
Floyd, Alex C: 9040
Flynn, Sheryl: 8020
Fogleman, Connor: 4530
Foley, Olivia: 1130
Fonarow, Gregg: 9025
Fong, Arwen: 8015
Fonseca, Luciana LM: 4515
Fontaine, Leslie: 7660
Fontanese, Matthew: 7750
Foo, Jamie: 3115
Foradori, Megan A: 1490
Ford, Cassandra: 3585, 7700
Ford, James: 7705
Forrester, Sarah N: 3180
Fors, Stefan: 1450, 2060
Fortinsky, Richard H: 1275, 2495, 3210, 7740
Fosnight, Sue: 7440, 7475, 7900
Foster, Brandon: 3150
Foster, Justin: 8015
Foster, Norman L: 3175, 4070
Foster, Thomas C: 1445
Fountain, Brent: 4040
Fountain, William A: 7305
Fowler, Nicole: 4230, 8020
Fowler Davis, Sally: 3060
Fox, Aimee: 7480, 7900
Fox, Jennifer: 1035, 4310
Fox Ludden, Emily: 7465
Foxe, John: 8000
Foy, Capri G: 1315
Fracachan Cabrera, Monica: 8000
Fraiman, Paule Sarah: 8010
Francis, Sarah L: 7350
Francis- Edoziuno, Confidence: 8015
Franck, Maximilien: 7010
Frank, Robynn: 3345
Franzone, Monica S: 3515
Franzosa, Emily: 1395, 2430, 3105, 3485, 4480, 7045, 7420
Fraser, Renee: 7200
Fraser, Sarah: 1010, 2510
Frasier, Pamela: 8010
Fratiglioni, Laura: 3035
Frazier, Cleothia: 3235
Frazier, Robbin H: 2155
Frazier, Trivia: 1500
Freburger, Janet K: 7330
Freddolino, Paul P: 3415, 7745
Frederick, Jared: 7690
Fredolini, Claudia: 7010
Fredriksen-Goldsen, Karen I: 2420, 2540, 4495
Fredriksen-Goldsen, Karen: 8010
Freedman, Vicki: 3165, 7030, 8025
Freeland, Kerri S: 8015
Freeman, Shannon: 2360, 3205
Freer, Elinor: 9010
Freidus, Andrea: 8010
Freitag, Callie J: 3065, 4475, 7120
Freitas, Samuel: 7010
Frenkel Toledo, Silvi: 1245
Friberg-Felsted, Katarina: 3195, 3465, 4040
Fried, Terri R: 2305
Friedland, Hannah: 7460
Friedman, Ari B: 3550, 4220, 8015
Friedman, Ben: 7720
Friedmann, Erika: 3050, 8020
Friis, Marlene: 8020
Frimpong, Eric: 7240, 7405, 7635
Frissen, Laura: 4345
Fristedt, Sofi: 7155
Fritz, Julie: 9010
Froberg, Ruthann: 1145
Frogner, Bianca: 7545
Frohmberg, Eric J: 3100
Fromenthal, Ashley L: 1210
Fromknecht, Catharine Q: 7125
Fruhauf, Christine A: 3265, 3575
Fry, Carrie: 3075
Fry, Liam: 3240
Fry, Noelle: 7410, 7900
Fu, Jason: 7465, 8010
Fu, Liang: 8085
Fu, Lillian X: 3355
Fu, Longdi: 8010
Fu, Sunyang: 3135
Fu, WeiJie: 4535
Fu, Xiaoyu: 7235, 7610
Fu, Yu: 1195
Fudolig, Miguel: 4535
Fugate, Matthew (Brent): 4235
Fugate-Whitlock, Elizabeth: 2325, 3495, 8010
Fujihara, Satoko: 7490
Fujimoto, Kayo: 1105
Fujita, Koji: 7275, 7695
Fujiwara, Yoshinori: 7205, 7540, 7695
Fukuda, David H: 7205, 7640, 7735, 8015
Fukui, Sakiko: 7340, 7510
Fukushima, Kiyoko: 7405
Fukuzawa, Ai: 7360
Fulay, Aarohee: 8015
Fuller, Heather: 3405, 7025, 7070, 7090, 7605, 7745
Fuller, Patrice M: 3140
Fuller, Shannon: 2520
Fuller-Thomson, Elysia G: 7025
Fuller-Thomson, Esme: 7025
Fulmer, Terry: 4355
Fulzele, Sadanand: 7210, 8015
Fung, Chi Tat: 4195, 8015
Fung, Constance: 8015
Fung, Helene H.: 1375, 3480, 4020, 7355, 7610, 8000
Fung, Helene Hoi-Lam: 9035
Fung, Nicole L: 1375
Funk, Laura: 2495
Furht, Borko: 7360, 7610
Furman, David: 2530
Furstenberg, Frank: 7350
Furuya, Alex: 4550
Fusca, Laura A: 8010
Futterman, Andrew: 7150

G

Gaballah, Nada A: 1155
Gaber, Sophie N: 1555
Gabralla, Sarra: 8015
Gabriel, Kelley Pettee: 7720
Gabrio, Andrea: 4345
Gad, Dustin: 8020
Gadbois, Emily: 4460
Gaeta, Laura: 1050
Gaglio, Marisa: 8000
Gagne, Danielle: 3495
Gagnon, Christine: 4490
Gahche, Jaime: 8015
Gaillard, Trudy: 2330
Gaines, Bryan F: 1060, 4010
Galambos, Colleen: 2555
Galanza, William Son: 7155
Galasko, Douglas: 7005, 8015
Galatioto, Jessica: 7140
Galik, Elizabeth: 4090, 8020
Gallagher, Michael: 7040
Gallagher-Thompson, Dolores L: 1135, 8040
Gallant, Kayla O: 7105
Gallistl, Vera: 2565, 3315, 4275
Gallo, Joseph: 3570, 7615, 8000
Galloway, Rebecca: 3340
Galucia, Natalie: 3310, 8010
Galvin, James: 4430, 8015
Gamache, Seana: 2130
Gamaldo, Alyssa A: 1250, 2055, 2330, 7735, 8000, 8020
Gamaldo, Charlene: 7735
Gamble, Julia: 2145
Gamper, Mary Jo: 2085
Gan, Daniel R Y: 3200, 4320, 8010, 8020
Ganbat, Uyanga: 7205
Ganci, Aaron: 2410
Gandy, Stacey: 7250
Gandy, Tyler: 2360
Gangnon, Ronald: 7160
Gangula, Sushma: 7540
Gangupantula, Pratima: 2535, 7530
Gans, Hannah: 1010
Gao, Ashley: 8020
Gao, Bruce: 8000
Gao, Courtney: 8085
Gao, Fengli: 7470
Gao, Haihua: 7670
Gao, Hongxin: 2465
Gao, Oude: 4550
Gao, Shangde: 1445
Gao, Sibo: 7045, 7540
Gao, Siyu: 2150, 3270
Gao, Sujuan: 4440
Gao, Wei: 7030
Gao, Xianqi: 7355, 8015
Gaona, Carlos: 7735
Garbarino, Valentina R: 3020
Garber, Johnathon: 7300
Garberson, Lisa A: 2500
Garbin, Alexander J: 2135, 4215

García, Catherine: 1320, 3235, 4015, 8020
García De La Garza, Ángel: 2160
Garces Torres, Ana Sophia: 7065, 8020
Garcia, Alexandra: 8010
Garcia, Andrea: 8025
Garcia, Ivis: 1295
Garcia, Jose M: 7305
Garcia, Lizbeth: 7005
Garcia, Marc A: 2120
Garcia, Mary Ellen: 8010, 8015
Garcia, Michael A: 1540
Garcia, Reagan E: 1240
Garcia, Thelma: 4170
Garcia Marquez, Grecia Gabriella: 1420
Garcia Marquez, Grecia G: 8000
Garcia Morales, Emmanuel E: 1055, 1440
Garcia-Aymerich, Judith: 2170
Garcia-Chanes, Rosa: 3030
Garcia-Davis, Sandra: 2545, 8010, 8070
Gardener, Hannah: 8085
Gareri, Michele: 7440, 7475, 7900
Gargiulo, Paolo: 2240, 7305
Garner, Charity: 8020
Garner, Kayla M: 7455
Garnett, Anna: 4270
Garrison, Brianna V: 2260
Garrity, Katherine: 7140
Garrow, George: 7410
Gary-Webb, Tiffany L: 3375
Gaskin, Darrell: 3030
Gassoumis, Zachary D: 2001, 2150, 2375, 7405
Gaster, Barak: 1430
Gaston, Harriett: 7690
Gately, Megan: 3395
Gatenio-Hefling, Orly: 1435
Gatz, Margaret: 4375
Gaugler, Joseph E: 1260, 2015, 2155, 2350, 3025, 3130, 3425, 3450, 4235, 7640, 7685, 8020
Gauss, C. Heath: 7360, 7630
Gautam, Ramraj: 1050, 2440
Gawryluk, Jodie R: 7525
Gay, Emma: 1240
Gayda, Mathieu: 4490
Gaye, Angela: 7030
Ge, Huini: 7510
Ge, Mei-Ling: 8015
Ge, Shaoqing: 2190
Gea-Sánchez, Montserrat: 4255
Gee, Nancy R: 3050, 8020
Gehling, Jacklyn A: 1020
Geissal, Erik D: 7350
Geisser, Sophia R: 1070, 1210, 3240
Geldhof, John: 7730
Gelfond, Jonathan: 4310
Gelinas, isabelle: 3185
Gell, Nancy: 2450, 7655
Gelmon, Sherril B: 4235
Gendron, Tracey: 1010, 8000
George, Claudene: 8000
George, Daniel: 3380
George, Kiran: 7000
George, Mollie J: 8000
George, Regina A: 3240, 7065, 7685
George, Rickey E: 8015
Gerald, Melissa: 3420
Gergis, Mary: 7475, 7900
Gerken, Danielle R: 8010
Germain, Cassandra M: 8000
Germine, Laura T: 4515
Gerstorf, Denis: 4065, 4500, 4525, 7030, 7210, 7535, 8020
Gertin, Robin: 7055
Gettel, Cameron: 1130, 8010
Getz, Marci J: 3100
Gharib, Sina A: 7305
Ghazi Saidi, Ladan: 2205, 7465
Ghezzi, Elisa: 3305
Ghiasi, Akbar: 1425, 1550
Ghimire, Saruna: 4015
Ghoraani, Behnaz: 7610
Ghose, Sarah M: 8010
Ghose, Urmimala: 3040, 4525
Ghosh, Bhaswati: 4170, 8000
Ghosh, Neeta: 7530, 7905
Ghosh, Ritam: 7155
Giannotto, Emily L: 7225
Giardino, Elizabeth: 7420
Giasson, Hannah L: 3080
Gibbon, Samuel: 7425, 7930
Gibbs, Lisa: 2340, 7405
Gibson, Allison: 3370, 7545, 7905, 8020
Gibson, Regina v: 8015
Giffuni, Jamie: 7080, 7315, 7720, 8015
Gigi, Amateau: 1010
Gil, Efrat: 1435, 7300
Gil, Heidi: 1345, 4165
Gilani, Owais: 2045
Gilbert, Lauren R: 3485
Gilchrist, Laura: 7000
Gilfix, Talia: 7355
Gill, Amarjot S: 7435, 7915
Gill, Jessica: 8000
Gill, Thomas: 2020, 3340
Gilleard, Chris: 3015
Gillespie-Heyman, Sarah: 7475
Gilligan, Megan: 3055, 3165, 7005, 7265
Gilliland, Jaime: 2295
Gilmore-Bykovskyi, Andrea L: 3425, 4530, 7200, 8010
Gilsanz, Paola: 7535
Gil-Silva, Mauricio: 8000
Gimm, Gilbert: 2415, 7270
Gingerelli, Toni: 3570
Ginggeaw, Sangduan: 4465
Ginn, Brandi: 3175, 4450
Ginoux, Laura: 7635
Ginsberg, Charles: 3330
Giordani, Bruno: 8000
Giordano, Gregory: 3115
Giordano, Stephanie J: 4245
Giorgio, Luciana: 8000
Giraldo Santiago, Natalia: 1345, 2315
Girgus, Joan: 7235
Girling, Laura: 2015
Gislason, Magnus Kjartan: 2240, 7305
Gitelman, Darren R: 7425, 7930, 8070
Gitlin, Laura N: 1260, 3395, 4130, 4340, 7685, 8020, 9010
Gjestsen, Martha: 8015
Glaser, Karen: 1475
Glass, Lauren: 1520
Gleason, Carey E: 2190, 2335
Gleason, Kelly T: 2085
Gleason, Lauren: 2340
Glennie, Kristen S: 1005, 7075
Glickman, Adam: 4290
Glicksman, Allen: 3550
Gliebus, Gediminas: 9010
Glockner, Katherine: 4310
Gloger, Elana M: 9010
Glover, Crystal M: 4025
Glymour, M Maria: 1545, 7535, 9010
Glynn, Jennifer: 8015
Glynn, Nancy W: 1240, 1410, 2035, 7060
Godin, Judith: 8000
Goel, Sanchit E: 4180
Goerzen, Maya: 3465
Gogia, Rhea: 7530
Goldberg, Allon: 7300
Golden, Blair P: 3335, 4530
Goldfeld, Keith: 3010
Golding, CJ: 7250
Goldman, Alyssa: 1140, 3405
Goldman, Dana P: 1390
Goldstein, Karen: 4530
Goldtooth, Amanda: 1525, 8000, 8020
Goler, Timothy: 1140
Golithadka, Kaushik: 1060, 4010
Gomes Goncalves, Natalia: 1320
Göbl, Linda: 7680
Gomez, Melissa: 8010
Gomez, Morgan: 8000
Gomez, Rowena G: 2440, 8000
Gomez, Scarlett Lin: 2020
Gómez Cáceres, Bárbara: 8000
Gonçalves, Rui: 8010, 8015
Gondo, Yasuyuki: 1385, 2470, 7115, 7750, 8020
Gong, Jessica: 4440, 7005
Gong, Ke: 8000
Gong, Ni: 7080
Gong, Zengliang: 1230
Gongloor, Pratik: 7400, 7920, 8015
Gonyea, Judith G: 7215
González, Hector: 7455
Gonzales, Ernest: 2335, 2470, 8000, 8020
Gonzales, Gilbert: 3075
Gonzales, Mitzi: 4210, 7500, 7735, 7930
Gonzales, Samantha: 7540, 7735
Gonzalez, Amanda: 8010
Gonzalez, Anais Claire: 7210
Gonzalez, Andrea: 7000
Gonzalez, Hector: 7540
Gonzalez, Richard: 3540, 8025
Good, Mary K: 1395
Goodman, Claire: 4255
Goodman, Jeri: 4360
Goodman, Octavia K: 9025
Goodpaster, Bret: 1240, 4310
Goodrich, Grant: 2355
Goodwin, Laurance: 7440
Goodwin, Richard: 8000
Gooneratne, Nalaka: 7525

Gopalakrishnan, Nisha P: 8010
Gorbenko, Ksenia: 8010, 8010
Gorbunova, Galina: 7200
Gordon, Barbara: 7340, 7730
Gordon, Dawn L: 3100
Gordon, Emma: 1025
Gordon, Kate: 3565
Gordon, Kristina C: 8020
Gordon, Stacey: 2470
Gordon, Taryn: 3140
Gordon-Larsen, Penny: 7275
Gore, Janelle: 4175, 4390
Gore, John C: 7010
Gore, Shweta: 7720, 8020
Gorelik, Aaron: 8010
Gorla, Vandita: 8000
Goronzy, Jorg: 3160
Gorospe, Myriam: 7305
Gorzelle, Gregg: 7465
Gorzycki, Emily: 1265
Goss, Arianna P: 7200
Gossner, Jacob D: 4435, 7065, 7270, 8020
Gotch, Chad M: 7350
Gothard, Sarah: 1125, 3140, 7425
Gothe, Neha: 7545
Goto, Jun: 7420
Gou, Yang: 7315
Gould, Christine E: 1135, 2280, 3110, 7135, 7235, 7480, 7900
Gourley, Amy N: 7605
Gourley, Keone: 3205
Gouveia, Gillian: 8000
Goyal, Riya: 7425, 7905
Gozalo, Pedro L: 4460, 7200, 7295
Gozalo, Pedro: 8010
Grünjes, Carlotta E: 7535
Grabanski, Morgan: 8000
Grabowski, David C.: 8020, 8055
Grace, Rachel: 7360
Grady, Alexandra: 7100
Grady, James: 2495
Graf, Allyson S: 3450, 7345
Graf, Peter: 8000
Graff, Julie N: 7020, 7205
Graham, Cameron: 7335
Graham, Carrie L: 4510
Graham, David: 7260
Graham, Eileen K: 1570, 4375, 4480, 7455
Graham, Karen L: 2215
Graham, Laura A: 8010
Graham, Wan Chen K: 3545
Graham-Engeland, Jennifer E: 4405, 4515, 7210, 7455, 8020
Gramani-Say, Karina: 7225, 7655
Granbom, Marianne: 1285
Grande, Giulia: 4315, 7010
Grandy, Scott: 8000
Granger, Teresa Ann: 8015
Grant, Claire: 4200, 8020
Grant, Elizabeth: 8015
Grant, Jeremy G: 1495
Grant, Nicole: 4300
Gras, Laura Z: 7330
Graupner, Jeffrey: 2340, 8020
Gravenstein, Stefan: 7030, 8010
Gray, Jullie: 2495
Gray, Shelly: 7320
Green, Ariel R: 2085
Green, Bev: 1265, 9020
Green, David M.: 7205
Green, Dr. Robin R: 1365
Green, Gavin: 8020
Green, Krystal: 7350
Green, Makenna R: 8010
Green, Michael: 7625
Green, Natalie: 2470
Greenberg, Barry: 3070
Greenberg, Jeremiah J: 8015
Greenberg, Jonathan: 2315
Greenberg, Phyllis A: 7250
Greene, Barry: 7635
Greene, Celeste M: 8070
Greenfield, Annabelle: 1165
Greenfield, Emily A: 3340
Greenfield, Jennifer C: 2480
Greenwald, Blaine: 7695
Greenwood-Hickman, Mikael Anne: 1265, 7720, 8010, 9020
Gregg, Jeffrey J: 2280
Gregor, Neil: 7080
Gregorio, Mario: 7465, 8010
Gregory, Sarah: 7425, 7930
Greig, Erin E: 2510
Greiner, Chieko: 8015
Grenier, Amanda: 4275
Grewal, Kiren: 7200
Griffin, Hannah: 8010
Griffin, Joan M: 1475, 4415
Griffin, Rebekah: 7750
Griffith, Candace: 7030
Griffith, Christopher X: 7695, 8020
Griffith, Kathleen: 8000
Griffith, Lauren E: 7030
Griffiths, Leroy: 8015
Griffiths, Patricia C: 8020
Griggs, Stephanie: 2015, 7270
Grigorovich, Alisa: 3315, 4445
Grilli, Matthew: 8020
Grimes, Darla: 7145
Grimm, Kevin: 4065, 4500, 8040
Griner, Stacey: 7700
Grineski, Sara E: 7420, 7455
Grisales, Maria: 7425, 7630
Griswold, Michael E: 1000
Grittner, Alison L: 2395, 2560
Groden, Sheryl: 7155, 7740
Grodsky, Eric: 1095, 7230, 7350
Grol-Prokopczyk, Hanna: 7225
Gronlund, Carina: 2040
Grooms, Jevay: 8000
Groover, Kimberly: 1015
Gross, Alden: 1290, 2515, 3070, 3335, 4380, 4440, 7015, 7225
Grossberg, George: 8020
Groulx, Mark: 3205
Groves, Angela: 1465, 2330
Growney, Claire M: 1485, 1575
Grubbs, Allison: 1130
Grubbs, Brandon: 1265, 7075
Gruber-Baldini, Ann: 2015, 3495, 8010, 8095
Grudzen, Corita: 3010
Grudzien, Adrienne: 7540, 7735
Gruenewald, Tara L: 4375, 4505
Gu, Danan: 7155
Gu, Yian: 1025, 1580
Guan, Ting: 7020
Guan, Weihua: 7010
Gubner, Jennie M: 4480
Gubrium, Aline: 2555
Gudnason, Vilmundur: 2040
Gudzune, Kimberly: 7710
Guerbaai, Raphaelle-Ashley: 4255
Guerra, Gabriella: 2565
Guest, M. Aaron: 2245, 2455, 3080, 3560, 4040, 4320, 8000, 9000
Guevara, Nathalie: 2520
Gugliucci, Marilyn R: 2490, 3195, 8010
Guha, Deyaan: 8000
Guidarelli, Carolyn: 7205
Guidry, John A: 7110
Guilcher, Sara: 8015
Gujjala, Vikas A: 7005
Gumikiriza, Joy Louise: 1190
Gunsalus, Paul: 7235
Gunzler, Douglas: 7235, 7690, 8000
Guo, Crystal: 4340
Guo, Guifang: 1355
Guo, Jia-Wen: 4520
Guo, Man: 4110, 7235, 7700, 8050
Guo, Niting: 4065
Guo, Xiufang: 7425
Guo, Yingqi: 3200
Guo, Yu: 3270
Guo, Yuxiang: 8015
Gupta, Aashish: 9035
Gupta, Avinash: 8010, 8020
Gupta, Ayush: 8015
Gupta, Ravi: 7470
Gupta, Shivani: 1425, 1550, 7510
Gupta, Sonu Kumar: 7210
Gurchiek, Reed D: 8015
Gurlu, Merve: 3050, 8015, 8020
Gurung, Shreemouna: 2560
Gurupur, Varadraj: 8015
Gurven, Michael: 7010
Gurvich, Olga: 1265
Gur-Yaish, Nurit: 4075, 7265, 7400, 7920
Gusmano, Michael K: 1360
Gustafson, Deborah: 8010
Gustafson, Sally: 7455
Gustat, Jeanette: 8020
Gustave-Schelling, Kyla: 7075
Gustavson, Allison M: 3130, 4415
Gustavson, Daniel: 8020
Gutierrez, Angela: 1045, 1495, 3475, 4535, 7535, 7540
Gutierrez, Eduardo: 7000
Gutierrez, Jose: 8085
Gutierrez, Mariela: 1170
Gutierrez, Sirena: 7235, 9000
Gutierrez, Victoria: 8010
Gutierrez-Ramirez, Paulina: 4130, 7270, 7660
Gutman, Gloria M: 7155, 7405
Gutman, Roee: 3240
Gwathmey, TanYa M: 1315

### H

Ha, Jung-Hwa: 1375, 4070
Haas, Jennifer: 8015
Haase, Claudia: 3355

Haber, Abigail: 8000
Håberg, Asta K: 2425, 7060
Haberstroh, Julia: 1555
Hacker, J David: 4035
Hacker, Sarah D: 7080
Hackett, Katherine: 3250, 7630
Haddad, Yara: 1235
Hadidi, Niloufar N: 1265
Hadlandsmyth, Katherine: 7205
Hadley, Lesca: 1065
Hadsell, Killian: 7680
Hadson, Kim: 8010, 8045
Hafner, Camryn: 2175, 7480
Hagaman, Ashley: 8010
Hagemann, Lauren: 4060, 4330
Hagen-Lillevik, Synneva: 2060
Hägg, Sara: 7010
Haggar, Katherine: 3190
Haglund, Kristin: 2555
Hahm, Bridget: 2145
Hahne, Jessica: 7055
Hahn-Goldberg, Shoshana: 8010
Haigh, Sylvia: 7250
Haim, Todd: 1500
Haines, Terry P: 2145, 4100
Hair, Nicole: 1280
Haire, Cameron: 7205
Haitsuka, Kilohana: 8010
Haji Hosseini, Sogol: 2395, 4320, 7155
Hajjar, Emily R: 7020
Hakanson, Megan: 1520
Hakian, Mia: 8000
Halasz, Ildiko: 3335
Halaszynski, Jaime: 8015
Haldar, Srabani: 3165, 7065
Halder, Puja: 3055
Hale, Jo: 3530
Haley, William: 2370, 3495, 7065, 7070, 7140
Halilaj, Eni: 8000
Hall, Charles B: 4185
Hall, Katherine S: 2345, 7080, 7155
Hall, LaToya N: 2155
Hall, Lauren: 2430, 3485, 7045
Hall, Leighanna N: 7000
Hall, Lisa C: 7480
Halloway, Shannon: 7615
Halpern, Scott D: 8015
Halpin, Sean N: 2445
Halvorsen, Cal: 2425, 3310, 4140, 4320, 7355, 8020
Ham, Carolyn R: 4510
Hamada, Fumiko: 8020
Hamann, Kate: 1465
Hamel, Kate: 1050
Hameleers, Niels: 7295
Hamer, Mark: 1245
Hamers, Jan PH: 2140, 4255, 7295, 7685, 8015
Hamill, Randi: 8010
Hamilton, Jill B: 8020
Hamilton, Karyn L: 7075
Hamilton, Lucas J: 4295, 9015
Hamler, Tyrone: 7700, 7935
Hamlin, Abbey M: 9000
Hamm, Jeremy M: 1525, 7030, 7455, 8020
Hammad, Suzanne: 1155
Hammers, Dustin: 8000
Hammill, Bradley G: 7625
Hampton, Marsha: 2055, 8020
Hamrick, Mark: 7285
Han, Chengming: 1170, 7140, 7420
Han, Duke: 1370, 2350, 3220, 7495, 7930
Han, Gang: 4130, 4340
Han, Hae-Ra: 1440
Han, Jayoung: 8015
Han, Jimin: 7275, 8015
Han, Ling: 7060
Han, Sae Hwang: 1405, 4400, 7690, 8020
Han, Shuo: 3470
Han, Soojeong: 8015, 8040
Han, Sunghei: 8010
Han, Xianlin: 4170
Han, Yang: 7030
Hanaoka, Hideaki: 7270
Hancock, Cynthia: 8010
Hancock, David W: 7205, 7405
Hancock, Jeffrey T: 8020
Hancock, Kevin: 2480
Hancock, Laura M: 4300
Hand, Brittany: 4475
Hand, Michelle D: 2370, 4540
Handa, Satoru: 1505
Handique, Swasati: 7005, 8010
Handra, Amy: 7265
Handy, Todd: 7720
Hanes, Douglas W: 3015, 4450, 7100
Hanna, Karim: 1355
Hannan, Marian T: 2080
Hansen, Abigail: 3185
Hansen, Jared: 2545
Hansen, Maria Swennergren: 4075
Hansmann, Kellia J: 7160
Hanson, Angela: 8000
Hanson, Emily J: 8000
Hanson, Laura: 8015
Hanson, Leah R: 7455
Hao, Qiukui: 7655, 7720
Hao, Ying: 8000
Happ, Mary Beth: 2415
Happel, Christine: 2070, 3080
Haque, Sabrina: 7155
Harada, Ann SM: 1390
Harada, Ko: 7260
Harding, Rachel: 8010
Hardy-Smith, Oliver: 2330
Harkins, Kristin: 8015
Harrell, Erin R: 1045, 7750
Harrington, Charlene: 8015
Harrington, Christina: 3315
Harrington, Erin E: 4280, 4515, 7200, 7455, 8020
Harrington, Laura B: 8010
Harris, Christina: 7350
Harris, Daniel: 7030
Harris, Jenny: 2465
Harris, John: 7510, 8010
Harris, Katherine: 9030
Harris, Maude: 7440, 7935
Harris, Rebekah: 3335
Harris, Robin: 7720
Harris, Steven: 1020
Harrison, Sean Lee: 7145
Harrison, Tracie C: 7700
Harris-Speight, Jasmine: 3575
Hart, Dani: 2555
Hart, Julia: 8010
Hart, Laura A: 1435
Hart, Sara E: 3465, 7065
Hartley, Gregory W: 7330
Hartmann, Christine W: 1070, 3240, 7110, 7250, 7420, 7685
Hartnell, Lisa M: 8000
Hartung, Johanna: 4185
Haruo, Emy: 1470
Harvey, Idethia Shevon S: 7440, 7935
Harvey, Kelsey: 3315
Harvey, Shanelle: 7055
Hasche, Leslie: 4115, 7650, 7720
Hasegawa, Nanako: 8015
Hash, Kristina M: 8000
Hashizume, Yumi: 7125
Haskins, Anne: 7010
Haslam, Lynn: 9040
Hasnani, Madison: 7440
Hass, Zachary: 2245
Hassell, Susan J: 4450, 7545
Hassenstab, Jason: 4515
Hasten, Lauren N: 7545, 7905
Hastings, Susan N: 1130, 2145, 2180, 7685
Hasworth, Serena: 3560
Hata, Hiroshi: 8015
Hata, Toshiki: 7275
Hatami, Fatemeh: 3455
Hatanaka, Sho: 8015
Hatch, Maddy: 7110
Hathaway, Wendy: 7730
Hatten, Christina: 7695
Haunch, Kirsty J: 4255, 7295, 7520
Hauptschein, Max: 2310
Hausdorff, Jeffrey: 1235
Haverhals, Leah M: 3485, 8015, 9025, 9040
Hawes, Frances: 3450
Hawks, Cate: 3565
Hawks, Zoe W: 4515
Hawley, Chelsea: 2345
Hawthorne, Timothy L: 2300, 7025
Hayajneh, Audai A: 7435
Hayashida, Cullen T: 7145
Hayat, Yumna: 7465, 7910
Hayden, Kathleen M: 1390
Hayek, Roee: 1245, 2240
Hayes, Kaley N: 7030
Hayes-Larson, Eleanor: 1545, 7535
Haynes, Laura: 1580
Hayslip, Bert: 7265
Hayslip, Natalie: 8000
Hayward, Mark D.: 3370
Hayward, Matt: 8085
Hazelett, Susan: 7440
Hazlewood, Hudson: 7470
Hazuda, Helen: 8015, 8050
He, Chanchan: 7630
He, Daqing: 1355, 2355
He, Haiyan: 2255, 2500, 4055
He, Jasmin: 2150, 4365
He, Leo: 3415
He, Lingxiao: 1365, 2500, 7305
He, Ranran: 7070
He, Rendong: 7695, 8015
He, Xiaona: 7030
He, Xin: 7060
He, Yao: 4070
He, Zhe: 1355
He, Ziyi: 3480
Head, Denise: 7720
Heath, Sarah: 7720
Heaton, Felicia: 2140
Hebblethwaite, Shannon: 3320
Hebert, James: 2080, 7315

Hebpurn, Kenneth: 2295
Hecht, Crystal A: 7605
Heckhausen, Jutta: 4525
Hedayati, Niloofar: 4320
Heffner, Kathi L: 2315, 3045, 9010
Heidenreich, Paul: 9025
Heimler, Stephanie R: 4170
Hein, Maria: 7650
Heinz, Melinda: 1385, 7265, 7350
Helbostad, Jorunn L: 2170
Heldin, Paraskevi: 8000
Heldreth, Courtney: 3315
Helgertz, Jonas: 4035
Helmly, Victoria: 3420
Helsabeck, Nathan P: 7490, 7935
Hemmy, Laura: 1485, 7145
Hemrajani, Girish: 8010
Henderson, Alexandria: 9030
Henderson, Charles: 4060
Henderson, Wendy: 8015
Hendricksen, Meghan: 7420
Hendricks-Ferguson, Verna: 4080, 7520
Hendrix, Cristina: 4450, 7270
Hendry, Anne: 7215
Hengerer, David: 1370
Heniff, Erin M: 8035
Henion, Amy: 7630
Henke, Maria LG: 1005, 2250
Henneman, Lucas: 4035
Henning-Smith, Carrie: 4105
Henricks, Evan: 2535
Henry, Kyle: 7155
Henry, Maureen: 1200
Hensley, Robert B: 7115
Hentges, Rochelle: 4085
Hentnik, Haley B: 8015
Heo, Jinmoo: 3050, 4505, 7070, 7280, 8080
Heo, Seok-Jae: 8015
Hepburn, Kenneth: 1125, 2295, 3045, 4165
Hepple, Russell T: 2080
Herd, Pamela: 1280, 3225
Herman, Hattie: 2570
Hermann, Carla P: 4335
Hermanns, Melinda: 4240
Hernández, Belinda: 8060
Hernández de Jesús, Alma: 3175, 4450, 7325
Hernández Gamboa, Rebeca: 1535
Hernandez, Beatriz: 7715
Hernandez, Bianca: 7425, 7905
Hernandez, Christopher: 4520, 8020
Hernandez, Elise: 3490
Hernandez, Elizabeth: 7410, 7900
Hernandez, Ester: 8000
Hernandez, Fernando: 7470
Hernandez, Frank: 7005
Hernandez, Herb Howard C: 8015
Hernandez, Jessica A: 1510
Hernandez, Jessica: 1510
Hernandez, Raymond: 2465
Hernandez, Rolando: 7210
Hernandez Arciga, Ulalume: 8000
Hernandez Cervantes, Laura E: 7010
Hernandez-Ochoa, Erick: 7305
Hernandez-Tejada, Melba A: 2520
Herpich, Catrin: 7315
Herpst, Jessica L: 7005, 7465
Herreid-O'Neill, Anders: 8010
Herrick, Tamara H: 3565
Herrig, Shelby: 3215
Herrmann, Francois R: 1220
Hertel, Johannes: 7010
Hertzog, Christopher: 7225
Her-Xiong, Youhung: 4445, 7600
Herzog, Monica Anderson: 4060
Heselton, Hyeon Jung (Judith): 8020
Hesgrove, Brandon W: 7545
Heston-Mullins, Jennifer: 7435
Hetherington-Rauth, Megan: 7060
Heuer, Sierra: 8025
Hewitt, Elizabeth: 8010
Hews, Ellis J: 8020
Hewson, Jennifer: 3465
Heyn, Patricia C: 1300, 2455, 3460, 3505
Heys, Jodi L: 7065
Hiatt, Shirin O: 7440
Hicken, Bret L: 2430
Hicken, Margaret: 7350
Hickman, James: 7425, 7630
Hickman, Ronald L: 2015
Hickman, Susan: 4440, 8010, 9040
Hicks Patrick, Julie: 8000
Higbie, Anna: 8020
Highberg, Nels P: 2175
Hilderbrand, Zoey: 8070
Hilgeman, Michelle M: 1070, 1080, 1210, 3240, 7250, 7685
Hill, Carl V.: 3180, 3475
Hill, Cristal: 8000
Hill, Heather: 7120, 8010
Hill, Jordan R: 7005
Hill, Julianna: 7480, 7900
Hill, Kyrsten C: 4405
Hill, Kyrsten: 8000
Hill, Linda: 7080
Hill, Nikki: 1525, 4280, 7630
Hill, Patrick L: 8000, 8020, 8080
Hill-Ashford, Yolanda: 7030
Hilmer, Sarah N: 3520
Hilsabeck, Robin C: 1355, 2355
Hiltz, Sydney: 7720
Himes, Dani: 2185, 8010, 8010, 8020
Himes, McKenzie: 7200
Himmelfarb, Cheryl Dennison: 7440, 7500
Himmelsbach, Ines: 7480
Hinderberger, Brian: 3285
Hinrichs, Raegan: 7000
Hinrichs-Kinney, Lauren: 2135
Hinson, Justin: 3190
Hinton, Allyshia V: 8015
Hinyard, Leslie: 4080
Hirano, Hirohiko: 7205, 7540, 8015
Hirdes, John P.: 7620
Hirsch, Ben: 3485
Hirsch, Bruce: 8010
Hirsch, Jana A: 3375, 8020
Hirsch, Jen: 3370, 8010
Hirschman, Karen: 3300, 3385, 7435, 7535, 8010
Hirsch-Matsioulas, Orit: 3465, 4040
Hisamatstu, Yasushi: 7400, 7920
Hites, Kenneth W: 2175
Hiyama, Atsushi: 8015
Hladek, Melissa deCardi: 2010, 3150, 4420, 8000
Hladyshau, Siarhei: 8000
Ho, Alvin K K: 4390
Ho, Anita: 2565, 4435
Ho, Cheuk Yin: 4195
Ho, Chin Wei: 7155
Ho, Dorian: 2180
Ho, Rainbow Tin Hung: 2520, 7725
Ho, Simon: 8010
Ho, Tuong-Vi: 2205
Ho, Yuen Wan: 7725, 8020
Hoban, Luke: 7290
Hoch, Jackson: 8010
Hochberg, Marc: 7010, 7440
Hochman, Yael: 3465, 4040
Hodes, Richard J: 1340, 2210
Hodgins, Maddison L: 8000
Hodgson, Nancy: 3215, 4200, 7525, 8015, 8020
Hoel, Sydney: 4530, 7005, 7270, 8020
Hoeppner, Bettina B: 2315, 3250
Hof, Patrick R: 8000
Hoffarth, Phillip: 8020
Hoffman, Adar: 1220
Hoffman, Elise V: 2450
Hoffman, Geoffrey: 7030
Hoffman, Jessica: 8000
Hoffman, Rashelle: 2360, 3285, 8060
Hoffman, Yaakov S: 1565
Hofstra, Emma: 8010
Hogan, David B: 3465, 7030
Hohman, Timothy J: 7015
Holaday, Louisa: 7535
Ho-Lastimosa, Ilima: 8020
Holben, Rachel: 7695
Holcombe, Sven A: 1435
Holden, Erika: 9020
Holden, Richard J: 2410
Holland, Alexandra: 7025
Holland, Carol A: 3060
Holland, Diane: 1475, 4415
Holland, John: 3100
Holland, Sydney N: 8000
Hollandsworth Reed, Jenn: 4235
Holley, Lyn M: 1310, 2175, 3345, 3585, 8010
Hollingshaus, Mike: 4520
Hollingsworth, Holly: 7260
Hollis, Linda: 1050
Hollman, Nicholas: 7330
Hollmann, Peter: 7200
Holloway, Jeremy C: 2130
Holm, Johanna B: 7210
Holman, E. Alison: 7030
Holmes, Holly M: 3135
Holmes, Rachel: 7210, 7715
Holmes, Sarah D: 1070, 2000, 3255, 8020
Holt, Nigel: 8015
Holteng, Lise Birgitte: 8015
Holtzer, Roee: 1150
Homann, Jan: 7030, 7210

Homayouni, Ramin: 8010, 9050
Hommel, Erin: 7400, 7920, 8015
Honda, Ayumi: 7030
Honda, Sumihisa: 7030
Hong, Chenlu: 4065
Hong, Dahye: 7080, 7275, 7630 8015
Hong, Dongfang: 7350, 7735
Hong, Keung-Ja: 7460
Hong, Michin: 3545, 7425, 7630, 7935
Hong, Philip: 7545
Hong, Sungjae: 4420, 8020, 9050
Hong, Yinglu: 4535
Honig, Lawrence S: 1025
Honore, Chelsea: 7425, 7630
Hood, Michelle M: 1365
Hoodin, Derek: 1015
Hoogendijk, Emiel O: 2230
Hoover, Julia: 7210
Hooyman, Andrew: 7325
Hope, Peter: 4300, 7150
Hopfer, Elizabeth: 8010
Hopkins, Lauren: 7735
Hopp, Faith Pratt: 8010
Hopper, Shawna: 7720
Hoppmann, Christiane: 3480, 7535, 7725, 8000, 8020
Horbal, Steven R: 1435
Horhota, Michelle: 1010
Horiuchi, Takashi: 8015
Horn, Amanda J: 3170
Horn Mallers, Melanie: 7475
Horne, Kensley M: 4370
Horowitz, Chloe S: 1525
Horstman, Molly: 4340, 4415, 7680
Hort, Jakub: 8000
Horwitz, Shelley: 3265
Hoselton, Jill H: 2560
Hosking, Diane: 3185
Hossain, MD Sarafat: 7055
Hossain, Mohammad S: 7065, 7215
Hossain, Mohammad D: 8075
Hossain, Sayeem: 7000
Hotz, V. J: 4050
Hou, Erin: 7110
Hou, Su-I: 2100, 7460, 7925, 8010, 8015
Hou, Zhaoyi: 7425, 7930
Hougaard, Benjamin T: 8000
Houge, Angela: 7410
Hough, Katrina A: 2520
Houlihan, Daniel: 7510
Houmard, Joseph: 1410
Houston, Denise K: 1390, 1410, 2505, 7710, 8015
Hoverman, Adam: 8020
Hovland, Cynthia A: 7040, 7475, 7900
Howard, Annie Green: 7275
Howard, Brittni: 8010
Howe, Clarissa: 7455
Howe, Judith L: 2280, 2345, 4150
Howe, Matthew: 7040
Howe, Melissa J K: 8010
Howe, Rebecca: 8000, 8010
Howell, Britteny M: 7480
Howell, Cynthia: 3170
Howington, Marshall: 4005
Howlett, Susan E: 3520, 8000
Hoy-Ellis, Charles P: 2420, 4495
Hoyt, Margaret: 8010
Hren, Katie: 7350
Hristidis, Vagelis: 4060
Hsieh, Jenny Yi-Chen: 7625
Hsieh, Jessica: 4365, 7480, 7900
Hsieh, Katherine K: 8015
Hsiung, Ging-Yuek R: 3335, 8015
Hsu, Chun-Liang: 1150, 7720
Hsu, Clarissa: 3010, 4230
Hsu, Erh-Chi: 8010, 8015
Hsu, Fang-Chi: 2505, 7710, 8000
Hsu, Hui-chuan: 7735
Hsu, Jean W: 2235
Hsu, Joanne: 7050
HSU, Sheng-Chin: 8010
Hsu, Wan-Chen: 7540
Hsu, Wanhsiang: 3255
Hsu, Wan-Ling: 7465
Hu, Elise: 8020
Hu, Grace: 7750
Hu, Jing: 8010
Hu, Jinxiang: 7635
Hu, Luxin: 1190, 7265
Hu, Mengyao: 8025
Hu, Mingyue: 1245
Hu, Qijia: 7005
Hu, Rita X: 2305, 2475, 3040, 3400
Hu, Susan C.: 7540
Hu, Ting: 7120
Hu, Xiuying: 1355
Hu, Yi-Ling: 8015
Hu, Yu: 1435
Hu, Yuanyuan: 7145
Hu, Yu-Ching: 7525
Hu, Yue: 3480
Hu, Yuexia: 1090
Hu, Zhan: 7605
Hua, Alice: 8020
Hua, Cassandra: 1535, 2185, 8010
Hua, Yindong: 3415
Huan, Tianwen: 8020, 8055
Huang, Alison: 1055, 2035, 8015
Huang, Alison J: 1245
Huang, Andrew W: 1530
Huang, Bin: 2190, 8015
Huang, Chenshan: 7450, 7930
Huang, Chenxi: 1115
Huang, Chongmei: 2040, 2255, 2500, 3330, 4055, 7440, 7440
Huang, Feng: 7275, 7710
Huang, Helen: 8015
Huang, Hsien-Hao: 8000
Huang, Liming: 4200, 7665
Huang, Min-Hui: 8010
Huang, Nuan-Ching: 7540
Huang, Qiyuan: 7670
Huang, Ro: 1475, 4415
Huang, Rui: 8015
Huang, Siou-Tang: 7670
Huang, Tianyi: 9020
Huang, Weidong: 1355
Huang, Wei-Jia: 7275
Huang, Xianging: 8000
Huang, Xiaoming: 7710
Huang, Yan: 7145
Huang, Yi-Jia: 7280
Huang, Yiqing: 7625
Huang, Zewen: 3480, 7255
Huang, Zideng: 2050
Hubbart, Chase: 4310
Huber, Lesa: 3530
Huberty, Jen: 1345
Hubig, Nina: 2355
Hubner, Sarah: 7675
Huckfeldt, Peter J: 1390
Hudes, Seth: 7720
Huebschmann, Amy: 8020
Hueluer, Gizem: 4185, 4525, 7355, 7535
Hugenschmidt, Christina: 7490
Hughes, Jaime M: 2145, 2475, 7685
Hughes, MacKenzie L: 7225
Hughes, Mark: 2540
Hughes, Michelle: 7440
Hughes, Tiffany F: 1485, 7145
Hughes, Timothy: 2220, 3180, 7010, 7015, 8020
Huh, Jimi: 4375
Huh-Yoo, Jina: 4220
Hui, Katherine: 7320
Huijsman, Robbert: 7445
Huisingh-Scheetz, Megan: 2570, 3150, 7205
Hule, Sunil S: 7430
Hulko, Wendy: 4025, 8010
Humber, Marika B: 3110, 7135
Humbert, Andrew: 7080
Hundza, Sandra: 7260, 7525
Hung, Arthur: 7020
Hung, Lillian: 1115, 2410, 2530, 3205, 7465, 7510, 7750, 7910, 8010, 8015, 8020
Hung, William: 2430, 8010
Hunt, Angela: 8015
Hunt, Lauren: 2020, 4080
Hunter, Heather: 7700
Hunter, Paulette V: 4195, 7055
Hunter, Ruth: 3125
Hunter, Sarah: 3170
Huo, Meng: 7005
Huo, Qun: 8015
Huo, Shutong: 2020
Huo, Zhiguang: 1445
Huot, Suzanne: 7300
Huppert, Theodore J: 2510
Hurd, Laura C: 7300
Hursh, Colleen: 2345
Hurtt, Barbekka: 7720
Huseth-Zosel, Andrea: 7090
Husser, Erica: 7410
Huth, Megan K: 1030
Hutto, Lashawn: 1465, 7350
Huxhold, Oliver: 2025, 4525
Huyen, Do Thi Thu: 7355, 8015
Huynh, Duong: 8015
Huynh, Mia: 7005
Huynh, Stephanie: 8025
Huynh, Tai: 8010
Huynh, Thinh: 7720
Hwan Chung, Tae: 7305
Hwang, Billie: 7625
Hwang, Boram: 7430
Hwang, Jeongeun: 8015
Hwang, Mina: 7235
Hwang, Misun: 7360
Hwang, Phoebe W: 8020
Hwang, Sinwoo: 7715, 7750
Hwang, Ula: 8010
Hwang, Yeji: 7235, 7435, 7630

Hyde, Eleanor R A: 4490
Hyde, Rebecca: 7055
Hyer, J. Madison: 4475
Hyer, Madison: 2445
Hyun, Jinshil: 7145, 7225
Hyun, Kate: 7155

**I**

Iaboni, Andrea: 4445, 9040
Iacob, Eli: 3500, 4520, 7425, 7905
Ibrao, Miquela: 7055
Idler, Ellen: 7530
Idu, Abisola: 8010
Iemoto, Naoki: 8015
Igarashi, Heidi: 7030
Igawa, Hitoshi: 7095
Igbokwe, Beatrice Chinwe: 4000
Igbokwe, Chima Charles: 4000
Ignácio Sossai, Mariana: 7225, 7655
Ihara, Emily S: 2415, 3510, 4540, 7270
Ihara, Kazushige: 7205, 7540
Iizuka, Ai: 1265, 7680
Ikebe, Kazunori: 7105, 7115
Ikeda, Naoki: 4135
Ikesu, Ryo: 1545, 7535
Ikeuchi, Tomoko: 7145
Iktilat, Khalil: 7695, 9040
Ikuta, Kasumi: 7510
Ilardo, Joan L: 7105, 7640
Iloputaife, Ike: 4535, 7450, 8000
Iluobe, Victory: 7000
Ilyas, Nazish: 4100
Im, Eun-Ok: 3370, 7270
Im, Jennifer: 8010
Im, Sua: 3050, 7280
Imamura, Keigo: 7205, 7540
Imms, Phoebe: 4310
In Der Schmitten, Jürgen: 2255
Inagaki, Hiroki: 7350, 7640
Indran, Nicole: 1425, 3015, 7155
Infurna, Frank J: 1190, 1450, 4065, 4500, 7265
Inger, Jacob: 7680
Ingle, Pilar: 7650
Ingram, Cory: 4415
Ingvalson, Stephanie: 8020
Inoue, Kosuke: 7535
Inoue, Megumi: 2370, 2415, 4190, 7095, 7270
Inouye, Sharon K: 4530
Intrator, Orna: 2430, 2545, 8020, 8055
Iob, Eleonora: 2365
Irani, Elliane: 2015, 7270
Irimia, Andrei: 1580, 4310
Irish, Stephanie: 7425
Isacson, Michal: 7695
Isales, Carlos: 7210
Isenberg, A. Lisette: 8015
Isenberg, Avital: 4530
Ishii, Keiko: 7340
Ishitake, Tatsuya: 7400, 7920
Ishiwata, Eric: 3575
Ishizaki, Tatsuro: 7115
Islaya, Cesz: 7490
Isola, Jose: 4310
Ito, Daisuke: 8020
Ito, Hiro: 4435
Ito, Kae: 7640
Ito, Koki: 7680
Itoh, Sakiko: 7750
Itotani, Keisuke: 7420
Iveniuk, James: 1110, 3410
Ivey, Kimberly: 4510
Iwarsson, Susanne: 1285, 3170, 7155
Iwasaki, Michiko: 7150
Iyer, Gitanjali: 1015
Iyer, Sandhya: 8010
Izumi, Seiko: 7440

**J**

Jaber, Sausan: 8000
Jablonski, Rita: 2375, 4045, 7525 8000
Jacelon, Cynthia: 7630, 8015, 8020
Jacklin, Kristen: 2335, 2395
Jackson, Ajani: 8020
Jackson, Alexandra: 2295
Jackson, Barbara: 8020
Jackson, Christian: 7350
Jackson, Christie: 4160
Jackson, Christine R: 4040
Jackson, Eva MJ: 7425, 7910
Jackson, Gary B: 4040
Jackson, Grace: 7010
Jackson, Heide: 7040
Jackson, Kathryn: 1570, 3220, 7455
Jackson, Kelley: 7360
Jackson, Kimethria L: 2550
Jackson, Lynn: 3370, 7465
Jackson, Phronie: 7030
Jackson, W. Clay: 8020
Jacob, Alexandra E: 7285
Jacob, Riya: 3010
Jacobs, Jeremy M: 7060
Jacobs, Josephine C: 2190
Jacobs, Leslie: 7425, 7930, 8070
Jacobs, M. Lindsey: 1210, 7070, 7455, 7610, 8015
Jacobs, Moyosoreoluwa: 7630
Jacobsen, Erin: 1485, 7145
Jacobson, Mireille: 7630
Jacova Chenoweth, Claudia: 1020
Jadczak, Agathe Daria: 7275
Jaeggi, Susanne M: 4490, 8000
Jaen, Carlos: 8050
Jafari, Mahtab: 7525
Jahoor, Farook: 2080
Jain, Abhinav: 3160
Jain, Felipe A: 4130, 7270, 7660
Jain, Jennifer C: 8020
Jain, Paavani: 8090
Jain, Shalini: 7210, 8015
Jajko, Shannon: 2450
Jakicic, John M: 1390
Jalil, Tanjina: 3365
Jallorina, Bianca: 7210
Jaman, Rafeeul: 8015
James, Bev: 3200
James, Bryan: 1570, 7205
James, Clara E: 4490
James, Dara L: 3015, 4010, 7285
James, Jennifer: 3420
James, Shirley A: 7480
Jan, Muhammad Tanveer: 7610
Jandric, Jakov: 2425
Janes, Heather: 7480, 7900
Janevic, Mary: 1155, 7030
Jang, Ah Ram: 7095
Jang, HaeJin: 8020
Jang, Heejung: 1525, 7630
Jang, Il-Young: 7305
Jang, Jae Young: 8000, 8010, 8015
Jang, Jeein: 2205, 3545, 7350, 8015, 8020
Jang, Jieun: 3135
Jang, Jinwoo: 7360, 7425
Jang, Jiwon: 8000
Jang, Soeun: 7155, 7340, 8010
Jang, Soog-nang: 7275
Jang, Woo: 8085
Jang, Yuri: 1140, 3440, 7640, 8000, 8020
Janke, Megan: 7545
Jankowski, Catherine: 7360
Jannati, Ali: 7425, 7635, 7930
Jansen, Carl-Philipp: 2170
Jansen, Taylor: 2480, 3265, 4105, 7065, 7240, 7635, 7695, 7700
Janssen, Leah M: 4510
Jantzi, Micaela: 7620
Jao, Ying-Ling: 7040, 7200, 8015, 9015
Jarke, Juliane: 3315
Jarmul, Cynthia: 4330
Jaros, Maria: 7650
Jarrín, Olga F: 1380, 4485
Jarrott, Shannon E: 2130, 2270, 3265, 7510
Jarvis-Powers, Robin: 7425
Jaskson, Christie: 4160
Jasper, Heinrich: 2310
Jasper, Lisa: 8010
Javier, Noelle Marie Coloma: 8010
Jawadi, Sagrika: 1370
Jayakody, Oshadi: 7750, 8000
Jazwinski, S. Michal: 7225
Jeamjitvibool, Thanakrit: 1505
Jean Jacques, Wildjena: 7110
Jeanblanc, Alexandra: 7265, 7660
Jee, Young Min: 8020
Jefferson, Rebeca C: 1505
Jeffroy-Meynard, Anne-Celine: 7200
Jehu, Deborah A: 8015
Jen, Sarah: 4250, 8010
Jenkins, Emerald: 1225, 2010, 2230
Jennings, Lee: 7480
Jennings, Stephen: 7080, 7155
Jensen, Christy J: 4470
Jensen, Leif: 4105
Jensen, Megan: 8015
Jensen-Battaglia, Marielle: 7420, 7920
Jeon, Ashley: 7010
Jeon, Gyeahyung: 4265
Jeon, Jaemin: 7695
Jeon, Sangha: 1350
Jeon, Yein: 8015
Jeong, Chung Hyeon: 7030
Jeong, Haelim: 7440
Jeong, Jihyeong: 7240
Jeong, Mijin: 7630
Jeong, Sol-ah: 8015
Jesmin, Syeda S: 1485, 7125
Jester, Dylan: 2185
Jeung, Jihong: 7035
Ji, Linying: 1250, 2055
Ji, Meihua: 7410
Ji, Minghui: 8000

Ji, Soo-Yeon: 7630, 7935
Ji, Sunghwan: 7305
Ji, Xiayan: 7145
Jia, HongFei: 7520, 7910, 9045
Jia, Jie: 7630
Jia, Lile: 8020
Jia, Shuli: 8015
Jia, Xueqing: 8015
Jiang, Da: 2520, 3480, 7255, 7725, 8000, 8020
Jiang, Jiabei: 7035, 7520
Jiang, Kening: 1055, 2525
Jiang, Lin: 1335, 2355
Jiang, Nan: 3305, 3540, 7235, 7630
Jiang, Nisi: 4310
Jiang, Shuangke: 4490
Jiang, Xiao-han: 1560
Jiang, Xinye: 7710
Jiang, Yan: 7425, 7930
Jiang, Yanping: 1380, 3245, 3440, 4365, 8020, 8050
Jiang, Yun: 7360
Jiang, Zhe: 4060
Jiangqiong, Li: 7465
Jimenez, Andreina: 8035, 8070
Jimenez, Daniel: 7540, 7735
Jiminez, Marcia P: 3180
Jin, Benjamin: 8000
Jin, Guoping: 1170
Jin, Hao: 3270
Jin, Haomiao: 2465
Jin, Liangyi: 3505
Jin, Qiao: 1355
Jin, Shiow: 7060
Jin, Tingan: 8010
Jin, Tongtong: 7265
Jin, Xianglan: 7465
Jin, Xiaotong: 7470, 7905
Jin, Yaping: 7440
Jing, Liwei: 7280
Jing, Xia: 2355
Jivraj, Stephen: 1450
João Forjaz, Maria: 4045
Joannette, Tania Valentine: 7645
Joblin, Celie: 7490
Johanning, Jason M: 3435
Johansson, Linda: 4010, 4335, 8015
John, Karen: 3345
John, Samantha: 7240
Johns, Michelle: 1110
Johnson, Alisa J: 1445
Johnson, Alisha H: 1530, 9025
Johnson, Benjamin B: 7605
Johnson, Eileen: 1090, 1240, 2220
Johnson, Elma B: 1260, 2155, 4235, 7640
Johnson, Florence U: 7665
Johnson, Ian M: 3170, 8080
Johnson, Jada: 1075
Johnson, Julene K: 7085
Johnson, Keith: 7500, 7930
Johnson, Khrystal: 8010
Johnson, Kim G: 7425, 7930, 8070
Johnson, Kimson E: 7630, 8020
Johnson, Maddie: 1260
johnson, mallory l: 8015
Johnson, Melanie L: 1485
Johnson, Michael P: 4460, 8010
Johnson, Nina: 4440
Johnson, Richard W: 2420
Johnson, Shawn M: 7465
Johnson, Theodore M: 1430
Johnson, Thomas: 4215
Johnson, Yuuko M: 2200
Johnson Nicholson, Meneka C: 1385
Johnson-Jennings, Michelle: 4110
Johnson-Toliver, Stephanie: 3140
Johnston, Elizabeth: 7060
Johnston, Joseph: 8010
Johnstone, Sara Lynn: 8010
Joiner, Raquael J: 3165
Jokisch, Mario R.: 7680
Jolliff, Anna: 7135
Jones, C. Allyson: 8010
Jones, Christine D: 4460, 8010
Jones, Clarence: 1265
Jones, Damon: 7145
Jones, David: 7520
Jones, Jennifer M: 7140
Jones, Liz: 4255
Jones, Nancy: 4215
Jones, Paige: 1535
Jones, Patricia: 1340, 2475
Jones, Richard: 3335, 4380
Jones, Sarah E: 4270, 7660
Jones, Shannon: 1295
Jones, Valerie: 4205
Jones Weiss, Deborah: 8020
Jones-Cobb, Brittany R: 2260
Jones-Locklear, Jennifer: 8015, 8020
Jonsson, Oskar: 3170, 7155
Jonsson, Palmi: 2040
Joo, Jinhui: 2315
Joo, Susanna: 7360
Jopp, Daniela S: 1220
Jordan, Aydan: 8000
Jordan, Claire: 1575
Jordan, Jacqueline: 1230
Jordan, Kerry J: 7040
Jor'dan, Azizah: 4535
Jordre, Becca: 8015
Joseph, Rodney P: 8020
Joshi, Sama: 8020
Joya, Mikaela: 7210
Ju, Catherine H: 7115, 7490
Ju, Wonkyu: 7015
Juang, Wang-Chuan: 7110, 7400
Juckett, Lisa: 7435
Judd, Suzanne: 8015
Judge, Katherine S: 1265, 4200, 8020
Juhasz, Audrey: 4435, 7270
Jump, Alayna: 7425
Jump, Robin: 2345
Jun, Hee-jin: 7300
Jun, Jung Sim: 7020, 7235
June, Andrea: 2440
Jung, Bock-Young: 8015
Jung, Dukyoo: 7005
Jung, Hailey H: 4495, 8010
Jung, Heeeun: 8000, 8010, 8015
Jung, Inhye: 7155, 7410, 7535
Jung, Lanee: 4515
Jung, Mi Sook: 7265, 8000, 8010, 8015
Jung, Miyeon: 8000
Jung, Wonkyung: 1365, 2010, 8015
Jung, Yeongin: 7470, 7905
Jung, Yunkyung: 7275
Junghaenel, Doerte U.: 2465
Jurczak, Michael: 8000
Juris, Jill: 1185, 2130
Jurivich, Donald: 8020
Jurkowski, Elaine T: 7480
Justice, Jamie N: 1330, 3045
Jutkowitz, Eric: 3535, 4245, 4340, 8010
Juturu, Preeti: 7510
Jylha, Marja: 7530
Jylhävä, Juulia: 7010

K

Kaapu, Kristi: 4110
Kaasalainen, Sharon: 4195, 7055
Kabalan, Mayssan: 1155
Kabaya, Sonoko: 8015
Kabil, Gül G: 7205
Kabir, Ameera: 4300
Kabo, Felix: 4325
Kabua, Philmar M: 8000, 8015
Kackley, Madison: 8000
Kadi, Selma: 2060
Kado, Deborah: 1575
Kadowaki, Laura: 7740
Kaeberlein, Matt: 7010
Kafle, Bharat: 3055
Kahana, Eva: 1070, 1140, 4430, 7660
Kahsay, Eskira: 3235
Kai, Ichiro: 7490
Kaiksow, Farah A: 3335, 4530
Kaileh, Mary: 7210
Kailley, Jaya N: 7430
Kain, Nichole: 2460, 8010
Kainradl, Anna-Christina: 2320, 7085
Kaizi-Lutu, Marc: 1255
Kajitani, Shinya: 7130
Kaka, Baasil M: 3475
Kakara, Ramakrishna S: 1235
Kakulavar, Avinash: 7530
Kakuta, Momoho: 7115
Kalantari, Saleh: 3060
Kales, Helen C: 4130, 4340
Kalesnikava, Viktoryia A: 3245, 7695
Kaluna, Lia: 3140
Kalvesmaki, Andrea F: 2415, 2545
Kalyani, Rita: 7010, 7710
Kamalyan, Lily: 3065
Kamanga, Foster: 7660
Kamath, Vidyulata: 4440, 7285
Kameyama, Mayumi: 8015
Kamide, Kei: 7115, 8020
Kaminski, Margi: 4325
Kaminski, Naftali: 4170
Kan, Denise: 7440
Kananen, Laura: 2460
Kane, Alice E: 3520, 7275
Kane, Kevin: 7695, 7935, 8010
Kane, Tanya: 1155
Kaneshiro, Jasmine: 2530
Kang, Bada: 7080, 7275, 7630, 7640, 8015
Kang, Chaeeun: 7275, 8015
Kang, Chaeryon: 4315
Kang, Hun: 7680
Kang, Jee eun: 4405, 4515
Kang, Ji young: 4265
Kang, JungHee: 3320
Kang, Miseon: 7155, 7410, 7535
Kang, Ning: 3050, 7680, 8085
Kang, Rui: 1215, 8010
Kang, Sarah: 8010
Kang, Simeon C: 7480, 7900

Kang, Sophia: 3350
Kang, Suk-Young: 7360
Kang, Sungwan: 7430
Kang, Sunny: 7515
Kang, Yixin: 2240
Kang, Youjeong: 8000
Kang, Yu Jin: 1230
Kanter, Jeremy: 7070
Kao, Hsueh-Fen S: 1175
Kapadia, Farzana: 4225
Kapilan, Kaya: 2510
Kaplan, Celia C: 1245
Kaplan, Daniel B: 3175
Kapoor, Akshat: 7360
Kappler, Caitlin B: 2145, 7685
Karakida, Maki: 2205, 8010, 8015
Karakose, Selin: 7245, 7350
Karam, Rita: 7350, 7475
Karas, Marta: 8015
Karasik, Rona J: 2490, 7480
Karawani, Hanin: 3085
Karbakhsh, Mojgan: 7405
Kårelind, Fanny: 4010
Karczewski, Hannah A: 7420, 7920
Karel, Michele: 1080, 7695
Karere, Genesio: 8000
Karikari, Thomas K: 3375
Kariya, Hitomi: 3065
Karki, Dinesh: 7485
Karlawish, Jason: 7205, 8015
Karliner, Leah S: 3010, 4230, 7325
Karlsen, John: 8010
Karmacharya, Isha: 4015
Karon, Sara: 4305
Karr, Daniel W: 7665
Kartoz, Connie R: 1505
Karve, Aadit: 7210
Karvonen-Gutierrez, Carrie: 1365, 1435
Kasahara, Sachiko: 7600
Kasarskis, Edward J: 7245
Kasenetz, Whitney: 7610
Kaskie, Brian P: 3115, 3310, 3495, 4395, 8075
Kasler, Kamryn: 1160
Kaspar, Roman: 1285
Kasperovich, Natalia: 8010
Kassay, Abraham: 7540
Kassem, Moustapha: 7010
Kastner, Keri: 2480, 3255, 7510, 7915
Kasuga, Ayaka: 8020
Katagiri, Keiko: 2100, 7030, 7540
Katare, Bhagyashree: 4115, 7660, 8015
Kats, Allyson: 4055, 7630
Katsumata, Asako: 4135, 7405
Katz, Ben: 4315, 7615, 8020
Katz, Mindy J: 2160, 7225, 7455, 7630
Katz, Ruth: 4040
Katz, Stephen: 2265, 4275
Kauenhowen, Cornelia: 1480, 2440
Kaufman, Brystana G: 1475, 2180, 4415
Kaufmann, Christopher N: 1255, 1445, 2290, 7225
Kaur, Jagbir: 7465
Kaushal, Navin: 3050, 3235
Kautz, Tiffany: 7735
Kavanaugh, Melinda S: 2415
Kavcic, Voyko: 8000
Kawai, Hisashi: 1505, 7205, 7540
Kawakami, Keilyn L: 7055
Kawakami, Shinpei: 8015
Kawasaki, Teruaki: 8010
Kay, Denis G: 8015
Kaya, Alaattin: 7005, 7210, 8000
Kaye, Jeffrey: 3140, 3285, 7020, 7735, 8020
Kaye, Lenard W: 7415
Kayser, Jay: 8020
Keane, Connor Thomas Francisco: 8090
Keating, Norah: 1120
Keatings, Margaret: 4255
Keaulana-Scott, Samantha: 8020
Kebbede, Liyat Y: 8000
Keefe, Bronwyn: 2455, 7475, 7650
Keefe, Janice: 2495, 7420
Keefe, Kate: 1345
Keenan, Teresa A: 1085
Kehne, Theresa H: 1480, 7200
Kehrer-Dunlap, Abigail: 7655
Keisari, Shoshi: 8010
Keiser, Cassie: 1160
Kelekar, Uma: 1235, 3505
Keleman, Audrey: 7655, 8015
Kelle, Nadiya: 2025
Keller, Abiola: 2555
Keller, Michelle S: 7320
Keller, Valerie: 2320
Kellett, Kathy G: 7740
Kelley, Amy S: 1340, 2210, 3145
Kelley, Heather: 8020
Kelley, Jessica A: 1180, 2350, 3065, 3385, 3425
Kelley, Johnna L: 7695
Kelley, Lynette: 2430
Kellogg, Dean Lundt: 3350
Kelly, Christine: 4450
Kelly, Christopher M: 2275, 4290, 7510
Kelly, CJ: 7010
Kelly, Kathleen: 2200
Kelly, Megan: 8015
Kelly, Monica R: 7735
Kemp, Blakelee: 2120
Kemp, Candace L: 4290, 8010, 8020, 8070
Kemp, Elizabeth C: 2485
Kendall, Jaiden: 7300
Kendzor, Darla E: 7330
Kennedy, Katherine A: 1080, 4460, 7250, 7545
Kennedy, Kimberly A: 1410
Kennedy, Meaghan: 2430, 8010
Kennedy, Quinn: 7715
kennedy, Scott: 7075
Kennedy, Teri: 2495
Kennedy, Winston: 8015
Kennedy-Malone, Laurie: 7275
Kennel, Victoria: 8015
Kent-Thomas, Kaylin: 7350
Keny, Christina: 4045
Kenyhercz, Michael: 7235
Kerac, Marko: 4455
Keri, Ben: 8000
Kern, Kristopher C: 1325
Kerper, Shaye A: 3045
Kerr, Haiming: 7305
Kersten, Alan W: 7225
Kessler, Claudette M: 1250
Kestle, Dorothy: 2395
Ketcher, Dana: 2335, 2395
Ketel, Christian: 9050
Key, Kent: 3170
Kezios, Katrina: 1545
Khadeer, Mohammed: 8000
Khadka, Aayush: 2020
Khairallah, Ramzi J: 8000
Khakharia, Anjali: 7220
Khalighi, Mehraneh: 3435
Khalikov, Marla: 8010, 8015
Khalil, Mohammad: 7000
Khalilian, Mohammad: 1480
Khamzina, Madina: 8000
Khan, Aishah: 2355
Khan, Maham: 4110, 4315, 8010
Khan, Mushira M: 1215, 4015, 7505, 7935
Khan, Nadia: 7665
Khan, Nibraas: 7155
Khan, Sadiya: 8000
Khani, Saber: 1280
Kheroua, Sirine: 8020
Khetir, Imene: 7645
Khoo, Shuna S.: 8020
Khoo, Shuna Shi Ann: 7155
Khor, Ern Chern: 7155
Khov, Brian: 7015
Khundmiri, Syed: 7000
Khvatova, Elizabeth: 2145
Kiani, Shirin: 1175
Kidd, Bethany: 7155
Kidner, Madison: 2325
Kiefer, Jamie: 8000
Kieffer, Elana F: 2460, 3485
Kiel, Douglas P: 1275, 2080
Kiely, Kim: 3185
Kietzman, Kathryn G: 7510
Kiiti, Krungkraipetch: 7460
Kilaberia, Tina R: 1270, 7145
Kilcrease, Amanda K: 1060
Kilgore, Zach: 1295, 4265
Kilkey, Majella: 3550
Killian, Emily A: 2185, 3485
Killian, Tim S: 2550
Kilmer, Greta: 7065
Kilpatrick, Abbey: 7435
Kilpatrick, Abigail: 7155
Kilpela, Lisa: 7720
Kim, Andy J: 7225
Kim, Boah: 7740, 8010
Kim, Boeun: 3140, 7445, 7465, 7935
Kim, BoRin: 1295, 7030, 7265, 7290, 7630, 7665, 8010
Kim, Chang-O: 7275
Kim, Dae: 2080
Kim, Dae Hyun: 3135, 4225, 7080, 7275, 7280, 8015
Kim, Daejin: 4325
Kim, DaEun: 8000
Kim, Dahee: 2300, 7025, 8000, 8015, 8020
Kim, Dahye: 3545
Kim, Daniel: 9030
Kim, Do Yun: 7545
Kim, Dongmi: 3370, 7270
Kim, Dylan: 7750
Kim, Eric S: 1545, 4550
Kim, Eunbea: 4060, 4095
Kim, Eunhye: 7030
Kim, Eunjung: 3055
Kim, Eunkung: 7150
Kim, EunKyo: 7295, 7715
Kim, Eunsaem: 7275, 8015
Kim, Eunyoung: 7310

Kim, Gee: 7470
Kim, Giyeon: 7225, 7275, 7535
Kim, Gwang Suk: 7260, 7275, 7290, 7295, 7680, 8015
Kim, Ha Neul: 2265, 7745
Kim, Haesung: 8010
Kim, Haeun: 8000
Kim, Haklyoung: 7430
Kim, Haneul: 3270
Kim, Hansol: 7725
Kim, Heewon: 8010
Kim, Hokon: 7440
Kim, Hyejoong: 7720
Kim, Hye-Mi: 7460
Kim, Hyosin: 1380, 4485
Kim, Hyun Jung: 7705, 7720, 8020
Kim, Hyungjin Myra: 7490
Kim, Hyun-Jun: 2540, 4495, 8010
Kim, Hyunmi: 8010
Kim, Jaehyun: 8015
Kim, Jaein: 3370
Kim, Jasmyn: 8080
Kim, Jay: 8015
Kim, Jayeong: 7435
Kim, Jeehoon: 7355, 7625
Kim, Jennifer Ivy: 7080, 7275, 7630, 8015
Kim, Jenny: 1140
Kim, Jeongsuk: 3440
Kim, Jeung Hyun: 3030, 8020
Kim, Jeungkun: 7360
Kim, Jinhee: 8015
Kim, Jinho: 3360
Kim, Jinyoung: 4535
Kim, Jiyeon: 1395
Kim, Julie S: 7405, 7660
Kim, Jung Hyun: 9010
Kim, Jung Ki: 1455
Kim, Jung Yeon: 7645
Kim, Junhyoung: 7750, 8015, 8015
Kim, Kahyun: 7005
Kim, Keunyoung: 7015
Kim, Kiyeon: 7310
Kim, Korinek: 4050
Kim, Kwang-il: 8015
Kim, Kyeongmo: 7630, 7935
Kim, Kyungmin: 1360, 2065, 3545, 7005, 7460, 7720, 8000
Kim, Layoung: 7260, 7275, 7290, 7680
Kim, Manjun: 2265
Kim, May: 7280
Kim, Meeryoung: 7095
Kim, Miji: 7275, 8000, 8010, 8015
Kim, Min Hee: 2020
Kim, Min Hee: 2020
Kim, Min Jung: 8015, 8020
Kim, Minsub: 8015
Kim, Nahyun: 2100, 7460, 7540
Kim, Namhee: 7275, 7290, 7680
Kim, Nani: 7145
Kim, Nayeon: 7040
Kim, Oksim: 7440
Kim, Patricia S: 1395
Kim, Richard: 8015
Kim, Sangkyu: 7225
Kim, Sarah: 8000
Kim, Seo Young: 8010
Kim, Seohyeon: 8020
Kim, Seokmin: 1475
Kim, Seon: 7535, 7630, 7935
Kim, Seoyoun: 2425, 7355, 8020
Kim, Seoyoung: 8010
Kim, Sewon M: 7270
Kim, Seyoung: 8000
Kim, Sohee: 2375
Kim, Sohyun: 1455, 3400, 4045
Kim, Soo Hyun: 7055
Kim, Steffi M: 3005, 3345, 3585, 4015, 4320, 8070
Kim, Su Hyun: 7705, 8085
Kim, Su-Hwan: 3125
Kim, Suk-hee: 2455
Kim, Sukyung: 7275, 8015
Kim, Sun Ju: 7220
Kim, Suyoung: 8020
Kim, Taeeun: 7265
Kim, Tasha: 4420
Kim, Yeonju: 7360, 7750
Kim, Yeri: 8010
Kim, Yesol: 7360, 7750
Kim, Yijung K: 7640, 8020
Kim, Yongseop: 7750
Kim, Yoon Chung: 1070, 2000, 3565
Kim, Youngran: 3135
Kim, Yubeen: 8015
Kim, Yulri: 1070, 4060
Kimball, Patricia: 2150
Kim-Knauss, Yaeji: 1375
Kimmel, Jacob C: 2310
Kimmel, Susan: 8020
Kimpel, Christine C: 9050
Kimzey, Michelle: 4240
Kincaid, Reilly: 7265
Kincaid, William: 3340
Kindratt, Tiffany B: 3055, 7530
King, Abby C: 4000
King, Angelique: 1190
King, Anne E: 4160
King, Barbara J: 4075, 8015
King, Christina D: 3045, 4170
King, Deborah: 4230
King, Jessica: 3570
King, Natalie: 4255
Kinkade, Emily E: 7605
Kinnear, Lana B: 7255
Kinney, Jefferson: 4535
Kinoshita, Ayae: 8010
Kinosian, Bruce: 2545, 4535
Kinugawa, Arisa: 1115
Kinzig, Elizabeth: 8020
Kiosses, Dimitris: 7630
Kirby, Mackenzie E: 7475
Kirch, Matthias: 1085
Kircher, Julie A: 7255
Kirchner, Kathleen A: 7005, 7465
Kirk, Jeanette W: 4075
Kirkland, Jim: 8000
Kirsch, Jaclyn: 2195
Kirschner, Kristina: 3325
Kirsh, Jaclyn: 1105
Kirvalidze, Mariam: 4045, 7215
Kishimoto, Kyoko: 7480
Kissell, Paul E: 7640
Kitago, Moe: 7680
Kitamura, Akihiko: 7275
Kitamura, Koji: 1505
Kitayama, Atsushi: 7420
Kitayama, Jun: 7095
Kitchen, Courtney: 1130
Kitchen Andren, Katherine: 7490
Kitchens, Katherine: 2195, 4400
Kittle, Krystal R: 2015, 2225, 3190
Kittleman, Cory: 7530
Kitt-Lewis, Erin: 2140
Kitto, Elizabeth S: 4005
Kitzman, Dalane: 7710
Kiwak, Eliza: 7085
Kizony, Rachel: 7300
Klaiber, Patrick: 4405, 4410
Klank, Christina: 7480
Klasen, Lisa Joanne: 2025
Klatt, Maryanna D.: 7490, 7935
Klenczar, Brittany: 2225, 3490
Klenk, Jochen: 2170
Klepacz, Laura M: 1525, 7030, 7455
Kleszynski, Keith: 7480, 8015
Kleykamp, Bethea A: 9040
Kliegel, Matthias: 4490
Klimstra, Marc: 7260
Kline, Caitlyn: 7625
Kline, Chelsea: 3000, 7005
Kline, Christopher E: 1365
Klinedinst, Tara C: 7330
Klinger, Christopher: 7480, 7900
Klokgieters, Silvia: 1120
Klopack, Eric T: 3095, 8060
Klug, Marilyn: 8020
Klunk, William: 9000
Knauft, Katherine M: 7540
Kneale, Dylan: 1110
Knebl, Janice: 1065, 3510
Knecht-Fredo, Jennifer: 7410
Knickerbocker, Kelly J: 1265, 7075
Knight, Erik L: 7210
Knoefel, Frank: 3285
Knoepke, Christopher: 3320
Knox, Sara: 1075
Knutson, Kristen: 2290
Ko, Eunjung: 7660, 8020
Ko, Fred C: 3435
Ko, Jung Eun: 3440
Ko, Linda K: 1140, 1480, 7200
Ko, Sunghyun: 8010
Ko, Woorim: 4070
Ko, Young: 8010
Koçer, Rüya: 2565
Kobayashi, Lindsay: 1290, 2515, 3145, 4380, 7140, 7420
Kobayashi, Tetsuro: 7360
Kobayashi-Cuya, Kimi E: 1265
Kobylarz, Fred A: 4485
Koch, Jana: 7610
Koch, Mary Kate: 7020
Kociolek, Judyta: 2300
Koehn, Sharon D: 8010
Koenigshoff, Melanie: 2435
Koeuth, Sokha: 4340
Koffer, Rachel E: 1350, 3080, 4010, 8020
Koffer, Rachel: 8000
Koffski, Maya J: 4235
Koget, Jennifer: 8015
Koh, YoungBin: 8010
Kohl, Gianna: 8000, 8035
Kohlbrenner, Briana L: 7495
Kohler, Hans-Peter V: 1280, 2425
Kohler, Iliana V: 1280
Kohli, Amarpreet S: 7680
Kohli, Maulika: 7675
Kohli, Nidhi: 3490

Kohlrausch, Estela: 3155
Kohon, Jacklyn: 2185, 4510, 8010
Koike, Teruhiko: 7315
Koios, Daniela: 7110
Koirala, Binu: 7440, 7500
Koizumi, Naoru: 2370
Kojima, Narumi: 8015
Kojis, Daniel: 7500, 7930
Kokinakos, Angela: 8010
Kolanowski, Ann M: 2140
Kole, Debarati: 3405, 7070
Koletar, Susan L: 7285
Kollasserry, Felix Joy: 9000
Kolpakov Nikitin, Stanislav: 7200
Kolpina, Lola: 3465, 4045, 8020
Komalasari, Renata K: 4315
Komisar, Harriet: 1085
Kong, Dexia: 4115, 7035, 7290, 8020
Kong, Jooyoung: 2001, 3225, 7660
Kong, Weijuan: 7640
Konlan, Kennedy Diema: 8010
Kono, Ayumi: 7080, 7105
Kontor, Okyere D: 8000
Kontos, Pia: 4445, 7040, 7055
Koon, Lyndsie: 8020
Kooshiar, Hadi: 8015
Kopera-Frye, Karen: 3345, 3585
Koppel, Sjaan: 3455
Kops, William J: 1480, 2440
Koren, Chaya: 3465
Korer, Burton: 8015
Korfage, Ida J: 2255
Korinek, Kim: 1485, 8020
Korlat, Selma: 2300
Kornadt, Anna E: 4020, 7610
Koronowski, Kevin: 7000
Kosar, Cyrus: 1230, 8075
Koss, Catheryn: 1145
Kossowska-Kuhn, Dorota: 8000
Kostelic, Amy: 1480
Kosteniuk, Julie: 7135
Koster, Annemarie: 1245
Koszalinski, Rebecca S: 2300, 7610
Kotecho, Messay G: 7540
Kothari, Meeraj: 4550
Kotkowski, Kornelia: 4255
Köttl, Hanna: 3425
Kotwal, Ashwin: 2020, 2520
Kou, Minghao: 7460, 7715
Kouame, Gail: 8010
Koufacos, Nicholas: 2280
Kouts, Holly: 1490
Kowal, Paul: 8015
Kowalchik, Kalei H: 1080, 8090
Koyama, Toshihiro: 7260
Kozlov, Elissa: 7110, 7360
Kozlov, Elissa K: 8035
Krämer, Michael D: 1215
Kraemer, Brian C: 7310
Kraemer, Kyle: 8020
Krafft, Jennifer: 3575
Kramer, B Josea: 2280, 7730
Kramer, Jenna: 7350, 7475
Kramer, Joel: 8020
Kramer, Philip A: 2080, 4005
Kraschnewski, Jennifer: 1080
Krassikova, Alexandra: 4255, 8015
Krauss, Melissa: 7260, 8015
Kravchenko, Julia: 3010, 3085, 7300, 7620
Kravljaca, Nikolina: 4295
Kreimer, Simion: 8000
Krekula, Clary: 2425
Krenek-Schneider, Brittany: 9050
Kreps, Gary L: 7360
Kriebernegg, Ulla: 2320, 3320, 4285, 7085
Kringle-Baer, Kirsten: 2325
Kritchevsky, Stephen: 1090, 1240, 1410, 2080, 2220, 2505, 3045, 4005, 7015, 7060, 8000
Kroenke, Kurt: 4440
Krok-Schoen, Jessica L: 2445, 3220, 7265
Kroplewski, Renee: 8020
Kropp, Denise: 7475, 7900
Krueger, Tillmann HC: 4490
Krull, Drew A: 8085
Krysko, Alyssa: 7140
Krywe, April: 8060
Ku, Hok Bun: 7530
Ku, Te-Lien: 7705
Ku, Vivian: 7535
Kuai, Zheng: 1435
Kuan, Yi-Chun: 7735
Kuan, Yun-Han: 8015
Kuang, Jiahao: 7710
Kubzansky, Laura: 4440, 4550, 7275
Kucharska-Newton, Anna: 2525, 4440, 7060, 7630, 8015
Kuchel, George A: 1275, 2495, 3210, 4350
Kuehn, Kevin: 7660
Kuhl, Melanie: 7360
Kuhl, Olivia: 8015
Kühn, Simone: 7210
Kuipers, Allison L: 7010, 7625
Kulkarni, Snehal S: 4055
Kullgren, Jeff: 1085
Kulminski, Alexander: 1025
Kuluski, Kerry: 8015
Kum, Cleopatra: 1465, 3455
Kum, Crescentia: 3455
Kum, Flavius: 3455
Kum, Mirabelle: 3455
Kuma, Mischa: 4370
Kumar, Amiritha: 8020
Kumar, Amit: 1075, 4385
Kumar, Chandrika: 7475
Kumar, Premranjan: 1420, 2080, 2235
Kumar, Sandeep: 7210
Kumar, Vivek: 7210
Kumari, Neha: 1195
Kumthekar, Nihar: 7680
Kuniholm, Mark: 8010
Kunik, Mark E: 4340
Kunkel, Miranda: 1160
Kuntajak, Premgamon: 7725
Kunzel, Carol: 8010
Kuo, Chun-Tung: 7460
Kuo, Fang-Lin: 4110, 7655
Kuo, Han-Yueh: 7080
Kuo, Tony: 7015, 7200
Kuo, Tsuann: 7040, 7055, 7070, 7280, 7335
Kuo, Yong-Fang: 2090, 4095, 7405, 8010
Kupferschmidt, Anthony, L.: 8010
Kurth, Maria: 2385, 4405, 7030
Kuryk, Kaitlyn: 2495, 7420
Kurzman, Alissa: 7325
Kusmaul, Nancy: 2000, 2470
Kuspinar, Ayse: 7655
Kutney-Lee, Ann: 1160, 4195
Kuy, Kakada: 4050
Kuzmik, Ashley: 3230
Kvale, Elizabeth: 2090
Kwak, Jacqueline R: 1440
Kwak, Judy J: 8020
Kwak, Jung: 2090
Kwak, Min Ji: 3135
Kwiatek, Susan: 8015
Kwiecien, Robert: 8015
Kwoh, kent: 8010
Kwok, Jojo: 2520
Kwon, Ester J: 7310
Kwon, Eunsun: 4265, 8010, 8010, 8010
Kwon, Ilan: 7695
Kwon, Jenny: 7295
Kwon, Simona C: 8095
Kwon, Soonhyung: 3440, 4365
Kwon, Sooyoung: 8015
Kwong, Katherine: 3515

L

Labbé, Angéline: 8020
Lachman, Margie E: 1525, 4065, 4500, 7455, 7610
Lachmann, Mark: 7530
Lachs, David: 7205
Lachs, Mark: 1270, 2150, 4365, 7205
LaCoss, Jennifer: 8010, 8015
LaCroix, Andrea Z: 8010
Ladiges, Warren: 7075, 8000
Laffer, Alex: 7475
Laflamme, Maeva: 7475
Lafontant, Kworweinski: 3055, 7640, 7720, 7735, 8000, 8015
Laforest, Justin: 7720
Lagacé, Martine: 3320
Lahar, Cindy J: 7415
LaHue, Sara C: 2530
Lai, An Chi: 7040
Lai, Daniel W.L.: 2050, 2150, 8050
Lai, Hsiu-Yun: 7080
Lai, Patrick HL: 2555
Lai, Patrick Ho Lam: 4140, 7355
Lai, Philip: 3550
Lai, Wenhua: 3165, 7065
Lai, Wen-Hua: 2015
Laird, Barrett: 7680
Laiton Jimenez, Laura: 7455
Lajambe, Lexie: 7720
Lakatta, Edward G: 8015
Lake, Jordan: 7285
Lakomý, Martin: 3360
Lakomy, Martin: 3190
Lakshmanan, Sarada S: 8020
Lalani, Nasreen: 4115, 7660, 8015
Lalwani, Anil K: 7140
lam, Jamie: 8015
Lam, Julia: 4100
Lam, Justin W: 3415, 8015
Lam, Michael: 7015
Lam, Michelle: 8015
Lam, Phillip: 9025
Lamba, Saanvi: 7360, 8010, 8070
Lambrou, Nickolas: 2335
Lampe, Nik M: 3075, 9030

Lampenfeld, Nathan: 2480
Lampkin, Cheryl L: 1085
Lanba, Asheesh: 7680
Landman, Bennett A: 2510
Landvoigt Schmitt, Brenda: 8000
Landwehr, Samantha: 8015
Landy, Ania: 3370
Lane, Nancy: 4005
Lane-Nelson, Nia R: 8080
Lang, Frieder R: 1375
Langa, Kenneth: 1290, 2515, 3145, 4380, 7350, 7490, 9010
Lange-Maia, Brittney: 1235
Langsetmo, Lisa: 4055, 7630
Langton, Lynn: 1370, 2150, 3270
Langworthy, Benjamin: 4245
Lanzi, Alyssa M: 3000
Lanzkron, Sophie: 8000
Lapane, Kate: 7060, 7275, 7320, 8015
Lapidus, Jodi: 7300, 8000
LaPierre, Tracey: 2500
Lapins, Allison: 7220
Lapointe, Mia: 8020
Lariviere-Chartier, Julien: 3285
Larkin, Marni J: 7330
Larkina, Marina: 7525, 7630, 7725
Larnyo, Ebenezer: 7020, 9000
LaRocca, Thomas: 7075
LaRowe, Lisa R: 9020
Larsen, Matthew W: 8015
Larson, Carol: 1575
Larson, Eric B: 3065
Larson, Janet L: 7435
Laskow, Thomas: 1205
Lasseter, Amanda: 8000
Latham, Amanda: 7075
Latham-Mintus, Kenzie: 3065, 8000
Latimer, Abigail: 3320, 7215
Lau, Terence K: 7695
Lauber, McKenzie: 7270
Lauck, Jen: 8020
Lauderdale, Diane S: 1110
Laukka, Erika Jonsson: 4315
Launer, Lenore: 2040, 9020
Laura, Bradbury: 7640
Laura Oliveira Dias, Ana: 7225
Laurienti, Paul J: 1150
Lavangu, Vaishali: 3235
Lavari, Kavin: 7270
Lavoie, Patrick: 7645
Law, Makenna: 8000
Lawrence, Aneesah: 8020
Lawrence, Joanna: 7750
Lawrence, Molly: 4515, 7040
Lawton, Alexander: 2520
Lawton, Dylan RY: 7080
Lay, Jennifer: 7725, 8000, 8020
Layman, Shannon: 2415, 7270
Le, Bao Ngoc: 8025
Le, Christine: 7005
Le, Elaine T: 4430
Le, Minh Quan: 7455
Le, Qun: 3245, 7260, 7695, 7935, 8010, 8050
Leach, Bethany: 8010
Leach, Carrie: 4320
Leach, Susan J: 7330, 8015
Leath, Seanna: 8080
Leavey, Gerard F: 2055
LeBrasseur, Nathan: 2005, 2435, 4005
Ledermann, Thomas: 7350
Lee, Adrian KC: 1175
Lee, Ahrim: 8010, 8015
Lee, Alexandra K: 3330
Lee, Boram: 9030
Lee, Chae Man: 3265, 7065, 7240, 7740
Lee, Chaejeong: 2260
Lee, Chaiwoo: 7065
Lee, Chanam: 7040
Lee, Cheryl A: 7525
Lee, Chioun: 1540
Lee, Chiyoung: 8010, 8015
Lee, Christopher S: 7440
Lee, Chung-Sheng: 7275
Lee, Daehyun: 8000, 8010, 8015, 8015
Lee, Dahyeon: 8015
Lee, Ellen E: 4180
Lee, Eun Jeong: 1140
Lee, Eunju: 3545, 7005
Lee, Gina: 1385, 3225, 3405, 4095
Lee, Haerim: 7265
Lee, Hajin: 8020
Lee, Hanna: 7205, 7440
Lee, Harold H: 1545, 8045
Lee, Heather: 8010
Lee, Hedwig E: 7350
Lee, Hee Y: 8020
Lee, Hi-Woo: 1140
Lee, Hyangkyu: 8010, 8015
Lee, Hye Soo: 3415, 8000
Lee, Hyeyeon: 7275
Lee, Hyo Jung: 7095, 7155, 7265, 7410, 7535
Lee, Hyunjoo: 3200
Lee, Hyunmin: 8000
Lee, Inheok: 7280
Lee, Insuk: 8015
Lee, Insup: 7145
Lee, Jae Jun: 7260, 7275, 7290, 7680
Lee, Jaesung: 7155
Lee, Jane: 2470
Lee, Jean Mui Hua: 8015
Lee, Jennifer A: 3010
Lee, Jenny Jinyoung: 8010
Lee, JeongEun: 1070, 1165, 1335, 3575, 4060, 4095, 7145
Lee, Ji Hyun: 1495, 7355
Lee, Ji Yeon: 7260, 7290, 7640, 7680, 7750
Lee, Jieun: 2145
Lee, Jinkook: 2040, 2515, 3125, 3335
Lee, Jongwon: 8000
Lee, Joseph H: 1025
Lee, Juhee: 7310
Lee, Juhyeong: 7225
Lee, June: 7145
Lee, Juneyoung: 1460
Lee, Jung-Ah: 1165, 2340, 3460, 7300, 7660, 7705
Lee, Jungjoo: 7750
Lee, Kathy: 7155, 7340
Lee, Kyoung Hag: 7020, 7235
Lee, Kyuho: 7695
Lee, Kyung Eun: 4505, 7070
Lee, Kyung Hee: 7295, 7420, 7645, 7750
Lee, Kyungmi: 8010
Lee, Kyuri: 2550, 7005, 7245
Lee, Lewina O: 4375
Lee, Lewis: 7440
Lee, Matthew: 8045
Lee, Mi-Jeong: 7625
Lee, Mina: 7545
Lee, Minyong: 8010
Lee, Mitchell: 7010
Lee, Myungeun: 7295, 8015
Lee, Paul: 4300
Lee, Pei-An: 4215
Lee, Rokbit Sanghee: 7280
Lee, Sang E: 3545, 7425
Lee, Sei J: 3330, 4230
Lee, Seolah: 7155, 7265, 7410, 7535
Lee, Seonhwa: 3190, 8085
Lee, Seungkeun: 8020
Lee, Shinduk: 2060
Lee, Soo-Kyoung: 8015
Lee, Soomi: 1255, 1350, 2290, 3275, 3525, 7130, 7455, 7735, 8000, 8020
Lee, Sophia: 7265
Lee, Sun Ah: 7255, 7360
Lee, Sung-Jin: 8010
Lee, Sungkyu: 8010
Lee, Tae-Ho: 8020
Lee, Ya-Wen: 7510, 8010
Lee, Yeong Ju: 7430
Lee, Yeonjung: 1100, 1195, 7605, 8000, 8010, 8020, 8045
Lee, Yeonjung J: 4445, 7600, 8000, 8000
Lee, Yeonjung Jane: 1310
Lee, Yeon-Shim: 7425, 7500
Lee, Yeonwoo: 8015
Lee, Yoojin: 8020
Lee, Yookyong: 8010
Lee, Youjung: 7410
Lee, Young-Shin: 7300, 7705
Lee, Yunhwan: 7275, 8015
Lee, Yura: 7070, 7425, 7540, 7930, 8090, 9010
Leedahl, Skye N: 1185, 2130
Lees Haggerty, Kristin: 1260, 1270, 4355
Lee-Turner, Meosia: 7030
Leff, Bruce: 8010
Leger, Kate A: 7255
Leggett, Amanda N: 2015, 3165, 7030, 7065
Lehning, Amanda: 1295
Lei, Lianlian: 7490
Leiderman, Alexis: 7140
Leinbach, Carla B: 8000, 8010
Leis, Aleda: 1435
Leiser, Scott F: 4005
Leist, Anja K: 9010
Lekan, Deborah: 7275
Lekhak, Nirmala: 1140, 7660
Leland, Natalie E: 4100, 7080
Lemack, Bergen: 1065
Lemonde, Manon: 3010
Lemus, Armando: 7000
Lenchik, Leon A: 2505
Leng, Xiaoyan I: 1410, 4005
Lentz, Jeffrey: 8020
Leonard, Chelsea: 8010
Leonard, Delaney: 8020
Leong, I. Tek: 1380
Leopold, David: 7680
Lepore, Michael J: 1315, 2000, 2350, 3565, 4465
Lero, Christi M: 7310

LeRoy, Angie: 9030
Lervick, Dana: 3115
Lester, Barbara R: 8010
Leung, Angela: 7680, 7695
Leung, Lok Man: 4365, 8020
Leung, Peggy: 8015
Leung, Vania: 7270
Leutwyler, Heather C: 1530, 2530
Levan, Darius T: 8040
Levasseur, Melanie: 1100, 3320, 4270, 8020
Levchenko, Yuliana: 1230
Levenson, Robert W: 3285, 3355, 8020, 8040
Levin, Michael: 4435, 7270, 8020
Levine, David: 8010
Levine, Meredith: 8010
Levine, Stacie: 7250
Levinsky, Michal: 2025
Levitan, Eric: 2485
Levy, Becca R: 2305, 3165, 4235
Levy, Cari: 8010, 9040
Levy, Helen: 7490
Levy, Sorah: 3090, 3255, 4090, 8020
Lewis, Charity T: 7140
Lewis, Frances M: 8015
Lewis, Hannah M: 7605
Lewis, Jordan P: 2335, 3005, 3345, 3585, 4015, 4320
Lewis, Jordan: 8070
Lewis, Lisa M: 7440, 7925
Lewis, Nathan A: 8000
Lewis, Nicole: 7660
Lewis, Stuart: 2150
Lewis Thames, Marquita: 4480, 4370
Leykum, Luci: 2545, 8070
Li, Aaron C: 8010
Li, Ashlee: 7425
Li, Brookelle: 3175
Li, Chen: 2500
Li, Chenxi: 2525, 7090, 7630
Li, Chien-Ching: 7080, 8020
Li, Chihua: 4115
Li, Chunxiao: 2040, 2500, 7080
Li, Dan: 7430
Li, Daniel: 7285
Li, Donna: 7315
Li, Fan: 3370
Li, Han: 7075
Li, Hong: 7450, 7630, 7930
Li, Hongyu: 1355
Li, Huiling: 9030
Li, Jiajia: 8010, 8085
Li, Jianwen: 8020
Li, Jiaying: 8015
Li, Jing: 3145
Li, Jingxi: 3090, 7510, 7705
Li, Jingyi: 2125
Li, Joyce: 3520
Li, Juan: 2465, 7630
Li, Judy: 7085
Li, Junxin: 1535, 2465, 3335, 4010, 7525, 7695, 8015
Li, Ke: 1290, 3440, 7030
Li, Lingsheng: 4045
Li, Linyan: 8035
Li, Lun: 1100, 4445, 7445, 7740, 8010, 8045
Li, Lydia: 1105, 2260, 3440
Li, Meiyi: 2030
Li, Melody: 8015
Li, Mengchi: 2465, 3335, 4010
Li, Mengting: 8050
Li, Mingyang: 7455
Li, Na: 7275, 7710
Li, Niying: 2065, 7200
Li, Pingnan: 8010
Li, Polly Wai Chi: 7005, 7040
Li, Qi: 1245
Li, Qianyuan: 1235, 2255, 2465
Li, Qing: 7725
Li, Ruixi: 4310
Li, Shuang: 7400, 7920, 8015
Li, Shuwen: 7265
Li, Tao: 7080
Li, Tianyuan: 2030, 3480, 4065, 7610
Li, Wei: 7285
Li, Wenjun: 3245, 7260, 7695, 7935, 8010
Li, Xiaoang: 7320
Li, Xiaoli: 7200, 7425, 7910
Li, Xiaoqian: 7455, 7925
Li, Xiaoyouxiang: 8020
Li, Xinyue: 8020
Li, Xiuli: 7425, 7930
Li, Xuena: 7080
Li, Yaming: 4195
Li, Yan: 3540
Li, Yaxin: 7080
Li, Yiang: 3410
Li, Yichao: 7230
Li, Ying-Chun: 7110, 7400, 7490
Li, Yingru: 8015
Li, Yixia: 3010
Li, Yongmei: 8010
Li, Yu S: 3355
Li, Yuanbin: 7440
Li, Yuanyuan: 3270
Li, Yuekang: 7535
Li, Yuelin: 7275, 7640
Li, Yun: 1090
Li, Zhijiang: 7630
Li, Zhuochun: 1355
Li, Ziyi: 8000
Liang, Bo: 8010
Liang, ChangPu: 7735
Liang, Jersey: 7265
Liang, Jiaming: 1335
Liang, Jianling: 7615
Liang, Nana: 7670
Liang, Peichao: 3320
Liang, Shiwei: 7320
Liang, Wukaiyang: 7075
Liang, Yanjing: 2125, 7415, 7610, 7900
Liao, Gerald Y: 7075
Liao, Hsiao-Wen: 8000
Liao, Jinhui: 4005, 7275
Liao, Yanru: 8020
Liao, Yo-Jen K: 7040, 7200, 8015, 9015
Liao, Zih-Yong: 4110, 7655
Liberalesso Neri, Anita: 8015
Lichtenberg, Peter A: 2155, 3270, 7630, 8000
Liebegott, Ryan K: 8010
Lieber, Sarah B: 7080
Liebman, Michael: 3550
Liebzeit, Daniel: 8020
Lien, Zoe H: 8010
Lifshitz, Rinat: 3530
Light, Michael: 7215
Lighthall, Nichole R: 4420, 7640, 8000, 8015
Liljas, Anne: 7215
Lill, Christina: 7210
Lim, Aaron C: 7495, 7930
Lim, Angelica: 2410, 2530
Lim, Arum: 7440, 7500
Lim, Celestine: 8015
Lim, Emily: 8015
Lim, In-Taek: 8010
Lim, Jun Pei: 8015
Lim, Junyub: 8000
Lim, Kelvin O: 1485, 7145
Lim, Kian Lee: 7150
Lim, Mihye: 7095
Lim, Nahyun: 8000, 8010, 8015
Lim, Wee Shiong: 8015
Lim, Yejin: 4070
Lim, Youn-Hee: 3125
Limaye, Seema: 7250
Lim-Soh, Jeremy: 1255, 2065, 3025, 3545
Lin, Chia Jou: 7040
Lin, Ching-Huang: 7275
Lin, Feng Vankee L: 1135, 7015
Lin, Feng-Chang: 3585, 7535
Lin, Frank R: 1055, 1440, 2525
Lin, Gloria J: 4390
Lin, Haiqun: 1380, 4485
Lin, Hongjing: 7330
Lin, Hui-Yi: 8020
Lin, I-Fen: 4050
Lin, Kueiyu Joshua: 3135
Lin, Ming-Jen: 7510
Lin, Qing: 7000
Lin, Rong: 7450, 7930
Lin, Rose: 9010
Lin, Shih-Yin: 7645, 8020
Lin, Siyang: 7275, 7710
Lin, Vankee: 7450
Lin, Vivian: 8015
Lin, Xin Yao: 2355, 3415, 4420, 7360
Lin, Yen: 7125
Lin, Yi-Hsiu: 7280, 7335
Lin, Yunann: 8000
Lin, Zhao Xiang: 7025
Lin, Zhixuan: 9035
Lin, Zhiyong: 7145
Lin, Zhuoer: 2120, 3165, 7055, 7465, 7925, 8055
Linares, Jackeline: 3190
Lincoln, Karen D: 1060, 4010
Lindauer, Allison: 1125, 3045, 3285, 7425
Lindberg, Brian W: 2245, 2390, 3260, 3390, 4395
Lindberg, Jimmy: 8015
Lindeman, Cliff: 8010
Linden, Wolfgang: 8020
Lindgren, Aleksandra L: 3275
Lindquist, Lee: 4480, 7005, 7420, 7490
Lindsay, Rebecca: 7030
Lindsay, Ryan: 7490
Ling, Shari M: 4230, 8030
Linge, Jennifer: 4310
Lingham, Jayanthi O: 3550
Lingler, Jennifer: 7200
Link, Benjamin: 2200
Linton, Elizabeth: 8000
Lipat, Ania: 1445
Lipsitz, Lewis: 2040, 2080, 2350, 4535, 7450
Lipson, Debra J: 1315
Lipton, Richard B: 2160, 7225, 7455
Liskovec, Leona: 9030
Liston, Dorion: 7715

Lithgow, Gordon: 7010
Little, Kevin: 1490
Little, Natalie: 7300
Littleton, Tenesha: 7535
Litzelman, Kristin: 4095, 7610
Liu, Bingyang: 7315
Liu, Bochu: 4430
Liu, Chang: 7620, 7655, 7720, 8015
Liu, Chiung-ju (CJ): 7205
Liu, Chun: 2080, 2235
Liu, Chunyu: 1255, 7360, 8015
Liu, Fangyu: 8015
Liu, Guangtian: 7280
Liu, Huan: 7510, 7915
Liu, Huangqin: 7670
Liu, Hui: 1110, 3410, 4455
Liu, Jingwen: 7030
Liu, Jingyun: 7275
Liu, Jinming: 7470
Liu, Jinxiu: 7425, 7930
Liu, Jinyu: 1105, 4030, 7055
Liu, Jue: 7350
Liu, Kehan: 2040, 2500, 4055
Liu, Li: 1355
Liu, Lili: 7620, 7630, 7905
Liu, Liyun: 2120
Liu, Longjian: 7620
Liu, Mengjing: 3415
Liu, Mengyu: 8010
Liu, Michelle: 1535
Liu, Min: 3050, 7680
Liu, Minhui: 1235, 1365, 2040, 2255, 2465, 2500, 2530, 3330, 4055, 7080, 7155, 7440
Liu, Pi Ju: 1200
Liu, Qian: 1355
Liu, Qianqian: 4310
Liu, Qimin: 3165
Liu, Rui: 8010
Liu, Ruotong: 7270
Liu, Sofia: 1535, 2465, 3335
Liu, Tao: 7430
Liu, Tianyin: 7640
Liu, Wen: 2550, 7005, 7205, 7245
Liu, Xiaohui: 7670
Liu, Xiaoye: 2030, 3480
Liu, Xiaoyu: 7200, 7425, 7910
Liu, Xinran: 7300
Liu, Xueying: 1245
Liu, Yaqian: 7680
Liu, Yating: 3330
Liu, Yi: 7655, 8015
Liu, Yichun: 3255
Liu, Yifan: 1535, 3570
Liu, Yin: 2065, 7005, 7030, 8020
Liu, Yuanyuan: 7035, 7265, 7520
Liu, Yuchen: 7275, 7280
Liu, Yujin: 1355
Liu, Yuqi: 3200
Liu, Zeyu: 2300
Liu, Zhilin: 4430
Liu, Zhiwei: 3330
Liu, Zhong-Xu: 7455
Liu, Zuyun: 8015, 8055
Liu Woronko, Susan: 4320
Liu-Ambrose, Teresa: 1150, 1365, 1535, 2510, 3335, 7030, 7455, 7720, 8015
Livingston, Melvin: 4355
Livingston, Rachel: 8020
Livingston, Raechel: 1525, 8000, 8020
Lkhamsuren, Zuchi: 1140
Llaneza, Danielle H: 7360
Lloyd, Althea: 3110
Lloyd, Amanda J: 8015
Lloyd, Patricia: 8015
Lo, On-Yee (Amy): 4215
Lo, Temmy L. T.: 7725
Lo-Ciganic, Wei-Hsuan: 2145
Löckenhoff, Corinna: 8020
Locklear, Jessica: 8015
Locklear, Keenya: 8020
Lockman, Jeffrey: 7690
Lodha, Neha: 7630
Loeckenhoff, Corinna: 7690
Loerzel, Victoria: 4420, 7260
Loewenstein, David: 8010
Loewenthal, Julia: 3150
Logan, David: 3455
Logue Cook, Rachel N: 1175, 3085, 7205, 7610
Loh, Kah Poh: 7420, 7920
Lohman, Matthew C: 1535, 7315, 7425, 7930
Lohmar, Sabine: 7115, 7220, 7490
Lomeli, Sindy: 7725, 8010
London, Andrew S: 2385
Long, Chengxu: 1475
Long, Erin: 3565
Long, Monica: 2340, 3175, 7250
Longley, Hannah: 7680
Loomer, Lacey: 7295
Loosen, Julia: 2085
Lopes, Mario: 8010, 8015
Lopes De Oliveira, Thais: 8015
Lopez, Bruna: 2555, 4015, 4140
Lopez, Janet: 8010, 8015
Lopez, Katherine I: 1480
Lopez, Maria J: 4485
Lopez, Ruth Palan: 4045, 8015
Lopez-Ortega, Mariana: 3030
López Anuarbe, Mónika: 1045
LoPilato, Danielle M: 3245, 7695, 7935
Loranger, Elisabeth: 4490
Lord, Justin: 1550, 3255
Loskot, Taylor: 4120, 4180
Lotufo, Paulo A: 1320
Lou, Christine Wei-Mien: 7030
Lou, Qian: 7655, 7720
Lou, Vivian Weiqun: 1115, 4115, 7080, 7150, 7235, 7290, 7640, 7695, 7750, 8020, 8040
Lou, Yifan: 1105, 2305, 4030, 4505, 7055, 7245
Louis-Charles, Willencia: 8000, 8010, 8020
Lourie, Michael: 7470
Louwagie, Katie W: 3130
Lovasi, Gina S: 3375
Love, Jill W: 7325
Love, Madeleine: 7720
Lowers, Jane: 1200, 4395
Lox, Dennis M: 7410
Lu, Chuan: 7655
Lü, Jiaojiao: 4535
Lu, Jiaying: 8000
Lu, Jiehua: 8000
Lu, Lingyi: 7015
Lu, Minjie: 7355
Lu, Peiyi: 4115, 7035, 7290
Lu, Shiyu: 3200, 8000, 8010, 8020, 8035
Lu, Siyao: 4505, 7485
Lu, Xiaomin: 2495
Lu, Yi: 7690
Lu, Yifei: 1000
Lu, Yu: 7660
Lu, Yu-Lan: 7280
Lu, Zhiyong: 1355
Lubben, Jim: 1225, 2055, 4190
Luborsky, Mark R: 7440
Lucas, Iaah: 8015
Lucas, Ruth: 8015
Lucca, Kaitlyn: 1125, 7220
Lucero, Louis: 8020
Lucero, Robert J: 8015
Luchetti, Martina: 7245, 7350
Luchsinger, José: 3180
Luci, Katherine S: 4060, 7695, 8015
Luehrsen, Samantha: 8010
Lui, Li-Yung: 4005
Luiz de Melo, Mariana: 7225, 7655
Lujintanon, Sita: 7540
Luke, Miles: 9040
Luk-Jones, Susanna: 1065
Lum, Hillary: 4215
Lum, Terry Yat Sang: 3200, 8010
Luna, Catherine: 2360
Lunardini, Melissa: 2410
Lunetta, Kathryn: 2160, 7735, 8010
Lunger, Judith: 2310
Luo, Hao: 1455
Luo, Huabin: 3305
Luo, Jing: 1570
Luo, Liying: 2120, 7350
Luo, Minxia: 4185
Luo, Wei: 7430
Luo, Yanan: 4065
Luo, Yuqian: 1365, 2530, 7155
Luo, Yuting: 7630
Luo, Zhehui: 2525, 7090, 7630
Luo, Zhimeng: 1355, 2355
Luong, Gloria: 7255
Lusardi, Michelle M: 7330
Lusignani, Maura: 7245
Luskey, Valerie E: 7070, 7455, 7610
Luth, Elizabeth A: 3220, 3500, 4360, 4550, 8035
Luther, Stephen: 2145
Lutsey, Pamela: 4440
Luu, Britney: 8010, 8015
Luxton, India: 3515
Luy, Luis: 2295
Luz, Helena: 7465
Luzum, Geske: 7060
Lv, Yalin: 2465, 7630
Lwere, Kamada: 1190
Ly, Timothy K: 1325
Lydon, Elizabeth A: 4270, 7015, 7630, 7660, 8020
Lynch, David H: 3585, 7275, 7535
Lynch, Doug: 8015
Lynch, Erin: 9040
Lynch, Morgan: 3220, 7725
Lynch, Scott M: 7625
Lyndes, Kathryn: 8020
Lynn, Freda: 2185
Lyons, Jason: 2085
Lyons, Karen S: 2555, 3285, 4165, 7020, 7245
Lyons, Sophia: 7005

Lyu, Benshuai: 7430
Lyu, Jiyoung: 7275, 7460

**M**

Ma, Chenjuan: 4360
Ma, Flora: 1325
Ma, Guoyong: 3270
Ma, Hao: 7460, 7715
Ma, Jessica E: 2180
Ma, Jiahui: 7330
MA, Ke: 8010
Ma, Kris (Pui Kwan): 7490, 7935
Ma, Lina: 3085, 3350, 7205
Ma, Mengyao: 7510
Ma, Mingyue: 7690
Ma, Stefan: 3290
Ma, Xinlin: 4430
Ma, Yan: 7060
Ma, Yu: 7515
Ma, Zhihao: 8020
Mülleder, Michael: 7210
MacArthur, Rodger: 7285
Macchia, Lucía: 4440
Macdonald, Birthe: 4525
MacDonald, Elizabeth: 7695
MacDonald, Geoff: 1215
MacDonald, Stuart WS: 7260, 7525
Mace, Ryan A: 2315, 3250
MacGillivray, Thomas: 7425, 7930
Mach, John: 3520
Machinga-Asaolu, Rujeko O: 3275, 7405
Mack, David L: 7075
Mack, Melanie: 4490
MacKay-Dunn, Marion: 7750
MacLeod, Tim: 7325
Macnaughton, Eric: 7040
MacNeil, Andie: 4365, 7025, 7405
MacPherson, Andrew: 2390, 3260
MacTavish, Erica: 7055
Madden, Kenneth: 1365, 7535, 7720, 8020
Madhavan, Sidharth: 4170
Madrigal, Caroline B: 2550, 3240, 8075
Maeda, Harumi: 1480
Magaziner, Jay: 1275, 1410, 7010
Magder, Laurence S: 8010
Magid, Kate: 4460, 8010
Magnani, Jared W: 7330
Magnotta, Vincent: 7735
Magrath, Priscilla A: 8020
Magrini, Mitchel: 8060
Mahajan, Bhushan: 7360
Mahanna, Allexis: 7480
Mahapatra, Gargi: 2235, 7010, 7425
Maher, Brion: 4005
Mahmood, Atiya: 2395, 2560, 4320, 8000
Mahmoud, Kafayat: 4000, 4330
Mahmoudi, Elham: 1495, 8075
Mahmoudi, Parsa: 7640
Mahoney, Jane E: 2155
Mahoney, Jeannette R: 2525
Mai, An: 7200
Mai, Chunyan: 7610
Maiden, Robert J: 2250, 3495
Mailhot, Tanya: 7645
Maillard, Pauline: 4430
Mair, Christina F: 3375
Mair, Christine A: 4050, 4190, 8010, 8020
Majón Valpuesta, Dolores: 1100, 4270
Majid, Tabassum: 3340
Major, Chloe': 7000
Mak, Jonathan K. L.: 7010
Mak, Wingyun: 8010
Makaroun, Lena K: 4195, 8015
Makeen, Mutasim: 4530
Makowski, Maryam L: 1135
Malatyali, Ayse: 7200, 7260
Malcolm, Paulina M: 7040
Malesevic, Nebojsa: 7155
Malheiro, Grace E M: 1070
Malhotra, Rahul: 1095, 1255, 2065, 3025, 3290, 7205, 7300, 8015
Mallender, William: 7750
Mallett, Christopher A: 7040
Malone, Carolyn A: 2305
Maltsev, Alexander V: 8000
Mameno, Tomoaki: 7105
Mami, Gwendolyn: 8020
Mamo, Sara K: 7140
Mandrekar, Jay M: 4415
Manduca-Barone, Alessandro: 4445
Mandujano, Melissa: 2520
Manheim, Chelsea: 8015, 9025, 9040
Mani, Sneha Sarah: 4380, 9035
Manini, Todd M: 1445, 8015
Manly, Jennifer J: 4375, 7350
Mann, Catherine M: 7055
Mann, Jim: 2410, 2530, 7465, 7750, 7910
Manna, Martina: 7275
Mannen, Delia: 7005
Mannheim, Ittay: 3315, 3425, 3530
Manning, John: 8000
Manning, Lydia K: 3450
Manning, Mark: 1495, 9000
Manning, Wendy D: 3065
Manocha, Gunjan: 8020
Manor, Brad: 2040, 4215, 4535
Mansfield, Tyler: 1090
Mansion, Natalie: 4205
Mansoor, Marrium: 7615
Mansour, Amer: 1425
Manternach, Celine M: 7265, 7460
Mao, Hui-Fen: 8010
Mao, Jieyu: 8055
Mao, Junyan: 1355
Mao, Weiyu: 3305, 3505, 8010
Mao, Ziling: 7060
Mar, Amanda B: 7005
Maraganore, Demetrius M: 7425, 7930
Maras, Jessica C: 7255
Marcel, Solange M: 7600
Marchiano, Joseph S: 7440
Marcinek, David J: 1240, 7075, 7210, 7625
Marcus, Michael S: 4545
Marcus, Robin L: 1275
Marcus, Steven: 8055
Mardini, Mamoun: 1445
Marengoni, Alessandra: 7060
Marfeo, Elizabeth: 7135, 7490
Margot-Cattin, Isabel: 1555
Margrett, Jennifer: 8020
Margulis, Brianna: 8010
Marie, Damien: 4490
Mariko, Hawa: 1060
Marin, Omar A: 7215
Marini, Christina: 2385, 7070
Marino, Francesca R: 8015
Marino, Victoria R: 4375
Mark, Katrina: 7060
Markaki, Adelais: 8000
Markides, Kyriakos: 1495, 4070, 7035
Markot, Michael: 3335
Marks, Suzette: 7040
Markwardt, Sheila: 8010
Marnfeldt, Kelly A: 7520
Marottoli, Richard: 7085, 7410, 7900
Marquez, Stephanie Lyn: 8020
Marriott, Deanna J: 7435
Marron, Megan M: 7710, 8000
Marrone, Nicole: 1440
Marrone-Lloyd, Kenzi: 7000
Marrs, Sarah: 1145, 1270, 3510
Mars, Lori: 2001
Marshall, Barbara: 3315
Marshall, Shawn: 3185
Marsiske, Michael: 4490
Martens, Lindsey: 8020
Martens, Nancy L: 8020
Martey, Ebenezer: 8010
Martignetti, Lucas: 3010, 8010
Martikainen, Aleksiina M E: 2365
Martin, Daniel: 2085
Martin, Diane: 8015
Martin, Jennifer: 7660, 7735, 8000
Martin, Jessica: 4060
Martin, Melanie: 8020
Martin, Mike: 1220
Martin, Peter: 1385, 2255, 4095, 7115, 7355, 7610, 8000, 9045
Martin, Summer: 3465
Martin Ginis, Kathleen: 2525
Martinchek, Michelle: 2345
Martindale-Adams, Jennifer L: 4060
Martinez, Adrian: 7210
Martinez, Angelica: 3175
Martinez, Ariza: 8015
Martinez, Daniel E: 8010
Martinez, Erica: 7205
Martinez, Leah: 8000
Martinez, Leilani S: 2520
Martinez, Margarita: 4165, 8000, 8020
Martinez, Nicolina: 8015
Martinez, Suzanna: 8025
Martinez, Vanessa: 1185
Martinez Amezcua, Pablo: 2035, 2525, 7540
Martinez Lujan, Lazaro: 7720
Martinos, Fadi: 3115
Martins-Klein, Bruna: 7255
Martire, Lynn: 4405, 7145, 7455
Marts Skoglund, Eva: 7010
Maruki-Uchida, Hiroko: 8015
Marx, Hannah: 1020

Marx, Katherine A: 4130, 7005, 7465, 7685
Marx, Ruth: 8000
Mas, Rafael J: 7080
Masaki, Harue: 7400, 7505, 7910, 7920
Masaki, Kamal: 1385, 8000
Masarwa, Malek: 7265
Masdeu, Joseph: 2235
Masella, Norine: 7055
Masliah, Eliezer: 1340
Maslow, Katie: 1030
Mason, Hannah: 1165, 7065
Mason, Stephanie: 7420
Mason, Tyara R: 7080
Masotti, Barbara: 1220
Massart, Mylynda: 3045
Massimo, Lauren: 8020
Mast, Benjamin: 7660
Mastel-Smith, Beth A: 4240
Master, Lindsay: 1250
Masternak, Michal M: 8000
Masters, Julie L: 2490, 8000
Masui, Yukie: 2470, 7115, 8020
Matai, Lavina: 8010
Matarazzo, Richelle: 8010
Mateo-Abad, Maider: 4045
Mateos, Jose T: 4255
Mather, Harriet: 4460
Mather, Mara: 7225
Mathershed, Amor: 8020
Mathias, Vincent F.: 4270, 7660
Mathis, Lindsey M: 1075, 4385, 8010, 8065
Mathis, Michael: 1435
Matlock, Daniel: 8020
Matos-Moreno, Amilcar: 2475, 4050
Matsumoto, Yoshiko: 1480
Matsunaga, Hiroko: 7695
Matsuzaki, Yuriko: 7680
Matta Gallero, Constanza Tamara: 7635
Mattam, Ashwin: 8010
Mattek, Nora: 3140
Matters, Doreen: 4450
Matthews, Alicia: 7080
Matthews, Ian: 8000
Mattie, Johanne: 7155
Matz, Christina: 2555, 4140
Mau, Theresa: 2080
Mauck, Erin: 8020
Mauldin, Rebecca L: 1105, 2260, 4345, 7725, 8010
Maulod, Ad: 1015, 2065, 2185
Maupin, Danny: 2465
Mausbach, Brent: 1335, 7660
Maust, Donovan: 7490, 8010, 8055
Maxfield, Molly: 3015, 4010, 4320, 8020
Maxwell, Cathy A: 1265, 7075, 7155, 7435
Maxwell, Hillary: 3185
May, Jennifer T: 7100
May, Terri M: 7205
May, Jr, Alfred J: 3100
Mayahara, Masako: 2370
Mayeda, Elizabeth Rose: 1545, 4035, 4375, 4380, 7535
Mayer, Jamie: 8010
Maynard, Quentin: 7055
Mays, Allison: 2520
Maza, Valentina: 9030
Mazzola, Paolo: 7275
Mbam, Kingsley C: 7070, 7490
Mbamba, Crispin R: 7335
Mbanefo, Onyinye: 8010
McAdams DeMarco, Mara: 2010, 3150, 4420
McAllum, Kirstie: 7635
McAndrews, Carolyn: 7160
McAtee, Robin E: 7325, 7650
McCabe, Janet: 7295
McCall, Jennifer R.: 8015
McCart, Elizabeth: 7210
McCarthy, Ellen: 3135, 7080
McCarthy, Kate: 1265
McCarthy, Megan: 1165
McCarthy, Michael J: 1525, 4165, 8000, 8020
McCauley, Kathleen: 3300, 7535, 8010
McClay, Joseph L: 7715
McClean, Melissa: 1080
McCleary, Lynn: 4175, 7055
McClellan, Angela: 2205, 3320, 7335, 7740
McClure, Jennifer B: 1265, 9020
McClure, Leslie A: 4340
McConnell, Eleanor S: 2000, 2205
McConnell, Kelly: 2295
McConnell, William R: 3190
McCormack, Brendan: 7215
McCormack, Mary E: 1490
McCorvey, Stephanie: 8010
McCoy, Megan C: 1525, 3080, 4165, 8000, 8020
McCoy, Tessa: 7005
McCoy, Thomas: 7275
McCrae, Christina: 1135, 1405, 3055, 3360, 7500
McCreedy, Ellen: 1115, 1530, 3240, 8010, 8015
McCrory, Cathal: 8060
McCullough, Louise D: 3470
McCullough, Megan: 7685
McCune, Teagan: 7635
McCurry, Susan M: 7735, 8010
McDaniel, Kennedy: 2010, 4420
McDaniel, Laura E: 8000, 8015
McDarby, Meghan: 7055
McDermott, Cara: 7470
McDermott, Kate: 2315
McDevitt, Ross A: 7210
McDonald, Claire: 7065
McDonald, Margaret V: 1395, 3105, 4360
McDonald, Shelley R: 8015
McDonnell, Jenny L: 7530
McDonough, Christine M: 1275
McDonough, John: 4170
McDonough, Meghan H: 3465
McDowell, Cynthia: 7525
McElfish, Pearl: 8000, 8015
McElroy, Jane A: 7100
McEvoy, Sarah: 8020
McFadden, Angela: 8015
McFarland, Malcolm: 2300
McGarry, Kathleen: 3145
McGee, Blake T: 1230
McGee Lawrence, Meghan: 7285
McGhan, Gwen: 3465
McGill, Robert M: 9020
McGilton, Katherine: 4255, 8015
McGinity, Ashley C: 8010, 8015
McGinley, Jacqueline: 7410
McGinty, Patrick: 3585
McGough, Ellen: 7655
McGovern, Justine: 4540
McGovney, Kevin: 1405, 3360, 7500
McGowan, Joseph: 8010
McGrath, Lydia: 8015
McGrath, Ryan: 1525
McGuire, Katrina: 7720
McGuire, Lisa: 3100, 4235, 4390, 7200, 7205
McHale, Jenna R: 7465
McHugh, Molly: 8010
McIntosh, Nathalie: 1080
McIntyre, Madeline: 1145, 3510, 8000, 8010
McIntyre, Thomas: 7630
McKay, Sandra: 8015
McKay, Tara: 1170, 3075, 8080, 9030
McKelvey, Miechelle: 2205
McKenna, Kevin: 2090
McKenzie, Colin: 7130
McKenzie, Michael R: 7465
McKibbin, Christine: 7440, 7490
McKiddy, Sarah: 2115, 2450, 4285, 7415, 7900
McKinley, Catherine: 4110
McKinnon, Monette L: 3215
McKneely, Katelyn: 7045
McLaren, Jaye: 3395, 9020
McLaughlin, Sara: 1105
McLaughlin, Sara J: 8010
McLean, Elisabeth: 1335, 3385, 4435, 7425, 9015
McLin, Maia: 7610
McMullen, Tara: 1315, 3090, 8030
McMurray, Josephine J: 3315
McNally, James: 8090
McNamara, Kelsey C: 8000
McNeish, Brendan: 2220
McNellis, Cynthia: 8010
McNicoll, Lynn: 7070
McPeak, Ashley: 4470
McPherson, Rachel: 4090
McPhillips, Emily A: 8015
McPhillips, Miranda V: 1535, 3335, 7525
McVey, Ava: 1185, 8000
Meadows, Jeffrey: 8020
Mealer, Meredith L: 7155
Meara, Ellen R: 7000
Mechanic-Hamilton, Dawn: 8020
Medcalf, Robyn: 7490
Medders, Laura: 7220, 8015
Medina, David: 8000
Medina, Jose T: 3035
Medlin, Austin: 1455, 2085
Medrano, Valeria: 8015
Meek, Paula M: 7440
Meghelli, Ilan: 8010, 8020
Mehboob, Gul Rukh: 8075
Mehl, Matthias: 8020
Mehrabi, Fereshteh: 1225, 2230
Mehrjerd, Ameneh: 7010
Mehta, Neil K: 4455, 7230, 7300, 8000
Mehta, Rhea: 1535, 3035
Mei, Yongxia: 3330
Meier, Diane: 2520

Meier, Helen CS: 1495, 8060, 9000
Meijers, Judith MM: 4255, 8015
Meinertz, Naomi R: 1165, 3055, 3575
Mejia, Shannon T: 1570, 4420, 7720, 8000, 8020, 9050
Mejia Guevara, Ivan: 4035
Melero-Dominguez, Maria: 4130, 7660
Melgar Castilo, Andrea I: 8065
Mell, Johnathan: 7490
Mellett, Katlyn: 8010
Melo, Rosa: 7465
Melo, Ruth C: 4465, 8015
Memmer, Nicole: 1040
Mena, Emily: 4345
Menard, Alixe: 1010
Menashe, Aviad: 7600
Mendes de Leon, Carlos F: 1155, 3540, 8020
Mendez, Benjamin: 3150
Mendez, Jennifer: 1050
Mendez, Therese M: 7315
Mendez Campos, Barbara: 2555, 3275
Mendoza, Edna Patricia: 9050
Meneilly, Graydon: 7205
Meng, Hongdao: 2370, 3365, 7455
Meng, Lingsong: 1445
Menne, Heather L: 2070, 2495, 3565, 7145, 7295
Menne, Heather: 8010
Mensik, Ashley: 8020
Mentch, Heather: 2480
Mera, Alejandra L: 7215
Mercan, Murat A: 7160
Mergenthaler, Andreas: 1100
Mernitz, Sara: 1540
Merrilees, Jennifer: 3285
Merrill, David A: 7425, 7930
Merrill, Ray M: 8015
Merss, Kristin: 7200
Merten, Natascha: 1000, 4300
Mertz, Kathryn: 7695, 7695
Mertz, Laurel: 7050, 7085
Metcalf, Judith: 8015
Meyer, Ilan H: 1110
Meyer, Kylie: 2375
Meyer, Oanh: 4430, 8025
Meyers, Gabrielle: 7020
Meynadasy, Melissa: 8020
Meynet, Stéphanie: 1100
Meza, Erika: 1495
Mezuk, Briana: 3235, 3540, 7490, 7695
Mgabhi, Philile: 7150, 7735
Mi, Hong: 2185, 3270, 7230, 7295, 7510, 7740, 7915, 8010
Miao, Hongyu: 2240
Miceli, Chelsea: 7340
Michael, Yvonne L: 8020
Michaels, LeAnn: 4160
Michaud, Veronique: 7425, 7630
Micheel, Frank: 1100
Michel Pinto, Antonio F: 7425
Michelson, Lisa: 7275
Mick, Paul: 4300, 7635
Mickus, Maureen A: 7510
Middleton, Laura E: 8015
Miglani, Jasroop K: 8000
Migliaccio, John N: 4545
Migneault, Deborah: 7740
Migovich, Miroslava: 7155
Miguel-Cruz, Antonio: 7620, 7630, 7905
Mijkovic, Iva: 2220
Mikal, Jude P: 1485, 7145
Mike, Leigh Ann: 4470
Mikels, Joseph A: 3355
Milan, Rebecca A: 7420
Milani, Sadaf A: 1495, 4095, 7025, 7235
Milberg, William: 3335
Mildrum Chana, Sofia: 7700
Milidonis, Mary: 3585
Miljkovic, Iva: 2220, 7010, 8000
Millar, Courtney: 2080, 7450, 8000
Miller, Edward A: 1360, 3495, 4305, 7295, 7670
Miller, Emily: 7700, 7935
Miller, Jacqueline: 2265
Miller, Julie: 1370, 2001, 2420, 3145, 4140
Miller, Justin B: 8055
Miller, Katherine: 1170, 1395, 2500, 3570, 4520, 7005, 7470
Miller, Kenneth L: 7330
Miller, Kristin A: 3575
Miller, Lindsay: 3330
Miller, Lyndsey M: 3285, 4160, 4165
Miller, Maggi: 1045, 7425, 7930
Miller, Mary Beth: 1405, 7500
Miller, Matthew J: 3010
Miller, Matthew: 4430
Miller, Melicent: 4510
Miller, Michael E: 1090, 1410, 2220, 2505
Miller, Nancy: 7515
Miller, Richard: 8000
Miller, Sara E: 7675
Miller, Sarah J: 7115
Miller, Steven J: 1530
Miller, Vivian J: 7505
Miller-Winder, Amber: 7005, 7420
Mills, Sarah: 2090
Mills, Whitney L: 1080, 3425, 7250, 7545, 8020
Milnamow, Mary: 2265, 7155, 8025
Mimbs, Hope: 1010, 7160
Min, Hae Youn: 4505, 7070
Minard, Charles G: 1420, 2080, 2235
Minch, Kendra: 8010
Mindlis, Irina: 7270, 7630, 8020
Miner, Jennifer: 7065
Mingo, Chivon A: 2245
Minor, Bryan J: 2360
Mintz, Yonatan: 7510
Minyo, Morgan J: 1030, 1265
Mion, Lorraine C: 7435
Mir, Nabiel A: 7205
Miranda, Jacob F: 7685
Miran-Khan, Karim: 1365
Mirarchi, Kelly A: 7150
Mireles, Josh: 8050
Mirjan, Nadia: 7200
Mirza, Mansha: 8015
Mirza, Noeman: 4025, 8010
Mirza, Raza M: 7480, 7900
Mishra, Sidharth P: 7210
Misonow, Julia: 4345
Missell-Gray, Rachel: 4400
Mitchell, Barbara A: 4445, 7445, 7740
Mitchell, Calli O: 7680
Mitchell, Colter: 8060
Mitchell, Jacob: 7235
Mitchell, Jamie: 3170
Mitchell, Lauren L: 3130
Mitchell, Mary: 1470
Mitchell, Michael N: 7735, 8015
Mitchell, Uchechi A: 3180
Mitschke, Diane: 2195
Mitsova, Diana M: 4430
Mittal, Kanika: 2340
Mittelman, Mary S: 1380, 3130
Mitzner, Tracy L: 1040, 3415
Miwa, Hiroyasu: 7750
Mixon, Amanda: 8035
Miyae, Yamakawa: 7330
MIYAMA, Tomoko: 8015
Miyamae, Fumiko: 7350
MIYASHITA, Mariko: 8015
Miyawaki, Christina E: 2205, 3320, 4070, 7335, 7740
Miyazaki, Atsuko: 1205, 8015
Mizell, Nate: 7530
Mkuu, Rahma: 7440, 7935
Mo, Cen: 1365
Moaddel, Ruin: 8000
Mobley, Catherine: 2355
Mobley, Taylor M: 4035, 4375
Mochel, Amy: 7250
Moczygemba, Walter: 2105
Modig, Karin: 7510, 7915
Modrek, Sepideh: 4035, 8020
Moeller, Kelly: 1520
Moen, Maria D: 3090
Moffitt, Chad: 3175
Moffitt, Terrie E: 2220
Moghekar, Abhay: 3070
Mogle, Jacqueline: 1080, 1250, 1525, 2160, 4280, 4515, 7255, 7630
Mohamed, Leena: 8015
Mohamed Mansoor, Mishfak Abdullah: 8000
Mohammed, Hajir: 8015
Mohankumar, Rakshitha: 1325
Mohanty, Sanjay: 3335
Mohd Farhan, Nur ‘Atiqah: 2065
Mohile, Supriya: 7420, 7920
Mohr, David C.: 7545
Mohr, lynn L: 3500
Moineddin, Rahim: 7300
Mois, George: 8010, 8020, 8020
Mokuau, Noreen: 4015
Molholm, Sophie: 8000
Molina, Anthony: 1435, 2235, 2435, 4170, 4210, 7010, 7425, 8000
Molina Hermosilla, Irene: 3150
Molinari, Victor: 7355
Molly, Delzio: 3010
Molnar, Lisa J: 3455
Molton, Ivan: 7240
Monaghan-Geernaert, Pamela: 4435
Monane, Mark: 7425, 7930, 8070
Mondol, Swastika: 8020

Mongoven, Jennifer: 7065
Monin, Joan K: 1345, 2075, 2305, 3285, 4165, 7245, 7270, 8010, 8020
Monroe, Kelly: 1275
Monroe, Stephanie: 3030
Monroe-Lord, Lillie: 7030
Montano, Monty: 1330
Montayre, Jed: 7680
Monteiro, Andréa: 7040
Montepare, Joann M: 1310, 2440, 3120, 3310, 3515, 4240, 7415
Montgomery, Aoyjai P: 8000
Montori, Victor M: 8010
Montoro-Rodriguez, Julian: 7265
Montoya, Ana: 8015
Monzio Compagnoni, Matteo: 7275
Moo, Lauren R: 2430, 3110, 7135, 7420, 7490, 8010, 9020
Moody Jones, Beth: 8015
Moon, Chooza: 7735
Moon, Heehyul: 1045, 7425, 7500
Moone, Rajean: 1520, 2225, 2535, 3490
Moonen, Esmee: 8015
Mooney, Aimee: 1125, 7425
Mooney, Laken A: 8000
Mooney, Scott: 7360
Moore, Alison A: 3065, 3115
Moore, Ann Zenobia: 4170, 7720
Moore, Kari A: 8020
Moore, Miranda A: 7220
Moore, Raeanne: 7660
Moore, Ryan C: 8020
Moore, Susan L: 3575
Moored, Kyle D: 1240, 2045, 3375
Moorman, Sara M: 1140, 1280, 2001, 3410, 4400
Moorthy, Arsha: 7075
Mor, Maria: 4195
Mor, Vincent: 3240, 7030
Morales, Joseph: 3515
Morales Hernandez, Beatriz: 4475
Moran, Ryan: 7080
Morante, Mariana: 4290
Moreau, Kerrie: 1410
Moreines, Laura T: 1395
Morello-Frosch, Rachel: 4035
Moreno, Julie A: 7075
Moreno, Monica: 2305, 4200
Moreno, Sylvain: 3060
Morgan, David: 7425
Morgan, Debra G: 7135
Morgan, Jennifer: 4520
Morgan, Jennifer Craft: 4290, 8010, 8020, 8070, 4290
Morgan, Kerri: 7260
Morgan, Robert O: 3240
Morgan, Stephanie: 7435
Morhardt, Darby: 1125, 4480, 7220
Moriarty, Haley: 8015
Moriarty, Helene: 7290
Moriarty, Sheila: 7250
Morin, Brittany: 2020
Morin, Ruth: 3010
Morisaki, Naoko: 7105
Moritz, Robert: 8000
Moriyama, Yoko: 7490
Mork, Paul Jarle: 7005
Morka, Angela: 7085
Moro, Manuel: 4310
Morris, Jada L: 7080
Morris, John C: 4515, 7720, 8015
Morris, Stephen: 4195
Morrison, Christopher D.: 8000
Morrison, Jake: 4325
Morrison, Lindsay: 3465
Morrison, Wanda: 7480
Morriss, Emily P: 1495
Morrow, Isaiah: 7635
Morrow, Jason: 7530
Morrow-Howell, Nancy: 7535, 8000, 8010
Mortenson, Ben: 2410, 2530, 7300
Morton, Cynthia: 3090
Morton, Elizabeth R: 8010
Morton Padovano, Cynthia: 2330
Moser, Debra K: 3320, 7215
Moses, Ellen Sue: 4165
Mosesso, Kelly M: 7625
Mosley, Thomas H: 1000, 4440, 7060
Mosqueda, Laura: 1270, 2001, 2150, 2375, 4355, 7495
Moss, Benjamin: 4170
Moss, Daniel: 1185, 8020
Moss, David: 3285
Moss, Karen O: 2415, 7490, 7935
Moss, Mark: 3305
Motilla, Ronaldo A: 2405
Moura Maurmann, Rafael: 8000
Mourany, Lyla: 7235
Mousavi, Seyedeh Leila: 7210
Moustafa, Sara A: 1155
Mouton, Charles P: 7405
Moxley, Jerad: 7080, 8020
Moy, Marilyn: 7720
Moye, Jennifer: 1325, 2345, 2385, 3425, 8020
Mpofu, Elias: 3085, 8080
Mroczek, Daniel: 1570, 4375, 4480
Mroz, Emily L: 1345, 2305, 7055, 7310
Mroz, Tracy M: 2500, 4100, 4360, 7080, 7545, 8010
Mu, Christina: 3275
Muñoz, Elizabeth: 2330, 7225, 7485, 7925
Mudar, Raksha A: 1405, 3415, 4270, 7015, 7270, 7455, 7630, 7660, 8015, 8020
Mudrazija, Stipica: 3030, 7470
Mueller, Alexandra: 2230
Mueller, Christine: 4125
Mueller, Kim: 3425
Mueller, Megan: 8020
Mueller, Ranell L: 7610
Muench, Ulrike: 8055
Muir, Michael: 7010
Mukherjee, Bhramar: 1435
Mukherjee, Namrata: 8010
Mukherjee, Shalini: 1565
Mukhtar, Yerkyebulan: 1140
Mulcahy, John: 7425
Mulhorn, Kristine A: 2470, 7230, 7680
Mulkey, Heather: 8010
Muller, Chandra: 1095, 7230, 7350
Mulligan, Kate: 8020
Mulligan, Michael: 7625
Mumby, Rachel A: 1115
Mumma, Kara T: 3415
Mun, Kyoungmi: 7430
Muneoka, Shelley: 7055
Mungas, Dan: 7535
Muniz Terrera, Graciela: 4535, 7425, 7535, 7540, 7930
Munly, Kelly A: 7690
Munnell, Alicia H: 2420
Munoz, Elizabeth: 3095
Munoz, Richard: 2545, 8010, 8070
Murabito, Joanne M: 2160, 7360, 7735, 8010, 8015
Murali, Komal Patel: 4360
Muramatsu, Naoko: 8020
Murawski, Alaine: 7005, 7420, 7490
Murayama, Hiroshi: 4190, 7350, 8015
Murillo, Fernando I: 3420
Muroff, Jordana: 7475
Murphey, Yi L: 8025
Murphy, Blake: 3285, 8060
Murphy, Rachel A: 7535, 8020
Murphy, Sara C: 1065, 7080
Murphy, Terrence: 7040
Murray, Andrew: 1160
Murray, James: 7325
Murray, Sarah: 7665, 8015
Murthy, Venkatesh L: 7710, 8000
Murton, Andrew J: 1130
Musa, Najma A: 7480
Musci, Rashelle: 7615
Musi, Nicolas: 2505, 4210
Musich, Madison: 1135
Musil, Carol: 7265, 7660
Muskin, Ryan: 8020
Muss, Hyman B: 8000
Mussaad, Ahmed: 8015
Mustafa, Dina: 7320
Mutchler, Jan E: 1475, 7485
Muthupillai, Raja: 2080
Mutthina, Ramesh: 7425, 7910
Mwanda, Sheila: 8015
Mwangi, Eunice Muthoni: 4380
Mwendwa, Denee: 8020
Myagmarjav, Sugarmaa: 1140
Myers, Janet A: 2520

N

Na, Annalisa: 9010
Nabong, Jennifer: 1490
Nabors, Emily B: 1005
Nadarajan, Gayathri Devi D/O: 8015
Nadash, Pamela: 1360, 4115, 7490, 7670
Nadobnik, Halina: 2565
Nadorff, Danielle Kristen: 1360, 3360, 7610
Nagarajan, Niranjani: 4380
Nagel, Corey L.: 3035, 7300, 7700
Naglie, Gary: 3185
Nagururu, Nimesh V: 2080
Nah, Suyoung: 8020
Naidoo, Nirmala: 8015
Naik, Aanand D: 2075, 4340, 7215
Naim, Sigal Pearl: 3465, 4040

Nair, Anil: 8015
Nair, Malini: 8015
Nair, Sangeeta: 2220
Nair, Tripti: 8000
Nair, Uma: 7500
Nakad, Lynn: 1530
Nakagawa, Kazuma: 8000
Nakagawa, Takeshi: 2470, 7115, 7490, 8015, 8020
Nakamura, Masahide: 8015
Nakanishi, Miharu: 2255, 7640
Nam, Stephanie J: 4535
Namasivayam-MacDonald, Ashwini: 2060, 3500
Nambisan, Priya: 7070, 7360, 7425, 7540, 7930
Namrouti, Amina: 7125
Namukasa, Juliet: 8010
Nandigam, Maanasa: 7085
Nanjiba, Nushaiba: 2395
Napoleon, Betty: 1490
Narine, Donnette R: 7350, 7475
Nash, Princess: 3240
Nashef-Hamuda, Reem: 3015, 7130
Nasiri, Rojan: 4320
Nasr, Samer: 7730
Nasrallah, Ilya: 2220
Nathan, Kobi: 8035
Nathaniel, Thomas: 8000
Natsume, Yukie: 7315
Naud, Daniel: 1100, 3320
Navos, Melanie: 7440
Naylor, Mary: 3300, 7435, 7535, 8010
Nazario-Acevedo, Jaminette M: 1505
Nduka, Chinelo: 7600
Neale Furneaux, Heather K: 7040
Nearing, Kathryn A: 1415, 7155, 8020
Nebeker, Johanthan: 8030
Nece, Patricia: 3580
Nederostek, Kate: 3100
Needham, Belinda: 1290, 2045
Neelamegam, Malinee: 2445, 7700, 8000
Ne'eman, Ari: 7000
Neer, Emory: 8010
Neff, Donna F: 2330
Neiberg, Rebecca H: 1090, 1390, 1410
Neiderman, Adrianna C: 7225, 7500
Neidre, Daria: 2295
Neill, Kathryn: 7650
Neilson, Lee: 7310
Neiman, Joseph: 4530
Neises, Kristen: 7220
Neller, Sarah A: 2395, 3385, 7085
Nelson, Christi L: 2540
Nelson, Elizabeth: 3065
Nelson, James F: 3350, 4310
Nelson, Matt: 4510, 7295
Nelson, Niccole A: 3165, 7255
Nelson, Suchitra: 3340
Nelson-Faulconer, Jennifer: 2460
Nelson-Twakor, Elizabeth: 8015
Nemati, Donya: 3050, 3235
Nemeth, Samuel: 8020, 8035
Nemmers, Natasha L: 1335, 2015, 3165, 7065
Nemoto, Kiyotaka: 8015
Nemoto, Miyuki: 8015
Neretti, Nicola: 3020
Neri, Mel: 8025
Nessen, Sarah: 8015
Nesser, Whitney: 7680, 7725
Nesvet, Matthew: 7110
Neubauer, Noelannah: 7630, 7905
Neugebauer, Benjamin: 9015
Neugebauer, Volker: 1335, 3385, 4435, 7065, 7425, 9015
Neupert, Shevaun D: 1565, 4020, 4405, 7610
Newby, Launa: 3140
Newman, Anne: 1240, 1410, 2080, 2220, 2510, 4005, 7060, 7710, 8000
Newman, Anne B: 8020
Newman, David: 7360, 7425, 7610
Newman, John C: 1460, 2530, 3210, 4170, 7075, 8000
Newman-Casey, Paula Anne: 7205
Newmeyer, Natalie: 8015
Newsham, Tina: 2130, 2325, 3265, 3495, 8010
Newsom, Jason T: 3035
Ng, Amy C C: 4390
Ng, Angus Jun Jie: 8015
Ng, Boon Peng: 8015
Ng, Crystal: 8020
Ng, Peter H F: 8020
Ng, Reuben: 1425, 2270, 3015, 7155
Ng, Yee To: 4185
Ngo, Victoria: 3110, 7490
Ngugi, Anthony: 4380
Nguyen, An T: 3300
Nguyen, Andrew H: 9020
Nguyen, Anh Thy H: 7040
Nguyen, Ann W: 1060, 2055, 3045, 4190
Nguyen, Annie L: 3115, 7495, 7930
Nguyen, Annie: 8000
Nguyen, Boi-san: 8025
Nguyen, Duy: 8010
Nguyen, Emerald: 4085
Nguyen, Hoang: 4070
Nguyen, Jade: 7510
Nguyen, Kim N: 2205
Nguyen, Kristine: 7225
Nguyen, Manh Hung: 8020
Nguyen, Nhan: 7145
Nguyen, Thien: 8015
Nguyen, Tho: 7620, 7655, 7720, 8015
Nguyen, Tiffany: 7525
Nguyen, Trang TH: 1100
Nguyen, Uyen-Sa D T: 9050
Nguyen, Victor H: 7085
Nhan, James: 8000
Nhpang, RoiSan: 7700
Ni, Pengsheng: 7420
Ni Leime, Aine: 2425
Nicholas, Lauren H: 3145
Nichols, Emma: 3335, 3540
Nichols, Linda O: 4060
Nichols, Madeline J: 4280, 7730
Nicholson, Jody: 4280
Nicklas, Barbara J: 1240, 1410, 2485, 2505
Nicklett, Emily J: 4475
Nickrent, Megan S: 8020
Nicolay, Sarah: 7470, 8020
Nicosia, Francesca M: 1245, 3010
Nidadavolu, Lolita S: 4005
Nie, Hao: 7075
Niedoba, Simone: 1555
Nielsen, Karen: 1230
Nielsen, Kelli: 7000
Nielsen, Lisbeth: 1340, 4500
Nielsen, Natalia N: 7060
Nieman, Carrie L: 1440, 4300
Niimi, Yukari: 8015
Nikahd, Melica: 2445, 4475
Nikitin, Jana: 1375, 2300, 7245
Nikzad, Amir: 7695
Nikzad-Terhune, Katherina: 3450, 4240, 8010
Ning, Yanhua: 7640
Nishi, Mariko: 7695
Nishida, Yoshifumi: 1505, 7260
Nishikitani, Mariko: 7430
Nishimura, Jun: 7295
Nishita, Christy: 3565, 8010
Nishitani, Megan: 7010
Nishiyama, Hiroyuki: 8015
Nittinger, Sophia: 7440, 7935
Nix, Caitlyn A: 4120
Nizza, Megan: 3090, 7475
Njoku, Crystal: 7150
Nketsiah, Ebow T: 8020
Nkimbeng, Manka: 1465, 2015, 3025, 7640
Nnemnbeng, Jeannys F: 8010
Noble, Cheryl: 3515
Noble, Molly E: 1160, 8010
Nofuji, Yu: 7275, 8015
Noguchi-Watanabe, Maiko: 7295, 7340, 7510
Noh, Hyunjin: 7440
Nolte, Julia: 8020
Nomura, Ayano: 7260
Nomura, Mitsunori: 4170, 7075
Nonaka, Kumiko: 8015
Noor, Mirza Ishrat: 8000
Norberto Pereira, Graziele: 7225, 7655
Norgaard, Martin: 8010
Noriega de la Colina, Adrian: 3050
Noriega-Makarskyy, Daisy: 7495, 7930
Norling, Amani M: 8000
Norman, Kristina: 7315
Norris, Grace: 1070, 2410, 7465, 7910
Norris, Hayden S: 7300
Norris, Keith C: 3475
Norstrand, Lu: 3495
Nortey, Rose T: 7070
Norton, Edward: 3145, 7490
Norton, Sara: 8010
Nothelle, Stephanie: 2085, 4300
Noureddine, Sarah: 8000
Nouri, Mohammadjavad: 3205
Nouryan, Christian: 8010, 8015
Novek, Sheila: 7040
Nowakowski, Alexandra C H: 3075
Nowels, Molly: 7110
Nowicki, Scott: 7060
Nowinski, Cindy: 4230
Noy, Roy: 8010
Noyer, Emily: 7660, 8020
Numata, Keizo: 7105

Nutakor, Jonathan A: 7020
Nwakasi, Candi: 3475, 7600
Nweke, Chizobam: 7600
Nwoke, Mary Basil: 4000
Nyang'au, Getrude K: 3055
Nyarko, Albert Junior: 8015
Nyarko, Veneranda: 8000
Nyrop, Kirsten: 8000

**O**

Obando, Viviana: 8015
Obeid, Jihad: 2355
Oberdier, Matt T: 8015
Obhi, Hardeep: 1385
Obioha, Chidiebere Wisdom: 4000
O'Brien, Charles: 8015
O'Brien, Erin: 8020
O'Brien, Jennifer L: 7610
O'Brien, Kyra: 8015
Obuchi, Shuichi: 7205, 7490, 7540
Ocanas, Sarah R: 4310
O'Connell, Jeremy D: 1500
O'Connell, Megan: 7030
O'Connor, Maureen: 9020
O'Connor, Melissa: 7055, 7150, 7360
O'Conor, Rachel: 4480
Odden, Michelle C: 8010, 8015
O'Dea, Eireann: 3200, 4445, 7155, 7445
Odeyemi, Emmanuel: 4000
Odlum, Michelle: 1465
Odo, Obinna Casmir: 3475
O'Donnel, Arden: 1345
ODonnell, Arden E: 7215
O'Donnell, Caitlin: 7000
O'Donnell, Kari: 7700, 7935, 8000
O'Donnell, Kennedy: 3565
Oduro, Elisha B: 8010, 8015
Offerman, Jens: 7155
Ofori, Edward: 8015
Ofstedal, Mary Beth: 7265, 7525
Ogawa, Elisa F: 3335
Ogawa, Kenichi: 7400, 7920
Ogawa, Madoka: 8020
Ogawa, Susumu: 1265
Ogg, Jordan D: 7320
Ogle, Destiny M: 7070, 7265
O'Grady, Brandon B: 7300
O'Grady, Thomas: 3255
Ogugu, Everlyne: 2200
Ogunade, Temi: 8020
OGUNGBILE, Adedayo J: 8075
Ogunjesa, Babatope: 7145
Oh, Chorong: 9010
Oh, Esther S: 1440, 4005, 4300
Oh, Eunna: 7640
Oh, Gahee: 3135, 7080
Oh, Kyeung Mi: 1165
Oh, Oonjee: 7270
Oh, Patricia: 7415
Oh, Sarah Soyeon: 7630
Oh, Seungjun: 8015
Oh, Yun taek: 1475, 7050, 7130
OHala, Max: 3490
O'Hanlon, Ann M: 7315
Ohene, Eric: 8075
O'Hern, Steve: 3455
Ohgi, Sayuri: 2045
Ohmer, Mary L: 1290
Ohta, Takahisa: 8015
Ojelabi, Olanike: 4355
Ojo, Eunice: 7260
Okada, Jamil: 8015
Okamura, Tsuyoshi: 7350, 7680, 8015
Okechi, Benard Chibuike: 4000
Okereke, Olivia I: 2080, 2315, 3250, 4550
Okine, Joana: 7095
Okolie, Tochukwu J: 2495, 8010
Okoye, Ifeoma J: 8065
Okoye, Safiyyah M: 3275
Okpoebo, Amanda: 8010
Okrah, Albert K: 8015
Okubo, Suguru: 7490
Okun, Sarit: 1190
Okura, Tomohiro: 8015
Oladejo, Dianne: 4480
Oladimeji, Abolade: 8000
Olanrewaju, Sherif A: 7535
Olds, Danielle: 2500
O'Leary, Bryant: 7260
O'Leary, Bryant J: 8020
O'Leary, Meghan: 7125
Olivari, Benjamin S: 4390, 7200, 7205
Oliveira, Iara B: 4305
Olivieri-Mui, Brianne: 3135, 4225, 7080, 8015
Olmo, Jeana: 7035
Olmos, Marina: 7055
Olsen, Bonnie J: 2150, 4355, 8055
Olson, Sarah: 2080
Oltmanns, Thomas: 8010
Olvera, Alfredo: 7400, 7920
Olvera, Alice Gavarrete: 2020
Olvera, Charlie: 7005, 7420, 7490
O'Malley, Kelly: 2370, 2385, 3425, 8020
O'Malley, Pearse: 3115
O'Malley, Terrance: 3090
O'Mathuna, Donal: 8020
Ome, Blessing Nneka: 4000
O'Muircheartaigh, Colm: 1110
Omura, John: 7205
Ondanat Veetil, Shyam Krishnan: 9020
O'Neil, GeVonne C: 4100
O'neil, Jahn: 7000
O'Neil, Kelly: 7420
O'Neill, Margaret: 2425
Ong, Anthony: 1570
Ong, Chong Yau: 8015
Ong, Lawrence: 8015
Ong, Liane: 7040
Ong, Lydia Q: 4405
Ong, Marcus Eng Hock: 8015
Ono, Keiko: 7055, 8010
Onyechi, Chimaoge: 2235
Operario, Don: 8015
Oppong, Kwaku Duah: 2090
Opresko, Patricia: 3020
Orange, Joseph B: 1070, 2410, 7635
Oravecz, Zita: 8020
Orchard, Carole A: 7525
Orewa, Gregory N: 1425, 1550, 3255
Orkaby, Ariela R: 8015
Orkibi, Hod: 8020
Orlovsky, Irina: 7255
Ornago, Alice M: 4315, 7010
Ornstein, Katherine: 2155, 2230, 3570, 4460, 4520, 7470
Orr, Miranda E: 3020
Orr, Timothy: 3020
Orsega-Smith, Elizabeth: 7545
Orsulic-Jeras, Silvia: 7465, 8020
Ortega, Alexandra: 8010
Ortega Hernández, María: 3190
Ortenberg, Elia K: 4085
Ortiz, Hector L: 7495
Ortiz, Jahmar: 2175
Ortolá, Rosario: 3330
Orwig, Denise: 1275, 1535, 2015, 3035, 7010, 8095
Ory, Marcia G: 4130, 4340
Osae-Oppong, Edward: 3115, 7280
Osahon, Ob W: 2235
Osborne, Danielle: 7200
Osborne, Jason W: 7145
Osei, Evans A: 8015
O'Shields, Jay: 7155
Oskarsson, Bjorn: 2200
Osmond, Claudia: 7480, 7900
Osokpo, Onome H: 3300, 7440, 7535, 7925, 8010
Osorio, Jose: 3330
Osorio, Leslie: 7530
Osso, Francesco: 1165
Ost-Mor, Sharon: 8010, 8020
Ostrem, Nicholas: 3485
Ostrom, Ethan L: 7210
Osuka, Yosuke: 8015
O'Sullivan, Kelly: 2295, 7350, 7530
O'Sullivan, Roger: 1225, 1225, 2055
Osuna, Margarita Maria M: 1320
Oswald, Austin G: 2540, 4250, 4445, 4495
Oswald, Frank: 1555, 3155
Ota, Kazuki: 8015
Ota, Miho: 8015
Otake, Eriko: 8015
Otewa, Meshack W: 2115, 3140
Otgon, Saranchuluun: 1140
Otsuki, Naoko: 7080, 7105
Ott, Brian R: 7320
Ott, Corilyn: 3240
Ou, Alison Y.T.: 8050, 2050
Ou, Yinuo: 2465, 7630
Ouellet, Jennifer: 7410, 7900
Ouyang, Meishuo: 3085
Ouyang, Zheng: 4270
Oveisgharan, Shahram: 1235
Overbeck, Kevin: 8010
Owen, Donna C: 7080
Owens, Becky: 4320
Owens, Joshua: 2020
Owens, Victoria: 8010
Owino, Jonix: 2195, 7030
Owsiany, Montgomery T: 7115, 7490
Oxley, Jennie: 3455
Oyales, Carmelina F: 2405
Oyesanya, Tolu: 4530
Oz, Naci: 7210

**P**

Pa, Judy: 7750, 8015
Pärtel Araújo, João: 7465
Paccagnella, Omar: 7035
Pace, Doug: 3535

Pack, Allison: 4480
Padala, Kalpana P: 7360, 7630
Padala, Prasad R: 7360, 7630
Pagan, Jose: 3540
Page, Kyle S: 1080, 7695
Paguirigan, Mark Ryan B: 7205
Pahlen, Shandell: 4095
Pahor, Anja: 8000
Pal, Jay: 7625
Palavicini, Juan Pablo: 1130, 7000
Palena Hall, Elizabeth: 8030
Palgi, Yuval: 4020
Palimaru, Alina: 3340
Palleschi, Mary K: 3335
Pallis, Maryssa: 4115, 7670
Palmberg, Kyle: 7455
Palta, Priya: 1000, 2525
Paluch, Rocco A: 1275
Pan, Minmin: 4440
Pan, Tiffany: 8020
Pan, Xi: 3505
Pan, Yinghao: 7425, 7930
Pan, Yu-Ten: 7055
Panda, Satchidananda: 2005, 4210, 7015
Pandey, Atul Kumar: 7470
Pandsheno, Sepehr: 4320
Panesar, Simran: 8000
Pang, Vincy Wing Sze: 7640
Panos, Raquel: 3460
Paola Gomezjurado Gonzalez, Laura: 1575
Paoletta, Camille: 7265
Paone, Deborah: 2010
Papazyan, Anna: 7735
Pappa, Sara T: 1300, 3460
Pappadis, Monique R: 4355, 7405
Parekh, Rupal M: 1105, 8010
Parella, Morgan: 3235
Pargai, Dhruv: 8010
Parikh, Alexander: 8085
Parikh, Romil R: 3090, 4245
Park, Bohye: 1140
Park, Byoung-Jun: 7460
Park, Caroline: 7480, 7900
Park, Chan Mi: 3135, 7080
Park, Chang Gi: 7080, 8015
Park, Cherrie: 7605
Park, Chung Hyuk: 7295, 8015
Park, Elyse R: 7680, 8015
Park, Esther: 8015
Park, Eunyoung: 7265, 8000, 8010, 8015
Park, Hayoung: 7360
Park, Hee Man: 8045
Park, Hyeran: 7310
Park, Hyunki: 8010
Park, Jeongmin: 7350
Park, Ji Yeong: 7420
Park, Jisung: 7005
Park, Joanne R: 1440
Park, Joo Young: 8000
Park, Joonhyeog: 7030
Park, Joon-Hyuk: 4420, 7205, 7620, 7640, 7720, 7750, 8015, 8020
Park, Jun Ho: 8020
Park, Junmin: 7605, 8020
Park, Juyoung: 1140
Park, Juyoung: 2240
Park, Juyoung: 3440, 7640
Park, Juyoung: 8010, 8015, 8020
Park, Min Kyung: 7260, 7275, 7290, 7680, 8015
Park, Min Kyung: 8015, 9010
Park, Moonkyoung: 7355, 8015
Park, Nan Sook: 1140, 3440
Park, Pil: 8015
Park, Rinseo: 4525
Park, Saeun: 7010
Park, Seeun: 8065
Park, Serim: 8015
Park, Sojung: 1295, 3200, 4265, 7030, 7290, 7630, 7665, 8010
Park, Soobin: 3200, 7150, 7310, 7630, 8010
Park, Soobin: 8010, 1540
Park, Sooyoun: 2295
Park, Sooyoung: 7150
Park, Sophie: 4355
Park, Suah: 7735
Park, Suhyun: 7295, 8015
Park, Sujeong: 3360
Park, Sung S: 1475, 2425, 7490
Park, Sung Ji: 1380
Park, Yanghyun: 3275
Park, Yujin: 3125
Parker, Kelly RB: 1525, 8020
Parker, Lauren: 4130, 8015
Parker, Patricia: 7055
Parker, Tassy: 2335
Parker Oliver, Debra: 7270, 7490, 7665
Parker-Autry, Candace: 4350
Parkes, Christopher: 7360, 7630
Parkhideh, Arianna: 3150
Parkhitko, Andrey: 8000
Parrish, Elise: 2500
Parrott, Kathleen: 8010
Parsons, Michael: 2300
Paruchuri, Nishita: 8020
Pascoal, Tharick: 3375, 9000
Pascoe, Kelley M: 1480, 7200
Pascual-Leone, Alvaro: 7355, 7425, 7635, 7930
Pasdo, Allison: 1265
Passamonte, Michela: 7275
Passos, Joao: 3020, 4210
Patano, Arienne: 8020
Patel, Aditi H: 8020
Patel, Anup Dharmendrakumar: 9050
Patel, Himalaya: 2410
Patel, Isha: 8020
Patel, Kushang: 2450, 7655
Patel, Maulik R: 1265, 7075
Patel, Mehul: 8020
Patel, Nikil: 7320
Patel, Purvi: 2145
Patel, Raj: 4100
Patel, Rhianna: 7720
Patel, Roshni: 7200, 7205
Patel, Sweta: 2185, 7295
Pathiravasan, Chathurangi: 7360, 8015
Patil, Shilpa: 7700
Patille, Rachelle: 2560
Patino, Joshua: 8015
Patrician, Patricia A: 8000
Patrick, Julie Hicks: 1360
Patskanick, Taylor: 1095, 3245, 7090, 7610
Patterson, Davis: 2500
Patterson, kayla: 4420
Patterson, Logan: 1190
Patterson, Mary: 8015
Patterson, Sarah E: 3165
Patti, Gary J: 1025
Pattiam Giriprakash, Pavithran: 8055
Patusco, Rachael: 7455
Patyal, Pankaj: 7465, 8000
Pau, Massimiliano: 1235
Paudel, Anju: 3230
Paul, Anirban: 8000
Paul, Constanca: 8015
Paul, Robert H: 4225
Paulsen, Adam J: 4300
Pauly, Theresa: 4410, 7510, 7915, 8020
Paun, Olimpia: 2215, 3500
Pavlik, Jan: 8000
Payne, Emma: 2410
Payne, Laura L: 7545
Payonk, Cassie: 8015
Peacock, Kimberly: 2295
Peak, Katherine D.: 4160, 7270
Pearman, Ann: 7225
Pecina, Susana: 7455
Pecker, Lydia: 8000
Peckham, Allie: 3015, 4010, 9000
Pedersen, Maja: 3005
Pedersen, Mette Merete: 4075
Peduzzi, Peter N: 3340
Peel, Emily: 7215
Peeples, Amanda D: 3110, 7135, 9025
Peeples, Landon B: 1210
Pei, Heming: 4550, 8010, 8085
Pei, Lijun: 8085
Pei, Xingtong: 1475
Pei, Yalian: 8020
Pei, Yaolin: 1380, 3305, 3505, 4030
Peinado-Martinez, Ignacio: 3150
Peine, Alexander: 3315
Pelcher, Lindsay: 9025
Pelkmans, Jordan L: 2015
Pellerin, Jim: 4110
Pemmasani, Deepishka: 3050, 3235
Pena Dias, Jenny: 7285
Pence, Brandt D: 8000
Pendergrast, Claire: 4105, 7145
Peng, Changmin: 1360, 8050
Peng, Huamao: 7725, 7745
Peng, Siyun: 2120
Peng, Wenting: 1235, 1365, 2040, 2255, 2530, 4055, 7080, 7155
Peng, Xinyuan: 3480, 7725
Penger, Susanne: 1555
Peniche, Jorge: 1320
Pennavaria, Adam: 8010
Penning, Nehle: 8010
Pepin, Marc: 7080
Pepin, Renèe: 8020
Perales-Puchalt, Jaime: 8000
Peralta, Sawyer: 8000, 8000, 8000
Perazza, Lais: 8015
Percival, Nicole: 7465
Perepelkin, Jason: 4440
Perepezko, Kate M: 2190, 3575
Perera, Rohini: 7420
Perera, Subashan: 4215
Perez, Adriana: 7000
Perez, Hector: 7620

Perez, Rene: 4475
Perez, Sasha: 3105
Perez-Aguilar, Giselle: 1245
Perez-Jolles, Monica: 8020
Perez-Rodriguez, Rodrigo: 3150
Perissinotto, Carla: 2520
Perkhounkova, Yelena: 7650
Perkins, Anthony J: 8020
Perkins, Cade: 7005
Perkins, Connie J: 7065
Perkins, Fatima: 1265
Perkins, Molly M: 8020
Perkins, Rebekah: 3465
Perone, Angela K: 2245, 3365, 7540
Perreault, Elisabeth: 4325
Perry, Brea: 2120, 8025, 9015
Perry, Marieke: 7640
Perry, Tam E: 1295, 2155, 2560, 3170, 4265
Perryman, Collin: 7455
Persson, Annelie: 8020
Perzynski, Adam T: 7235, 7690
Perzynski, Adam: 8000
Peschin, Sue: 8020
Peters, Kelly: 4375
Peters, Volletta: 3010
Peterson, Colleen M: 3130, 7520, 8020
Peterson, Courtney: 2005
Peterson, Daniel: 8015, 9015
Peterson, Krysta: 3380
Peterson, Lindsay: 1230, 2185, 2370, 3485, 3495, 7435, 7455
Peterson, Matthew: 8015
Peterson, Tina L: 7065
Petito, Lucia: 9030
Petroff, Lisa: 7720
Petrovic, Milenko: 8015
Petrovsky, Darina: 3500
Pettigrew, Corinne: 2525, 3070, 3555, 7015, 7200
Pettis, Jennifer L: 2485, 8000, 8015
Pevie, Noah: 8010
Pfeifer, Valeria: 8020
Pflieger, Lance: 8000
Pfoh, Elizabeth: 7235, 8000
Pfund, Gabrielle N: 1570, 8000
Pham, Anh: 8010
Pham, Jason: 8025
Pham, Julie Q: 8020
Pham, Thuy: 8000
Pham, Vivian: 7525
Phang, Howard J: 1435, 7010, 8000
Phatak, Vaishali: 2205, 3285
Phelan, Elizabeth: 1060, 4470, 7655
Phianhasin, Lalipat: 8000
Philippi Jr., H. George: 8010
Phillip, Jude: 4005
Phillips, Casey: 1465
Phillips, Christine: 1185, 7735, 8000, 8015, 8020
Phillips, Karon: 1315
Phillips, Kristin: 7320
Phillips, Mirelle: 4370
Phillips, Natalie A: 4300
Phillips, Patrick C: 7010
Phillips, Stuart: 4260
Phillips, Susan: 2060
Phillipson, Chris: 1180, 4275
Phinney, Alison: 3370, 4435, 7040
Phung, Quynh: 1190
Piaseu, Noppawan: 8015, 8015
Piazza, Jennifer R: 7000, 7475
Picchiello, Matthew C: 8000
Piccinin, Andrea M: 8010
Pichardo, Abel: 1335
Pichora-Fuller, M. Kathleen: 4300, 7225, 7635
Pickering, Andrew M: 8020
Pickering, Carolyn E: 1510, 2215, 4355, 7040
Pickett, Andrew C: 3190, 7005, 8020
Piechota, Amanda: 7245
Piedra, Lissette M: 8010
Pien, Chien-Jung: 7080
Pierce, Matthew J: 7030, 7455
Piersol, Catherine: 4340
Pike, James R: 1000, 1055, 4440, 7140
Pike, Kenneth C: 1400
Pilkinton, Patricia: 8015
Pillemer, Karl: 2150, 2300, 4365, 7270
Pilling, Luke C: 9015
Pimentel, Camilla B: 2430, 7110, 8010
Pinardi, Elena: 4315, 7010
Ping, Yongjing: 2065, 3025
Pino, Lilia D: 1490
Pinto, A Alex: 4300
Pinto, Jayant: 7060
Pirone, Judi: 2450
Pisu, Maria: 7495
Pitawanakwat, Karen: 2395
Pituch, Keenan: 7015, 7260, 8020
Pitzer, Kyle: 1165, 7490
Plasencia, Rosa: 4245
Plassman, Brenda: 2090, 3280, 9030
Platt, Katherine: 2555
Pleasants, Hanna: 7060
Pless Kaiser, Anica: 2385
Plotzke, Michael R: 2180
Plummer, Narcissa: 4225
Plunkett, Laura: 7110
Plys, Evan: 1345, 4370, 8000, 8020
Poblete, Ronald: 4235
Podber, Naomi: 4505
Podosek, Alexandra: 7475
Poe, Abigail: 7615
Poettcker, Karli R: 8020
Poghosyan, Lusine: 8010
Pohl, Janet: 7260
Pohnert, Anne M: 1490
Polacek, Laura: 2385
Polenick, Courtney A: 1200, 7440, 8020
Poliakov, Oleksandr: 7505
Poljanska, Ewa: 4310
Polk, Donna: 3345
Polsky, Daniel: 4460, 7470
Polychronopoulou, Efstathia: 8010
Pomeroy, Mary Louise: 1225, 2230
Pond, Brittney: 4025, 7665, 8025
Ponsiglione, Alfonso Maria: 2240, 7305
Ponstein, Klarissa: 7685
Pontiff, Mattie: 2135
Poon, Leonard: 1385
Pope, Natalie E: 3340
Pope, Natalie D: 7065, 7215
Popejoy, Lori L: 1530, 8010
Popelsky, Braden K: 7300, 7720
Popescu, Mihail: 8015
Popivker, David: 8020
Popov, Vladimir: 7075
Porter, Ben: 4120
Porter, Laura: 4470
Porter, Martha: 7740
Porter, Michelle M: 1480, 2440, 3185, 3310, 4000
Porter Starr, Kathryn N: 2485, 8015
Portz, Jennifer D: 2085, 4370, 7065
Pospisilova, Marie: 2425
Post, Emily K: 7235, 7685
Pot, Anna: 7255
Potter, Claire: 2425
Powell, Danielle: 4300, 7140
Powell, Georgia: 1105
Powell, Kimberly: 8015
Powell, Laura: 8000
Powell, Leonard: 8010
Powell, Wizdom: 4165
Powell-Cope, Gail: 2145
Power, Shannon R: 8010
Powers, Dave: 2085
Powers, Melissa: 4040
Powers, Sara M: 1030, 7465, 8020
Pradhan, Rohit: 1425, 1550, 7510
Pranoto, Inez K A: 7320
Prasad, Anyah: 3065, 3075, 8080
Praska, Nicole A: 7510, 7640
Preble, Edward: 1370
Preece, Natasha: 9000
Preisser, John S: 1520
Pressler, Susan: 8000
Preston, A'mie: 7360
Pretzer-Aboff, Ingrid: 8010
Preuss, Benedikt: 2140
Prevratil, Michael: 8020
Price, Andrea: 4510
Price, Ashley N: 7205
Price, Brenda: 1295
Price, Catherine C: 1445
Price, Quentin: 8000
Price, Sherry: 7315
Prichett, Laura: 2230
Prieto, Lucas R: 7345
Prina, Matthew: 2365, 3125
Prine, Halle: 8000
Pritchard, Kevin T: 3135
Pritchard, Sharon: 7635
Prizment, Anna: 7010
Probst, Karen: 7655
Procter-Gray, Elizabeth: 3245, 7260, 7695, 7935
Procter-Gray, Elizabeth: 8010
Prophater, Lorna: 7465
Prosperi, Mattia: 8015
Prosser, Dina: 7010
Prud'hommeaux, Emily: 4235
Pruskowski, Jennifer A: 2345, 7645
Prusynski, Rachel A: 4100, 7080, 8010
Psenka, Riley: 7680
Pu, Debbie: 2145
Pu, Fan: 8055
Puangtong, Inchai: 7460
Pucciarelli, Gianluca: 7245
Pudelek, Kelly: 8010

Puga, Frank: 2375, 7285, 7425, 7615, 7700, 7930
Pugh, Mary Jo: 2545
Pulkki, Jutta: 2460
Punke, Elizabeth LA: 7440, 7490
Puterman, Eli: 3210
Putnam, Michelle: 4135, 4475, 7260
Puts, Martine: 4175, 8015
Putzier, Alexandra: 7075
Puurveen, Gloria: 7510, 7915
Pynoos, Jon: 1005

**Q**
Qato, Dima M: 9020
Qi, Andy: 8000
Qi, Bingbing: 8010
Qi, Lu: 7460, 7715
Qi, Xiang: 1380, 3305, 3505, 4030, 7630, 8000, 8025
Qi, Yan: 1355
Qian, Wenqing: 7525
Qian, Yiqing: 2230, 3570
Qian, Yong: 9015
Qian, Yuting: 3370, 7490
Qin, Tian: 8025
Qin, Weidi: 1060, 2020, 3045, 3245, 4190, 4430, 7490
Qin, Yue: 2001, 3225
Qin, Zeyu: 7330
Qiu, Qian: 2450
Qiu, Xiao: 1535
Qiu, Xichenhui: 7625, 7710, 8000
Qiu, Yiming: 7695
Qu, Doria: 8020
Qu, Shan: 7065, 7270, 8015
Qu, Xiaomin: 3505
Quach, Emma: 7110, 7420
Quach, Ha-Linh: 1255, 2065
Quach, Lien: 8015, 9050
Quashie, Nekehia T: 2515, 8000
Quiñones, Ana: 3035, 3540, 4160, 7270, 7300, 8010
Quiñones-Cordero, Maria: 3045
Quiben, Myla: 7330
Quigley, Molly: 7355
Quiles, Penelope: 7000
Quillen, Ellen E: 3045
Quinlan, Olga: 2370
Quinn, Ryan: 3385
Quintero, Laura: 2060

**R**
Rababa, Mohammad: 7320
Rabalais, Jennifer: 4450
Rabin, Laura A: 2160
Rabinowitz, Jill: 1255, 3070
Rachmuth, Lisa: 2001
Raciti, Audrieanna E: 7510, 7910
Radeck, Leon: 1040
Rademaker, Alfred: 9040
Rafoul, Bahaa: 3085
Ragab, Ahmed: 2160, 7735, 8010
Ragavan, Maya: 3045
Raghavachari, Nalini: 1025
Rahemi, Zahra: 3175
Rahman, Ashikur: 4100
Raj, Minakshi: 2060, 4270, 7270, 7660
Rajabali, Saima: 8000
Rajendra, Anil: 3330
Raji, Mukaila: 2090, 4095, 7405, 8010
Rakel, Barbara: 7205
Rakotoarivelo, Harivony: 4550
Ralser, Markus: 7210
Ralston, Margaret: 4040
Ram, Nilam: 1575, 4525
Ramaiyer, Malini: 4045
Ramamurthi, Hema: 8095
Raman, Rema: 8000
Ramdass, Jeff V: 7490
Ramirez, Maggie: 2125
Ramirez Gomez, Liliana: 4130, 7270, 7660
Ramirez Mantilla, Mariana: 8000
Ramirez-Matias, Julliana: 2310
Ramirez-Zohfeld, Vanessa: 7005, 7420, 7490
Ramos, Carmen: 8000
Ramos, Manuel: 8010
Ramos, Sofhia V: 4310, 8020
Randa-Beaulieu, Cari: 1555, 3205, 7510, 7915
Randall, Kevin: 7150
Rane Levendovszky, Swati: 8000
Rangan, Apoorva: 1575
Rank, Jennifer: 3380
Rantanen, Taina: 1285
Rao, Smitha: 2070, 2195, 3485
Rapp, Stephen R: 3180
Rappaport, Noa: 8000
Rataj, Alison C: 2000, 3075, 4270, 4450, 7320
Rathakrishnan, Sharmini: 3105
Rathbun, Alan M: 7010, 7440
Ratnayake, Aneeka: 4225
Rauer, Amy: 4525, 7070
Rauktis, Mary E: 1290
Rauzi, Michelle R: 1275, 2135, 7155
Raval, Aasha D: 8035, 8070
Ravdin, Lisa: 7630
Ravel, Jacques: 7060, 7210
Ravi, Vishnu: 8015
Ravindran, Malcolm S: 1015, 2185
Rayan-Gharra, Nosaiba: 7400, 7920, 8010
Raye-Brown, Sharnette: 7405
Raymo, James: 7265
Raza, Sakeena: 7070
Razo, Christian: 7040
Ream, Molly: 2385
Reardon, Claire: 1480
Reavis, Kelly M: 7310
Rebadulla, Hannah: 8070
Rebok, George W: 4490, 9000
Recenti, Marco: 2240, 7305
Recker, Amy: 3240
Reckrey, Jennifer: 3105, 3570, 4480, 8010, 8070
Reczek, Rin: 7225
Redd, Tara: 7425, 7910
Reddy, Ann: 1530, 3240, 8010, 8015
Redmond, Leslie: 7480
Reed, Alexandra M: 7200
Reed, David: 1520
Reed, May J: 8000
Reed, Nicholas: 1055, 2525, 4300, 7140
Reeds, Dominic: 1275
Reese, Alexandria C: 3375
Reese, Elaina: 4070
Reese, Jordan: 4130, 4340
Reese-Melancon, Celinda: 7065, 7225, 8020
Reeves, Mathew: 2525
Regan, Claire: 8010
Regier, Natalie G: 8000
Regitz-Zagrosek, Vera: 7030
Regos-Stewart, Dalia: 7200
Rehkopf, David: 1575, 4035, 7350, 8020
Rehman, Haseeb: 8020
Reid, Colleen: 4430
Reid, Katrina: 7105
Reid, Kieran: 3350
Reid, M. Carrington: 7080, 7270, 7630, 8020
Reif, John: 2300
Reifsnyder, JoAnne: 2140
Reiko, Kanaya: 7330
Reilly, Elizabeth: 8010, 8015
Rein, David: 1115
Reinhartz, Alice: 4490
Rejeski, W. Jack Jack: 1410
Rekant, Julie S: 7720
Relerford, Raven: 7005
Remillard, Elena T: 3415, 9025
Ren, Cheng: 7030
Ren, Haopu (Lily): 1115, 2410, 7750, 8015
Ren, Jinnan: 7070, 8020
Ren, Lily: 3205
Ren, Yuanyi: 7605
Rendell, Peter: 8020
Renn, Brenna N: 1325, 7235, 7685
Rensema, Elspeth: 2450
Renzetti, Lucrezia: 4100
Research Team, TACAD: 7270
Resendes, Kelly: 2370
Resnick, Barbara: 3050, 3255, 3555, 4090, 4205, 7040, 7720, 8020
Resnick, Susan M: 2510
Reuben, David: 3340
Rey, Sergio: 4095
Reyes, Jimmy A: 3550, 4205
Reyes, Joel: 8020
Reyes, Mariana A: 7015, 7200
Reynolds, Addam S: 4400
Reynolds, Chandra A: 4095, 7225, 8020
Reynolds, Courtney: 1145, 7405
Reynolds, Tera: 1070
Reynolds, Zahra: 4225
Rezapour, Rezvaneh: 4220
Rhee, Min-Kyoung: 1140
Rhoads, Kristoffer: 4285
Rhodes, Annie S: 4290, 4510
Rhodes, David: 8015
Rhodes, Ramona: 8015
Rhodes, Shanae L: 9010
Riang'a, Roselyter M: 4380
Rianon, Nahid: 3135
Ribeiro, Oscar: 7745
Ricciardi, Carlo: 2240, 7305
Rice, Elise: 1345
Rice, Ian: 9025
Rice, Jordyn: 1365
Rice, Ketra: 1235
Richard, Patrick: 3030
Richards, Emily A: 2045
Richards, Kathy C: 3240

Richards, Nicole: 7005, 7540
Richards Adams, Ingrid K: 7490, 7935
Richardson, Daniel: 8035
Richardson, Julie: 7655
Richardson, Katy: 7440
Richardson, Lena Rebecca: 2560
Richman, Alice: 7530
Richmond-Rakerd, Leah: 7695
Richter, David: 1215
Rickenbach, Elizabeth: 2130
Riding, Bethany: 7480, 7900
Riegel, Barbara: 3385, 7440, 7925
Riffin, Catherine: 8020
Rigdon, Joseph: 1090
Rigotti, Nancy A: 8015
Riklon, Sheldon: 8000, 8015
Riley, Jessica: 2430, 8010
Rimar, Alexander: 4100
Riminchan, Anna: 2565
Ring, Lia: 8010
Rink, Andrea: 7410, 7900
Ripperger, Hayley: 4315
Rippon, Isla R: 1225
Risacher, Shannon: 8000
Riser, Tiffany: 2010, 4360, 7445, 7935
Ristl, Christina: 2300
Ritchey, Katherine C: 2135, 3435, 4285, 7205
Ritchie, Christine: 1345, 2315, 3250, 4370, 9020
Ritsema, Chad: 4325
Ritter, Aaron: 3010
Ritter, Ashley Z: 3550, 7290
Rivas, Daniel: 8020
RIvas, Jennifer: 7695
Rivera-Hernandez, Maricruz: 1075, 1195, 8010, 8020
Rivers, Mia: 7000
Rizzo, Samantha M: 7640
Rizzo, Victoria: 8010
Rizzuto, Debora: 4315
Roa Diaz, Stephanie: 4170, 8000, 8000
Roache, Tavia: 8020
Robbins, Ronna: 2505
Roberson, Donna: 7510
Robert, Stephanie: 7160, 7660
Roberto, Karen A: 3190, 7660
Roberts, Amy R: 1270
Roberts, Angela: 9040
Roberts, Jackson A: 8085
Roberts, Jimmie E: 8015
Roberts, Mitchell: 8015
Roberts, Pamela S: 3300
Roberts, Scott: 1085
Roberts, Shelby: 3430, 7005, 7425, 7910
Roberts, Summer: 7415
Roberts, Tonya: 2550, 3090, 7510, 7705, 8010
Robertson, Frank: 1020
Robertson, Neil: 3325
Robillard, Ashley: 2325
Robillard, Julie M: 2565, 7430
Robins, Amy: 3570
Robinson, Kristen: 2070, 7205
Robinson, Laura: 9010
Robinson, Max: 8000
Robinson, Randee: 1400
Robinson, Renee: 8010
Robinson, Stephanie: 7720
Robinson-Lane, Sheria: 7030, 7665
Robison, Julie: 2495, 7740
Roche-Dean, Maria V: 1045, 1465, 4475
Rochon, Elizabeth: 4370
Rochon, Paula A: 3520, 8010
Rockette, Ra'Shida S: 7000
Rockwood, Kenneth: 7275, 8000
Rodill, Zena: 3075
Rodríguez-Artalejo, Fernando: 3330
Rodríguez-Blázquez, Carmen: 4045
Rodrigues, Annette E: 8010
Rodrigues, Ricardo: 2060
Rodriguez, Jacob: 7010
Rodriguez, Misha: 3550
Rodriguez, Rachel L: 2280
Rodriguez, Robert A: 8010
Rodriguez, Tania M: 7135
Rodriguez Colmenares, Nury A: 7425, 7930
Rodriguez Tiburcio, Alan: 8010
Rodriguez-Galan, Marta: 7445
Rodriguez-Manas, Leocadio: 3150
Rodriquez, Jason: 7490
Roe, Lauren S: 7060
Roecke, Christina: 1220, 4185
Roeland, Eric J: 7020, 7205
Rogalski, Emily: 9040
Rogers, Geoff: 2150
Rogers, Megan: 4510
Rogers, Wendy A: 1040, 3415, 4130, 4270, 7360, 7660, 8000, 8010, 8015, 8020, 8035, 9025
Roh, Soonhee: 7425, 7500
Rojas-Saunero, L. Paloma: 1545, 4375
Rojina, Janet A: 1480, 7200
Rolbiecki, Abigail: 8000, 8010
Rollandi, Isabel: 4365
Romañach Álvarez, Lourdes S: 7485, 7925
Roman, Anna: 1190
Roman, Carly: 8010
Romanelli, Meghan: 4495
Romero Saenz, Janette: 7285
Rompilla, David B: 3355
Rong, Jian: 7360, 8015
Ronis-Tobin, Victor: 8010
Rook, Erin: 3490
Rooks, Ronica N: 1415, 8000
Rooney, Alicia L: 3215
Roopsawang, Inthira: 7205, 7440
Root, Eve Z: 7055, 7475
Roque, Leslye: 7080
Roque, Nelson: 7750, 8000, 8020, 9020
Rorai, Vanessa O: 2155, 3170
Rosa, Paola: 8015
Rosales, Anrea: 2565
Rosales-Soto, Giovanni: 7305
Rosano, Caterina: 1055, 1240, 2220, 2510, 7640, 8000
Rosas, Ivan: 4170
Rose, Andrew: 7150
Rose, Grace: 7720
Rose, Jeff: 1295
Rose, Karen M: 7490, 7660, 7935, 8020
Rose, Liam: 7080
Rose, Tara: 8015
Roseman, Jeffrey M: 2160
Rosen, Anthony E: 1270
Rosen, Sonja: 2520, 7320, 7350
Rosen, Tony: 4195, 4355, 7405
Rosenberg, Dori E: 1265, 7720, 8010, 9020
Rosenblatt, Lucy: 4165
Rosenbloom, Michael H: 3510
Rosendaal, Nicole: 3535
Rosenstein, Benjamin EM: 2535, 7645
Rosland, Ann-Marie: 4195
Ross, Allison D: 8000
Ross, Lesley A: 1185, 1250, 2055, 2330, 7735, 8000, 8015, 8020
Ross, Sarah: 1065
Rosselli, Mónica: 7360, 7425
Rosser, B.R. Simon: 2225, 3490
Rossi, Martina: 7305
Rossnagel, Emma: 3205
Rosso, Andrea L: 1240, 2510, 3375, 4215, 7300, 8000, 8015
Rosteius, Katharina: 4345
Rotblatt, Lindsay J: 7005
Rote, Sunshine: 1495, 3440, 8020
Rotenberg, Shlomit: 1205, 8085
Roth, David L: 1255, 3130, 7225
Roth, Jennifer C: 4060
Rothenberg, Craig: 1130
Rothermund, Klaus: 1375, 4020
Rothgang, Heinz: 2140
Rouse, Sasha S: 2065
Rousseau, Julie: 2340, 7405
Routh, Brianna N: 8020
Rowan, Nicholas R: 2080
Rowe, John: 7350
Rowell, Londi: 3235
Roy, Arka: 4190
Roy, Brita: 7535
Roy, Jason: 4485
Roy, Mishy: 3510
Royo, Sofia: 3280
Royse, Sarah K: 3375
Rozario, Philip: 8010
Rozenberg, Aliza: 7060
Ruan, Iris Xiaoyu: 7640
Ruan, Lei: 7080
Rubin, Leah: 8020
Rubinstein, Stephanie R: 7460, 8020
Rubio, Olivia C: 3150, 4420
Rubovits, Eve: 7270
Rubtsova, Anna: 8015
Rudek, Michelle: 8000
Rudoler, David: 7295
Rudolph, Catherine S: 7350
Ruggiano, Nicole: 4060, 7095, 8000
Ruggles, Steven: 4035
Ruiz, Diana: 7360, 7675, 8010, 8070
Ruksakulpiwat, Suebsarn: 8000
Rule, Payton D: 7745, 8000

Rundek, Tatjana: 8085
Ruopp, Marcus: 8020
Rupp, Hayley: 3585
Ruppar, Todd: 3500
Rupper, Randall: 4520
Rupprecht, Fiona S: 1375, 2300, 7245
Rush, Jonathan: 1525, 4180
Rushing, Baylie: 7720
Ruskin, Andrea: 7475
Rusmevichientong, Pimbucha: 7000
Russell, Andrea: 4480
Russell, David: 7740
Russell, Kristy: 3100
Russell, Luke T: 4165
Russo, Frank A: 7225
Ruth, Terrance: 3275
Rutledge, Emily: 7630, 7905
Rutledge, Jocelyn A: 4410
Ryan, Alice: 7010, 7440, 8000
Ryan, Healey: 7465
Ryff, Carol D: 8000
Ryu, Byeongju: 1295, 4140, 4265, 7290
Ryu, HyeRim: 1080, 7695
Ryu, Jungsu: 7535
Ryvicker, Miriam: 4360

**S**

Sabatini, Serena: 1285
Sabbatini, Amber: 8010
Sabol, Jacqueline: 7410
Sacco, Alessandra: 8000
Sacco, Paul: 7020
Sachs, Greg: 4440
Sadarangani, Tina: 4130
Sadeq, Nasreen: 3495
Sadeqi, Farima: 8000
Sadler, Richard C: 4430
Sadruddin, Sabeen: 7270
Saenz, Joseph: 1320, 2515, 4455, 8000, 9000
Saffel-Shrier, Susan: 7315
Sagehorn, Mallory A P: 3375, 7420
Sagong, Hae: 7095, 7275
Saha, Troyee: 7155
Sahab, Lujain: 7060
Sahawneh, Fursan: 4000
Sahli, Michelle W: 7155
Sahni, Shivani: 2080
Said, Adam: 8000
Saikia, Nandita: 1195
Sailors, Olivia: 8015
Saint Onge, Jarron: 4330
Saito, Taeko: 7400, 7920
Saito, Yasuhiko: 3290, 7205, 7265
saiyed, sahebi: 8020
Sakai, Tomomichi: 7275
Sakal, Collin: 8020
Sakamoto, Mariko: 3370, 7510
Sakata, Kei: 7130
Salama, Mohamed: 1155
Salari, Sonia L: 7030
Salas, Joanne: 7275
Salbach, Nancy M: 7300
Salcido De Pablo, Pamela: 7400, 7920
Sales Martinez, Sabrina: 7540, 7735
Saliba, Debra: 4100, 9040
Saligan, Leorey: 2445
Salinas Navarro, Sheila A: 7725, 8010, 8020
Salisbury, Dereck L: 7015, 7450, 8020
Salive, Marcel: 1390
Salminen, Lauren: 7495, 7930
Salmon, David: 1485
Salner, Andrew: 2295
Saltvedt, Ingvild: 7060
Salvo, Erin: 2150, 7405
Salzman, Talia: 2510
Samelli, Alessandra G: 1320
Samper-Ternent, Rafael: 1320, 2075, 3540, 7215
Samson, Andrew: 7040
Samuel, Linda: 8000
Samuelson, Brady: 7275, 7275
Samus, Quincy M: 2015, 2155, 3215, 4100, 7005, 7465
San Roman, Irene: 1395
Sanabria Véaz, Karla: 7155
Sanches Yassuda, Mônica: 8015
Sanchez, David: 7405
Sanchez, Travis: 8015
Sanchez, Victoria: 7140
Sanchez-Contreras, Monica: 7075
Sanchez-Reilly, Sandra: 8010, 8015
Sanders, Edie C: 8000, 8020
Sanders, Lisbeth: 8020
Sanders, Margaret: 7475, 7900
Sandhu, Sahir: 7210
Sandoval, Jacqueline A: 8010
Sandoval, Moises H: 3290
Sandoval Garrido, Felipe A: 7605
Sandri, Tina: 8010
Sands, Laura: 2495, 4110, 4315, 7680, 8010
Sands, Talyah: 3430, 1260
Sanford, Claudia: 1295, 4265
Sanford, Jon A: 8010, 8020
Sangrar, Ruheena: 8085
Sanner, Haley K: 7285
Santaella, Paulina: 8010
Santaella-Tafolla, Paulina: 7510
Santana, Layla Katharine: 7455
Santangelo, Kelly: 7075
Santilli, Josie N: 1185, 2130
Santioni, Margaret: 7720
Santora, Kenneth: 1340, 2210
Santos, Suzanne T: 4465
Santos-Lozada, Alexis R: 1350, 4050, 7350
Santoso, Kate: 7645
Santoyo-Olsson, Jasmine: 3175
Sanz Simon, Sharon: 7350
Sapir, Adi: 3465, 4040
Sapkota, Krishna P: 3055
Sappington, Erica: 8015
Sapra, Mamta: 4060
Saraboon, Natthayawadee: 7440
Sardina, Angie L: 8020
Sargent, Lana: 3565, 4510
Sarkar, Nilanjan: 7155, 7435
Sarma, Anya: 2410
Sasai, Hiroyuki: 7205, 7540
Sasdi, Olivia: 7215
Sasser, Jennifer R: 1175, 7415, 8010
Sathish, Tanish: 8000
Sathler Tavares Batistoni, Samila: 8015
Sato, Kana: 4135
Satoh, Kana: 4135
Sauer, Jennifer H: 7125
Sauerteig-Rolston, Madison R: 3405
Saunders, Michele J: 3505
Saunders, Pamela A: 2275, 3365
Saunders, Stephanie: 7655
Sausman, Kathryn: 8015
Savage, Rachel D: 8010
Savard, Grace M: 3375, 8020
Savary, Patricia E: 8015
Saville, Neika N: 2340
Savin, Douglas: 8010
Savla, Jyoti: 3190, 4060, 7660, 8020
Savundranayagam, Marie Y: 1070, 2410, 7465, 7635, 7910
Saw, Anne: 7490, 7935
Sawyer, Linda: 8015
sayles, anijah: 7000
Saylor, Martha Abshire: 2230
Scales, Kezia: 2500, 3570, 4290
Scambray, Kiana A: 1495, 9015
Scandalis, Lina: 8000
Scarpelli, Michael: 7695
Sceppa, Carmen: 2110
Scerpella, Daniel L: 4130
Schüz, Benjamin: 4345
Schaaf, George W: 3045
Schaefer, Adam: 2300
Schaefer, Caroline: 3135
Schaefer, Cathy A: 1545
Schaefer, Sydney Y: 7200, 7325, 9015
Schafer, Donna E: 2250
Schafer, Marissa: 3400
Schafer, Markus: 3410
Schaff, Branden: 4120
Schaffer, Sigourney: 4320
Scharlach, Andrew E: 1195
Schechtman, Kenneth B: 1275
Scheffer, Julian A: 3285, 8040
Schenck, Sabine: 7440, 7490
Schepens Niemiec, Stacey: 3165
Schere, Laura D: 8020
Scherr, Susan: 8020
Scherrer, Jeffery: 7275
Scheunemann, Leslie P: 3045, 4530
Schexnayder, Julie: 7285
Schierer, Allison: 7005
Schilling, Birgit: 2435, 3045, 4170
Schilling, Oliver K: 1285
Schiloski, Kylie A: 1525, 7455
Schilt, Kristen: 1110
Schiltz, Nicholas K: 1490
Schilz, Jodi: 8015
Schindel Martin, Lori: 4445
Schlag, Karen E: 4355, 7405
Schlomann, Anna: 1040
Schmidt, Laura: 8015
Schmidt, Steven: 1285, 3170, 7155
Schmiedeler, Anthony: 1135
Schmitter-Edgecombe, Maureen J: 2360
Schneider, Augusto: 8000

Schneider, Claudia: 8000
Schneider, Kevin: 2530
Schneider, Stefan: 2465, 3335
Schneider, Sue: 3575
Schneider, Sydnie: 1335, 3385, 4435, 7065, 7425
Schoenborn, Nancy: 4220
Schoenfeld, Elinor R: 3265, 3415
Schoenhofen Sharp, Emily: 1215
Scholes, Shaun: 2365, 3125
Scholler, Emily: 8015
Schomburg, Lutz: 7210
Schousboe, John: 4055, 7630
Schrack, Jennifer: 8000
Schrack, Jennifer A: 1240, 1255, 2035, 2525, 3070, 7540, 8015
Schrager, Sarina: 4160
Schramm, Eric: 7360
Schrauf, Robert: 3550
Schriner, Samuel E: 7010
Schroepfer, Tracy A: 4080
Schubert, Carla R: 4300
Schuh, Madyson: 4510
Schuller, Jeanne: 2010
Schulman-Green, Dena: 4360
Schultz, January: 7040
Schultz, Leslie: 2300
Schumacher, Alexander T: 1215
Schumacher, John: 2535, 3495, 8010
Schumacher, Linus: 3325
Schumann, Annette: 1070, 2410, 7635
Schumm, Phil: 1110
Schurr, Matthew: 7235, 7685
Schutte, Debra: 8020
Schwarte, Liz: 7200
Schwartz, Abby J: 7530
Schwartz, Andrea W: 8015
Schwartz, Anna: 1175
Schwartz, Heather: 8020
Schwartz, Tatum A: 3065
Schwarze-Taufiq, Tiara: 7320
Schwingel, Andiara: 7145
Scior, Katrina: 8000
Scott, Cathy B: 8000
Scott, Gregory: 7310
Scott, Julia A: 7200
Scott, Julia C: 7695
Scott, Stacey B: 4185, 4280, 4410, 8020
Scott, Stephanie L: 7540, 7735
Scott, Victoria: 1535
Scott-Poe, Deneisha S: 7005
Scow, Laura: 2525
Scrivano, Rachel: 2130
Scroggins, Laila: 7000
Scully, Madeleine: 7680
Seago, Elayna: 8020
Seal, Susan D: 4040
Seaman, Aaron: 1395, 7465, 7685
Seaman, Ilona: 1490
Seaman, Jennifer B: 7200
Seaton, Thomasina: 8000, 8020
Sebastiao, Emerson: 7260
Seblova, Dominika: 4375
Seckin, Gul: 7360
Sedaghat, Sanaz: 7010
Sedey-Seitz, Cory: 1415
Sedikides, Constantine: 7255
Sedore, Christine: 7010
Seetharaman, Kishore: 1555, 3205
Segal, Daniel L.: 7115
Segal-Gidan, Freddi: 1430
Segawa, Dai: 7420
Segel-Karpas, Dikla: 1565, 8010
Segerstrom, Suzanne C: 7245, 8020, 9010
Segundo, Joahana: 9000
Seidel, Elizabeth: 8020
Seidenfeld, Justine: 2090
Seigel, Zachary W: 4370
Seino, Satoshi: 7275
Seitz, Aaron R: 8000
Sekhar, Rajagopal V: 1420, 2080, 2235
Sekhon, Vishaldeep: 1255, 7225
Seliger, Stephen L.: 3330
Seligman, Benjamin: 4055, 7275, 7300
Selmanoff, Mollie: 4530
Selovin, Fabio: 1375
Seltzer, Judith A: 4050
Selzler, Katherine J: 7325
Semba, Richard: 7010
Semelka, Charles T: 1390
Sen, Ranjan: 7210
Senadheera, Chatura: 8000
Seo, Jinsil: 8015
Seo, Jisu: 7275, 7640
Seo, Minhwa: 3050
Seong, Minju: 7535
Seplaki, Christopher: 7030, 7420, 7920
Ser, Sarah E: 8015
Sereika, Susan M: 7200
Serina, Peter: 1530, 8010
Serna, Myrna: 8010
Serra, Monica C: 2345, 2505, 7720
Serrano-Rubio, Luis: 7735
Sesek, Greg: 8010
Seshadri, Gokul: 2460, 7010
Seshadri, Sudha: 7500, 7930
Setoguchi, Soko: 4485
Severance, Jennifer J: 1065, 3510, 7080
Sevi, Barış: 4535, 7540
Seward, Morgan: 1345, 8000, 8020
Seward, Tanya: 7065
Sexson, Kathryn: 7065
Sexton, Athena: 1185
Shaaban, C. Elizabeth: 3375
Shade, Marcia: 1500, 4205
Shadmi, Efrat: 4075, 7265, 7400, 7920
Shaffer, Tasha M: 8020
Shafir, Irit: 1565
Shah, Avani A: 7450
Shah, Manish N: 3010, 4530
Shah, Ravi V: 7710, 8000
Shah, Sameer B: 7310
Shahid, Kainat: 9025
Shahriar, Kamal Md: 7315
Shaid, Elizabeth: 3300, 7535, 8010
Shaji, Chacko K: 2235
Shakir, Madiha: 7425, 7910
Shakya, Shamatree: 7615
Shakya, Sunita: 8010
Shaleha, Rinanda Rizky Amalia: 7750, 8000
Shalev, Idan: 7010, 7455, 7490
Shallcross, Amanda J: 1140
Shamas, Tracy: 7475
Shan, Liang: 2375
Shan, Xiaorong: 4035
Shan, Yong: 2090, 4095
Shands, Rachel: 1520
Shang, Shaomei: 7430
Shang, Yongxin: 3165
Shankar, Janki: 2050
Shannon, Kay: 7520
Shannon, Zoe Y D: 7140
Shardell, Michelle: 1535, 3035, 3330, 4385, 7010, 7210, 7285, 7440, 8015
Sharma, Anisha: 7065, 8020
Sharma, Anjali: 8010, 8015
Sharma, Dinesh: 3330
Sharma, Hari: 8075
Sharma, Isha: 7470
Sharma, Rashmi: 7530, 7905
Sharma, Shakshi: 7010, 8000, 8015, 8015, 8015
Sharninghausen, Jody: 7085
Sharon, Tal: 7025
Sharp, Bethanie: 8020
Sharp, Lisa: 7615
Sharpp, Tara J: 1050
Sharrett, Richey: 4440
Shastri, Vinita: 7480, 7900
Shatswell, L. Elizabeth: 7530
Shaughnessy, Marianne: 2280, 2345
Shavit, Yochai Z: 1575
Shaw, Amy L: 4355, 7405
Shaw, Clarissa: 1530, 7400, 7650
Shaw, Pamela A: 8010
Shayya, Ashley: 3255
Shealy, Carlisle: 2275, 2495
Shean, John: 3430, 7425, 7910
Sheardova, Katerina: 8000
Shearon, Jennifer: 7720
Shearston, Jenni A: 4035, 4375
Sheehan, Connor: 8000
Sheets, Kerry: 4055, 7630
Shelar, Shatakshi: 7660
Sheldon, Kira G: 8035
Sheldon, Marisa: 2070, 2195, 3080
Shellman, Juliette M: 7335
Shen, Bin: 4535
Shen, Huei-Wern: 7445, 7700
Shen, Jenny: 8035
Shen, Jiahao: 4195
Shen, Joseph: 8015
Shen, Karen: 1395, 8070
Shen, Lingyi: 3505, 9015
Shen, Nicola: 8015
Shen, Xinyue: 3480
Shen, Yi: 1175
Shen, Yiyi: 7015
Shen, Yuqi: 1250
Shende, Shraddha: 1405, 7015, 7455
Sheng, Xiaoming: 4520
Shenk, Dena: 8010
Shenoy, Shivani: 8035, 8070
Shenoy, Vineet: 8015
Shepherd-Banigan, Megan: 2090, 7005
Sherman, Aurora M: 8020
Sherwood, Chet C: 8000
Shi, Huidong: 7285
Shi, Jing: 7640
Shi, Junrong: 8000
Shi, Kewei: 7305
Shi, Sandra: 3135, 7080
Shi, Yang: 4110

Shi, Yanhong: 7450, 7930
Shiba, Koichiro: 7355
Shibeika, Sahar: 1475
Shibuya, Kyohei: 8015
Shida, Takashi: 8015
Shields, Stephen L: 3215
Shields, Timothy M: 3375
Shieu, Bianca: 7510, 8010
Shiferaw, Tsion: 8020
Shih, Regina A: 3340, 3570, 4245
Shih, Yao-Chi: 7095
Shilvock, Abigail R: 8015
Shim, Eun-Jung: 2365
Shim, Soyeon: 3050
Shin, Donghee: 7535
Shin, Esther: 7290, 7665, 8010
Shin, Haeun: 7460
Shin, Hyeyeon: 8020
Shin, Hyung Eun: 8000, 8010, 8015
Shin, Jinhee: 7080, 8010
Shin, Jiyoung: 7640, 7680
Shin, Juh Hyun: 7295, 8015
Shin, So Young: 7295
Shin, Soogyung: 7005
Shin, Sun-ui: 7265, 8000
Shin, Yeonsoo: 7275
Shin, YooGyung: 7275, 8015
Shin, Yumi: 1375, 7275
Shippee, Tetyana P: 1520, 2225, 3090, 3490, 4245, 4395, 4445
Shiraishi, Natsue: 8015
Shires, Deirdre: 7345
Shirk, Steven D: 3110, 7490
Shiroma, Kristina: 2265, 4330
Shiu, Chian-Huei: 7110
Shivappa, Nitin: 2080
Shoji, Kristy: 1080
Shore, Amanda: 4100
Shorr, Ronald I: 2145
Shovali, Tamar: 1185, 4240
Showalter, John: 7425, 7930
Showalter, Kathryn: 3275, 7405
Shrestha, Aman: 3055, 3330
Shrestha, Hom L: 8055
Shrestha, Lucy: 8055
Shrestha, Srishti: 2525, 4440, 7060, 7630
Shrira, Amit: 1565, 2001, 4020
Shrivastava, Jyotsna: 8000
Shtrahman, Matthew: 7210
Shukla, Rohit: 7210
Shulman, Lisa M: 8010
Shulyaev, Ksenya: 3015, 4075, 7265, 7400, 7920, 8090
Shuman, Sari B: 1260
Shuman, Stephen K: 3305
Shuman, Valerie: 4215
Shune, Samantha: 2060, 3500
Si, Wanwan: 7295, 7740
Siamdoust, Shahrzad: 9030
Siby, Ninette: 7530
Sicinski, Kamil: 3225
Sidani, Souraya: 4255
Siddiqui, Aziza: 7485
Sidle, Erin: 8010, 8015
Siebecker, Megan: 7135, 7670, 7695, 7700
Siedner, Mark J: 4225
Siedner, Mark: 8015
Siegel, Elena: 2200
Siegfried, Alexa: 1425
Sigurdardottir, Sigurveig: 2040
Silberman, Susan Lynn: 7110
Silbermann, Rebecca: 7020
Sillito, Carrie LeFevre: 7030
Silva, Jennifer: 8015
Silva, Susan: 7270
Silva Arbex Borim, Flávia: 8015
Silva de sá, Mel: 7225, 7655
Silvasstar, Josh: 7065
Silver, Michelle: 7130
Silver, Roxane Cohen: 7030
Silverman-Martin, Wendie: 8000
Silverstein, Hannah: 4000
Silverstein, Merril: 3030, 3190, 3360, 7235, 7610, 8085
Silverstein, Nina M: 1305, 3265, 3310, 7065, 7240
Silveyra, Eduardo: 7700
Sim, Hyebeen: 8010
Simard, Pascale: 8020
Simmons, Cassandra: 2060
Simmons, Sandra: 8035
Simning, Adam: 8020, 8055
Simon, Bala: 3430
Simon, Darby M: 7660, 8020
Simon, Judy: 8015
Simon, Kennedy N: 2330
Simon, Lena G: 2330
Simon, Lena: 8000, 8020
Simone, Patti M: 7200
Simonsick, Eleanor M: 1255, 3050, 3070, 3330, 7090, 7630, 7710, 7720, 8015
Simpson, Deborah: 7480
Simpson, Elizabeth: 1360, 4305, 7670
Simpson, Kit: 1075
Sin, Mo-kyung: 2160, 3555, 9025
Sin, Nancy L: 4405
Singer, Dianne: 1085
Singer, Jonathan: 1335, 3385, 4435, 7005, 7065, 7425, 9015
Singh, Amaya: 9040
Singh, Amit: 7210
Singh, Nandini: 2520
Singh, Roshni: 7675, 8010, 8070
Singh, Satya N: 4065
Singleton, Andrew: 8000
Singleton, Meki: 2180, 8020
Singsank, Katie T: 7240
Sinke, Christopher: 4490
Siperstein, Gary: 7490
Siqueira, Vitor: 7260
Siren, Anu: 3455
Sirey, Jo Anne: 4365
Sison, Stephanie M: 8015
Sison, Stephanie Denise M: 3135, 7080
Sisti, Anthony: 3240
Siu, Albert: 7535, 8010
Siudzinski, Jayne: 8010
Sivakumar, Nikita: 4005
Sivers-Teixeira, Theresa: 8055
Sixsmith, Andrew: 3060
Sixsmith, Judith: 1015, 8010
Sjöberg, Linnea: 3035
Skalla, Lesley A: 2180
Skarphol, Tricia: 1520, 2225, 4445
Skemp, Eleanor: 8000
Skinner, Jonathan: 3145
Skinner, Kelly: 2360
Skipper, Antonius D: 7150, 8010
Skirbekk, Vegard: 2425
Skoblow, Hanamori F: 3055
Skovgaard, Asmus Cosmos: 7010
Skowronski, Jordan: 8020
Skrajner, Michael: 7465
Skrzynski, Carillon J: 3115
Slaug, Björn: 1285
Slaven, James: 7465
Sliwinski, Martin J: 4405, 4515, 7225, 7455, 8020
Sloan, Caroline: 7470
Sloan, Kylie: 7410
Sloane, Philip: 1520, 2105
Slossberg, Rebecca K: 4100
Sloup, Sharon: 3240
Slowey, AJ: 8020
Small, Brent: 7070, 8000
Smichenko, Juliana: 4075, 7400, 7920
Smiley, Adrianne: 1270
Smilovsky, Kyle: 3315
Smith, Alexander K: 4480
Smith, Alexandra: 3055
Smith, Amanda: 2370
Smith, Amelia: 3090
Smith, Brian N: 2385
Smith, Brian: 8000
Smith, Brigid: 3485
Smith, Caroline: 7000
Smith, Claire: 7130
Smith, Eleanor: 3270
Smith, Eric: 7030
Smith, Gregory C: 1190, 3360, 7265
Smith, Hannah: 9040
Smith, Jacqui: 1210, 2025, 4525, 7050, 7130, 7335, 7525, 7630, 7690, 7725
Smith, Jamie M: 4300, 4360, 4460
Smith, Jason: 3220, 4440
Smith, Jennifer: 1435
Smith, Jennifer L: 3355, 7050, 7085, 7720, 8010
Smith, Julie: 7480
Smith, Kate: 1070, 3240
Smith, Kelly M: 1230
Smith, Kelly: 8000
Smith, Ken R: 4520
Smith, LaTonya: 7350, 7540, 7910
Smith, Laura T: 3575
Smith, Lenora W: 8015
Smith, Lindsey: 3535, 8010, 8010, 8010, 8020
Smith, Louisa H: 4225
Smith, Matthew Lee: 1190, 7145, 8000, 8020
Smith, Patrick: 9010
Smith, Sarah K: 2530
Smith, Tracey: 7205
Smith Hinders, Julie: 7350
Smith Monroe, Suzanne C: 3380
Smith Slingerland, Kerri: 8010
Smithroat, Noemi: 3215
Smithwick, Megan: 7225
Smyles, Hanna: 7100
Smyre, Patricia M: 1315
Sneed, Rodlescia S: 1540, 2425, 4070, 7265, 7735
Snigurska, Urszula A: 8015
Snipe, Brandon: 4420
Snitz, Beth E: 7720, 9000
Snodgrass, Josh: 8015

Snow, A. Lynn: 1070, 1080, 1210, 3240, 7250, 7685
Snyder, Scott: 7680, 7725
So, Rina: 3125
Sodhi, Jaspreet K: 7300
Sodums, Devin J: 7040
Soerensen, Mette: 7010
Soke, Lisa A: 8015
Sokolova, Alexandra O: 7020, 7205
Sokolsky, Oleg: 7145
Sol, Ketlyne: 1045, 1495, 3400, 7535
Soldan, Anja: 2525, 3070, 3555, 7015
Sole, Jorge L: 7405
Solis-Herrera, Carolina: 1460
Solomon, Annie: 8010
Solomon, Philip: 8015
Solorzano, Emily: 3485, 7045
Solway, Erica: 1085
Somerville, Ceara: 7485
Sommers, Delaney D: 7150
Sommet, Nicolas: 4455, 7535
Son, Claudia: 1325
Son, Jung Yoen: 7435
Son, Rynnie Cin Ee: 8020
Sone, Chikako: 8015
Song, Guojie: 7605
Song, Huiru: 8010
Song, Jieun: 8000, 8020
Song, Lixin: 7535, 8015, 8050, 8085
Song, Max: 4220
Song, Minju: 3185, 7005
Song, Qian: 3495, 7235, 8015
Song, Rhayun: 7275, 7640
Song, Shulin: 8000
Song, Woojin: 8020
Song, Xiaowei: 7275
Song, Yeonsu: 2345, 7660, 7735
Song, Yixiao: 7640
Song, Yuting: 1245, 4195
Song, Yuxiao: 1130
Soni, Albin: 2410, 8010
Sonnega, Amanda: 3540, 7050, 7525, 8020
Sonnega, John R: 8020
Sonnenfeld, Mandi: 4340
Sonnentag, David: 4530
Sonney, Jennifer: 7465
Sopheab, Heng: 8015
Sörensen, Silvia: 3045, 4400, 7240, 8020
Sorra, Joann: 7545
Sosnowski, David: 4005
Sothers, Hanna: 8000
Sou, Ka Lon: 2100
Soulie, Brianna: 1010, 7160
Soumyanath, Amala: 7320
Sourbati, Maria: 2565
Sousa, Carlos: 8010
Sousa, Sarah: 2360
Southerland, Jodi L.: 8000, 8020
Souza, Anita M: 1400
Spangler, Hillary: 3585, 7275, 7535
Sparks, Sarah: 3385, 9015
Spartano, Nicole: 7500, 7930
Spears, Isaiah: 8010
Spears, Katie S: 3100, 4390, 7065
Speers, Alex: 7320
Sperber, Nina: 8015
Spetz, Joanne: 3105, 8055
Spilsbury, Karen: 2140, 4255, 7295, 7520
Spini, Dario: 4455, 7535
Spira, Adam P: 1255, 3070, 7615
Spiro, Avron: 2385
Spiro III, Avron: 4375
Spofford, Victoria: 8015
Spradley, Laura: 7650
Sprague, Briana N: 7625
Sprague, Karalin: 3490
Spring, Lauren: 2495, 7420
Springer, Jenna: 7735
springer, Shmuel: 1245, 2240
Springstein, Tabea: 3355
Squillace, Marie: 4305
Squires, Allison P: 7535
Sridhar, Aditya: 4005
Srikanth, Velandai: 4100
Srisuk, Wasana: 8015
Srivastava, Ankita: 7455
Srivastava, Sanjana: 4420
Srivastava, Shobhit: 7605
St. John, Phil: 7060
St. Louis, Renée M: 3455, 8020
Stahlmann, Melanie: 1220
Staley, Mark: 2490
Stallard, Eric: 1025
Stalter, Lily: 2190
Stam, Elisabeth J: 3065, 3530, 4270
Stamatakis, Emmanuel: 1245
Stamenkovic, Ceda: 8000
Stanger, Madeline: 3115
Stangl, Morgan: 4110, 7235, 7700
Stankovic, John A: 8020
Stanley, Jennifer T: 4000, 4295
Stanmore, Emma: 2530
Stansbury, Kim L: 3275
Stark, Susan: 4475, 7260, 7655, 8000, 8015
Staton, Ashley: 4510
Stavrakopolous, Dawn: 2370
Stawski, Robert S: 4280, 4410
Stearns, Melanie A: 1135, 1405, 3360, 7500
Stechuchak, Karen M: 2145
Steckenrider, Janie: 7345
Steege, Linsey M: 4075, 8015
Steele, Joel S: 3285
Stefanacci, Richard: 4535
Stefanucci, Jeanine: 3175
Steffens, David C: 2495
Stein, Gary L: 2225
Stein, Ryan G: 7720, 8015
Steinman, Audrey J: 2215
Steinman, Bernard A.: 1005, 7140
Steinman, Kenneth J: 1270, 4110
Steinman, Lesley: 1470, 2010
Steinmetz, Jaimie D: 7040
Stellman, Jeanne M: 2385
Stellman, Steven D: 2385
Stephan, Abigail T: 1185, 2245, 7735, 8015, 8020
Stephan, Yannick: 7350
Stephen, Kelly: 2145
Stephens, Caroline E: 4520
Stephens, Elizabeth: 8000
Stephens, Elizabeth B: 8000
Stephens, Jacquelyn: 3355
Stephens, Kevin: 8015
Stephenson, Jane: 8000, 8020
Stepniak, Catherine: 3165
Steptoe, Andrew: 2365, 3050, 3095, 4440, 4500, 7005
Sterling, Madeline: 8015
Stern, Julia: 1215
Stern, Yaakov: 2425
Sterns, Harvey L: 2250, 2440
Stessman, Jochanan: 7060
Stessman-Lande, Irit: 7060
Stetten, Nichole: 8010
Stevens, Alan B: 3340, 4130, 4340, 7680
Stevens, Erin: 2445
Stevens-Lapsley, Jennifer E: 1275, 2135, 7155
Steward, Andrew T: 8090
Stewart, Anne L: 2460
Stewart, Heather G: 7405
Stewart, Heaven: 8020
Stewart, Ian: 3260
Stewart, Meagan: 7070
Sthur, Nicole: 8000
Stickel, Ariana M: 7000, 7455, 7540
Still, Catherine: 1455, 4530, 7270
Stinchcombe, Arne: 7100
Stites, Shana: 1280, 8020
St-Martin, Genevieve: 7465
Stocks, Jane: 8020
Stockwell, Robert: 8000
Stodola, Allyson: 8010, 8020
Stoeckle, Rebecca J: 1260
Stoffel, Devon: 8060
Stojanovic, Marta: 7720
Stokes, Jeffrey: 1360, 2205, 3065, 3075, 3495, 3530, 3545, 4270, 7245, 8015
Stolle-Wahl, Claudia: 2140
Stoltenberg, Peggy: 3575
Stone, Alison: 8010
Stone, Katie L,: 1255, 2290
Stone, Kimberly: 7535
Stone, Robyn: 4265, 4395, 7135
Stone, Sarah M: 9020
Stone, Sasheen T.: 7425, 7500
Stone-Bury, Lisa E: 7115
Stout, Jeffrey R: 4420, 7205, 7640, 7735, 8015, 8020
Stout, Michael B: 4310
Stoyles, Sydnee: 7020, 7205
Stradford, Joy: 8015
Stradtman, Megan: 7205
Strand, Bjørn Heine: 2425, 7005
Strath, Larissa: 1445
Stratton, Lauren: 2105, 4200, 8010
Straughan, Paulin: 7050
Strayer, Andrea: 1455, 4530, 7270
Streed, Carl: 4225
Strickland-Hughes, Carla M: 7250, 8020
Strickland-Yanick, Leslie M: 8015
Stringer, Wilma: 3170
Stringfellow, Mary Kris: 8010
Stripling, Ashley: 7460
Strobach, Tilo: 4490
Strohl, Katyayani: 7530, 7905

Strommen, Jane: 7725
Strong, Jessica V: 7065, 7150
Strong, Randy: 4310
Strotmeyer, Elsa: 1090, 1410, 7060, 8015
Stroud, Jared: 8020
Strough, JoNell: 8000
Struble, Laura M: 7435
Stuart, Nicole S: 4405
Stubbs, Brianna J: 8000
Stuckenschneider, Tim: 8015
Studenski, Stephanie L: 2510
Stuifbergen, Alexa: 7145, 7435
Stump, Timothy: 4195
Sturdevant, Diana L: 2550
Sturmlechner, Ines: 3160
Stypinska, Justyna Monika: 2565, 3315
Su, Mengxuan: 7355
Su, Tai-Te: 7300
Su, Wei-Hsuan: 7010
Su, Yan: 2530, 8015
Su, Yan-Jhu: 1405, 4270, 7070, 7095, 7240, 7735, 8015
Suanet, Bianca: 2025
Suarez, Jethro Raphael M: 3055, 7620, 7640, 7720, 7735, 7750, 8015, 8020
Subbalakshmi, Koduvayur: 4180
Suberry, Assaf: 1190, 2030
Sucic, Joseph F: 7300
Suda, Hitoshi: 4135
Sudore, Rebecca L: 2255, 3175
Sue Mangalindan, Ruby: 8000
Suemoto, Claudia Kimie: 1320
Suen, Jonathan J: 1440
Suganuma, Ippei: 7420
Sugar, Judith A: 2250
Suggs, Andy: 7700
Sugimori, Haruka: 4050
Sugiyama, Mika: 7350, 7640
Suh, Heather: 2550, 7005, 7245
Suitor, J. Jill: 3165, 7265
Sukoff Rizzo, Stacey J: 8000
Suliburk, James: 2080
Sullivan, Barbara: 3340
Sullivan, Daniel: 8020
Sullivan, Darcy: 2500
Sullivan, Dennis: 7480, 8015
Sullivan, Jennifer L: 3425
Sullivan, Jennifer: 8020
Sullivan, Kevin J: 1000
Sullivan, Suzanne S: 7055, 7435, 7915
Sultana, Shabiha: 7285
Summapund, Jenny: 3340
Sun, Changxian: 7545, 7915
Sun, Eric: 2310
Sun, Fei: 1355, 2355, 3270, 8020
Sun, Haosen: 1405, 3410, 8025
Sun, Huaman: 7350
Sun, Kevin: 2190
Sun, Na: 1535, 4385, 8010
Sun, Nianzi: 8020
Sun, Nuo: 2165
Sun, Peter C: 8010
Sun, Shuimei: 7010
Sun, Tongxin: 7035, 7265, 7520,: 8010
Sun, Winnie: 3010, 7295, 8010
Sun, Xin: 3025, 7605
Sun, Ying: 8015
Sun, Yue: 2160, 7750
Sun, Yue Yang: 2030, 3480
Sunada, Junko: 7295
Sundby, Jonathan M: 8020
Sundstrom, Kim: 7630
Sung, Pildoo: 7145
Sunil, Surya: 8015
Sun-Mitchell, Shan: 1455, 4045
Supiano, Katherine P: 4520, 7425, 7640, 7905
Supiyev, Adil: 4535, 7535, 7540
Surachman, Agus: 1350
Suriaga, Armiel: 3460
Sussman, Tamara: 2560, 4195, 7055
Sutin, Angelina R.: 7245, 7350
Sutphin, Brandy: 3430
Sutphin, George L: 4005, 7010
Suzuki, Haruno: 7745
Suzuki, Hiroyuki: 1265, 7680
Suzuki, Lisa: 8085
Svec, Joseph: 1070, 1165, 1335, 4060, 7145
Svedberg, Pia: 2365
Sverdrup, Karen: 7005
Svitak, Aleta: 7455
Svynarenko, Radion: 3385
Swafford, Riley J: 8015
Swaim, Kathryn E: 4405
Swanson, Breanne M: 7350
Swanson, Helena: 2440
Swanson, Leslie M: 1365
Swanson, Sarah R: 7660
Swinkels, Joukje: 1120
Swint, Shelton: 8000
Sy, Maimouna: 4370
Syed, Ayesha: 2105
Syed, Orchee: 4500
Syed, Quratulain: 1200, 2345
Sylvers, Dominique: 7350
Syme, Maggie: 7355
Syros, Reiley: 4205
Szanton, Sarah L: 1365, 2010, 3150, 3500, 4420, 7445, 7935, 8000
Sze Him Isaac, Leung: 7490
Szerszen, Anita: 7025
Szmulewitz, Russell: 7205
Szymanski, Benjamin R: 7695
Szymanski, Gabrielle: 2480
Szymkowski, Amanda: 7480
Szypulski, Evan: 8010

**T**

T, Muhammad: 3505
Taani, Murad H: 7070, 7425, 7540, 7930
Taffet, George E: 1420, 2080, 2235, 3210, 7450, 8000
Taha, Hash Brown: 9000
Tahmaseb, Jasmin T: 8090
Tai, Daria: 7720, 8015
Tai, Joe: 1015
Takagi, Emiko: 7265
Takahashi, Tomoya: 1265
Takase, Mai: 7420, 8015
Takeda, Kazuya: 7270, 8000
Takeuchi, Masumi: 2470, 7360
Takeuchi, Satoko: 7105
Takeya, Yasushi: 7330, 8055
Talamantes, Michael: 7650
Talavera-Garza, Liza: 7425, 7935
Talmage, Alexis: 1160
Talton, Jennifer: 1410
Talwar, Sonia: 4200
Tam, Roger: 1150, 3335
Tambor, Ellen: 1260
Tamburri, Nicholas: 7525
Tan, Annabel: 8015
Tan, Brian: 7675
Tan, Cai Ning: 8015
Tan, Chia Jie: 7200
Tan, Edina YQ: 7205
Tan, Micah: 7050
Tan, Nasya S: 7140
Tan, Xiangchun: 7060
Tanaka, Tiffany Suchada: 2460
Tanaka, Toshiko: 4170
Tanbonliong, Gretchen: 3580, 7110
Tang, Fei: 7675
Tang, Fengyan: 1170, 1290, 1380, 3245, 3440, 4365, 8020, 8050
Tang, Foong Yee Vivien: 8010
Tang, Jennifer: 3200
Tang, Lingzi: 8000
Tang, Siyuan: 4055
Tang, Vera Mun Yu: 4050, 7640
Tang, Victoria: 4045
Tang, Weihong: 7010
Tang, Wen: 8015
Tang, Xiaotong: 2185
Tang, Xueting: 9000
Tang, Yimin: 2530
Tang, Yiting: 1560
Tangen, Gro Gujord: 7005
Tanglai, Wirampa: 1505
Taniguchi, Yu: 7275
Tanji, Tarin T: 7055
Tanley, Jordan: 3180
Tanner, Jeremy A: 4225
Tanner, Kamaryn: 7010
Tanner, Laura: 1320
Tanoue, Leslie: 7055
Tantipinichwong, Nicha: 7320
Tapola, Ella: 2460
Tappen, Ruth M: 3460, 7360, 7425, 7610
Tarabochia, Dawn: 7260, 8020
Tarantini, Stefano: 8015
Tarbox, Nathan: 2265
Tarpey, Thaddeus: 7465
Tarraf, Wassim: 1495, 2120, 7455, 7540, 7630, 8010, 9015, 9050
Tate, Alex: 7215
Tate, Channing E: 8020
Tate, Judith A: 7155, 7435, 7660
Tate, Rachel: 8055
Tate, Rio S: 8000
Tate, Robert: 7060
Tavares, Karina: 8015
Tawodzera, Obert: 3550
Tay, Djin: 4520
Tayade, Arti: 7155
Tayari Ashtiani, Reza: 3410
Taylor, Brent: 4055
Taylor, Christopher: 7200
Taylor, Corliss: 3150
Taylor, Harry: 1225, 2055, 3045, 3180, 4190
Taylor, Janelle S: 3065

Taylor, Janiece: 1365, 2230, 8000, 8015
Taylor, Jean O: 7425, 7465, 7905
Taylor, Miles G: 1280, 3540, 3555
Taylor, Morgan K: 1525, 7455, 7610
Taylor, Philip: 1080, 1360, 4290
Taylor, Tanya: 3240
Taylor, Zachary J: 1265
Taylor, Zachary E: 4410
Tchkonia, Tamara: 8000
Teachman, Gail: 7465, 7910
Teaster, Pamela B: 1200, 2275, 2495, 4135, 8010
Tebay, Jordan: 7440, 7500
Tedder, Jamie: 8015
Tee, Silvia: 7480, 7900
Teerawichitchainan, Bussarawan Puk: 4050
Teichman, Samantha: 2395
Teixeira, Laetitia: 7745
Tejada Arias, Karla: 7410, 7900
Teles, Soraia: 8015
Tell, Eileen J: 7490
Telonidis, Jacqueline: 4520, 8020
Templeman, Maureen E: 7065
Teng, Chiao-Hsin: 8035
Tennenbaum, Louis: 8020
Tennyson, Robert L: 7210, 8020
Teno, Joan: 3535
Tenquist, Miriam: 1450
Teodoro Maciel, Leticia: 7225, 7655
Teply, Abby L: 7440, 7490
Tepper, Lynn M: 8010
Terashima, Mikiko: 3170
Terhorst, Lauren: 7330
Terkpertey, Eric: 2220
Terracciano, Antonio: 7245, 7350
Terry, Christine: 1265
Terry, James Greg: 2220, 8000
Terveer, Katy: 1415, 8065
Tesar, Sarah: 8010
Teshome, Abraham Z: 7515, 7610
Testad, Ingelin: 8015
Tester, Griff: 8050
Tha, Soe Han A: 2520
Thacker, Ayush: 4370
Thalil, Muhammad: 1255, 7605, 8000
Thayer, Erin K: 7405, 8055
Thayer, Rachel: 7225, 7500
Theeke, Laurie A: 1465, 7350, 7540, 7910
Thiamwong, Ladda: 2300, 3055, 4315, 4420, 7025, 7200, 7205, 7260, 7620, 7640, 7655, 7720, 7735, 7750, 8000, 8015, 8020
Thibault, Emma: 7500, 7930
Thibodeaux, Brett: 8000
Thiel, Susie: 1480
Thierry, Amy D: 2330, 3000, 3180
Thin-Elk, Serene: 7425, 7500
Thingstad, Pernille: 7060
Thoma, Mary C: 8015
Thomas, Amanda S: 1095
Thomas, Amy: 3435, 7205
Thomas, Kali: 2155, 2185, 3535, 4460, 8010
Thomas, Karuna S: 8000
Thomas, Kelsey R: 7005, 8010, 8015
Thomas, Lisa: 7360
Thomas, Neil W D: 3285
Thomas, Nikita: 3150
Thomas, Zoe: 8020
Thomas Hebdon, Megan C: 4330
Thomas Tobin, Courtney S: 3475, 7540
Thomeer, Mieke B: 7225
Thompson, Alisha R: 3275
Thompson, Anya: 7150
Thompson, Atalie C: 1090, 2220
Thompson, Carl A: 4255
Thompson, Devin A: 8020
Thompson, Edward H: 7150
Thompson, Genevieve: 7055
Thompson, Hilaire: 4220, 7655
Thompson, Karl: 7425, 7930
Thompson, Katherine: 2340, 8020
Thompson, LaDora V: 8015
Thompson, Lena: 3005, 3345, 3485, 3585, 4015, 4320, 8070
Thompson, Louisa I: 4515, 7040
Thompson, Meghan: 2450
Thompson, Roy A: 1530, 7535
Thompson, Veronica: 9025
Thorne, Adrienna: 2265
Thorne, Christopher B: 3330
Thorpe, Carolyn: 4195
Thorpe, Joshua M.: 2180
Thorpe, Roland J: 2020, 2055, 3180, 3475, 4485, 7455, 7700, 1250, 2190, 4490
Thuemling, Teresa N: 2410, 4530
Thunell, Johanna: 2500
Thyagarajan, Bharat: 2460, 7010, 7060
Tian, Felicia F.: 1195
Tian, Junyan: 2330, 8000
Tian, Kun: 8010
Tian, Qu: 3070
Tian, Shubo: 1355
Tian, Teresa: 2510, 3330, 7090, 7720, 8000, 9015
Tian, Xin: 1090
Tian, Yuke: 1520
Tibbitts, Deanne: 7020
Tibirica, Lize: 3115
Tice, Abigail: 8010, 8015
Tiede, Ben: 8015
Tiemeier, Henning: 4550
Tien, Hsaio: 7275
Tien, Phyllis: 8010
Tillmann, Jacob: 7320
Timmerman, Christa: 3515
Tingvold, Laila: 1195
Tingvold, Maiken: 7610
Tintle, Nathan: 7615
Tipton, Mary E: 3485
Tiwari, Anjali: 7630
Tnesh, Basma B: 7530
To, Louis: 2150, 4365
Toba, Kenji: 7680
Tobón, Diana P: 2510
Tobey-Moore, Leah: 7325, 7650
Tobin, Karin: 2230
Tobyne, Sean: 7425, 7635, 7930
Tocchi, Christine: 1105
Toda, Eiji: 7410
Toh, Wei Xing: 7155
Toledo, Anna B: 8020
Toliver, Joy: 1500
Toll, Kara M: 4370
Tom, Sarah: 2425
Tomarevska, Olena S: 7505
Tomasini, Sergio Luiz V: 3155
Tompkins, Catherine J: 2415, 3510, 7270
Tong, Heling: 2240
Tong, Hongmei: 2050
Tong, Jialiang: 1355
Tong, Juncheng: 1215, 2030
Tong, Yao: 4225
Tong, Yuying: 3360
Tonkikh, Orly: 2200
Tonui, Betty: 7505
Toohey, Ann M: 3465
Topping, Michael S: 3225
Torke, Alexia: 4440, 7465
Toro-Serey, Claudio: 7425, 7930
Torres, Jacqueline M: 2020, 8015
Torres, Karen: 1480, 7200
Torres, Sandra: 1360, 3550
Torres, Stacy: 1140
Toseef, Mohammad Usama: 7530, 8010, 9050
Toshiko, Tanaka: 8000
Tosun, Duygu: 7005
Toth, Federico: 7515, 7915
Toto, Pam: 2190
Totten, Annette M: 4160
Touchinsky, Susan Martin: 7160
Towe, Maria: 8010
Towell, Michelle: 7750
Tower, Leslie: 8000
Towns, Juell: 3140
Townsend, Lisa: 8020
Towsley, Gail L: 1160, 2395
Toyama, Itsuko Kanamoto: 7505
Toyokawa, Noriko: 3560
Trabant, Marieke: 7075
Tracy, Brian L: 3115
Tracy, Eunjin: 3055
Tracy, Karen K: 8015
Traeger, Lara: 7680
Trainum, Katie: 7435
Tramujas Vasconcellos Neumann, Lycia: 3155, 8010
Tran, Dylan T: 3475
Tran, Melissa K: 1275, 2135, 7155
Tran, My Duyen L: 8095
Tran, Nathaniel M: 3075
Tran, Phoebe: 1060
Tran, Toan: 8020
Tran, Viet-Thi: 8010
Trane, Ralph Moeller: 3225
Trapido, Edward J. J: 8020
Traver, Anthony C: 1295, 2070, 2195
Travers, Jasmine: 8010, 8035, 8070
Travison, Thomas: 2040
Traywick, LaVona: 4540
Treat, Anny: 3255, 7510, 7545, 7915
Tremblay, Roma: 8015
Tremont, Geoffrey: 7630
Tretyakova, Natalia Y: 2165
Trevisan, Caterina: 3035
Trexberg, Kaitlin M: 7490

Trimble, Shawn X: 4370
Trimboli, Tracy: 3265
Trinitas, Joseph: 4080
Trinkaus, Eva-Maria: 2320, 4285
Trinquart, Ludovic: 7360
Triolo, Federico: 3035
Tripathi, Alok: 7285
Trittschuh, Emily H: 7675
Trivedi, Ranak: 2190, 2545, 3110, 7135
Trojanczyk, Carrie: 2335
Troost, Jonathan: 7065
Troosters, Thierry: 2170
Trost, Alexander: 4100
Trottier, Lise: 1100
Troutman, Brooke: 1105, 7265
Troutman-Jordan, Meredith: 1270
Trubenstein, Paige: 4095
Trubits, Em F: 7270
Trudeau, Scott A: 1005, 3395
Trujillo Tanner, Corinna M: 7035
Trumbo, Jami: 1440
Truskinovsky, Yulya: 7030
Tsai, Chih-TIng (Gina): 7300
Tsai, Feng-Jen: 7735
Tsai, Jenny Hsin-Chun: 1400
Tsai, Kai-Li: 7360
Tsai, Pao-Feng: 7275
Tschoe, Marianne: 7490
Tse, Dwight: 4020, 7725, 8020
Tseng, Chien-Yu: 1255
Tseng, Tung-Sung: 8020
Tseng, Winston: 7035
Tsimpida, Dialechti: 1170, 1360, 3195
Tsitsipatis, Dimitrios: 7305
Tsosie, Nathania: 2335
Tsotsoros, Cindy: 1495, 2130, 8015
Tsou, Hsin-Ying: 7055
Tsuchiya, Kazumi: 3180, 4190
Tsui, Iona: 1195
Tsui, Ronald: 4365
Tsukada, Noriko: 4135, 7295, 7405
Tsuker, Sophia: 2015, 3165, 7065
Tsukiashi, Motoki: 8015
Tsuzuki, Takamasa: 7315
Tsygankov, Denis: 8000
Tu, Wanzhu: 4195, 7465
Tu, Xueting: 4300
Tu, Yufang: 7055, 7150, 7360
Tucker, Brittany: 3270
Tucker, David: 8015
Tucker, Gretchen G: 1070, 2015, 4195
Tucker, Julia E: 1405, 4400
Tucker, She'Lon: 7435
Tucker-Roghi, Gina C: 2535, 3395
Tuckowski, Angela: 4005
Tuffier, Stéphane: 3125
Tuft, Samantha E: 1145, 7405, 7470
Tuladhar, Lirisha: 3055, 3565
Tulloh, Anna: 2260, 4345, 7725
Tumin, Dmitry: 7080
Tunalilar, Ozcan: 1230, 2185, 4510, 9045
Tunningley, Joan: 8010
Tuokko, Holly: 3185
Tupper, Emily: 1125, 7425
Tupper, Susan: 4195
Turcotte, Samuel: 8020
Turgeon, Jacques: 7425, 7630
Turiano, Nicholas A: 7070, 7115
Turkelson, Angela: 4525, 7660, 8020
Turner, Anne M: 7425, 7465, 7905
Turner, Chris: 8000
Turner, Grace: 3245
Turner, Jennifer R: 1525, 4280, 7255
Turner, Natalie: 3315, 8010
Turner, Pamela R: 3575
Turner, Rachael: 7065, 8020
Turner, Robert W: 1125
Turner, Seymour: 8020
Turner, Shelbie: 2305, 7270
Turner, Sidney: 3505
Tursi, Mark: 8015
Twardzik, Erica: 2525, 3070, 8065
Tyagi, Pranjal: 7360, 7730, 8010
Tyler, Emily R: 7545
Tzemah-Shahar, Roy: 1435, 7300, 7600, 7695, 9040
Tzeng, Huey-Ming: 2090
Tziarli, Dimitra: 8020

U

Ubaque, Lina Paola Buitrago: 8015
Uchida, Hayato: 7420
Uddin, Jalal: 3490
Udeh, Kingsley: 3475, 8010
Udenta, Mesomachi: 7000
Udo, Charles: 7700
Uehara, Tamiko: 4135
Ueki, Kaori: 8015
Ujano-De Motta, Lexus: 8010
Ukraintseva, Svetlana V: 1025, 7010, 7075, 7200, 7210, 7715
Ulitsa, Natalie: 7420
Umar, Tooba: 8015
Umberson, Debra: 1540
Umegaki, Hiroyuki: 7275
Umoh, Mfon: 2230
Undem, Teri: 7090, 7745
Unger, Kathryn: 7655
Unick, George: 8010
Unni, Vivek: 7310
Unroe, Kathleen: 1160, 4195, 7110, 8010, 8015, 9040
Unsworth, Julia: 8010
Upal, Shaneen: 7495
Upton, Rachel D: 7205
Ura, Chiaki: 7350, 7680
Urbanski, Dana: 1175, 3130
Urias, Uriel: 8010, 8015
Uribe, Alondra: 7700
Urrutia-Pujana, Leixuri: 7540
Usami, Satoshi: 7640
Utz, Rebecca: 3500, 4520, 7425, 7905

V

Vaccaro, Joan A: 2330
Vaddi, Prerana: 8000
Vafaei, Afshin: 2060, 7465, 7910
Vafiadis, Dorothea K: 3580, 7110
Vahedi, Vanessa: 7630, 7905
Valdivia, Nathaly: 1425
Vale, Michael T: 1010
Valenti, Korijna: 2225
Valentine-Graves, Mariah: 8015
Valenzano, Dario Riccardo: 3470, 4210
Vallejo, Rose: 8015
Valletta, Martina: 4315, 7010
Van, Mary: 7465, 7910
Van Boekel, Leonieke: 3425
Van Bogart, Karina: 4180, 4515
Van Camp, Bennett: 8000
Van den Block, Lieve: 2255
van den Broek-Altenburg,, Eline: 8000
van der Steen, Jenny T: 2255, 7640
van der Willik, Odette: 3045, 4210
van Dijk, Aileen: 8015
Van Eck, Richard: 8020
Van Gameren, Mateja: 1010
Van Haitsma, Kimberly: 1160
Van Houtven, Courtney H: 1475, 2090, 2180, 4530
Van Ooteghem, Karen: 2170
Van Orden, Kimberly A: 3110, 4120, 4180
Van Raamsdonk, Jeremy M: 8020
Van Scov, Lauren J: 1080
Van Son, Catherine R: 7155, 7465
Van Vleet, Bryce A: 7025, 7070, 7090
Van Vleet, Samuel: 7750, 8015
van Wijngaarden, Jeroen: 7445
van Zaalen, Yvonne: 3425
Vance, David E: 7285
VanCleef, Tricia R: 7625
Vandawalker, Melissa: 1015, 7420
Vanderboom, Catherine: 1475, 4415
Vanderlaan, Courtney: 1175
VanderWeele, Tyler: 1545, 4550
VanderWerf, Jess A: 2490, 7340
VandeVrede, Lawren A: 8070
VandeWeerd, Carla: 8015
Vang, Cindy: 4445, 7600
VanHaitsma, Kimberly: 1080
VanPutten, Rachel: 3110
Varadhan, Ravi: 1205, 7620
Vargas, Andrea-Paula R: 7720
Varley, Allyson: 3330
Varma, Hiren: 7200
Varma, Vijay R: 2045
Varshney, Ishan: 2340
Vasilevskis, Eduard E: 4530
Vasquez, Elizabeth: 3460, 8000, 8010
Vass, Emma L: 7000
Vasylenko, Maksim: 2325
Veeramah, Krishna: 8020
Vega, Irving E: 2020
Veilleux, Megan: 8020
Velazquez, Ana E: 7700
Velazquez, Enilda M: 8020

Velez-Ortiz, Daniel: 8020
Veliz, Philip: 7360
Velk, Lace J: 7440
Vellani, Shirin: 4255, 9040
Vellone, Ercole: 7245
Venditti, Elizabeth M: 7300
Venegas, Maria D: 7135
Venema, Dawn: 8015
Venkata, Pruthvinath Jeripity: 8015
Venkatesh, Arjun: 1130
Venna, Venugopal: 1460
Ventakesh, Manasa: 2190
Ventura, Juan D: 7700
Venugopal, Surya: 7310
Verbeek, Hilde: 2140, 4345
Verdery, Ashton: 4050
Verdin, Eric: 8000
Vereijken, Beatrix: 2170
Vergara, Kristine E: 7000
Vergenz, Laura: 7200
Vergez, Sasha: 4360
Verghese, Joe: 7750
Verma, Ambika: 7465, 8000
Verma, Mansi: 1535
Versey, H. Shellae: 1295
Verstaen, Alice: 7675
Vetrano, Davide Liborio: 3035, 3330, 4315, 7010, 7060
Vetter, Valentin M: 7030, 7210
Veturi, Yogasudha: 7455
Viceconte, Madeleine: 4205
Vichos, Theodoros: 8000
Vick, Judith: 2090, 2180, 4530
Vickburg, Jonathan: 2520
Victor, Christina R: 1225, 2055
Vidal, Jennifer Landeta: 2555
Videla, Emma: 7030
Vieira, Candice: 8085
Vieira, Edgar Ramos: 7610
Viel, Isabella C: 7720
Vig, Elizabeth K: 3065
Vijinski, Jon Parr: 7040
Villalba-Mora, Elena: 3150
Villar, Michelle: 7360
Villareal, Dennis T: 1330
Villemagne, Victor L: 3375
Vincent, Thomas: 4490
Vincent Duro, Marlon: 8020
Vinson, Latrice D: 1205
Virgin, Nicole: 8020
Visaria, Abhijit: 3290, 8015
Visser, Mandy: 2255
Visvanathan, Pallavi: 1140
Visvanathan, Renuka: 7275
Vivek, Sithara: 7010
Vivoda, Jonathon M: 3455
Vluggen, Stan: 8015
Vo, Quynh: 8010
Voegtline, Kristin: 2080
Vogelsang, Eric: 4400
Vogelsmeier, Amy A: 1530, 9025
Voils, Corrine I: 2190
Volek, Jeff: 8000
Volpi, Elena: 1275, 7400, 7920, 8015, 8015, 8050
Von Ah, Diane: 2445
von Bastian, Claudia C: 4490
von Gunten, Armin: 1220
von Hacht, Markus: 7260
Vonderheide, Clara: 2315
Vos, Carol M: 7435
Voss, Michelle: 7735
Voyich, Jovanka M: 7210
Vranceanu, Ana-Maria: 1345, 2315, 3250, 4370, 8000, 8020, 8025
Vrinceanu, Tudor: 4490
Vrlkjan, Brenda: 3185
Vu, Thi: 8020
Vu, Thi Hoang: 7245, 7270
Vyavahare, Sagar: 7210
Vytniorgu, Richard: 1015

### W

Wada, Mineko: 7270, 8000
Wada, Tadashi: 4135
Waddell, Wendy: 7440
Waddington, Emily: 7465
Wadsworth, Sally J: 4095, 7225
Wagenknecht, Lynne: 1390, 7015
Wagg, Adrian: 8000
Wagle, Sampada: 4115, 8015
Wagner, Brandon: 7350
Wagner, Johanna: 8000
Wagner, Melanie: 2025
Wagner, Richard K: 8000
Wagner, Todd: 7080
Wagshul, Mark E: 1150
Wagster, Molly: 1340
Wahl, Anne L: 7640
Wahl, Hans-Werner: 1040, 1285, 1555, 7680
Waite, Elinor E: 7255
Waite, Linda J: 1110, 3410
Wakui, Tomoko: 7490, 7750, 8020
Waldinger, Robert J: 4375
Waldron, Danielle: 3530
Walker, Jenny A: 4390, 7065, 7200, 7205
Walker, Kealie J: 3035
Walker, Keenan: 1000, 7010, 7015
Walker, Teresa J: 7235, 7685
Walkner, Tammy J: 1395, 7665
Walkup, Michael P: 1390
Wall, Lucas A: 7440
Wallace, Brandy H: 7700
Wallace, Bruce: 3285
Wallace, Cara L: 4080, 7055, 7520
Wallace, Gail: 4490
Wallace, Meredith: 2290
Wallhagen, Margaret: 7140, 7745
Wallsworth, Christine: 2530, 7465, 7910
Walsemann, Katrina: 1280, 7040
Walser, Tracie: 3385
Walsh, Christine A: 2395, 2560
Walsh, Judith: 4230
Walsh, Maureen A: 7075
Walsh, Sarah: 8045
Walston, Jeremy D: 1205, 2570, 7305, 8000
Walters, Monica E: 1495, 2045, 9015
Walton, Allison: 8020
Walton, Mary: 1185
Waltz, Thomas J: 3110
Wan, Adrian H. Y.: 7725
Wan, Debby: 2150, 4365
Wan, Lu: 4315
Wan, Qiaoqin: 8015
Wan, Rachel: 7465
Wandee, Chanchanok: 8020
Wang, Adrienne M: 7075
Wang, Anni: 7245
Wang, Can: 8020
Wang, Chen-Pin: 7735
Wang, Chih-Hsuan: 7275
Wang, Claire L: 2145, 3200, 4010, 4320
Wang, Connor: 1130
Wang, Cuiling: 2160
Wang, Danny: 7425, 7615, 7930
Wang, Dingyue: 4450, 7270
Wang, Dongmin: 3050, 7680
Wang, Emily: 7535
Wang, Ethan B: 1440
Wang, Fei: 1060
Wang, Fei: 4310
Wang, Feng: 7010
Wang, Ge: 7285
Wang, Gene: 3285, 8040
Wang, Gwenneth: 4360
Wang, Hang: 3070
Wang, Haoqing: 4310
Wang, Haowei: 7140
Wang, Haowei: 8020
Wang, Helen: 7510
Wang, Hongning: 8020
Wang, Hsiu-Jung: 8010
Wang, Huijuan: 7670
Wang, Inga: 7205
Wang, Jia: 7640, 7690
Wang, Jiangxia: 7015
Wang, Jianye: 3350
Wang, Jing: 1380, 2000, 3305, 4030
Wang, Jing: 7315
Wang, Jing: 8010, 9000
Wang, Jing: 9030
Wang, Jingxuan: 1545
Wang, Jinjiao: 8035
Wang, Jiuzhou: 4430
Wang, Jouchieh: 7040
Wang, Julian: 7200, 8015, 9015
Wang, Junqiao: 9000
Wang, Juwen: 4455
Wang, Kaipeng: 4115, 7055, 8020
Wang, Katherine: 1380
Wang, Kuan-Yuan (Ken): 8015
Wang, Kun: 3030, 7155, 8010
Wang, Libin: 7010
Wang, Liru: 7355
Wang, Lu: 4220
Wang, Mei-Chen: 8010
Wang, Mengru: 3510
Wang, Mengya: 2060
Wang, Moyao: 8015
Wang, Mu-Cyun: 7080
Wang, Nan: 7275
Wang, Natalie: 4300
Wang, Ning: 4180
Wang, Peng: 8020
Wang, Qinchuan: 7305
Wang, Qingwei: 7490, 7515, 7925, 8095, 9045
Wang, Qun: 7625
Wang, Rui: 7280
Wang, Ruifeng: 7620
Wang, Shanshan: 7490
Wang, Shuangshuang: 7270
Wang, Shuo: 7010
Wang, Shuomin: 1235, 2255, 2465, 4055
Wang, Siqi: 4470
WANG, Siqiang: 8075
Wang, Siqing: 8010
Wang, Suyi: 7355

Wang, Tianhao: 1235
Wang, Weijun: 7245
Wang, Wenjin: 7040, 8020
Wang, Xi: 7540
Wang, Xia: 3270
Wang, Xiao (Joyce): 3535
Wang, Xiaochuan: 7105
Wang, Xiaomeng: 7235
Wang, Xin: 7610
Wang, Xuan: 7460, 7715
Wang, Xuedong: 8015
Wang, Xuzhi: 7360, 8015
Wang, Yan: 1445
Wang, Yanxin: 4055
Wang, Yanyan: 1090
Wang, Yaping: 7350
Wang, Yi: 4110, 7235, 7445
Wang, Yi: 7630
Wang, Yi: 7700
Wang, Yilin: 1355
Wang, Ying: 7420, 7920
Wang, Yiqi: 3480
Wang, Yixuan: 7055
Wang, Youhua: 8000
Wang, Yue: 3050
Wang, Yueting: 1485, 7145
Wang, Zheng: 4245
Wang, Zhi-wen: 2160, 4070, 7235, 7355, 7750
Wang, Zhong: 8090
Wang, Zih-Ling: 2090, 2115, 2450, 7465
Wanigatunga, Amal A: 2035, 3070, 8015
Wanigatunga, Sarah K: 3070
Wanlass, Jase: 7035
Ward, Angela: 3100
Ward, Chesney J: 7745
Ward, Heather A: 4440
Ward, Mark: 1290
Ward, Rachel: 3335
Warne, Donald K.: 7425, 7500
Warner, Lisa M: 2520
Warner, Lynn: 1465
Warren, Connor: 9025
Warren, John R: 1095, 4035, 4375, 7230, 7350
Warren, Teresa S: 7015
Warren-Walker, Alina: 8015
Warry, Wayne: 2335, 2395
Washburn, Stephanie: 8000
Washington, Ashley: 7135
Washington, Karla T: 1165, 2085, 4080, 4220, 7270, 7490, 7665, 8000, 8010
Washington, Tiffany R: 4175
Washington, Tracey: 1080
Wasik, Sophia: 2520
Watanabe, Kengo: 8000
Watanabe, Kentaro: 7750
Watanabe, Yuichi: 3200, 4190
Watanabe, Yuki: 7420
Waterhouse, Jodi L: 1415, 3465, 8020
Waters, Leland: 3510, 4290, 4470
Watkins, Michele: 1295, 4265
Watman, Deborah: 3105, 4480, 8010
Watts, Amber: 4315
Waugh, Kellie: 1260
Weathers, Brandy: 8000, 8000
Weaver, Ashley A: 2505
Weaver, France: 8045
Weaver, Raven H: 1190, 2180, 2295, 2325, 7350, 7530
Webb, Fern J: 2330
Webb, Jason: 7020
Webb, Sara: 7685, 8015
Webel, Allison: 2450
Weber, Daniela: 7315
Weber, Emma: 7660, 8020
Weber, Madeline K: 7205
Webster, Amy: 2145, 8015
Webster, Melinda J: 4130, 4340
Webster, Micah T: 8000
Webster, Noah J: 1045, 1495, 2025, 3040, 7720, 9000
Weech-Maldonado, Robert: 1425, 1550, 3255
Weese, Richelle: 7040
Wehage, Mattea: 7050
Wehry, Susan: 8010, 8015
Wei, Amy: 2205
Wei, Changning: 7625, 7710
Wei, Duo Helen: 3515, 7425, 7905
Wei, Hui: 2465
Wei, Jeanne Y: 7010, 7210, 7465, 8000, 8015
Wei, Kai: 7010, 7235
Wei, Wenxing: 1060, 7530
Wei, Yehua Dennis: 7420
Weil, Joyce: 1015
Weimar, Lisa S: 7735
Weinberg, Gil: 8010
Weiner, Bryan: 8010
Weir, David R: 2515
Weiser, Sheri: 8020
Weiskittle, Rachel: 7510, 7640, 8020
Weiss, Charlotte R: 1290, 7435
Weiss, Jenna: 4165
Weiss, Jordan: 1575
Weiss, Samantha: 9030
Weissberger, Gali H: 2001, 7495, 7930
Weith, Joanne: 7480, 7900
Weitman, Cassidy: 7030
Weitzel, Kelsey J: 3575
Weiyun, Chen: 7435
Wek, Aleksandra: 2085
Weldrick, Rachel: 2395, 2560
Welhaf, Matthew S: 4515
Wells, Jenna L: 3355, 8020, 8040
Welsh-Bohmer, Kathleen A: 3280
Wen, Chiung-Jung: 7275
Wen, Juncheng: 7035
Wen, Ming: 7230, 7420
Wen, Wan: 2265, 4420, 7505, 7720
Wen, Xi: 9030
Wende, Marilyn: 1045
Wendel-Hummell, Carrie L: 2500
Weng, Hui-Ching: 7460, 8000
Wenhan, Zhang: 1475
Wenzel, Arlett: 7110
Werbeloff, Merle: 2060
Werner, Nicole E: 1455, 2085, 2410, 4370, 4530, 7005, 7135, 7270, 8020
West, Jessica S: 7625
Westcott, James S: 1415
Westerlind, Bjorn: 8015
Westlake, Frances: 2135
Westmore, Megan: 2260, 4345, 7725
Westra, Jordan: 7405
Wethington, Elaine: 3040
Wettstein, Gal: 2420
Wettstein, Markus: 1375, 4065, 4500, 7035
Weuve, Jennifer: 4380
Weyrauch, Karen: 7325
Wezeman, Jackson: 7075, 8000
Whaikid, Phatcharaphon: 8015
Whalen, Stacey: 8010
Wharton, Whitney: 2225
Wheaton, Felicia V: 3235
Wheeler, Pariya: 7285
Wherry, Sarah J: 2345
Whetung, Cliff: 2335, 2470
Whitaker, Janice: 3380
Whitaker, Kara: 7735
Whitbourne, Susan Krauss: 3310, 3465
White, Bess: 7205
White, Carole L: 2295
White, Christian: 8010
White, Julie: 8010
White, Katie: 2070
White, Kina: 2480, 3100, 7240
White, Lane: 8010
White, Laura: 8010
White, Lillian A: 7005
White, Lindsay LY: 7000
White, Rebecca E: 3060
White, Stephanie: 8040
Whiteside, Phoebe G: 9025
Whitfield, Keith E.: 2190, 7455
Whitman, Kathrine: 8000, 8020
Whitmer, Rachel: 7535
Whitmore, Kim: 7660
Whitney, Robin: 2200, 8010
Whitson, Heather: 8015, 8020
Whitten, Justin M: 7260, 8020
Wicklein Sanchez, Heather: 7415, 7900
Wickline, Sierra: 9030
Wickson-Griffiths, Abigail: 4195, 7055
Wickwire, Emerson M: 1255
Widener, Michael: 4430
Widjaja, Nathan: 1095
Wiedrick, Jack: 7310
Wiemers, Emily: 4050, 7030
Wiersma Strauss, Anna: 4050
Wiese, Lisa A: 2360, 7200, 7260
Wiggins, Arika: 7440, 7935
Wihry, David C: 8010, 8015
Wijk, Helle: 4010
Wilber, Kathleen: 2150, 2375, 3525, 4150, 7725, 8010
Wild, Ellen: 4415
Wild, Tess: 8020
Wilder, Jocelyn: 4505
Wilder, Jocelyn R: 9020
Wildschut, Tim: 7255
Wiley, Christopher: 2165
Wilgenburg, Brian: 7210
Wilk, Adam: 7530
Wilkie, Rachel Z: 3245
Wilkins, James M: 3135
Wilkinson, Lindsay R: 8000
Wilks, Hannah: 4515
Wilks, Scott E: 3275
Willard, Meredith A: 7070
Willcox, Bradley: 1385, 8000

Willgerodt, Mayumi: 4470
Williams, Adrian: 7070
Williams, Amanda L: 8070
Williams, Brie: 3420, 7535
Williams, Cara: 8010
Williams, Cedric: 8020
Williams, Cierra: 8015, 8020
Williams, Claire M: 7725, 8010
Williams, Crystal Warren: 8070
Williams, Dylan: 7005
Williams, Ginger: 3575
Williams, Ishan: 7005
Williams, Ivan: 4050
Williams, Kelvin L: 1315
Williams, Kirk B: 7205
Williams, Kristine N: 1530, 4475, 7635, 7650
Williams, Micah: 8000
Williams, Mikaela: 2395
Williams, Shellie: 7250
Williams, Victoria J: 3225
Williamson, Christin: 1415
Williamson, Jeff D: 1090
Williamson, Marsha: 8015
Williman, Marty: 8020
Willis, Crysta: 1265
Willis, Matthew L: 7225
Willis, Sherry L: 4490
Willroth, Emily C: 7745
Wills, Celia E: 2415
Wilson, Annalee: 7650
Wilson, Chandra: 3100
Wilson, Deborah: 2010
Wilson, Jeff: 8000
Wilson, Kimberley J: 8010
Wilson, Sanjaya: 8010
Wilson, Stephanie J: 8020
Wilson, Tracey: 8015
Wilson, Traci L: 2070, 7465, 7700
Winberry, Joseph W: 3075, 8010
Windham, B. Gwen: 1000, 2525
Winesberry, Nehemiah Jude: 2460
Wing, Rena R: 1390
Wingo, Anna: 7055
Wingood, Mariana: 7720
Wininger, Claire: 8020
Winship, Jodi M: 4205
Winstead, Vicki: 1510
Winters, Emily D: 7005
Winters-Stone, Kerri M: 7020, 7205
Wirth, Maria: 1375, 4020
Wise, Jenni: 8020
Wisnivesky, Juan: 7630
Wister, Andrew: 1100, 4445, 7155, 7445, 7720, 7740, 8045
Witmeier, Kelsey N.: 8020
Wittenberg, Eve: 7275, 7280
Wittenberg, Grace F: 8010
Wittenberg, Nir: 8020
Wittich, Walter: 4300, 7635
Wittie, Michael: 8030
Witzel, Dakota D: 1525, 2385, 4280, 4405, 4410
Wladkowski, Stephanie P: 4080, 7055, 7520
Woehrmann, Anne: 7050
Wohns, Nicolai: 4285
Wojczynski, Mary K: 1025, 7010, 7060
Wolf, Jack: 4245
Wolf, Karsten D: 2140
Wolf, Michael S: 4230, 4480
Wolfe, Joseph D: 7225
Wolff, Jennifer L: 2085, 2230, 3570, 4300
Wolf-Ostermann, Karin: 2140, 4345
Wolfson, Christina: 7030
Wolinsky, Steven: 7285
Wolk, Megan W: 8010, 8020
Womack, Dana: 7440
Won, Chang Won: 8000, 8010, 8015, 8015
Won, Sarah: 8000
Wong, Alice C.: 8050
Wong, Alina Clarise: 2100
Wong, Chelsea: 4225, 8015
Wong, Dipsy H. S.: 8020
Wong, Edwin: 8010
Wong, Jason: 2120, 3360, 3410, 3480, 4505
Wong, Joey: 2530, 3205, 7465, 7750, 7910, 8010
Wong, Ka Kit: 7540
Wong, Karen: 7510
Wong, Karen Lok Yi: 1115, 7465, 7910, 8010
Wong, Kayla: 7085
Wong, Lily: 2410, 2530, 7465, 7750, 7910
Wong, Loong Mun: 3545
Wong, Meghan: 8000
Wong, Rebeca: 1320, 4500, 7140, 7235
Wong, Roger: 1425, 2185, 9015
Wong, Sarah: 8015
Wong, Yun Leng: 1485, 7145
Wong, Yunjie: 1015, 2185
Woo, Seoyoon: 7440
Wood, Julie K: 7325, 8015
Wood, Kristie A: 7270
Wood, Leila: 1510, 4355, 7405
Wood, Michele M: 7000
Wood, Stacey: 1370
Woodhouse, Mark: 1390, 1520
Woods, Ashley: 8015
Woodward, Amanda T: 8010
Woodworth, Emily: 1345
Woolever, David: 7635
Woolson, Sandra: 2180, 8015
Worschech, Florian: 4490
Worthington, Amber K: 7480
Wouters, Eveline JM: 3425
Wright, Bernadette: 8010
Wright, Eric R: 8020
Wright, Kathy D: 2415, 2550, 7490, 7660, 7935, 8020
Wright, Leslie A: 1415
Wright, Matthew R: 3065
Wright, Molly A: 7210, 7455
Wright, Morgan M: 2225, 3490
Wright, Rebecca: 7055
Wright, Regina: 2330
Wrigley-Field, Elizabeth: 4035
Wu, Barry: 7410, 7900
Wu, Bei: 1380, 2095, 2205, 3305, 3505, 3540, 4030, 7535, 7630, 8000, 8010, 8025, 9000, 9030
Wu, Chao-Yi: 8000
Wu, Chenkai: 4005, 4055, 7275
Wu, Deqing: 7715
Wu, Dongmei: 7235
Wu, Haorui: 3585
Wu, Heng: 7275
Wu, Horng-Shiuann: 8000
Wu, Huiping: 1355
Wu, Jiajun: 1575, 4420
Wu, Jia-Rong: 3320
Wu, Jingchuan: 8020
Wu, Jingxuan: 7725, 7745
Wu, Judy Z: 7275
Wu, Junhui: 7430
Wu, Kaili: 7470, 7905
Wu, Kevin Chien-Chang: 7460
Wu, Kuan-Ching: 7465, 8015
Wu, Mingche: 2085
Wu, MJ Mingche: 4300
Wu, Peter C: 7305
Wu, Rachel: 7135
Wu, Serena: 7525
Wu, Shaohui: 4360
Wu, Sherry: 1470
Wu, Shih-Cyuan: 7510, 7525, 7915
Wu, Tiffany: 8015
Wu, Xucheng: 8015
Wu, Yafei: 2255
Wu, Yanyan: 8000
Wu, YingHua: 8000
Wu, Yingyan: 4380
Wu, Yiwen: 4020
Wu, Yuxia: 3270, 7740
Wu, Zixuan: 7490, 7925, 8095, 9045
Wunderlich, Tiffany: 3485, 7685
Wyatt, Riley: 8020
Wylie, Molly: 7135
Wyman, Mary F: 2190
Wyte-Lake, Tamar: 3485, 4160, 7045

X

Xaio, Xaviera: 2190
Xi, Yueming: 1405
Xia, Lu: 7305
Xia, Nan: 7080
Xia, Weiyi R: 4485
Xia, Wenran: 7445
Xia, Xiaohu: 8000
Xia, Xin: 1090
Xiang, Xiaoling: 2410, 4115
Xiao, Chunhong: 4045, 7285, 8000, 8010
Xiao, Huimin: 7670
Xiao, Michelle: 8015
Xiao, Yexuan: 7235, 7630
Xicota, Laura Vila: 1025
Xie, Bo: 1355, 2245, 2355
Xie, Jinchen: 2125
Xie, Kathy: 2220
Xie, Rui: 2300, 4420, 7025, 7200, 7205, 7260, 7620, 7655, 7720, 8015, 8020
Xie, Serena Jinchen: 2125
Xie, Wenyi: 3585, 7535
Xie, Yuqing: 1090
Xie, Zongxing: 3415
Xing, Yurong: 7545, 7915
Xiong, Xueying: 7035

Xiong, Yuan: 7345
Xu, Chunjuan: 1230
Xu, Emily L: 3105
Xu, Hanzhang: 2205, 3505, 7205, 7625, 8015
Xu, Jiahui: 7350
Xu, Jianhong: 4005, 4055, 7275
Xu, Jianhui (Frank): 4460
Xu, Jingjing: 2125
Xu, Kaidi: 7620
Xu, Ling: 1105, 2050, 7005, 7265, 8010
Xu, Lucy: 2310
Xu, Mengjiao: 1245
Xu, Mingming: 1475
Xu, Qin: 8000
Xu, Qingwen: 1115, 7540
Xu, Richard: 8000
Xu, Shicheng: 8020
Xu, Shu: 7140, 8015, 8020
Xu, Sophie Zhijing: 8010, 9030
Xu, Ting: 8000
Xu, Wenqian: 7430
Xu, Xiao: 7265
Xu, Ying: 7235, 7610
Xu, Yvonne Yiru: 7040
Xu, Ziyao: 1535
Xu, Ziying: 4170
Xue, Dandan: 7005
Xue, Qian-Li: 1205, 2010, 2465, 3150, 8015
Xue, Tingzhong (Michelle): 2205
Xue, Wei- Lin: 7405

**Y**

Yabluchanskiy, Andriy: 8015
Yadav, Hariom: 3470, 7210, 8000, 8015
Yadav, Shweta: 8000
Yaffe, Kristine: 2220, 8015
Yahirun, Jenjira: 8010
Yaisikana, Mo'e: 7540
Yakong, Vida N: 4175
Yam, Hawking: 3130
Yamada, Cody: 2225
Yamada, Yuji: 7260
Yamaguchi, Satoshi: 7640
Yamaguchi, Yuko: 8015
Yamahana, Reiko: 7295
yamakawa, Miyae: 8055
Yamamoto, Hiroshi: 7400, 7920
Yamamoto, Ryohei: 7080, 7105
Yamamoto, Yasuhiro: 8015
Yamashita, Takashi: 1505, 1535, 2470, 3035, 7350, 7475
Yan, Changmin: 4205
Yan, Elsie: 2150, 4365, 7530, 8020
Yan, Jinhua: 7075
Yan, Mengzhao: 1005, 3385, 7725, 8010
Yan, Shibin: 1015
Yan, Xiting: 4170
Yan, Yuanjiao: 7450, 7930
Yan, Zi: 3025
Yancura, Loriena A: 8020
Yang, Annabelle R: 8010
Yang, Betsy: 2345, 7480, 7900
Yang, Chengfengyi: 8015
Yang, Chih-Hsiang: 8000
Yang, Chun-Ting: 3135
Yang, Eunjin: 7750, 8020
Yang, Frances M: 4475, 7650
Yang, Huili: 1235, 7440
Yang, Iris S: 8020
Yang, Jen-Hao: 7305
Yang, Kaite: 7235
Yang, KwangSoo: 7360, 7425
Yang, Liu: 7330
Yang, Mia: 3215
Yang, Michelle: 7400, 7920, 8015
Yang, Minhee: 7655, 7715
Yang, Po-Lung: 7080
Yang, Qing: 7625
Yang, Seonkyeong: 2145
Yang, Shuhui: 7425, 7930
Yang, Siqi: 4115, 7660, 8015
Yang, Wei: 1475
Yang, Xiali: 2500
Yang, Xiaoping: 7670
Yang, Xuebing: 8015
Yang, Yang: 1395, 3570, 8070
Yang, Yanping: 7010
Yang, Yijian: 4195, 8015
Yang, Yingqi: 8000
Yang, You Lee: 3370
Yang, Yuanyuan: 8010
Yang, Yue: 8015
Yang, Yulin: 2020, 7225
Yang, Yuxuan: 7335
Yang, Zhen: 7075
Yang, Zhongfang: 3505, 8010
Yang, Ziying: 7040
Yank, Veronica: 4045
Yankeelov, Pamela: 4470, 7340, 7730
Yanover, Chen: 8015
Yao, Kaichen: 8010
Yao, Li: 7505, 7910
Yao, Shanshan: 7710, 8000
Yao, Yao: 7300, 8015
Yarrarapu, Siva Naga Srinivas: 8010
Yasar, Sevil: 4300
Yashin, Anatoliy: 1025, 7075, 7200, 7210, 7300, 7715
Yashkin, Arseniy: 3010, 7075, 7200, 7300, 7620
Yasin, Reham: 7275
Yassine, Hussein: 8020
Yasumoto, Saori: 2470, 8020, 8020
Yauch, John: 7510, 7915
Yauk, Jessica: 2370, 3365, 7435
Ye, Fan: 3265, 3415
Ye, Haiger: 2565
Ye, Jing: 4435
Ye, Minzhi: 3360
Ye, Qiaofeng: 7010
Ye, Yan: 7280
Yedavalli, Anjali: 7270
Yee, Claire I: 3285
Yeh, Jarmin: 8025
Yen, Pei-Shan: 7080
Yenko, Ira: 1325
Yentes, Jennifer: 8000
Yeo, Audrey: 8015
Yeo, Hyesu: 7545
Yeom, Hyun-E: 7265, 8000, 8010, 8015
Yeoman, Carl J: 7210
Yeong, Joe: 7010
Yeung, Dannii Y.: 2520, 4390, 7745, 8000
Yeung, Lydia: 8015
Yi, Grace: 8000
Yi, Mo: 4070
Yi, Stella S: 8095
Yi, Zhiqi: 8020
Yigit, Oykum E: 7160
Yildiz, Mustafa: 1510, 2215, 7040
Yim, Cynthia: 8075
Yin, Cheng: 3085
Yin, Jiamin: 7030
Yin, Jianing: 7520
Yin, Lijuan: 8020
Yin, Michael: 8010
Yin, Qingqing: 7300
Yin, Xuecheng: 3165
Yin, Zenong: 8050
Ying, Gelan Lana: 2020
Ylitalo, Kelly R: 1365, 7300, 7720
Yockey, Robert: 7080
Yoder, Maria: 2140
Yokoyama, Sae: 8020
Yokoyama, Yuri: 7275, 7695, 8015
Yon, Yongjie: 1195
Yonashiro-Cho, Jeanine: 2001, 2375, 7405
Yoneda, Tomiko: 1570, 7205
Yoo, Aejung: 3545
Yoo, Haeun: 8000
Yoo, Leeho: 7005
Yook, Geun Sil: 8010
Yoon, Caroline: 2500
Yoon, Dokyung: 8000
Yoon, Jieun: 8015
Yoon, Jong Sung: 2360
Yoon, Joonsik: 8000
Yoon, Seolah: 7275, 8015, 8015
Yoquinto, Lucas: 3145
Yorgason, Jeremy: 2385, 7035
Yoshida, Hiroto: 7680
Yoshikawa, Aya: 8000
Yoshimoto, Kukuna: 4015
You, Jin: 3480
Young, Colin: 4235
Young, Dominic: 7265
Young, Heather M: 2200, 8010
Young, Jessica: 7320
Young, Khristina: 8000
Young, Lisa: 1530
Young, Meghan: 3380
Young, Vanessa: 7735
Young, Yuchi: 3255, 7055, 7460, 7925
Yount, Naomi: 7545
Yous, Marie-Lee: 7510
Yousefzadeh, Matt: 3160
Yow, W.Quin: 1405, 2100, 7455, 7925
Yu, Chang: 7225
Yu, Chen-Wei: 3355
Yu, Chin Wing: 7300
Yu, Doris Sau Fung: 1560, 7005, 7040, 8015
Yu, Fang: 7015, 7450, 8020, 8035
Yu, Heshuo: 7055
Yu, Hsiao-Wei: 7525
Yu, Jiao: 2020, 4430, 7630, 8055
Yu, Kexin: 7005, 8000
Yu, Oliver Hang Kin: 8020
Yu, Wanting: 2040, 4215, 4535
Yu, Wenshan: 7335, 7525
Yu, Xinfang: 3405
Yu, Xuexin: 1290, 2515, 9000

Yu, Yue: 7630
Yu, Zhan: 1115, 7540
Yuan, Jialin: 7670
Yuan, Rong: 8000
Yuan, Yaqun: 4440, 7060, 7090, 7630
Yuan, Yin: 7275, 7710
Yuan, Yiyang: 7275, 8015
Yuan, Yue: 3165
Yue, Hao: 4535
Yue, Yiwei: 1255, 3070
Yuelling, Thomas J: 8020
Yueter, Sheila: 7200
Yuh, Ahhyun: 7145
Yun, Hyunkyung: 1115, 1230, 8075
Yun, Seon Kyung: 8010
Yunfan, Wu: 7330
Yung, Hiu Kwan Esther: 8075
Yurekli, Hulya: 7160
Yurkiv, Halyna: 4270
Yus, Roberto: 1070
Yusuf, Khalid: 8010
Yuwen, Weichao: 2125

Z

Zabielaite, Meda: 8000
Zaborenko, Callie: 4455
Zaccaria, Daniele: 1220
Zadok, Erez: 3265
Zafar, Anum: 1380, 4485
Zaguri-Greener, Dalit: 4045
Zahodne, Laura: 1045, 1290, 1495, 2045, 3040, 7535, 8025
Zakrajsek, Jennifer S: 3455, 8020
Zaller, Nickolas: 3420
Zaman, Eman: 7105
Zamarripa, Estefania: 8015
Zambrano Garza, Elizabeth: 7535, 8020
Zammitti, Melissa G: 1020, 7630
Zampino, Marta: 8015
Zamrini, Edward: 2160
Zan, Hua: 8020
Zanasi, Francesca: 3025
Zang, Emma: 2120, 2305, 4505, 7485
Zanier, Nicole: 3455, 8020
Zaninotto, Paola: 2365, 2460, 3125
Zanjani, Faika: 3565, 8000
Zúñiga, Franziska: 4255
Zarit, Steven H: 3525, 4010, 4165, 4335, 7005
Zaritsky, Rebecca: 4485
Zarzaur, Ben L: 3335
Zaslavsky, Oleg: 2530, 7465, 8015
Zauszniewski, Jaclene A: 7265, 7660
Zavala, Daisy V: 4185, 8020
Zea, Miranda: 4130, 7270, 7660
Zecevic, Aleksandra A: 1050, 7200
Zei, Paul: 3330
Zeinali, Nahid: 8040
Zeitler, Katie: 7465, 7905
Zeki Al Hazzouri, Adina: 1545
Zelinski, Elizabeth: 7460, 8000, 8020
Zell, Angela: 7105, 7640
Zeng, Derong: 7200, 8010
Zeng, Shengyin: 7080
Zeng, Xiaoyi: 7655
Zeng, Ziwei: 4195
Zettel-Watson, Laura: 7475
Zhai, Xiangyu: 1560, 8015
Zhan, Rongfang: 8080
Zhan, Shiyin: 8015
Zhang, Anna Yan: 7460
Zhang, Bo: 1570
Zhang, Cuntai: 3350, 7075, 7080
Zhang, Haihong: 1235, 3330
Zhang, Hengyuan: 7080, 7420
Zhang, Jiaan: 8000, 8020
Zhang, Jianguo: 7080
Zhang, Jiawei: 3125
Zhang, Jing: 3325
Zhang, Jing: 7280
Zhang, Jingwen: 1450
Zhang, Meina: 7735
Zhang, Meng: 3245, 7695, 7935
Zhang, Nan: 8010
Zhang, Peiling: 7425, 7930
Zhang, Peiyuan: 4195, 7020, 7055
Zhang, Pengyuan (Kelvin): 2515, 4380
Zhang, Qingqing: 3330
Zhang, Qiong: 7035, 7265
Zhang, Ruiqi: 2255
Zhang, Ruohan: 1575, 4420
Zhang, Shenghao: 4420
Zhang, Shiyang: 7540, 8020
Zhang, Shuo: 4030
Zhang, Sifeng: 7420
Zhang, Sixun: 3330
Zhang, Ting: 7515
Zhang, Wei: 3305, 8015
Zhang, Wencheng: 4455, 7535
Zhang, Wenjun: 4030
Zhang, Wenyu: 7410
Zhang, Wuyang: 2035, 7140
Zhang, Xiaomin: 7010, 7210, 7465, 8000
Zhang, Xin: 7605, 8000
Zhang, Xing: 2515
Zhang, Xiping: 8000
Zhang, Xuefeng: 8000
Zhang, Xuemin: 3270
Zhang, Yalu: 8010
Zhang, Yan: 7080
Zhang, Yanrong: 2465
Zhang, Yaqing: 7430
Zhang, Yichen: 8000
Zhang, Yichi: 3135
Zhang, Yiming: 7320
Zhang, Youjuan: 8095
Zhang, Yuan S: 2515, 4380, 4445
Zhang, Yuankai: 7360, 8015
Zhang, Yuchen: 7200
Zhang, Yufeng: 4535
Zhang, Yukai: 2030, 3480
Zhang, Yuping: 7425, 7930
Zhang, Zhang: 9030
Zhang, Zhe: 7420
Zhang, Zhenmei: 3270
Zhang, Zhenxiang: 3330
Zhang, Zheyuan: 7720
Zhang, Ziwei: 3490
Zhang, Ziying: 4345
Zhang, Ziying: 7040
Zhao, Dan: 8000, 8010
Zhao, Fengzhi: 7490, 7925
Zhao, Hui: 2460, 7065
Zhao, Jin: 3270
Zhao, Karrie: 8015
Zhao, Lirong: 8000
Zhao, Mengke: 3360
Zhao, Qianhua: 9000
Zhao, Shangang: 7000
Zhao, Shibei: 7110, 7420
Zhao, Wei: 1580
Zhao, Xinyi: 4115
Zhao, Yong: 7750, 8015
Zhao, Yuanchang: 3385
Zhen, Zhihong: 3505
Zheng, Anqing: 7225, 8020
Zheng, Diane: 8010
Zheng, Lidan: 8020
Zheng, Mingyang: 8010
Zheng, Qianyan: 7655
Zheng, Shimin: 8020
Zheng, Xujuan: 7710
Zheng, Yaguang: 1380, 7630, 8000, 8010
Zheng, Yu: 2040, 7080
Zhong, Chuwen: 3245, 7695
Zhong, Lily: 8015
Zhong, Selena: 1110, 8010
Zhong, YunYing: 2300, 7025, 8015, 8020
Zhou, Dan: 8015
Zhou, Jia-jia: 1215
Zhou, Jiayi: 1455
Zhou, Juliet: 4375
Zhou, Junhong: 4215, 4535
Zhou, Kangyi: 8020
Zhou, Leyi: 3365, 7030, 7540
Zhou, Lulin: 7020
Zhou, Nan: 1180, 7420
Zhou, Shally: 8020
Zhou, Shandong: 8010
Zhou, Shuai: 8090
Zhou, Tianqi: 3360
Zhou, Yu: 1560
Zhou, Yuanjin: 1060, 7655
Zhou, Yunshu: 1115
Zhou, Zexi: 4180, 7045, 7735
Zhu, Dantong: 7275
Zhu, Haoran: 7280
Zhu, Hongge: 3270
Zhu, Jiafeng: 1205
Zhu, Jie: 8020
Zhu, Junmin: 2255
Zhu, Ling: 7520, 7910
Zhu, Pengli: 7275, 7710
Zhu, Ruowei: 7320
Zhu, Shida: 7080
Zhu, Shijun: 8015, 8020
Zhu, Weiwei: 1265, 8010
Zhu, Xi: 2215
Zhu, Xiang: 8000
Zhu, Xiaofan: 4110
Zhu, Xiaoqian: 4440
Zhu, Yiwen: 3035
Zhu, Yujun: 7665, 8010
Zhu, Yun: 8000
Zhu, Zheng: 3305, 3505, 4030
Zhuang, Jia: 7615
Zhuang, Jie: 7615
Zhuang, Tianchi: 8000
Zieschang, Tania: 8015
Zimbinski, William J: 7455
Zimmer, Chantelle: 3465
Zimmer, Rachel: 8015

Zimmer, Zachary: 4050, 8020
Zimmerman, Elizabeth: 1490
Zimmerman, Luke: 1070
Zimmerman, M. Bridget: 7205
Zimmerman, Scott C: 1545
Zimmerman, Sheryl: 1520, 2105, 2480, 3000, 8010, 9040
Zimmermann, Jaroslava: 1285
Zisberg, Anna: 3465, 4040, 4045, 4075, 7265, 7400, 7920, 8010, 8020
Zlatar, Zvinka Z: 7720
Zmuda, Joseph M: 7010, 7060, 7625
Zoaktafi, Mojgan: 2360
Zólyomi, Eszter: 2060
Zonderman, Alan B: 1250, 2055, 2330
Zonderman, Alan: 8000
Zonker, Christina: 1125, 7425
Zonsius, Mary C: 1490
Zou, Baiming: 7535
Zou, Ning: 1355, 2355
Zubatsky, Max J: 8020
Zuber, Mohammed: 7200
Zuelsdorff, Megan: 2335
Zuendel, Matthias: 2140
Zullig, Leah L: 7685
Zuniga, Franziska: 4465
Zuo, Dongmei: 7060
Zuraw, Matthew: 7005, 7135
Zurek, Alina: 8010
Zurek, Grzegorz: 8010
Zwakhalen, Sandra MG: 4255
Zweibaum, David: 9015
