# Correction to: CHALLENGES AND KEY ASPECTS OF COLLABORATION BETWEEN VISITING NURSES AND CARE MANAGERS IN JAPAN

**DOI:** 10.1093/geroni/igaf060

**Published:** 2025-06-12

This is a correction to: Keiko Ono, CHALLENGES AND KEY ASPECTS OF COLLABORATION BETWEEN VISITING NURSES AND CARE MANAGERS IN JAPAN, *Innovation in Aging*, Volume 8, Issue Supplement_1, December 2024, Page 731, https://doi.org/10.1093/geroni/igae098.2384

Upon original publication, this abstract was mistakenly assigned an incorrect Digital Object Identifier (DOI) in the Full GSA 2024 Abstract Book PDF of Volume 8, Issue Supplement_1, December 2024, resulting in duplication of a different abstract’s DOI.

Additionally, this abstract was omitted from the HTML version of this issue.

These errors have now been corrected and the abstract has been republished at https://doi.org/10.1093/geroni/igae098.2384.

